# Supplementary material for: Macroevolutionary dynamics of gene family gain and loss along multicellular eukaryotic lineages
Source: Nat Commun. 2024 Mar 26;15:2663. doi: 10.1038/s41467-024-47017-w (PMC10966110; doi:10.1038/s41467-024-47017-w)
Supplement: Supplementary file 23 — Supplementary Dataset 18 [file 41467_2024_47017_MOESM23_ESM.pdf]

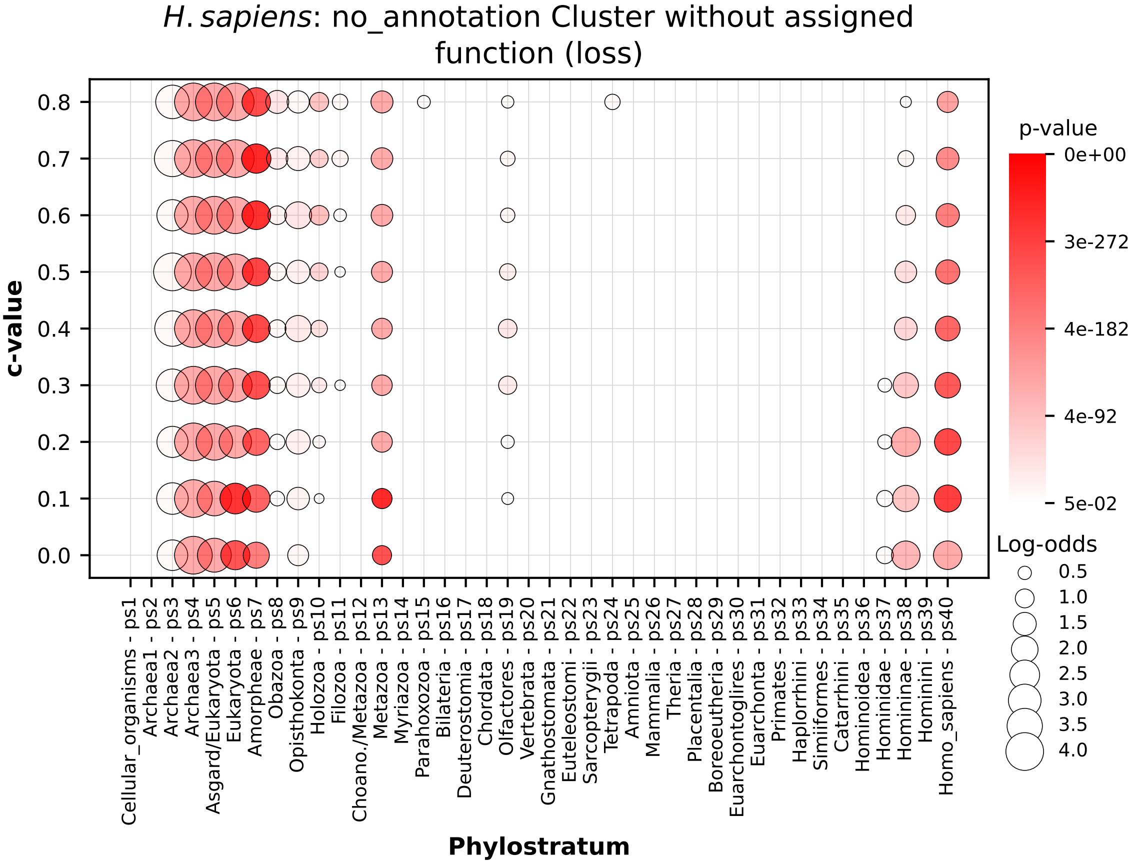

$H. sapiens$: no_annotation Cluster without assigned function (loss)
c-value
0.8
0.7
0.6
0.5
0.4
0.3
0.2
0.1
0.0
Phylostratum
Cellular_organisms - ps1
Archaea1 - ps2
Archaea2 - ps3
Archaea3 - ps4
Asgard/Eukaryota - ps5
Eukaryota - ps6
Amorpheae - ps7
Obazoa - ps8
Opisthokonta - ps9
Holozoa - ps10
Filozoa - ps11
Choano./Metazoa - ps12
Metazoa - ps13
Myriazoa - ps14
Parahoxozoa - ps15
Bilateria - ps16
Deuterostomia - ps17
Chordata - ps18
Olfactores - ps19
Vertebrata - ps20
Gnathostomata - ps21
Euteleostomi - ps22
Sarcopterygii - ps23
Tetrapoda - ps24
Amniota - ps25
Mammalia - ps26
Theria - ps27
Placentalia - ps28
Boreoeutheria - ps29
Euarchontoglires - ps30
Euarchonta - ps31
Primates - ps32
Haplorrhini - ps33
Simiiformes - ps34
Catarrhini - ps35
Hominoidea - ps36
Hominidae - ps37
Homininae - ps38
Hominini - ps39
Homo_sapiens - ps40
p-value
0e+00
3e-272
4e-182
4e-92
5e-02
Log-odds
0.5
1.0
1.5
2.0
2.5
3.0
3.5
4.0

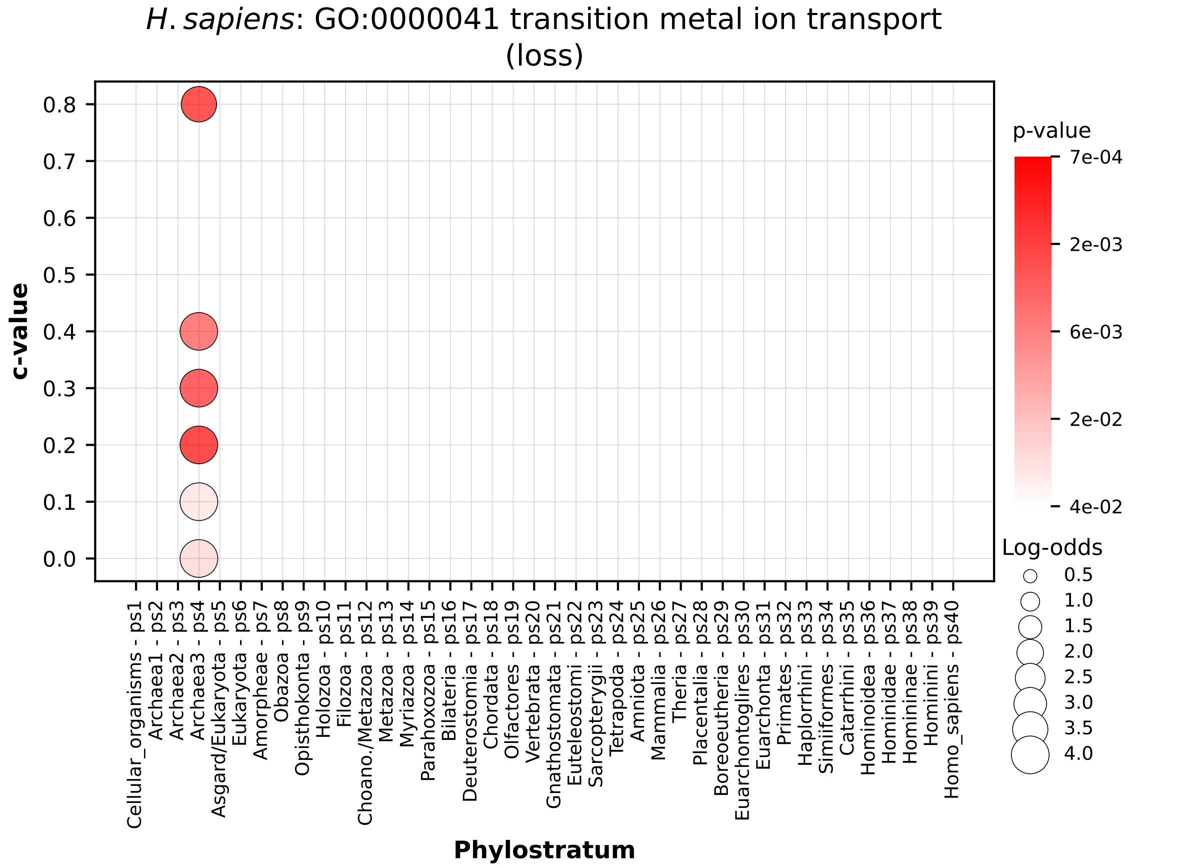

H. sapiens: GO:0000041 transition metal ion transport
(loss)
c-value
0.8
0.7
0.6
0.5
0.4
0.3
0.2
0.1
0.0
p-value
7e-04
2e-03
6e-03
2e-02
4e-02
Log-odds
0.5
1.0
1.5
2.0
2.5
3.0
3.5
4.0
Cellular_organisms - ps1
Archaea1 - ps2
Archaea2 - ps3
Archaea3 - ps4
Asgard/Eukaryota - ps5
Eukaryota - ps6
Amorpheae - ps7
Obazoa - ps8
Opisthokonta - ps9
Holozoa - ps10
Filozoa - ps11
Choano./Metazoa - ps12
Metazoa - ps13
Myriazoa - ps14
Parahoxozoa - ps15
Bilateria - ps16
Deuterostomia - ps17
Chordata - ps18
Olfactores - ps19
Vertebrata - ps20
Gnathostomata - ps21
Euteleostomi - ps22
Sarcopterygii - ps23
Tetrapoda - ps24
Amniota - ps25
Mammalia - ps26
Theria - ps27
Placentalia - ps28
Boreoeutheria - ps29
Euarchontoglires - ps30
Euarchonta - ps31
Primates - ps32
Haplorrhini - ps33
Simiiformes - ps34
Catarrhini - ps35
Hominoidea - ps36
Hominidae - ps37
Homininae - ps38
Hominini - ps39
Homo_sapiens - ps40
Phylostratum

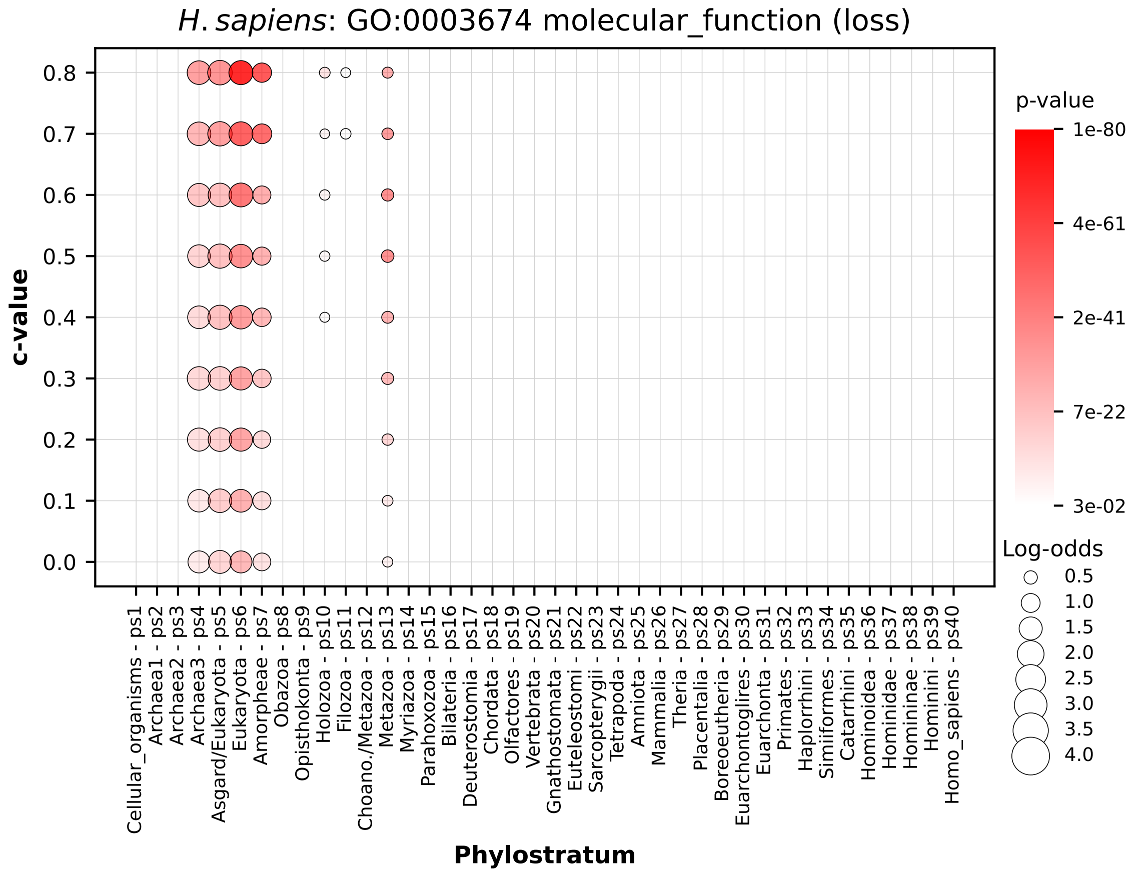
H. sapiens: GO:0003674 molecular_function (loss)
c-value
0.8
0.7
0.6
0.5
0.4
0.3
0.2
0.1
0.0
Phylostratum
Cellular_organisms - ps1
Archaea1 - ps2
Archaea2 - ps3
Archaea3 - ps4
Asgard/Eukaryota - ps5
Eukaryota - ps6
Amorpheae - ps7
Obazoa - ps8
Opisthokonta - ps9
Holozoa - ps10
Filozoa - ps11
Choano./Metazoa - ps12
Metazoa - ps13
Myriazoa - ps14
Parahoxozoa - ps15
Bilateria - ps16
Deuterostomia - ps17
Chordata - ps18
Olfactores - ps19
Vertebrata - ps20
Gnathostomata - ps21
Euteleostomi - ps22
Sarcopterygii - ps23
Tetrapoda - ps24
Amniota - ps25
Mammalia - ps26
Theria - ps27
Placentalia - ps28
Boreoeutheria - ps29
Euarchontoglires - ps30
Euarchonta - ps31
Primates - ps32
Haplorrhini - ps33
Simiiformes - ps34
Catarrhini - ps35
Hominoidea - ps36
Hominidae - ps37
Homininae - ps38
Hominini - ps39
Homo_sapiens - ps40
p-value
1e-80
4e-61
2e-41
7e-22
3e-02
Log-odds
0.5
1.0
1.5
2.0
2.5
3.0
3.5
4.0

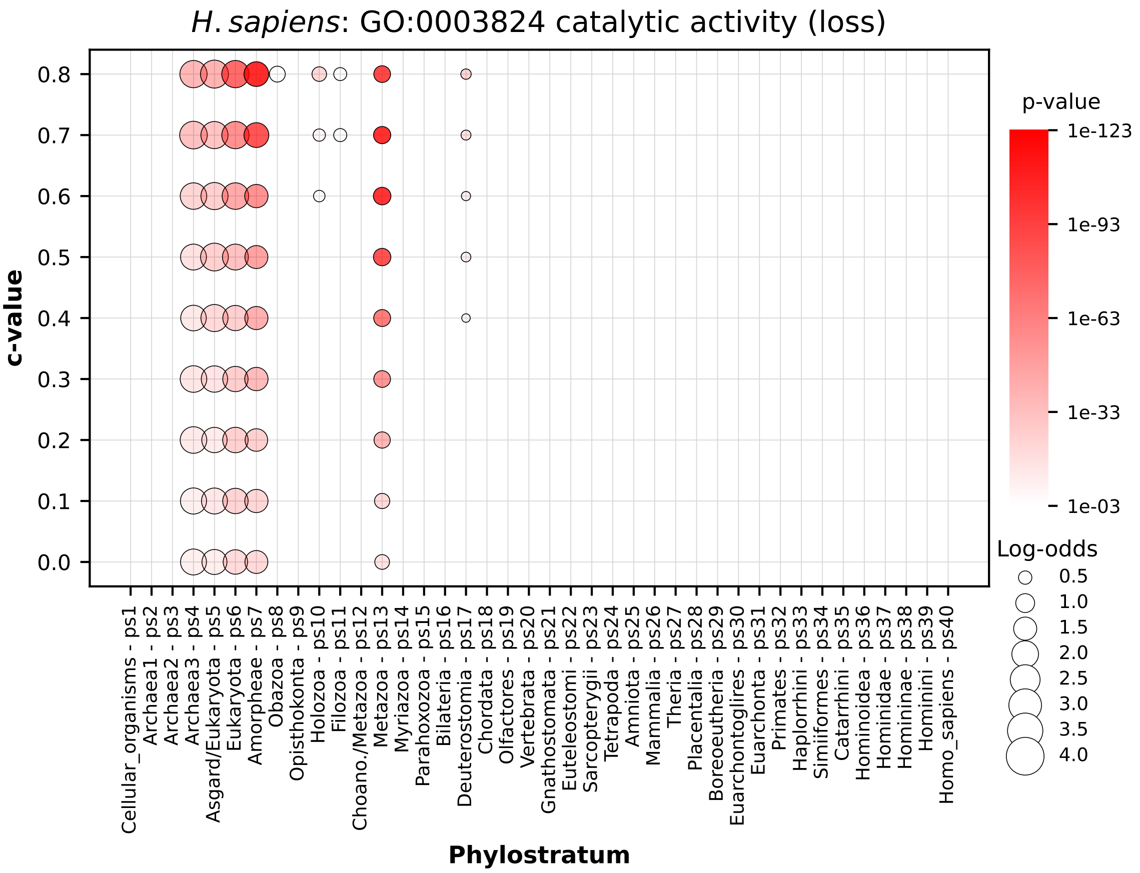

H. sapiens: GO:0003824 catalytic activity (loss)
c-value
0.8
0.7
0.6
0.5
0.4
0.3
0.2
0.1
0.0
Phylostratum
Cellular_organisms - ps1
Archaea1 - ps2
Archaea2 - ps3
Archaea3 - ps4
Asgard/Eukaryota - ps5
Eukaryota - ps6
Amorpheae - ps7
Obazoa - ps8
Opisthokonta - ps9
Holozoa - ps10
Filozoa - ps11
Choano./Metazoa - ps12
Metazoa - ps13
Myriazoa - ps14
Parahoxozoa - ps15
Bilateria - ps16
Deuterostomia - ps17
Chordata - ps18
Olfactores - ps19
Vertebrata - ps20
Gnathostomata - ps21
Euteleostomi - ps22
Sarcopterygii - ps23
Tetrapoda - ps24
Amniota - ps25
Mammalia - ps26
Theria - ps27
Placentalia - ps28
Boreoeutheria - ps29
Euarchontoglires - ps30
Euarchonta - ps31
Primates - ps32
Haplorrhini - ps33
Simiiformes - ps34
Catarrhini - ps35
Hominoidea - ps36
Hominidae - ps37
Homininae - ps38
Hominini - ps39
Homo_sapiens - ps40
p-value
1e-123
1e-93
1e-63
1e-33
1e-03
Log-odds
0.5
1.0
1.5
2.0
2.5
3.0
3.5
4.0

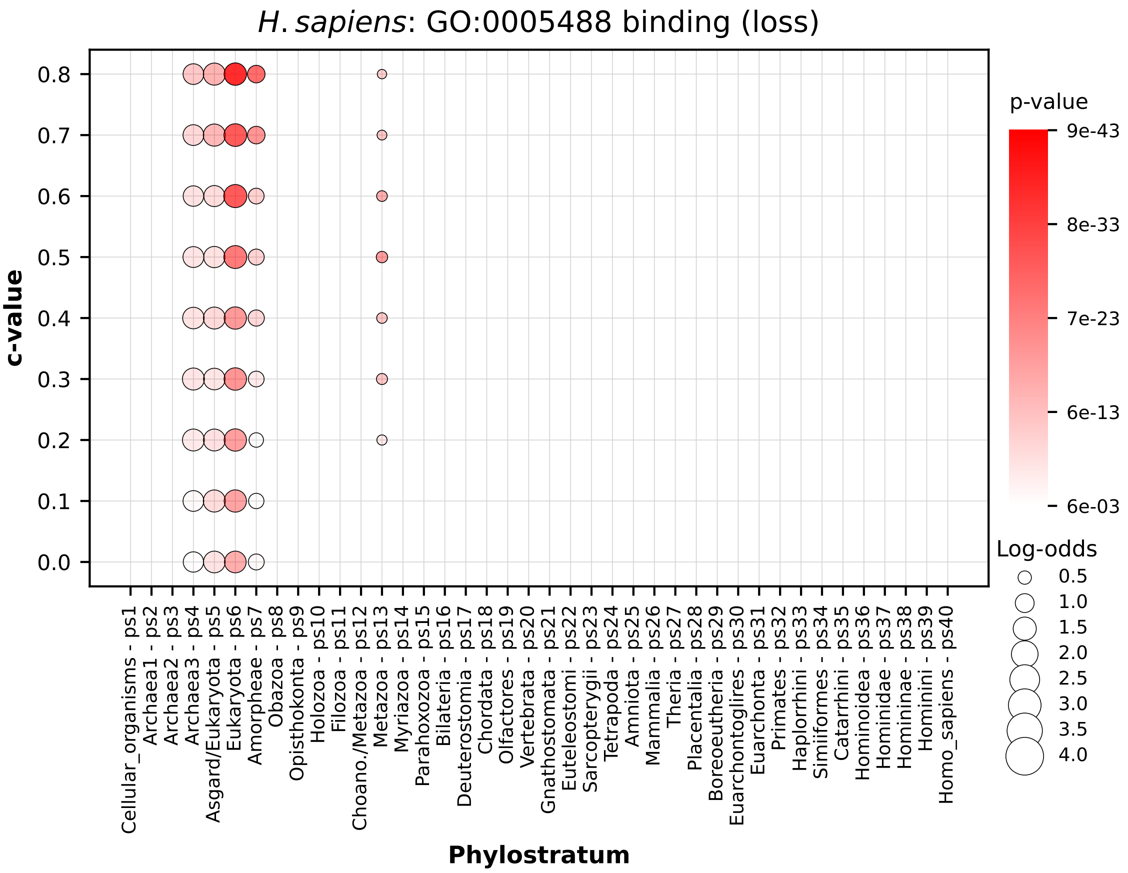

H. sapiens: GO:0005488 binding (loss)
c-value
0.0
0.1
0.2
0.3
0.4
0.5
0.6
0.7
0.8
p-value
9e-43
8e-33
7e-23
6e-13
6e-03
Log-odds
0.5
1.0
1.5
2.0
2.5
3.0
3.5
4.0
Cellular_organisms - ps1
Archaea1 - ps2
Archaea2 - ps3
Archaea3 - ps4
Asgard/Eukaryota - ps5
Eukaryota - ps6
Amorpheae - ps7
Obazoa - ps8
Opisthokonta - ps9
Holozoa - ps10
Filozoa - ps11
Choano./Metazoa - ps12
Metazoa - ps13
Myriazoa - ps14
Parahoxozoa - ps15
Bilateria - ps16
Deuterostomia - ps17
Chordata - ps18
Olfactores - ps19
Vertebrata - ps20
Gnathostomata - ps21
Euteleostomi - ps22
Sarcopterygii - ps23
Tetrapoda - ps24
Amniota - ps25
Mammalia - ps26
Theria - ps27
Placentalia - ps28
Boreoeutheria - ps29
Euarchontoglires - ps30
Euarchonta - ps31
Primates - ps32
Haplorrhini - ps33
Simiiformes - ps34
Catarrhini - ps35
Hominoidea - ps36
Hominidae - ps37
Homininae - ps38
Hominini - ps39
Homo_sapiens - ps40
Phylostratum

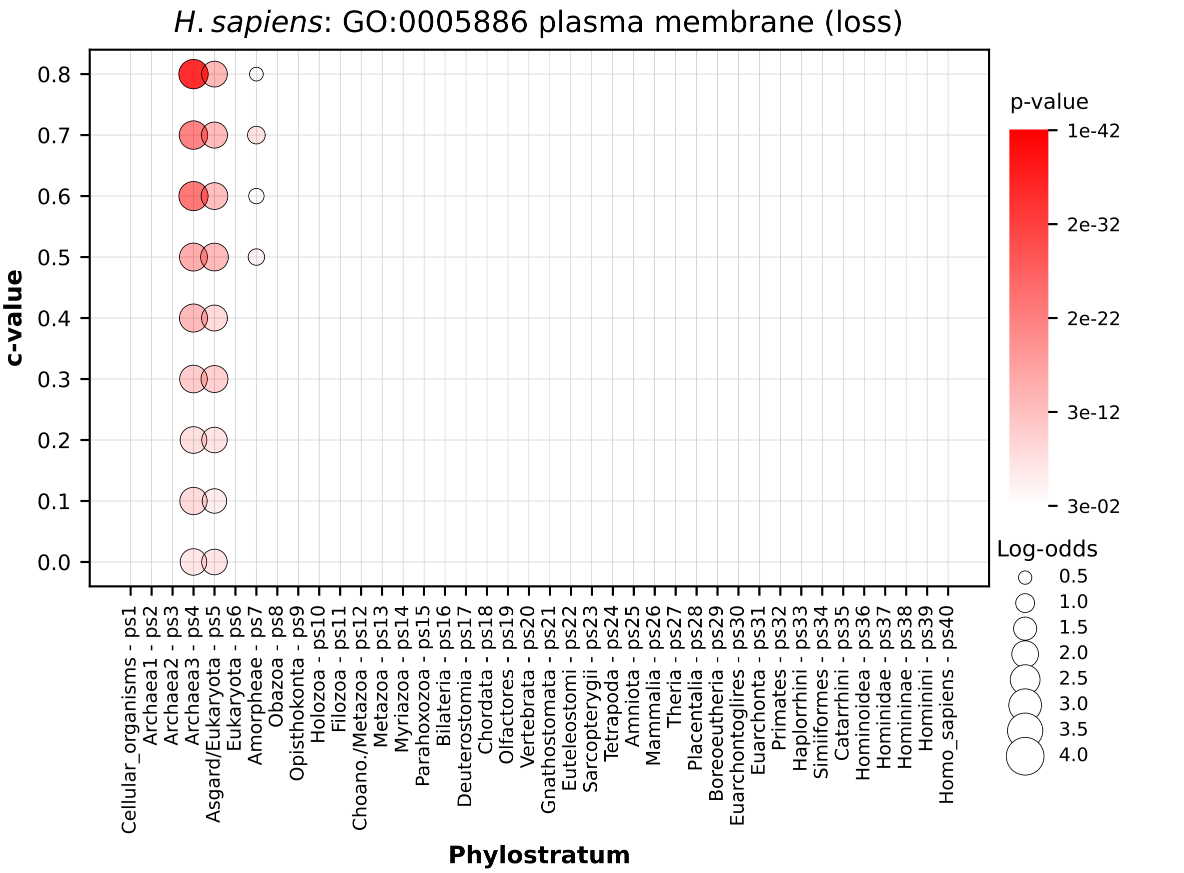

H. sapiens: GO:0005886 plasma membrane (loss)
c-value
0.0
0.1
0.2
0.3
0.4
0.5
0.6
0.7
0.8
Phylostratum
Cellular_organisms - ps1
Archaea1 - ps2
Archaea2 - ps3
Archaea3 - ps4
Asgard/Eukaryota - ps5
Eukaryota - ps6
Amorpheae - ps7
Obazoa - ps8
Opisthokonta - ps9
Holozoa - ps10
Filozoa - ps11
Choano./Metazoa - ps12
Metazoa - ps13
Myriazoa - ps14
Parahoxozoa - ps15
Bilateria - ps16
Deuterostomia - ps17
Chordata - ps18
Olfactores - ps19
Vertebrata - ps20
Gnathostomata - ps21
Euteleostomi - ps22
Sarcopterygii - ps23
Tetrapoda - ps24
Amniota - ps25
Mammalia - ps26
Theria - ps27
Placentalia - ps28
Boreoeutheria - ps29
Euarchontoglires - ps30
Euarchonta - ps31
Primates - ps32
Haplorrhini - ps33
Simiiformes - ps34
Catarrhini - ps35
Hominoidea - ps36
Hominidae - ps37
Homininae - ps38
Hominini - ps39
Homo_sapiens - ps40
p-value
1e-42
2e-32
2e-22
3e-12
3e-02
Log-odds
0.5
1.0
1.5
2.0
2.5
3.0
3.5
4.0

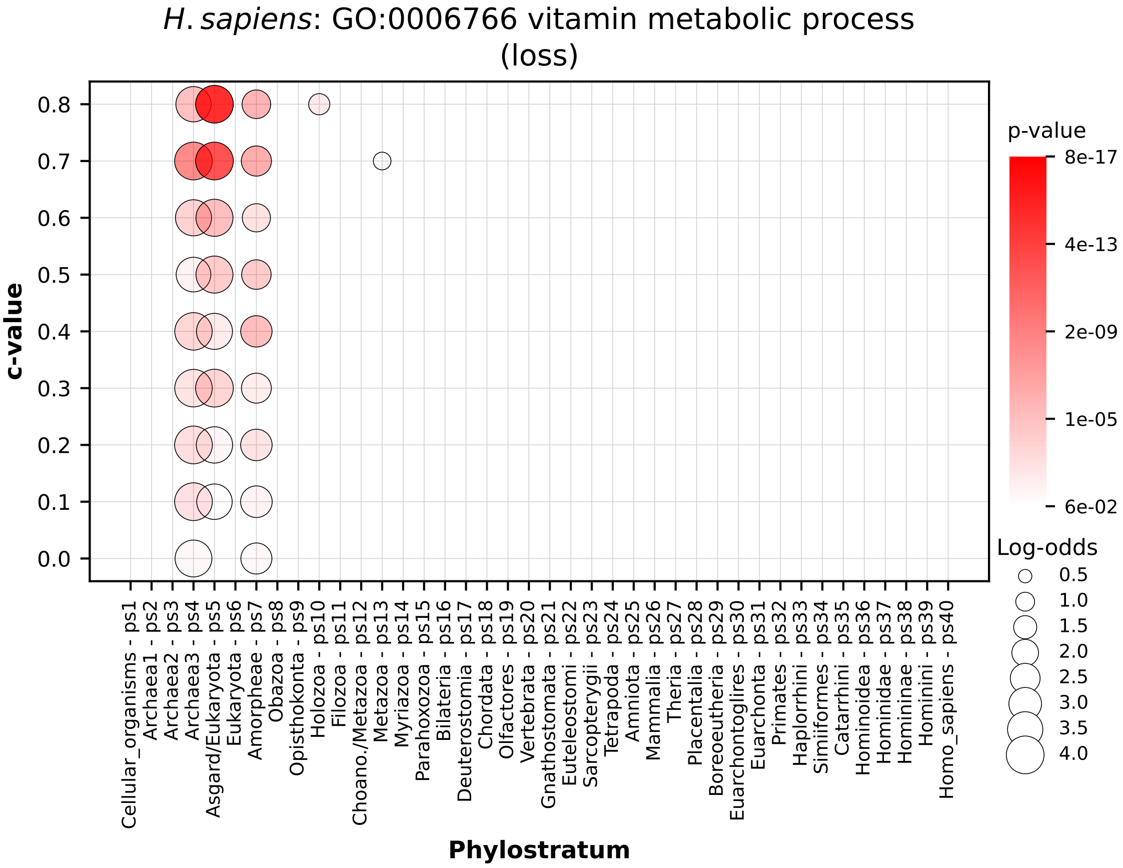

H. sapiens: GO:0006766 vitamin metabolic process
(loss)
c-value
0.8
0.7
0.6
0.5
0.4
0.3
0.2
0.1
0.0
p-value
8e-17
4e-13
2e-09
1e-05
6e-02
Log-odds
0.5
1.0
1.5
2.0
2.5
3.0
3.5
4.0
Cellular_organisms - ps1
Archaea1 - ps2
Archaea2 - ps3
Archaea3 - ps4
Asgard/Eukaryota - ps5
Eukaryota - ps6
Amorpheae - ps7
Obazoa - ps8
Opisthokonta - ps9
Holozoa - ps10
Filozoa - ps11
Choano./Metazoa - ps12
Metazoa - ps13
Myriazoa - ps14
Parahoxozoa - ps15
Bilateria - ps16
Deuterostomia - ps17
Chordata - ps18
Olfactores - ps19
Vertebrata - ps20
Gnathostomata - ps21
Euteleostomi - ps22
Sarcopterygii - ps23
Tetrapoda - ps24
Amniota - ps25
Mammalia - ps26
Theria - ps27
Placentalia - ps28
Boreoeutheria - ps29
Euarchontoglires - ps30
Euarchonta - ps31
Primates - ps32
Haplorrhini - ps33
Simiiformes - ps34
Catarrhini - ps35
Hominoidea - ps36
Hominidae - ps37
Homininae - ps38
Hominini - ps39
Homo_sapiens - ps40
Phylostratum

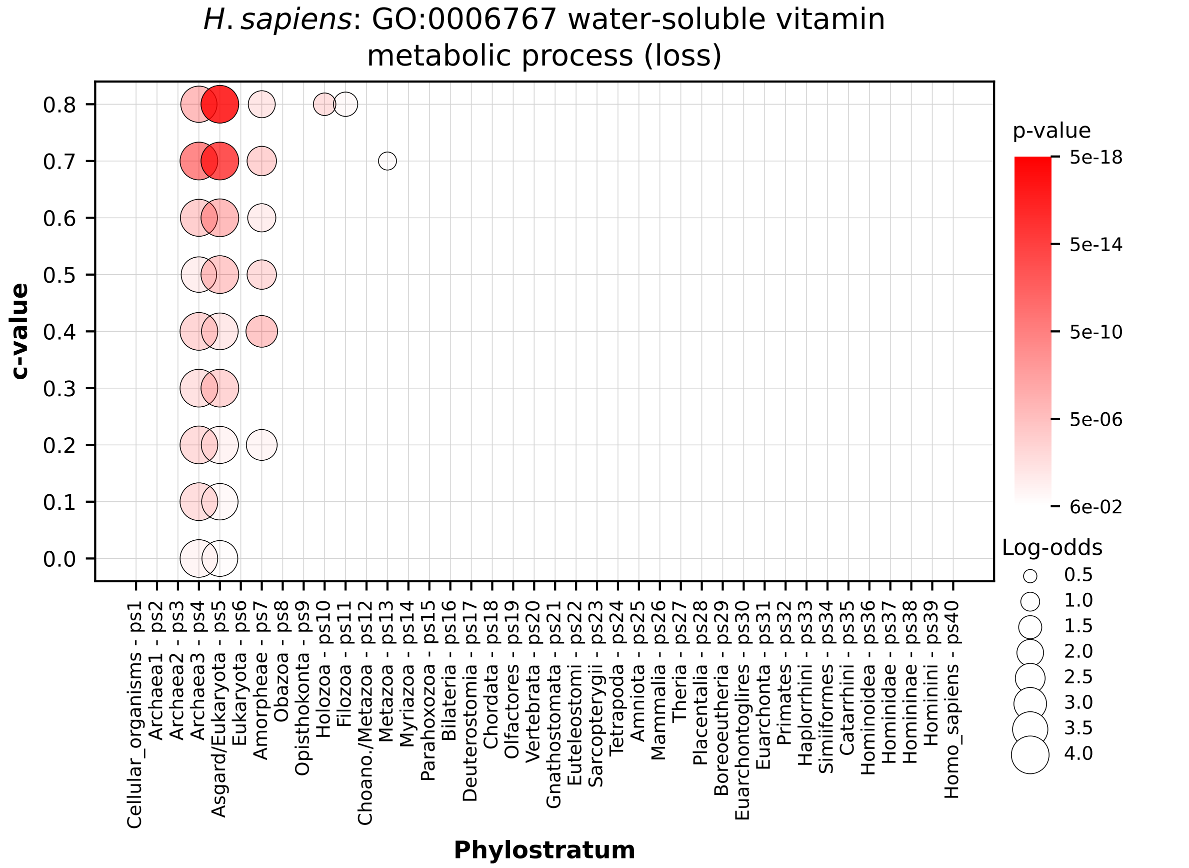
H. sapiens: GO:0006767 water-soluble vitamin metabolic process (loss)
c-value
0.8
0.7
0.6
0.5
0.4
0.3
0.2
0.1
0.0
p-value
5e-18
5e-14
5e-10
5e-06
6e-02
Log-odds
0.5
1.0
1.5
2.0
2.5
3.0
3.5
4.0
Cellular_organisms - ps1
Archaea1 - ps2
Archaea2 - ps3
Archaea3 - ps4
Asgard/Eukaryota - ps5
Eukaryota - ps6
Amorpheae - ps7
Obazoa - ps8
Opisthokonta - ps9
Holozoa - ps10
Filozoa - ps11
Choano./Metazoa - ps12
Metazoa - ps13
Myriazoa - ps14
Parahoxozoa - ps15
Bilateria - ps16
Deuterostomia - ps17
Chordata - ps18
Olfactores - ps19
Vertebrata - ps20
Gnathostomata - ps21
Euteleostomi - ps22
Sarcopterygii - ps23
Tetrapoda - ps24
Amniota - ps25
Mammalia - ps26
Theria - ps27
Placentalia - ps28
Boreoeutheria - ps29
Euarchontoglires - ps30
Euarchonta - ps31
Primates - ps32
Haplorrhini - ps33
Simiiformes - ps34
Catarrhini - ps35
Hominoidea - ps36
Hominidae - ps37
Homininae - ps38
Hominini - ps39
Homo_sapiens - ps40
Phylostratum

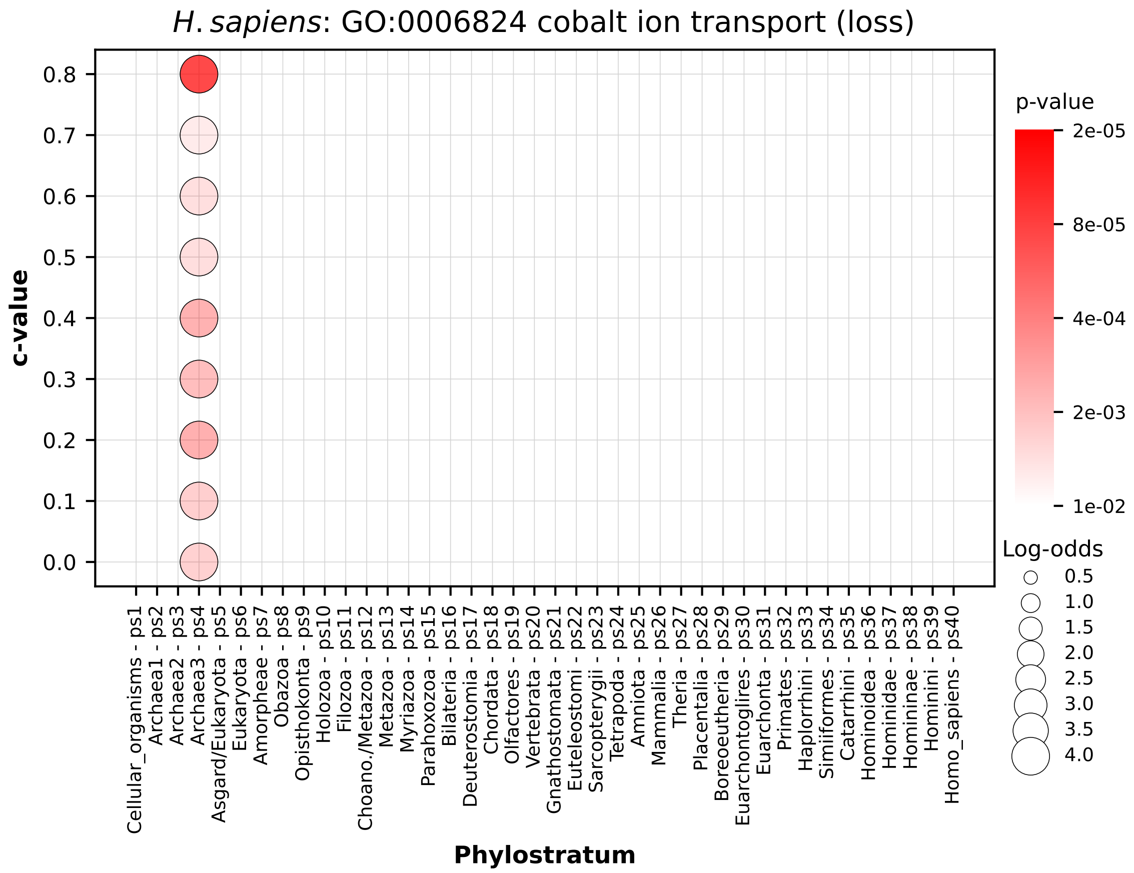

H. sapiens: GO:0006824 cobalt ion transport (loss)
c-value
0.8
0.7
0.6
0.5
0.4
0.3
0.2
0.1
0.0
p-value
2e-05
8e-05
4e-04
2e-03
1e-02
Log-odds
0.5
1.0
1.5
2.0
2.5
3.0
3.5
4.0
Cellular_organisms - ps1
Archaea1 - ps2
Archaea2 - ps3
Archaea3 - ps4
Asgard/Eukaryota - ps5
Eukaryota - ps6
Amorpheae - ps7
Obazoa - ps8
Opisthokonta - ps9
Holozoa - ps10
Filozoa - ps11
Choano./Metazoa - ps12
Metazoa - ps13
Myriazoa - ps14
Parahoxozoa - ps15
Bilateria - ps16
Deuterostomia - ps17
Chordata - ps18
Olfactores - ps19
Vertebrata - ps20
Gnathostomata - ps21
Euteleostomi - ps22
Sarcopterygii - ps23
Tetrapoda - ps24
Amniota - ps25
Mammalia - ps26
Theria - ps27
Placentalia - ps28
Boreoeutheria - ps29
Euarchontoglires - ps30
Euarchonta - ps31
Primates - ps32
Haplorrhini - ps33
Simiiformes - ps34
Catarrhini - ps35
Hominoidea - ps36
Hominidae - ps37
Homininae - ps38
Hominini - ps39
Homo_sapiens - ps40
Phylostratum

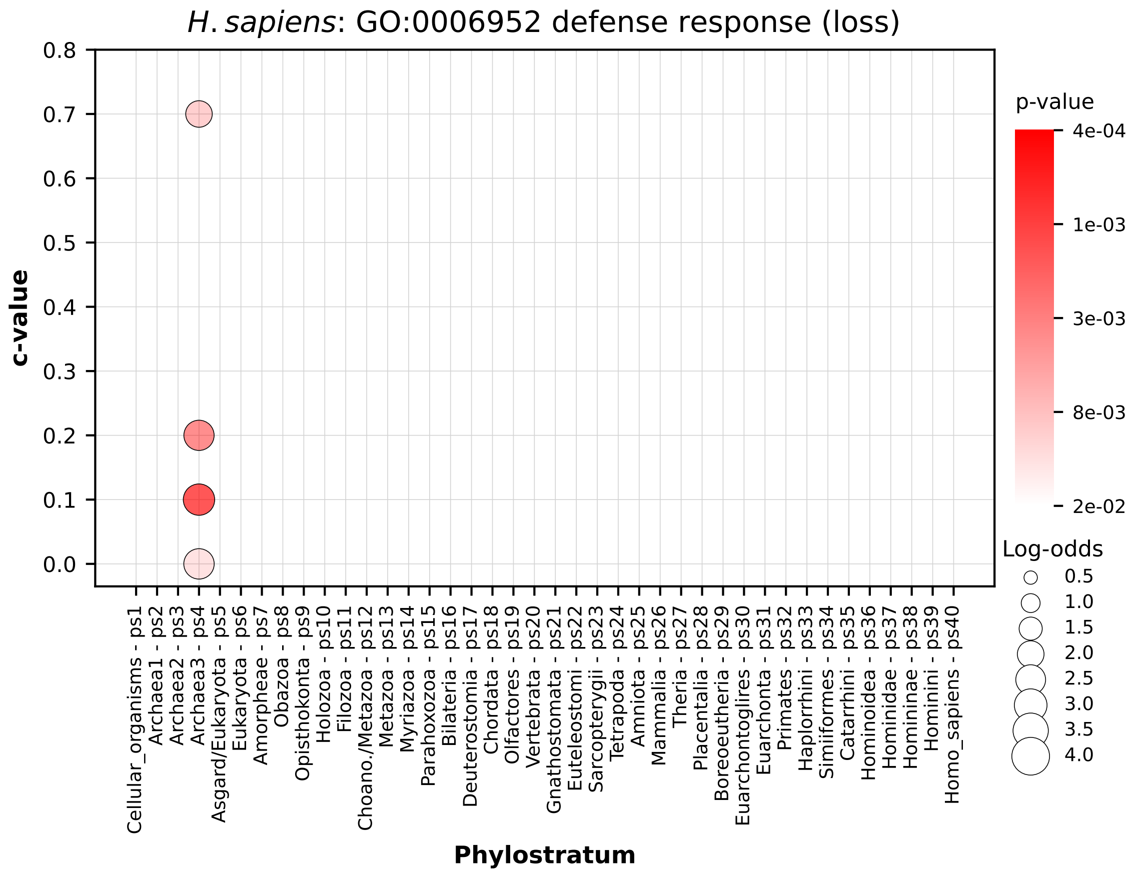

H. sapiens: GO:0006952 defense response (loss)
c-value
0.0
0.1
0.2
0.3
0.4
0.5
0.6
0.7
0.8
Phylostratum
Cellular_organisms - ps1
Archaea1 - ps2
Archaea2 - ps3
Archaea3 - ps4
Asgard/Eukaryota - ps5
Eukaryota - ps6
Amorpheae - ps7
Obazoa - ps8
Opisthokonta - ps9
Holozoa - ps10
Filozoa - ps11
Choano./Metazoa - ps12
Metazoa - ps13
Myriazoa - ps14
Parahoxozoa - ps15
Bilateria - ps16
Deuterostomia - ps17
Chordata - ps18
Olfactores - ps19
Vertebrata - ps20
Gnathostomata - ps21
Euteleostomi - ps22
Sarcopterygii - ps23
Tetrapoda - ps24
Amniota - ps25
Mammalia - ps26
Theria - ps27
Placentalia - ps28
Boreoeutheria - ps29
Euarchontoglires - ps30
Euarchonta - ps31
Primates - ps32
Haplorrhini - ps33
Simiiformes - ps34
Catarrhini - ps35
Hominoidea - ps36
Hominidae - ps37
Homininae - ps38
Hominini - ps39
Homo_sapiens - ps40
p-value
4e-04
1e-03
3e-03
8e-03
2e-02
Log-odds
0.5
1.0
1.5
2.0
2.5
3.0
3.5
4.0

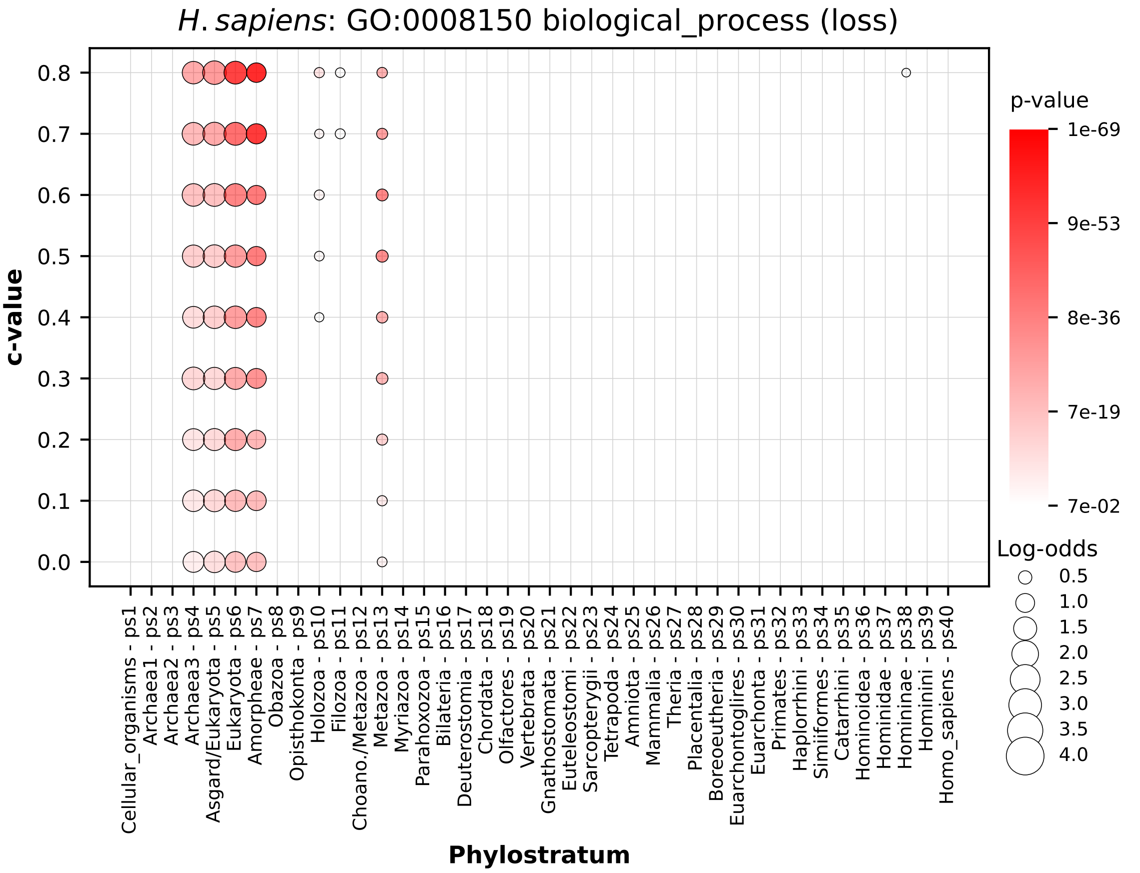

H. sapiens: GO:0008150 biological_process (loss)
c-value
0.8
0.7
0.6
0.5
0.4
0.3
0.2
0.1
0.0
p-value
1e-69
9e-53
8e-36
7e-19
7e-02
Log-odds
0.5
1.0
1.5
2.0
2.5
3.0
3.5
4.0
Cellular_organisms - ps1
Archaea1 - ps2
Archaea2 - ps3
Archaea3 - ps4
Asgard/Eukaryota - ps5
Eukaryota - ps6
Amorpheae - ps7
Obazoa - ps8
Opisthokonta - ps9
Holozoa - ps10
Filozoa - ps11
Choano./Metazoa - ps12
Metazoa - ps13
Myriazoa - ps14
Parahoxozoa - ps15
Bilateria - ps16
Deuterostomia - ps17
Chordata - ps18
Olfactores - ps19
Vertebrata - ps20
Gnathostomata - ps21
Euteleostomi - ps22
Sarcopterygii - ps23
Tetrapoda - ps24
Amniota - ps25
Mammalia - ps26
Theria - ps27
Placentalia - ps28
Boreoeutheria - ps29
Euarchontoglires - ps30
Euarchonta - ps31
Primates - ps32
Haplorrhini - ps33
Simiiformes - ps34
Catarrhini - ps35
Hominoidea - ps36
Hominidae - ps37
Homininae - ps38
Hominini - ps39
Homo_sapiens - ps40
Phylostratum

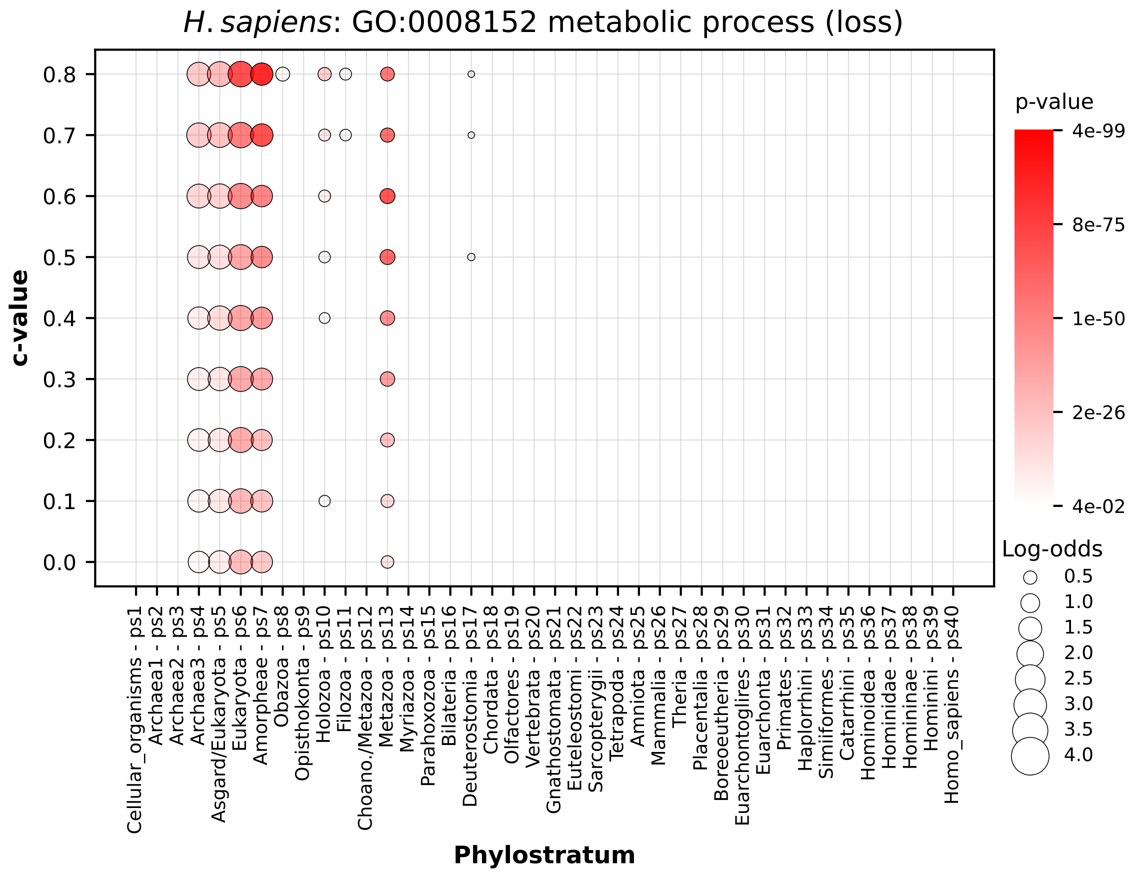

H. sapiens: GO:0008152 metabolic process (loss)
c-value
0.8
0.7
0.6
0.5
0.4
0.3
0.2
0.1
0.0
p-value
4e-99
8e-75
1e-50
2e-26
4e-02
Log-odds
0.5
1.0
1.5
2.0
2.5
3.0
3.5
4.0
Cellular_organisms - ps1
Archaea1 - ps2
Archaea2 - ps3
Archaea3 - ps4
Asgard/Eukaryota - ps5
Eukaryota - ps6
Amorpheae - ps7
Obazoa - ps8
Opisthokonta - ps9
Holozoa - ps10
Filozoa - ps11
Choano./Metazoa - ps12
Metazoa - ps13
Myriazoa - ps14
Parahoxozoa - ps15
Bilateria - ps16
Deuterostomia - ps17
Chordata - ps18
Olfactores - ps19
Vertebrata - ps20
Gnathostomata - ps21
Euteleostomi - ps22
Sarcopterygii - ps23
Tetrapoda - ps24
Amniota - ps25
Mammalia - ps26
Theria - ps27
Placentalia - ps28
Boreoeutheria - ps29
Euarchontoglires - ps30
Euarchonta - ps31
Primates - ps32
Haplorrhini - ps33
Simiiformes - ps34
Catarrhini - ps35
Hominoidea - ps36
Hominidae - ps37
Homininae - ps38
Hominini - ps39
Homo_sapiens - ps40
Phylostratum

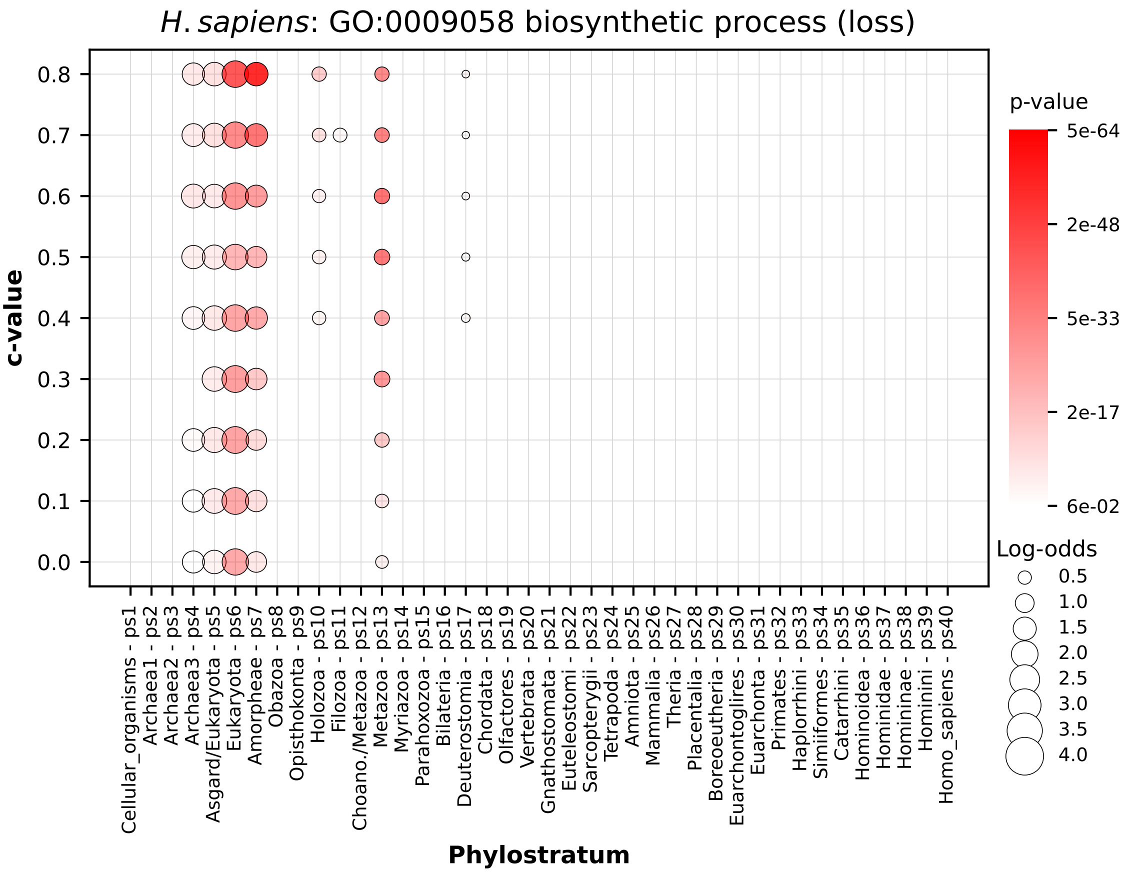
H. sapiens: GO:0009058 biosynthetic process (loss)
c-value
0.8
0.7
0.6
0.5
0.4
0.3
0.2
0.1
0.0
p-value
5e-64
2e-48
5e-33
2e-17
6e-02
Log-odds
0.5
1.0
1.5
2.0
2.5
3.0
3.5
4.0
Cellular_organisms - ps1
Archaea1 - ps2
Archaea2 - ps3
Archaea3 - ps4
Asgard/Eukaryota - ps5
Eukaryota - ps6
Amorpheae - ps7
Obazoa - ps8
Opisthokonta - ps9
Holozoa - ps10
Filozoa - ps11
Choano./Metazoa - ps12
Metazoa - ps13
Myriazoa - ps14
Parahoxozoa - ps15
Bilateria - ps16
Deuterostomia - ps17
Chordata - ps18
Olfactores - ps19
Vertebrata - ps20
Gnathostomata - ps21
Euteleostomi - ps22
Sarcopterygii - ps23
Tetrapoda - ps24
Amniota - ps25
Mammalia - ps26
Theria - ps27
Placentalia - ps28
Boreoeutheria - ps29
Euarchontoglires - ps30
Euarchonta - ps31
Primates - ps32
Haplorrhini - ps33
Simiiformes - ps34
Catarrhini - ps35
Hominoidea - ps36
Hominidae - ps37
Homininae - ps38
Hominini - ps39
Homo_sapiens - ps40
Phylostratum

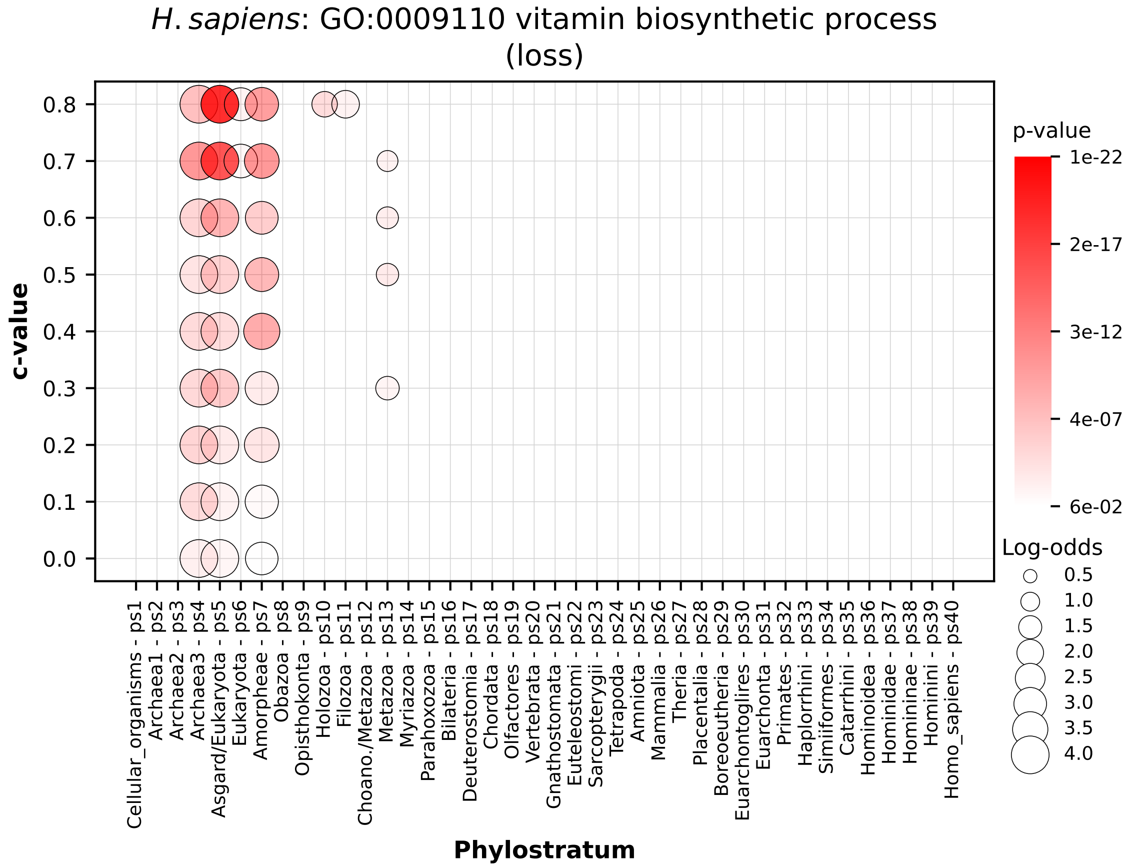

H. sapiens: GO:0009110 vitamin biosynthetic process
(loss)
c-value
0.8
0.7
0.6
0.5
0.4
0.3
0.2
0.1
0.0
p-value
1e-22
2e-17
3e-12
4e-07
6e-02
Log-odds
0.5
1.0
1.5
2.0
2.5
3.0
3.5
4.0
Cellular_organisms - ps1
Archaea1 - ps2
Archaea2 - ps3
Archaea3 - ps4
Asgard/Eukaryota - ps5
Eukaryota - ps6
Amorpheae - ps7
Obazoa - ps8
Opisthokonta - ps9
Holozoa - ps10
Filozoa - ps11
Choano./Metazoa - ps12
Metazoa - ps13
Myriazoa - ps14
Parahoxozoa - ps15
Bilateria - ps16
Deuterostomia - ps17
Chordata - ps18
Olfactores - ps19
Vertebrata - ps20
Gnathostomata - ps21
Euteleostomi - ps22
Sarcopterygii - ps23
Tetrapoda - ps24
Amniota - ps25
Mammalia - ps26
Theria - ps27
Placentalia - ps28
Boreoeutheria - ps29
Euarchontoglires - ps30
Euarchonta - ps31
Primates - ps32
Haplorrhini - ps33
Simiiformes - ps34
Catarrhini - ps35
Hominoidea - ps36
Hominidae - ps37
Homininae - ps38
Hominini - ps39
Homo_sapiens - ps40
Phylostratum

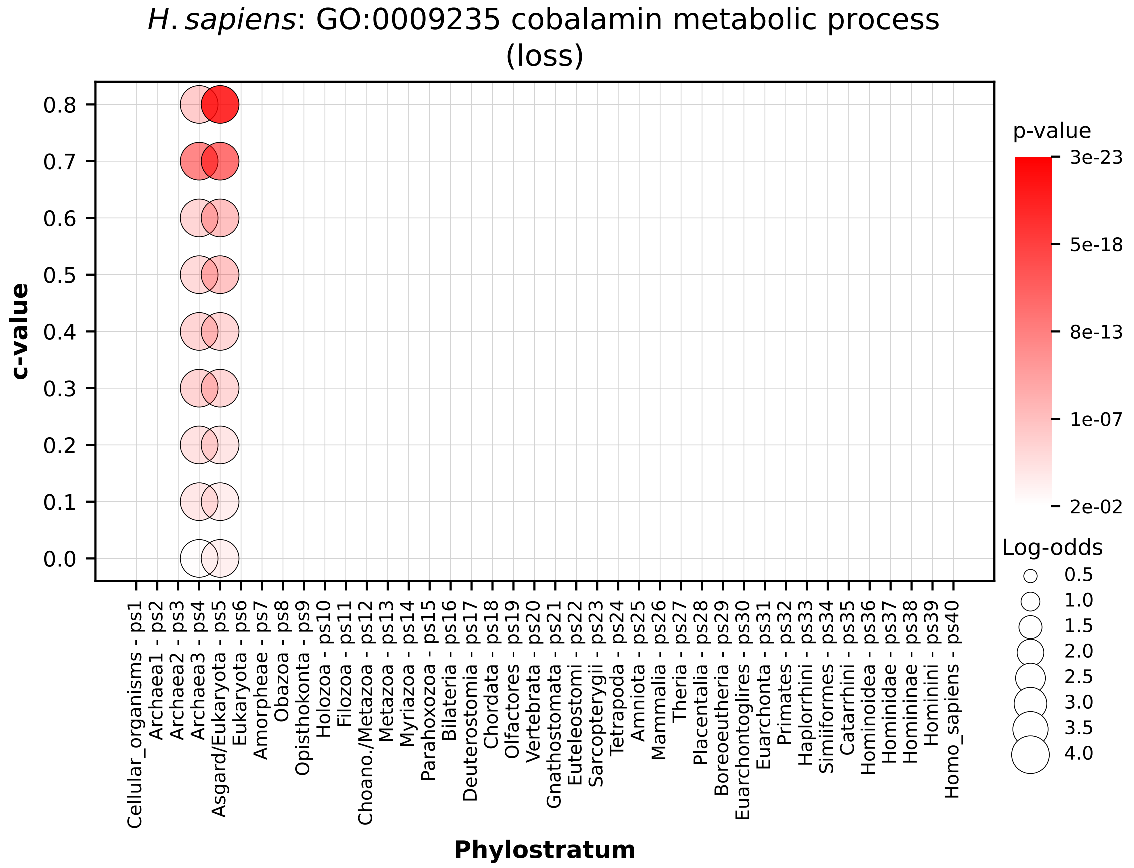

H. sapiens: GO:0009235 cobalamin metabolic process
(loss)
c-value
0.8
0.7
0.6
0.5
0.4
0.3
0.2
0.1
0.0
p-value
3e-23
5e-18
8e-13
1e-07
2e-02
Log-odds
0.5
1.0
1.5
2.0
2.5
3.0
3.5
4.0
Cellular_organisms - ps1
Archaea1 - ps2
Archaea2 - ps3
Archaea3 - ps4
Asgard/Eukaryota - ps5
Eukaryota - ps6
Amorpheae - ps7
Obazoa - ps8
Opisthokonta - ps9
Holozoa - ps10
Filozoa - ps11
Choano./Metazoa - ps12
Metazoa - ps13
Myriazoa - ps14
Parahoxozoa - ps15
Bilateria - ps16
Deuterostomia - ps17
Chordata - ps18
Olfactores - ps19
Vertebrata - ps20
Gnathostomata - ps21
Euteleostomi - ps22
Sarcopterygii - ps23
Tetrapoda - ps24
Amniota - ps25
Mammalia - ps26
Theria - ps27
Placentalia - ps28
Boreoeutheria - ps29
Euarchontoglires - ps30
Euarchonta - ps31
Primates - ps32
Haplorrhini - ps33
Simiiformes - ps34
Catarrhini - ps35
Hominoidea - ps36
Hominidae - ps37
Homininae - ps38
Hominini - ps39
Homo_sapiens - ps40
Phylostratum

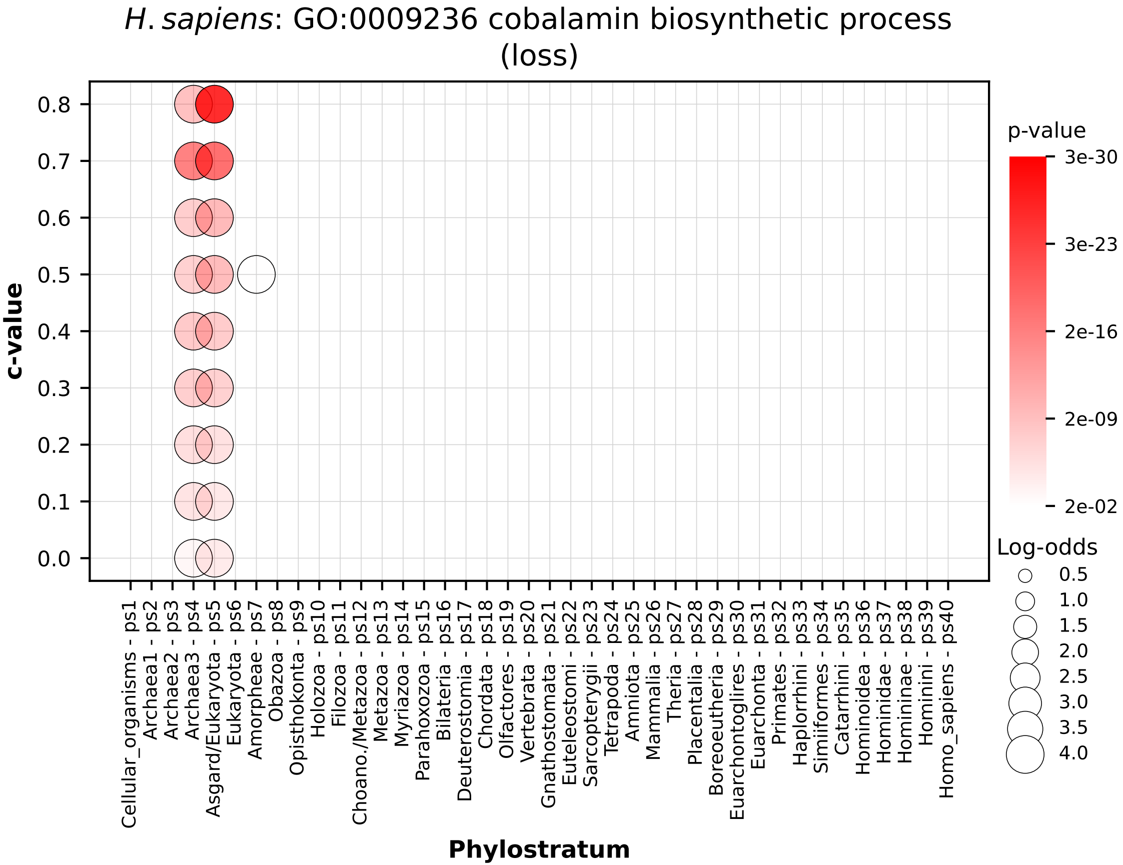

H. sapiens: GO:0009236 cobalamin biosynthetic process
(loss)
c-value
0.8
0.7
0.6
0.5
0.4
0.3
0.2
0.1
0.0
Phylostratum
Cellular_organisms - ps1
Archaea1 - ps2
Archaea2 - ps3
Archaea3 - ps4
Asgard/Eukaryota - ps5
Eukaryota - ps6
Amorpheae - ps7
Obazoa - ps8
Opisthokonta - ps9
Holozoa - ps10
Filozoa - ps11
Choano./Metazoa - ps12
Metazoa - ps13
Myriazoa - ps14
Parahoxozoa - ps15
Bilateria - ps16
Deuterostomia - ps17
Chordata - ps18
Olfactores - ps19
Vertebrata - ps20
Gnathostomata - ps21
Euteleostomi - ps22
Sarcopterygii - ps23
Tetrapoda - ps24
Amniota - ps25
Mammalia - ps26
Theria - ps27
Placentalia - ps28
Boreoeutheria - ps29
Euarchontoglires - ps30
Euarchonta - ps31
Primates - ps32
Haplorrhini - ps33
Simiiformes - ps34
Catarrhini - ps35
Hominoidea - ps36
Hominidae - ps37
Homininae - ps38
Hominini - ps39
Homo_sapiens - ps40
p-value
3e-30
3e-23
2e-16
2e-09
2e-02
Log-odds
0.5
1.0
1.5
2.0
2.5
3.0
3.5
4.0

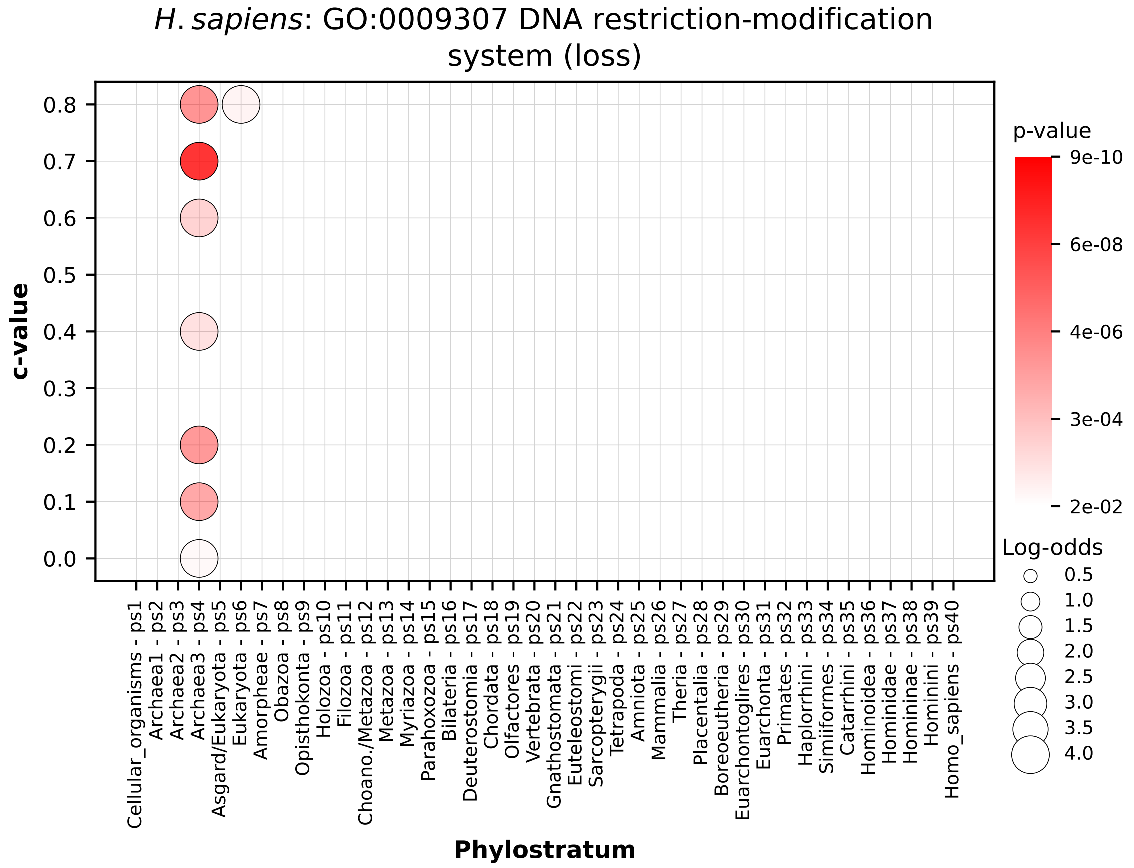

H. sapiens: GO:0009307 DNA restriction-modification system (loss)
c-value
0.0
0.1
0.2
0.3
0.4
0.5
0.6
0.7
0.8
Phylostratum
Cellular_organisms - ps1
Archaea1 - ps2
Archaea2 - ps3
Archaea3 - ps4
Asgard/Eukaryota - ps5
Eukaryota - ps6
Amorpheae - ps7
Obazoa - ps8
Opisthokonta - ps9
Holozoa - ps10
Filozoa - ps11
Choano./Metazoa - ps12
Metazoa - ps13
Myriazoa - ps14
Parahoxozoa - ps15
Bilateria - ps16
Deuterostomia - ps17
Chordata - ps18
Olfactores - ps19
Vertebrata - ps20
Gnathostomata - ps21
Euteleostomi - ps22
Sarcopterygii - ps23
Tetrapoda - ps24
Amniota - ps25
Mammalia - ps26
Theria - ps27
Placentalia - ps28
Boreoeutheria - ps29
Euarchontoglires - ps30
Euarchonta - ps31
Primates - ps32
Haplorrhini - ps33
Simiiformes - ps34
Catarrhini - ps35
Hominoidea - ps36
Hominidae - ps37
Homininae - ps38
Hominini - ps39
Homo_sapiens - ps40
p-value
9e-10
6e-08
4e-06
3e-04
2e-02
Log-odds
0.5
1.0
1.5
2.0
2.5
3.0
3.5
4.0

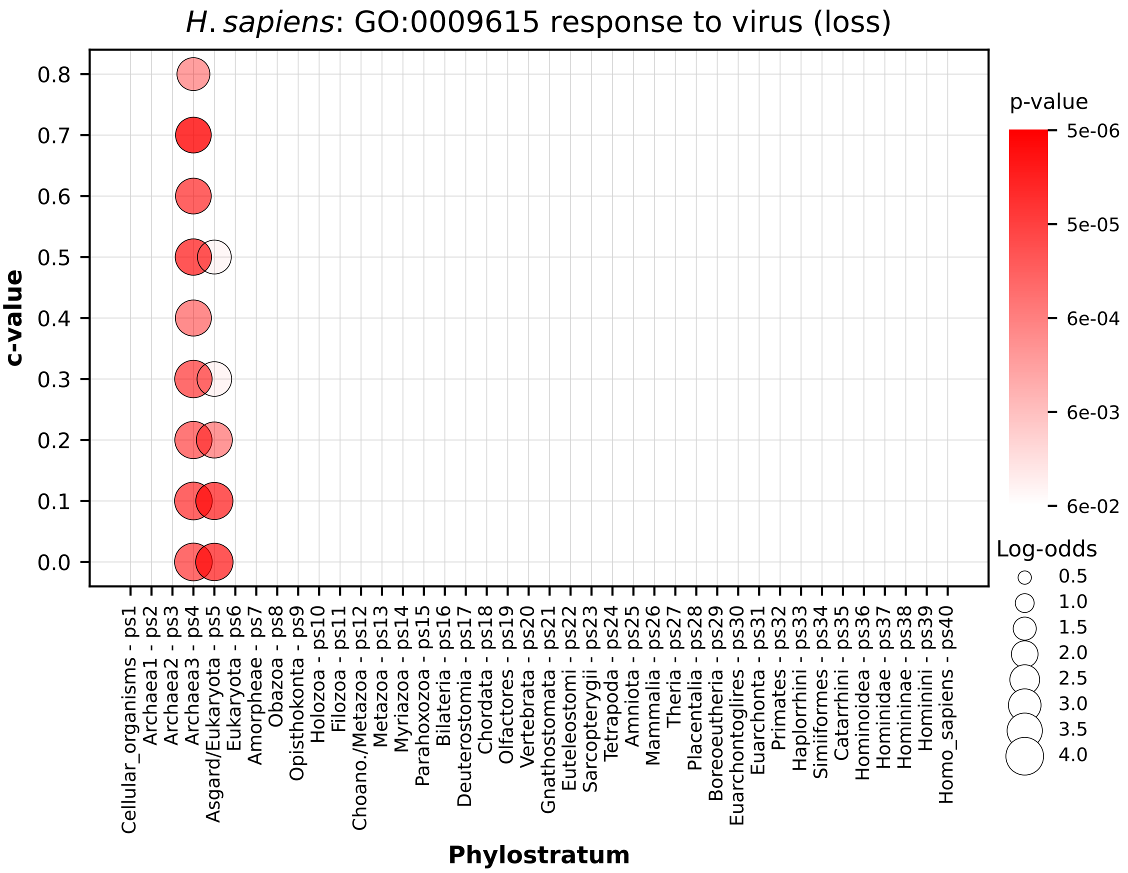

H. sapiens: GO:0009615 response to virus (loss)
c-value
0.8
0.7
0.6
0.5
0.4
0.3
0.2
0.1
0.0
Phylostratum
Cellular_organisms - ps1
Archaea1 - ps2
Archaea2 - ps3
Archaea3 - ps4
Asgard/Eukaryota - ps5
Eukaryota - ps6
Amorpheae - ps7
Obazoa - ps8
Opisthokonta - ps9
Holozoa - ps10
Filozoa - ps11
Choano./Metazoa - ps12
Metazoa - ps13
Myriazoa - ps14
Parahoxozoa - ps15
Bilateria - ps16
Deuterostomia - ps17
Chordata - ps18
Olfactores - ps19
Vertebrata - ps20
Gnathostomata - ps21
Euteleostomi - ps22
Sarcopterygii - ps23
Tetrapoda - ps24
Amniota - ps25
Mammalia - ps26
Theria - ps27
Placentalia - ps28
Boreoeutheria - ps29
Euarchontoglires - ps30
Euarchonta - ps31
Primates - ps32
Haplorrhini - ps33
Simiiformes - ps34
Catarrhini - ps35
Hominoidea - ps36
Hominidae - ps37
Homininae - ps38
Hominini - ps39
Homo_sapiens - ps40
p-value
5e-06
5e-05
6e-04
6e-03
6e-02
Log-odds
0.5
1.0
1.5
2.0
2.5
3.0
3.5
4.0

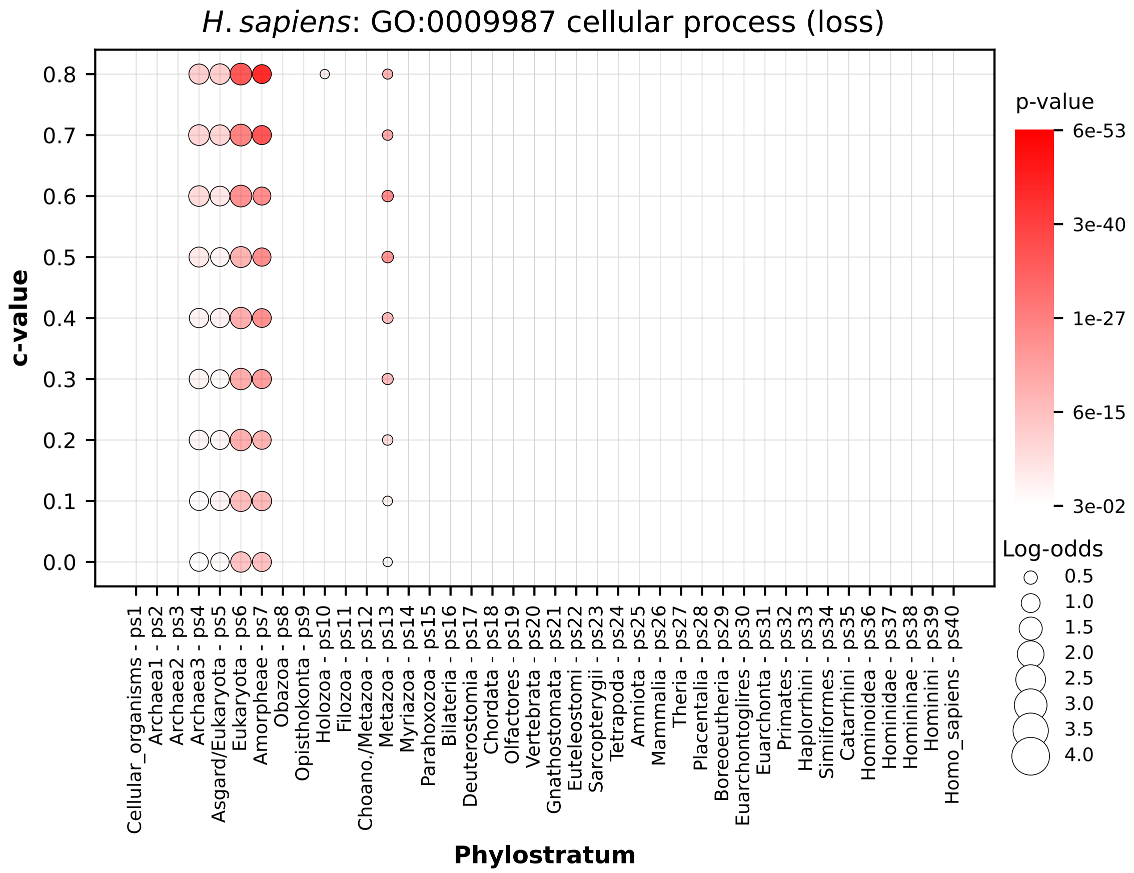

H. sapiens: GO:0009987 cellular process (loss)
c-value
0.8
0.7
0.6
0.5
0.4
0.3
0.2
0.1
0.0
Phylostratum
Cellular_organisms - ps1
Archaea1 - ps2
Archaea2 - ps3
Archaea3 - ps4
Asgard/Eukaryota - ps5
Eukaryota - ps6
Amorpheae - ps7
Obazoa - ps8
Opisthokonta - ps9
Holozoa - ps10
Filozoa - ps11
Choano./Metazoa - ps12
Metazoa - ps13
Myriazoa - ps14
Parahoxozoa - ps15
Bilateria - ps16
Deuterostomia - ps17
Chordata - ps18
Olfactores - ps19
Vertebrata - ps20
Gnathostomata - ps21
Euteleostomi - ps22
Sarcopterygii - ps23
Tetrapoda - ps24
Amniota - ps25
Mammalia - ps26
Theria - ps27
Placentalia - ps28
Boreoeutheria - ps29
Euarchontoglires - ps30
Euarchonta - ps31
Primates - ps32
Haplorrhini - ps33
Simiiformes - ps34
Catarrhini - ps35
Hominoidea - ps36
Hominidae - ps37
Homininae - ps38
Hominini - ps39
Homo_sapiens - ps40
p-value
6e-53
3e-40
1e-27
6e-15
3e-02
Log-odds
0.5
1.0
1.5
2.0
2.5
3.0
3.5
4.0

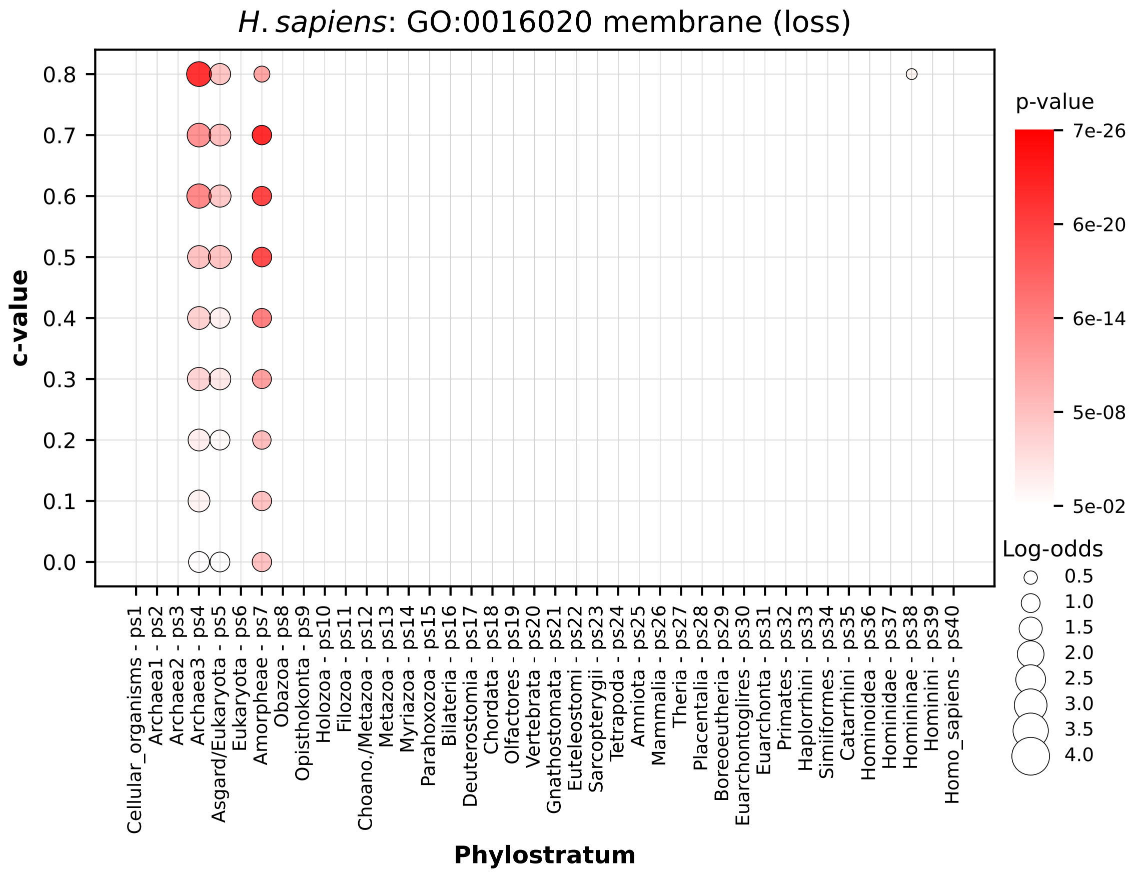

H. sapiens: GO:0016020 membrane (loss)
c-value
0.8
0.7
0.6
0.5
0.4
0.3
0.2
0.1
0.0
Phylostratum
Cellular_organisms - ps1
Archaea1 - ps2
Archaea2 - ps3
Archaea3 - ps4
Asgard/Eukaryota - ps5
Eukaryota - ps6
Amorpheae - ps7
Obazoa - ps8
Opisthokonta - ps9
Holozoa - ps10
Filozoa - ps11
Choano./Metazoa - ps12
Metazoa - ps13
Myriazoa - ps14
Parahoxozoa - ps15
Bilateria - ps16
Deuterostomia - ps17
Chordata - ps18
Olfactores - ps19
Vertebrata - ps20
Gnathostomata - ps21
Euteleostomi - ps22
Sarcopterygii - ps23
Tetrapoda - ps24
Amniota - ps25
Mammalia - ps26
Theria - ps27
Placentalia - ps28
Boreoeutheria - ps29
Euarchontoglires - ps30
Euarchonta - ps31
Primates - ps32
Haplorrhini - ps33
Simiiformes - ps34
Catarrhini - ps35
Hominoidea - ps36
Hominidae - ps37
Homininae - ps38
Hominini - ps39
Homo_sapiens - ps40
p-value
7e-26
6e-20
6e-14
5e-08
5e-02
Log-odds
0.5
1.0
1.5
2.0
2.5
3.0
3.5
4.0

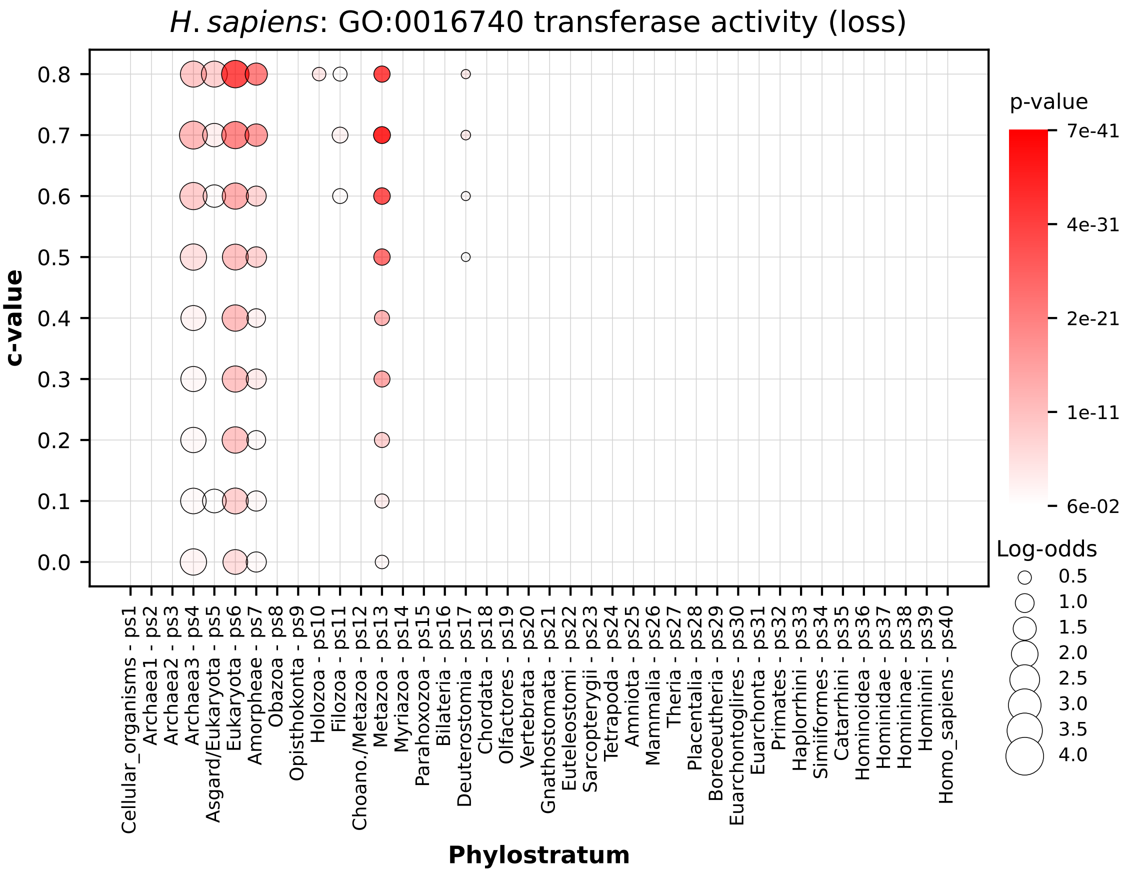
H. sapiens: GO:0016740 transferase activity (loss)
c-value
0.0
0.1
0.2
0.3
0.4
0.5
0.6
0.7
0.8
Phylostratum
Cellular_organisms - ps1
Archaea1 - ps2
Archaea2 - ps3
Archaea3 - ps4
Asgard/Eukaryota - ps5
Eukaryota - ps6
Amorpheae - ps7
Obazoa - ps8
Opisthokonta - ps9
Holozoa - ps10
Filozoa - ps11
Choano./Metazoa - ps12
Metazoa - ps13
Myriazoa - ps14
Parahoxozoa - ps15
Bilateria - ps16
Deuterostomia - ps17
Chordata - ps18
Olfactores - ps19
Vertebrata - ps20
Gnathostomata - ps21
Euteleostomi - ps22
Sarcopterygii - ps23
Tetrapoda - ps24
Amniota - ps25
Mammalia - ps26
Theria - ps27
Placentalia - ps28
Boreoeutheria - ps29
Euarchontoglires - ps30
Euarchonta - ps31
Primates - ps32
Haplorrhini - ps33
Simiiformes - ps34
Catarrhini - ps35
Hominoidea - ps36
Hominidae - ps37
Homininae - ps38
Hominini - ps39
Homo_sapiens - ps40
p-value
7e-41
4e-31
2e-21
1e-11
6e-02
Log-odds
0.5
1.0
1.5
2.0
2.5
3.0
3.5
4.0

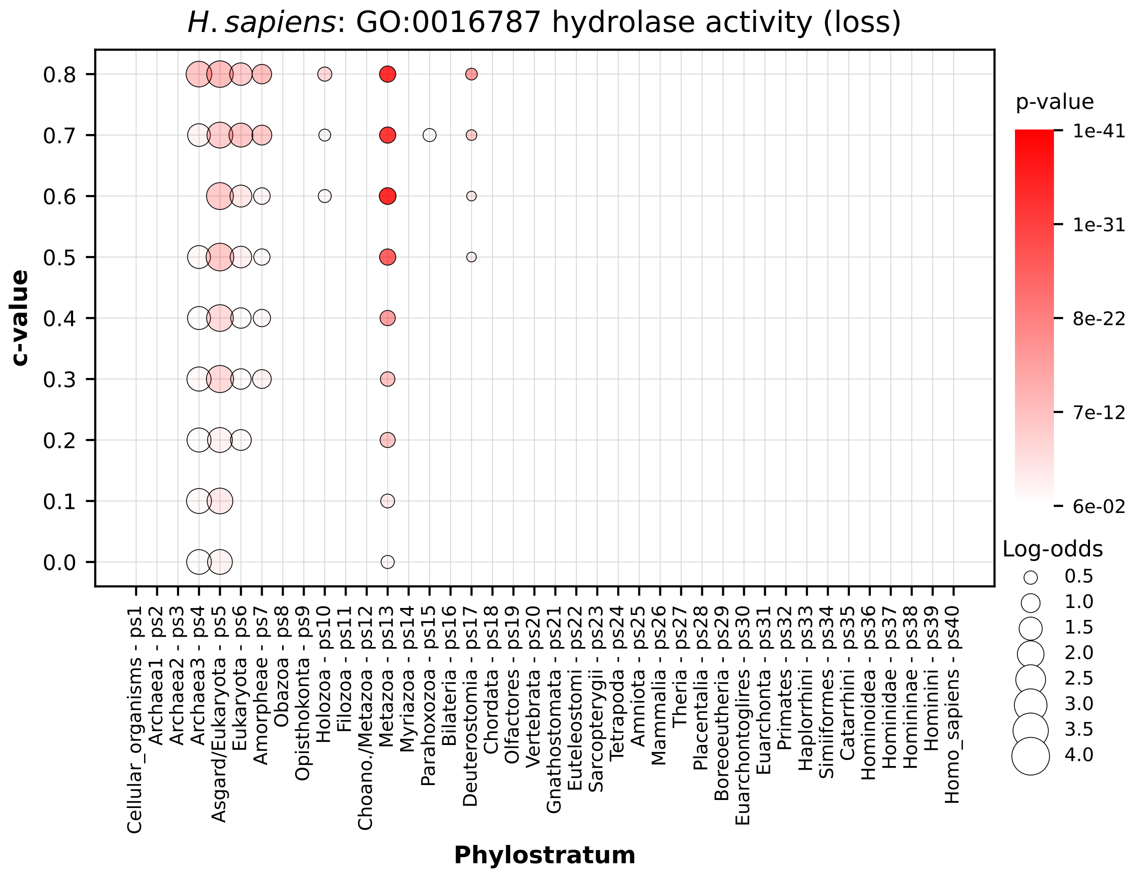

H. sapiens: GO:0016787 hydrolase activity (loss)
c-value
0.8
0.7
0.6
0.5
0.4
0.3
0.2
0.1
0.0
Phylostratum
Cellular_organisms - ps1
Archaea1 - ps2
Archaea2 - ps3
Archaea3 - ps4
Asgard/Eukaryota - ps5
Eukaryota - ps6
Amorpheae - ps7
Obazoa - ps8
Opisthokonta - ps9
Holozoa - ps10
Filozoa - ps11
Choano./Metazoa - ps12
Metazoa - ps13
Myriazoa - ps14
Parahoxozoa - ps15
Bilateria - ps16
Deuterostomia - ps17
Chordata - ps18
Olfactores - ps19
Vertebrata - ps20
Gnathostomata - ps21
Euteleostomi - ps22
Sarcopterygii - ps23
Tetrapoda - ps24
Amniota - ps25
Mammalia - ps26
Theria - ps27
Placentalia - ps28
Boreoeutheria - ps29
Euarchontoglires - ps30
Euarchonta - ps31
Primates - ps32
Haplorrhini - ps33
Simiiformes - ps34
Catarrhini - ps35
Hominoidea - ps36
Hominidae - ps37
Homininae - ps38
Hominini - ps39
Homo_sapiens - ps40
p-value
1e-41
1e-31
8e-22
7e-12
6e-02
Log-odds
0.5
1.0
1.5
2.0
2.5
3.0
3.5
4.0

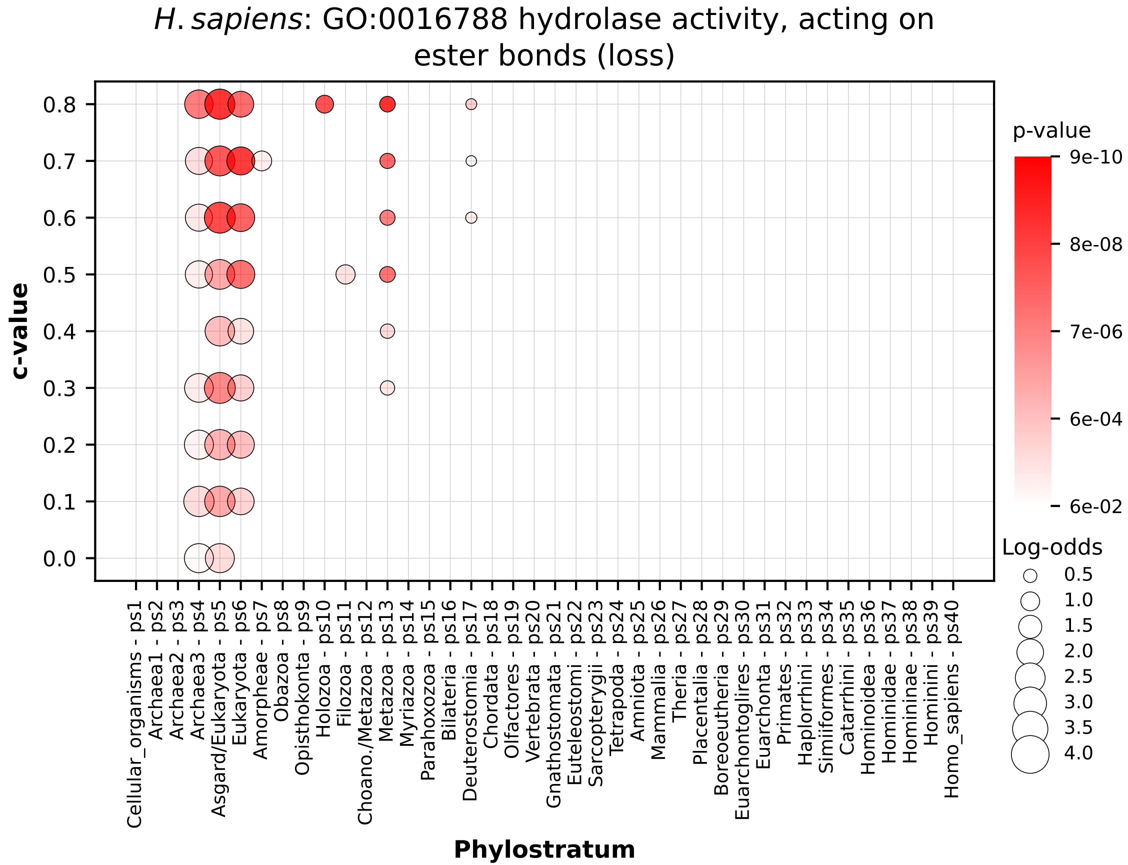
H. sapiens: GO:0016788 hydrolase activity, acting on ester bonds (loss)
c-value
0.8
0.7
0.6
0.5
0.4
0.3
0.2
0.1
0.0
Phylostratum
Cellular_organisms - ps1
Archaea1 - ps2
Archaea2 - ps3
Archaea3 - ps4
Asgard/Eukaryota - ps5
Eukaryota - ps6
Amorpheae - ps7
Obazoa - ps8
Opisthokonta - ps9
Holozoa - ps10
Filozoa - ps11
Choano./Metazoa - ps12
Metazoa - ps13
Myriazoa - ps14
Parahoxozoa - ps15
Bilateria - ps16
Deuterostomia - ps17
Chordata - ps18
Olfactores - ps19
Vertebrata - ps20
Gnathostomata - ps21
Euteleostomi - ps22
Sarcopterygii - ps23
Tetrapoda - ps24
Amniota - ps25
Mammalia - ps26
Theria - ps27
Placentalia - ps28
Boreoeutheria - ps29
Euarchontoglires - ps30
Euarchonta - ps31
Primates - ps32
Haplorrhini - ps33
Simiiformes - ps34
Catarrhini - ps35
Hominoidea - ps36
Hominidae - ps37
Homininae - ps38
Hominini - ps39
Homo_sapiens - ps40
p-value
9e-10
8e-08
7e-06
6e-04
6e-02
Log-odds
0.5
1.0
1.5
2.0
2.5
3.0
3.5
4.0

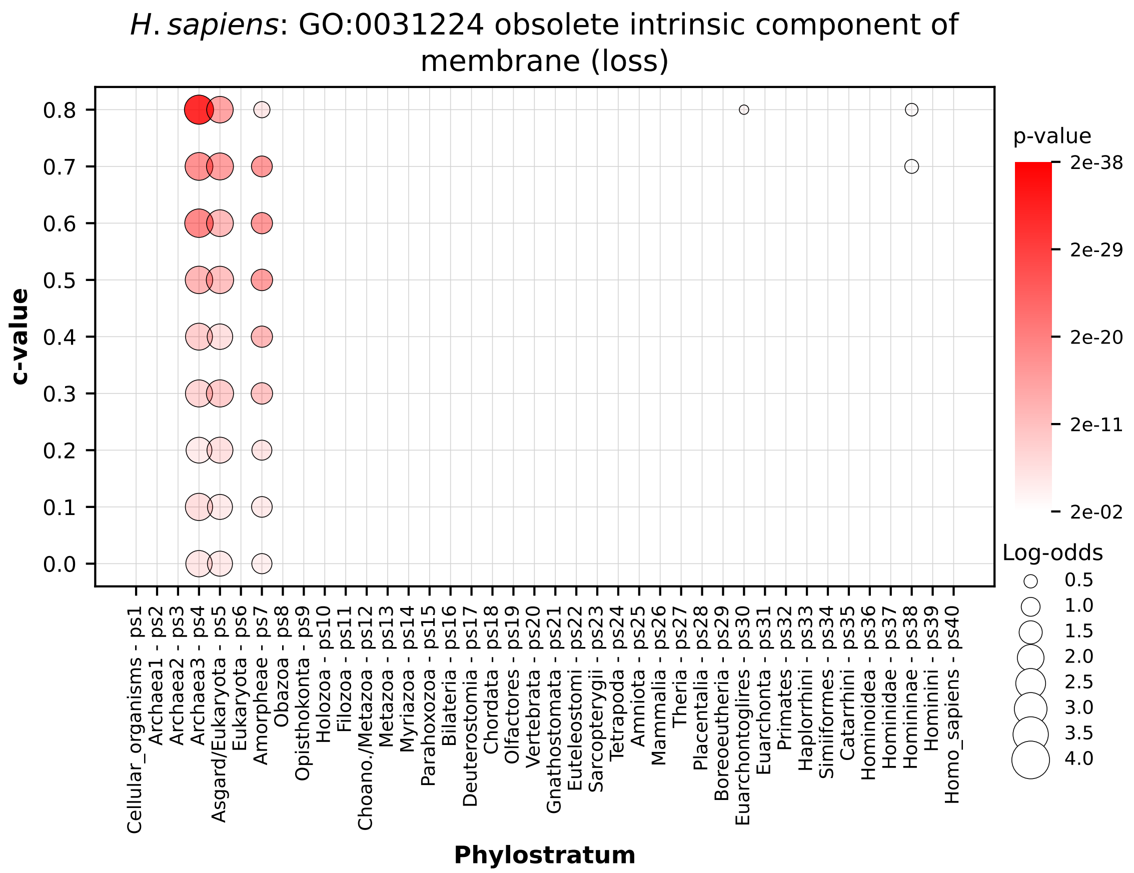

H. sapiens: GO:0031224 obsolete intrinsic component of membrane (loss)
c-value
0.8
0.7
0.6
0.5
0.4
0.3
0.2
0.1
0.0
Phylostratum
Cellular_organisms - ps1
Archaea1 - ps2
Archaea2 - ps3
Archaea3 - ps4
Asgard/Eukaryota - ps5
Eukaryota - ps6
Amorpheae - ps7
Obazoa - ps8
Opisthokonta - ps9
Holozoa - ps10
Filozoa - ps11
Choano./Metazoa - ps12
Metazoa - ps13
Myriazoa - ps14
Parahoxozoa - ps15
Bilateria - ps16
Deuterostomia - ps17
Chordata - ps18
Olfactores - ps19
Vertebrata - ps20
Gnathostomata - ps21
Euteleostomi - ps22
Sarcopterygii - ps23
Tetrapoda - ps24
Amniota - ps25
Mammalia - ps26
Theria - ps27
Placentalia - ps28
Boreoeutheria - ps29
Euarchontoglires - ps30
Euarchonta - ps31
Primates - ps32
Haplorrhini - ps33
Simiiformes - ps34
Catarrhini - ps35
Hominoidea - ps36
Hominidae - ps37
Homininae - ps38
Hominini - ps39
Homo_sapiens - ps40
p-value
2e-38
2e-29
2e-20
2e-11
2e-02
Log-odds
0.5
1.0
1.5
2.0
2.5
3.0
3.5
4.0

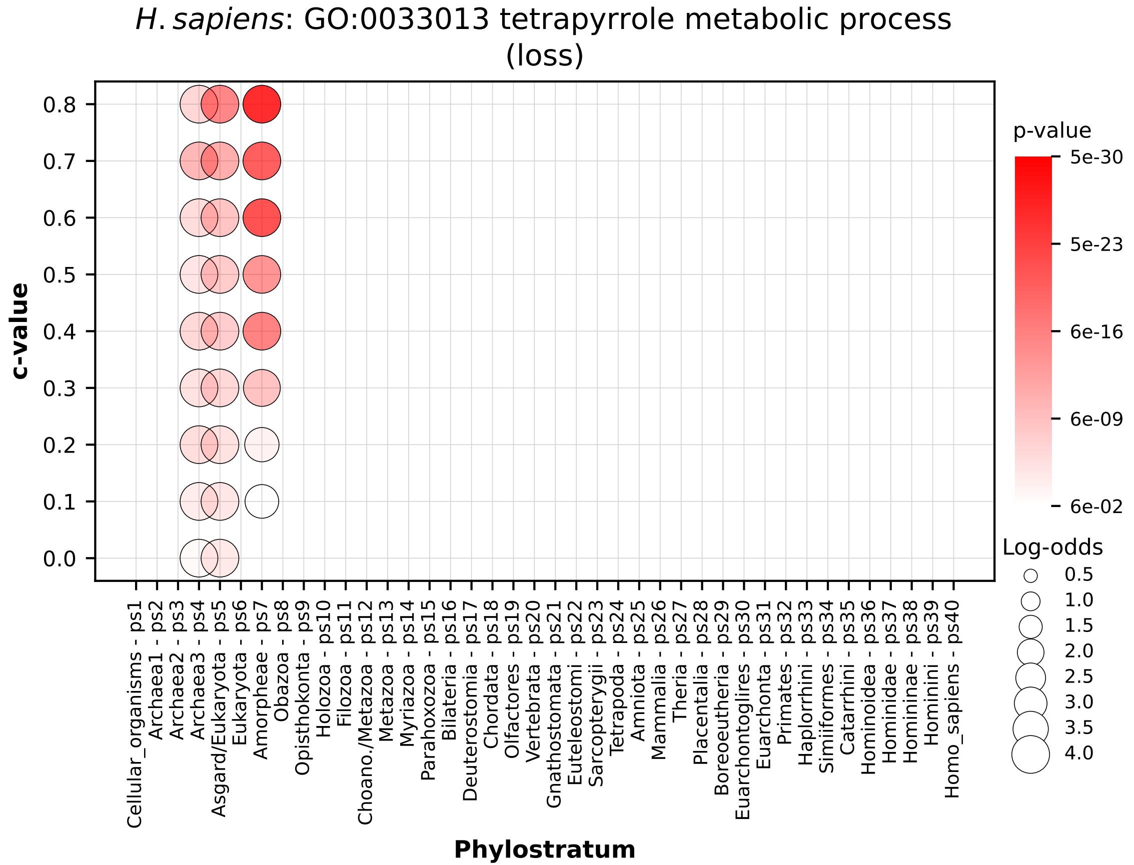

H. sapiens: GO:0033013 tetrapyrrole metabolic process (loss)
c-value
0.8
0.7
0.6
0.5
0.4
0.3
0.2
0.1
0.0
p-value
5e-30
5e-23
6e-16
6e-09
6e-02
Log-odds
0.5
1.0
1.5
2.0
2.5
3.0
3.5
4.0
Cellular_organisms - ps1
Archaea1 - ps2
Archaea2 - ps3
Archaea3 - ps4
Asgard/Eukaryota - ps5
Eukaryota - ps6
Amorpheae - ps7
Obazoa - ps8
Opisthokonta - ps9
Holozoa - ps10
Filozoa - ps11
Choano./Metazoa - ps12
Metazoa - ps13
Myriazoa - ps14
Parahoxozoa - ps15
Bilateria - ps16
Deuterostomia - ps17
Chordata - ps18
Olfactores - ps19
Vertebrata - ps20
Gnathostomata - ps21
Euteleostomi - ps22
Sarcopterygii - ps23
Tetrapoda - ps24
Amniota - ps25
Mammalia - ps26
Theria - ps27
Placentalia - ps28
Boreoeutheria - ps29
Euarchontoglires - ps30
Euarchonta - ps31
Primates - ps32
Haplorrhini - ps33
Simiiformes - ps34
Catarrhini - ps35
Hominoidea - ps36
Hominidae - ps37
Homininae - ps38
Hominini - ps39
Homo_sapiens - ps40
Phylostratum

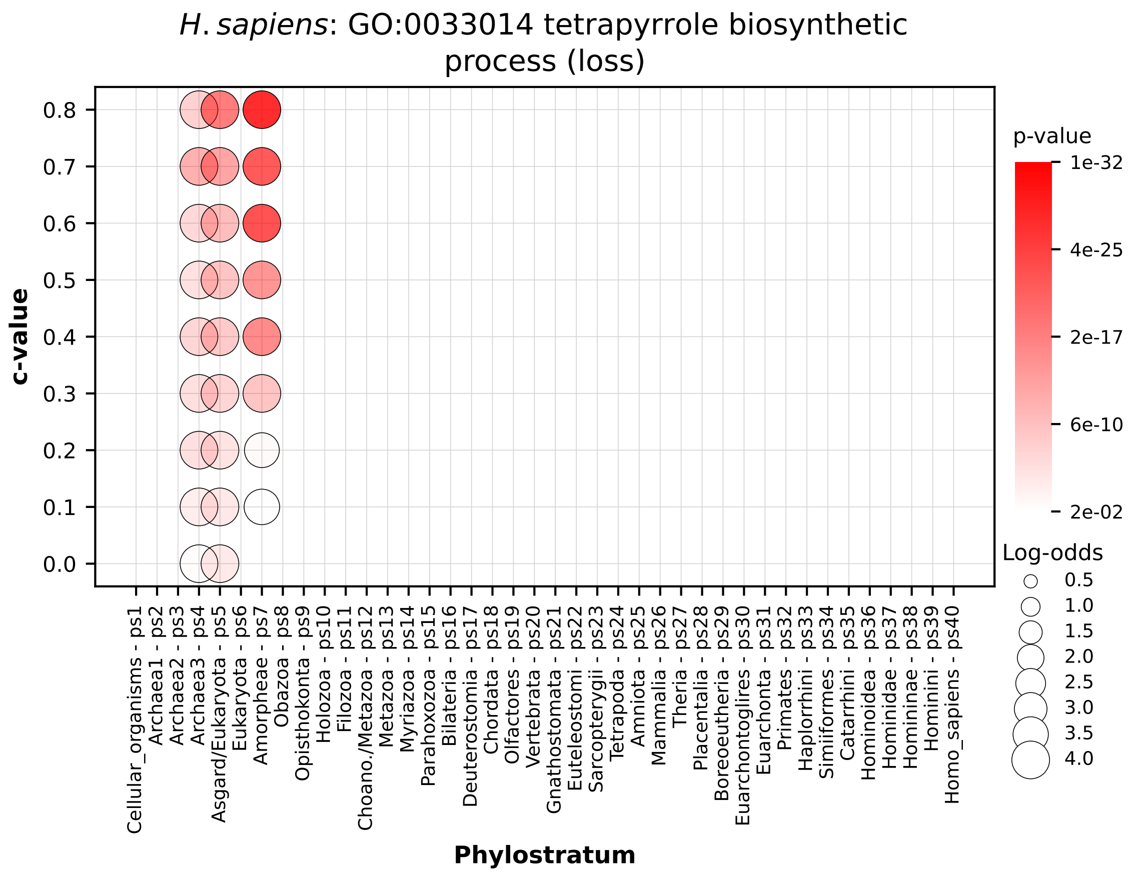

H. sapiens: GO:0033014 tetrapyrrole biosynthetic process (loss)
c-value
0.8
0.7
0.6
0.5
0.4
0.3
0.2
0.1
0.0
Phylostratum
Cellular_organisms - ps1
Archaea1 - ps2
Archaea2 - ps3
Archaea3 - ps4
Asgard/Eukaryota - ps5
Eukaryota - ps6
Amorpheae - ps7
Obazoa - ps8
Opisthokonta - ps9
Holozoa - ps10
Filozoa - ps11
Choano./Metazoa - ps12
Metazoa - ps13
Myriazoa - ps14
Parahoxozoa - ps15
Bilateria - ps16
Deuterostomia - ps17
Chordata - ps18
Olfactores - ps19
Vertebrata - ps20
Gnathostomata - ps21
Euteleostomi - ps22
Sarcopterygii - ps23
Tetrapoda - ps24
Amniota - ps25
Mammalia - ps26
Theria - ps27
Placentalia - ps28
Boreoeutheria - ps29
Euarchontoglires - ps30
Euarchonta - ps31
Primates - ps32
Haplorrhini - ps33
Simiiformes - ps34
Catarrhini - ps35
Hominoidea - ps36
Hominidae - ps37
Homininae - ps38
Hominini - ps39
Homo_sapiens - ps40
p-value
1e-32
4e-25
2e-17
6e-10
2e-02
Log-odds
0.5
1.0
1.5
2.0
2.5
3.0
3.5
4.0

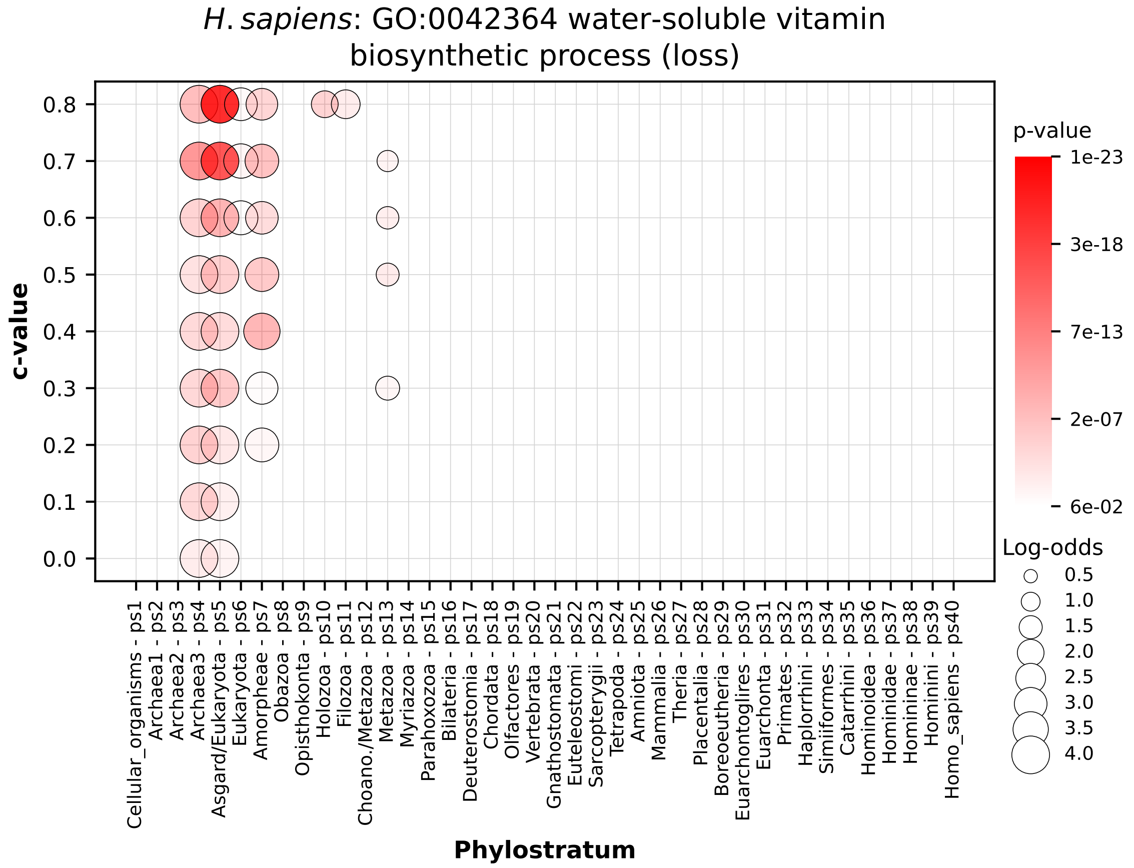

H. sapiens: GO:0042364 water-soluble vitamin biosynthetic process (loss)
c-value
0.8
0.7
0.6
0.5
0.4
0.3
0.2
0.1
0.0
p-value
1e-23
3e-18
7e-13
2e-07
6e-02
Log-odds
0.5
1.0
1.5
2.0
2.5
3.0
3.5
4.0
Cellular_organisms - ps1
Archaea1 - ps2
Archaea2 - ps3
Archaea3 - ps4
Asgard/Eukaryota - ps5
Eukaryota - ps6
Amorpheae - ps7
Obazoa - ps8
Opisthokonta - ps9
Holozoa - ps10
Filozoa - ps11
Choano./Metazoa - ps12
Metazoa - ps13
Myriazoa - ps14
Parahoxozoa - ps15
Bilateria - ps16
Deuterostomia - ps17
Chordata - ps18
Olfactores - ps19
Vertebrata - ps20
Gnathostomata - ps21
Euteleostomi - ps22
Sarcopterygii - ps23
Tetrapoda - ps24
Amniota - ps25
Mammalia - ps26
Theria - ps27
Placentalia - ps28
Boreoeutheria - ps29
Euarchontoglires - ps30
Euarchonta - ps31
Primates - ps32
Haplorrhini - ps33
Simiiformes - ps34
Catarrhini - ps35
Hominoidea - ps36
Hominidae - ps37
Homininae - ps38
Hominini - ps39
Homo_sapiens - ps40
Phylostratum

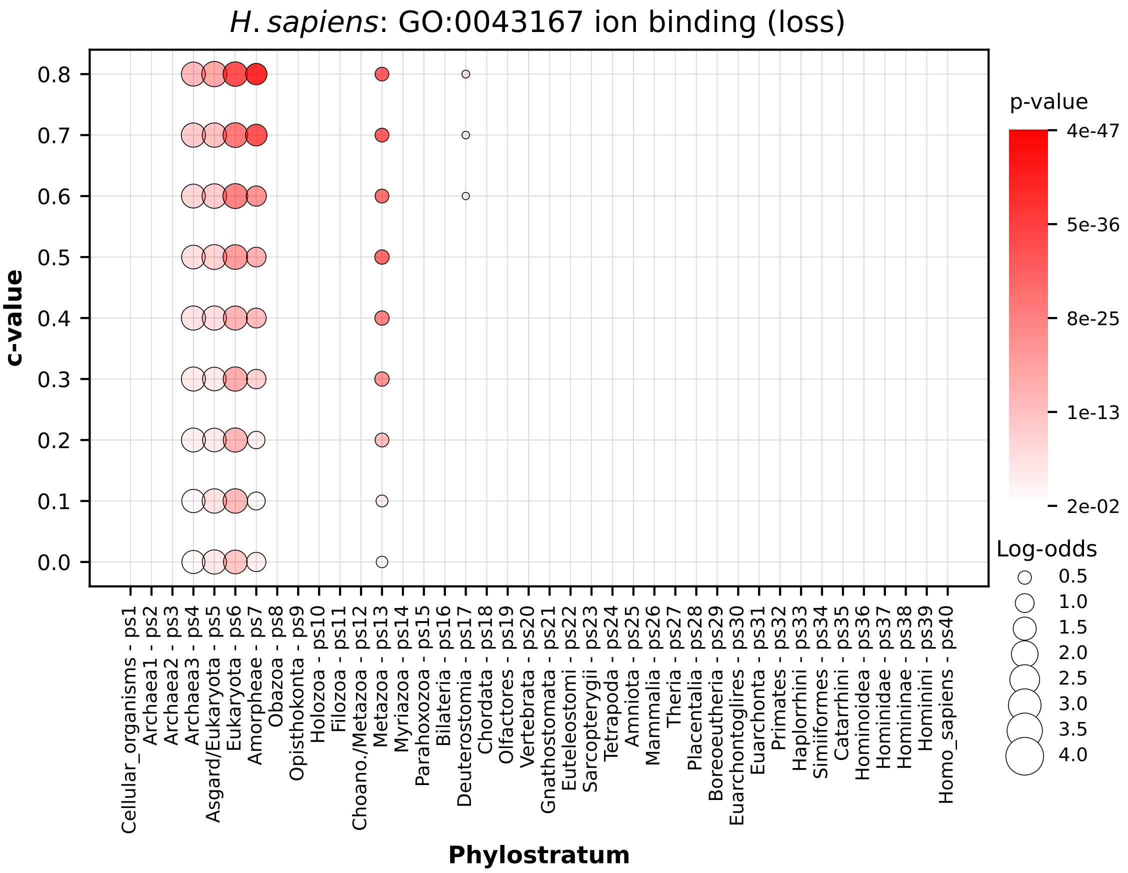

H. sapiens: GO:0043167 ion binding (loss)
c-value
Phylostratum
p-value
4e-47
5e-36
8e-25
1e-13
2e-02
Log-odds
0.5
1.0
1.5
2.0
2.5
3.0
3.5
4.0
Cellular_organisms - ps1
Archaea1 - ps2
Archaea2 - ps3
Archaea3 - ps4
Asgard/Eukaryota - ps5
Eukaryota - ps6
Amorpheae - ps7
Obazoa - ps8
Opisthokonta - ps9
Holozoa - ps10
Filozoa - ps11
Choano./Metazoa - ps12
Metazoa - ps13
Myriazoa - ps14
Parahoxozoa - ps15
Bilateria - ps16
Deuterostomia - ps17
Chordata - ps18
Olfactores - ps19
Vertebrata - ps20
Gnathostomata - ps21
Euteleostomi - ps22
Sarcopterygii - ps23
Tetrapoda - ps24
Amniota - ps25
Mammalia - ps26
Theria - ps27
Placentalia - ps28
Boreoeutheria - ps29
Euarchontoglires - ps30
Euarchonta - ps31
Primates - ps32
Haplorrhini - ps33
Simiiformes - ps34
Catarrhini - ps35
Hominoidea - ps36
Hominidae - ps37
Homininae - ps38
Hominini - ps39
Homo_sapiens - ps40

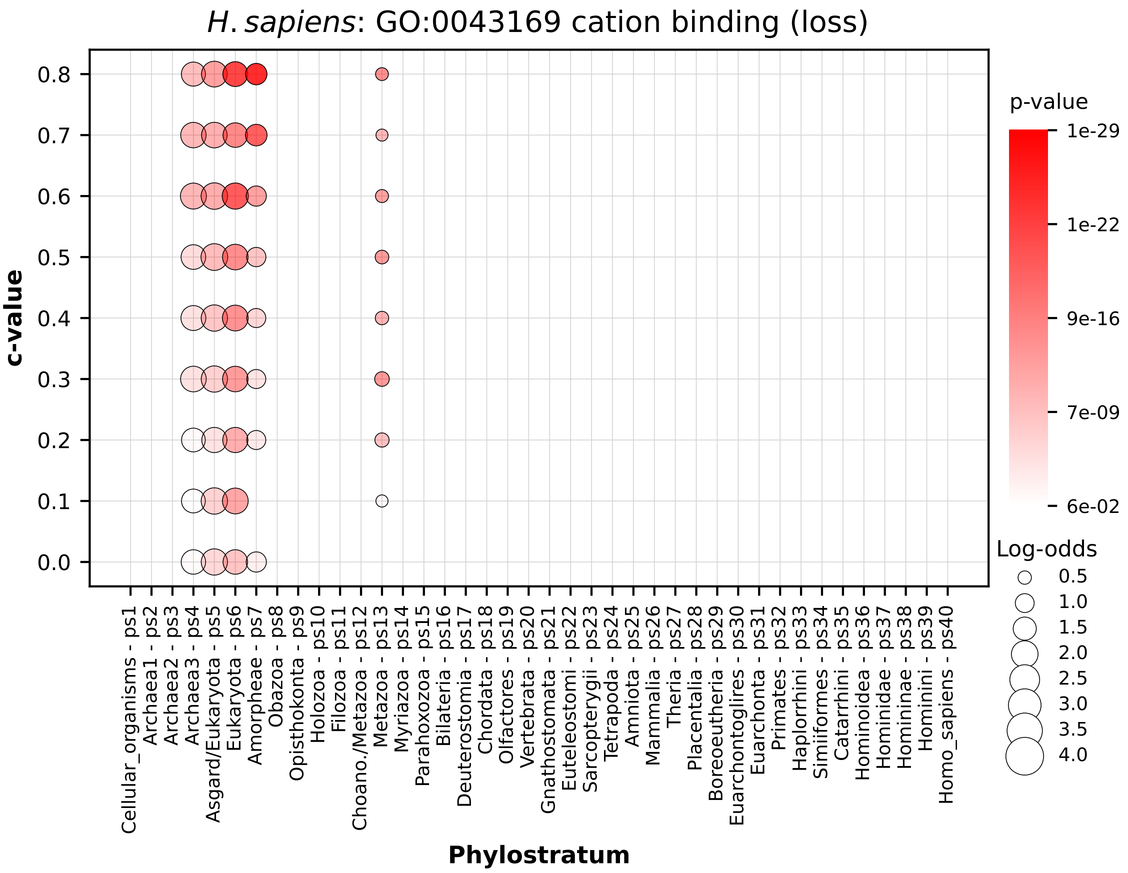

H. sapiens: GO:0043169 cation binding (loss)
c-value
0.0
0.1
0.2
0.3
0.4
0.5
0.6
0.7
0.8
Phylostratum
Cellular_organisms - ps1
Archaea1 - ps2
Archaea2 - ps3
Archaea3 - ps4
Asgard/Eukaryota - ps5
Eukaryota - ps6
Amorpheae - ps7
Obazoa - ps8
Opisthokonta - ps9
Holozoa - ps10
Filozoa - ps11
Choano./Metazoa - ps12
Metazoa - ps13
Myriazoa - ps14
Parahoxozoa - ps15
Bilateria - ps16
Deuterostomia - ps17
Chordata - ps18
Olfactores - ps19
Vertebrata - ps20
Gnathostomata - ps21
Euteleostomi - ps22
Sarcopterygii - ps23
Tetrapoda - ps24
Amniota - ps25
Mammalia - ps26
Theria - ps27
Placentalia - ps28
Boreoeutheria - ps29
Euarchontoglires - ps30
Euarchonta - ps31
Primates - ps32
Haplorrhini - ps33
Simiiformes - ps34
Catarrhini - ps35
Hominoidea - ps36
Hominidae - ps37
Homininae - ps38
Hominini - ps39
Homo_sapiens - ps40
p-value
1e-29
1e-22
9e-16
7e-09
6e-02
Log-odds
0.5
1.0
1.5
2.0
2.5
3.0
3.5
4.0

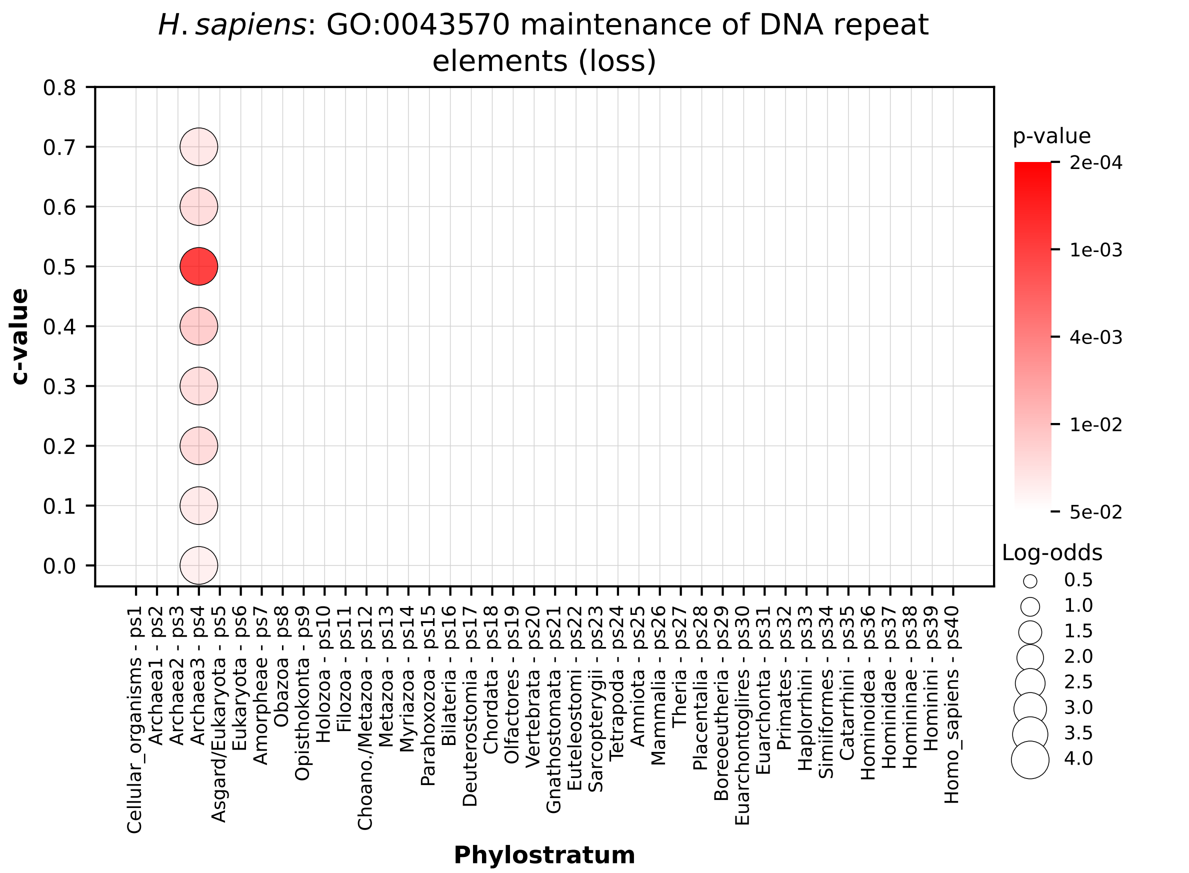

H. sapiens: GO:0043570 maintenance of DNA repeat elements (loss)
c-value
0.8
0.7
0.6
0.5
0.4
0.3
0.2
0.1
0.0
p-value
2e-04
1e-03
4e-03
1e-02
5e-02
Log-odds
0.5
1.0
1.5
2.0
2.5
3.0
3.5
4.0
Cellular_organisms - ps1
Archaea1 - ps2
Archaea2 - ps3
Archaea3 - ps4
Asgard/Eukaryota - ps5
Eukaryota - ps6
Amorpheae - ps7
Obazoa - ps8
Opisthokonta - ps9
Holozoa - ps10
Filozoa - ps11
Choano./Metazoa - ps12
Metazoa - ps13
Myriazoa - ps14
Parahoxozoa - ps15
Bilateria - ps16
Deuterostomia - ps17
Chordata - ps18
Olfactores - ps19
Vertebrata - ps20
Gnathostomata - ps21
Euteleostomi - ps22
Sarcopterygii - ps23
Tetrapoda - ps24
Amniota - ps25
Mammalia - ps26
Theria - ps27
Placentalia - ps28
Boreoeutheria - ps29
Euarchontoglires - ps30
Euarchonta - ps31
Primates - ps32
Haplorrhini - ps33
Simiiformes - ps34
Catarrhini - ps35
Hominoidea - ps36
Hominidae - ps37
Homininae - ps38
Hominini - ps39
Homo_sapiens - ps40
Phylostratum

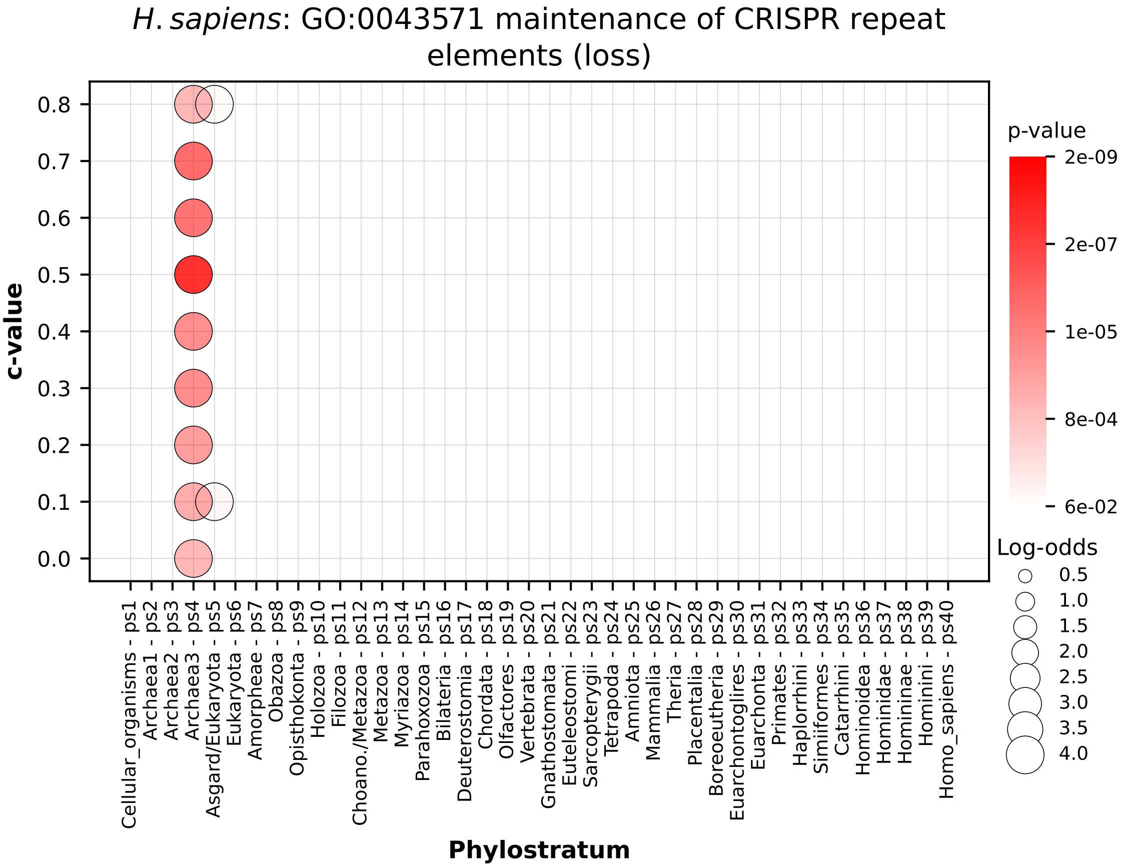

H. sapiens: GO:0043571 maintenance of CRISPR repeat elements (loss)
c-value
0.8
0.7
0.6
0.5
0.4
0.3
0.2
0.1
0.0
p-value
2e-09
2e-07
1e-05
8e-04
6e-02
Log-odds
0.5
1.0
1.5
2.0
2.5
3.0
3.5
4.0
Cellular_organisms - ps1
Archaea1 - ps2
Archaea2 - ps3
Archaea3 - ps4
Asgard/Eukaryota - ps5
Eukaryota - ps6
Amorpheae - ps7
Obazoa - ps8
Opisthokonta - ps9
Holozoa - ps10
Filozoa - ps11
Choano./Metazoa - ps12
Metazoa - ps13
Myriazoa - ps14
Parahoxozoa - ps15
Bilateria - ps16
Deuterostomia - ps17
Chordata - ps18
Olfactores - ps19
Vertebrata - ps20
Gnathostomata - ps21
Euteleostomi - ps22
Sarcopterygii - ps23
Tetrapoda - ps24
Amniota - ps25
Mammalia - ps26
Theria - ps27
Placentalia - ps28
Boreoeutheria - ps29
Euarchontoglires - ps30
Euarchonta - ps31
Primates - ps32
Haplorrhini - ps33
Simiiformes - ps34
Catarrhini - ps35
Hominoidea - ps36
Hominidae - ps37
Homininae - ps38
Hominini - ps39
Homo_sapiens - ps40
Phylostratum

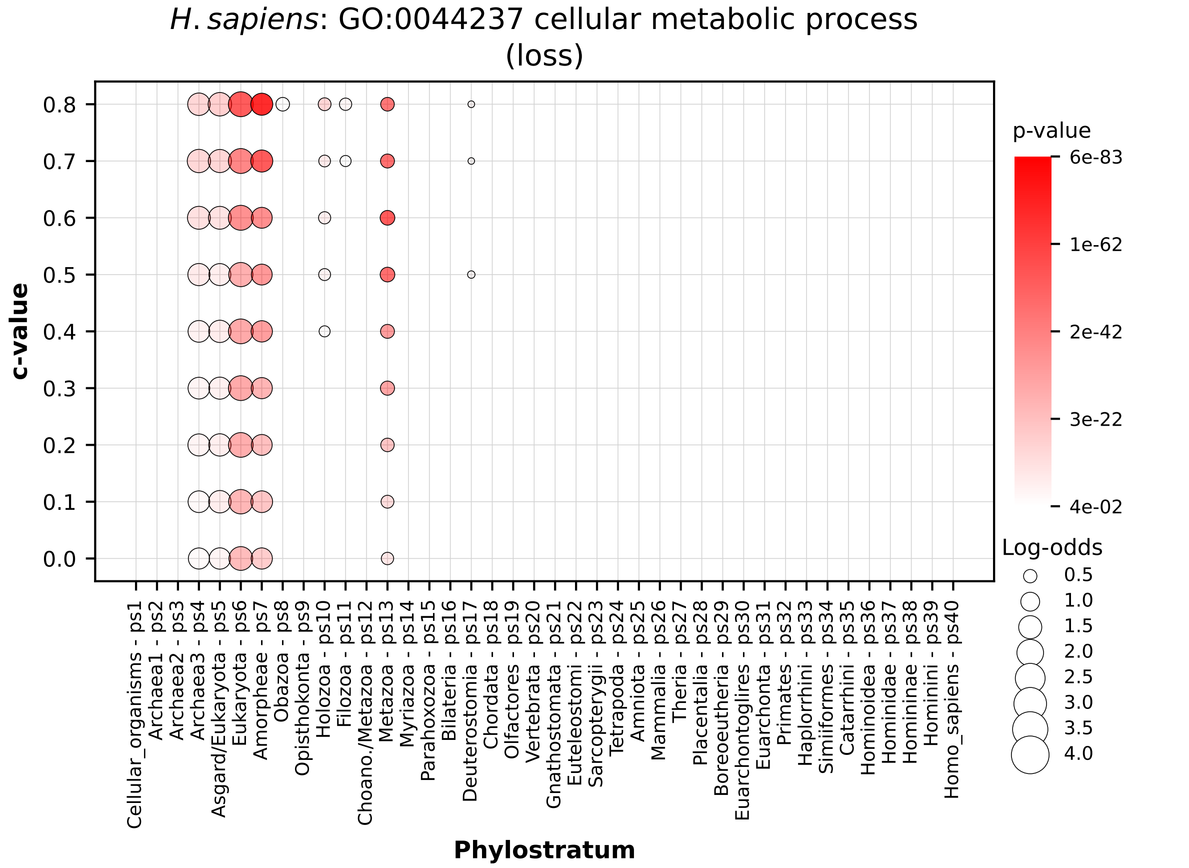
H. sapiens: GO:0044237 cellular metabolic process
(loss)
c-value
0.8
0.7
0.6
0.5
0.4
0.3
0.2
0.1
0.0
Phylostratum
Cellular_organisms - ps1
Archaea1 - ps2
Archaea2 - ps3
Archaea3 - ps4
Asgard/Eukaryota - ps5
Eukaryota - ps6
Amorpheae - ps7
Obazoa - ps8
Opisthokonta - ps9
Holozoa - ps10
Filozoa - ps11
Choano./Metazoa - ps12
Metazoa - ps13
Myriazoa - ps14
Parahoxozoa - ps15
Bilateria - ps16
Deuterostomia - ps17
Chordata - ps18
Olfactores - ps19
Vertebrata - ps20
Gnathostomata - ps21
Euteleostomi - ps22
Sarcopterygii - ps23
Tetrapoda - ps24
Amniota - ps25
Mammalia - ps26
Theria - ps27
Placentalia - ps28
Boreoeutheria - ps29
Euarchontoglires - ps30
Euarchonta - ps31
Primates - ps32
Haplorrhini - ps33
Simiiformes - ps34
Catarrhini - ps35
Hominoidea - ps36
Hominidae - ps37
Homininae - ps38
Hominini - ps39
Homo_sapiens - ps40
p-value
6e-83
1e-62
2e-42
3e-22
4e-02
Log-odds
0.5
1.0
1.5
2.0
2.5
3.0
3.5
4.0

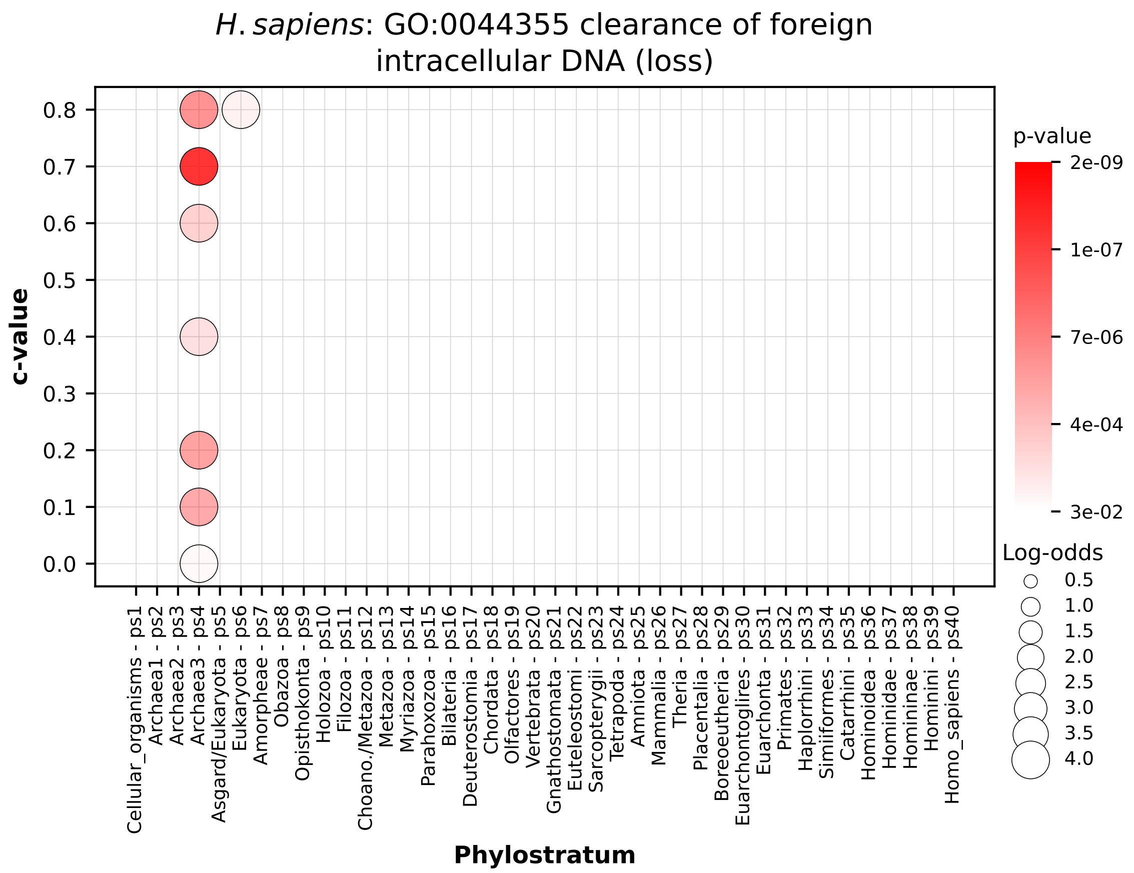

H. sapiens: GO:0044355 clearance of foreign intracellular DNA (loss)
c-value
0.8
0.7
0.6
0.5
0.4
0.3
0.2
0.1
0.0
p-value
2e-09
1e-07
7e-06
4e-04
3e-02
Log-odds
0.5
1.0
1.5
2.0
2.5
3.0
3.5
4.0
Cellular_organisms - ps1
Archaea1 - ps2
Archaea2 - ps3
Archaea3 - ps4
Asgard/Eukaryota - ps5
Eukaryota - ps6
Amorpheae - ps7
Obazoa - ps8
Opisthokonta - ps9
Holozoa - ps10
Filozoa - ps11
Choano./Metazoa - ps12
Metazoa - ps13
Myriazoa - ps14
Parahoxozoa - ps15
Bilateria - ps16
Deuterostomia - ps17
Chordata - ps18
Olfactores - ps19
Vertebrata - ps20
Gnathostomata - ps21
Euteleostomi - ps22
Sarcopterygii - ps23
Tetrapoda - ps24
Amniota - ps25
Mammalia - ps26
Theria - ps27
Placentalia - ps28
Boreoeutheria - ps29
Euarchontoglires - ps30
Euarchonta - ps31
Primates - ps32
Haplorrhini - ps33
Simiiformes - ps34
Catarrhini - ps35
Hominoidea - ps36
Hominidae - ps37
Homininae - ps38
Hominini - ps39
Homo_sapiens - ps40
Phylostratum

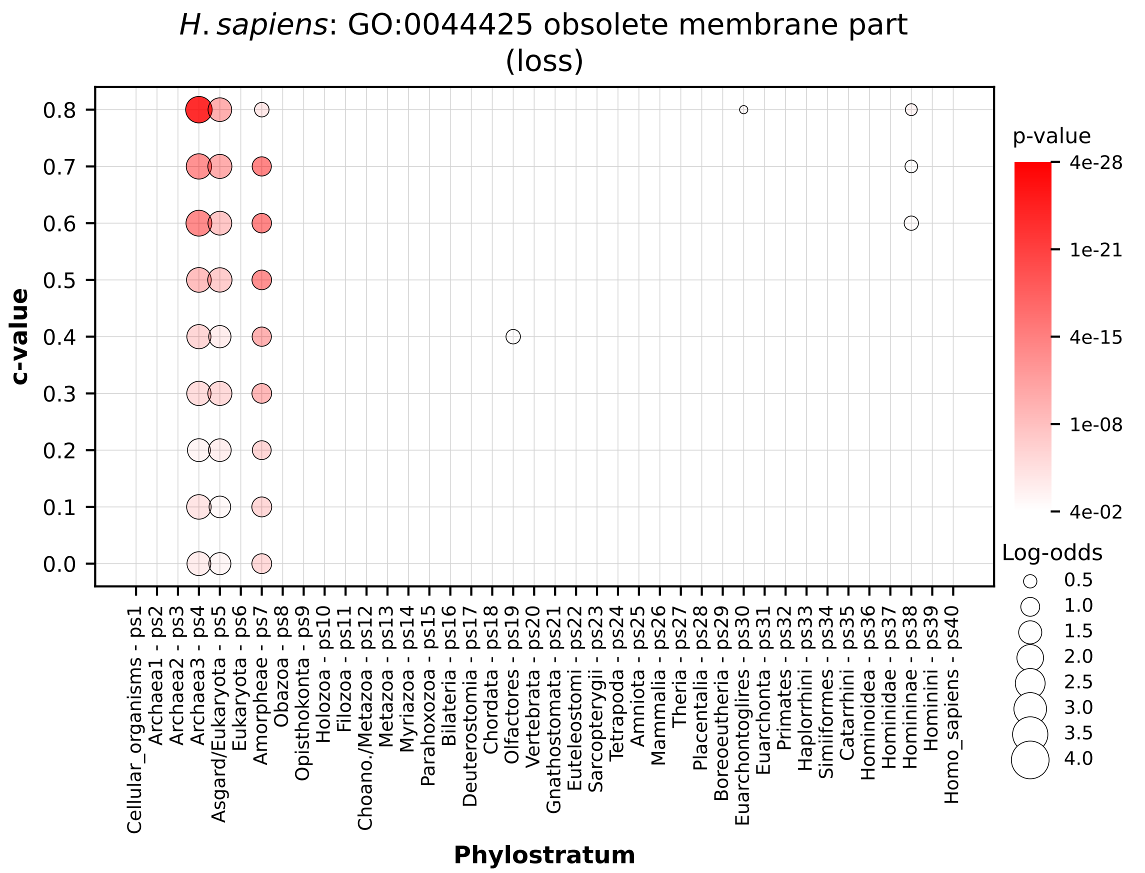

H. sapiens: GO:0044425 obsolete membrane part
(loss)
c-value
0.8
0.7
0.6
0.5
0.4
0.3
0.2
0.1
0.0
p-value
4e-28
1e-21
4e-15
1e-08
4e-02
Log-odds
0.5
1.0
1.5
2.0
2.5
3.0
3.5
4.0
Cellular_organisms - ps1
Archaea1 - ps2
Archaea2 - ps3
Archaea3 - ps4
Asgard/Eukaryota - ps5
Eukaryota - ps6
Amorpheae - ps7
Obazoa - ps8
Opisthokonta - ps9
Holozoa - ps10
Filozoa - ps11
Choano./Metazoa - ps12
Metazoa - ps13
Myriazoa - ps14
Parahoxozoa - ps15
Bilateria - ps16
Deuterostomia - ps17
Chordata - ps18
Olfactores - ps19
Vertebrata - ps20
Gnathostomata - ps21
Euteleostomi - ps22
Sarcopterygii - ps23
Tetrapoda - ps24
Amniota - ps25
Mammalia - ps26
Theria - ps27
Placentalia - ps28
Boreoeutheria - ps29
Euarchontoglires - ps30
Euarchonta - ps31
Primates - ps32
Haplorrhini - ps33
Simiiformes - ps34
Catarrhini - ps35
Hominoidea - ps36
Hominidae - ps37
Homininae - ps38
Hominini - ps39
Homo_sapiens - ps40
Phylostratum

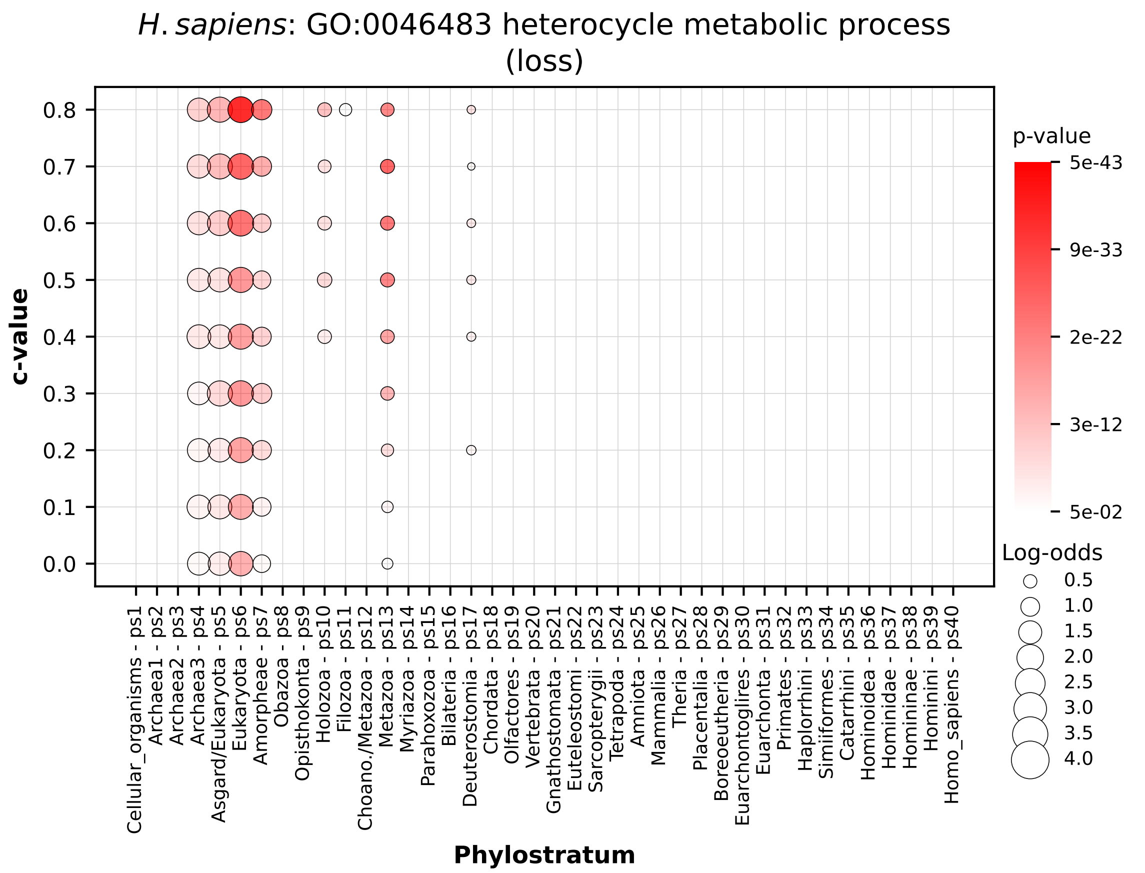

H. sapiens: GO:0046483 heterocycle metabolic process
(loss)
c-value
0.8
0.7
0.6
0.5
0.4
0.3
0.2
0.1
0.0
p-value
5e-43
9e-33
2e-22
3e-12
5e-02
Log-odds
0.5
1.0
1.5
2.0
2.5
3.0
3.5
4.0
Cellular_organisms - ps1
Archaea1 - ps2
Archaea2 - ps3
Archaea3 - ps4
Asgard/Eukaryota - ps5
Eukaryota - ps6
Amorpheae - ps7
Obazoa - ps8
Opisthokonta - ps9
Holozoa - ps10
Filozoa - ps11
Choano./Metazoa - ps12
Metazoa - ps13
Myriazoa - ps14
Parahoxozoa - ps15
Bilateria - ps16
Deuterostomia - ps17
Chordata - ps18
Olfactores - ps19
Vertebrata - ps20
Gnathostomata - ps21
Euteleostomi - ps22
Sarcopterygii - ps23
Tetrapoda - ps24
Amniota - ps25
Mammalia - ps26
Theria - ps27
Placentalia - ps28
Boreoeutheria - ps29
Euarchontoglires - ps30
Euarchonta - ps31
Primates - ps32
Haplorrhini - ps33
Simiiformes - ps34
Catarrhini - ps35
Hominoidea - ps36
Hominidae - ps37
Homininae - ps38
Hominini - ps39
Homo_sapiens - ps40
Phylostratum

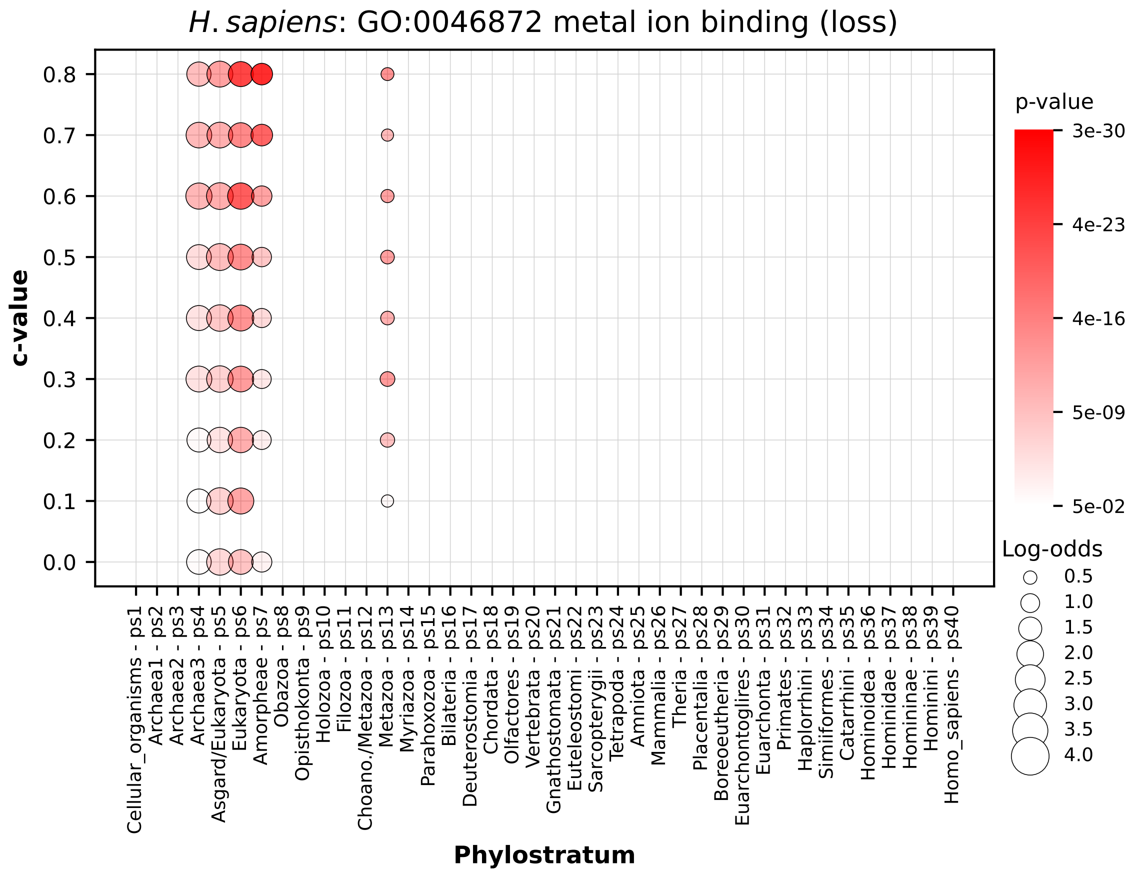

H. sapiens: GO:0046872 metal ion binding (loss)
c-value
0.0
0.1
0.2
0.3
0.4
0.5
0.6
0.7
0.8
Phylostratum
Cellular_organisms - ps1
Archaea1 - ps2
Archaea2 - ps3
Archaea3 - ps4
Asgard/Eukaryota - ps5
Eukaryota - ps6
Amorpheae - ps7
Obazoa - ps8
Opisthokonta - ps9
Holozoa - ps10
Filozoa - ps11
Choano./Metazoa - ps12
Metazoa - ps13
Myriazoa - ps14
Parahoxozoa - ps15
Bilateria - ps16
Deuterostomia - ps17
Chordata - ps18
Olfactores - ps19
Vertebrata - ps20
Gnathostomata - ps21
Euteleostomi - ps22
Sarcopterygii - ps23
Tetrapoda - ps24
Amniota - ps25
Mammalia - ps26
Theria - ps27
Placentalia - ps28
Boreoeutheria - ps29
Euarchontoglires - ps30
Euarchonta - ps31
Primates - ps32
Haplorrhini - ps33
Simiiformes - ps34
Catarrhini - ps35
Hominoidea - ps36
Hominidae - ps37
Homininae - ps38
Hominini - ps39
Homo_sapiens - ps40
p-value
3e-30
4e-23
4e-16
5e-09
5e-02
Log-odds
0.5
1.0
1.5
2.0
2.5
3.0
3.5
4.0

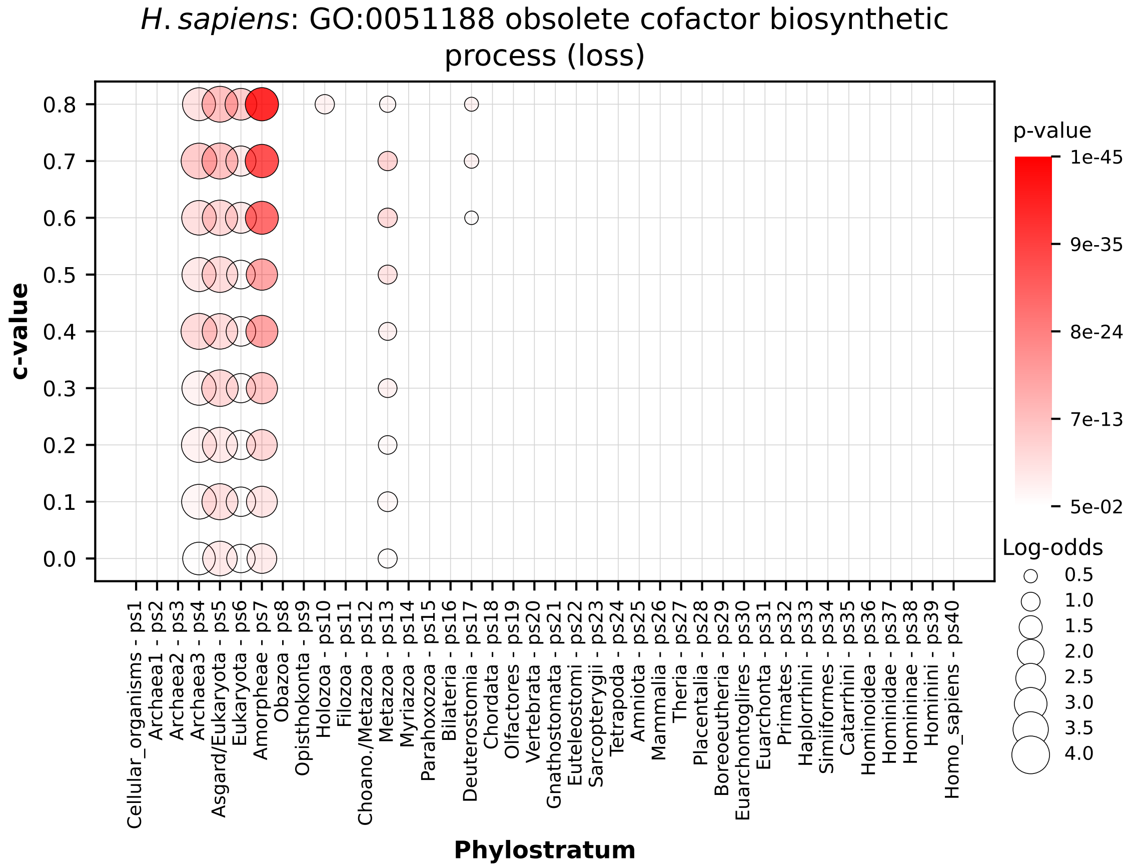

H. sapiens: GO:0051188 obsolete cofactor biosynthetic process (loss)
c-value
0.8
0.7
0.6
0.5
0.4
0.3
0.2
0.1
0.0
p-value
1e-45
9e-35
8e-24
7e-13
5e-02
Log-odds
0.5
1.0
1.5
2.0
2.5
3.0
3.5
4.0
Cellular_organisms - ps1
Archaea1 - ps2
Archaea2 - ps3
Archaea3 - ps4
Asgard/Eukaryota - ps5
Eukaryota - ps6
Amorpheae - ps7
Obazoa - ps8
Opisthokonta - ps9
Holozoa - ps10
Filozoa - ps11
Choano./Metazoa - ps12
Metazoa - ps13
Myriazoa - ps14
Parahoxozoa - ps15
Bilateria - ps16
Deuterostomia - ps17
Chordata - ps18
Olfactores - ps19
Vertebrata - ps20
Gnathostomata - ps21
Euteleostomi - ps22
Sarcopterygii - ps23
Tetrapoda - ps24
Amniota - ps25
Mammalia - ps26
Theria - ps27
Placentalia - ps28
Boreoeutheria - ps29
Euarchontoglires - ps30
Euarchonta - ps31
Primates - ps32
Haplorrhini - ps33
Simiiformes - ps34
Catarrhini - ps35
Hominoidea - ps36
Hominidae - ps37
Homininae - ps38
Hominini - ps39
Homo_sapiens - ps40
Phylostratum

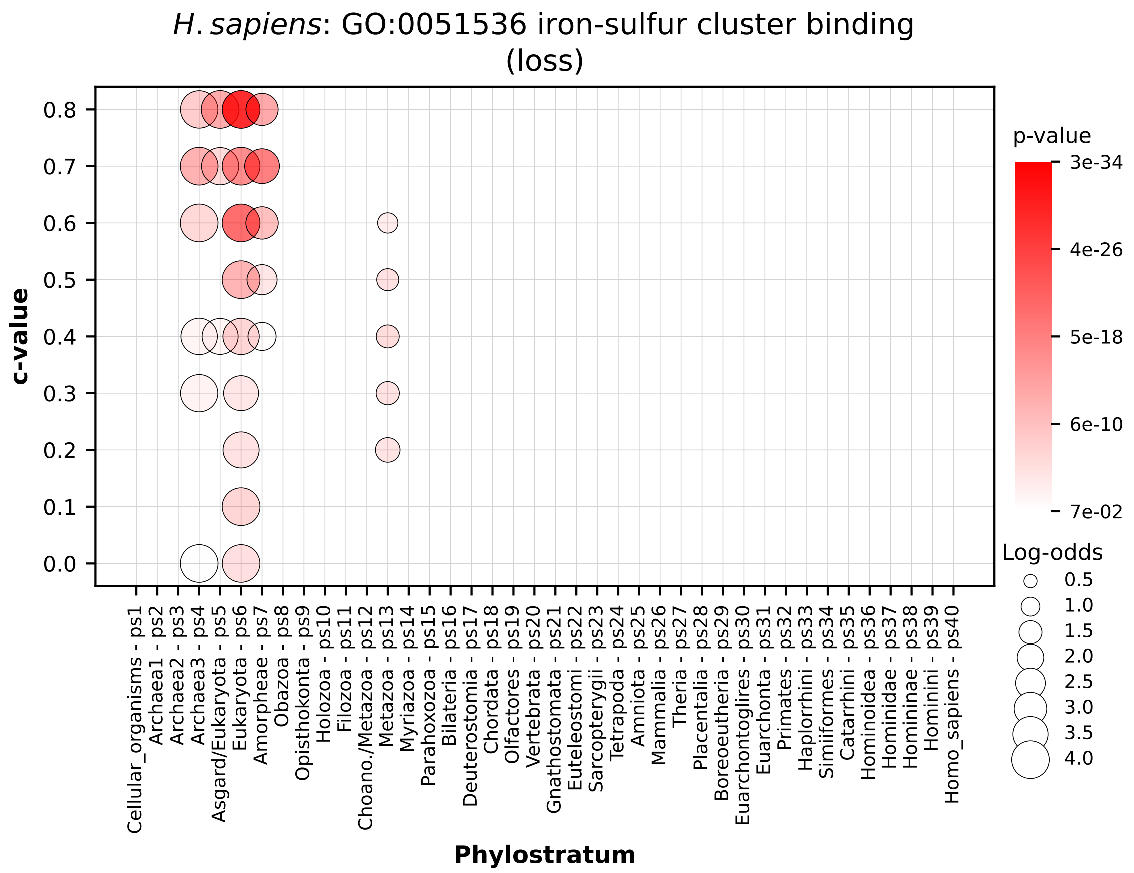

H. sapiens: GO:0051536 iron-sulfur cluster binding
(loss)
c-value
0.0
0.1
0.2
0.3
0.4
0.5
0.6
0.7
0.8
Phylostratum
Cellular_organisms - ps1
Archaea1 - ps2
Archaea2 - ps3
Archaea3 - ps4
Asgard/Eukaryota - ps5
Eukaryota - ps6
Amorpheae - ps7
Obazoa - ps8
Opisthokonta - ps9
Holozoa - ps10
Filozoa - ps11
Choano./Metazoa - ps12
Metazoa - ps13
Myriazoa - ps14
Parahoxozoa - ps15
Bilateria - ps16
Deuterostomia - ps17
Chordata - ps18
Olfactores - ps19
Vertebrata - ps20
Gnathostomata - ps21
Euteleostomi - ps22
Sarcopterygii - ps23
Tetrapoda - ps24
Amniota - ps25
Mammalia - ps26
Theria - ps27
Placentalia - ps28
Boreoeutheria - ps29
Euarchontoglires - ps30
Euarchonta - ps31
Primates - ps32
Haplorrhini - ps33
Simiiformes - ps34
Catarrhini - ps35
Hominoidea - ps36
Hominidae - ps37
Homininae - ps38
Hominini - ps39
Homo_sapiens - ps40
p-value
3e-34
4e-26
5e-18
6e-10
7e-02
Log-odds
0.5
1.0
1.5
2.0
2.5
3.0
3.5
4.0

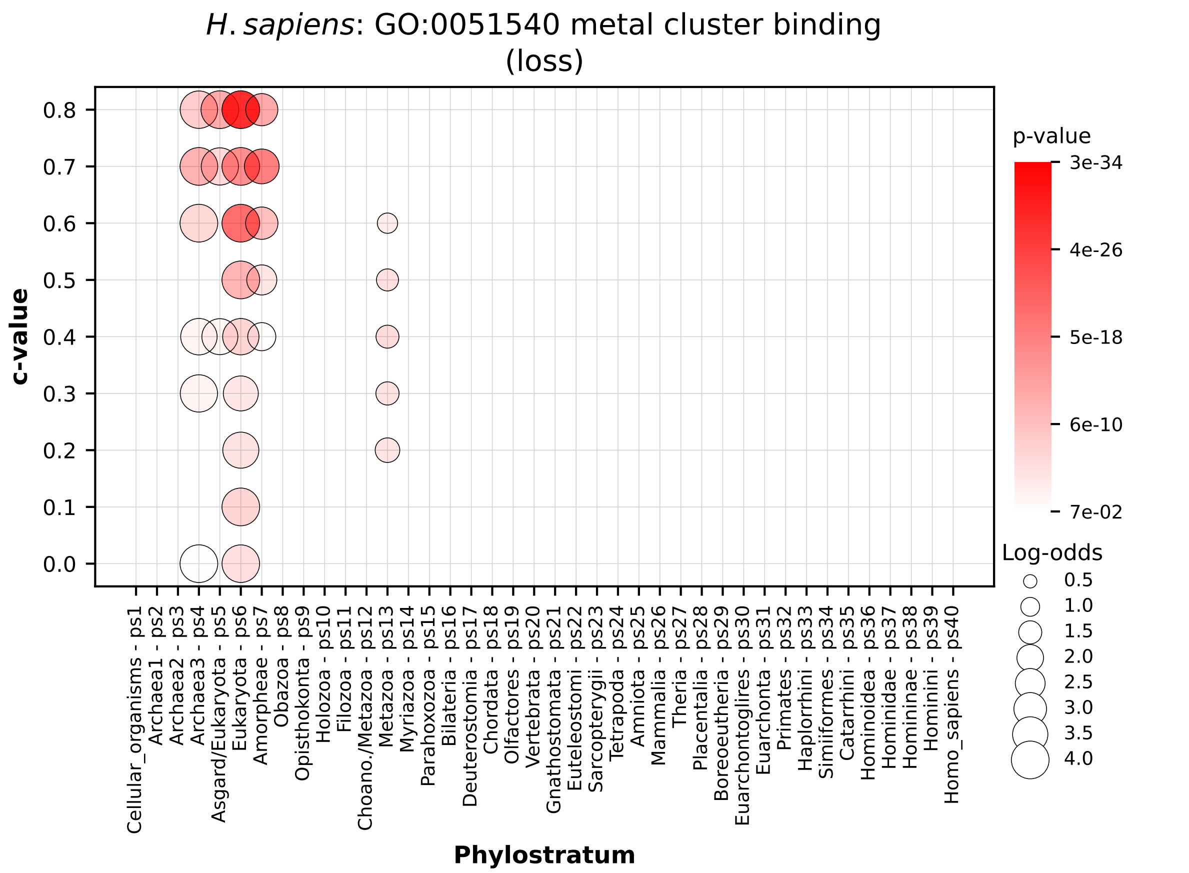
H. sapiens: GO:0051540 metal cluster binding
(loss)
c-value
0.8
0.7
0.6
0.5
0.4
0.3
0.2
0.1
0.0
p-value
3e-34
4e-26
5e-18
6e-10
7e-02
Log-odds
0.5
1.0
1.5
2.0
2.5
3.0
3.5
4.0
Cellular_organisms - ps1
Archaea1 - ps2
Archaea2 - ps3
Archaea3 - ps4
Asgard/Eukaryota - ps5
Eukaryota - ps6
Amorpheae - ps7
Obazoa - ps8
Opisthokonta - ps9
Holozoa - ps10
Filozoa - ps11
Choano./Metazoa - ps12
Metazoa - ps13
Myriazoa - ps14
Parahoxozoa - ps15
Bilateria - ps16
Deuterostomia - ps17
Chordata - ps18
Olfactores - ps19
Vertebrata - ps20
Gnathostomata - ps21
Euteleostomi - ps22
Sarcopterygii - ps23
Tetrapoda - ps24
Amniota - ps25
Mammalia - ps26
Theria - ps27
Placentalia - ps28
Boreoeutheria - ps29
Euarchontoglires - ps30
Euarchonta - ps31
Primates - ps32
Haplorrhini - ps33
Simiiformes - ps34
Catarrhini - ps35
Hominoidea - ps36
Hominidae - ps37
Homininae - ps38
Hominini - ps39
Homo_sapiens - ps40
Phylostratum

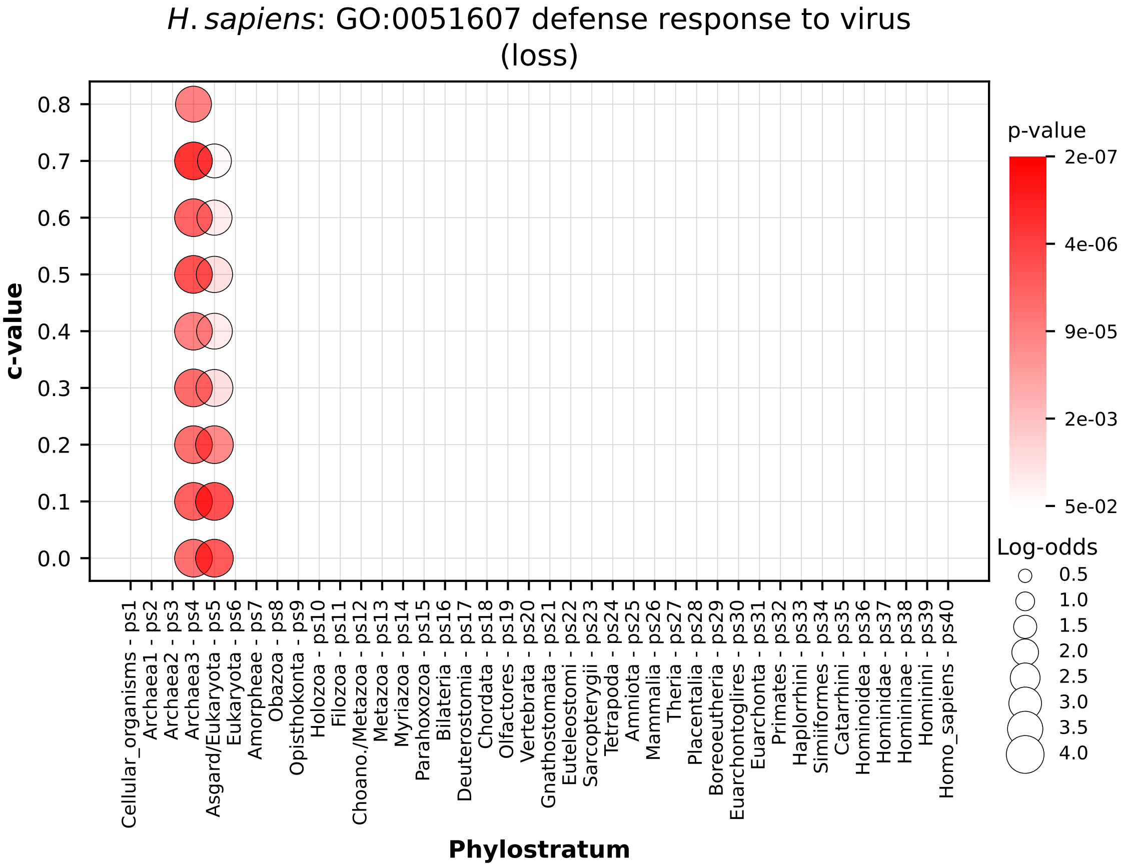

H. sapiens: GO:0051607 defense response to virus
(loss)
c-value
0.8
0.7
0.6
0.5
0.4
0.3
0.2
0.1
0.0
p-value
2e-07
4e-06
9e-05
2e-03
5e-02
Log-odds
0.5
1.0
1.5
2.0
2.5
3.0
3.5
4.0
Cellular_organisms - ps1
Archaea1 - ps2
Archaea2 - ps3
Archaea3 - ps4
Asgard/Eukaryota - ps5
Eukaryota - ps6
Amorpheae - ps7
Obazoa - ps8
Opisthokonta - ps9
Holozoa - ps10
Filozoa - ps11
Choano./Metazoa - ps12
Metazoa - ps13
Myriazoa - ps14
Parahoxozoa - ps15
Bilateria - ps16
Deuterostomia - ps17
Chordata - ps18
Olfactores - ps19
Vertebrata - ps20
Gnathostomata - ps21
Euteleostomi - ps22
Sarcopterygii - ps23
Tetrapoda - ps24
Amniota - ps25
Mammalia - ps26
Theria - ps27
Placentalia - ps28
Boreoeutheria - ps29
Euarchontoglires - ps30
Euarchonta - ps31
Primates - ps32
Haplorrhini - ps33
Simiiformes - ps34
Catarrhini - ps35
Hominoidea - ps36
Hominidae - ps37
Homininae - ps38
Hominini - ps39
Homo_sapiens - ps40
Phylostratum

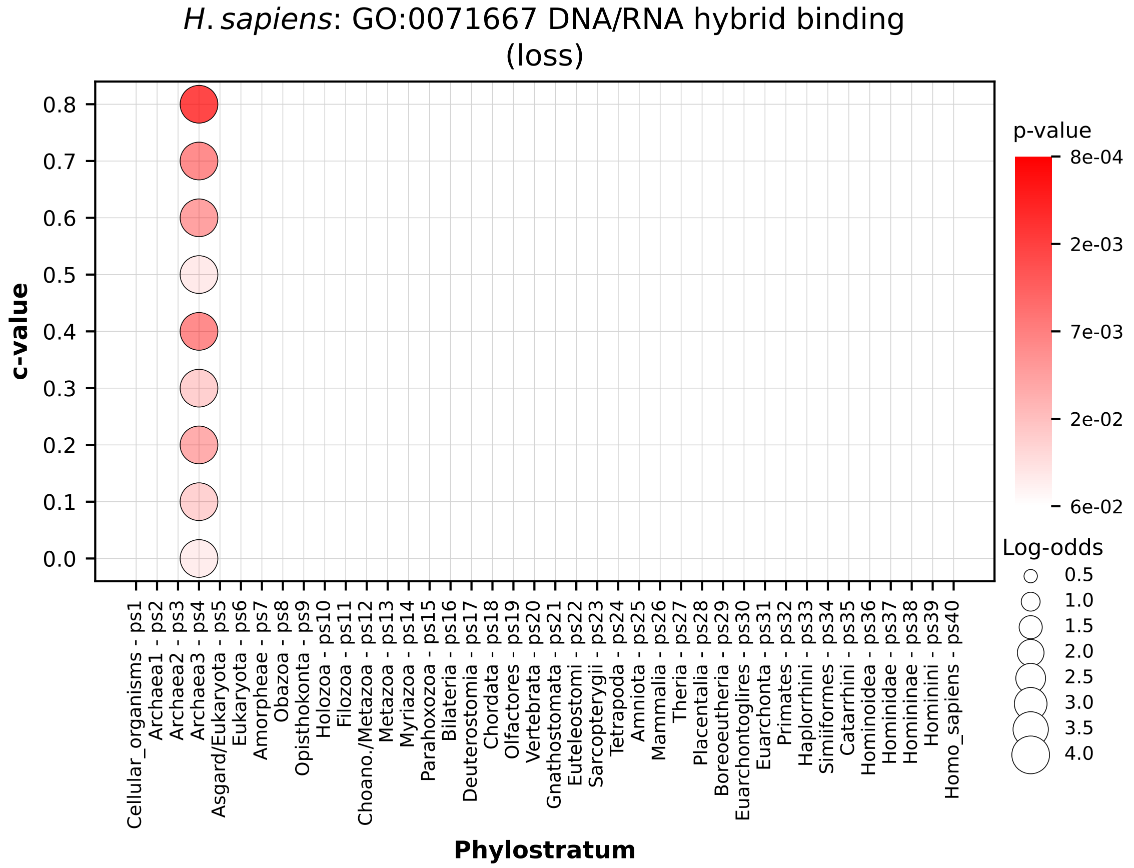

H. sapiens: GO:0071667 DNA/RNA hybrid binding
(loss)
c-value
0.8
0.7
0.6
0.5
0.4
0.3
0.2
0.1
0.0
p-value
8e-04
2e-03
7e-03
2e-02
6e-02
Log-odds
0.5
1.0
1.5
2.0
2.5
3.0
3.5
4.0
Cellular_organisms - ps1
Archaea1 - ps2
Archaea2 - ps3
Archaea3 - ps4
Asgard/Eukaryota - ps5
Eukaryota - ps6
Amorpheae - ps7
Obazoa - ps8
Opisthokonta - ps9
Holozoa - ps10
Filozoa - ps11
Choano./Metazoa - ps12
Metazoa - ps13
Myriazoa - ps14
Parahoxozoa - ps15
Bilateria - ps16
Deuterostomia - ps17
Chordata - ps18
Olfactores - ps19
Vertebrata - ps20
Gnathostomata - ps21
Euteleostomi - ps22
Sarcopterygii - ps23
Tetrapoda - ps24
Amniota - ps25
Mammalia - ps26
Theria - ps27
Placentalia - ps28
Boreoeutheria - ps29
Euarchontoglires - ps30
Euarchonta - ps31
Primates - ps32
Haplorrhini - ps33
Simiiformes - ps34
Catarrhini - ps35
Hominoidea - ps36
Hominidae - ps37
Homininae - ps38
Hominini - ps39
Homo_sapiens - ps40
Phylostratum

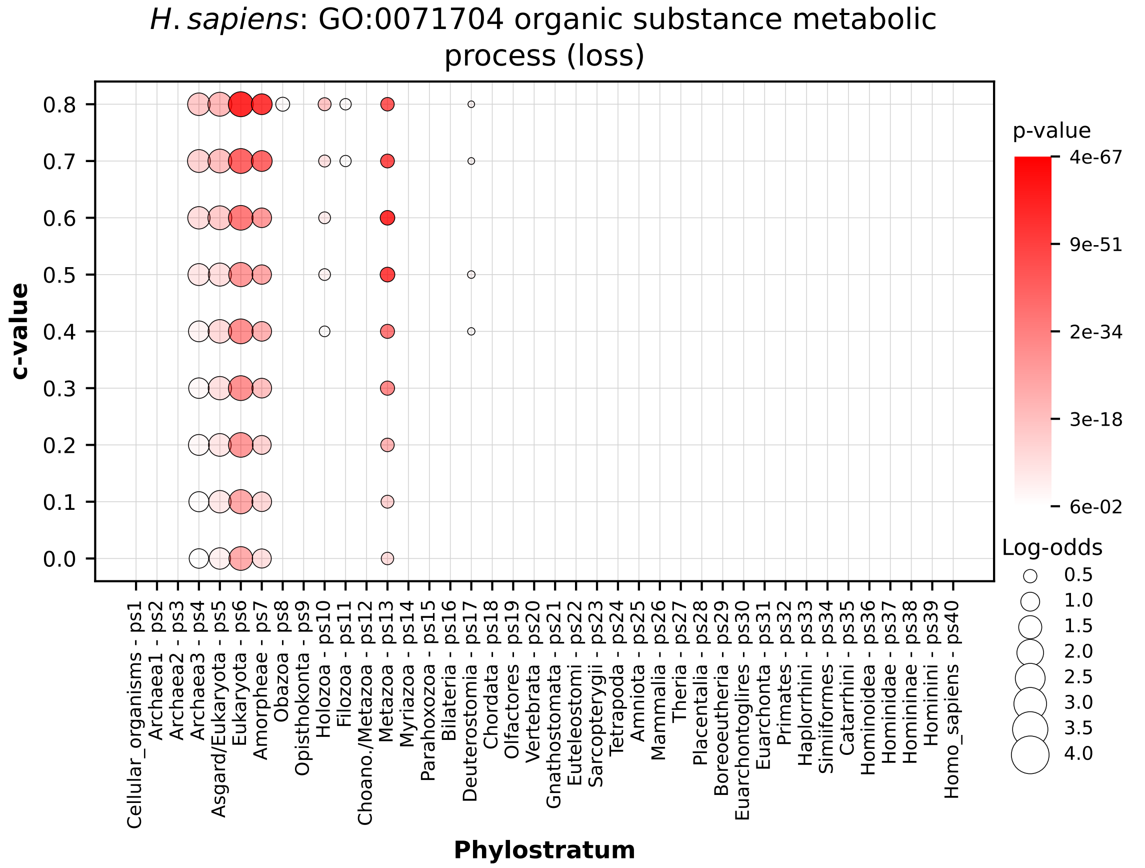

H. sapiens: GO:0071704 organic substance metabolic process (loss)
c-value
0.8
0.7
0.6
0.5
0.4
0.3
0.2
0.1
0.0
Phylostratum
Cellular_organisms - ps1
Archaea1 - ps2
Archaea2 - ps3
Archaea3 - ps4
Asgard/Eukaryota - ps5
Eukaryota - ps6
Amorpheae - ps7
Obazoa - ps8
Opisthokonta - ps9
Holozoa - ps10
Filozoa - ps11
Choano./Metazoa - ps12
Metazoa - ps13
Myriazoa - ps14
Parahoxozoa - ps15
Bilateria - ps16
Deuterostomia - ps17
Chordata - ps18
Olfactores - ps19
Vertebrata - ps20
Gnathostomata - ps21
Euteleostomi - ps22
Sarcopterygii - ps23
Tetrapoda - ps24
Amniota - ps25
Mammalia - ps26
Theria - ps27
Placentalia - ps28
Boreoeutheria - ps29
Euarchontoglires - ps30
Euarchonta - ps31
Primates - ps32
Haplorrhini - ps33
Simiiformes - ps34
Catarrhini - ps35
Hominoidea - ps36
Hominidae - ps37
Homininae - ps38
Hominini - ps39
Homo_sapiens - ps40
p-value
4e-67
9e-51
2e-34
3e-18
6e-02
Log-odds
0.5
1.0
1.5
2.0
2.5
3.0
3.5
4.0

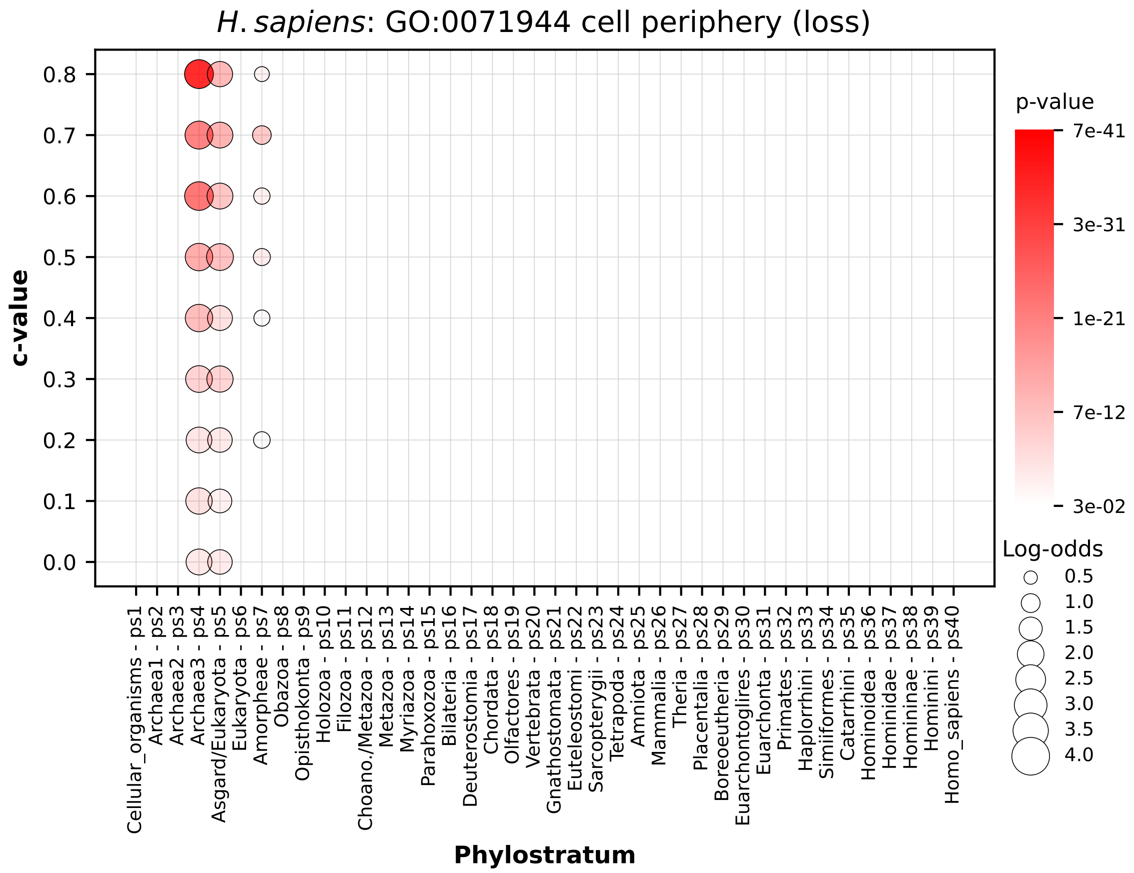

H. sapiens: GO:0071944 cell periphery (loss)
c-value
0.8
0.7
0.6
0.5
0.4
0.3
0.2
0.1
0.0
p-value
7e-41
3e-31
1e-21
7e-12
3e-02
Log-odds
0.5
1.0
1.5
2.0
2.5
3.0
3.5
4.0
Cellular_organisms - ps1
Archaea1 - ps2
Archaea2 - ps3
Archaea3 - ps4
Asgard/Eukaryota - ps5
Eukaryota - ps6
Amorpheae - ps7
Obazoa - ps8
Opisthokonta - ps9
Holozoa - ps10
Filozoa - ps11
Choano./Metazoa - ps12
Metazoa - ps13
Myriazoa - ps14
Parahoxozoa - ps15
Bilateria - ps16
Deuterostomia - ps17
Chordata - ps18
Olfactores - ps19
Vertebrata - ps20
Gnathostomata - ps21
Euteleostomi - ps22
Sarcopterygii - ps23
Tetrapoda - ps24
Amniota - ps25
Mammalia - ps26
Theria - ps27
Placentalia - ps28
Boreoeutheria - ps29
Euarchontoglires - ps30
Euarchonta - ps31
Primates - ps32
Haplorrhini - ps33
Simiiformes - ps34
Catarrhini - ps35
Hominoidea - ps36
Hominidae - ps37
Homininae - ps38
Hominini - ps39
Homo_sapiens - ps40
Phylostratum

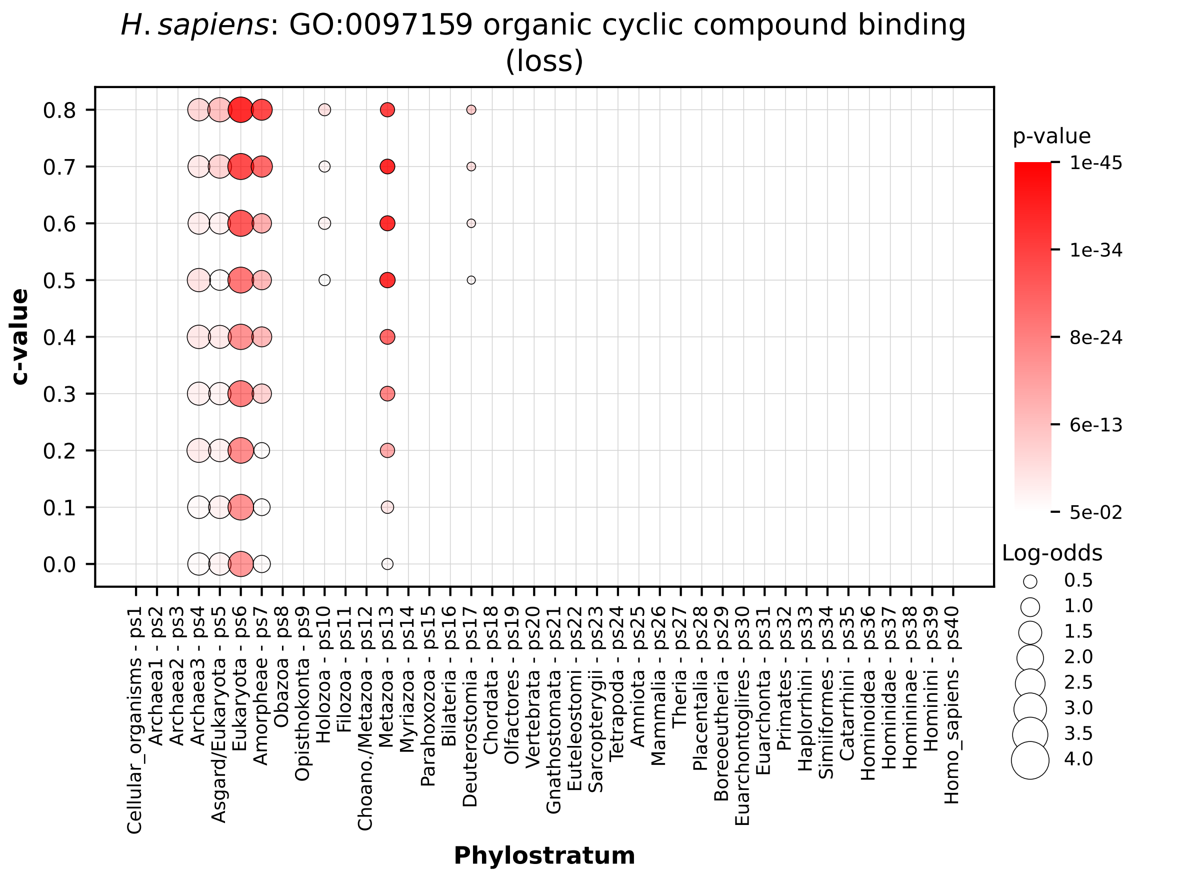

H. sapiens: GO:0097159 organic cyclic compound binding (loss)
c-value
0.0
0.1
0.2
0.3
0.4
0.5
0.6
0.7
0.8
Phylostratum
Cellular_organisms - ps1
Archaea1 - ps2
Archaea2 - ps3
Archaea3 - ps4
Asgard/Eukaryota - ps5
Eukaryota - ps6
Amorpheae - ps7
Obazoa - ps8
Opisthokonta - ps9
Holozoa - ps10
Filozoa - ps11
Choano./Metazoa - ps12
Metazoa - ps13
Myriazoa - ps14
Parahoxozoa - ps15
Bilateria - ps16
Deuterostomia - ps17
Chordata - ps18
Olfactores - ps19
Vertebrata - ps20
Gnathostomata - ps21
Euteleostomi - ps22
Sarcopterygii - ps23
Tetrapoda - ps24
Amniota - ps25
Mammalia - ps26
Theria - ps27
Placentalia - ps28
Boreoeutheria - ps29
Euarchontoglires - ps30
Euarchonta - ps31
Primates - ps32
Haplorrhini - ps33
Simiiformes - ps34
Catarrhini - ps35
Hominoidea - ps36
Hominidae - ps37
Homininae - ps38
Hominini - ps39
Homo_sapiens - ps40
p-value
1e-45
1e-34
8e-24
6e-13
5e-02
Log-odds
0.5
1.0
1.5
2.0
2.5
3.0
3.5
4.0

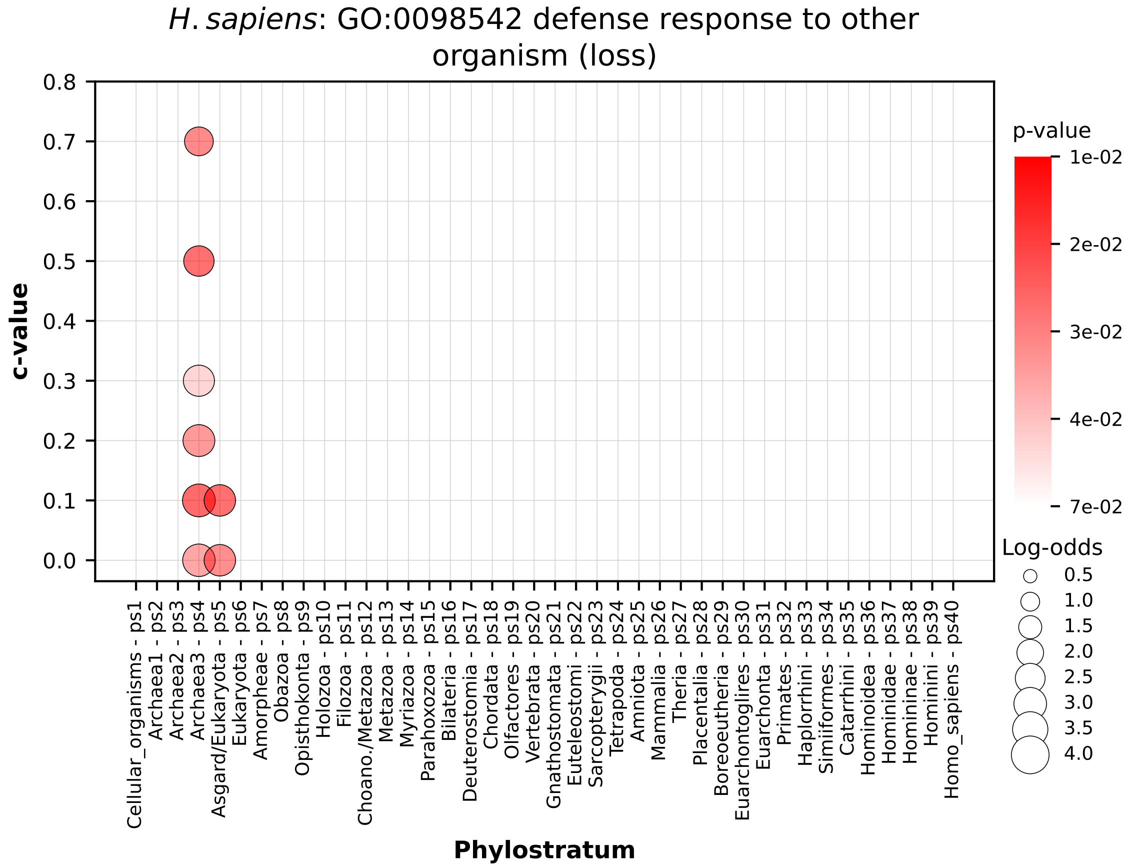

H. sapiens: GO:0098542 defense response to other organism (loss)
c-value
0.8
0.7
0.6
0.5
0.4
0.3
0.2
0.1
0.0
Phylostratum
Cellular_organisms - ps1
Archaea1 - ps2
Archaea2 - ps3
Archaea3 - ps4
Asgard/Eukaryota - ps5
Eukaryota - ps6
Amorpheae - ps7
Obazoa - ps8
Opisthokonta - ps9
Holozoa - ps10
Filozoa - ps11
Choano./Metazoa - ps12
Metazoa - ps13
Myriazoa - ps14
Parahoxozoa - ps15
Bilateria - ps16
Deuterostomia - ps17
Chordata - ps18
Olfactores - ps19
Vertebrata - ps20
Gnathostomata - ps21
Euteleostomi - ps22
Sarcopterygii - ps23
Tetrapoda - ps24
Amniota - ps25
Mammalia - ps26
Theria - ps27
Placentalia - ps28
Boreoeutheria - ps29
Euarchontoglires - ps30
Euarchonta - ps31
Primates - ps32
Haplorrhini - ps33
Simiiformes - ps34
Catarrhini - ps35
Hominoidea - ps36
Hominidae - ps37
Homininae - ps38
Hominini - ps39
Homo_sapiens - ps40
p-value
1e-02
2e-02
3e-02
4e-02
7e-02
Log-odds
0.5
1.0
1.5
2.0
2.5
3.0
3.5
4.0

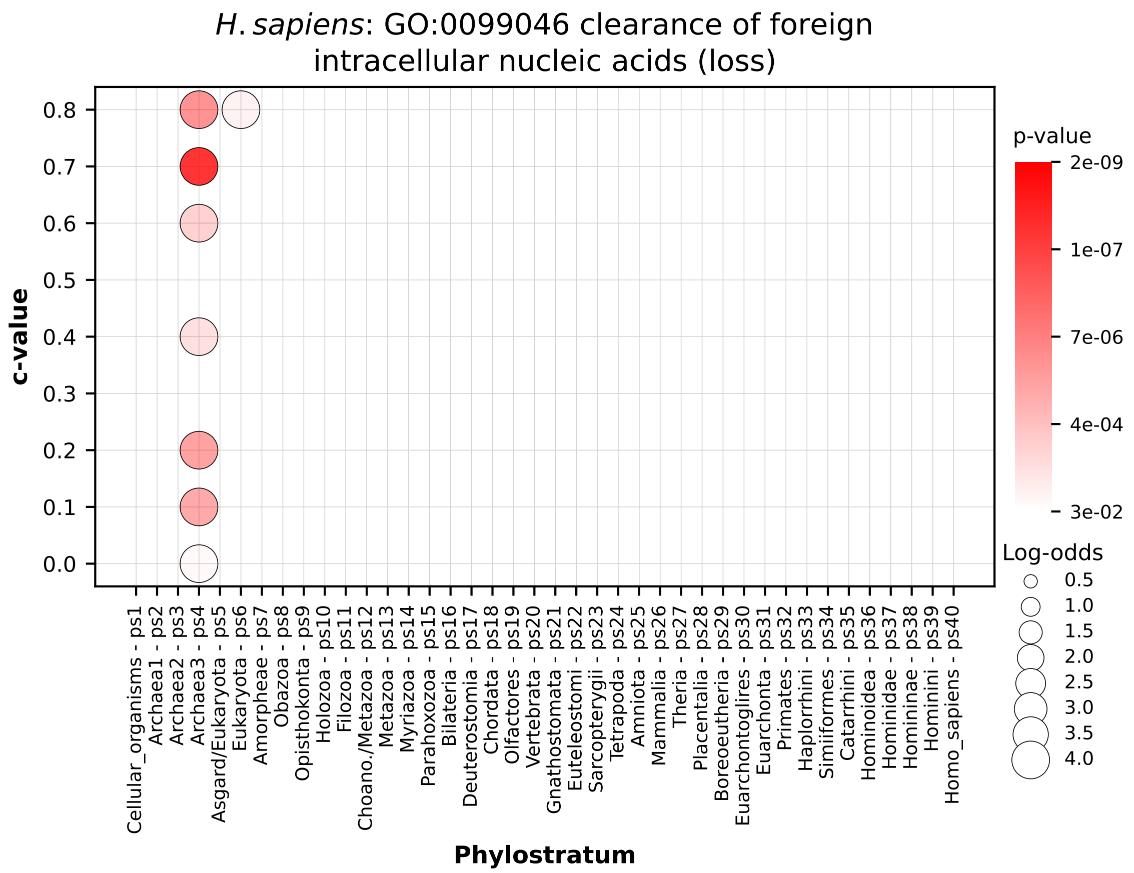

H. sapiens: GO:0099046 clearance of foreign intracellular nucleic acids (loss)
c-value
0.8
0.7
0.6
0.5
0.4
0.3
0.2
0.1
0.0
p-value
2e-09
1e-07
7e-06
4e-04
3e-02
Log-odds
0.5
1.0
1.5
2.0
2.5
3.0
3.5
4.0
Cellular_organisms - ps1
Archaea1 - ps2
Archaea2 - ps3
Archaea3 - ps4
Asgard/Eukaryota - ps5
Eukaryota - ps6
Amorpheae - ps7
Obazoa - ps8
Opisthokonta - ps9
Holozoa - ps10
Filozoa - ps11
Choano./Metazoa - ps12
Metazoa - ps13
Myriazoa - ps14
Parahoxozoa - ps15
Bilateria - ps16
Deuterostomia - ps17
Chordata - ps18
Olfactores - ps19
Vertebrata - ps20
Gnathostomata - ps21
Euteleostomi - ps22
Sarcopterygii - ps23
Tetrapoda - ps24
Amniota - ps25
Mammalia - ps26
Theria - ps27
Placentalia - ps28
Boreoeutheria - ps29
Euarchontoglires - ps30
Euarchonta - ps31
Primates - ps32
Haplorrhini - ps33
Simiiformes - ps34
Catarrhini - ps35
Hominoidea - ps36
Hominidae - ps37
Homininae - ps38
Hominini - ps39
Homo_sapiens - ps40
Phylostratum

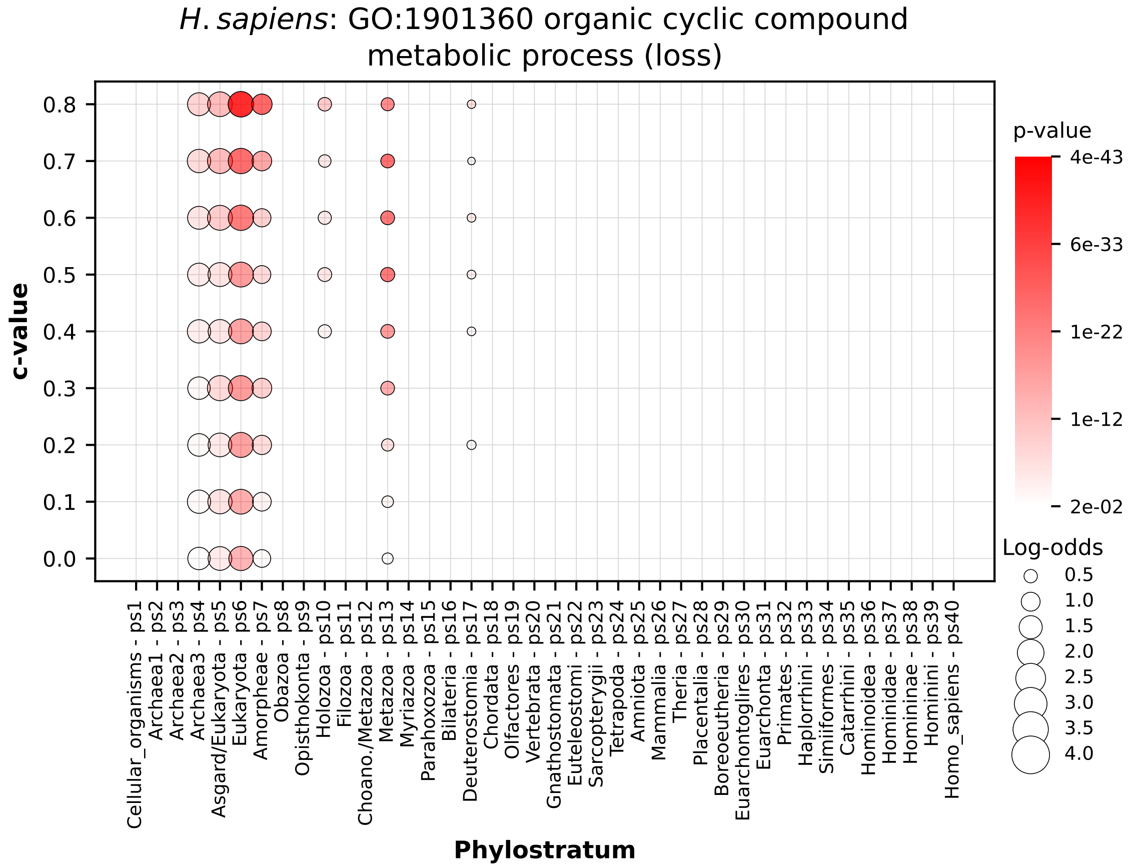

H. sapiens: GO:1901360 organic cyclic compound metabolic process (loss)
c-value
0.0
0.1
0.2
0.3
0.4
0.5
0.6
0.7
0.8
Phylostratum
Cellular_organisms - ps1
Archaea1 - ps2
Archaea2 - ps3
Archaea3 - ps4
Asgard/Eukaryota - ps5
Eukaryota - ps6
Amorpheae - ps7
Obazoa - ps8
Opisthokonta - ps9
Holozoa - ps10
Filozoa - ps11
Choano./Metazoa - ps12
Metazoa - ps13
Myriazoa - ps14
Parahoxozoa - ps15
Bilateria - ps16
Deuterostomia - ps17
Chordata - ps18
Olfactores - ps19
Vertebrata - ps20
Gnathostomata - ps21
Euteleostomi - ps22
Sarcopterygii - ps23
Tetrapoda - ps24
Amniota - ps25
Mammalia - ps26
Theria - ps27
Placentalia - ps28
Boreoeutheria - ps29
Euarchontoglires - ps30
Euarchonta - ps31
Primates - ps32
Haplorrhini - ps33
Simiiformes - ps34
Catarrhini - ps35
Hominoidea - ps36
Hominidae - ps37
Homininae - ps38
Hominini - ps39
Homo_sapiens - ps40
p-value
4e-43
6e-33
1e-22
1e-12
2e-02
Log-odds
0.5
1.0
1.5
2.0
2.5
3.0
3.5
4.0

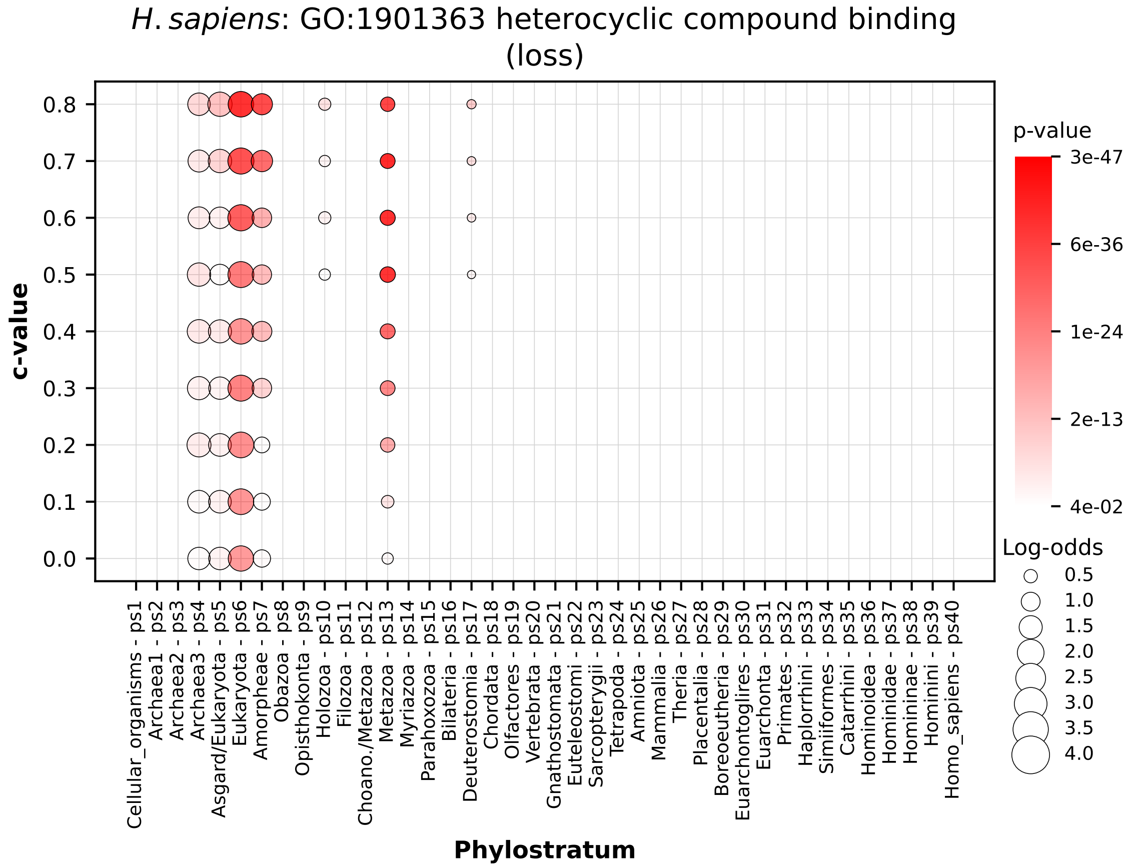

H. sapiens: GO:1901363 heterocyclic compound binding (loss)
c-value
0.8
0.7
0.6
0.5
0.4
0.3
0.2
0.1
0.0
Phylostratum
Cellular_organisms - ps1
Archaea1 - ps2
Archaea2 - ps3
Archaea3 - ps4
Asgard/Eukaryota - ps5
Eukaryota - ps6
Amorpheae - ps7
Obazoa - ps8
Opisthokonta - ps9
Holozoa - ps10
Filozoa - ps11
Choano./Metazoa - ps12
Metazoa - ps13
Myriazoa - ps14
Parahoxozoa - ps15
Bilateria - ps16
Deuterostomia - ps17
Chordata - ps18
Olfactores - ps19
Vertebrata - ps20
Gnathostomata - ps21
Euteleostomi - ps22
Sarcopterygii - ps23
Tetrapoda - ps24
Amniota - ps25
Mammalia - ps26
Theria - ps27
Placentalia - ps28
Boreoeutheria - ps29
Euarchontoglires - ps30
Euarchonta - ps31
Primates - ps32
Haplorrhini - ps33
Simiiformes - ps34
Catarrhini - ps35
Hominoidea - ps36
Hominidae - ps37
Homininae - ps38
Hominini - ps39
Homo_sapiens - ps40
p-value
3e-47
6e-36
1e-24
2e-13
4e-02
Log-odds
0.5
1.0
1.5
2.0
2.5
3.0
3.5
4.0

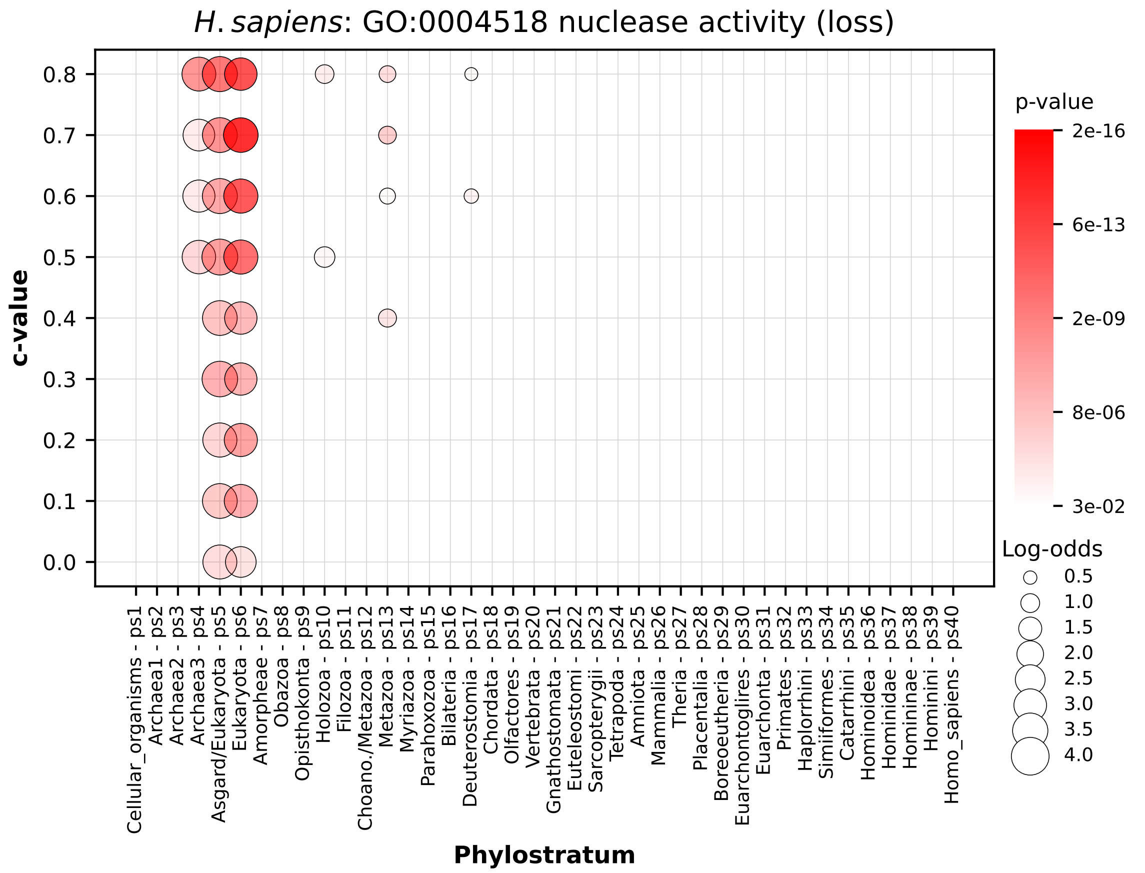

H. sapiens: GO:0004518 nuclease activity (loss)
c-value
Phylostratum
p-value
2e-16
6e-13
2e-09
8e-06
3e-02
Log-odds
0.5
1.0
1.5
2.0
2.5
3.0
3.5
4.0
0.0
0.1
0.2
0.3
0.4
0.5
0.6
0.7
0.8
Cellular_organisms - ps1
Archaea1 - ps2
Archaea2 - ps3
Archaea3 - ps4
Asgard/Eukaryota - ps5
Eukaryota - ps6
Amorpheae - ps7
Obazoa - ps8
Opisthokonta - ps9
Holozoa - ps10
Filozoa - ps11
Choano./Metazoa - ps12
Metazoa - ps13
Myriazoa - ps14
Parahoxozoa - ps15
Bilateria - ps16
Deuterostomia - ps17
Chordata - ps18
Olfactores - ps19
Vertebrata - ps20
Gnathostomata - ps21
Euteleostomi - ps22
Sarcopterygii - ps23
Tetrapoda - ps24
Amniota - ps25
Mammalia - ps26
Theria - ps27
Placentalia - ps28
Boreoeutheria - ps29
Euarchontoglires - ps30
Euarchonta - ps31
Primates - ps32
Haplorrhini - ps33
Simiiformes - ps34
Catarrhini - ps35
Hominoidea - ps36
Hominidae - ps37
Homininae - ps38
Hominini - ps39
Homo_sapiens - ps40

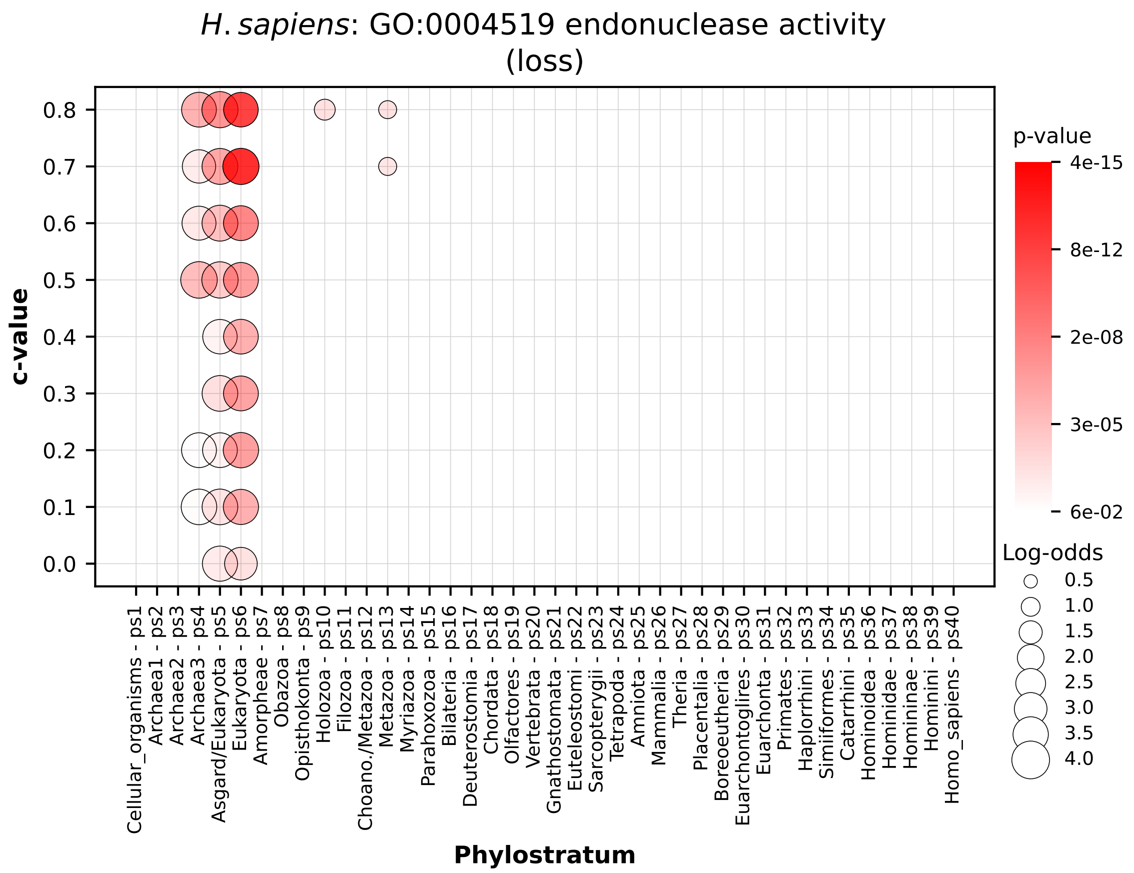
H. sapiens: GO:0004519 endonuclease activity
(loss)
c-value
0.8
0.7
0.6
0.5
0.4
0.3
0.2
0.1
0.0
p-value
4e-15
8e-12
2e-08
3e-05
6e-02
Log-odds
0.5
1.0
1.5
2.0
2.5
3.0
3.5
4.0
Cellular_organisms - ps1
Archaea1 - ps2
Archaea2 - ps3
Archaea3 - ps4
Asgard/Eukaryota - ps5
Eukaryota - ps6
Amorpheae - ps7
Obazoa - ps8
Opisthokonta - ps9
Holozoa - ps10
Filozoa - ps11
Choano./Metazoa - ps12
Metazoa - ps13
Myriazoa - ps14
Parahoxozoa - ps15
Bilateria - ps16
Deuterostomia - ps17
Chordata - ps18
Olfactores - ps19
Vertebrata - ps20
Gnathostomata - ps21
Euteleostomi - ps22
Sarcopterygii - ps23
Tetrapoda - ps24
Amniota - ps25
Mammalia - ps26
Theria - ps27
Placentalia - ps28
Boreoeutheria - ps29
Euarchontoglires - ps30
Euarchonta - ps31
Primates - ps32
Haplorrhini - ps33
Simiiformes - ps34
Catarrhini - ps35
Hominoidea - ps36
Hominidae - ps37
Homininae - ps38
Hominini - ps39
Homo_sapiens - ps40
Phylostratum

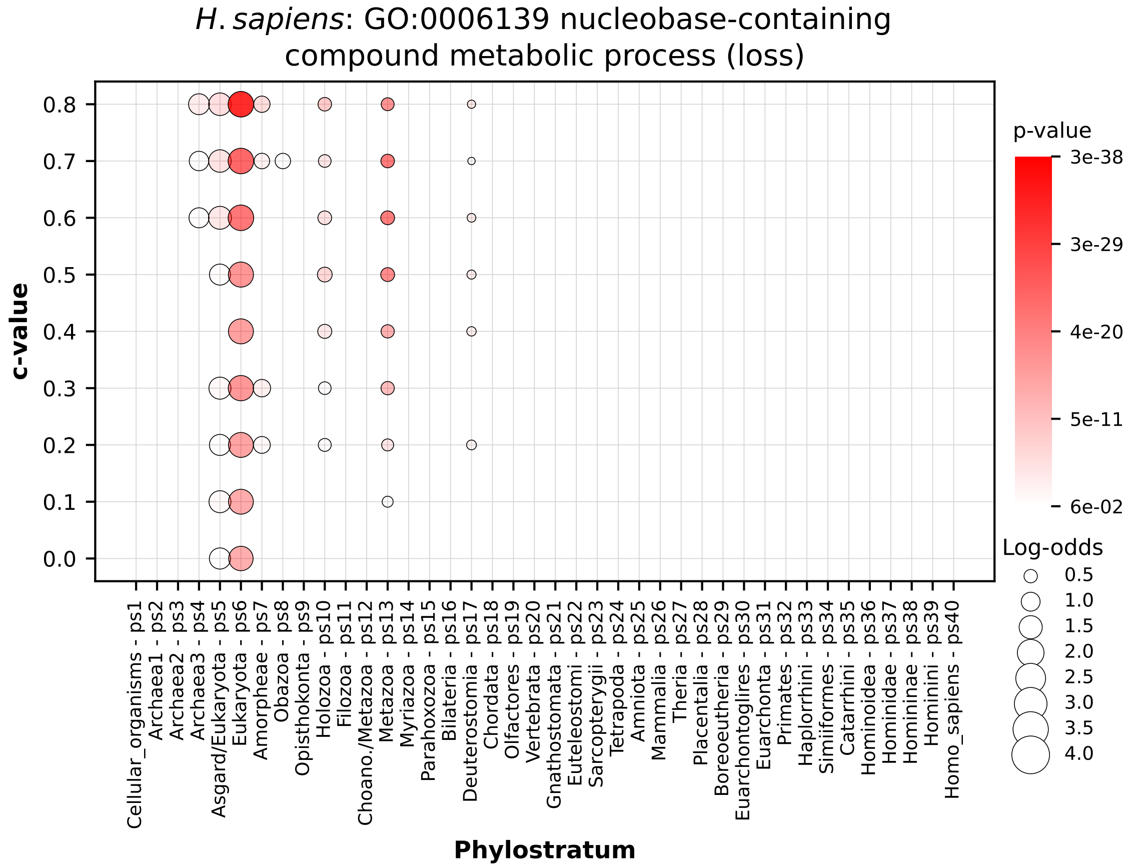
H. sapiens: GO:0006139 nucleobase-containing compound metabolic process (loss)
c-value
0.8
0.7
0.6
0.5
0.4
0.3
0.2
0.1
0.0
p-value
3e-38
3e-29
4e-20
5e-11
6e-02
Log-odds
0.5
1.0
1.5
2.0
2.5
3.0
3.5
4.0
Cellular_organisms - ps1
Archaea1 - ps2
Archaea2 - ps3
Archaea3 - ps4
Asgard/Eukaryota - ps5
Eukaryota - ps6
Amorpheae - ps7
Obazoa - ps8
Opisthokonta - ps9
Holozoa - ps10
Filozoa - ps11
Choano./Metazoa - ps12
Metazoa - ps13
Myriazoa - ps14
Parahoxozoa - ps15
Bilateria - ps16
Deuterostomia - ps17
Chordata - ps18
Olfactores - ps19
Vertebrata - ps20
Gnathostomata - ps21
Euteleostomi - ps22
Sarcopterygii - ps23
Tetrapoda - ps24
Amniota - ps25
Mammalia - ps26
Theria - ps27
Placentalia - ps28
Boreoeutheria - ps29
Euarchontoglires - ps30
Euarchonta - ps31
Primates - ps32
Haplorrhini - ps33
Simiiformes - ps34
Catarrhini - ps35
Hominoidea - ps36
Hominidae - ps37
Homininae - ps38
Hominini - ps39
Homo_sapiens - ps40
Phylostratum

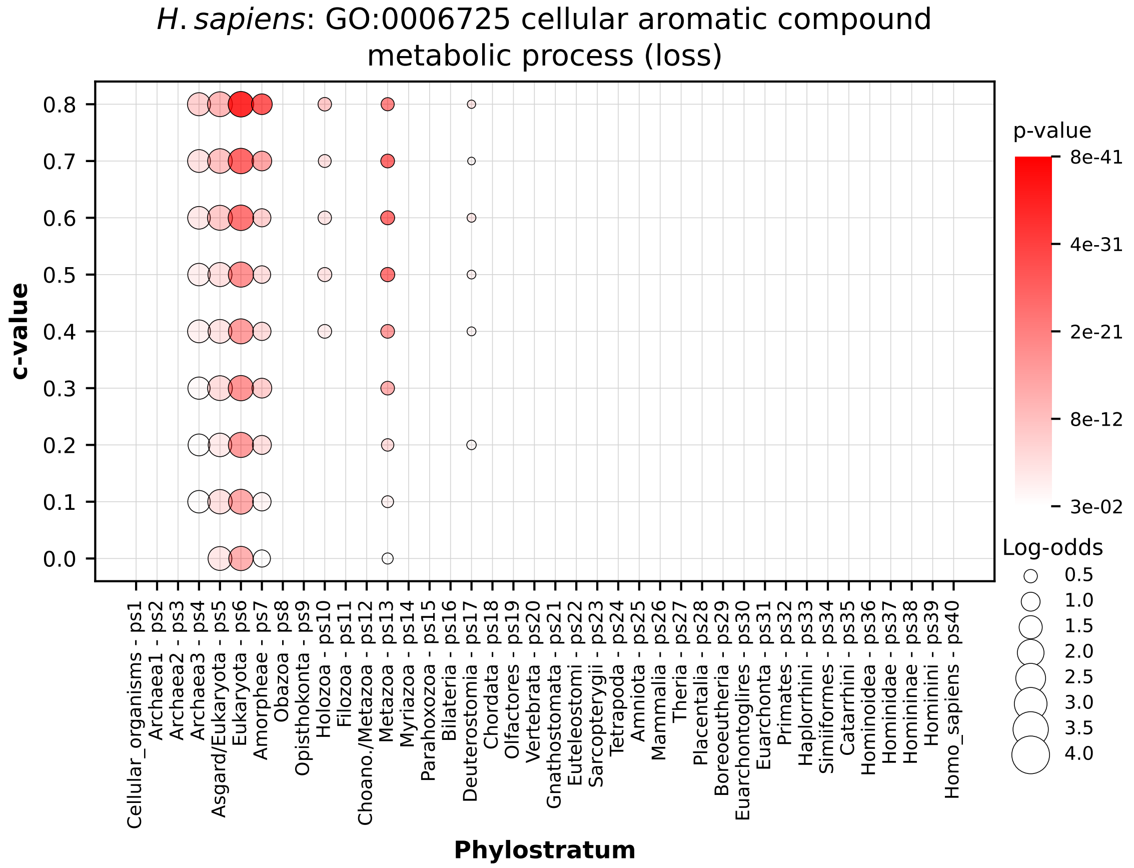

H. sapiens: GO:0006725 cellular aromatic compound metabolic process (loss)
c-value
0.8
0.7
0.6
0.5
0.4
0.3
0.2
0.1
0.0
p-value
8e-41
4e-31
2e-21
8e-12
3e-02
Log-odds
0.5
1.0
1.5
2.0
2.5
3.0
3.5
4.0
Cellular_organisms - ps1
Archaea1 - ps2
Archaea2 - ps3
Archaea3 - ps4
Asgard/Eukaryota - ps5
Eukaryota - ps6
Amorpheae - ps7
Obazoa - ps8
Opisthokonta - ps9
Holozoa - ps10
Filozoa - ps11
Choano./Metazoa - ps12
Metazoa - ps13
Myriazoa - ps14
Parahoxozoa - ps15
Bilateria - ps16
Deuterostomia - ps17
Chordata - ps18
Olfactores - ps19
Vertebrata - ps20
Gnathostomata - ps21
Euteleostomi - ps22
Sarcopterygii - ps23
Tetrapoda - ps24
Amniota - ps25
Mammalia - ps26
Theria - ps27
Placentalia - ps28
Boreoeutheria - ps29
Euarchontoglires - ps30
Euarchonta - ps31
Primates - ps32
Haplorrhini - ps33
Simiiformes - ps34
Catarrhini - ps35
Hominoidea - ps36
Hominidae - ps37
Homininae - ps38
Hominini - ps39
Homo_sapiens - ps40
Phylostratum

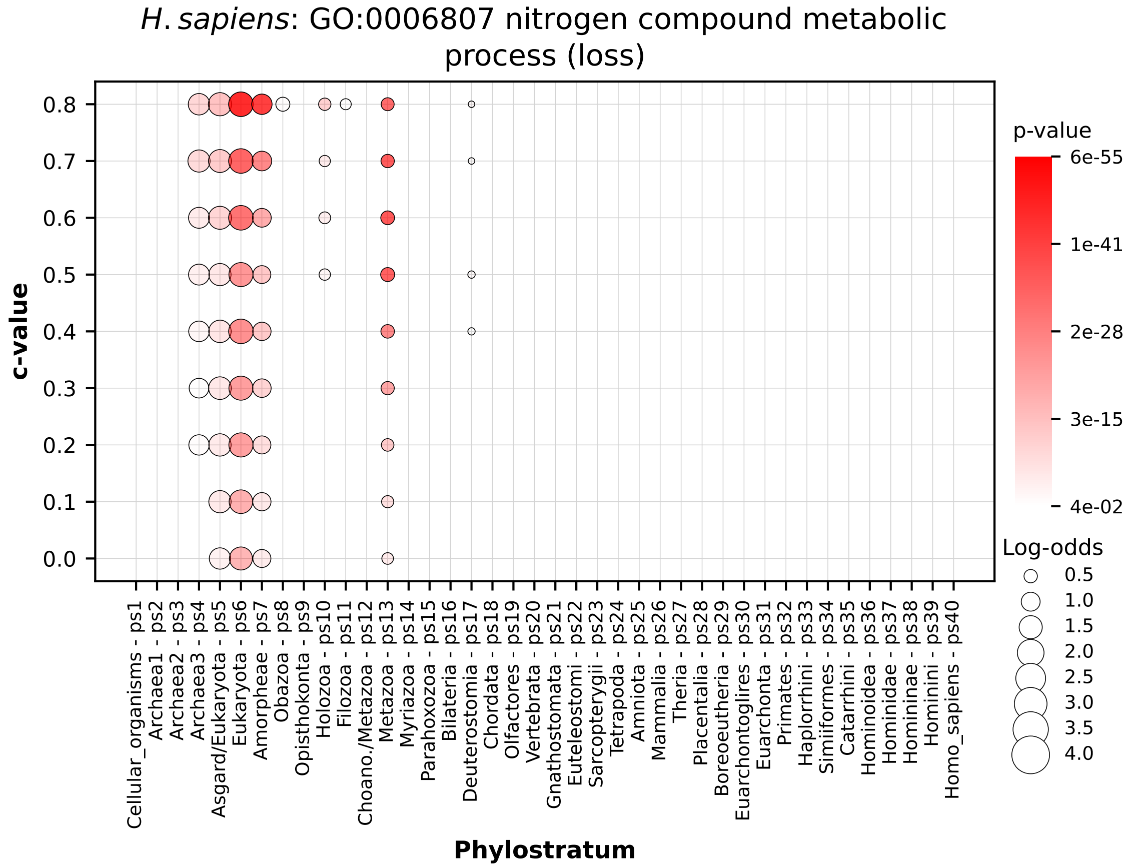
H. sapiens: GO:0006807 nitrogen compound metabolic process (loss)
c-value
Phylostratum
p-value
6e-55
1e-41
2e-28
3e-15
4e-02
Log-odds
0.5
1.0
1.5
2.0
2.5
3.0
3.5
4.0
Cellular_organisms - ps1
Archaea1 - ps2
Archaea2 - ps3
Archaea3 - ps4
Asgard/Eukaryota - ps5
Eukaryota - ps6
Amorpheae - ps7
Obazoa - ps8
Opisthokonta - ps9
Holozoa - ps10
Filozoa - ps11
Choano./Metazoa - ps12
Metazoa - ps13
Myriazoa - ps14
Parahoxozoa - ps15
Bilateria - ps16
Deuterostomia - ps17
Chordata - ps18
Olfactores - ps19
Vertebrata - ps20
Gnathostomata - ps21
Euteleostomi - ps22
Sarcopterygii - ps23
Tetrapoda - ps24
Amniota - ps25
Mammalia - ps26
Theria - ps27
Placentalia - ps28
Boreoeutheria - ps29
Euarchontoglires - ps30
Euarchonta - ps31
Primates - ps32
Haplorrhini - ps33
Simiiformes - ps34
Catarrhini - ps35
Hominoidea - ps36
Hominidae - ps37
Homininae - ps38
Hominini - ps39
Homo_sapiens - ps40

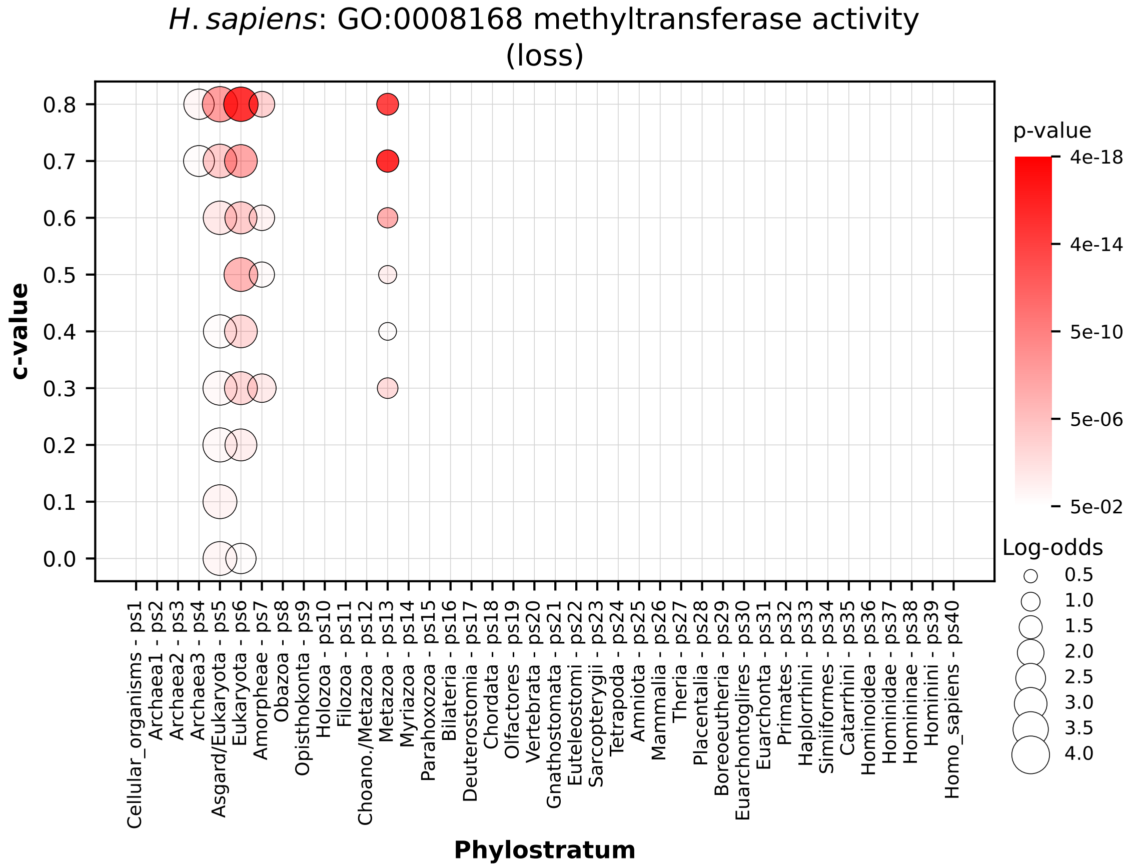

H. sapiens: GO:0008168 methyltransferase activity
(loss)
c-value
0.8
0.7
0.6
0.5
0.4
0.3
0.2
0.1
0.0
Phylostratum
Cellular_organisms - ps1
Archaea1 - ps2
Archaea2 - ps3
Archaea3 - ps4
Asgard/Eukaryota - ps5
Eukaryota - ps6
Amorpheae - ps7
Obazoa - ps8
Opisthokonta - ps9
Holozoa - ps10
Filozoa - ps11
Choano./Metazoa - ps12
Metazoa - ps13
Myriazoa - ps14
Parahoxozoa - ps15
Bilateria - ps16
Deuterostomia - ps17
Chordata - ps18
Olfactores - ps19
Vertebrata - ps20
Gnathostomata - ps21
Euteleostomi - ps22
Sarcopterygii - ps23
Tetrapoda - ps24
Amniota - ps25
Mammalia - ps26
Theria - ps27
Placentalia - ps28
Boreoeutheria - ps29
Euarchontoglires - ps30
Euarchonta - ps31
Primates - ps32
Haplorrhini - ps33
Simiiformes - ps34
Catarrhini - ps35
Hominoidea - ps36
Hominidae - ps37
Homininae - ps38
Hominini - ps39
Homo_sapiens - ps40
p-value
4e-18
4e-14
5e-10
5e-06
5e-02
Log-odds
0.5
1.0
1.5
2.0
2.5
3.0
3.5
4.0

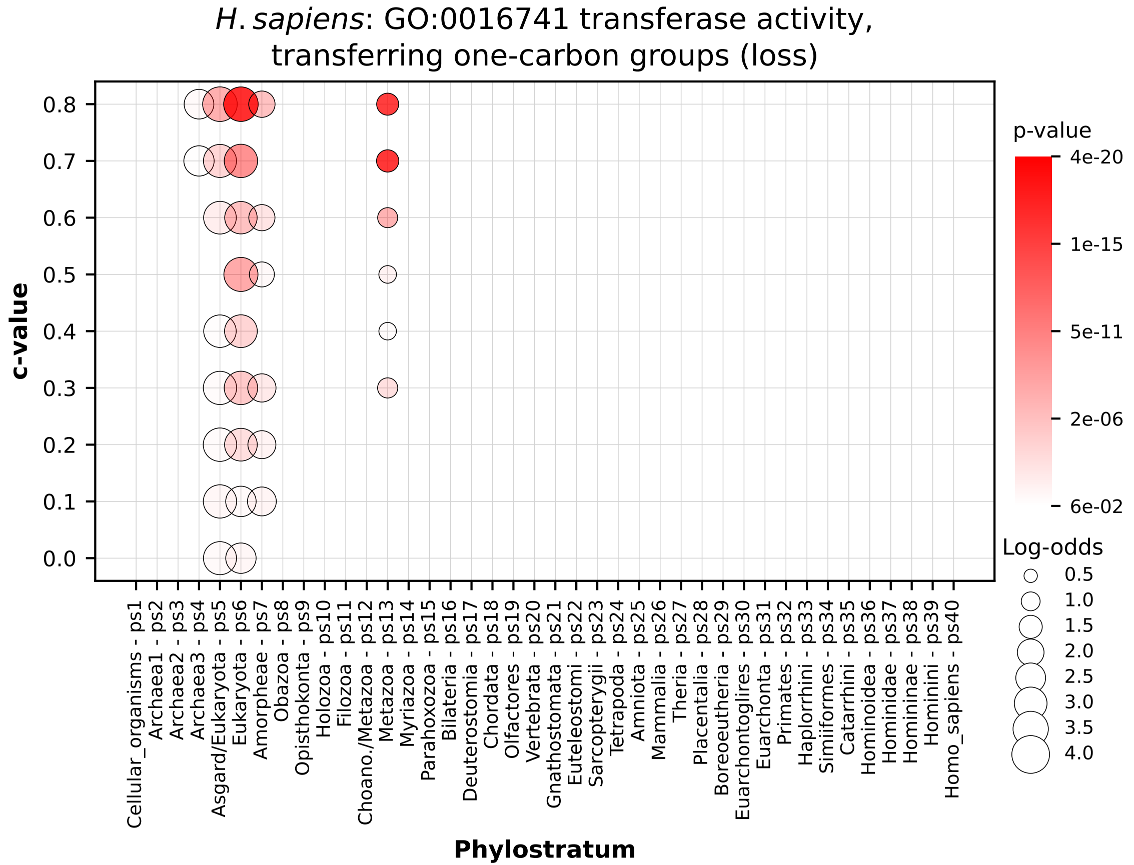

H. sapiens: GO:0016741 transferase activity, transferring one-carbon groups (loss)
c-value
0.8
0.7
0.6
0.5
0.4
0.3
0.2
0.1
0.0
Phylostratum
Cellular_organisms - ps1
Archaea1 - ps2
Archaea2 - ps3
Archaea3 - ps4
Asgard/Eukaryota - ps5
Eukaryota - ps6
Amorpheae - ps7
Obazoa - ps8
Opisthokonta - ps9
Holozoa - ps10
Filozoa - ps11
Choano./Metazoa - ps12
Metazoa - ps13
Myriazoa - ps14
Parahoxozoa - ps15
Bilateria - ps16
Deuterostomia - ps17
Chordata - ps18
Olfactores - ps19
Vertebrata - ps20
Gnathostomata - ps21
Euteleostomi - ps22
Sarcopterygii - ps23
Tetrapoda - ps24
Amniota - ps25
Mammalia - ps26
Theria - ps27
Placentalia - ps28
Boreoeutheria - ps29
Euarchontoglires - ps30
Euarchonta - ps31
Primates - ps32
Haplorrhini - ps33
Simiiformes - ps34
Catarrhini - ps35
Hominoidea - ps36
Hominidae - ps37
Homininae - ps38
Hominini - ps39
Homo_sapiens - ps40
p-value
4e-20
1e-15
5e-11
2e-06
6e-02
Log-odds
0.5
1.0
1.5
2.0
2.5
3.0
3.5
4.0

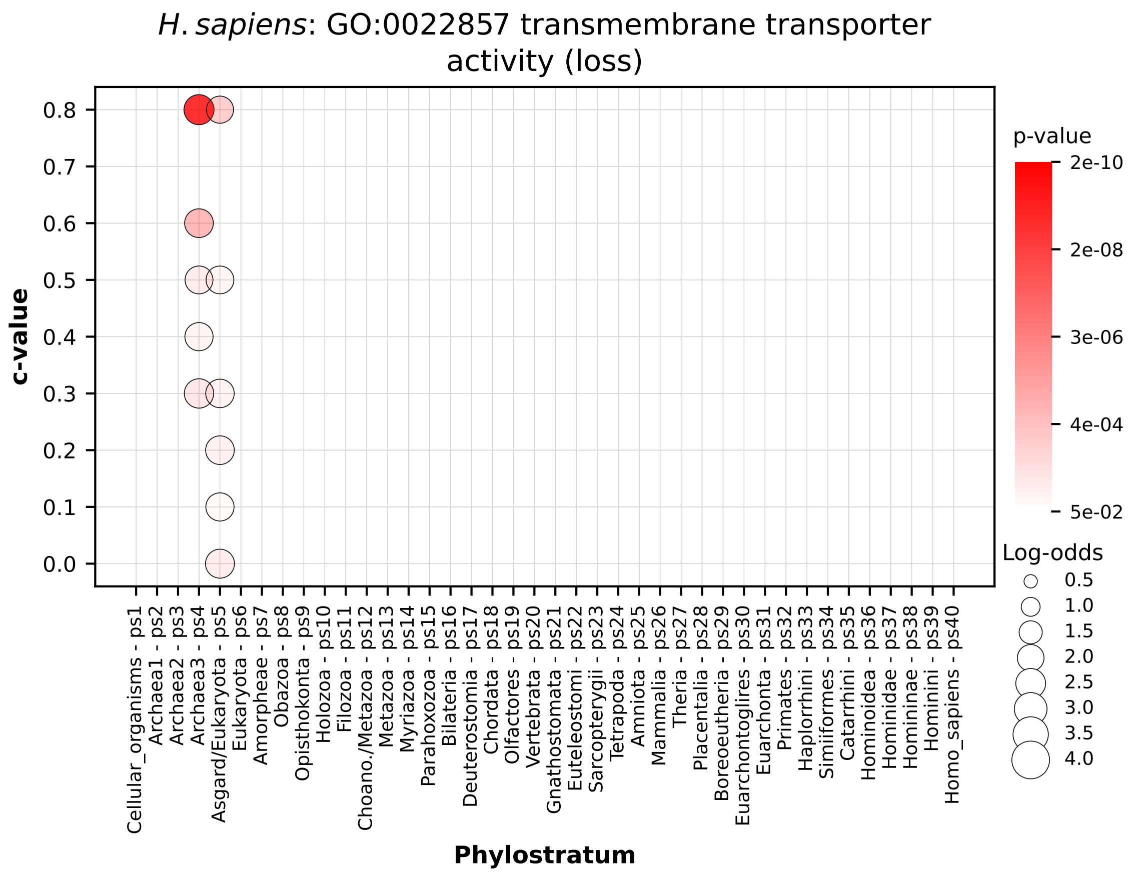

H. sapiens: GO:0022857 transmembrane transporter activity (loss)
c-value
0.8
0.7
0.6
0.5
0.4
0.3
0.2
0.1
0.0
p-value
2e-10
2e-08
3e-06
4e-04
5e-02
Log-odds
0.5
1.0
1.5
2.0
2.5
3.0
3.5
4.0
Cellular_organisms - ps1
Archaea1 - ps2
Archaea2 - ps3
Archaea3 - ps4
Asgard/Eukaryota - ps5
Eukaryota - ps6
Amorpheae - ps7
Obazoa - ps8
Opisthokonta - ps9
Holozoa - ps10
Filozoa - ps11
Choano./Metazoa - ps12
Metazoa - ps13
Myriazoa - ps14
Parahoxozoa - ps15
Bilateria - ps16
Deuterostomia - ps17
Chordata - ps18
Olfactores - ps19
Vertebrata - ps20
Gnathostomata - ps21
Euteleostomi - ps22
Sarcopterygii - ps23
Tetrapoda - ps24
Amniota - ps25
Mammalia - ps26
Theria - ps27
Placentalia - ps28
Boreoeutheria - ps29
Euarchontoglires - ps30
Euarchonta - ps31
Primates - ps32
Haplorrhini - ps33
Simiiformes - ps34
Catarrhini - ps35
Hominoidea - ps36
Hominidae - ps37
Homininae - ps38
Hominini - ps39
Homo_sapiens - ps40
Phylostratum

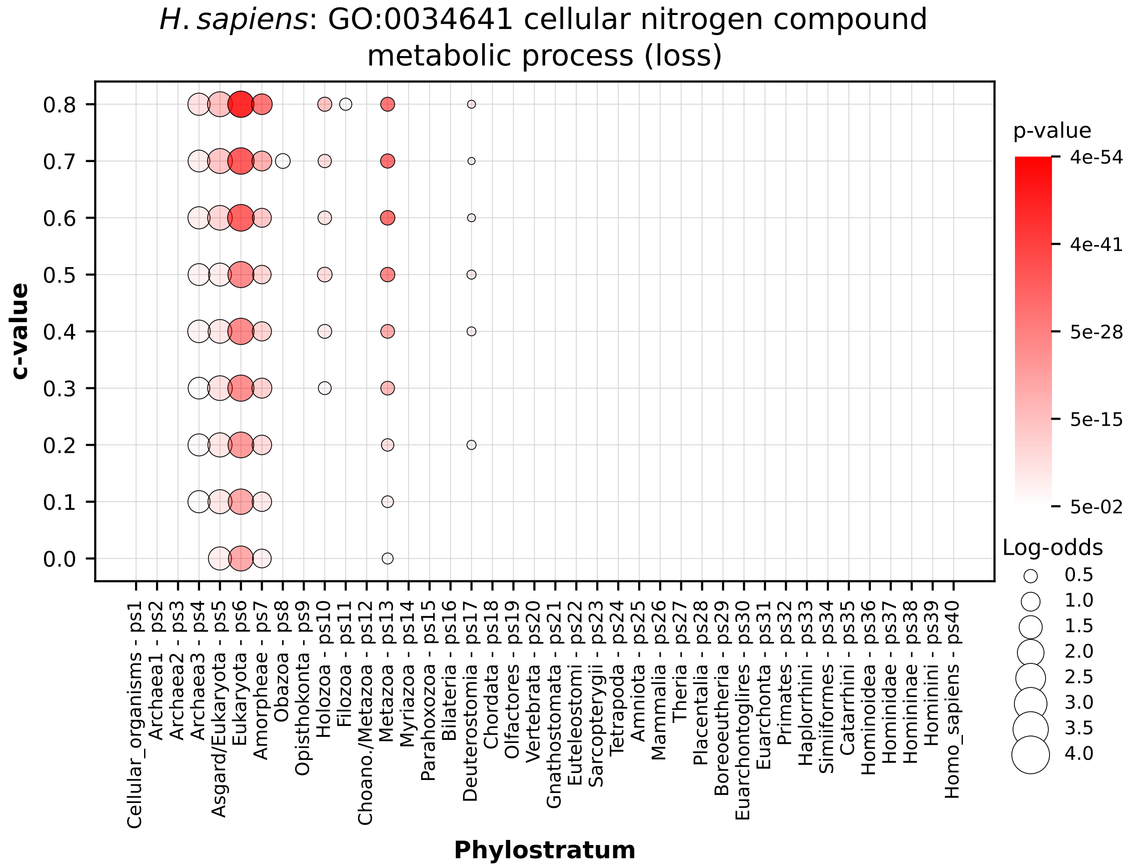

H. sapiens: GO:0034641 cellular nitrogen compound metabolic process (loss)
c-value
0.8
0.7
0.6
0.5
0.4
0.3
0.2
0.1
0.0
p-value
4e-54
4e-41
5e-28
5e-15
5e-02
Log-odds
0.5
1.0
1.5
2.0
2.5
3.0
3.5
4.0
Cellular_organisms - ps1
Archaea1 - ps2
Archaea2 - ps3
Archaea3 - ps4
Asgard/Eukaryota - ps5
Eukaryota - ps6
Amorpheae - ps7
Obazoa - ps8
Opisthokonta - ps9
Holozoa - ps10
Filozoa - ps11
Choano./Metazoa - ps12
Metazoa - ps13
Myriazoa - ps14
Parahoxozoa - ps15
Bilateria - ps16
Deuterostomia - ps17
Chordata - ps18
Olfactores - ps19
Vertebrata - ps20
Gnathostomata - ps21
Euteleostomi - ps22
Sarcopterygii - ps23
Tetrapoda - ps24
Amniota - ps25
Mammalia - ps26
Theria - ps27
Placentalia - ps28
Boreoeutheria - ps29
Euarchontoglires - ps30
Euarchonta - ps31
Primates - ps32
Haplorrhini - ps33
Simiiformes - ps34
Catarrhini - ps35
Hominoidea - ps36
Hominidae - ps37
Homininae - ps38
Hominini - ps39
Homo_sapiens - ps40
Phylostratum

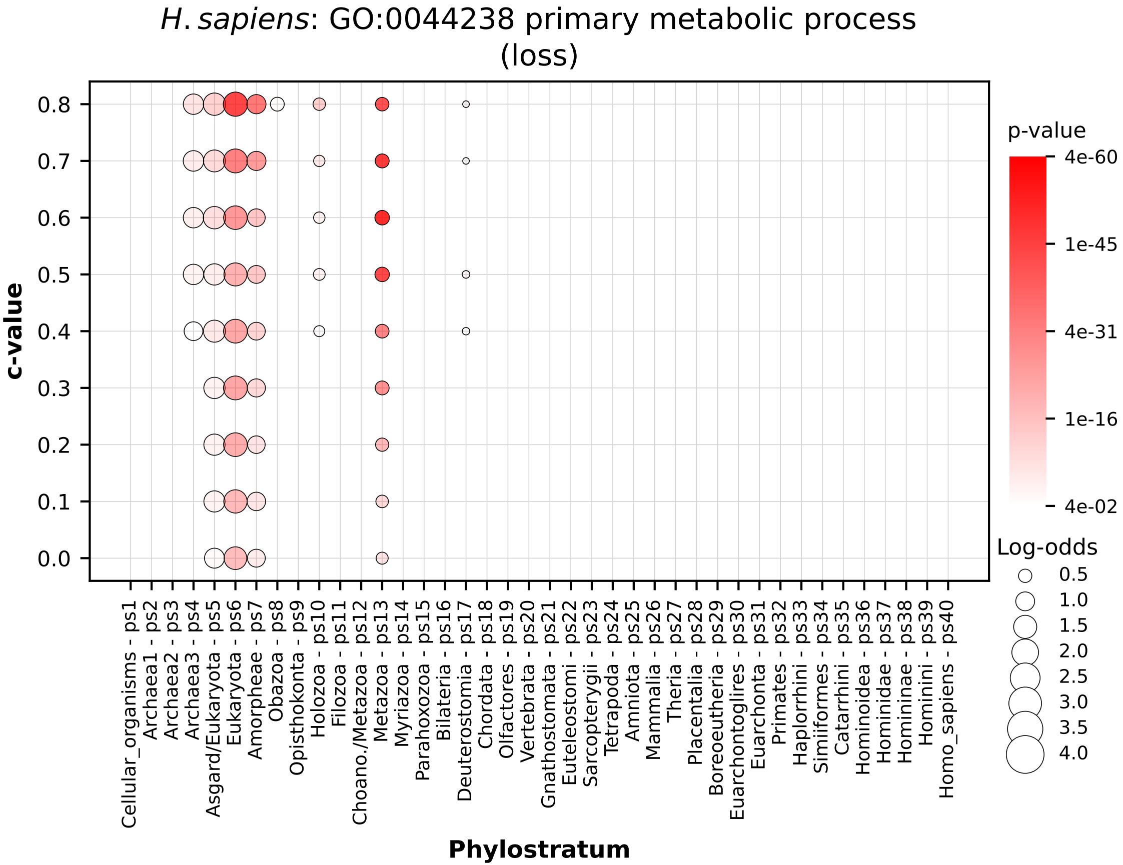

H. sapiens: GO:0044238 primary metabolic process
(loss)
c-value
0.8
0.7
0.6
0.5
0.4
0.3
0.2
0.1
0.0
p-value
4e-60
1e-45
4e-31
1e-16
4e-02
Log-odds
0.5
1.0
1.5
2.0
2.5
3.0
3.5
4.0
Cellular_organisms - ps1
Archaea1 - ps2
Archaea2 - ps3
Archaea3 - ps4
Asgard/Eukaryota - ps5
Eukaryota - ps6
Amorpheae - ps7
Obazoa - ps8
Opisthokonta - ps9
Holozoa - ps10
Filozoa - ps11
Choano./Metazoa - ps12
Metazoa - ps13
Myriazoa - ps14
Parahoxozoa - ps15
Bilateria - ps16
Deuterostomia - ps17
Chordata - ps18
Olfactores - ps19
Vertebrata - ps20
Gnathostomata - ps21
Euteleostomi - ps22
Sarcopterygii - ps23
Tetrapoda - ps24
Amniota - ps25
Mammalia - ps26
Theria - ps27
Placentalia - ps28
Boreoeutheria - ps29
Euarchontoglires - ps30
Euarchonta - ps31
Primates - ps32
Haplorrhini - ps33
Simiiformes - ps34
Catarrhini - ps35
Hominoidea - ps36
Hominidae - ps37
Homininae - ps38
Hominini - ps39
Homo_sapiens - ps40
Phylostratum

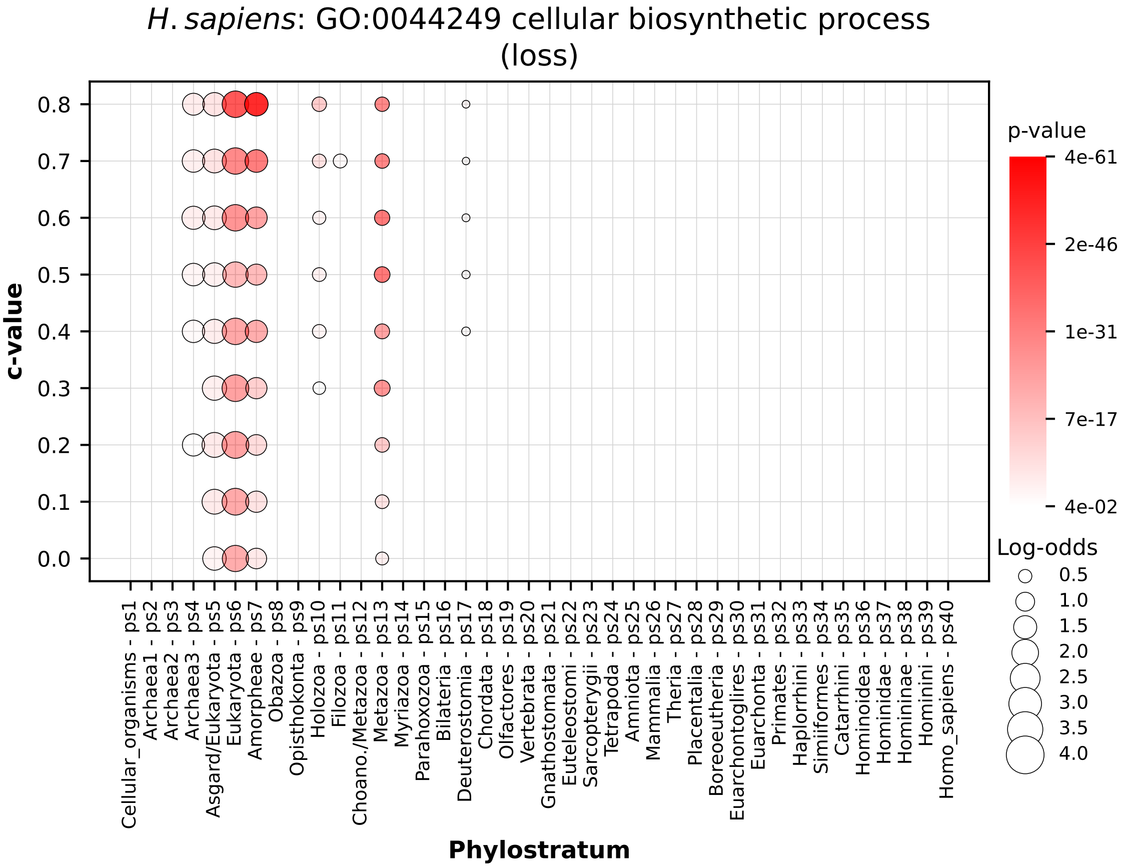

H. sapiens: GO:0044249 cellular biosynthetic process
(loss)
c-value
0.8
0.7
0.6
0.5
0.4
0.3
0.2
0.1
0.0
p-value
4e-61
2e-46
1e-31
7e-17
4e-02
Log-odds
0.5
1.0
1.5
2.0
2.5
3.0
3.5
4.0
Cellular_organisms - ps1
Archaea1 - ps2
Archaea2 - ps3
Archaea3 - ps4
Asgard/Eukaryota - ps5
Eukaryota - ps6
Amorpheae - ps7
Obazoa - ps8
Opisthokonta - ps9
Holozoa - ps10
Filozoa - ps11
Choano./Metazoa - ps12
Metazoa - ps13
Myriazoa - ps14
Parahoxozoa - ps15
Bilateria - ps16
Deuterostomia - ps17
Chordata - ps18
Olfactores - ps19
Vertebrata - ps20
Gnathostomata - ps21
Euteleostomi - ps22
Sarcopterygii - ps23
Tetrapoda - ps24
Amniota - ps25
Mammalia - ps26
Theria - ps27
Placentalia - ps28
Boreoeutheria - ps29
Euarchontoglires - ps30
Euarchonta - ps31
Primates - ps32
Haplorrhini - ps33
Simiiformes - ps34
Catarrhini - ps35
Hominoidea - ps36
Hominidae - ps37
Homininae - ps38
Hominini - ps39
Homo_sapiens - ps40
Phylostratum

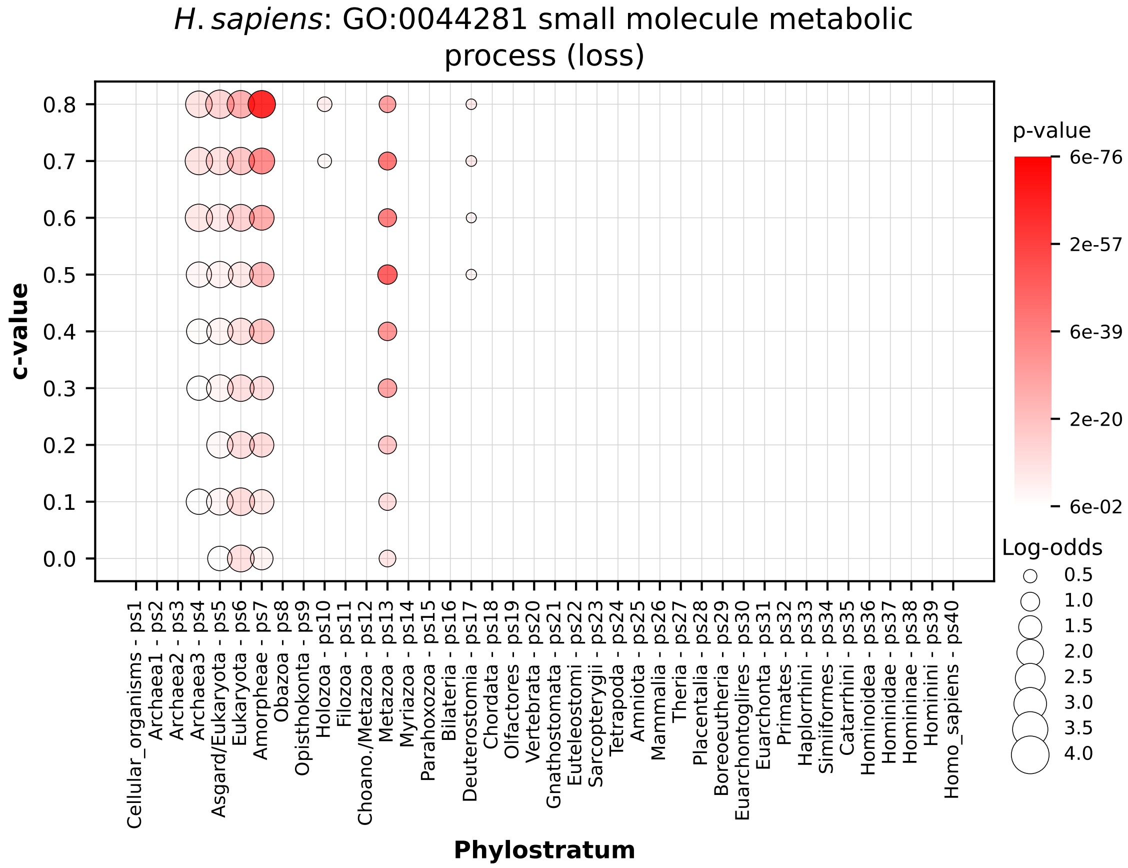
H. sapiens: GO:0044281 small molecule metabolic process (loss)
c-value
0.8
0.7
0.6
0.5
0.4
0.3
0.2
0.1
0.0
Phylostratum
Cellular_organisms - ps1
Archaea1 - ps2
Archaea2 - ps3
Archaea3 - ps4
Asgard/Eukaryota - ps5
Eukaryota - ps6
Amorpheae - ps7
Obazoa - ps8
Opisthokonta - ps9
Holozoa - ps10
Filozoa - ps11
Choano./Metazoa - ps12
Metazoa - ps13
Myriazoa - ps14
Parahoxozoa - ps15
Bilateria - ps16
Deuterostomia - ps17
Chordata - ps18
Olfactores - ps19
Vertebrata - ps20
Gnathostomata - ps21
Euteleostomi - ps22
Sarcopterygii - ps23
Tetrapoda - ps24
Amniota - ps25
Mammalia - ps26
Theria - ps27
Placentalia - ps28
Boreoeutheria - ps29
Euarchontoglires - ps30
Euarchonta - ps31
Primates - ps32
Haplorrhini - ps33
Simiiformes - ps34
Catarrhini - ps35
Hominoidea - ps36
Hominidae - ps37
Homininae - ps38
Hominini - ps39
Homo_sapiens - ps40
p-value
6e-76
2e-57
6e-39
2e-20
6e-02
Log-odds
0.5
1.0
1.5
2.0
2.5
3.0
3.5
4.0

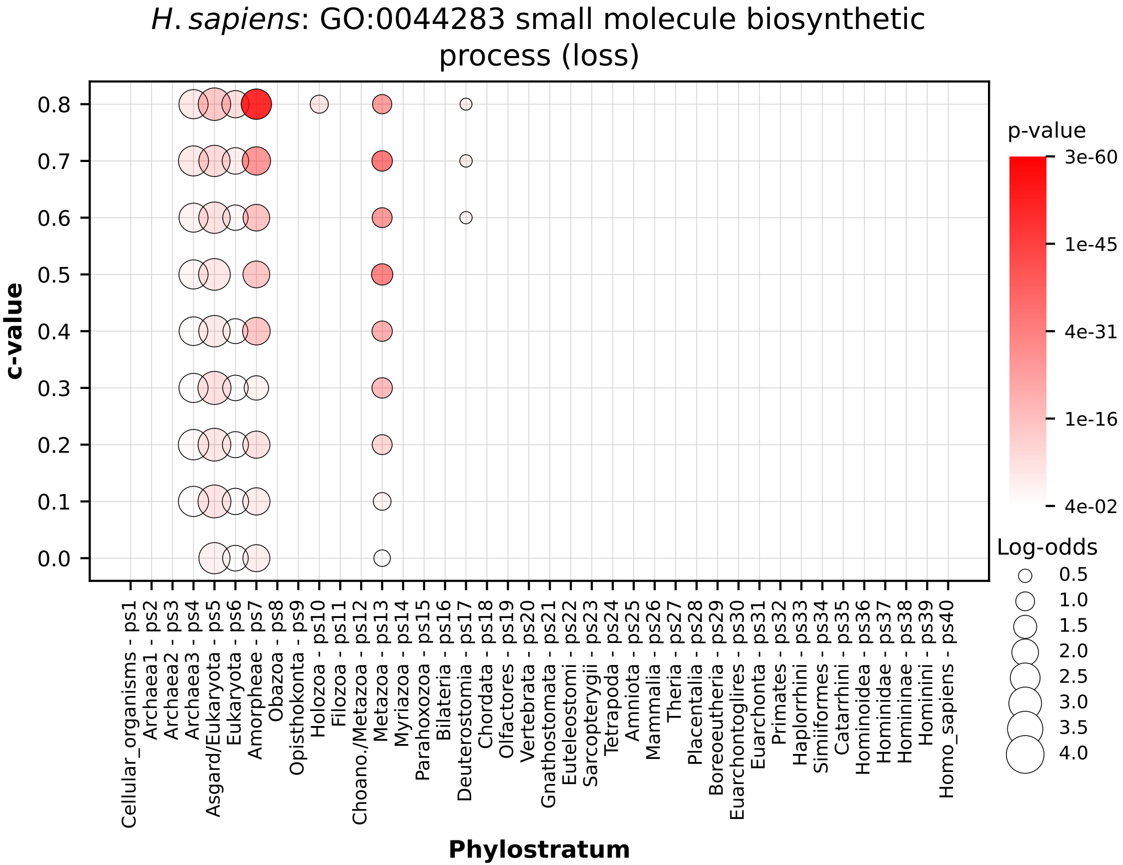

H. sapiens: GO:0044283 small molecule biosynthetic process (loss)
c-value
0.8
0.7
0.6
0.5
0.4
0.3
0.2
0.1
0.0
Phylostratum
Cellular_organisms - ps1
Archaea1 - ps2
Archaea2 - ps3
Archaea3 - ps4
Asgard/Eukaryota - ps5
Eukaryota - ps6
Amorpheae - ps7
Obazoa - ps8
Opisthokonta - ps9
Holozoa - ps10
Filozoa - ps11
Choano./Metazoa - ps12
Metazoa - ps13
Myriazoa - ps14
Parahoxozoa - ps15
Bilateria - ps16
Deuterostomia - ps17
Chordata - ps18
Olfactores - ps19
Vertebrata - ps20
Gnathostomata - ps21
Euteleostomi - ps22
Sarcopterygii - ps23
Tetrapoda - ps24
Amniota - ps25
Mammalia - ps26
Theria - ps27
Placentalia - ps28
Boreoeutheria - ps29
Euarchontoglires - ps30
Euarchonta - ps31
Primates - ps32
Haplorrhini - ps33
Simiiformes - ps34
Catarrhini - ps35
Hominoidea - ps36
Hominidae - ps37
Homininae - ps38
Hominini - ps39
Homo_sapiens - ps40
p-value
3e-60
1e-45
4e-31
1e-16
4e-02
Log-odds
0.5
1.0
1.5
2.0
2.5
3.0
3.5
4.0

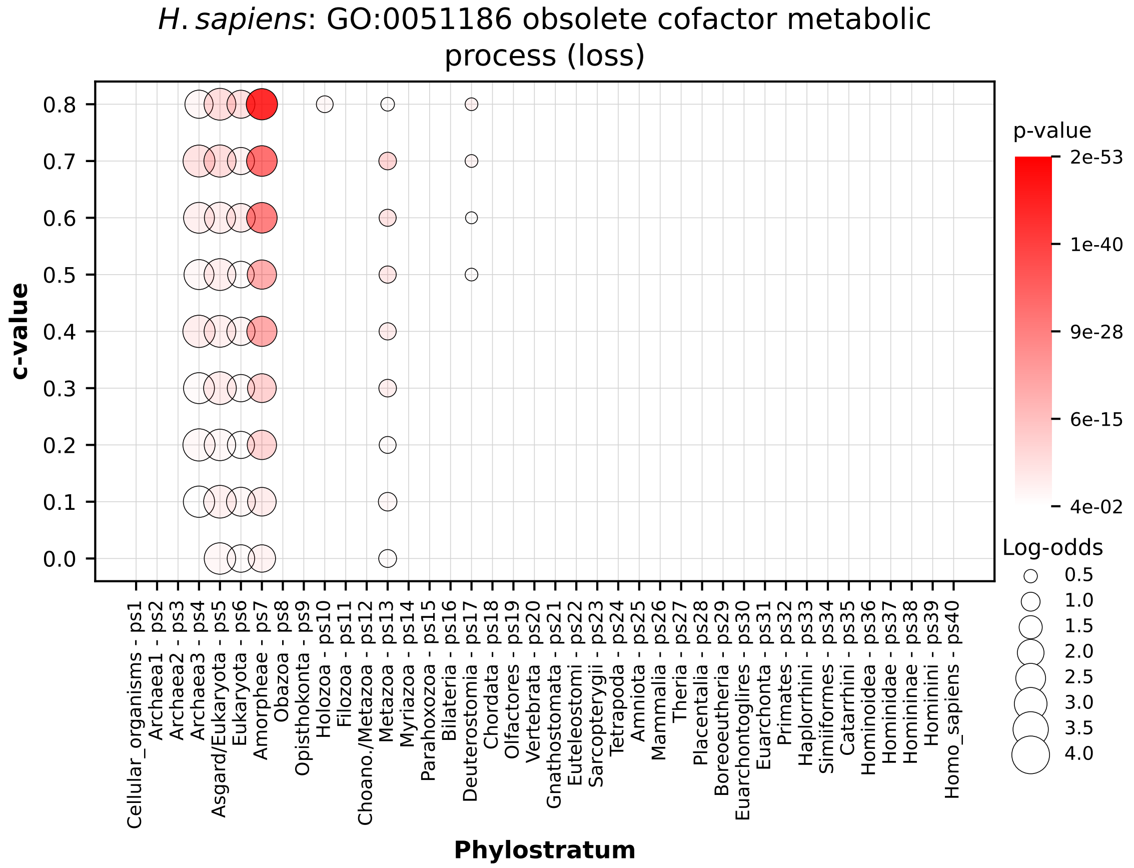

H. sapiens: GO:0051186 obsolete cofactor metabolic process (loss)
c-value
0.8
0.7
0.6
0.5
0.4
0.3
0.2
0.1
0.0
p-value
2e-53
1e-40
9e-28
6e-15
4e-02
Log-odds
0.5
1.0
1.5
2.0
2.5
3.0
3.5
4.0
Cellular_organisms - ps1
Archaea1 - ps2
Archaea2 - ps3
Archaea3 - ps4
Asgard/Eukaryota - ps5
Eukaryota - ps6
Amorpheae - ps7
Obazoa - ps8
Opisthokonta - ps9
Holozoa - ps10
Filozoa - ps11
Choano./Metazoa - ps12
Metazoa - ps13
Myriazoa - ps14
Parahoxozoa - ps15
Bilateria - ps16
Deuterostomia - ps17
Chordata - ps18
Olfactores - ps19
Vertebrata - ps20
Gnathostomata - ps21
Euteleostomi - ps22
Sarcopterygii - ps23
Tetrapoda - ps24
Amniota - ps25
Mammalia - ps26
Theria - ps27
Placentalia - ps28
Boreoeutheria - ps29
Euarchontoglires - ps30
Euarchonta - ps31
Primates - ps32
Haplorrhini - ps33
Simiiformes - ps34
Catarrhini - ps35
Hominoidea - ps36
Hominidae - ps37
Homininae - ps38
Hominini - ps39
Homo_sapiens - ps40
Phylostratum

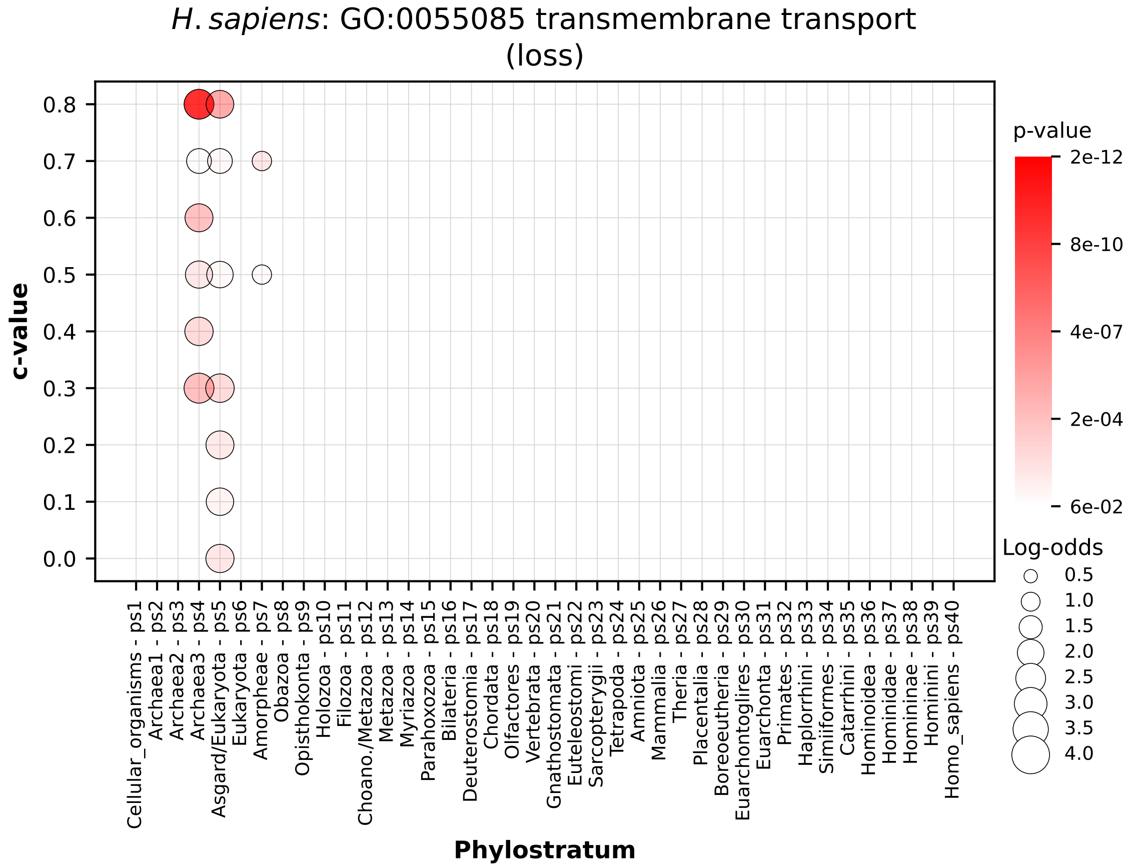

H. sapiens: GO:0055085 transmembrane transport
(loss)
c-value
0.8
0.7
0.6
0.5
0.4
0.3
0.2
0.1
0.0
Cellular_organisms - ps1
Archaea1 - ps2
Archaea2 - ps3
Archaea3 - ps4
Asgard/Eukaryota - ps5
Eukaryota - ps6
Amorpheae - ps7
Obazoa - ps8
Opisthokonta - ps9
Holozoa - ps10
Filozoa - ps11
Choano./Metazoa - ps12
Metazoa - ps13
Myriazoa - ps14
Parahoxozoa - ps15
Bilateria - ps16
Deuterostomia - ps17
Chordata - ps18
Olfactores - ps19
Vertebrata - ps20
Gnathostomata - ps21
Euteleostomi - ps22
Sarcopterygii - ps23
Tetrapoda - ps24
Amniota - ps25
Mammalia - ps26
Theria - ps27
Placentalia - ps28
Boreoeutheria - ps29
Euarchontoglires - ps30
Euarchonta - ps31
Primates - ps32
Haplorrhini - ps33
Simiiformes - ps34
Catarrhini - ps35
Hominoidea - ps36
Hominidae - ps37
Homininae - ps38
Hominini - ps39
Homo_sapiens - ps40
Phylostratum
p-value
2e-12
8e-10
4e-07
2e-04
6e-02
Log-odds
0.5
1.0
1.5
2.0
2.5
3.0
3.5
4.0

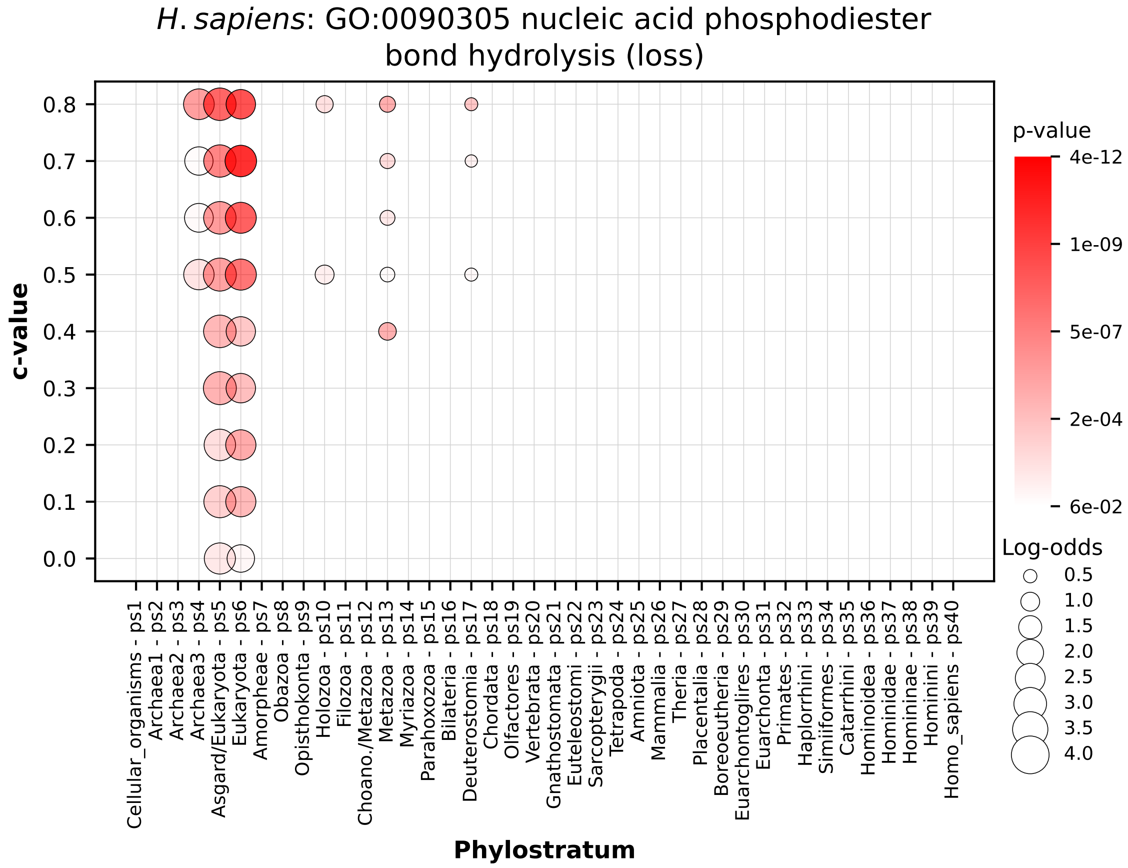

H. sapiens: GO:0090305 nucleic acid phosphodiester bond hydrolysis (loss)
c-value
0.8
0.7
0.6
0.5
0.4
0.3
0.2
0.1
0.0
p-value
4e-12
1e-09
5e-07
2e-04
6e-02
Log-odds
0.5
1.0
1.5
2.0
2.5
3.0
3.5
4.0
Cellular_organisms - ps1
Archaea1 - ps2
Archaea2 - ps3
Archaea3 - ps4
Asgard/Eukaryota - ps5
Eukaryota - ps6
Amorpheae - ps7
Obazoa - ps8
Opisthokonta - ps9
Holozoa - ps10
Filozoa - ps11
Choano./Metazoa - ps12
Metazoa - ps13
Myriazoa - ps14
Parahoxozoa - ps15
Bilateria - ps16
Deuterostomia - ps17
Chordata - ps18
Olfactores - ps19
Vertebrata - ps20
Gnathostomata - ps21
Euteleostomi - ps22
Sarcopterygii - ps23
Tetrapoda - ps24
Amniota - ps25
Mammalia - ps26
Theria - ps27
Placentalia - ps28
Boreoeutheria - ps29
Euarchontoglires - ps30
Euarchonta - ps31
Primates - ps32
Haplorrhini - ps33
Simiiformes - ps34
Catarrhini - ps35
Hominoidea - ps36
Hominidae - ps37
Homininae - ps38
Hominini - ps39
Homo_sapiens - ps40
Phylostratum

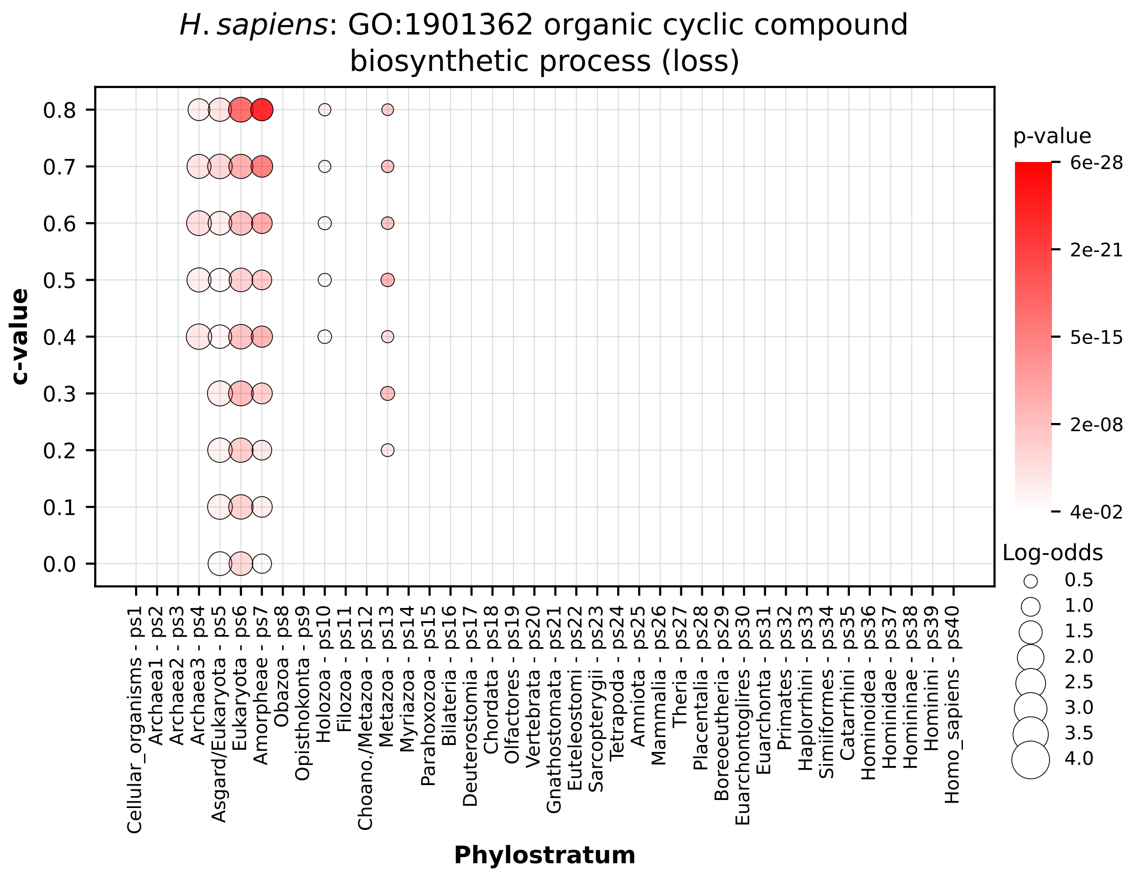
H. sapiens: GO:1901362 organic cyclic compound biosynthetic process (loss)
c-value
Phylostratum
p-value
6e-28
2e-21
5e-15
2e-08
4e-02
Log-odds
0.5
1.0
1.5
2.0
2.5
3.0
3.5
4.0
Cellular_organisms - ps1
Archaea1 - ps2
Archaea2 - ps3
Archaea3 - ps4
Asgard/Eukaryota - ps5
Eukaryota - ps6
Amorpheae - ps7
Obazoa - ps8
Opisthokonta - ps9
Holozoa - ps10
Filozoa - ps11
Choano./Metazoa - ps12
Metazoa - ps13
Myriazoa - ps14
Parahoxozoa - ps15
Bilateria - ps16
Deuterostomia - ps17
Chordata - ps18
Olfactores - ps19
Vertebrata - ps20
Gnathostomata - ps21
Euteleostomi - ps22
Sarcopterygii - ps23
Tetrapoda - ps24
Amniota - ps25
Mammalia - ps26
Theria - ps27
Placentalia - ps28
Boreoeutheria - ps29
Euarchontoglires - ps30
Euarchonta - ps31
Primates - ps32
Haplorrhini - ps33
Simiiformes - ps34
Catarrhini - ps35
Hominoidea - ps36
Hominidae - ps37
Homininae - ps38
Hominini - ps39
Homo_sapiens - ps40

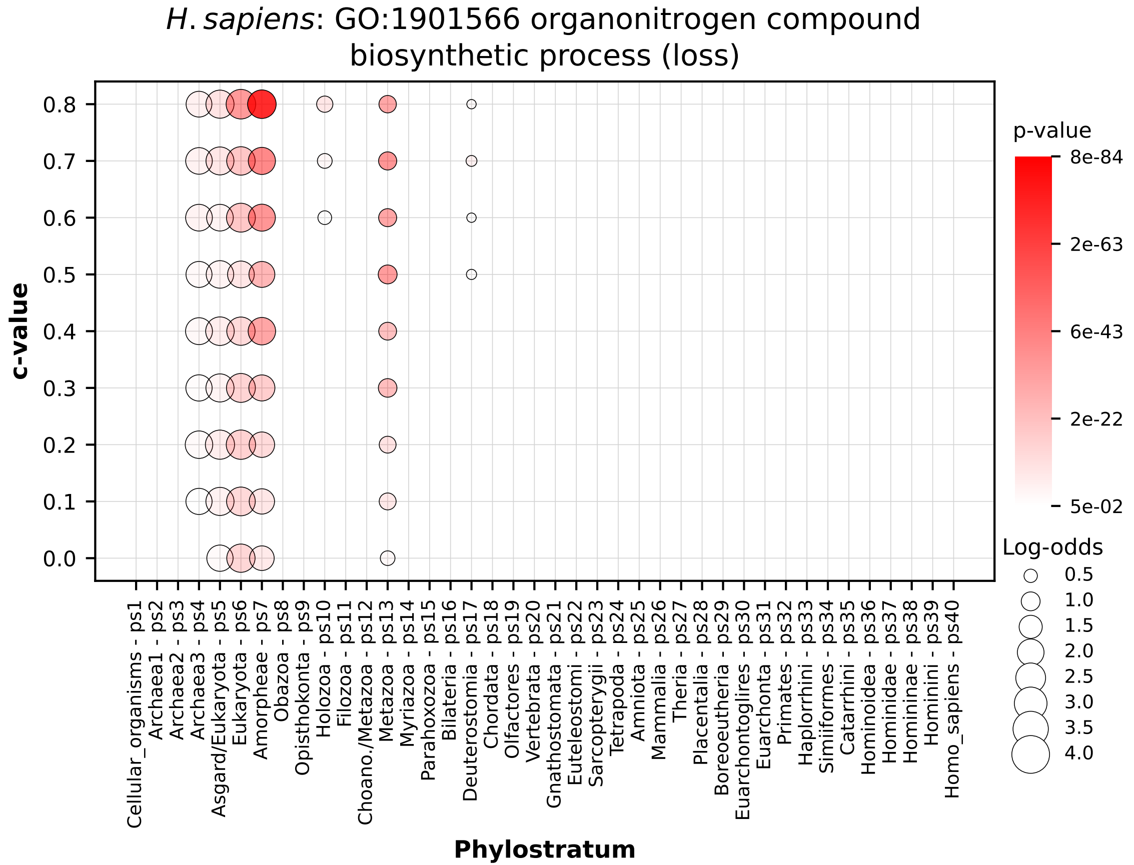

H. sapiens: GO:1901566 organonitrogen compound biosynthetic process (loss)
c-value
0.8
0.7
0.6
0.5
0.4
0.3
0.2
0.1
0.0
p-value
8e-84
2e-63
6e-43
2e-22
5e-02
Log-odds
0.5
1.0
1.5
2.0
2.5
3.0
3.5
4.0
Phylostratum
Cellular_organisms - ps1
Archaea1 - ps2
Archaea2 - ps3
Archaea3 - ps4
Asgard/Eukaryota - ps5
Eukaryota - ps6
Amorpheae - ps7
Obazoa - ps8
Opisthokonta - ps9
Holozoa - ps10
Filozoa - ps11
Choano./Metazoa - ps12
Metazoa - ps13
Myriazoa - ps14
Parahoxozoa - ps15
Bilateria - ps16
Deuterostomia - ps17
Chordata - ps18
Olfactores - ps19
Vertebrata - ps20
Gnathostomata - ps21
Euteleostomi - ps22
Sarcopterygii - ps23
Tetrapoda - ps24
Amniota - ps25
Mammalia - ps26
Theria - ps27
Placentalia - ps28
Boreoeutheria - ps29
Euarchontoglires - ps30
Euarchonta - ps31
Primates - ps32
Haplorrhini - ps33
Simiiformes - ps34
Catarrhini - ps35
Hominoidea - ps36
Hominidae - ps37
Homininae - ps38
Hominini - ps39
Homo_sapiens - ps40

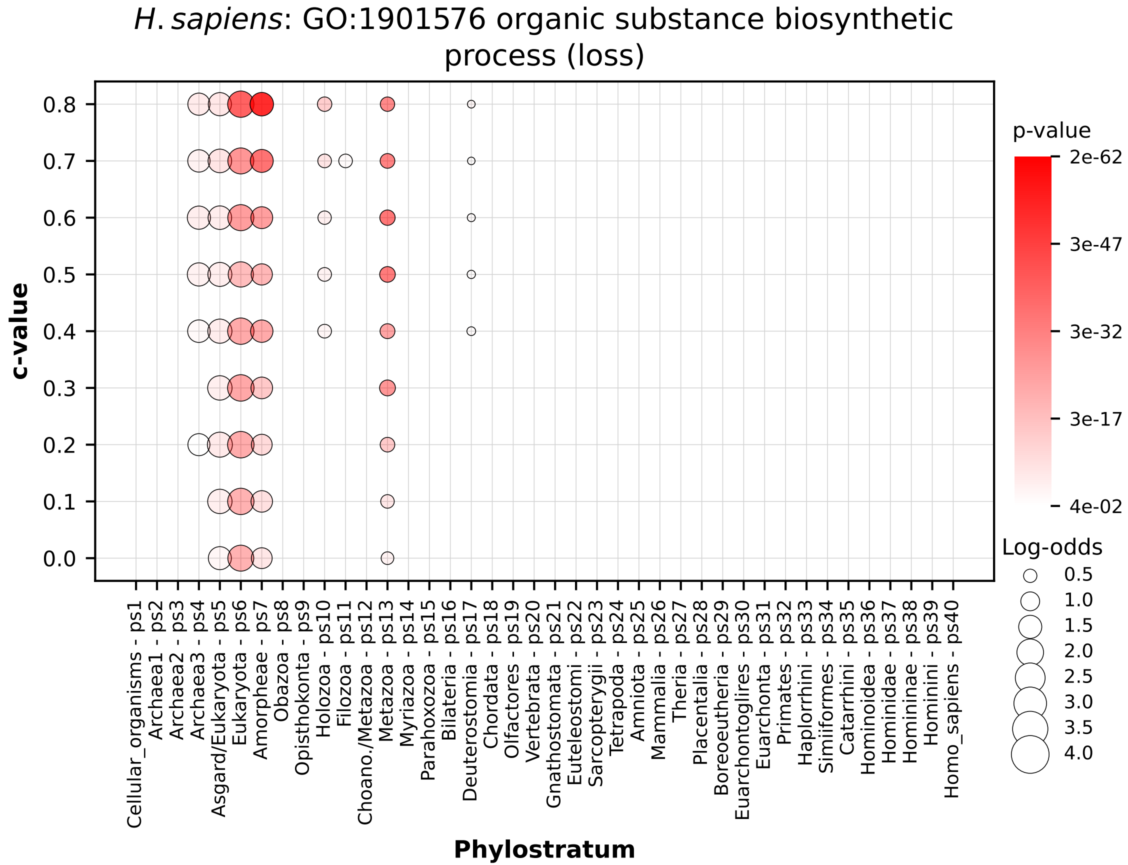

H. sapiens: GO:1901576 organic substance biosynthetic process (loss)
c-value
0.8
0.7
0.6
0.5
0.4
0.3
0.2
0.1
0.0
Phylostratum
Cellular_organisms - ps1
Archaea1 - ps2
Archaea2 - ps3
Archaea3 - ps4
Asgard/Eukaryota - ps5
Eukaryota - ps6
Amorpheae - ps7
Obazoa - ps8
Opisthokonta - ps9
Holozoa - ps10
Filozoa - ps11
Choano./Metazoa - ps12
Metazoa - ps13
Myriazoa - ps14
Parahoxozoa - ps15
Bilateria - ps16
Deuterostomia - ps17
Chordata - ps18
Olfactores - ps19
Vertebrata - ps20
Gnathostomata - ps21
Euteleostomi - ps22
Sarcopterygii - ps23
Tetrapoda - ps24
Amniota - ps25
Mammalia - ps26
Theria - ps27
Placentalia - ps28
Boreoeutheria - ps29
Euarchontoglires - ps30
Euarchonta - ps31
Primates - ps32
Haplorrhini - ps33
Simiiformes - ps34
Catarrhini - ps35
Hominoidea - ps36
Hominidae - ps37
Homininae - ps38
Hominini - ps39
Homo_sapiens - ps40
p-value
2e-62
3e-47
3e-32
3e-17
4e-02
Log-odds
0.5
1.0
1.5
2.0
2.5
3.0
3.5
4.0

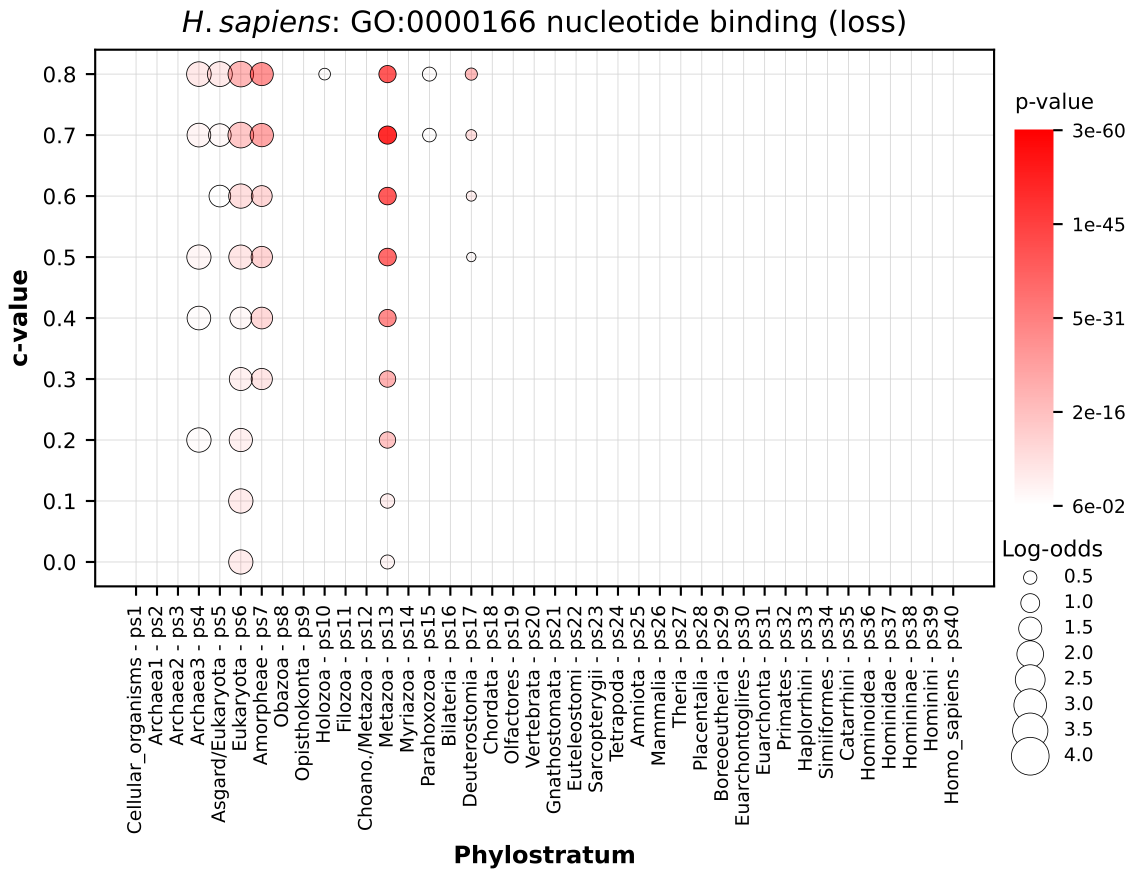
H. sapiens: GO:0000166 nucleotide binding (loss)
c-value
0.8
0.7
0.6
0.5
0.4
0.3
0.2
0.1
0.0
Phylostratum
Cellular_organisms - ps1
Archaea1 - ps2
Archaea2 - ps3
Archaea3 - ps4
Asgard/Eukaryota - ps5
Eukaryota - ps6
Amorpheae - ps7
Obazoa - ps8
Opisthokonta - ps9
Holozoa - ps10
Filozoa - ps11
Choano./Metazoa - ps12
Metazoa - ps13
Myriazoa - ps14
Parahoxozoa - ps15
Bilateria - ps16
Deuterostomia - ps17
Chordata - ps18
Olfactores - ps19
Vertebrata - ps20
Gnathostomata - ps21
Euteleostomi - ps22
Sarcopterygii - ps23
Tetrapoda - ps24
Amniota - ps25
Mammalia - ps26
Theria - ps27
Placentalia - ps28
Boreoeutheria - ps29
Euarchontoglires - ps30
Euarchonta - ps31
Primates - ps32
Haplorrhini - ps33
Simiiformes - ps34
Catarrhini - ps35
Hominoidea - ps36
Hominidae - ps37
Homininae - ps38
Hominini - ps39
Homo_sapiens - ps40
p-value
3e-60
1e-45
5e-31
2e-16
6e-02
Log-odds
0.5
1.0
1.5
2.0
2.5
3.0
3.5
4.0

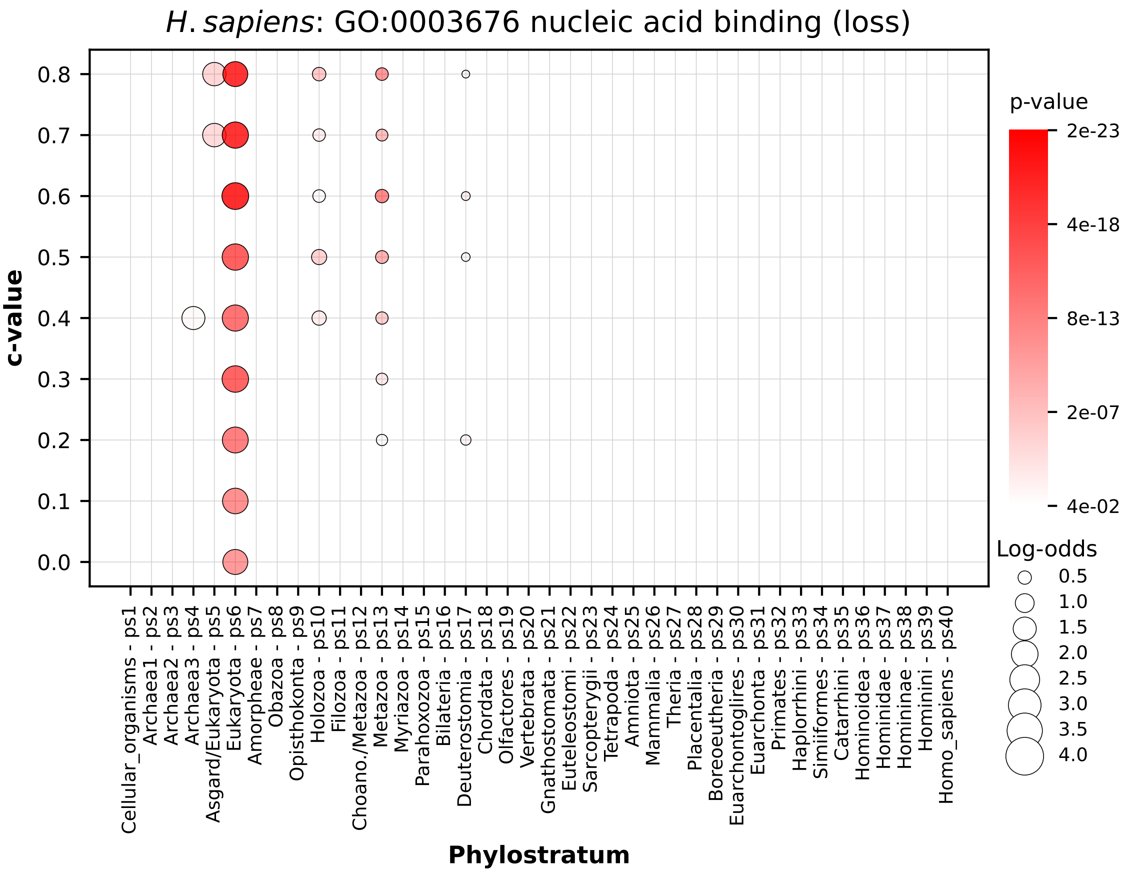

H. sapiens: GO:0003676 nucleic acid binding (loss)
c-value
0.8
0.7
0.6
0.5
0.4
0.3
0.2
0.1
0.0
p-value
2e-23
4e-18
8e-13
2e-07
4e-02
Log-odds
0.5
1.0
1.5
2.0
2.5
3.0
3.5
4.0
Cellular_organisms - ps1
Archaea1 - ps2
Archaea2 - ps3
Archaea3 - ps4
Asgard/Eukaryota - ps5
Eukaryota - ps6
Amorpheae - ps7
Obazoa - ps8
Opisthokonta - ps9
Holozoa - ps10
Filozoa - ps11
Choano./Metazoa - ps12
Metazoa - ps13
Myriazoa - ps14
Parahoxozoa - ps15
Bilateria - ps16
Deuterostomia - ps17
Chordata - ps18
Olfactores - ps19
Vertebrata - ps20
Gnathostomata - ps21
Euteleostomi - ps22
Sarcopterygii - ps23
Tetrapoda - ps24
Amniota - ps25
Mammalia - ps26
Theria - ps27
Placentalia - ps28
Boreoeutheria - ps29
Euarchontoglires - ps30
Euarchonta - ps31
Primates - ps32
Haplorrhini - ps33
Simiiformes - ps34
Catarrhini - ps35
Hominoidea - ps36
Hominidae - ps37
Homininae - ps38
Hominini - ps39
Homo_sapiens - ps40
Phylostratum

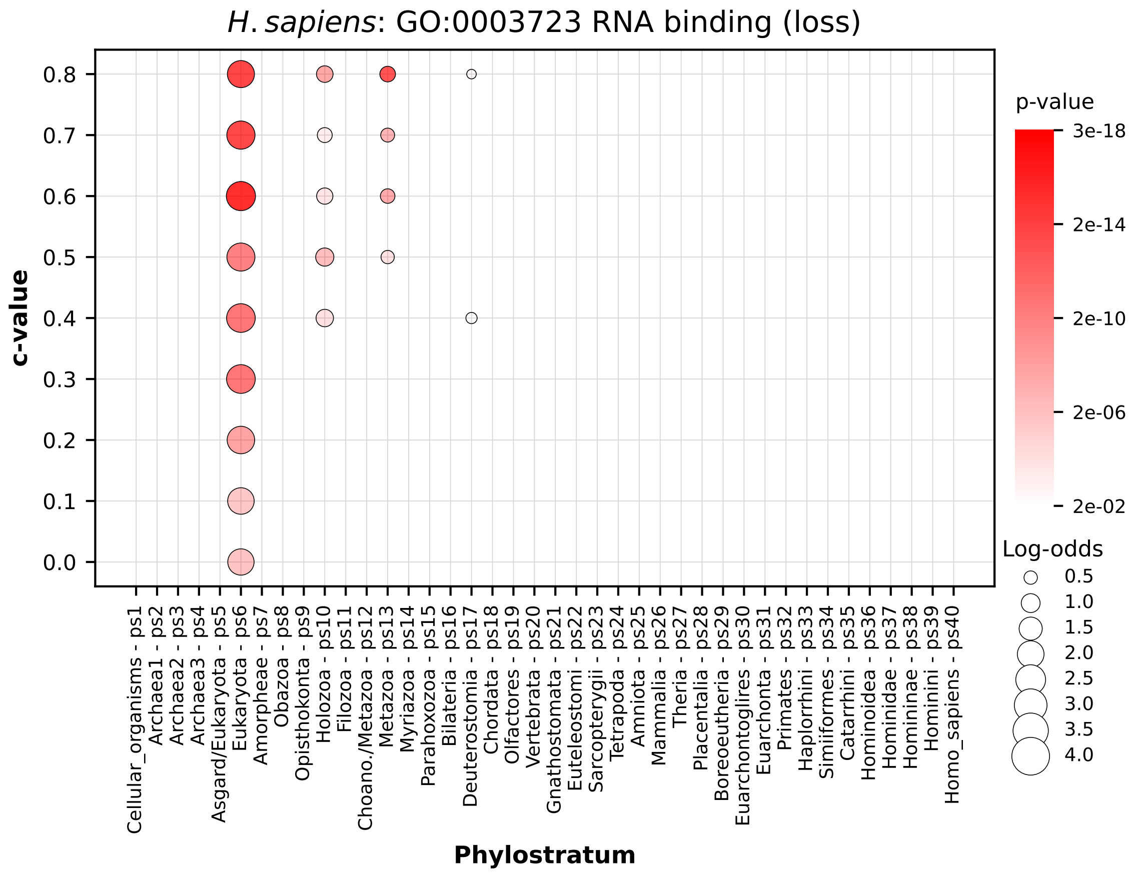

H. sapiens: GO:0003723 RNA binding (loss)
c-value
0.8
0.7
0.6
0.5
0.4
0.3
0.2
0.1
0.0
p-value
3e-18
2e-14
2e-10
2e-06
2e-02
Log-odds
0.5
1.0
1.5
2.0
2.5
3.0
3.5
4.0
Cellular_organisms - ps1
Archaea1 - ps2
Archaea2 - ps3
Archaea3 - ps4
Asgard/Eukaryota - ps5
Eukaryota - ps6
Amorpheae - ps7
Obazoa - ps8
Opisthokonta - ps9
Holozoa - ps10
Filozoa - ps11
Choano./Metazoa - ps12
Metazoa - ps13
Myriazoa - ps14
Parahoxozoa - ps15
Bilateria - ps16
Deuterostomia - ps17
Chordata - ps18
Olfactores - ps19
Vertebrata - ps20
Gnathostomata - ps21
Euteleostomi - ps22
Sarcopterygii - ps23
Tetrapoda - ps24
Amniota - ps25
Mammalia - ps26
Theria - ps27
Placentalia - ps28
Boreoeutheria - ps29
Euarchontoglires - ps30
Euarchonta - ps31
Primates - ps32
Haplorrhini - ps33
Simiiformes - ps34
Catarrhini - ps35
Hominoidea - ps36
Hominidae - ps37
Homininae - ps38
Hominini - ps39
Homo_sapiens - ps40
Phylostratum

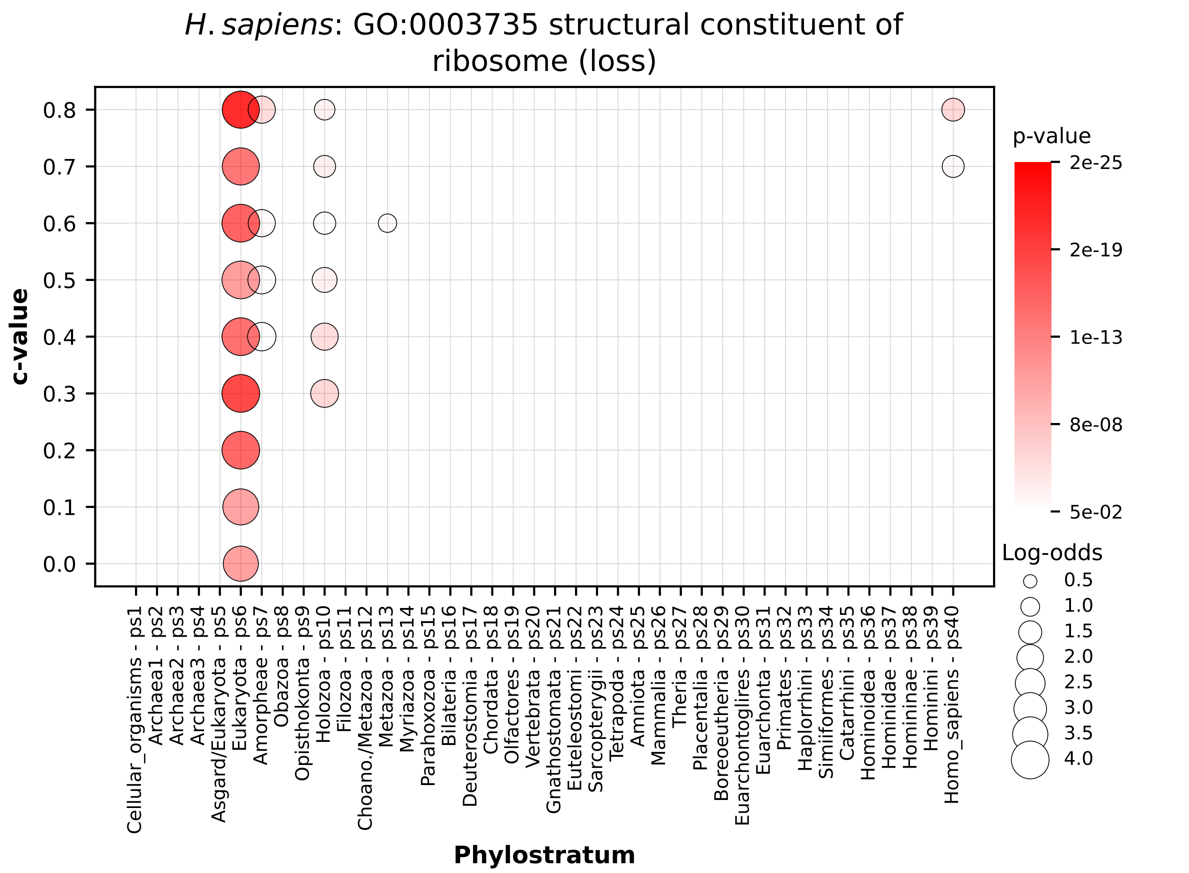

H. sapiens: GO:0003735 structural constituent of ribosome (loss)
c-value
0.8
0.7
0.6
0.5
0.4
0.3
0.2
0.1
0.0
Phylostratum
Cellular_organisms - ps1
Archaea1 - ps2
Archaea2 - ps3
Archaea3 - ps4
Asgard/Eukaryota - ps5
Eukaryota - ps6
Amorpheae - ps7
Obazoa - ps8
Opisthokonta - ps9
Holozoa - ps10
Filozoa - ps11
Choano./Metazoa - ps12
Metazoa - ps13
Myriazoa - ps14
Parahoxozoa - ps15
Bilateria - ps16
Deuterostomia - ps17
Chordata - ps18
Olfactores - ps19
Vertebrata - ps20
Gnathostomata - ps21
Euteleostomi - ps22
Sarcopterygii - ps23
Tetrapoda - ps24
Amniota - ps25
Mammalia - ps26
Theria - ps27
Placentalia - ps28
Boreoeutheria - ps29
Euarchontoglires - ps30
Euarchonta - ps31
Primates - ps32
Haplorrhini - ps33
Simiiformes - ps34
Catarrhini - ps35
Hominoidea - ps36
Hominidae - ps37
Homininae - ps38
Hominini - ps39
Homo_sapiens - ps40
p-value
2e-25
2e-19
1e-13
8e-08
5e-02
Log-odds
0.5
1.0
1.5
2.0
2.5
3.0
3.5
4.0

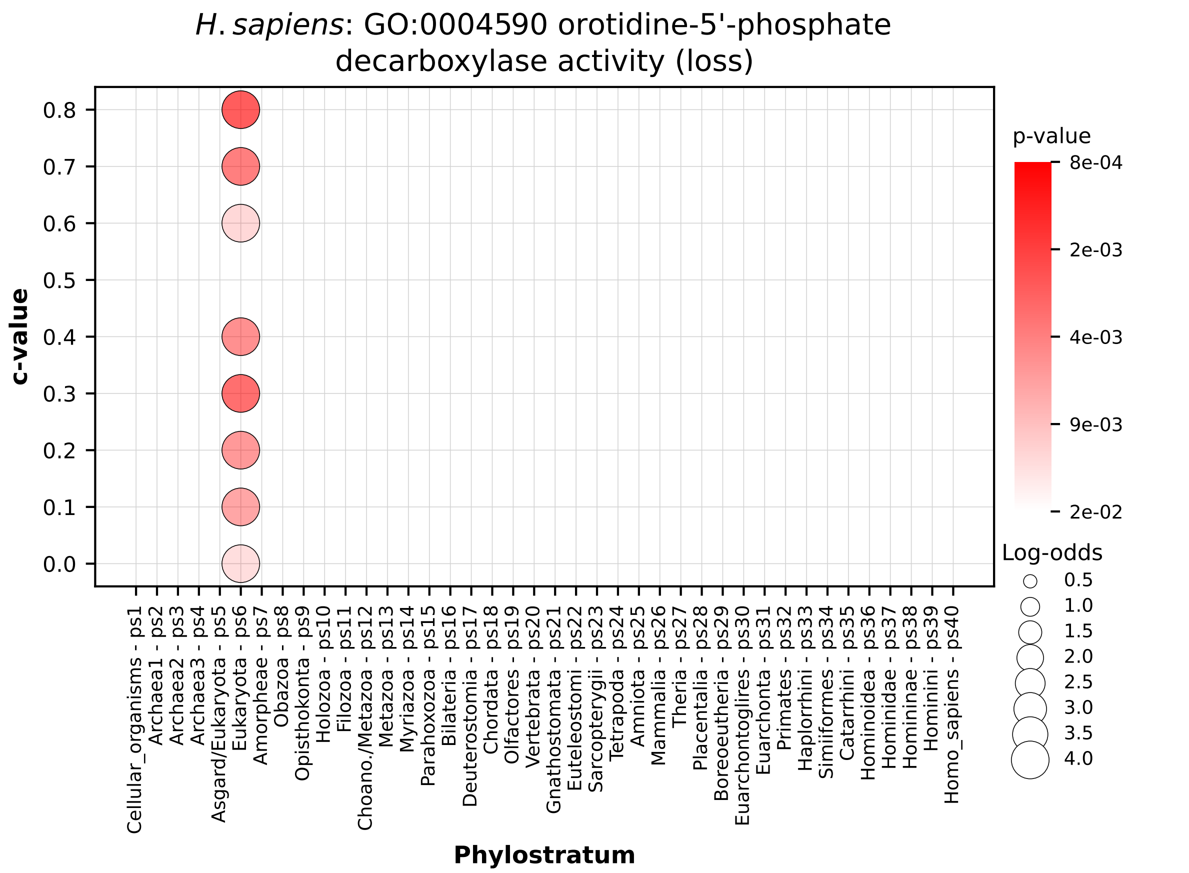

H. sapiens: GO:0004590 orotidine-5'-phosphate decarboxylase activity (loss)
c-value
0.8
0.7
0.6
0.5
0.4
0.3
0.2
0.1
0.0
p-value
8e-04
2e-03
4e-03
9e-03
2e-02
Log-odds
0.5
1.0
1.5
2.0
2.5
3.0
3.5
4.0
Cellular_organisms - ps1
Archaea1 - ps2
Archaea2 - ps3
Archaea3 - ps4
Asgard/Eukaryota - ps5
Eukaryota - ps6
Amorpheae - ps7
Obazoa - ps8
Opisthokonta - ps9
Holozoa - ps10
Filozoa - ps11
Choano./Metazoa - ps12
Metazoa - ps13
Myriazoa - ps14
Parahoxozoa - ps15
Bilateria - ps16
Deuterostomia - ps17
Chordata - ps18
Olfactores - ps19
Vertebrata - ps20
Gnathostomata - ps21
Euteleostomi - ps22
Sarcopterygii - ps23
Tetrapoda - ps24
Amniota - ps25
Mammalia - ps26
Theria - ps27
Placentalia - ps28
Boreoeutheria - ps29
Euarchontoglires - ps30
Euarchonta - ps31
Primates - ps32
Haplorrhini - ps33
Simiiformes - ps34
Catarrhini - ps35
Hominoidea - ps36
Hominidae - ps37
Homininae - ps38
Hominini - ps39
Homo_sapiens - ps40
Phylostratum

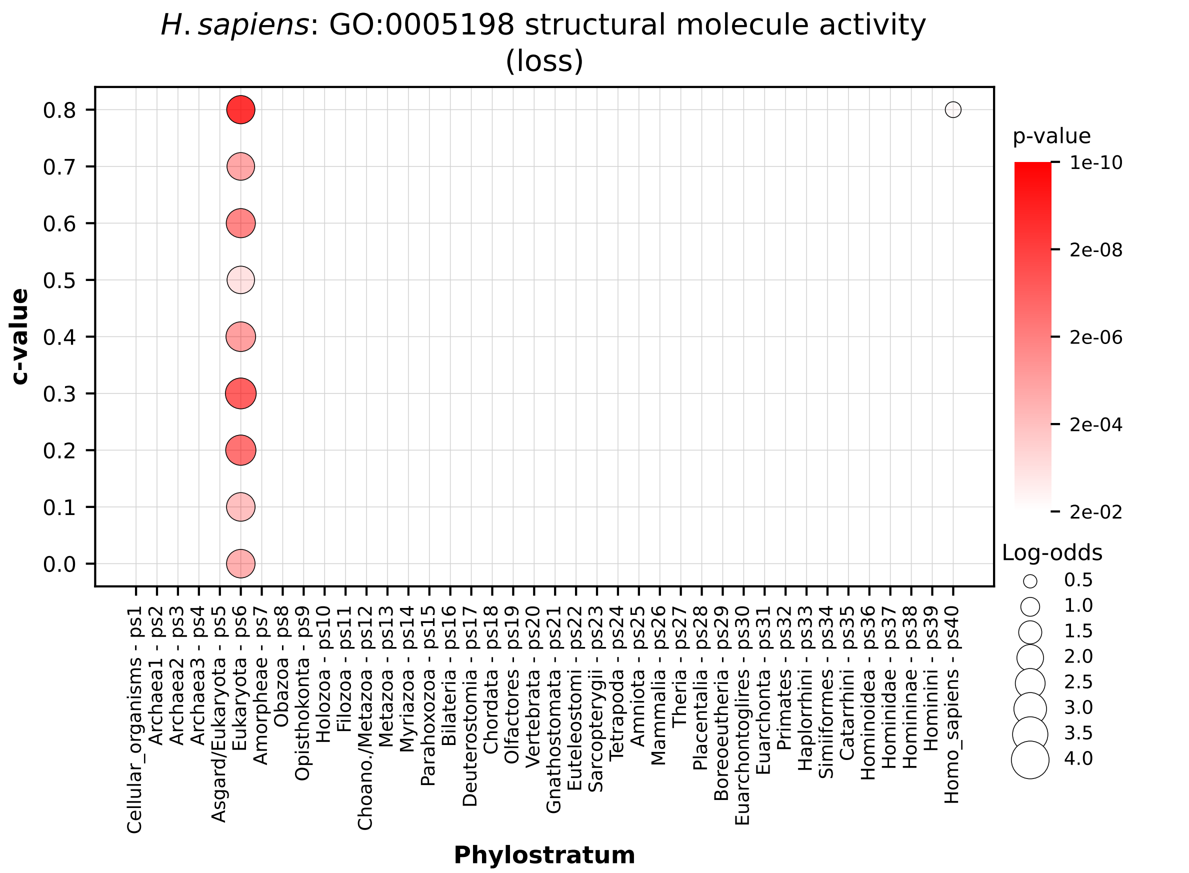

H. sapiens: GO:0005198 structural molecule activity
(loss)
c-value
0.0
0.1
0.2
0.3
0.4
0.5
0.6
0.7
0.8
Phylostratum
Cellular_organisms - ps1
Archaea1 - ps2
Archaea2 - ps3
Archaea3 - ps4
Asgard/Eukaryota - ps5
Eukaryota - ps6
Amorpheae - ps7
Obazoa - ps8
Opisthokonta - ps9
Holozoa - ps10
Filozoa - ps11
Choano./Metazoa - ps12
Metazoa - ps13
Myriazoa - ps14
Parahoxozoa - ps15
Bilateria - ps16
Deuterostomia - ps17
Chordata - ps18
Olfactores - ps19
Vertebrata - ps20
Gnathostomata - ps21
Euteleostomi - ps22
Sarcopterygii - ps23
Tetrapoda - ps24
Amniota - ps25
Mammalia - ps26
Theria - ps27
Placentalia - ps28
Boreoeutheria - ps29
Euarchontoglires - ps30
Euarchonta - ps31
Primates - ps32
Haplorrhini - ps33
Simiiformes - ps34
Catarrhini - ps35
Hominoidea - ps36
Hominidae - ps37
Homininae - ps38
Hominini - ps39
Homo_sapiens - ps40
p-value
1e-10
2e-08
2e-06
2e-04
2e-02
Log-odds
0.5
1.0
1.5
2.0
2.5
3.0
3.5
4.0

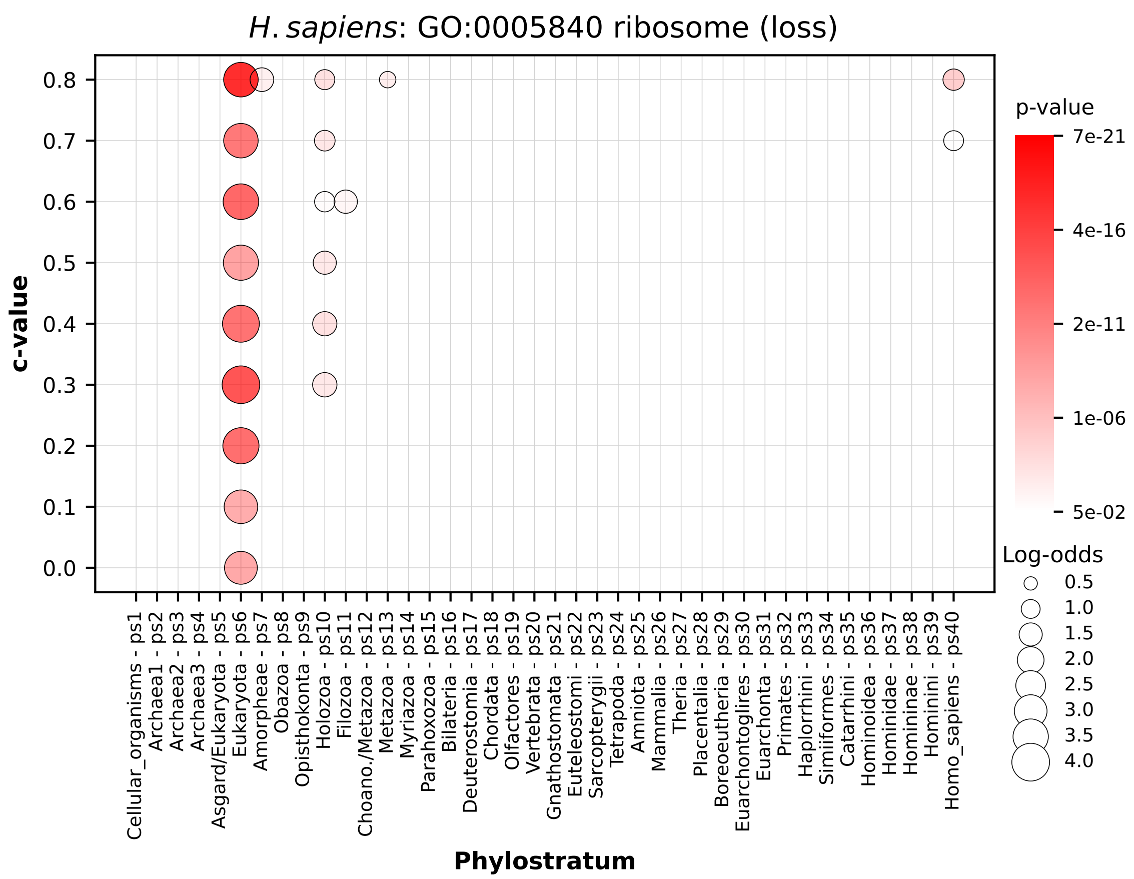

H. sapiens: GO:0005840 ribosome (loss)
c-value
0.8
0.7
0.6
0.5
0.4
0.3
0.2
0.1
0.0
p-value
7e-21
4e-16
2e-11
1e-06
5e-02
Log-odds
0.5
1.0
1.5
2.0
2.5
3.0
3.5
4.0
Cellular_organisms - ps1
Archaea1 - ps2
Archaea2 - ps3
Archaea3 - ps4
Asgard/Eukaryota - ps5
Eukaryota - ps6
Amorpheae - ps7
Obazoa - ps8
Opisthokonta - ps9
Holozoa - ps10
Filozoa - ps11
Choano./Metazoa - ps12
Metazoa - ps13
Myriazoa - ps14
Parahoxozoa - ps15
Bilateria - ps16
Deuterostomia - ps17
Chordata - ps18
Olfactores - ps19
Vertebrata - ps20
Gnathostomata - ps21
Euteleostomi - ps22
Sarcopterygii - ps23
Tetrapoda - ps24
Amniota - ps25
Mammalia - ps26
Theria - ps27
Placentalia - ps28
Boreoeutheria - ps29
Euarchontoglires - ps30
Euarchonta - ps31
Primates - ps32
Haplorrhini - ps33
Simiiformes - ps34
Catarrhini - ps35
Hominoidea - ps36
Hominidae - ps37
Homininae - ps38
Hominini - ps39
Homo_sapiens - ps40
Phylostratum

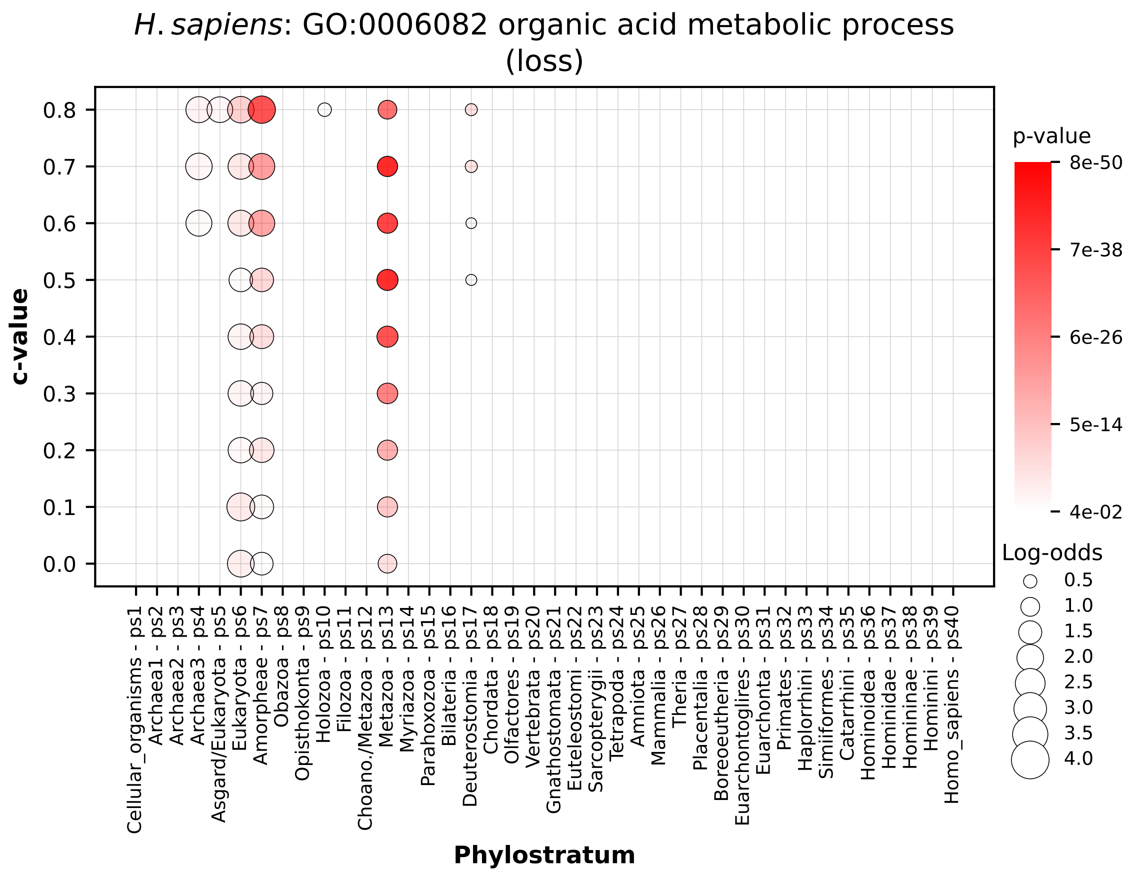

H. sapiens: GO:0006082 organic acid metabolic process
(loss)
c-value
0.8
0.7
0.6
0.5
0.4
0.3
0.2
0.1
0.0
Phylostratum
Cellular_organisms - ps1
Archaea1 - ps2
Archaea2 - ps3
Archaea3 - ps4
Asgard/Eukaryota - ps5
Eukaryota - ps6
Amorpheae - ps7
Obazoa - ps8
Opisthokonta - ps9
Holozoa - ps10
Filozoa - ps11
Choano./Metazoa - ps12
Metazoa - ps13
Myriazoa - ps14
Parahoxozoa - ps15
Bilateria - ps16
Deuterostomia - ps17
Chordata - ps18
Olfactores - ps19
Vertebrata - ps20
Gnathostomata - ps21
Euteleostomi - ps22
Sarcopterygii - ps23
Tetrapoda - ps24
Amniota - ps25
Mammalia - ps26
Theria - ps27
Placentalia - ps28
Boreoeutheria - ps29
Euarchontoglires - ps30
Euarchonta - ps31
Primates - ps32
Haplorrhini - ps33
Simiiformes - ps34
Catarrhini - ps35
Hominoidea - ps36
Hominidae - ps37
Homininae - ps38
Hominini - ps39
Homo_sapiens - ps40
p-value
8e-50
7e-38
6e-26
5e-14
4e-02
Log-odds
0.5
1.0
1.5
2.0
2.5
3.0
3.5
4.0

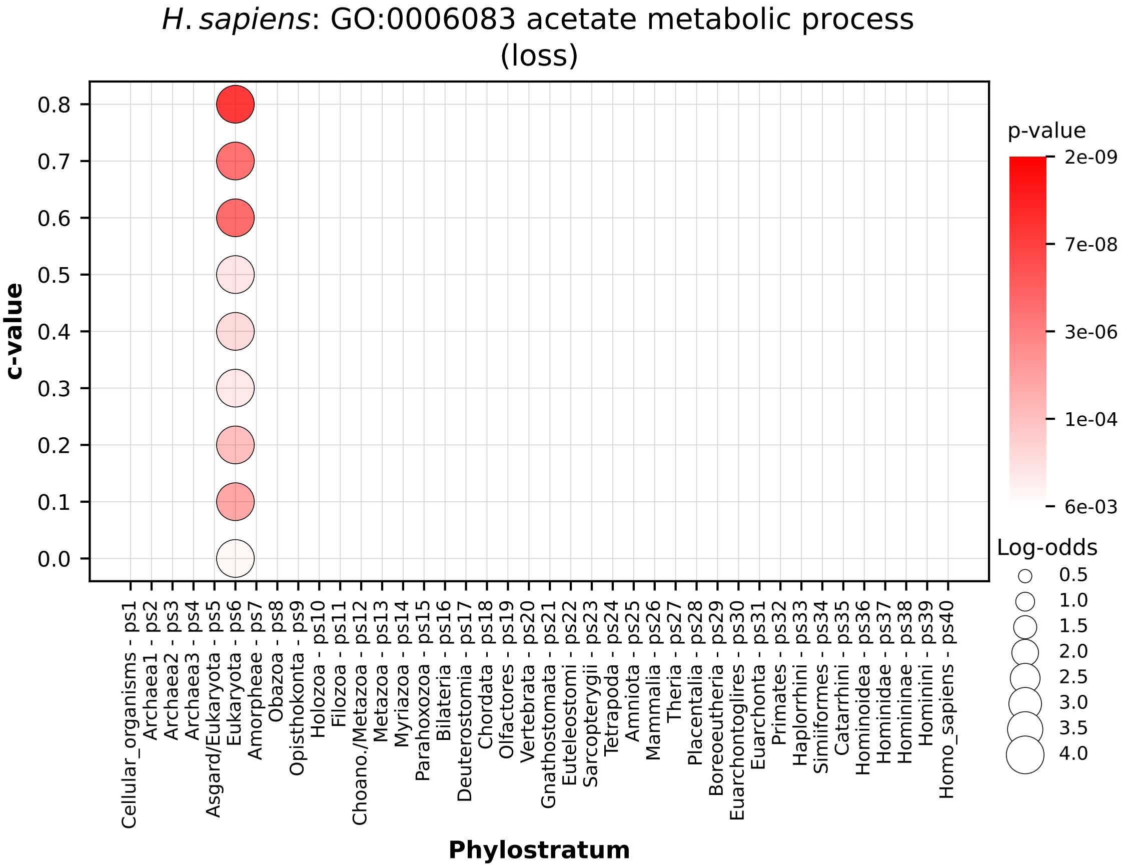

H. sapiens: GO:0006083 acetate metabolic process
(loss)
c-value
0.8
0.7
0.6
0.5
0.4
0.3
0.2
0.1
0.0
Phylostratum
Cellular_organisms - ps1
Archaea1 - ps2
Archaea2 - ps3
Archaea3 - ps4
Asgard/Eukaryota - ps5
Eukaryota - ps6
Amorpheae - ps7
Obazoa - ps8
Opisthokonta - ps9
Holozoa - ps10
Filozoa - ps11
Choano./Metazoa - ps12
Metazoa - ps13
Myriazoa - ps14
Parahoxozoa - ps15
Bilateria - ps16
Deuterostomia - ps17
Chordata - ps18
Olfactores - ps19
Vertebrata - ps20
Gnathostomata - ps21
Euteleostomi - ps22
Sarcopterygii - ps23
Tetrapoda - ps24
Amniota - ps25
Mammalia - ps26
Theria - ps27
Placentalia - ps28
Boreoeutheria - ps29
Euarchontoglires - ps30
Euarchonta - ps31
Primates - ps32
Haplorrhini - ps33
Simiiformes - ps34
Catarrhini - ps35
Hominoidea - ps36
Hominidae - ps37
Homininae - ps38
Hominini - ps39
Homo_sapiens - ps40
p-value
2e-09
7e-08
3e-06
1e-04
6e-03
Log-odds
0.5
1.0
1.5
2.0
2.5
3.0
3.5
4.0

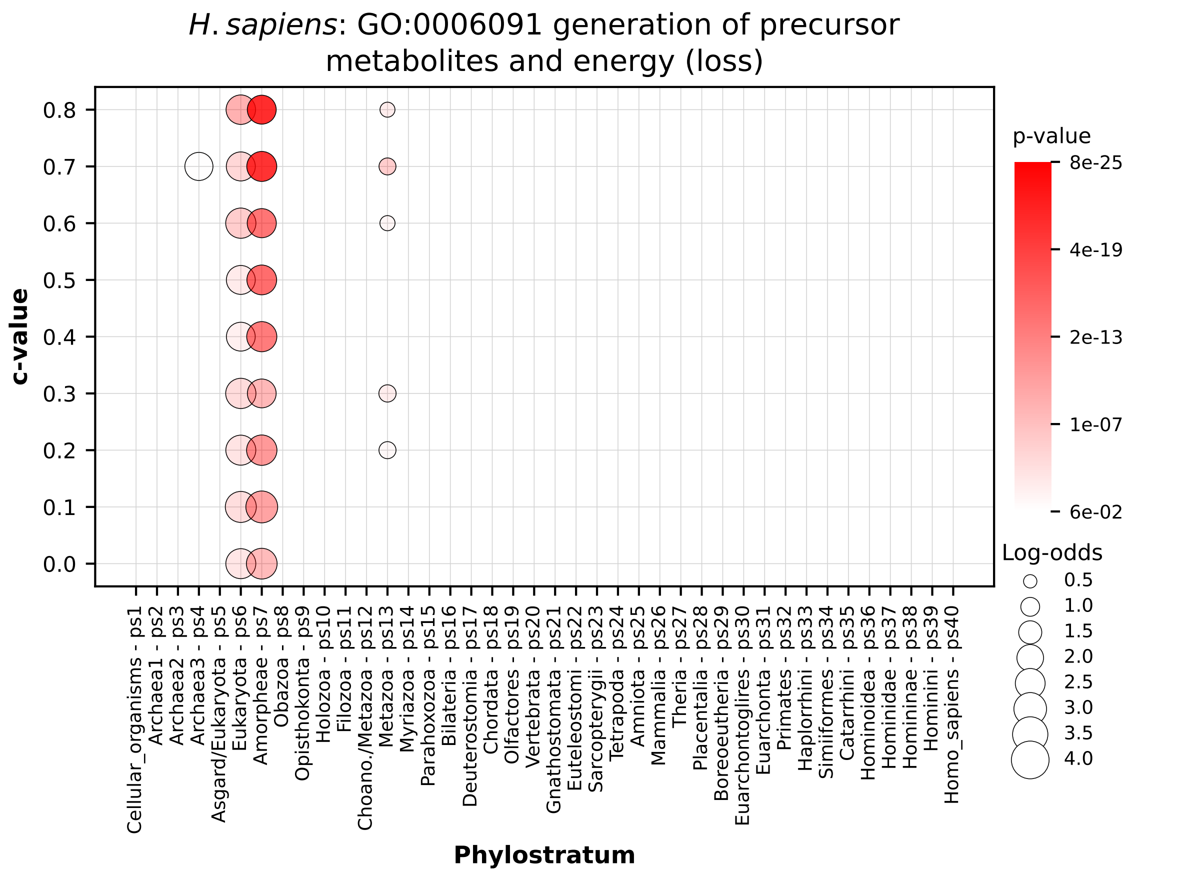

H. sapiens: GO:0006091 generation of precursor metabolites and energy (loss)
c-value
0.8
0.7
0.6
0.5
0.4
0.3
0.2
0.1
0.0
Phylostratum
Cellular_organisms - ps1
Archaea1 - ps2
Archaea2 - ps3
Archaea3 - ps4
Asgard/Eukaryota - ps5
Eukaryota - ps6
Amorpheae - ps7
Obazoa - ps8
Opisthokonta - ps9
Holozoa - ps10
Filozoa - ps11
Choano./Metazoa - ps12
Metazoa - ps13
Myriazoa - ps14
Parahoxozoa - ps15
Bilateria - ps16
Deuterostomia - ps17
Chordata - ps18
Olfactores - ps19
Vertebrata - ps20
Gnathostomata - ps21
Euteleostomi - ps22
Sarcopterygii - ps23
Tetrapoda - ps24
Amniota - ps25
Mammalia - ps26
Theria - ps27
Placentalia - ps28
Boreoeutheria - ps29
Euarchontoglires - ps30
Euarchonta - ps31
Primates - ps32
Haplorrhini - ps33
Simiiformes - ps34
Catarrhini - ps35
Hominoidea - ps36
Hominidae - ps37
Homininae - ps38
Hominini - ps39
Homo_sapiens - ps40
p-value
8e-25
4e-19
2e-13
1e-07
6e-02
Log-odds
0.5
1.0
1.5
2.0
2.5
3.0
3.5
4.0

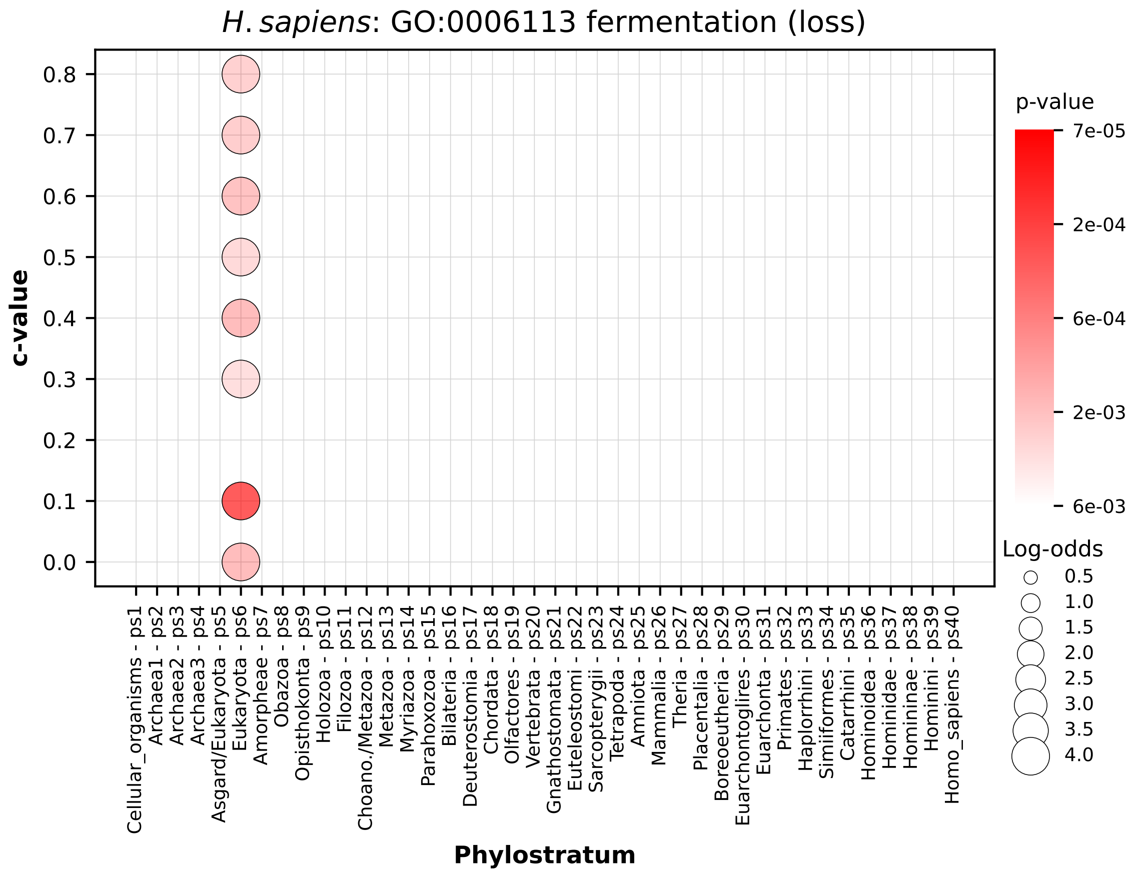
H. sapiens: GO:0006113 fermentation (loss)
c-value
0.8
0.7
0.6
0.5
0.4
0.3
0.2
0.1
0.0
Phylostratum
Cellular_organisms - ps1
Archaea1 - ps2
Archaea2 - ps3
Archaea3 - ps4
Asgard/Eukaryota - ps5
Eukaryota - ps6
Amorpheae - ps7
Obazoa - ps8
Opisthokonta - ps9
Holozoa - ps10
Filozoa - ps11
Choano./Metazoa - ps12
Metazoa - ps13
Myriazoa - ps14
Parahoxozoa - ps15
Bilateria - ps16
Deuterostomia - ps17
Chordata - ps18
Olfactores - ps19
Vertebrata - ps20
Gnathostomata - ps21
Euteleostomi - ps22
Sarcopterygii - ps23
Tetrapoda - ps24
Amniota - ps25
Mammalia - ps26
Theria - ps27
Placentalia - ps28
Boreoeutheria - ps29
Euarchontoglires - ps30
Euarchonta - ps31
Primates - ps32
Haplorrhini - ps33
Simiiformes - ps34
Catarrhini - ps35
Hominoidea - ps36
Hominidae - ps37
Homininae - ps38
Hominini - ps39
Homo_sapiens - ps40
p-value
7e-05
2e-04
6e-04
2e-03
6e-03
Log-odds
0.5
1.0
1.5
2.0
2.5
3.0
3.5
4.0

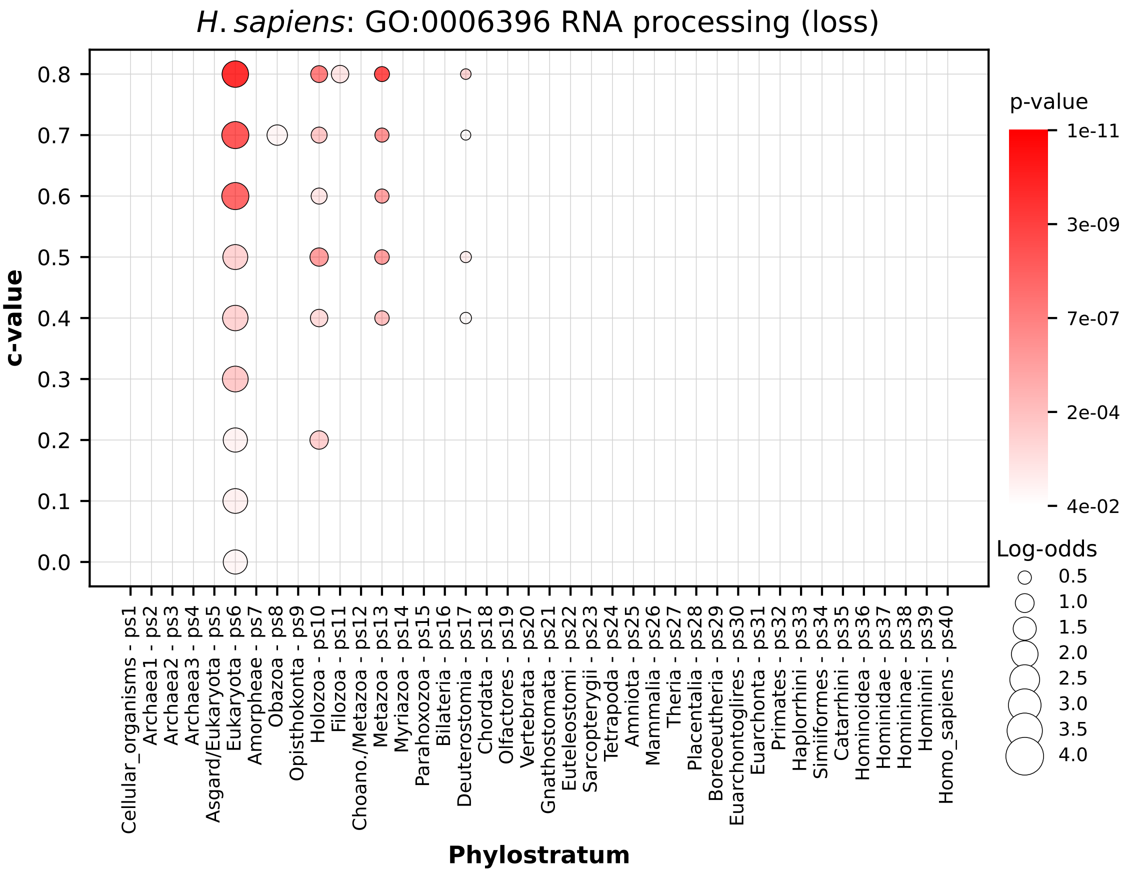

H. sapiens: GO:0006396 RNA processing (loss)
c-value
Phylostratum
p-value
1e-11
3e-09
7e-07
2e-04
4e-02
Log-odds
0.5
1.0
1.5
2.0
2.5
3.0
3.5
4.0
Cellular_organisms - ps1
Archaea1 - ps2
Archaea2 - ps3
Archaea3 - ps4
Asgard/Eukaryota - ps5
Eukaryota - ps6
Amorpheae - ps7
Obazoa - ps8
Opisthokonta - ps9
Holozoa - ps10
Filozoa - ps11
Choano./Metazoa - ps12
Metazoa - ps13
Myriazoa - ps14
Parahoxozoa - ps15
Bilateria - ps16
Deuterostomia - ps17
Chordata - ps18
Olfactores - ps19
Vertebrata - ps20
Gnathostomata - ps21
Euteleostomi - ps22
Sarcopterygii - ps23
Tetrapoda - ps24
Amniota - ps25
Mammalia - ps26
Theria - ps27
Placentalia - ps28
Boreoeutheria - ps29
Euarchontoglires - ps30
Euarchonta - ps31
Primates - ps32
Haplorrhini - ps33
Simiiformes - ps34
Catarrhini - ps35
Hominoidea - ps36
Hominidae - ps37
Homininae - ps38
Hominini - ps39
Homo_sapiens - ps40

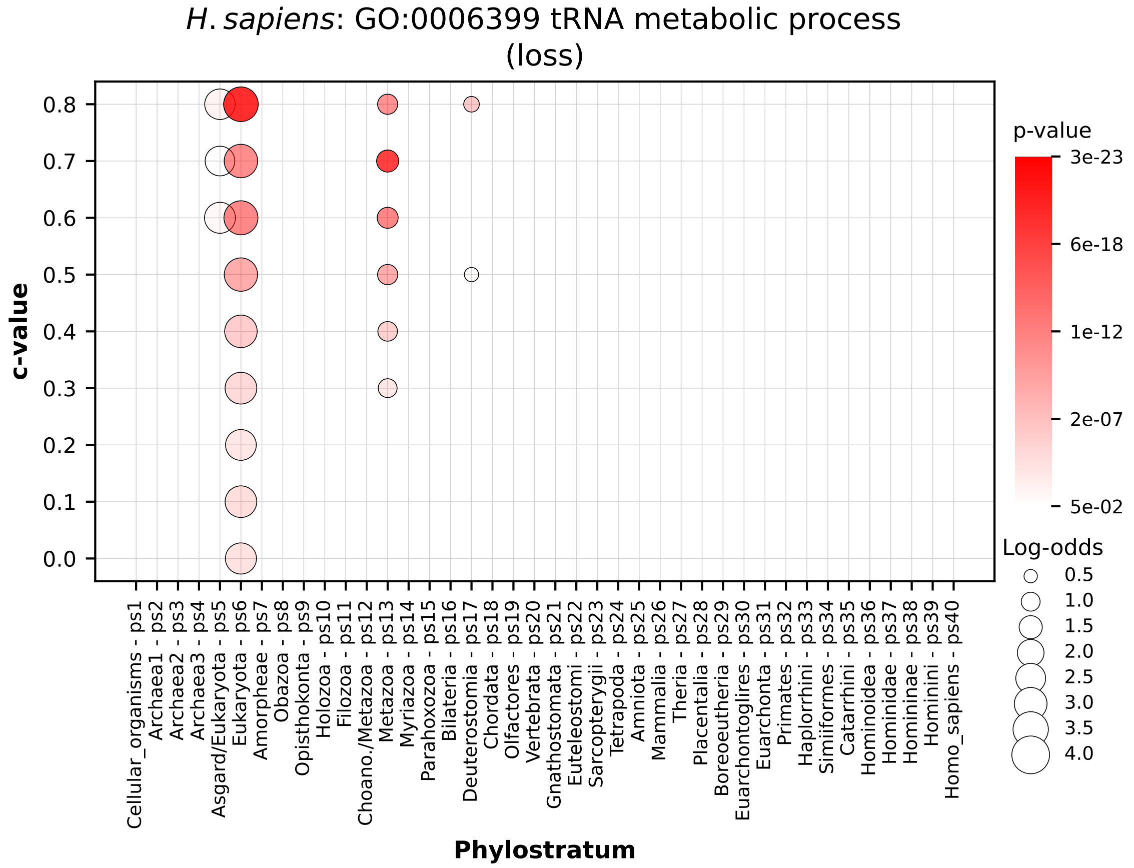

H. sapiens: GO:0006399 tRNA metabolic process (loss)
c-value
0.8
0.7
0.6
0.5
0.4
0.3
0.2
0.1
0.0
p-value
3e-23
6e-18
1e-12
2e-07
5e-02
Log-odds
0.5
1.0
1.5
2.0
2.5
3.0
3.5
4.0
Cellular_organisms - ps1
Archaea1 - ps2
Archaea2 - ps3
Archaea3 - ps4
Asgard/Eukaryota - ps5
Eukaryota - ps6
Amorpheae - ps7
Obazoa - ps8
Opisthokonta - ps9
Holozoa - ps10
Filozoa - ps11
Choano./Metazoa - ps12
Metazoa - ps13
Myriazoa - ps14
Parahoxozoa - ps15
Bilateria - ps16
Deuterostomia - ps17
Chordata - ps18
Olfactores - ps19
Vertebrata - ps20
Gnathostomata - ps21
Euteleostomi - ps22
Sarcopterygii - ps23
Tetrapoda - ps24
Amniota - ps25
Mammalia - ps26
Theria - ps27
Placentalia - ps28
Boreoeutheria - ps29
Euarchontoglires - ps30
Euarchonta - ps31
Primates - ps32
Haplorrhini - ps33
Simiiformes - ps34
Catarrhini - ps35
Hominoidea - ps36
Hominidae - ps37
Homininae - ps38
Hominini - ps39
Homo_sapiens - ps40
Phylostratum

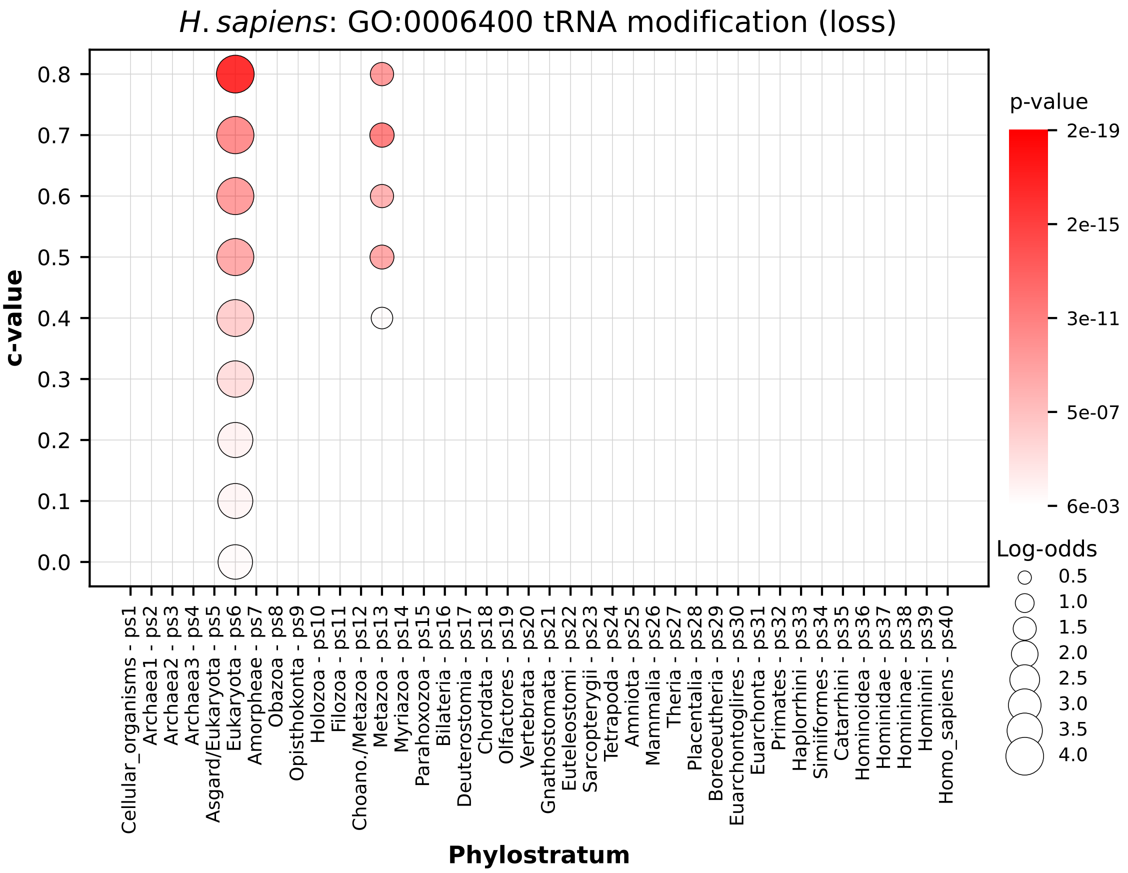
H. sapiens: GO:0006400 tRNA modification (loss)
c-value
0.8
0.7
0.6
0.5
0.4
0.3
0.2
0.1
0.0
Phylostratum
Cellular_organisms - ps1
Archaea1 - ps2
Archaea2 - ps3
Archaea3 - ps4
Asgard/Eukaryota - ps5
Eukaryota - ps6
Amorpheae - ps7
Obazoa - ps8
Opisthokonta - ps9
Holozoa - ps10
Filozoa - ps11
Choano./Metazoa - ps12
Metazoa - ps13
Myriazoa - ps14
Parahoxozoa - ps15
Bilateria - ps16
Deuterostomia - ps17
Chordata - ps18
Olfactores - ps19
Vertebrata - ps20
Gnathostomata - ps21
Euteleostomi - ps22
Sarcopterygii - ps23
Tetrapoda - ps24
Amniota - ps25
Mammalia - ps26
Theria - ps27
Placentalia - ps28
Boreoeutheria - ps29
Euarchontoglires - ps30
Euarchonta - ps31
Primates - ps32
Haplorrhini - ps33
Simiiformes - ps34
Catarrhini - ps35
Hominoidea - ps36
Hominidae - ps37
Homininae - ps38
Hominini - ps39
Homo_sapiens - ps40
p-value
2e-19
2e-15
3e-11
5e-07
6e-03
Log-odds
0.5
1.0
1.5
2.0
2.5
3.0
3.5
4.0

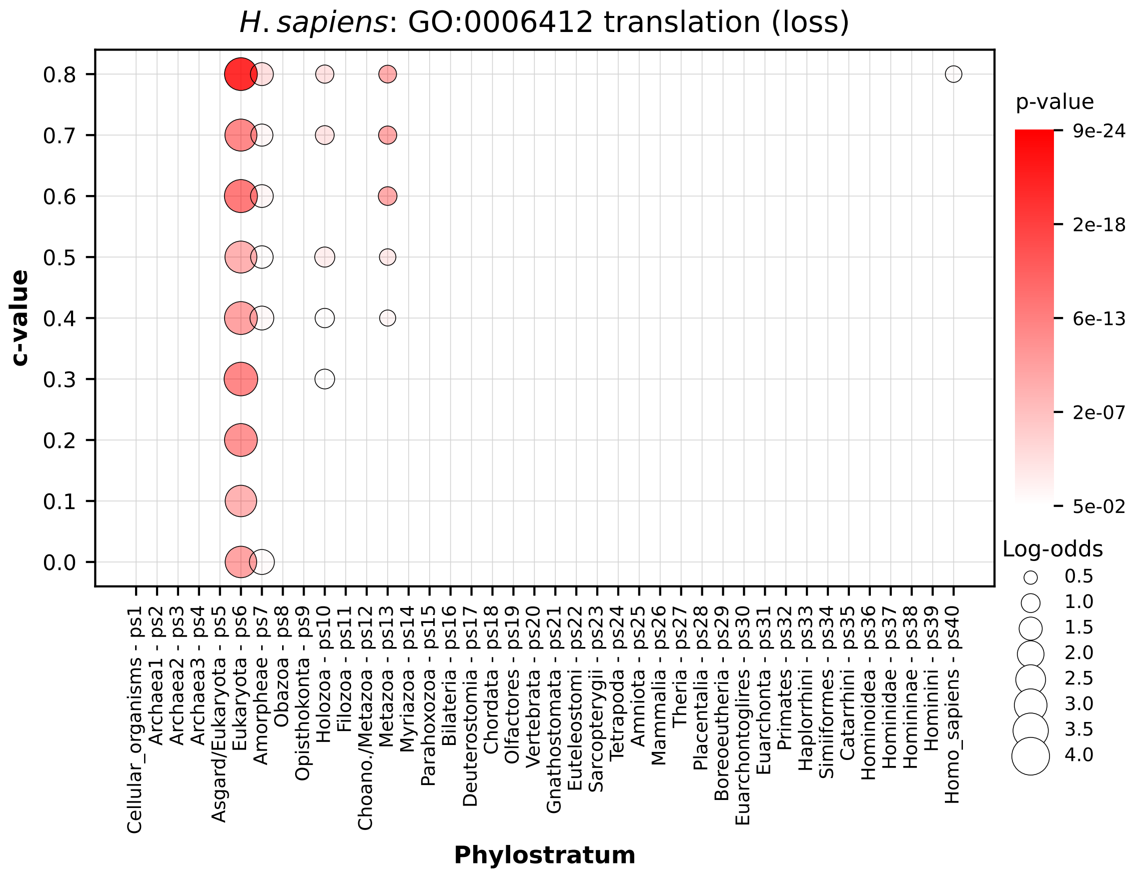
H. sapiens: GO:0006412 translation (loss)
c-value
0.8
0.7
0.6
0.5
0.4
0.3
0.2
0.1
0.0
Phylostratum
Cellular_organisms - ps1
Archaea1 - ps2
Archaea2 - ps3
Archaea3 - ps4
Asgard/Eukaryota - ps5
Eukaryota - ps6
Amorpheae - ps7
Obazoa - ps8
Opisthokonta - ps9
Holozoa - ps10
Filozoa - ps11
Choano./Metazoa - ps12
Metazoa - ps13
Myriazoa - ps14
Parahoxozoa - ps15
Bilateria - ps16
Deuterostomia - ps17
Chordata - ps18
Olfactores - ps19
Vertebrata - ps20
Gnathostomata - ps21
Euteleostomi - ps22
Sarcopterygii - ps23
Tetrapoda - ps24
Amniota - ps25
Mammalia - ps26
Theria - ps27
Placentalia - ps28
Boreoeutheria - ps29
Euarchontoglires - ps30
Euarchonta - ps31
Primates - ps32
Haplorrhini - ps33
Simiiformes - ps34
Catarrhini - ps35
Hominoidea - ps36
Hominidae - ps37
Homininae - ps38
Hominini - ps39
Homo_sapiens - ps40
p-value
9e-24
2e-18
6e-13
2e-07
5e-02
Log-odds
0.5
1.0
1.5
2.0
2.5
3.0
3.5
4.0

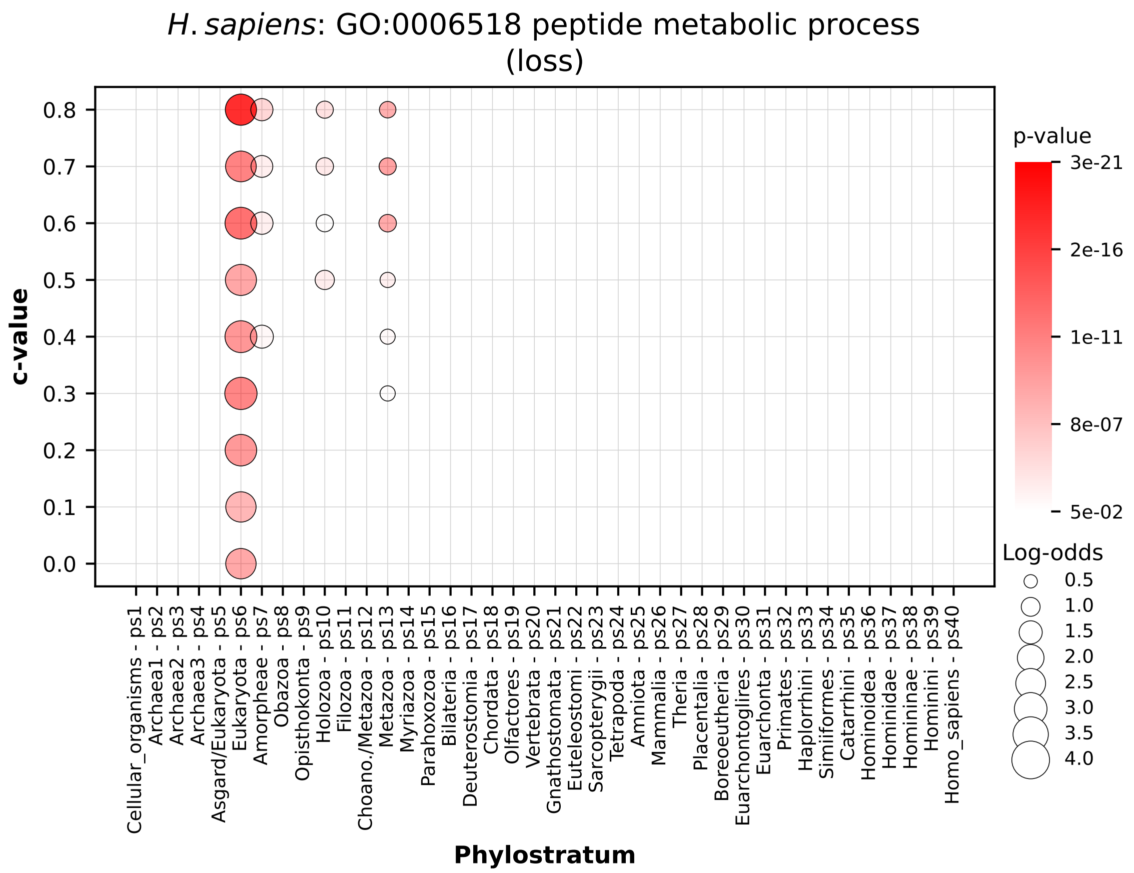

H. sapiens: GO:0006518 peptide metabolic process
(loss)
c-value
0.8
0.7
0.6
0.5
0.4
0.3
0.2
0.1
0.0
Phylostratum
Cellular_organisms - ps1
Archaea1 - ps2
Archaea2 - ps3
Archaea3 - ps4
Asgard/Eukaryota - ps5
Eukaryota - ps6
Amorpheae - ps7
Obazoa - ps8
Opisthokonta - ps9
Holozoa - ps10
Filozoa - ps11
Choano./Metazoa - ps12
Metazoa - ps13
Myriazoa - ps14
Parahoxozoa - ps15
Bilateria - ps16
Deuterostomia - ps17
Chordata - ps18
Olfactores - ps19
Vertebrata - ps20
Gnathostomata - ps21
Euteleostomi - ps22
Sarcopterygii - ps23
Tetrapoda - ps24
Amniota - ps25
Mammalia - ps26
Theria - ps27
Placentalia - ps28
Boreoeutheria - ps29
Euarchontoglires - ps30
Euarchonta - ps31
Primates - ps32
Haplorrhini - ps33
Simiiformes - ps34
Catarrhini - ps35
Hominoidea - ps36
Hominidae - ps37
Homininae - ps38
Hominini - ps39
Homo_sapiens - ps40
p-value
3e-21
2e-16
1e-11
8e-07
5e-02
Log-odds
0.5
1.0
1.5
2.0
2.5
3.0
3.5
4.0

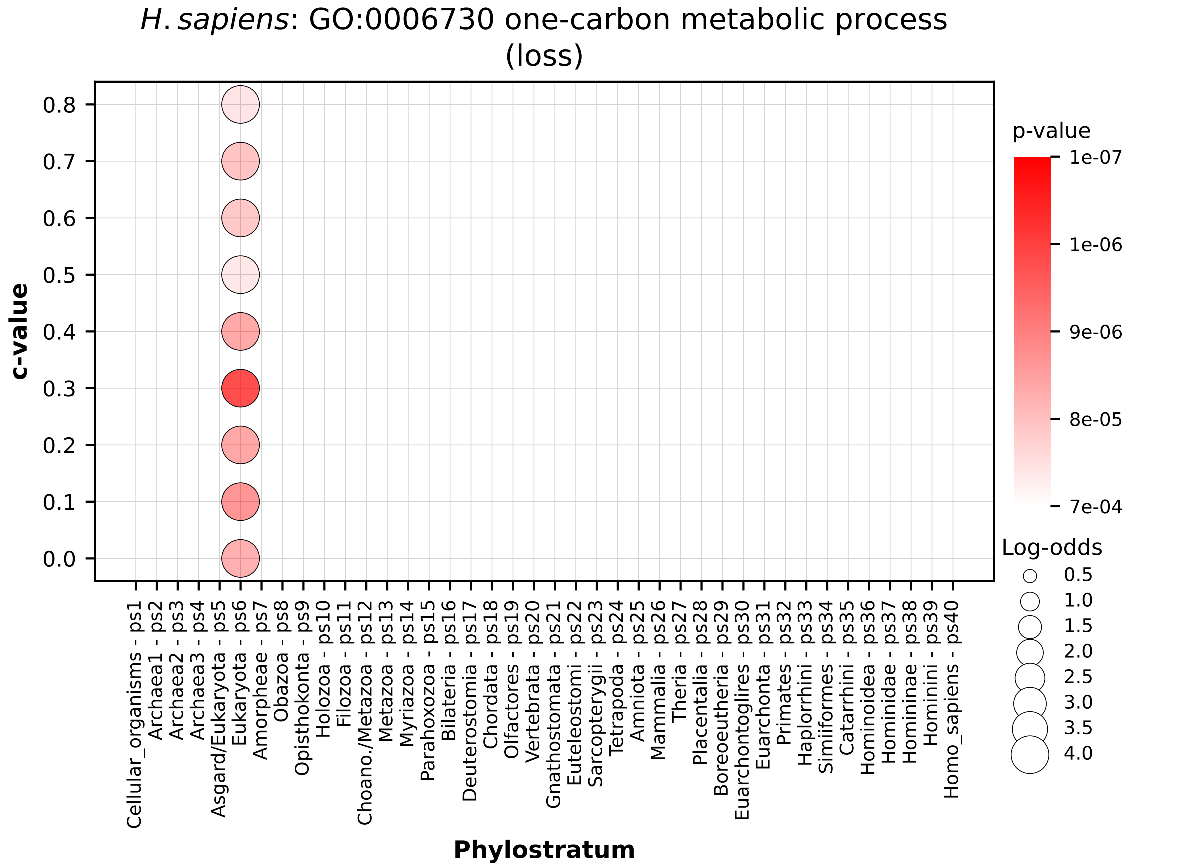

H. sapiens: GO:0006730 one-carbon metabolic process (loss)
c-value
0.8
0.7
0.6
0.5
0.4
0.3
0.2
0.1
0.0
p-value
1e-07
1e-06
9e-06
8e-05
7e-04
Log-odds
0.5
1.0
1.5
2.0
2.5
3.0
3.5
4.0
Cellular_organisms - ps1
Archaea1 - ps2
Archaea2 - ps3
Archaea3 - ps4
Asgard/Eukaryota - ps5
Eukaryota - ps6
Amorpheae - ps7
Obazoa - ps8
Opisthokonta - ps9
Holozoa - ps10
Filozoa - ps11
Choano./Metazoa - ps12
Metazoa - ps13
Myriazoa - ps14
Parahoxozoa - ps15
Bilateria - ps16
Deuterostomia - ps17
Chordata - ps18
Olfactores - ps19
Vertebrata - ps20
Gnathostomata - ps21
Euteleostomi - ps22
Sarcopterygii - ps23
Tetrapoda - ps24
Amniota - ps25
Mammalia - ps26
Theria - ps27
Placentalia - ps28
Boreoeutheria - ps29
Euarchontoglires - ps30
Euarchonta - ps31
Primates - ps32
Haplorrhini - ps33
Simiiformes - ps34
Catarrhini - ps35
Hominoidea - ps36
Hominidae - ps37
Homininae - ps38
Hominini - ps39
Homo_sapiens - ps40
Phylostratum

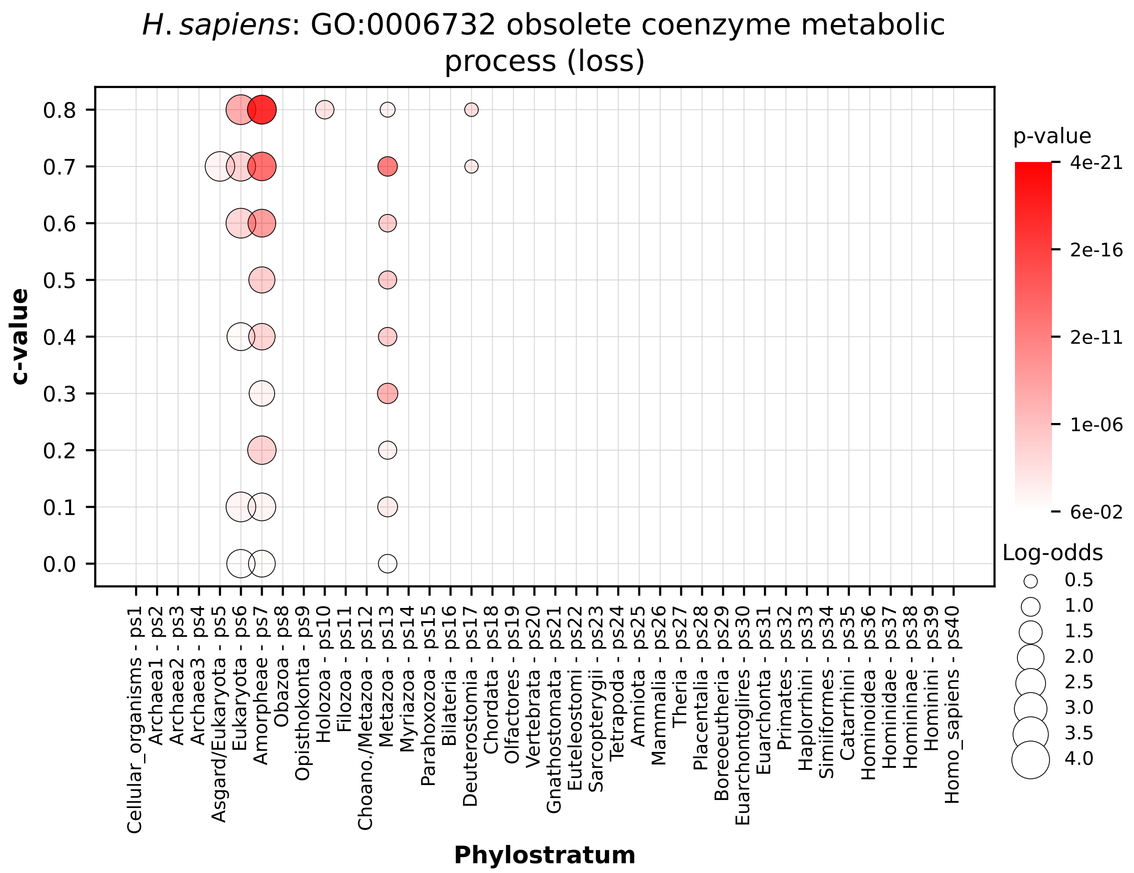
H. sapiens: GO:0006732 obsolete coenzyme metabolic process (loss)
c-value
0.8
0.7
0.6
0.5
0.4
0.3
0.2
0.1
0.0
Phylostratum
Cellular_organisms - ps1
Archaea1 - ps2
Archaea2 - ps3
Archaea3 - ps4
Asgard/Eukaryota - ps5
Eukaryota - ps6
Amorpheae - ps7
Obazoa - ps8
Opisthokonta - ps9
Holozoa - ps10
Filozoa - ps11
Choano./Metazoa - ps12
Metazoa - ps13
Myriazoa - ps14
Parahoxozoa - ps15
Bilateria - ps16
Deuterostomia - ps17
Chordata - ps18
Olfactores - ps19
Vertebrata - ps20
Gnathostomata - ps21
Euteleostomi - ps22
Sarcopterygii - ps23
Tetrapoda - ps24
Amniota - ps25
Mammalia - ps26
Theria - ps27
Placentalia - ps28
Boreoeutheria - ps29
Euarchontoglires - ps30
Euarchonta - ps31
Primates - ps32
Haplorrhini - ps33
Simiiformes - ps34
Catarrhini - ps35
Hominoidea - ps36
Hominidae - ps37
Homininae - ps38
Hominini - ps39
Homo_sapiens - ps40
p-value
4e-21
2e-16
2e-11
1e-06
6e-02
Log-odds
0.5
1.0
1.5
2.0
2.5
3.0
3.5
4.0

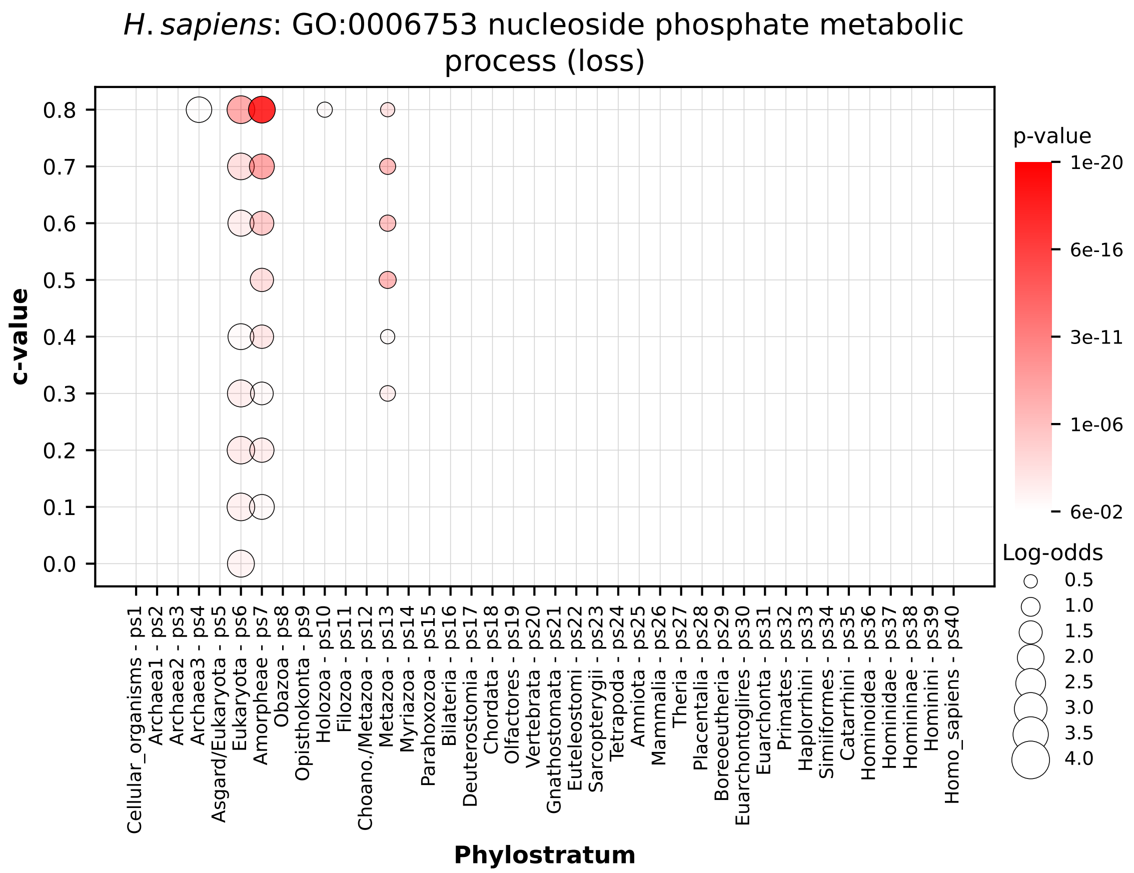

H. sapiens: GO:0006753 nucleoside phosphate metabolic process (loss)
c-value
0.0
0.1
0.2
0.3
0.4
0.5
0.6
0.7
0.8
Phylostratum
Cellular_organisms - ps1
Archaea1 - ps2
Archaea2 - ps3
Archaea3 - ps4
Asgard/Eukaryota - ps5
Eukaryota - ps6
Amorpheae - ps7
Obazoa - ps8
Opisthokonta - ps9
Holozoa - ps10
Filozoa - ps11
Choano./Metazoa - ps12
Metazoa - ps13
Myriazoa - ps14
Parahoxozoa - ps15
Bilateria - ps16
Deuterostomia - ps17
Chordata - ps18
Olfactores - ps19
Vertebrata - ps20
Gnathostomata - ps21
Euteleostomi - ps22
Sarcopterygii - ps23
Tetrapoda - ps24
Amniota - ps25
Mammalia - ps26
Theria - ps27
Placentalia - ps28
Boreoeutheria - ps29
Euarchontoglires - ps30
Euarchonta - ps31
Primates - ps32
Haplorrhini - ps33
Simiiformes - ps34
Catarrhini - ps35
Hominoidea - ps36
Hominidae - ps37
Homininae - ps38
Hominini - ps39
Homo_sapiens - ps40
p-value
1e-20
6e-16
3e-11
1e-06
6e-02
Log-odds
0.5
1.0
1.5
2.0
2.5
3.0
3.5
4.0

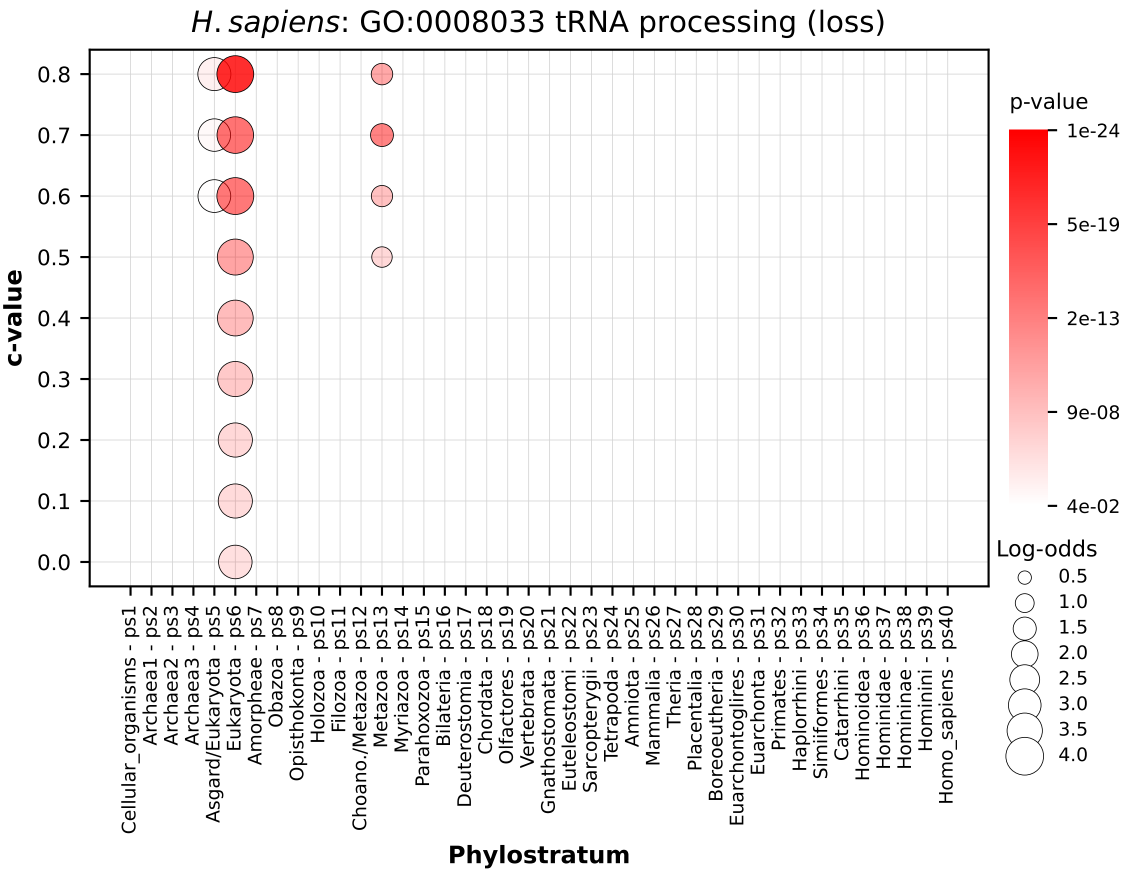

H. sapiens: GO:0008033 tRNA processing (loss)
c-value
0.8
0.7
0.6
0.5
0.4
0.3
0.2
0.1
0.0
Phylostratum
Cellular_organisms - ps1
Archaea1 - ps2
Archaea2 - ps3
Archaea3 - ps4
Asgard/Eukaryota - ps5
Eukaryota - ps6
Amorpheae - ps7
Obazoa - ps8
Opisthokonta - ps9
Holozoa - ps10
Filozoa - ps11
Choano./Metazoa - ps12
Metazoa - ps13
Myriazoa - ps14
Parahoxozoa - ps15
Bilateria - ps16
Deuterostomia - ps17
Chordata - ps18
Olfactores - ps19
Vertebrata - ps20
Gnathostomata - ps21
Euteleostomi - ps22
Sarcopterygii - ps23
Tetrapoda - ps24
Amniota - ps25
Mammalia - ps26
Theria - ps27
Placentalia - ps28
Boreoeutheria - ps29
Euarchontoglires - ps30
Euarchonta - ps31
Primates - ps32
Haplorrhini - ps33
Simiiformes - ps34
Catarrhini - ps35
Hominoidea - ps36
Hominidae - ps37
Homininae - ps38
Hominini - ps39
Homo_sapiens - ps40
p-value
1e-24
5e-19
2e-13
9e-08
4e-02
Log-odds
0.5
1.0
1.5
2.0
2.5
3.0
3.5
4.0

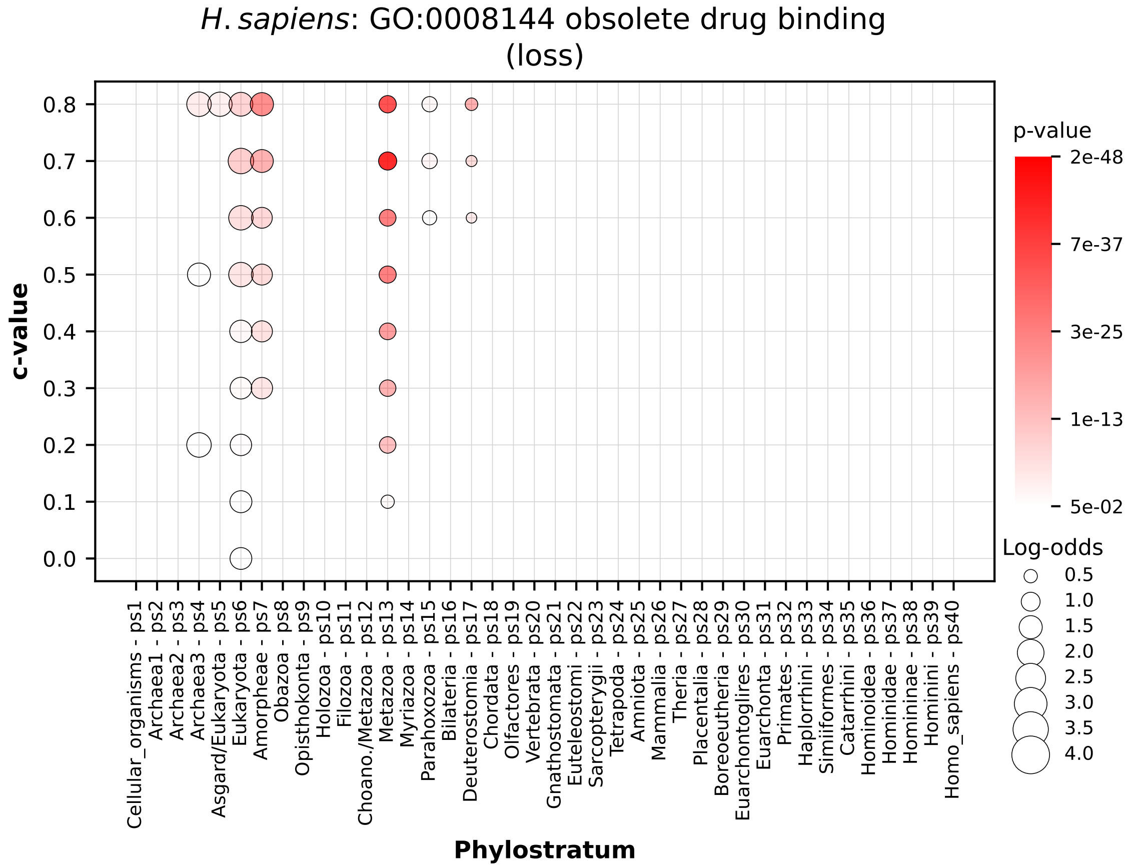

H. sapiens: GO:0008144 obsolete drug binding
(loss)
c-value
0.8
0.7
0.6
0.5
0.4
0.3
0.2
0.1
0.0
p-value
2e-48
7e-37
3e-25
1e-13
5e-02
Log-odds
0.5
1.0
1.5
2.0
2.5
3.0
3.5
4.0
Cellular_organisms - ps1
Archaea1 - ps2
Archaea2 - ps3
Archaea3 - ps4
Asgard/Eukaryota - ps5
Eukaryota - ps6
Amorpheae - ps7
Obazoa - ps8
Opisthokonta - ps9
Holozoa - ps10
Filozoa - ps11
Choano./Metazoa - ps12
Metazoa - ps13
Myriazoa - ps14
Parahoxozoa - ps15
Bilateria - ps16
Deuterostomia - ps17
Chordata - ps18
Olfactores - ps19
Vertebrata - ps20
Gnathostomata - ps21
Euteleostomi - ps22
Sarcopterygii - ps23
Tetrapoda - ps24
Amniota - ps25
Mammalia - ps26
Theria - ps27
Placentalia - ps28
Boreoeutheria - ps29
Euarchontoglires - ps30
Euarchonta - ps31
Primates - ps32
Haplorrhini - ps33
Simiiformes - ps34
Catarrhini - ps35
Hominoidea - ps36
Hominidae - ps37
Homininae - ps38
Hominini - ps39
Homo_sapiens - ps40
Phylostratum

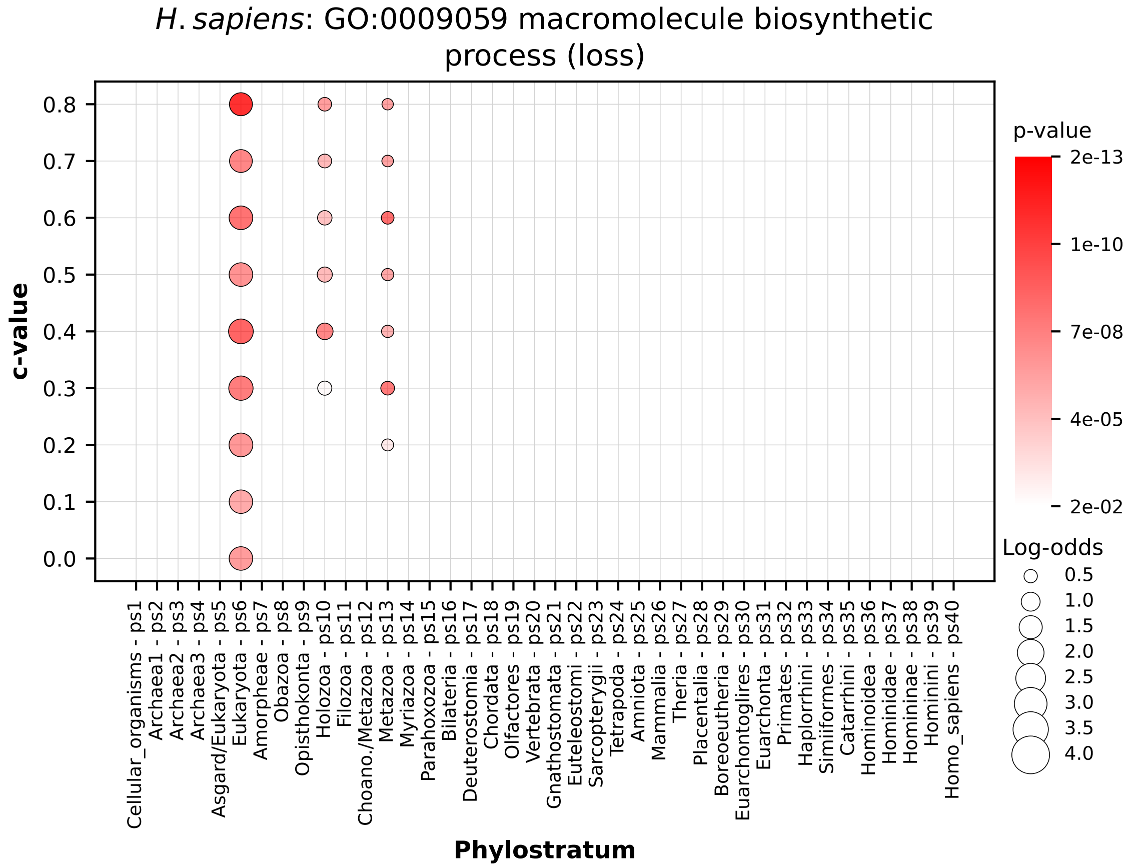

H. sapiens: GO:0009059 macromolecule biosynthetic process (loss)
c-value
0.0
0.1
0.2
0.3
0.4
0.5
0.6
0.7
0.8
p-value
2e-13
1e-10
7e-08
4e-05
2e-02
Log-odds
0.5
1.0
1.5
2.0
2.5
3.0
3.5
4.0
Cellular_organisms - ps1
Archaea1 - ps2
Archaea2 - ps3
Archaea3 - ps4
Asgard/Eukaryota - ps5
Eukaryota - ps6
Amorpheae - ps7
Obazoa - ps8
Opisthokonta - ps9
Holozoa - ps10
Filozoa - ps11
Choano./Metazoa - ps12
Metazoa - ps13
Myriazoa - ps14
Parahoxozoa - ps15
Bilateria - ps16
Deuterostomia - ps17
Chordata - ps18
Olfactores - ps19
Vertebrata - ps20
Gnathostomata - ps21
Euteleostomi - ps22
Sarcopterygii - ps23
Tetrapoda - ps24
Amniota - ps25
Mammalia - ps26
Theria - ps27
Placentalia - ps28
Boreoeutheria - ps29
Euarchontoglires - ps30
Euarchonta - ps31
Primates - ps32
Haplorrhini - ps33
Simiiformes - ps34
Catarrhini - ps35
Hominoidea - ps36
Hominidae - ps37
Homininae - ps38
Hominini - ps39
Homo_sapiens - ps40
Phylostratum

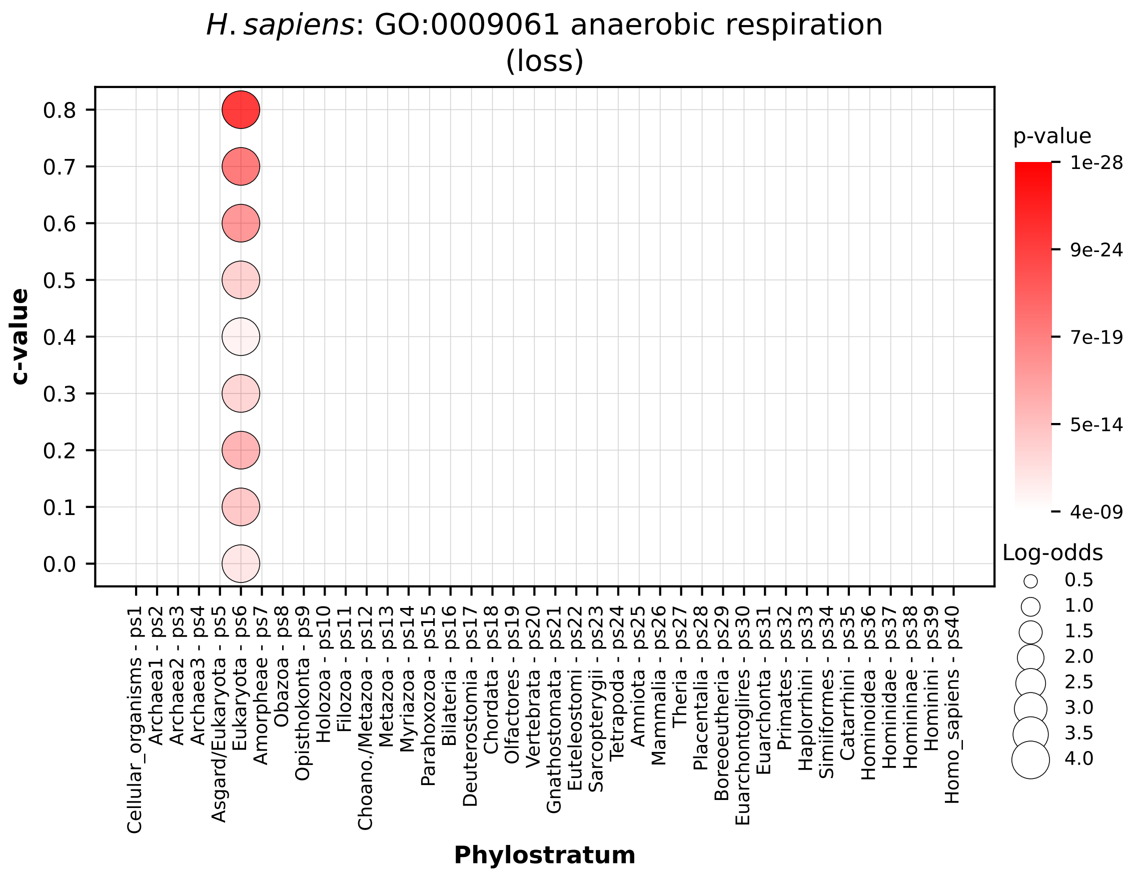

H. sapiens: GO:0009061 anaerobic respiration
(loss)
c-value
0.0
0.1
0.2
0.3
0.4
0.5
0.6
0.7
0.8
p-value
1e-28
9e-24
7e-19
5e-14
4e-09
Log-odds
0.5
1.0
1.5
2.0
2.5
3.0
3.5
4.0
Cellular_organisms - ps1
Archaea1 - ps2
Archaea2 - ps3
Archaea3 - ps4
Asgard/Eukaryota - ps5
Eukaryota - ps6
Amorpheae - ps7
Obazoa - ps8
Opisthokonta - ps9
Holozoa - ps10
Filozoa - ps11
Choano./Metazoa - ps12
Metazoa - ps13
Myriazoa - ps14
Parahoxozoa - ps15
Bilateria - ps16
Deuterostomia - ps17
Chordata - ps18
Olfactores - ps19
Vertebrata - ps20
Gnathostomata - ps21
Euteleostomi - ps22
Sarcopterygii - ps23
Tetrapoda - ps24
Amniota - ps25
Mammalia - ps26
Theria - ps27
Placentalia - ps28
Boreoeutheria - ps29
Euarchontoglires - ps30
Euarchonta - ps31
Primates - ps32
Haplorrhini - ps33
Simiiformes - ps34
Catarrhini - ps35
Hominoidea - ps36
Hominidae - ps37
Homininae - ps38
Hominini - ps39
Homo_sapiens - ps40
Phylostratum

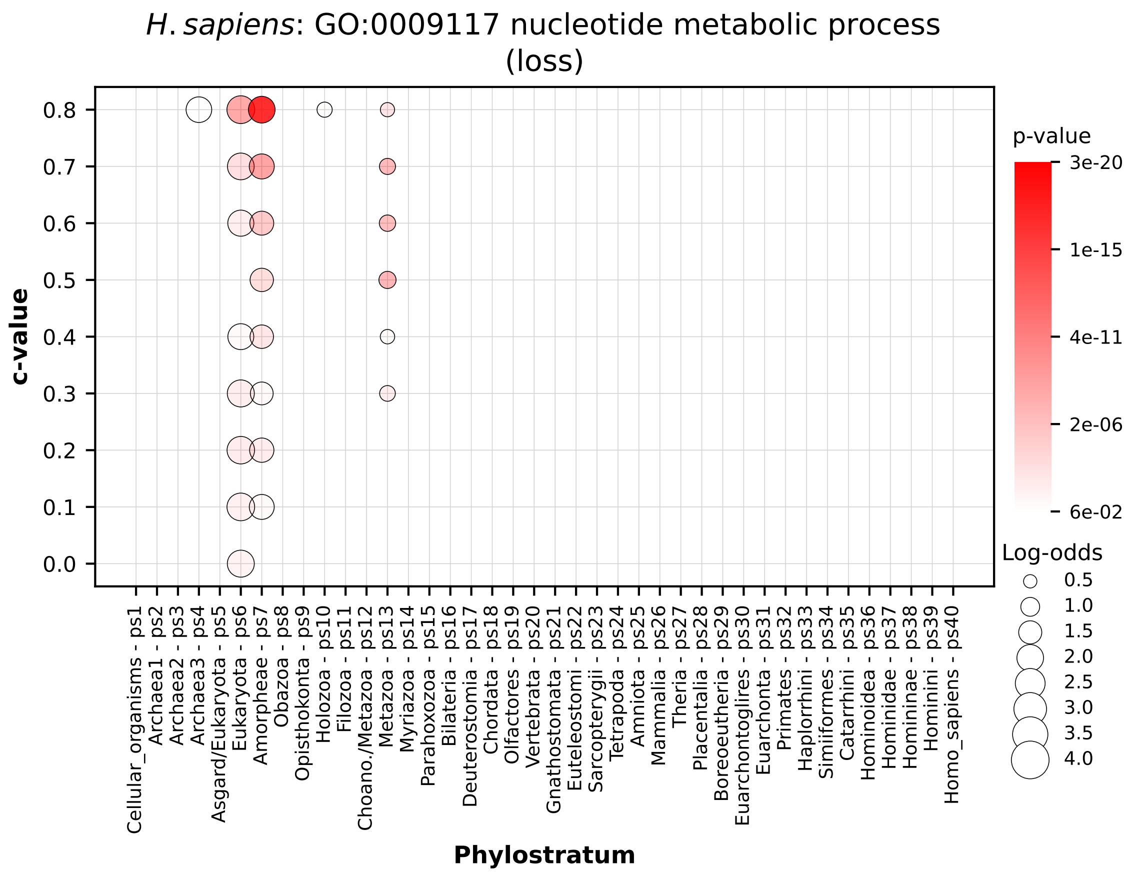

H. sapiens: GO:0009117 nucleotide metabolic process
(loss)
c-value
0.8
0.7
0.6
0.5
0.4
0.3
0.2
0.1
0.0
Phylostratum
Cellular_organisms - ps1
Archaea1 - ps2
Archaea2 - ps3
Archaea3 - ps4
Asgard/Eukaryota - ps5
Eukaryota - ps6
Amorpheae - ps7
Obazoa - ps8
Opisthokonta - ps9
Holozoa - ps10
Filozoa - ps11
Choano./Metazoa - ps12
Metazoa - ps13
Myriazoa - ps14
Parahoxozoa - ps15
Bilateria - ps16
Deuterostomia - ps17
Chordata - ps18
Olfactores - ps19
Vertebrata - ps20
Gnathostomata - ps21
Euteleostomi - ps22
Sarcopterygii - ps23
Tetrapoda - ps24
Amniota - ps25
Mammalia - ps26
Theria - ps27
Placentalia - ps28
Boreoeutheria - ps29
Euarchontoglires - ps30
Euarchonta - ps31
Primates - ps32
Haplorrhini - ps33
Simiiformes - ps34
Catarrhini - ps35
Hominoidea - ps36
Hominidae - ps37
Homininae - ps38
Hominini - ps39
Homo_sapiens - ps40
p-value
3e-20
1e-15
4e-11
2e-06
6e-02
Log-odds
0.5
1.0
1.5
2.0
2.5
3.0
3.5
4.0

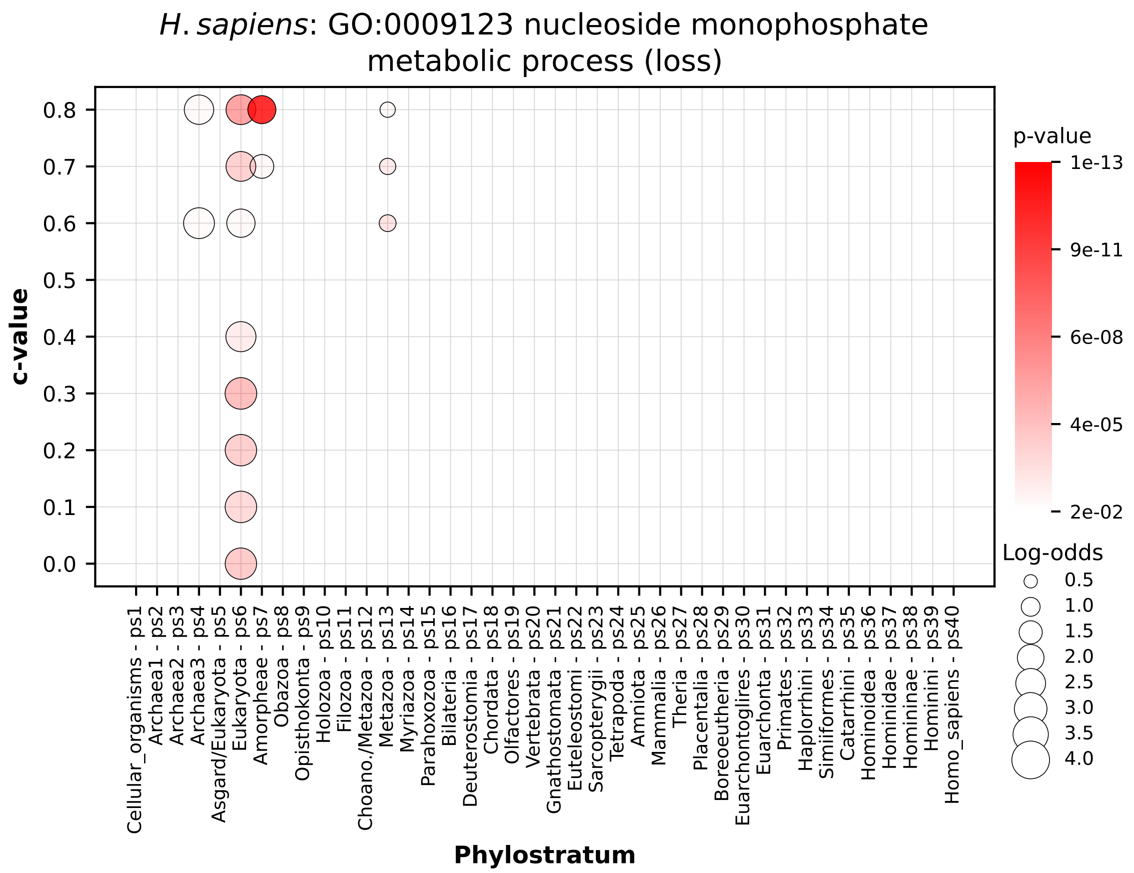

H. sapiens: GO:0009123 nucleoside monophosphate metabolic process (loss)
c-value
0.8
0.7
0.6
0.5
0.4
0.3
0.2
0.1
0.0
Phylostratum
Cellular_organisms - ps1
Archaea1 - ps2
Archaea2 - ps3
Archaea3 - ps4
Asgard/Eukaryota - ps5
Eukaryota - ps6
Amorpheae - ps7
Obazoa - ps8
Opisthokonta - ps9
Holozoa - ps10
Filozoa - ps11
Choano./Metazoa - ps12
Metazoa - ps13
Myriazoa - ps14
Parahoxozoa - ps15
Bilateria - ps16
Deuterostomia - ps17
Chordata - ps18
Olfactores - ps19
Vertebrata - ps20
Gnathostomata - ps21
Euteleostomi - ps22
Sarcopterygii - ps23
Tetrapoda - ps24
Amniota - ps25
Mammalia - ps26
Theria - ps27
Placentalia - ps28
Boreoeutheria - ps29
Euarchontoglires - ps30
Euarchonta - ps31
Primates - ps32
Haplorrhini - ps33
Simiiformes - ps34
Catarrhini - ps35
Hominoidea - ps36
Hominidae - ps37
Homininae - ps38
Hominini - ps39
Homo_sapiens - ps40
p-value
1e-13
9e-11
6e-08
4e-05
2e-02
Log-odds
0.5
1.0
1.5
2.0
2.5
3.0
3.5
4.0

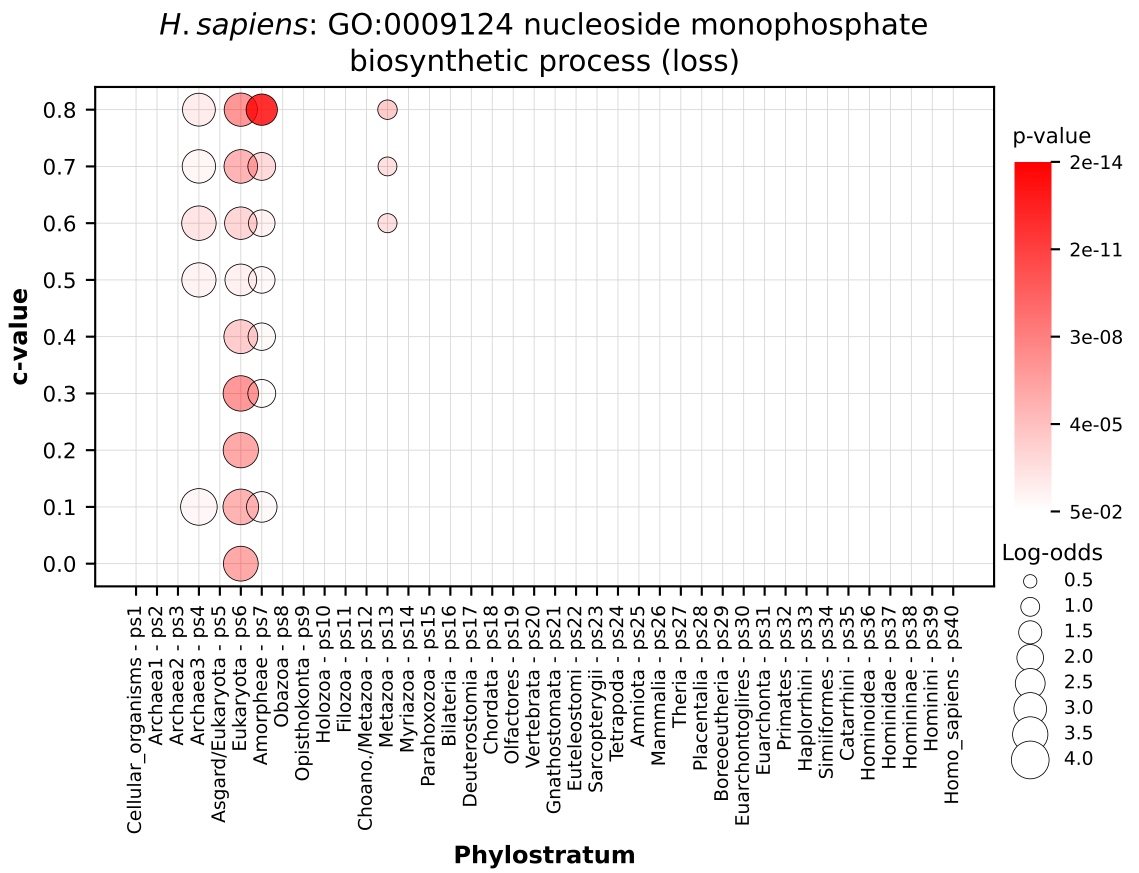

H. sapiens: GO:0009124 nucleoside monophosphate biosynthetic process (loss)
c-value
0.8
0.7
0.6
0.5
0.4
0.3
0.2
0.1
0.0
Phylostratum
Cellular_organisms - ps1
Archaea1 - ps2
Archaea2 - ps3
Archaea3 - ps4
Asgard/Eukaryota - ps5
Eukaryota - ps6
Amorpheae - ps7
Obazoa - ps8
Opisthokonta - ps9
Holozoa - ps10
Filozoa - ps11
Choano./Metazoa - ps12
Metazoa - ps13
Myriazoa - ps14
Parahoxozoa - ps15
Bilateria - ps16
Deuterostomia - ps17
Chordata - ps18
Olfactores - ps19
Vertebrata - ps20
Gnathostomata - ps21
Euteleostomi - ps22
Sarcopterygii - ps23
Tetrapoda - ps24
Amniota - ps25
Mammalia - ps26
Theria - ps27
Placentalia - ps28
Boreoeutheria - ps29
Euarchontoglires - ps30
Euarchonta - ps31
Primates - ps32
Haplorrhini - ps33
Simiiformes - ps34
Catarrhini - ps35
Hominoidea - ps36
Hominidae - ps37
Homininae - ps38
Hominini - ps39
Homo_sapiens - ps40
p-value
2e-14
2e-11
3e-08
4e-05
5e-02
Log-odds
0.5
1.0
1.5
2.0
2.5
3.0
3.5
4.0

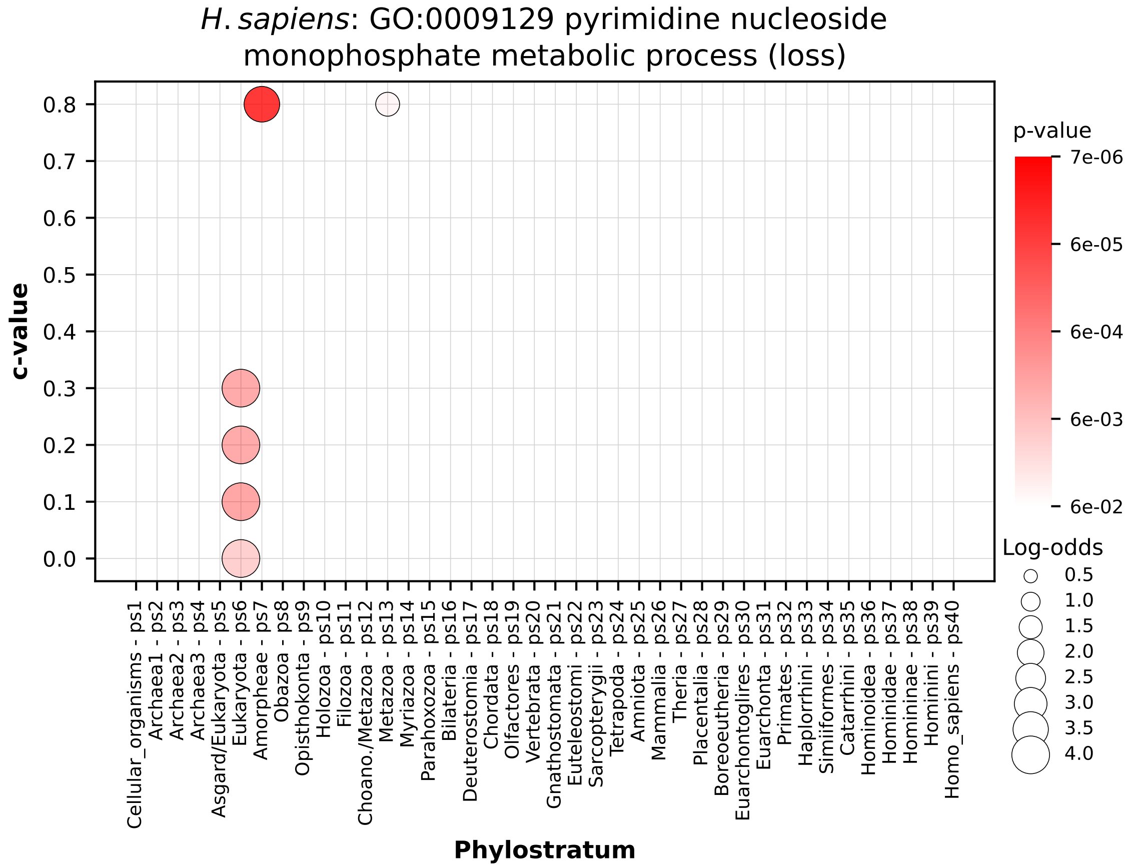
H. sapiens: GO:0009129 pyrimidine nucleoside monophosphate metabolic process (loss)
c-value
0.0
0.1
0.2
0.3
0.4
0.5
0.6
0.7
0.8
p-value
7e-06
6e-05
6e-04
6e-03
6e-02
Log-odds
0.5
1.0
1.5
2.0
2.5
3.0
3.5
4.0
Cellular_organisms - ps1
Archaea1 - ps2
Archaea2 - ps3
Archaea3 - ps4
Asgard/Eukaryota - ps5
Eukaryota - ps6
Amorpheae - ps7
Obazoa - ps8
Opisthokonta - ps9
Holozoa - ps10
Filozoa - ps11
Choano./Metazoa - ps12
Metazoa - ps13
Myriazoa - ps14
Parahoxozoa - ps15
Bilateria - ps16
Deuterostomia - ps17
Chordata - ps18
Olfactores - ps19
Vertebrata - ps20
Gnathostomata - ps21
Euteleostomi - ps22
Sarcopterygii - ps23
Tetrapoda - ps24
Amniota - ps25
Mammalia - ps26
Theria - ps27
Placentalia - ps28
Boreoeutheria - ps29
Euarchontoglires - ps30
Euarchonta - ps31
Primates - ps32
Haplorrhini - ps33
Simiiformes - ps34
Catarrhini - ps35
Hominoidea - ps36
Hominidae - ps37
Homininae - ps38
Hominini - ps39
Homo_sapiens - ps40
Phylostratum

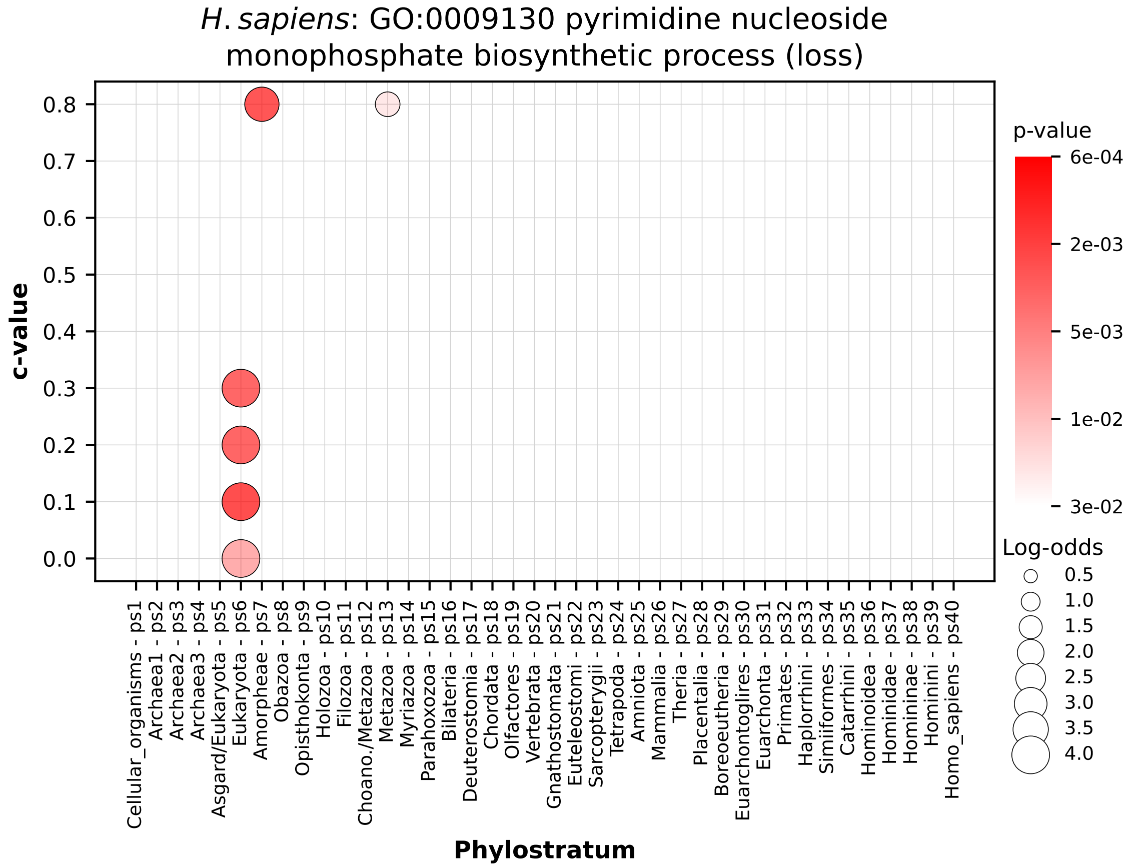

H. sapiens: GO:0009130 pyrimidine nucleoside monophosphate biosynthetic process (loss)
c-value
0.8
0.7
0.6
0.5
0.4
0.3
0.2
0.1
0.0
p-value
6e-04
2e-03
5e-03
1e-02
3e-02
Log-odds
0.5
1.0
1.5
2.0
2.5
3.0
3.5
4.0
Cellular_organisms - ps1
Archaea1 - ps2
Archaea2 - ps3
Archaea3 - ps4
Asgard/Eukaryota - ps5
Eukaryota - ps6
Amorpheae - ps7
Obazoa - ps8
Opisthokonta - ps9
Holozoa - ps10
Filozoa - ps11
Choano./Metazoa - ps12
Metazoa - ps13
Myriazoa - ps14
Parahoxozoa - ps15
Bilateria - ps16
Deuterostomia - ps17
Chordata - ps18
Olfactores - ps19
Vertebrata - ps20
Gnathostomata - ps21
Euteleostomi - ps22
Sarcopterygii - ps23
Tetrapoda - ps24
Amniota - ps25
Mammalia - ps26
Theria - ps27
Placentalia - ps28
Boreoeutheria - ps29
Euarchontoglires - ps30
Euarchonta - ps31
Primates - ps32
Haplorrhini - ps33
Simiiformes - ps34
Catarrhini - ps35
Hominoidea - ps36
Hominidae - ps37
Homininae - ps38
Hominini - ps39
Homo_sapiens - ps40
Phylostratum

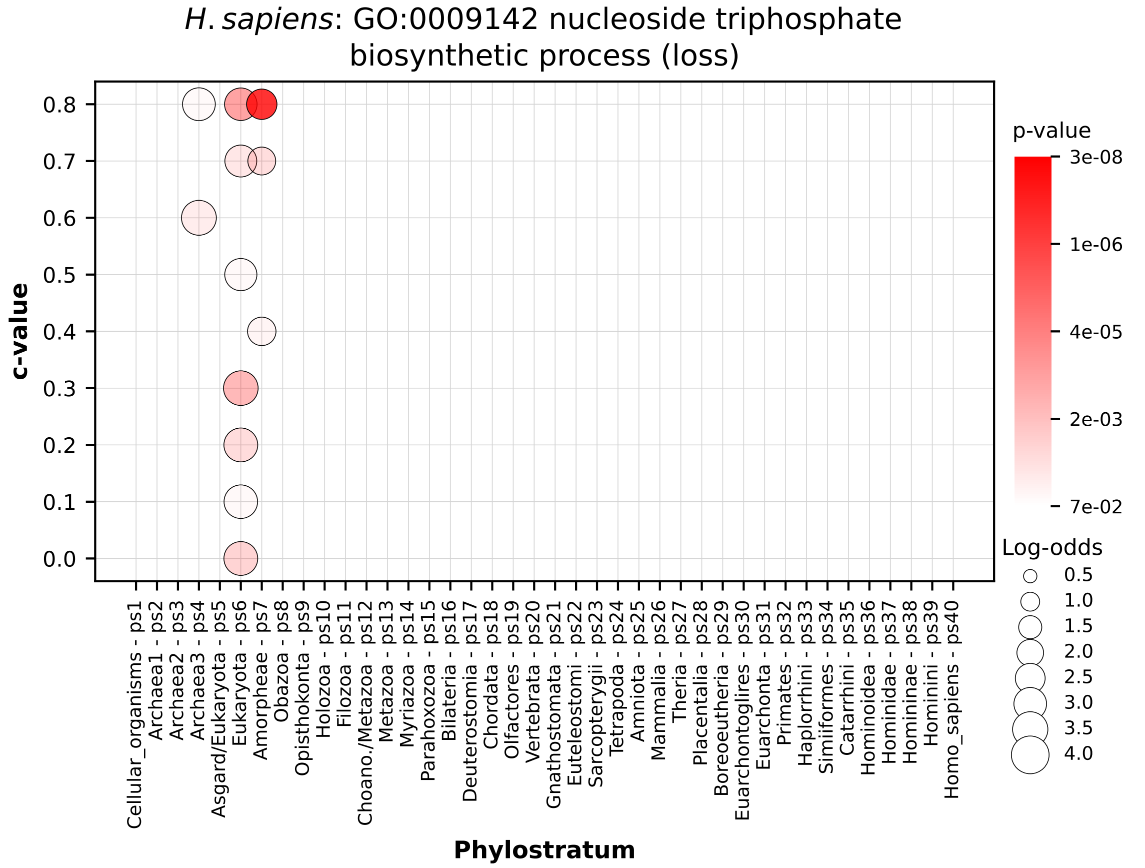

H. sapiens: GO:0009142 nucleoside triphosphate biosynthetic process (loss)
c-value
0.8
0.7
0.6
0.5
0.4
0.3
0.2
0.1
0.0
p-value
3e-08
1e-06
4e-05
2e-03
7e-02
Log-odds
0.5
1.0
1.5
2.0
2.5
3.0
3.5
4.0
Cellular_organisms - ps1
Archaea1 - ps2
Archaea2 - ps3
Archaea3 - ps4
Asgard/Eukaryota - ps5
Eukaryota - ps6
Amorpheae - ps7
Obazoa - ps8
Opisthokonta - ps9
Holozoa - ps10
Filozoa - ps11
Choano./Metazoa - ps12
Metazoa - ps13
Myriazoa - ps14
Parahoxozoa - ps15
Bilateria - ps16
Deuterostomia - ps17
Chordata - ps18
Olfactores - ps19
Vertebrata - ps20
Gnathostomata - ps21
Euteleostomi - ps22
Sarcopterygii - ps23
Tetrapoda - ps24
Amniota - ps25
Mammalia - ps26
Theria - ps27
Placentalia - ps28
Boreoeutheria - ps29
Euarchontoglires - ps30
Euarchonta - ps31
Primates - ps32
Haplorrhini - ps33
Simiiformes - ps34
Catarrhini - ps35
Hominoidea - ps36
Hominidae - ps37
Homininae - ps38
Hominini - ps39
Homo_sapiens - ps40
Phylostratum

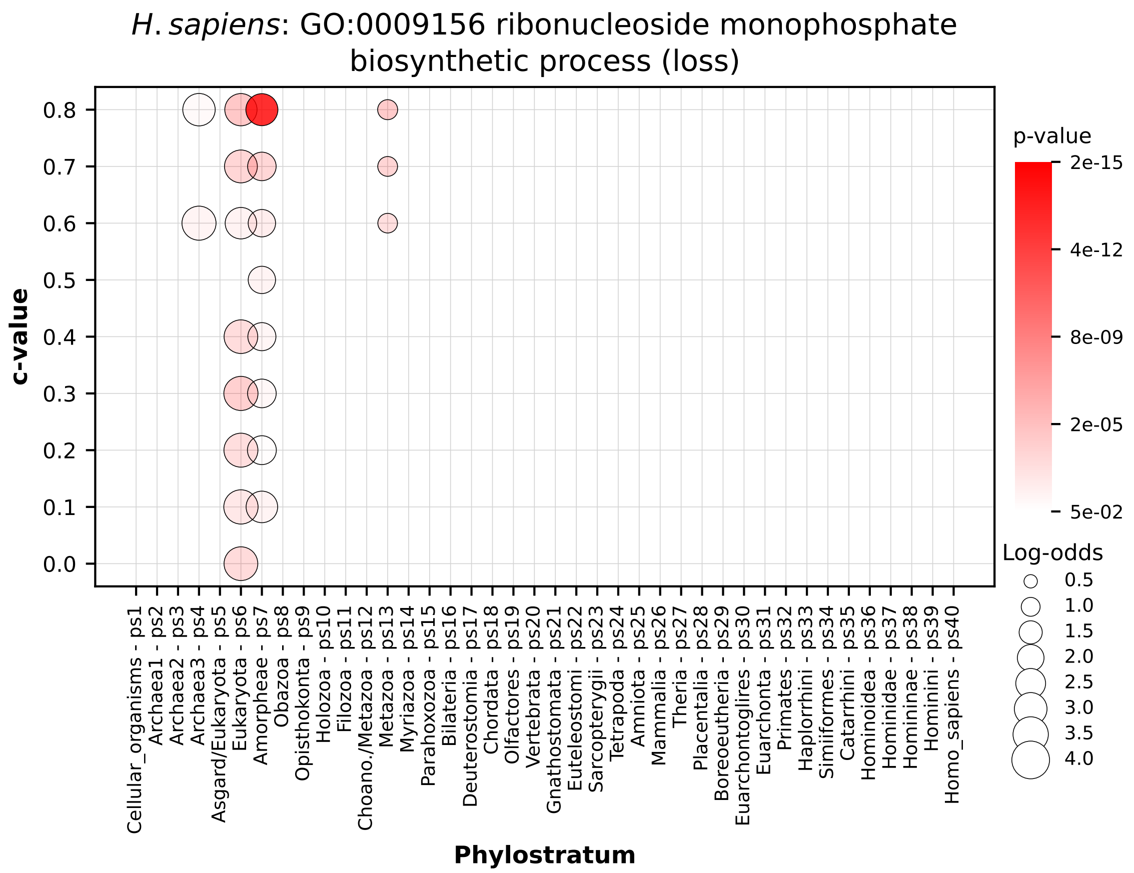

H. sapiens: GO:0009156 ribonucleoside monophosphate biosynthetic process (loss)
c-value
0.8
0.7
0.6
0.5
0.4
0.3
0.2
0.1
0.0
p-value
2e-15
4e-12
8e-09
2e-05
5e-02
Log-odds
0.5
1.0
1.5
2.0
2.5
3.0
3.5
4.0
Cellular_organisms - ps1
Archaea1 - ps2
Archaea2 - ps3
Archaea3 - ps4
Asgard/Eukaryota - ps5
Eukaryota - ps6
Amorpheae - ps7
Obazoa - ps8
Opisthokonta - ps9
Holozoa - ps10
Filozoa - ps11
Choano./Metazoa - ps12
Metazoa - ps13
Myriazoa - ps14
Parahoxozoa - ps15
Bilateria - ps16
Deuterostomia - ps17
Chordata - ps18
Olfactores - ps19
Vertebrata - ps20
Gnathostomata - ps21
Euteleostomi - ps22
Sarcopterygii - ps23
Tetrapoda - ps24
Amniota - ps25
Mammalia - ps26
Theria - ps27
Placentalia - ps28
Boreoeutheria - ps29
Euarchontoglires - ps30
Euarchonta - ps31
Primates - ps32
Haplorrhini - ps33
Simiiformes - ps34
Catarrhini - ps35
Hominoidea - ps36
Hominidae - ps37
Homininae - ps38
Hominini - ps39
Homo_sapiens - ps40
Phylostratum

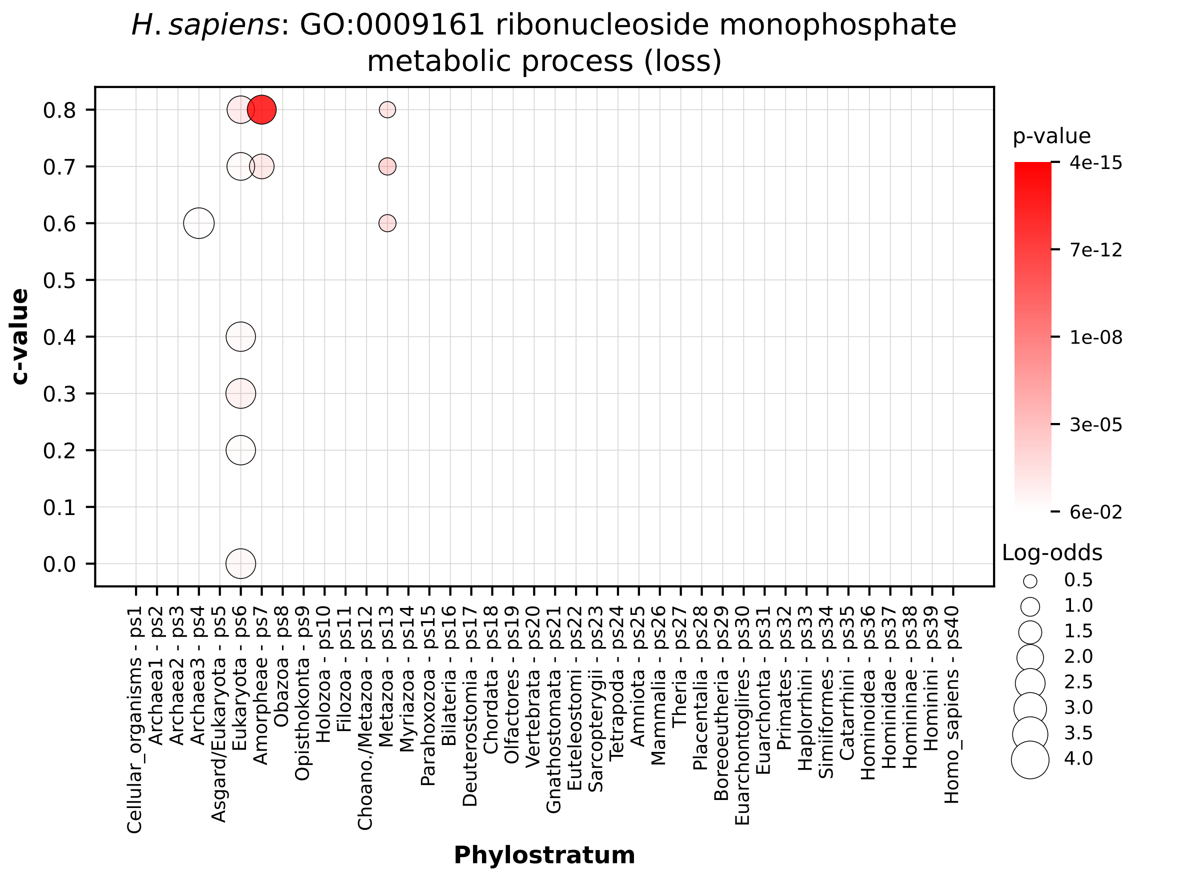

H. sapiens: GO:0009161 ribonucleoside monophosphate metabolic process (loss)
c-value
0.8
0.7
0.6
0.5
0.4
0.3
0.2
0.1
0.0
p-value
4e-15
7e-12
1e-08
3e-05
6e-02
Log-odds
0.5
1.0
1.5
2.0
2.5
3.0
3.5
4.0
Cellular_organisms - ps1
Archaea1 - ps2
Archaea2 - ps3
Archaea3 - ps4
Asgard/Eukaryota - ps5
Eukaryota - ps6
Amorpheae - ps7
Obazoa - ps8
Opisthokonta - ps9
Holozoa - ps10
Filozoa - ps11
Choano./Metazoa - ps12
Metazoa - ps13
Myriazoa - ps14
Parahoxozoa - ps15
Bilateria - ps16
Deuterostomia - ps17
Chordata - ps18
Olfactores - ps19
Vertebrata - ps20
Gnathostomata - ps21
Euteleostomi - ps22
Sarcopterygii - ps23
Tetrapoda - ps24
Amniota - ps25
Mammalia - ps26
Theria - ps27
Placentalia - ps28
Boreoeutheria - ps29
Euarchontoglires - ps30
Euarchonta - ps31
Primates - ps32
Haplorrhini - ps33
Simiiformes - ps34
Catarrhini - ps35
Hominoidea - ps36
Hominidae - ps37
Homininae - ps38
Hominini - ps39
Homo_sapiens - ps40
Phylostratum

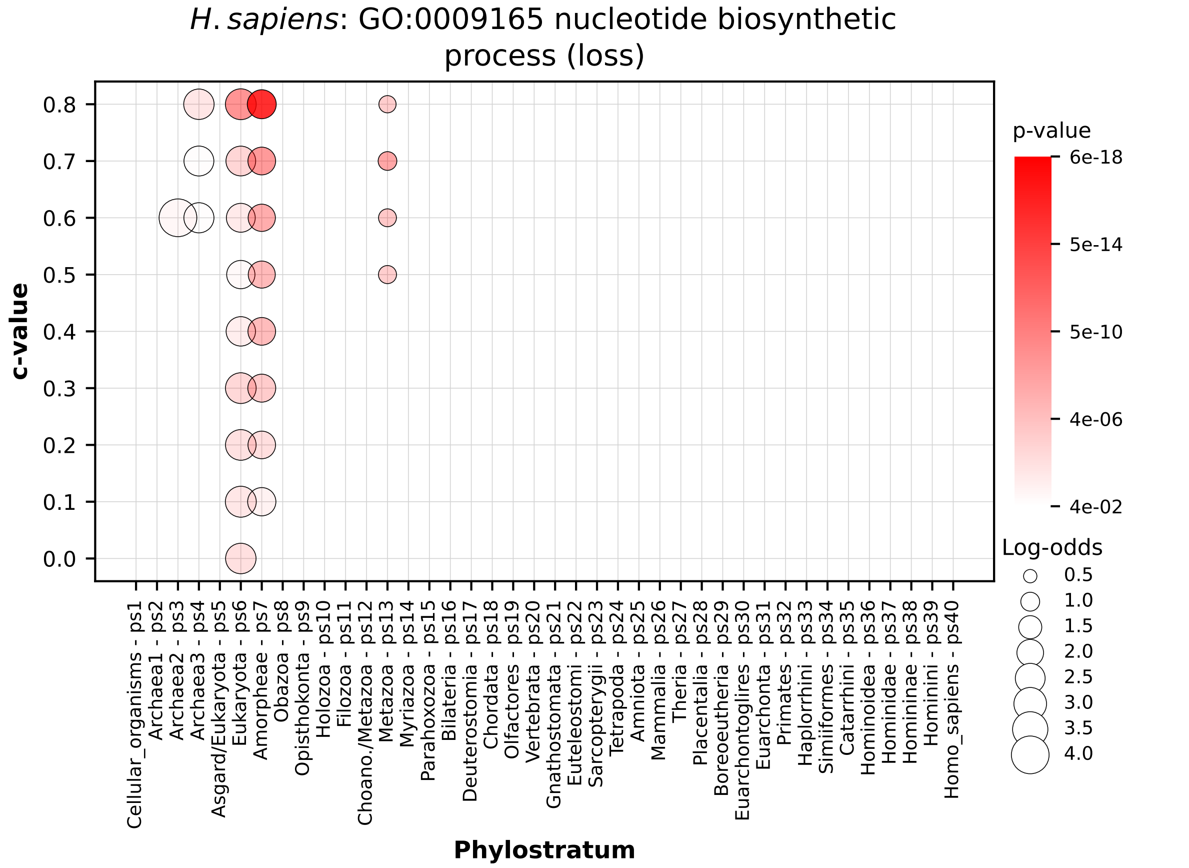

H. sapiens: GO:0009165 nucleotide biosynthetic process (loss)
c-value
0.8
0.7
0.6
0.5
0.4
0.3
0.2
0.1
0.0
p-value
6e-18
5e-14
5e-10
4e-06
4e-02
Log-odds
0.5
1.0
1.5
2.0
2.5
3.0
3.5
4.0
Cellular_organisms - ps1
Archaea1 - ps2
Archaea2 - ps3
Archaea3 - ps4
Asgard/Eukaryota - ps5
Eukaryota - ps6
Amorpheae - ps7
Obazoa - ps8
Opisthokonta - ps9
Holozoa - ps10
Filozoa - ps11
Choano./Metazoa - ps12
Metazoa - ps13
Myriazoa - ps14
Parahoxozoa - ps15
Bilateria - ps16
Deuterostomia - ps17
Chordata - ps18
Olfactores - ps19
Vertebrata - ps20
Gnathostomata - ps21
Euteleostomi - ps22
Sarcopterygii - ps23
Tetrapoda - ps24
Amniota - ps25
Mammalia - ps26
Theria - ps27
Placentalia - ps28
Boreoeutheria - ps29
Euarchontoglires - ps30
Euarchonta - ps31
Primates - ps32
Haplorrhini - ps33
Simiiformes - ps34
Catarrhini - ps35
Hominoidea - ps36
Hominidae - ps37
Homininae - ps38
Hominini - ps39
Homo_sapiens - ps40
Phylostratum

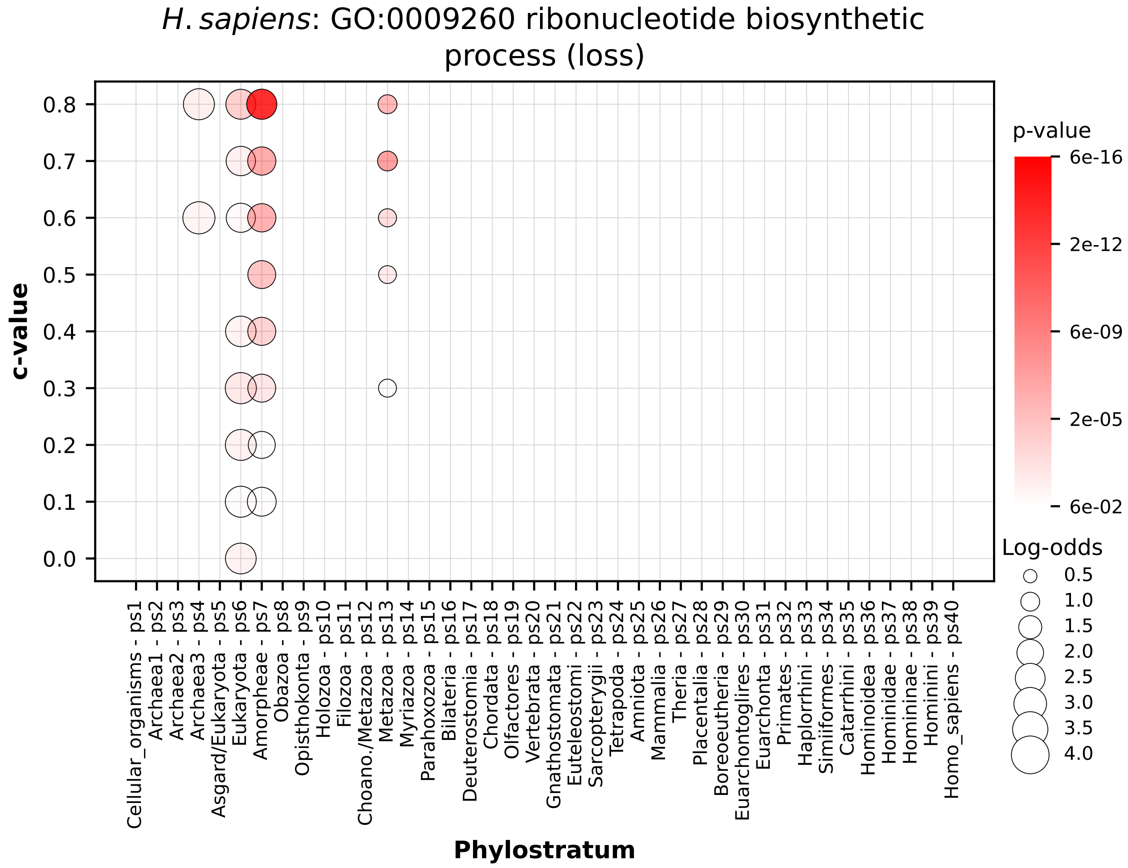

H. sapiens: GO:0009260 ribonucleotide biosynthetic process (loss)
c-value
0.8
0.7
0.6
0.5
0.4
0.3
0.2
0.1
0.0
p-value
6e-16
2e-12
6e-09
2e-05
6e-02
Log-odds
0.5
1.0
1.5
2.0
2.5
3.0
3.5
4.0
Cellular_organisms - ps1
Archaea1 - ps2
Archaea2 - ps3
Archaea3 - ps4
Asgard/Eukaryota - ps5
Eukaryota - ps6
Amorpheae - ps7
Obazoa - ps8
Opisthokonta - ps9
Holozoa - ps10
Filozoa - ps11
Choano./Metazoa - ps12
Metazoa - ps13
Myriazoa - ps14
Parahoxozoa - ps15
Bilateria - ps16
Deuterostomia - ps17
Chordata - ps18
Olfactores - ps19
Vertebrata - ps20
Gnathostomata - ps21
Euteleostomi - ps22
Sarcopterygii - ps23
Tetrapoda - ps24
Amniota - ps25
Mammalia - ps26
Theria - ps27
Placentalia - ps28
Boreoeutheria - ps29
Euarchontoglires - ps30
Euarchonta - ps31
Primates - ps32
Haplorrhini - ps33
Simiiformes - ps34
Catarrhini - ps35
Hominoidea - ps36
Hominidae - ps37
Homininae - ps38
Hominini - ps39
Homo_sapiens - ps40
Phylostratum

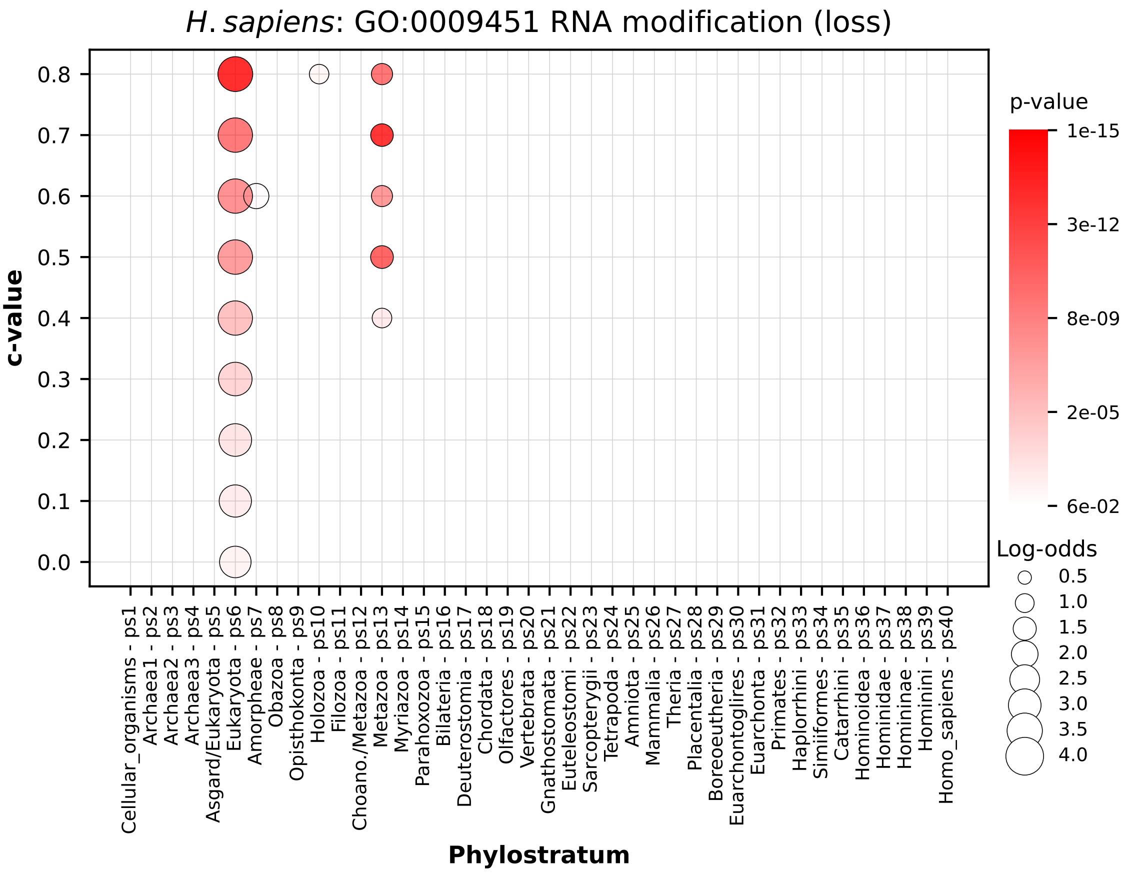

H. sapiens: GO:0009451 RNA modification (loss)
c-value
0.8
0.7
0.6
0.5
0.4
0.3
0.2
0.1
0.0
p-value
1e-15
3e-12
8e-09
2e-05
6e-02
Log-odds
0.5
1.0
1.5
2.0
2.5
3.0
3.5
4.0
Cellular_organisms - ps1
Archaea1 - ps2
Archaea2 - ps3
Archaea3 - ps4
Asgard/Eukaryota - ps5
Eukaryota - ps6
Amorpheae - ps7
Obazoa - ps8
Opisthokonta - ps9
Holozoa - ps10
Filozoa - ps11
Choano./Metazoa - ps12
Metazoa - ps13
Myriazoa - ps14
Parahoxozoa - ps15
Bilateria - ps16
Deuterostomia - ps17
Chordata - ps18
Olfactores - ps19
Vertebrata - ps20
Gnathostomata - ps21
Euteleostomi - ps22
Sarcopterygii - ps23
Tetrapoda - ps24
Amniota - ps25
Mammalia - ps26
Theria - ps27
Placentalia - ps28
Boreoeutheria - ps29
Euarchontoglires - ps30
Euarchonta - ps31
Primates - ps32
Haplorrhini - ps33
Simiiformes - ps34
Catarrhini - ps35
Hominoidea - ps36
Hominidae - ps37
Homininae - ps38
Hominini - ps39
Homo_sapiens - ps40
Phylostratum

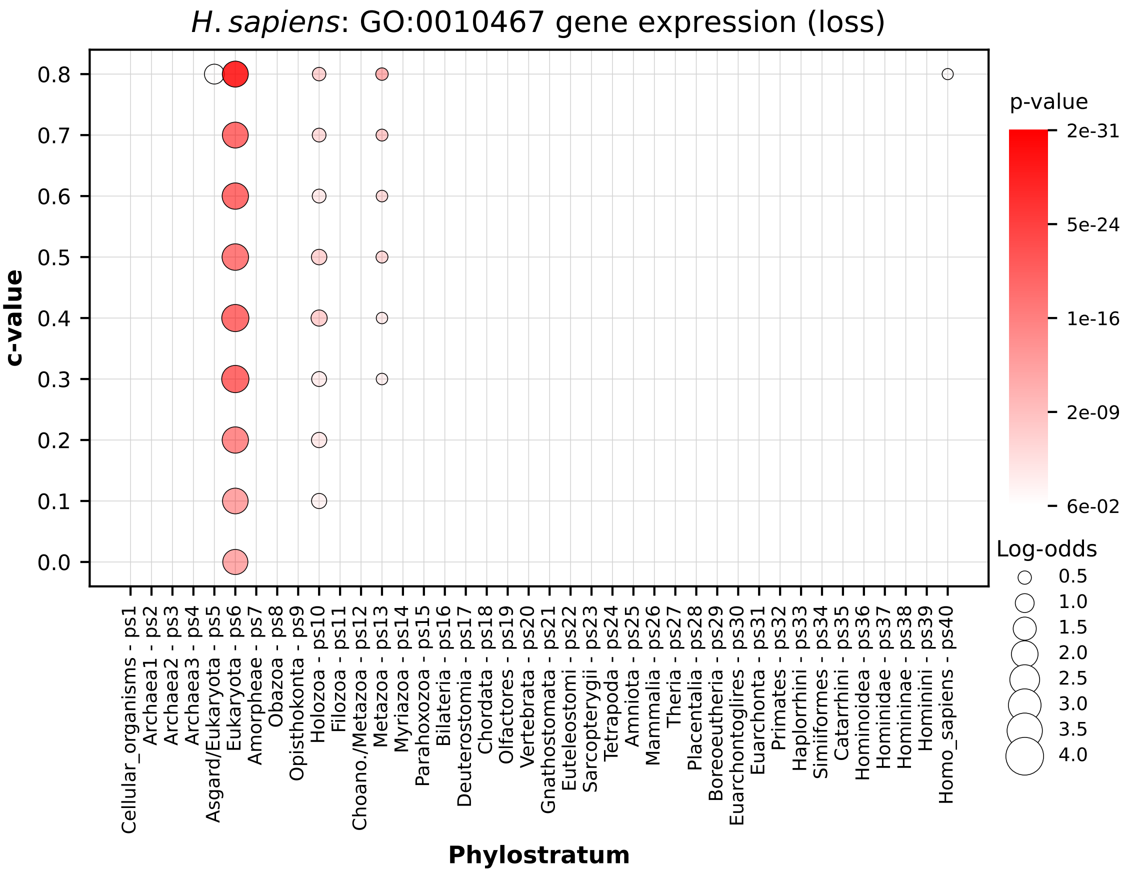

H. sapiens: GO:0010467 gene expression (loss)
c-value
0.0
0.1
0.2
0.3
0.4
0.5
0.6
0.7
0.8
Phylostratum
Cellular_organisms - ps1
Archaea1 - ps2
Archaea2 - ps3
Archaea3 - ps4
Asgard/Eukaryota - ps5
Eukaryota - ps6
Amorpheae - ps7
Obazoa - ps8
Opisthokonta - ps9
Holozoa - ps10
Filozoa - ps11
Choano./Metazoa - ps12
Metazoa - ps13
Myriazoa - ps14
Parahoxozoa - ps15
Bilateria - ps16
Deuterostomia - ps17
Chordata - ps18
Olfactores - ps19
Vertebrata - ps20
Gnathostomata - ps21
Euteleostomi - ps22
Sarcopterygii - ps23
Tetrapoda - ps24
Amniota - ps25
Mammalia - ps26
Theria - ps27
Placentalia - ps28
Boreoeutheria - ps29
Euarchontoglires - ps30
Euarchonta - ps31
Primates - ps32
Haplorrhini - ps33
Simiiformes - ps34
Catarrhini - ps35
Hominoidea - ps36
Hominidae - ps37
Homininae - ps38
Hominini - ps39
Homo_sapiens - ps40
p-value
2e-31
5e-24
1e-16
2e-09
6e-02
Log-odds
0.5
1.0
1.5
2.0
2.5
3.0
3.5
4.0

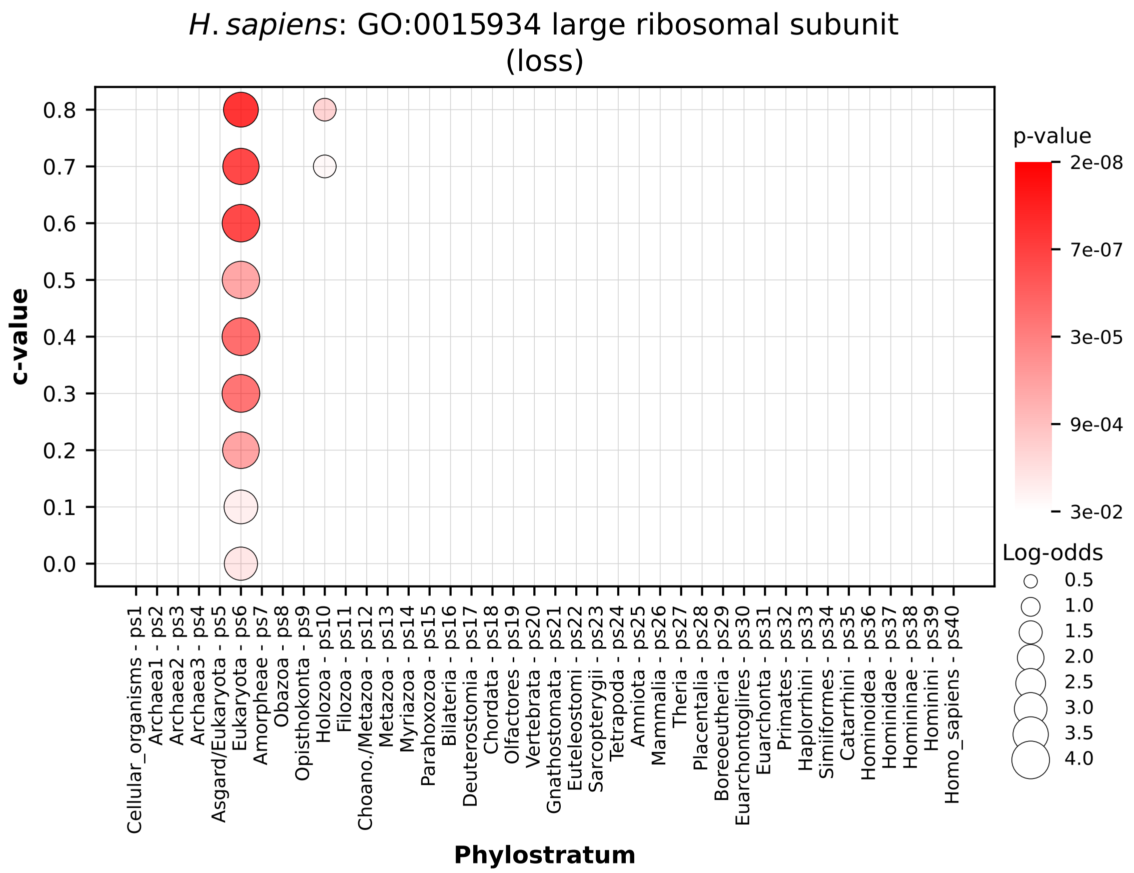

H. sapiens: GO:0015934 large ribosomal subunit (loss)
c-value
0.8
0.7
0.6
0.5
0.4
0.3
0.2
0.1
0.0
Phylostratum
Cellular_organisms - ps1
Archaea1 - ps2
Archaea2 - ps3
Archaea3 - ps4
Asgard/Eukaryota - ps5
Eukaryota - ps6
Amorpheae - ps7
Obazoa - ps8
Opisthokonta - ps9
Holozoa - ps10
Filozoa - ps11
Choano./Metazoa - ps12
Metazoa - ps13
Myriazoa - ps14
Parahoxozoa - ps15
Bilateria - ps16
Deuterostomia - ps17
Chordata - ps18
Olfactores - ps19
Vertebrata - ps20
Gnathostomata - ps21
Euteleostomi - ps22
Sarcopterygii - ps23
Tetrapoda - ps24
Amniota - ps25
Mammalia - ps26
Theria - ps27
Placentalia - ps28
Boreoeutheria - ps29
Euarchontoglires - ps30
Euarchonta - ps31
Primates - ps32
Haplorrhini - ps33
Simiiformes - ps34
Catarrhini - ps35
Hominoidea - ps36
Hominidae - ps37
Homininae - ps38
Hominini - ps39
Homo_sapiens - ps40
p-value
2e-08
7e-07
3e-05
9e-04
3e-02
Log-odds
0.5
1.0
1.5
2.0
2.5
3.0
3.5
4.0

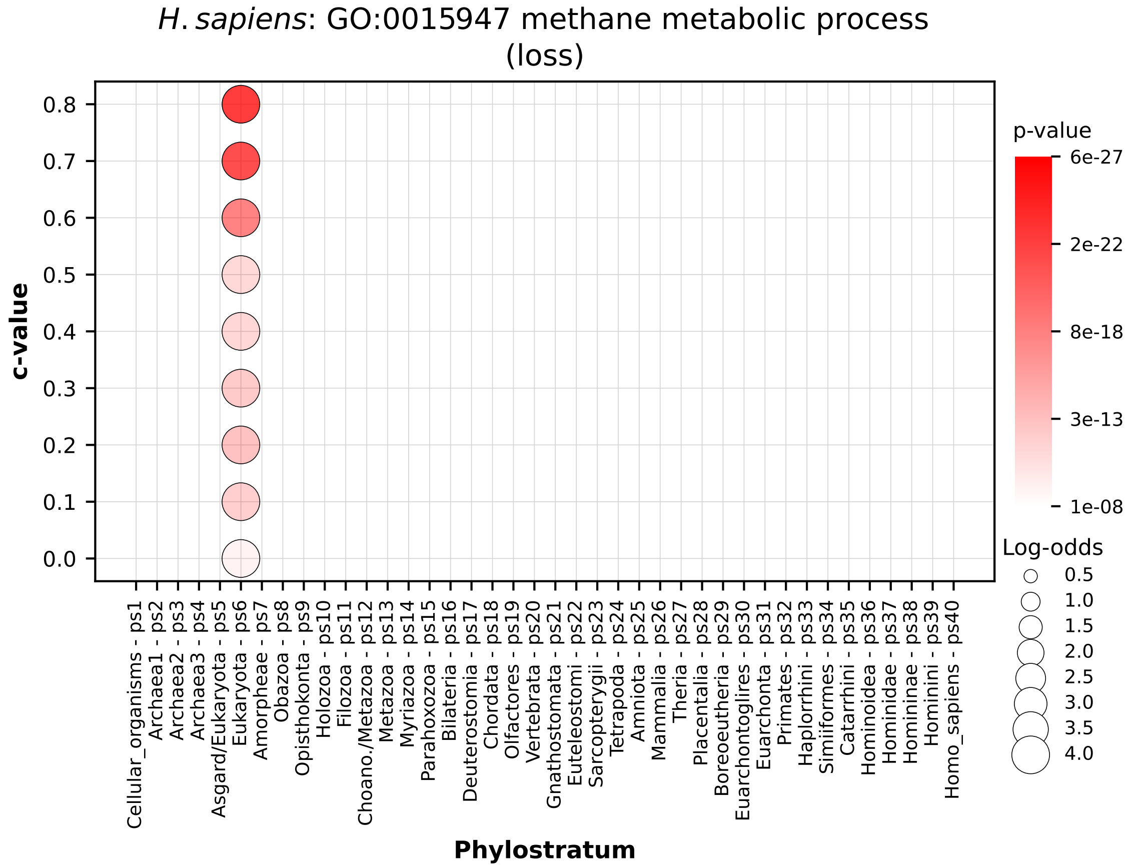

H. sapiens: GO:0015947 methane metabolic process
(loss)
c-value
0.0
0.1
0.2
0.3
0.4
0.5
0.6
0.7
0.8
p-value
6e-27
2e-22
8e-18
3e-13
1e-08
Log-odds
0.5
1.0
1.5
2.0
2.5
3.0
3.5
4.0
Cellular_organisms - ps1
Archaea1 - ps2
Archaea2 - ps3
Archaea3 - ps4
Asgard/Eukaryota - ps5
Eukaryota - ps6
Amorpheae - ps7
Obazoa - ps8
Opisthokonta - ps9
Holozoa - ps10
Filozoa - ps11
Choano./Metazoa - ps12
Metazoa - ps13
Myriazoa - ps14
Parahoxozoa - ps15
Bilateria - ps16
Deuterostomia - ps17
Chordata - ps18
Olfactores - ps19
Vertebrata - ps20
Gnathostomata - ps21
Euteleostomi - ps22
Sarcopterygii - ps23
Tetrapoda - ps24
Amniota - ps25
Mammalia - ps26
Theria - ps27
Placentalia - ps28
Boreoeutheria - ps29
Euarchontoglires - ps30
Euarchonta - ps31
Primates - ps32
Haplorrhini - ps33
Simiiformes - ps34
Catarrhini - ps35
Hominoidea - ps36
Hominidae - ps37
Homininae - ps38
Hominini - ps39
Homo_sapiens - ps40
Phylostratum

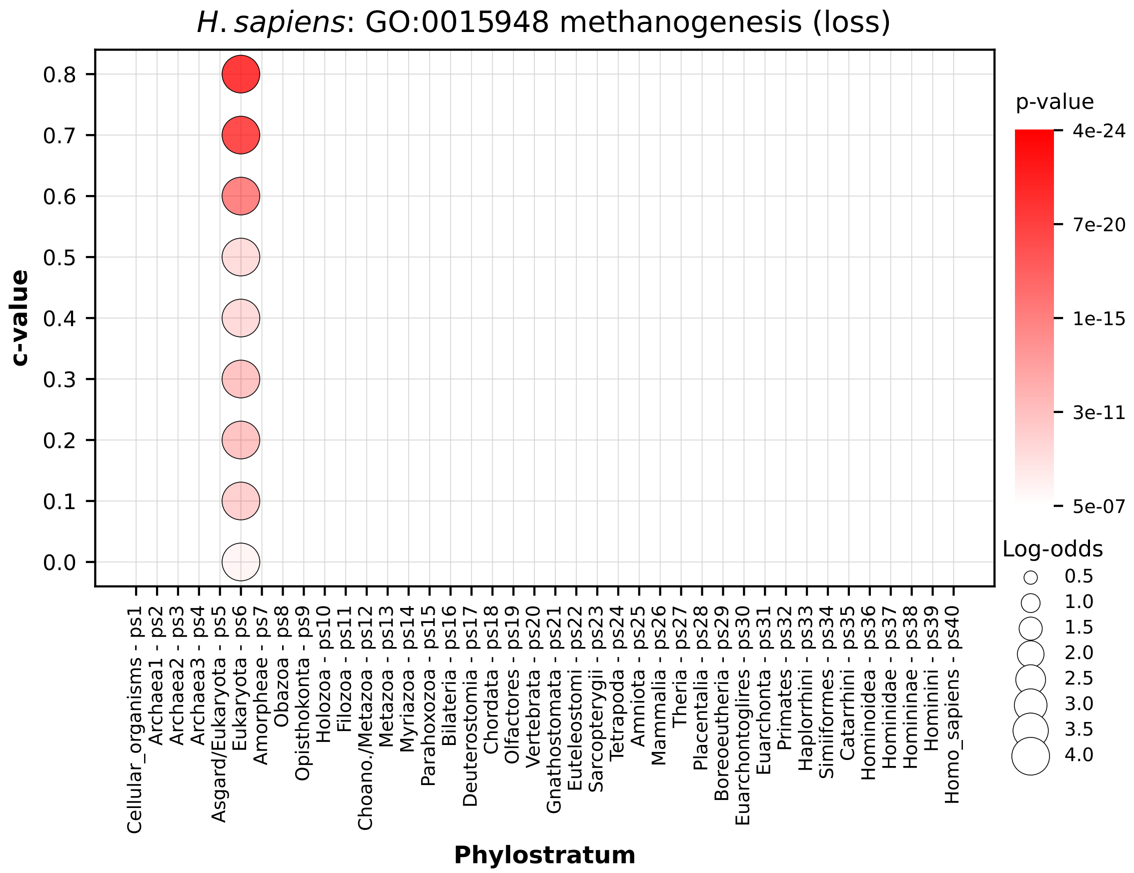

H. sapiens: GO:0015948 methanogenesis (loss)
c-value
0.8
0.7
0.6
0.5
0.4
0.3
0.2
0.1
0.0
p-value
4e-24
7e-20
1e-15
3e-11
5e-07
Log-odds
0.5
1.0
1.5
2.0
2.5
3.0
3.5
4.0
Cellular_organisms - ps1
Archaea1 - ps2
Archaea2 - ps3
Archaea3 - ps4
Asgard/Eukaryota - ps5
Eukaryota - ps6
Amorpheae - ps7
Obazoa - ps8
Opisthokonta - ps9
Holozoa - ps10
Filozoa - ps11
Choano./Metazoa - ps12
Metazoa - ps13
Myriazoa - ps14
Parahoxozoa - ps15
Bilateria - ps16
Deuterostomia - ps17
Chordata - ps18
Olfactores - ps19
Vertebrata - ps20
Gnathostomata - ps21
Euteleostomi - ps22
Sarcopterygii - ps23
Tetrapoda - ps24
Amniota - ps25
Mammalia - ps26
Theria - ps27
Placentalia - ps28
Boreoeutheria - ps29
Euarchontoglires - ps30
Euarchonta - ps31
Primates - ps32
Haplorrhini - ps33
Simiiformes - ps34
Catarrhini - ps35
Hominoidea - ps36
Hominidae - ps37
Homininae - ps38
Hominini - ps39
Homo_sapiens - ps40
Phylostratum

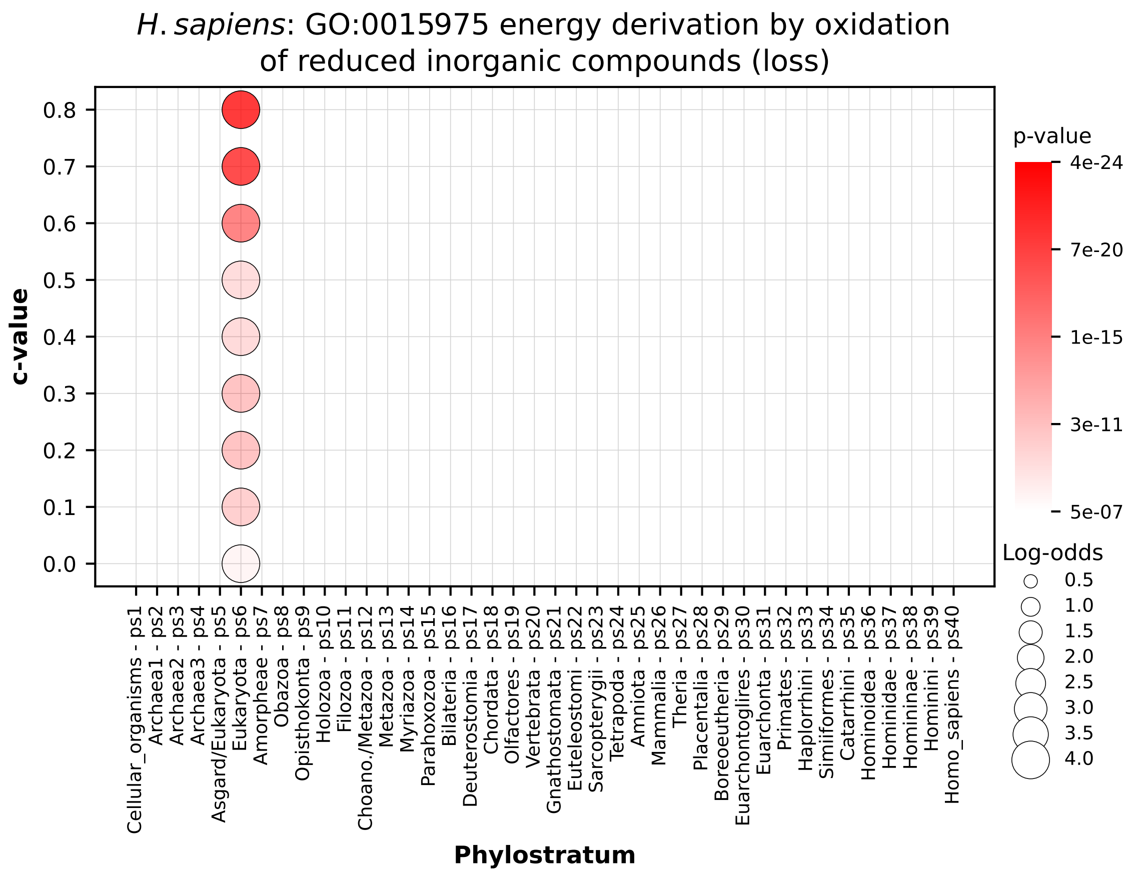

H. sapiens: GO:0015975 energy derivation by oxidation of reduced inorganic compounds (loss)
c-value
0.0
0.1
0.2
0.3
0.4
0.5
0.6
0.7
0.8
Phylostratum
Cellular_organisms - ps1
Archaea1 - ps2
Archaea2 - ps3
Archaea3 - ps4
Asgard/Eukaryota - ps5
Eukaryota - ps6
Amorpheae - ps7
Obazoa - ps8
Opisthokonta - ps9
Holozoa - ps10
Filozoa - ps11
Choano./Metazoa - ps12
Metazoa - ps13
Myriazoa - ps14
Parahoxozoa - ps15
Bilateria - ps16
Deuterostomia - ps17
Chordata - ps18
Olfactores - ps19
Vertebrata - ps20
Gnathostomata - ps21
Euteleostomi - ps22
Sarcopterygii - ps23
Tetrapoda - ps24
Amniota - ps25
Mammalia - ps26
Theria - ps27
Placentalia - ps28
Boreoeutheria - ps29
Euarchontoglires - ps30
Euarchonta - ps31
Primates - ps32
Haplorrhini - ps33
Simiiformes - ps34
Catarrhini - ps35
Hominoidea - ps36
Hominidae - ps37
Homininae - ps38
Hominini - ps39
Homo_sapiens - ps40
p-value
4e-24
7e-20
1e-15
3e-11
5e-07
Log-odds
0.5
1.0
1.5
2.0
2.5
3.0
3.5
4.0

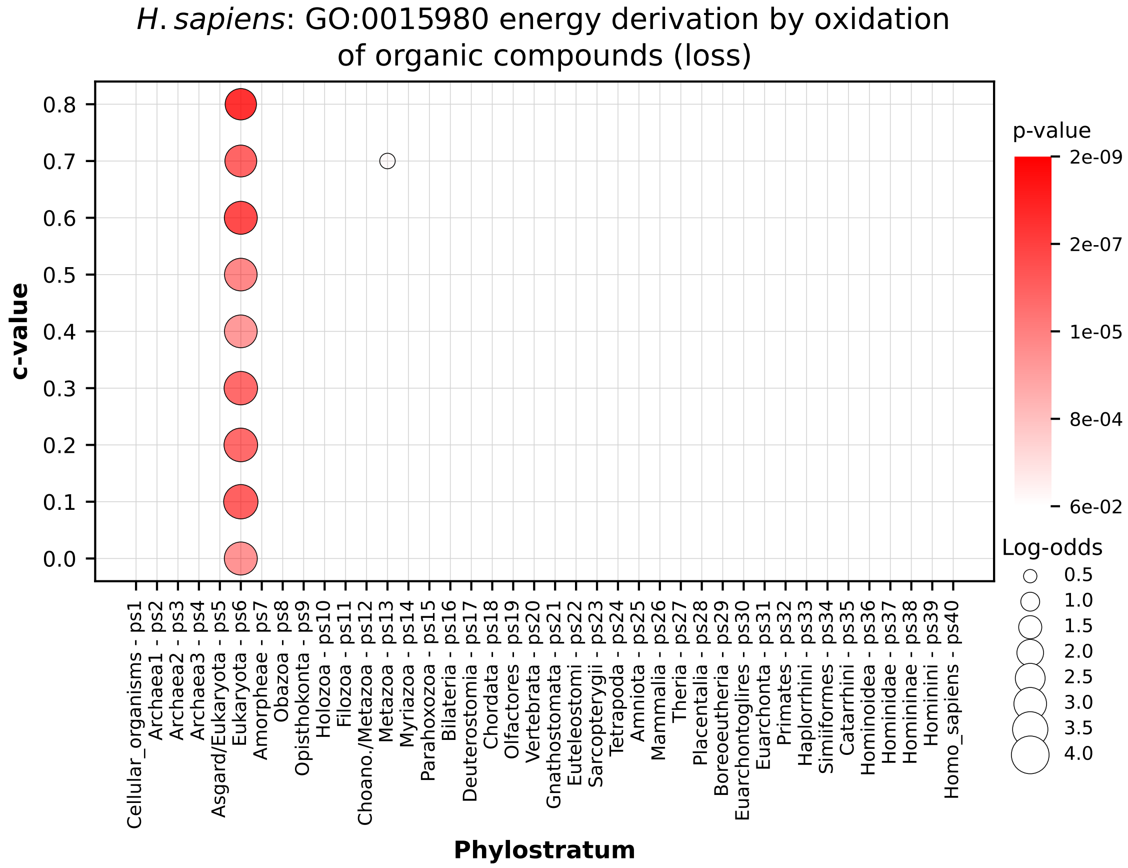

H. sapiens: GO:0015980 energy derivation by oxidation of organic compounds (loss)
c-value
0.8
0.7
0.6
0.5
0.4
0.3
0.2
0.1
0.0
Phylostratum
Cellular_organisms - ps1
Archaea1 - ps2
Archaea2 - ps3
Archaea3 - ps4
Asgard/Eukaryota - ps5
Eukaryota - ps6
Amorpheae - ps7
Obazoa - ps8
Opisthokonta - ps9
Holozoa - ps10
Filozoa - ps11
Choano./Metazoa - ps12
Metazoa - ps13
Myriazoa - ps14
Parahoxozoa - ps15
Bilateria - ps16
Deuterostomia - ps17
Chordata - ps18
Olfactores - ps19
Vertebrata - ps20
Gnathostomata - ps21
Euteleostomi - ps22
Sarcopterygii - ps23
Tetrapoda - ps24
Amniota - ps25
Mammalia - ps26
Theria - ps27
Placentalia - ps28
Boreoeutheria - ps29
Euarchontoglires - ps30
Euarchonta - ps31
Primates - ps32
Haplorrhini - ps33
Simiiformes - ps34
Catarrhini - ps35
Hominoidea - ps36
Hominidae - ps37
Homininae - ps38
Hominini - ps39
Homo_sapiens - ps40
p-value
2e-09
2e-07
1e-05
8e-04
6e-02
Log-odds
0.5
1.0
1.5
2.0
2.5
3.0
3.5
4.0

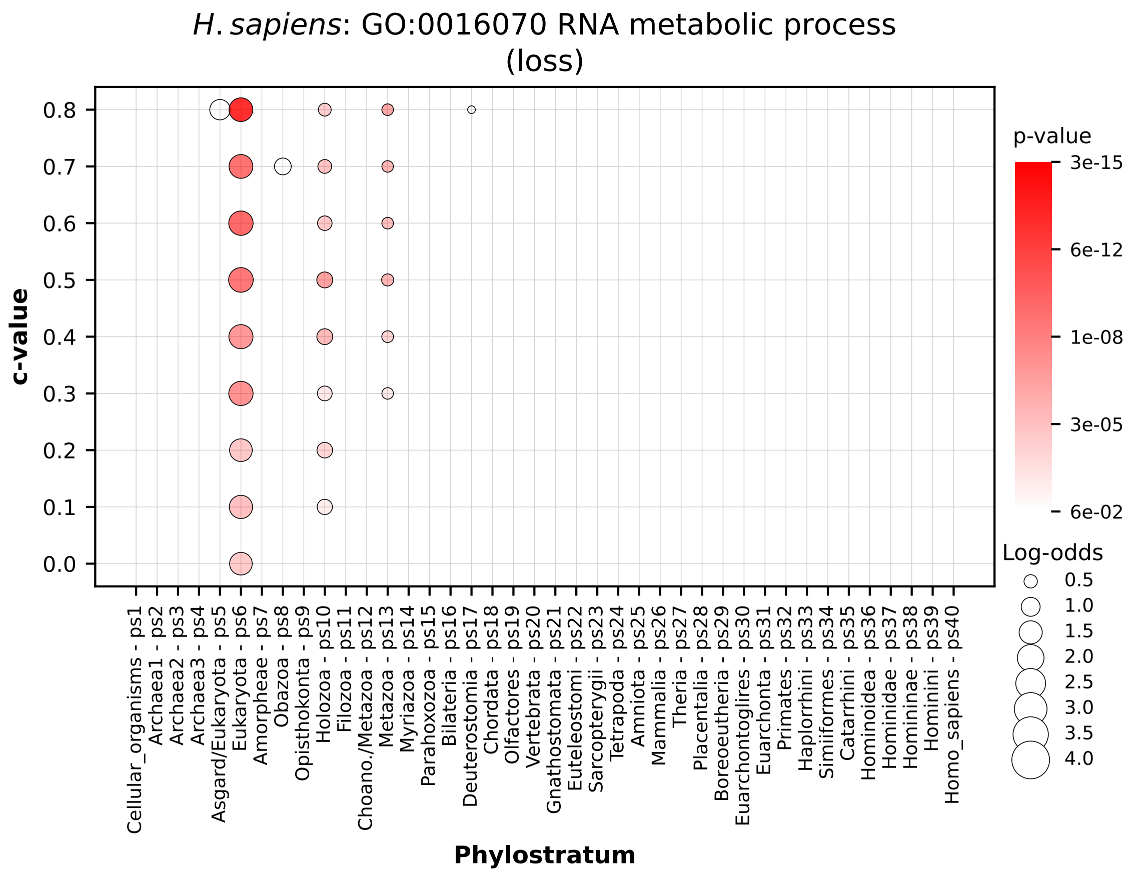

H. sapiens: GO:0016070 RNA metabolic process
(loss)
c-value
0.8
0.7
0.6
0.5
0.4
0.3
0.2
0.1
0.0
p-value
3e-15
6e-12
1e-08
3e-05
6e-02
Log-odds
0.5
1.0
1.5
2.0
2.5
3.0
3.5
4.0
Cellular_organisms - ps1
Archaea1 - ps2
Archaea2 - ps3
Archaea3 - ps4
Asgard/Eukaryota - ps5
Eukaryota - ps6
Amorpheae - ps7
Obazoa - ps8
Opisthokonta - ps9
Holozoa - ps10
Filozoa - ps11
Choano./Metazoa - ps12
Metazoa - ps13
Myriazoa - ps14
Parahoxozoa - ps15
Bilateria - ps16
Deuterostomia - ps17
Chordata - ps18
Olfactores - ps19
Vertebrata - ps20
Gnathostomata - ps21
Euteleostomi - ps22
Sarcopterygii - ps23
Tetrapoda - ps24
Amniota - ps25
Mammalia - ps26
Theria - ps27
Placentalia - ps28
Boreoeutheria - ps29
Euarchontoglires - ps30
Euarchonta - ps31
Primates - ps32
Haplorrhini - ps33
Simiiformes - ps34
Catarrhini - ps35
Hominoidea - ps36
Hominidae - ps37
Homininae - ps38
Hominini - ps39
Homo_sapiens - ps40
Phylostratum

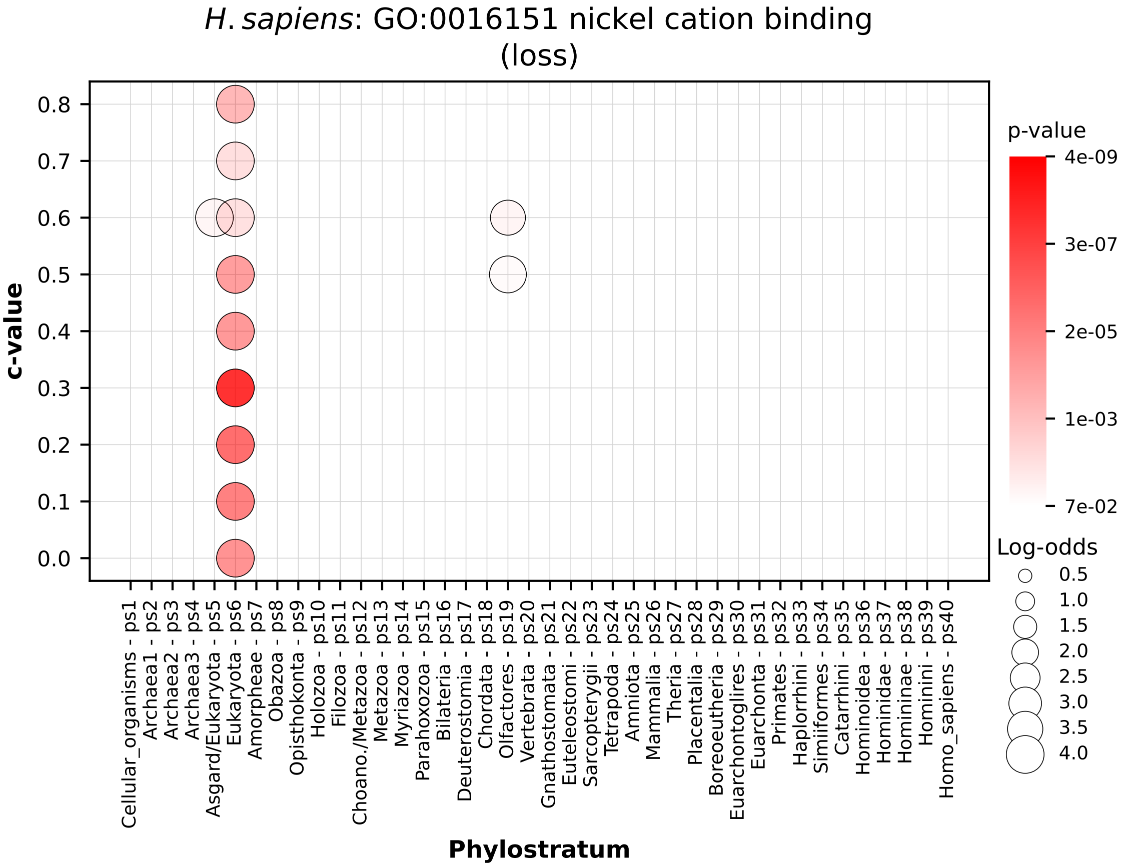
H. sapiens: GO:0016151 nickel cation binding
(loss)
c-value
0.8
0.7
0.6
0.5
0.4
0.3
0.2
0.1
0.0
Phylostratum
Cellular_organisms - ps1
Archaea1 - ps2
Archaea2 - ps3
Archaea3 - ps4
Asgard/Eukaryota - ps5
Eukaryota - ps6
Amorpheae - ps7
Obazoa - ps8
Opisthokonta - ps9
Holozoa - ps10
Filozoa - ps11
Choano./Metazoa - ps12
Metazoa - ps13
Myriazoa - ps14
Parahoxozoa - ps15
Bilateria - ps16
Deuterostomia - ps17
Chordata - ps18
Olfactores - ps19
Vertebrata - ps20
Gnathostomata - ps21
Euteleostomi - ps22
Sarcopterygii - ps23
Tetrapoda - ps24
Amniota - ps25
Mammalia - ps26
Theria - ps27
Placentalia - ps28
Boreoeutheria - ps29
Euarchontoglires - ps30
Euarchonta - ps31
Primates - ps32
Haplorrhini - ps33
Simiiformes - ps34
Catarrhini - ps35
Hominoidea - ps36
Hominidae - ps37
Homininae - ps38
Hominini - ps39
Homo_sapiens - ps40
p-value
4e-09
3e-07
2e-05
1e-03
7e-02
Log-odds
0.5
1.0
1.5
2.0
2.5
3.0
3.5
4.0

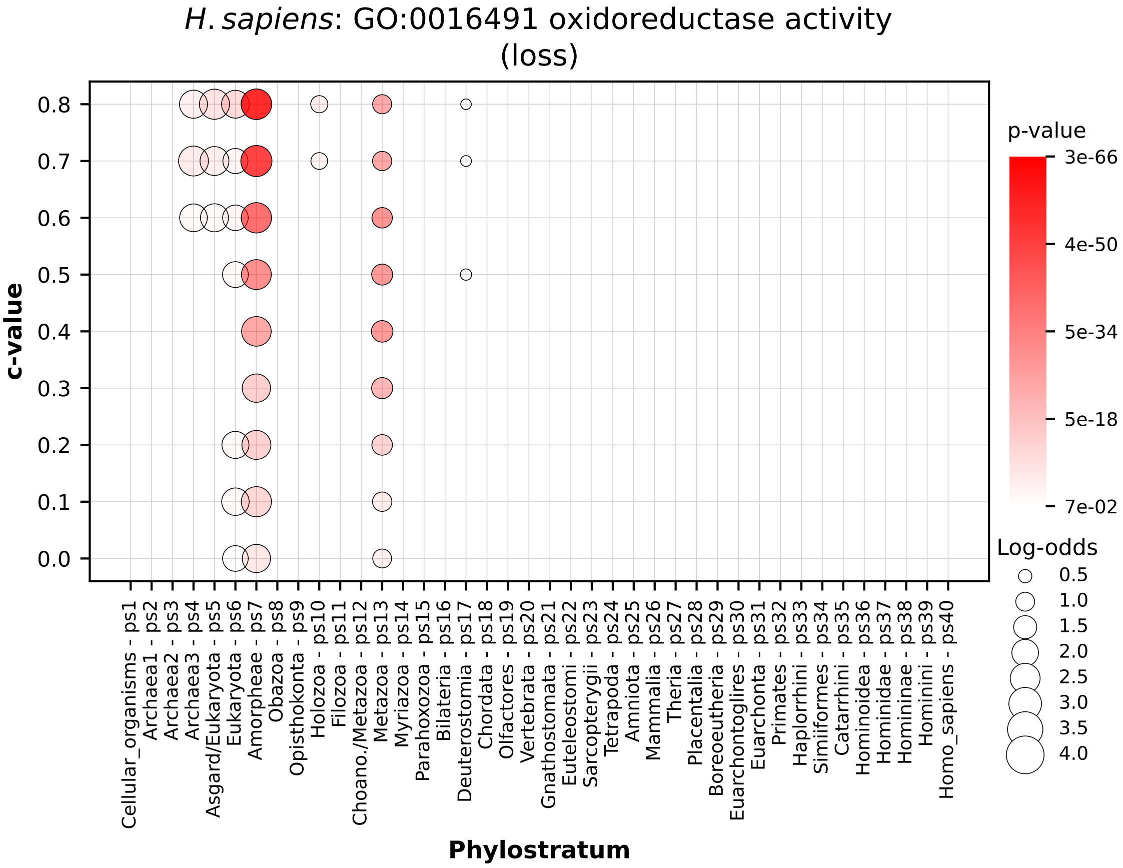
H. sapiens: GO:0016491 oxidoreductase activity
(loss)
c-value
0.8
0.7
0.6
0.5
0.4
0.3
0.2
0.1
0.0
p-value
3e-66
4e-50
5e-34
5e-18
7e-02
Log-odds
0.5
1.0
1.5
2.0
2.5
3.0
3.5
4.0
Cellular_organisms - ps1
Archaea1 - ps2
Archaea2 - ps3
Archaea3 - ps4
Asgard/Eukaryota - ps5
Eukaryota - ps6
Amorpheae - ps7
Obazoa - ps8
Opisthokonta - ps9
Holozoa - ps10
Filozoa - ps11
Choano./Metazoa - ps12
Metazoa - ps13
Myriazoa - ps14
Parahoxozoa - ps15
Bilateria - ps16
Deuterostomia - ps17
Chordata - ps18
Olfactores - ps19
Vertebrata - ps20
Gnathostomata - ps21
Euteleostomi - ps22
Sarcopterygii - ps23
Tetrapoda - ps24
Amniota - ps25
Mammalia - ps26
Theria - ps27
Placentalia - ps28
Boreoeutheria - ps29
Euarchontoglires - ps30
Euarchonta - ps31
Primates - ps32
Haplorrhini - ps33
Simiiformes - ps34
Catarrhini - ps35
Hominoidea - ps36
Hominidae - ps37
Homininae - ps38
Hominini - ps39
Homo_sapiens - ps40
Phylostratum

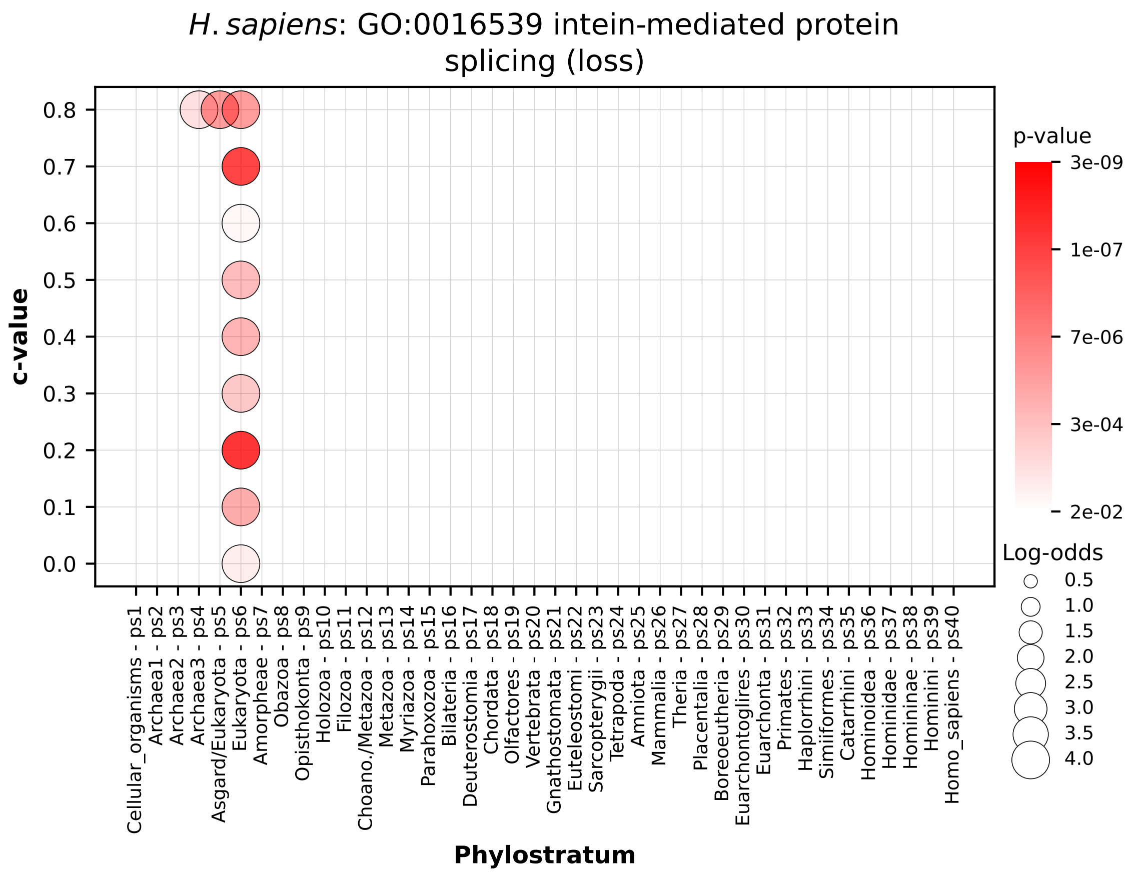

H. sapiens: GO:0016539 intein-mediated protein splicing (loss)
c-value
0.8
0.7
0.6
0.5
0.4
0.3
0.2
0.1
0.0
p-value
3e-09
1e-07
7e-06
3e-04
2e-02
Log-odds
0.5
1.0
1.5
2.0
2.5
3.0
3.5
4.0
Cellular_organisms - ps1
Archaea1 - ps2
Archaea2 - ps3
Archaea3 - ps4
Asgard/Eukaryota - ps5
Eukaryota - ps6
Amorpheae - ps7
Obazoa - ps8
Opisthokonta - ps9
Holozoa - ps10
Filozoa - ps11
Choano./Metazoa - ps12
Metazoa - ps13
Myriazoa - ps14
Parahoxozoa - ps15
Bilateria - ps16
Deuterostomia - ps17
Chordata - ps18
Olfactores - ps19
Vertebrata - ps20
Gnathostomata - ps21
Euteleostomi - ps22
Sarcopterygii - ps23
Tetrapoda - ps24
Amniota - ps25
Mammalia - ps26
Theria - ps27
Placentalia - ps28
Boreoeutheria - ps29
Euarchontoglires - ps30
Euarchonta - ps31
Primates - ps32
Haplorrhini - ps33
Simiiformes - ps34
Catarrhini - ps35
Hominoidea - ps36
Hominidae - ps37
Homininae - ps38
Hominini - ps39
Homo_sapiens - ps40
Phylostratum

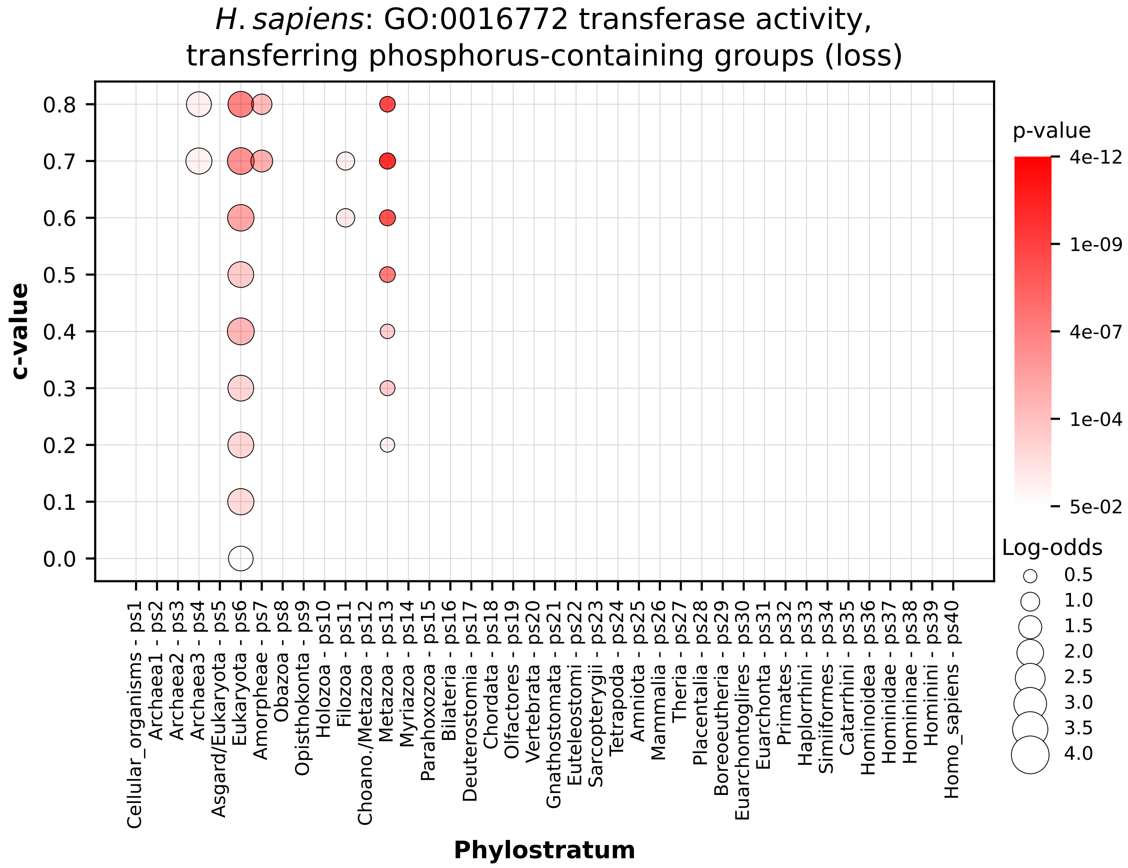
H. sapiens: GO:0016772 transferase activity, transferring phosphorus-containing groups (loss)
c-value
0.8
0.7
0.6
0.5
0.4
0.3
0.2
0.1
0.0
Phylostratum
Cellular_organisms - ps1
Archaea1 - ps2
Archaea2 - ps3
Archaea3 - ps4
Asgard/Eukaryota - ps5
Eukaryota - ps6
Amorpheae - ps7
Obazoa - ps8
Opisthokonta - ps9
Holozoa - ps10
Filozoa - ps11
Choano./Metazoa - ps12
Metazoa - ps13
Myriazoa - ps14
Parahoxozoa - ps15
Bilateria - ps16
Deuterostomia - ps17
Chordata - ps18
Olfactores - ps19
Vertebrata - ps20
Gnathostomata - ps21
Euteleostomi - ps22
Sarcopterygii - ps23
Tetrapoda - ps24
Amniota - ps25
Mammalia - ps26
Theria - ps27
Placentalia - ps28
Boreoeutheria - ps29
Euarchontoglires - ps30
Euarchonta - ps31
Primates - ps32
Haplorrhini - ps33
Simiiformes - ps34
Catarrhini - ps35
Hominoidea - ps36
Hominidae - ps37
Homininae - ps38
Hominini - ps39
Homo_sapiens - ps40
p-value
4e-12
1e-09
4e-07
1e-04
5e-02
Log-odds
0.5
1.0
1.5
2.0
2.5
3.0
3.5
4.0

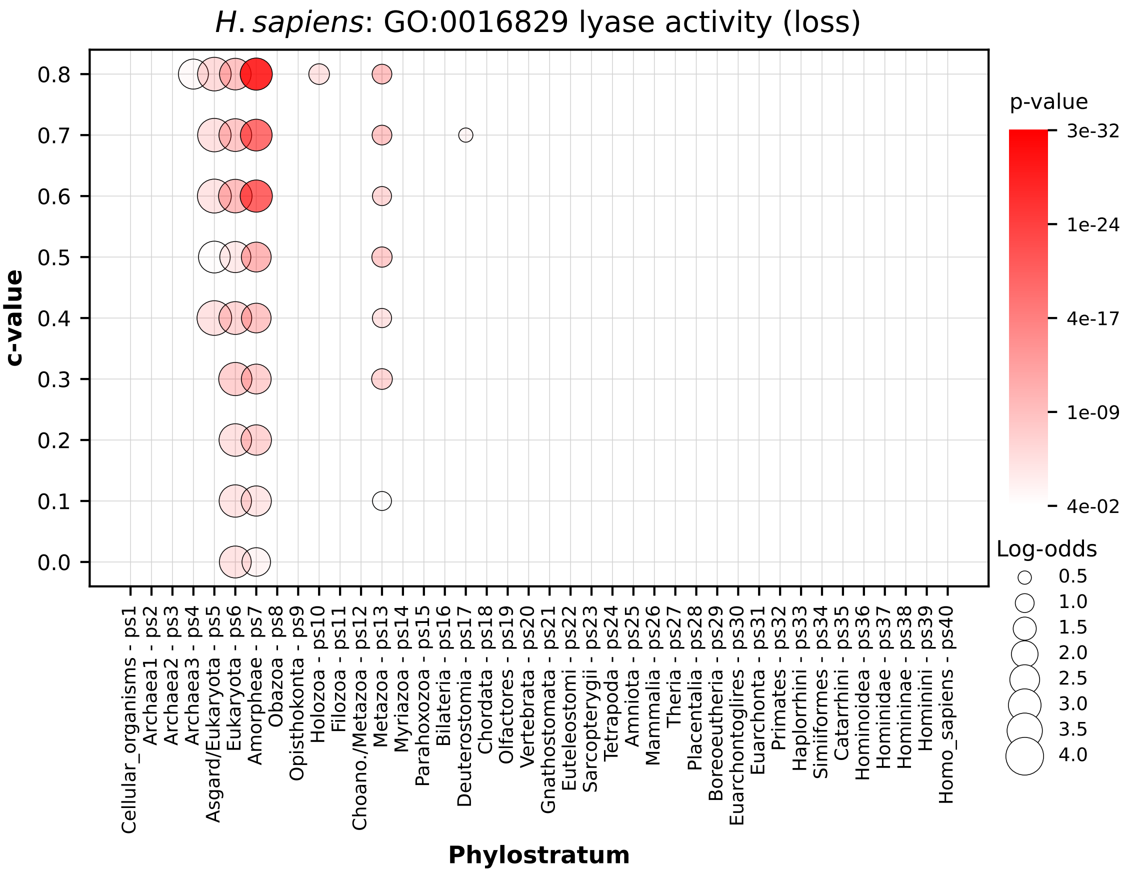

H. sapiens: GO:0016829 lyase activity (loss)
c-value
0.8
0.7
0.6
0.5
0.4
0.3
0.2
0.1
0.0
Phylostratum
Cellular_organisms - ps1
Archaea1 - ps2
Archaea2 - ps3
Archaea3 - ps4
Asgard/Eukaryota - ps5
Eukaryota - ps6
Amorpheae - ps7
Obazoa - ps8
Opisthokonta - ps9
Holozoa - ps10
Filozoa - ps11
Choano./Metazoa - ps12
Metazoa - ps13
Myriazoa - ps14
Parahoxozoa - ps15
Bilateria - ps16
Deuterostomia - ps17
Chordata - ps18
Olfactores - ps19
Vertebrata - ps20
Gnathostomata - ps21
Euteleostomi - ps22
Sarcopterygii - ps23
Tetrapoda - ps24
Amniota - ps25
Mammalia - ps26
Theria - ps27
Placentalia - ps28
Boreoeutheria - ps29
Euarchontoglires - ps30
Euarchonta - ps31
Primates - ps32
Haplorrhini - ps33
Simiiformes - ps34
Catarrhini - ps35
Hominoidea - ps36
Hominidae - ps37
Homininae - ps38
Hominini - ps39
Homo_sapiens - ps40
p-value
3e-32
1e-24
4e-17
1e-09
4e-02
Log-odds
0.5
1.0
1.5
2.0
2.5
3.0
3.5
4.0

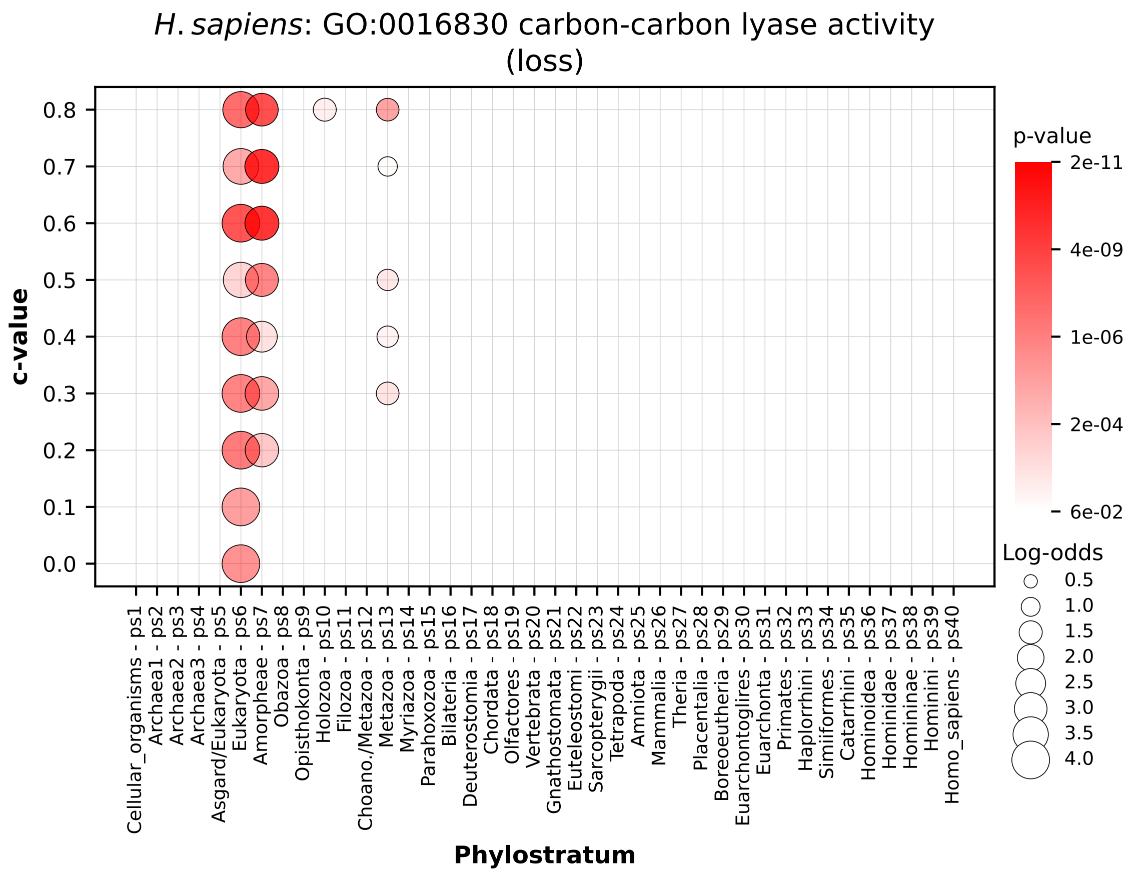

H. sapiens: GO:0016830 carbon-carbon lyase activity
(loss)
c-value
0.8
0.7
0.6
0.5
0.4
0.3
0.2
0.1
0.0
Phylostratum
Cellular_organisms - ps1
Archaea1 - ps2
Archaea2 - ps3
Archaea3 - ps4
Asgard/Eukaryota - ps5
Eukaryota - ps6
Amorpheae - ps7
Obazoa - ps8
Opisthokonta - ps9
Holozoa - ps10
Filozoa - ps11
Choano./Metazoa - ps12
Metazoa - ps13
Myriazoa - ps14
Parahoxozoa - ps15
Bilateria - ps16
Deuterostomia - ps17
Chordata - ps18
Olfactores - ps19
Vertebrata - ps20
Gnathostomata - ps21
Euteleostomi - ps22
Sarcopterygii - ps23
Tetrapoda - ps24
Amniota - ps25
Mammalia - ps26
Theria - ps27
Placentalia - ps28
Boreoeutheria - ps29
Euarchontoglires - ps30
Euarchonta - ps31
Primates - ps32
Haplorrhini - ps33
Simiiformes - ps34
Catarrhini - ps35
Hominoidea - ps36
Hominidae - ps37
Homininae - ps38
Hominini - ps39
Homo_sapiens - ps40
p-value
2e-11
4e-09
1e-06
2e-04
6e-02
Log-odds
0.5
1.0
1.5
2.0
2.5
3.0
3.5
4.0

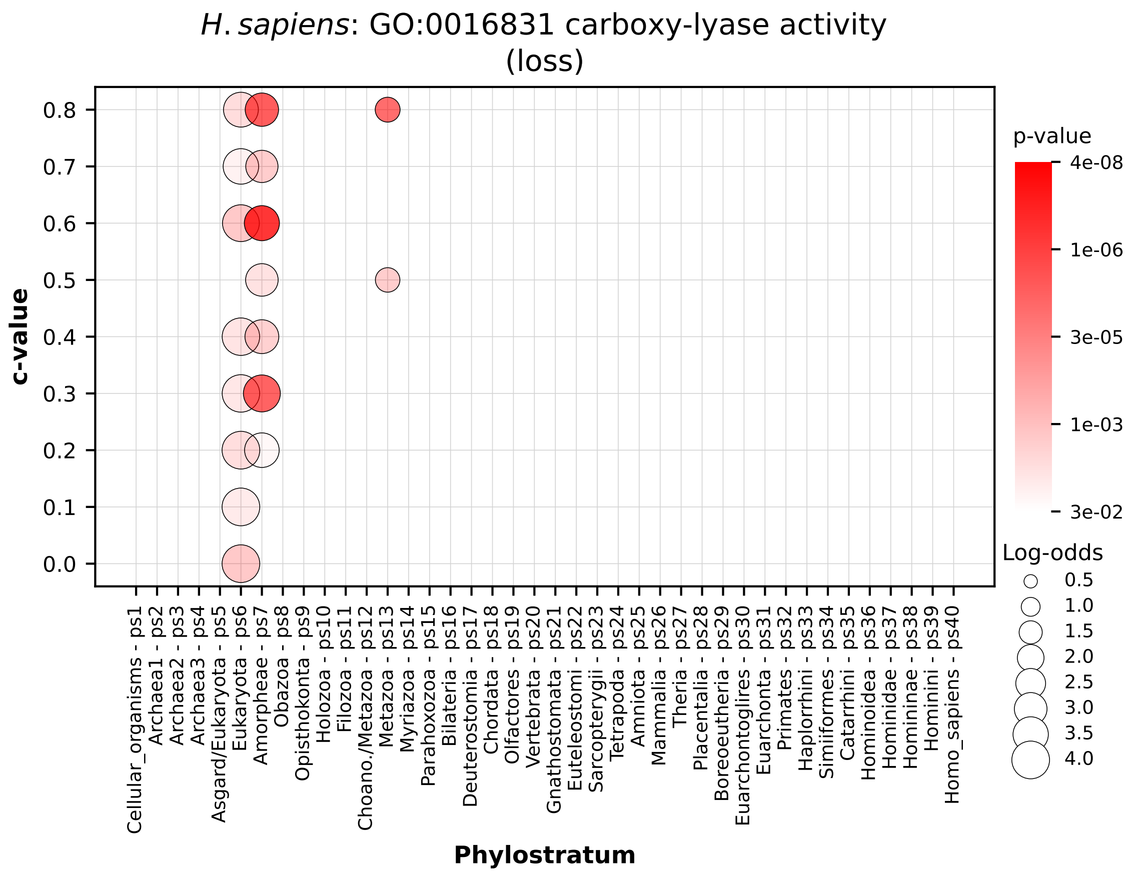

H. sapiens: GO:0016831 carboxy-lyase activity
(loss)
c-value
0.8
0.7
0.6
0.5
0.4
0.3
0.2
0.1
0.0
p-value
4e-08
1e-06
3e-05
1e-03
3e-02
Log-odds
0.5
1.0
1.5
2.0
2.5
3.0
3.5
4.0
Cellular_organisms - ps1
Archaea1 - ps2
Archaea2 - ps3
Archaea3 - ps4
Asgard/Eukaryota - ps5
Eukaryota - ps6
Amorpheae - ps7
Obazoa - ps8
Opisthokonta - ps9
Holozoa - ps10
Filozoa - ps11
Choano./Metazoa - ps12
Metazoa - ps13
Myriazoa - ps14
Parahoxozoa - ps15
Bilateria - ps16
Deuterostomia - ps17
Chordata - ps18
Olfactores - ps19
Vertebrata - ps20
Gnathostomata - ps21
Euteleostomi - ps22
Sarcopterygii - ps23
Tetrapoda - ps24
Amniota - ps25
Mammalia - ps26
Theria - ps27
Placentalia - ps28
Boreoeutheria - ps29
Euarchontoglires - ps30
Euarchonta - ps31
Primates - ps32
Haplorrhini - ps33
Simiiformes - ps34
Catarrhini - ps35
Hominoidea - ps36
Hominidae - ps37
Homininae - ps38
Hominini - ps39
Homo_sapiens - ps40
Phylostratum

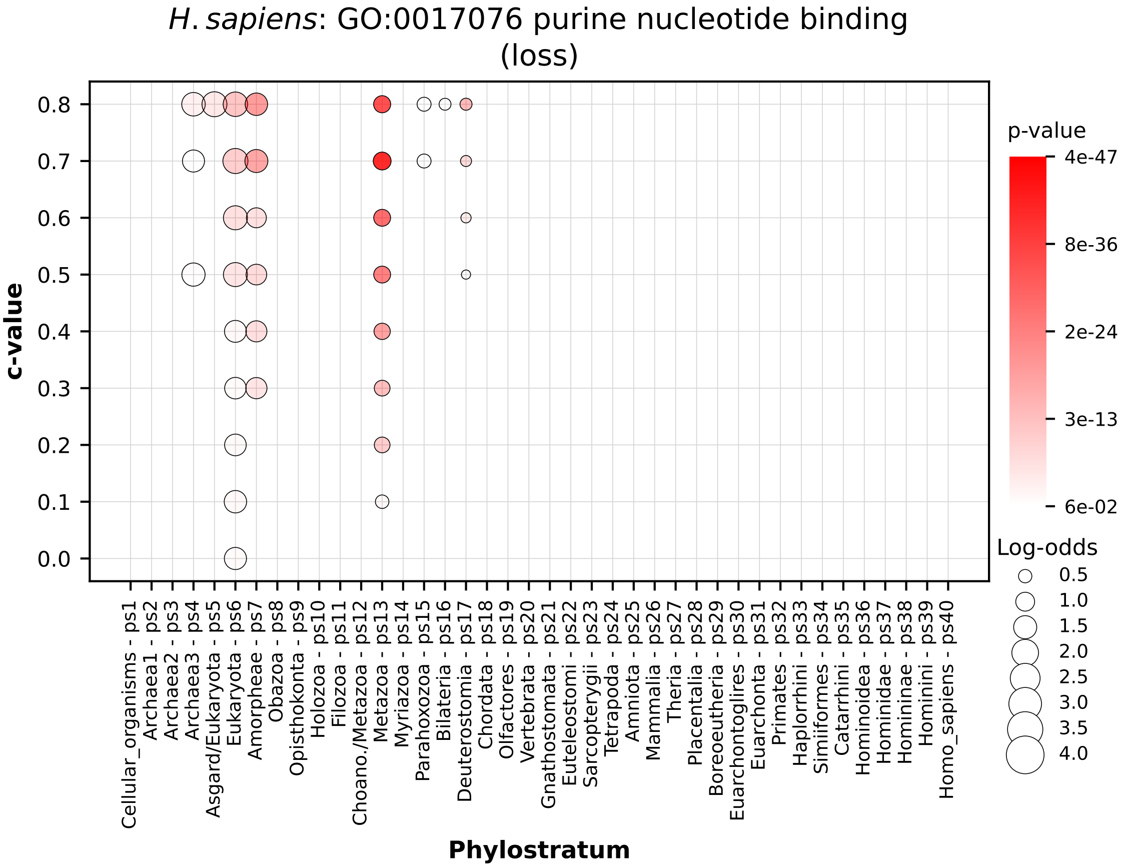

H. sapiens: GO:0017076 purine nucleotide binding
(loss)
c-value
0.0
0.1
0.2
0.3
0.4
0.5
0.6
0.7
0.8
Phylostratum
Cellular_organisms - ps1
Archaea1 - ps2
Archaea2 - ps3
Archaea3 - ps4
Asgard/Eukaryota - ps5
Eukaryota - ps6
Amorpheae - ps7
Obazoa - ps8
Opisthokonta - ps9
Holozoa - ps10
Filozoa - ps11
Choano./Metazoa - ps12
Metazoa - ps13
Myriazoa - ps14
Parahoxozoa - ps15
Bilateria - ps16
Deuterostomia - ps17
Chordata - ps18
Olfactores - ps19
Vertebrata - ps20
Gnathostomata - ps21
Euteleostomi - ps22
Sarcopterygii - ps23
Tetrapoda - ps24
Amniota - ps25
Mammalia - ps26
Theria - ps27
Placentalia - ps28
Boreoeutheria - ps29
Euarchontoglires - ps30
Euarchonta - ps31
Primates - ps32
Haplorrhini - ps33
Simiiformes - ps34
Catarrhini - ps35
Hominoidea - ps36
Hominidae - ps37
Homininae - ps38
Hominini - ps39
Homo_sapiens - ps40
p-value
4e-47
8e-36
2e-24
3e-13
6e-02
Log-odds
0.5
1.0
1.5
2.0
2.5
3.0
3.5
4.0

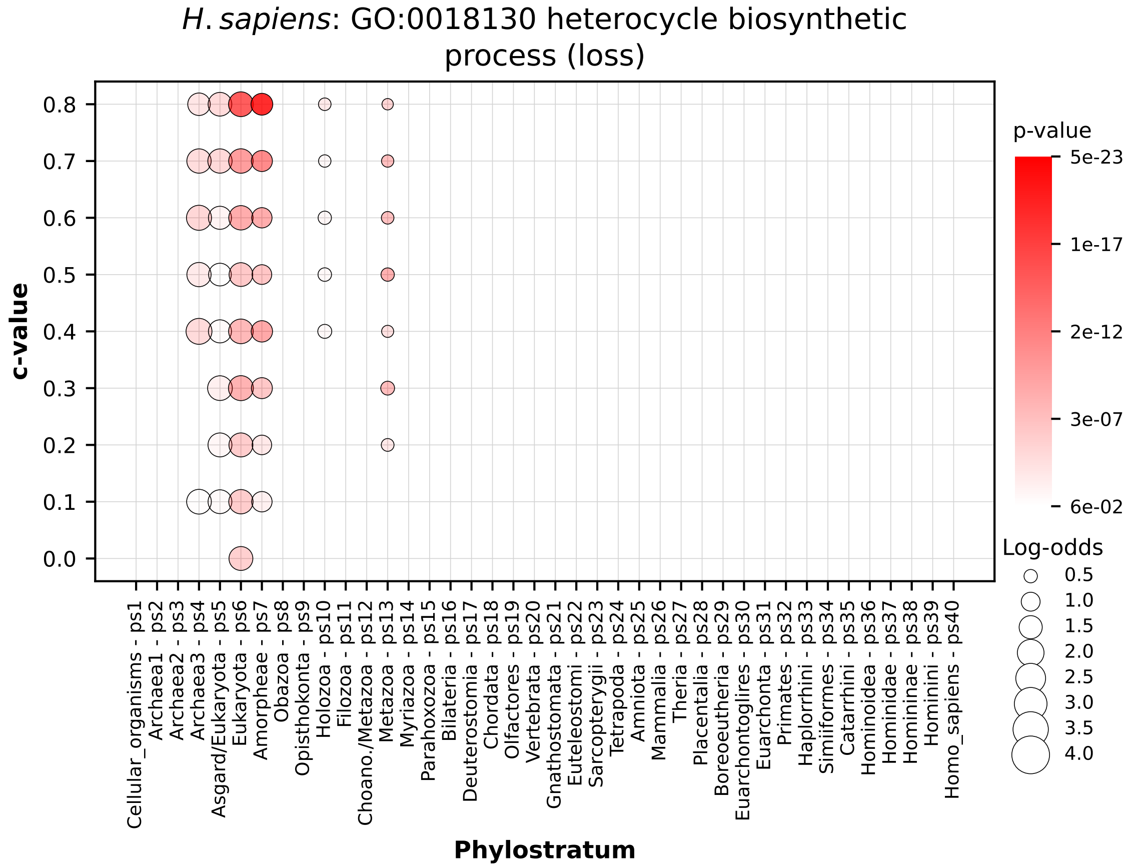

H. sapiens: GO:0018130 heterocycle biosynthetic process (loss)
c-value
0.8
0.7
0.6
0.5
0.4
0.3
0.2
0.1
0.0
Phylostratum
Cellular_organisms - ps1
Archaea1 - ps2
Archaea2 - ps3
Archaea3 - ps4
Asgard/Eukaryota - ps5
Eukaryota - ps6
Amorpheae - ps7
Obazoa - ps8
Opisthokonta - ps9
Holozoa - ps10
Filozoa - ps11
Choano./Metazoa - ps12
Metazoa - ps13
Myriazoa - ps14
Parahoxozoa - ps15
Bilateria - ps16
Deuterostomia - ps17
Chordata - ps18
Olfactores - ps19
Vertebrata - ps20
Gnathostomata - ps21
Euteleostomi - ps22
Sarcopterygii - ps23
Tetrapoda - ps24
Amniota - ps25
Mammalia - ps26
Theria - ps27
Placentalia - ps28
Boreoeutheria - ps29
Euarchontoglires - ps30
Euarchonta - ps31
Primates - ps32
Haplorrhini - ps33
Simiiformes - ps34
Catarrhini - ps35
Hominoidea - ps36
Hominidae - ps37
Homininae - ps38
Hominini - ps39
Homo_sapiens - ps40
p-value
5e-23
1e-17
2e-12
3e-07
6e-02
Log-odds
0.5
1.0
1.5
2.0
2.5
3.0
3.5
4.0

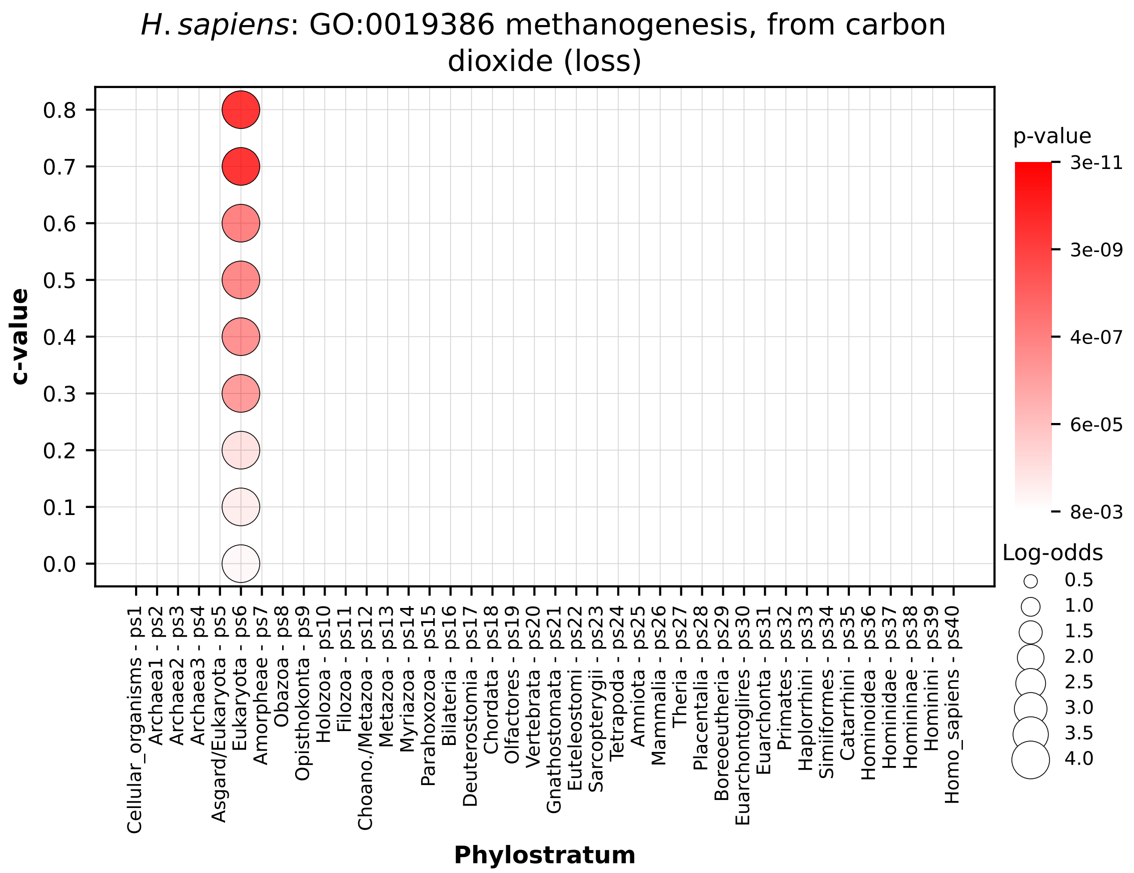

H. sapiens: GO:0019386 methanogenesis, from carbon dioxide (loss)
c-value
0.0
0.1
0.2
0.3
0.4
0.5
0.6
0.7
0.8
p-value
3e-11
3e-09
4e-07
6e-05
8e-03
Log-odds
0.5
1.0
1.5
2.0
2.5
3.0
3.5
4.0
Cellular_organisms - ps1
Archaea1 - ps2
Archaea2 - ps3
Archaea3 - ps4
Asgard/Eukaryota - ps5
Eukaryota - ps6
Amorpheae - ps7
Obazoa - ps8
Opisthokonta - ps9
Holozoa - ps10
Filozoa - ps11
Choano./Metazoa - ps12
Metazoa - ps13
Myriazoa - ps14
Parahoxozoa - ps15
Bilateria - ps16
Deuterostomia - ps17
Chordata - ps18
Olfactores - ps19
Vertebrata - ps20
Gnathostomata - ps21
Euteleostomi - ps22
Sarcopterygii - ps23
Tetrapoda - ps24
Amniota - ps25
Mammalia - ps26
Theria - ps27
Placentalia - ps28
Boreoeutheria - ps29
Euarchontoglires - ps30
Euarchonta - ps31
Primates - ps32
Haplorrhini - ps33
Simiiformes - ps34
Catarrhini - ps35
Hominoidea - ps36
Hominidae - ps37
Homininae - ps38
Hominini - ps39
Homo_sapiens - ps40
Phylostratum

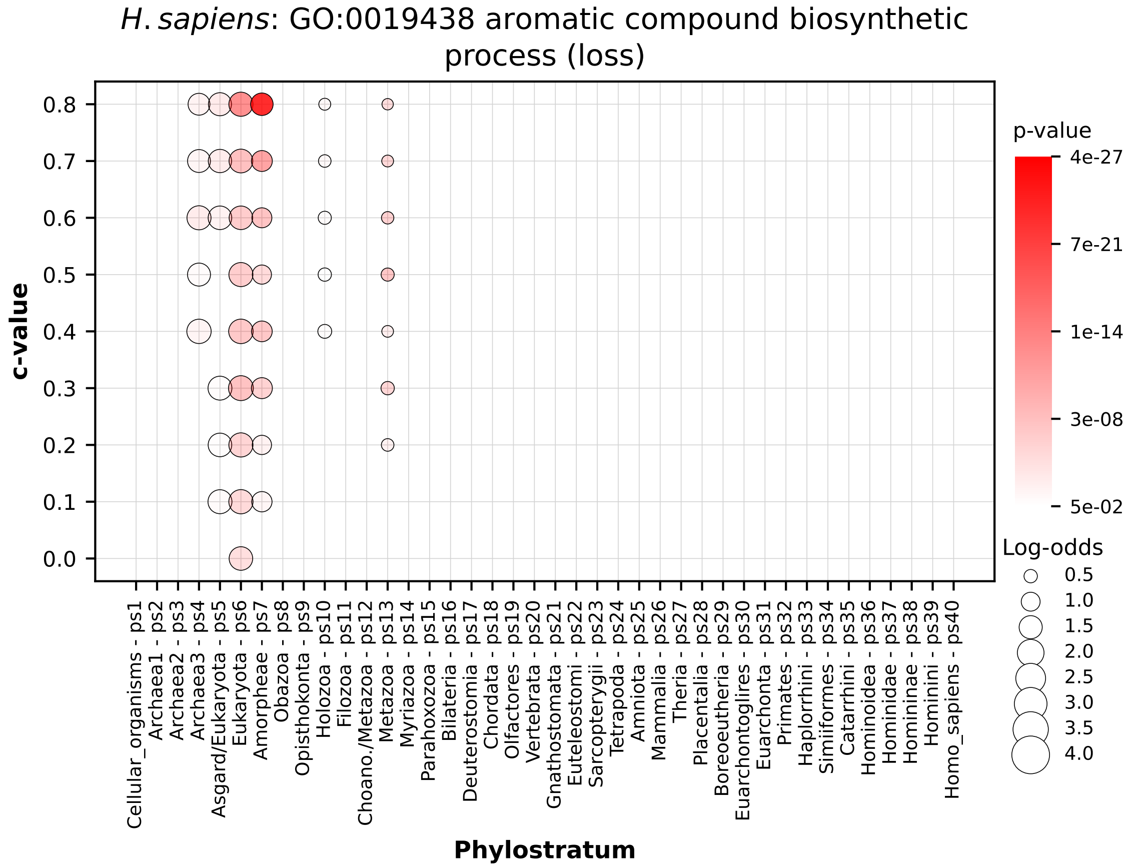
H. sapiens: GO:0019438 aromatic compound biosynthetic process (loss)
c-value
0.8
0.7
0.6
0.5
0.4
0.3
0.2
0.1
0.0
Phylostratum
Cellular_organisms - ps1
Archaea1 - ps2
Archaea2 - ps3
Archaea3 - ps4
Asgard/Eukaryota - ps5
Eukaryota - ps6
Amorpheae - ps7
Obazoa - ps8
Opisthokonta - ps9
Holozoa - ps10
Filozoa - ps11
Choano./Metazoa - ps12
Metazoa - ps13
Myriazoa - ps14
Parahoxozoa - ps15
Bilateria - ps16
Deuterostomia - ps17
Chordata - ps18
Olfactores - ps19
Vertebrata - ps20
Gnathostomata - ps21
Euteleostomi - ps22
Sarcopterygii - ps23
Tetrapoda - ps24
Amniota - ps25
Mammalia - ps26
Theria - ps27
Placentalia - ps28
Boreoeutheria - ps29
Euarchontoglires - ps30
Euarchonta - ps31
Primates - ps32
Haplorrhini - ps33
Simiiformes - ps34
Catarrhini - ps35
Hominoidea - ps36
Hominidae - ps37
Homininae - ps38
Hominini - ps39
Homo_sapiens - ps40
p-value
4e-27
7e-21
1e-14
3e-08
5e-02
Log-odds
0.5
1.0
1.5
2.0
2.5
3.0
3.5
4.0

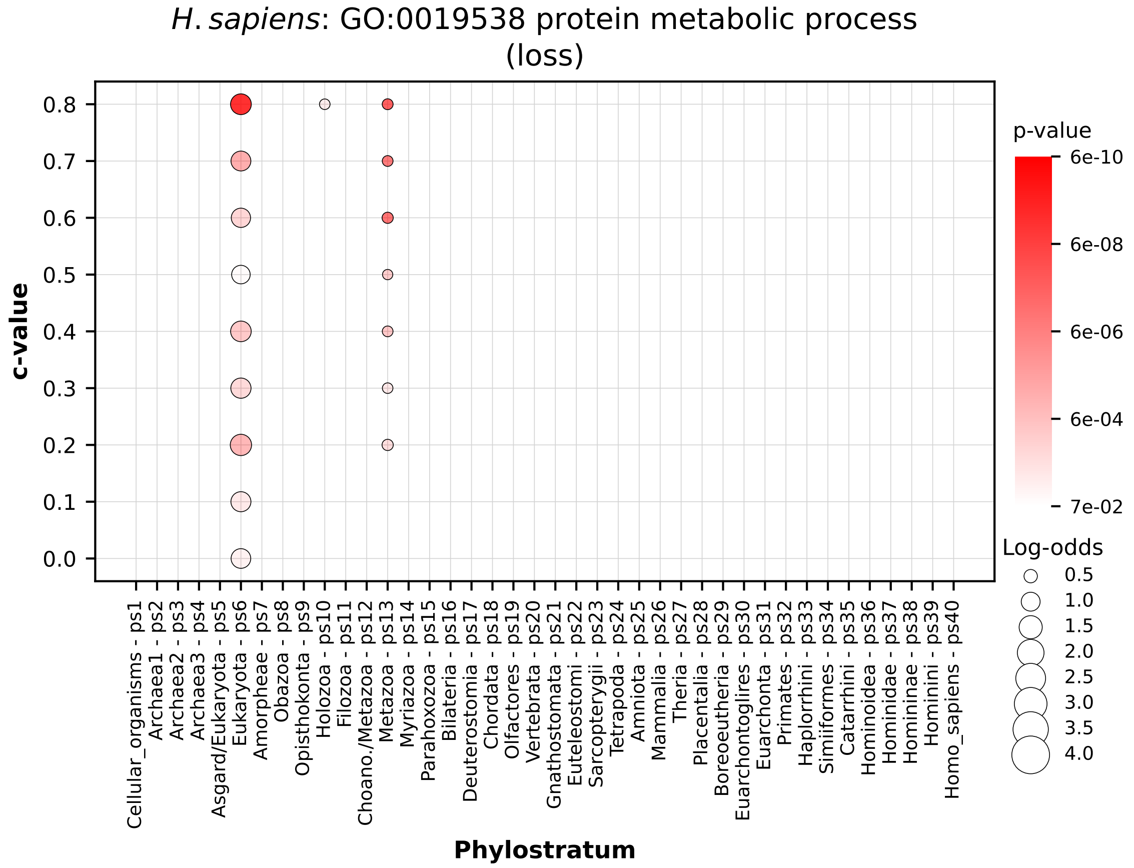

H. sapiens: GO:0019538 protein metabolic process
(loss)
c-value
0.8
0.7
0.6
0.5
0.4
0.3
0.2
0.1
0.0
Phylostratum
Cellular_organisms - ps1
Archaea1 - ps2
Archaea2 - ps3
Archaea3 - ps4
Asgard/Eukaryota - ps5
Eukaryota - ps6
Amorpheae - ps7
Obazoa - ps8
Opisthokonta - ps9
Holozoa - ps10
Filozoa - ps11
Choano./Metazoa - ps12
Metazoa - ps13
Myriazoa - ps14
Parahoxozoa - ps15
Bilateria - ps16
Deuterostomia - ps17
Chordata - ps18
Olfactores - ps19
Vertebrata - ps20
Gnathostomata - ps21
Euteleostomi - ps22
Sarcopterygii - ps23
Tetrapoda - ps24
Amniota - ps25
Mammalia - ps26
Theria - ps27
Placentalia - ps28
Boreoeutheria - ps29
Euarchontoglires - ps30
Euarchonta - ps31
Primates - ps32
Haplorrhini - ps33
Simiiformes - ps34
Catarrhini - ps35
Hominoidea - ps36
Hominidae - ps37
Homininae - ps38
Hominini - ps39
Homo_sapiens - ps40
p-value
6e-10
6e-08
6e-06
6e-04
7e-02
Log-odds
0.5
1.0
1.5
2.0
2.5
3.0
3.5
4.0

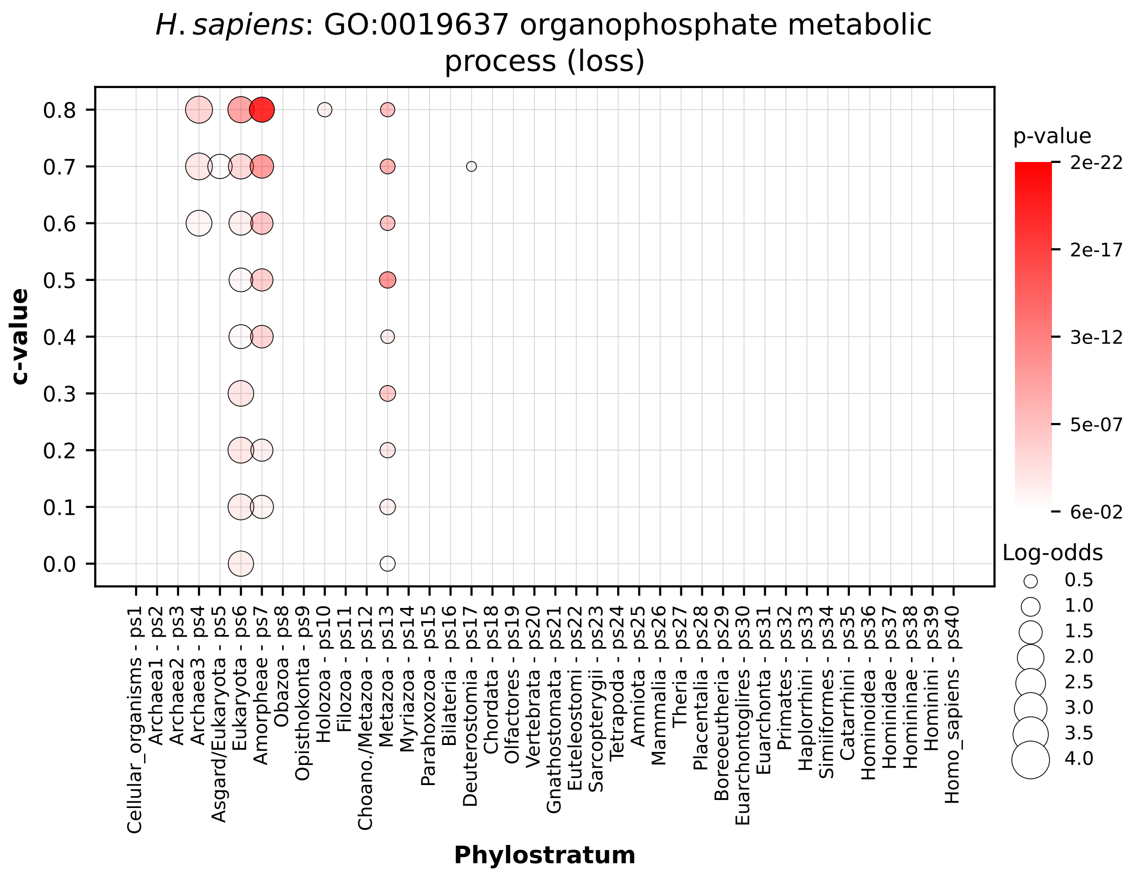

H. sapiens: GO:0019637 organophosphate metabolic process (loss)
c-value
0.8
0.7
0.6
0.5
0.4
0.3
0.2
0.1
0.0
p-value
2e-22
2e-17
3e-12
5e-07
6e-02
Log-odds
0.5
1.0
1.5
2.0
2.5
3.0
3.5
4.0
Cellular_organisms - ps1
Archaea1 - ps2
Archaea2 - ps3
Archaea3 - ps4
Asgard/Eukaryota - ps5
Eukaryota - ps6
Amorpheae - ps7
Obazoa - ps8
Opisthokonta - ps9
Holozoa - ps10
Filozoa - ps11
Choano./Metazoa - ps12
Metazoa - ps13
Myriazoa - ps14
Parahoxozoa - ps15
Bilateria - ps16
Deuterostomia - ps17
Chordata - ps18
Olfactores - ps19
Vertebrata - ps20
Gnathostomata - ps21
Euteleostomi - ps22
Sarcopterygii - ps23
Tetrapoda - ps24
Amniota - ps25
Mammalia - ps26
Theria - ps27
Placentalia - ps28
Boreoeutheria - ps29
Euarchontoglires - ps30
Euarchonta - ps31
Primates - ps32
Haplorrhini - ps33
Simiiformes - ps34
Catarrhini - ps35
Hominoidea - ps36
Hominidae - ps37
Homininae - ps38
Hominini - ps39
Homo_sapiens - ps40
Phylostratum

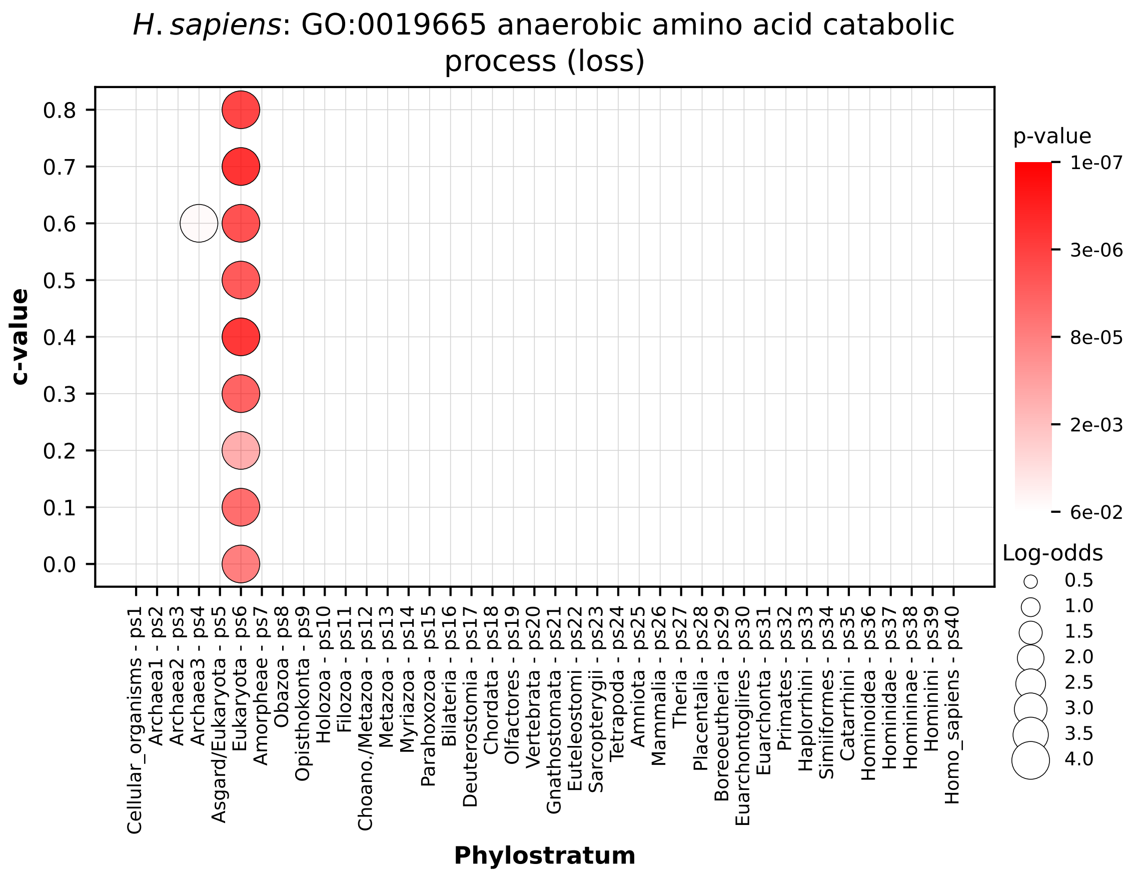

H. sapiens: GO:0019665 anaerobic amino acid catabolic process (loss)
c-value
0.8
0.7
0.6
0.5
0.4
0.3
0.2
0.1
0.0
p-value
1e-07
3e-06
8e-05
2e-03
6e-02
Log-odds
0.5
1.0
1.5
2.0
2.5
3.0
3.5
4.0
Cellular_organisms - ps1
Archaea1 - ps2
Archaea2 - ps3
Archaea3 - ps4
Asgard/Eukaryota - ps5
Eukaryota - ps6
Amorpheae - ps7
Obazoa - ps8
Opisthokonta - ps9
Holozoa - ps10
Filozoa - ps11
Choano./Metazoa - ps12
Metazoa - ps13
Myriazoa - ps14
Parahoxozoa - ps15
Bilateria - ps16
Deuterostomia - ps17
Chordata - ps18
Olfactores - ps19
Vertebrata - ps20
Gnathostomata - ps21
Euteleostomi - ps22
Sarcopterygii - ps23
Tetrapoda - ps24
Amniota - ps25
Mammalia - ps26
Theria - ps27
Placentalia - ps28
Boreoeutheria - ps29
Euarchontoglires - ps30
Euarchonta - ps31
Primates - ps32
Haplorrhini - ps33
Simiiformes - ps34
Catarrhini - ps35
Hominoidea - ps36
Hominidae - ps37
Homininae - ps38
Hominini - ps39
Homo_sapiens - ps40
Phylostratum

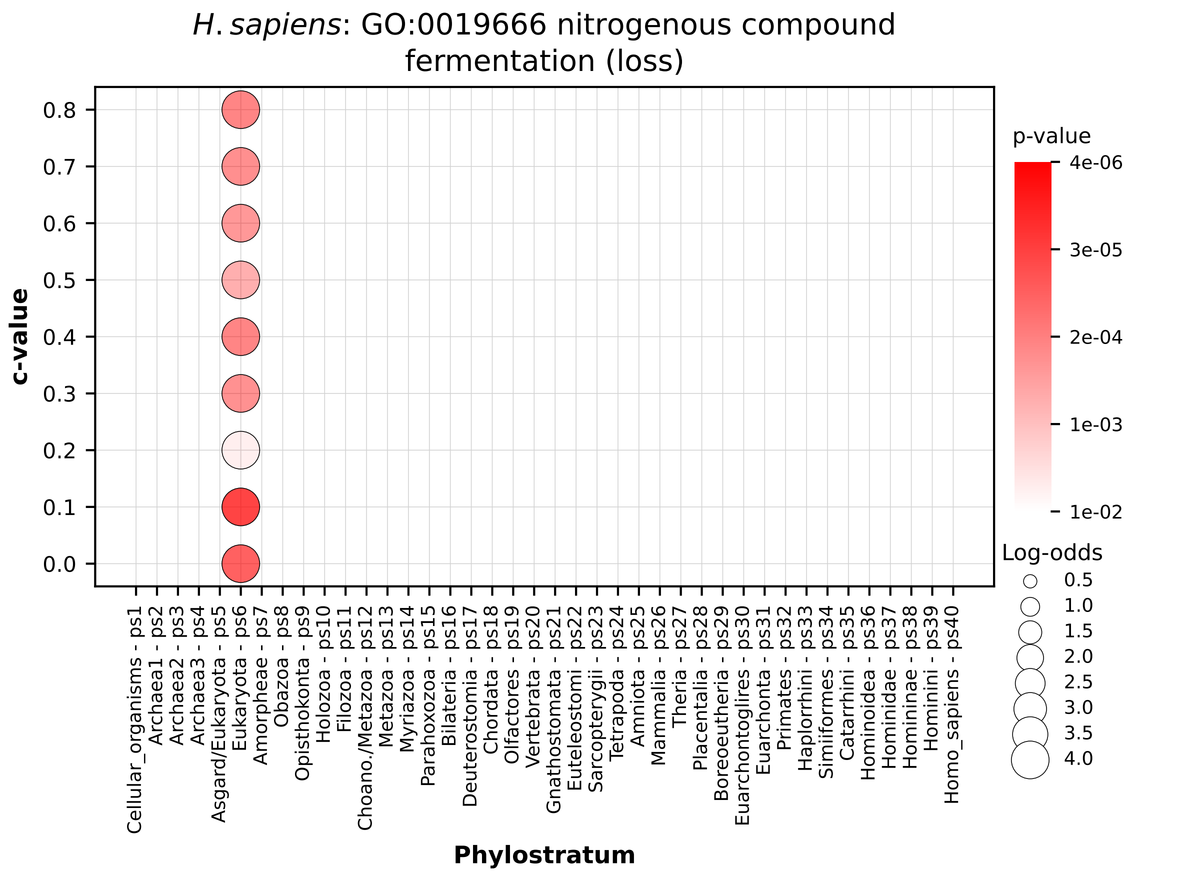
H. sapiens: GO:0019666 nitrogenous compound fermentation (loss)
c-value
0.0
0.1
0.2
0.3
0.4
0.5
0.6
0.7
0.8
Phylostratum
Cellular_organisms - ps1
Archaea1 - ps2
Archaea2 - ps3
Archaea3 - ps4
Asgard/Eukaryota - ps5
Eukaryota - ps6
Amorpheae - ps7
Obazoa - ps8
Opisthokonta - ps9
Holozoa - ps10
Filozoa - ps11
Choano./Metazoa - ps12
Metazoa - ps13
Myriazoa - ps14
Parahoxozoa - ps15
Bilateria - ps16
Deuterostomia - ps17
Chordata - ps18
Olfactores - ps19
Vertebrata - ps20
Gnathostomata - ps21
Euteleostomi - ps22
Sarcopterygii - ps23
Tetrapoda - ps24
Amniota - ps25
Mammalia - ps26
Theria - ps27
Placentalia - ps28
Boreoeutheria - ps29
Euarchontoglires - ps30
Euarchonta - ps31
Primates - ps32
Haplorrhini - ps33
Simiiformes - ps34
Catarrhini - ps35
Hominoidea - ps36
Hominidae - ps37
Homininae - ps38
Hominini - ps39
Homo_sapiens - ps40
p-value
4e-06
3e-05
2e-04
1e-03
1e-02
Log-odds
0.5
1.0
1.5
2.0
2.5
3.0
3.5
4.0

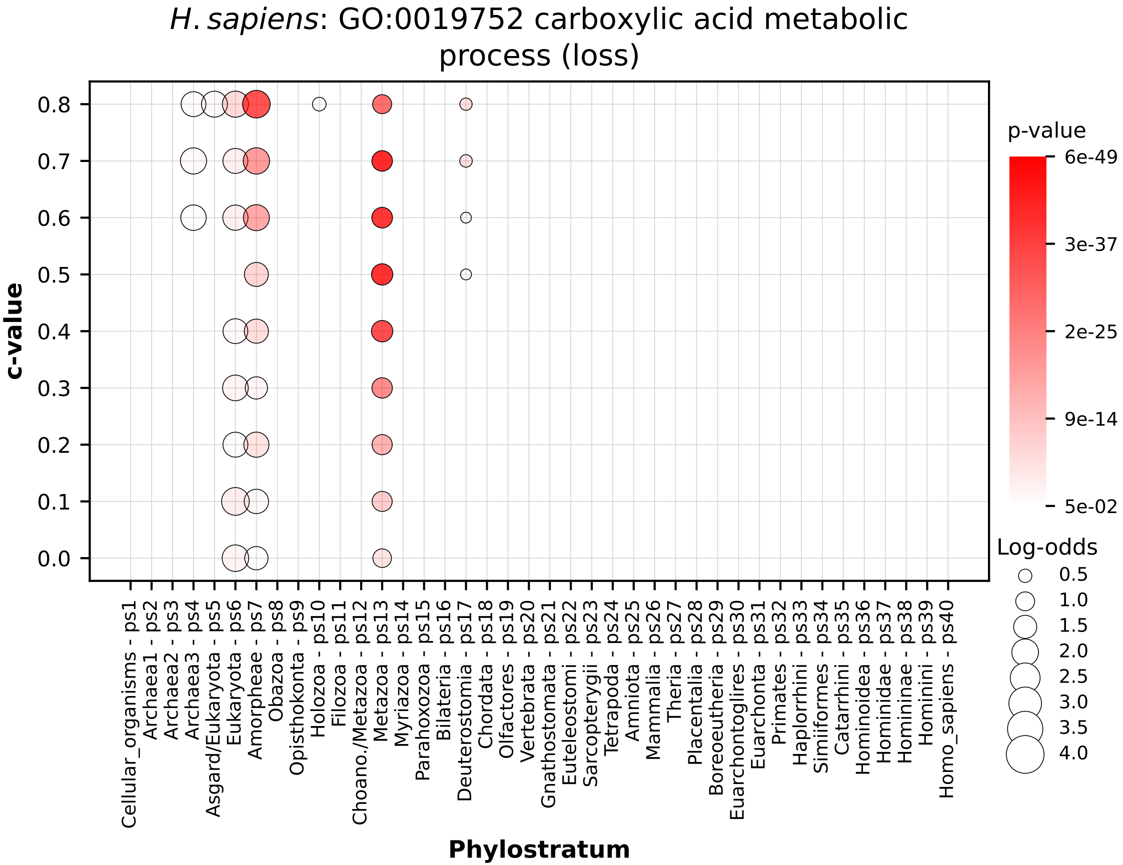

H. sapiens: GO:0019752 carboxylic acid metabolic process (loss)
c-value
0.8
0.7
0.6
0.5
0.4
0.3
0.2
0.1
0.0
Phylostratum
Cellular_organisms - ps1
Archaea1 - ps2
Archaea2 - ps3
Archaea3 - ps4
Asgard/Eukaryota - ps5
Eukaryota - ps6
Amorpheae - ps7
Obazoa - ps8
Opisthokonta - ps9
Holozoa - ps10
Filozoa - ps11
Choano./Metazoa - ps12
Metazoa - ps13
Myriazoa - ps14
Parahoxozoa - ps15
Bilateria - ps16
Deuterostomia - ps17
Chordata - ps18
Olfactores - ps19
Vertebrata - ps20
Gnathostomata - ps21
Euteleostomi - ps22
Sarcopterygii - ps23
Tetrapoda - ps24
Amniota - ps25
Mammalia - ps26
Theria - ps27
Placentalia - ps28
Boreoeutheria - ps29
Euarchontoglires - ps30
Euarchonta - ps31
Primates - ps32
Haplorrhini - ps33
Simiiformes - ps34
Catarrhini - ps35
Hominoidea - ps36
Hominidae - ps37
Homininae - ps38
Hominini - ps39
Homo_sapiens - ps40
p-value
6e-49
3e-37
2e-25
9e-14
5e-02
Log-odds
0.5
1.0
1.5
2.0
2.5
3.0
3.5
4.0

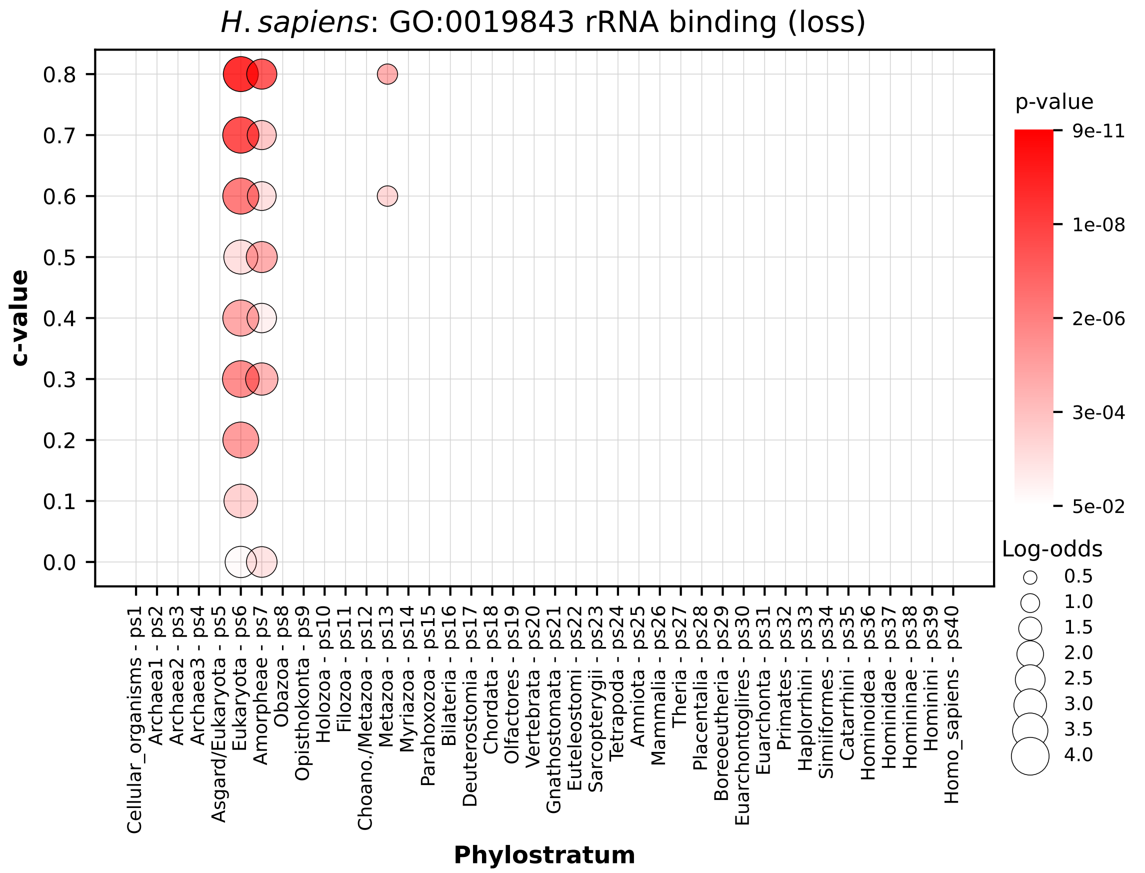

H. sapiens: GO:0019843 rRNA binding (loss)
c-value
0.0
0.1
0.2
0.3
0.4
0.5
0.6
0.7
0.8
p-value
9e-11
1e-08
2e-06
3e-04
5e-02
Log-odds
0.5
1.0
1.5
2.0
2.5
3.0
3.5
4.0
Cellular_organisms - ps1
Archaea1 - ps2
Archaea2 - ps3
Archaea3 - ps4
Asgard/Eukaryota - ps5
Eukaryota - ps6
Amorpheae - ps7
Obazoa - ps8
Opisthokonta - ps9
Holozoa - ps10
Filozoa - ps11
Choano./Metazoa - ps12
Metazoa - ps13
Myriazoa - ps14
Parahoxozoa - ps15
Bilateria - ps16
Deuterostomia - ps17
Chordata - ps18
Olfactores - ps19
Vertebrata - ps20
Gnathostomata - ps21
Euteleostomi - ps22
Sarcopterygii - ps23
Tetrapoda - ps24
Amniota - ps25
Mammalia - ps26
Theria - ps27
Placentalia - ps28
Boreoeutheria - ps29
Euarchontoglires - ps30
Euarchonta - ps31
Primates - ps32
Haplorrhini - ps33
Simiiformes - ps34
Catarrhini - ps35
Hominoidea - ps36
Hominidae - ps37
Homininae - ps38
Hominini - ps39
Homo_sapiens - ps40
Phylostratum

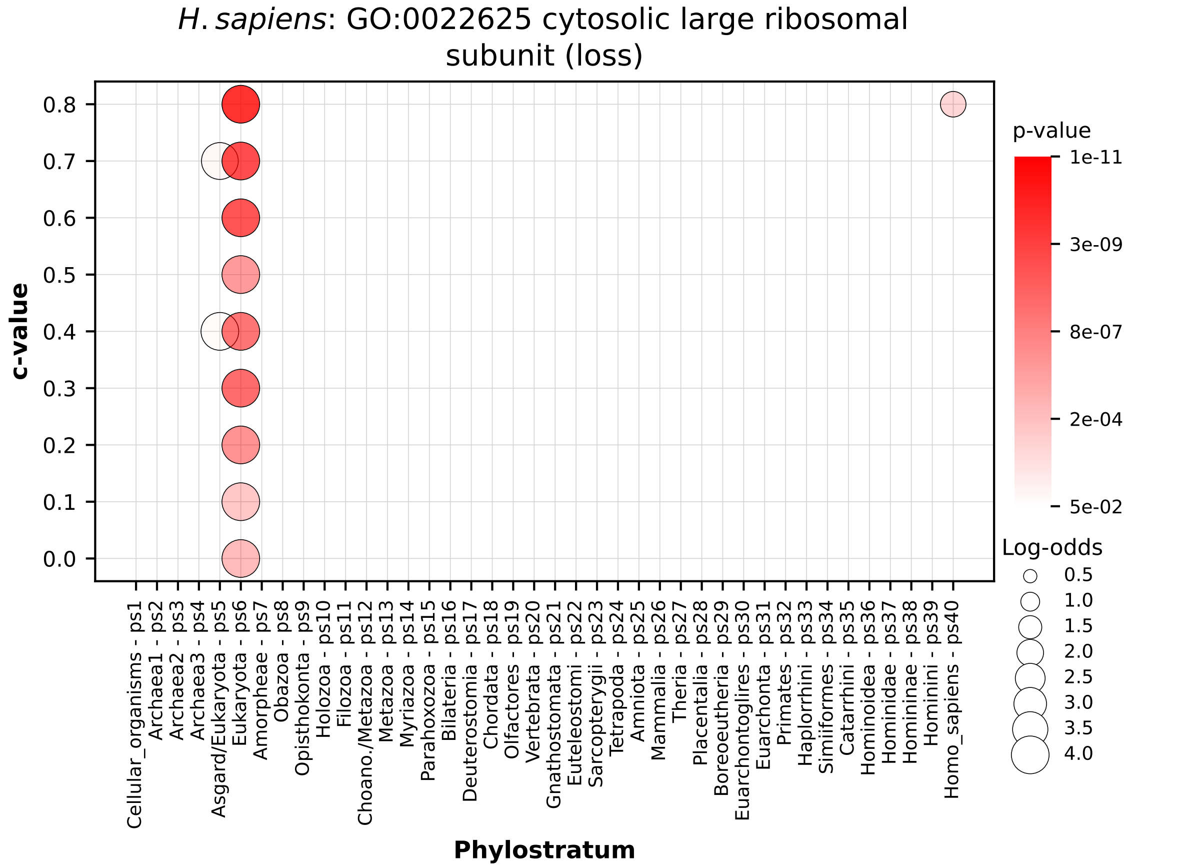
H. sapiens: GO:0022625 cytosolic large ribosomal subunit (loss)
c-value
0.8
0.7
0.6
0.5
0.4
0.3
0.2
0.1
0.0
p-value
1e-11
3e-09
8e-07
2e-04
5e-02
Log-odds
0.5
1.0
1.5
2.0
2.5
3.0
3.5
4.0
Cellular_organisms - ps1
Archaea1 - ps2
Archaea2 - ps3
Archaea3 - ps4
Asgard/Eukaryota - ps5
Eukaryota - ps6
Amorpheae - ps7
Obazoa - ps8
Opisthokonta - ps9
Holozoa - ps10
Filozoa - ps11
Choano./Metazoa - ps12
Metazoa - ps13
Myriazoa - ps14
Parahoxozoa - ps15
Bilateria - ps16
Deuterostomia - ps17
Chordata - ps18
Olfactores - ps19
Vertebrata - ps20
Gnathostomata - ps21
Euteleostomi - ps22
Sarcopterygii - ps23
Tetrapoda - ps24
Amniota - ps25
Mammalia - ps26
Theria - ps27
Placentalia - ps28
Boreoeutheria - ps29
Euarchontoglires - ps30
Euarchonta - ps31
Primates - ps32
Haplorrhini - ps33
Simiiformes - ps34
Catarrhini - ps35
Hominoidea - ps36
Hominidae - ps37
Homininae - ps38
Hominini - ps39
Homo_sapiens - ps40
Phylostratum

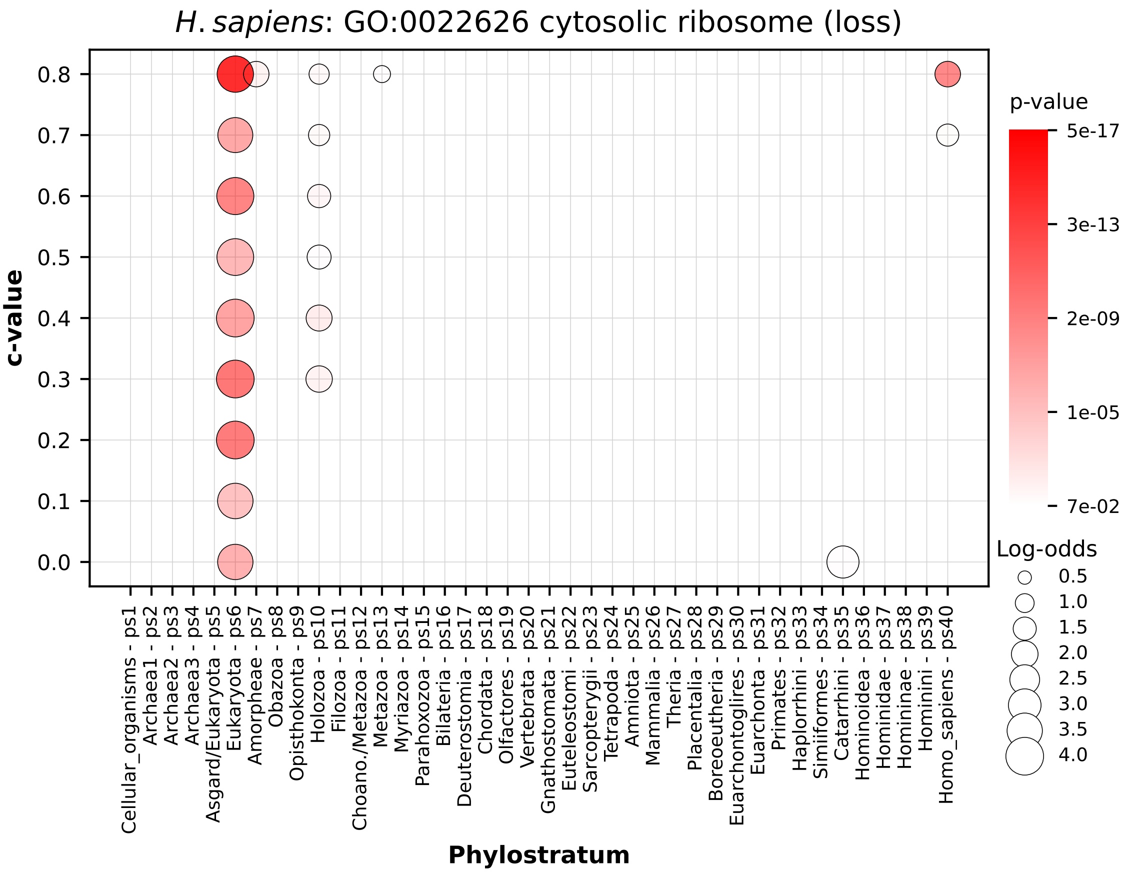

H. sapiens: GO:0022626 cytosolic ribosome (loss)
c-value
0.0
0.1
0.2
0.3
0.4
0.5
0.6
0.7
0.8
Phylostratum
Cellular_organisms - ps1
Archaea1 - ps2
Archaea2 - ps3
Archaea3 - ps4
Asgard/Eukaryota - ps5
Eukaryota - ps6
Amorpheae - ps7
Obazoa - ps8
Opisthokonta - ps9
Holozoa - ps10
Filozoa - ps11
Choano./Metazoa - ps12
Metazoa - ps13
Myriazoa - ps14
Parahoxozoa - ps15
Bilateria - ps16
Deuterostomia - ps17
Chordata - ps18
Olfactores - ps19
Vertebrata - ps20
Gnathostomata - ps21
Euteleostomi - ps22
Sarcopterygii - ps23
Tetrapoda - ps24
Amniota - ps25
Mammalia - ps26
Theria - ps27
Placentalia - ps28
Boreoeutheria - ps29
Euarchontoglires - ps30
Euarchonta - ps31
Primates - ps32
Haplorrhini - ps33
Simiiformes - ps34
Catarrhini - ps35
Hominoidea - ps36
Hominidae - ps37
Homininae - ps38
Hominini - ps39
Homo_sapiens - ps40
p-value
5e-17
3e-13
2e-09
1e-05
7e-02
Log-odds
0.5
1.0
1.5
2.0
2.5
3.0
3.5
4.0

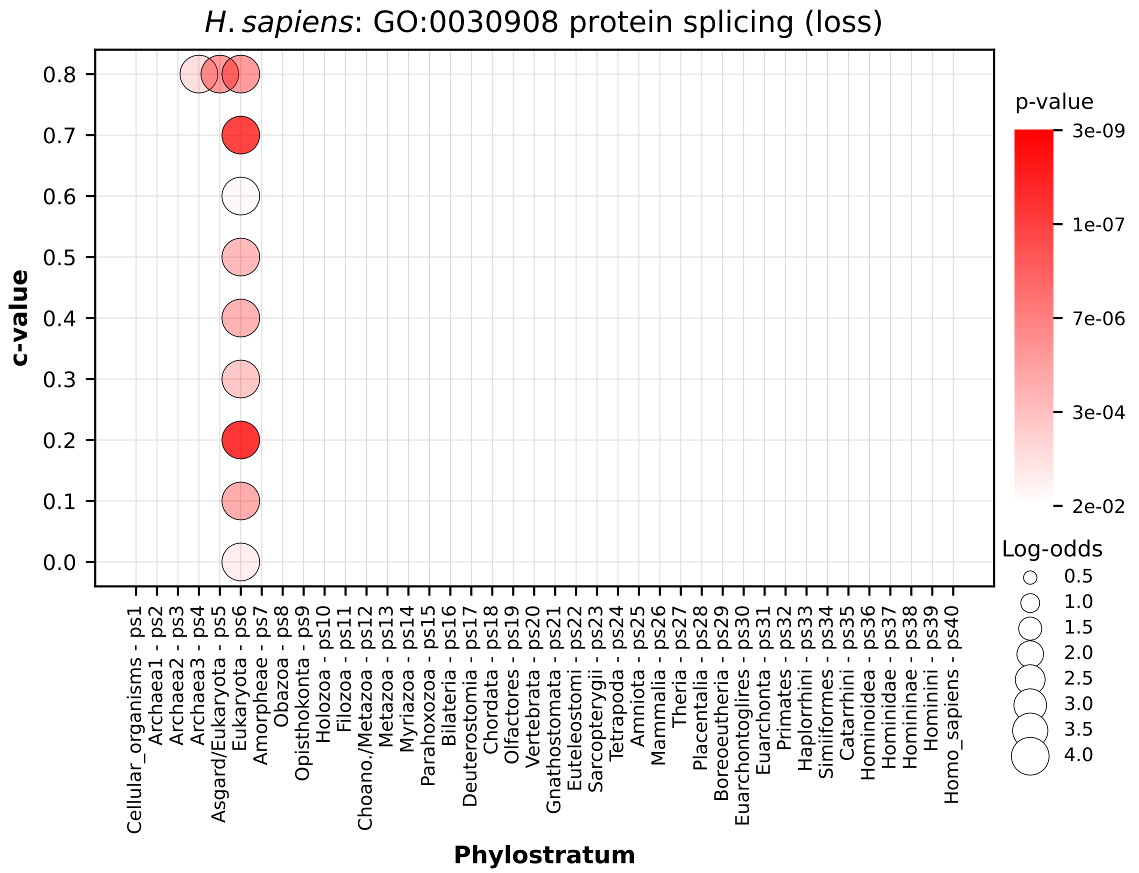

H. sapiens: GO:0030908 protein splicing (loss)
c-value
0.0
0.1
0.2
0.3
0.4
0.5
0.6
0.7
0.8
Phylostratum
Cellular_organisms - ps1
Archaea1 - ps2
Archaea2 - ps3
Archaea3 - ps4
Asgard/Eukaryota - ps5
Eukaryota - ps6
Amorpheae - ps7
Obazoa - ps8
Opisthokonta - ps9
Holozoa - ps10
Filozoa - ps11
Choano./Metazoa - ps12
Metazoa - ps13
Myriazoa - ps14
Parahoxozoa - ps15
Bilateria - ps16
Deuterostomia - ps17
Chordata - ps18
Olfactores - ps19
Vertebrata - ps20
Gnathostomata - ps21
Euteleostomi - ps22
Sarcopterygii - ps23
Tetrapoda - ps24
Amniota - ps25
Mammalia - ps26
Theria - ps27
Placentalia - ps28
Boreoeutheria - ps29
Euarchontoglires - ps30
Euarchonta - ps31
Primates - ps32
Haplorrhini - ps33
Simiiformes - ps34
Catarrhini - ps35
Hominoidea - ps36
Hominidae - ps37
Homininae - ps38
Hominini - ps39
Homo_sapiens - ps40
p-value
3e-09
1e-07
7e-06
3e-04
2e-02
Log-odds
0.5
1.0
1.5
2.0
2.5
3.0
3.5
4.0

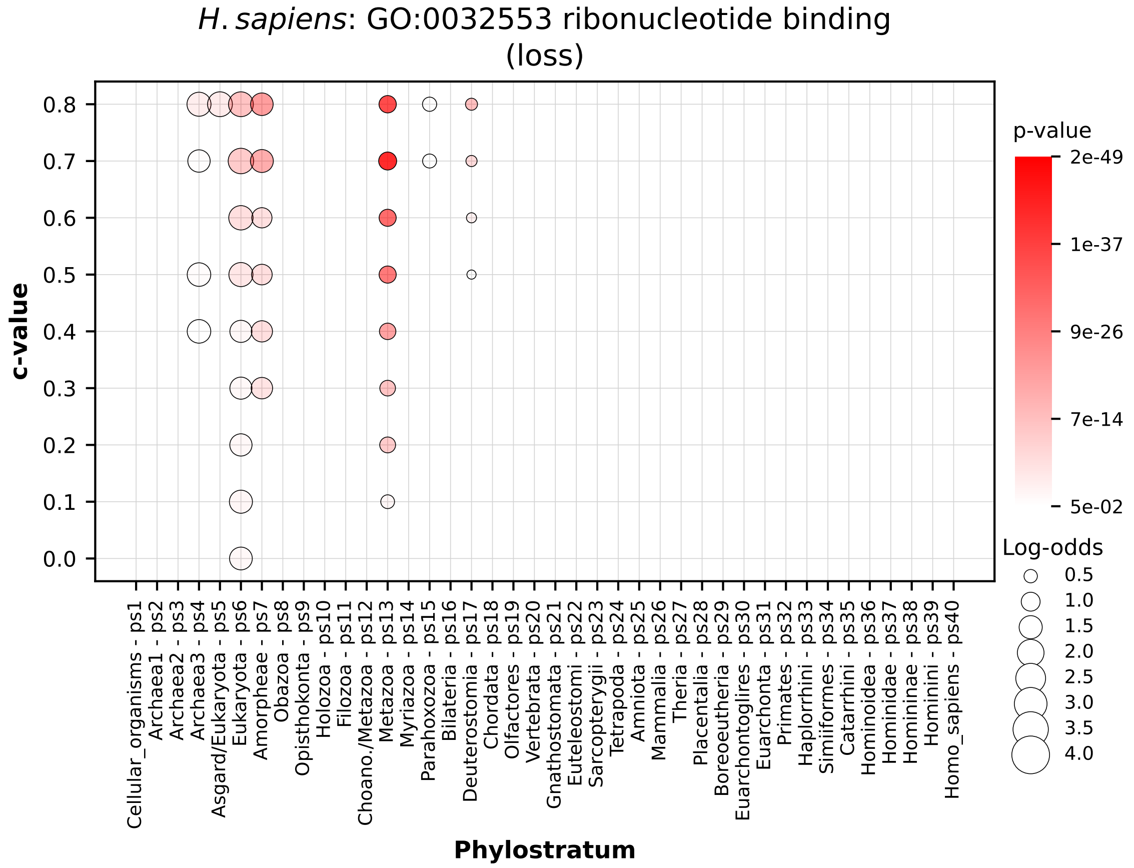
H. sapiens: GO:0032553 ribonucleotide binding
(loss)
c-value
0.8
0.7
0.6
0.5
0.4
0.3
0.2
0.1
0.0
p-value
2e-49
1e-37
9e-26
7e-14
5e-02
Log-odds
0.5
1.0
1.5
2.0
2.5
3.0
3.5
4.0
Cellular_organisms - ps1
Archaea1 - ps2
Archaea2 - ps3
Archaea3 - ps4
Asgard/Eukaryota - ps5
Eukaryota - ps6
Amorpheae - ps7
Obazoa - ps8
Opisthokonta - ps9
Holozoa - ps10
Filozoa - ps11
Choano./Metazoa - ps12
Metazoa - ps13
Myriazoa - ps14
Parahoxozoa - ps15
Bilateria - ps16
Deuterostomia - ps17
Chordata - ps18
Olfactores - ps19
Vertebrata - ps20
Gnathostomata - ps21
Euteleostomi - ps22
Sarcopterygii - ps23
Tetrapoda - ps24
Amniota - ps25
Mammalia - ps26
Theria - ps27
Placentalia - ps28
Boreoeutheria - ps29
Euarchontoglires - ps30
Euarchonta - ps31
Primates - ps32
Haplorrhini - ps33
Simiiformes - ps34
Catarrhini - ps35
Hominoidea - ps36
Hominidae - ps37
Homininae - ps38
Hominini - ps39
Homo_sapiens - ps40
Phylostratum

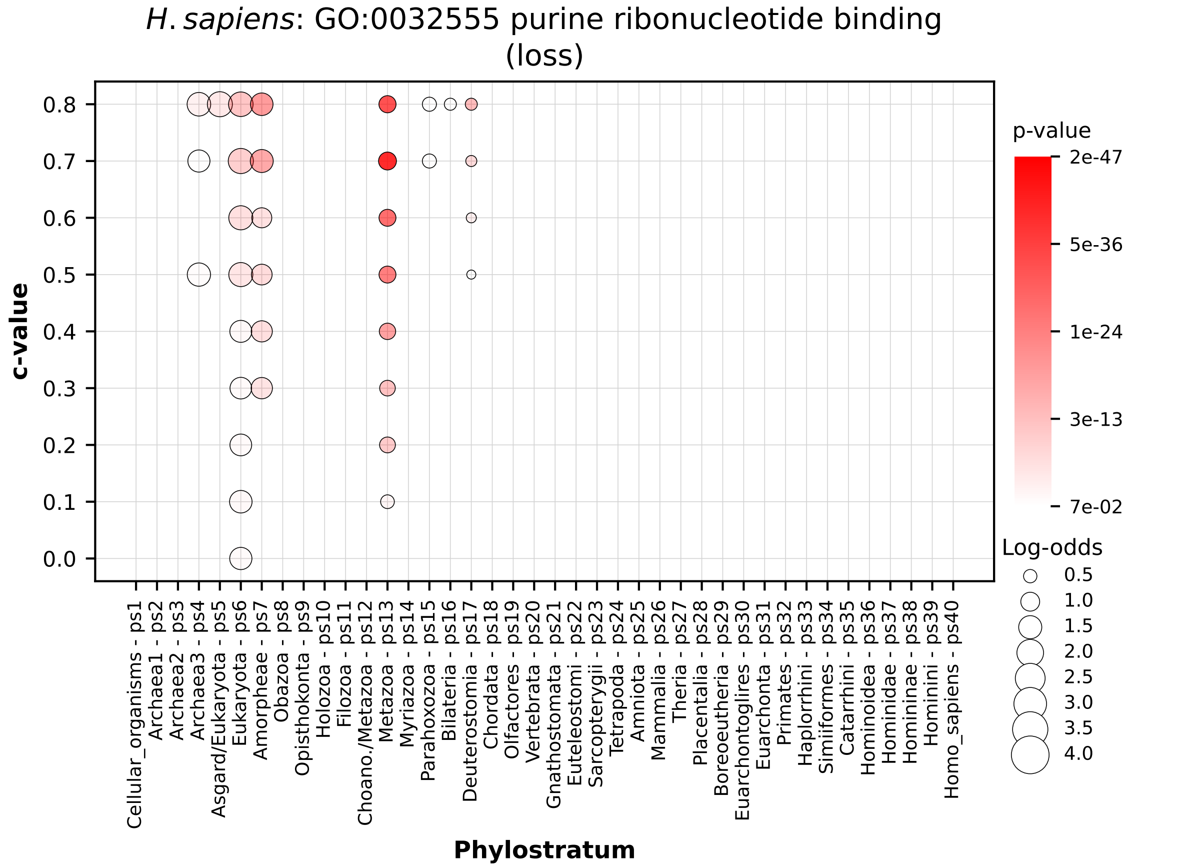
H. sapiens: GO:0032555 purine ribonucleotide binding
(loss)
c-value
0.8
0.7
0.6
0.5
0.4
0.3
0.2
0.1
0.0
p-value
2e-47
5e-36
1e-24
3e-13
7e-02
Log-odds
0.5
1.0
1.5
2.0
2.5
3.0
3.5
4.0
Cellular_organisms - ps1
Archaea1 - ps2
Archaea2 - ps3
Archaea3 - ps4
Asgard/Eukaryota - ps5
Eukaryota - ps6
Amorpheae - ps7
Obazoa - ps8
Opisthokonta - ps9
Holozoa - ps10
Filozoa - ps11
Choano./Metazoa - ps12
Metazoa - ps13
Myriazoa - ps14
Parahoxozoa - ps15
Bilateria - ps16
Deuterostomia - ps17
Chordata - ps18
Olfactores - ps19
Vertebrata - ps20
Gnathostomata - ps21
Euteleostomi - ps22
Sarcopterygii - ps23
Tetrapoda - ps24
Amniota - ps25
Mammalia - ps26
Theria - ps27
Placentalia - ps28
Boreoeutheria - ps29
Euarchontoglires - ps30
Euarchonta - ps31
Primates - ps32
Haplorrhini - ps33
Simiiformes - ps34
Catarrhini - ps35
Hominoidea - ps36
Hominidae - ps37
Homininae - ps38
Hominini - ps39
Homo_sapiens - ps40
Phylostratum

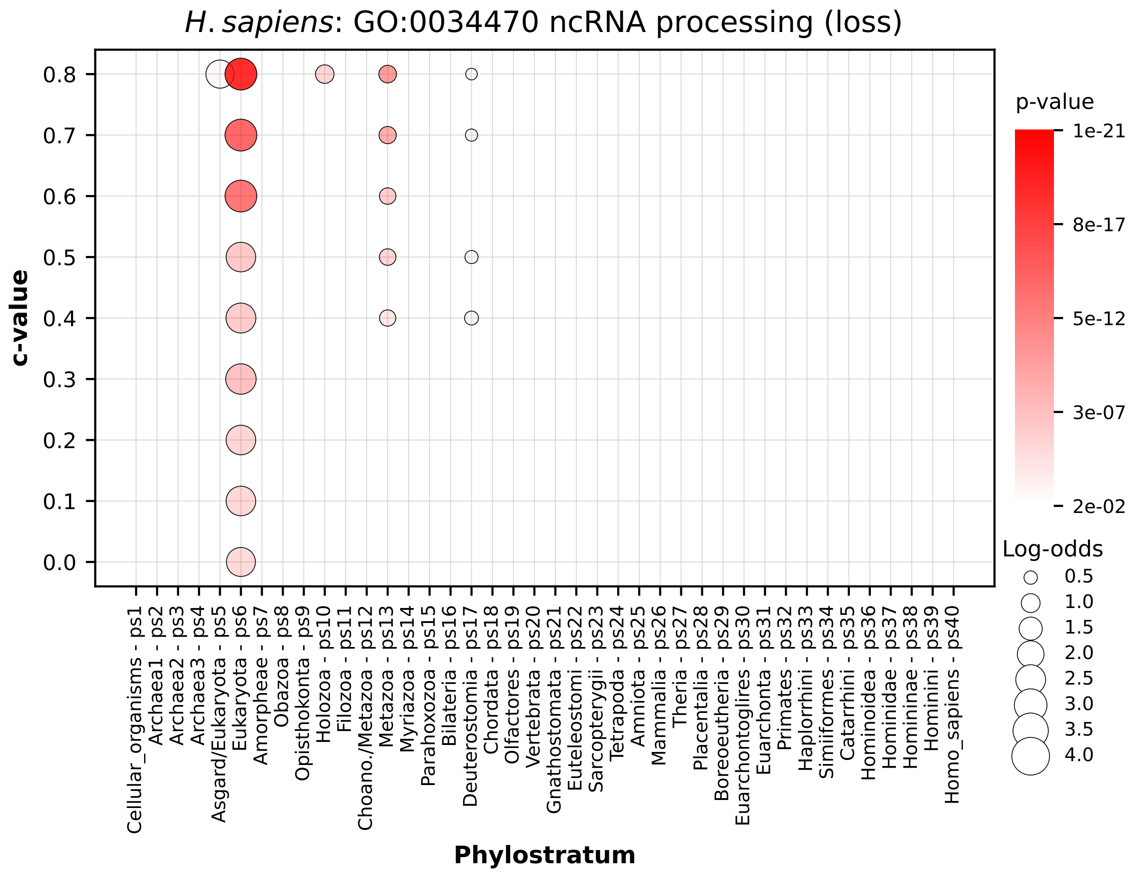

H. sapiens: GO:0034470 ncRNA processing (loss)
c-value
0.0
0.1
0.2
0.3
0.4
0.5
0.6
0.7
0.8
p-value
1e-21
8e-17
5e-12
3e-07
2e-02
Log-odds
0.5
1.0
1.5
2.0
2.5
3.0
3.5
4.0
Cellular_organisms - ps1
Archaea1 - ps2
Archaea2 - ps3
Archaea3 - ps4
Asgard/Eukaryota - ps5
Eukaryota - ps6
Amorpheae - ps7
Obazoa - ps8
Opisthokonta - ps9
Holozoa - ps10
Filozoa - ps11
Choano./Metazoa - ps12
Metazoa - ps13
Myriazoa - ps14
Parahoxozoa - ps15
Bilateria - ps16
Deuterostomia - ps17
Chordata - ps18
Olfactores - ps19
Vertebrata - ps20
Gnathostomata - ps21
Euteleostomi - ps22
Sarcopterygii - ps23
Tetrapoda - ps24
Amniota - ps25
Mammalia - ps26
Theria - ps27
Placentalia - ps28
Boreoeutheria - ps29
Euarchontoglires - ps30
Euarchonta - ps31
Primates - ps32
Haplorrhini - ps33
Simiiformes - ps34
Catarrhini - ps35
Hominoidea - ps36
Hominidae - ps37
Homininae - ps38
Hominini - ps39
Homo_sapiens - ps40
Phylostratum

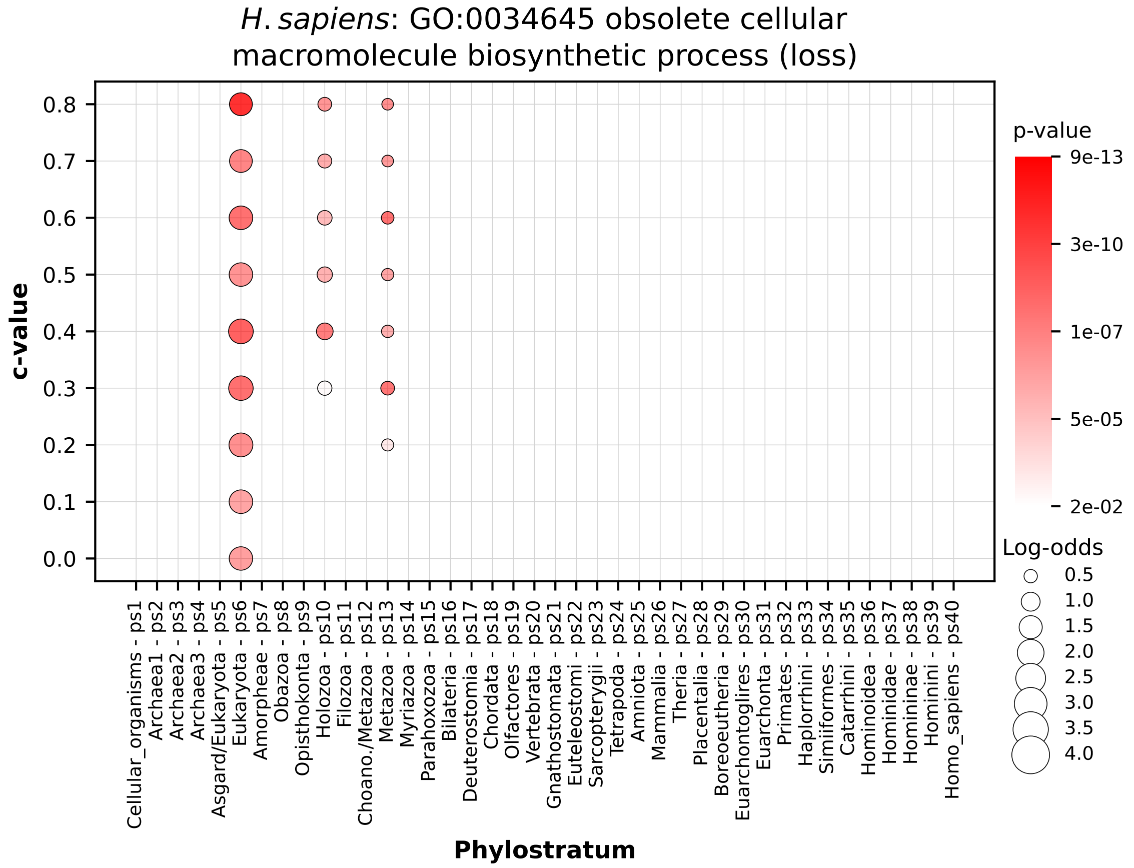
H. sapiens: GO:0034645 obsolete cellular macromolecule biosynthetic process (loss)
c-value
0.8
0.7
0.6
0.5
0.4
0.3
0.2
0.1
0.0
Phylostratum
Cellular_organisms - ps1
Archaea1 - ps2
Archaea2 - ps3
Archaea3 - ps4
Asgard/Eukaryota - ps5
Eukaryota - ps6
Amorpheae - ps7
Obazoa - ps8
Opisthokonta - ps9
Holozoa - ps10
Filozoa - ps11
Choano./Metazoa - ps12
Metazoa - ps13
Myriazoa - ps14
Parahoxozoa - ps15
Bilateria - ps16
Deuterostomia - ps17
Chordata - ps18
Olfactores - ps19
Vertebrata - ps20
Gnathostomata - ps21
Euteleostomi - ps22
Sarcopterygii - ps23
Tetrapoda - ps24
Amniota - ps25
Mammalia - ps26
Theria - ps27
Placentalia - ps28
Boreoeutheria - ps29
Euarchontoglires - ps30
Euarchonta - ps31
Primates - ps32
Haplorrhini - ps33
Simiiformes - ps34
Catarrhini - ps35
Hominoidea - ps36
Hominidae - ps37
Homininae - ps38
Hominini - ps39
Homo_sapiens - ps40
p-value
9e-13
3e-10
1e-07
5e-05
2e-02
Log-odds
0.5
1.0
1.5
2.0
2.5
3.0
3.5
4.0

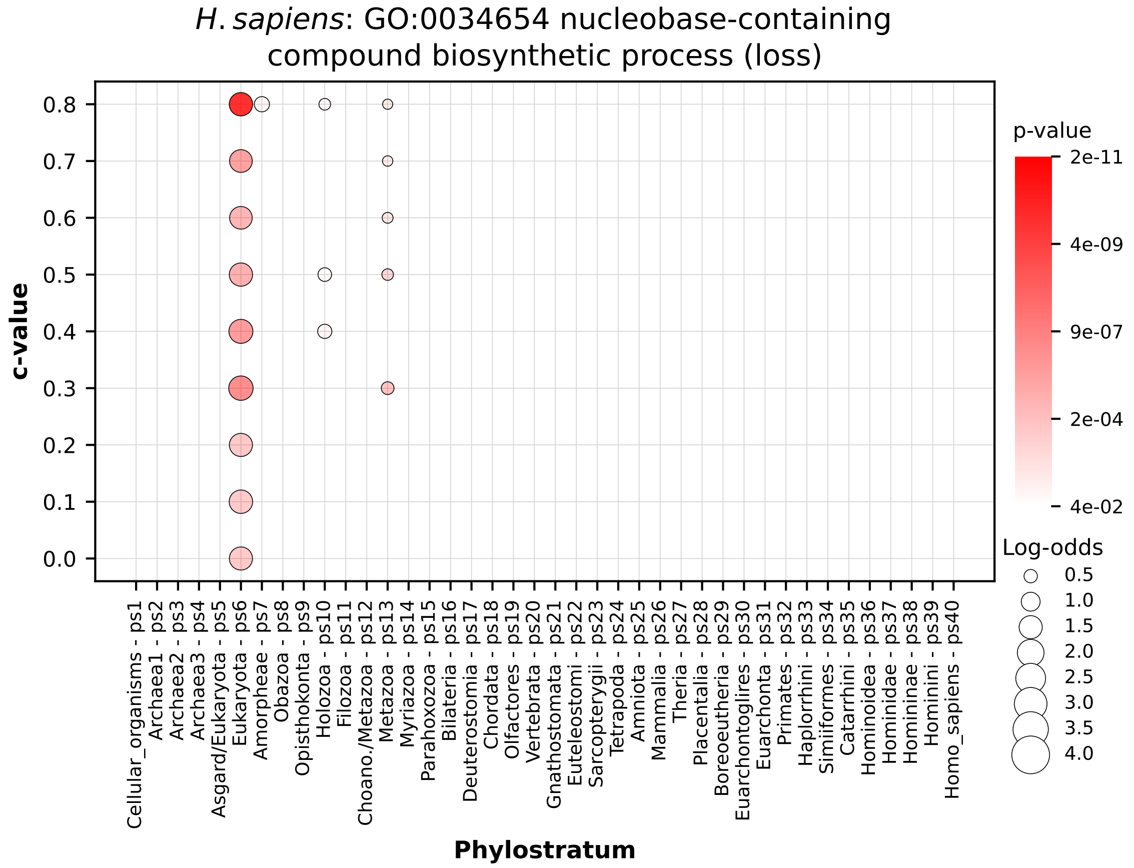

H. sapiens: GO:0034654 nucleobase-containing compound biosynthetic process (loss)
c-value
0.8
0.7
0.6
0.5
0.4
0.3
0.2
0.1
0.0
p-value
2e-11
4e-09
9e-07
2e-04
4e-02
Log-odds
0.5
1.0
1.5
2.0
2.5
3.0
3.5
4.0
Cellular_organisms - ps1
Archaea1 - ps2
Archaea2 - ps3
Archaea3 - ps4
Asgard/Eukaryota - ps5
Eukaryota - ps6
Amorpheae - ps7
Obazoa - ps8
Opisthokonta - ps9
Holozoa - ps10
Filozoa - ps11
Choano./Metazoa - ps12
Metazoa - ps13
Myriazoa - ps14
Parahoxozoa - ps15
Bilateria - ps16
Deuterostomia - ps17
Chordata - ps18
Olfactores - ps19
Vertebrata - ps20
Gnathostomata - ps21
Euteleostomi - ps22
Sarcopterygii - ps23
Tetrapoda - ps24
Amniota - ps25
Mammalia - ps26
Theria - ps27
Placentalia - ps28
Boreoeutheria - ps29
Euarchontoglires - ps30
Euarchonta - ps31
Primates - ps32
Haplorrhini - ps33
Simiiformes - ps34
Catarrhini - ps35
Hominoidea - ps36
Hominidae - ps37
Homininae - ps38
Hominini - ps39
Homo_sapiens - ps40
Phylostratum

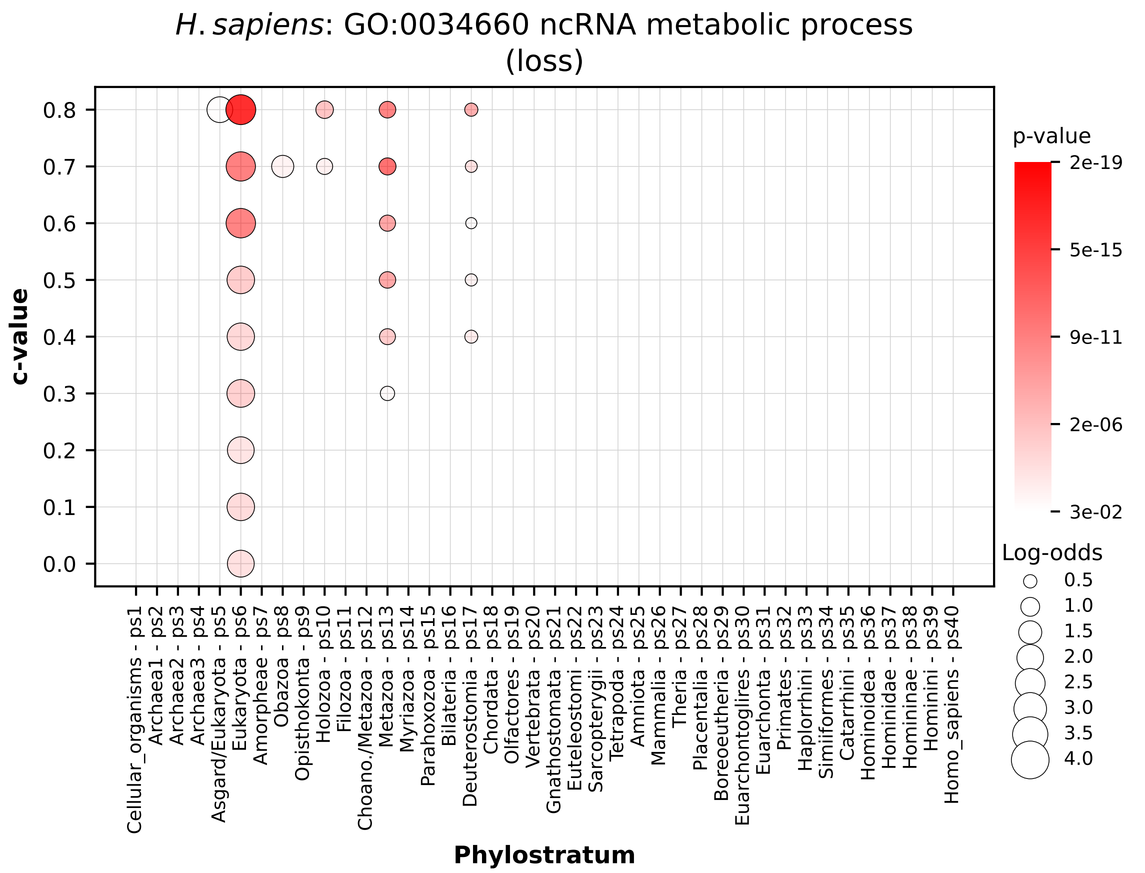

H. sapiens: GO:0034660 ncRNA metabolic process
(loss)
c-value
0.8
0.7
0.6
0.5
0.4
0.3
0.2
0.1
0.0
p-value
2e-19
5e-15
9e-11
2e-06
3e-02
Log-odds
0.5
1.0
1.5
2.0
2.5
3.0
3.5
4.0
Cellular_organisms - ps1
Archaea1 - ps2
Archaea2 - ps3
Archaea3 - ps4
Asgard/Eukaryota - ps5
Eukaryota - ps6
Amorpheae - ps7
Obazoa - ps8
Opisthokonta - ps9
Holozoa - ps10
Filozoa - ps11
Choano./Metazoa - ps12
Metazoa - ps13
Myriazoa - ps14
Parahoxozoa - ps15
Bilateria - ps16
Deuterostomia - ps17
Chordata - ps18
Olfactores - ps19
Vertebrata - ps20
Gnathostomata - ps21
Euteleostomi - ps22
Sarcopterygii - ps23
Tetrapoda - ps24
Amniota - ps25
Mammalia - ps26
Theria - ps27
Placentalia - ps28
Boreoeutheria - ps29
Euarchontoglires - ps30
Euarchonta - ps31
Primates - ps32
Haplorrhini - ps33
Simiiformes - ps34
Catarrhini - ps35
Hominoidea - ps36
Hominidae - ps37
Homininae - ps38
Hominini - ps39
Homo_sapiens - ps40
Phylostratum

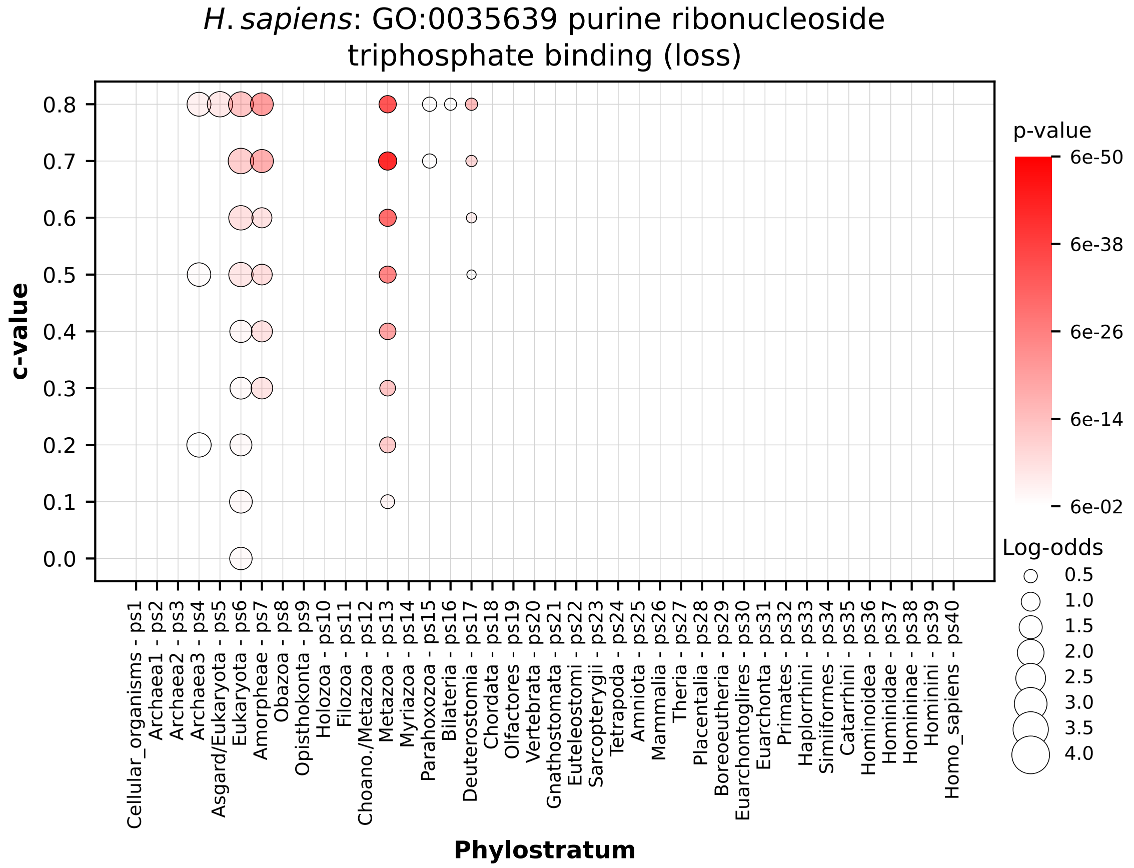

H. sapiens: GO:0035639 purine ribonucleoside triphosphate binding (loss)
c-value
0.8
0.7
0.6
0.5
0.4
0.3
0.2
0.1
0.0
Phylostratum
Cellular_organisms - ps1
Archaea1 - ps2
Archaea2 - ps3
Archaea3 - ps4
Asgard/Eukaryota - ps5
Eukaryota - ps6
Amorpheae - ps7
Obazoa - ps8
Opisthokonta - ps9
Holozoa - ps10
Filozoa - ps11
Choano./Metazoa - ps12
Metazoa - ps13
Myriazoa - ps14
Parahoxozoa - ps15
Bilateria - ps16
Deuterostomia - ps17
Chordata - ps18
Olfactores - ps19
Vertebrata - ps20
Gnathostomata - ps21
Euteleostomi - ps22
Sarcopterygii - ps23
Tetrapoda - ps24
Amniota - ps25
Mammalia - ps26
Theria - ps27
Placentalia - ps28
Boreoeutheria - ps29
Euarchontoglires - ps30
Euarchonta - ps31
Primates - ps32
Haplorrhini - ps33
Simiiformes - ps34
Catarrhini - ps35
Hominoidea - ps36
Hominidae - ps37
Homininae - ps38
Hominini - ps39
Homo_sapiens - ps40
p-value
6e-50
6e-38
6e-26
6e-14
6e-02
Log-odds
0.5
1.0
1.5
2.0
2.5
3.0
3.5
4.0

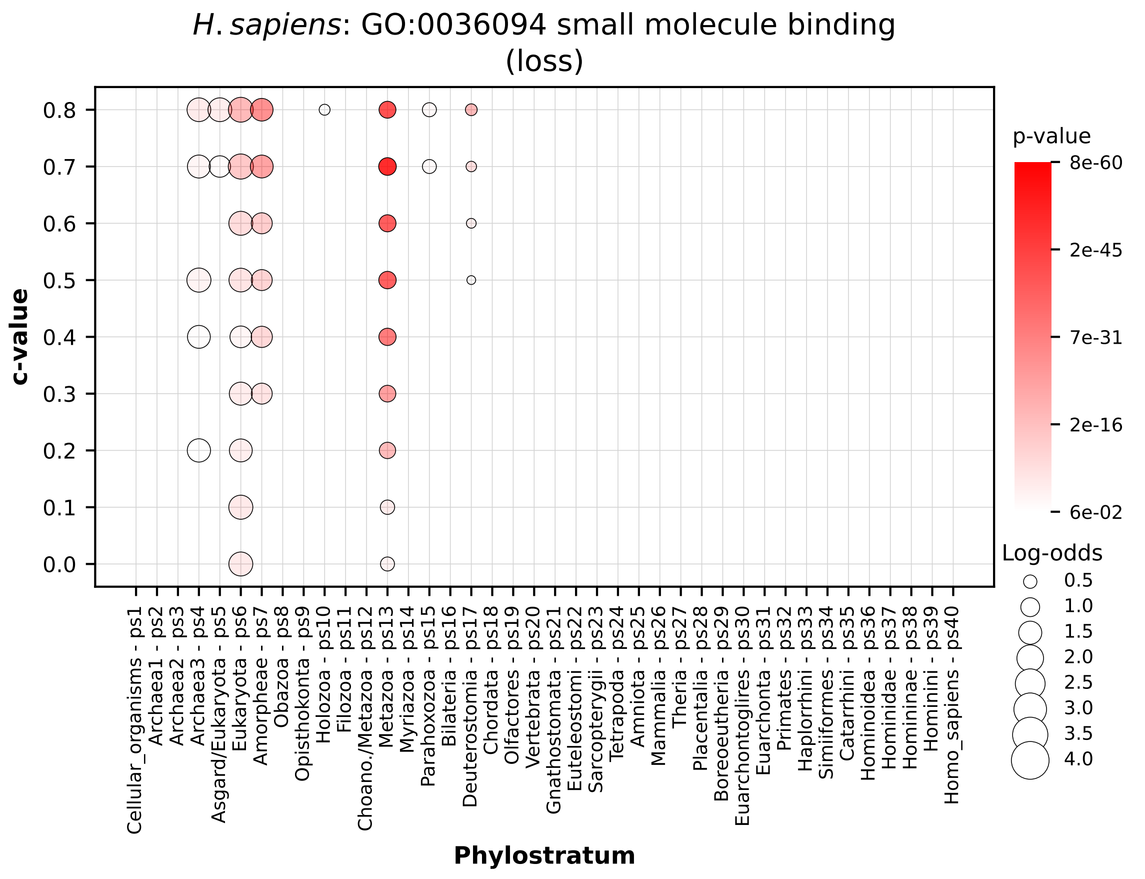

$H. sapiens$: GO:0036094 small molecule binding (loss)
c-value
0.8
0.7
0.6
0.5
0.4
0.3
0.2
0.1
0.0
Phylostratum
Cellular_organisms - ps1
Archaea1 - ps2
Archaea2 - ps3
Archaea3 - ps4
Asgard/Eukaryota - ps5
Eukaryota - ps6
Amorpheae - ps7
Obazoa - ps8
Opisthokonta - ps9
Holozoa - ps10
Filozoa - ps11
Choano./Metazoa - ps12
Metazoa - ps13
Myriazoa - ps14
Parahoxozoa - ps15
Bilateria - ps16
Deuterostomia - ps17
Chordata - ps18
Olfactores - ps19
Vertebrata - ps20
Gnathostomata - ps21
Euteleostomi - ps22
Sarcopterygii - ps23
Tetrapoda - ps24
Amniota - ps25
Mammalia - ps26
Theria - ps27
Placentalia - ps28
Boreoeutheria - ps29
Euarchontoglires - ps30
Euarchonta - ps31
Primates - ps32
Haplorrhini - ps33
Simiiformes - ps34
Catarrhini - ps35
Hominoidea - ps36
Hominidae - ps37
Homininae - ps38
Hominini - ps39
Homo_sapiens - ps40
p-value
8e-60
2e-45
7e-31
2e-16
6e-02
Log-odds
0.5
1.0
1.5
2.0
2.5
3.0
3.5
4.0

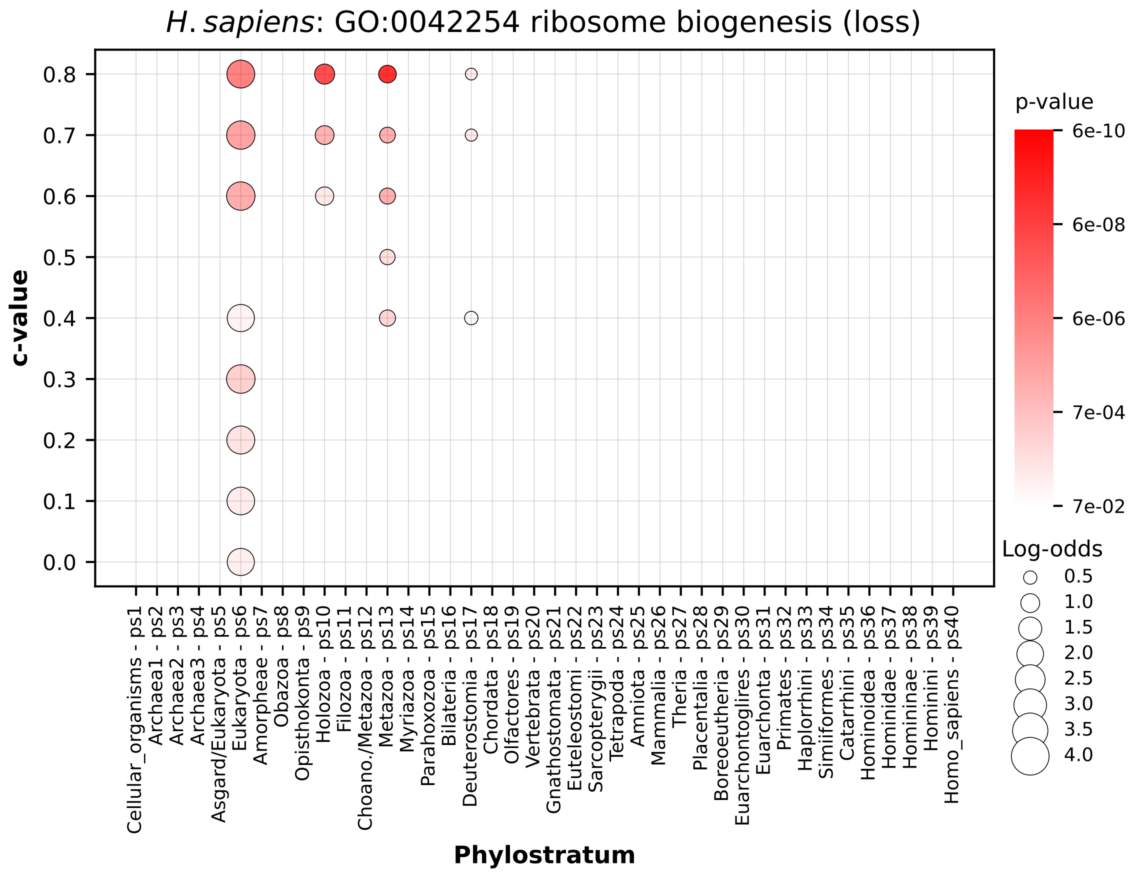

H. sapiens: GO:0042254 ribosome biogenesis (loss)
c-value
0.0
0.1
0.2
0.3
0.4
0.5
0.6
0.7
0.8
Phylostratum
Cellular_organisms - ps1
Archaea1 - ps2
Archaea2 - ps3
Archaea3 - ps4
Asgard/Eukaryota - ps5
Eukaryota - ps6
Amorpheae - ps7
Obazoa - ps8
Opisthokonta - ps9
Holozoa - ps10
Filozoa - ps11
Choano./Metazoa - ps12
Metazoa - ps13
Myriazoa - ps14
Parahoxozoa - ps15
Bilateria - ps16
Deuterostomia - ps17
Chordata - ps18
Olfactores - ps19
Vertebrata - ps20
Gnathostomata - ps21
Euteleostomi - ps22
Sarcopterygii - ps23
Tetrapoda - ps24
Amniota - ps25
Mammalia - ps26
Theria - ps27
Placentalia - ps28
Boreoeutheria - ps29
Euarchontoglires - ps30
Euarchonta - ps31
Primates - ps32
Haplorrhini - ps33
Simiiformes - ps34
Catarrhini - ps35
Hominoidea - ps36
Hominidae - ps37
Homininae - ps38
Hominini - ps39
Homo_sapiens - ps40
p-value
6e-10
6e-08
6e-06
7e-04
7e-02
Log-odds
0.5
1.0
1.5
2.0
2.5
3.0
3.5
4.0

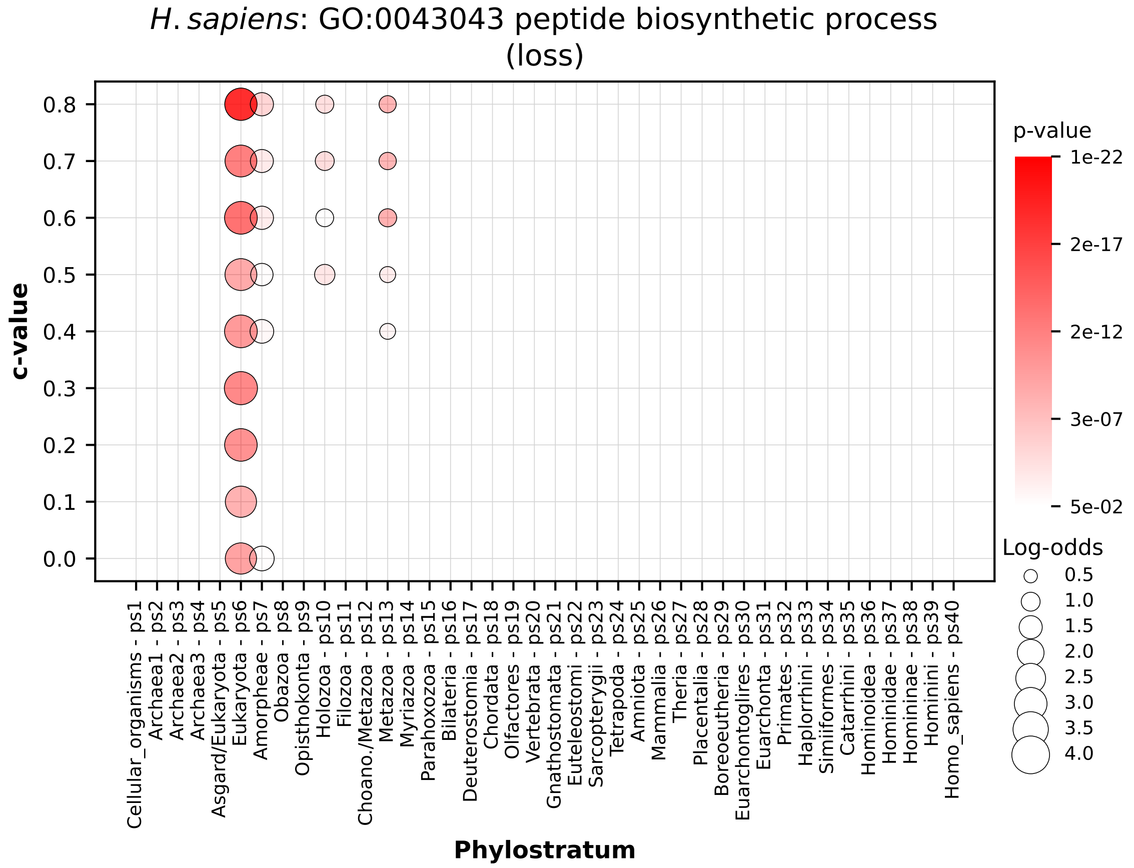

H. sapiens: GO:0043043 peptide biosynthetic process
(loss)
c-value
0.8
0.7
0.6
0.5
0.4
0.3
0.2
0.1
0.0
p-value
1e-22
2e-17
2e-12
3e-07
5e-02
Log-odds
0.5
1.0
1.5
2.0
2.5
3.0
3.5
4.0
Cellular_organisms - ps1
Archaea1 - ps2
Archaea2 - ps3
Archaea3 - ps4
Asgard/Eukaryota - ps5
Eukaryota - ps6
Amorpheae - ps7
Obazoa - ps8
Opisthokonta - ps9
Holozoa - ps10
Filozoa - ps11
Choano./Metazoa - ps12
Metazoa - ps13
Myriazoa - ps14
Parahoxozoa - ps15
Bilateria - ps16
Deuterostomia - ps17
Chordata - ps18
Olfactores - ps19
Vertebrata - ps20
Gnathostomata - ps21
Euteleostomi - ps22
Sarcopterygii - ps23
Tetrapoda - ps24
Amniota - ps25
Mammalia - ps26
Theria - ps27
Placentalia - ps28
Boreoeutheria - ps29
Euarchontoglires - ps30
Euarchonta - ps31
Primates - ps32
Haplorrhini - ps33
Simiiformes - ps34
Catarrhini - ps35
Hominoidea - ps36
Hominidae - ps37
Homininae - ps38
Hominini - ps39
Homo_sapiens - ps40
Phylostratum

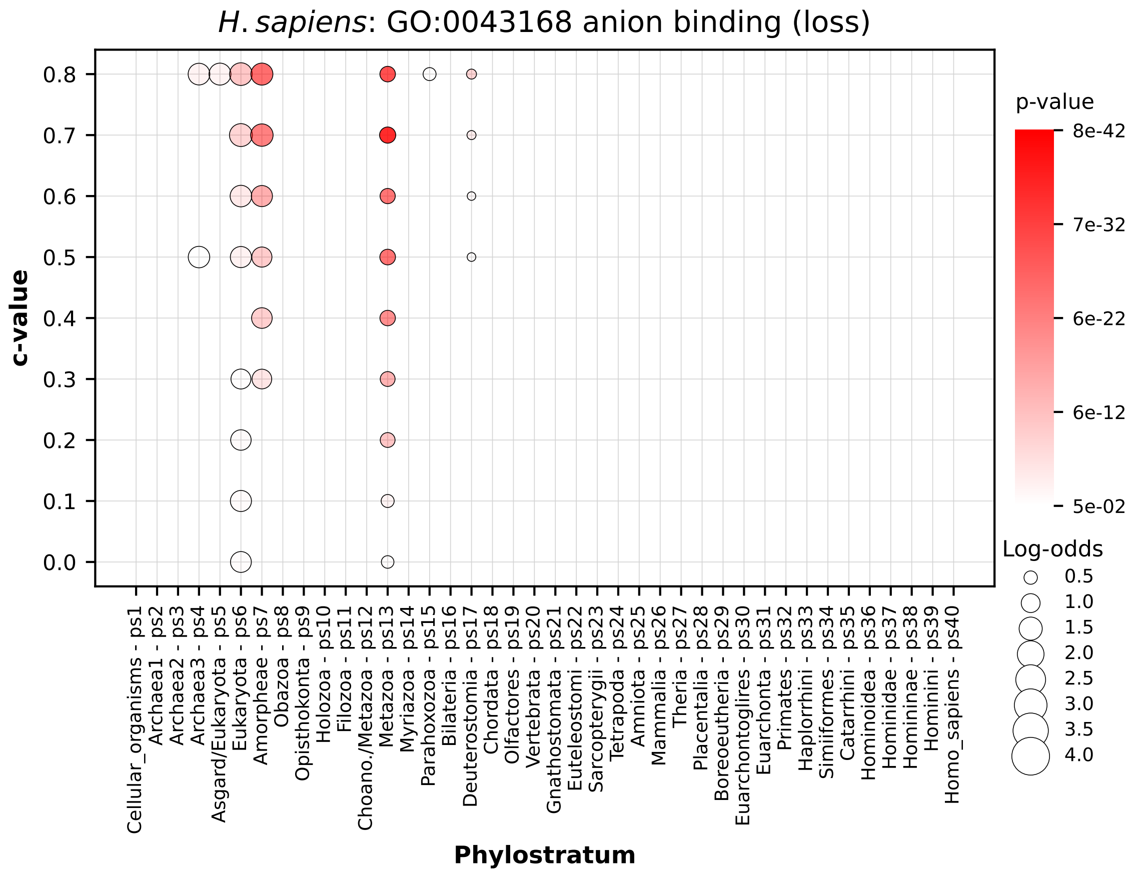

H. sapiens: GO:0043168 anion binding (loss)
c-value
0.8
0.7
0.6
0.5
0.4
0.3
0.2
0.1
0.0
p-value
8e-42
7e-32
6e-22
6e-12
5e-02
Log-odds
0.5
1.0
1.5
2.0
2.5
3.0
3.5
4.0
Cellular_organisms - ps1
Archaea1 - ps2
Archaea2 - ps3
Archaea3 - ps4
Asgard/Eukaryota - ps5
Eukaryota - ps6
Amorpheae - ps7
Obazoa - ps8
Opisthokonta - ps9
Holozoa - ps10
Filozoa - ps11
Choano./Metazoa - ps12
Metazoa - ps13
Myriazoa - ps14
Parahoxozoa - ps15
Bilateria - ps16
Deuterostomia - ps17
Chordata - ps18
Olfactores - ps19
Vertebrata - ps20
Gnathostomata - ps21
Euteleostomi - ps22
Sarcopterygii - ps23
Tetrapoda - ps24
Amniota - ps25
Mammalia - ps26
Theria - ps27
Placentalia - ps28
Boreoeutheria - ps29
Euarchontoglires - ps30
Euarchonta - ps31
Primates - ps32
Haplorrhini - ps33
Simiiformes - ps34
Catarrhini - ps35
Hominoidea - ps36
Hominidae - ps37
Homininae - ps38
Hominini - ps39
Homo_sapiens - ps40
Phylostratum

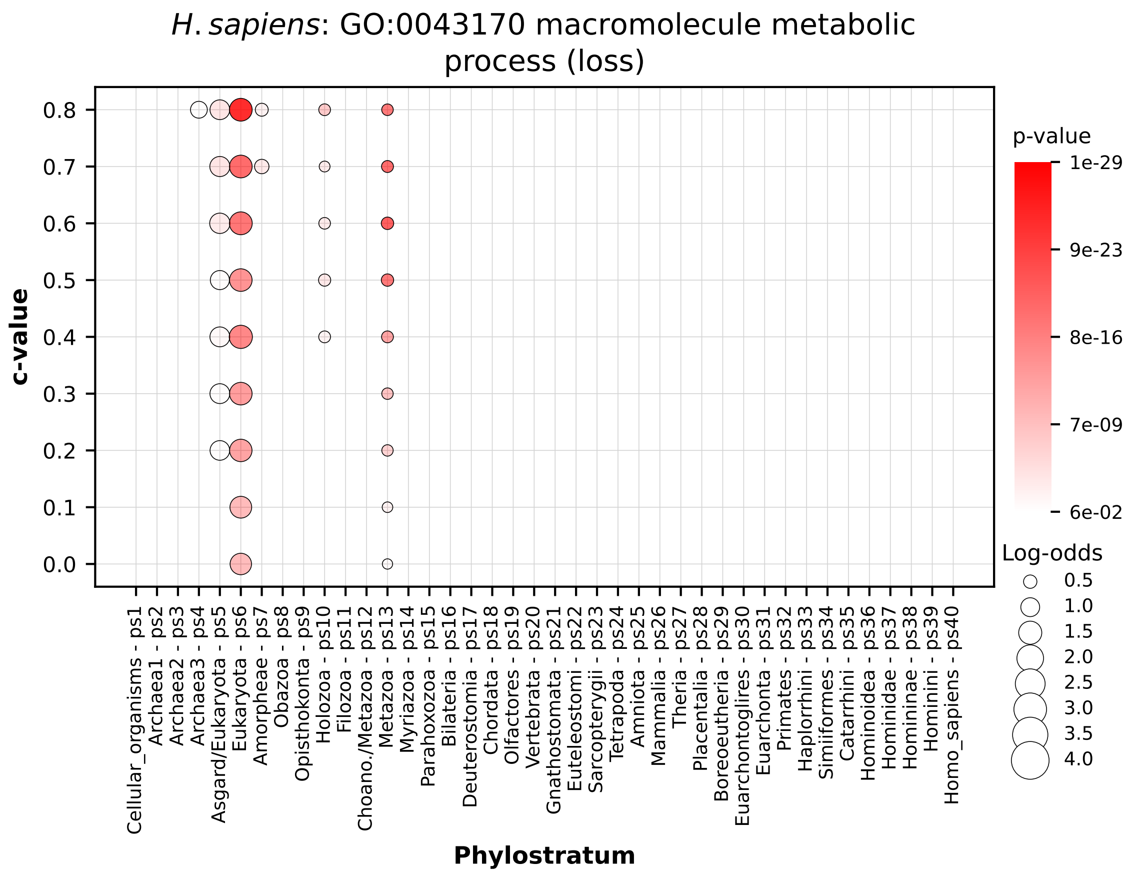

H. sapiens: GO:0043170 macromolecule metabolic process (loss)
c-value
0.8
0.7
0.6
0.5
0.4
0.3
0.2
0.1
0.0
p-value
1e-29
9e-23
8e-16
7e-09
6e-02
Log-odds
0.5
1.0
1.5
2.0
2.5
3.0
3.5
4.0
Cellular_organisms - ps1
Archaea1 - ps2
Archaea2 - ps3
Archaea3 - ps4
Asgard/Eukaryota - ps5
Eukaryota - ps6
Amorpheae - ps7
Obazoa - ps8
Opisthokonta - ps9
Holozoa - ps10
Filozoa - ps11
Choano./Metazoa - ps12
Metazoa - ps13
Myriazoa - ps14
Parahoxozoa - ps15
Bilateria - ps16
Deuterostomia - ps17
Chordata - ps18
Olfactores - ps19
Vertebrata - ps20
Gnathostomata - ps21
Euteleostomi - ps22
Sarcopterygii - ps23
Tetrapoda - ps24
Amniota - ps25
Mammalia - ps26
Theria - ps27
Placentalia - ps28
Boreoeutheria - ps29
Euarchontoglires - ps30
Euarchonta - ps31
Primates - ps32
Haplorrhini - ps33
Simiiformes - ps34
Catarrhini - ps35
Hominoidea - ps36
Hominidae - ps37
Homininae - ps38
Hominini - ps39
Homo_sapiens - ps40
Phylostratum

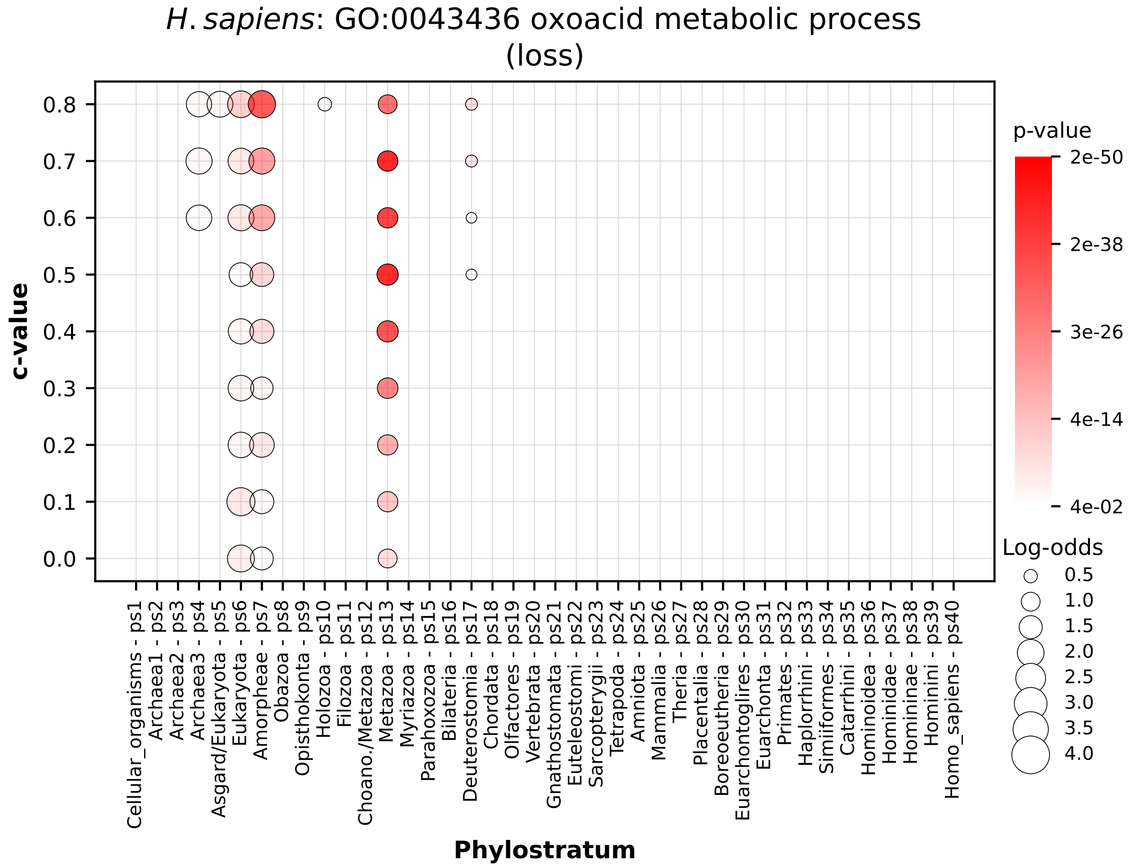

H. sapiens: GO:0043436 oxoacid metabolic process
(loss)
c-value
0.8
0.7
0.6
0.5
0.4
0.3
0.2
0.1
0.0
Phylostratum
Cellular_organisms - ps1
Archaea1 - ps2
Archaea2 - ps3
Archaea3 - ps4
Asgard/Eukaryota - ps5
Eukaryota - ps6
Amorpheae - ps7
Obazoa - ps8
Opisthokonta - ps9
Holozoa - ps10
Filozoa - ps11
Choano./Metazoa - ps12
Metazoa - ps13
Myriazoa - ps14
Parahoxozoa - ps15
Bilateria - ps16
Deuterostomia - ps17
Chordata - ps18
Olfactores - ps19
Vertebrata - ps20
Gnathostomata - ps21
Euteleostomi - ps22
Sarcopterygii - ps23
Tetrapoda - ps24
Amniota - ps25
Mammalia - ps26
Theria - ps27
Placentalia - ps28
Boreoeutheria - ps29
Euarchontoglires - ps30
Euarchonta - ps31
Primates - ps32
Haplorrhini - ps33
Simiiformes - ps34
Catarrhini - ps35
Hominoidea - ps36
Hominidae - ps37
Homininae - ps38
Hominini - ps39
Homo_sapiens - ps40
p-value
2e-50
2e-38
3e-26
4e-14
4e-02
Log-odds
0.5
1.0
1.5
2.0
2.5
3.0
3.5
4.0

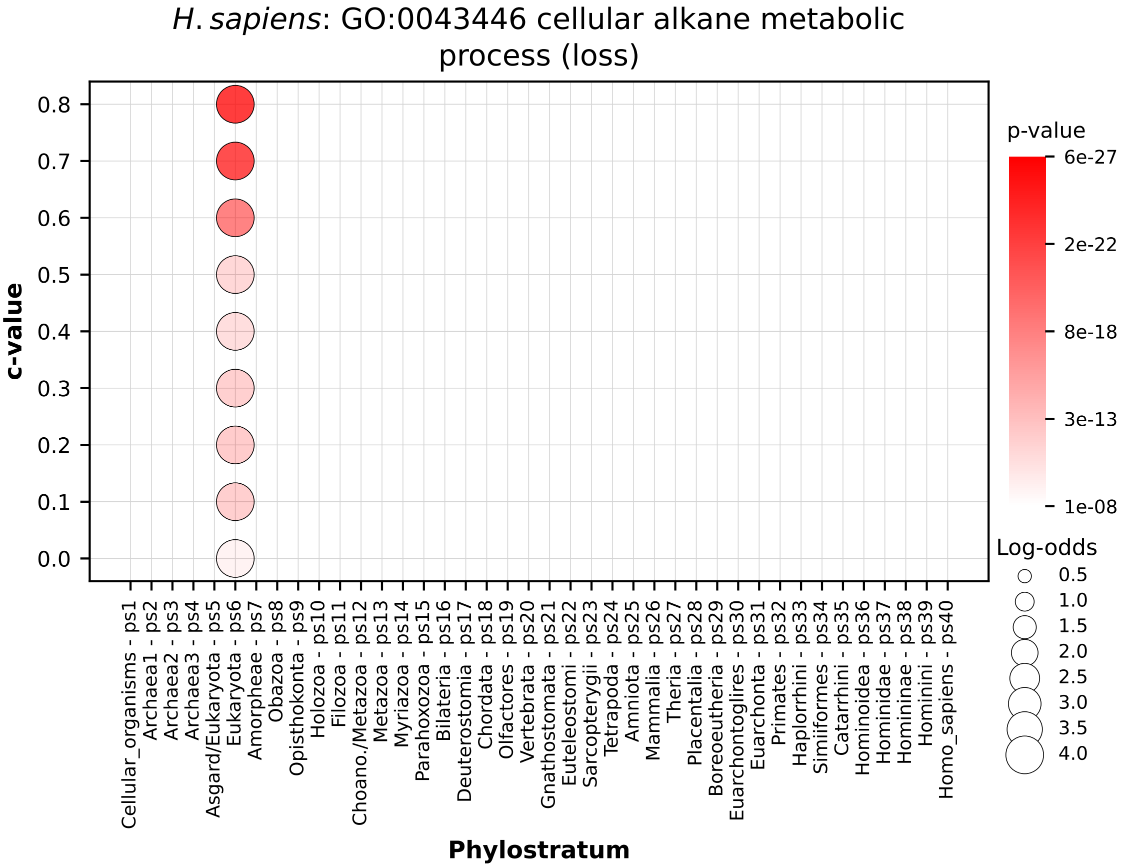
H. sapiens: GO:0043446 cellular alkane metabolic process (loss)
c-value
0.8
0.7
0.6
0.5
0.4
0.3
0.2
0.1
0.0
p-value
6e-27
2e-22
8e-18
3e-13
1e-08
Log-odds
0.5
1.0
1.5
2.0
2.5
3.0
3.5
4.0
Cellular_organisms - ps1
Archaea1 - ps2
Archaea2 - ps3
Archaea3 - ps4
Asgard/Eukaryota - ps5
Eukaryota - ps6
Amorpheae - ps7
Obazoa - ps8
Opisthokonta - ps9
Holozoa - ps10
Filozoa - ps11
Choano./Metazoa - ps12
Metazoa - ps13
Myriazoa - ps14
Parahoxozoa - ps15
Bilateria - ps16
Deuterostomia - ps17
Chordata - ps18
Olfactores - ps19
Vertebrata - ps20
Gnathostomata - ps21
Euteleostomi - ps22
Sarcopterygii - ps23
Tetrapoda - ps24
Amniota - ps25
Mammalia - ps26
Theria - ps27
Placentalia - ps28
Boreoeutheria - ps29
Euarchontoglires - ps30
Euarchonta - ps31
Primates - ps32
Haplorrhini - ps33
Simiiformes - ps34
Catarrhini - ps35
Hominoidea - ps36
Hominidae - ps37
Homininae - ps38
Hominini - ps39
Homo_sapiens - ps40
Phylostratum

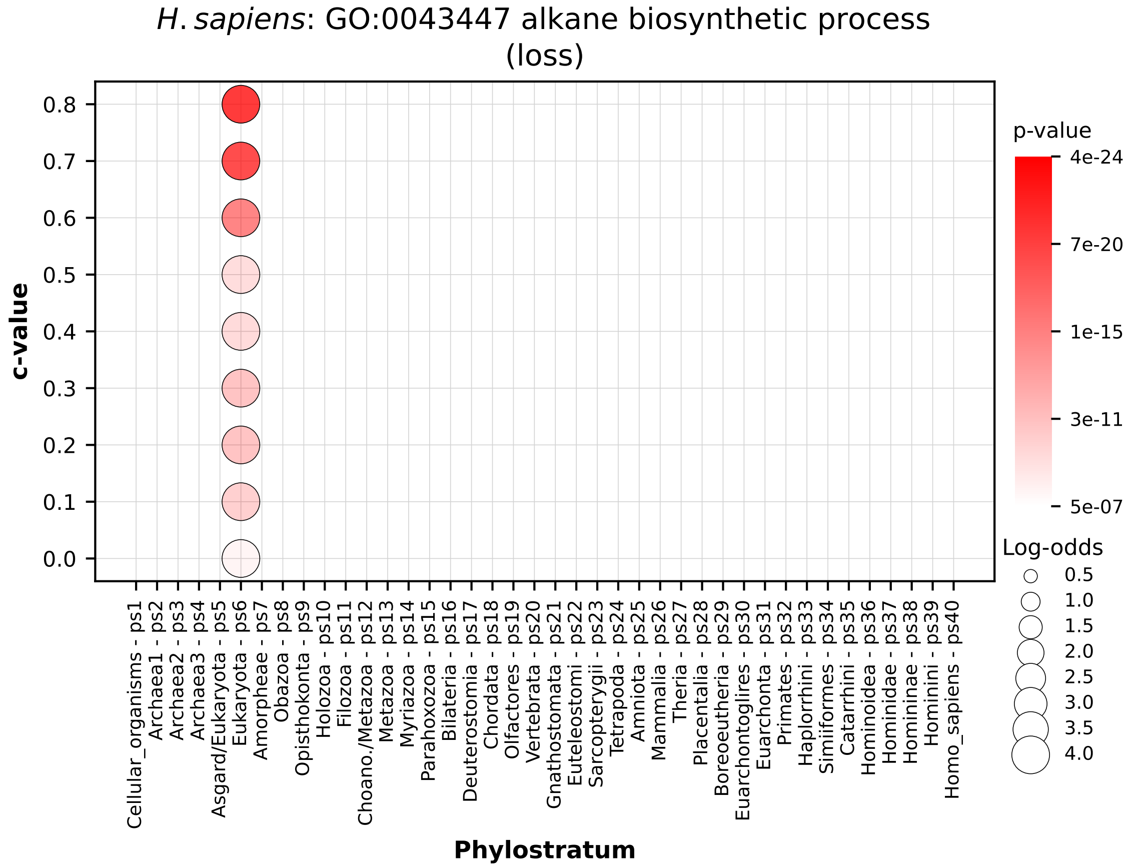
H. sapiens: GO:0043447 alkane biosynthetic process
(loss)
c-value
0.8
0.7
0.6
0.5
0.4
0.3
0.2
0.1
0.0
p-value
4e-24
7e-20
1e-15
3e-11
5e-07
Log-odds
0.5
1.0
1.5
2.0
2.5
3.0
3.5
4.0
Cellular_organisms - ps1
Archaea1 - ps2
Archaea2 - ps3
Archaea3 - ps4
Asgard/Eukaryota - ps5
Eukaryota - ps6
Amorpheae - ps7
Obazoa - ps8
Opisthokonta - ps9
Holozoa - ps10
Filozoa - ps11
Choano./Metazoa - ps12
Metazoa - ps13
Myriazoa - ps14
Parahoxozoa - ps15
Bilateria - ps16
Deuterostomia - ps17
Chordata - ps18
Olfactores - ps19
Vertebrata - ps20
Gnathostomata - ps21
Euteleostomi - ps22
Sarcopterygii - ps23
Tetrapoda - ps24
Amniota - ps25
Mammalia - ps26
Theria - ps27
Placentalia - ps28
Boreoeutheria - ps29
Euarchontoglires - ps30
Euarchonta - ps31
Primates - ps32
Haplorrhini - ps33
Simiiformes - ps34
Catarrhini - ps35
Hominoidea - ps36
Hominidae - ps37
Homininae - ps38
Hominini - ps39
Homo_sapiens - ps40
Phylostratum

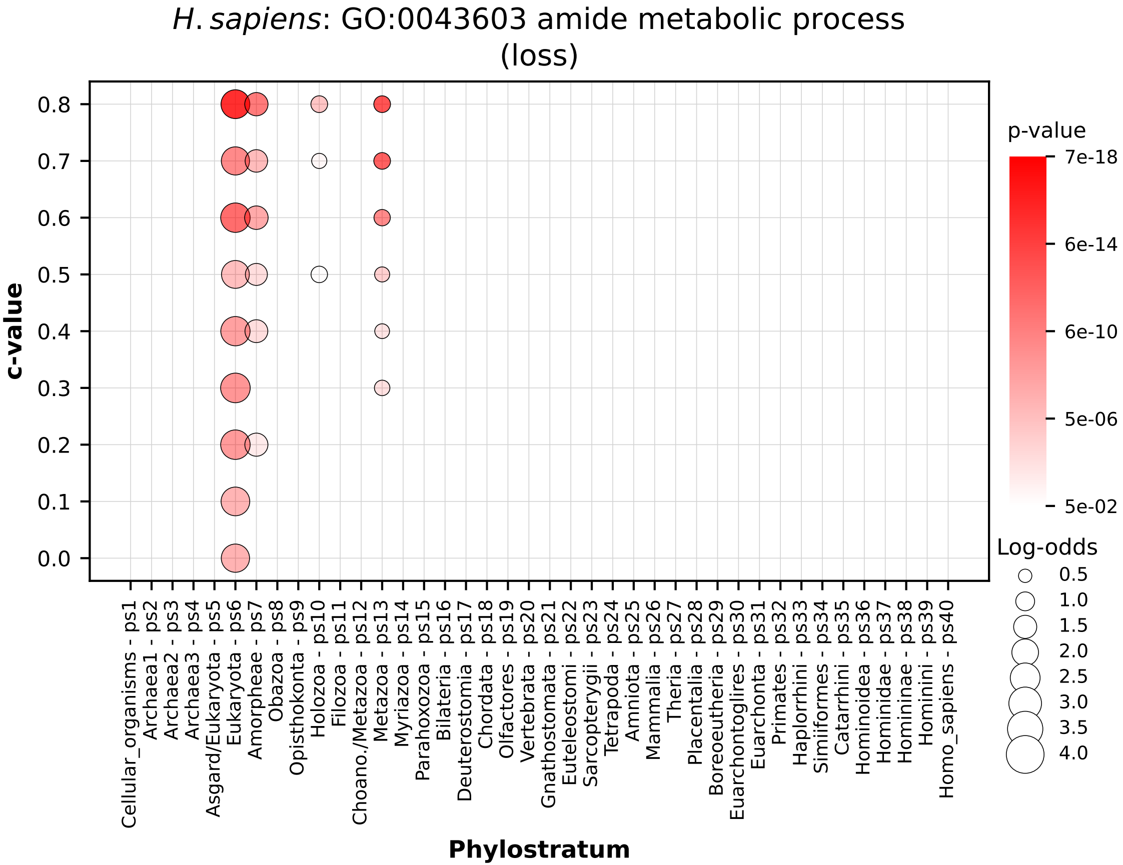

H. sapiens: GO:0043603 amide metabolic process
(loss)
c-value
0.8
0.7
0.6
0.5
0.4
0.3
0.2
0.1
0.0
Phylostratum
Cellular_organisms - ps1
Archaea1 - ps2
Archaea2 - ps3
Archaea3 - ps4
Asgard/Eukaryota - ps5
Eukaryota - ps6
Amorpheae - ps7
Obazoa - ps8
Opisthokonta - ps9
Holozoa - ps10
Filozoa - ps11
Choano./Metazoa - ps12
Metazoa - ps13
Myriazoa - ps14
Parahoxozoa - ps15
Bilateria - ps16
Deuterostomia - ps17
Chordata - ps18
Olfactores - ps19
Vertebrata - ps20
Gnathostomata - ps21
Euteleostomi - ps22
Sarcopterygii - ps23
Tetrapoda - ps24
Amniota - ps25
Mammalia - ps26
Theria - ps27
Placentalia - ps28
Boreoeutheria - ps29
Euarchontoglires - ps30
Euarchonta - ps31
Primates - ps32
Haplorrhini - ps33
Simiiformes - ps34
Catarrhini - ps35
Hominoidea - ps36
Hominidae - ps37
Homininae - ps38
Hominini - ps39
Homo_sapiens - ps40
p-value
7e-18
6e-14
6e-10
5e-06
5e-02
Log-odds
0.5
1.0
1.5
2.0
2.5
3.0
3.5
4.0

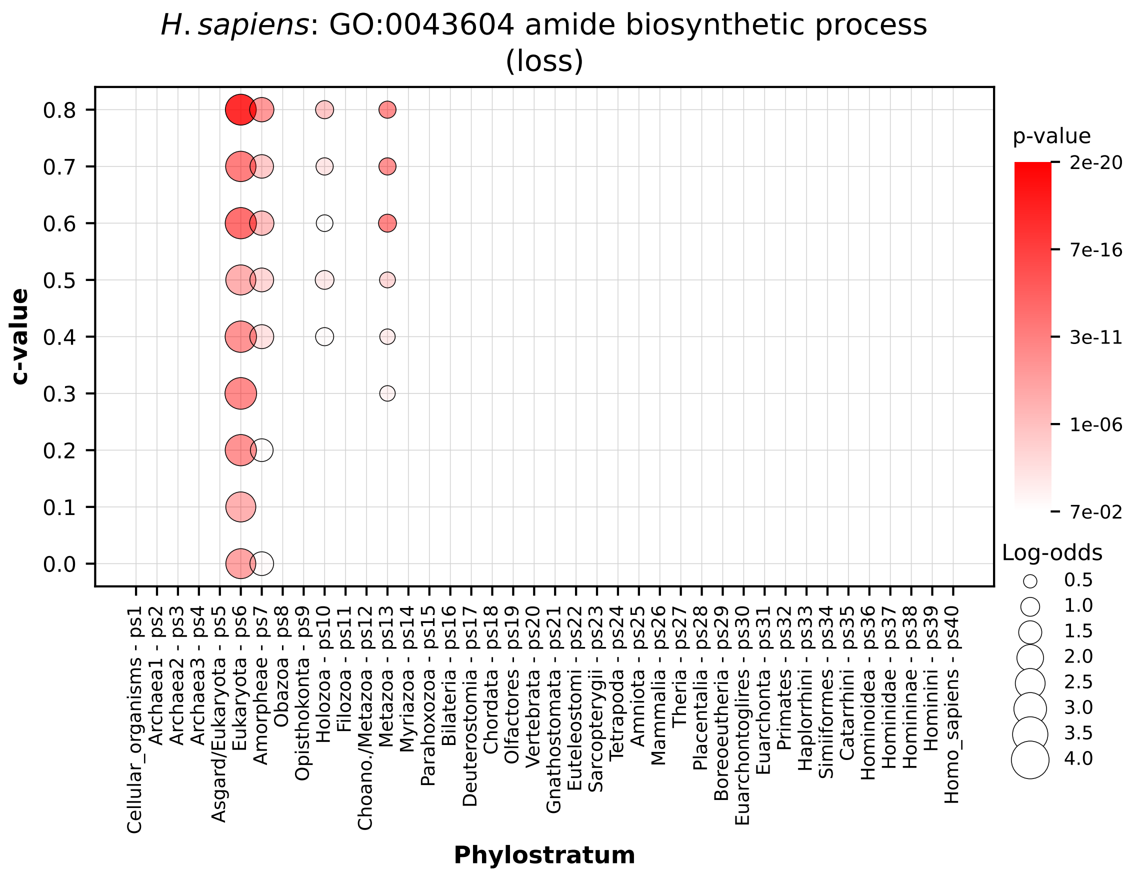

H. sapiens: GO:0043604 amide biosynthetic process (loss)
c-value
0.0
0.1
0.2
0.3
0.4
0.5
0.6
0.7
0.8
Phylostratum
Cellular_organisms - ps1
Archaea1 - ps2
Archaea2 - ps3
Archaea3 - ps4
Asgard/Eukaryota - ps5
Eukaryota - ps6
Amorpheae - ps7
Obazoa - ps8
Opisthokonta - ps9
Holozoa - ps10
Filozoa - ps11
Choano./Metazoa - ps12
Metazoa - ps13
Myriazoa - ps14
Parahoxozoa - ps15
Bilateria - ps16
Deuterostomia - ps17
Chordata - ps18
Olfactores - ps19
Vertebrata - ps20
Gnathostomata - ps21
Euteleostomi - ps22
Sarcopterygii - ps23
Tetrapoda - ps24
Amniota - ps25
Mammalia - ps26
Theria - ps27
Placentalia - ps28
Boreoeutheria - ps29
Euarchontoglires - ps30
Euarchonta - ps31
Primates - ps32
Haplorrhini - ps33
Simiiformes - ps34
Catarrhini - ps35
Hominoidea - ps36
Hominidae - ps37
Homininae - ps38
Hominini - ps39
Homo_sapiens - ps40
p-value
2e-20
7e-16
3e-11
1e-06
7e-02
Log-odds
0.5
1.0
1.5
2.0
2.5
3.0
3.5
4.0

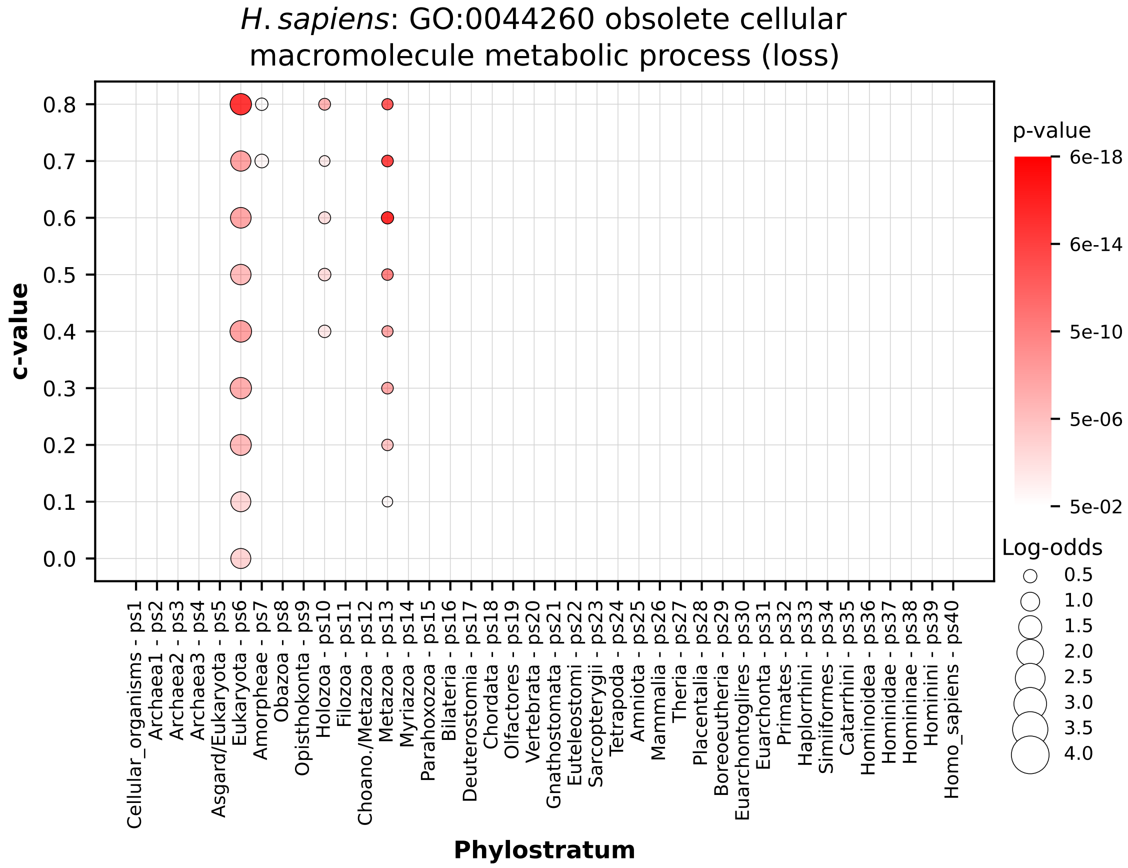

H. sapiens: GO:0044260 obsolete cellular macromolecule metabolic process (loss)
c-value
0.8
0.7
0.6
0.5
0.4
0.3
0.2
0.1
0.0
Phylostratum
Cellular_organisms - ps1
Archaea1 - ps2
Archaea2 - ps3
Archaea3 - ps4
Asgard/Eukaryota - ps5
Eukaryota - ps6
Amorpheae - ps7
Obazoa - ps8
Opisthokonta - ps9
Holozoa - ps10
Filozoa - ps11
Choano./Metazoa - ps12
Metazoa - ps13
Myriazoa - ps14
Parahoxozoa - ps15
Bilateria - ps16
Deuterostomia - ps17
Chordata - ps18
Olfactores - ps19
Vertebrata - ps20
Gnathostomata - ps21
Euteleostomi - ps22
Sarcopterygii - ps23
Tetrapoda - ps24
Amniota - ps25
Mammalia - ps26
Theria - ps27
Placentalia - ps28
Boreoeutheria - ps29
Euarchontoglires - ps30
Euarchonta - ps31
Primates - ps32
Haplorrhini - ps33
Simiiformes - ps34
Catarrhini - ps35
Hominoidea - ps36
Hominidae - ps37
Homininae - ps38
Hominini - ps39
Homo_sapiens - ps40
p-value
6e-18
6e-14
5e-10
5e-06
5e-02
Log-odds
0.5
1.0
1.5
2.0
2.5
3.0
3.5
4.0

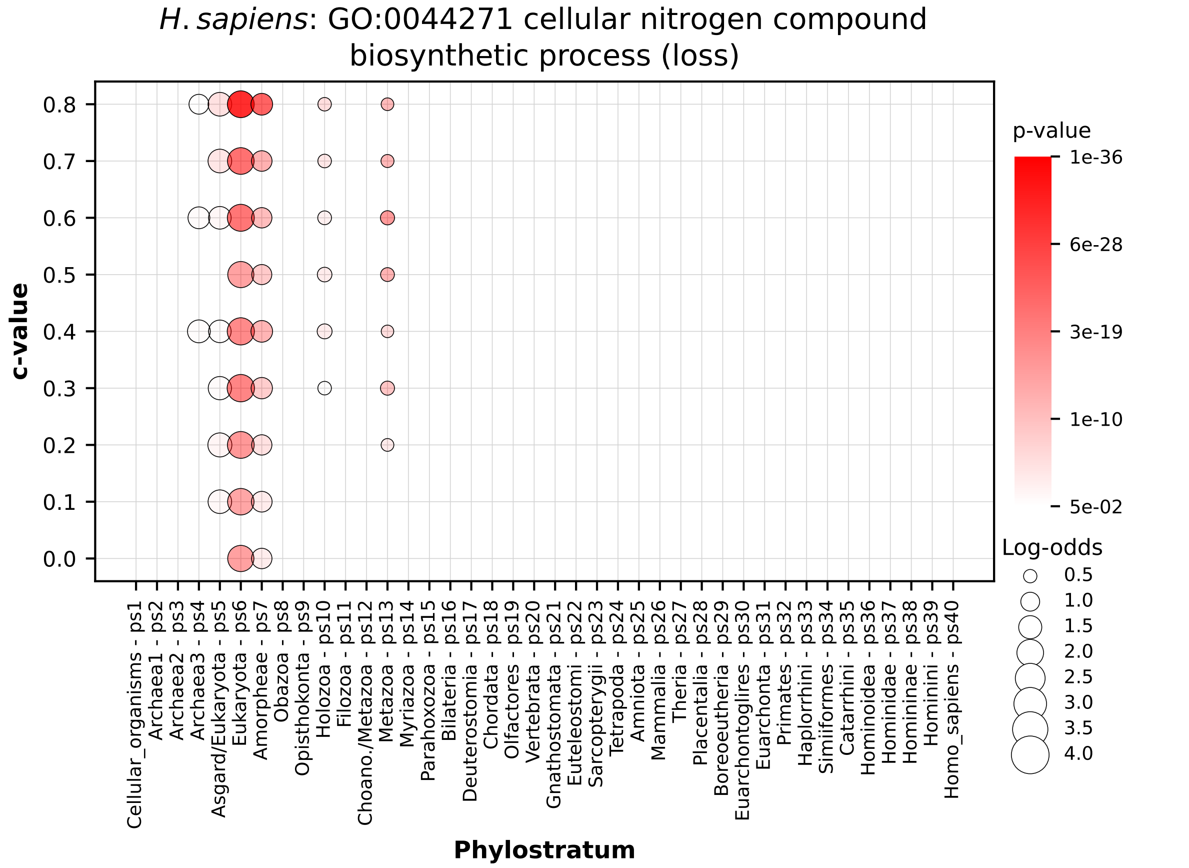

H. sapiens: GO:0044271 cellular nitrogen compound biosynthetic process (loss)
c-value
0.8
0.7
0.6
0.5
0.4
0.3
0.2
0.1
0.0
p-value
1e-36
6e-28
3e-19
1e-10
5e-02
Log-odds
0.5
1.0
1.5
2.0
2.5
3.0
3.5
4.0
Cellular_organisms - ps1
Archaea1 - ps2
Archaea2 - ps3
Archaea3 - ps4
Asgard/Eukaryota - ps5
Eukaryota - ps6
Amorpheae - ps7
Obazoa - ps8
Opisthokonta - ps9
Holozoa - ps10
Filozoa - ps11
Choano./Metazoa - ps12
Metazoa - ps13
Myriazoa - ps14
Parahoxozoa - ps15
Bilateria - ps16
Deuterostomia - ps17
Chordata - ps18
Olfactores - ps19
Vertebrata - ps20
Gnathostomata - ps21
Euteleostomi - ps22
Sarcopterygii - ps23
Tetrapoda - ps24
Amniota - ps25
Mammalia - ps26
Theria - ps27
Placentalia - ps28
Boreoeutheria - ps29
Euarchontoglires - ps30
Euarchonta - ps31
Primates - ps32
Haplorrhini - ps33
Simiiformes - ps34
Catarrhini - ps35
Hominoidea - ps36
Hominidae - ps37
Homininae - ps38
Hominini - ps39
Homo_sapiens - ps40
Phylostratum

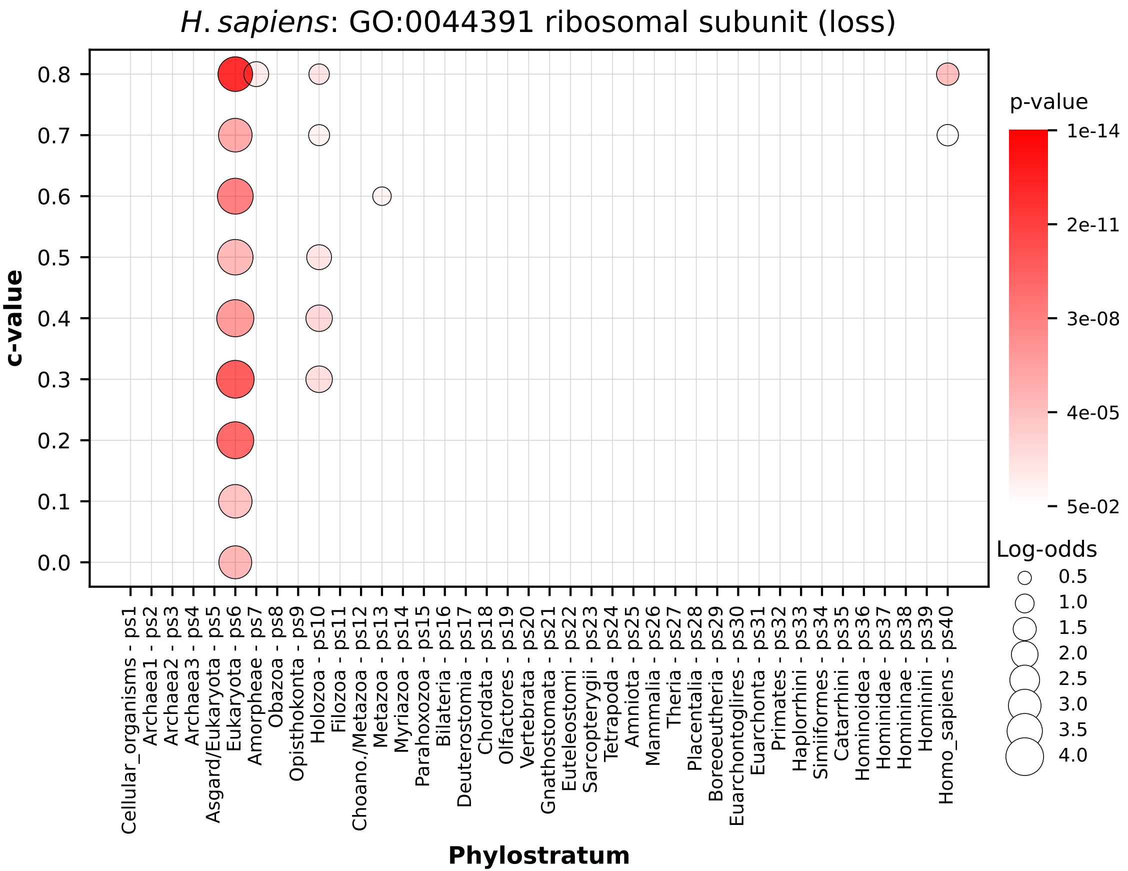
H. sapiens: GO:0044391 ribosomal subunit (loss)
c-value
Phylostratum
p-value
1e-14
2e-11
3e-08
4e-05
5e-02
Log-odds
0.5
1.0
1.5
2.0
2.5
3.0
3.5
4.0
Cellular_organisms - ps1
Archaea1 - ps2
Archaea2 - ps3
Archaea3 - ps4
Asgard/Eukaryota - ps5
Eukaryota - ps6
Amorpheae - ps7
Obazoa - ps8
Opisthokonta - ps9
Holozoa - ps10
Filozoa - ps11
Choano./Metazoa - ps12
Metazoa - ps13
Myriazoa - ps14
Parahoxozoa - ps15
Bilateria - ps16
Deuterostomia - ps17
Chordata - ps18
Olfactores - ps19
Vertebrata - ps20
Gnathostomata - ps21
Euteleostomi - ps22
Sarcopterygii - ps23
Tetrapoda - ps24
Amniota - ps25
Mammalia - ps26
Theria - ps27
Placentalia - ps28
Boreoeutheria - ps29
Euarchontoglires - ps30
Euarchonta - ps31
Primates - ps32
Haplorrhini - ps33
Simiiformes - ps34
Catarrhini - ps35
Hominoidea - ps36
Hominidae - ps37
Homininae - ps38
Hominini - ps39
Homo_sapiens - ps40

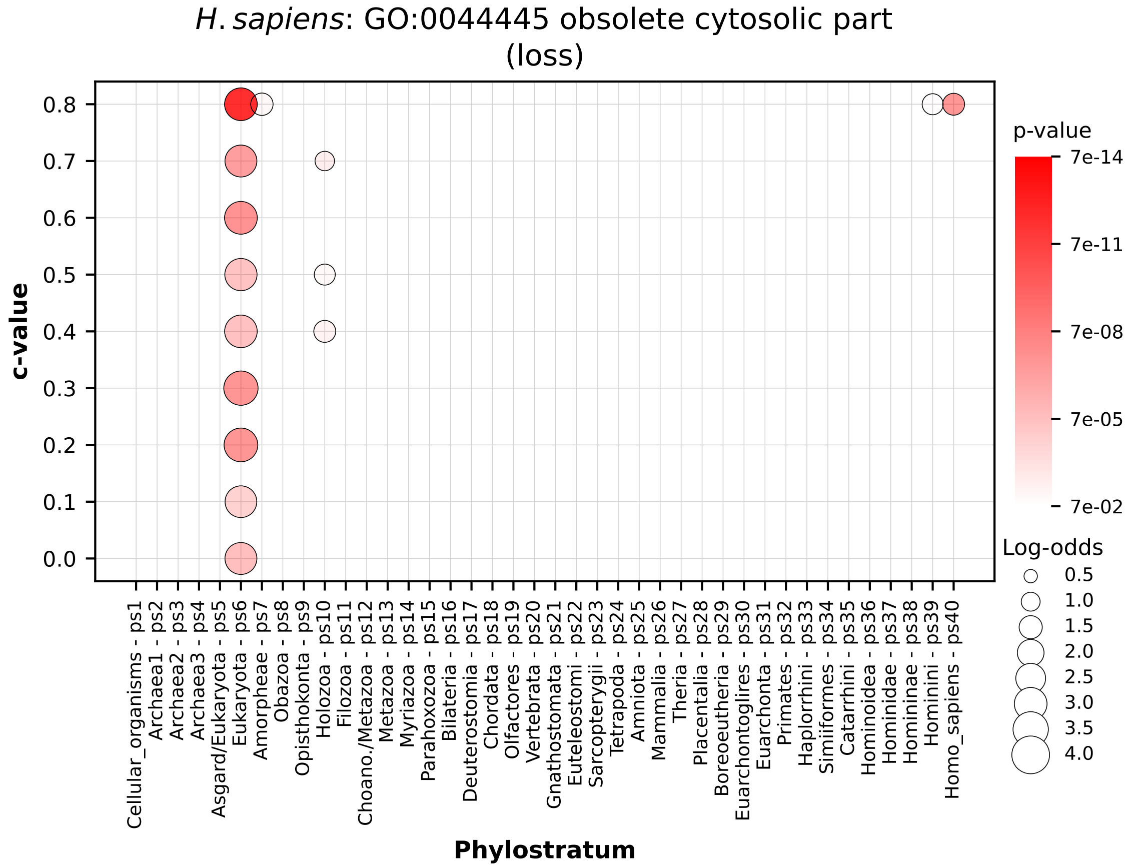

H. sapiens: GO:0044445 obsolete cytosolic part
(loss)
c-value
0.8
0.7
0.6
0.5
0.4
0.3
0.2
0.1
0.0
p-value
7e-14
7e-11
7e-08
7e-05
7e-02
Log-odds
0.5
1.0
1.5
2.0
2.5
3.0
3.5
4.0
Cellular_organisms - ps1
Archaea1 - ps2
Archaea2 - ps3
Archaea3 - ps4
Asgard/Eukaryota - ps5
Eukaryota - ps6
Amorpheae - ps7
Obazoa - ps8
Opisthokonta - ps9
Holozoa - ps10
Filozoa - ps11
Choano./Metazoa - ps12
Metazoa - ps13
Myriazoa - ps14
Parahoxozoa - ps15
Bilateria - ps16
Deuterostomia - ps17
Chordata - ps18
Olfactores - ps19
Vertebrata - ps20
Gnathostomata - ps21
Euteleostomi - ps22
Sarcopterygii - ps23
Tetrapoda - ps24
Amniota - ps25
Mammalia - ps26
Theria - ps27
Placentalia - ps28
Boreoeutheria - ps29
Euarchontoglires - ps30
Euarchonta - ps31
Primates - ps32
Haplorrhini - ps33
Simiiformes - ps34
Catarrhini - ps35
Hominoidea - ps36
Hominidae - ps37
Homininae - ps38
Hominini - ps39
Homo_sapiens - ps40
Phylostratum

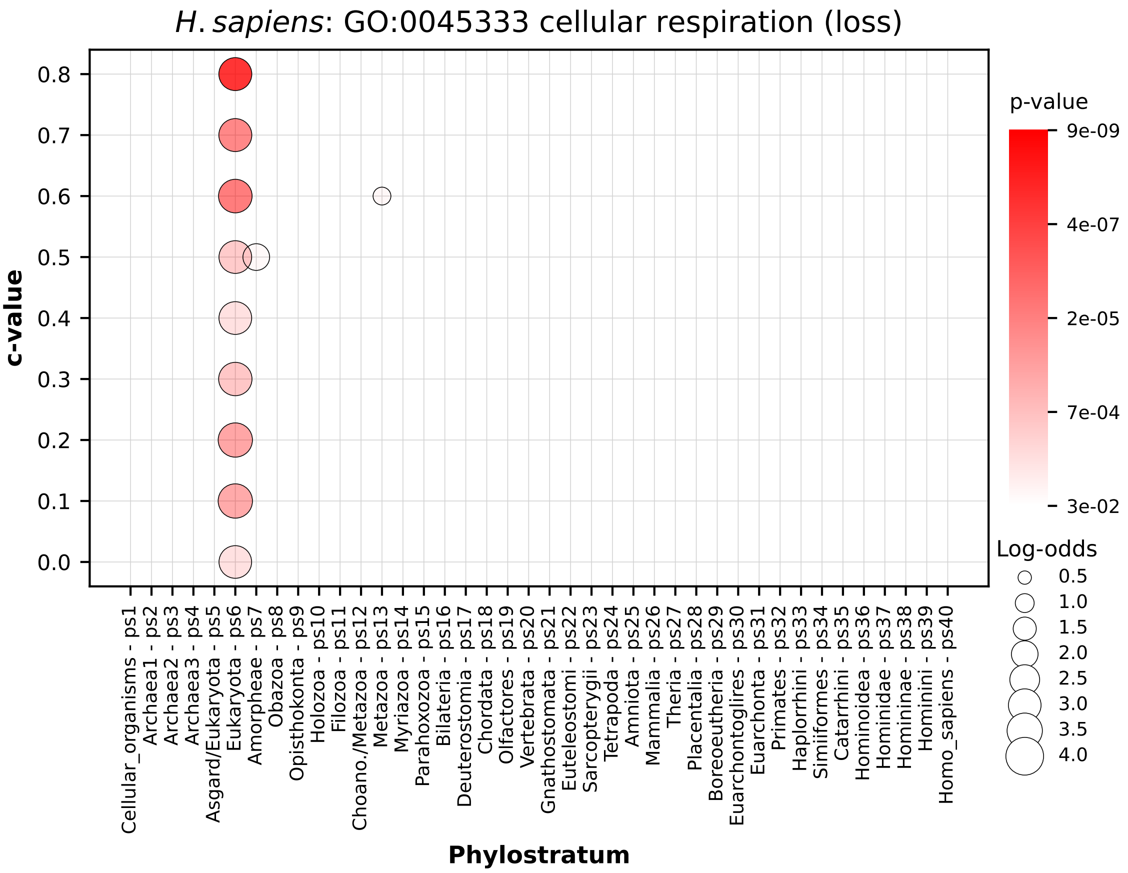
H. sapiens: GO:0045333 cellular respiration (loss)
c-value
0.0
0.1
0.2
0.3
0.4
0.5
0.6
0.7
0.8
p-value
9e-09
4e-07
2e-05
7e-04
3e-02
Log-odds
0.5
1.0
1.5
2.0
2.5
3.0
3.5
4.0
Cellular_organisms - ps1
Archaea1 - ps2
Archaea2 - ps3
Archaea3 - ps4
Asgard/Eukaryota - ps5
Eukaryota - ps6
Amorpheae - ps7
Obazoa - ps8
Opisthokonta - ps9
Holozoa - ps10
Filozoa - ps11
Choano./Metazoa - ps12
Metazoa - ps13
Myriazoa - ps14
Parahoxozoa - ps15
Bilateria - ps16
Deuterostomia - ps17
Chordata - ps18
Olfactores - ps19
Vertebrata - ps20
Gnathostomata - ps21
Euteleostomi - ps22
Sarcopterygii - ps23
Tetrapoda - ps24
Amniota - ps25
Mammalia - ps26
Theria - ps27
Placentalia - ps28
Boreoeutheria - ps29
Euarchontoglires - ps30
Euarchonta - ps31
Primates - ps32
Haplorrhini - ps33
Simiiformes - ps34
Catarrhini - ps35
Hominoidea - ps36
Hominidae - ps37
Homininae - ps38
Hominini - ps39
Homo_sapiens - ps40
Phylostratum

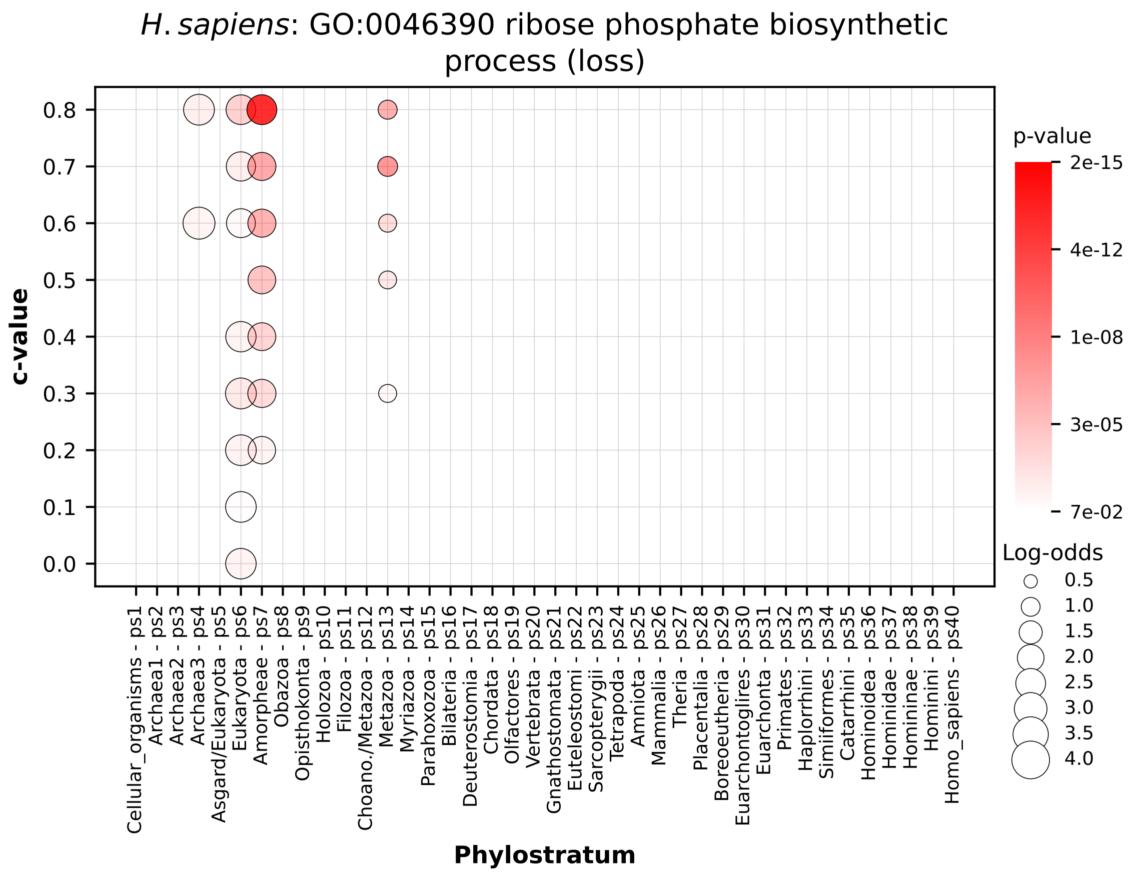

H. sapiens: GO:0046390 ribose phosphate biosynthetic process (loss)
c-value
0.8
0.7
0.6
0.5
0.4
0.3
0.2
0.1
0.0
Phylostratum
Cellular_organisms - ps1
Archaea1 - ps2
Archaea2 - ps3
Archaea3 - ps4
Asgard/Eukaryota - ps5
Eukaryota - ps6
Amorpheae - ps7
Obazoa - ps8
Opisthokonta - ps9
Holozoa - ps10
Filozoa - ps11
Choano./Metazoa - ps12
Metazoa - ps13
Myriazoa - ps14
Parahoxozoa - ps15
Bilateria - ps16
Deuterostomia - ps17
Chordata - ps18
Olfactores - ps19
Vertebrata - ps20
Gnathostomata - ps21
Euteleostomi - ps22
Sarcopterygii - ps23
Tetrapoda - ps24
Amniota - ps25
Mammalia - ps26
Theria - ps27
Placentalia - ps28
Boreoeutheria - ps29
Euarchontoglires - ps30
Euarchonta - ps31
Primates - ps32
Haplorrhini - ps33
Simiiformes - ps34
Catarrhini - ps35
Hominoidea - ps36
Hominidae - ps37
Homininae - ps38
Hominini - ps39
Homo_sapiens - ps40
p-value
2e-15
4e-12
1e-08
3e-05
7e-02
Log-odds
0.5
1.0
1.5
2.0
2.5
3.0
3.5
4.0

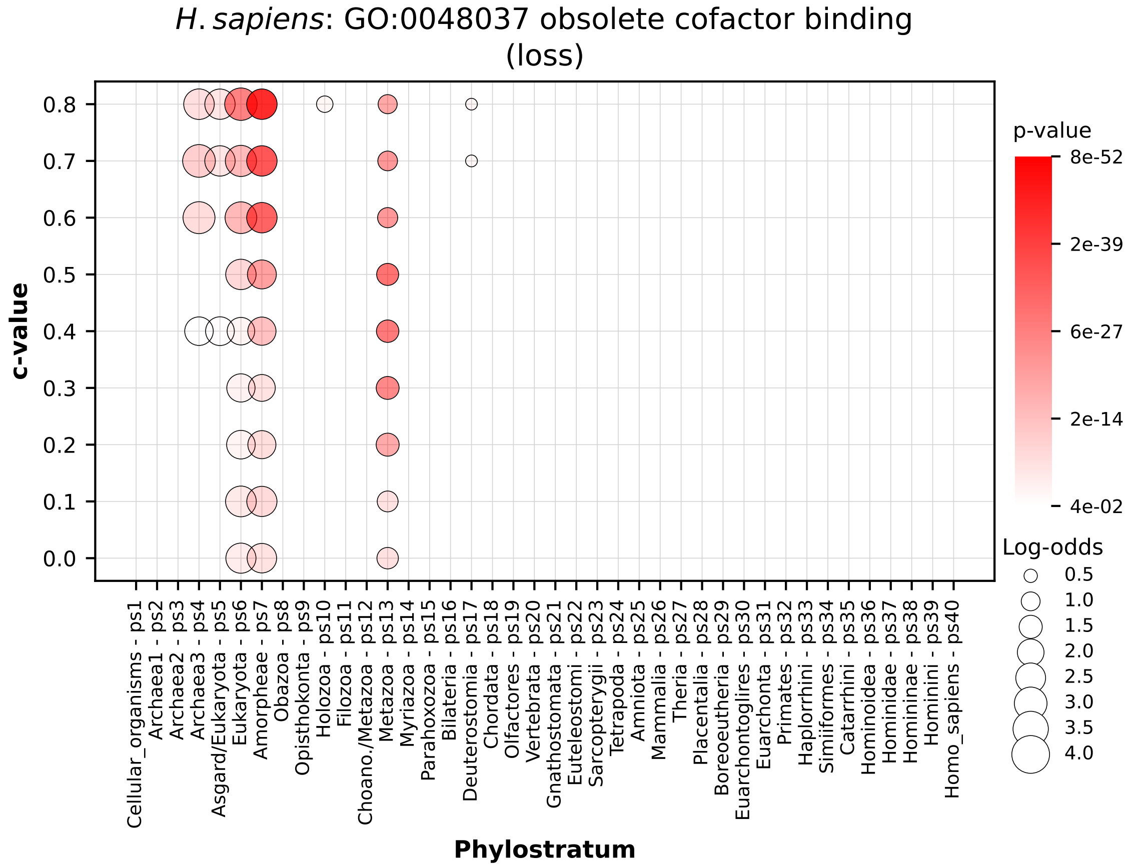

H. sapiens: GO:0048037 obsolete cofactor binding
(loss)
c-value
0.8
0.7
0.6
0.5
0.4
0.3
0.2
0.1
0.0
p-value
8e-52
2e-39
6e-27
2e-14
4e-02
Log-odds
0.5
1.0
1.5
2.0
2.5
3.0
3.5
4.0
Cellular_organisms - ps1
Archaea1 - ps2
Archaea2 - ps3
Archaea3 - ps4
Asgard/Eukaryota - ps5
Eukaryota - ps6
Amorpheae - ps7
Obazoa - ps8
Opisthokonta - ps9
Holozoa - ps10
Filozoa - ps11
Choano./Metazoa - ps12
Metazoa - ps13
Myriazoa - ps14
Parahoxozoa - ps15
Bilateria - ps16
Deuterostomia - ps17
Chordata - ps18
Olfactores - ps19
Vertebrata - ps20
Gnathostomata - ps21
Euteleostomi - ps22
Sarcopterygii - ps23
Tetrapoda - ps24
Amniota - ps25
Mammalia - ps26
Theria - ps27
Placentalia - ps28
Boreoeutheria - ps29
Euarchontoglires - ps30
Euarchonta - ps31
Primates - ps32
Haplorrhini - ps33
Simiiformes - ps34
Catarrhini - ps35
Hominoidea - ps36
Hominidae - ps37
Homininae - ps38
Hominini - ps39
Homo_sapiens - ps40
Phylostratum

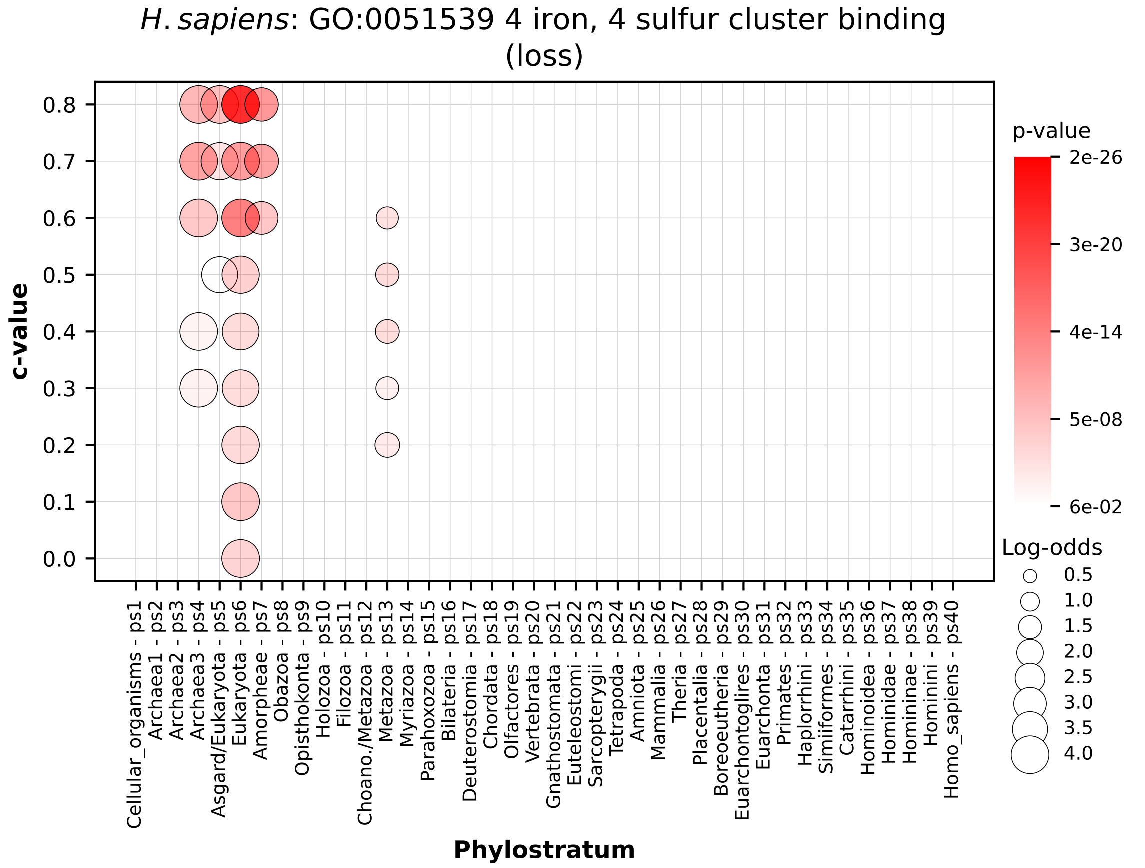

H. sapiens: GO:0051539 4 iron, 4 sulfur cluster binding (loss)
c-value
0.8
0.7
0.6
0.5
0.4
0.3
0.2
0.1
0.0
Phylostratum
Cellular_organisms - ps1
Archaea1 - ps2
Archaea2 - ps3
Archaea3 - ps4
Asgard/Eukaryota - ps5
Eukaryota - ps6
Amorpheae - ps7
Obazoa - ps8
Opisthokonta - ps9
Holozoa - ps10
Filozoa - ps11
Choano./Metazoa - ps12
Metazoa - ps13
Myriazoa - ps14
Parahoxozoa - ps15
Bilateria - ps16
Deuterostomia - ps17
Chordata - ps18
Olfactores - ps19
Vertebrata - ps20
Gnathostomata - ps21
Euteleostomi - ps22
Sarcopterygii - ps23
Tetrapoda - ps24
Amniota - ps25
Mammalia - ps26
Theria - ps27
Placentalia - ps28
Boreoeutheria - ps29
Euarchontoglires - ps30
Euarchonta - ps31
Primates - ps32
Haplorrhini - ps33
Simiiformes - ps34
Catarrhini - ps35
Hominoidea - ps36
Hominidae - ps37
Homininae - ps38
Hominini - ps39
Homo_sapiens - ps40
p-value
2e-26
3e-20
4e-14
5e-08
6e-02
Log-odds
0.5
1.0
1.5
2.0
2.5
3.0
3.5
4.0

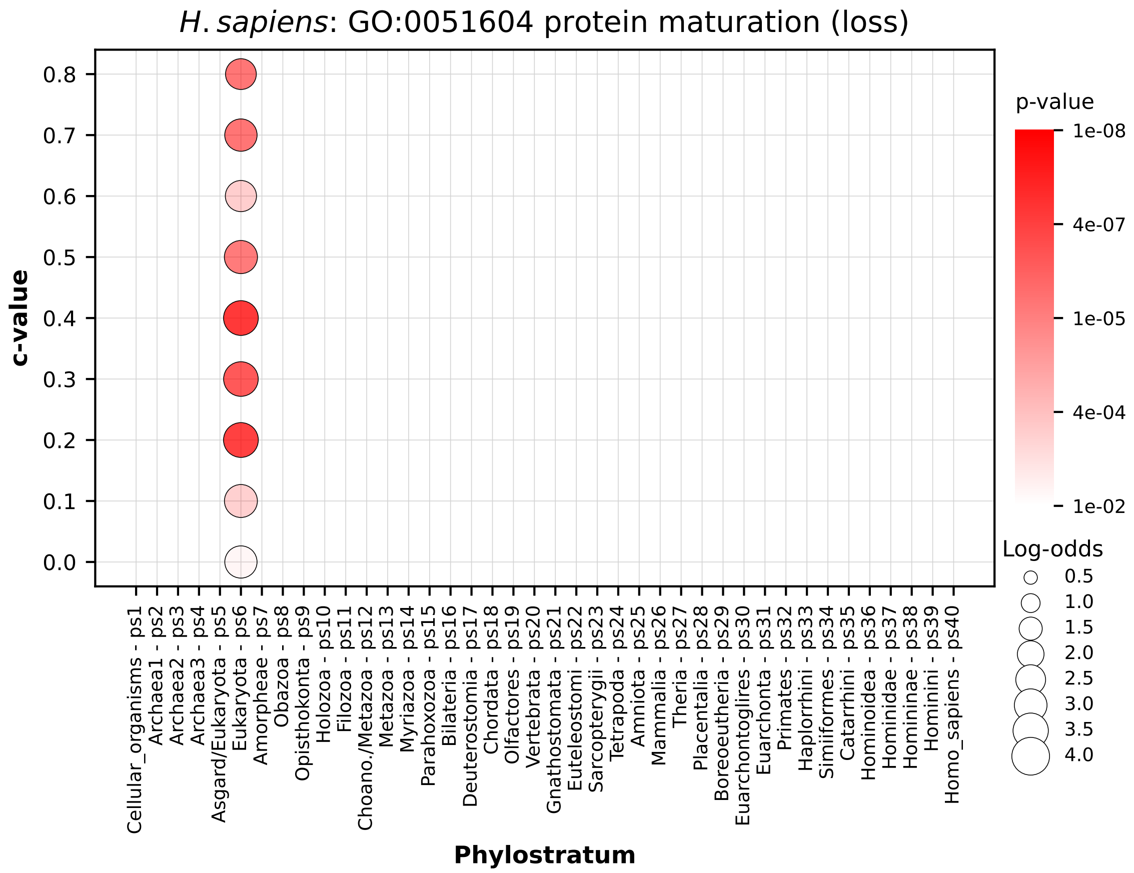

H. sapiens: GO:0051604 protein maturation (loss)
c-value
0.0
0.1
0.2
0.3
0.4
0.5
0.6
0.7
0.8
Phylostratum
Cellular_organisms - ps1
Archaea1 - ps2
Archaea2 - ps3
Archaea3 - ps4
Asgard/Eukaryota - ps5
Eukaryota - ps6
Amorpheae - ps7
Obazoa - ps8
Opisthokonta - ps9
Holozoa - ps10
Filozoa - ps11
Choano./Metazoa - ps12
Metazoa - ps13
Myriazoa - ps14
Parahoxozoa - ps15
Bilateria - ps16
Deuterostomia - ps17
Chordata - ps18
Olfactores - ps19
Vertebrata - ps20
Gnathostomata - ps21
Euteleostomi - ps22
Sarcopterygii - ps23
Tetrapoda - ps24
Amniota - ps25
Mammalia - ps26
Theria - ps27
Placentalia - ps28
Boreoeutheria - ps29
Euarchontoglires - ps30
Euarchonta - ps31
Primates - ps32
Haplorrhini - ps33
Simiiformes - ps34
Catarrhini - ps35
Hominoidea - ps36
Hominidae - ps37
Homininae - ps38
Hominini - ps39
Homo_sapiens - ps40
p-value
1e-08
4e-07
1e-05
4e-04
1e-02
Log-odds
0.5
1.0
1.5
2.0
2.5
3.0
3.5
4.0

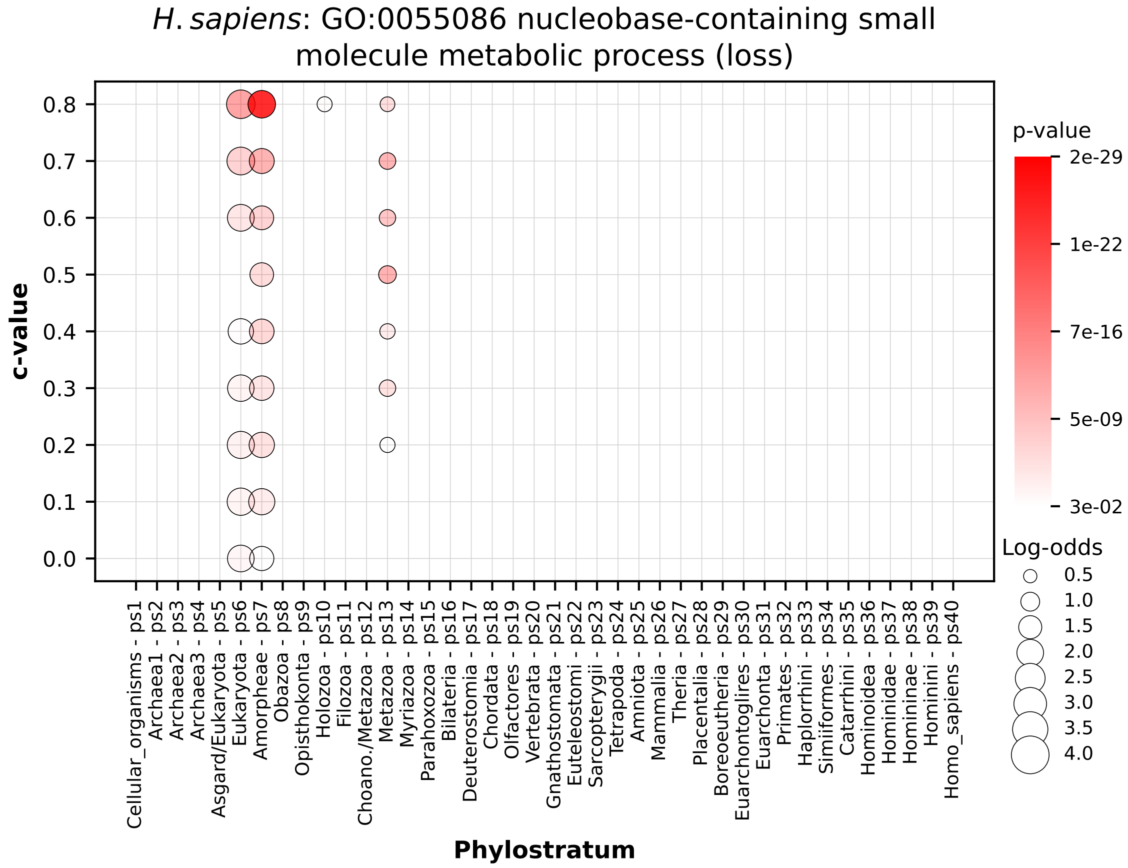
H. sapiens: GO:0055086 nucleobase-containing small molecule metabolic process (loss)
c-value
0.8
0.7
0.6
0.5
0.4
0.3
0.2
0.1
0.0
Phylostratum
Cellular_organisms - ps1
Archaea1 - ps2
Archaea2 - ps3
Archaea3 - ps4
Asgard/Eukaryota - ps5
Eukaryota - ps6
Amorpheae - ps7
Obazoa - ps8
Opisthokonta - ps9
Holozoa - ps10
Filozoa - ps11
Choano./Metazoa - ps12
Metazoa - ps13
Myriazoa - ps14
Parahoxozoa - ps15
Bilateria - ps16
Deuterostomia - ps17
Chordata - ps18
Olfactores - ps19
Vertebrata - ps20
Gnathostomata - ps21
Euteleostomi - ps22
Sarcopterygii - ps23
Tetrapoda - ps24
Amniota - ps25
Mammalia - ps26
Theria - ps27
Placentalia - ps28
Boreoeutheria - ps29
Euarchontoglires - ps30
Euarchonta - ps31
Primates - ps32
Haplorrhini - ps33
Simiiformes - ps34
Catarrhini - ps35
Hominoidea - ps36
Hominidae - ps37
Homininae - ps38
Hominini - ps39
Homo_sapiens - ps40
p-value
2e-29
1e-22
7e-16
5e-09
3e-02
Log-odds
0.5
1.0
1.5
2.0
2.5
3.0
3.5
4.0

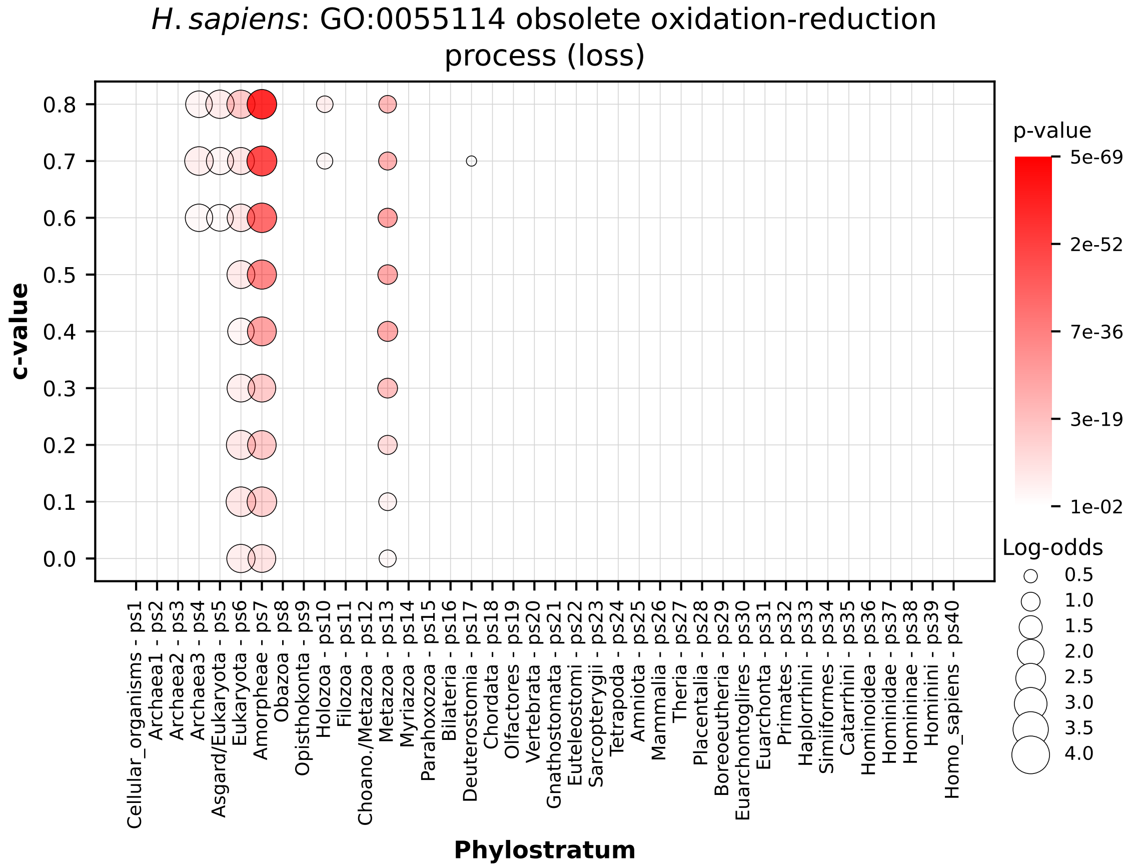

H. sapiens: GO:0055114 obsolete oxidation-reduction process (loss)
c-value
0.0
0.1
0.2
0.3
0.4
0.5
0.6
0.7
0.8
p-value
5e-69
2e-52
7e-36
3e-19
1e-02
Log-odds
0.5
1.0
1.5
2.0
2.5
3.0
3.5
4.0
Cellular_organisms - ps1
Archaea1 - ps2
Archaea2 - ps3
Archaea3 - ps4
Asgard/Eukaryota - ps5
Eukaryota - ps6
Amorpheae - ps7
Obazoa - ps8
Opisthokonta - ps9
Holozoa - ps10
Filozoa - ps11
Choano./Metazoa - ps12
Metazoa - ps13
Myriazoa - ps14
Parahoxozoa - ps15
Bilateria - ps16
Deuterostomia - ps17
Chordata - ps18
Olfactores - ps19
Vertebrata - ps20
Gnathostomata - ps21
Euteleostomi - ps22
Sarcopterygii - ps23
Tetrapoda - ps24
Amniota - ps25
Mammalia - ps26
Theria - ps27
Placentalia - ps28
Boreoeutheria - ps29
Euarchontoglires - ps30
Euarchonta - ps31
Primates - ps32
Haplorrhini - ps33
Simiiformes - ps34
Catarrhini - ps35
Hominoidea - ps36
Hominidae - ps37
Homininae - ps38
Hominini - ps39
Homo_sapiens - ps40
Phylostratum

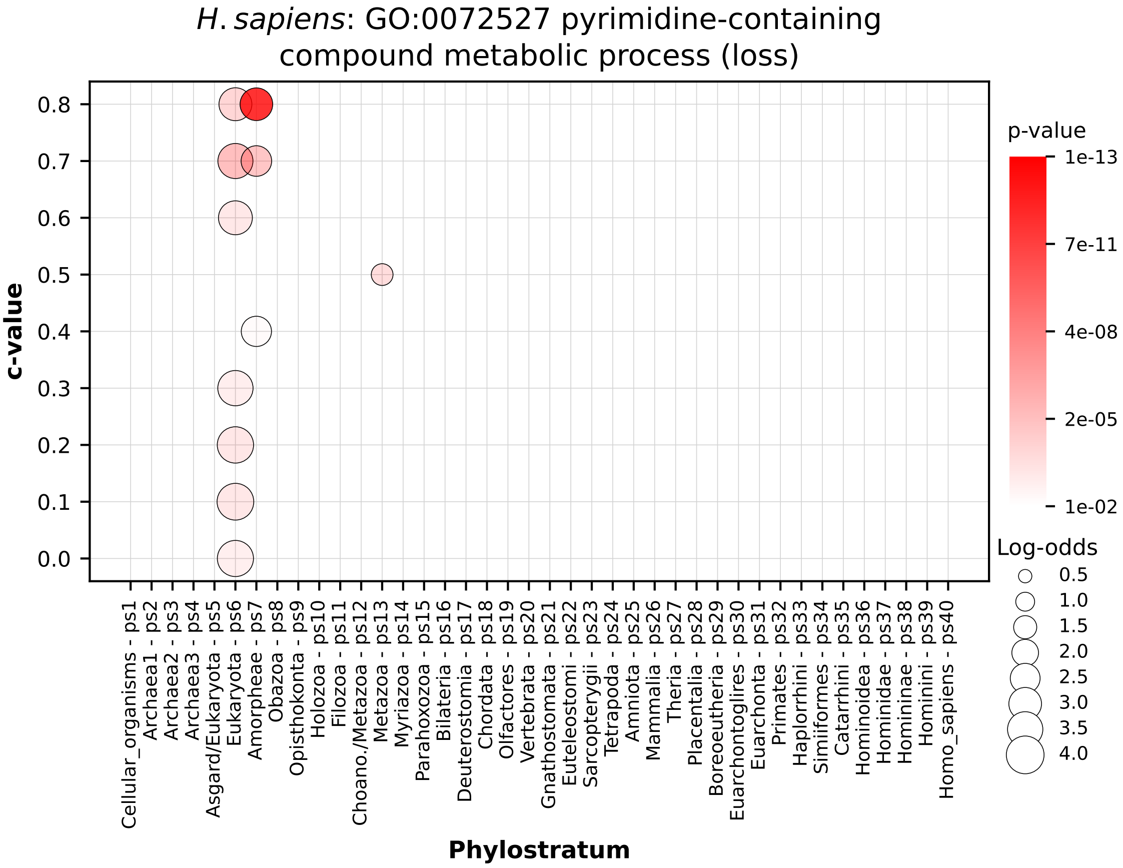
H. sapiens: GO:0072527 pyrimidine-containing compound metabolic process (loss)
c-value
0.0
0.1
0.2
0.3
0.4
0.5
0.6
0.7
0.8
Phylostratum
Cellular_organisms - ps1
Archaea1 - ps2
Archaea2 - ps3
Archaea3 - ps4
Asgard/Eukaryota - ps5
Eukaryota - ps6
Amorpheae - ps7
Obazoa - ps8
Opisthokonta - ps9
Holozoa - ps10
Filozoa - ps11
Choano./Metazoa - ps12
Metazoa - ps13
Myriazoa - ps14
Parahoxozoa - ps15
Bilateria - ps16
Deuterostomia - ps17
Chordata - ps18
Olfactores - ps19
Vertebrata - ps20
Gnathostomata - ps21
Euteleostomi - ps22
Sarcopterygii - ps23
Tetrapoda - ps24
Amniota - ps25
Mammalia - ps26
Theria - ps27
Placentalia - ps28
Boreoeutheria - ps29
Euarchontoglires - ps30
Euarchonta - ps31
Primates - ps32
Haplorrhini - ps33
Simiiformes - ps34
Catarrhini - ps35
Hominoidea - ps36
Hominidae - ps37
Homininae - ps38
Hominini - ps39
Homo_sapiens - ps40
p-value
1e-13
7e-11
4e-08
2e-05
1e-02
Log-odds
0.5
1.0
1.5
2.0
2.5
3.0
3.5
4.0

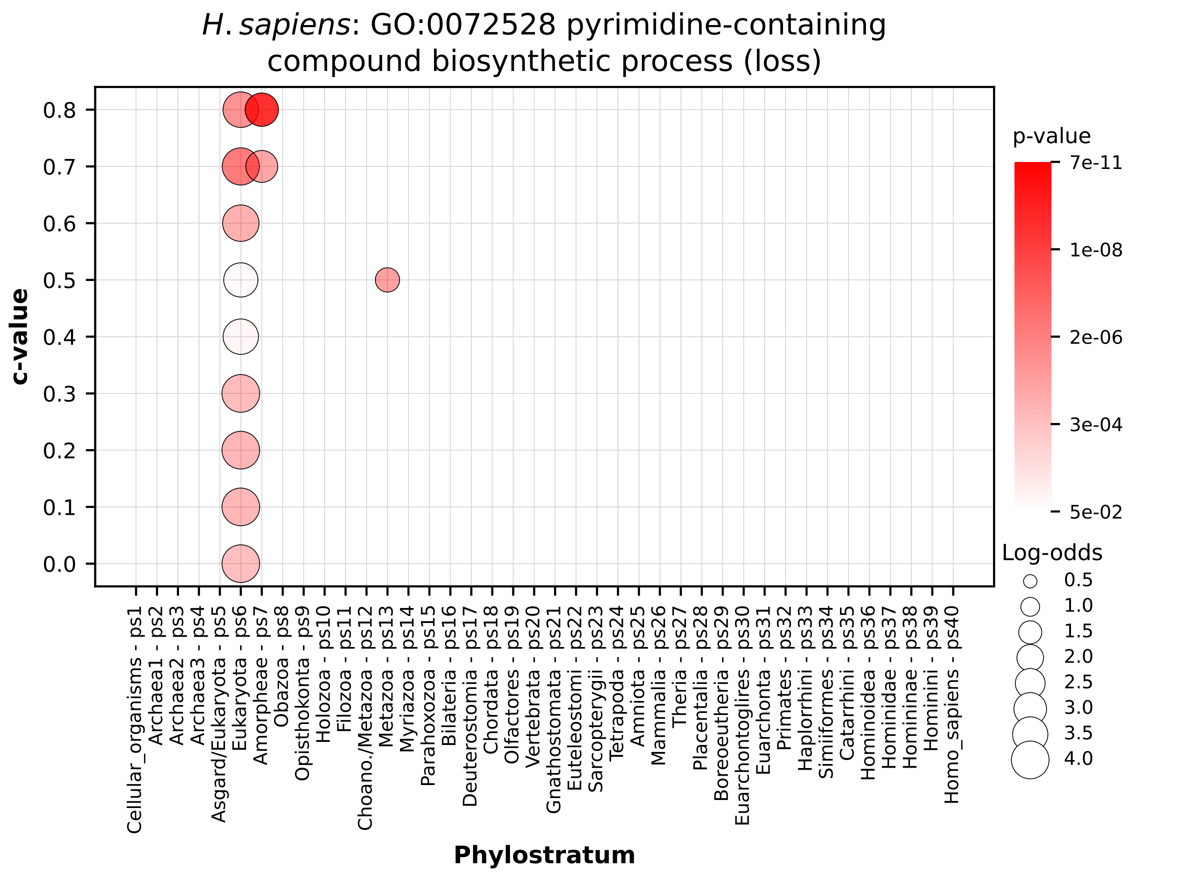

H. sapiens: GO:0072528 pyrimidine-containing compound biosynthetic process (loss)
c-value
0.0
0.1
0.2
0.3
0.4
0.5
0.6
0.7
0.8
p-value
7e-11
1e-08
2e-06
3e-04
5e-02
Log-odds
0.5
1.0
1.5
2.0
2.5
3.0
3.5
4.0
Cellular_organisms - ps1
Archaea1 - ps2
Archaea2 - ps3
Archaea3 - ps4
Asgard/Eukaryota - ps5
Eukaryota - ps6
Amorpheae - ps7
Obazoa - ps8
Opisthokonta - ps9
Holozoa - ps10
Filozoa - ps11
Choano./Metazoa - ps12
Metazoa - ps13
Myriazoa - ps14
Parahoxozoa - ps15
Bilateria - ps16
Deuterostomia - ps17
Chordata - ps18
Olfactores - ps19
Vertebrata - ps20
Gnathostomata - ps21
Euteleostomi - ps22
Sarcopterygii - ps23
Tetrapoda - ps24
Amniota - ps25
Mammalia - ps26
Theria - ps27
Placentalia - ps28
Boreoeutheria - ps29
Euarchontoglires - ps30
Euarchonta - ps31
Primates - ps32
Haplorrhini - ps33
Simiiformes - ps34
Catarrhini - ps35
Hominoidea - ps36
Hominidae - ps37
Homininae - ps38
Hominini - ps39
Homo_sapiens - ps40
Phylostratum

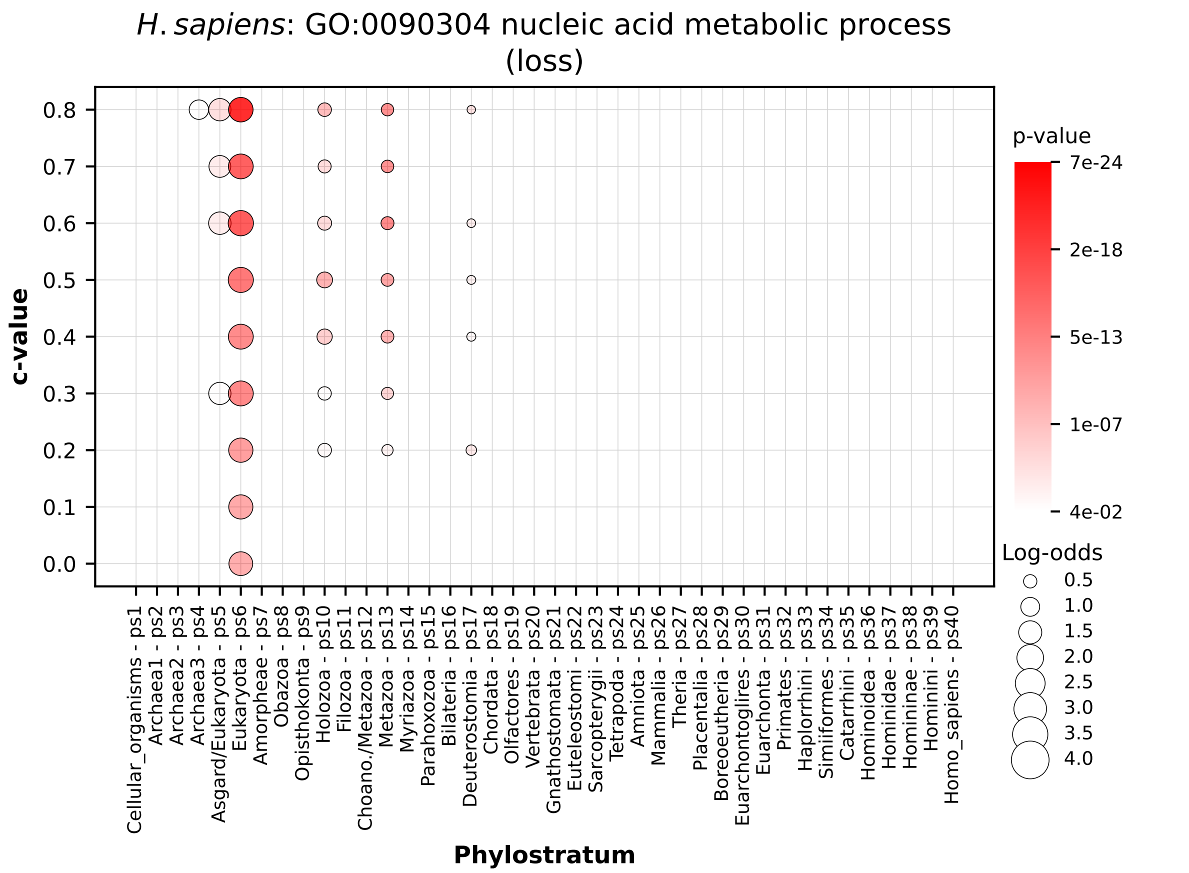

H. sapiens: GO:0090304 nucleic acid metabolic process
(loss)
c-value
0.8
0.7
0.6
0.5
0.4
0.3
0.2
0.1
0.0
p-value
7e-24
2e-18
5e-13
1e-07
4e-02
Log-odds
0.5
1.0
1.5
2.0
2.5
3.0
3.5
4.0
Cellular_organisms - ps1
Archaea1 - ps2
Archaea2 - ps3
Archaea3 - ps4
Asgard/Eukaryota - ps5
Eukaryota - ps6
Amorpheae - ps7
Obazoa - ps8
Opisthokonta - ps9
Holozoa - ps10
Filozoa - ps11
Choano./Metazoa - ps12
Metazoa - ps13
Myriazoa - ps14
Parahoxozoa - ps15
Bilateria - ps16
Deuterostomia - ps17
Chordata - ps18
Olfactores - ps19
Vertebrata - ps20
Gnathostomata - ps21
Euteleostomi - ps22
Sarcopterygii - ps23
Tetrapoda - ps24
Amniota - ps25
Mammalia - ps26
Theria - ps27
Placentalia - ps28
Boreoeutheria - ps29
Euarchontoglires - ps30
Euarchonta - ps31
Primates - ps32
Haplorrhini - ps33
Simiiformes - ps34
Catarrhini - ps35
Hominoidea - ps36
Hominidae - ps37
Homininae - ps38
Hominini - ps39
Homo_sapiens - ps40
Phylostratum

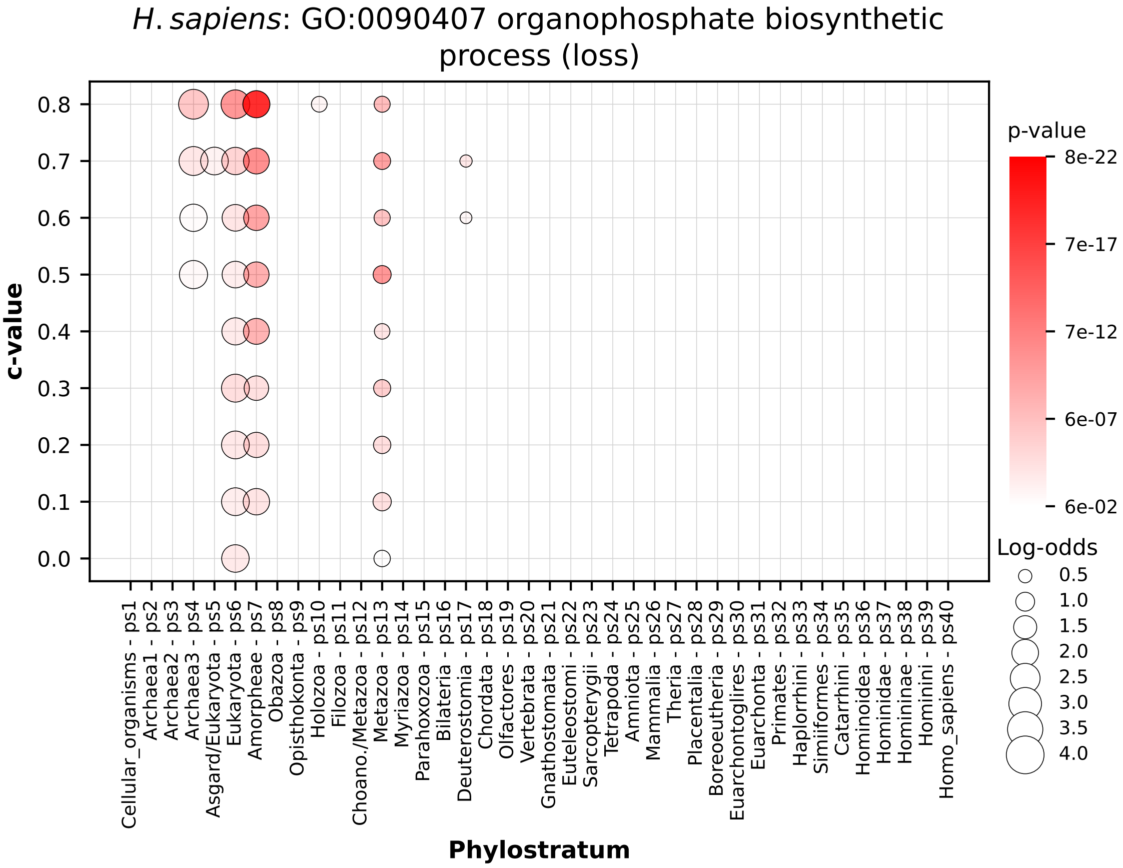

H. sapiens: GO:0090407 organophosphate biosynthetic process (loss)
c-value
0.8
0.7
0.6
0.5
0.4
0.3
0.2
0.1
0.0
Phylostratum
Cellular_organisms - ps1
Archaea1 - ps2
Archaea2 - ps3
Archaea3 - ps4
Asgard/Eukaryota - ps5
Eukaryota - ps6
Amorpheae - ps7
Obazoa - ps8
Opisthokonta - ps9
Holozoa - ps10
Filozoa - ps11
Choano./Metazoa - ps12
Metazoa - ps13
Myriazoa - ps14
Parahoxozoa - ps15
Bilateria - ps16
Deuterostomia - ps17
Chordata - ps18
Olfactores - ps19
Vertebrata - ps20
Gnathostomata - ps21
Euteleostomi - ps22
Sarcopterygii - ps23
Tetrapoda - ps24
Amniota - ps25
Mammalia - ps26
Theria - ps27
Placentalia - ps28
Boreoeutheria - ps29
Euarchontoglires - ps30
Euarchonta - ps31
Primates - ps32
Haplorrhini - ps33
Simiiformes - ps34
Catarrhini - ps35
Hominoidea - ps36
Hominidae - ps37
Homininae - ps38
Hominini - ps39
Homo_sapiens - ps40
p-value
8e-22
7e-17
7e-12
6e-07
6e-02
Log-odds
0.5
1.0
1.5
2.0
2.5
3.0
3.5
4.0

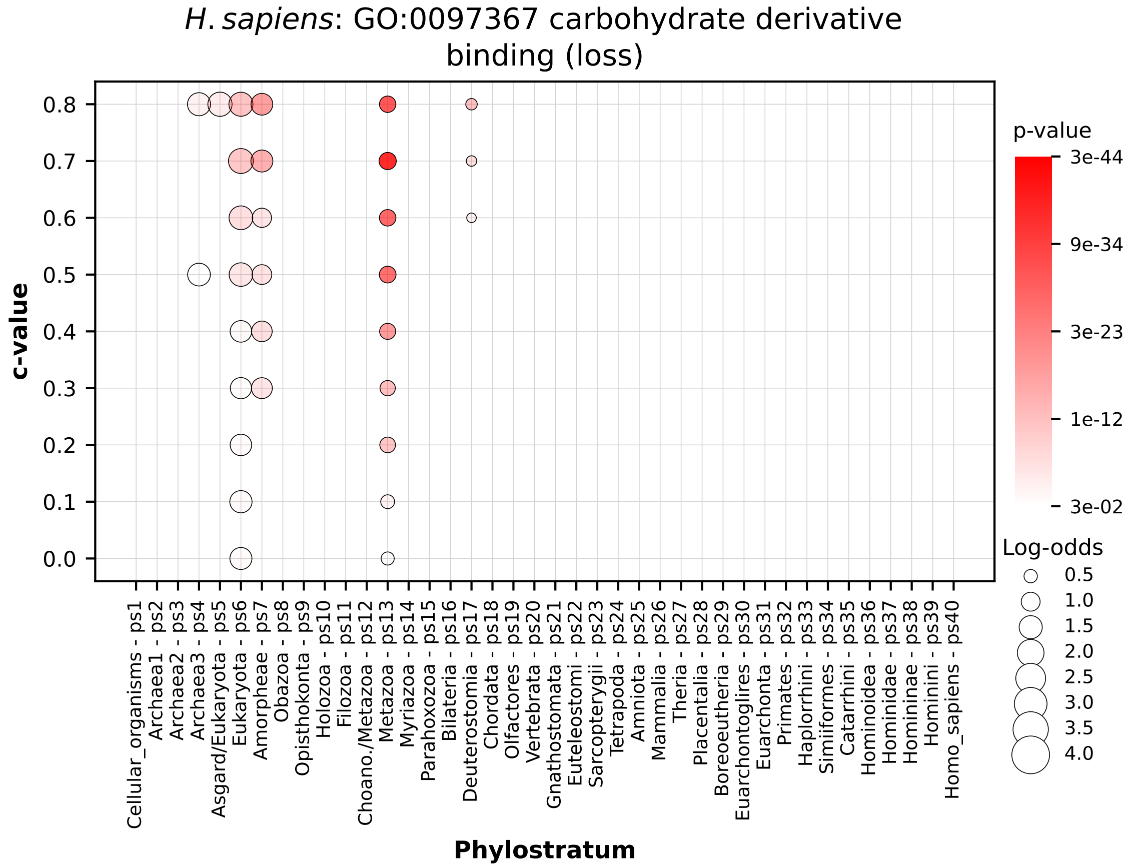
H. sapiens: GO:0097367 carbohydrate derivative binding (loss)
c-value
0.8
0.7
0.6
0.5
0.4
0.3
0.2
0.1
0.0
Phylostratum
Cellular_organisms - ps1
Archaea1 - ps2
Archaea2 - ps3
Archaea3 - ps4
Asgard/Eukaryota - ps5
Eukaryota - ps6
Amorpheae - ps7
Obazoa - ps8
Opisthokonta - ps9
Holozoa - ps10
Filozoa - ps11
Choano./Metazoa - ps12
Metazoa - ps13
Myriazoa - ps14
Parahoxozoa - ps15
Bilateria - ps16
Deuterostomia - ps17
Chordata - ps18
Olfactores - ps19
Vertebrata - ps20
Gnathostomata - ps21
Euteleostomi - ps22
Sarcopterygii - ps23
Tetrapoda - ps24
Amniota - ps25
Mammalia - ps26
Theria - ps27
Placentalia - ps28
Boreoeutheria - ps29
Euarchontoglires - ps30
Euarchonta - ps31
Primates - ps32
Haplorrhini - ps33
Simiiformes - ps34
Catarrhini - ps35
Hominoidea - ps36
Hominidae - ps37
Homininae - ps38
Hominini - ps39
Homo_sapiens - ps40
p-value
3e-44
9e-34
3e-23
1e-12
3e-02
Log-odds
0.5
1.0
1.5
2.0
2.5
3.0
3.5
4.0

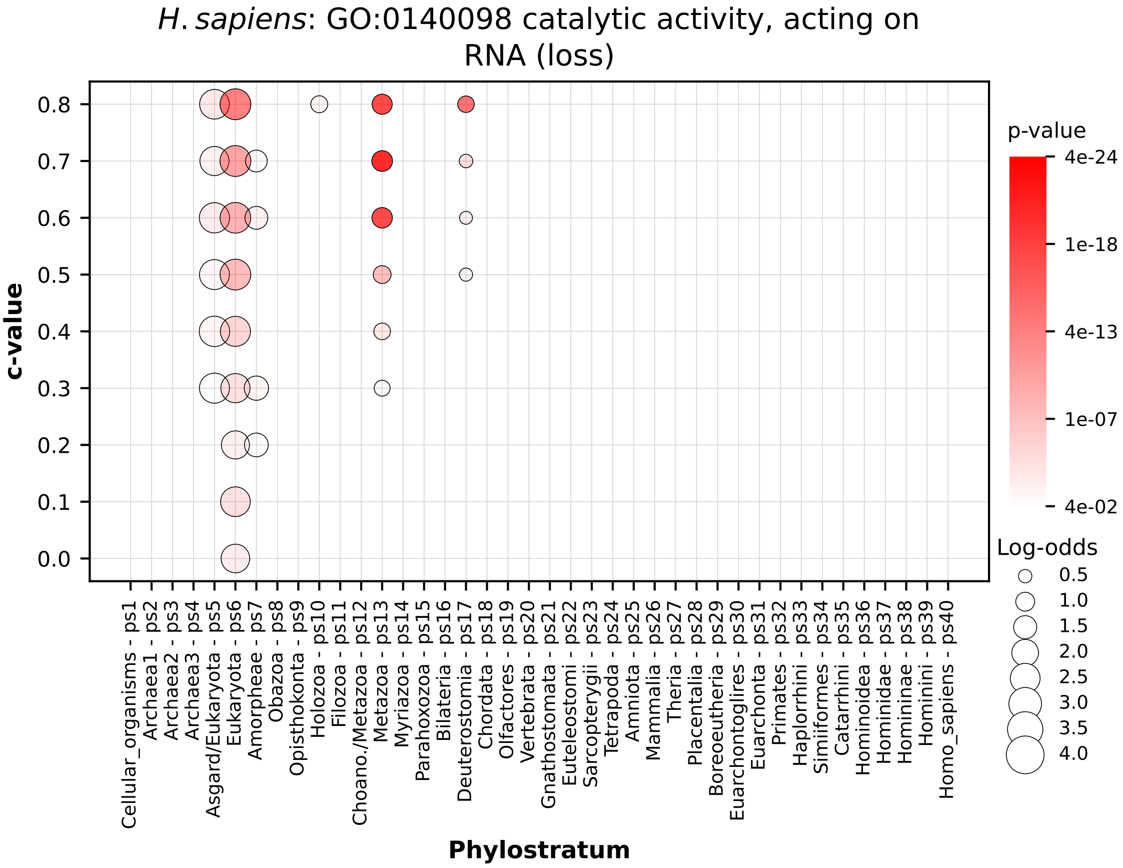

H. sapiens: GO:0140098 catalytic activity, acting on RNA (loss)
c-value
0.8
0.7
0.6
0.5
0.4
0.3
0.2
0.1
0.0
p-value
4e-24
1e-18
4e-13
1e-07
4e-02
Log-odds
0.5
1.0
1.5
2.0
2.5
3.0
3.5
4.0
Cellular_organisms - ps1
Archaea1 - ps2
Archaea2 - ps3
Archaea3 - ps4
Asgard/Eukaryota - ps5
Eukaryota - ps6
Amorpheae - ps7
Obazoa - ps8
Opisthokonta - ps9
Holozoa - ps10
Filozoa - ps11
Choano./Metazoa - ps12
Metazoa - ps13
Myriazoa - ps14
Parahoxozoa - ps15
Bilateria - ps16
Deuterostomia - ps17
Chordata - ps18
Olfactores - ps19
Vertebrata - ps20
Gnathostomata - ps21
Euteleostomi - ps22
Sarcopterygii - ps23
Tetrapoda - ps24
Amniota - ps25
Mammalia - ps26
Theria - ps27
Placentalia - ps28
Boreoeutheria - ps29
Euarchontoglires - ps30
Euarchonta - ps31
Primates - ps32
Haplorrhini - ps33
Simiiformes - ps34
Catarrhini - ps35
Hominoidea - ps36
Hominidae - ps37
Homininae - ps38
Hominini - ps39
Homo_sapiens - ps40
Phylostratum

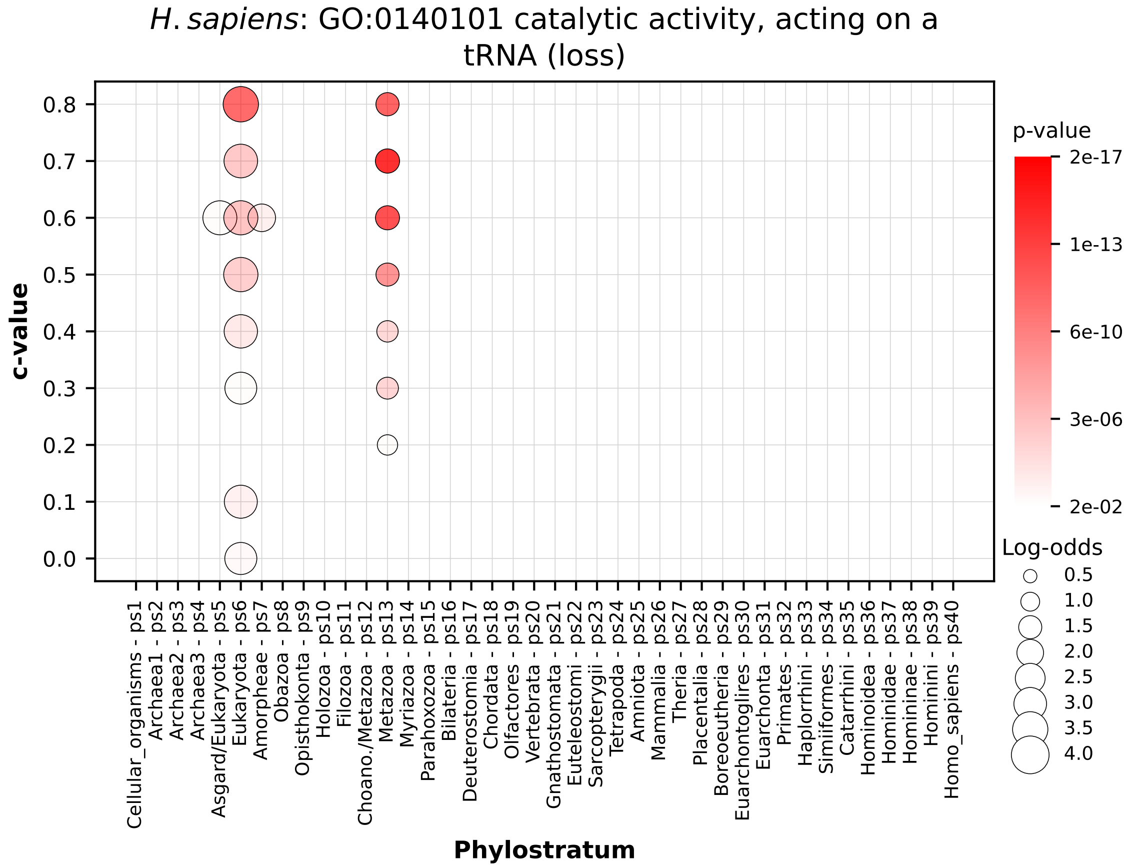

H. sapiens: GO:0140101 catalytic activity, acting on a tRNA (loss)
c-value
0.0
0.1
0.2
0.3
0.4
0.5
0.6
0.7
0.8
Phylostratum
Cellular_organisms - ps1
Archaea1 - ps2
Archaea2 - ps3
Archaea3 - ps4
Asgard/Eukaryota - ps5
Eukaryota - ps6
Amorpheae - ps7
Obazoa - ps8
Opisthokonta - ps9
Holozoa - ps10
Filozoa - ps11
Choano./Metazoa - ps12
Metazoa - ps13
Myriazoa - ps14
Parahoxozoa - ps15
Bilateria - ps16
Deuterostomia - ps17
Chordata - ps18
Olfactores - ps19
Vertebrata - ps20
Gnathostomata - ps21
Euteleostomi - ps22
Sarcopterygii - ps23
Tetrapoda - ps24
Amniota - ps25
Mammalia - ps26
Theria - ps27
Placentalia - ps28
Boreoeutheria - ps29
Euarchontoglires - ps30
Euarchonta - ps31
Primates - ps32
Haplorrhini - ps33
Simiiformes - ps34
Catarrhini - ps35
Hominoidea - ps36
Hominidae - ps37
Homininae - ps38
Hominini - ps39
Homo_sapiens - ps40
p-value
2e-17
1e-13
6e-10
3e-06
2e-02
Log-odds
0.5
1.0
1.5
2.0
2.5
3.0
3.5
4.0

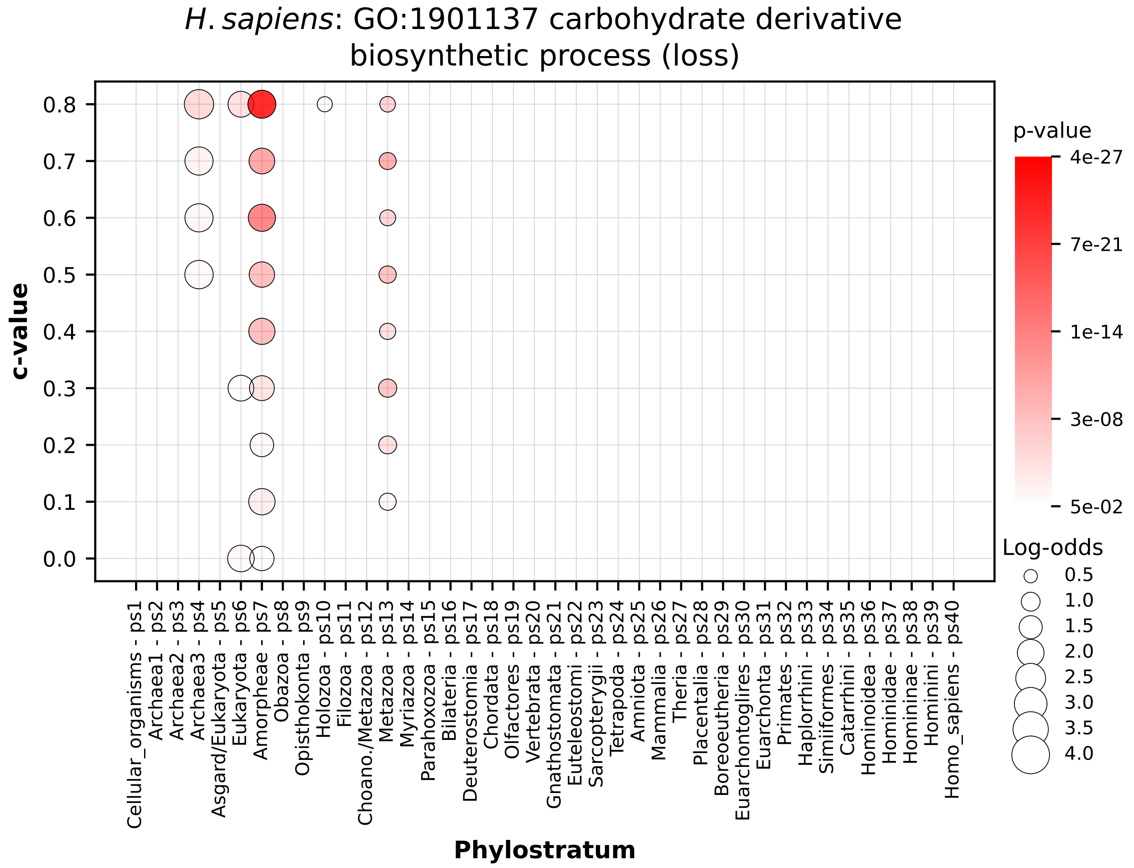

H. sapiens: GO:1901137 carbohydrate derivative biosynthetic process (loss)
c-value
0.8
0.7
0.6
0.5
0.4
0.3
0.2
0.1
0.0
Phylostratum
Cellular_organisms - ps1
Archaea1 - ps2
Archaea2 - ps3
Archaea3 - ps4
Asgard/Eukaryota - ps5
Eukaryota - ps6
Amorpheae - ps7
Obazoa - ps8
Opisthokonta - ps9
Holozoa - ps10
Filozoa - ps11
Choano./Metazoa - ps12
Metazoa - ps13
Myriazoa - ps14
Parahoxozoa - ps15
Bilateria - ps16
Deuterostomia - ps17
Chordata - ps18
Olfactores - ps19
Vertebrata - ps20
Gnathostomata - ps21
Euteleostomi - ps22
Sarcopterygii - ps23
Tetrapoda - ps24
Amniota - ps25
Mammalia - ps26
Theria - ps27
Placentalia - ps28
Boreoeutheria - ps29
Euarchontoglires - ps30
Euarchonta - ps31
Primates - ps32
Haplorrhini - ps33
Simiiformes - ps34
Catarrhini - ps35
Hominoidea - ps36
Hominidae - ps37
Homininae - ps38
Hominini - ps39
Homo_sapiens - ps40
p-value
4e-27
7e-21
1e-14
3e-08
5e-02
Log-odds
0.5
1.0
1.5
2.0
2.5
3.0
3.5
4.0

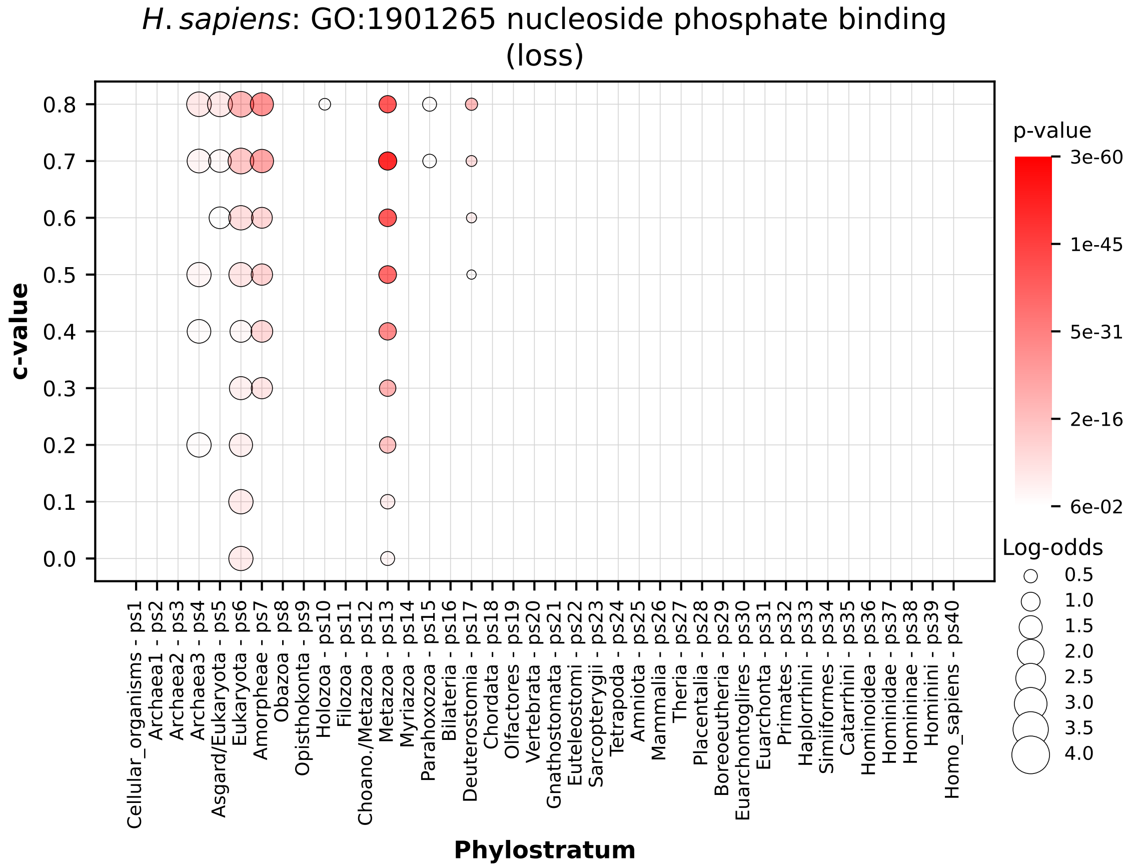

H. sapiens: GO:1901265 nucleoside phosphate binding (loss)
c-value
0.8
0.7
0.6
0.5
0.4
0.3
0.2
0.1
0.0
p-value
3e-60
1e-45
5e-31
2e-16
6e-02
Log-odds
0.5
1.0
1.5
2.0
2.5
3.0
3.5
4.0
Cellular_organisms - ps1
Archaea1 - ps2
Archaea2 - ps3
Archaea3 - ps4
Asgard/Eukaryota - ps5
Eukaryota - ps6
Amorpheae - ps7
Obazoa - ps8
Opisthokonta - ps9
Holozoa - ps10
Filozoa - ps11
Choano./Metazoa - ps12
Metazoa - ps13
Myriazoa - ps14
Parahoxozoa - ps15
Bilateria - ps16
Deuterostomia - ps17
Chordata - ps18
Olfactores - ps19
Vertebrata - ps20
Gnathostomata - ps21
Euteleostomi - ps22
Sarcopterygii - ps23
Tetrapoda - ps24
Amniota - ps25
Mammalia - ps26
Theria - ps27
Placentalia - ps28
Boreoeutheria - ps29
Euarchontoglires - ps30
Euarchonta - ps31
Primates - ps32
Haplorrhini - ps33
Simiiformes - ps34
Catarrhini - ps35
Hominoidea - ps36
Hominidae - ps37
Homininae - ps38
Hominini - ps39
Homo_sapiens - ps40
Phylostratum

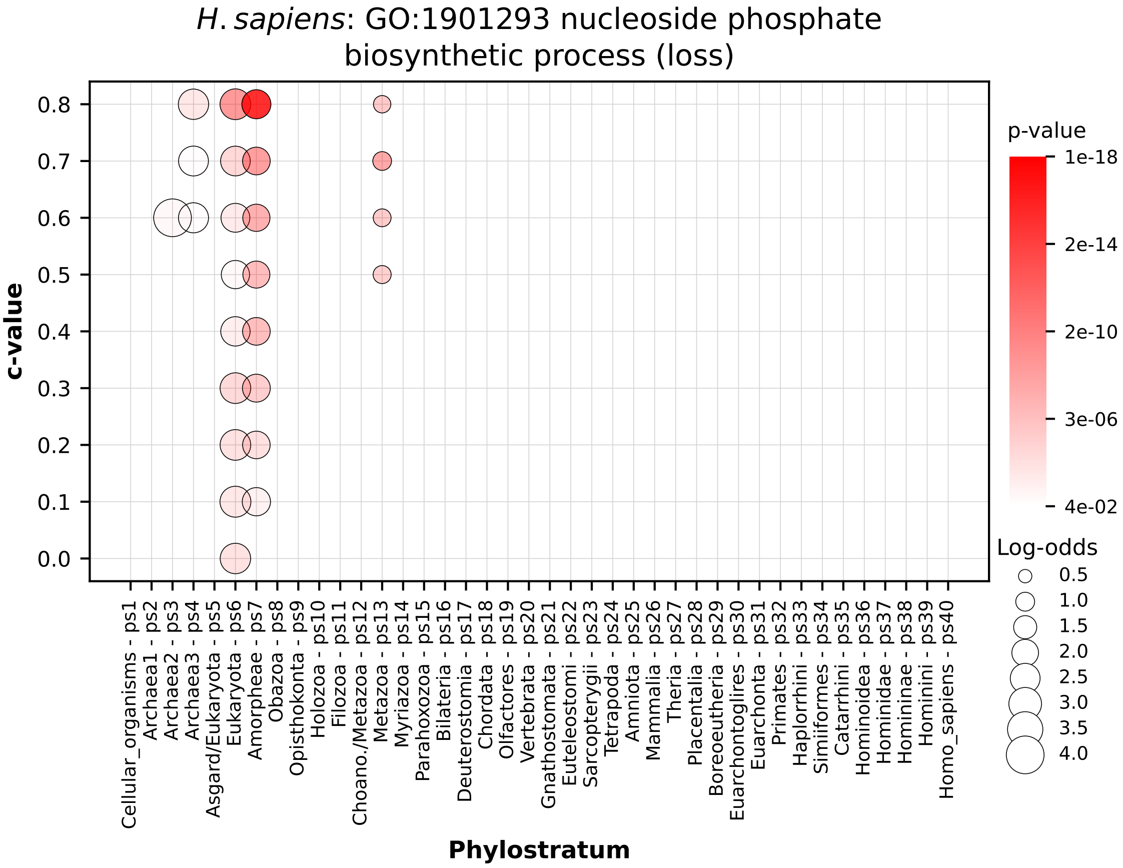

H. sapiens: GO:1901293 nucleoside phosphate biosynthetic process (loss)
c-value
0.8
0.7
0.6
0.5
0.4
0.3
0.2
0.1
0.0
Phylostratum
Cellular_organisms - ps1
Archaea1 - ps2
Archaea2 - ps3
Archaea3 - ps4
Asgard/Eukaryota - ps5
Eukaryota - ps6
Amorpheae - ps7
Obazoa - ps8
Opisthokonta - ps9
Holozoa - ps10
Filozoa - ps11
Choano./Metazoa - ps12
Metazoa - ps13
Myriazoa - ps14
Parahoxozoa - ps15
Bilateria - ps16
Deuterostomia - ps17
Chordata - ps18
Olfactores - ps19
Vertebrata - ps20
Gnathostomata - ps21
Euteleostomi - ps22
Sarcopterygii - ps23
Tetrapoda - ps24
Amniota - ps25
Mammalia - ps26
Theria - ps27
Placentalia - ps28
Boreoeutheria - ps29
Euarchontoglires - ps30
Euarchonta - ps31
Primates - ps32
Haplorrhini - ps33
Simiiformes - ps34
Catarrhini - ps35
Hominoidea - ps36
Hominidae - ps37
Homininae - ps38
Hominini - ps39
Homo_sapiens - ps40
p-value
1e-18
2e-14
2e-10
3e-06
4e-02
Log-odds
0.5
1.0
1.5
2.0
2.5
3.0
3.5
4.0

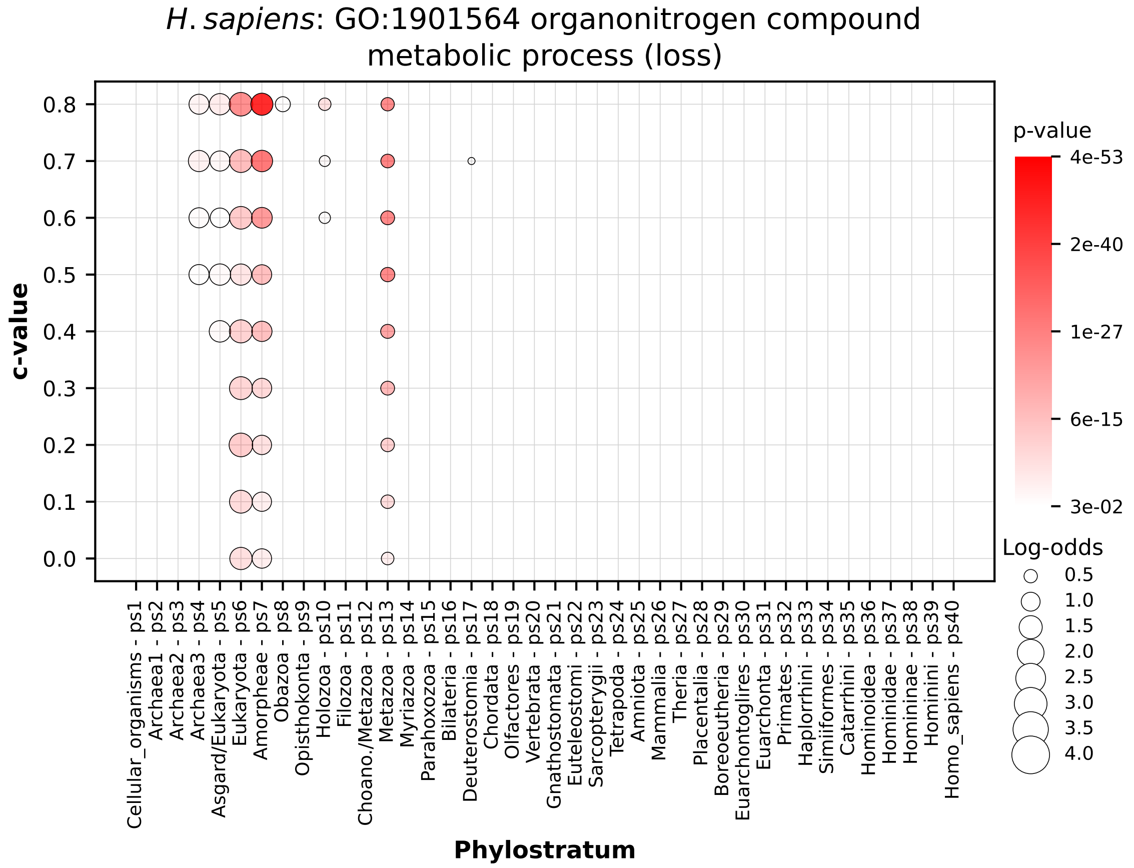
H. sapiens: GO:1901564 organonitrogen compound metabolic process (loss)
c-value
0.8
0.7
0.6
0.5
0.4
0.3
0.2
0.1
0.0
Phylostratum
Cellular_organisms - ps1
Archaea1 - ps2
Archaea2 - ps3
Archaea3 - ps4
Asgard/Eukaryota - ps5
Eukaryota - ps6
Amorpheae - ps7
Obazoa - ps8
Opisthokonta - ps9
Holozoa - ps10
Filozoa - ps11
Choano./Metazoa - ps12
Metazoa - ps13
Myriazoa - ps14
Parahoxozoa - ps15
Bilateria - ps16
Deuterostomia - ps17
Chordata - ps18
Olfactores - ps19
Vertebrata - ps20
Gnathostomata - ps21
Euteleostomi - ps22
Sarcopterygii - ps23
Tetrapoda - ps24
Amniota - ps25
Mammalia - ps26
Theria - ps27
Placentalia - ps28
Boreoeutheria - ps29
Euarchontoglires - ps30
Euarchonta - ps31
Primates - ps32
Haplorrhini - ps33
Simiiformes - ps34
Catarrhini - ps35
Hominoidea - ps36
Hominidae - ps37
Homininae - ps38
Hominini - ps39
Homo_sapiens - ps40
p-value
4e-53
2e-40
1e-27
6e-15
3e-02
Log-odds
0.5
1.0
1.5
2.0
2.5
3.0
3.5
4.0

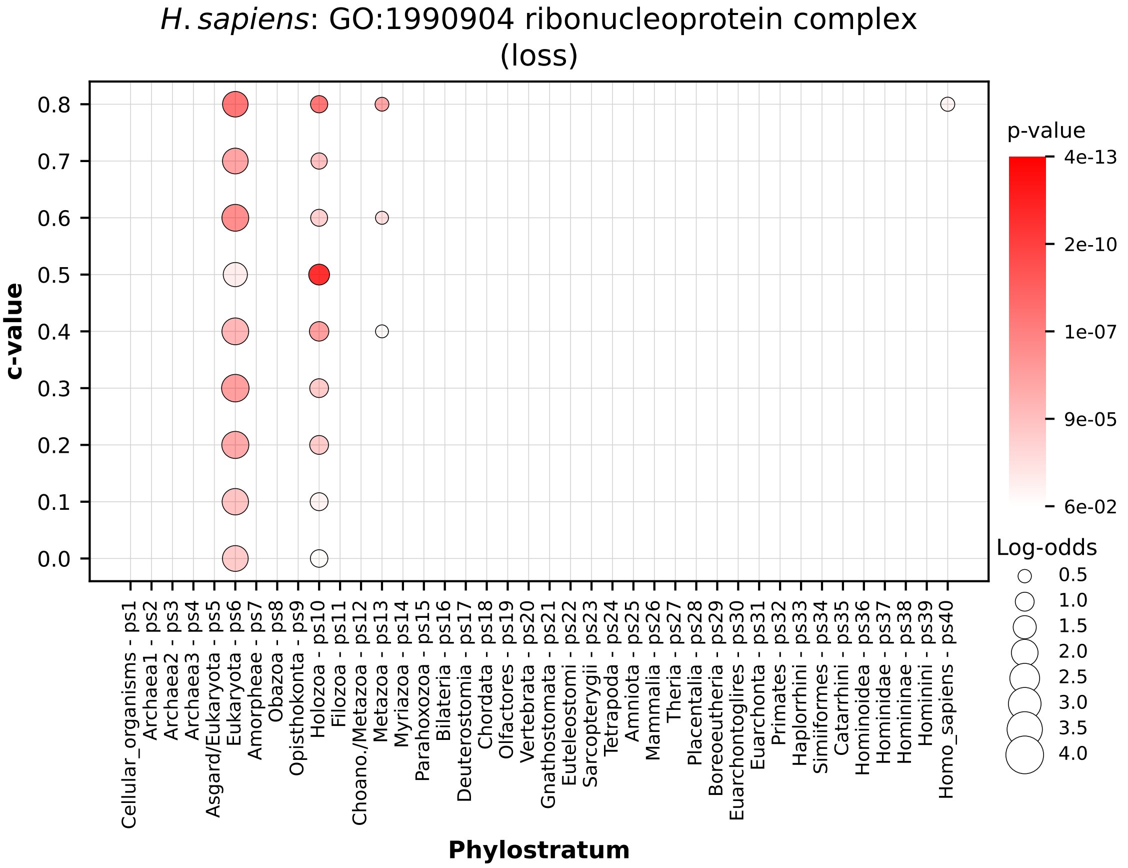
H. sapiens: GO:1990904 ribonucleoprotein complex
(loss)
c-value
0.8
0.7
0.6
0.5
0.4
0.3
0.2
0.1
0.0
Phylostratum
Cellular_organisms - ps1
Archaea1 - ps2
Archaea2 - ps3
Archaea3 - ps4
Asgard/Eukaryota - ps5
Eukaryota - ps6
Amorpheae - ps7
Obazoa - ps8
Opisthokonta - ps9
Holozoa - ps10
Filozoa - ps11
Choano./Metazoa - ps12
Metazoa - ps13
Myriazoa - ps14
Parahoxozoa - ps15
Bilateria - ps16
Deuterostomia - ps17
Chordata - ps18
Olfactores - ps19
Vertebrata - ps20
Gnathostomata - ps21
Euteleostomi - ps22
Sarcopterygii - ps23
Tetrapoda - ps24
Amniota - ps25
Mammalia - ps26
Theria - ps27
Placentalia - ps28
Boreoeutheria - ps29
Euarchontoglires - ps30
Euarchonta - ps31
Primates - ps32
Haplorrhini - ps33
Simiiformes - ps34
Catarrhini - ps35
Hominoidea - ps36
Hominidae - ps37
Homininae - ps38
Hominini - ps39
Homo_sapiens - ps40
p-value
4e-13
2e-10
1e-07
9e-05
6e-02
Log-odds
0.5
1.0
1.5
2.0
2.5
3.0
3.5
4.0

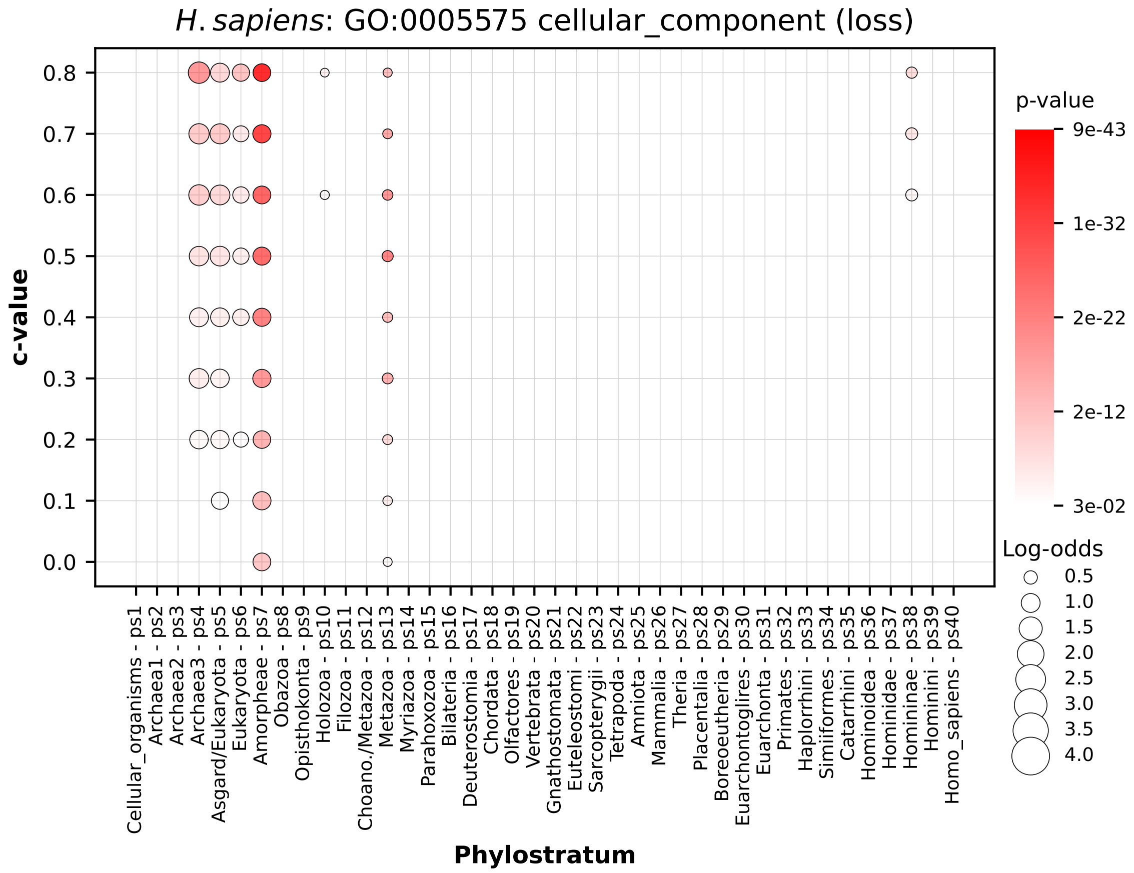

H. sapiens: GO:0005575 cellular_component (loss)
c-value
0.8
0.7
0.6
0.5
0.4
0.3
0.2
0.1
0.0
p-value
9e-43
1e-32
2e-22
2e-12
3e-02
Log-odds
0.5
1.0
1.5
2.0
2.5
3.0
3.5
4.0
Cellular_organisms - ps1
Archaea1 - ps2
Archaea2 - ps3
Archaea3 - ps4
Asgard/Eukaryota - ps5
Eukaryota - ps6
Amorpheae - ps7
Obazoa - ps8
Opisthokonta - ps9
Holozoa - ps10
Filozoa - ps11
Choano./Metazoa - ps12
Metazoa - ps13
Myriazoa - ps14
Parahoxozoa - ps15
Bilateria - ps16
Deuterostomia - ps17
Chordata - ps18
Olfactores - ps19
Vertebrata - ps20
Gnathostomata - ps21
Euteleostomi - ps22
Sarcopterygii - ps23
Tetrapoda - ps24
Amniota - ps25
Mammalia - ps26
Theria - ps27
Placentalia - ps28
Boreoeutheria - ps29
Euarchontoglires - ps30
Euarchonta - ps31
Primates - ps32
Haplorrhini - ps33
Simiiformes - ps34
Catarrhini - ps35
Hominoidea - ps36
Hominidae - ps37
Homininae - ps38
Hominini - ps39
Homo_sapiens - ps40
Phylostratum

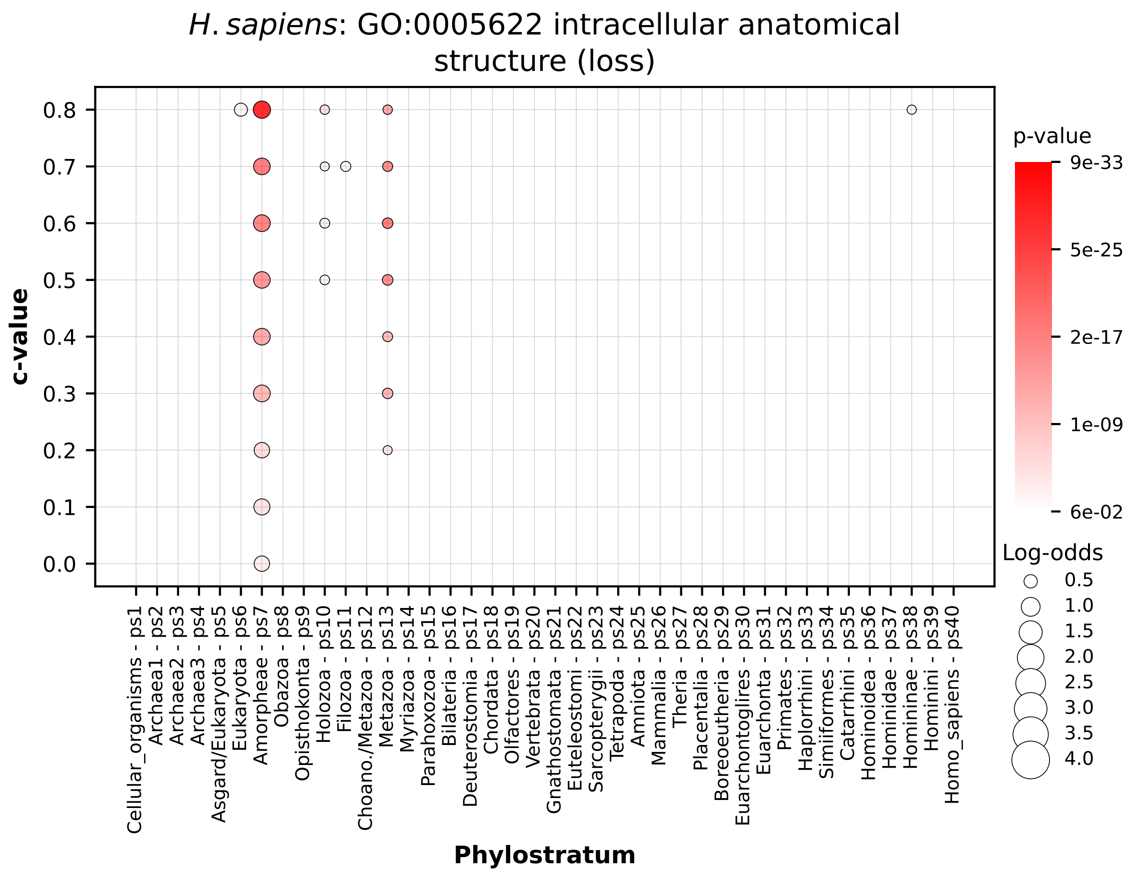
H. sapiens: GO:0005622 intracellular anatomical structure (loss)
c-value
0.8
0.7
0.6
0.5
0.4
0.3
0.2
0.1
0.0
Phylostratum
Cellular_organisms - ps1
Archaea1 - ps2
Archaea2 - ps3
Archaea3 - ps4
Asgard/Eukaryota - ps5
Eukaryota - ps6
Amorpheae - ps7
Obazoa - ps8
Opisthokonta - ps9
Holozoa - ps10
Filozoa - ps11
Choano./Metazoa - ps12
Metazoa - ps13
Myriazoa - ps14
Parahoxozoa - ps15
Bilateria - ps16
Deuterostomia - ps17
Chordata - ps18
Olfactores - ps19
Vertebrata - ps20
Gnathostomata - ps21
Euteleostomi - ps22
Sarcopterygii - ps23
Tetrapoda - ps24
Amniota - ps25
Mammalia - ps26
Theria - ps27
Placentalia - ps28
Boreoeutheria - ps29
Euarchontoglires - ps30
Euarchonta - ps31
Primates - ps32
Haplorrhini - ps33
Simiiformes - ps34
Catarrhini - ps35
Hominoidea - ps36
Hominidae - ps37
Homininae - ps38
Hominini - ps39
Homo_sapiens - ps40
p-value
9e-33
5e-25
2e-17
1e-09
6e-02
Log-odds
0.5
1.0
1.5
2.0
2.5
3.0
3.5
4.0

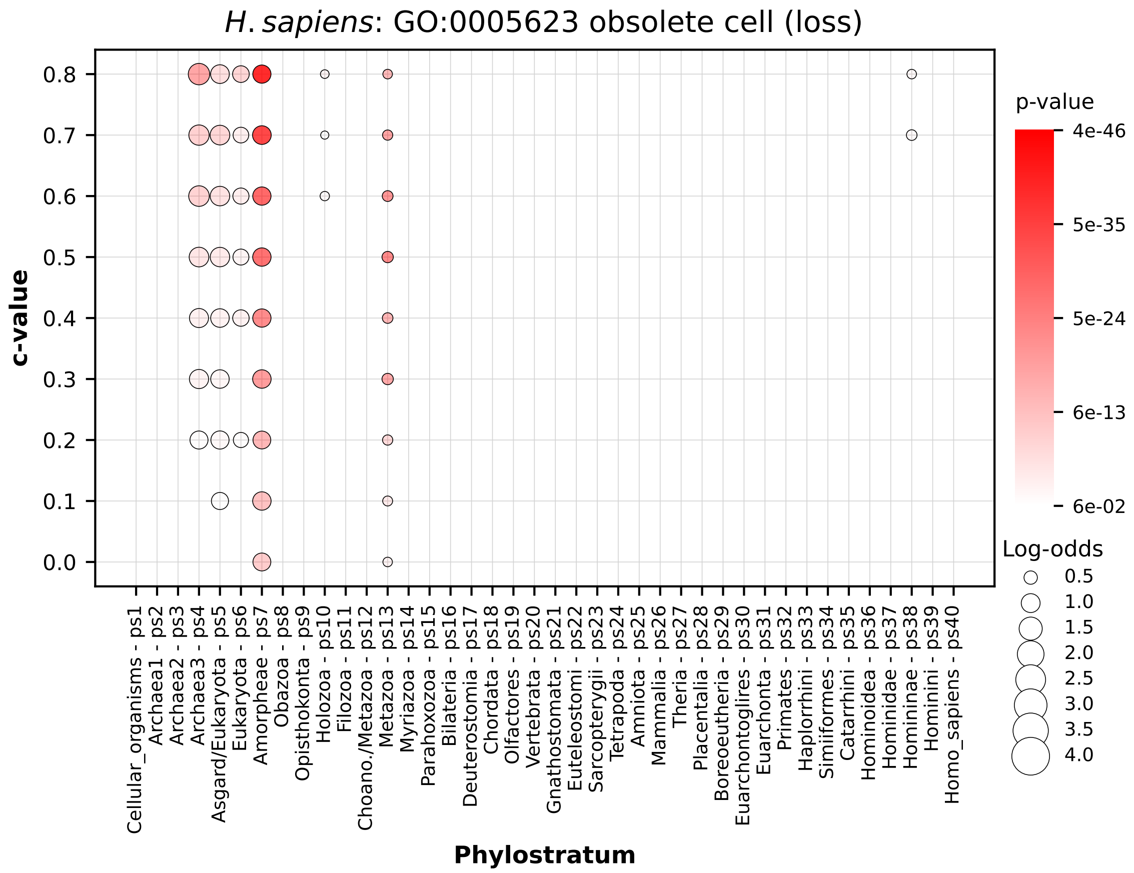
H. sapiens: GO:0005623 obsolete cell (loss)
c-value
0.0
0.1
0.2
0.3
0.4
0.5
0.6
0.7
0.8
Phylostratum
Cellular_organisms - ps1
Archaea1 - ps2
Archaea2 - ps3
Archaea3 - ps4
Asgard/Eukaryota - ps5
Eukaryota - ps6
Amorpheae - ps7
Obazoa - ps8
Opisthokonta - ps9
Holozoa - ps10
Filozoa - ps11
Choano./Metazoa - ps12
Metazoa - ps13
Myriazoa - ps14
Parahoxozoa - ps15
Bilateria - ps16
Deuterostomia - ps17
Chordata - ps18
Olfactores - ps19
Vertebrata - ps20
Gnathostomata - ps21
Euteleostomi - ps22
Sarcopterygii - ps23
Tetrapoda - ps24
Amniota - ps25
Mammalia - ps26
Theria - ps27
Placentalia - ps28
Boreoeutheria - ps29
Euarchontoglires - ps30
Euarchonta - ps31
Primates - ps32
Haplorrhini - ps33
Simiiformes - ps34
Catarrhini - ps35
Hominoidea - ps36
Hominidae - ps37
Homininae - ps38
Hominini - ps39
Homo_sapiens - ps40
p-value
4e-46
5e-35
5e-24
6e-13
6e-02
Log-odds
0.5
1.0
1.5
2.0
2.5
3.0
3.5
4.0

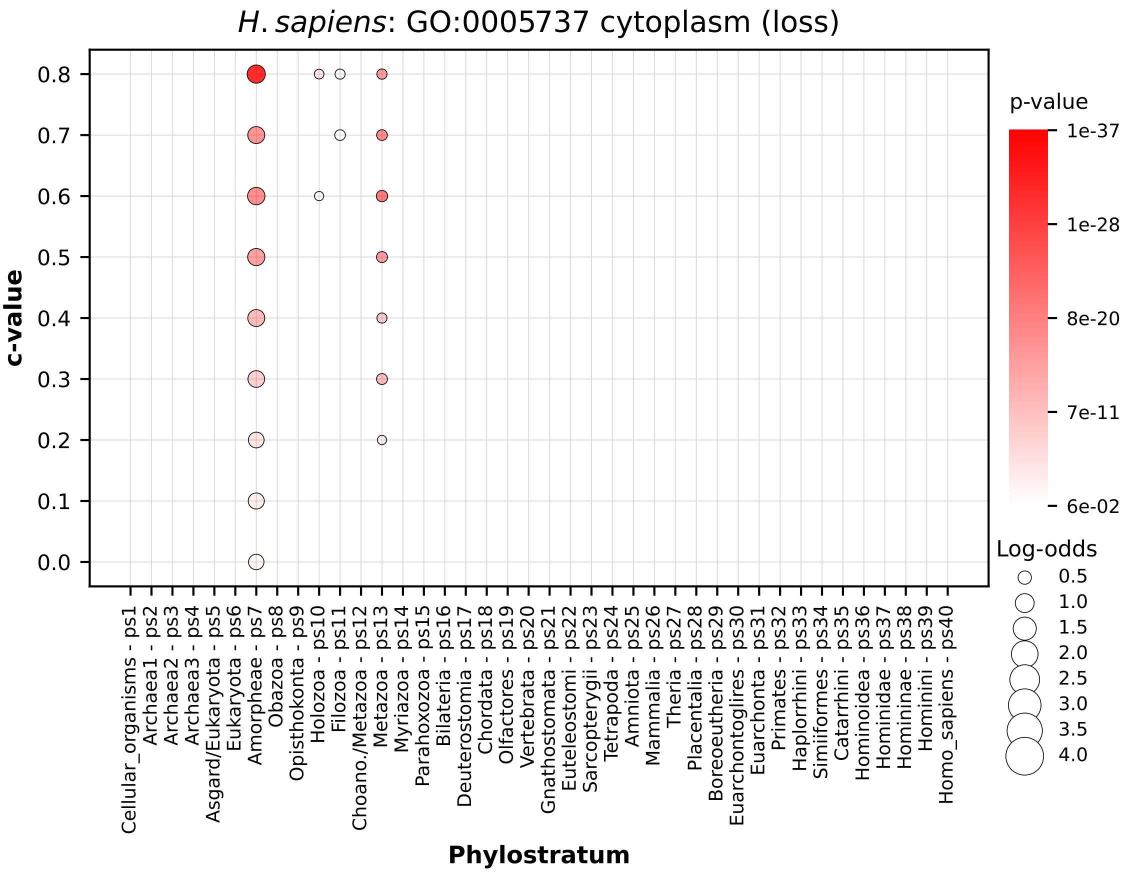

H. sapiens: GO:0005737 cytoplasm (loss)
c-value
0.8
0.7
0.6
0.5
0.4
0.3
0.2
0.1
0.0
Phylostratum
Cellular_organisms - ps1
Archaea1 - ps2
Archaea2 - ps3
Archaea3 - ps4
Asgard/Eukaryota - ps5
Eukaryota - ps6
Amorpheae - ps7
Obazoa - ps8
Opisthokonta - ps9
Holozoa - ps10
Filozoa - ps11
Choano./Metazoa - ps12
Metazoa - ps13
Myriazoa - ps14
Parahoxozoa - ps15
Bilateria - ps16
Deuterostomia - ps17
Chordata - ps18
Olfactores - ps19
Vertebrata - ps20
Gnathostomata - ps21
Euteleostomi - ps22
Sarcopterygii - ps23
Tetrapoda - ps24
Amniota - ps25
Mammalia - ps26
Theria - ps27
Placentalia - ps28
Boreoeutheria - ps29
Euarchontoglires - ps30
Euarchonta - ps31
Primates - ps32
Haplorrhini - ps33
Simiiformes - ps34
Catarrhini - ps35
Hominoidea - ps36
Hominidae - ps37
Homininae - ps38
Hominini - ps39
Homo_sapiens - ps40
p-value
1e-37
1e-28
8e-20
7e-11
6e-02
Log-odds
0.5
1.0
1.5
2.0
2.5
3.0
3.5
4.0

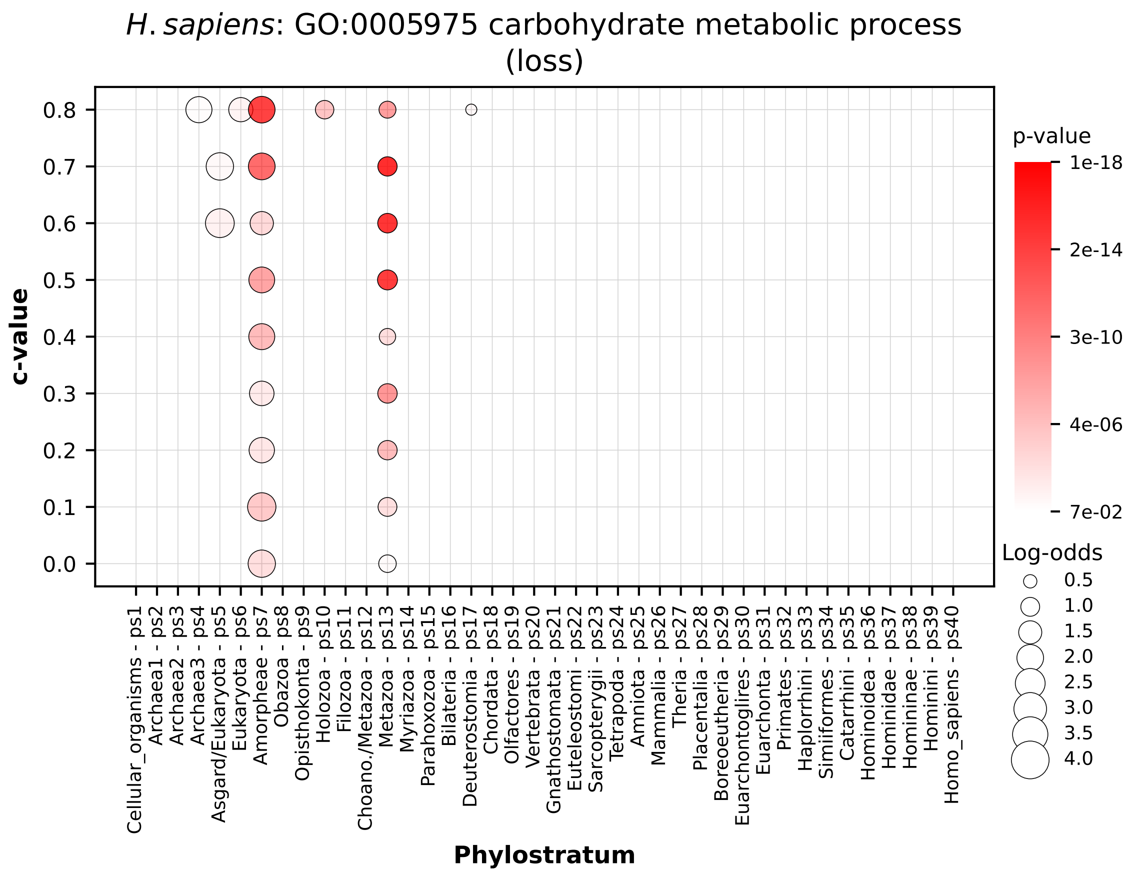

H. sapiens: GO:0005975 carbohydrate metabolic process
(loss)
c-value
0.8
0.7
0.6
0.5
0.4
0.3
0.2
0.1
0.0
Phylostratum
Cellular_organisms - ps1
Archaea1 - ps2
Archaea2 - ps3
Archaea3 - ps4
Asgard/Eukaryota - ps5
Eukaryota - ps6
Amorpheae - ps7
Obazoa - ps8
Opisthokonta - ps9
Holozoa - ps10
Filozoa - ps11
Choano./Metazoa - ps12
Metazoa - ps13
Myriazoa - ps14
Parahoxozoa - ps15
Bilateria - ps16
Deuterostomia - ps17
Chordata - ps18
Olfactores - ps19
Vertebrata - ps20
Gnathostomata - ps21
Euteleostomi - ps22
Sarcopterygii - ps23
Tetrapoda - ps24
Amniota - ps25
Mammalia - ps26
Theria - ps27
Placentalia - ps28
Boreoeutheria - ps29
Euarchontoglires - ps30
Euarchonta - ps31
Primates - ps32
Haplorrhini - ps33
Simiiformes - ps34
Catarrhini - ps35
Hominoidea - ps36
Hominidae - ps37
Homininae - ps38
Hominini - ps39
Homo_sapiens - ps40
p-value
1e-18
2e-14
3e-10
4e-06
7e-02
Log-odds
0.5
1.0
1.5
2.0
2.5
3.0
3.5
4.0

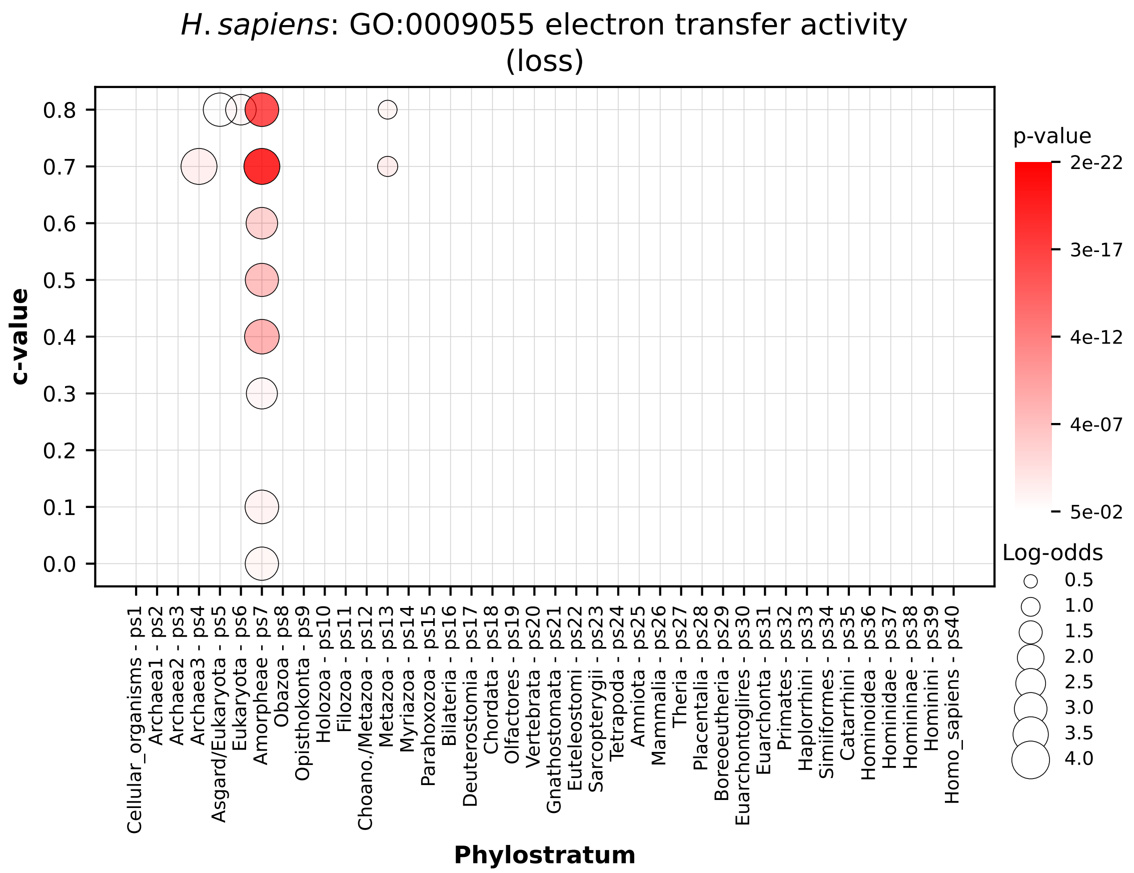

H. sapiens: GO:0009055 electron transfer activity (loss)
c-value
0.8
0.7
0.6
0.5
0.4
0.3
0.2
0.1
0.0
p-value
2e-22
3e-17
4e-12
4e-07
5e-02
Log-odds
0.5
1.0
1.5
2.0
2.5
3.0
3.5
4.0
Cellular_organisms - ps1
Archaea1 - ps2
Archaea2 - ps3
Archaea3 - ps4
Asgard/Eukaryota - ps5
Eukaryota - ps6
Amorpheae - ps7
Obazoa - ps8
Opisthokonta - ps9
Holozoa - ps10
Filozoa - ps11
Choano./Metazoa - ps12
Metazoa - ps13
Myriazoa - ps14
Parahoxozoa - ps15
Bilateria - ps16
Deuterostomia - ps17
Chordata - ps18
Olfactores - ps19
Vertebrata - ps20
Gnathostomata - ps21
Euteleostomi - ps22
Sarcopterygii - ps23
Tetrapoda - ps24
Amniota - ps25
Mammalia - ps26
Theria - ps27
Placentalia - ps28
Boreoeutheria - ps29
Euarchontoglires - ps30
Euarchonta - ps31
Primates - ps32
Haplorrhini - ps33
Simiiformes - ps34
Catarrhini - ps35
Hominoidea - ps36
Hominidae - ps37
Homininae - ps38
Hominini - ps39
Homo_sapiens - ps40
Phylostratum

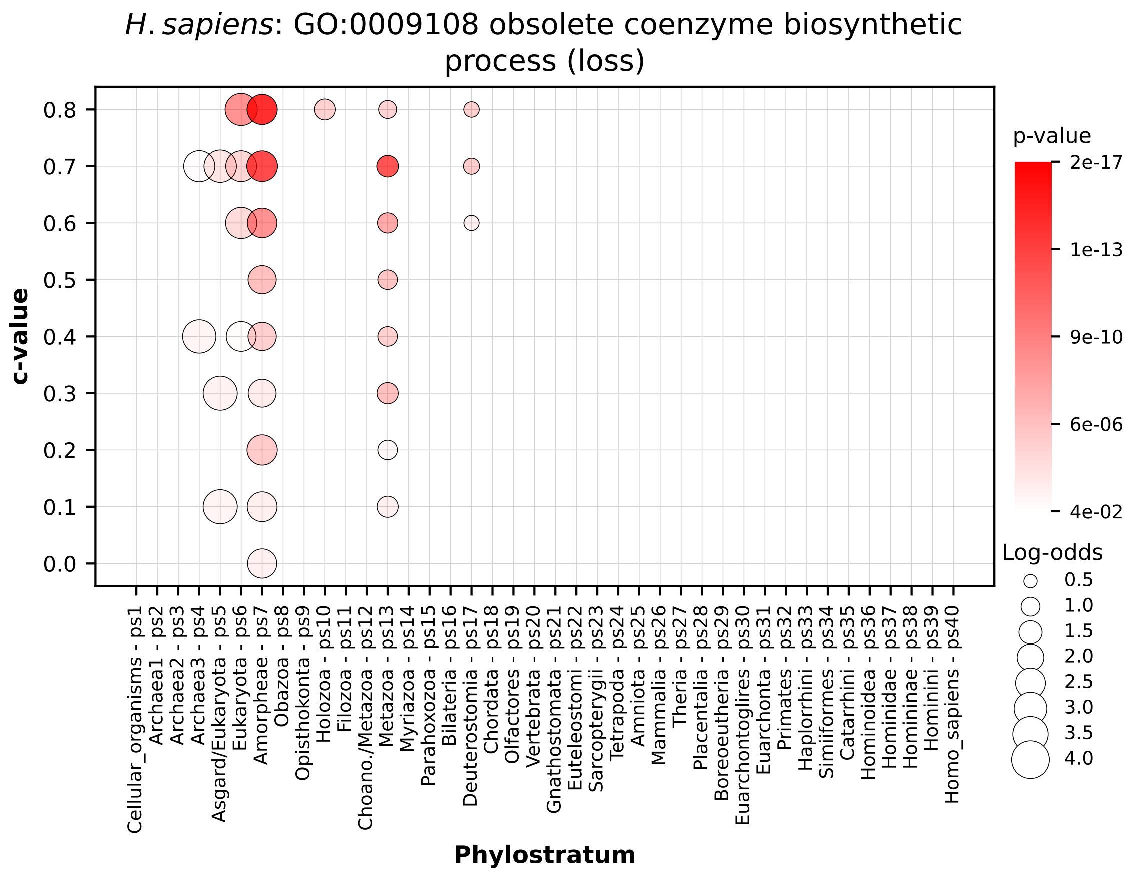
H. sapiens: GO:0009108 obsolete coenzyme biosynthetic process (loss)
c-value
0.8
0.7
0.6
0.5
0.4
0.3
0.2
0.1
0.0
p-value
2e-17
1e-13
9e-10
6e-06
4e-02
Log-odds
0.5
1.0
1.5
2.0
2.5
3.0
3.5
4.0
Cellular_organisms - ps1
Archaea1 - ps2
Archaea2 - ps3
Archaea3 - ps4
Asgard/Eukaryota - ps5
Eukaryota - ps6
Amorpheae - ps7
Obazoa - ps8
Opisthokonta - ps9
Holozoa - ps10
Filozoa - ps11
Choano./Metazoa - ps12
Metazoa - ps13
Myriazoa - ps14
Parahoxozoa - ps15
Bilateria - ps16
Deuterostomia - ps17
Chordata - ps18
Olfactores - ps19
Vertebrata - ps20
Gnathostomata - ps21
Euteleostomi - ps22
Sarcopterygii - ps23
Tetrapoda - ps24
Amniota - ps25
Mammalia - ps26
Theria - ps27
Placentalia - ps28
Boreoeutheria - ps29
Euarchontoglires - ps30
Euarchonta - ps31
Primates - ps32
Haplorrhini - ps33
Simiiformes - ps34
Catarrhini - ps35
Hominoidea - ps36
Hominidae - ps37
Homininae - ps38
Hominini - ps39
Homo_sapiens - ps40
Phylostratum

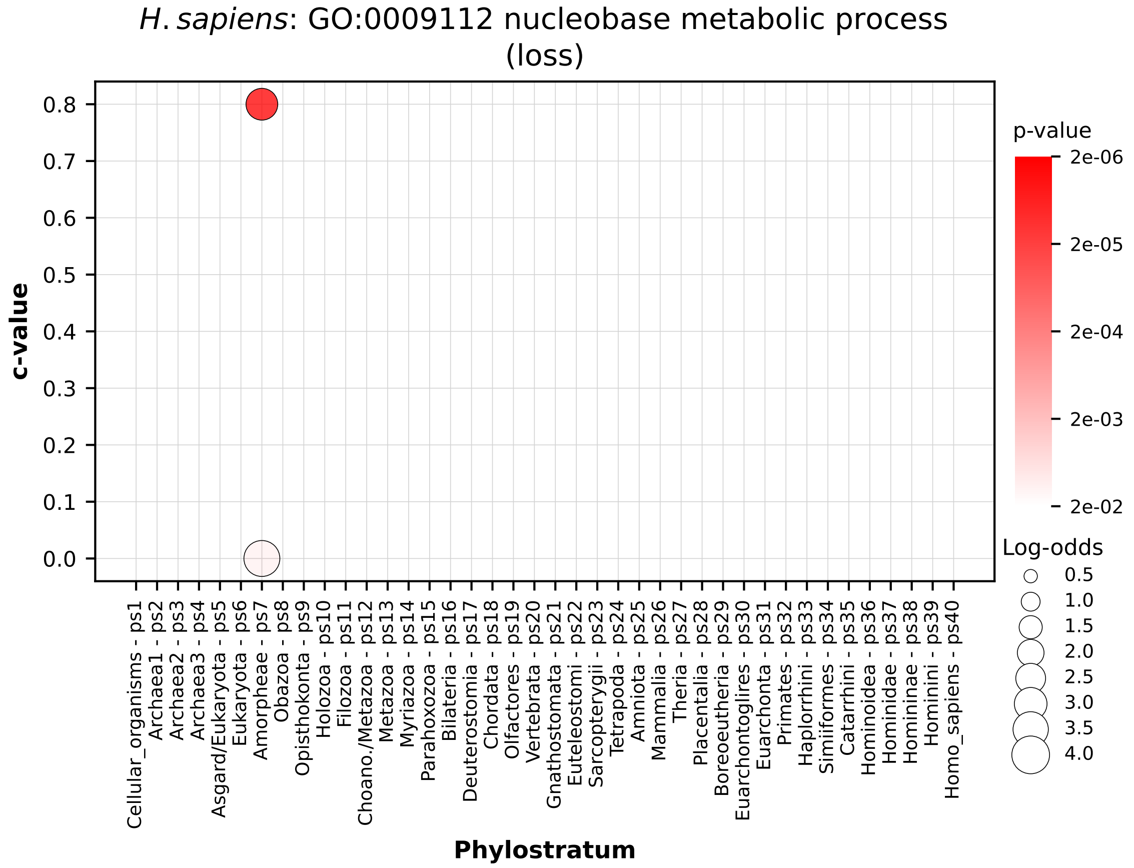
H. sapiens: GO:0009112 nucleobase metabolic process
(loss)
c-value
0.8
0.7
0.6
0.5
0.4
0.3
0.2
0.1
0.0
p-value
2e-06
2e-05
2e-04
2e-03
2e-02
Log-odds
0.5
1.0
1.5
2.0
2.5
3.0
3.5
4.0
Cellular_organisms - ps1
Archaea1 - ps2
Archaea2 - ps3
Archaea3 - ps4
Asgard/Eukaryota - ps5
Eukaryota - ps6
Amorpheae - ps7
Obazoa - ps8
Opisthokonta - ps9
Holozoa - ps10
Filozoa - ps11
Choano./Metazoa - ps12
Metazoa - ps13
Myriazoa - ps14
Parahoxozoa - ps15
Bilateria - ps16
Deuterostomia - ps17
Chordata - ps18
Olfactores - ps19
Vertebrata - ps20
Gnathostomata - ps21
Euteleostomi - ps22
Sarcopterygii - ps23
Tetrapoda - ps24
Amniota - ps25
Mammalia - ps26
Theria - ps27
Placentalia - ps28
Boreoeutheria - ps29
Euarchontoglires - ps30
Euarchonta - ps31
Primates - ps32
Haplorrhini - ps33
Simiiformes - ps34
Catarrhini - ps35
Hominoidea - ps36
Hominidae - ps37
Homininae - ps38
Hominini - ps39
Homo_sapiens - ps40
Phylostratum

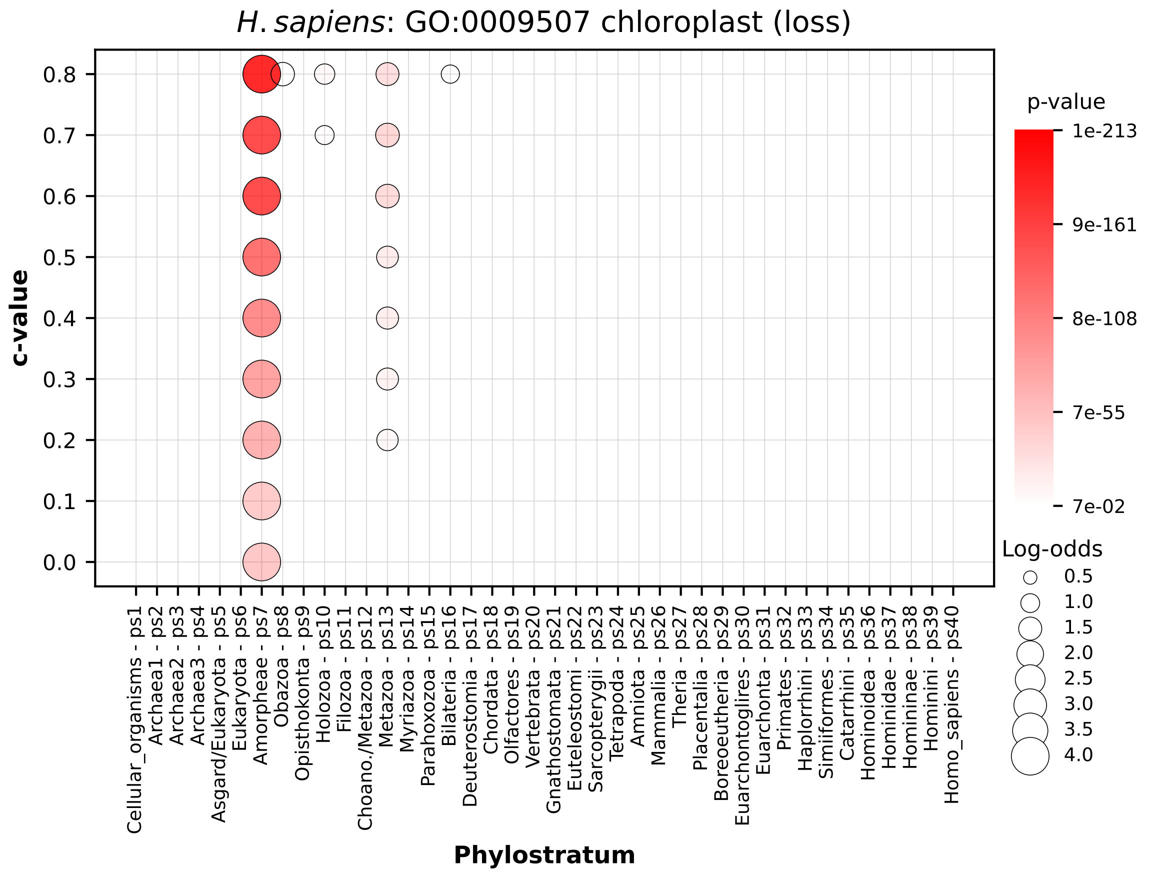
H. sapiens: GO:0009507 chloroplast (loss)
c-value
0.8
0.7
0.6
0.5
0.4
0.3
0.2
0.1
0.0
Phylostratum
Cellular_organisms - ps1
Archaea1 - ps2
Archaea2 - ps3
Archaea3 - ps4
Asgard/Eukaryota - ps5
Eukaryota - ps6
Amorpheae - ps7
Obazoa - ps8
Opisthokonta - ps9
Holozoa - ps10
Filozoa - ps11
Choano./Metazoa - ps12
Metazoa - ps13
Myriazoa - ps14
Parahoxozoa - ps15
Bilateria - ps16
Deuterostomia - ps17
Chordata - ps18
Olfactores - ps19
Vertebrata - ps20
Gnathostomata - ps21
Euteleostomi - ps22
Sarcopterygii - ps23
Tetrapoda - ps24
Amniota - ps25
Mammalia - ps26
Theria - ps27
Placentalia - ps28
Boreoeutheria - ps29
Euarchontoglires - ps30
Euarchonta - ps31
Primates - ps32
Haplorrhini - ps33
Simiiformes - ps34
Catarrhini - ps35
Hominoidea - ps36
Hominidae - ps37
Homininae - ps38
Hominini - ps39
Homo_sapiens - ps40
p-value
1e-213
9e-161
8e-108
7e-55
7e-02
Log-odds
0.5
1.0
1.5
2.0
2.5
3.0
3.5
4.0

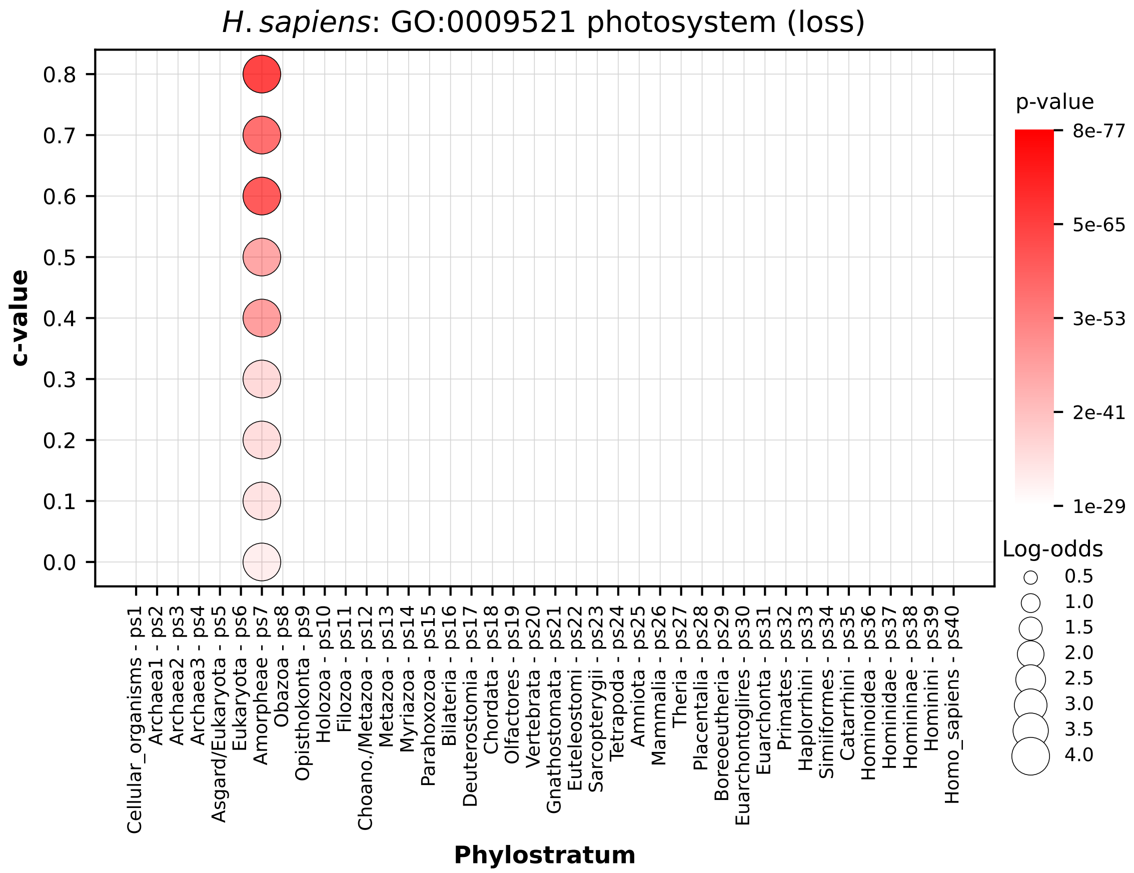

H. sapiens: GO:0009521 photosystem (loss)
c-value
0.8
0.7
0.6
0.5
0.4
0.3
0.2
0.1
0.0
p-value
8e-77
5e-65
3e-53
2e-41
1e-29
Log-odds
0.5
1.0
1.5
2.0
2.5
3.0
3.5
4.0
Cellular_organisms - ps1
Archaea1 - ps2
Archaea2 - ps3
Archaea3 - ps4
Asgard/Eukaryota - ps5
Eukaryota - ps6
Amorpheae - ps7
Obazoa - ps8
Opisthokonta - ps9
Holozoa - ps10
Filozoa - ps11
Choano./Metazoa - ps12
Metazoa - ps13
Myriazoa - ps14
Parahoxozoa - ps15
Bilateria - ps16
Deuterostomia - ps17
Chordata - ps18
Olfactores - ps19
Vertebrata - ps20
Gnathostomata - ps21
Euteleostomi - ps22
Sarcopterygii - ps23
Tetrapoda - ps24
Amniota - ps25
Mammalia - ps26
Theria - ps27
Placentalia - ps28
Boreoeutheria - ps29
Euarchontoglires - ps30
Euarchonta - ps31
Primates - ps32
Haplorrhini - ps33
Simiiformes - ps34
Catarrhini - ps35
Hominoidea - ps36
Hominidae - ps37
Homininae - ps38
Hominini - ps39
Homo_sapiens - ps40
Phylostratum

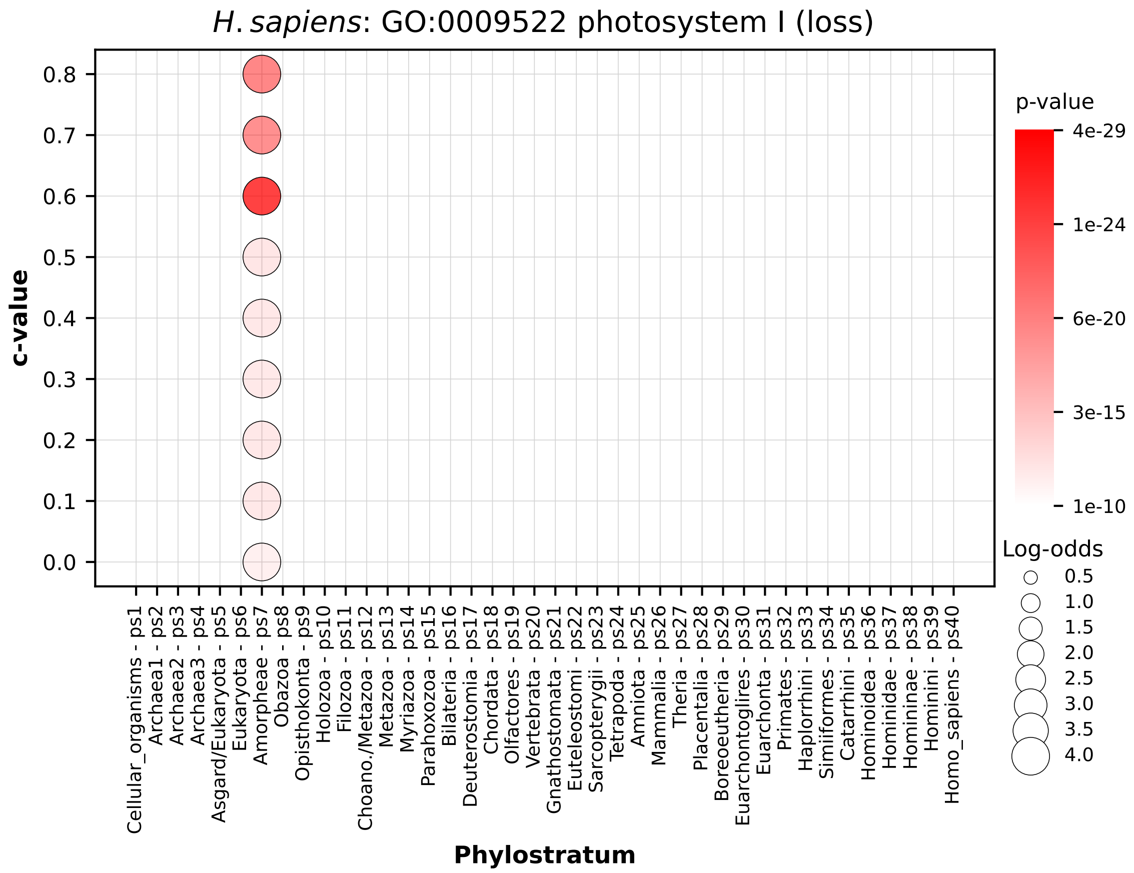

H. sapiens: GO:0009522 photosystem I (loss)
c-value
0.0
0.1
0.2
0.3
0.4
0.5
0.6
0.7
0.8
Phylostratum
Cellular_organisms - ps1
Archaea1 - ps2
Archaea2 - ps3
Archaea3 - ps4
Asgard/Eukaryota - ps5
Eukaryota - ps6
Amorpheae - ps7
Obazoa - ps8
Opisthokonta - ps9
Holozoa - ps10
Filozoa - ps11
Choano./Metazoa - ps12
Metazoa - ps13
Myriazoa - ps14
Parahoxozoa - ps15
Bilateria - ps16
Deuterostomia - ps17
Chordata - ps18
Olfactores - ps19
Vertebrata - ps20
Gnathostomata - ps21
Euteleostomi - ps22
Sarcopterygii - ps23
Tetrapoda - ps24
Amniota - ps25
Mammalia - ps26
Theria - ps27
Placentalia - ps28
Boreoeutheria - ps29
Euarchontoglires - ps30
Euarchonta - ps31
Primates - ps32
Haplorrhini - ps33
Simiiformes - ps34
Catarrhini - ps35
Hominoidea - ps36
Hominidae - ps37
Homininae - ps38
Hominini - ps39
Homo_sapiens - ps40
p-value
4e-29
1e-24
6e-20
3e-15
1e-10
Log-odds
0.5
1.0
1.5
2.0
2.5
3.0
3.5
4.0

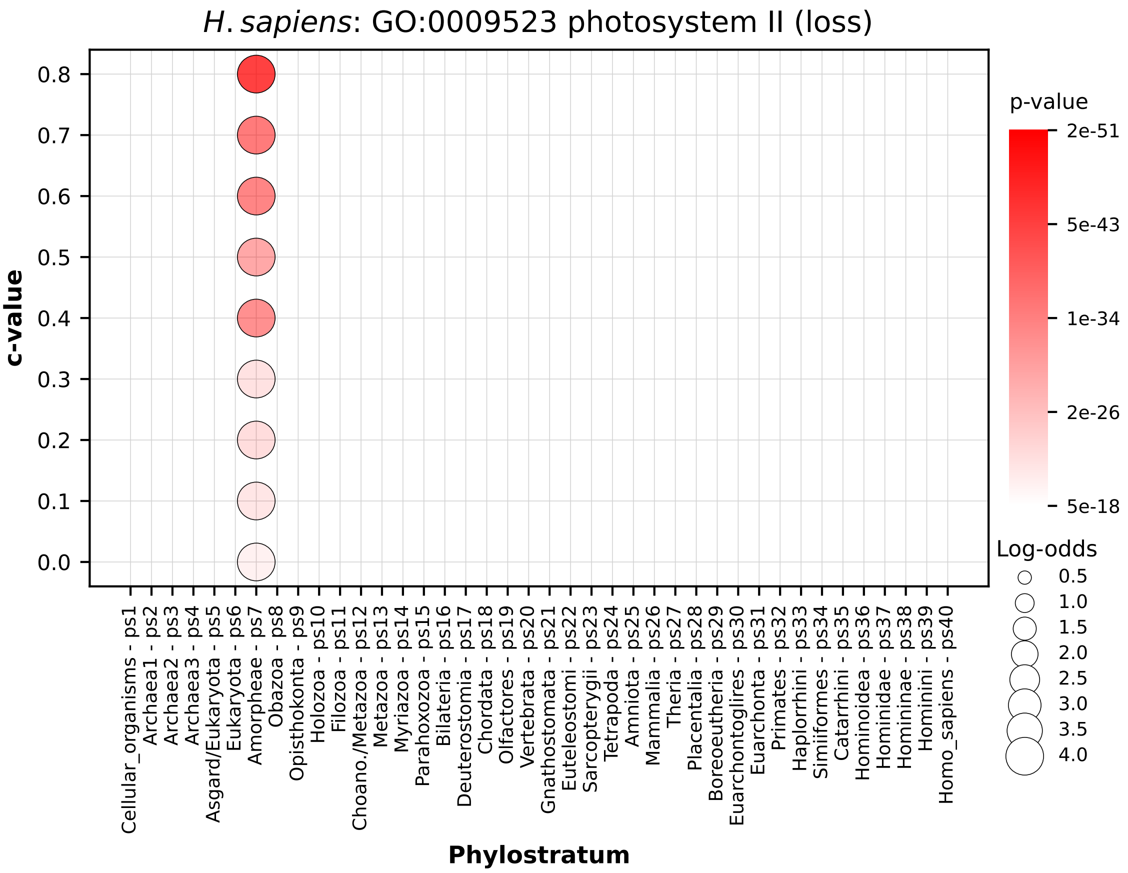
H. sapiens: GO:0009523 photosystem II (loss)
c-value
0.8
0.7
0.6
0.5
0.4
0.3
0.2
0.1
0.0
p-value
2e-51
5e-43
1e-34
2e-26
5e-18
Log-odds
0.5
1.0
1.5
2.0
2.5
3.0
3.5
4.0
Cellular_organisms - ps1
Archaea1 - ps2
Archaea2 - ps3
Archaea3 - ps4
Asgard/Eukaryota - ps5
Eukaryota - ps6
Amorpheae - ps7
Obazoa - ps8
Opisthokonta - ps9
Holozoa - ps10
Filozoa - ps11
Choano./Metazoa - ps12
Metazoa - ps13
Myriazoa - ps14
Parahoxozoa - ps15
Bilateria - ps16
Deuterostomia - ps17
Chordata - ps18
Olfactores - ps19
Vertebrata - ps20
Gnathostomata - ps21
Euteleostomi - ps22
Sarcopterygii - ps23
Tetrapoda - ps24
Amniota - ps25
Mammalia - ps26
Theria - ps27
Placentalia - ps28
Boreoeutheria - ps29
Euarchontoglires - ps30
Euarchonta - ps31
Primates - ps32
Haplorrhini - ps33
Simiiformes - ps34
Catarrhini - ps35
Hominoidea - ps36
Hominidae - ps37
Homininae - ps38
Hominini - ps39
Homo_sapiens - ps40
Phylostratum

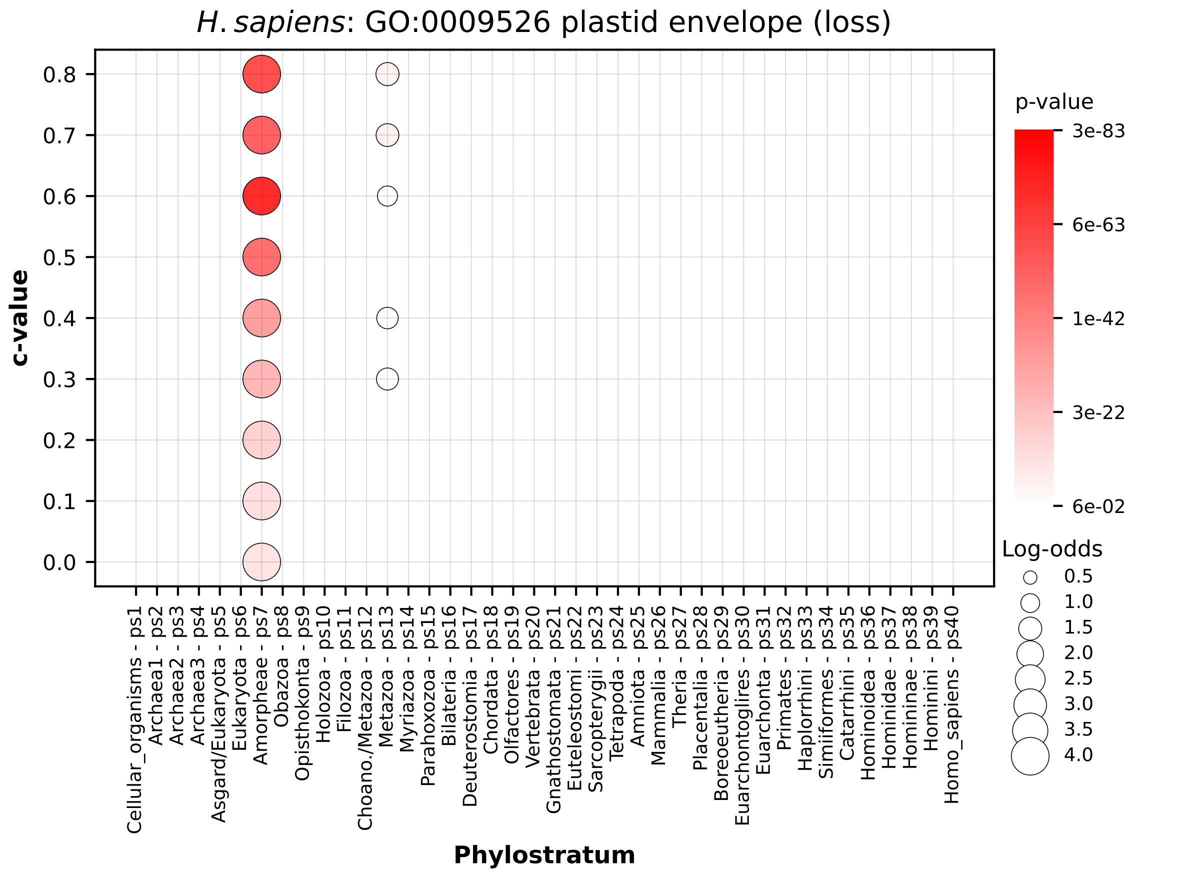

H. sapiens: GO:0009526 plastid envelope (loss)
c-value
0.8
0.7
0.6
0.5
0.4
0.3
0.2
0.1
0.0
Phylostratum
Cellular_organisms - ps1
Archaea1 - ps2
Archaea2 - ps3
Archaea3 - ps4
Asgard/Eukaryota - ps5
Eukaryota - ps6
Amorpheae - ps7
Obazoa - ps8
Opisthokonta - ps9
Holozoa - ps10
Filozoa - ps11
Choano./Metazoa - ps12
Metazoa - ps13
Myriazoa - ps14
Parahoxozoa - ps15
Bilateria - ps16
Deuterostomia - ps17
Chordata - ps18
Olfactores - ps19
Vertebrata - ps20
Gnathostomata - ps21
Euteleostomi - ps22
Sarcopterygii - ps23
Tetrapoda - ps24
Amniota - ps25
Mammalia - ps26
Theria - ps27
Placentalia - ps28
Boreoeutheria - ps29
Euarchontoglires - ps30
Euarchonta - ps31
Primates - ps32
Haplorrhini - ps33
Simiiformes - ps34
Catarrhini - ps35
Hominoidea - ps36
Hominidae - ps37
Homininae - ps38
Hominini - ps39
Homo_sapiens - ps40
p-value
3e-83
6e-63
1e-42
3e-22
6e-02
Log-odds
0.5
1.0
1.5
2.0
2.5
3.0
3.5
4.0

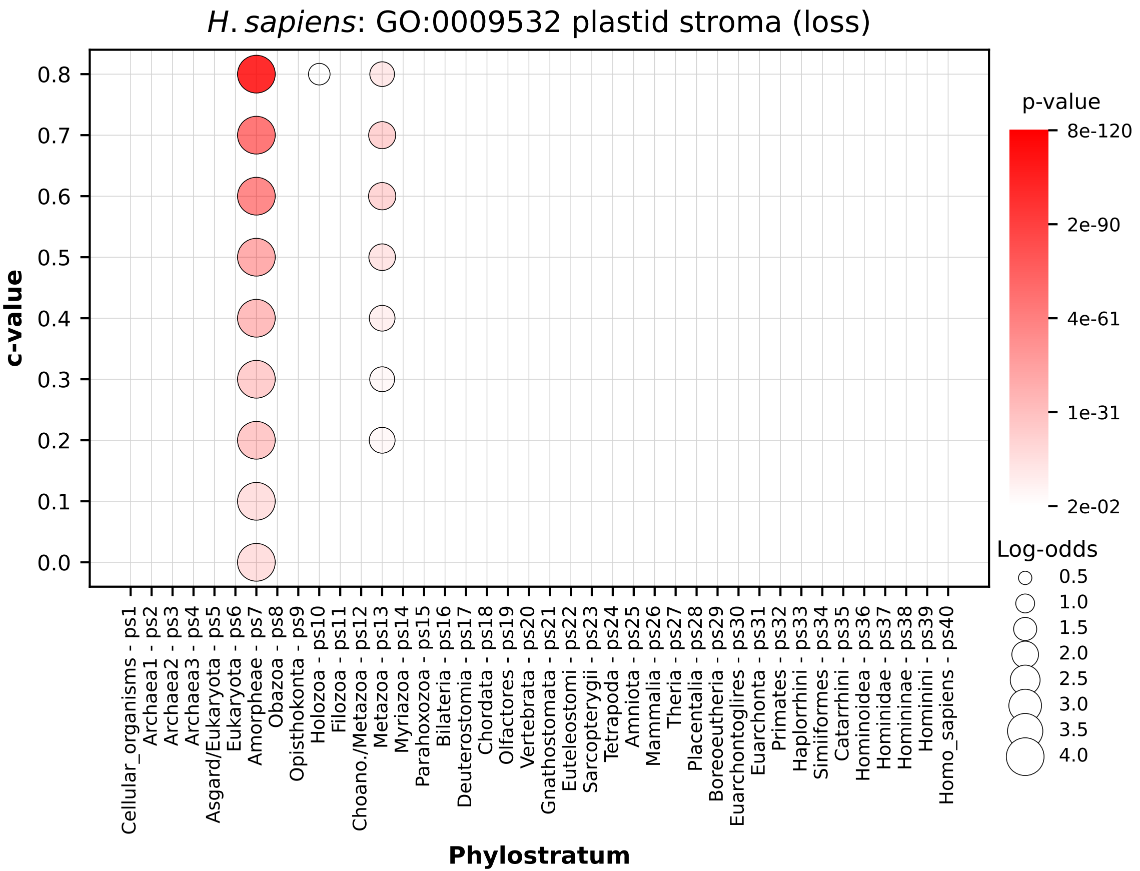
H. sapiens: GO:0009532 plastid stroma (loss)
c-value
0.8
0.7
0.6
0.5
0.4
0.3
0.2
0.1
0.0
Phylostratum
Cellular_organisms - ps1
Archaea1 - ps2
Archaea2 - ps3
Archaea3 - ps4
Asgard/Eukaryota - ps5
Eukaryota - ps6
Amorpheae - ps7
Obazoa - ps8
Opisthokonta - ps9
Holozoa - ps10
Filozoa - ps11
Choano./Metazoa - ps12
Metazoa - ps13
Myriazoa - ps14
Parahoxozoa - ps15
Bilateria - ps16
Deuterostomia - ps17
Chordata - ps18
Olfactores - ps19
Vertebrata - ps20
Gnathostomata - ps21
Euteleostomi - ps22
Sarcopterygii - ps23
Tetrapoda - ps24
Amniota - ps25
Mammalia - ps26
Theria - ps27
Placentalia - ps28
Boreoeutheria - ps29
Euarchontoglires - ps30
Euarchonta - ps31
Primates - ps32
Haplorrhini - ps33
Simiiformes - ps34
Catarrhini - ps35
Hominoidea - ps36
Hominidae - ps37
Homininae - ps38
Hominini - ps39
Homo_sapiens - ps40
p-value
8e-120
2e-90
4e-61
1e-31
2e-02
Log-odds
0.5
1.0
1.5
2.0
2.5
3.0
3.5
4.0

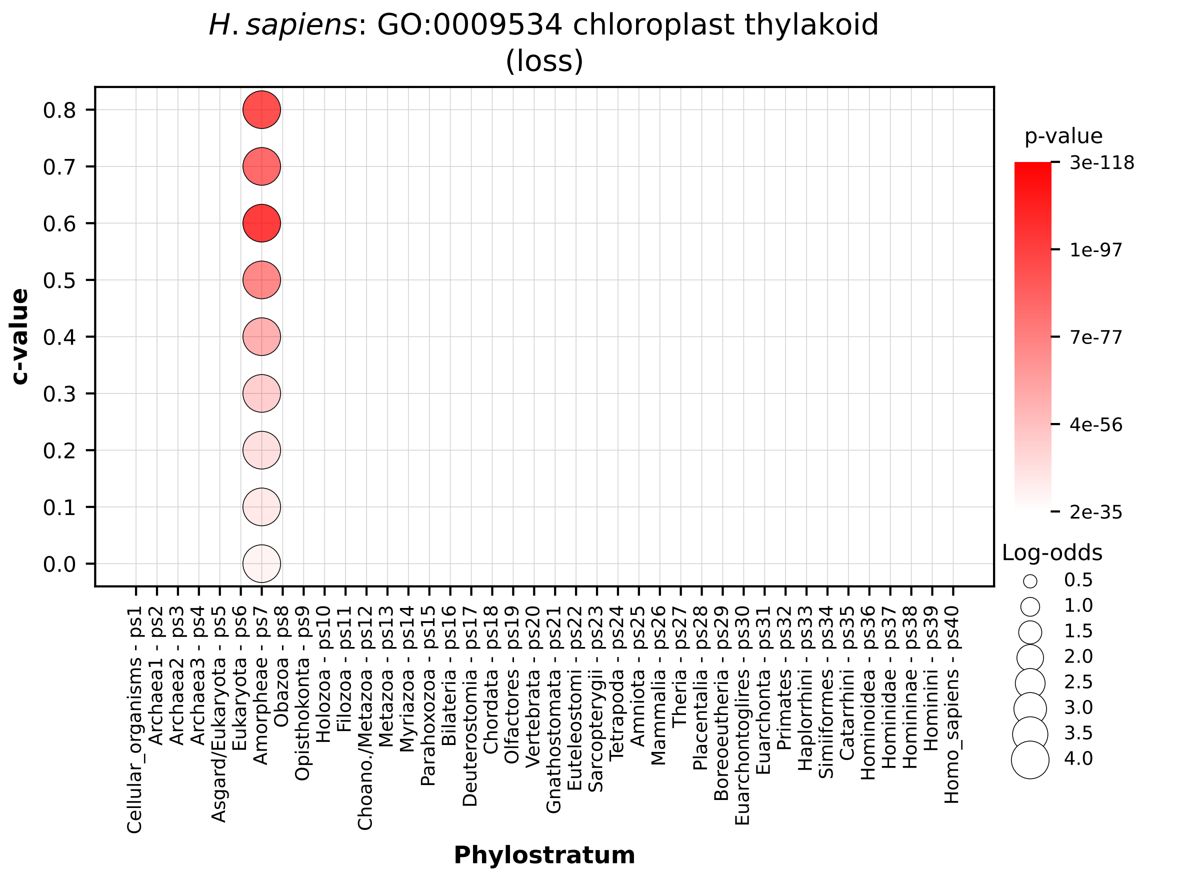

H. sapiens: GO:0009534 chloroplast thylakoid
(loss)
c-value
0.8
0.7
0.6
0.5
0.4
0.3
0.2
0.1
0.0
p-value
3e-118
1e-97
7e-77
4e-56
2e-35
Log-odds
0.5
1.0
1.5
2.0
2.5
3.0
3.5
4.0
Cellular_organisms - ps1
Archaea1 - ps2
Archaea2 - ps3
Archaea3 - ps4
Asgard/Eukaryota - ps5
Eukaryota - ps6
Amorpheae - ps7
Obazoa - ps8
Opisthokonta - ps9
Holozoa - ps10
Filozoa - ps11
Choano./Metazoa - ps12
Metazoa - ps13
Myriazoa - ps14
Parahoxozoa - ps15
Bilateria - ps16
Deuterostomia - ps17
Chordata - ps18
Olfactores - ps19
Vertebrata - ps20
Gnathostomata - ps21
Euteleostomi - ps22
Sarcopterygii - ps23
Tetrapoda - ps24
Amniota - ps25
Mammalia - ps26
Theria - ps27
Placentalia - ps28
Boreoeutheria - ps29
Euarchontoglires - ps30
Euarchonta - ps31
Primates - ps32
Haplorrhini - ps33
Simiiformes - ps34
Catarrhini - ps35
Hominoidea - ps36
Hominidae - ps37
Homininae - ps38
Hominini - ps39
Homo_sapiens - ps40
Phylostratum

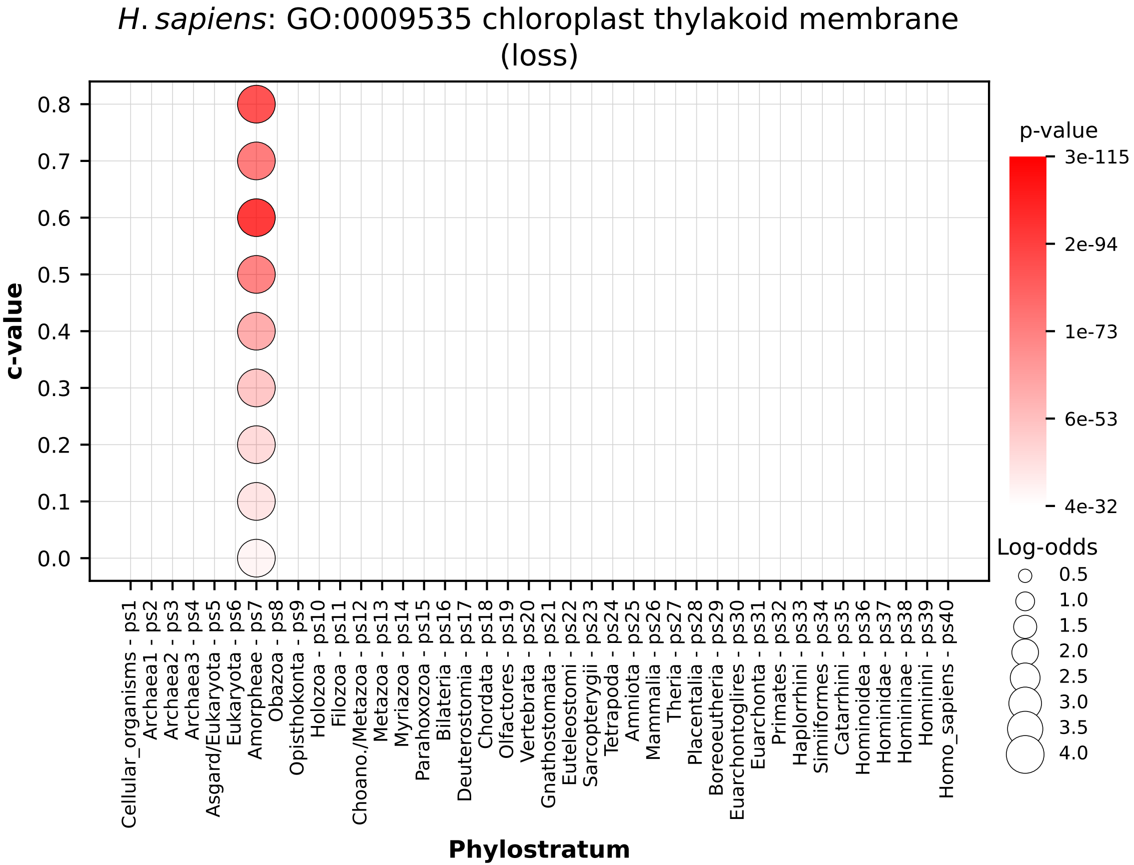

H. sapiens: GO:0009535 chloroplast thylakoid membrane (loss)
c-value
0.8
0.7
0.6
0.5
0.4
0.3
0.2
0.1
0.0
p-value
3e-115
2e-94
1e-73
6e-53
4e-32
Log-odds
0.5
1.0
1.5
2.0
2.5
3.0
3.5
4.0
Cellular_organisms - ps1
Archaea1 - ps2
Archaea2 - ps3
Archaea3 - ps4
Asgard/Eukaryota - ps5
Eukaryota - ps6
Amorpheae - ps7
Obazoa - ps8
Opisthokonta - ps9
Holozoa - ps10
Filozoa - ps11
Choano./Metazoa - ps12
Metazoa - ps13
Myriazoa - ps14
Parahoxozoa - ps15
Bilateria - ps16
Deuterostomia - ps17
Chordata - ps18
Olfactores - ps19
Vertebrata - ps20
Gnathostomata - ps21
Euteleostomi - ps22
Sarcopterygii - ps23
Tetrapoda - ps24
Amniota - ps25
Mammalia - ps26
Theria - ps27
Placentalia - ps28
Boreoeutheria - ps29
Euarchontoglires - ps30
Euarchonta - ps31
Primates - ps32
Haplorrhini - ps33
Simiiformes - ps34
Catarrhini - ps35
Hominoidea - ps36
Hominidae - ps37
Homininae - ps38
Hominini - ps39
Homo_sapiens - ps40
Phylostratum

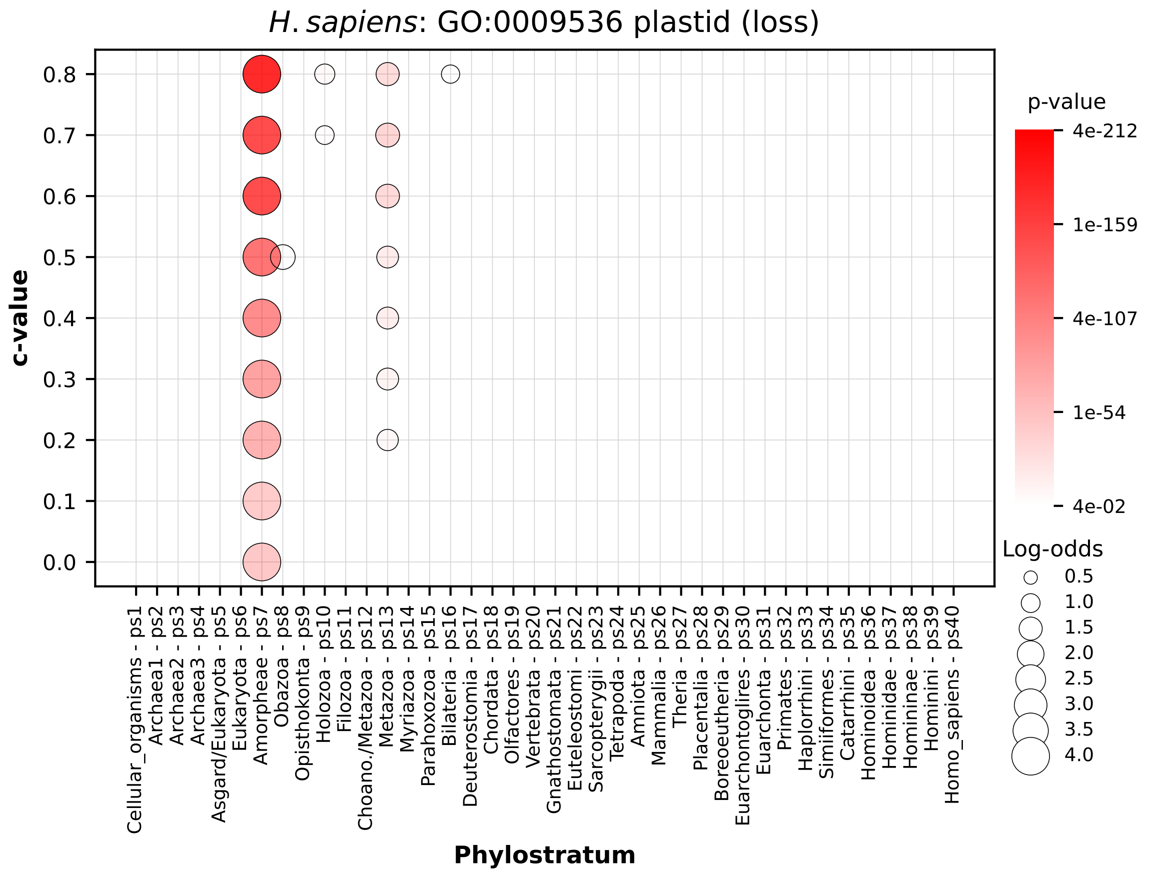

H. sapiens: GO:0009536 plastid (loss)
c-value
0.8
0.7
0.6
0.5
0.4
0.3
0.2
0.1
0.0
Phylostratum
Cellular_organisms - ps1
Archaea1 - ps2
Archaea2 - ps3
Archaea3 - ps4
Asgard/Eukaryota - ps5
Eukaryota - ps6
Amorpheae - ps7
Obazoa - ps8
Opisthokonta - ps9
Holozoa - ps10
Filozoa - ps11
Choano./Metazoa - ps12
Metazoa - ps13
Myriazoa - ps14
Parahoxozoa - ps15
Bilateria - ps16
Deuterostomia - ps17
Chordata - ps18
Olfactores - ps19
Vertebrata - ps20
Gnathostomata - ps21
Euteleostomi - ps22
Sarcopterygii - ps23
Tetrapoda - ps24
Amniota - ps25
Mammalia - ps26
Theria - ps27
Placentalia - ps28
Boreoeutheria - ps29
Euarchontoglires - ps30
Euarchonta - ps31
Primates - ps32
Haplorrhini - ps33
Simiiformes - ps34
Catarrhini - ps35
Hominoidea - ps36
Hominidae - ps37
Homininae - ps38
Hominini - ps39
Homo_sapiens - ps40
p-value
4e-212
1e-159
4e-107
1e-54
4e-02
Log-odds
0.5
1.0
1.5
2.0
2.5
3.0
3.5
4.0

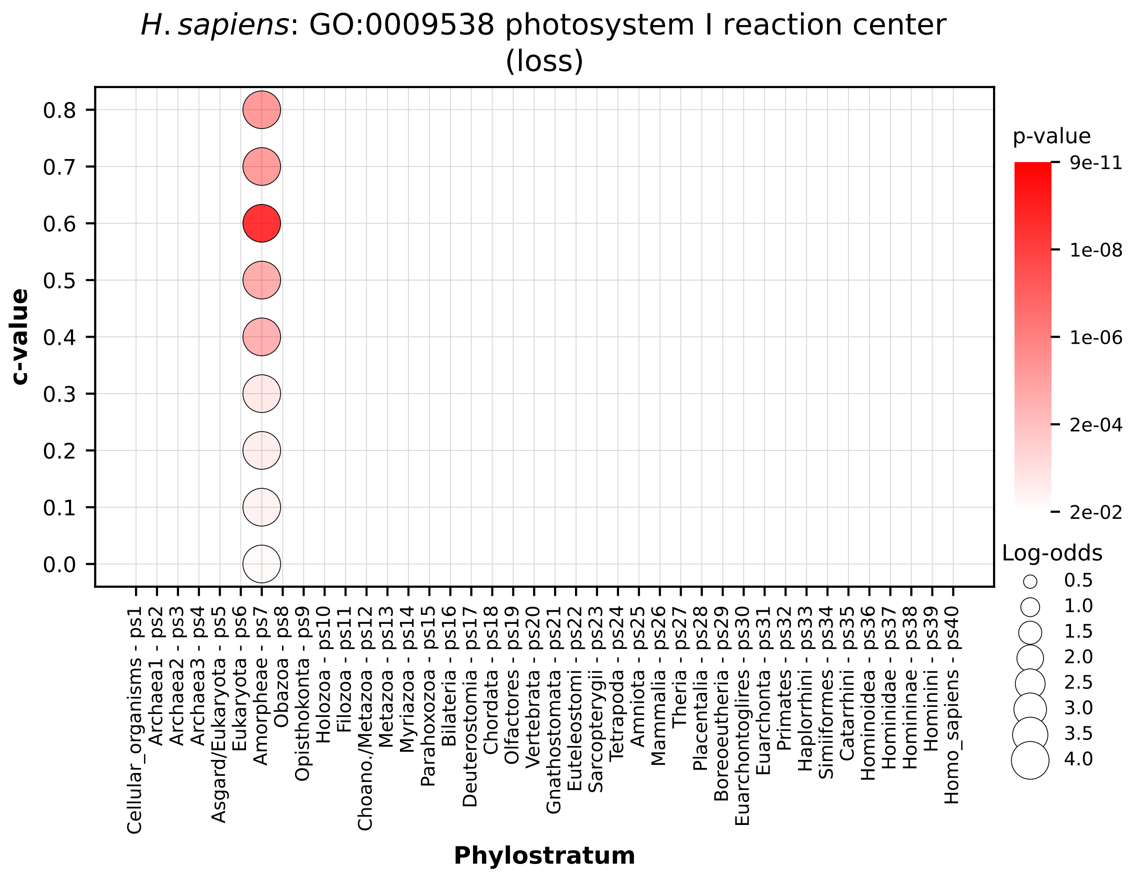

H. sapiens: GO:0009538 photosystem I reaction center
(loss)
c-value
0.8
0.7
0.6
0.5
0.4
0.3
0.2
0.1
0.0
p-value
9e-11
1e-08
1e-06
2e-04
2e-02
Log-odds
0.5
1.0
1.5
2.0
2.5
3.0
3.5
4.0
Cellular_organisms - ps1
Archaea1 - ps2
Archaea2 - ps3
Archaea3 - ps4
Asgard/Eukaryota - ps5
Eukaryota - ps6
Amorpheae - ps7
Obazoa - ps8
Opisthokonta - ps9
Holozoa - ps10
Filozoa - ps11
Choano./Metazoa - ps12
Metazoa - ps13
Myriazoa - ps14
Parahoxozoa - ps15
Bilateria - ps16
Deuterostomia - ps17
Chordata - ps18
Olfactores - ps19
Vertebrata - ps20
Gnathostomata - ps21
Euteleostomi - ps22
Sarcopterygii - ps23
Tetrapoda - ps24
Amniota - ps25
Mammalia - ps26
Theria - ps27
Placentalia - ps28
Boreoeutheria - ps29
Euarchontoglires - ps30
Euarchonta - ps31
Primates - ps32
Haplorrhini - ps33
Simiiformes - ps34
Catarrhini - ps35
Hominoidea - ps36
Hominidae - ps37
Homininae - ps38
Hominini - ps39
Homo_sapiens - ps40
Phylostratum

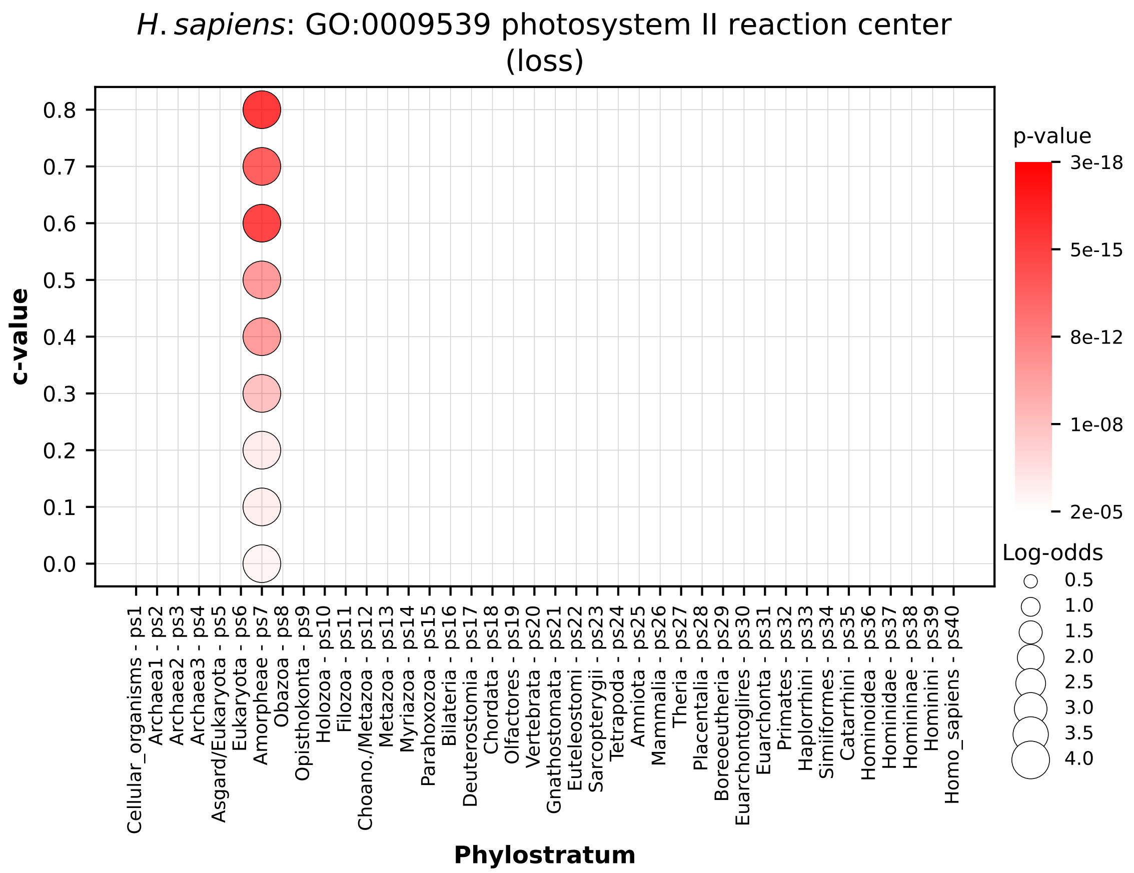

H. sapiens: GO:0009539 photosystem II reaction center (loss)
c-value
0.8
0.7
0.6
0.5
0.4
0.3
0.2
0.1
0.0
p-value
3e-18
5e-15
8e-12
1e-08
2e-05
Log-odds
0.5
1.0
1.5
2.0
2.5
3.0
3.5
4.0
Cellular_organisms - ps1
Archaea1 - ps2
Archaea2 - ps3
Archaea3 - ps4
Asgard/Eukaryota - ps5
Eukaryota - ps6
Amorpheae - ps7
Obazoa - ps8
Opisthokonta - ps9
Holozoa - ps10
Filozoa - ps11
Choano./Metazoa - ps12
Metazoa - ps13
Myriazoa - ps14
Parahoxozoa - ps15
Bilateria - ps16
Deuterostomia - ps17
Chordata - ps18
Olfactores - ps19
Vertebrata - ps20
Gnathostomata - ps21
Euteleostomi - ps22
Sarcopterygii - ps23
Tetrapoda - ps24
Amniota - ps25
Mammalia - ps26
Theria - ps27
Placentalia - ps28
Boreoeutheria - ps29
Euarchontoglires - ps30
Euarchonta - ps31
Primates - ps32
Haplorrhini - ps33
Simiiformes - ps34
Catarrhini - ps35
Hominoidea - ps36
Hominidae - ps37
Homininae - ps38
Hominini - ps39
Homo_sapiens - ps40
Phylostratum

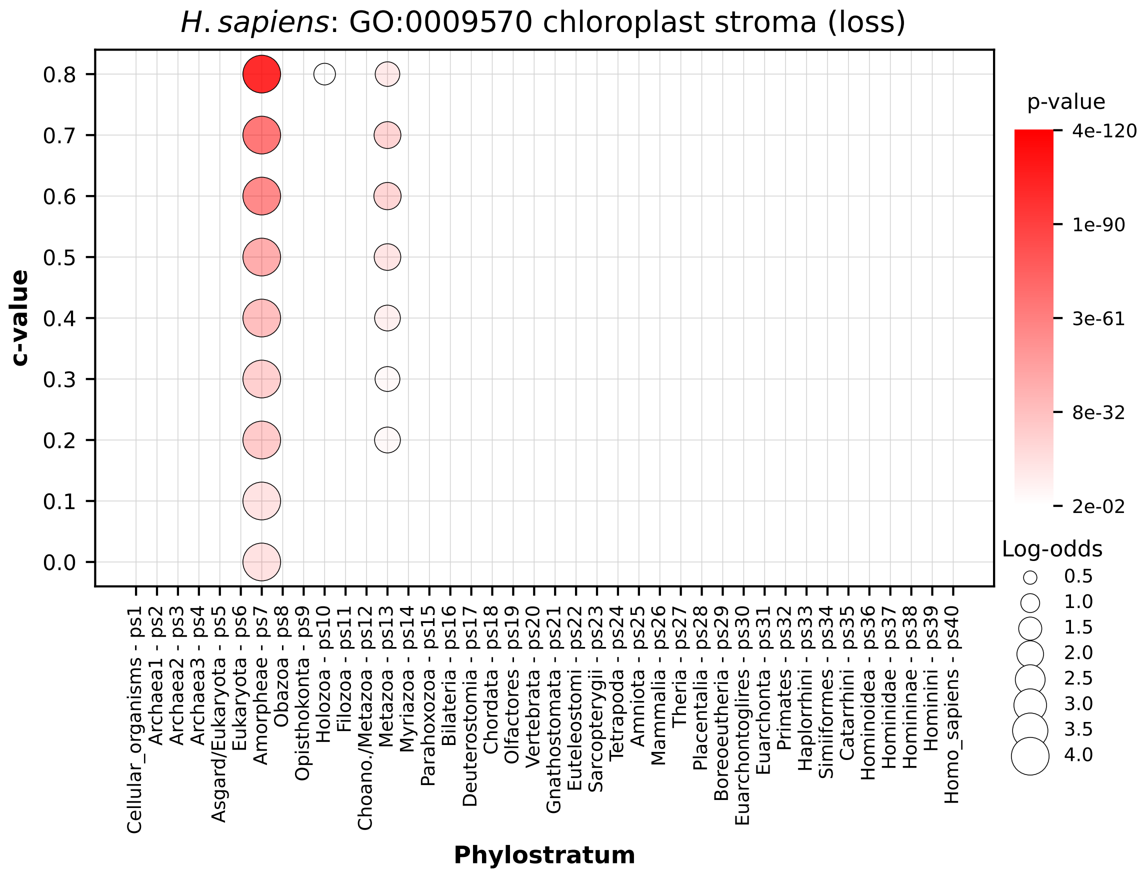

H. sapiens: GO:0009570 chloroplast stroma (loss)
c-value
0.8
0.7
0.6
0.5
0.4
0.3
0.2
0.1
0.0
p-value
4e-120
1e-90
3e-61
8e-32
2e-02
Log-odds
0.5
1.0
1.5
2.0
2.5
3.0
3.5
4.0
Cellular_organisms - ps1
Archaea1 - ps2
Archaea2 - ps3
Archaea3 - ps4
Asgard/Eukaryota - ps5
Eukaryota - ps6
Amorpheae - ps7
Obazoa - ps8
Opisthokonta - ps9
Holozoa - ps10
Filozoa - ps11
Choano./Metazoa - ps12
Metazoa - ps13
Myriazoa - ps14
Parahoxozoa - ps15
Bilateria - ps16
Deuterostomia - ps17
Chordata - ps18
Olfactores - ps19
Vertebrata - ps20
Gnathostomata - ps21
Euteleostomi - ps22
Sarcopterygii - ps23
Tetrapoda - ps24
Amniota - ps25
Mammalia - ps26
Theria - ps27
Placentalia - ps28
Boreoeutheria - ps29
Euarchontoglires - ps30
Euarchonta - ps31
Primates - ps32
Haplorrhini - ps33
Simiiformes - ps34
Catarrhini - ps35
Hominoidea - ps36
Hominidae - ps37
Homininae - ps38
Hominini - ps39
Homo_sapiens - ps40
Phylostratum

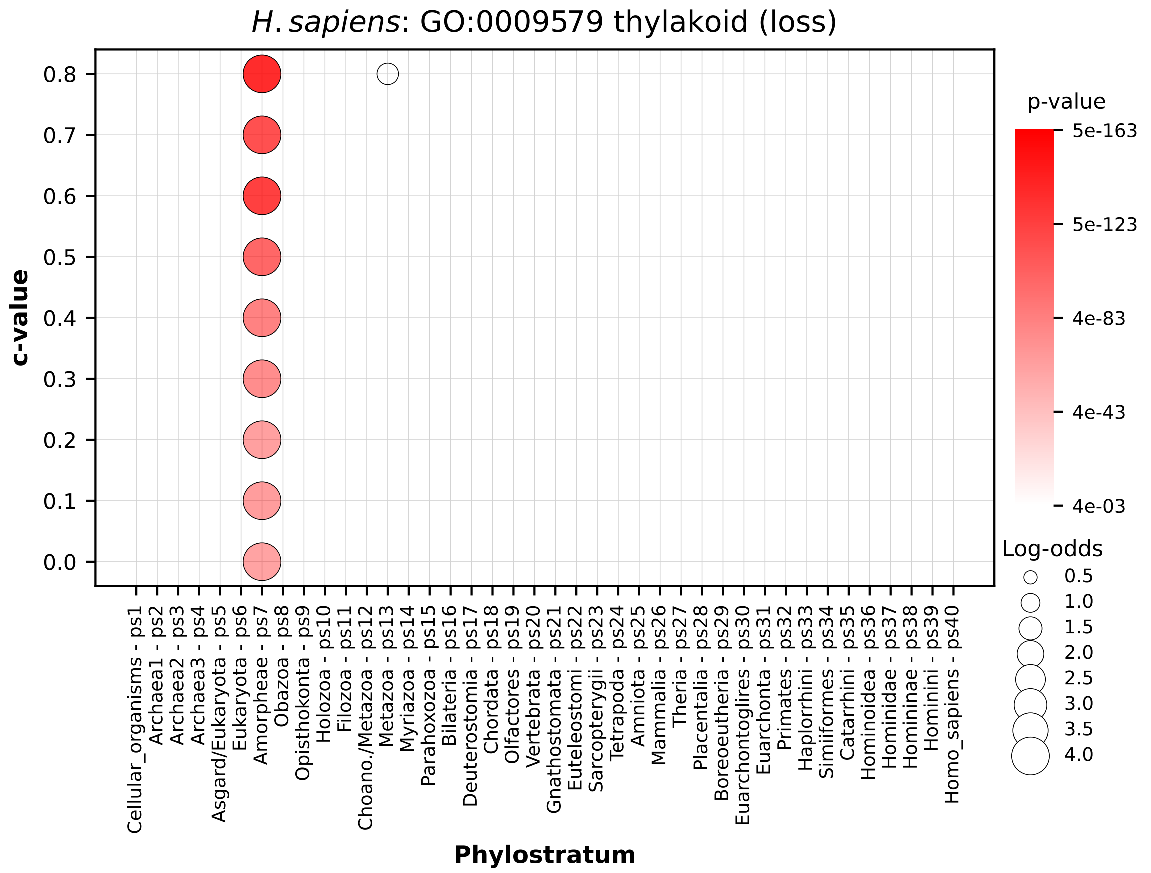

H. sapiens: GO:0009579 thylakoid (loss)
c-value
0.8
0.7
0.6
0.5
0.4
0.3
0.2
0.1
0.0
Phylostratum
Cellular_organisms - ps1
Archaea1 - ps2
Archaea2 - ps3
Archaea3 - ps4
Asgard/Eukaryota - ps5
Eukaryota - ps6
Amorpheae - ps7
Obazoa - ps8
Opisthokonta - ps9
Holozoa - ps10
Filozoa - ps11
Choano./Metazoa - ps12
Metazoa - ps13
Myriazoa - ps14
Parahoxozoa - ps15
Bilateria - ps16
Deuterostomia - ps17
Chordata - ps18
Olfactores - ps19
Vertebrata - ps20
Gnathostomata - ps21
Euteleostomi - ps22
Sarcopterygii - ps23
Tetrapoda - ps24
Amniota - ps25
Mammalia - ps26
Theria - ps27
Placentalia - ps28
Boreoeutheria - ps29
Euarchontoglires - ps30
Euarchonta - ps31
Primates - ps32
Haplorrhini - ps33
Simiiformes - ps34
Catarrhini - ps35
Hominoidea - ps36
Hominidae - ps37
Homininae - ps38
Hominini - ps39
Homo_sapiens - ps40
p-value
5e-163
5e-123
4e-83
4e-43
4e-03
Log-odds
0.5
1.0
1.5
2.0
2.5
3.0
3.5
4.0

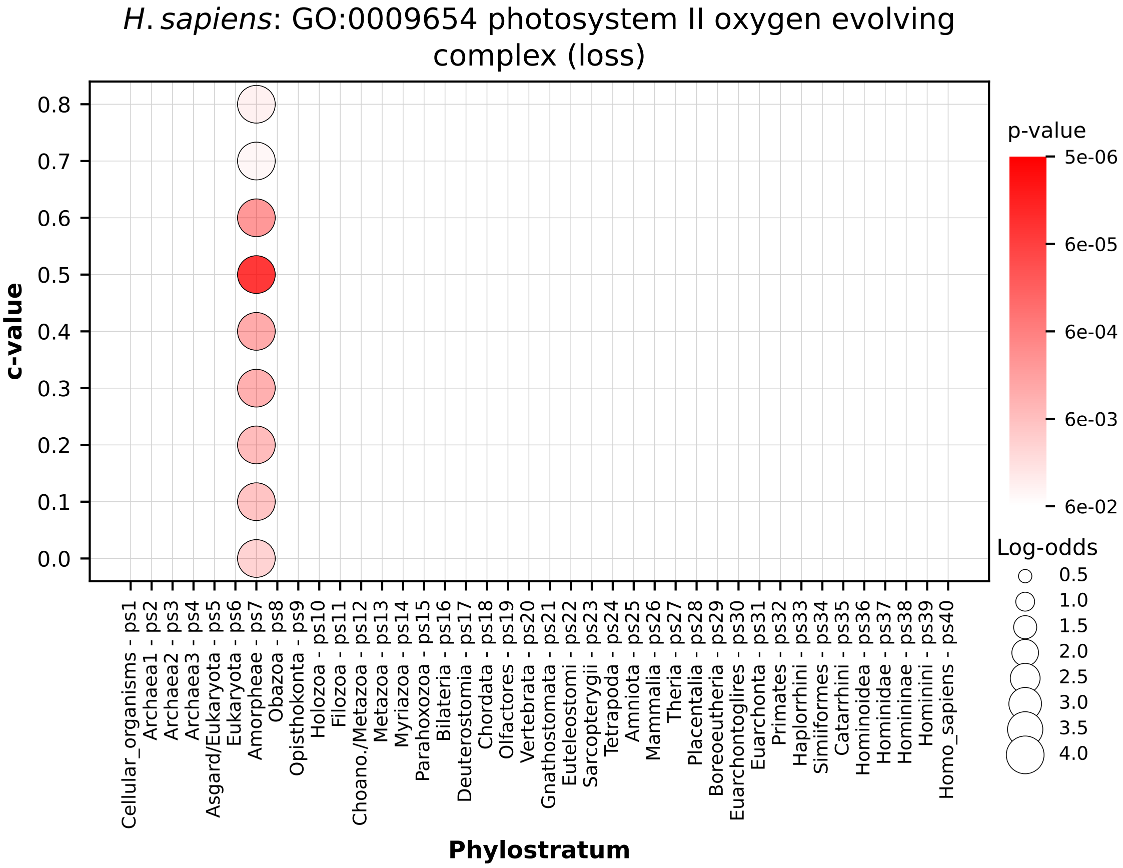
H. sapiens: GO:0009654 photosystem II oxygen evolving complex (loss)
c-value
0.0
0.1
0.2
0.3
0.4
0.5
0.6
0.7
0.8
Phylostratum
Cellular_organisms - ps1
Archaea1 - ps2
Archaea2 - ps3
Archaea3 - ps4
Asgard/Eukaryota - ps5
Eukaryota - ps6
Amorpheae - ps7
Obazoa - ps8
Opisthokonta - ps9
Holozoa - ps10
Filozoa - ps11
Choano./Metazoa - ps12
Metazoa - ps13
Myriazoa - ps14
Parahoxozoa - ps15
Bilateria - ps16
Deuterostomia - ps17
Chordata - ps18
Olfactores - ps19
Vertebrata - ps20
Gnathostomata - ps21
Euteleostomi - ps22
Sarcopterygii - ps23
Tetrapoda - ps24
Amniota - ps25
Mammalia - ps26
Theria - ps27
Placentalia - ps28
Boreoeutheria - ps29
Euarchontoglires - ps30
Euarchonta - ps31
Primates - ps32
Haplorrhini - ps33
Simiiformes - ps34
Catarrhini - ps35
Hominoidea - ps36
Hominidae - ps37
Homininae - ps38
Hominini - ps39
Homo_sapiens - ps40
p-value
5e-06
6e-05
6e-04
6e-03
6e-02
Log-odds
0.5
1.0
1.5
2.0
2.5
3.0
3.5
4.0

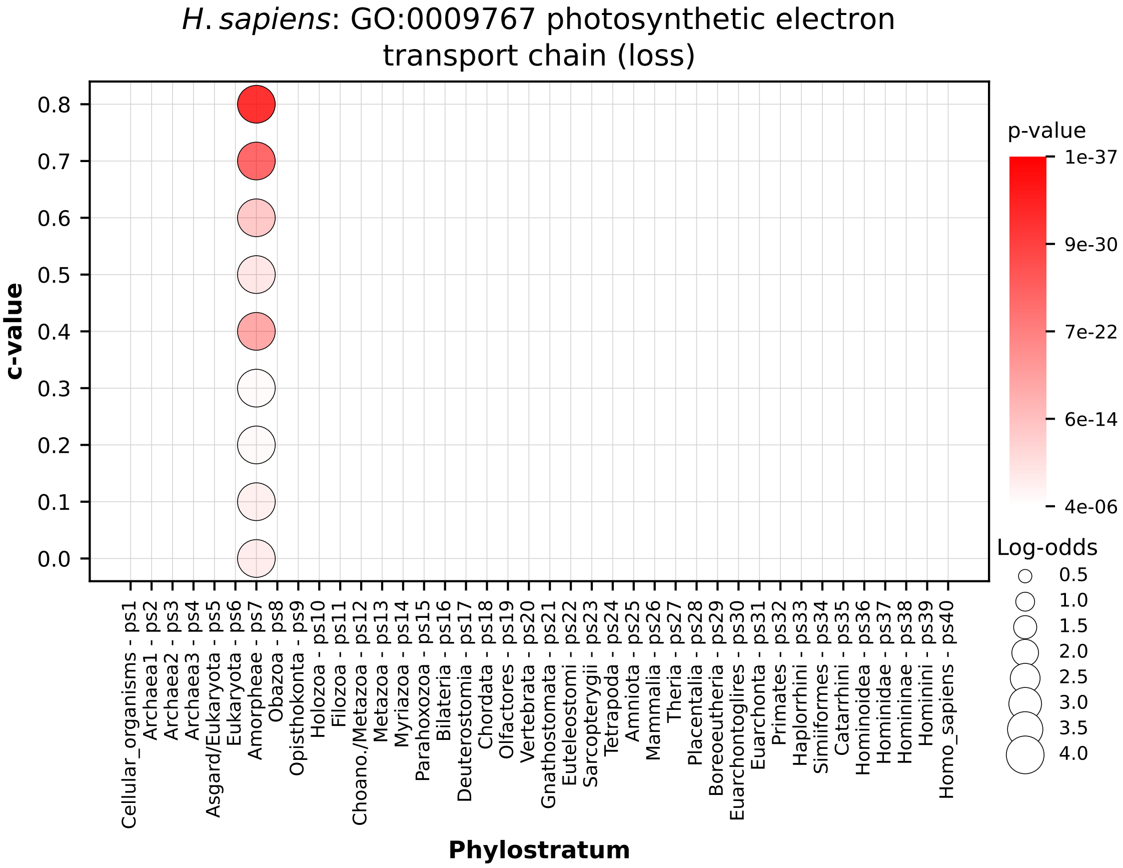

H. sapiens: GO:0009767 photosynthetic electron transport chain (loss)
c-value
0.8
0.7
0.6
0.5
0.4
0.3
0.2
0.1
0.0
p-value
1e-37
9e-30
7e-22
6e-14
4e-06
Log-odds
0.5
1.0
1.5
2.0
2.5
3.0
3.5
4.0
Cellular_organisms - ps1
Archaea1 - ps2
Archaea2 - ps3
Archaea3 - ps4
Asgard/Eukaryota - ps5
Eukaryota - ps6
Amorpheae - ps7
Obazoa - ps8
Opisthokonta - ps9
Holozoa - ps10
Filozoa - ps11
Choano./Metazoa - ps12
Metazoa - ps13
Myriazoa - ps14
Parahoxozoa - ps15
Bilateria - ps16
Deuterostomia - ps17
Chordata - ps18
Olfactores - ps19
Vertebrata - ps20
Gnathostomata - ps21
Euteleostomi - ps22
Sarcopterygii - ps23
Tetrapoda - ps24
Amniota - ps25
Mammalia - ps26
Theria - ps27
Placentalia - ps28
Boreoeutheria - ps29
Euarchontoglires - ps30
Euarchonta - ps31
Primates - ps32
Haplorrhini - ps33
Simiiformes - ps34
Catarrhini - ps35
Hominoidea - ps36
Hominidae - ps37
Homininae - ps38
Hominini - ps39
Homo_sapiens - ps40
Phylostratum

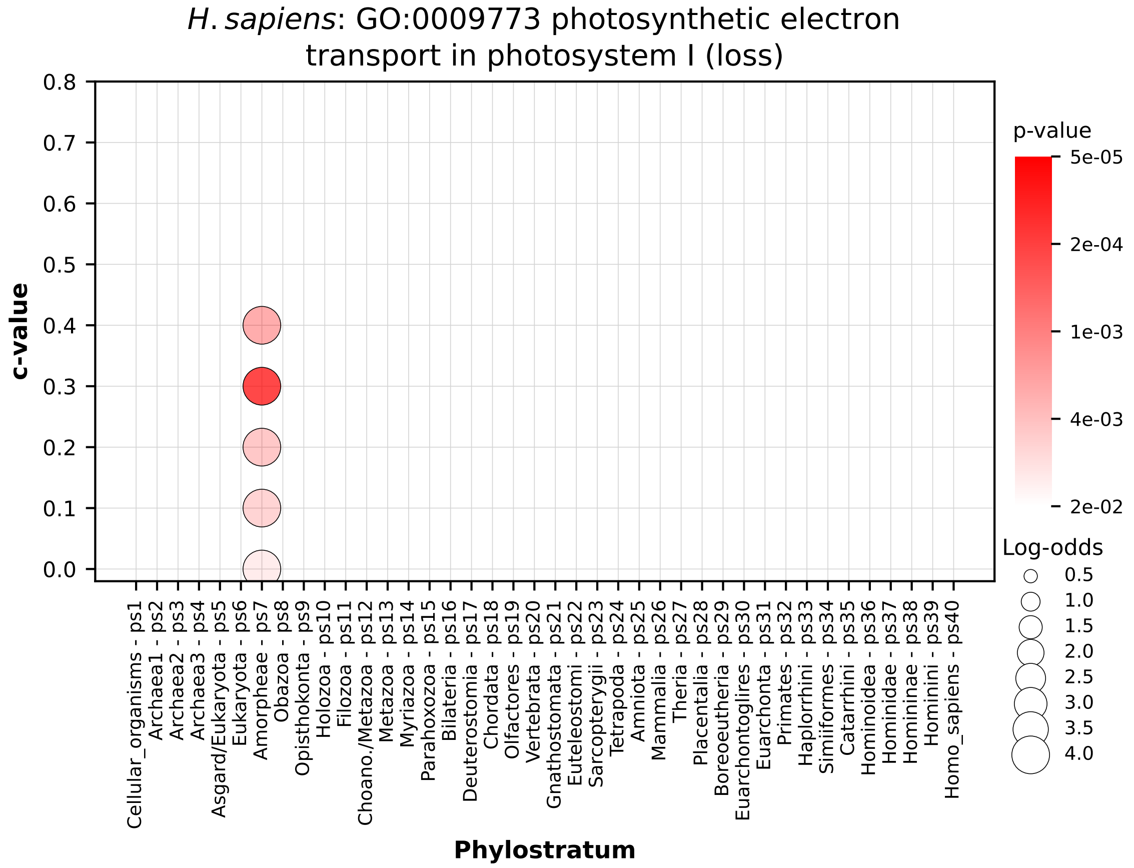
H. sapiens: GO:0009773 photosynthetic electron transport in photosystem I (loss)
c-value
0.0
0.1
0.2
0.3
0.4
0.5
0.6
0.7
0.8
Phylostratum
Cellular_organisms - ps1
Archaea1 - ps2
Archaea2 - ps3
Archaea3 - ps4
Asgard/Eukaryota - ps5
Eukaryota - ps6
Amorpheae - ps7
Obazoa - ps8
Opisthokonta - ps9
Holozoa - ps10
Filozoa - ps11
Choano./Metazoa - ps12
Metazoa - ps13
Myriazoa - ps14
Parahoxozoa - ps15
Bilateria - ps16
Deuterostomia - ps17
Chordata - ps18
Olfactores - ps19
Vertebrata - ps20
Gnathostomata - ps21
Euteleostomi - ps22
Sarcopterygii - ps23
Tetrapoda - ps24
Amniota - ps25
Mammalia - ps26
Theria - ps27
Placentalia - ps28
Boreoeutheria - ps29
Euarchontoglires - ps30
Euarchonta - ps31
Primates - ps32
Haplorrhini - ps33
Simiiformes - ps34
Catarrhini - ps35
Hominoidea - ps36
Hominidae - ps37
Homininae - ps38
Hominini - ps39
Homo_sapiens - ps40
p-value
5e-05
2e-04
1e-03
4e-03
2e-02
Log-odds
0.5
1.0
1.5
2.0
2.5
3.0
3.5
4.0

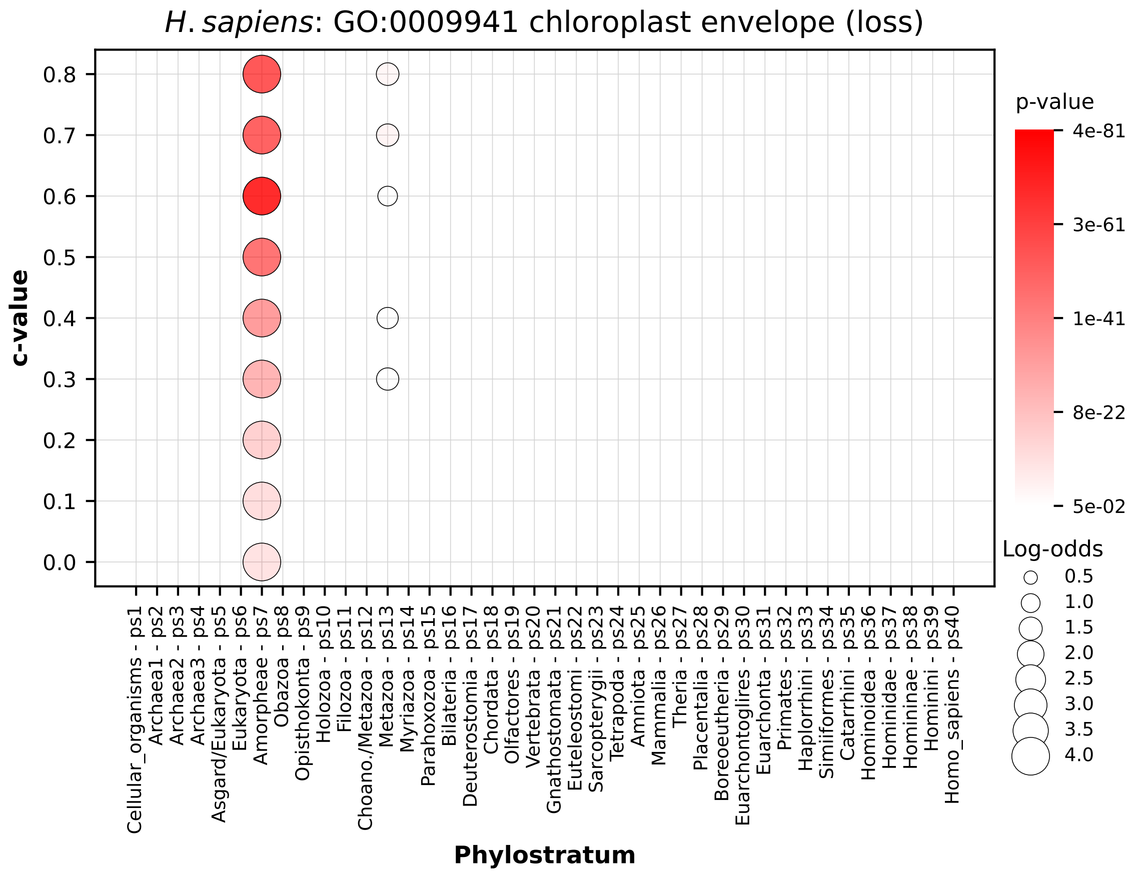

H. sapiens: GO:0009941 chloroplast envelope (loss)
c-value
0.8
0.7
0.6
0.5
0.4
0.3
0.2
0.1
0.0
p-value
4e-81
3e-61
1e-41
8e-22
5e-02
Log-odds
0.5
1.0
1.5
2.0
2.5
3.0
3.5
4.0
Cellular_organisms - ps1
Archaea1 - ps2
Archaea2 - ps3
Archaea3 - ps4
Asgard/Eukaryota - ps5
Eukaryota - ps6
Amorpheae - ps7
Obazoa - ps8
Opisthokonta - ps9
Holozoa - ps10
Filozoa - ps11
Choano./Metazoa - ps12
Metazoa - ps13
Myriazoa - ps14
Parahoxozoa - ps15
Bilateria - ps16
Deuterostomia - ps17
Chordata - ps18
Olfactores - ps19
Vertebrata - ps20
Gnathostomata - ps21
Euteleostomi - ps22
Sarcopterygii - ps23
Tetrapoda - ps24
Amniota - ps25
Mammalia - ps26
Theria - ps27
Placentalia - ps28
Boreoeutheria - ps29
Euarchontoglires - ps30
Euarchonta - ps31
Primates - ps32
Haplorrhini - ps33
Simiiformes - ps34
Catarrhini - ps35
Hominoidea - ps36
Hominidae - ps37
Homininae - ps38
Hominini - ps39
Homo_sapiens - ps40
Phylostratum

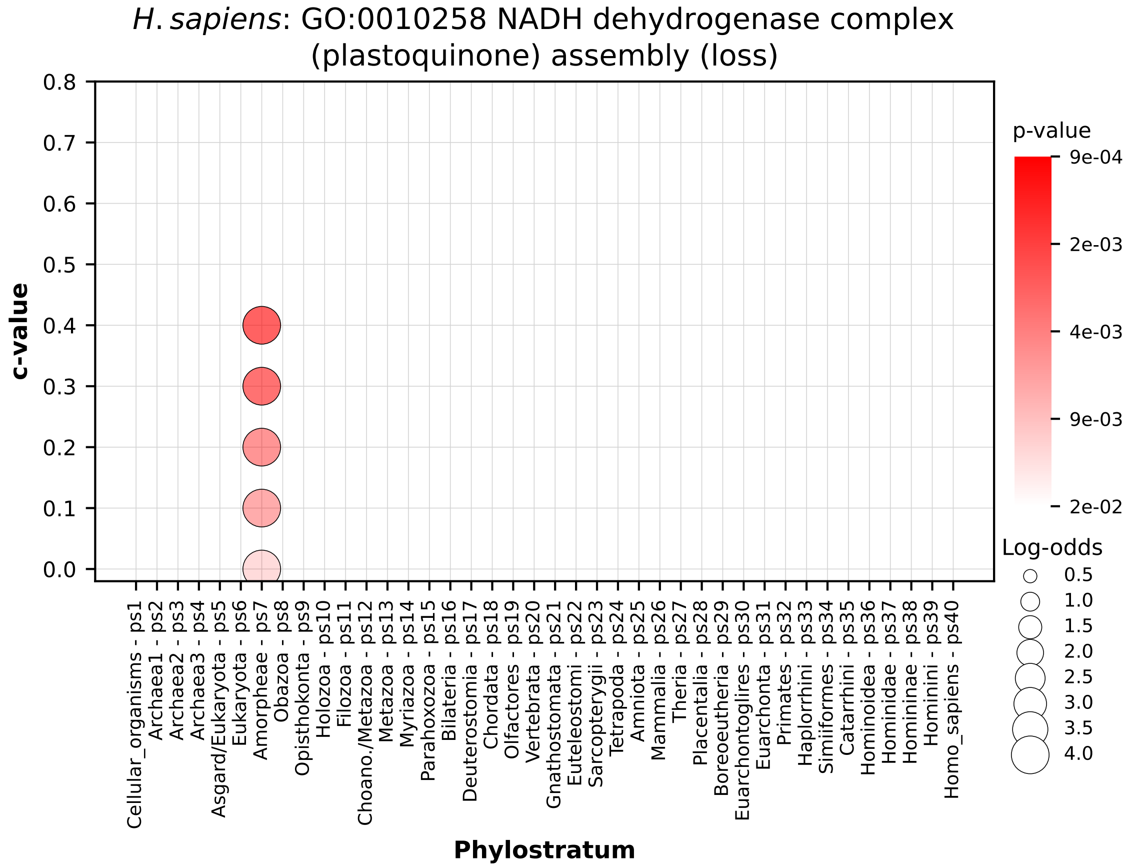

H. sapiens: GO:0010258 NADH dehydrogenase complex (plastoquinone) assembly (loss)
c-value
0.8
0.7
0.6
0.5
0.4
0.3
0.2
0.1
0.0
p-value
9e-04
2e-03
4e-03
9e-03
2e-02
Log-odds
0.5
1.0
1.5
2.0
2.5
3.0
3.5
4.0
Cellular_organisms - ps1
Archaea1 - ps2
Archaea2 - ps3
Archaea3 - ps4
Asgard/Eukaryota - ps5
Eukaryota - ps6
Amorpheae - ps7
Obazoa - ps8
Opisthokonta - ps9
Holozoa - ps10
Filozoa - ps11
Choano./Metazoa - ps12
Metazoa - ps13
Myriazoa - ps14
Parahoxozoa - ps15
Bilateria - ps16
Deuterostomia - ps17
Chordata - ps18
Olfactores - ps19
Vertebrata - ps20
Gnathostomata - ps21
Euteleostomi - ps22
Sarcopterygii - ps23
Tetrapoda - ps24
Amniota - ps25
Mammalia - ps26
Theria - ps27
Placentalia - ps28
Boreoeutheria - ps29
Euarchontoglires - ps30
Euarchonta - ps31
Primates - ps32
Haplorrhini - ps33
Simiiformes - ps34
Catarrhini - ps35
Hominoidea - ps36
Hominidae - ps37
Homininae - ps38
Hominini - ps39
Homo_sapiens - ps40
Phylostratum

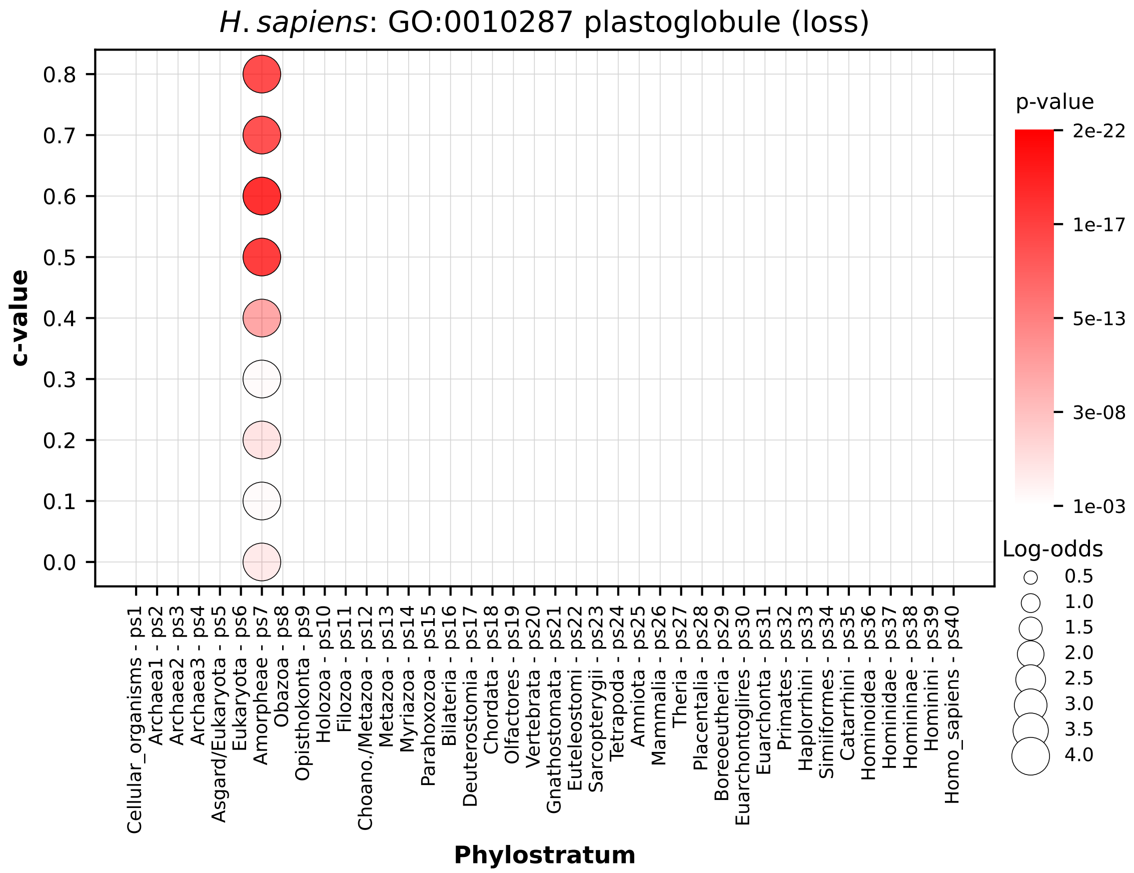

H. sapiens: GO:0010287 plastoglobule (loss)
c-value
0.0
0.1
0.2
0.3
0.4
0.5
0.6
0.7
0.8
p-value
2e-22
1e-17
5e-13
3e-08
1e-03
Log-odds
0.5
1.0
1.5
2.0
2.5
3.0
3.5
4.0
Cellular_organisms - ps1
Archaea1 - ps2
Archaea2 - ps3
Archaea3 - ps4
Asgard/Eukaryota - ps5
Eukaryota - ps6
Amorpheae - ps7
Obazoa - ps8
Opisthokonta - ps9
Holozoa - ps10
Filozoa - ps11
Choano./Metazoa - ps12
Metazoa - ps13
Myriazoa - ps14
Parahoxozoa - ps15
Bilateria - ps16
Deuterostomia - ps17
Chordata - ps18
Olfactores - ps19
Vertebrata - ps20
Gnathostomata - ps21
Euteleostomi - ps22
Sarcopterygii - ps23
Tetrapoda - ps24
Amniota - ps25
Mammalia - ps26
Theria - ps27
Placentalia - ps28
Boreoeutheria - ps29
Euarchontoglires - ps30
Euarchonta - ps31
Primates - ps32
Haplorrhini - ps33
Simiiformes - ps34
Catarrhini - ps35
Hominoidea - ps36
Hominidae - ps37
Homininae - ps38
Hominini - ps39
Homo_sapiens - ps40
Phylostratum

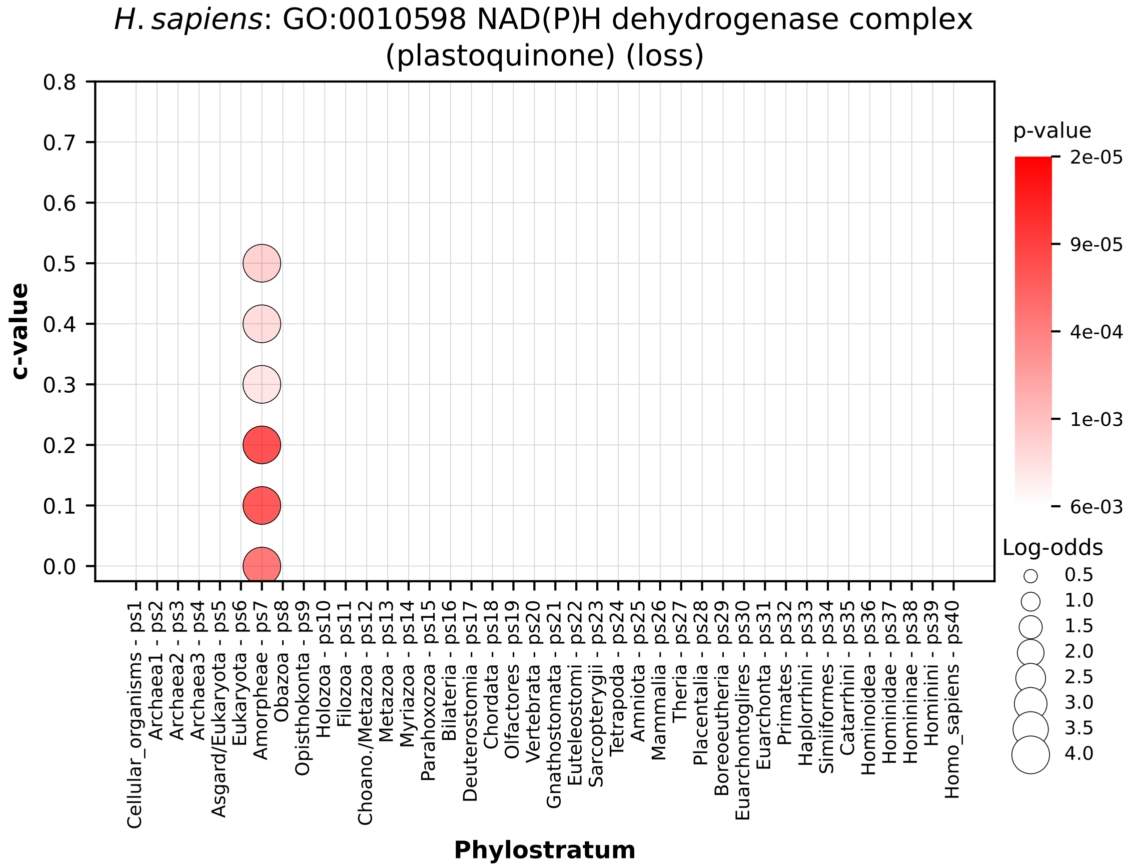

H. sapiens: GO:0010598 NAD(P)H dehydrogenase complex (plastoquinone) (loss)
c-value
0.8
0.7
0.6
0.5
0.4
0.3
0.2
0.1
0.0
Phylostratum
Cellular_organisms - ps1
Archaea1 - ps2
Archaea2 - ps3
Archaea3 - ps4
Asgard/Eukaryota - ps5
Eukaryota - ps6
Amorpheae - ps7
Obazoa - ps8
Opisthokonta - ps9
Holozoa - ps10
Filozoa - ps11
Choano./Metazoa - ps12
Metazoa - ps13
Myriazoa - ps14
Parahoxozoa - ps15
Bilateria - ps16
Deuterostomia - ps17
Chordata - ps18
Olfactores - ps19
Vertebrata - ps20
Gnathostomata - ps21
Euteleostomi - ps22
Sarcopterygii - ps23
Tetrapoda - ps24
Amniota - ps25
Mammalia - ps26
Theria - ps27
Placentalia - ps28
Boreoeutheria - ps29
Euarchontoglires - ps30
Euarchonta - ps31
Primates - ps32
Haplorrhini - ps33
Simiiformes - ps34
Catarrhini - ps35
Hominoidea - ps36
Hominidae - ps37
Homininae - ps38
Hominini - ps39
Homo_sapiens - ps40
p-value
2e-05
9e-05
4e-04
1e-03
6e-03
Log-odds
0.5
1.0
1.5
2.0
2.5
3.0
3.5
4.0

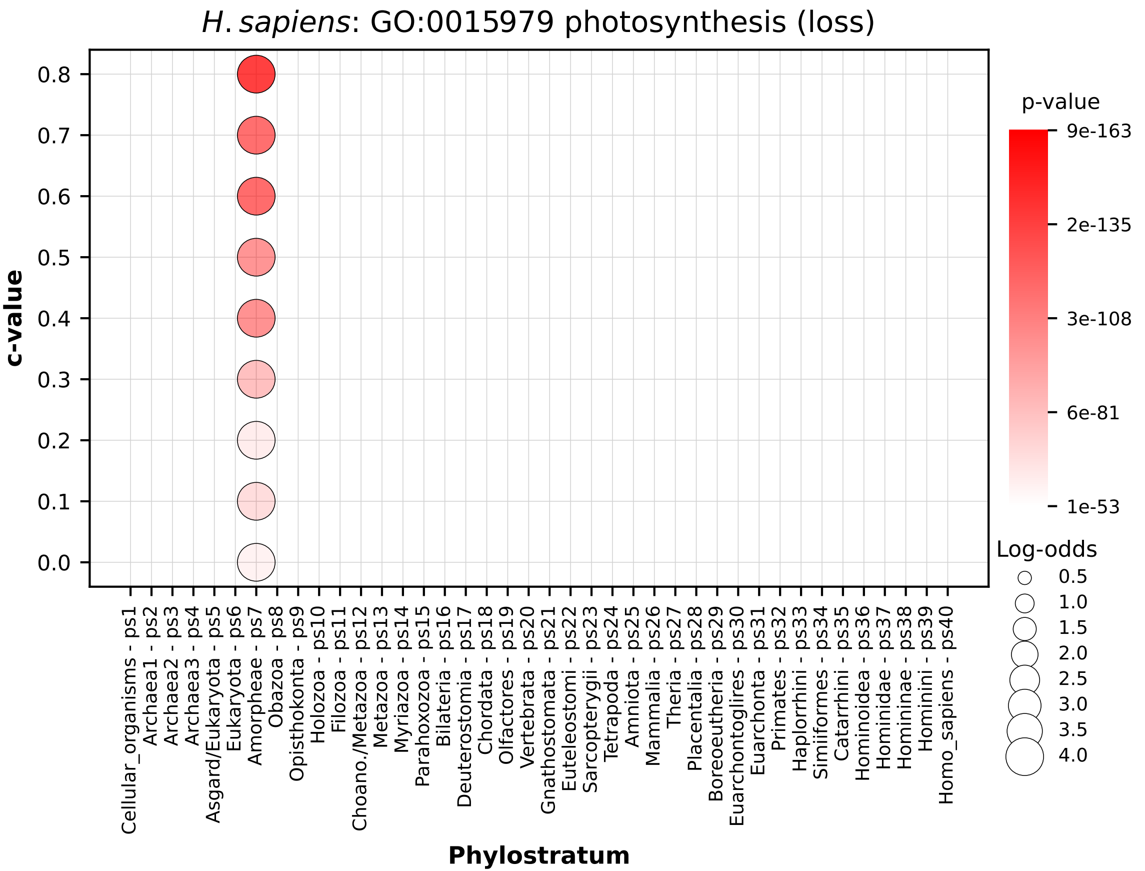

H. sapiens: GO:0015979 photosynthesis (loss)
c-value
0.0
0.1
0.2
0.3
0.4
0.5
0.6
0.7
0.8
Phylostratum
Cellular_organisms - ps1
Archaea1 - ps2
Archaea2 - ps3
Archaea3 - ps4
Asgard/Eukaryota - ps5
Eukaryota - ps6
Amorpheae - ps7
Obazoa - ps8
Opisthokonta - ps9
Holozoa - ps10
Filozoa - ps11
Choano./Metazoa - ps12
Metazoa - ps13
Myriazoa - ps14
Parahoxozoa - ps15
Bilateria - ps16
Deuterostomia - ps17
Chordata - ps18
Olfactores - ps19
Vertebrata - ps20
Gnathostomata - ps21
Euteleostomi - ps22
Sarcopterygii - ps23
Tetrapoda - ps24
Amniota - ps25
Mammalia - ps26
Theria - ps27
Placentalia - ps28
Boreoeutheria - ps29
Euarchontoglires - ps30
Euarchonta - ps31
Primates - ps32
Haplorrhini - ps33
Simiiformes - ps34
Catarrhini - ps35
Hominoidea - ps36
Hominidae - ps37
Homininae - ps38
Hominini - ps39
Homo_sapiens - ps40
p-value
9e-163
2e-135
3e-108
6e-81
1e-53
Log-odds
0.5
1.0
1.5
2.0
2.5
3.0
3.5
4.0

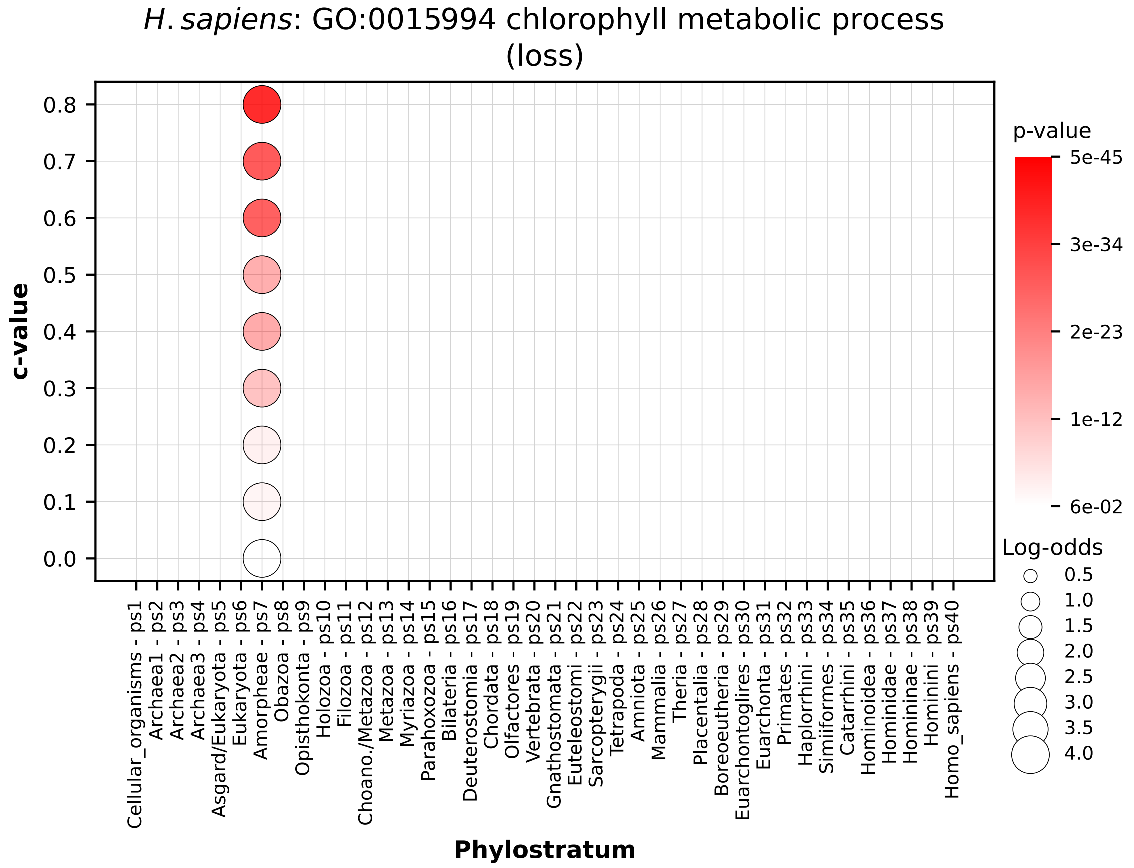

H. sapiens: GO:0015994 chlorophyll metabolic process
(loss)
c-value
0.8
0.7
0.6
0.5
0.4
0.3
0.2
0.1
0.0
p-value
5e-45
3e-34
2e-23
1e-12
6e-02
Log-odds
0.5
1.0
1.5
2.0
2.5
3.0
3.5
4.0
Cellular_organisms - ps1
Archaea1 - ps2
Archaea2 - ps3
Archaea3 - ps4
Asgard/Eukaryota - ps5
Eukaryota - ps6
Amorpheae - ps7
Obazoa - ps8
Opisthokonta - ps9
Holozoa - ps10
Filozoa - ps11
Choano./Metazoa - ps12
Metazoa - ps13
Myriazoa - ps14
Parahoxozoa - ps15
Bilateria - ps16
Deuterostomia - ps17
Chordata - ps18
Olfactores - ps19
Vertebrata - ps20
Gnathostomata - ps21
Euteleostomi - ps22
Sarcopterygii - ps23
Tetrapoda - ps24
Amniota - ps25
Mammalia - ps26
Theria - ps27
Placentalia - ps28
Boreoeutheria - ps29
Euarchontoglires - ps30
Euarchonta - ps31
Primates - ps32
Haplorrhini - ps33
Simiiformes - ps34
Catarrhini - ps35
Hominoidea - ps36
Hominidae - ps37
Homininae - ps38
Hominini - ps39
Homo_sapiens - ps40
Phylostratum

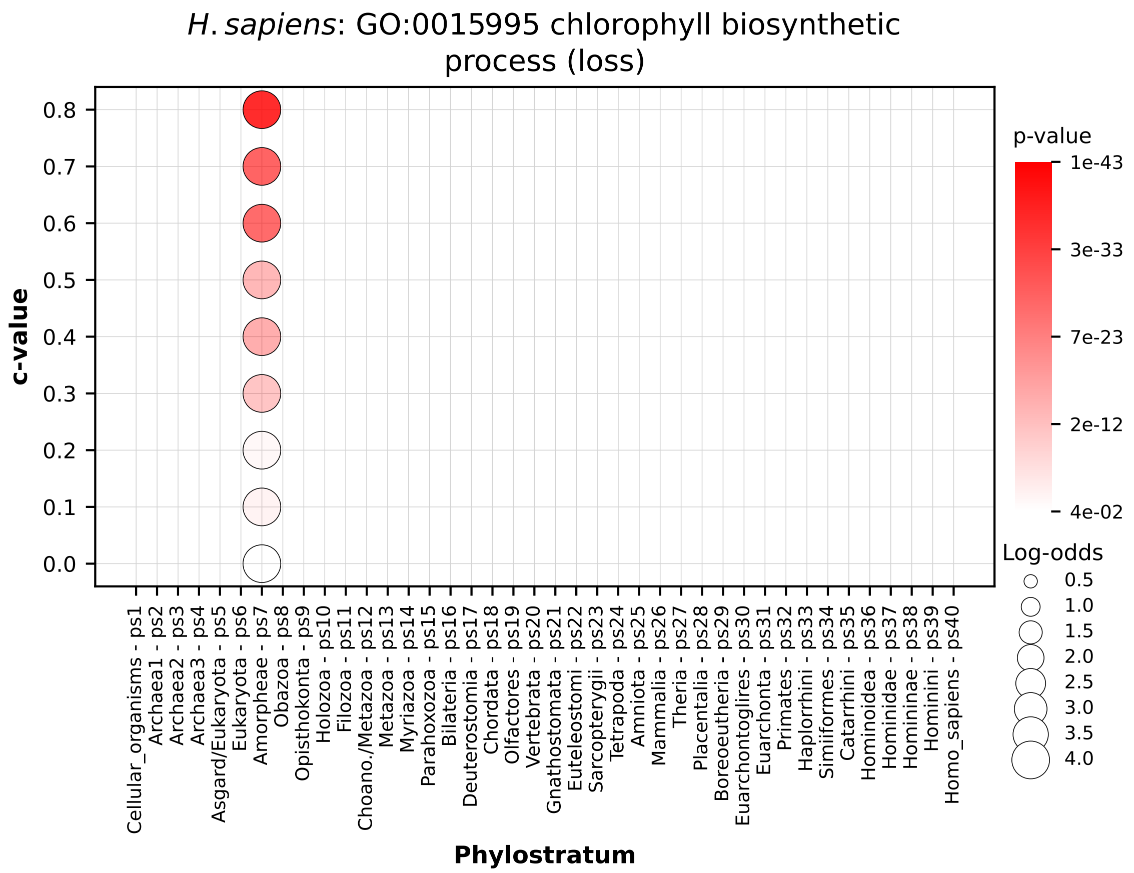
H. sapiens: GO:0015995 chlorophyll biosynthetic process (loss)
c-value
0.8
0.7
0.6
0.5
0.4
0.3
0.2
0.1
0.0
p-value
1e-43
3e-33
7e-23
2e-12
4e-02
Log-odds
0.5
1.0
1.5
2.0
2.5
3.0
3.5
4.0
Cellular_organisms - ps1
Archaea1 - ps2
Archaea2 - ps3
Archaea3 - ps4
Asgard/Eukaryota - ps5
Eukaryota - ps6
Amorpheae - ps7
Obazoa - ps8
Opisthokonta - ps9
Holozoa - ps10
Filozoa - ps11
Choano./Metazoa - ps12
Metazoa - ps13
Myriazoa - ps14
Parahoxozoa - ps15
Bilateria - ps16
Deuterostomia - ps17
Chordata - ps18
Olfactores - ps19
Vertebrata - ps20
Gnathostomata - ps21
Euteleostomi - ps22
Sarcopterygii - ps23
Tetrapoda - ps24
Amniota - ps25
Mammalia - ps26
Theria - ps27
Placentalia - ps28
Boreoeutheria - ps29
Euarchontoglires - ps30
Euarchonta - ps31
Primates - ps32
Haplorrhini - ps33
Simiiformes - ps34
Catarrhini - ps35
Hominoidea - ps36
Hominidae - ps37
Homininae - ps38
Hominini - ps39
Homo_sapiens - ps40
Phylostratum

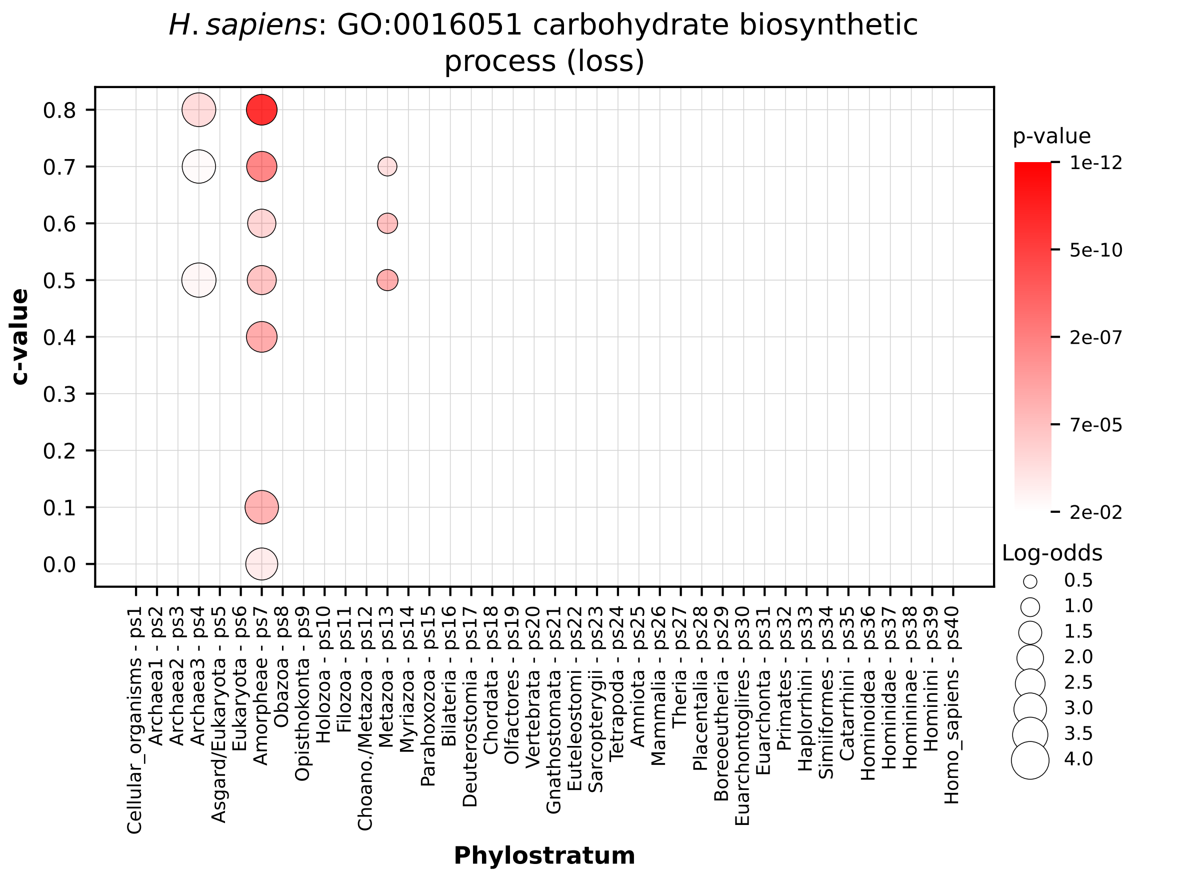
H. sapiens: GO:0016051 carbohydrate biosynthetic process (loss)
c-value
0.8
0.7
0.6
0.5
0.4
0.3
0.2
0.1
0.0
Phylostratum
Cellular_organisms - ps1
Archaea1 - ps2
Archaea2 - ps3
Archaea3 - ps4
Asgard/Eukaryota - ps5
Eukaryota - ps6
Amorpheae - ps7
Obazoa - ps8
Opisthokonta - ps9
Holozoa - ps10
Filozoa - ps11
Choano./Metazoa - ps12
Metazoa - ps13
Myriazoa - ps14
Parahoxozoa - ps15
Bilateria - ps16
Deuterostomia - ps17
Chordata - ps18
Olfactores - ps19
Vertebrata - ps20
Gnathostomata - ps21
Euteleostomi - ps22
Sarcopterygii - ps23
Tetrapoda - ps24
Amniota - ps25
Mammalia - ps26
Theria - ps27
Placentalia - ps28
Boreoeutheria - ps29
Euarchontoglires - ps30
Euarchonta - ps31
Primates - ps32
Haplorrhini - ps33
Simiiformes - ps34
Catarrhini - ps35
Hominoidea - ps36
Hominidae - ps37
Homininae - ps38
Hominini - ps39
Homo_sapiens - ps40
p-value
1e-12
5e-10
2e-07
7e-05
2e-02
Log-odds
0.5
1.0
1.5
2.0
2.5
3.0
3.5
4.0

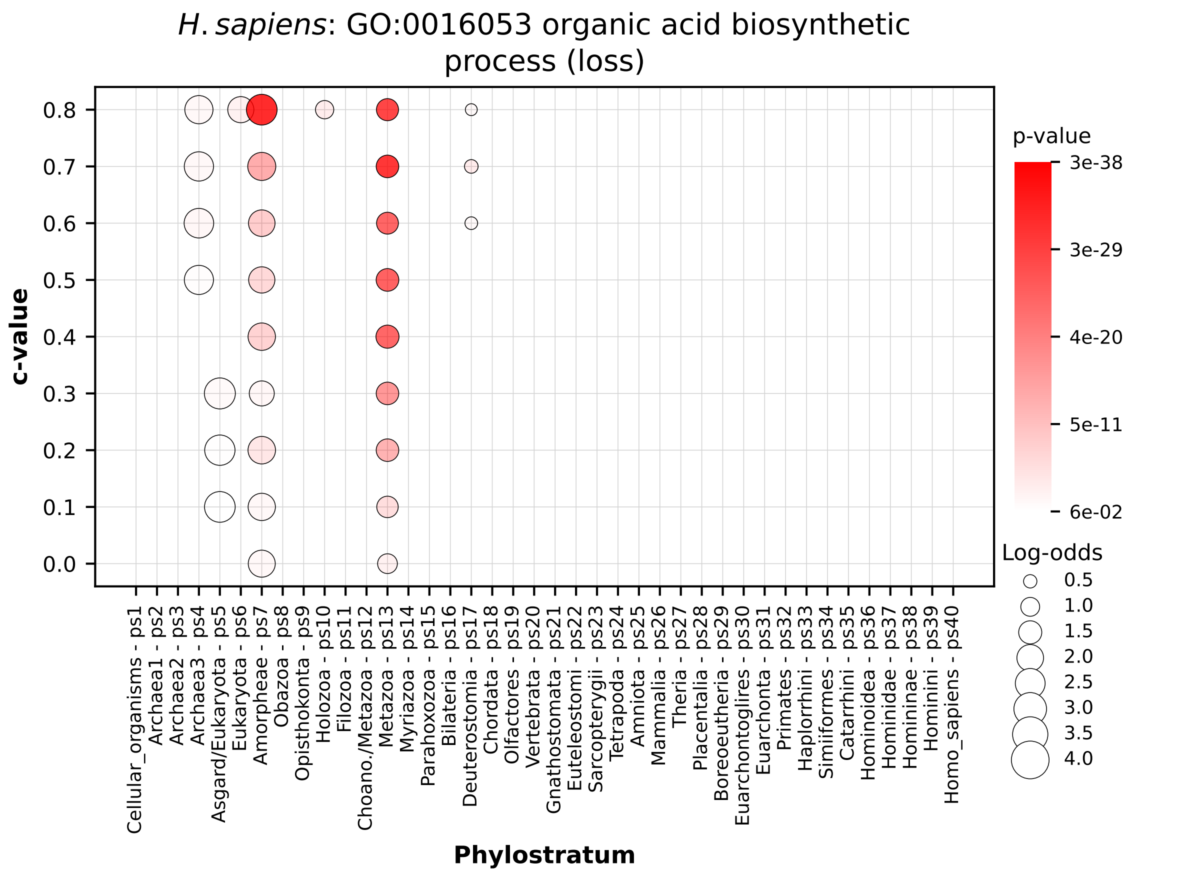

H. sapiens: GO:0016053 organic acid biosynthetic process (loss)
c-value
0.8
0.7
0.6
0.5
0.4
0.3
0.2
0.1
0.0
p-value
3e-38
3e-29
4e-20
5e-11
6e-02
Log-odds
0.5
1.0
1.5
2.0
2.5
3.0
3.5
4.0
Cellular_organisms - ps1
Archaea1 - ps2
Archaea2 - ps3
Archaea3 - ps4
Asgard/Eukaryota - ps5
Eukaryota - ps6
Amorpheae - ps7
Obazoa - ps8
Opisthokonta - ps9
Holozoa - ps10
Filozoa - ps11
Choano./Metazoa - ps12
Metazoa - ps13
Myriazoa - ps14
Parahoxozoa - ps15
Bilateria - ps16
Deuterostomia - ps17
Chordata - ps18
Olfactores - ps19
Vertebrata - ps20
Gnathostomata - ps21
Euteleostomi - ps22
Sarcopterygii - ps23
Tetrapoda - ps24
Amniota - ps25
Mammalia - ps26
Theria - ps27
Placentalia - ps28
Boreoeutheria - ps29
Euarchontoglires - ps30
Euarchonta - ps31
Primates - ps32
Haplorrhini - ps33
Simiiformes - ps34
Catarrhini - ps35
Hominoidea - ps36
Hominidae - ps37
Homininae - ps38
Hominini - ps39
Homo_sapiens - ps40
Phylostratum

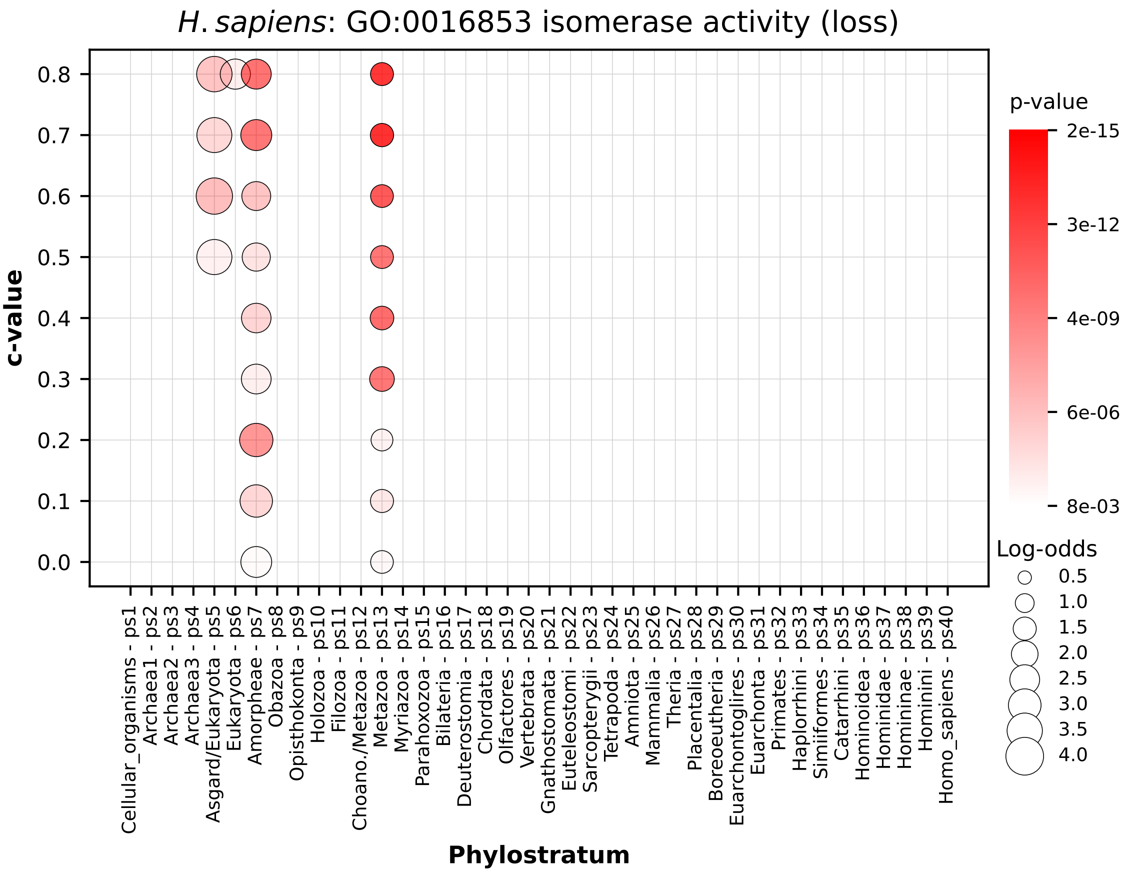

H. sapiens: GO:0016853 isomerase activity (loss)
c-value
0.0
0.1
0.2
0.3
0.4
0.5
0.6
0.7
0.8
Phylostratum
Cellular_organisms - ps1
Archaea1 - ps2
Archaea2 - ps3
Archaea3 - ps4
Asgard/Eukaryota - ps5
Eukaryota - ps6
Amorpheae - ps7
Obazoa - ps8
Opisthokonta - ps9
Holozoa - ps10
Filozoa - ps11
Choano./Metazoa - ps12
Metazoa - ps13
Myriazoa - ps14
Parahoxozoa - ps15
Bilateria - ps16
Deuterostomia - ps17
Chordata - ps18
Olfactores - ps19
Vertebrata - ps20
Gnathostomata - ps21
Euteleostomi - ps22
Sarcopterygii - ps23
Tetrapoda - ps24
Amniota - ps25
Mammalia - ps26
Theria - ps27
Placentalia - ps28
Boreoeutheria - ps29
Euarchontoglires - ps30
Euarchonta - ps31
Primates - ps32
Haplorrhini - ps33
Simiiformes - ps34
Catarrhini - ps35
Hominoidea - ps36
Hominidae - ps37
Homininae - ps38
Hominini - ps39
Homo_sapiens - ps40
p-value
2e-15
3e-12
4e-09
6e-06
8e-03
Log-odds
0.5
1.0
1.5
2.0
2.5
3.0
3.5
4.0

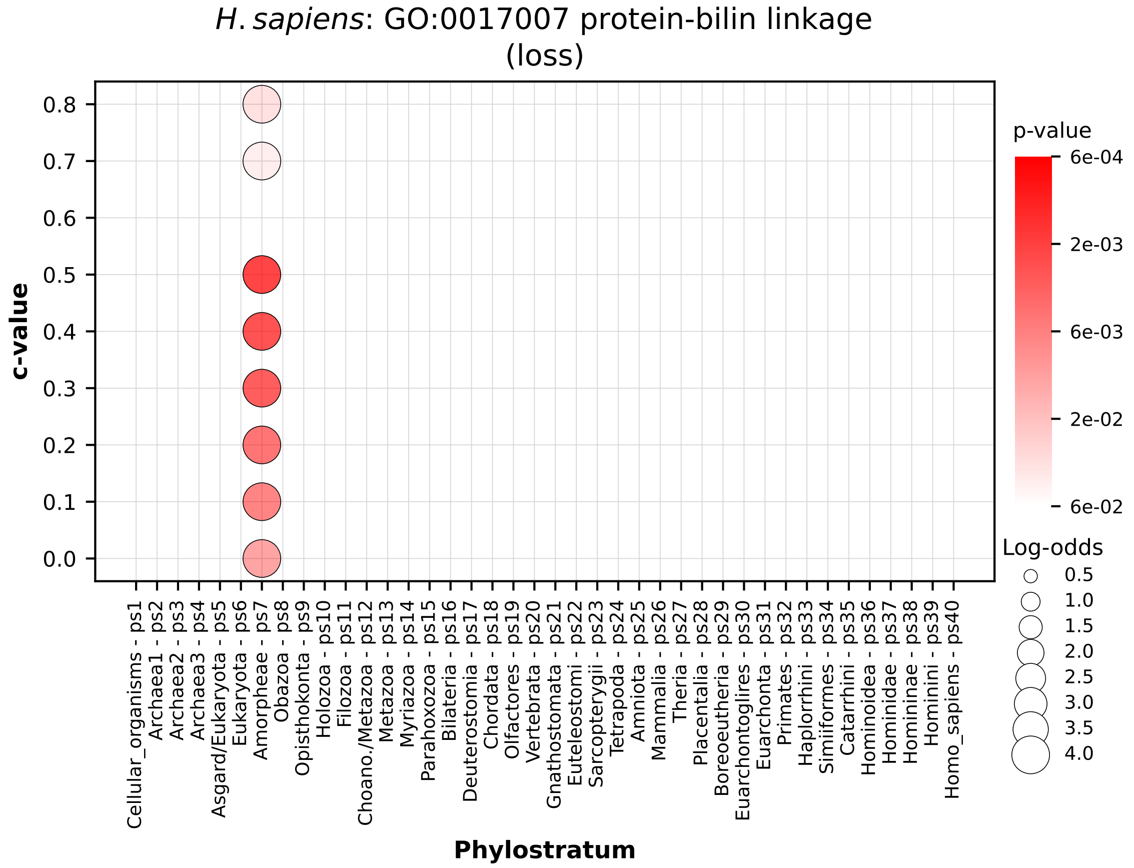

H. sapiens: GO:0017007 protein-bilin linkage
(loss)
c-value
0.8
0.7
0.6
0.5
0.4
0.3
0.2
0.1
0.0
p-value
6e-04
2e-03
6e-03
2e-02
6e-02
Log-odds
0.5
1.0
1.5
2.0
2.5
3.0
3.5
4.0
Cellular_organisms - ps1
Archaea1 - ps2
Archaea2 - ps3
Archaea3 - ps4
Asgard/Eukaryota - ps5
Eukaryota - ps6
Amorpheae - ps7
Obazoa - ps8
Opisthokonta - ps9
Holozoa - ps10
Filozoa - ps11
Choano./Metazoa - ps12
Metazoa - ps13
Myriazoa - ps14
Parahoxozoa - ps15
Bilateria - ps16
Deuterostomia - ps17
Chordata - ps18
Olfactores - ps19
Vertebrata - ps20
Gnathostomata - ps21
Euteleostomi - ps22
Sarcopterygii - ps23
Tetrapoda - ps24
Amniota - ps25
Mammalia - ps26
Theria - ps27
Placentalia - ps28
Boreoeutheria - ps29
Euarchontoglires - ps30
Euarchonta - ps31
Primates - ps32
Haplorrhini - ps33
Simiiformes - ps34
Catarrhini - ps35
Hominoidea - ps36
Hominidae - ps37
Homininae - ps38
Hominini - ps39
Homo_sapiens - ps40
Phylostratum

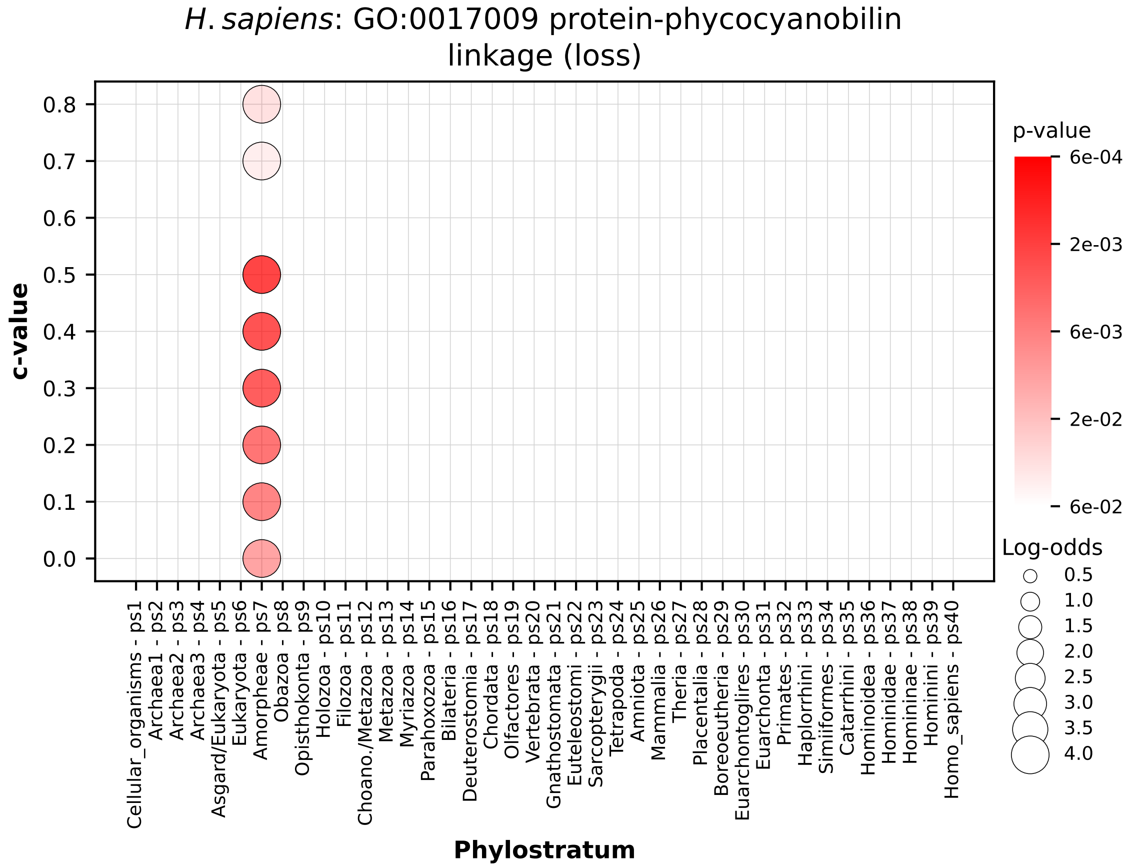

H. sapiens: GO:0017009 protein-phycocyanobilin linkage (loss)
c-value
0.8
0.7
0.6
0.5
0.4
0.3
0.2
0.1
0.0
p-value
6e-04
2e-03
6e-03
2e-02
6e-02
Log-odds
0.5
1.0
1.5
2.0
2.5
3.0
3.5
4.0
Cellular_organisms - ps1
Archaea1 - ps2
Archaea2 - ps3
Archaea3 - ps4
Asgard/Eukaryota - ps5
Eukaryota - ps6
Amorpheae - ps7
Obazoa - ps8
Opisthokonta - ps9
Holozoa - ps10
Filozoa - ps11
Choano./Metazoa - ps12
Metazoa - ps13
Myriazoa - ps14
Parahoxozoa - ps15
Bilateria - ps16
Deuterostomia - ps17
Chordata - ps18
Olfactores - ps19
Vertebrata - ps20
Gnathostomata - ps21
Euteleostomi - ps22
Sarcopterygii - ps23
Tetrapoda - ps24
Amniota - ps25
Mammalia - ps26
Theria - ps27
Placentalia - ps28
Boreoeutheria - ps29
Euarchontoglires - ps30
Euarchonta - ps31
Primates - ps32
Haplorrhini - ps33
Simiiformes - ps34
Catarrhini - ps35
Hominoidea - ps36
Hominidae - ps37
Homininae - ps38
Hominini - ps39
Homo_sapiens - ps40
Phylostratum

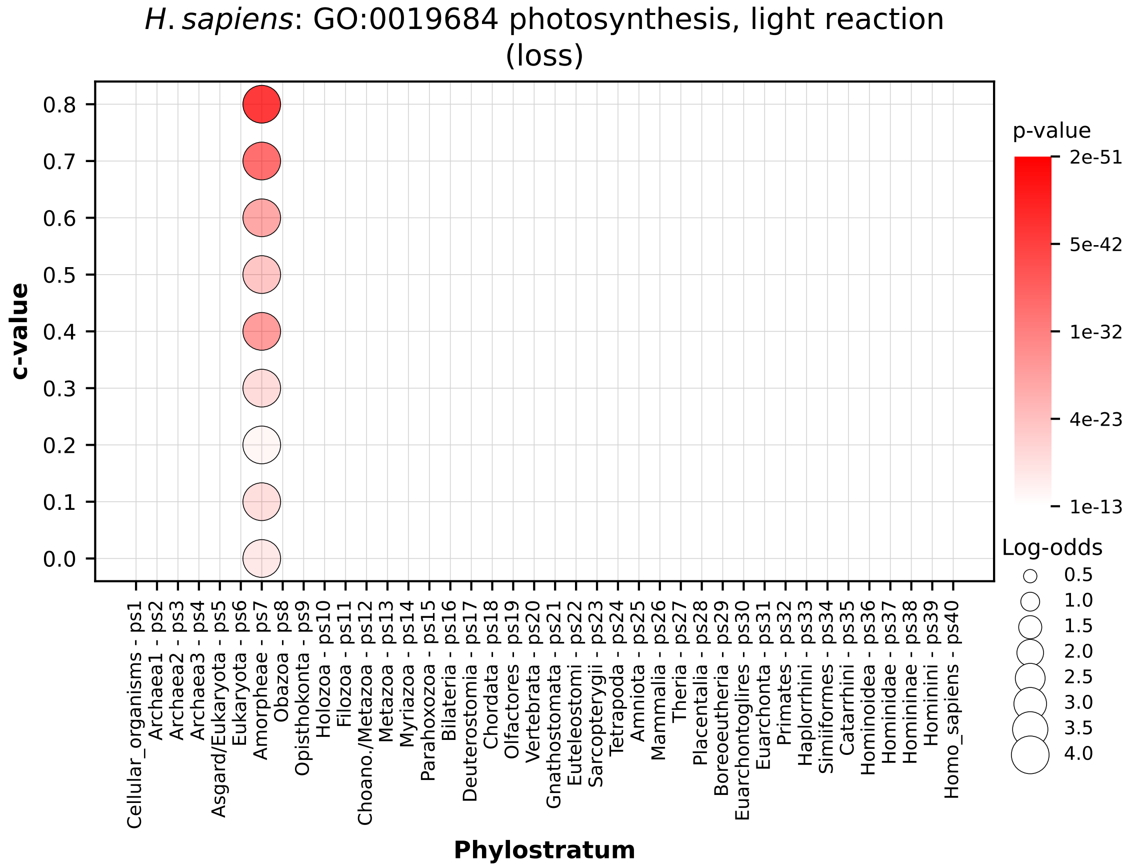

H. sapiens: GO:0019684 photosynthesis, light reaction
(loss)
c-value
0.0
0.1
0.2
0.3
0.4
0.5
0.6
0.7
0.8
Phylostratum
Cellular_organisms - ps1
Archaea1 - ps2
Archaea2 - ps3
Archaea3 - ps4
Asgard/Eukaryota - ps5
Eukaryota - ps6
Amorpheae - ps7
Obazoa - ps8
Opisthokonta - ps9
Holozoa - ps10
Filozoa - ps11
Choano./Metazoa - ps12
Metazoa - ps13
Myriazoa - ps14
Parahoxozoa - ps15
Bilateria - ps16
Deuterostomia - ps17
Chordata - ps18
Olfactores - ps19
Vertebrata - ps20
Gnathostomata - ps21
Euteleostomi - ps22
Sarcopterygii - ps23
Tetrapoda - ps24
Amniota - ps25
Mammalia - ps26
Theria - ps27
Placentalia - ps28
Boreoeutheria - ps29
Euarchontoglires - ps30
Euarchonta - ps31
Primates - ps32
Haplorrhini - ps33
Simiiformes - ps34
Catarrhini - ps35
Hominoidea - ps36
Hominidae - ps37
Homininae - ps38
Hominini - ps39
Homo_sapiens - ps40
p-value
2e-51
5e-42
1e-32
4e-23
1e-13
Log-odds
0.5
1.0
1.5
2.0
2.5
3.0
3.5
4.0

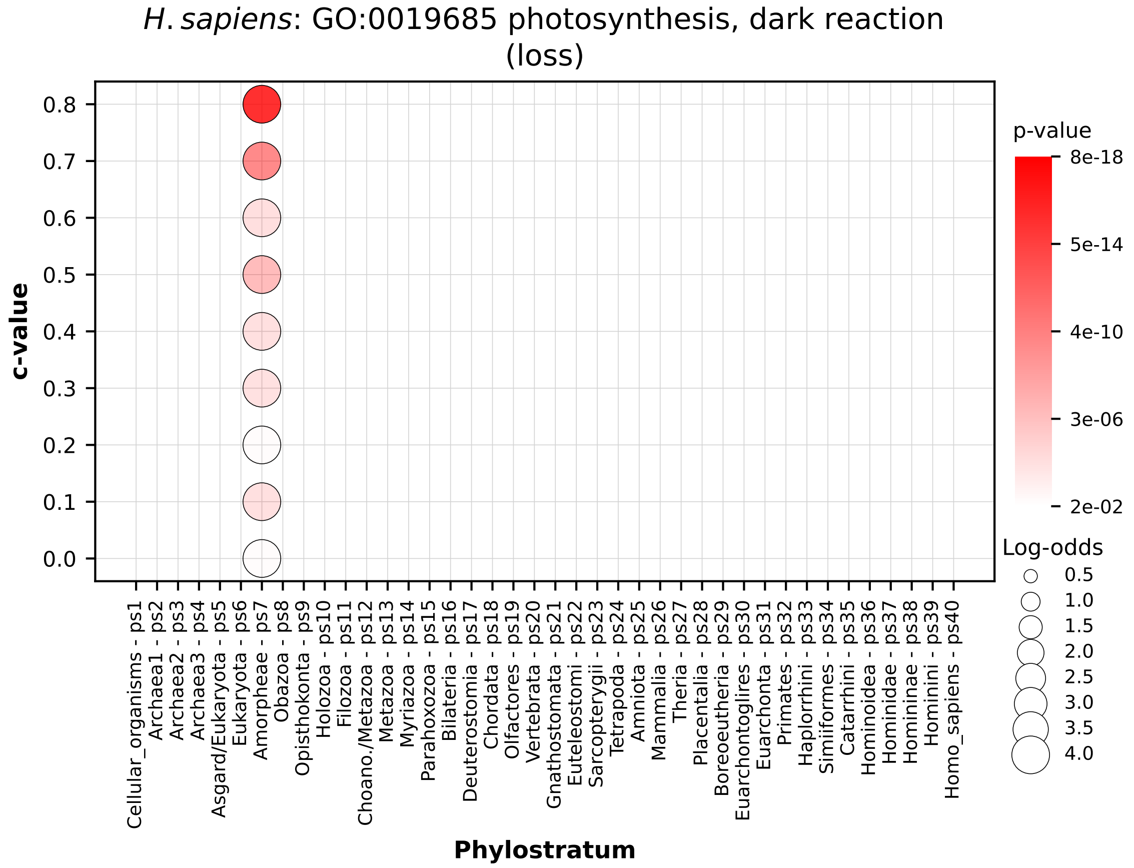

H. sapiens: GO:0019685 photosynthesis, dark reaction (loss)
c-value
0.8
0.7
0.6
0.5
0.4
0.3
0.2
0.1
0.0
Phylostratum
Cellular_organisms - ps1
Archaea1 - ps2
Archaea2 - ps3
Archaea3 - ps4
Asgard/Eukaryota - ps5
Eukaryota - ps6
Amorpheae - ps7
Obazoa - ps8
Opisthokonta - ps9
Holozoa - ps10
Filozoa - ps11
Choano./Metazoa - ps12
Metazoa - ps13
Myriazoa - ps14
Parahoxozoa - ps15
Bilateria - ps16
Deuterostomia - ps17
Chordata - ps18
Olfactores - ps19
Vertebrata - ps20
Gnathostomata - ps21
Euteleostomi - ps22
Sarcopterygii - ps23
Tetrapoda - ps24
Amniota - ps25
Mammalia - ps26
Theria - ps27
Placentalia - ps28
Boreoeutheria - ps29
Euarchontoglires - ps30
Euarchonta - ps31
Primates - ps32
Haplorrhini - ps33
Simiiformes - ps34
Catarrhini - ps35
Hominoidea - ps36
Hominidae - ps37
Homininae - ps38
Hominini - ps39
Homo_sapiens - ps40
p-value
8e-18
5e-14
4e-10
3e-06
2e-02
Log-odds
0.5
1.0
1.5
2.0
2.5
3.0
3.5
4.0

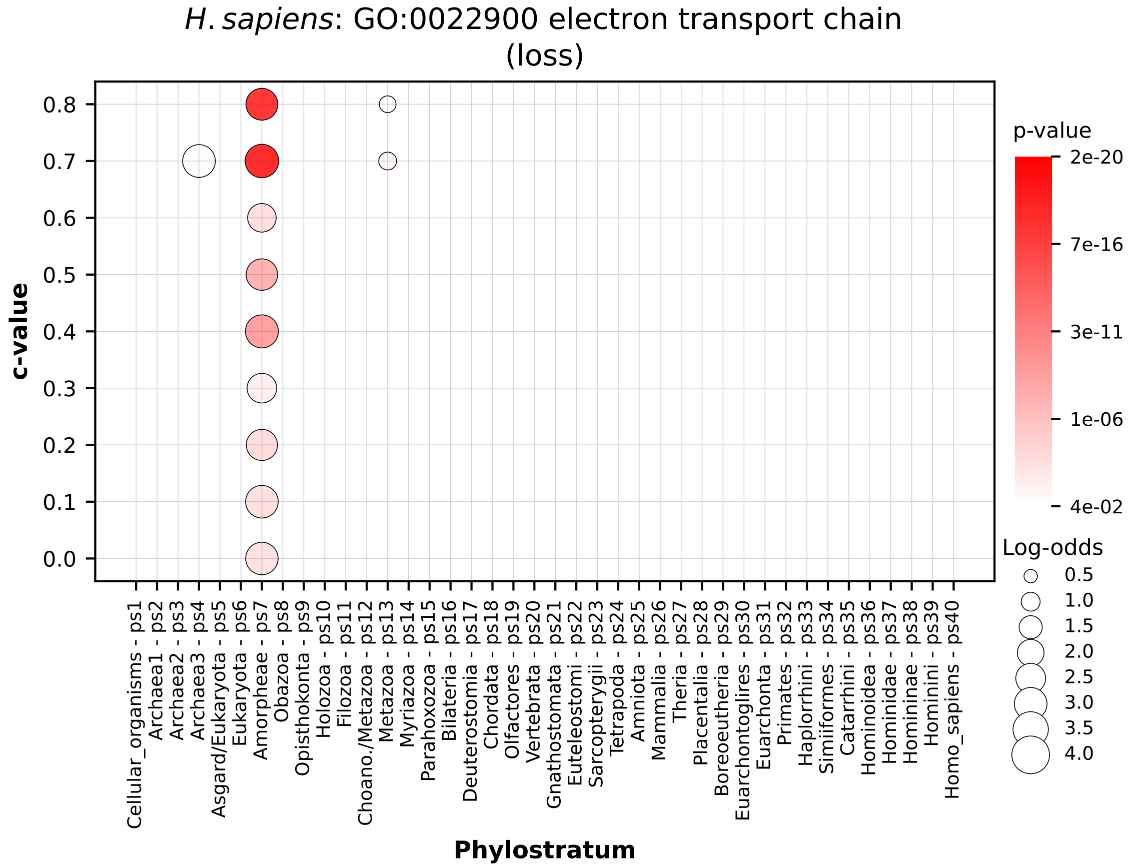
H. sapiens: GO:0022900 electron transport chain (loss)
c-value
0.8
0.7
0.6
0.5
0.4
0.3
0.2
0.1
0.0
Phylostratum
Cellular_organisms - ps1
Archaea1 - ps2
Archaea2 - ps3
Archaea3 - ps4
Asgard/Eukaryota - ps5
Eukaryota - ps6
Amorpheae - ps7
Obazoa - ps8
Opisthokonta - ps9
Holozoa - ps10
Filozoa - ps11
Choano./Metazoa - ps12
Metazoa - ps13
Myriazoa - ps14
Parahoxozoa - ps15
Bilateria - ps16
Deuterostomia - ps17
Chordata - ps18
Olfactores - ps19
Vertebrata - ps20
Gnathostomata - ps21
Euteleostomi - ps22
Sarcopterygii - ps23
Tetrapoda - ps24
Amniota - ps25
Mammalia - ps26
Theria - ps27
Placentalia - ps28
Boreoeutheria - ps29
Euarchontoglires - ps30
Euarchonta - ps31
Primates - ps32
Haplorrhini - ps33
Simiiformes - ps34
Catarrhini - ps35
Hominoidea - ps36
Hominidae - ps37
Homininae - ps38
Hominini - ps39
Homo_sapiens - ps40
p-value
2e-20
7e-16
3e-11
1e-06
4e-02
Log-odds
0.5
1.0
1.5
2.0
2.5
3.0
3.5
4.0

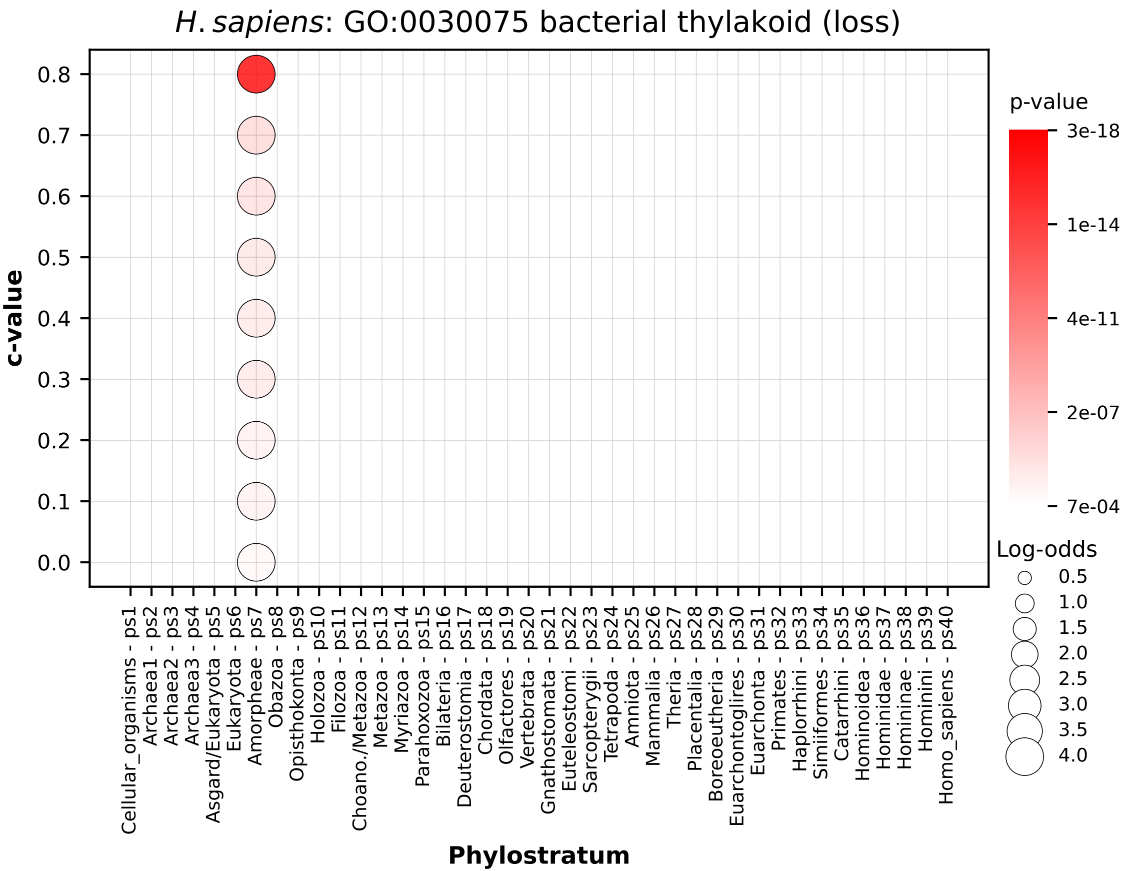

H. sapiens: GO:0030075 bacterial thylakoid (loss)
c-value
0.8
0.7
0.6
0.5
0.4
0.3
0.2
0.1
0.0
Phylostratum
Cellular_organisms - ps1
Archaea1 - ps2
Archaea2 - ps3
Archaea3 - ps4
Asgard/Eukaryota - ps5
Eukaryota - ps6
Amorpheae - ps7
Obazoa - ps8
Opisthokonta - ps9
Holozoa - ps10
Filozoa - ps11
Choano./Metazoa - ps12
Metazoa - ps13
Myriazoa - ps14
Parahoxozoa - ps15
Bilateria - ps16
Deuterostomia - ps17
Chordata - ps18
Olfactores - ps19
Vertebrata - ps20
Gnathostomata - ps21
Euteleostomi - ps22
Sarcopterygii - ps23
Tetrapoda - ps24
Amniota - ps25
Mammalia - ps26
Theria - ps27
Placentalia - ps28
Boreoeutheria - ps29
Euarchontoglires - ps30
Euarchonta - ps31
Primates - ps32
Haplorrhini - ps33
Simiiformes - ps34
Catarrhini - ps35
Hominoidea - ps36
Hominidae - ps37
Homininae - ps38
Hominini - ps39
Homo_sapiens - ps40
p-value
3e-18
1e-14
4e-11
2e-07
7e-04
Log-odds
0.5
1.0
1.5
2.0
2.5
3.0
3.5
4.0

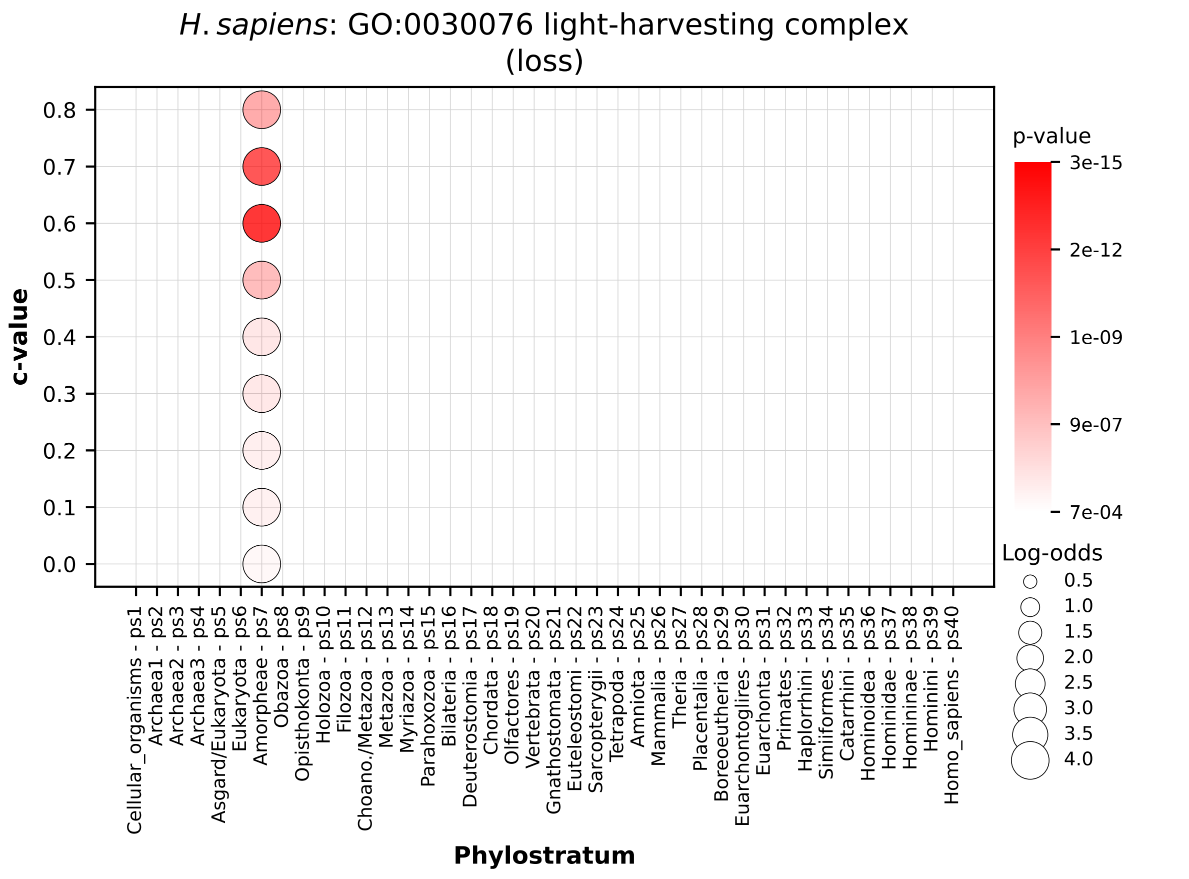

H. sapiens: GO:0030076 light-harvesting complex
(loss)
c-value
0.8
0.7
0.6
0.5
0.4
0.3
0.2
0.1
0.0
p-value
3e-15
2e-12
1e-09
9e-07
7e-04
Log-odds
0.5
1.0
1.5
2.0
2.5
3.0
3.5
4.0
Cellular_organisms - ps1
Archaea1 - ps2
Archaea2 - ps3
Archaea3 - ps4
Asgard/Eukaryota - ps5
Eukaryota - ps6
Amorpheae - ps7
Obazoa - ps8
Opisthokonta - ps9
Holozoa - ps10
Filozoa - ps11
Choano./Metazoa - ps12
Metazoa - ps13
Myriazoa - ps14
Parahoxozoa - ps15
Bilateria - ps16
Deuterostomia - ps17
Chordata - ps18
Olfactores - ps19
Vertebrata - ps20
Gnathostomata - ps21
Euteleostomi - ps22
Sarcopterygii - ps23
Tetrapoda - ps24
Amniota - ps25
Mammalia - ps26
Theria - ps27
Placentalia - ps28
Boreoeutheria - ps29
Euarchontoglires - ps30
Euarchonta - ps31
Primates - ps32
Haplorrhini - ps33
Simiiformes - ps34
Catarrhini - ps35
Hominoidea - ps36
Hominidae - ps37
Homininae - ps38
Hominini - ps39
Homo_sapiens - ps40
Phylostratum

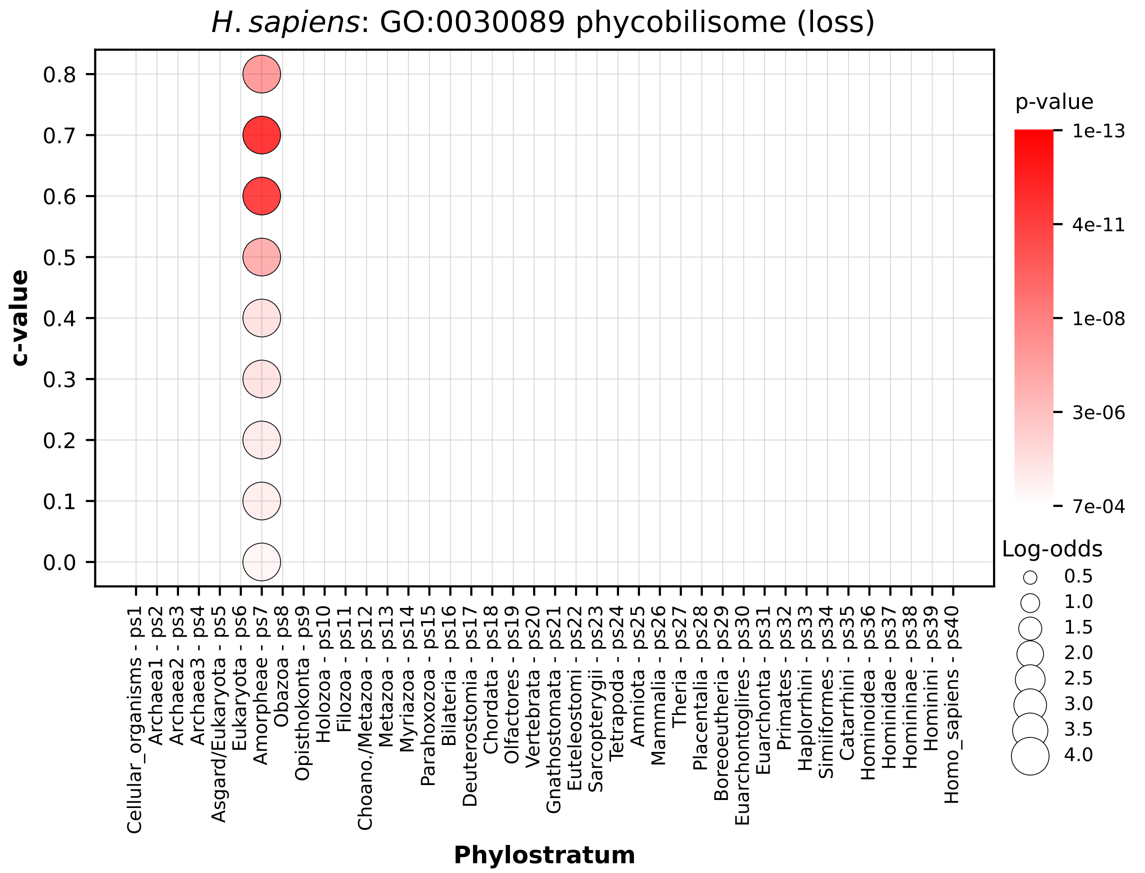

H. sapiens: GO:0030089 phycobilisome (loss)
c-value
0.0
0.1
0.2
0.3
0.4
0.5
0.6
0.7
0.8
Phylostratum
Cellular_organisms - ps1
Archaea1 - ps2
Archaea2 - ps3
Archaea3 - ps4
Asgard/Eukaryota - ps5
Eukaryota - ps6
Amorpheae - ps7
Obazoa - ps8
Opisthokonta - ps9
Holozoa - ps10
Filozoa - ps11
Choano./Metazoa - ps12
Metazoa - ps13
Myriazoa - ps14
Parahoxozoa - ps15
Bilateria - ps16
Deuterostomia - ps17
Chordata - ps18
Olfactores - ps19
Vertebrata - ps20
Gnathostomata - ps21
Euteleostomi - ps22
Sarcopterygii - ps23
Tetrapoda - ps24
Amniota - ps25
Mammalia - ps26
Theria - ps27
Placentalia - ps28
Boreoeutheria - ps29
Euarchontoglires - ps30
Euarchonta - ps31
Primates - ps32
Haplorrhini - ps33
Simiiformes - ps34
Catarrhini - ps35
Hominoidea - ps36
Hominidae - ps37
Homininae - ps38
Hominini - ps39
Homo_sapiens - ps40
p-value
1e-13
4e-11
1e-08
3e-06
7e-04
Log-odds
0.5
1.0
1.5
2.0
2.5
3.0
3.5
4.0

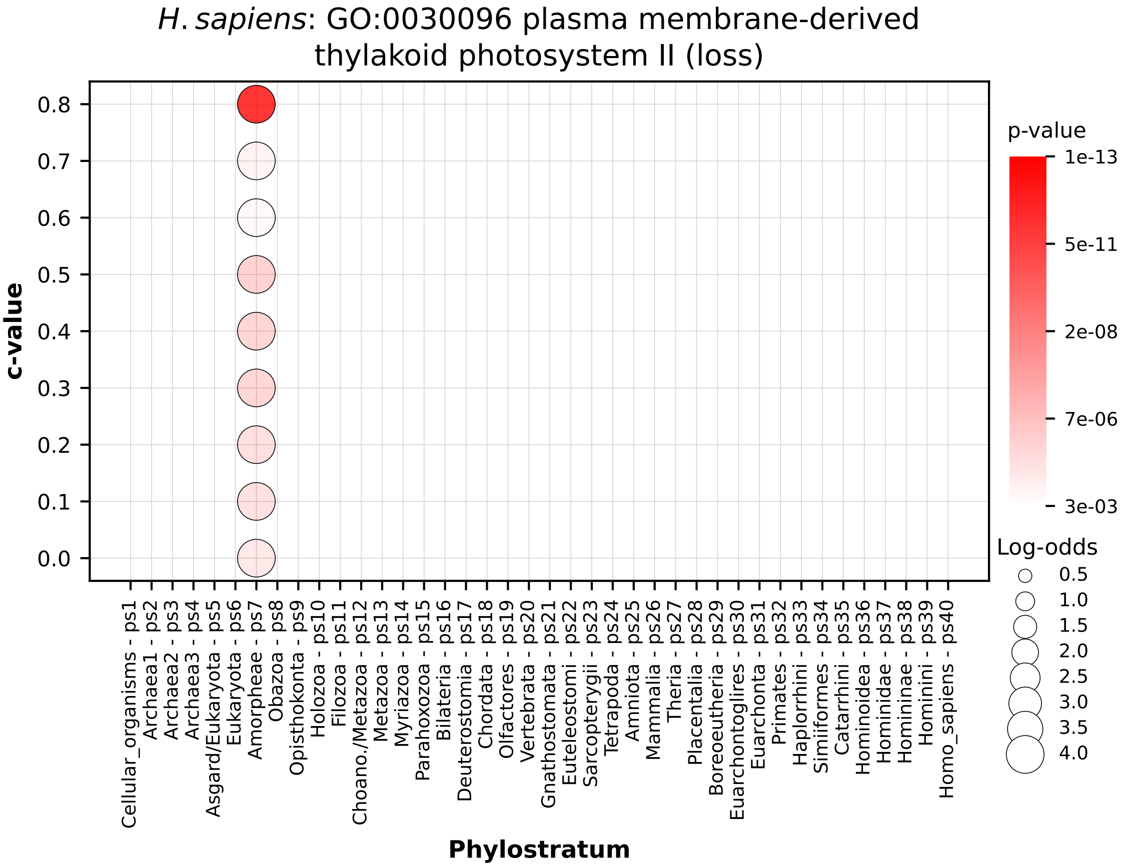

H. sapiens: GO:0030096 plasma membrane-derived thylakoid photosystem II (loss)
c-value
0.8
0.7
0.6
0.5
0.4
0.3
0.2
0.1
0.0
p-value
1e-13
5e-11
2e-08
7e-06
3e-03
Log-odds
0.5
1.0
1.5
2.0
2.5
3.0
3.5
4.0
Cellular_organisms - ps1
Archaea1 - ps2
Archaea2 - ps3
Archaea3 - ps4
Asgard/Eukaryota - ps5
Eukaryota - ps6
Amorpheae - ps7
Obazoa - ps8
Opisthokonta - ps9
Holozoa - ps10
Filozoa - ps11
Choano./Metazoa - ps12
Metazoa - ps13
Myriazoa - ps14
Parahoxozoa - ps15
Bilateria - ps16
Deuterostomia - ps17
Chordata - ps18
Olfactores - ps19
Vertebrata - ps20
Gnathostomata - ps21
Euteleostomi - ps22
Sarcopterygii - ps23
Tetrapoda - ps24
Amniota - ps25
Mammalia - ps26
Theria - ps27
Placentalia - ps28
Boreoeutheria - ps29
Euarchontoglires - ps30
Euarchonta - ps31
Primates - ps32
Haplorrhini - ps33
Simiiformes - ps34
Catarrhini - ps35
Hominoidea - ps36
Hominidae - ps37
Homininae - ps38
Hominini - ps39
Homo_sapiens - ps40
Phylostratum

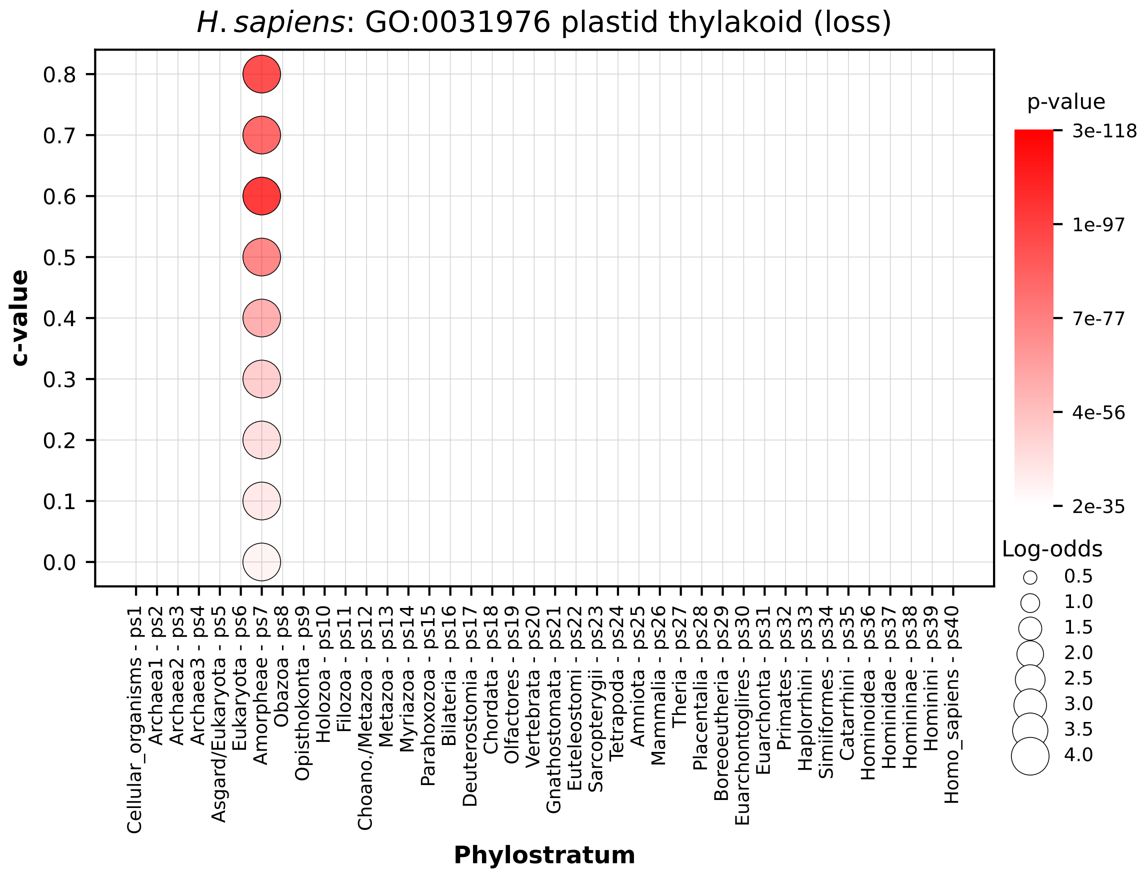
H. sapiens: GO:0031976 plastid thylakoid (loss)
c-value
0.0
0.1
0.2
0.3
0.4
0.5
0.6
0.7
0.8
Phylostratum
Cellular_organisms - ps1
Archaea1 - ps2
Archaea2 - ps3
Archaea3 - ps4
Asgard/Eukaryota - ps5
Eukaryota - ps6
Amorpheae - ps7
Obazoa - ps8
Opisthokonta - ps9
Holozoa - ps10
Filozoa - ps11
Choano./Metazoa - ps12
Metazoa - ps13
Myriazoa - ps14
Parahoxozoa - ps15
Bilateria - ps16
Deuterostomia - ps17
Chordata - ps18
Olfactores - ps19
Vertebrata - ps20
Gnathostomata - ps21
Euteleostomi - ps22
Sarcopterygii - ps23
Tetrapoda - ps24
Amniota - ps25
Mammalia - ps26
Theria - ps27
Placentalia - ps28
Boreoeutheria - ps29
Euarchontoglires - ps30
Euarchonta - ps31
Primates - ps32
Haplorrhini - ps33
Simiiformes - ps34
Catarrhini - ps35
Hominoidea - ps36
Hominidae - ps37
Homininae - ps38
Hominini - ps39
Homo_sapiens - ps40
p-value
3e-118
1e-97
7e-77
4e-56
2e-35
Log-odds
0.5
1.0
1.5
2.0
2.5
3.0
3.5
4.0

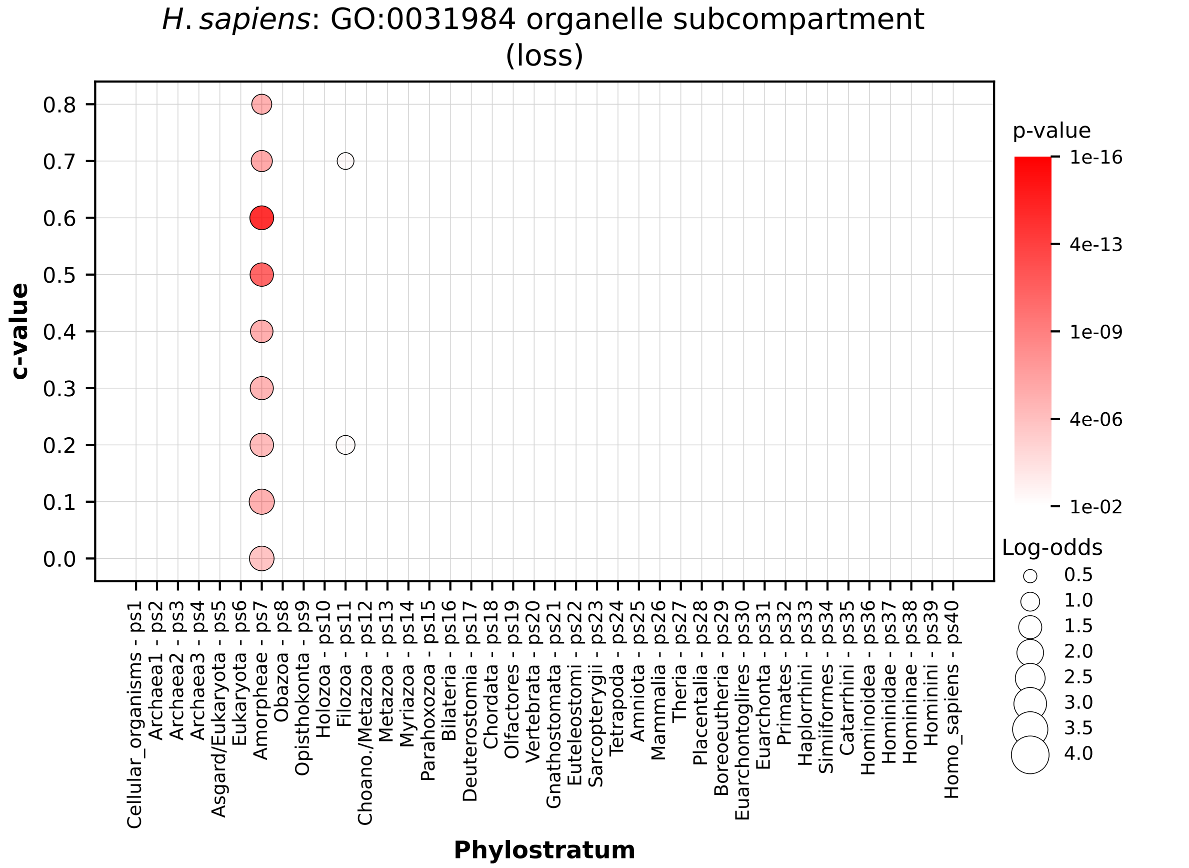

H. sapiens: GO:0031984 organelle subcompartment
(loss)
c-value
0.8
0.7
0.6
0.5
0.4
0.3
0.2
0.1
0.0
Phylostratum
Cellular_organisms - ps1
Archaea1 - ps2
Archaea2 - ps3
Archaea3 - ps4
Asgard/Eukaryota - ps5
Eukaryota - ps6
Amorpheae - ps7
Obazoa - ps8
Opisthokonta - ps9
Holozoa - ps10
Filozoa - ps11
Choano./Metazoa - ps12
Metazoa - ps13
Myriazoa - ps14
Parahoxozoa - ps15
Bilateria - ps16
Deuterostomia - ps17
Chordata - ps18
Olfactores - ps19
Vertebrata - ps20
Gnathostomata - ps21
Euteleostomi - ps22
Sarcopterygii - ps23
Tetrapoda - ps24
Amniota - ps25
Mammalia - ps26
Theria - ps27
Placentalia - ps28
Boreoeutheria - ps29
Euarchontoglires - ps30
Euarchonta - ps31
Primates - ps32
Haplorrhini - ps33
Simiiformes - ps34
Catarrhini - ps35
Hominoidea - ps36
Hominidae - ps37
Homininae - ps38
Hominini - ps39
Homo_sapiens - ps40
p-value
1e-16
4e-13
1e-09
4e-06
1e-02
Log-odds
0.5
1.0
1.5
2.0
2.5
3.0
3.5
4.0

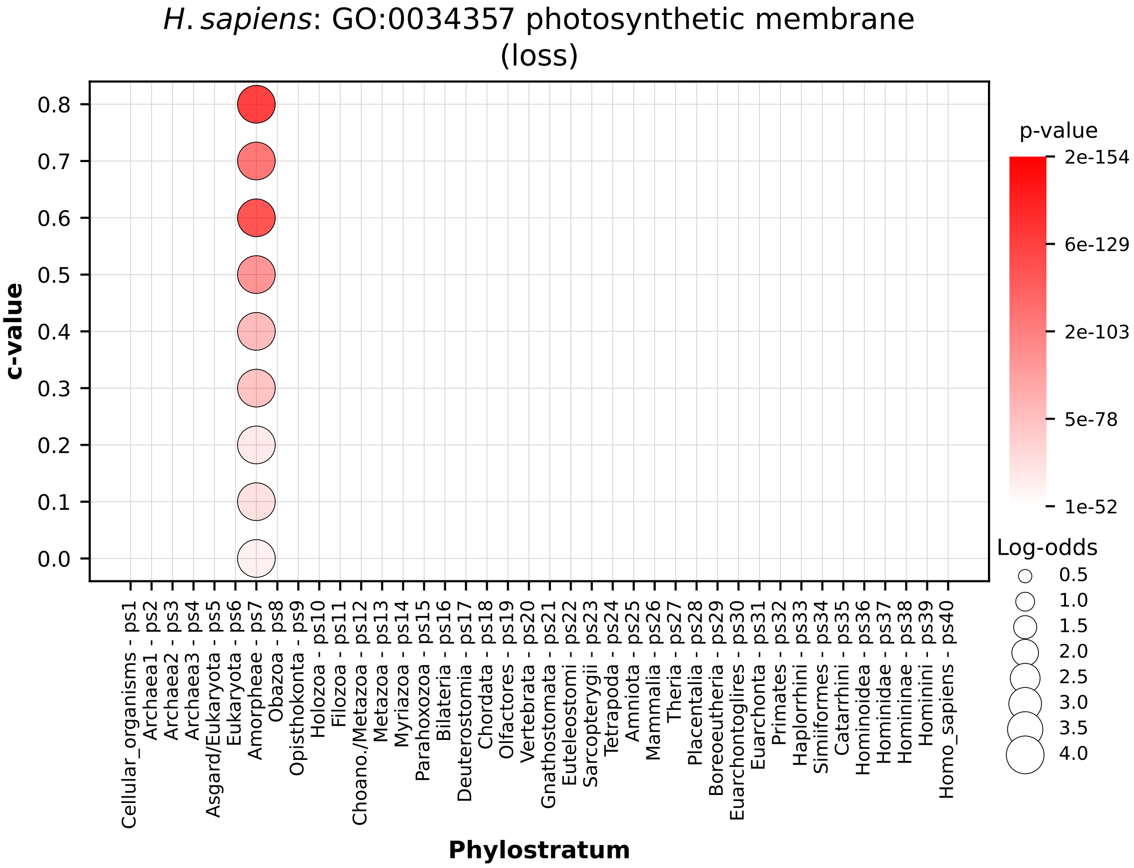

H. sapiens: GO:0034357 photosynthetic membrane
(loss)
c-value
0.8
0.7
0.6
0.5
0.4
0.3
0.2
0.1
0.0
p-value
2e-154
6e-129
2e-103
5e-78
1e-52
Log-odds
0.5
1.0
1.5
2.0
2.5
3.0
3.5
4.0
Cellular_organisms - ps1
Archaea1 - ps2
Archaea2 - ps3
Archaea3 - ps4
Asgard/Eukaryota - ps5
Eukaryota - ps6
Amorpheae - ps7
Obazoa - ps8
Opisthokonta - ps9
Holozoa - ps10
Filozoa - ps11
Choano./Metazoa - ps12
Metazoa - ps13
Myriazoa - ps14
Parahoxozoa - ps15
Bilateria - ps16
Deuterostomia - ps17
Chordata - ps18
Olfactores - ps19
Vertebrata - ps20
Gnathostomata - ps21
Euteleostomi - ps22
Sarcopterygii - ps23
Tetrapoda - ps24
Amniota - ps25
Mammalia - ps26
Theria - ps27
Placentalia - ps28
Boreoeutheria - ps29
Euarchontoglires - ps30
Euarchonta - ps31
Primates - ps32
Haplorrhini - ps33
Simiiformes - ps34
Catarrhini - ps35
Hominoidea - ps36
Hominidae - ps37
Homininae - ps38
Hominini - ps39
Homo_sapiens - ps40
Phylostratum

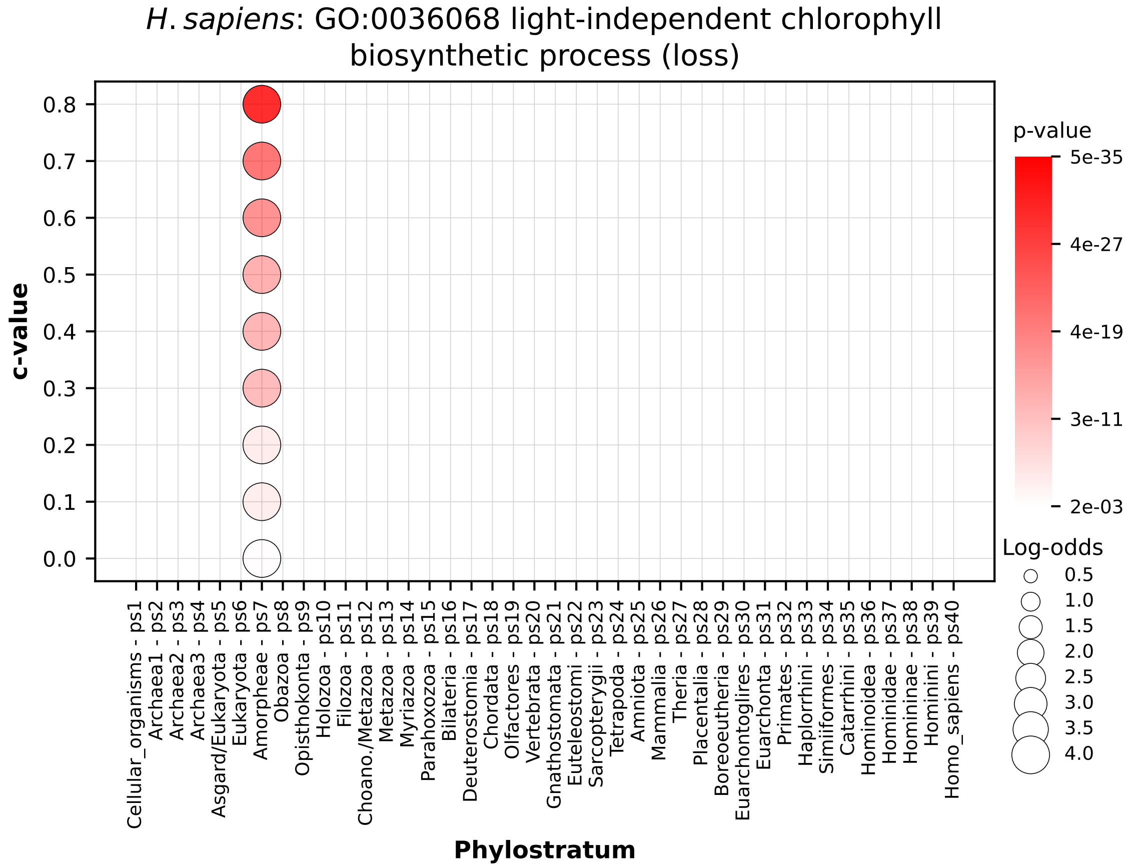
H. sapiens: GO:0036068 light-independent chlorophyll biosynthetic process (loss)
c-value
0.0
0.1
0.2
0.3
0.4
0.5
0.6
0.7
0.8
p-value
5e-35
4e-27
4e-19
3e-11
2e-03
Log-odds
0.5
1.0
1.5
2.0
2.5
3.0
3.5
4.0
Cellular_organisms - ps1
Archaea1 - ps2
Archaea2 - ps3
Archaea3 - ps4
Asgard/Eukaryota - ps5
Eukaryota - ps6
Amorpheae - ps7
Obazoa - ps8
Opisthokonta - ps9
Holozoa - ps10
Filozoa - ps11
Choano./Metazoa - ps12
Metazoa - ps13
Myriazoa - ps14
Parahoxozoa - ps15
Bilateria - ps16
Deuterostomia - ps17
Chordata - ps18
Olfactores - ps19
Vertebrata - ps20
Gnathostomata - ps21
Euteleostomi - ps22
Sarcopterygii - ps23
Tetrapoda - ps24
Amniota - ps25
Mammalia - ps26
Theria - ps27
Placentalia - ps28
Boreoeutheria - ps29
Euarchontoglires - ps30
Euarchonta - ps31
Primates - ps32
Haplorrhini - ps33
Simiiformes - ps34
Catarrhini - ps35
Hominoidea - ps36
Hominidae - ps37
Homininae - ps38
Hominini - ps39
Homo_sapiens - ps40
Phylostratum

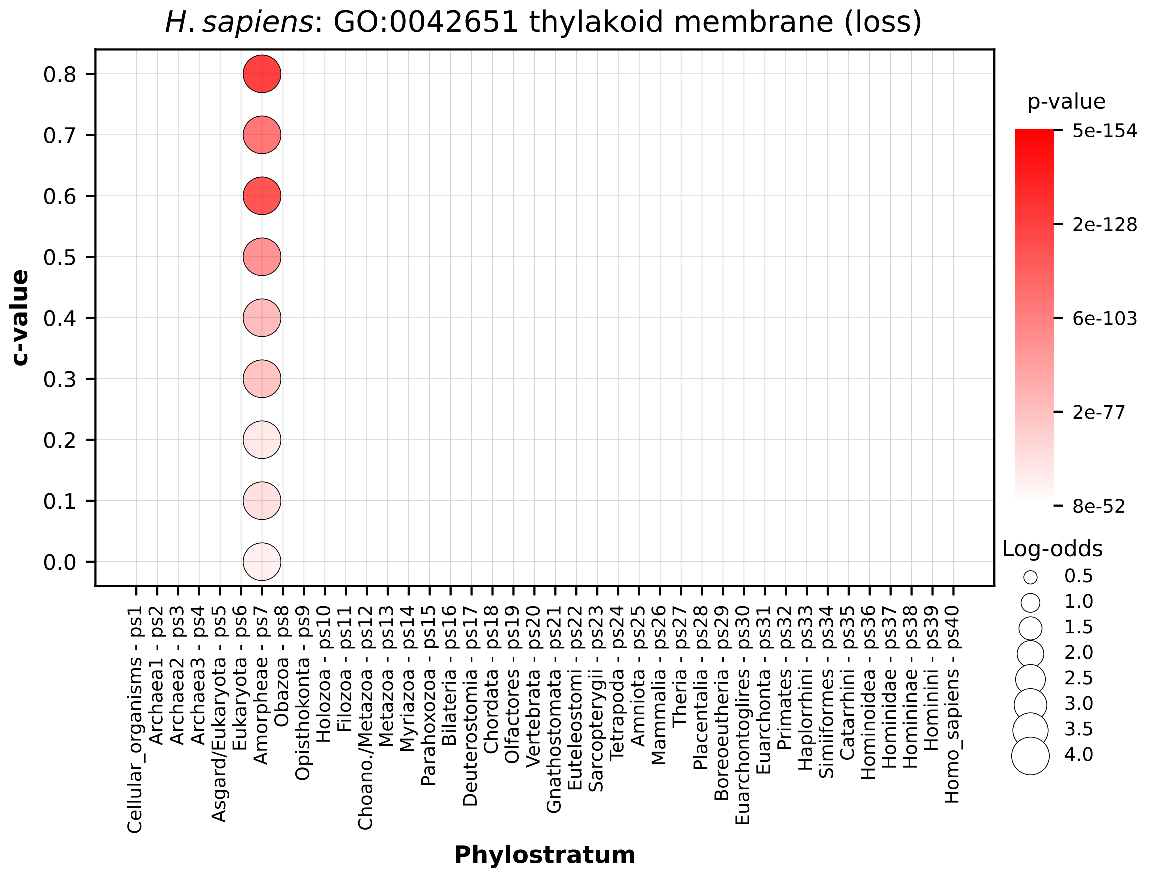

H. sapiens: GO:0042651 thylakoid membrane (loss)
c-value
0.8
0.7
0.6
0.5
0.4
0.3
0.2
0.1
0.0
p-value
5e-154
2e-128
6e-103
2e-77
8e-52
Log-odds
0.5
1.0
1.5
2.0
2.5
3.0
3.5
4.0
Cellular_organisms - ps1
Archaea1 - ps2
Archaea2 - ps3
Archaea3 - ps4
Asgard/Eukaryota - ps5
Eukaryota - ps6
Amorpheae - ps7
Obazoa - ps8
Opisthokonta - ps9
Holozoa - ps10
Filozoa - ps11
Choano./Metazoa - ps12
Metazoa - ps13
Myriazoa - ps14
Parahoxozoa - ps15
Bilateria - ps16
Deuterostomia - ps17
Chordata - ps18
Olfactores - ps19
Vertebrata - ps20
Gnathostomata - ps21
Euteleostomi - ps22
Sarcopterygii - ps23
Tetrapoda - ps24
Amniota - ps25
Mammalia - ps26
Theria - ps27
Placentalia - ps28
Boreoeutheria - ps29
Euarchontoglires - ps30
Euarchonta - ps31
Primates - ps32
Haplorrhini - ps33
Simiiformes - ps34
Catarrhini - ps35
Hominoidea - ps36
Hominidae - ps37
Homininae - ps38
Hominini - ps39
Homo_sapiens - ps40
Phylostratum

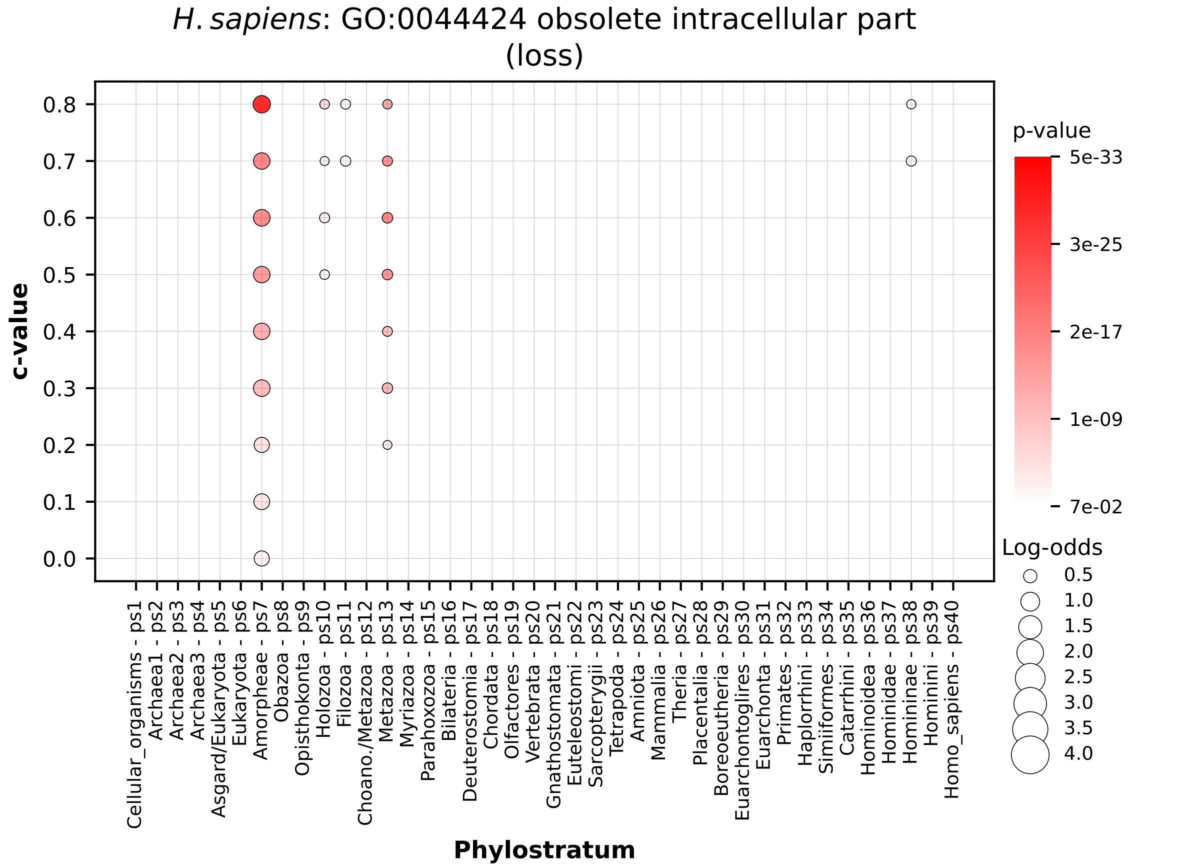

H. sapiens: GO:0044424 obsolete intracellular part
(loss)
c-value
0.8
0.7
0.6
0.5
0.4
0.3
0.2
0.1
0.0
p-value
5e-33
3e-25
2e-17
1e-09
7e-02
Log-odds
0.5
1.0
1.5
2.0
2.5
3.0
3.5
4.0
Cellular_organisms - ps1
Archaea1 - ps2
Archaea2 - ps3
Archaea3 - ps4
Asgard/Eukaryota - ps5
Eukaryota - ps6
Amorpheae - ps7
Obazoa - ps8
Opisthokonta - ps9
Holozoa - ps10
Filozoa - ps11
Choano./Metazoa - ps12
Metazoa - ps13
Myriazoa - ps14
Parahoxozoa - ps15
Bilateria - ps16
Deuterostomia - ps17
Chordata - ps18
Olfactores - ps19
Vertebrata - ps20
Gnathostomata - ps21
Euteleostomi - ps22
Sarcopterygii - ps23
Tetrapoda - ps24
Amniota - ps25
Mammalia - ps26
Theria - ps27
Placentalia - ps28
Boreoeutheria - ps29
Euarchontoglires - ps30
Euarchonta - ps31
Primates - ps32
Haplorrhini - ps33
Simiiformes - ps34
Catarrhini - ps35
Hominoidea - ps36
Hominidae - ps37
Homininae - ps38
Hominini - ps39
Homo_sapiens - ps40
Phylostratum

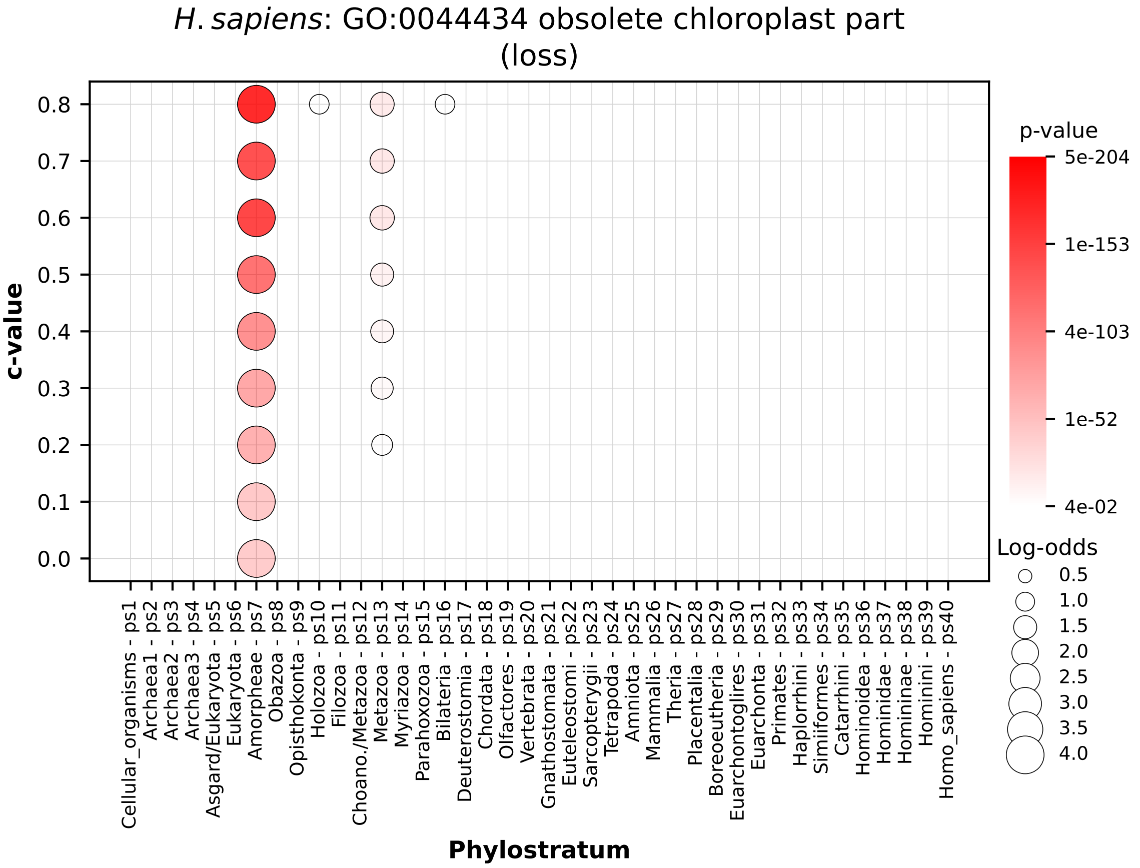

H. sapiens: GO:0044434 obsolete chloroplast part
(loss)
c-value
0.8
0.7
0.6
0.5
0.4
0.3
0.2
0.1
0.0
Phylostratum
Cellular_organisms - ps1
Archaea1 - ps2
Archaea2 - ps3
Archaea3 - ps4
Asgard/Eukaryota - ps5
Eukaryota - ps6
Amorpheae - ps7
Obazoa - ps8
Opisthokonta - ps9
Holozoa - ps10
Filozoa - ps11
Choano./Metazoa - ps12
Metazoa - ps13
Myriazoa - ps14
Parahoxozoa - ps15
Bilateria - ps16
Deuterostomia - ps17
Chordata - ps18
Olfactores - ps19
Vertebrata - ps20
Gnathostomata - ps21
Euteleostomi - ps22
Sarcopterygii - ps23
Tetrapoda - ps24
Amniota - ps25
Mammalia - ps26
Theria - ps27
Placentalia - ps28
Boreoeutheria - ps29
Euarchontoglires - ps30
Euarchonta - ps31
Primates - ps32
Haplorrhini - ps33
Simiiformes - ps34
Catarrhini - ps35
Hominoidea - ps36
Hominidae - ps37
Homininae - ps38
Hominini - ps39
Homo_sapiens - ps40
p-value
5e-204
1e-153
4e-103
1e-52
4e-02
Log-odds
0.5
1.0
1.5
2.0
2.5
3.0
3.5
4.0

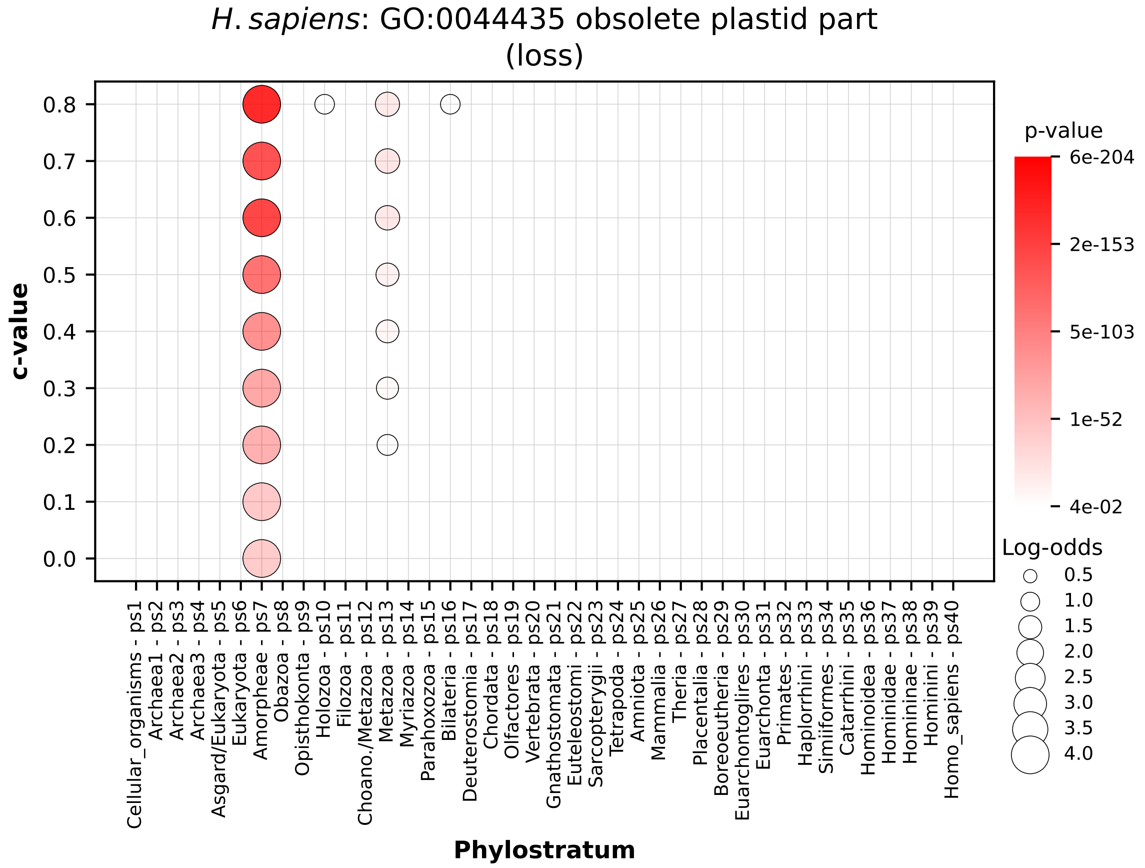

H. sapiens: GO:0044435 obsolete plastid part
(loss)
c-value
0.8
0.7
0.6
0.5
0.4
0.3
0.2
0.1
0.0
p-value
6e-204
2e-153
5e-103
1e-52
4e-02
Log-odds
0.5
1.0
1.5
2.0
2.5
3.0
3.5
4.0
Cellular_organisms - ps1
Archaea1 - ps2
Archaea2 - ps3
Archaea3 - ps4
Asgard/Eukaryota - ps5
Eukaryota - ps6
Amorpheae - ps7
Obazoa - ps8
Opisthokonta - ps9
Holozoa - ps10
Filozoa - ps11
Choano./Metazoa - ps12
Metazoa - ps13
Myriazoa - ps14
Parahoxozoa - ps15
Bilateria - ps16
Deuterostomia - ps17
Chordata - ps18
Olfactores - ps19
Vertebrata - ps20
Gnathostomata - ps21
Euteleostomi - ps22
Sarcopterygii - ps23
Tetrapoda - ps24
Amniota - ps25
Mammalia - ps26
Theria - ps27
Placentalia - ps28
Boreoeutheria - ps29
Euarchontoglires - ps30
Euarchonta - ps31
Primates - ps32
Haplorrhini - ps33
Simiiformes - ps34
Catarrhini - ps35
Hominoidea - ps36
Hominidae - ps37
Homininae - ps38
Hominini - ps39
Homo_sapiens - ps40
Phylostratum

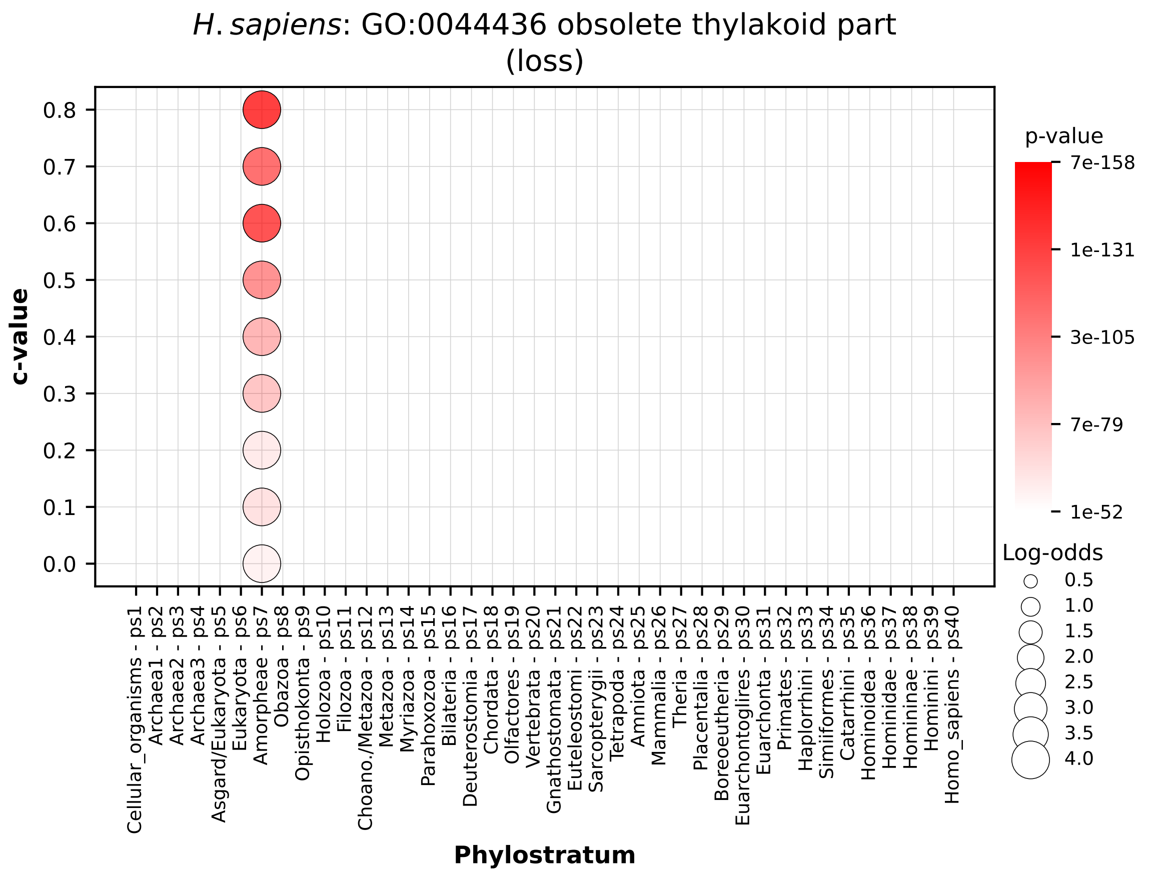

H. sapiens: GO:0044436 obsolete thylakoid part
(loss)
c-value
0.8
0.7
0.6
0.5
0.4
0.3
0.2
0.1
0.0
p-value
7e-158
1e-131
3e-105
7e-79
1e-52
Log-odds
0.5
1.0
1.5
2.0
2.5
3.0
3.5
4.0
Cellular_organisms - ps1
Archaea1 - ps2
Archaea2 - ps3
Archaea3 - ps4
Asgard/Eukaryota - ps5
Eukaryota - ps6
Amorpheae - ps7
Obazoa - ps8
Opisthokonta - ps9
Holozoa - ps10
Filozoa - ps11
Choano./Metazoa - ps12
Metazoa - ps13
Myriazoa - ps14
Parahoxozoa - ps15
Bilateria - ps16
Deuterostomia - ps17
Chordata - ps18
Olfactores - ps19
Vertebrata - ps20
Gnathostomata - ps21
Euteleostomi - ps22
Sarcopterygii - ps23
Tetrapoda - ps24
Amniota - ps25
Mammalia - ps26
Theria - ps27
Placentalia - ps28
Boreoeutheria - ps29
Euarchontoglires - ps30
Euarchonta - ps31
Primates - ps32
Haplorrhini - ps33
Simiiformes - ps34
Catarrhini - ps35
Hominoidea - ps36
Hominidae - ps37
Homininae - ps38
Hominini - ps39
Homo_sapiens - ps40
Phylostratum

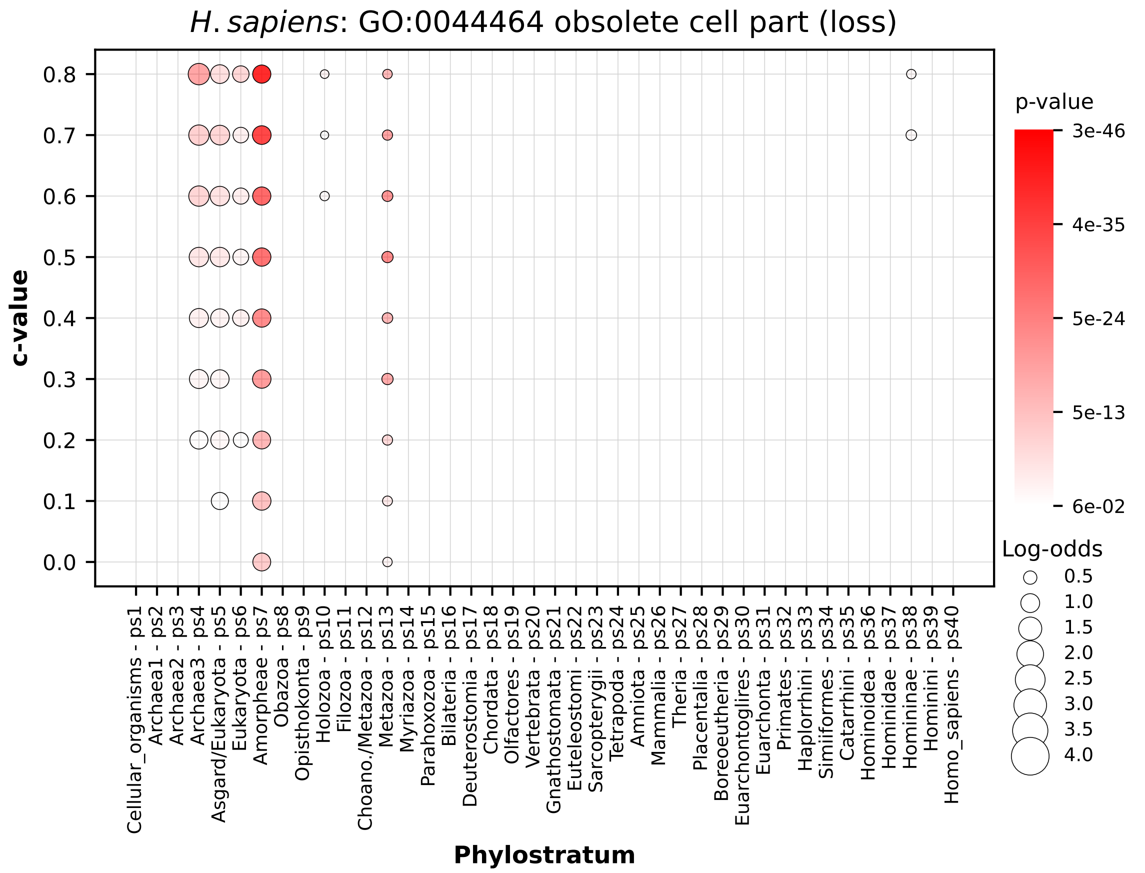

H. sapiens: GO:0044464 obsolete cell part (loss)
c-value
0.8
0.7
0.6
0.5
0.4
0.3
0.2
0.1
0.0
p-value
3e-46
4e-35
5e-24
5e-13
6e-02
Log-odds
0.5
1.0
1.5
2.0
2.5
3.0
3.5
4.0
Cellular_organisms - ps1
Archaea1 - ps2
Archaea2 - ps3
Archaea3 - ps4
Asgard/Eukaryota - ps5
Eukaryota - ps6
Amorpheae - ps7
Obazoa - ps8
Opisthokonta - ps9
Holozoa - ps10
Filozoa - ps11
Choano./Metazoa - ps12
Metazoa - ps13
Myriazoa - ps14
Parahoxozoa - ps15
Bilateria - ps16
Deuterostomia - ps17
Chordata - ps18
Olfactores - ps19
Vertebrata - ps20
Gnathostomata - ps21
Euteleostomi - ps22
Sarcopterygii - ps23
Tetrapoda - ps24
Amniota - ps25
Mammalia - ps26
Theria - ps27
Placentalia - ps28
Boreoeutheria - ps29
Euarchontoglires - ps30
Euarchonta - ps31
Primates - ps32
Haplorrhini - ps33
Simiiformes - ps34
Catarrhini - ps35
Hominoidea - ps36
Hominidae - ps37
Homininae - ps38
Hominini - ps39
Homo_sapiens - ps40
Phylostratum

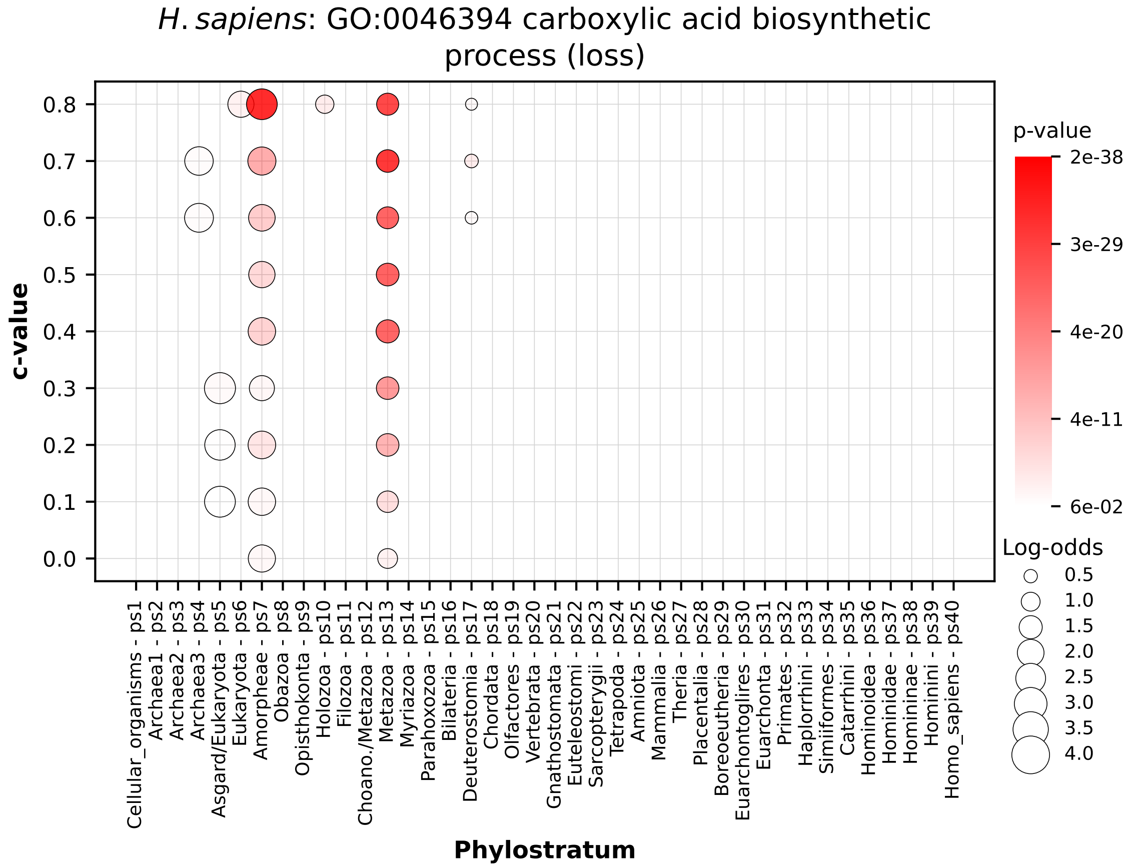

H. sapiens: GO:0046394 carboxylic acid biosynthetic process (loss)
c-value
0.8
0.7
0.6
0.5
0.4
0.3
0.2
0.1
0.0
Phylostratum
Cellular_organisms - ps1
Archaea1 - ps2
Archaea2 - ps3
Archaea3 - ps4
Asgard/Eukaryota - ps5
Eukaryota - ps6
Amorpheae - ps7
Obazoa - ps8
Opisthokonta - ps9
Holozoa - ps10
Filozoa - ps11
Choano./Metazoa - ps12
Metazoa - ps13
Myriazoa - ps14
Parahoxozoa - ps15
Bilateria - ps16
Deuterostomia - ps17
Chordata - ps18
Olfactores - ps19
Vertebrata - ps20
Gnathostomata - ps21
Euteleostomi - ps22
Sarcopterygii - ps23
Tetrapoda - ps24
Amniota - ps25
Mammalia - ps26
Theria - ps27
Placentalia - ps28
Boreoeutheria - ps29
Euarchontoglires - ps30
Euarchonta - ps31
Primates - ps32
Haplorrhini - ps33
Simiiformes - ps34
Catarrhini - ps35
Hominoidea - ps36
Hominidae - ps37
Homininae - ps38
Hominini - ps39
Homo_sapiens - ps40
p-value
2e-38
3e-29
4e-20
4e-11
6e-02
Log-odds
0.5
1.0
1.5
2.0
2.5
3.0
3.5
4.0

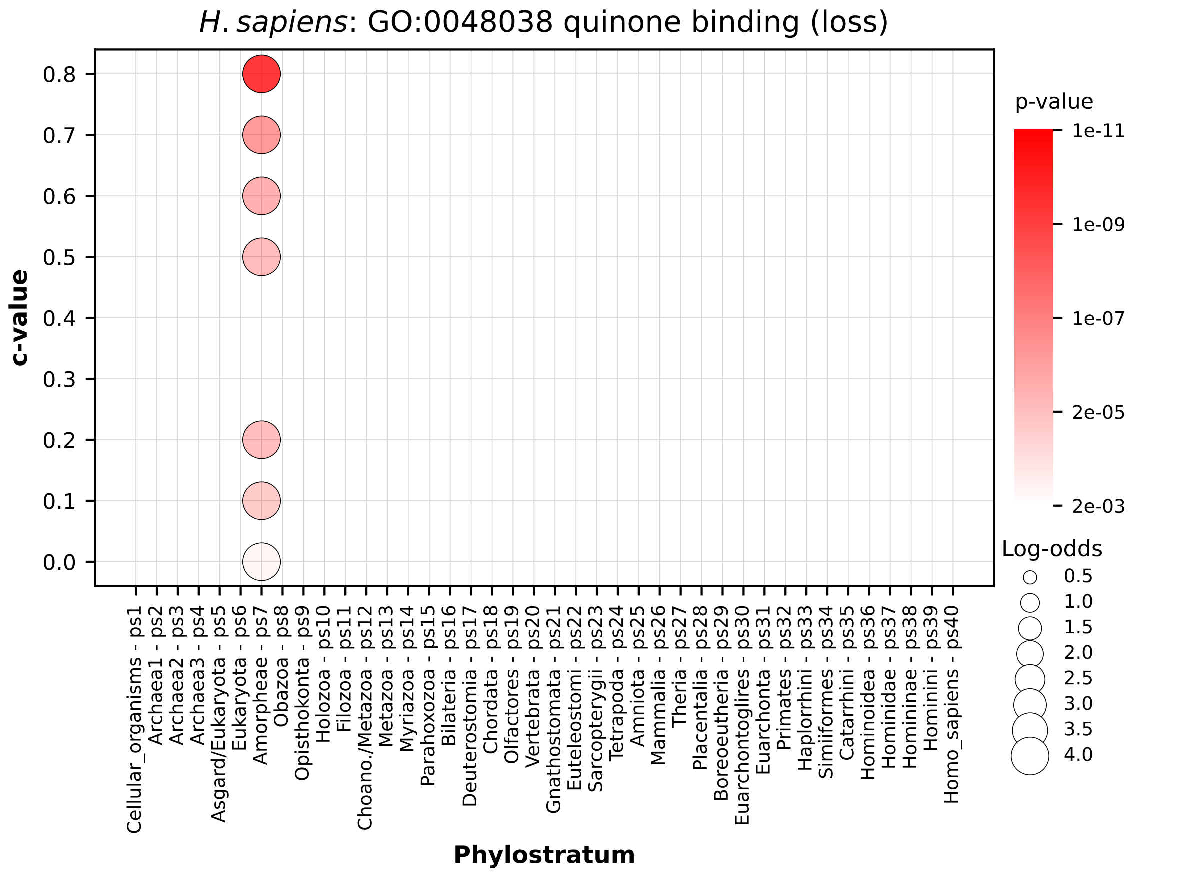

H. sapiens: GO:0048038 quinone binding (loss)
c-value
0.8
0.7
0.6
0.5
0.4
0.3
0.2
0.1
0.0
Cellular_organisms - ps1
Archaea1 - ps2
Archaea2 - ps3
Archaea3 - ps4
Asgard/Eukaryota - ps5
Eukaryota - ps6
Amorpheae - ps7
Obazoa - ps8
Opisthokonta - ps9
Holozoa - ps10
Filozoa - ps11
Choano./Metazoa - ps12
Metazoa - ps13
Myriazoa - ps14
Parahoxozoa - ps15
Bilateria - ps16
Deuterostomia - ps17
Chordata - ps18
Olfactores - ps19
Vertebrata - ps20
Gnathostomata - ps21
Euteleostomi - ps22
Sarcopterygii - ps23
Tetrapoda - ps24
Amniota - ps25
Mammalia - ps26
Theria - ps27
Placentalia - ps28
Boreoeutheria - ps29
Euarchontoglires - ps30
Euarchonta - ps31
Primates - ps32
Haplorrhini - ps33
Simiiformes - ps34
Catarrhini - ps35
Hominoidea - ps36
Hominidae - ps37
Homininae - ps38
Hominini - ps39
Homo_sapiens - ps40
Phylostratum
p-value
1e-11
1e-09
1e-07
2e-05
2e-03
Log-odds
0.5
1.0
1.5
2.0
2.5
3.0
3.5
4.0

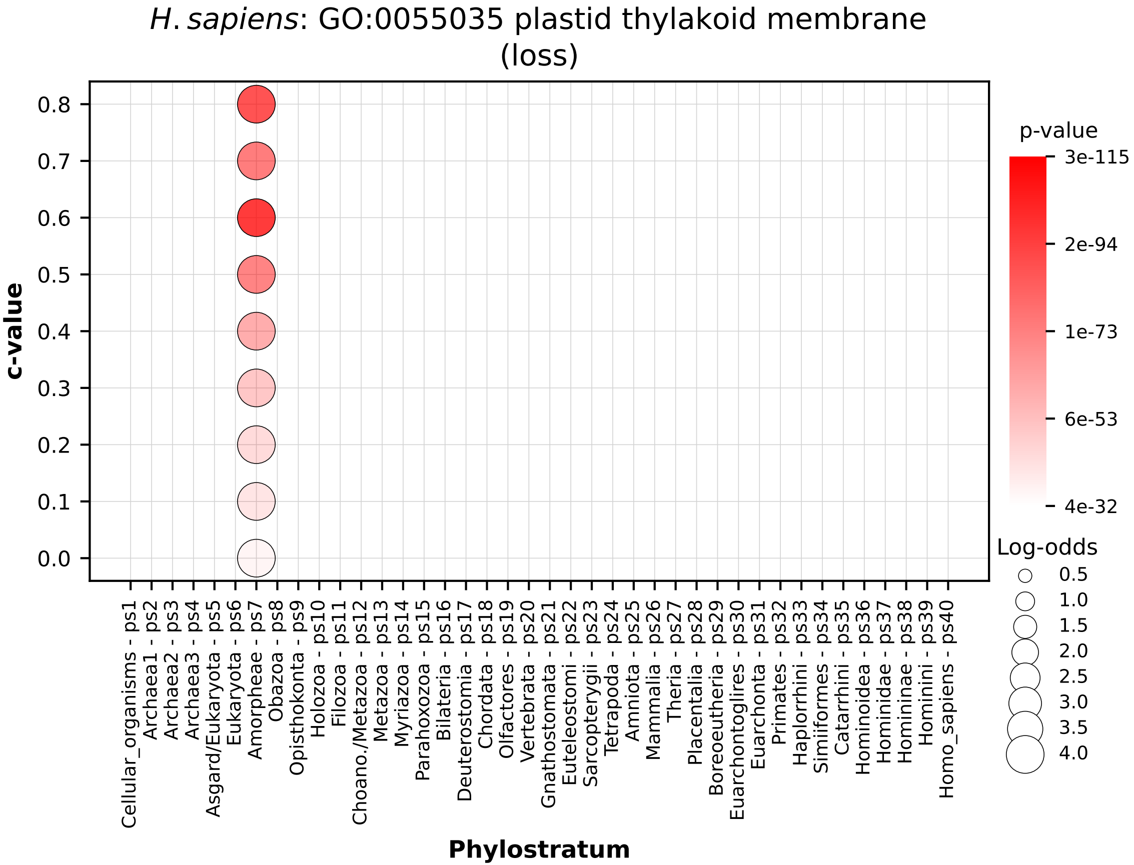
H. sapiens: GO:0055035 plastid thylakoid membrane
(loss)
c-value
0.8
0.7
0.6
0.5
0.4
0.3
0.2
0.1
0.0
p-value
3e-115
2e-94
1e-73
6e-53
4e-32
Log-odds
0.5
1.0
1.5
2.0
2.5
3.0
3.5
4.0
Cellular_organisms - ps1
Archaea1 - ps2
Archaea2 - ps3
Archaea3 - ps4
Asgard/Eukaryota - ps5
Eukaryota - ps6
Amorpheae - ps7
Obazoa - ps8
Opisthokonta - ps9
Holozoa - ps10
Filozoa - ps11
Choano./Metazoa - ps12
Metazoa - ps13
Myriazoa - ps14
Parahoxozoa - ps15
Bilateria - ps16
Deuterostomia - ps17
Chordata - ps18
Olfactores - ps19
Vertebrata - ps20
Gnathostomata - ps21
Euteleostomi - ps22
Sarcopterygii - ps23
Tetrapoda - ps24
Amniota - ps25
Mammalia - ps26
Theria - ps27
Placentalia - ps28
Boreoeutheria - ps29
Euarchontoglires - ps30
Euarchonta - ps31
Primates - ps32
Haplorrhini - ps33
Simiiformes - ps34
Catarrhini - ps35
Hominoidea - ps36
Hominidae - ps37
Homininae - ps38
Hominini - ps39
Homo_sapiens - ps40
Phylostratum

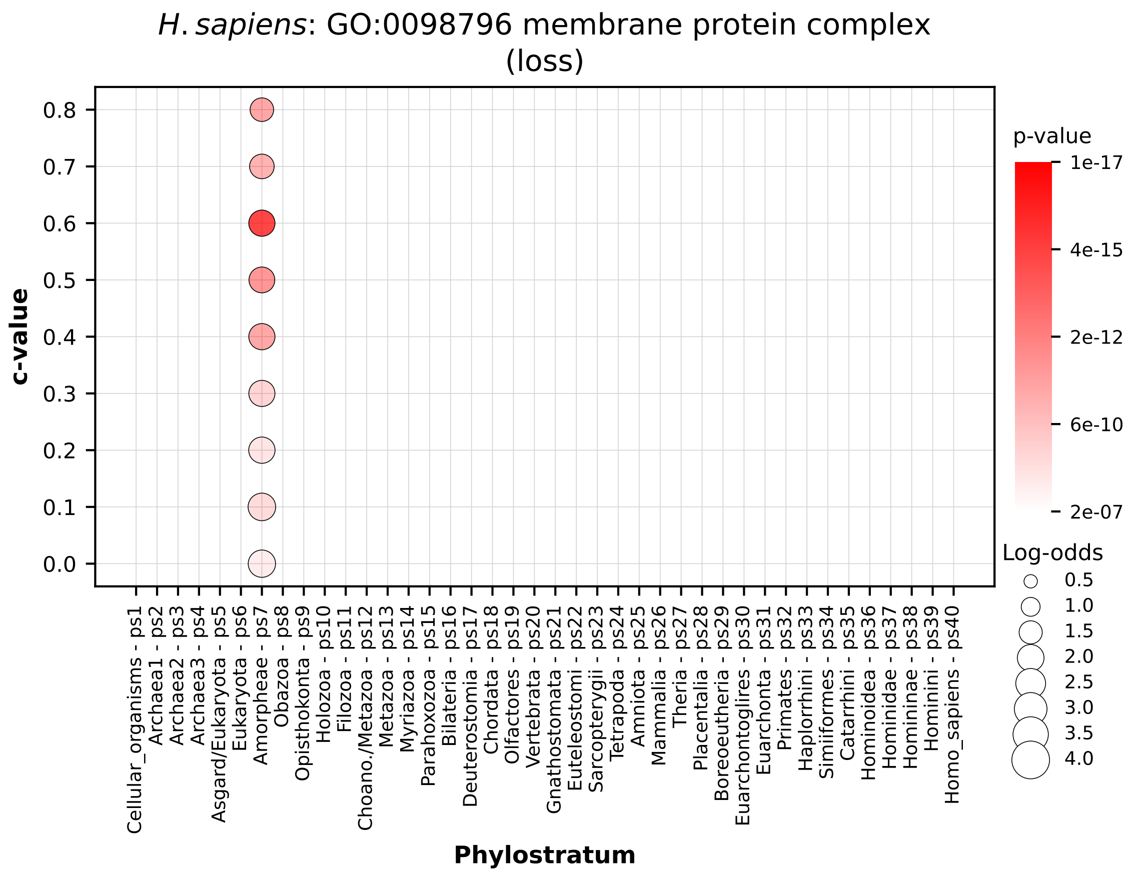

H. sapiens: GO:0098796 membrane protein complex (loss)
c-value
0.8
0.7
0.6
0.5
0.4
0.3
0.2
0.1
0.0
p-value
1e-17
4e-15
2e-12
6e-10
2e-07
Log-odds
0.5
1.0
1.5
2.0
2.5
3.0
3.5
4.0
Cellular_organisms - ps1
Archaea1 - ps2
Archaea2 - ps3
Archaea3 - ps4
Asgard/Eukaryota - ps5
Eukaryota - ps6
Amorpheae - ps7
Obazoa - ps8
Opisthokonta - ps9
Holozoa - ps10
Filozoa - ps11
Choano./Metazoa - ps12
Metazoa - ps13
Myriazoa - ps14
Parahoxozoa - ps15
Bilateria - ps16
Deuterostomia - ps17
Chordata - ps18
Olfactores - ps19
Vertebrata - ps20
Gnathostomata - ps21
Euteleostomi - ps22
Sarcopterygii - ps23
Tetrapoda - ps24
Amniota - ps25
Mammalia - ps26
Theria - ps27
Placentalia - ps28
Boreoeutheria - ps29
Euarchontoglires - ps30
Euarchonta - ps31
Primates - ps32
Haplorrhini - ps33
Simiiformes - ps34
Catarrhini - ps35
Hominoidea - ps36
Hominidae - ps37
Homininae - ps38
Hominini - ps39
Homo_sapiens - ps40
Phylostratum

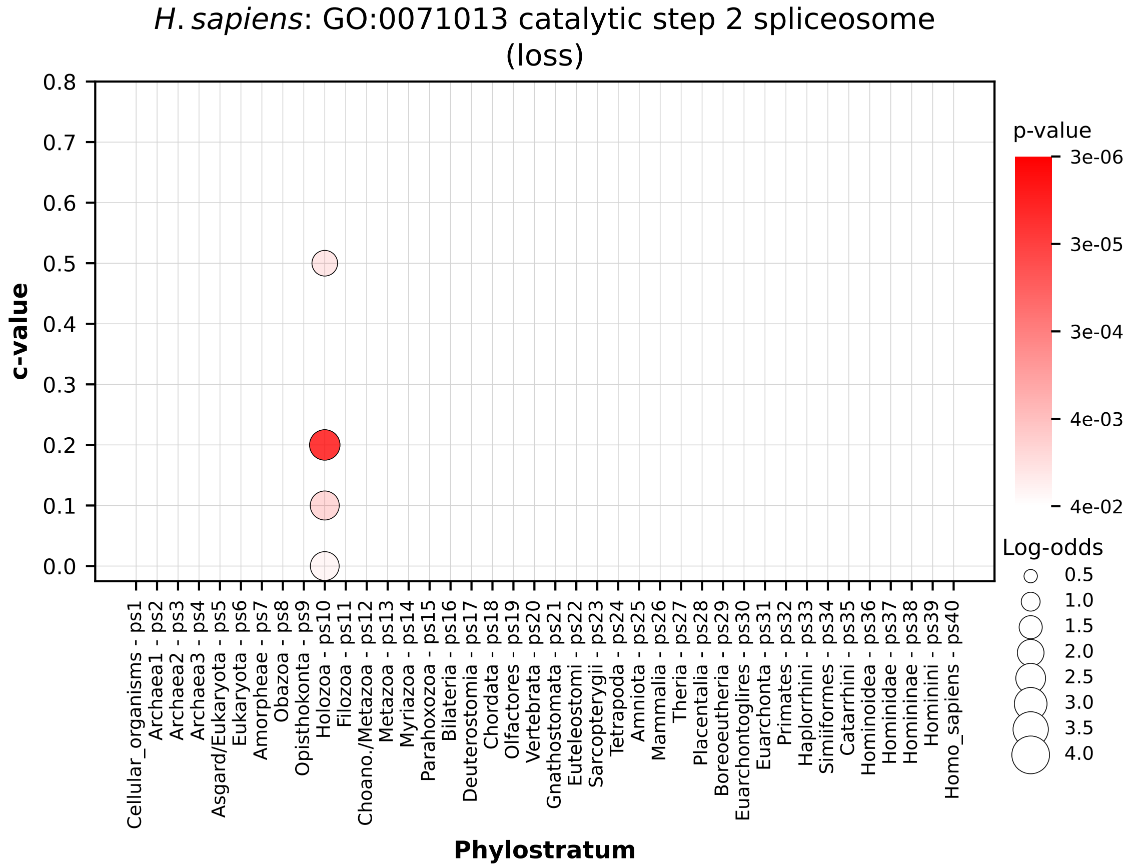

H. sapiens: GO:0071013 catalytic step 2 spliceosome (loss)
c-value
0.0
0.1
0.2
0.3
0.4
0.5
0.6
0.7
0.8
Phylostratum
Cellular_organisms - ps1
Archaea1 - ps2
Archaea2 - ps3
Archaea3 - ps4
Asgard/Eukaryota - ps5
Eukaryota - ps6
Amorpheae - ps7
Obazoa - ps8
Opisthokonta - ps9
Holozoa - ps10
Filozoa - ps11
Choano./Metazoa - ps12
Metazoa - ps13
Myriazoa - ps14
Parahoxozoa - ps15
Bilateria - ps16
Deuterostomia - ps17
Chordata - ps18
Olfactores - ps19
Vertebrata - ps20
Gnathostomata - ps21
Euteleostomi - ps22
Sarcopterygii - ps23
Tetrapoda - ps24
Amniota - ps25
Mammalia - ps26
Theria - ps27
Placentalia - ps28
Boreoeutheria - ps29
Euarchontoglires - ps30
Euarchonta - ps31
Primates - ps32
Haplorrhini - ps33
Simiiformes - ps34
Catarrhini - ps35
Hominoidea - ps36
Hominidae - ps37
Homininae - ps38
Hominini - ps39
Homo_sapiens - ps40
p-value
3e-06
3e-05
3e-04
4e-03
4e-02
Log-odds
0.5
1.0
1.5
2.0
2.5
3.0
3.5
4.0

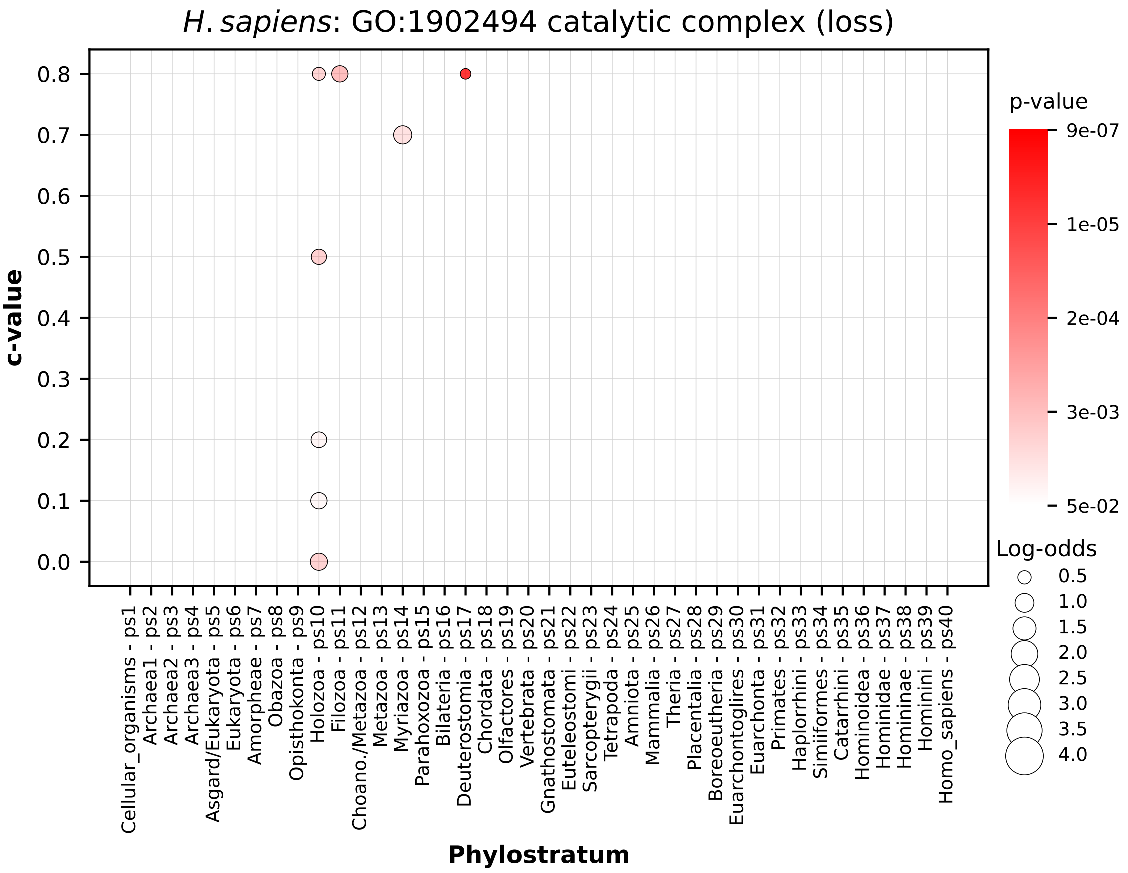

H. sapiens: GO:1902494 catalytic complex (loss)
c-value
0.0
0.1
0.2
0.3
0.4
0.5
0.6
0.7
0.8
Phylostratum
Cellular_organisms - ps1
Archaea1 - ps2
Archaea2 - ps3
Archaea3 - ps4
Asgard/Eukaryota - ps5
Eukaryota - ps6
Amorpheae - ps7
Obazoa - ps8
Opisthokonta - ps9
Holozoa - ps10
Filozoa - ps11
Choano./Metazoa - ps12
Metazoa - ps13
Myriazoa - ps14
Parahoxozoa - ps15
Bilateria - ps16
Deuterostomia - ps17
Chordata - ps18
Olfactores - ps19
Vertebrata - ps20
Gnathostomata - ps21
Euteleostomi - ps22
Sarcopterygii - ps23
Tetrapoda - ps24
Amniota - ps25
Mammalia - ps26
Theria - ps27
Placentalia - ps28
Boreoeutheria - ps29
Euarchontoglires - ps30
Euarchonta - ps31
Primates - ps32
Haplorrhini - ps33
Simiiformes - ps34
Catarrhini - ps35
Hominoidea - ps36
Hominidae - ps37
Homininae - ps38
Hominini - ps39
Homo_sapiens - ps40
p-value
9e-07
1e-05
2e-04
3e-03
5e-02
Log-odds
0.5
1.0
1.5
2.0
2.5
3.0
3.5
4.0

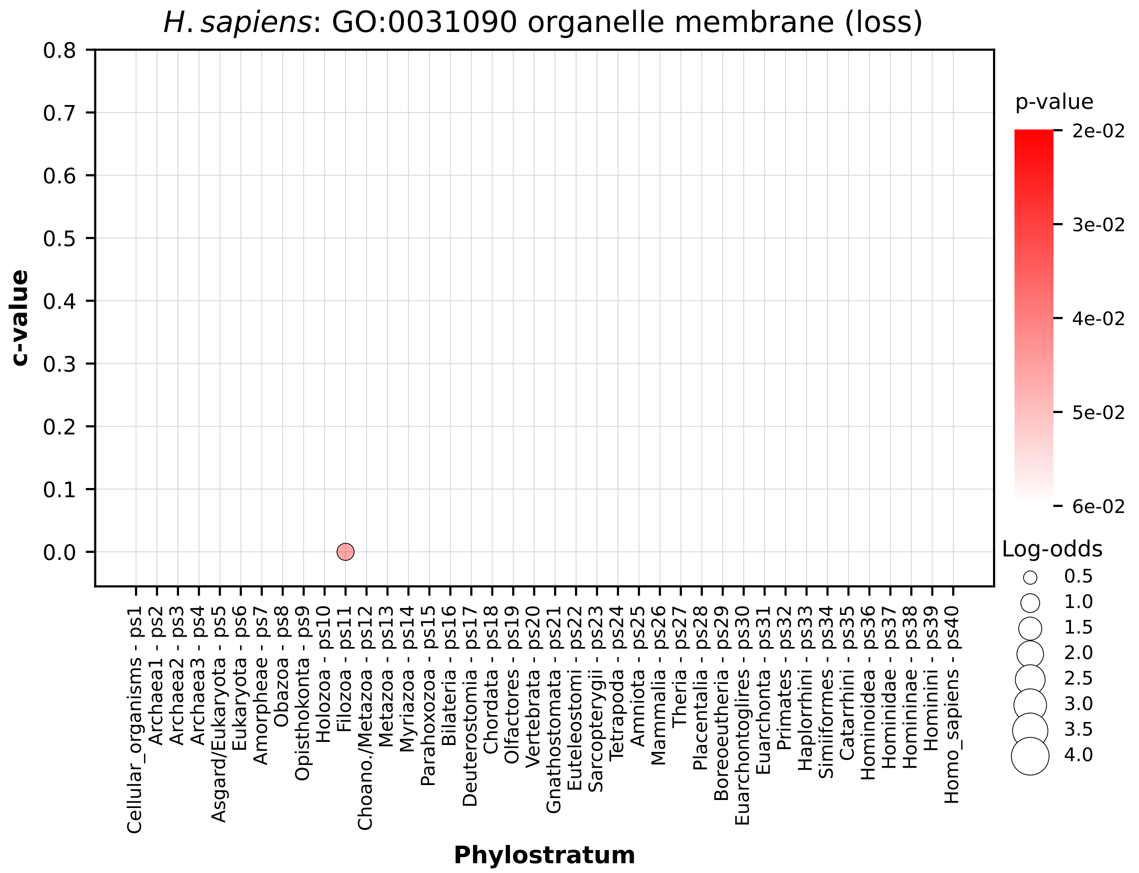

H. sapiens: GO:0031090 organelle membrane (loss)
c-value
0.8
0.7
0.6
0.5
0.4
0.3
0.2
0.1
0.0
Phylostratum
Cellular_organisms - ps1
Archaea1 - ps2
Archaea2 - ps3
Archaea3 - ps4
Asgard/Eukaryota - ps5
Eukaryota - ps6
Amorpheae - ps7
Obazoa - ps8
Opisthokonta - ps9
Holozoa - ps10
Filozoa - ps11
Choano./Metazoa - ps12
Metazoa - ps13
Myriazoa - ps14
Parahoxozoa - ps15
Bilateria - ps16
Deuterostomia - ps17
Chordata - ps18
Olfactores - ps19
Vertebrata - ps20
Gnathostomata - ps21
Euteleostomi - ps22
Sarcopterygii - ps23
Tetrapoda - ps24
Amniota - ps25
Mammalia - ps26
Theria - ps27
Placentalia - ps28
Boreoeutheria - ps29
Euarchontoglires - ps30
Euarchonta - ps31
Primates - ps32
Haplorrhini - ps33
Simiiformes - ps34
Catarrhini - ps35
Hominoidea - ps36
Hominidae - ps37
Homininae - ps38
Hominini - ps39
Homo_sapiens - ps40
p-value
2e-02
3e-02
4e-02
5e-02
6e-02
Log-odds
0.5
1.0
1.5
2.0
2.5
3.0
3.5
4.0

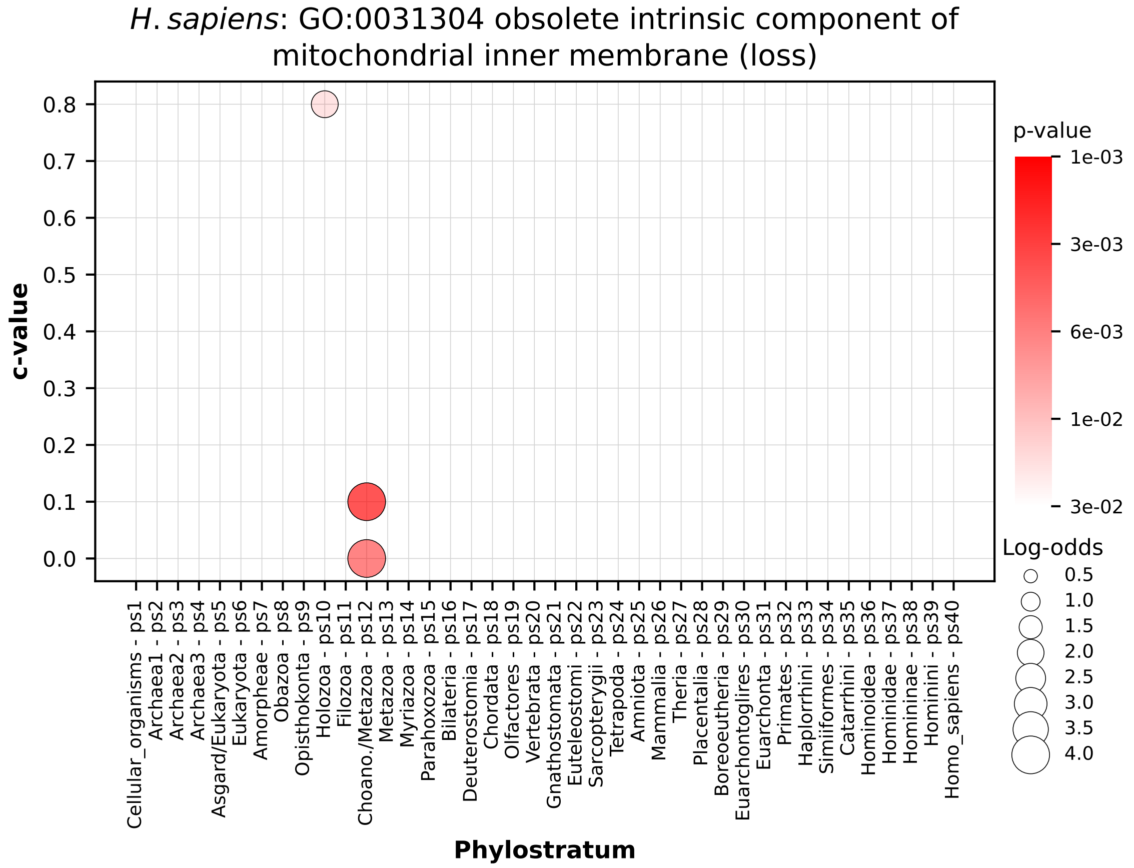

H. sapiens: GO:0031304 obsolete intrinsic component of mitochondrial inner membrane (loss)
c-value
0.8
0.7
0.6
0.5
0.4
0.3
0.2
0.1
0.0
p-value
1e-03
3e-03
6e-03
1e-02
3e-02
Log-odds
0.5
1.0
1.5
2.0
2.5
3.0
3.5
4.0
Cellular_organisms - ps1
Archaea1 - ps2
Archaea2 - ps3
Archaea3 - ps4
Asgard/Eukaryota - ps5
Eukaryota - ps6
Amorpheae - ps7
Obazoa - ps8
Opisthokonta - ps9
Holozoa - ps10
Filozoa - ps11
Choano./Metazoa - ps12
Metazoa - ps13
Myriazoa - ps14
Parahoxozoa - ps15
Bilateria - ps16
Deuterostomia - ps17
Chordata - ps18
Olfactores - ps19
Vertebrata - ps20
Gnathostomata - ps21
Euteleostomi - ps22
Sarcopterygii - ps23
Tetrapoda - ps24
Amniota - ps25
Mammalia - ps26
Theria - ps27
Placentalia - ps28
Boreoeutheria - ps29
Euarchontoglires - ps30
Euarchonta - ps31
Primates - ps32
Haplorrhini - ps33
Simiiformes - ps34
Catarrhini - ps35
Hominoidea - ps36
Hominidae - ps37
Homininae - ps38
Hominini - ps39
Homo_sapiens - ps40
Phylostratum

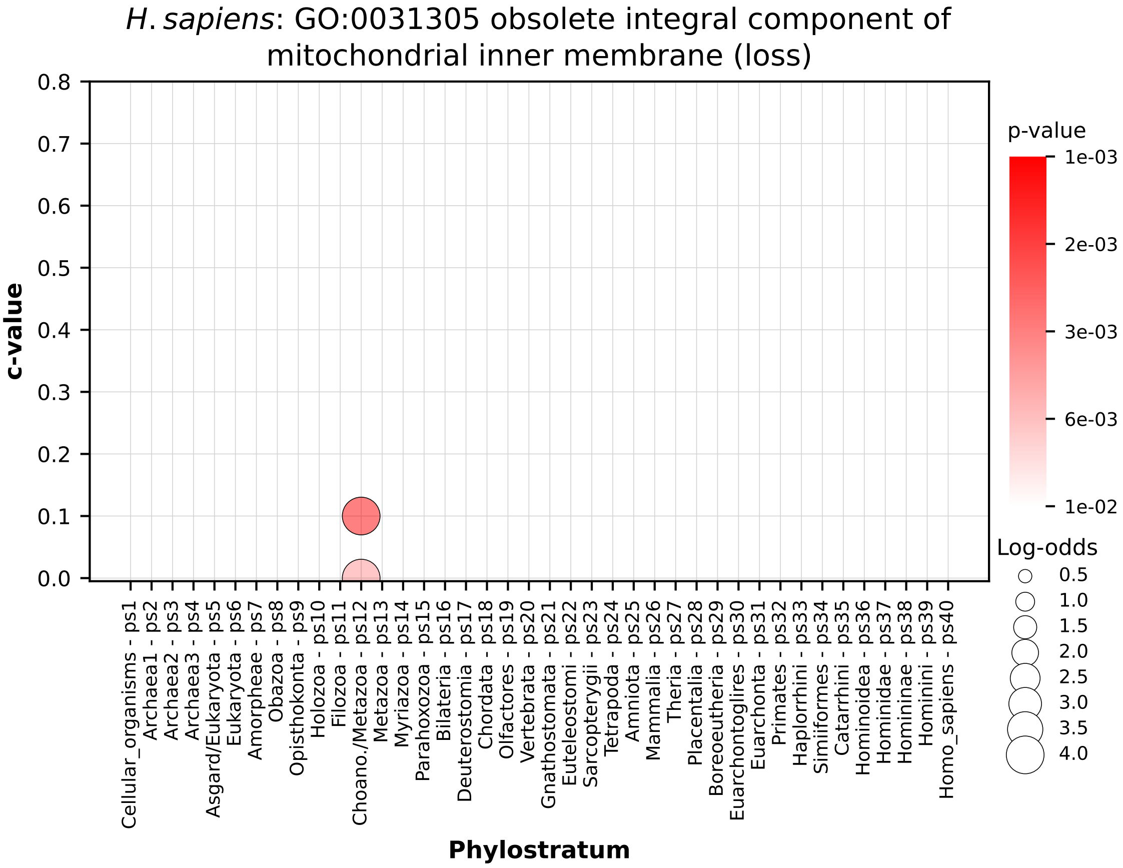

H. sapiens: GO:0031305 obsolete integral component of mitochondrial inner membrane (loss)
c-value
0.8
0.7
0.6
0.5
0.4
0.3
0.2
0.1
0.0
p-value
1e-03
2e-03
3e-03
6e-03
1e-02
Log-odds
0.5
1.0
1.5
2.0
2.5
3.0
3.5
4.0
Cellular_organisms - ps1
Archaea1 - ps2
Archaea2 - ps3
Archaea3 - ps4
Asgard/Eukaryota - ps5
Eukaryota - ps6
Amorpheae - ps7
Obazoa - ps8
Opisthokonta - ps9
Holozoa - ps10
Filozoa - ps11
Choano./Metazoa - ps12
Metazoa - ps13
Myriazoa - ps14
Parahoxozoa - ps15
Bilateria - ps16
Deuterostomia - ps17
Chordata - ps18
Olfactores - ps19
Vertebrata - ps20
Gnathostomata - ps21
Euteleostomi - ps22
Sarcopterygii - ps23
Tetrapoda - ps24
Amniota - ps25
Mammalia - ps26
Theria - ps27
Placentalia - ps28
Boreoeutheria - ps29
Euarchontoglires - ps30
Euarchonta - ps31
Primates - ps32
Haplorrhini - ps33
Simiiformes - ps34
Catarrhini - ps35
Hominoidea - ps36
Hominidae - ps37
Homininae - ps38
Hominini - ps39
Homo_sapiens - ps40
Phylostratum

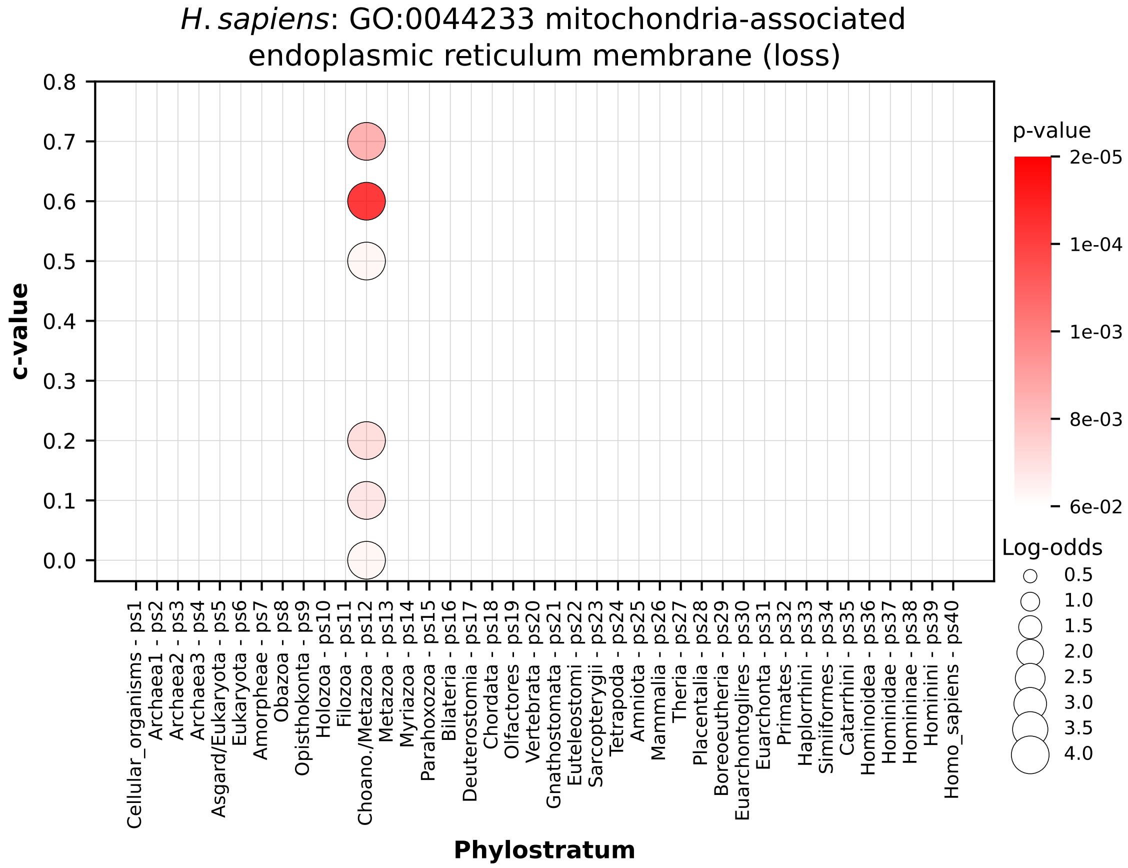

H. sapiens: GO:0044233 mitochondria-associated endoplasmic reticulum membrane (loss)
c-value
0.0
0.1
0.2
0.3
0.4
0.5
0.6
0.7
0.8
Phylostratum
Cellular_organisms - ps1
Archaea1 - ps2
Archaea2 - ps3
Archaea3 - ps4
Asgard/Eukaryota - ps5
Eukaryota - ps6
Amorpheae - ps7
Obazoa - ps8
Opisthokonta - ps9
Holozoa - ps10
Filozoa - ps11
Choano./Metazoa - ps12
Metazoa - ps13
Myriazoa - ps14
Parahoxozoa - ps15
Bilateria - ps16
Deuterostomia - ps17
Chordata - ps18
Olfactores - ps19
Vertebrata - ps20
Gnathostomata - ps21
Euteleostomi - ps22
Sarcopterygii - ps23
Tetrapoda - ps24
Amniota - ps25
Mammalia - ps26
Theria - ps27
Placentalia - ps28
Boreoeutheria - ps29
Euarchontoglires - ps30
Euarchonta - ps31
Primates - ps32
Haplorrhini - ps33
Simiiformes - ps34
Catarrhini - ps35
Hominoidea - ps36
Hominidae - ps37
Homininae - ps38
Hominini - ps39
Homo_sapiens - ps40
p-value
2e-05
1e-04
1e-03
8e-03
6e-02
Log-odds
0.5
1.0
1.5
2.0
2.5
3.0
3.5
4.0

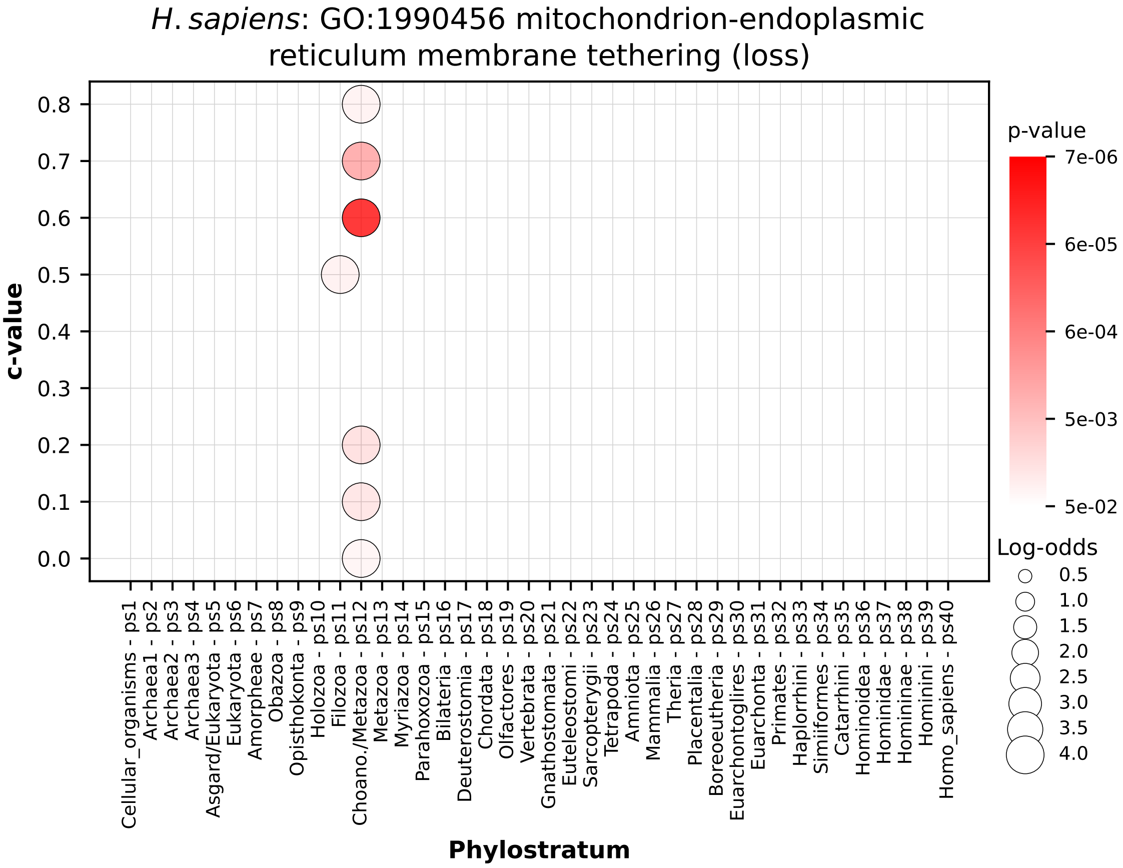

H. sapiens: GO:1990456 mitochondrion-endoplasmic reticulum membrane tethering (loss)
c-value
0.8
0.7
0.6
0.5
0.4
0.3
0.2
0.1
0.0
p-value
7e-06
6e-05
6e-04
5e-03
5e-02
Log-odds
0.5
1.0
1.5
2.0
2.5
3.0
3.5
4.0
Cellular_organisms - ps1
Archaea1 - ps2
Archaea2 - ps3
Archaea3 - ps4
Asgard/Eukaryota - ps5
Eukaryota - ps6
Amorpheae - ps7
Obazoa - ps8
Opisthokonta - ps9
Holozoa - ps10
Filozoa - ps11
Choano./Metazoa - ps12
Metazoa - ps13
Myriazoa - ps14
Parahoxozoa - ps15
Bilateria - ps16
Deuterostomia - ps17
Chordata - ps18
Olfactores - ps19
Vertebrata - ps20
Gnathostomata - ps21
Euteleostomi - ps22
Sarcopterygii - ps23
Tetrapoda - ps24
Amniota - ps25
Mammalia - ps26
Theria - ps27
Placentalia - ps28
Boreoeutheria - ps29
Euarchontoglires - ps30
Euarchonta - ps31
Primates - ps32
Haplorrhini - ps33
Simiiformes - ps34
Catarrhini - ps35
Hominoidea - ps36
Hominidae - ps37
Homininae - ps38
Hominini - ps39
Homo_sapiens - ps40
Phylostratum

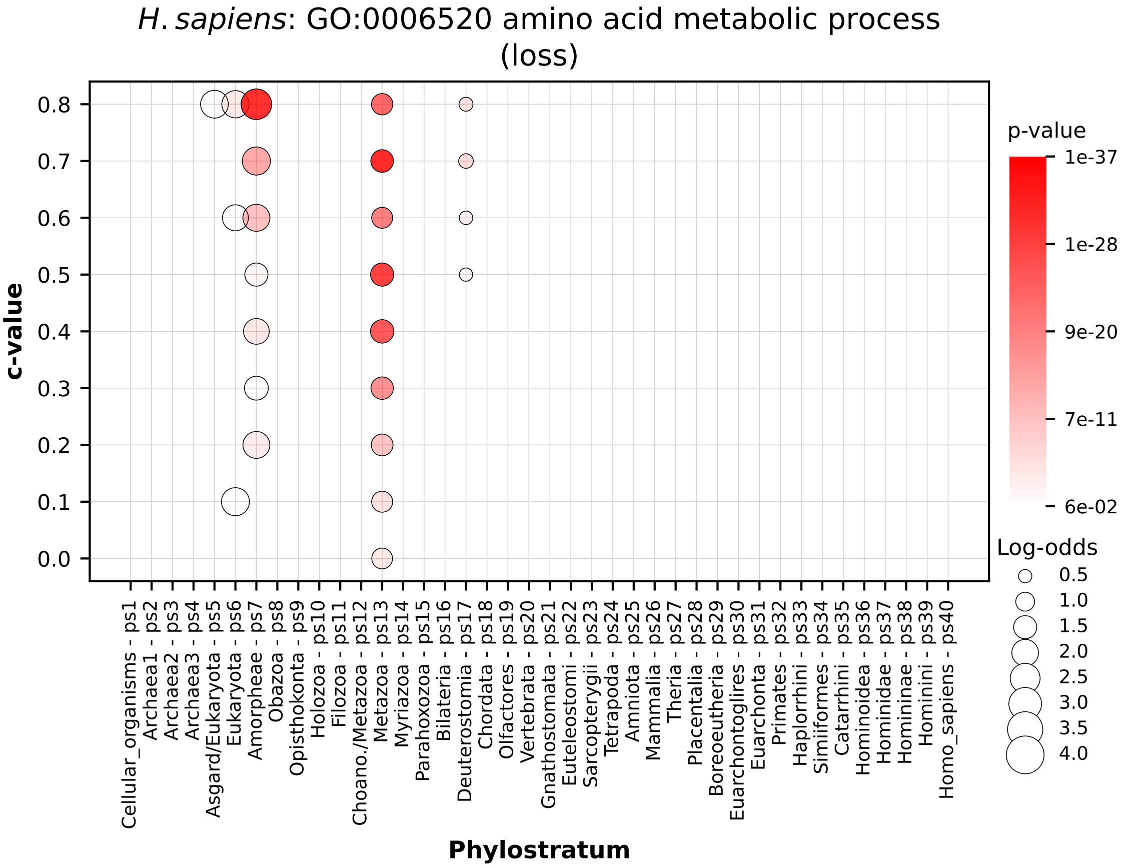

H. sapiens: GO:0006520 amino acid metabolic process
(loss)
c-value
0.8
0.7
0.6
0.5
0.4
0.3
0.2
0.1
0.0
Phylostratum
Cellular_organisms - ps1
Archaea1 - ps2
Archaea2 - ps3
Archaea3 - ps4
Asgard/Eukaryota - ps5
Eukaryota - ps6
Amorpheae - ps7
Obazoa - ps8
Opisthokonta - ps9
Holozoa - ps10
Filozoa - ps11
Choano./Metazoa - ps12
Metazoa - ps13
Myriazoa - ps14
Parahoxozoa - ps15
Bilateria - ps16
Deuterostomia - ps17
Chordata - ps18
Olfactores - ps19
Vertebrata - ps20
Gnathostomata - ps21
Euteleostomi - ps22
Sarcopterygii - ps23
Tetrapoda - ps24
Amniota - ps25
Mammalia - ps26
Theria - ps27
Placentalia - ps28
Boreoeutheria - ps29
Euarchontoglires - ps30
Euarchonta - ps31
Primates - ps32
Haplorrhini - ps33
Simiiformes - ps34
Catarrhini - ps35
Hominoidea - ps36
Hominidae - ps37
Homininae - ps38
Hominini - ps39
Homo_sapiens - ps40
p-value
1e-37
1e-28
9e-20
7e-11
6e-02
Log-odds
0.5
1.0
1.5
2.0
2.5
3.0
3.5
4.0

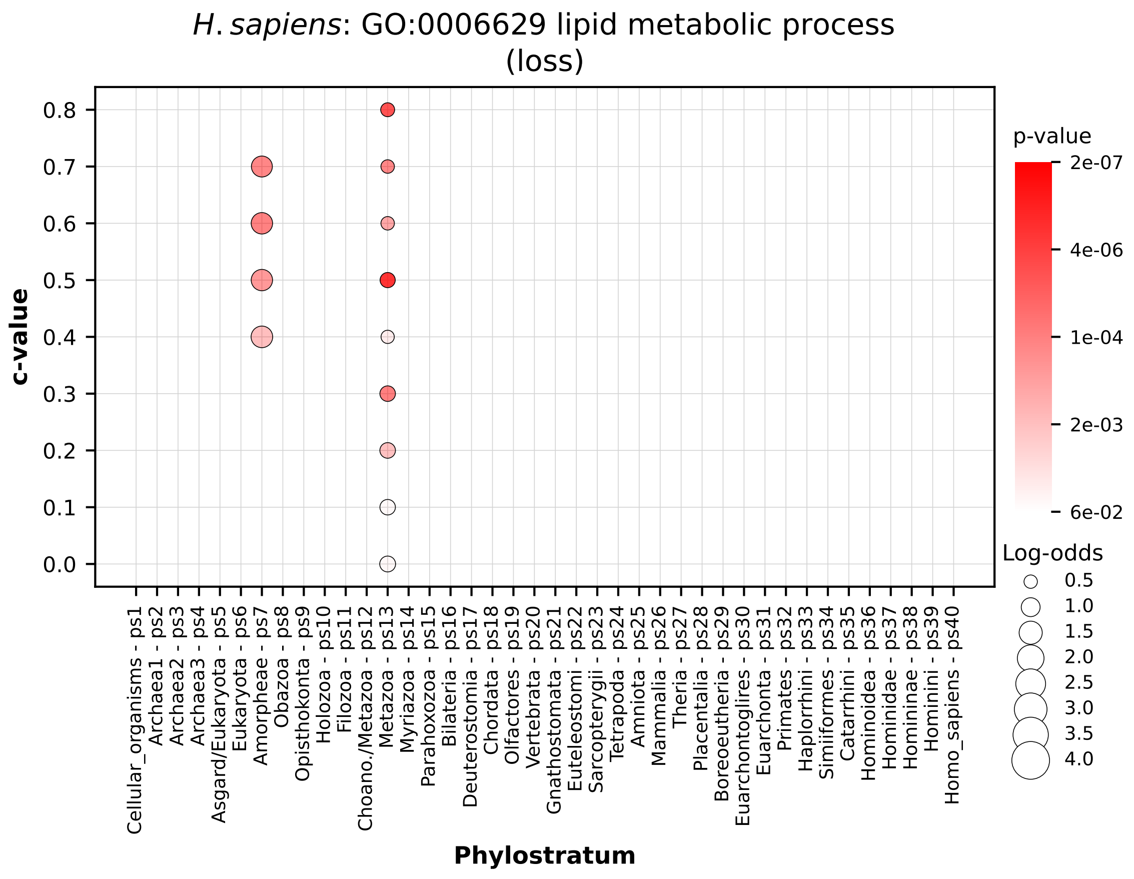

H. sapiens: GO:0006629 lipid metabolic process
(loss)
c-value
0.0
0.1
0.2
0.3
0.4
0.5
0.6
0.7
0.8
p-value
2e-07
4e-06
1e-04
2e-03
6e-02
Log-odds
0.5
1.0
1.5
2.0
2.5
3.0
3.5
4.0
Cellular_organisms - ps1
Archaea1 - ps2
Archaea2 - ps3
Archaea3 - ps4
Asgard/Eukaryota - ps5
Eukaryota - ps6
Amorpheae - ps7
Obazoa - ps8
Opisthokonta - ps9
Holozoa - ps10
Filozoa - ps11
Choano./Metazoa - ps12
Metazoa - ps13
Myriazoa - ps14
Parahoxozoa - ps15
Bilateria - ps16
Deuterostomia - ps17
Chordata - ps18
Olfactores - ps19
Vertebrata - ps20
Gnathostomata - ps21
Euteleostomi - ps22
Sarcopterygii - ps23
Tetrapoda - ps24
Amniota - ps25
Mammalia - ps26
Theria - ps27
Placentalia - ps28
Boreoeutheria - ps29
Euarchontoglires - ps30
Euarchonta - ps31
Primates - ps32
Haplorrhini - ps33
Simiiformes - ps34
Catarrhini - ps35
Hominoidea - ps36
Hominidae - ps37
Homininae - ps38
Hominini - ps39
Homo_sapiens - ps40
Phylostratum

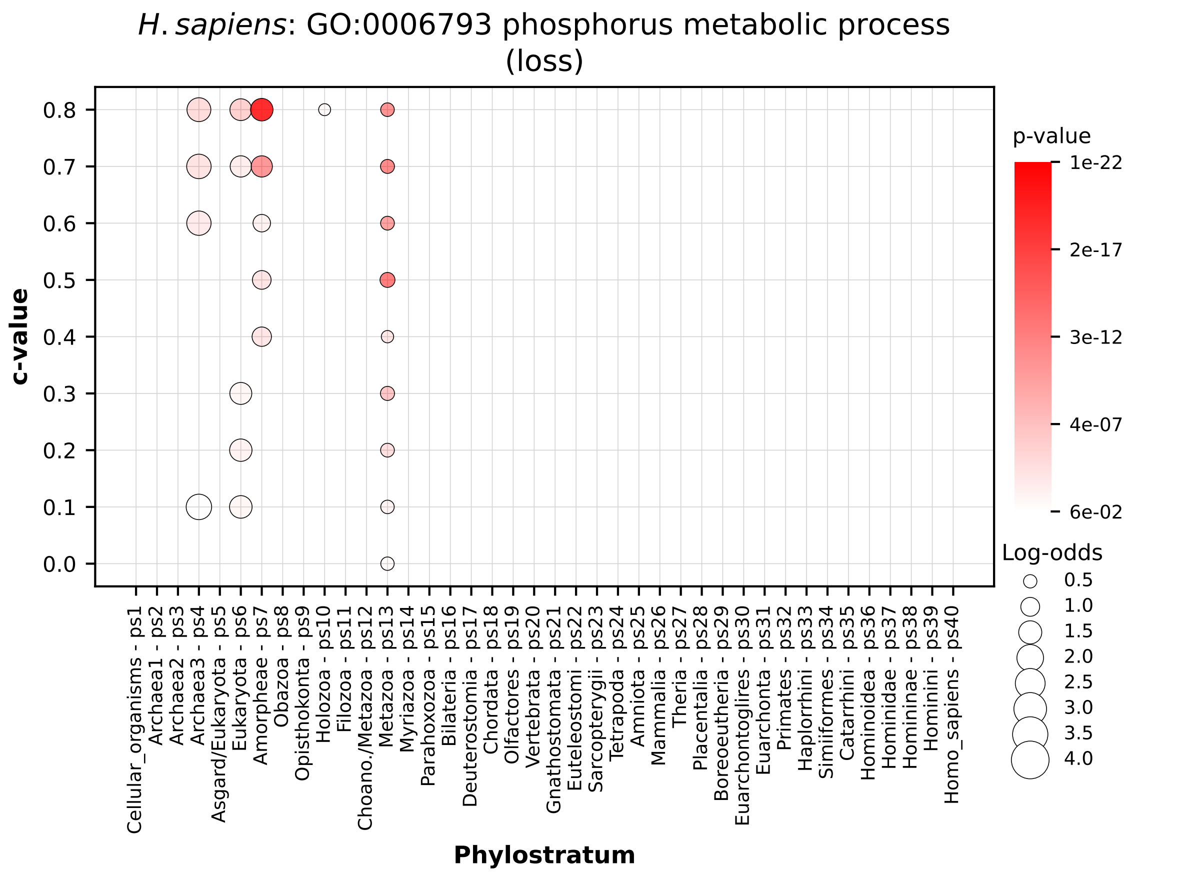
H. sapiens: GO:0006793 phosphorus metabolic process
(loss)
c-value
0.0
0.1
0.2
0.3
0.4
0.5
0.6
0.7
0.8
Phylostratum
Cellular_organisms - ps1
Archaea1 - ps2
Archaea2 - ps3
Archaea3 - ps4
Asgard/Eukaryota - ps5
Eukaryota - ps6
Amorpheae - ps7
Obazoa - ps8
Opisthokonta - ps9
Holozoa - ps10
Filozoa - ps11
Choano./Metazoa - ps12
Metazoa - ps13
Myriazoa - ps14
Parahoxozoa - ps15
Bilateria - ps16
Deuterostomia - ps17
Chordata - ps18
Olfactores - ps19
Vertebrata - ps20
Gnathostomata - ps21
Euteleostomi - ps22
Sarcopterygii - ps23
Tetrapoda - ps24
Amniota - ps25
Mammalia - ps26
Theria - ps27
Placentalia - ps28
Boreoeutheria - ps29
Euarchontoglires - ps30
Euarchonta - ps31
Primates - ps32
Haplorrhini - ps33
Simiiformes - ps34
Catarrhini - ps35
Hominoidea - ps36
Hominidae - ps37
Homininae - ps38
Hominini - ps39
Homo_sapiens - ps40
p-value
1e-22
2e-17
3e-12
4e-07
6e-02
Log-odds
0.5
1.0
1.5
2.0
2.5
3.0
3.5
4.0

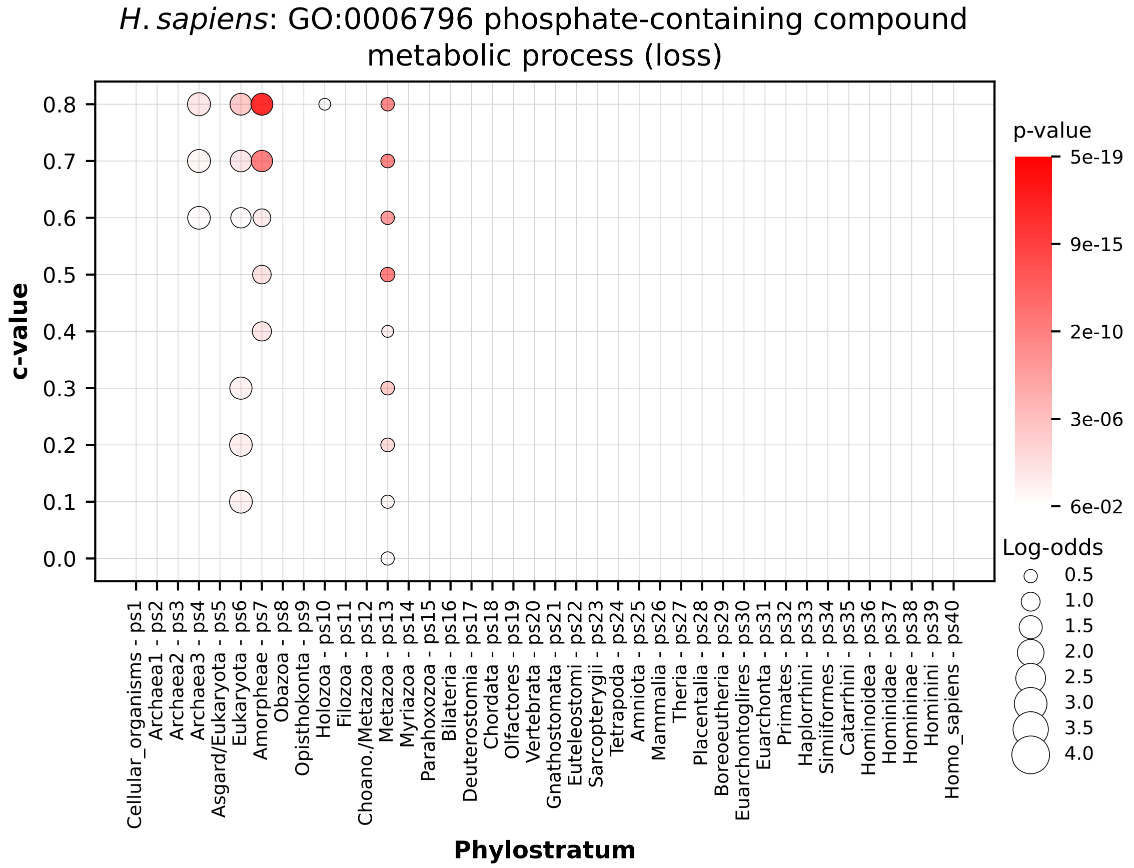
H. sapiens: GO:0006796 phosphate-containing compound metabolic process (loss)
c-value
0.0
0.1
0.2
0.3
0.4
0.5
0.6
0.7
0.8
p-value
5e-19
9e-15
2e-10
3e-06
6e-02
Log-odds
0.5
1.0
1.5
2.0
2.5
3.0
3.5
4.0
Phylostratum
Cellular_organisms - ps1
Archaea1 - ps2
Archaea2 - ps3
Archaea3 - ps4
Asgard/Eukaryota - ps5
Eukaryota - ps6
Amorpheae - ps7
Obazoa - ps8
Opisthokonta - ps9
Holozoa - ps10
Filozoa - ps11
Choano./Metazoa - ps12
Metazoa - ps13
Myriazoa - ps14
Parahoxozoa - ps15
Bilateria - ps16
Deuterostomia - ps17
Chordata - ps18
Olfactores - ps19
Vertebrata - ps20
Gnathostomata - ps21
Euteleostomi - ps22
Sarcopterygii - ps23
Tetrapoda - ps24
Amniota - ps25
Mammalia - ps26
Theria - ps27
Placentalia - ps28
Boreoeutheria - ps29
Euarchontoglires - ps30
Euarchonta - ps31
Primates - ps32
Haplorrhini - ps33
Simiiformes - ps34
Catarrhini - ps35
Hominoidea - ps36
Hominidae - ps37
Homininae - ps38
Hominini - ps39
Homo_sapiens - ps40

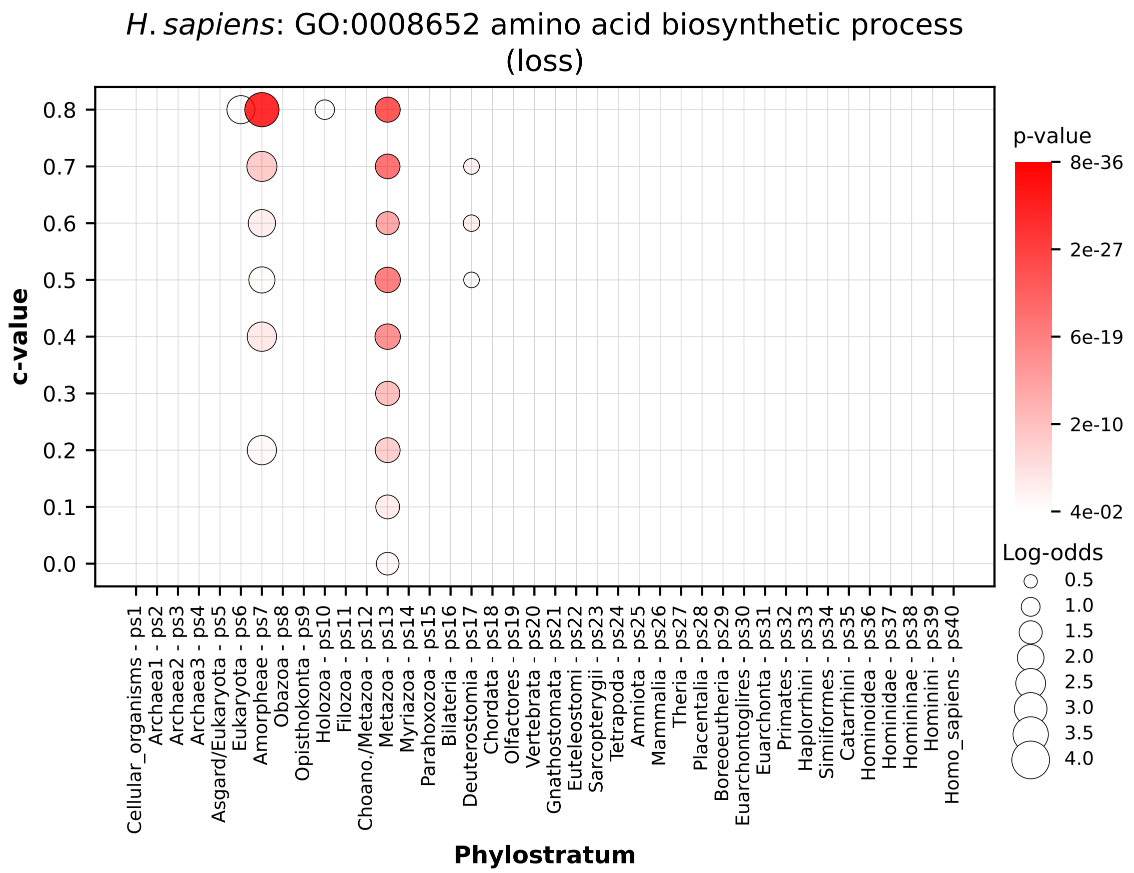

H. sapiens: GO:0008652 amino acid biosynthetic process (loss)
c-value
0.8
0.7
0.6
0.5
0.4
0.3
0.2
0.1
0.0
Phylostratum
Cellular_organisms - ps1
Archaea1 - ps2
Archaea2 - ps3
Archaea3 - ps4
Asgard/Eukaryota - ps5
Eukaryota - ps6
Amorpheae - ps7
Obazoa - ps8
Opisthokonta - ps9
Holozoa - ps10
Filozoa - ps11
Choano./Metazoa - ps12
Metazoa - ps13
Myriazoa - ps14
Parahoxozoa - ps15
Bilateria - ps16
Deuterostomia - ps17
Chordata - ps18
Olfactores - ps19
Vertebrata - ps20
Gnathostomata - ps21
Euteleostomi - ps22
Sarcopterygii - ps23
Tetrapoda - ps24
Amniota - ps25
Mammalia - ps26
Theria - ps27
Placentalia - ps28
Boreoeutheria - ps29
Euarchontoglires - ps30
Euarchonta - ps31
Primates - ps32
Haplorrhini - ps33
Simiiformes - ps34
Catarrhini - ps35
Hominoidea - ps36
Hominidae - ps37
Homininae - ps38
Hominini - ps39
Homo_sapiens - ps40
p-value
8e-36
2e-27
6e-19
2e-10
4e-02
Log-odds
0.5
1.0
1.5
2.0
2.5
3.0
3.5
4.0

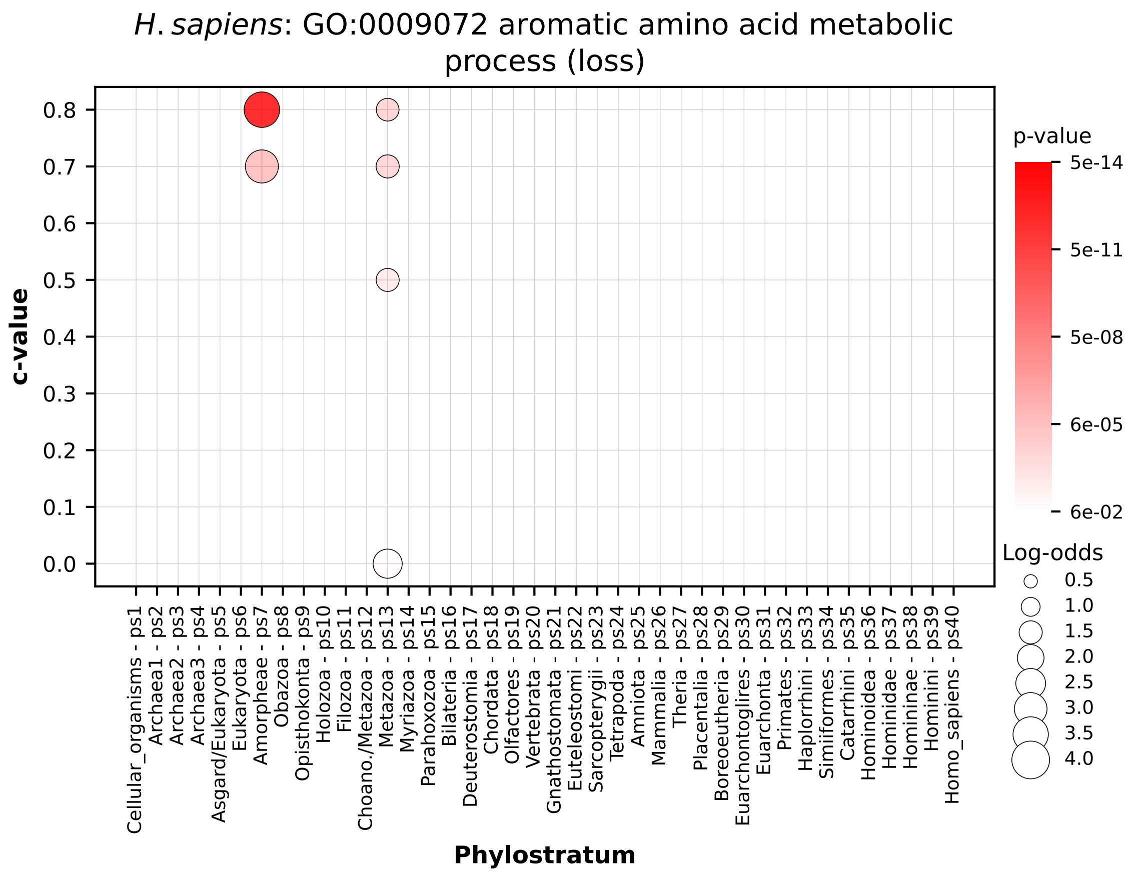

H. sapiens: GO:0009072 aromatic amino acid metabolic process (loss)
c-value
0.0
0.1
0.2
0.3
0.4
0.5
0.6
0.7
0.8
Phylostratum
Cellular_organisms - ps1
Archaea1 - ps2
Archaea2 - ps3
Archaea3 - ps4
Asgard/Eukaryota - ps5
Eukaryota - ps6
Amorpheae - ps7
Obazoa - ps8
Opisthokonta - ps9
Holozoa - ps10
Filozoa - ps11
Choano./Metazoa - ps12
Metazoa - ps13
Myriazoa - ps14
Parahoxozoa - ps15
Bilateria - ps16
Deuterostomia - ps17
Chordata - ps18
Olfactores - ps19
Vertebrata - ps20
Gnathostomata - ps21
Euteleostomi - ps22
Sarcopterygii - ps23
Tetrapoda - ps24
Amniota - ps25
Mammalia - ps26
Theria - ps27
Placentalia - ps28
Boreoeutheria - ps29
Euarchontoglires - ps30
Euarchonta - ps31
Primates - ps32
Haplorrhini - ps33
Simiiformes - ps34
Catarrhini - ps35
Hominoidea - ps36
Hominidae - ps37
Homininae - ps38
Hominini - ps39
Homo_sapiens - ps40
p-value
5e-14
5e-11
5e-08
6e-05
6e-02
Log-odds
0.5
1.0
1.5
2.0
2.5
3.0
3.5
4.0

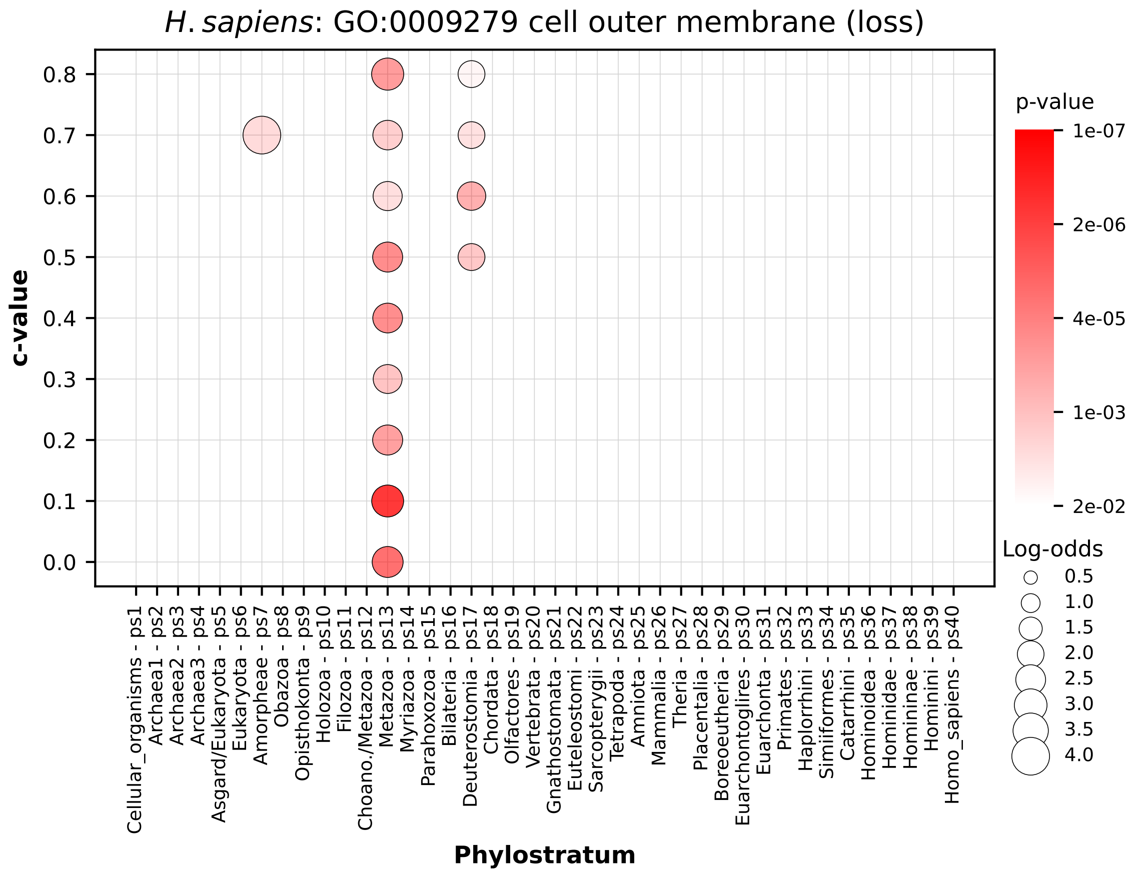

H. sapiens: GO:0009279 cell outer membrane (loss)
c-value
0.8
0.7
0.6
0.5
0.4
0.3
0.2
0.1
0.0
Phylostratum
Cellular_organisms - ps1
Archaea1 - ps2
Archaea2 - ps3
Archaea3 - ps4
Asgard/Eukaryota - ps5
Eukaryota - ps6
Amorpheae - ps7
Obazoa - ps8
Opisthokonta - ps9
Holozoa - ps10
Filozoa - ps11
Choano./Metazoa - ps12
Metazoa - ps13
Myriazoa - ps14
Parahoxozoa - ps15
Bilateria - ps16
Deuterostomia - ps17
Chordata - ps18
Olfactores - ps19
Vertebrata - ps20
Gnathostomata - ps21
Euteleostomi - ps22
Sarcopterygii - ps23
Tetrapoda - ps24
Amniota - ps25
Mammalia - ps26
Theria - ps27
Placentalia - ps28
Boreoeutheria - ps29
Euarchontoglires - ps30
Euarchonta - ps31
Primates - ps32
Haplorrhini - ps33
Simiiformes - ps34
Catarrhini - ps35
Hominoidea - ps36
Hominidae - ps37
Homininae - ps38
Hominini - ps39
Homo_sapiens - ps40
p-value
1e-07
2e-06
4e-05
1e-03
2e-02
Log-odds
0.5
1.0
1.5
2.0
2.5
3.0
3.5
4.0

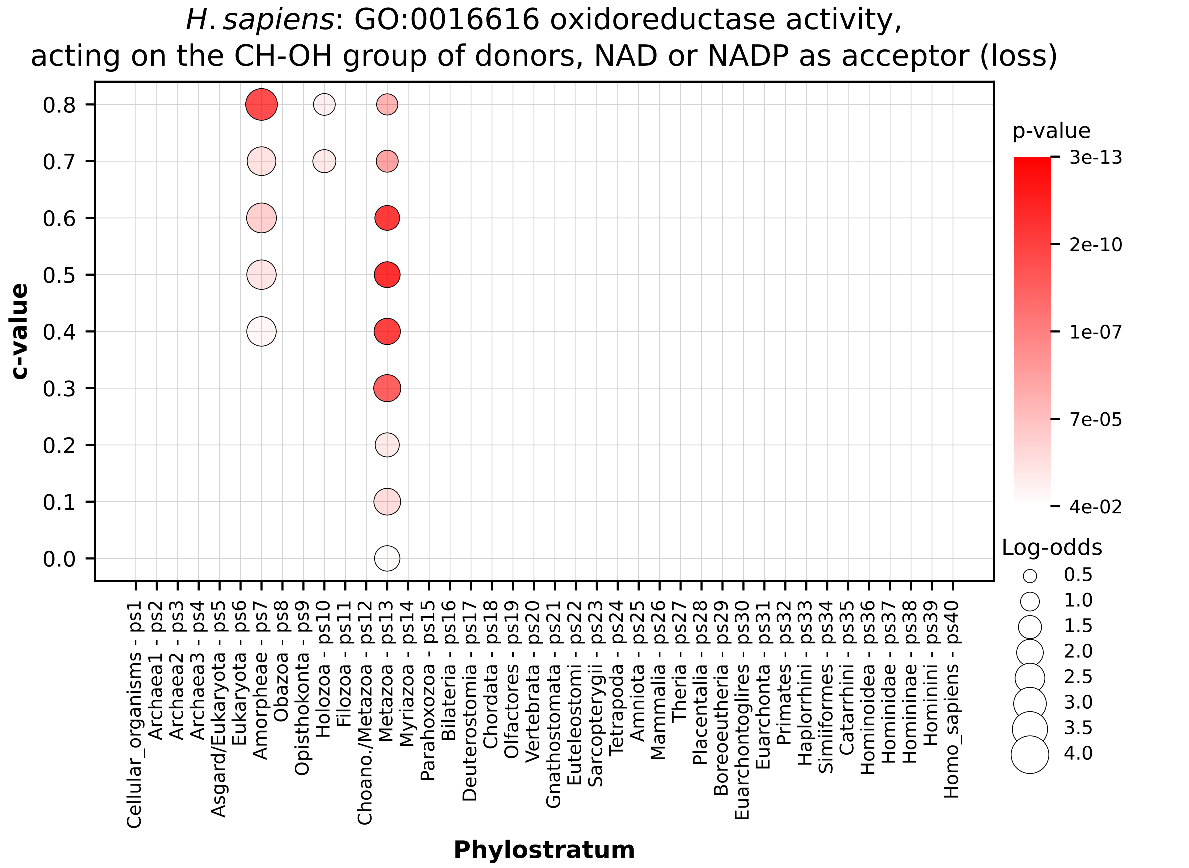

H. sapiens: GO:0016616 oxidoreductase activity,
acting on the CH-OH group of donors, NAD or NADP as acceptor (loss)
c-value
0.0
0.1
0.2
0.3
0.4
0.5
0.6
0.7
0.8
Phylostratum
Cellular_organisms - ps1
Archaea1 - ps2
Archaea2 - ps3
Archaea3 - ps4
Asgard/Eukaryota - ps5
Eukaryota - ps6
Amorpheae - ps7
Obazoa - ps8
Opisthokonta - ps9
Holozoa - ps10
Filozoa - ps11
Choano./Metazoa - ps12
Metazoa - ps13
Myriazoa - ps14
Parahoxozoa - ps15
Bilateria - ps16
Deuterostomia - ps17
Chordata - ps18
Olfactores - ps19
Vertebrata - ps20
Gnathostomata - ps21
Euteleostomi - ps22
Sarcopterygii - ps23
Tetrapoda - ps24
Amniota - ps25
Mammalia - ps26
Theria - ps27
Placentalia - ps28
Boreoeutheria - ps29
Euarchontoglires - ps30
Euarchonta - ps31
Primates - ps32
Haplorrhini - ps33
Simiiformes - ps34
Catarrhini - ps35
Hominoidea - ps36
Hominidae - ps37
Homininae - ps38
Hominini - ps39
Homo_sapiens - ps40
p-value
3e-13
2e-10
1e-07
7e-05
4e-02
Log-odds
0.5
1.0
1.5
2.0
2.5
3.0
3.5
4.0

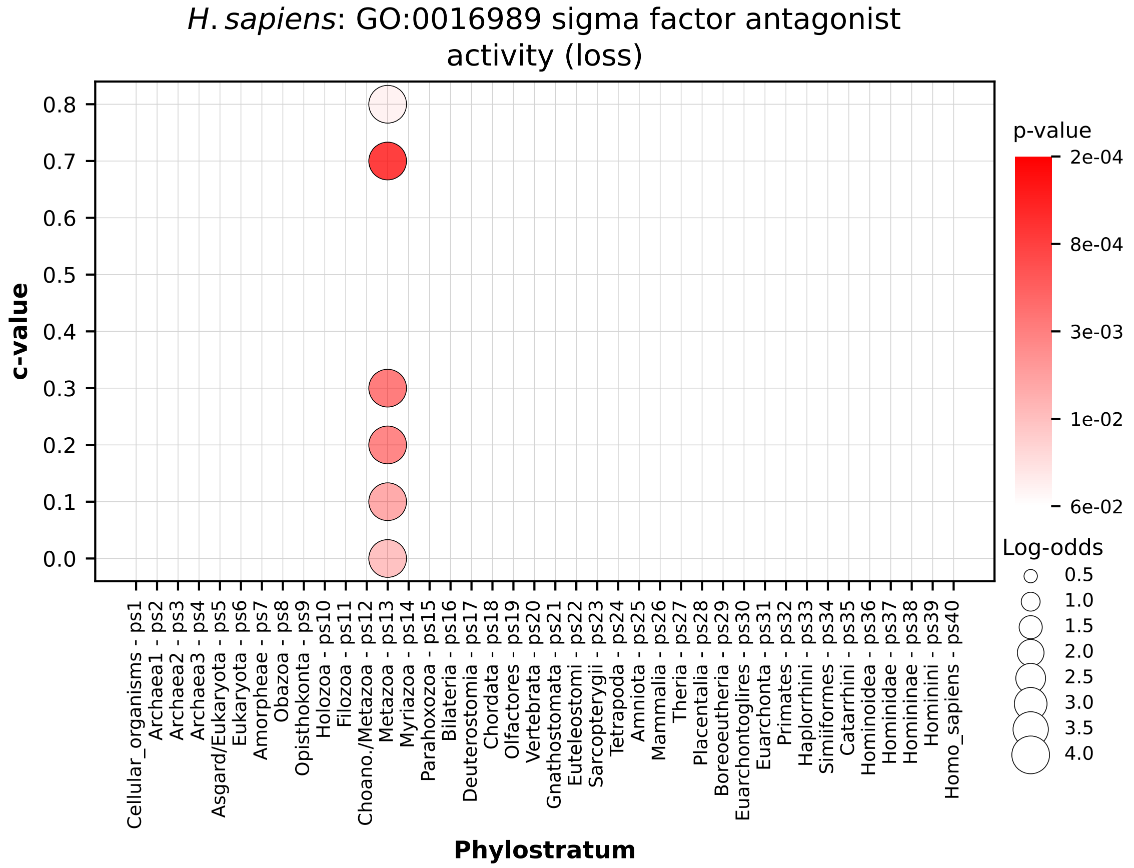

H. sapiens: GO:0016989 sigma factor antagonist activity (loss)
c-value
0.8
0.7
0.6
0.5
0.4
0.3
0.2
0.1
0.0
p-value
2e-04
8e-04
3e-03
1e-02
6e-02
Log-odds
0.5
1.0
1.5
2.0
2.5
3.0
3.5
4.0
Cellular_organisms - ps1
Archaea1 - ps2
Archaea2 - ps3
Archaea3 - ps4
Asgard/Eukaryota - ps5
Eukaryota - ps6
Amorpheae - ps7
Obazoa - ps8
Opisthokonta - ps9
Holozoa - ps10
Filozoa - ps11
Choano./Metazoa - ps12
Metazoa - ps13
Myriazoa - ps14
Parahoxozoa - ps15
Bilateria - ps16
Deuterostomia - ps17
Chordata - ps18
Olfactores - ps19
Vertebrata - ps20
Gnathostomata - ps21
Euteleostomi - ps22
Sarcopterygii - ps23
Tetrapoda - ps24
Amniota - ps25
Mammalia - ps26
Theria - ps27
Placentalia - ps28
Boreoeutheria - ps29
Euarchontoglires - ps30
Euarchonta - ps31
Primates - ps32
Haplorrhini - ps33
Simiiformes - ps34
Catarrhini - ps35
Hominoidea - ps36
Hominidae - ps37
Homininae - ps38
Hominini - ps39
Homo_sapiens - ps40
Phylostratum

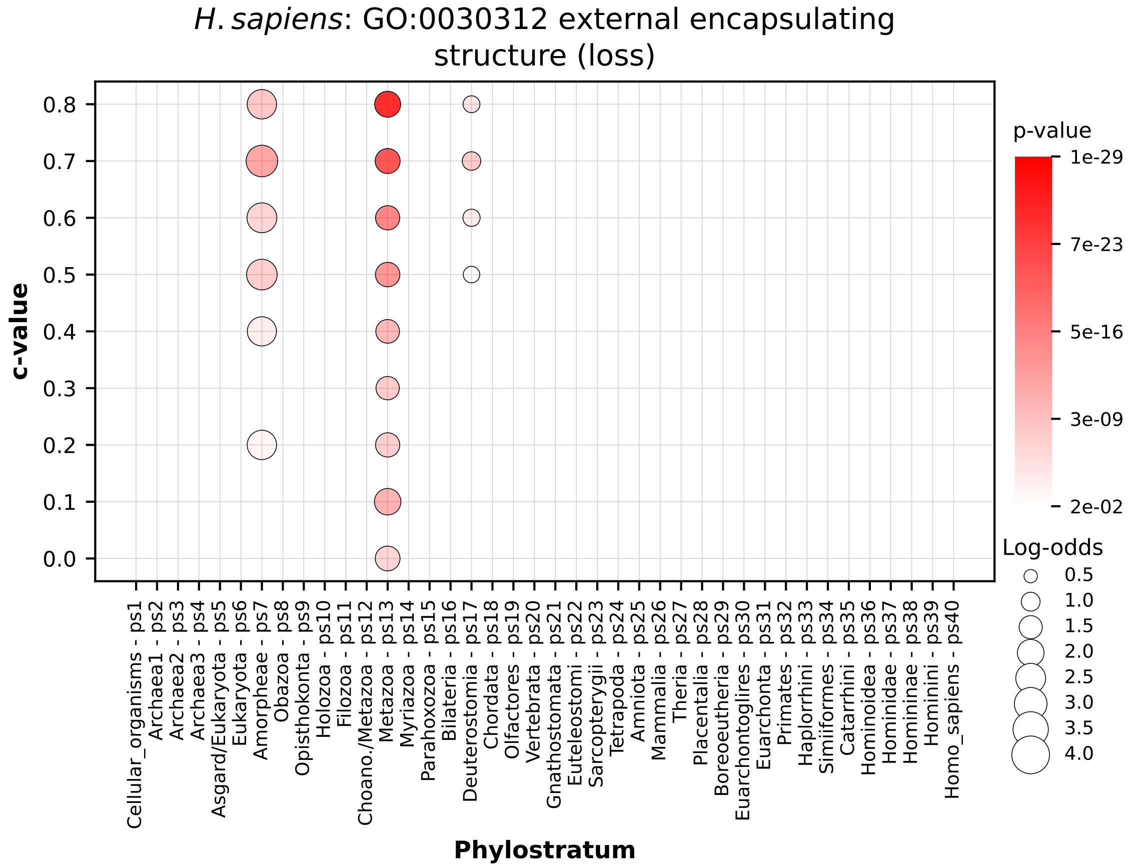

H. sapiens: GO:0030312 external encapsulating structure (loss)
c-value
Phylostratum
p-value
1e-29
7e-23
5e-16
3e-09
2e-02
Log-odds
0.5
1.0
1.5
2.0
2.5
3.0
3.5
4.0
Cellular_organisms - ps1
Archaea1 - ps2
Archaea2 - ps3
Archaea3 - ps4
Asgard/Eukaryota - ps5
Eukaryota - ps6
Amorpheae - ps7
Obazoa - ps8
Opisthokonta - ps9
Holozoa - ps10
Filozoa - ps11
Choano./Metazoa - ps12
Metazoa - ps13
Myriazoa - ps14
Parahoxozoa - ps15
Bilateria - ps16
Deuterostomia - ps17
Chordata - ps18
Olfactores - ps19
Vertebrata - ps20
Gnathostomata - ps21
Euteleostomi - ps22
Sarcopterygii - ps23
Tetrapoda - ps24
Amniota - ps25
Mammalia - ps26
Theria - ps27
Placentalia - ps28
Boreoeutheria - ps29
Euarchontoglires - ps30
Euarchonta - ps31
Primates - ps32
Haplorrhini - ps33
Simiiformes - ps34
Catarrhini - ps35
Hominoidea - ps36
Hominidae - ps37
Homininae - ps38
Hominini - ps39
Homo_sapiens - ps40

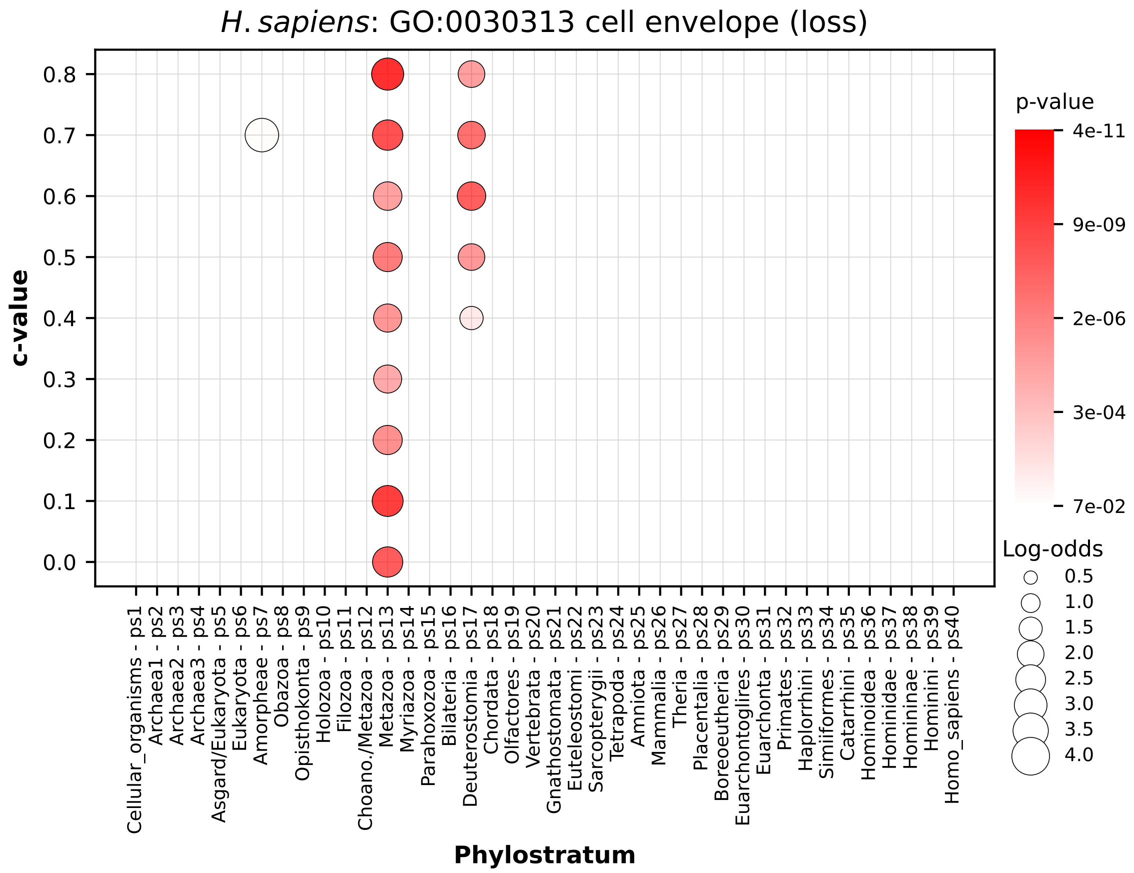

H. sapiens: GO:0030313 cell envelope (loss)
c-value
0.0
0.1
0.2
0.3
0.4
0.5
0.6
0.7
0.8
Phylostratum
Cellular_organisms - ps1
Archaea1 - ps2
Archaea2 - ps3
Archaea3 - ps4
Asgard/Eukaryota - ps5
Eukaryota - ps6
Amorpheae - ps7
Obazoa - ps8
Opisthokonta - ps9
Holozoa - ps10
Filozoa - ps11
Choano./Metazoa - ps12
Metazoa - ps13
Myriazoa - ps14
Parahoxozoa - ps15
Bilateria - ps16
Deuterostomia - ps17
Chordata - ps18
Olfactores - ps19
Vertebrata - ps20
Gnathostomata - ps21
Euteleostomi - ps22
Sarcopterygii - ps23
Tetrapoda - ps24
Amniota - ps25
Mammalia - ps26
Theria - ps27
Placentalia - ps28
Boreoeutheria - ps29
Euarchontoglires - ps30
Euarchonta - ps31
Primates - ps32
Haplorrhini - ps33
Simiiformes - ps34
Catarrhini - ps35
Hominoidea - ps36
Hominidae - ps37
Homininae - ps38
Hominini - ps39
Homo_sapiens - ps40
p-value
4e-11
9e-09
2e-06
3e-04
7e-02
Log-odds
0.5
1.0
1.5
2.0
2.5
3.0
3.5
4.0

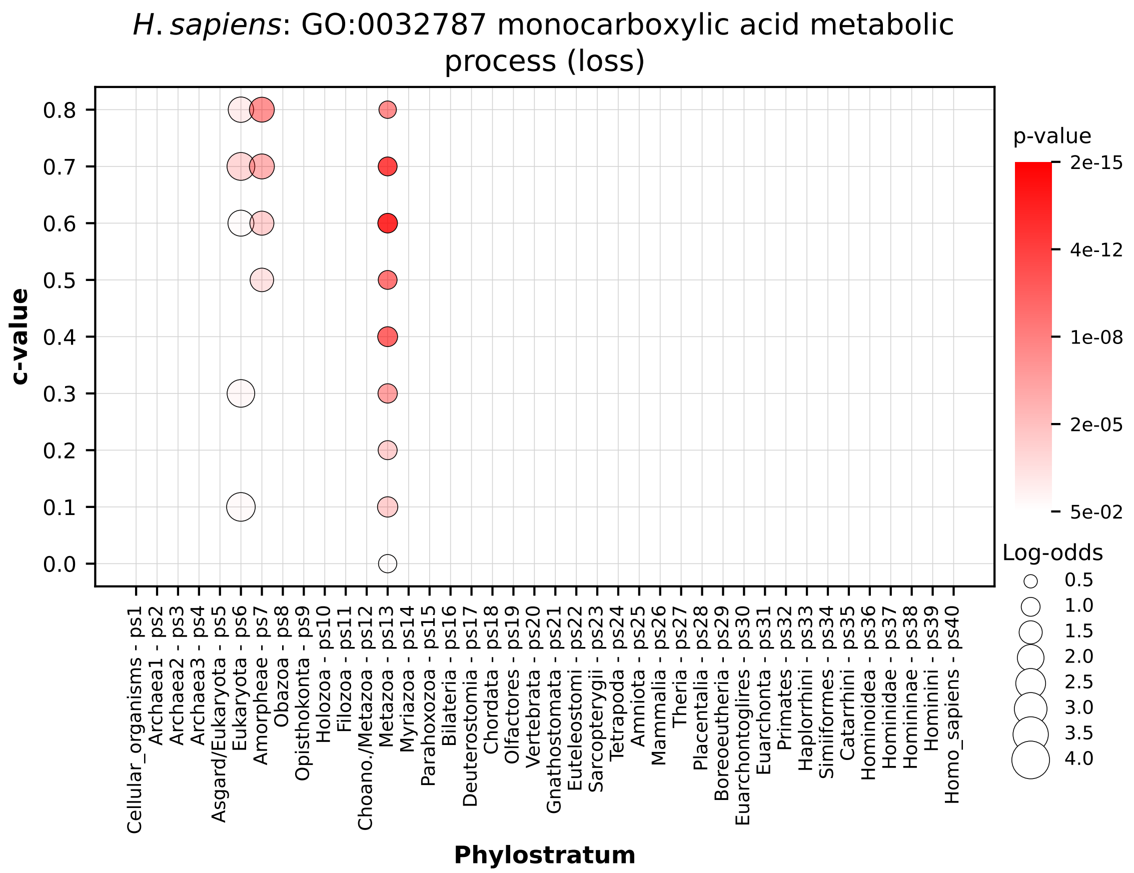
H. sapiens: GO:0032787 monocarboxylic acid metabolic process (loss)
c-value
0.8
0.7
0.6
0.5
0.4
0.3
0.2
0.1
0.0
Phylostratum
Cellular_organisms - ps1
Archaea1 - ps2
Archaea2 - ps3
Archaea3 - ps4
Asgard/Eukaryota - ps5
Eukaryota - ps6
Amorpheae - ps7
Obazoa - ps8
Opisthokonta - ps9
Holozoa - ps10
Filozoa - ps11
Choano./Metazoa - ps12
Metazoa - ps13
Myriazoa - ps14
Parahoxozoa - ps15
Bilateria - ps16
Deuterostomia - ps17
Chordata - ps18
Olfactores - ps19
Vertebrata - ps20
Gnathostomata - ps21
Euteleostomi - ps22
Sarcopterygii - ps23
Tetrapoda - ps24
Amniota - ps25
Mammalia - ps26
Theria - ps27
Placentalia - ps28
Boreoeutheria - ps29
Euarchontoglires - ps30
Euarchonta - ps31
Primates - ps32
Haplorrhini - ps33
Simiiformes - ps34
Catarrhini - ps35
Hominoidea - ps36
Hominidae - ps37
Homininae - ps38
Hominini - ps39
Homo_sapiens - ps40
p-value
2e-15
4e-12
1e-08
2e-05
5e-02
Log-odds
0.5
1.0
1.5
2.0
2.5
3.0
3.5
4.0

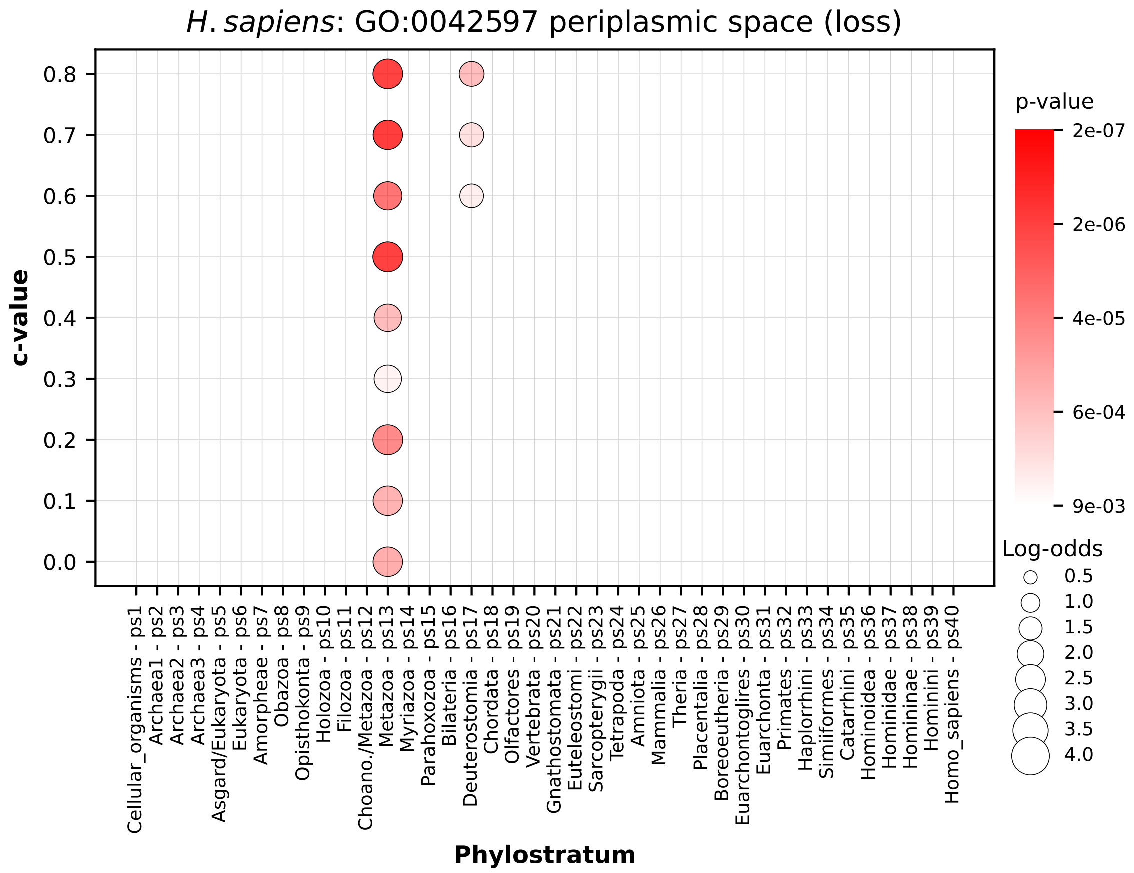

H. sapiens: GO:0042597 periplasmic space (loss)
c-value
0.0
0.1
0.2
0.3
0.4
0.5
0.6
0.7
0.8
p-value
2e-07
2e-06
4e-05
6e-04
9e-03
Log-odds
0.5
1.0
1.5
2.0
2.5
3.0
3.5
4.0
Cellular_organisms - ps1
Archaea1 - ps2
Archaea2 - ps3
Archaea3 - ps4
Asgard/Eukaryota - ps5
Eukaryota - ps6
Amorpheae - ps7
Obazoa - ps8
Opisthokonta - ps9
Holozoa - ps10
Filozoa - ps11
Choano./Metazoa - ps12
Metazoa - ps13
Myriazoa - ps14
Parahoxozoa - ps15
Bilateria - ps16
Deuterostomia - ps17
Chordata - ps18
Olfactores - ps19
Vertebrata - ps20
Gnathostomata - ps21
Euteleostomi - ps22
Sarcopterygii - ps23
Tetrapoda - ps24
Amniota - ps25
Mammalia - ps26
Theria - ps27
Placentalia - ps28
Boreoeutheria - ps29
Euarchontoglires - ps30
Euarchonta - ps31
Primates - ps32
Haplorrhini - ps33
Simiiformes - ps34
Catarrhini - ps35
Hominoidea - ps36
Hominidae - ps37
Homininae - ps38
Hominini - ps39
Homo_sapiens - ps40
Phylostratum

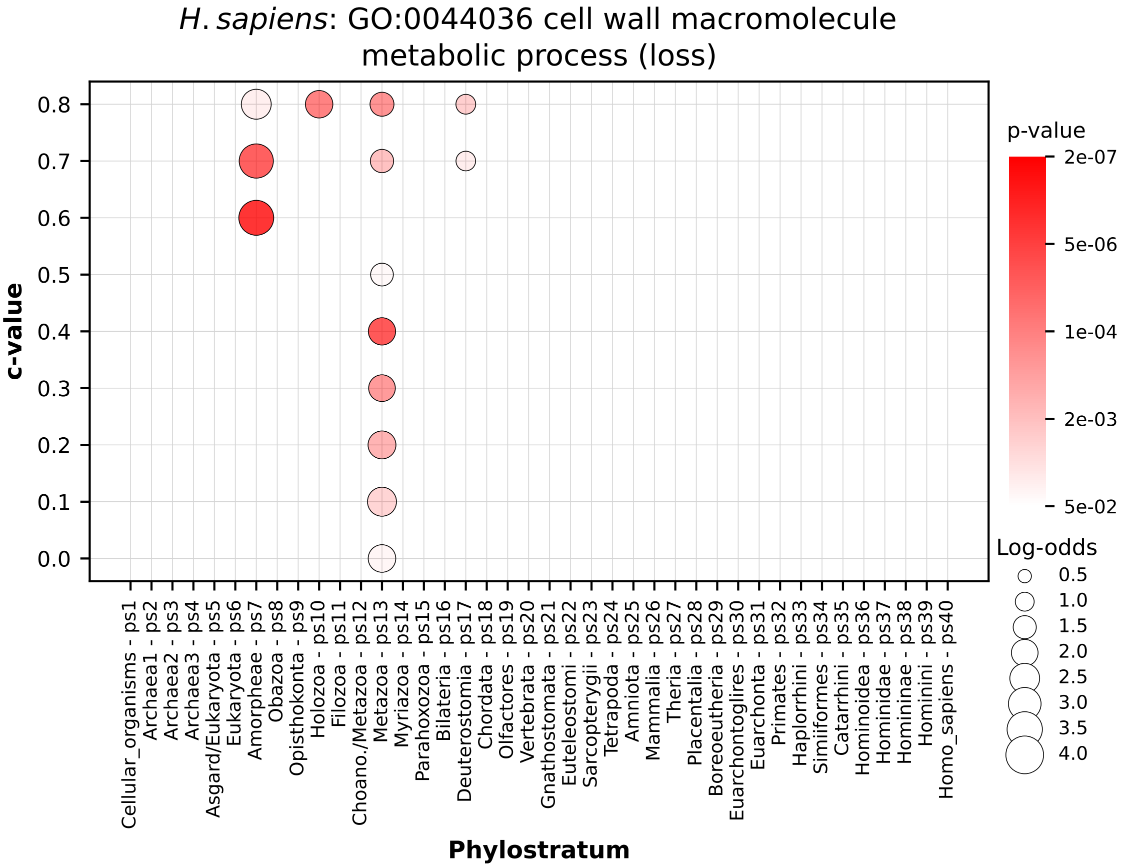

H. sapiens: GO:0044036 cell wall macromolecule metabolic process (loss)
c-value
0.8
0.7
0.6
0.5
0.4
0.3
0.2
0.1
0.0
Phylostratum
Cellular_organisms - ps1
Archaea1 - ps2
Archaea2 - ps3
Archaea3 - ps4
Asgard/Eukaryota - ps5
Eukaryota - ps6
Amorpheae - ps7
Obazoa - ps8
Opisthokonta - ps9
Holozoa - ps10
Filozoa - ps11
Choano./Metazoa - ps12
Metazoa - ps13
Myriazoa - ps14
Parahoxozoa - ps15
Bilateria - ps16
Deuterostomia - ps17
Chordata - ps18
Olfactores - ps19
Vertebrata - ps20
Gnathostomata - ps21
Euteleostomi - ps22
Sarcopterygii - ps23
Tetrapoda - ps24
Amniota - ps25
Mammalia - ps26
Theria - ps27
Placentalia - ps28
Boreoeutheria - ps29
Euarchontoglires - ps30
Euarchonta - ps31
Primates - ps32
Haplorrhini - ps33
Simiiformes - ps34
Catarrhini - ps35
Hominoidea - ps36
Hominidae - ps37
Homininae - ps38
Hominini - ps39
Homo_sapiens - ps40
p-value
2e-07
5e-06
1e-04
2e-03
5e-02
Log-odds
0.5
1.0
1.5
2.0
2.5
3.0
3.5
4.0

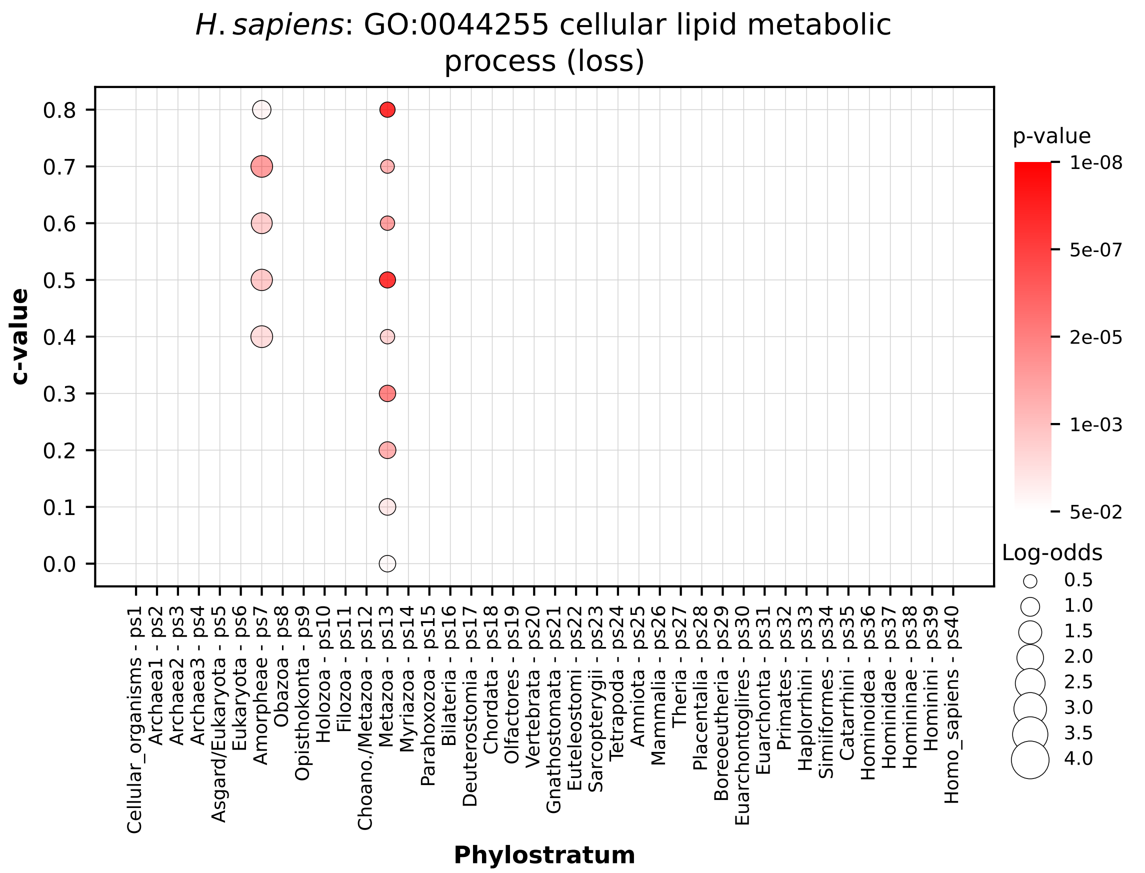
H. sapiens: GO:0044255 cellular lipid metabolic process (loss)
c-value
0.8
0.7
0.6
0.5
0.4
0.3
0.2
0.1
0.0
p-value
1e-08
5e-07
2e-05
1e-03
5e-02
Log-odds
0.5
1.0
1.5
2.0
2.5
3.0
3.5
4.0
Cellular_organisms - ps1
Archaea1 - ps2
Archaea2 - ps3
Archaea3 - ps4
Asgard/Eukaryota - ps5
Eukaryota - ps6
Amorpheae - ps7
Obazoa - ps8
Opisthokonta - ps9
Holozoa - ps10
Filozoa - ps11
Choano./Metazoa - ps12
Metazoa - ps13
Myriazoa - ps14
Parahoxozoa - ps15
Bilateria - ps16
Deuterostomia - ps17
Chordata - ps18
Olfactores - ps19
Vertebrata - ps20
Gnathostomata - ps21
Euteleostomi - ps22
Sarcopterygii - ps23
Tetrapoda - ps24
Amniota - ps25
Mammalia - ps26
Theria - ps27
Placentalia - ps28
Boreoeutheria - ps29
Euarchontoglires - ps30
Euarchonta - ps31
Primates - ps32
Haplorrhini - ps33
Simiiformes - ps34
Catarrhini - ps35
Hominoidea - ps36
Hominidae - ps37
Homininae - ps38
Hominini - ps39
Homo_sapiens - ps40
Phylostratum

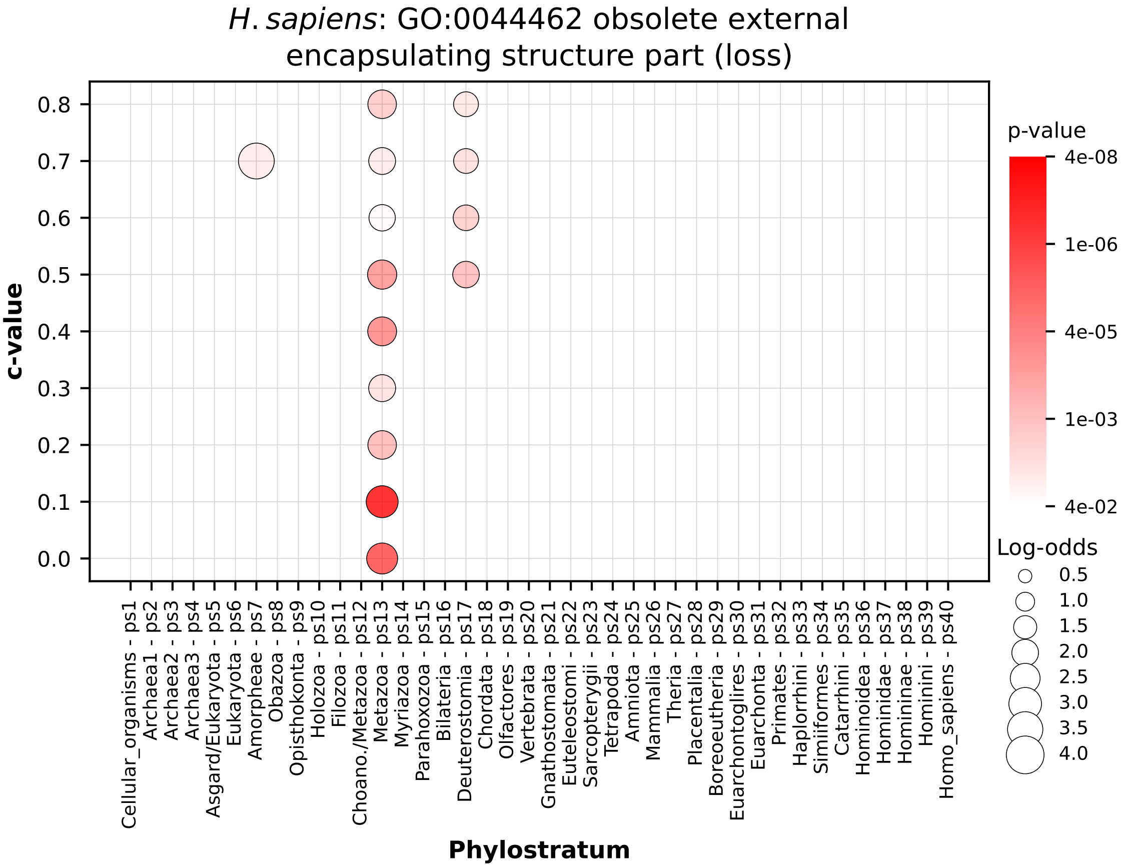

H. sapiens: GO:0044462 obsolete external encapsulating structure part (loss)
c-value
0.8
0.7
0.6
0.5
0.4
0.3
0.2
0.1
0.0
p-value
4e-08
1e-06
4e-05
1e-03
4e-02
Log-odds
0.5
1.0
1.5
2.0
2.5
3.0
3.5
4.0
Cellular_organisms - ps1
Archaea1 - ps2
Archaea2 - ps3
Archaea3 - ps4
Asgard/Eukaryota - ps5
Eukaryota - ps6
Amorpheae - ps7
Obazoa - ps8
Opisthokonta - ps9
Holozoa - ps10
Filozoa - ps11
Choano./Metazoa - ps12
Metazoa - ps13
Myriazoa - ps14
Parahoxozoa - ps15
Bilateria - ps16
Deuterostomia - ps17
Chordata - ps18
Olfactores - ps19
Vertebrata - ps20
Gnathostomata - ps21
Euteleostomi - ps22
Sarcopterygii - ps23
Tetrapoda - ps24
Amniota - ps25
Mammalia - ps26
Theria - ps27
Placentalia - ps28
Boreoeutheria - ps29
Euarchontoglires - ps30
Euarchonta - ps31
Primates - ps32
Haplorrhini - ps33
Simiiformes - ps34
Catarrhini - ps35
Hominoidea - ps36
Hominidae - ps37
Homininae - ps38
Hominini - ps39
Homo_sapiens - ps40
Phylostratum

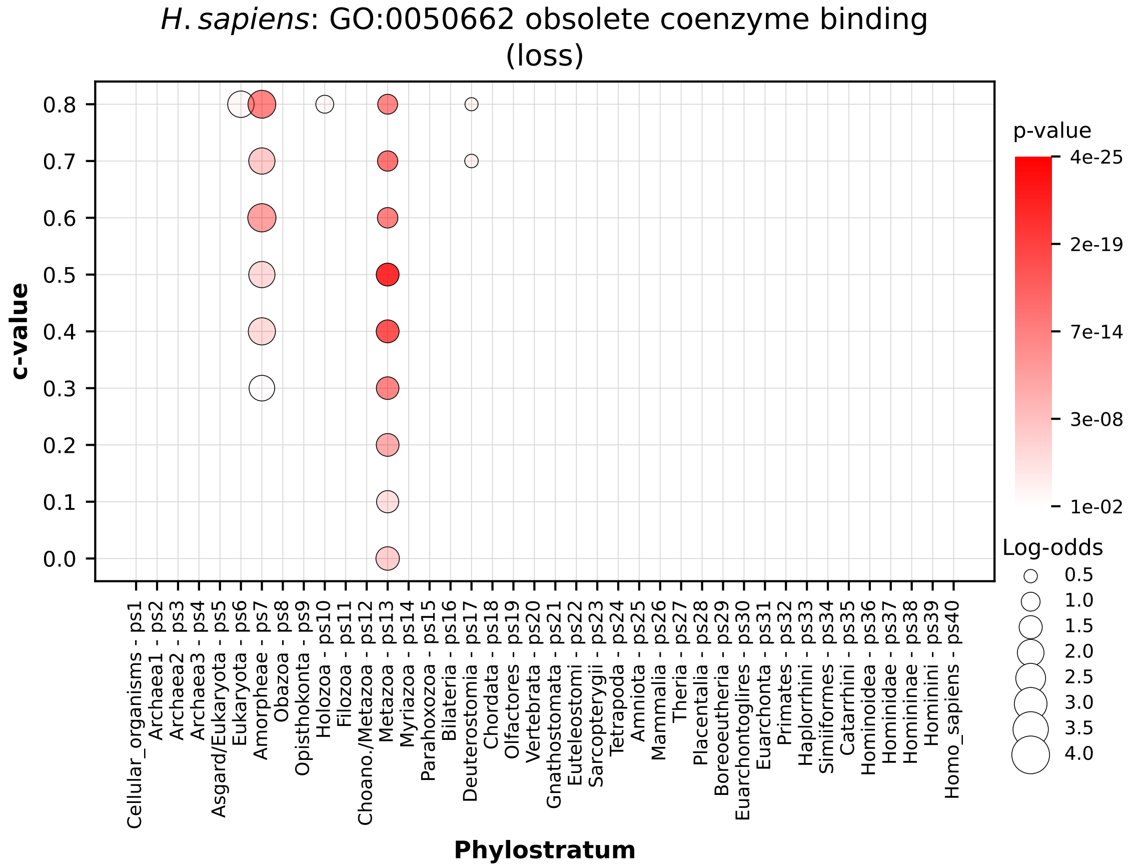

H. sapiens: GO:0050662 obsolete coenzyme binding
(loss)
c-value
0.8
0.7
0.6
0.5
0.4
0.3
0.2
0.1
0.0
p-value
4e-25
2e-19
7e-14
3e-08
1e-02
Log-odds
0.5
1.0
1.5
2.0
2.5
3.0
3.5
4.0
Cellular_organisms - ps1
Archaea1 - ps2
Archaea2 - ps3
Archaea3 - ps4
Asgard/Eukaryota - ps5
Eukaryota - ps6
Amorpheae - ps7
Obazoa - ps8
Opisthokonta - ps9
Holozoa - ps10
Filozoa - ps11
Choano./Metazoa - ps12
Metazoa - ps13
Myriazoa - ps14
Parahoxozoa - ps15
Bilateria - ps16
Deuterostomia - ps17
Chordata - ps18
Olfactores - ps19
Vertebrata - ps20
Gnathostomata - ps21
Euteleostomi - ps22
Sarcopterygii - ps23
Tetrapoda - ps24
Amniota - ps25
Mammalia - ps26
Theria - ps27
Placentalia - ps28
Boreoeutheria - ps29
Euarchontoglires - ps30
Euarchonta - ps31
Primates - ps32
Haplorrhini - ps33
Simiiformes - ps34
Catarrhini - ps35
Hominoidea - ps36
Hominidae - ps37
Homininae - ps38
Hominini - ps39
Homo_sapiens - ps40
Phylostratum

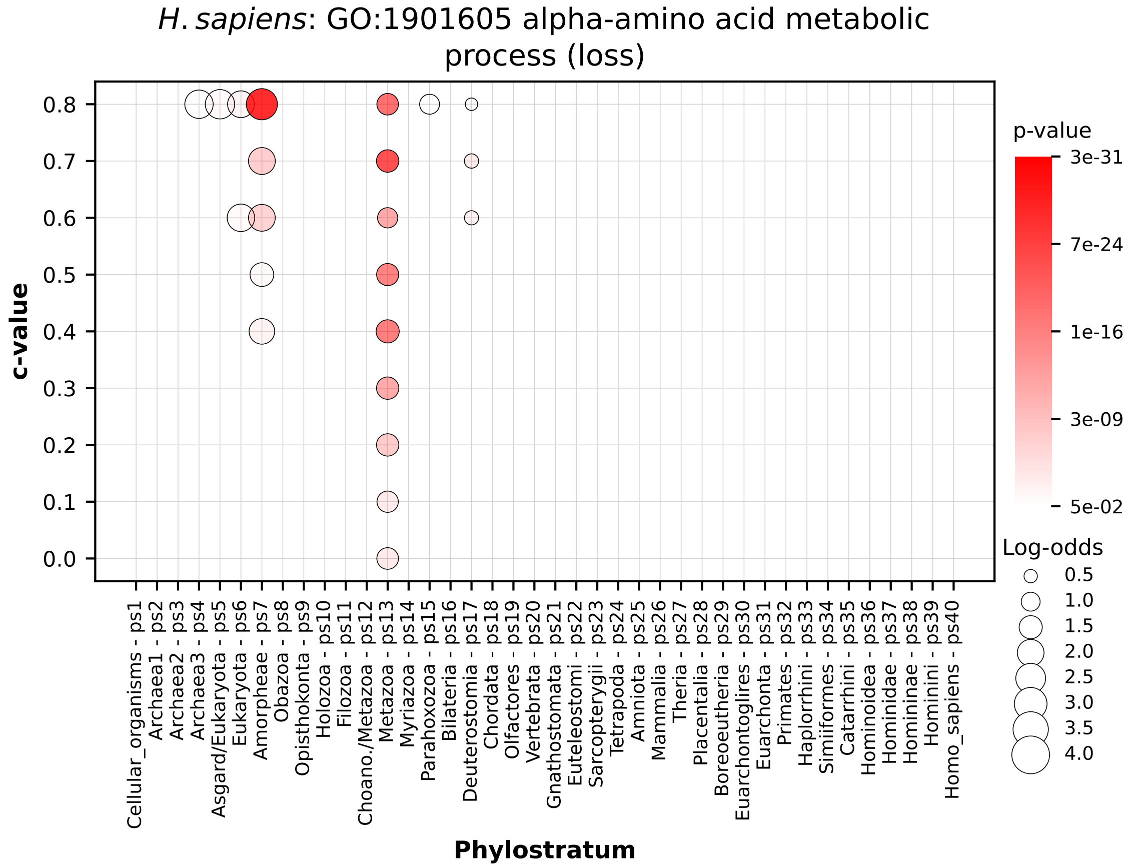

H. sapiens: GO:1901605 alpha-amino acid metabolic process (loss)
c-value
Phylostratum
p-value
3e-31
7e-24
1e-16
3e-09
5e-02
Log-odds
0.5
1.0
1.5
2.0
2.5
3.0
3.5
4.0
Cellular_organisms - ps1
Archaea1 - ps2
Archaea2 - ps3
Archaea3 - ps4
Asgard/Eukaryota - ps5
Eukaryota - ps6
Amorpheae - ps7
Obazoa - ps8
Opisthokonta - ps9
Holozoa - ps10
Filozoa - ps11
Choano./Metazoa - ps12
Metazoa - ps13
Myriazoa - ps14
Parahoxozoa - ps15
Bilateria - ps16
Deuterostomia - ps17
Chordata - ps18
Olfactores - ps19
Vertebrata - ps20
Gnathostomata - ps21
Euteleostomi - ps22
Sarcopterygii - ps23
Tetrapoda - ps24
Amniota - ps25
Mammalia - ps26
Theria - ps27
Placentalia - ps28
Boreoeutheria - ps29
Euarchontoglires - ps30
Euarchonta - ps31
Primates - ps32
Haplorrhini - ps33
Simiiformes - ps34
Catarrhini - ps35
Hominoidea - ps36
Hominidae - ps37
Homininae - ps38
Hominini - ps39
Homo_sapiens - ps40

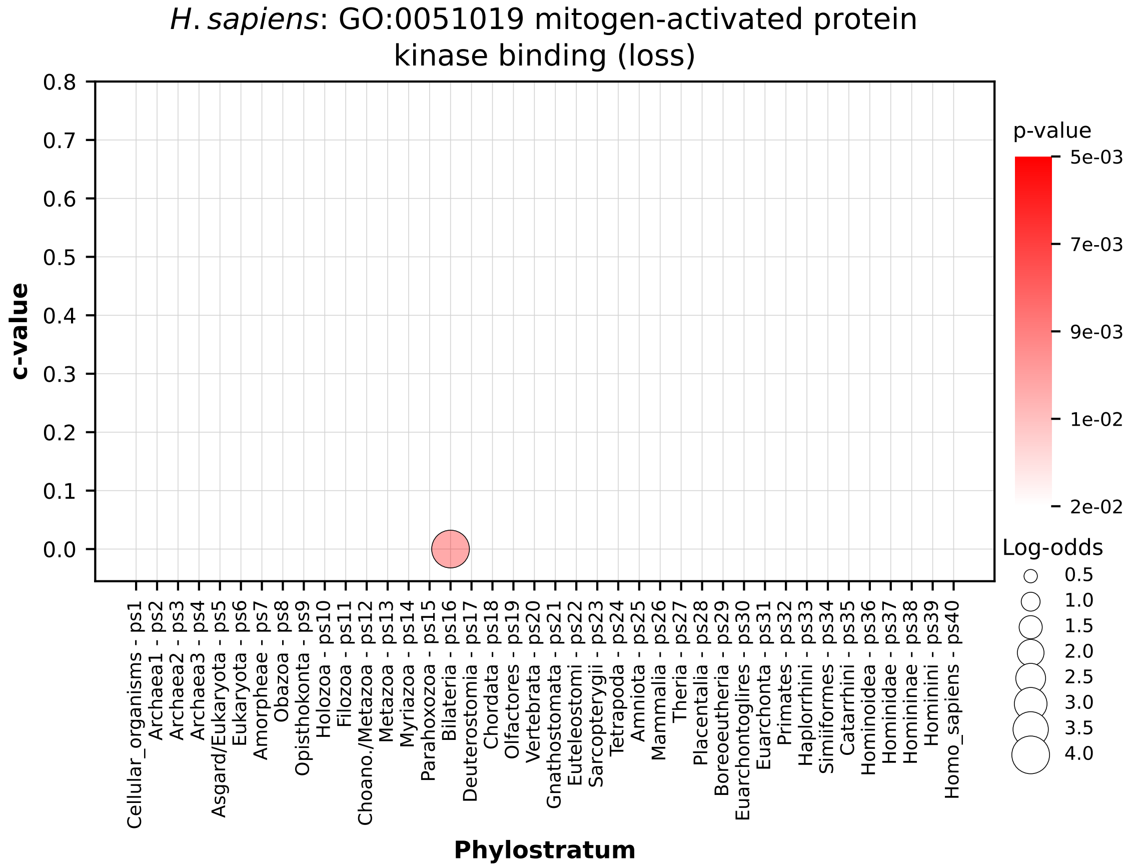

H. sapiens: GO:0051019 mitogen-activated protein kinase binding (loss)
c-value
0.8
0.7
0.6
0.5
0.4
0.3
0.2
0.1
0.0
p-value
5e-03
7e-03
9e-03
1e-02
2e-02
Log-odds
0.5
1.0
1.5
2.0
2.5
3.0
3.5
4.0
Cellular_organisms - ps1
Archaea1 - ps2
Archaea2 - ps3
Archaea3 - ps4
Asgard/Eukaryota - ps5
Eukaryota - ps6
Amorpheae - ps7
Obazoa - ps8
Opisthokonta - ps9
Holozoa - ps10
Filozoa - ps11
Choano./Metazoa - ps12
Metazoa - ps13
Myriazoa - ps14
Parahoxozoa - ps15
Bilateria - ps16
Deuterostomia - ps17
Chordata - ps18
Olfactores - ps19
Vertebrata - ps20
Gnathostomata - ps21
Euteleostomi - ps22
Sarcopterygii - ps23
Tetrapoda - ps24
Amniota - ps25
Mammalia - ps26
Theria - ps27
Placentalia - ps28
Boreoeutheria - ps29
Euarchontoglires - ps30
Euarchonta - ps31
Primates - ps32
Haplorrhini - ps33
Simiiformes - ps34
Catarrhini - ps35
Hominoidea - ps36
Hominidae - ps37
Homininae - ps38
Hominini - ps39
Homo_sapiens - ps40
Phylostratum

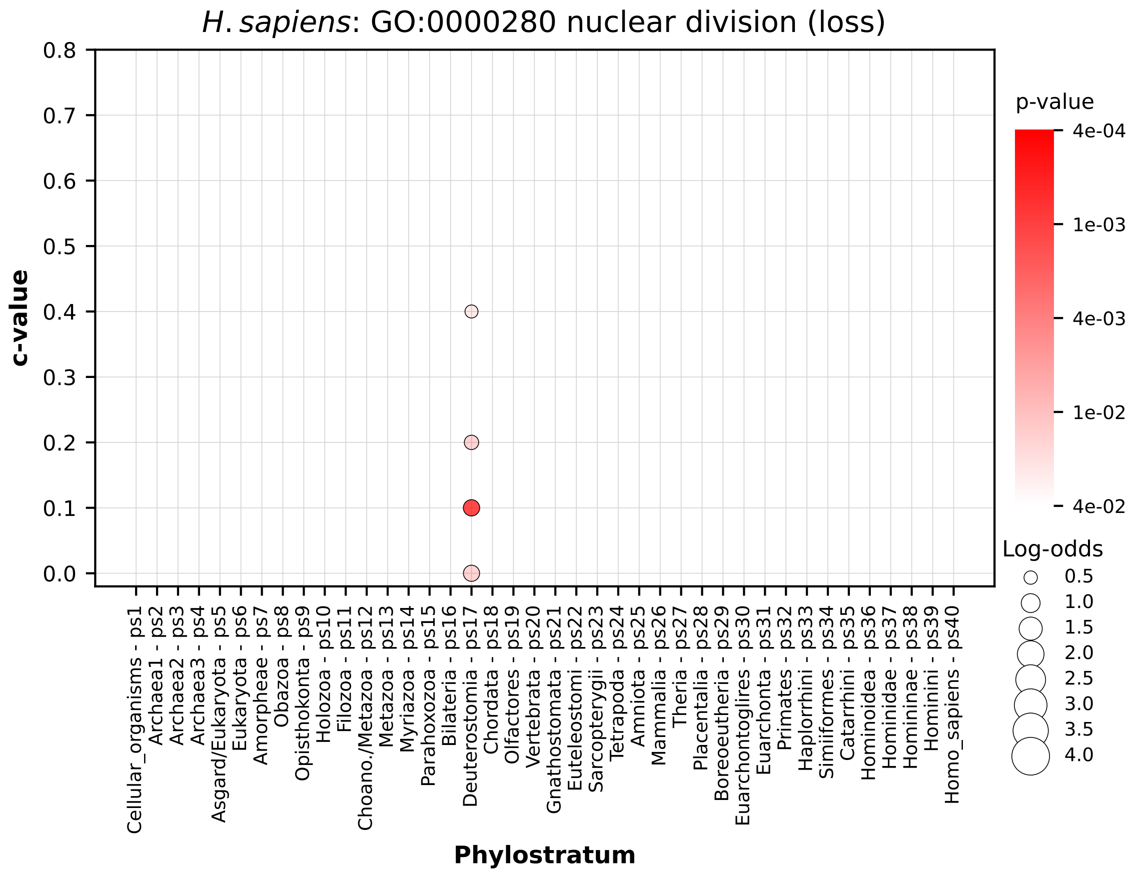

H. sapiens: GO:0000280 nuclear division (loss)
c-value
0.8
0.7
0.6
0.5
0.4
0.3
0.2
0.1
0.0
Phylostratum
Cellular_organisms - ps1
Archaea1 - ps2
Archaea2 - ps3
Archaea3 - ps4
Asgard/Eukaryota - ps5
Eukaryota - ps6
Amorpheae - ps7
Obazoa - ps8
Opisthokonta - ps9
Holozoa - ps10
Filozoa - ps11
Choano./Metazoa - ps12
Metazoa - ps13
Myriazoa - ps14
Parahoxozoa - ps15
Bilateria - ps16
Deuterostomia - ps17
Chordata - ps18
Olfactores - ps19
Vertebrata - ps20
Gnathostomata - ps21
Euteleostomi - ps22
Sarcopterygii - ps23
Tetrapoda - ps24
Amniota - ps25
Mammalia - ps26
Theria - ps27
Placentalia - ps28
Boreoeutheria - ps29
Euarchontoglires - ps30
Euarchonta - ps31
Primates - ps32
Haplorrhini - ps33
Simiiformes - ps34
Catarrhini - ps35
Hominoidea - ps36
Hominidae - ps37
Homininae - ps38
Hominini - ps39
Homo_sapiens - ps40
p-value
4e-04
1e-03
4e-03
1e-02
4e-02
Log-odds
0.5
1.0
1.5
2.0
2.5
3.0
3.5
4.0

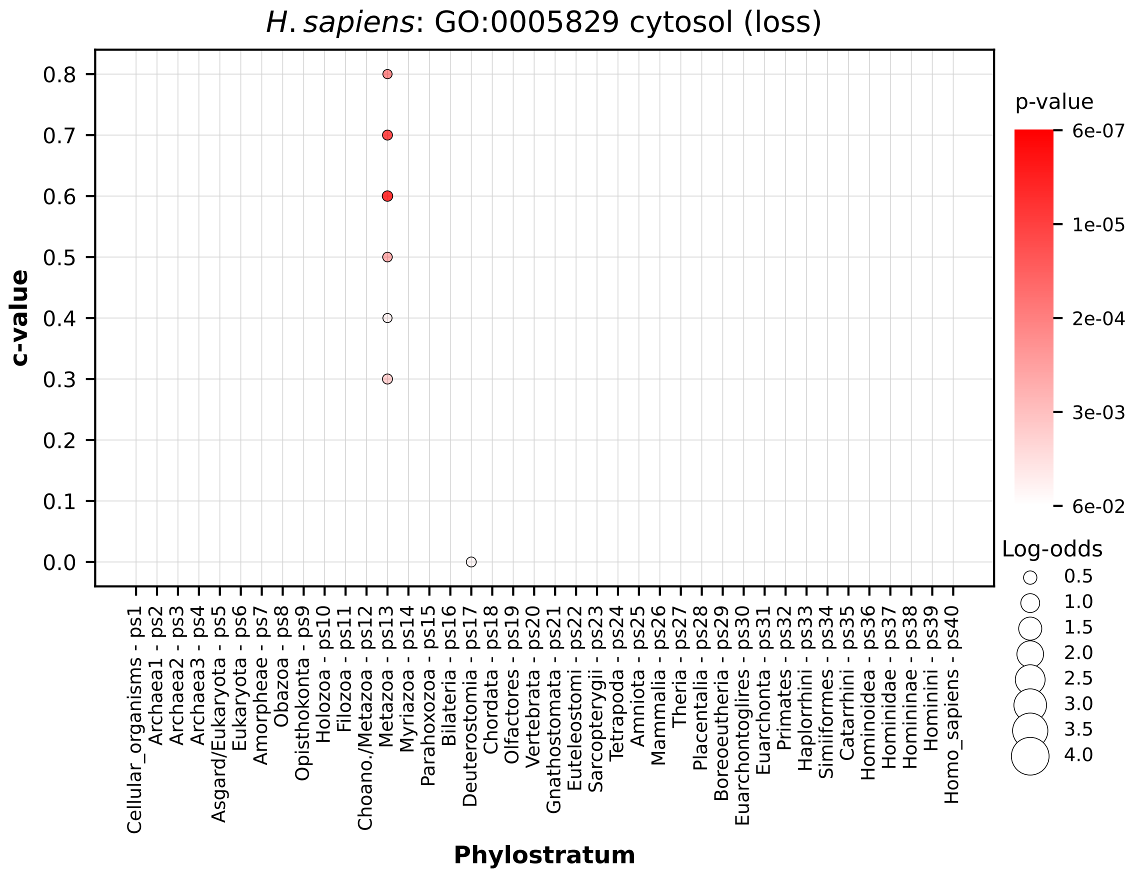

H. sapiens: GO:0005829 cytosol (loss)
c-value
Phylostratum
p-value
6e-07
1e-05
2e-04
3e-03
6e-02
Log-odds
0.5
1.0
1.5
2.0
2.5
3.0
3.5
4.0
Cellular_organisms - ps1
Archaea1 - ps2
Archaea2 - ps3
Archaea3 - ps4
Asgard/Eukaryota - ps5
Eukaryota - ps6
Amorpheae - ps7
Obazoa - ps8
Opisthokonta - ps9
Holozoa - ps10
Filozoa - ps11
Choano./Metazoa - ps12
Metazoa - ps13
Myriazoa - ps14
Parahoxozoa - ps15
Bilateria - ps16
Deuterostomia - ps17
Chordata - ps18
Olfactores - ps19
Vertebrata - ps20
Gnathostomata - ps21
Euteleostomi - ps22
Sarcopterygii - ps23
Tetrapoda - ps24
Amniota - ps25
Mammalia - ps26
Theria - ps27
Placentalia - ps28
Boreoeutheria - ps29
Euarchontoglires - ps30
Euarchonta - ps31
Primates - ps32
Haplorrhini - ps33
Simiiformes - ps34
Catarrhini - ps35
Hominoidea - ps36
Hominidae - ps37
Homininae - ps38
Hominini - ps39
Homo_sapiens - ps40

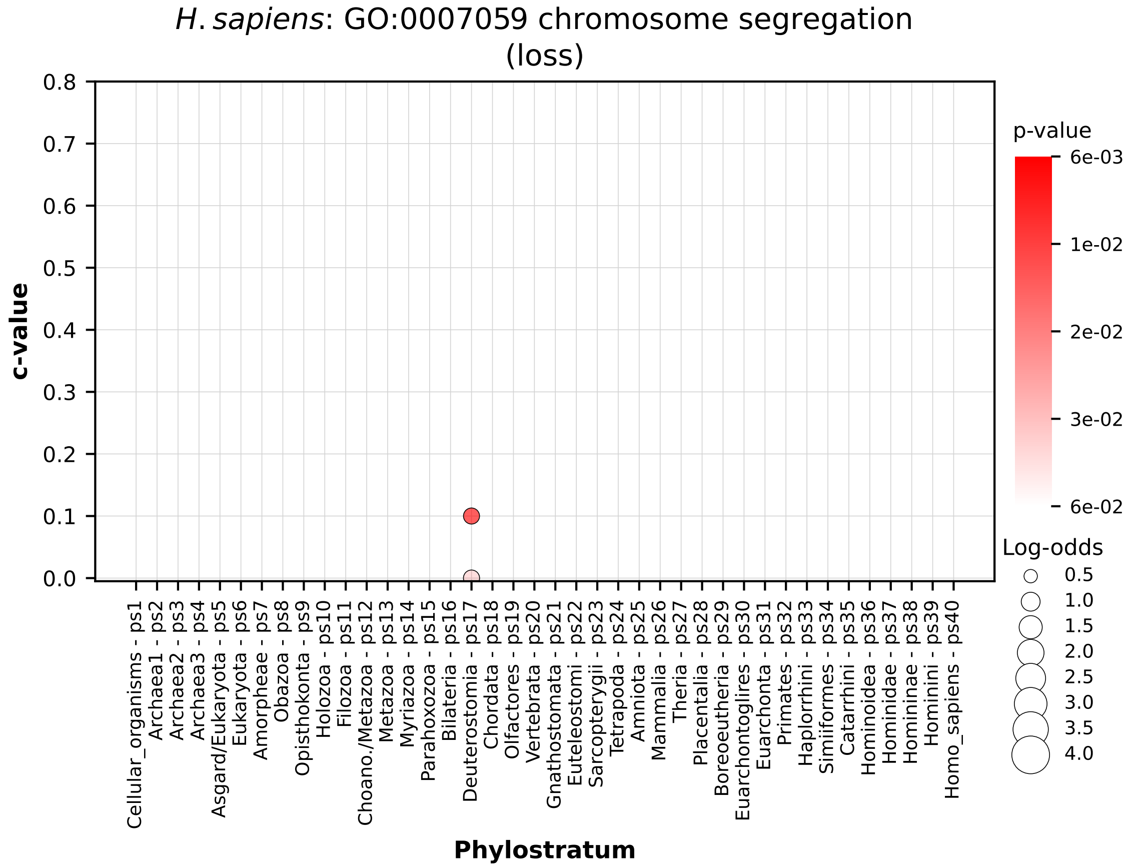

H. sapiens: GO:0007059 chromosome segregation
(loss)
c-value
0.8
0.7
0.6
0.5
0.4
0.3
0.2
0.1
0.0
p-value
6e-03
1e-02
2e-02
3e-02
6e-02
Log-odds
0.5
1.0
1.5
2.0
2.5
3.0
3.5
4.0
Cellular_organisms - ps1
Archaea1 - ps2
Archaea2 - ps3
Archaea3 - ps4
Asgard/Eukaryota - ps5
Eukaryota - ps6
Amorpheae - ps7
Obazoa - ps8
Opisthokonta - ps9
Holozoa - ps10
Filozoa - ps11
Choano./Metazoa - ps12
Metazoa - ps13
Myriazoa - ps14
Parahoxozoa - ps15
Bilateria - ps16
Deuterostomia - ps17
Chordata - ps18
Olfactores - ps19
Vertebrata - ps20
Gnathostomata - ps21
Euteleostomi - ps22
Sarcopterygii - ps23
Tetrapoda - ps24
Amniota - ps25
Mammalia - ps26
Theria - ps27
Placentalia - ps28
Boreoeutheria - ps29
Euarchontoglires - ps30
Euarchonta - ps31
Primates - ps32
Haplorrhini - ps33
Simiiformes - ps34
Catarrhini - ps35
Hominoidea - ps36
Hominidae - ps37
Homininae - ps38
Hominini - ps39
Homo_sapiens - ps40
Phylostratum

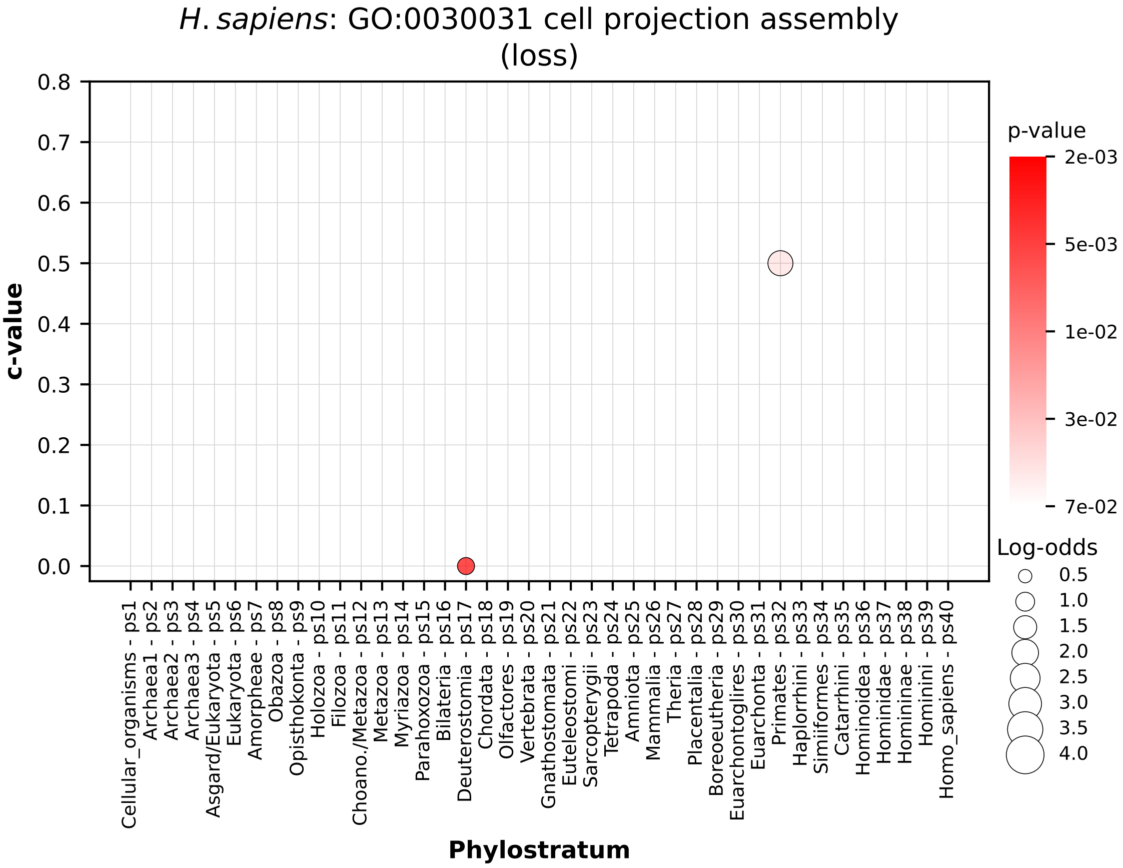

H. sapiens: GO:0030031 cell projection assembly (loss)
c-value
0.8
0.7
0.6
0.5
0.4
0.3
0.2
0.1
0.0
p-value
2e-03
5e-03
1e-02
3e-02
7e-02
Log-odds
0.5
1.0
1.5
2.0
2.5
3.0
3.5
4.0
Cellular_organisms - ps1
Archaea1 - ps2
Archaea2 - ps3
Archaea3 - ps4
Asgard/Eukaryota - ps5
Eukaryota - ps6
Amorpheae - ps7
Obazoa - ps8
Opisthokonta - ps9
Holozoa - ps10
Filozoa - ps11
Choano./Metazoa - ps12
Metazoa - ps13
Myriazoa - ps14
Parahoxozoa - ps15
Bilateria - ps16
Deuterostomia - ps17
Chordata - ps18
Olfactores - ps19
Vertebrata - ps20
Gnathostomata - ps21
Euteleostomi - ps22
Sarcopterygii - ps23
Tetrapoda - ps24
Amniota - ps25
Mammalia - ps26
Theria - ps27
Placentalia - ps28
Boreoeutheria - ps29
Euarchontoglires - ps30
Euarchonta - ps31
Primates - ps32
Haplorrhini - ps33
Simiiformes - ps34
Catarrhini - ps35
Hominoidea - ps36
Hominidae - ps37
Homininae - ps38
Hominini - ps39
Homo_sapiens - ps40
Phylostratum

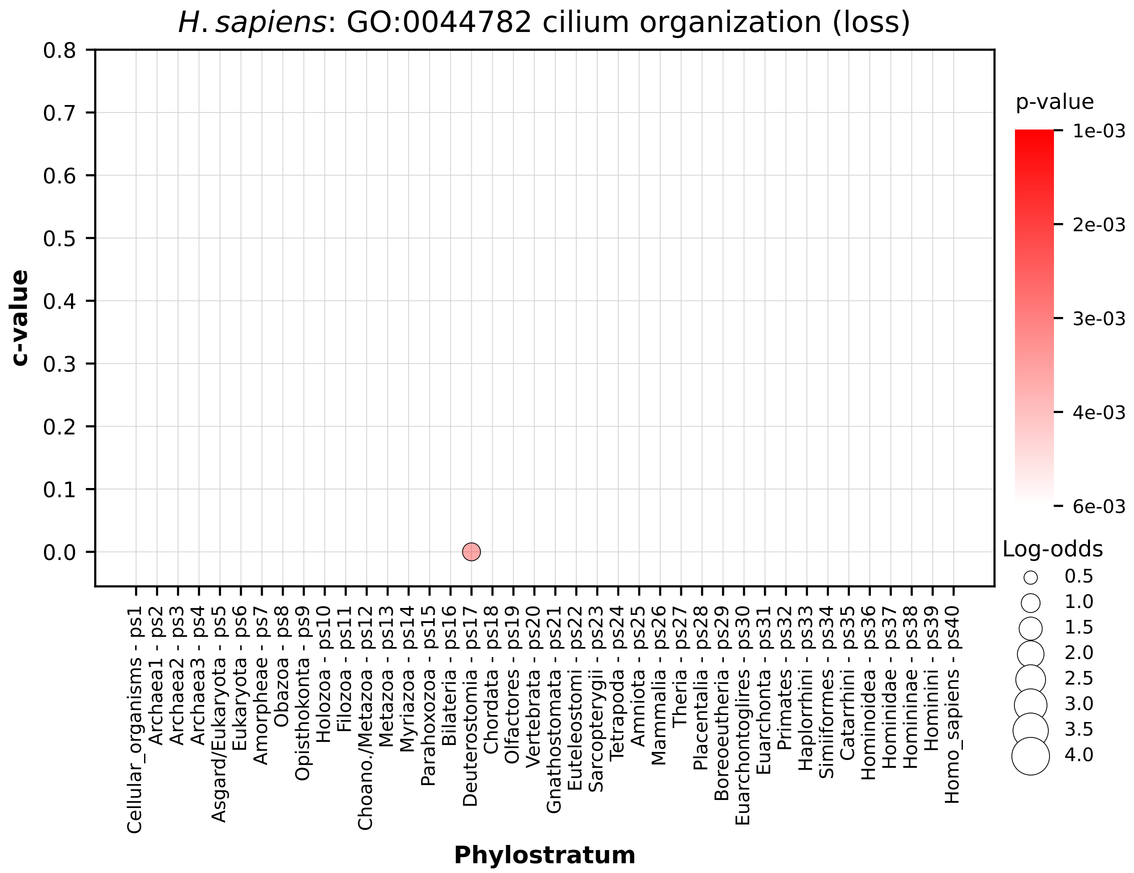

H. sapiens: GO:0044782 cilium organization (loss)
c-value
0.8
0.7
0.6
0.5
0.4
0.3
0.2
0.1
0.0
p-value
1e-03
2e-03
3e-03
4e-03
6e-03
Log-odds
0.5
1.0
1.5
2.0
2.5
3.0
3.5
4.0
Cellular_organisms - ps1
Archaea1 - ps2
Archaea2 - ps3
Archaea3 - ps4
Asgard/Eukaryota - ps5
Eukaryota - ps6
Amorpheae - ps7
Obazoa - ps8
Opisthokonta - ps9
Holozoa - ps10
Filozoa - ps11
Choano./Metazoa - ps12
Metazoa - ps13
Myriazoa - ps14
Parahoxozoa - ps15
Bilateria - ps16
Deuterostomia - ps17
Chordata - ps18
Olfactores - ps19
Vertebrata - ps20
Gnathostomata - ps21
Euteleostomi - ps22
Sarcopterygii - ps23
Tetrapoda - ps24
Amniota - ps25
Mammalia - ps26
Theria - ps27
Placentalia - ps28
Boreoeutheria - ps29
Euarchontoglires - ps30
Euarchonta - ps31
Primates - ps32
Haplorrhini - ps33
Simiiformes - ps34
Catarrhini - ps35
Hominoidea - ps36
Hominidae - ps37
Homininae - ps38
Hominini - ps39
Homo_sapiens - ps40
Phylostratum

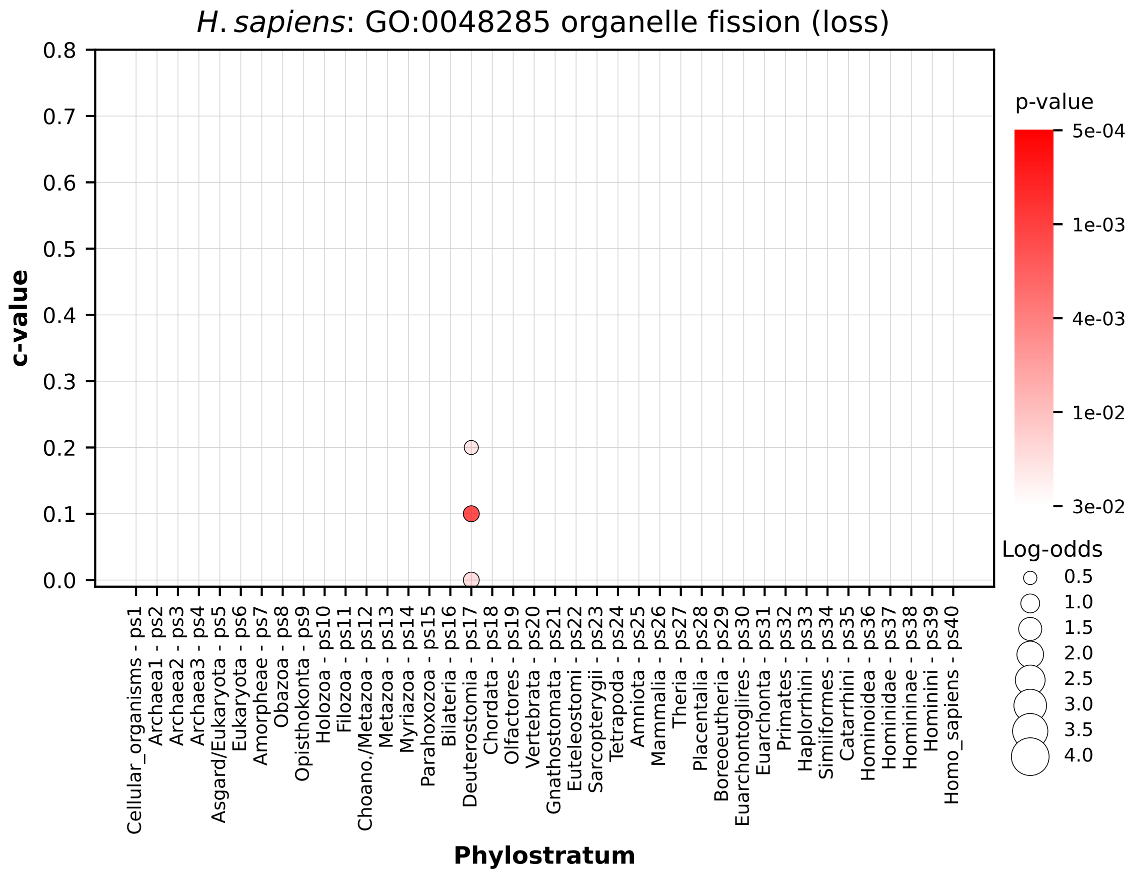
H. sapiens: GO:0048285 organelle fission (loss)
c-value
0.8
0.7
0.6
0.5
0.4
0.3
0.2
0.1
0.0
Phylostratum
Cellular_organisms - ps1
Archaea1 - ps2
Archaea2 - ps3
Archaea3 - ps4
Asgard/Eukaryota - ps5
Eukaryota - ps6
Amorpheae - ps7
Obazoa - ps8
Opisthokonta - ps9
Holozoa - ps10
Filozoa - ps11
Choano./Metazoa - ps12
Metazoa - ps13
Myriazoa - ps14
Parahoxozoa - ps15
Bilateria - ps16
Deuterostomia - ps17
Chordata - ps18
Olfactores - ps19
Vertebrata - ps20
Gnathostomata - ps21
Euteleostomi - ps22
Sarcopterygii - ps23
Tetrapoda - ps24
Amniota - ps25
Mammalia - ps26
Theria - ps27
Placentalia - ps28
Boreoeutheria - ps29
Euarchontoglires - ps30
Euarchonta - ps31
Primates - ps32
Haplorrhini - ps33
Simiiformes - ps34
Catarrhini - ps35
Hominoidea - ps36
Hominidae - ps37
Homininae - ps38
Hominini - ps39
Homo_sapiens - ps40
p-value
5e-04
1e-03
4e-03
1e-02
3e-02
Log-odds
0.5
1.0
1.5
2.0
2.5
3.0
3.5
4.0

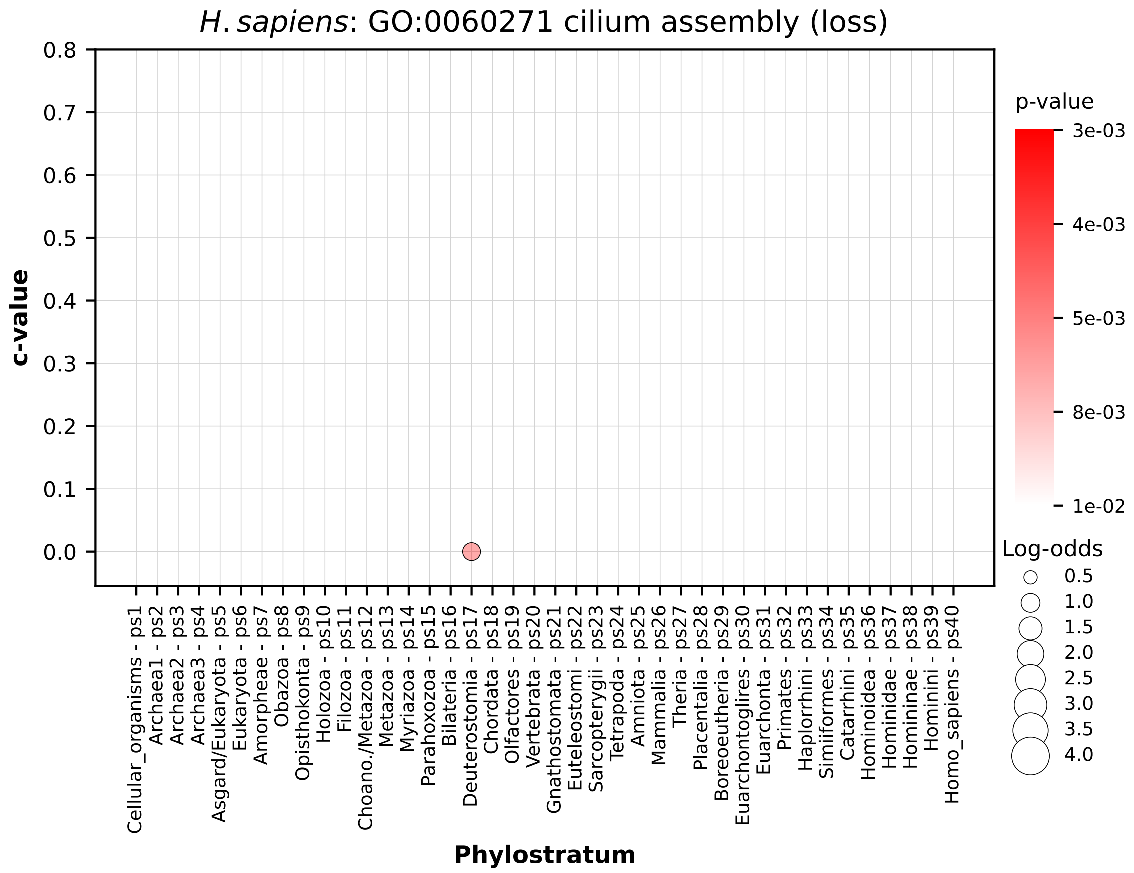

H. sapiens: GO:0060271 cilium assembly (loss)
c-value
0.8
0.7
0.6
0.5
0.4
0.3
0.2
0.1
0.0
p-value
3e-03
4e-03
5e-03
8e-03
1e-02
Log-odds
0.5
1.0
1.5
2.0
2.5
3.0
3.5
4.0
Cellular_organisms - ps1
Archaea1 - ps2
Archaea2 - ps3
Archaea3 - ps4
Asgard/Eukaryota - ps5
Eukaryota - ps6
Amorpheae - ps7
Obazoa - ps8
Opisthokonta - ps9
Holozoa - ps10
Filozoa - ps11
Choano./Metazoa - ps12
Metazoa - ps13
Myriazoa - ps14
Parahoxozoa - ps15
Bilateria - ps16
Deuterostomia - ps17
Chordata - ps18
Olfactores - ps19
Vertebrata - ps20
Gnathostomata - ps21
Euteleostomi - ps22
Sarcopterygii - ps23
Tetrapoda - ps24
Amniota - ps25
Mammalia - ps26
Theria - ps27
Placentalia - ps28
Boreoeutheria - ps29
Euarchontoglires - ps30
Euarchonta - ps31
Primates - ps32
Haplorrhini - ps33
Simiiformes - ps34
Catarrhini - ps35
Hominoidea - ps36
Hominidae - ps37
Homininae - ps38
Hominini - ps39
Homo_sapiens - ps40
Phylostratum

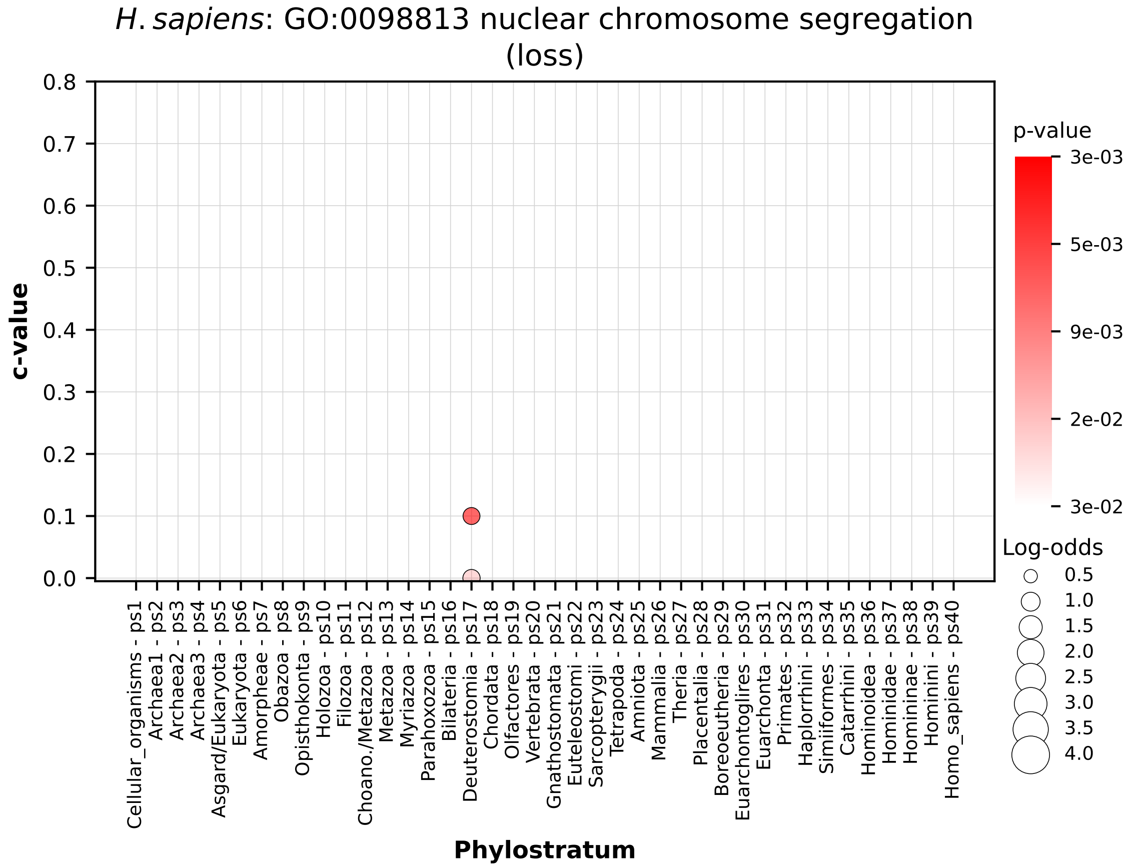

H. sapiens: GO:0098813 nuclear chromosome segregation (loss)
c-value
0.0
0.1
0.2
0.3
0.4
0.5
0.6
0.7
0.8
Phylostratum
Cellular_organisms - ps1
Archaea1 - ps2
Archaea2 - ps3
Archaea3 - ps4
Asgard/Eukaryota - ps5
Eukaryota - ps6
Amorpheae - ps7
Obazoa - ps8
Opisthokonta - ps9
Holozoa - ps10
Filozoa - ps11
Choano./Metazoa - ps12
Metazoa - ps13
Myriazoa - ps14
Parahoxozoa - ps15
Bilateria - ps16
Deuterostomia - ps17
Chordata - ps18
Olfactores - ps19
Vertebrata - ps20
Gnathostomata - ps21
Euteleostomi - ps22
Sarcopterygii - ps23
Tetrapoda - ps24
Amniota - ps25
Mammalia - ps26
Theria - ps27
Placentalia - ps28
Boreoeutheria - ps29
Euarchontoglires - ps30
Euarchonta - ps31
Primates - ps32
Haplorrhini - ps33
Simiiformes - ps34
Catarrhini - ps35
Hominoidea - ps36
Hominidae - ps37
Homininae - ps38
Hominini - ps39
Homo_sapiens - ps40
p-value
3e-03
5e-03
9e-03
2e-02
3e-02
Log-odds
0.5
1.0
1.5
2.0
2.5
3.0
3.5
4.0

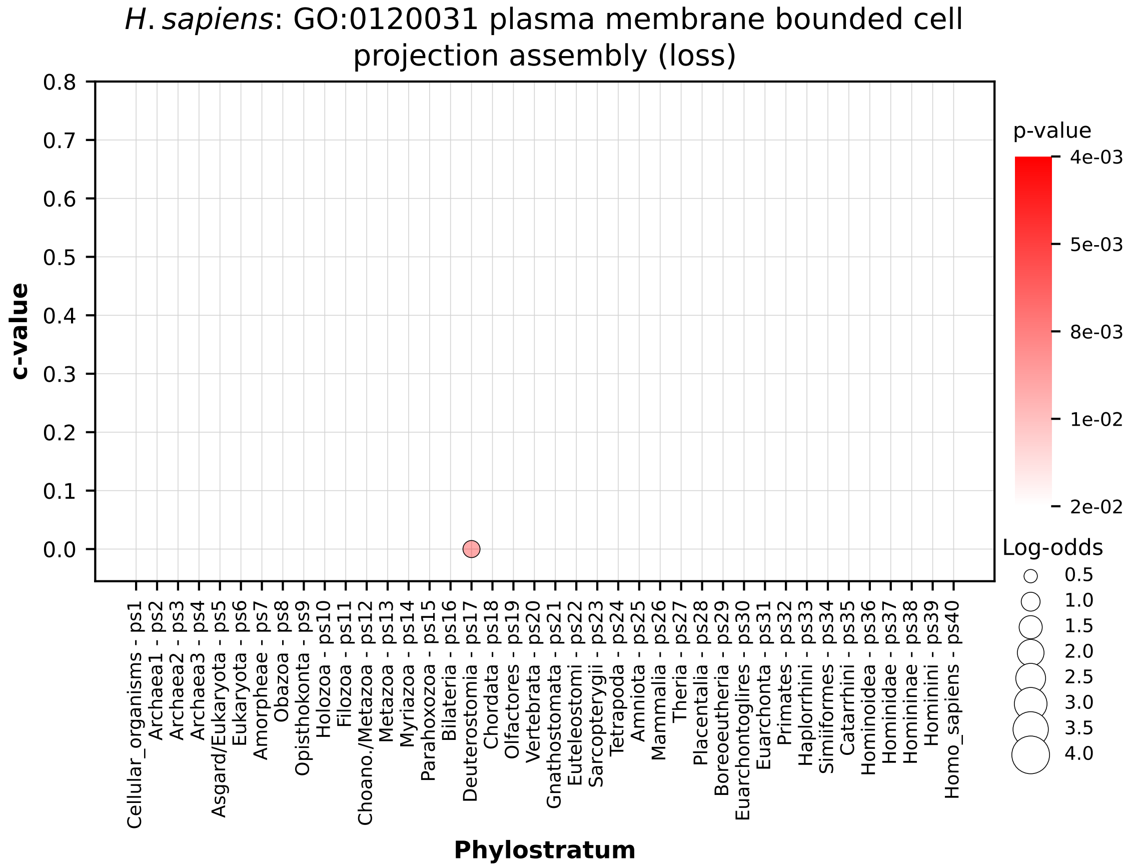

H. sapiens: GO:0120031 plasma membrane bounded cell projection assembly (loss)
c-value
0.8
0.7
0.6
0.5
0.4
0.3
0.2
0.1
0.0
Phylostratum
Cellular_organisms - ps1
Archaea1 - ps2
Archaea2 - ps3
Archaea3 - ps4
Asgard/Eukaryota - ps5
Eukaryota - ps6
Amorpheae - ps7
Obazoa - ps8
Opisthokonta - ps9
Holozoa - ps10
Filozoa - ps11
Choano./Metazoa - ps12
Metazoa - ps13
Myriazoa - ps14
Parahoxozoa - ps15
Bilateria - ps16
Deuterostomia - ps17
Chordata - ps18
Olfactores - ps19
Vertebrata - ps20
Gnathostomata - ps21
Euteleostomi - ps22
Sarcopterygii - ps23
Tetrapoda - ps24
Amniota - ps25
Mammalia - ps26
Theria - ps27
Placentalia - ps28
Boreoeutheria - ps29
Euarchontoglires - ps30
Euarchonta - ps31
Primates - ps32
Haplorrhini - ps33
Simiiformes - ps34
Catarrhini - ps35
Hominoidea - ps36
Hominidae - ps37
Homininae - ps38
Hominini - ps39
Homo_sapiens - ps40
p-value
4e-03
5e-03
8e-03
1e-02
2e-02
Log-odds
0.5
1.0
1.5
2.0
2.5
3.0
3.5
4.0

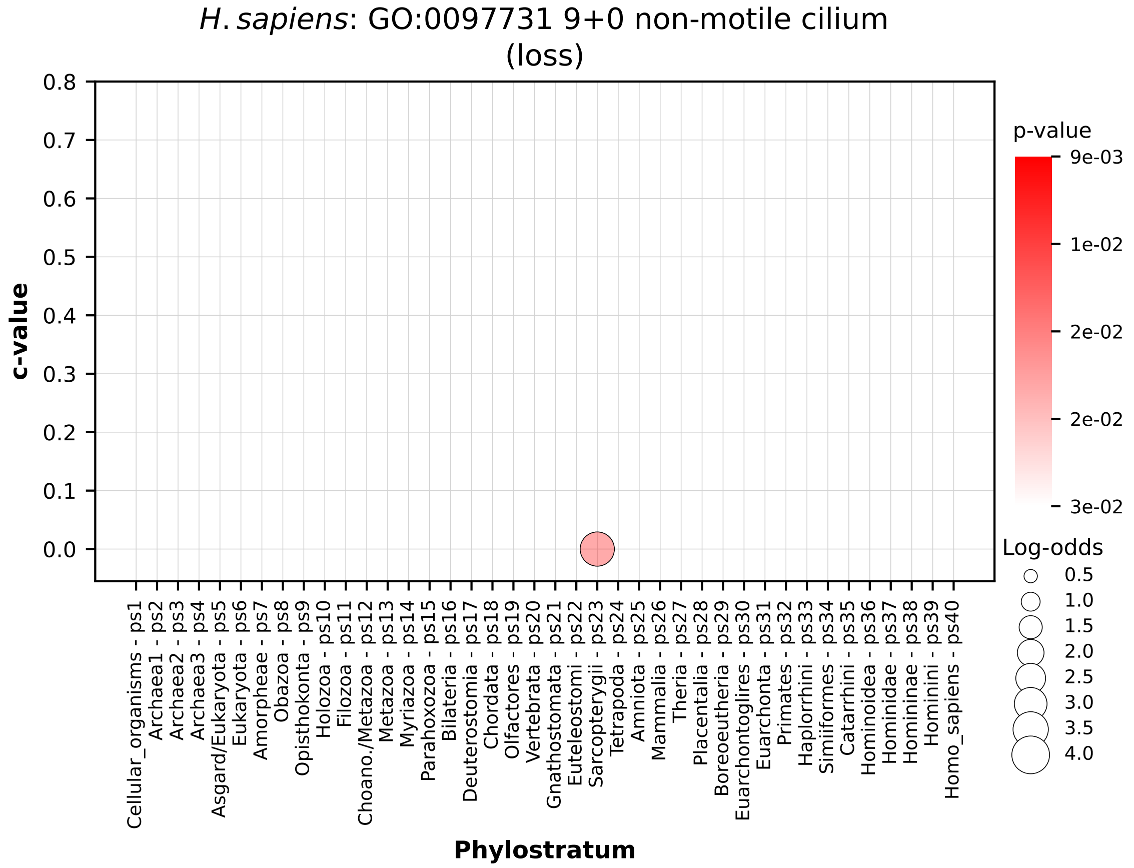

H. sapiens: GO:0097731 9+0 non-motile cilium
(loss)
c-value
0.8
0.7
0.6
0.5
0.4
0.3
0.2
0.1
0.0
p-value
9e-03
1e-02
2e-02
2e-02
3e-02
Log-odds
0.5
1.0
1.5
2.0
2.5
3.0
3.5
4.0
Cellular_organisms - ps1
Archaea1 - ps2
Archaea2 - ps3
Archaea3 - ps4
Asgard/Eukaryota - ps5
Eukaryota - ps6
Amorpheae - ps7
Obazoa - ps8
Opisthokonta - ps9
Holozoa - ps10
Filozoa - ps11
Choano./Metazoa - ps12
Metazoa - ps13
Myriazoa - ps14
Parahoxozoa - ps15
Bilateria - ps16
Deuterostomia - ps17
Chordata - ps18
Olfactores - ps19
Vertebrata - ps20
Gnathostomata - ps21
Euteleostomi - ps22
Sarcopterygii - ps23
Tetrapoda - ps24
Amniota - ps25
Mammalia - ps26
Theria - ps27
Placentalia - ps28
Boreoeutheria - ps29
Euarchontoglires - ps30
Euarchonta - ps31
Primates - ps32
Haplorrhini - ps33
Simiiformes - ps34
Catarrhini - ps35
Hominoidea - ps36
Hominidae - ps37
Homininae - ps38
Hominini - ps39
Homo_sapiens - ps40
Phylostratum

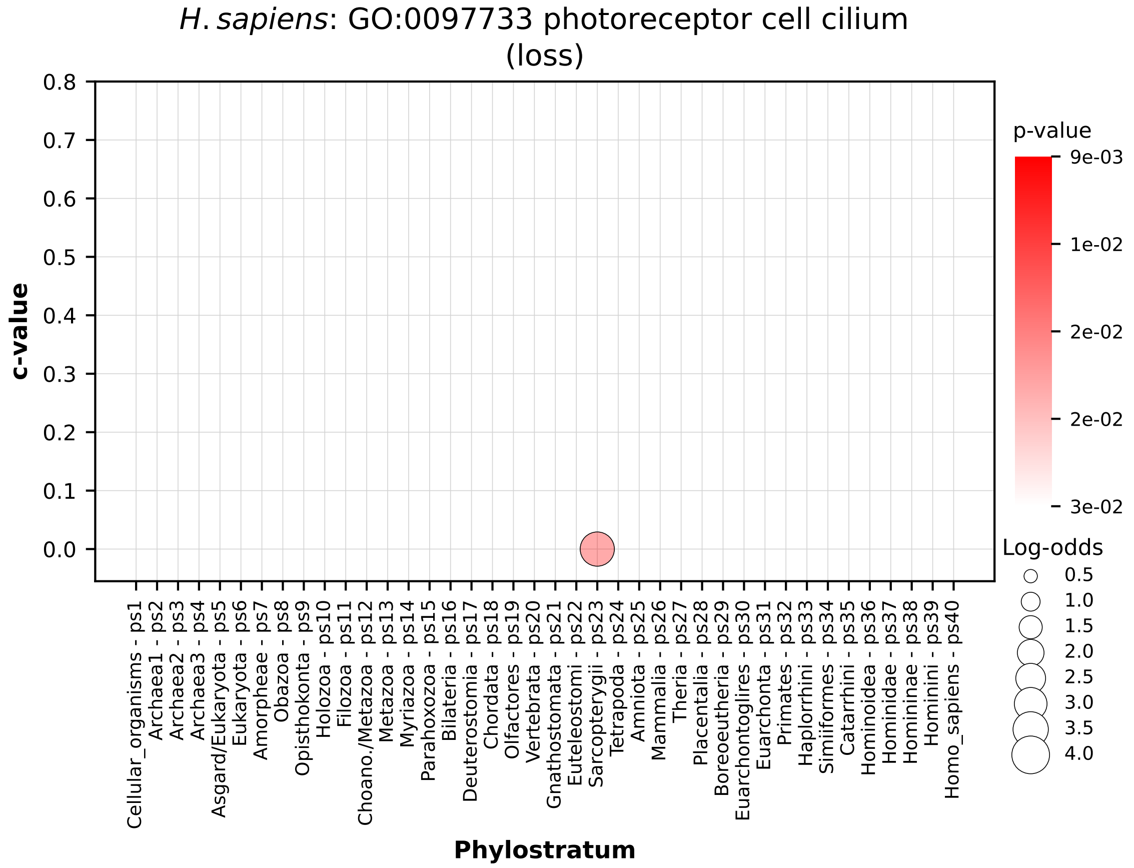

H. sapiens: GO:0097733 photoreceptor cell cilium
(loss)
c-value
0.8
0.7
0.6
0.5
0.4
0.3
0.2
0.1
0.0
p-value
9e-03
1e-02
2e-02
2e-02
3e-02
Log-odds
0.5
1.0
1.5
2.0
2.5
3.0
3.5
4.0
Cellular_organisms - ps1
Archaea1 - ps2
Archaea2 - ps3
Archaea3 - ps4
Asgard/Eukaryota - ps5
Eukaryota - ps6
Amorpheae - ps7
Obazoa - ps8
Opisthokonta - ps9
Holozoa - ps10
Filozoa - ps11
Choano./Metazoa - ps12
Metazoa - ps13
Myriazoa - ps14
Parahoxozoa - ps15
Bilateria - ps16
Deuterostomia - ps17
Chordata - ps18
Olfactores - ps19
Vertebrata - ps20
Gnathostomata - ps21
Euteleostomi - ps22
Sarcopterygii - ps23
Tetrapoda - ps24
Amniota - ps25
Mammalia - ps26
Theria - ps27
Placentalia - ps28
Boreoeutheria - ps29
Euarchontoglires - ps30
Euarchonta - ps31
Primates - ps32
Haplorrhini - ps33
Simiiformes - ps34
Catarrhini - ps35
Hominoidea - ps36
Hominidae - ps37
Homininae - ps38
Hominini - ps39
Homo_sapiens - ps40
Phylostratum

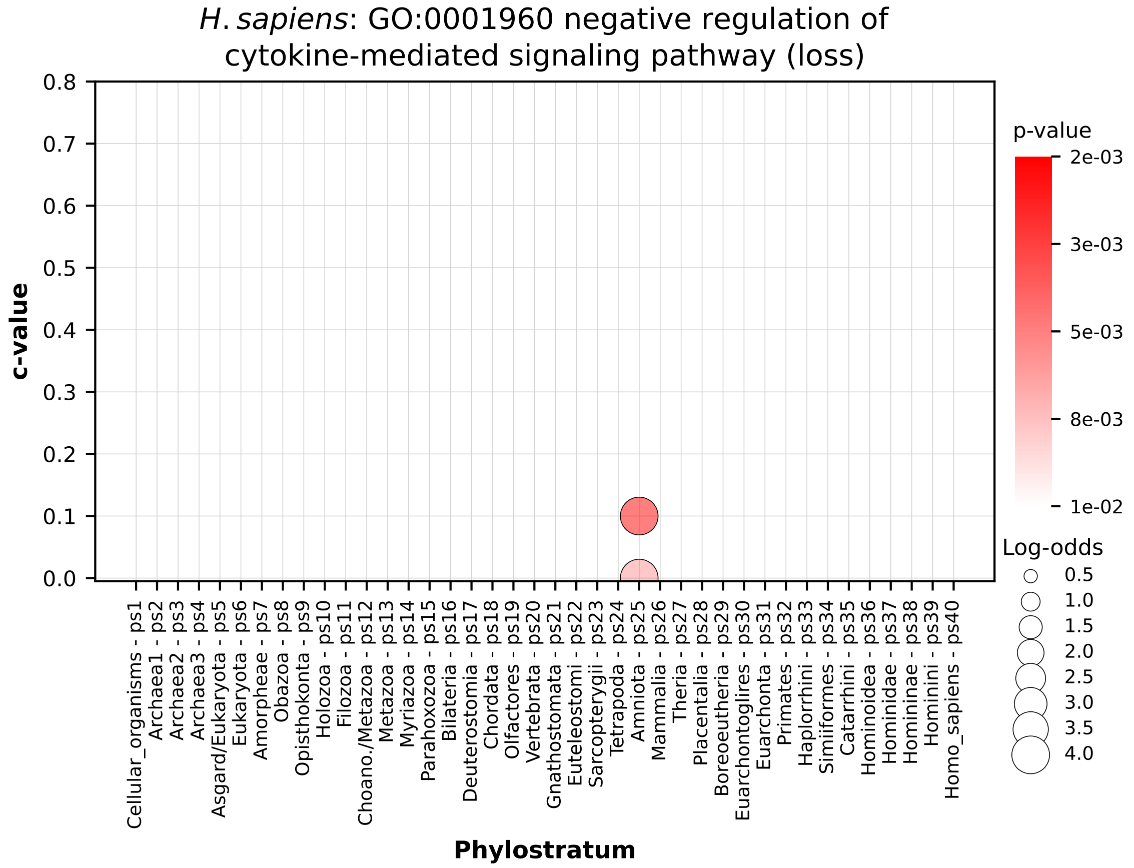

H. sapiens: GO:0001960 negative regulation of cytokine-mediated signaling pathway (loss)
c-value
0.8
0.7
0.6
0.5
0.4
0.3
0.2
0.1
0.0
p-value
2e-03
3e-03
5e-03
8e-03
1e-02
Log-odds
0.5
1.0
1.5
2.0
2.5
3.0
3.5
4.0
Cellular_organisms - ps1
Archaea1 - ps2
Archaea2 - ps3
Archaea3 - ps4
Asgard/Eukaryota - ps5
Eukaryota - ps6
Amorpheae - ps7
Obazoa - ps8
Opisthokonta - ps9
Holozoa - ps10
Filozoa - ps11
Choano./Metazoa - ps12
Metazoa - ps13
Myriazoa - ps14
Parahoxozoa - ps15
Bilateria - ps16
Deuterostomia - ps17
Chordata - ps18
Olfactores - ps19
Vertebrata - ps20
Gnathostomata - ps21
Euteleostomi - ps22
Sarcopterygii - ps23
Tetrapoda - ps24
Amniota - ps25
Mammalia - ps26
Theria - ps27
Placentalia - ps28
Boreoeutheria - ps29
Euarchontoglires - ps30
Euarchonta - ps31
Primates - ps32
Haplorrhini - ps33
Simiiformes - ps34
Catarrhini - ps35
Hominoidea - ps36
Hominidae - ps37
Homininae - ps38
Hominini - ps39
Homo_sapiens - ps40
Phylostratum

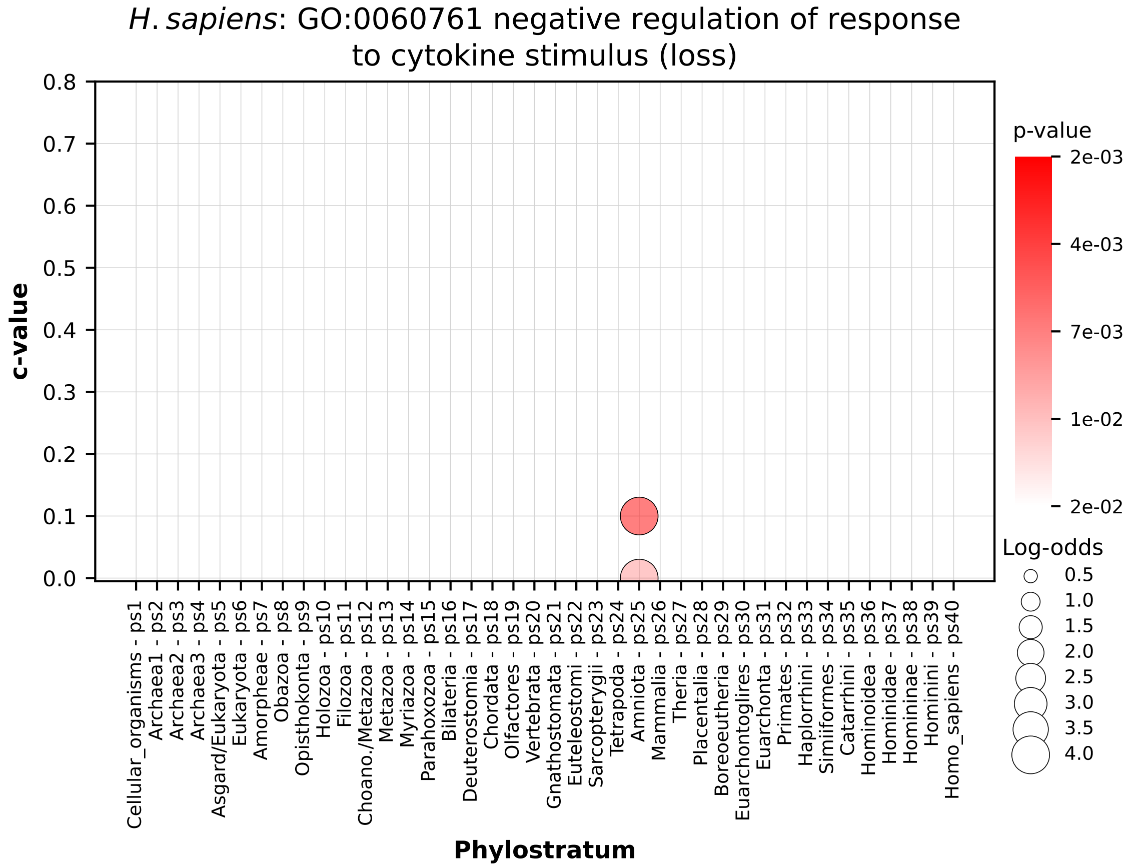

H. sapiens: GO:0060761 negative regulation of response to cytokine stimulus (loss)
c-value
0.0
0.1
0.2
0.3
0.4
0.5
0.6
0.7
0.8
Phylostratum
Cellular_organisms - ps1
Archaea1 - ps2
Archaea2 - ps3
Archaea3 - ps4
Asgard/Eukaryota - ps5
Eukaryota - ps6
Amorpheae - ps7
Obazoa - ps8
Opisthokonta - ps9
Holozoa - ps10
Filozoa - ps11
Choano./Metazoa - ps12
Metazoa - ps13
Myriazoa - ps14
Parahoxozoa - ps15
Bilateria - ps16
Deuterostomia - ps17
Chordata - ps18
Olfactores - ps19
Vertebrata - ps20
Gnathostomata - ps21
Euteleostomi - ps22
Sarcopterygii - ps23
Tetrapoda - ps24
Amniota - ps25
Mammalia - ps26
Theria - ps27
Placentalia - ps28
Boreoeutheria - ps29
Euarchontoglires - ps30
Euarchonta - ps31
Primates - ps32
Haplorrhini - ps33
Simiiformes - ps34
Catarrhini - ps35
Hominoidea - ps36
Hominidae - ps37
Homininae - ps38
Hominini - ps39
Homo_sapiens - ps40
p-value
2e-03
4e-03
7e-03
1e-02
2e-02
Log-odds
0.5
1.0
1.5
2.0
2.5
3.0
3.5
4.0

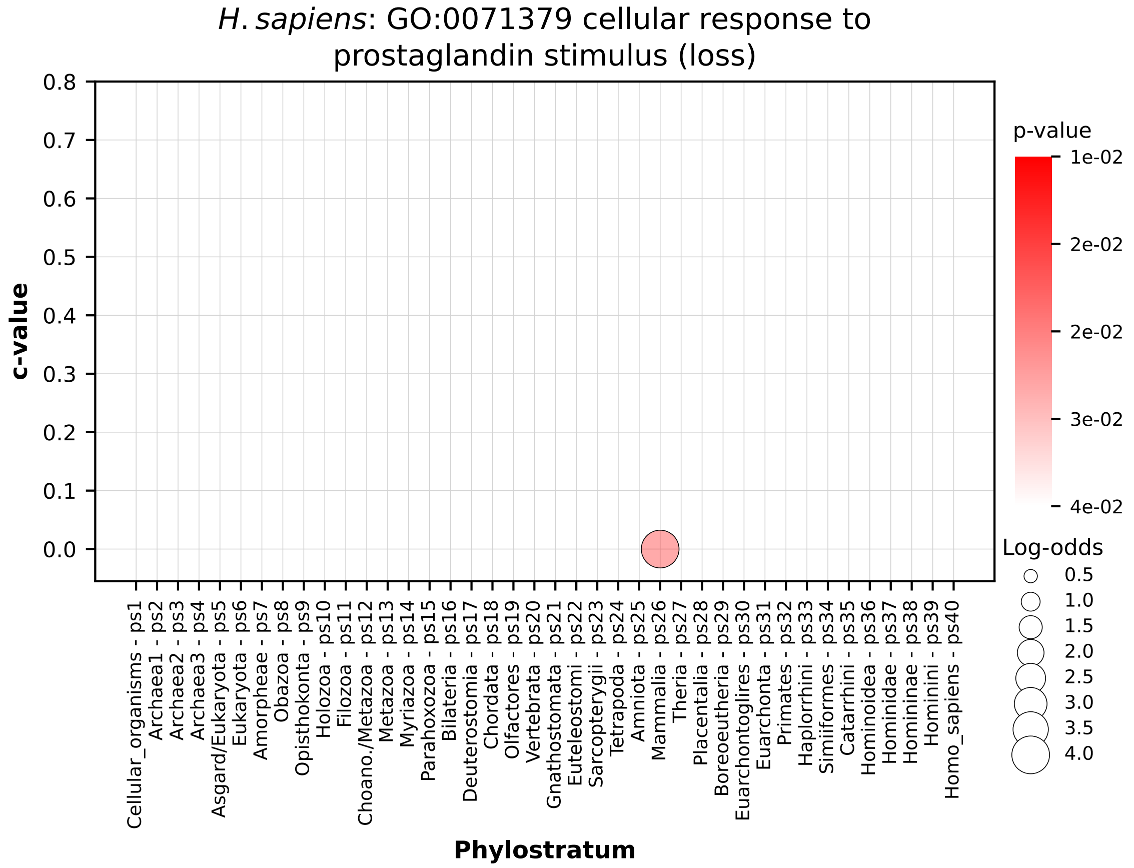

H. sapiens: GO:0071379 cellular response to prostaglandin stimulus (loss)
c-value
0.8
0.7
0.6
0.5
0.4
0.3
0.2
0.1
0.0
p-value
1e-02
2e-02
2e-02
3e-02
4e-02
Log-odds
0.5
1.0
1.5
2.0
2.5
3.0
3.5
4.0
Cellular_organisms - ps1
Archaea1 - ps2
Archaea2 - ps3
Archaea3 - ps4
Asgard/Eukaryota - ps5
Eukaryota - ps6
Amorpheae - ps7
Obazoa - ps8
Opisthokonta - ps9
Holozoa - ps10
Filozoa - ps11
Choano./Metazoa - ps12
Metazoa - ps13
Myriazoa - ps14
Parahoxozoa - ps15
Bilateria - ps16
Deuterostomia - ps17
Chordata - ps18
Olfactores - ps19
Vertebrata - ps20
Gnathostomata - ps21
Euteleostomi - ps22
Sarcopterygii - ps23
Tetrapoda - ps24
Amniota - ps25
Mammalia - ps26
Theria - ps27
Placentalia - ps28
Boreoeutheria - ps29
Euarchontoglires - ps30
Euarchonta - ps31
Primates - ps32
Haplorrhini - ps33
Similformes - ps34
Catarrhini - ps35
Hominoidea - ps36
Hominidae - ps37
Homininae - ps38
Hominini - ps39
Homo_sapiens - ps40
Phylostratum

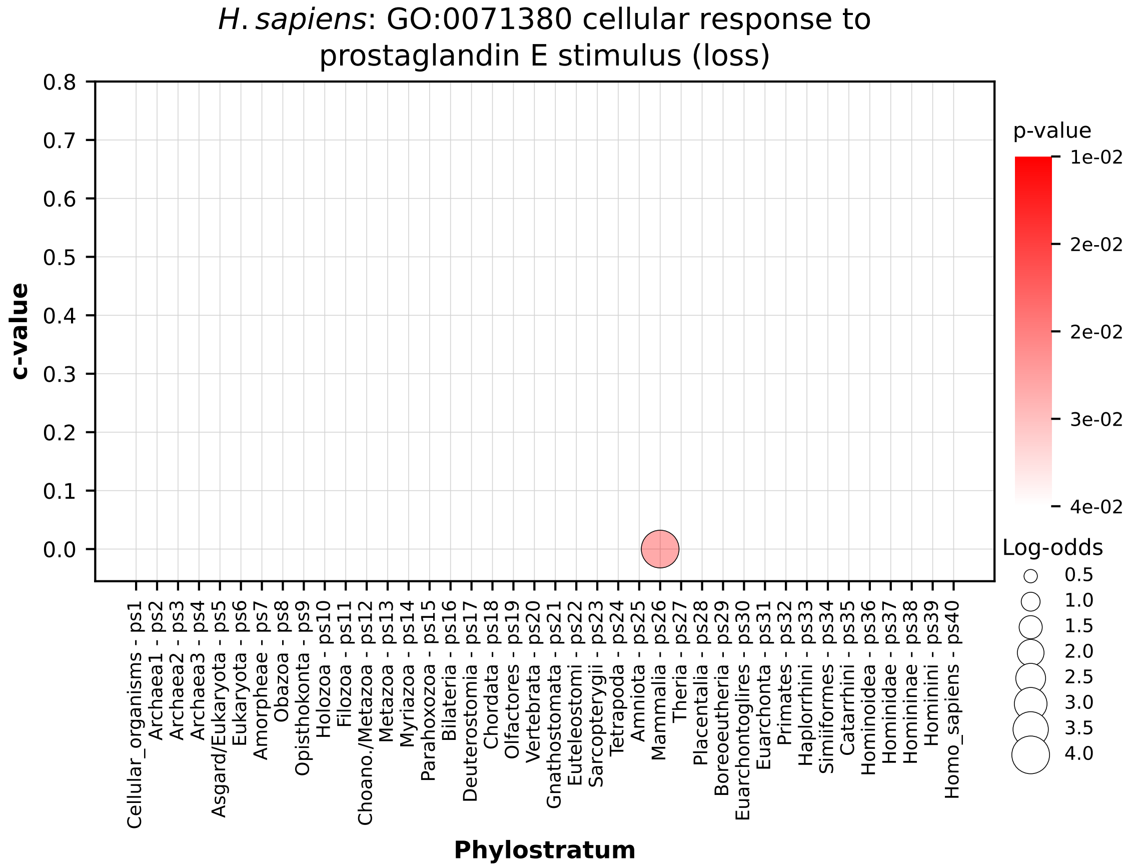

H. sapiens: GO:0071380 cellular response to prostaglandin E stimulus (loss)
c-value
0.8
0.7
0.6
0.5
0.4
0.3
0.2
0.1
0.0
Phylostratum
Cellular_organisms - ps1
Archaea1 - ps2
Archaea2 - ps3
Archaea3 - ps4
Asgard/Eukaryota - ps5
Eukaryota - ps6
Amorpheae - ps7
Obazoa - ps8
Opisthokonta - ps9
Holozoa - ps10
Filozoa - ps11
Choano./Metazoa - ps12
Metazoa - ps13
Myriazoa - ps14
Parahoxozoa - ps15
Bilateria - ps16
Deuterostomia - ps17
Chordata - ps18
Olfactores - ps19
Vertebrata - ps20
Gnathostomata - ps21
Euteleostomi - ps22
Sarcopterygii - ps23
Tetrapoda - ps24
Amniota - ps25
Mammalia - ps26
Theria - ps27
Placentalia - ps28
Boreoeutheria - ps29
Euarchontoglires - ps30
Euarchonta - ps31
Primates - ps32
Haplorrhini - ps33
Simiiformes - ps34
Catarrhini - ps35
Hominoidea - ps36
Hominidae - ps37
Homininae - ps38
Hominini - ps39
Homo_sapiens - ps40
p-value
1e-02
2e-02
2e-02
3e-02
4e-02
Log-odds
0.5
1.0
1.5
2.0
2.5
3.0
3.5
4.0

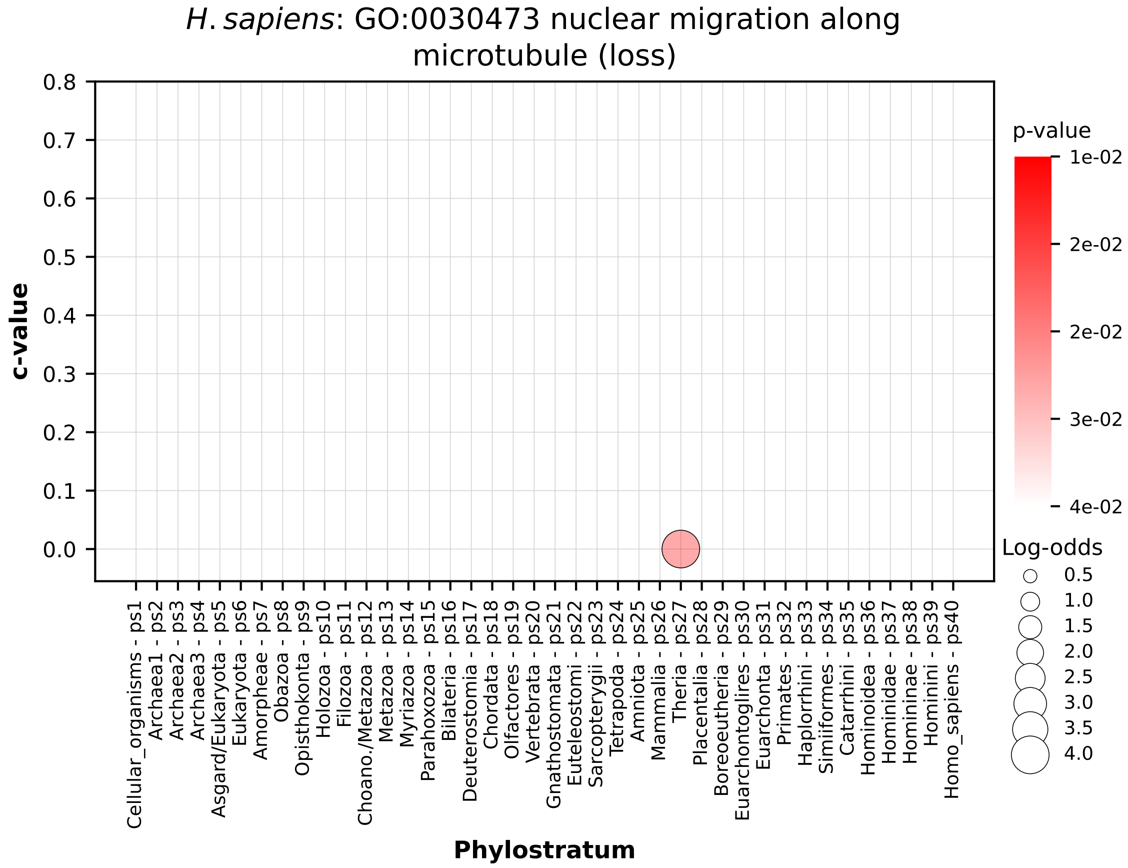

H. sapiens: GO:0030473 nuclear migration along microtubule (loss)
c-value
0.8
0.7
0.6
0.5
0.4
0.3
0.2
0.1
0.0
Phylostratum
Cellular_organisms - ps1
Archaea1 - ps2
Archaea2 - ps3
Archaea3 - ps4
Asgard/Eukaryota - ps5
Eukaryota - ps6
Amorpheae - ps7
Obazoa - ps8
Opisthokonta - ps9
Holozoa - ps10
Filozoa - ps11
Choano./Metazoa - ps12
Metazoa - ps13
Myriazoa - ps14
Parahoxozoa - ps15
Bilateria - ps16
Deuterostomia - ps17
Chordata - ps18
Olfactores - ps19
Vertebrata - ps20
Gnathostomata - ps21
Euteleostomi - ps22
Sarcopterygii - ps23
Tetrapoda - ps24
Amniota - ps25
Mammalia - ps26
Theria - ps27
Placentalia - ps28
Boreoeutheria - ps29
Euarchontoglires - ps30
Euarchonta - ps31
Primates - ps32
Haplorrhini - ps33
Simiiformes - ps34
Catarrhini - ps35
Hominoidea - ps36
Hominidae - ps37
Homininae - ps38
Hominini - ps39
Homo_sapiens - ps40
p-value
1e-02
2e-02
2e-02
3e-02
4e-02
Log-odds
0.5
1.0
1.5
2.0
2.5
3.0
3.5
4.0

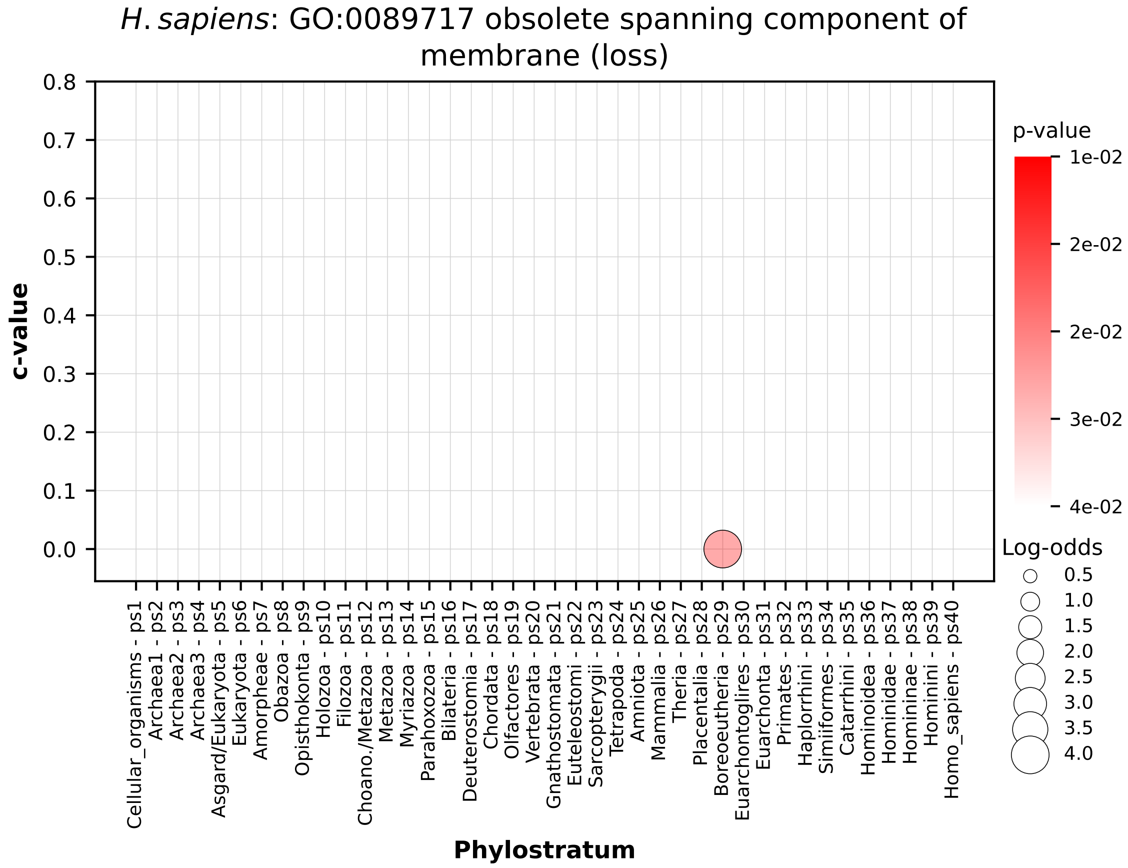

H. sapiens: GO:0089717 obsolete spanning component of membrane (loss)
c-value
0.8
0.7
0.6
0.5
0.4
0.3
0.2
0.1
0.0
Phylostratum
Cellular_organisms - ps1
Archaea1 - ps2
Archaea2 - ps3
Archaea3 - ps4
Asgard/Eukaryota - ps5
Eukaryota - ps6
Amorpheae - ps7
Obazoa - ps8
Opisthokonta - ps9
Holozoa - ps10
Filozoa - ps11
Choano./Metazoa - ps12
Metazoa - ps13
Myriazoa - ps14
Parahoxozoa - ps15
Bilateria - ps16
Deuterostomia - ps17
Chordata - ps18
Olfactores - ps19
Vertebrata - ps20
Gnathostomata - ps21
Euteleostomi - ps22
Sarcopterygii - ps23
Tetrapoda - ps24
Amniota - ps25
Mammalia - ps26
Theria - ps27
Placentalia - ps28
Boreoeutheria - ps29
Euarchontoglires - ps30
Euarchonta - ps31
Primates - ps32
Haplorrhini - ps33
Simiiformes - ps34
Catarrhini - ps35
Hominoidea - ps36
Hominidae - ps37
Homininae - ps38
Hominini - ps39
Homo_sapiens - ps40
p-value
1e-02
2e-02
2e-02
3e-02
4e-02
Log-odds
0.5
1.0
1.5
2.0
2.5
3.0
3.5
4.0

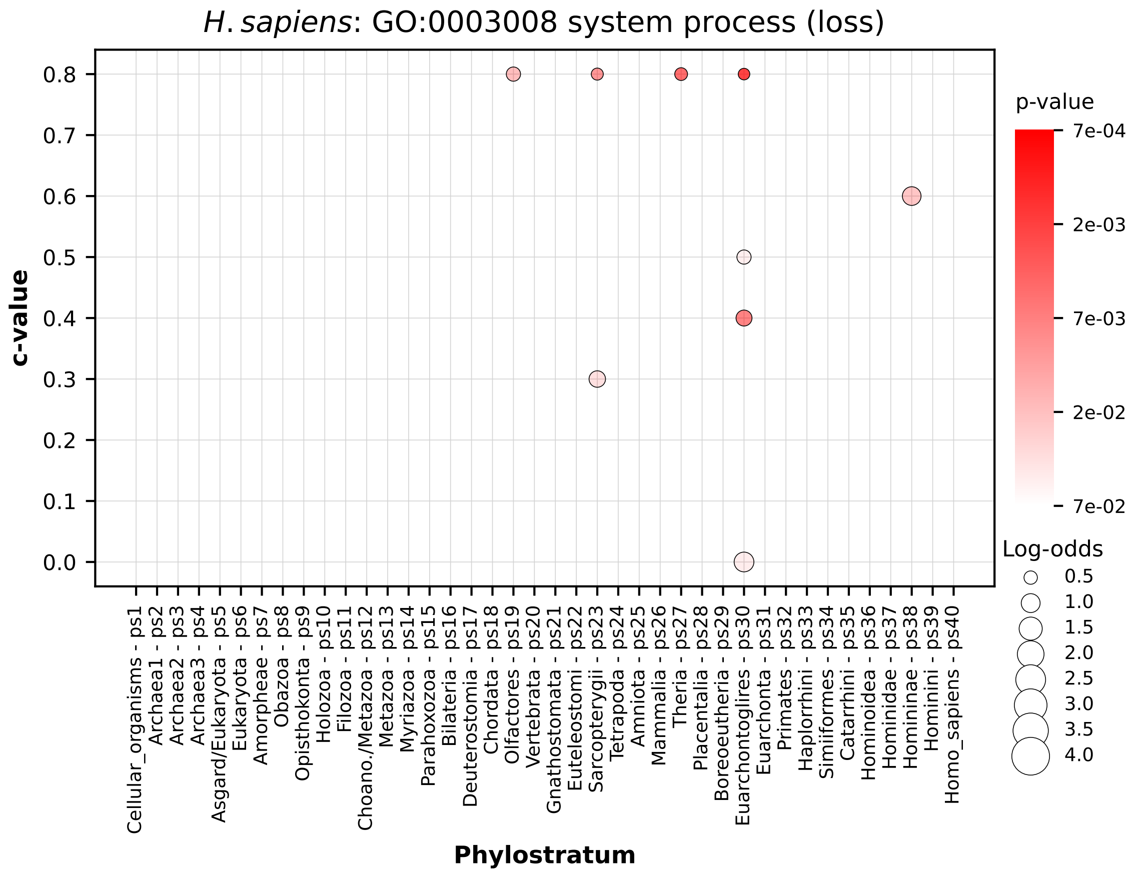
H. sapiens: GO:0003008 system process (loss)
c-value
0.8
0.7
0.6
0.5
0.4
0.3
0.2
0.1
0.0
p-value
7e-04
2e-03
7e-03
2e-02
7e-02
Log-odds
0.5
1.0
1.5
2.0
2.5
3.0
3.5
4.0
Cellular_organisms - ps1
Archaea1 - ps2
Archaea2 - ps3
Archaea3 - ps4
Asgard/Eukaryota - ps5
Eukaryota - ps6
Amorpheae - ps7
Obazoa - ps8
Opisthokonta - ps9
Holozoa - ps10
Filozoa - ps11
Choano./Metazoa - ps12
Metazoa - ps13
Myriazoa - ps14
Parahoxozoa - ps15
Bilateria - ps16
Deuterostomia - ps17
Chordata - ps18
Olfactores - ps19
Vertebrata - ps20
Gnathostomata - ps21
Euteleostomi - ps22
Sarcopterygii - ps23
Tetrapoda - ps24
Amniota - ps25
Mammalia - ps26
Theria - ps27
Placentalia - ps28
Boreoeutheria - ps29
Euarchontoglires - ps30
Euarchonta - ps31
Primates - ps32
Haplorrhini - ps33
Simiiformes - ps34
Catarrhini - ps35
Hominoidea - ps36
Hominidae - ps37
Homininae - ps38
Hominini - ps39
Homo_sapiens - ps40
Phylostratum

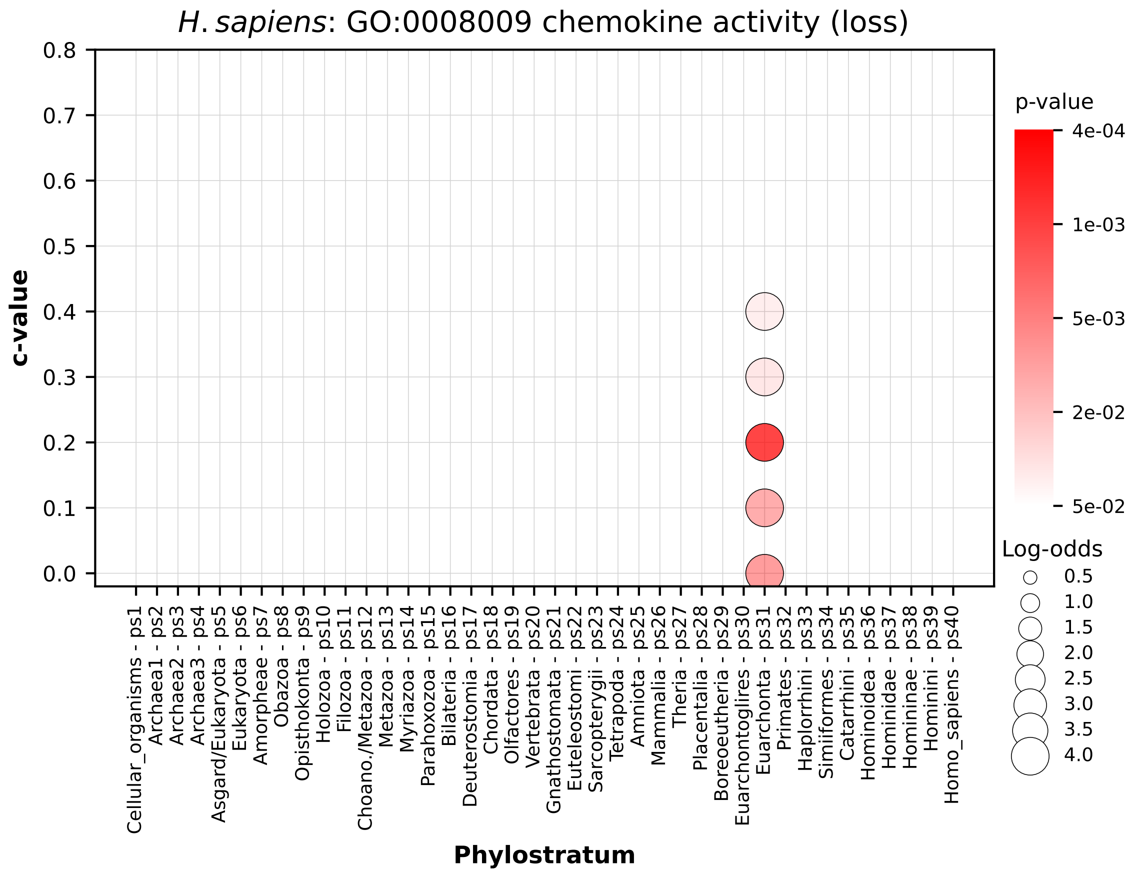

H. sapiens: GO:0008009 chemokine activity (loss)
c-value
0.8
0.7
0.6
0.5
0.4
0.3
0.2
0.1
0.0
p-value
4e-04
1e-03
5e-03
2e-02
5e-02
Log-odds
0.5
1.0
1.5
2.0
2.5
3.0
3.5
4.0
Cellular_organisms - ps1
Archaea1 - ps2
Archaea2 - ps3
Archaea3 - ps4
Asgard/Eukaryota - ps5
Eukaryota - ps6
Amorpheae - ps7
Obazoa - ps8
Opisthokonta - ps9
Holozoa - ps10
Filozoa - ps11
Choano./Metazoa - ps12
Metazoa - ps13
Myriazoa - ps14
Parahoxozoa - ps15
Bilateria - ps16
Deuterostomia - ps17
Chordata - ps18
Olfactores - ps19
Vertebrata - ps20
Gnathostomata - ps21
Euteleostomi - ps22
Sarcopterygii - ps23
Tetrapoda - ps24
Amniota - ps25
Mammalia - ps26
Theria - ps27
Placentalia - ps28
Boreoeutheria - ps29
Euarchontoglires - ps30
Euarchonta - ps31
Primates - ps32
Haplorrhini - ps33
Simiiformes - ps34
Catarrhini - ps35
Hominoidea - ps36
Hominidae - ps37
Homininae - ps38
Hominini - ps39
Homo_sapiens - ps40
Phylostratum

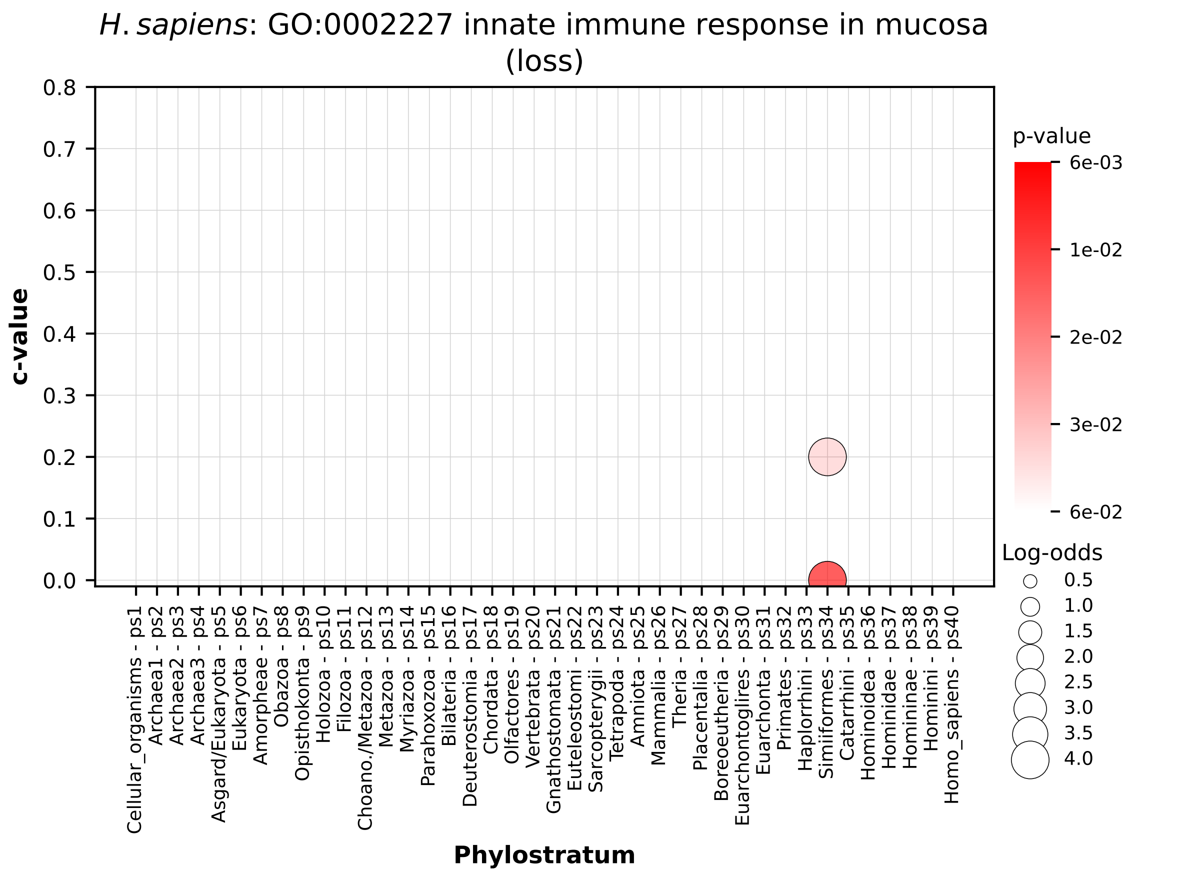

H. sapiens: GO:0002227 innate immune response in mucosa (loss)
c-value
0.8
0.7
0.6
0.5
0.4
0.3
0.2
0.1
0.0
p-value
6e-03
1e-02
2e-02
3e-02
6e-02
Log-odds
0.5
1.0
1.5
2.0
2.5
3.0
3.5
4.0
Cellular_organisms - ps1
Archaea1 - ps2
Archaea2 - ps3
Archaea3 - ps4
Asgard/Eukaryota - ps5
Eukaryota - ps6
Amorpheae - ps7
Obazoa - ps8
Opisthokonta - ps9
Holozoa - ps10
Filozoa - ps11
Choano./Metazoa - ps12
Metazoa - ps13
Myriazoa - ps14
Parahoxozoa - ps15
Bilateria - ps16
Deuterostomia - ps17
Chordata - ps18
Olfactores - ps19
Vertebrata - ps20
Gnathostomata - ps21
Euteleostomi - ps22
Sarcopterygii - ps23
Tetrapoda - ps24
Amniota - ps25
Mammalia - ps26
Theria - ps27
Placentalia - ps28
Boreoeutheria - ps29
Euarchontoglires - ps30
Euarchonta - ps31
Primates - ps32
Haplorrhini - ps33
Simiiformes - ps34
Catarrhini - ps35
Hominoidea - ps36
Hominidae - ps37
Homininae - ps38
Hominini - ps39
Homo_sapiens - ps40
Phylostratum

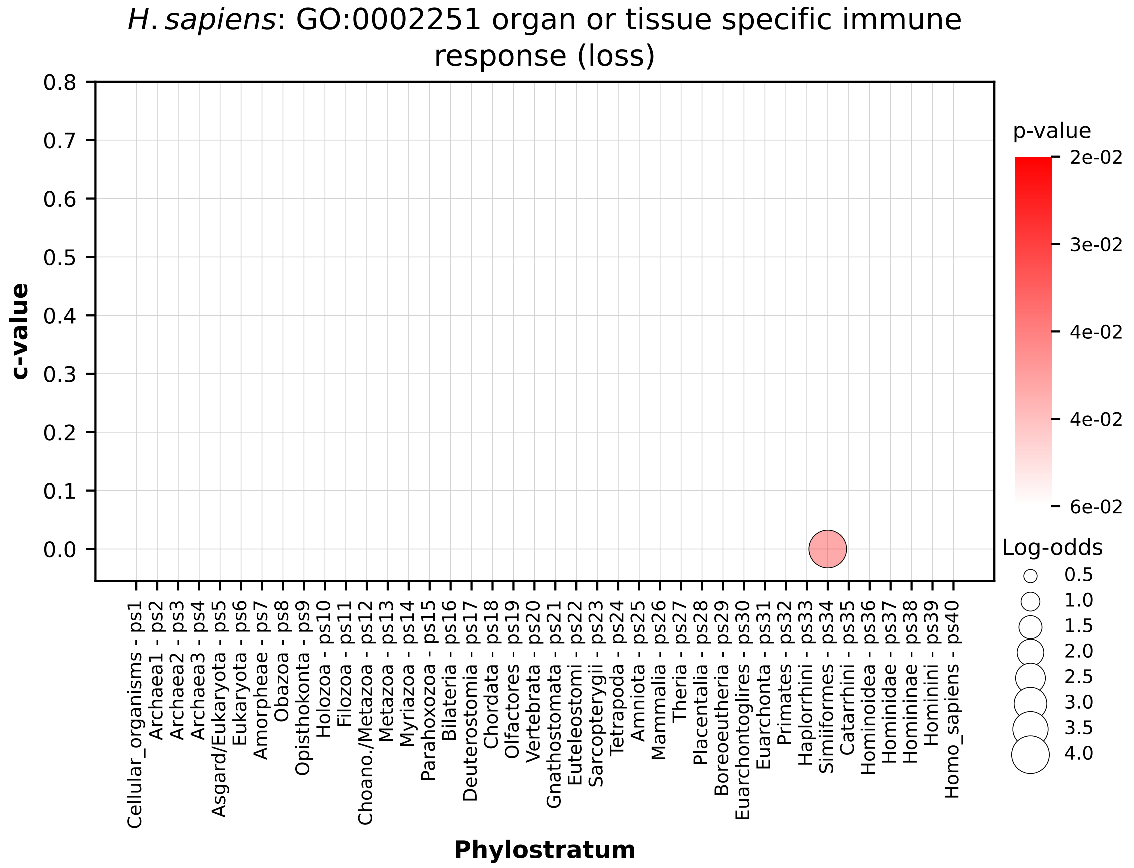
H. sapiens: GO:0002251 organ or tissue specific immune response (loss)
c-value
0.8
0.7
0.6
0.5
0.4
0.3
0.2
0.1
0.0
Phylostratum
Cellular_organisms - ps1
Archaea1 - ps2
Archaea2 - ps3
Archaea3 - ps4
Asgard/Eukaryota - ps5
Eukaryota - ps6
Amorpheae - ps7
Obazoa - ps8
Opisthokonta - ps9
Holozoa - ps10
Filozoa - ps11
Choano./Metazoa - ps12
Metazoa - ps13
Myriazoa - ps14
Parahoxozoa - ps15
Bilateria - ps16
Deuterostomia - ps17
Chordata - ps18
Olfactores - ps19
Vertebrata - ps20
Gnathostomata - ps21
Euteleostomi - ps22
Sarcopterygii - ps23
Tetrapoda - ps24
Amniota - ps25
Mammalia - ps26
Theria - ps27
Placentalia - ps28
Boreoeutheria - ps29
Euarchontoglires - ps30
Euarchonta - ps31
Primates - ps32
Haplorrhini - ps33
Simiiformes - ps34
Catarrhini - ps35
Hominoidea - ps36
Hominidae - ps37
Homininae - ps38
Hominini - ps39
Homo_sapiens - ps40
p-value
2e-02
3e-02
4e-02
4e-02
6e-02
Log-odds
0.5
1.0
1.5
2.0
2.5
3.0
3.5
4.0

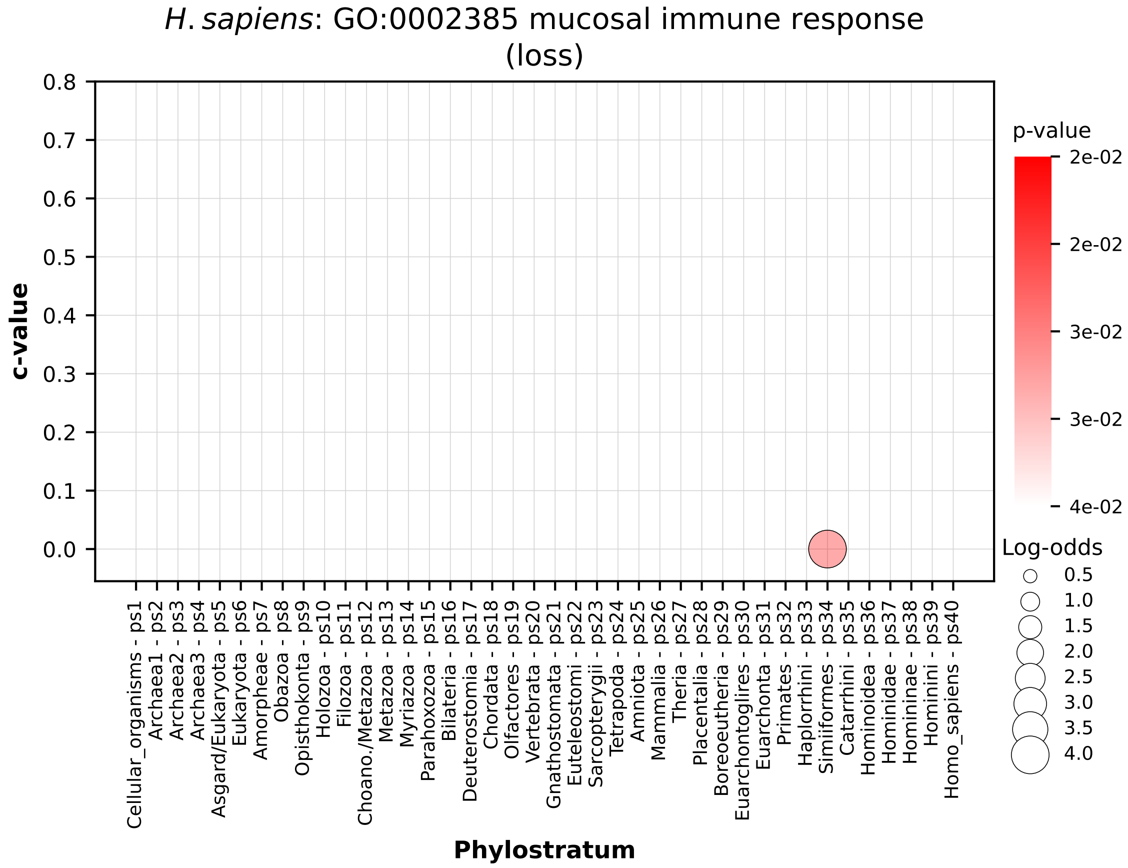

H. sapiens: GO:0002385 mucosal immune response
(loss)
c-value
0.8
0.7
0.6
0.5
0.4
0.3
0.2
0.1
0.0
Phylostratum
Cellular_organisms - ps1
Archaea1 - ps2
Archaea2 - ps3
Archaea3 - ps4
Asgard/Eukaryota - ps5
Eukaryota - ps6
Amorpheae - ps7
Obazoa - ps8
Opisthokonta - ps9
Holozoa - ps10
Filozoa - ps11
Choano./Metazoa - ps12
Metazoa - ps13
Myriazoa - ps14
Parahoxozoa - ps15
Bilateria - ps16
Deuterostomia - ps17
Chordata - ps18
Olfactores - ps19
Vertebrata - ps20
Gnathostomata - ps21
Euteleostomi - ps22
Sarcopterygii - ps23
Tetrapoda - ps24
Amniota - ps25
Mammalia - ps26
Theria - ps27
Placentalia - ps28
Boreoeutheria - ps29
Euarchontoglires - ps30
Euarchonta - ps31
Primates - ps32
Haplorrhini - ps33
Similformes - ps34
Catarrhini - ps35
Hominoidea - ps36
Hominidae - ps37
Homininae - ps38
Hominini - ps39
Homo_sapiens - ps40
p-value
2e-02
2e-02
3e-02
3e-02
4e-02
Log-odds
0.5
1.0
1.5
2.0
2.5
3.0
3.5
4.0

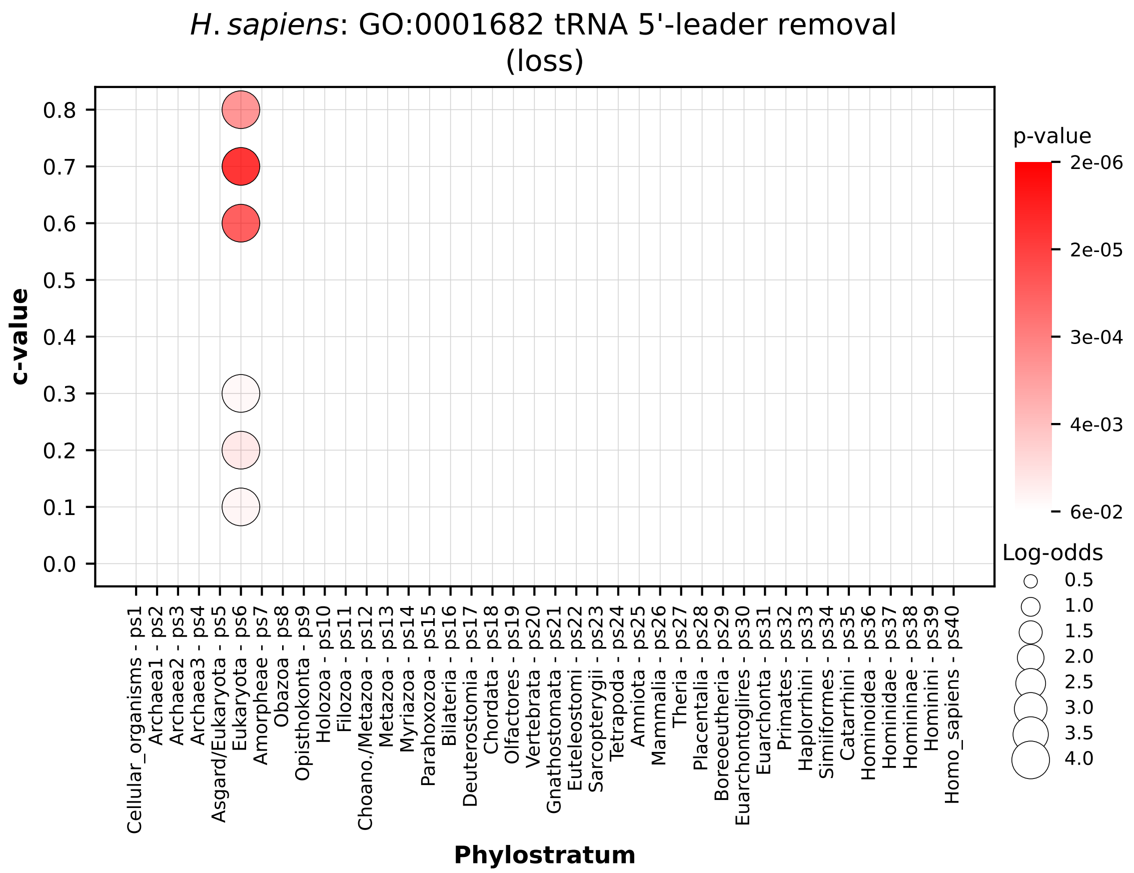

H. sapiens: GO:0001682 tRNA 5'-leader removal
(loss)
c-value
0.0
0.1
0.2
0.3
0.4
0.5
0.6
0.7
0.8
Phylostratum
Cellular_organisms - ps1
Archaea1 - ps2
Archaea2 - ps3
Archaea3 - ps4
Asgard/Eukaryota - ps5
Eukaryota - ps6
Amorpheae - ps7
Obazoa - ps8
Opisthokonta - ps9
Holozoa - ps10
Filozoa - ps11
Choano./Metazoa - ps12
Metazoa - ps13
Myriazoa - ps14
Parahoxozoa - ps15
Bilateria - ps16
Deuterostomia - ps17
Chordata - ps18
Olfactores - ps19
Vertebrata - ps20
Gnathostomata - ps21
Euteleostomi - ps22
Sarcopterygii - ps23
Tetrapoda - ps24
Amniota - ps25
Mammalia - ps26
Theria - ps27
Placentalia - ps28
Boreoeutheria - ps29
Euarchontoglires - ps30
Euarchonta - ps31
Primates - ps32
Haplorrhini - ps33
Simiiformes - ps34
Catarrhini - ps35
Hominoidea - ps36
Hominidae - ps37
Homininae - ps38
Hominini - ps39
Homo_sapiens - ps40
p-value
2e-06
2e-05
3e-04
4e-03
6e-02
Log-odds
0.5
1.0
1.5
2.0
2.5
3.0
3.5
4.0

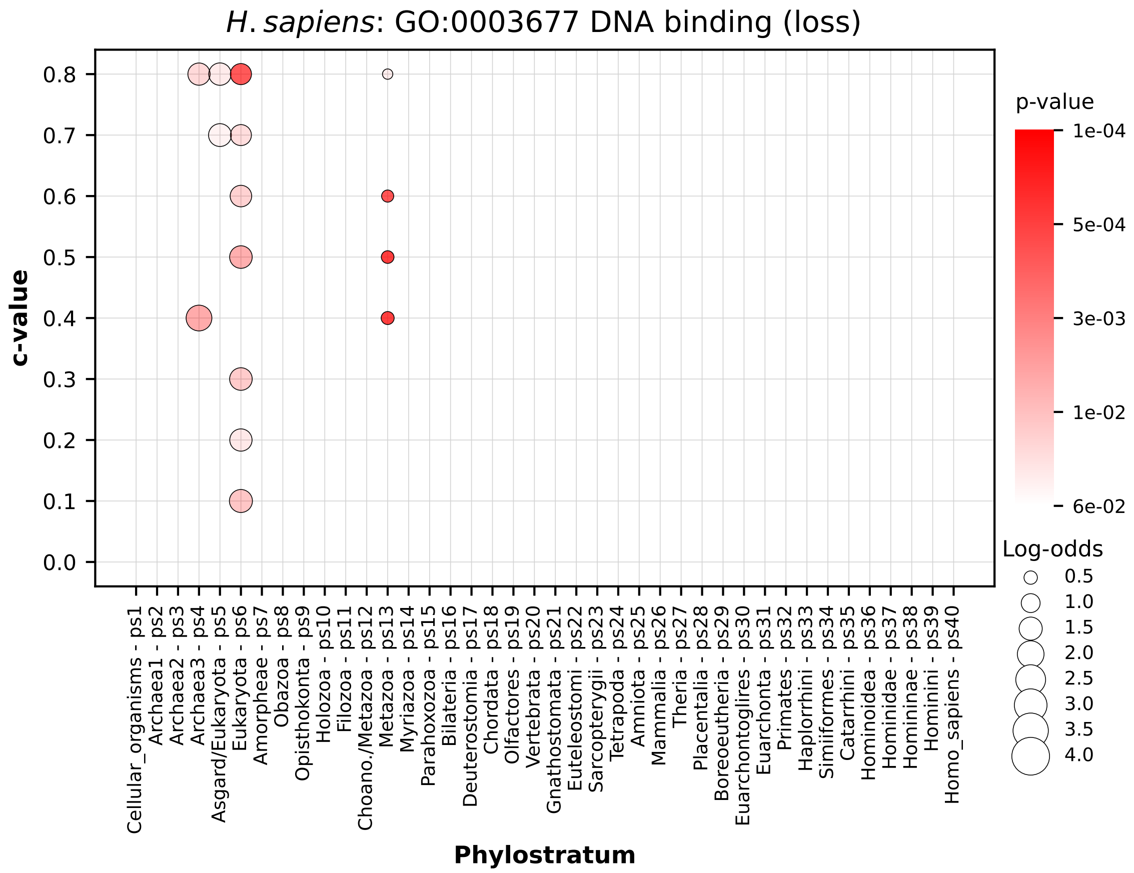

H. sapiens: GO:0003677 DNA binding (loss)
c-value
0.8
0.7
0.6
0.5
0.4
0.3
0.2
0.1
0.0
p-value
1e-04
5e-04
3e-03
1e-02
6e-02
Log-odds
0.5
1.0
1.5
2.0
2.5
3.0
3.5
4.0
Cellular_organisms - ps1
Archaea1 - ps2
Archaea2 - ps3
Archaea3 - ps4
Asgard/Eukaryota - ps5
Eukaryota - ps6
Amorpheae - ps7
Obazoa - ps8
Opisthokonta - ps9
Holozoa - ps10
Filozoa - ps11
Choano./Metazoa - ps12
Metazoa - ps13
Myriazoa - ps14
Parahoxozoa - ps15
Bilateria - ps16
Deuterostomia - ps17
Chordata - ps18
Olfactores - ps19
Vertebrata - ps20
Gnathostomata - ps21
Euteleostomi - ps22
Sarcopterygii - ps23
Tetrapoda - ps24
Amniota - ps25
Mammalia - ps26
Theria - ps27
Placentalia - ps28
Boreoeutheria - ps29
Euarchontoglires - ps30
Euarchonta - ps31
Primates - ps32
Haplorrhini - ps33
Simiiformes - ps34
Catarrhini - ps35
Hominoidea - ps36
Hominidae - ps37
Homininae - ps38
Hominini - ps39
Homo_sapiens - ps40
Phylostratum

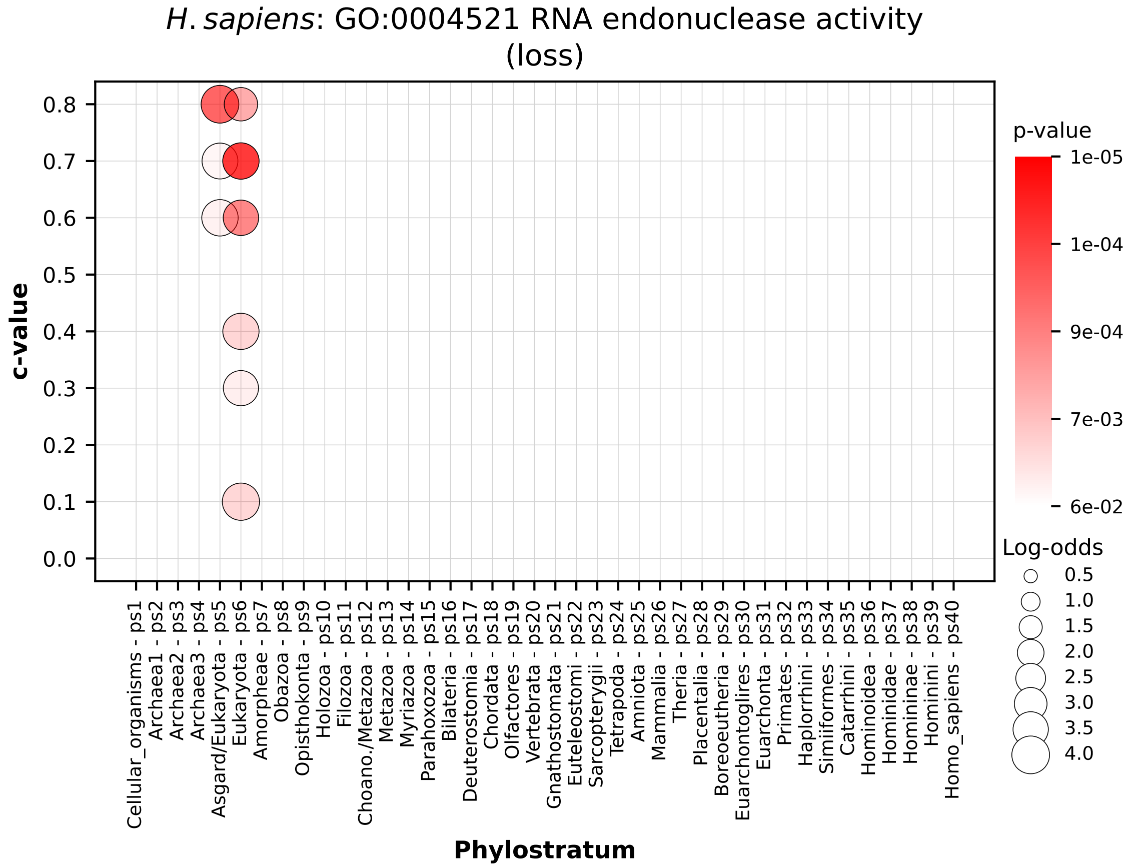

H. sapiens: GO:0004521 RNA endonuclease activity (loss)
c-value
0.8
0.7
0.6
0.5
0.4
0.3
0.2
0.1
0.0
Phylostratum
Cellular_organisms - ps1
Archaea1 - ps2
Archaea2 - ps3
Archaea3 - ps4
Asgard/Eukaryota - ps5
Eukaryota - ps6
Amorpheae - ps7
Obazoa - ps8
Opisthokonta - ps9
Holozoa - ps10
Filozoa - ps11
Choano./Metazoa - ps12
Metazoa - ps13
Myriazoa - ps14
Parahoxozoa - ps15
Bilateria - ps16
Deuterostomia - ps17
Chordata - ps18
Olfactores - ps19
Vertebrata - ps20
Gnathostomata - ps21
Euteleostomi - ps22
Sarcopterygii - ps23
Tetrapoda - ps24
Amniota - ps25
Mammalia - ps26
Theria - ps27
Placentalia - ps28
Boreoeutheria - ps29
Euarchontoglires - ps30
Euarchonta - ps31
Primates - ps32
Haplorrhini - ps33
Simiiformes - ps34
Catarrhini - ps35
Hominoidea - ps36
Hominidae - ps37
Homininae - ps38
Hominini - ps39
Homo_sapiens - ps40
p-value
1e-05
1e-04
9e-04
7e-03
6e-02
Log-odds
0.5
1.0
1.5
2.0
2.5
3.0
3.5
4.0

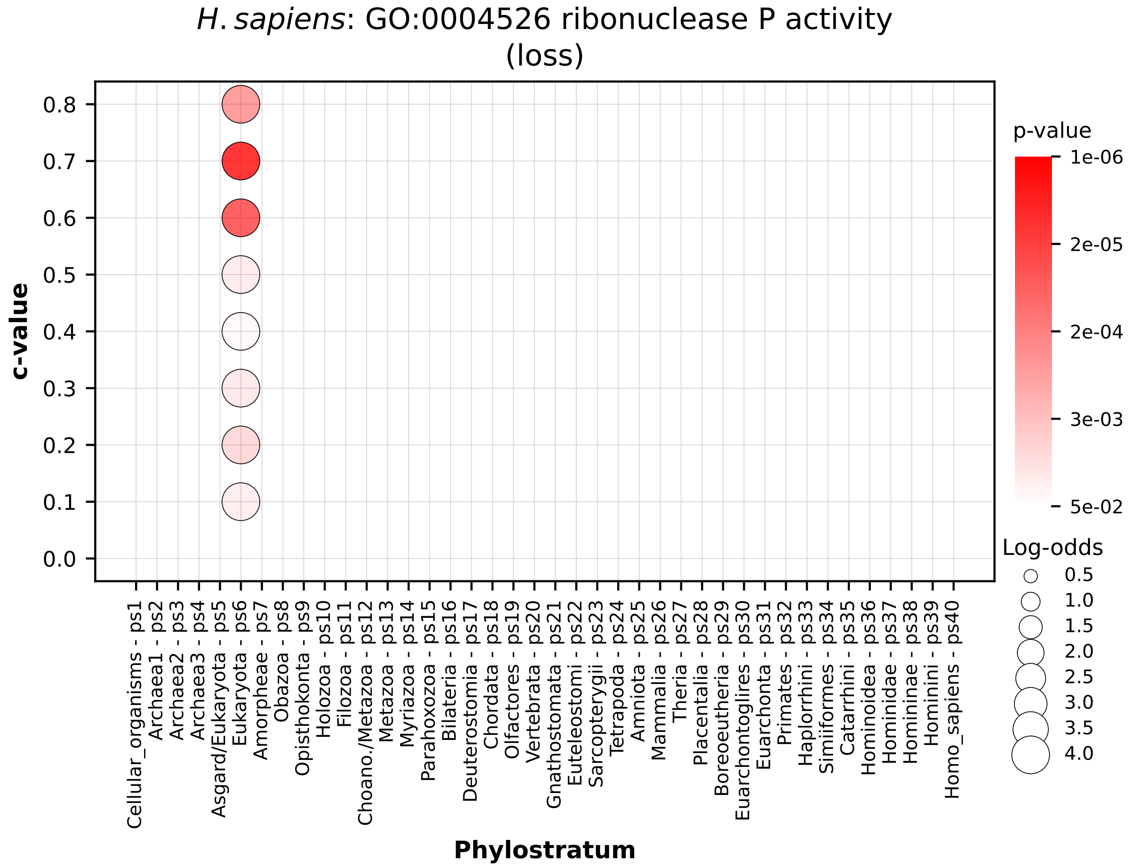
H. sapiens: GO:0004526 ribonuclease P activity
(loss)
c-value
0.0
0.1
0.2
0.3
0.4
0.5
0.6
0.7
0.8
p-value
1e-06
2e-05
2e-04
3e-03
5e-02
Log-odds
0.5
1.0
1.5
2.0
2.5
3.0
3.5
4.0
Cellular_organisms - ps1
Archaea1 - ps2
Archaea2 - ps3
Archaea3 - ps4
Asgard/Eukaryota - ps5
Eukaryota - ps6
Amorpheae - ps7
Obazoa - ps8
Opisthokonta - ps9
Holozoa - ps10
Filozoa - ps11
Choano./Metazoa - ps12
Metazoa - ps13
Myriazoa - ps14
Parahoxozoa - ps15
Bilateria - ps16
Deuterostomia - ps17
Chordata - ps18
Olfactores - ps19
Vertebrata - ps20
Gnathostomata - ps21
Euteleostomi - ps22
Sarcopterygii - ps23
Tetrapoda - ps24
Amniota - ps25
Mammalia - ps26
Theria - ps27
Placentalia - ps28
Boreoeutheria - ps29
Euarchontoglires - ps30
Euarchonta - ps31
Primates - ps32
Haplorrhini - ps33
Simiiformes - ps34
Catarrhini - ps35
Hominoidea - ps36
Hominidae - ps37
Homininae - ps38
Hominini - ps39
Homo_sapiens - ps40
Phylostratum

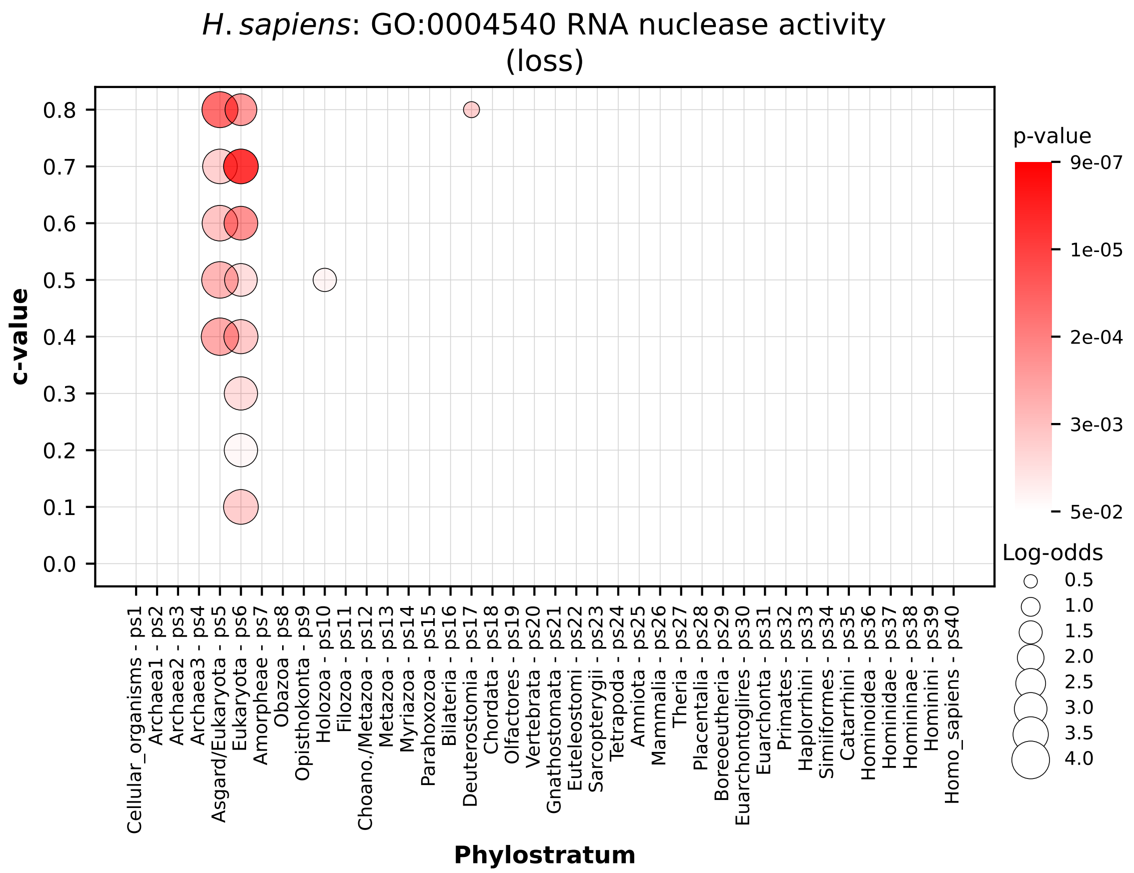

H. sapiens: GO:0004540 RNA nuclease activity
(loss)
c-value
0.0
0.1
0.2
0.3
0.4
0.5
0.6
0.7
0.8
Phylostratum
Cellular_organisms - ps1
Archaea1 - ps2
Archaea2 - ps3
Archaea3 - ps4
Asgard/Eukaryota - ps5
Eukaryota - ps6
Amorpheae - ps7
Obazoa - ps8
Opisthokonta - ps9
Holozoa - ps10
Filozoa - ps11
Choano./Metazoa - ps12
Metazoa - ps13
Myriazoa - ps14
Parahoxozoa - ps15
Bilateria - ps16
Deuterostomia - ps17
Chordata - ps18
Olfactores - ps19
Vertebrata - ps20
Gnathostomata - ps21
Euteleostomi - ps22
Sarcopterygii - ps23
Tetrapoda - ps24
Amniota - ps25
Mammalia - ps26
Theria - ps27
Placentalia - ps28
Boreoeutheria - ps29
Euarchontoglires - ps30
Euarchonta - ps31
Primates - ps32
Haplorrhini - ps33
Simiiformes - ps34
Catarrhini - ps35
Hominoidea - ps36
Hominidae - ps37
Homininae - ps38
Hominini - ps39
Homo_sapiens - ps40
p-value
9e-07
1e-05
2e-04
3e-03
5e-02
Log-odds
0.5
1.0
1.5
2.0
2.5
3.0
3.5
4.0

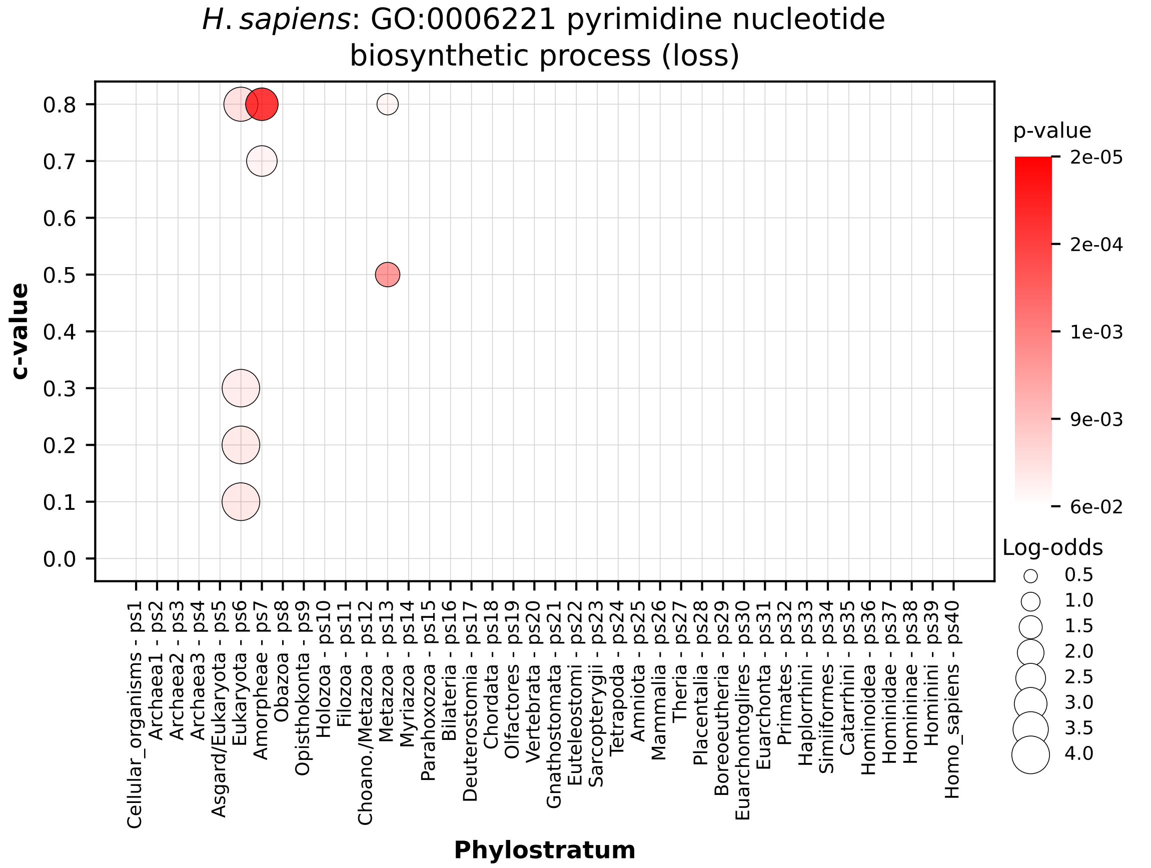

H. sapiens: GO:0006221 pyrimidine nucleotide biosynthetic process (loss)
c-value
0.8
0.7
0.6
0.5
0.4
0.3
0.2
0.1
0.0
p-value
2e-05
2e-04
1e-03
9e-03
6e-02
Log-odds
0.5
1.0
1.5
2.0
2.5
3.0
3.5
4.0
Cellular_organisms - ps1
Archaea1 - ps2
Archaea2 - ps3
Archaea3 - ps4
Asgard/Eukaryota - ps5
Eukaryota - ps6
Amorpheae - ps7
Obazoa - ps8
Opisthokonta - ps9
Holozoa - ps10
Filozoa - ps11
Choano./Metazoa - ps12
Metazoa - ps13
Myriazoa - ps14
Parahoxozoa - ps15
Bilateria - ps16
Deuterostomia - ps17
Chordata - ps18
Olfactores - ps19
Vertebrata - ps20
Gnathostomata - ps21
Euteleostomi - ps22
Sarcopterygii - ps23
Tetrapoda - ps24
Amniota - ps25
Mammalia - ps26
Theria - ps27
Placentalia - ps28
Boreoeutheria - ps29
Euarchontoglires - ps30
Euarchonta - ps31
Primates - ps32
Haplorrhini - ps33
Simiiformes - ps34
Catarrhini - ps35
Hominoidea - ps36
Hominidae - ps37
Homininae - ps38
Hominini - ps39
Homo_sapiens - ps40
Phylostratum

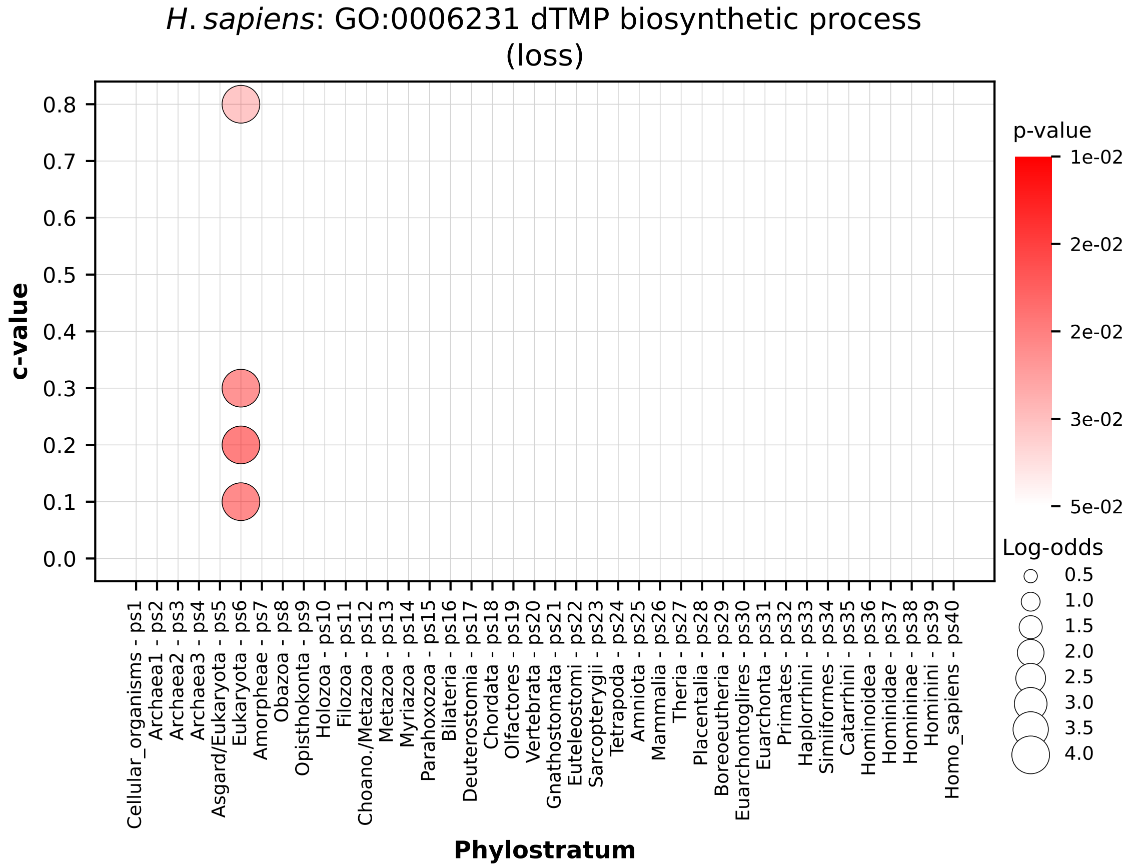

H. sapiens: GO:0006231 dTMP biosynthetic process (loss)
c-value
0.8
0.7
0.6
0.5
0.4
0.3
0.2
0.1
0.0
p-value
1e-02
2e-02
2e-02
3e-02
5e-02
Log-odds
0.5
1.0
1.5
2.0
2.5
3.0
3.5
4.0
Cellular_organisms - ps1
Archaea1 - ps2
Archaea2 - ps3
Archaea3 - ps4
Asgard/Eukaryota - ps5
Eukaryota - ps6
Amorpheae - ps7
Obazoa - ps8
Opisthokonta - ps9
Holozoa - ps10
Filozoa - ps11
Choano./Metazoa - ps12
Metazoa - ps13
Myriazoa - ps14
Parahoxozoa - ps15
Bilateria - ps16
Deuterostomia - ps17
Chordata - ps18
Olfactores - ps19
Vertebrata - ps20
Gnathostomata - ps21
Euteleostomi - ps22
Sarcopterygii - ps23
Tetrapoda - ps24
Amniota - ps25
Mammalia - ps26
Theria - ps27
Placentalia - ps28
Boreoeutheria - ps29
Euarchontoglires - ps30
Euarchonta - ps31
Primates - ps32
Haplorrhini - ps33
Simiiformes - ps34
Catarrhini - ps35
Hominoidea - ps36
Hominidae - ps37
Homininae - ps38
Hominini - ps39
Homo_sapiens - ps40
Phylostratum

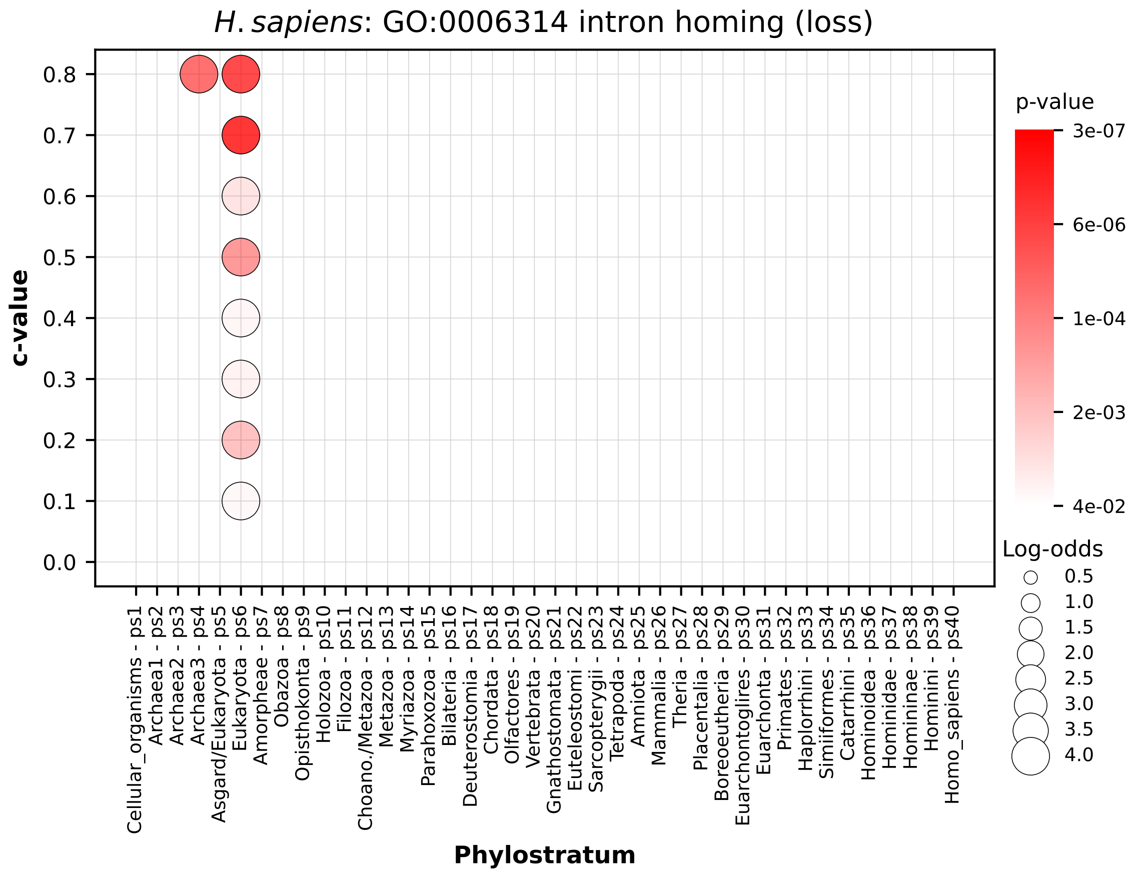
H. sapiens: GO:0006314 intron homing (loss)
c-value
0.0
0.1
0.2
0.3
0.4
0.5
0.6
0.7
0.8
p-value
3e-07
6e-06
1e-04
2e-03
4e-02
Log-odds
0.5
1.0
1.5
2.0
2.5
3.0
3.5
4.0
Cellular_organisms - ps1
Archaea1 - ps2
Archaea2 - ps3
Archaea3 - ps4
Asgard/Eukaryota - ps5
Eukaryota - ps6
Amorpheae - ps7
Obazoa - ps8
Opisthokonta - ps9
Holozoa - ps10
Filozoa - ps11
Choano./Metazoa - ps12
Metazoa - ps13
Myriazoa - ps14
Parahoxozoa - ps15
Bilateria - ps16
Deuterostomia - ps17
Chordata - ps18
Olfactores - ps19
Vertebrata - ps20
Gnathostomata - ps21
Euteleostomi - ps22
Sarcopterygii - ps23
Tetrapoda - ps24
Amniota - ps25
Mammalia - ps26
Theria - ps27
Placentalia - ps28
Boreoeutheria - ps29
Euarchontoglires - ps30
Euarchonta - ps31
Primates - ps32
Haplorrhini - ps33
Simiiformes - ps34
Catarrhini - ps35
Hominoidea - ps36
Hominidae - ps37
Homininae - ps38
Hominini - ps39
Homo_sapiens - ps40
Phylostratum

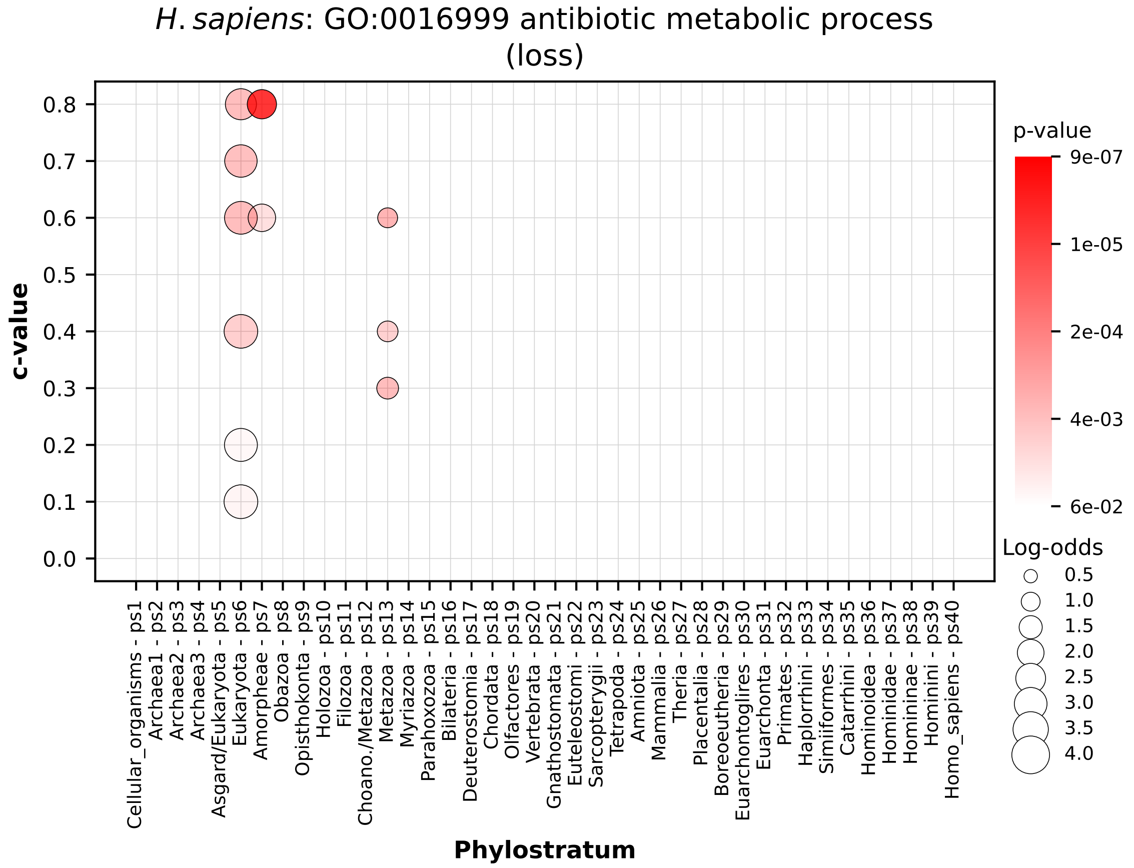

H. sapiens: GO:0016999 antibiotic metabolic process
(loss)
c-value
0.0
0.1
0.2
0.3
0.4
0.5
0.6
0.7
0.8
Phylostratum
Cellular_organisms - ps1
Archaea1 - ps2
Archaea2 - ps3
Archaea3 - ps4
Asgard/Eukaryota - ps5
Eukaryota - ps6
Amorpheae - ps7
Obazoa - ps8
Opisthokonta - ps9
Holozoa - ps10
Filozoa - ps11
Choano./Metazoa - ps12
Metazoa - ps13
Myriazoa - ps14
Parahoxozoa - ps15
Bilateria - ps16
Deuterostomia - ps17
Chordata - ps18
Olfactores - ps19
Vertebrata - ps20
Gnathostomata - ps21
Euteleostomi - ps22
Sarcopterygii - ps23
Tetrapoda - ps24
Amniota - ps25
Mammalia - ps26
Theria - ps27
Placentalia - ps28
Boreoeutheria - ps29
Euarchontoglires - ps30
Euarchonta - ps31
Primates - ps32
Haplorrhini - ps33
Simiiformes - ps34
Catarrhini - ps35
Hominoidea - ps36
Hominidae - ps37
Homininae - ps38
Hominini - ps39
Homo_sapiens - ps40
p-value
9e-07
1e-05
2e-04
4e-03
6e-02
Log-odds
0.5
1.0
1.5
2.0
2.5
3.0
3.5
4.0

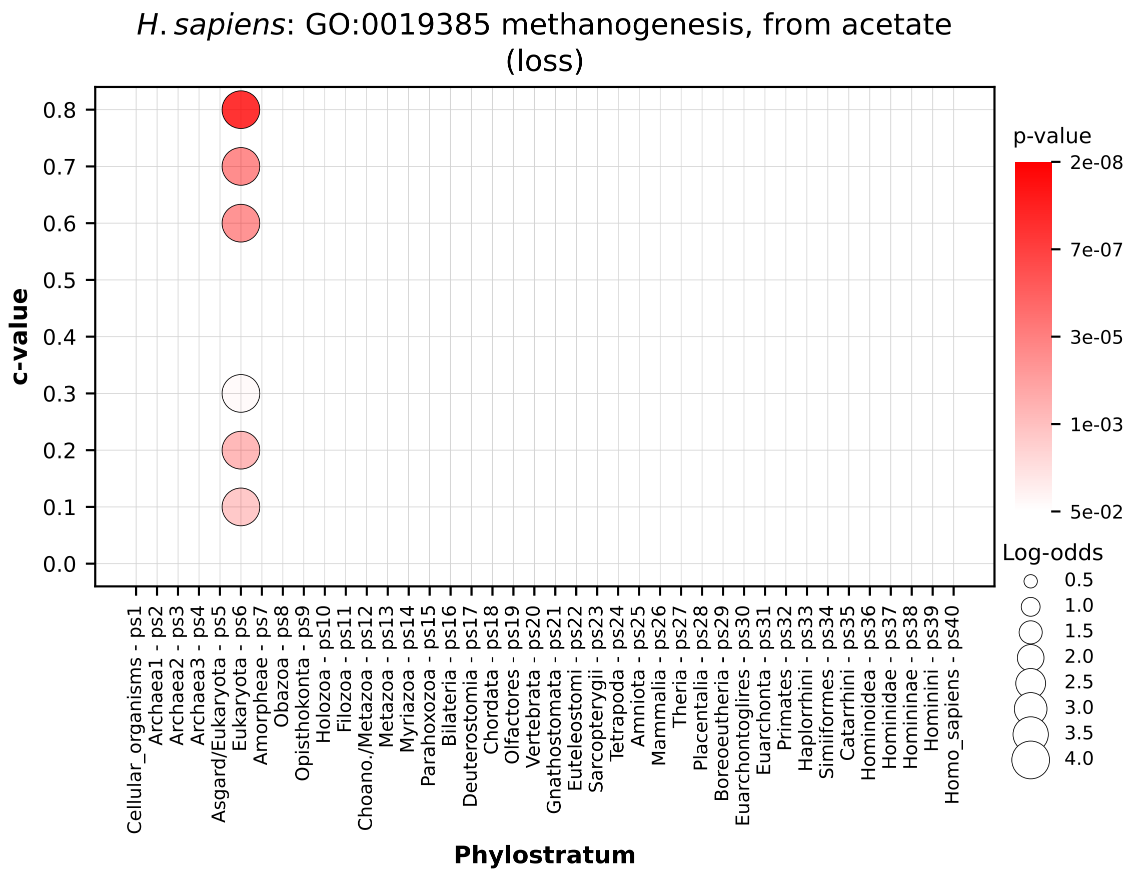

H. sapiens: GO:0019385 methanogenesis, from acetate (loss)
c-value
0.8
0.7
0.6
0.5
0.4
0.3
0.2
0.1
0.0
p-value
2e-08
7e-07
3e-05
1e-03
5e-02
Log-odds
0.5
1.0
1.5
2.0
2.5
3.0
3.5
4.0
Cellular_organisms - ps1
Archaea1 - ps2
Archaea2 - ps3
Archaea3 - ps4
Asgard/Eukaryota - ps5
Eukaryota - ps6
Amorpheae - ps7
Obazoa - ps8
Opisthokonta - ps9
Holozoa - ps10
Filozoa - ps11
Choano./Metazoa - ps12
Metazoa - ps13
Myriazoa - ps14
Parahoxozoa - ps15
Bilateria - ps16
Deuterostomia - ps17
Chordata - ps18
Olfactores - ps19
Vertebrata - ps20
Gnathostomata - ps21
Euteleostomi - ps22
Sarcopterygii - ps23
Tetrapoda - ps24
Amniota - ps25
Mammalia - ps26
Theria - ps27
Placentalia - ps28
Boreoeutheria - ps29
Euarchontoglires - ps30
Euarchonta - ps31
Primates - ps32
Haplorrhini - ps33
Simiiformes - ps34
Catarrhini - ps35
Hominoidea - ps36
Hominidae - ps37
Homininae - ps38
Hominini - ps39
Homo_sapiens - ps40
Phylostratum

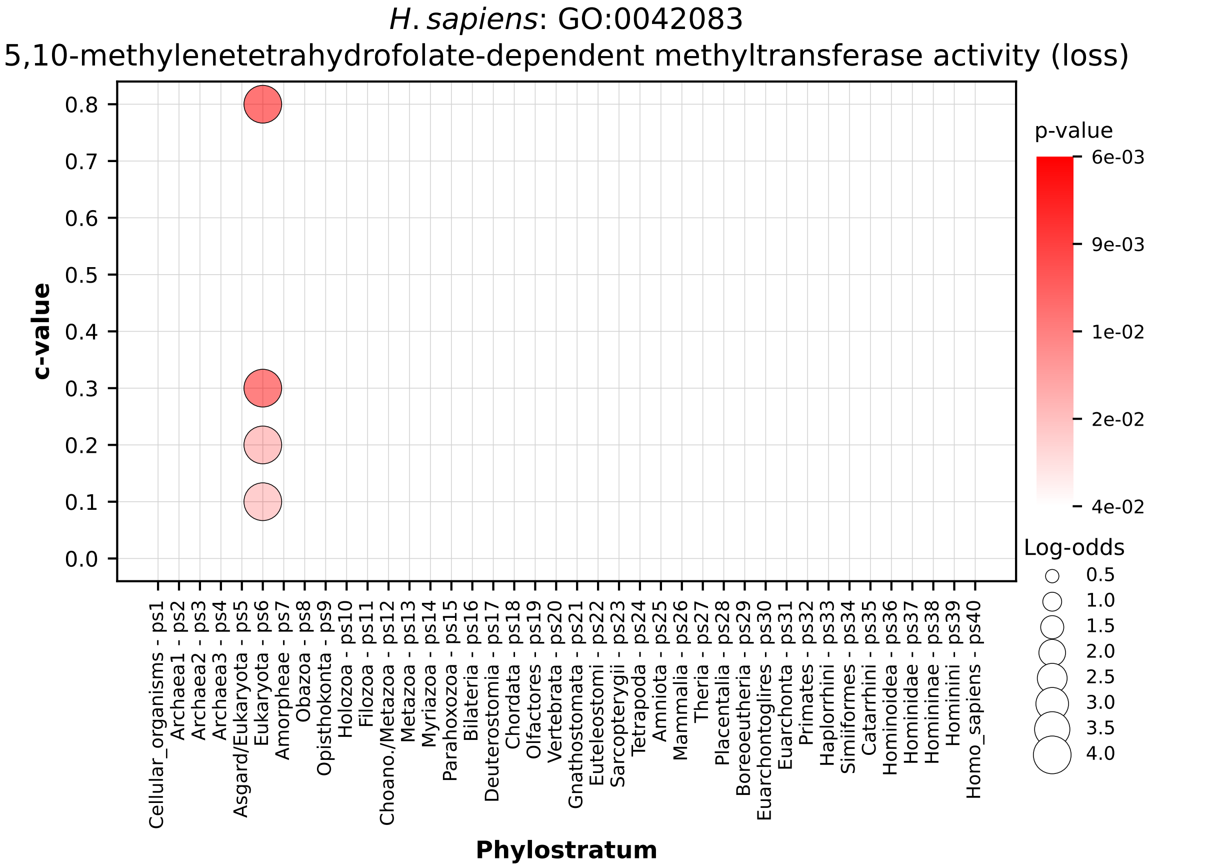

H. sapiens: GO:0042083
5,10-methylenetetrahydrofolate-dependent methyltransferase activity (loss)
c-value
0.8
0.7
0.6
0.5
0.4
0.3
0.2
0.1
0.0
p-value
6e-03
9e-03
1e-02
2e-02
4e-02
Log-odds
0.5
1.0
1.5
2.0
2.5
3.0
3.5
4.0
Cellular_organisms - ps1
Archaea1 - ps2
Archaea2 - ps3
Archaea3 - ps4
Asgard/Eukaryota - ps5
Eukaryota - ps6
Amorpheae - ps7
Obazoa - ps8
Opisthokonta - ps9
Holozoa - ps10
Filozoa - ps11
Choano./Metazoa - ps12
Metazoa - ps13
Myriazoa - ps14
Parahoxozoa - ps15
Bilateria - ps16
Deuterostomia - ps17
Chordata - ps18
Olfactores - ps19
Vertebrata - ps20
Gnathostomata - ps21
Euteleostomi - ps22
Sarcopterygii - ps23
Tetrapoda - ps24
Amniota - ps25
Mammalia - ps26
Theria - ps27
Placentalia - ps28
Boreoeutheria - ps29
Euarchontoglires - ps30
Euarchonta - ps31
Primates - ps32
Haplorrhini - ps33
Simiiformes - ps34
Catarrhini - ps35
Hominoidea - ps36
Hominidae - ps37
Homininae - ps38
Hominini - ps39
Homo_sapiens - ps40
Phylostratum

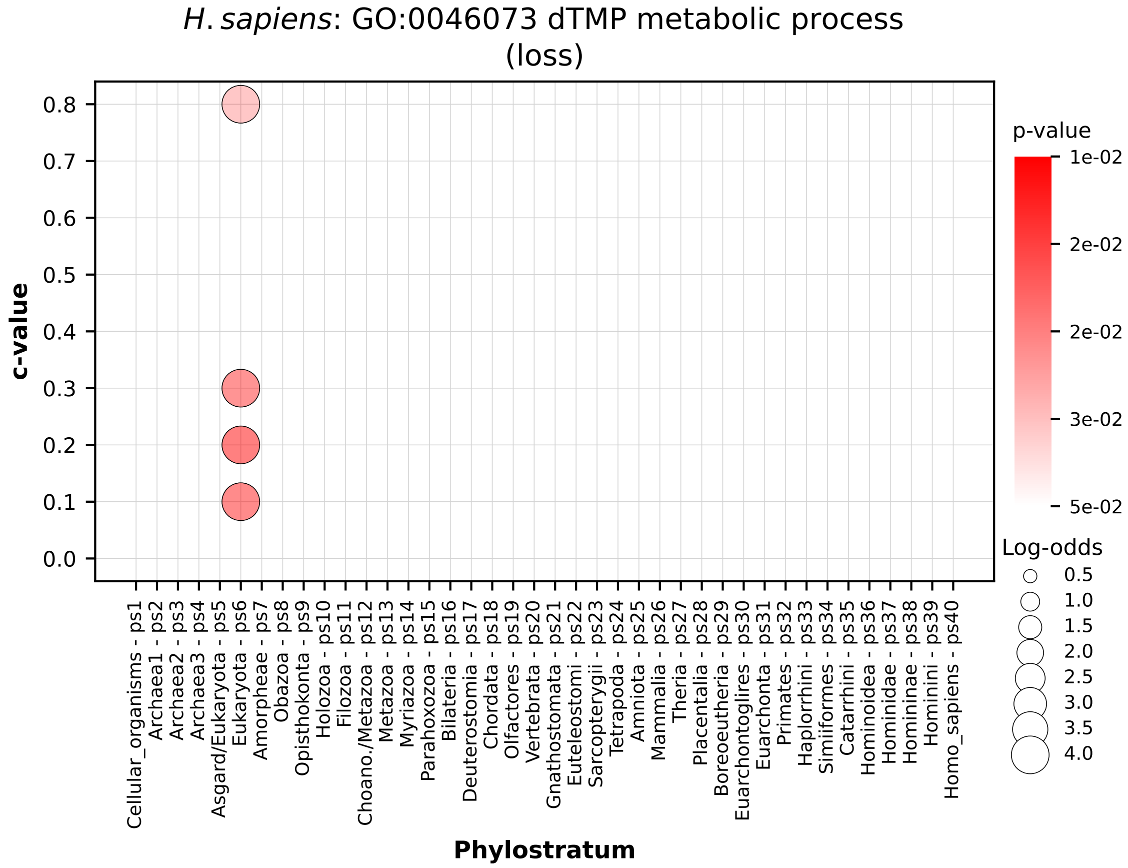

H. sapiens: GO:0046073 dTMP metabolic process
(loss)
c-value
0.8
0.7
0.6
0.5
0.4
0.3
0.2
0.1
0.0
Phylostratum
Cellular_organisms - ps1
Archaea1 - ps2
Archaea2 - ps3
Archaea3 - ps4
Asgard/Eukaryota - ps5
Eukaryota - ps6
Amorpheae - ps7
Obazoa - ps8
Opisthokonta - ps9
Holozoa - ps10
Filozoa - ps11
Choano./Metazoa - ps12
Metazoa - ps13
Myriazoa - ps14
Parahoxozoa - ps15
Bilateria - ps16
Deuterostomia - ps17
Chordata - ps18
Olfactores - ps19
Vertebrata - ps20
Gnathostomata - ps21
Euteleostomi - ps22
Sarcopterygii - ps23
Tetrapoda - ps24
Amniota - ps25
Mammalia - ps26
Theria - ps27
Placentalia - ps28
Boreoeutheria - ps29
Euarchontoglires - ps30
Euarchonta - ps31
Primates - ps32
Haplorrhini - ps33
Simiiformes - ps34
Catarrhini - ps35
Hominoidea - ps36
Hominidae - ps37
Homininae - ps38
Hominini - ps39
Homo_sapiens - ps40
p-value
1e-02
2e-02
2e-02
3e-02
5e-02
Log-odds
0.5
1.0
1.5
2.0
2.5
3.0
3.5
4.0

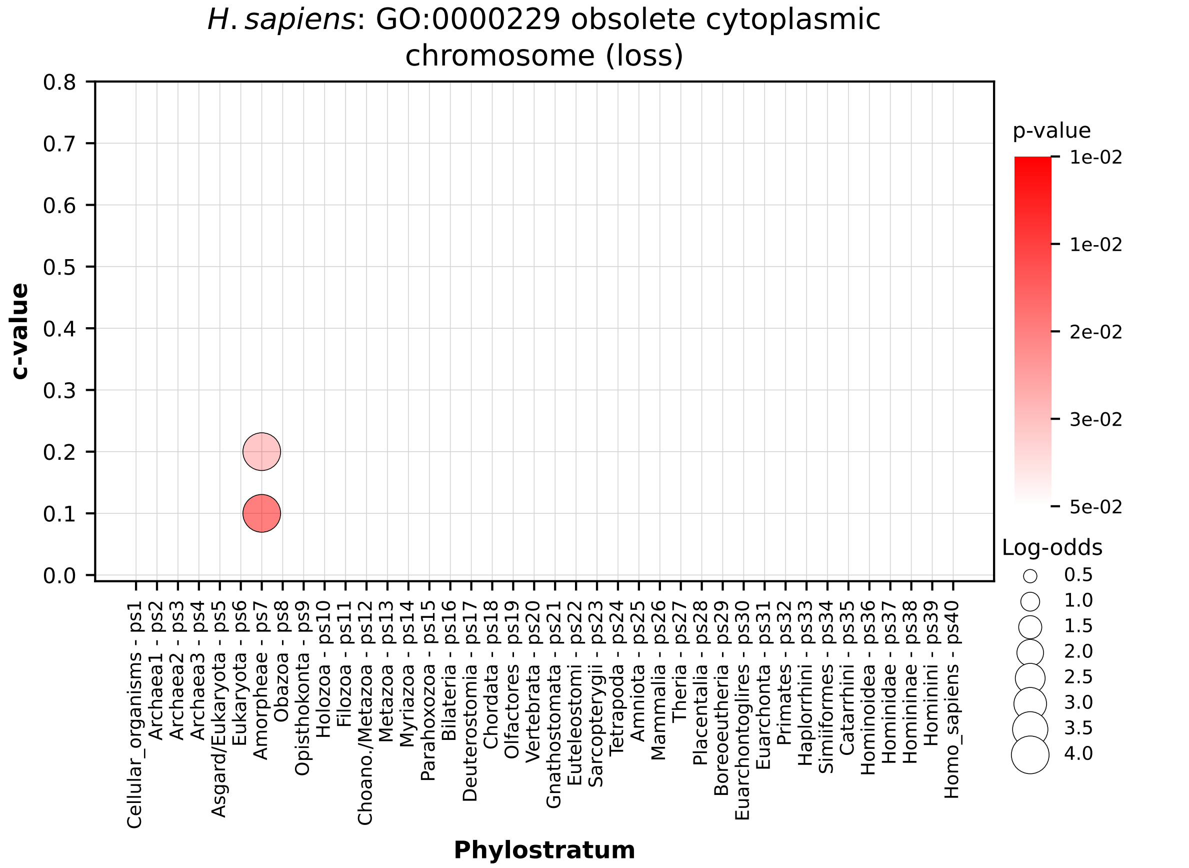

H. sapiens: GO:0000229 obsolete cytoplasmic chromosome (loss)
c-value
0.8
0.7
0.6
0.5
0.4
0.3
0.2
0.1
0.0
Phylostratum
Cellular_organisms - ps1
Archaea1 - ps2
Archaea2 - ps3
Archaea3 - ps4
Asgard/Eukaryota - ps5
Eukaryota - ps6
Amorpheae - ps7
Obazoa - ps8
Opisthokonta - ps9
Holozoa - ps10
Filozoa - ps11
Choano./Metazoa - ps12
Metazoa - ps13
Myriazoa - ps14
Parahoxozoa - ps15
Bilateria - ps16
Deuterostomia - ps17
Chordata - ps18
Olfactores - ps19
Vertebrata - ps20
Gnathostomata - ps21
Euteleostomi - ps22
Sarcopterygii - ps23
Tetrapoda - ps24
Amniota - ps25
Mammalia - ps26
Theria - ps27
Placentalia - ps28
Boreoeutheria - ps29
Euarchontoglires - ps30
Euarchonta - ps31
Primates - ps32
Haplorrhini - ps33
Simiiformes - ps34
Catarrhini - ps35
Hominoidea - ps36
Hominidae - ps37
Homininae - ps38
Hominini - ps39
Homo_sapiens - ps40
p-value
1e-02
1e-02
2e-02
3e-02
5e-02
Log-odds
0.5
1.0
1.5
2.0
2.5
3.0
3.5
4.0

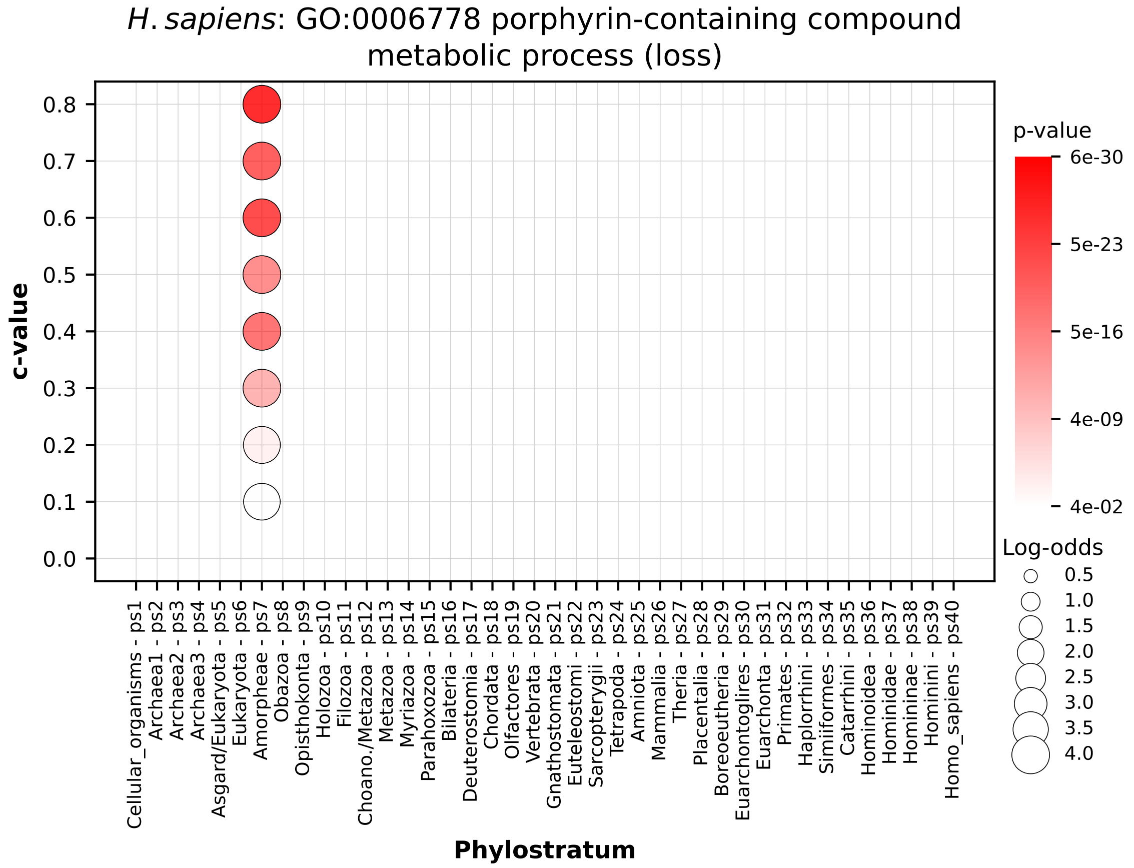

H. sapiens: GO:0006778 porphyrin-containing compound metabolic process (loss)
c-value
0.0
0.1
0.2
0.3
0.4
0.5
0.6
0.7
0.8
Phylostratum
Cellular_organisms - ps1
Archaea1 - ps2
Archaea2 - ps3
Archaea3 - ps4
Asgard/Eukaryota - ps5
Eukaryota - ps6
Amorpheae - ps7
Obazoa - ps8
Opisthokonta - ps9
Holozoa - ps10
Filozoa - ps11
Choano./Metazoa - ps12
Metazoa - ps13
Myriazoa - ps14
Parahoxozoa - ps15
Bilateria - ps16
Deuterostomia - ps17
Chordata - ps18
Olfactores - ps19
Vertebrata - ps20
Gnathostomata - ps21
Euteleostomi - ps22
Sarcopterygii - ps23
Tetrapoda - ps24
Amniota - ps25
Mammalia - ps26
Theria - ps27
Placentalia - ps28
Boreoeutheria - ps29
Euarchontoglires - ps30
Euarchonta - ps31
Primates - ps32
Haplorrhini - ps33
Simiiformes - ps34
Catarrhini - ps35
Hominoidea - ps36
Hominidae - ps37
Homininae - ps38
Hominini - ps39
Homo_sapiens - ps40
p-value
6e-30
5e-23
5e-16
4e-09
4e-02
Log-odds
0.5
1.0
1.5
2.0
2.5
3.0
3.5
4.0

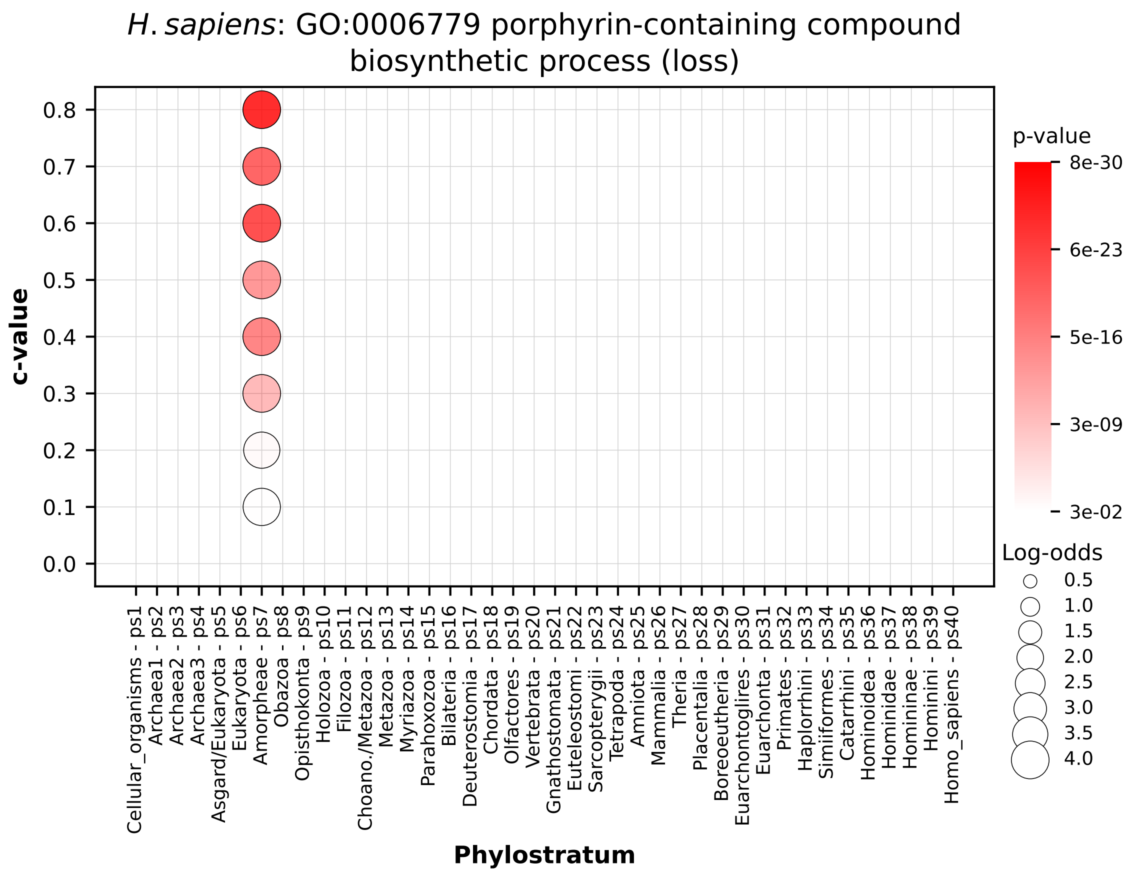

H. sapiens: GO:0006779 porphyrin-containing compound biosynthetic process (loss)
c-value
0.0
0.1
0.2
0.3
0.4
0.5
0.6
0.7
0.8
p-value
8e-30
6e-23
5e-16
3e-09
3e-02
Log-odds
0.5
1.0
1.5
2.0
2.5
3.0
3.5
4.0
Cellular_organisms - ps1
Archaea1 - ps2
Archaea2 - ps3
Archaea3 - ps4
Asgard/Eukaryota - ps5
Eukaryota - ps6
Amorpheae - ps7
Obazoa - ps8
Opisthokonta - ps9
Holozoa - ps10
Filozoa - ps11
Choano./Metazoa - ps12
Metazoa - ps13
Myriazoa - ps14
Parahoxozoa - ps15
Bilateria - ps16
Deuterostomia - ps17
Chordata - ps18
Olfactores - ps19
Vertebrata - ps20
Gnathostomata - ps21
Euteleostomi - ps22
Sarcopterygii - ps23
Tetrapoda - ps24
Amniota - ps25
Mammalia - ps26
Theria - ps27
Placentalia - ps28
Boreoeutheria - ps29
Euarchontoglires - ps30
Euarchonta - ps31
Primates - ps32
Haplorrhini - ps33
Simiiformes - ps34
Catarrhini - ps35
Hominoidea - ps36
Hominidae - ps37
Homininae - ps38
Hominini - ps39
Homo_sapiens - ps40
Phylostratum

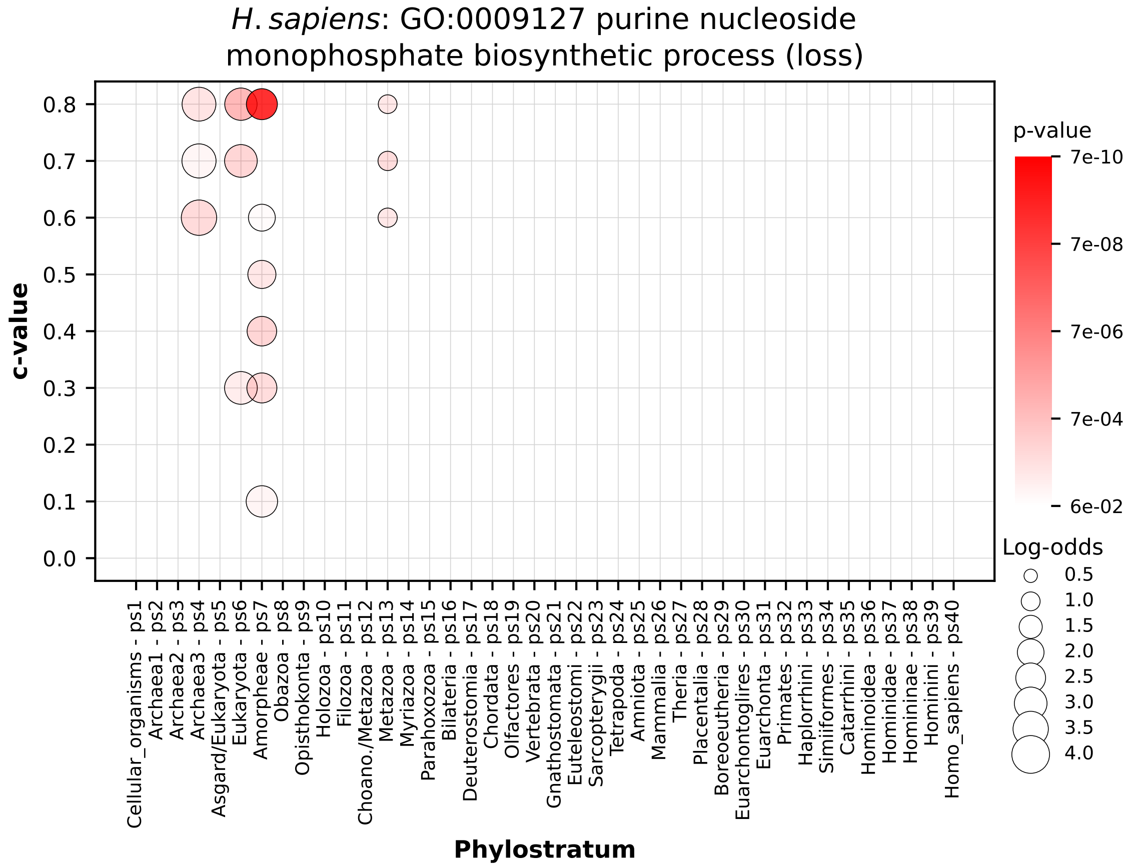

$H. sapiens$: GO:0009127 purine nucleoside monophosphate biosynthetic process (loss)
c-value
0.8
0.7
0.6
0.5
0.4
0.3
0.2
0.1
0.0
Phylostratum
Cellular_organisms - ps1
Archaea1 - ps2
Archaea2 - ps3
Archaea3 - ps4
Asgard/Eukaryota - ps5
Eukaryota - ps6
Amorpheae - ps7
Obazoa - ps8
Opisthokonta - ps9
Holozoa - ps10
Filozoa - ps11
Choano./Metazoa - ps12
Metazoa - ps13
Myriazoa - ps14
Parahoxozoa - ps15
Bilateria - ps16
Deuterostomia - ps17
Chordata - ps18
Olfactores - ps19
Vertebrata - ps20
Gnathostomata - ps21
Euteleostomi - ps22
Sarcopterygii - ps23
Tetrapoda - ps24
Amniota - ps25
Mammalia - ps26
Theria - ps27
Placentalia - ps28
Boreoeutheria - ps29
Euarchontoglires - ps30
Euarchonta - ps31
Primates - ps32
Haplorrhini - ps33
Simiiformes - ps34
Catarrhini - ps35
Hominoidea - ps36
Hominidae - ps37
Homininae - ps38
Hominini - ps39
Homo_sapiens - ps40
p-value
7e-10
7e-08
7e-06
7e-04
6e-02
Log-odds
0.5
1.0
1.5
2.0
2.5
3.0
3.5
4.0

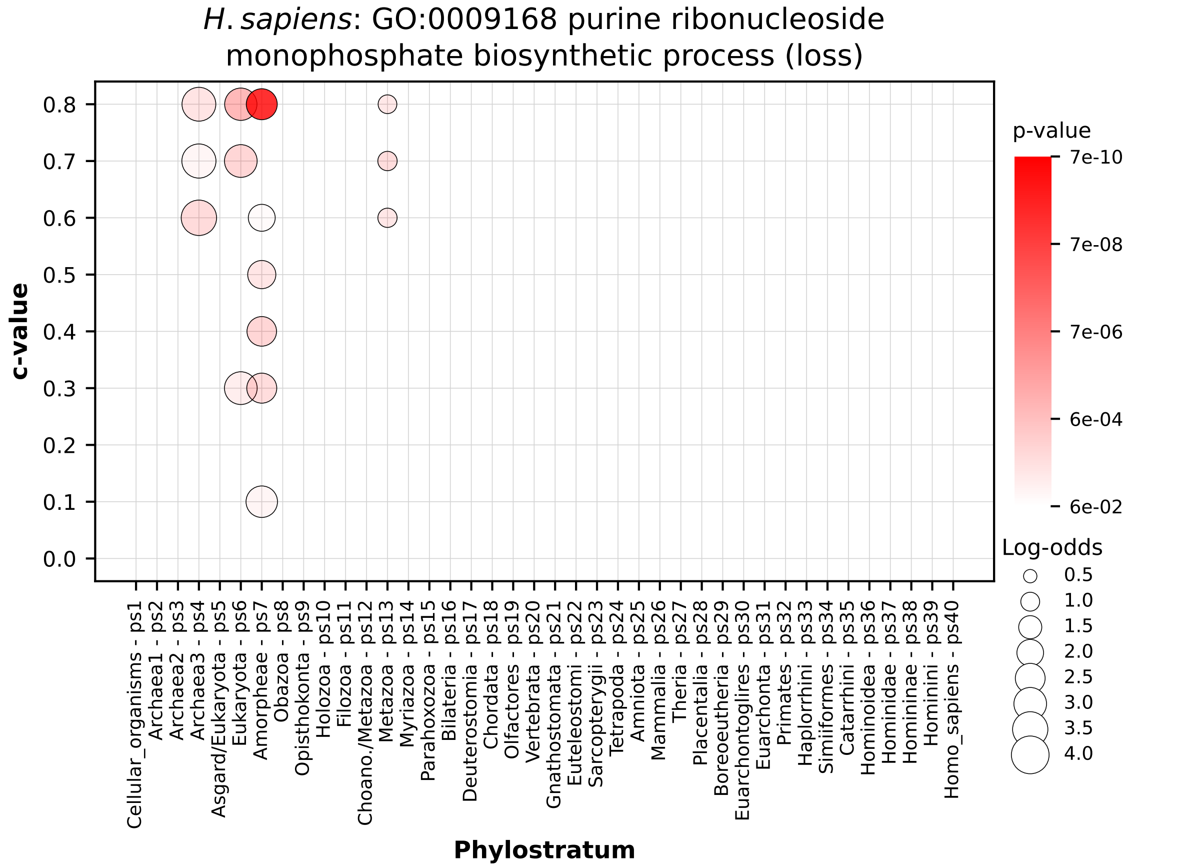
H. sapiens: GO:0009168 purine ribonucleoside monophosphate biosynthetic process (loss)
c-value
0.8
0.7
0.6
0.5
0.4
0.3
0.2
0.1
0.0
Phylostratum
Cellular_organisms - ps1
Archaea1 - ps2
Archaea2 - ps3
Archaea3 - ps4
Asgard/Eukaryota - ps5
Eukaryota - ps6
Amorpheae - ps7
Obazoa - ps8
Opisthokonta - ps9
Holozoa - ps10
Filozoa - ps11
Choano./Metazoa - ps12
Metazoa - ps13
Myriazoa - ps14
Parahoxozoa - ps15
Bilateria - ps16
Deuterostomia - ps17
Chordata - ps18
Olfactores - ps19
Vertebrata - ps20
Gnathostomata - ps21
Euteleostomi - ps22
Sarcopterygii - ps23
Tetrapoda - ps24
Amniota - ps25
Mammalia - ps26
Theria - ps27
Placentalia - ps28
Boreoeutheria - ps29
Euarchontoglires - ps30
Euarchonta - ps31
Primates - ps32
Haplorrhini - ps33
Simiiformes - ps34
Catarrhini - ps35
Hominoidea - ps36
Hominidae - ps37
Homininae - ps38
Hominini - ps39
Homo_sapiens - ps40
p-value
7e-10
7e-08
7e-06
6e-04
6e-02
Log-odds
0.5
1.0
1.5
2.0
2.5
3.0
3.5
4.0

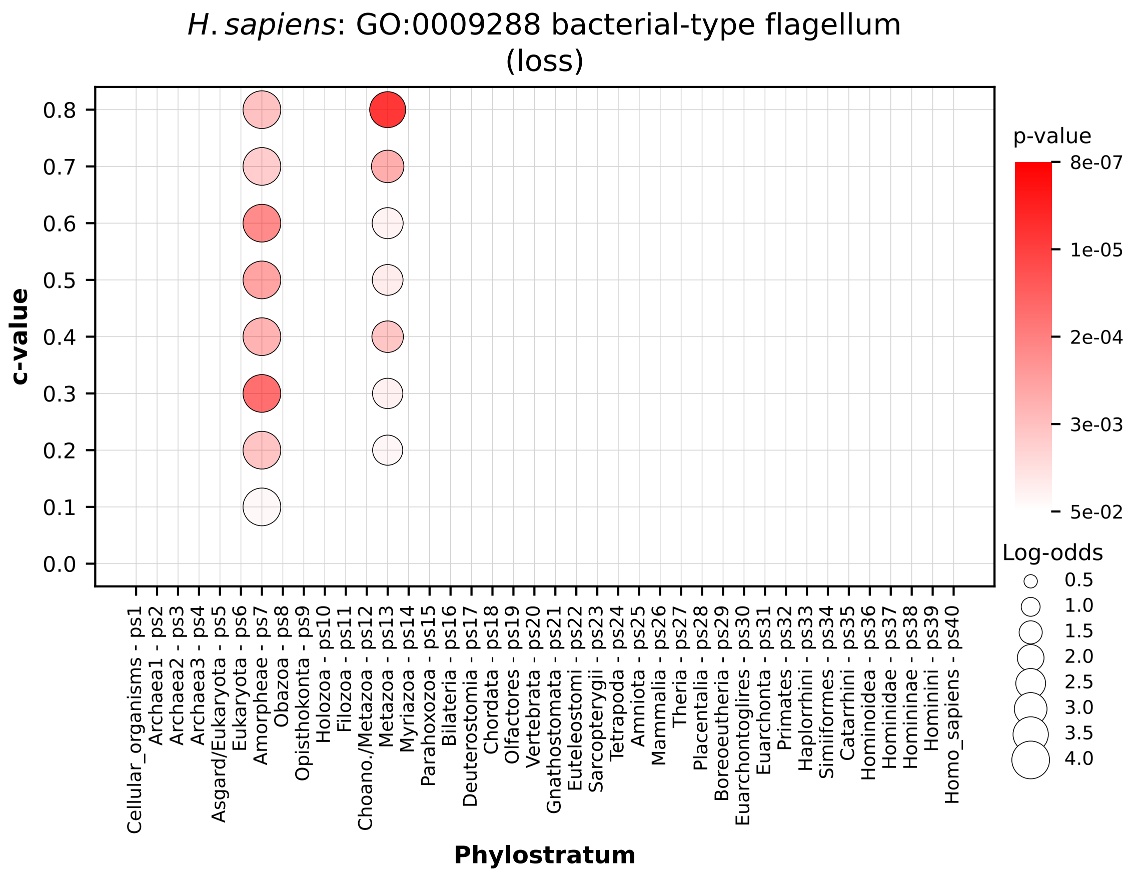
H. sapiens: GO:0009288 bacterial-type flagellum
(loss)
c-value
0.0
0.1
0.2
0.3
0.4
0.5
0.6
0.7
0.8
Phylostratum
Cellular_organisms - ps1
Archaea1 - ps2
Archaea2 - ps3
Archaea3 - ps4
Asgard/Eukaryota - ps5
Eukaryota - ps6
Amorpheae - ps7
Obazoa - ps8
Opisthokonta - ps9
Holozoa - ps10
Filozoa - ps11
Choano./Metazoa - ps12
Metazoa - ps13
Myriazoa - ps14
Parahoxozoa - ps15
Bilateria - ps16
Deuterostomia - ps17
Chordata - ps18
Olfactores - ps19
Vertebrata - ps20
Gnathostomata - ps21
Euteleostomi - ps22
Sarcopterygii - ps23
Tetrapoda - ps24
Amniota - ps25
Mammalia - ps26
Theria - ps27
Placentalia - ps28
Boreoeutheria - ps29
Euarchontoglires - ps30
Euarchonta - ps31
Primates - ps32
Haplorrhini - ps33
Simiiformes - ps34
Catarrhini - ps35
Hominoidea - ps36
Hominidae - ps37
Homininae - ps38
Hominini - ps39
Homo_sapiens - ps40
p-value
8e-07
1e-05
2e-04
3e-03
5e-02
Log-odds
0.5
1.0
1.5
2.0
2.5
3.0
3.5
4.0

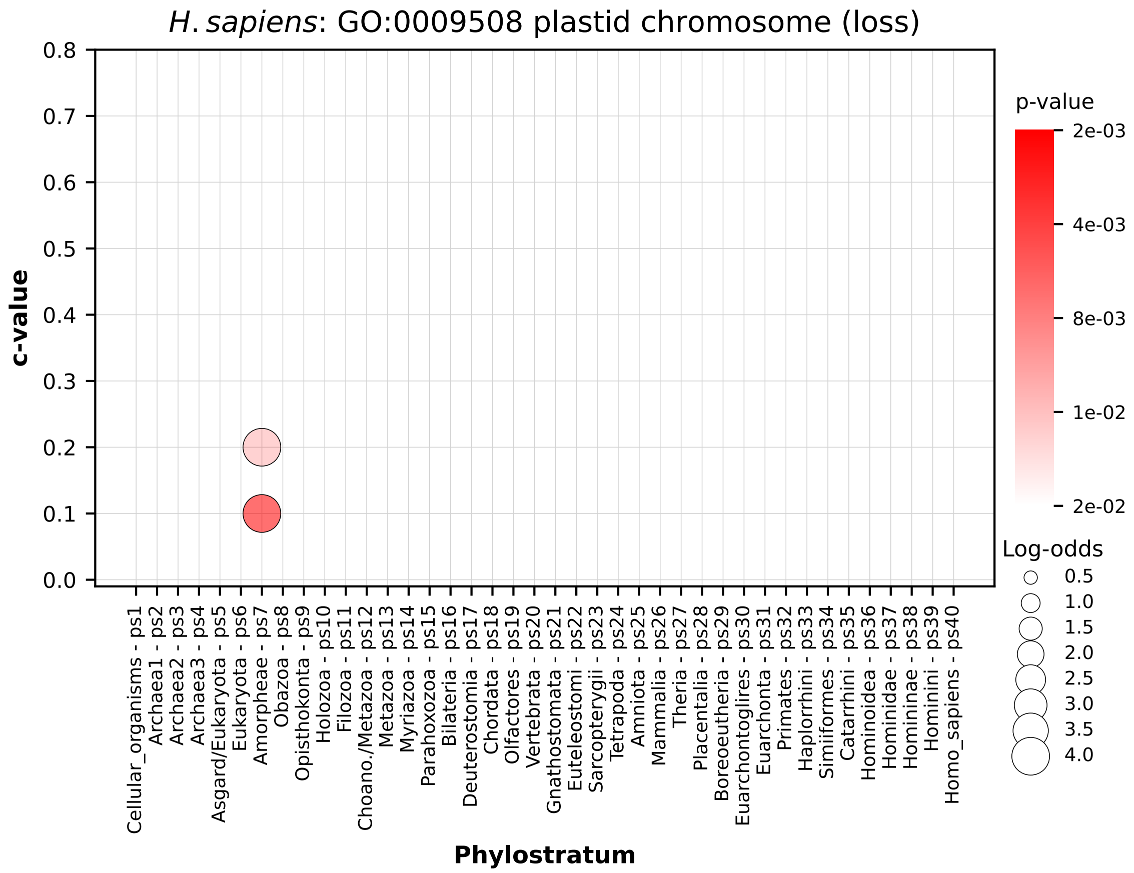

H. sapiens: GO:0009508 plastid chromosome (loss)
c-value
0.0
0.1
0.2
0.3
0.4
0.5
0.6
0.7
0.8
Phylostratum
Cellular_organisms - ps1
Archaea1 - ps2
Archaea2 - ps3
Archaea3 - ps4
Asgard/Eukaryota - ps5
Eukaryota - ps6
Amorpheae - ps7
Obazoa - ps8
Opisthokonta - ps9
Holozoa - ps10
Filozoa - ps11
Choano./Metazoa - ps12
Metazoa - ps13
Myriazoa - ps14
Parahoxozoa - ps15
Bilateria - ps16
Deuterostomia - ps17
Chordata - ps18
Olfactores - ps19
Vertebrata - ps20
Gnathostomata - ps21
Euteleostomi - ps22
Sarcopterygii - ps23
Tetrapoda - ps24
Amniota - ps25
Mammalia - ps26
Theria - ps27
Placentalia - ps28
Boreoeutheria - ps29
Euarchontoglires - ps30
Euarchonta - ps31
Primates - ps32
Haplorrhini - ps33
Simiiformes - ps34
Catarrhini - ps35
Hominoidea - ps36
Hominidae - ps37
Homininae - ps38
Hominini - ps39
Homo_sapiens - ps40
p-value
2e-03
4e-03
8e-03
1e-02
2e-02
Log-odds
0.5
1.0
1.5
2.0
2.5
3.0
3.5
4.0

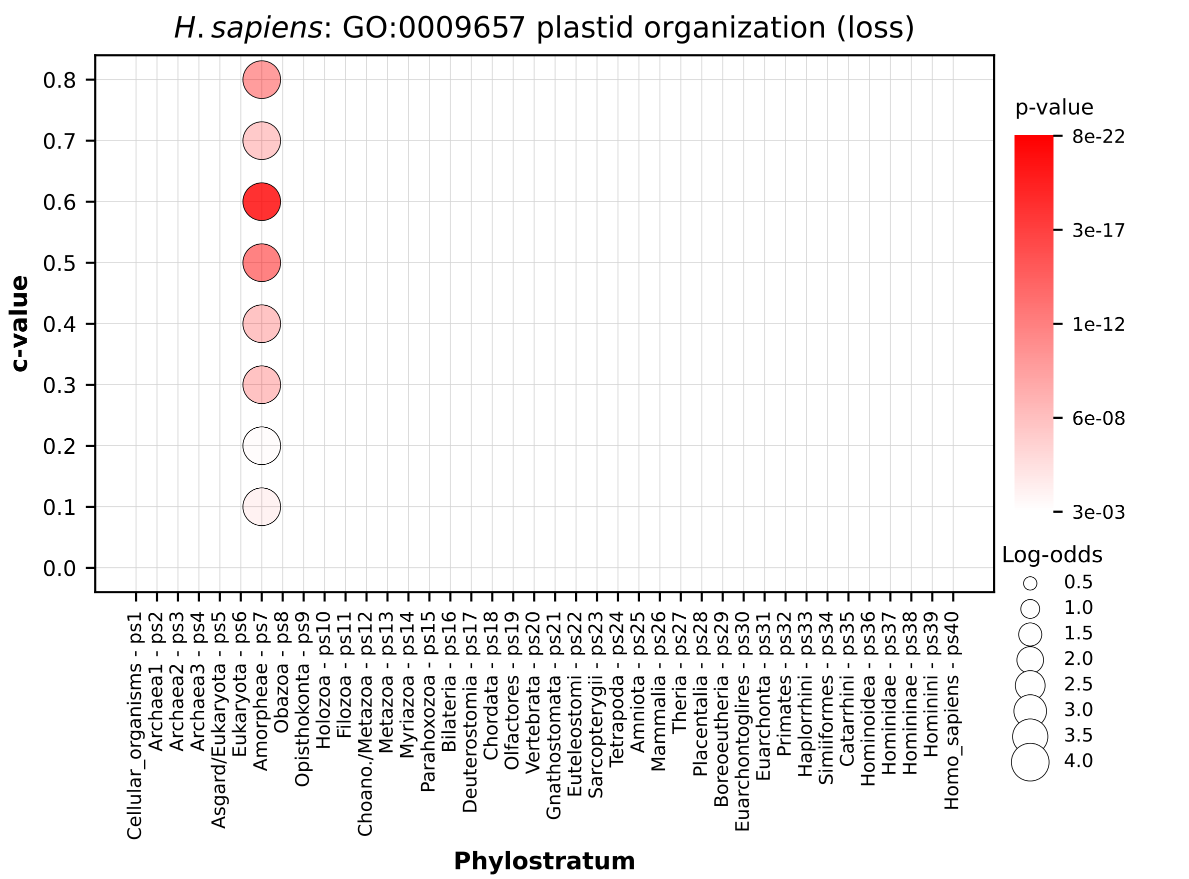

H. sapiens: GO:0009657 plastid organization (loss)
c-value
0.8
0.7
0.6
0.5
0.4
0.3
0.2
0.1
0.0
p-value
8e-22
3e-17
1e-12
6e-08
3e-03
Log-odds
0.5
1.0
1.5
2.0
2.5
3.0
3.5
4.0
Cellular_organisms - ps1
Archaea1 - ps2
Archaea2 - ps3
Archaea3 - ps4
Asgard/Eukaryota - ps5
Eukaryota - ps6
Amorpheae - ps7
Obazoa - ps8
Opisthokonta - ps9
Holozoa - ps10
Filozoa - ps11
Choano./Metazoa - ps12
Metazoa - ps13
Myriazoa - ps14
Parahoxozoa - ps15
Bilateria - ps16
Deuterostomia - ps17
Chordata - ps18
Olfactores - ps19
Vertebrata - ps20
Gnathostomata - ps21
Euteleostomi - ps22
Sarcopterygii - ps23
Tetrapoda - ps24
Amniota - ps25
Mammalia - ps26
Theria - ps27
Placentalia - ps28
Boreoeutheria - ps29
Euarchontoglires - ps30
Euarchonta - ps31
Primates - ps32
Haplorrhini - ps33
Simiiformes - ps34
Catarrhini - ps35
Hominoidea - ps36
Hominidae - ps37
Homininae - ps38
Hominini - ps39
Homo_sapiens - ps40
Phylostratum

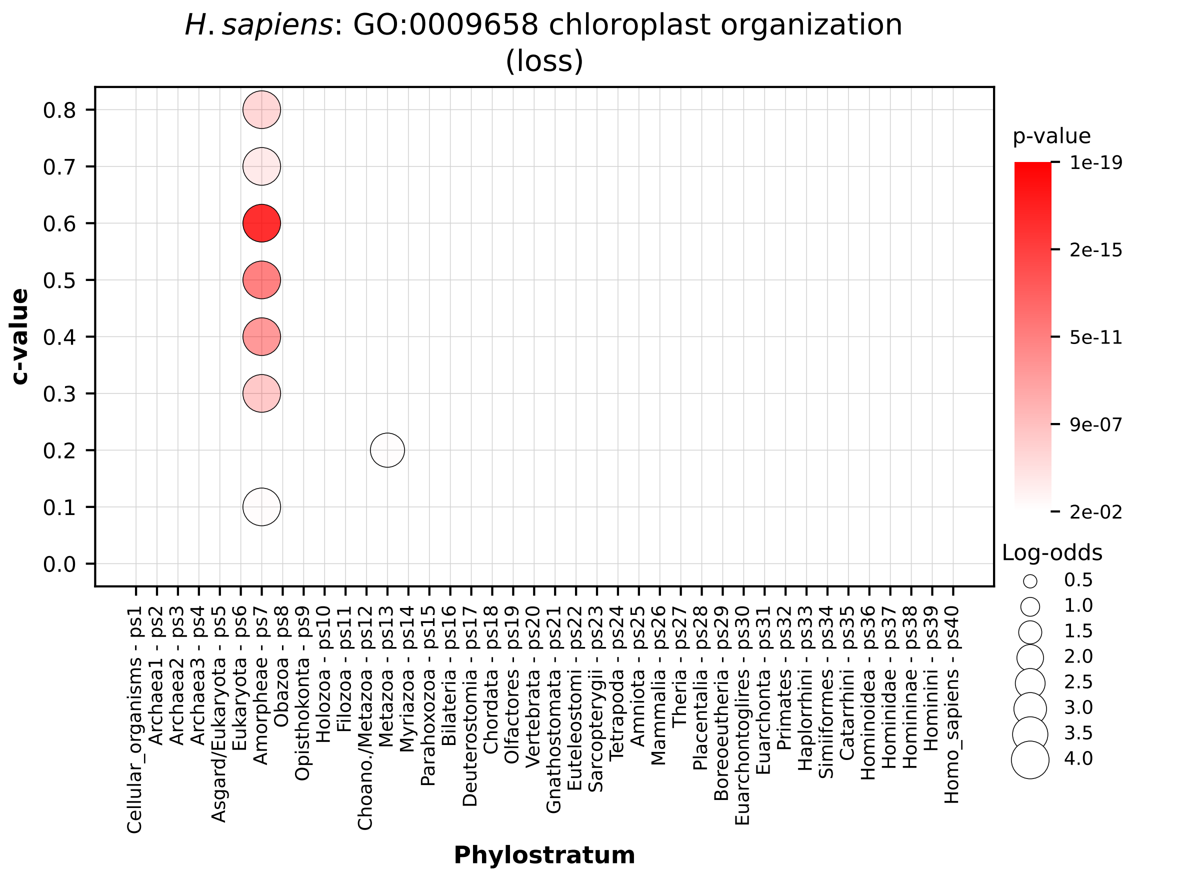

H. sapiens: GO:0009658 chloroplast organization
(loss)
c-value
0.8
0.7
0.6
0.5
0.4
0.3
0.2
0.1
0.0
Phylostratum
Cellular_organisms - ps1
Archaea1 - ps2
Archaea2 - ps3
Archaea3 - ps4
Asgard/Eukaryota - ps5
Eukaryota - ps6
Amorpheae - ps7
Obazoa - ps8
Opisthokonta - ps9
Holozoa - ps10
Filozoa - ps11
Choano./Metazoa - ps12
Metazoa - ps13
Myriazoa - ps14
Parahoxozoa - ps15
Bilateria - ps16
Deuterostomia - ps17
Chordata - ps18
Olfactores - ps19
Vertebrata - ps20
Gnathostomata - ps21
Euteleostomi - ps22
Sarcopterygii - ps23
Tetrapoda - ps24
Amniota - ps25
Mammalia - ps26
Theria - ps27
Placentalia - ps28
Boreoeutheria - ps29
Euarchontoglires - ps30
Euarchonta - ps31
Primates - ps32
Haplorrhini - ps33
Simiiformes - ps34
Catarrhini - ps35
Hominoidea - ps36
Hominidae - ps37
Homininae - ps38
Hominini - ps39
Homo_sapiens - ps40
p-value
1e-19
2e-15
5e-11
9e-07
2e-02
Log-odds
0.5
1.0
1.5
2.0
2.5
3.0
3.5
4.0

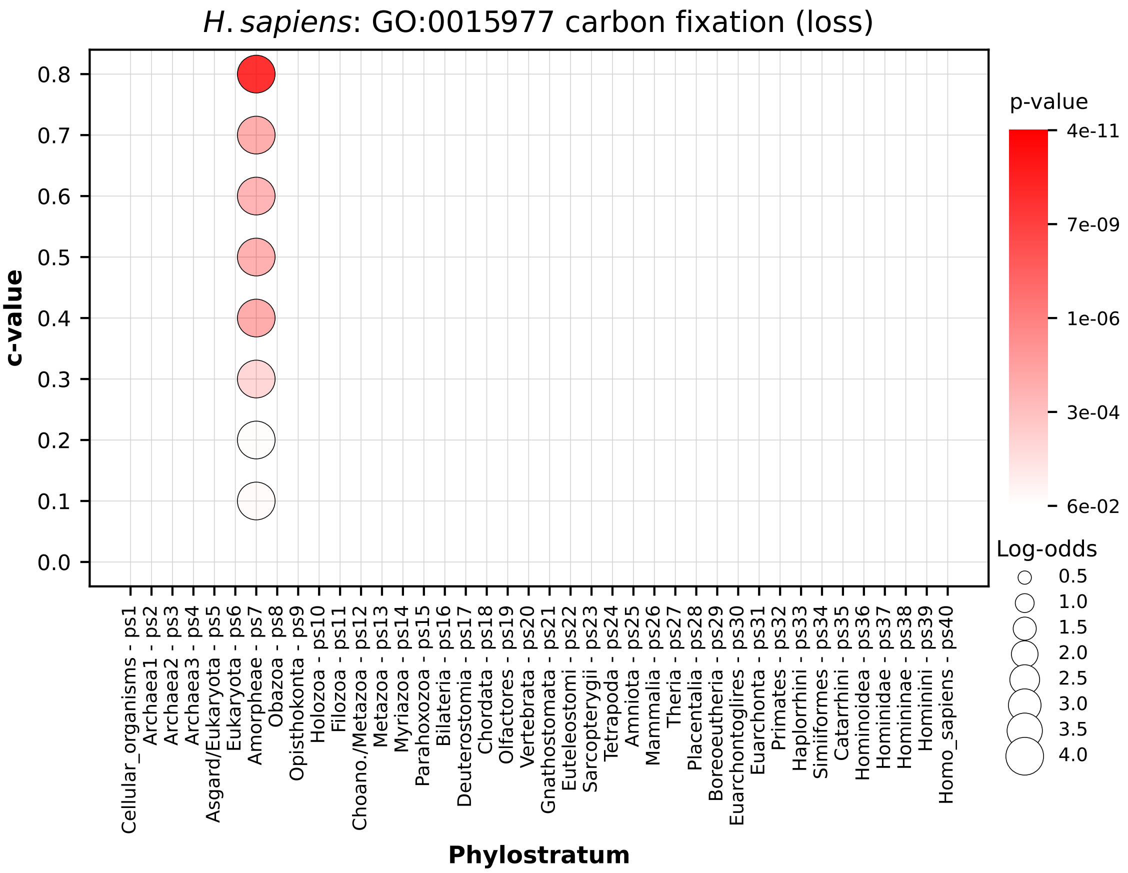
H. sapiens: GO:0015977 carbon fixation (loss)
c-value
0.0
0.1
0.2
0.3
0.4
0.5
0.6
0.7
0.8
Phylostratum
Cellular_organisms - ps1
Archaea1 - ps2
Archaea2 - ps3
Archaea3 - ps4
Asgard/Eukaryota - ps5
Eukaryota - ps6
Amorpheae - ps7
Obazoa - ps8
Opisthokonta - ps9
Holozoa - ps10
Filozoa - ps11
Choano./Metazoa - ps12
Metazoa - ps13
Myriazoa - ps14
Parahoxozoa - ps15
Bilateria - ps16
Deuterostomia - ps17
Chordata - ps18
Olfactores - ps19
Vertebrata - ps20
Gnathostomata - ps21
Euteleostomi - ps22
Sarcopterygii - ps23
Tetrapoda - ps24
Amniota - ps25
Mammalia - ps26
Theria - ps27
Placentalia - ps28
Boreoeutheria - ps29
Euarchontoglires - ps30
Euarchonta - ps31
Primates - ps32
Haplorrhini - ps33
Simiiformes - ps34
Catarrhini - ps35
Hominoidea - ps36
Hominidae - ps37
Homininae - ps38
Hominini - ps39
Homo_sapiens - ps40
p-value
4e-11
7e-09
1e-06
3e-04
6e-02
Log-odds
0.5
1.0
1.5
2.0
2.5
3.0
3.5
4.0

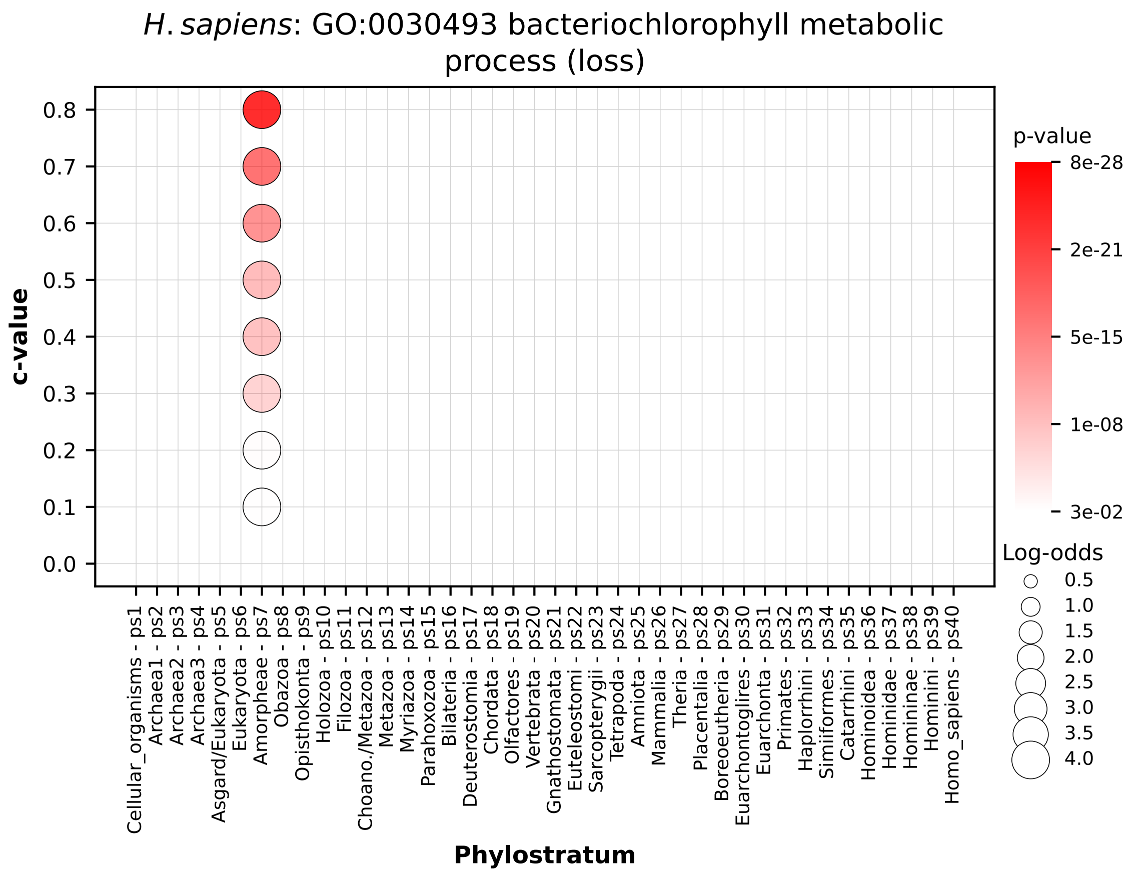

H. sapiens: GO:0030493 bacteriochlorophyll metabolic process (loss)
c-value
0.0
0.1
0.2
0.3
0.4
0.5
0.6
0.7
0.8
Phylostratum
Cellular_organisms - ps1
Archaea1 - ps2
Archaea2 - ps3
Archaea3 - ps4
Asgard/Eukaryota - ps5
Eukaryota - ps6
Amorpheae - ps7
Obazoa - ps8
Opisthokonta - ps9
Holozoa - ps10
Filozoa - ps11
Choano./Metazoa - ps12
Metazoa - ps13
Myriazoa - ps14
Parahoxozoa - ps15
Bilateria - ps16
Deuterostomia - ps17
Chordata - ps18
Olfactores - ps19
Vertebrata - ps20
Gnathostomata - ps21
Euteleostomi - ps22
Sarcopterygii - ps23
Tetrapoda - ps24
Amniota - ps25
Mammalia - ps26
Theria - ps27
Placentalia - ps28
Boreoeutheria - ps29
Euarchontoglires - ps30
Euarchonta - ps31
Primates - ps32
Haplorrhini - ps33
Simiiformes - ps34
Catarrhini - ps35
Hominoidea - ps36
Hominidae - ps37
Homininae - ps38
Hominini - ps39
Homo_sapiens - ps40
p-value
8e-28
2e-21
5e-15
1e-08
3e-02
Log-odds
0.5
1.0
1.5
2.0
2.5
3.0
3.5
4.0

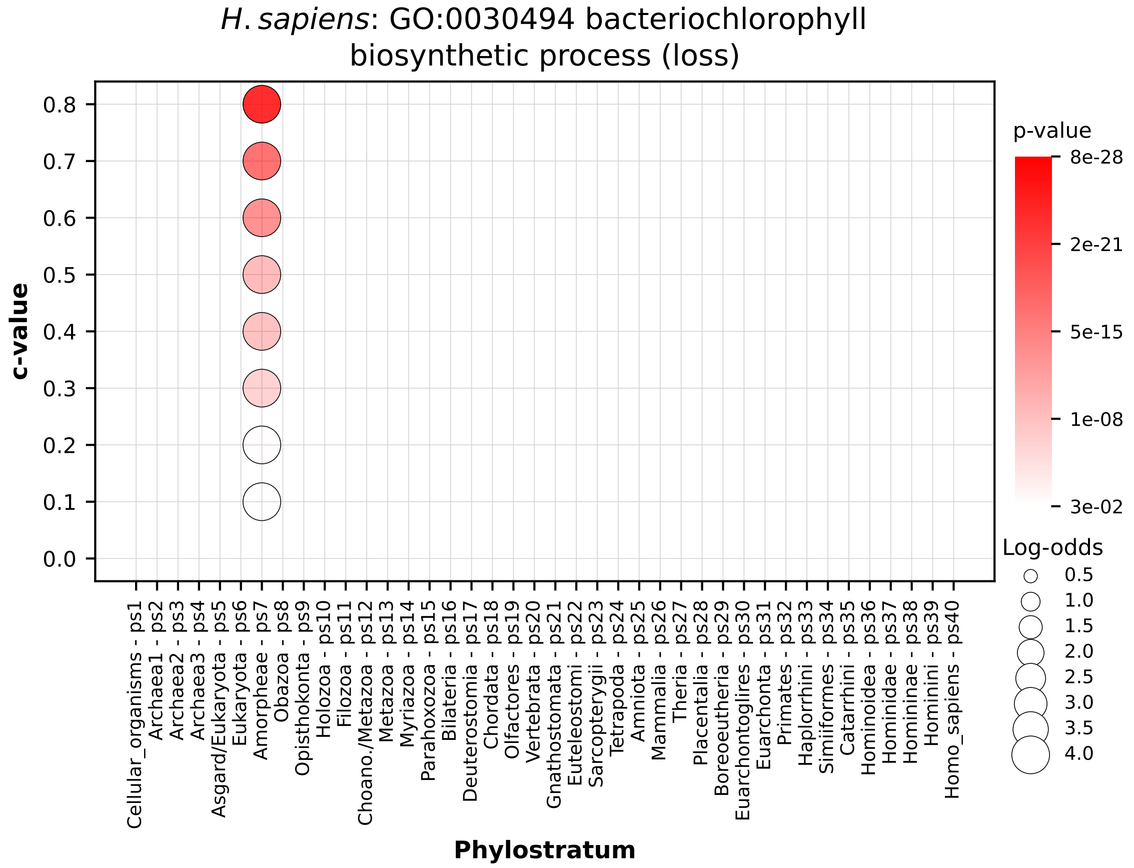

H. sapiens: GO:0030494 bacteriochlorophyll biosynthetic process (loss)
c-value
0.8
0.7
0.6
0.5
0.4
0.3
0.2
0.1
0.0
Phylostratum
Cellular_organisms - ps1
Archaea1 - ps2
Archaea2 - ps3
Archaea3 - ps4
Asgard/Eukaryota - ps5
Eukaryota - ps6
Amorpheae - ps7
Obazoa - ps8
Opisthokonta - ps9
Holozoa - ps10
Filozoa - ps11
Choano./Metazoa - ps12
Metazoa - ps13
Myriazoa - ps14
Parahoxozoa - ps15
Bilateria - ps16
Deuterostomia - ps17
Chordata - ps18
Olfactores - ps19
Vertebrata - ps20
Gnathostomata - ps21
Euteleostomi - ps22
Sarcopterygii - ps23
Tetrapoda - ps24
Amniota - ps25
Mammalia - ps26
Theria - ps27
Placentalia - ps28
Boreoeutheria - ps29
Euarchontoglires - ps30
Euarchonta - ps31
Primates - ps32
Haplorrhini - ps33
Simiiformes - ps34
Catarrhini - ps35
Hominoidea - ps36
Hominidae - ps37
Homininae - ps38
Hominini - ps39
Homo_sapiens - ps40
p-value
8e-28
2e-21
5e-15
1e-08
3e-02
Log-odds
0.5
1.0
1.5
2.0
2.5
3.0
3.5
4.0

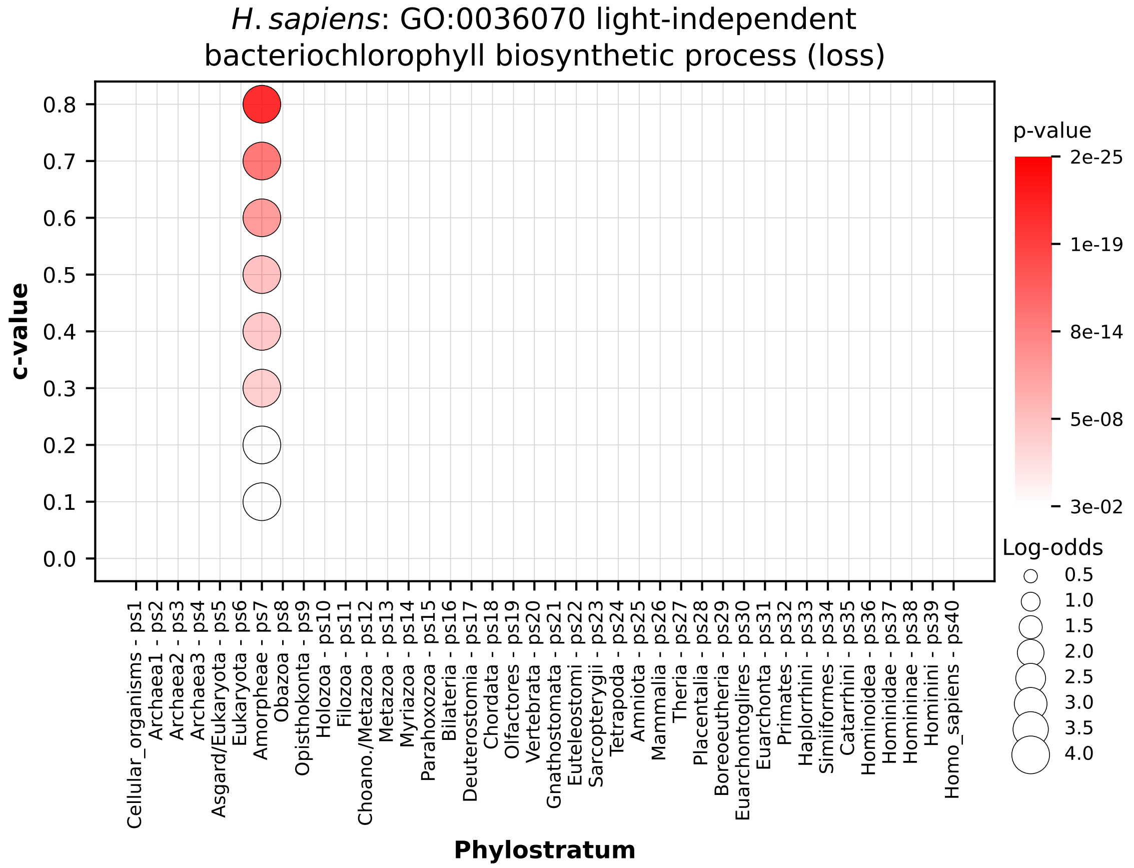

H. sapiens: GO:0036070 light-independent bacteriochlorophyll biosynthetic process (loss)
c-value
0.8
0.7
0.6
0.5
0.4
0.3
0.2
0.1
0.0
p-value
2e-25
1e-19
8e-14
5e-08
3e-02
Log-odds
0.5
1.0
1.5
2.0
2.5
3.0
3.5
4.0
Cellular_organisms - ps1
Archaea1 - ps2
Archaea2 - ps3
Archaea3 - ps4
Asgard/Eukaryota - ps5
Eukaryota - ps6
Amorpheae - ps7
Obazoa - ps8
Opisthokonta - ps9
Holozoa - ps10
Filozoa - ps11
Choano./Metazoa - ps12
Metazoa - ps13
Myriazoa - ps14
Parahoxozoa - ps15
Bilateria - ps16
Deuterostomia - ps17
Chordata - ps18
Olfactores - ps19
Vertebrata - ps20
Gnathostomata - ps21
Euteleostomi - ps22
Sarcopterygii - ps23
Tetrapoda - ps24
Amniota - ps25
Mammalia - ps26
Theria - ps27
Placentalia - ps28
Boreoeutheria - ps29
Euarchontoglires - ps30
Euarchonta - ps31
Primates - ps32
Haplorrhini - ps33
Simiiformes - ps34
Catarrhini - ps35
Hominoidea - ps36
Hominidae - ps37
Homininae - ps38
Hominini - ps39
Homo_sapiens - ps40
Phylostratum

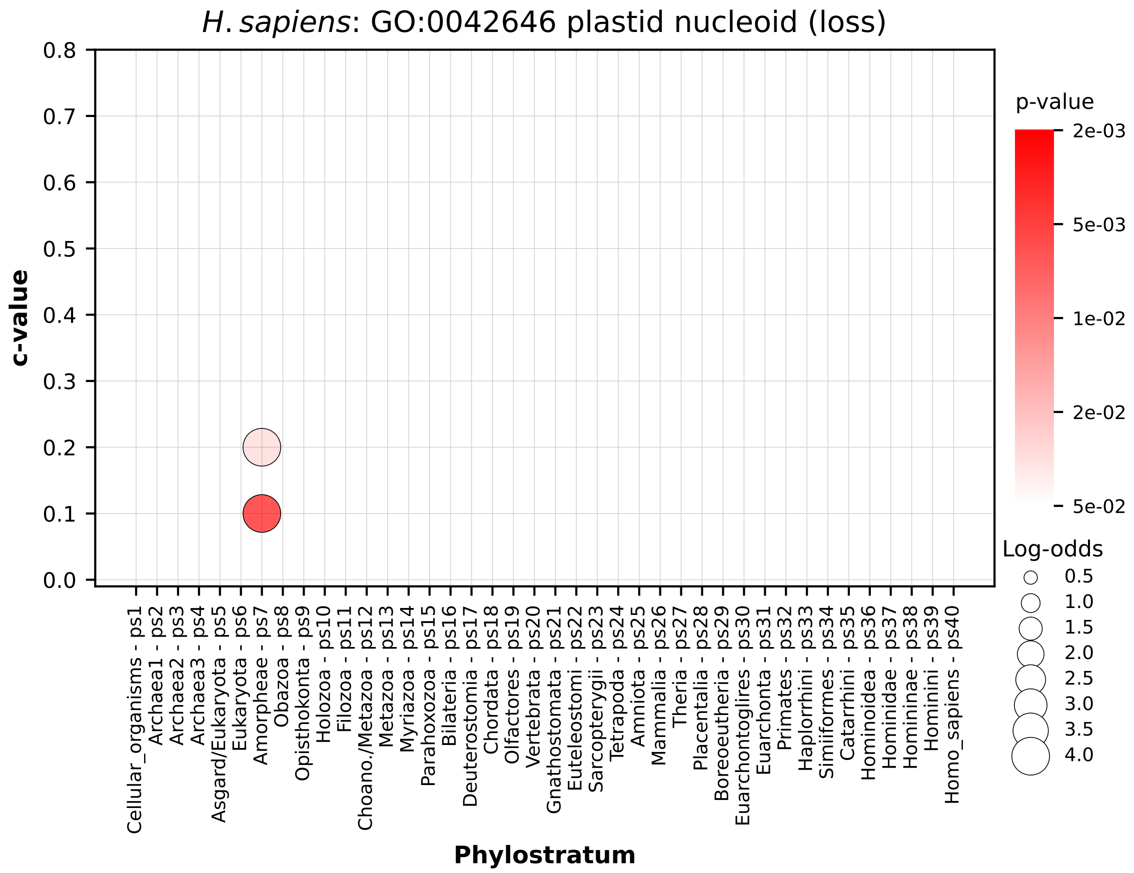
H. sapiens: GO:0042646 plastid nucleoid (loss)
c-value
0.0
0.1
0.2
0.3
0.4
0.5
0.6
0.7
0.8
Phylostratum
Cellular_organisms - ps1
Archaea1 - ps2
Archaea2 - ps3
Archaea3 - ps4
Asgard/Eukaryota - ps5
Eukaryota - ps6
Amorpheae - ps7
Obazoa - ps8
Opisthokonta - ps9
Holozoa - ps10
Filozoa - ps11
Choano./Metazoa - ps12
Metazoa - ps13
Myriazoa - ps14
Parahoxozoa - ps15
Bilateria - ps16
Deuterostomia - ps17
Chordata - ps18
Olfactores - ps19
Vertebrata - ps20
Gnathostomata - ps21
Euteleostomi - ps22
Sarcopterygii - ps23
Tetrapoda - ps24
Amniota - ps25
Mammalia - ps26
Theria - ps27
Placentalia - ps28
Boreoeutheria - ps29
Euarchontoglires - ps30
Euarchonta - ps31
Primates - ps32
Haplorrhini - ps33
Simiiformes - ps34
Catarrhini - ps35
Hominoidea - ps36
Hominidae - ps37
Homininae - ps38
Hominini - ps39
Homo_sapiens - ps40
p-value
2e-03
5e-03
1e-02
2e-02
5e-02
Log-odds
0.5
1.0
1.5
2.0
2.5
3.0
3.5
4.0

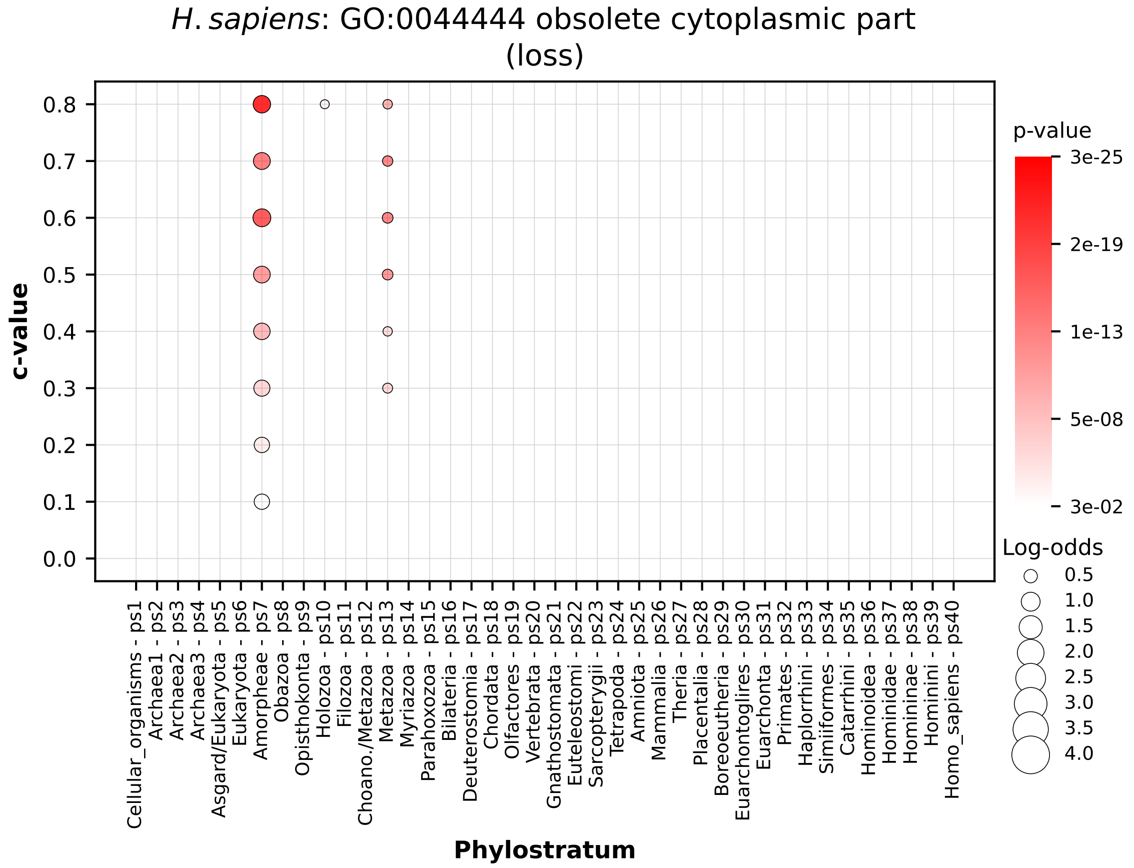
H. sapiens: GO:0044444 obsolete cytoplasmic part
(loss)
c-value
0.8
0.7
0.6
0.5
0.4
0.3
0.2
0.1
0.0
Phylostratum
Cellular_organisms - ps1
Archaea1 - ps2
Archaea2 - ps3
Archaea3 - ps4
Asgard/Eukaryota - ps5
Eukaryota - ps6
Amorpheae - ps7
Obazoa - ps8
Opisthokonta - ps9
Holozoa - ps10
Filozoa - ps11
Choano./Metazoa - ps12
Metazoa - ps13
Myriazoa - ps14
Parahoxozoa - ps15
Bilateria - ps16
Deuterostomia - ps17
Chordata - ps18
Olfactores - ps19
Vertebrata - ps20
Gnathostomata - ps21
Euteleostomi - ps22
Sarcopterygii - ps23
Tetrapoda - ps24
Amniota - ps25
Mammalia - ps26
Theria - ps27
Placentalia - ps28
Boreoeutheria - ps29
Euarchontoglires - ps30
Euarchonta - ps31
Primates - ps32
Haplorrhini - ps33
Simiiformes - ps34
Catarrhini - ps35
Hominoidea - ps36
Hominidae - ps37
Homininae - ps38
Hominini - ps39
Homo_sapiens - ps40
p-value
3e-25
2e-19
1e-13
5e-08
3e-02
Log-odds
0.5
1.0
1.5
2.0
2.5
3.0
3.5
4.0

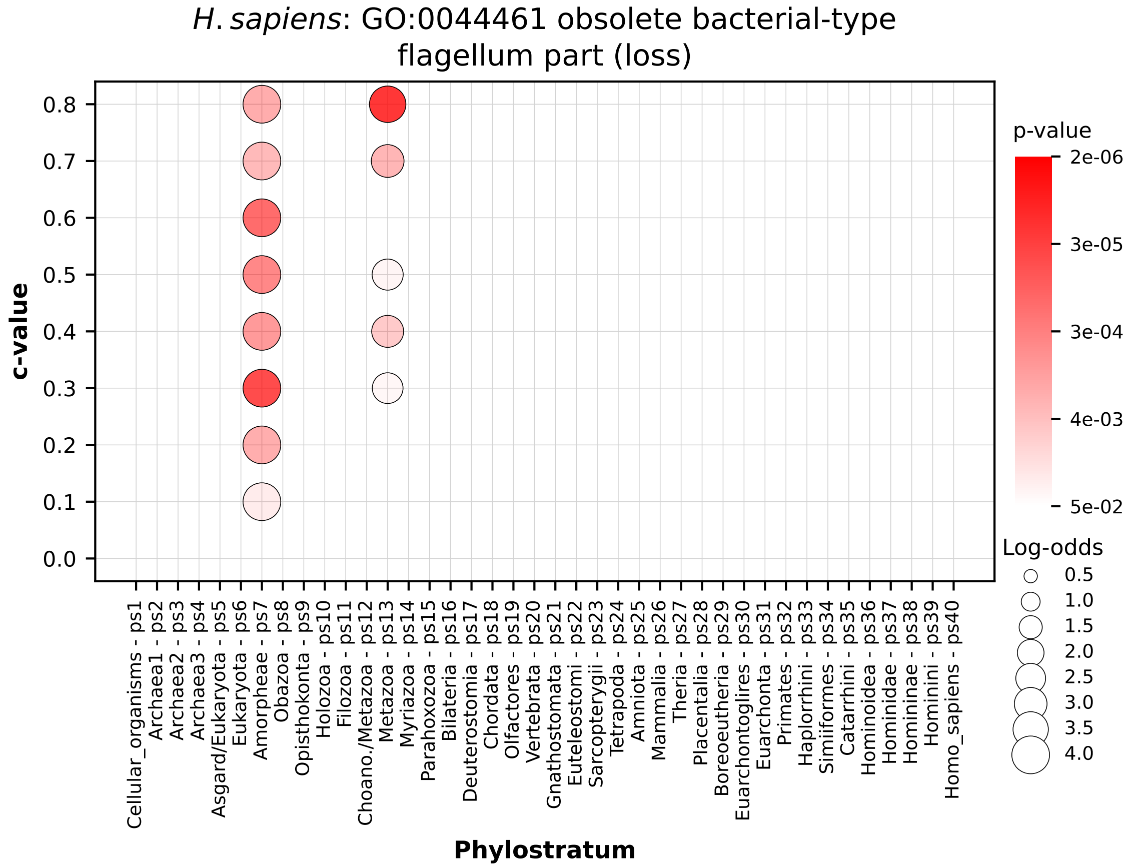
H. sapiens: GO:0044461 obsolete bacterial-type flagellum part (loss)
c-value
0.0
0.1
0.2
0.3
0.4
0.5
0.6
0.7
0.8
Phylostratum
Cellular_organisms - ps1
Archaea1 - ps2
Archaea2 - ps3
Archaea3 - ps4
Asgard/Eukaryota - ps5
Eukaryota - ps6
Amorpheae - ps7
Obazoa - ps8
Opisthokonta - ps9
Holozoa - ps10
Filozoa - ps11
Choano./Metazoa - ps12
Metazoa - ps13
Myriazoa - ps14
Parahoxozoa - ps15
Bilateria - ps16
Deuterostomia - ps17
Chordata - ps18
Olfactores - ps19
Vertebrata - ps20
Gnathostomata - ps21
Euteleostomi - ps22
Sarcopterygii - ps23
Tetrapoda - ps24
Amniota - ps25
Mammalia - ps26
Theria - ps27
Placentalia - ps28
Boreoeutheria - ps29
Euarchontoglires - ps30
Euarchonta - ps31
Primates - ps32
Haplorrhini - ps33
Simiiformes - ps34
Catarrhini - ps35
Hominoidea - ps36
Hominidae - ps37
Homininae - ps38
Hominini - ps39
Homo_sapiens - ps40
p-value
2e-06
3e-05
3e-04
4e-03
5e-02
Log-odds
0.5
1.0
1.5
2.0
2.5
3.0
3.5
4.0

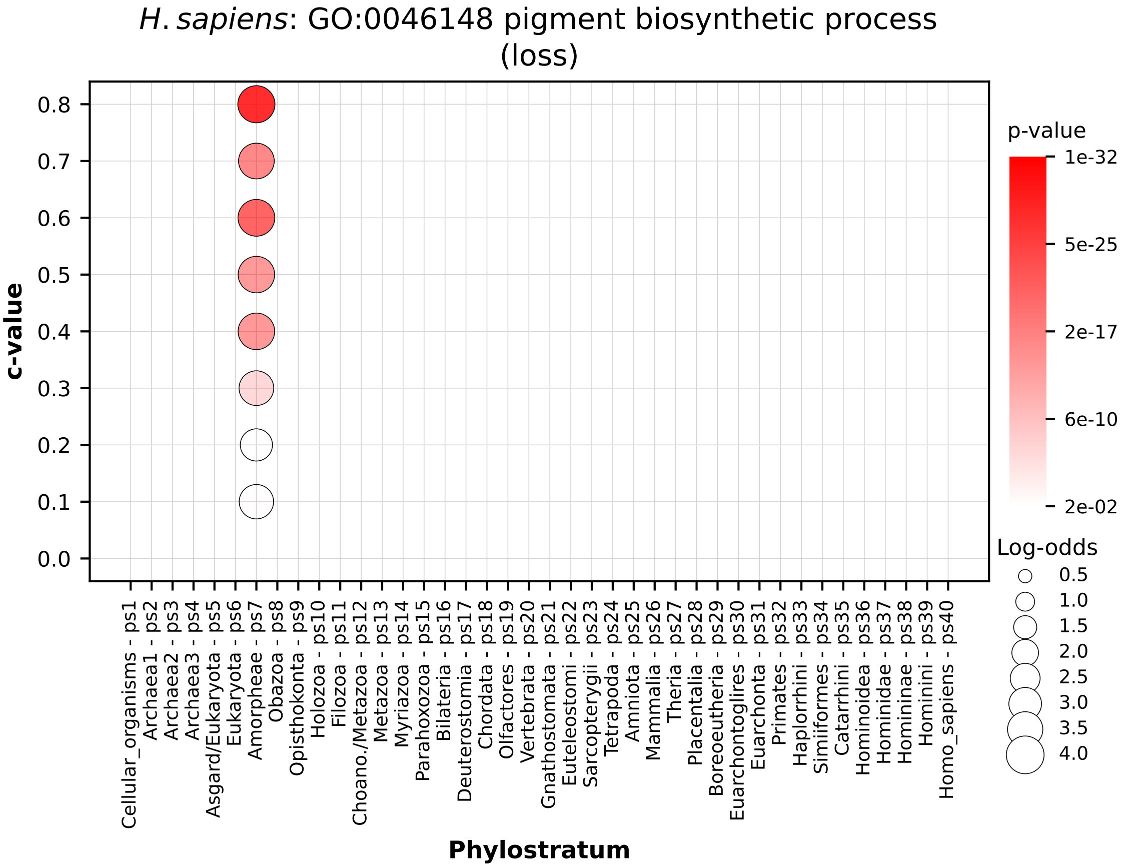

H. sapiens: GO:0046148 pigment biosynthetic process
(loss)
c-value
0.8
0.7
0.6
0.5
0.4
0.3
0.2
0.1
0.0
p-value
1e-32
5e-25
2e-17
6e-10
2e-02
Log-odds
0.5
1.0
1.5
2.0
2.5
3.0
3.5
4.0
Cellular_organisms - ps1
Archaea1 - ps2
Archaea2 - ps3
Archaea3 - ps4
Asgard/Eukaryota - ps5
Eukaryota - ps6
Amorpheae - ps7
Obazoa - ps8
Opisthokonta - ps9
Holozoa - ps10
Filozoa - ps11
Choano./Metazoa - ps12
Metazoa - ps13
Myriazoa - ps14
Parahoxozoa - ps15
Bilateria - ps16
Deuterostomia - ps17
Chordata - ps18
Olfactores - ps19
Vertebrata - ps20
Gnathostomata - ps21
Euteleostomi - ps22
Sarcopterygii - ps23
Tetrapoda - ps24
Amniota - ps25
Mammalia - ps26
Theria - ps27
Placentalia - ps28
Boreoeutheria - ps29
Euarchontoglires - ps30
Euarchonta - ps31
Primates - ps32
Haplorrhini - ps33
Simiiformes - ps34
Catarrhini - ps35
Hominoidea - ps36
Hominidae - ps37
Homininae - ps38
Hominini - ps39
Homo_sapiens - ps40
Phylostratum

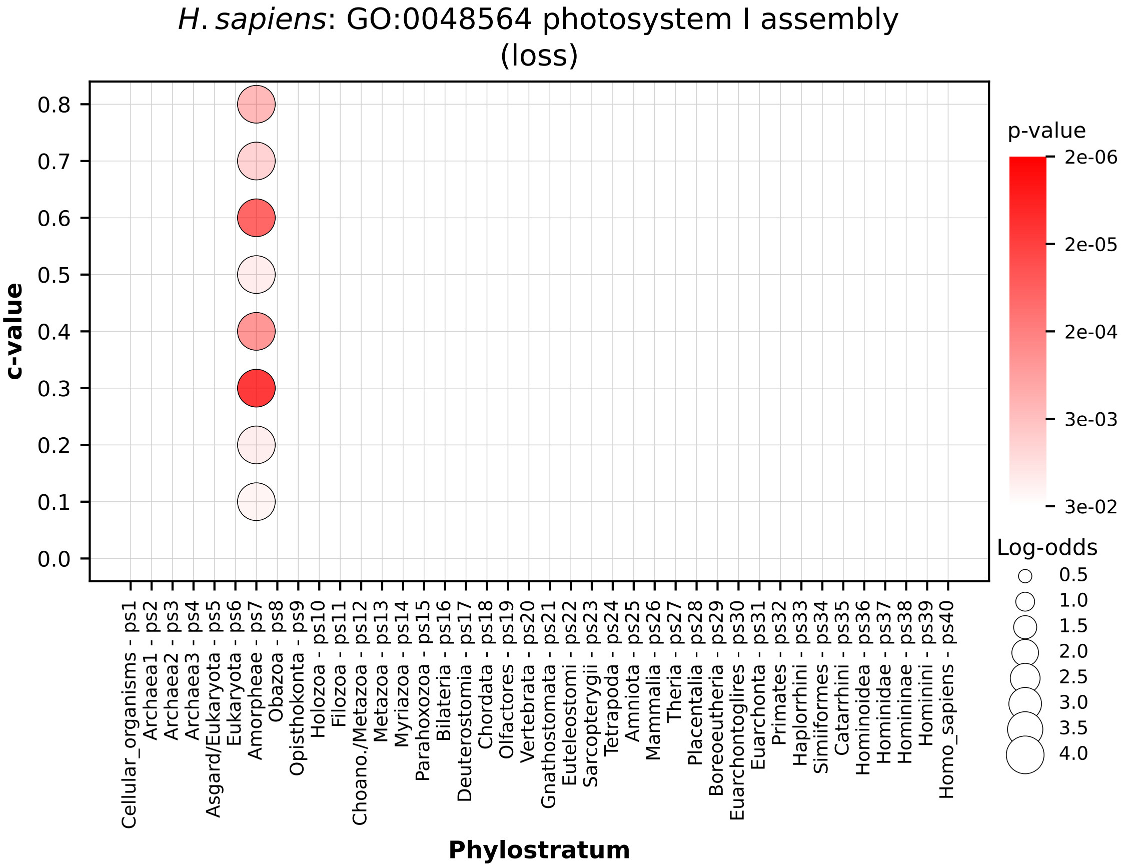

H. sapiens: GO:0048564 photosystem I assembly (loss)
c-value
0.8
0.7
0.6
0.5
0.4
0.3
0.2
0.1
0.0
p-value
2e-06
2e-05
2e-04
3e-03
3e-02
Log-odds
0.5
1.0
1.5
2.0
2.5
3.0
3.5
4.0
Cellular_organisms - ps1
Archaea1 - ps2
Archaea2 - ps3
Archaea3 - ps4
Asgard/Eukaryota - ps5
Eukaryota - ps6
Amorpheae - ps7
Obazoa - ps8
Opisthokonta - ps9
Holozoa - ps10
Filozoa - ps11
Choano./Metazoa - ps12
Metazoa - ps13
Myriazoa - ps14
Parahoxozoa - ps15
Bilateria - ps16
Deuterostomia - ps17
Chordata - ps18
Olfactores - ps19
Vertebrata - ps20
Gnathostomata - ps21
Euteleostomi - ps22
Sarcopterygii - ps23
Tetrapoda - ps24
Amniota - ps25
Mammalia - ps26
Theria - ps27
Placentalia - ps28
Boreoeutheria - ps29
Euarchontoglires - ps30
Euarchonta - ps31
Primates - ps32
Haplorrhini - ps33
Simiiformes - ps34
Catarrhini - ps35
Hominoidea - ps36
Hominidae - ps37
Homininae - ps38
Hominini - ps39
Homo_sapiens - ps40
Phylostratum

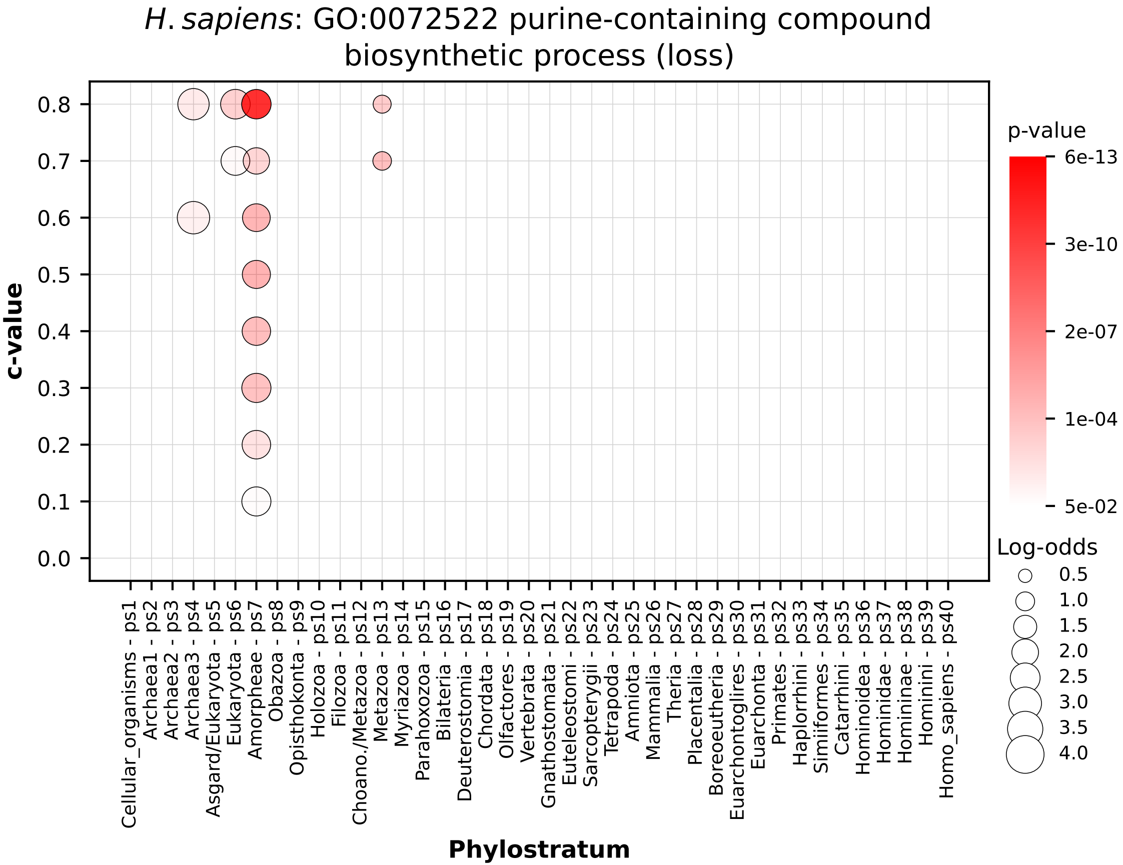
H. sapiens: GO:0072522 purine-containing compound biosynthetic process (loss)
c-value
0.0
0.1
0.2
0.3
0.4
0.5
0.6
0.7
0.8
Phylostratum
Cellular_organisms - ps1
Archaea1 - ps2
Archaea2 - ps3
Archaea3 - ps4
Asgard/Eukaryota - ps5
Eukaryota - ps6
Amorpheae - ps7
Obazoa - ps8
Opisthokonta - ps9
Holozoa - ps10
Filozoa - ps11
Choano./Metazoa - ps12
Metazoa - ps13
Myriazoa - ps14
Parahoxozoa - ps15
Bilateria - ps16
Deuterostomia - ps17
Chordata - ps18
Olfactores - ps19
Vertebrata - ps20
Gnathostomata - ps21
Euteleostomi - ps22
Sarcopterygii - ps23
Tetrapoda - ps24
Amniota - ps25
Mammalia - ps26
Theria - ps27
Placentalia - ps28
Boreoeutheria - ps29
Euarchontoglires - ps30
Euarchonta - ps31
Primates - ps32
Haplorrhini - ps33
Simiiformes - ps34
Catarrhini - ps35
Hominoidea - ps36
Hominidae - ps37
Homininae - ps38
Hominini - ps39
Homo_sapiens - ps40
p-value
6e-13
3e-10
2e-07
1e-04
5e-02
Log-odds
0.5
1.0
1.5
2.0
2.5
3.0
3.5
4.0

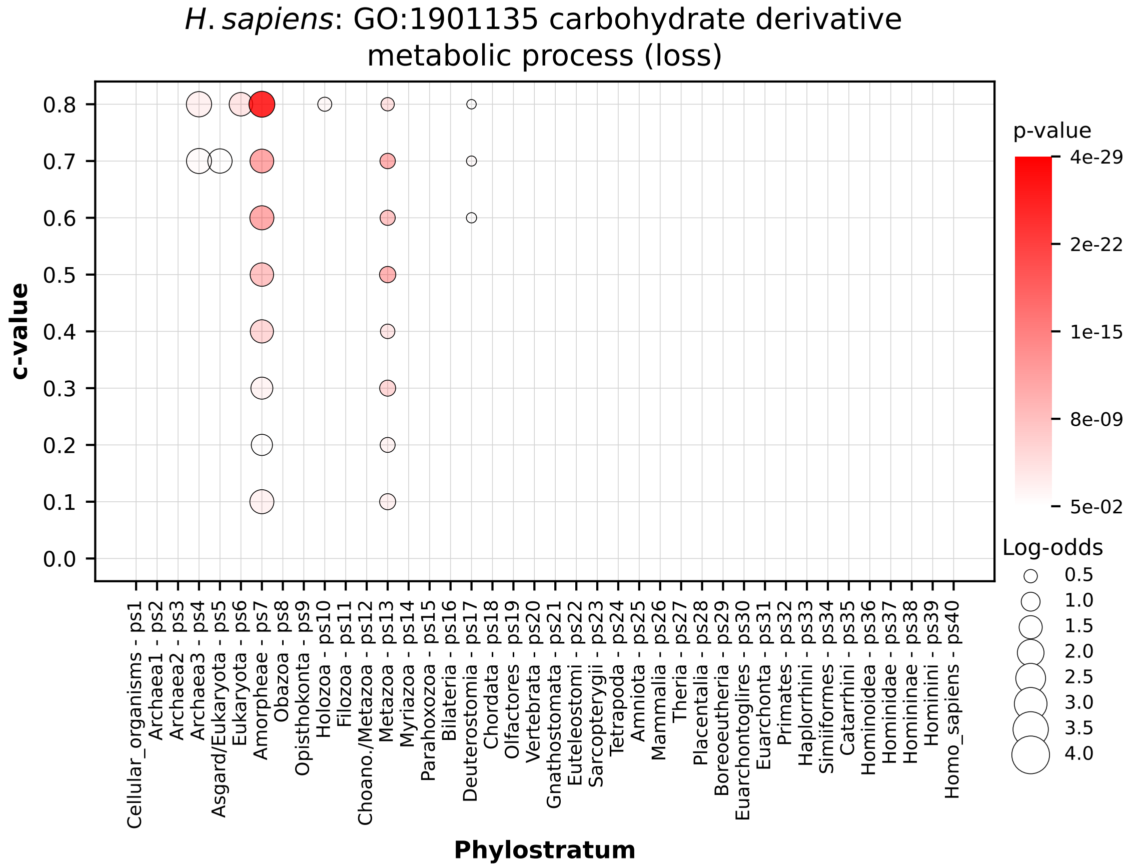

H. sapiens: GO:1901135 carbohydrate derivative metabolic process (loss)
c-value
Phylostratum
p-value
4e-29
2e-22
1e-15
8e-09
5e-02
Log-odds
0.5
1.0
1.5
2.0
2.5
3.0
3.5
4.0
Cellular_organisms - ps1
Archaea1 - ps2
Archaea2 - ps3
Archaea3 - ps4
Asgard/Eukaryota - ps5
Eukaryota - ps6
Amorpheae - ps7
Obazoa - ps8
Opisthokonta - ps9
Holozoa - ps10
Filozoa - ps11
Choano./Metazoa - ps12
Metazoa - ps13
Myriazoa - ps14
Parahoxozoa - ps15
Bilateria - ps16
Deuterostomia - ps17
Chordata - ps18
Olfactores - ps19
Vertebrata - ps20
Gnathostomata - ps21
Euteleostomi - ps22
Sarcopterygii - ps23
Tetrapoda - ps24
Amniota - ps25
Mammalia - ps26
Theria - ps27
Placentalia - ps28
Boreoeutheria - ps29
Euarchontoglires - ps30
Euarchonta - ps31
Primates - ps32
Haplorrhini - ps33
Simiiformes - ps34
Catarrhini - ps35
Hominoidea - ps36
Hominidae - ps37
Homininae - ps38
Hominini - ps39
Homo_sapiens - ps40

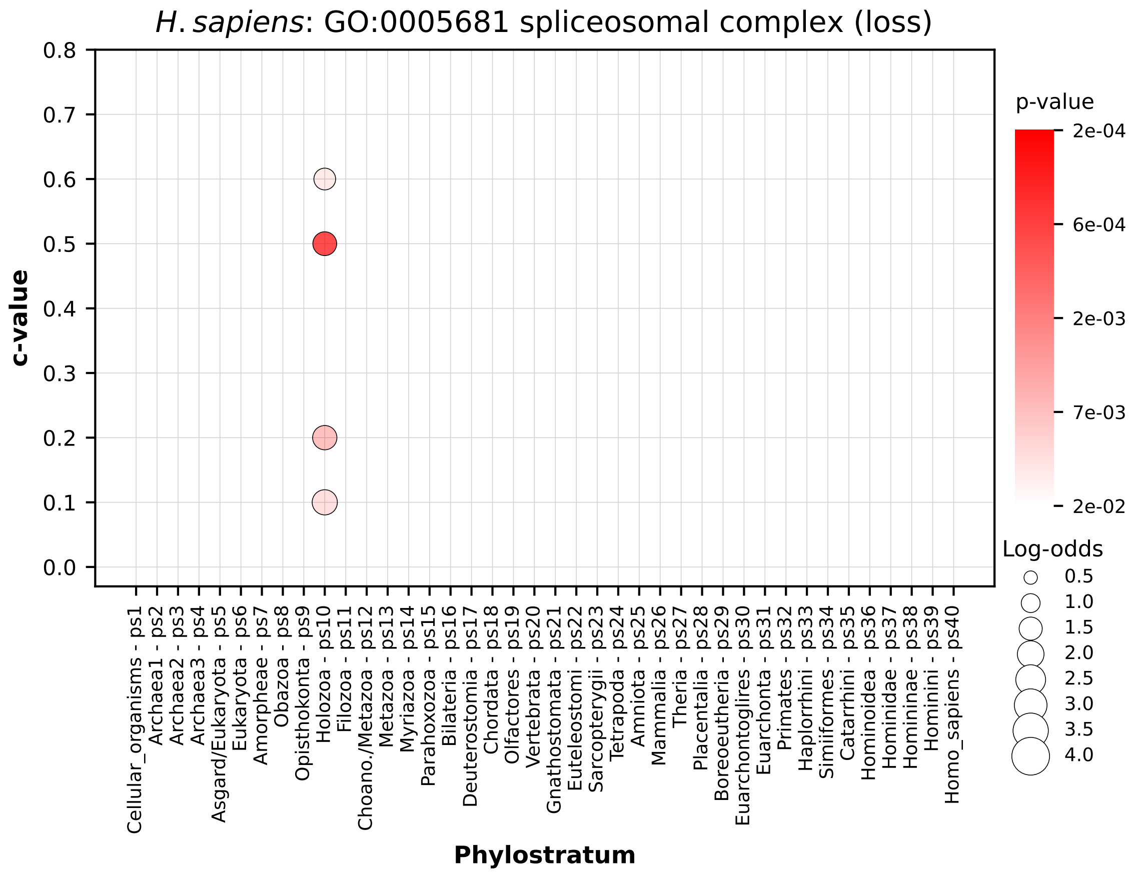

H. sapiens: GO:0005681 spliceosomal complex (loss)
c-value
0.8
0.7
0.6
0.5
0.4
0.3
0.2
0.1
0.0
p-value
2e-04
6e-04
2e-03
7e-03
2e-02
Log-odds
0.5
1.0
1.5
2.0
2.5
3.0
3.5
4.0
Cellular_organisms - ps1
Archaea1 - ps2
Archaea2 - ps3
Archaea3 - ps4
Asgard/Eukaryota - ps5
Eukaryota - ps6
Amorpheae - ps7
Obazoa - ps8
Opisthokonta - ps9
Holozoa - ps10
Filozoa - ps11
Choano./Metazoa - ps12
Metazoa - ps13
Myriazoa - ps14
Parahoxozoa - ps15
Bilateria - ps16
Deuterostomia - ps17
Chordata - ps18
Olfactores - ps19
Vertebrata - ps20
Gnathostomata - ps21
Euteleostomi - ps22
Sarcopterygii - ps23
Tetrapoda - ps24
Amniota - ps25
Mammalia - ps26
Theria - ps27
Placentalia - ps28
Boreoeutheria - ps29
Euarchontoglires - ps30
Euarchonta - ps31
Primates - ps32
Haplorrhini - ps33
Simiiformes - ps34
Catarrhini - ps35
Hominoidea - ps36
Hominidae - ps37
Homininae - ps38
Hominini - ps39
Homo_sapiens - ps40
Phylostratum

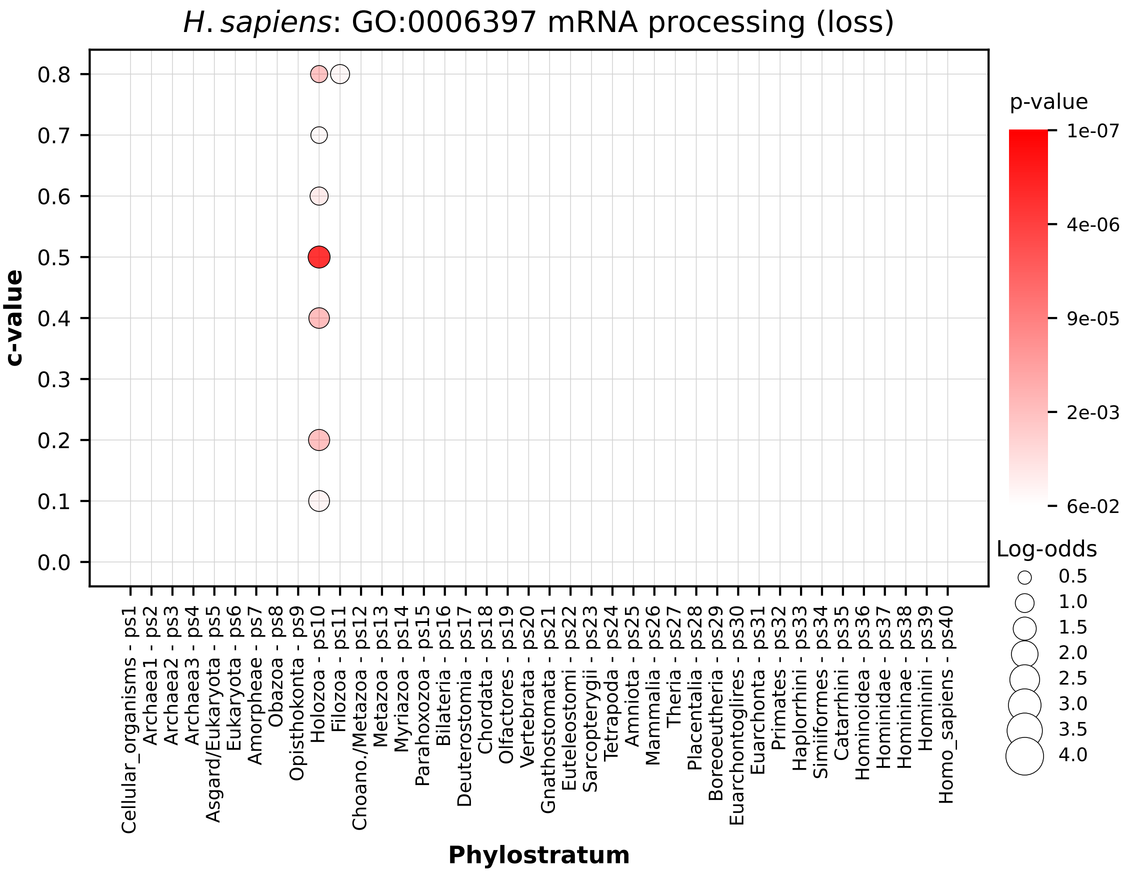

H. sapiens: GO:0006397 mRNA processing (loss)
c-value
0.0
0.1
0.2
0.3
0.4
0.5
0.6
0.7
0.8
Phylostratum
Cellular_organisms - ps1
Archaea1 - ps2
Archaea2 - ps3
Archaea3 - ps4
Asgard/Eukaryota - ps5
Eukaryota - ps6
Amorpheae - ps7
Obazoa - ps8
Opisthokonta - ps9
Holozoa - ps10
Filozoa - ps11
Choano./Metazoa - ps12
Metazoa - ps13
Myriazoa - ps14
Parahoxozoa - ps15
Bilateria - ps16
Deuterostomia - ps17
Chordata - ps18
Olfactores - ps19
Vertebrata - ps20
Gnathostomata - ps21
Euteleostomi - ps22
Sarcopterygii - ps23
Tetrapoda - ps24
Amniota - ps25
Mammalia - ps26
Theria - ps27
Placentalia - ps28
Boreoeutheria - ps29
Euarchontoglires - ps30
Euarchonta - ps31
Primates - ps32
Haplorrhini - ps33
Simiiformes - ps34
Catarrhini - ps35
Hominoidea - ps36
Hominidae - ps37
Homininae - ps38
Hominini - ps39
Homo_sapiens - ps40
p-value
1e-07
4e-06
9e-05
2e-03
6e-02
Log-odds
0.5
1.0
1.5
2.0
2.5
3.0
3.5
4.0

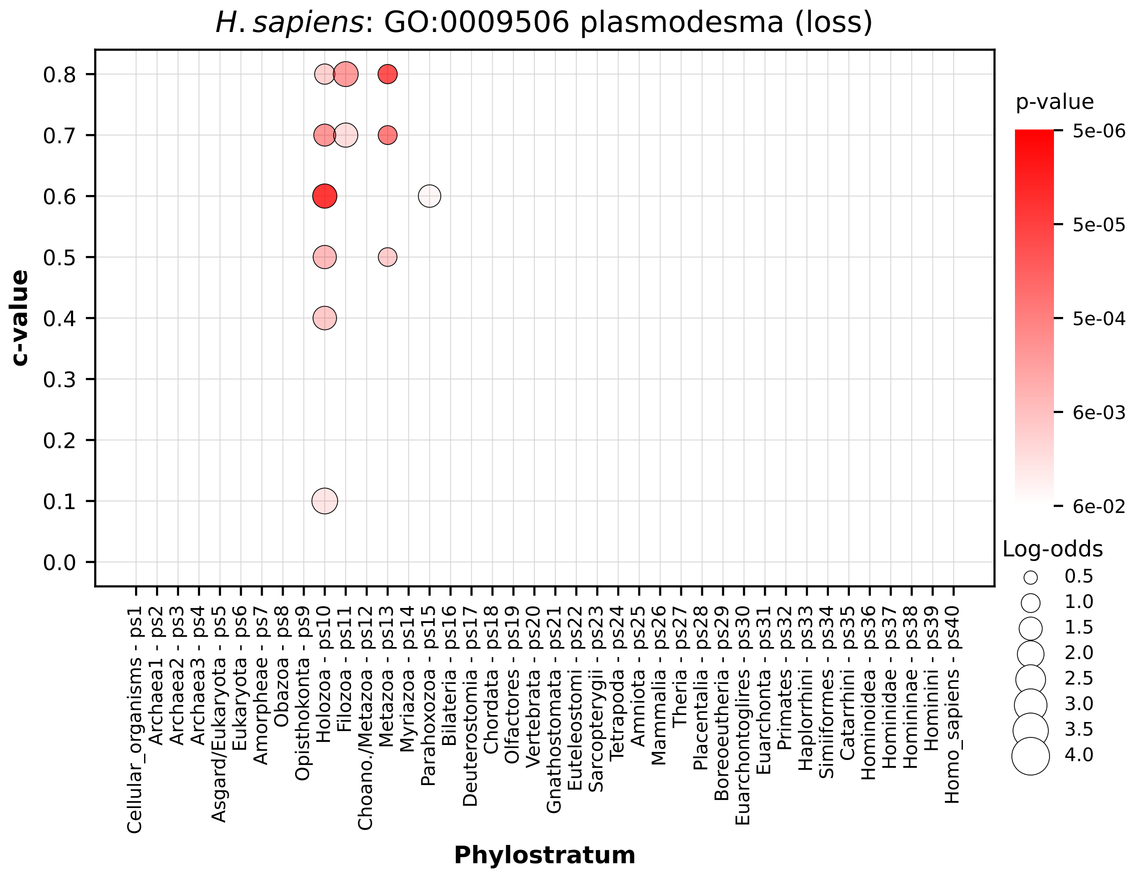

H. sapiens: GO:0009506 plasmodesma (loss)
c-value
0.8
0.7
0.6
0.5
0.4
0.3
0.2
0.1
0.0
p-value
5e-06
5e-05
5e-04
6e-03
6e-02
Log-odds
0.5
1.0
1.5
2.0
2.5
3.0
3.5
4.0
Cellular_organisms - ps1
Archaea1 - ps2
Archaea2 - ps3
Archaea3 - ps4
Asgard/Eukaryota - ps5
Eukaryota - ps6
Amorpheae - ps7
Obazoa - ps8
Opisthokonta - ps9
Holozoa - ps10
Filozoa - ps11
Choano./Metazoa - ps12
Metazoa - ps13
Myriazoa - ps14
Parahoxozoa - ps15
Bilateria - ps16
Deuterostomia - ps17
Chordata - ps18
Olfactores - ps19
Vertebrata - ps20
Gnathostomata - ps21
Euteleostomi - ps22
Sarcopterygii - ps23
Tetrapoda - ps24
Amniota - ps25
Mammalia - ps26
Theria - ps27
Placentalia - ps28
Boreoeutheria - ps29
Euarchontoglires - ps30
Euarchonta - ps31
Primates - ps32
Haplorrhini - ps33
Simiiformes - ps34
Catarrhini - ps35
Hominoidea - ps36
Hominidae - ps37
Homininae - ps38
Hominini - ps39
Homo_sapiens - ps40
Phylostratum

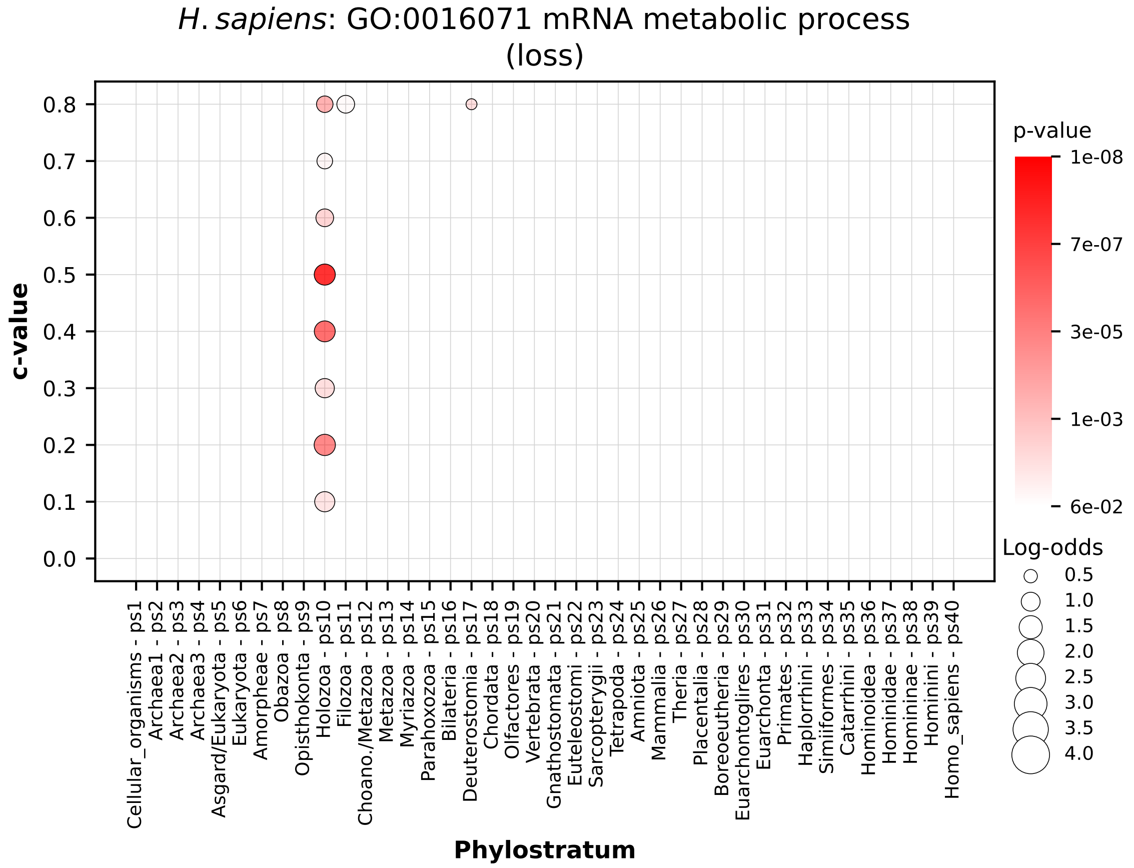

H. sapiens: GO:0016071 mRNA metabolic process
(loss)
c-value
0.8
0.7
0.6
0.5
0.4
0.3
0.2
0.1
0.0
Phylostratum
Cellular_organisms - ps1
Archaea1 - ps2
Archaea2 - ps3
Archaea3 - ps4
Asgard/Eukaryota - ps5
Eukaryota - ps6
Amorpheae - ps7
Obazoa - ps8
Opisthokonta - ps9
Holozoa - ps10
Filozoa - ps11
Choano./Metazoa - ps12
Metazoa - ps13
Myriazoa - ps14
Parahoxozoa - ps15
Bilateria - ps16
Deuterostomia - ps17
Chordata - ps18
Olfactores - ps19
Vertebrata - ps20
Gnathostomata - ps21
Euteleostomi - ps22
Sarcopterygii - ps23
Tetrapoda - ps24
Amniota - ps25
Mammalia - ps26
Theria - ps27
Placentalia - ps28
Boreoeutheria - ps29
Euarchontoglires - ps30
Euarchonta - ps31
Primates - ps32
Haplorrhini - ps33
Simiiformes - ps34
Catarrhini - ps35
Hominoidea - ps36
Hominidae - ps37
Homininae - ps38
Hominini - ps39
Homo_sapiens - ps40
p-value
1e-08
7e-07
3e-05
1e-03
6e-02
Log-odds
0.5
1.0
1.5
2.0
2.5
3.0
3.5
4.0

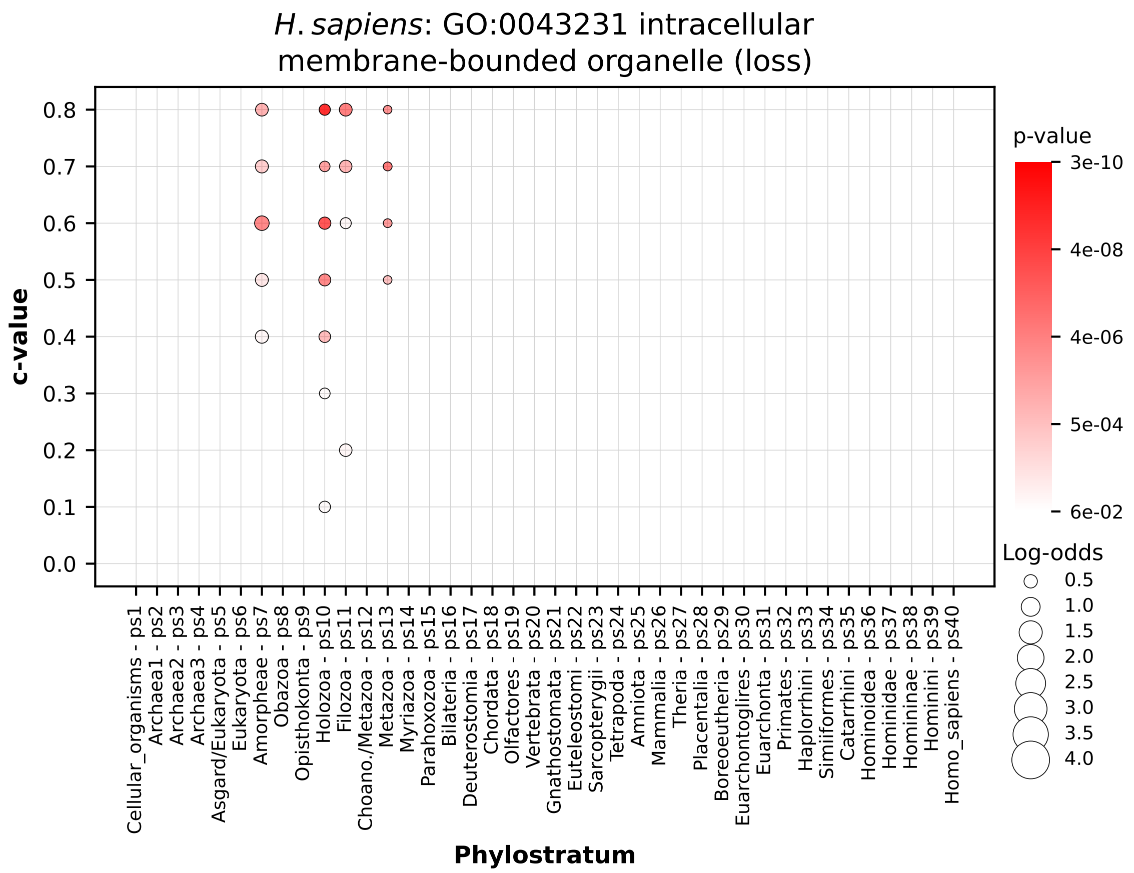

H. sapiens: GO:0043231 intracellular membrane-bounded organelle (loss)
c-value
0.8
0.7
0.6
0.5
0.4
0.3
0.2
0.1
0.0
Phylostratum
Cellular_organisms - ps1
Archaea1 - ps2
Archaea2 - ps3
Archaea3 - ps4
Asgard/Eukaryota - ps5
Eukaryota - ps6
Amorpheae - ps7
Obazoa - ps8
Opisthokonta - ps9
Holozoa - ps10
Filozoa - ps11
Choano./Metazoa - ps12
Metazoa - ps13
Myriazoa - ps14
Parahoxozoa - ps15
Bilateria - ps16
Deuterostomia - ps17
Chordata - ps18
Olfactores - ps19
Vertebrata - ps20
Gnathostomata - ps21
Euteleostomi - ps22
Sarcopterygii - ps23
Tetrapoda - ps24
Amniota - ps25
Mammalia - ps26
Theria - ps27
Placentalia - ps28
Boreoeutheria - ps29
Euarchontoglires - ps30
Euarchonta - ps31
Primates - ps32
Haplorrhini - ps33
Simiiformes - ps34
Catarrhini - ps35
Hominoidea - ps36
Hominidae - ps37
Homininae - ps38
Hominini - ps39
Homo_sapiens - ps40
p-value
3e-10
4e-08
4e-06
5e-04
6e-02
Log-odds
0.5
1.0
1.5
2.0
2.5
3.0
3.5
4.0

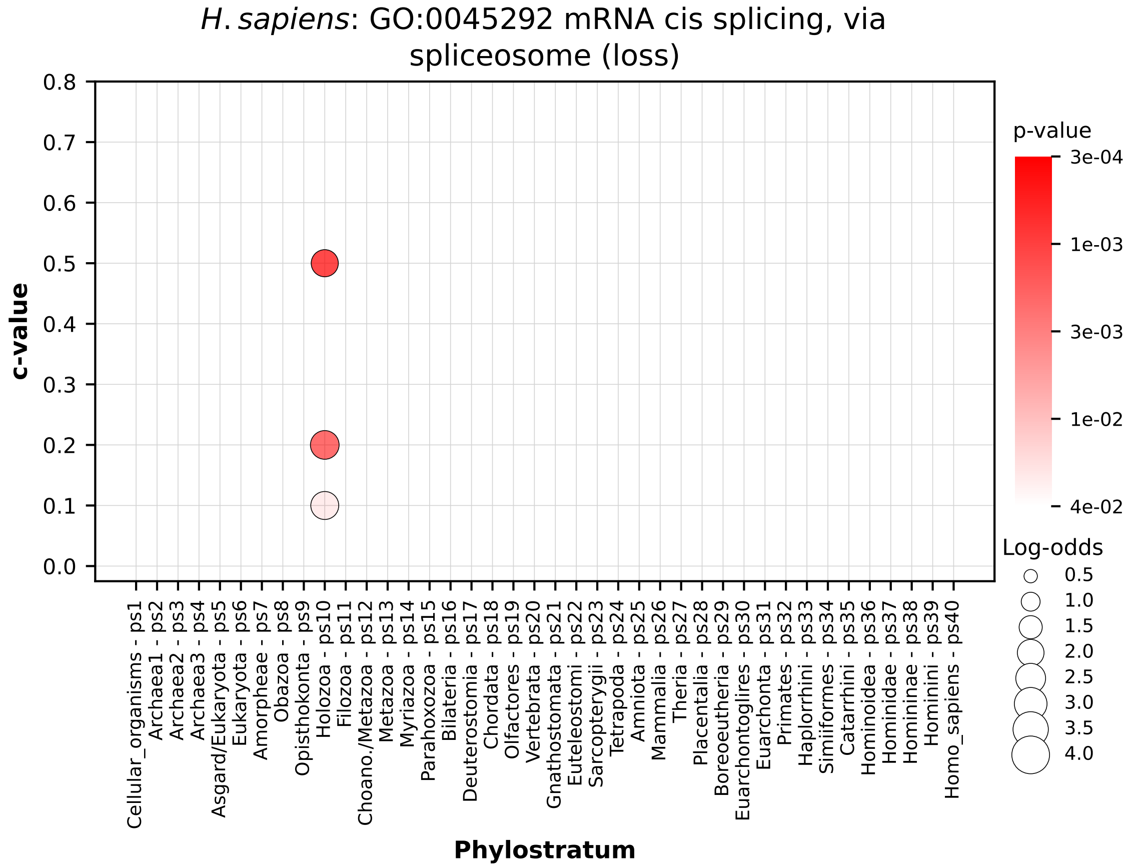

H. sapiens: GO:0045292 mRNA cis splicing, via spliceosome (loss)
c-value
0.8
0.7
0.6
0.5
0.4
0.3
0.2
0.1
0.0
Phylostratum
Cellular_organisms - ps1
Archaea1 - ps2
Archaea2 - ps3
Archaea3 - ps4
Asgard/Eukaryota - ps5
Eukaryota - ps6
Amorpheae - ps7
Obazoa - ps8
Opisthokonta - ps9
Holozoa - ps10
Filozoa - ps11
Choano./Metazoa - ps12
Metazoa - ps13
Myriazoa - ps14
Parahoxozoa - ps15
Bilateria - ps16
Deuterostomia - ps17
Chordata - ps18
Olfactores - ps19
Vertebrata - ps20
Gnathostomata - ps21
Euteleostomi - ps22
Sarcopterygii - ps23
Tetrapoda - ps24
Amniota - ps25
Mammalia - ps26
Theria - ps27
Placentalia - ps28
Boreoeutheria - ps29
Euarchontoglires - ps30
Euarchonta - ps31
Primates - ps32
Haplorrhini - ps33
Simiiformes - ps34
Catarrhini - ps35
Hominoidea - ps36
Hominidae - ps37
Homininae - ps38
Hominini - ps39
Homo_sapiens - ps40
p-value
3e-04
1e-03
3e-03
1e-02
4e-02
Log-odds
0.5
1.0
1.5
2.0
2.5
3.0
3.5
4.0

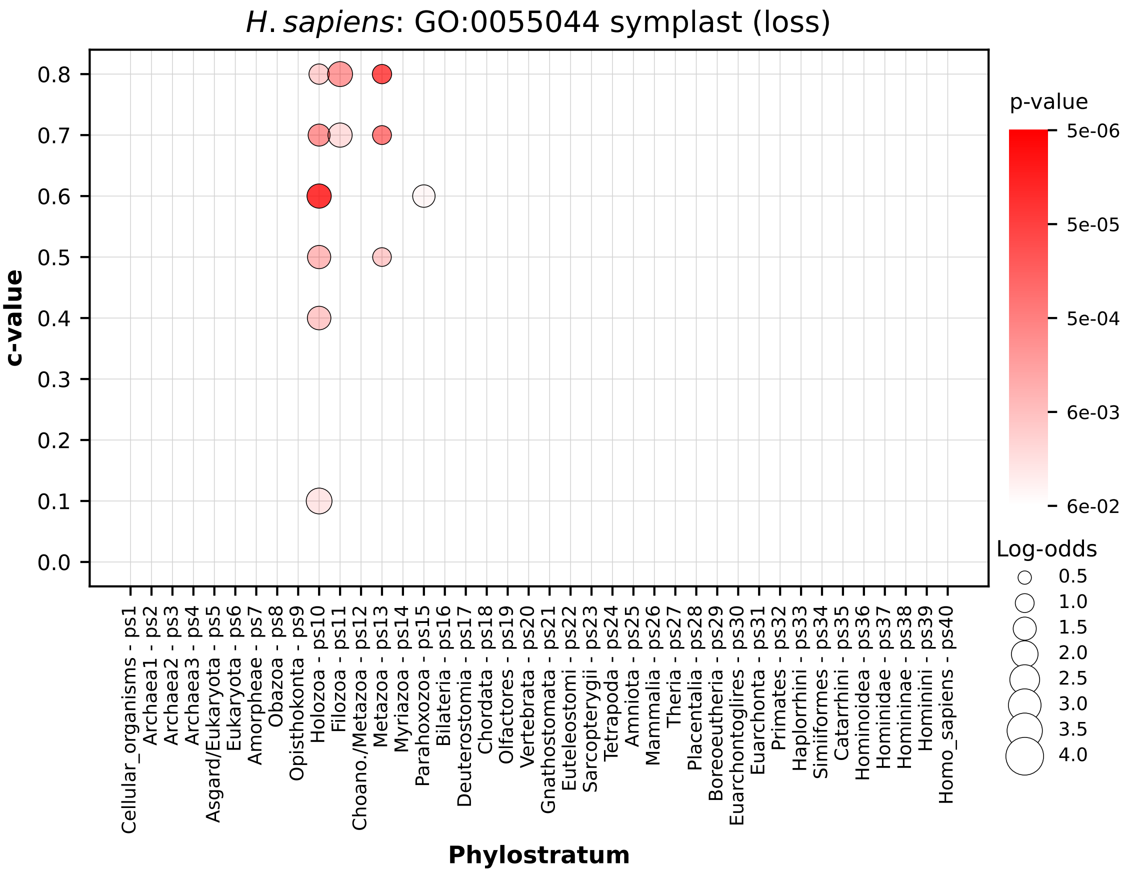
H. sapiens: GO:0055044 symplast (loss)
c-value
0.8
0.7
0.6
0.5
0.4
0.3
0.2
0.1
0.0
Phylostratum
Cellular_organisms - ps1
Archaea1 - ps2
Archaea2 - ps3
Archaea3 - ps4
Asgard/Eukaryota - ps5
Eukaryota - ps6
Amorpheae - ps7
Obazoa - ps8
Opisthokonta - ps9
Holozoa - ps10
Filozoa - ps11
Choano./Metazoa - ps12
Metazoa - ps13
Myriazoa - ps14
Parahoxozoa - ps15
Bilateria - ps16
Deuterostomia - ps17
Chordata - ps18
Olfactores - ps19
Vertebrata - ps20
Gnathostomata - ps21
Euteleostomi - ps22
Sarcopterygii - ps23
Tetrapoda - ps24
Amniota - ps25
Mammalia - ps26
Theria - ps27
Placentalia - ps28
Boreoeutheria - ps29
Euarchontoglires - ps30
Euarchonta - ps31
Primates - ps32
Haplorrhini - ps33
Simiiformes - ps34
Catarrhini - ps35
Hominoidea - ps36
Hominidae - ps37
Homininae - ps38
Hominini - ps39
Homo_sapiens - ps40
p-value
5e-06
5e-05
5e-04
6e-03
6e-02
Log-odds
0.5
1.0
1.5
2.0
2.5
3.0
3.5
4.0

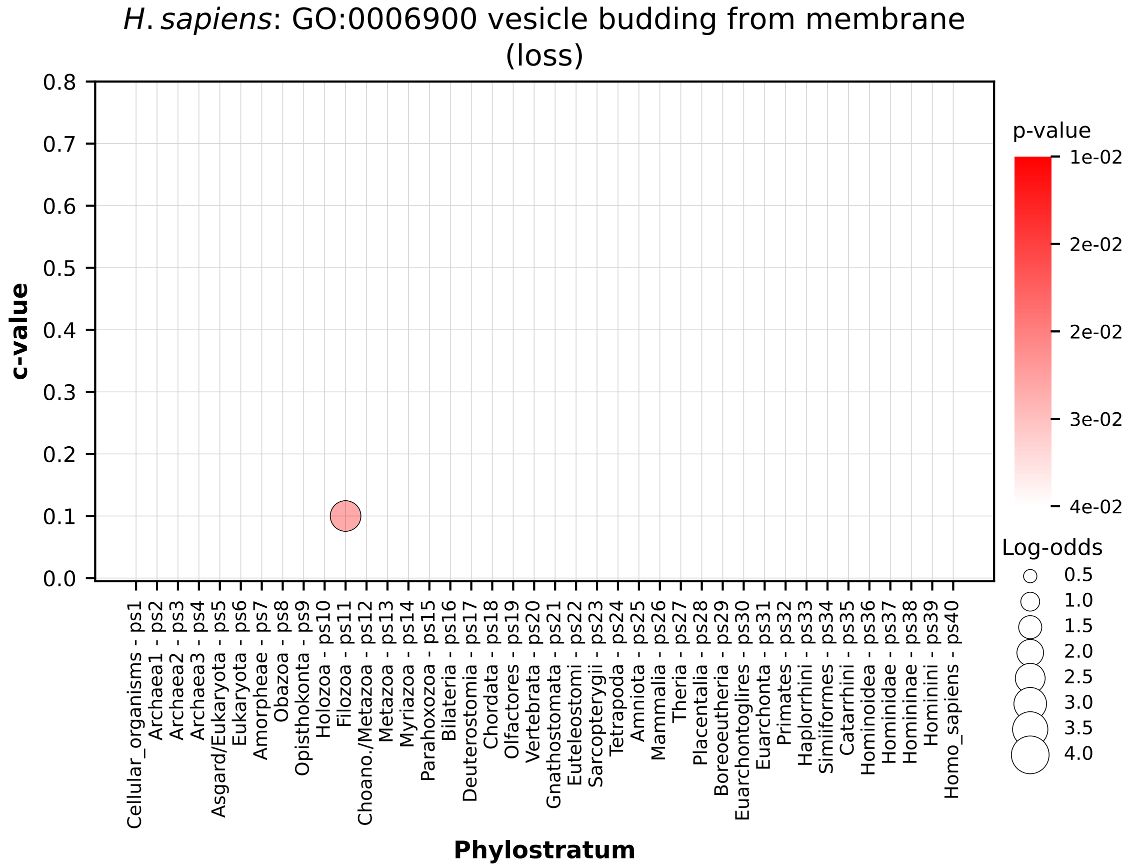

H. sapiens: GO:0006900 vesicle budding from membrane
(loss)
c-value
0.8
0.7
0.6
0.5
0.4
0.3
0.2
0.1
0.0
Phylostratum
Cellular_organisms - ps1
Archaea1 - ps2
Archaea2 - ps3
Archaea3 - ps4
Asgard/Eukaryota - ps5
Eukaryota - ps6
Amorpheae - ps7
Obazoa - ps8
Opisthokonta - ps9
Holozoa - ps10
Filozoa - ps11
Choano./Metazoa - ps12
Metazoa - ps13
Myriazoa - ps14
Parahoxozoa - ps15
Bilateria - ps16
Deuterostomia - ps17
Chordata - ps18
Olfactores - ps19
Vertebrata - ps20
Gnathostomata - ps21
Euteleostomi - ps22
Sarcopterygii - ps23
Tetrapoda - ps24
Amniota - ps25
Mammalia - ps26
Theria - ps27
Placentalia - ps28
Boreoeutheria - ps29
Euarchontoglires - ps30
Euarchonta - ps31
Primates - ps32
Haplorrhini - ps33
Simiiformes - ps34
Catarrhini - ps35
Hominoidea - ps36
Hominidae - ps37
Homininae - ps38
Hominini - ps39
Homo_sapiens - ps40
p-value
1e-02
2e-02
2e-02
3e-02
4e-02
Log-odds
0.5
1.0
1.5
2.0
2.5
3.0
3.5
4.0

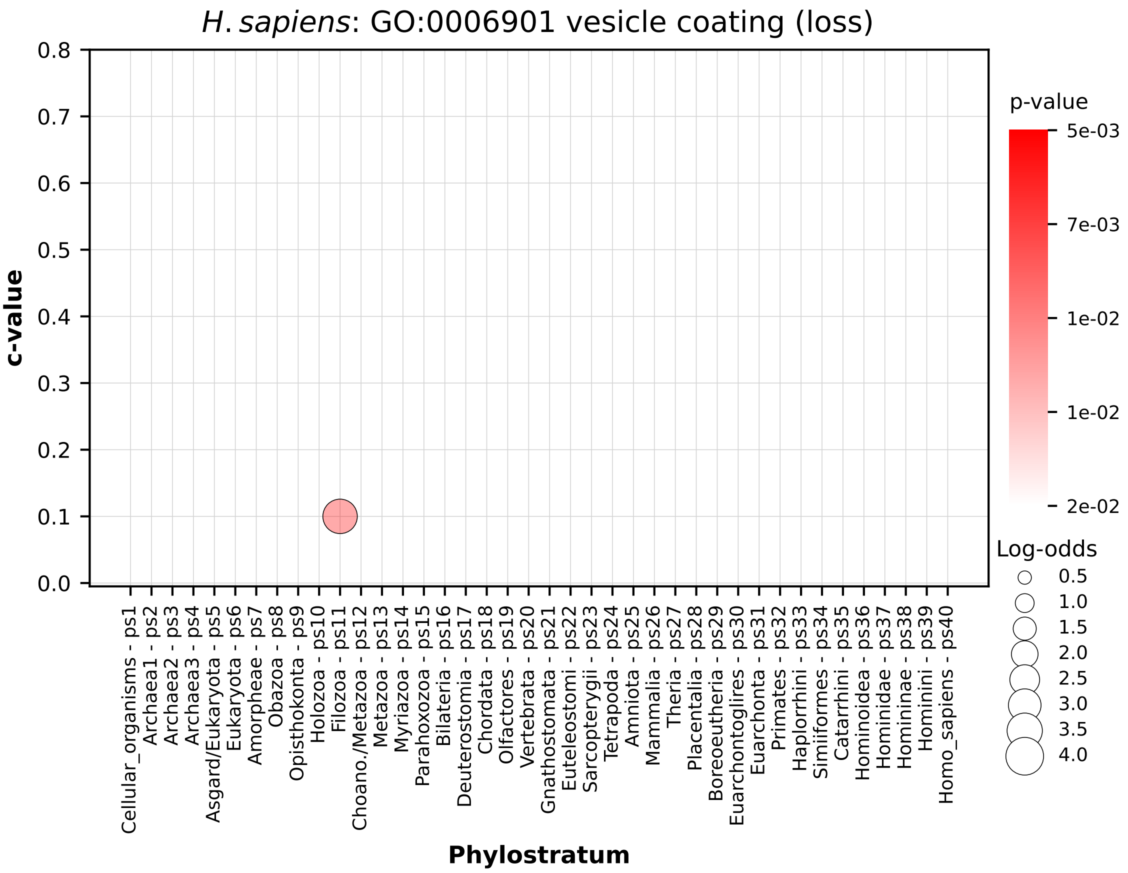

H. sapiens: GO:0006901 vesicle coating (loss)
c-value
0.8
0.7
0.6
0.5
0.4
0.3
0.2
0.1
0.0
p-value
5e-03
7e-03
1e-02
1e-02
2e-02
Log-odds
0.5
1.0
1.5
2.0
2.5
3.0
3.5
4.0
Cellular_organisms - ps1
Archaea1 - ps2
Archaea2 - ps3
Archaea3 - ps4
Asgard/Eukaryota - ps5
Eukaryota - ps6
Amorpheae - ps7
Obazoa - ps8
Opisthokonta - ps9
Holozoa - ps10
Filozoa - ps11
Choano./Metazoa - ps12
Metazoa - ps13
Myriazoa - ps14
Parahoxozoa - ps15
Bilateria - ps16
Deuterostomia - ps17
Chordata - ps18
Olfactores - ps19
Vertebrata - ps20
Gnathostomata - ps21
Euteleostomi - ps22
Sarcopterygii - ps23
Tetrapoda - ps24
Amniota - ps25
Mammalia - ps26
Theria - ps27
Placentalia - ps28
Boreoeutheria - ps29
Euarchontoglires - ps30
Euarchonta - ps31
Primates - ps32
Haplorrhini - ps33
Simiiformes - ps34
Catarrhini - ps35
Hominoidea - ps36
Hominidae - ps37
Homininae - ps38
Hominini - ps39
Homo_sapiens - ps40
Phylostratum

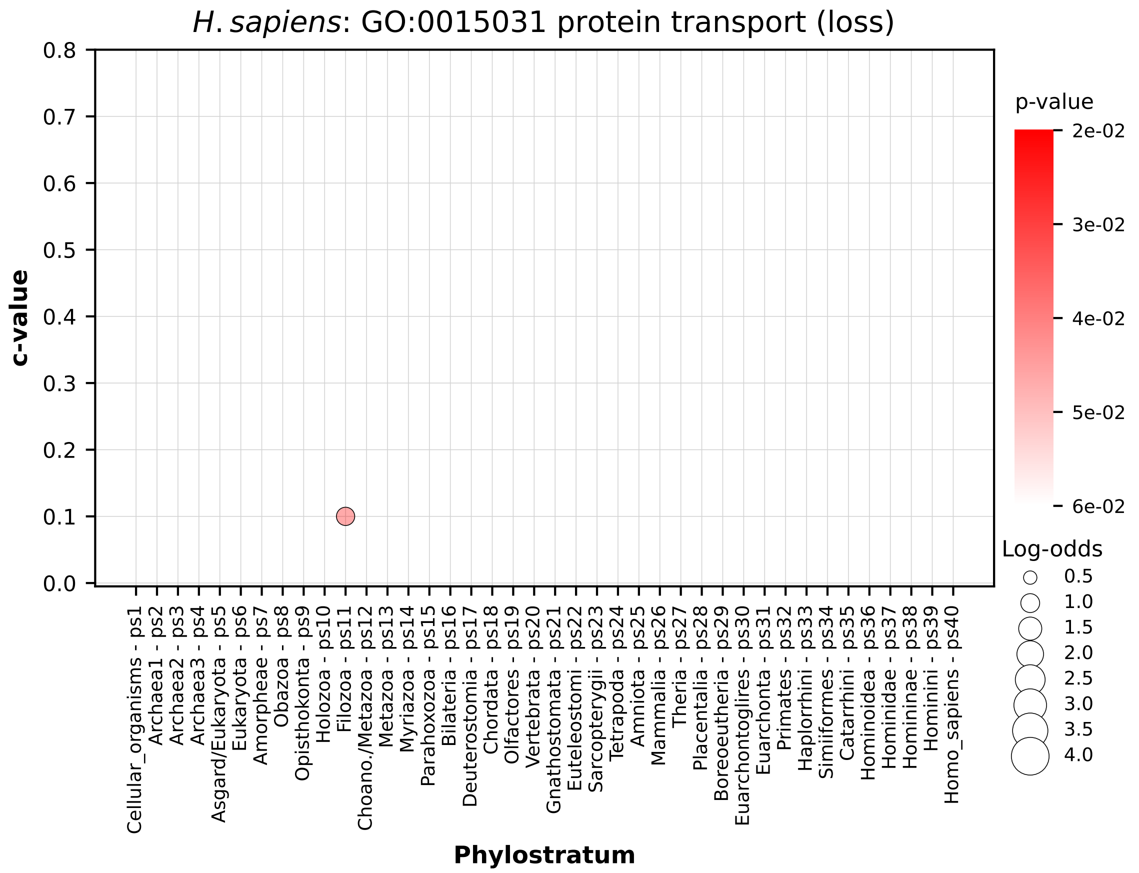
H. sapiens: GO:0015031 protein transport (loss)
c-value
0.0
0.1
0.2
0.3
0.4
0.5
0.6
0.7
0.8
p-value
2e-02
3e-02
4e-02
5e-02
6e-02
Log-odds
0.5
1.0
1.5
2.0
2.5
3.0
3.5
4.0
Cellular_organisms - ps1
Archaea1 - ps2
Archaea2 - ps3
Archaea3 - ps4
Asgard/Eukaryota - ps5
Eukaryota - ps6
Amorpheae - ps7
Obazoa - ps8
Opisthokonta - ps9
Holozoa - ps10
Filozoa - ps11
Choano./Metazoa - ps12
Metazoa - ps13
Myriazoa - ps14
Parahoxozoa - ps15
Bilateria - ps16
Deuterostomia - ps17
Chordata - ps18
Olfactores - ps19
Vertebrata - ps20
Gnathostomata - ps21
Euteleostomi - ps22
Sarcopterygii - ps23
Tetrapoda - ps24
Amniota - ps25
Mammalia - ps26
Theria - ps27
Placentalia - ps28
Boreoeutheria - ps29
Euarchontoglires - ps30
Euarchonta - ps31
Primates - ps32
Haplorrhini - ps33
Simiiformes - ps34
Catarrhini - ps35
Hominoidea - ps36
Hominidae - ps37
Homininae - ps38
Hominini - ps39
Homo_sapiens - ps40
Phylostratum

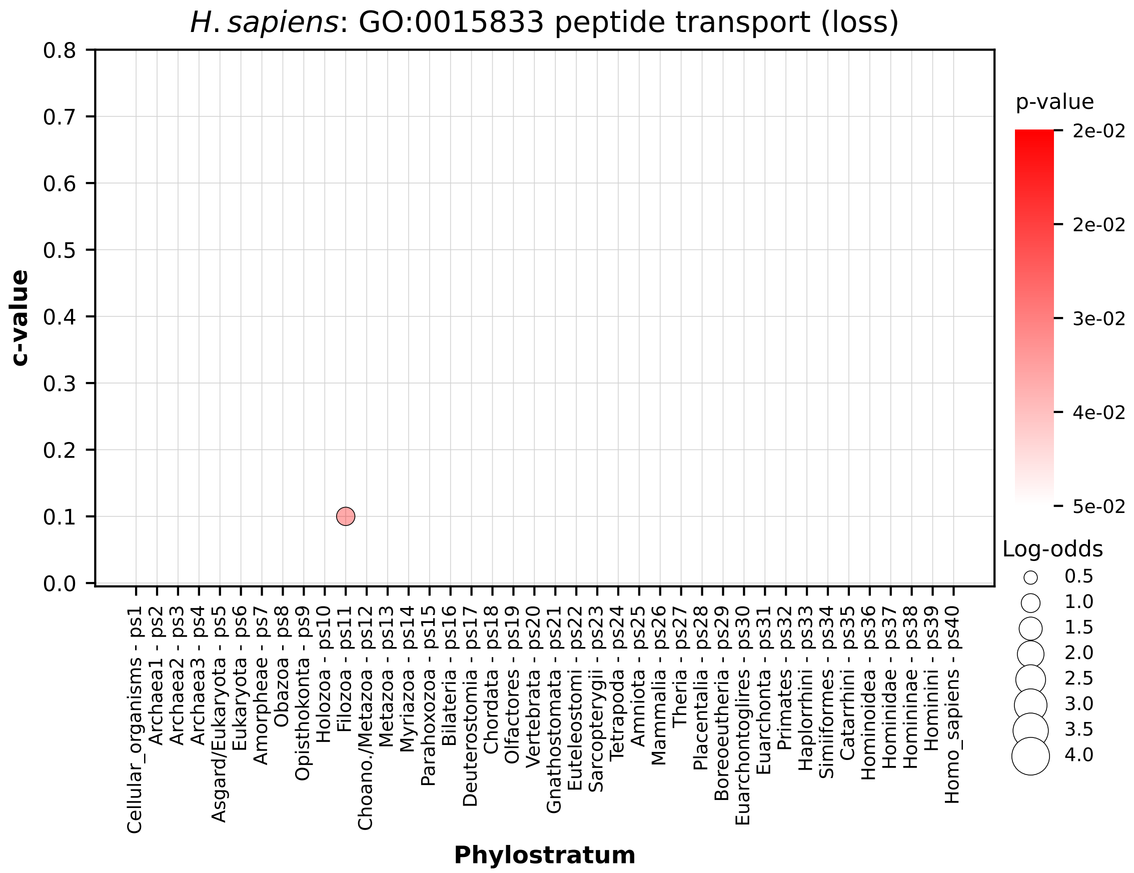

H. sapiens: GO:0015833 peptide transport (loss)
c-value
0.0
0.1
0.2
0.3
0.4
0.5
0.6
0.7
0.8
Phylostratum
Cellular_organisms - ps1
Archaea1 - ps2
Archaea2 - ps3
Archaea3 - ps4
Asgard/Eukaryota - ps5
Eukaryota - ps6
Amorpheae - ps7
Obazoa - ps8
Opisthokonta - ps9
Holozoa - ps10
Filozoa - ps11
Choano./Metazoa - ps12
Metazoa - ps13
Myriazoa - ps14
Parahoxozoa - ps15
Bilateria - ps16
Deuterostomia - ps17
Chordata - ps18
Olfactores - ps19
Vertebrata - ps20
Gnathostomata - ps21
Euteleostomi - ps22
Sarcopterygii - ps23
Tetrapoda - ps24
Amniota - ps25
Mammalia - ps26
Theria - ps27
Placentalia - ps28
Boreoeutheria - ps29
Euarchontoglires - ps30
Euarchonta - ps31
Primates - ps32
Haplorrhini - ps33
Simiiformes - ps34
Catarrhini - ps35
Hominoidea - ps36
Hominidae - ps37
Homininae - ps38
Hominini - ps39
Homo_sapiens - ps40
p-value
2e-02
2e-02
3e-02
4e-02
5e-02
Log-odds
0.5
1.0
1.5
2.0
2.5
3.0
3.5
4.0

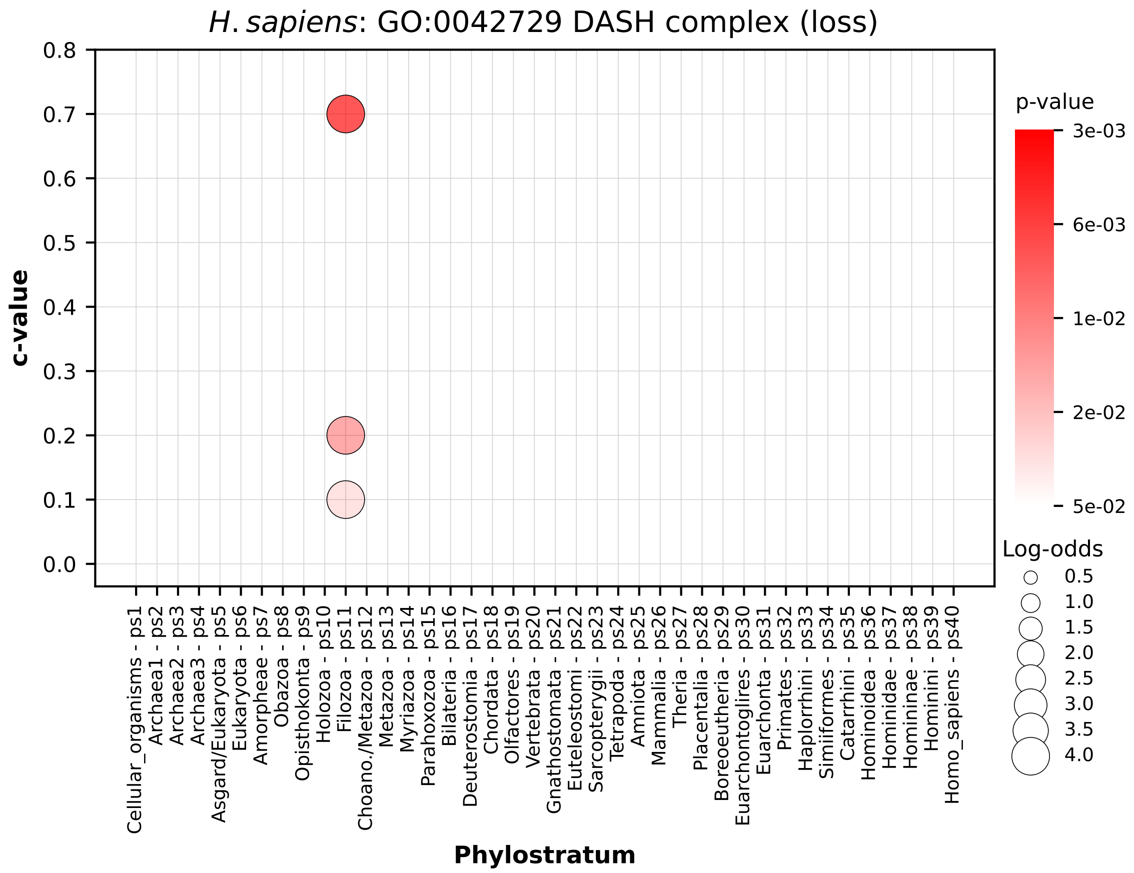

H. sapiens: GO:0042729 DASH complex (loss)
c-value
0.8
0.7
0.6
0.5
0.4
0.3
0.2
0.1
0.0
p-value
3e-03
6e-03
1e-02
2e-02
5e-02
Log-odds
0.5
1.0
1.5
2.0
2.5
3.0
3.5
4.0
Cellular_organisms - ps1
Archaea1 - ps2
Archaea2 - ps3
Archaea3 - ps4
Asgard/Eukaryota - ps5
Eukaryota - ps6
Amorpheae - ps7
Obazoa - ps8
Opisthokonta - ps9
Holozoa - ps10
Filozoa - ps11
Choano./Metazoa - ps12
Metazoa - ps13
Myriazoa - ps14
Parahoxozoa - ps15
Bilateria - ps16
Deuterostomia - ps17
Chordata - ps18
Olfactores - ps19
Vertebrata - ps20
Gnathostomata - ps21
Euteleostomi - ps22
Sarcopterygii - ps23
Tetrapoda - ps24
Amniota - ps25
Mammalia - ps26
Theria - ps27
Placentalia - ps28
Boreoeutheria - ps29
Euarchontoglires - ps30
Euarchonta - ps31
Primates - ps32
Haplorrhini - ps33
Simiiformes - ps34
Catarrhini - ps35
Hominoidea - ps36
Hominidae - ps37
Homininae - ps38
Hominini - ps39
Homo_sapiens - ps40
Phylostratum

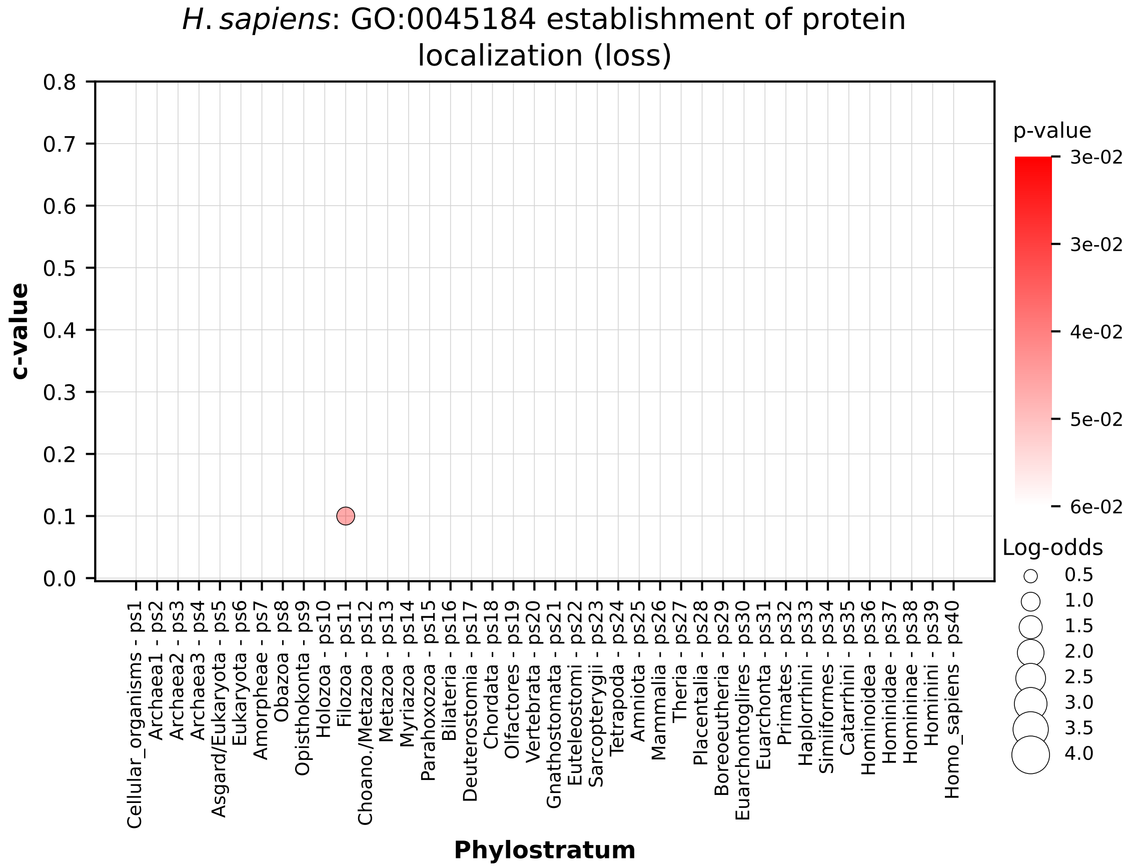

H. sapiens: GO:0045184 establishment of protein localization (loss)
c-value
0.8
0.7
0.6
0.5
0.4
0.3
0.2
0.1
0.0
Phylostratum
Cellular_organisms - ps1
Archaea1 - ps2
Archaea2 - ps3
Archaea3 - ps4
Asgard/Eukaryota - ps5
Eukaryota - ps6
Amorpheae - ps7
Obazoa - ps8
Opisthokonta - ps9
Holozoa - ps10
Filozoa - ps11
Choano./Metazoa - ps12
Metazoa - ps13
Myriazoa - ps14
Parahoxozoa - ps15
Bilateria - ps16
Deuterostomia - ps17
Chordata - ps18
Olfactores - ps19
Vertebrata - ps20
Gnathostomata - ps21
Euteleostomi - ps22
Sarcopterygii - ps23
Tetrapoda - ps24
Amniota - ps25
Mammalia - ps26
Theria - ps27
Placentalia - ps28
Boreoeutheria - ps29
Euarchontoglires - ps30
Euarchonta - ps31
Primates - ps32
Haplorrhini - ps33
Simiiformes - ps34
Catarrhini - ps35
Hominoidea - ps36
Hominidae - ps37
Homininae - ps38
Hominini - ps39
Homo_sapiens - ps40
p-value
3e-02
3e-02
4e-02
5e-02
6e-02
Log-odds
0.5
1.0
1.5
2.0
2.5
3.0
3.5
4.0

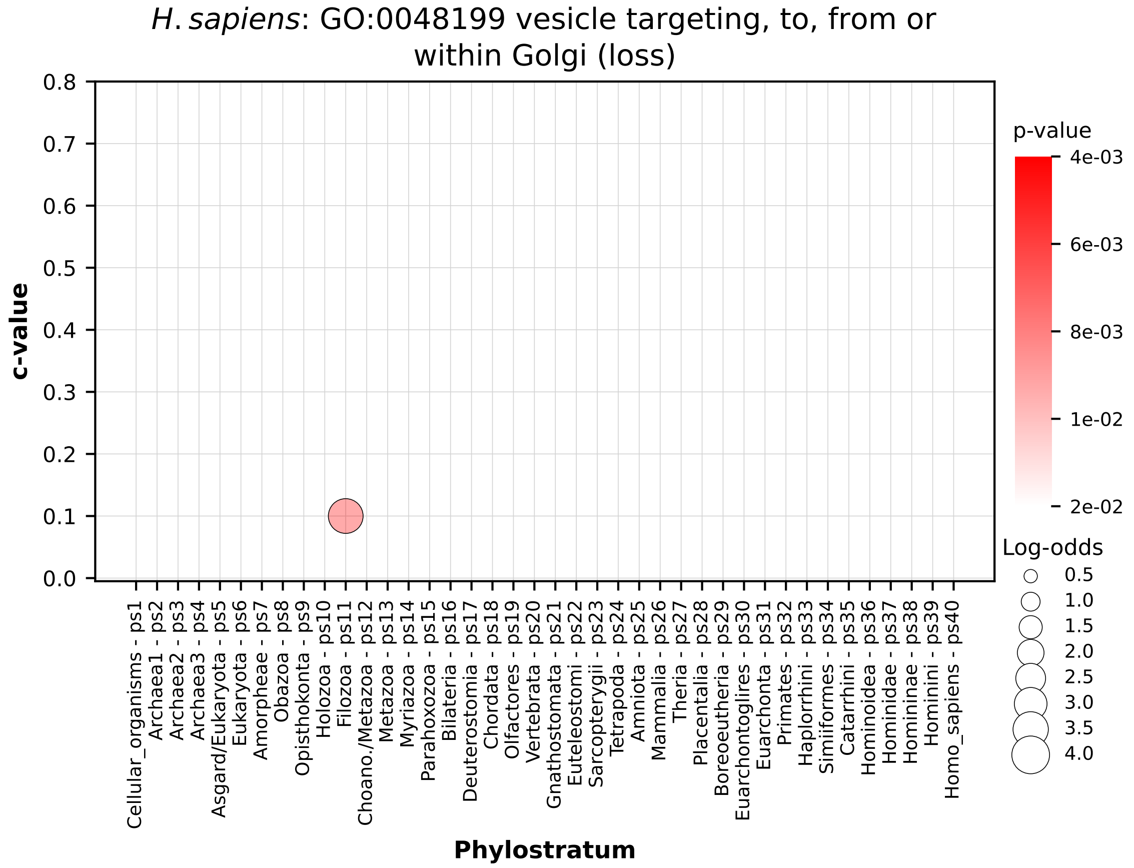

H. sapiens: GO:0048199 vesicle targeting, to, from or within Golgi (loss)
c-value
0.8
0.7
0.6
0.5
0.4
0.3
0.2
0.1
0.0
p-value
4e-03
6e-03
8e-03
1e-02
2e-02
Log-odds
0.5
1.0
1.5
2.0
2.5
3.0
3.5
4.0
Cellular_organisms - ps1
Archaea1 - ps2
Archaea2 - ps3
Archaea3 - ps4
Asgard/Eukaryota - ps5
Eukaryota - ps6
Amorpheae - ps7
Obazoa - ps8
Opisthokonta - ps9
Holozoa - ps10
Filozoa - ps11
Choano./Metazoa - ps12
Metazoa - ps13
Myriazoa - ps14
Parahoxozoa - ps15
Bilateria - ps16
Deuterostomia - ps17
Chordata - ps18
Olfactores - ps19
Vertebrata - ps20
Gnathostomata - ps21
Euteleostomi - ps22
Sarcopterygii - ps23
Tetrapoda - ps24
Amniota - ps25
Mammalia - ps26
Theria - ps27
Placentalia - ps28
Boreoeutheria - ps29
Euarchontoglires - ps30
Euarchonta - ps31
Primates - ps32
Haplorrhini - ps33
Simiiformes - ps34
Catarrhini - ps35
Hominoidea - ps36
Hominidae - ps37
Homininae - ps38
Hominini - ps39
Homo_sapiens - ps40
Phylostratum

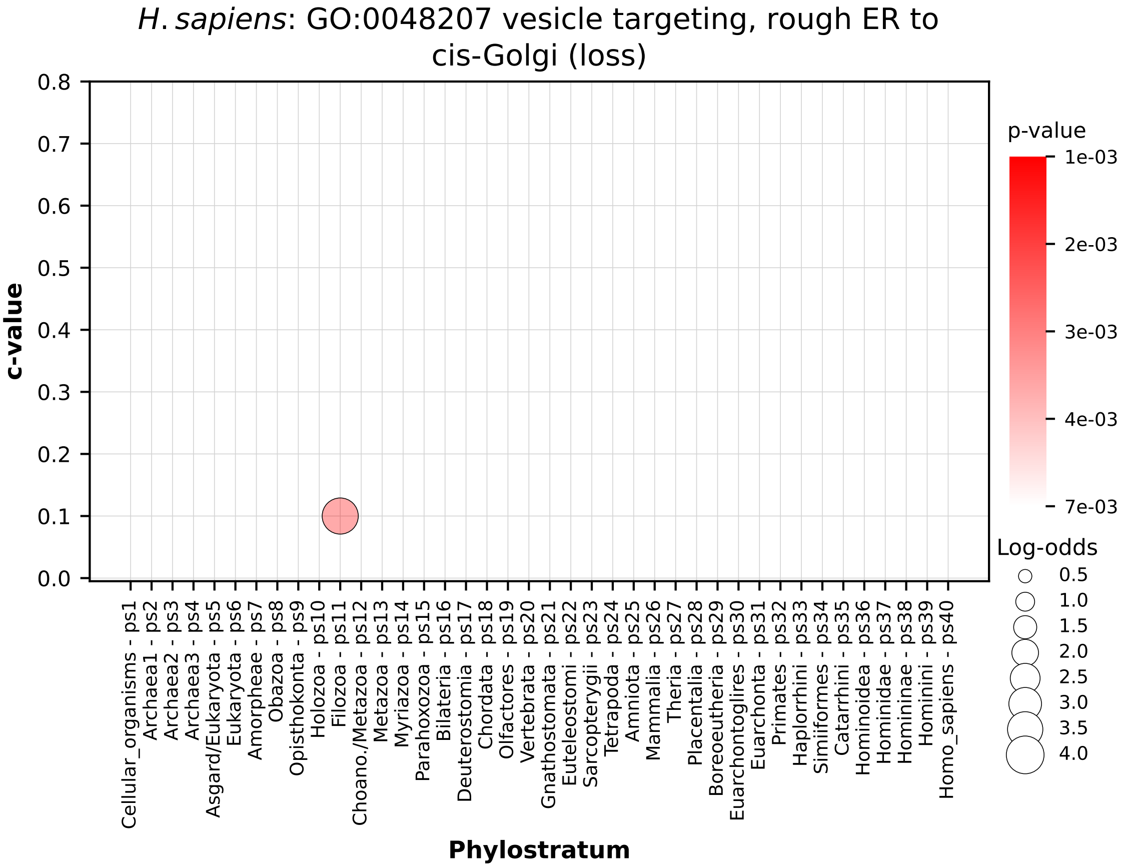

H. sapiens: GO:0048207 vesicle targeting, rough ER to cis-Golgi (loss)
c-value
0.0
0.1
0.2
0.3
0.4
0.5
0.6
0.7
0.8
Phylostratum
Cellular_organisms - ps1
Archaea1 - ps2
Archaea2 - ps3
Archaea3 - ps4
Asgard/Eukaryota - ps5
Eukaryota - ps6
Amorpheae - ps7
Obazoa - ps8
Opisthokonta - ps9
Holozoa - ps10
Filozoa - ps11
Choano./Metazoa - ps12
Metazoa - ps13
Myriazoa - ps14
Parahoxozoa - ps15
Bilateria - ps16
Deuterostomia - ps17
Chordata - ps18
Olfactores - ps19
Vertebrata - ps20
Gnathostomata - ps21
Euteleostomi - ps22
Sarcopterygii - ps23
Tetrapoda - ps24
Amniota - ps25
Mammalia - ps26
Theria - ps27
Placentalia - ps28
Boreoeutheria - ps29
Euarchontoglires - ps30
Euarchonta - ps31
Primates - ps32
Haplorrhini - ps33
Simiiformes - ps34
Catarrhini - ps35
Hominoidea - ps36
Hominidae - ps37
Homininae - ps38
Hominini - ps39
Homo_sapiens - ps40
p-value
1e-03
2e-03
3e-03
4e-03
7e-03
Log-odds
0.5
1.0
1.5
2.0
2.5
3.0
3.5
4.0

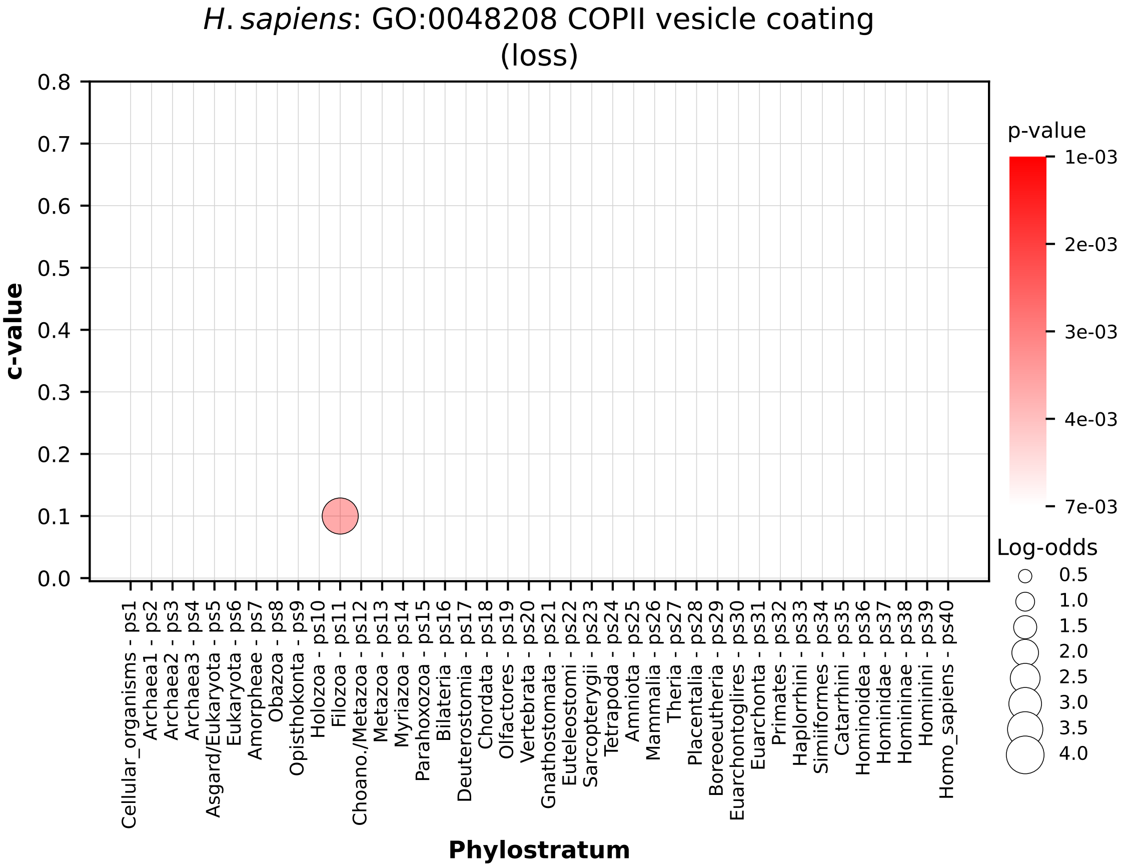

H. sapiens: GO:0048208 COPII vesicle coating (loss)
c-value
0.0
0.1
0.2
0.3
0.4
0.5
0.6
0.7
0.8
Phylostratum
Cellular_organisms - ps1
Archaea1 - ps2
Archaea2 - ps3
Archaea3 - ps4
Asgard/Eukaryota - ps5
Eukaryota - ps6
Amorpheae - ps7
Obazoa - ps8
Opisthokonta - ps9
Holozoa - ps10
Filozoa - ps11
Choano./Metazoa - ps12
Metazoa - ps13
Myriazoa - ps14
Parahoxozoa - ps15
Bilateria - ps16
Deuterostomia - ps17
Chordata - ps18
Olfactores - ps19
Vertebrata - ps20
Gnathostomata - ps21
Euteleostomi - ps22
Sarcopterygii - ps23
Tetrapoda - ps24
Amniota - ps25
Mammalia - ps26
Theria - ps27
Placentalia - ps28
Boreoeutheria - ps29
Euarchontoglires - ps30
Euarchonta - ps31
Primates - ps32
Haplorrhini - ps33
Simiiformes - ps34
Catarrhini - ps35
Hominoidea - ps36
Hominidae - ps37
Homininae - ps38
Hominini - ps39
Homo_sapiens - ps40
p-value
1e-03
2e-03
3e-03
4e-03
7e-03
Log-odds
0.5
1.0
1.5
2.0
2.5
3.0
3.5
4.0

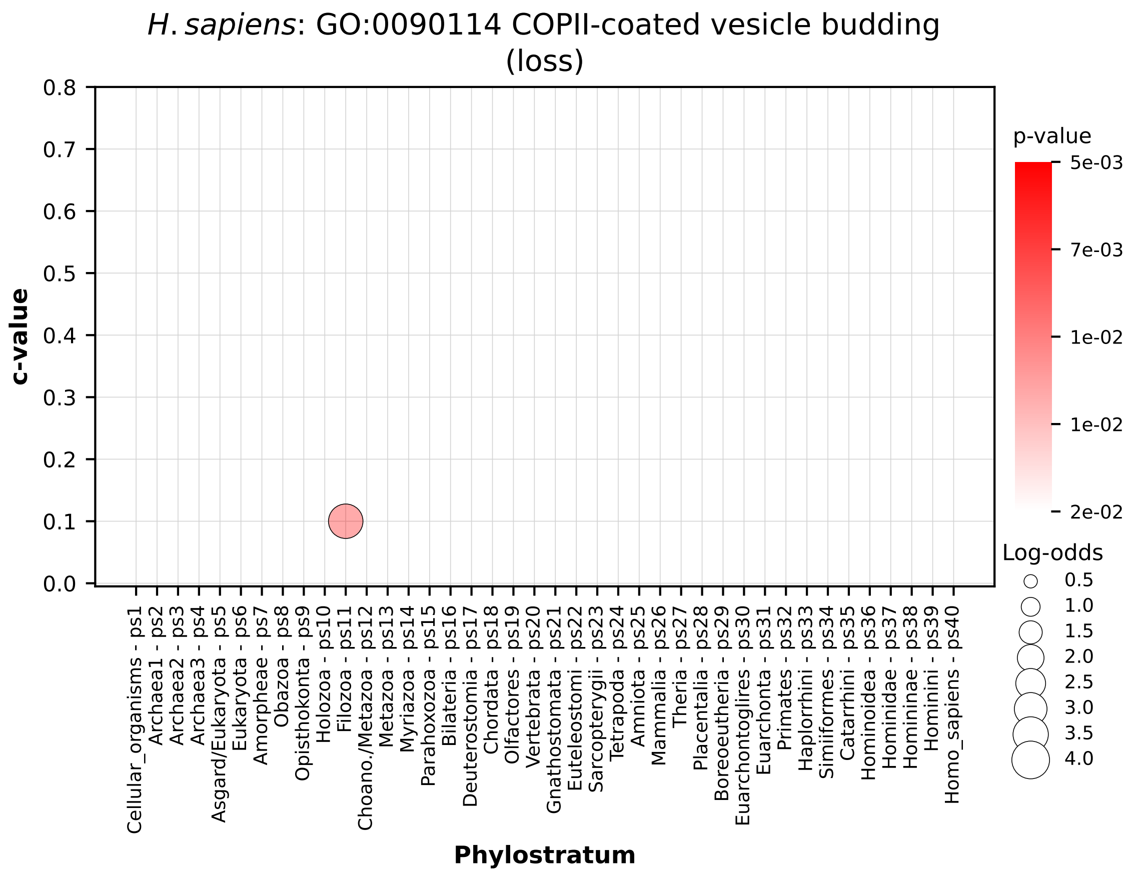
H. sapiens: GO:0090114 COPII-coated vesicle budding
(loss)
c-value
0.0
0.1
0.2
0.3
0.4
0.5
0.6
0.7
0.8
p-value
5e-03
7e-03
1e-02
1e-02
2e-02
Log-odds
0.5
1.0
1.5
2.0
2.5
3.0
3.5
4.0
Cellular_organisms - ps1
Archaea1 - ps2
Archaea2 - ps3
Archaea3 - ps4
Asgard/Eukaryota - ps5
Eukaryota - ps6
Amorpheae - ps7
Obazoa - ps8
Opisthokonta - ps9
Holozoa - ps10
Filozoa - ps11
Choano./Metazoa - ps12
Metazoa - ps13
Myriazoa - ps14
Parahoxozoa - ps15
Bilateria - ps16
Deuterostomia - ps17
Chordata - ps18
Olfactores - ps19
Vertebrata - ps20
Gnathostomata - ps21
Euteleostomi - ps22
Sarcopterygii - ps23
Tetrapoda - ps24
Amniota - ps25
Mammalia - ps26
Theria - ps27
Placentalia - ps28
Boreoeutheria - ps29
Euarchontoglires - ps30
Euarchonta - ps31
Primates - ps32
Haplorrhini - ps33
Simiiformes - ps34
Catarrhini - ps35
Hominoidea - ps36
Hominidae - ps37
Homininae - ps38
Hominini - ps39
Homo_sapiens - ps40
Phylostratum

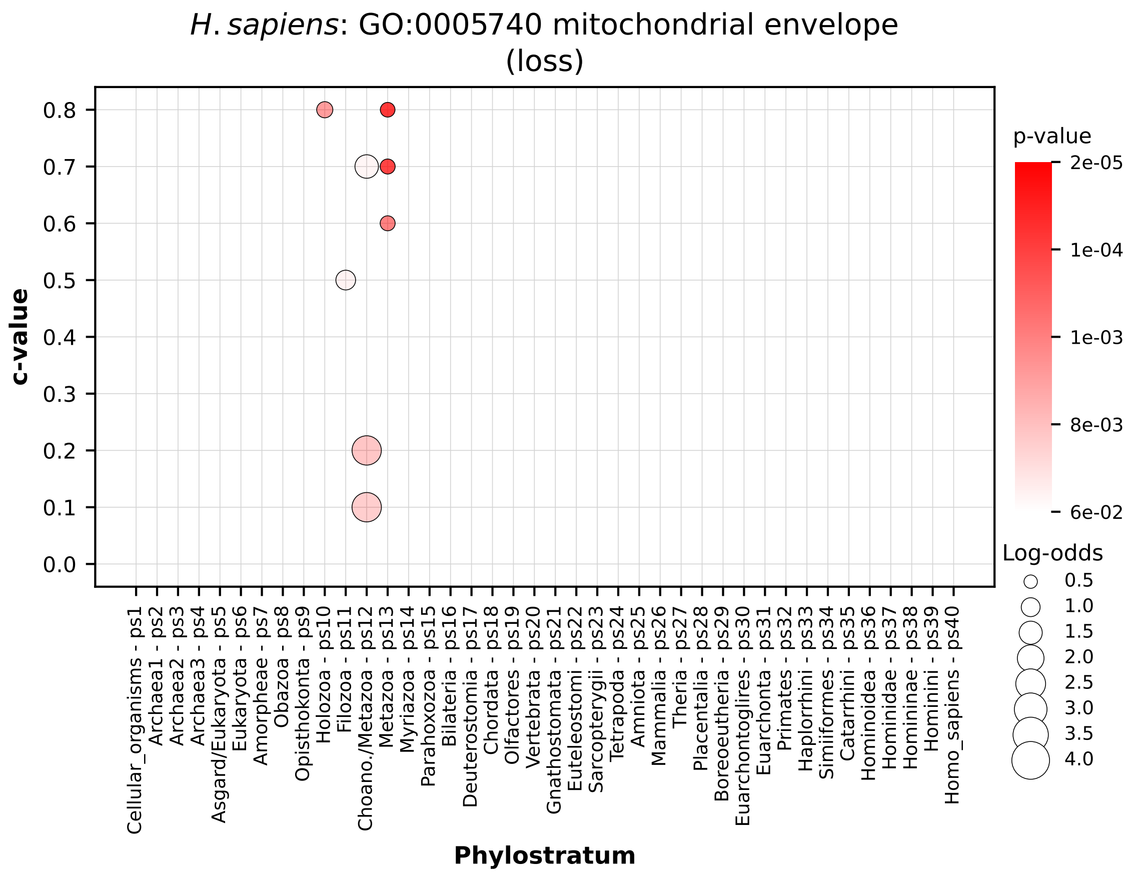

H. sapiens: GO:0005740 mitochondrial envelope
(loss)
c-value
0.8
0.7
0.6
0.5
0.4
0.3
0.2
0.1
0.0
p-value
2e-05
1e-04
1e-03
8e-03
6e-02
Log-odds
0.5
1.0
1.5
2.0
2.5
3.0
3.5
4.0
Cellular_organisms - ps1
Archaea1 - ps2
Archaea2 - ps3
Archaea3 - ps4
Asgard/Eukaryota - ps5
Eukaryota - ps6
Amorpheae - ps7
Obazoa - ps8
Opisthokonta - ps9
Holozoa - ps10
Filozoa - ps11
Choano./Metazoa - ps12
Metazoa - ps13
Myriazoa - ps14
Parahoxozoa - ps15
Bilateria - ps16
Deuterostomia - ps17
Chordata - ps18
Olfactores - ps19
Vertebrata - ps20
Gnathostomata - ps21
Euteleostomi - ps22
Sarcopterygii - ps23
Tetrapoda - ps24
Amniota - ps25
Mammalia - ps26
Theria - ps27
Placentalia - ps28
Boreoeutheria - ps29
Euarchontoglires - ps30
Euarchonta - ps31
Primates - ps32
Haplorrhini - ps33
Simiiformes - ps34
Catarrhini - ps35
Hominoidea - ps36
Hominidae - ps37
Homininae - ps38
Hominini - ps39
Homo_sapiens - ps40
Phylostratum

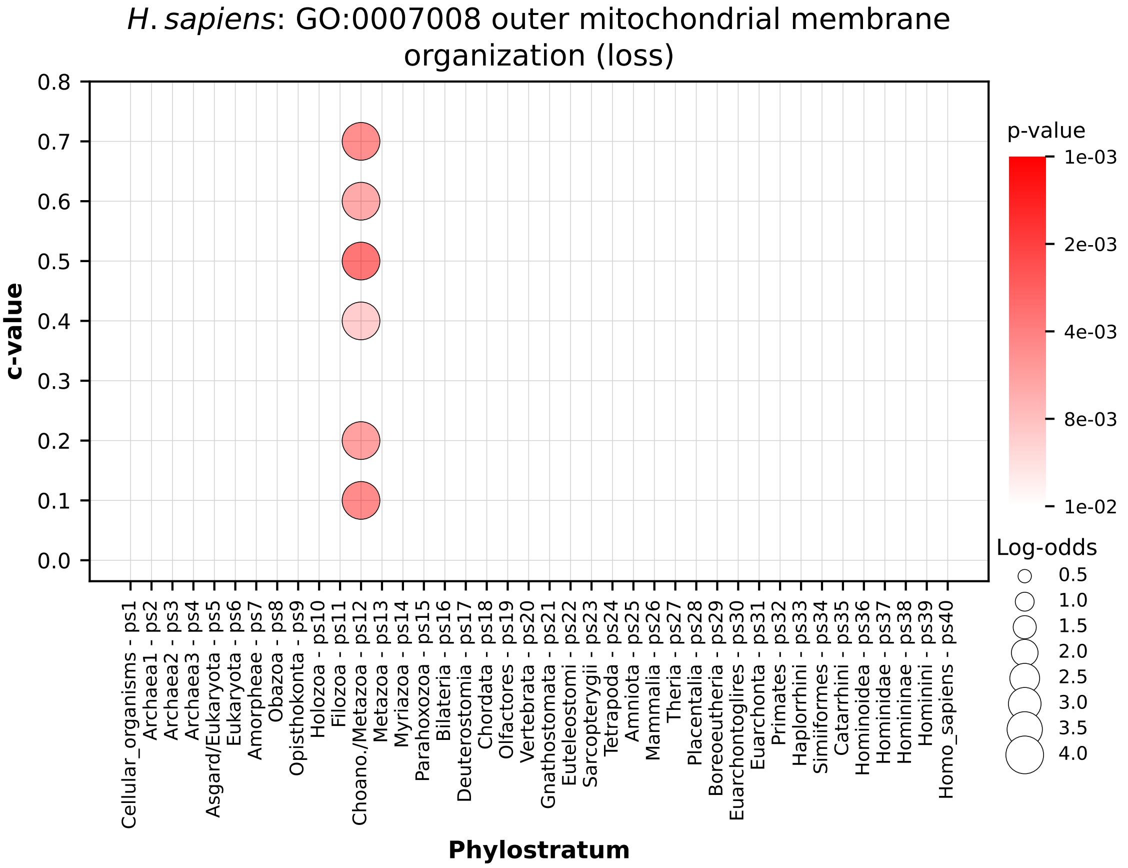

H. sapiens: GO:0007008 outer mitochondrial membrane organization (loss)
c-value
0.8
0.7
0.6
0.5
0.4
0.3
0.2
0.1
0.0
Phylostratum
Cellular_organisms - ps1
Archaea1 - ps2
Archaea2 - ps3
Archaea3 - ps4
Asgard/Eukaryota - ps5
Eukaryota - ps6
Amorpheae - ps7
Obazoa - ps8
Opisthokonta - ps9
Holozoa - ps10
Filozoa - ps11
Choano./Metazoa - ps12
Metazoa - ps13
Myriazoa - ps14
Parahoxozoa - ps15
Bilateria - ps16
Deuterostomia - ps17
Chordata - ps18
Olfactores - ps19
Vertebrata - ps20
Gnathostomata - ps21
Euteleostomi - ps22
Sarcopterygii - ps23
Tetrapoda - ps24
Amniota - ps25
Mammalia - ps26
Theria - ps27
Placentalia - ps28
Boreoeutheria - ps29
Euarchontoglires - ps30
Euarchonta - ps31
Primates - ps32
Haplorrhini - ps33
Simiiformes - ps34
Catarrhini - ps35
Hominoidea - ps36
Hominidae - ps37
Homininae - ps38
Hominini - ps39
Homo_sapiens - ps40
p-value
1e-03
2e-03
4e-03
8e-03
1e-02
Log-odds
0.5
1.0
1.5
2.0
2.5
3.0
3.5
4.0

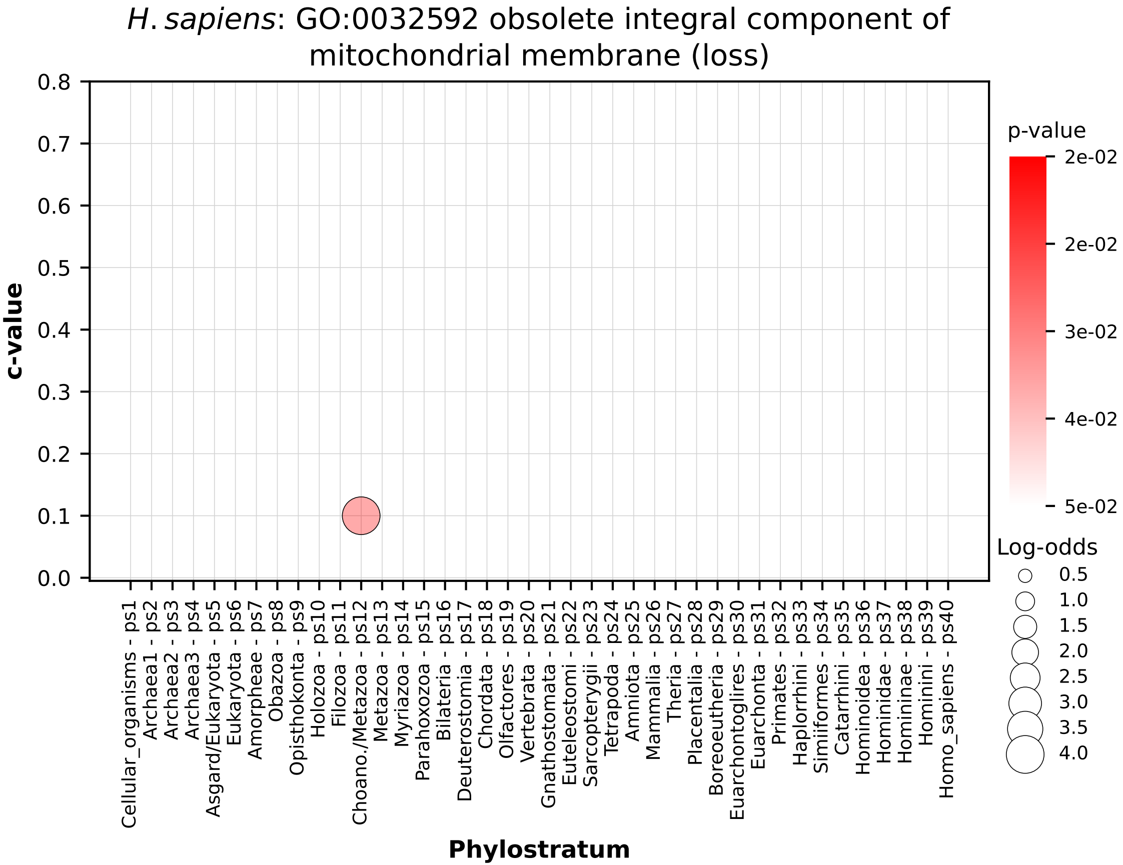

H. sapiens: GO:0032592 obsolete integral component of mitochondrial membrane (loss)
c-value
0.8
0.7
0.6
0.5
0.4
0.3
0.2
0.1
0.0
Phylostratum
Cellular_organisms - ps1
Archaea1 - ps2
Archaea2 - ps3
Archaea3 - ps4
Asgard/Eukaryota - ps5
Eukaryota - ps6
Amorpheae - ps7
Obazoa - ps8
Opisthokonta - ps9
Holozoa - ps10
Filozoa - ps11
Choano./Metazoa - ps12
Metazoa - ps13
Myriazoa - ps14
Parahoxozoa - ps15
Bilateria - ps16
Deuterostomia - ps17
Chordata - ps18
Olfactores - ps19
Vertebrata - ps20
Gnathostomata - ps21
Euteleostomi - ps22
Sarcopterygii - ps23
Tetrapoda - ps24
Amniota - ps25
Mammalia - ps26
Theria - ps27
Placentalia - ps28
Boreoeutheria - ps29
Euarchontoglires - ps30
Euarchonta - ps31
Primates - ps32
Haplorrhini - ps33
Simiiformes - ps34
Catarrhini - ps35
Hominoidea - ps36
Hominidae - ps37
Homininae - ps38
Hominini - ps39
Homo_sapiens - ps40
p-value
2e-02
2e-02
3e-02
4e-02
5e-02
Log-odds
0.5
1.0
1.5
2.0
2.5
3.0
3.5
4.0

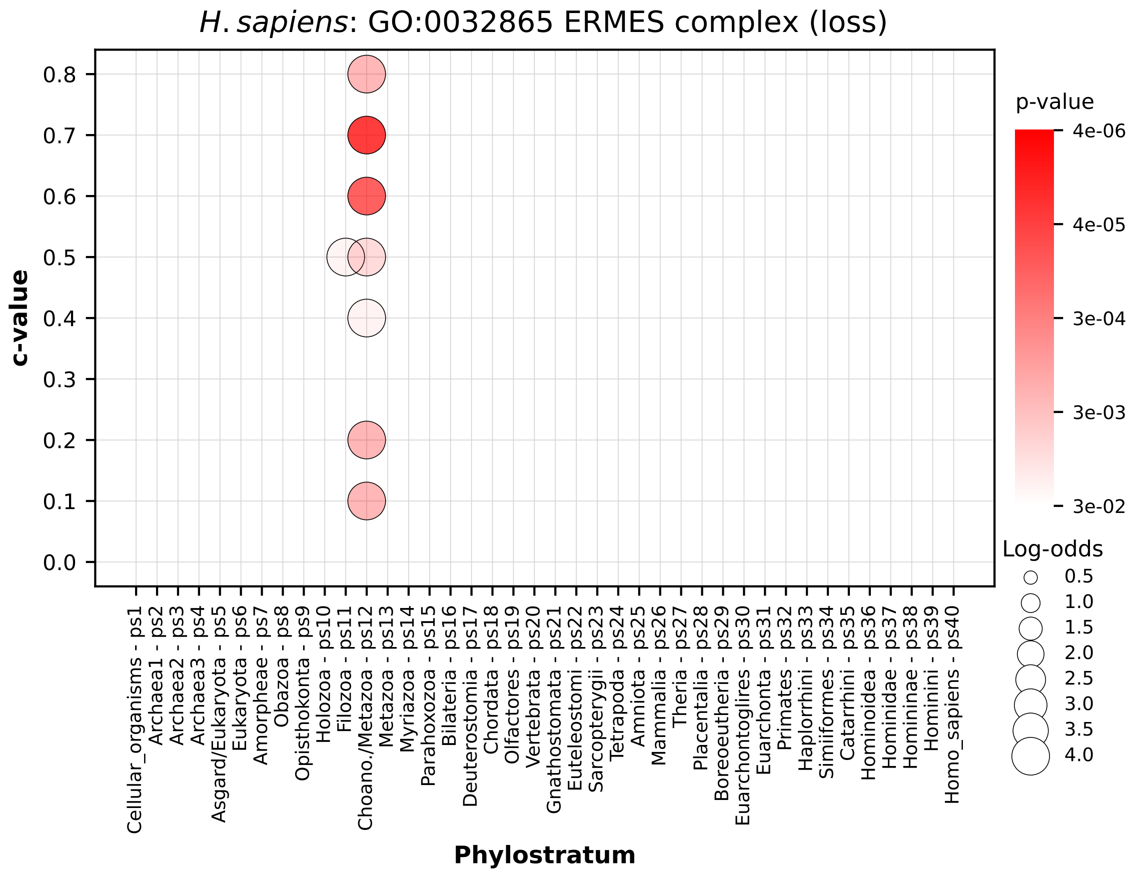
H. sapiens: GO:0032865 ERMES complex (loss)
c-value
0.0
0.1
0.2
0.3
0.4
0.5
0.6
0.7
0.8
Phylostratum
Cellular_organisms - ps1
Archaea1 - ps2
Archaea2 - ps3
Archaea3 - ps4
Asgard/Eukaryota - ps5
Eukaryota - ps6
Amorpheae - ps7
Obazoa - ps8
Opisthokonta - ps9
Holozoa - ps10
Filozoa - ps11
Choano./Metazoa - ps12
Metazoa - ps13
Myriazoa - ps14
Parahoxozoa - ps15
Bilateria - ps16
Deuterostomia - ps17
Chordata - ps18
Olfactores - ps19
Vertebrata - ps20
Gnathostomata - ps21
Euteleostomi - ps22
Sarcopterygii - ps23
Tetrapoda - ps24
Amniota - ps25
Mammalia - ps26
Theria - ps27
Placentalia - ps28
Boreoeutheria - ps29
Euarchontoglires - ps30
Euarchonta - ps31
Primates - ps32
Haplorrhini - ps33
Simiiformes - ps34
Catarrhini - ps35
Hominoidea - ps36
Hominidae - ps37
Homininae - ps38
Hominini - ps39
Homo_sapiens - ps40
p-value
4e-06
4e-05
3e-04
3e-03
3e-02
Log-odds
0.5
1.0
1.5
2.0
2.5
3.0
3.5
4.0

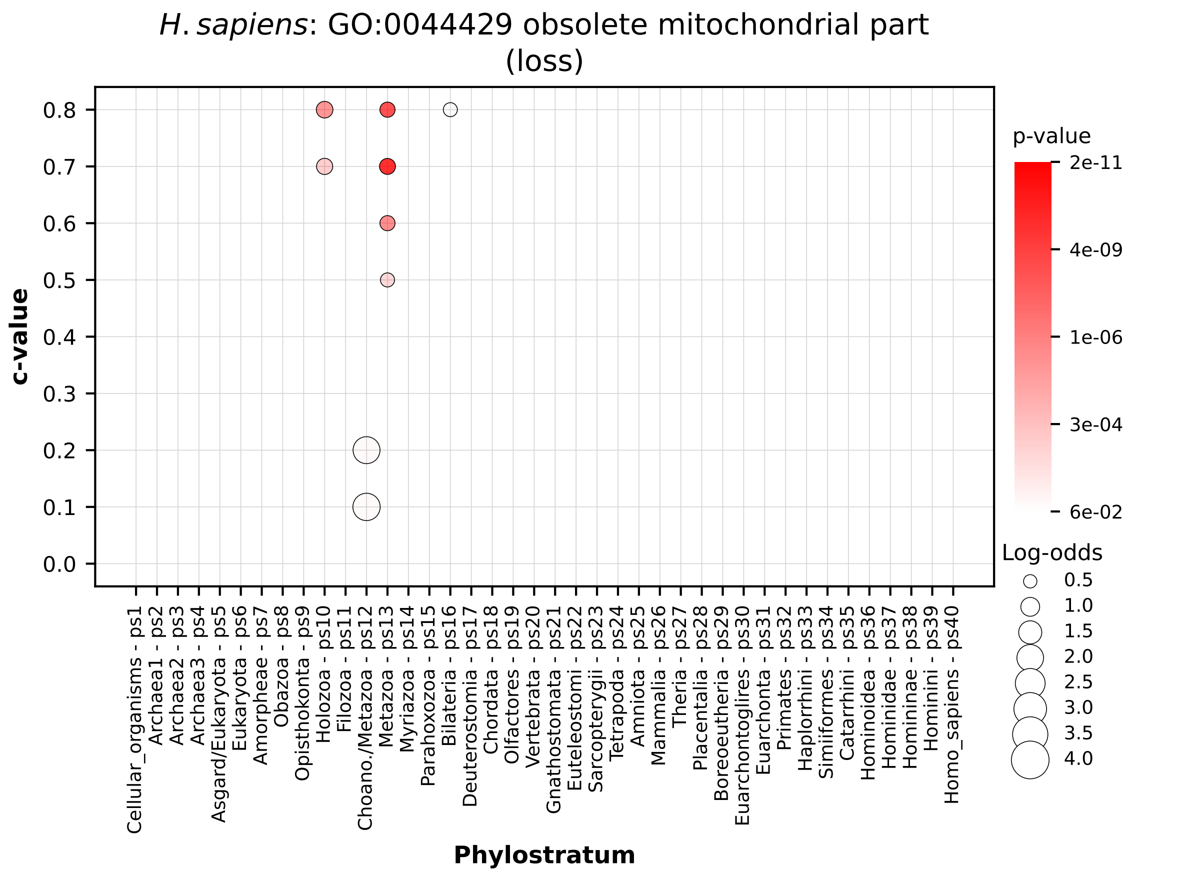

H. sapiens: GO:0044429 obsolete mitochondrial part
(loss)
c-value
0.8
0.7
0.6
0.5
0.4
0.3
0.2
0.1
0.0
p-value
2e-11
4e-09
1e-06
3e-04
6e-02
Log-odds
0.5
1.0
1.5
2.0
2.5
3.0
3.5
4.0
Cellular_organisms - ps1
Archaea1 - ps2
Archaea2 - ps3
Archaea3 - ps4
Asgard/Eukaryota - ps5
Eukaryota - ps6
Amorpheae - ps7
Obazoa - ps8
Opisthokonta - ps9
Holozoa - ps10
Filozoa - ps11
Choano./Metazoa - ps12
Metazoa - ps13
Myriazoa - ps14
Parahoxozoa - ps15
Bilateria - ps16
Deuterostomia - ps17
Chordata - ps18
Olfactores - ps19
Vertebrata - ps20
Gnathostomata - ps21
Euteleostomi - ps22
Sarcopterygii - ps23
Tetrapoda - ps24
Amniota - ps25
Mammalia - ps26
Theria - ps27
Placentalia - ps28
Boreoeutheria - ps29
Euarchontoglires - ps30
Euarchonta - ps31
Primates - ps32
Haplorrhini - ps33
Simiiformes - ps34
Catarrhini - ps35
Hominoidea - ps36
Hominidae - ps37
Homininae - ps38
Hominini - ps39
Homo_sapiens - ps40
Phylostratum

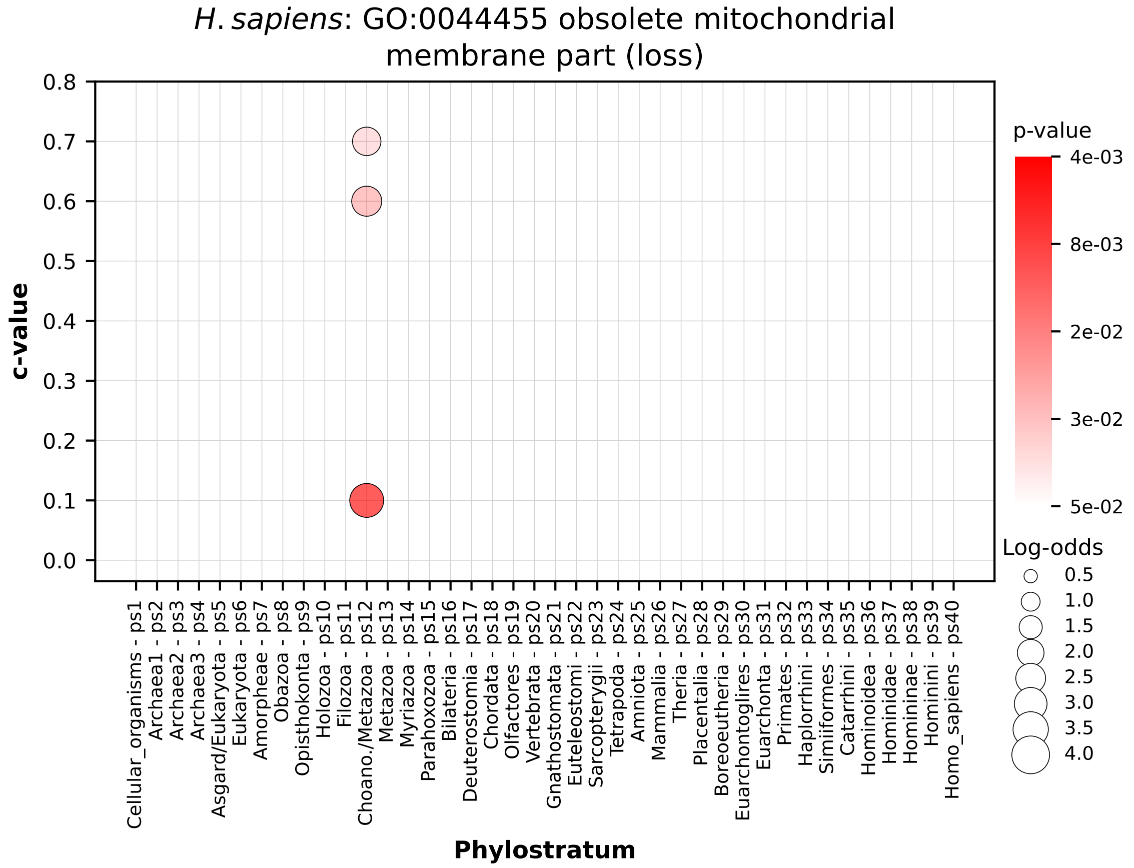

H. sapiens: GO:0044455 obsolete mitochondrial membrane part (loss)
c-value
0.0
0.1
0.2
0.3
0.4
0.5
0.6
0.7
0.8
Phylostratum
Cellular_organisms - ps1
Archaea1 - ps2
Archaea2 - ps3
Archaea3 - ps4
Asgard/Eukaryota - ps5
Eukaryota - ps6
Amorpheae - ps7
Obazoa - ps8
Opisthokonta - ps9
Holozoa - ps10
Filozoa - ps11
Choano./Metazoa - ps12
Metazoa - ps13
Myriazoa - ps14
Parahoxozoa - ps15
Bilateria - ps16
Deuterostomia - ps17
Chordata - ps18
Olfactores - ps19
Vertebrata - ps20
Gnathostomata - ps21
Euteleostomi - ps22
Sarcopterygii - ps23
Tetrapoda - ps24
Amniota - ps25
Mammalia - ps26
Theria - ps27
Placentalia - ps28
Boreoeutheria - ps29
Euarchontoglires - ps30
Euarchonta - ps31
Primates - ps32
Haplorrhini - ps33
Simiiformes - ps34
Catarrhini - ps35
Hominoidea - ps36
Hominidae - ps37
Homininae - ps38
Hominini - ps39
Homo_sapiens - ps40
p-value
4e-03
8e-03
2e-02
3e-02
5e-02
Log-odds
0.5
1.0
1.5
2.0
2.5
3.0
3.5
4.0

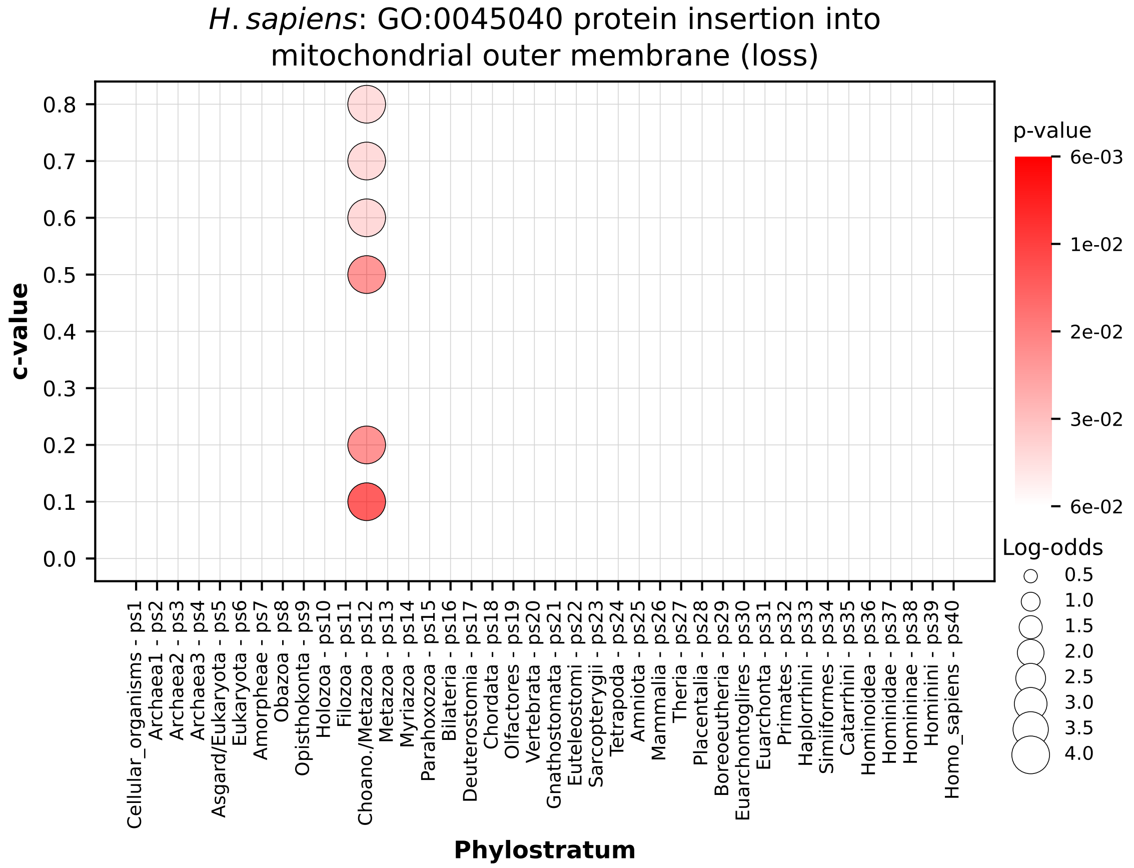

H. sapiens: GO:0045040 protein insertion into mitochondrial outer membrane (loss)
c-value
0.0
0.1
0.2
0.3
0.4
0.5
0.6
0.7
0.8
Phylostratum
Cellular_organisms - ps1
Archaea1 - ps2
Archaea2 - ps3
Archaea3 - ps4
Asgard/Eukaryota - ps5
Eukaryota - ps6
Amorpheae - ps7
Obazoa - ps8
Opisthokonta - ps9
Holozoa - ps10
Filozoa - ps11
Choano./Metazoa - ps12
Metazoa - ps13
Myriazoa - ps14
Parahoxozoa - ps15
Bilateria - ps16
Deuterostomia - ps17
Chordata - ps18
Olfactores - ps19
Vertebrata - ps20
Gnathostomata - ps21
Euteleostomi - ps22
Sarcopterygii - ps23
Tetrapoda - ps24
Amniota - ps25
Mammalia - ps26
Theria - ps27
Placentalia - ps28
Boreoeutheria - ps29
Euarchontoglires - ps30
Euarchonta - ps31
Primates - ps32
Haplorrhini - ps33
Simiiformes - ps34
Catarrhini - ps35
Hominoidea - ps36
Hominidae - ps37
Homininae - ps38
Hominini - ps39
Homo_sapiens - ps40
p-value
6e-03
1e-02
2e-02
3e-02
6e-02
Log-odds
0.5
1.0
1.5
2.0
2.5
3.0
3.5
4.0

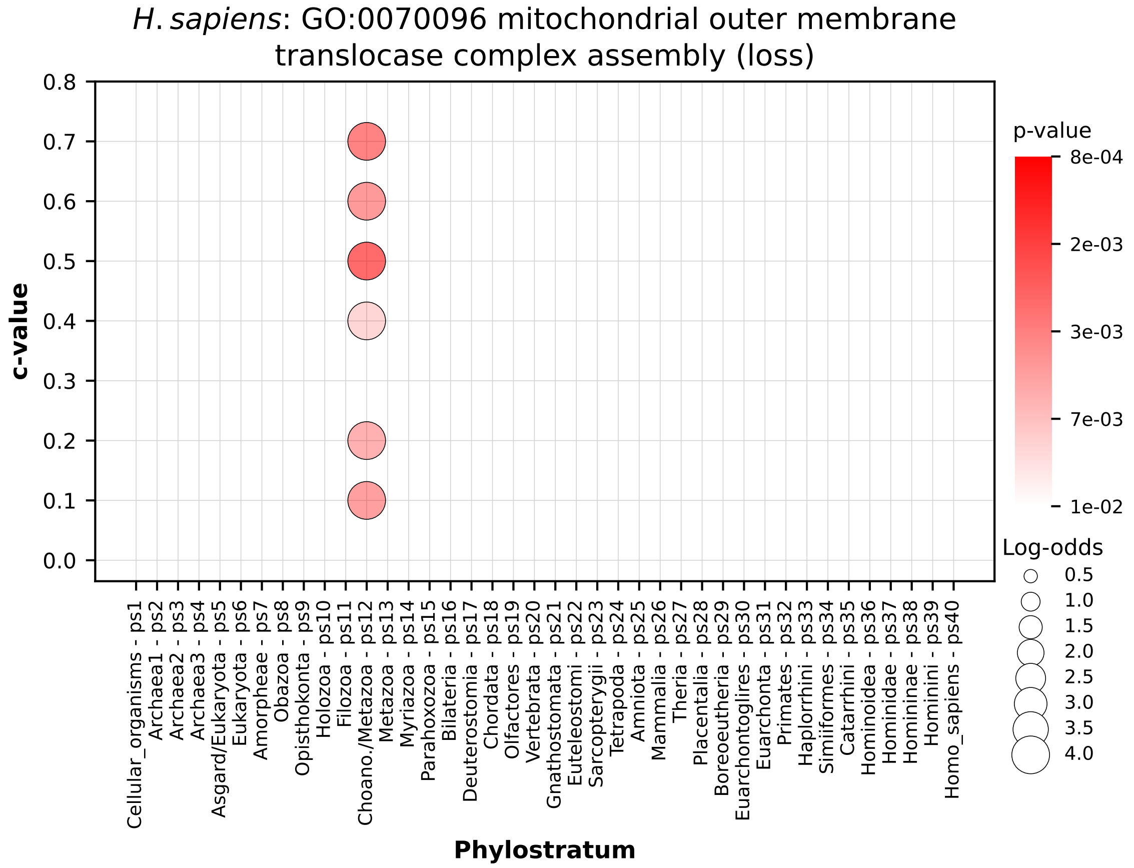

H. sapiens: GO:0070096 mitochondrial outer membrane translocase complex assembly (loss)
c-value
0.0
0.1
0.2
0.3
0.4
0.5
0.6
0.7
0.8
Phylostratum
Cellular_organisms - ps1
Archaea1 - ps2
Archaea2 - ps3
Archaea3 - ps4
Asgard/Eukaryota - ps5
Eukaryota - ps6
Amorpheae - ps7
Obazoa - ps8
Opisthokonta - ps9
Holozoa - ps10
Filozoa - ps11
Choano./Metazoa - ps12
Metazoa - ps13
Myriazoa - ps14
Parahoxozoa - ps15
Bilateria - ps16
Deuterostomia - ps17
Chordata - ps18
Olfactores - ps19
Vertebrata - ps20
Gnathostomata - ps21
Euteleostomi - ps22
Sarcopterygii - ps23
Tetrapoda - ps24
Amniota - ps25
Mammalia - ps26
Theria - ps27
Placentalia - ps28
Boreoeutheria - ps29
Euarchontoglires - ps30
Euarchonta - ps31
Primates - ps32
Haplorrhini - ps33
Simiiformes - ps34
Catarrhini - ps35
Hominoidea - ps36
Hominidae - ps37
Homininae - ps38
Hominini - ps39
Homo_sapiens - ps40
p-value
8e-04
2e-03
3e-03
7e-03
1e-02
Log-odds
0.5
1.0
1.5
2.0
2.5
3.0
3.5
4.0

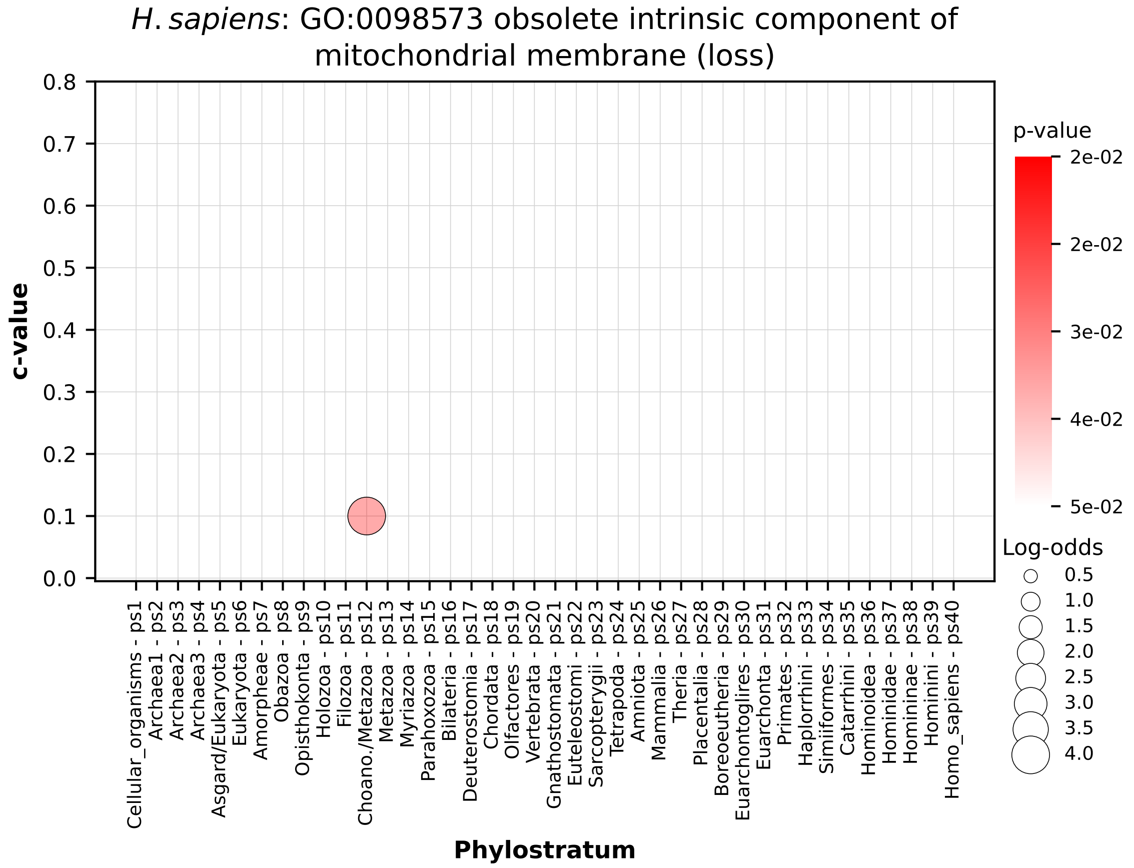

H. sapiens: GO:0098573 obsolete intrinsic component of mitochondrial membrane (loss)
c-value
0.8
0.7
0.6
0.5
0.4
0.3
0.2
0.1
0.0
Cellular_organisms - ps1
Archaea1 - ps2
Archaea2 - ps3
Archaea3 - ps4
Asgard/Eukaryota - ps5
Eukaryota - ps6
Amorpheae - ps7
Obazoa - ps8
Opisthokonta - ps9
Holozoa - ps10
Filozoa - ps11
Choano./Metazoa - ps12
Metazoa - ps13
Myriazoa - ps14
Parahoxozoa - ps15
Bilateria - ps16
Deuterostomia - ps17
Chordata - ps18
Olfactores - ps19
Vertebrata - ps20
Gnathostomata - ps21
Euteleostomi - ps22
Sarcopterygii - ps23
Tetrapoda - ps24
Amniota - ps25
Mammalia - ps26
Theria - ps27
Placentalia - ps28
Boreoeutheria - ps29
Euarchontoglires - ps30
Euarchonta - ps31
Primates - ps32
Haplorrhini - ps33
Simiiformes - ps34
Catarrhini - ps35
Hominoidea - ps36
Hominidae - ps37
Homininae - ps38
Hominini - ps39
Homo_sapiens - ps40
Phylostratum
p-value
2e-02
2e-02
3e-02
4e-02
5e-02
Log-odds
0.5
1.0
1.5
2.0
2.5
3.0
3.5
4.0

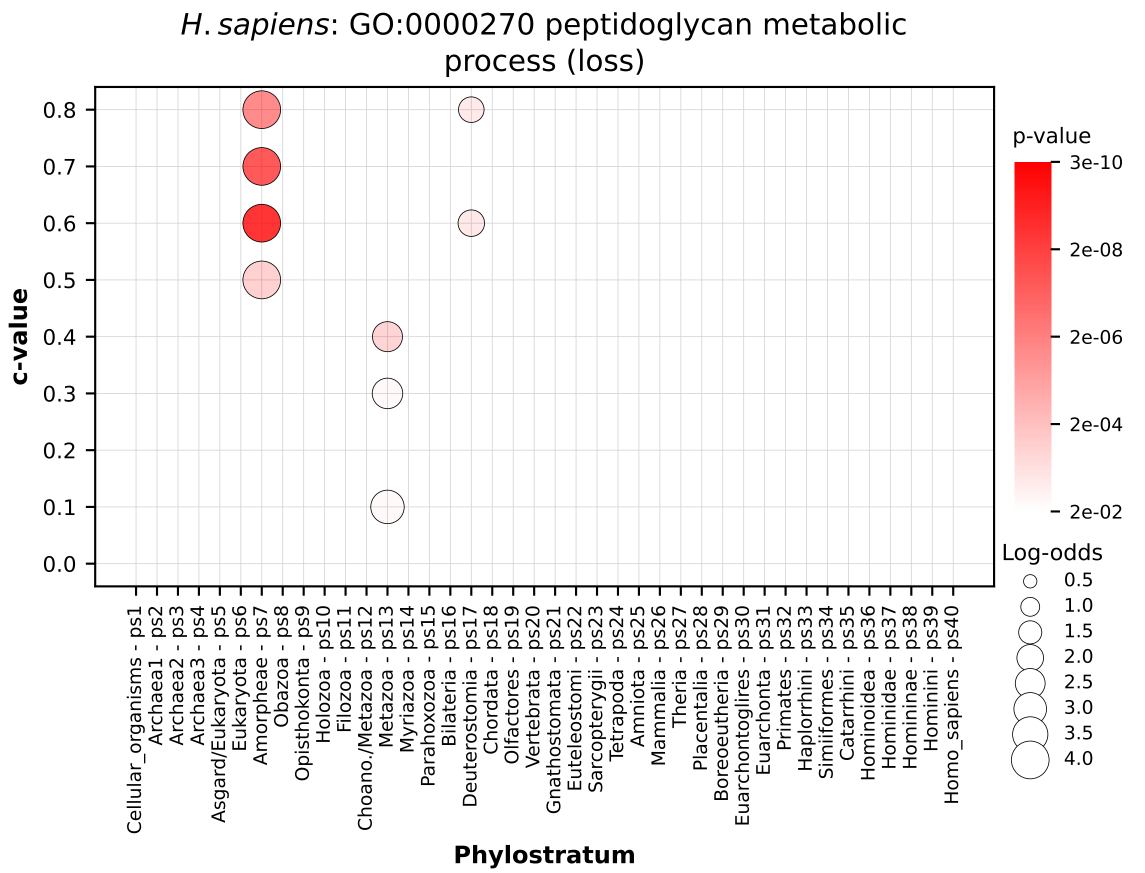
H. sapiens: GO:0000270 peptidoglycan metabolic process (loss)
c-value
0.0
0.1
0.2
0.3
0.4
0.5
0.6
0.7
0.8
Phylostratum
Cellular_organisms - ps1
Archaea1 - ps2
Archaea2 - ps3
Archaea3 - ps4
Asgard/Eukaryota - ps5
Eukaryota - ps6
Amorpheae - ps7
Obazoa - ps8
Opisthokonta - ps9
Holozoa - ps10
Filozoa - ps11
Choano./Metazoa - ps12
Metazoa - ps13
Myriazoa - ps14
Parahoxozoa - ps15
Bilateria - ps16
Deuterostomia - ps17
Chordata - ps18
Olfactores - ps19
Vertebrata - ps20
Gnathostomata - ps21
Euteleostomi - ps22
Sarcopterygii - ps23
Tetrapoda - ps24
Amniota - ps25
Mammalia - ps26
Theria - ps27
Placentalia - ps28
Boreoeutheria - ps29
Euarchontoglires - ps30
Euarchonta - ps31
Primates - ps32
Haplorrhini - ps33
Simiiformes - ps34
Catarrhini - ps35
Hominoidea - ps36
Hominidae - ps37
Homininae - ps38
Hominini - ps39
Homo_sapiens - ps40
p-value
3e-10
2e-08
2e-06
2e-04
2e-02
Log-odds
0.5
1.0
1.5
2.0
2.5
3.0
3.5
4.0

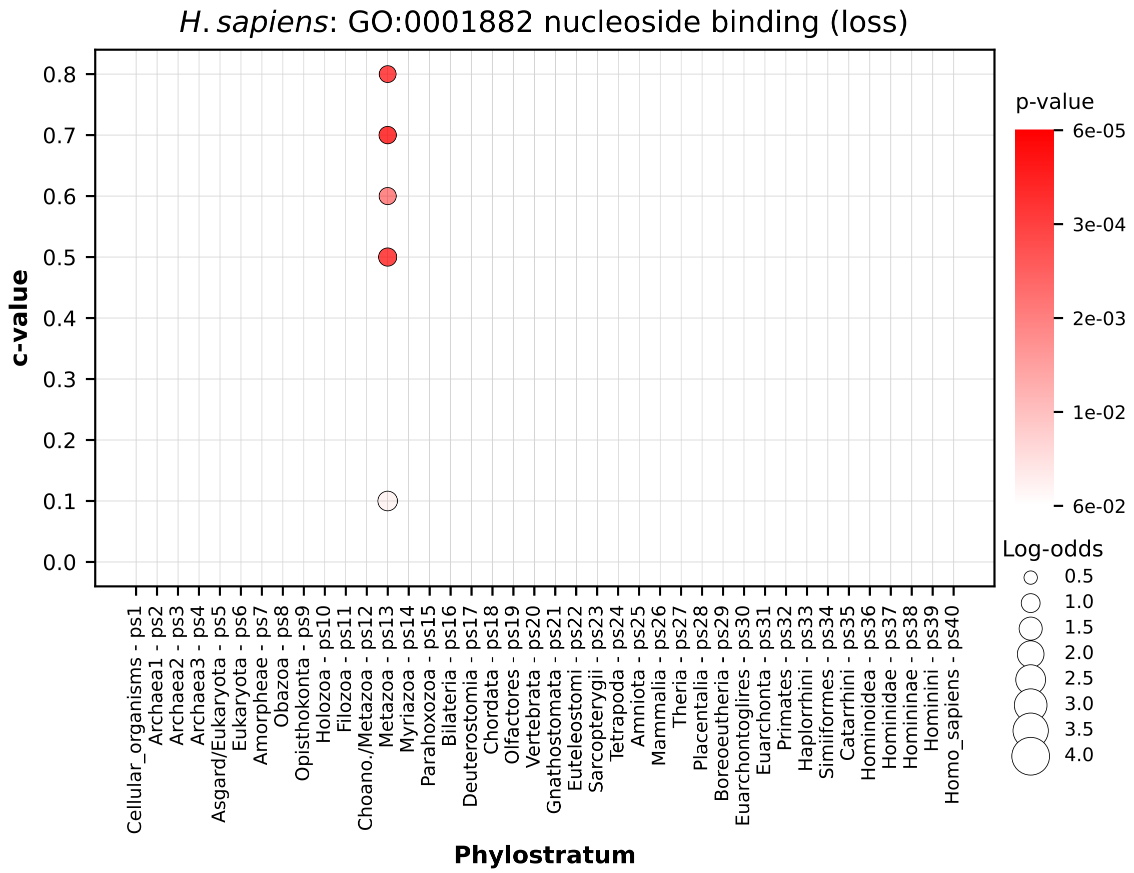

H. sapiens: GO:0001882 nucleoside binding (loss)
c-value
0.8
0.7
0.6
0.5
0.4
0.3
0.2
0.1
0.0
Phylostratum
Cellular_organisms - ps1
Archaea1 - ps2
Archaea2 - ps3
Archaea3 - ps4
Asgard/Eukaryota - ps5
Eukaryota - ps6
Amorpheae - ps7
Obazoa - ps8
Opisthokonta - ps9
Holozoa - ps10
Filozoa - ps11
Choano./Metazoa - ps12
Metazoa - ps13
Myriazoa - ps14
Parahoxozoa - ps15
Bilateria - ps16
Deuterostomia - ps17
Chordata - ps18
Olfactores - ps19
Vertebrata - ps20
Gnathostomata - ps21
Euteleostomi - ps22
Sarcopterygii - ps23
Tetrapoda - ps24
Amniota - ps25
Mammalia - ps26
Theria - ps27
Placentalia - ps28
Boreoeutheria - ps29
Euarchontoglires - ps30
Euarchonta - ps31
Primates - ps32
Haplorrhini - ps33
Simiiformes - ps34
Catarrhini - ps35
Hominoidea - ps36
Hominidae - ps37
Homininae - ps38
Hominini - ps39
Homo_sapiens - ps40
p-value
6e-05
3e-04
2e-03
1e-02
6e-02
Log-odds
0.5
1.0
1.5
2.0
2.5
3.0
3.5
4.0

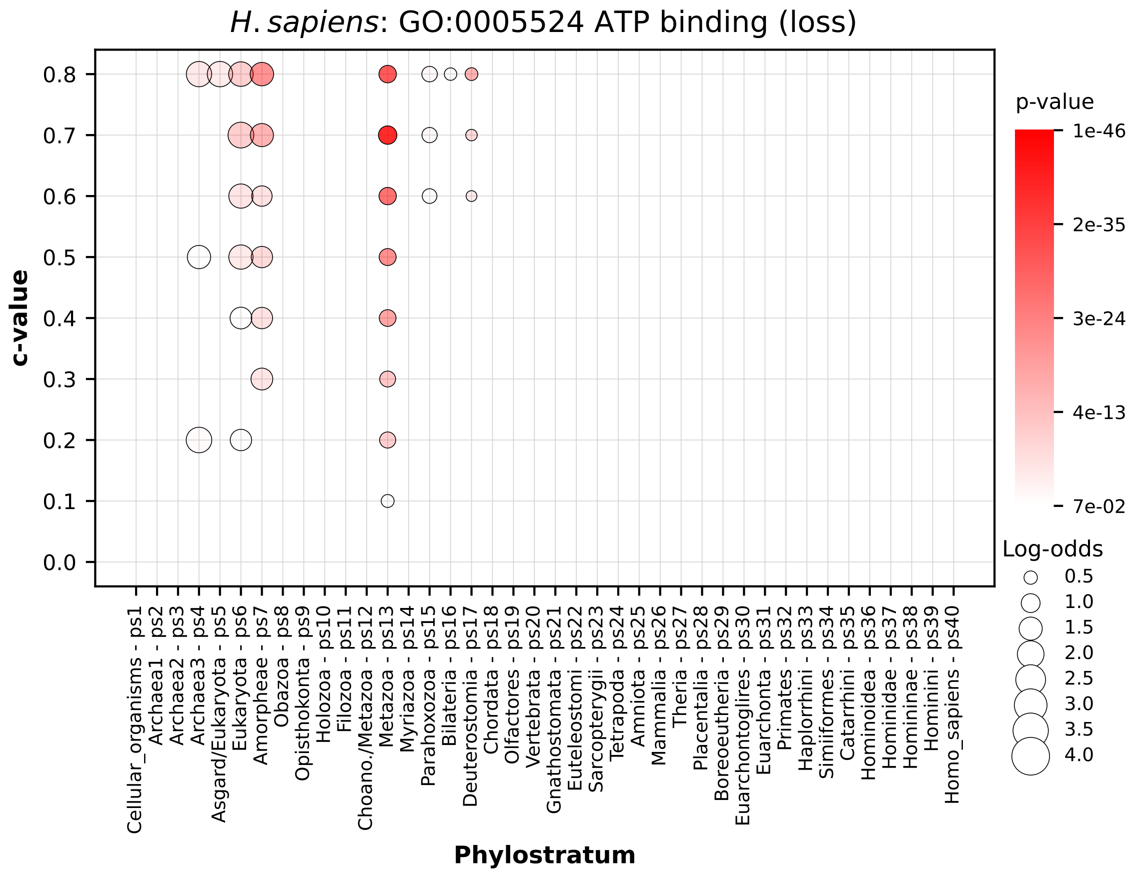

H. sapiens: GO:0005524 ATP binding (loss)
c-value
0.0
0.1
0.2
0.3
0.4
0.5
0.6
0.7
0.8
Phylostratum
Cellular_organisms - ps1
Archaea1 - ps2
Archaea2 - ps3
Archaea3 - ps4
Asgard/Eukaryota - ps5
Eukaryota - ps6
Amorpheae - ps7
Obazoa - ps8
Opisthokonta - ps9
Holozoa - ps10
Filozoa - ps11
Choano./Metazoa - ps12
Metazoa - ps13
Myriazoa - ps14
Parahoxozoa - ps15
Bilateria - ps16
Deuterostomia - ps17
Chordata - ps18
Olfactores - ps19
Vertebrata - ps20
Gnathostomata - ps21
Euteleostomi - ps22
Sarcopterygii - ps23
Tetrapoda - ps24
Amniota - ps25
Mammalia - ps26
Theria - ps27
Placentalia - ps28
Boreoeutheria - ps29
Euarchontoglires - ps30
Euarchonta - ps31
Primates - ps32
Haplorrhini - ps33
Simiiformes - ps34
Catarrhini - ps35
Hominoidea - ps36
Hominidae - ps37
Homininae - ps38
Hominini - ps39
Homo_sapiens - ps40
p-value
1e-46
2e-35
3e-24
4e-13
7e-02
Log-odds
0.5
1.0
1.5
2.0
2.5
3.0
3.5
4.0

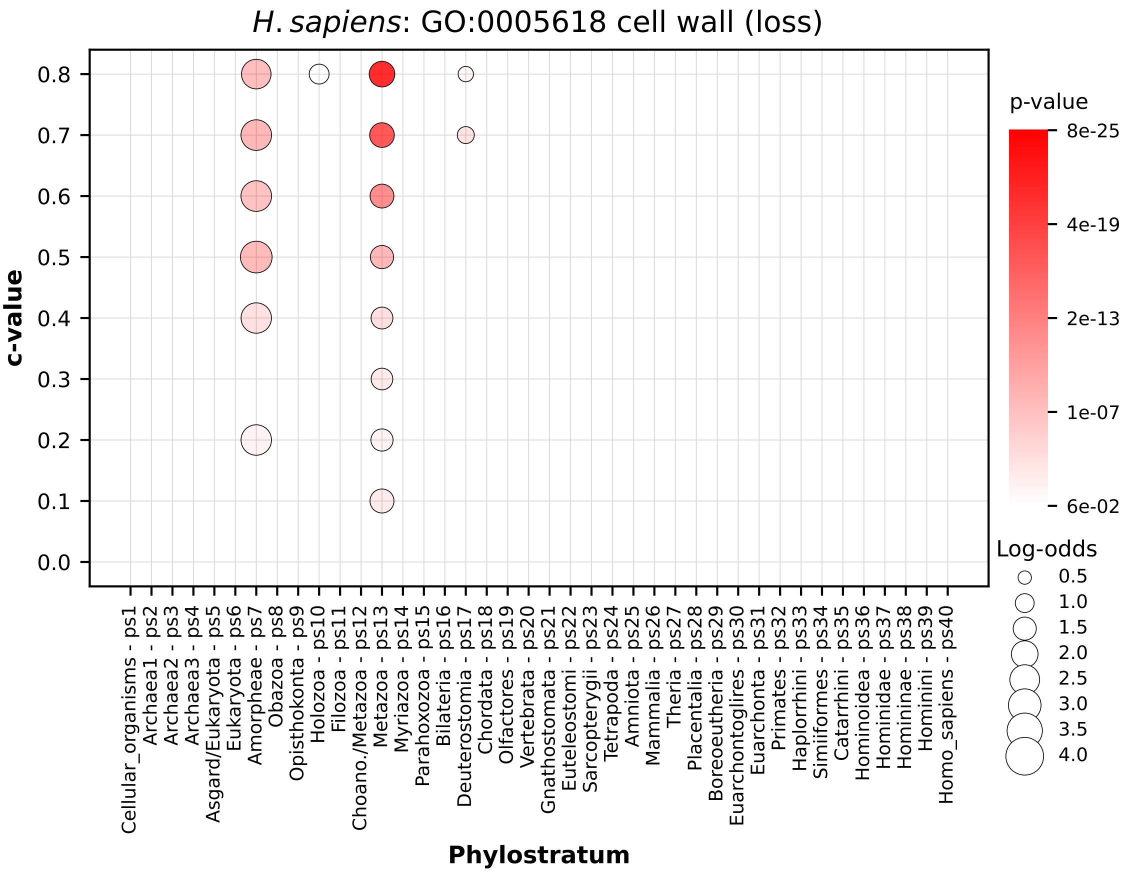

H. sapiens: GO:0005618 cell wall (loss)
c-value
0.8
0.7
0.6
0.5
0.4
0.3
0.2
0.1
0.0
Phylostratum
Cellular_organisms - ps1
Archaea1 - ps2
Archaea2 - ps3
Archaea3 - ps4
Asgard/Eukaryota - ps5
Eukaryota - ps6
Amorpheae - ps7
Obazoa - ps8
Opisthokonta - ps9
Holozoa - ps10
Filozoa - ps11
Choano./Metazoa - ps12
Metazoa - ps13
Myriazoa - ps14
Parahoxozoa - ps15
Bilateria - ps16
Deuterostomia - ps17
Chordata - ps18
Olfactores - ps19
Vertebrata - ps20
Gnathostomata - ps21
Euteleostomi - ps22
Sarcopterygii - ps23
Tetrapoda - ps24
Amniota - ps25
Mammalia - ps26
Theria - ps27
Placentalia - ps28
Boreoeutheria - ps29
Euarchontoglires - ps30
Euarchonta - ps31
Primates - ps32
Haplorrhini - ps33
Simiiformes - ps34
Catarrhini - ps35
Hominoidea - ps36
Hominidae - ps37
Homininae - ps38
Hominini - ps39
Homo_sapiens - ps40
p-value
8e-25
4e-19
2e-13
1e-07
6e-02
Log-odds
0.5
1.0
1.5
2.0
2.5
3.0
3.5
4.0

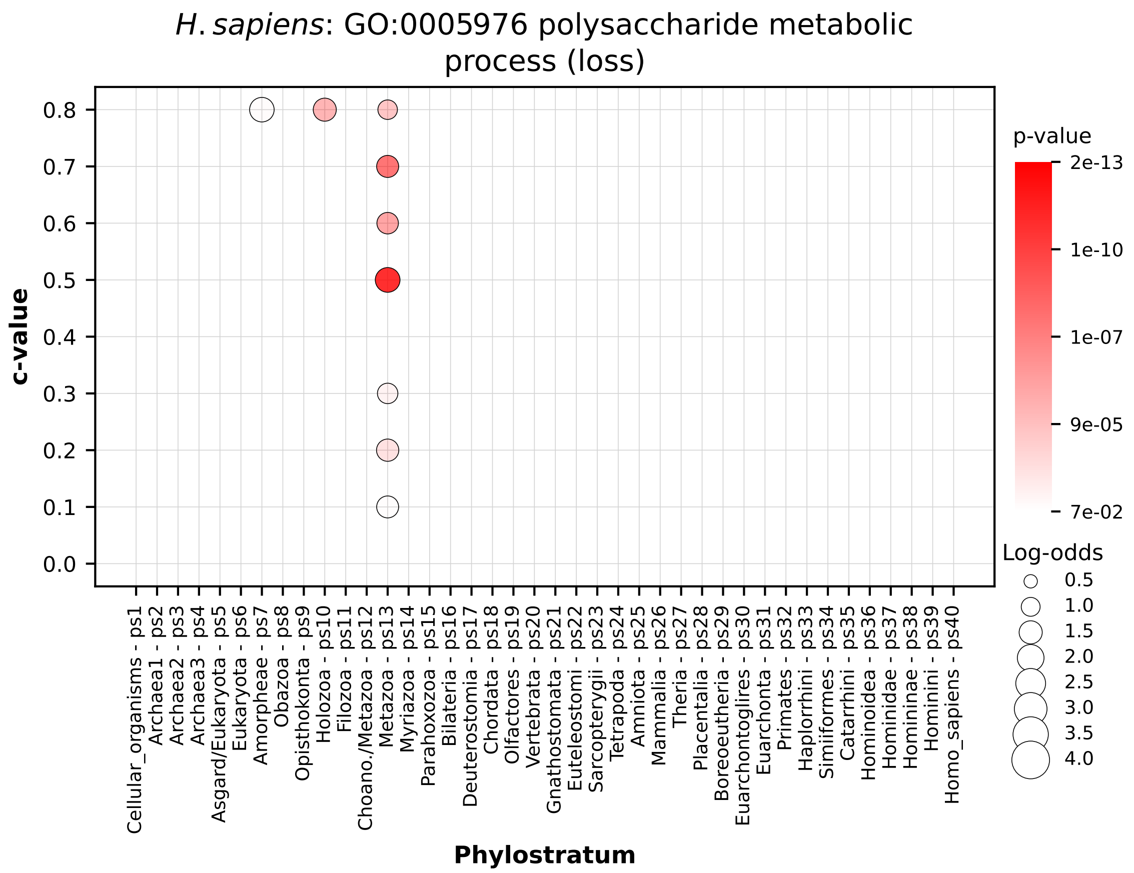
H. sapiens: GO:0005976 polysaccharide metabolic process (loss)
c-value
0.8
0.7
0.6
0.5
0.4
0.3
0.2
0.1
0.0
p-value
2e-13
1e-10
1e-07
9e-05
7e-02
Log-odds
0.5
1.0
1.5
2.0
2.5
3.0
3.5
4.0
Cellular_organisms - ps1
Archaea1 - ps2
Archaea2 - ps3
Archaea3 - ps4
Asgard/Eukaryota - ps5
Eukaryota - ps6
Amorpheae - ps7
Obazoa - ps8
Opisthokonta - ps9
Holozoa - ps10
Filozoa - ps11
Choano./Metazoa - ps12
Metazoa - ps13
Myriazoa - ps14
Parahoxozoa - ps15
Bilateria - ps16
Deuterostomia - ps17
Chordata - ps18
Olfactores - ps19
Vertebrata - ps20
Gnathostomata - ps21
Euteleostomi - ps22
Sarcopterygii - ps23
Tetrapoda - ps24
Amniota - ps25
Mammalia - ps26
Theria - ps27
Placentalia - ps28
Boreoeutheria - ps29
Euarchontoglires - ps30
Euarchonta - ps31
Primates - ps32
Haplorrhini - ps33
Simiiformes - ps34
Catarrhini - ps35
Hominoidea - ps36
Hominidae - ps37
Homininae - ps38
Hominini - ps39
Homo_sapiens - ps40
Phylostratum

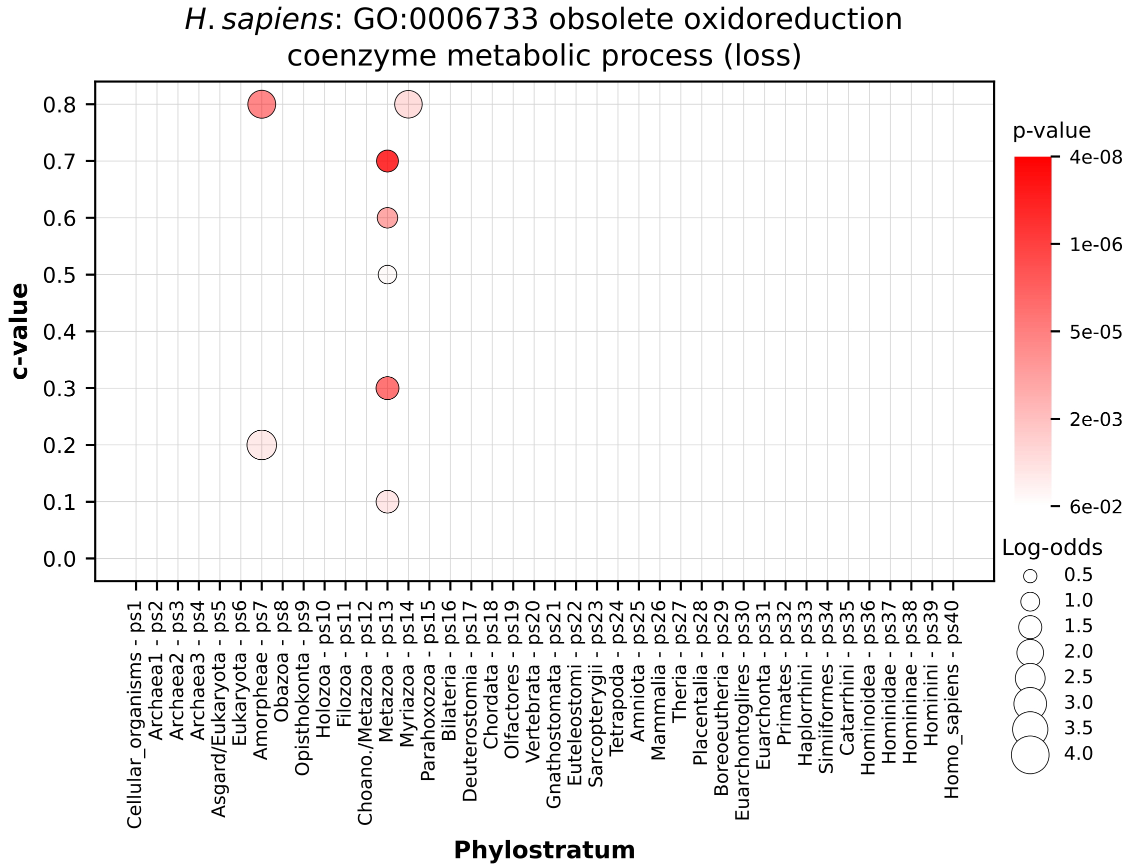

H. sapiens: GO:0006733 obsolete oxidoreduction coenzyme metabolic process (loss)
c-value
0.8
0.7
0.6
0.5
0.4
0.3
0.2
0.1
0.0
p-value
4e-08
1e-06
5e-05
2e-03
6e-02
Log-odds
0.5
1.0
1.5
2.0
2.5
3.0
3.5
4.0
Cellular_organisms - ps1
Archaea1 - ps2
Archaea2 - ps3
Archaea3 - ps4
Asgard/Eukaryota - ps5
Eukaryota - ps6
Amorpheae - ps7
Obazoa - ps8
Opisthokonta - ps9
Holozoa - ps10
Filozoa - ps11
Choano./Metazoa - ps12
Metazoa - ps13
Myriazoa - ps14
Parahoxozoa - ps15
Bilateria - ps16
Deuterostomia - ps17
Chordata - ps18
Olfactores - ps19
Vertebrata - ps20
Gnathostomata - ps21
Euteleostomi - ps22
Sarcopterygii - ps23
Tetrapoda - ps24
Amniota - ps25
Mammalia - ps26
Theria - ps27
Placentalia - ps28
Boreoeutheria - ps29
Euarchontoglires - ps30
Euarchonta - ps31
Primates - ps32
Haplorrhini - ps33
Simiiformes - ps34
Catarrhini - ps35
Hominoidea - ps36
Hominidae - ps37
Homininae - ps38
Hominini - ps39
Homo_sapiens - ps40
Phylostratum

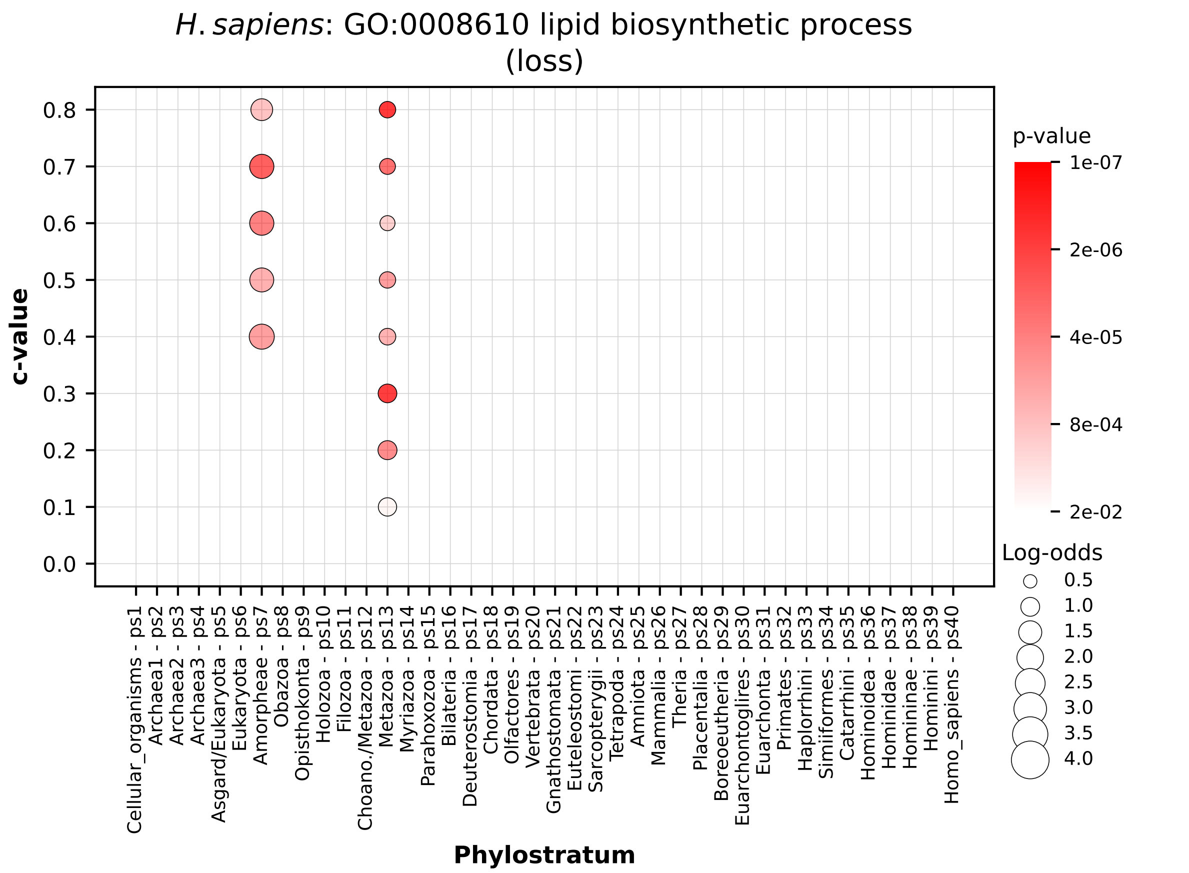

H. sapiens: GO:0008610 lipid biosynthetic process
(loss)
c-value
0.8
0.7
0.6
0.5
0.4
0.3
0.2
0.1
0.0
p-value
1e-07
2e-06
4e-05
8e-04
2e-02
Log-odds
0.5
1.0
1.5
2.0
2.5
3.0
3.5
4.0
Cellular_organisms - ps1
Archaea1 - ps2
Archaea2 - ps3
Archaea3 - ps4
Asgard/Eukaryota - ps5
Eukaryota - ps6
Amorpheae - ps7
Obazoa - ps8
Opisthokonta - ps9
Holozoa - ps10
Filozoa - ps11
Choano./Metazoa - ps12
Metazoa - ps13
Myriazoa - ps14
Parahoxozoa - ps15
Bilateria - ps16
Deuterostomia - ps17
Chordata - ps18
Olfactores - ps19
Vertebrata - ps20
Gnathostomata - ps21
Euteleostomi - ps22
Sarcopterygii - ps23
Tetrapoda - ps24
Amniota - ps25
Mammalia - ps26
Theria - ps27
Placentalia - ps28
Boreoeutheria - ps29
Euarchontoglires - ps30
Euarchonta - ps31
Primates - ps32
Haplorrhini - ps33
Simiiformes - ps34
Catarrhini - ps35
Hominoidea - ps36
Hominidae - ps37
Homininae - ps38
Hominini - ps39
Homo_sapiens - ps40
Phylostratum

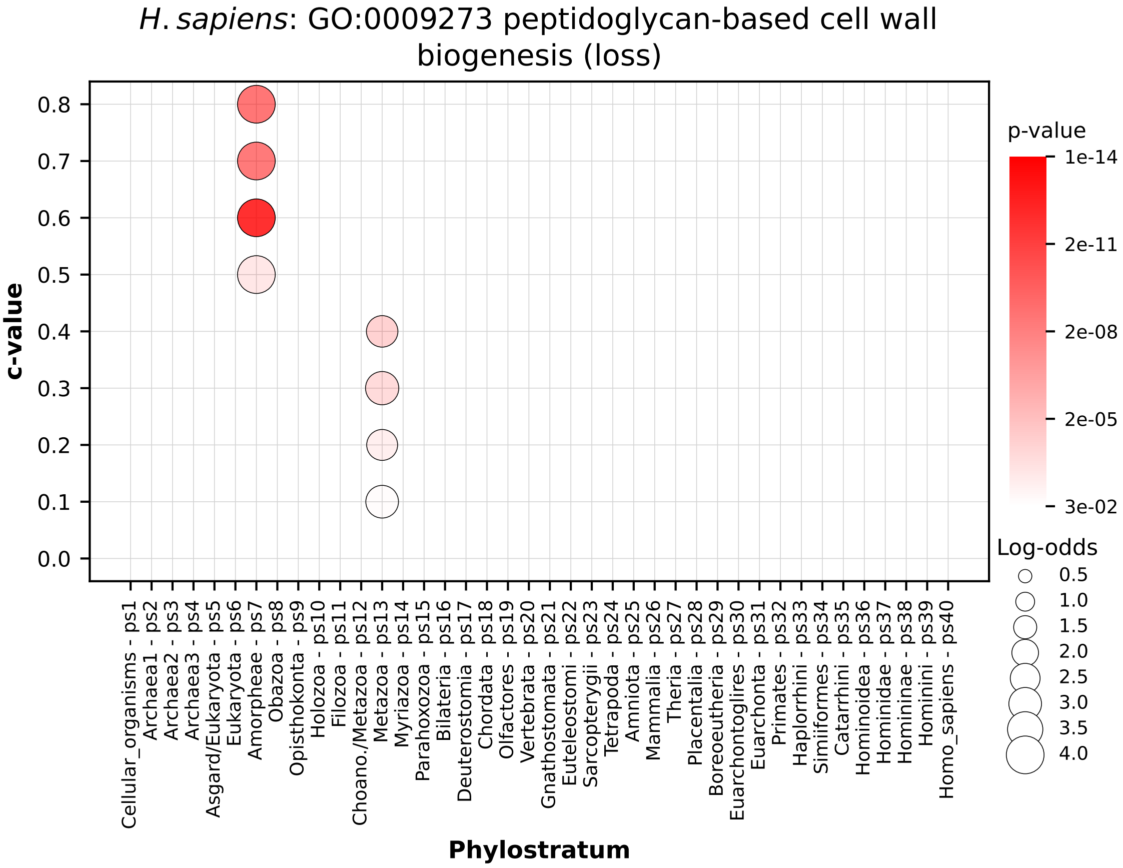

H. sapiens: GO:0009273 peptidoglycan-based cell wall biogenesis (loss)
c-value
0.0
0.1
0.2
0.3
0.4
0.5
0.6
0.7
0.8
Phylostratum
Cellular_organisms - ps1
Archaea1 - ps2
Archaea2 - ps3
Archaea3 - ps4
Asgard/Eukaryota - ps5
Eukaryota - ps6
Amorpheae - ps7
Obazoa - ps8
Opisthokonta - ps9
Holozoa - ps10
Filozoa - ps11
Choano./Metazoa - ps12
Metazoa - ps13
Myriazoa - ps14
Parahoxozoa - ps15
Bilateria - ps16
Deuterostomia - ps17
Chordata - ps18
Olfactores - ps19
Vertebrata - ps20
Gnathostomata - ps21
Euteleostomi - ps22
Sarcopterygii - ps23
Tetrapoda - ps24
Amniota - ps25
Mammalia - ps26
Theria - ps27
Placentalia - ps28
Boreoeutheria - ps29
Euarchontoglires - ps30
Euarchonta - ps31
Primates - ps32
Haplorrhini - ps33
Simiiformes - ps34
Catarrhini - ps35
Hominoidea - ps36
Hominidae - ps37
Homininae - ps38
Hominini - ps39
Homo_sapiens - ps40
p-value
1e-14
2e-11
2e-08
2e-05
3e-02
Log-odds
0.5
1.0
1.5
2.0
2.5
3.0
3.5
4.0

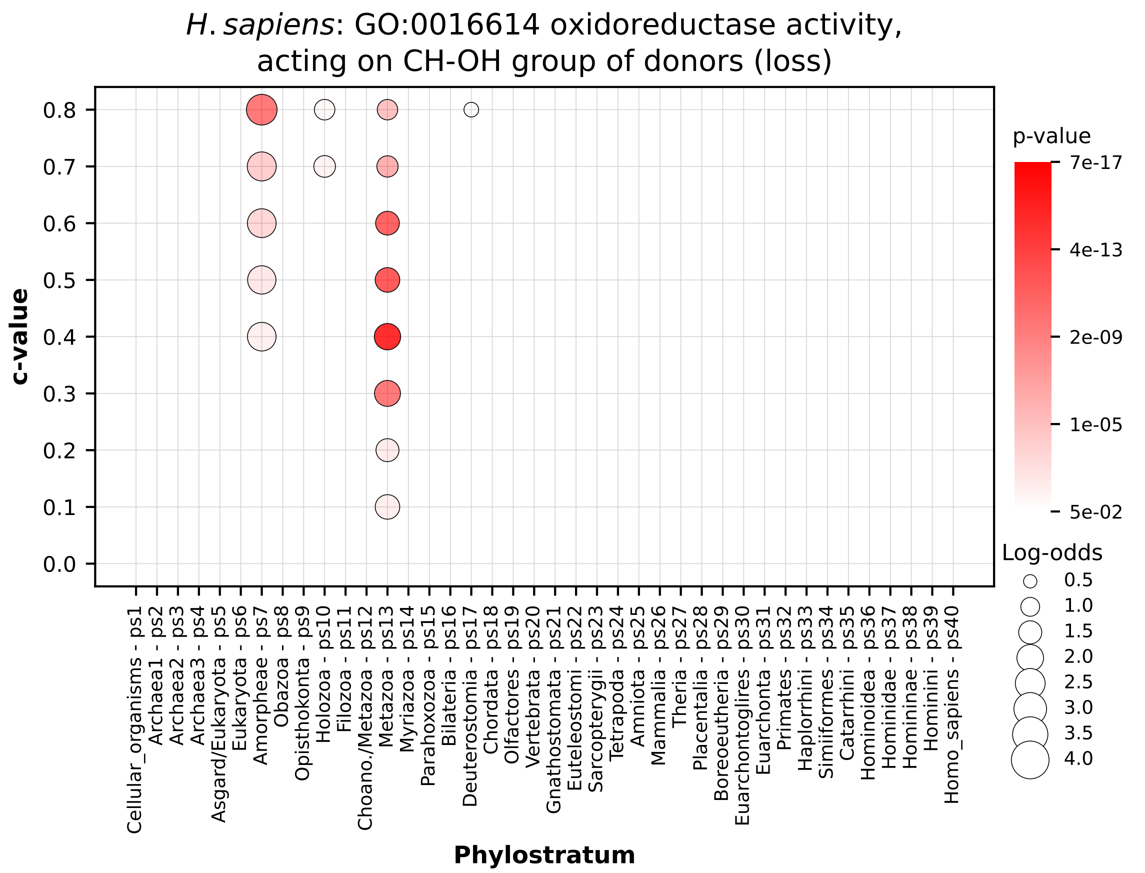

H. sapiens: GO:0016614 oxidoreductase activity,
acting on CH-OH group of donors (loss)
c-value
0.8
0.7
0.6
0.5
0.4
0.3
0.2
0.1
0.0
p-value
7e-17
4e-13
2e-09
1e-05
5e-02
Log-odds
0.5
1.0
1.5
2.0
2.5
3.0
3.5
4.0
Cellular_organisms - ps1
Archaea1 - ps2
Archaea2 - ps3
Archaea3 - ps4
Asgard/Eukaryota - ps5
Eukaryota - ps6
Amorpheae - ps7
Obazoa - ps8
Opisthokonta - ps9
Holozoa - ps10
Filozoa - ps11
Choano./Metazoa - ps12
Metazoa - ps13
Myriazoa - ps14
Parahoxozoa - ps15
Bilateria - ps16
Deuterostomia - ps17
Chordata - ps18
Olfactores - ps19
Vertebrata - ps20
Gnathostomata - ps21
Euteleostomi - ps22
Sarcopterygii - ps23
Tetrapoda - ps24
Amniota - ps25
Mammalia - ps26
Theria - ps27
Placentalia - ps28
Boreoeutheria - ps29
Euarchontoglires - ps30
Euarchonta - ps31
Primates - ps32
Haplorrhini - ps33
Simiiformes - ps34
Catarrhini - ps35
Hominoidea - ps36
Hominidae - ps37
Homininae - ps38
Hominini - ps39
Homo_sapiens - ps40
Phylostratum

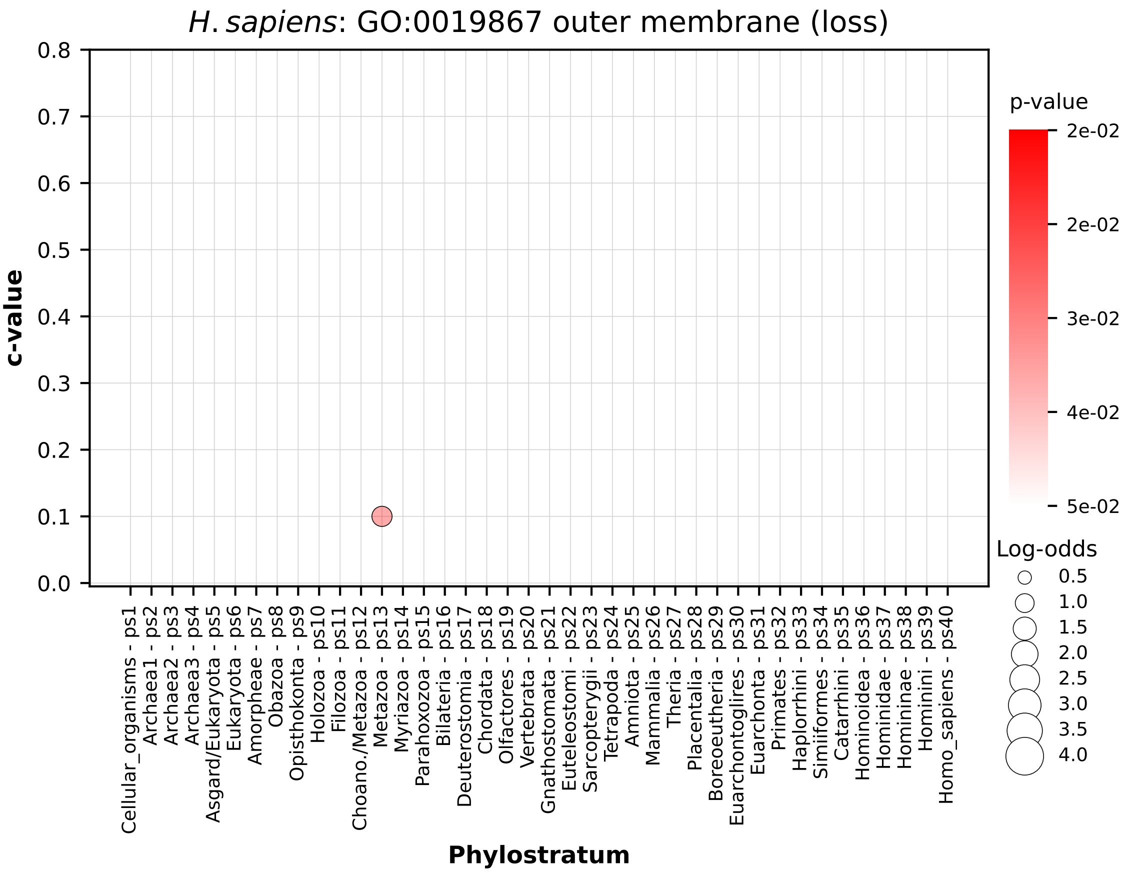

H. sapiens: GO:0019867 outer membrane (loss)
c-value
0.0
0.1
0.2
0.3
0.4
0.5
0.6
0.7
0.8
Phylostratum
Cellular_organisms - ps1
Archaea1 - ps2
Archaea2 - ps3
Archaea3 - ps4
Asgard/Eukaryota - ps5
Eukaryota - ps6
Amorpheae - ps7
Obazoa - ps8
Opisthokonta - ps9
Holozoa - ps10
Filozoa - ps11
Choano./Metazoa - ps12
Metazoa - ps13
Myriazoa - ps14
Parahoxozoa - ps15
Bilateria - ps16
Deuterostomia - ps17
Chordata - ps18
Olfactores - ps19
Vertebrata - ps20
Gnathostomata - ps21
Euteleostomi - ps22
Sarcopterygii - ps23
Tetrapoda - ps24
Amniota - ps25
Mammalia - ps26
Theria - ps27
Placentalia - ps28
Boreoeutheria - ps29
Euarchontoglires - ps30
Euarchonta - ps31
Primates - ps32
Haplorrhini - ps33
Simiiformes - ps34
Catarrhini - ps35
Hominoidea - ps36
Hominidae - ps37
Homininae - ps38
Hominini - ps39
Homo_sapiens - ps40
p-value
2e-02
2e-02
3e-02
4e-02
5e-02
Log-odds
0.5
1.0
1.5
2.0
2.5
3.0
3.5
4.0

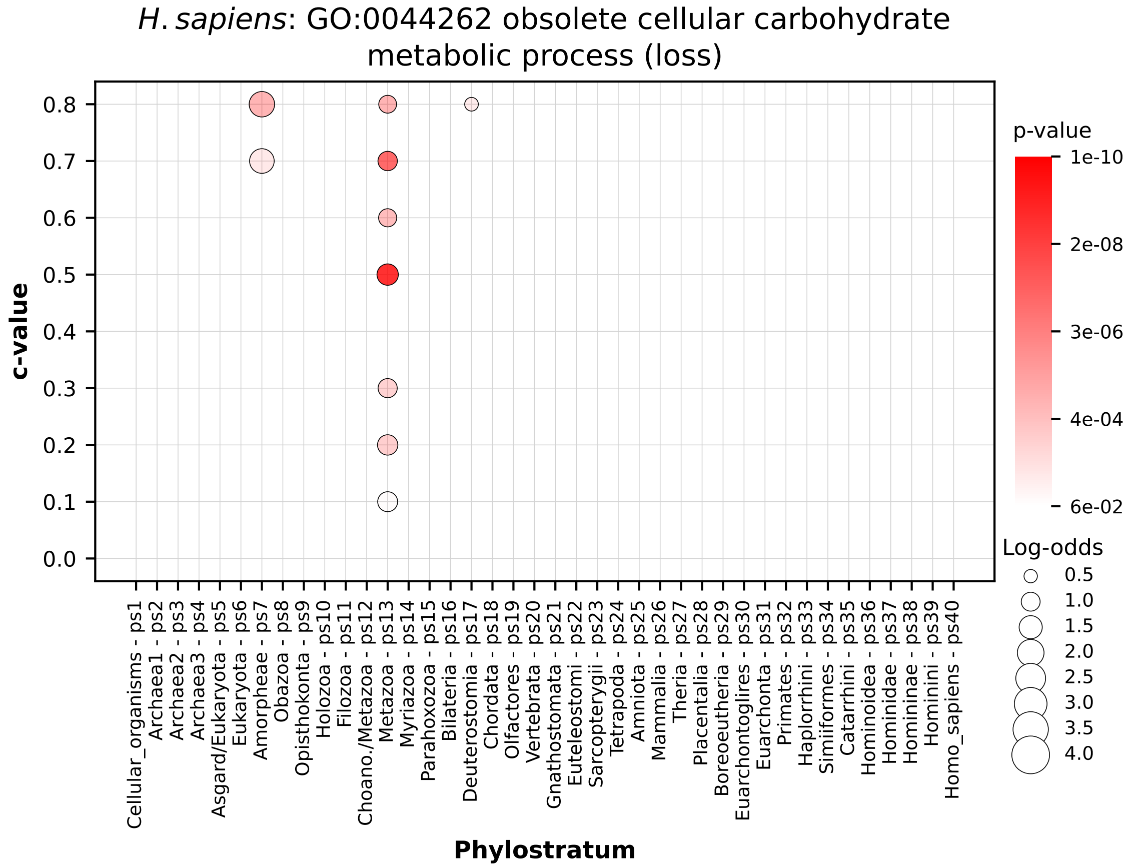

H. sapiens: GO:0044262 obsolete cellular carbohydrate metabolic process (loss)
c-value
0.0
0.1
0.2
0.3
0.4
0.5
0.6
0.7
0.8
p-value
1e-10
2e-08
3e-06
4e-04
6e-02
Log-odds
0.5
1.0
1.5
2.0
2.5
3.0
3.5
4.0
Cellular_organisms - ps1
Archaea1 - ps2
Archaea2 - ps3
Archaea3 - ps4
Asgard/Eukaryota - ps5
Eukaryota - ps6
Amorpheae - ps7
Obazoa - ps8
Opisthokonta - ps9
Holozoa - ps10
Filozoa - ps11
Choano./Metazoa - ps12
Metazoa - ps13
Myriazoa - ps14
Parahoxozoa - ps15
Bilateria - ps16
Deuterostomia - ps17
Chordata - ps18
Olfactores - ps19
Vertebrata - ps20
Gnathostomata - ps21
Euteleostomi - ps22
Sarcopterygii - ps23
Tetrapoda - ps24
Amniota - ps25
Mammalia - ps26
Theria - ps27
Placentalia - ps28
Boreoeutheria - ps29
Euarchontoglires - ps30
Euarchonta - ps31
Primates - ps32
Haplorrhini - ps33
Simiiformes - ps34
Catarrhini - ps35
Hominoidea - ps36
Hominidae - ps37
Homininae - ps38
Hominini - ps39
Homo_sapiens - ps40
Phylostratum

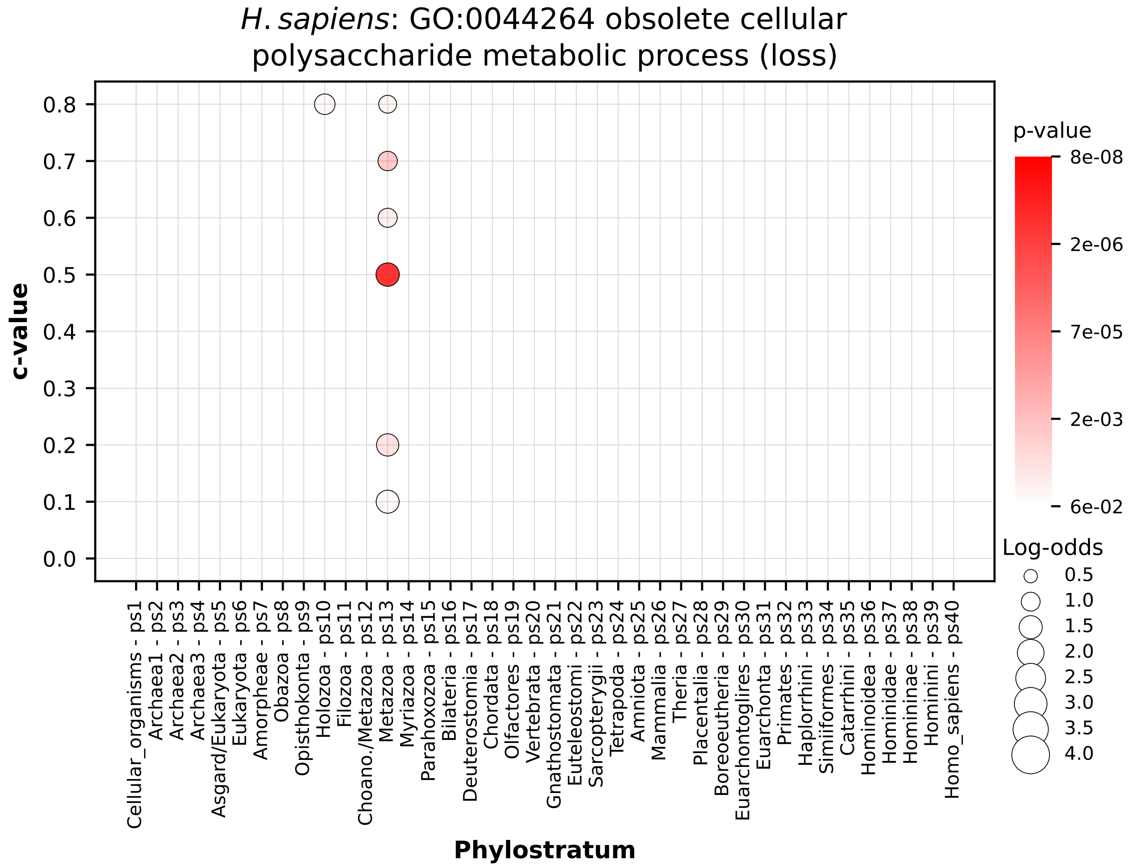

H. sapiens: GO:0044264 obsolete cellular polysaccharide metabolic process (loss)
c-value
0.8
0.7
0.6
0.5
0.4
0.3
0.2
0.1
0.0
p-value
8e-08
2e-06
7e-05
2e-03
6e-02
Log-odds
0.5
1.0
1.5
2.0
2.5
3.0
3.5
4.0
Cellular_organisms - ps1
Archaea1 - ps2
Archaea2 - ps3
Archaea3 - ps4
Asgard/Eukaryota - ps5
Eukaryota - ps6
Amorpheae - ps7
Obazoa - ps8
Opisthokonta - ps9
Holozoa - ps10
Filozoa - ps11
Choano./Metazoa - ps12
Metazoa - ps13
Myriazoa - ps14
Parahoxozoa - ps15
Bilateria - ps16
Deuterostomia - ps17
Chordata - ps18
Olfactores - ps19
Vertebrata - ps20
Gnathostomata - ps21
Euteleostomi - ps22
Sarcopterygii - ps23
Tetrapoda - ps24
Amniota - ps25
Mammalia - ps26
Theria - ps27
Placentalia - ps28
Boreoeutheria - ps29
Euarchontoglires - ps30
Euarchonta - ps31
Primates - ps32
Haplorrhini - ps33
Simiiformes - ps34
Catarrhini - ps35
Hominoidea - ps36
Hominidae - ps37
Homininae - ps38
Hominini - ps39
Homo_sapiens - ps40
Phylostratum

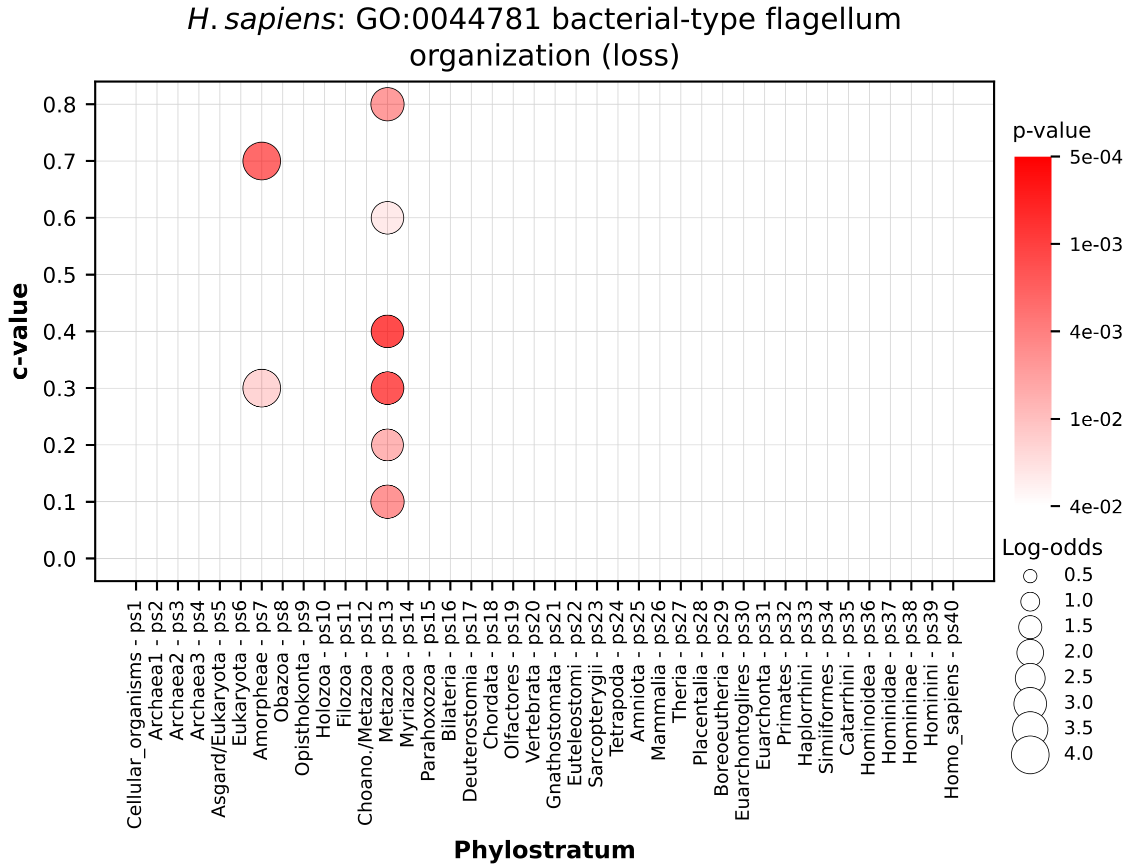

H. sapiens: GO:0044781 bacterial-type flagellum organization (loss)
c-value
0.0
0.1
0.2
0.3
0.4
0.5
0.6
0.7
0.8
Phylostratum
Cellular_organisms - ps1
Archaea1 - ps2
Archaea2 - ps3
Archaea3 - ps4
Asgard/Eukaryota - ps5
Eukaryota - ps6
Amorpheae - ps7
Obazoa - ps8
Opisthokonta - ps9
Holozoa - ps10
Filozoa - ps11
Choano./Metazoa - ps12
Metazoa - ps13
Myriazoa - ps14
Parahoxozoa - ps15
Bilateria - ps16
Deuterostomia - ps17
Chordata - ps18
Olfactores - ps19
Vertebrata - ps20
Gnathostomata - ps21
Euteleostomi - ps22
Sarcopterygii - ps23
Tetrapoda - ps24
Amniota - ps25
Mammalia - ps26
Theria - ps27
Placentalia - ps28
Boreoeutheria - ps29
Euarchontoglires - ps30
Euarchonta - ps31
Primates - ps32
Haplorrhini - ps33
Simiiformes - ps34
Catarrhini - ps35
Hominoidea - ps36
Hominidae - ps37
Homininae - ps38
Hominini - ps39
Homo_sapiens - ps40
p-value
5e-04
1e-03
4e-03
1e-02
4e-02
Log-odds
0.5
1.0
1.5
2.0
2.5
3.0
3.5
4.0

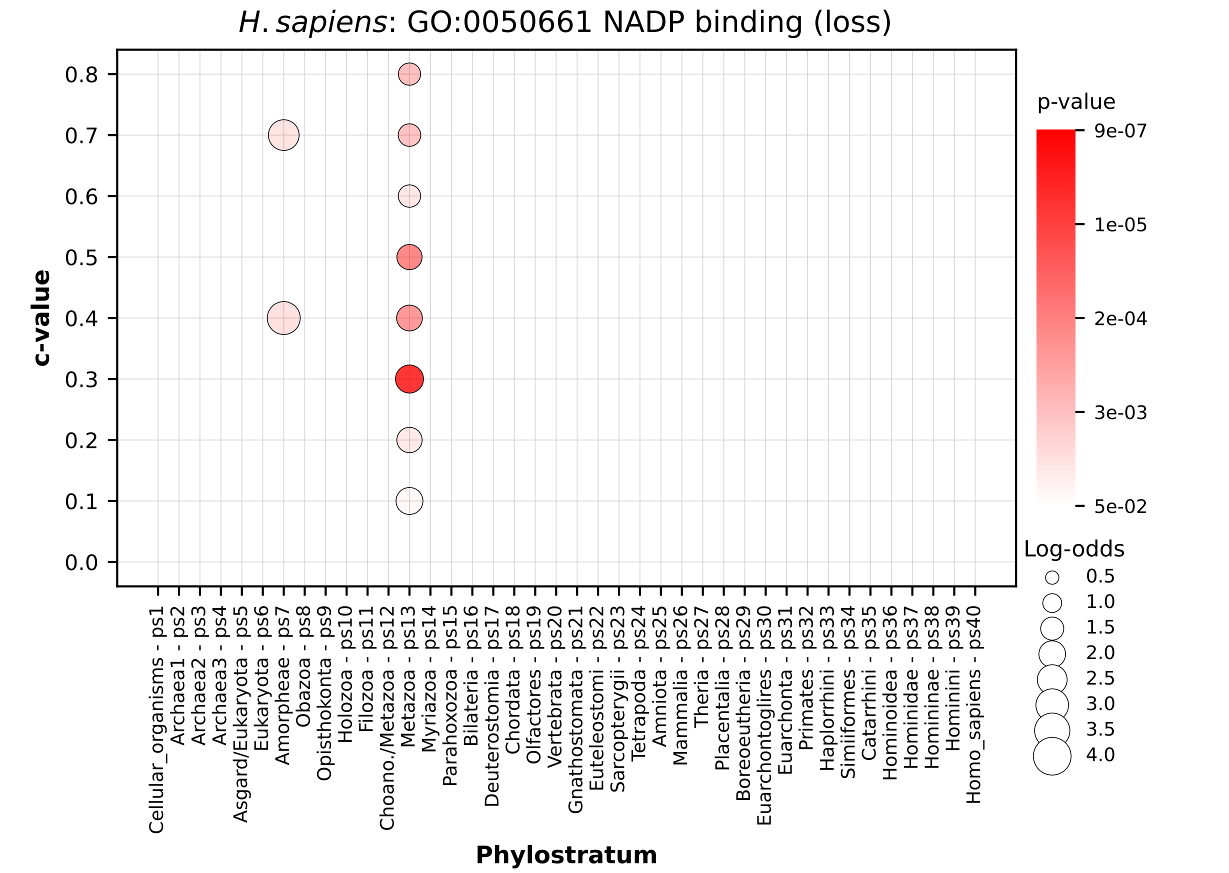

H. sapiens: GO:0050661 NADP binding (loss)
c-value
0.0
0.1
0.2
0.3
0.4
0.5
0.6
0.7
0.8
p-value
9e-07
1e-05
2e-04
3e-03
5e-02
Log-odds
0.5
1.0
1.5
2.0
2.5
3.0
3.5
4.0
Cellular_organisms - ps1
Archaea1 - ps2
Archaea2 - ps3
Archaea3 - ps4
Asgard/Eukaryota - ps5
Eukaryota - ps6
Amorpheae - ps7
Obazoa - ps8
Opisthokonta - ps9
Holozoa - ps10
Filozoa - ps11
Choano./Metazoa - ps12
Metazoa - ps13
Myriazoa - ps14
Parahoxozoa - ps15
Bilateria - ps16
Deuterostomia - ps17
Chordata - ps18
Olfactores - ps19
Vertebrata - ps20
Gnathostomata - ps21
Euteleostomi - ps22
Sarcopterygii - ps23
Tetrapoda - ps24
Amniota - ps25
Mammalia - ps26
Theria - ps27
Placentalia - ps28
Boreoeutheria - ps29
Euarchontoglires - ps30
Euarchonta - ps31
Primates - ps32
Haplorrhini - ps33
Simiiformes - ps34
Catarrhini - ps35
Hominoidea - ps36
Hominidae - ps37
Homininae - ps38
Hominini - ps39
Homo_sapiens - ps40
Phylostratum

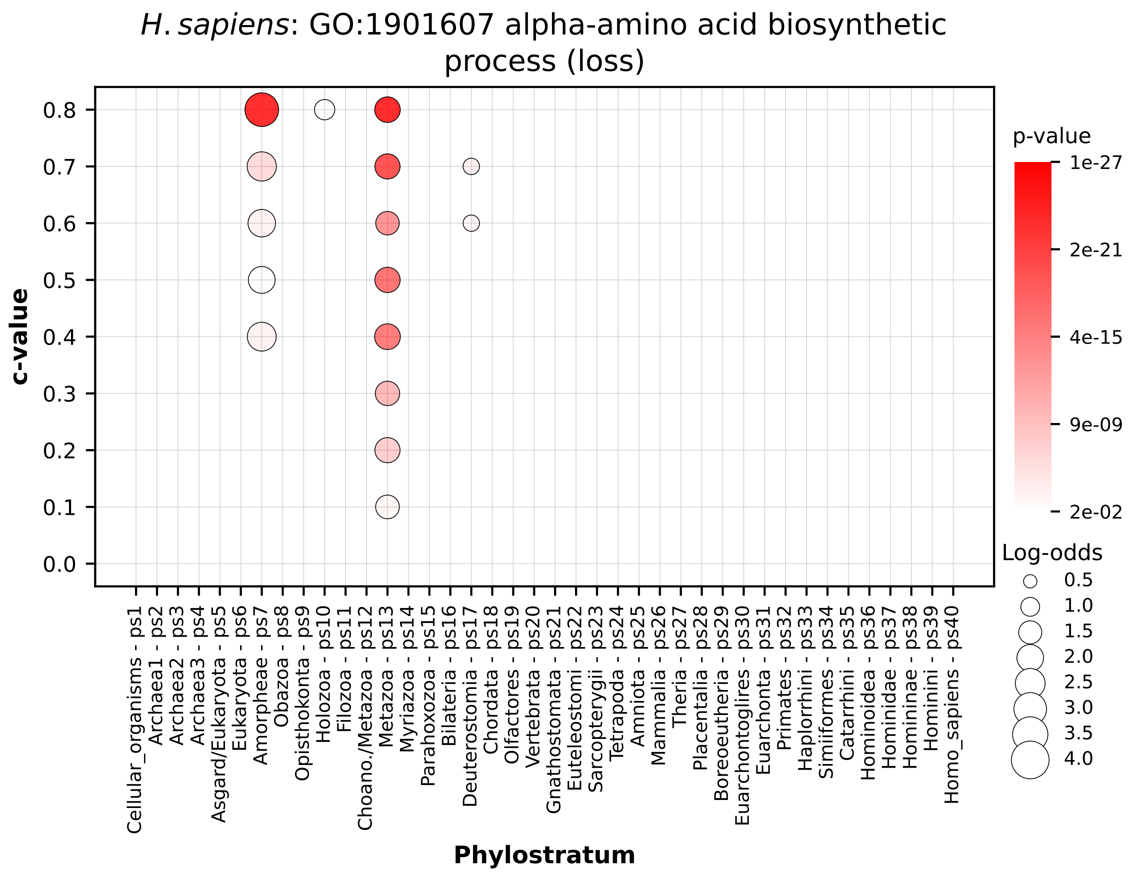

H. sapiens: GO:1901607 alpha-amino acid biosynthetic process (loss)
c-value
0.0
0.1
0.2
0.3
0.4
0.5
0.6
0.7
0.8
Phylostratum
Cellular_organisms - ps1
Archaea1 - ps2
Archaea2 - ps3
Archaea3 - ps4
Asgard/Eukaryota - ps5
Eukaryota - ps6
Amorpheae - ps7
Obazoa - ps8
Opisthokonta - ps9
Holozoa - ps10
Filozoa - ps11
Choano./Metazoa - ps12
Metazoa - ps13
Myriazoa - ps14
Parahoxozoa - ps15
Bilateria - ps16
Deuterostomia - ps17
Chordata - ps18
Olfactores - ps19
Vertebrata - ps20
Gnathostomata - ps21
Euteleostomi - ps22
Sarcopterygii - ps23
Tetrapoda - ps24
Amniota - ps25
Mammalia - ps26
Theria - ps27
Placentalia - ps28
Boreoeutheria - ps29
Euarchontoglires - ps30
Euarchonta - ps31
Primates - ps32
Haplorrhini - ps33
Simiiformes - ps34
Catarrhini - ps35
Hominoidea - ps36
Hominidae - ps37
Homininae - ps38
Hominini - ps39
Homo_sapiens - ps40
p-value
1e-27
2e-21
4e-15
9e-09
2e-02
Log-odds
0.5
1.0
1.5
2.0
2.5
3.0
3.5
4.0

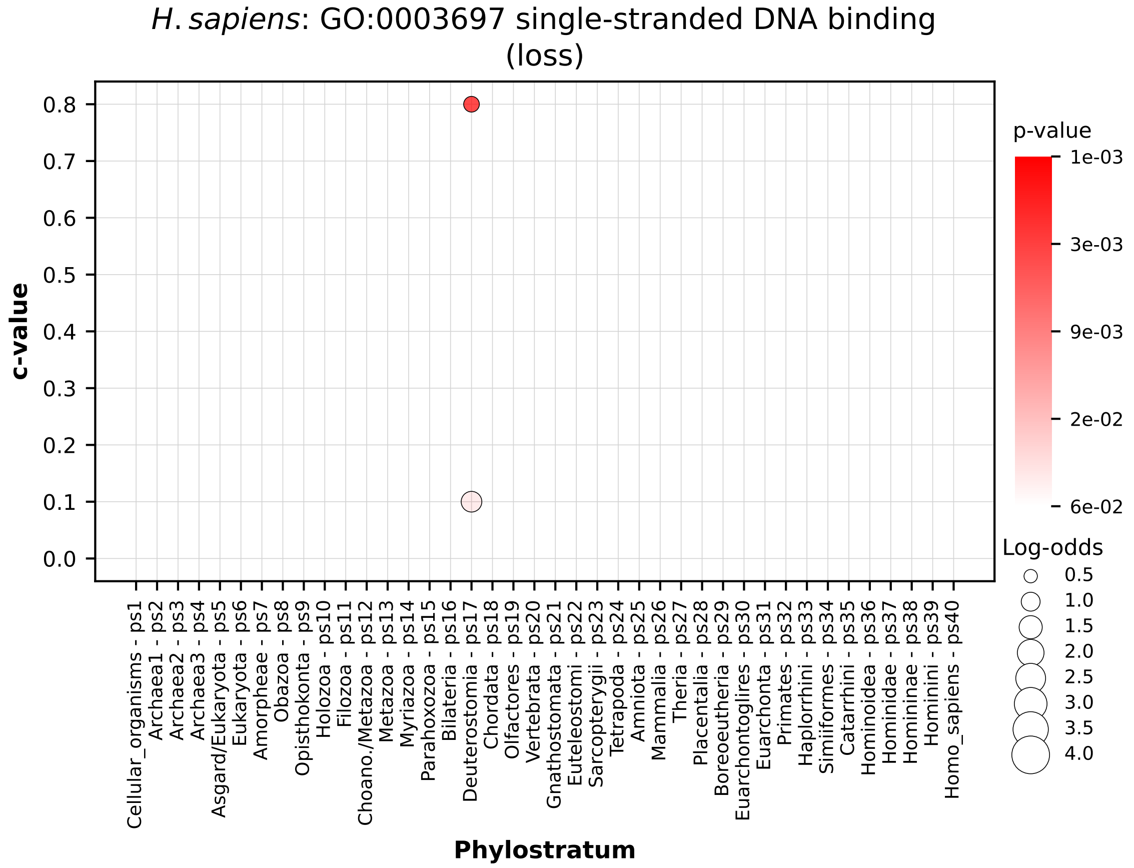
H. sapiens: GO:0003697 single-stranded DNA binding
(loss)
c-value
0.0
0.1
0.2
0.3
0.4
0.5
0.6
0.7
0.8
p-value
1e-03
3e-03
9e-03
2e-02
6e-02
Log-odds
0.5
1.0
1.5
2.0
2.5
3.0
3.5
4.0
Cellular_organisms - ps1
Archaea1 - ps2
Archaea2 - ps3
Archaea3 - ps4
Asgard/Eukaryota - ps5
Eukaryota - ps6
Amorpheae - ps7
Obazoa - ps8
Opisthokonta - ps9
Holozoa - ps10
Filozoa - ps11
Choano./Metazoa - ps12
Metazoa - ps13
Myriazoa - ps14
Parahoxozoa - ps15
Bilateria - ps16
Deuterostomia - ps17
Chordata - ps18
Olfactores - ps19
Vertebrata - ps20
Gnathostomata - ps21
Euteleostomi - ps22
Sarcopterygii - ps23
Tetrapoda - ps24
Amniota - ps25
Mammalia - ps26
Theria - ps27
Placentalia - ps28
Boreoeutheria - ps29
Euarchontoglires - ps30
Euarchonta - ps31
Primates - ps32
Haplorrhini - ps33
Simiiformes - ps34
Catarrhini - ps35
Hominoidea - ps36
Hominidae - ps37
Homininae - ps38
Hominini - ps39
Homo_sapiens - ps40
Phylostratum

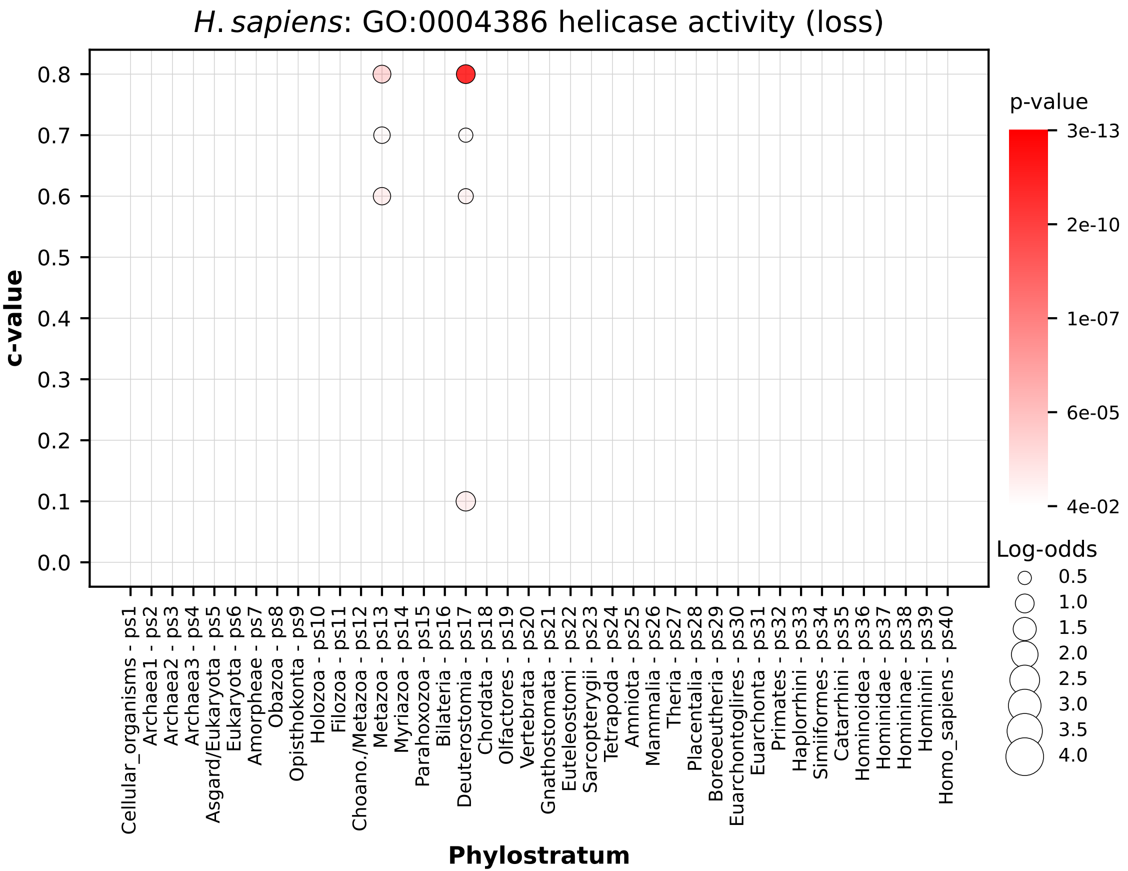

H. sapiens: GO:0004386 helicase activity (loss)
c-value
0.8
0.7
0.6
0.5
0.4
0.3
0.2
0.1
0.0
Phylostratum
Cellular_organisms - ps1
Archaea1 - ps2
Archaea2 - ps3
Archaea3 - ps4
Asgard/Eukaryota - ps5
Eukaryota - ps6
Amorpheae - ps7
Obazoa - ps8
Opisthokonta - ps9
Holozoa - ps10
Filozoa - ps11
Choano./Metazoa - ps12
Metazoa - ps13
Myriazoa - ps14
Parahoxozoa - ps15
Bilateria - ps16
Deuterostomia - ps17
Chordata - ps18
Olfactores - ps19
Vertebrata - ps20
Gnathostomata - ps21
Euteleostomi - ps22
Sarcopterygii - ps23
Tetrapoda - ps24
Amniota - ps25
Mammalia - ps26
Theria - ps27
Placentalia - ps28
Boreoeutheria - ps29
Euarchontoglires - ps30
Euarchonta - ps31
Primates - ps32
Haplorrhini - ps33
Simiiformes - ps34
Catarrhini - ps35
Hominoidea - ps36
Hominidae - ps37
Homininae - ps38
Hominini - ps39
Homo_sapiens - ps40
p-value
3e-13
2e-10
1e-07
6e-05
4e-02
Log-odds
0.5
1.0
1.5
2.0
2.5
3.0
3.5
4.0

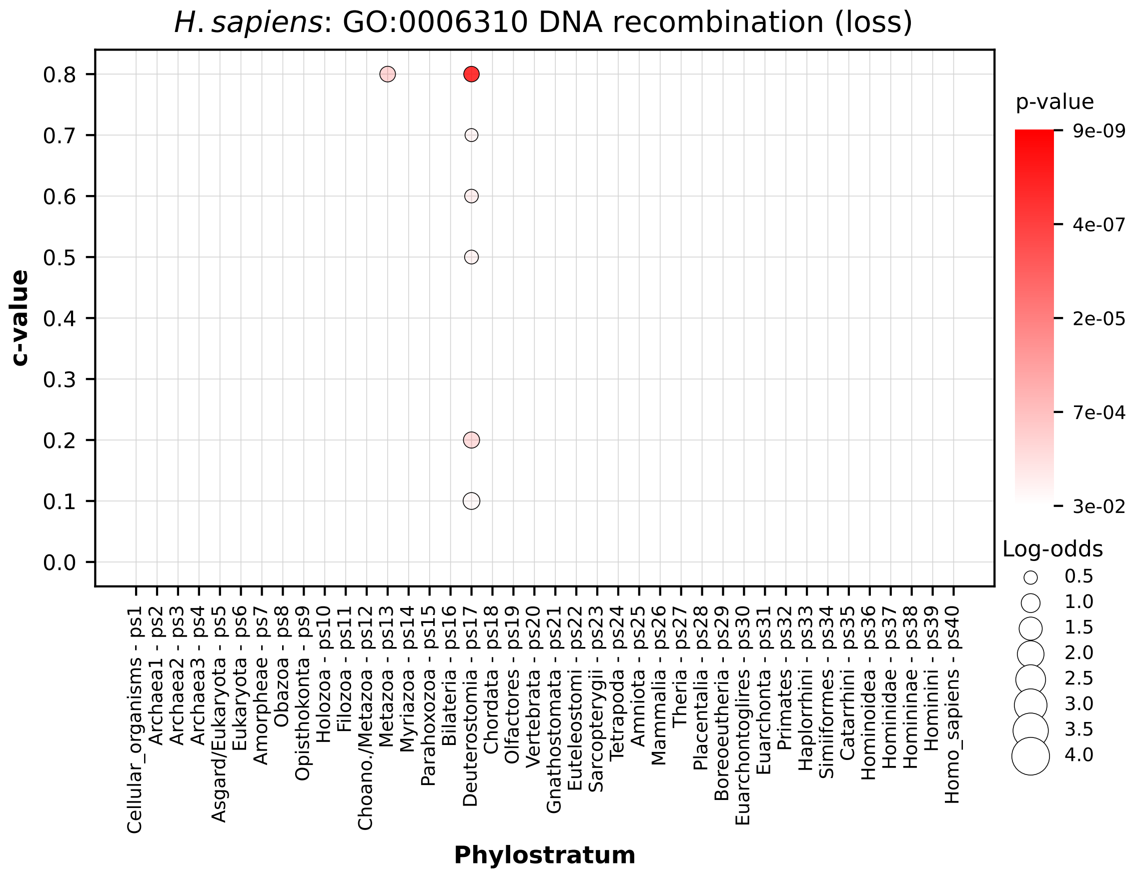

H. sapiens: GO:0006310 DNA recombination (loss)
c-value
0.0
0.1
0.2
0.3
0.4
0.5
0.6
0.7
0.8
p-value
9e-09
4e-07
2e-05
7e-04
3e-02
Log-odds
0.5
1.0
1.5
2.0
2.5
3.0
3.5
4.0
Cellular_organisms - ps1
Archaea1 - ps2
Archaea2 - ps3
Archaea3 - ps4
Asgard/Eukaryota - ps5
Eukaryota - ps6
Amorpheae - ps7
Obazoa - ps8
Opisthokonta - ps9
Holozoa - ps10
Filozoa - ps11
Choano./Metazoa - ps12
Metazoa - ps13
Myriazoa - ps14
Parahoxozoa - ps15
Bilateria - ps16
Deuterostomia - ps17
Chordata - ps18
Olfactores - ps19
Vertebrata - ps20
Gnathostomata - ps21
Euteleostomi - ps22
Sarcopterygii - ps23
Tetrapoda - ps24
Amniota - ps25
Mammalia - ps26
Theria - ps27
Placentalia - ps28
Boreoeutheria - ps29
Euarchontoglires - ps30
Euarchonta - ps31
Primates - ps32
Haplorrhini - ps33
Simiiformes - ps34
Catarrhini - ps35
Hominoidea - ps36
Hominidae - ps37
Homininae - ps38
Hominini - ps39
Homo_sapiens - ps40
Phylostratum

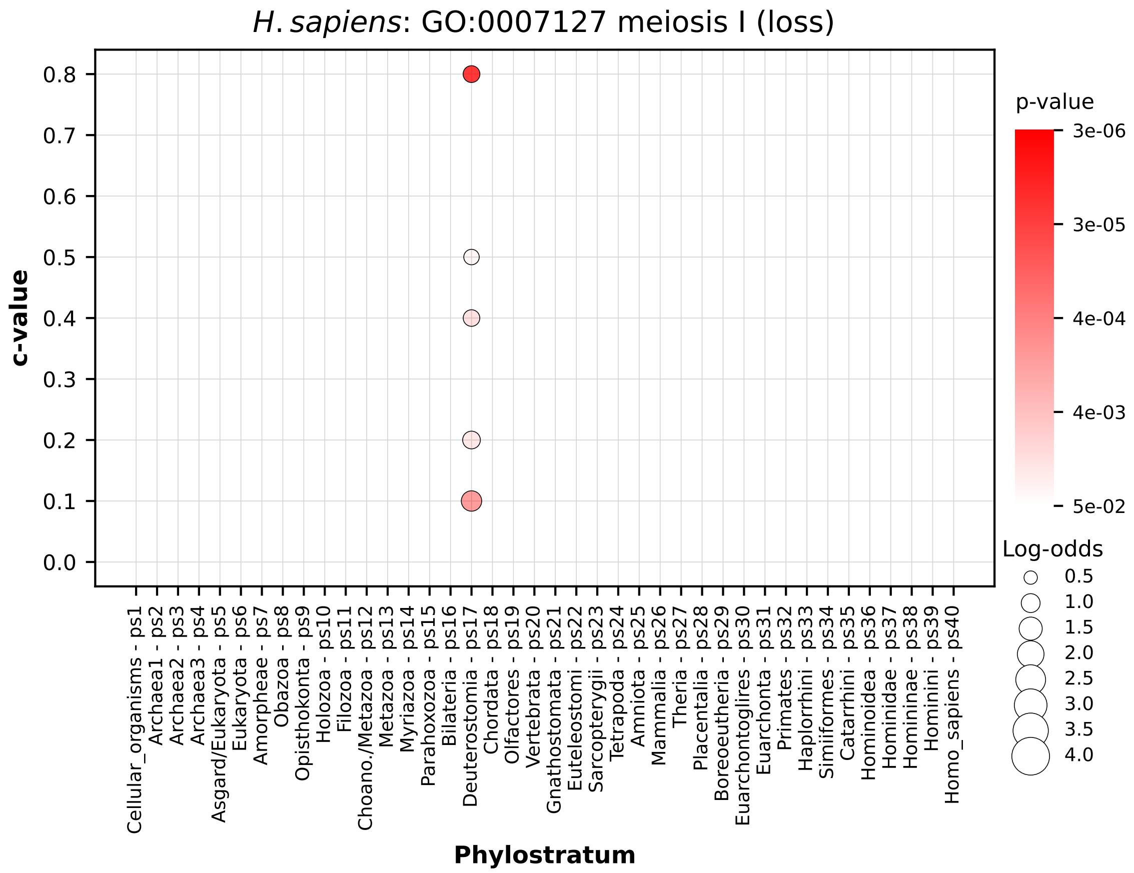

H. sapiens: GO:0007127 meiosis I (loss)
c-value
0.0
0.1
0.2
0.3
0.4
0.5
0.6
0.7
0.8
Phylostratum
Cellular_organisms - ps1
Archaea1 - ps2
Archaea2 - ps3
Archaea3 - ps4
Asgard/Eukaryota - ps5
Eukaryota - ps6
Amorpheae - ps7
Obazoa - ps8
Opisthokonta - ps9
Holozoa - ps10
Filozoa - ps11
Choano./Metazoa - ps12
Metazoa - ps13
Myriazoa - ps14
Parahoxozoa - ps15
Bilateria - ps16
Deuterostomia - ps17
Chordata - ps18
Olfactores - ps19
Vertebrata - ps20
Gnathostomata - ps21
Euteleostomi - ps22
Sarcopterygii - ps23
Tetrapoda - ps24
Amniota - ps25
Mammalia - ps26
Theria - ps27
Placentalia - ps28
Boreoeutheria - ps29
Euarchontoglires - ps30
Euarchonta - ps31
Primates - ps32
Haplorrhini - ps33
Simiiformes - ps34
Catarrhini - ps35
Hominoidea - ps36
Hominidae - ps37
Homininae - ps38
Hominini - ps39
Homo_sapiens - ps40
p-value
3e-06
3e-05
4e-04
4e-03
5e-02
Log-odds
0.5
1.0
1.5
2.0
2.5
3.0
3.5
4.0

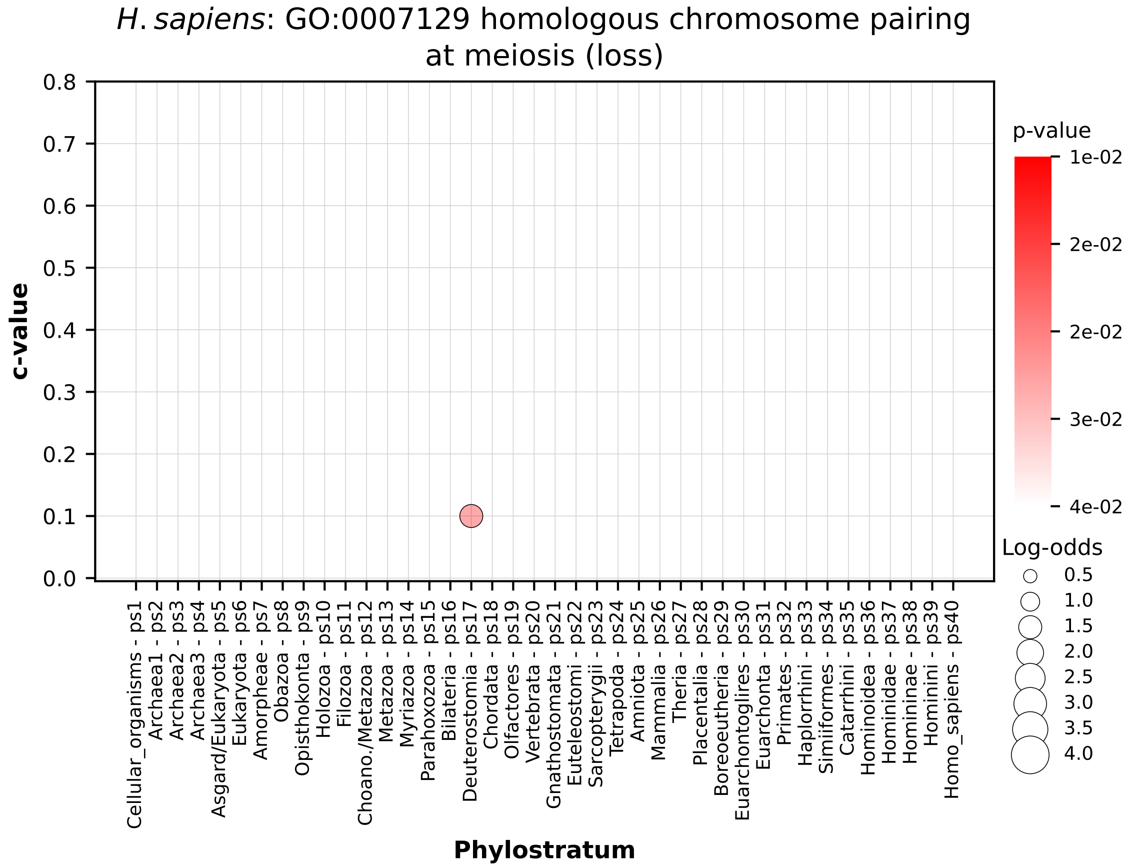

H. sapiens: GO:0007129 homologous chromosome pairing at meiosis (loss)
c-value
0.8
0.7
0.6
0.5
0.4
0.3
0.2
0.1
0.0
p-value
1e-02
2e-02
2e-02
3e-02
4e-02
Log-odds
0.5
1.0
1.5
2.0
2.5
3.0
3.5
4.0
Cellular_organisms - ps1
Archaea1 - ps2
Archaea2 - ps3
Archaea3 - ps4
Asgard/Eukaryota - ps5
Eukaryota - ps6
Amorpheae - ps7
Obazoa - ps8
Opisthokonta - ps9
Holozoa - ps10
Filozoa - ps11
Choano./Metazoa - ps12
Metazoa - ps13
Myriazoa - ps14
Parahoxozoa - ps15
Bilateria - ps16
Deuterostomia - ps17
Chordata - ps18
Olfactores - ps19
Vertebrata - ps20
Gnathostomata - ps21
Euteleostomi - ps22
Sarcopterygii - ps23
Tetrapoda - ps24
Amniota - ps25
Mammalia - ps26
Theria - ps27
Placentalia - ps28
Boreoeutheria - ps29
Euarchontoglires - ps30
Euarchonta - ps31
Primates - ps32
Haplorrhini - ps33
Simiiformes - ps34
Catarrhini - ps35
Hominoidea - ps36
Hominidae - ps37
Homininae - ps38
Hominini - ps39
Homo_sapiens - ps40
Phylostratum

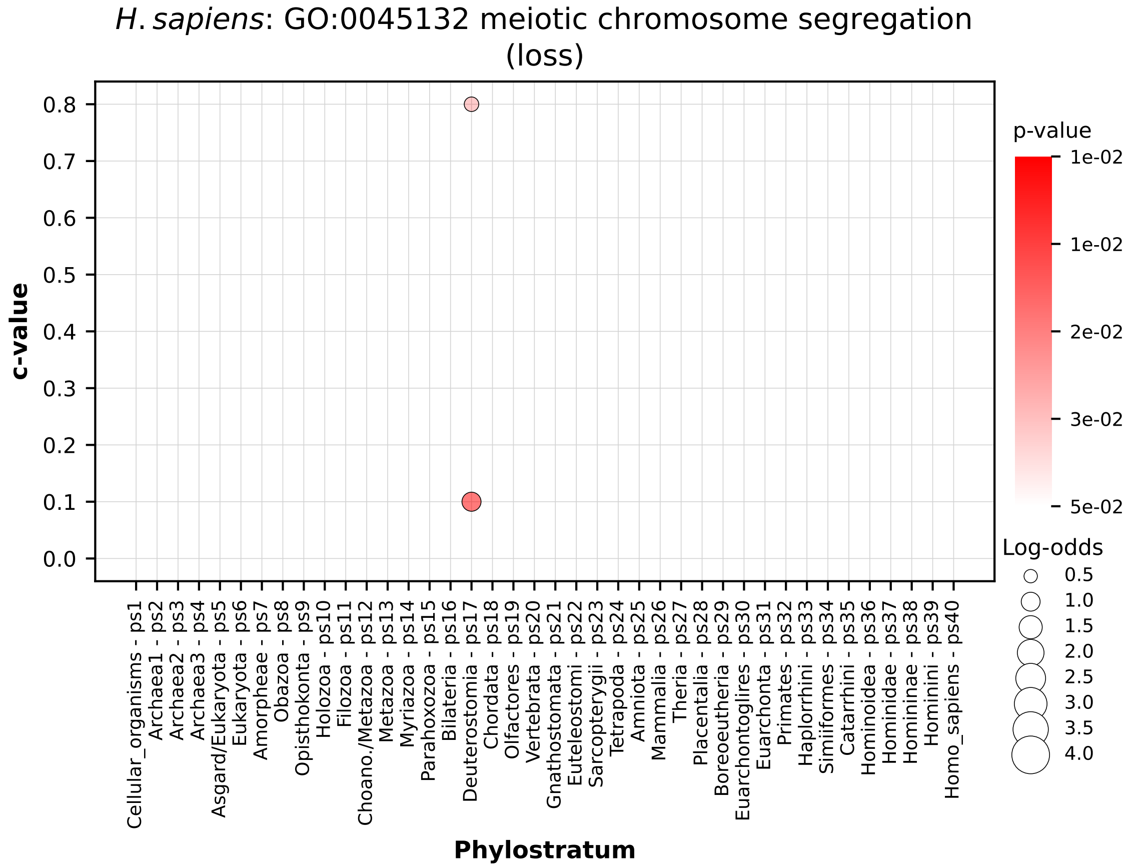
H. sapiens: GO:0045132 meiotic chromosome segregation (loss)
c-value
0.0
0.1
0.2
0.3
0.4
0.5
0.6
0.7
0.8
Phylostratum
Cellular_organisms - ps1
Archaea1 - ps2
Archaea2 - ps3
Archaea3 - ps4
Asgard/Eukaryota - ps5
Eukaryota - ps6
Amorpheae - ps7
Obazoa - ps8
Opisthokonta - ps9
Holozoa - ps10
Filozoa - ps11
Choano./Metazoa - ps12
Metazoa - ps13
Myriazoa - ps14
Parahoxozoa - ps15
Bilateria - ps16
Deuterostomia - ps17
Chordata - ps18
Olfactores - ps19
Vertebrata - ps20
Gnathostomata - ps21
Euteleostomi - ps22
Sarcopterygii - ps23
Tetrapoda - ps24
Amniota - ps25
Mammalia - ps26
Theria - ps27
Placentalia - ps28
Boreoeutheria - ps29
Euarchontoglires - ps30
Euarchonta - ps31
Primates - ps32
Haplorrhini - ps33
Simiiformes - ps34
Catarrhini - ps35
Hominoidea - ps36
Hominidae - ps37
Homininae - ps38
Hominini - ps39
Homo_sapiens - ps40
p-value
1e-02
1e-02
2e-02
3e-02
5e-02
Log-odds
0.5
1.0
1.5
2.0
2.5
3.0
3.5
4.0

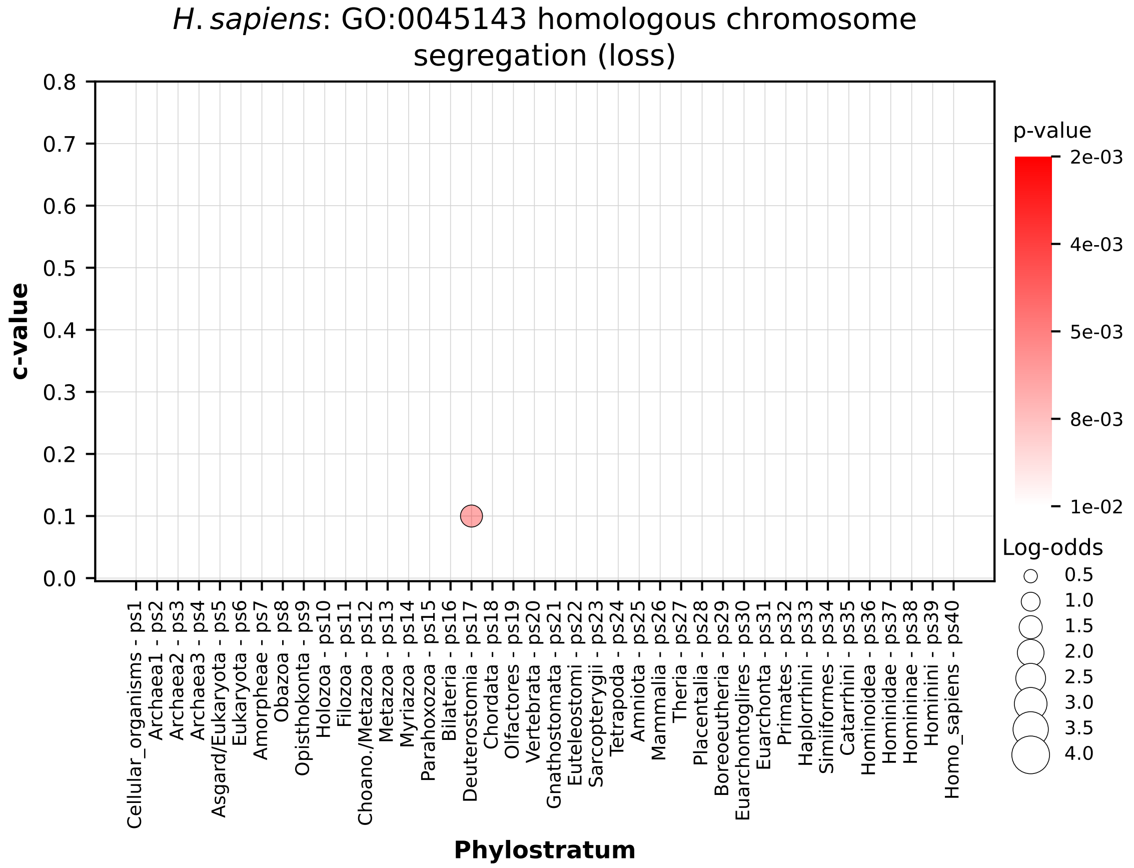

H. sapiens: GO:0045143 homologous chromosome segregation (loss)
c-value
0.0
0.1
0.2
0.3
0.4
0.5
0.6
0.7
0.8
Phylostratum
Cellular_organisms - ps1
Archaea1 - ps2
Archaea2 - ps3
Archaea3 - ps4
Asgard/Eukaryota - ps5
Eukaryota - ps6
Amorpheae - ps7
Obazoa - ps8
Opisthokonta - ps9
Holozoa - ps10
Filozoa - ps11
Choano./Metazoa - ps12
Metazoa - ps13
Myriazoa - ps14
Parahoxozoa - ps15
Bilateria - ps16
Deuterostomia - ps17
Chordata - ps18
Olfactores - ps19
Vertebrata - ps20
Gnathostomata - ps21
Euteleostomi - ps22
Sarcopterygii - ps23
Tetrapoda - ps24
Amniota - ps25
Mammalia - ps26
Theria - ps27
Placentalia - ps28
Boreoeutheria - ps29
Euarchontoglires - ps30
Euarchonta - ps31
Primates - ps32
Haplorrhini - ps33
Simiiformes - ps34
Catarrhini - ps35
Hominoidea - ps36
Hominidae - ps37
Homininae - ps38
Hominini - ps39
Homo_sapiens - ps40
p-value
2e-03
4e-03
5e-03
8e-03
1e-02
Log-odds
0.5
1.0
1.5
2.0
2.5
3.0
3.5
4.0

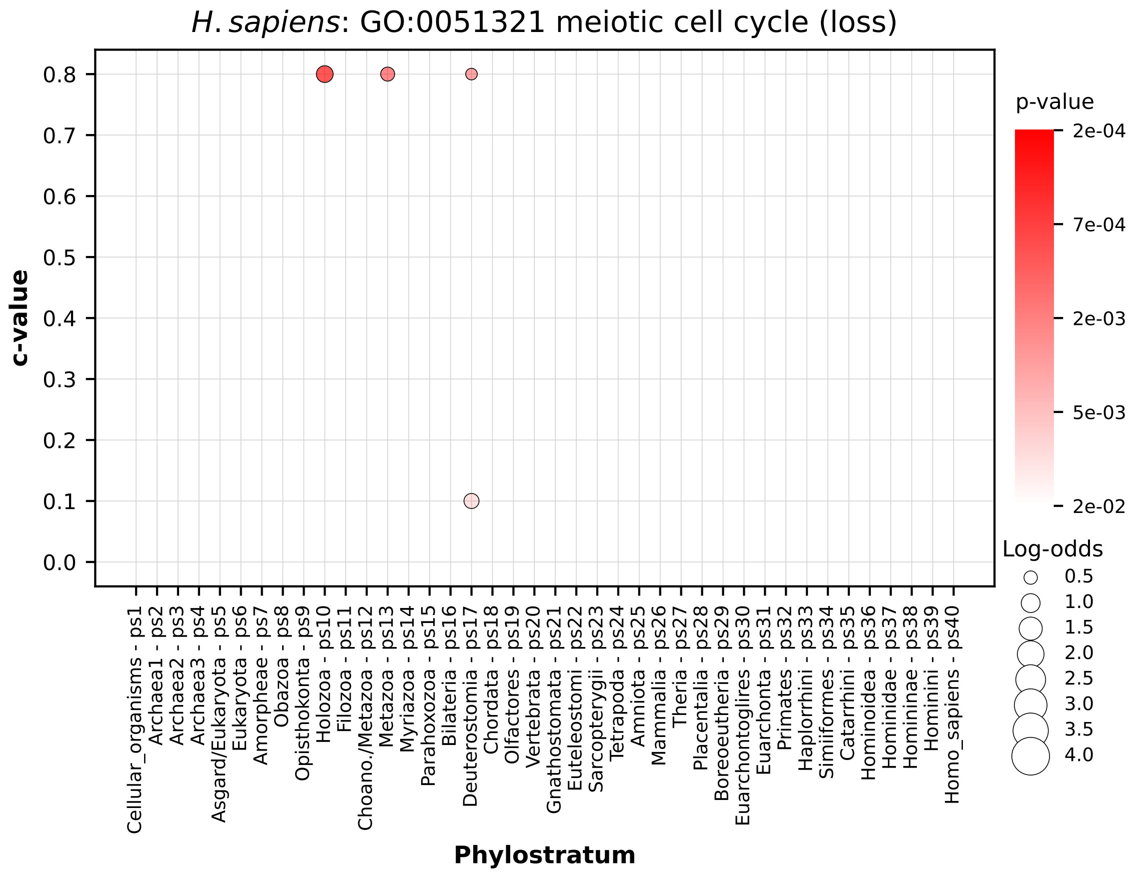

H. sapiens: GO:0051321 meiotic cell cycle (loss)
c-value
0.0
0.1
0.2
0.3
0.4
0.5
0.6
0.7
0.8
Phylostratum
Cellular_organisms - ps1
Archaea1 - ps2
Archaea2 - ps3
Archaea3 - ps4
Asgard/Eukaryota - ps5
Eukaryota - ps6
Amorpheae - ps7
Obazoa - ps8
Opisthokonta - ps9
Holozoa - ps10
Filozoa - ps11
Choano./Metazoa - ps12
Metazoa - ps13
Myriazoa - ps14
Parahoxozoa - ps15
Bilateria - ps16
Deuterostomia - ps17
Chordata - ps18
Olfactores - ps19
Vertebrata - ps20
Gnathostomata - ps21
Euteleostomi - ps22
Sarcopterygii - ps23
Tetrapoda - ps24
Amniota - ps25
Mammalia - ps26
Theria - ps27
Placentalia - ps28
Boreoeutheria - ps29
Euarchontoglires - ps30
Euarchonta - ps31
Primates - ps32
Haplorrhini - ps33
Simiiformes - ps34
Catarrhini - ps35
Hominoidea - ps36
Hominidae - ps37
Homininae - ps38
Hominini - ps39
Homo_sapiens - ps40
p-value
2e-04
7e-04
2e-03
5e-03
2e-02
Log-odds
0.5
1.0
1.5
2.0
2.5
3.0
3.5
4.0

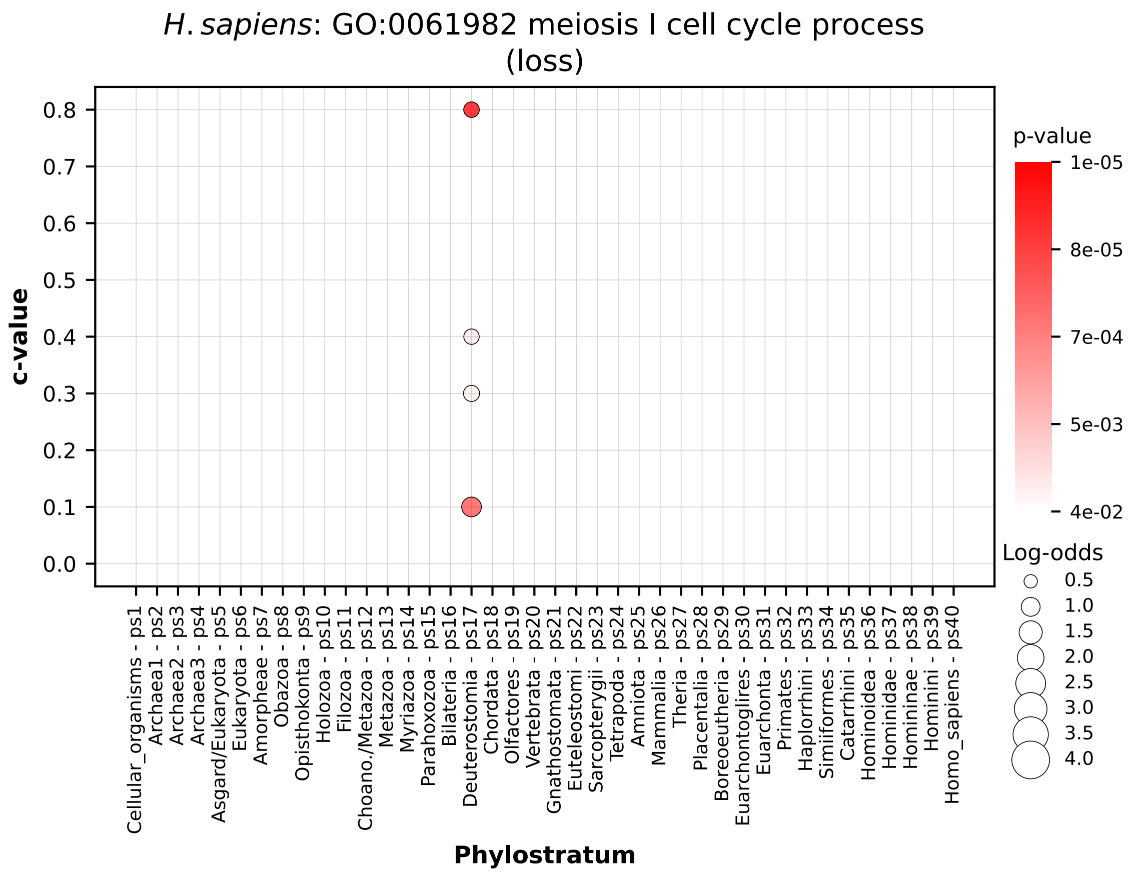

H. sapiens: GO:0061982 meiosis I cell cycle process (loss)
c-value
0.0
0.1
0.2
0.3
0.4
0.5
0.6
0.7
0.8
Phylostratum
Cellular_organisms - ps1
Archaea1 - ps2
Archaea2 - ps3
Archaea3 - ps4
Asgard/Eukaryota - ps5
Eukaryota - ps6
Amorpheae - ps7
Obazoa - ps8
Opisthokonta - ps9
Holozoa - ps10
Filozoa - ps11
Choano./Metazoa - ps12
Metazoa - ps13
Myriazoa - ps14
Parahoxozoa - ps15
Bilateria - ps16
Deuterostomia - ps17
Chordata - ps18
Olfactores - ps19
Vertebrata - ps20
Gnathostomata - ps21
Euteleostomi - ps22
Sarcopterygii - ps23
Tetrapoda - ps24
Amniota - ps25
Mammalia - ps26
Theria - ps27
Placentalia - ps28
Boreoeutheria - ps29
Euarchontoglires - ps30
Euarchonta - ps31
Primates - ps32
Haplorrhini - ps33
Simiiformes - ps34
Catarrhini - ps35
Hominoidea - ps36
Hominidae - ps37
Homininae - ps38
Hominini - ps39
Homo_sapiens - ps40
p-value
1e-05
8e-05
7e-04
5e-03
4e-02
Log-odds
0.5
1.0
1.5
2.0
2.5
3.0
3.5
4.0

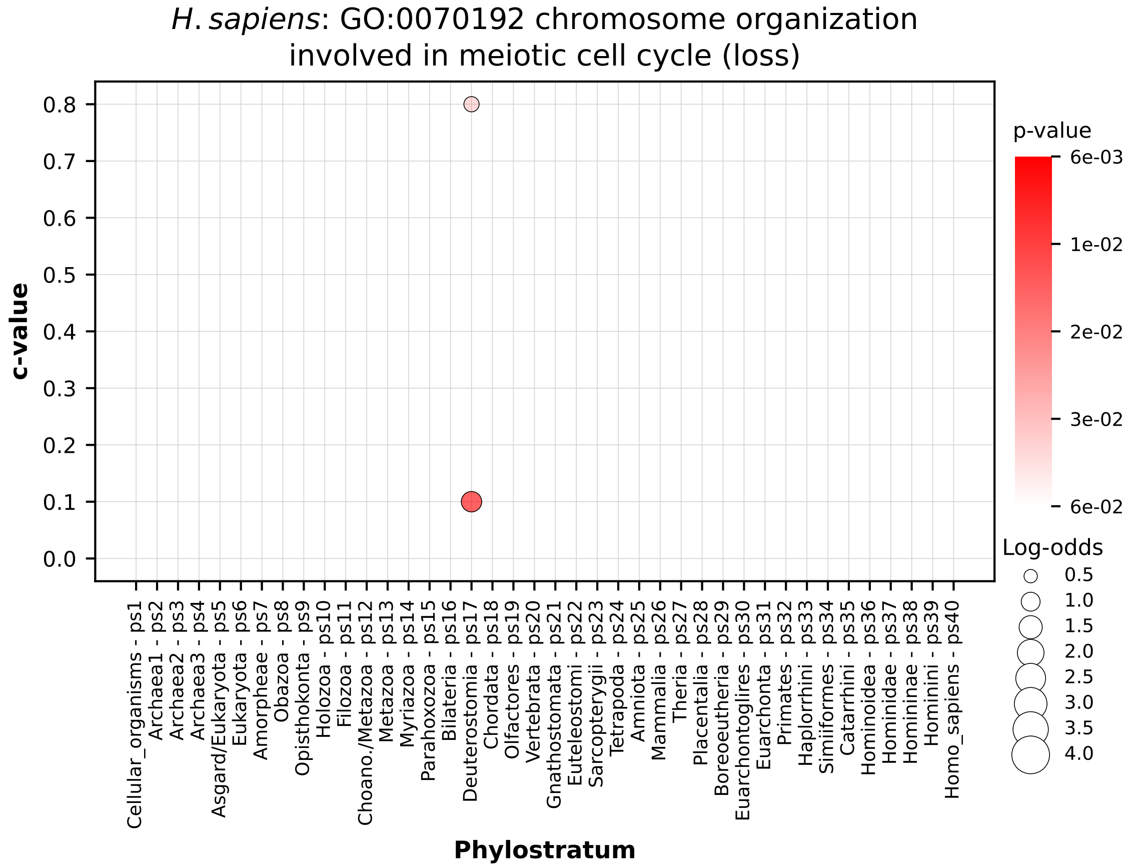
H. sapiens: GO:0070192 chromosome organization involved in meiotic cell cycle (loss)
c-value
0.0
0.1
0.2
0.3
0.4
0.5
0.6
0.7
0.8
p-value
6e-03
1e-02
2e-02
3e-02
6e-02
Log-odds
0.5
1.0
1.5
2.0
2.5
3.0
3.5
4.0
Cellular_organisms - ps1
Archaea1 - ps2
Archaea2 - ps3
Archaea3 - ps4
Asgard/Eukaryota - ps5
Eukaryota - ps6
Amorpheae - ps7
Obazoa - ps8
Opisthokonta - ps9
Holozoa - ps10
Filozoa - ps11
Choano./Metazoa - ps12
Metazoa - ps13
Myriazoa - ps14
Parahoxozoa - ps15
Bilateria - ps16
Deuterostomia - ps17
Chordata - ps18
Olfactores - ps19
Vertebrata - ps20
Gnathostomata - ps21
Euteleostomi - ps22
Sarcopterygii - ps23
Tetrapoda - ps24
Amniota - ps25
Mammalia - ps26
Theria - ps27
Placentalia - ps28
Boreoeutheria - ps29
Euarchontoglires - ps30
Euarchonta - ps31
Primates - ps32
Haplorrhini - ps33
Simiiformes - ps34
Catarrhini - ps35
Hominoidea - ps36
Hominidae - ps37
Homininae - ps38
Hominini - ps39
Homo_sapiens - ps40
Phylostratum

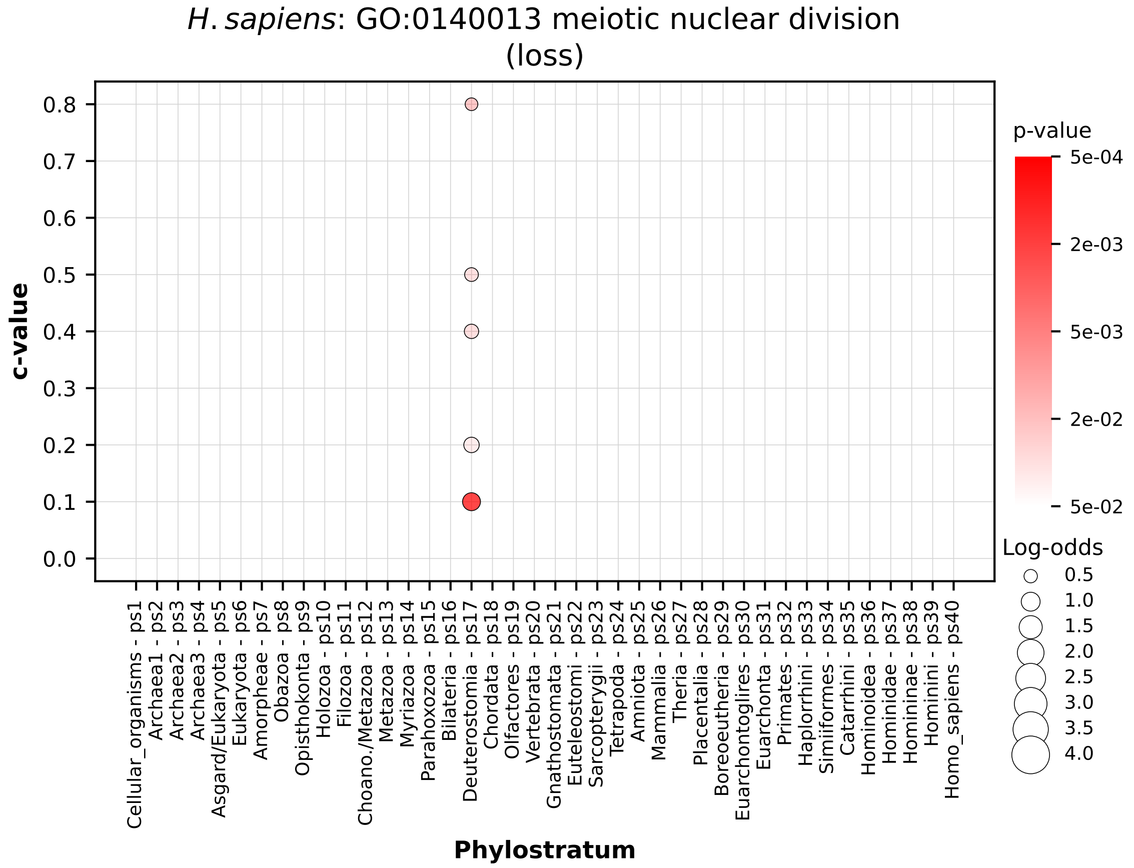
H. sapiens: GO:0140013 meiotic nuclear division (loss)
c-value
0.0
0.1
0.2
0.3
0.4
0.5
0.6
0.7
0.8
Phylostratum
Cellular_organisms - ps1
Archaea1 - ps2
Archaea2 - ps3
Archaea3 - ps4
Asgard/Eukaryota - ps5
Eukaryota - ps6
Amorpheae - ps7
Obazoa - ps8
Opisthokonta - ps9
Holozoa - ps10
Filozoa - ps11
Choano./Metazoa - ps12
Metazoa - ps13
Myriazoa - ps14
Parahoxozoa - ps15
Bilateria - ps16
Deuterostomia - ps17
Chordata - ps18
Olfactores - ps19
Vertebrata - ps20
Gnathostomata - ps21
Euteleostomi - ps22
Sarcopterygii - ps23
Tetrapoda - ps24
Amniota - ps25
Mammalia - ps26
Theria - ps27
Placentalia - ps28
Boreoeutheria - ps29
Euarchontoglires - ps30
Euarchonta - ps31
Primates - ps32
Haplorrhini - ps33
Simiiformes - ps34
Catarrhini - ps35
Hominoidea - ps36
Hominidae - ps37
Homininae - ps38
Hominini - ps39
Homo_sapiens - ps40
p-value
5e-04
2e-03
5e-03
2e-02
5e-02
Log-odds
0.5
1.0
1.5
2.0
2.5
3.0
3.5
4.0

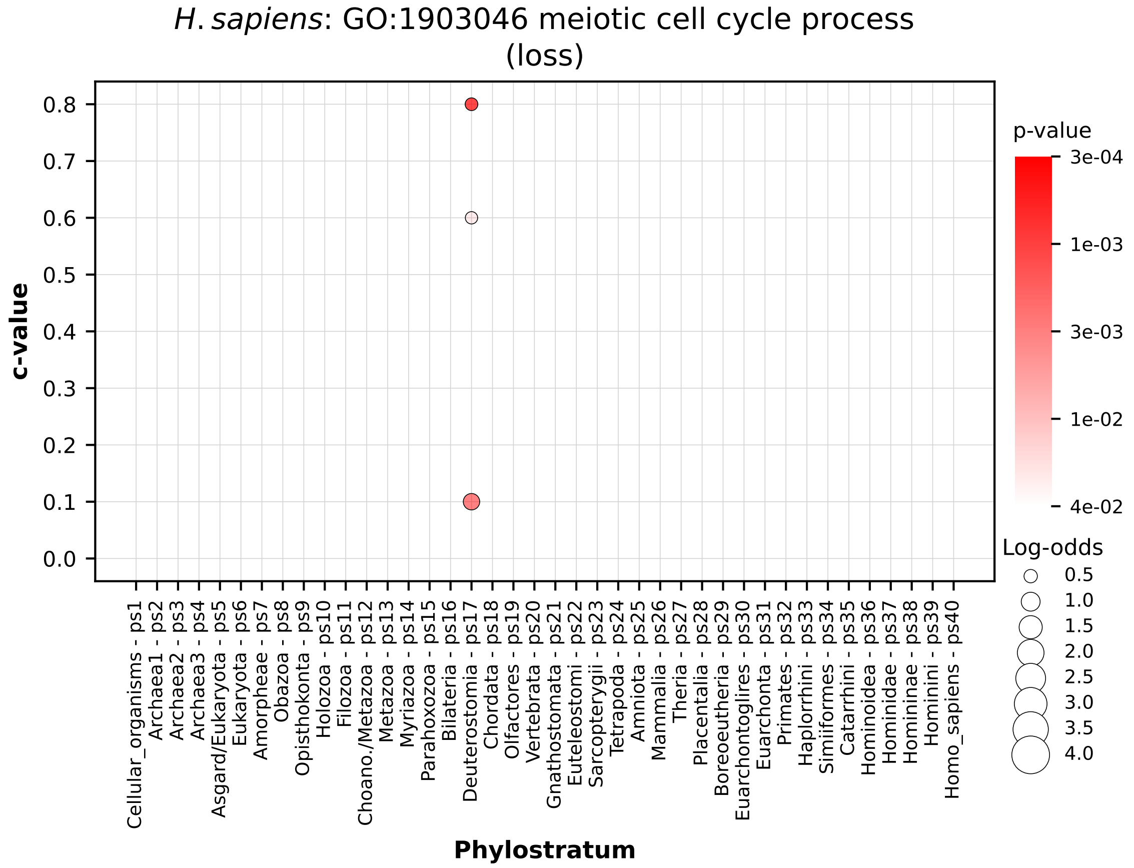

H. sapiens: GO:1903046 meiotic cell cycle process
(loss)
c-value
0.0
0.1
0.2
0.3
0.4
0.5
0.6
0.7
0.8
Phylostratum
Cellular_organisms - ps1
Archaea1 - ps2
Archaea2 - ps3
Archaea3 - ps4
Asgard/Eukaryota - ps5
Eukaryota - ps6
Amorpheae - ps7
Obazoa - ps8
Opisthokonta - ps9
Holozoa - ps10
Filozoa - ps11
Choano./Metazoa - ps12
Metazoa - ps13
Myriazoa - ps14
Parahoxozoa - ps15
Bilateria - ps16
Deuterostomia - ps17
Chordata - ps18
Olfactores - ps19
Vertebrata - ps20
Gnathostomata - ps21
Euteleostomi - ps22
Sarcopterygii - ps23
Tetrapoda - ps24
Amniota - ps25
Mammalia - ps26
Theria - ps27
Placentalia - ps28
Boreoeutheria - ps29
Euarchontoglires - ps30
Euarchonta - ps31
Primates - ps32
Haplorrhini - ps33
Simiiformes - ps34
Catarrhini - ps35
Hominoidea - ps36
Hominidae - ps37
Homininae - ps38
Hominini - ps39
Homo_sapiens - ps40
p-value
3e-04
1e-03
3e-03
1e-02
4e-02
Log-odds
0.5
1.0
1.5
2.0
2.5
3.0
3.5
4.0

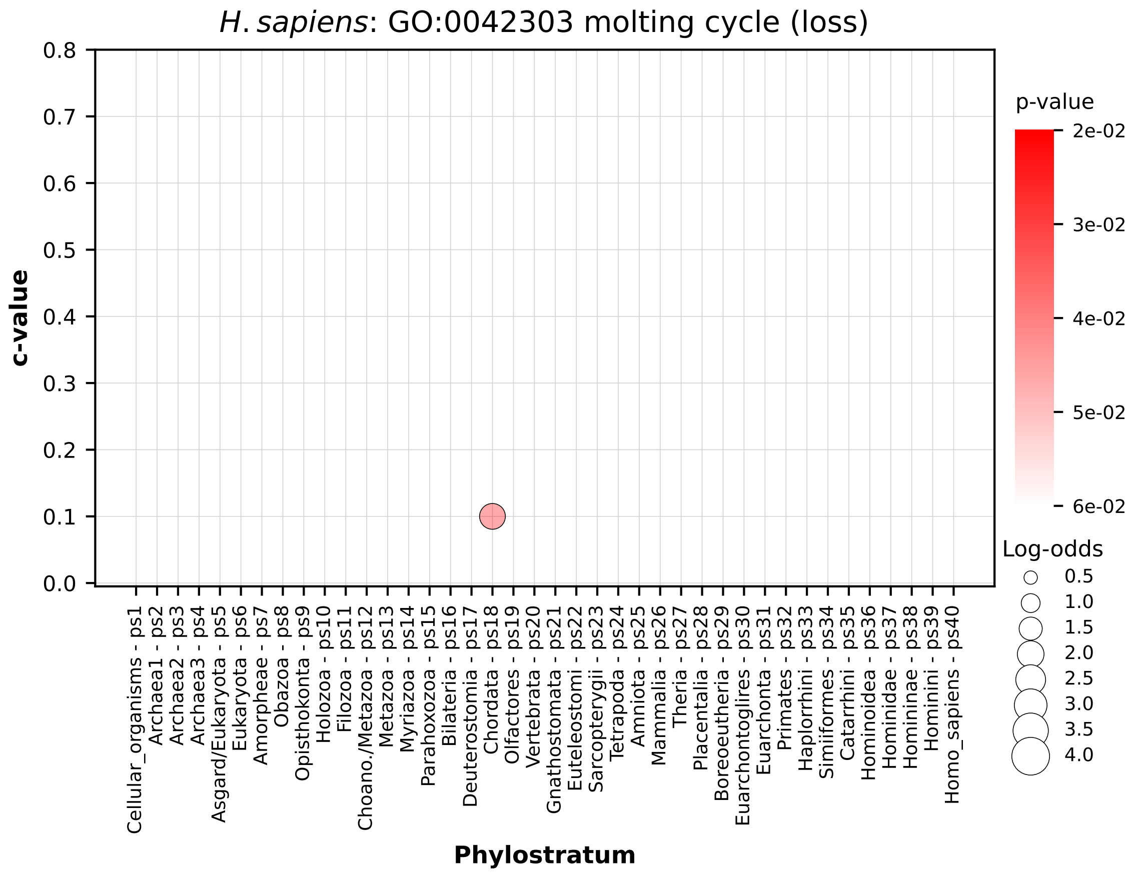

H. sapiens: GO:0042303 molting cycle (loss)
c-value
0.8
0.7
0.6
0.5
0.4
0.3
0.2
0.1
0.0
p-value
2e-02
3e-02
4e-02
5e-02
6e-02
Log-odds
0.5
1.0
1.5
2.0
2.5
3.0
3.5
4.0
Cellular_organisms - ps1
Archaea1 - ps2
Archaea2 - ps3
Archaea3 - ps4
Asgard/Eukaryota - ps5
Eukaryota - ps6
Amorpheae - ps7
Obazoa - ps8
Opisthokonta - ps9
Holozoa - ps10
Filozoa - ps11
Choano./Metazoa - ps12
Metazoa - ps13
Myriazoa - ps14
Parahoxozoa - ps15
Bilateria - ps16
Deuterostomia - ps17
Chordata - ps18
Olfactores - ps19
Vertebrata - ps20
Gnathostomata - ps21
Euteleostomi - ps22
Sarcopterygii - ps23
Tetrapoda - ps24
Amniota - ps25
Mammalia - ps26
Theria - ps27
Placentalia - ps28
Boreoeutheria - ps29
Euarchontoglires - ps30
Euarchonta - ps31
Primates - ps32
Haplorrhini - ps33
Simiiformes - ps34
Catarrhini - ps35
Hominoidea - ps36
Hominidae - ps37
Homininae - ps38
Hominini - ps39
Homo_sapiens - ps40
Phylostratum

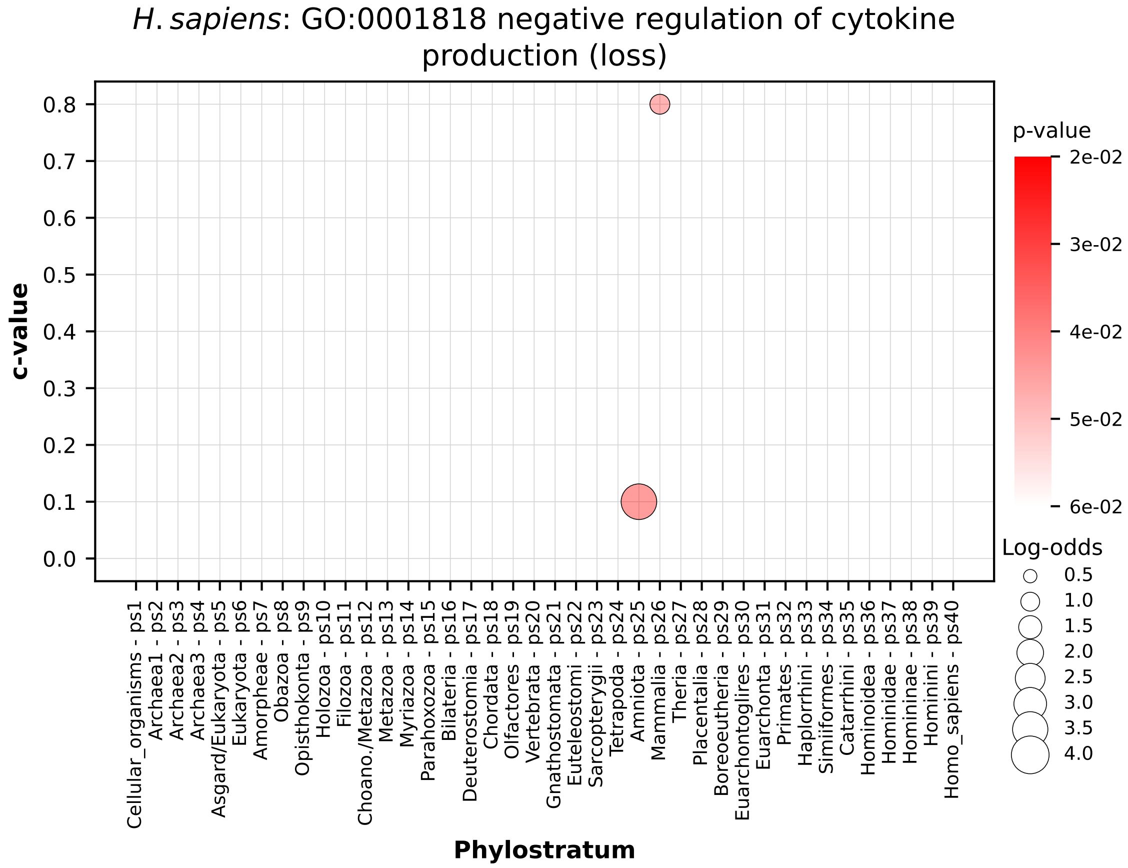

H. sapiens: GO:0001818 negative regulation of cytokine production (loss)
c-value
0.8
0.7
0.6
0.5
0.4
0.3
0.2
0.1
0.0
p-value
2e-02
3e-02
4e-02
5e-02
6e-02
Log-odds
0.5
1.0
1.5
2.0
2.5
3.0
3.5
4.0
Cellular_organisms - ps1
Archaea1 - ps2
Archaea2 - ps3
Archaea3 - ps4
Asgard/Eukaryota - ps5
Eukaryota - ps6
Amorpheae - ps7
Obazoa - ps8
Opisthokonta - ps9
Holozoa - ps10
Filozoa - ps11
Choano./Metazoa - ps12
Metazoa - ps13
Myriazoa - ps14
Parahoxozoa - ps15
Bilateria - ps16
Deuterostomia - ps17
Chordata - ps18
Olfactores - ps19
Vertebrata - ps20
Gnathostomata - ps21
Euteleostomi - ps22
Sarcopterygii - ps23
Tetrapoda - ps24
Amniota - ps25
Mammalia - ps26
Theria - ps27
Placentalia - ps28
Boreoeutheria - ps29
Euarchontoglires - ps30
Euarchonta - ps31
Primates - ps32
Haplorrhini - ps33
Simiiformes - ps34
Catarrhini - ps35
Hominoidea - ps36
Hominidae - ps37
Homininae - ps38
Hominini - ps39
Homo_sapiens - ps40
Phylostratum

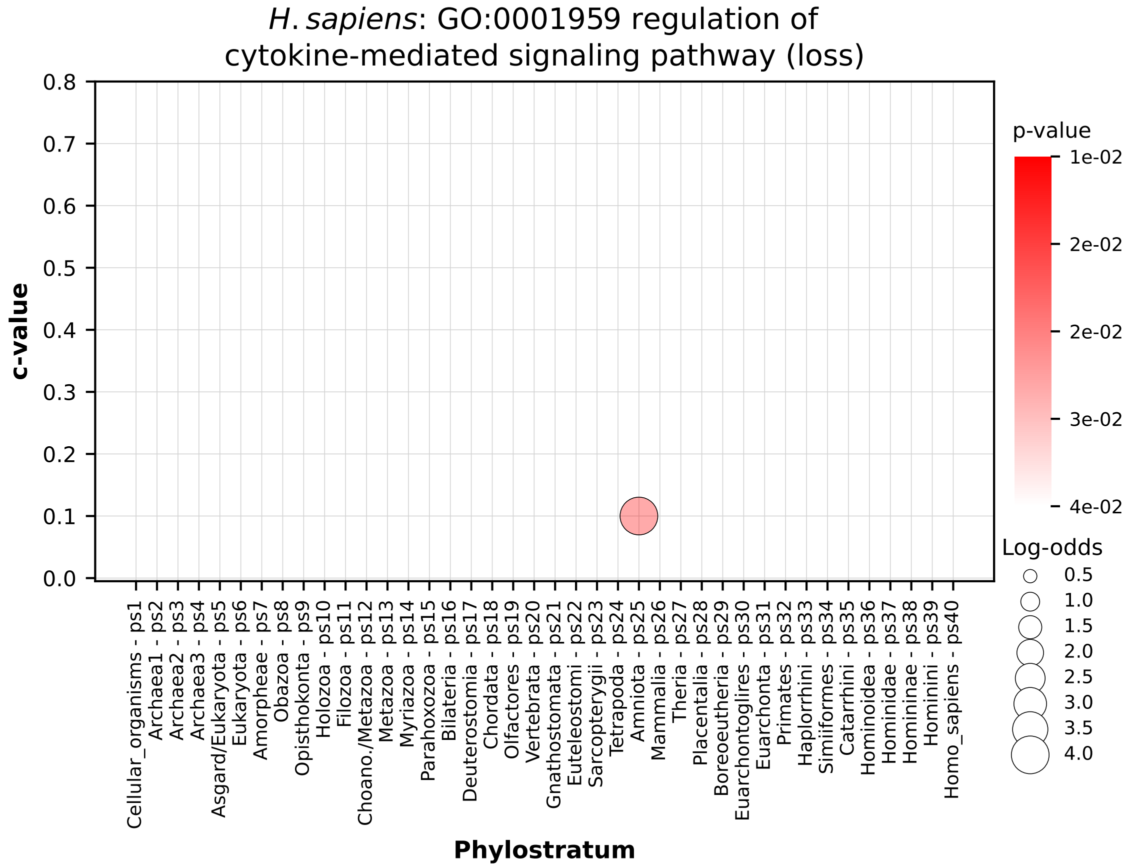

H. sapiens: GO:0001959 regulation of cytokine-mediated signaling pathway (loss)
c-value
0.0
0.1
0.2
0.3
0.4
0.5
0.6
0.7
0.8
Phylostratum
Cellular_organisms - ps1
Archaea1 - ps2
Archaea2 - ps3
Archaea3 - ps4
Asgard/Eukaryota - ps5
Eukaryota - ps6
Amorpheae - ps7
Obazoa - ps8
Opisthokonta - ps9
Holozoa - ps10
Filozoa - ps11
Choano./Metazoa - ps12
Metazoa - ps13
Myriazoa - ps14
Parahoxozoa - ps15
Bilateria - ps16
Deuterostomia - ps17
Chordata - ps18
Olfactores - ps19
Vertebrata - ps20
Gnathostomata - ps21
Euteleostomi - ps22
Sarcopterygii - ps23
Tetrapoda - ps24
Amniota - ps25
Mammalia - ps26
Theria - ps27
Placentalia - ps28
Boreoeutheria - ps29
Euarchontoglires - ps30
Euarchonta - ps31
Primates - ps32
Haplorrhini - ps33
Simiiformes - ps34
Catarrhini - ps35
Hominoidea - ps36
Hominidae - ps37
Homininae - ps38
Hominini - ps39
Homo_sapiens - ps40
p-value
1e-02
2e-02
2e-02
3e-02
4e-02
Log-odds
0.5
1.0
1.5
2.0
2.5
3.0
3.5
4.0

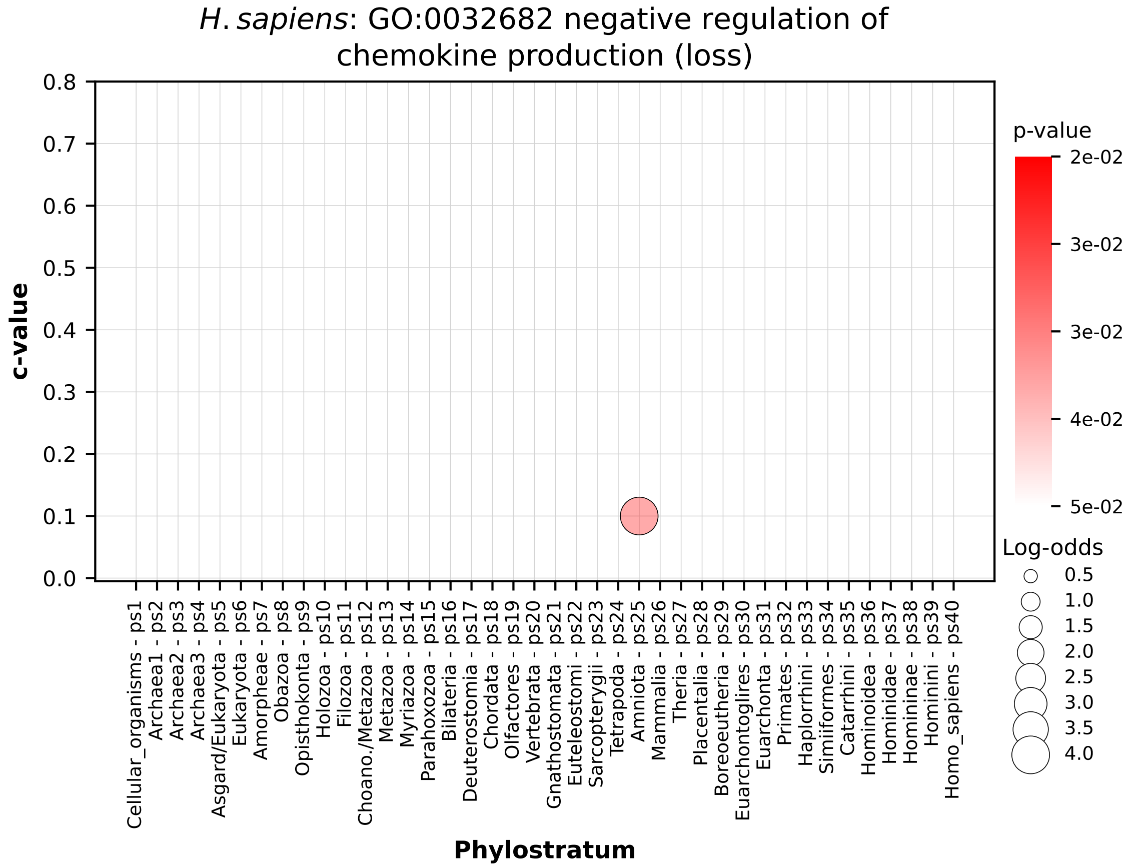

H. sapiens: GO:0032682 negative regulation of chemokine production (loss)
c-value
0.8
0.7
0.6
0.5
0.4
0.3
0.2
0.1
0.0
p-value
2e-02
3e-02
3e-02
4e-02
5e-02
Log-odds
0.5
1.0
1.5
2.0
2.5
3.0
3.5
4.0
Cellular_organisms - ps1
Archaea1 - ps2
Archaea2 - ps3
Archaea3 - ps4
Asgard/Eukaryota - ps5
Eukaryota - ps6
Amorpheae - ps7
Obazoa - ps8
Opisthokonta - ps9
Holozoa - ps10
Filozoa - ps11
Choano./Metazoa - ps12
Metazoa - ps13
Myriazoa - ps14
Parahoxozoa - ps15
Bilateria - ps16
Deuterostomia - ps17
Chordata - ps18
Olfactores - ps19
Vertebrata - ps20
Gnathostomata - ps21
Euteleostomi - ps22
Sarcopterygii - ps23
Tetrapoda - ps24
Amniota - ps25
Mammalia - ps26
Theria - ps27
Placentalia - ps28
Boreoeutheria - ps29
Euarchontoglires - ps30
Euarchonta - ps31
Primates - ps32
Haplorrhini - ps33
Simiiformes - ps34
Catarrhini - ps35
Hominoidea - ps36
Hominidae - ps37
Homininae - ps38
Hominini - ps39
Homo_sapiens - ps40
Phylostratum

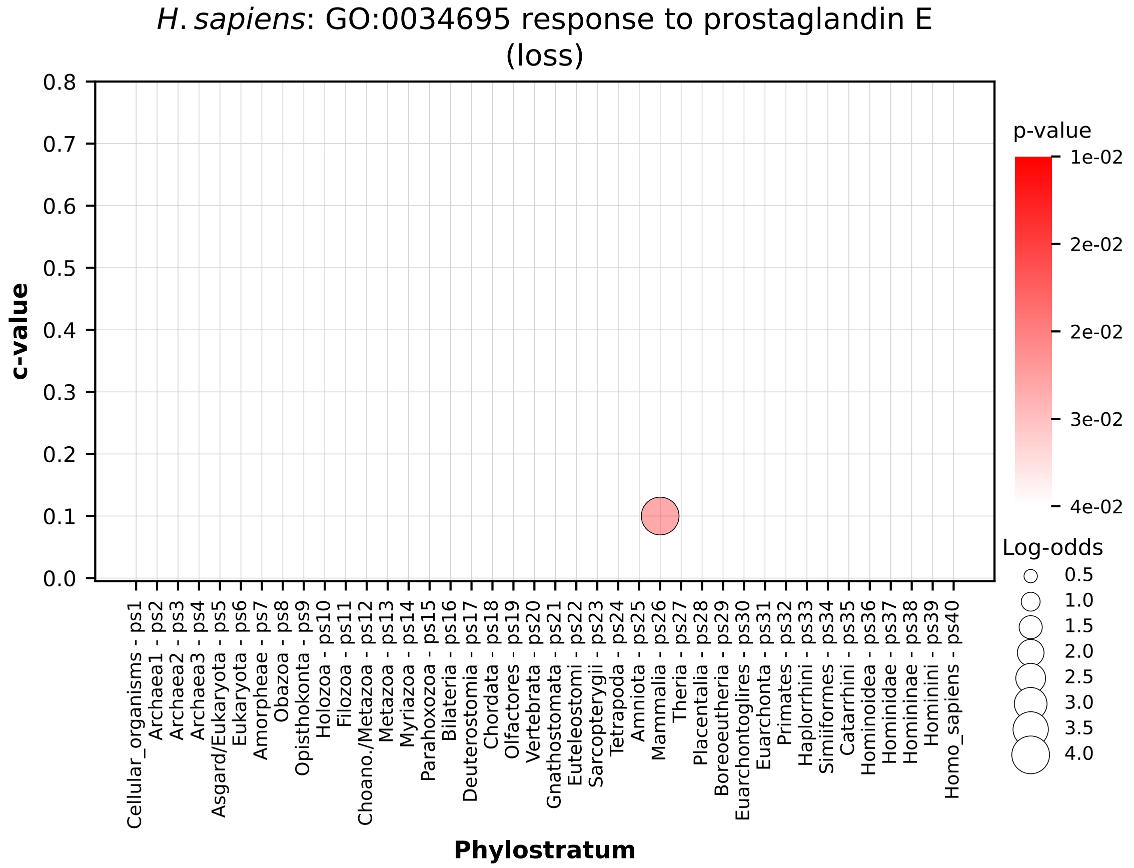

H. sapiens: GO:0034695 response to prostaglandin E (loss)
c-value
0.0
0.1
0.2
0.3
0.4
0.5
0.6
0.7
0.8
Phylostratum
Cellular_organisms - ps1
Archaea1 - ps2
Archaea2 - ps3
Archaea3 - ps4
Asgard/Eukaryota - ps5
Eukaryota - ps6
Amorpheae - ps7
Obazoa - ps8
Opisthokonta - ps9
Holozoa - ps10
Filozoa - ps11
Choano./Metazoa - ps12
Metazoa - ps13
Myriazoa - ps14
Parahoxozoa - ps15
Bilateria - ps16
Deuterostomia - ps17
Chordata - ps18
Olfactores - ps19
Vertebrata - ps20
Gnathostomata - ps21
Euteleostomi - ps22
Sarcopterygii - ps23
Tetrapoda - ps24
Amniota - ps25
Mammalia - ps26
Theria - ps27
Placentalia - ps28
Boreoeutheria - ps29
Euarchontoglires - ps30
Euarchonta - ps31
Primates - ps32
Haplorrhini - ps33
Simiiformes - ps34
Catarrhini - ps35
Hominoidea - ps36
Hominidae - ps37
Homininae - ps38
Hominini - ps39
Homo_sapiens - ps40
p-value
1e-02
2e-02
2e-02
3e-02
4e-02
Log-odds
0.5
1.0
1.5
2.0
2.5
3.0
3.5
4.0

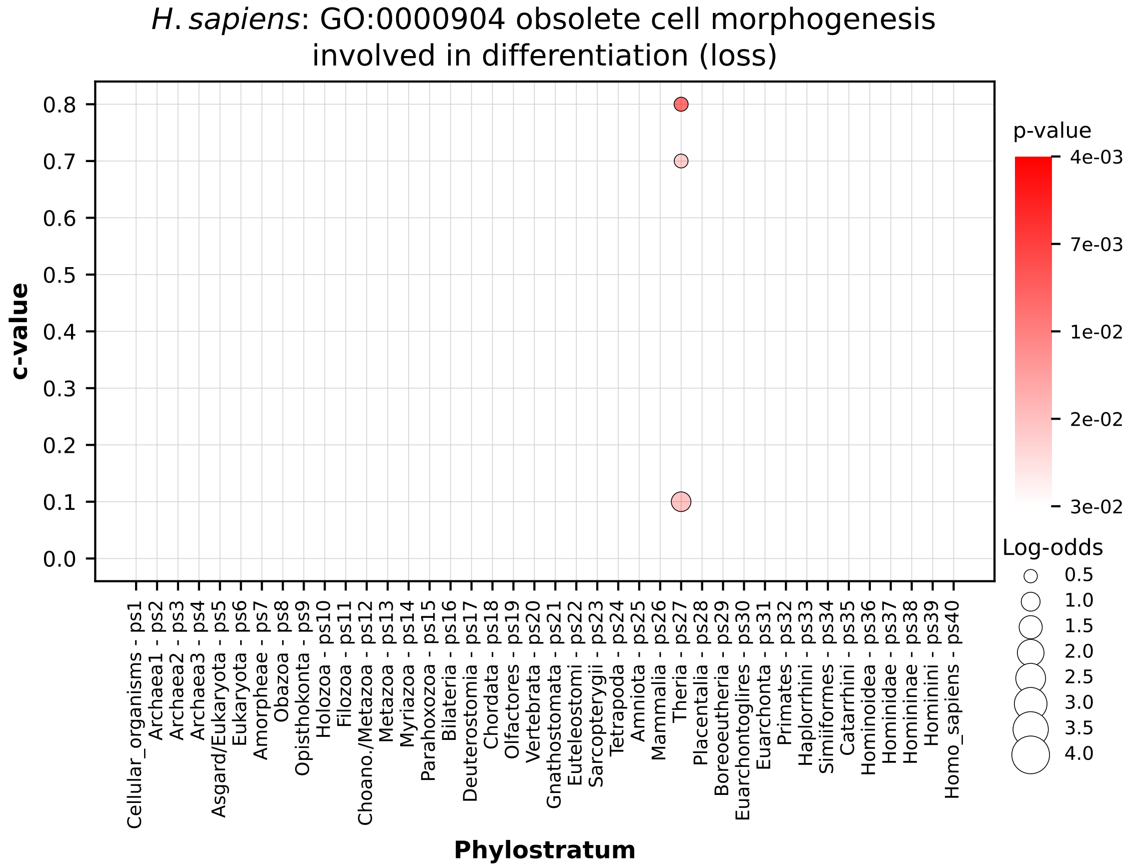

H. sapiens: GO:0000904 obsolete cell morphogenesis involved in differentiation (loss)
c-value
0.8
0.7
0.6
0.5
0.4
0.3
0.2
0.1
0.0
p-value
4e-03
7e-03
1e-02
2e-02
3e-02
Log-odds
0.5
1.0
1.5
2.0
2.5
3.0
3.5
4.0
Cellular_organisms - ps1
Archaea1 - ps2
Archaea2 - ps3
Archaea3 - ps4
Asgard/Eukaryota - ps5
Eukaryota - ps6
Amorpheae - ps7
Obazoa - ps8
Opisthokonta - ps9
Holozoa - ps10
Filozoa - ps11
Choano./Metazoa - ps12
Metazoa - ps13
Myriazoa - ps14
Parahoxozoa - ps15
Bilateria - ps16
Deuterostomia - ps17
Chordata - ps18
Olfactores - ps19
Vertebrata - ps20
Gnathostomata - ps21
Euteleostomi - ps22
Sarcopterygii - ps23
Tetrapoda - ps24
Amniota - ps25
Mammalia - ps26
Theria - ps27
Placentalia - ps28
Boreoeutheria - ps29
Euarchontoglires - ps30
Euarchonta - ps31
Primates - ps32
Haplorrhini - ps33
Simiiformes - ps34
Catarrhini - ps35
Hominoidea - ps36
Hominidae - ps37
Homininae - ps38
Hominini - ps39
Homo_sapiens - ps40
Phylostratum

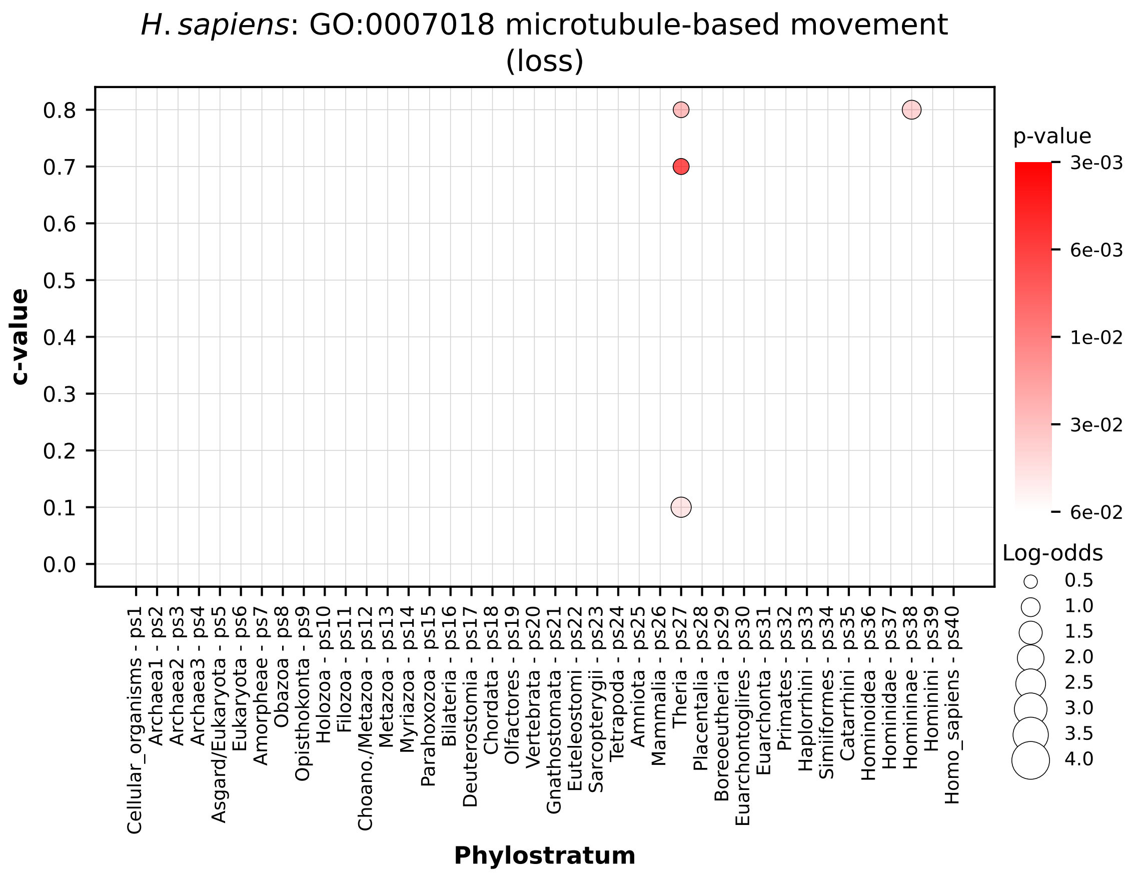

H. sapiens: GO:0007018 microtubule-based movement (loss)
c-value
0.0
0.1
0.2
0.3
0.4
0.5
0.6
0.7
0.8
Phylostratum
Cellular_organisms - ps1
Archaea1 - ps2
Archaea2 - ps3
Archaea3 - ps4
Asgard/Eukaryota - ps5
Eukaryota - ps6
Amorpheae - ps7
Obazoa - ps8
Opisthokonta - ps9
Holozoa - ps10
Filozoa - ps11
Choano./Metazoa - ps12
Metazoa - ps13
Myriazoa - ps14
Parahoxozoa - ps15
Bilateria - ps16
Deuterostomia - ps17
Chordata - ps18
Olfactores - ps19
Vertebrata - ps20
Gnathostomata - ps21
Euteleostomi - ps22
Sarcopterygii - ps23
Tetrapoda - ps24
Amniota - ps25
Mammalia - ps26
Theria - ps27
Placentalia - ps28
Boreoeutheria - ps29
Euarchontoglires - ps30
Euarchonta - ps31
Primates - ps32
Haplorrhini - ps33
Simiiformes - ps34
Catarrhini - ps35
Hominoidea - ps36
Hominidae - ps37
Homininae - ps38
Hominini - ps39
Homo_sapiens - ps40
p-value
3e-03
6e-03
1e-02
3e-02
6e-02
Log-odds
0.5
1.0
1.5
2.0
2.5
3.0
3.5
4.0

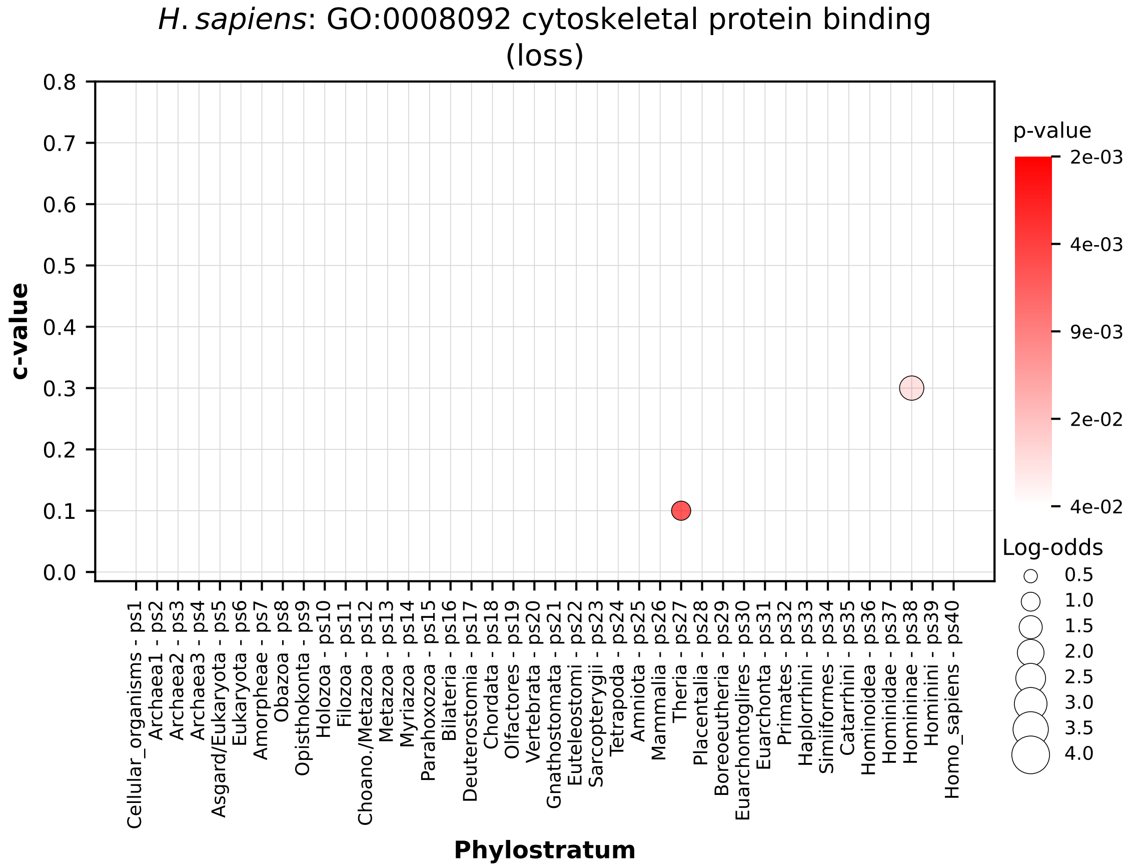

H. sapiens: GO:0008092 cytoskeletal protein binding
(loss)
c-value
0.0
0.1
0.2
0.3
0.4
0.5
0.6
0.7
0.8
Phylostratum
Cellular_organisms - ps1
Archaea1 - ps2
Archaea2 - ps3
Archaea3 - ps4
Asgard/Eukaryota - ps5
Eukaryota - ps6
Amorpheae - ps7
Obazoa - ps8
Opisthokonta - ps9
Holozoa - ps10
Filozoa - ps11
Choano./Metazoa - ps12
Metazoa - ps13
Myriazoa - ps14
Parahoxozoa - ps15
Bilateria - ps16
Deuterostomia - ps17
Chordata - ps18
Olfactores - ps19
Vertebrata - ps20
Gnathostomata - ps21
Euteleostomi - ps22
Sarcopterygii - ps23
Tetrapoda - ps24
Amniota - ps25
Mammalia - ps26
Theria - ps27
Placentalia - ps28
Boreoeutheria - ps29
Euarchontoglires - ps30
Euarchonta - ps31
Primates - ps32
Haplorrhini - ps33
Simiiformes - ps34
Catarrhini - ps35
Hominoidea - ps36
Hominidae - ps37
Homininae - ps38
Hominini - ps39
Homo_sapiens - ps40
p-value
2e-03
4e-03
9e-03
2e-02
4e-02
Log-odds
0.5
1.0
1.5
2.0
2.5
3.0
3.5
4.0

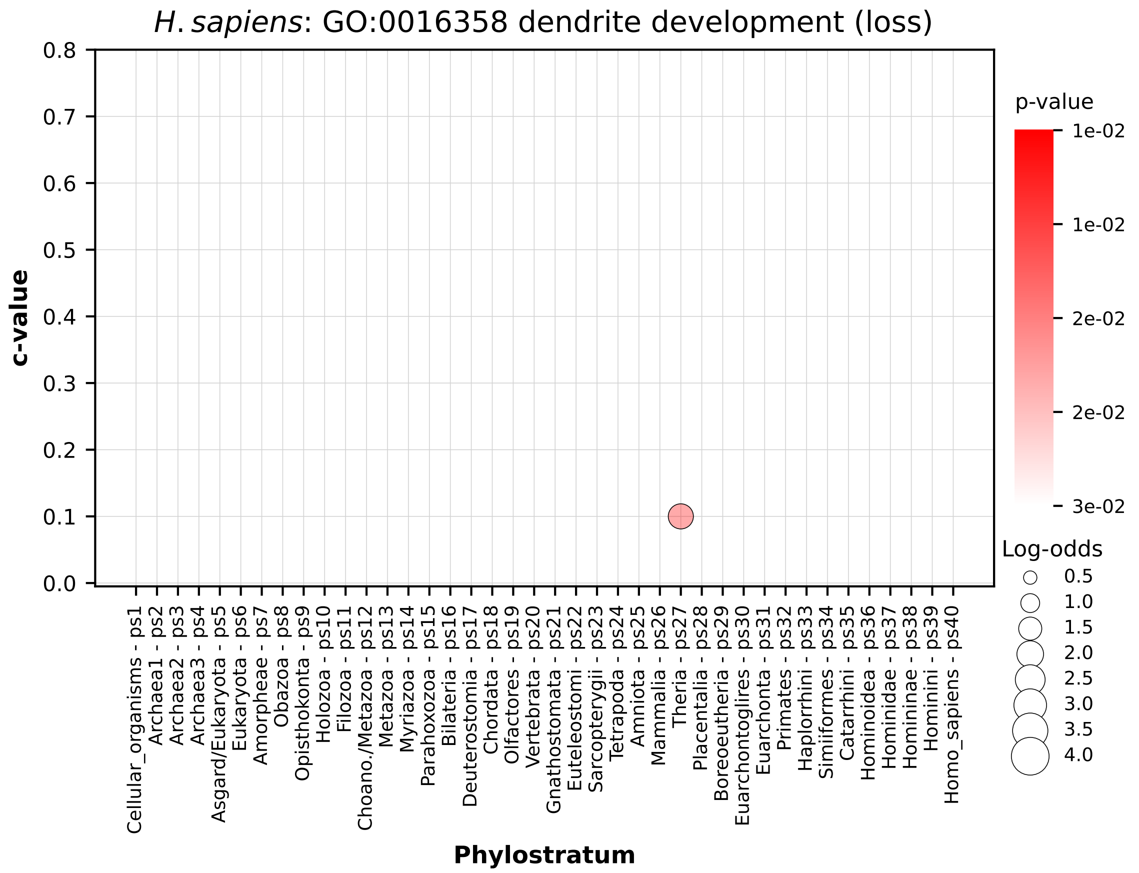

H. sapiens: GO:0016358 dendrite development (loss)
c-value
0.0
0.1
0.2
0.3
0.4
0.5
0.6
0.7
0.8
Phylostratum
Cellular_organisms - ps1
Archaea1 - ps2
Archaea2 - ps3
Archaea3 - ps4
Asgard/Eukaryota - ps5
Eukaryota - ps6
Amorpheae - ps7
Obazoa - ps8
Opisthokonta - ps9
Holozoa - ps10
Filozoa - ps11
Choano./Metazoa - ps12
Metazoa - ps13
Myriazoa - ps14
Parahoxozoa - ps15
Bilateria - ps16
Deuterostomia - ps17
Chordata - ps18
Olfactores - ps19
Vertebrata - ps20
Gnathostomata - ps21
Euteleostomi - ps22
Sarcopterygii - ps23
Tetrapoda - ps24
Amniota - ps25
Mammalia - ps26
Theria - ps27
Placentalia - ps28
Boreoeutheria - ps29
Euarchontoglires - ps30
Euarchonta - ps31
Primates - ps32
Haplorrhini - ps33
Simiiformes - ps34
Catarrhini - ps35
Hominoidea - ps36
Hominidae - ps37
Homininae - ps38
Hominini - ps39
Homo_sapiens - ps40
p-value
1e-02
1e-02
2e-02
2e-02
3e-02
Log-odds
0.5
1.0
1.5
2.0
2.5
3.0
3.5
4.0

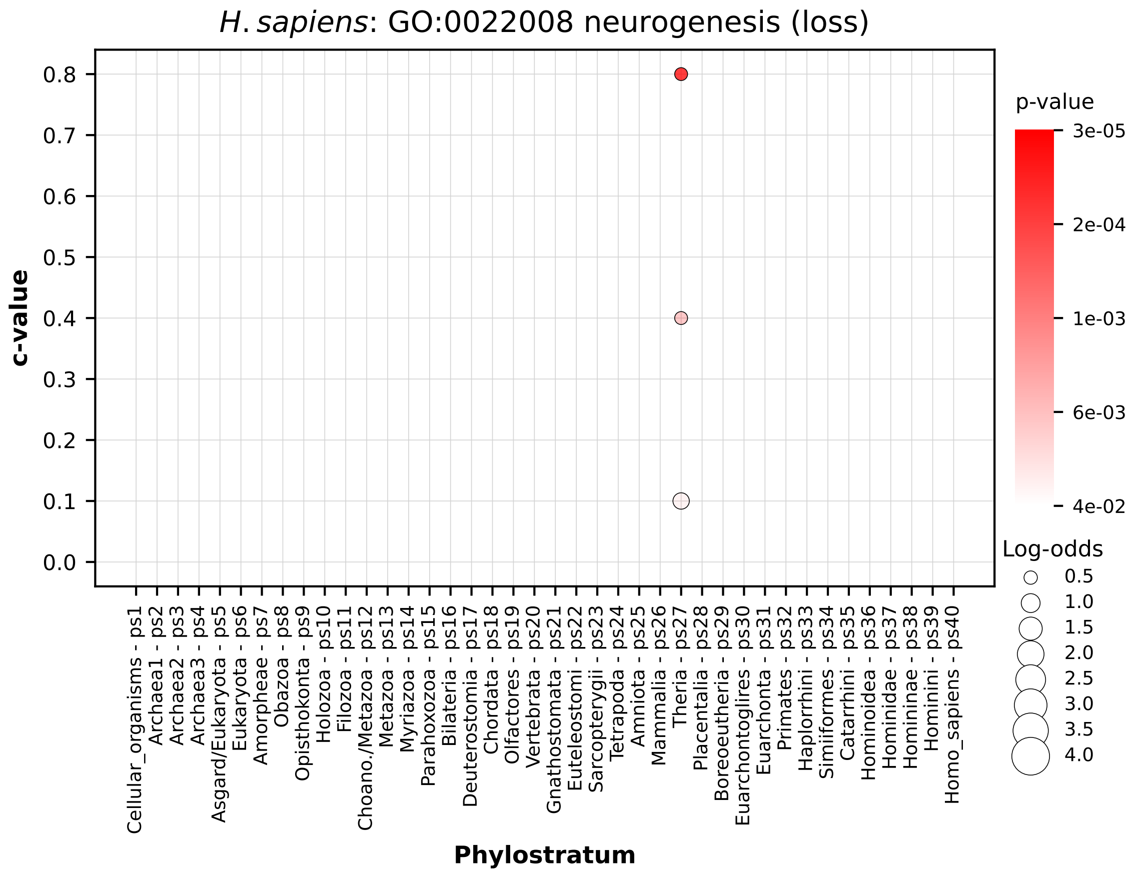

H. sapiens: GO:0022008 neurogenesis (loss)
c-value
0.8
0.7
0.6
0.5
0.4
0.3
0.2
0.1
0.0
p-value
3e-05
2e-04
1e-03
6e-03
4e-02
Log-odds
0.5
1.0
1.5
2.0
2.5
3.0
3.5
4.0
Cellular_organisms - ps1
Archaea1 - ps2
Archaea2 - ps3
Archaea3 - ps4
Asgard/Eukaryota - ps5
Eukaryota - ps6
Amorpheae - ps7
Obazoa - ps8
Opisthokonta - ps9
Holozoa - ps10
Filozoa - ps11
Choano./Metazoa - ps12
Metazoa - ps13
Myriazoa - ps14
Parahoxozoa - ps15
Bilateria - ps16
Deuterostomia - ps17
Chordata - ps18
Olfactores - ps19
Vertebrata - ps20
Gnathostomata - ps21
Euteleostomi - ps22
Sarcopterygii - ps23
Tetrapoda - ps24
Amniota - ps25
Mammalia - ps26
Theria - ps27
Placentalia - ps28
Boreoeutheria - ps29
Euarchontoglires - ps30
Euarchonta - ps31
Primates - ps32
Haplorrhini - ps33
Simiiformes - ps34
Catarrhini - ps35
Hominoidea - ps36
Hominidae - ps37
Homininae - ps38
Hominini - ps39
Homo_sapiens - ps40
Phylostratum

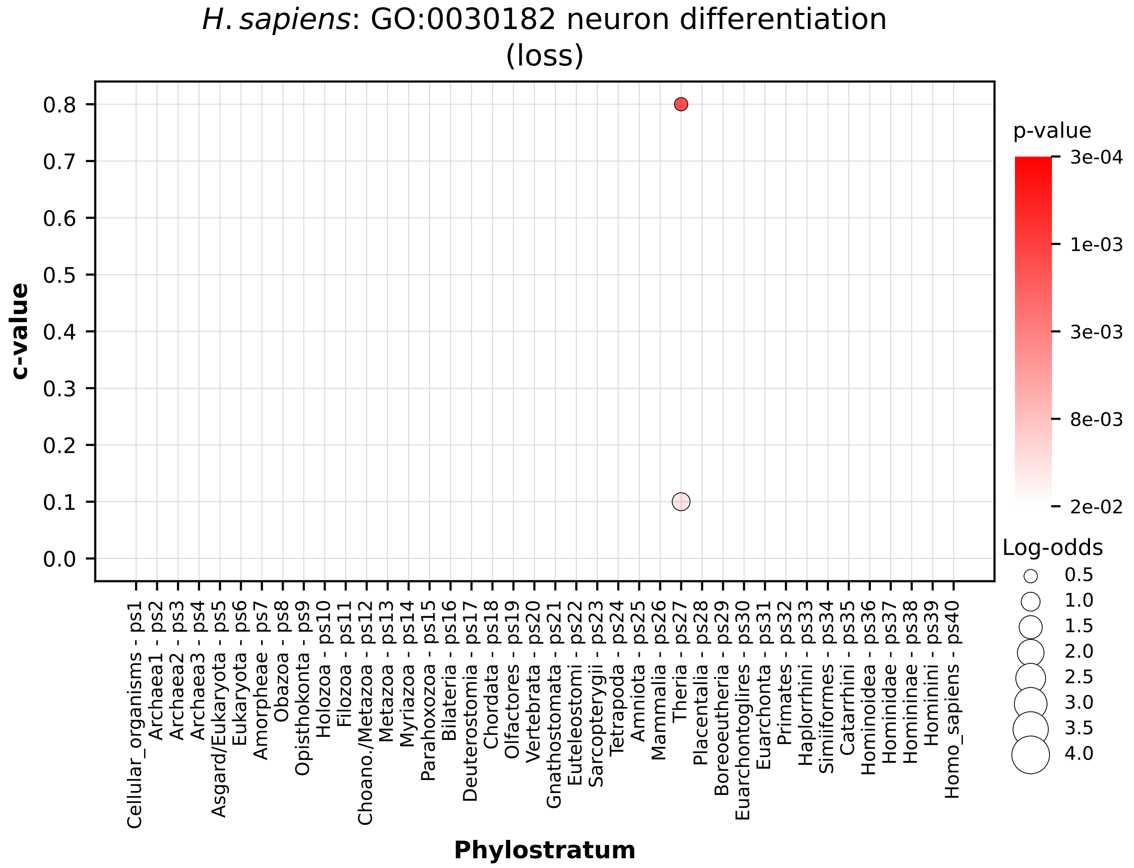

H. sapiens: GO:0030182 neuron differentiation
(loss)
c-value
0.8
0.7
0.6
0.5
0.4
0.3
0.2
0.1
0.0
p-value
3e-04
1e-03
3e-03
8e-03
2e-02
Log-odds
0.5
1.0
1.5
2.0
2.5
3.0
3.5
4.0
Cellular_organisms - ps1
Archaea1 - ps2
Archaea2 - ps3
Archaea3 - ps4
Asgard/Eukaryota - ps5
Eukaryota - ps6
Amorpheae - ps7
Obazoa - ps8
Opisthokonta - ps9
Holozoa - ps10
Filozoa - ps11
Choano./Metazoa - ps12
Metazoa - ps13
Myriazoa - ps14
Parahoxozoa - ps15
Bilateria - ps16
Deuterostomia - ps17
Chordata - ps18
Olfactores - ps19
Vertebrata - ps20
Gnathostomata - ps21
Euteleostomi - ps22
Sarcopterygii - ps23
Tetrapoda - ps24
Amniota - ps25
Mammalia - ps26
Theria - ps27
Placentalia - ps28
Boreoeutheria - ps29
Euarchontoglires - ps30
Euarchonta - ps31
Primates - ps32
Haplorrhini - ps33
Simiiformes - ps34
Catarrhini - ps35
Hominoidea - ps36
Hominidae - ps37
Homininae - ps38
Hominini - ps39
Homo_sapiens - ps40
Phylostratum

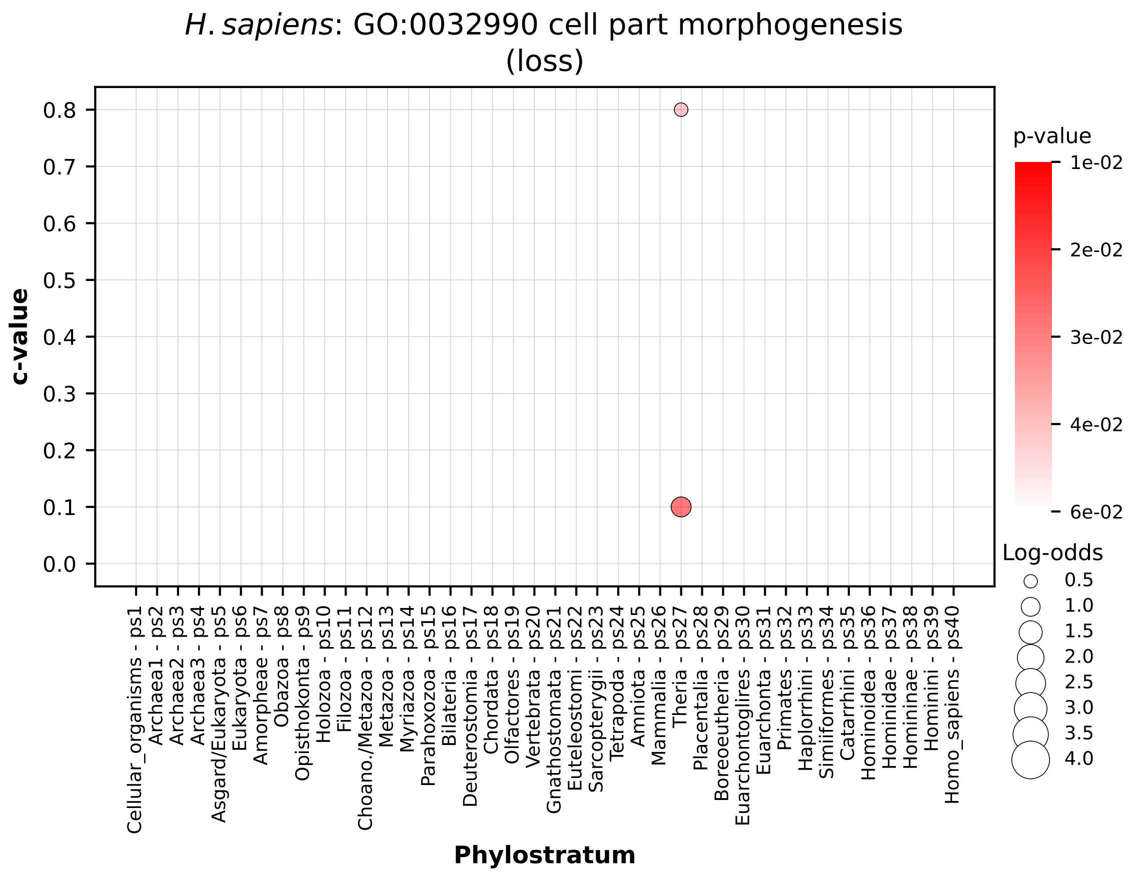

H. sapiens: GO:0032990 cell part morphogenesis
(loss)
c-value
0.0
0.1
0.2
0.3
0.4
0.5
0.6
0.7
0.8
Phylostratum
Cellular_organisms - ps1
Archaea1 - ps2
Archaea2 - ps3
Archaea3 - ps4
Asgard/Eukaryota - ps5
Eukaryota - ps6
Amorpheae - ps7
Obazoa - ps8
Opisthokonta - ps9
Holozoa - ps10
Filozoa - ps11
Choano./Metazoa - ps12
Metazoa - ps13
Myriazoa - ps14
Parahoxozoa - ps15
Bilateria - ps16
Deuterostomia - ps17
Chordata - ps18
Olfactores - ps19
Vertebrata - ps20
Gnathostomata - ps21
Euteleostomi - ps22
Sarcopterygii - ps23
Tetrapoda - ps24
Amniota - ps25
Mammalia - ps26
Theria - ps27
Placentalia - ps28
Boreoeutheria - ps29
Euarchontoglires - ps30
Euarchonta - ps31
Primates - ps32
Haplorrhini - ps33
Simiiformes - ps34
Catarrhini - ps35
Hominoidea - ps36
Hominidae - ps37
Homininae - ps38
Hominini - ps39
Homo_sapiens - ps40
p-value
1e-02
2e-02
3e-02
4e-02
6e-02
Log-odds
0.5
1.0
1.5
2.0
2.5
3.0
3.5
4.0

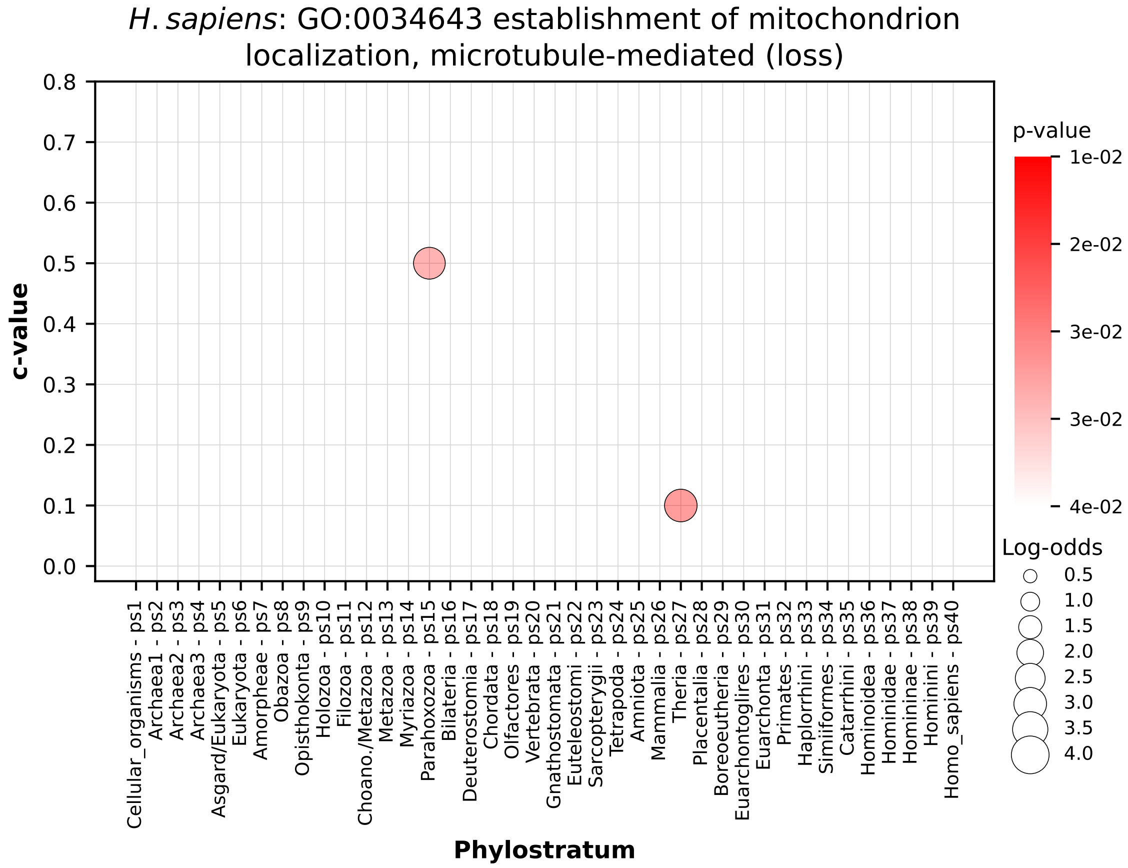

H. sapiens: GO:0034643 establishment of mitochondrion localization, microtubule-mediated (loss)
c-value
0.0
0.1
0.2
0.3
0.4
0.5
0.6
0.7
0.8
Phylostratum
Cellular_organisms - ps1
Archaea1 - ps2
Archaea2 - ps3
Archaea3 - ps4
Asgard/Eukaryota - ps5
Eukaryota - ps6
Amorpheae - ps7
Obazoa - ps8
Opisthokonta - ps9
Holozoa - ps10
Filozoa - ps11
Choano./Metazoa - ps12
Metazoa - ps13
Myriazoa - ps14
Parahoxozoa - ps15
Bilateria - ps16
Deuterostomia - ps17
Chordata - ps18
Olfactores - ps19
Vertebrata - ps20
Gnathostomata - ps21
Euteleostomi - ps22
Sarcopterygii - ps23
Tetrapoda - ps24
Amniota - ps25
Mammalia - ps26
Theria - ps27
Placentalia - ps28
Boreoeutheria - ps29
Euarchontoglires - ps30
Euarchonta - ps31
Primates - ps32
Haplorrhini - ps33
Simiiformes - ps34
Catarrhini - ps35
Hominoidea - ps36
Hominidae - ps37
Homininae - ps38
Hominini - ps39
Homo_sapiens - ps40
p-value
1e-02
2e-02
3e-02
3e-02
4e-02
Log-odds
0.5
1.0
1.5
2.0
2.5
3.0
3.5
4.0

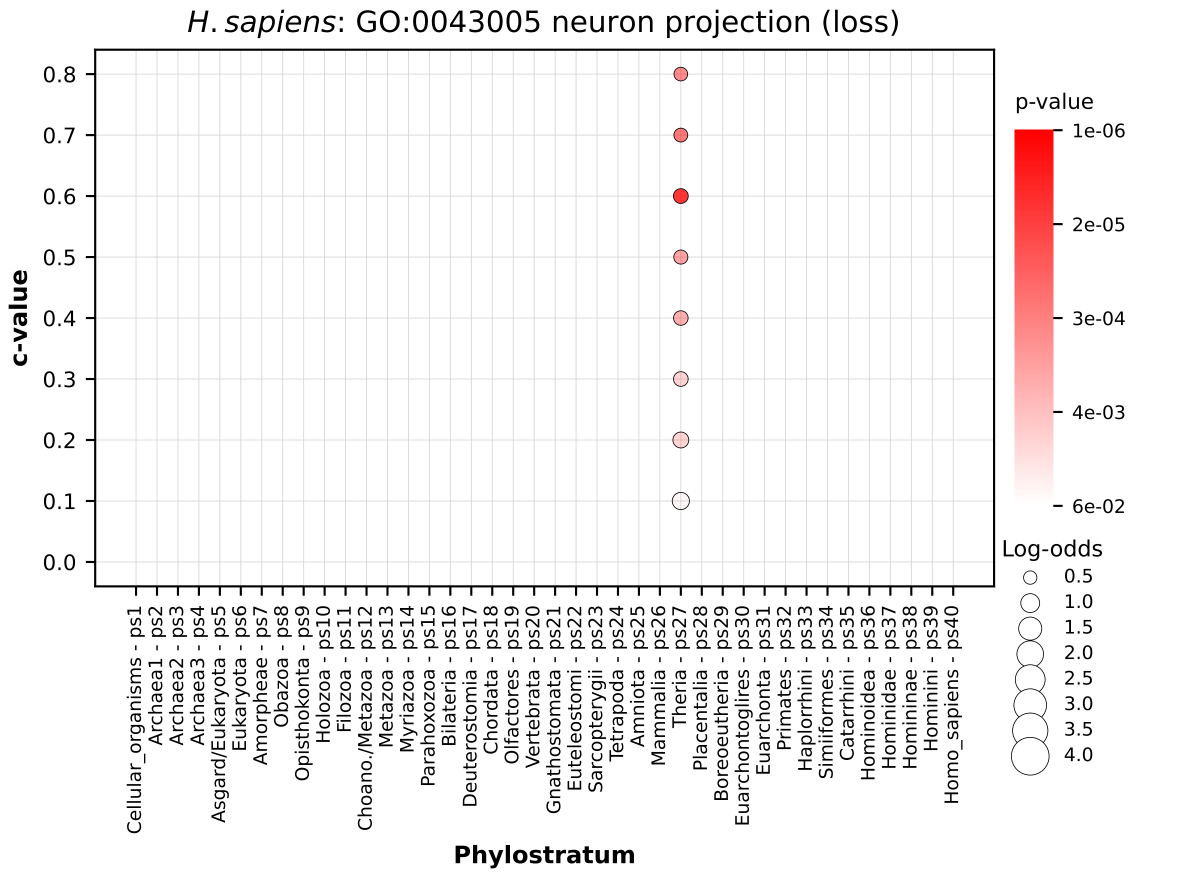

H. sapiens: GO:0043005 neuron projection (loss)
c-value
0.0
0.1
0.2
0.3
0.4
0.5
0.6
0.7
0.8
Phylostratum
Cellular_organisms - ps1
Archaea1 - ps2
Archaea2 - ps3
Archaea3 - ps4
Asgard/Eukaryota - ps5
Eukaryota - ps6
Amorpheae - ps7
Obazoa - ps8
Opisthokonta - ps9
Holozoa - ps10
Filozoa - ps11
Choano./Metazoa - ps12
Metazoa - ps13
Myriazoa - ps14
Parahoxozoa - ps15
Bilateria - ps16
Deuterostomia - ps17
Chordata - ps18
Olfactores - ps19
Vertebrata - ps20
Gnathostomata - ps21
Euteleostomi - ps22
Sarcopterygii - ps23
Tetrapoda - ps24
Amniota - ps25
Mammalia - ps26
Theria - ps27
Placentalia - ps28
Boreoeutheria - ps29
Euarchontoglires - ps30
Euarchonta - ps31
Primates - ps32
Haplorrhini - ps33
Simiiformes - ps34
Catarrhini - ps35
Hominoidea - ps36
Hominidae - ps37
Homininae - ps38
Hominini - ps39
Homo_sapiens - ps40
p-value
1e-06
2e-05
3e-04
4e-03
6e-02
Log-odds
0.5
1.0
1.5
2.0
2.5
3.0
3.5
4.0

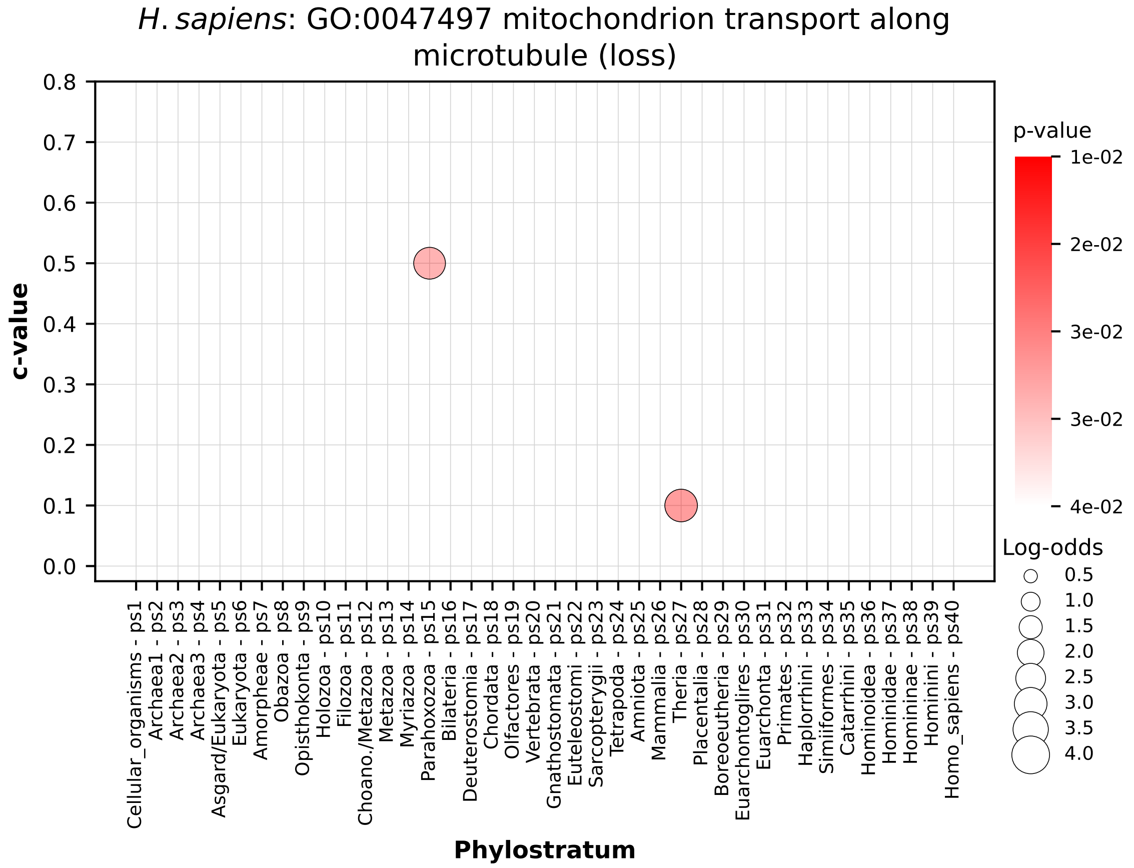

H. sapiens: GO:0047497 mitochondrion transport along microtubule (loss)
c-value
0.0
0.1
0.2
0.3
0.4
0.5
0.6
0.7
0.8
Phylostratum
Cellular_organisms - ps1
Archaea1 - ps2
Archaea2 - ps3
Archaea3 - ps4
Asgard/Eukaryota - ps5
Eukaryota - ps6
Amorpheae - ps7
Obazoa - ps8
Opisthokonta - ps9
Holozoa - ps10
Filozoa - ps11
Choano./Metazoa - ps12
Metazoa - ps13
Myriazoa - ps14
Parahoxozoa - ps15
Bilateria - ps16
Deuterostomia - ps17
Chordata - ps18
Olfactores - ps19
Vertebrata - ps20
Gnathostomata - ps21
Euteleostomi - ps22
Sarcopterygii - ps23
Tetrapoda - ps24
Amniota - ps25
Mammalia - ps26
Theria - ps27
Placentalia - ps28
Boreoeutheria - ps29
Euarchontoglires - ps30
Euarchonta - ps31
Primates - ps32
Haplorrhini - ps33
Simiiformes - ps34
Catarrhini - ps35
Hominoidea - ps36
Hominidae - ps37
Homininae - ps38
Hominini - ps39
Homo_sapiens - ps40
p-value
1e-02
2e-02
3e-02
3e-02
4e-02
Log-odds
0.5
1.0
1.5
2.0
2.5
3.0
3.5
4.0

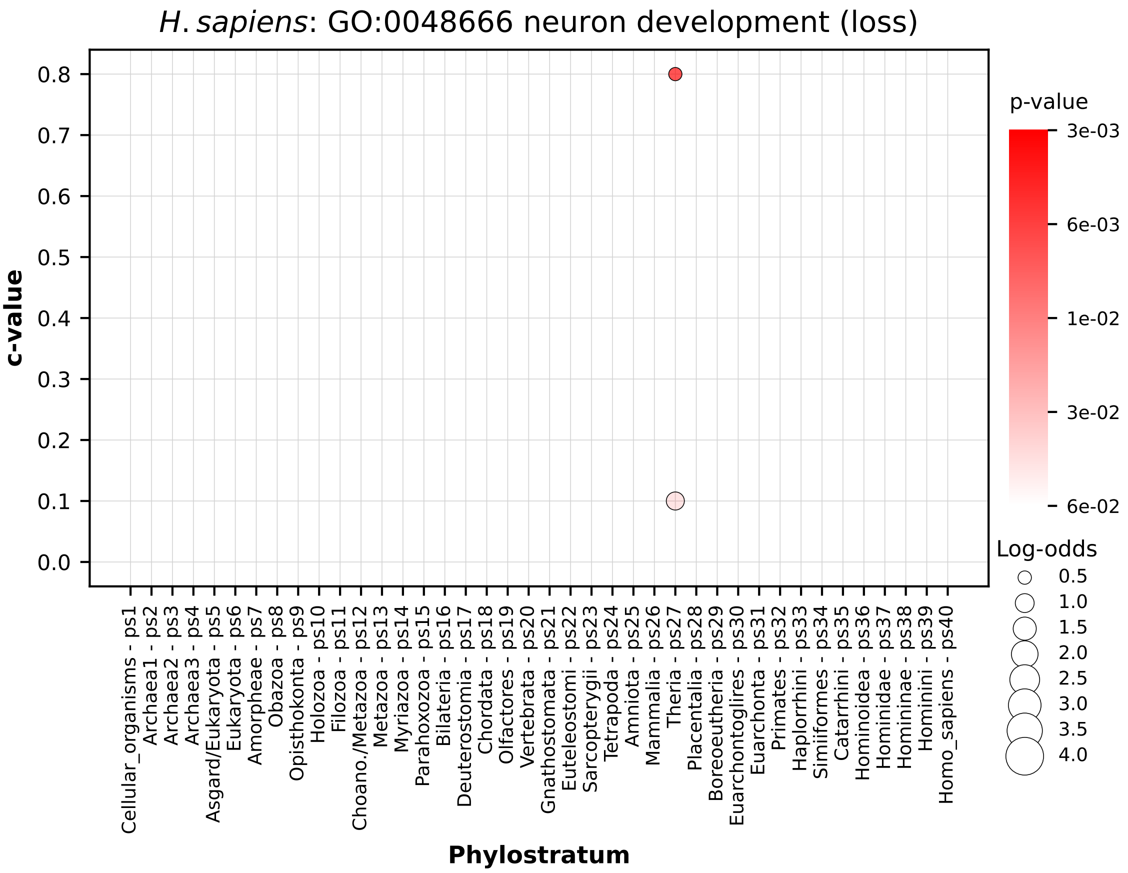

H. sapiens: GO:0048666 neuron development (loss)
c-value
0.0
0.1
0.2
0.3
0.4
0.5
0.6
0.7
0.8
Phylostratum
Cellular_organisms - ps1
Archaea1 - ps2
Archaea2 - ps3
Archaea3 - ps4
Asgard/Eukaryota - ps5
Eukaryota - ps6
Amorpheae - ps7
Obazoa - ps8
Opisthokonta - ps9
Holozoa - ps10
Filozoa - ps11
Choano./Metazoa - ps12
Metazoa - ps13
Myriazoa - ps14
Parahoxozoa - ps15
Bilateria - ps16
Deuterostomia - ps17
Chordata - ps18
Olfactores - ps19
Vertebrata - ps20
Gnathostomata - ps21
Euteleostomi - ps22
Sarcopterygii - ps23
Tetrapoda - ps24
Amniota - ps25
Mammalia - ps26
Theria - ps27
Placentalia - ps28
Boreoeutheria - ps29
Euarchontoglires - ps30
Euarchonta - ps31
Primates - ps32
Haplorrhini - ps33
Simiiformes - ps34
Catarrhini - ps35
Hominoidea - ps36
Hominidae - ps37
Homininae - ps38
Hominini - ps39
Homo_sapiens - ps40
p-value
3e-03
6e-03
1e-02
3e-02
6e-02
Log-odds
0.5
1.0
1.5
2.0
2.5
3.0
3.5
4.0

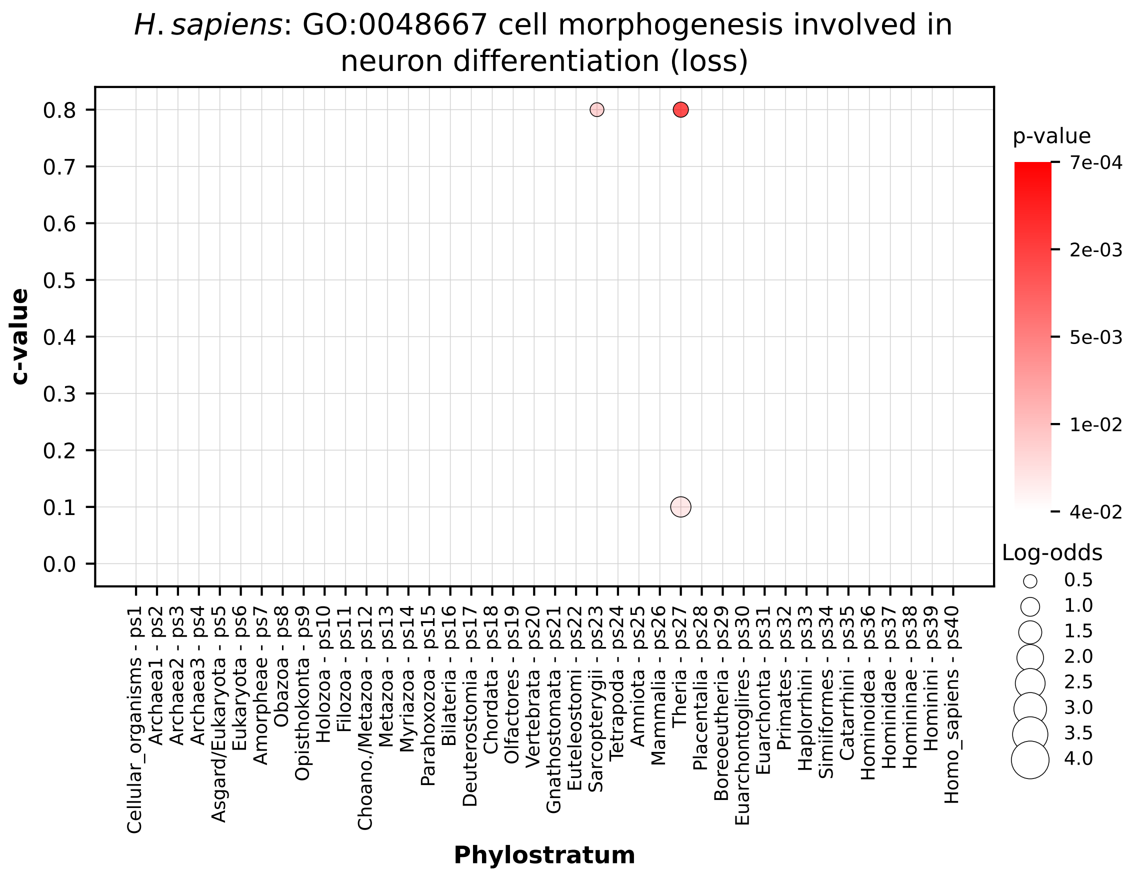

H. sapiens: GO:0048667 cell morphogenesis involved in neuron differentiation (loss)
c-value
0.0
0.1
0.2
0.3
0.4
0.5
0.6
0.7
0.8
p-value
7e-04
2e-03
5e-03
1e-02
4e-02
Log-odds
0.5
1.0
1.5
2.0
2.5
3.0
3.5
4.0
Cellular_organisms - ps1
Archaea1 - ps2
Archaea2 - ps3
Archaea3 - ps4
Asgard/Eukaryota - ps5
Eukaryota - ps6
Amorpheae - ps7
Obazoa - ps8
Opisthokonta - ps9
Holozoa - ps10
Filozoa - ps11
Choano./Metazoa - ps12
Metazoa - ps13
Myriazoa - ps14
Parahoxozoa - ps15
Bilateria - ps16
Deuterostomia - ps17
Chordata - ps18
Olfactores - ps19
Vertebrata - ps20
Gnathostomata - ps21
Euteleostomi - ps22
Sarcopterygii - ps23
Tetrapoda - ps24
Amniota - ps25
Mammalia - ps26
Theria - ps27
Placentalia - ps28
Boreoeutheria - ps29
Euarchontoglires - ps30
Euarchonta - ps31
Primates - ps32
Haplorrhini - ps33
Simiiformes - ps34
Catarrhini - ps35
Hominoidea - ps36
Hominidae - ps37
Homininae - ps38
Hominini - ps39
Homo_sapiens - ps40
Phylostratum

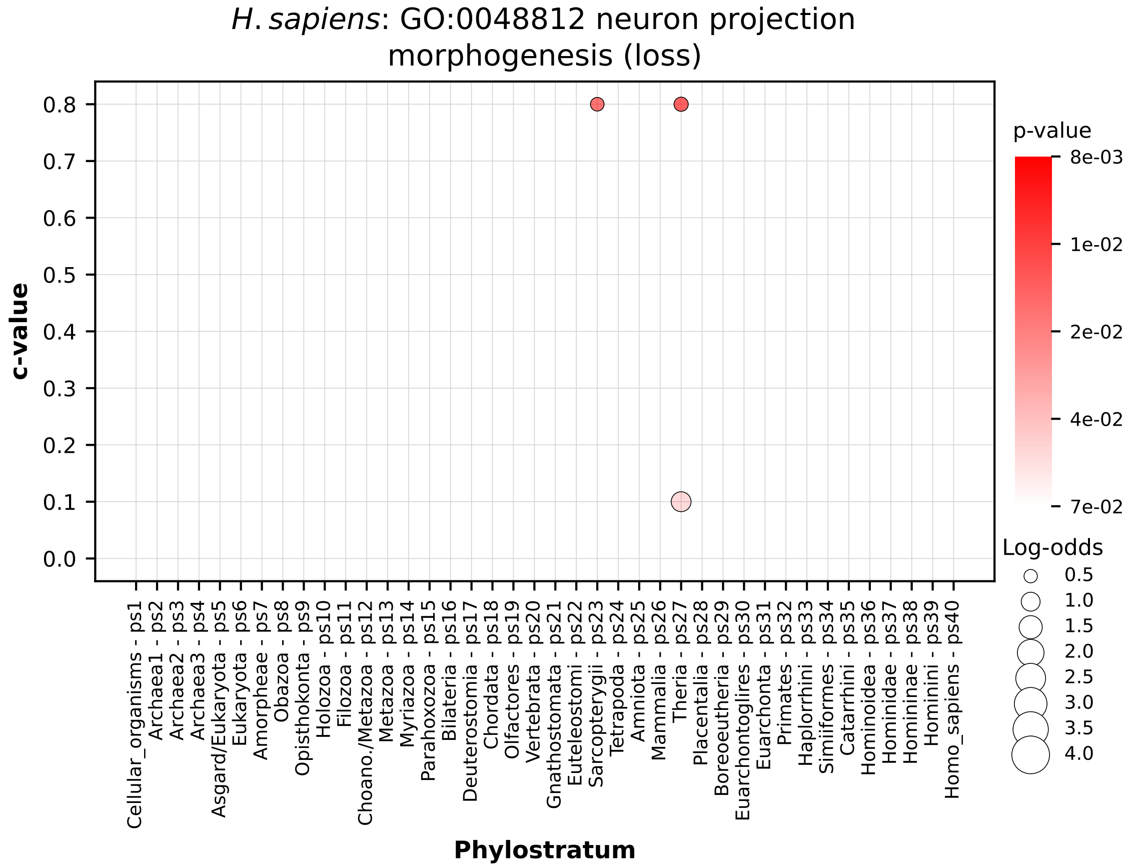
H. sapiens: GO:0048812 neuron projection morphogenesis (loss)
c-value
0.0
0.1
0.2
0.3
0.4
0.5
0.6
0.7
0.8
Phylostratum
Cellular_organisms - ps1
Archaea1 - ps2
Archaea2 - ps3
Archaea3 - ps4
Asgard/Eukaryota - ps5
Eukaryota - ps6
Amorpheae - ps7
Obazoa - ps8
Opisthokonta - ps9
Holozoa - ps10
Filozoa - ps11
Choano./Metazoa - ps12
Metazoa - ps13
Myriazoa - ps14
Parahoxozoa - ps15
Bilateria - ps16
Deuterostomia - ps17
Chordata - ps18
Olfactores - ps19
Vertebrata - ps20
Gnathostomata - ps21
Euteleostomi - ps22
Sarcopterygii - ps23
Tetrapoda - ps24
Amniota - ps25
Mammalia - ps26
Theria - ps27
Placentalia - ps28
Boreoeutheria - ps29
Euarchontoglires - ps30
Euarchonta - ps31
Primates - ps32
Haplorrhini - ps33
Simiiformes - ps34
Catarrhini - ps35
Hominoidea - ps36
Hominidae - ps37
Homininae - ps38
Hominini - ps39
Homo_sapiens - ps40
p-value
8e-03
1e-02
2e-02
4e-02
7e-02
Log-odds
0.5
1.0
1.5
2.0
2.5
3.0
3.5
4.0

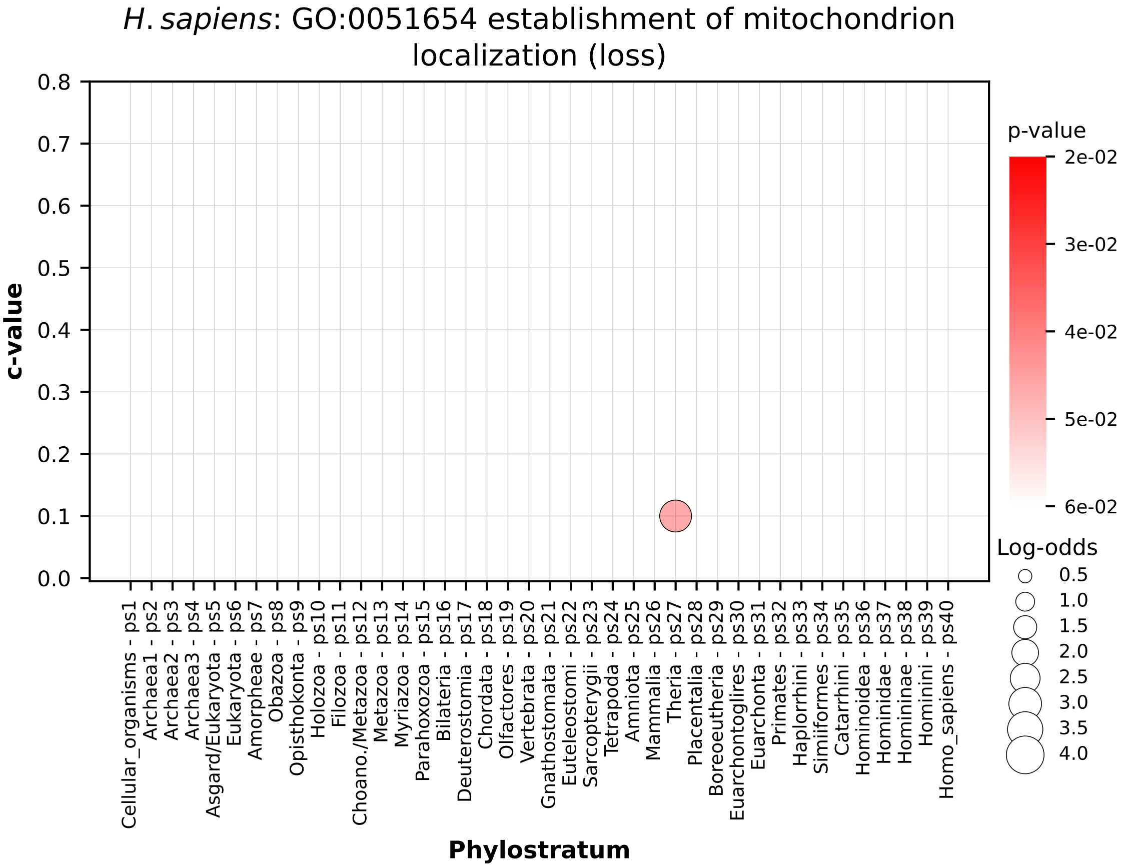
H. sapiens: GO:0051654 establishment of mitochondrion localization (loss)
c-value
0.8
0.7
0.6
0.5
0.4
0.3
0.2
0.1
0.0
Phylostratum
Cellular_organisms - ps1
Archaea1 - ps2
Archaea2 - ps3
Archaea3 - ps4
Asgard/Eukaryota - ps5
Eukaryota - ps6
Amorpheae - ps7
Obazoa - ps8
Opisthokonta - ps9
Holozoa - ps10
Filozoa - ps11
Choano./Metazoa - ps12
Metazoa - ps13
Myriazoa - ps14
Parahoxozoa - ps15
Bilateria - ps16
Deuterostomia - ps17
Chordata - ps18
Olfactores - ps19
Vertebrata - ps20
Gnathostomata - ps21
Euteleostomi - ps22
Sarcopterygii - ps23
Tetrapoda - ps24
Amniota - ps25
Mammalia - ps26
Theria - ps27
Placentalia - ps28
Boreoeutheria - ps29
Euarchontoglires - ps30
Euarchonta - ps31
Primates - ps32
Haplorrhini - ps33
Simiiformes - ps34
Catarrhini - ps35
Hominoidea - ps36
Hominidae - ps37
Homininae - ps38
Hominini - ps39
Homo_sapiens - ps40
p-value
2e-02
3e-02
4e-02
5e-02
6e-02
Log-odds
0.5
1.0
1.5
2.0
2.5
3.0
3.5
4.0

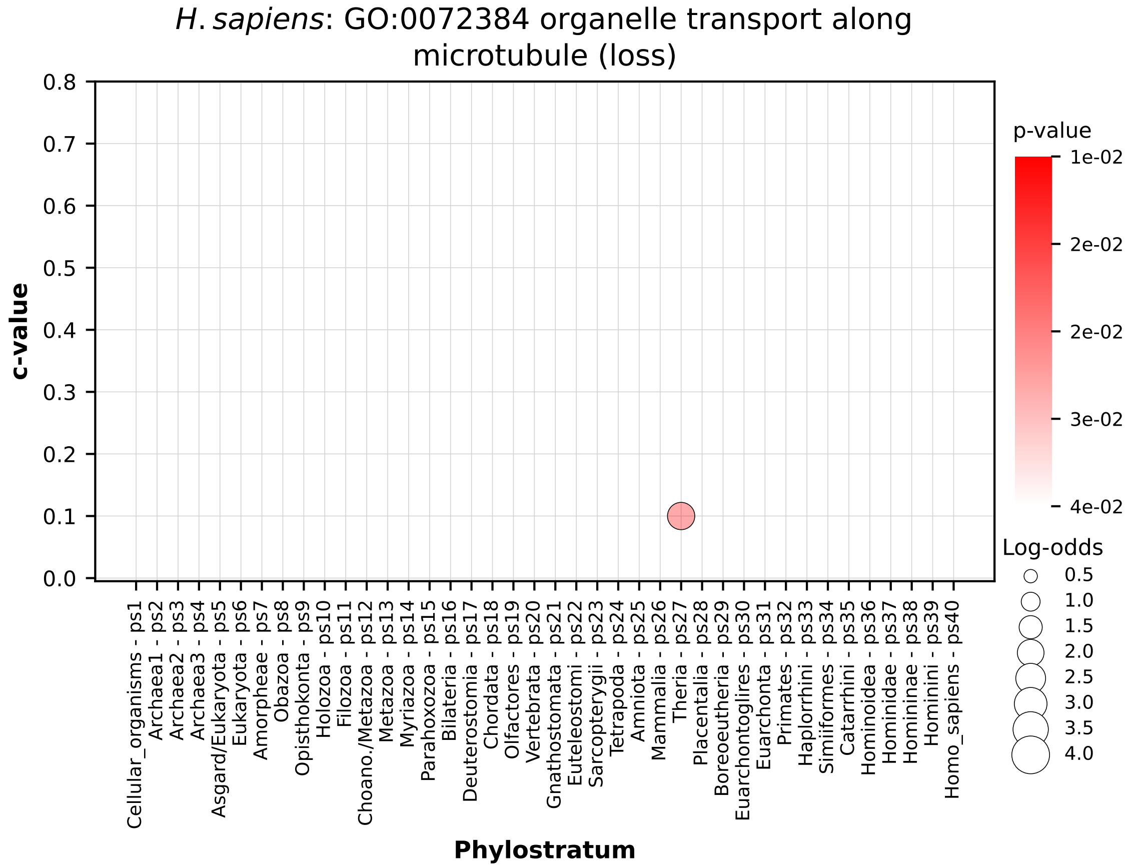

H. sapiens: GO:0072384 organelle transport along microtubule (loss)
c-value
0.0
0.1
0.2
0.3
0.4
0.5
0.6
0.7
0.8
Phylostratum
Cellular_organisms - ps1
Archaea1 - ps2
Archaea2 - ps3
Archaea3 - ps4
Asgard/Eukaryota - ps5
Eukaryota - ps6
Amorpheae - ps7
Obazoa - ps8
Opisthokonta - ps9
Holozoa - ps10
Filozoa - ps11
Choano./Metazoa - ps12
Metazoa - ps13
Myriazoa - ps14
Parahoxozoa - ps15
Bilateria - ps16
Deuterostomia - ps17
Chordata - ps18
Olfactores - ps19
Vertebrata - ps20
Gnathostomata - ps21
Euteleostomi - ps22
Sarcopterygii - ps23
Tetrapoda - ps24
Amniota - ps25
Mammalia - ps26
Theria - ps27
Placentalia - ps28
Boreoeutheria - ps29
Euarchontoglires - ps30
Euarchonta - ps31
Primates - ps32
Haplorrhini - ps33
Simiiformes - ps34
Catarrhini - ps35
Hominoidea - ps36
Hominidae - ps37
Homininae - ps38
Hominini - ps39
Homo_sapiens - ps40
p-value
1e-02
2e-02
2e-02
3e-02
4e-02
Log-odds
0.5
1.0
1.5
2.0
2.5
3.0
3.5
4.0

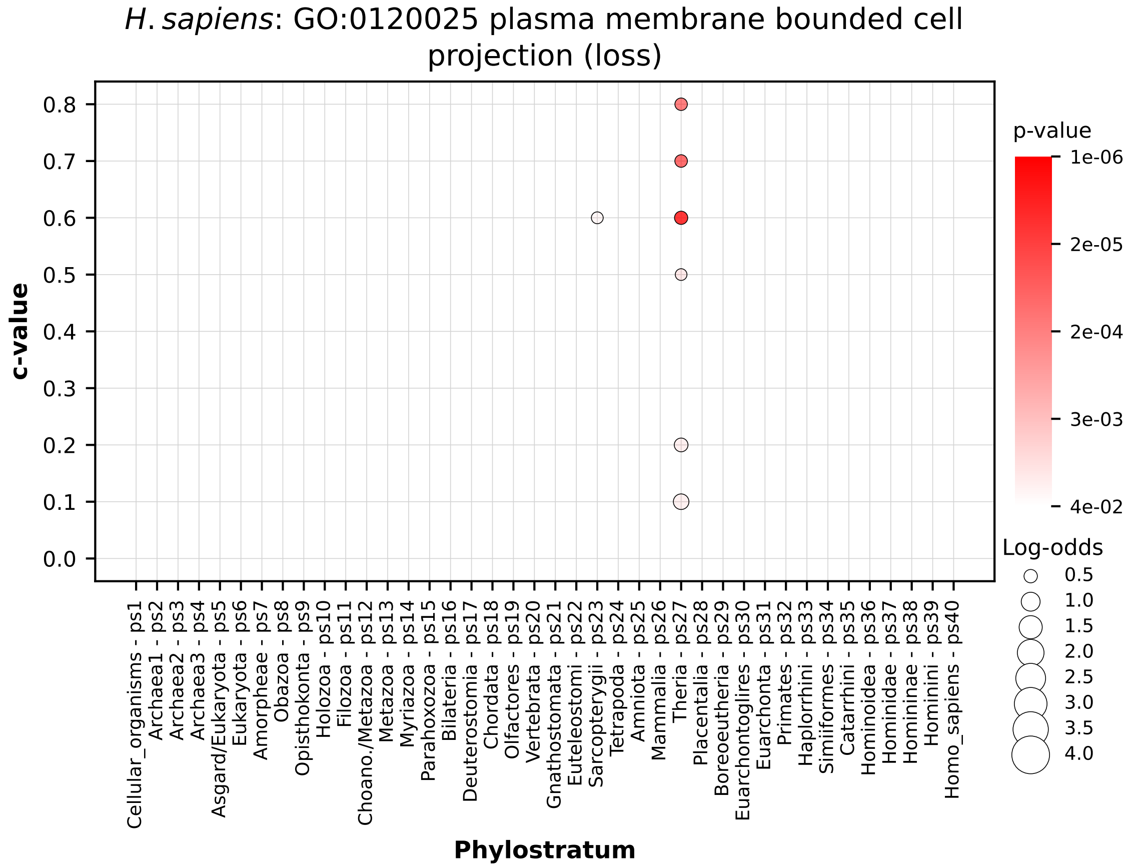
H. sapiens: GO:0120025 plasma membrane bounded cell projection (loss)
c-value
0.8
0.7
0.6
0.5
0.4
0.3
0.2
0.1
0.0
p-value
1e-06
2e-05
2e-04
3e-03
4e-02
Log-odds
0.5
1.0
1.5
2.0
2.5
3.0
3.5
4.0
Cellular_organisms - ps1
Archaea1 - ps2
Archaea2 - ps3
Archaea3 - ps4
Asgard/Eukaryota - ps5
Eukaryota - ps6
Amorpheae - ps7
Obazoa - ps8
Opisthokonta - ps9
Holozoa - ps10
Filozoa - ps11
Choano./Metazoa - ps12
Metazoa - ps13
Myriazoa - ps14
Parahoxozoa - ps15
Bilateria - ps16
Deuterostomia - ps17
Chordata - ps18
Olfactores - ps19
Vertebrata - ps20
Gnathostomata - ps21
Euteleostomi - ps22
Sarcopterygii - ps23
Tetrapoda - ps24
Amniota - ps25
Mammalia - ps26
Theria - ps27
Placentalia - ps28
Boreoeutheria - ps29
Euarchontoglires - ps30
Euarchonta - ps31
Primates - ps32
Haplorrhini - ps33
Simiiformes - ps34
Catarrhini - ps35
Hominoidea - ps36
Hominidae - ps37
Homininae - ps38
Hominini - ps39
Homo_sapiens - ps40
Phylostratum

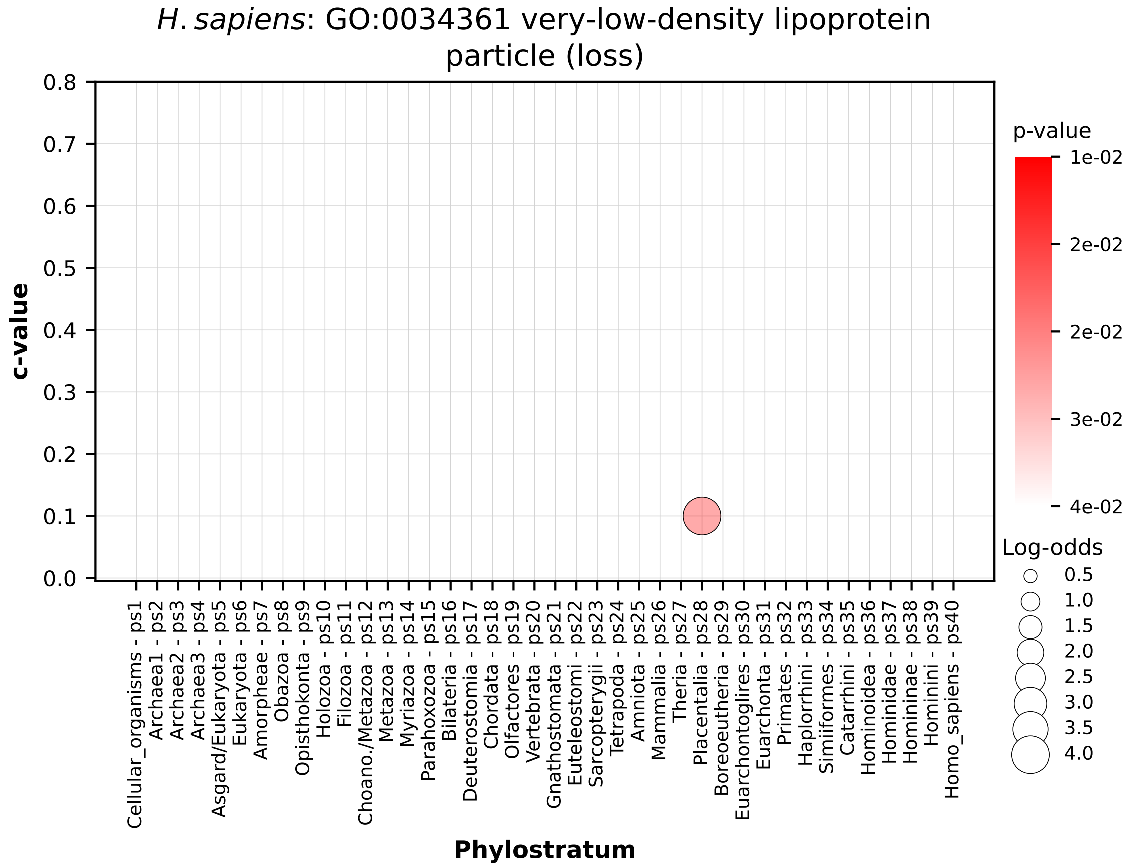
H. sapiens: GO:0034361 very-low-density lipoprotein particle (loss)
c-value
0.0
0.1
0.2
0.3
0.4
0.5
0.6
0.7
0.8
Phylostratum
Cellular_organisms - ps1
Archaea1 - ps2
Archaea2 - ps3
Archaea3 - ps4
Asgard/Eukaryota - ps5
Eukaryota - ps6
Amorpheae - ps7
Obazoa - ps8
Opisthokonta - ps9
Holozoa - ps10
Filozoa - ps11
Choano./Metazoa - ps12
Metazoa - ps13
Myriazoa - ps14
Parahoxozoa - ps15
Bilateria - ps16
Deuterostomia - ps17
Chordata - ps18
Olfactores - ps19
Vertebrata - ps20
Gnathostomata - ps21
Euteleostomi - ps22
Sarcopterygii - ps23
Tetrapoda - ps24
Amniota - ps25
Mammalia - ps26
Theria - ps27
Placentalia - ps28
Boreoeutheria - ps29
Euarchontoglires - ps30
Euarchonta - ps31
Primates - ps32
Haplorrhini - ps33
Simiiformes - ps34
Catarrhini - ps35
Hominoidea - ps36
Hominidae - ps37
Homininae - ps38
Hominini - ps39
Homo_sapiens - ps40
p-value
1e-02
2e-02
2e-02
3e-02
4e-02
Log-odds
0.5
1.0
1.5
2.0
2.5
3.0
3.5
4.0

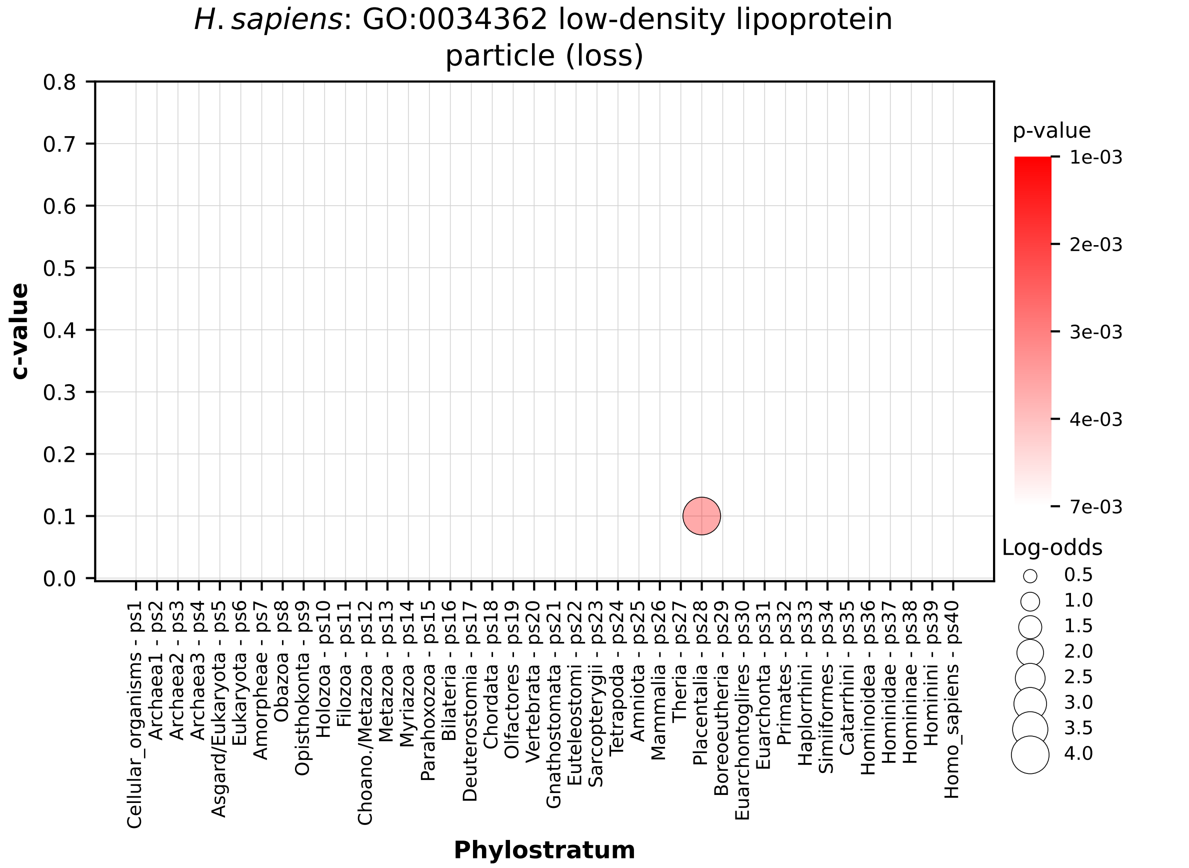
H. sapiens: GO:0034362 low-density lipoprotein particle (loss)
c-value
Phylostratum
p-value
1e-03
2e-03
3e-03
4e-03
7e-03
Log-odds
0.5
1.0
1.5
2.0
2.5
3.0
3.5
4.0
0.0
0.1
0.2
0.3
0.4
0.5
0.6
0.7
0.8
Cellular_organisms - ps1
Archaea1 - ps2
Archaea2 - ps3
Archaea3 - ps4
Asgard/Eukaryota - ps5
Eukaryota - ps6
Amorpheae - ps7
Obazoa - ps8
Opisthokonta - ps9
Holozoa - ps10
Filozoa - ps11
Choano./Metazoa - ps12
Metazoa - ps13
Myriazoa - ps14
Parahoxozoa - ps15
Bilateria - ps16
Deuterostomia - ps17
Chordata - ps18
Olfactores - ps19
Vertebrata - ps20
Gnathostomata - ps21
Euteleostomi - ps22
Sarcopterygii - ps23
Tetrapoda - ps24
Amniota - ps25
Mammalia - ps26
Theria - ps27
Placentalia - ps28
Boreoeutheria - ps29
Euarchontoglires - ps30
Euarchonta - ps31
Primates - ps32
Haplorrhini - ps33
Simiiformes - ps34
Catarrhini - ps35
Hominoidea - ps36
Hominidae - ps37
Homininae - ps38
Hominini - ps39
Homo_sapiens - ps40

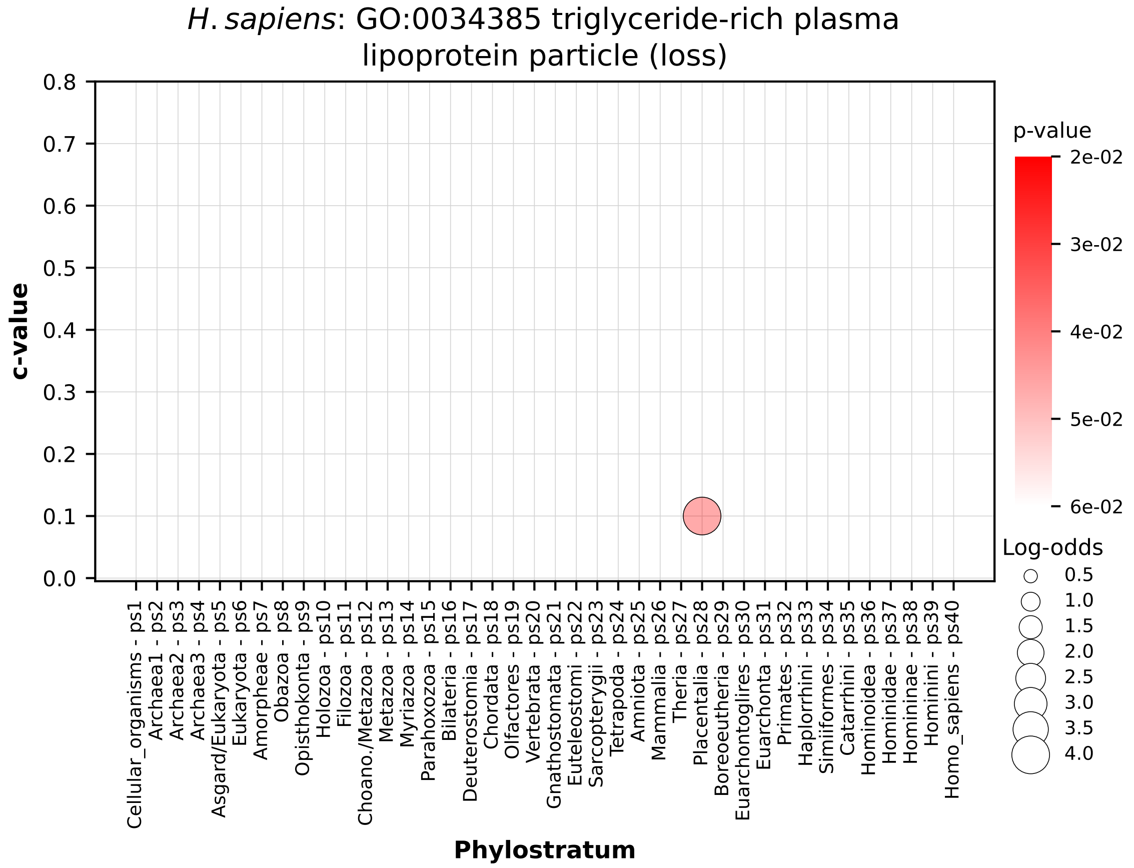

H. sapiens: GO:0034385 triglyceride-rich plasma lipoprotein particle (loss)
c-value
0.8
0.7
0.6
0.5
0.4
0.3
0.2
0.1
0.0
p-value
2e-02
3e-02
4e-02
5e-02
6e-02
Log-odds
0.5
1.0
1.5
2.0
2.5
3.0
3.5
4.0
Cellular_organisms - ps1
Archaea1 - ps2
Archaea2 - ps3
Archaea3 - ps4
Asgard/Eukaryota - ps5
Eukaryota - ps6
Amorpheae - ps7
Obazoa - ps8
Opisthokonta - ps9
Holozoa - ps10
Filozoa - ps11
Choano./Metazoa - ps12
Metazoa - ps13
Myriazoa - ps14
Parahoxozoa - ps15
Bilateria - ps16
Deuterostomia - ps17
Chordata - ps18
Olfactores - ps19
Vertebrata - ps20
Gnathostomata - ps21
Euteleostomi - ps22
Sarcopterygii - ps23
Tetrapoda - ps24
Amniota - ps25
Mammalia - ps26
Theria - ps27
Placentalia - ps28
Boreoeutheria - ps29
Euarchontoglires - ps30
Euarchonta - ps31
Primates - ps32
Haplorrhini - ps33
Simiiformes - ps34
Catarrhini - ps35
Hominoidea - ps36
Hominidae - ps37
Homininae - ps38
Hominini - ps39
Homo_sapiens - ps40
Phylostratum

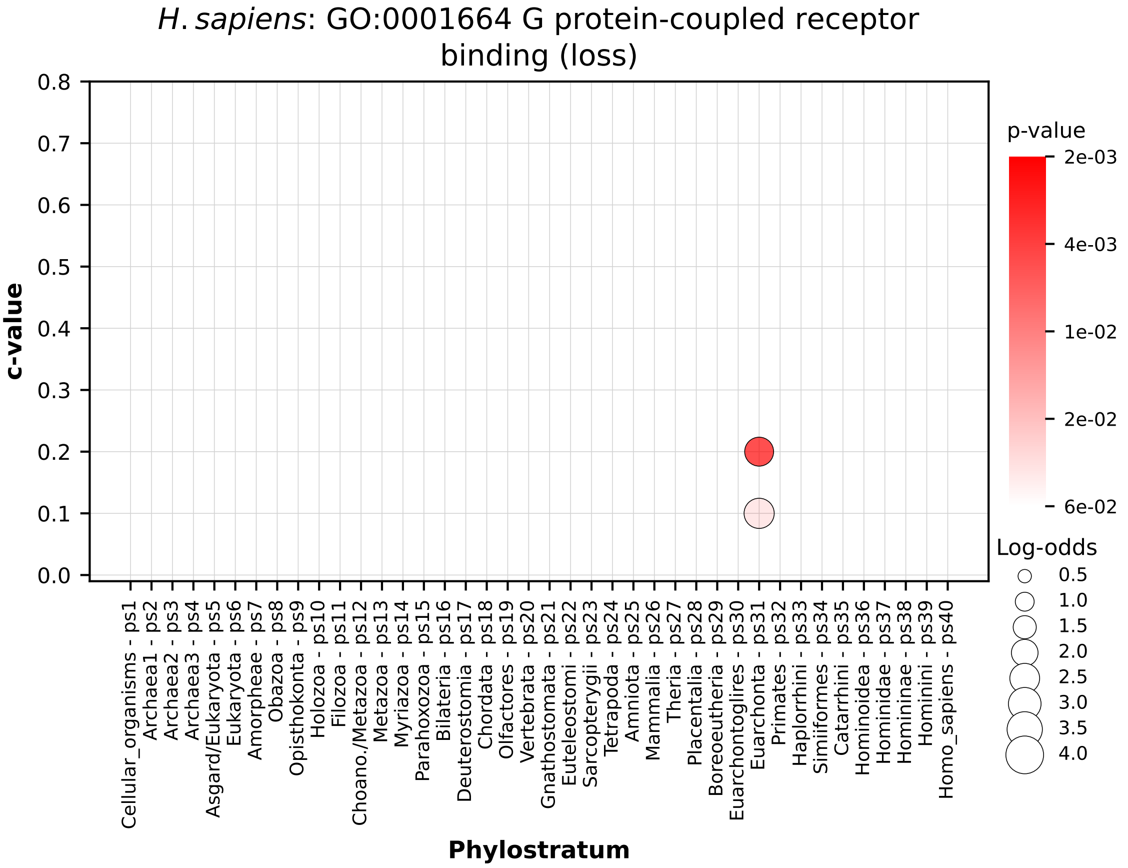
H. sapiens: GO:0001664 G protein-coupled receptor binding (loss)
c-value
0.0
0.1
0.2
0.3
0.4
0.5
0.6
0.7
0.8
Phylostratum
Cellular_organisms - ps1
Archaea1 - ps2
Archaea2 - ps3
Archaea3 - ps4
Asgard/Eukaryota - ps5
Eukaryota - ps6
Amorpheae - ps7
Obazoa - ps8
Opisthokonta - ps9
Holozoa - ps10
Filozoa - ps11
Choano./Metazoa - ps12
Metazoa - ps13
Myriazoa - ps14
Parahoxozoa - ps15
Bilateria - ps16
Deuterostomia - ps17
Chordata - ps18
Olfactores - ps19
Vertebrata - ps20
Gnathostomata - ps21
Euteleostomi - ps22
Sarcopterygii - ps23
Tetrapoda - ps24
Amniota - ps25
Mammalia - ps26
Theria - ps27
Placentalia - ps28
Boreoeutheria - ps29
Euarchontoglires - ps30
Euarchonta - ps31
Primates - ps32
Haplorrhini - ps33
Simiiformes - ps34
Catarrhini - ps35
Hominoidea - ps36
Hominidae - ps37
Homininae - ps38
Hominini - ps39
Homo_sapiens - ps40
p-value
2e-03
4e-03
1e-02
2e-02
6e-02
Log-odds
0.5
1.0
1.5
2.0
2.5
3.0
3.5
4.0

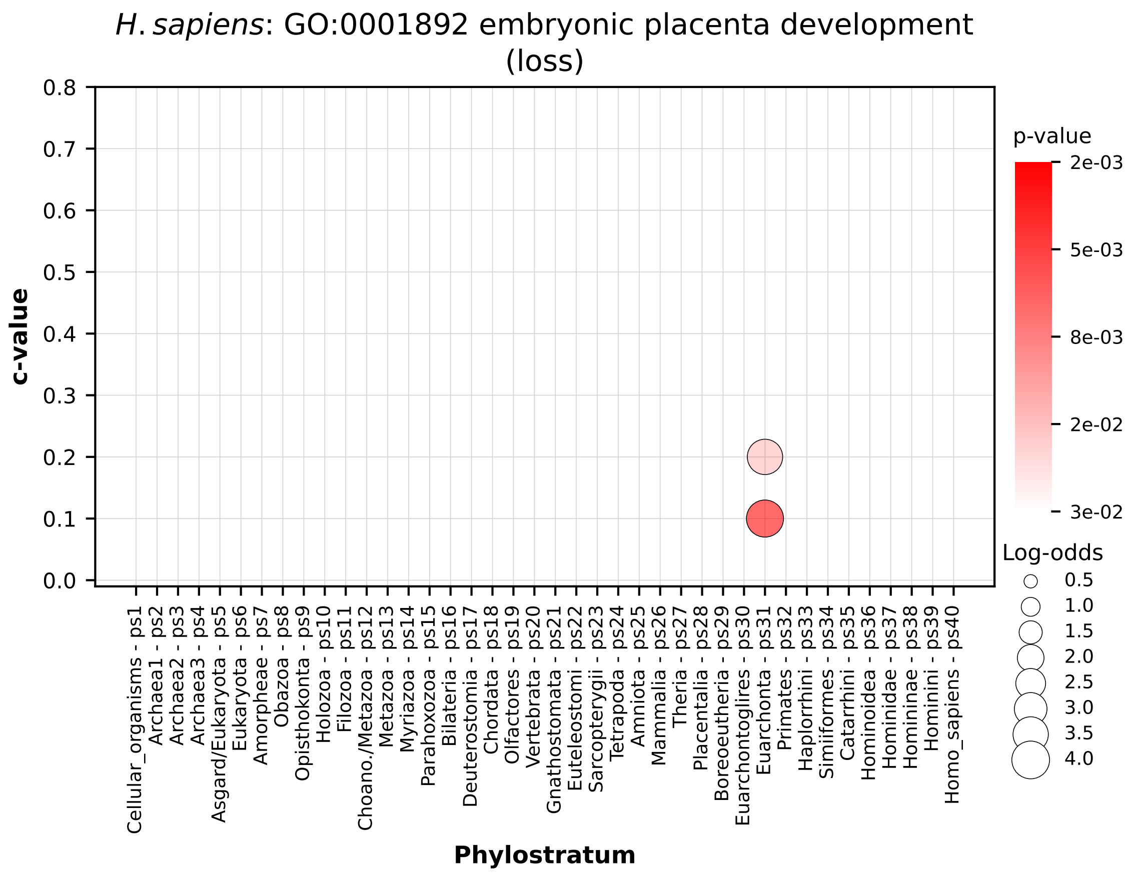

H. sapiens: GO:0001892 embryonic placenta development (loss)
c-value
0.0
0.1
0.2
0.3
0.4
0.5
0.6
0.7
0.8
Phylostratum
Cellular_organisms - ps1
Archaea1 - ps2
Archaea2 - ps3
Archaea3 - ps4
Asgard/Eukaryota - ps5
Eukaryota - ps6
Amorpheae - ps7
Obazoa - ps8
Opisthokonta - ps9
Holozoa - ps10
Filozoa - ps11
Choano./Metazoa - ps12
Metazoa - ps13
Myriazoa - ps14
Parahoxozoa - ps15
Bilateria - ps16
Deuterostomia - ps17
Chordata - ps18
Olfactores - ps19
Vertebrata - ps20
Gnathostomata - ps21
Euteleostomi - ps22
Sarcopterygii - ps23
Tetrapoda - ps24
Amniota - ps25
Mammalia - ps26
Theria - ps27
Placentalia - ps28
Boreoeutheria - ps29
Euarchontoglires - ps30
Euarchonta - ps31
Primates - ps32
Haplorrhini - ps33
Simiiformes - ps34
Catarrhini - ps35
Hominoidea - ps36
Hominidae - ps37
Homininae - ps38
Hominini - ps39
Homo_sapiens - ps40
p-value
2e-03
5e-03
8e-03
2e-02
3e-02
Log-odds
0.5
1.0
1.5
2.0
2.5
3.0
3.5
4.0

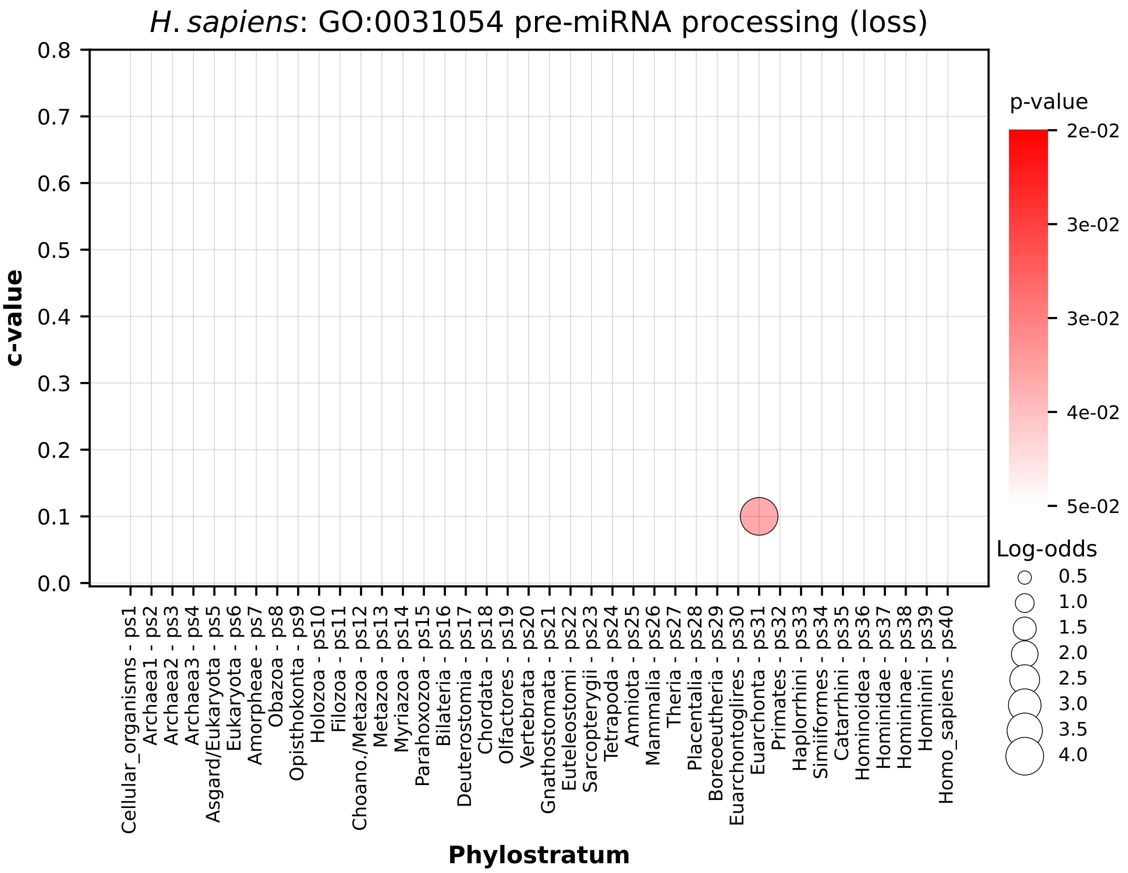

H. sapiens: GO:0031054 pre-miRNA processing (loss)
c-value
0.0
0.1
0.2
0.3
0.4
0.5
0.6
0.7
0.8
p-value
2e-02
3e-02
3e-02
4e-02
5e-02
Log-odds
0.5
1.0
1.5
2.0
2.5
3.0
3.5
4.0
Cellular_organisms - ps1
Archaea1 - ps2
Archaea2 - ps3
Archaea3 - ps4
Asgard/Eukaryota - ps5
Eukaryota - ps6
Amorpheae - ps7
Obazoa - ps8
Opisthokonta - ps9
Holozoa - ps10
Filozoa - ps11
Choano./Metazoa - ps12
Metazoa - ps13
Myriazoa - ps14
Parahoxozoa - ps15
Bilateria - ps16
Deuterostomia - ps17
Chordata - ps18
Olfactores - ps19
Vertebrata - ps20
Gnathostomata - ps21
Euteleostomi - ps22
Sarcopterygii - ps23
Tetrapoda - ps24
Amniota - ps25
Mammalia - ps26
Theria - ps27
Placentalia - ps28
Boreoeutheria - ps29
Euarchontoglires - ps30
Euarchonta - ps31
Primates - ps32
Haplorrhini - ps33
Simiiformes - ps34
Catarrhini - ps35
Hominoidea - ps36
Hominidae - ps37
Homininae - ps38
Hominini - ps39
Homo_sapiens - ps40
Phylostratum

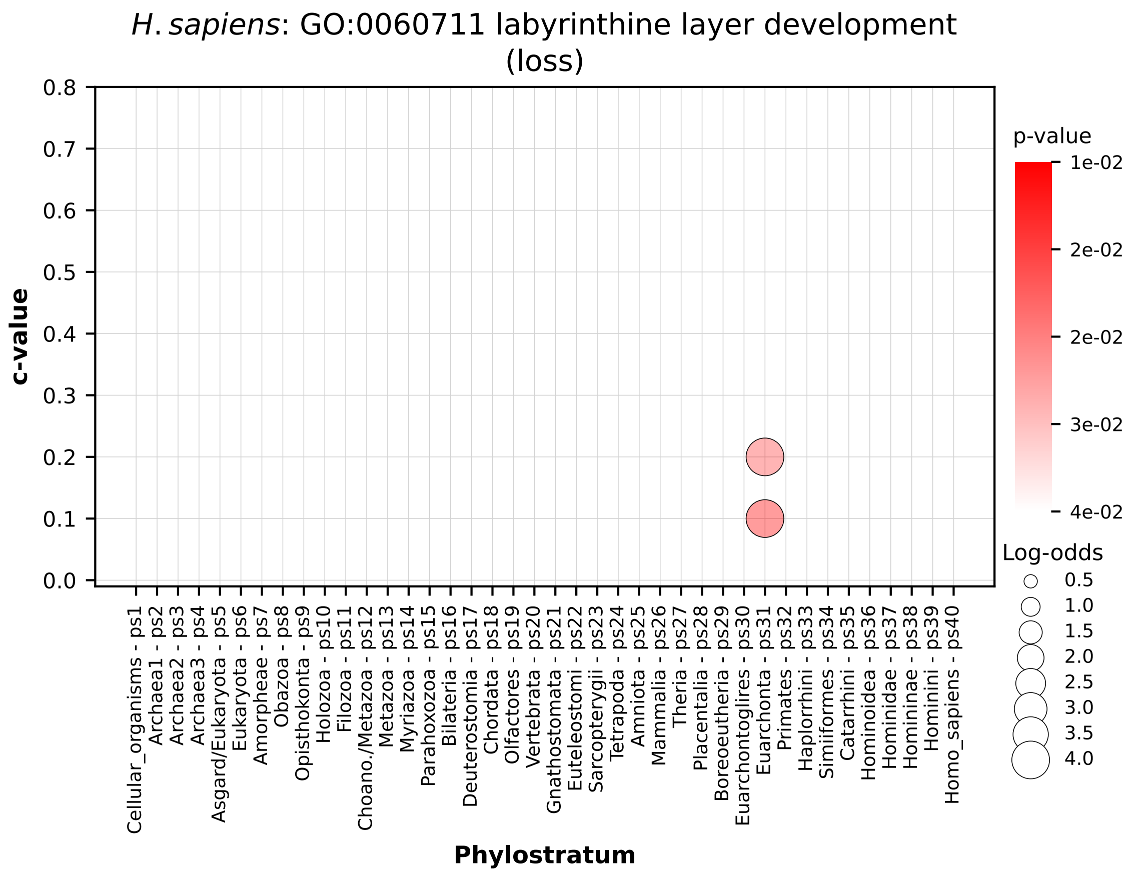

H. sapiens: GO:0060711 labyrinthine layer development
(loss)
c-value
0.8
0.7
0.6
0.5
0.4
0.3
0.2
0.1
0.0
p-value
1e-02
2e-02
2e-02
3e-02
4e-02
Log-odds
0.5
1.0
1.5
2.0
2.5
3.0
3.5
4.0
Cellular_organisms - ps1
Archaea1 - ps2
Archaea2 - ps3
Archaea3 - ps4
Asgard/Eukaryota - ps5
Eukaryota - ps6
Amorpheae - ps7
Obazoa - ps8
Opisthokonta - ps9
Holozoa - ps10
Filozoa - ps11
Choano./Metazoa - ps12
Metazoa - ps13
Myriazoa - ps14
Parahoxozoa - ps15
Bilateria - ps16
Deuterostomia - ps17
Chordata - ps18
Olfactores - ps19
Vertebrata - ps20
Gnathostomata - ps21
Euteleostomi - ps22
Sarcopterygii - ps23
Tetrapoda - ps24
Amniota - ps25
Mammalia - ps26
Theria - ps27
Placentalia - ps28
Boreoeutheria - ps29
Euarchontoglires - ps30
Euarchonta - ps31
Primates - ps32
Haplorrhini - ps33
Simiiformes - ps34
Catarrhini - ps35
Hominoidea - ps36
Hominidae - ps37
Homininae - ps38
Hominini - ps39
Homo_sapiens - ps40
Phylostratum

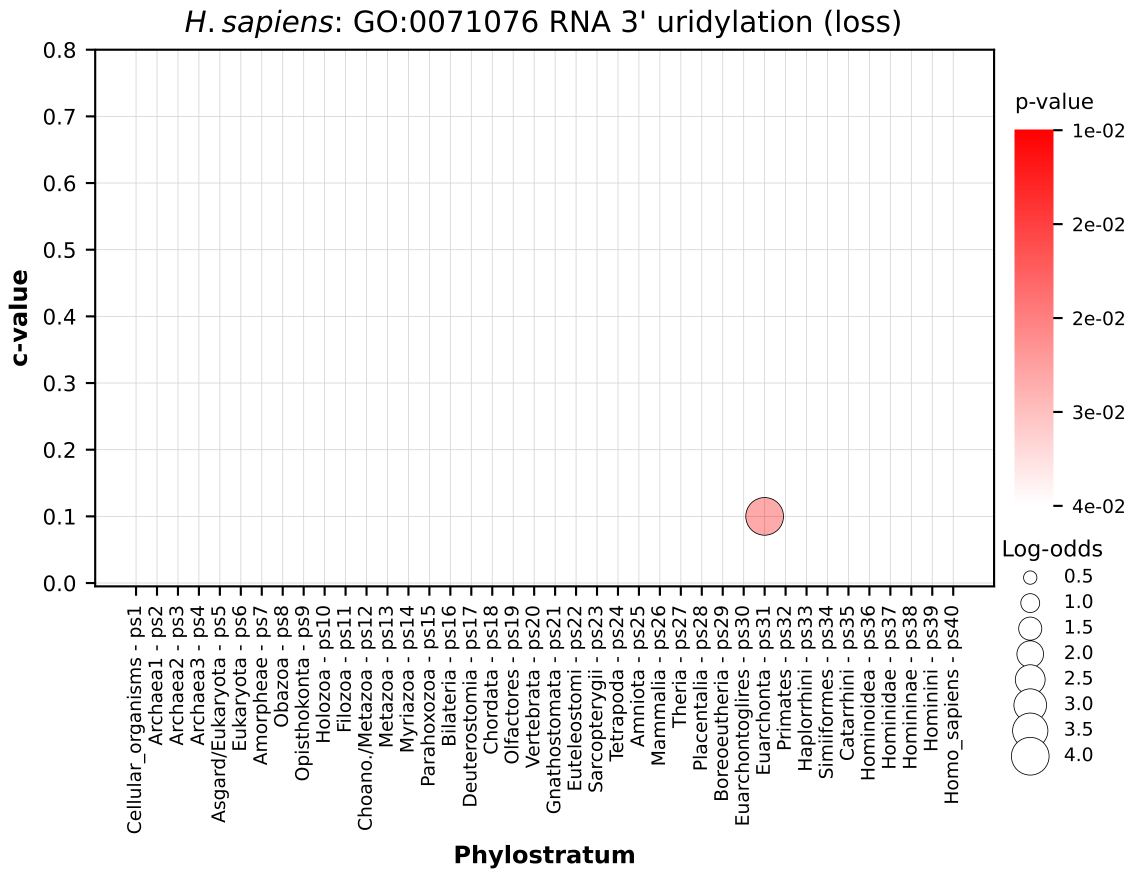

H. sapiens: GO:0071076 RNA 3' uridylation (loss)
c-value
0.8
0.7
0.6
0.5
0.4
0.3
0.2
0.1
0.0
Phylostratum
Cellular_organisms - ps1
Archaea1 - ps2
Archaea2 - ps3
Archaea3 - ps4
Asgard/Eukaryota - ps5
Eukaryota - ps6
Amorpheae - ps7
Obazoa - ps8
Opisthokonta - ps9
Holozoa - ps10
Filozoa - ps11
Choano./Metazoa - ps12
Metazoa - ps13
Myriazoa - ps14
Parahoxozoa - ps15
Bilateria - ps16
Deuterostomia - ps17
Chordata - ps18
Olfactores - ps19
Vertebrata - ps20
Gnathostomata - ps21
Euteleostomi - ps22
Sarcopterygii - ps23
Tetrapoda - ps24
Amniota - ps25
Mammalia - ps26
Theria - ps27
Placentalia - ps28
Boreoeutheria - ps29
Euarchontoglires - ps30
Euarchonta - ps31
Primates - ps32
Haplorrhini - ps33
Similformes - ps34
Catarrhini - ps35
Hominoidea - ps36
Hominidae - ps37
Homininae - ps38
Hominini - ps39
Homo_sapiens - ps40
p-value
1e-02
2e-02
2e-02
3e-02
4e-02
Log-odds
0.5
1.0
1.5
2.0
2.5
3.0
3.5
4.0

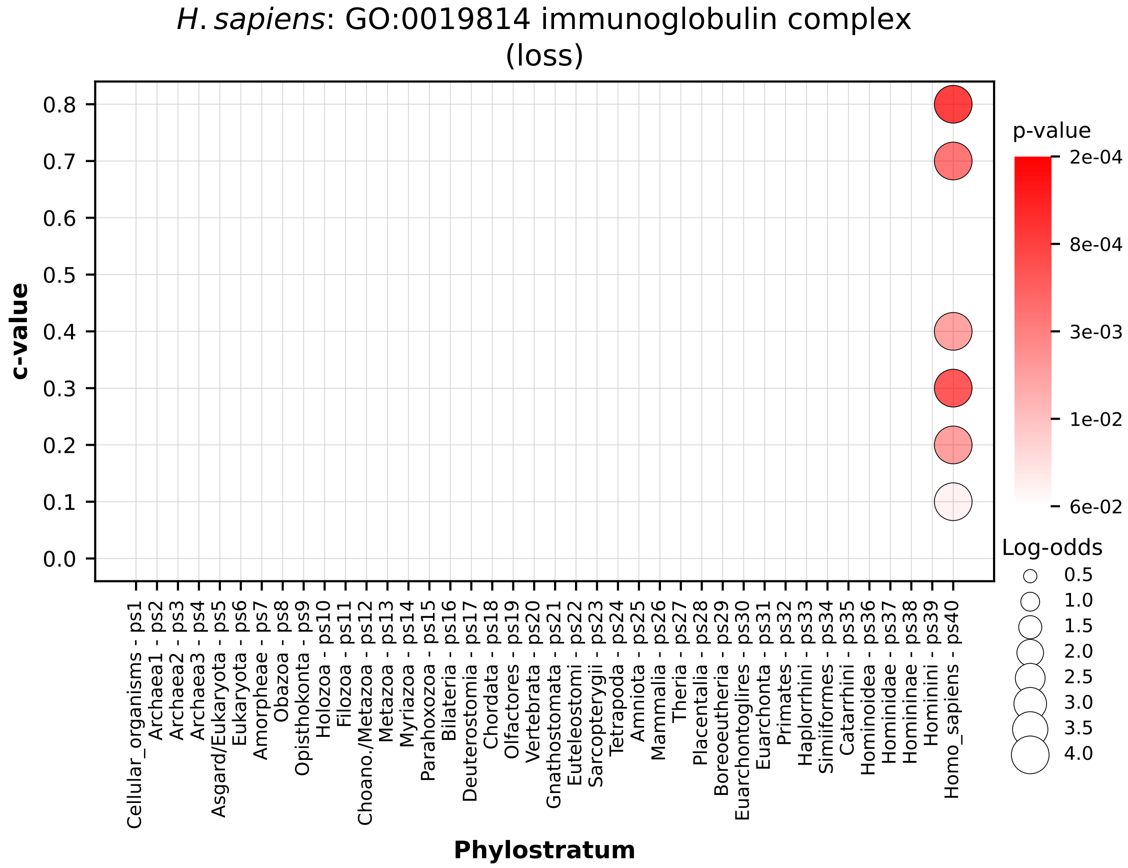

H. sapiens: GO:0019814 immunoglobulin complex
(loss)
c-value
0.8
0.7
0.6
0.5
0.4
0.3
0.2
0.1
0.0
p-value
2e-04
8e-04
3e-03
1e-02
6e-02
Log-odds
0.5
1.0
1.5
2.0
2.5
3.0
3.5
4.0
Cellular_organisms - ps1
Archaea1 - ps2
Archaea2 - ps3
Archaea3 - ps4
Asgard/Eukaryota - ps5
Eukaryota - ps6
Amorpheae - ps7
Obazoa - ps8
Opisthokonta - ps9
Holozoa - ps10
Filozoa - ps11
Choano./Metazoa - ps12
Metazoa - ps13
Myriazoa - ps14
Parahoxozoa - ps15
Bilateria - ps16
Deuterostomia - ps17
Chordata - ps18
Olfactores - ps19
Vertebrata - ps20
Gnathostomata - ps21
Euteleostomi - ps22
Sarcopterygii - ps23
Tetrapoda - ps24
Amniota - ps25
Mammalia - ps26
Theria - ps27
Placentalia - ps28
Boreoeutheria - ps29
Euarchontoglires - ps30
Euarchonta - ps31
Primates - ps32
Haplorrhini - ps33
Simiiformes - ps34
Catarrhini - ps35
Hominoidea - ps36
Hominidae - ps37
Homininae - ps38
Hominini - ps39
Homo_sapiens - ps40
Phylostratum

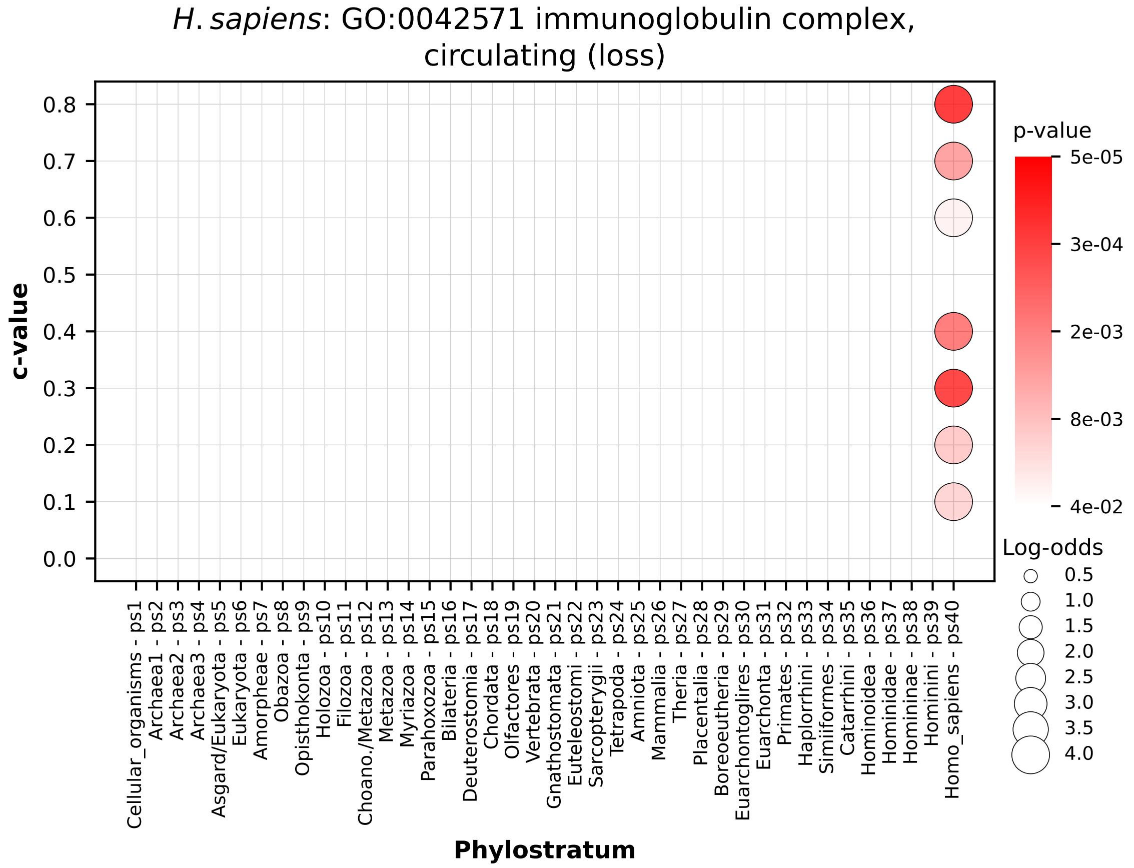

H. sapiens: GO:0042571 immunoglobulin complex, circulating (loss)
c-value
0.0
0.1
0.2
0.3
0.4
0.5
0.6
0.7
0.8
Phylostratum
Cellular_organisms - ps1
Archaea1 - ps2
Archaea2 - ps3
Archaea3 - ps4
Asgard/Eukaryota - ps5
Eukaryota - ps6
Amorpheae - ps7
Obazoa - ps8
Opisthokonta - ps9
Holozoa - ps10
Filozoa - ps11
Choano./Metazoa - ps12
Metazoa - ps13
Myriazoa - ps14
Parahoxozoa - ps15
Bilateria - ps16
Deuterostomia - ps17
Chordata - ps18
Olfactores - ps19
Vertebrata - ps20
Gnathostomata - ps21
Euteleostomi - ps22
Sarcopterygii - ps23
Tetrapoda - ps24
Amniota - ps25
Mammalia - ps26
Theria - ps27
Placentalia - ps28
Boreoeutheria - ps29
Euarchontoglires - ps30
Euarchonta - ps31
Primates - ps32
Haplorrhini - ps33
Simiiformes - ps34
Catarrhini - ps35
Hominoidea - ps36
Hominidae - ps37
Homininae - ps38
Hominini - ps39
Homo_sapiens - ps40
p-value
5e-05
3e-04
2e-03
8e-03
4e-02
Log-odds
0.5
1.0
1.5
2.0
2.5
3.0
3.5
4.0

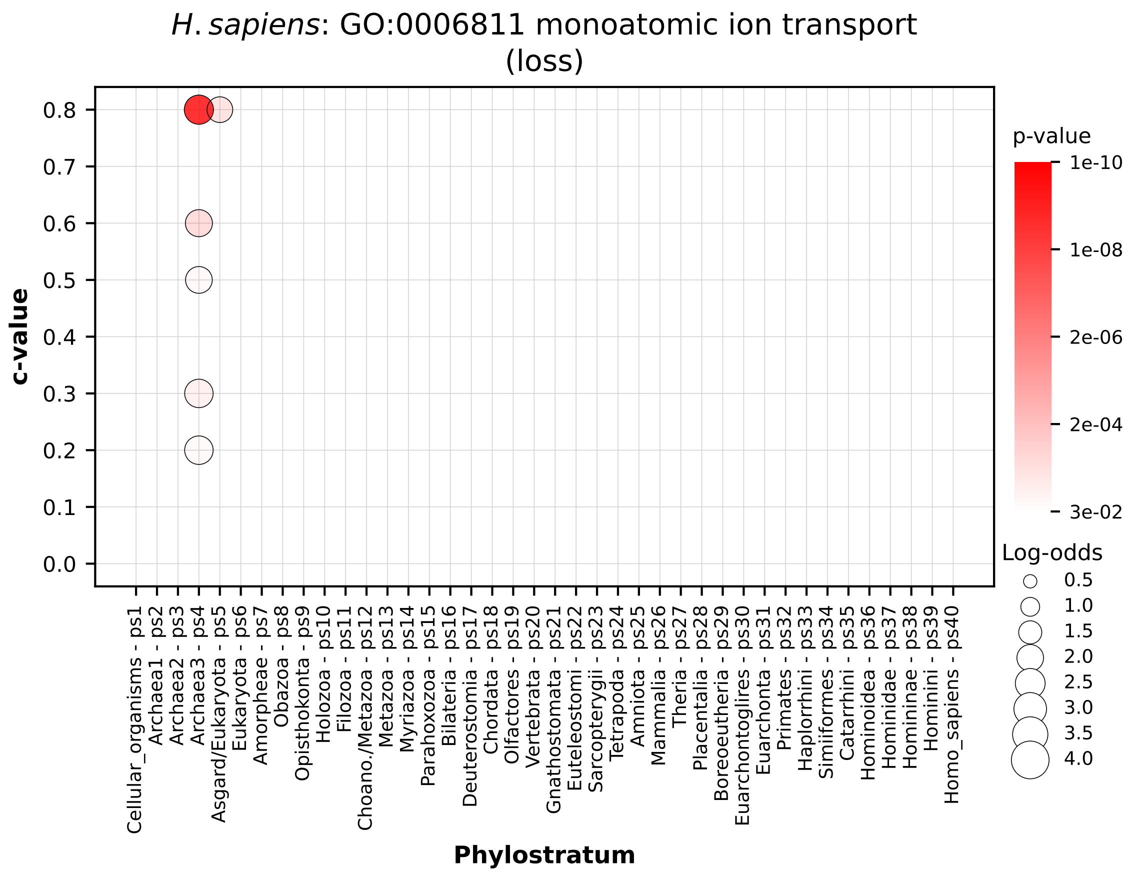

H. sapiens: GO:0006811 monoatomic ion transport
(loss)
c-value
0.0
0.1
0.2
0.3
0.4
0.5
0.6
0.7
0.8
Phylostratum
Cellular_organisms - ps1
Archaea1 - ps2
Archaea2 - ps3
Archaea3 - ps4
Asgard/Eukaryota - ps5
Eukaryota - ps6
Amorpheae - ps7
Obazoa - ps8
Opisthokonta - ps9
Holozoa - ps10
Filozoa - ps11
Choano./Metazoa - ps12
Metazoa - ps13
Myriazoa - ps14
Parahoxozoa - ps15
Bilateria - ps16
Deuterostomia - ps17
Chordata - ps18
Olfactores - ps19
Vertebrata - ps20
Gnathostomata - ps21
Euteleostomi - ps22
Sarcopterygii - ps23
Tetrapoda - ps24
Amniota - ps25
Mammalia - ps26
Theria - ps27
Placentalia - ps28
Boreoeutheria - ps29
Euarchontoglires - ps30
Euarchonta - ps31
Primates - ps32
Haplorrhini - ps33
Simiiformes - ps34
Catarrhini - ps35
Hominoidea - ps36
Hominidae - ps37
Homininae - ps38
Hominini - ps39
Homo_sapiens - ps40
p-value
1e-10
1e-08
2e-06
2e-04
3e-02
Log-odds
0.5
1.0
1.5
2.0
2.5
3.0
3.5
4.0

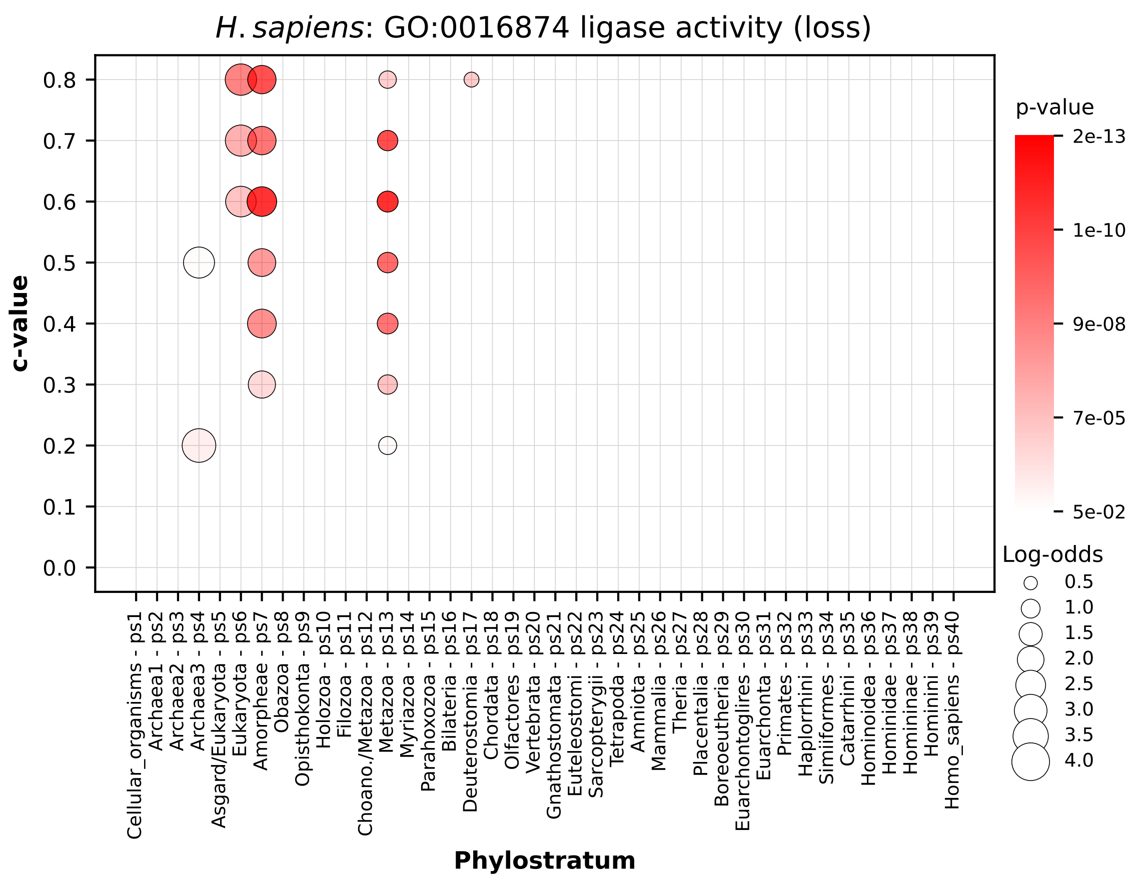
H. sapiens: GO:0016874 ligase activity (loss)
c-value
0.8
0.7
0.6
0.5
0.4
0.3
0.2
0.1
0.0
p-value
2e-13
1e-10
9e-08
7e-05
5e-02
Log-odds
0.5
1.0
1.5
2.0
2.5
3.0
3.5
4.0
Cellular_organisms - ps1
Archaea1 - ps2
Archaea2 - ps3
Archaea3 - ps4
Asgard/Eukaryota - ps5
Eukaryota - ps6
Amorpheae - ps7
Obazoa - ps8
Opisthokonta - ps9
Holozoa - ps10
Filozoa - ps11
Choano./Metazoa - ps12
Metazoa - ps13
Myriazoa - ps14
Parahoxozoa - ps15
Bilateria - ps16
Deuterostomia - ps17
Chordata - ps18
Olfactores - ps19
Vertebrata - ps20
Gnathostomata - ps21
Euteleostomi - ps22
Sarcopterygii - ps23
Tetrapoda - ps24
Amniota - ps25
Mammalia - ps26
Theria - ps27
Placentalia - ps28
Boreoeutheria - ps29
Euarchontoglires - ps30
Euarchonta - ps31
Primates - ps32
Haplorrhini - ps33
Simiiformes - ps34
Catarrhini - ps35
Hominoidea - ps36
Hominidae - ps37
Homininae - ps38
Hominini - ps39
Homo_sapiens - ps40
Phylostratum

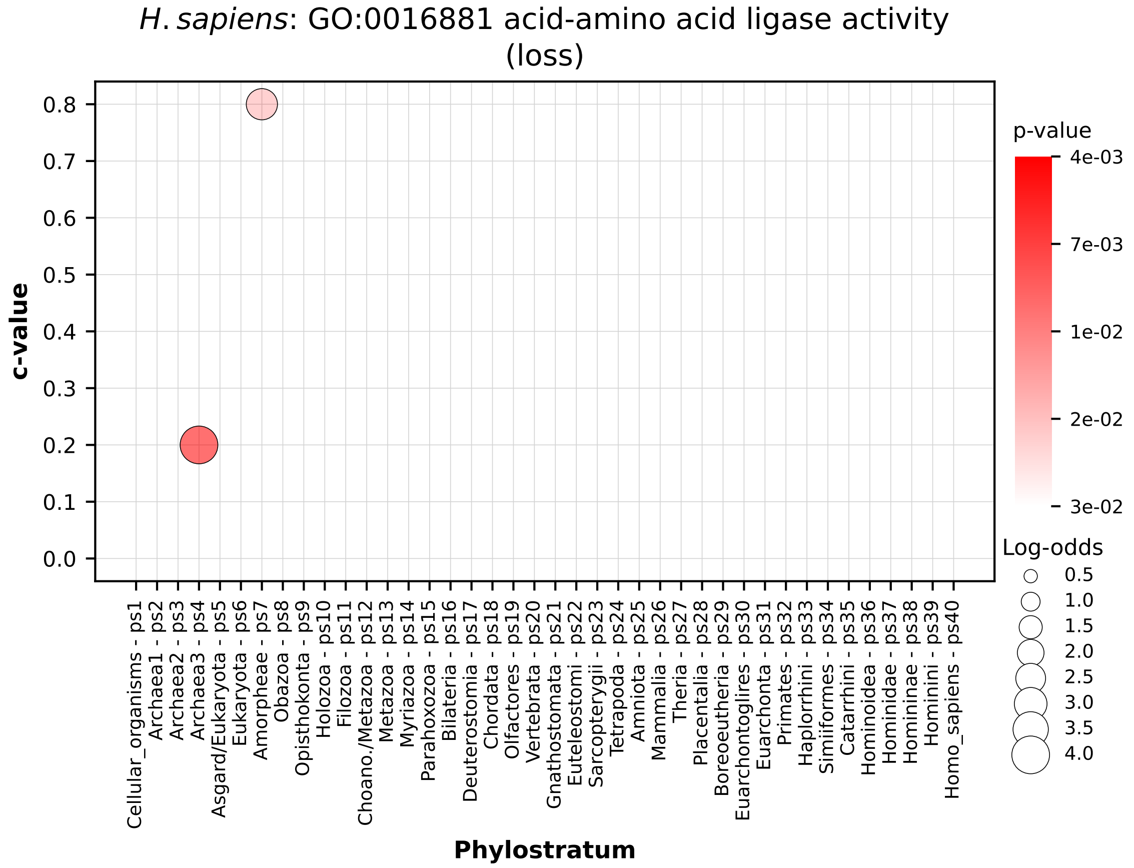

H. sapiens: GO:0016881 acid-amino acid ligase activity (loss)
c-value
0.8
0.7
0.6
0.5
0.4
0.3
0.2
0.1
0.0
p-value
4e-03
7e-03
1e-02
2e-02
3e-02
Log-odds
0.5
1.0
1.5
2.0
2.5
3.0
3.5
4.0
Cellular_organisms - ps1
Archaea1 - ps2
Archaea2 - ps3
Archaea3 - ps4
Asgard/Eukaryota - ps5
Eukaryota - ps6
Amorpheae - ps7
Obazoa - ps8
Opisthokonta - ps9
Holozoa - ps10
Filozoa - ps11
Choano./Metazoa - ps12
Metazoa - ps13
Myriazoa - ps14
Parahoxozoa - ps15
Bilateria - ps16
Deuterostomia - ps17
Chordata - ps18
Olfactores - ps19
Vertebrata - ps20
Gnathostomata - ps21
Euteleostomi - ps22
Sarcopterygii - ps23
Tetrapoda - ps24
Amniota - ps25
Mammalia - ps26
Theria - ps27
Placentalia - ps28
Boreoeutheria - ps29
Euarchontoglires - ps30
Euarchonta - ps31
Primates - ps32
Haplorrhini - ps33
Simiiformes - ps34
Catarrhini - ps35
Hominoidea - ps36
Hominidae - ps37
Homininae - ps38
Hominini - ps39
Homo_sapiens - ps40
Phylostratum

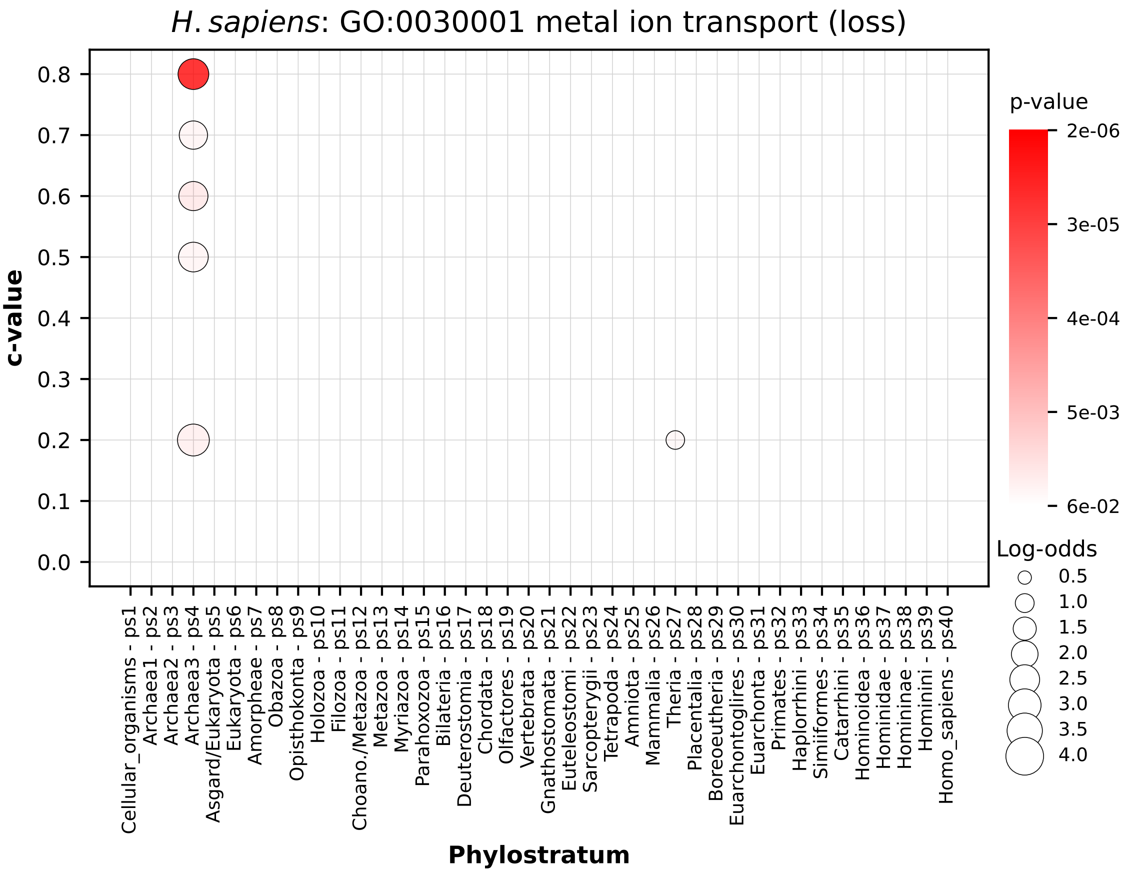
H. sapiens: GO:0030001 metal ion transport (loss)
c-value
0.8
0.7
0.6
0.5
0.4
0.3
0.2
0.1
0.0
p-value
2e-06
3e-05
4e-04
5e-03
6e-02
Log-odds
0.5
1.0
1.5
2.0
2.5
3.0
3.5
4.0
Cellular_organisms - ps1
Archaea1 - ps2
Archaea2 - ps3
Archaea3 - ps4
Asgard/Eukaryota - ps5
Eukaryota - ps6
Amorpheae - ps7
Obazoa - ps8
Opisthokonta - ps9
Holozoa - ps10
Filozoa - ps11
Choano./Metazoa - ps12
Metazoa - ps13
Myriazoa - ps14
Parahoxozoa - ps15
Bilateria - ps16
Deuterostomia - ps17
Chordata - ps18
Olfactores - ps19
Vertebrata - ps20
Gnathostomata - ps21
Euteleostomi - ps22
Sarcopterygii - ps23
Tetrapoda - ps24
Amniota - ps25
Mammalia - ps26
Theria - ps27
Placentalia - ps28
Boreoeutheria - ps29
Euarchontoglires - ps30
Euarchonta - ps31
Primates - ps32
Haplorrhini - ps33
Simiiformes - ps34
Catarrhini - ps35
Hominoidea - ps36
Hominidae - ps37
Homininae - ps38
Hominini - ps39
Homo_sapiens - ps40
Phylostratum

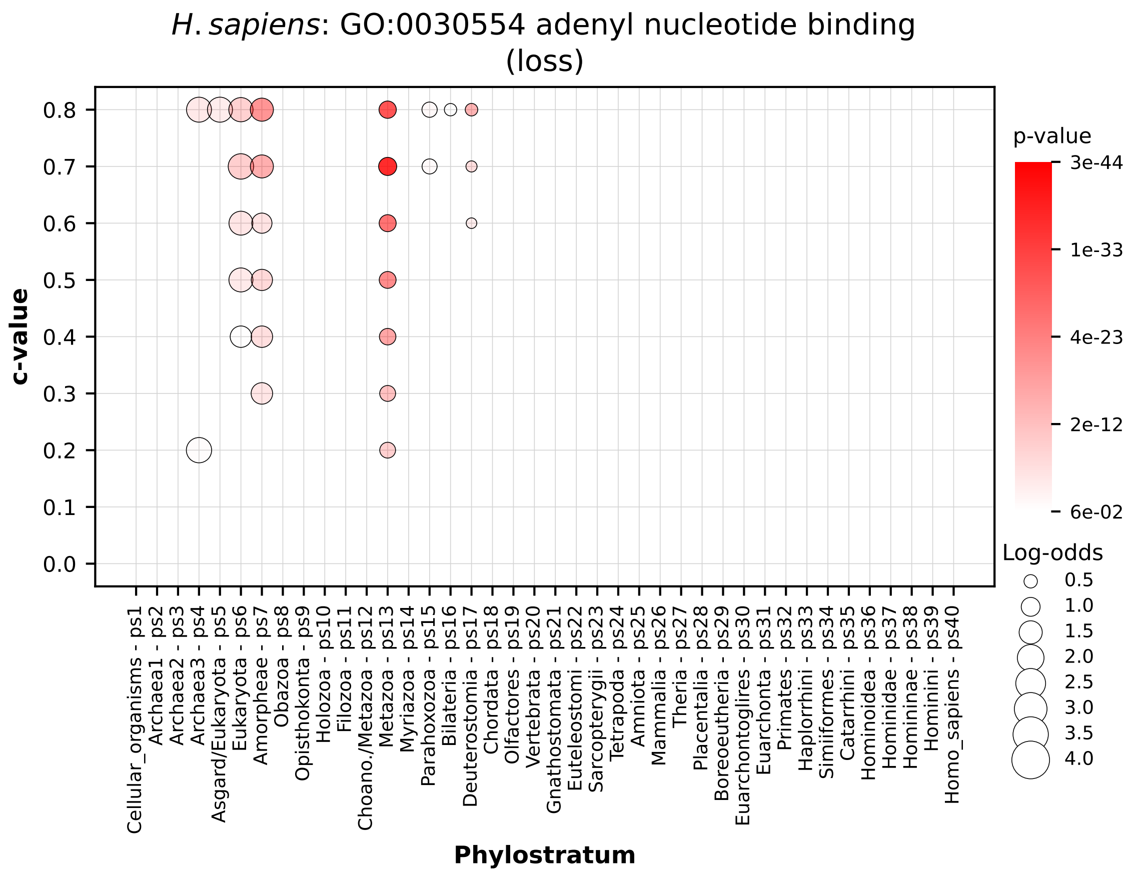
H. sapiens: GO:0030554 adenyl nucleotide binding
(loss)
c-value
0.8
0.7
0.6
0.5
0.4
0.3
0.2
0.1
0.0
p-value
3e-44
1e-33
4e-23
2e-12
6e-02
Log-odds
0.5
1.0
1.5
2.0
2.5
3.0
3.5
4.0
Cellular_organisms - ps1
Archaea1 - ps2
Archaea2 - ps3
Archaea3 - ps4
Asgard/Eukaryota - ps5
Eukaryota - ps6
Amorpheae - ps7
Obazoa - ps8
Opisthokonta - ps9
Holozoa - ps10
Filozoa - ps11
Choano./Metazoa - ps12
Metazoa - ps13
Myriazoa - ps14
Parahoxozoa - ps15
Bilateria - ps16
Deuterostomia - ps17
Chordata - ps18
Olfactores - ps19
Vertebrata - ps20
Gnathostomata - ps21
Euteleostomi - ps22
Sarcopterygii - ps23
Tetrapoda - ps24
Amniota - ps25
Mammalia - ps26
Theria - ps27
Placentalia - ps28
Boreoeutheria - ps29
Euarchontoglires - ps30
Euarchonta - ps31
Primates - ps32
Haplorrhini - ps33
Simiiformes - ps34
Catarrhini - ps35
Hominoidea - ps36
Hominidae - ps37
Homininae - ps38
Hominini - ps39
Homo_sapiens - ps40
Phylostratum

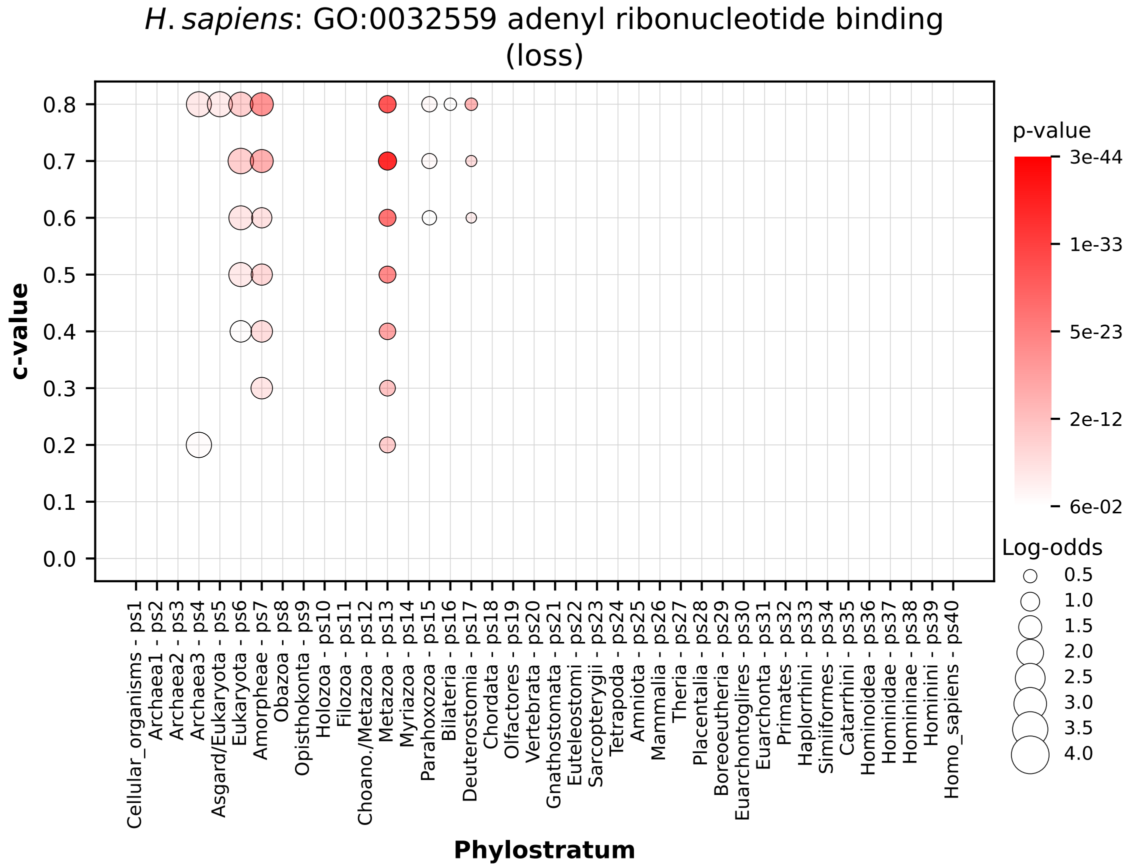

H. sapiens: GO:0032559 adenyl ribonucleotide binding
(loss)
c-value
0.8
0.7
0.6
0.5
0.4
0.3
0.2
0.1
0.0
p-value
3e-44
1e-33
5e-23
2e-12
6e-02
Log-odds
0.5
1.0
1.5
2.0
2.5
3.0
3.5
4.0
Cellular_organisms - ps1
Archaea1 - ps2
Archaea2 - ps3
Archaea3 - ps4
Asgard/Eukaryota - ps5
Eukaryota - ps6
Amorpheae - ps7
Obazoa - ps8
Opisthokonta - ps9
Holozoa - ps10
Filozoa - ps11
Choano./Metazoa - ps12
Metazoa - ps13
Myriazoa - ps14
Parahoxozoa - ps15
Bilateria - ps16
Deuterostomia - ps17
Chordata - ps18
Olfactores - ps19
Vertebrata - ps20
Gnathostomata - ps21
Euteleostomi - ps22
Sarcopterygii - ps23
Tetrapoda - ps24
Amniota - ps25
Mammalia - ps26
Theria - ps27
Placentalia - ps28
Boreoeutheria - ps29
Euarchontoglires - ps30
Euarchonta - ps31
Primates - ps32
Haplorrhini - ps33
Simiiformes - ps34
Catarrhini - ps35
Hominoidea - ps36
Hominidae - ps37
Homininae - ps38
Hominini - ps39
Homo_sapiens - ps40
Phylostratum

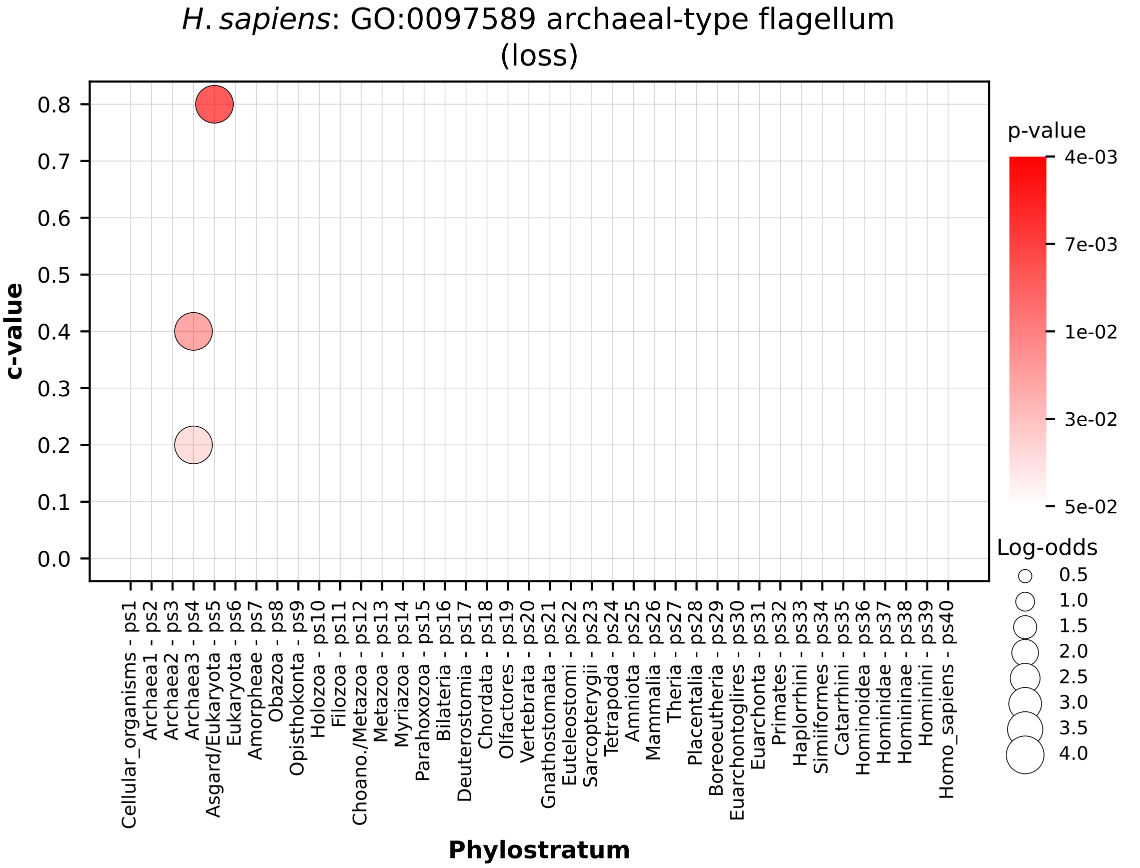

H. sapiens: GO:0097589 archaeal-type flagellum
(loss)
c-value
0.0
0.1
0.2
0.3
0.4
0.5
0.6
0.7
0.8
Phylostratum
Cellular_organisms - ps1
Archaea1 - ps2
Archaea2 - ps3
Archaea3 - ps4
Asgard/Eukaryota - ps5
Eukaryota - ps6
Amorpheae - ps7
Obazoa - ps8
Opisthokonta - ps9
Holozoa - ps10
Filozoa - ps11
Choano./Metazoa - ps12
Metazoa - ps13
Myriazoa - ps14
Parahoxozoa - ps15
Bilateria - ps16
Deuterostomia - ps17
Chordata - ps18
Olfactores - ps19
Vertebrata - ps20
Gnathostomata - ps21
Euteleostomi - ps22
Sarcopterygii - ps23
Tetrapoda - ps24
Amniota - ps25
Mammalia - ps26
Theria - ps27
Placentalia - ps28
Boreoeutheria - ps29
Euarchontoglires - ps30
Euarchonta - ps31
Primates - ps32
Haplorrhini - ps33
Simiiformes - ps34
Catarrhini - ps35
Hominoidea - ps36
Hominidae - ps37
Homininae - ps38
Hominini - ps39
Homo_sapiens - ps40
p-value
4e-03
7e-03
1e-02
3e-02
5e-02
Log-odds
0.5
1.0
1.5
2.0
2.5
3.0
3.5
4.0

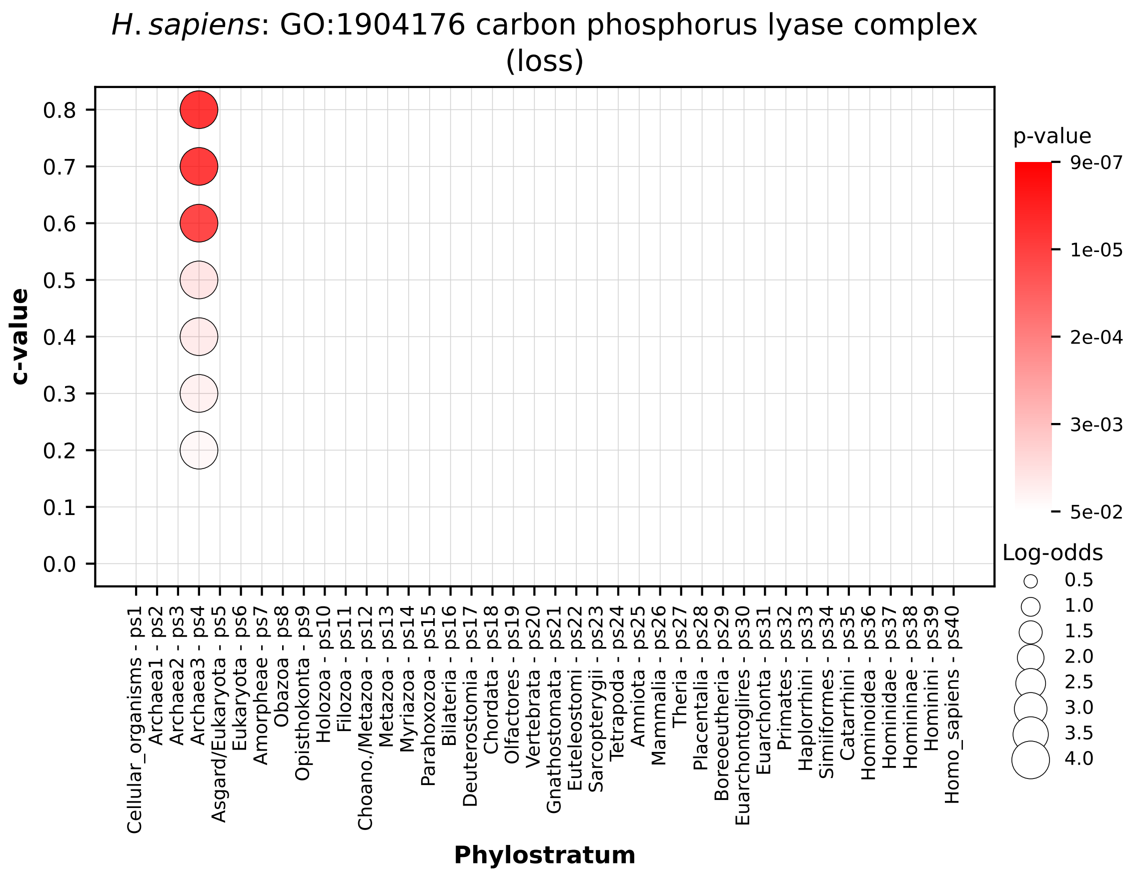

H. sapiens: GO:1904176 carbon phosphorus lyase complex (loss)
c-value
0.0
0.1
0.2
0.3
0.4
0.5
0.6
0.7
0.8
Phylostratum
Cellular_organisms - ps1
Archaea1 - ps2
Archaea2 - ps3
Archaea3 - ps4
Asgard/Eukaryota - ps5
Eukaryota - ps6
Amorpheae - ps7
Obazoa - ps8
Opisthokonta - ps9
Holozoa - ps10
Filozoa - ps11
Choano./Metazoa - ps12
Metazoa - ps13
Myriazoa - ps14
Parahoxozoa - ps15
Bilateria - ps16
Deuterostomia - ps17
Chordata - ps18
Olfactores - ps19
Vertebrata - ps20
Gnathostomata - ps21
Euteleostomi - ps22
Sarcopterygii - ps23
Tetrapoda - ps24
Amniota - ps25
Mammalia - ps26
Theria - ps27
Placentalia - ps28
Boreoeutheria - ps29
Euarchontoglires - ps30
Euarchonta - ps31
Primates - ps32
Haplorrhini - ps33
Simiiformes - ps34
Catarrhini - ps35
Hominoidea - ps36
Hominidae - ps37
Homininae - ps38
Hominini - ps39
Homo_sapiens - ps40
p-value
9e-07
1e-05
2e-04
3e-03
5e-02
Log-odds
0.5
1.0
1.5
2.0
2.5
3.0
3.5
4.0

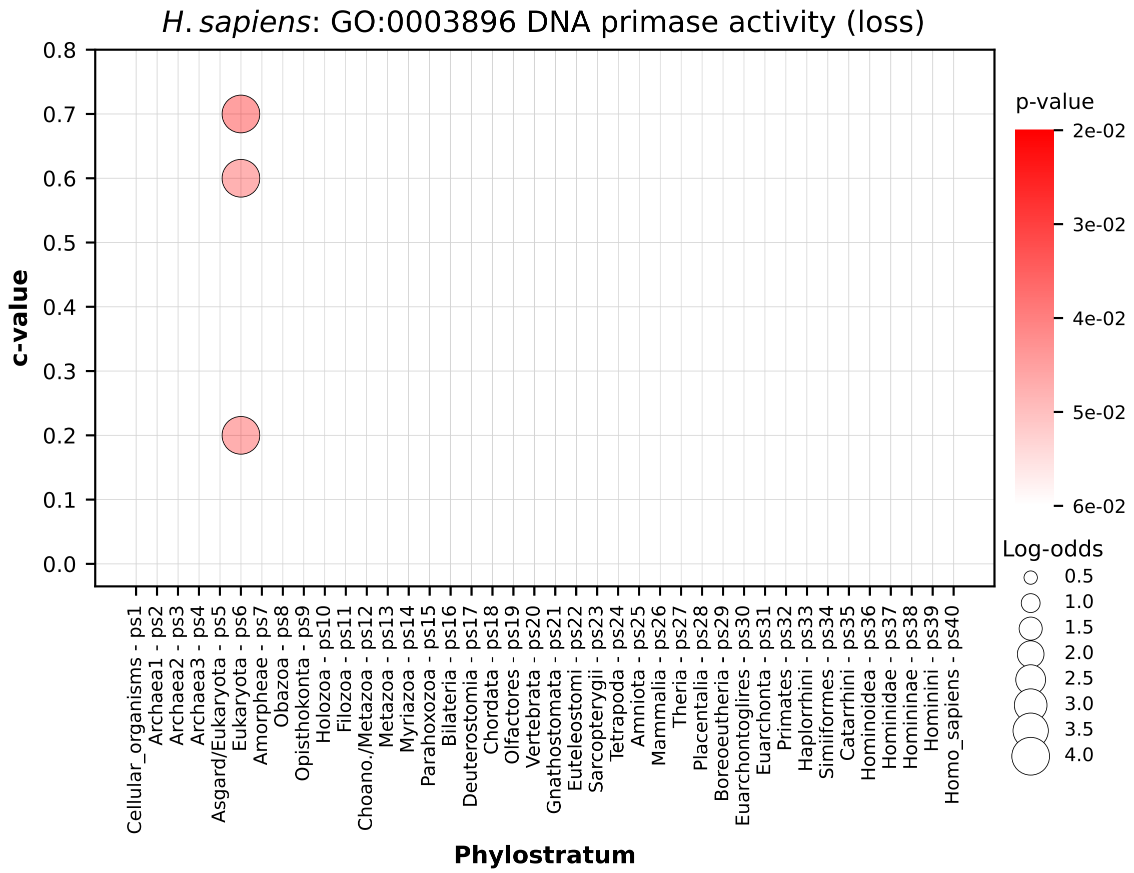

H. sapiens: GO:0003896 DNA primase activity (loss)
c-value
0.0
0.1
0.2
0.3
0.4
0.5
0.6
0.7
0.8
Phylostratum
Cellular_organisms - ps1
Archaea1 - ps2
Archaea2 - ps3
Archaea3 - ps4
Asgard/Eukaryota - ps5
Eukaryota - ps6
Amorpheae - ps7
Obazoa - ps8
Opisthokonta - ps9
Holozoa - ps10
Filozoa - ps11
Choano./Metazoa - ps12
Metazoa - ps13
Myriazoa - ps14
Parahoxozoa - ps15
Bilateria - ps16
Deuterostomia - ps17
Chordata - ps18
Olfactores - ps19
Vertebrata - ps20
Gnathostomata - ps21
Euteleostomi - ps22
Sarcopterygii - ps23
Tetrapoda - ps24
Amniota - ps25
Mammalia - ps26
Theria - ps27
Placentalia - ps28
Boreoeutheria - ps29
Euarchontoglires - ps30
Euarchonta - ps31
Primates - ps32
Haplorrhini - ps33
Simiiformes - ps34
Catarrhini - ps35
Hominoidea - ps36
Hominidae - ps37
Homininae - ps38
Hominini - ps39
Homo_sapiens - ps40
p-value
2e-02
3e-02
4e-02
5e-02
6e-02
Log-odds
0.5
1.0
1.5
2.0
2.5
3.0
3.5
4.0

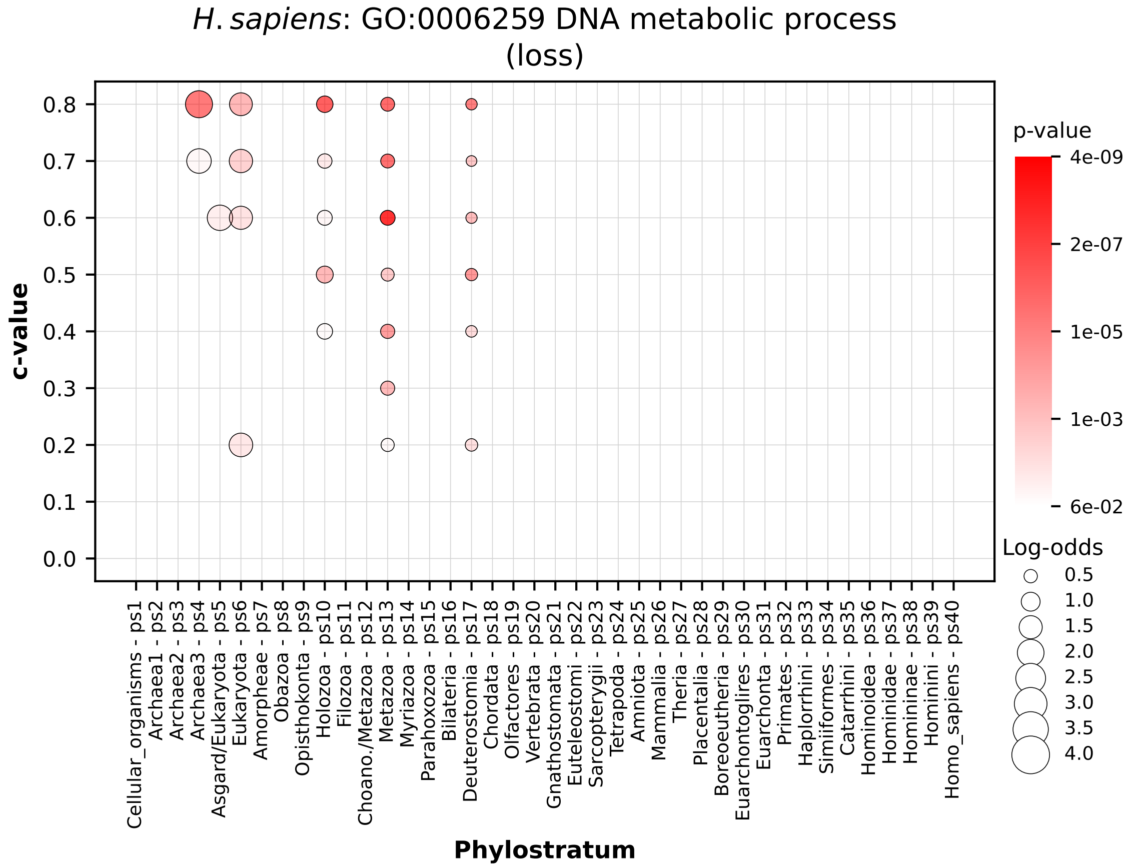

H. sapiens: GO:0006259 DNA metabolic process
(loss)
c-value
0.8
0.7
0.6
0.5
0.4
0.3
0.2
0.1
0.0
Phylostratum
Cellular_organisms - ps1
Archaea1 - ps2
Archaea2 - ps3
Archaea3 - ps4
Asgard/Eukaryota - ps5
Eukaryota - ps6
Amorpheae - ps7
Obazoa - ps8
Opisthokonta - ps9
Holozoa - ps10
Filozoa - ps11
Choano./Metazoa - ps12
Metazoa - ps13
Myriazoa - ps14
Parahoxozoa - ps15
Bilateria - ps16
Deuterostomia - ps17
Chordata - ps18
Olfactores - ps19
Vertebrata - ps20
Gnathostomata - ps21
Euteleostomi - ps22
Sarcopterygii - ps23
Tetrapoda - ps24
Amniota - ps25
Mammalia - ps26
Theria - ps27
Placentalia - ps28
Boreoeutheria - ps29
Euarchontoglires - ps30
Euarchonta - ps31
Primates - ps32
Haplorrhini - ps33
Simiiformes - ps34
Catarrhini - ps35
Hominoidea - ps36
Hominidae - ps37
Homininae - ps38
Hominini - ps39
Homo_sapiens - ps40
p-value
4e-09
2e-07
1e-05
1e-03
6e-02
Log-odds
0.5
1.0
1.5
2.0
2.5
3.0
3.5
4.0

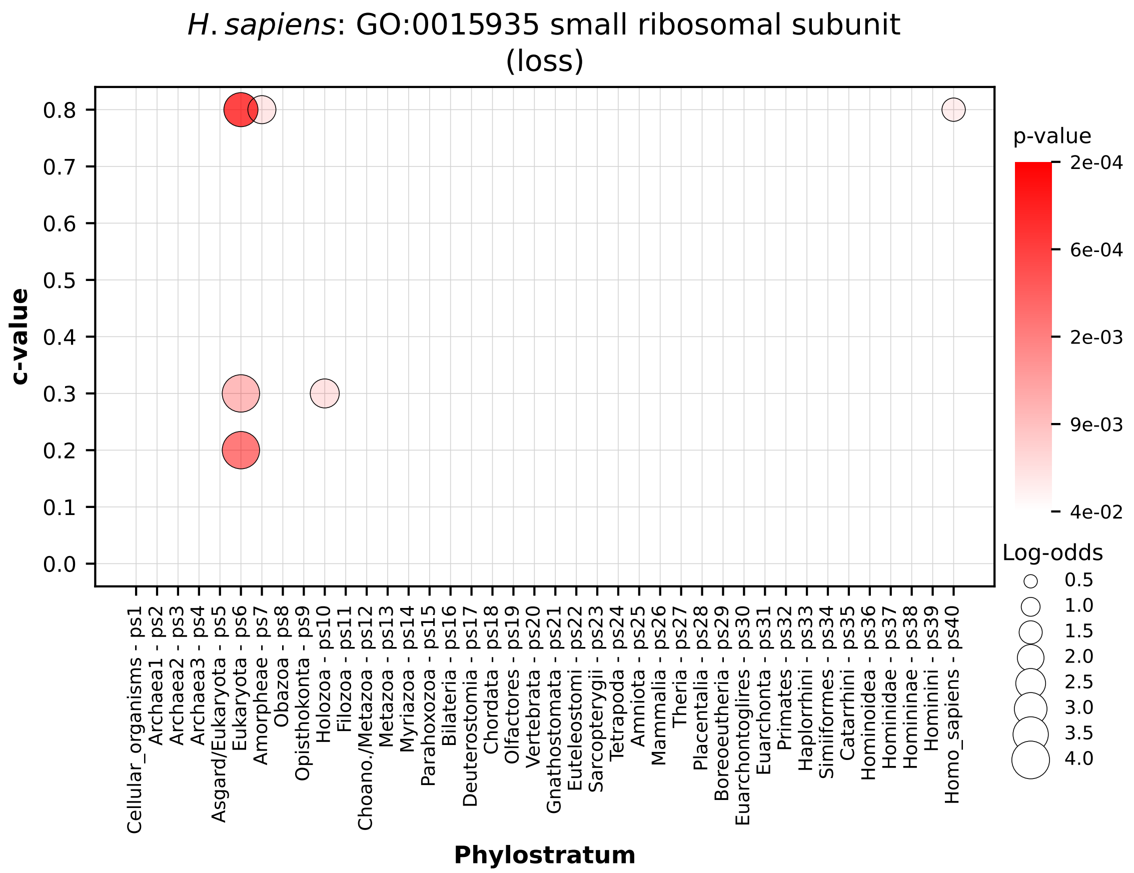

H. sapiens: GO:0015935 small ribosomal subunit
(loss)
c-value
0.0
0.1
0.2
0.3
0.4
0.5
0.6
0.7
0.8
p-value
2e-04
6e-04
2e-03
9e-03
4e-02
Log-odds
0.5
1.0
1.5
2.0
2.5
3.0
3.5
4.0
Cellular_organisms - ps1
Archaea1 - ps2
Archaea2 - ps3
Archaea3 - ps4
Asgard/Eukaryota - ps5
Eukaryota - ps6
Amorpheae - ps7
Obazoa - ps8
Opisthokonta - ps9
Holozoa - ps10
Filozoa - ps11
Choano./Metazoa - ps12
Metazoa - ps13
Myriazoa - ps14
Parahoxozoa - ps15
Bilateria - ps16
Deuterostomia - ps17
Chordata - ps18
Olfactores - ps19
Vertebrata - ps20
Gnathostomata - ps21
Euteleostomi - ps22
Sarcopterygii - ps23
Tetrapoda - ps24
Amniota - ps25
Mammalia - ps26
Theria - ps27
Placentalia - ps28
Boreoeutheria - ps29
Euarchontoglires - ps30
Euarchonta - ps31
Primates - ps32
Haplorrhini - ps33
Simiiformes - ps34
Catarrhini - ps35
Hominoidea - ps36
Hominidae - ps37
Homininae - ps38
Hominini - ps39
Homo_sapiens - ps40
Phylostratum

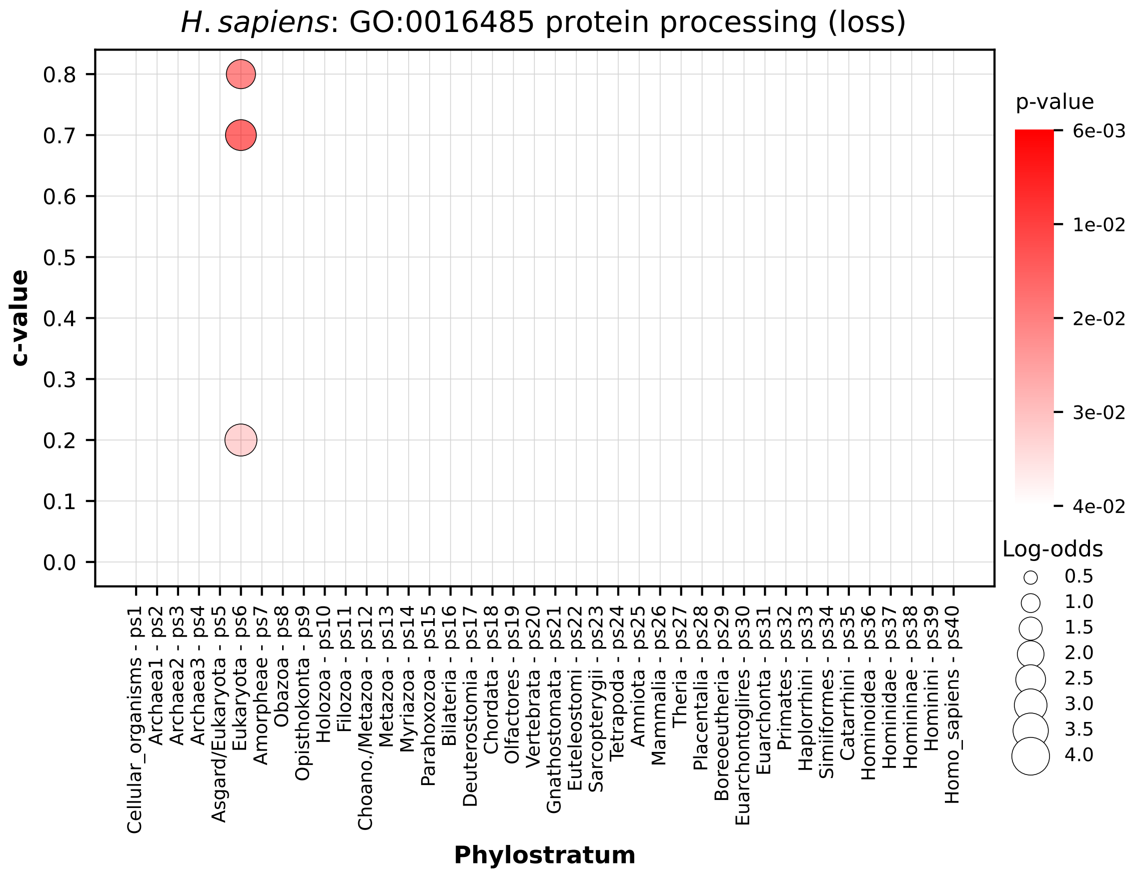

H. sapiens: GO:0016485 protein processing (loss)
c-value
0.8
0.7
0.6
0.5
0.4
0.3
0.2
0.1
0.0
Phylostratum
Cellular_organisms - ps1
Archaea1 - ps2
Archaea2 - ps3
Archaea3 - ps4
Asgard/Eukaryota - ps5
Eukaryota - ps6
Amorpheae - ps7
Obazoa - ps8
Opisthokonta - ps9
Holozoa - ps10
Filozoa - ps11
Choano./Metazoa - ps12
Metazoa - ps13
Myriazoa - ps14
Parahoxozoa - ps15
Bilateria - ps16
Deuterostomia - ps17
Chordata - ps18
Olfactores - ps19
Vertebrata - ps20
Gnathostomata - ps21
Euteleostomi - ps22
Sarcopterygii - ps23
Tetrapoda - ps24
Amniota - ps25
Mammalia - ps26
Theria - ps27
Placentalia - ps28
Boreoeutheria - ps29
Euarchontoglires - ps30
Euarchonta - ps31
Primates - ps32
Haplorrhini - ps33
Simiiformes - ps34
Catarrhini - ps35
Hominoidea - ps36
Hominidae - ps37
Homininae - ps38
Hominini - ps39
Homo_sapiens - ps40
p-value
6e-03
1e-02
2e-02
3e-02
4e-02
Log-odds
0.5
1.0
1.5
2.0
2.5
3.0
3.5
4.0

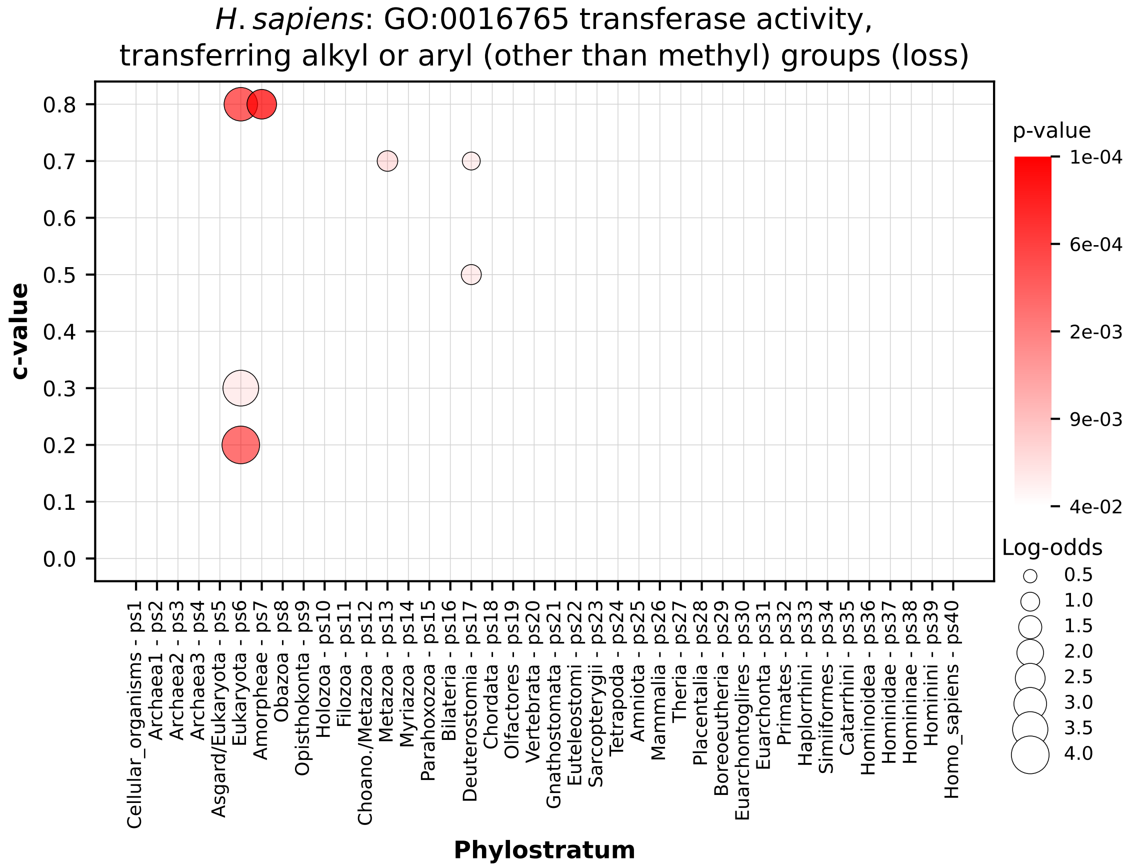
$H. sapiens$: GO:0016765 transferase activity, transferring alkyl or aryl (other than methyl) groups (loss)
c-value
0.0
0.1
0.2
0.3
0.4
0.5
0.6
0.7
0.8
Phylostratum
Cellular_organisms - ps1
Archaea1 - ps2
Archaea2 - ps3
Archaea3 - ps4
Asgard/Eukaryota - ps5
Eukaryota - ps6
Amorpheae - ps7
Obazoa - ps8
Opisthokonta - ps9
Holozoa - ps10
Filozoa - ps11
Choano./Metazoa - ps12
Metazoa - ps13
Myriazoa - ps14
Parahoxozoa - ps15
Bilateria - ps16
Deuterostomia - ps17
Chordata - ps18
Olfactores - ps19
Vertebrata - ps20
Gnathostomata - ps21
Euteleostomi - ps22
Sarcopterygii - ps23
Tetrapoda - ps24
Amniota - ps25
Mammalia - ps26
Theria - ps27
Placentalia - ps28
Boreoeutheria - ps29
Euarchontoglires - ps30
Euarchonta - ps31
Primates - ps32
Haplorrhini - ps33
Simiiformes - ps34
Catarrhini - ps35
Hominoidea - ps36
Hominidae - ps37
Homininae - ps38
Hominini - ps39
Homo_sapiens - ps40
p-value
1e-04
6e-04
2e-03
9e-03
4e-02
Log-odds
0.5
1.0
1.5
2.0
2.5
3.0
3.5
4.0

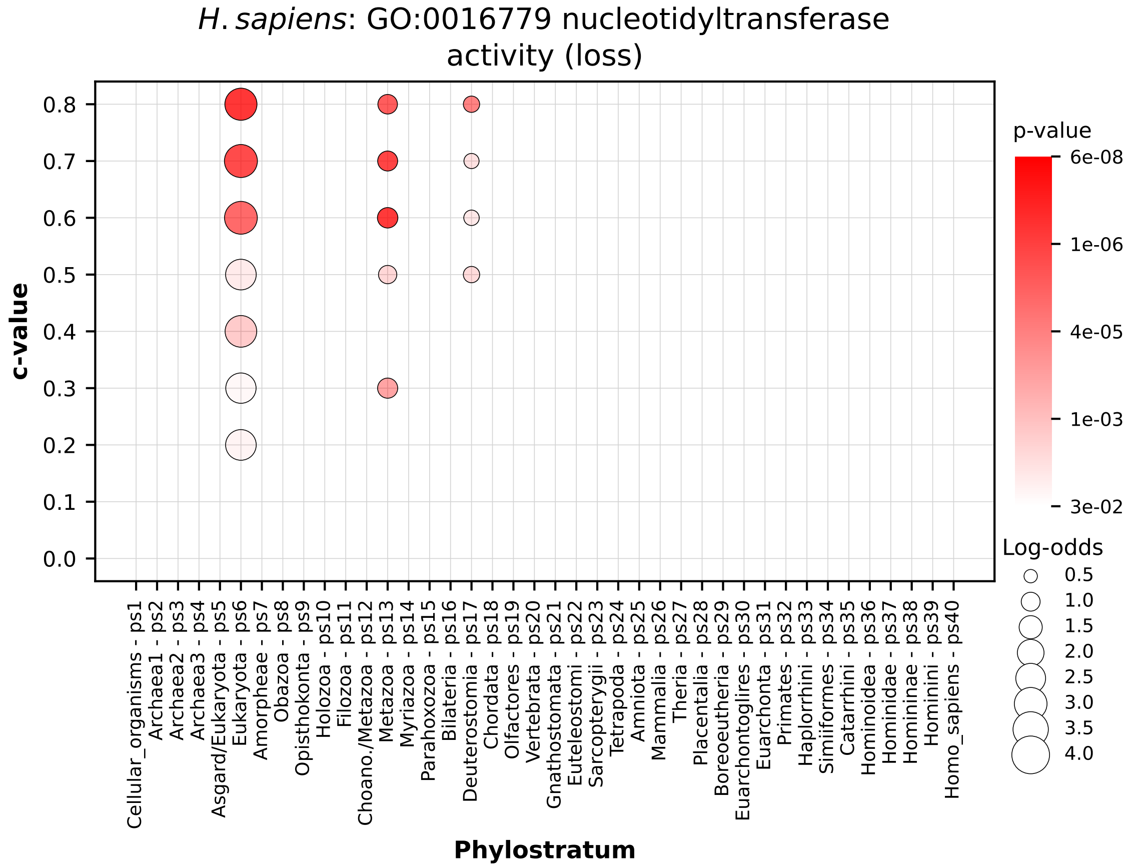

H. sapiens: GO:0016779 nucleotidyltransferase activity (loss)
c-value
0.8
0.7
0.6
0.5
0.4
0.3
0.2
0.1
0.0
p-value
6e-08
1e-06
4e-05
1e-03
3e-02
Log-odds
0.5
1.0
1.5
2.0
2.5
3.0
3.5
4.0
Cellular_organisms - ps1
Archaea1 - ps2
Archaea2 - ps3
Archaea3 - ps4
Asgard/Eukaryota - ps5
Eukaryota - ps6
Amorpheae - ps7
Obazoa - ps8
Opisthokonta - ps9
Holozoa - ps10
Filozoa - ps11
Choano./Metazoa - ps12
Metazoa - ps13
Myriazoa - ps14
Parahoxozoa - ps15
Bilateria - ps16
Deuterostomia - ps17
Chordata - ps18
Olfactores - ps19
Vertebrata - ps20
Gnathostomata - ps21
Euteleostomi - ps22
Sarcopterygii - ps23
Tetrapoda - ps24
Amniota - ps25
Mammalia - ps26
Theria - ps27
Placentalia - ps28
Boreoeutheria - ps29
Euarchontoglires - ps30
Euarchonta - ps31
Primates - ps32
Haplorrhini - ps33
Simiiformes - ps34
Catarrhini - ps35
Hominoidea - ps36
Hominidae - ps37
Homininae - ps38
Hominini - ps39
Homo_sapiens - ps40
Phylostratum

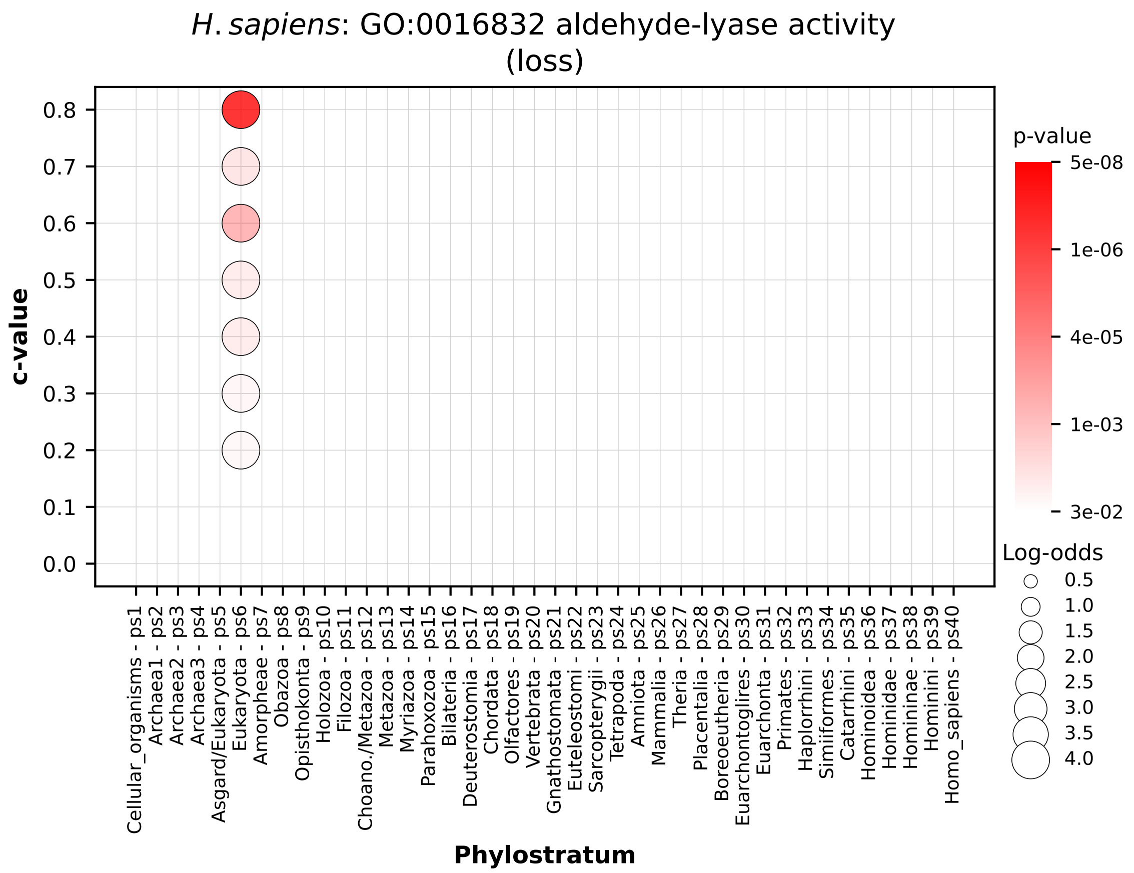

H. sapiens: GO:0016832 aldehyde-lyase activity
(loss)
c-value
0.8
0.7
0.6
0.5
0.4
0.3
0.2
0.1
0.0
Phylostratum
Cellular_organisms - ps1
Archaea1 - ps2
Archaea2 - ps3
Archaea3 - ps4
Asgard/Eukaryota - ps5
Eukaryota - ps6
Amorpheae - ps7
Obazoa - ps8
Opisthokonta - ps9
Holozoa - ps10
Filozoa - ps11
Choano./Metazoa - ps12
Metazoa - ps13
Myriazoa - ps14
Parahoxozoa - ps15
Bilateria - ps16
Deuterostomia - ps17
Chordata - ps18
Olfactores - ps19
Vertebrata - ps20
Gnathostomata - ps21
Euteleostomi - ps22
Sarcopterygii - ps23
Tetrapoda - ps24
Amniota - ps25
Mammalia - ps26
Theria - ps27
Placentalia - ps28
Boreoeutheria - ps29
Euarchontoglires - ps30
Euarchonta - ps31
Primates - ps32
Haplorrhini - ps33
Simiiformes - ps34
Catarrhini - ps35
Hominoidea - ps36
Hominidae - ps37
Homininae - ps38
Hominini - ps39
Homo_sapiens - ps40
p-value
5e-08
1e-06
4e-05
1e-03
3e-02
Log-odds
0.5
1.0
1.5
2.0
2.5
3.0
3.5
4.0

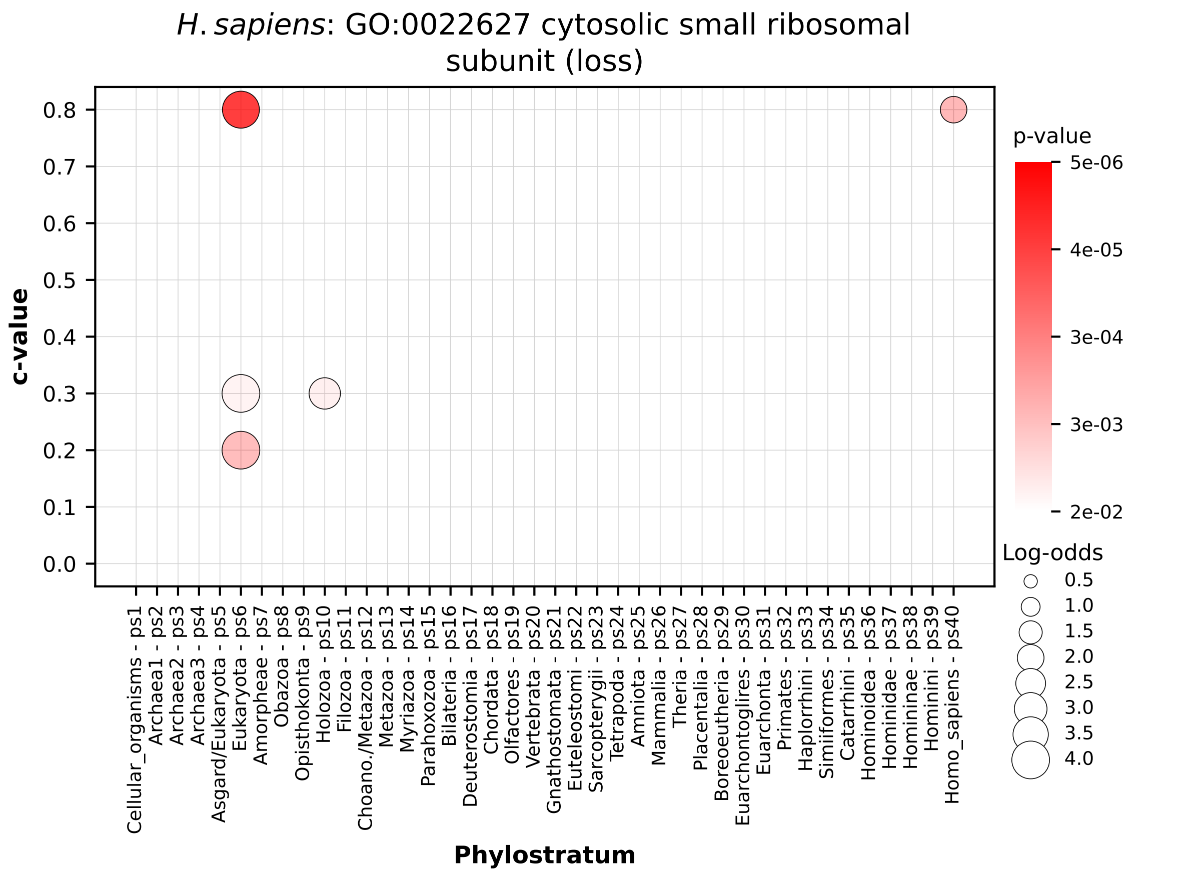
H. sapiens: GO:0022627 cytosolic small ribosomal subunit (loss)
c-value
0.8
0.7
0.6
0.5
0.4
0.3
0.2
0.1
0.0
p-value
5e-06
4e-05
3e-04
3e-03
2e-02
Log-odds
0.5
1.0
1.5
2.0
2.5
3.0
3.5
4.0
Cellular_organisms - ps1
Archaea1 - ps2
Archaea2 - ps3
Archaea3 - ps4
Asgard/Eukaryota - ps5
Eukaryota - ps6
Amorpheae - ps7
Obazoa - ps8
Opisthokonta - ps9
Holozoa - ps10
Filozoa - ps11
Choano./Metazoa - ps12
Metazoa - ps13
Myriazoa - ps14
Parahoxozoa - ps15
Bilateria - ps16
Deuterostomia - ps17
Chordata - ps18
Olfactores - ps19
Vertebrata - ps20
Gnathostomata - ps21
Euteleostomi - ps22
Sarcopterygii - ps23
Tetrapoda - ps24
Amniota - ps25
Mammalia - ps26
Theria - ps27
Placentalia - ps28
Boreoeutheria - ps29
Euarchontoglires - ps30
Euarchonta - ps31
Primates - ps32
Haplorrhini - ps33
Simiiformes - ps34
Catarrhini - ps35
Hominoidea - ps36
Hominidae - ps37
Homininae - ps38
Hominini - ps39
Homo_sapiens - ps40
Phylostratum

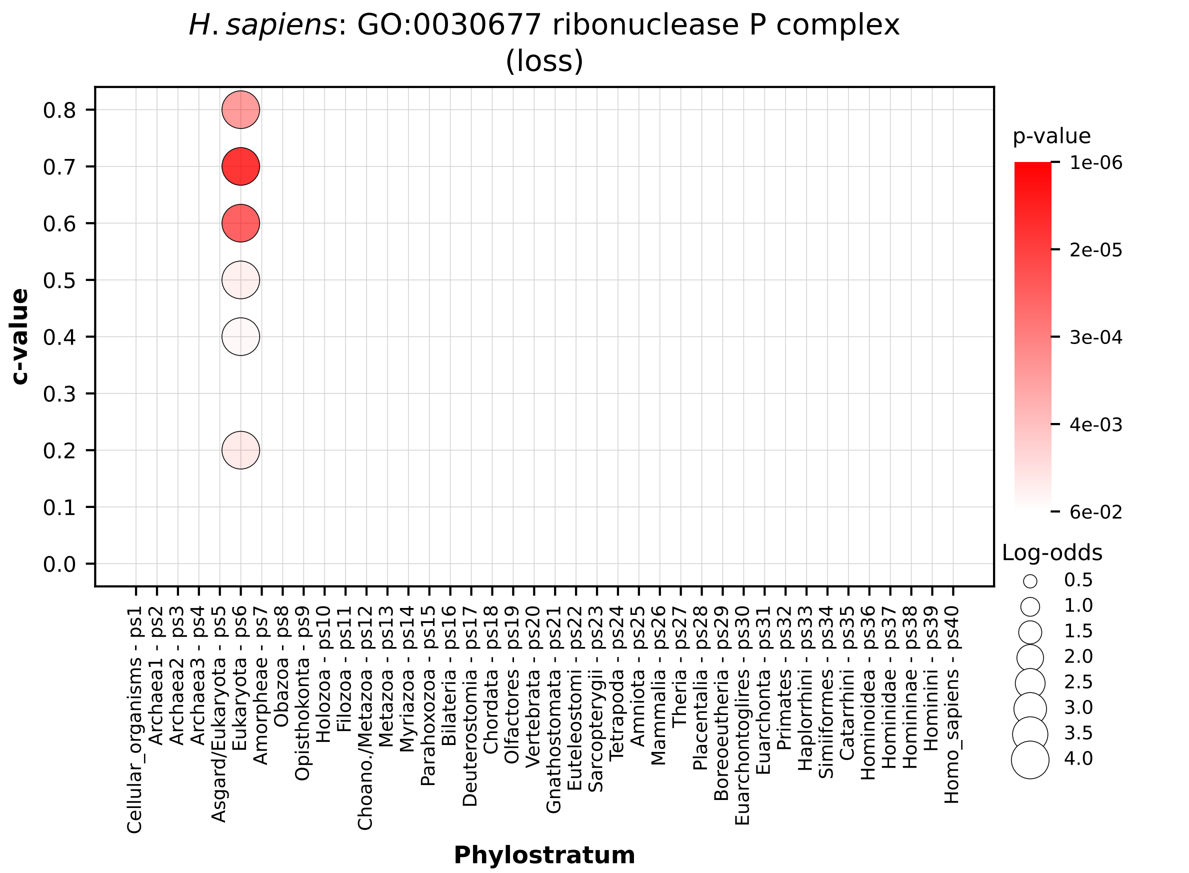

H. sapiens: GO:0030677 ribonuclease P complex
(loss)
c-value
0.0
0.1
0.2
0.3
0.4
0.5
0.6
0.7
0.8
p-value
1e-06
2e-05
3e-04
4e-03
6e-02
Log-odds
0.5
1.0
1.5
2.0
2.5
3.0
3.5
4.0
Cellular_organisms - ps1
Archaea1 - ps2
Archaea2 - ps3
Archaea3 - ps4
Asgard/Eukaryota - ps5
Eukaryota - ps6
Amorpheae - ps7
Obazoa - ps8
Opisthokonta - ps9
Holozoa - ps10
Filozoa - ps11
Choano./Metazoa - ps12
Metazoa - ps13
Myriazoa - ps14
Parahoxozoa - ps15
Bilateria - ps16
Deuterostomia - ps17
Chordata - ps18
Olfactores - ps19
Vertebrata - ps20
Gnathostomata - ps21
Euteleostomi - ps22
Sarcopterygii - ps23
Tetrapoda - ps24
Amniota - ps25
Mammalia - ps26
Theria - ps27
Placentalia - ps28
Boreoeutheria - ps29
Euarchontoglires - ps30
Euarchonta - ps31
Primates - ps32
Haplorrhini - ps33
Simiiformes - ps34
Catarrhini - ps35
Hominoidea - ps36
Hominidae - ps37
Homininae - ps38
Hominini - ps39
Homo_sapiens - ps40
Phylostratum

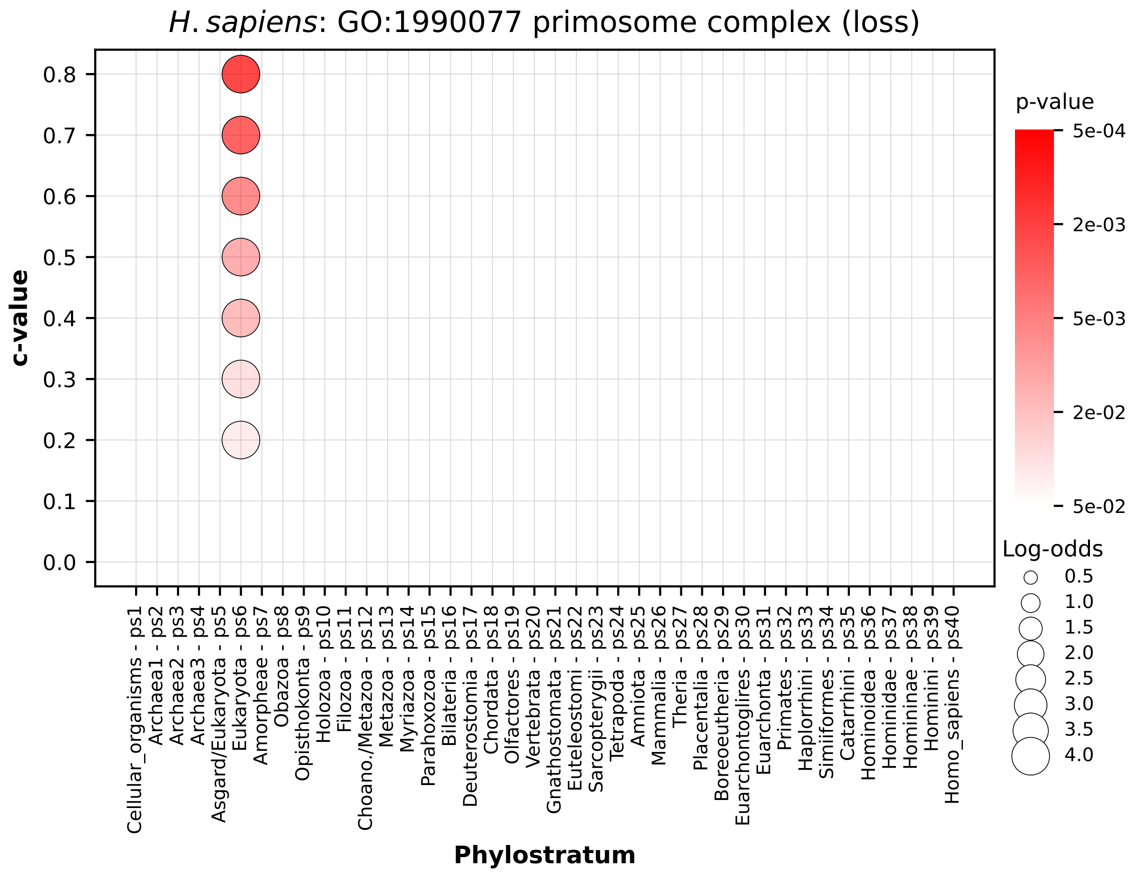

H. sapiens: GO:1990077 primosome complex (loss)
c-value
0.8
0.7
0.6
0.5
0.4
0.3
0.2
0.1
0.0
Phylostratum
Cellular_organisms - ps1
Archaea1 - ps2
Archaea2 - ps3
Archaea3 - ps4
Asgard/Eukaryota - ps5
Eukaryota - ps6
Amorpheae - ps7
Obazoa - ps8
Opisthokonta - ps9
Holozoa - ps10
Filozoa - ps11
Choano./Metazoa - ps12
Metazoa - ps13
Myriazoa - ps14
Parahoxozoa - ps15
Bilateria - ps16
Deuterostomia - ps17
Chordata - ps18
Olfactores - ps19
Vertebrata - ps20
Gnathostomata - ps21
Euteleostomi - ps22
Sarcopterygii - ps23
Tetrapoda - ps24
Amniota - ps25
Mammalia - ps26
Theria - ps27
Placentalia - ps28
Boreoeutheria - ps29
Euarchontoglires - ps30
Euarchonta - ps31
Primates - ps32
Haplorrhini - ps33
Simiiformes - ps34
Catarrhini - ps35
Hominoidea - ps36
Hominidae - ps37
Homininae - ps38
Hominini - ps39
Homo_sapiens - ps40
p-value
5e-04
2e-03
5e-03
2e-02
5e-02
Log-odds
0.5
1.0
1.5
2.0
2.5
3.0
3.5
4.0

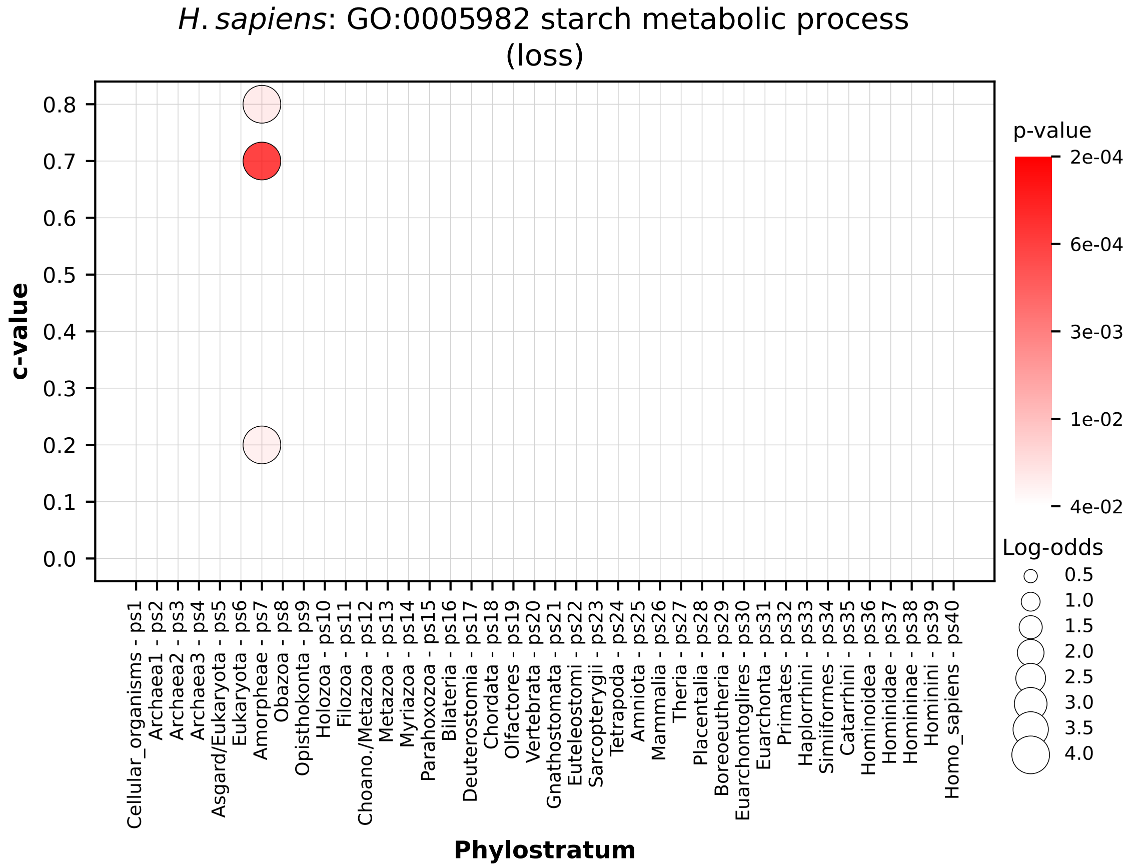

H. sapiens: GO:0005982 starch metabolic process
(loss)
c-value
0.8
0.7
0.6
0.5
0.4
0.3
0.2
0.1
0.0
p-value
2e-04
6e-04
3e-03
1e-02
4e-02
Log-odds
0.5
1.0
1.5
2.0
2.5
3.0
3.5
4.0
Cellular_organisms - ps1
Archaea1 - ps2
Archaea2 - ps3
Archaea3 - ps4
Asgard/Eukaryota - ps5
Eukaryota - ps6
Amorpheae - ps7
Obazoa - ps8
Opisthokonta - ps9
Holozoa - ps10
Filozoa - ps11
Choano./Metazoa - ps12
Metazoa - ps13
Myriazoa - ps14
Parahoxozoa - ps15
Bilateria - ps16
Deuterostomia - ps17
Chordata - ps18
Olfactores - ps19
Vertebrata - ps20
Gnathostomata - ps21
Euteleostomi - ps22
Sarcopterygii - ps23
Tetrapoda - ps24
Amniota - ps25
Mammalia - ps26
Theria - ps27
Placentalia - ps28
Boreoeutheria - ps29
Euarchontoglires - ps30
Euarchonta - ps31
Primates - ps32
Haplorrhini - ps33
Simiiformes - ps34
Catarrhini - ps35
Hominoidea - ps36
Hominidae - ps37
Homininae - ps38
Hominini - ps39
Homo_sapiens - ps40
Phylostratum

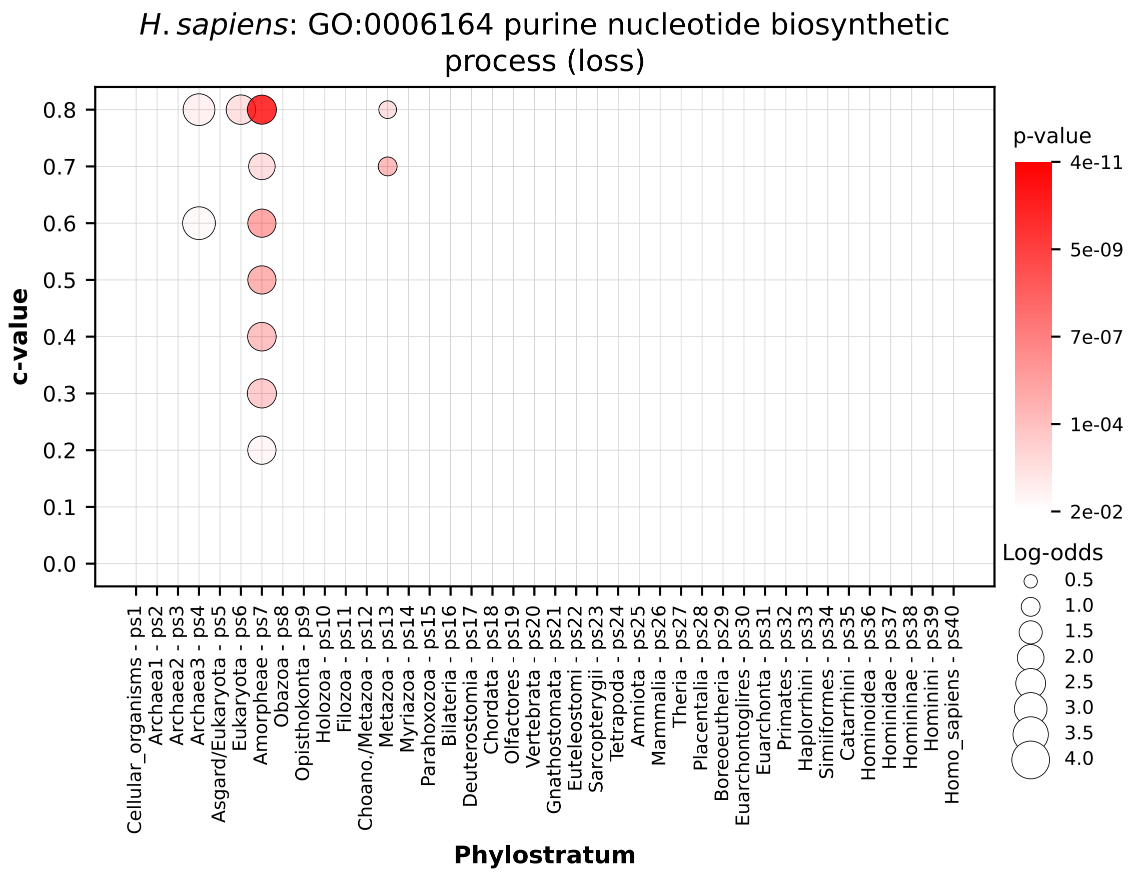
H. sapiens: GO:0006164 purine nucleotide biosynthetic process (loss)
c-value
0.8
0.7
0.6
0.5
0.4
0.3
0.2
0.1
0.0
p-value
4e-11
5e-09
7e-07
1e-04
2e-02
Log-odds
0.5
1.0
1.5
2.0
2.5
3.0
3.5
4.0
Cellular_organisms - ps1
Archaea1 - ps2
Archaea2 - ps3
Archaea3 - ps4
Asgard/Eukaryota - ps5
Eukaryota - ps6
Amorpheae - ps7
Obazoa - ps8
Opisthokonta - ps9
Holozoa - ps10
Filozoa - ps11
Choano./Metazoa - ps12
Metazoa - ps13
Myriazoa - ps14
Parahoxozoa - ps15
Bilateria - ps16
Deuterostomia - ps17
Chordata - ps18
Olfactores - ps19
Vertebrata - ps20
Gnathostomata - ps21
Euteleostomi - ps22
Sarcopterygii - ps23
Tetrapoda - ps24
Amniota - ps25
Mammalia - ps26
Theria - ps27
Placentalia - ps28
Boreoeutheria - ps29
Euarchontoglires - ps30
Euarchonta - ps31
Primates - ps32
Haplorrhini - ps33
Simiiformes - ps34
Catarrhini - ps35
Hominoidea - ps36
Hominidae - ps37
Homininae - ps38
Hominini - ps39
Homo_sapiens - ps40
Phylostratum

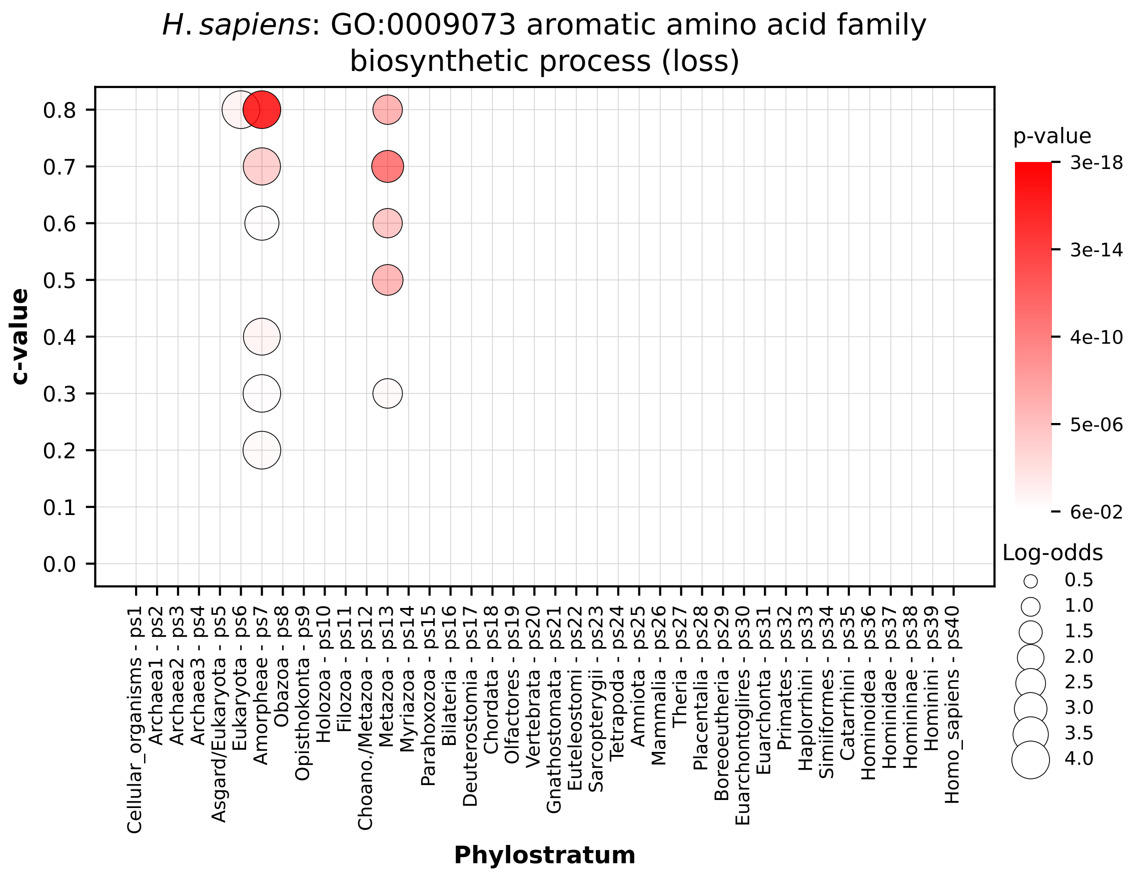
H. sapiens: GO:0009073 aromatic amino acid family biosynthetic process (loss)
c-value
0.8
0.7
0.6
0.5
0.4
0.3
0.2
0.1
0.0
p-value
3e-18
3e-14
4e-10
5e-06
6e-02
Log-odds
0.5
1.0
1.5
2.0
2.5
3.0
3.5
4.0
Cellular_organisms - ps1
Archaea1 - ps2
Archaea2 - ps3
Archaea3 - ps4
Asgard/Eukaryota - ps5
Eukaryota - ps6
Amorpheae - ps7
Obazoa - ps8
Opisthokonta - ps9
Holozoa - ps10
Filozoa - ps11
Choano./Metazoa - ps12
Metazoa - ps13
Myriazoa - ps14
Parahoxozoa - ps15
Bilateria - ps16
Deuterostomia - ps17
Chordata - ps18
Olfactores - ps19
Vertebrata - ps20
Gnathostomata - ps21
Euteleostomi - ps22
Sarcopterygii - ps23
Tetrapoda - ps24
Amniota - ps25
Mammalia - ps26
Theria - ps27
Placentalia - ps28
Boreoeutheria - ps29
Euarchontoglires - ps30
Euarchonta - ps31
Primates - ps32
Haplorrhini - ps33
Simiiformes - ps34
Catarrhini - ps35
Hominoidea - ps36
Hominidae - ps37
Homininae - ps38
Hominini - ps39
Homo_sapiens - ps40
Phylostratum

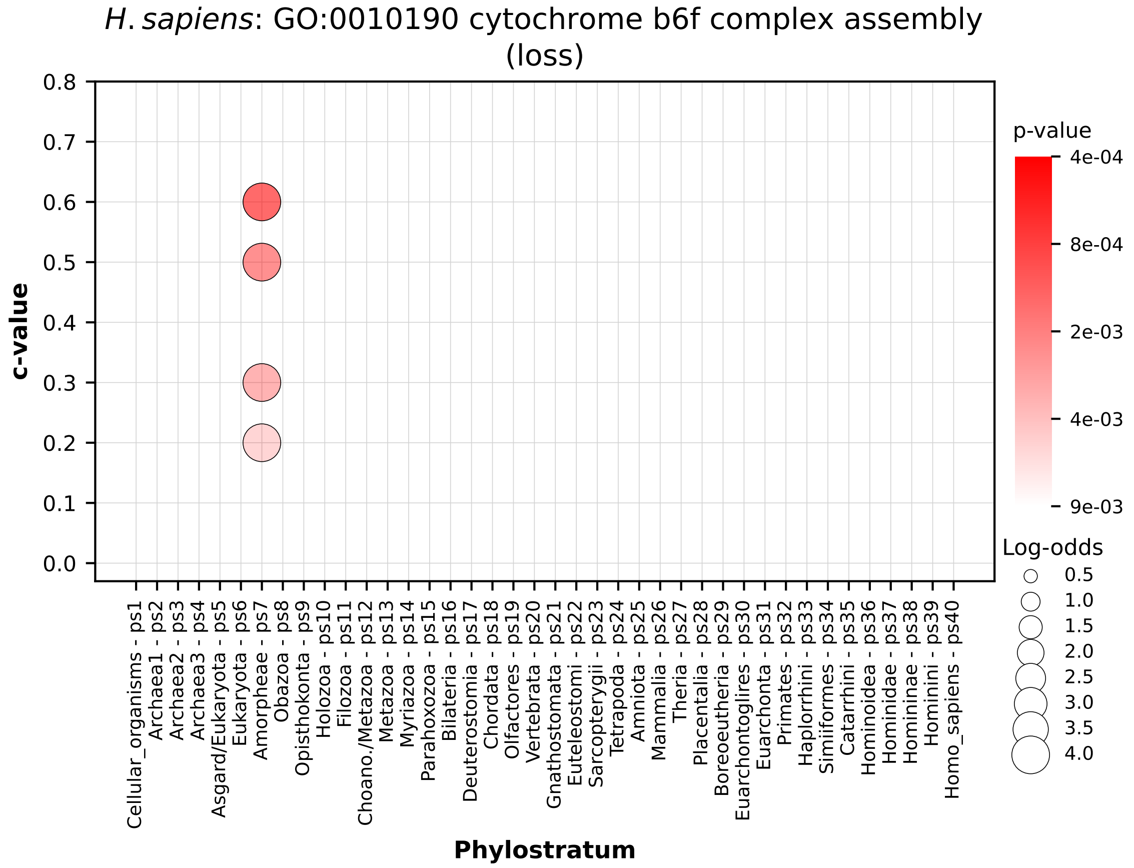
H. sapiens: GO:0010190 cytochrome b6f complex assembly (loss)
c-value
0.0
0.1
0.2
0.3
0.4
0.5
0.6
0.7
0.8
p-value
4e-04
8e-04
2e-03
4e-03
9e-03
Log-odds
0.5
1.0
1.5
2.0
2.5
3.0
3.5
4.0
Cellular_organisms - ps1
Archaea1 - ps2
Archaea2 - ps3
Archaea3 - ps4
Asgard/Eukaryota - ps5
Eukaryota - ps6
Amorpheae - ps7
Obazoa - ps8
Opisthokonta - ps9
Holozoa - ps10
Filozoa - ps11
Choano./Metazoa - ps12
Metazoa - ps13
Myriazoa - ps14
Parahoxozoa - ps15
Bilateria - ps16
Deuterostomia - ps17
Chordata - ps18
Olfactores - ps19
Vertebrata - ps20
Gnathostomata - ps21
Euteleostomi - ps22
Sarcopterygii - ps23
Tetrapoda - ps24
Amniota - ps25
Mammalia - ps26
Theria - ps27
Placentalia - ps28
Boreoeutheria - ps29
Euarchontoglires - ps30
Euarchonta - ps31
Primates - ps32
Haplorrhini - ps33
Simiiformes - ps34
Catarrhini - ps35
Hominoidea - ps36
Hominidae - ps37
Homininae - ps38
Hominini - ps39
Homo_sapiens - ps40
Phylostratum

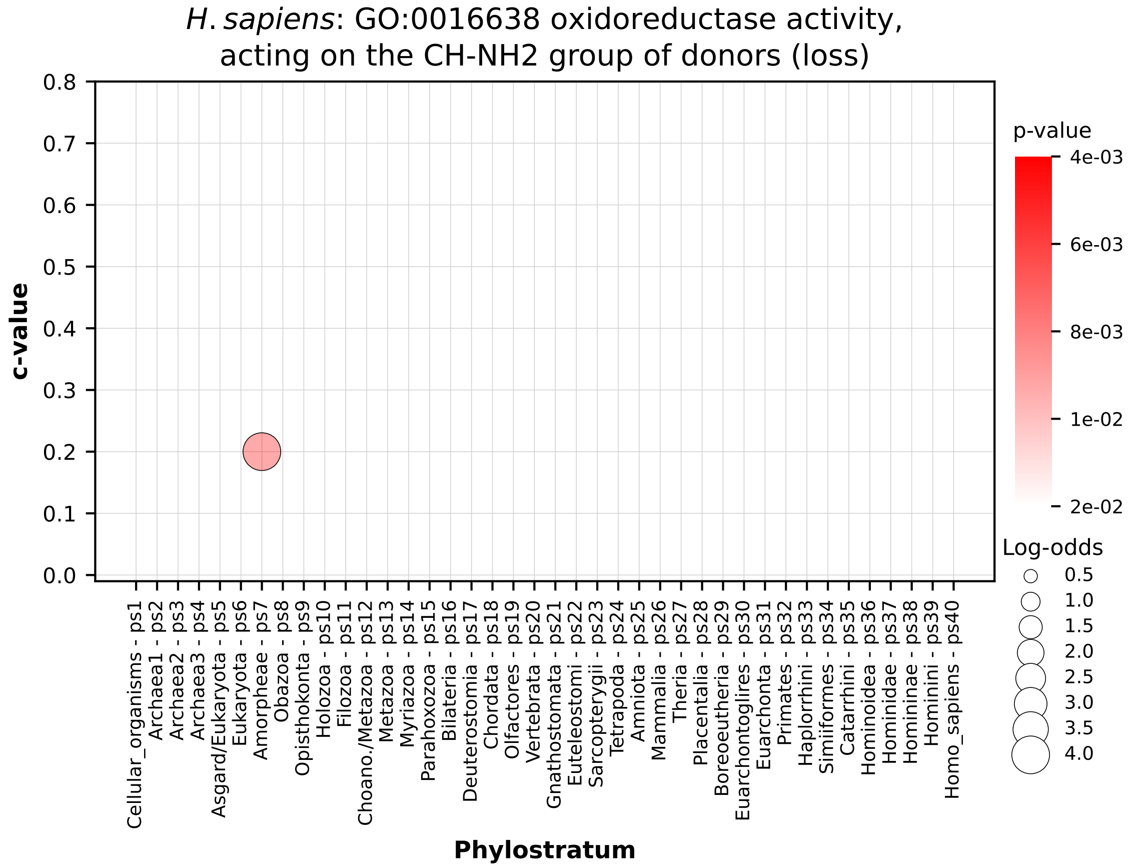

H. sapiens: GO:0016638 oxidoreductase activity, acting on the CH-NH2 group of donors (loss)
c-value
0.0
0.1
0.2
0.3
0.4
0.5
0.6
0.7
0.8
Phylostratum
Cellular_organisms - ps1
Archaea1 - ps2
Archaea2 - ps3
Archaea3 - ps4
Asgard/Eukaryota - ps5
Eukaryota - ps6
Amorpheae - ps7
Obazoa - ps8
Opisthokonta - ps9
Holozoa - ps10
Filozoa - ps11
Choano./Metazoa - ps12
Metazoa - ps13
Myriazoa - ps14
Parahoxozoa - ps15
Bilateria - ps16
Deuterostomia - ps17
Chordata - ps18
Olfactores - ps19
Vertebrata - ps20
Gnathostomata - ps21
Euteleostomi - ps22
Sarcopterygii - ps23
Tetrapoda - ps24
Amniota - ps25
Mammalia - ps26
Theria - ps27
Placentalia - ps28
Boreoeutheria - ps29
Euarchontoglires - ps30
Euarchonta - ps31
Primates - ps32
Haplorrhini - ps33
Simiiformes - ps34
Catarrhini - ps35
Hominoidea - ps36
Hominidae - ps37
Homininae - ps38
Hominini - ps39
Homo_sapiens - ps40
p-value
4e-03
6e-03
8e-03
1e-02
2e-02
Log-odds
0.5
1.0
1.5
2.0
2.5
3.0
3.5
4.0

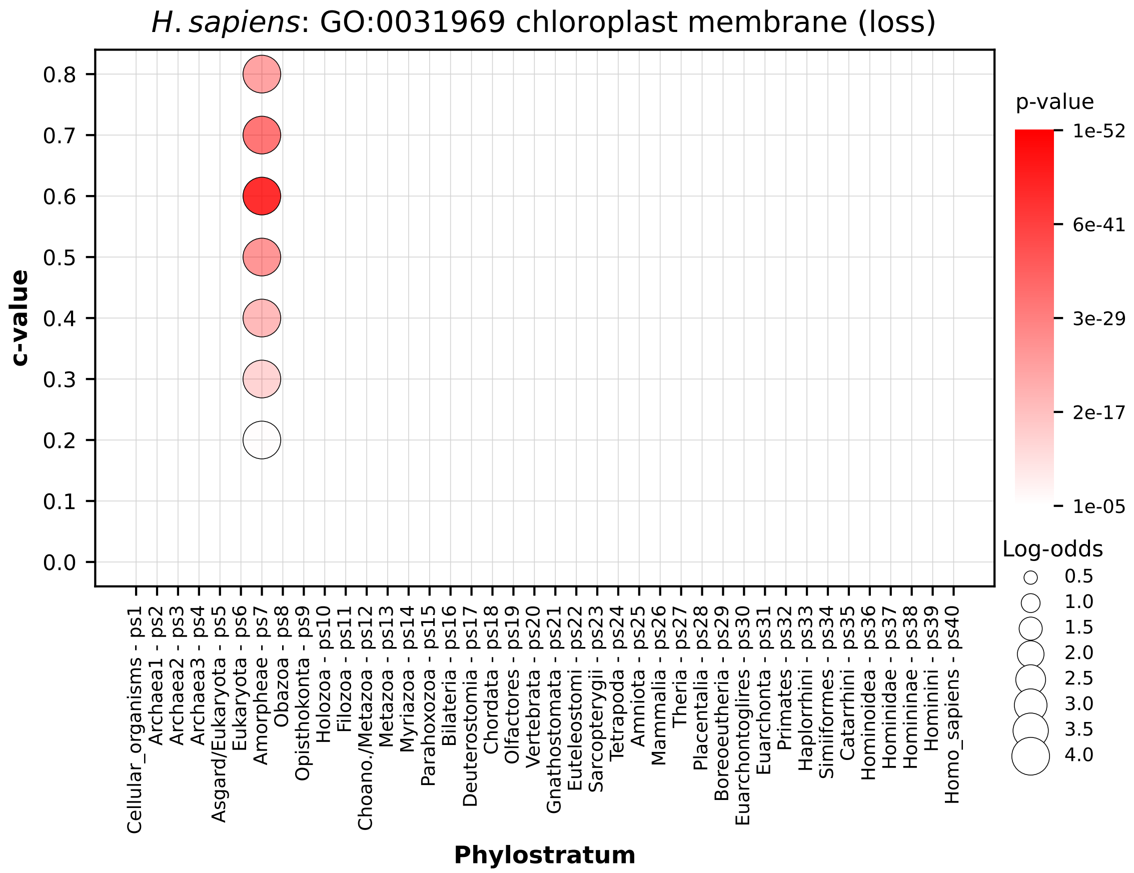

H. sapiens: GO:0031969 chloroplast membrane (loss)
c-value
0.8
0.7
0.6
0.5
0.4
0.3
0.2
0.1
0.0
Phylostratum
Cellular_organisms - ps1
Archaea1 - ps2
Archaea2 - ps3
Archaea3 - ps4
Asgard/Eukaryota - ps5
Eukaryota - ps6
Amorpheae - ps7
Obazoa - ps8
Opisthokonta - ps9
Holozoa - ps10
Filozoa - ps11
Choano./Metazoa - ps12
Metazoa - ps13
Myriazoa - ps14
Parahoxozoa - ps15
Bilateria - ps16
Deuterostomia - ps17
Chordata - ps18
Olfactores - ps19
Vertebrata - ps20
Gnathostomata - ps21
Euteleostomi - ps22
Sarcopterygii - ps23
Tetrapoda - ps24
Amniota - ps25
Mammalia - ps26
Theria - ps27
Placentalia - ps28
Boreoeutheria - ps29
Euarchontoglires - ps30
Euarchonta - ps31
Primates - ps32
Haplorrhini - ps33
Simiiformes - ps34
Catarrhini - ps35
Hominoidea - ps36
Hominidae - ps37
Homininae - ps38
Hominini - ps39
Homo_sapiens - ps40
p-value
1e-52
6e-41
3e-29
2e-17
1e-05
Log-odds
0.5
1.0
1.5
2.0
2.5
3.0
3.5
4.0

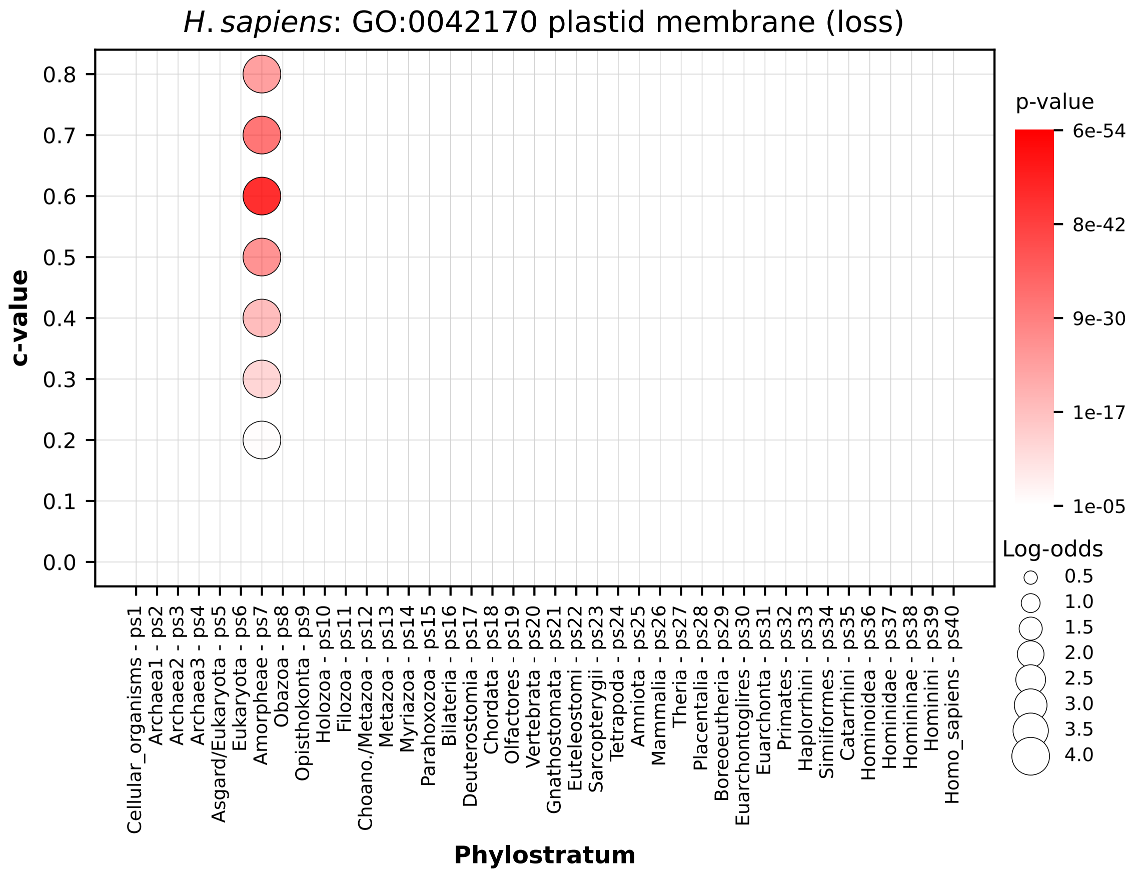

H. sapiens: GO:0042170 plastid membrane (loss)
c-value
0.8
0.7
0.6
0.5
0.4
0.3
0.2
0.1
0.0
p-value
6e-54
8e-42
9e-30
1e-17
1e-05
Log-odds
0.5
1.0
1.5
2.0
2.5
3.0
3.5
4.0
Cellular_organisms - ps1
Archaea1 - ps2
Archaea2 - ps3
Archaea3 - ps4
Asgard/Eukaryota - ps5
Eukaryota - ps6
Amorpheae - ps7
Obazoa - ps8
Opisthokonta - ps9
Holozoa - ps10
Filozoa - ps11
Choano./Metazoa - ps12
Metazoa - ps13
Myriazoa - ps14
Parahoxozoa - ps15
Bilateria - ps16
Deuterostomia - ps17
Chordata - ps18
Olfactores - ps19
Vertebrata - ps20
Gnathostomata - ps21
Euteleostomi - ps22
Sarcopterygii - ps23
Tetrapoda - ps24
Amniota - ps25
Mammalia - ps26
Theria - ps27
Placentalia - ps28
Boreoeutheria - ps29
Euarchontoglires - ps30
Euarchonta - ps31
Primates - ps32
Haplorrhini - ps33
Simiiformes - ps34
Catarrhini - ps35
Hominoidea - ps36
Hominidae - ps37
Homininae - ps38
Hominini - ps39
Homo_sapiens - ps40
Phylostratum

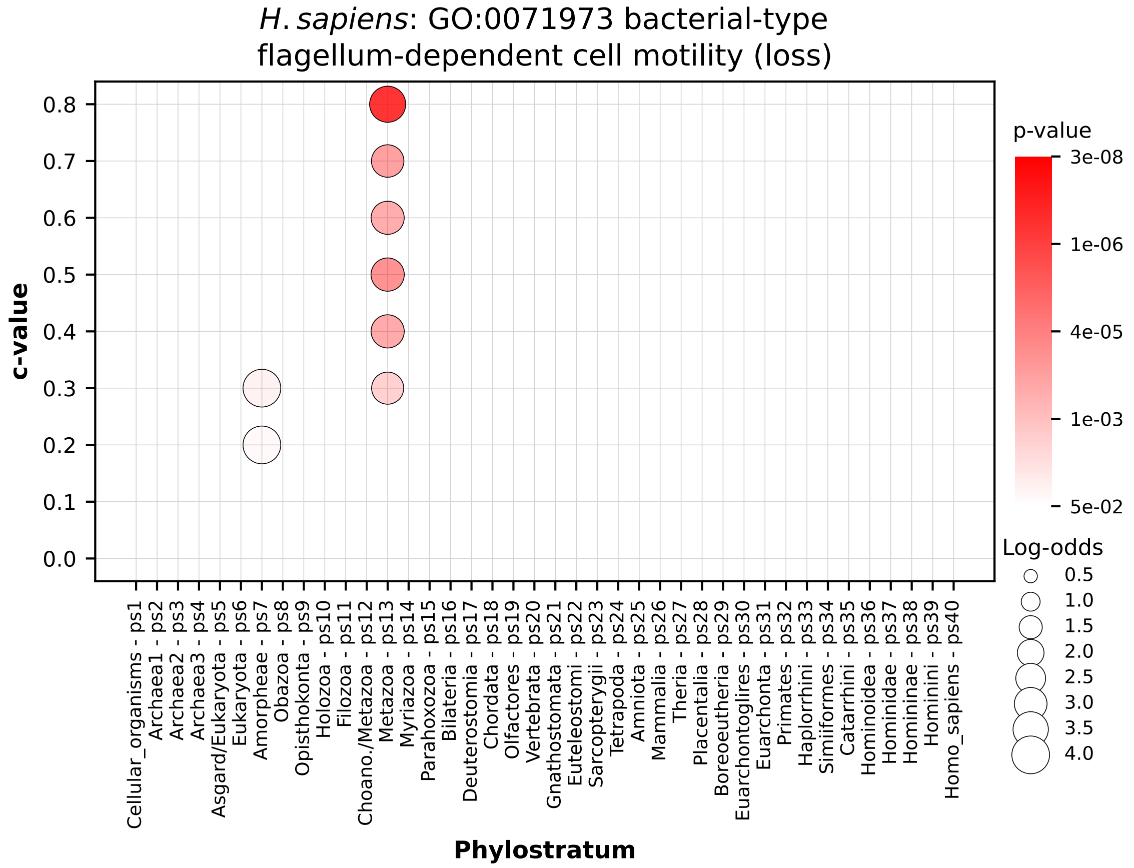

H. sapiens: GO:0071973 bacterial-type flagellum-dependent cell motility (loss)
c-value
0.8
0.7
0.6
0.5
0.4
0.3
0.2
0.1
0.0
Phylostratum
Cellular_organisms - ps1
Archaea1 - ps2
Archaea2 - ps3
Archaea3 - ps4
Asgard/Eukaryota - ps5
Eukaryota - ps6
Amorpheae - ps7
Obazoa - ps8
Opisthokonta - ps9
Holozoa - ps10
Filozoa - ps11
Choano./Metazoa - ps12
Metazoa - ps13
Myriazoa - ps14
Parahoxozoa - ps15
Bilateria - ps16
Deuterostomia - ps17
Chordata - ps18
Olfactores - ps19
Vertebrata - ps20
Gnathostomata - ps21
Euteleostomi - ps22
Sarcopterygii - ps23
Tetrapoda - ps24
Amniota - ps25
Mammalia - ps26
Theria - ps27
Placentalia - ps28
Boreoeutheria - ps29
Euarchontoglires - ps30
Euarchonta - ps31
Primates - ps32
Haplorrhini - ps33
Simiiformes - ps34
Catarrhini - ps35
Hominoidea - ps36
Hominidae - ps37
Homininae - ps38
Hominini - ps39
Homo_sapiens - ps40
p-value
3e-08
1e-06
4e-05
1e-03
5e-02
Log-odds
0.5
1.0
1.5
2.0
2.5
3.0
3.5
4.0

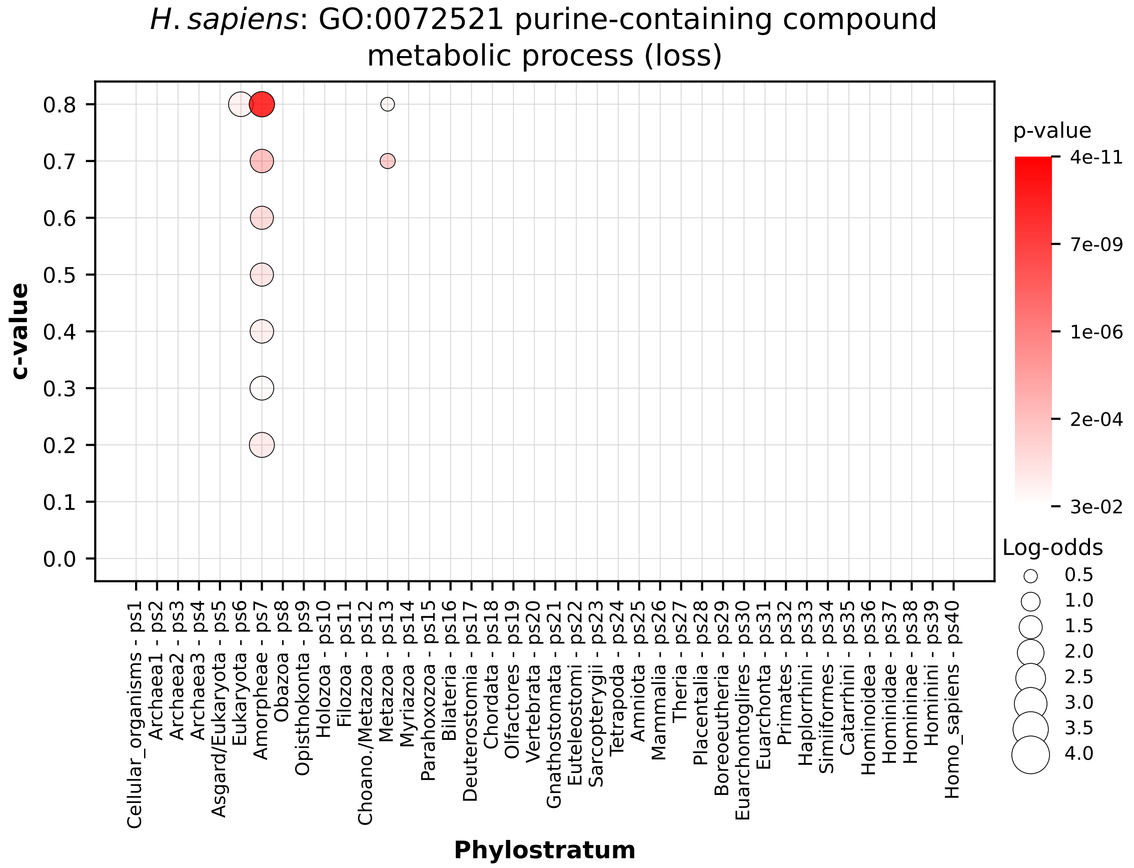
H. sapiens: GO:0072521 purine-containing compound metabolic process (loss)
c-value
0.8
0.7
0.6
0.5
0.4
0.3
0.2
0.1
0.0
Phylostratum
Cellular_organisms - ps1
Archaea1 - ps2
Archaea2 - ps3
Archaea3 - ps4
Asgard/Eukaryota - ps5
Eukaryota - ps6
Amorpheae - ps7
Obazoa - ps8
Opisthokonta - ps9
Holozoa - ps10
Filozoa - ps11
Choano./Metazoa - ps12
Metazoa - ps13
Myriazoa - ps14
Parahoxozoa - ps15
Bilateria - ps16
Deuterostomia - ps17
Chordata - ps18
Olfactores - ps19
Vertebrata - ps20
Gnathostomata - ps21
Euteleostomi - ps22
Sarcopterygii - ps23
Tetrapoda - ps24
Amniota - ps25
Mammalia - ps26
Theria - ps27
Placentalia - ps28
Boreoeutheria - ps29
Euarchontoglires - ps30
Euarchonta - ps31
Primates - ps32
Haplorrhini - ps33
Simiiformes - ps34
Catarrhini - ps35
Hominoidea - ps36
Hominidae - ps37
Homininae - ps38
Hominini - ps39
Homo_sapiens - ps40
p-value
4e-11
7e-09
1e-06
2e-04
3e-02
Log-odds
0.5
1.0
1.5
2.0
2.5
3.0
3.5
4.0

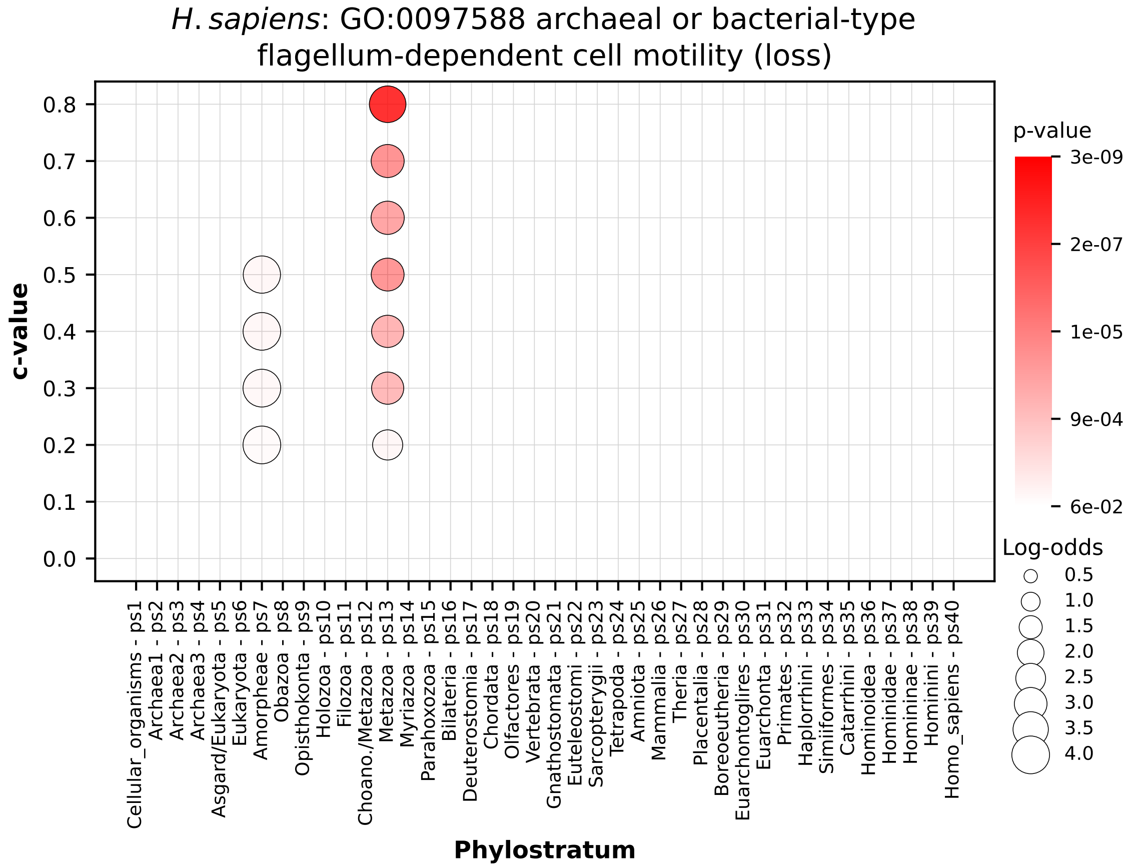

H. sapiens: GO:0097588 archaeal or bacterial-type flagellum-dependent cell motility (loss)
c-value
0.8
0.7
0.6
0.5
0.4
0.3
0.2
0.1
0.0
p-value
3e-09
2e-07
1e-05
9e-04
6e-02
Log-odds
0.5
1.0
1.5
2.0
2.5
3.0
3.5
4.0
Cellular_organisms - ps1
Archaea1 - ps2
Archaea2 - ps3
Archaea3 - ps4
Asgard/Eukaryota - ps5
Eukaryota - ps6
Amorpheae - ps7
Obazoa - ps8
Opisthokonta - ps9
Holozoa - ps10
Filozoa - ps11
Choano./Metazoa - ps12
Metazoa - ps13
Myriazoa - ps14
Parahoxozoa - ps15
Bilateria - ps16
Deuterostomia - ps17
Chordata - ps18
Olfactores - ps19
Vertebrata - ps20
Gnathostomata - ps21
Euteleostomi - ps22
Sarcopterygii - ps23
Tetrapoda - ps24
Amniota - ps25
Mammalia - ps26
Theria - ps27
Placentalia - ps28
Boreoeutheria - ps29
Euarchontoglires - ps30
Euarchonta - ps31
Primates - ps32
Haplorrhini - ps33
Simiiformes - ps34
Catarrhini - ps35
Hominoidea - ps36
Hominidae - ps37
Homininae - ps38
Hominini - ps39
Homo_sapiens - ps40
Phylostratum

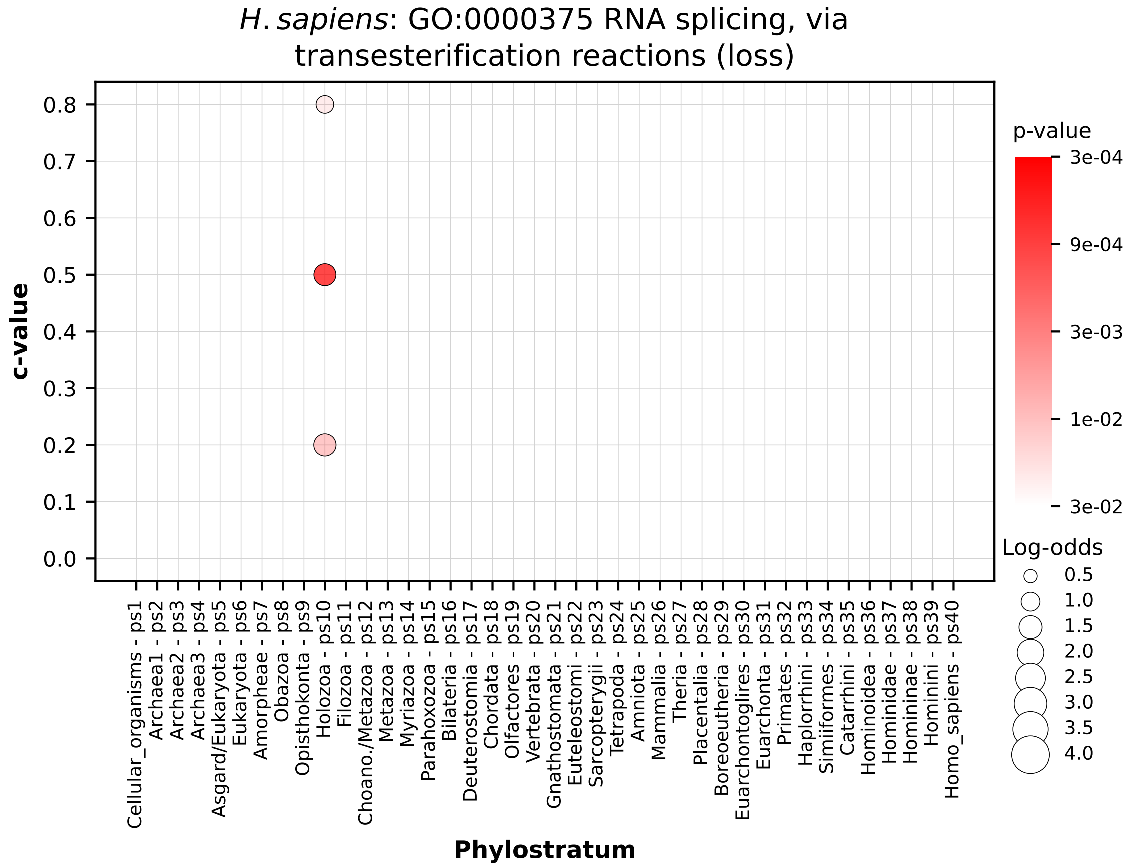
H. sapiens: GO:0000375 RNA splicing, via transesterification reactions (loss)
c-value
0.0
0.1
0.2
0.3
0.4
0.5
0.6
0.7
0.8
Phylostratum
Cellular_organisms - ps1
Archaea1 - ps2
Archaea2 - ps3
Archaea3 - ps4
Asgard/Eukaryota - ps5
Eukaryota - ps6
Amorpheae - ps7
Obazoa - ps8
Opisthokonta - ps9
Holozoa - ps10
Filozoa - ps11
Choano./Metazoa - ps12
Metazoa - ps13
Myriazoa - ps14
Parahoxozoa - ps15
Bilateria - ps16
Deuterostomia - ps17
Chordata - ps18
Olfactores - ps19
Vertebrata - ps20
Gnathostomata - ps21
Euteleostomi - ps22
Sarcopterygii - ps23
Tetrapoda - ps24
Amniota - ps25
Mammalia - ps26
Theria - ps27
Placentalia - ps28
Boreoeutheria - ps29
Euarchontoglires - ps30
Euarchonta - ps31
Primates - ps32
Haplorrhini - ps33
Simiiformes - ps34
Catarrhini - ps35
Hominoidea - ps36
Hominidae - ps37
Homininae - ps38
Hominini - ps39
Homo_sapiens - ps40
p-value
3e-04
9e-04
3e-03
1e-02
3e-02
Log-odds
0.5
1.0
1.5
2.0
2.5
3.0
3.5
4.0

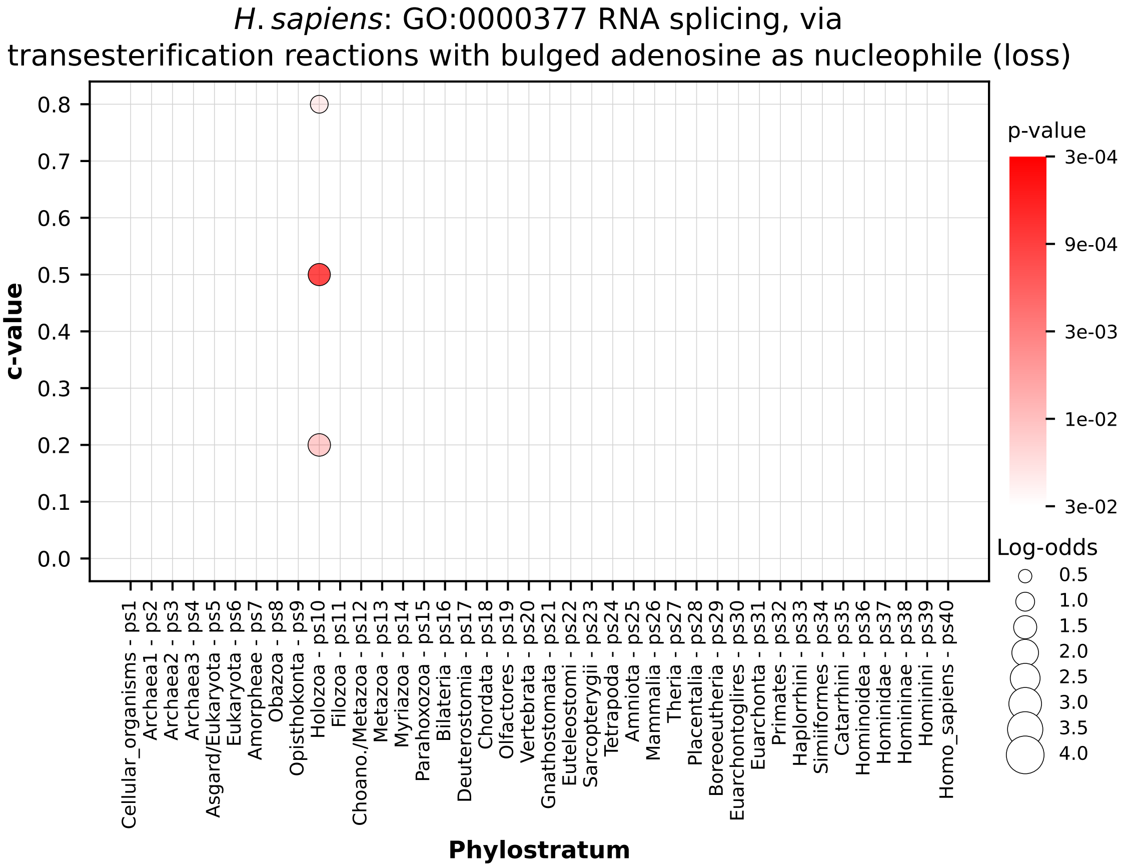
H. sapiens: GO:0000377 RNA splicing, via
transesterification reactions with bulged adenosine as nucleophile (loss)
c-value
0.8
0.7
0.6
0.5
0.4
0.3
0.2
0.1
0.0
p-value
3e-04
9e-04
3e-03
1e-02
3e-02
Log-odds
0.5
1.0
1.5
2.0
2.5
3.0
3.5
4.0
Cellular_organisms - ps1
Archaea1 - ps2
Archaea2 - ps3
Archaea3 - ps4
Asgard/Eukaryota - ps5
Eukaryota - ps6
Amorpheae - ps7
Obazoa - ps8
Opisthokonta - ps9
Holozoa - ps10
Filozoa - ps11
Choano./Metazoa - ps12
Metazoa - ps13
Myriazoa - ps14
Parahoxozoa - ps15
Bilateria - ps16
Deuterostomia - ps17
Chordata - ps18
Olfactores - ps19
Vertebrata - ps20
Gnathostomata - ps21
Euteleostomi - ps22
Sarcopterygii - ps23
Tetrapoda - ps24
Amniota - ps25
Mammalia - ps26
Theria - ps27
Placentalia - ps28
Boreoeutheria - ps29
Euarchontoglires - ps30
Euarchonta - ps31
Primates - ps32
Haplorrhini - ps33
Simiiformes - ps34
Catarrhini - ps35
Hominoidea - ps36
Hominidae - ps37
Homininae - ps38
Hominini - ps39
Homo_sapiens - ps40
Phylostratum

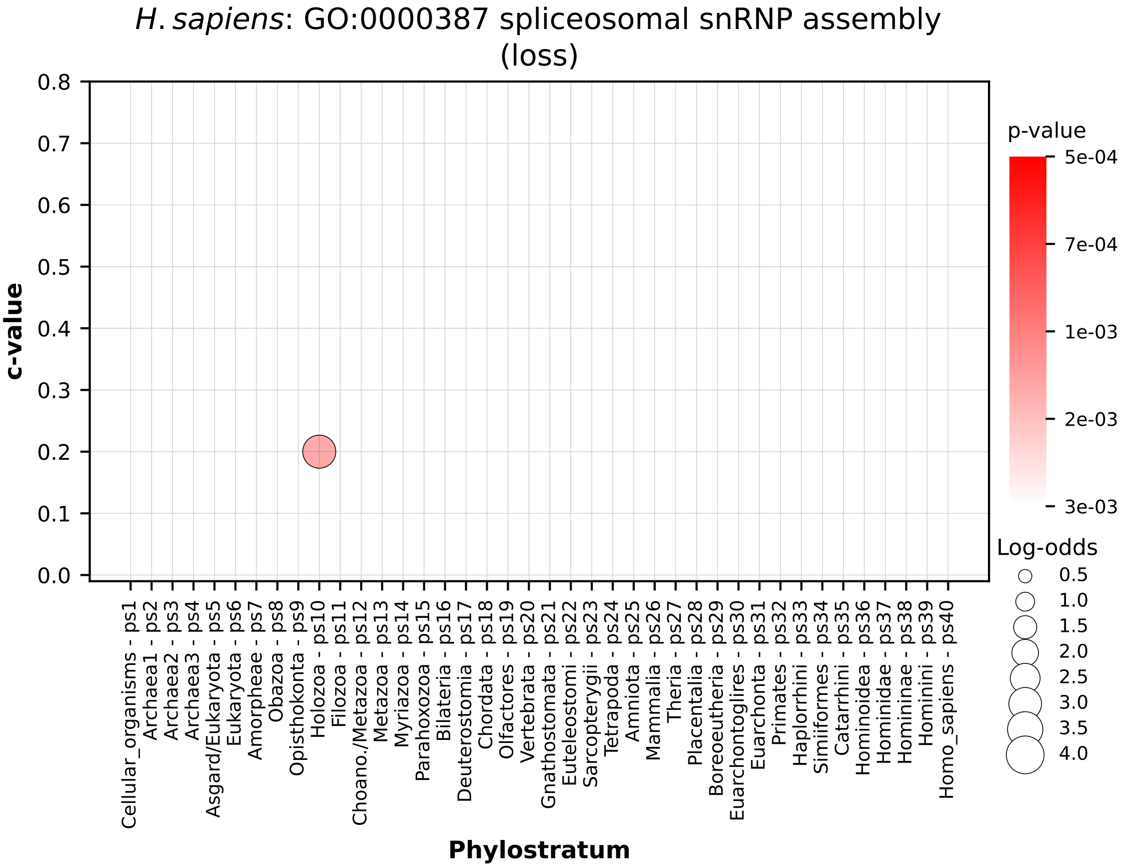

H. sapiens: GO:0000387 spliceosomal snRNP assembly (loss)
c-value
0.8
0.7
0.6
0.5
0.4
0.3
0.2
0.1
0.0
Phylostratum
Cellular_organisms - ps1
Archaea1 - ps2
Archaea2 - ps3
Archaea3 - ps4
Asgard/Eukaryota - ps5
Eukaryota - ps6
Amorpheae - ps7
Obazoa - ps8
Opisthokonta - ps9
Holozoa - ps10
Filozoa - ps11
Choano./Metazoa - ps12
Metazoa - ps13
Myriazoa - ps14
Parahoxozoa - ps15
Bilateria - ps16
Deuterostomia - ps17
Chordata - ps18
Olfactores - ps19
Vertebrata - ps20
Gnathostomata - ps21
Euteleostomi - ps22
Sarcopterygii - ps23
Tetrapoda - ps24
Amniota - ps25
Mammalia - ps26
Theria - ps27
Placentalia - ps28
Boreoeutheria - ps29
Euarchontoglires - ps30
Euarchonta - ps31
Primates - ps32
Haplorrhini - ps33
Simiiformes - ps34
Catarrhini - ps35
Hominoidea - ps36
Hominidae - ps37
Homininae - ps38
Hominini - ps39
Homo_sapiens - ps40
p-value
5e-04
7e-04
1e-03
2e-03
3e-03
Log-odds
0.5
1.0
1.5
2.0
2.5
3.0
3.5
4.0

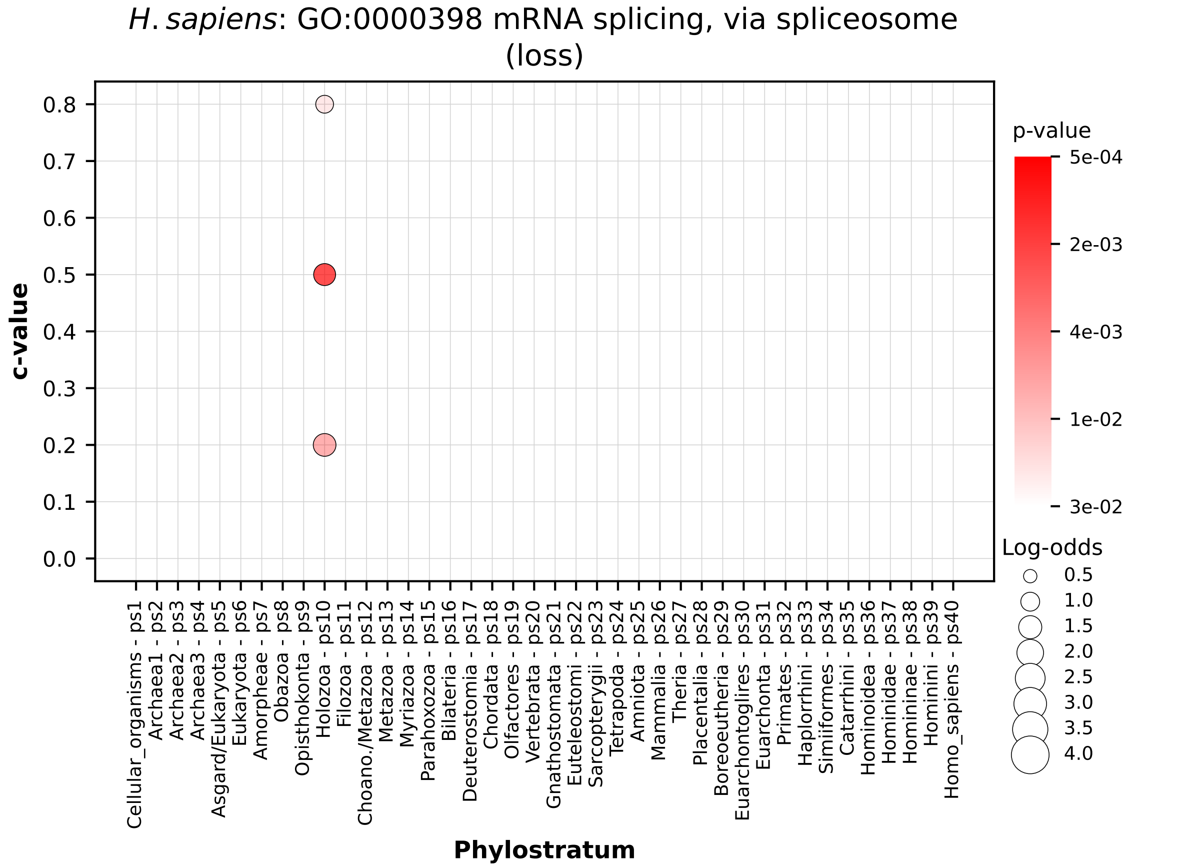
H. sapiens: GO:0000398 mRNA splicing, via spliceosome (loss)
c-value
0.8
0.7
0.6
0.5
0.4
0.3
0.2
0.1
0.0
p-value
5e-04
2e-03
4e-03
1e-02
3e-02
Log-odds
0.5
1.0
1.5
2.0
2.5
3.0
3.5
4.0
Cellular_organisms - ps1
Archaea1 - ps2
Archaea2 - ps3
Archaea3 - ps4
Asgard/Eukaryota - ps5
Eukaryota - ps6
Amorpheae - ps7
Obazoa - ps8
Opisthokonta - ps9
Holozoa - ps10
Filozoa - ps11
Choano./Metazoa - ps12
Metazoa - ps13
Myriazoa - ps14
Parahoxozoa - ps15
Bilateria - ps16
Deuterostomia - ps17
Chordata - ps18
Olfactores - ps19
Vertebrata - ps20
Gnathostomata - ps21
Euteleostomi - ps22
Sarcopterygii - ps23
Tetrapoda - ps24
Amniota - ps25
Mammalia - ps26
Theria - ps27
Placentalia - ps28
Boreoeutheria - ps29
Euarchontoglires - ps30
Euarchonta - ps31
Primates - ps32
Haplorrhini - ps33
Simiiformes - ps34
Catarrhini - ps35
Hominoidea - ps36
Hominidae - ps37
Homininae - ps38
Hominini - ps39
Homo_sapiens - ps40
Phylostratum

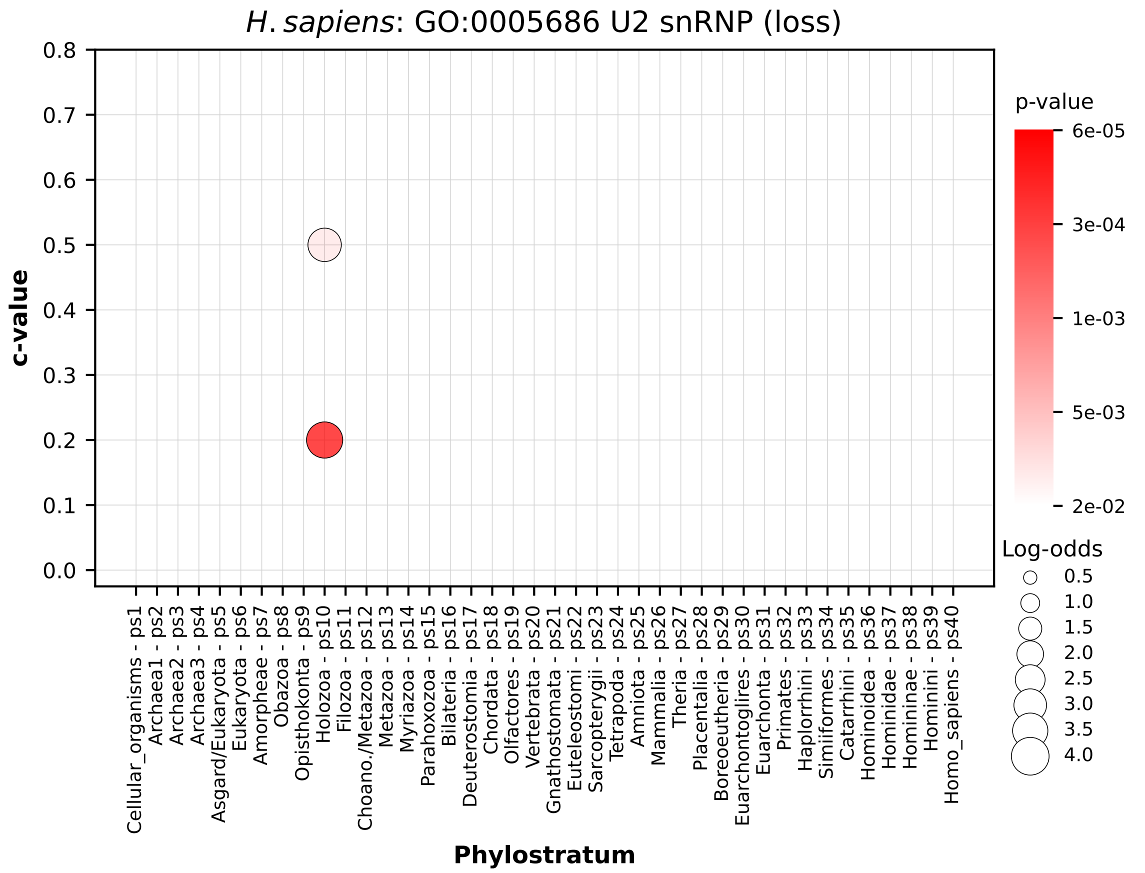

H. sapiens: GO:0005686 U2 snRNP (loss)
c-value
0.0
0.1
0.2
0.3
0.4
0.5
0.6
0.7
0.8
Cellular_organisms - ps1
Archaea1 - ps2
Archaea2 - ps3
Archaea3 - ps4
Asgard/Eukaryota - ps5
Eukaryota - ps6
Amorpheae - ps7
Obazoa - ps8
Opisthokonta - ps9
Holozoa - ps10
Filozoa - ps11
Choano./Metazoa - ps12
Metazoa - ps13
Myriazoa - ps14
Parahoxozoa - ps15
Bilateria - ps16
Deuterostomia - ps17
Chordata - ps18
Olfactores - ps19
Vertebrata - ps20
Gnathostomata - ps21
Euteleostomi - ps22
Sarcopterygii - ps23
Tetrapoda - ps24
Amniota - ps25
Mammalia - ps26
Theria - ps27
Placentalia - ps28
Boreoeutheria - ps29
Euarchontoglires - ps30
Euarchonta - ps31
Primates - ps32
Haplorrhini - ps33
Simiiformes - ps34
Catarrhini - ps35
Hominoidea - ps36
Hominidae - ps37
Homininae - ps38
Hominini - ps39
Homo_sapiens - ps40
Phylostratum
p-value
6e-05
3e-04
1e-03
5e-03
2e-02
Log-odds
0.5
1.0
1.5
2.0
2.5
3.0
3.5
4.0

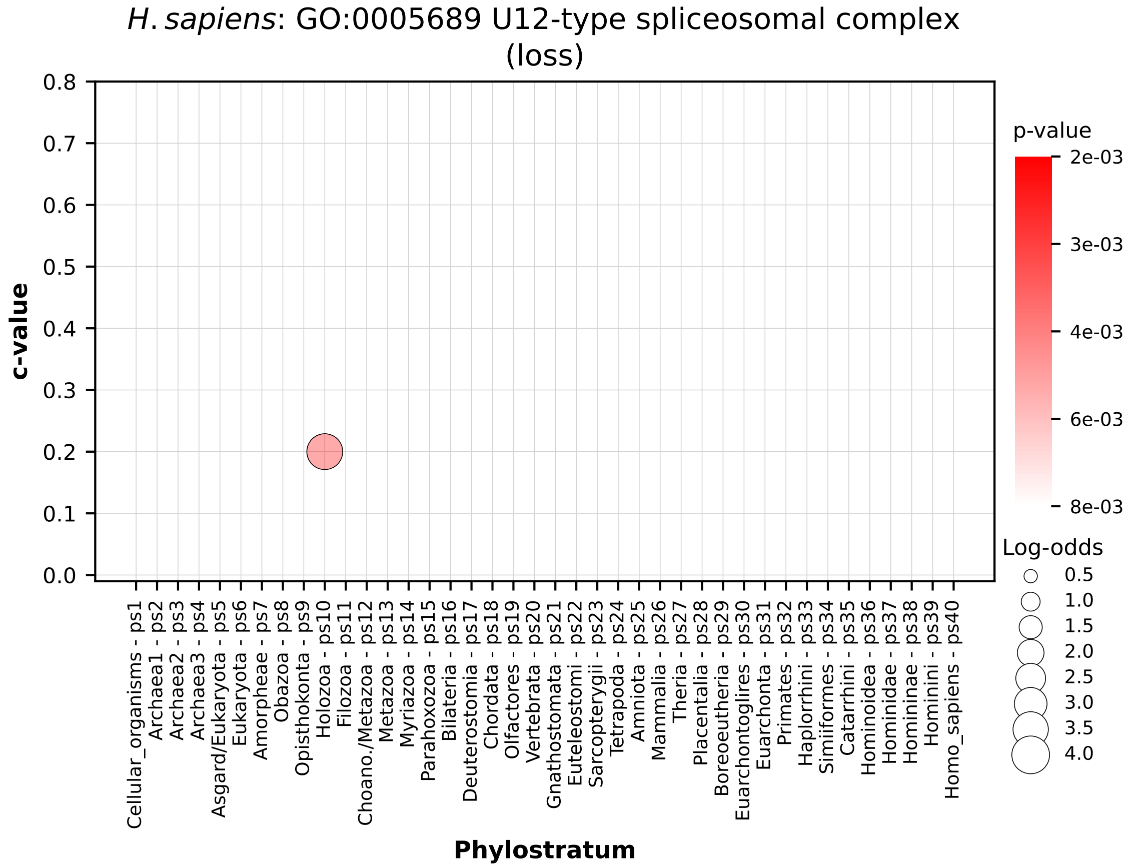

H. sapiens: GO:0005689 U12-type spliceosomal complex
(loss)
c-value
0.8
0.7
0.6
0.5
0.4
0.3
0.2
0.1
0.0
Cellular_organisms - ps1
Archaea1 - ps2
Archaea2 - ps3
Archaea3 - ps4
Asgard/Eukaryota - ps5
Eukaryota - ps6
Amorpheae - ps7
Obazoa - ps8
Opisthokonta - ps9
Holozoa - ps10
Filozoa - ps11
Choano./Metazoa - ps12
Metazoa - ps13
Myriazoa - ps14
Parahoxozoa - ps15
Bilateria - ps16
Deuterostomia - ps17
Chordata - ps18
Olfactores - ps19
Vertebrata - ps20
Gnathostomata - ps21
Euteleostomi - ps22
Sarcopterygii - ps23
Tetrapoda - ps24
Amniota - ps25
Mammalia - ps26
Theria - ps27
Placentalia - ps28
Boreoeutheria - ps29
Euarchontoglires - ps30
Euarchonta - ps31
Primates - ps32
Haplorrhini - ps33
Simiiformes - ps34
Catarrhini - ps35
Hominoidea - ps36
Hominidae - ps37
Homininae - ps38
Hominini - ps39
Homo_sapiens - ps40
Phylostratum
p-value
2e-03
3e-03
4e-03
6e-03
8e-03
Log-odds
0.5
1.0
1.5
2.0
2.5
3.0
3.5
4.0

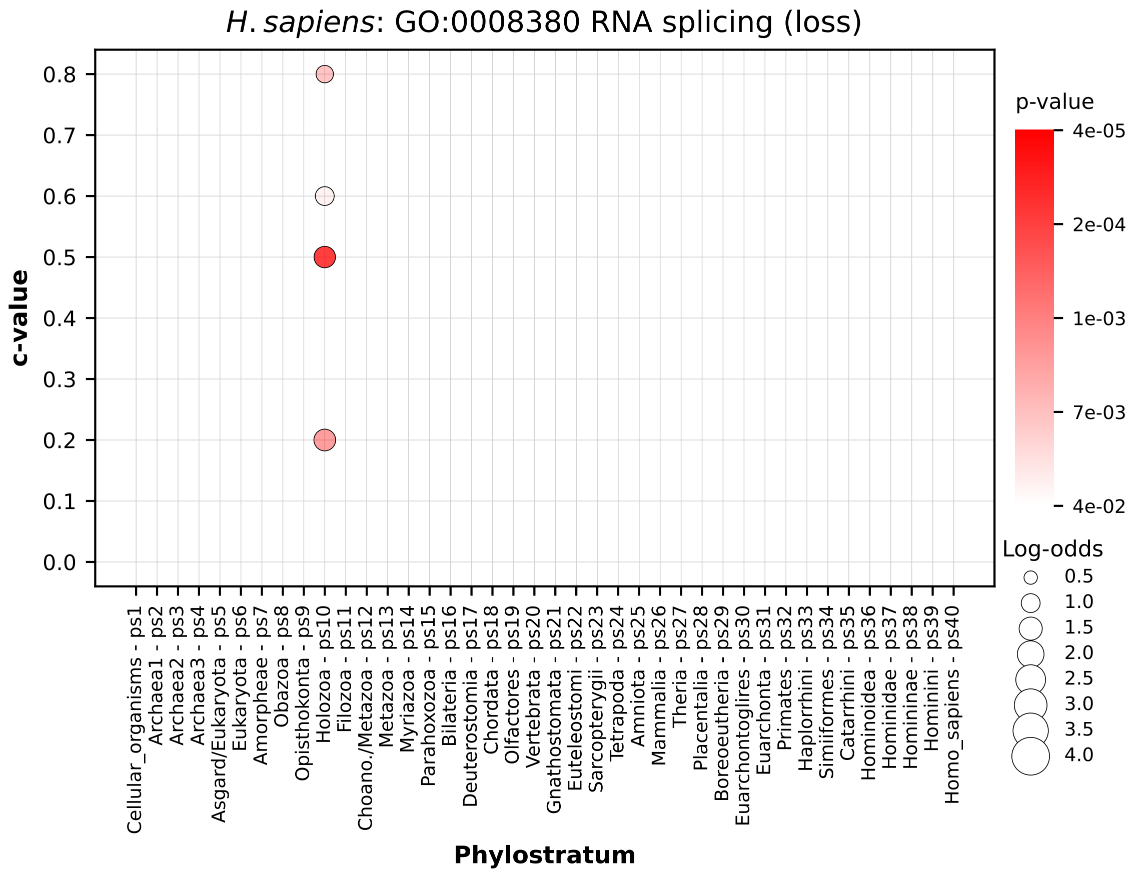

H. sapiens: GO:0008380 RNA splicing (loss)
c-value
0.0
0.1
0.2
0.3
0.4
0.5
0.6
0.7
0.8
Phylostratum
Cellular_organisms - ps1
Archaea1 - ps2
Archaea2 - ps3
Archaea3 - ps4
Asgard/Eukaryota - ps5
Eukaryota - ps6
Amorpheae - ps7
Obazoa - ps8
Opisthokonta - ps9
Holozoa - ps10
Filozoa - ps11
Choano./Metazoa - ps12
Metazoa - ps13
Myriazoa - ps14
Parahoxozoa - ps15
Bilateria - ps16
Deuterostomia - ps17
Chordata - ps18
Olfactores - ps19
Vertebrata - ps20
Gnathostomata - ps21
Euteleostomi - ps22
Sarcopterygii - ps23
Tetrapoda - ps24
Amniota - ps25
Mammalia - ps26
Theria - ps27
Placentalia - ps28
Boreoeutheria - ps29
Euarchontoglires - ps30
Euarchonta - ps31
Primates - ps32
Haplorrhini - ps33
Simiiformes - ps34
Catarrhini - ps35
Hominoidea - ps36
Hominidae - ps37
Homininae - ps38
Hominini - ps39
Homo_sapiens - ps40
p-value
4e-05
2e-04
1e-03
7e-03
4e-02
Log-odds
0.5
1.0
1.5
2.0
2.5
3.0
3.5
4.0

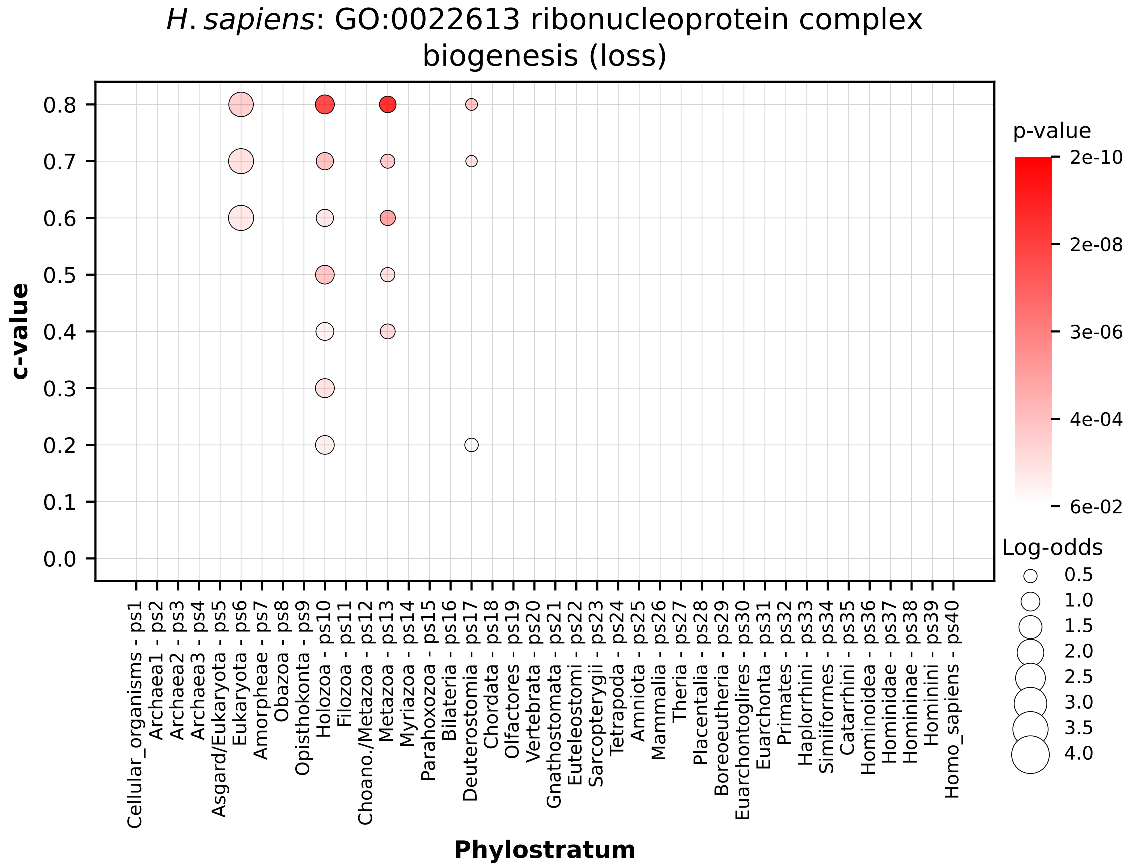

H. sapiens: GO:0022613 ribonucleoprotein complex biogenesis (loss)
c-value
0.8
0.7
0.6
0.5
0.4
0.3
0.2
0.1
0.0
p-value
2e-10
2e-08
3e-06
4e-04
6e-02
Log-odds
0.5
1.0
1.5
2.0
2.5
3.0
3.5
4.0
Cellular_organisms - ps1
Archaea1 - ps2
Archaea2 - ps3
Archaea3 - ps4
Asgard/Eukaryota - ps5
Eukaryota - ps6
Amorpheae - ps7
Obazoa - ps8
Opisthokonta - ps9
Holozoa - ps10
Filozoa - ps11
Choano./Metazoa - ps12
Metazoa - ps13
Myriazoa - ps14
Parahoxozoa - ps15
Bilateria - ps16
Deuterostomia - ps17
Chordata - ps18
Olfactores - ps19
Vertebrata - ps20
Gnathostomata - ps21
Euteleostomi - ps22
Sarcopterygii - ps23
Tetrapoda - ps24
Amniota - ps25
Mammalia - ps26
Theria - ps27
Placentalia - ps28
Boreoeutheria - ps29
Euarchontoglires - ps30
Euarchonta - ps31
Primates - ps32
Haplorrhini - ps33
Simiiformes - ps34
Catarrhini - ps35
Hominoidea - ps36
Hominidae - ps37
Homininae - ps38
Hominini - ps39
Homo_sapiens - ps40
Phylostratum

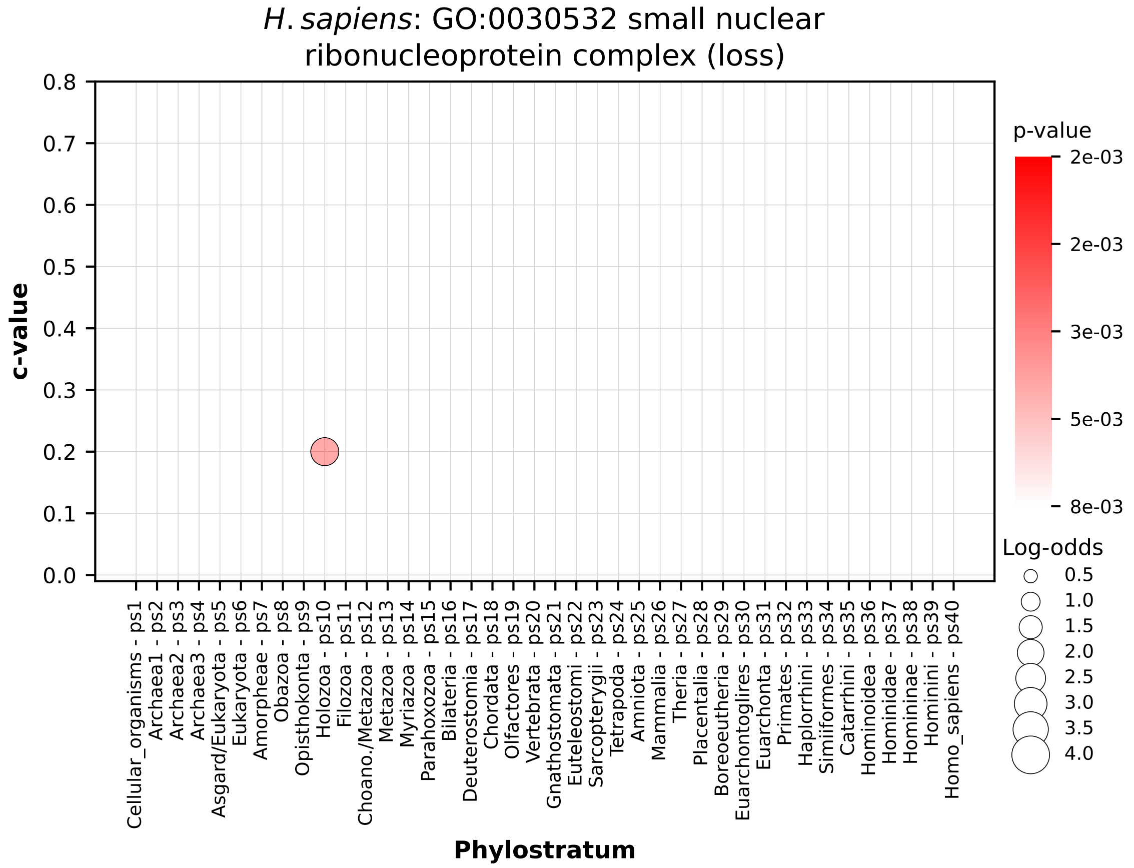

H. sapiens: GO:0030532 small nuclear ribonucleoprotein complex (loss)
c-value
0.0
0.1
0.2
0.3
0.4
0.5
0.6
0.7
0.8
Phylostratum
Cellular_organisms - ps1
Archaea1 - ps2
Archaea2 - ps3
Archaea3 - ps4
Asgard/Eukaryota - ps5
Eukaryota - ps6
Amorpheae - ps7
Obazoa - ps8
Opisthokonta - ps9
Holozoa - ps10
Filozoa - ps11
Choano./Metazoa - ps12
Metazoa - ps13
Myriazoa - ps14
Parahoxozoa - ps15
Bilateria - ps16
Deuterostomia - ps17
Chordata - ps18
Olfactores - ps19
Vertebrata - ps20
Gnathostomata - ps21
Euteleostomi - ps22
Sarcopterygii - ps23
Tetrapoda - ps24
Amniota - ps25
Mammalia - ps26
Theria - ps27
Placentalia - ps28
Boreoeutheria - ps29
Euarchontoglires - ps30
Euarchonta - ps31
Primates - ps32
Haplorrhini - ps33
Simiiformes - ps34
Catarrhini - ps35
Hominoidea - ps36
Hominidae - ps37
Homininae - ps38
Hominini - ps39
Homo_sapiens - ps40
p-value
2e-03
2e-03
3e-03
5e-03
8e-03
Log-odds
0.5
1.0
1.5
2.0
2.5
3.0
3.5
4.0

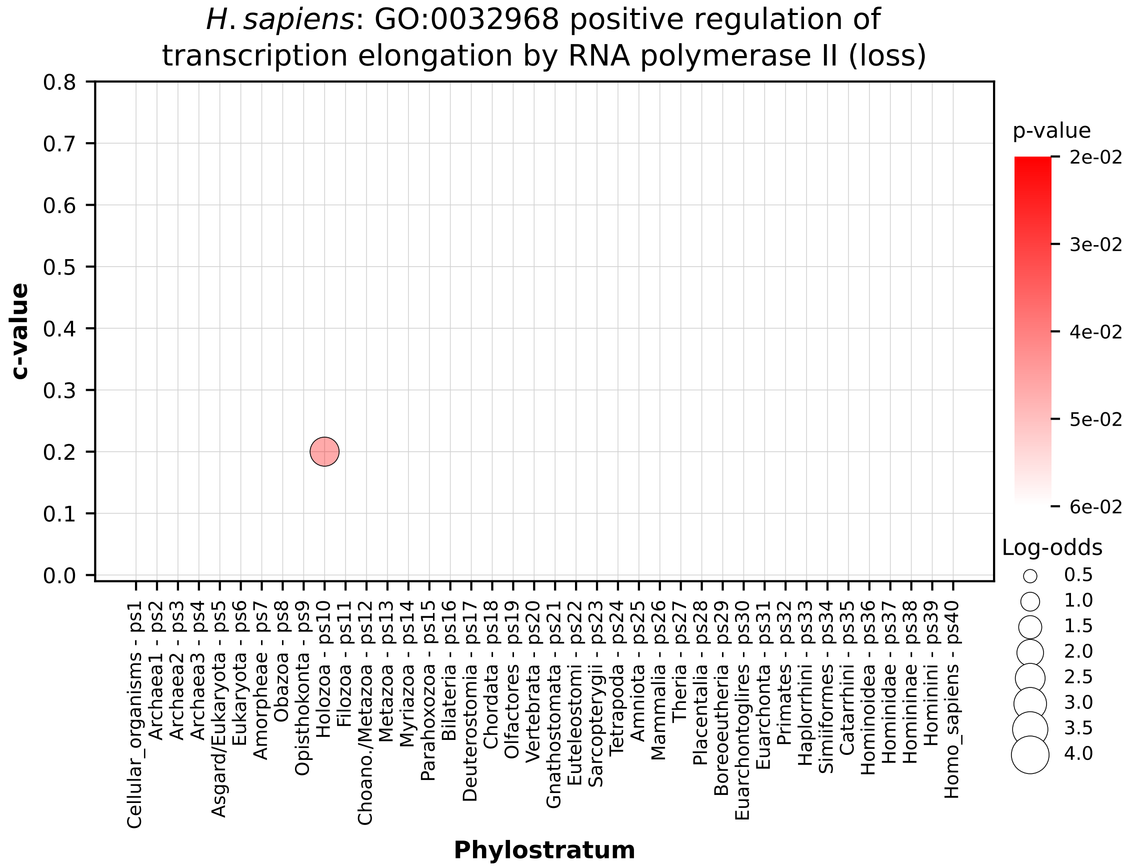

H. sapiens: GO:0032968 positive regulation of transcription elongation by RNA polymerase II (loss)
c-value
0.8
0.7
0.6
0.5
0.4
0.3
0.2
0.1
0.0
p-value
2e-02
3e-02
4e-02
5e-02
6e-02
Log-odds
0.5
1.0
1.5
2.0
2.5
3.0
3.5
4.0
Cellular_organisms - ps1
Archaea1 - ps2
Archaea2 - ps3
Archaea3 - ps4
Asgard/Eukaryota - ps5
Eukaryota - ps6
Amorpheae - ps7
Obazoa - ps8
Opisthokonta - ps9
Holozoa - ps10
Filozoa - ps11
Choano./Metazoa - ps12
Metazoa - ps13
Myriazoa - ps14
Parahoxozoa - ps15
Bilateria - ps16
Deuterostomia - ps17
Chordata - ps18
Olfactores - ps19
Vertebrata - ps20
Gnathostomata - ps21
Euteleostomi - ps22
Sarcopterygii - ps23
Tetrapoda - ps24
Amniota - ps25
Mammalia - ps26
Theria - ps27
Placentalia - ps28
Boreoeutheria - ps29
Euarchontoglires - ps30
Euarchonta - ps31
Primates - ps32
Haplorrhini - ps33
Simiiformes - ps34
Catarrhini - ps35
Hominoidea - ps36
Hominidae - ps37
Homininae - ps38
Hominini - ps39
Homo_sapiens - ps40
Phylostratum

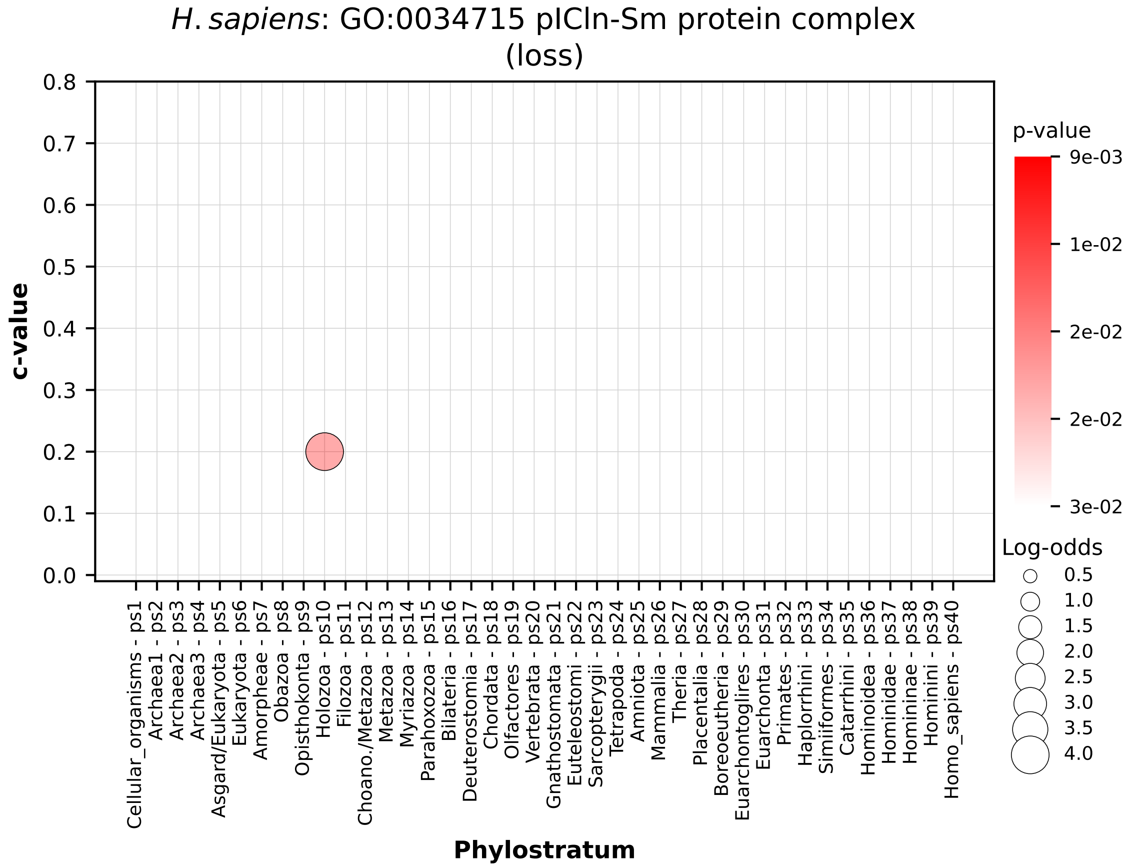
H. sapiens: GO:0034715 pICln-Sm protein complex
(loss)
c-value
0.8
0.7
0.6
0.5
0.4
0.3
0.2
0.1
0.0
p-value
9e-03
1e-02
2e-02
2e-02
3e-02
Log-odds
0.5
1.0
1.5
2.0
2.5
3.0
3.5
4.0
Cellular_organisms - ps1
Archaea1 - ps2
Archaea2 - ps3
Archaea3 - ps4
Asgard/Eukaryota - ps5
Eukaryota - ps6
Amorpheae - ps7
Obazoa - ps8
Opisthokonta - ps9
Holozoa - ps10
Filozoa - ps11
Choano./Metazoa - ps12
Metazoa - ps13
Myriazoa - ps14
Parahoxozoa - ps15
Bilateria - ps16
Deuterostomia - ps17
Chordata - ps18
Olfactores - ps19
Vertebrata - ps20
Gnathostomata - ps21
Euteleostomi - ps22
Sarcopterygii - ps23
Tetrapoda - ps24
Amniota - ps25
Mammalia - ps26
Theria - ps27
Placentalia - ps28
Boreoeutheria - ps29
Euarchontoglires - ps30
Euarchonta - ps31
Primates - ps32
Haplorrhini - ps33
Simiiformes - ps34
Catarrhini - ps35
Hominoidea - ps36
Hominidae - ps37
Homininae - ps38
Hominini - ps39
Homo_sapiens - ps40
Phylostratum

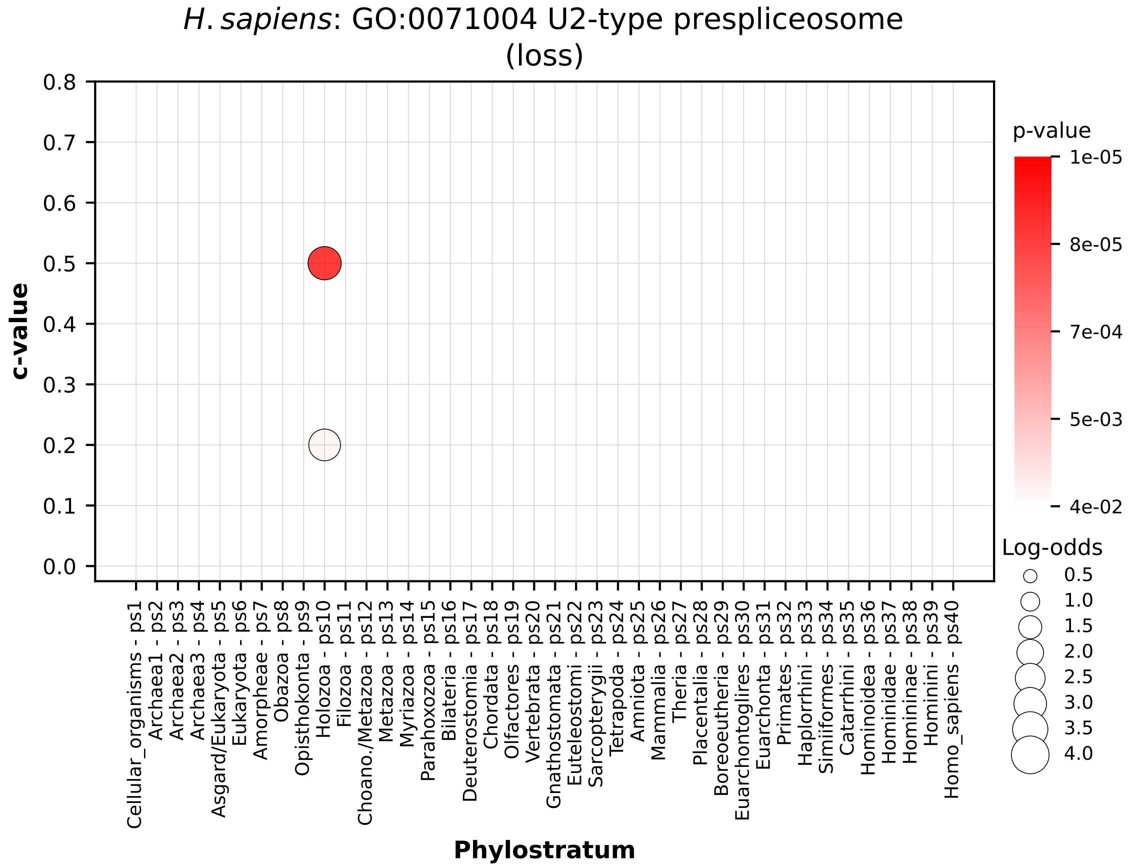

H. sapiens: GO:0071004 U2-type prespliceosome
(loss)
c-value
0.8
0.7
0.6
0.5
0.4
0.3
0.2
0.1
0.0
p-value
1e-05
8e-05
7e-04
5e-03
4e-02
Log-odds
0.5
1.0
1.5
2.0
2.5
3.0
3.5
4.0
Cellular_organisms - ps1
Archaea1 - ps2
Archaea2 - ps3
Archaea3 - ps4
Asgard/Eukaryota - ps5
Eukaryota - ps6
Amorpheae - ps7
Obazoa - ps8
Opisthokonta - ps9
Holozoa - ps10
Filozoa - ps11
Choano./Metazoa - ps12
Metazoa - ps13
Myriazoa - ps14
Parahoxozoa - ps15
Bilateria - ps16
Deuterostomia - ps17
Chordata - ps18
Olfactores - ps19
Vertebrata - ps20
Gnathostomata - ps21
Euteleostomi - ps22
Sarcopterygii - ps23
Tetrapoda - ps24
Amniota - ps25
Mammalia - ps26
Theria - ps27
Placentalia - ps28
Boreoeutheria - ps29
Euarchontoglires - ps30
Euarchonta - ps31
Primates - ps32
Haplorrhini - ps33
Simiiformes - ps34
Catarrhini - ps35
Hominoidea - ps36
Hominidae - ps37
Homininae - ps38
Hominini - ps39
Homo_sapiens - ps40
Phylostratum

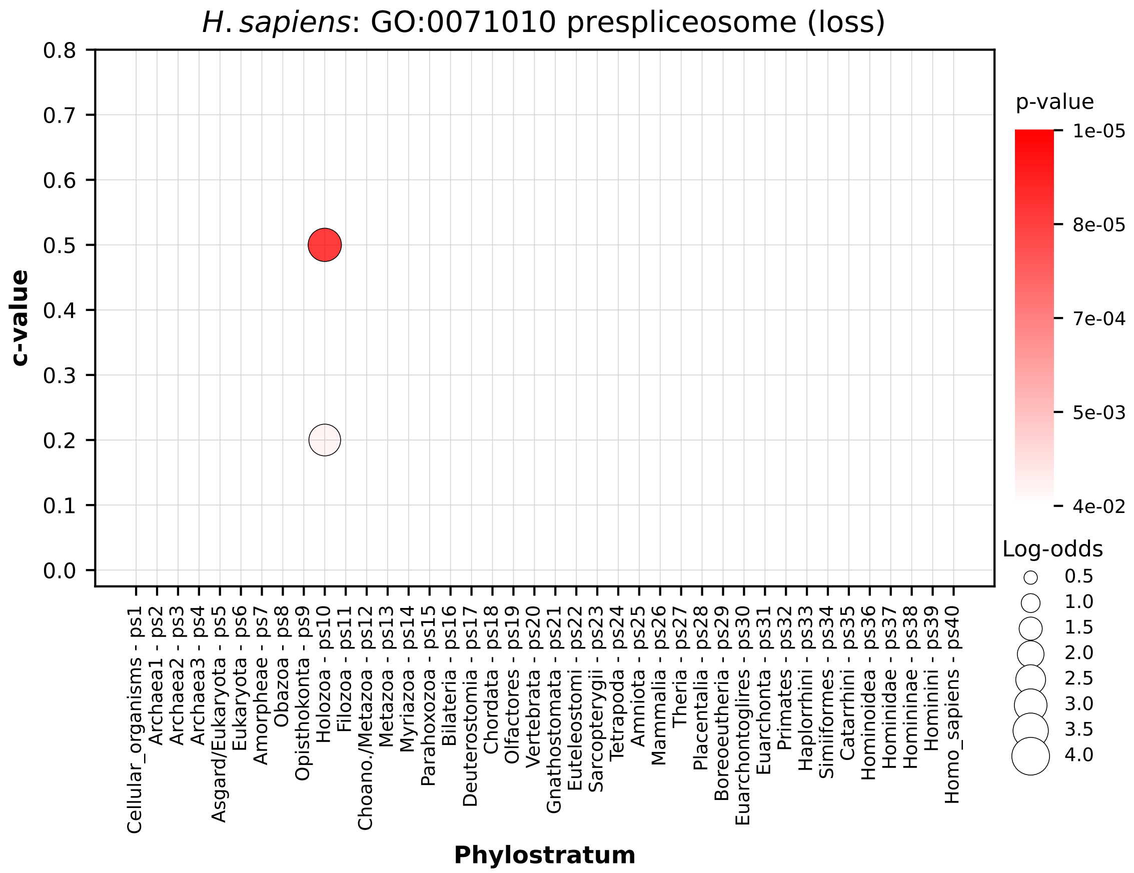
H. sapiens: GO:0071010 prespliceosome (loss)
c-value
0.8
0.7
0.6
0.5
0.4
0.3
0.2
0.1
0.0
p-value
1e-05
8e-05
7e-04
5e-03
4e-02
Log-odds
0.5
1.0
1.5
2.0
2.5
3.0
3.5
4.0
Cellular_organisms - ps1
Archaea1 - ps2
Archaea2 - ps3
Archaea3 - ps4
Asgard/Eukaryota - ps5
Eukaryota - ps6
Amorpheae - ps7
Obazoa - ps8
Opisthokonta - ps9
Holozoa - ps10
Filozoa - ps11
Choano./Metazoa - ps12
Metazoa - ps13
Myriazoa - ps14
Parahoxozoa - ps15
Bilateria - ps16
Deuterostomia - ps17
Chordata - ps18
Olfactores - ps19
Vertebrata - ps20
Gnathostomata - ps21
Euteleostomi - ps22
Sarcopterygii - ps23
Tetrapoda - ps24
Amniota - ps25
Mammalia - ps26
Theria - ps27
Placentalia - ps28
Boreoeutheria - ps29
Euarchontoglires - ps30
Euarchonta - ps31
Primates - ps32
Haplorrhini - ps33
Simiiformes - ps34
Catarrhini - ps35
Hominoidea - ps36
Hominidae - ps37
Homininae - ps38
Hominini - ps39
Homo_sapiens - ps40
Phylostratum

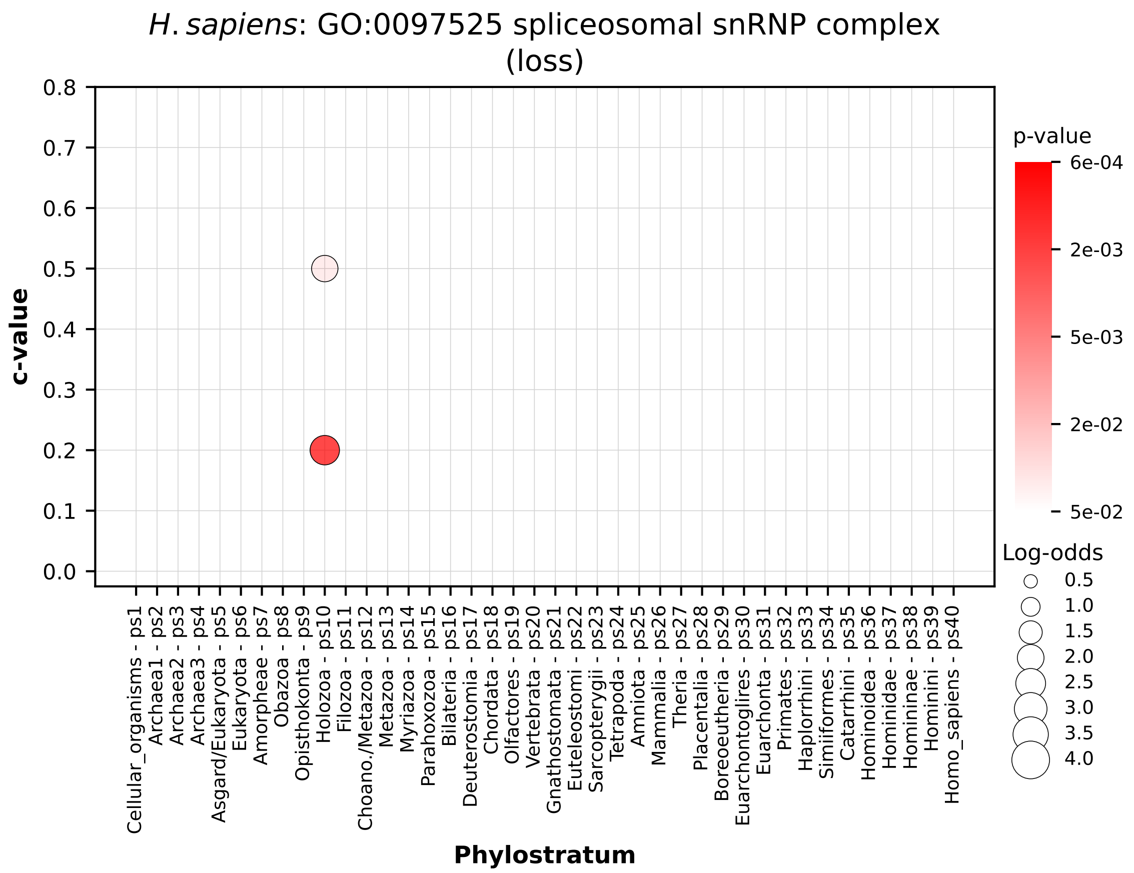

H. sapiens: GO:0097525 spliceosomal snRNP complex
(loss)
c-value
0.8
0.7
0.6
0.5
0.4
0.3
0.2
0.1
0.0
Phylostratum
Cellular_organisms - ps1
Archaea1 - ps2
Archaea2 - ps3
Archaea3 - ps4
Asgard/Eukaryota - ps5
Eukaryota - ps6
Amorpheae - ps7
Obazoa - ps8
Opisthokonta - ps9
Holozoa - ps10
Filozoa - ps11
Choano./Metazoa - ps12
Metazoa - ps13
Myriazoa - ps14
Parahoxozoa - ps15
Bilateria - ps16
Deuterostomia - ps17
Chordata - ps18
Olfactores - ps19
Vertebrata - ps20
Gnathostomata - ps21
Euteleostomi - ps22
Sarcopterygii - ps23
Tetrapoda - ps24
Amniota - ps25
Mammalia - ps26
Theria - ps27
Placentalia - ps28
Boreoeutheria - ps29
Euarchontoglires - ps30
Euarchonta - ps31
Primates - ps32
Haplorrhini - ps33
Simiiformes - ps34
Catarrhini - ps35
Hominoidea - ps36
Hominidae - ps37
Homininae - ps38
Hominini - ps39
Homo_sapiens - ps40
p-value
6e-04
2e-03
5e-03
2e-02
5e-02
Log-odds
0.5
1.0
1.5
2.0
2.5
3.0
3.5
4.0

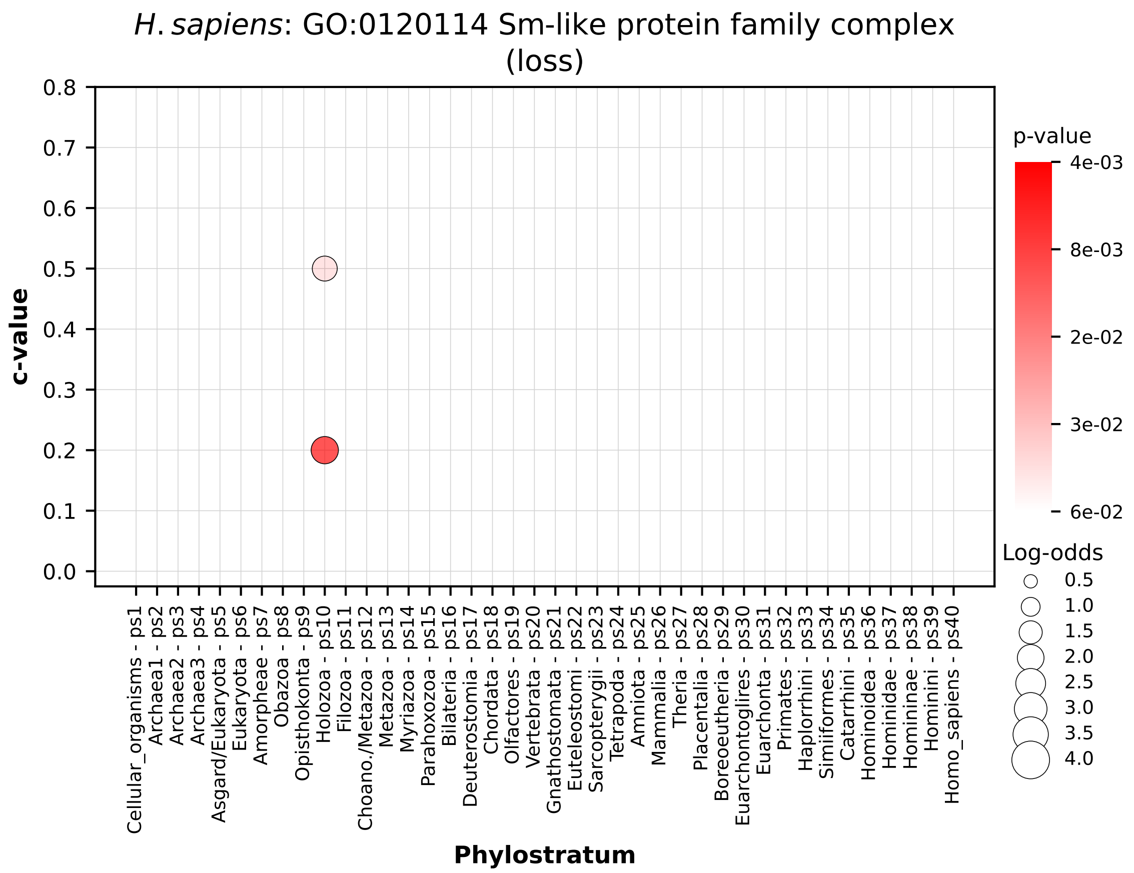

H. sapiens: GO:0120114 Sm-like protein family complex
(loss)
c-value
0.0
0.1
0.2
0.3
0.4
0.5
0.6
0.7
0.8
Phylostratum
Cellular_organisms - ps1
Archaea1 - ps2
Archaea2 - ps3
Archaea3 - ps4
Asgard/Eukaryota - ps5
Eukaryota - ps6
Amorpheae - ps7
Obazoa - ps8
Opisthokonta - ps9
Holozoa - ps10
Filozoa - ps11
Choano./Metazoa - ps12
Metazoa - ps13
Myriazoa - ps14
Parahoxozoa - ps15
Bilateria - ps16
Deuterostomia - ps17
Chordata - ps18
Olfactores - ps19
Vertebrata - ps20
Gnathostomata - ps21
Euteleostomi - ps22
Sarcopterygii - ps23
Tetrapoda - ps24
Amniota - ps25
Mammalia - ps26
Theria - ps27
Placentalia - ps28
Boreoeutheria - ps29
Euarchontoglires - ps30
Euarchonta - ps31
Primates - ps32
Haplorrhini - ps33
Simiiformes - ps34
Catarrhini - ps35
Hominoidea - ps36
Hominidae - ps37
Homininae - ps38
Hominini - ps39
Homo_sapiens - ps40
p-value
4e-03
8e-03
2e-02
3e-02
6e-02
Log-odds
0.5
1.0
1.5
2.0
2.5
3.0
3.5
4.0

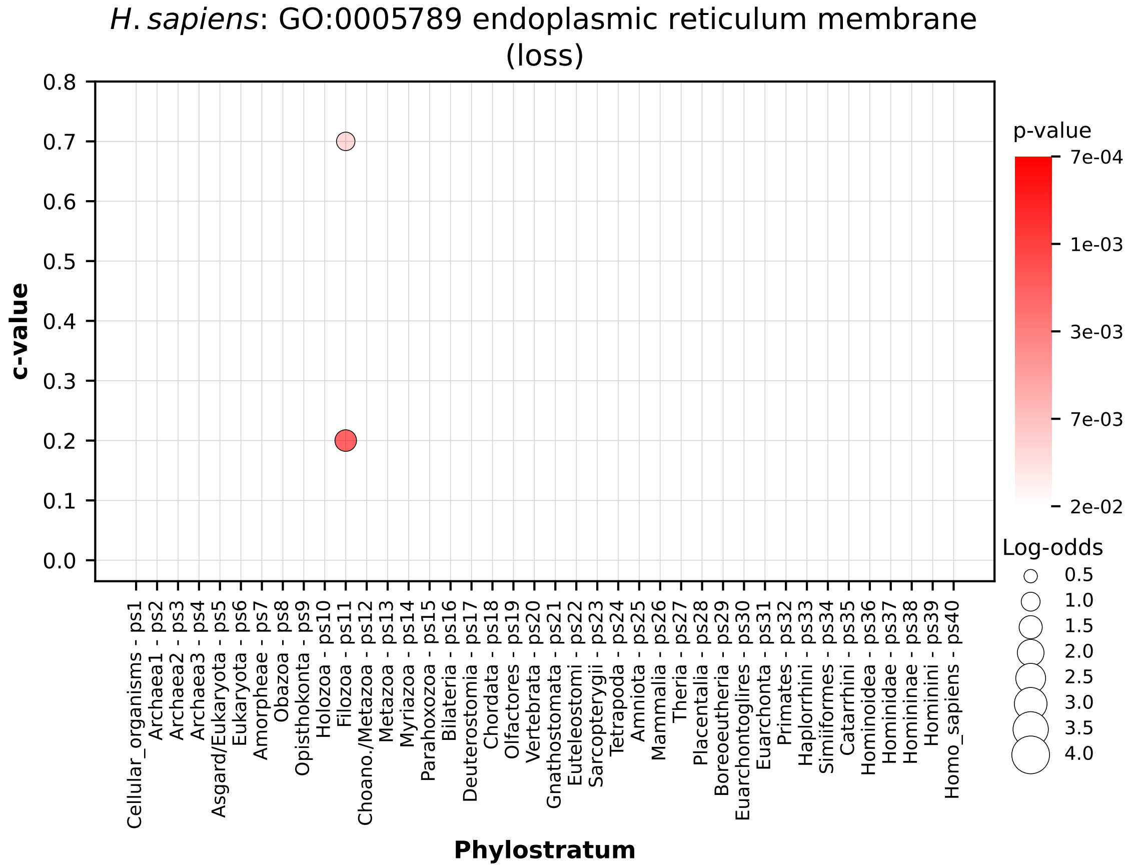

H. sapiens: GO:0005789 endoplasmic reticulum membrane (loss)
c-value
0.0
0.1
0.2
0.3
0.4
0.5
0.6
0.7
0.8
Phylostratum
Cellular_organisms - ps1
Archaea1 - ps2
Archaea2 - ps3
Archaea3 - ps4
Asgard/Eukaryota - ps5
Eukaryota - ps6
Amorpheae - ps7
Obazoa - ps8
Opisthokonta - ps9
Holozoa - ps10
Filozoa - ps11
Choano./Metazoa - ps12
Metazoa - ps13
Myriazoa - ps14
Parahoxozoa - ps15
Bilateria - ps16
Deuterostomia - ps17
Chordata - ps18
Olfactores - ps19
Vertebrata - ps20
Gnathostomata - ps21
Euteleostomi - ps22
Sarcopterygii - ps23
Tetrapoda - ps24
Amniota - ps25
Mammalia - ps26
Theria - ps27
Placentalia - ps28
Boreoeutheria - ps29
Euarchontoglires - ps30
Euarchonta - ps31
Primates - ps32
Haplorrhini - ps33
Simiiformes - ps34
Catarrhini - ps35
Hominoidea - ps36
Hominidae - ps37
Homininae - ps38
Hominini - ps39
Homo_sapiens - ps40
p-value
7e-04
1e-03
3e-03
7e-03
2e-02
Log-odds
0.5
1.0
1.5
2.0
2.5
3.0
3.5
4.0

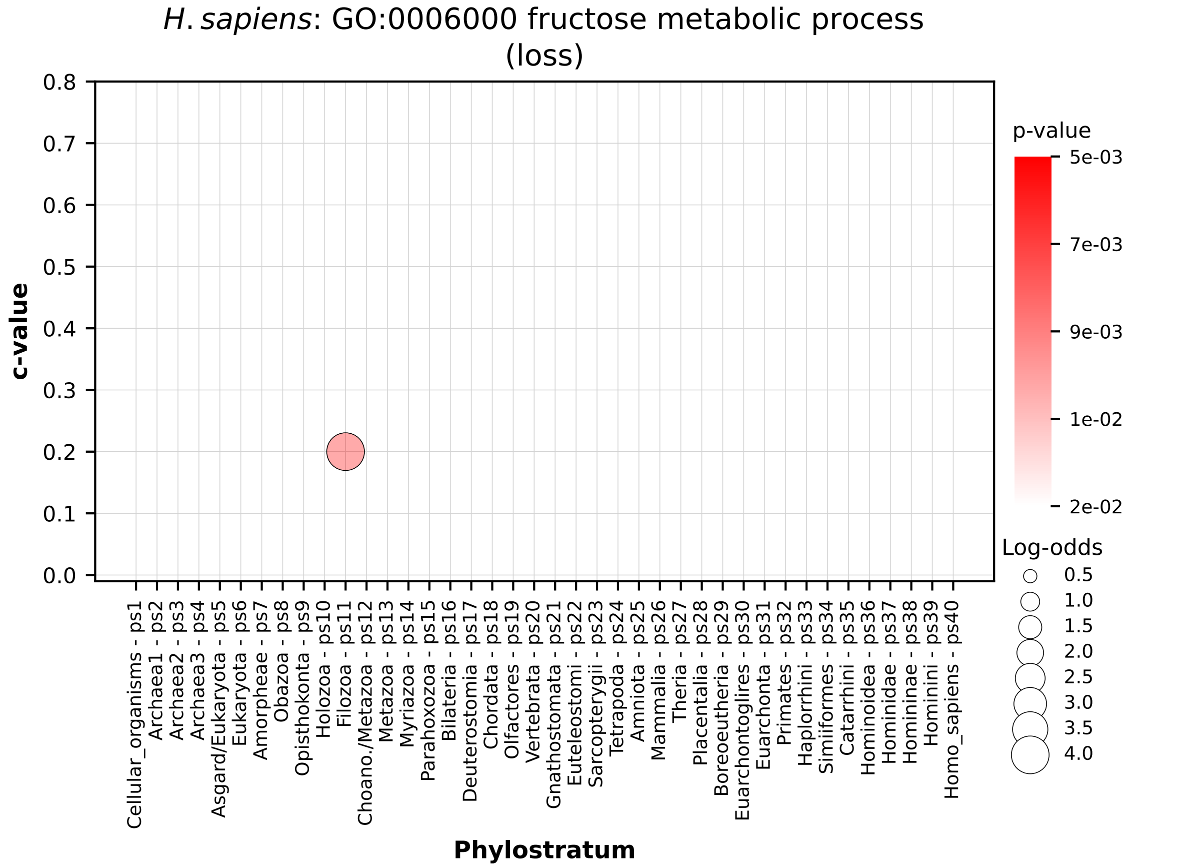

H. sapiens: GO:0006000 fructose metabolic process
(loss)
c-value
0.8
0.7
0.6
0.5
0.4
0.3
0.2
0.1
0.0
p-value
5e-03
7e-03
9e-03
1e-02
2e-02
Log-odds
0.5
1.0
1.5
2.0
2.5
3.0
3.5
4.0
Cellular_organisms - ps1
Archaea1 - ps2
Archaea2 - ps3
Archaea3 - ps4
Asgard/Eukaryota - ps5
Eukaryota - ps6
Amorpheae - ps7
Obazoa - ps8
Opisthokonta - ps9
Holozoa - ps10
Filozoa - ps11
Choano./Metazoa - ps12
Metazoa - ps13
Myriazoa - ps14
Parahoxozoa - ps15
Bilateria - ps16
Deuterostomia - ps17
Chordata - ps18
Olfactores - ps19
Vertebrata - ps20
Gnathostomata - ps21
Euteleostomi - ps22
Sarcopterygii - ps23
Tetrapoda - ps24
Amniota - ps25
Mammalia - ps26
Theria - ps27
Placentalia - ps28
Boreoeutheria - ps29
Euarchontoglires - ps30
Euarchonta - ps31
Primates - ps32
Haplorrhini - ps33
Simiiformes - ps34
Catarrhini - ps35
Hominoidea - ps36
Hominidae - ps37
Homininae - ps38
Hominini - ps39
Homo_sapiens - ps40
Phylostratum

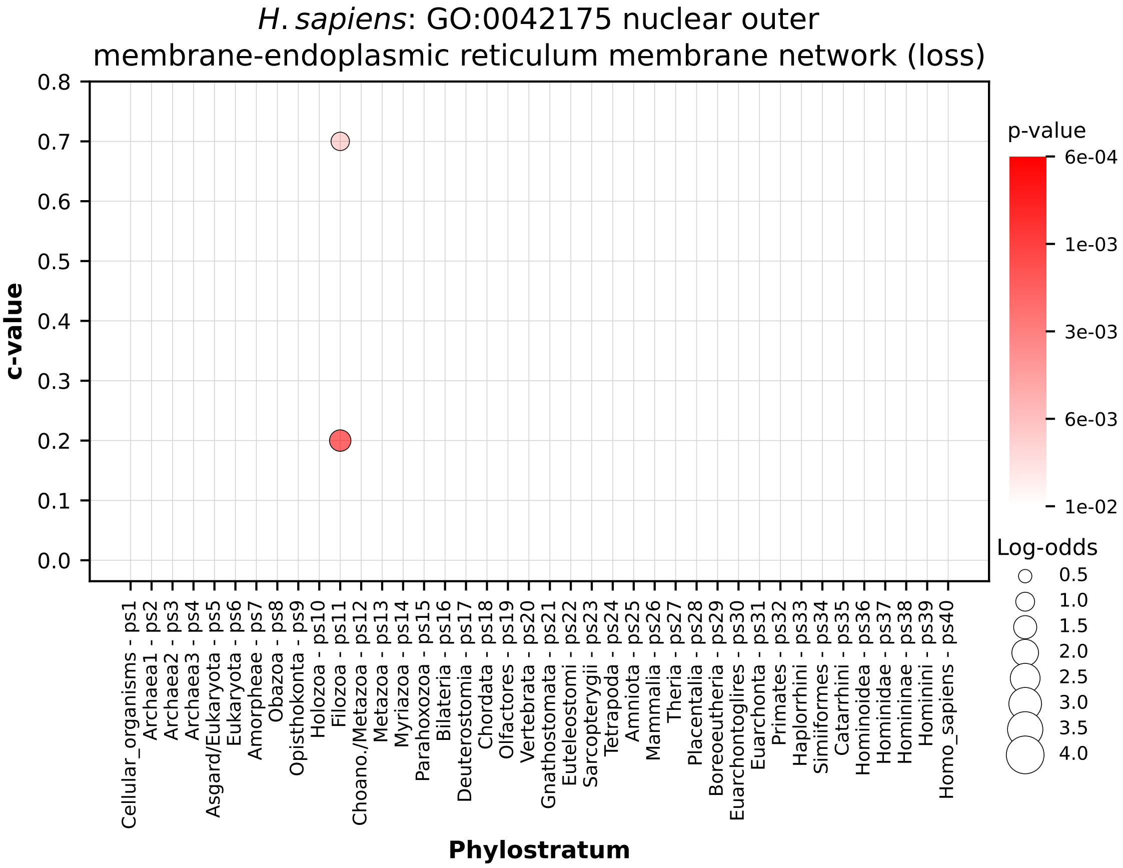

H. sapiens: GO:0042175 nuclear outer membrane-endoplasmic reticulum membrane network (loss)
c-value
0.8
0.7
0.6
0.5
0.4
0.3
0.2
0.1
0.0
p-value
6e-04
1e-03
3e-03
6e-03
1e-02
Log-odds
0.5
1.0
1.5
2.0
2.5
3.0
3.5
4.0
Cellular_organisms - ps1
Archaea1 - ps2
Archaea2 - ps3
Archaea3 - ps4
Asgard/Eukaryota - ps5
Eukaryota - ps6
Amorpheae - ps7
Obazoa - ps8
Opisthokonta - ps9
Holozoa - ps10
Filozoa - ps11
Choano./Metazoa - ps12
Metazoa - ps13
Myriazoa - ps14
Parahoxozoa - ps15
Bilateria - ps16
Deuterostomia - ps17
Chordata - ps18
Olfactores - ps19
Vertebrata - ps20
Gnathostomata - ps21
Euteleostomi - ps22
Sarcopterygii - ps23
Tetrapoda - ps24
Amniota - ps25
Mammalia - ps26
Theria - ps27
Placentalia - ps28
Boreoeutheria - ps29
Euarchontoglires - ps30
Euarchonta - ps31
Primates - ps32
Haplorrhini - ps33
Simiiformes - ps34
Catarrhini - ps35
Hominoidea - ps36
Hominidae - ps37
Homininae - ps38
Hominini - ps39
Homo_sapiens - ps40
Phylostratum

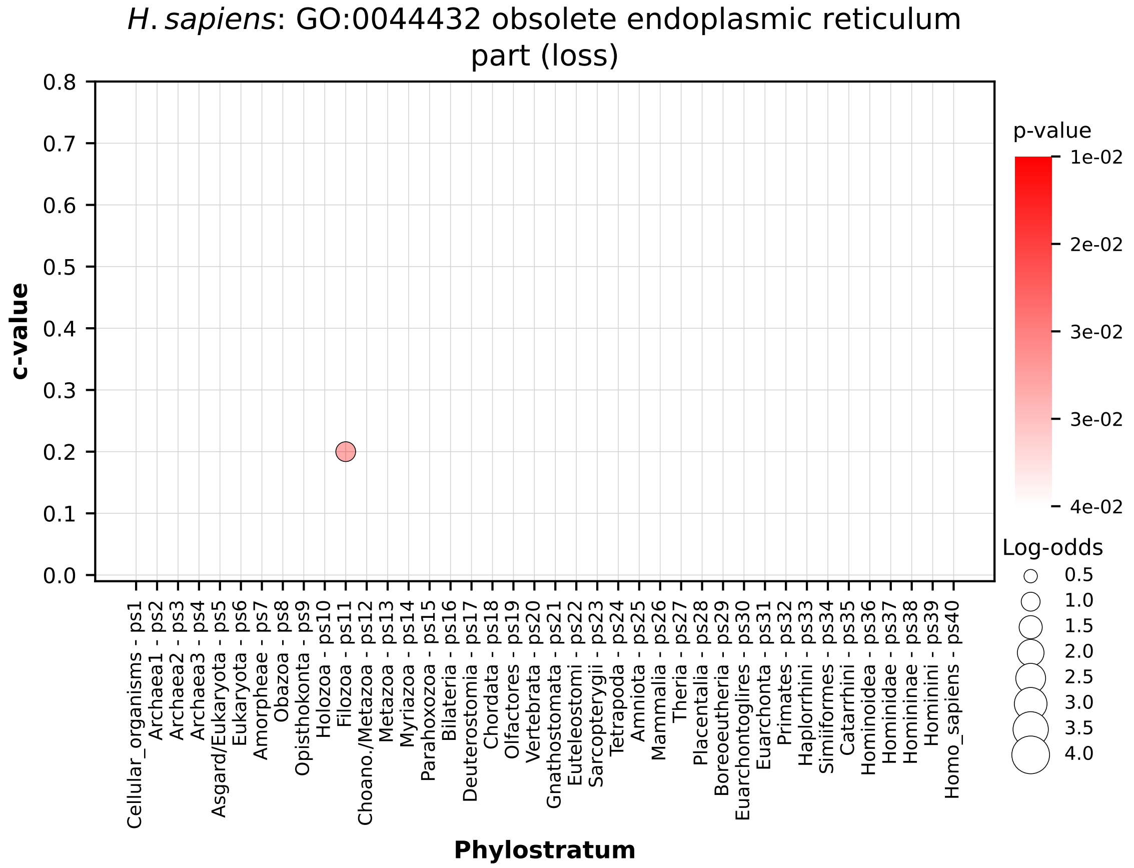

H. sapiens: GO:0044432 obsolete endoplasmic reticulum part (loss)
c-value
0.0
0.1
0.2
0.3
0.4
0.5
0.6
0.7
0.8
Phylostratum
Cellular_organisms - ps1
Archaea1 - ps2
Archaea2 - ps3
Archaea3 - ps4
Asgard/Eukaryota - ps5
Eukaryota - ps6
Amorpheae - ps7
Obazoa - ps8
Opisthokonta - ps9
Holozoa - ps10
Filozoa - ps11
Choano./Metazoa - ps12
Metazoa - ps13
Myriazoa - ps14
Parahoxozoa - ps15
Bilateria - ps16
Deuterostomia - ps17
Chordata - ps18
Olfactores - ps19
Vertebrata - ps20
Gnathostomata - ps21
Euteleostomi - ps22
Sarcopterygii - ps23
Tetrapoda - ps24
Amniota - ps25
Mammalia - ps26
Theria - ps27
Placentalia - ps28
Boreoeutheria - ps29
Euarchontoglires - ps30
Euarchonta - ps31
Primates - ps32
Haplorrhini - ps33
Simiiformes - ps34
Catarrhini - ps35
Hominoidea - ps36
Hominidae - ps37
Homininae - ps38
Hominini - ps39
Homo_sapiens - ps40
p-value
1e-02
2e-02
3e-02
3e-02
4e-02
Log-odds
0.5
1.0
1.5
2.0
2.5
3.0
3.5
4.0

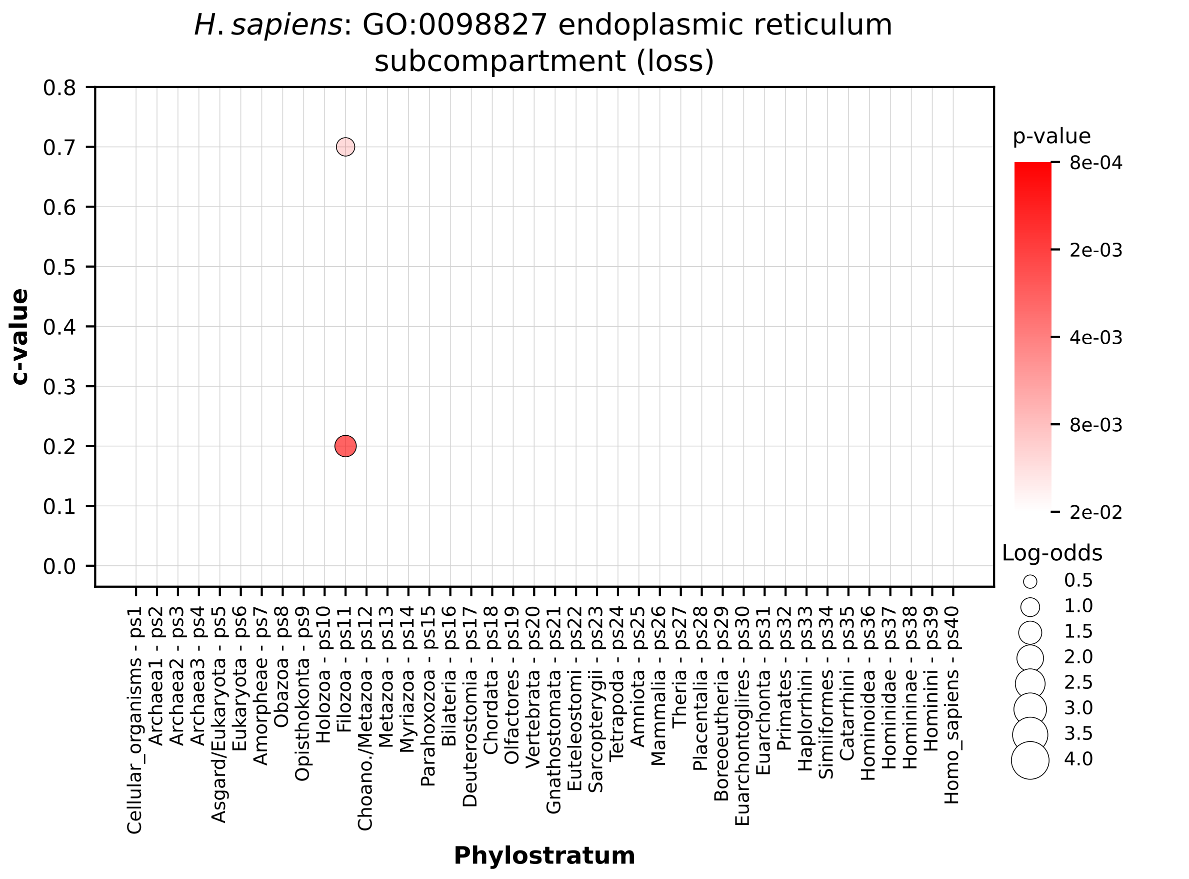
H. sapiens: GO:0098827 endoplasmic reticulum subcompartment (loss)
c-value
0.0
0.1
0.2
0.3
0.4
0.5
0.6
0.7
0.8
Phylostratum
Cellular_organisms - ps1
Archaea1 - ps2
Archaea2 - ps3
Archaea3 - ps4
Asgard/Eukaryota - ps5
Eukaryota - ps6
Amorpheae - ps7
Obazoa - ps8
Opisthokonta - ps9
Holozoa - ps10
Filozoa - ps11
Choano./Metazoa - ps12
Metazoa - ps13
Myriazoa - ps14
Parahoxozoa - ps15
Bilateria - ps16
Deuterostomia - ps17
Chordata - ps18
Olfactores - ps19
Vertebrata - ps20
Gnathostomata - ps21
Euteleostomi - ps22
Sarcopterygii - ps23
Tetrapoda - ps24
Amniota - ps25
Mammalia - ps26
Theria - ps27
Placentalia - ps28
Boreoeutheria - ps29
Euarchontoglires - ps30
Euarchonta - ps31
Primates - ps32
Haplorrhini - ps33
Simiiformes - ps34
Catarrhini - ps35
Hominoidea - ps36
Hominidae - ps37
Homininae - ps38
Hominini - ps39
Homo_sapiens - ps40
p-value
8e-04
2e-03
4e-03
8e-03
2e-02
Log-odds
0.5
1.0
1.5
2.0
2.5
3.0
3.5
4.0

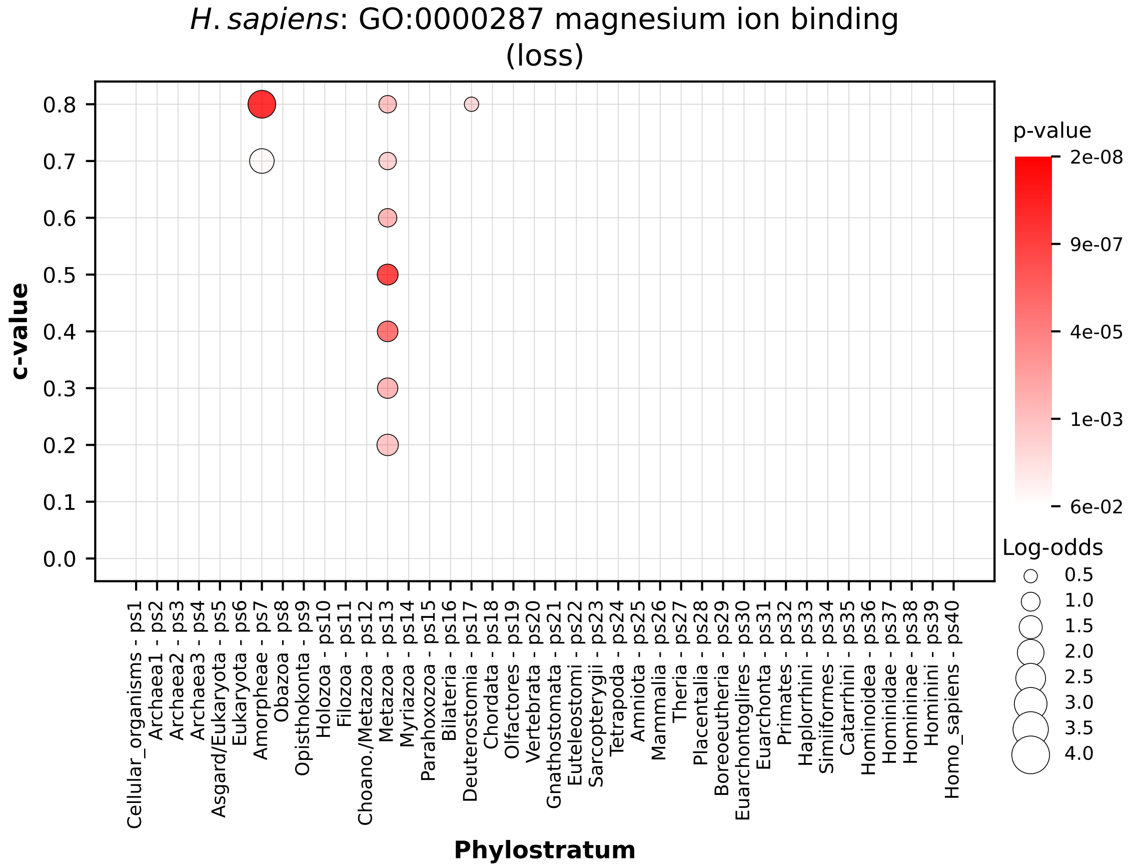
H. sapiens: GO:0000287 magnesium ion binding
(loss)
c-value
0.8
0.7
0.6
0.5
0.4
0.3
0.2
0.1
0.0
p-value
2e-08
9e-07
4e-05
1e-03
6e-02
Log-odds
0.5
1.0
1.5
2.0
2.5
3.0
3.5
4.0
Cellular_organisms - ps1
Archaea1 - ps2
Archaea2 - ps3
Archaea3 - ps4
Asgard/Eukaryota - ps5
Eukaryota - ps6
Amorpheae - ps7
Obazoa - ps8
Opisthokonta - ps9
Holozoa - ps10
Filozoa - ps11
Choano./Metazoa - ps12
Metazoa - ps13
Myriazoa - ps14
Parahoxozoa - ps15
Bilateria - ps16
Deuterostomia - ps17
Chordata - ps18
Olfactores - ps19
Vertebrata - ps20
Gnathostomata - ps21
Euteleostomi - ps22
Sarcopterygii - ps23
Tetrapoda - ps24
Amniota - ps25
Mammalia - ps26
Theria - ps27
Placentalia - ps28
Boreoeutheria - ps29
Euarchontoglires - ps30
Euarchonta - ps31
Primates - ps32
Haplorrhini - ps33
Simiiformes - ps34
Catarrhini - ps35
Hominoidea - ps36
Hominidae - ps37
Homininae - ps38
Hominini - ps39
Homo_sapiens - ps40
Phylostratum

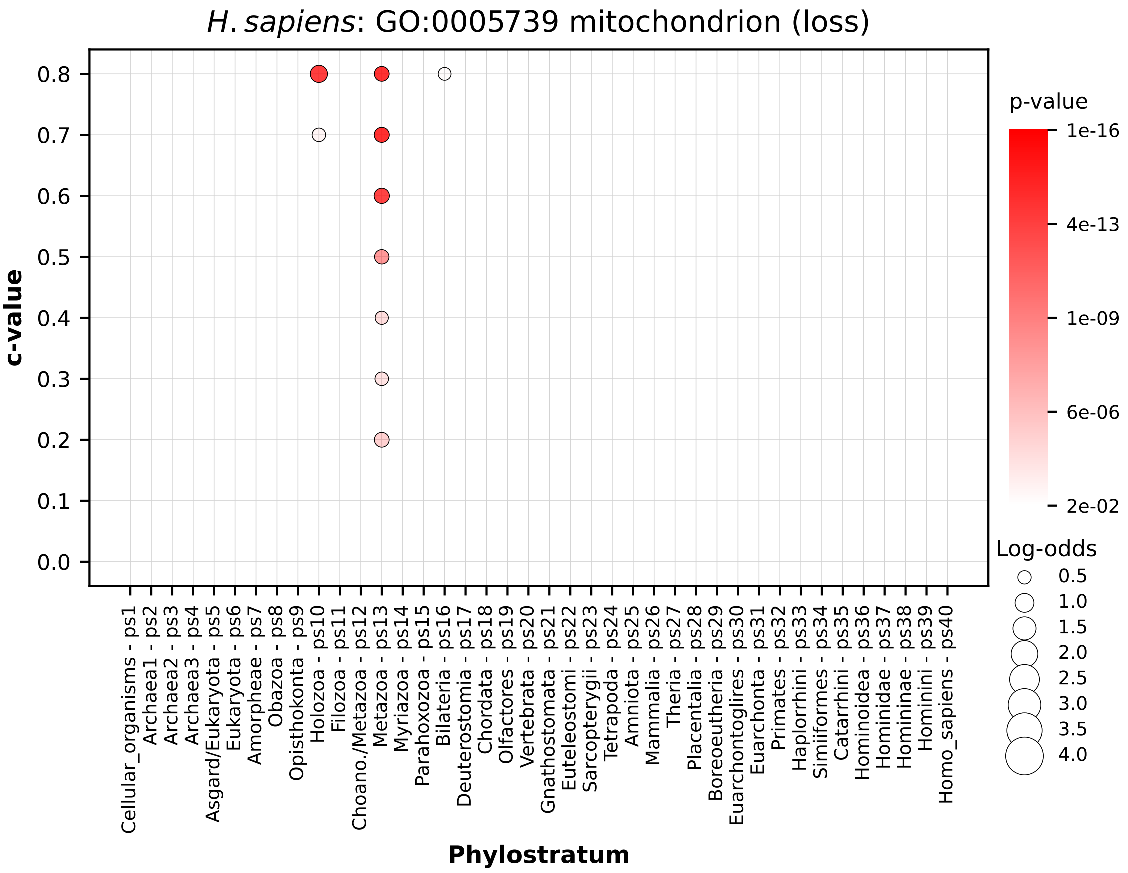

H. sapiens: GO:0005739 mitochondrion (loss)
c-value
0.8
0.7
0.6
0.5
0.4
0.3
0.2
0.1
0.0
p-value
1e-16
4e-13
1e-09
6e-06
2e-02
Log-odds
0.5
1.0
1.5
2.0
2.5
3.0
3.5
4.0
Cellular_organisms - ps1
Archaea1 - ps2
Archaea2 - ps3
Archaea3 - ps4
Asgard/Eukaryota - ps5
Eukaryota - ps6
Amorpheae - ps7
Obazoa - ps8
Opisthokonta - ps9
Holozoa - ps10
Filozoa - ps11
Choano./Metazoa - ps12
Metazoa - ps13
Myriazoa - ps14
Parahoxozoa - ps15
Bilateria - ps16
Deuterostomia - ps17
Chordata - ps18
Olfactores - ps19
Vertebrata - ps20
Gnathostomata - ps21
Euteleostomi - ps22
Sarcopterygii - ps23
Tetrapoda - ps24
Amniota - ps25
Mammalia - ps26
Theria - ps27
Placentalia - ps28
Boreoeutheria - ps29
Euarchontoglires - ps30
Euarchonta - ps31
Primates - ps32
Haplorrhini - ps33
Simiiformes - ps34
Catarrhini - ps35
Hominoidea - ps36
Hominidae - ps37
Homininae - ps38
Hominini - ps39
Homo_sapiens - ps40
Phylostratum

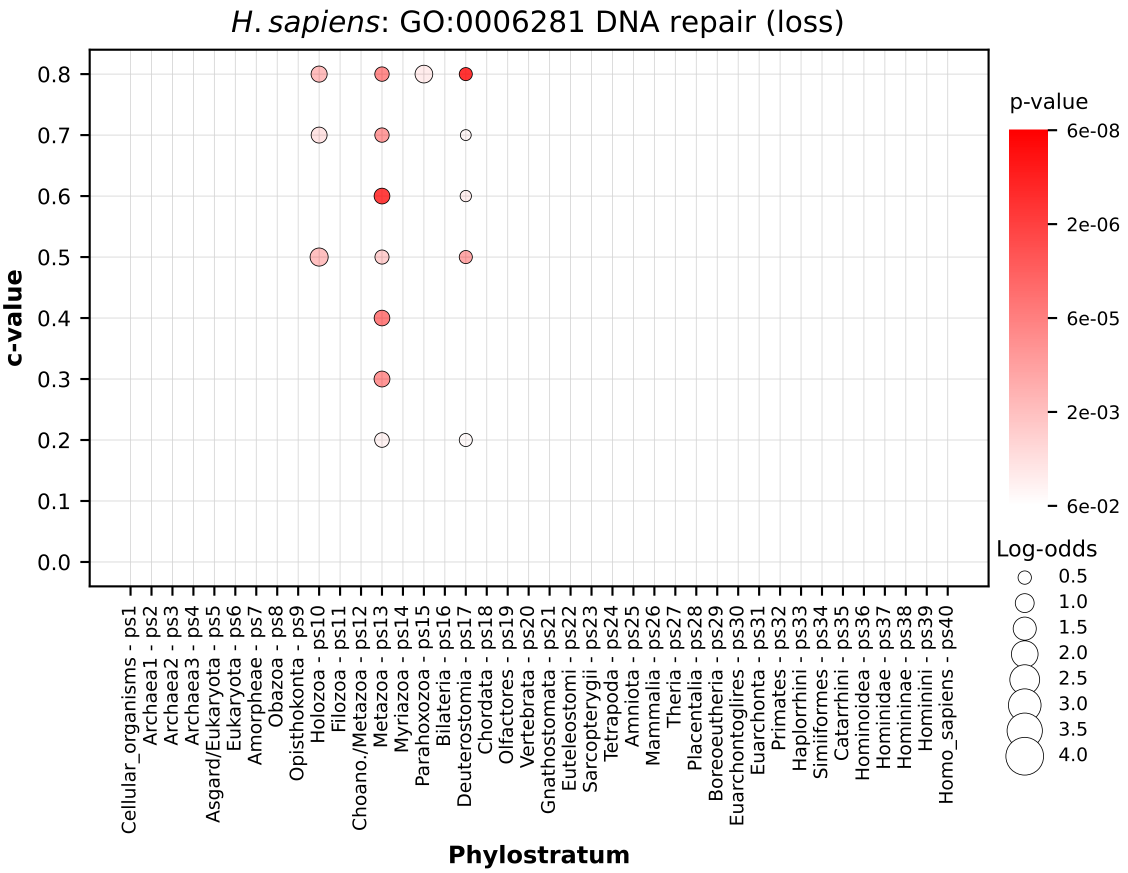

H. sapiens: GO:0006281 DNA repair (loss)
c-value
0.8
0.7
0.6
0.5
0.4
0.3
0.2
0.1
0.0
p-value
6e-08
2e-06
6e-05
2e-03
6e-02
Log-odds
0.5
1.0
1.5
2.0
2.5
3.0
3.5
4.0
Cellular_organisms - ps1
Archaea1 - ps2
Archaea2 - ps3
Archaea3 - ps4
Asgard/Eukaryota - ps5
Eukaryota - ps6
Amorpheae - ps7
Obazoa - ps8
Opisthokonta - ps9
Holozoa - ps10
Filozoa - ps11
Choano./Metazoa - ps12
Metazoa - ps13
Myriazoa - ps14
Parahoxozoa - ps15
Bilateria - ps16
Deuterostomia - ps17
Chordata - ps18
Olfactores - ps19
Vertebrata - ps20
Gnathostomata - ps21
Euteleostomi - ps22
Sarcopterygii - ps23
Tetrapoda - ps24
Amniota - ps25
Mammalia - ps26
Theria - ps27
Placentalia - ps28
Boreoeutheria - ps29
Euarchontoglires - ps30
Euarchonta - ps31
Primates - ps32
Haplorrhini - ps33
Simiiformes - ps34
Catarrhini - ps35
Hominoidea - ps36
Hominidae - ps37
Homininae - ps38
Hominini - ps39
Homo_sapiens - ps40
Phylostratum

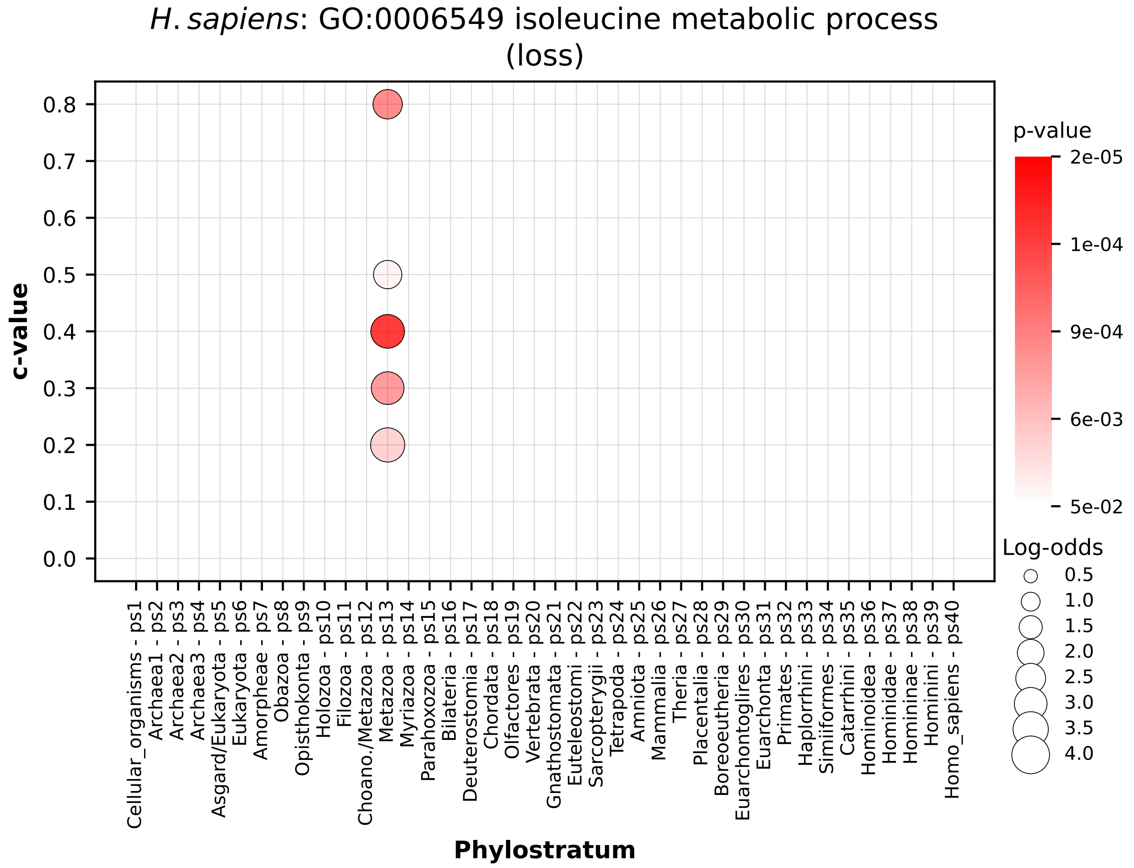
H. sapiens: GO:0006549 isoleucine metabolic process
(loss)
c-value
0.0
0.1
0.2
0.3
0.4
0.5
0.6
0.7
0.8
Phylostratum
Cellular_organisms - ps1
Archaea1 - ps2
Archaea2 - ps3
Archaea3 - ps4
Asgard/Eukaryota - ps5
Eukaryota - ps6
Amorpheae - ps7
Obazoa - ps8
Opisthokonta - ps9
Holozoa - ps10
Filozoa - ps11
Choano./Metazoa - ps12
Metazoa - ps13
Myriazoa - ps14
Parahoxozoa - ps15
Bilateria - ps16
Deuterostomia - ps17
Chordata - ps18
Olfactores - ps19
Vertebrata - ps20
Gnathostomata - ps21
Euteleostomi - ps22
Sarcopterygii - ps23
Tetrapoda - ps24
Amniota - ps25
Mammalia - ps26
Theria - ps27
Placentalia - ps28
Boreoeutheria - ps29
Euarchontoglires - ps30
Euarchonta - ps31
Primates - ps32
Haplorrhini - ps33
Simiiformes - ps34
Catarrhini - ps35
Hominoidea - ps36
Hominidae - ps37
Homininae - ps38
Hominini - ps39
Homo_sapiens - ps40
p-value
2e-05
1e-04
9e-04
6e-03
5e-02
Log-odds
0.5
1.0
1.5
2.0
2.5
3.0
3.5
4.0

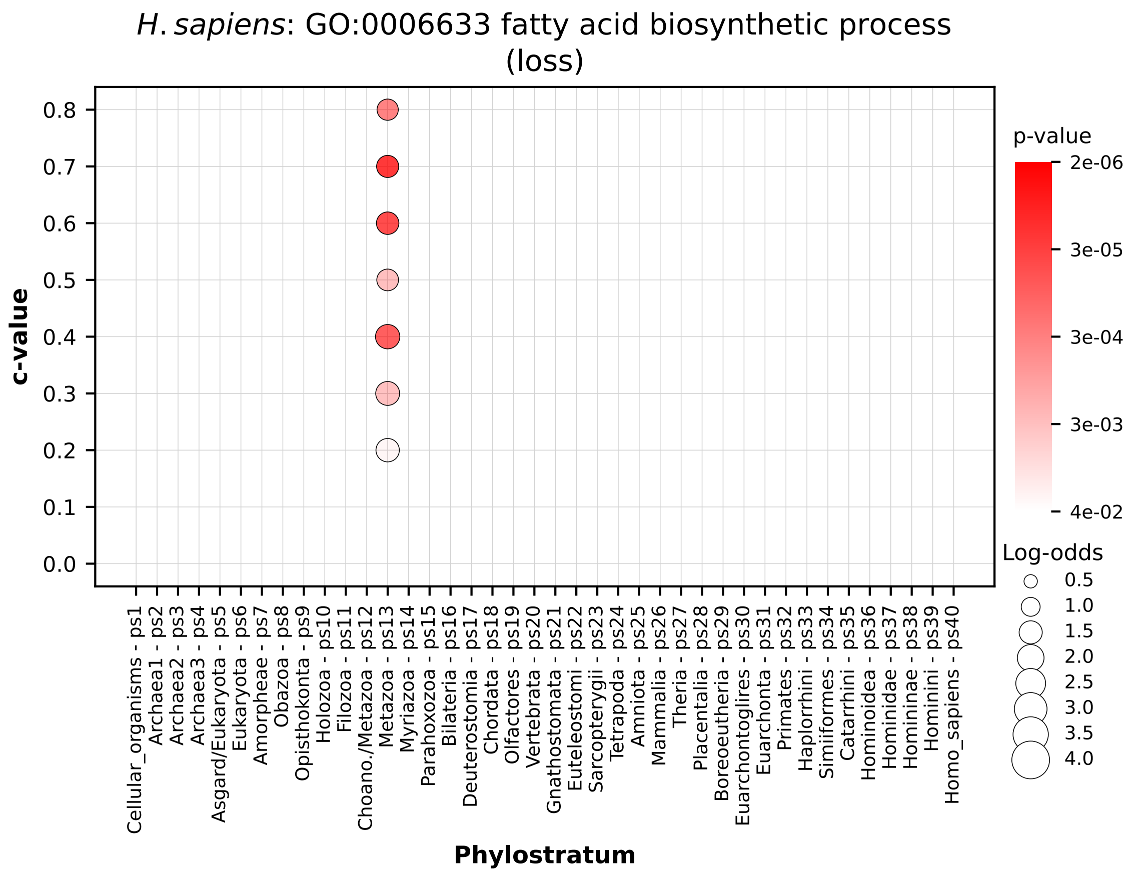

H. sapiens: GO:0006633 fatty acid biosynthetic process
(loss)
c-value
0.0
0.1
0.2
0.3
0.4
0.5
0.6
0.7
0.8
Phylostratum
Cellular_organisms - ps1
Archaea1 - ps2
Archaea2 - ps3
Archaea3 - ps4
Asgard/Eukaryota - ps5
Eukaryota - ps6
Amorpheae - ps7
Obazoa - ps8
Opisthokonta - ps9
Holozoa - ps10
Filozoa - ps11
Choano./Metazoa - ps12
Metazoa - ps13
Myriazoa - ps14
Parahoxozoa - ps15
Bilateria - ps16
Deuterostomia - ps17
Chordata - ps18
Olfactores - ps19
Vertebrata - ps20
Gnathostomata - ps21
Euteleostomi - ps22
Sarcopterygii - ps23
Tetrapoda - ps24
Amniota - ps25
Mammalia - ps26
Theria - ps27
Placentalia - ps28
Boreoeutheria - ps29
Euarchontoglires - ps30
Euarchonta - ps31
Primates - ps32
Haplorrhini - ps33
Simiiformes - ps34
Catarrhini - ps35
Hominoidea - ps36
Hominidae - ps37
Homininae - ps38
Hominini - ps39
Homo_sapiens - ps40
p-value
2e-06
3e-05
3e-04
3e-03
4e-02
Log-odds
0.5
1.0
1.5
2.0
2.5
3.0
3.5
4.0

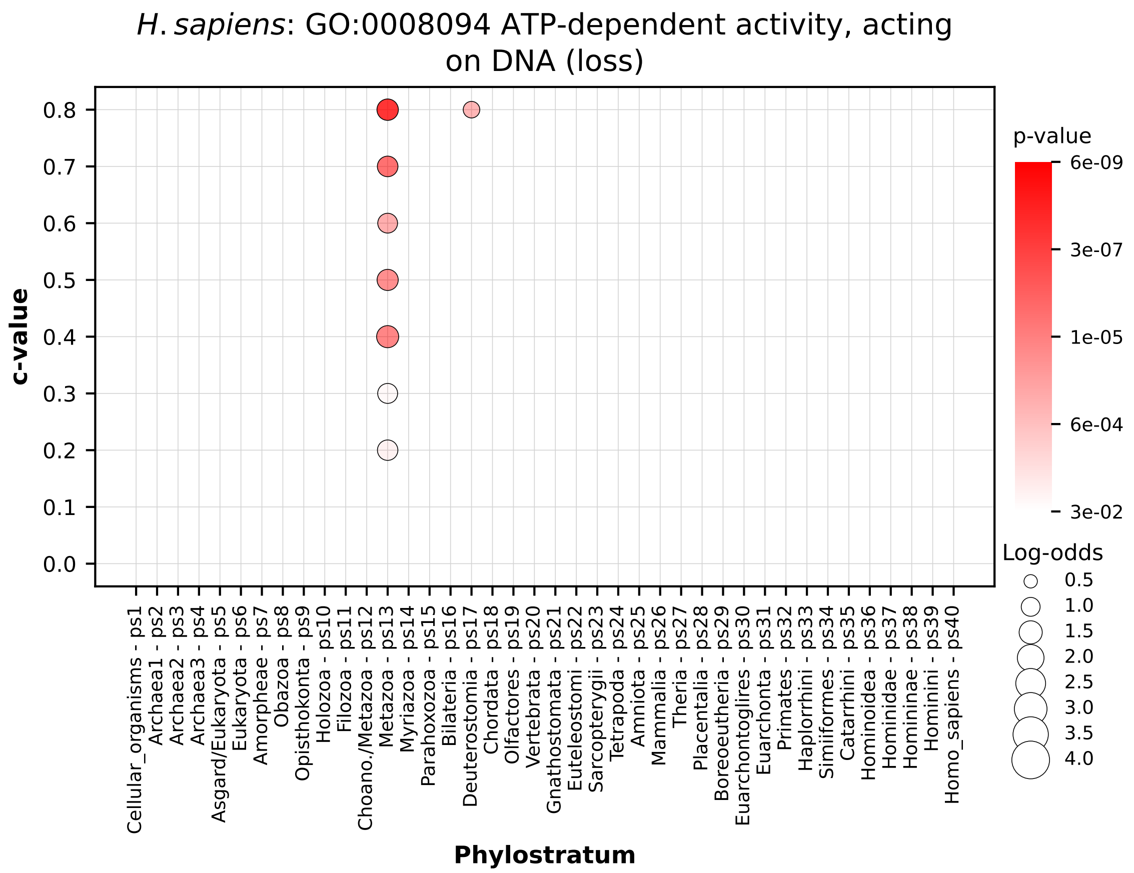

H. sapiens: GO:0008094 ATP-dependent activity, acting on DNA (loss)
c-value
0.0
0.1
0.2
0.3
0.4
0.5
0.6
0.7
0.8
Phylostratum
Cellular_organisms - ps1
Archaea1 - ps2
Archaea2 - ps3
Archaea3 - ps4
Asgard/Eukaryota - ps5
Eukaryota - ps6
Amorpheae - ps7
Obazoa - ps8
Opisthokonta - ps9
Holozoa - ps10
Filozoa - ps11
Choano./Metazoa - ps12
Metazoa - ps13
Myriazoa - ps14
Parahoxozoa - ps15
Bilateria - ps16
Deuterostomia - ps17
Chordata - ps18
Olfactores - ps19
Vertebrata - ps20
Gnathostomata - ps21
Euteleostomi - ps22
Sarcopterygii - ps23
Tetrapoda - ps24
Amniota - ps25
Mammalia - ps26
Theria - ps27
Placentalia - ps28
Boreoeutheria - ps29
Euarchontoglires - ps30
Euarchonta - ps31
Primates - ps32
Haplorrhini - ps33
Simiiformes - ps34
Catarrhini - ps35
Hominoidea - ps36
Hominidae - ps37
Homininae - ps38
Hominini - ps39
Homo_sapiens - ps40
p-value
6e-09
3e-07
1e-05
6e-04
3e-02
Log-odds
0.5
1.0
1.5
2.0
2.5
3.0
3.5
4.0

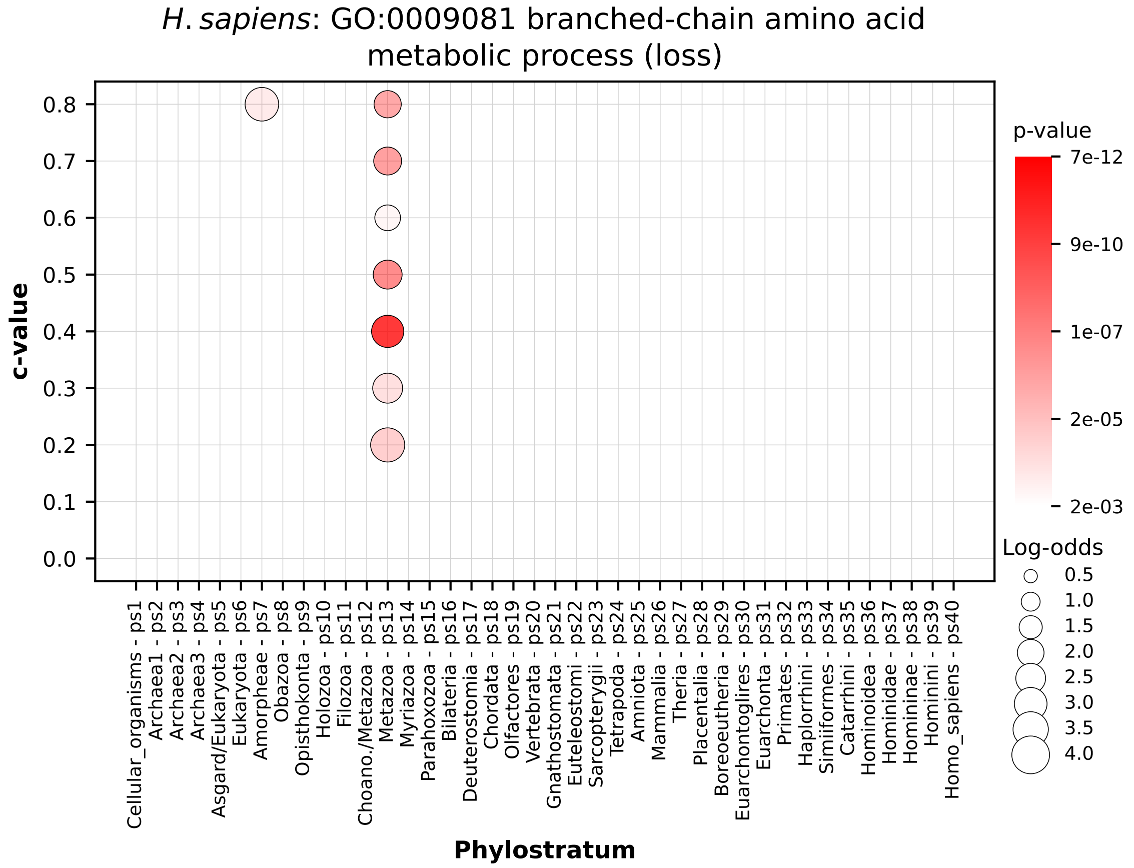

H. sapiens: GO:0009081 branched-chain amino acid metabolic process (loss)
c-value
0.8
0.7
0.6
0.5
0.4
0.3
0.2
0.1
0.0
p-value
7e-12
9e-10
1e-07
2e-05
2e-03
Log-odds
0.5
1.0
1.5
2.0
2.5
3.0
3.5
4.0
Cellular_organisms - ps1
Archaea1 - ps2
Archaea2 - ps3
Archaea3 - ps4
Asgard/Eukaryota - ps5
Eukaryota - ps6
Amorpheae - ps7
Obazoa - ps8
Opisthokonta - ps9
Holozoa - ps10
Filozoa - ps11
Choano./Metazoa - ps12
Metazoa - ps13
Myriazoa - ps14
Parahoxozoa - ps15
Bilateria - ps16
Deuterostomia - ps17
Chordata - ps18
Olfactores - ps19
Vertebrata - ps20
Gnathostomata - ps21
Euteleostomi - ps22
Sarcopterygii - ps23
Tetrapoda - ps24
Amniota - ps25
Mammalia - ps26
Theria - ps27
Placentalia - ps28
Boreoeutheria - ps29
Euarchontoglires - ps30
Euarchonta - ps31
Primates - ps32
Haplorrhini - ps33
Simiiformes - ps34
Catarrhini - ps35
Hominoidea - ps36
Hominidae - ps37
Homininae - ps38
Hominini - ps39
Homo_sapiens - ps40
Phylostratum

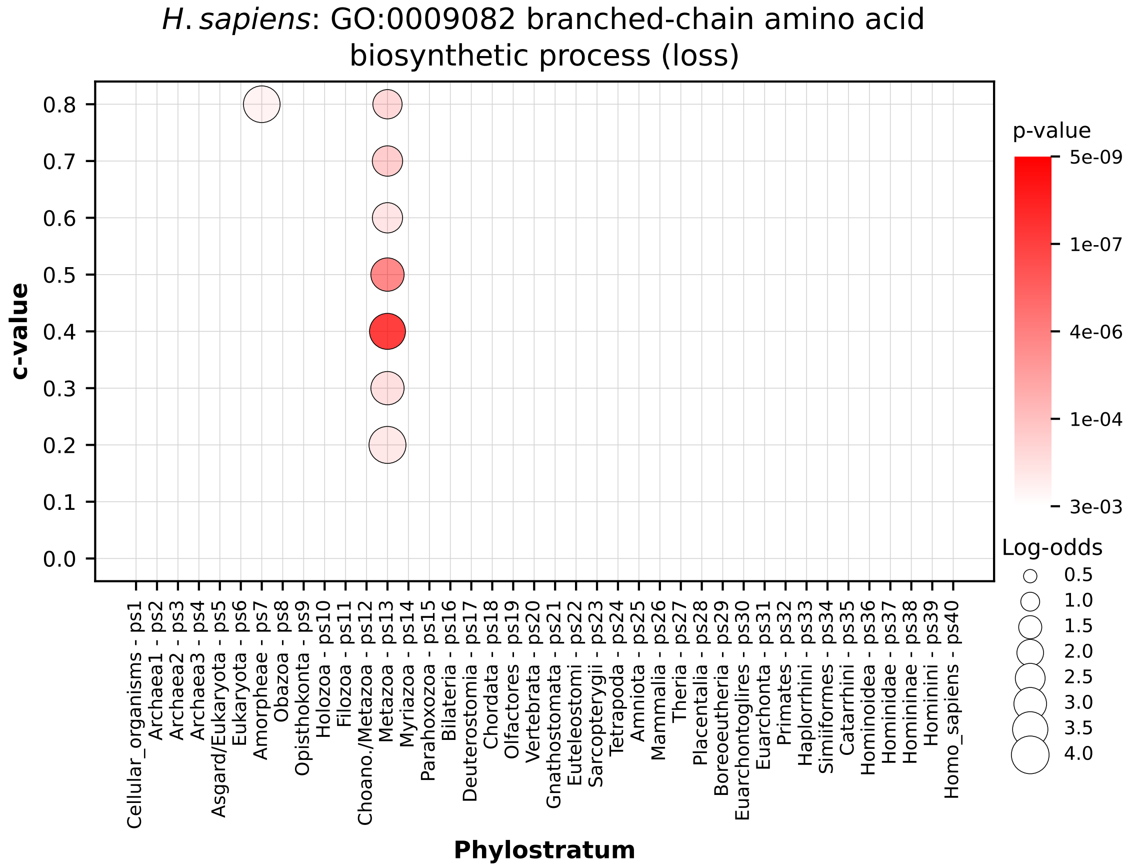
H. sapiens: GO:0009082 branched-chain amino acid biosynthetic process (loss)
c-value
0.8
0.7
0.6
0.5
0.4
0.3
0.2
0.1
0.0
p-value
5e-09
1e-07
4e-06
1e-04
3e-03
Log-odds
0.5
1.0
1.5
2.0
2.5
3.0
3.5
4.0
Cellular_organisms - ps1
Archaea1 - ps2
Archaea2 - ps3
Archaea3 - ps4
Asgard/Eukaryota - ps5
Eukaryota - ps6
Amorpheae - ps7
Obazoa - ps8
Opisthokonta - ps9
Holozoa - ps10
Filozoa - ps11
Choano./Metazoa - ps12
Metazoa - ps13
Myriazoa - ps14
Parahoxozoa - ps15
Bilateria - ps16
Deuterostomia - ps17
Chordata - ps18
Olfactores - ps19
Vertebrata - ps20
Gnathostomata - ps21
Euteleostomi - ps22
Sarcopterygii - ps23
Tetrapoda - ps24
Amniota - ps25
Mammalia - ps26
Theria - ps27
Placentalia - ps28
Boreoeutheria - ps29
Euarchontoglires - ps30
Euarchonta - ps31
Primates - ps32
Haplorrhini - ps33
Simiiformes - ps34
Catarrhini - ps35
Hominoidea - ps36
Hominidae - ps37
Homininae - ps38
Hominini - ps39
Homo_sapiens - ps40
Phylostratum

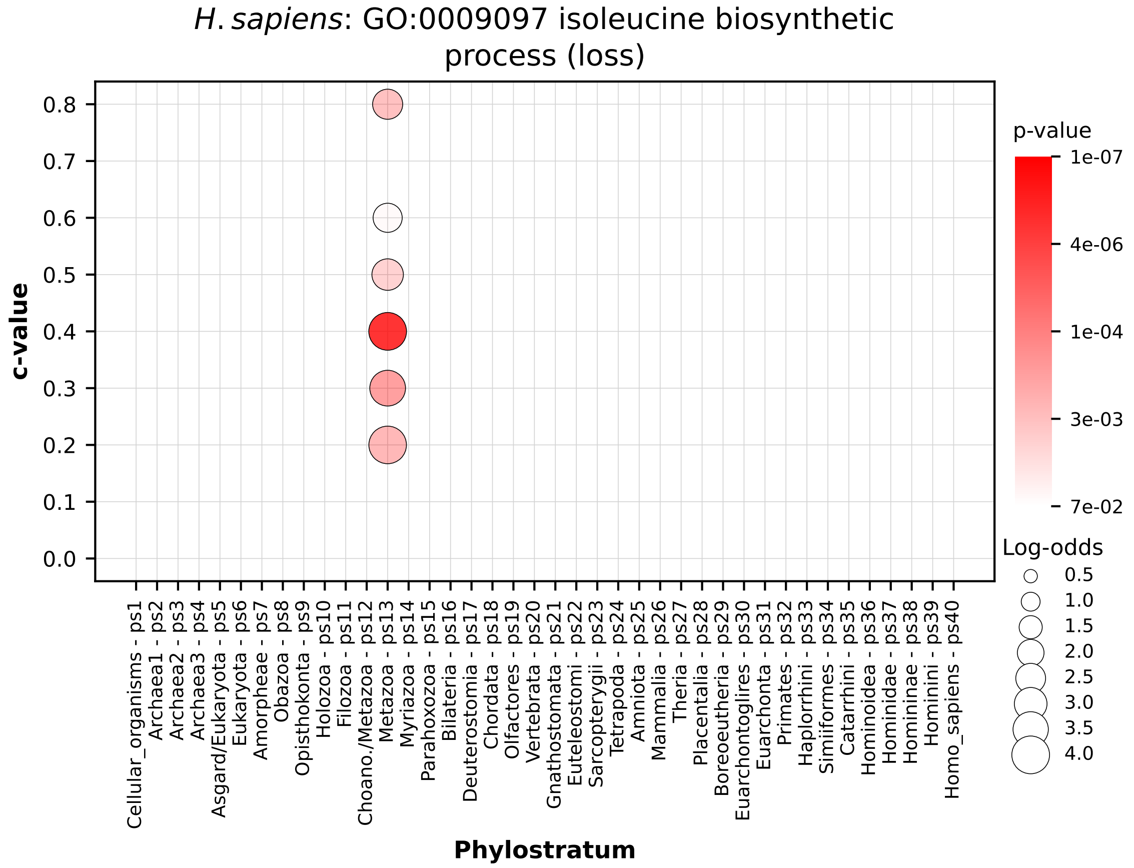

H. sapiens: GO:0009097 isoleucine biosynthetic process (loss)
c-value
0.8
0.7
0.6
0.5
0.4
0.3
0.2
0.1
0.0
Phylostratum
Cellular_organisms - ps1
Archaea1 - ps2
Archaea2 - ps3
Archaea3 - ps4
Asgard/Eukaryota - ps5
Eukaryota - ps6
Amorpheae - ps7
Obazoa - ps8
Opisthokonta - ps9
Holozoa - ps10
Filozoa - ps11
Choano./Metazoa - ps12
Metazoa - ps13
Myriazoa - ps14
Parahoxozoa - ps15
Bilateria - ps16
Deuterostomia - ps17
Chordata - ps18
Olfactores - ps19
Vertebrata - ps20
Gnathostomata - ps21
Euteleostomi - ps22
Sarcopterygii - ps23
Tetrapoda - ps24
Amniota - ps25
Mammalia - ps26
Theria - ps27
Placentalia - ps28
Boreoeutheria - ps29
Euarchontoglires - ps30
Euarchonta - ps31
Primates - ps32
Haplorrhini - ps33
Simiiformes - ps34
Catarrhini - ps35
Hominoidea - ps36
Hominidae - ps37
Homininae - ps38
Hominini - ps39
Homo_sapiens - ps40
p-value
1e-07
4e-06
1e-04
3e-03
7e-02
Log-odds
0.5
1.0
1.5
2.0
2.5
3.0
3.5
4.0

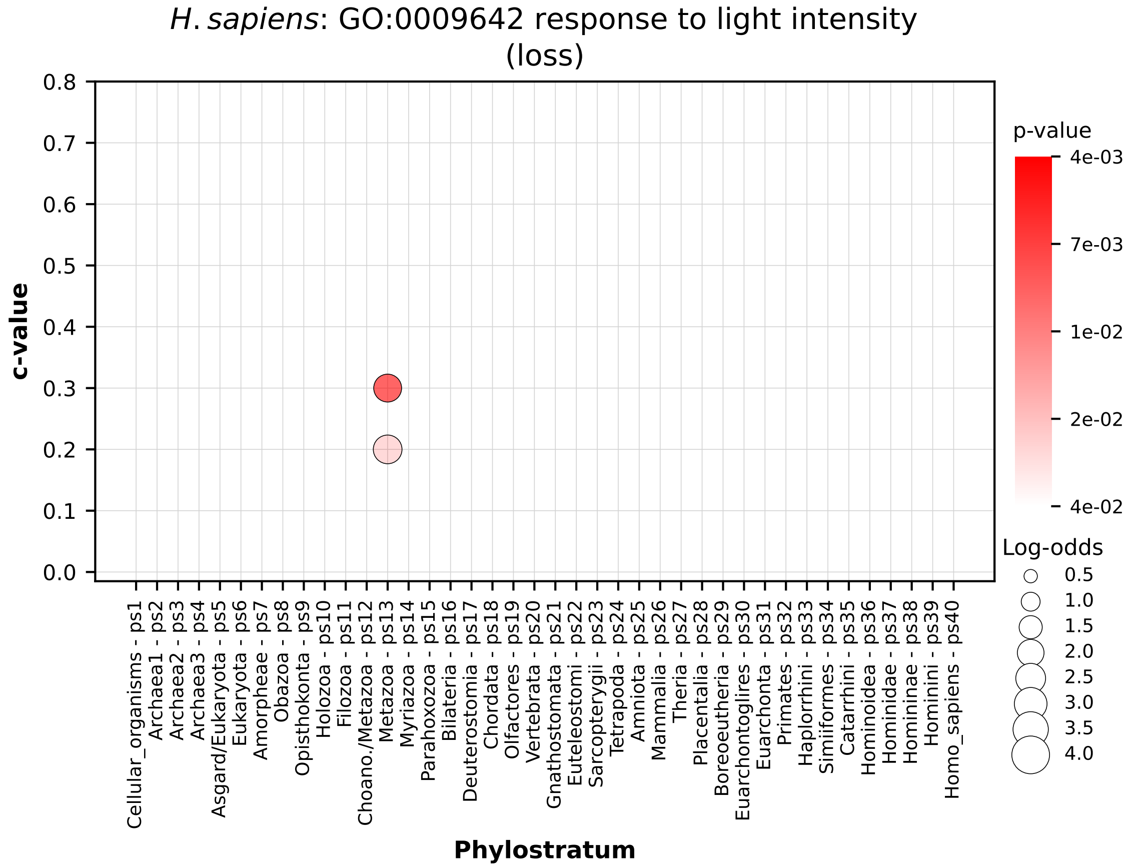

H. sapiens: GO:0009642 response to light intensity
(loss)
c-value
0.0
0.1
0.2
0.3
0.4
0.5
0.6
0.7
0.8
p-value
4e-03
7e-03
1e-02
2e-02
4e-02
Log-odds
0.5
1.0
1.5
2.0
2.5
3.0
3.5
4.0
Cellular_organisms - ps1
Archaea1 - ps2
Archaea2 - ps3
Archaea3 - ps4
Asgard/Eukaryota - ps5
Eukaryota - ps6
Amorpheae - ps7
Obazoa - ps8
Opisthokonta - ps9
Holozoa - ps10
Filozoa - ps11
Choano./Metazoa - ps12
Metazoa - ps13
Myriazoa - ps14
Parahoxozoa - ps15
Bilateria - ps16
Deuterostomia - ps17
Chordata - ps18
Olfactores - ps19
Vertebrata - ps20
Gnathostomata - ps21
Euteleostomi - ps22
Sarcopterygii - ps23
Tetrapoda - ps24
Amniota - ps25
Mammalia - ps26
Theria - ps27
Placentalia - ps28
Boreoeutheria - ps29
Euarchontoglires - ps30
Euarchonta - ps31
Primates - ps32
Haplorrhini - ps33
Simiiformes - ps34
Catarrhini - ps35
Hominoidea - ps36
Hominidae - ps37
Homininae - ps38
Hominini - ps39
Homo_sapiens - ps40
Phylostratum

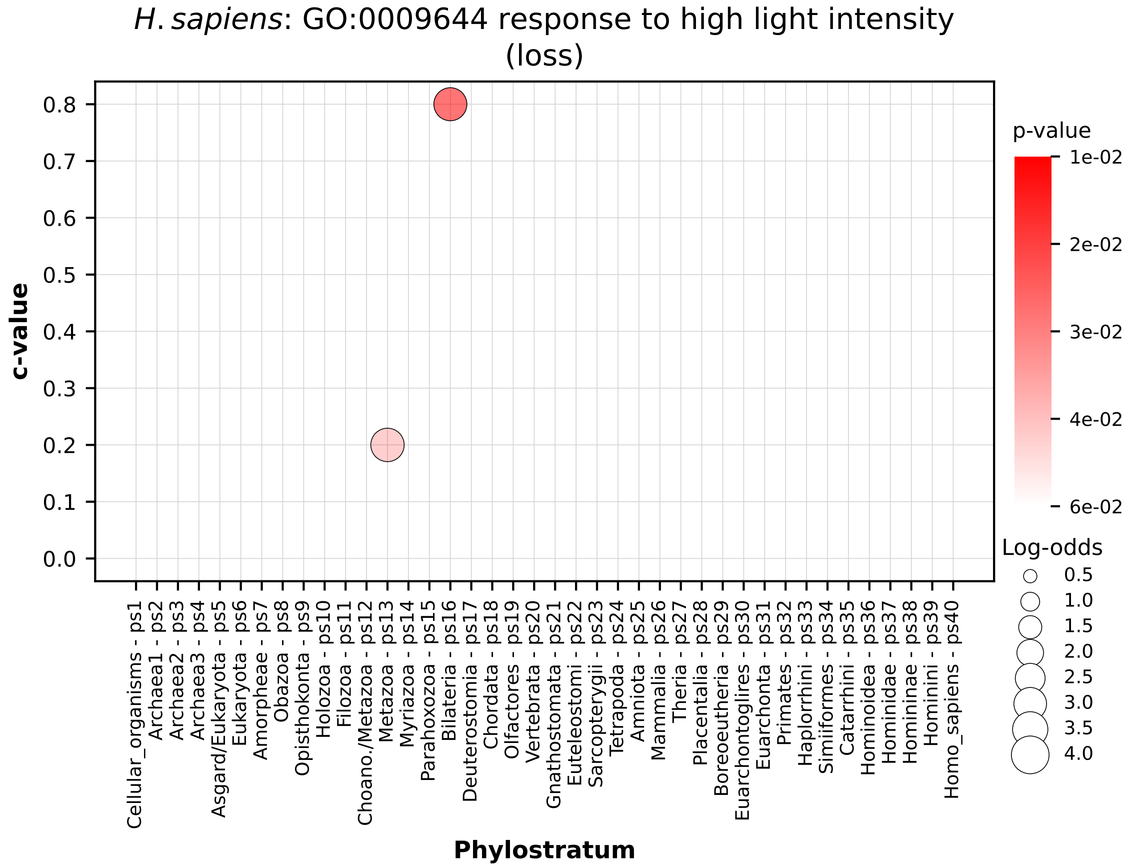

H. sapiens: GO:0009644 response to high light intensity (loss)
c-value
0.0
0.1
0.2
0.3
0.4
0.5
0.6
0.7
0.8
p-value
1e-02
2e-02
3e-02
4e-02
6e-02
Log-odds
0.5
1.0
1.5
2.0
2.5
3.0
3.5
4.0
Cellular_organisms - ps1
Archaea1 - ps2
Archaea2 - ps3
Archaea3 - ps4
Asgard/Eukaryota - ps5
Eukaryota - ps6
Amorpheae - ps7
Obazoa - ps8
Opisthokonta - ps9
Holozoa - ps10
Filozoa - ps11
Choano./Metazoa - ps12
Metazoa - ps13
Myriazoa - ps14
Parahoxozoa - ps15
Bilateria - ps16
Deuterostomia - ps17
Chordata - ps18
Olfactores - ps19
Vertebrata - ps20
Gnathostomata - ps21
Euteleostomi - ps22
Sarcopterygii - ps23
Tetrapoda - ps24
Amniota - ps25
Mammalia - ps26
Theria - ps27
Placentalia - ps28
Boreoeutheria - ps29
Euarchontoglires - ps30
Euarchonta - ps31
Primates - ps32
Haplorrhini - ps33
Simiiformes - ps34
Catarrhini - ps35
Hominoidea - ps36
Hominidae - ps37
Homininae - ps38
Hominini - ps39
Homo_sapiens - ps40
Phylostratum

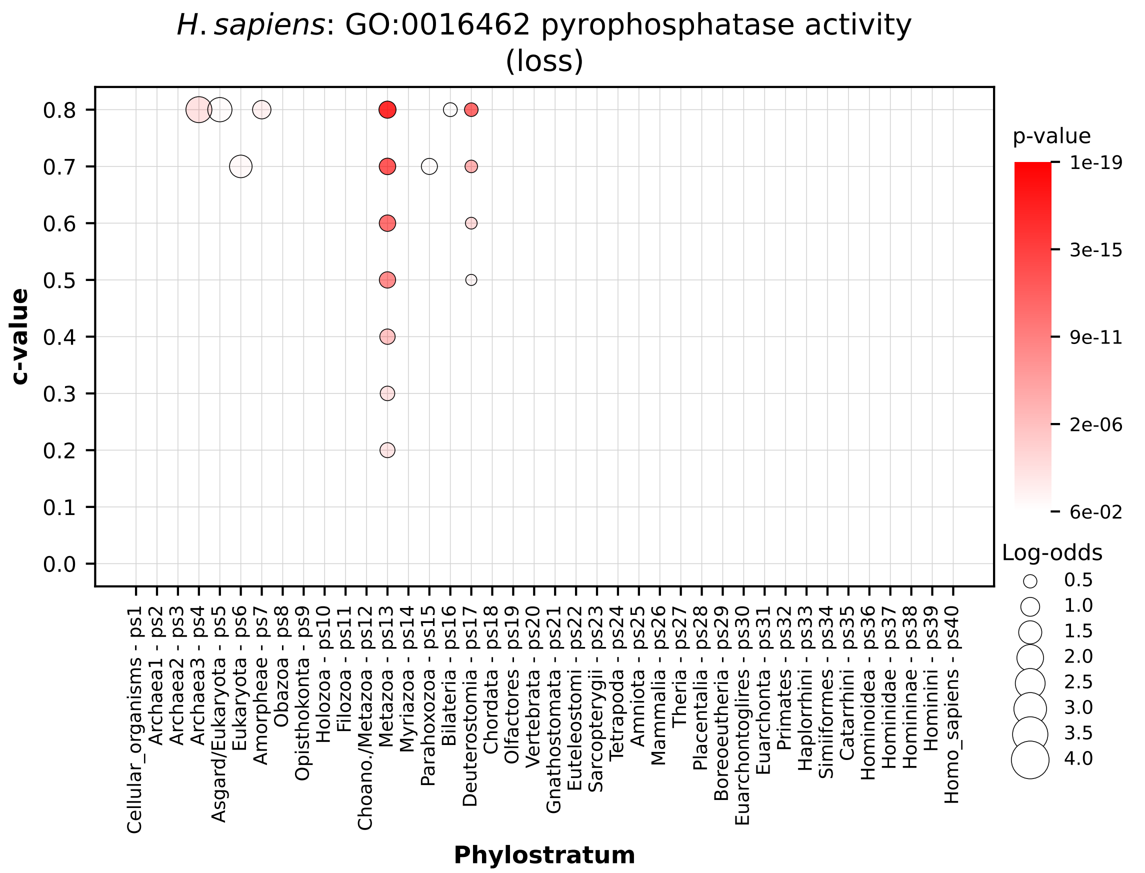

H. sapiens: GO:0016462 pyrophosphatase activity
(loss)
c-value
0.8
0.7
0.6
0.5
0.4
0.3
0.2
0.1
0.0
p-value
1e-19
3e-15
9e-11
2e-06
6e-02
Log-odds
0.5
1.0
1.5
2.0
2.5
3.0
3.5
4.0
Cellular_organisms - ps1
Archaea1 - ps2
Archaea2 - ps3
Archaea3 - ps4
Asgard/Eukaryota - ps5
Eukaryota - ps6
Amorpheae - ps7
Obazoa - ps8
Opisthokonta - ps9
Holozoa - ps10
Filozoa - ps11
Choano./Metazoa - ps12
Metazoa - ps13
Myriazoa - ps14
Parahoxozoa - ps15
Bilateria - ps16
Deuterostomia - ps17
Chordata - ps18
Olfactores - ps19
Vertebrata - ps20
Gnathostomata - ps21
Euteleostomi - ps22
Sarcopterygii - ps23
Tetrapoda - ps24
Amniota - ps25
Mammalia - ps26
Theria - ps27
Placentalia - ps28
Boreoeutheria - ps29
Euarchontoglires - ps30
Euarchonta - ps31
Primates - ps32
Haplorrhini - ps33
Simiiformes - ps34
Catarrhini - ps35
Hominoidea - ps36
Hominidae - ps37
Homininae - ps38
Hominini - ps39
Homo_sapiens - ps40
Phylostratum

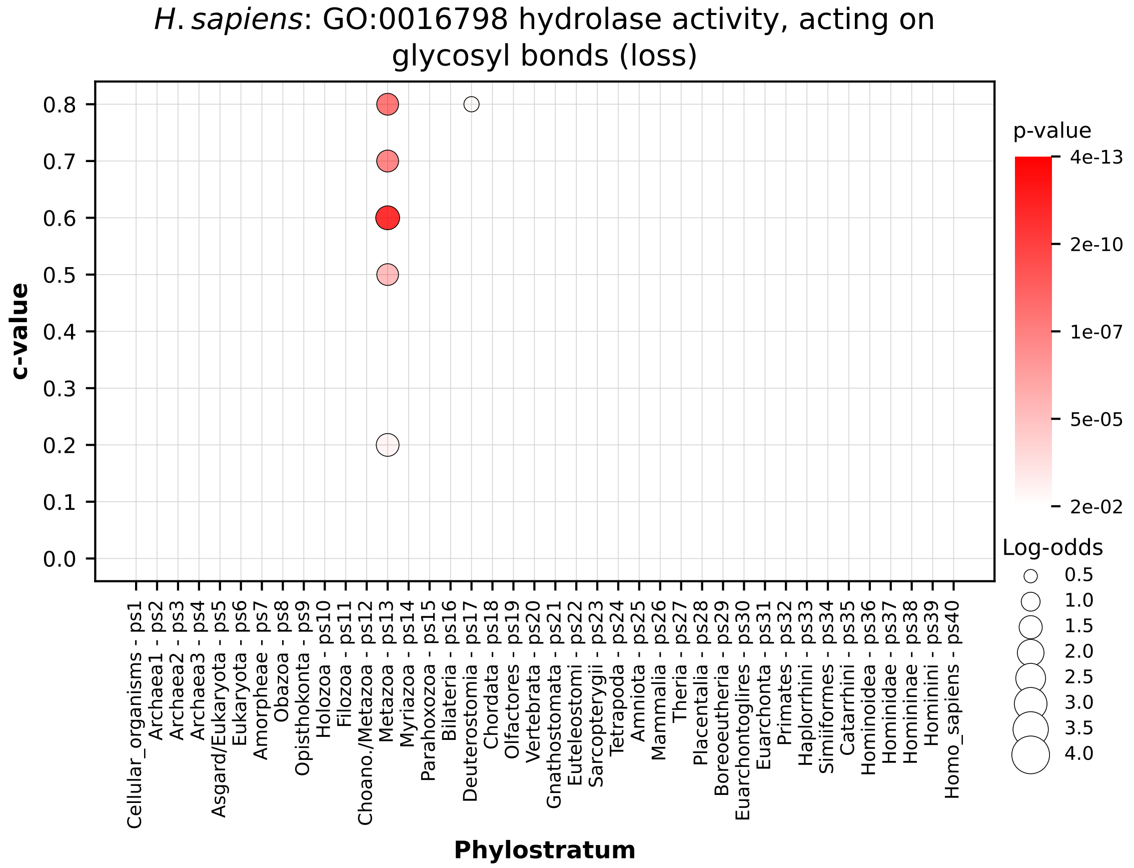
H. sapiens: GO:0016798 hydrolase activity, acting on glycosyl bonds (loss)
c-value
0.0
0.1
0.2
0.3
0.4
0.5
0.6
0.7
0.8
p-value
4e-13
2e-10
1e-07
5e-05
2e-02
Log-odds
0.5
1.0
1.5
2.0
2.5
3.0
3.5
4.0
Cellular_organisms - ps1
Archaea1 - ps2
Archaea2 - ps3
Archaea3 - ps4
Asgard/Eukaryota - ps5
Eukaryota - ps6
Amorpheae - ps7
Obazoa - ps8
Opisthokonta - ps9
Holozoa - ps10
Filozoa - ps11
Choano./Metazoa - ps12
Metazoa - ps13
Myriazoa - ps14
Parahoxozoa - ps15
Bilateria - ps16
Deuterostomia - ps17
Chordata - ps18
Olfactores - ps19
Vertebrata - ps20
Gnathostomata - ps21
Euteleostomi - ps22
Sarcopterygii - ps23
Tetrapoda - ps24
Amniota - ps25
Mammalia - ps26
Theria - ps27
Placentalia - ps28
Boreoeutheria - ps29
Euarchontoglires - ps30
Euarchonta - ps31
Primates - ps32
Haplorrhini - ps33
Simiiformes - ps34
Catarrhini - ps35
Hominoidea - ps36
Hominidae - ps37
Homininae - ps38
Hominini - ps39
Homo_sapiens - ps40
Phylostratum

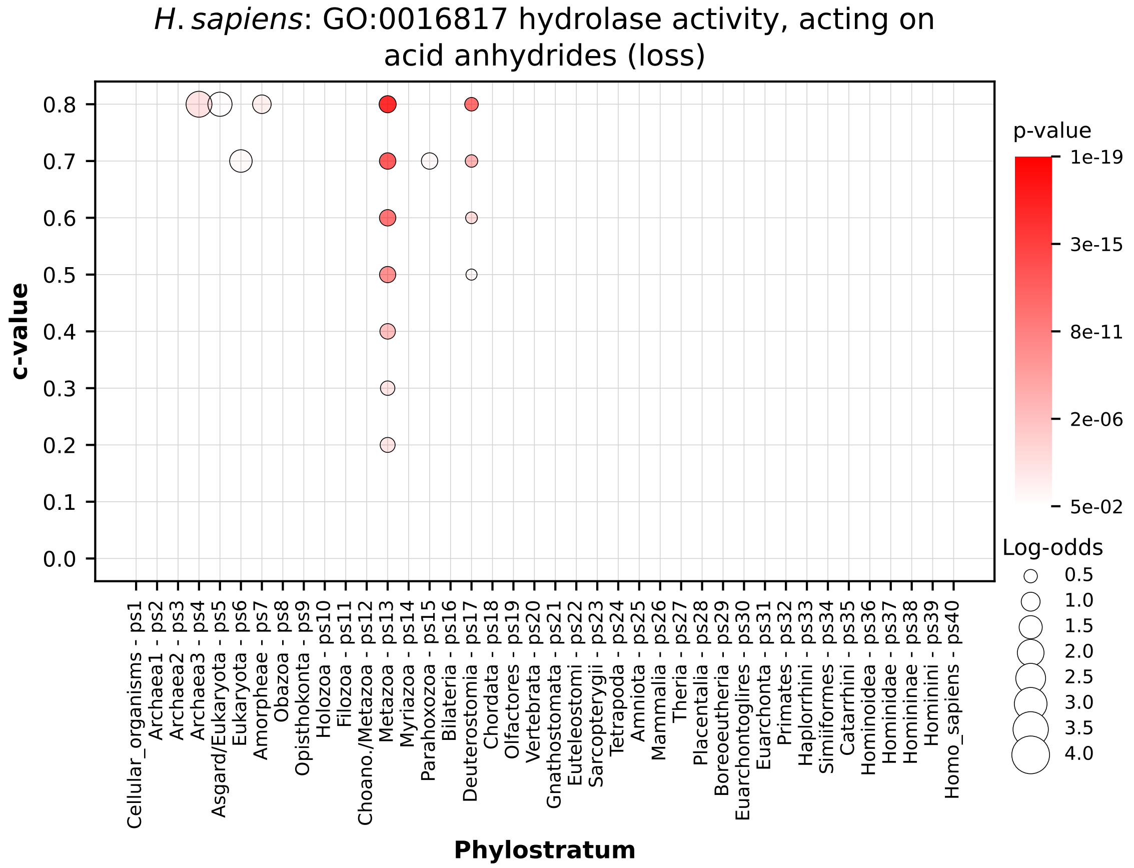

H. sapiens: GO:0016817 hydrolase activity, acting on acid anhydrides (loss)
c-value
0.8
0.7
0.6
0.5
0.4
0.3
0.2
0.1
0.0
Phylostratum
Cellular_organisms - ps1
Archaea1 - ps2
Archaea2 - ps3
Archaea3 - ps4
Asgard/Eukaryota - ps5
Eukaryota - ps6
Amorpheae - ps7
Obazoa - ps8
Opisthokonta - ps9
Holozoa - ps10
Filozoa - ps11
Choano./Metazoa - ps12
Metazoa - ps13
Myriazoa - ps14
Parahoxozoa - ps15
Bilateria - ps16
Deuterostomia - ps17
Chordata - ps18
Olfactores - ps19
Vertebrata - ps20
Gnathostomata - ps21
Euteleostomi - ps22
Sarcopterygii - ps23
Tetrapoda - ps24
Amniota - ps25
Mammalia - ps26
Theria - ps27
Placentalia - ps28
Boreoeutheria - ps29
Euarchontoglires - ps30
Euarchonta - ps31
Primates - ps32
Haplorrhini - ps33
Simiiformes - ps34
Catarrhini - ps35
Hominoidea - ps36
Hominidae - ps37
Homininae - ps38
Hominini - ps39
Homo_sapiens - ps40
p-value
1e-19
3e-15
8e-11
2e-06
5e-02
Log-odds
0.5
1.0
1.5
2.0
2.5
3.0
3.5
4.0

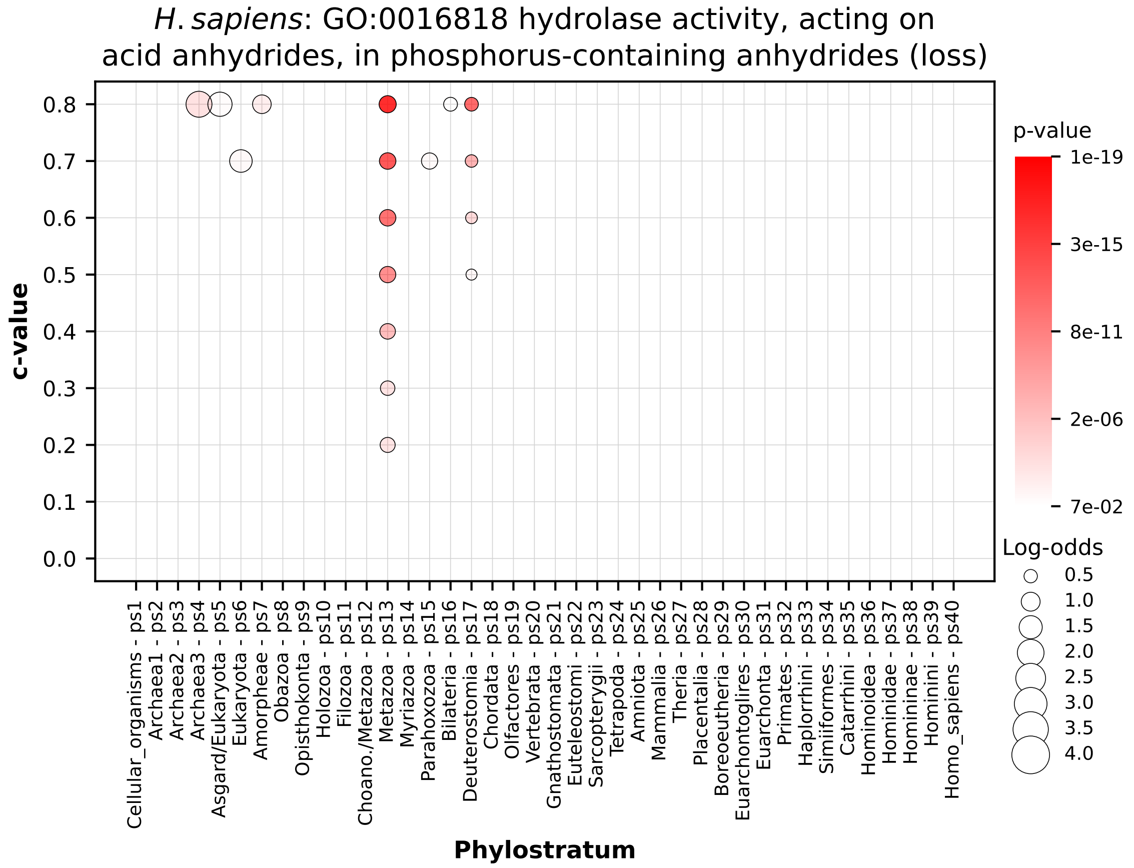

H. sapiens: GO:0016818 hydrolase activity, acting on acid anhydrides, in phosphorus-containing anhydrides (loss)
c-value
0.0
0.1
0.2
0.3
0.4
0.5
0.6
0.7
0.8
Phylostratum
Cellular_organisms - ps1
Archaea1 - ps2
Archaea2 - ps3
Archaea3 - ps4
Asgard/Eukaryota - ps5
Eukaryota - ps6
Amorpheae - ps7
Obazoa - ps8
Opisthokonta - ps9
Holozoa - ps10
Filozoa - ps11
Choano./Metazoa - ps12
Metazoa - ps13
Myriazoa - ps14
Parahoxozoa - ps15
Bilateria - ps16
Deuterostomia - ps17
Chordata - ps18
Olfactores - ps19
Vertebrata - ps20
Gnathostomata - ps21
Euteleostomi - ps22
Sarcopterygii - ps23
Tetrapoda - ps24
Amniota - ps25
Mammalia - ps26
Theria - ps27
Placentalia - ps28
Boreoeutheria - ps29
Euarchontoglires - ps30
Euarchonta - ps31
Primates - ps32
Haplorrhini - ps33
Simiiformes - ps34
Catarrhini - ps35
Hominoidea - ps36
Hominidae - ps37
Homininae - ps38
Hominini - ps39
Homo_sapiens - ps40
p-value
1e-19
3e-15
8e-11
2e-06
7e-02
Log-odds
0.5
1.0
1.5
2.0
2.5
3.0
3.5
4.0

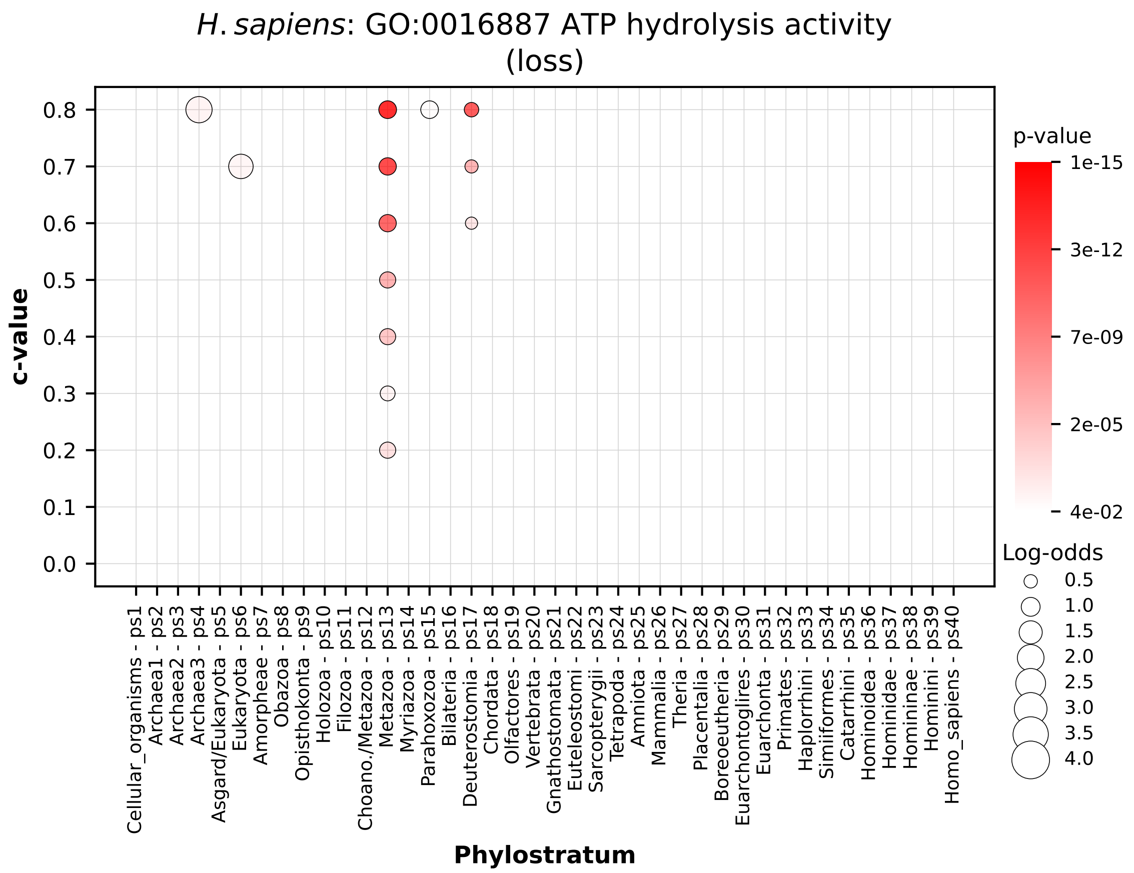

H. sapiens: GO:0016887 ATP hydrolysis activity
(loss)
c-value
0.0
0.1
0.2
0.3
0.4
0.5
0.6
0.7
0.8
p-value
1e-15
3e-12
7e-09
2e-05
4e-02
Log-odds
0.5
1.0
1.5
2.0
2.5
3.0
3.5
4.0
Cellular_organisms - ps1
Archaea1 - ps2
Archaea2 - ps3
Archaea3 - ps4
Asgard/Eukaryota - ps5
Eukaryota - ps6
Amorpheae - ps7
Obazoa - ps8
Opisthokonta - ps9
Holozoa - ps10
Filozoa - ps11
Choano./Metazoa - ps12
Metazoa - ps13
Myriazoa - ps14
Parahoxozoa - ps15
Bilateria - ps16
Deuterostomia - ps17
Chordata - ps18
Olfactores - ps19
Vertebrata - ps20
Gnathostomata - ps21
Euteleostomi - ps22
Sarcopterygii - ps23
Tetrapoda - ps24
Amniota - ps25
Mammalia - ps26
Theria - ps27
Placentalia - ps28
Boreoeutheria - ps29
Euarchontoglires - ps30
Euarchonta - ps31
Primates - ps32
Haplorrhini - ps33
Simiiformes - ps34
Catarrhini - ps35
Hominoidea - ps36
Hominidae - ps37
Homininae - ps38
Hominini - ps39
Homo_sapiens - ps40
Phylostratum

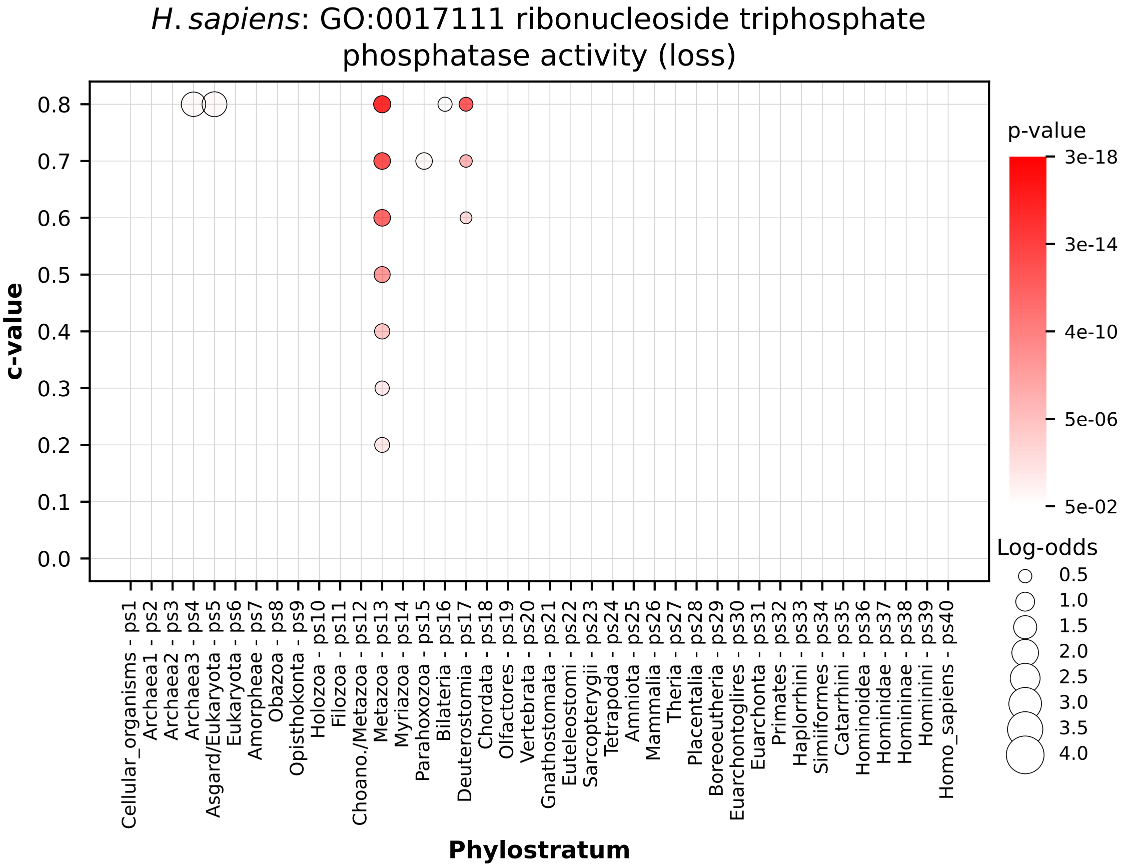

H. sapiens: GO:0017111 ribonucleoside triphosphate phosphatase activity (loss)
c-value
0.0
0.1
0.2
0.3
0.4
0.5
0.6
0.7
0.8
Phylostratum
Cellular_organisms - ps1
Archaea1 - ps2
Archaea2 - ps3
Archaea3 - ps4
Asgard/Eukaryota - ps5
Eukaryota - ps6
Amorpheae - ps7
Obazoa - ps8
Opisthokonta - ps9
Holozoa - ps10
Filozoa - ps11
Choano./Metazoa - ps12
Metazoa - ps13
Myriazoa - ps14
Parahoxozoa - ps15
Bilateria - ps16
Deuterostomia - ps17
Chordata - ps18
Olfactores - ps19
Vertebrata - ps20
Gnathostomata - ps21
Euteleostomi - ps22
Sarcopterygii - ps23
Tetrapoda - ps24
Amniota - ps25
Mammalia - ps26
Theria - ps27
Placentalia - ps28
Boreoeutheria - ps29
Euarchontoglires - ps30
Euarchonta - ps31
Primates - ps32
Haplorrhini - ps33
Simiiformes - ps34
Catarrhini - ps35
Hominoidea - ps36
Hominidae - ps37
Homininae - ps38
Hominini - ps39
Homo_sapiens - ps40
p-value
3e-18
3e-14
4e-10
5e-06
5e-02
Log-odds
0.5
1.0
1.5
2.0
2.5
3.0
3.5
4.0

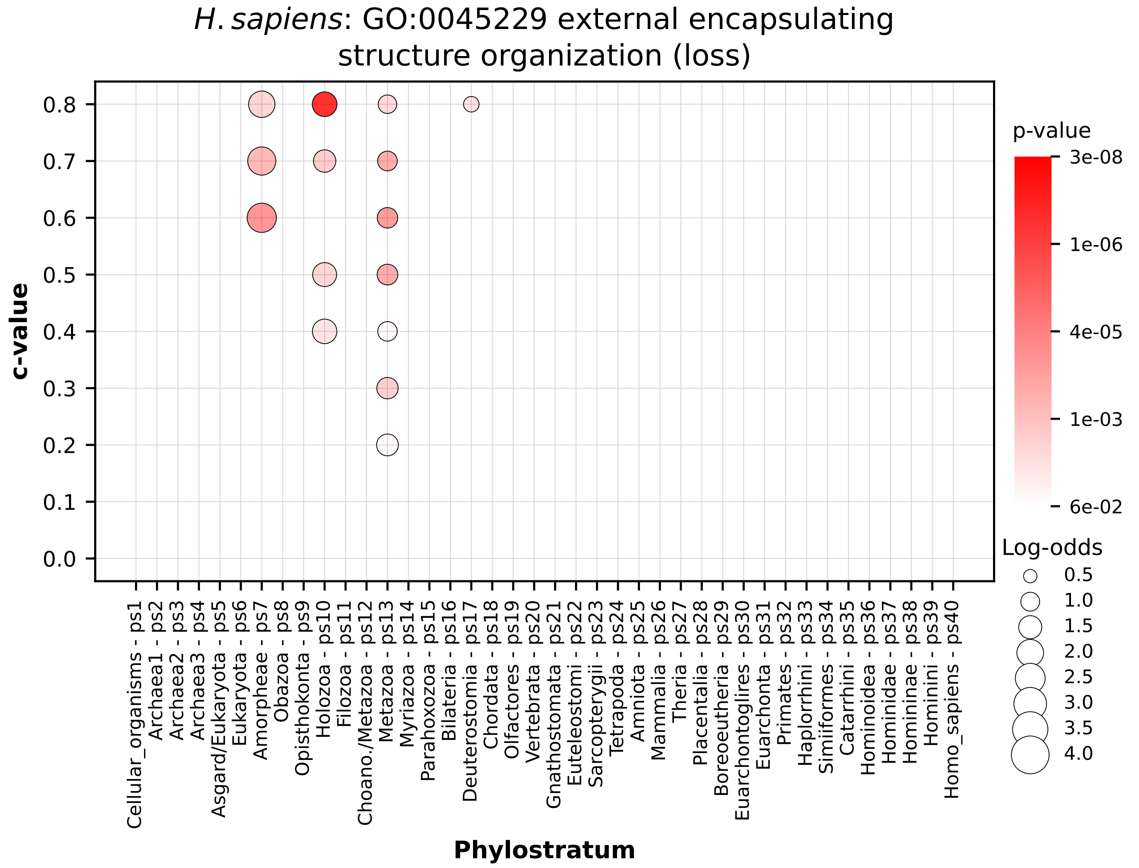

H. sapiens: GO:0045229 external encapsulating structure organization (loss)
c-value
0.8
0.7
0.6
0.5
0.4
0.3
0.2
0.1
0.0
Phylostratum
Cellular_organisms - ps1
Archaea1 - ps2
Archaea2 - ps3
Archaea3 - ps4
Asgard/Eukaryota - ps5
Eukaryota - ps6
Amorpheae - ps7
Obazoa - ps8
Opisthokonta - ps9
Holozoa - ps10
Filozoa - ps11
Choano./Metazoa - ps12
Metazoa - ps13
Myriazoa - ps14
Parahoxozoa - ps15
Bilateria - ps16
Deuterostomia - ps17
Chordata - ps18
Olfactores - ps19
Vertebrata - ps20
Gnathostomata - ps21
Euteleostomi - ps22
Sarcopterygii - ps23
Tetrapoda - ps24
Amniota - ps25
Mammalia - ps26
Theria - ps27
Placentalia - ps28
Boreoeutheria - ps29
Euarchontoglires - ps30
Euarchonta - ps31
Primates - ps32
Haplorrhini - ps33
Simiiformes - ps34
Catarrhini - ps35
Hominoidea - ps36
Hominidae - ps37
Homininae - ps38
Hominini - ps39
Homo_sapiens - ps40
p-value
3e-08
1e-06
4e-05
1e-03
6e-02
Log-odds
0.5
1.0
1.5
2.0
2.5
3.0
3.5
4.0

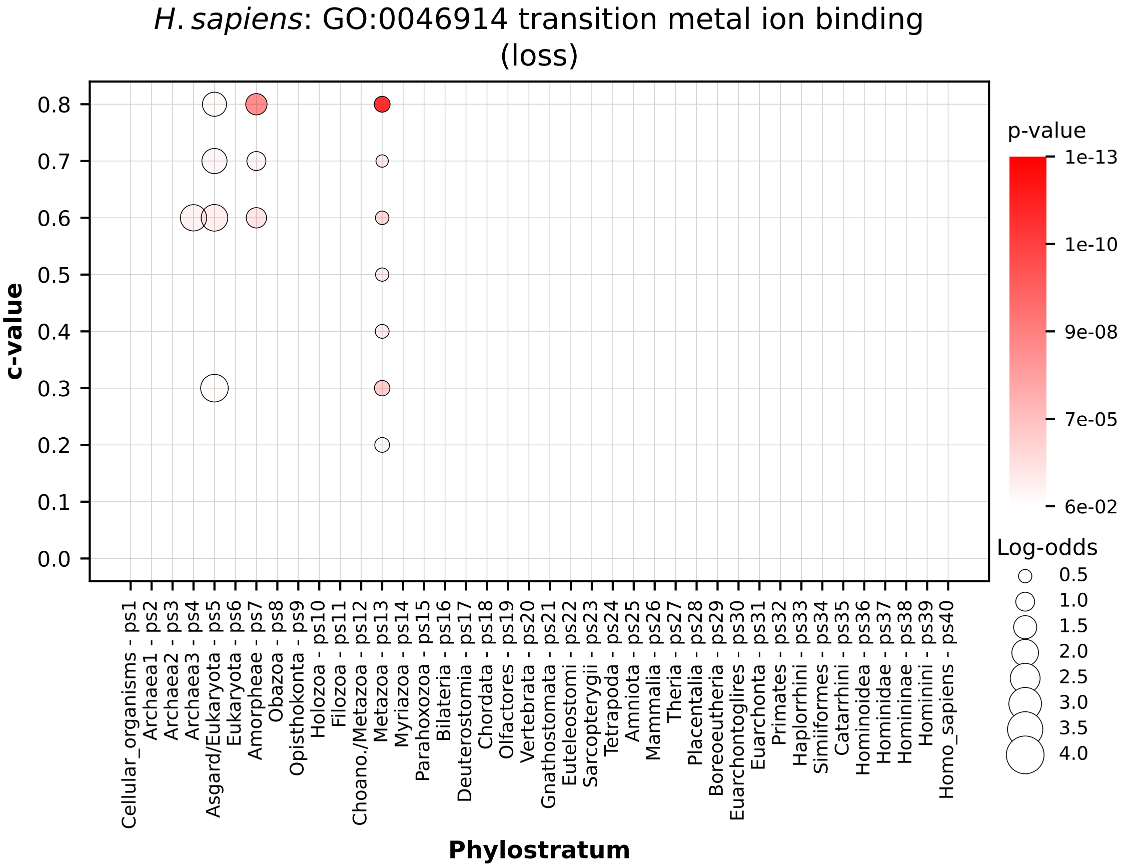

H. sapiens: GO:0046914 transition metal ion binding (loss)
c-value
0.8
0.7
0.6
0.5
0.4
0.3
0.2
0.1
0.0
Phylostratum
Cellular_organisms - ps1
Archaea1 - ps2
Archaea2 - ps3
Archaea3 - ps4
Asgard/Eukaryota - ps5
Eukaryota - ps6
Amorpheae - ps7
Obazoa - ps8
Opisthokonta - ps9
Holozoa - ps10
Filozoa - ps11
Choano./Metazoa - ps12
Metazoa - ps13
Myriazoa - ps14
Parahoxozoa - ps15
Bilateria - ps16
Deuterostomia - ps17
Chordata - ps18
Olfactores - ps19
Vertebrata - ps20
Gnathostomata - ps21
Euteleostomi - ps22
Sarcopterygii - ps23
Tetrapoda - ps24
Amniota - ps25
Mammalia - ps26
Theria - ps27
Placentalia - ps28
Boreoeutheria - ps29
Euarchontoglires - ps30
Euarchonta - ps31
Primates - ps32
Haplorrhini - ps33
Simiiformes - ps34
Catarrhini - ps35
Hominoidea - ps36
Hominidae - ps37
Homininae - ps38
Hominini - ps39
Homo_sapiens - ps40
p-value
1e-13
1e-10
9e-08
7e-05
6e-02
Log-odds
0.5
1.0
1.5
2.0
2.5
3.0
3.5
4.0

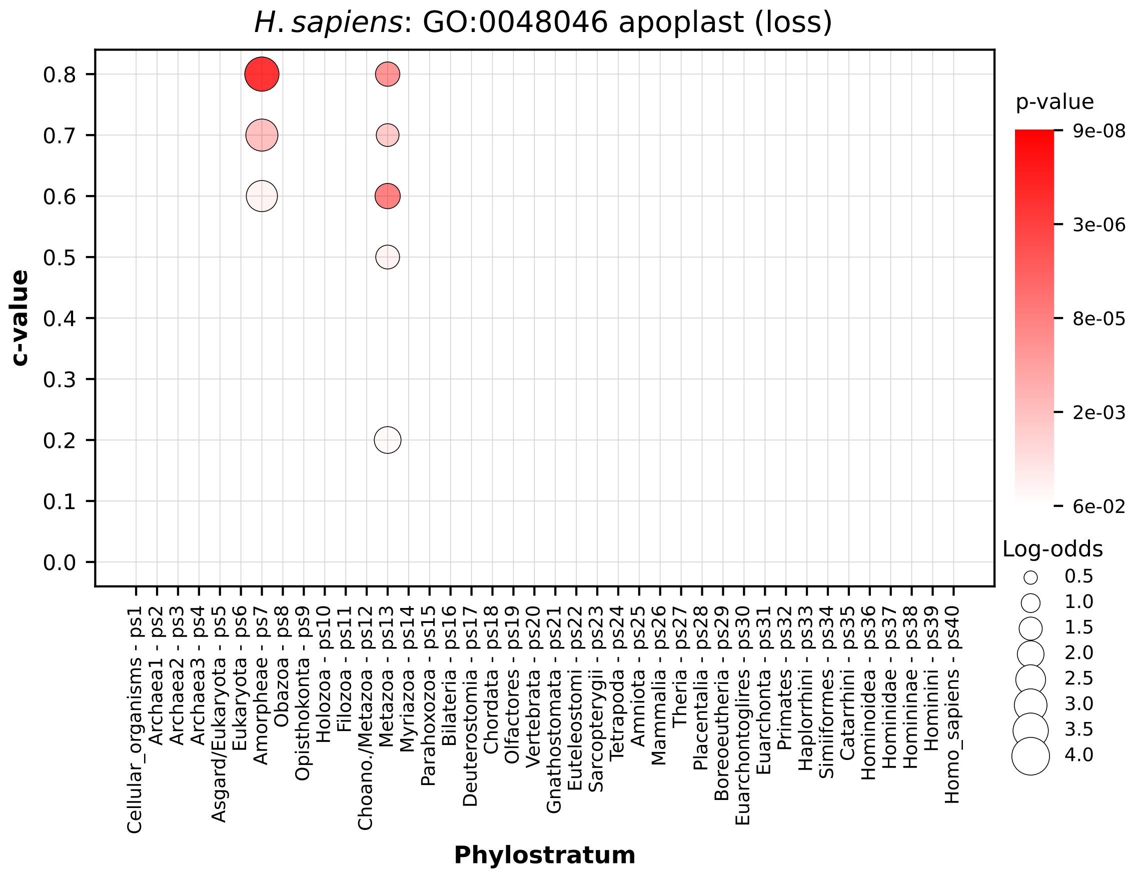

H. sapiens: GO:0048046 apoplast (loss)
c-value
0.8
0.7
0.6
0.5
0.4
0.3
0.2
0.1
0.0
p-value
9e-08
3e-06
8e-05
2e-03
6e-02
Log-odds
0.5
1.0
1.5
2.0
2.5
3.0
3.5
4.0
Cellular_organisms - ps1
Archaea1 - ps2
Archaea2 - ps3
Archaea3 - ps4
Asgard/Eukaryota - ps5
Eukaryota - ps6
Amorpheae - ps7
Obazoa - ps8
Opisthokonta - ps9
Holozoa - ps10
Filozoa - ps11
Choano./Metazoa - ps12
Metazoa - ps13
Myriazoa - ps14
Parahoxozoa - ps15
Bilateria - ps16
Deuterostomia - ps17
Chordata - ps18
Olfactores - ps19
Vertebrata - ps20
Gnathostomata - ps21
Euteleostomi - ps22
Sarcopterygii - ps23
Tetrapoda - ps24
Amniota - ps25
Mammalia - ps26
Theria - ps27
Placentalia - ps28
Boreoeutheria - ps29
Euarchontoglires - ps30
Euarchonta - ps31
Primates - ps32
Haplorrhini - ps33
Simiiformes - ps34
Catarrhini - ps35
Hominoidea - ps36
Hominidae - ps37
Homininae - ps38
Hominini - ps39
Homo_sapiens - ps40
Phylostratum

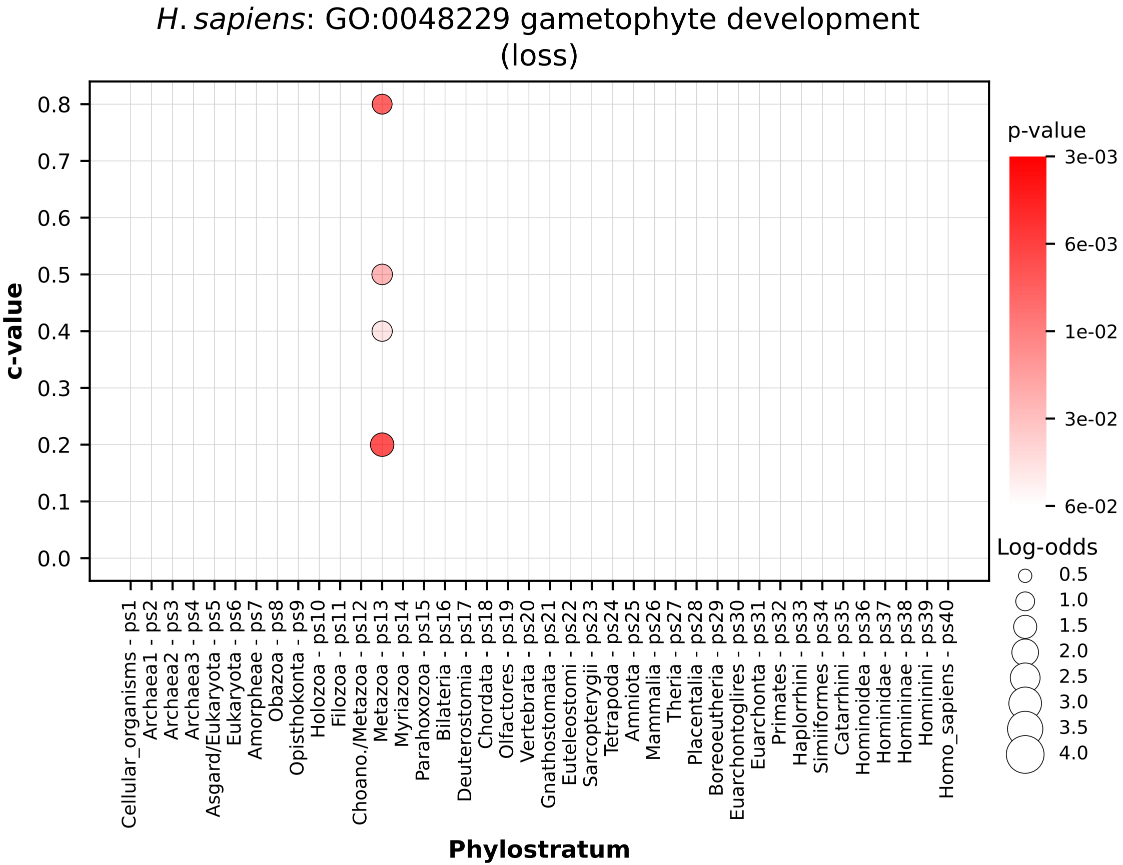

H. sapiens: GO:0048229 gametophyte development
(loss)
c-value
0.0
0.1
0.2
0.3
0.4
0.5
0.6
0.7
0.8
Phylostratum
Cellular_organisms - ps1
Archaea1 - ps2
Archaea2 - ps3
Archaea3 - ps4
Asgard/Eukaryota - ps5
Eukaryota - ps6
Amorpheae - ps7
Obazoa - ps8
Opisthokonta - ps9
Holozoa - ps10
Filozoa - ps11
Choano./Metazoa - ps12
Metazoa - ps13
Myriazoa - ps14
Parahoxozoa - ps15
Bilateria - ps16
Deuterostomia - ps17
Chordata - ps18
Olfactores - ps19
Vertebrata - ps20
Gnathostomata - ps21
Euteleostomi - ps22
Sarcopterygii - ps23
Tetrapoda - ps24
Amniota - ps25
Mammalia - ps26
Theria - ps27
Placentalia - ps28
Boreoeutheria - ps29
Euarchontoglires - ps30
Euarchonta - ps31
Primates - ps32
Haplorrhini - ps33
Simiiformes - ps34
Catarrhini - ps35
Hominoidea - ps36
Hominidae - ps37
Homininae - ps38
Hominini - ps39
Homo_sapiens - ps40
p-value
3e-03
6e-03
1e-02
3e-02
6e-02
Log-odds
0.5
1.0
1.5
2.0
2.5
3.0
3.5
4.0

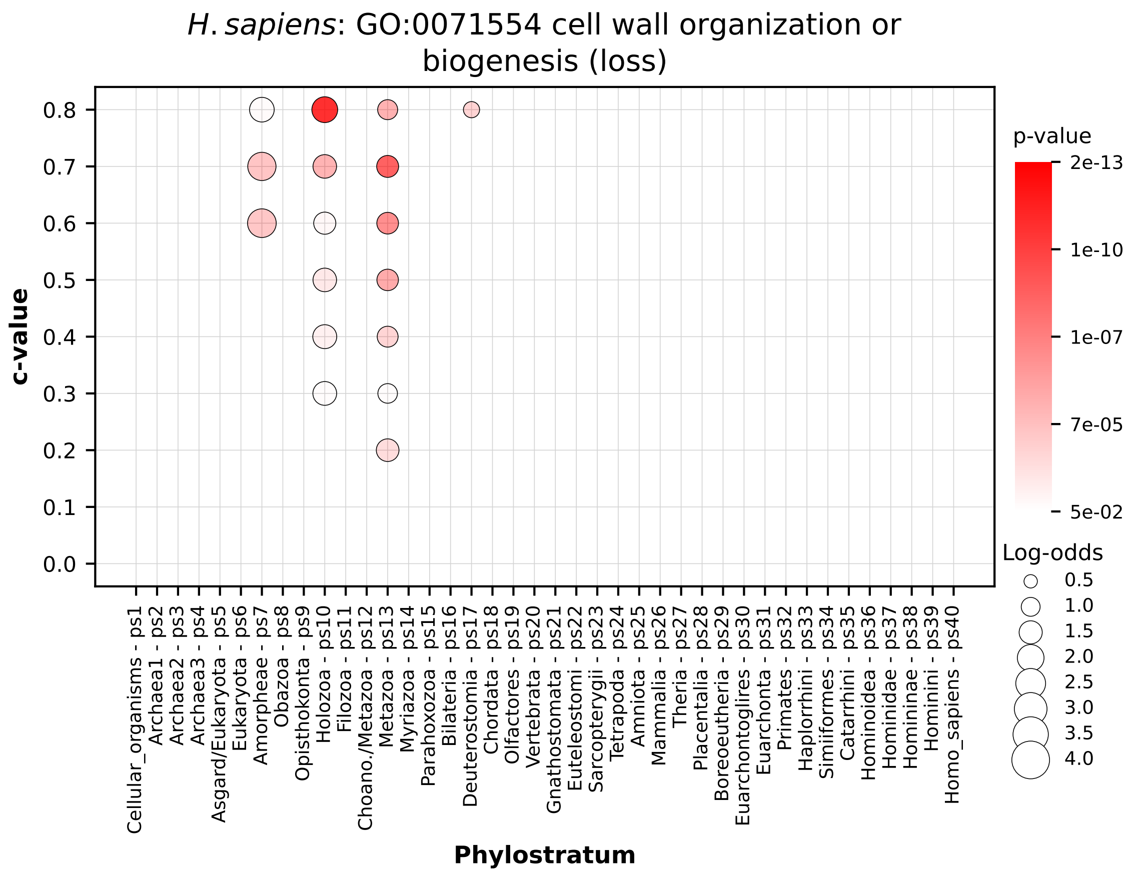
H. sapiens: GO:0071554 cell wall organization or biogenesis (loss)
c-value
0.8
0.7
0.6
0.5
0.4
0.3
0.2
0.1
0.0
Phylostratum
Cellular_organisms - ps1
Archaea1 - ps2
Archaea2 - ps3
Archaea3 - ps4
Asgard/Eukaryota - ps5
Eukaryota - ps6
Amorpheae - ps7
Obazoa - ps8
Opisthokonta - ps9
Holozoa - ps10
Filozoa - ps11
Choano./Metazoa - ps12
Metazoa - ps13
Myriazoa - ps14
Parahoxozoa - ps15
Bilateria - ps16
Deuterostomia - ps17
Chordata - ps18
Olfactores - ps19
Vertebrata - ps20
Gnathostomata - ps21
Euteleostomi - ps22
Sarcopterygii - ps23
Tetrapoda - ps24
Amniota - ps25
Mammalia - ps26
Theria - ps27
Placentalia - ps28
Boreoeutheria - ps29
Euarchontoglires - ps30
Euarchonta - ps31
Primates - ps32
Haplorrhini - ps33
Simiiformes - ps34
Catarrhini - ps35
Hominoidea - ps36
Hominidae - ps37
Homininae - ps38
Hominini - ps39
Homo_sapiens - ps40
p-value
2e-13
1e-10
1e-07
7e-05
5e-02
Log-odds
0.5
1.0
1.5
2.0
2.5
3.0
3.5
4.0

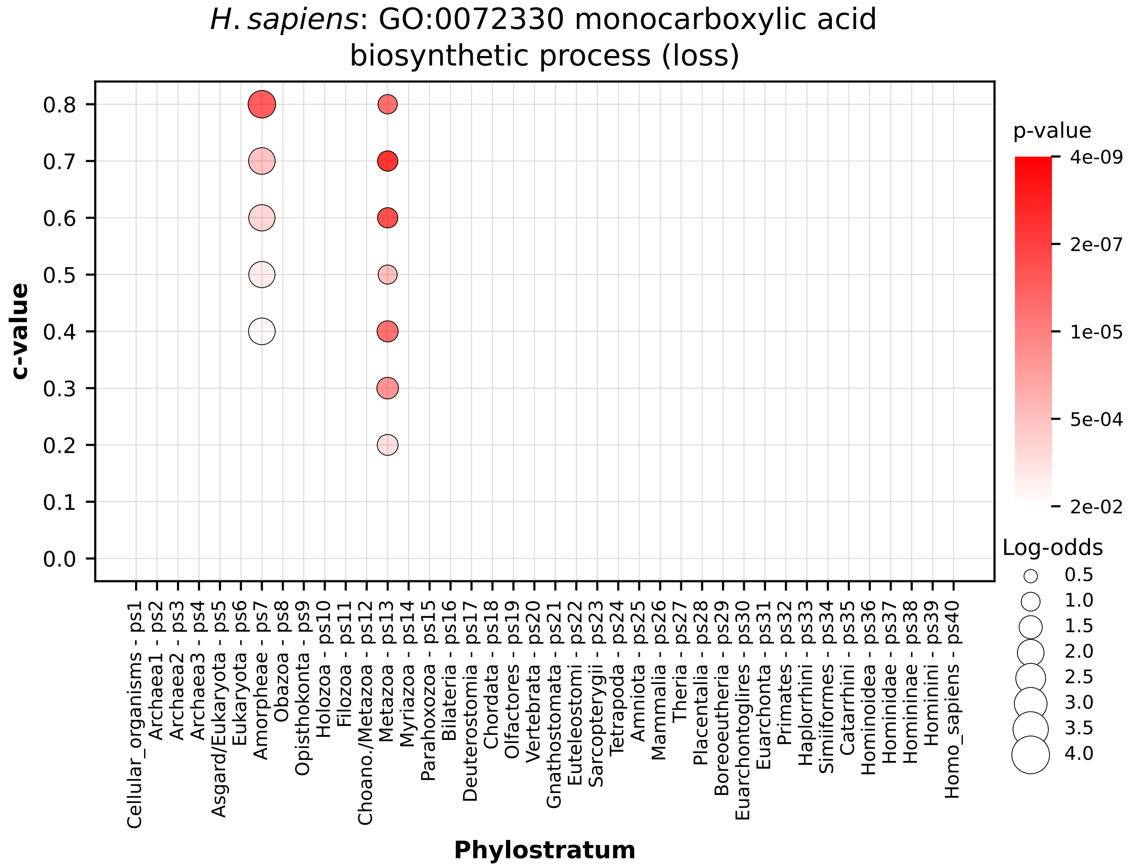

H. sapiens: GO:0072330 monocarboxylic acid biosynthetic process (loss)
c-value
0.8
0.7
0.6
0.5
0.4
0.3
0.2
0.1
0.0
Phylostratum
Cellular_organisms - ps1
Archaea1 - ps2
Archaea2 - ps3
Archaea3 - ps4
Asgard/Eukaryota - ps5
Eukaryota - ps6
Amorpheae - ps7
Obazoa - ps8
Opisthokonta - ps9
Holozoa - ps10
Filozoa - ps11
Choano./Metazoa - ps12
Metazoa - ps13
Myriazoa - ps14
Parahoxozoa - ps15
Bilateria - ps16
Deuterostomia - ps17
Chordata - ps18
Olfactores - ps19
Vertebrata - ps20
Gnathostomata - ps21
Euteleostomi - ps22
Sarcopterygii - ps23
Tetrapoda - ps24
Amniota - ps25
Mammalia - ps26
Theria - ps27
Placentalia - ps28
Boreoeutheria - ps29
Euarchontoglires - ps30
Euarchonta - ps31
Primates - ps32
Haplorrhini - ps33
Simiiformes - ps34
Catarrhini - ps35
Hominoidea - ps36
Hominidae - ps37
Homininae - ps38
Hominini - ps39
Homo_sapiens - ps40
p-value
4e-09
2e-07
1e-05
5e-04
2e-02
Log-odds
0.5
1.0
1.5
2.0
2.5
3.0
3.5
4.0

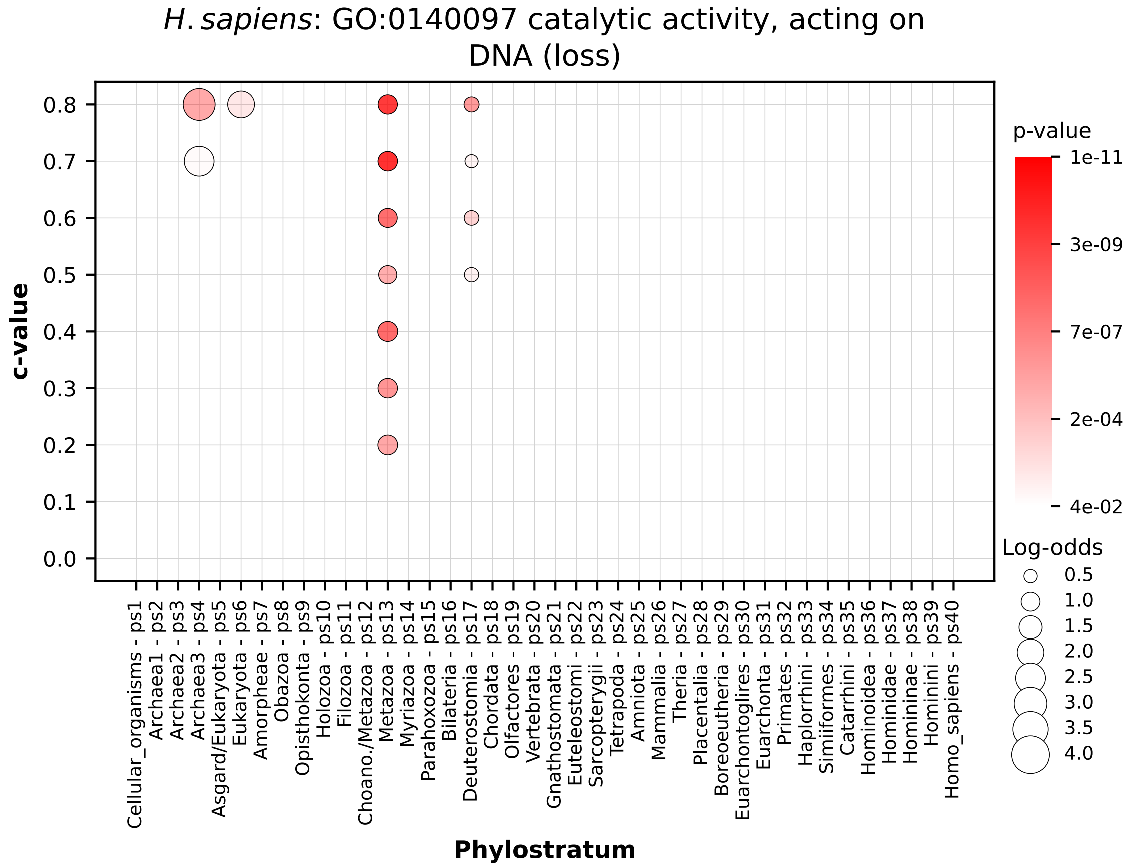

H. sapiens: GO:0140097 catalytic activity, acting on DNA (loss)
c-value
0.8
0.7
0.6
0.5
0.4
0.3
0.2
0.1
0.0
p-value
1e-11
3e-09
7e-07
2e-04
4e-02
Log-odds
0.5
1.0
1.5
2.0
2.5
3.0
3.5
4.0
Cellular_organisms - ps1
Archaea1 - ps2
Archaea2 - ps3
Archaea3 - ps4
Asgard/Eukaryota - ps5
Eukaryota - ps6
Amorpheae - ps7
Obazoa - ps8
Opisthokonta - ps9
Holozoa - ps10
Filozoa - ps11
Choano./Metazoa - ps12
Metazoa - ps13
Myriazoa - ps14
Parahoxozoa - ps15
Bilateria - ps16
Deuterostomia - ps17
Chordata - ps18
Olfactores - ps19
Vertebrata - ps20
Gnathostomata - ps21
Euteleostomi - ps22
Sarcopterygii - ps23
Tetrapoda - ps24
Amniota - ps25
Mammalia - ps26
Theria - ps27
Placentalia - ps28
Boreoeutheria - ps29
Euarchontoglires - ps30
Euarchonta - ps31
Primates - ps32
Haplorrhini - ps33
Simiiformes - ps34
Catarrhini - ps35
Hominoidea - ps36
Hominidae - ps37
Homininae - ps38
Hominini - ps39
Homo_sapiens - ps40
Phylostratum

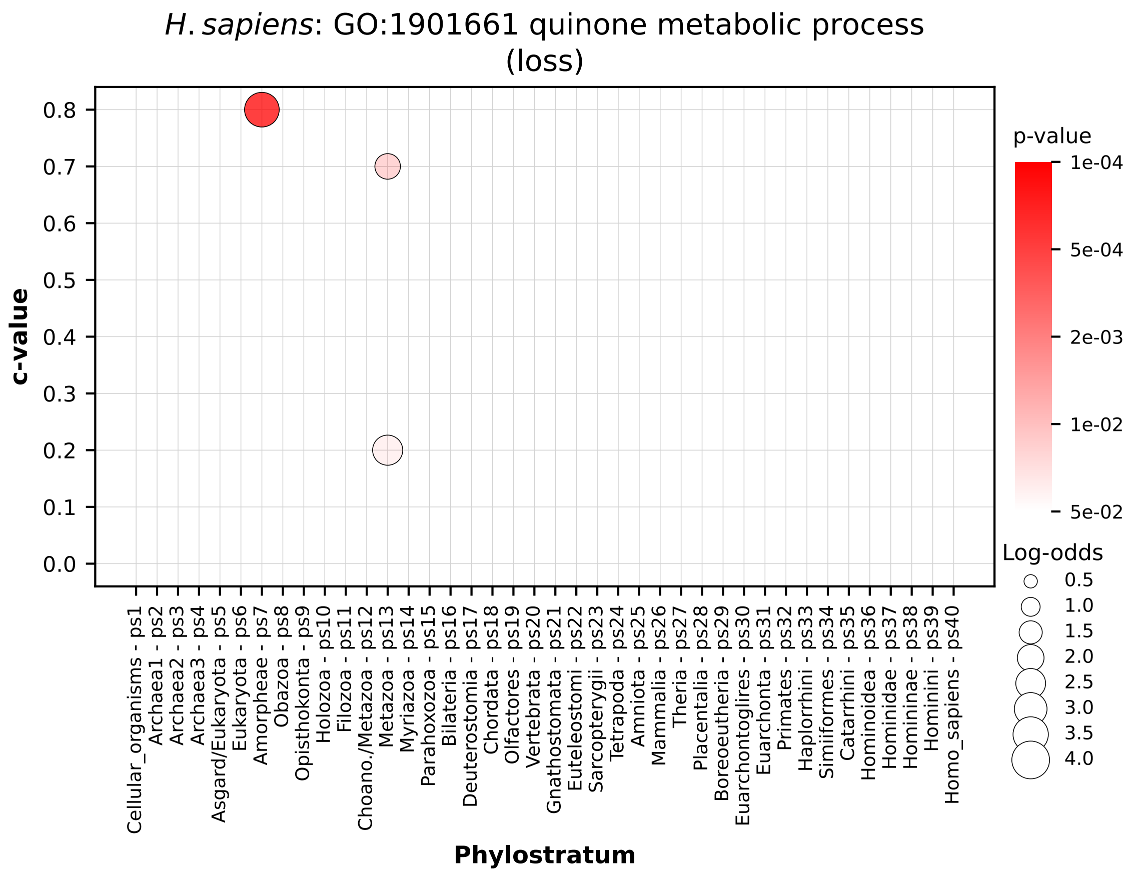

H. sapiens: GO:1901661 quinone metabolic process
(loss)
c-value
0.0
0.1
0.2
0.3
0.4
0.5
0.6
0.7
0.8
p-value
1e-04
5e-04
2e-03
1e-02
5e-02
Log-odds
0.5
1.0
1.5
2.0
2.5
3.0
3.5
4.0
Cellular_organisms - ps1
Archaea1 - ps2
Archaea2 - ps3
Archaea3 - ps4
Asgard/Eukaryota - ps5
Eukaryota - ps6
Amorpheae - ps7
Obazoa - ps8
Opisthokonta - ps9
Holozoa - ps10
Filozoa - ps11
Choano./Metazoa - ps12
Metazoa - ps13
Myriazoa - ps14
Parahoxozoa - ps15
Bilateria - ps16
Deuterostomia - ps17
Chordata - ps18
Olfactores - ps19
Vertebrata - ps20
Gnathostomata - ps21
Euteleostomi - ps22
Sarcopterygii - ps23
Tetrapoda - ps24
Amniota - ps25
Mammalia - ps26
Theria - ps27
Placentalia - ps28
Boreoeutheria - ps29
Euarchontoglires - ps30
Euarchonta - ps31
Primates - ps32
Haplorrhini - ps33
Simiiformes - ps34
Catarrhini - ps35
Hominoidea - ps36
Hominidae - ps37
Homininae - ps38
Hominini - ps39
Homo_sapiens - ps40
Phylostratum

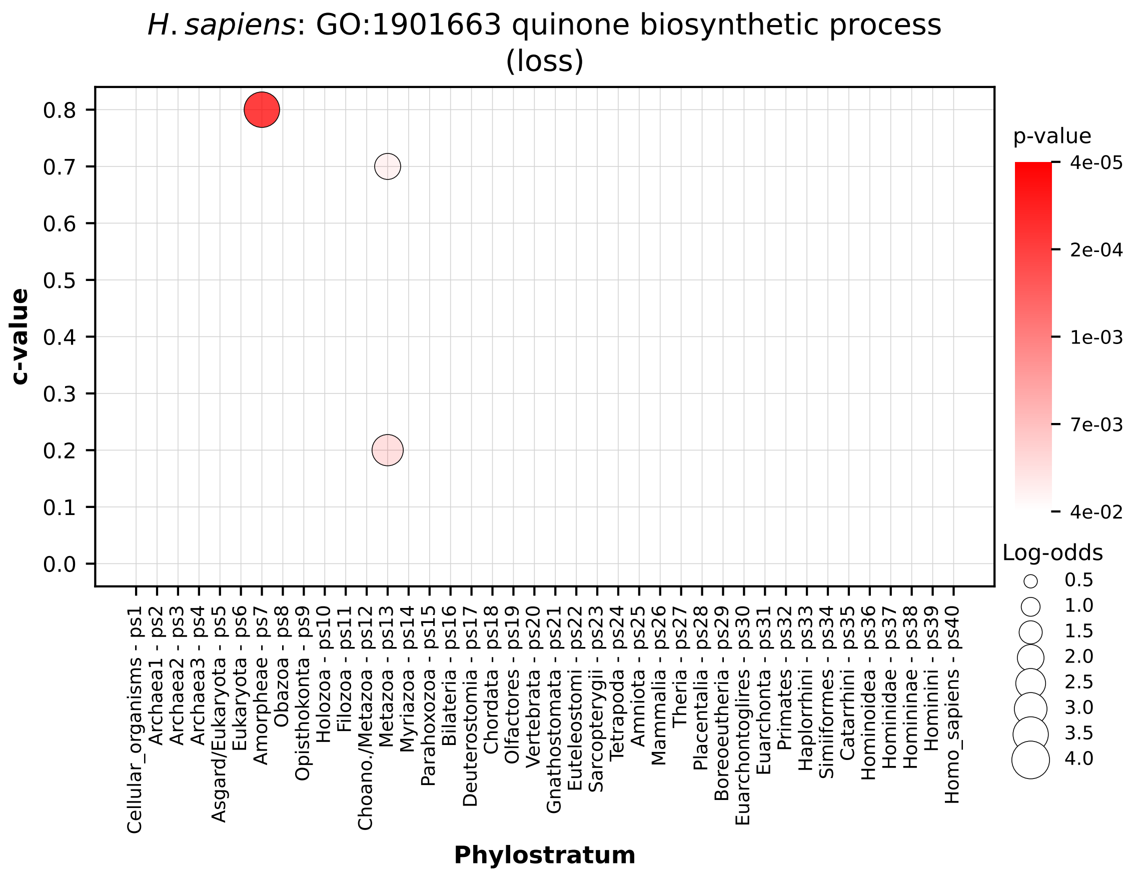
H. sapiens: GO:1901663 quinone biosynthetic process (loss)
c-value
0.0
0.1
0.2
0.3
0.4
0.5
0.6
0.7
0.8
Phylostratum
Cellular_organisms - ps1
Archaea1 - ps2
Archaea2 - ps3
Archaea3 - ps4
Asgard/Eukaryota - ps5
Eukaryota - ps6
Amorpheae - ps7
Obazoa - ps8
Opisthokonta - ps9
Holozoa - ps10
Filozoa - ps11
Choano./Metazoa - ps12
Metazoa - ps13
Myriazoa - ps14
Parahoxozoa - ps15
Bilateria - ps16
Deuterostomia - ps17
Chordata - ps18
Olfactores - ps19
Vertebrata - ps20
Gnathostomata - ps21
Euteleostomi - ps22
Sarcopterygii - ps23
Tetrapoda - ps24
Amniota - ps25
Mammalia - ps26
Theria - ps27
Placentalia - ps28
Boreoeutheria - ps29
Euarchontoglires - ps30
Euarchonta - ps31
Primates - ps32
Haplorrhini - ps33
Simiiformes - ps34
Catarrhini - ps35
Hominoidea - ps36
Hominidae - ps37
Homininae - ps38
Hominini - ps39
Homo_sapiens - ps40
p-value
4e-05
2e-04
1e-03
7e-03
4e-02
Log-odds
0.5
1.0
1.5
2.0
2.5
3.0
3.5
4.0

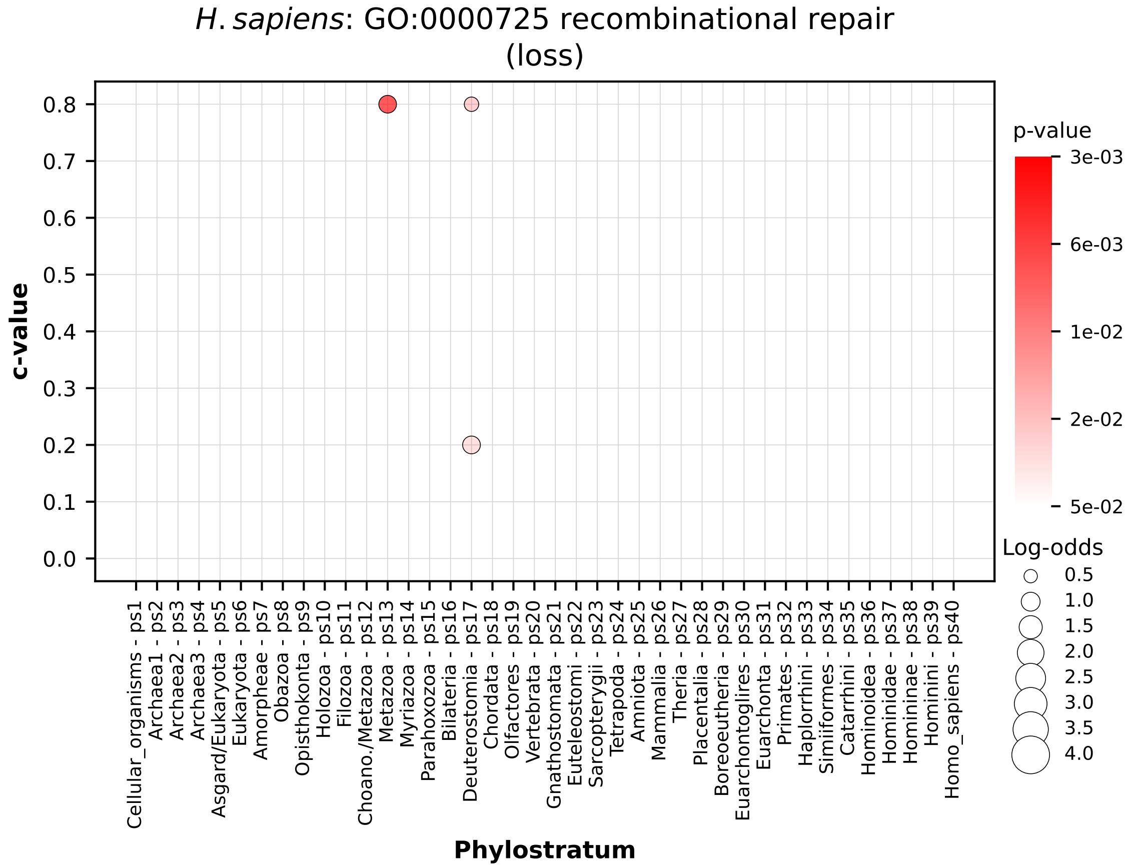

H. sapiens: GO:0000725 recombinational repair
(loss)
c-value
0.0
0.1
0.2
0.3
0.4
0.5
0.6
0.7
0.8
p-value
3e-03
6e-03
1e-02
2e-02
5e-02
Log-odds
0.5
1.0
1.5
2.0
2.5
3.0
3.5
4.0
Cellular_organisms - ps1
Archaea1 - ps2
Archaea2 - ps3
Archaea3 - ps4
Asgard/Eukaryota - ps5
Eukaryota - ps6
Amorpheae - ps7
Obazoa - ps8
Opisthokonta - ps9
Holozoa - ps10
Filozoa - ps11
Choano./Metazoa - ps12
Metazoa - ps13
Myriazoa - ps14
Parahoxozoa - ps15
Bilateria - ps16
Deuterostomia - ps17
Chordata - ps18
Olfactores - ps19
Vertebrata - ps20
Gnathostomata - ps21
Euteleostomi - ps22
Sarcopterygii - ps23
Tetrapoda - ps24
Amniota - ps25
Mammalia - ps26
Theria - ps27
Placentalia - ps28
Boreoeutheria - ps29
Euarchontoglires - ps30
Euarchonta - ps31
Primates - ps32
Haplorrhini - ps33
Simiiformes - ps34
Catarrhini - ps35
Hominoidea - ps36
Hominidae - ps37
Homininae - ps38
Hominini - ps39
Homo_sapiens - ps40
Phylostratum

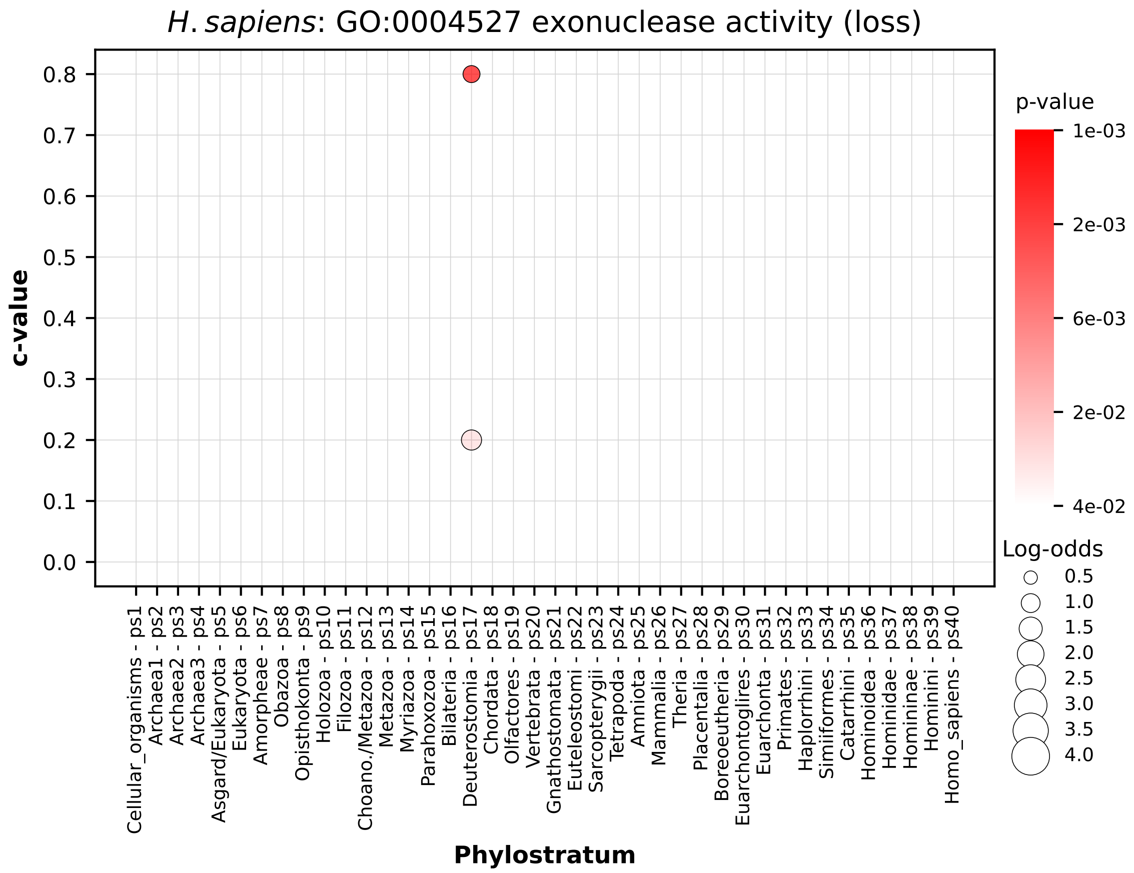

H. sapiens: GO:0004527 exonuclease activity (loss)
c-value
0.8
0.7
0.6
0.5
0.4
0.3
0.2
0.1
0.0
p-value
1e-03
2e-03
6e-03
2e-02
4e-02
Log-odds
0.5
1.0
1.5
2.0
2.5
3.0
3.5
4.0
Cellular_organisms - ps1
Archaea1 - ps2
Archaea2 - ps3
Archaea3 - ps4
Asgard/Eukaryota - ps5
Eukaryota - ps6
Amorpheae - ps7
Obazoa - ps8
Opisthokonta - ps9
Holozoa - ps10
Filozoa - ps11
Choano./Metazoa - ps12
Metazoa - ps13
Myriazoa - ps14
Parahoxozoa - ps15
Bilateria - ps16
Deuterostomia - ps17
Chordata - ps18
Olfactores - ps19
Vertebrata - ps20
Gnathostomata - ps21
Euteleostomi - ps22
Sarcopterygii - ps23
Tetrapoda - ps24
Amniota - ps25
Mammalia - ps26
Theria - ps27
Placentalia - ps28
Boreoeutheria - ps29
Euarchontoglires - ps30
Euarchonta - ps31
Primates - ps32
Haplorrhini - ps33
Simiiformes - ps34
Catarrhini - ps35
Hominoidea - ps36
Hominidae - ps37
Homininae - ps38
Hominini - ps39
Homo_sapiens - ps40
Phylostratum

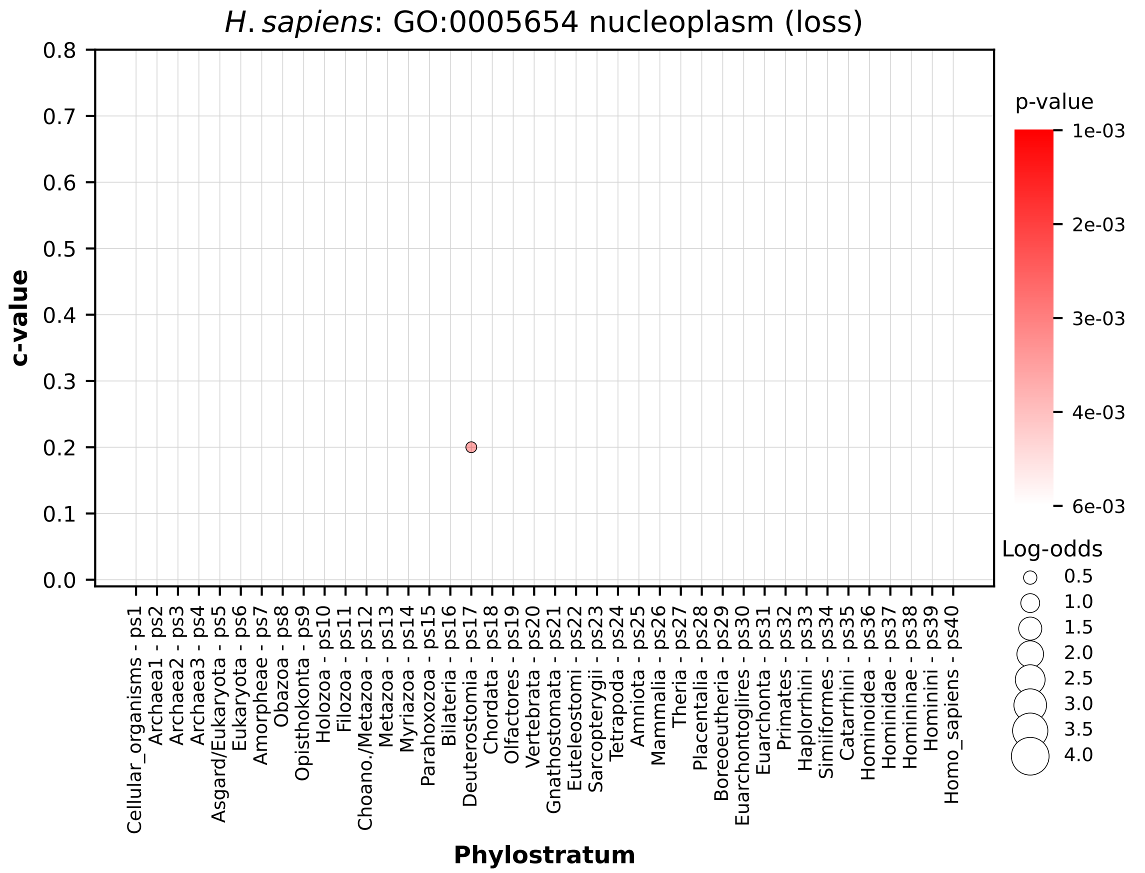

H. sapiens: GO:0005654 nucleoplasm (loss)
c-value
0.8
0.7
0.6
0.5
0.4
0.3
0.2
0.1
0.0
Phylostratum
Cellular_organisms - ps1
Archaea1 - ps2
Archaea2 - ps3
Archaea3 - ps4
Asgard/Eukaryota - ps5
Eukaryota - ps6
Amorpheae - ps7
Obazoa - ps8
Opisthokonta - ps9
Holozoa - ps10
Filozoa - ps11
Choano./Metazoa - ps12
Metazoa - ps13
Myriazoa - ps14
Parahoxozoa - ps15
Bilateria - ps16
Deuterostomia - ps17
Chordata - ps18
Olfactores - ps19
Vertebrata - ps20
Gnathostomata - ps21
Euteleostomi - ps22
Sarcopterygii - ps23
Tetrapoda - ps24
Amniota - ps25
Mammalia - ps26
Theria - ps27
Placentalia - ps28
Boreoeutheria - ps29
Euarchontoglires - ps30
Euarchonta - ps31
Primates - ps32
Haplorrhini - ps33
Simiiformes - ps34
Catarrhini - ps35
Hominoidea - ps36
Hominidae - ps37
Homininae - ps38
Hominini - ps39
Homo_sapiens - ps40
p-value
1e-03
2e-03
3e-03
4e-03
6e-03
Log-odds
0.5
1.0
1.5
2.0
2.5
3.0
3.5
4.0

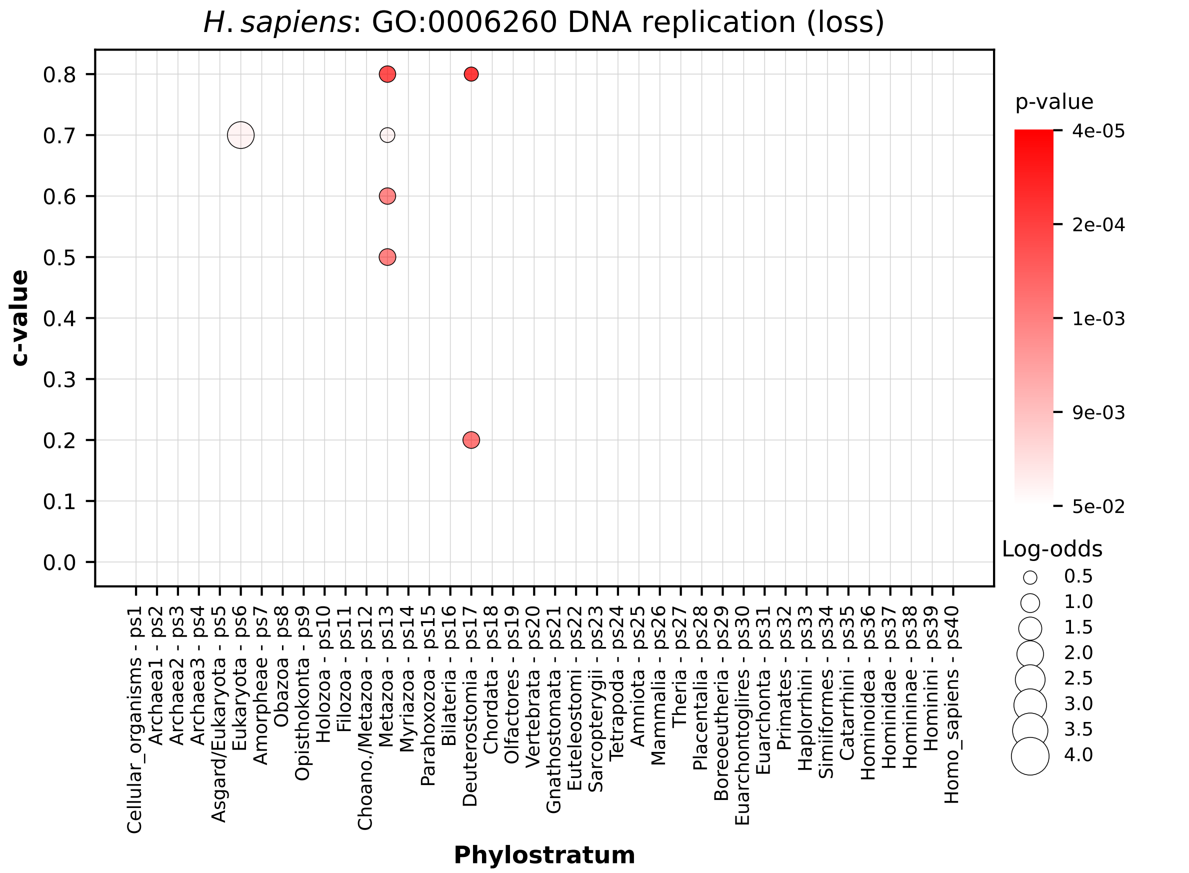

H. sapiens: GO:0006260 DNA replication (loss)
c-value
0.8
0.7
0.6
0.5
0.4
0.3
0.2
0.1
0.0
p-value
4e-05
2e-04
1e-03
9e-03
5e-02
Log-odds
0.5
1.0
1.5
2.0
2.5
3.0
3.5
4.0
Cellular_organisms - ps1
Archaea1 - ps2
Archaea2 - ps3
Archaea3 - ps4
Asgard/Eukaryota - ps5
Eukaryota - ps6
Amorpheae - ps7
Obazoa - ps8
Opisthokonta - ps9
Holozoa - ps10
Filozoa - ps11
Choano./Metazoa - ps12
Metazoa - ps13
Myriazoa - ps14
Parahoxozoa - ps15
Bilateria - ps16
Deuterostomia - ps17
Chordata - ps18
Olfactores - ps19
Vertebrata - ps20
Gnathostomata - ps21
Euteleostomi - ps22
Sarcopterygii - ps23
Tetrapoda - ps24
Amniota - ps25
Mammalia - ps26
Theria - ps27
Placentalia - ps28
Boreoeutheria - ps29
Euarchontoglires - ps30
Euarchonta - ps31
Primates - ps32
Haplorrhini - ps33
Simiiformes - ps34
Catarrhini - ps35
Hominoidea - ps36
Hominidae - ps37
Homininae - ps38
Hominini - ps39
Homo_sapiens - ps40
Phylostratum

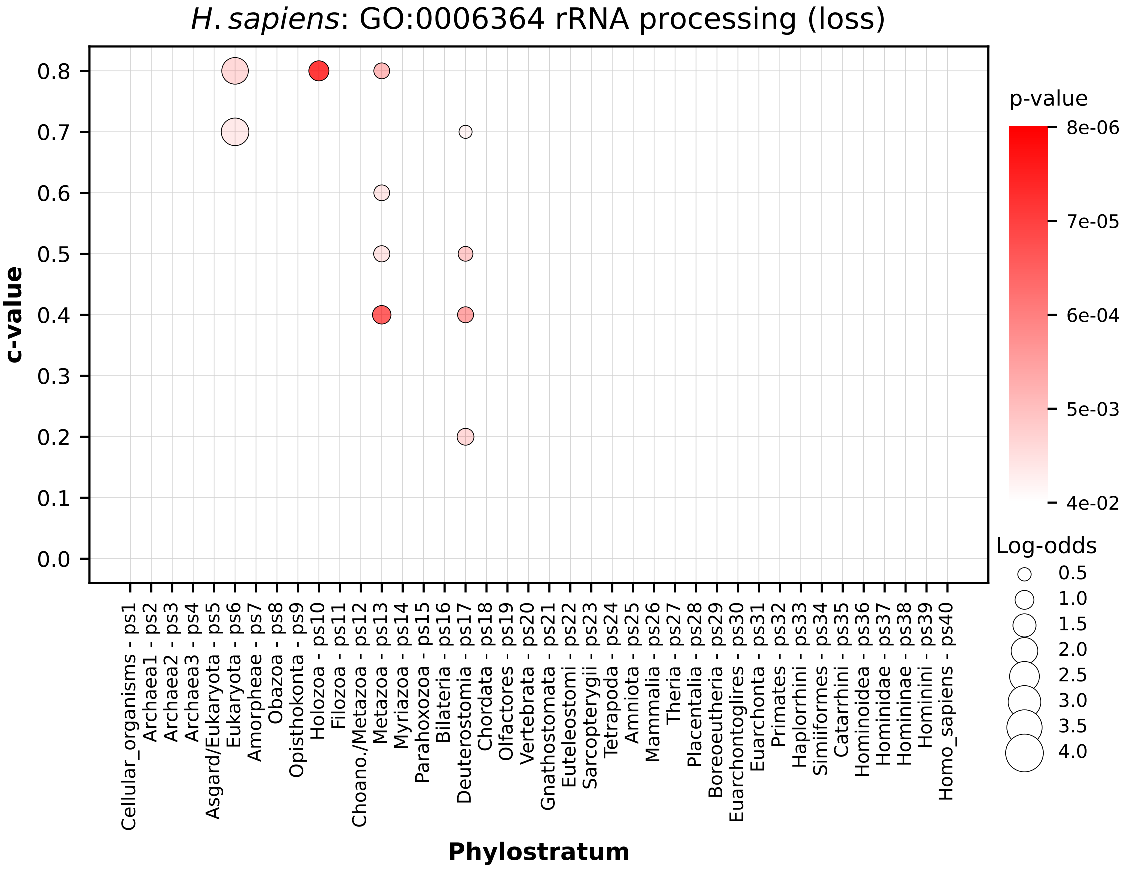
H. sapiens: GO:0006364 rRNA processing (loss)
c-value
0.8
0.7
0.6
0.5
0.4
0.3
0.2
0.1
0.0
Phylostratum
Cellular_organisms - ps1
Archaea1 - ps2
Archaea2 - ps3
Archaea3 - ps4
Asgard/Eukaryota - ps5
Eukaryota - ps6
Amorpheae - ps7
Obazoa - ps8
Opisthokonta - ps9
Holozoa - ps10
Filozoa - ps11
Choano./Metazoa - ps12
Metazoa - ps13
Myriazoa - ps14
Parahoxozoa - ps15
Bilateria - ps16
Deuterostomia - ps17
Chordata - ps18
Olfactores - ps19
Vertebrata - ps20
Gnathostomata - ps21
Euteleostomi - ps22
Sarcopterygii - ps23
Tetrapoda - ps24
Amniota - ps25
Mammalia - ps26
Theria - ps27
Placentalia - ps28
Boreoeutheria - ps29
Euarchontoglires - ps30
Euarchonta - ps31
Primates - ps32
Haplorrhini - ps33
Simiiformes - ps34
Catarrhini - ps35
Hominoidea - ps36
Hominidae - ps37
Homininae - ps38
Hominini - ps39
Homo_sapiens - ps40
p-value
8e-06
7e-05
6e-04
5e-03
4e-02
Log-odds
0.5
1.0
1.5
2.0
2.5
3.0
3.5
4.0

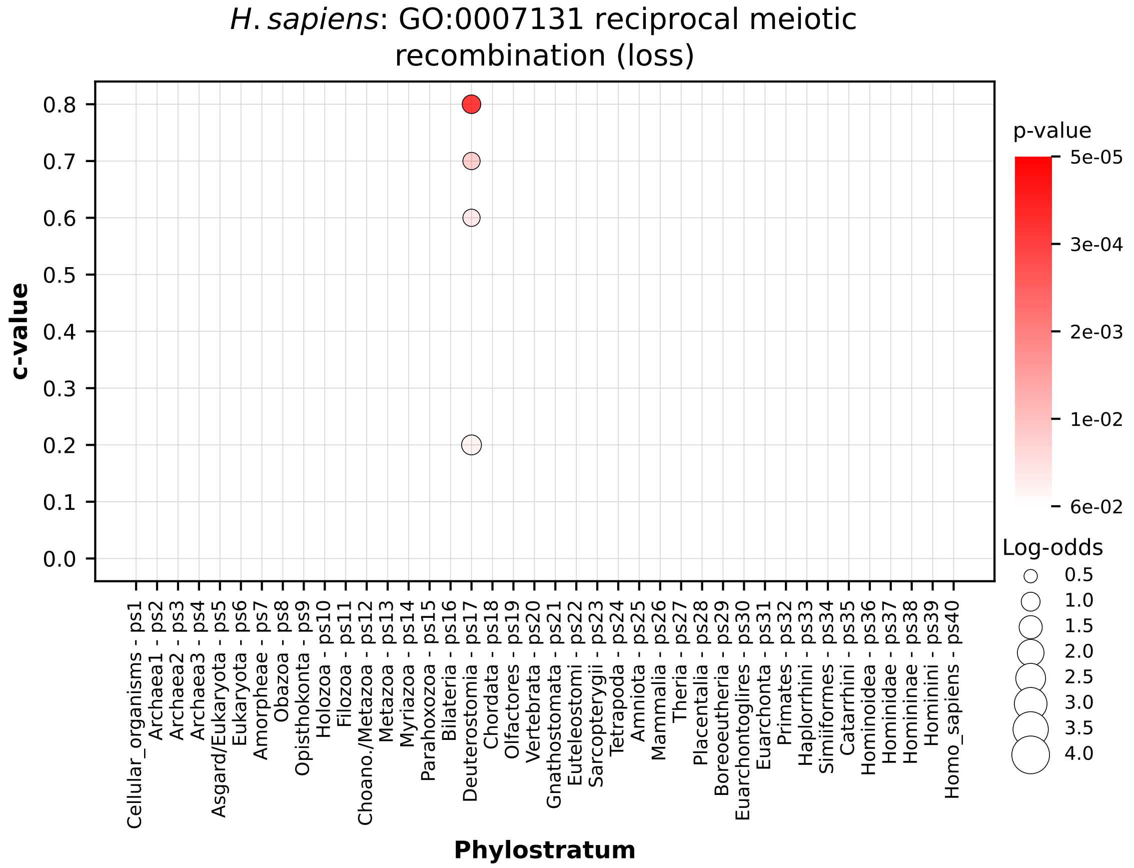

H. sapiens: GO:0007131 reciprocal meiotic recombination (loss)
c-value
0.8
0.7
0.6
0.5
0.4
0.3
0.2
0.1
0.0
p-value
5e-05
3e-04
2e-03
1e-02
6e-02
Log-odds
0.5
1.0
1.5
2.0
2.5
3.0
3.5
4.0
Cellular_organisms - ps1
Archaea1 - ps2
Archaea2 - ps3
Archaea3 - ps4
Asgard/Eukaryota - ps5
Eukaryota - ps6
Amorpheae - ps7
Obazoa - ps8
Opisthokonta - ps9
Holozoa - ps10
Filozoa - ps11
Choano./Metazoa - ps12
Metazoa - ps13
Myriazoa - ps14
Parahoxozoa - ps15
Bilateria - ps16
Deuterostomia - ps17
Chordata - ps18
Olfactores - ps19
Vertebrata - ps20
Gnathostomata - ps21
Euteleostomi - ps22
Sarcopterygii - ps23
Tetrapoda - ps24
Amniota - ps25
Mammalia - ps26
Theria - ps27
Placentalia - ps28
Boreoeutheria - ps29
Euarchontoglires - ps30
Euarchonta - ps31
Primates - ps32
Haplorrhini - ps33
Simiiformes - ps34
Catarrhini - ps35
Hominoidea - ps36
Hominidae - ps37
Homininae - ps38
Hominini - ps39
Homo_sapiens - ps40
Phylostratum

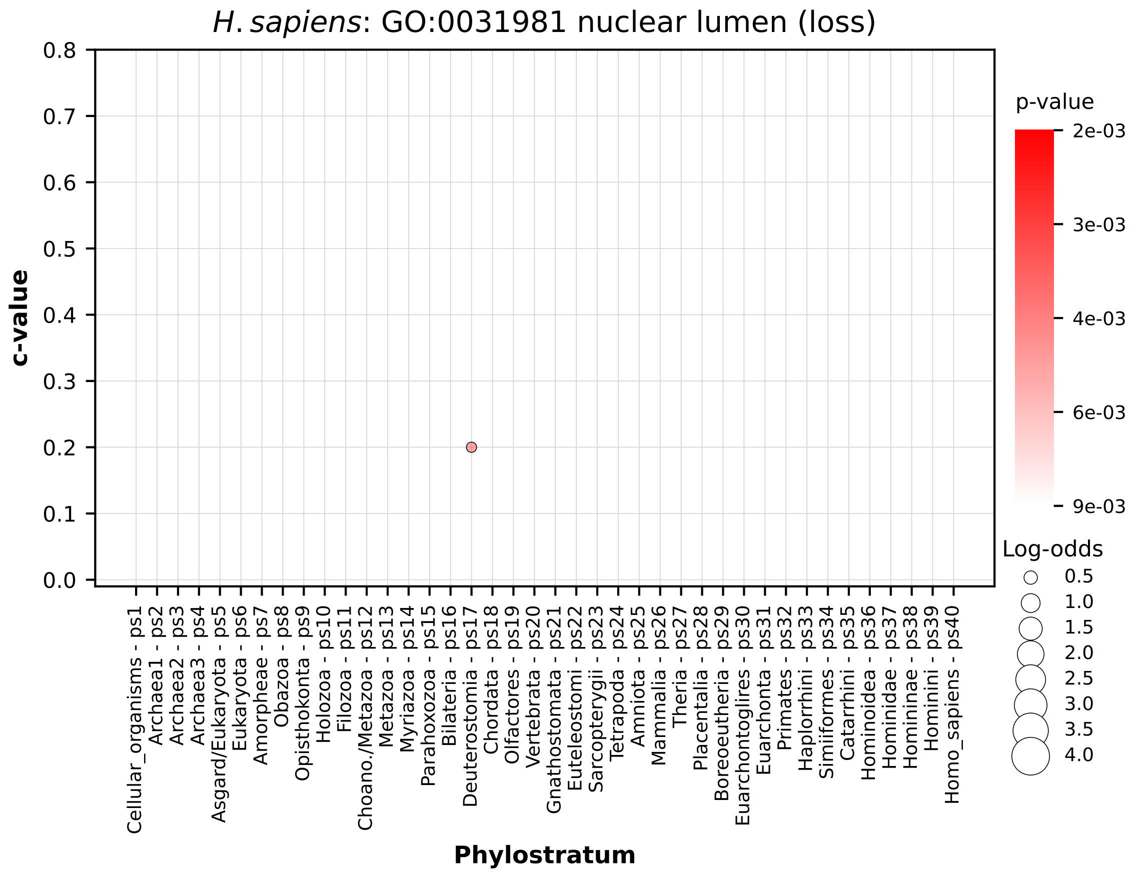

H. sapiens: GO:0031981 nuclear lumen (loss)
c-value
0.8
0.7
0.6
0.5
0.4
0.3
0.2
0.1
0.0
p-value
2e-03
3e-03
4e-03
6e-03
9e-03
Log-odds
0.5
1.0
1.5
2.0
2.5
3.0
3.5
4.0
Cellular_organisms - ps1
Archaea1 - ps2
Archaea2 - ps3
Archaea3 - ps4
Asgard/Eukaryota - ps5
Eukaryota - ps6
Amorpheae - ps7
Obazoa - ps8
Opisthokonta - ps9
Holozoa - ps10
Filozoa - ps11
Choano./Metazoa - ps12
Metazoa - ps13
Myriazoa - ps14
Parahoxozoa - ps15
Bilateria - ps16
Deuterostomia - ps17
Chordata - ps18
Olfactores - ps19
Vertebrata - ps20
Gnathostomata - ps21
Euteleostomi - ps22
Sarcopterygii - ps23
Tetrapoda - ps24
Amniota - ps25
Mammalia - ps26
Theria - ps27
Placentalia - ps28
Boreoeutheria - ps29
Euarchontoglires - ps30
Euarchonta - ps31
Primates - ps32
Haplorrhini - ps33
Simiiformes - ps34
Catarrhini - ps35
Hominoidea - ps36
Hominidae - ps37
Homininae - ps38
Hominini - ps39
Homo_sapiens - ps40
Phylostratum

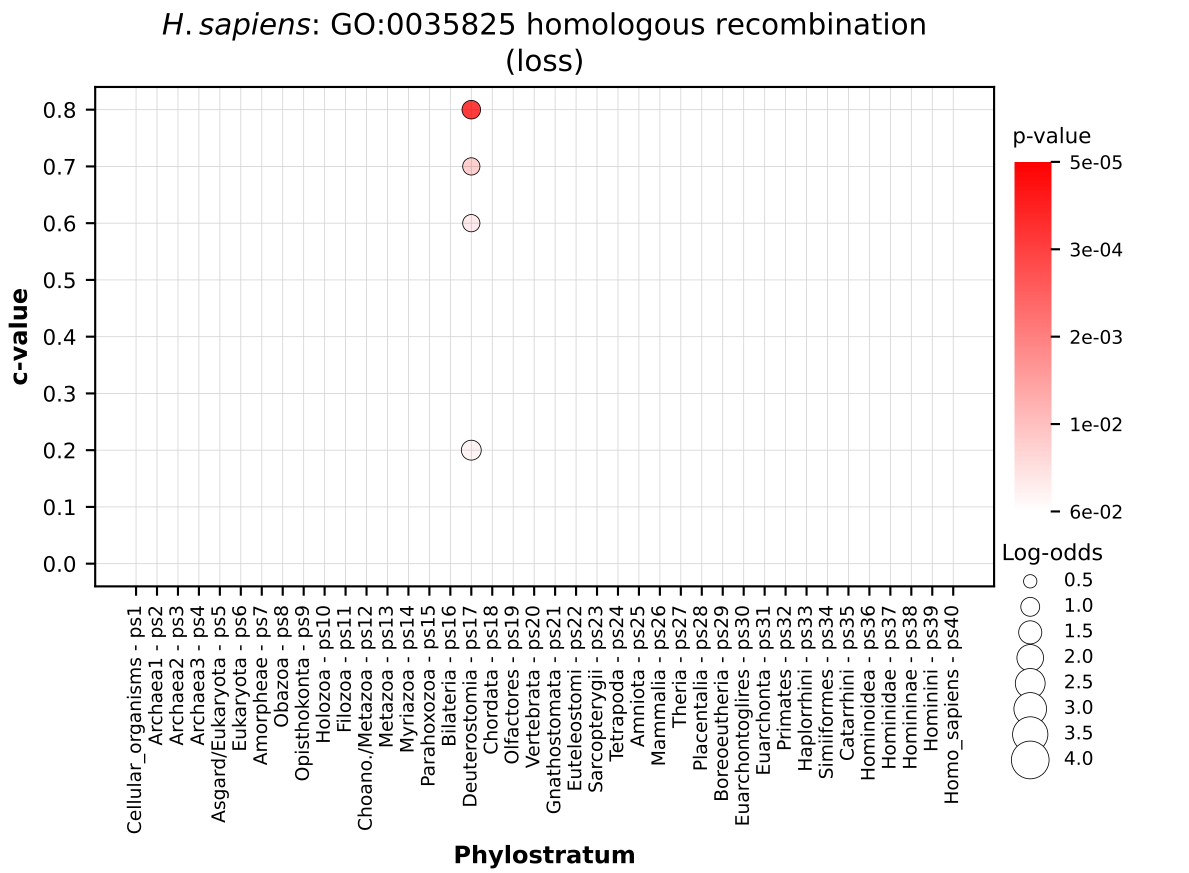

H. sapiens: GO:0035825 homologous recombination
(loss)
c-value
0.8
0.7
0.6
0.5
0.4
0.3
0.2
0.1
0.0
Phylostratum
Cellular_organisms - ps1
Archaea1 - ps2
Archaea2 - ps3
Archaea3 - ps4
Asgard/Eukaryota - ps5
Eukaryota - ps6
Amorpheae - ps7
Obazoa - ps8
Opisthokonta - ps9
Holozoa - ps10
Filozoa - ps11
Choano./Metazoa - ps12
Metazoa - ps13
Myriazoa - ps14
Parahoxozoa - ps15
Bilateria - ps16
Deuterostomia - ps17
Chordata - ps18
Olfactores - ps19
Vertebrata - ps20
Gnathostomata - ps21
Euteleostomi - ps22
Sarcopterygii - ps23
Tetrapoda - ps24
Amniota - ps25
Mammalia - ps26
Theria - ps27
Placentalia - ps28
Boreoeutheria - ps29
Euarchontoglires - ps30
Euarchonta - ps31
Primates - ps32
Haplorrhini - ps33
Simiiformes - ps34
Catarrhini - ps35
Hominoidea - ps36
Hominidae - ps37
Homininae - ps38
Hominini - ps39
Homo_sapiens - ps40
p-value
5e-05
3e-04
2e-03
1e-02
6e-02
Log-odds
0.5
1.0
1.5
2.0
2.5
3.0
3.5
4.0

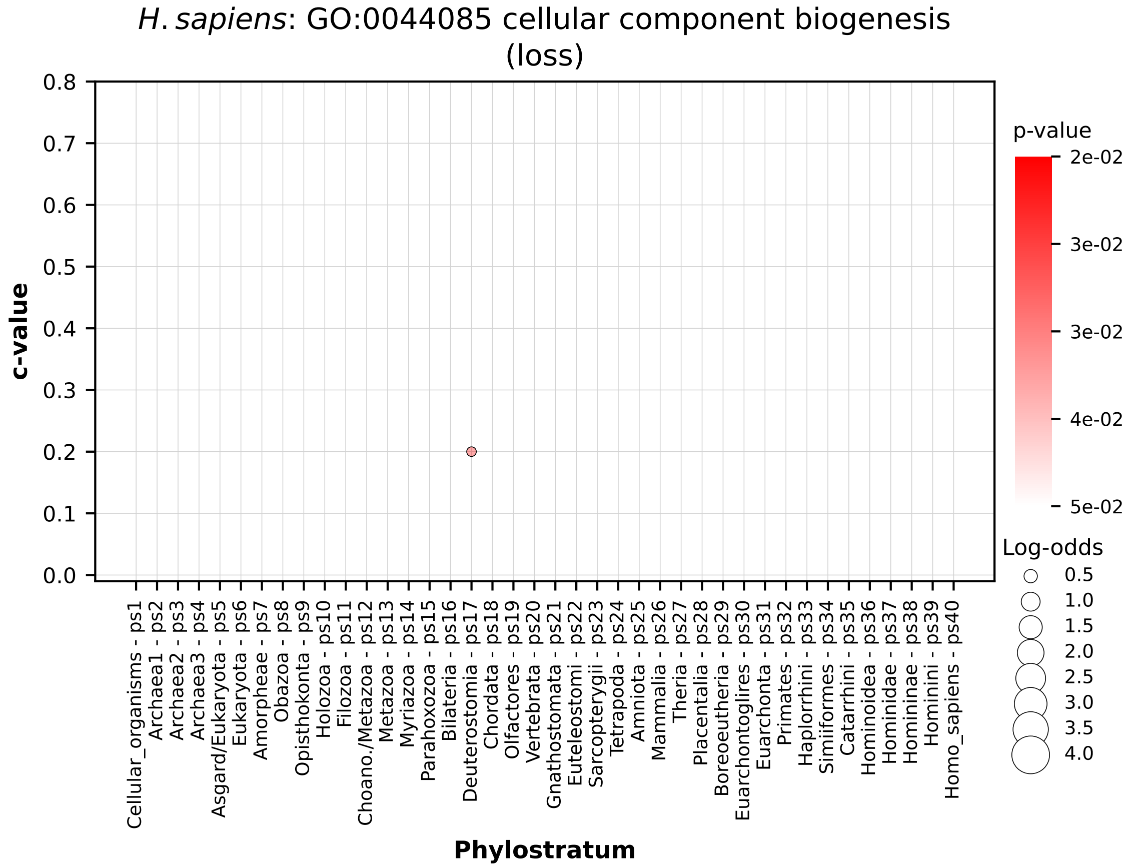

H. sapiens: GO:0044085 cellular component biogenesis
(loss)
c-value
0.8
0.7
0.6
0.5
0.4
0.3
0.2
0.1
0.0
p-value
2e-02
3e-02
3e-02
4e-02
5e-02
Log-odds
0.5
1.0
1.5
2.0
2.5
3.0
3.5
4.0
Cellular_organisms - ps1
Archaea1 - ps2
Archaea2 - ps3
Archaea3 - ps4
Asgard/Eukaryota - ps5
Eukaryota - ps6
Amorpheae - ps7
Obazoa - ps8
Opisthokonta - ps9
Holozoa - ps10
Filozoa - ps11
Choano./Metazoa - ps12
Metazoa - ps13
Myriazoa - ps14
Parahoxozoa - ps15
Bilateria - ps16
Deuterostomia - ps17
Chordata - ps18
Olfactores - ps19
Vertebrata - ps20
Gnathostomata - ps21
Euteleostomi - ps22
Sarcopterygii - ps23
Tetrapoda - ps24
Amniota - ps25
Mammalia - ps26
Theria - ps27
Placentalia - ps28
Boreoeutheria - ps29
Euarchontoglires - ps30
Euarchonta - ps31
Primates - ps32
Haplorrhini - ps33
Simiiformes - ps34
Catarrhini - ps35
Hominoidea - ps36
Hominidae - ps37
Homininae - ps38
Hominini - ps39
Homo_sapiens - ps40
Phylostratum

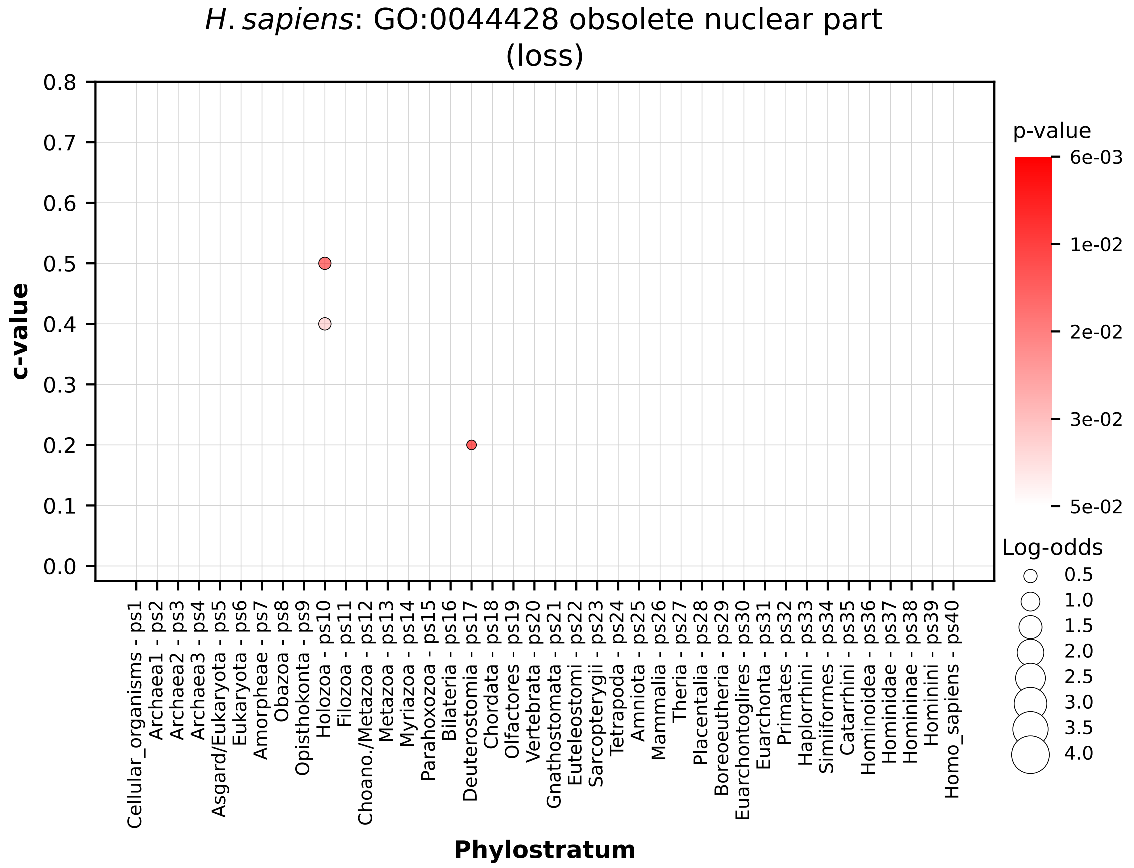

H. sapiens: GO:0044428 obsolete nuclear part
(loss)
c-value
0.8
0.7
0.6
0.5
0.4
0.3
0.2
0.1
0.0
p-value
6e-03
1e-02
2e-02
3e-02
5e-02
Log-odds
0.5
1.0
1.5
2.0
2.5
3.0
3.5
4.0
Cellular_organisms - ps1
Archaea1 - ps2
Archaea2 - ps3
Archaea3 - ps4
Asgard/Eukaryota - ps5
Eukaryota - ps6
Amorpheae - ps7
Obazoa - ps8
Opisthokonta - ps9
Holozoa - ps10
Filozoa - ps11
Choano./Metazoa - ps12
Metazoa - ps13
Myriazoa - ps14
Parahoxozoa - ps15
Bilateria - ps16
Deuterostomia - ps17
Chordata - ps18
Olfactores - ps19
Vertebrata - ps20
Gnathostomata - ps21
Euteleostomi - ps22
Sarcopterygii - ps23
Tetrapoda - ps24
Amniota - ps25
Mammalia - ps26
Theria - ps27
Placentalia - ps28
Boreoeutheria - ps29
Euarchontoglires - ps30
Euarchonta - ps31
Primates - ps32
Haplorrhini - ps33
Simiiformes - ps34
Catarrhini - ps35
Hominoidea - ps36
Hominidae - ps37
Homininae - ps38
Hominini - ps39
Homo_sapiens - ps40
Phylostratum

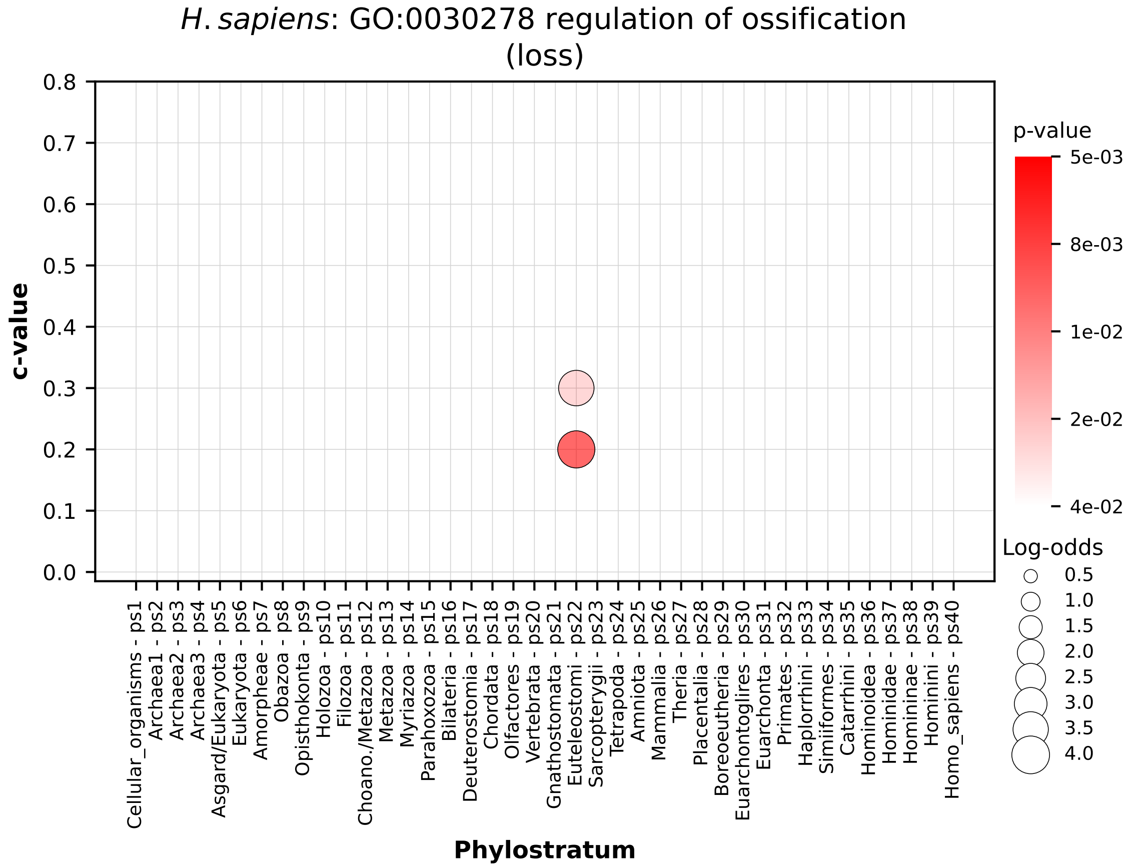

H. sapiens: GO:0030278 regulation of ossification
(loss)
c-value
0.8
0.7
0.6
0.5
0.4
0.3
0.2
0.1
0.0
Phylostratum
Cellular_organisms - ps1
Archaea1 - ps2
Archaea2 - ps3
Archaea3 - ps4
Asgard/Eukaryota - ps5
Eukaryota - ps6
Amorpheae - ps7
Obazoa - ps8
Opisthokonta - ps9
Holozoa - ps10
Filozoa - ps11
Choano./Metazoa - ps12
Metazoa - ps13
Myriazoa - ps14
Parahoxozoa - ps15
Bilateria - ps16
Deuterostomia - ps17
Chordata - ps18
Olfactores - ps19
Vertebrata - ps20
Gnathostomata - ps21
Euteleostomi - ps22
Sarcopterygii - ps23
Tetrapoda - ps24
Amniota - ps25
Mammalia - ps26
Theria - ps27
Placentalia - ps28
Boreoeutheria - ps29
Euarchontoglires - ps30
Euarchonta - ps31
Primates - ps32
Haplorrhini - ps33
Simiiformes - ps34
Catarrhini - ps35
Hominoidea - ps36
Hominidae - ps37
Homininae - ps38
Hominini - ps39
Homo_sapiens - ps40
p-value
5e-03
8e-03
1e-02
2e-02
4e-02
Log-odds
0.5
1.0
1.5
2.0
2.5
3.0
3.5
4.0

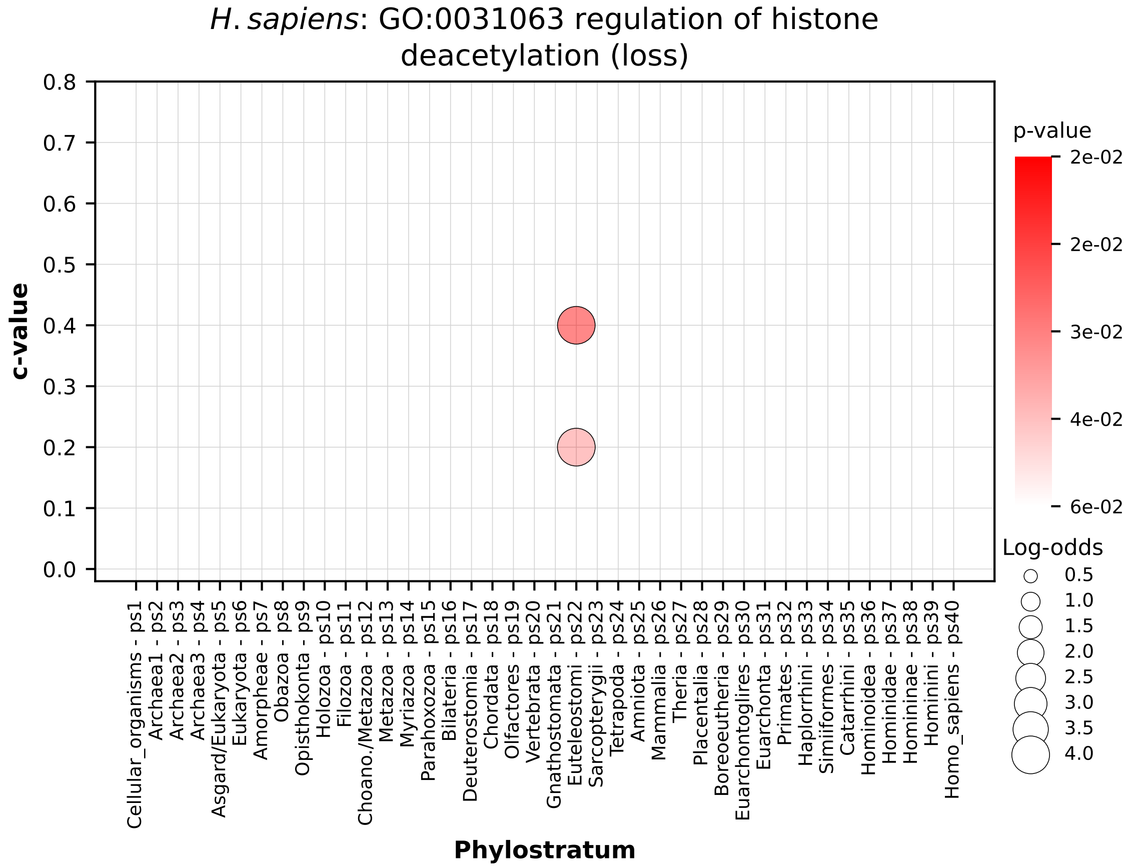
H. sapiens: GO:0031063 regulation of histone deacetylation (loss)
c-value
0.0
0.1
0.2
0.3
0.4
0.5
0.6
0.7
0.8
Phylostratum
Cellular_organisms - ps1
Archaea1 - ps2
Archaea2 - ps3
Archaea3 - ps4
Asgard/Eukaryota - ps5
Eukaryota - ps6
Amorpheae - ps7
Obazoa - ps8
Opisthokonta - ps9
Holozoa - ps10
Filozoa - ps11
Choano./Metazoa - ps12
Metazoa - ps13
Myriazoa - ps14
Parahoxozoa - ps15
Bilateria - ps16
Deuterostomia - ps17
Chordata - ps18
Olfactores - ps19
Vertebrata - ps20
Gnathostomata - ps21
Euteleostomi - ps22
Sarcopterygii - ps23
Tetrapoda - ps24
Amniota - ps25
Mammalia - ps26
Theria - ps27
Placentalia - ps28
Boreoeutheria - ps29
Euarchontoglires - ps30
Euarchonta - ps31
Primates - ps32
Haplorrhini - ps33
Simiiformes - ps34
Catarrhini - ps35
Hominoidea - ps36
Hominidae - ps37
Homininae - ps38
Hominini - ps39
Homo_sapiens - ps40
p-value
2e-02
2e-02
3e-02
4e-02
6e-02
Log-odds
0.5
1.0
1.5
2.0
2.5
3.0
3.5
4.0

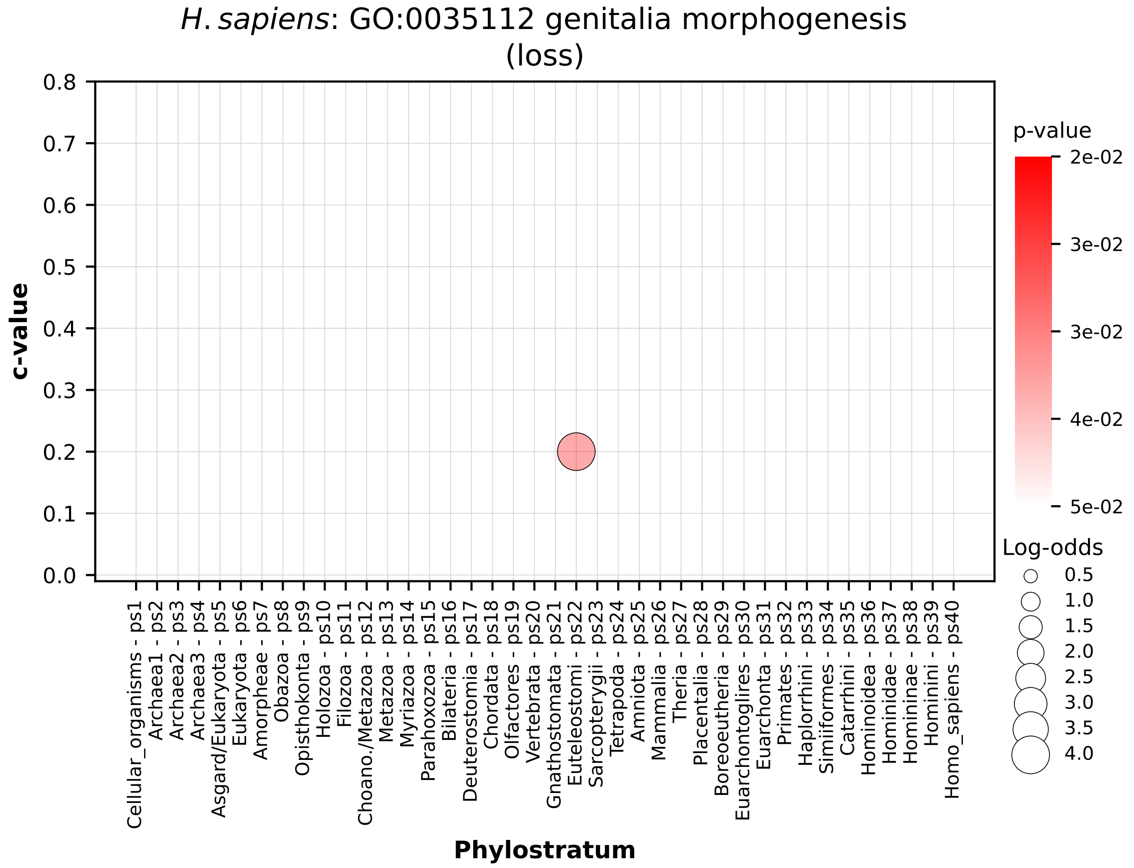
H. sapiens: GO:0035112 genitalia morphogenesis
(loss)
c-value
0.8
0.7
0.6
0.5
0.4
0.3
0.2
0.1
0.0
p-value
2e-02
3e-02
3e-02
4e-02
5e-02
Log-odds
0.5
1.0
1.5
2.0
2.5
3.0
3.5
4.0
Cellular_organisms - ps1
Archaea1 - ps2
Archaea2 - ps3
Archaea3 - ps4
Asgard/Eukaryota - ps5
Eukaryota - ps6
Amorpheae - ps7
Obazoa - ps8
Opisthokonta - ps9
Holozoa - ps10
Filozoa - ps11
Choano./Metazoa - ps12
Metazoa - ps13
Myriazoa - ps14
Parahoxozoa - ps15
Bilateria - ps16
Deuterostomia - ps17
Chordata - ps18
Olfactores - ps19
Vertebrata - ps20
Gnathostomata - ps21
Euteleostomi - ps22
Sarcopterygii - ps23
Tetrapoda - ps24
Amniota - ps25
Mammalia - ps26
Theria - ps27
Placentalia - ps28
Boreoeutheria - ps29
Euarchontoglires - ps30
Euarchonta - ps31
Primates - ps32
Haplorrhini - ps33
Simiiformes - ps34
Catarrhini - ps35
Hominoidea - ps36
Hominidae - ps37
Homininae - ps38
Hominini - ps39
Homo_sapiens - ps40
Phylostratum

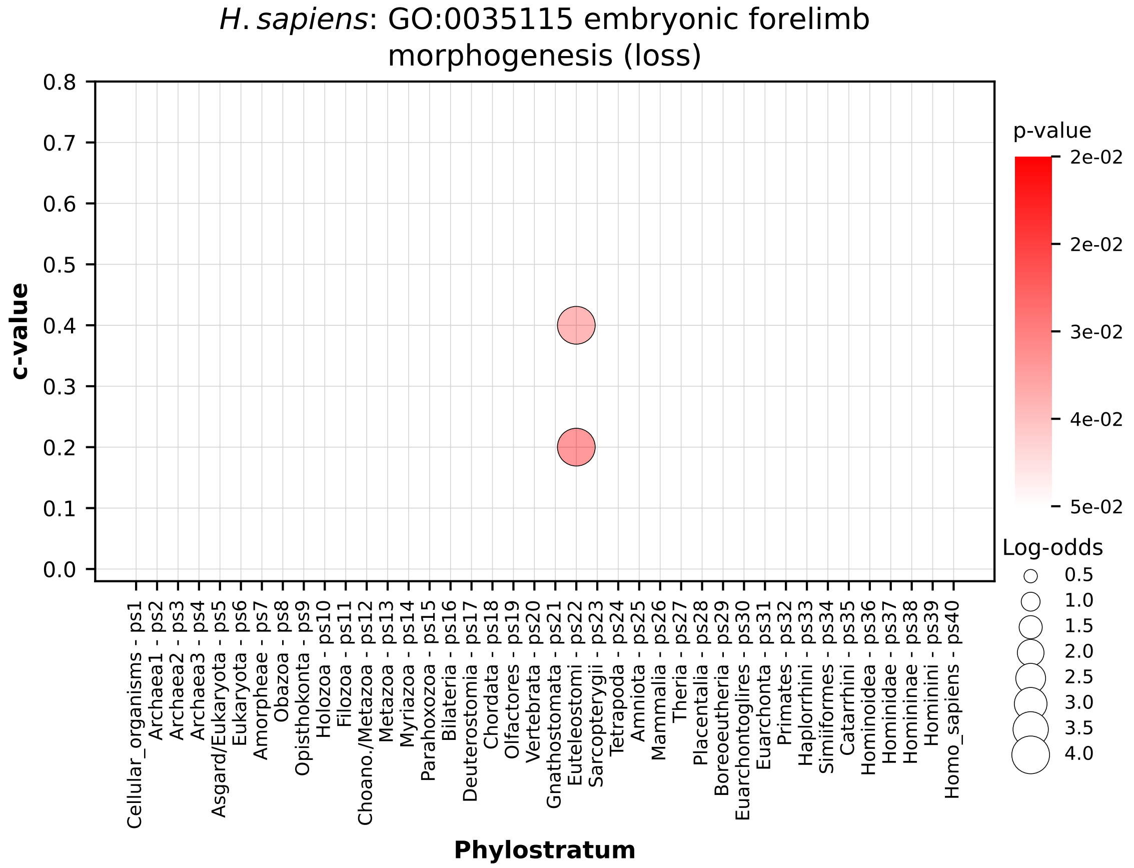

H. sapiens: GO:0035115 embryonic forelimb morphogenesis (loss)
c-value
0.8
0.7
0.6
0.5
0.4
0.3
0.2
0.1
0.0
Phylostratum
Cellular_organisms - ps1
Archaea1 - ps2
Archaea2 - ps3
Archaea3 - ps4
Asgard/Eukaryota - ps5
Eukaryota - ps6
Amorpheae - ps7
Obazoa - ps8
Opisthokonta - ps9
Holozoa - ps10
Filozoa - ps11
Choano./Metazoa - ps12
Metazoa - ps13
Myriazoa - ps14
Parahoxozoa - ps15
Bilateria - ps16
Deuterostomia - ps17
Chordata - ps18
Olfactores - ps19
Vertebrata - ps20
Gnathostomata - ps21
Euteleostomi - ps22
Sarcopterygii - ps23
Tetrapoda - ps24
Amniota - ps25
Mammalia - ps26
Theria - ps27
Placentalia - ps28
Boreoeutheria - ps29
Euarchontoglires - ps30
Euarchonta - ps31
Primates - ps32
Haplorrhini - ps33
Simiiformes - ps34
Catarrhini - ps35
Hominoidea - ps36
Hominidae - ps37
Homininae - ps38
Hominini - ps39
Homo_sapiens - ps40
p-value
2e-02
2e-02
3e-02
4e-02
5e-02
Log-odds
0.5
1.0
1.5
2.0
2.5
3.0
3.5
4.0

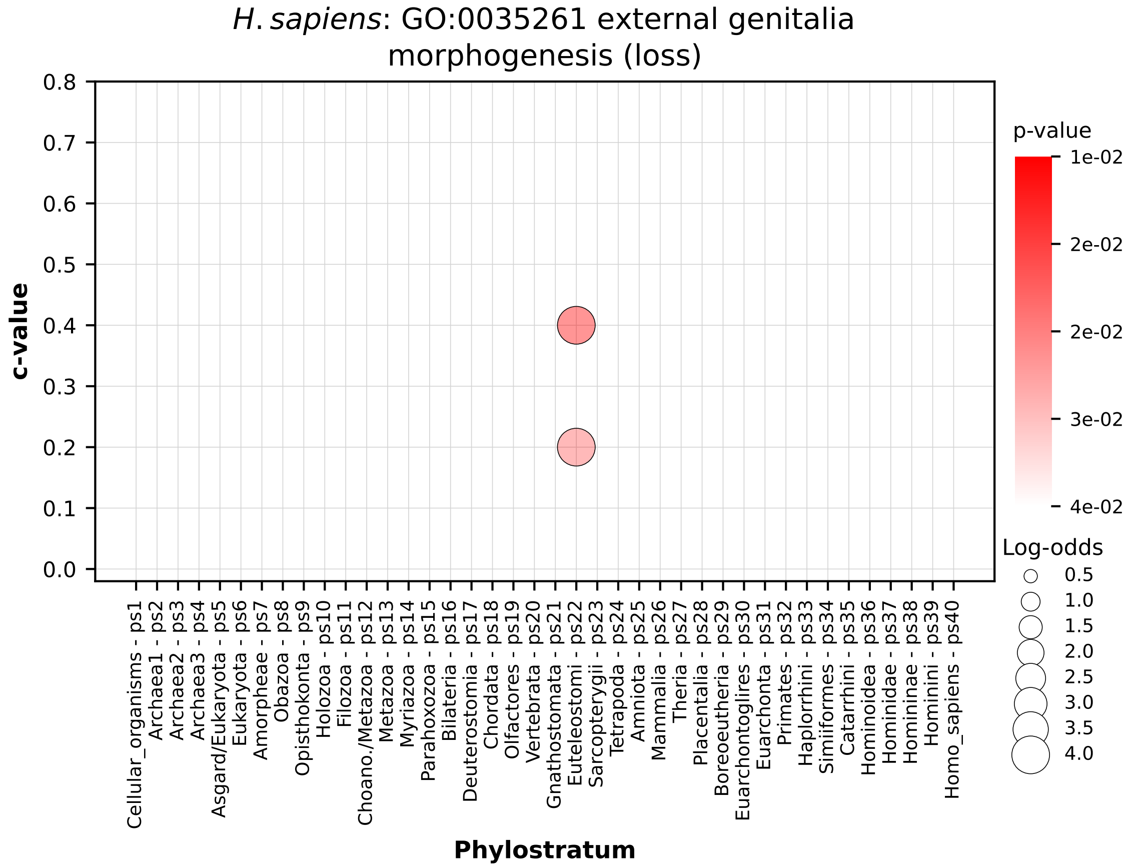

H. sapiens: GO:0035261 external genitalia morphogenesis (loss)
c-value
0.8
0.7
0.6
0.5
0.4
0.3
0.2
0.1
0.0
p-value
1e-02
2e-02
2e-02
3e-02
4e-02
Log-odds
0.5
1.0
1.5
2.0
2.5
3.0
3.5
4.0
Cellular_organisms - ps1
Archaea1 - ps2
Archaea2 - ps3
Archaea3 - ps4
Asgard/Eukaryota - ps5
Eukaryota - ps6
Amorpheae - ps7
Obazoa - ps8
Opisthokonta - ps9
Holozoa - ps10
Filozoa - ps11
Choano./Metazoa - ps12
Metazoa - ps13
Myriazoa - ps14
Parahoxozoa - ps15
Bilateria - ps16
Deuterostomia - ps17
Chordata - ps18
Olfactores - ps19
Vertebrata - ps20
Gnathostomata - ps21
Euteleostomi - ps22
Sarcopterygii - ps23
Tetrapoda - ps24
Amniota - ps25
Mammalia - ps26
Theria - ps27
Placentalia - ps28
Boreoeutheria - ps29
Euarchontoglires - ps30
Euarchonta - ps31
Primates - ps32
Haplorrhini - ps33
Simiiformes - ps34
Catarrhini - ps35
Hominoidea - ps36
Hominidae - ps37
Homininae - ps38
Hominini - ps39
Homo_sapiens - ps40
Phylostratum

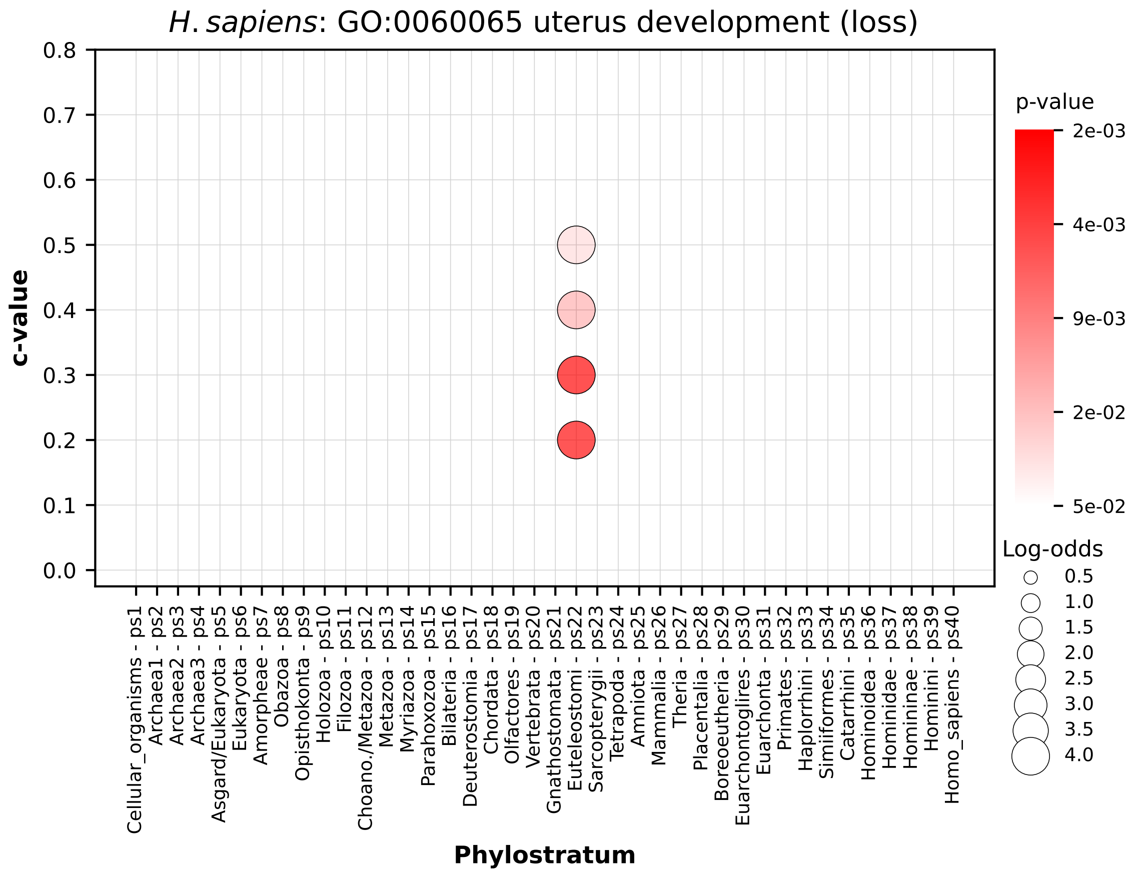

H. sapiens: GO:0060065 uterus development (loss)
c-value
0.0
0.1
0.2
0.3
0.4
0.5
0.6
0.7
0.8
Phylostratum
Cellular_organisms - ps1
Archaea1 - ps2
Archaea2 - ps3
Archaea3 - ps4
Asgard/Eukaryota - ps5
Eukaryota - ps6
Amorpheae - ps7
Obazoa - ps8
Opisthokonta - ps9
Holozoa - ps10
Filozoa - ps11
Choano./Metazoa - ps12
Metazoa - ps13
Myriazoa - ps14
Parahoxozoa - ps15
Bilateria - ps16
Deuterostomia - ps17
Chordata - ps18
Olfactores - ps19
Vertebrata - ps20
Gnathostomata - ps21
Euteleostomi - ps22
Sarcopterygii - ps23
Tetrapoda - ps24
Amniota - ps25
Mammalia - ps26
Theria - ps27
Placentalia - ps28
Boreoeutheria - ps29
Euarchontoglires - ps30
Euarchonta - ps31
Primates - ps32
Haplorrhini - ps33
Simiiformes - ps34
Catarrhini - ps35
Hominoidea - ps36
Hominidae - ps37
Homininae - ps38
Hominini - ps39
Homo_sapiens - ps40
p-value
2e-03
4e-03
9e-03
2e-02
5e-02
Log-odds
0.5
1.0
1.5
2.0
2.5
3.0
3.5
4.0

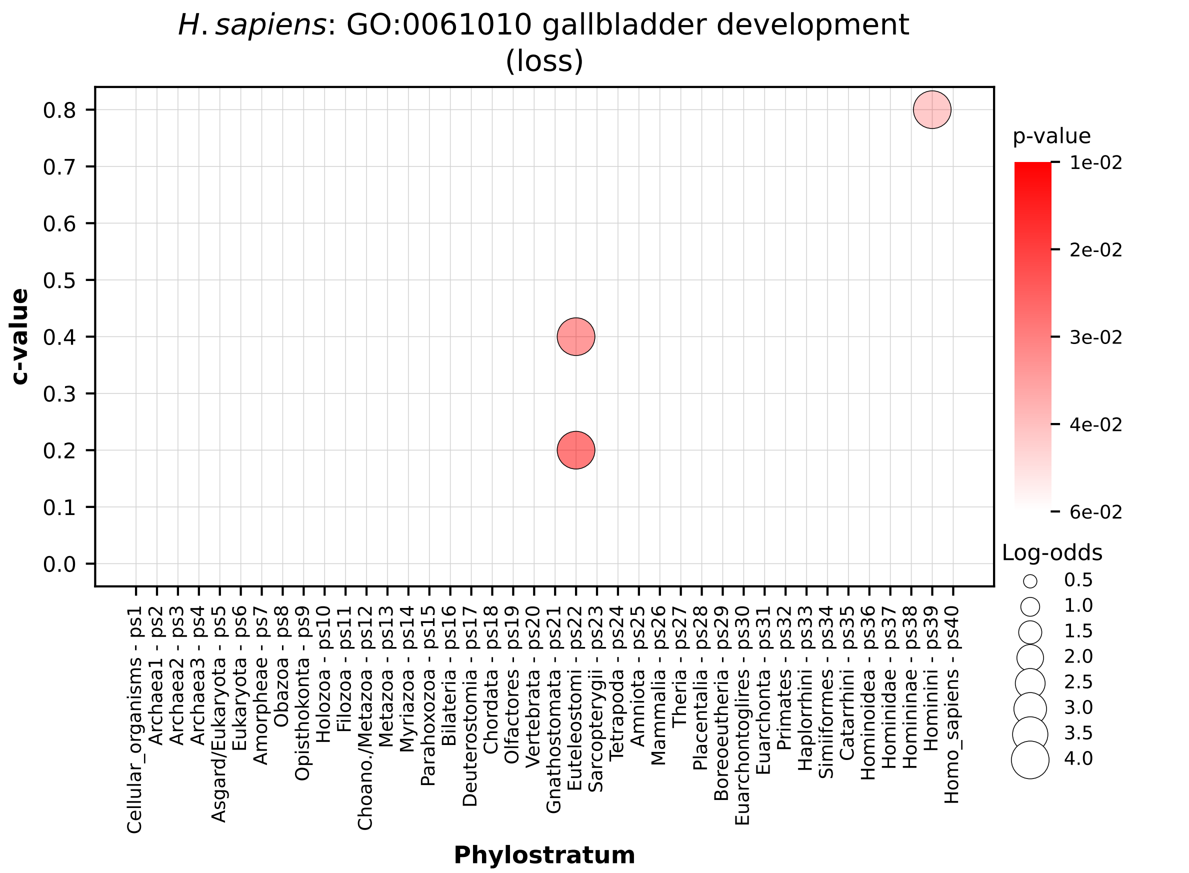
H. sapiens: GO:0061010 gallbladder development
(loss)
c-value
0.8
0.7
0.6
0.5
0.4
0.3
0.2
0.1
0.0
p-value
1e-02
2e-02
3e-02
4e-02
6e-02
Log-odds
0.5
1.0
1.5
2.0
2.5
3.0
3.5
4.0
Cellular_organisms - ps1
Archaea1 - ps2
Archaea2 - ps3
Archaea3 - ps4
Asgard/Eukaryota - ps5
Eukaryota - ps6
Amorpheae - ps7
Obazoa - ps8
Opisthokonta - ps9
Holozoa - ps10
Filozoa - ps11
Choano./Metazoa - ps12
Metazoa - ps13
Myriazoa - ps14
Parahoxozoa - ps15
Bilateria - ps16
Deuterostomia - ps17
Chordata - ps18
Olfactores - ps19
Vertebrata - ps20
Gnathostomata - ps21
Euteleostomi - ps22
Sarcopterygii - ps23
Tetrapoda - ps24
Amniota - ps25
Mammalia - ps26
Theria - ps27
Placentalia - ps28
Boreoeutheria - ps29
Euarchontoglires - ps30
Euarchonta - ps31
Primates - ps32
Haplorrhini - ps33
Simiiformes - ps34
Catarrhini - ps35
Hominoidea - ps36
Hominidae - ps37
Homininae - ps38
Hominini - ps39
Homo_sapiens - ps40
Phylostratum

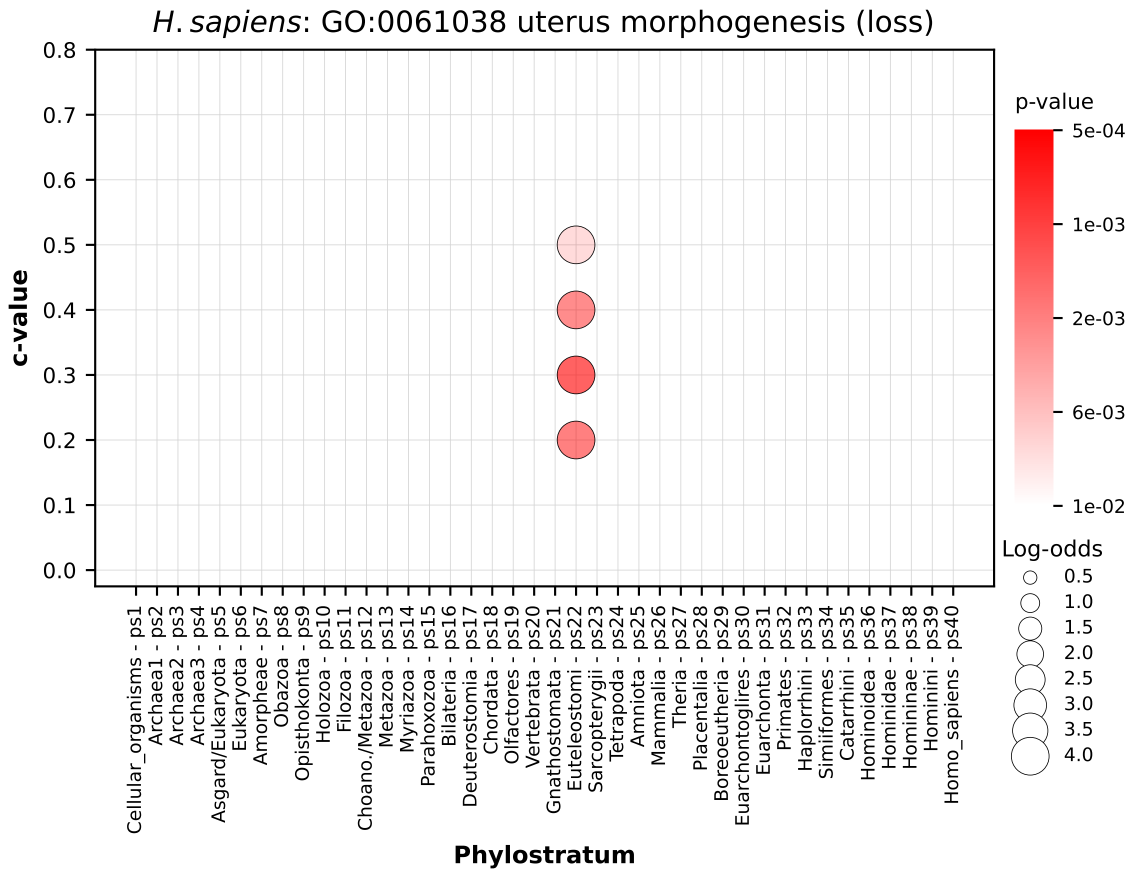

H. sapiens: GO:0061038 uterus morphogenesis (loss)
c-value
0.8
0.7
0.6
0.5
0.4
0.3
0.2
0.1
0.0
p-value
5e-04
1e-03
2e-03
6e-03
1e-02
Log-odds
0.5
1.0
1.5
2.0
2.5
3.0
3.5
4.0
Cellular_organisms - ps1
Archaea1 - ps2
Archaea2 - ps3
Archaea3 - ps4
Asgard/Eukaryota - ps5
Eukaryota - ps6
Amorpheae - ps7
Obazoa - ps8
Opisthokonta - ps9
Holozoa - ps10
Filozoa - ps11
Choano./Metazoa - ps12
Metazoa - ps13
Myriazoa - ps14
Parahoxozoa - ps15
Bilateria - ps16
Deuterostomia - ps17
Chordata - ps18
Olfactores - ps19
Vertebrata - ps20
Gnathostomata - ps21
Euteleostomi - ps22
Sarcopterygii - ps23
Tetrapoda - ps24
Amniota - ps25
Mammalia - ps26
Theria - ps27
Placentalia - ps28
Boreoeutheria - ps29
Euarchontoglires - ps30
Euarchonta - ps31
Primates - ps32
Haplorrhini - ps33
Simiiformes - ps34
Catarrhini - ps35
Hominoidea - ps36
Hominidae - ps37
Homininae - ps38
Hominini - ps39
Homo_sapiens - ps40
Phylostratum

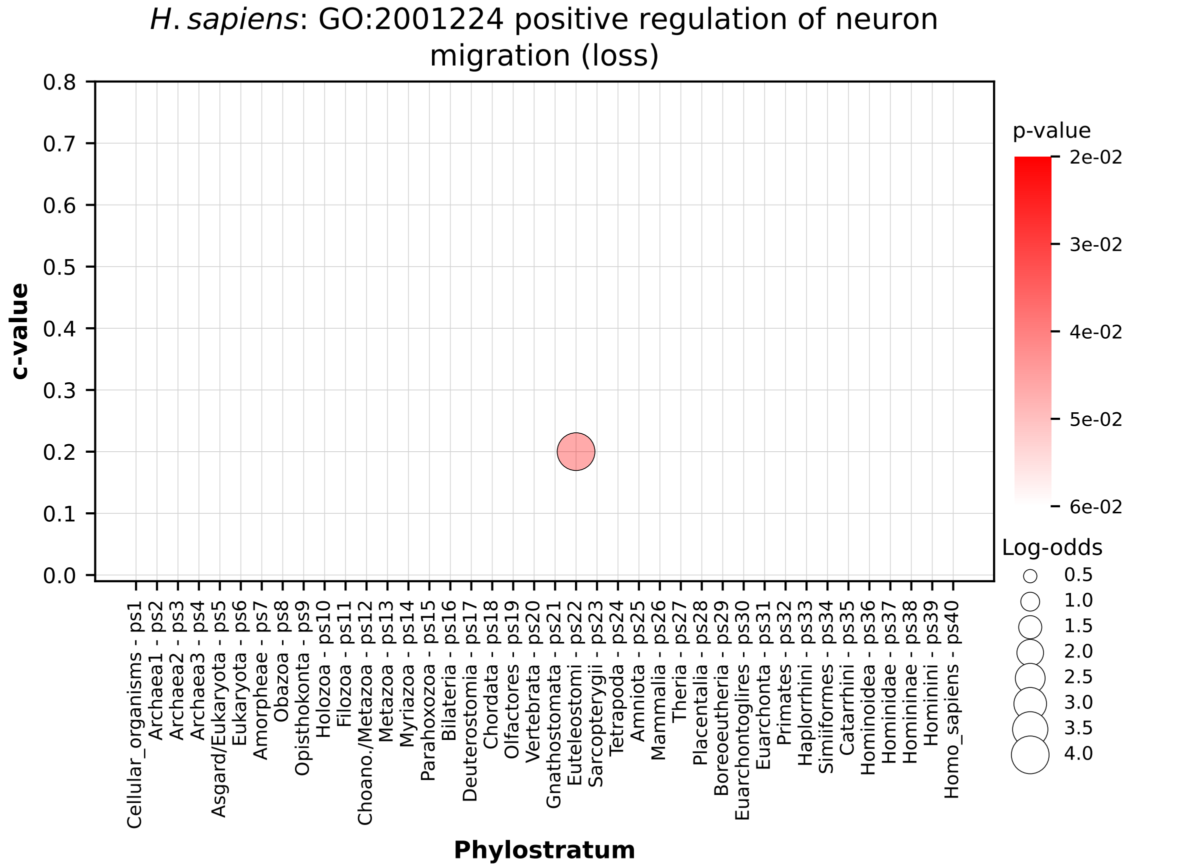

H. sapiens: GO:2001224 positive regulation of neuron migration (loss)
c-value
0.0
0.1
0.2
0.3
0.4
0.5
0.6
0.7
0.8
Phylostratum
Cellular_organisms - ps1
Archaea1 - ps2
Archaea2 - ps3
Archaea3 - ps4
Asgard/Eukaryota - ps5
Eukaryota - ps6
Amorpheae - ps7
Obazoa - ps8
Opisthokonta - ps9
Holozoa - ps10
Filozoa - ps11
Choano./Metazoa - ps12
Metazoa - ps13
Myriazoa - ps14
Parahoxozoa - ps15
Bilateria - ps16
Deuterostomia - ps17
Chordata - ps18
Olfactores - ps19
Vertebrata - ps20
Gnathostomata - ps21
Euteleostomi - ps22
Sarcopterygii - ps23
Tetrapoda - ps24
Amniota - ps25
Mammalia - ps26
Theria - ps27
Placentalia - ps28
Boreoeutheria - ps29
Euarchontoglires - ps30
Euarchonta - ps31
Primates - ps32
Haplorrhini - ps33
Simiiformes - ps34
Catarrhini - ps35
Hominoidea - ps36
Hominidae - ps37
Homininae - ps38
Hominini - ps39
Homo_sapiens - ps40
p-value
2e-02
3e-02
4e-02
5e-02
6e-02
Log-odds
0.5
1.0
1.5
2.0
2.5
3.0
3.5
4.0

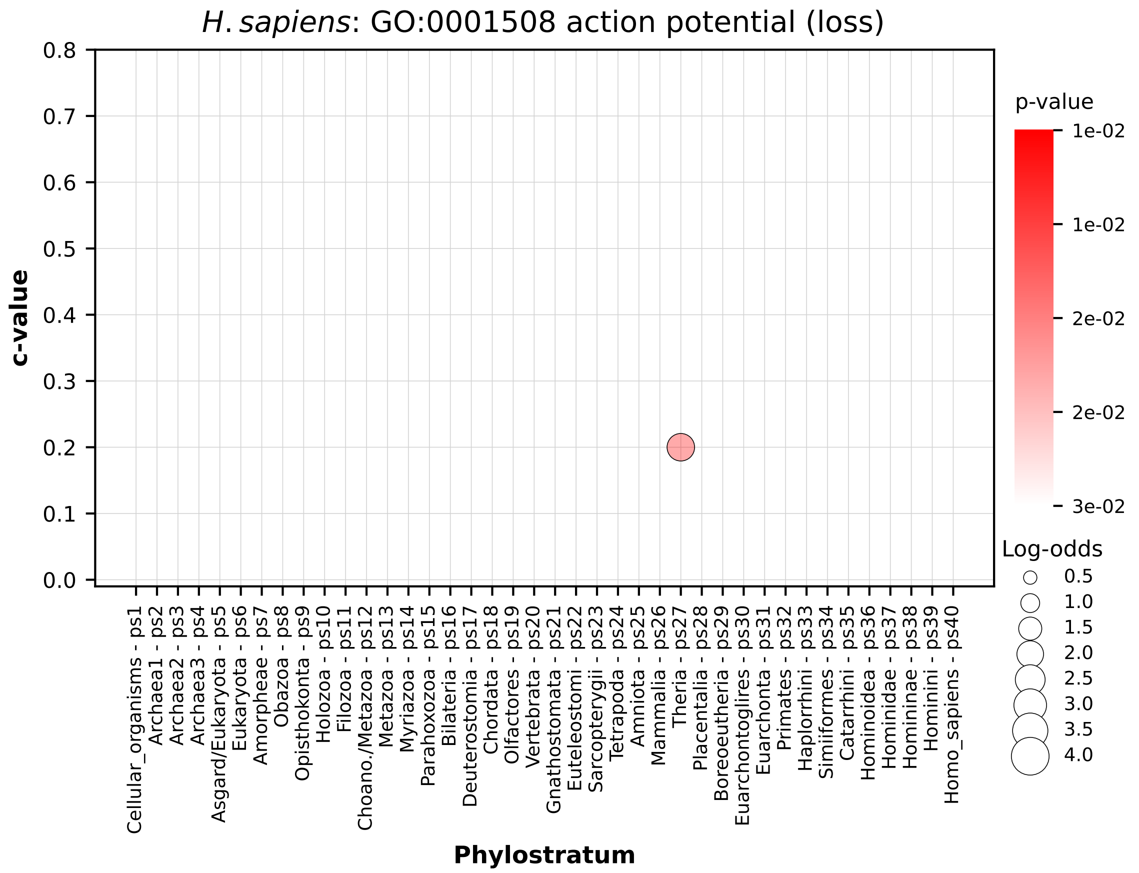

H. sapiens: GO:0001508 action potential (loss)
c-value
0.0
0.1
0.2
0.3
0.4
0.5
0.6
0.7
0.8
Phylostratum
Cellular_organisms - ps1
Archaea1 - ps2
Archaea2 - ps3
Archaea3 - ps4
Asgard/Eukaryota - ps5
Eukaryota - ps6
Amorpheae - ps7
Obazoa - ps8
Opisthokonta - ps9
Holozoa - ps10
Filozoa - ps11
Choano./Metazoa - ps12
Metazoa - ps13
Myriazoa - ps14
Parahoxozoa - ps15
Bilateria - ps16
Deuterostomia - ps17
Chordata - ps18
Olfactores - ps19
Vertebrata - ps20
Gnathostomata - ps21
Euteleostomi - ps22
Sarcopterygii - ps23
Tetrapoda - ps24
Amniota - ps25
Mammalia - ps26
Theria - ps27
Placentalia - ps28
Boreoeutheria - ps29
Euarchontoglires - ps30
Euarchonta - ps31
Primates - ps32
Haplorrhini - ps33
Simiiformes - ps34
Catarrhini - ps35
Hominoidea - ps36
Hominidae - ps37
Homininae - ps38
Hominini - ps39
Homo_sapiens - ps40
p-value
1e-02
1e-02
2e-02
2e-02
3e-02
Log-odds
0.5
1.0
1.5
2.0
2.5
3.0
3.5
4.0

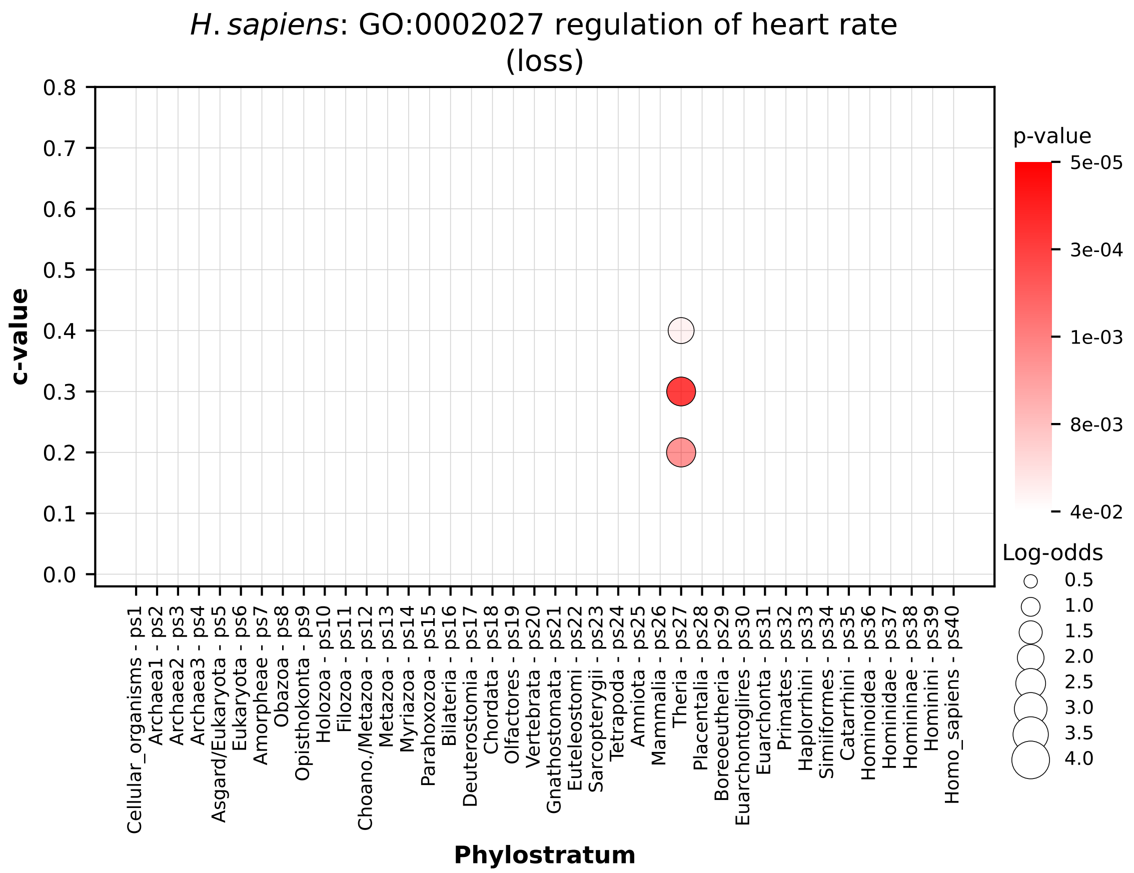

H. sapiens: GO:0002027 regulation of heart rate
(loss)
c-value
0.8
0.7
0.6
0.5
0.4
0.3
0.2
0.1
0.0
p-value
5e-05
3e-04
1e-03
8e-03
4e-02
Log-odds
0.5
1.0
1.5
2.0
2.5
3.0
3.5
4.0
Cellular_organisms - ps1
Archaea1 - ps2
Archaea2 - ps3
Archaea3 - ps4
Asgard/Eukaryota - ps5
Eukaryota - ps6
Amorpheae - ps7
Obazoa - ps8
Opisthokonta - ps9
Holozoa - ps10
Filozoa - ps11
Choano./Metazoa - ps12
Metazoa - ps13
Myriazoa - ps14
Parahoxozoa - ps15
Bilateria - ps16
Deuterostomia - ps17
Chordata - ps18
Olfactores - ps19
Vertebrata - ps20
Gnathostomata - ps21
Euteleostomi - ps22
Sarcopterygii - ps23
Tetrapoda - ps24
Amniota - ps25
Mammalia - ps26
Theria - ps27
Placentalia - ps28
Boreoeutheria - ps29
Euarchontoglires - ps30
Euarchonta - ps31
Primates - ps32
Haplorrhini - ps33
Simiiformes - ps34
Catarrhini - ps35
Hominoidea - ps36
Hominidae - ps37
Homininae - ps38
Hominini - ps39
Homo_sapiens - ps40
Phylostratum

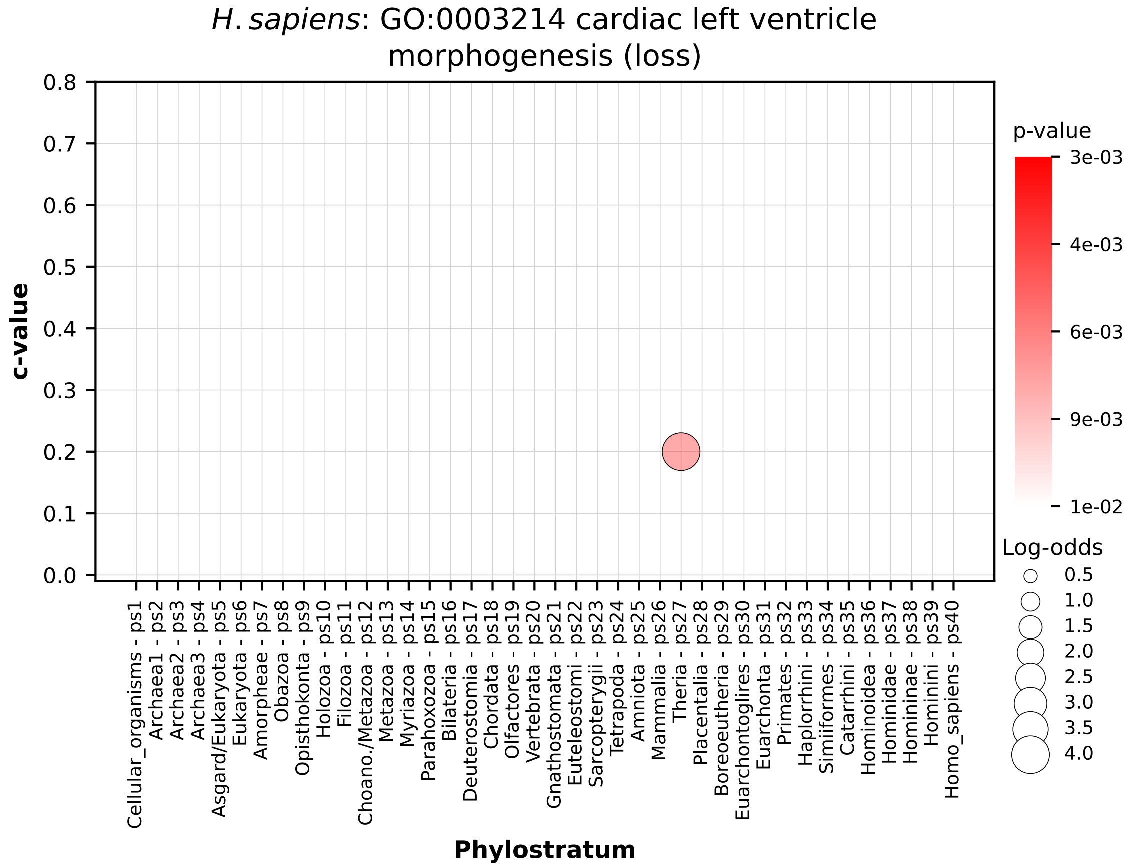

H. sapiens: GO:0003214 cardiac left ventricle morphogenesis (loss)
c-value
0.0
0.1
0.2
0.3
0.4
0.5
0.6
0.7
0.8
Phylostratum
Cellular_organisms - ps1
Archaea1 - ps2
Archaea2 - ps3
Archaea3 - ps4
Asgard/Eukaryota - ps5
Eukaryota - ps6
Amorpheae - ps7
Obazoa - ps8
Opisthokonta - ps9
Holozoa - ps10
Filozoa - ps11
Choano./Metazoa - ps12
Metazoa - ps13
Myriazoa - ps14
Parahoxozoa - ps15
Bilateria - ps16
Deuterostomia - ps17
Chordata - ps18
Olfactores - ps19
Vertebrata - ps20
Gnathostomata - ps21
Euteleostomi - ps22
Sarcopterygii - ps23
Tetrapoda - ps24
Amniota - ps25
Mammalia - ps26
Theria - ps27
Placentalia - ps28
Boreoeutheria - ps29
Euarchontoglires - ps30
Euarchonta - ps31
Primates - ps32
Haplorrhini - ps33
Simiiformes - ps34
Catarrhini - ps35
Hominoidea - ps36
Hominidae - ps37
Homininae - ps38
Hominini - ps39
Homo_sapiens - ps40
p-value
3e-03
4e-03
6e-03
9e-03
1e-02
Log-odds
0.5
1.0
1.5
2.0
2.5
3.0
3.5
4.0

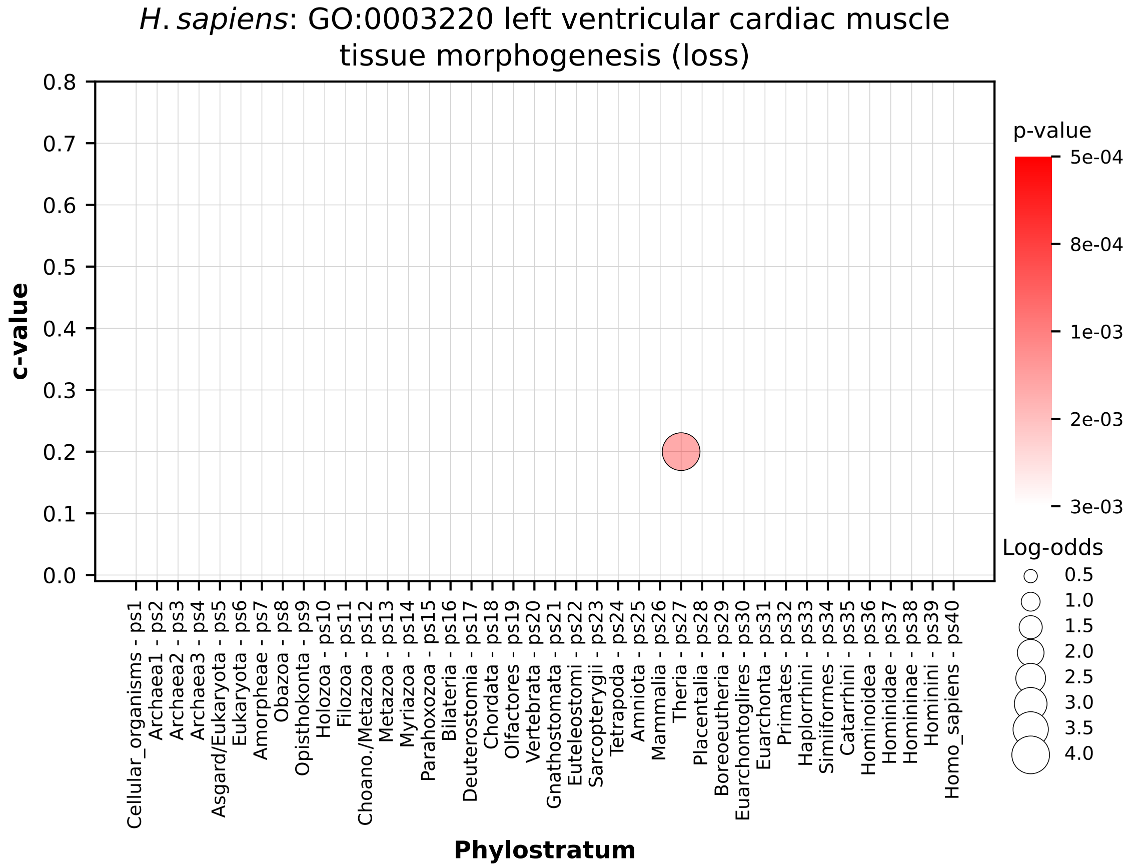

H. sapiens: GO:0003220 left ventricular cardiac muscle tissue morphogenesis (loss)
c-value
0.8
0.7
0.6
0.5
0.4
0.3
0.2
0.1
0.0
Phylostratum
Cellular_organisms - ps1
Archaea1 - ps2
Archaea2 - ps3
Archaea3 - ps4
Asgard/Eukaryota - ps5
Eukaryota - ps6
Amorpheae - ps7
Obazoa - ps8
Opisthokonta - ps9
Holozoa - ps10
Filozoa - ps11
Choano./Metazoa - ps12
Metazoa - ps13
Myriazoa - ps14
Parahoxozoa - ps15
Bilateria - ps16
Deuterostomia - ps17
Chordata - ps18
Olfactores - ps19
Vertebrata - ps20
Gnathostomata - ps21
Euteleostomi - ps22
Sarcopterygii - ps23
Tetrapoda - ps24
Amniota - ps25
Mammalia - ps26
Theria - ps27
Placentalia - ps28
Boreoeutheria - ps29
Euarchontoglires - ps30
Euarchonta - ps31
Primates - ps32
Haplorrhini - ps33
Simiiformes - ps34
Catarrhini - ps35
Hominoidea - ps36
Hominidae - ps37
Homininae - ps38
Hominini - ps39
Homo_sapiens - ps40
p-value
5e-04
8e-04
1e-03
2e-03
3e-03
Log-odds
0.5
1.0
1.5
2.0
2.5
3.0
3.5
4.0

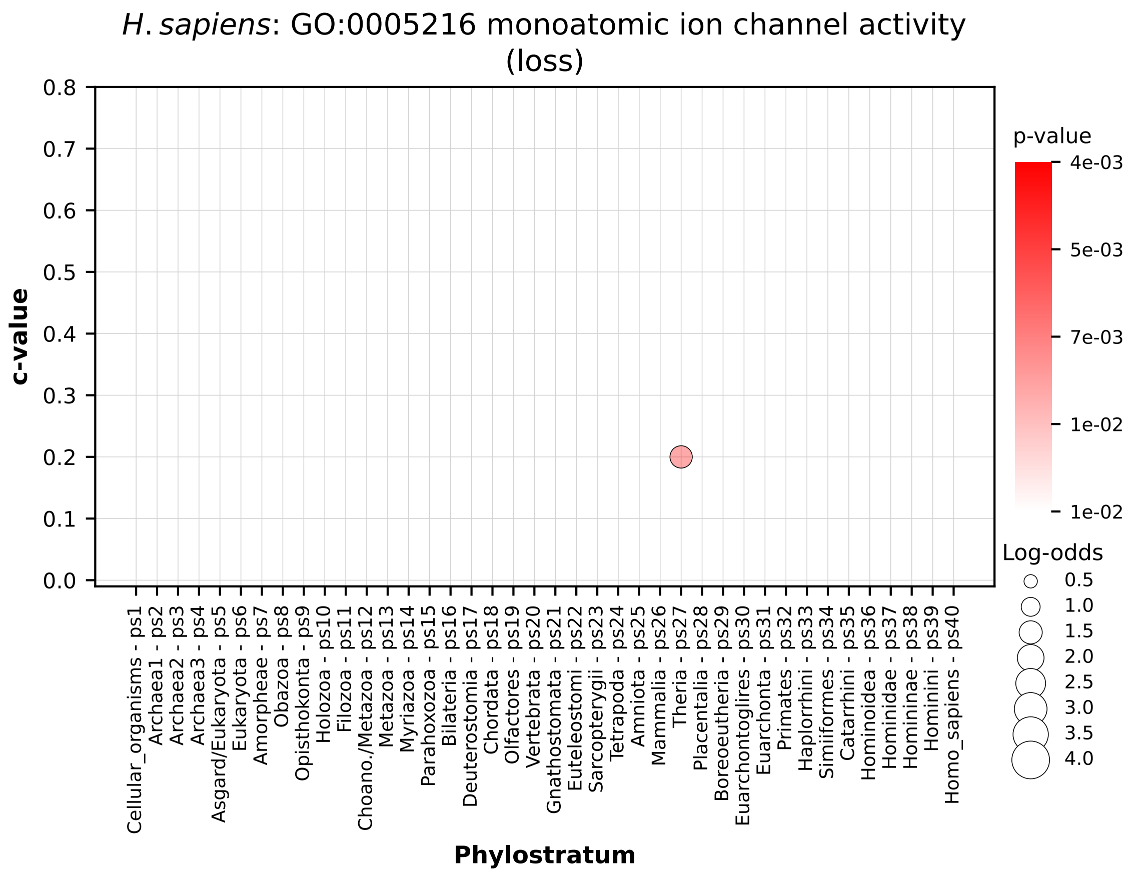

H. sapiens: GO:0005216 monoatomic ion channel activity (loss)
c-value
0.8
0.7
0.6
0.5
0.4
0.3
0.2
0.1
0.0
Phylostratum
Cellular_organisms - ps1
Archaea1 - ps2
Archaea2 - ps3
Archaea3 - ps4
Asgard/Eukaryota - ps5
Eukaryota - ps6
Amorpheae - ps7
Obazoa - ps8
Opisthokonta - ps9
Holozoa - ps10
Filozoa - ps11
Choano./Metazoa - ps12
Metazoa - ps13
Myriazoa - ps14
Parahoxozoa - ps15
Bilateria - ps16
Deuterostomia - ps17
Chordata - ps18
Olfactores - ps19
Vertebrata - ps20
Gnathostomata - ps21
Euteleostomi - ps22
Sarcopterygii - ps23
Tetrapoda - ps24
Amniota - ps25
Mammalia - ps26
Theria - ps27
Placentalia - ps28
Boreoeutheria - ps29
Euarchontoglires - ps30
Euarchonta - ps31
Primates - ps32
Haplorrhini - ps33
Simiiformes - ps34
Catarrhini - ps35
Hominoidea - ps36
Hominidae - ps37
Homininae - ps38
Hominini - ps39
Homo_sapiens - ps40
p-value
4e-03
5e-03
7e-03
1e-02
1e-02
Log-odds
0.5
1.0
1.5
2.0
2.5
3.0
3.5
4.0

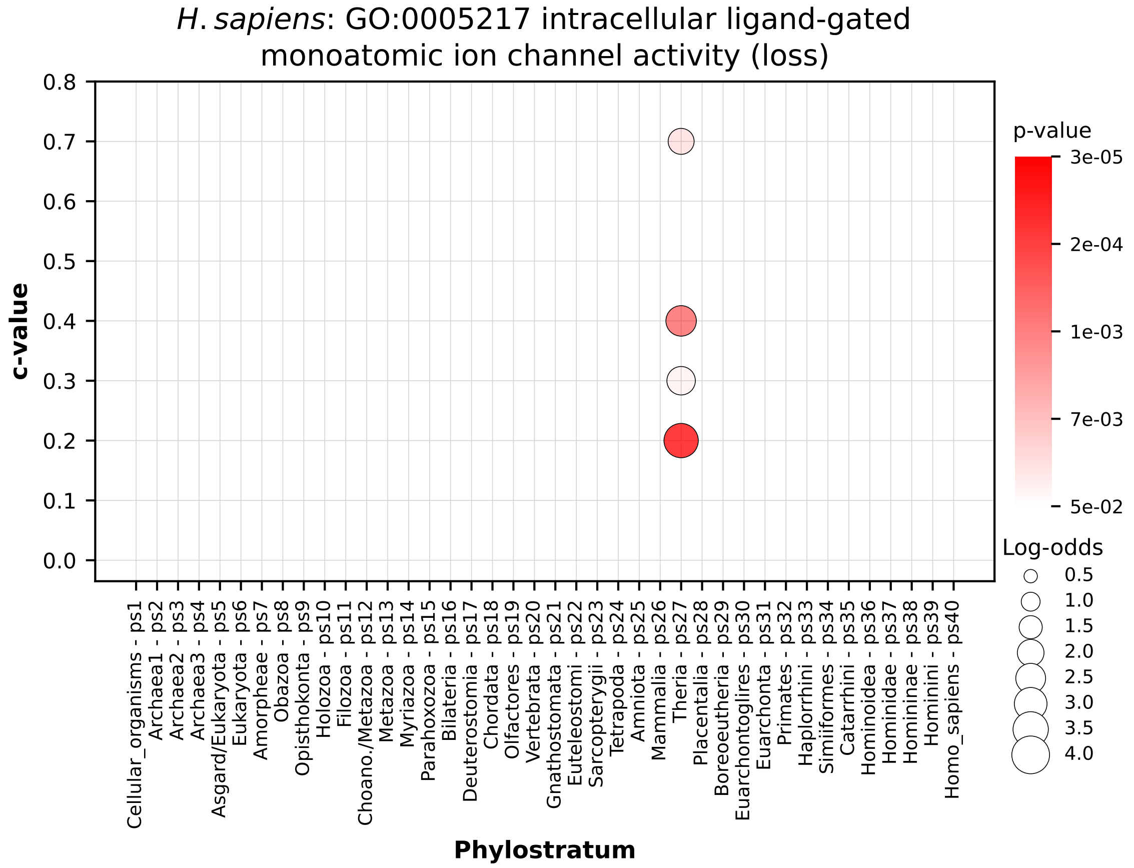

H. sapiens: GO:0005217 intracellular ligand-gated monoatomic ion channel activity (loss)
c-value
0.8
0.7
0.6
0.5
0.4
0.3
0.2
0.1
0.0
p-value
3e-05
2e-04
1e-03
7e-03
5e-02
Log-odds
0.5
1.0
1.5
2.0
2.5
3.0
3.5
4.0
Cellular_organisms - ps1
Archaea1 - ps2
Archaea2 - ps3
Archaea3 - ps4
Asgard/Eukaryota - ps5
Eukaryota - ps6
Amorpheae - ps7
Obazoa - ps8
Opisthokonta - ps9
Holozoa - ps10
Filozoa - ps11
Choano./Metazoa - ps12
Metazoa - ps13
Myriazoa - ps14
Parahoxozoa - ps15
Bilateria - ps16
Deuterostomia - ps17
Chordata - ps18
Olfactores - ps19
Vertebrata - ps20
Gnathostomata - ps21
Euteleostomi - ps22
Sarcopterygii - ps23
Tetrapoda - ps24
Amniota - ps25
Mammalia - ps26
Theria - ps27
Placentalia - ps28
Boreoeutheria - ps29
Euarchontoglires - ps30
Euarchonta - ps31
Primates - ps32
Haplorrhini - ps33
Simiiformes - ps34
Catarrhini - ps35
Hominoidea - ps36
Hominidae - ps37
Homininae - ps38
Hominini - ps39
Homo_sapiens - ps40
Phylostratum

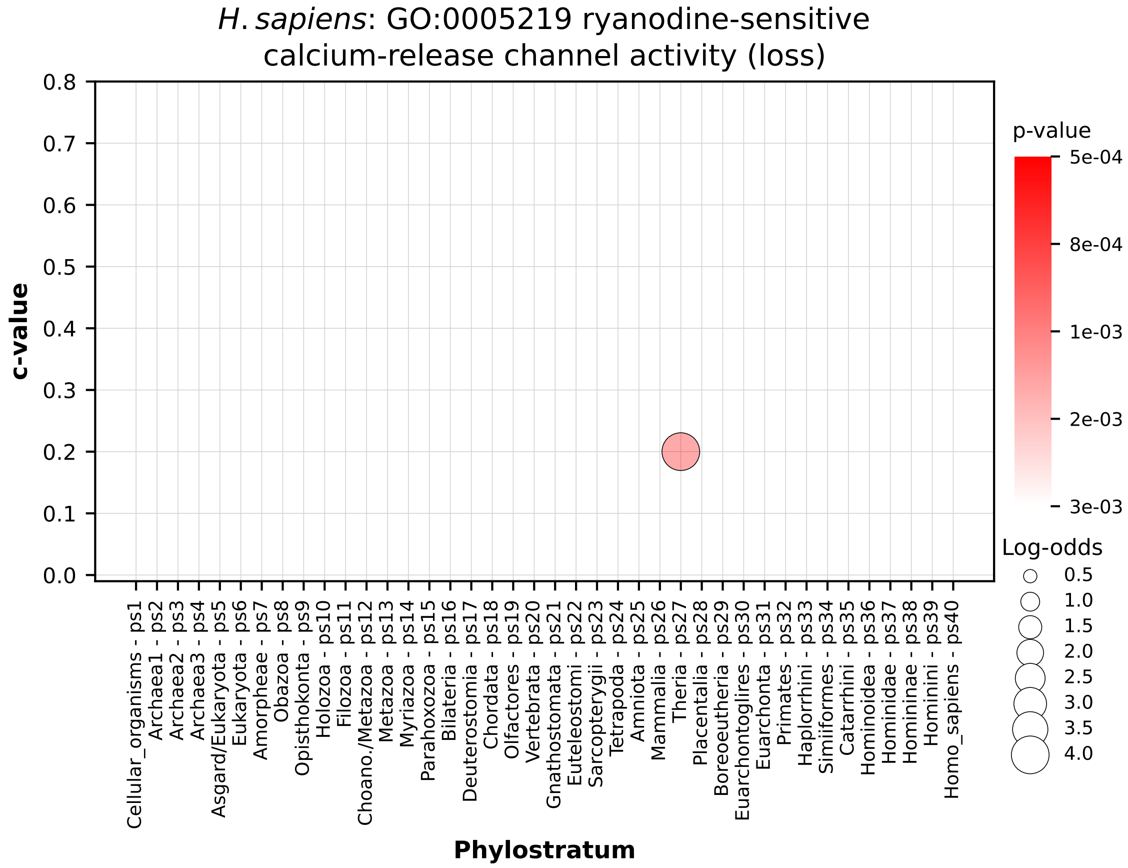

H. sapiens: GO:0005219 ryanodine-sensitive calcium-release channel activity (loss)
c-value
0.8
0.7
0.6
0.5
0.4
0.3
0.2
0.1
0.0
p-value
5e-04
8e-04
1e-03
2e-03
3e-03
Log-odds
0.5
1.0
1.5
2.0
2.5
3.0
3.5
4.0
Cellular_organisms - ps1
Archaea1 - ps2
Archaea2 - ps3
Archaea3 - ps4
Asgard/Eukaryota - ps5
Eukaryota - ps6
Amorpheae - ps7
Obazoa - ps8
Opisthokonta - ps9
Holozoa - ps10
Filozoa - ps11
Choano./Metazoa - ps12
Metazoa - ps13
Myriazoa - ps14
Parahoxozoa - ps15
Bilateria - ps16
Deuterostomia - ps17
Chordata - ps18
Olfactores - ps19
Vertebrata - ps20
Gnathostomata - ps21
Euteleostomi - ps22
Sarcopterygii - ps23
Tetrapoda - ps24
Amniota - ps25
Mammalia - ps26
Theria - ps27
Placentalia - ps28
Boreoeutheria - ps29
Euarchontoglires - ps30
Euarchonta - ps31
Primates - ps32
Haplorrhini - ps33
Simiiformes - ps34
Catarrhini - ps35
Hominoidea - ps36
Hominidae - ps37
Homininae - ps38
Hominini - ps39
Homo_sapiens - ps40
Phylostratum

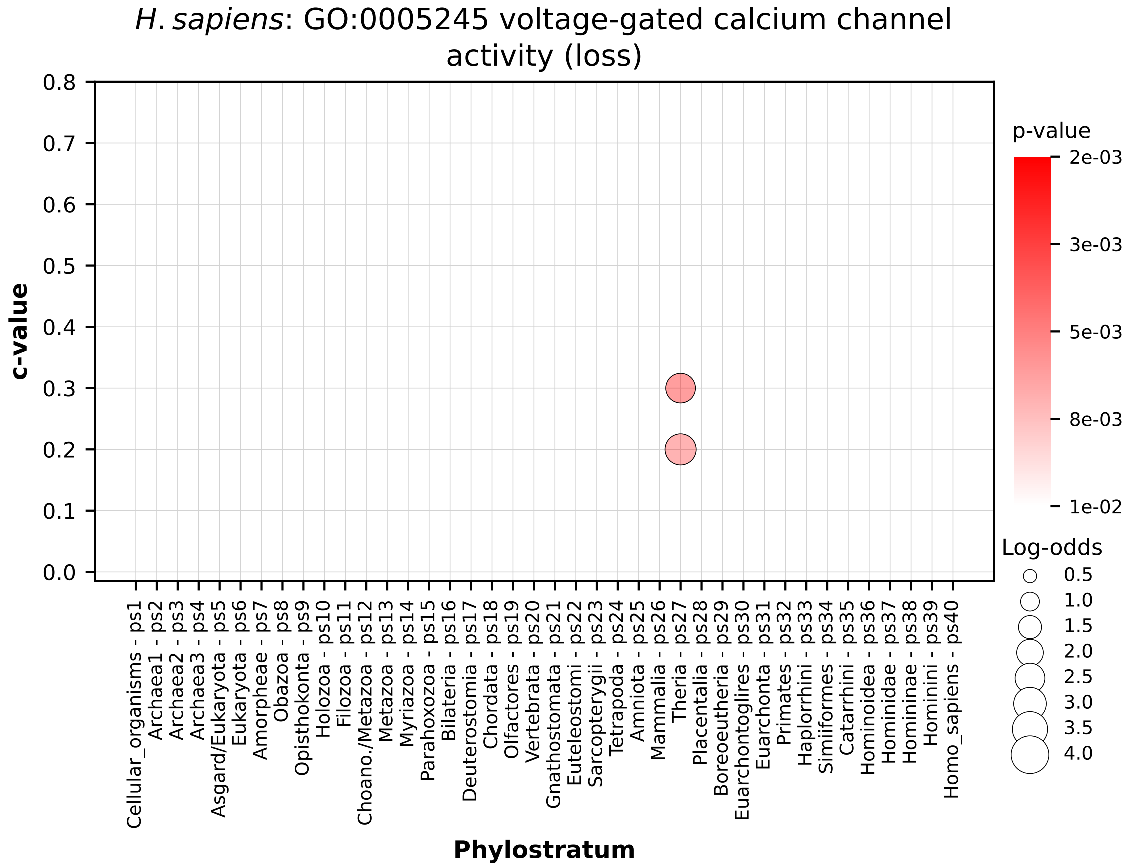

H. sapiens: GO:0005245 voltage-gated calcium channel activity (loss)
c-value
0.8
0.7
0.6
0.5
0.4
0.3
0.2
0.1
0.0
Cellular_organisms - ps1
Archaea1 - ps2
Archaea2 - ps3
Archaea3 - ps4
Asgard/Eukaryota - ps5
Eukaryota - ps6
Amorpheae - ps7
Obazoa - ps8
Opisthokonta - ps9
Holozoa - ps10
Filozoa - ps11
Choano./Metazoa - ps12
Metazoa - ps13
Myriazoa - ps14
Parahoxozoa - ps15
Bilateria - ps16
Deuterostomia - ps17
Chordata - ps18
Olfactores - ps19
Vertebrata - ps20
Gnathostomata - ps21
Euteleostomi - ps22
Sarcopterygii - ps23
Tetrapoda - ps24
Amniota - ps25
Mammalia - ps26
Theria - ps27
Placentalia - ps28
Boreoeutheria - ps29
Euarchontoglires - ps30
Euarchonta - ps31
Primates - ps32
Haplorrhini - ps33
Simiiformes - ps34
Catarrhini - ps35
Hominoidea - ps36
Hominidae - ps37
Homininae - ps38
Hominini - ps39
Homo_sapiens - ps40
Phylostratum
p-value
2e-03
3e-03
5e-03
8e-03
1e-02
Log-odds
0.5
1.0
1.5
2.0
2.5
3.0
3.5
4.0

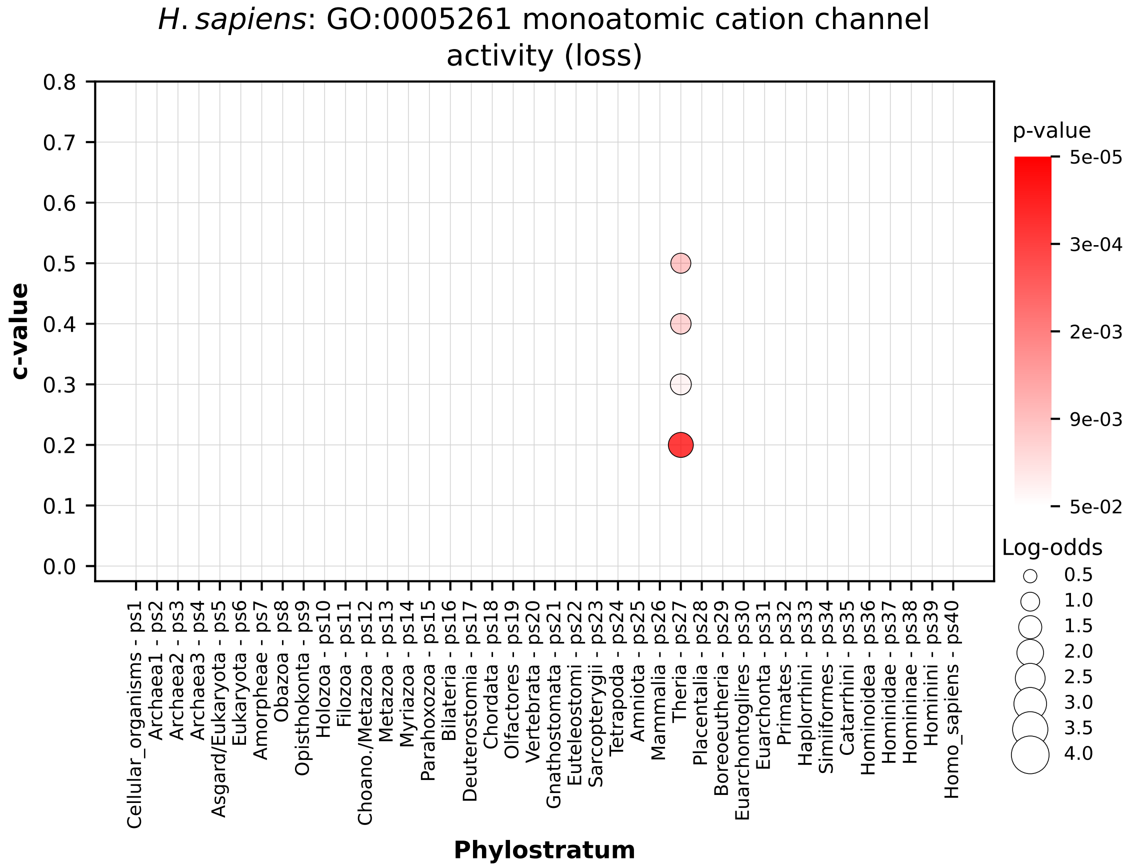
H. sapiens: GO:0005261 monoatomic cation channel activity (loss)
c-value
0.8
0.7
0.6
0.5
0.4
0.3
0.2
0.1
0.0
p-value
5e-05
3e-04
2e-03
9e-03
5e-02
Log-odds
0.5
1.0
1.5
2.0
2.5
3.0
3.5
4.0
Cellular_organisms - ps1
Archaea1 - ps2
Archaea2 - ps3
Archaea3 - ps4
Asgard/Eukaryota - ps5
Eukaryota - ps6
Amorpheae - ps7
Obazoa - ps8
Opisthokonta - ps9
Holozoa - ps10
Filozoa - ps11
Choano./Metazoa - ps12
Metazoa - ps13
Myriazoa - ps14
Parahoxozoa - ps15
Bilateria - ps16
Deuterostomia - ps17
Chordata - ps18
Olfactores - ps19
Vertebrata - ps20
Gnathostomata - ps21
Euteleostomi - ps22
Sarcopterygii - ps23
Tetrapoda - ps24
Amniota - ps25
Mammalia - ps26
Theria - ps27
Placentalia - ps28
Boreoeutheria - ps29
Euarchontoglires - ps30
Euarchonta - ps31
Primates - ps32
Haplorrhini - ps33
Simiiformes - ps34
Catarrhini - ps35
Hominoidea - ps36
Hominidae - ps37
Homininae - ps38
Hominini - ps39
Homo_sapiens - ps40
Phylostratum

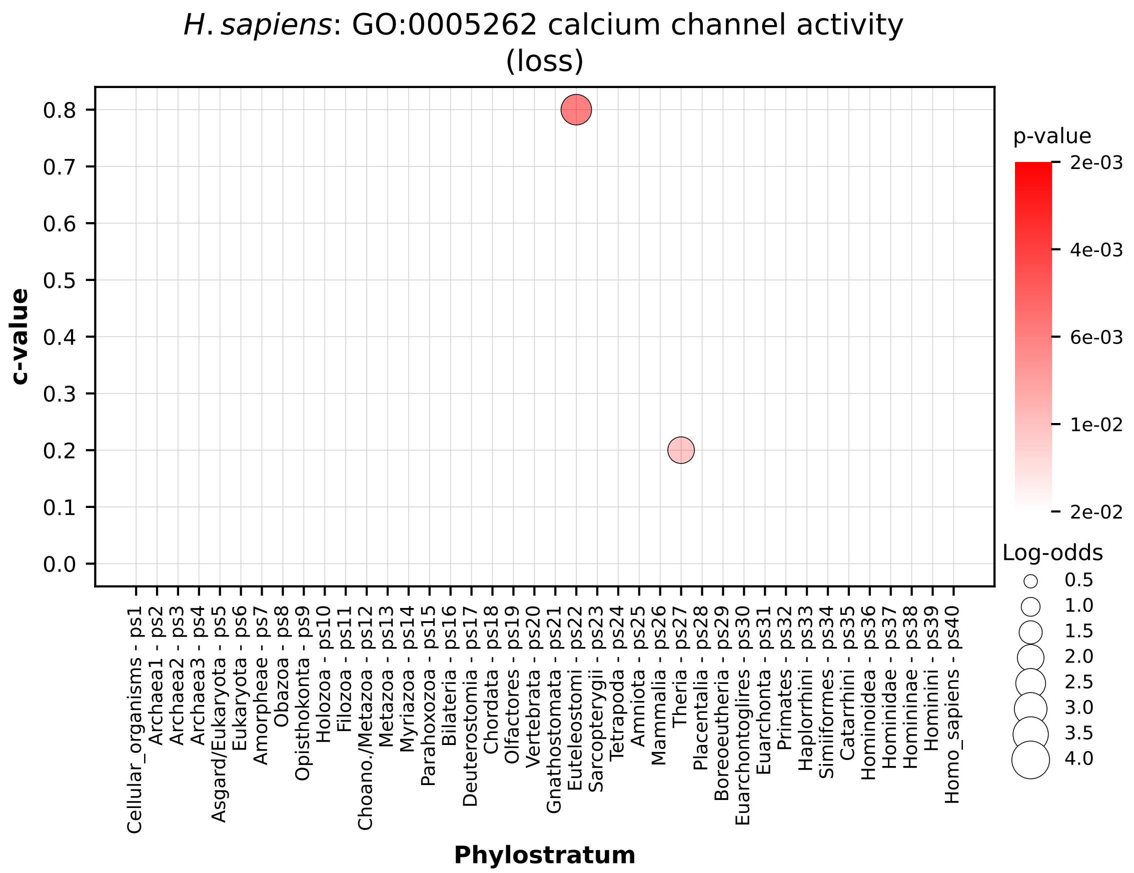
H. sapiens: GO:0005262 calcium channel activity
(loss)
c-value
0.0
0.1
0.2
0.3
0.4
0.5
0.6
0.7
0.8
p-value
2e-03
4e-03
6e-03
1e-02
2e-02
Log-odds
0.5
1.0
1.5
2.0
2.5
3.0
3.5
4.0
Cellular_organisms - ps1
Archaea1 - ps2
Archaea2 - ps3
Archaea3 - ps4
Asgard/Eukaryota - ps5
Eukaryota - ps6
Amorpheae - ps7
Obazoa - ps8
Opisthokonta - ps9
Holozoa - ps10
Filozoa - ps11
Choano./Metazoa - ps12
Metazoa - ps13
Myriazoa - ps14
Parahoxozoa - ps15
Bilateria - ps16
Deuterostomia - ps17
Chordata - ps18
Olfactores - ps19
Vertebrata - ps20
Gnathostomata - ps21
Euteleostomi - ps22
Sarcopterygii - ps23
Tetrapoda - ps24
Amniota - ps25
Mammalia - ps26
Theria - ps27
Placentalia - ps28
Boreoeutheria - ps29
Euarchontoglires - ps30
Euarchonta - ps31
Primates - ps32
Haplorrhini - ps33
Simiiformes - ps34
Catarrhini - ps35
Hominoidea - ps36
Hominidae - ps37
Homininae - ps38
Hominini - ps39
Homo_sapiens - ps40
Phylostratum

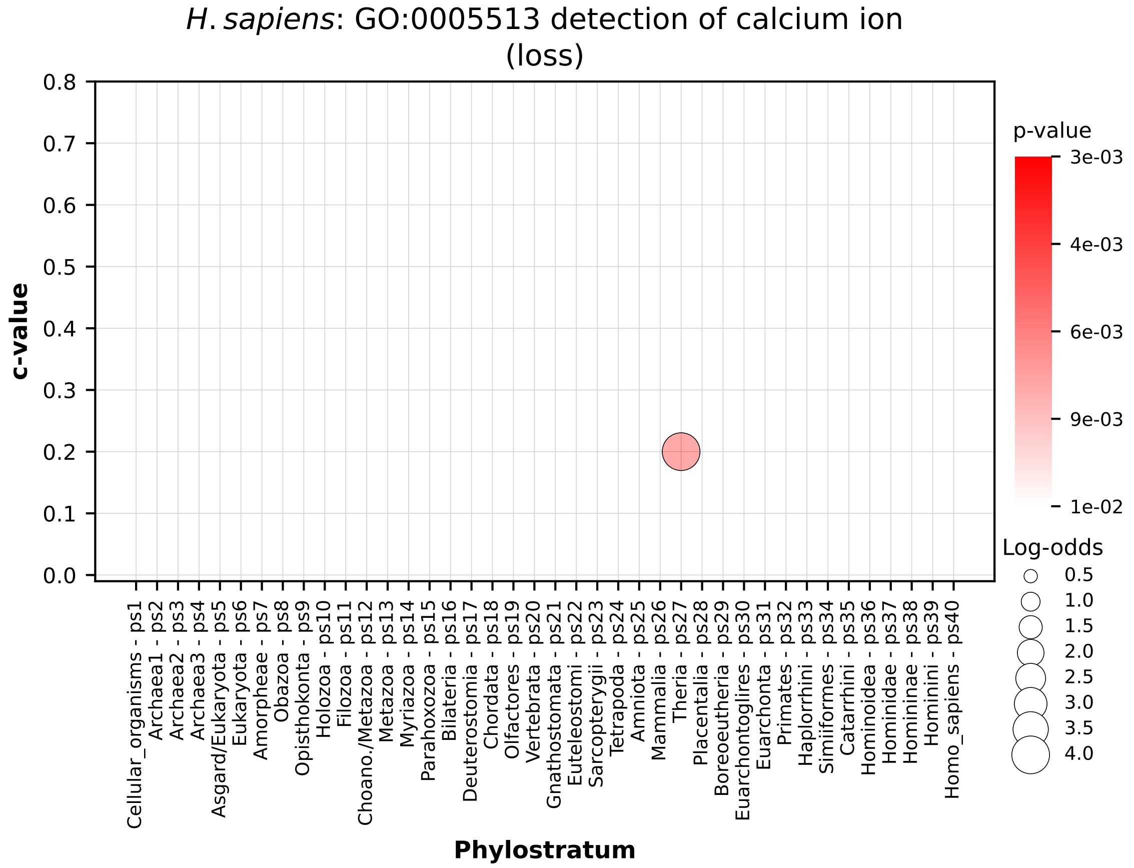
H. sapiens: GO:0005513 detection of calcium ion (loss)
c-value
Phylostratum
p-value
3e-03
4e-03
6e-03
9e-03
1e-02
Log-odds
0.5
1.0
1.5
2.0
2.5
3.0
3.5
4.0
Cellular_organisms - ps1
Archaea1 - ps2
Archaea2 - ps3
Archaea3 - ps4
Asgard/Eukaryota - ps5
Eukaryota - ps6
Amorpheae - ps7
Obazoa - ps8
Opisthokonta - ps9
Holozoa - ps10
Filozoa - ps11
Choano./Metazoa - ps12
Metazoa - ps13
Myriazoa - ps14
Parahoxozoa - ps15
Bilateria - ps16
Deuterostomia - ps17
Chordata - ps18
Olfactores - ps19
Vertebrata - ps20
Gnathostomata - ps21
Euteleostomi - ps22
Sarcopterygii - ps23
Tetrapoda - ps24
Amniota - ps25
Mammalia - ps26
Theria - ps27
Placentalia - ps28
Boreoeutheria - ps29
Euarchontoglires - ps30
Euarchonta - ps31
Primates - ps32
Haplorrhini - ps33
Simiiformes - ps34
Catarrhini - ps35
Hominoidea - ps36
Hominidae - ps37
Homininae - ps38
Hominini - ps39
Homo_sapiens - ps40

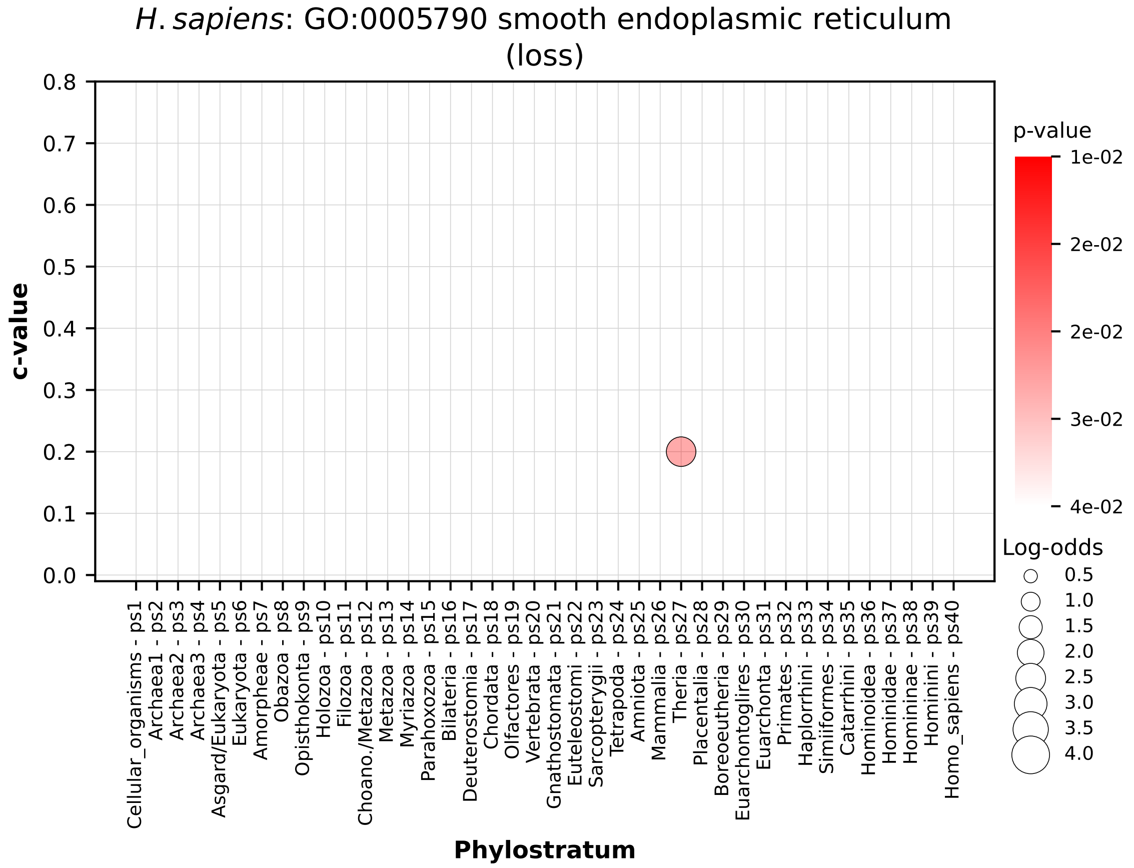
H. sapiens: GO:0005790 smooth endoplasmic reticulum
(loss)
c-value
0.8
0.7
0.6
0.5
0.4
0.3
0.2
0.1
0.0
p-value
1e-02
2e-02
2e-02
3e-02
4e-02
Log-odds
0.5
1.0
1.5
2.0
2.5
3.0
3.5
4.0
Cellular_organisms - ps1
Archaea1 - ps2
Archaea2 - ps3
Archaea3 - ps4
Asgard/Eukaryota - ps5
Eukaryota - ps6
Amorpheae - ps7
Obazoa - ps8
Opisthokonta - ps9
Holozoa - ps10
Filozoa - ps11
Choano./Metazoa - ps12
Metazoa - ps13
Myriazoa - ps14
Parahoxozoa - ps15
Bilateria - ps16
Deuterostomia - ps17
Chordata - ps18
Olfactores - ps19
Vertebrata - ps20
Gnathostomata - ps21
Euteleostomi - ps22
Sarcopterygii - ps23
Tetrapoda - ps24
Amniota - ps25
Mammalia - ps26
Theria - ps27
Placentalia - ps28
Boreoeutheria - ps29
Euarchontoglires - ps30
Euarchonta - ps31
Primates - ps32
Haplorrhini - ps33
Simiiformes - ps34
Catarrhini - ps35
Hominoidea - ps36
Hominidae - ps37
Homininae - ps38
Hominini - ps39
Homo_sapiens - ps40
Phylostratum

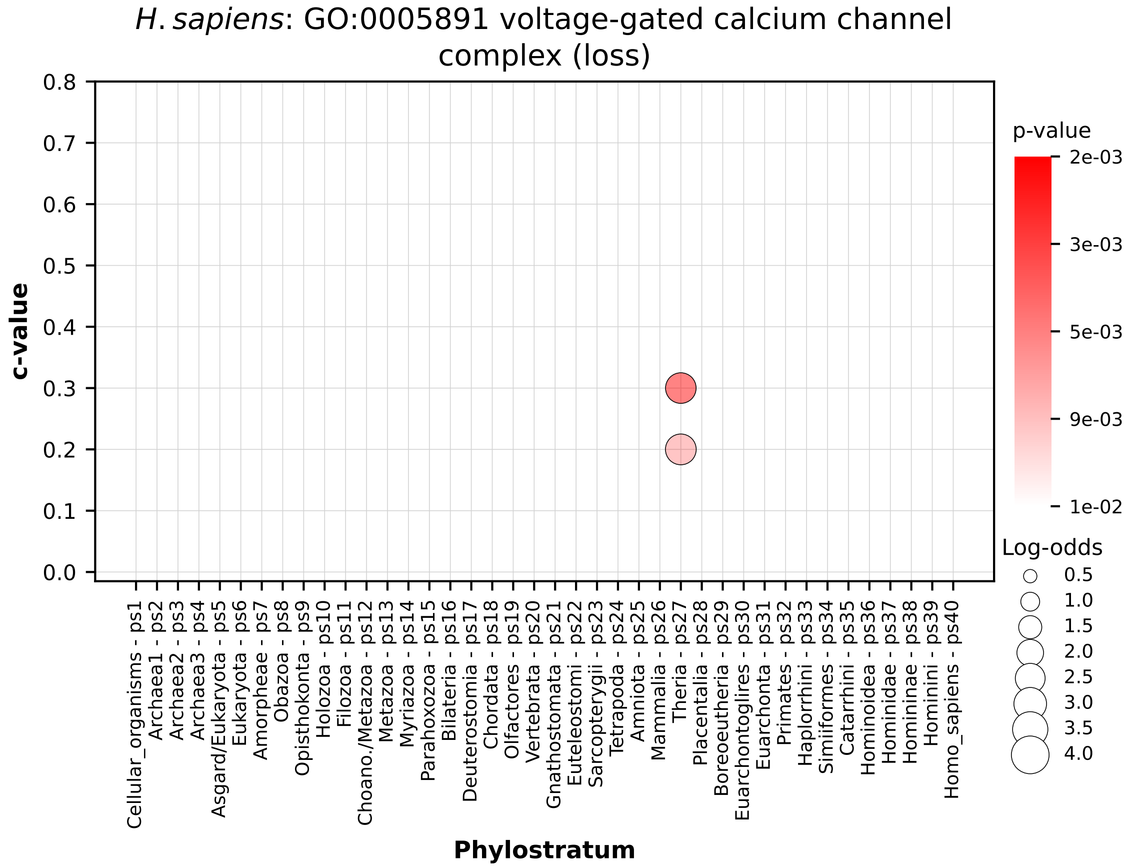

H. sapiens: GO:0005891 voltage-gated calcium channel complex (loss)
c-value
0.0
0.1
0.2
0.3
0.4
0.5
0.6
0.7
0.8
Phylostratum
Cellular_organisms - ps1
Archaea1 - ps2
Archaea2 - ps3
Archaea3 - ps4
Asgard/Eukaryota - ps5
Eukaryota - ps6
Amorpheae - ps7
Obazoa - ps8
Opisthokonta - ps9
Holozoa - ps10
Filozoa - ps11
Choano./Metazoa - ps12
Metazoa - ps13
Myriazoa - ps14
Parahoxozoa - ps15
Bilateria - ps16
Deuterostomia - ps17
Chordata - ps18
Olfactores - ps19
Vertebrata - ps20
Gnathostomata - ps21
Euteleostomi - ps22
Sarcopterygii - ps23
Tetrapoda - ps24
Amniota - ps25
Mammalia - ps26
Theria - ps27
Placentalia - ps28
Boreoeutheria - ps29
Euarchontoglires - ps30
Euarchonta - ps31
Primates - ps32
Haplorrhini - ps33
Simiiformes - ps34
Catarrhini - ps35
Hominoidea - ps36
Hominidae - ps37
Homininae - ps38
Hominini - ps39
Homo_sapiens - ps40
p-value
2e-03
3e-03
5e-03
9e-03
1e-02
Log-odds
0.5
1.0
1.5
2.0
2.5
3.0
3.5
4.0

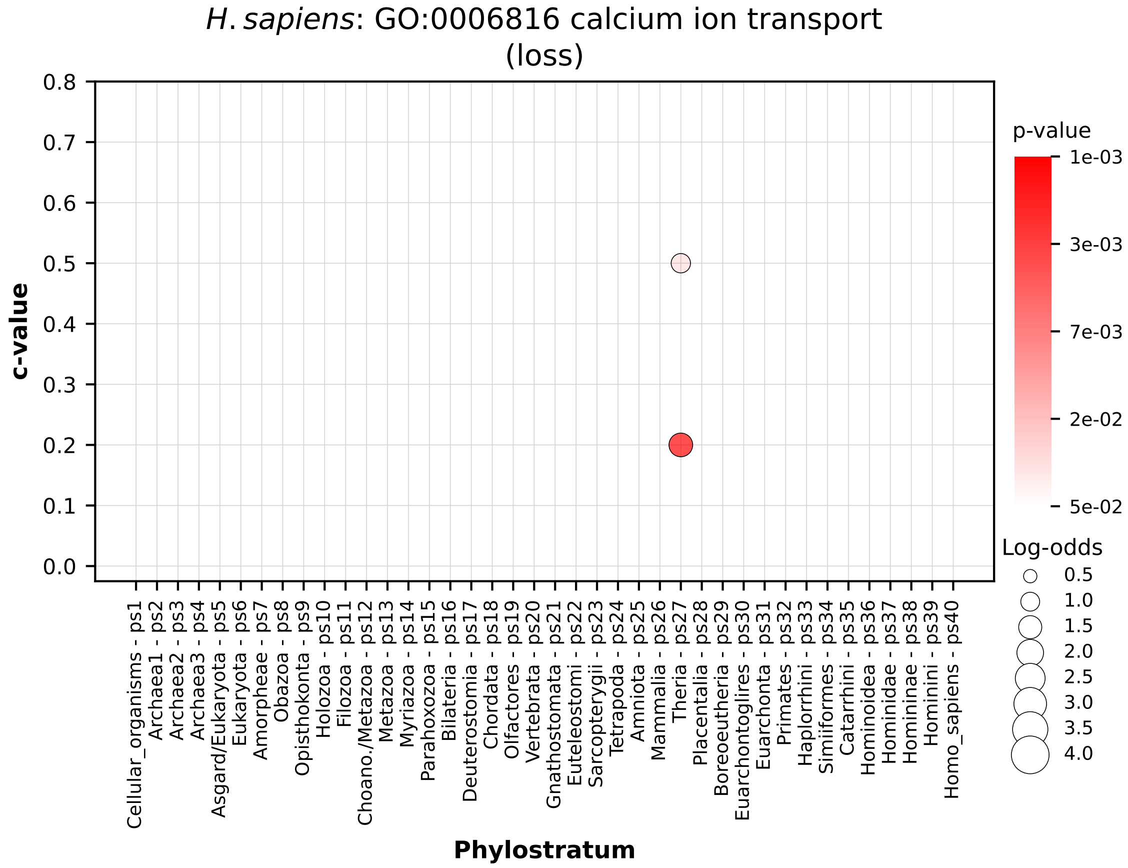

H. sapiens: GO:0006816 calcium ion transport
(loss)
c-value
0.0
0.1
0.2
0.3
0.4
0.5
0.6
0.7
0.8
Phylostratum
Cellular_organisms - ps1
Archaea1 - ps2
Archaea2 - ps3
Archaea3 - ps4
Asgard/Eukaryota - ps5
Eukaryota - ps6
Amorpheae - ps7
Obazoa - ps8
Opisthokonta - ps9
Holozoa - ps10
Filozoa - ps11
Choano./Metazoa - ps12
Metazoa - ps13
Myriazoa - ps14
Parahoxozoa - ps15
Bilateria - ps16
Deuterostomia - ps17
Chordata - ps18
Olfactores - ps19
Vertebrata - ps20
Gnathostomata - ps21
Euteleostomi - ps22
Sarcopterygii - ps23
Tetrapoda - ps24
Amniota - ps25
Mammalia - ps26
Theria - ps27
Placentalia - ps28
Boreoeutheria - ps29
Euarchontoglires - ps30
Euarchonta - ps31
Primates - ps32
Haplorrhini - ps33
Simiiformes - ps34
Catarrhini - ps35
Hominoidea - ps36
Hominidae - ps37
Homininae - ps38
Hominini - ps39
Homo_sapiens - ps40
p-value
1e-03
3e-03
7e-03
2e-02
5e-02
Log-odds
0.5
1.0
1.5
2.0
2.5
3.0
3.5
4.0

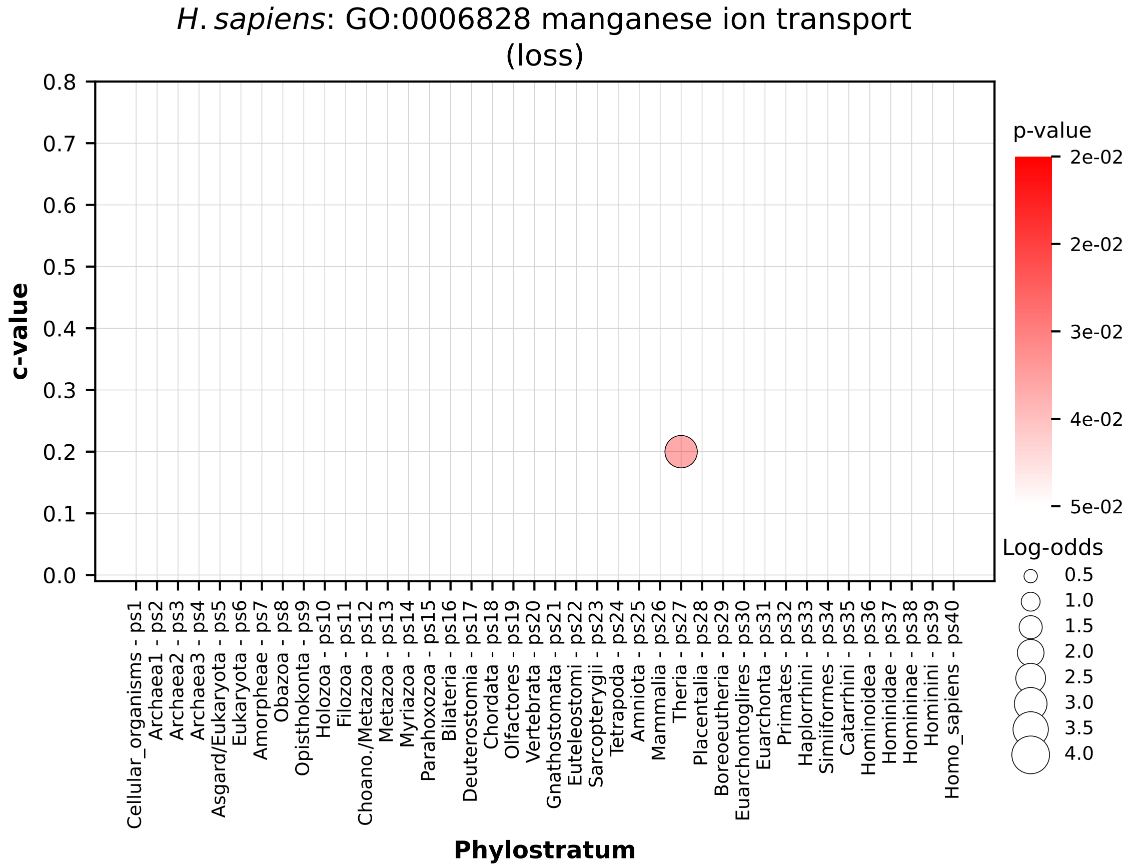
H. sapiens: GO:0006828 manganese ion transport
(loss)
c-value
0.8
0.7
0.6
0.5
0.4
0.3
0.2
0.1
0.0
Phylostratum
Cellular_organisms - ps1
Archaea1 - ps2
Archaea2 - ps3
Archaea3 - ps4
Asgard/Eukaryota - ps5
Eukaryota - ps6
Amorpheae - ps7
Obazoa - ps8
Opisthokonta - ps9
Holozoa - ps10
Filozoa - ps11
Choano./Metazoa - ps12
Metazoa - ps13
Myriazoa - ps14
Parahoxozoa - ps15
Bilateria - ps16
Deuterostomia - ps17
Chordata - ps18
Olfactores - ps19
Vertebrata - ps20
Gnathostomata - ps21
Euteleostomi - ps22
Sarcopterygii - ps23
Tetrapoda - ps24
Amniota - ps25
Mammalia - ps26
Theria - ps27
Placentalia - ps28
Boreoeutheria - ps29
Euarchontoglires - ps30
Euarchonta - ps31
Primates - ps32
Haplorrhini - ps33
Simiiformes - ps34
Catarrhini - ps35
Hominoidea - ps36
Hominidae - ps37
Homininae - ps38
Hominini - ps39
Homo_sapiens - ps40
p-value
2e-02
2e-02
3e-02
4e-02
5e-02
Log-odds
0.5
1.0
1.5
2.0
2.5
3.0
3.5
4.0

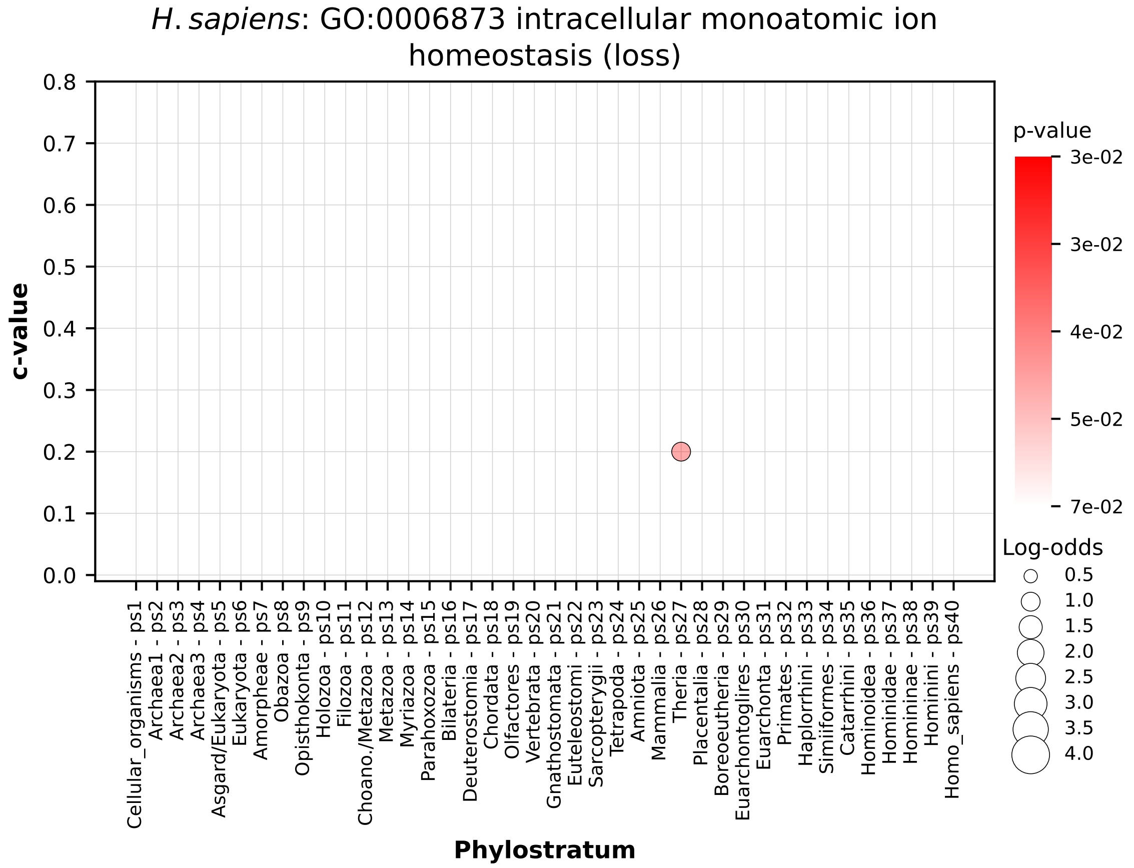
H. sapiens: GO:0006873 intracellular monoatomic ion homeostasis (loss)
c-value
0.0
0.1
0.2
0.3
0.4
0.5
0.6
0.7
0.8
Phylostratum
Cellular_organisms - ps1
Archaea1 - ps2
Archaea2 - ps3
Archaea3 - ps4
Asgard/Eukaryota - ps5
Eukaryota - ps6
Amorpheae - ps7
Obazoa - ps8
Opisthokonta - ps9
Holozoa - ps10
Filozoa - ps11
Choano./Metazoa - ps12
Metazoa - ps13
Myriazoa - ps14
Parahoxozoa - ps15
Bilateria - ps16
Deuterostomia - ps17
Chordata - ps18
Olfactores - ps19
Vertebrata - ps20
Gnathostomata - ps21
Euteleostomi - ps22
Sarcopterygii - ps23
Tetrapoda - ps24
Amniota - ps25
Mammalia - ps26
Theria - ps27
Placentalia - ps28
Boreoeutheria - ps29
Euarchontoglires - ps30
Euarchonta - ps31
Primates - ps32
Haplorrhini - ps33
Simiiformes - ps34
Catarrhini - ps35
Hominoidea - ps36
Hominidae - ps37
Homininae - ps38
Hominini - ps39
Homo_sapiens - ps40
p-value
3e-02
3e-02
4e-02
5e-02
7e-02
Log-odds
0.5
1.0
1.5
2.0
2.5
3.0
3.5
4.0

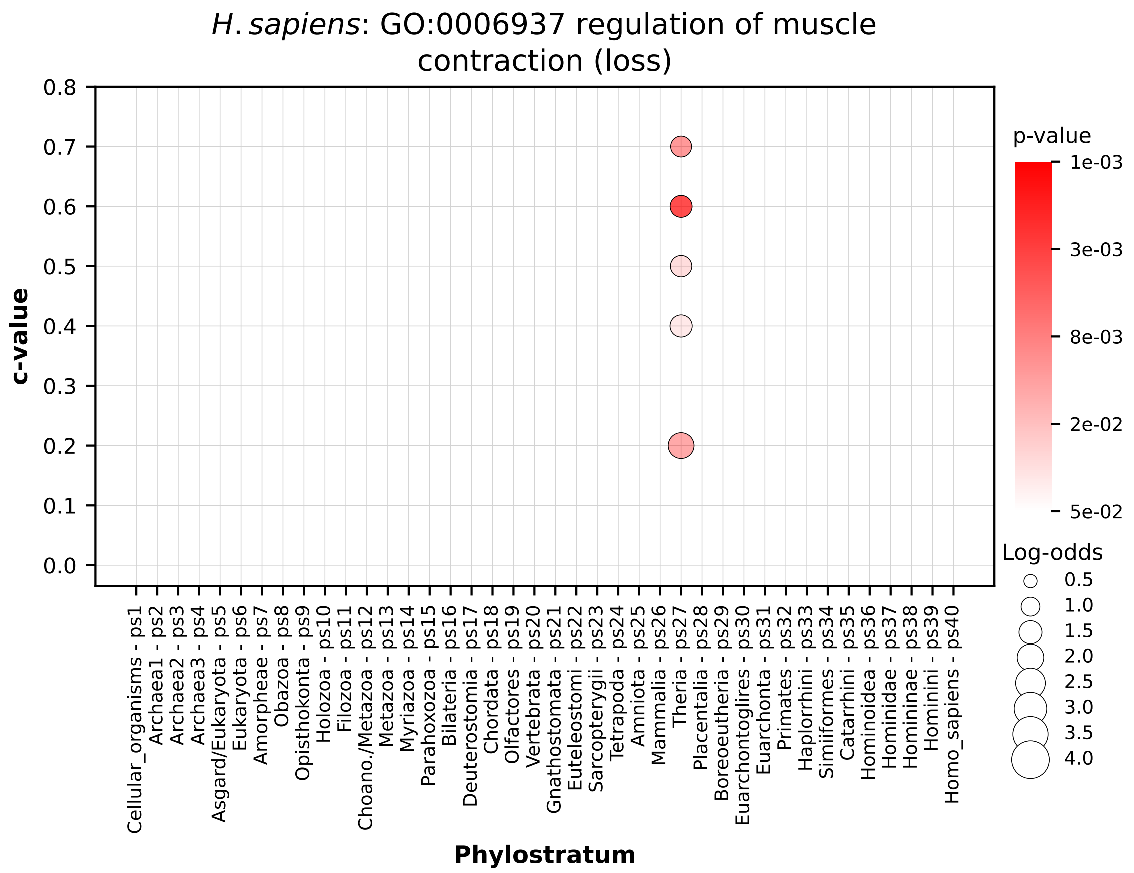

H. sapiens: GO:0006937 regulation of muscle contraction (loss)
c-value
0.0
0.1
0.2
0.3
0.4
0.5
0.6
0.7
0.8
Phylostratum
Cellular_organisms - ps1
Archaea1 - ps2
Archaea2 - ps3
Archaea3 - ps4
Asgard/Eukaryota - ps5
Eukaryota - ps6
Amorpheae - ps7
Obazoa - ps8
Opisthokonta - ps9
Holozoa - ps10
Filozoa - ps11
Choano./Metazoa - ps12
Metazoa - ps13
Myriazoa - ps14
Parahoxozoa - ps15
Bilateria - ps16
Deuterostomia - ps17
Chordata - ps18
Olfactores - ps19
Vertebrata - ps20
Gnathostomata - ps21
Euteleostomi - ps22
Sarcopterygii - ps23
Tetrapoda - ps24
Amniota - ps25
Mammalia - ps26
Theria - ps27
Placentalia - ps28
Boreoeutheria - ps29
Euarchontoglires - ps30
Euarchonta - ps31
Primates - ps32
Haplorrhini - ps33
Simiiformes - ps34
Catarrhini - ps35
Hominoidea - ps36
Hominidae - ps37
Homininae - ps38
Hominini - ps39
Homo_sapiens - ps40
p-value
1e-03
3e-03
8e-03
2e-02
5e-02
Log-odds
0.5
1.0
1.5
2.0
2.5
3.0
3.5
4.0

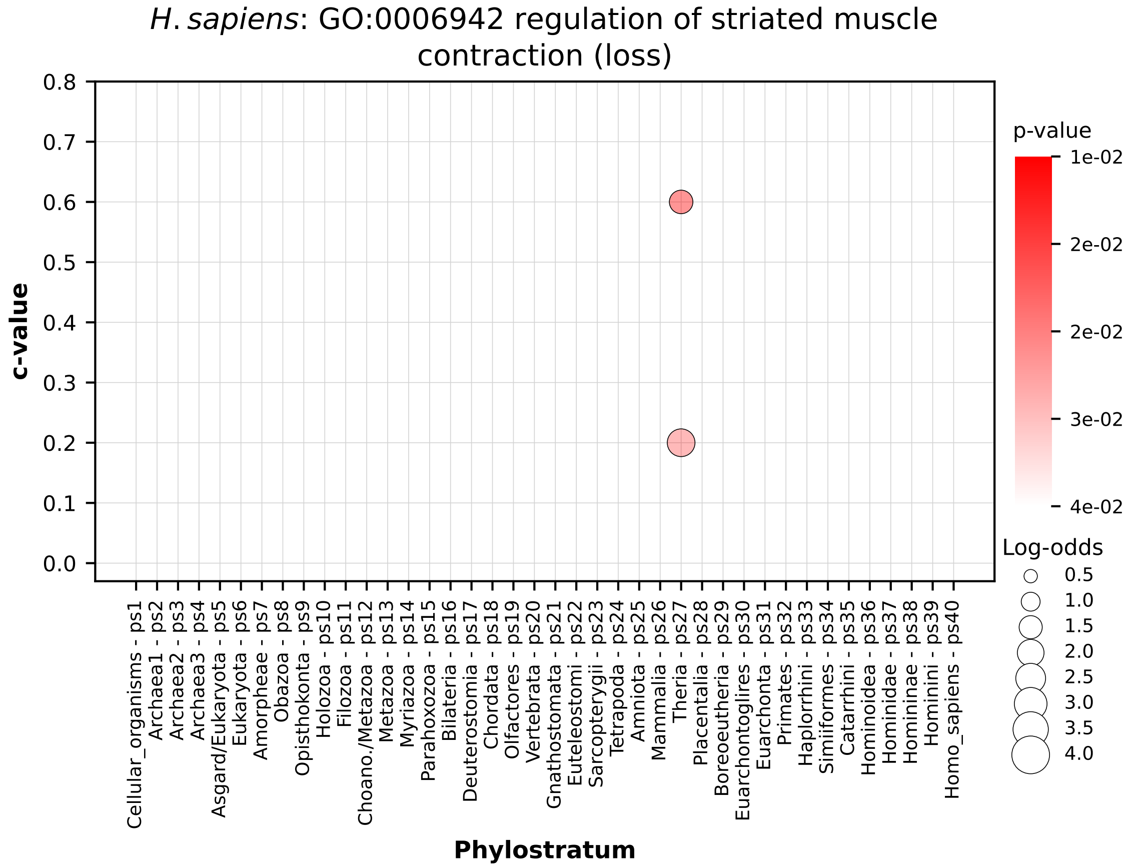

H. sapiens: GO:0006942 regulation of striated muscle contraction (loss)
c-value
0.8
0.7
0.6
0.5
0.4
0.3
0.2
0.1
0.0
Phylostratum
Cellular_organisms - ps1
Archaea1 - ps2
Archaea2 - ps3
Archaea3 - ps4
Asgard/Eukaryota - ps5
Eukaryota - ps6
Amorpheae - ps7
Obazoa - ps8
Opisthokonta - ps9
Holozoa - ps10
Filozoa - ps11
Choano./Metazoa - ps12
Metazoa - ps13
Myriazoa - ps14
Parahoxozoa - ps15
Bilateria - ps16
Deuterostomia - ps17
Chordata - ps18
Olfactores - ps19
Vertebrata - ps20
Gnathostomata - ps21
Euteleostomi - ps22
Sarcopterygii - ps23
Tetrapoda - ps24
Amniota - ps25
Mammalia - ps26
Theria - ps27
Placentalia - ps28
Boreoeutheria - ps29
Euarchontoglires - ps30
Euarchonta - ps31
Primates - ps32
Haplorrhini - ps33
Simiiformes - ps34
Catarrhini - ps35
Hominoidea - ps36
Hominidae - ps37
Homininae - ps38
Hominini - ps39
Homo_sapiens - ps40
p-value
1e-02
2e-02
2e-02
3e-02
4e-02
Log-odds
0.5
1.0
1.5
2.0
2.5
3.0
3.5
4.0

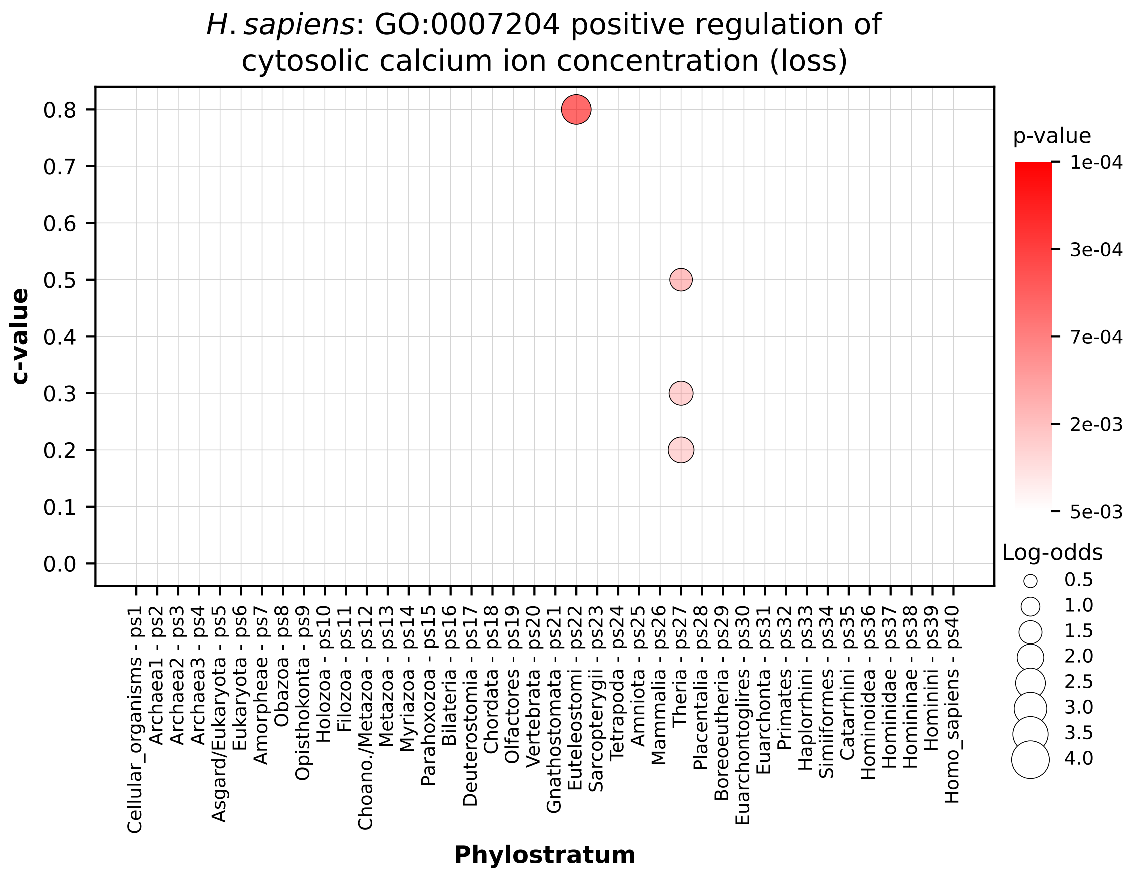

H. sapiens: GO:0007204 positive regulation of cytosolic calcium ion concentration (loss)
c-value
Phylostratum
p-value
1e-04
3e-04
7e-04
2e-03
5e-03
Log-odds
0.5
1.0
1.5
2.0
2.5
3.0
3.5
4.0
Cellular_organisms - ps1
Archaea1 - ps2
Archaea2 - ps3
Archaea3 - ps4
Asgard/Eukaryota - ps5
Eukaryota - ps6
Amorpheae - ps7
Obazoa - ps8
Opisthokonta - ps9
Holozoa - ps10
Filozoa - ps11
Choano./Metazoa - ps12
Metazoa - ps13
Myriazoa - ps14
Parahoxozoa - ps15
Bilateria - ps16
Deuterostomia - ps17
Chordata - ps18
Olfactores - ps19
Vertebrata - ps20
Gnathostomata - ps21
Euteleostomi - ps22
Sarcopterygii - ps23
Tetrapoda - ps24
Amniota - ps25
Mammalia - ps26
Theria - ps27
Placentalia - ps28
Boreoeutheria - ps29
Euarchontoglires - ps30
Euarchonta - ps31
Primates - ps32
Haplorrhini - ps33
Simiiformes - ps34
Catarrhini - ps35
Hominoidea - ps36
Hominidae - ps37
Homininae - ps38
Hominini - ps39
Homo_sapiens - ps40

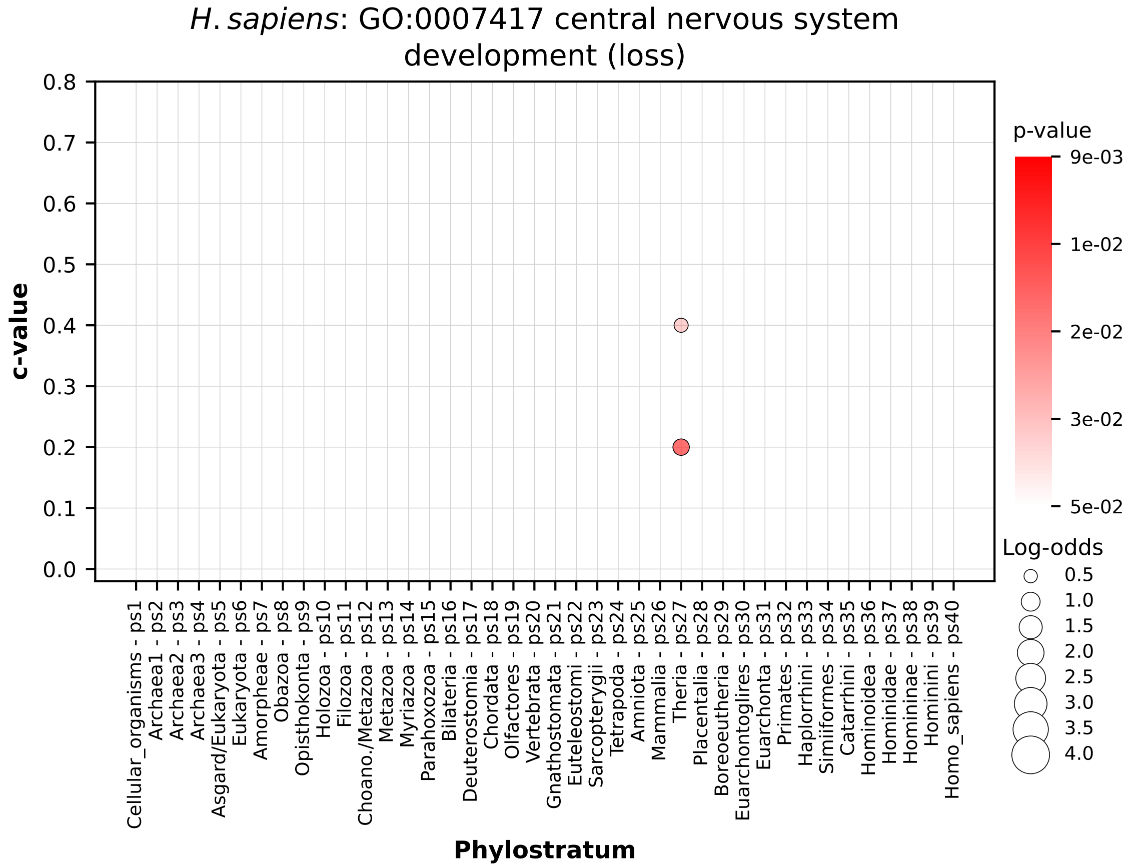
H. sapiens: GO:0007417 central nervous system development (loss)
c-value
0.8
0.7
0.6
0.5
0.4
0.3
0.2
0.1
0.0
p-value
9e-03
1e-02
2e-02
3e-02
5e-02
Log-odds
0.5
1.0
1.5
2.0
2.5
3.0
3.5
4.0
Cellular_organisms - ps1
Archaea1 - ps2
Archaea2 - ps3
Archaea3 - ps4
Asgard/Eukaryota - ps5
Eukaryota - ps6
Amorpheae - ps7
Obazoa - ps8
Opisthokonta - ps9
Holozoa - ps10
Filozoa - ps11
Choano./Metazoa - ps12
Metazoa - ps13
Myriazoa - ps14
Parahoxozoa - ps15
Bilateria - ps16
Deuterostomia - ps17
Chordata - ps18
Olfactores - ps19
Vertebrata - ps20
Gnathostomata - ps21
Euteleostomi - ps22
Sarcopterygii - ps23
Tetrapoda - ps24
Amniota - ps25
Mammalia - ps26
Theria - ps27
Placentalia - ps28
Boreoeutheria - ps29
Euarchontoglires - ps30
Euarchonta - ps31
Primates - ps32
Haplorrhini - ps33
Simiiformes - ps34
Catarrhini - ps35
Hominoidea - ps36
Hominidae - ps37
Homininae - ps38
Hominini - ps39
Homo_sapiens - ps40
Phylostratum

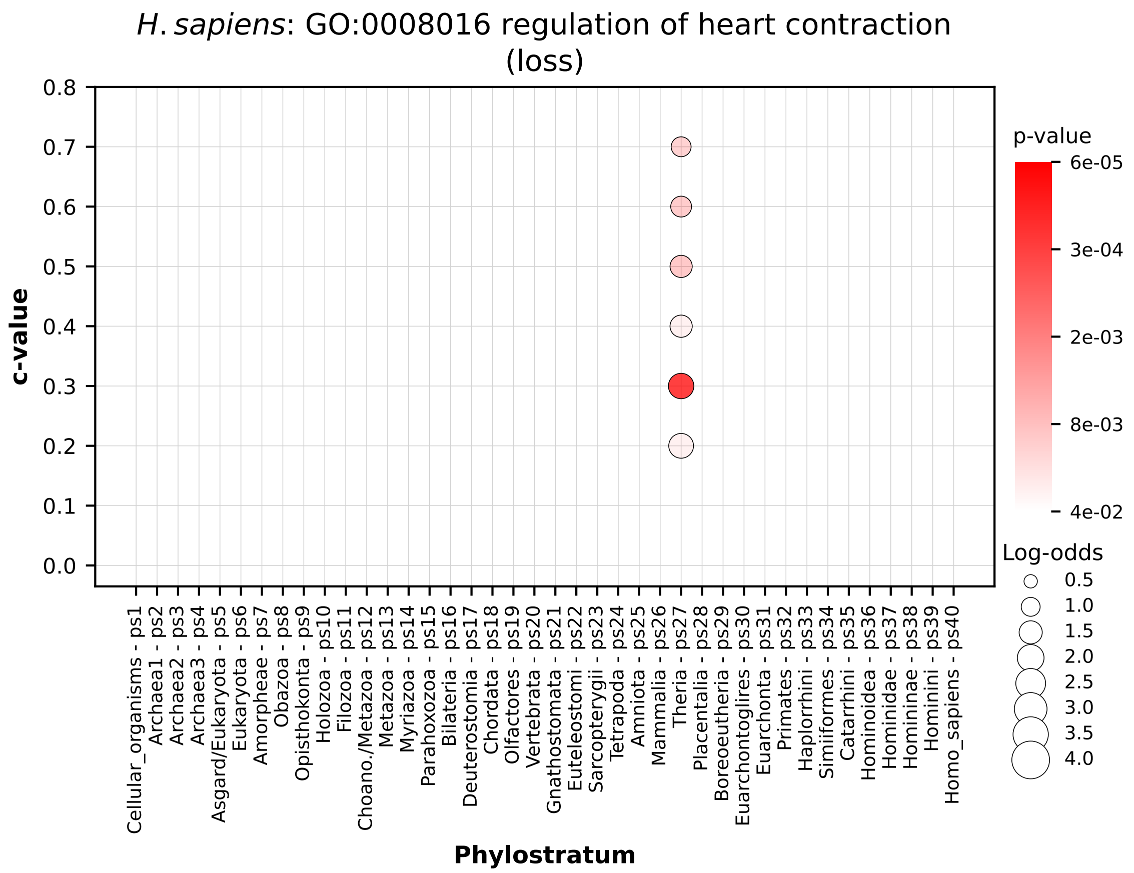

H. sapiens: GO:0008016 regulation of heart contraction (loss)
c-value
0.8
0.7
0.6
0.5
0.4
0.3
0.2
0.1
0.0
Phylostratum
Cellular_organisms - ps1
Archaea1 - ps2
Archaea2 - ps3
Archaea3 - ps4
Asgard/Eukaryota - ps5
Eukaryota - ps6
Amorpheae - ps7
Obazoa - ps8
Opisthokonta - ps9
Holozoa - ps10
Filozoa - ps11
Choano./Metazoa - ps12
Metazoa - ps13
Myriazoa - ps14
Parahoxozoa - ps15
Bilateria - ps16
Deuterostomia - ps17
Chordata - ps18
Olfactores - ps19
Vertebrata - ps20
Gnathostomata - ps21
Euteleostomi - ps22
Sarcopterygii - ps23
Tetrapoda - ps24
Amniota - ps25
Mammalia - ps26
Theria - ps27
Placentalia - ps28
Boreoeutheria - ps29
Euarchontoglires - ps30
Euarchonta - ps31
Primates - ps32
Haplorrhini - ps33
Simiiformes - ps34
Catarrhini - ps35
Hominoidea - ps36
Hominidae - ps37
Homininae - ps38
Hominini - ps39
Homo_sapiens - ps40
p-value
6e-05
3e-04
2e-03
8e-03
4e-02
Log-odds
0.5
1.0
1.5
2.0
2.5
3.0
3.5
4.0

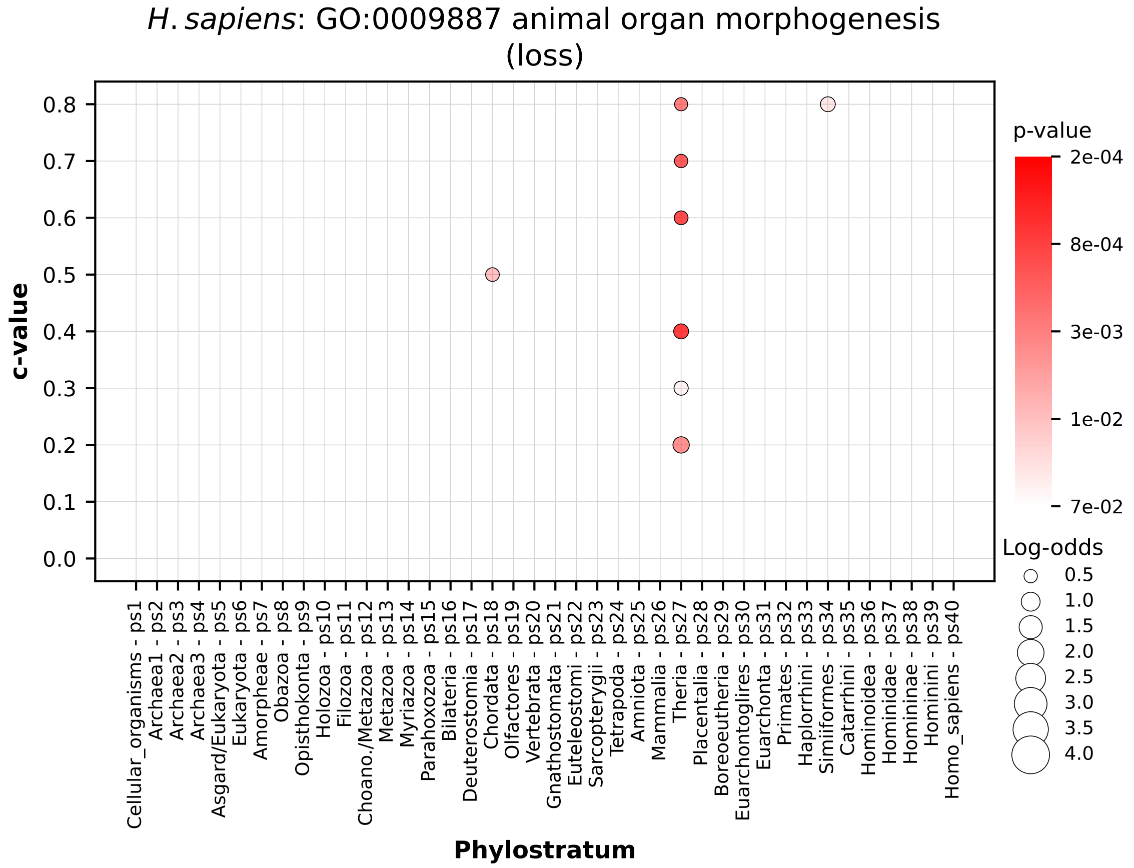

H. sapiens: GO:0009887 animal organ morphogenesis (loss)
c-value
0.0
0.1
0.2
0.3
0.4
0.5
0.6
0.7
0.8
Phylostratum
Cellular_organisms - ps1
Archaea1 - ps2
Archaea2 - ps3
Archaea3 - ps4
Asgard/Eukaryota - ps5
Eukaryota - ps6
Amorpheae - ps7
Obazoa - ps8
Opisthokonta - ps9
Holozoa - ps10
Filozoa - ps11
Choano./Metazoa - ps12
Metazoa - ps13
Myriazoa - ps14
Parahoxozoa - ps15
Bilateria - ps16
Deuterostomia - ps17
Chordata - ps18
Olfactores - ps19
Vertebrata - ps20
Gnathostomata - ps21
Euteleostomi - ps22
Sarcopterygii - ps23
Tetrapoda - ps24
Amniota - ps25
Mammalia - ps26
Theria - ps27
Placentalia - ps28
Boreoeutheria - ps29
Euarchontoglires - ps30
Euarchonta - ps31
Primates - ps32
Haplorrhini - ps33
Simiiformes - ps34
Catarrhini - ps35
Hominoidea - ps36
Hominidae - ps37
Homininae - ps38
Hominini - ps39
Homo_sapiens - ps40
p-value
2e-04
8e-04
3e-03
1e-02
7e-02
Log-odds
0.5
1.0
1.5
2.0
2.5
3.0
3.5
4.0

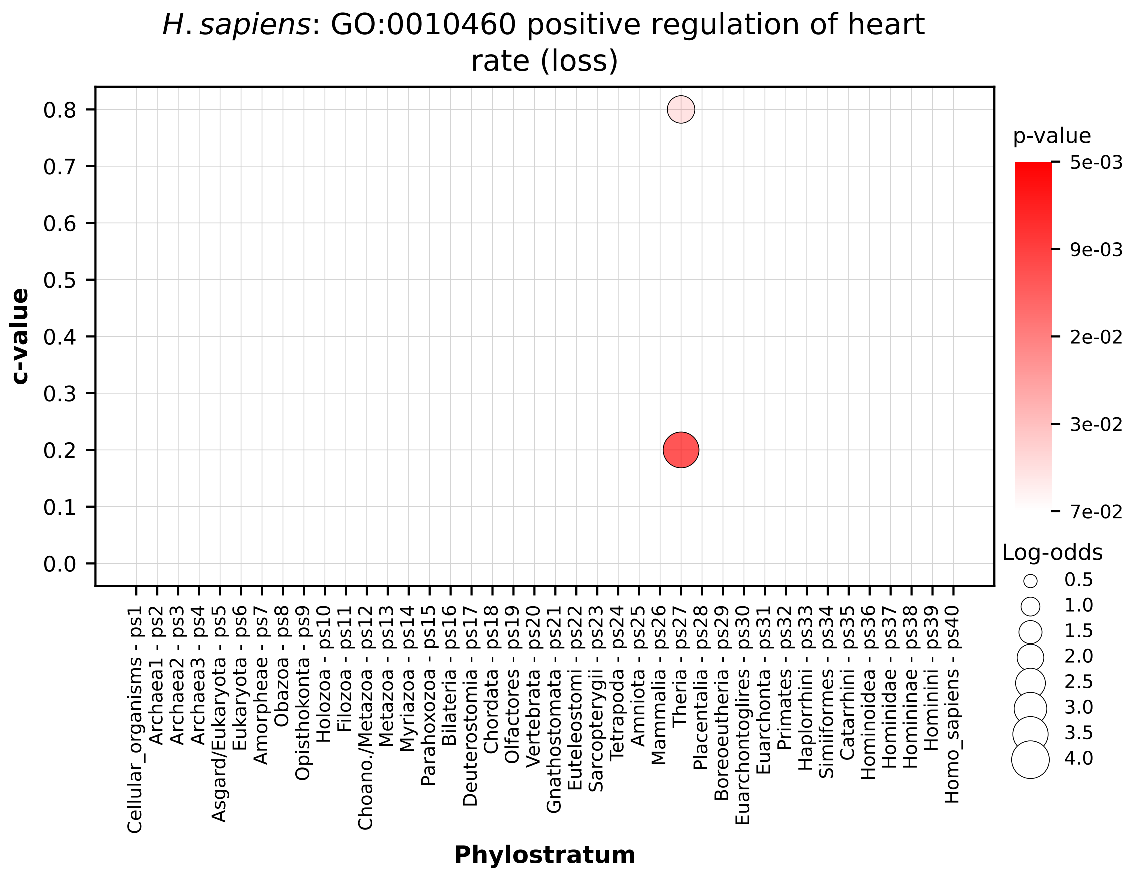

H. sapiens: GO:0010460 positive regulation of heart rate (loss)
c-value
0.0
0.1
0.2
0.3
0.4
0.5
0.6
0.7
0.8
Phylostratum
Cellular_organisms - ps1
Archaea1 - ps2
Archaea2 - ps3
Archaea3 - ps4
Asgard/Eukaryota - ps5
Eukaryota - ps6
Amorpheae - ps7
Obazoa - ps8
Opisthokonta - ps9
Holozoa - ps10
Filozoa - ps11
Choano./Metazoa - ps12
Metazoa - ps13
Myriazoa - ps14
Parahoxozoa - ps15
Bilateria - ps16
Deuterostomia - ps17
Chordata - ps18
Olfactores - ps19
Vertebrata - ps20
Gnathostomata - ps21
Euteleostomi - ps22
Sarcopterygii - ps23
Tetrapoda - ps24
Amniota - ps25
Mammalia - ps26
Theria - ps27
Placentalia - ps28
Boreoeutheria - ps29
Euarchontoglires - ps30
Euarchonta - ps31
Primates - ps32
Haplorrhini - ps33
Simiiformes - ps34
Catarrhini - ps35
Hominoidea - ps36
Hominidae - ps37
Homininae - ps38
Hominini - ps39
Homo_sapiens - ps40
p-value
5e-03
9e-03
2e-02
3e-02
7e-02
Log-odds
0.5
1.0
1.5
2.0
2.5
3.0
3.5
4.0

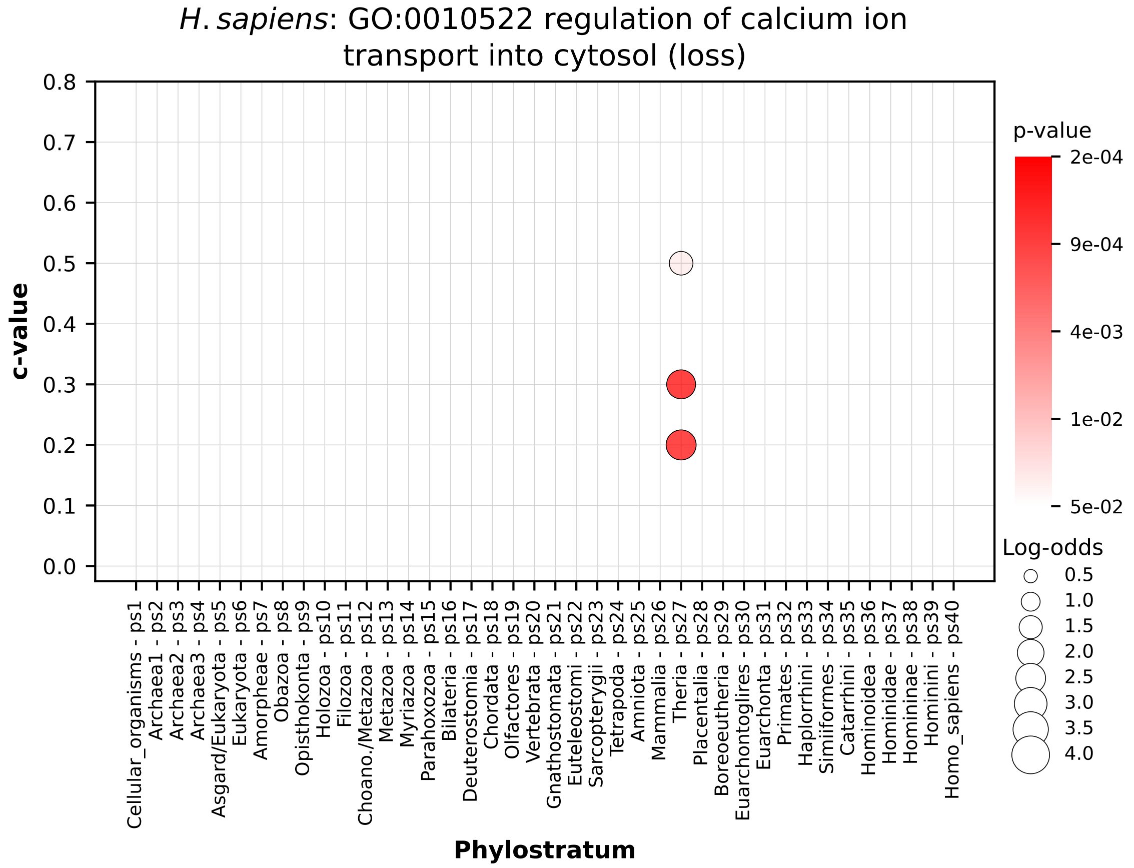

H. sapiens: GO:0010522 regulation of calcium ion transport into cytosol (loss)
c-value
0.8
0.7
0.6
0.5
0.4
0.3
0.2
0.1
0.0
Phylostratum
Cellular_organisms - ps1
Archaea1 - ps2
Archaea2 - ps3
Archaea3 - ps4
Asgard/Eukaryota - ps5
Eukaryota - ps6
Amorpheae - ps7
Obazoa - ps8
Opisthokonta - ps9
Holozoa - ps10
Filozoa - ps11
Choano./Metazoa - ps12
Metazoa - ps13
Myriazoa - ps14
Parahoxozoa - ps15
Bilateria - ps16
Deuterostomia - ps17
Chordata - ps18
Olfactores - ps19
Vertebrata - ps20
Gnathostomata - ps21
Euteleostomi - ps22
Sarcopterygii - ps23
Tetrapoda - ps24
Amniota - ps25
Mammalia - ps26
Theria - ps27
Placentalia - ps28
Boreoeutheria - ps29
Euarchontoglires - ps30
Euarchonta - ps31
Primates - ps32
Haplorrhini - ps33
Simiiformes - ps34
Catarrhini - ps35
Hominoidea - ps36
Hominidae - ps37
Homininae - ps38
Hominini - ps39
Homo_sapiens - ps40
p-value
2e-04
9e-04
4e-03
1e-02
5e-02
Log-odds
0.5
1.0
1.5
2.0
2.5
3.0
3.5
4.0

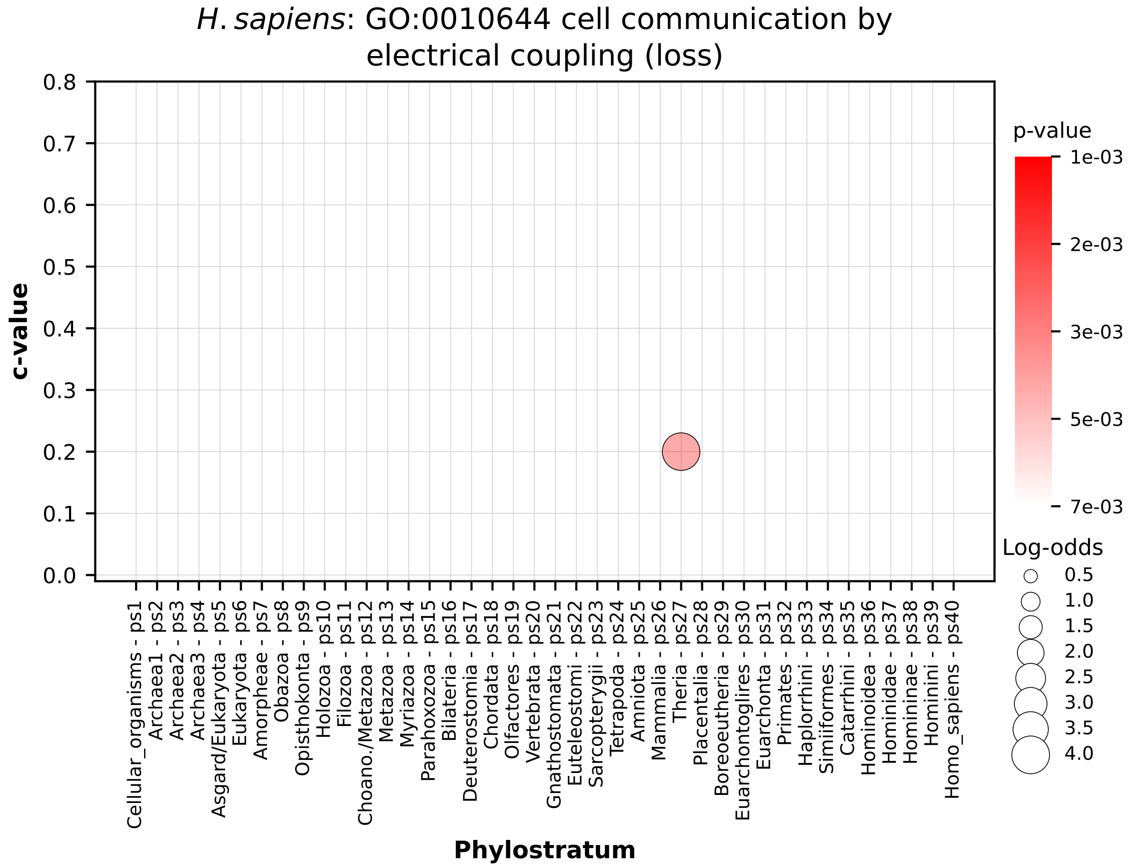

H. sapiens: GO:0010644 cell communication by electrical coupling (loss)
c-value
0.0
0.1
0.2
0.3
0.4
0.5
0.6
0.7
0.8
Phylostratum
Cellular_organisms - ps1
Archaea1 - ps2
Archaea2 - ps3
Archaea3 - ps4
Asgard/Eukaryota - ps5
Eukaryota - ps6
Amorpheae - ps7
Obazoa - ps8
Opisthokonta - ps9
Holozoa - ps10
Filozoa - ps11
Choano./Metazoa - ps12
Metazoa - ps13
Myriazoa - ps14
Parahoxozoa - ps15
Bilateria - ps16
Deuterostomia - ps17
Chordata - ps18
Olfactores - ps19
Vertebrata - ps20
Gnathostomata - ps21
Euteleostomi - ps22
Sarcopterygii - ps23
Tetrapoda - ps24
Amniota - ps25
Mammalia - ps26
Theria - ps27
Placentalia - ps28
Boreoeutheria - ps29
Euarchontoglires - ps30
Euarchonta - ps31
Primates - ps32
Haplorrhini - ps33
Simiiformes - ps34
Catarrhini - ps35
Hominoidea - ps36
Hominidae - ps37
Homininae - ps38
Hominini - ps39
Homo_sapiens - ps40
p-value
1e-03
2e-03
3e-03
5e-03
7e-03
Log-odds
0.5
1.0
1.5
2.0
2.5
3.0
3.5
4.0

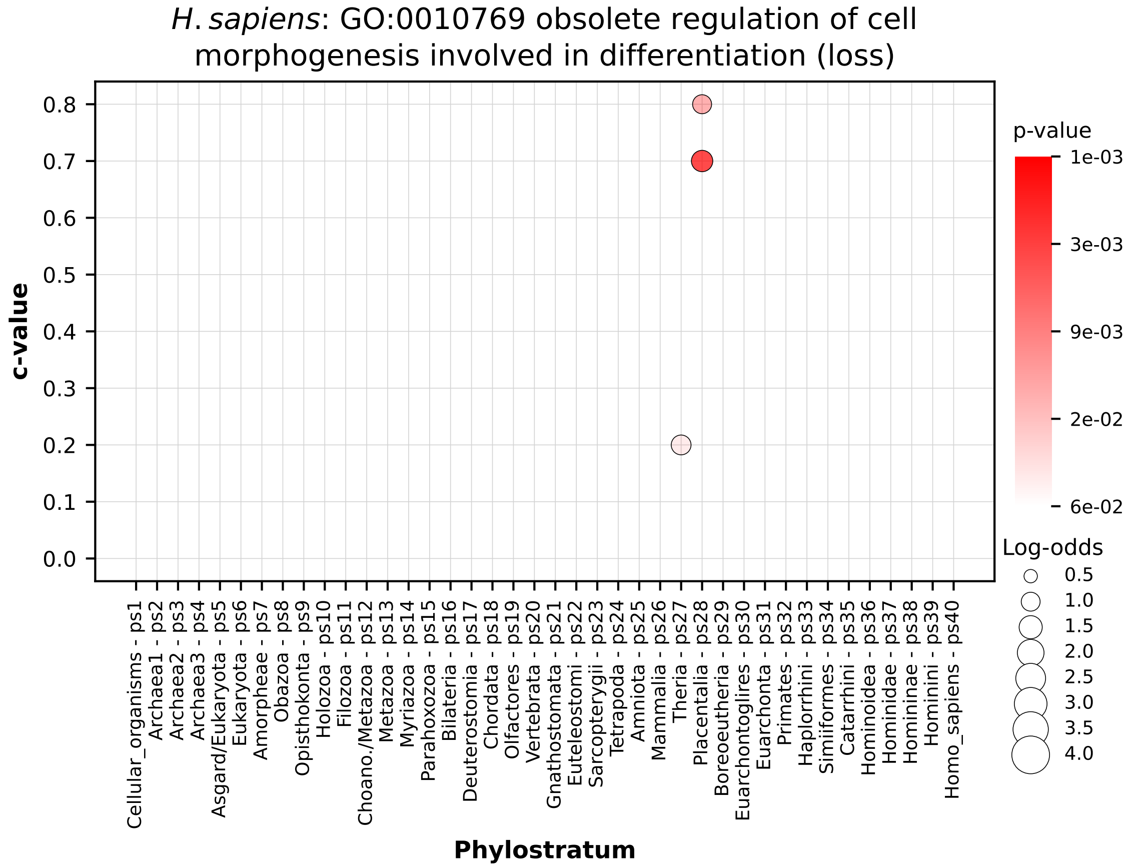

H. sapiens: GO:0010769 obsolete regulation of cell morphogenesis involved in differentiation (loss)
c-value
0.0
0.1
0.2
0.3
0.4
0.5
0.6
0.7
0.8
Phylostratum
Cellular_organisms - ps1
Archaea1 - ps2
Archaea2 - ps3
Archaea3 - ps4
Asgard/Eukaryota - ps5
Eukaryota - ps6
Amorpheae - ps7
Obazoa - ps8
Opisthokonta - ps9
Holozoa - ps10
Filozoa - ps11
Choano./Metazoa - ps12
Metazoa - ps13
Myriazoa - ps14
Parahoxozoa - ps15
Bilateria - ps16
Deuterostomia - ps17
Chordata - ps18
Olfactores - ps19
Vertebrata - ps20
Gnathostomata - ps21
Euteleostomi - ps22
Sarcopterygii - ps23
Tetrapoda - ps24
Amniota - ps25
Mammalia - ps26
Theria - ps27
Placentalia - ps28
Boreoeutheria - ps29
Euarchontoglires - ps30
Euarchonta - ps31
Primates - ps32
Haplorrhini - ps33
Simiiformes - ps34
Catarrhini - ps35
Hominoidea - ps36
Hominidae - ps37
Homininae - ps38
Hominini - ps39
Homo_sapiens - ps40
p-value
1e-03
3e-03
9e-03
2e-02
6e-02
Log-odds
0.5
1.0
1.5
2.0
2.5
3.0
3.5
4.0

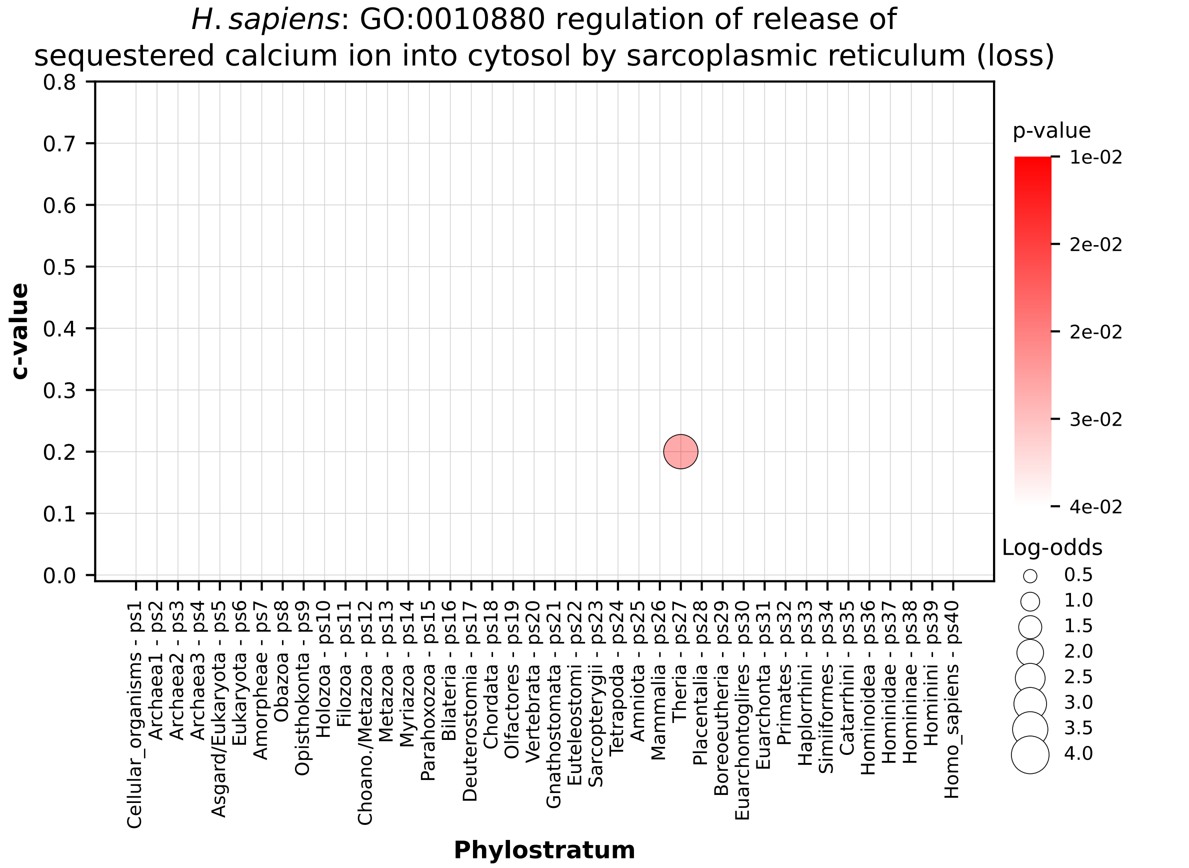
H. sapiens: GO:0010880 regulation of release of sequestered calcium ion into cytosol by sarcoplasmic reticulum (loss)
c-value
0.0
0.1
0.2
0.3
0.4
0.5
0.6
0.7
0.8
Phylostratum
Cellular_organisms - ps1
Archaea1 - ps2
Archaea2 - ps3
Archaea3 - ps4
Asgard/Eukaryota - ps5
Eukaryota - ps6
Amorpheae - ps7
Obazoa - ps8
Opisthokonta - ps9
Holozoa - ps10
Filozoa - ps11
Choano./Metazoa - ps12
Metazoa - ps13
Myriazoa - ps14
Parahoxozoa - ps15
Bilateria - ps16
Deuterostomia - ps17
Chordata - ps18
Olfactores - ps19
Vertebrata - ps20
Gnathostomata - ps21
Euteleostomi - ps22
Sarcopterygii - ps23
Tetrapoda - ps24
Amniota - ps25
Mammalia - ps26
Theria - ps27
Placentalia - ps28
Boreoeutheria - ps29
Euarchontoglires - ps30
Euarchonta - ps31
Primates - ps32
Haplorrhini - ps33
Simiiformes - ps34
Catarrhini - ps35
Hominoidea - ps36
Hominidae - ps37
Homininae - ps38
Hominini - ps39
Homo_sapiens - ps40
p-value
1e-02
2e-02
2e-02
3e-02
4e-02
Log-odds
0.5
1.0
1.5
2.0
2.5
3.0
3.5
4.0

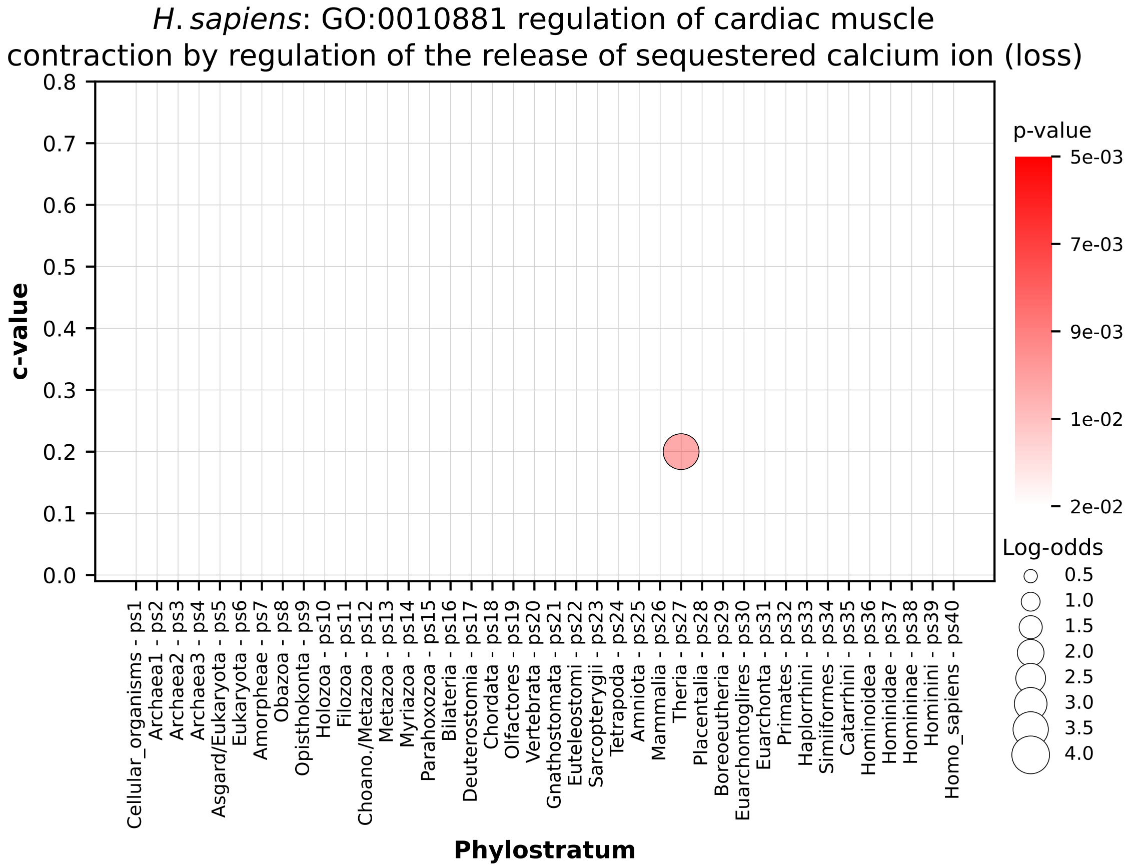

H. sapiens: GO:0010881 regulation of cardiac muscle contraction by regulation of the release of sequestered calcium ion (loss)
c-value
0.8
0.7
0.6
0.5
0.4
0.3
0.2
0.1
0.0
Phylostratum
Cellular_organisms - ps1
Archaea1 - ps2
Archaea2 - ps3
Archaea3 - ps4
Asgard/Eukaryota - ps5
Eukaryota - ps6
Amorpheae - ps7
Obazoa - ps8
Opisthokonta - ps9
Holozoa - ps10
Filozoa - ps11
Choano./Metazoa - ps12
Metazoa - ps13
Myriazoa - ps14
Parahoxozoa - ps15
Bilateria - ps16
Deuterostomia - ps17
Chordata - ps18
Olfactores - ps19
Vertebrata - ps20
Gnathostomata - ps21
Euteleostomi - ps22
Sarcopterygii - ps23
Tetrapoda - ps24
Amniota - ps25
Mammalia - ps26
Theria - ps27
Placentalia - ps28
Boreoeutheria - ps29
Euarchontoglires - ps30
Euarchonta - ps31
Primates - ps32
Haplorrhini - ps33
Simiiformes - ps34
Catarrhini - ps35
Hominoidea - ps36
Hominidae - ps37
Homininae - ps38
Hominini - ps39
Homo_sapiens - ps40
p-value
5e-03
7e-03
9e-03
1e-02
2e-02
Log-odds
0.5
1.0
1.5
2.0
2.5
3.0
3.5
4.0

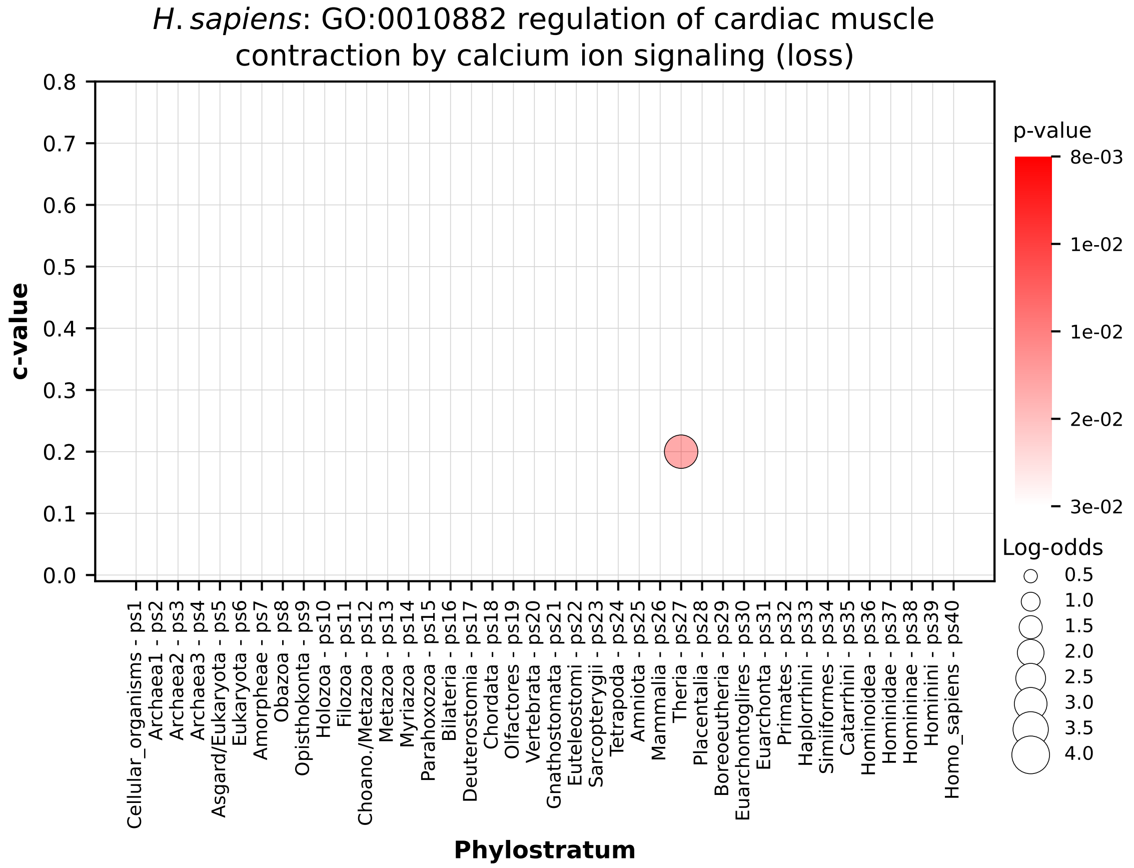

H. sapiens: GO:0010882 regulation of cardiac muscle contraction by calcium ion signaling (loss)
c-value
0.8
0.7
0.6
0.5
0.4
0.3
0.2
0.1
0.0
Phylostratum
Cellular_organisms - ps1
Archaea1 - ps2
Archaea2 - ps3
Archaea3 - ps4
Asgard/Eukaryota - ps5
Eukaryota - ps6
Amorpheae - ps7
Obazoa - ps8
Opisthokonta - ps9
Holozoa - ps10
Filozoa - ps11
Choano./Metazoa - ps12
Metazoa - ps13
Myriazoa - ps14
Parahoxozoa - ps15
Bilateria - ps16
Deuterostomia - ps17
Chordata - ps18
Olfactores - ps19
Vertebrata - ps20
Gnathostomata - ps21
Euteleostomi - ps22
Sarcopterygii - ps23
Tetrapoda - ps24
Amniota - ps25
Mammalia - ps26
Theria - ps27
Placentalia - ps28
Boreoeutheria - ps29
Euarchontoglires - ps30
Euarchonta - ps31
Primates - ps32
Haplorrhini - ps33
Simiiformes - ps34
Catarrhini - ps35
Hominoidea - ps36
Hominidae - ps37
Homininae - ps38
Hominini - ps39
Homo_sapiens - ps40
p-value
8e-03
1e-02
1e-02
2e-02
3e-02
Log-odds
0.5
1.0
1.5
2.0
2.5
3.0
3.5
4.0

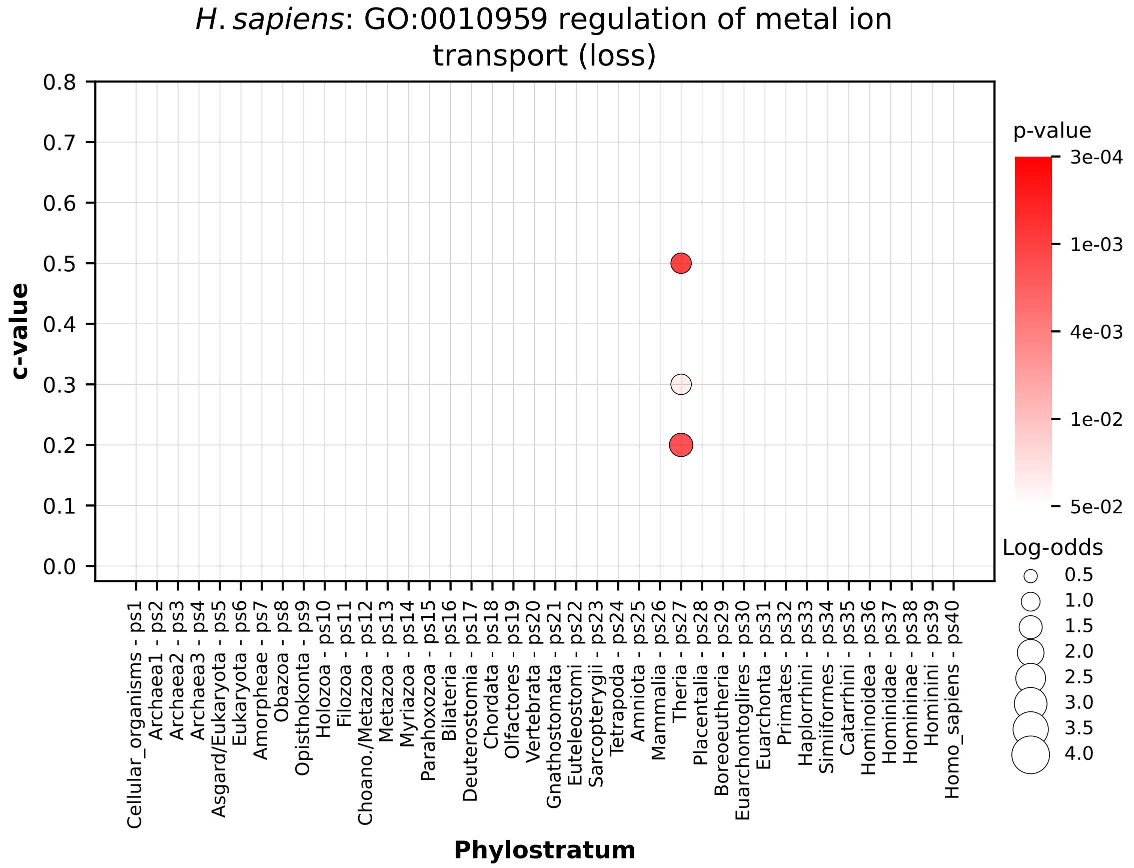

H. sapiens: GO:0010959 regulation of metal ion transport (loss)
c-value
0.8
0.7
0.6
0.5
0.4
0.3
0.2
0.1
0.0
Phylostratum
Cellular_organisms - ps1
Archaea1 - ps2
Archaea2 - ps3
Archaea3 - ps4
Asgard/Eukaryota - ps5
Eukaryota - ps6
Amorpheae - ps7
Obazoa - ps8
Opisthokonta - ps9
Holozoa - ps10
Filozoa - ps11
Choano./Metazoa - ps12
Metazoa - ps13
Myriazoa - ps14
Parahoxozoa - ps15
Bilateria - ps16
Deuterostomia - ps17
Chordata - ps18
Olfactores - ps19
Vertebrata - ps20
Gnathostomata - ps21
Euteleostomi - ps22
Sarcopterygii - ps23
Tetrapoda - ps24
Amniota - ps25
Mammalia - ps26
Theria - ps27
Placentalia - ps28
Boreoeutheria - ps29
Euarchontoglires - ps30
Euarchonta - ps31
Primates - ps32
Haplorrhini - ps33
Simiiformes - ps34
Catarrhini - ps35
Hominoidea - ps36
Hominidae - ps37
Homininae - ps38
Hominini - ps39
Homo_sapiens - ps40
p-value
3e-04
1e-03
4e-03
1e-02
5e-02
Log-odds
0.5
1.0
1.5
2.0
2.5
3.0
3.5
4.0

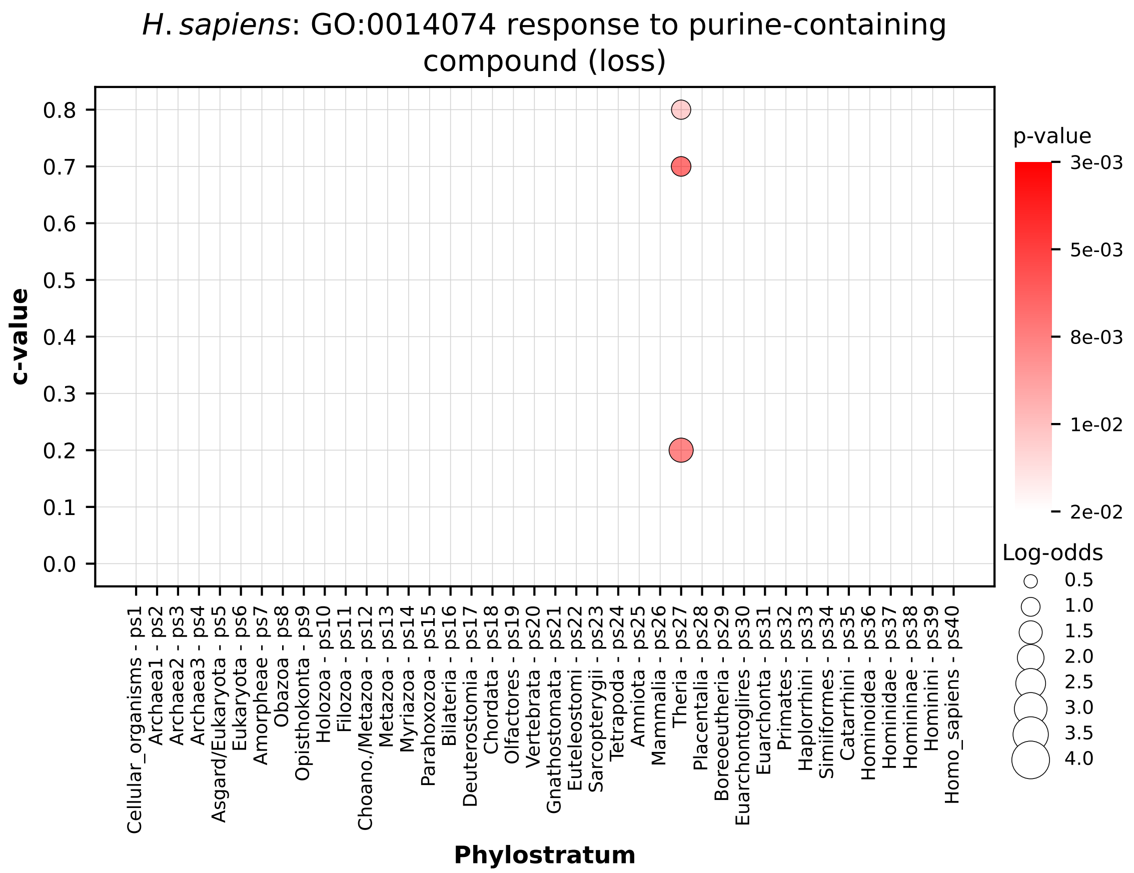

H. sapiens: GO:0014074 response to purine-containing compound (loss)
c-value
0.8
0.7
0.6
0.5
0.4
0.3
0.2
0.1
0.0
p-value
3e-03
5e-03
8e-03
1e-02
2e-02
Log-odds
0.5
1.0
1.5
2.0
2.5
3.0
3.5
4.0
Cellular_organisms - ps1
Archaea1 - ps2
Archaea2 - ps3
Archaea3 - ps4
Asgard/Eukaryota - ps5
Eukaryota - ps6
Amorpheae - ps7
Obazoa - ps8
Opisthokonta - ps9
Holozoa - ps10
Filozoa - ps11
Choano./Metazoa - ps12
Metazoa - ps13
Myriazoa - ps14
Parahoxozoa - ps15
Bilateria - ps16
Deuterostomia - ps17
Chordata - ps18
Olfactores - ps19
Vertebrata - ps20
Gnathostomata - ps21
Euteleostomi - ps22
Sarcopterygii - ps23
Tetrapoda - ps24
Amniota - ps25
Mammalia - ps26
Theria - ps27
Placentalia - ps28
Boreoeutheria - ps29
Euarchontoglires - ps30
Euarchonta - ps31
Primates - ps32
Haplorrhini - ps33
Simiiformes - ps34
Catarrhini - ps35
Hominoidea - ps36
Hominidae - ps37
Homininae - ps38
Hominini - ps39
Homo_sapiens - ps40
Phylostratum

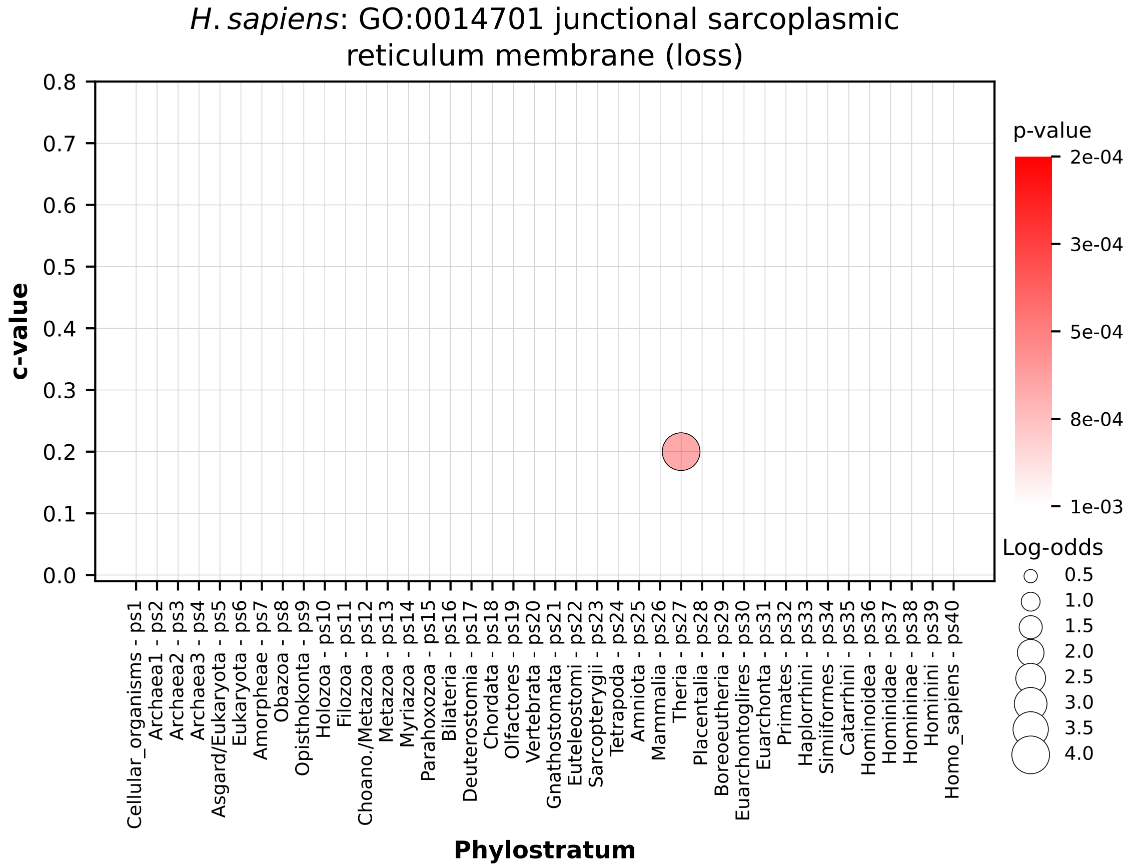

H. sapiens: GO:0014701 junctional sarcoplasmic reticulum membrane (loss)
c-value
0.8
0.7
0.6
0.5
0.4
0.3
0.2
0.1
0.0
p-value
2e-04
3e-04
5e-04
8e-04
1e-03
Log-odds
0.5
1.0
1.5
2.0
2.5
3.0
3.5
4.0
Cellular_organisms - ps1
Archaea1 - ps2
Archaea2 - ps3
Archaea3 - ps4
Asgard/Eukaryota - ps5
Eukaryota - ps6
Amorpheae - ps7
Obazoa - ps8
Opisthokonta - ps9
Holozoa - ps10
Filozoa - ps11
Choano./Metazoa - ps12
Metazoa - ps13
Myriazoa - ps14
Parahoxozoa - ps15
Bilateria - ps16
Deuterostomia - ps17
Chordata - ps18
Olfactores - ps19
Vertebrata - ps20
Gnathostomata - ps21
Euteleostomi - ps22
Sarcopterygii - ps23
Tetrapoda - ps24
Amniota - ps25
Mammalia - ps26
Theria - ps27
Placentalia - ps28
Boreoeutheria - ps29
Euarchontoglires - ps30
Euarchonta - ps31
Primates - ps32
Haplorrhini - ps33
Simiiformes - ps34
Catarrhini - ps35
Hominoidea - ps36
Hominidae - ps37
Homininae - ps38
Hominini - ps39
Homo_sapiens - ps40
Phylostratum

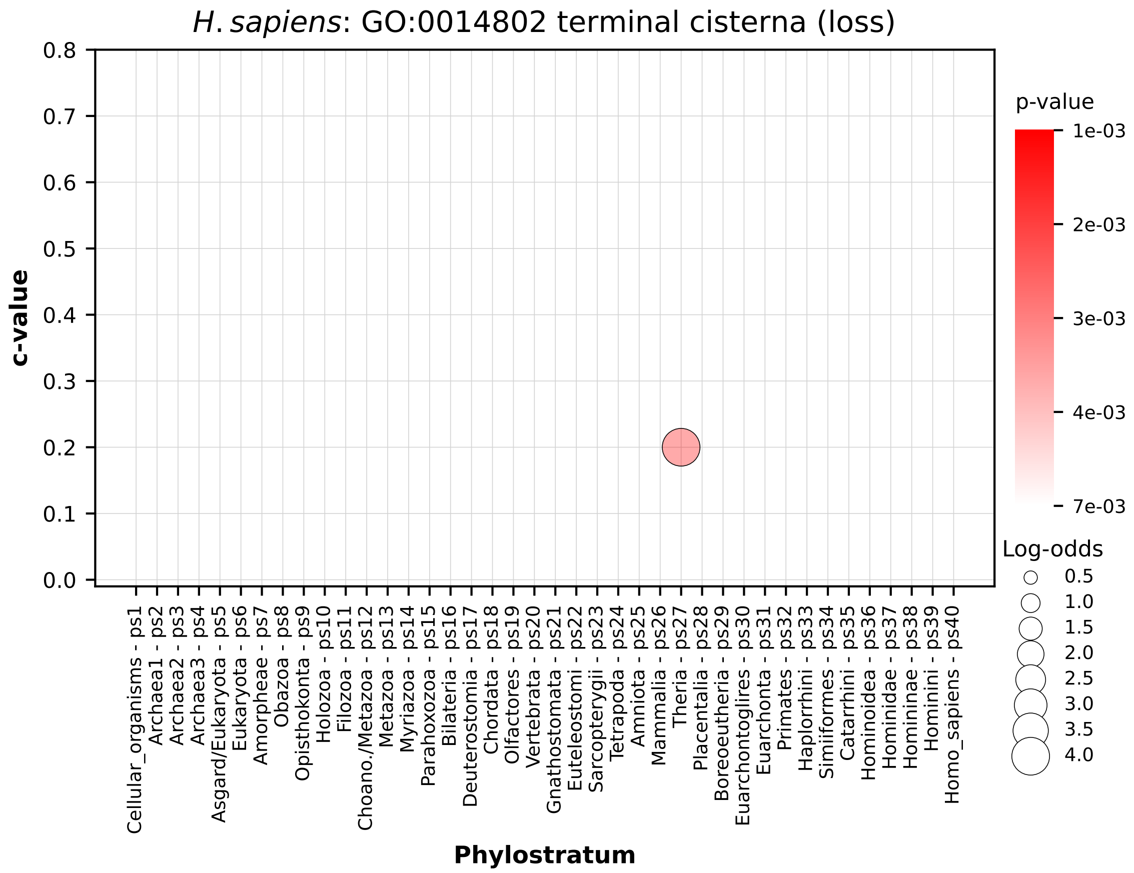

H. sapiens: GO:0014802 terminal cisterna (loss)
c-value
0.0
0.1
0.2
0.3
0.4
0.5
0.6
0.7
0.8
Phylostratum
Cellular_organisms - ps1
Archaea1 - ps2
Archaea2 - ps3
Archaea3 - ps4
Asgard/Eukaryota - ps5
Eukaryota - ps6
Amorpheae - ps7
Obazoa - ps8
Opisthokonta - ps9
Holozoa - ps10
Filozoa - ps11
Choano./Metazoa - ps12
Metazoa - ps13
Myriazoa - ps14
Parahoxozoa - ps15
Bilateria - ps16
Deuterostomia - ps17
Chordata - ps18
Olfactores - ps19
Vertebrata - ps20
Gnathostomata - ps21
Euteleostomi - ps22
Sarcopterygii - ps23
Tetrapoda - ps24
Amniota - ps25
Mammalia - ps26
Theria - ps27
Placentalia - ps28
Boreoeutheria - ps29
Euarchontoglires - ps30
Euarchonta - ps31
Primates - ps32
Haplorrhini - ps33
Simiiformes - ps34
Catarrhini - ps35
Hominoidea - ps36
Hominidae - ps37
Homininae - ps38
Hominini - ps39
Homo_sapiens - ps40
p-value
1e-03
2e-03
3e-03
4e-03
7e-03
Log-odds
0.5
1.0
1.5
2.0
2.5
3.0
3.5
4.0

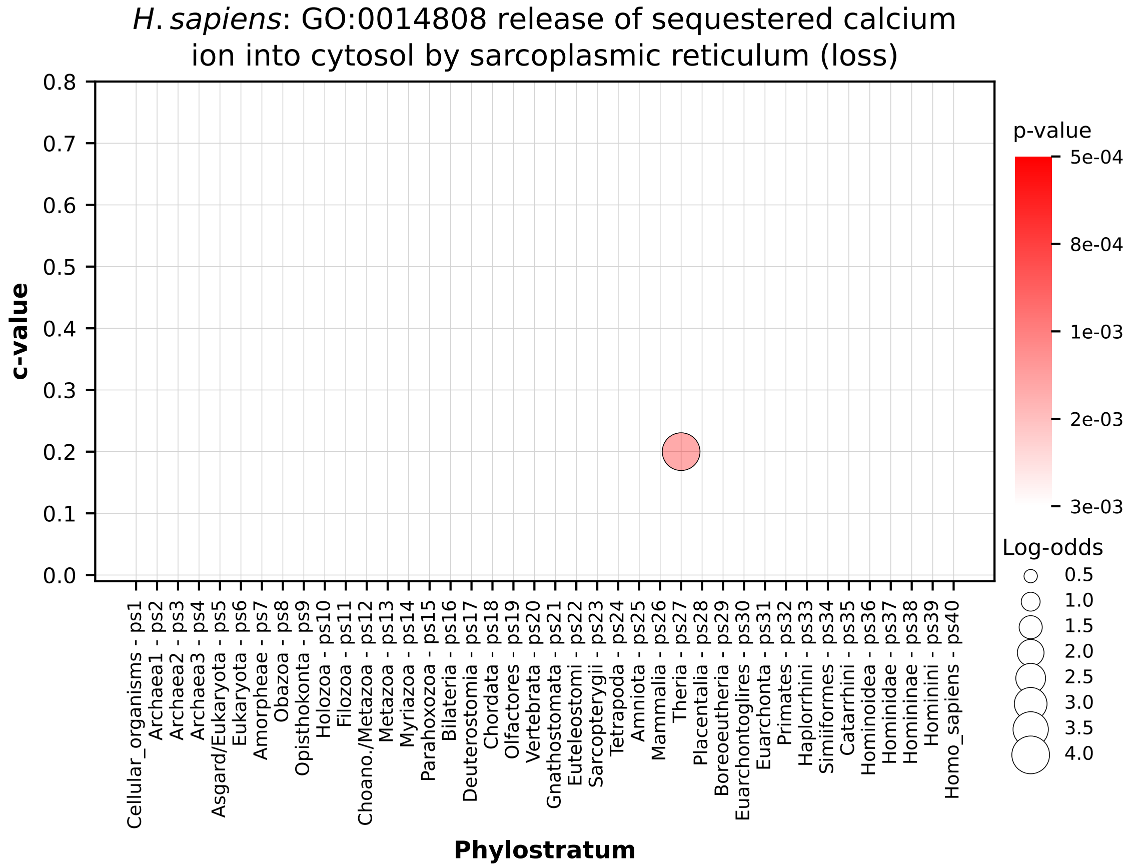

H. sapiens: GO:0014808 release of sequestered calcium ion into cytosol by sarcoplasmic reticulum (loss)
c-value
0.0
0.1
0.2
0.3
0.4
0.5
0.6
0.7
0.8
Phylostratum
Cellular_organisms - ps1
Archaea1 - ps2
Archaea2 - ps3
Archaea3 - ps4
Asgard/Eukaryota - ps5
Eukaryota - ps6
Amorpheae - ps7
Obazoa - ps8
Opisthokonta - ps9
Holozoa - ps10
Filozoa - ps11
Choano./Metazoa - ps12
Metazoa - ps13
Myriazoa - ps14
Parahoxozoa - ps15
Bilateria - ps16
Deuterostomia - ps17
Chordata - ps18
Olfactores - ps19
Vertebrata - ps20
Gnathostomata - ps21
Euteleostomi - ps22
Sarcopterygii - ps23
Tetrapoda - ps24
Amniota - ps25
Mammalia - ps26
Theria - ps27
Placentalia - ps28
Boreoeutheria - ps29
Euarchontoglires - ps30
Euarchonta - ps31
Primates - ps32
Haplorrhini - ps33
Simiiformes - ps34
Catarrhini - ps35
Hominoidea - ps36
Hominidae - ps37
Homininae - ps38
Hominini - ps39
Homo_sapiens - ps40
p-value
5e-04
8e-04
1e-03
2e-03
3e-03
Log-odds
0.5
1.0
1.5
2.0
2.5
3.0
3.5
4.0

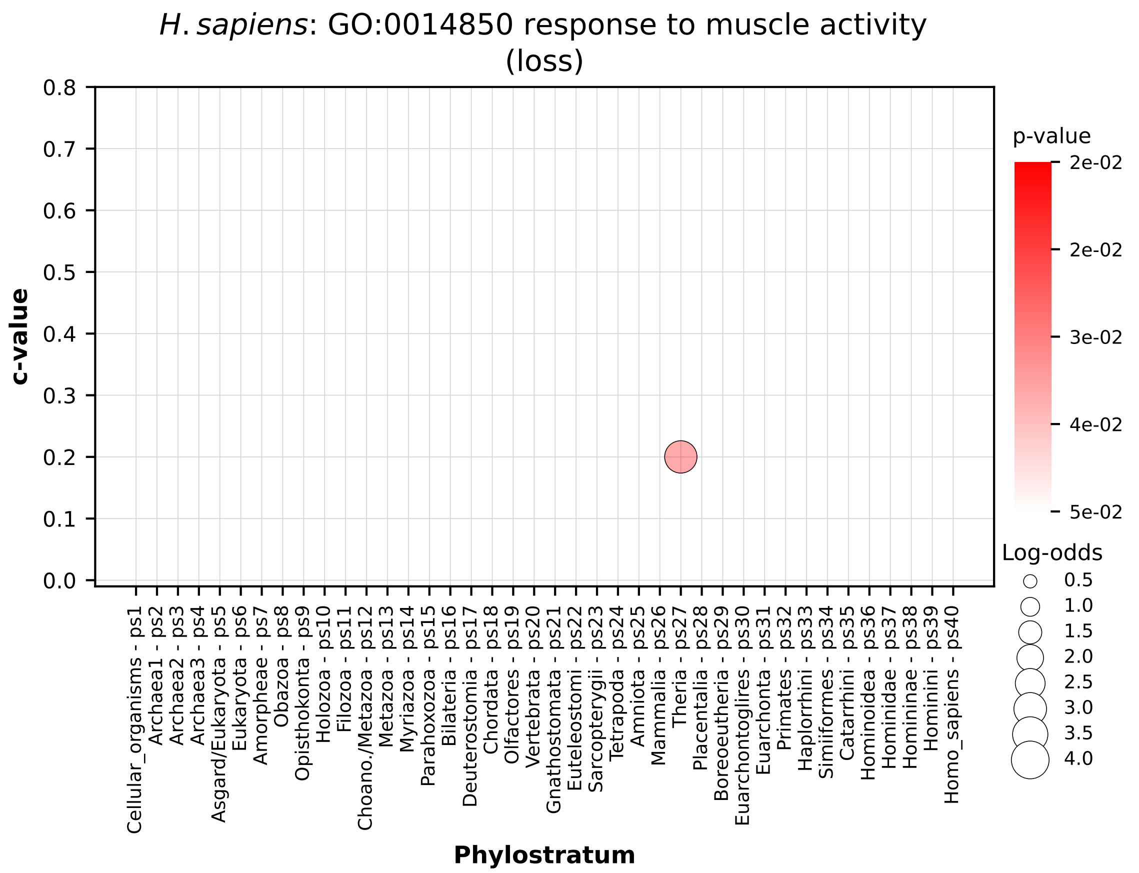
H. sapiens: GO:0014850 response to muscle activity
(loss)
c-value
0.0
0.1
0.2
0.3
0.4
0.5
0.6
0.7
0.8
Phylostratum
Cellular_organisms - ps1
Archaea1 - ps2
Archaea2 - ps3
Archaea3 - ps4
Asgard/Eukaryota - ps5
Eukaryota - ps6
Amorpheae - ps7
Obazoa - ps8
Opisthokonta - ps9
Holozoa - ps10
Filozoa - ps11
Choano./Metazoa - ps12
Metazoa - ps13
Myriazoa - ps14
Parahoxozoa - ps15
Bilateria - ps16
Deuterostomia - ps17
Chordata - ps18
Olfactores - ps19
Vertebrata - ps20
Gnathostomata - ps21
Euteleostomi - ps22
Sarcopterygii - ps23
Tetrapoda - ps24
Amniota - ps25
Mammalia - ps26
Theria - ps27
Placentalia - ps28
Boreoeutheria - ps29
Euarchontoglires - ps30
Euarchonta - ps31
Primates - ps32
Haplorrhini - ps33
Simiiformes - ps34
Catarrhini - ps35
Hominoidea - ps36
Hominidae - ps37
Homininae - ps38
Hominini - ps39
Homo_sapiens - ps40
p-value
2e-02
2e-02
3e-02
4e-02
5e-02
Log-odds
0.5
1.0
1.5
2.0
2.5
3.0
3.5
4.0

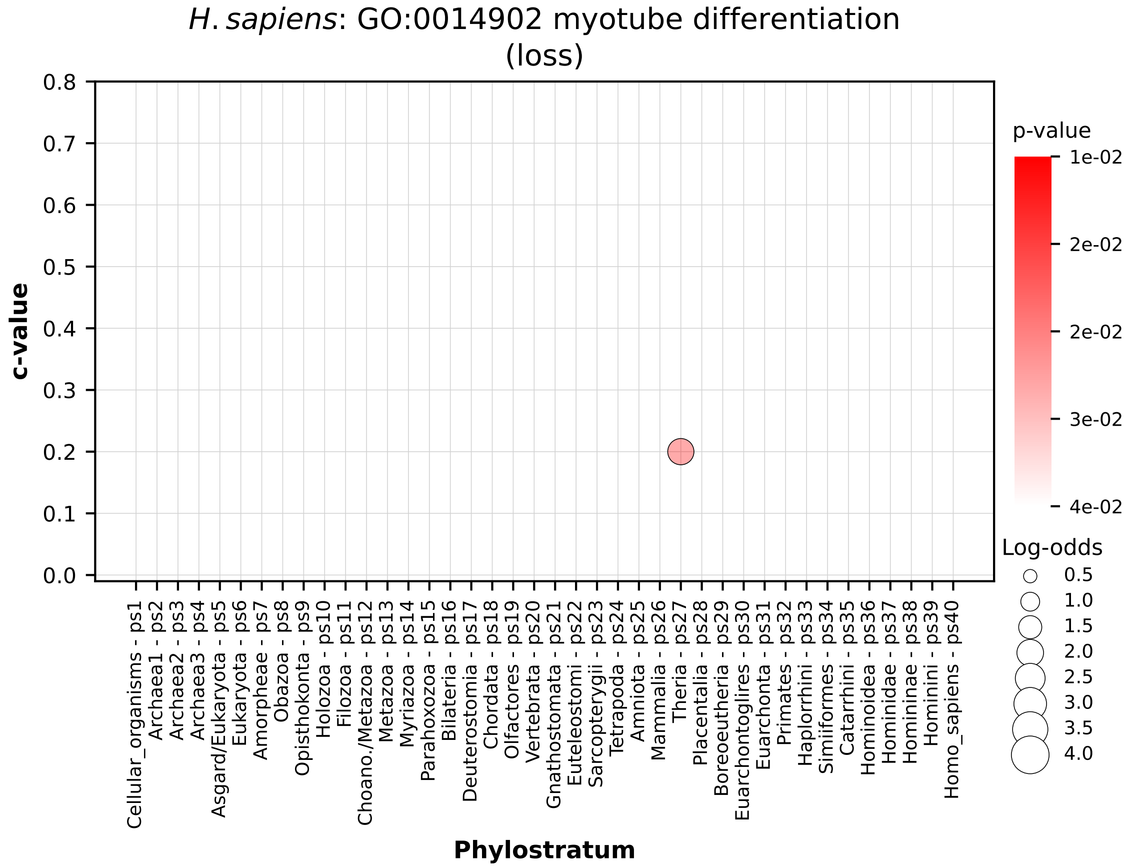

H. sapiens: GO:0014902 myotube differentiation
(loss)
c-value
0.8
0.7
0.6
0.5
0.4
0.3
0.2
0.1
0.0
p-value
1e-02
2e-02
2e-02
3e-02
4e-02
Log-odds
0.5
1.0
1.5
2.0
2.5
3.0
3.5
4.0
Cellular_organisms - ps1
Archaea1 - ps2
Archaea2 - ps3
Archaea3 - ps4
Asgard/Eukaryota - ps5
Eukaryota - ps6
Amorpheae - ps7
Obazoa - ps8
Opisthokonta - ps9
Holozoa - ps10
Filozoa - ps11
Choano./Metazoa - ps12
Metazoa - ps13
Myriazoa - ps14
Parahoxozoa - ps15
Bilateria - ps16
Deuterostomia - ps17
Chordata - ps18
Olfactores - ps19
Vertebrata - ps20
Gnathostomata - ps21
Euteleostomi - ps22
Sarcopterygii - ps23
Tetrapoda - ps24
Amniota - ps25
Mammalia - ps26
Theria - ps27
Placentalia - ps28
Boreoeutheria - ps29
Euarchontoglires - ps30
Euarchonta - ps31
Primates - ps32
Haplorrhini - ps33
Simiiformes - ps34
Catarrhini - ps35
Hominoidea - ps36
Hominidae - ps37
Homininae - ps38
Hominini - ps39
Homo_sapiens - ps40
Phylostratum

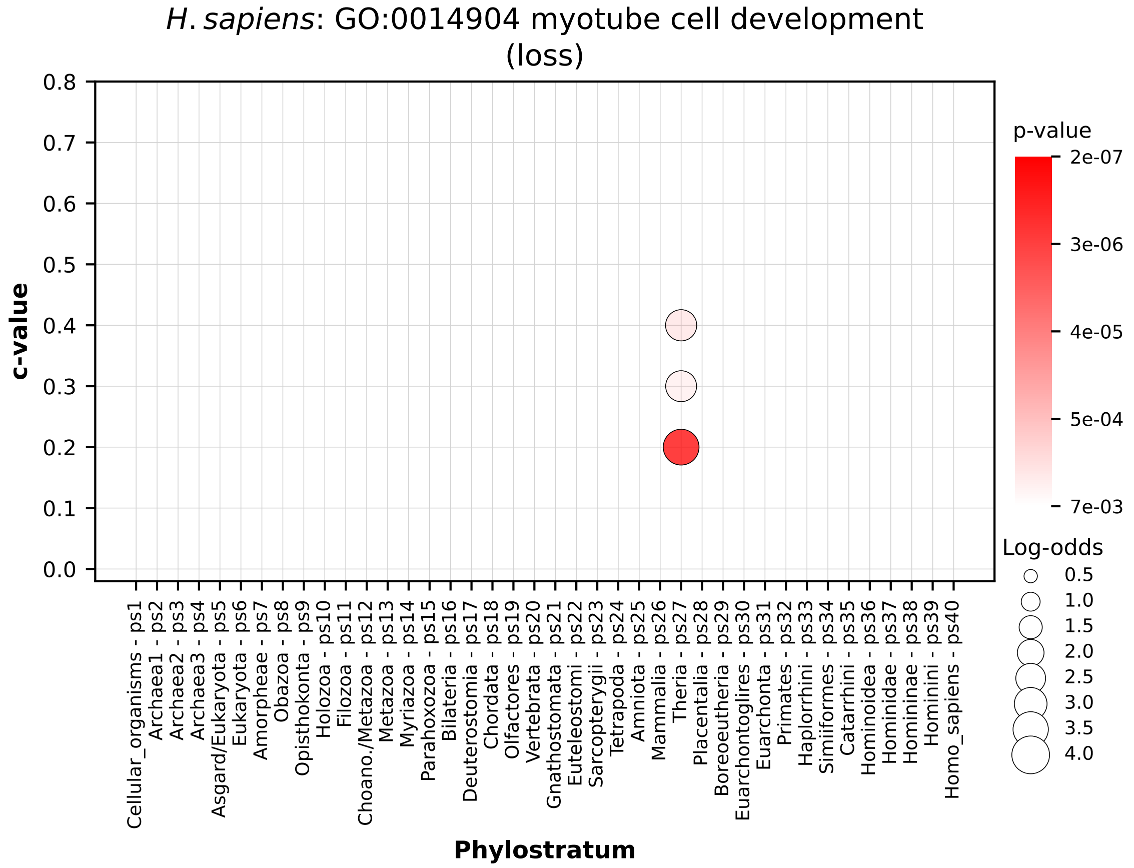
H. sapiens: GO:0014904 myotube cell development
(loss)
c-value
0.0
0.1
0.2
0.3
0.4
0.5
0.6
0.7
0.8
Phylostratum
Cellular_organisms - ps1
Archaea1 - ps2
Archaea2 - ps3
Archaea3 - ps4
Asgard/Eukaryota - ps5
Eukaryota - ps6
Amorpheae - ps7
Obazoa - ps8
Opisthokonta - ps9
Holozoa - ps10
Filozoa - ps11
Choano./Metazoa - ps12
Metazoa - ps13
Myriazoa - ps14
Parahoxozoa - ps15
Bilateria - ps16
Deuterostomia - ps17
Chordata - ps18
Olfactores - ps19
Vertebrata - ps20
Gnathostomata - ps21
Euteleostomi - ps22
Sarcopterygii - ps23
Tetrapoda - ps24
Amniota - ps25
Mammalia - ps26
Theria - ps27
Placentalia - ps28
Boreoeutheria - ps29
Euarchontoglires - ps30
Euarchonta - ps31
Primates - ps32
Haplorrhini - ps33
Simiiformes - ps34
Catarrhini - ps35
Hominoidea - ps36
Hominidae - ps37
Homininae - ps38
Hominini - ps39
Homo_sapiens - ps40
p-value
2e-07
3e-06
4e-05
5e-04
7e-03
Log-odds
0.5
1.0
1.5
2.0
2.5
3.0
3.5
4.0

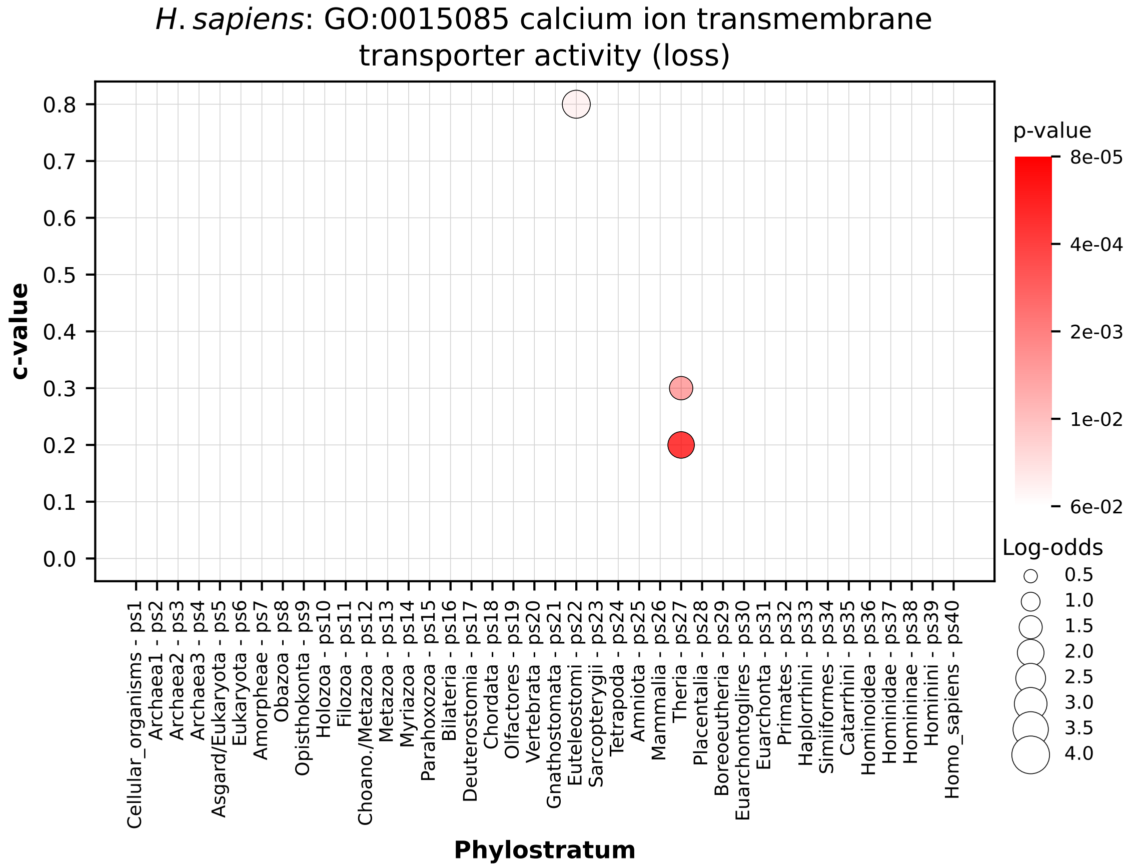
H. sapiens: GO:0015085 calcium ion transmembrane transporter activity (loss)
c-value
0.8
0.7
0.6
0.5
0.4
0.3
0.2
0.1
0.0
p-value
8e-05
4e-04
2e-03
1e-02
6e-02
Log-odds
0.5
1.0
1.5
2.0
2.5
3.0
3.5
4.0
Cellular_organisms - ps1
Archaea1 - ps2
Archaea2 - ps3
Archaea3 - ps4
Asgard/Eukaryota - ps5
Eukaryota - ps6
Amorpheae - ps7
Obazoa - ps8
Opisthokonta - ps9
Holozoa - ps10
Filozoa - ps11
Choano./Metazoa - ps12
Metazoa - ps13
Myriazoa - ps14
Parahoxozoa - ps15
Bilateria - ps16
Deuterostomia - ps17
Chordata - ps18
Olfactores - ps19
Vertebrata - ps20
Gnathostomata - ps21
Euteleostomi - ps22
Sarcopterygii - ps23
Tetrapoda - ps24
Amniota - ps25
Mammalia - ps26
Theria - ps27
Placentalia - ps28
Boreoeutheria - ps29
Euarchontoglires - ps30
Euarchonta - ps31
Primates - ps32
Haplorrhini - ps33
Simiiformes - ps34
Catarrhini - ps35
Hominoidea - ps36
Hominidae - ps37
Homininae - ps38
Hominini - ps39
Homo_sapiens - ps40
Phylostratum

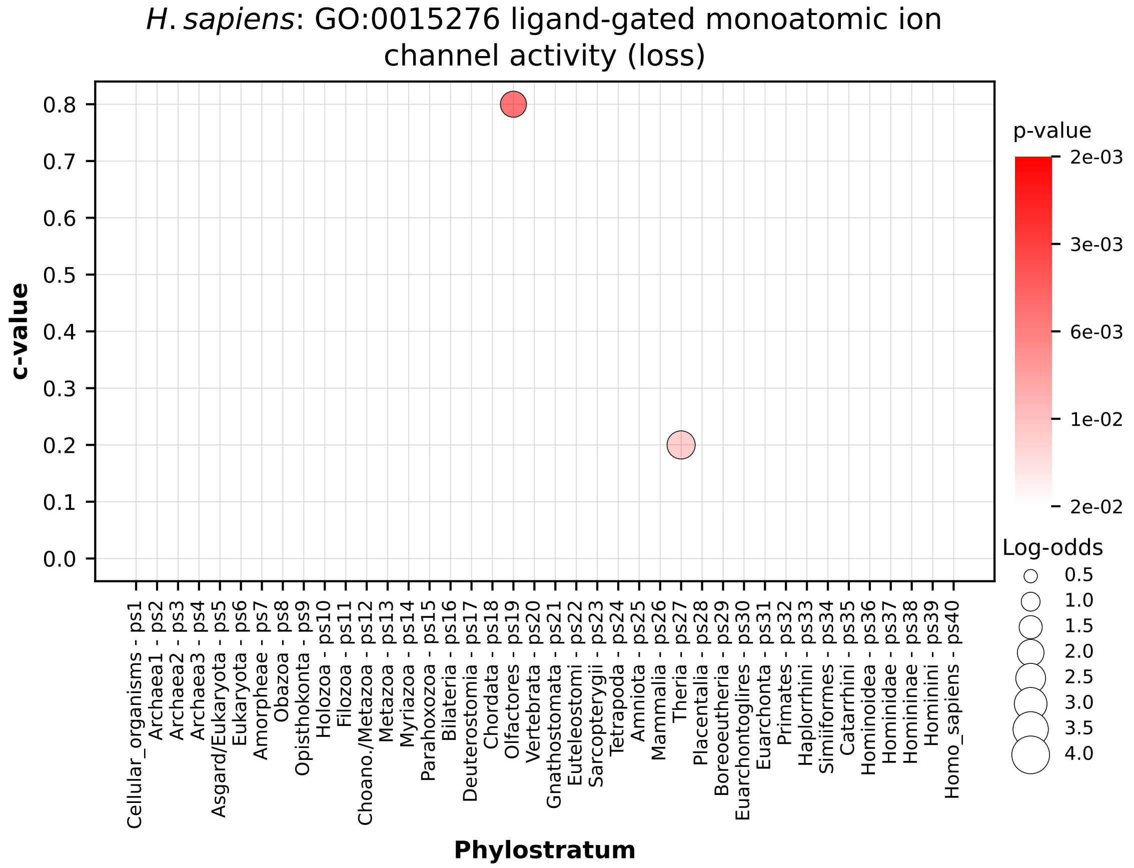

H. sapiens: GO:0015276 ligand-gated monoatomic ion channel activity (loss)
c-value
0.0
0.1
0.2
0.3
0.4
0.5
0.6
0.7
0.8
Phylostratum
Cellular_organisms - ps1
Archaea1 - ps2
Archaea2 - ps3
Archaea3 - ps4
Asgard/Eukaryota - ps5
Eukaryota - ps6
Amorpheae - ps7
Obazoa - ps8
Opisthokonta - ps9
Holozoa - ps10
Filozoa - ps11
Choano./Metazoa - ps12
Metazoa - ps13
Myriazoa - ps14
Parahoxozoa - ps15
Bilateria - ps16
Deuterostomia - ps17
Chordata - ps18
Olfactores - ps19
Vertebrata - ps20
Gnathostomata - ps21
Euteleostomi - ps22
Sarcopterygii - ps23
Tetrapoda - ps24
Amniota - ps25
Mammalia - ps26
Theria - ps27
Placentalia - ps28
Boreoeutheria - ps29
Euarchontoglires - ps30
Euarchonta - ps31
Primates - ps32
Haplorrhini - ps33
Simiiformes - ps34
Catarrhini - ps35
Hominoidea - ps36
Hominidae - ps37
Homininae - ps38
Hominini - ps39
Homo_sapiens - ps40
p-value
2e-03
3e-03
6e-03
1e-02
2e-02
Log-odds
0.5
1.0
1.5
2.0
2.5
3.0
3.5
4.0

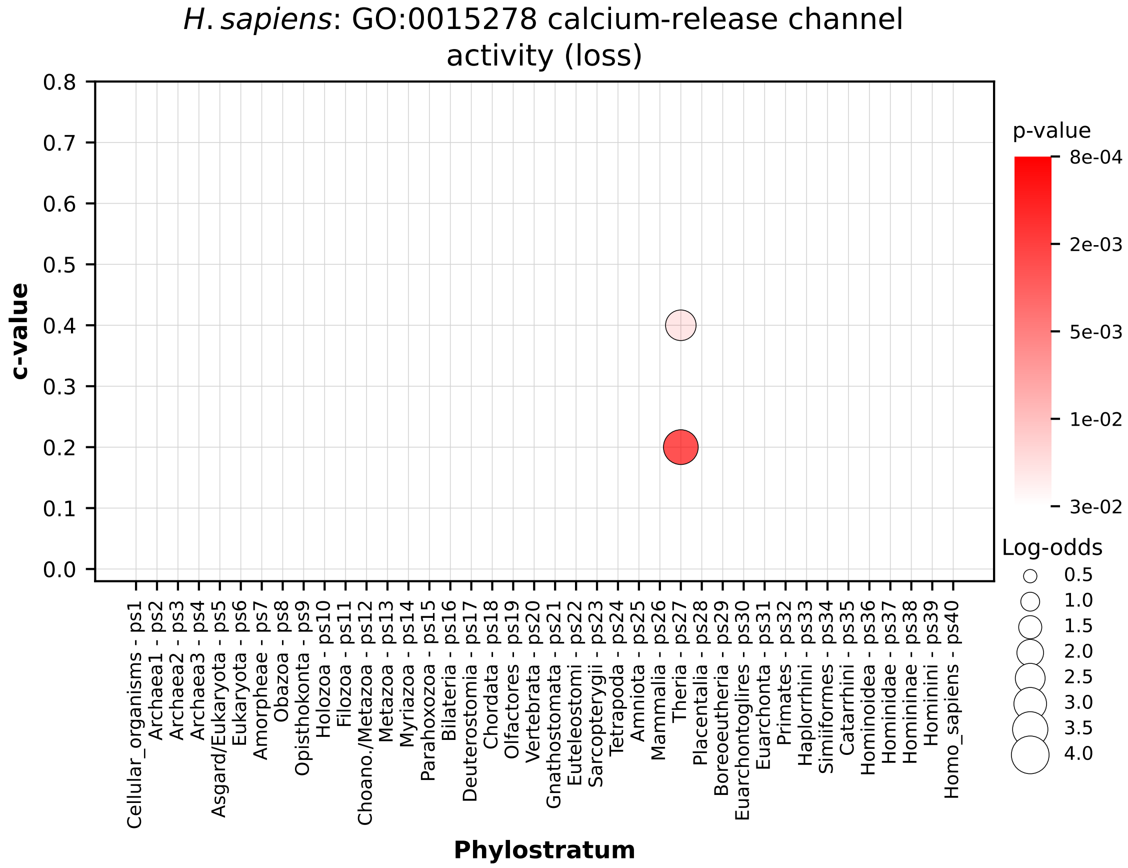

H. sapiens: GO:0015278 calcium-release channel activity (loss)
c-value
0.0
0.1
0.2
0.3
0.4
0.5
0.6
0.7
0.8
Phylostratum
Cellular_organisms - ps1
Archaea1 - ps2
Archaea2 - ps3
Archaea3 - ps4
Asgard/Eukaryota - ps5
Eukaryota - ps6
Amorpheae - ps7
Obazoa - ps8
Opisthokonta - ps9
Holozoa - ps10
Filozoa - ps11
Choano./Metazoa - ps12
Metazoa - ps13
Myriazoa - ps14
Parahoxozoa - ps15
Bilateria - ps16
Deuterostomia - ps17
Chordata - ps18
Olfactores - ps19
Vertebrata - ps20
Gnathostomata - ps21
Euteleostomi - ps22
Sarcopterygii - ps23
Tetrapoda - ps24
Amniota - ps25
Mammalia - ps26
Theria - ps27
Placentalia - ps28
Boreoeutheria - ps29
Euarchontoglires - ps30
Euarchonta - ps31
Primates - ps32
Haplorrhini - ps33
Simiiformes - ps34
Catarrhini - ps35
Hominoidea - ps36
Hominidae - ps37
Homininae - ps38
Hominini - ps39
Homo_sapiens - ps40
p-value
8e-04
2e-03
5e-03
1e-02
3e-02
Log-odds
0.5
1.0
1.5
2.0
2.5
3.0
3.5
4.0

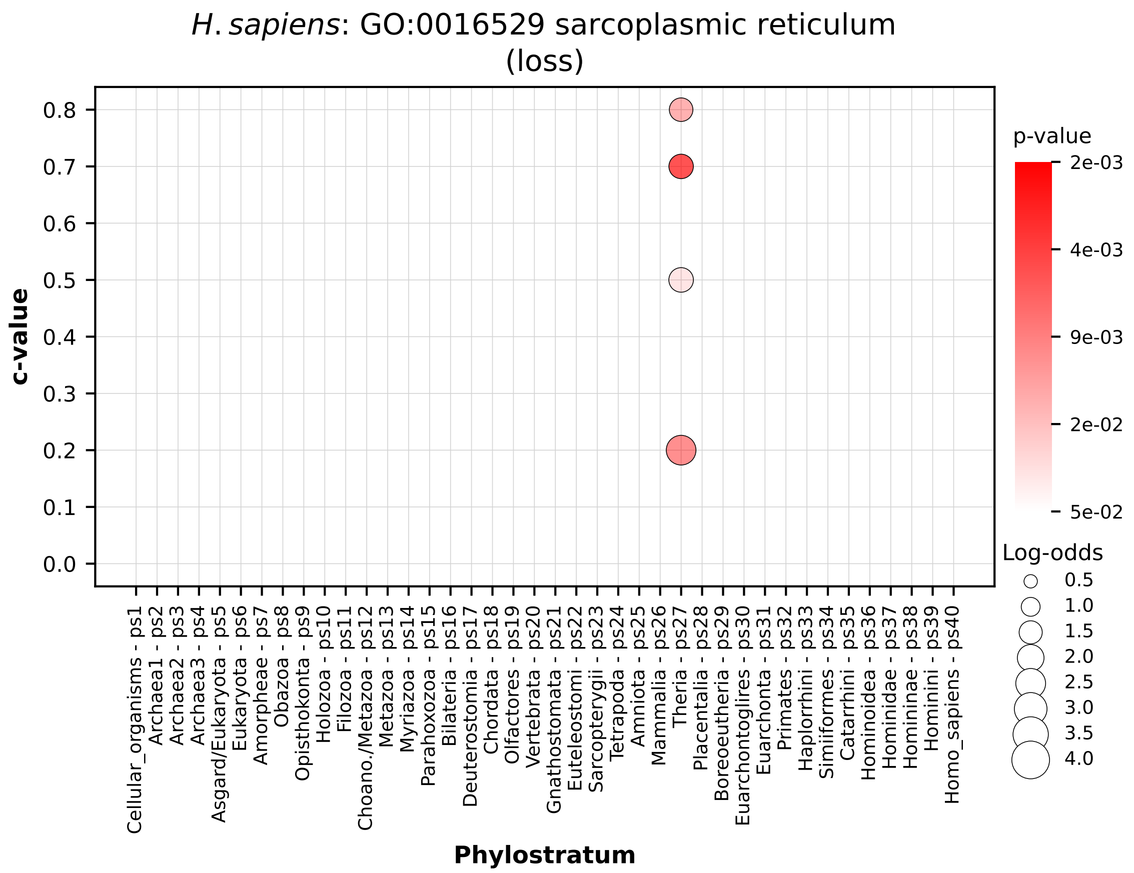

H. sapiens: GO:0016529 sarcoplasmic reticulum
(loss)
c-value
0.0
0.1
0.2
0.3
0.4
0.5
0.6
0.7
0.8
Phylostratum
Cellular_organisms - ps1
Archaea1 - ps2
Archaea2 - ps3
Archaea3 - ps4
Asgard/Eukaryota - ps5
Eukaryota - ps6
Amorpheae - ps7
Obazoa - ps8
Opisthokonta - ps9
Holozoa - ps10
Filozoa - ps11
Choano./Metazoa - ps12
Metazoa - ps13
Myriazoa - ps14
Parahoxozoa - ps15
Bilateria - ps16
Deuterostomia - ps17
Chordata - ps18
Olfactores - ps19
Vertebrata - ps20
Gnathostomata - ps21
Euteleostomi - ps22
Sarcopterygii - ps23
Tetrapoda - ps24
Amniota - ps25
Mammalia - ps26
Theria - ps27
Placentalia - ps28
Boreoeutheria - ps29
Euarchontoglires - ps30
Euarchonta - ps31
Primates - ps32
Haplorrhini - ps33
Simiiformes - ps34
Catarrhini - ps35
Hominoidea - ps36
Hominidae - ps37
Homininae - ps38
Hominini - ps39
Homo_sapiens - ps40
p-value
2e-03
4e-03
9e-03
2e-02
5e-02
Log-odds
0.5
1.0
1.5
2.0
2.5
3.0
3.5
4.0

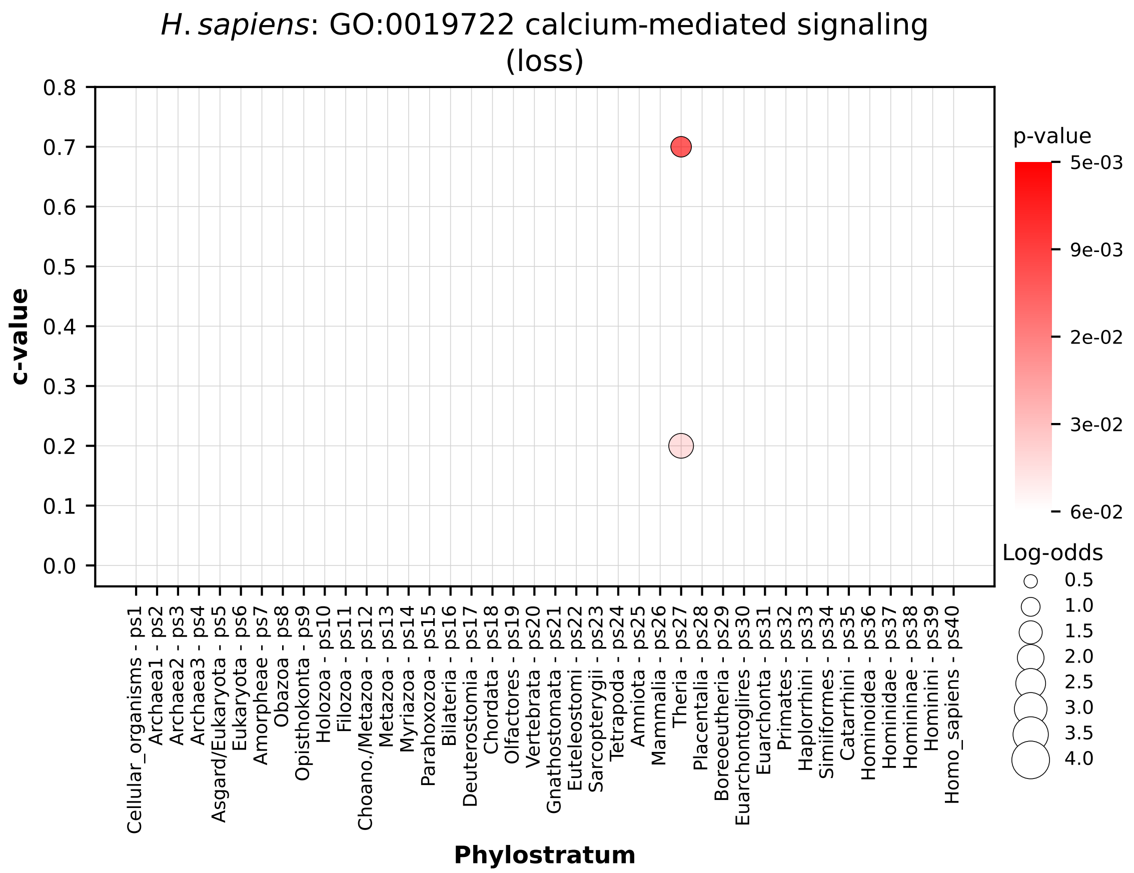
H. sapiens: GO:0019722 calcium-mediated signaling
(loss)
c-value
0.8
0.7
0.6
0.5
0.4
0.3
0.2
0.1
0.0
p-value
5e-03
9e-03
2e-02
3e-02
6e-02
Log-odds
0.5
1.0
1.5
2.0
2.5
3.0
3.5
4.0
Cellular_organisms - ps1
Archaea1 - ps2
Archaea2 - ps3
Archaea3 - ps4
Asgard/Eukaryota - ps5
Eukaryota - ps6
Amorpheae - ps7
Obazoa - ps8
Opisthokonta - ps9
Holozoa - ps10
Filozoa - ps11
Choano./Metazoa - ps12
Metazoa - ps13
Myriazoa - ps14
Parahoxozoa - ps15
Bilateria - ps16
Deuterostomia - ps17
Chordata - ps18
Olfactores - ps19
Vertebrata - ps20
Gnathostomata - ps21
Euteleostomi - ps22
Sarcopterygii - ps23
Tetrapoda - ps24
Amniota - ps25
Mammalia - ps26
Theria - ps27
Placentalia - ps28
Boreoeutheria - ps29
Euarchontoglires - ps30
Euarchonta - ps31
Primates - ps32
Haplorrhini - ps33
Simiiformes - ps34
Catarrhini - ps35
Hominoidea - ps36
Hominidae - ps37
Homininae - ps38
Hominini - ps39
Homo_sapiens - ps40
Phylostratum

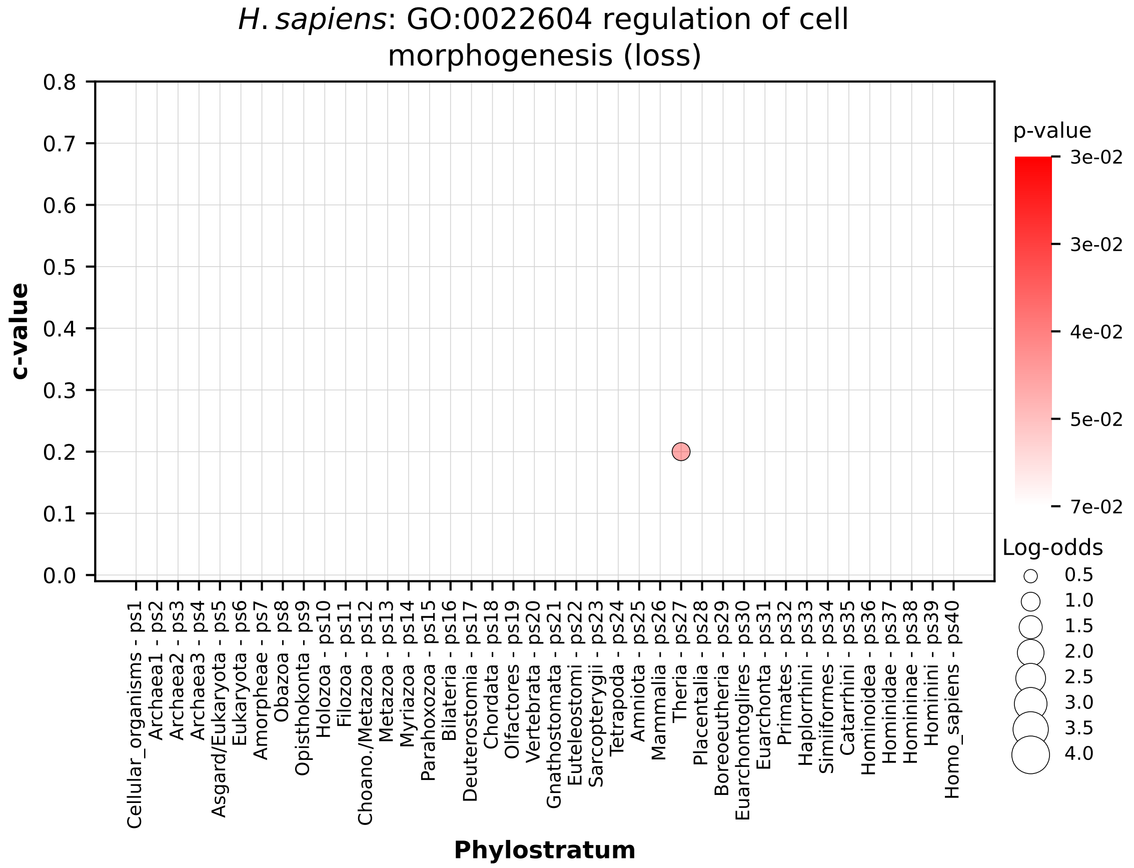

H. sapiens: GO:0022604 regulation of cell morphogenesis (loss)
c-value
0.0
0.1
0.2
0.3
0.4
0.5
0.6
0.7
0.8
Phylostratum
Cellular_organisms - ps1
Archaea1 - ps2
Archaea2 - ps3
Archaea3 - ps4
Asgard/Eukaryota - ps5
Eukaryota - ps6
Amorpheae - ps7
Obazoa - ps8
Opisthokonta - ps9
Holozoa - ps10
Filozoa - ps11
Choano./Metazoa - ps12
Metazoa - ps13
Myriazoa - ps14
Parahoxozoa - ps15
Bilateria - ps16
Deuterostomia - ps17
Chordata - ps18
Olfactores - ps19
Vertebrata - ps20
Gnathostomata - ps21
Euteleostomi - ps22
Sarcopterygii - ps23
Tetrapoda - ps24
Amniota - ps25
Mammalia - ps26
Theria - ps27
Placentalia - ps28
Boreoeutheria - ps29
Euarchontoglires - ps30
Euarchonta - ps31
Primates - ps32
Haplorrhini - ps33
Simiiformes - ps34
Catarrhini - ps35
Hominoidea - ps36
Hominidae - ps37
Homininae - ps38
Hominini - ps39
Homo_sapiens - ps40
p-value
3e-02
3e-02
4e-02
5e-02
7e-02
Log-odds
0.5
1.0
1.5
2.0
2.5
3.0
3.5
4.0

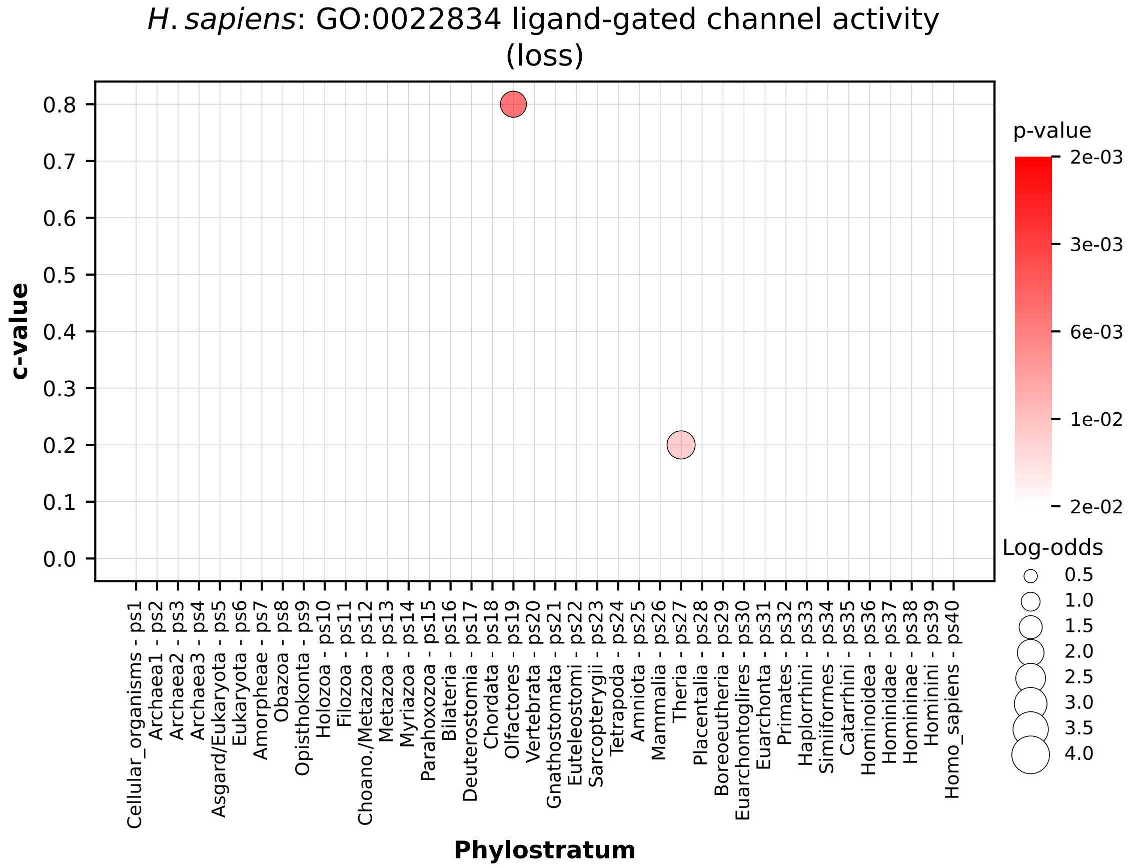

H. sapiens: GO:0022834 ligand-gated channel activity
(loss)
c-value
0.0
0.1
0.2
0.3
0.4
0.5
0.6
0.7
0.8
Phylostratum
Cellular_organisms - ps1
Archaea1 - ps2
Archaea2 - ps3
Archaea3 - ps4
Asgard/Eukaryota - ps5
Eukaryota - ps6
Amorpheae - ps7
Obazoa - ps8
Opisthokonta - ps9
Holozoa - ps10
Filozoa - ps11
Choano./Metazoa - ps12
Metazoa - ps13
Myriazoa - ps14
Parahoxozoa - ps15
Bilateria - ps16
Deuterostomia - ps17
Chordata - ps18
Olfactores - ps19
Vertebrata - ps20
Gnathostomata - ps21
Euteleostomi - ps22
Sarcopterygii - ps23
Tetrapoda - ps24
Amniota - ps25
Mammalia - ps26
Theria - ps27
Placentalia - ps28
Boreoeutheria - ps29
Euarchontoglires - ps30
Euarchonta - ps31
Primates - ps32
Haplorrhini - ps33
Simiiformes - ps34
Catarrhini - ps35
Hominoidea - ps36
Hominidae - ps37
Homininae - ps38
Hominini - ps39
Homo_sapiens - ps40
p-value
2e-03
3e-03
6e-03
1e-02
2e-02
Log-odds
0.5
1.0
1.5
2.0
2.5
3.0
3.5
4.0

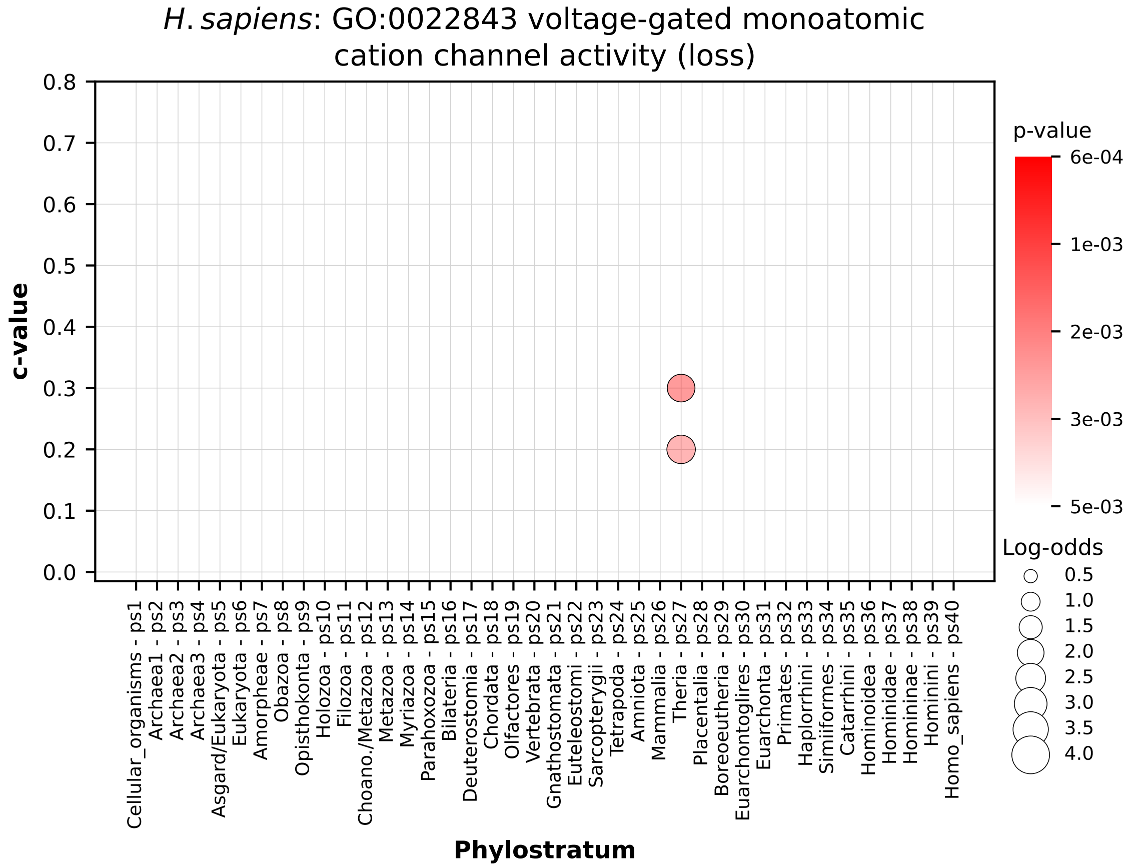

H. sapiens: GO:0022843 voltage-gated monoatomic cation channel activity (loss)
c-value
0.8
0.7
0.6
0.5
0.4
0.3
0.2
0.1
0.0
p-value
6e-04
1e-03
2e-03
3e-03
5e-03
Log-odds
0.5
1.0
1.5
2.0
2.5
3.0
3.5
4.0
Cellular_organisms - ps1
Archaea1 - ps2
Archaea2 - ps3
Archaea3 - ps4
Asgard/Eukaryota - ps5
Eukaryota - ps6
Amorpheae - ps7
Obazoa - ps8
Opisthokonta - ps9
Holozoa - ps10
Filozoa - ps11
Choano./Metazoa - ps12
Metazoa - ps13
Myriazoa - ps14
Parahoxozoa - ps15
Bilateria - ps16
Deuterostomia - ps17
Chordata - ps18
Olfactores - ps19
Vertebrata - ps20
Gnathostomata - ps21
Euteleostomi - ps22
Sarcopterygii - ps23
Tetrapoda - ps24
Amniota - ps25
Mammalia - ps26
Theria - ps27
Placentalia - ps28
Boreoeutheria - ps29
Euarchontoglires - ps30
Euarchonta - ps31
Primates - ps32
Haplorrhini - ps33
Simiiformes - ps34
Catarrhini - ps35
Hominoidea - ps36
Hominidae - ps37
Homininae - ps38
Hominini - ps39
Homo_sapiens - ps40
Phylostratum

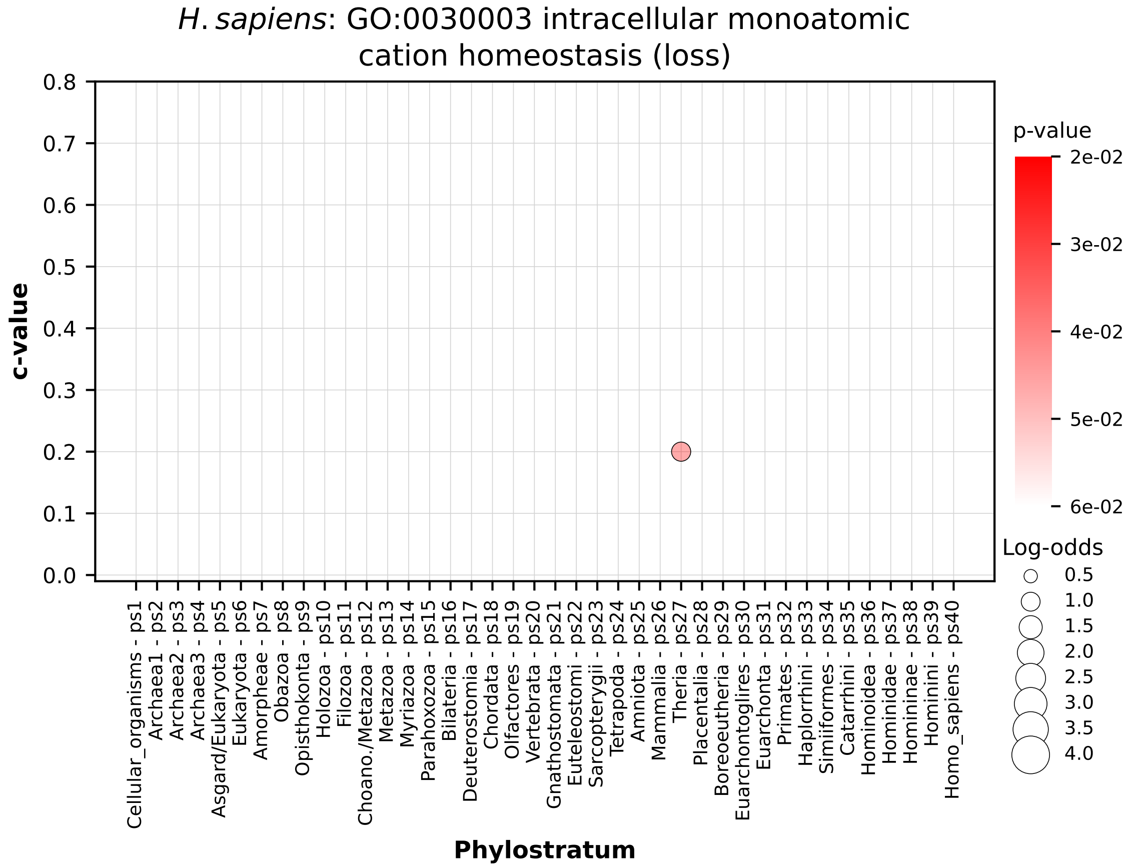

H. sapiens: GO:0030003 intracellular monoatomic cation homeostasis (loss)
c-value
0.8
0.7
0.6
0.5
0.4
0.3
0.2
0.1
0.0
p-value
2e-02
3e-02
4e-02
5e-02
6e-02
Log-odds
0.5
1.0
1.5
2.0
2.5
3.0
3.5
4.0
Cellular_organisms - ps1
Archaea1 - ps2
Archaea2 - ps3
Archaea3 - ps4
Asgard/Eukaryota - ps5
Eukaryota - ps6
Amorpheae - ps7
Obazoa - ps8
Opisthokonta - ps9
Holozoa - ps10
Filozoa - ps11
Choano./Metazoa - ps12
Metazoa - ps13
Myriazoa - ps14
Parahoxozoa - ps15
Bilateria - ps16
Deuterostomia - ps17
Chordata - ps18
Olfactores - ps19
Vertebrata - ps20
Gnathostomata - ps21
Euteleostomi - ps22
Sarcopterygii - ps23
Tetrapoda - ps24
Amniota - ps25
Mammalia - ps26
Theria - ps27
Placentalia - ps28
Boreoeutheria - ps29
Euarchontoglires - ps30
Euarchonta - ps31
Primates - ps32
Haplorrhini - ps33
Simiiformes - ps34
Catarrhini - ps35
Hominoidea - ps36
Hominidae - ps37
Homininae - ps38
Hominini - ps39
Homo_sapiens - ps40
Phylostratum

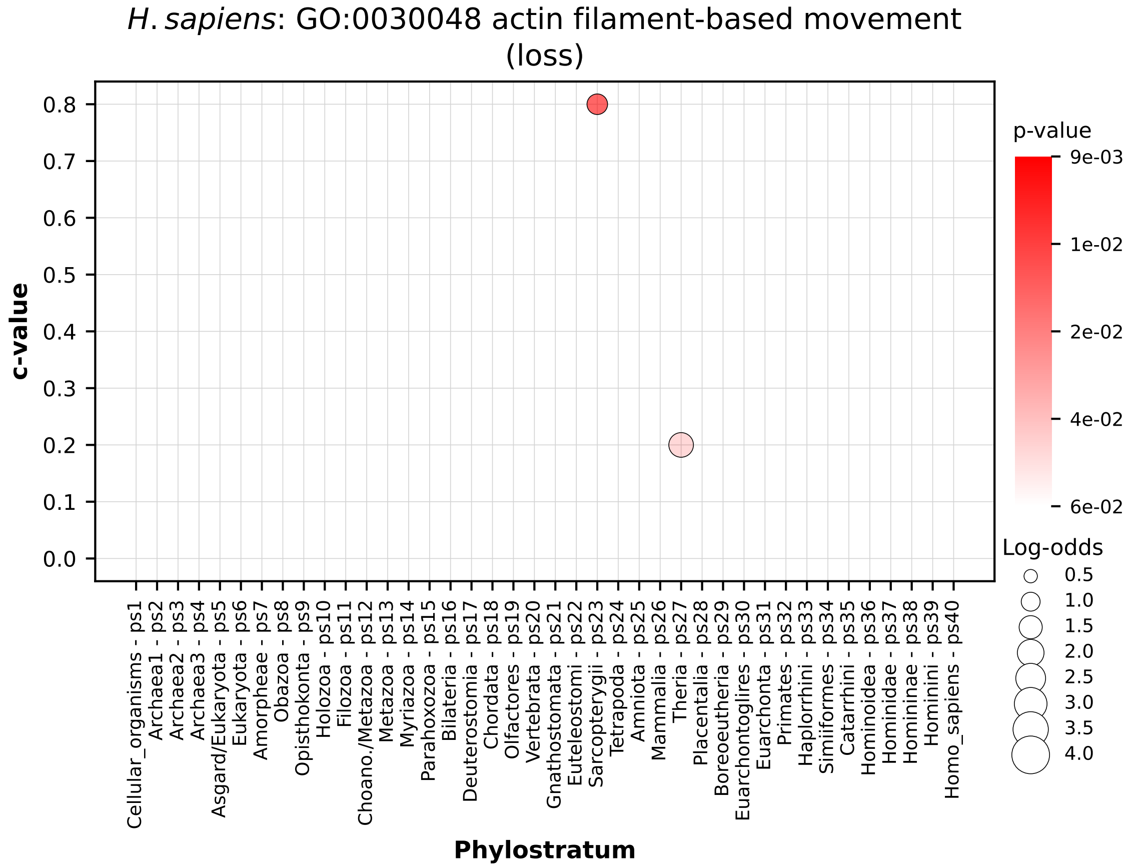

H. sapiens: GO:0030048 actin filament-based movement (loss)
c-value
0.0
0.1
0.2
0.3
0.4
0.5
0.6
0.7
0.8
Phylostratum
Cellular_organisms - ps1
Archaea1 - ps2
Archaea2 - ps3
Archaea3 - ps4
Asgard/Eukaryota - ps5
Eukaryota - ps6
Amorpheae - ps7
Obazoa - ps8
Opisthokonta - ps9
Holozoa - ps10
Filozoa - ps11
Choano./Metazoa - ps12
Metazoa - ps13
Myriazoa - ps14
Parahoxozoa - ps15
Bilateria - ps16
Deuterostomia - ps17
Chordata - ps18
Olfactores - ps19
Vertebrata - ps20
Gnathostomata - ps21
Euteleostomi - ps22
Sarcopterygii - ps23
Tetrapoda - ps24
Amniota - ps25
Mammalia - ps26
Theria - ps27
Placentalia - ps28
Boreoeutheria - ps29
Euarchontoglires - ps30
Euarchonta - ps31
Primates - ps32
Haplorrhini - ps33
Simiiformes - ps34
Catarrhini - ps35
Hominoidea - ps36
Hominidae - ps37
Homininae - ps38
Hominini - ps39
Homo_sapiens - ps40
p-value
9e-03
1e-02
2e-02
4e-02
6e-02
Log-odds
0.5
1.0
1.5
2.0
2.5
3.0
3.5
4.0

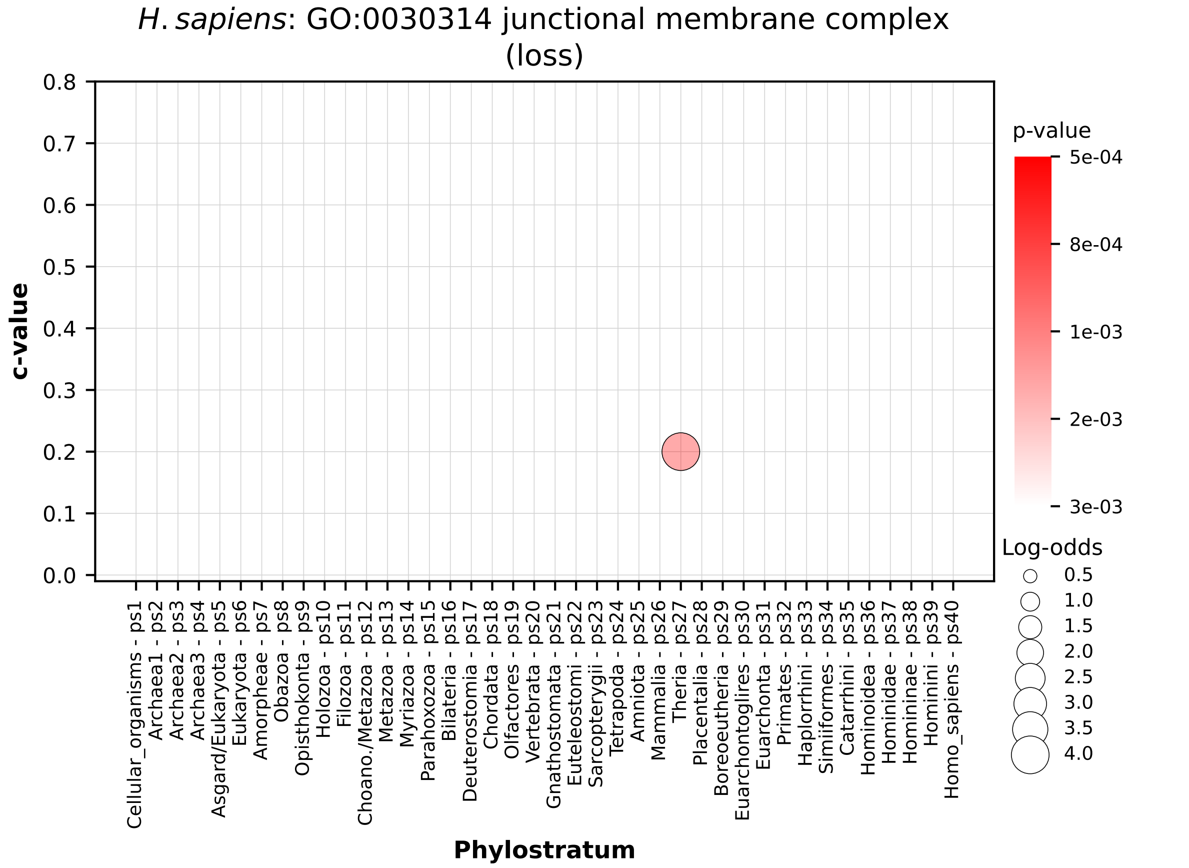

H. sapiens: GO:0030314 junctional membrane complex (loss)
c-value
0.8
0.7
0.6
0.5
0.4
0.3
0.2
0.1
0.0
p-value
5e-04
8e-04
1e-03
2e-03
3e-03
Log-odds
0.5
1.0
1.5
2.0
2.5
3.0
3.5
4.0
Cellular_organisms - ps1
Archaea1 - ps2
Archaea2 - ps3
Archaea3 - ps4
Asgard/Eukaryota - ps5
Eukaryota - ps6
Amorpheae - ps7
Obazoa - ps8
Opisthokonta - ps9
Holozoa - ps10
Filozoa - ps11
Choano./Metazoa - ps12
Metazoa - ps13
Myriazoa - ps14
Parahoxozoa - ps15
Bilateria - ps16
Deuterostomia - ps17
Chordata - ps18
Olfactores - ps19
Vertebrata - ps20
Gnathostomata - ps21
Euteleostomi - ps22
Sarcopterygii - ps23
Tetrapoda - ps24
Amniota - ps25
Mammalia - ps26
Theria - ps27
Placentalia - ps28
Boreoeutheria - ps29
Euarchontoglires - ps30
Euarchonta - ps31
Primates - ps32
Haplorrhini - ps33
Simiiformes - ps34
Catarrhini - ps35
Hominoidea - ps36
Hominidae - ps37
Homininae - ps38
Hominini - ps39
Homo_sapiens - ps40
Phylostratum

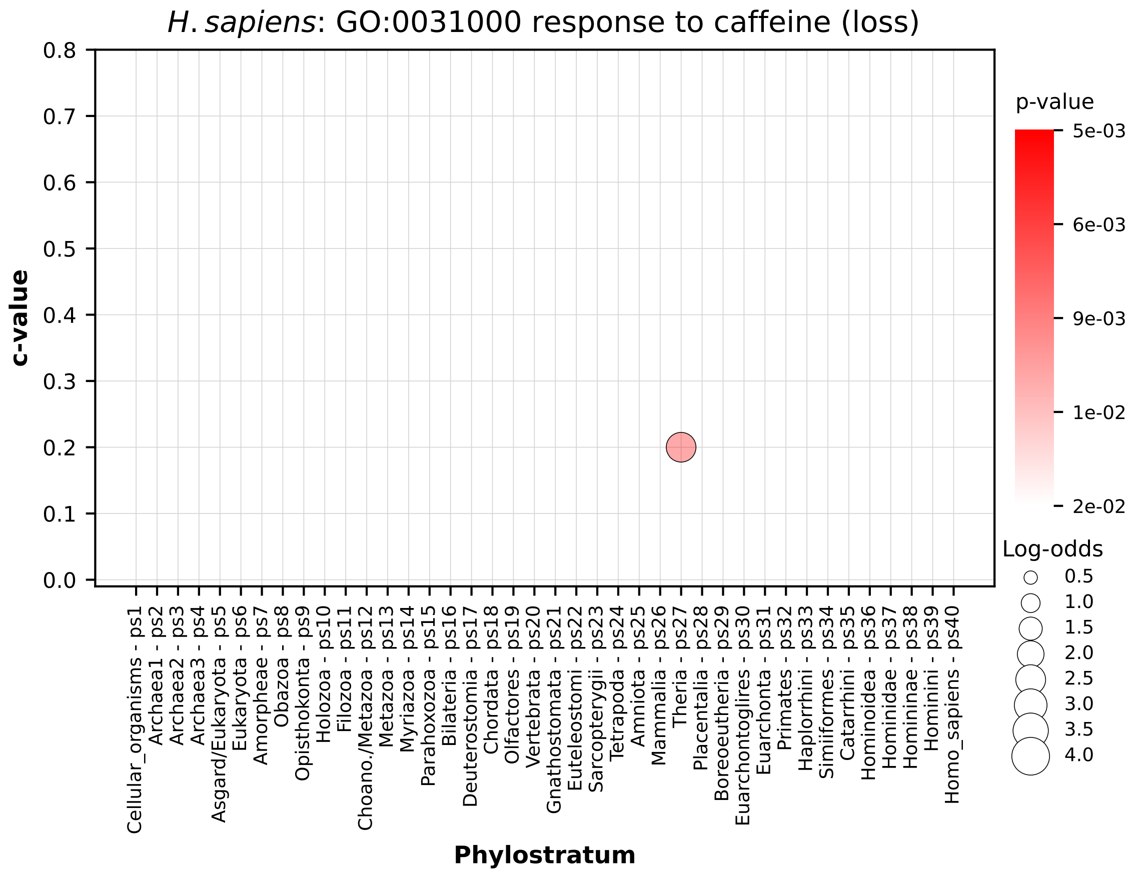

H. sapiens: GO:0031000 response to caffeine (loss)
c-value
0.0
0.1
0.2
0.3
0.4
0.5
0.6
0.7
0.8
Phylostratum
Cellular_organisms - ps1
Archaea1 - ps2
Archaea2 - ps3
Archaea3 - ps4
Asgard/Eukaryota - ps5
Eukaryota - ps6
Amorpheae - ps7
Obazoa - ps8
Opisthokonta - ps9
Holozoa - ps10
Filozoa - ps11
Choano./Metazoa - ps12
Metazoa - ps13
Myriazoa - ps14
Parahoxozoa - ps15
Bilateria - ps16
Deuterostomia - ps17
Chordata - ps18
Olfactores - ps19
Vertebrata - ps20
Gnathostomata - ps21
Euteleostomi - ps22
Sarcopterygii - ps23
Tetrapoda - ps24
Amniota - ps25
Mammalia - ps26
Theria - ps27
Placentalia - ps28
Boreoeutheria - ps29
Euarchontoglires - ps30
Euarchonta - ps31
Primates - ps32
Haplorrhini - ps33
Simiiformes - ps34
Catarrhini - ps35
Hominoidea - ps36
Hominidae - ps37
Homininae - ps38
Hominini - ps39
Homo_sapiens - ps40
p-value
5e-03
6e-03
9e-03
1e-02
2e-02
Log-odds
0.5
1.0
1.5
2.0
2.5
3.0
3.5
4.0

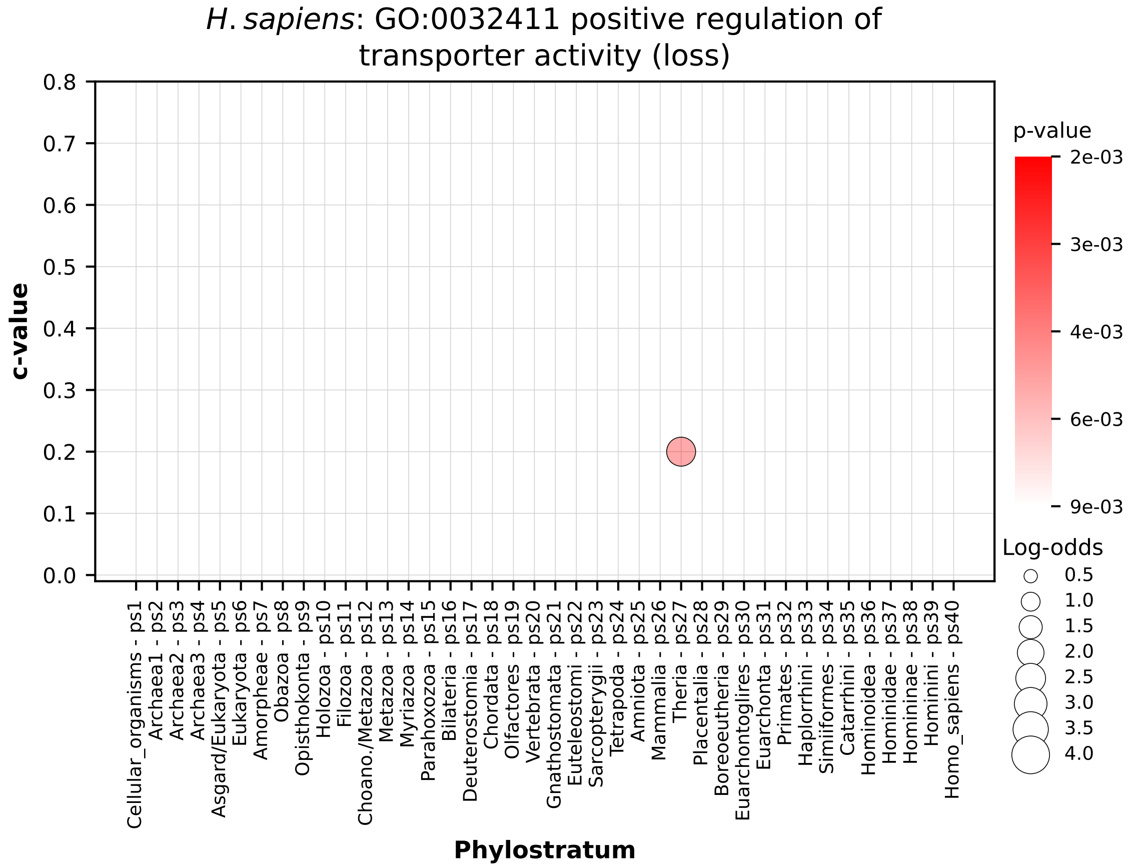

H. sapiens: GO:0032411 positive regulation of transporter activity (loss)
c-value
0.0
0.1
0.2
0.3
0.4
0.5
0.6
0.7
0.8
Phylostratum
Cellular_organisms - ps1
Archaea1 - ps2
Archaea2 - ps3
Archaea3 - ps4
Asgard/Eukaryota - ps5
Eukaryota - ps6
Amorpheae - ps7
Obazoa - ps8
Opisthokonta - ps9
Holozoa - ps10
Filozoa - ps11
Choano./Metazoa - ps12
Metazoa - ps13
Myriazoa - ps14
Parahoxozoa - ps15
Bilateria - ps16
Deuterostomia - ps17
Chordata - ps18
Olfactores - ps19
Vertebrata - ps20
Gnathostomata - ps21
Euteleostomi - ps22
Sarcopterygii - ps23
Tetrapoda - ps24
Amniota - ps25
Mammalia - ps26
Theria - ps27
Placentalia - ps28
Boreoeutheria - ps29
Euarchontoglires - ps30
Euarchonta - ps31
Primates - ps32
Haplorrhini - ps33
Simiiformes - ps34
Catarrhini - ps35
Hominoidea - ps36
Hominidae - ps37
Homininae - ps38
Hominini - ps39
Homo_sapiens - ps40
p-value
2e-03
3e-03
4e-03
6e-03
9e-03
Log-odds
0.5
1.0
1.5
2.0
2.5
3.0
3.5
4.0

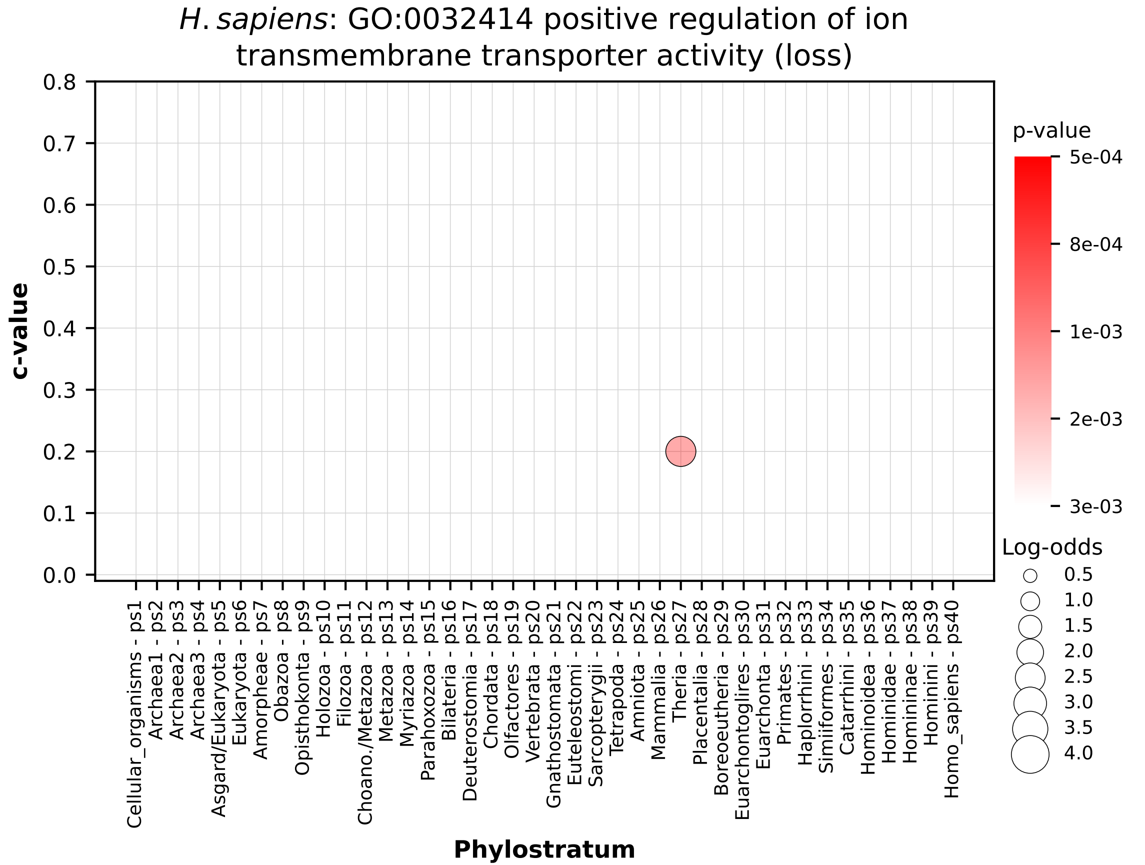
H. sapiens: GO:0032414 positive regulation of ion transmembrane transporter activity (loss)
c-value
0.8
0.7
0.6
0.5
0.4
0.3
0.2
0.1
0.0
p-value
5e-04
8e-04
1e-03
2e-03
3e-03
Log-odds
0.5
1.0
1.5
2.0
2.5
3.0
3.5
4.0
Cellular_organisms - ps1
Archaea1 - ps2
Archaea2 - ps3
Archaea3 - ps4
Asgard/Eukaryota - ps5
Eukaryota - ps6
Amorpheae - ps7
Obazoa - ps8
Opisthokonta - ps9
Holozoa - ps10
Filozoa - ps11
Choano./Metazoa - ps12
Metazoa - ps13
Myriazoa - ps14
Parahoxozoa - ps15
Bilateria - ps16
Deuterostomia - ps17
Chordata - ps18
Olfactores - ps19
Vertebrata - ps20
Gnathostomata - ps21
Euteleostomi - ps22
Sarcopterygii - ps23
Tetrapoda - ps24
Amniota - ps25
Mammalia - ps26
Theria - ps27
Placentalia - ps28
Boreoeutheria - ps29
Euarchontoglires - ps30
Euarchonta - ps31
Primates - ps32
Haplorrhini - ps33
Simiiformes - ps34
Catarrhini - ps35
Hominoidea - ps36
Hominidae - ps37
Homininae - ps38
Hominini - ps39
Homo_sapiens - ps40
Phylostratum

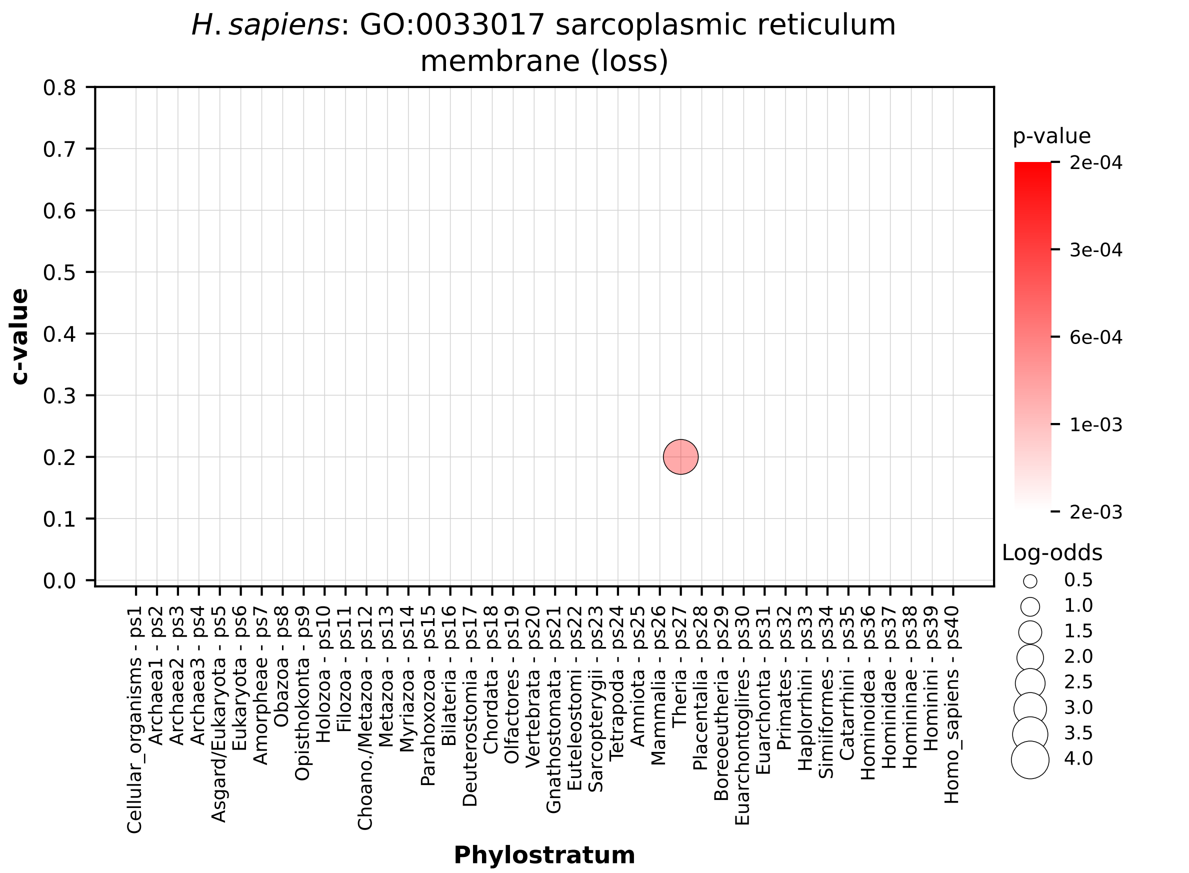

H. sapiens: GO:0033017 sarcoplasmic reticulum membrane (loss)
c-value
0.0
0.1
0.2
0.3
0.4
0.5
0.6
0.7
0.8
Phylostratum
Cellular_organisms - ps1
Archaea1 - ps2
Archaea2 - ps3
Archaea3 - ps4
Asgard/Eukaryota - ps5
Eukaryota - ps6
Amorpheae - ps7
Obazoa - ps8
Opisthokonta - ps9
Holozoa - ps10
Filozoa - ps11
Choano./Metazoa - ps12
Metazoa - ps13
Myriazoa - ps14
Parahoxozoa - ps15
Bilateria - ps16
Deuterostomia - ps17
Chordata - ps18
Olfactores - ps19
Vertebrata - ps20
Gnathostomata - ps21
Euteleostomi - ps22
Sarcopterygii - ps23
Tetrapoda - ps24
Amniota - ps25
Mammalia - ps26
Theria - ps27
Placentalia - ps28
Boreoeutheria - ps29
Euarchontoglires - ps30
Euarchonta - ps31
Primates - ps32
Haplorrhini - ps33
Simiiformes - ps34
Catarrhini - ps35
Hominoidea - ps36
Hominidae - ps37
Homininae - ps38
Hominini - ps39
Homo_sapiens - ps40
p-value
2e-04
3e-04
6e-04
1e-03
2e-03
Log-odds
0.5
1.0
1.5
2.0
2.5
3.0
3.5
4.0

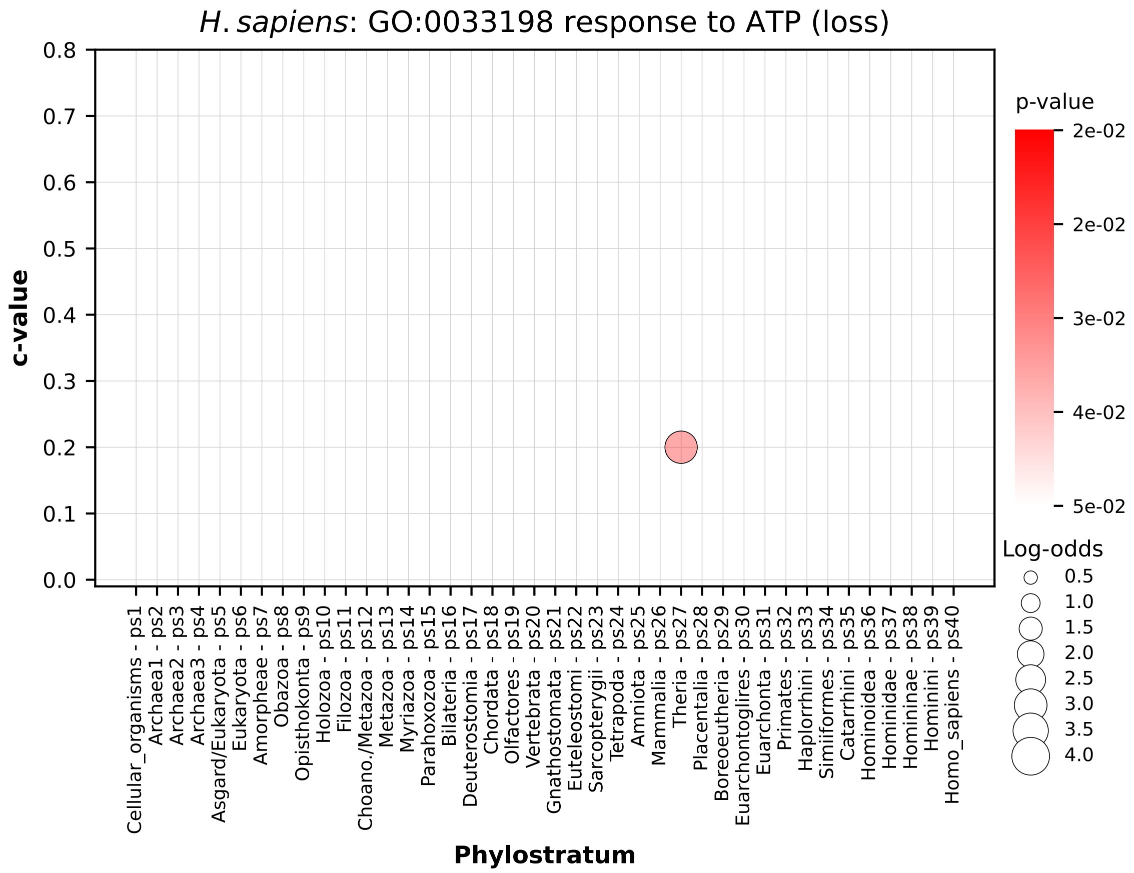

H. sapiens: GO:0033198 response to ATP (loss)
c-value
0.8
0.7
0.6
0.5
0.4
0.3
0.2
0.1
0.0
p-value
2e-02
2e-02
3e-02
4e-02
5e-02
Log-odds
0.5
1.0
1.5
2.0
2.5
3.0
3.5
4.0
Cellular_organisms - ps1
Archaea1 - ps2
Archaea2 - ps3
Archaea3 - ps4
Asgard/Eukaryota - ps5
Eukaryota - ps6
Amorpheae - ps7
Obazoa - ps8
Opisthokonta - ps9
Holozoa - ps10
Filozoa - ps11
Choano./Metazoa - ps12
Metazoa - ps13
Myriazoa - ps14
Parahoxozoa - ps15
Bilateria - ps16
Deuterostomia - ps17
Chordata - ps18
Olfactores - ps19
Vertebrata - ps20
Gnathostomata - ps21
Euteleostomi - ps22
Sarcopterygii - ps23
Tetrapoda - ps24
Amniota - ps25
Mammalia - ps26
Theria - ps27
Placentalia - ps28
Boreoeutheria - ps29
Euarchontoglires - ps30
Euarchonta - ps31
Primates - ps32
Haplorrhini - ps33
Simiiformes - ps34
Catarrhini - ps35
Hominoidea - ps36
Hominidae - ps37
Homininae - ps38
Hominini - ps39
Homo_sapiens - ps40
Phylostratum

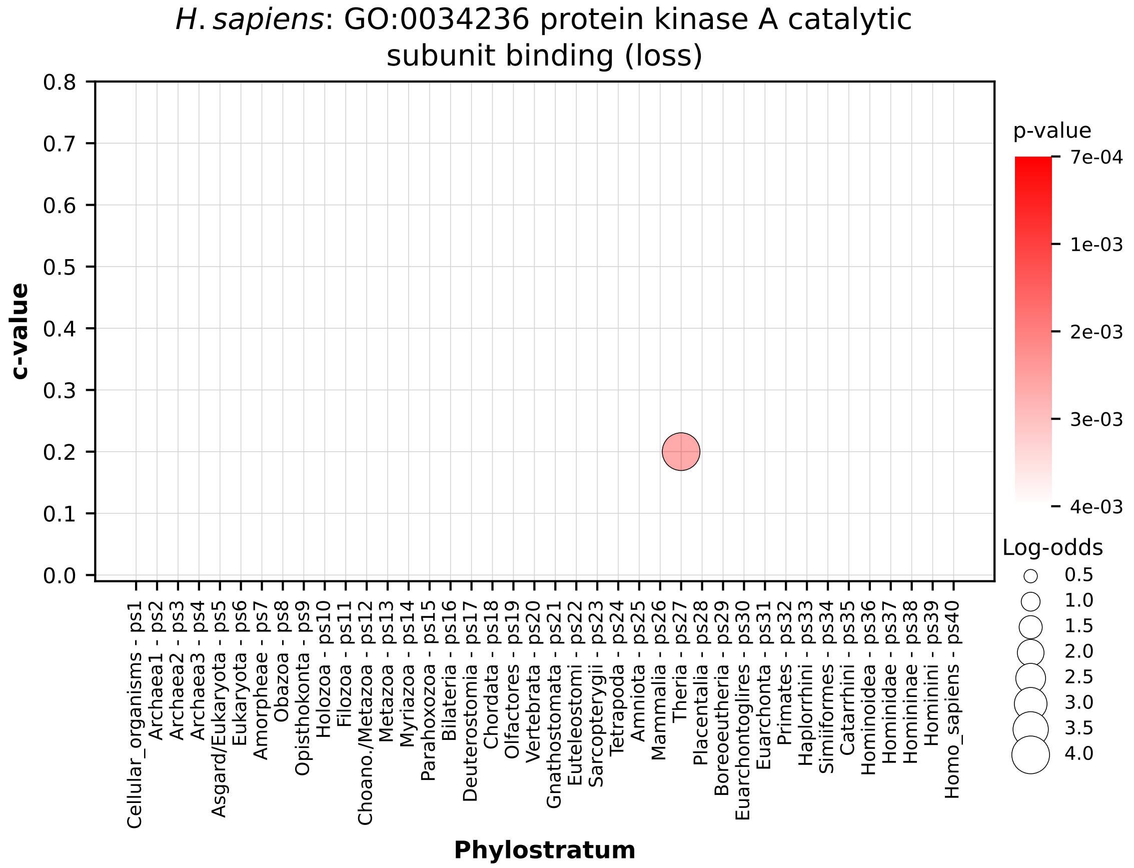

H. sapiens: GO:0034236 protein kinase A catalytic subunit binding (loss)
c-value
0.0
0.1
0.2
0.3
0.4
0.5
0.6
0.7
0.8
Phylostratum
Cellular_organisms - ps1
Archaea1 - ps2
Archaea2 - ps3
Archaea3 - ps4
Asgard/Eukaryota - ps5
Eukaryota - ps6
Amorpheae - ps7
Obazoa - ps8
Opisthokonta - ps9
Holozoa - ps10
Filozoa - ps11
Choano./Metazoa - ps12
Metazoa - ps13
Myriazoa - ps14
Parahoxozoa - ps15
Bilateria - ps16
Deuterostomia - ps17
Chordata - ps18
Olfactores - ps19
Vertebrata - ps20
Gnathostomata - ps21
Euteleostomi - ps22
Sarcopterygii - ps23
Tetrapoda - ps24
Amniota - ps25
Mammalia - ps26
Theria - ps27
Placentalia - ps28
Boreoeutheria - ps29
Euarchontoglires - ps30
Euarchonta - ps31
Primates - ps32
Haplorrhini - ps33
Simiiformes - ps34
Catarrhini - ps35
Hominoidea - ps36
Hominidae - ps37
Homininae - ps38
Hominini - ps39
Homo_sapiens - ps40
p-value
7e-04
1e-03
2e-03
3e-03
4e-03
Log-odds
0.5
1.0
1.5
2.0
2.5
3.0
3.5
4.0

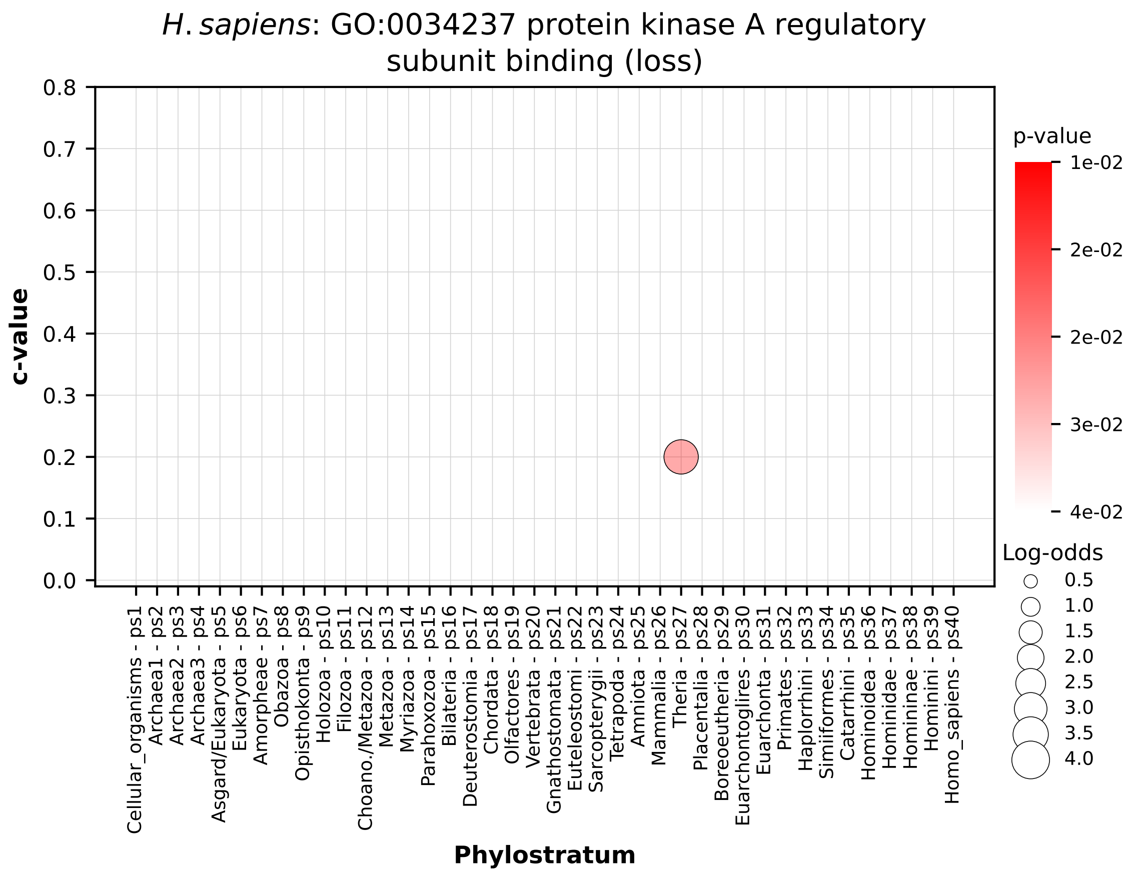

H. sapiens: GO:0034237 protein kinase A regulatory subunit binding (loss)
c-value
Phylostratum
p-value
1e-02
2e-02
2e-02
3e-02
4e-02
Log-odds
0.5
1.0
1.5
2.0
2.5
3.0
3.5
4.0
Cellular_organisms - ps1
Archaea1 - ps2
Archaea2 - ps3
Archaea3 - ps4
Asgard/Eukaryota - ps5
Eukaryota - ps6
Amorpheae - ps7
Obazoa - ps8
Opisthokonta - ps9
Holozoa - ps10
Filozoa - ps11
Choano./Metazoa - ps12
Metazoa - ps13
Myriazoa - ps14
Parahoxozoa - ps15
Bilateria - ps16
Deuterostomia - ps17
Chordata - ps18
Olfactores - ps19
Vertebrata - ps20
Gnathostomata - ps21
Euteleostomi - ps22
Sarcopterygii - ps23
Tetrapoda - ps24
Amniota - ps25
Mammalia - ps26
Theria - ps27
Placentalia - ps28
Boreoeutheria - ps29
Euarchontoglires - ps30
Euarchonta - ps31
Primates - ps32
Haplorrhini - ps33
Simiiformes - ps34
Catarrhini - ps35
Hominoidea - ps36
Hominidae - ps37
Homininae - ps38
Hominini - ps39
Homo_sapiens - ps40

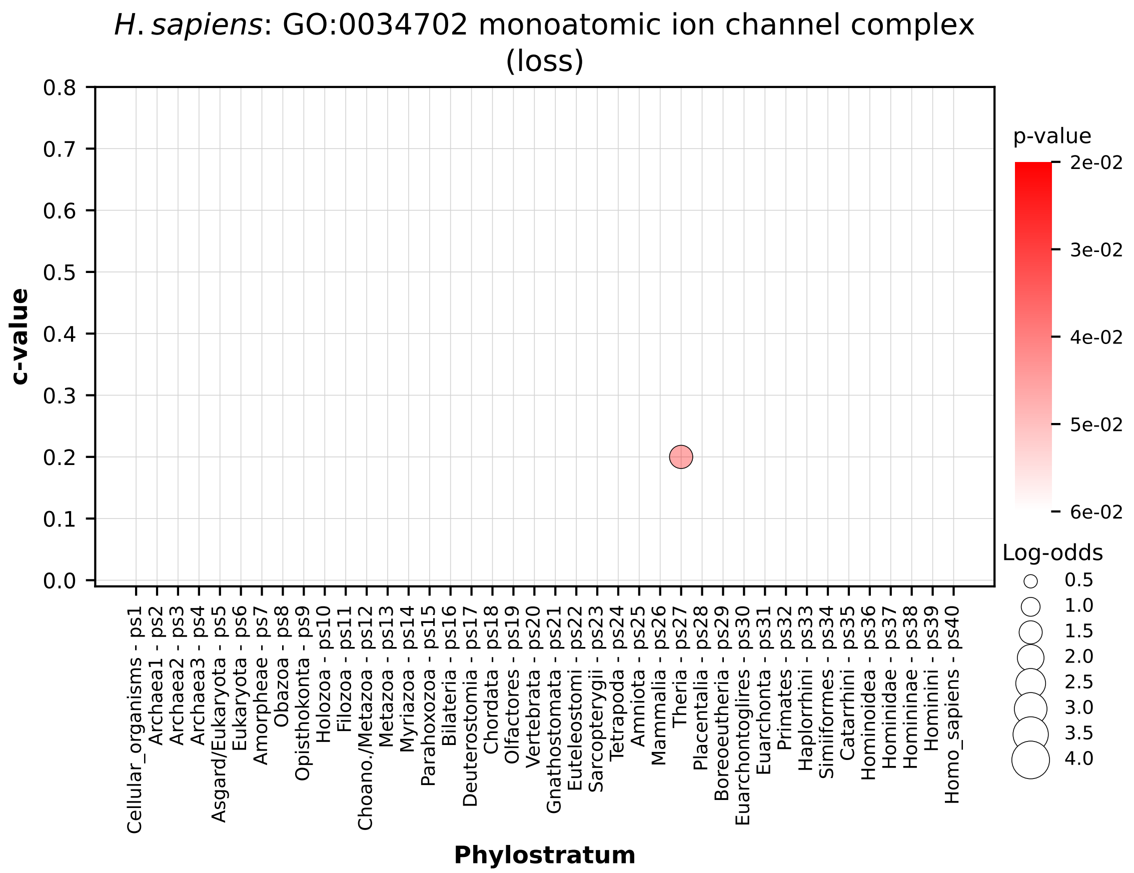

H. sapiens: GO:0034702 monoatomic ion channel complex
(loss)
c-value
0.0
0.1
0.2
0.3
0.4
0.5
0.6
0.7
0.8
p-value
2e-02
3e-02
4e-02
5e-02
6e-02
Log-odds
0.5
1.0
1.5
2.0
2.5
3.0
3.5
4.0
Cellular_organisms - ps1
Archaea1 - ps2
Archaea2 - ps3
Archaea3 - ps4
Asgard/Eukaryota - ps5
Eukaryota - ps6
Amorpheae - ps7
Obazoa - ps8
Opisthokonta - ps9
Holozoa - ps10
Filozoa - ps11
Choano./Metazoa - ps12
Metazoa - ps13
Myriazoa - ps14
Parahoxozoa - ps15
Bilateria - ps16
Deuterostomia - ps17
Chordata - ps18
Olfactores - ps19
Vertebrata - ps20
Gnathostomata - ps21
Euteleostomi - ps22
Sarcopterygii - ps23
Tetrapoda - ps24
Amniota - ps25
Mammalia - ps26
Theria - ps27
Placentalia - ps28
Boreoeutheria - ps29
Euarchontoglires - ps30
Euarchonta - ps31
Primates - ps32
Haplorrhini - ps33
Simiiformes - ps34
Catarrhini - ps35
Hominoidea - ps36
Hominidae - ps37
Homininae - ps38
Hominini - ps39
Homo_sapiens - ps40
Phylostratum

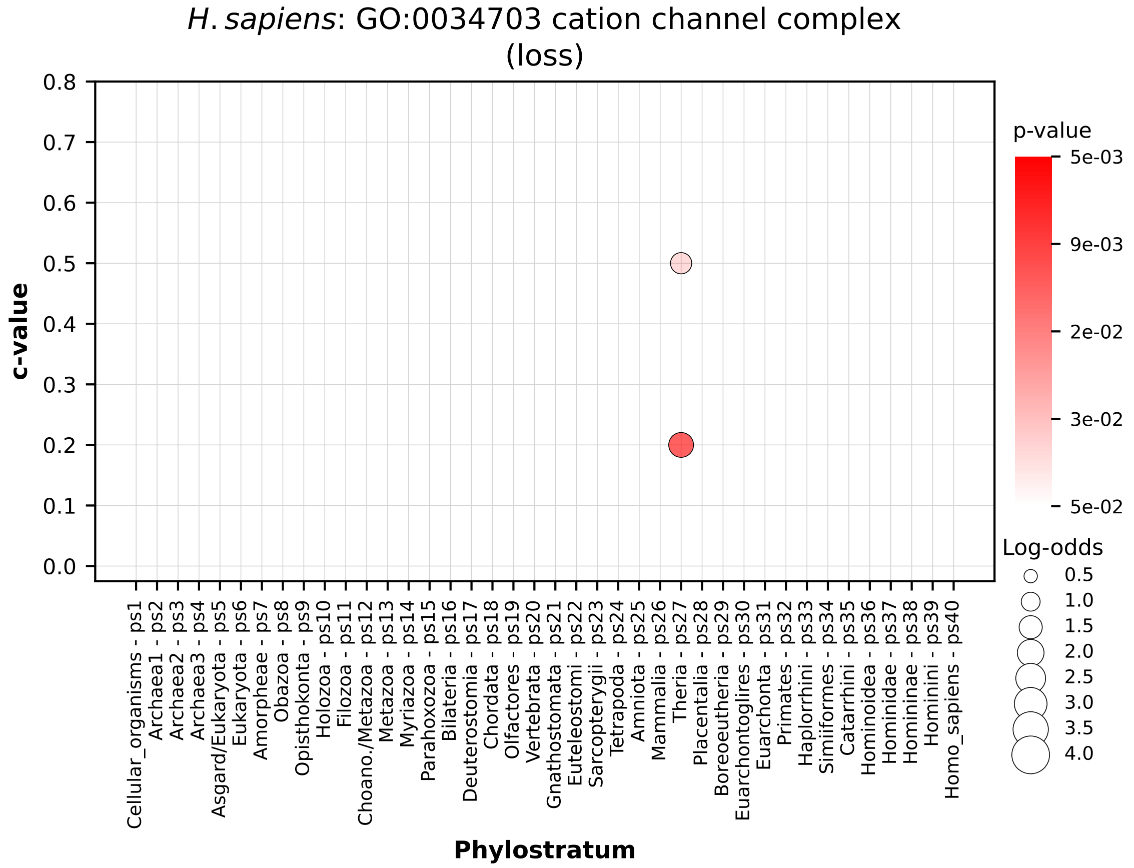
H. sapiens: GO:0034703 cation channel complex
(loss)
c-value
0.8
0.7
0.6
0.5
0.4
0.3
0.2
0.1
0.0
Phylostratum
Cellular_organisms - ps1
Archaea1 - ps2
Archaea2 - ps3
Archaea3 - ps4
Asgard/Eukaryota - ps5
Eukaryota - ps6
Amorpheae - ps7
Obazoa - ps8
Opisthokonta - ps9
Holozoa - ps10
Filozoa - ps11
Choano./Metazoa - ps12
Metazoa - ps13
Myriazoa - ps14
Parahoxozoa - ps15
Bilateria - ps16
Deuterostomia - ps17
Chordata - ps18
Olfactores - ps19
Vertebrata - ps20
Gnathostomata - ps21
Euteleostomi - ps22
Sarcopterygii - ps23
Tetrapoda - ps24
Amniota - ps25
Mammalia - ps26
Theria - ps27
Placentalia - ps28
Boreoeutheria - ps29
Euarchontoglires - ps30
Euarchonta - ps31
Primates - ps32
Haplorrhini - ps33
Simiiformes - ps34
Catarrhini - ps35
Hominoidea - ps36
Hominidae - ps37
Homininae - ps38
Hominini - ps39
Homo_sapiens - ps40
p-value
5e-03
9e-03
2e-02
3e-02
5e-02
Log-odds
0.5
1.0
1.5
2.0
2.5
3.0
3.5
4.0

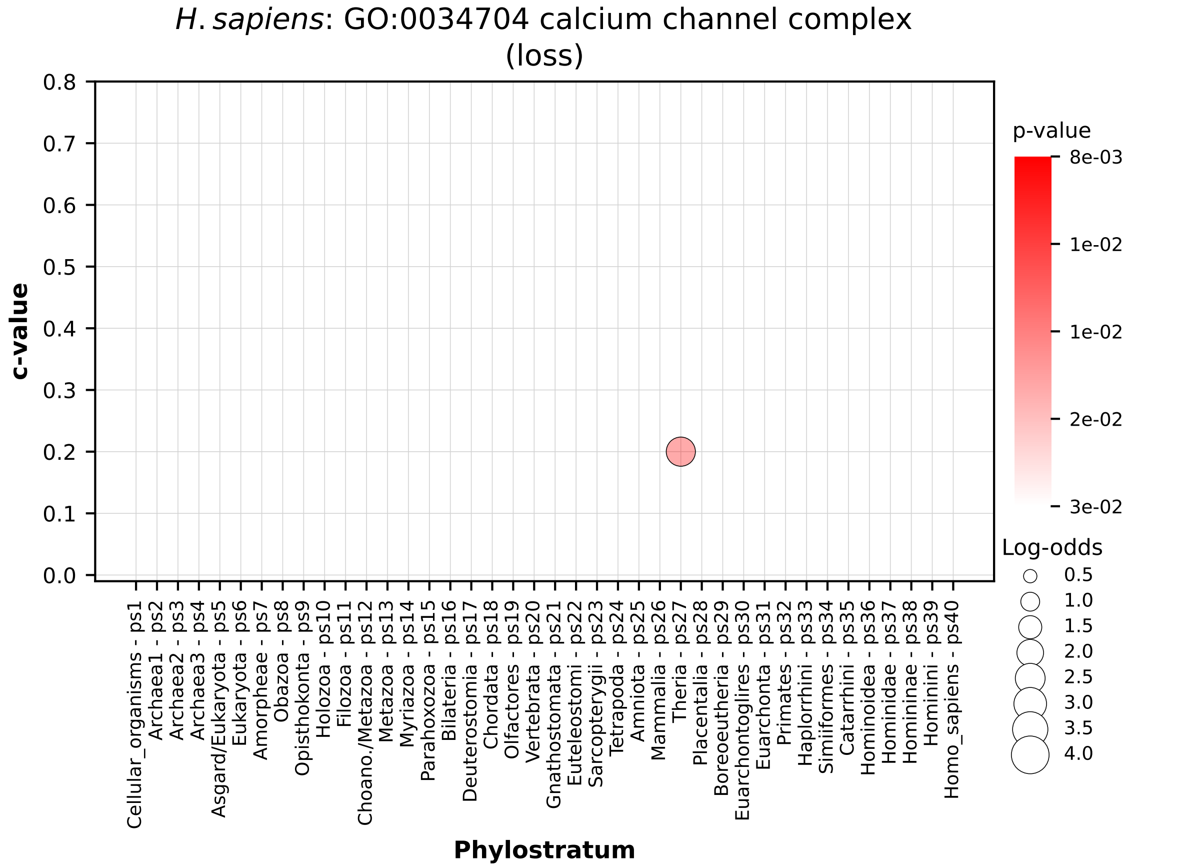

H. sapiens: GO:0034704 calcium channel complex
(loss)
c-value
0.8
0.7
0.6
0.5
0.4
0.3
0.2
0.1
0.0
Phylostratum
Cellular_organisms - ps1
Archaea1 - ps2
Archaea2 - ps3
Archaea3 - ps4
Asgard/Eukaryota - ps5
Eukaryota - ps6
Amorpheae - ps7
Obazoa - ps8
Opisthokonta - ps9
Holozoa - ps10
Filozoa - ps11
Choano./Metazoa - ps12
Metazoa - ps13
Myriazoa - ps14
Parahoxozoa - ps15
Bilateria - ps16
Deuterostomia - ps17
Chordata - ps18
Olfactores - ps19
Vertebrata - ps20
Gnathostomata - ps21
Euteleostomi - ps22
Sarcopterygii - ps23
Tetrapoda - ps24
Amniota - ps25
Mammalia - ps26
Theria - ps27
Placentalia - ps28
Boreoeutheria - ps29
Euarchontoglires - ps30
Euarchonta - ps31
Primates - ps32
Haplorrhini - ps33
Simiiformes - ps34
Catarrhini - ps35
Hominoidea - ps36
Hominidae - ps37
Homininae - ps38
Hominini - ps39
Homo_sapiens - ps40
p-value
8e-03
1e-02
1e-02
2e-02
3e-02
Log-odds
0.5
1.0
1.5
2.0
2.5
3.0
3.5
4.0

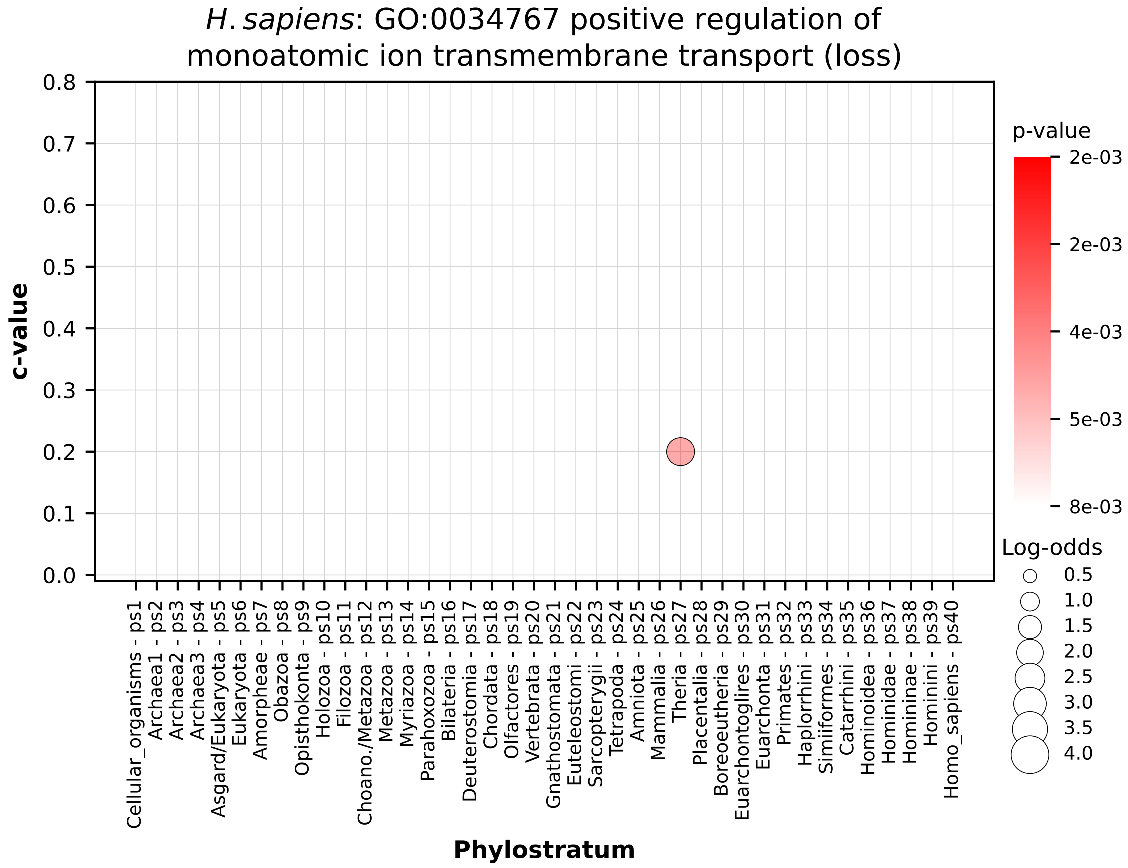

H. sapiens: GO:0034767 positive regulation of monoatomic ion transmembrane transport (loss)
c-value
0.8
0.7
0.6
0.5
0.4
0.3
0.2
0.1
0.0
p-value
2e-03
2e-03
4e-03
5e-03
8e-03
Log-odds
0.5
1.0
1.5
2.0
2.5
3.0
3.5
4.0
Cellular_organisms - ps1
Archaea1 - ps2
Archaea2 - ps3
Archaea3 - ps4
Asgard/Eukaryota - ps5
Eukaryota - ps6
Amorpheae - ps7
Obazoa - ps8
Opisthokonta - ps9
Holozoa - ps10
Filozoa - ps11
Choano./Metazoa - ps12
Metazoa - ps13
Myriazoa - ps14
Parahoxozoa - ps15
Bilateria - ps16
Deuterostomia - ps17
Chordata - ps18
Olfactores - ps19
Vertebrata - ps20
Gnathostomata - ps21
Euteleostomi - ps22
Sarcopterygii - ps23
Tetrapoda - ps24
Amniota - ps25
Mammalia - ps26
Theria - ps27
Placentalia - ps28
Boreoeutheria - ps29
Euarchontoglires - ps30
Euarchonta - ps31
Primates - ps32
Haplorrhini - ps33
Simiiformes - ps34
Catarrhini - ps35
Hominoidea - ps36
Hominidae - ps37
Homininae - ps38
Hominini - ps39
Homo_sapiens - ps40
Phylostratum

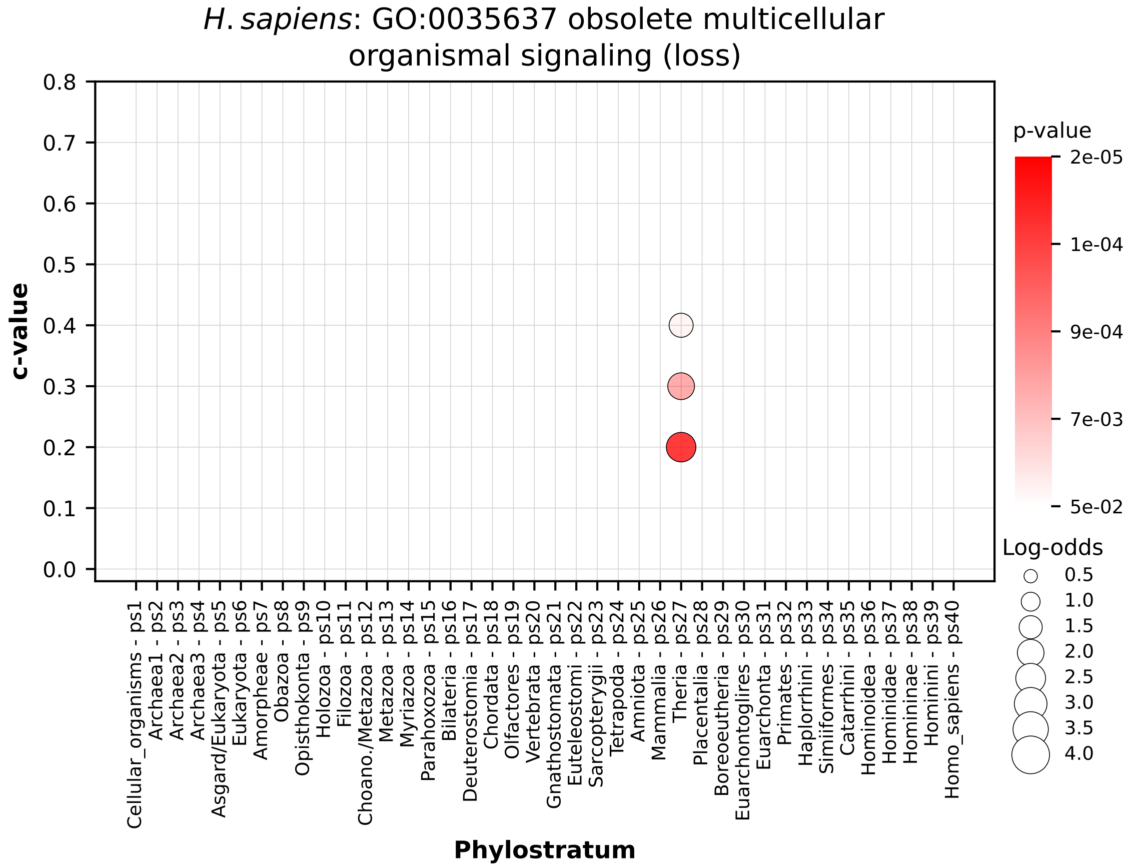

H. sapiens: GO:0035637 obsolete multicellular organismal signaling (loss)
c-value
Phylostratum
p-value
2e-05
1e-04
9e-04
7e-03
5e-02
Log-odds
0.5
1.0
1.5
2.0
2.5
3.0
3.5
4.0
Cellular_organisms - ps1
Archaea1 - ps2
Archaea2 - ps3
Archaea3 - ps4
Asgard/Eukaryota - ps5
Eukaryota - ps6
Amorpheae - ps7
Obazoa - ps8
Opisthokonta - ps9
Holozoa - ps10
Filozoa - ps11
Choano./Metazoa - ps12
Metazoa - ps13
Myriazoa - ps14
Parahoxozoa - ps15
Bilateria - ps16
Deuterostomia - ps17
Chordata - ps18
Olfactores - ps19
Vertebrata - ps20
Gnathostomata - ps21
Euteleostomi - ps22
Sarcopterygii - ps23
Tetrapoda - ps24
Amniota - ps25
Mammalia - ps26
Theria - ps27
Placentalia - ps28
Boreoeutheria - ps29
Euarchontoglires - ps30
Euarchonta - ps31
Primates - ps32
Haplorrhini - ps33
Simiiformes - ps34
Catarrhini - ps35
Hominoidea - ps36
Hominidae - ps37
Homininae - ps38
Hominini - ps39
Homo_sapiens - ps40

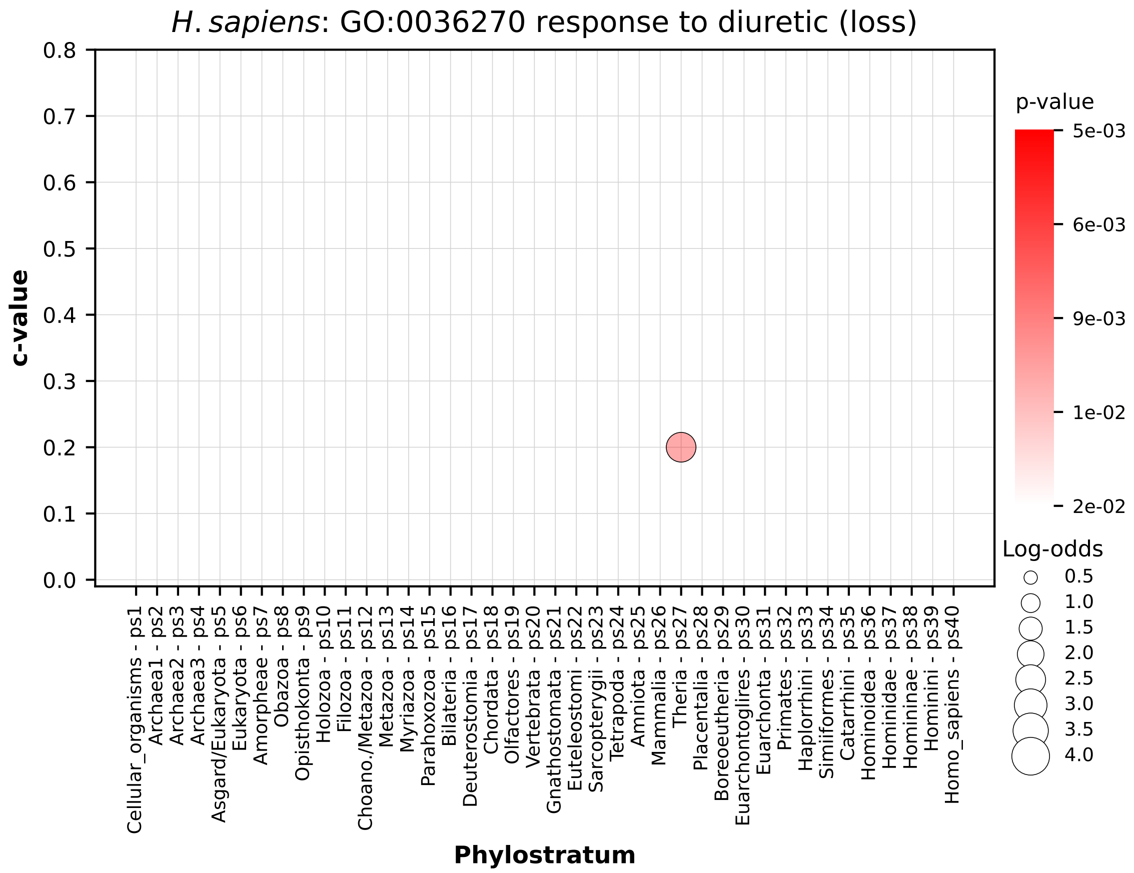

H. sapiens: GO:0036270 response to diuretic (loss)
c-value
0.8
0.7
0.6
0.5
0.4
0.3
0.2
0.1
0.0
Phylostratum
Cellular_organisms - ps1
Archaea1 - ps2
Archaea2 - ps3
Archaea3 - ps4
Asgard/Eukaryota - ps5
Eukaryota - ps6
Amorpheae - ps7
Obazoa - ps8
Opisthokonta - ps9
Holozoa - ps10
Filozoa - ps11
Choano./Metazoa - ps12
Metazoa - ps13
Myriazoa - ps14
Parahoxozoa - ps15
Bilateria - ps16
Deuterostomia - ps17
Chordata - ps18
Olfactores - ps19
Vertebrata - ps20
Gnathostomata - ps21
Euteleostomi - ps22
Sarcopterygii - ps23
Tetrapoda - ps24
Amniota - ps25
Mammalia - ps26
Theria - ps27
Placentalia - ps28
Boreoeutheria - ps29
Euarchontoglires - ps30
Euarchonta - ps31
Primates - ps32
Haplorrhini - ps33
Simiiformes - ps34
Catarrhini - ps35
Hominoidea - ps36
Hominidae - ps37
Homininae - ps38
Hominini - ps39
Homo_sapiens - ps40
p-value
5e-03
6e-03
9e-03
1e-02
2e-02
Log-odds
0.5
1.0
1.5
2.0
2.5
3.0
3.5
4.0

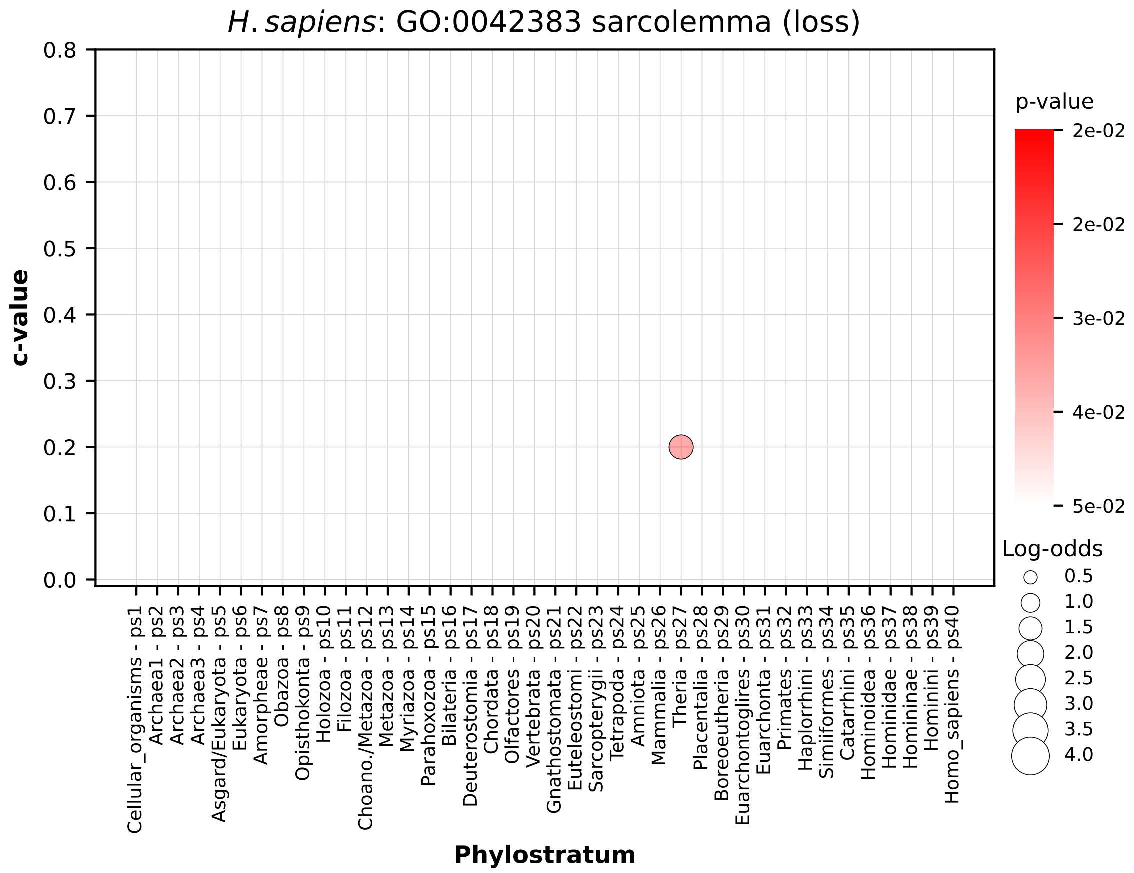

H. sapiens: GO:0042383 sarcolemma (loss)
c-value
0.0
0.1
0.2
0.3
0.4
0.5
0.6
0.7
0.8
Phylostratum
Cellular_organisms - ps1
Archaea1 - ps2
Archaea2 - ps3
Archaea3 - ps4
Asgard/Eukaryota - ps5
Eukaryota - ps6
Amorpheae - ps7
Obazoa - ps8
Opisthokonta - ps9
Holozoa - ps10
Filozoa - ps11
Choano./Metazoa - ps12
Metazoa - ps13
Myriazoa - ps14
Parahoxozoa - ps15
Bilateria - ps16
Deuterostomia - ps17
Chordata - ps18
Olfactores - ps19
Vertebrata - ps20
Gnathostomata - ps21
Euteleostomi - ps22
Sarcopterygii - ps23
Tetrapoda - ps24
Amniota - ps25
Mammalia - ps26
Theria - ps27
Placentalia - ps28
Boreoeutheria - ps29
Euarchontoglires - ps30
Euarchonta - ps31
Primates - ps32
Haplorrhini - ps33
Simiiformes - ps34
Catarrhini - ps35
Hominoidea - ps36
Hominidae - ps37
Homininae - ps38
Hominini - ps39
Homo_sapiens - ps40
p-value
2e-02
2e-02
3e-02
4e-02
5e-02
Log-odds
0.5
1.0
1.5
2.0
2.5
3.0
3.5
4.0

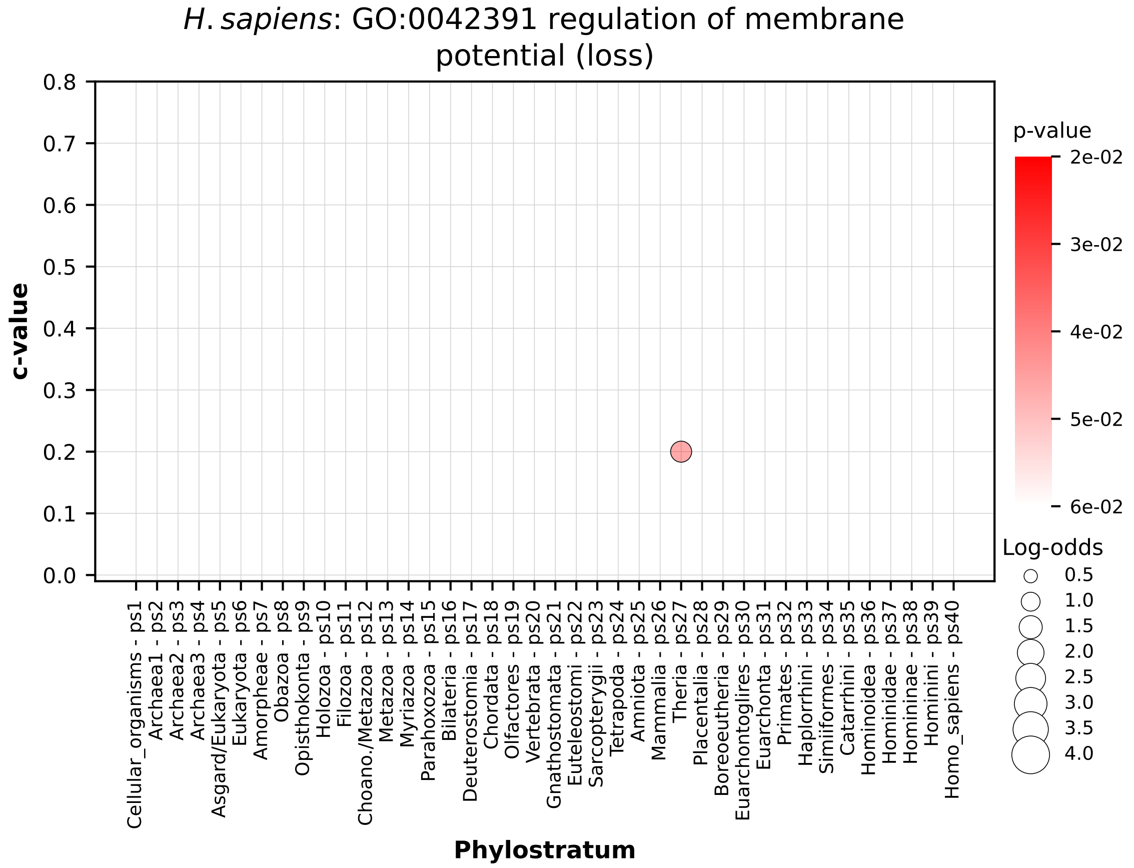

H. sapiens: GO:0042391 regulation of membrane potential (loss)
c-value
0.8
0.7
0.6
0.5
0.4
0.3
0.2
0.1
0.0
Phylostratum
Cellular_organisms - ps1
Archaea1 - ps2
Archaea2 - ps3
Archaea3 - ps4
Asgard/Eukaryota - ps5
Eukaryota - ps6
Amorpheae - ps7
Obazoa - ps8
Opisthokonta - ps9
Holozoa - ps10
Filozoa - ps11
Choano./Metazoa - ps12
Metazoa - ps13
Myriazoa - ps14
Parahoxozoa - ps15
Bilateria - ps16
Deuterostomia - ps17
Chordata - ps18
Olfactores - ps19
Vertebrata - ps20
Gnathostomata - ps21
Euteleostomi - ps22
Sarcopterygii - ps23
Tetrapoda - ps24
Amniota - ps25
Mammalia - ps26
Theria - ps27
Placentalia - ps28
Boreoeutheria - ps29
Euarchontoglires - ps30
Euarchonta - ps31
Primates - ps32
Haplorrhini - ps33
Simiiformes - ps34
Catarrhini - ps35
Hominoidea - ps36
Hominidae - ps37
Homininae - ps38
Hominini - ps39
Homo_sapiens - ps40
p-value
2e-02
3e-02
4e-02
5e-02
6e-02
Log-odds
0.5
1.0
1.5
2.0
2.5
3.0
3.5
4.0

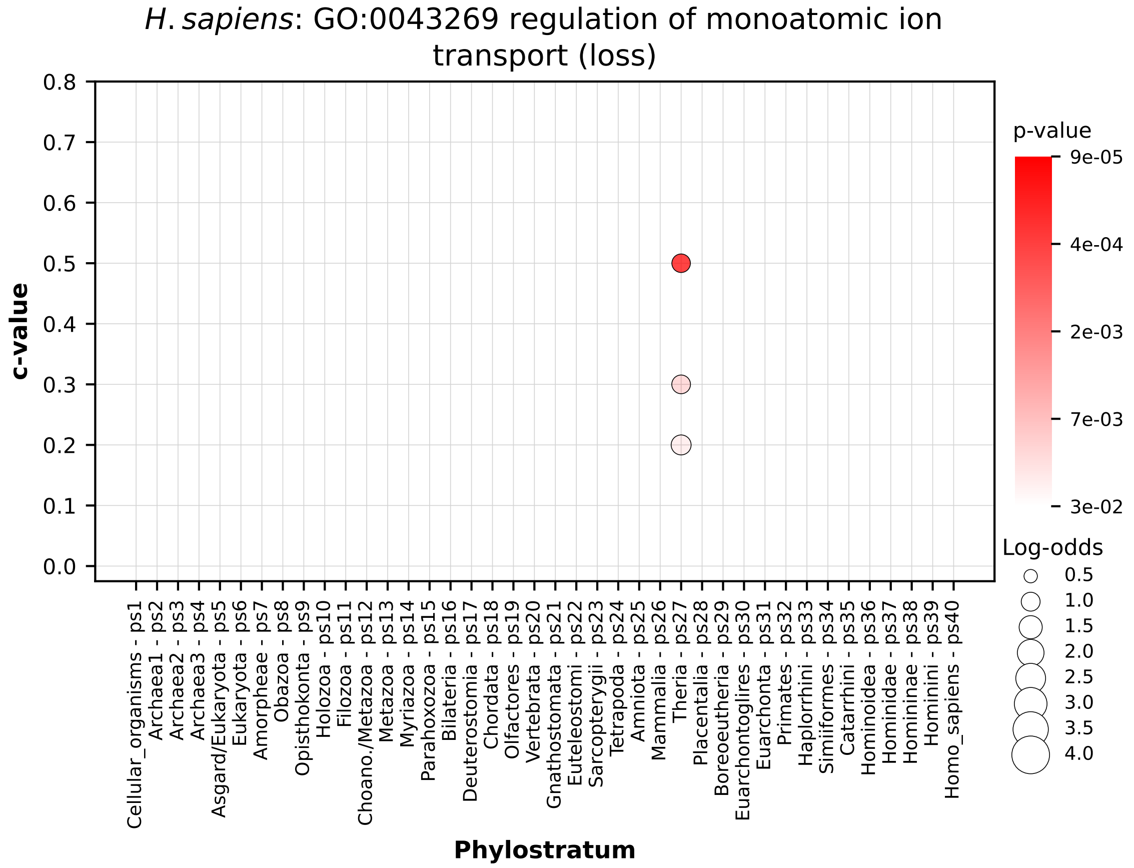

H. sapiens: GO:0043269 regulation of monoatomic ion transport (loss)
c-value
0.8
0.7
0.6
0.5
0.4
0.3
0.2
0.1
0.0
Phylostratum
Cellular_organisms - ps1
Archaea1 - ps2
Archaea2 - ps3
Archaea3 - ps4
Asgard/Eukaryota - ps5
Eukaryota - ps6
Amorpheae - ps7
Obazoa - ps8
Opisthokonta - ps9
Holozoa - ps10
Filozoa - ps11
Choano./Metazoa - ps12
Metazoa - ps13
Myriazoa - ps14
Parahoxozoa - ps15
Bilateria - ps16
Deuterostomia - ps17
Chordata - ps18
Olfactores - ps19
Vertebrata - ps20
Gnathostomata - ps21
Euteleostomi - ps22
Sarcopterygii - ps23
Tetrapoda - ps24
Amniota - ps25
Mammalia - ps26
Theria - ps27
Placentalia - ps28
Boreoeutheria - ps29
Euarchontoglires - ps30
Euarchonta - ps31
Primates - ps32
Haplorrhini - ps33
Simiiformes - ps34
Catarrhini - ps35
Hominoidea - ps36
Hominidae - ps37
Homininae - ps38
Hominini - ps39
Homo_sapiens - ps40
p-value
9e-05
4e-04
2e-03
7e-03
3e-02
Log-odds
0.5
1.0
1.5
2.0
2.5
3.0
3.5
4.0

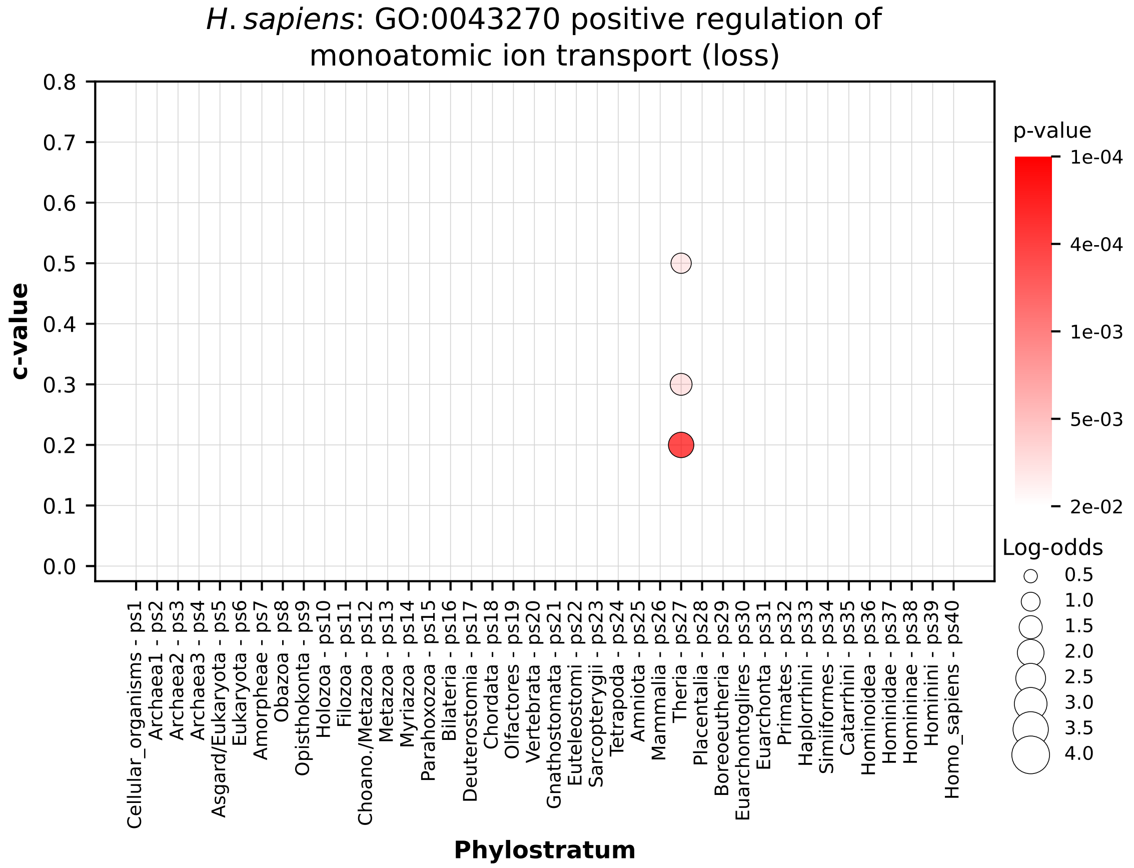

H. sapiens: GO:0043270 positive regulation of monoatomic ion transport (loss)
c-value
0.8
0.7
0.6
0.5
0.4
0.3
0.2
0.1
0.0
p-value
1e-04
4e-04
1e-03
5e-03
2e-02
Log-odds
0.5
1.0
1.5
2.0
2.5
3.0
3.5
4.0
Cellular_organisms - ps1
Archaea1 - ps2
Archaea2 - ps3
Archaea3 - ps4
Asgard/Eukaryota - ps5
Eukaryota - ps6
Amorpheae - ps7
Obazoa - ps8
Opisthokonta - ps9
Holozoa - ps10
Filozoa - ps11
Choano./Metazoa - ps12
Metazoa - ps13
Myriazoa - ps14
Parahoxozoa - ps15
Bilateria - ps16
Deuterostomia - ps17
Chordata - ps18
Olfactores - ps19
Vertebrata - ps20
Gnathostomata - ps21
Euteleostomi - ps22
Sarcopterygii - ps23
Tetrapoda - ps24
Amniota - ps25
Mammalia - ps26
Theria - ps27
Placentalia - ps28
Boreoeutheria - ps29
Euarchontoglires - ps30
Euarchonta - ps31
Primates - ps32
Haplorrhini - ps33
Simiiformes - ps34
Catarrhini - ps35
Hominoidea - ps36
Hominidae - ps37
Homininae - ps38
Hominini - ps39
Homo_sapiens - ps40
Phylostratum

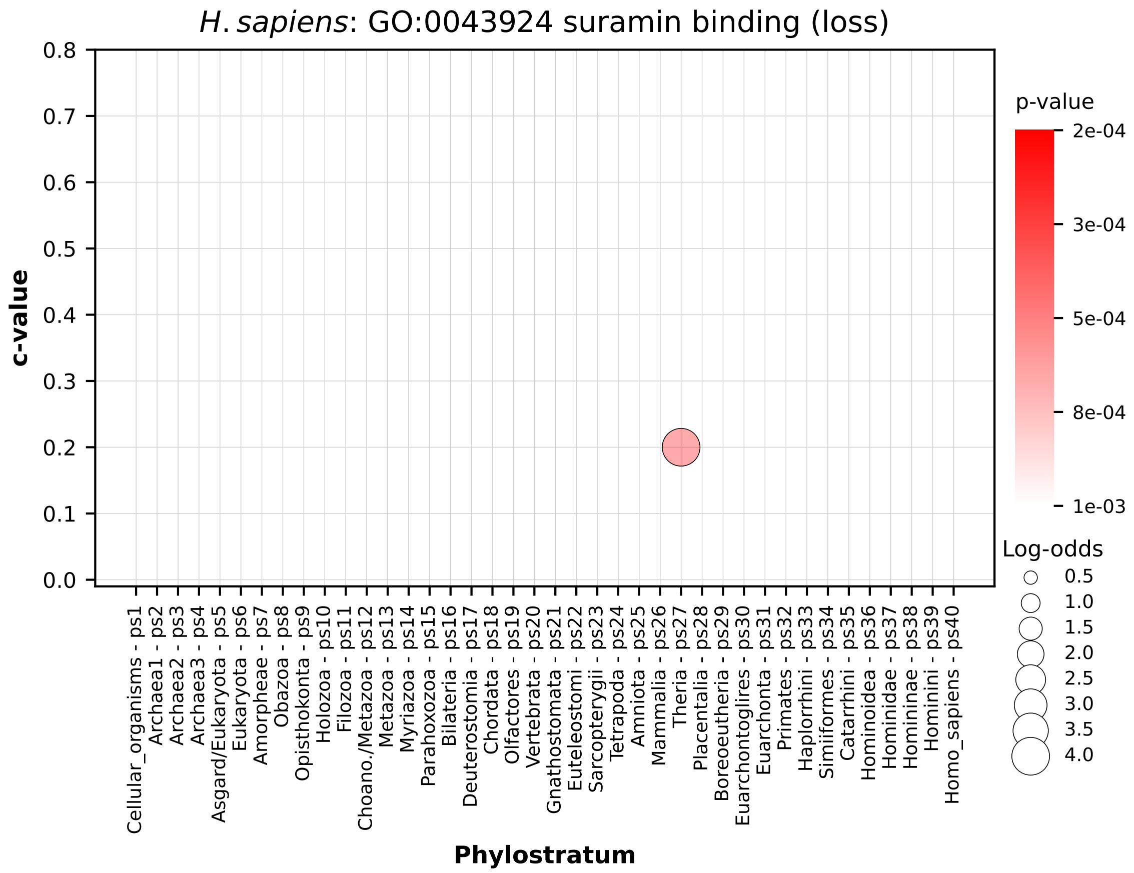

H. sapiens: GO:0043924 suramin binding (loss)
c-value
0.0
0.1
0.2
0.3
0.4
0.5
0.6
0.7
0.8
Phylostratum
Cellular_organisms - ps1
Archaea1 - ps2
Archaea2 - ps3
Archaea3 - ps4
Asgard/Eukaryota - ps5
Eukaryota - ps6
Amorpheae - ps7
Obazoa - ps8
Opisthokonta - ps9
Holozoa - ps10
Filozoa - ps11
Choano./Metazoa - ps12
Metazoa - ps13
Myriazoa - ps14
Parahoxozoa - ps15
Bilateria - ps16
Deuterostomia - ps17
Chordata - ps18
Olfactores - ps19
Vertebrata - ps20
Gnathostomata - ps21
Euteleostomi - ps22
Sarcopterygii - ps23
Tetrapoda - ps24
Amniota - ps25
Mammalia - ps26
Theria - ps27
Placentalia - ps28
Boreoeutheria - ps29
Euarchontoglires - ps30
Euarchonta - ps31
Primates - ps32
Haplorrhini - ps33
Simiiformes - ps34
Catarrhini - ps35
Hominoidea - ps36
Hominidae - ps37
Homininae - ps38
Hominini - ps39
Homo_sapiens - ps40
p-value
2e-04
3e-04
5e-04
8e-04
1e-03
Log-odds
0.5
1.0
1.5
2.0
2.5
3.0
3.5
4.0

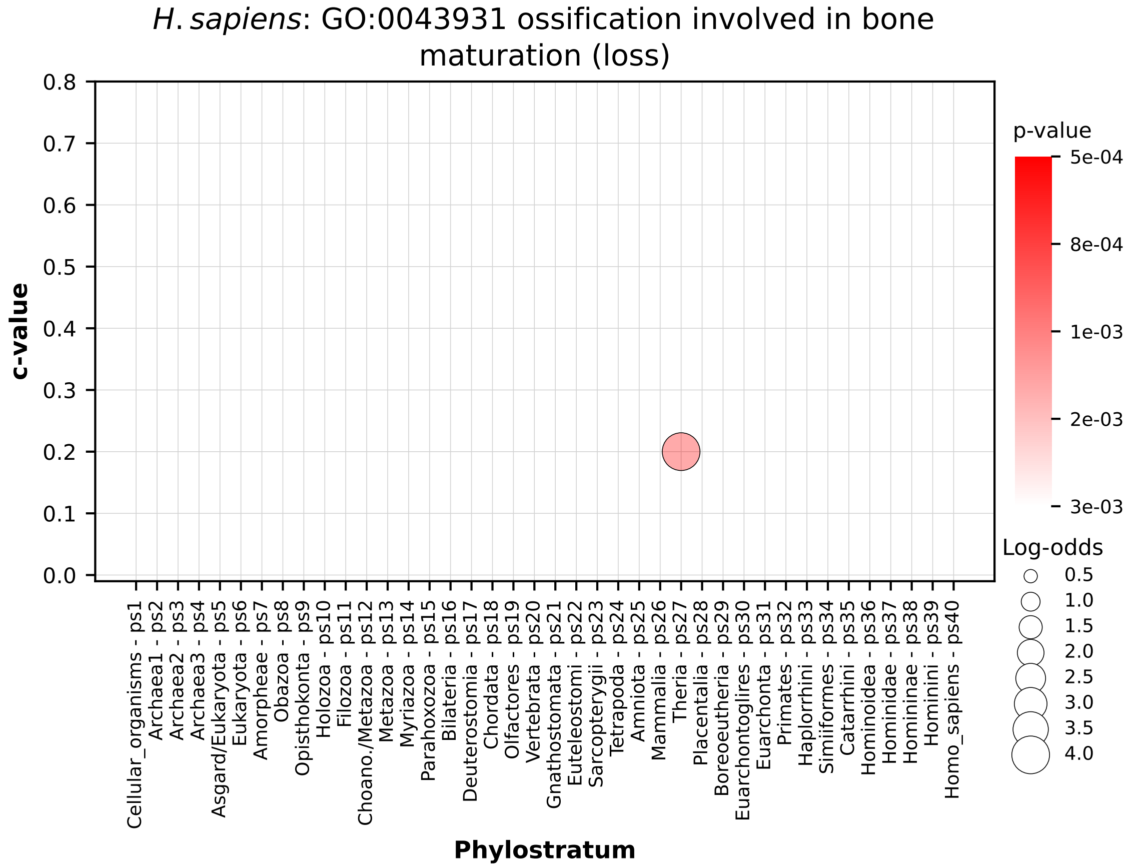

H. sapiens: GO:0043931 ossification involved in bone maturation (loss)
c-value
0.8
0.7
0.6
0.5
0.4
0.3
0.2
0.1
0.0
Phylostratum
Cellular_organisms - ps1
Archaea1 - ps2
Archaea2 - ps3
Archaea3 - ps4
Asgard/Eukaryota - ps5
Eukaryota - ps6
Amorpheae - ps7
Obazoa - ps8
Opisthokonta - ps9
Holozoa - ps10
Filozoa - ps11
Choano./Metazoa - ps12
Metazoa - ps13
Myriazoa - ps14
Parahoxozoa - ps15
Bilateria - ps16
Deuterostomia - ps17
Chordata - ps18
Olfactores - ps19
Vertebrata - ps20
Gnathostomata - ps21
Euteleostomi - ps22
Sarcopterygii - ps23
Tetrapoda - ps24
Amniota - ps25
Mammalia - ps26
Theria - ps27
Placentalia - ps28
Boreoeutheria - ps29
Euarchontoglires - ps30
Euarchonta - ps31
Primates - ps32
Haplorrhini - ps33
Simiiformes - ps34
Catarrhini - ps35
Hominoidea - ps36
Hominidae - ps37
Homininae - ps38
Hominini - ps39
Homo_sapiens - ps40
p-value
5e-04
8e-04
1e-03
2e-03
3e-03
Log-odds
0.5
1.0
1.5
2.0
2.5
3.0
3.5
4.0

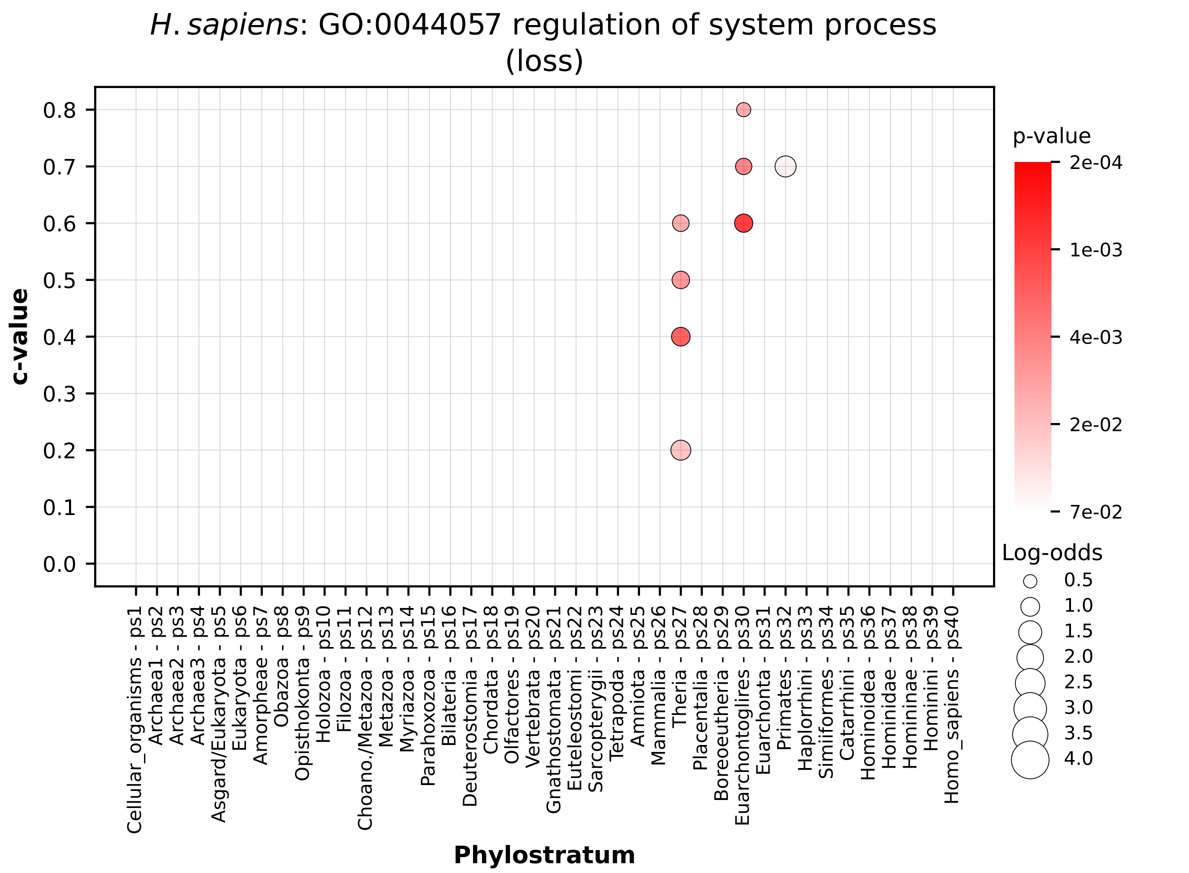

H. sapiens: GO:0044057 regulation of system process (loss)
c-value
0.8
0.7
0.6
0.5
0.4
0.3
0.2
0.1
0.0
Phylostratum
Cellular_organisms - ps1
Archaea1 - ps2
Archaea2 - ps3
Archaea3 - ps4
Asgard/Eukaryota - ps5
Eukaryota - ps6
Amorpheae - ps7
Obazoa - ps8
Opisthokonta - ps9
Holozoa - ps10
Filozoa - ps11
Choano./Metazoa - ps12
Metazoa - ps13
Myriazoa - ps14
Parahoxozoa - ps15
Bilateria - ps16
Deuterostomia - ps17
Chordata - ps18
Olfactores - ps19
Vertebrata - ps20
Gnathostomata - ps21
Euteleostomi - ps22
Sarcopterygii - ps23
Tetrapoda - ps24
Amniota - ps25
Mammalia - ps26
Theria - ps27
Placentalia - ps28
Boreoeutheria - ps29
Euarchontoglires - ps30
Euarchonta - ps31
Primates - ps32
Haplorrhini - ps33
Simiiformes - ps34
Catarrhini - ps35
Hominoidea - ps36
Hominidae - ps37
Homininae - ps38
Hominini - ps39
Homo_sapiens - ps40
p-value
2e-04
1e-03
4e-03
2e-02
7e-02
Log-odds
0.5
1.0
1.5
2.0
2.5
3.0
3.5
4.0

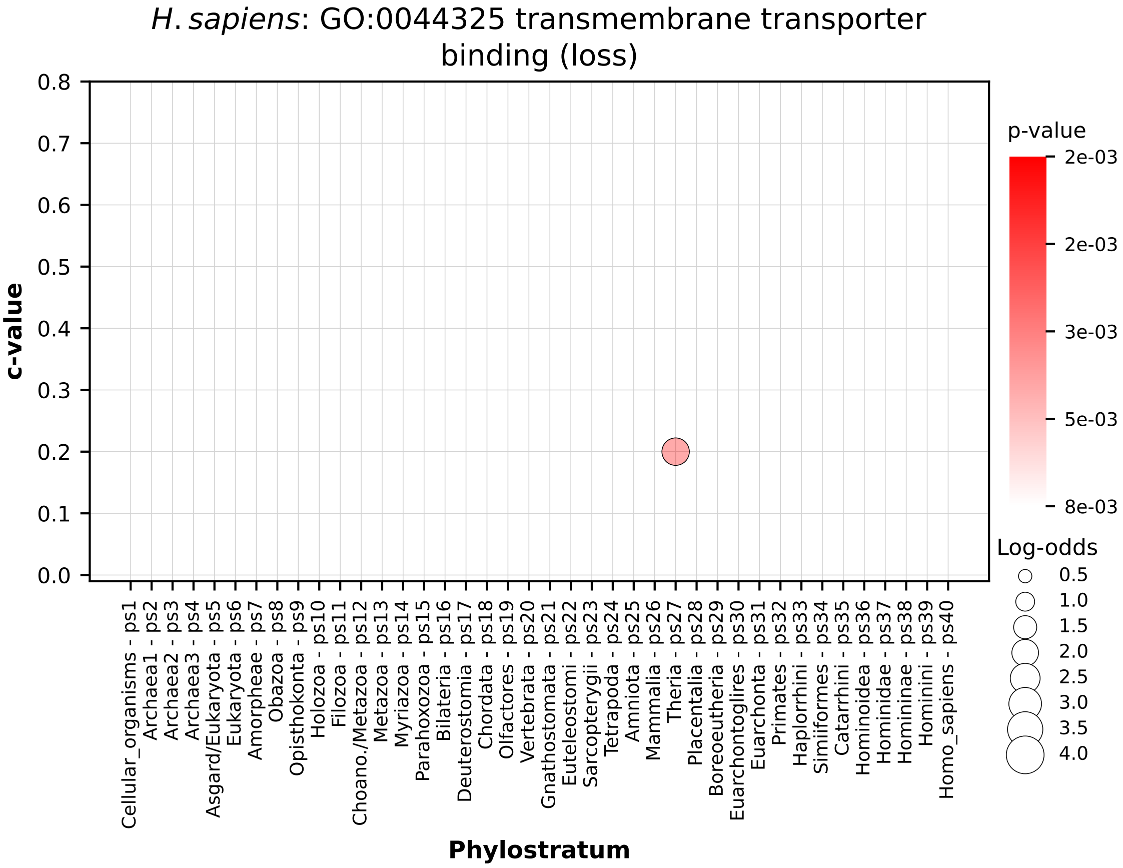

H. sapiens: GO:0044325 transmembrane transporter binding (loss)
c-value
0.8
0.7
0.6
0.5
0.4
0.3
0.2
0.1
0.0
p-value
2e-03
2e-03
3e-03
5e-03
8e-03
Log-odds
0.5
1.0
1.5
2.0
2.5
3.0
3.5
4.0
Cellular_organisms - ps1
Archaea1 - ps2
Archaea2 - ps3
Archaea3 - ps4
Asgard/Eukaryota - ps5
Eukaryota - ps6
Amorpheae - ps7
Obazoa - ps8
Opisthokonta - ps9
Holozoa - ps10
Filozoa - ps11
Choano./Metazoa - ps12
Metazoa - ps13
Myriazoa - ps14
Parahoxozoa - ps15
Bilateria - ps16
Deuterostomia - ps17
Chordata - ps18
Olfactores - ps19
Vertebrata - ps20
Gnathostomata - ps21
Euteleostomi - ps22
Sarcopterygii - ps23
Tetrapoda - ps24
Amniota - ps25
Mammalia - ps26
Theria - ps27
Placentalia - ps28
Boreoeutheria - ps29
Euarchontoglires - ps30
Euarchonta - ps31
Primates - ps32
Haplorrhini - ps33
Simiiformes - ps34
Catarrhini - ps35
Hominoidea - ps36
Hominidae - ps37
Homininae - ps38
Hominini - ps39
Homo_sapiens - ps40
Phylostratum

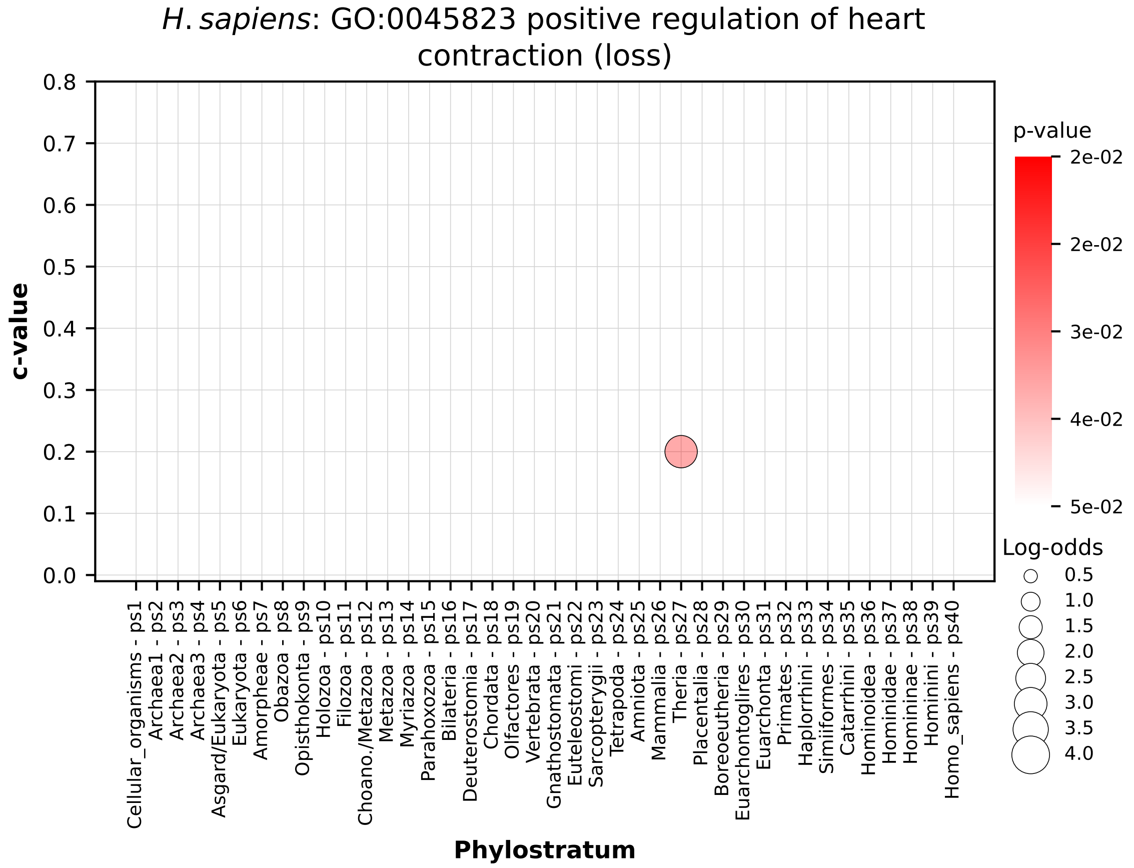

H. sapiens: GO:0045823 positive regulation of heart contraction (loss)
c-value
0.8
0.7
0.6
0.5
0.4
0.3
0.2
0.1
0.0
Phylostratum
Cellular_organisms - ps1
Archaea1 - ps2
Archaea2 - ps3
Archaea3 - ps4
Asgard/Eukaryota - ps5
Eukaryota - ps6
Amorpheae - ps7
Obazoa - ps8
Opisthokonta - ps9
Holozoa - ps10
Filozoa - ps11
Choano./Metazoa - ps12
Metazoa - ps13
Myriazoa - ps14
Parahoxozoa - ps15
Bilateria - ps16
Deuterostomia - ps17
Chordata - ps18
Olfactores - ps19
Vertebrata - ps20
Gnathostomata - ps21
Euteleostomi - ps22
Sarcopterygii - ps23
Tetrapoda - ps24
Amniota - ps25
Mammalia - ps26
Theria - ps27
Placentalia - ps28
Boreoeutheria - ps29
Euarchontoglires - ps30
Euarchonta - ps31
Primates - ps32
Haplorrhini - ps33
Simiiformes - ps34
Catarrhini - ps35
Hominoidea - ps36
Hominidae - ps37
Homininae - ps38
Hominini - ps39
Homo_sapiens - ps40
p-value
2e-02
2e-02
3e-02
4e-02
5e-02
Log-odds
0.5
1.0
1.5
2.0
2.5
3.0
3.5
4.0

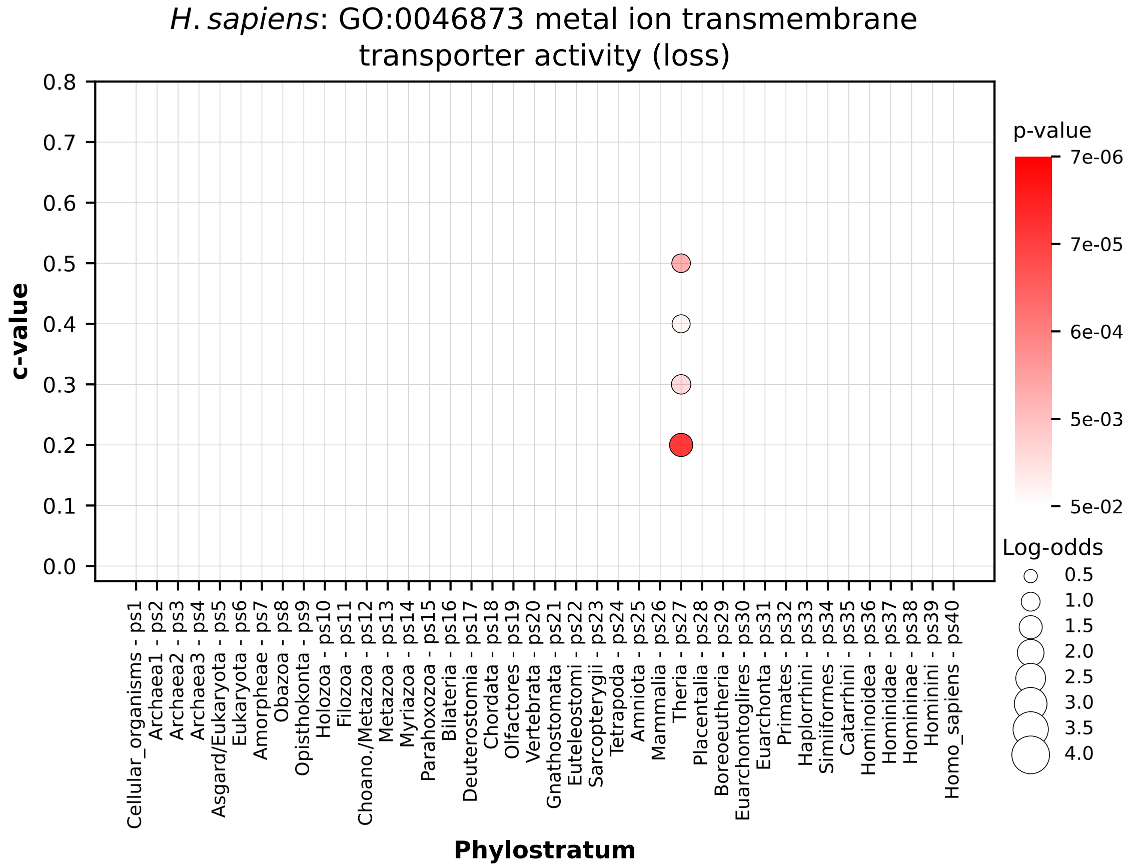

H. sapiens: GO:0046873 metal ion transmembrane transporter activity (loss)
c-value
0.8
0.7
0.6
0.5
0.4
0.3
0.2
0.1
0.0
p-value
7e-06
7e-05
6e-04
5e-03
5e-02
Log-odds
0.5
1.0
1.5
2.0
2.5
3.0
3.5
4.0
Cellular_organisms - ps1
Archaea1 - ps2
Archaea2 - ps3
Archaea3 - ps4
Asgard/Eukaryota - ps5
Eukaryota - ps6
Amorpheae - ps7
Obazoa - ps8
Opisthokonta - ps9
Holozoa - ps10
Filozoa - ps11
Choano./Metazoa - ps12
Metazoa - ps13
Myriazoa - ps14
Parahoxozoa - ps15
Bilateria - ps16
Deuterostomia - ps17
Chordata - ps18
Olfactores - ps19
Vertebrata - ps20
Gnathostomata - ps21
Euteleostomi - ps22
Sarcopterygii - ps23
Tetrapoda - ps24
Amniota - ps25
Mammalia - ps26
Theria - ps27
Placentalia - ps28
Boreoeutheria - ps29
Euarchontoglires - ps30
Euarchonta - ps31
Primates - ps32
Haplorrhini - ps33
Simiiformes - ps34
Catarrhini - ps35
Hominoidea - ps36
Hominidae - ps37
Homininae - ps38
Hominini - ps39
Homo_sapiens - ps40
Phylostratum

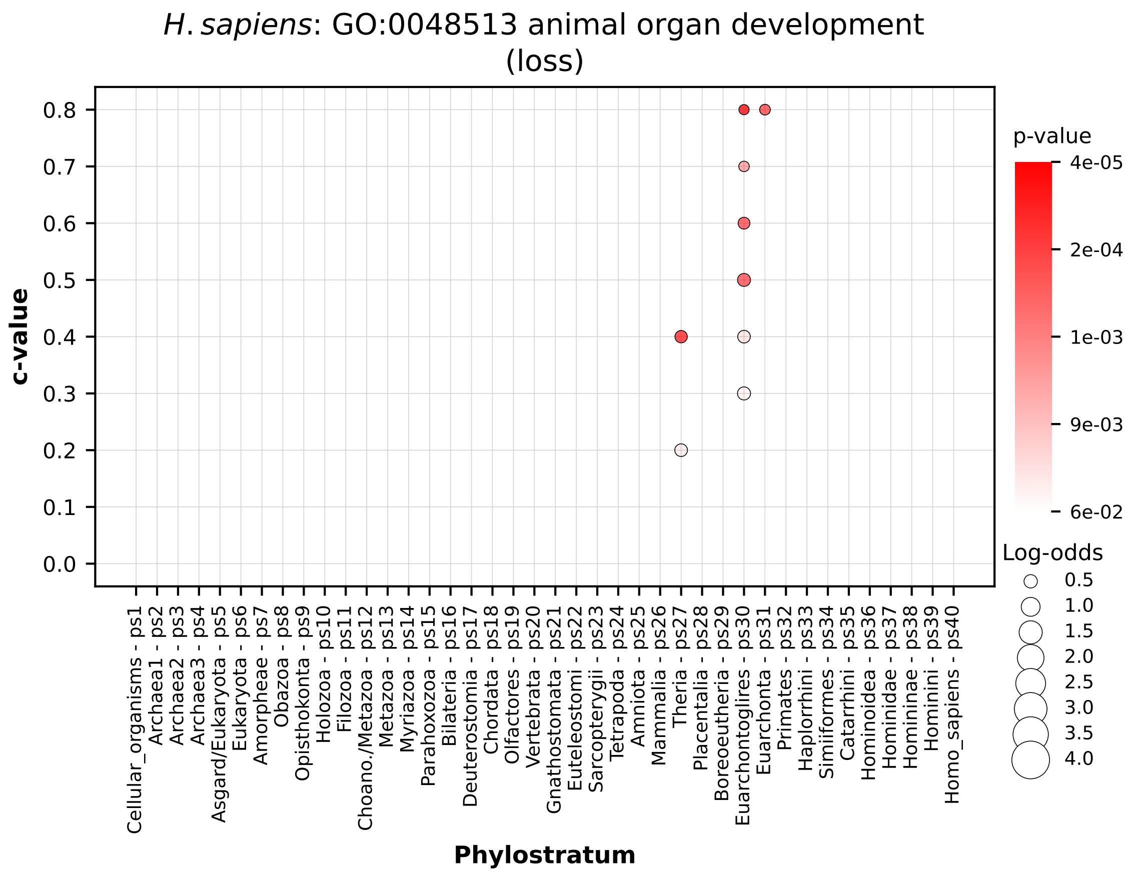

H. sapiens: GO:0048513 animal organ development
(loss)
c-value
0.0
0.1
0.2
0.3
0.4
0.5
0.6
0.7
0.8
Phylostratum
Cellular_organisms - ps1
Archaea1 - ps2
Archaea2 - ps3
Archaea3 - ps4
Asgard/Eukaryota - ps5
Eukaryota - ps6
Amorpheae - ps7
Obazoa - ps8
Opisthokonta - ps9
Holozoa - ps10
Filozoa - ps11
Choano./Metazoa - ps12
Metazoa - ps13
Myriazoa - ps14
Parahoxozoa - ps15
Bilateria - ps16
Deuterostomia - ps17
Chordata - ps18
Olfactores - ps19
Vertebrata - ps20
Gnathostomata - ps21
Euteleostomi - ps22
Sarcopterygii - ps23
Tetrapoda - ps24
Amniota - ps25
Mammalia - ps26
Theria - ps27
Placentalia - ps28
Boreoeutheria - ps29
Euarchontoglires - ps30
Euarchonta - ps31
Primates - ps32
Haplorrhini - ps33
Simiiformes - ps34
Catarrhini - ps35
Hominoidea - ps36
Hominidae - ps37
Homininae - ps38
Hominini - ps39
Homo_sapiens - ps40
p-value
4e-05
2e-04
1e-03
9e-03
6e-02
Log-odds
0.5
1.0
1.5
2.0
2.5
3.0
3.5
4.0

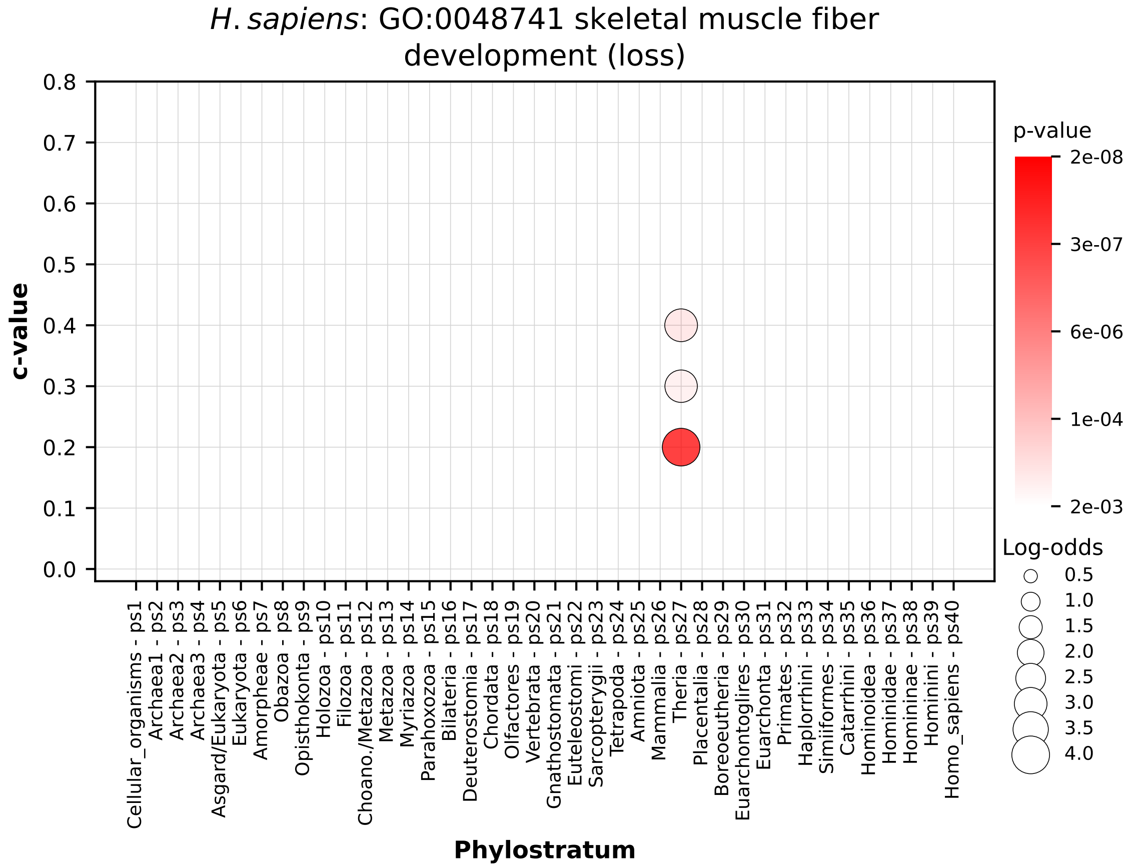

H. sapiens: GO:0048741 skeletal muscle fiber development (loss)
c-value
0.0
0.1
0.2
0.3
0.4
0.5
0.6
0.7
0.8
Phylostratum
Cellular_organisms - ps1
Archaea1 - ps2
Archaea2 - ps3
Archaea3 - ps4
Asgard/Eukaryota - ps5
Eukaryota - ps6
Amorpheae - ps7
Obazoa - ps8
Opisthokonta - ps9
Holozoa - ps10
Filozoa - ps11
Choano./Metazoa - ps12
Metazoa - ps13
Myriazoa - ps14
Parahoxozoa - ps15
Bilateria - ps16
Deuterostomia - ps17
Chordata - ps18
Olfactores - ps19
Vertebrata - ps20
Gnathostomata - ps21
Euteleostomi - ps22
Sarcopterygii - ps23
Tetrapoda - ps24
Amniota - ps25
Mammalia - ps26
Theria - ps27
Placentalia - ps28
Boreoeutheria - ps29
Euarchontoglires - ps30
Euarchonta - ps31
Primates - ps32
Haplorrhini - ps33
Simiiformes - ps34
Catarrhini - ps35
Hominoidea - ps36
Hominidae - ps37
Homininae - ps38
Hominini - ps39
Homo_sapiens - ps40
p-value
2e-08
3e-07
6e-06
1e-04
2e-03
Log-odds
0.5
1.0
1.5
2.0
2.5
3.0
3.5
4.0

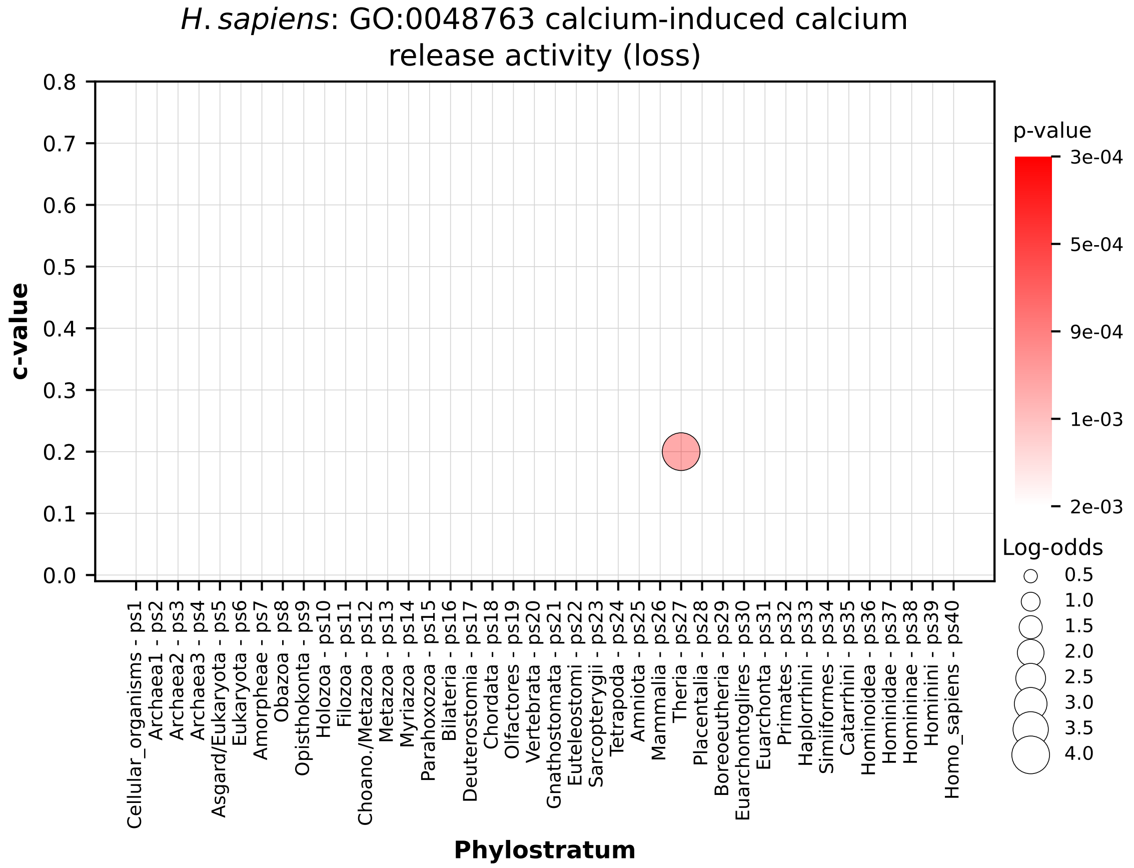
H. sapiens: GO:0048763 calcium-induced calcium release activity (loss)
c-value
0.0
0.1
0.2
0.3
0.4
0.5
0.6
0.7
0.8
Phylostratum
Cellular_organisms - ps1
Archaea1 - ps2
Archaea2 - ps3
Archaea3 - ps4
Asgard/Eukaryota - ps5
Eukaryota - ps6
Amorpheae - ps7
Obazoa - ps8
Opisthokonta - ps9
Holozoa - ps10
Filozoa - ps11
Choano./Metazoa - ps12
Metazoa - ps13
Myriazoa - ps14
Parahoxozoa - ps15
Bilateria - ps16
Deuterostomia - ps17
Chordata - ps18
Olfactores - ps19
Vertebrata - ps20
Gnathostomata - ps21
Euteleostomi - ps22
Sarcopterygii - ps23
Tetrapoda - ps24
Amniota - ps25
Mammalia - ps26
Theria - ps27
Placentalia - ps28
Boreoeutheria - ps29
Euarchontoglires - ps30
Euarchonta - ps31
Primates - ps32
Haplorrhini - ps33
Simiiformes - ps34
Catarrhini - ps35
Hominoidea - ps36
Hominidae - ps37
Homininae - ps38
Hominini - ps39
Homo_sapiens - ps40
p-value
3e-04
5e-04
9e-04
1e-03
2e-03
Log-odds
0.5
1.0
1.5
2.0
2.5
3.0
3.5
4.0

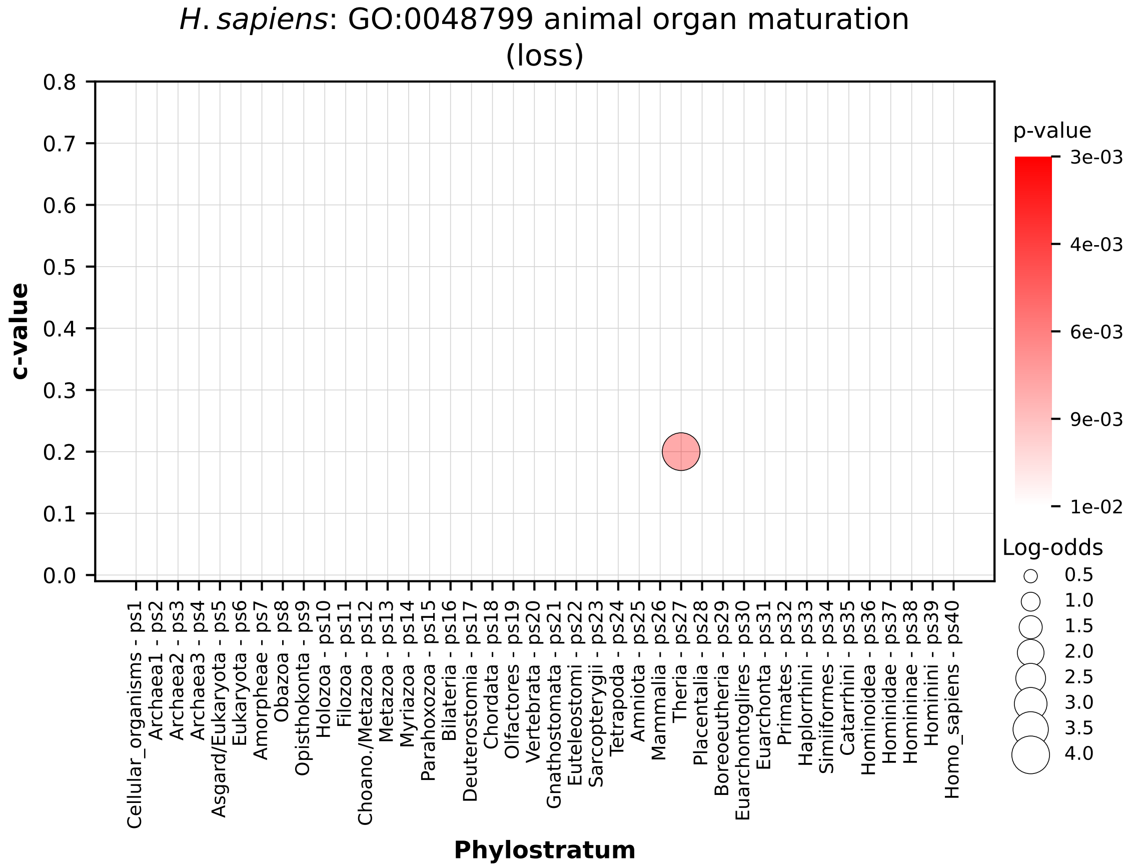

H. sapiens: GO:0048799 animal organ maturation
(loss)
c-value
0.0
0.1
0.2
0.3
0.4
0.5
0.6
0.7
0.8
Phylostratum
Cellular_organisms - ps1
Archaea1 - ps2
Archaea2 - ps3
Archaea3 - ps4
Asgard/Eukaryota - ps5
Eukaryota - ps6
Amorpheae - ps7
Obazoa - ps8
Opisthokonta - ps9
Holozoa - ps10
Filozoa - ps11
Choano./Metazoa - ps12
Metazoa - ps13
Myriazoa - ps14
Parahoxozoa - ps15
Bilateria - ps16
Deuterostomia - ps17
Chordata - ps18
Olfactores - ps19
Vertebrata - ps20
Gnathostomata - ps21
Euteleostomi - ps22
Sarcopterygii - ps23
Tetrapoda - ps24
Amniota - ps25
Mammalia - ps26
Theria - ps27
Placentalia - ps28
Boreoeutheria - ps29
Euarchontoglires - ps30
Euarchonta - ps31
Primates - ps32
Haplorrhini - ps33
Simiiformes - ps34
Catarrhini - ps35
Hominoidea - ps36
Hominidae - ps37
Homininae - ps38
Hominini - ps39
Homo_sapiens - ps40
p-value
3e-03
4e-03
6e-03
9e-03
1e-02
Log-odds
0.5
1.0
1.5
2.0
2.5
3.0
3.5
4.0

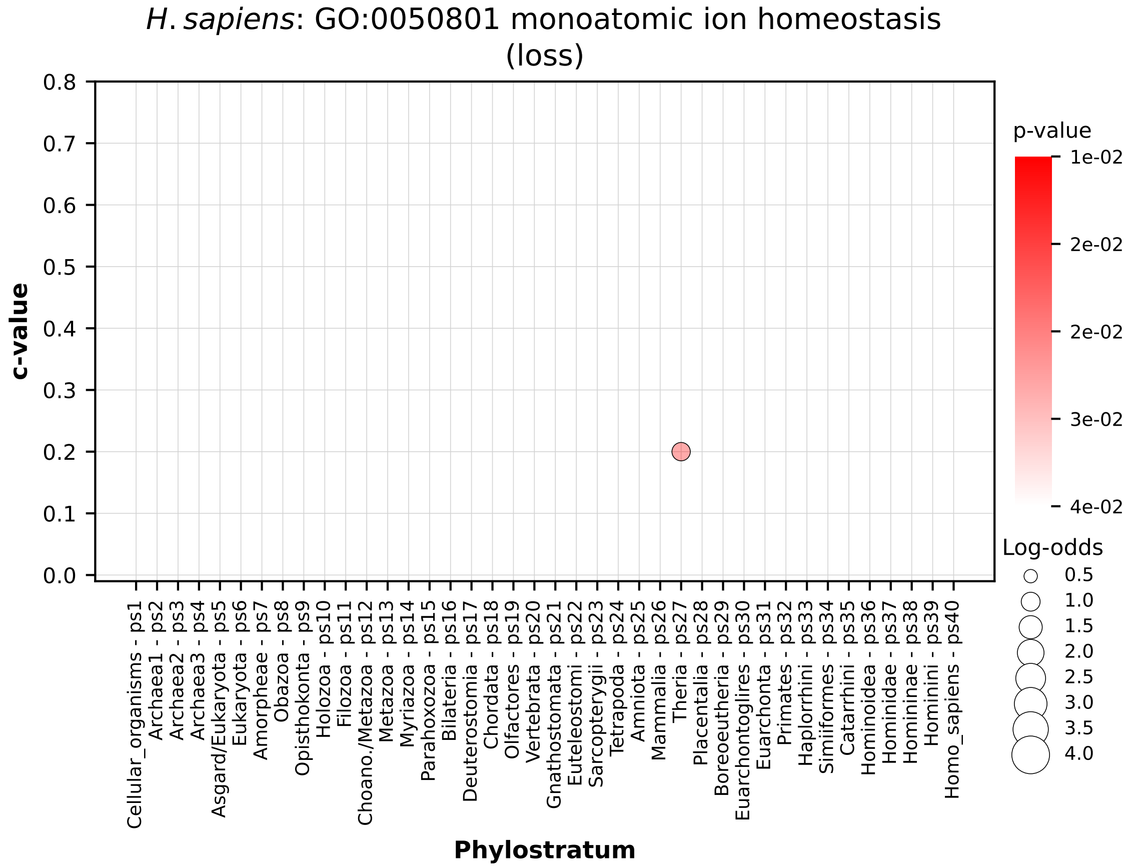
H. sapiens: GO:0050801 monoatomic ion homeostasis
(loss)
c-value
0.0
0.1
0.2
0.3
0.4
0.5
0.6
0.7
0.8
Phylostratum
Cellular_organisms - ps1
Archaea1 - ps2
Archaea2 - ps3
Archaea3 - ps4
Asgard/Eukaryota - ps5
Eukaryota - ps6
Amorpheae - ps7
Obazoa - ps8
Opisthokonta - ps9
Holozoa - ps10
Filozoa - ps11
Choano./Metazoa - ps12
Metazoa - ps13
Myriazoa - ps14
Parahoxozoa - ps15
Bilateria - ps16
Deuterostomia - ps17
Chordata - ps18
Olfactores - ps19
Vertebrata - ps20
Gnathostomata - ps21
Euteleostomi - ps22
Sarcopterygii - ps23
Tetrapoda - ps24
Amniota - ps25
Mammalia - ps26
Theria - ps27
Placentalia - ps28
Boreoeutheria - ps29
Euarchontoglires - ps30
Euarchonta - ps31
Primates - ps32
Haplorrhini - ps33
Simiiformes - ps34
Catarrhini - ps35
Hominoidea - ps36
Hominidae - ps37
Homininae - ps38
Hominini - ps39
Homo_sapiens - ps40
p-value
1e-02
2e-02
2e-02
3e-02
4e-02
Log-odds
0.5
1.0
1.5
2.0
2.5
3.0
3.5
4.0

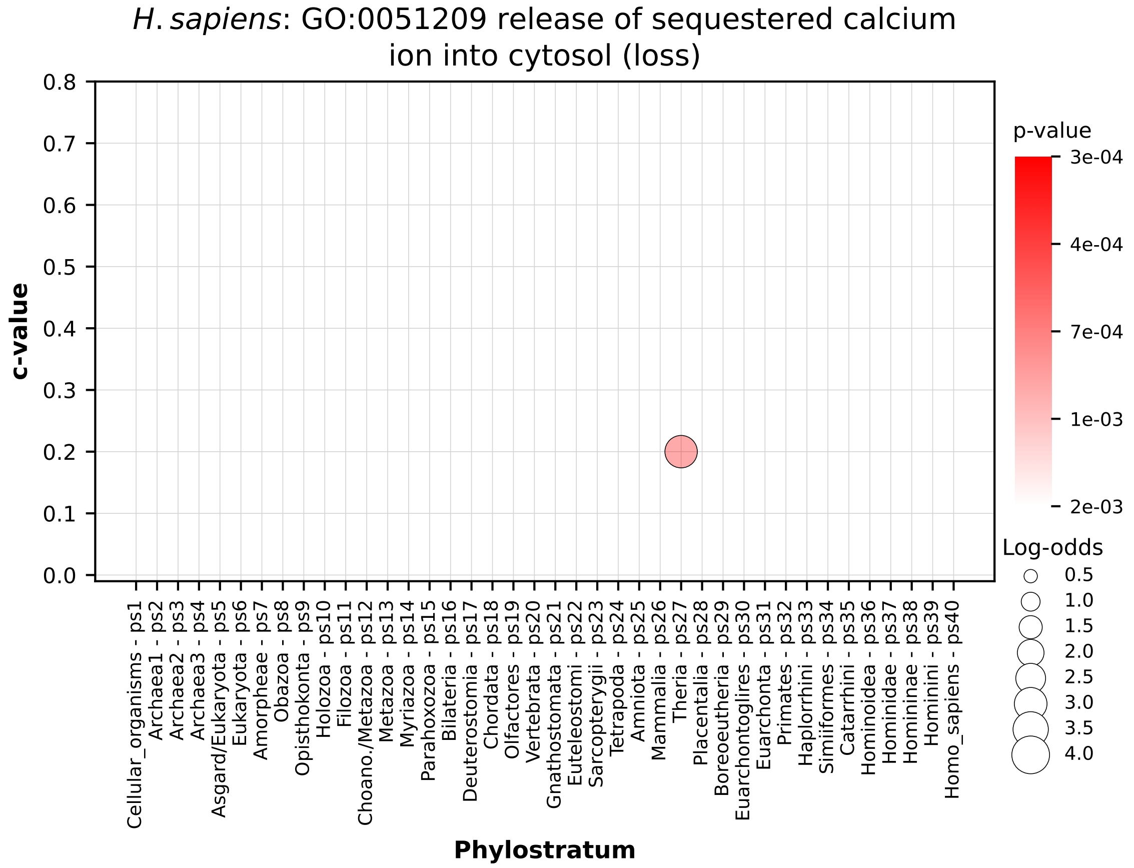

H. sapiens: GO:0051209 release of sequestered calcium ion into cytosol (loss)
c-value
0.8
0.7
0.6
0.5
0.4
0.3
0.2
0.1
0.0
Phylostratum
Cellular_organisms - ps1
Archaea1 - ps2
Archaea2 - ps3
Archaea3 - ps4
Asgard/Eukaryota - ps5
Eukaryota - ps6
Amorpheae - ps7
Obazoa - ps8
Opisthokonta - ps9
Holozoa - ps10
Filozoa - ps11
Choano./Metazoa - ps12
Metazoa - ps13
Myriazoa - ps14
Parahoxozoa - ps15
Bilateria - ps16
Deuterostomia - ps17
Chordata - ps18
Olfactores - ps19
Vertebrata - ps20
Gnathostomata - ps21
Euteleostomi - ps22
Sarcopterygii - ps23
Tetrapoda - ps24
Amniota - ps25
Mammalia - ps26
Theria - ps27
Placentalia - ps28
Boreoeutheria - ps29
Euarchontoglires - ps30
Euarchonta - ps31
Primates - ps32
Haplorrhini - ps33
Simiiformes - ps34
Catarrhini - ps35
Hominoidea - ps36
Hominidae - ps37
Homininae - ps38
Hominini - ps39
Homo_sapiens - ps40
p-value
3e-04
4e-04
7e-04
1e-03
2e-03
Log-odds
0.5
1.0
1.5
2.0
2.5
3.0
3.5
4.0

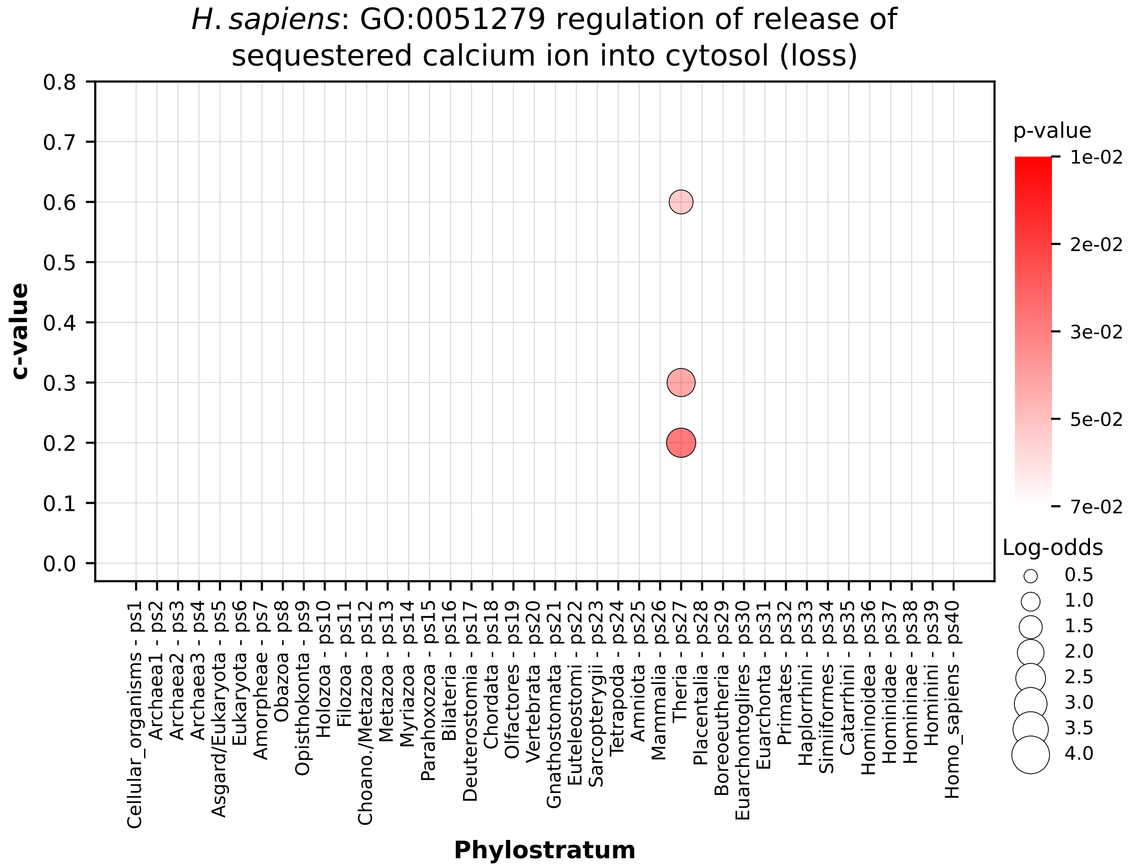

H. sapiens: GO:0051279 regulation of release of sequestered calcium ion into cytosol (loss)
c-value
0.0
0.1
0.2
0.3
0.4
0.5
0.6
0.7
0.8
p-value
1e-02
2e-02
3e-02
5e-02
7e-02
Log-odds
0.5
1.0
1.5
2.0
2.5
3.0
3.5
4.0
Cellular_organisms - ps1
Archaea1 - ps2
Archaea2 - ps3
Archaea3 - ps4
Asgard/Eukaryota - ps5
Eukaryota - ps6
Amorpheae - ps7
Obazoa - ps8
Opisthokonta - ps9
Holozoa - ps10
Filozoa - ps11
Choano./Metazoa - ps12
Metazoa - ps13
Myriazoa - ps14
Parahoxozoa - ps15
Bilateria - ps16
Deuterostomia - ps17
Chordata - ps18
Olfactores - ps19
Vertebrata - ps20
Gnathostomata - ps21
Euteleostomi - ps22
Sarcopterygii - ps23
Tetrapoda - ps24
Amniota - ps25
Mammalia - ps26
Theria - ps27
Placentalia - ps28
Boreoeutheria - ps29
Euarchontoglires - ps30
Euarchonta - ps31
Primates - ps32
Haplorrhini - ps33
Simiiformes - ps34
Catarrhini - ps35
Hominoidea - ps36
Hominidae - ps37
Homininae - ps38
Hominini - ps39
Homo_sapiens - ps40
Phylostratum

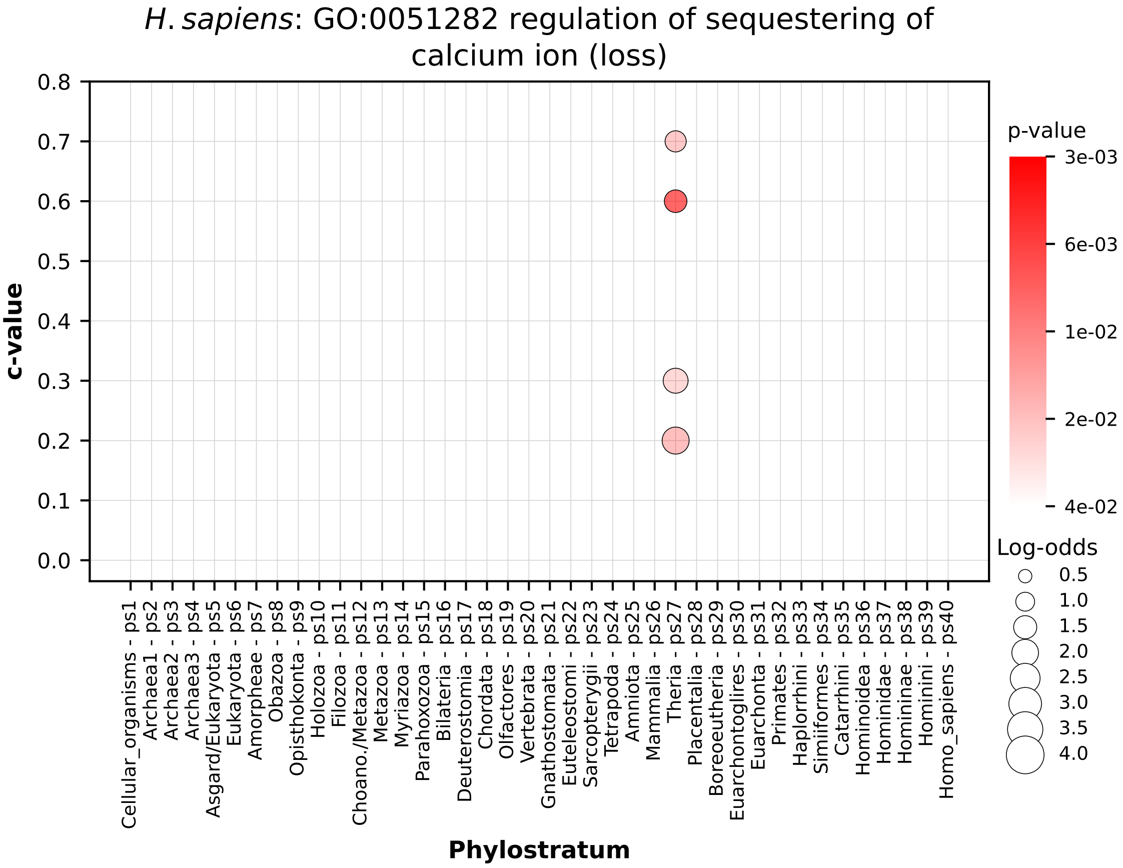

H. sapiens: GO:0051282 regulation of sequestering of calcium ion (loss)
c-value
0.8
0.7
0.6
0.5
0.4
0.3
0.2
0.1
0.0
p-value
3e-03
6e-03
1e-02
2e-02
4e-02
Log-odds
0.5
1.0
1.5
2.0
2.5
3.0
3.5
4.0
Cellular_organisms - ps1
Archaea1 - ps2
Archaea2 - ps3
Archaea3 - ps4
Asgard/Eukaryota - ps5
Eukaryota - ps6
Amorpheae - ps7
Obazoa - ps8
Opisthokonta - ps9
Holozoa - ps10
Filozoa - ps11
Choano./Metazoa - ps12
Metazoa - ps13
Myriazoa - ps14
Parahoxozoa - ps15
Bilateria - ps16
Deuterostomia - ps17
Chordata - ps18
Olfactores - ps19
Vertebrata - ps20
Gnathostomata - ps21
Euteleostomi - ps22
Sarcopterygii - ps23
Tetrapoda - ps24
Amniota - ps25
Mammalia - ps26
Theria - ps27
Placentalia - ps28
Boreoeutheria - ps29
Euarchontoglires - ps30
Euarchonta - ps31
Primates - ps32
Haplorrhini - ps33
Simiiformes - ps34
Catarrhini - ps35
Hominoidea - ps36
Hominidae - ps37
Homininae - ps38
Hominini - ps39
Homo_sapiens - ps40
Phylostratum

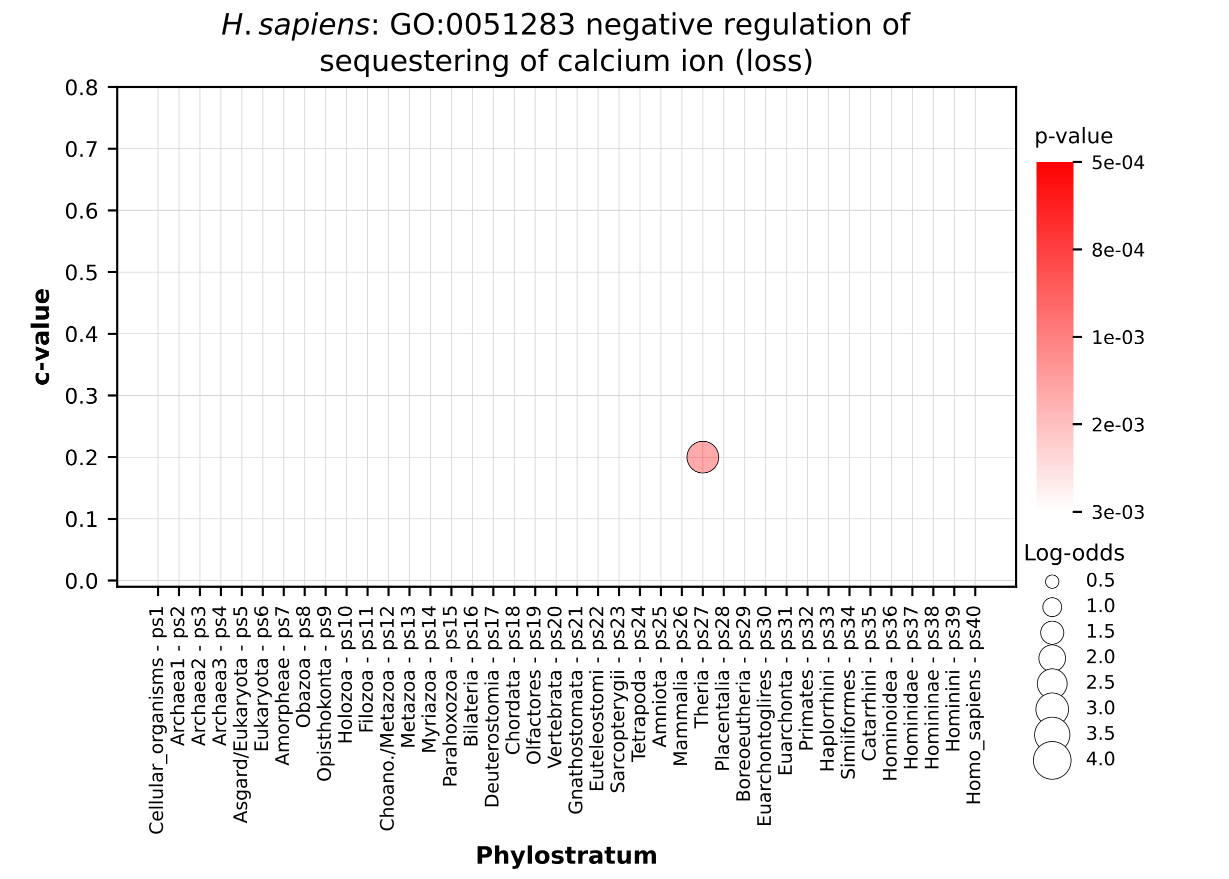

H. sapiens: GO:0051283 negative regulation of sequestering of calcium ion (loss)
c-value
0.0
0.1
0.2
0.3
0.4
0.5
0.6
0.7
0.8
Phylostratum
Cellular_organisms - ps1
Archaea1 - ps2
Archaea2 - ps3
Archaea3 - ps4
Asgard/Eukaryota - ps5
Eukaryota - ps6
Amorpheae - ps7
Obazoa - ps8
Opisthokonta - ps9
Holozoa - ps10
Filozoa - ps11
Choano./Metazoa - ps12
Metazoa - ps13
Myriazoa - ps14
Parahoxozoa - ps15
Bilateria - ps16
Deuterostomia - ps17
Chordata - ps18
Olfactores - ps19
Vertebrata - ps20
Gnathostomata - ps21
Euteleostomi - ps22
Sarcopterygii - ps23
Tetrapoda - ps24
Amniota - ps25
Mammalia - ps26
Theria - ps27
Placentalia - ps28
Boreoeutheria - ps29
Euarchontoglires - ps30
Euarchonta - ps31
Primates - ps32
Haplorrhini - ps33
Simiiformes - ps34
Catarrhini - ps35
Hominoidea - ps36
Hominidae - ps37
Homininae - ps38
Hominini - ps39
Homo_sapiens - ps40
p-value
5e-04
8e-04
1e-03
2e-03
3e-03
Log-odds
0.5
1.0
1.5
2.0
2.5
3.0
3.5
4.0

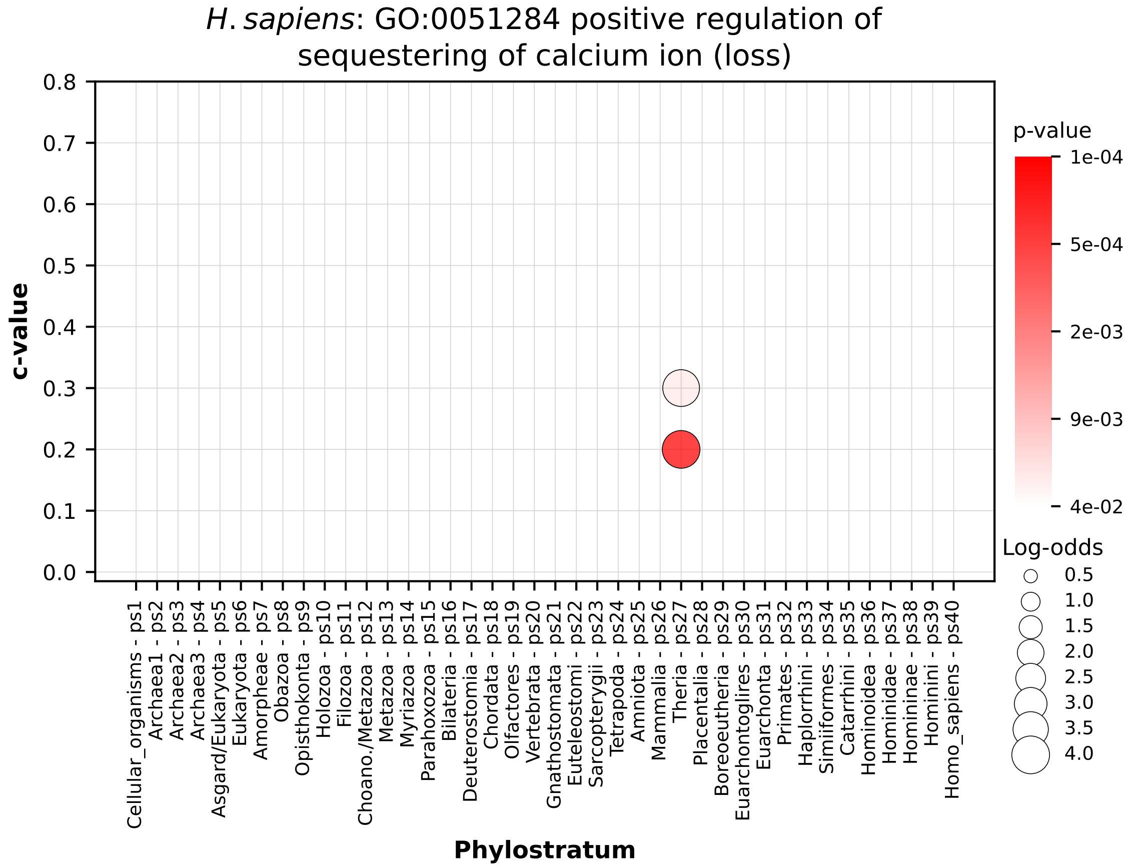
H. sapiens: GO:0051284 positive regulation of sequestering of calcium ion (loss)
c-value
0.0
0.1
0.2
0.3
0.4
0.5
0.6
0.7
0.8
Phylostratum
Cellular_organisms - ps1
Archaea1 - ps2
Archaea2 - ps3
Archaea3 - ps4
Asgard/Eukaryota - ps5
Eukaryota - ps6
Amorpheae - ps7
Obazoa - ps8
Opisthokonta - ps9
Holozoa - ps10
Filozoa - ps11
Choano./Metazoa - ps12
Metazoa - ps13
Myriazoa - ps14
Parahoxozoa - ps15
Bilateria - ps16
Deuterostomia - ps17
Chordata - ps18
Olfactores - ps19
Vertebrata - ps20
Gnathostomata - ps21
Euteleostomi - ps22
Sarcopterygii - ps23
Tetrapoda - ps24
Amniota - ps25
Mammalia - ps26
Theria - ps27
Placentalia - ps28
Boreoeutheria - ps29
Euarchontoglires - ps30
Euarchonta - ps31
Primates - ps32
Haplorrhini - ps33
Simiiformes - ps34
Catarrhini - ps35
Hominoidea - ps36
Hominidae - ps37
Homininae - ps38
Hominini - ps39
Homo_sapiens - ps40
p-value
1e-04
5e-04
2e-03
9e-03
4e-02
Log-odds
0.5
1.0
1.5
2.0
2.5
3.0
3.5
4.0

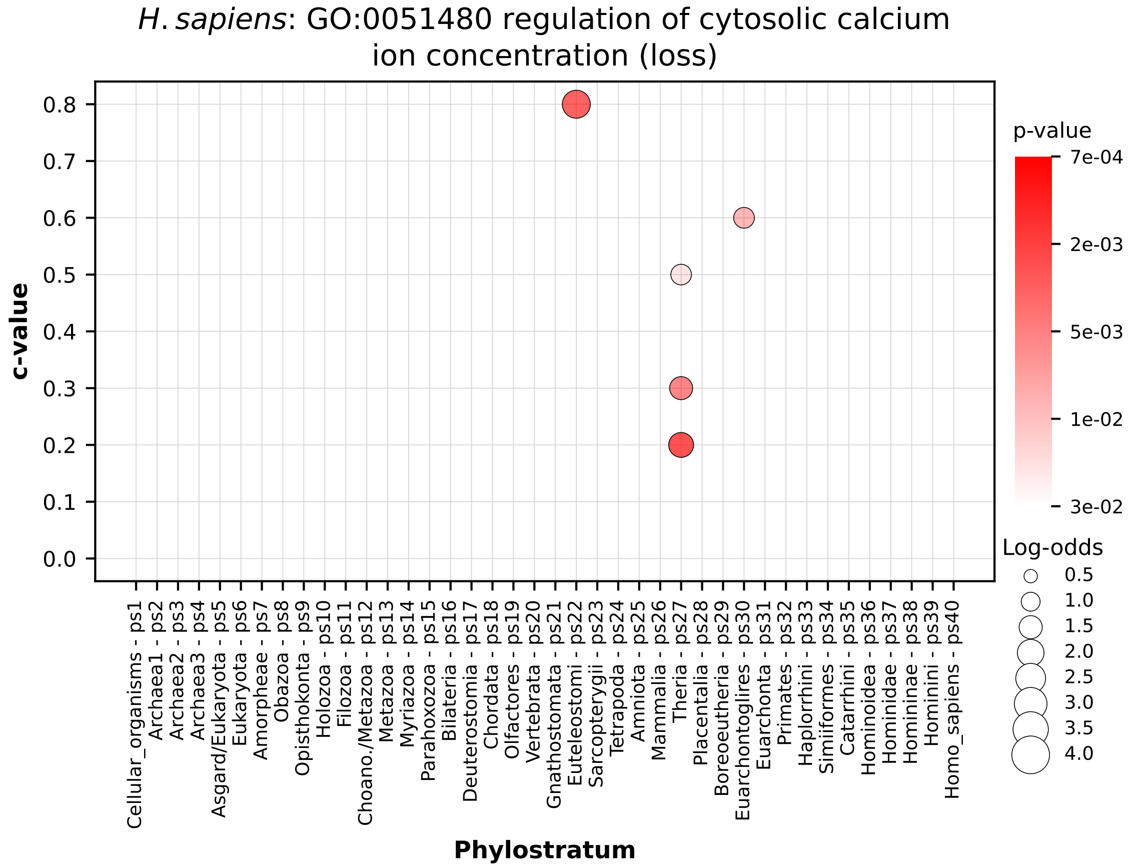

H. sapiens: GO:0051480 regulation of cytosolic calcium ion concentration (loss)
c-value
0.0
0.1
0.2
0.3
0.4
0.5
0.6
0.7
0.8
Phylostratum
Cellular_organisms - ps1
Archaea1 - ps2
Archaea2 - ps3
Archaea3 - ps4
Asgard/Eukaryota - ps5
Eukaryota - ps6
Amorpheae - ps7
Obazoa - ps8
Opisthokonta - ps9
Holozoa - ps10
Filozoa - ps11
Choano./Metazoa - ps12
Metazoa - ps13
Myriazoa - ps14
Parahoxozoa - ps15
Bilateria - ps16
Deuterostomia - ps17
Chordata - ps18
Olfactores - ps19
Vertebrata - ps20
Gnathostomata - ps21
Euteleostomi - ps22
Sarcopterygii - ps23
Tetrapoda - ps24
Amniota - ps25
Mammalia - ps26
Theria - ps27
Placentalia - ps28
Boreoeutheria - ps29
Euarchontoglires - ps30
Euarchonta - ps31
Primates - ps32
Haplorrhini - ps33
Simiiformes - ps34
Catarrhini - ps35
Hominoidea - ps36
Hominidae - ps37
Homininae - ps38
Hominini - ps39
Homo_sapiens - ps40
p-value
7e-04
2e-03
5e-03
1e-02
3e-02
Log-odds
0.5
1.0
1.5
2.0
2.5
3.0
3.5
4.0

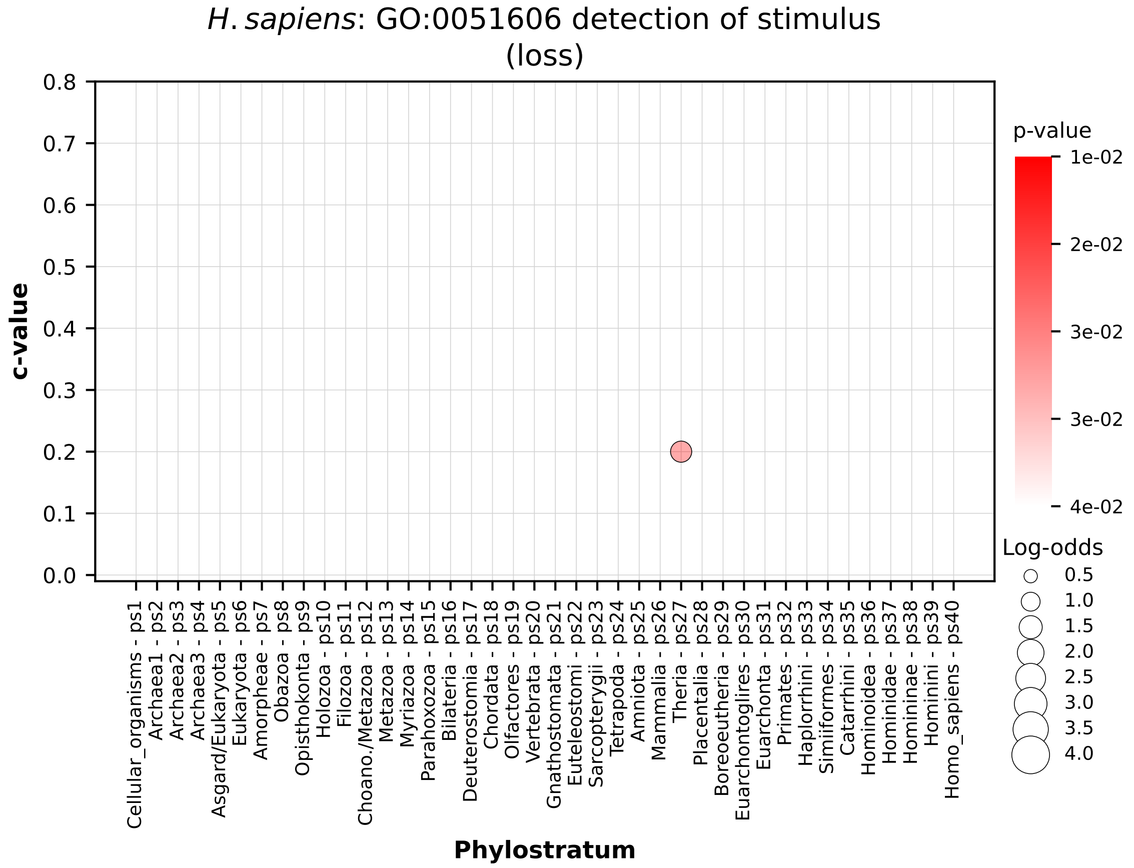
H. sapiens: GO:0051606 detection of stimulus
(loss)
c-value
0.8
0.7
0.6
0.5
0.4
0.3
0.2
0.1
0.0
p-value
1e-02
2e-02
3e-02
3e-02
4e-02
Log-odds
0.5
1.0
1.5
2.0
2.5
3.0
3.5
4.0
Cellular_organisms - ps1
Archaea1 - ps2
Archaea2 - ps3
Archaea3 - ps4
Asgard/Eukaryota - ps5
Eukaryota - ps6
Amorpheae - ps7
Obazoa - ps8
Opisthokonta - ps9
Holozoa - ps10
Filozoa - ps11
Choano./Metazoa - ps12
Metazoa - ps13
Myriazoa - ps14
Parahoxozoa - ps15
Bilateria - ps16
Deuterostomia - ps17
Chordata - ps18
Olfactores - ps19
Vertebrata - ps20
Gnathostomata - ps21
Euteleostomi - ps22
Sarcopterygii - ps23
Tetrapoda - ps24
Amniota - ps25
Mammalia - ps26
Theria - ps27
Placentalia - ps28
Boreoeutheria - ps29
Euarchontoglires - ps30
Euarchonta - ps31
Primates - ps32
Haplorrhini - ps33
Simiiformes - ps34
Catarrhini - ps35
Hominoidea - ps36
Hominidae - ps37
Homininae - ps38
Hominini - ps39
Homo_sapiens - ps40
Phylostratum

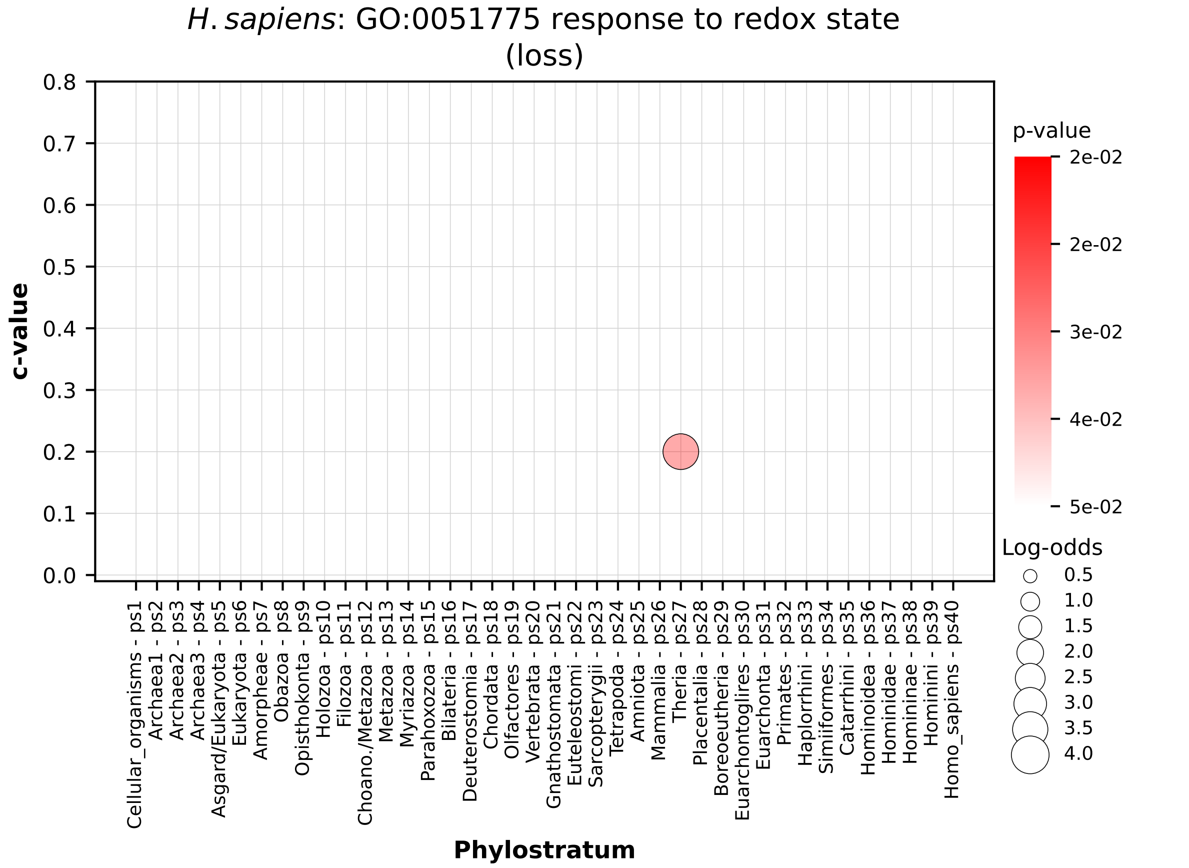

H. sapiens: GO:0051775 response to redox state
(loss)
c-value
0.8
0.7
0.6
0.5
0.4
0.3
0.2
0.1
0.0
Phylostratum
Cellular_organisms - ps1
Archaea1 - ps2
Archaea2 - ps3
Archaea3 - ps4
Asgard/Eukaryota - ps5
Eukaryota - ps6
Amorpheae - ps7
Obazoa - ps8
Opisthokonta - ps9
Holozoa - ps10
Filozoa - ps11
Choano./Metazoa - ps12
Metazoa - ps13
Myriazoa - ps14
Parahoxozoa - ps15
Bilateria - ps16
Deuterostomia - ps17
Chordata - ps18
Olfactores - ps19
Vertebrata - ps20
Gnathostomata - ps21
Euteleostomi - ps22
Sarcopterygii - ps23
Tetrapoda - ps24
Amniota - ps25
Mammalia - ps26
Theria - ps27
Placentalia - ps28
Boreoeutheria - ps29
Euarchontoglires - ps30
Euarchonta - ps31
Primates - ps32
Haplorrhini - ps33
Simiiformes - ps34
Catarrhini - ps35
Hominoidea - ps36
Hominidae - ps37
Homininae - ps38
Hominini - ps39
Homo_sapiens - ps40
p-value
2e-02
2e-02
3e-02
4e-02
5e-02
Log-odds
0.5
1.0
1.5
2.0
2.5
3.0
3.5
4.0

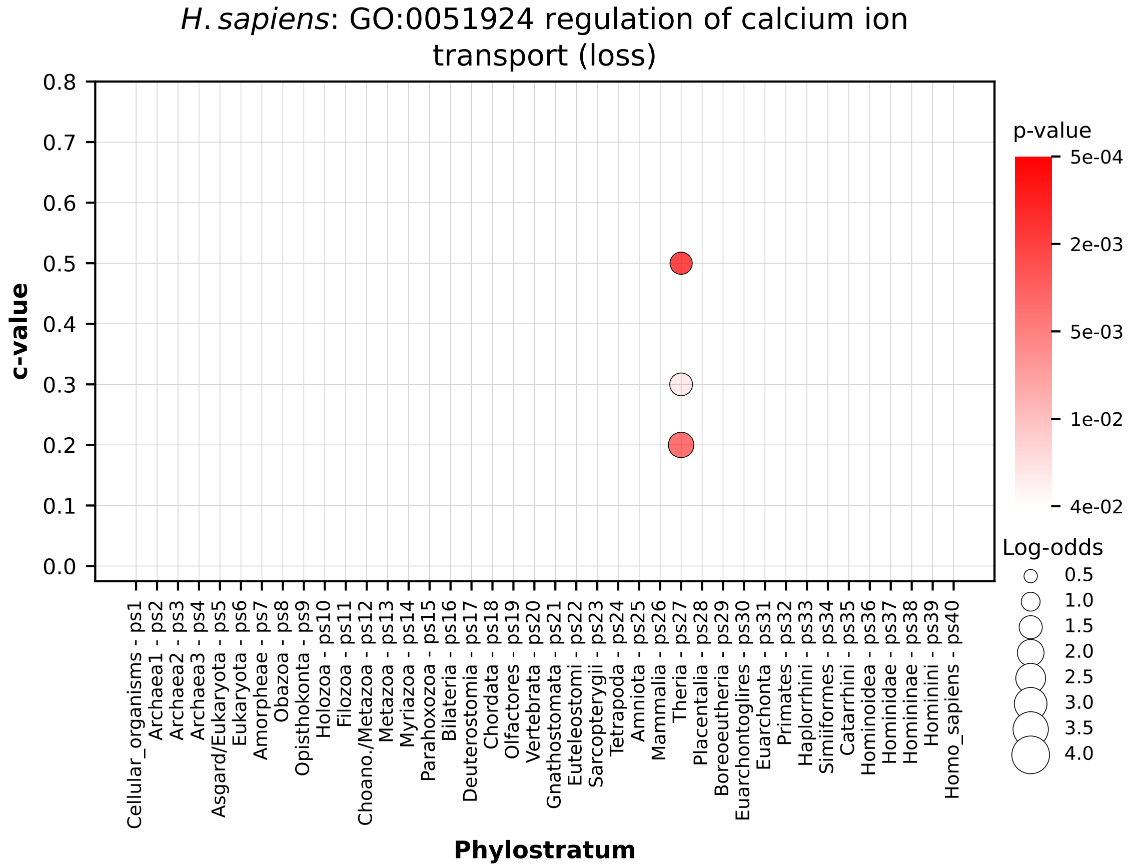
H. sapiens: GO:0051924 regulation of calcium ion transport (loss)
c-value
Phylostratum
p-value
5e-04
2e-03
5e-03
1e-02
4e-02
Log-odds
0.5
1.0
1.5
2.0
2.5
3.0
3.5
4.0
Cellular_organisms - ps1
Archaea1 - ps2
Archaea2 - ps3
Archaea3 - ps4
Asgard/Eukaryota - ps5
Eukaryota - ps6
Amorpheae - ps7
Obazoa - ps8
Opisthokonta - ps9
Holozoa - ps10
Filozoa - ps11
Choano./Metazoa - ps12
Metazoa - ps13
Myriazoa - ps14
Parahoxozoa - ps15
Bilateria - ps16
Deuterostomia - ps17
Chordata - ps18
Olfactores - ps19
Vertebrata - ps20
Gnathostomata - ps21
Euteleostomi - ps22
Sarcopterygii - ps23
Tetrapoda - ps24
Amniota - ps25
Mammalia - ps26
Theria - ps27
Placentalia - ps28
Boreoeutheria - ps29
Euarchontoglires - ps30
Euarchonta - ps31
Primates - ps32
Haplorrhini - ps33
Simiiformes - ps34
Catarrhini - ps35
Hominoidea - ps36
Hominidae - ps37
Homininae - ps38
Hominini - ps39
Homo_sapiens - ps40

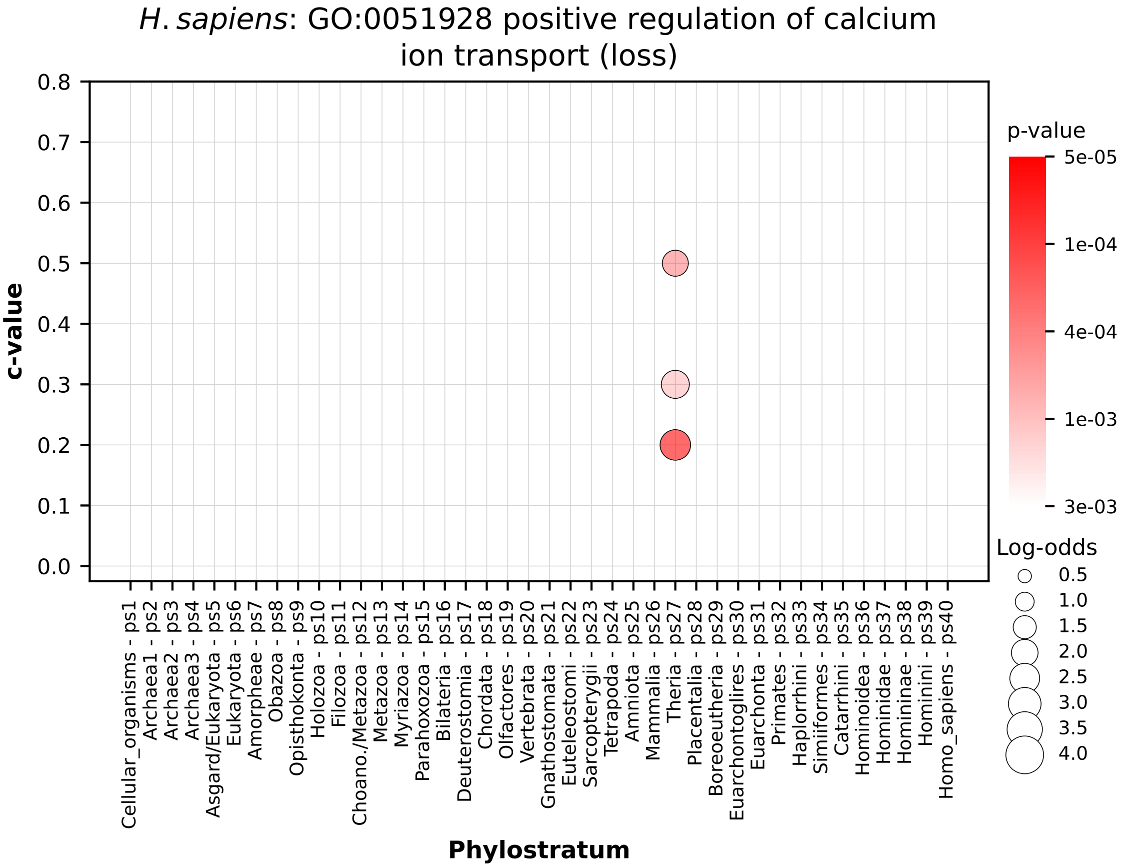

H. sapiens: GO:0051928 positive regulation of calcium ion transport (loss)
c-value
0.8
0.7
0.6
0.5
0.4
0.3
0.2
0.1
0.0
p-value
5e-05
1e-04
4e-04
1e-03
3e-03
Log-odds
0.5
1.0
1.5
2.0
2.5
3.0
3.5
4.0
Cellular_organisms - ps1
Archaea1 - ps2
Archaea2 - ps3
Archaea3 - ps4
Asgard/Eukaryota - ps5
Eukaryota - ps6
Amorpheae - ps7
Obazoa - ps8
Opisthokonta - ps9
Holozoa - ps10
Filozoa - ps11
Choano./Metazoa - ps12
Metazoa - ps13
Myriazoa - ps14
Parahoxozoa - ps15
Bilateria - ps16
Deuterostomia - ps17
Chordata - ps18
Olfactores - ps19
Vertebrata - ps20
Gnathostomata - ps21
Euteleostomi - ps22
Sarcopterygii - ps23
Tetrapoda - ps24
Amniota - ps25
Mammalia - ps26
Theria - ps27
Placentalia - ps28
Boreoeutheria - ps29
Euarchontoglires - ps30
Euarchonta - ps31
Primates - ps32
Haplorrhini - ps33
Simiiformes - ps34
Catarrhini - ps35
Hominoidea - ps36
Hominidae - ps37
Homininae - ps38
Hominini - ps39
Homo_sapiens - ps40
Phylostratum

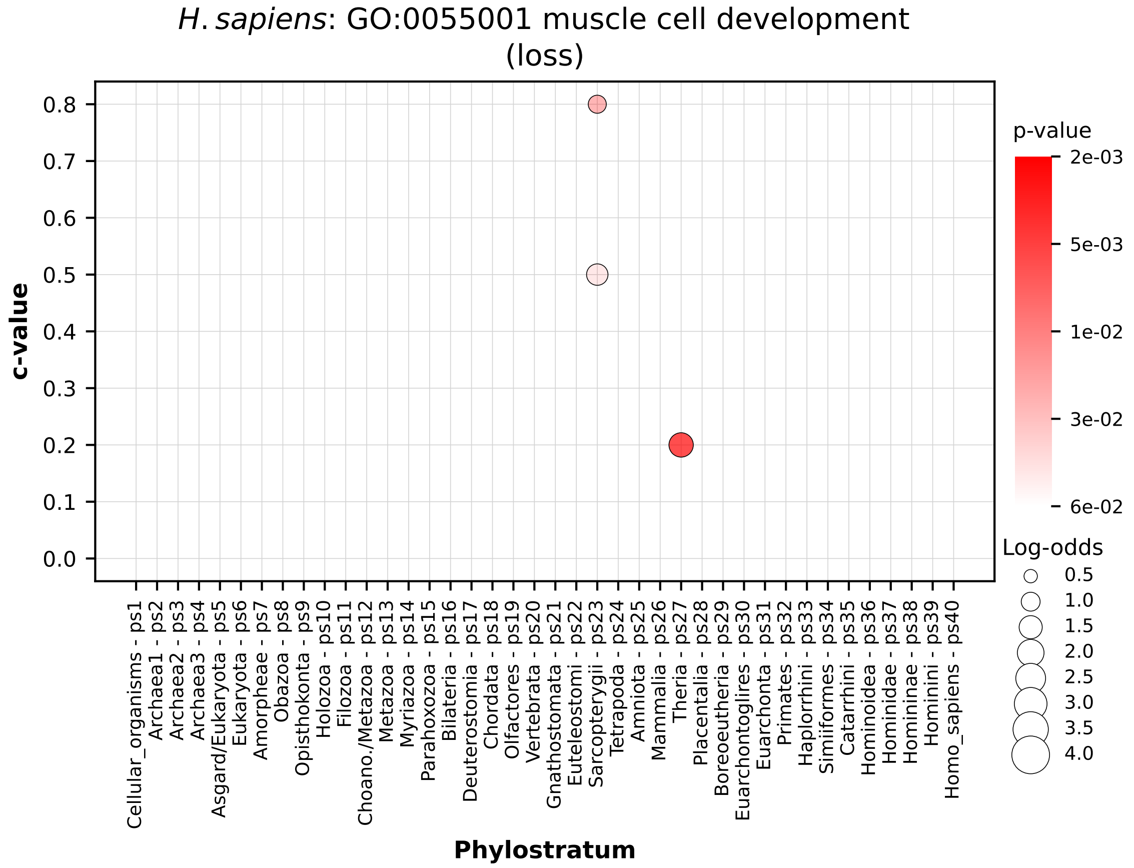

H. sapiens: GO:0055001 muscle cell development
(loss)
c-value
0.0
0.1
0.2
0.3
0.4
0.5
0.6
0.7
0.8
Phylostratum
Cellular_organisms - ps1
Archaea1 - ps2
Archaea2 - ps3
Archaea3 - ps4
Asgard/Eukaryota - ps5
Eukaryota - ps6
Amorpheae - ps7
Obazoa - ps8
Opisthokonta - ps9
Holozoa - ps10
Filozoa - ps11
Choano./Metazoa - ps12
Metazoa - ps13
Myriazoa - ps14
Parahoxozoa - ps15
Bilateria - ps16
Deuterostomia - ps17
Chordata - ps18
Olfactores - ps19
Vertebrata - ps20
Gnathostomata - ps21
Euteleostomi - ps22
Sarcopterygii - ps23
Tetrapoda - ps24
Amniota - ps25
Mammalia - ps26
Theria - ps27
Placentalia - ps28
Boreoeutheria - ps29
Euarchontoglires - ps30
Euarchonta - ps31
Primates - ps32
Haplorrhini - ps33
Simiiformes - ps34
Catarrhini - ps35
Hominoidea - ps36
Hominidae - ps37
Homininae - ps38
Hominini - ps39
Homo_sapiens - ps40
p-value
2e-03
5e-03
1e-02
3e-02
6e-02
Log-odds
0.5
1.0
1.5
2.0
2.5
3.0
3.5
4.0

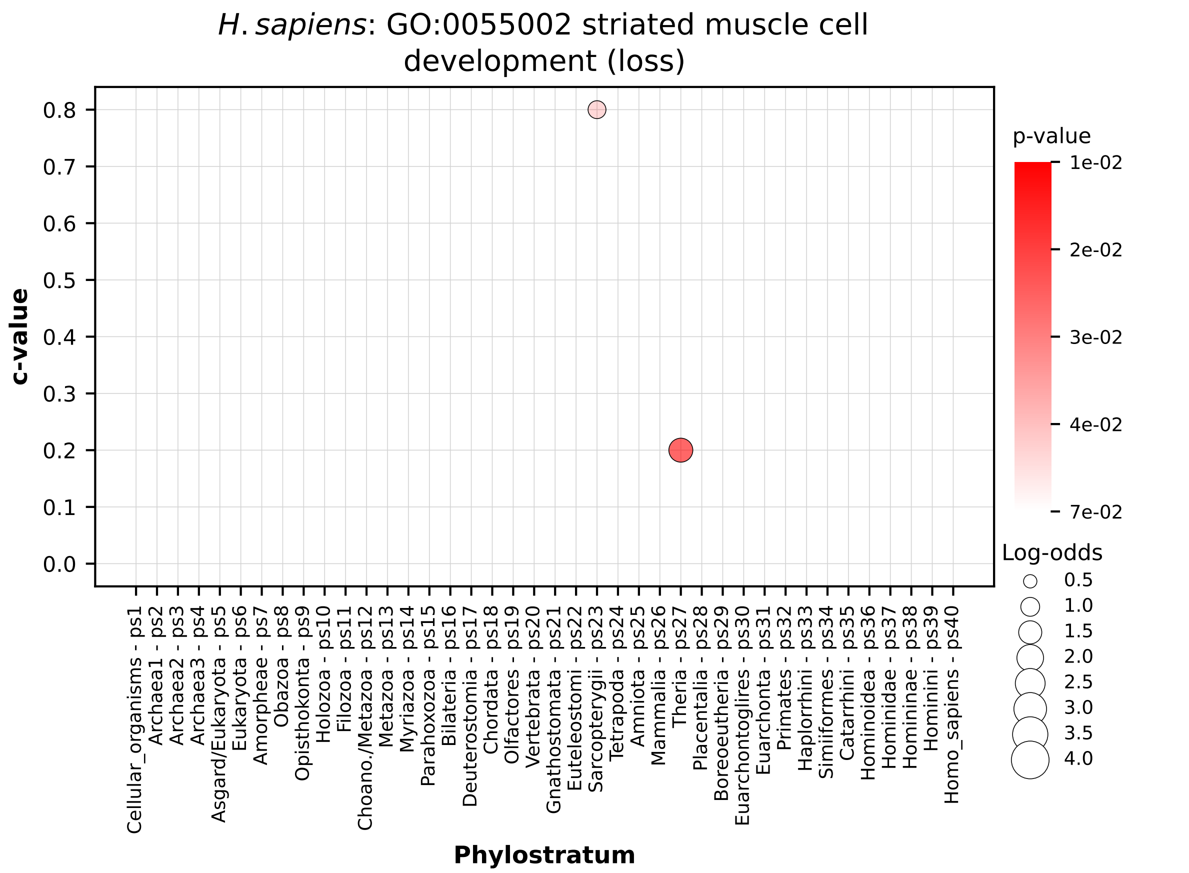

H. sapiens: GO:0055002 striated muscle cell development (loss)
c-value
0.8
0.7
0.6
0.5
0.4
0.3
0.2
0.1
0.0
p-value
1e-02
2e-02
3e-02
4e-02
7e-02
Log-odds
0.5
1.0
1.5
2.0
2.5
3.0
3.5
4.0
Cellular_organisms - ps1
Archaea1 - ps2
Archaea2 - ps3
Archaea3 - ps4
Asgard/Eukaryota - ps5
Eukaryota - ps6
Amorpheae - ps7
Obazoa - ps8
Opisthokonta - ps9
Holozoa - ps10
Filozoa - ps11
Choano./Metazoa - ps12
Metazoa - ps13
Myriazoa - ps14
Parahoxozoa - ps15
Bilateria - ps16
Deuterostomia - ps17
Chordata - ps18
Olfactores - ps19
Vertebrata - ps20
Gnathostomata - ps21
Euteleostomi - ps22
Sarcopterygii - ps23
Tetrapoda - ps24
Amniota - ps25
Mammalia - ps26
Theria - ps27
Placentalia - ps28
Boreoeutheria - ps29
Euarchontoglires - ps30
Euarchonta - ps31
Primates - ps32
Haplorrhini - ps33
Simiiformes - ps34
Catarrhini - ps35
Hominoidea - ps36
Hominidae - ps37
Homininae - ps38
Hominini - ps39
Homo_sapiens - ps40
Phylostratum

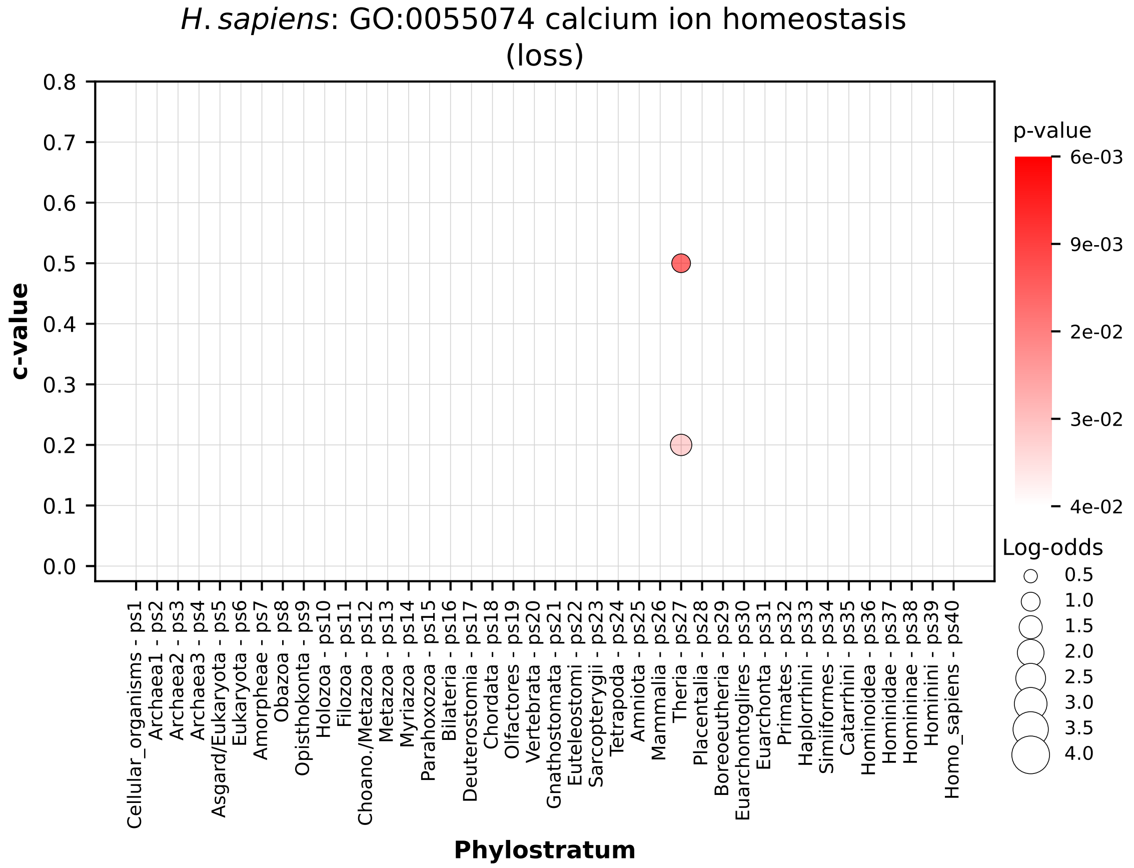
H. sapiens: GO:0055074 calcium ion homeostasis
(loss)
c-value
0.8
0.7
0.6
0.5
0.4
0.3
0.2
0.1
0.0
p-value
6e-03
9e-03
2e-02
3e-02
4e-02
Log-odds
0.5
1.0
1.5
2.0
2.5
3.0
3.5
4.0
Cellular_organisms - ps1
Archaea1 - ps2
Archaea2 - ps3
Archaea3 - ps4
Asgard/Eukaryota - ps5
Eukaryota - ps6
Amorpheae - ps7
Obazoa - ps8
Opisthokonta - ps9
Holozoa - ps10
Filozoa - ps11
Choano./Metazoa - ps12
Metazoa - ps13
Myriazoa - ps14
Parahoxozoa - ps15
Bilateria - ps16
Deuterostomia - ps17
Chordata - ps18
Olfactores - ps19
Vertebrata - ps20
Gnathostomata - ps21
Euteleostomi - ps22
Sarcopterygii - ps23
Tetrapoda - ps24
Amniota - ps25
Mammalia - ps26
Theria - ps27
Placentalia - ps28
Boreoeutheria - ps29
Euarchontoglires - ps30
Euarchonta - ps31
Primates - ps32
Haplorrhini - ps33
Simiiformes - ps34
Catarrhini - ps35
Hominoidea - ps36
Hominidae - ps37
Homininae - ps38
Hominini - ps39
Homo_sapiens - ps40
Phylostratum

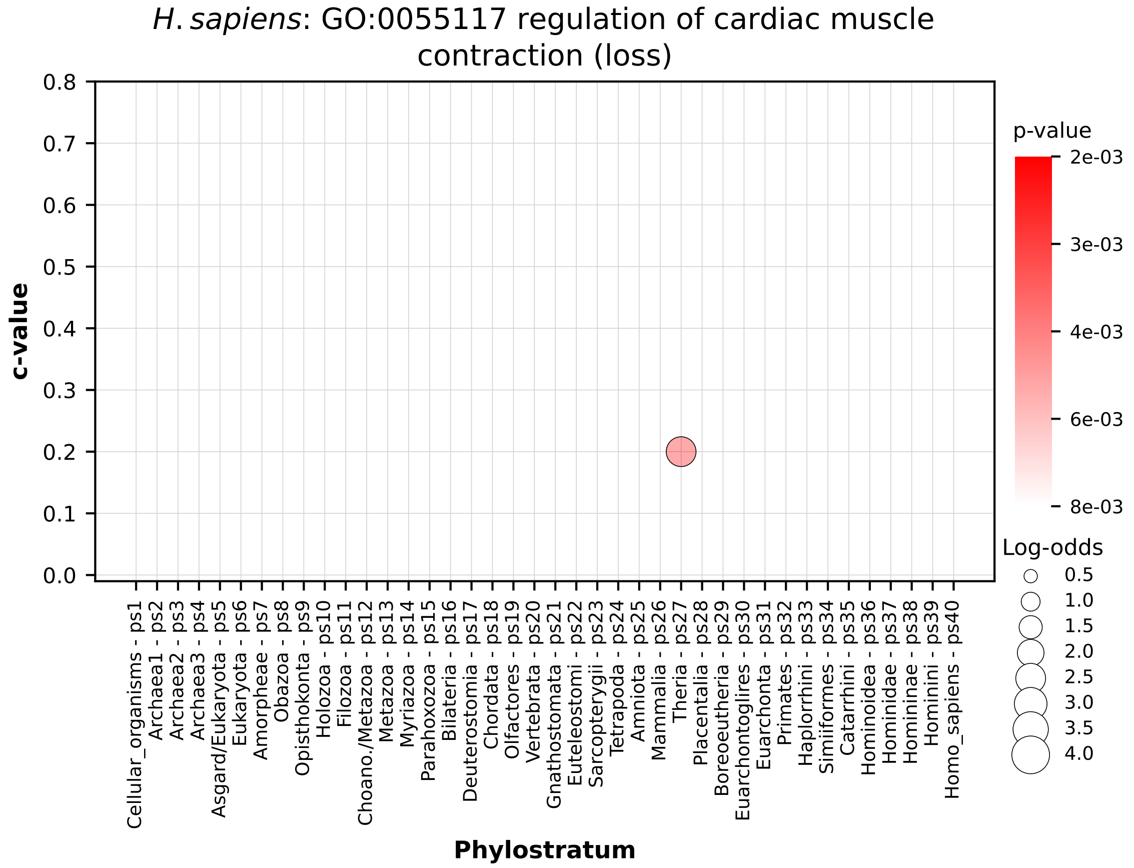

H. sapiens: GO:0055117 regulation of cardiac muscle contraction (loss)
c-value
0.0
0.1
0.2
0.3
0.4
0.5
0.6
0.7
0.8
Phylostratum
Cellular_organisms - ps1
Archaea1 - ps2
Archaea2 - ps3
Archaea3 - ps4
Asgard/Eukaryota - ps5
Eukaryota - ps6
Amorpheae - ps7
Obazoa - ps8
Opisthokonta - ps9
Holozoa - ps10
Filozoa - ps11
Choano./Metazoa - ps12
Metazoa - ps13
Myriazoa - ps14
Parahoxozoa - ps15
Bilateria - ps16
Deuterostomia - ps17
Chordata - ps18
Olfactores - ps19
Vertebrata - ps20
Gnathostomata - ps21
Euteleostomi - ps22
Sarcopterygii - ps23
Tetrapoda - ps24
Amniota - ps25
Mammalia - ps26
Theria - ps27
Placentalia - ps28
Boreoeutheria - ps29
Euarchontoglires - ps30
Euarchonta - ps31
Primates - ps32
Haplorrhini - ps33
Simiiformes - ps34
Catarrhini - ps35
Hominoidea - ps36
Hominidae - ps37
Homininae - ps38
Hominini - ps39
Homo_sapiens - ps40
p-value
2e-03
3e-03
4e-03
6e-03
8e-03
Log-odds
0.5
1.0
1.5
2.0
2.5
3.0
3.5
4.0

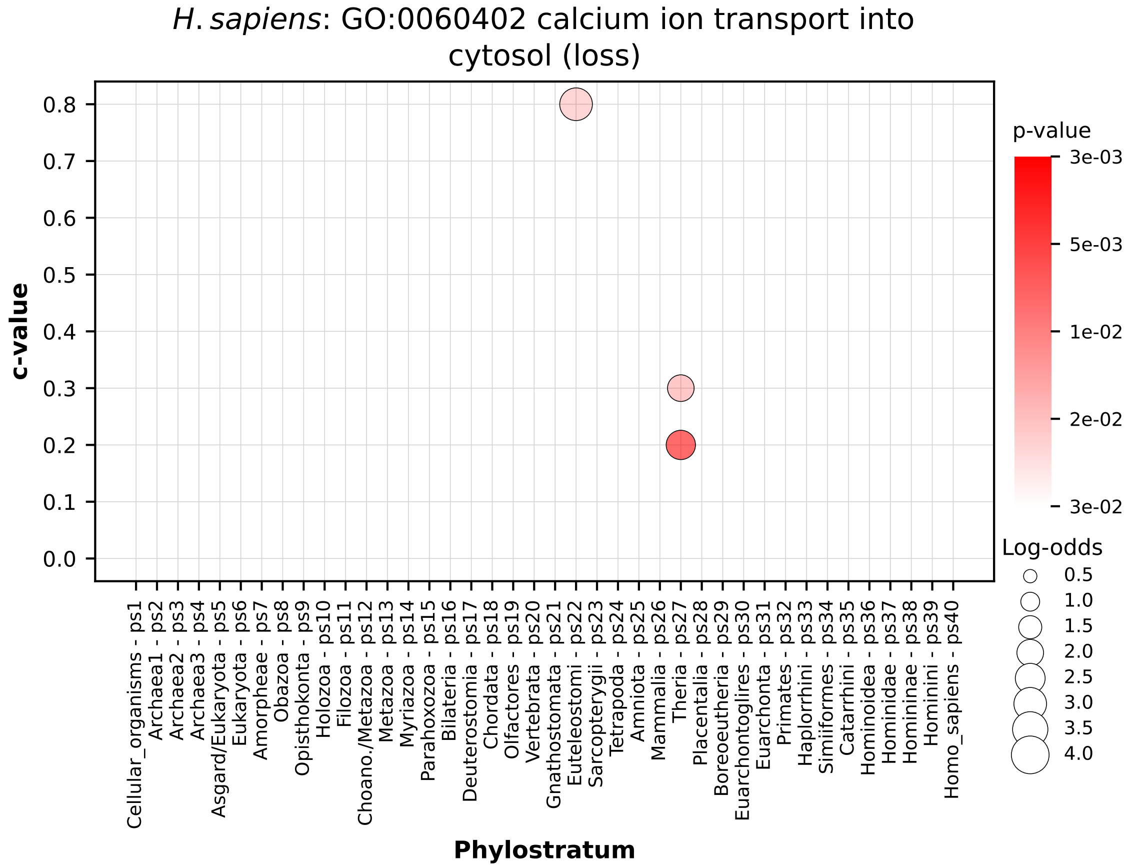

H. sapiens: GO:0060402 calcium ion transport into cytosol (loss)
c-value
0.0
0.1
0.2
0.3
0.4
0.5
0.6
0.7
0.8
Phylostratum
Cellular_organisms - ps1
Archaea1 - ps2
Archaea2 - ps3
Archaea3 - ps4
Asgard/Eukaryota - ps5
Eukaryota - ps6
Amorpheae - ps7
Obazoa - ps8
Opisthokonta - ps9
Holozoa - ps10
Filozoa - ps11
Choano./Metazoa - ps12
Metazoa - ps13
Myriazoa - ps14
Parahoxozoa - ps15
Bilateria - ps16
Deuterostomia - ps17
Chordata - ps18
Olfactores - ps19
Vertebrata - ps20
Gnathostomata - ps21
Euteleostomi - ps22
Sarcopterygii - ps23
Tetrapoda - ps24
Amniota - ps25
Mammalia - ps26
Theria - ps27
Placentalia - ps28
Boreoeutheria - ps29
Euarchontoglires - ps30
Euarchonta - ps31
Primates - ps32
Haplorrhini - ps33
Simiiformes - ps34
Catarrhini - ps35
Hominoidea - ps36
Hominidae - ps37
Homininae - ps38
Hominini - ps39
Homo_sapiens - ps40
p-value
3e-03
5e-03
1e-02
2e-02
3e-02
Log-odds
0.5
1.0
1.5
2.0
2.5
3.0
3.5
4.0

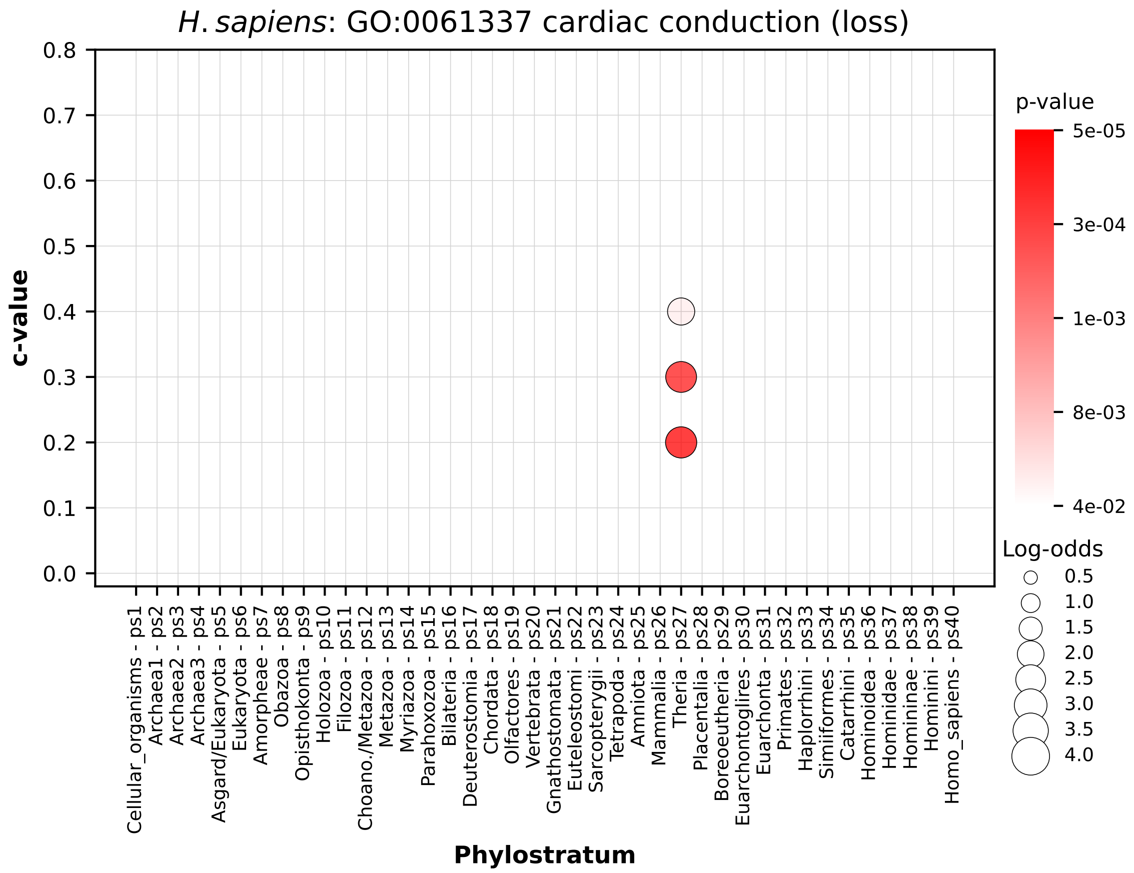

H. sapiens: GO:0061337 cardiac conduction (loss)
c-value
0.0
0.1
0.2
0.3
0.4
0.5
0.6
0.7
0.8
Phylostratum
Cellular_organisms - ps1
Archaea1 - ps2
Archaea2 - ps3
Archaea3 - ps4
Asgard/Eukaryota - ps5
Eukaryota - ps6
Amorpheae - ps7
Obazoa - ps8
Opisthokonta - ps9
Holozoa - ps10
Filozoa - ps11
Choano./Metazoa - ps12
Metazoa - ps13
Myriazoa - ps14
Parahoxozoa - ps15
Bilateria - ps16
Deuterostomia - ps17
Chordata - ps18
Olfactores - ps19
Vertebrata - ps20
Gnathostomata - ps21
Euteleostomi - ps22
Sarcopterygii - ps23
Tetrapoda - ps24
Amniota - ps25
Mammalia - ps26
Theria - ps27
Placentalia - ps28
Boreoeutheria - ps29
Euarchontoglires - ps30
Euarchonta - ps31
Primates - ps32
Haplorrhini - ps33
Simiiformes - ps34
Catarrhini - ps35
Hominoidea - ps36
Hominidae - ps37
Homininae - ps38
Hominini - ps39
Homo_sapiens - ps40
p-value
5e-05
3e-04
1e-03
8e-03
4e-02
Log-odds
0.5
1.0
1.5
2.0
2.5
3.0
3.5
4.0

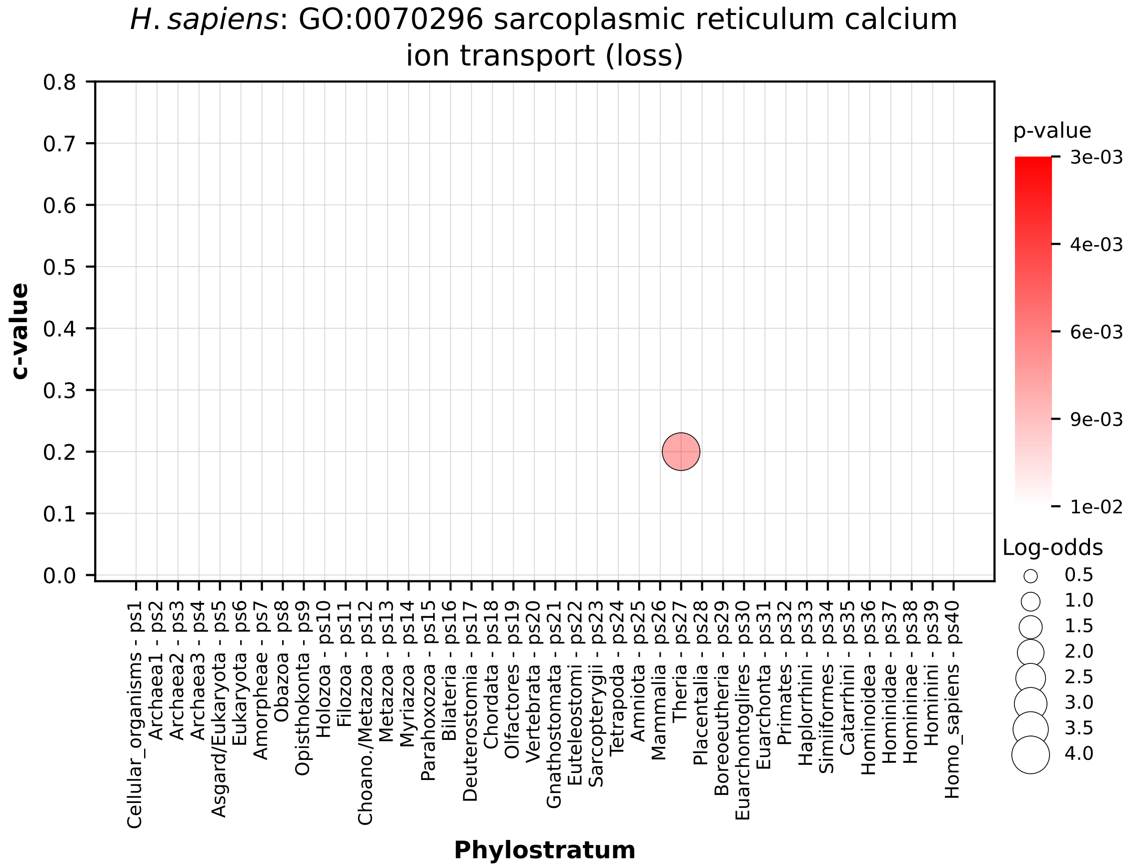
H. sapiens: GO:0070296 sarcoplasmic reticulum calcium ion transport (loss)
c-value
0.0
0.1
0.2
0.3
0.4
0.5
0.6
0.7
0.8
Phylostratum
Cellular_organisms - ps1
Archaea1 - ps2
Archaea2 - ps3
Archaea3 - ps4
Asgard/Eukaryota - ps5
Eukaryota - ps6
Amorpheae - ps7
Obazoa - ps8
Opisthokonta - ps9
Holozoa - ps10
Filozoa - ps11
Choano./Metazoa - ps12
Metazoa - ps13
Myriazoa - ps14
Parahoxozoa - ps15
Bilateria - ps16
Deuterostomia - ps17
Chordata - ps18
Olfactores - ps19
Vertebrata - ps20
Gnathostomata - ps21
Euteleostomi - ps22
Sarcopterygii - ps23
Tetrapoda - ps24
Amniota - ps25
Mammalia - ps26
Theria - ps27
Placentalia - ps28
Boreoeutheria - ps29
Euarchontoglires - ps30
Euarchonta - ps31
Primates - ps32
Haplorrhini - ps33
Simiiformes - ps34
Catarrhini - ps35
Hominoidea - ps36
Hominidae - ps37
Homininae - ps38
Hominini - ps39
Homo_sapiens - ps40
p-value
3e-03
4e-03
6e-03
9e-03
1e-02
Log-odds
0.5
1.0
1.5
2.0
2.5
3.0
3.5
4.0

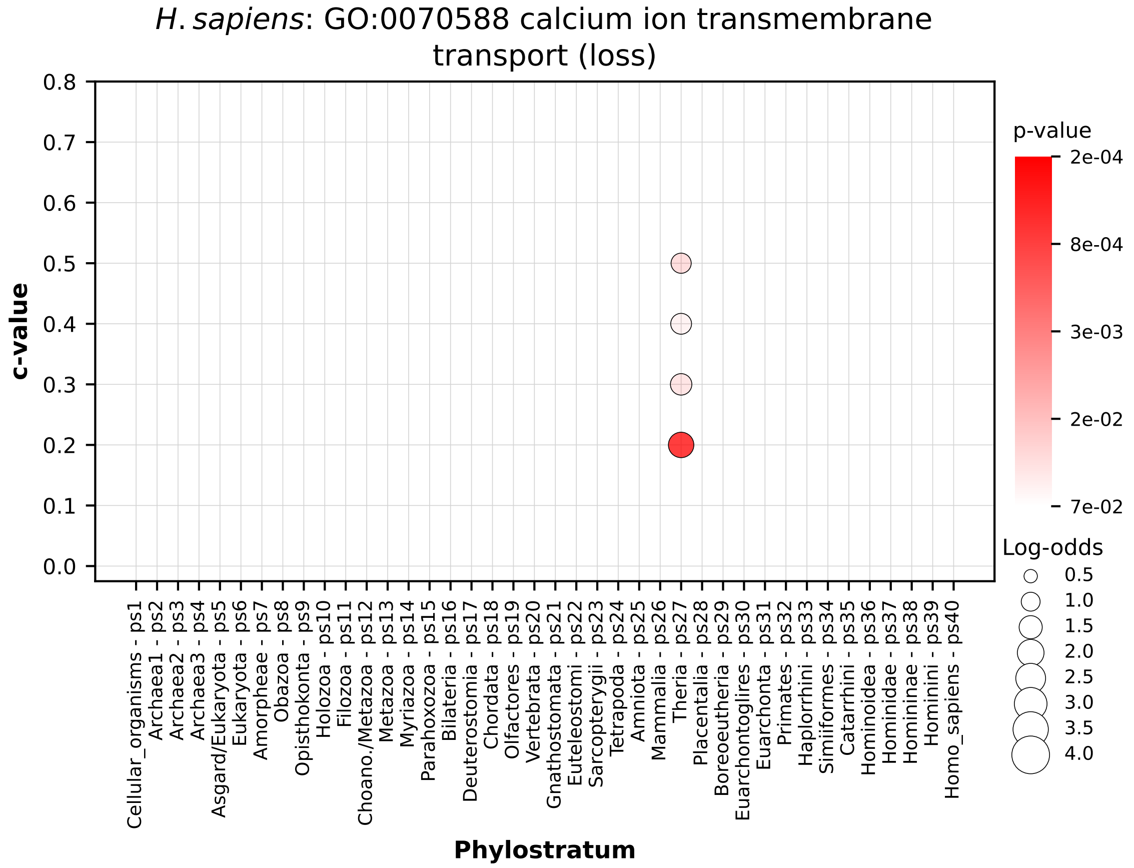
H. sapiens: GO:0070588 calcium ion transmembrane transport (loss)
c-value
0.8
0.7
0.6
0.5
0.4
0.3
0.2
0.1
0.0
p-value
2e-04
8e-04
3e-03
2e-02
7e-02
Log-odds
0.5
1.0
1.5
2.0
2.5
3.0
3.5
4.0
Cellular_organisms - ps1
Archaea1 - ps2
Archaea2 - ps3
Archaea3 - ps4
Asgard/Eukaryota - ps5
Eukaryota - ps6
Amorpheae - ps7
Obazoa - ps8
Opisthokonta - ps9
Holozoa - ps10
Filozoa - ps11
Choano./Metazoa - ps12
Metazoa - ps13
Myriazoa - ps14
Parahoxozoa - ps15
Bilateria - ps16
Deuterostomia - ps17
Chordata - ps18
Olfactores - ps19
Vertebrata - ps20
Gnathostomata - ps21
Euteleostomi - ps22
Sarcopterygii - ps23
Tetrapoda - ps24
Amniota - ps25
Mammalia - ps26
Theria - ps27
Placentalia - ps28
Boreoeutheria - ps29
Euarchontoglires - ps30
Euarchonta - ps31
Primates - ps32
Haplorrhini - ps33
Simiiformes - ps34
Catarrhini - ps35
Hominoidea - ps36
Hominidae - ps37
Homininae - ps38
Hominini - ps39
Homo_sapiens - ps40
Phylostratum

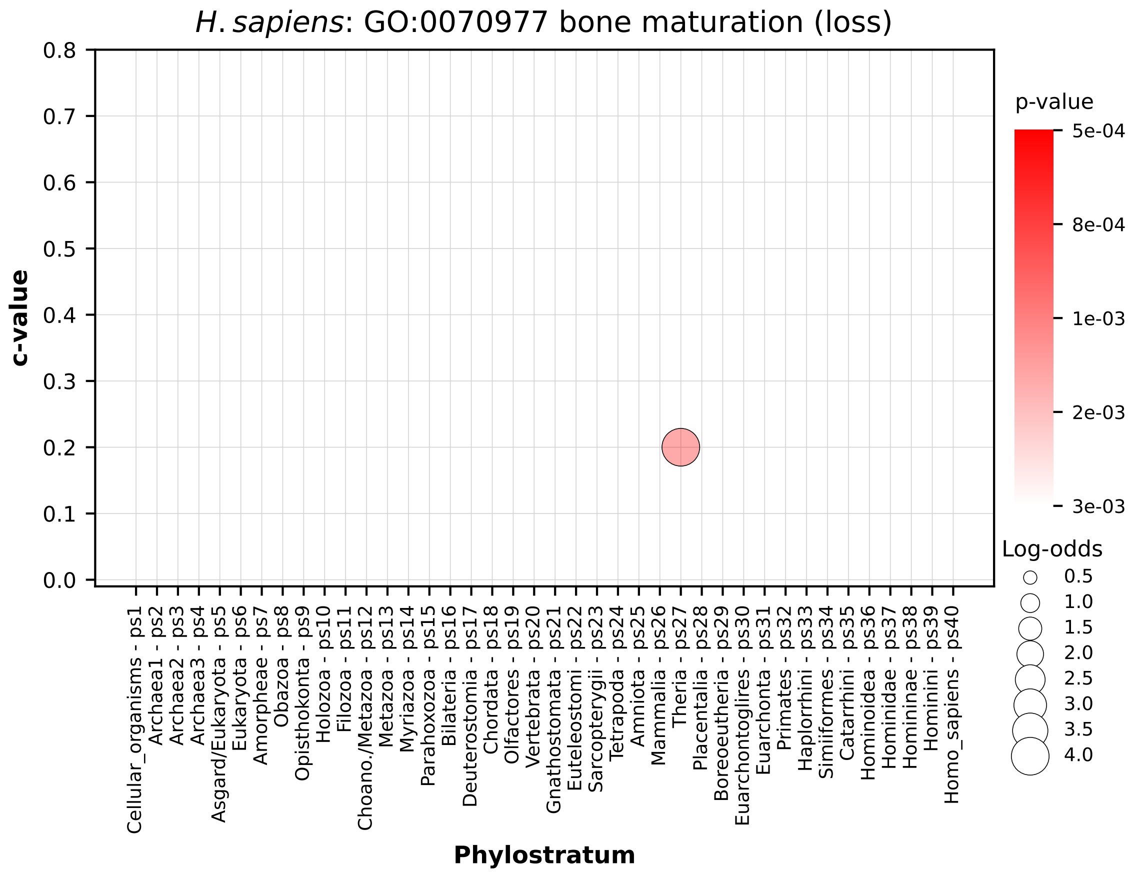

H. sapiens: GO:0070977 bone maturation (loss)
c-value
0.0
0.1
0.2
0.3
0.4
0.5
0.6
0.7
0.8
Phylostratum
Cellular_organisms - ps1
Archaea1 - ps2
Archaea2 - ps3
Archaea3 - ps4
Asgard/Eukaryota - ps5
Eukaryota - ps6
Amorpheae - ps7
Obazoa - ps8
Opisthokonta - ps9
Holozoa - ps10
Filozoa - ps11
Choano./Metazoa - ps12
Metazoa - ps13
Myriazoa - ps14
Parahoxozoa - ps15
Bilateria - ps16
Deuterostomia - ps17
Chordata - ps18
Olfactores - ps19
Vertebrata - ps20
Gnathostomata - ps21
Euteleostomi - ps22
Sarcopterygii - ps23
Tetrapoda - ps24
Amniota - ps25
Mammalia - ps26
Theria - ps27
Placentalia - ps28
Boreoeutheria - ps29
Euarchontoglires - ps30
Euarchonta - ps31
Primates - ps32
Haplorrhini - ps33
Simiiformes - ps34
Catarrhini - ps35
Hominoidea - ps36
Hominidae - ps37
Homininae - ps38
Hominini - ps39
Homo_sapiens - ps40
p-value
5e-04
8e-04
1e-03
2e-03
3e-03
Log-odds
0.5
1.0
1.5
2.0
2.5
3.0
3.5
4.0

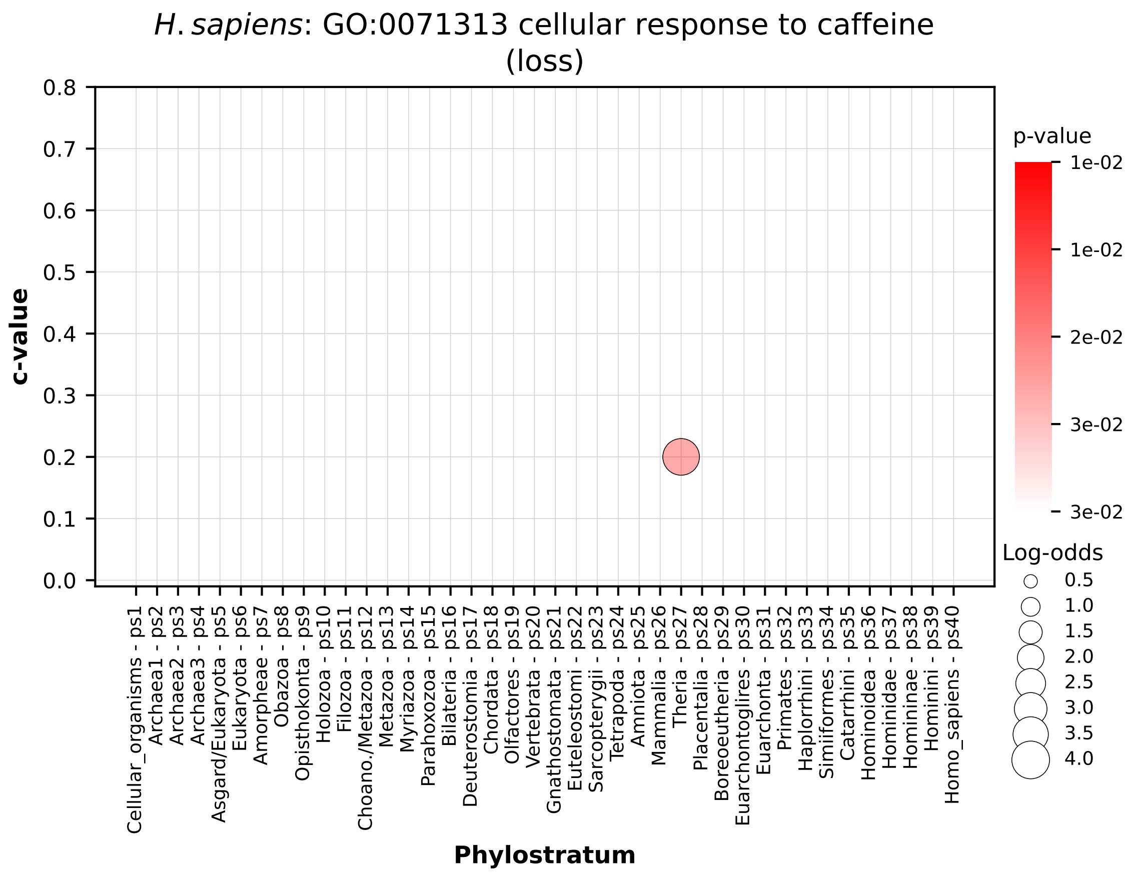
H. sapiens: GO:0071313 cellular response to caffeine
(loss)
c-value
0.0
0.1
0.2
0.3
0.4
0.5
0.6
0.7
0.8
Phylostratum
Cellular_organisms - ps1
Archaea1 - ps2
Archaea2 - ps3
Archaea3 - ps4
Asgard/Eukaryota - ps5
Eukaryota - ps6
Amorpheae - ps7
Obazoa - ps8
Opisthokonta - ps9
Holozoa - ps10
Filozoa - ps11
Choano./Metazoa - ps12
Metazoa - ps13
Myriazoa - ps14
Parahoxozoa - ps15
Bilateria - ps16
Deuterostomia - ps17
Chordata - ps18
Olfactores - ps19
Vertebrata - ps20
Gnathostomata - ps21
Euteleostomi - ps22
Sarcopterygii - ps23
Tetrapoda - ps24
Amniota - ps25
Mammalia - ps26
Theria - ps27
Placentalia - ps28
Boreoeutheria - ps29
Euarchontoglires - ps30
Euarchonta - ps31
Primates - ps32
Haplorrhini - ps33
Simiiformes - ps34
Catarrhini - ps35
Hominoidea - ps36
Hominidae - ps37
Homininae - ps38
Hominini - ps39
Homo_sapiens - ps40
p-value
1e-02
1e-02
2e-02
3e-02
3e-02
Log-odds
0.5
1.0
1.5
2.0
2.5
3.0
3.5
4.0

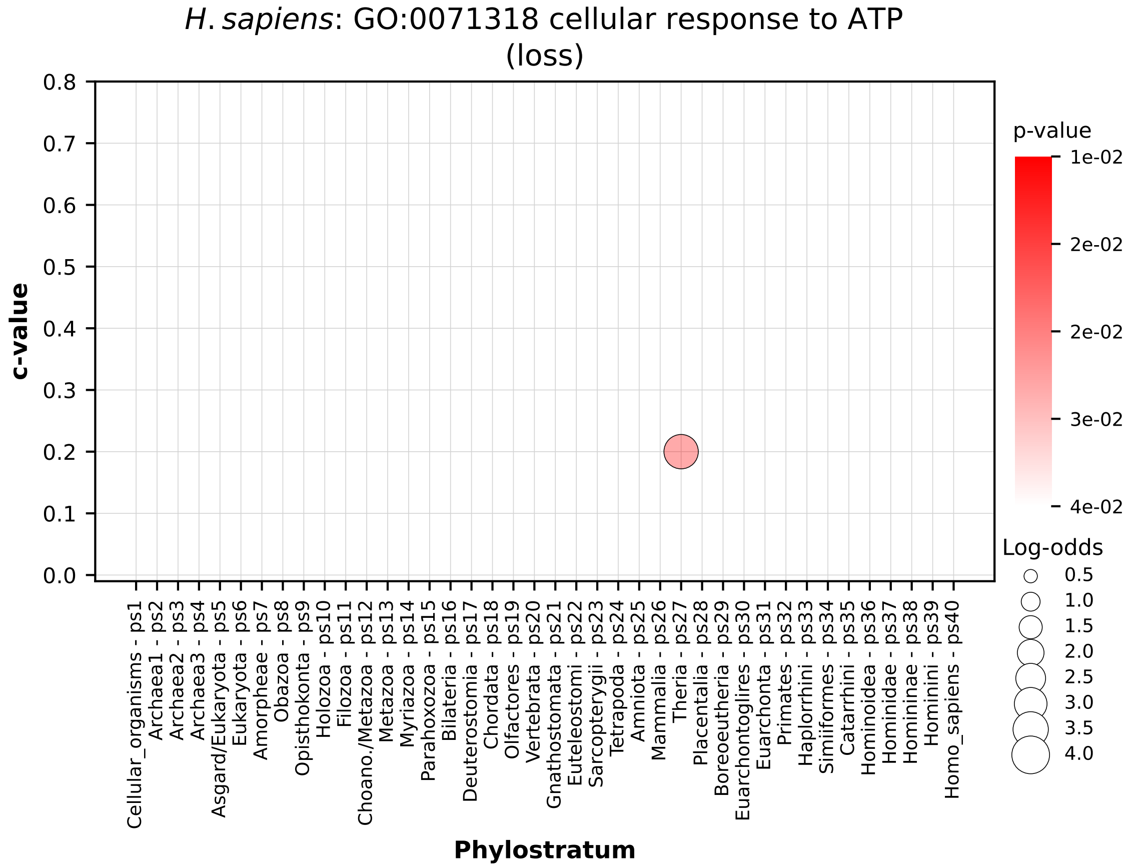

H. sapiens: GO:0071318 cellular response to ATP
(loss)
c-value
0.8
0.7
0.6
0.5
0.4
0.3
0.2
0.1
0.0
p-value
1e-02
2e-02
2e-02
3e-02
4e-02
Log-odds
0.5
1.0
1.5
2.0
2.5
3.0
3.5
4.0
Cellular_organisms - ps1
Archaea1 - ps2
Archaea2 - ps3
Archaea3 - ps4
Asgard/Eukaryota - ps5
Eukaryota - ps6
Amorpheae - ps7
Obazoa - ps8
Opisthokonta - ps9
Holozoa - ps10
Filozoa - ps11
Choano./Metazoa - ps12
Metazoa - ps13
Myriazoa - ps14
Parahoxozoa - ps15
Bilateria - ps16
Deuterostomia - ps17
Chordata - ps18
Olfactores - ps19
Vertebrata - ps20
Gnathostomata - ps21
Euteleostomi - ps22
Sarcopterygii - ps23
Tetrapoda - ps24
Amniota - ps25
Mammalia - ps26
Theria - ps27
Placentalia - ps28
Boreoeutheria - ps29
Euarchontoglires - ps30
Euarchonta - ps31
Primates - ps32
Haplorrhini - ps33
Simiiformes - ps34
Catarrhini - ps35
Hominoidea - ps36
Hominidae - ps37
Homininae - ps38
Hominini - ps39
Homo_sapiens - ps40
Phylostratum

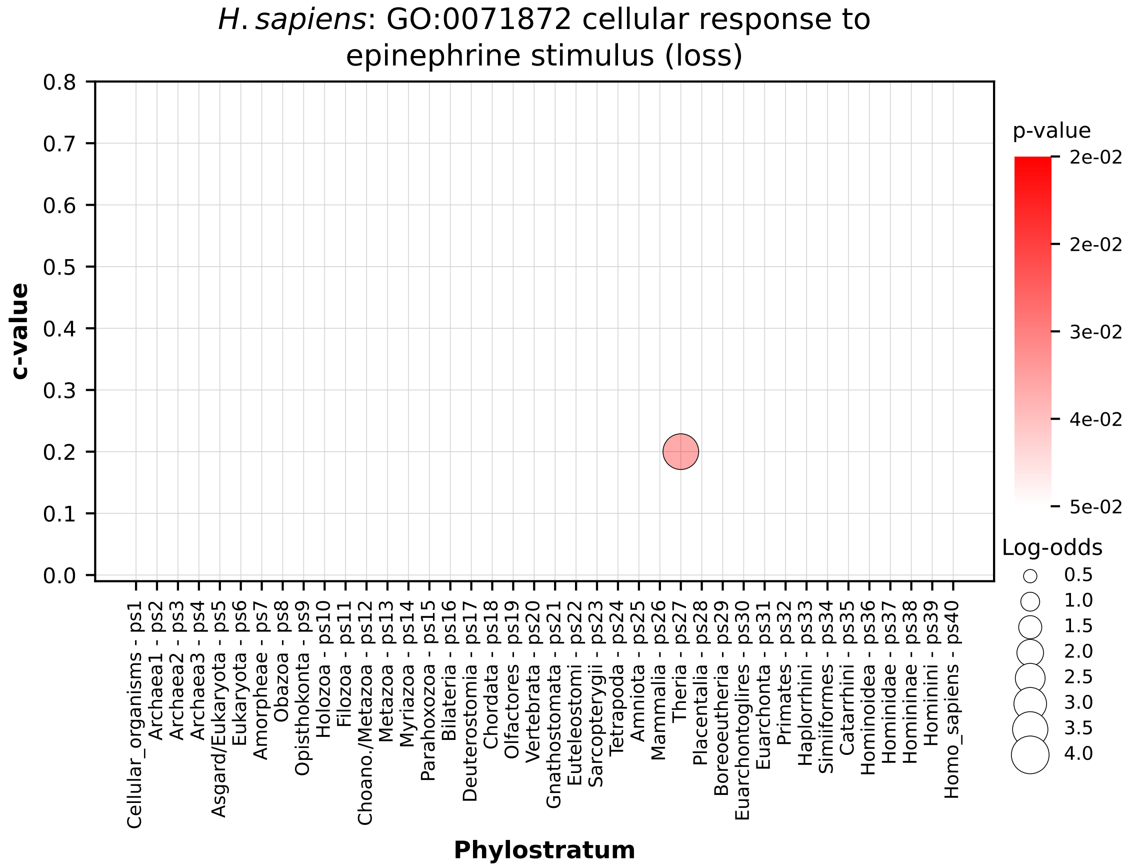

H. sapiens: GO:0071872 cellular response to epinephrine stimulus (loss)
c-value
0.0
0.1
0.2
0.3
0.4
0.5
0.6
0.7
0.8
Phylostratum
Cellular_organisms - ps1
Archaea1 - ps2
Archaea2 - ps3
Archaea3 - ps4
Asgard/Eukaryota - ps5
Eukaryota - ps6
Amorpheae - ps7
Obazoa - ps8
Opisthokonta - ps9
Holozoa - ps10
Filozoa - ps11
Choano./Metazoa - ps12
Metazoa - ps13
Myriazoa - ps14
Parahoxozoa - ps15
Bilateria - ps16
Deuterostomia - ps17
Chordata - ps18
Olfactores - ps19
Vertebrata - ps20
Gnathostomata - ps21
Euteleostomi - ps22
Sarcopterygii - ps23
Tetrapoda - ps24
Amniota - ps25
Mammalia - ps26
Theria - ps27
Placentalia - ps28
Boreoeutheria - ps29
Euarchontoglires - ps30
Euarchonta - ps31
Primates - ps32
Haplorrhini - ps33
Simiiformes - ps34
Catarrhini - ps35
Hominoidea - ps36
Hominidae - ps37
Homininae - ps38
Hominini - ps39
Homo_sapiens - ps40
p-value
2e-02
2e-02
3e-02
4e-02
5e-02
Log-odds
0.5
1.0
1.5
2.0
2.5
3.0
3.5
4.0

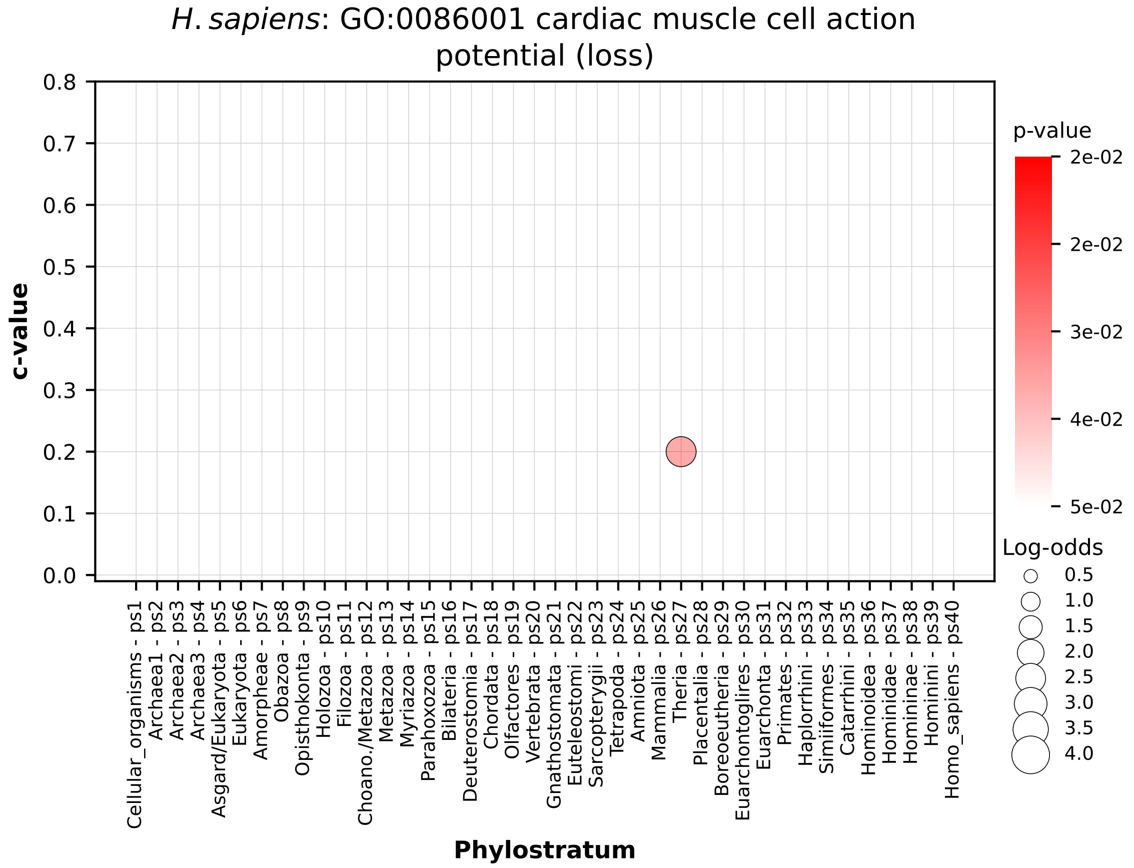
H. sapiens: GO:0086001 cardiac muscle cell action potential (loss)
c-value
0.8
0.7
0.6
0.5
0.4
0.3
0.2
0.1
0.0
p-value
2e-02
2e-02
3e-02
4e-02
5e-02
Log-odds
0.5
1.0
1.5
2.0
2.5
3.0
3.5
4.0
Cellular_organisms - ps1
Archaea1 - ps2
Archaea2 - ps3
Archaea3 - ps4
Asgard/Eukaryota - ps5
Eukaryota - ps6
Amorpheae - ps7
Obazoa - ps8
Opisthokonta - ps9
Holozoa - ps10
Filozoa - ps11
Choano./Metazoa - ps12
Metazoa - ps13
Myriazoa - ps14
Parahoxozoa - ps15
Bilateria - ps16
Deuterostomia - ps17
Chordata - ps18
Olfactores - ps19
Vertebrata - ps20
Gnathostomata - ps21
Euteleostomi - ps22
Sarcopterygii - ps23
Tetrapoda - ps24
Amniota - ps25
Mammalia - ps26
Theria - ps27
Placentalia - ps28
Boreoeutheria - ps29
Euarchontoglires - ps30
Euarchonta - ps31
Primates - ps32
Haplorrhini - ps33
Simiiformes - ps34
Catarrhini - ps35
Hominoidea - ps36
Hominidae - ps37
Homininae - ps38
Hominini - ps39
Homo_sapiens - ps40
Phylostratum

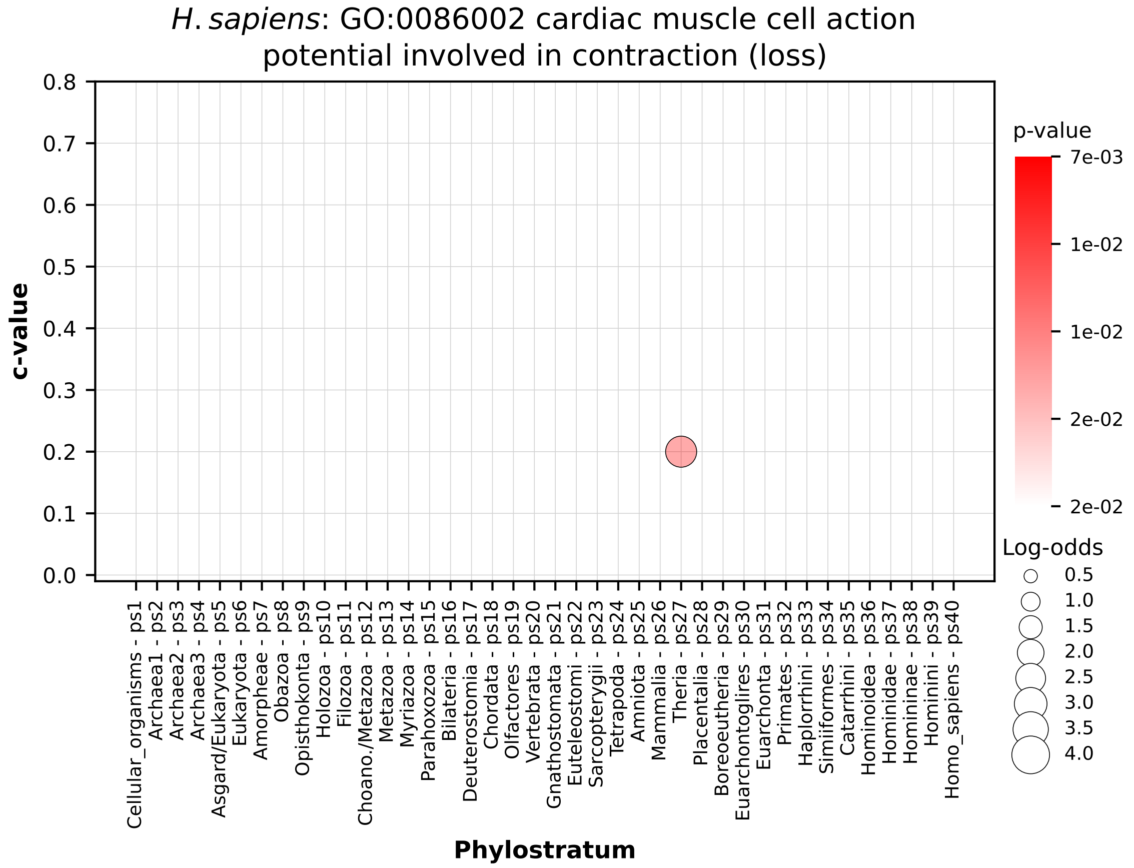
H. sapiens: GO:0086002 cardiac muscle cell action potential involved in contraction (loss)
c-value
0.0
0.1
0.2
0.3
0.4
0.5
0.6
0.7
0.8
Phylostratum
Cellular_organisms - ps1
Archaea1 - ps2
Archaea2 - ps3
Archaea3 - ps4
Asgard/Eukaryota - ps5
Eukaryota - ps6
Amorpheae - ps7
Obazoa - ps8
Opisthokonta - ps9
Holozoa - ps10
Filozoa - ps11
Choano./Metazoa - ps12
Metazoa - ps13
Myriazoa - ps14
Parahoxozoa - ps15
Bilateria - ps16
Deuterostomia - ps17
Chordata - ps18
Olfactores - ps19
Vertebrata - ps20
Gnathostomata - ps21
Euteleostomi - ps22
Sarcopterygii - ps23
Tetrapoda - ps24
Amniota - ps25
Mammalia - ps26
Theria - ps27
Placentalia - ps28
Boreoeutheria - ps29
Euarchontoglires - ps30
Euarchonta - ps31
Primates - ps32
Haplorrhini - ps33
Simiiformes - ps34
Catarrhini - ps35
Hominoidea - ps36
Hominidae - ps37
Homininae - ps38
Hominini - ps39
Homo_sapiens - ps40
p-value
7e-03
1e-02
1e-02
2e-02
2e-02
Log-odds
0.5
1.0
1.5
2.0
2.5
3.0
3.5
4.0

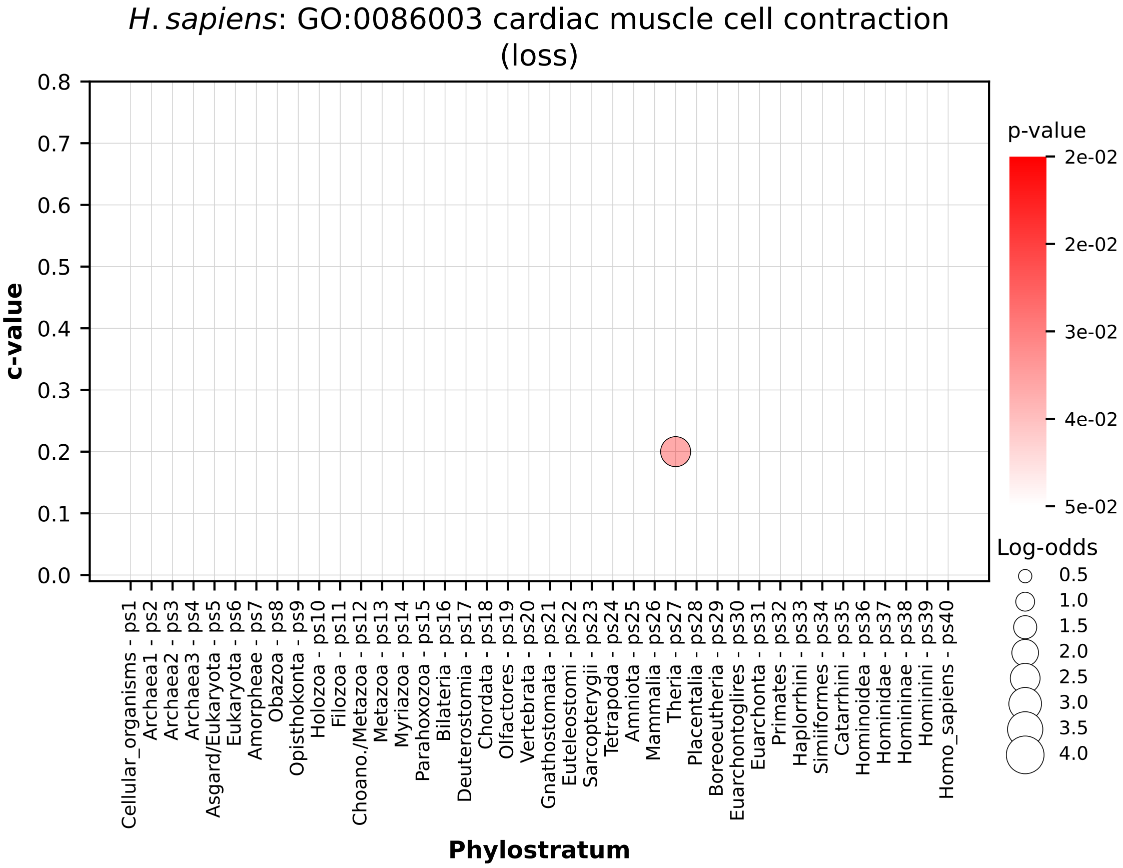
H. sapiens: GO:0086003 cardiac muscle cell contraction (loss)
c-value
0.8
0.7
0.6
0.5
0.4
0.3
0.2
0.1
0.0
Phylostratum
Cellular_organisms - ps1
Archaea1 - ps2
Archaea2 - ps3
Archaea3 - ps4
Asgard/Eukaryota - ps5
Eukaryota - ps6
Amorpheae - ps7
Obazoa - ps8
Opisthokonta - ps9
Holozoa - ps10
Filozoa - ps11
Choano./Metazoa - ps12
Metazoa - ps13
Myriazoa - ps14
Parahoxozoa - ps15
Bilateria - ps16
Deuterostomia - ps17
Chordata - ps18
Olfactores - ps19
Vertebrata - ps20
Gnathostomata - ps21
Euteleostomi - ps22
Sarcopterygii - ps23
Tetrapoda - ps24
Amniota - ps25
Mammalia - ps26
Theria - ps27
Placentalia - ps28
Boreoeutheria - ps29
Euarchontoglires - ps30
Euarchonta - ps31
Primates - ps32
Haplorrhini - ps33
Simiiformes - ps34
Catarrhini - ps35
Hominoidea - ps36
Hominidae - ps37
Homininae - ps38
Hominini - ps39
Homo_sapiens - ps40
p-value
2e-02
2e-02
3e-02
4e-02
5e-02
Log-odds
0.5
1.0
1.5
2.0
2.5
3.0
3.5
4.0

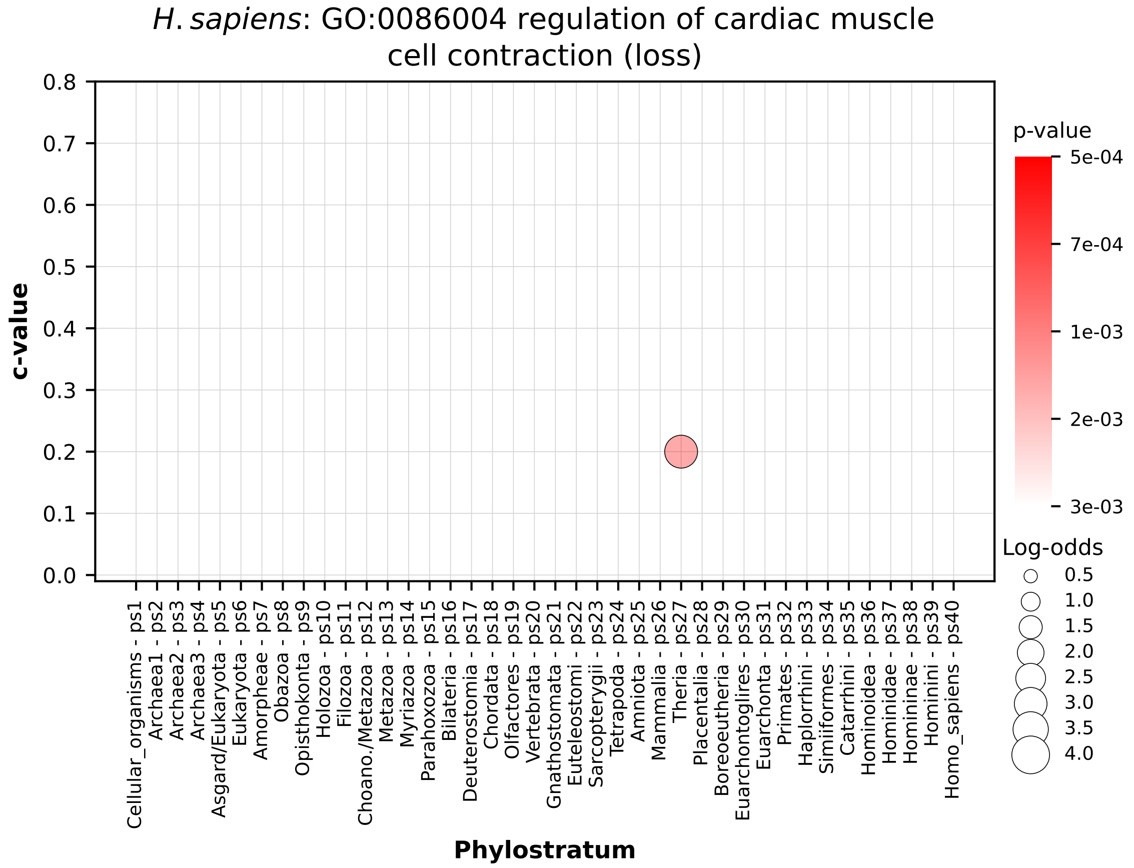

H. sapiens: GO:0086004 regulation of cardiac muscle cell contraction (loss)
c-value
0.8
0.7
0.6
0.5
0.4
0.3
0.2
0.1
0.0
p-value
5e-04
7e-04
1e-03
2e-03
3e-03
Log-odds
0.5
1.0
1.5
2.0
2.5
3.0
3.5
4.0
Cellular_organisms - ps1
Archaea1 - ps2
Archaea2 - ps3
Archaea3 - ps4
Asgard/Eukaryota - ps5
Eukaryota - ps6
Amorpheae - ps7
Obazoa - ps8
Opisthokonta - ps9
Holozoa - ps10
Filozoa - ps11
Choano./Metazoa - ps12
Metazoa - ps13
Myriazoa - ps14
Parahoxozoa - ps15
Bilateria - ps16
Deuterostomia - ps17
Chordata - ps18
Olfactores - ps19
Vertebrata - ps20
Gnathostomata - ps21
Euteleostomi - ps22
Sarcopterygii - ps23
Tetrapoda - ps24
Amniota - ps25
Mammalia - ps26
Theria - ps27
Placentalia - ps28
Boreoeutheria - ps29
Euarchontoglires - ps30
Euarchonta - ps31
Primates - ps32
Haplorrhini - ps33
Simiiformes - ps34
Catarrhini - ps35
Hominoidea - ps36
Hominidae - ps37
Homininae - ps38
Hominini - ps39
Homo_sapiens - ps40
Phylostratum

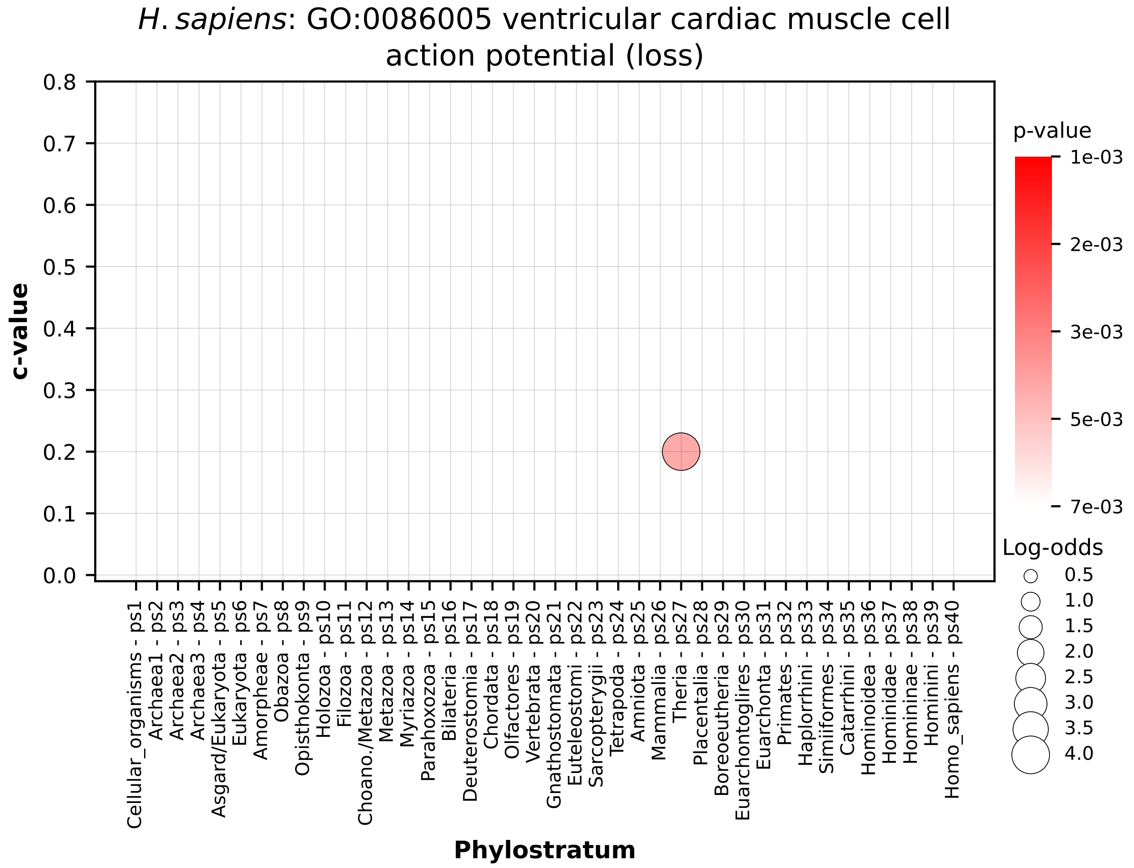

H. sapiens: GO:0086005 ventricular cardiac muscle cell action potential (loss)
c-value
0.0
0.1
0.2
0.3
0.4
0.5
0.6
0.7
0.8
Phylostratum
Cellular_organisms - ps1
Archaea1 - ps2
Archaea2 - ps3
Archaea3 - ps4
Asgard/Eukaryota - ps5
Eukaryota - ps6
Amorpheae - ps7
Obazoa - ps8
Opisthokonta - ps9
Holozoa - ps10
Filozoa - ps11
Choano./Metazoa - ps12
Metazoa - ps13
Myriazoa - ps14
Parahoxozoa - ps15
Bilateria - ps16
Deuterostomia - ps17
Chordata - ps18
Olfactores - ps19
Vertebrata - ps20
Gnathostomata - ps21
Euteleostomi - ps22
Sarcopterygii - ps23
Tetrapoda - ps24
Amniota - ps25
Mammalia - ps26
Theria - ps27
Placentalia - ps28
Boreoeutheria - ps29
Euarchontoglires - ps30
Euarchonta - ps31
Primates - ps32
Haplorrhini - ps33
Simiiformes - ps34
Catarrhini - ps35
Hominoidea - ps36
Hominidae - ps37
Homininae - ps38
Hominini - ps39
Homo_sapiens - ps40
p-value
1e-03
2e-03
3e-03
5e-03
7e-03
Log-odds
0.5
1.0
1.5
2.0
2.5
3.0
3.5
4.0

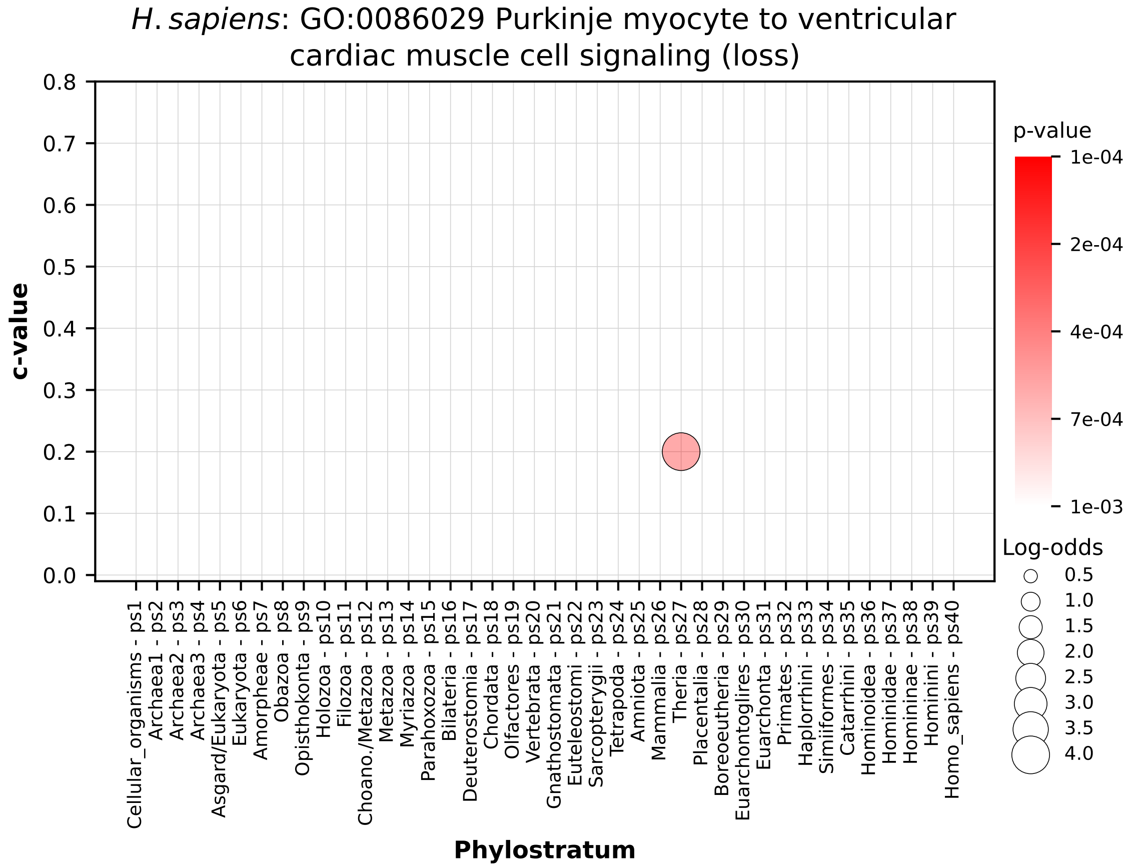

H. sapiens: GO:0086029 Purkinje myocyte to ventricular cardiac muscle cell signaling (loss)
c-value
0.8
0.7
0.6
0.5
0.4
0.3
0.2
0.1
0.0
Phylostratum
Cellular_organisms - ps1
Archaea1 - ps2
Archaea2 - ps3
Archaea3 - ps4
Asgard/Eukaryota - ps5
Eukaryota - ps6
Amorpheae - ps7
Obazoa - ps8
Opisthokonta - ps9
Holozoa - ps10
Filozoa - ps11
Choano./Metazoa - ps12
Metazoa - ps13
Myriazoa - ps14
Parahoxozoa - ps15
Bilateria - ps16
Deuterostomia - ps17
Chordata - ps18
Olfactores - ps19
Vertebrata - ps20
Gnathostomata - ps21
Euteleostomi - ps22
Sarcopterygii - ps23
Tetrapoda - ps24
Amniota - ps25
Mammalia - ps26
Theria - ps27
Placentalia - ps28
Boreoeutheria - ps29
Euarchontoglires - ps30
Euarchonta - ps31
Primates - ps32
Haplorrhini - ps33
Simiiformes - ps34
Catarrhini - ps35
Hominoidea - ps36
Hominidae - ps37
Homininae - ps38
Hominini - ps39
Homo_sapiens - ps40
p-value
1e-04
2e-04
4e-04
7e-04
1e-03
Log-odds
0.5
1.0
1.5
2.0
2.5
3.0
3.5
4.0

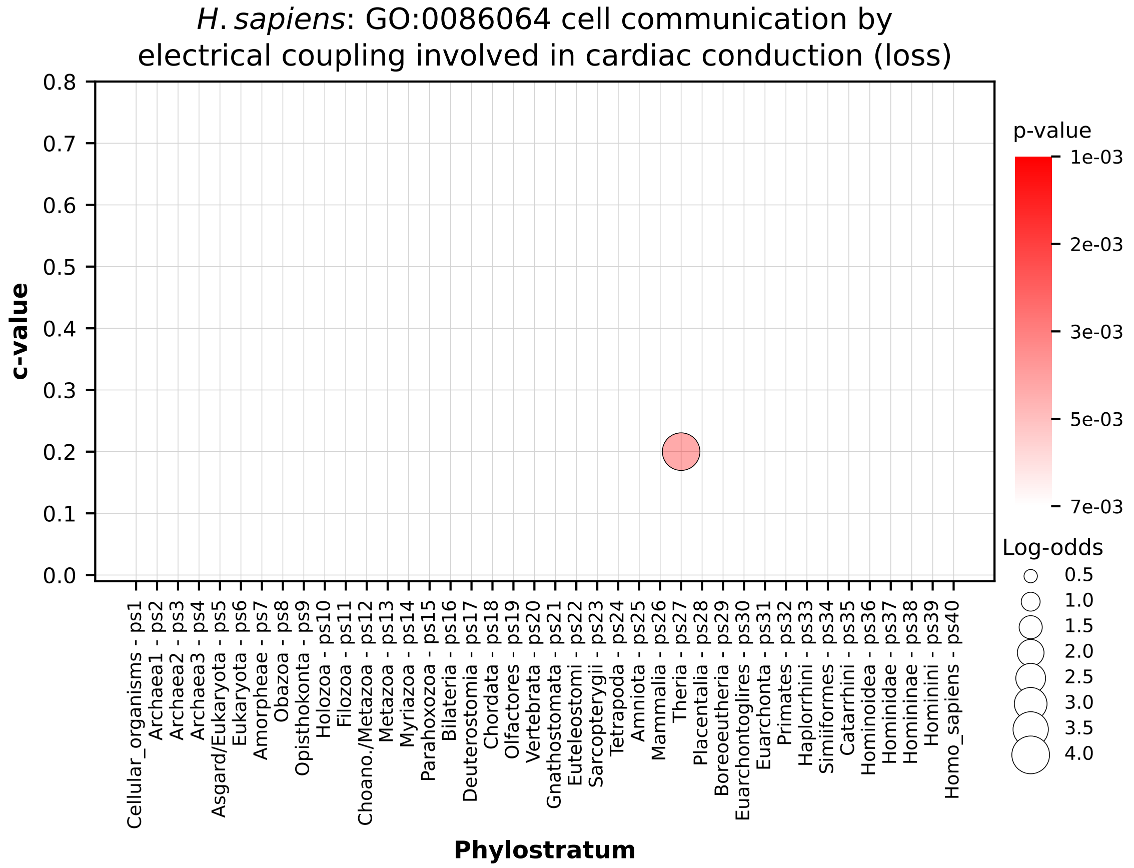
H. sapiens: GO:0086064 cell communication by electrical coupling involved in cardiac conduction (loss)
c-value
0.8
0.7
0.6
0.5
0.4
0.3
0.2
0.1
0.0
Phylostratum
Cellular_organisms - ps1
Archaea1 - ps2
Archaea2 - ps3
Archaea3 - ps4
Asgard/Eukaryota - ps5
Eukaryota - ps6
Amorpheae - ps7
Obazoa - ps8
Opisthokonta - ps9
Holozoa - ps10
Filozoa - ps11
Choano./Metazoa - ps12
Metazoa - ps13
Myriazoa - ps14
Parahoxozoa - ps15
Bilateria - ps16
Deuterostomia - ps17
Chordata - ps18
Olfactores - ps19
Vertebrata - ps20
Gnathostomata - ps21
Euteleostomi - ps22
Sarcopterygii - ps23
Tetrapoda - ps24
Amniota - ps25
Mammalia - ps26
Theria - ps27
Placentalia - ps28
Boreoeutheria - ps29
Euarchontoglires - ps30
Euarchonta - ps31
Primates - ps32
Haplorrhini - ps33
Simiiformes - ps34
Catarrhini - ps35
Hominoidea - ps36
Hominidae - ps37
Homininae - ps38
Hominini - ps39
Homo_sapiens - ps40
p-value
1e-03
2e-03
3e-03
5e-03
7e-03
Log-odds
0.5
1.0
1.5
2.0
2.5
3.0
3.5
4.0

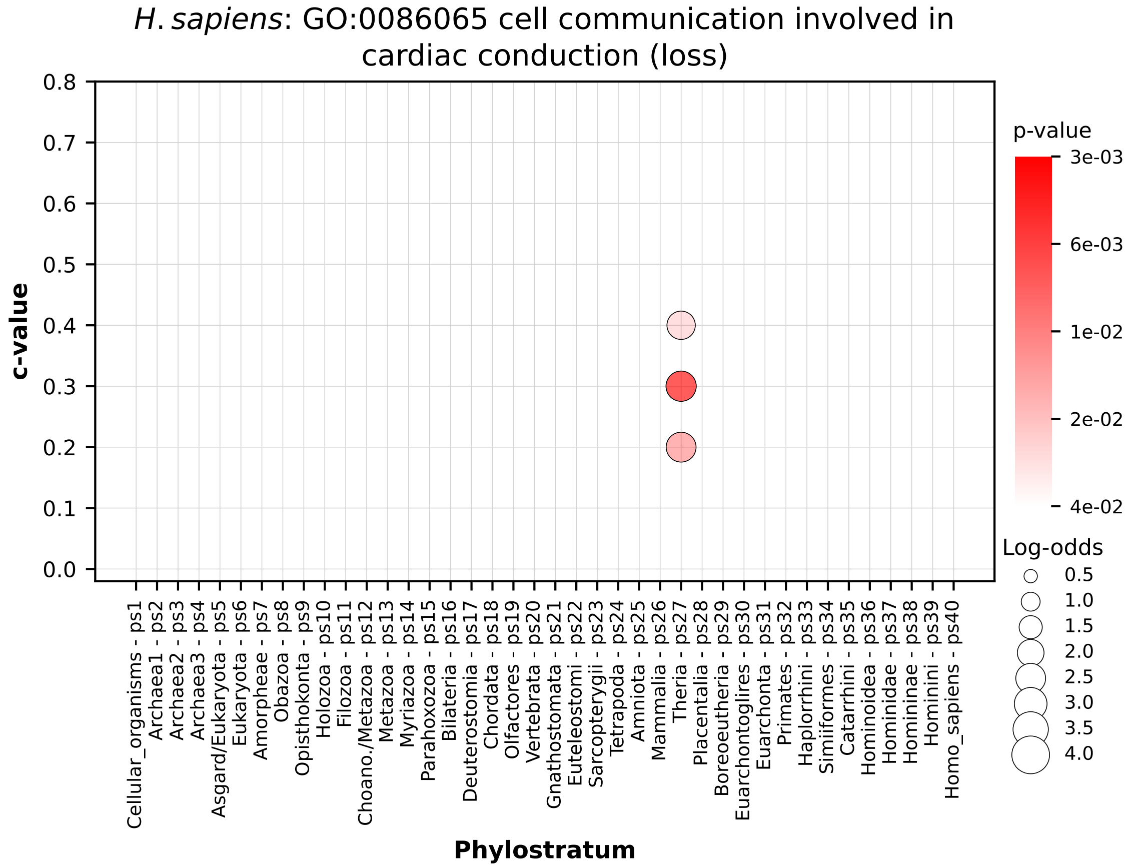

H. sapiens: GO:0086065 cell communication involved in cardiac conduction (loss)
c-value
0.8
0.7
0.6
0.5
0.4
0.3
0.2
0.1
0.0
Phylostratum
Cellular_organisms - ps1
Archaea1 - ps2
Archaea2 - ps3
Archaea3 - ps4
Asgard/Eukaryota - ps5
Eukaryota - ps6
Amorpheae - ps7
Obazoa - ps8
Opisthokonta - ps9
Holozoa - ps10
Filozoa - ps11
Choano./Metazoa - ps12
Metazoa - ps13
Myriazoa - ps14
Parahoxozoa - ps15
Bilateria - ps16
Deuterostomia - ps17
Chordata - ps18
Olfactores - ps19
Vertebrata - ps20
Gnathostomata - ps21
Euteleostomi - ps22
Sarcopterygii - ps23
Tetrapoda - ps24
Amniota - ps25
Mammalia - ps26
Theria - ps27
Placentalia - ps28
Boreoeutheria - ps29
Euarchontoglires - ps30
Euarchonta - ps31
Primates - ps32
Haplorrhini - ps33
Simiiformes - ps34
Catarrhini - ps35
Hominoidea - ps36
Hominidae - ps37
Homininae - ps38
Hominini - ps39
Homo_sapiens - ps40
p-value
3e-03
6e-03
1e-02
2e-02
4e-02
Log-odds
0.5
1.0
1.5
2.0
2.5
3.0
3.5
4.0

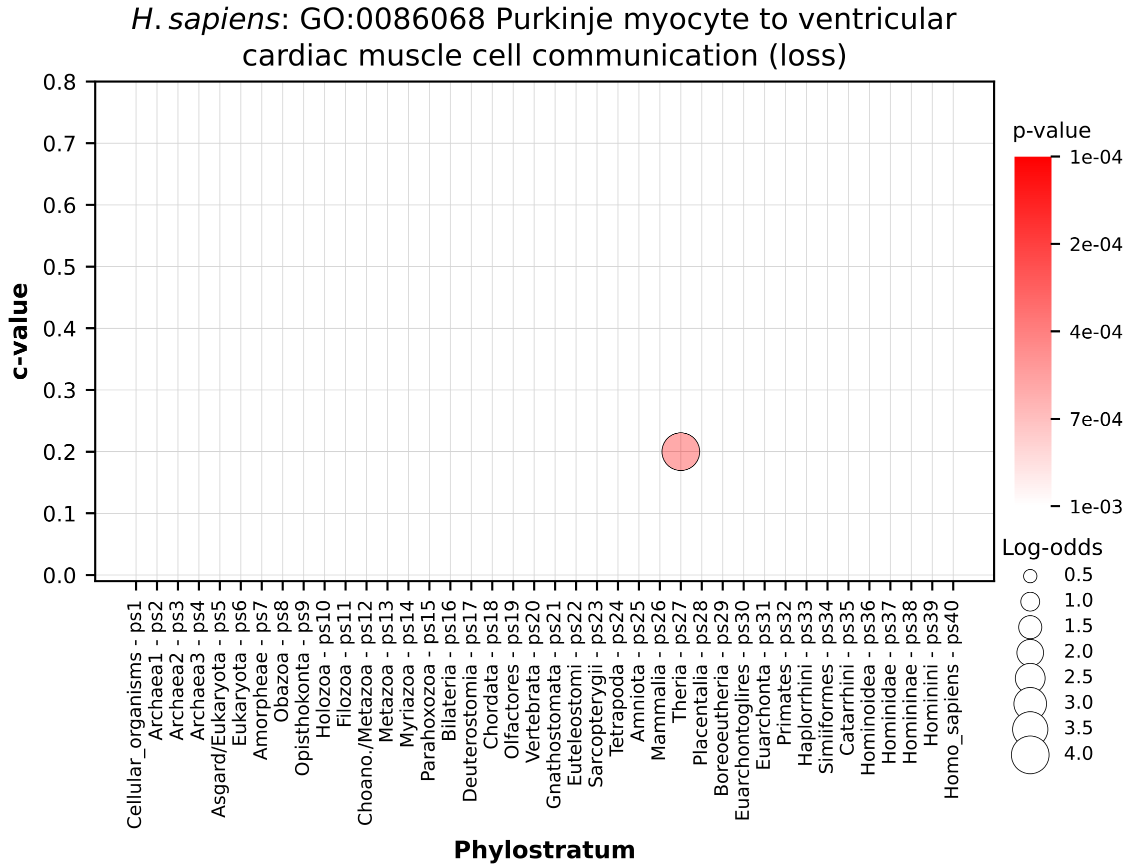

H. sapiens: GO:0086068 Purkinje myocyte to ventricular cardiac muscle cell communication (loss)
c-value
0.8
0.7
0.6
0.5
0.4
0.3
0.2
0.1
0.0
p-value
1e-04
2e-04
4e-04
7e-04
1e-03
Log-odds
0.5
1.0
1.5
2.0
2.5
3.0
3.5
4.0
Cellular_organisms - ps1
Archaea1 - ps2
Archaea2 - ps3
Archaea3 - ps4
Asgard/Eukaryota - ps5
Eukaryota - ps6
Amorpheae - ps7
Obazoa - ps8
Opisthokonta - ps9
Holozoa - ps10
Filozoa - ps11
Choano./Metazoa - ps12
Metazoa - ps13
Myriazoa - ps14
Parahoxozoa - ps15
Bilateria - ps16
Deuterostomia - ps17
Chordata - ps18
Olfactores - ps19
Vertebrata - ps20
Gnathostomata - ps21
Euteleostomi - ps22
Sarcopterygii - ps23
Tetrapoda - ps24
Amniota - ps25
Mammalia - ps26
Theria - ps27
Placentalia - ps28
Boreoeutheria - ps29
Euarchontoglires - ps30
Euarchonta - ps31
Primates - ps32
Haplorrhini - ps33
Simiiformes - ps34
Catarrhini - ps35
Hominoidea - ps36
Hominidae - ps37
Homininae - ps38
Hominini - ps39
Homo_sapiens - ps40
Phylostratum

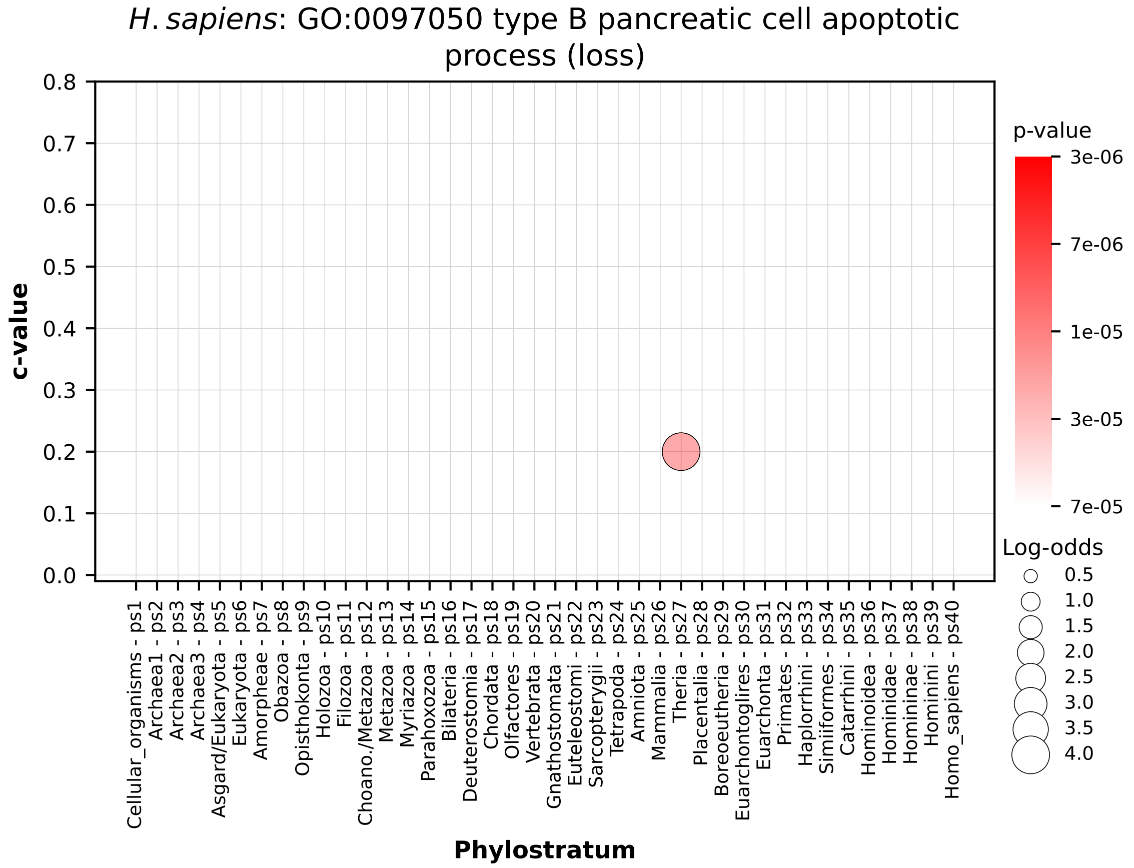

H. sapiens: GO:0097050 type B pancreatic cell apoptotic process (loss)
c-value
0.8
0.7
0.6
0.5
0.4
0.3
0.2
0.1
0.0
p-value
3e-06
7e-06
1e-05
3e-05
7e-05
Log-odds
0.5
1.0
1.5
2.0
2.5
3.0
3.5
4.0
Cellular_organisms - ps1
Archaea1 - ps2
Archaea2 - ps3
Archaea3 - ps4
Asgard/Eukaryota - ps5
Eukaryota - ps6
Amorpheae - ps7
Obazoa - ps8
Opisthokonta - ps9
Holozoa - ps10
Filozoa - ps11
Choano./Metazoa - ps12
Metazoa - ps13
Myriazoa - ps14
Parahoxozoa - ps15
Bilateria - ps16
Deuterostomia - ps17
Chordata - ps18
Olfactores - ps19
Vertebrata - ps20
Gnathostomata - ps21
Euteleostomi - ps22
Sarcopterygii - ps23
Tetrapoda - ps24
Amniota - ps25
Mammalia - ps26
Theria - ps27
Placentalia - ps28
Boreoeutheria - ps29
Euarchontoglires - ps30
Euarchonta - ps31
Primates - ps32
Haplorrhini - ps33
Simiiformes - ps34
Catarrhini - ps35
Hominoidea - ps36
Hominidae - ps37
Homininae - ps38
Hominini - ps39
Homo_sapiens - ps40
Phylostratum

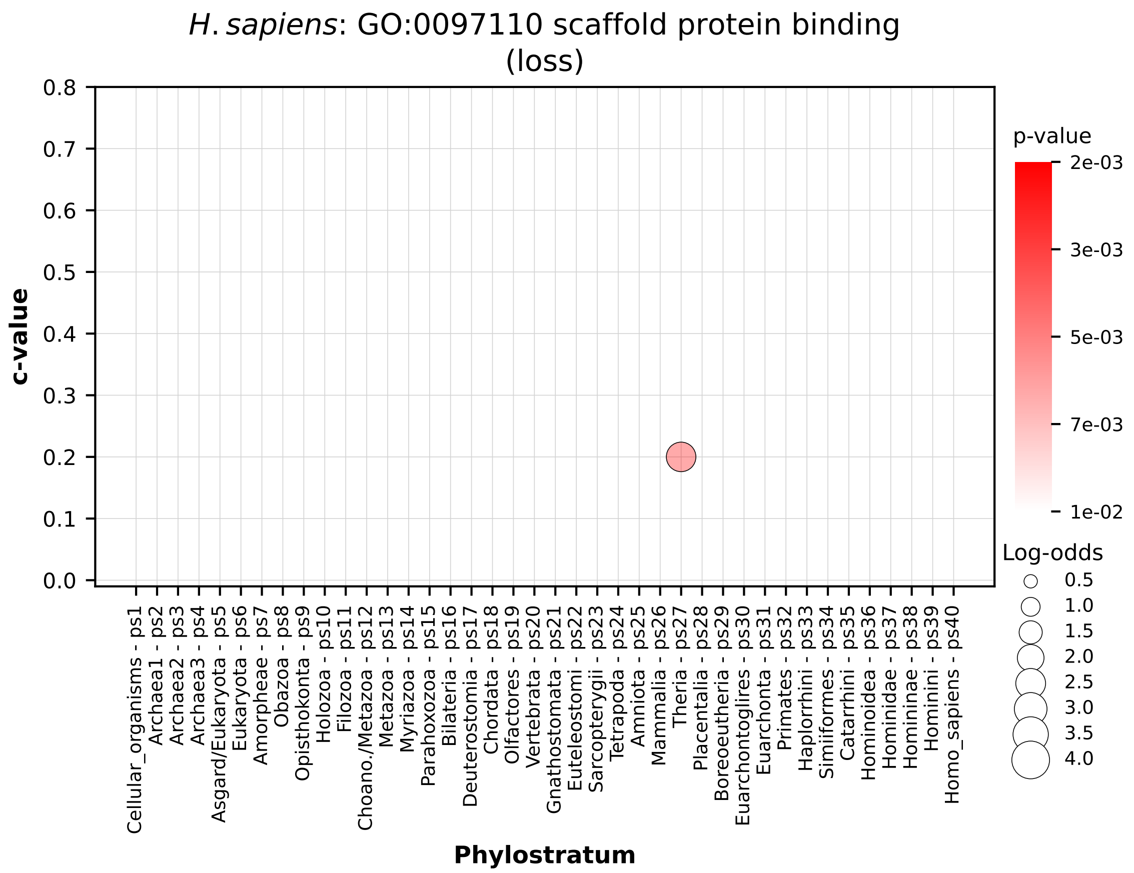

H. sapiens: GO:0097110 scaffold protein binding
(loss)
c-value
0.0
0.1
0.2
0.3
0.4
0.5
0.6
0.7
0.8
Phylostratum
Cellular_organisms - ps1
Archaea1 - ps2
Archaea2 - ps3
Archaea3 - ps4
Asgard/Eukaryota - ps5
Eukaryota - ps6
Amorpheae - ps7
Obazoa - ps8
Opisthokonta - ps9
Holozoa - ps10
Filozoa - ps11
Choano./Metazoa - ps12
Metazoa - ps13
Myriazoa - ps14
Parahoxozoa - ps15
Bilateria - ps16
Deuterostomia - ps17
Chordata - ps18
Olfactores - ps19
Vertebrata - ps20
Gnathostomata - ps21
Euteleostomi - ps22
Sarcopterygii - ps23
Tetrapoda - ps24
Amniota - ps25
Mammalia - ps26
Theria - ps27
Placentalia - ps28
Boreoeutheria - ps29
Euarchontoglires - ps30
Euarchonta - ps31
Primates - ps32
Haplorrhini - ps33
Simiiformes - ps34
Catarrhini - ps35
Hominoidea - ps36
Hominidae - ps37
Homininae - ps38
Hominini - ps39
Homo_sapiens - ps40
p-value
2e-03
3e-03
5e-03
7e-03
1e-02
Log-odds
0.5
1.0
1.5
2.0
2.5
3.0
3.5
4.0

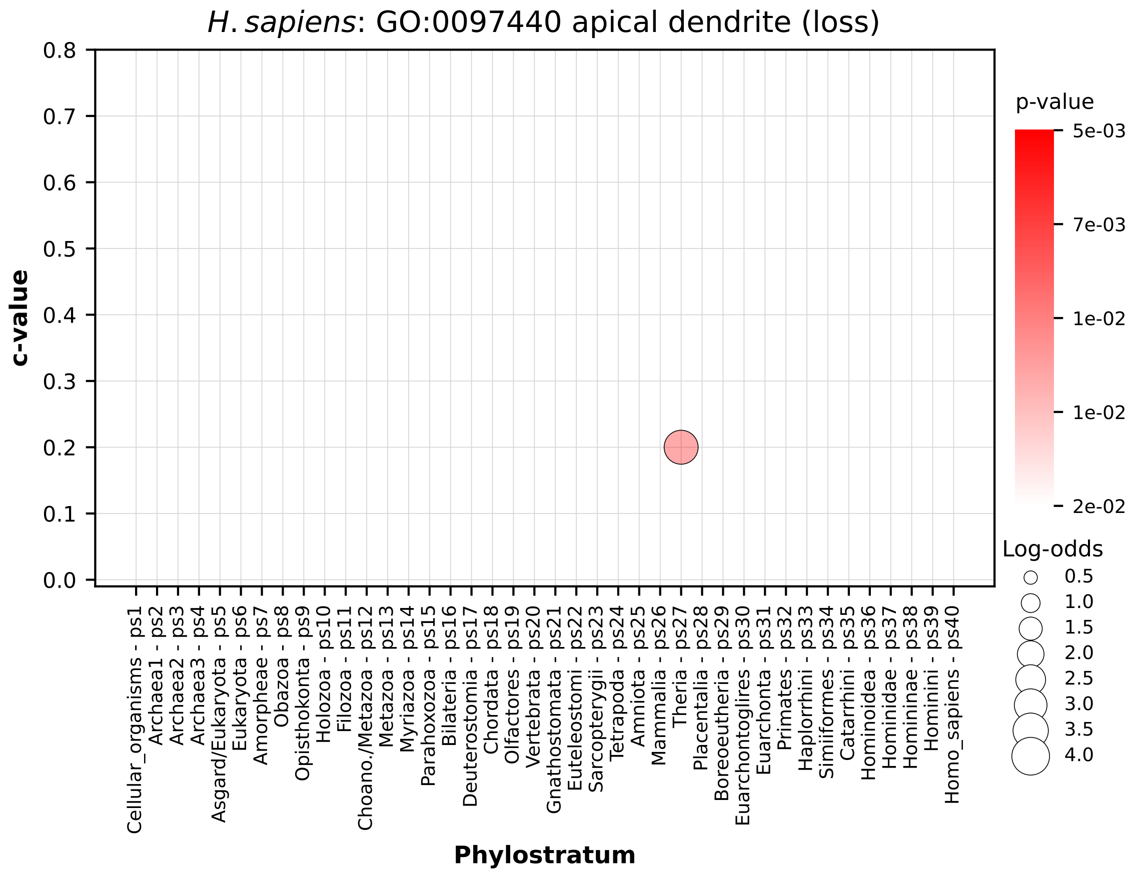

H. sapiens: GO:0097440 apical dendrite (loss)
c-value
0.8
0.7
0.6
0.5
0.4
0.3
0.2
0.1
0.0
Phylostratum
Cellular_organisms - ps1
Archaea1 - ps2
Archaea2 - ps3
Archaea3 - ps4
Asgard/Eukaryota - ps5
Eukaryota - ps6
Amorpheae - ps7
Obazoa - ps8
Opisthokonta - ps9
Holozoa - ps10
Filozoa - ps11
Choano./Metazoa - ps12
Metazoa - ps13
Myriazoa - ps14
Parahoxozoa - ps15
Bilateria - ps16
Deuterostomia - ps17
Chordata - ps18
Olfactores - ps19
Vertebrata - ps20
Gnathostomata - ps21
Euteleostomi - ps22
Sarcopterygii - ps23
Tetrapoda - ps24
Amniota - ps25
Mammalia - ps26
Theria - ps27
Placentalia - ps28
Boreoeutheria - ps29
Euarchontoglires - ps30
Euarchonta - ps31
Primates - ps32
Haplorrhini - ps33
Simiiformes - ps34
Catarrhini - ps35
Hominoidea - ps36
Hominidae - ps37
Homininae - ps38
Hominini - ps39
Homo_sapiens - ps40
p-value
5e-03
7e-03
1e-02
1e-02
2e-02
Log-odds
0.5
1.0
1.5
2.0
2.5
3.0
3.5
4.0

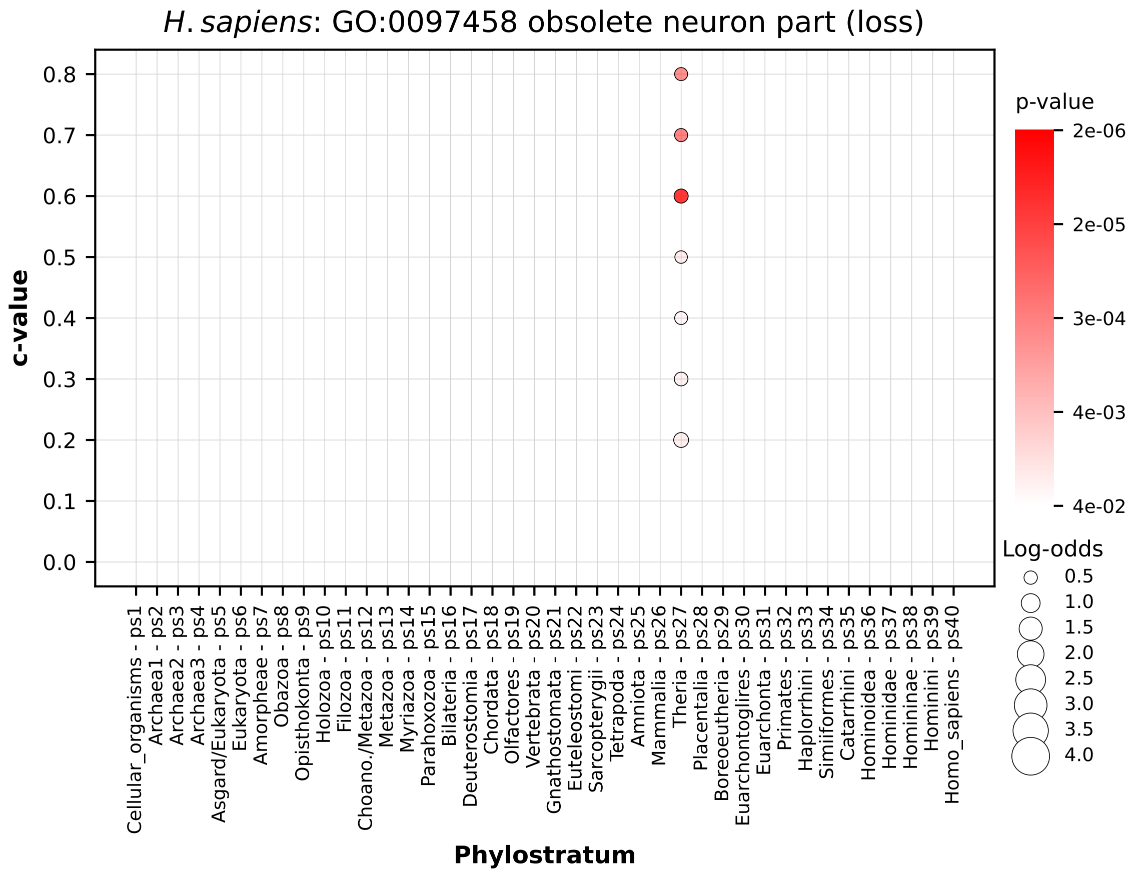

H. sapiens: GO:0097458 obsolete neuron part (loss)
c-value
0.8
0.7
0.6
0.5
0.4
0.3
0.2
0.1
0.0
p-value
2e-06
2e-05
3e-04
4e-03
4e-02
Log-odds
0.5
1.0
1.5
2.0
2.5
3.0
3.5
4.0
Cellular_organisms - ps1
Archaea1 - ps2
Archaea2 - ps3
Archaea3 - ps4
Asgard/Eukaryota - ps5
Eukaryota - ps6
Amorpheae - ps7
Obazoa - ps8
Opisthokonta - ps9
Holozoa - ps10
Filozoa - ps11
Choano./Metazoa - ps12
Metazoa - ps13
Myriazoa - ps14
Parahoxozoa - ps15
Bilateria - ps16
Deuterostomia - ps17
Chordata - ps18
Olfactores - ps19
Vertebrata - ps20
Gnathostomata - ps21
Euteleostomi - ps22
Sarcopterygii - ps23
Tetrapoda - ps24
Amniota - ps25
Mammalia - ps26
Theria - ps27
Placentalia - ps28
Boreoeutheria - ps29
Euarchontoglires - ps30
Euarchonta - ps31
Primates - ps32
Haplorrhini - ps33
Simiiformes - ps34
Catarrhini - ps35
Hominoidea - ps36
Hominidae - ps37
Homininae - ps38
Hominini - ps39
Homo_sapiens - ps40
Phylostratum

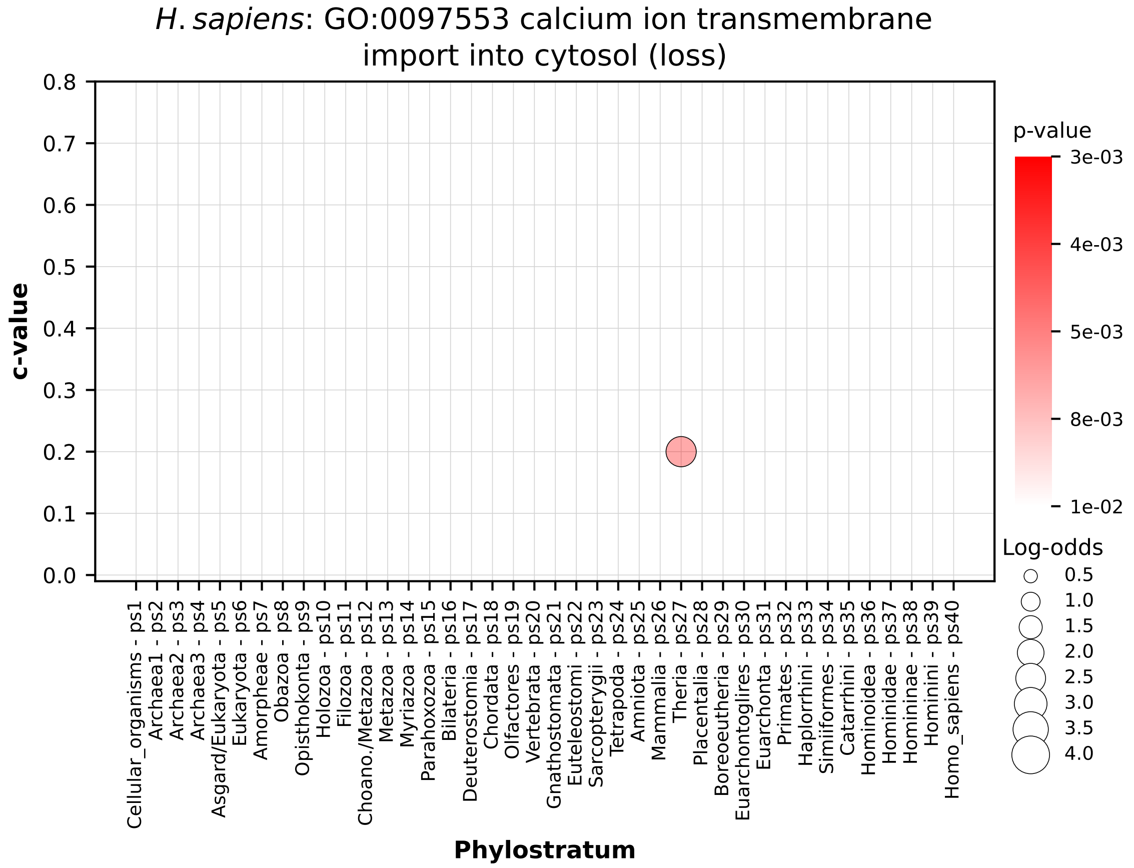
H. sapiens: GO:0097553 calcium ion transmembrane import into cytosol (loss)
c-value
0.0
0.1
0.2
0.3
0.4
0.5
0.6
0.7
0.8
Phylostratum
Cellular_organisms - ps1
Archaea1 - ps2
Archaea2 - ps3
Archaea3 - ps4
Asgard/Eukaryota - ps5
Eukaryota - ps6
Amorpheae - ps7
Obazoa - ps8
Opisthokonta - ps9
Holozoa - ps10
Filozoa - ps11
Choano./Metazoa - ps12
Metazoa - ps13
Myriazoa - ps14
Parahoxozoa - ps15
Bilateria - ps16
Deuterostomia - ps17
Chordata - ps18
Olfactores - ps19
Vertebrata - ps20
Gnathostomata - ps21
Euteleostomi - ps22
Sarcopterygii - ps23
Tetrapoda - ps24
Amniota - ps25
Mammalia - ps26
Theria - ps27
Placentalia - ps28
Boreoeutheria - ps29
Euarchontoglires - ps30
Euarchonta - ps31
Primates - ps32
Haplorrhini - ps33
Simiiformes - ps34
Catarrhini - ps35
Hominoidea - ps36
Hominidae - ps37
Homininae - ps38
Hominini - ps39
Homo_sapiens - ps40
p-value
3e-03
4e-03
5e-03
8e-03
1e-02
Log-odds
0.5
1.0
1.5
2.0
2.5
3.0
3.5
4.0

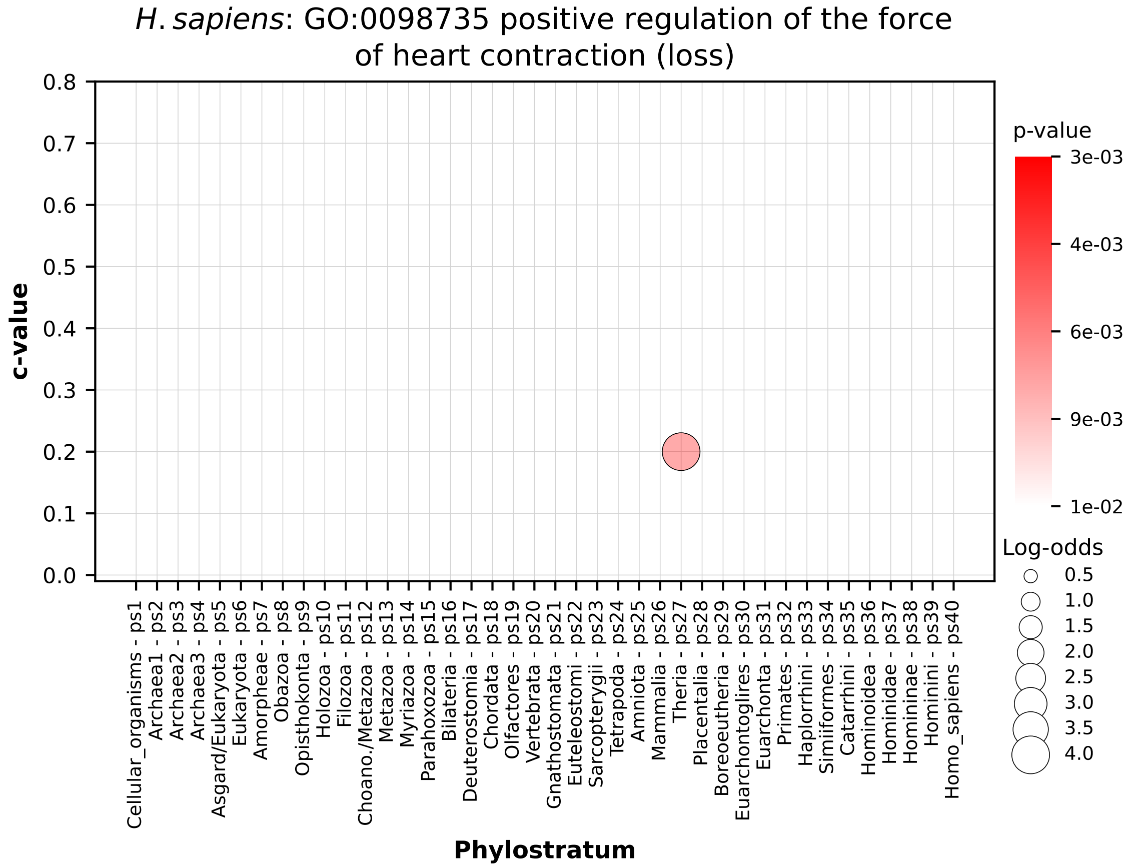

H. sapiens: GO:0098735 positive regulation of the force of heart contraction (loss)
c-value
0.8
0.7
0.6
0.5
0.4
0.3
0.2
0.1
0.0
Phylostratum
Cellular_organisms - ps1
Archaea1 - ps2
Archaea2 - ps3
Archaea3 - ps4
Asgard/Eukaryota - ps5
Eukaryota - ps6
Amorpheae - ps7
Obazoa - ps8
Opisthokonta - ps9
Holozoa - ps10
Filozoa - ps11
Choano./Metazoa - ps12
Metazoa - ps13
Myriazoa - ps14
Parahoxozoa - ps15
Bilateria - ps16
Deuterostomia - ps17
Chordata - ps18
Olfactores - ps19
Vertebrata - ps20
Gnathostomata - ps21
Euteleostomi - ps22
Sarcopterygii - ps23
Tetrapoda - ps24
Amniota - ps25
Mammalia - ps26
Theria - ps27
Placentalia - ps28
Boreoeutheria - ps29
Euarchontoglires - ps30
Euarchonta - ps31
Primates - ps32
Haplorrhini - ps33
Simiiformes - ps34
Catarrhini - ps35
Hominoidea - ps36
Hominidae - ps37
Homininae - ps38
Hominini - ps39
Homo_sapiens - ps40
p-value
3e-03
4e-03
6e-03
9e-03
1e-02
Log-odds
0.5
1.0
1.5
2.0
2.5
3.0
3.5
4.0

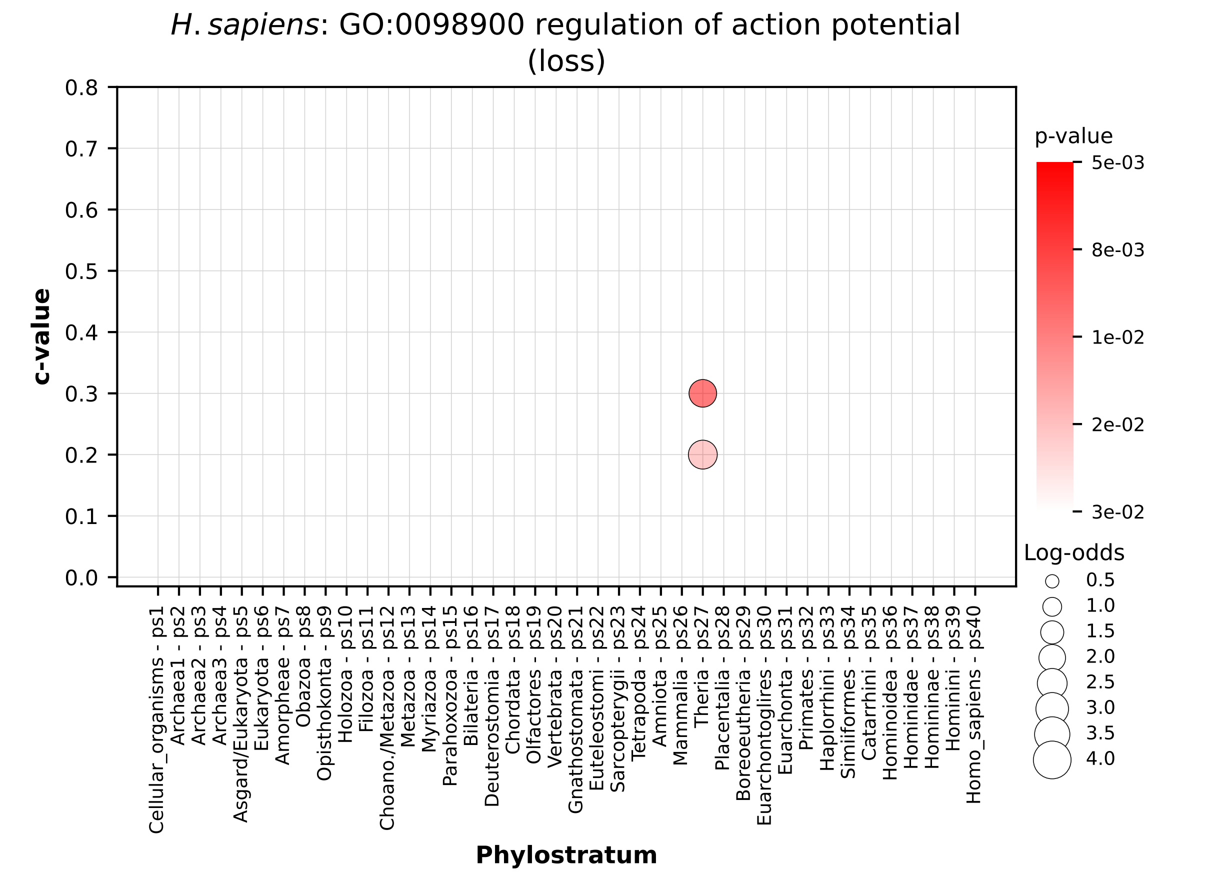

H. sapiens: GO:0098900 regulation of action potential (loss)
c-value
0.8
0.7
0.6
0.5
0.4
0.3
0.2
0.1
0.0
Phylostratum
Cellular_organisms - ps1
Archaea1 - ps2
Archaea2 - ps3
Archaea3 - ps4
Asgard/Eukaryota - ps5
Eukaryota - ps6
Amorpheae - ps7
Obazoa - ps8
Opisthokonta - ps9
Holozoa - ps10
Filozoa - ps11
Choano./Metazoa - ps12
Metazoa - ps13
Myriazoa - ps14
Parahoxozoa - ps15
Bilateria - ps16
Deuterostomia - ps17
Chordata - ps18
Olfactores - ps19
Vertebrata - ps20
Gnathostomata - ps21
Euteleostomi - ps22
Sarcopterygii - ps23
Tetrapoda - ps24
Amniota - ps25
Mammalia - ps26
Theria - ps27
Placentalia - ps28
Boreoeutheria - ps29
Euarchontoglires - ps30
Euarchonta - ps31
Primates - ps32
Haplorrhini - ps33
Simiiformes - ps34
Catarrhini - ps35
Hominoidea - ps36
Hominidae - ps37
Homininae - ps38
Hominini - ps39
Homo_sapiens - ps40
p-value
5e-03
8e-03
1e-02
2e-02
3e-02
Log-odds
0.5
1.0
1.5
2.0
2.5
3.0
3.5
4.0

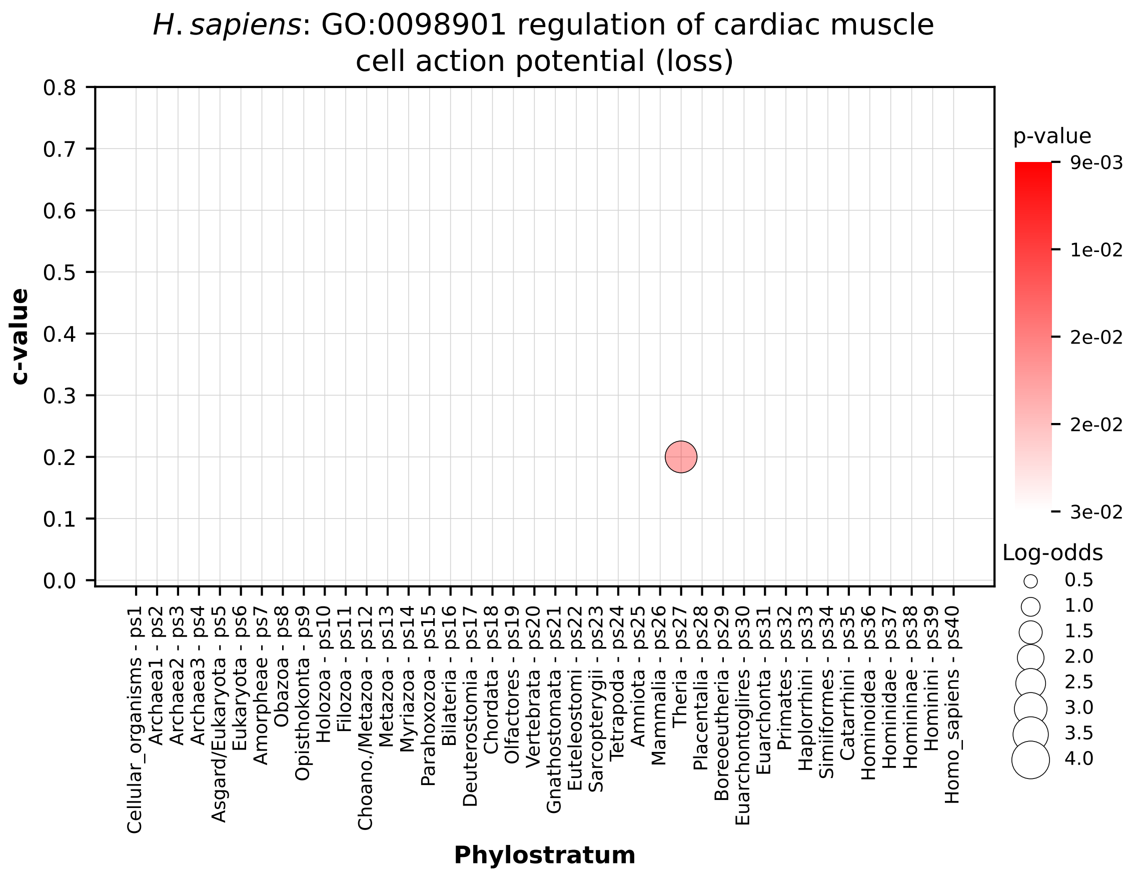
H. sapiens: GO:0098901 regulation of cardiac muscle cell action potential (loss)
c-value
0.0
0.1
0.2
0.3
0.4
0.5
0.6
0.7
0.8
Phylostratum
Cellular_organisms - ps1
Archaea1 - ps2
Archaea2 - ps3
Archaea3 - ps4
Asgard/Eukaryota - ps5
Eukaryota - ps6
Amorpheae - ps7
Obazoa - ps8
Opisthokonta - ps9
Holozoa - ps10
Filozoa - ps11
Choano./Metazoa - ps12
Metazoa - ps13
Myriazoa - ps14
Parahoxozoa - ps15
Bilateria - ps16
Deuterostomia - ps17
Chordata - ps18
Olfactores - ps19
Vertebrata - ps20
Gnathostomata - ps21
Euteleostomi - ps22
Sarcopterygii - ps23
Tetrapoda - ps24
Amniota - ps25
Mammalia - ps26
Theria - ps27
Placentalia - ps28
Boreoeutheria - ps29
Euarchontoglires - ps30
Euarchonta - ps31
Primates - ps32
Haplorrhini - ps33
Simiiformes - ps34
Catarrhini - ps35
Hominoidea - ps36
Hominidae - ps37
Homininae - ps38
Hominini - ps39
Homo_sapiens - ps40
p-value
9e-03
1e-02
2e-02
2e-02
3e-02
Log-odds
0.5
1.0
1.5
2.0
2.5
3.0
3.5
4.0

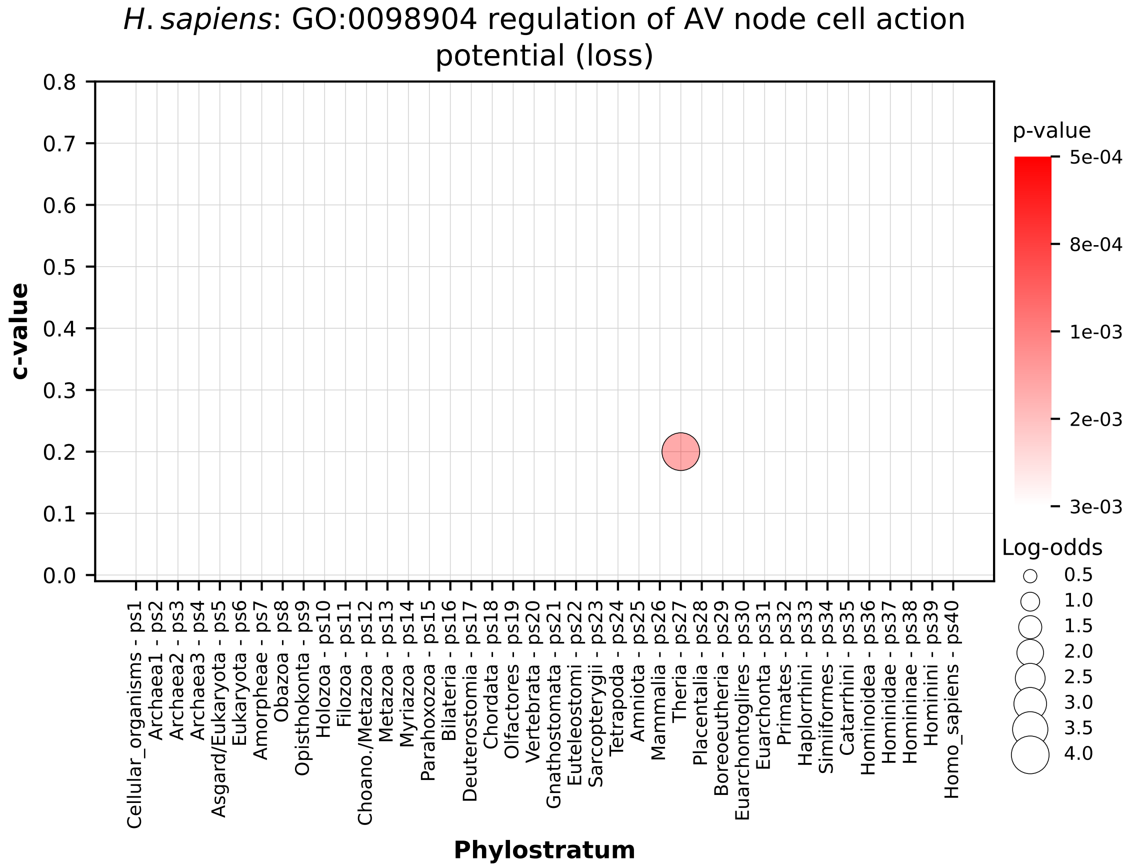

H. sapiens: GO:0098904 regulation of AV node cell action potential (loss)
c-value
0.8
0.7
0.6
0.5
0.4
0.3
0.2
0.1
0.0
Phylostratum
Cellular_organisms - ps1
Archaea1 - ps2
Archaea2 - ps3
Archaea3 - ps4
Asgard/Eukaryota - ps5
Eukaryota - ps6
Amorpheae - ps7
Obazoa - ps8
Opisthokonta - ps9
Holozoa - ps10
Filozoa - ps11
Choano./Metazoa - ps12
Metazoa - ps13
Myriazoa - ps14
Parahoxozoa - ps15
Bilateria - ps16
Deuterostomia - ps17
Chordata - ps18
Olfactores - ps19
Vertebrata - ps20
Gnathostomata - ps21
Euteleostomi - ps22
Sarcopterygii - ps23
Tetrapoda - ps24
Amniota - ps25
Mammalia - ps26
Theria - ps27
Placentalia - ps28
Boreoeutheria - ps29
Euarchontoglires - ps30
Euarchonta - ps31
Primates - ps32
Haplorrhini - ps33
Simiiformes - ps34
Catarrhini - ps35
Hominoidea - ps36
Hominidae - ps37
Homininae - ps38
Hominini - ps39
Homo_sapiens - ps40
p-value
5e-04
8e-04
1e-03
2e-03
3e-03
Log-odds
0.5
1.0
1.5
2.0
2.5
3.0
3.5
4.0

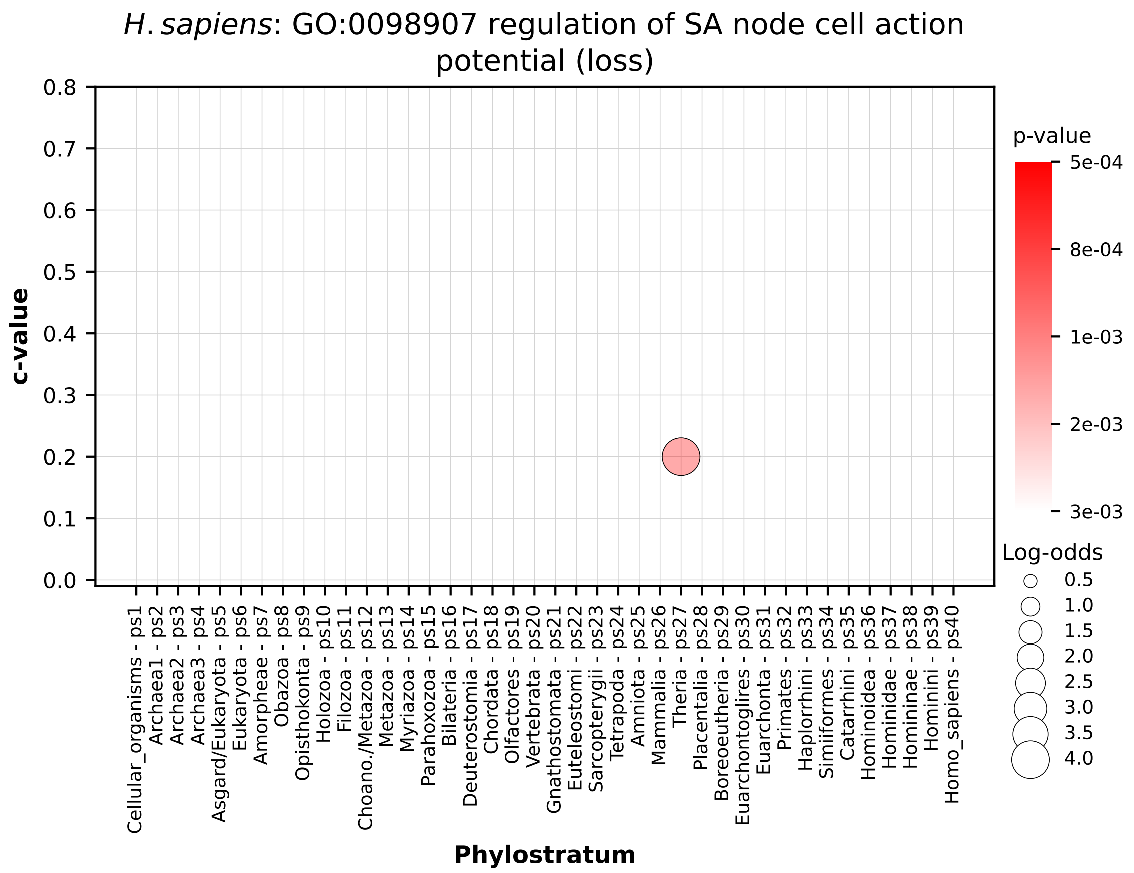

H. sapiens: GO:0098907 regulation of SA node cell action potential (loss)
c-value
0.8
0.7
0.6
0.5
0.4
0.3
0.2
0.1
0.0
Phylostratum
Cellular_organisms - ps1
Archaea1 - ps2
Archaea2 - ps3
Archaea3 - ps4
Asgard/Eukaryota - ps5
Eukaryota - ps6
Amorpheae - ps7
Obazoa - ps8
Opisthokonta - ps9
Holozoa - ps10
Filozoa - ps11
Choano./Metazoa - ps12
Metazoa - ps13
Myriazoa - ps14
Parahoxozoa - ps15
Bilateria - ps16
Deuterostomia - ps17
Chordata - ps18
Olfactores - ps19
Vertebrata - ps20
Gnathostomata - ps21
Euteleostomi - ps22
Sarcopterygii - ps23
Tetrapoda - ps24
Amniota - ps25
Mammalia - ps26
Theria - ps27
Placentalia - ps28
Boreoeutheria - ps29
Euarchontoglires - ps30
Euarchonta - ps31
Primates - ps32
Haplorrhini - ps33
Simiiformes - ps34
Catarrhini - ps35
Hominoidea - ps36
Hominidae - ps37
Homininae - ps38
Hominini - ps39
Homo_sapiens - ps40
p-value
5e-04
8e-04
1e-03
2e-03
3e-03
Log-odds
0.5
1.0
1.5
2.0
2.5
3.0
3.5
4.0

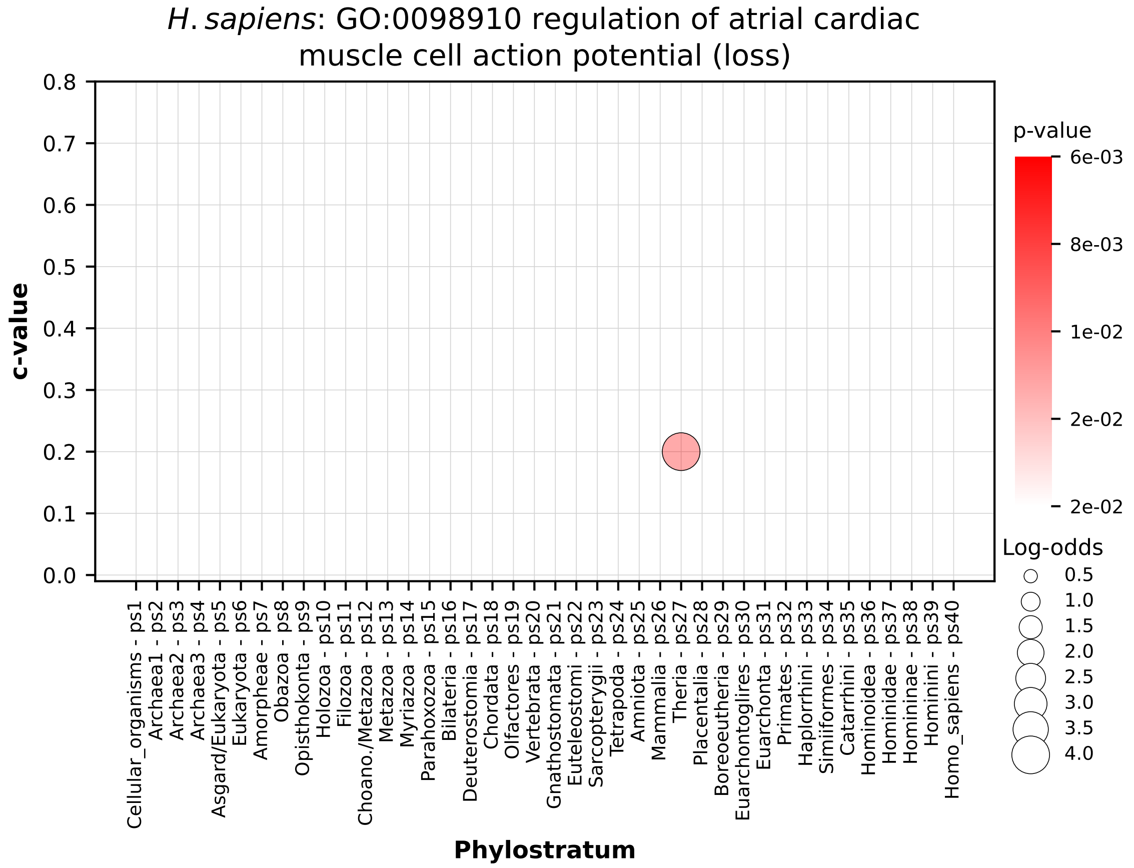

H. sapiens: GO:0098910 regulation of atrial cardiac muscle cell action potential (loss)
c-value
0.8
0.7
0.6
0.5
0.4
0.3
0.2
0.1
0.0
Phylostratum
Cellular_organisms - ps1
Archaea1 - ps2
Archaea2 - ps3
Archaea3 - ps4
Asgard/Eukaryota - ps5
Eukaryota - ps6
Amorpheae - ps7
Obazoa - ps8
Opisthokonta - ps9
Holozoa - ps10
Filozoa - ps11
Choano./Metazoa - ps12
Metazoa - ps13
Myriazoa - ps14
Parahoxozoa - ps15
Bilateria - ps16
Deuterostomia - ps17
Chordata - ps18
Olfactores - ps19
Vertebrata - ps20
Gnathostomata - ps21
Euteleostomi - ps22
Sarcopterygii - ps23
Tetrapoda - ps24
Amniota - ps25
Mammalia - ps26
Theria - ps27
Placentalia - ps28
Boreoeutheria - ps29
Euarchontoglires - ps30
Euarchonta - ps31
Primates - ps32
Haplorrhini - ps33
Simiiformes - ps34
Catarrhini - ps35
Hominoidea - ps36
Hominidae - ps37
Homininae - ps38
Hominini - ps39
Homo_sapiens - ps40
p-value
6e-03
8e-03
1e-02
2e-02
2e-02
Log-odds
0.5
1.0
1.5
2.0
2.5
3.0
3.5
4.0

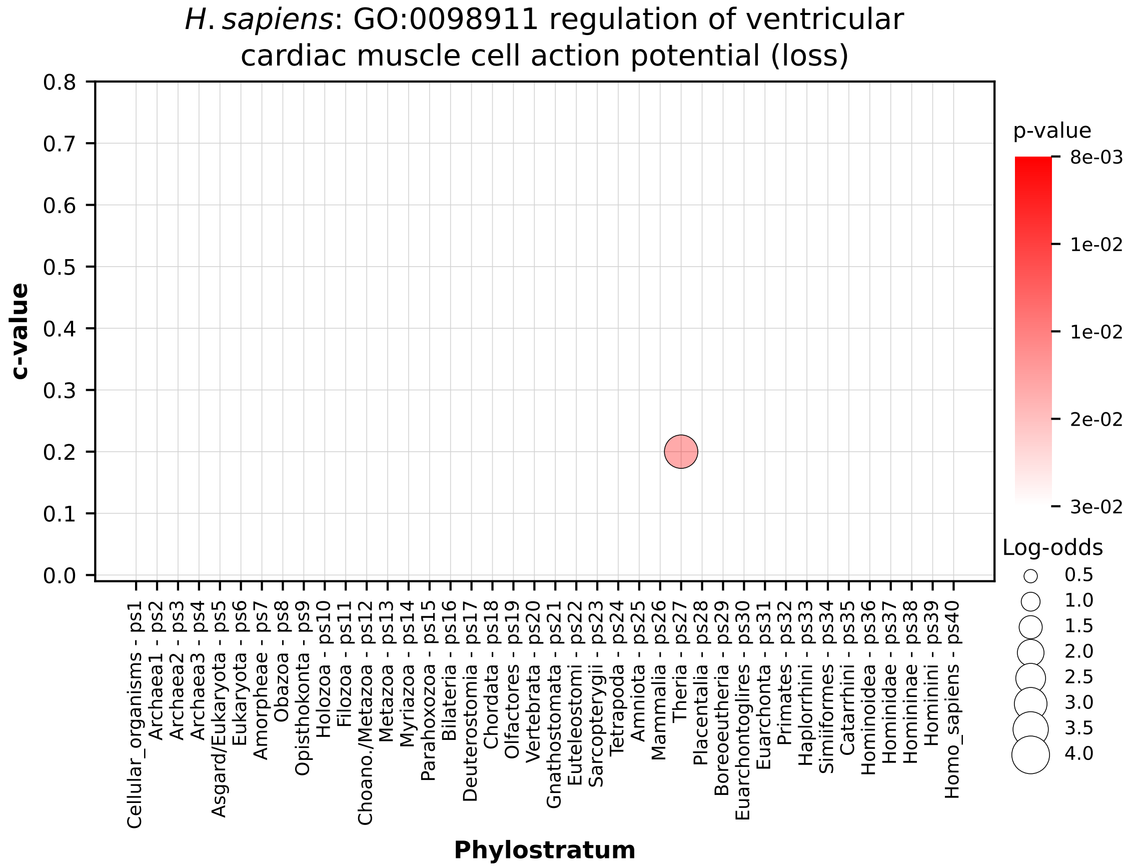

H. sapiens: GO:0098911 regulation of ventricular cardiac muscle cell action potential (loss)
c-value
0.8
0.7
0.6
0.5
0.4
0.3
0.2
0.1
0.0
Phylostratum
Cellular_organisms - ps1
Archaea1 - ps2
Archaea2 - ps3
Archaea3 - ps4
Asgard/Eukaryota - ps5
Eukaryota - ps6
Amorpheae - ps7
Obazoa - ps8
Opisthokonta - ps9
Holozoa - ps10
Filozoa - ps11
Choano./Metazoa - ps12
Metazoa - ps13
Myriazoa - ps14
Parahoxozoa - ps15
Bilateria - ps16
Deuterostomia - ps17
Chordata - ps18
Olfactores - ps19
Vertebrata - ps20
Gnathostomata - ps21
Euteleostomi - ps22
Sarcopterygii - ps23
Tetrapoda - ps24
Amniota - ps25
Mammalia - ps26
Theria - ps27
Placentalia - ps28
Boreoeutheria - ps29
Euarchontoglires - ps30
Euarchonta - ps31
Primates - ps32
Haplorrhini - ps33
Simiiformes - ps34
Catarrhini - ps35
Hominoidea - ps36
Hominidae - ps37
Homininae - ps38
Hominini - ps39
Homo_sapiens - ps40
p-value
8e-03
1e-02
1e-02
2e-02
3e-02
Log-odds
0.5
1.0
1.5
2.0
2.5
3.0
3.5
4.0

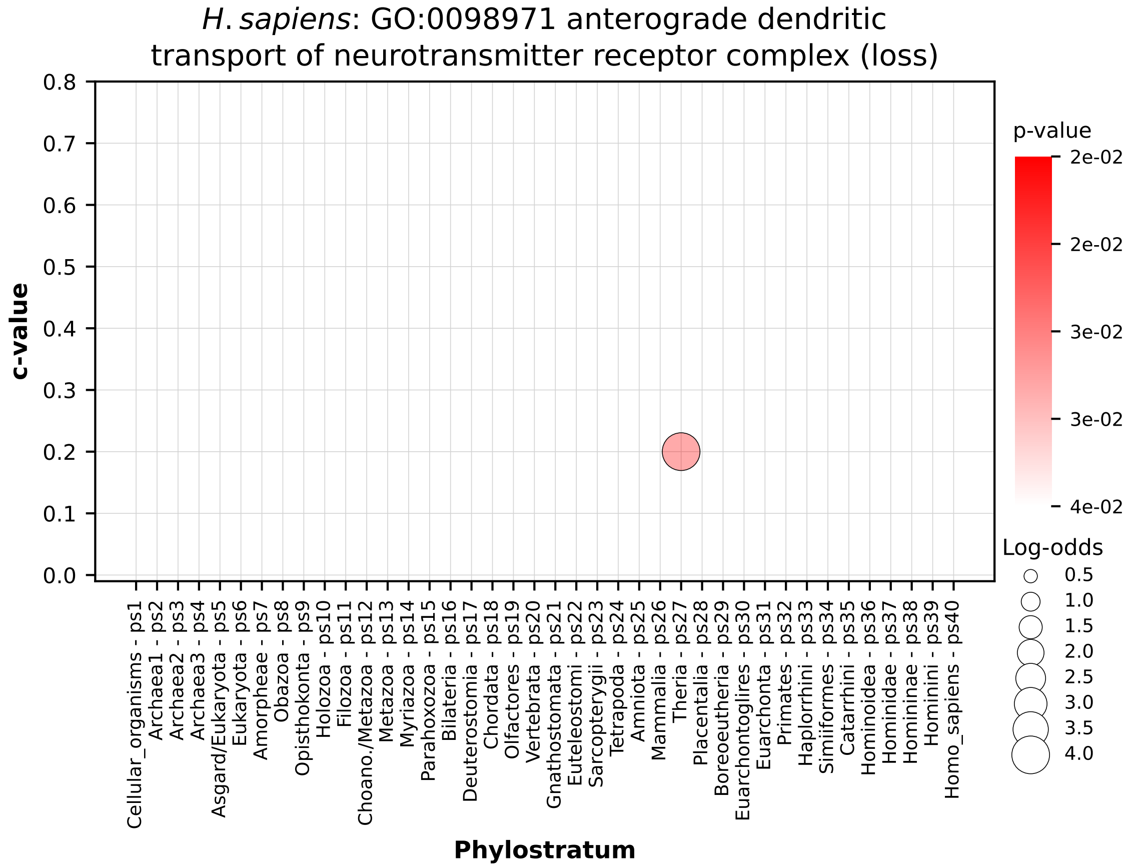

H. sapiens: GO:0098971 anterograde dendritic transport of neurotransmitter receptor complex (loss)
c-value
0.8
0.7
0.6
0.5
0.4
0.3
0.2
0.1
0.0
p-value
2e-02
2e-02
3e-02
3e-02
4e-02
Log-odds
0.5
1.0
1.5
2.0
2.5
3.0
3.5
4.0
Cellular_organisms - ps1
Archaea1 - ps2
Archaea2 - ps3
Archaea3 - ps4
Asgard/Eukaryota - ps5
Eukaryota - ps6
Amorpheae - ps7
Obazoa - ps8
Opisthokonta - ps9
Holozoa - ps10
Filozoa - ps11
Choano./Metazoa - ps12
Metazoa - ps13
Myriazoa - ps14
Parahoxozoa - ps15
Bilateria - ps16
Deuterostomia - ps17
Chordata - ps18
Olfactores - ps19
Vertebrata - ps20
Gnathostomata - ps21
Euteleostomi - ps22
Sarcopterygii - ps23
Tetrapoda - ps24
Amniota - ps25
Mammalia - ps26
Theria - ps27
Placentalia - ps28
Boreoeutheria - ps29
Euarchontoglires - ps30
Euarchonta - ps31
Primates - ps32
Haplorrhini - ps33
Simiiformes - ps34
Catarrhini - ps35
Hominoidea - ps36
Hominidae - ps37
Homininae - ps38
Hominini - ps39
Homo_sapiens - ps40
Phylostratum

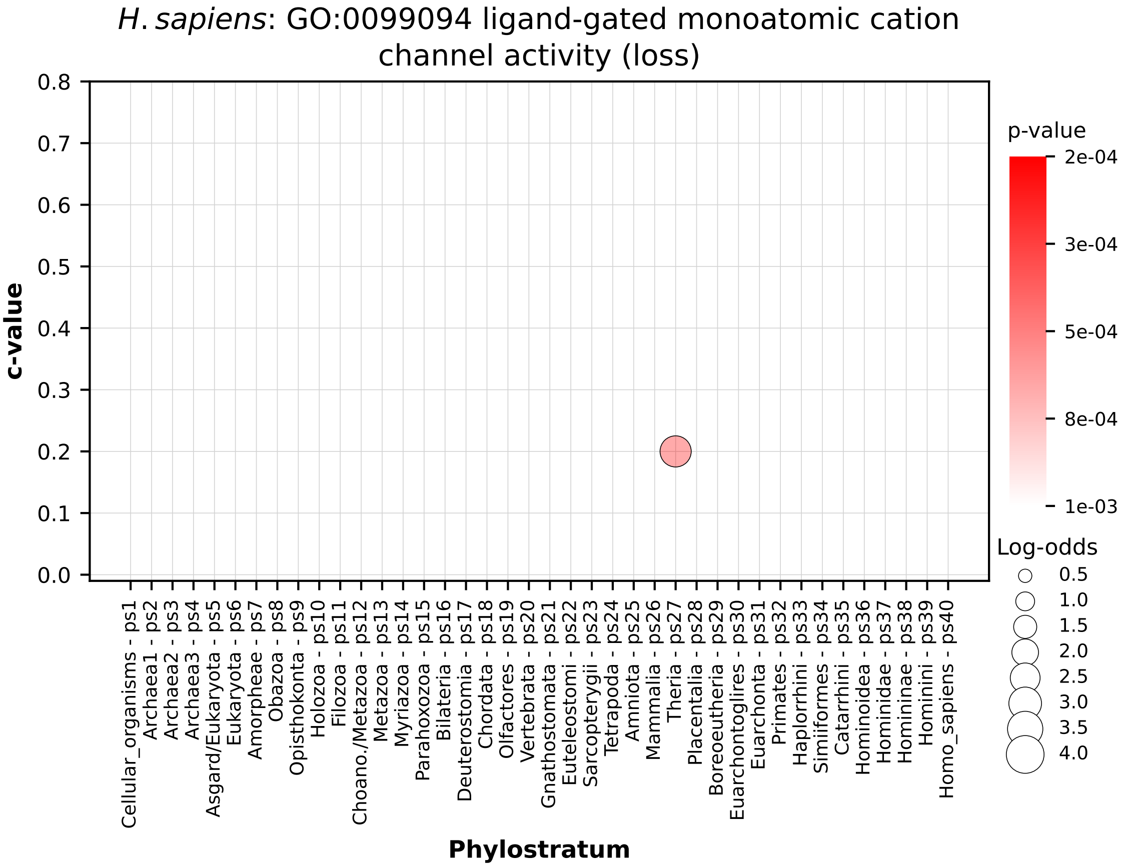

H. sapiens: GO:0099094 ligand-gated monoatomic cation channel activity (loss)
c-value
0.8
0.7
0.6
0.5
0.4
0.3
0.2
0.1
0.0
Phylostratum
Cellular_organisms - ps1
Archaea1 - ps2
Archaea2 - ps3
Archaea3 - ps4
Asgard/Eukaryota - ps5
Eukaryota - ps6
Amorpheae - ps7
Obazoa - ps8
Opisthokonta - ps9
Holozoa - ps10
Filozoa - ps11
Choano./Metazoa - ps12
Metazoa - ps13
Myriazoa - ps14
Parahoxozoa - ps15
Bilateria - ps16
Deuterostomia - ps17
Chordata - ps18
Olfactores - ps19
Vertebrata - ps20
Gnathostomata - ps21
Euteleostomi - ps22
Sarcopterygii - ps23
Tetrapoda - ps24
Amniota - ps25
Mammalia - ps26
Theria - ps27
Placentalia - ps28
Boreoeutheria - ps29
Euarchontoglires - ps30
Euarchonta - ps31
Primates - ps32
Haplorrhini - ps33
Simiiformes - ps34
Catarrhini - ps35
Hominoidea - ps36
Hominidae - ps37
Homininae - ps38
Hominini - ps39
Homo_sapiens - ps40
p-value
2e-04
3e-04
5e-04
8e-04
1e-03
Log-odds
0.5
1.0
1.5
2.0
2.5
3.0
3.5
4.0

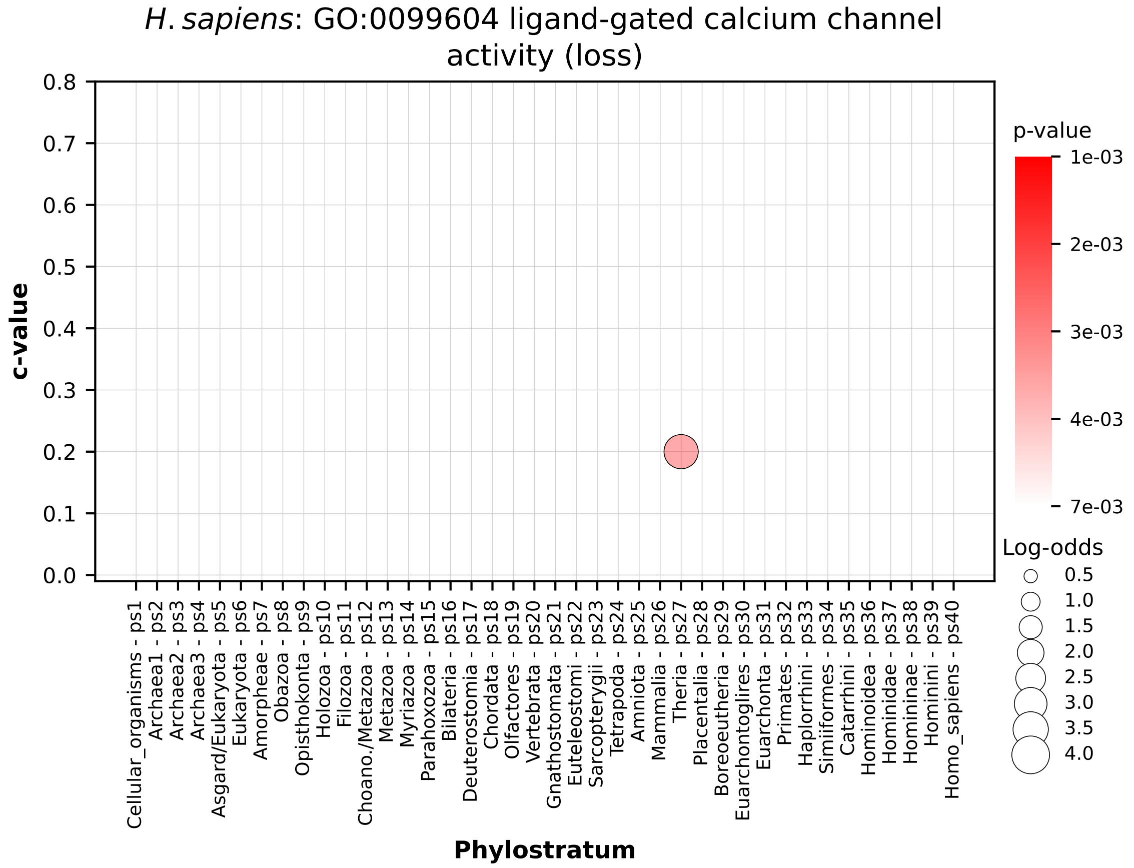

H. sapiens: GO:0099604 ligand-gated calcium channel activity (loss)
c-value
0.0
0.1
0.2
0.3
0.4
0.5
0.6
0.7
0.8
Phylostratum
Cellular_organisms - ps1
Archaea1 - ps2
Archaea2 - ps3
Archaea3 - ps4
Asgard/Eukaryota - ps5
Eukaryota - ps6
Amorpheae - ps7
Obazoa - ps8
Opisthokonta - ps9
Holozoa - ps10
Filozoa - ps11
Choano./Metazoa - ps12
Metazoa - ps13
Myriazoa - ps14
Parahoxozoa - ps15
Bilateria - ps16
Deuterostomia - ps17
Chordata - ps18
Olfactores - ps19
Vertebrata - ps20
Gnathostomata - ps21
Euteleostomi - ps22
Sarcopterygii - ps23
Tetrapoda - ps24
Amniota - ps25
Mammalia - ps26
Theria - ps27
Placentalia - ps28
Boreoeutheria - ps29
Euarchontoglires - ps30
Euarchonta - ps31
Primates - ps32
Haplorrhini - ps33
Simiiformes - ps34
Catarrhini - ps35
Hominoidea - ps36
Hominidae - ps37
Homininae - ps38
Hominini - ps39
Homo_sapiens - ps40
p-value
1e-03
2e-03
3e-03
4e-03
7e-03
Log-odds
0.5
1.0
1.5
2.0
2.5
3.0
3.5
4.0

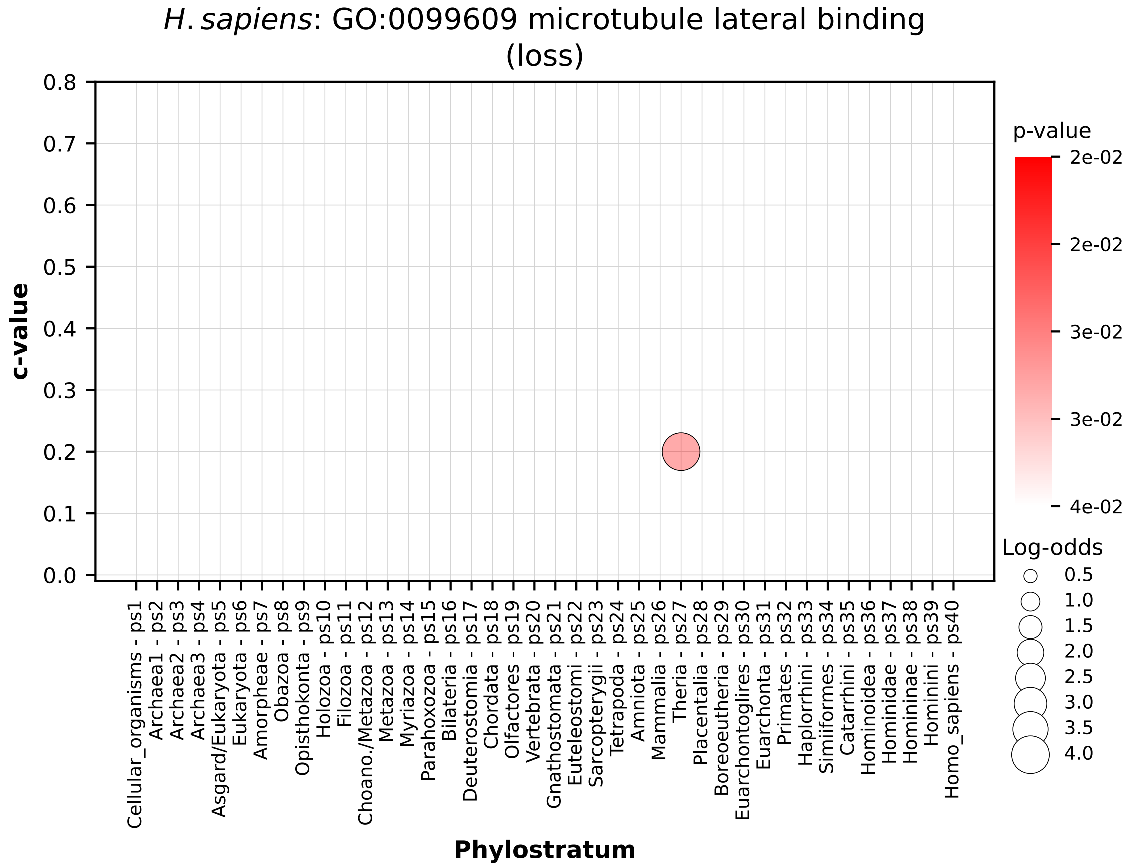

H. sapiens: GO:0099609 microtubule lateral binding (loss)
c-value
0.8
0.7
0.6
0.5
0.4
0.3
0.2
0.1
0.0
p-value
2e-02
2e-02
3e-02
3e-02
4e-02
Log-odds
0.5
1.0
1.5
2.0
2.5
3.0
3.5
4.0
Cellular_organisms - ps1
Archaea1 - ps2
Archaea2 - ps3
Archaea3 - ps4
Asgard/Eukaryota - ps5
Eukaryota - ps6
Amorpheae - ps7
Obazoa - ps8
Opisthokonta - ps9
Holozoa - ps10
Filozoa - ps11
Choano./Metazoa - ps12
Metazoa - ps13
Myriazoa - ps14
Parahoxozoa - ps15
Bilateria - ps16
Deuterostomia - ps17
Chordata - ps18
Olfactores - ps19
Vertebrata - ps20
Gnathostomata - ps21
Euteleostomi - ps22
Sarcopterygii - ps23
Tetrapoda - ps24
Amniota - ps25
Mammalia - ps26
Theria - ps27
Placentalia - ps28
Boreoeutheria - ps29
Euarchontoglires - ps30
Euarchonta - ps31
Primates - ps32
Haplorrhini - ps33
Simiiformes - ps34
Catarrhini - ps35
Hominoidea - ps36
Hominidae - ps37
Homininae - ps38
Hominini - ps39
Homo_sapiens - ps40
Phylostratum

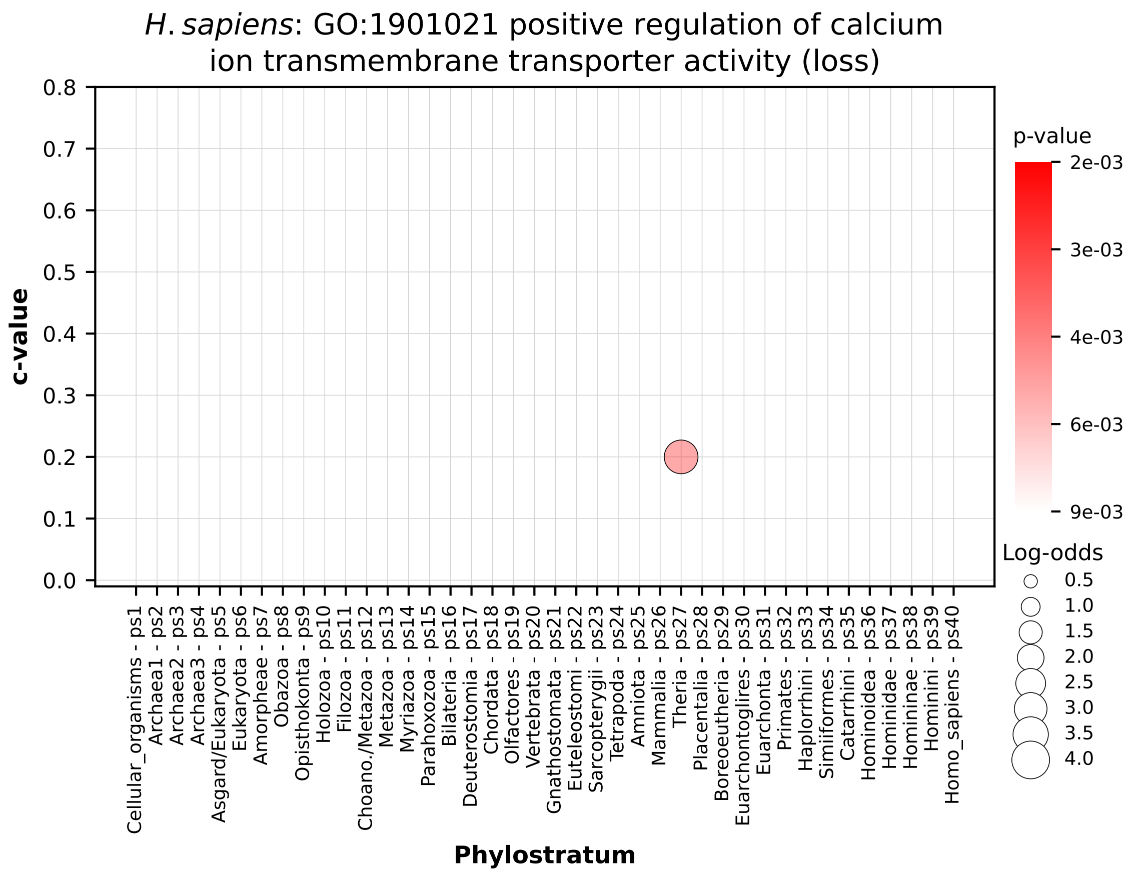

H. sapiens: GO:1901021 positive regulation of calcium ion transmembrane transporter activity (loss)
c-value
0.0
0.1
0.2
0.3
0.4
0.5
0.6
0.7
0.8
Phylostratum
Cellular_organisms - ps1
Archaea1 - ps2
Archaea2 - ps3
Archaea3 - ps4
Asgard/Eukaryota - ps5
Eukaryota - ps6
Amorpheae - ps7
Obazoa - ps8
Opisthokonta - ps9
Holozoa - ps10
Filozoa - ps11
Choano./Metazoa - ps12
Metazoa - ps13
Myriazoa - ps14
Parahoxozoa - ps15
Bilateria - ps16
Deuterostomia - ps17
Chordata - ps18
Olfactores - ps19
Vertebrata - ps20
Gnathostomata - ps21
Euteleostomi - ps22
Sarcopterygii - ps23
Tetrapoda - ps24
Amniota - ps25
Mammalia - ps26
Theria - ps27
Placentalia - ps28
Boreoeutheria - ps29
Euarchontoglires - ps30
Euarchonta - ps31
Primates - ps32
Haplorrhini - ps33
Simiiformes - ps34
Catarrhini - ps35
Hominoidea - ps36
Hominidae - ps37
Homininae - ps38
Hominini - ps39
Homo_sapiens - ps40
p-value
2e-03
3e-03
4e-03
6e-03
9e-03
Log-odds
0.5
1.0
1.5
2.0
2.5
3.0
3.5
4.0

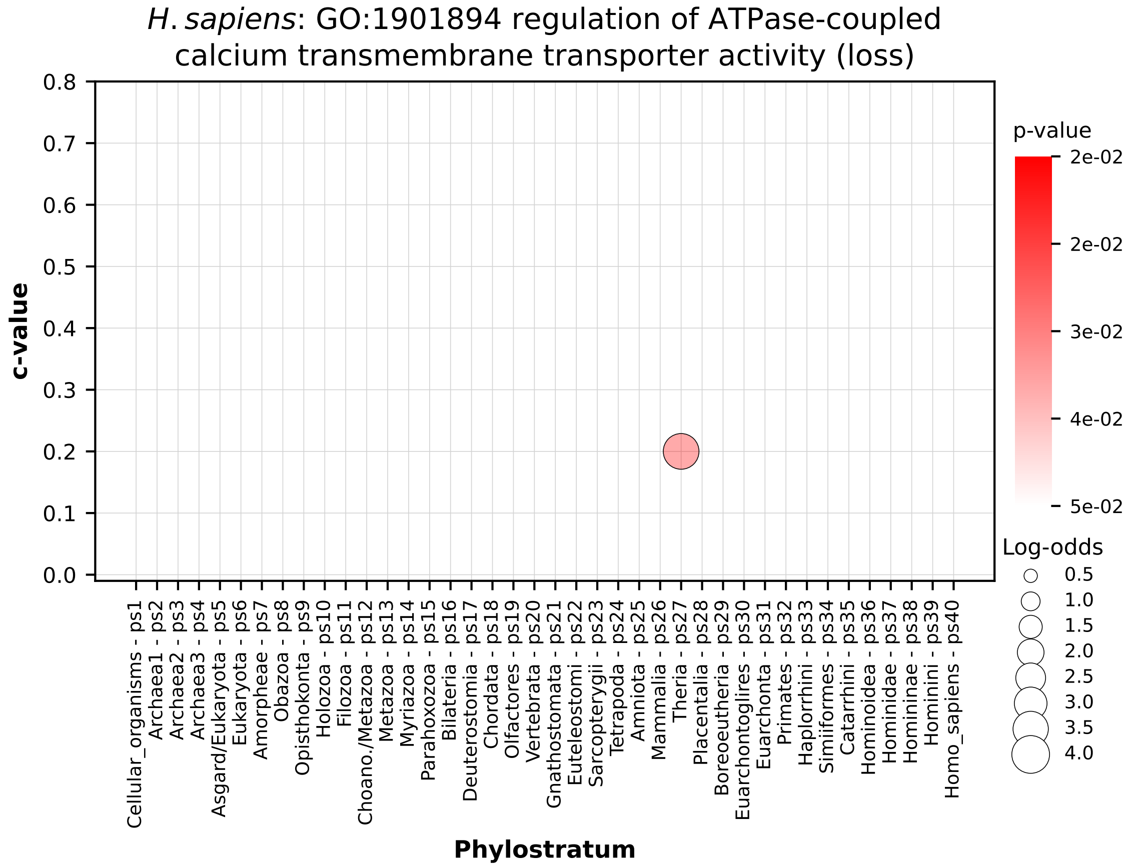

H. sapiens: GO:1901894 regulation of ATPase-coupled calcium transmembrane transporter activity (loss)
c-value
0.8
0.7
0.6
0.5
0.4
0.3
0.2
0.1
0.0
Phylostratum
Cellular_organisms - ps1
Archaea1 - ps2
Archaea2 - ps3
Archaea3 - ps4
Asgard/Eukaryota - ps5
Eukaryota - ps6
Amorpheae - ps7
Obazoa - ps8
Opisthokonta - ps9
Holozoa - ps10
Filozoa - ps11
Choano./Metazoa - ps12
Metazoa - ps13
Myriazoa - ps14
Parahoxozoa - ps15
Bilateria - ps16
Deuterostomia - ps17
Chordata - ps18
Olfactores - ps19
Vertebrata - ps20
Gnathostomata - ps21
Euteleostomi - ps22
Sarcopterygii - ps23
Tetrapoda - ps24
Amniota - ps25
Mammalia - ps26
Theria - ps27
Placentalia - ps28
Boreoeutheria - ps29
Euarchontoglires - ps30
Euarchonta - ps31
Primates - ps32
Haplorrhini - ps33
Simiiformes - ps34
Catarrhini - ps35
Hominoidea - ps36
Hominidae - ps37
Homininae - ps38
Hominini - ps39
Homo_sapiens - ps40
p-value
2e-02
2e-02
3e-02
4e-02
5e-02
Log-odds
0.5
1.0
1.5
2.0
2.5
3.0
3.5
4.0

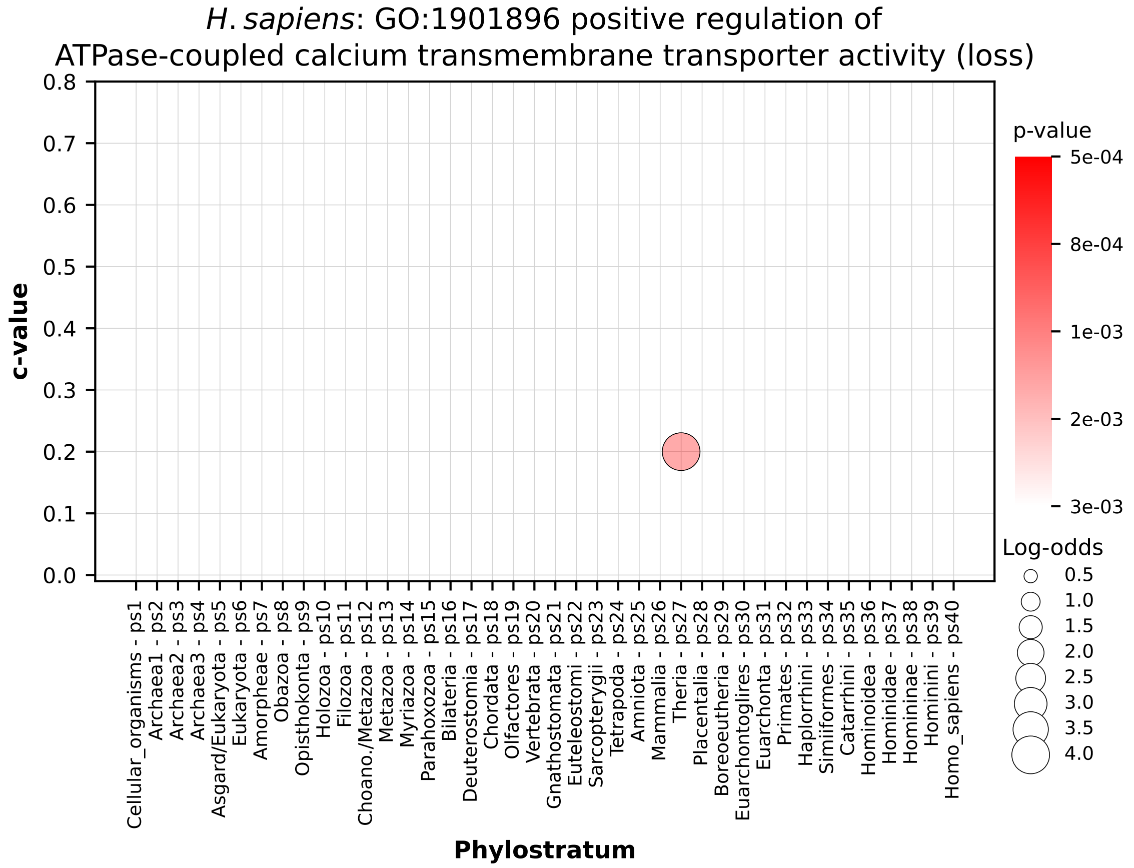

H. sapiens: GO:1901896 positive regulation of ATPase-coupled calcium transmembrane transporter activity (loss)
c-value
0.8
0.7
0.6
0.5
0.4
0.3
0.2
0.1
0.0
p-value
5e-04
8e-04
1e-03
2e-03
3e-03
Log-odds
0.5
1.0
1.5
2.0
2.5
3.0
3.5
4.0
Cellular_organisms - ps1
Archaea1 - ps2
Archaea2 - ps3
Archaea3 - ps4
Asgard/Eukaryota - ps5
Eukaryota - ps6
Amorpheae - ps7
Obazoa - ps8
Opisthokonta - ps9
Holozoa - ps10
Filozoa - ps11
Choano./Metazoa - ps12
Metazoa - ps13
Myriazoa - ps14
Parahoxozoa - ps15
Bilateria - ps16
Deuterostomia - ps17
Chordata - ps18
Olfactores - ps19
Vertebrata - ps20
Gnathostomata - ps21
Euteleostomi - ps22
Sarcopterygii - ps23
Tetrapoda - ps24
Amniota - ps25
Mammalia - ps26
Theria - ps27
Placentalia - ps28
Boreoeutheria - ps29
Euarchontoglires - ps30
Euarchonta - ps31
Primates - ps32
Haplorrhini - ps33
Simiiformes - ps34
Catarrhini - ps35
Hominoidea - ps36
Hominidae - ps37
Homininae - ps38
Hominini - ps39
Homo_sapiens - ps40
Phylostratum

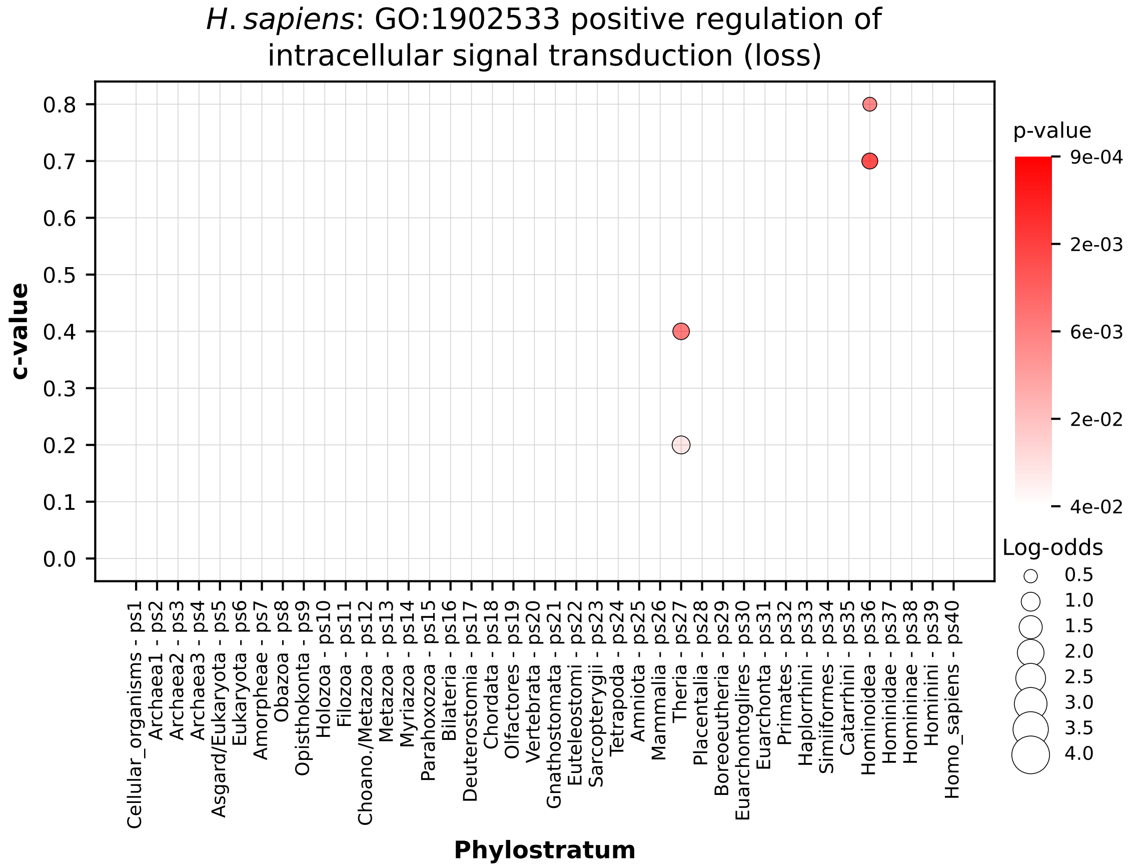

H. sapiens: GO:1902533 positive regulation of intracellular signal transduction (loss)
c-value
0.0
0.1
0.2
0.3
0.4
0.5
0.6
0.7
0.8
Phylostratum
Cellular_organisms - ps1
Archaea1 - ps2
Archaea2 - ps3
Archaea3 - ps4
Asgard/Eukaryota - ps5
Eukaryota - ps6
Amorpheae - ps7
Obazoa - ps8
Opisthokonta - ps9
Holozoa - ps10
Filozoa - ps11
Choano./Metazoa - ps12
Metazoa - ps13
Myriazoa - ps14
Parahoxozoa - ps15
Bilateria - ps16
Deuterostomia - ps17
Chordata - ps18
Olfactores - ps19
Vertebrata - ps20
Gnathostomata - ps21
Euteleostomi - ps22
Sarcopterygii - ps23
Tetrapoda - ps24
Amniota - ps25
Mammalia - ps26
Theria - ps27
Placentalia - ps28
Boreoeutheria - ps29
Euarchontoglires - ps30
Euarchonta - ps31
Primates - ps32
Haplorrhini - ps33
Simiiformes - ps34
Catarrhini - ps35
Hominoidea - ps36
Hominidae - ps37
Homininae - ps38
Hominini - ps39
Homo_sapiens - ps40
p-value
9e-04
2e-03
6e-03
2e-02
4e-02
Log-odds
0.5
1.0
1.5
2.0
2.5
3.0
3.5
4.0

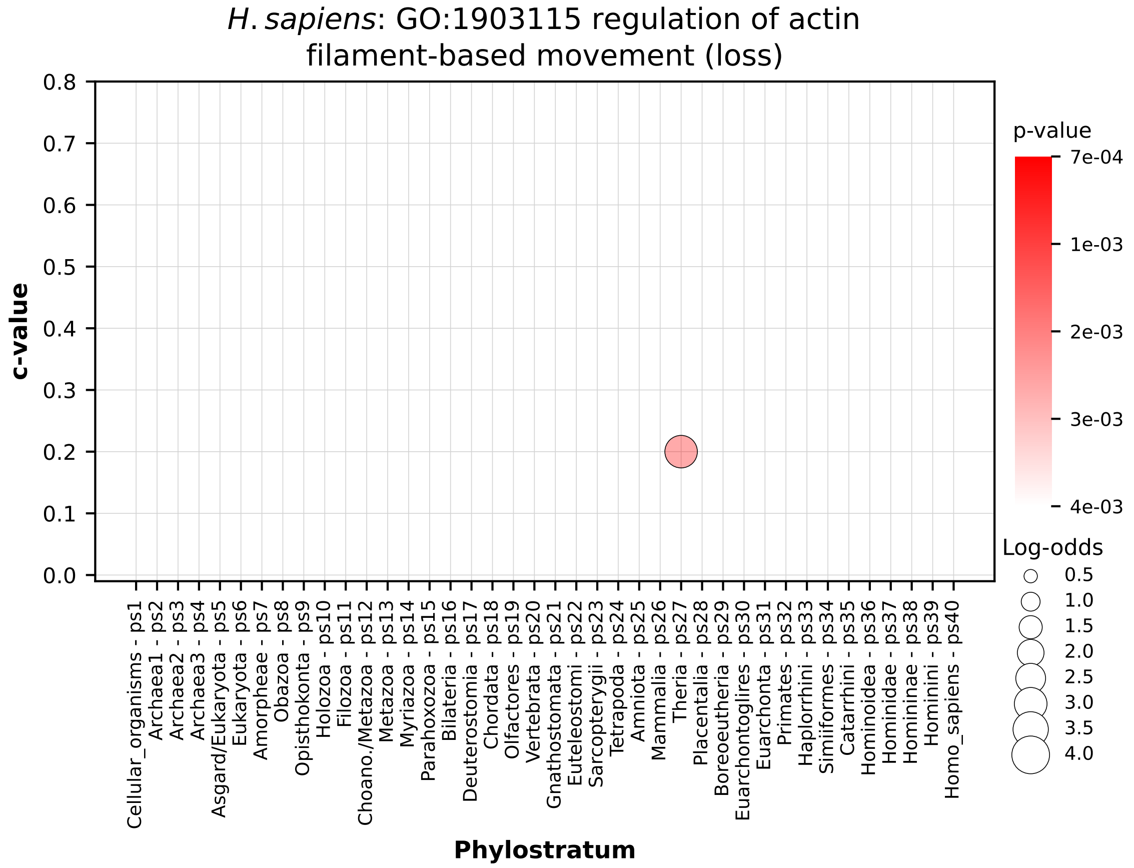

H. sapiens: GO:1903115 regulation of actin filament-based movement (loss)
c-value
0.8
0.7
0.6
0.5
0.4
0.3
0.2
0.1
0.0
Phylostratum
Cellular_organisms - ps1
Archaea1 - ps2
Archaea2 - ps3
Archaea3 - ps4
Asgard/Eukaryota - ps5
Eukaryota - ps6
Amorpheae - ps7
Obazoa - ps8
Opisthokonta - ps9
Holozoa - ps10
Filozoa - ps11
Choano./Metazoa - ps12
Metazoa - ps13
Myriazoa - ps14
Parahoxozoa - ps15
Bilateria - ps16
Deuterostomia - ps17
Chordata - ps18
Olfactores - ps19
Vertebrata - ps20
Gnathostomata - ps21
Euteleostomi - ps22
Sarcopterygii - ps23
Tetrapoda - ps24
Amniota - ps25
Mammalia - ps26
Theria - ps27
Placentalia - ps28
Boreoeutheria - ps29
Euarchontoglires - ps30
Euarchonta - ps31
Primates - ps32
Haplorrhini - ps33
Simiiformes - ps34
Catarrhini - ps35
Hominoidea - ps36
Hominidae - ps37
Homininae - ps38
Hominini - ps39
Homo_sapiens - ps40
p-value
7e-04
1e-03
2e-03
3e-03
4e-03
Log-odds
0.5
1.0
1.5
2.0
2.5
3.0
3.5
4.0

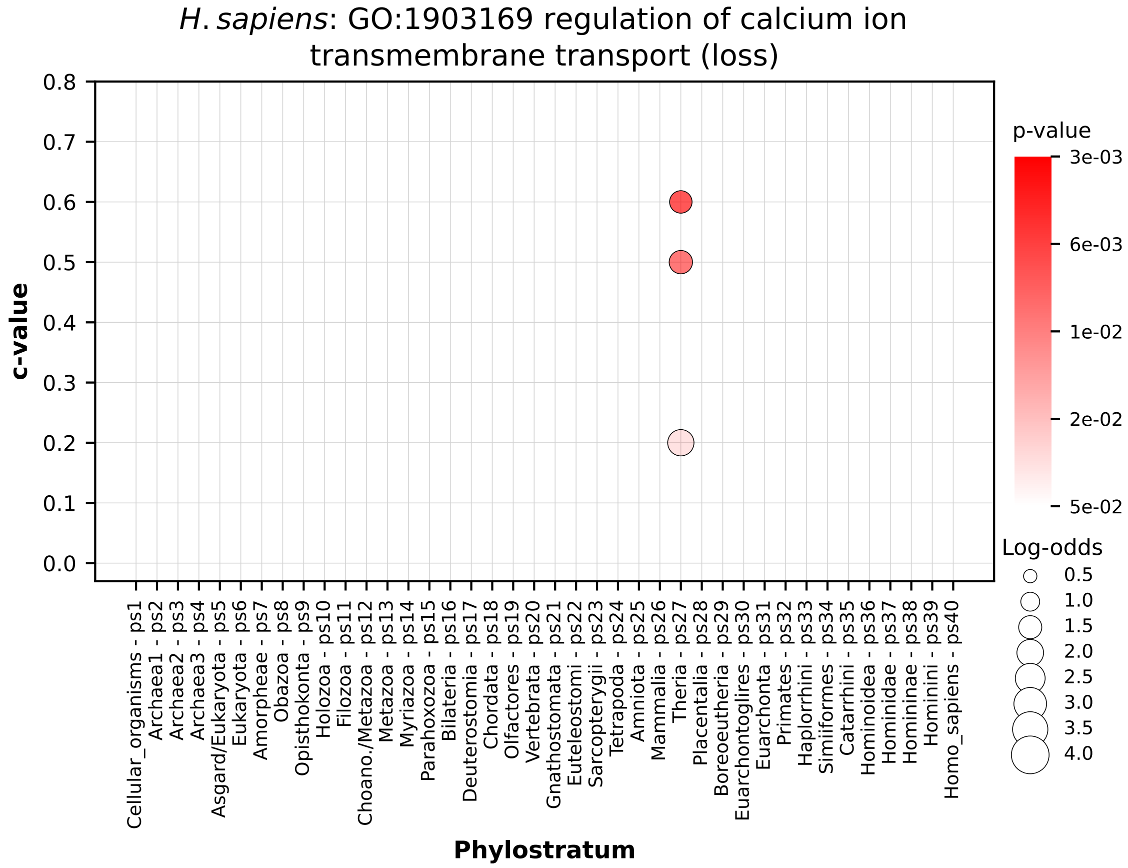
H. sapiens: GO:1903169 regulation of calcium ion transmembrane transport (loss)
c-value
0.8
0.7
0.6
0.5
0.4
0.3
0.2
0.1
0.0
p-value
3e-03
6e-03
1e-02
2e-02
5e-02
Log-odds
0.5
1.0
1.5
2.0
2.5
3.0
3.5
4.0
Cellular_organisms - ps1
Archaea1 - ps2
Archaea2 - ps3
Archaea3 - ps4
Asgard/Eukaryota - ps5
Eukaryota - ps6
Amorpheae - ps7
Obazoa - ps8
Opisthokonta - ps9
Holozoa - ps10
Filozoa - ps11
Choano./Metazoa - ps12
Metazoa - ps13
Myriazoa - ps14
Parahoxozoa - ps15
Bilateria - ps16
Deuterostomia - ps17
Chordata - ps18
Olfactores - ps19
Vertebrata - ps20
Gnathostomata - ps21
Euteleostomi - ps22
Sarcopterygii - ps23
Tetrapoda - ps24
Amniota - ps25
Mammalia - ps26
Theria - ps27
Placentalia - ps28
Boreoeutheria - ps29
Euarchontoglires - ps30
Euarchonta - ps31
Primates - ps32
Haplorrhini - ps33
Simiiformes - ps34
Catarrhini - ps35
Hominoidea - ps36
Hominidae - ps37
Homininae - ps38
Hominini - ps39
Homo_sapiens - ps40
Phylostratum

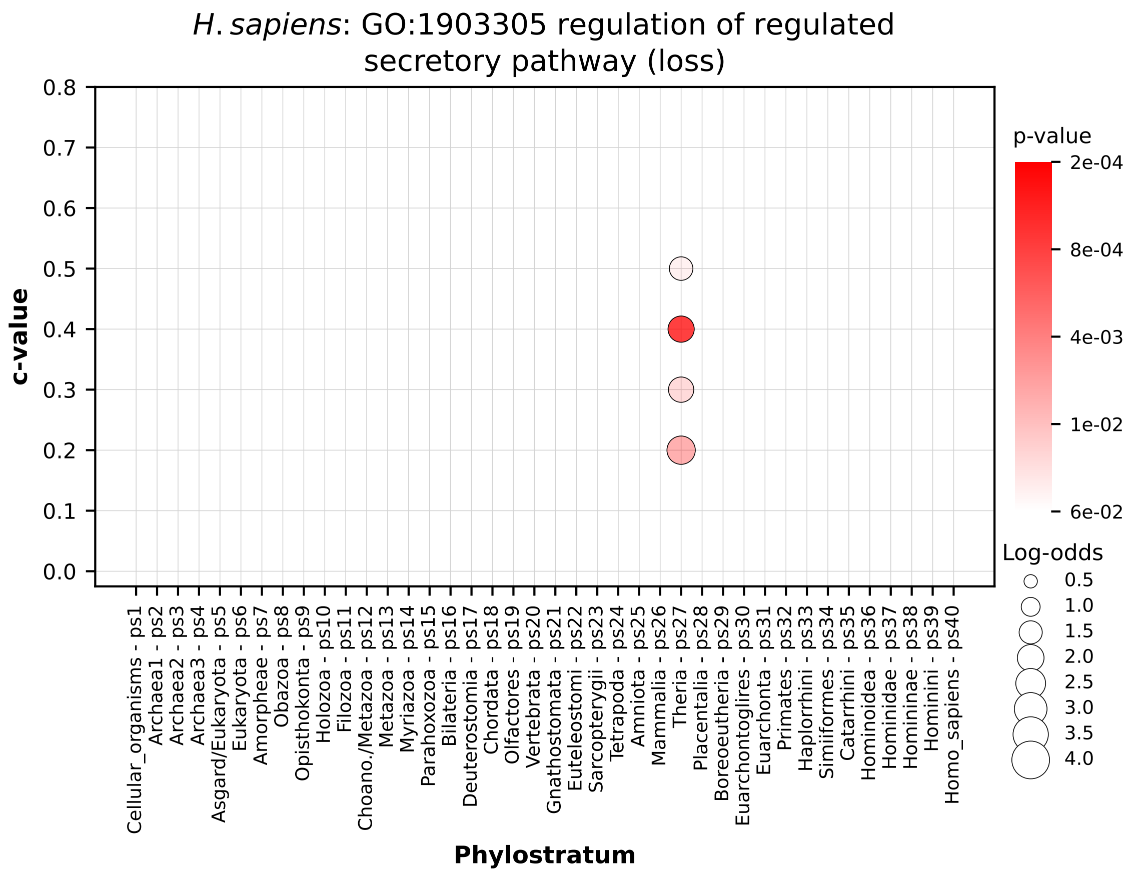

H. sapiens: GO:1903305 regulation of regulated secretory pathway (loss)
c-value
0.8
0.7
0.6
0.5
0.4
0.3
0.2
0.1
0.0
p-value
2e-04
8e-04
4e-03
1e-02
6e-02
Log-odds
0.5
1.0
1.5
2.0
2.5
3.0
3.5
4.0
Cellular_organisms - ps1
Archaea1 - ps2
Archaea2 - ps3
Archaea3 - ps4
Asgard/Eukaryota - ps5
Eukaryota - ps6
Amorpheae - ps7
Obazoa - ps8
Opisthokonta - ps9
Holozoa - ps10
Filozoa - ps11
Choano./Metazoa - ps12
Metazoa - ps13
Myriazoa - ps14
Parahoxozoa - ps15
Bilateria - ps16
Deuterostomia - ps17
Chordata - ps18
Olfactores - ps19
Vertebrata - ps20
Gnathostomata - ps21
Euteleostomi - ps22
Sarcopterygii - ps23
Tetrapoda - ps24
Amniota - ps25
Mammalia - ps26
Theria - ps27
Placentalia - ps28
Boreoeutheria - ps29
Euarchontoglires - ps30
Euarchonta - ps31
Primates - ps32
Haplorrhini - ps33
Simiiformes - ps34
Catarrhini - ps35
Hominoidea - ps36
Hominidae - ps37
Homininae - ps38
Hominini - ps39
Homo_sapiens - ps40
Phylostratum

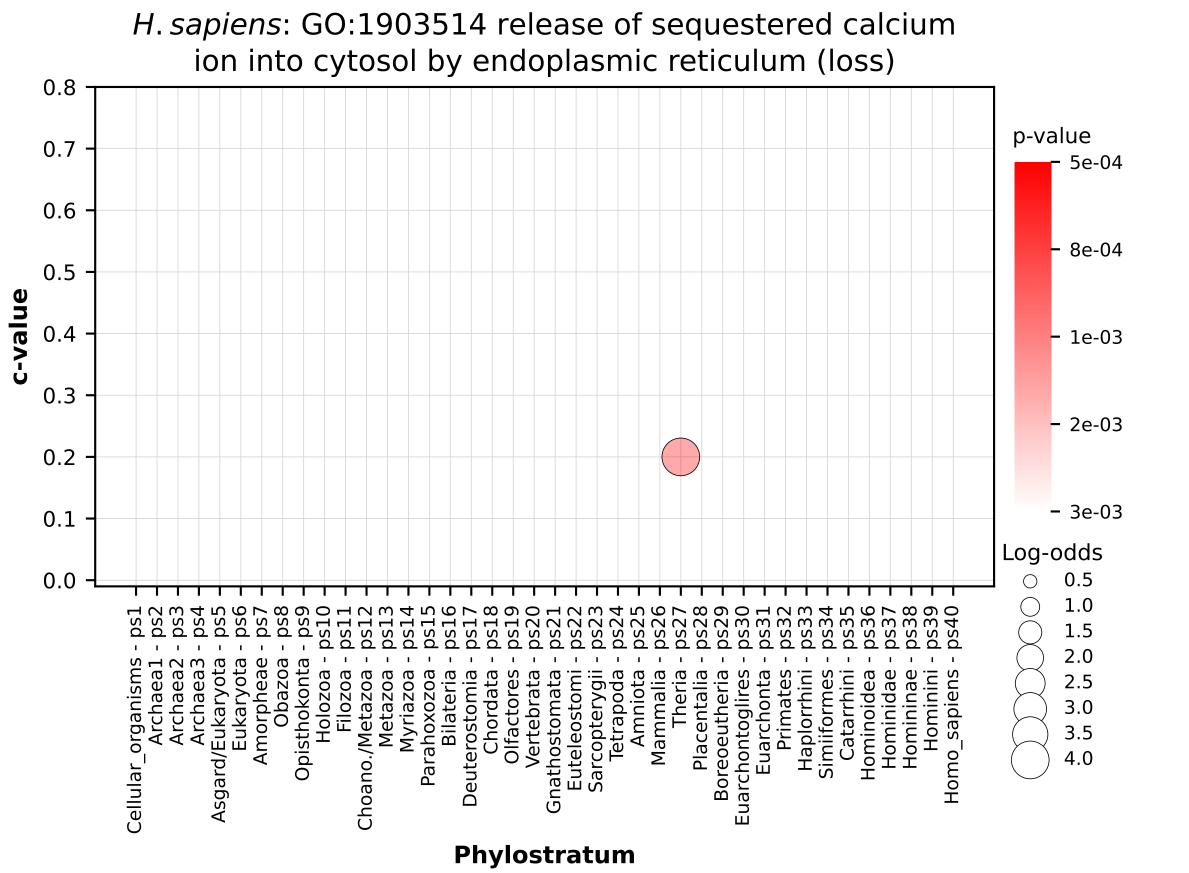
H. sapiens: GO:1903514 release of sequestered calcium ion into cytosol by endoplasmic reticulum (loss)
c-value
0.8
0.7
0.6
0.5
0.4
0.3
0.2
0.1
0.0
p-value
5e-04
8e-04
1e-03
2e-03
3e-03
Log-odds
0.5
1.0
1.5
2.0
2.5
3.0
3.5
4.0
Cellular_organisms - ps1
Archaea1 - ps2
Archaea2 - ps3
Archaea3 - ps4
Asgard/Eukaryota - ps5
Eukaryota - ps6
Amorpheae - ps7
Obazoa - ps8
Opisthokonta - ps9
Holozoa - ps10
Filozoa - ps11
Choano./Metazoa - ps12
Metazoa - ps13
Myriazoa - ps14
Parahoxozoa - ps15
Bilateria - ps16
Deuterostomia - ps17
Chordata - ps18
Olfactores - ps19
Vertebrata - ps20
Gnathostomata - ps21
Euteleostomi - ps22
Sarcopterygii - ps23
Tetrapoda - ps24
Amniota - ps25
Mammalia - ps26
Theria - ps27
Placentalia - ps28
Boreoeutheria - ps29
Euarchontoglires - ps30
Euarchonta - ps31
Primates - ps32
Haplorrhini - ps33
Simiiformes - ps34
Catarrhini - ps35
Hominoidea - ps36
Hominidae - ps37
Homininae - ps38
Hominini - ps39
Homo_sapiens - ps40
Phylostratum

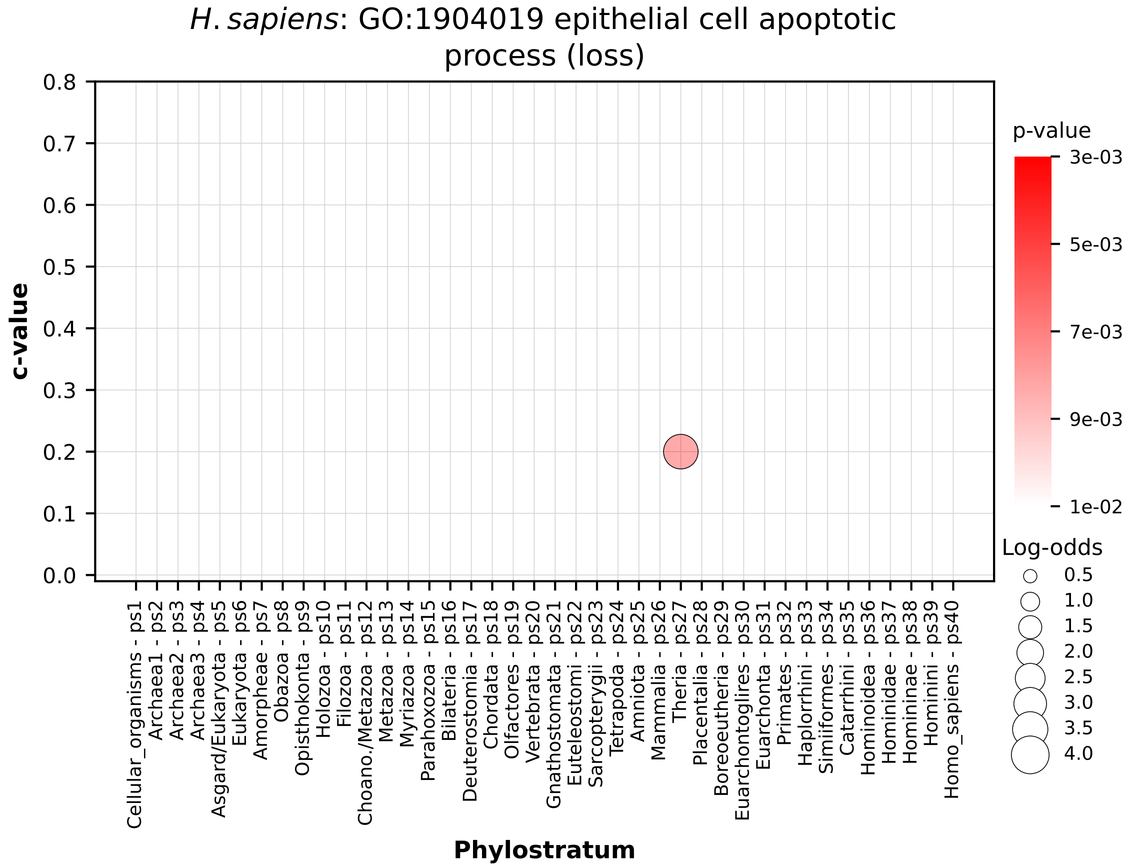

H. sapiens: GO:1904019 epithelial cell apoptotic process (loss)
c-value
0.0
0.1
0.2
0.3
0.4
0.5
0.6
0.7
0.8
Phylostratum
Cellular_organisms - ps1
Archaea1 - ps2
Archaea2 - ps3
Archaea3 - ps4
Asgard/Eukaryota - ps5
Eukaryota - ps6
Amorpheae - ps7
Obazoa - ps8
Opisthokonta - ps9
Holozoa - ps10
Filozoa - ps11
Choano./Metazoa - ps12
Metazoa - ps13
Myriazoa - ps14
Parahoxozoa - ps15
Bilateria - ps16
Deuterostomia - ps17
Chordata - ps18
Olfactores - ps19
Vertebrata - ps20
Gnathostomata - ps21
Euteleostomi - ps22
Sarcopterygii - ps23
Tetrapoda - ps24
Amniota - ps25
Mammalia - ps26
Theria - ps27
Placentalia - ps28
Boreoeutheria - ps29
Euarchontoglires - ps30
Euarchonta - ps31
Primates - ps32
Haplorrhini - ps33
Simiiformes - ps34
Catarrhini - ps35
Hominoidea - ps36
Hominidae - ps37
Homininae - ps38
Hominini - ps39
Homo_sapiens - ps40
p-value
3e-03
5e-03
7e-03
9e-03
1e-02
Log-odds
0.5
1.0
1.5
2.0
2.5
3.0
3.5
4.0

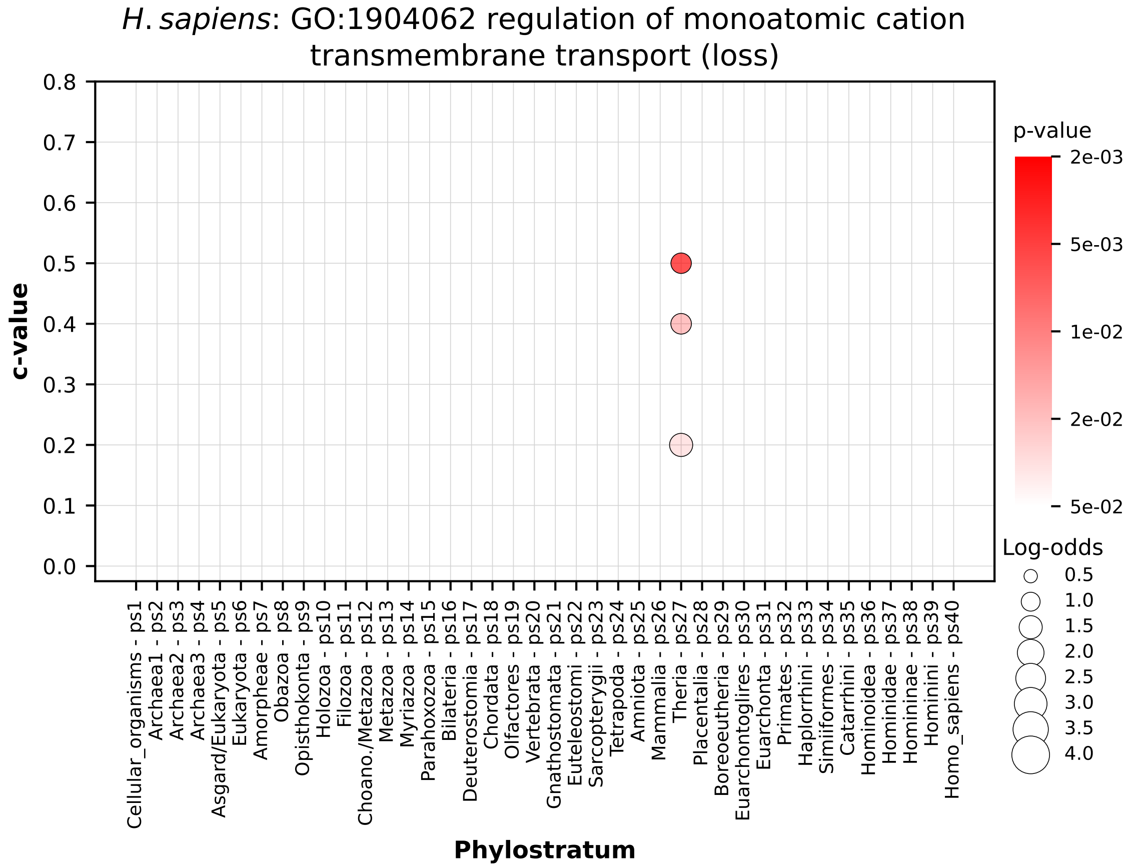

H. sapiens: GO:1904062 regulation of monoatomic cation transmembrane transport (loss)
c-value
0.0
0.1
0.2
0.3
0.4
0.5
0.6
0.7
0.8
p-value
2e-03
5e-03
1e-02
2e-02
5e-02
Log-odds
0.5
1.0
1.5
2.0
2.5
3.0
3.5
4.0
Cellular_organisms - ps1
Archaea1 - ps2
Archaea2 - ps3
Archaea3 - ps4
Asgard/Eukaryota - ps5
Eukaryota - ps6
Amorpheae - ps7
Obazoa - ps8
Opisthokonta - ps9
Holozoa - ps10
Filozoa - ps11
Choano./Metazoa - ps12
Metazoa - ps13
Myriazoa - ps14
Parahoxozoa - ps15
Bilateria - ps16
Deuterostomia - ps17
Chordata - ps18
Olfactores - ps19
Vertebrata - ps20
Gnathostomata - ps21
Euteleostomi - ps22
Sarcopterygii - ps23
Tetrapoda - ps24
Amniota - ps25
Mammalia - ps26
Theria - ps27
Placentalia - ps28
Boreoeutheria - ps29
Euarchontoglires - ps30
Euarchonta - ps31
Primates - ps32
Haplorrhini - ps33
Simiiformes - ps34
Catarrhini - ps35
Hominoidea - ps36
Hominidae - ps37
Homininae - ps38
Hominini - ps39
Homo_sapiens - ps40
Phylostratum

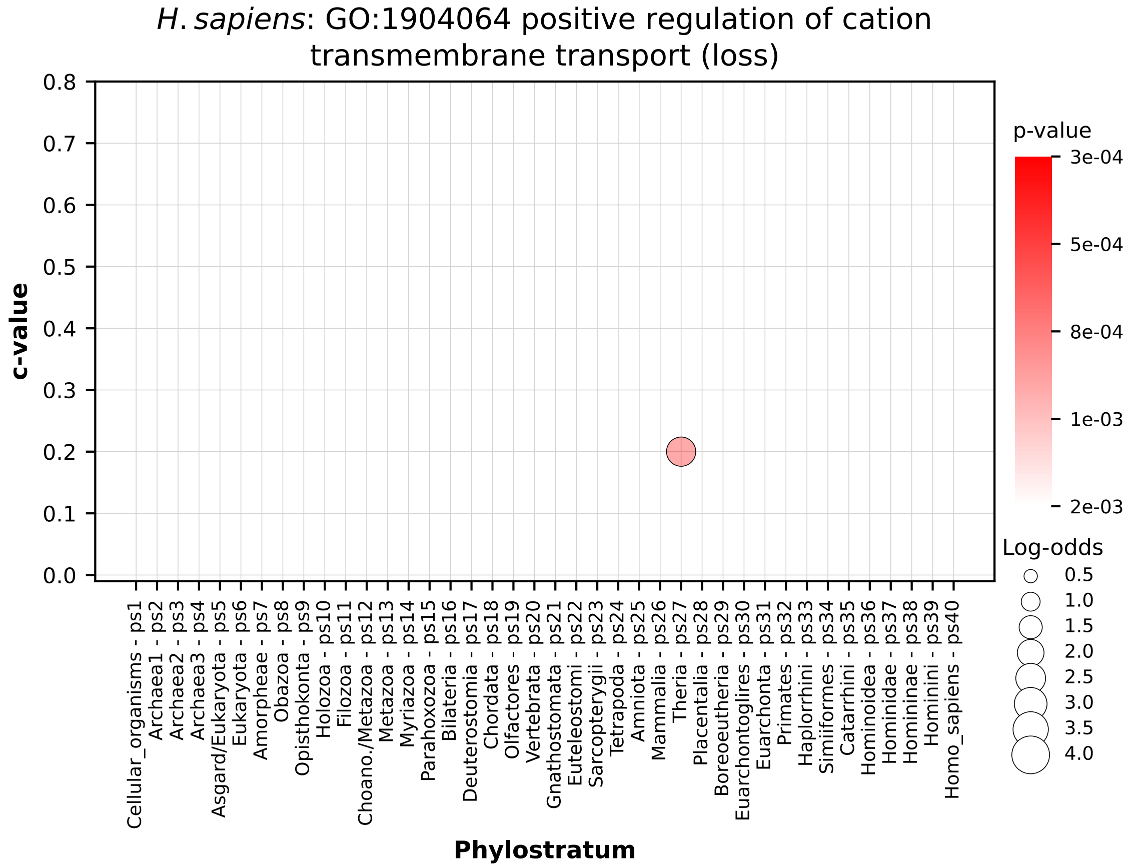
H. sapiens: GO:1904064 positive regulation of cation transmembrane transport (loss)
c-value
0.0
0.1
0.2
0.3
0.4
0.5
0.6
0.7
0.8
Phylostratum
Cellular_organisms - ps1
Archaea1 - ps2
Archaea2 - ps3
Archaea3 - ps4
Asgard/Eukaryota - ps5
Eukaryota - ps6
Amorpheae - ps7
Obazoa - ps8
Opisthokonta - ps9
Holozoa - ps10
Filozoa - ps11
Choano./Metazoa - ps12
Metazoa - ps13
Myriazoa - ps14
Parahoxozoa - ps15
Bilateria - ps16
Deuterostomia - ps17
Chordata - ps18
Olfactores - ps19
Vertebrata - ps20
Gnathostomata - ps21
Euteleostomi - ps22
Sarcopterygii - ps23
Tetrapoda - ps24
Amniota - ps25
Mammalia - ps26
Theria - ps27
Placentalia - ps28
Boreoeutheria - ps29
Euarchontoglires - ps30
Euarchonta - ps31
Primates - ps32
Haplorrhini - ps33
Simiiformes - ps34
Catarrhini - ps35
Hominoidea - ps36
Hominidae - ps37
Homininae - ps38
Hominini - ps39
Homo_sapiens - ps40
p-value
3e-04
5e-04
8e-04
1e-03
2e-03
Log-odds
0.5
1.0
1.5
2.0
2.5
3.0
3.5
4.0

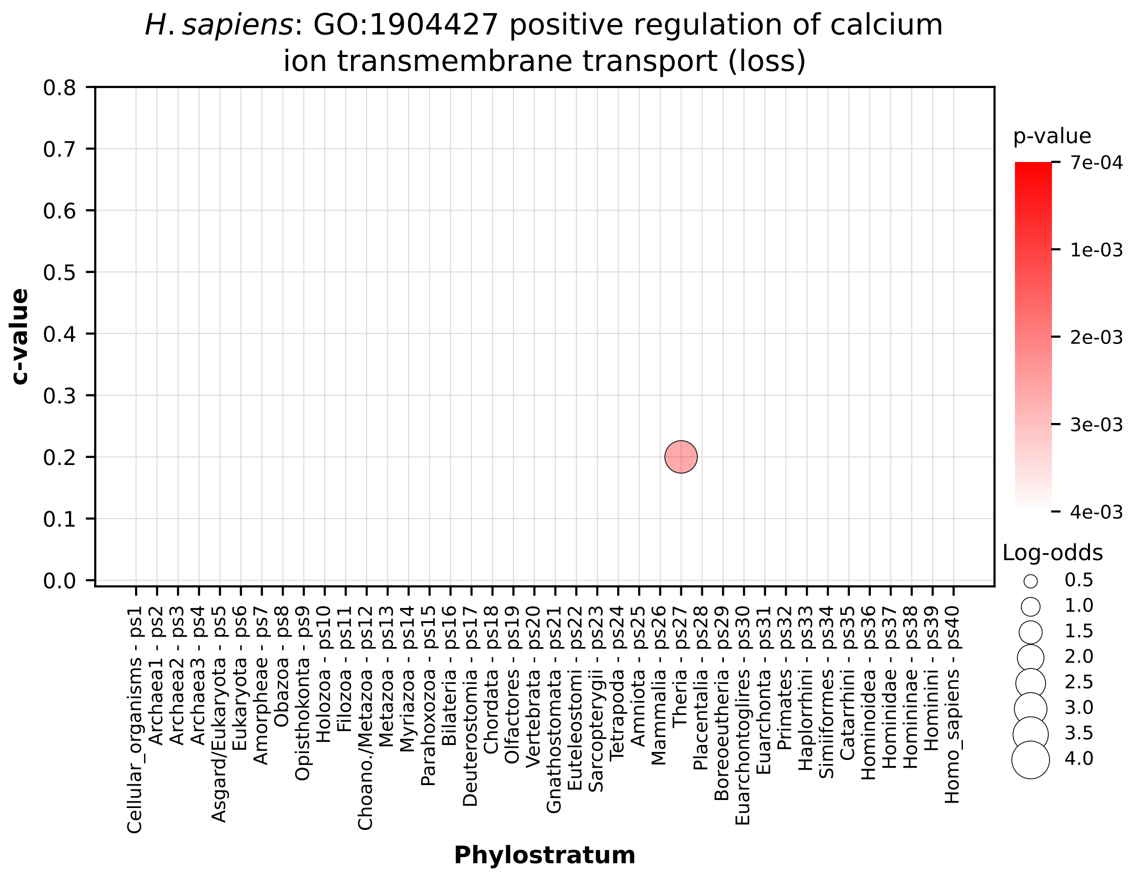

H. sapiens: GO:1904427 positive regulation of calcium ion transmembrane transport (loss)
c-value
0.0
0.1
0.2
0.3
0.4
0.5
0.6
0.7
0.8
Cellular_organisms - ps1
Archaea1 - ps2
Archaea2 - ps3
Archaea3 - ps4
Asgard/Eukaryota - ps5
Eukaryota - ps6
Amorpheae - ps7
Obazoa - ps8
Opisthokonta - ps9
Holozoa - ps10
Filozoa - ps11
Choano./Metazoa - ps12
Metazoa - ps13
Myriazoa - ps14
Parahoxozoa - ps15
Bilateria - ps16
Deuterostomia - ps17
Chordata - ps18
Olfactores - ps19
Vertebrata - ps20
Gnathostomata - ps21
Euteleostomi - ps22
Sarcopterygii - ps23
Tetrapoda - ps24
Amniota - ps25
Mammalia - ps26
Theria - ps27
Placentalia - ps28
Boreoeutheria - ps29
Euarchontoglires - ps30
Euarchonta - ps31
Primates - ps32
Haplorrhini - ps33
Simiiformes - ps34
Catarrhini - ps35
Hominoidea - ps36
Hominidae - ps37
Homininae - ps38
Hominini - ps39
Homo_sapiens - ps40
Phylostratum
p-value
7e-04
1e-03
2e-03
3e-03
4e-03
Log-odds
0.5
1.0
1.5
2.0
2.5
3.0
3.5
4.0

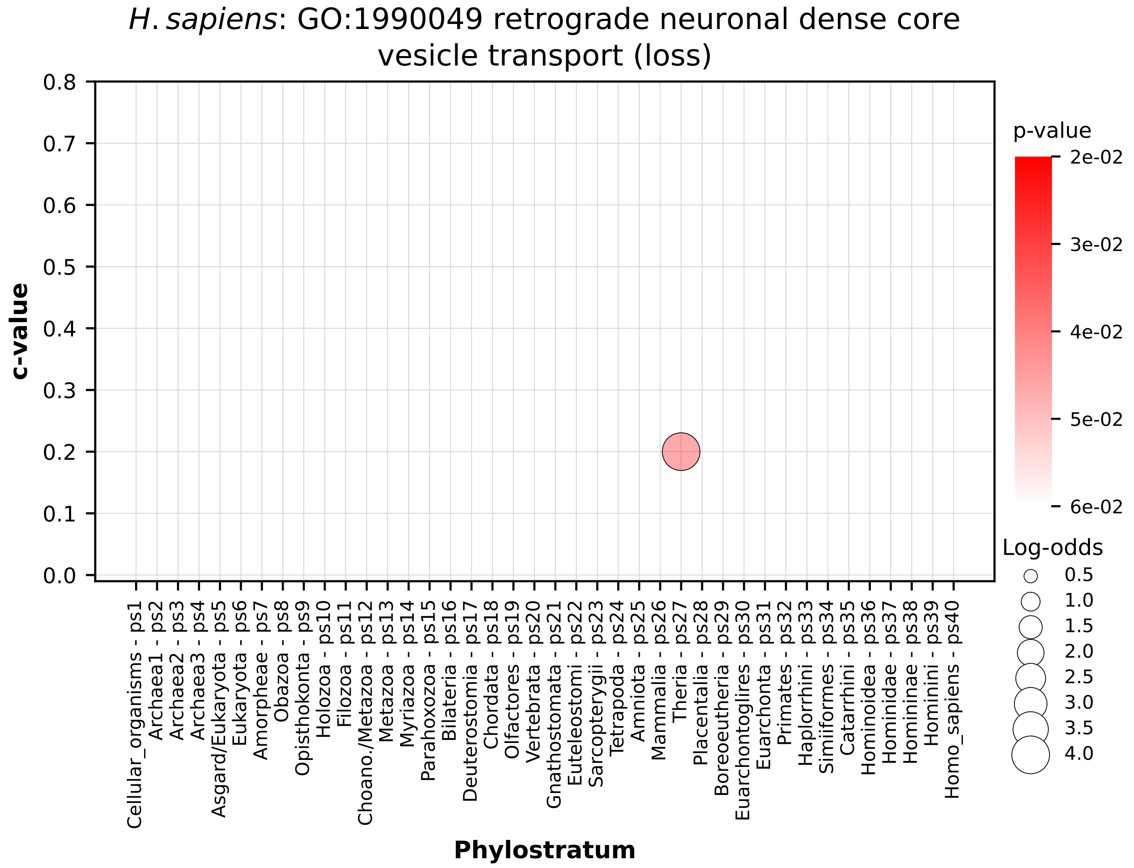

H. sapiens: GO:1990049 retrograde neuronal dense core vesicle transport (loss)
c-value
0.0
0.1
0.2
0.3
0.4
0.5
0.6
0.7
0.8
Phylostratum
Cellular_organisms - ps1
Archaea1 - ps2
Archaea2 - ps3
Archaea3 - ps4
Asgard/Eukaryota - ps5
Eukaryota - ps6
Amorpheae - ps7
Obazoa - ps8
Opisthokonta - ps9
Holozoa - ps10
Filozoa - ps11
Choano./Metazoa - ps12
Metazoa - ps13
Myriazoa - ps14
Parahoxozoa - ps15
Bilateria - ps16
Deuterostomia - ps17
Chordata - ps18
Olfactores - ps19
Vertebrata - ps20
Gnathostomata - ps21
Euteleostomi - ps22
Sarcopterygii - ps23
Tetrapoda - ps24
Amniota - ps25
Mammalia - ps26
Theria - ps27
Placentalia - ps28
Boreoeutheria - ps29
Euarchontoglires - ps30
Euarchonta - ps31
Primates - ps32
Haplorrhini - ps33
Simiiformes - ps34
Catarrhini - ps35
Hominoidea - ps36
Hominidae - ps37
Homininae - ps38
Hominini - ps39
Homo_sapiens - ps40
p-value
2e-02
3e-02
4e-02
5e-02
6e-02
Log-odds
0.5
1.0
1.5
2.0
2.5
3.0
3.5
4.0

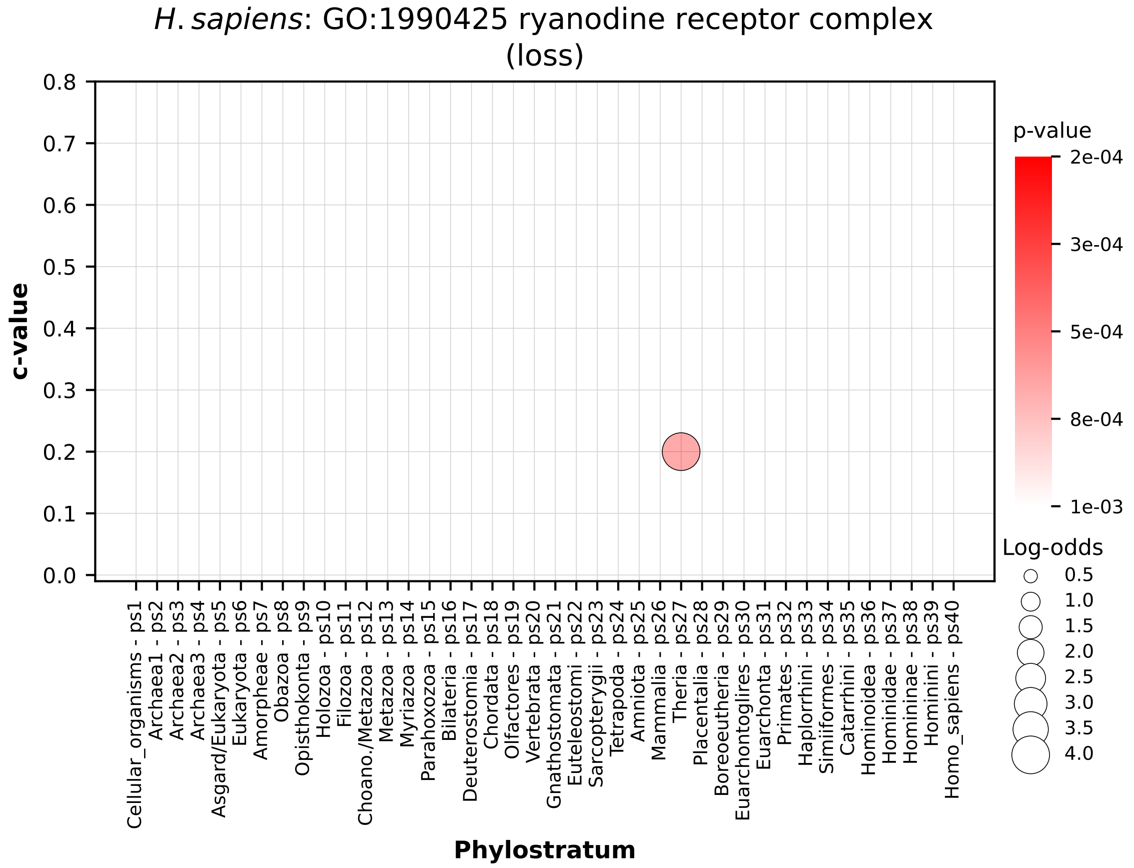

H. sapiens: GO:1990425 ryanodine receptor complex
(loss)
c-value
0.0
0.1
0.2
0.3
0.4
0.5
0.6
0.7
0.8
Phylostratum
Cellular_organisms - ps1
Archaea1 - ps2
Archaea2 - ps3
Archaea3 - ps4
Asgard/Eukaryota - ps5
Eukaryota - ps6
Amorpheae - ps7
Obazoa - ps8
Opisthokonta - ps9
Holozoa - ps10
Filozoa - ps11
Choano./Metazoa - ps12
Metazoa - ps13
Myriazoa - ps14
Parahoxozoa - ps15
Bilateria - ps16
Deuterostomia - ps17
Chordata - ps18
Olfactores - ps19
Vertebrata - ps20
Gnathostomata - ps21
Euteleostomi - ps22
Sarcopterygii - ps23
Tetrapoda - ps24
Amniota - ps25
Mammalia - ps26
Theria - ps27
Placentalia - ps28
Boreoeutheria - ps29
Euarchontoglires - ps30
Euarchonta - ps31
Primates - ps32
Haplorrhini - ps33
Simiiformes - ps34
Catarrhini - ps35
Hominoidea - ps36
Hominidae - ps37
Homininae - ps38
Hominini - ps39
Homo_sapiens - ps40
p-value
2e-04
3e-04
5e-04
8e-04
1e-03
Log-odds
0.5
1.0
1.5
2.0
2.5
3.0
3.5
4.0

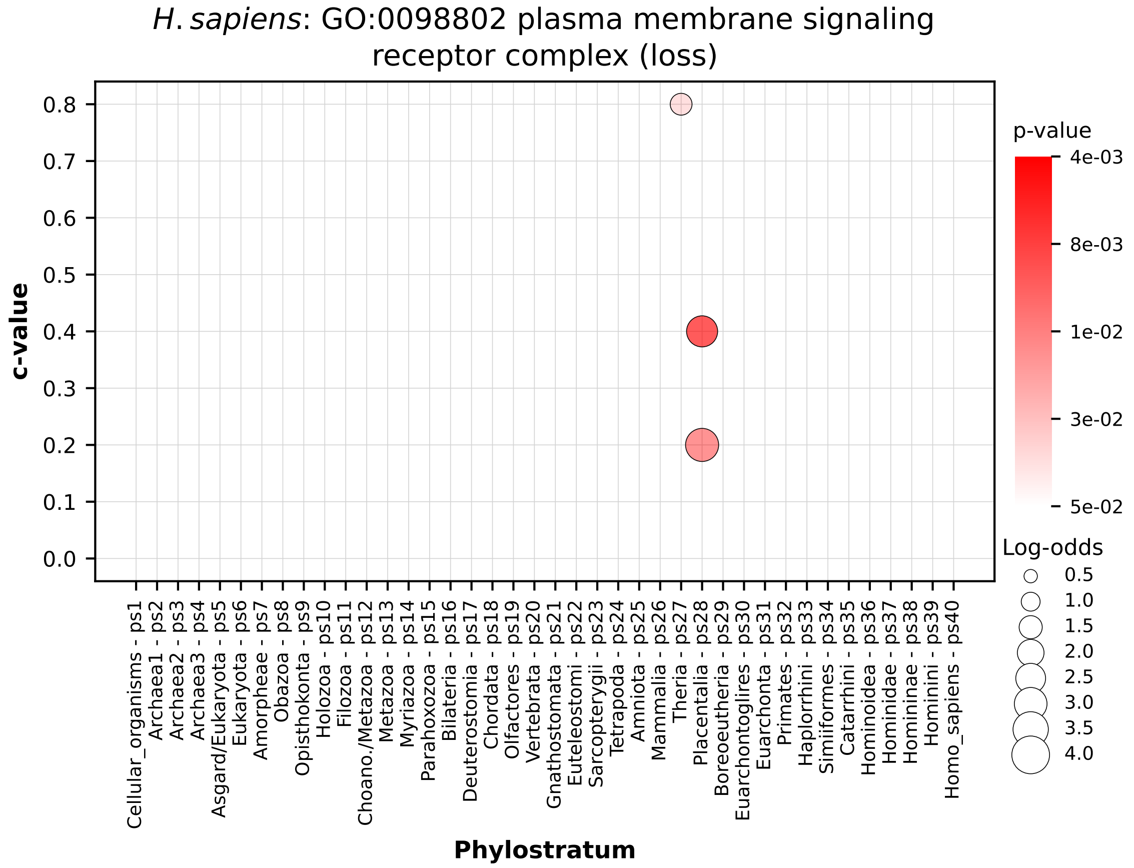

H. sapiens: GO:0098802 plasma membrane signaling receptor complex (loss)
c-value
0.0
0.1
0.2
0.3
0.4
0.5
0.6
0.7
0.8
Phylostratum
Cellular_organisms - ps1
Archaea1 - ps2
Archaea2 - ps3
Archaea3 - ps4
Asgard/Eukaryota - ps5
Eukaryota - ps6
Amorpheae - ps7
Obazoa - ps8
Opisthokonta - ps9
Holozoa - ps10
Filozoa - ps11
Choano./Metazoa - ps12
Metazoa - ps13
Myriazoa - ps14
Parahoxozoa - ps15
Bilateria - ps16
Deuterostomia - ps17
Chordata - ps18
Olfactores - ps19
Vertebrata - ps20
Gnathostomata - ps21
Euteleostomi - ps22
Sarcopterygii - ps23
Tetrapoda - ps24
Amniota - ps25
Mammalia - ps26
Theria - ps27
Placentalia - ps28
Boreoeutheria - ps29
Euarchontoglires - ps30
Euarchonta - ps31
Primates - ps32
Haplorrhini - ps33
Simiiformes - ps34
Catarrhini - ps35
Hominoidea - ps36
Hominidae - ps37
Homininae - ps38
Hominini - ps39
Homo_sapiens - ps40
p-value
4e-03
8e-03
1e-02
3e-02
5e-02
Log-odds
0.5
1.0
1.5
2.0
2.5
3.0
3.5
4.0

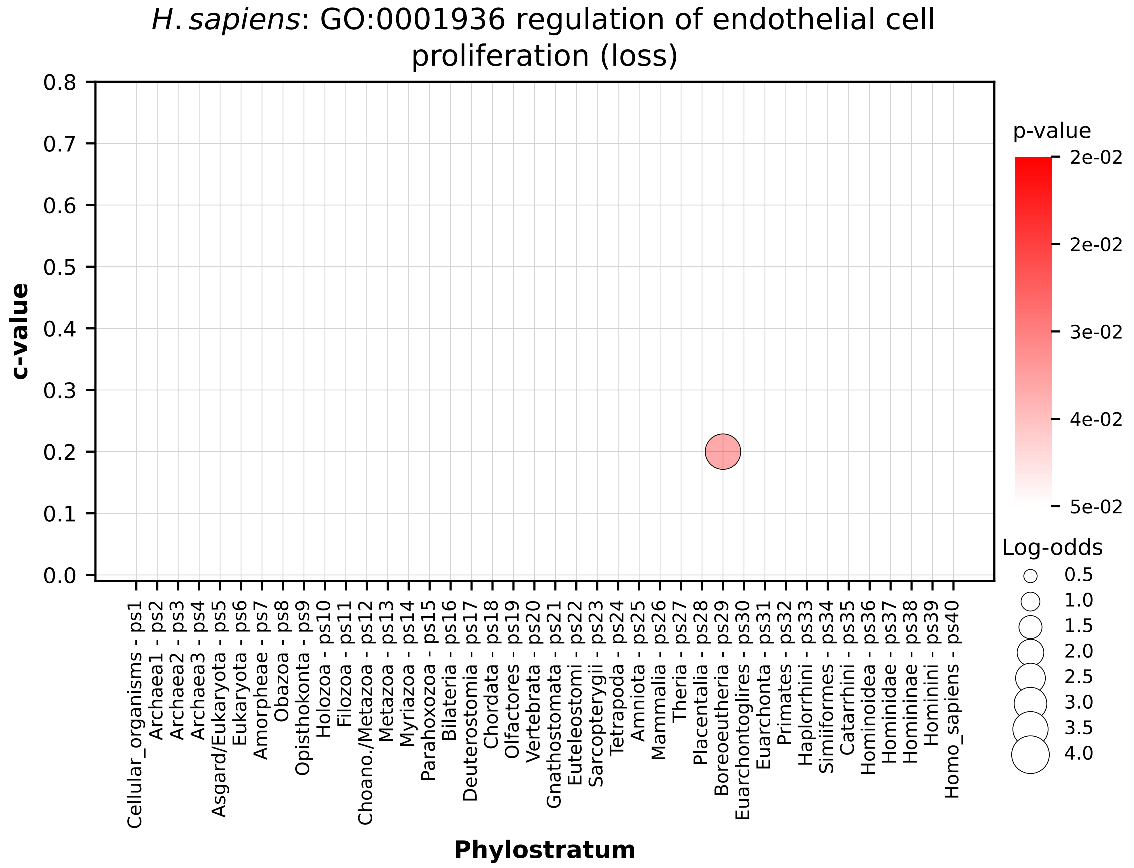

H. sapiens: GO:0001936 regulation of endothelial cell proliferation (loss)
c-value
0.8
0.7
0.6
0.5
0.4
0.3
0.2
0.1
0.0
p-value
2e-02
2e-02
3e-02
4e-02
5e-02
Log-odds
0.5
1.0
1.5
2.0
2.5
3.0
3.5
4.0
Cellular_organisms - ps1
Archaea1 - ps2
Archaea2 - ps3
Archaea3 - ps4
Asgard/Eukaryota - ps5
Eukaryota - ps6
Amorpheae - ps7
Obazoa - ps8
Opisthokonta - ps9
Holozoa - ps10
Filozoa - ps11
Choano./Metazoa - ps12
Metazoa - ps13
Myriazoa - ps14
Parahoxozoa - ps15
Bilateria - ps16
Deuterostomia - ps17
Chordata - ps18
Olfactores - ps19
Vertebrata - ps20
Gnathostomata - ps21
Euteleostomi - ps22
Sarcopterygii - ps23
Tetrapoda - ps24
Amniota - ps25
Mammalia - ps26
Theria - ps27
Placentalia - ps28
Boreoeutheria - ps29
Euarchontoglires - ps30
Euarchonta - ps31
Primates - ps32
Haplorrhini - ps33
Simiiformes - ps34
Catarrhini - ps35
Hominoidea - ps36
Hominidae - ps37
Homininae - ps38
Hominini - ps39
Homo_sapiens - ps40
Phylostratum

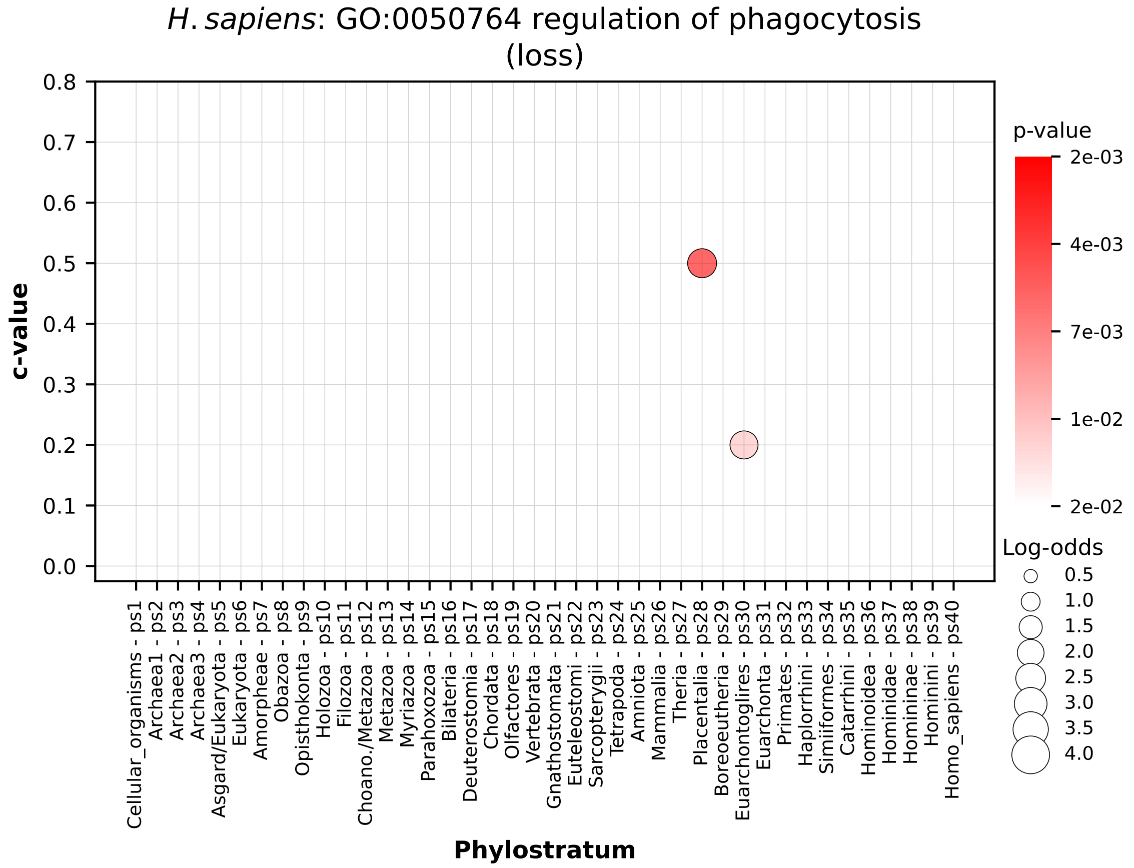

H. sapiens: GO:0050764 regulation of phagocytosis
(loss)
c-value
0.0
0.1
0.2
0.3
0.4
0.5
0.6
0.7
0.8
Phylostratum
Cellular_organisms - ps1
Archaea1 - ps2
Archaea2 - ps3
Archaea3 - ps4
Asgard/Eukaryota - ps5
Eukaryota - ps6
Amorpheae - ps7
Obazoa - ps8
Opisthokonta - ps9
Holozoa - ps10
Filozoa - ps11
Choano./Metazoa - ps12
Metazoa - ps13
Myriazoa - ps14
Parahoxozoa - ps15
Bilateria - ps16
Deuterostomia - ps17
Chordata - ps18
Olfactores - ps19
Vertebrata - ps20
Gnathostomata - ps21
Euteleostomi - ps22
Sarcopterygii - ps23
Tetrapoda - ps24
Amniota - ps25
Mammalia - ps26
Theria - ps27
Placentalia - ps28
Boreoeutheria - ps29
Euarchontoglires - ps30
Euarchonta - ps31
Primates - ps32
Haplorrhini - ps33
Simiiformes - ps34
Catarrhini - ps35
Hominoidea - ps36
Hominidae - ps37
Homininae - ps38
Hominini - ps39
Homo_sapiens - ps40
p-value
2e-03
4e-03
7e-03
1e-02
2e-02
Log-odds
0.5
1.0
1.5
2.0
2.5
3.0
3.5
4.0

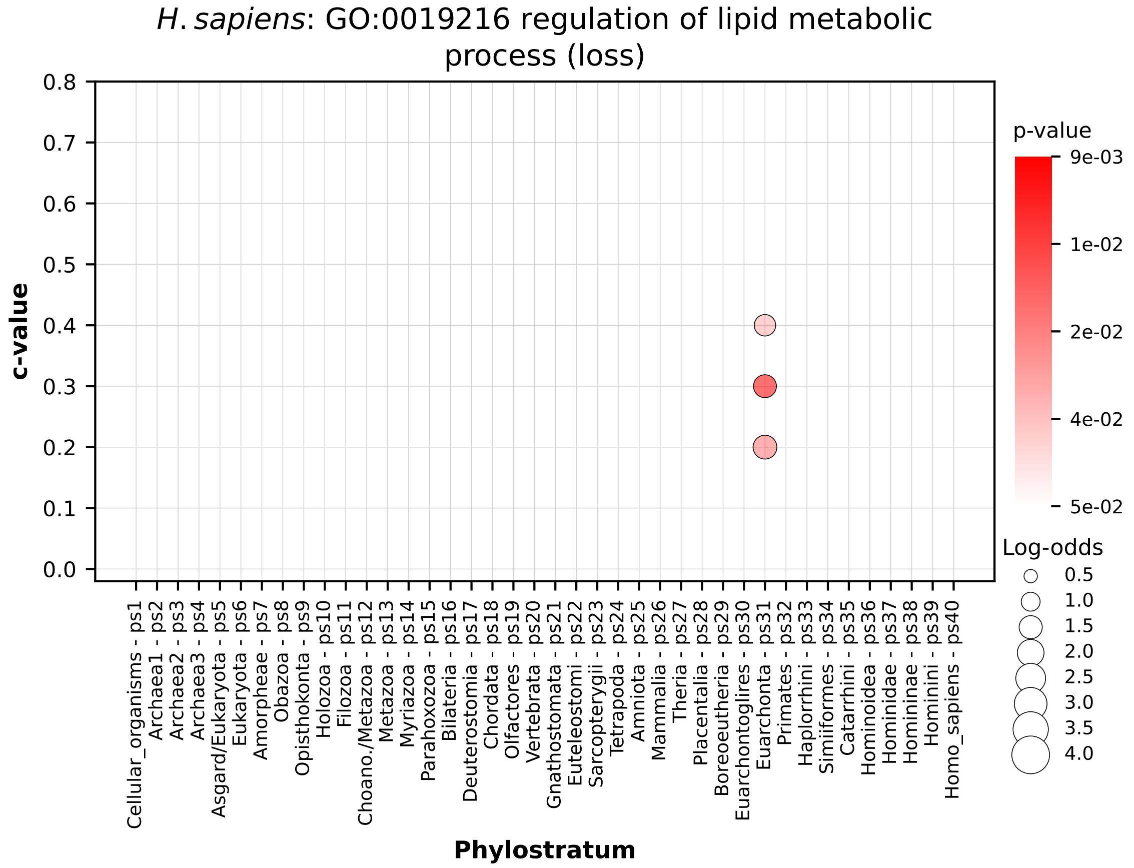

H. sapiens: GO:0019216 regulation of lipid metabolic process (loss)
c-value
0.8
0.7
0.6
0.5
0.4
0.3
0.2
0.1
0.0
Phylostratum
Cellular_organisms - ps1
Archaea1 - ps2
Archaea2 - ps3
Archaea3 - ps4
Asgard/Eukaryota - ps5
Eukaryota - ps6
Amorpheae - ps7
Obazoa - ps8
Opisthokonta - ps9
Holozoa - ps10
Filozoa - ps11
Choano./Metazoa - ps12
Metazoa - ps13
Myriazoa - ps14
Parahoxozoa - ps15
Bilateria - ps16
Deuterostomia - ps17
Chordata - ps18
Olfactores - ps19
Vertebrata - ps20
Gnathostomata - ps21
Euteleostomi - ps22
Sarcopterygii - ps23
Tetrapoda - ps24
Amniota - ps25
Mammalia - ps26
Theria - ps27
Placentalia - ps28
Boreoeutheria - ps29
Euarchontoglires - ps30
Euarchonta - ps31
Primates - ps32
Haplorrhini - ps33
Simiiformes - ps34
Catarrhini - ps35
Hominoidea - ps36
Hominidae - ps37
Homininae - ps38
Hominini - ps39
Homo_sapiens - ps40
p-value
9e-03
1e-02
2e-02
4e-02
5e-02
Log-odds
0.5
1.0
1.5
2.0
2.5
3.0
3.5
4.0

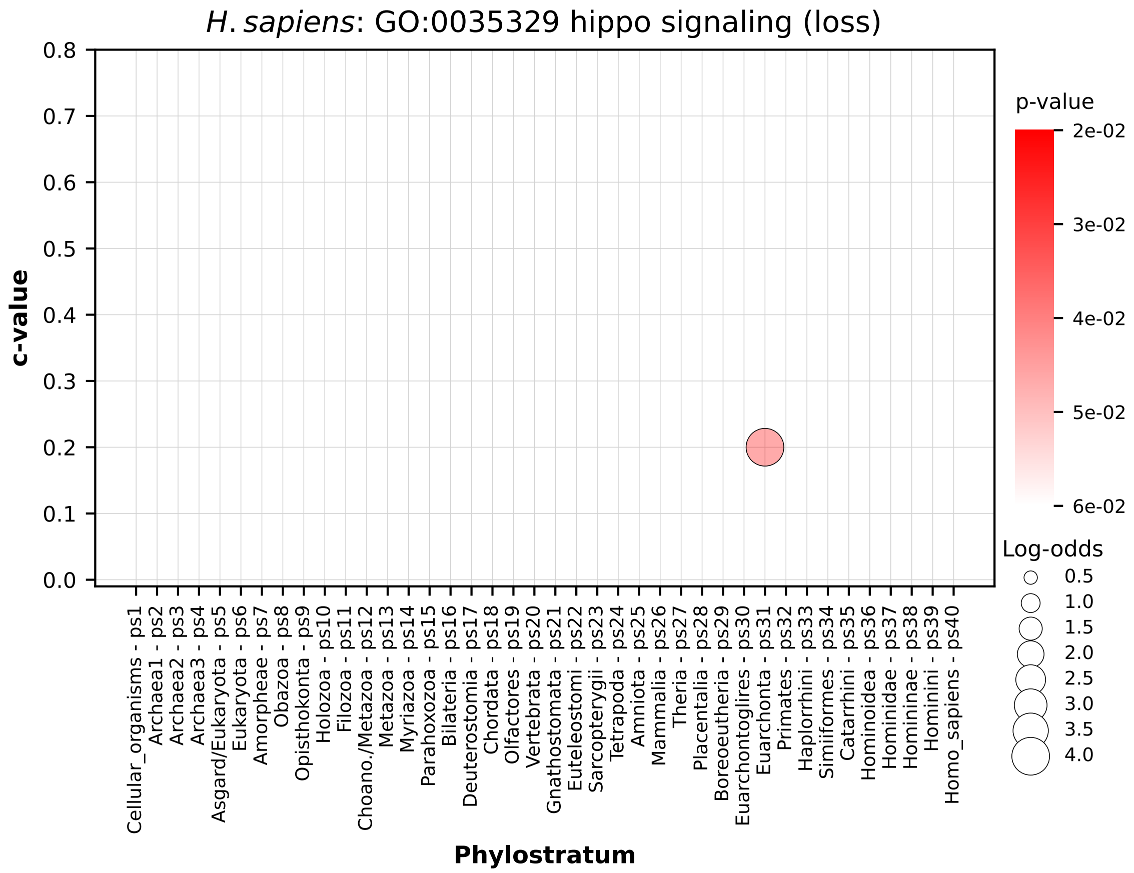

H. sapiens: GO:0035329 hippo signaling (loss)
c-value
0.0
0.1
0.2
0.3
0.4
0.5
0.6
0.7
0.8
Phylostratum
Cellular_organisms - ps1
Archaea1 - ps2
Archaea2 - ps3
Archaea3 - ps4
Asgard/Eukaryota - ps5
Eukaryota - ps6
Amorpheae - ps7
Obazoa - ps8
Opisthokonta - ps9
Holozoa - ps10
Filozoa - ps11
Choano./Metazoa - ps12
Metazoa - ps13
Myriazoa - ps14
Parahoxozoa - ps15
Bilateria - ps16
Deuterostomia - ps17
Chordata - ps18
Olfactores - ps19
Vertebrata - ps20
Gnathostomata - ps21
Euteleostomi - ps22
Sarcopterygii - ps23
Tetrapoda - ps24
Amniota - ps25
Mammalia - ps26
Theria - ps27
Placentalia - ps28
Boreoeutheria - ps29
Euarchontoglires - ps30
Euarchonta - ps31
Primates - ps32
Haplorrhini - ps33
Simiiformes - ps34
Catarrhini - ps35
Hominoidea - ps36
Hominidae - ps37
Homininae - ps38
Hominini - ps39
Homo_sapiens - ps40
p-value
2e-02
3e-02
4e-02
5e-02
6e-02
Log-odds
0.5
1.0
1.5
2.0
2.5
3.0
3.5
4.0

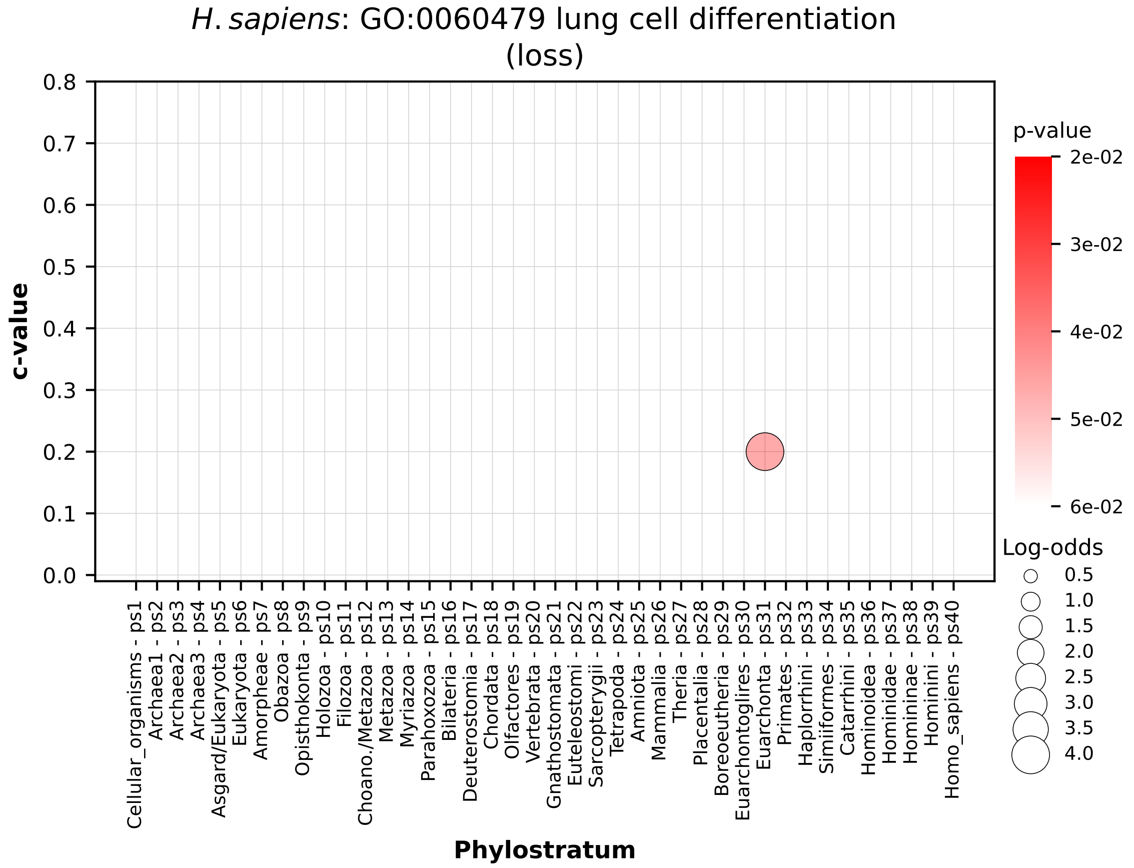

H. sapiens: GO:0060479 lung cell differentiation
(loss)
c-value
0.0
0.1
0.2
0.3
0.4
0.5
0.6
0.7
0.8
Phylostratum
Cellular_organisms - ps1
Archaea1 - ps2
Archaea2 - ps3
Archaea3 - ps4
Asgard/Eukaryota - ps5
Eukaryota - ps6
Amorpheae - ps7
Obazoa - ps8
Opisthokonta - ps9
Holozoa - ps10
Filozoa - ps11
Choano./Metazoa - ps12
Metazoa - ps13
Myriazoa - ps14
Parahoxozoa - ps15
Bilateria - ps16
Deuterostomia - ps17
Chordata - ps18
Olfactores - ps19
Vertebrata - ps20
Gnathostomata - ps21
Euteleostomi - ps22
Sarcopterygii - ps23
Tetrapoda - ps24
Amniota - ps25
Mammalia - ps26
Theria - ps27
Placentalia - ps28
Boreoeutheria - ps29
Euarchontoglires - ps30
Euarchonta - ps31
Primates - ps32
Haplorrhini - ps33
Simiiformes - ps34
Catarrhini - ps35
Hominoidea - ps36
Hominidae - ps37
Homininae - ps38
Hominini - ps39
Homo_sapiens - ps40
p-value
2e-02
3e-02
4e-02
5e-02
6e-02
Log-odds
0.5
1.0
1.5
2.0
2.5
3.0
3.5
4.0

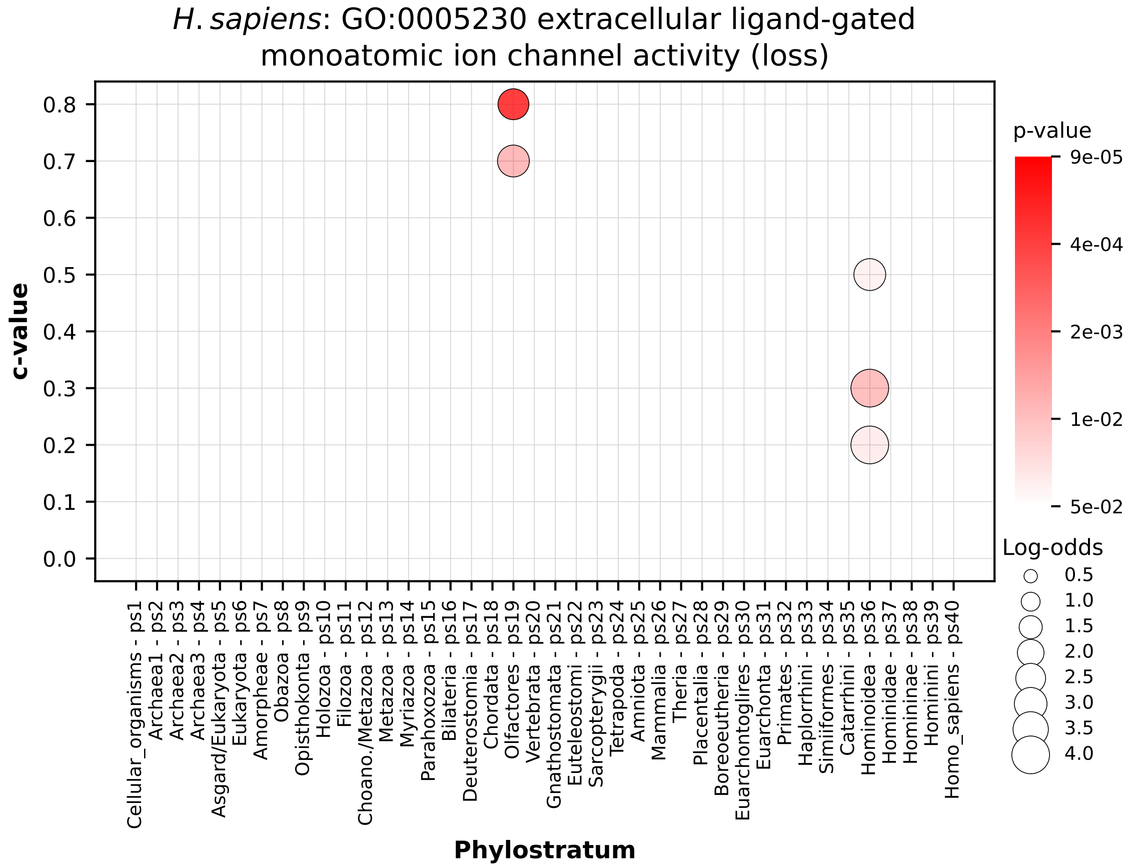
H. sapiens: GO:0005230 extracellular ligand-gated monoatomic ion channel activity (loss)
c-value
0.0
0.1
0.2
0.3
0.4
0.5
0.6
0.7
0.8
Phylostratum
Cellular_organisms - ps1
Archaea1 - ps2
Archaea2 - ps3
Archaea3 - ps4
Asgard/Eukaryota - ps5
Eukaryota - ps6
Amorpheae - ps7
Obazoa - ps8
Opisthokonta - ps9
Holozoa - ps10
Filozoa - ps11
Choano./Metazoa - ps12
Metazoa - ps13
Myriazoa - ps14
Parahoxozoa - ps15
Bilateria - ps16
Deuterostomia - ps17
Chordata - ps18
Olfactores - ps19
Vertebrata - ps20
Gnathostomata - ps21
Euteleostomi - ps22
Sarcopterygii - ps23
Tetrapoda - ps24
Amniota - ps25
Mammalia - ps26
Theria - ps27
Placentalia - ps28
Boreoeutheria - ps29
Euarchontoglires - ps30
Euarchonta - ps31
Primates - ps32
Haplorrhini - ps33
Simiiformes - ps34
Catarrhini - ps35
Hominoidea - ps36
Hominidae - ps37
Homininae - ps38
Hominini - ps39
Homo_sapiens - ps40
p-value
9e-05
4e-04
2e-03
1e-02
5e-02
Log-odds
0.5
1.0
1.5
2.0
2.5
3.0
3.5
4.0

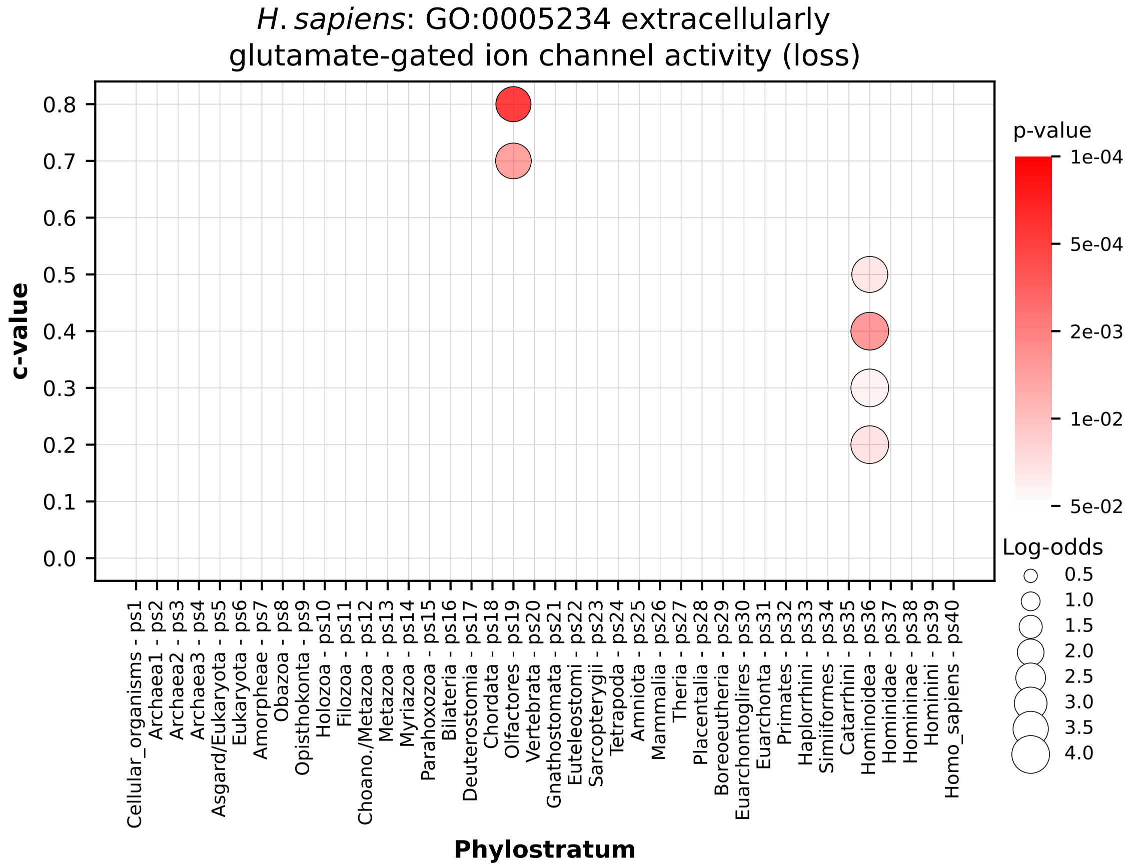

H. sapiens: GO:0005234 extracellularly glutamate-gated ion channel activity (loss)
c-value
0.8
0.7
0.6
0.5
0.4
0.3
0.2
0.1
0.0
p-value
1e-04
5e-04
2e-03
1e-02
5e-02
Log-odds
0.5
1.0
1.5
2.0
2.5
3.0
3.5
4.0
Cellular_organisms - ps1
Archaea1 - ps2
Archaea2 - ps3
Archaea3 - ps4
Asgard/Eukaryota - ps5
Eukaryota - ps6
Amorpheae - ps7
Obazoa - ps8
Opisthokonta - ps9
Holozoa - ps10
Filozoa - ps11
Choano./Metazoa - ps12
Metazoa - ps13
Myriazoa - ps14
Parahoxozoa - ps15
Bilateria - ps16
Deuterostomia - ps17
Chordata - ps18
Olfactores - ps19
Vertebrata - ps20
Gnathostomata - ps21
Euteleostomi - ps22
Sarcopterygii - ps23
Tetrapoda - ps24
Amniota - ps25
Mammalia - ps26
Theria - ps27
Placentalia - ps28
Boreoeutheria - ps29
Euarchontoglires - ps30
Euarchonta - ps31
Primates - ps32
Haplorrhini - ps33
Simiiformes - ps34
Catarrhini - ps35
Hominoidea - ps36
Hominidae - ps37
Homininae - ps38
Hominini - ps39
Homo_sapiens - ps40
Phylostratum

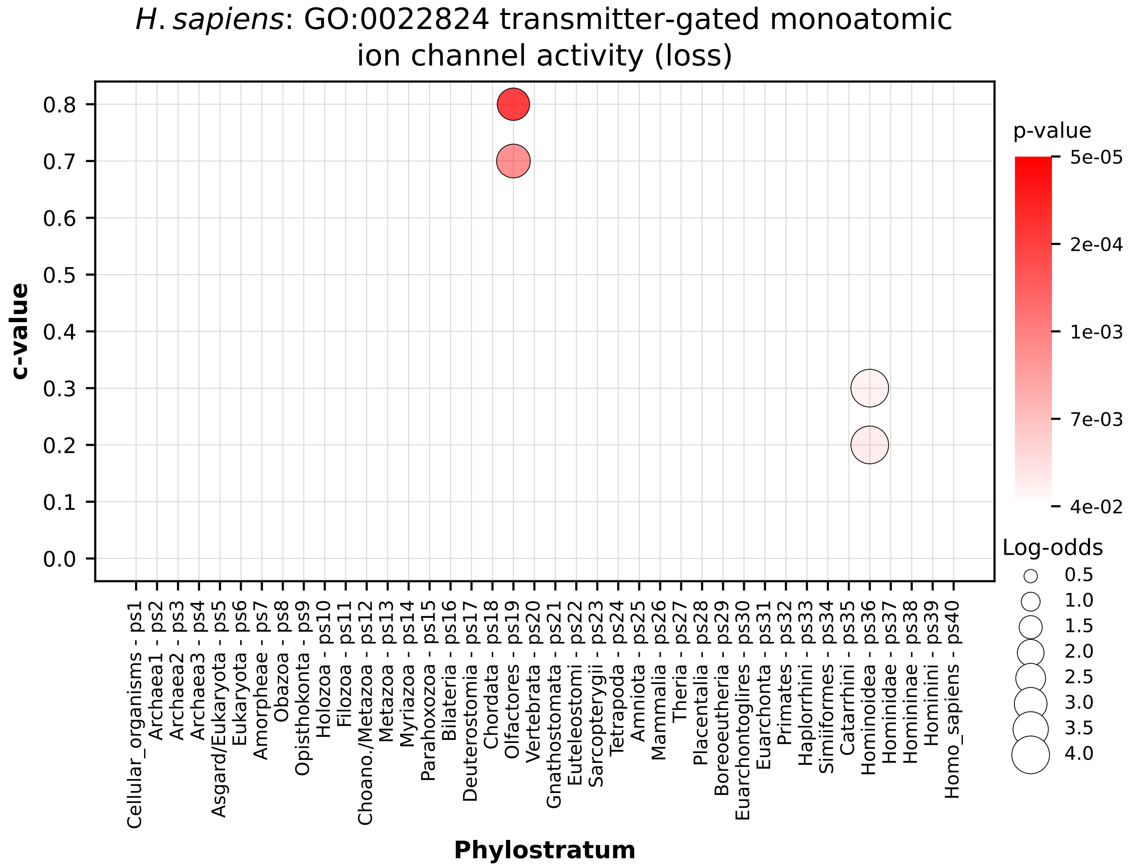

H. sapiens: GO:0022824 transmitter-gated monoatomic ion channel activity (loss)
c-value
0.8
0.7
0.6
0.5
0.4
0.3
0.2
0.1
0.0
p-value
5e-05
2e-04
1e-03
7e-03
4e-02
Log-odds
0.5
1.0
1.5
2.0
2.5
3.0
3.5
4.0
Cellular_organisms - ps1
Archaea1 - ps2
Archaea2 - ps3
Archaea3 - ps4
Asgard/Eukaryota - ps5
Eukaryota - ps6
Amorpheae - ps7
Obazoa - ps8
Opisthokonta - ps9
Holozoa - ps10
Filozoa - ps11
Choano./Metazoa - ps12
Metazoa - ps13
Myriazoa - ps14
Parahoxozoa - ps15
Bilateria - ps16
Deuterostomia - ps17
Chordata - ps18
Olfactores - ps19
Vertebrata - ps20
Gnathostomata - ps21
Euteleostomi - ps22
Sarcopterygii - ps23
Tetrapoda - ps24
Amniota - ps25
Mammalia - ps26
Theria - ps27
Placentalia - ps28
Boreoeutheria - ps29
Euarchontoglires - ps30
Euarchonta - ps31
Primates - ps32
Haplorrhini - ps33
Simiiformes - ps34
Catarrhini - ps35
Hominoidea - ps36
Hominidae - ps37
Homininae - ps38
Hominini - ps39
Homo_sapiens - ps40
Phylostratum

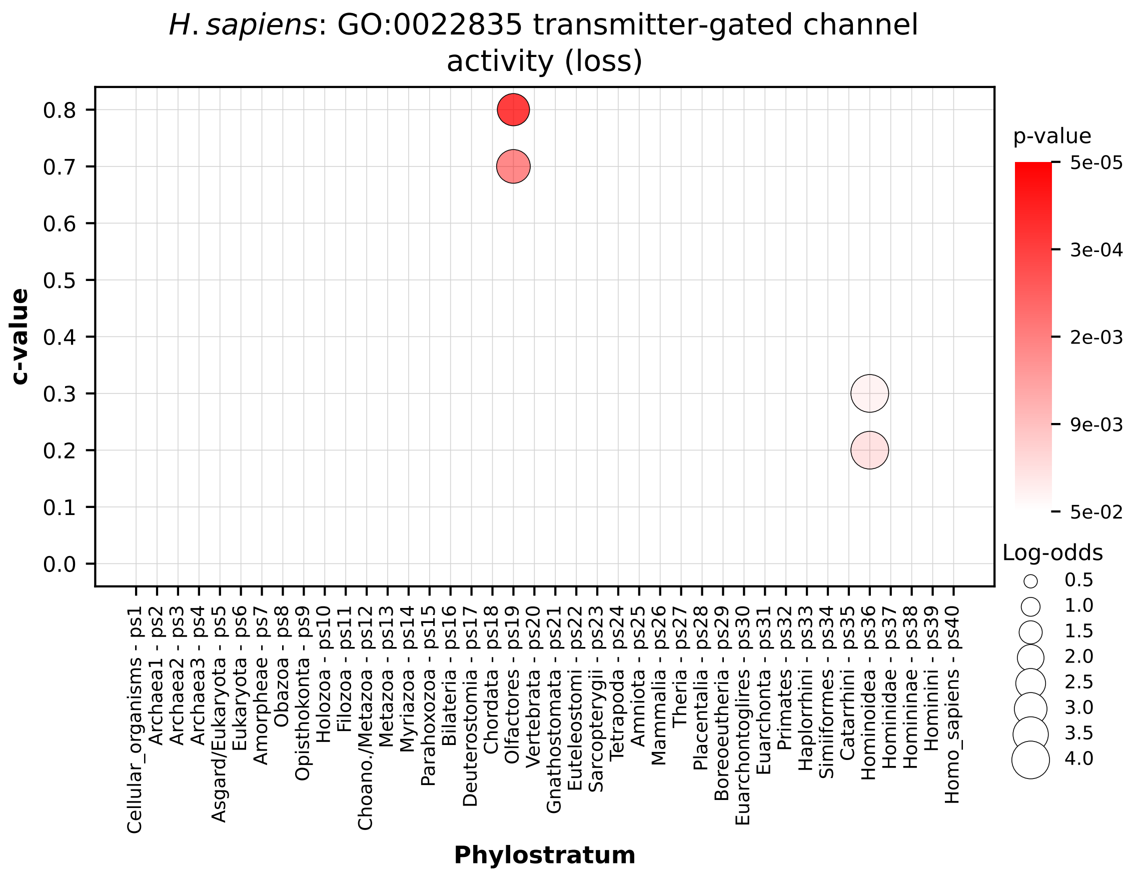

H. sapiens: GO:0022835 transmitter-gated channel activity (loss)
c-value
0.0
0.1
0.2
0.3
0.4
0.5
0.6
0.7
0.8
Phylostratum
Cellular_organisms - ps1
Archaea1 - ps2
Archaea2 - ps3
Archaea3 - ps4
Asgard/Eukaryota - ps5
Eukaryota - ps6
Amorpheae - ps7
Obazoa - ps8
Opisthokonta - ps9
Holozoa - ps10
Filozoa - ps11
Choano./Metazoa - ps12
Metazoa - ps13
Myriazoa - ps14
Parahoxozoa - ps15
Bilateria - ps16
Deuterostomia - ps17
Chordata - ps18
Olfactores - ps19
Vertebrata - ps20
Gnathostomata - ps21
Euteleostomi - ps22
Sarcopterygii - ps23
Tetrapoda - ps24
Amniota - ps25
Mammalia - ps26
Theria - ps27
Placentalia - ps28
Boreoeutheria - ps29
Euarchontoglires - ps30
Euarchonta - ps31
Primates - ps32
Haplorrhini - ps33
Simiiformes - ps34
Catarrhini - ps35
Hominoidea - ps36
Hominidae - ps37
Homininae - ps38
Hominini - ps39
Homo_sapiens - ps40
p-value
5e-05
3e-04
2e-03
9e-03
5e-02
Log-odds
0.5
1.0
1.5
2.0
2.5
3.0
3.5
4.0

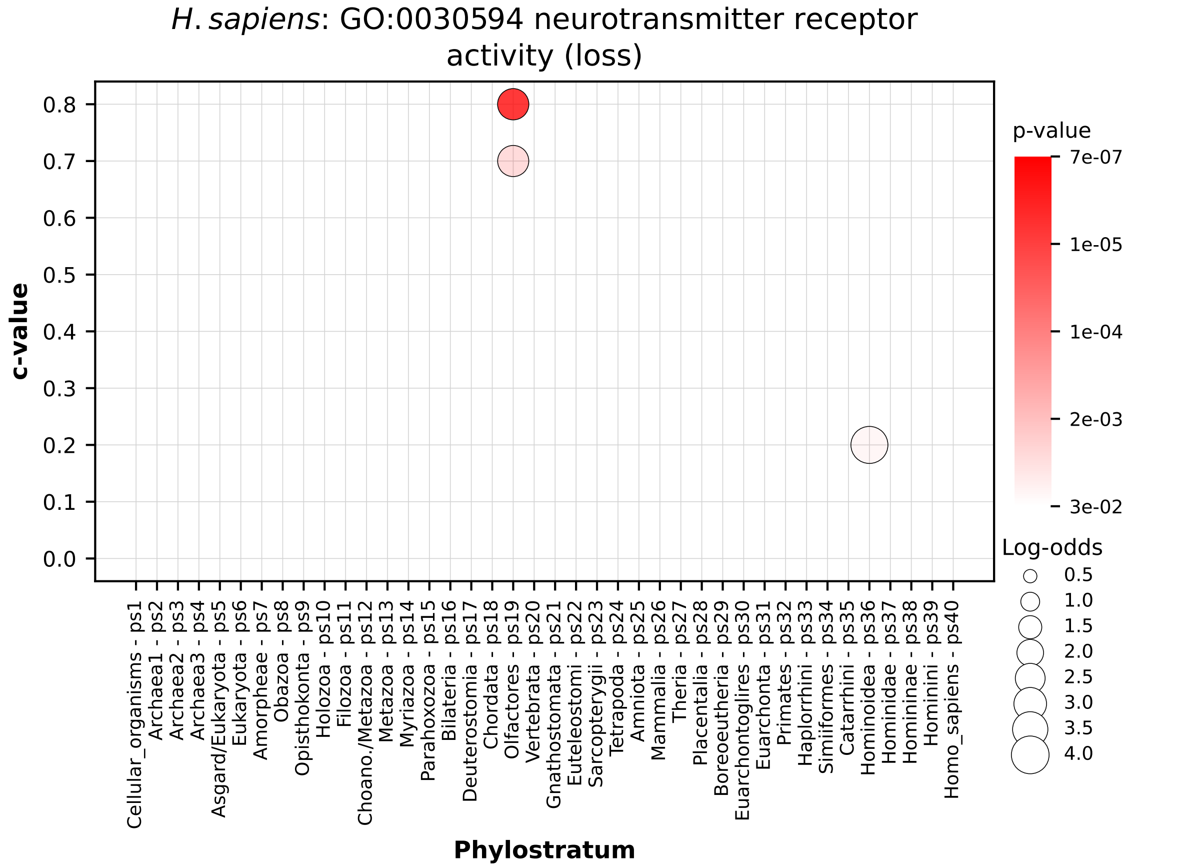

H. sapiens: GO:0030594 neurotransmitter receptor activity (loss)
c-value
0.8
0.7
0.6
0.5
0.4
0.3
0.2
0.1
0.0
p-value
7e-07
1e-05
1e-04
2e-03
3e-02
Log-odds
0.5
1.0
1.5
2.0
2.5
3.0
3.5
4.0
Cellular_organisms - ps1
Archaea1 - ps2
Archaea2 - ps3
Archaea3 - ps4
Asgard/Eukaryota - ps5
Eukaryota - ps6
Amorpheae - ps7
Obazoa - ps8
Opisthokonta - ps9
Holozoa - ps10
Filozoa - ps11
Choano./Metazoa - ps12
Metazoa - ps13
Myriazoa - ps14
Parahoxozoa - ps15
Bilateria - ps16
Deuterostomia - ps17
Chordata - ps18
Olfactores - ps19
Vertebrata - ps20
Gnathostomata - ps21
Euteleostomi - ps22
Sarcopterygii - ps23
Tetrapoda - ps24
Amniota - ps25
Mammalia - ps26
Theria - ps27
Placentalia - ps28
Boreoeutheria - ps29
Euarchontoglires - ps30
Euarchonta - ps31
Primates - ps32
Haplorrhini - ps33
Simiiformes - ps34
Catarrhini - ps35
Hominoidea - ps36
Hominidae - ps37
Homininae - ps38
Hominini - ps39
Homo_sapiens - ps40
Phylostratum

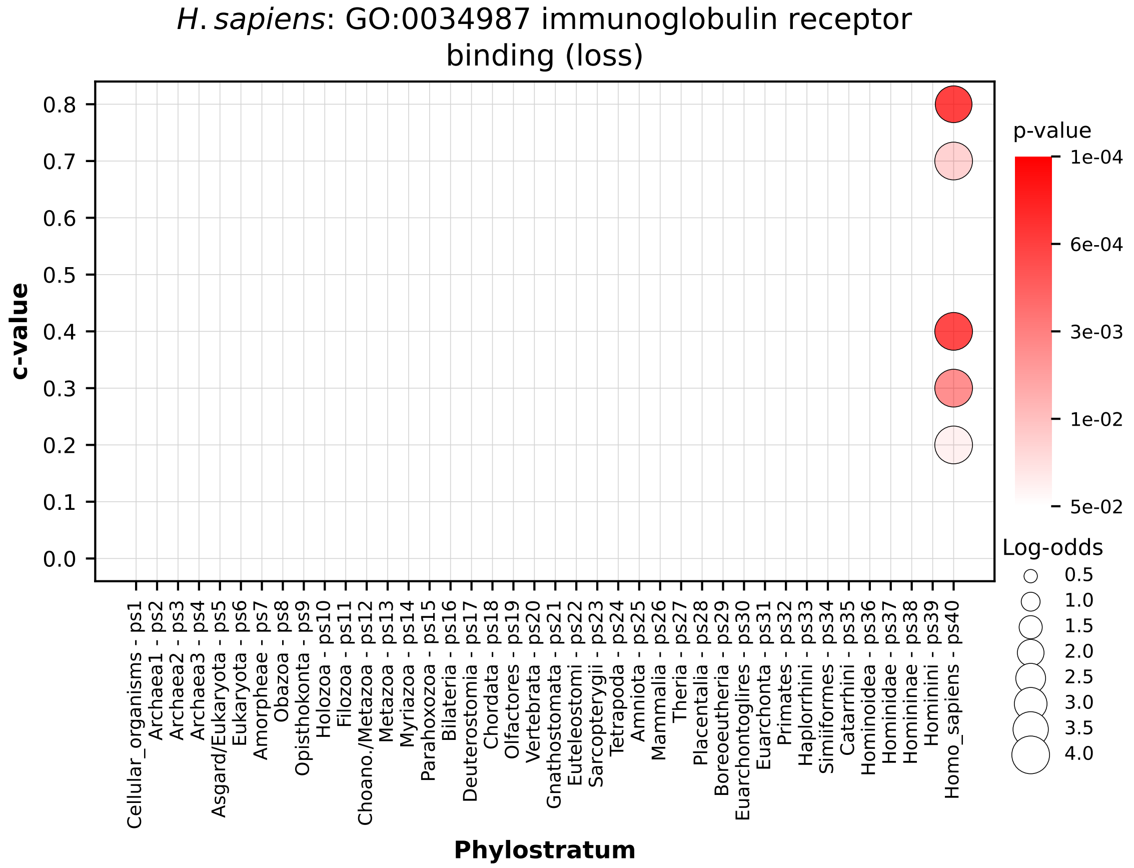

H. sapiens: GO:0034987 immunoglobulin receptor binding (loss)
c-value
0.0
0.1
0.2
0.3
0.4
0.5
0.6
0.7
0.8
Phylostratum
Cellular_organisms - ps1
Archaea1 - ps2
Archaea2 - ps3
Archaea3 - ps4
Asgard/Eukaryota - ps5
Eukaryota - ps6
Amorpheae - ps7
Obazoa - ps8
Opisthokonta - ps9
Holozoa - ps10
Filozoa - ps11
Choano./Metazoa - ps12
Metazoa - ps13
Myriazoa - ps14
Parahoxozoa - ps15
Bilateria - ps16
Deuterostomia - ps17
Chordata - ps18
Olfactores - ps19
Vertebrata - ps20
Gnathostomata - ps21
Euteleostomi - ps22
Sarcopterygii - ps23
Tetrapoda - ps24
Amniota - ps25
Mammalia - ps26
Theria - ps27
Placentalia - ps28
Boreoeutheria - ps29
Euarchontoglires - ps30
Euarchonta - ps31
Primates - ps32
Haplorrhini - ps33
Simiiformes - ps34
Catarrhini - ps35
Hominoidea - ps36
Hominidae - ps37
Homininae - ps38
Hominini - ps39
Homo_sapiens - ps40
p-value
1e-04
6e-04
3e-03
1e-02
5e-02
Log-odds
0.5
1.0
1.5
2.0
2.5
3.0
3.5
4.0

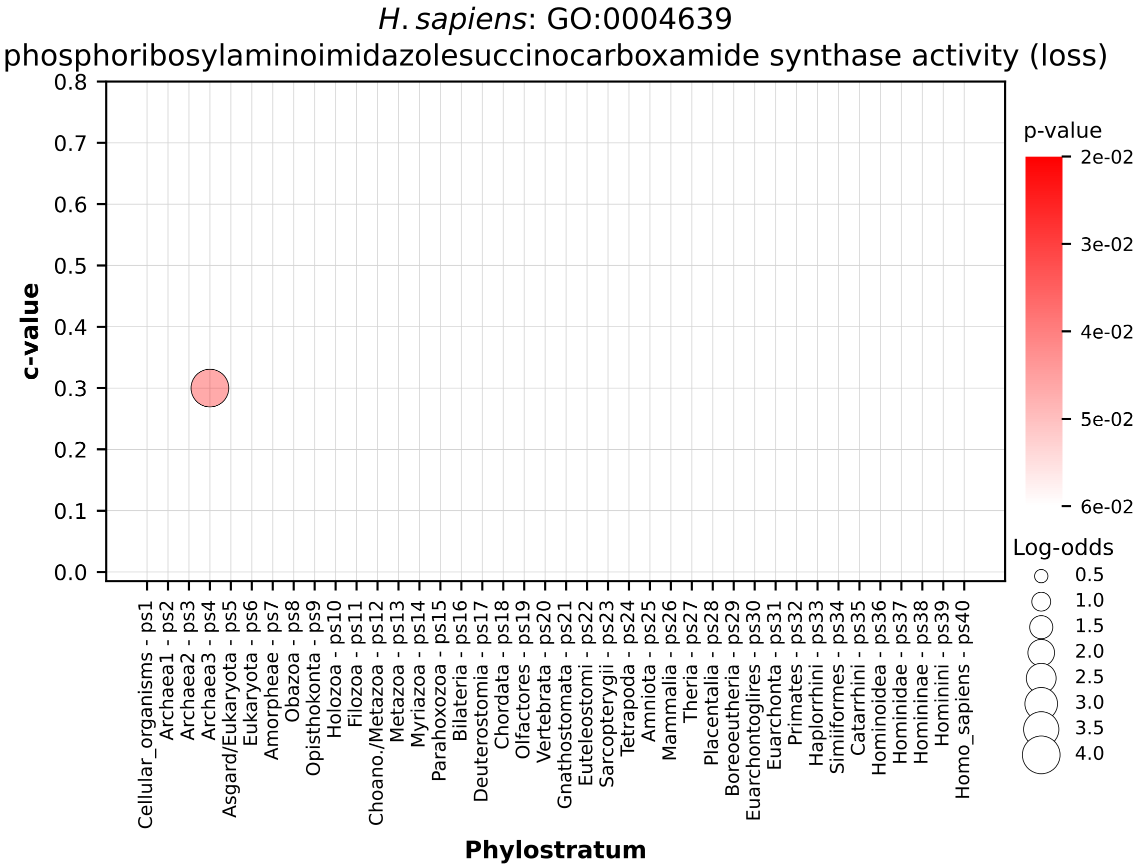

H. sapiens: GO:0004639
phosphoribosylaminoimidazolesuccinocarboxamide synthase activity (loss)
c-value
0.8
0.7
0.6
0.5
0.4
0.3
0.2
0.1
0.0
Phylostratum
Cellular_organisms - ps1
Archaea1 - ps2
Archaea2 - ps3
Archaea3 - ps4
Asgard/Eukaryota - ps5
Eukaryota - ps6
Amorpheae - ps7
Obazoa - ps8
Opisthokonta - ps9
Holozoa - ps10
Filozoa - ps11
Choano./Metazoa - ps12
Metazoa - ps13
Myriazoa - ps14
Parahoxozoa - ps15
Bilateria - ps16
Deuterostomia - ps17
Chordata - ps18
Olfactores - ps19
Vertebrata - ps20
Gnathostomata - ps21
Euteleostomi - ps22
Sarcopterygii - ps23
Tetrapoda - ps24
Amniota - ps25
Mammalia - ps26
Theria - ps27
Placentalia - ps28
Boreoeutheria - ps29
Euarchontoglires - ps30
Euarchonta - ps31
Primates - ps32
Haplorrhini - ps33
Simiiformes - ps34
Catarrhini - ps35
Hominoidea - ps36
Hominidae - ps37
Homininae - ps38
Hominini - ps39
Homo_sapiens - ps40
p-value
2e-02
3e-02
4e-02
5e-02
6e-02
Log-odds
0.5
1.0
1.5
2.0
2.5
3.0
3.5
4.0

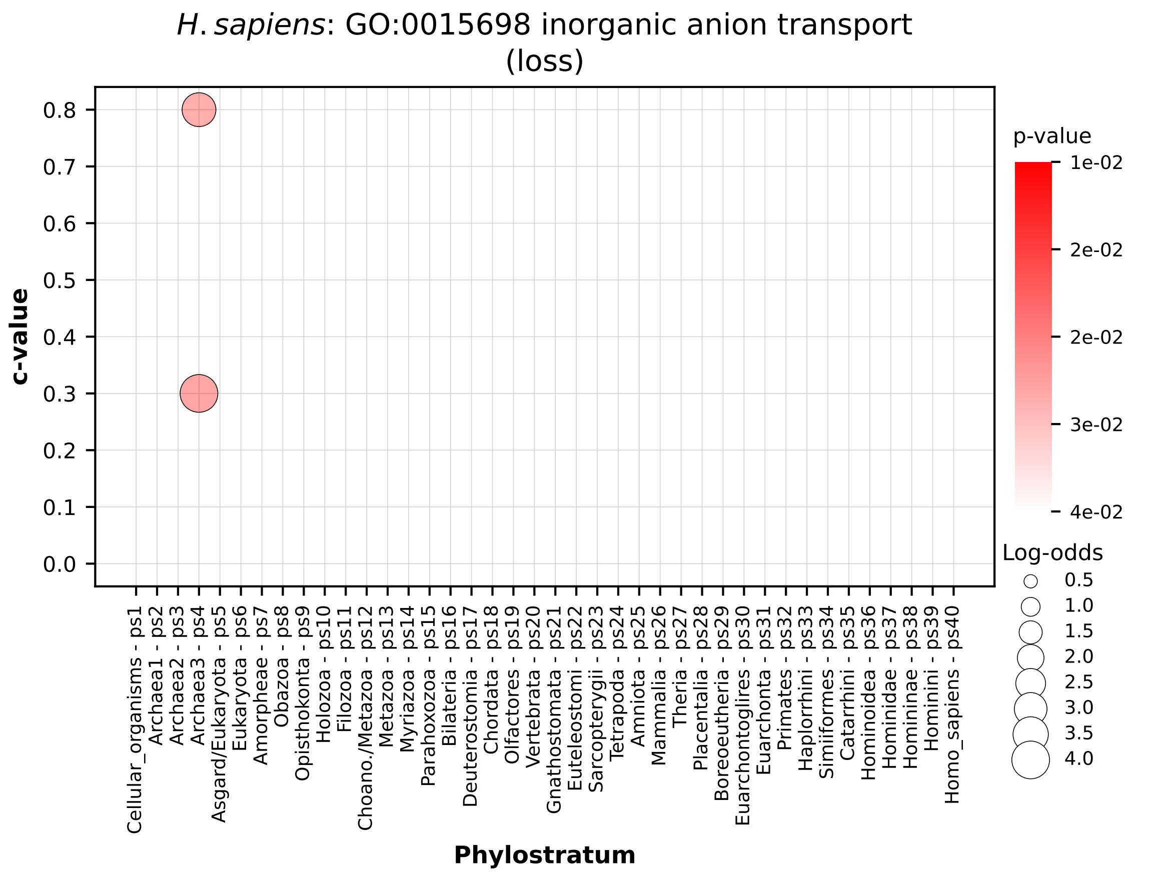

H. sapiens: GO:0015698 inorganic anion transport (loss)
c-value
0.8
0.7
0.6
0.5
0.4
0.3
0.2
0.1
0.0
Phylostratum
Cellular_organisms - ps1
Archaea1 - ps2
Archaea2 - ps3
Archaea3 - ps4
Asgard/Eukaryota - ps5
Eukaryota - ps6
Amorpheae - ps7
Obazoa - ps8
Opisthokonta - ps9
Holozoa - ps10
Filozoa - ps11
Choano./Metazoa - ps12
Metazoa - ps13
Myriazoa - ps14
Parahoxozoa - ps15
Bilateria - ps16
Deuterostomia - ps17
Chordata - ps18
Olfactores - ps19
Vertebrata - ps20
Gnathostomata - ps21
Euteleostomi - ps22
Sarcopterygii - ps23
Tetrapoda - ps24
Amniota - ps25
Mammalia - ps26
Theria - ps27
Placentalia - ps28
Boreoeutheria - ps29
Euarchontoglires - ps30
Euarchonta - ps31
Primates - ps32
Haplorrhini - ps33
Simiiformes - ps34
Catarrhini - ps35
Hominoidea - ps36
Hominidae - ps37
Homininae - ps38
Hominini - ps39
Homo_sapiens - ps40
p-value
1e-02
2e-02
2e-02
3e-02
4e-02
Log-odds
0.5
1.0
1.5
2.0
2.5
3.0
3.5
4.0

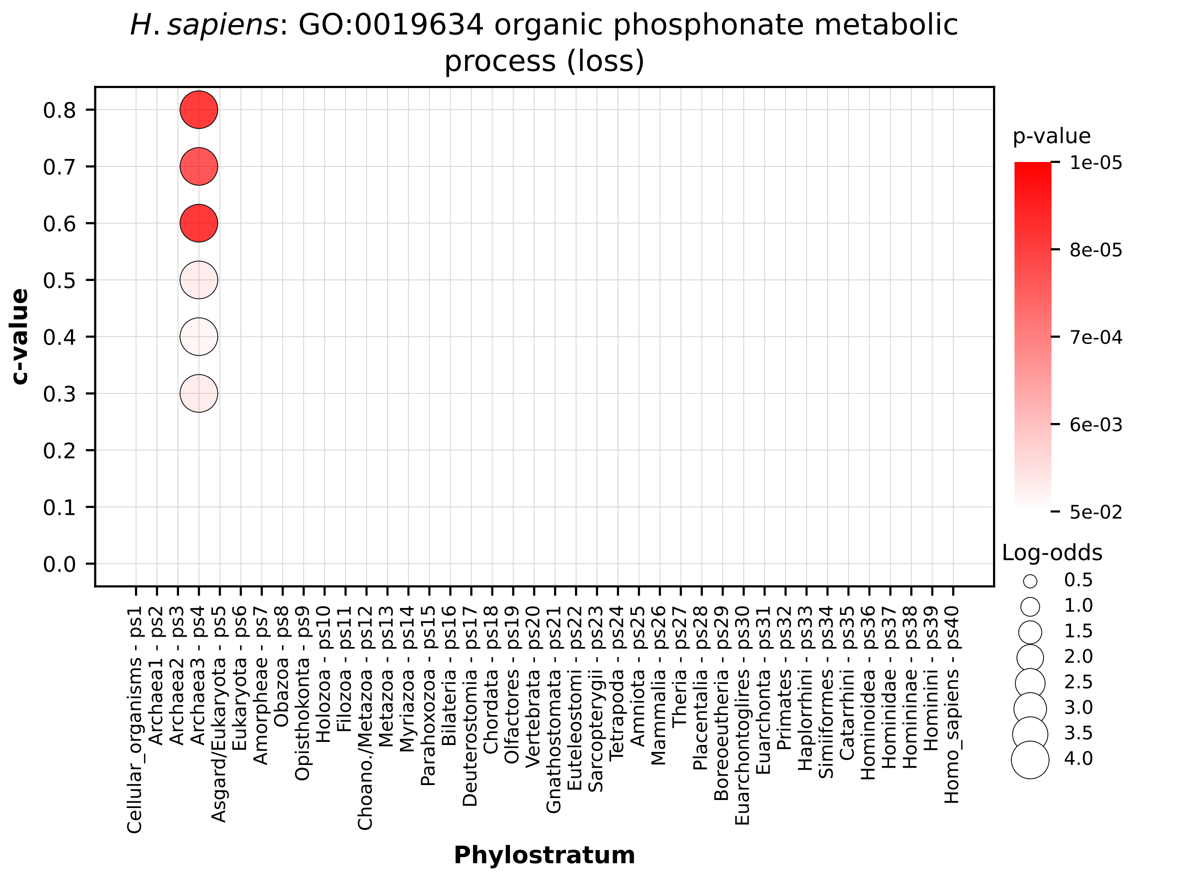

H. sapiens: GO:0019634 organic phosphonate metabolic process (loss)
c-value
0.0
0.1
0.2
0.3
0.4
0.5
0.6
0.7
0.8
Phylostratum
Cellular_organisms - ps1
Archaea1 - ps2
Archaea2 - ps3
Archaea3 - ps4
Asgard/Eukaryota - ps5
Eukaryota - ps6
Amorpheae - ps7
Obazoa - ps8
Opisthokonta - ps9
Holozoa - ps10
Filozoa - ps11
Choano./Metazoa - ps12
Metazoa - ps13
Myriazoa - ps14
Parahoxozoa - ps15
Bilateria - ps16
Deuterostomia - ps17
Chordata - ps18
Olfactores - ps19
Vertebrata - ps20
Gnathostomata - ps21
Euteleostomi - ps22
Sarcopterygii - ps23
Tetrapoda - ps24
Amniota - ps25
Mammalia - ps26
Theria - ps27
Placentalia - ps28
Boreoeutheria - ps29
Euarchontoglires - ps30
Euarchonta - ps31
Primates - ps32
Haplorrhini - ps33
Simiiformes - ps34
Catarrhini - ps35
Hominoidea - ps36
Hominidae - ps37
Homininae - ps38
Hominini - ps39
Homo_sapiens - ps40
p-value
1e-05
8e-05
7e-04
6e-03
5e-02
Log-odds
0.5
1.0
1.5
2.0
2.5
3.0
3.5
4.0

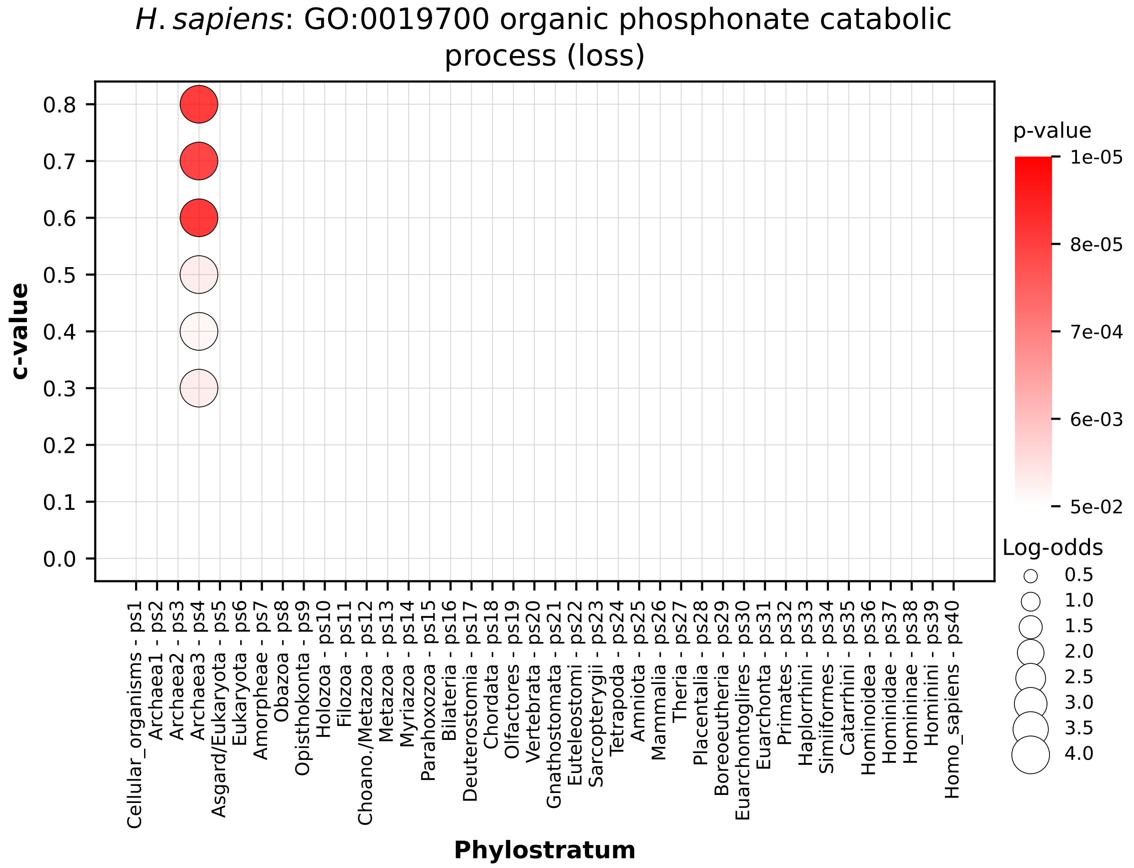

H. sapiens: GO:0019700 organic phosphonate catabolic process (loss)
c-value
0.8
0.7
0.6
0.5
0.4
0.3
0.2
0.1
0.0
Phylostratum
Cellular_organisms - ps1
Archaea1 - ps2
Archaea2 - ps3
Archaea3 - ps4
Asgard/Eukaryota - ps5
Eukaryota - ps6
Amorpheae - ps7
Obazoa - ps8
Opisthokonta - ps9
Holozoa - ps10
Filozoa - ps11
Choano./Metazoa - ps12
Metazoa - ps13
Myriazoa - ps14
Parahoxozoa - ps15
Bilateria - ps16
Deuterostomia - ps17
Chordata - ps18
Olfactores - ps19
Vertebrata - ps20
Gnathostomata - ps21
Euteleostomi - ps22
Sarcopterygii - ps23
Tetrapoda - ps24
Amniota - ps25
Mammalia - ps26
Theria - ps27
Placentalia - ps28
Boreoeutheria - ps29
Euarchontoglires - ps30
Euarchonta - ps31
Primates - ps32
Haplorrhini - ps33
Simiiformes - ps34
Catarrhini - ps35
Hominoidea - ps36
Hominidae - ps37
Homininae - ps38
Hominini - ps39
Homo_sapiens - ps40
p-value
1e-05
8e-05
7e-04
6e-03
5e-02
Log-odds
0.5
1.0
1.5
2.0
2.5
3.0
3.5
4.0

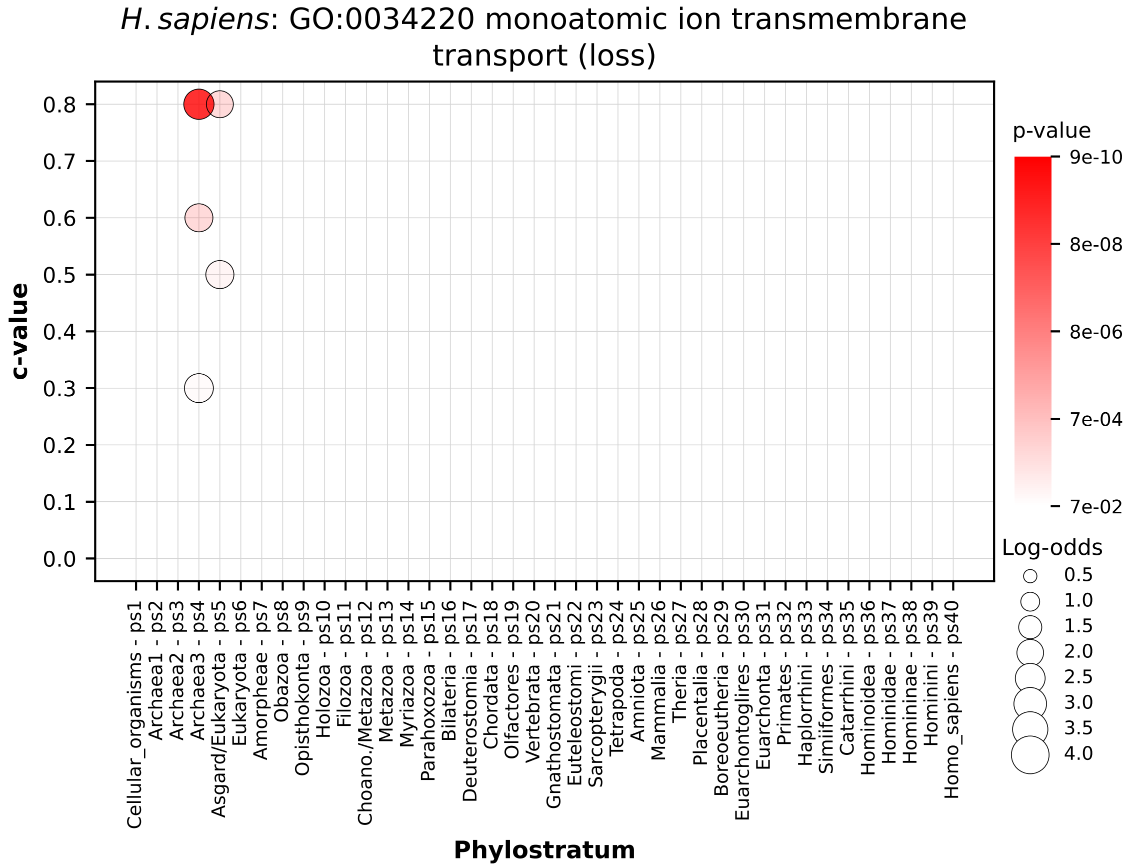

H. sapiens: GO:0034220 monoatomic ion transmembrane transport (loss)
c-value
0.8
0.7
0.6
0.5
0.4
0.3
0.2
0.1
0.0
Phylostratum
Cellular_organisms - ps1
Archaea1 - ps2
Archaea2 - ps3
Archaea3 - ps4
Asgard/Eukaryota - ps5
Eukaryota - ps6
Amorpheae - ps7
Obazoa - ps8
Opisthokonta - ps9
Holozoa - ps10
Filozoa - ps11
Choano./Metazoa - ps12
Metazoa - ps13
Myriazoa - ps14
Parahoxozoa - ps15
Bilateria - ps16
Deuterostomia - ps17
Chordata - ps18
Olfactores - ps19
Vertebrata - ps20
Gnathostomata - ps21
Euteleostomi - ps22
Sarcopterygii - ps23
Tetrapoda - ps24
Amniota - ps25
Mammalia - ps26
Theria - ps27
Placentalia - ps28
Boreoeutheria - ps29
Euarchontoglires - ps30
Euarchonta - ps31
Primates - ps32
Haplorrhini - ps33
Simiiformes - ps34
Catarrhini - ps35
Hominoidea - ps36
Hominidae - ps37
Homininae - ps38
Hominini - ps39
Homo_sapiens - ps40
p-value
9e-10
8e-08
8e-06
7e-04
7e-02
Log-odds
0.5
1.0
1.5
2.0
2.5
3.0
3.5
4.0

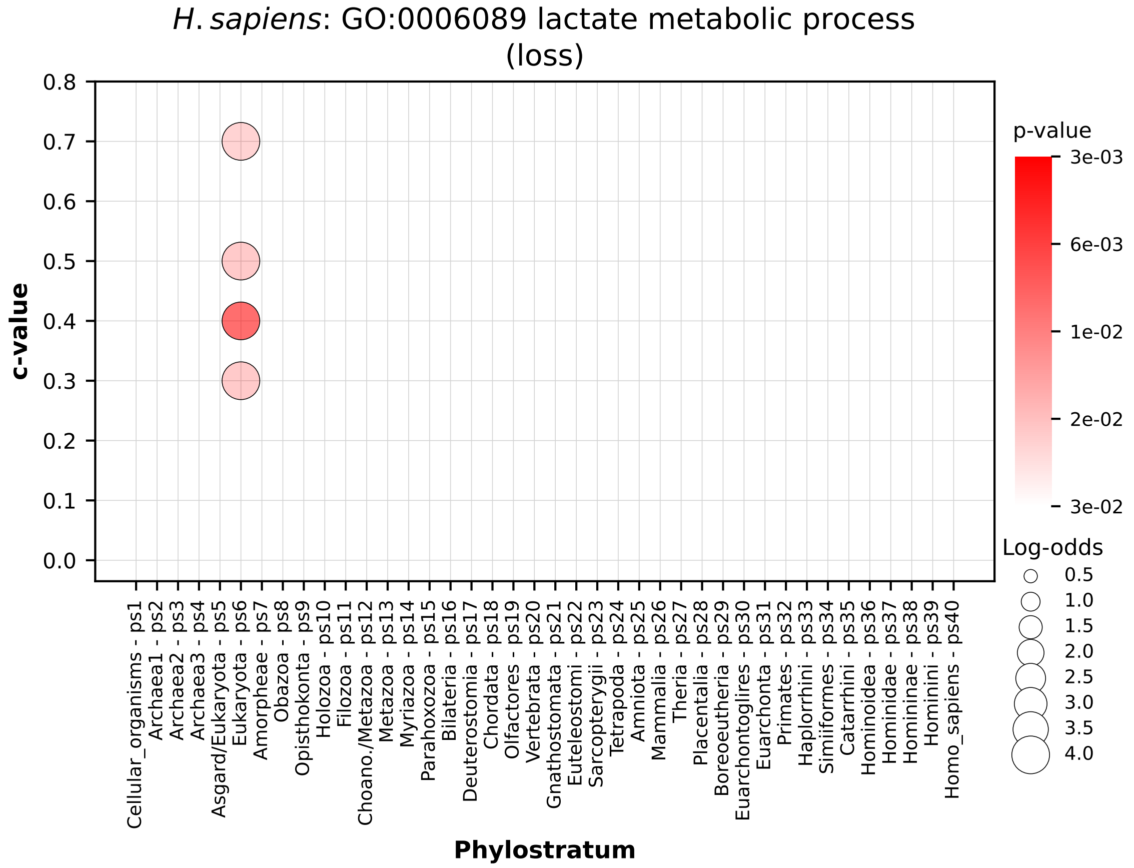

H. sapiens: GO:0006089 lactate metabolic process
(loss)
c-value
0.8
0.7
0.6
0.5
0.4
0.3
0.2
0.1
0.0
p-value
3e-03
6e-03
1e-02
2e-02
3e-02
Log-odds
0.5
1.0
1.5
2.0
2.5
3.0
3.5
4.0
Cellular_organisms - ps1
Archaea1 - ps2
Archaea2 - ps3
Archaea3 - ps4
Asgard/Eukaryota - ps5
Eukaryota - ps6
Amorpheae - ps7
Obazoa - ps8
Opisthokonta - ps9
Holozoa - ps10
Filozoa - ps11
Choano./Metazoa - ps12
Metazoa - ps13
Myriazoa - ps14
Parahoxozoa - ps15
Bilateria - ps16
Deuterostomia - ps17
Chordata - ps18
Olfactores - ps19
Vertebrata - ps20
Gnathostomata - ps21
Euteleostomi - ps22
Sarcopterygii - ps23
Tetrapoda - ps24
Amniota - ps25
Mammalia - ps26
Theria - ps27
Placentalia - ps28
Boreoeutheria - ps29
Euarchontoglires - ps30
Euarchonta - ps31
Primates - ps32
Haplorrhini - ps33
Simiiformes - ps34
Catarrhini - ps35
Hominoidea - ps36
Hominidae - ps37
Homininae - ps38
Hominini - ps39
Homo_sapiens - ps40
Phylostratum

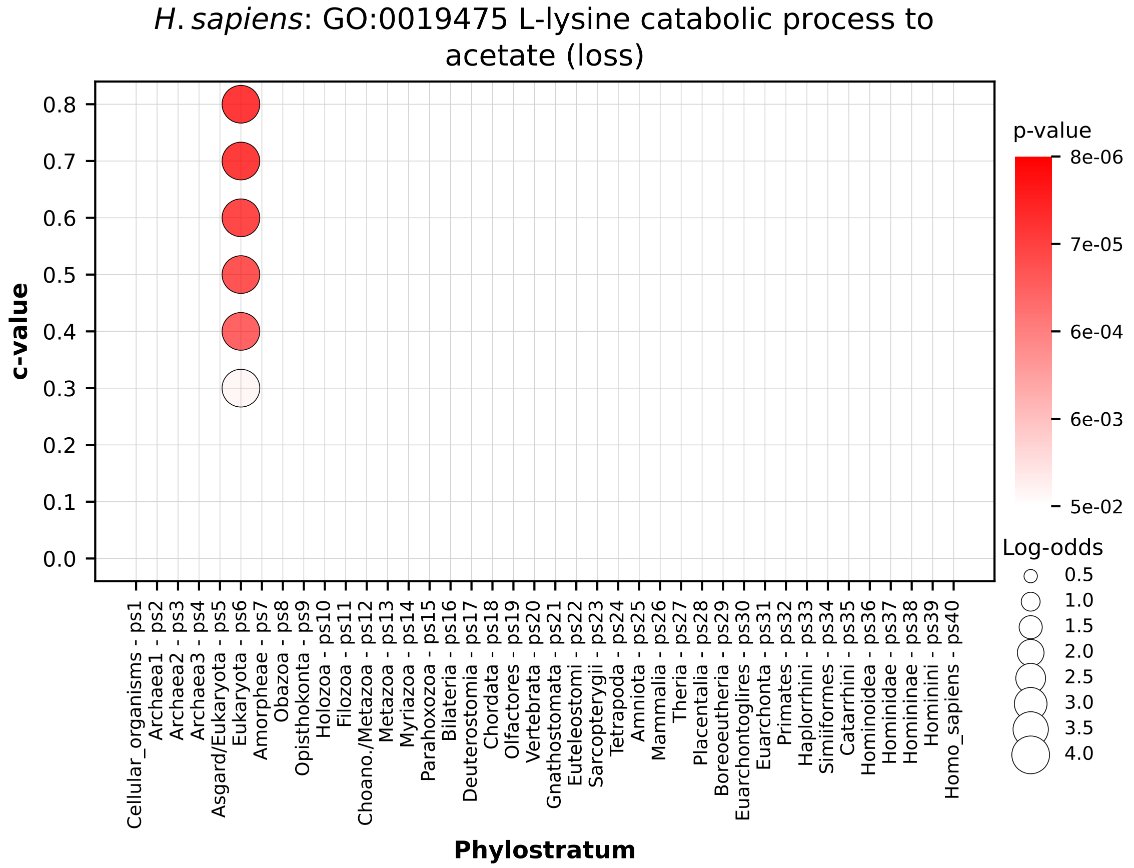

H. sapiens: GO:0019475 L-lysine catabolic process to acetate (loss)
c-value
0.0
0.1
0.2
0.3
0.4
0.5
0.6
0.7
0.8
Phylostratum
Cellular_organisms - ps1
Archaea1 - ps2
Archaea2 - ps3
Archaea3 - ps4
Asgard/Eukaryota - ps5
Eukaryota - ps6
Amorpheae - ps7
Obazoa - ps8
Opisthokonta - ps9
Holozoa - ps10
Filozoa - ps11
Choano./Metazoa - ps12
Metazoa - ps13
Myriazoa - ps14
Parahoxozoa - ps15
Bilateria - ps16
Deuterostomia - ps17
Chordata - ps18
Olfactores - ps19
Vertebrata - ps20
Gnathostomata - ps21
Euteleostomi - ps22
Sarcopterygii - ps23
Tetrapoda - ps24
Amniota - ps25
Mammalia - ps26
Theria - ps27
Placentalia - ps28
Boreoeutheria - ps29
Euarchontoglires - ps30
Euarchonta - ps31
Primates - ps32
Haplorrhini - ps33
Simiiformes - ps34
Catarrhini - ps35
Hominoidea - ps36
Hominidae - ps37
Homininae - ps38
Hominini - ps39
Homo_sapiens - ps40
p-value
8e-06
7e-05
6e-04
6e-03
5e-02
Log-odds
0.5
1.0
1.5
2.0
2.5
3.0
3.5
4.0

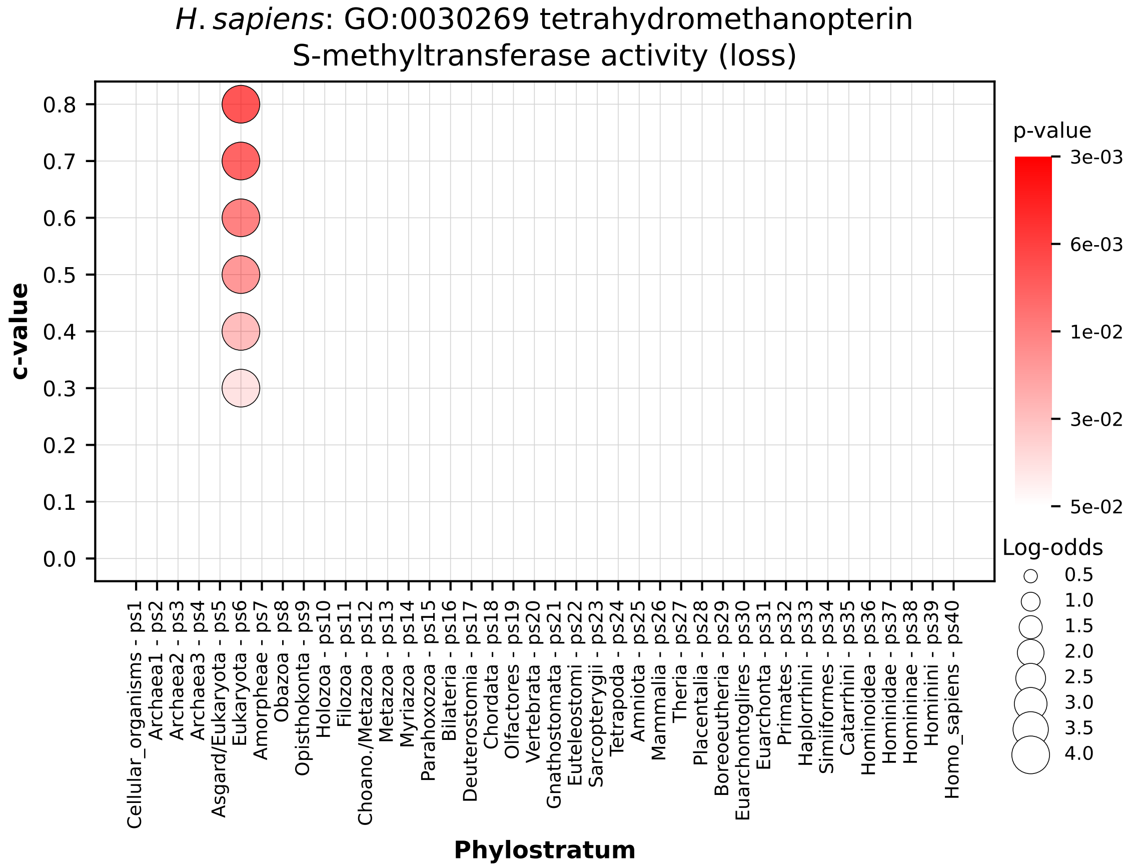

H. sapiens: GO:0030269 tetrahydromethanopterin S-methyltransferase activity (loss)
c-value
0.8
0.7
0.6
0.5
0.4
0.3
0.2
0.1
0.0
p-value
3e-03
6e-03
1e-02
3e-02
5e-02
Log-odds
0.5
1.0
1.5
2.0
2.5
3.0
3.5
4.0
Cellular_organisms - ps1
Archaea1 - ps2
Archaea2 - ps3
Archaea3 - ps4
Asgard/Eukaryota - ps5
Eukaryota - ps6
Amorpheae - ps7
Obazoa - ps8
Opisthokonta - ps9
Holozoa - ps10
Filozoa - ps11
Choano./Metazoa - ps12
Metazoa - ps13
Myriazoa - ps14
Parahoxozoa - ps15
Bilateria - ps16
Deuterostomia - ps17
Chordata - ps18
Olfactores - ps19
Vertebrata - ps20
Gnathostomata - ps21
Euteleostomi - ps22
Sarcopterygii - ps23
Tetrapoda - ps24
Amniota - ps25
Mammalia - ps26
Theria - ps27
Placentalia - ps28
Boreoeutheria - ps29
Euarchontoglires - ps30
Euarchonta - ps31
Primates - ps32
Haplorrhini - ps33
Simiiformes - ps34
Catarrhini - ps35
Hominoidea - ps36
Hominidae - ps37
Homininae - ps38
Hominini - ps39
Homo_sapiens - ps40
Phylostratum

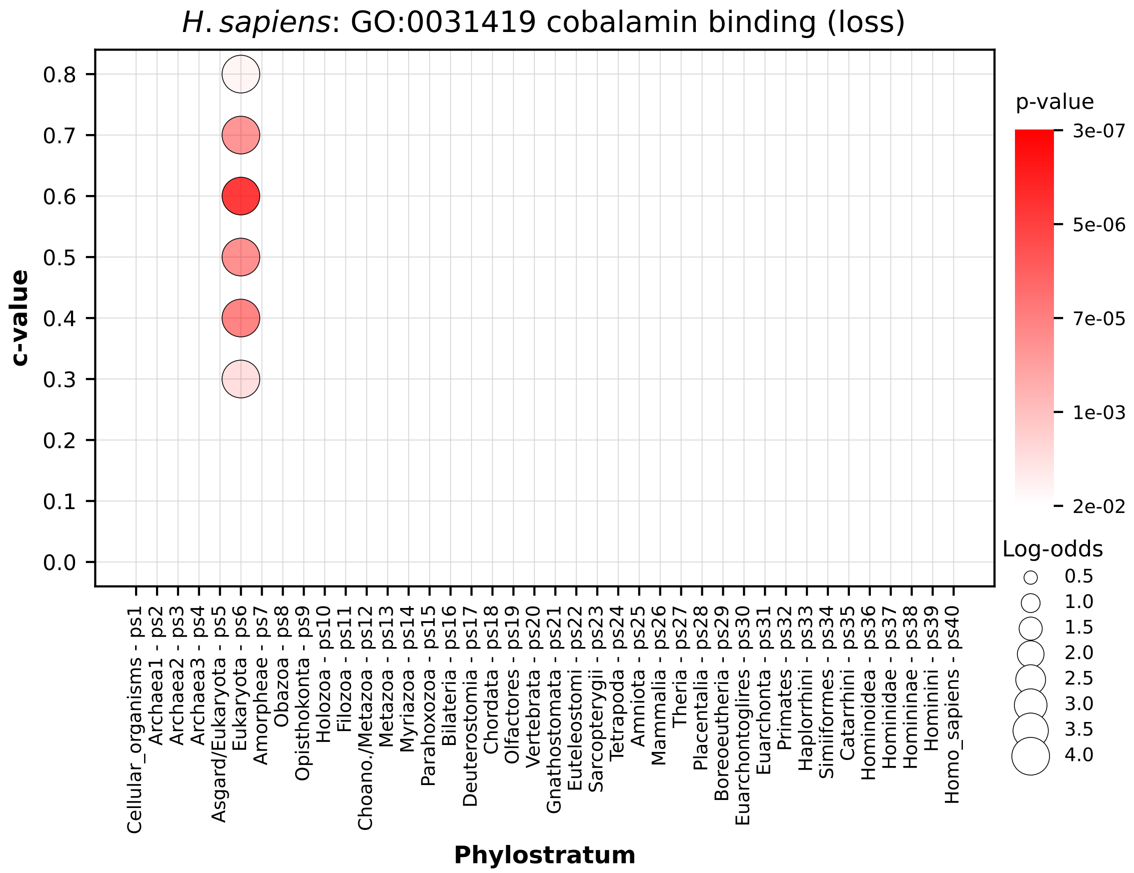

H. sapiens: GO:0031419 cobalamin binding (loss)
c-value
0.0
0.1
0.2
0.3
0.4
0.5
0.6
0.7
0.8
Phylostratum
Cellular_organisms - ps1
Archaea1 - ps2
Archaea2 - ps3
Archaea3 - ps4
Asgard/Eukaryota - ps5
Eukaryota - ps6
Amorpheae - ps7
Obazoa - ps8
Opisthokonta - ps9
Holozoa - ps10
Filozoa - ps11
Choano./Metazoa - ps12
Metazoa - ps13
Myriazoa - ps14
Parahoxozoa - ps15
Bilateria - ps16
Deuterostomia - ps17
Chordata - ps18
Olfactores - ps19
Vertebrata - ps20
Gnathostomata - ps21
Euteleostomi - ps22
Sarcopterygii - ps23
Tetrapoda - ps24
Amniota - ps25
Mammalia - ps26
Theria - ps27
Placentalia - ps28
Boreoeutheria - ps29
Euarchontoglires - ps30
Euarchonta - ps31
Primates - ps32
Haplorrhini - ps33
Simiiformes - ps34
Catarrhini - ps35
Hominoidea - ps36
Hominidae - ps37
Homininae - ps38
Hominini - ps39
Homo_sapiens - ps40
p-value
3e-07
5e-06
7e-05
1e-03
2e-02
Log-odds
0.5
1.0
1.5
2.0
2.5
3.0
3.5
4.0

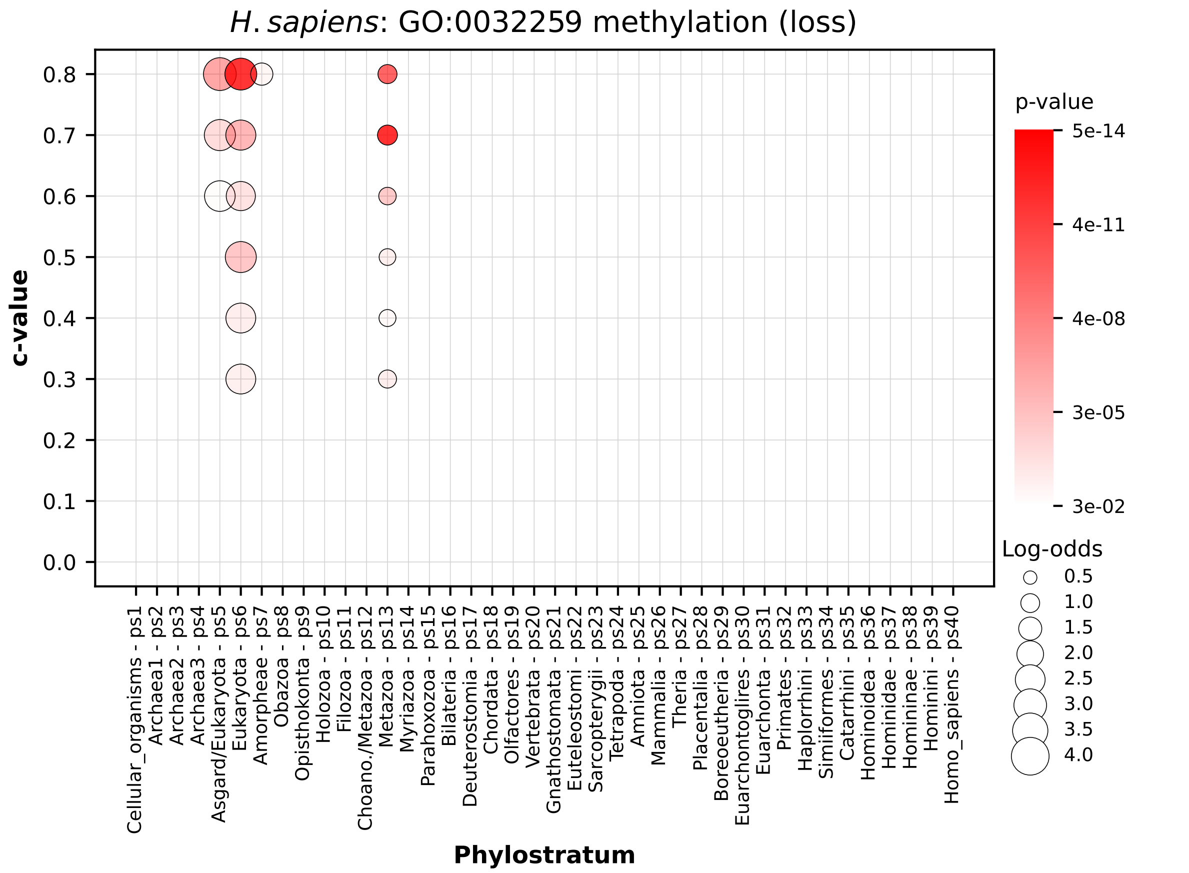

H. sapiens: GO:0032259 methylation (loss)
c-value
0.8
0.7
0.6
0.5
0.4
0.3
0.2
0.1
0.0
p-value
5e-14
4e-11
4e-08
3e-05
3e-02
Log-odds
0.5
1.0
1.5
2.0
2.5
3.0
3.5
4.0
Cellular_organisms - ps1
Archaea1 - ps2
Archaea2 - ps3
Archaea3 - ps4
Asgard/Eukaryota - ps5
Eukaryota - ps6
Amorpheae - ps7
Obazoa - ps8
Opisthokonta - ps9
Holozoa - ps10
Filozoa - ps11
Choano./Metazoa - ps12
Metazoa - ps13
Myriazoa - ps14
Parahoxozoa - ps15
Bilateria - ps16
Deuterostomia - ps17
Chordata - ps18
Olfactores - ps19
Vertebrata - ps20
Gnathostomata - ps21
Euteleostomi - ps22
Sarcopterygii - ps23
Tetrapoda - ps24
Amniota - ps25
Mammalia - ps26
Theria - ps27
Placentalia - ps28
Boreoeutheria - ps29
Euarchontoglires - ps30
Euarchonta - ps31
Primates - ps32
Haplorrhini - ps33
Simiiformes - ps34
Catarrhini - ps35
Hominoidea - ps36
Hominidae - ps37
Homininae - ps38
Hominini - ps39
Homo_sapiens - ps40
Phylostratum

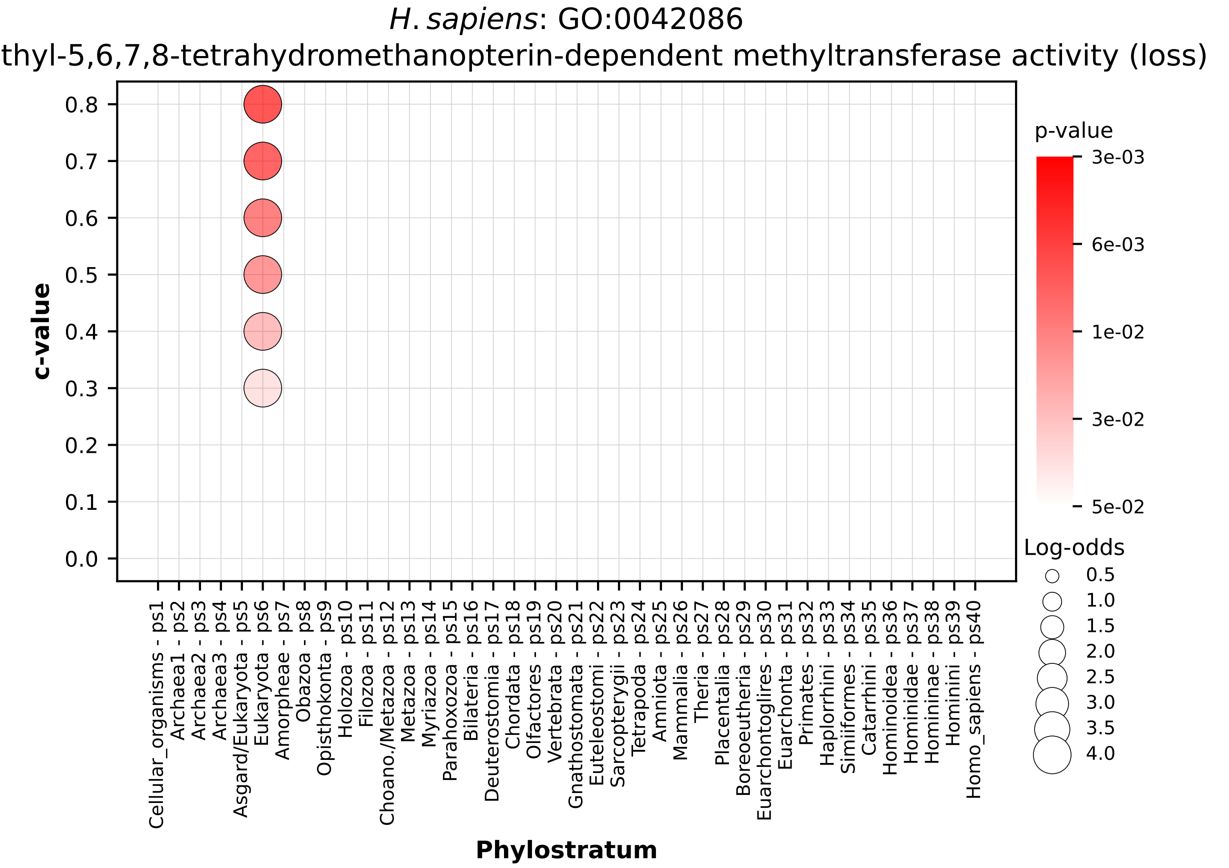
H. sapiens: GO:0042086
thyl-5,6,7,8-tetrahydromethanopterin-dependent methyltransferase activity (loss)
c-value
0.8
0.7
0.6
0.5
0.4
0.3
0.2
0.1
0.0
p-value
3e-03
6e-03
1e-02
3e-02
5e-02
Log-odds
0.5
1.0
1.5
2.0
2.5
3.0
3.5
4.0
Cellular_organisms - ps1
Archaea1 - ps2
Archaea2 - ps3
Archaea3 - ps4
Asgard/Eukaryota - ps5
Eukaryota - ps6
Amorpheae - ps7
Obazoa - ps8
Opisthokonta - ps9
Holozoa - ps10
Filozoa - ps11
Choano./Metazoa - ps12
Metazoa - ps13
Myriazoa - ps14
Parahoxozoa - ps15
Bilateria - ps16
Deuterostomia - ps17
Chordata - ps18
Olfactores - ps19
Vertebrata - ps20
Gnathostomata - ps21
Euteleostomi - ps22
Sarcopterygii - ps23
Tetrapoda - ps24
Amniota - ps25
Mammalia - ps26
Theria - ps27
Placentalia - ps28
Boreoeutheria - ps29
Euarchontoglires - ps30
Euarchonta - ps31
Primates - ps32
Haplorrhini - ps33
Simiiformes - ps34
Catarrhini - ps35
Hominoidea - ps36
Hominidae - ps37
Homininae - ps38
Hominini - ps39
Homo_sapiens - ps40
Phylostratum

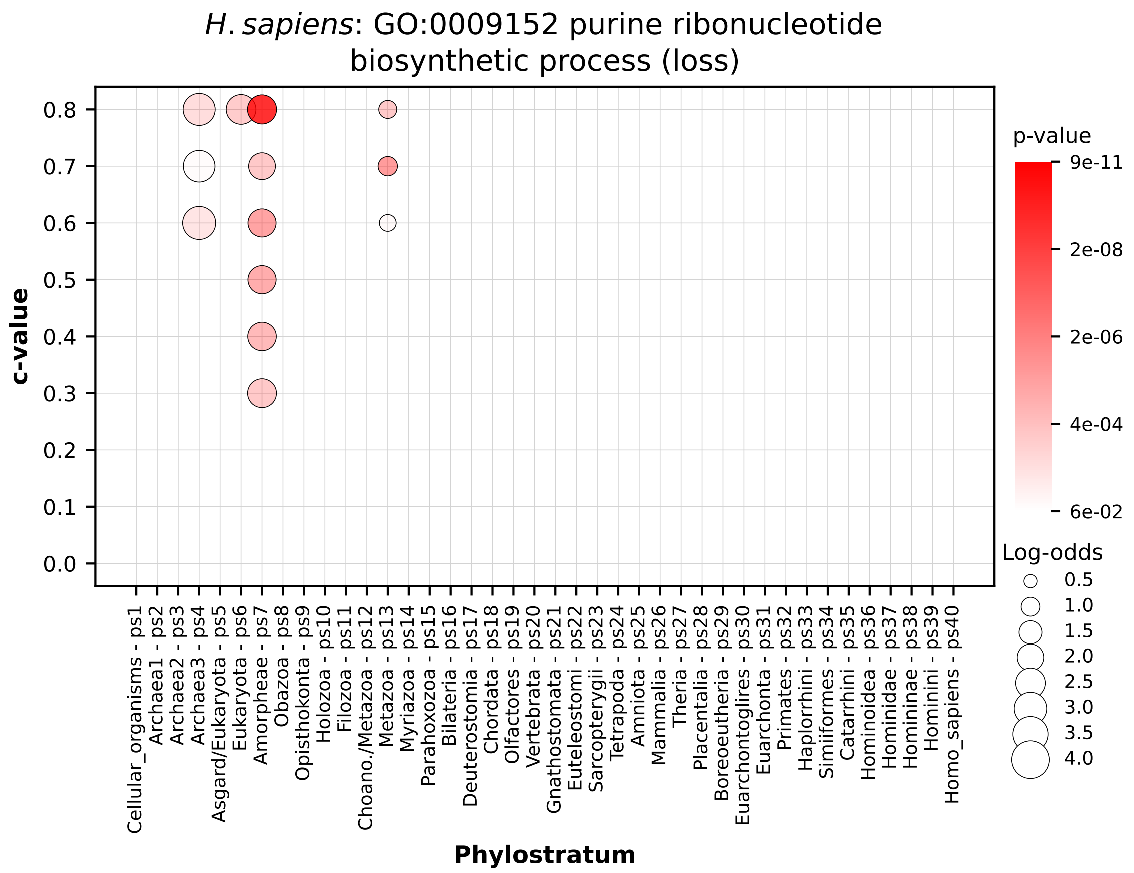

H. sapiens: GO:0009152 purine ribonucleotide biosynthetic process (loss)
c-value
0.8
0.7
0.6
0.5
0.4
0.3
0.2
0.1
0.0
p-value
9e-11
2e-08
2e-06
4e-04
6e-02
Log-odds
0.5
1.0
1.5
2.0
2.5
3.0
3.5
4.0
Cellular_organisms - ps1
Archaea1 - ps2
Archaea2 - ps3
Archaea3 - ps4
Asgard/Eukaryota - ps5
Eukaryota - ps6
Amorpheae - ps7
Obazoa - ps8
Opisthokonta - ps9
Holozoa - ps10
Filozoa - ps11
Choano./Metazoa - ps12
Metazoa - ps13
Myriazoa - ps14
Parahoxozoa - ps15
Bilateria - ps16
Deuterostomia - ps17
Chordata - ps18
Olfactores - ps19
Vertebrata - ps20
Gnathostomata - ps21
Euteleostomi - ps22
Sarcopterygii - ps23
Tetrapoda - ps24
Amniota - ps25
Mammalia - ps26
Theria - ps27
Placentalia - ps28
Boreoeutheria - ps29
Euarchontoglires - ps30
Euarchonta - ps31
Primates - ps32
Haplorrhini - ps33
Simiiformes - ps34
Catarrhini - ps35
Hominoidea - ps36
Hominidae - ps37
Homininae - ps38
Hominini - ps39
Homo_sapiens - ps40
Phylostratum

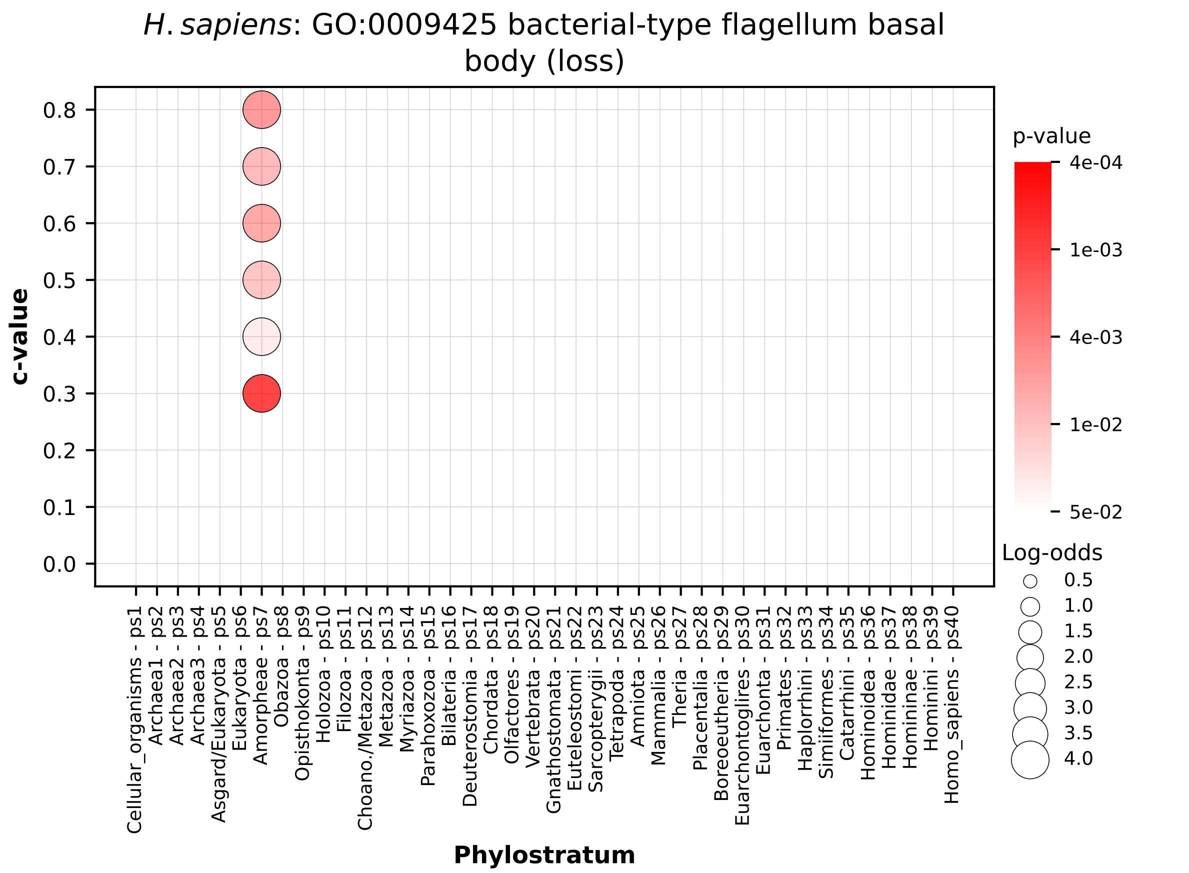

H. sapiens: GO:0009425 bacterial-type flagellum basal body (loss)
c-value
0.0
0.1
0.2
0.3
0.4
0.5
0.6
0.7
0.8
p-value
4e-04
1e-03
4e-03
1e-02
5e-02
Log-odds
0.5
1.0
1.5
2.0
2.5
3.0
3.5
4.0
Cellular_organisms - ps1
Archaea1 - ps2
Archaea2 - ps3
Archaea3 - ps4
Asgard/Eukaryota - ps5
Eukaryota - ps6
Amorpheae - ps7
Obazoa - ps8
Opisthokonta - ps9
Holozoa - ps10
Filozoa - ps11
Choano./Metazoa - ps12
Metazoa - ps13
Myriazoa - ps14
Parahoxozoa - ps15
Bilateria - ps16
Deuterostomia - ps17
Chordata - ps18
Olfactores - ps19
Vertebrata - ps20
Gnathostomata - ps21
Euteleostomi - ps22
Sarcopterygii - ps23
Tetrapoda - ps24
Amniota - ps25
Mammalia - ps26
Theria - ps27
Placentalia - ps28
Boreoeutheria - ps29
Euarchontoglires - ps30
Euarchonta - ps31
Primates - ps32
Haplorrhini - ps33
Simiiformes - ps34
Catarrhini - ps35
Hominoidea - ps36
Hominidae - ps37
Homininae - ps38
Hominini - ps39
Homo_sapiens - ps40
Phylostratum

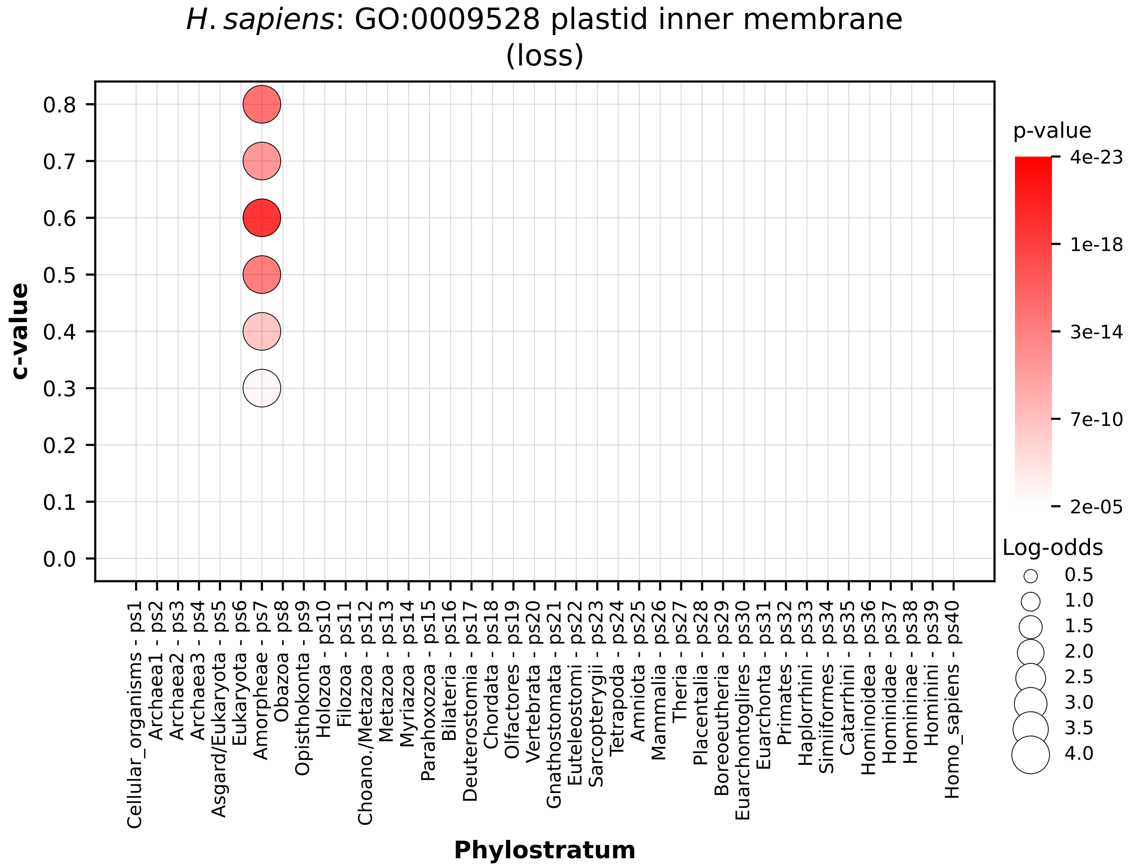

H. sapiens: GO:0009528 plastid inner membrane
(loss)
c-value
0.8
0.7
0.6
0.5
0.4
0.3
0.2
0.1
0.0
p-value
4e-23
1e-18
3e-14
7e-10
2e-05
Log-odds
0.5
1.0
1.5
2.0
2.5
3.0
3.5
4.0
Cellular_organisms - ps1
Archaea1 - ps2
Archaea2 - ps3
Archaea3 - ps4
Asgard/Eukaryota - ps5
Eukaryota - ps6
Amorpheae - ps7
Obazoa - ps8
Opisthokonta - ps9
Holozoa - ps10
Filozoa - ps11
Choano./Metazoa - ps12
Metazoa - ps13
Myriazoa - ps14
Parahoxozoa - ps15
Bilateria - ps16
Deuterostomia - ps17
Chordata - ps18
Olfactores - ps19
Vertebrata - ps20
Gnathostomata - ps21
Euteleostomi - ps22
Sarcopterygii - ps23
Tetrapoda - ps24
Amniota - ps25
Mammalia - ps26
Theria - ps27
Placentalia - ps28
Boreoeutheria - ps29
Euarchontoglires - ps30
Euarchonta - ps31
Primates - ps32
Haplorrhini - ps33
Simiiformes - ps34
Catarrhini - ps35
Hominoidea - ps36
Hominidae - ps37
Homininae - ps38
Hominini - ps39
Homo_sapiens - ps40
Phylostratum

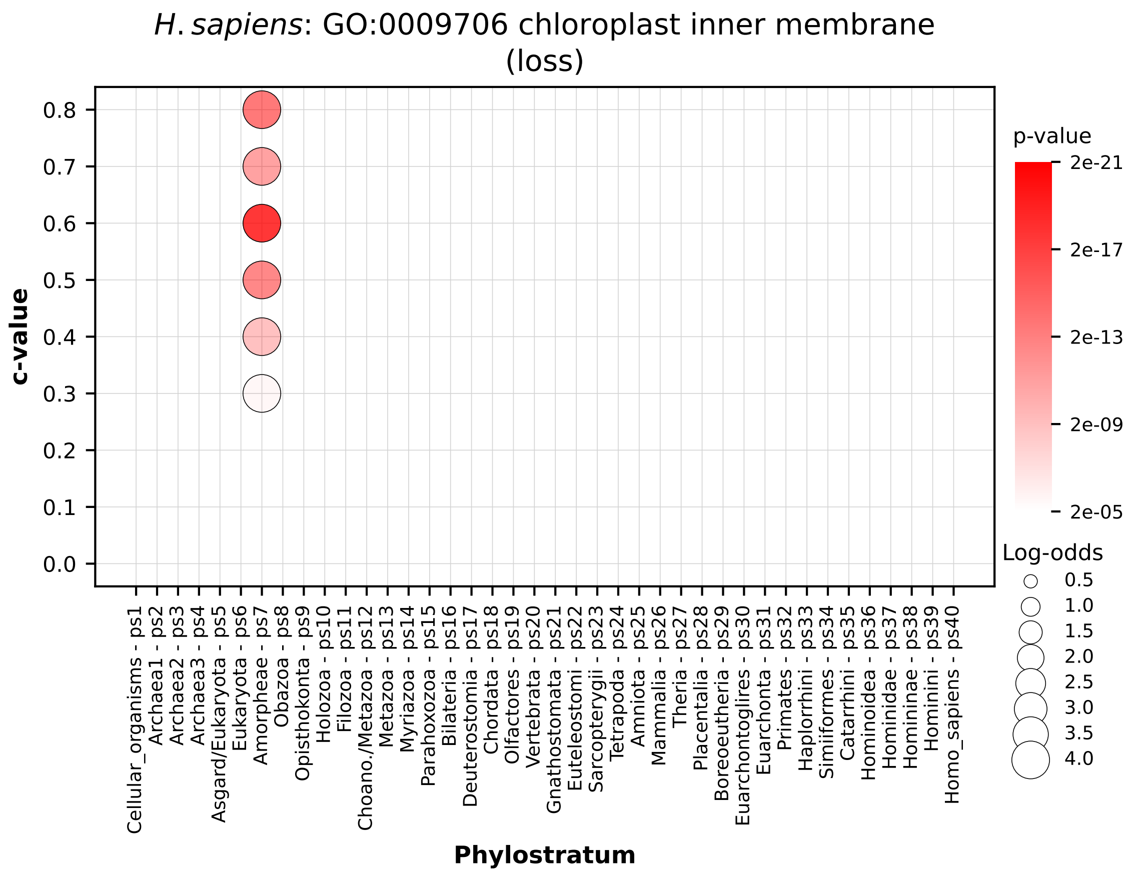

H. sapiens: GO:0009706 chloroplast inner membrane
(loss)
c-value
0.0
0.1
0.2
0.3
0.4
0.5
0.6
0.7
0.8
p-value
2e-21
2e-17
2e-13
2e-09
2e-05
Log-odds
0.5
1.0
1.5
2.0
2.5
3.0
3.5
4.0
Cellular_organisms - ps1
Archaea1 - ps2
Archaea2 - ps3
Archaea3 - ps4
Asgard/Eukaryota - ps5
Eukaryota - ps6
Amorpheae - ps7
Obazoa - ps8
Opisthokonta - ps9
Holozoa - ps10
Filozoa - ps11
Choano./Metazoa - ps12
Metazoa - ps13
Myriazoa - ps14
Parahoxozoa - ps15
Bilateria - ps16
Deuterostomia - ps17
Chordata - ps18
Olfactores - ps19
Vertebrata - ps20
Gnathostomata - ps21
Euteleostomi - ps22
Sarcopterygii - ps23
Tetrapoda - ps24
Amniota - ps25
Mammalia - ps26
Theria - ps27
Placentalia - ps28
Boreoeutheria - ps29
Euarchontoglires - ps30
Euarchonta - ps31
Primates - ps32
Haplorrhini - ps33
Simiiformes - ps34
Catarrhini - ps35
Hominoidea - ps36
Hominidae - ps37
Homininae - ps38
Hominini - ps39
Homo_sapiens - ps40
Phylostratum

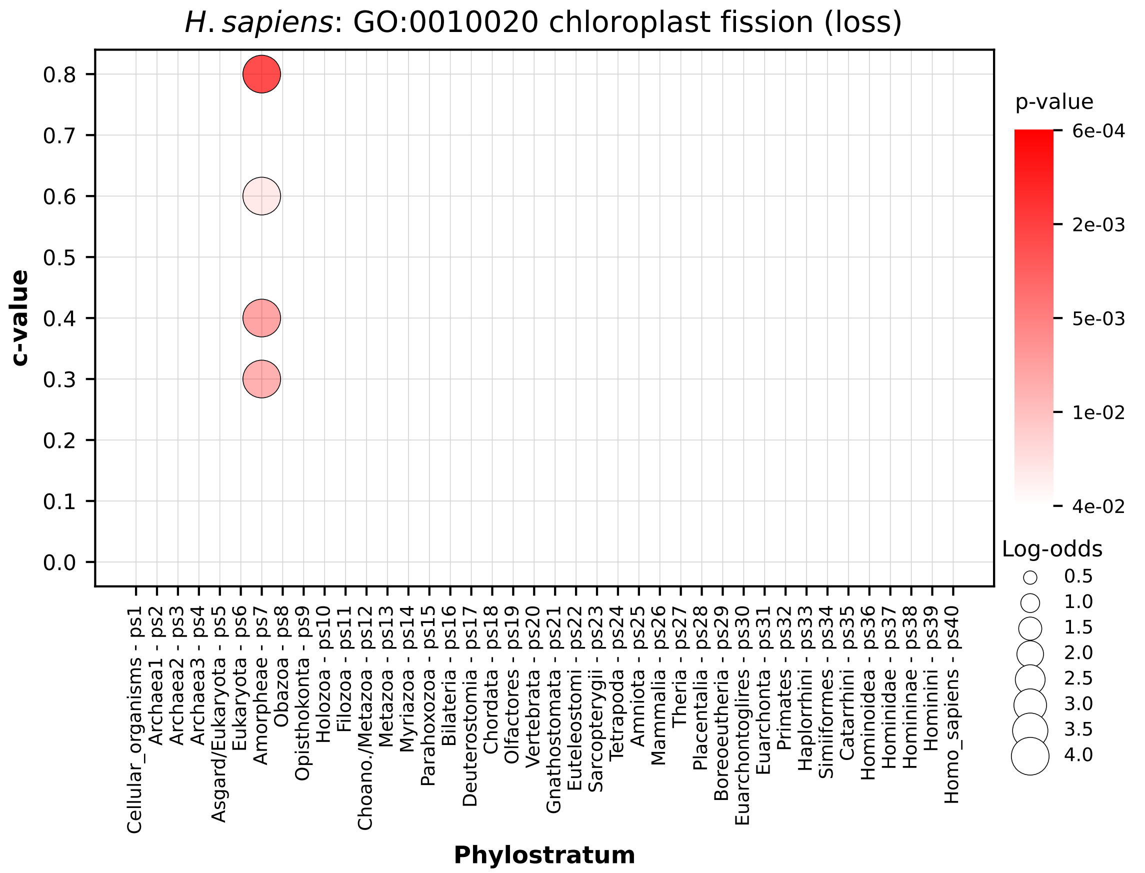

H. sapiens: GO:0010020 chloroplast fission (loss)
c-value
0.8
0.7
0.6
0.5
0.4
0.3
0.2
0.1
0.0
Phylostratum
Cellular_organisms - ps1
Archaea1 - ps2
Archaea2 - ps3
Archaea3 - ps4
Asgard/Eukaryota - ps5
Eukaryota - ps6
Amorpheae - ps7
Obazoa - ps8
Opisthokonta - ps9
Holozoa - ps10
Filozoa - ps11
Choano./Metazoa - ps12
Metazoa - ps13
Myriazoa - ps14
Parahoxozoa - ps15
Bilateria - ps16
Deuterostomia - ps17
Chordata - ps18
Olfactores - ps19
Vertebrata - ps20
Gnathostomata - ps21
Euteleostomi - ps22
Sarcopterygii - ps23
Tetrapoda - ps24
Amniota - ps25
Mammalia - ps26
Theria - ps27
Placentalia - ps28
Boreoeutheria - ps29
Euarchontoglires - ps30
Euarchonta - ps31
Primates - ps32
Haplorrhini - ps33
Simiiformes - ps34
Catarrhini - ps35
Hominoidea - ps36
Hominidae - ps37
Homininae - ps38
Hominini - ps39
Homo_sapiens - ps40
p-value
6e-04
2e-03
5e-03
1e-02
4e-02
Log-odds
0.5
1.0
1.5
2.0
2.5
3.0
3.5
4.0

H. sapiens: GO:0016851 magnesium chelatase activity (loss)

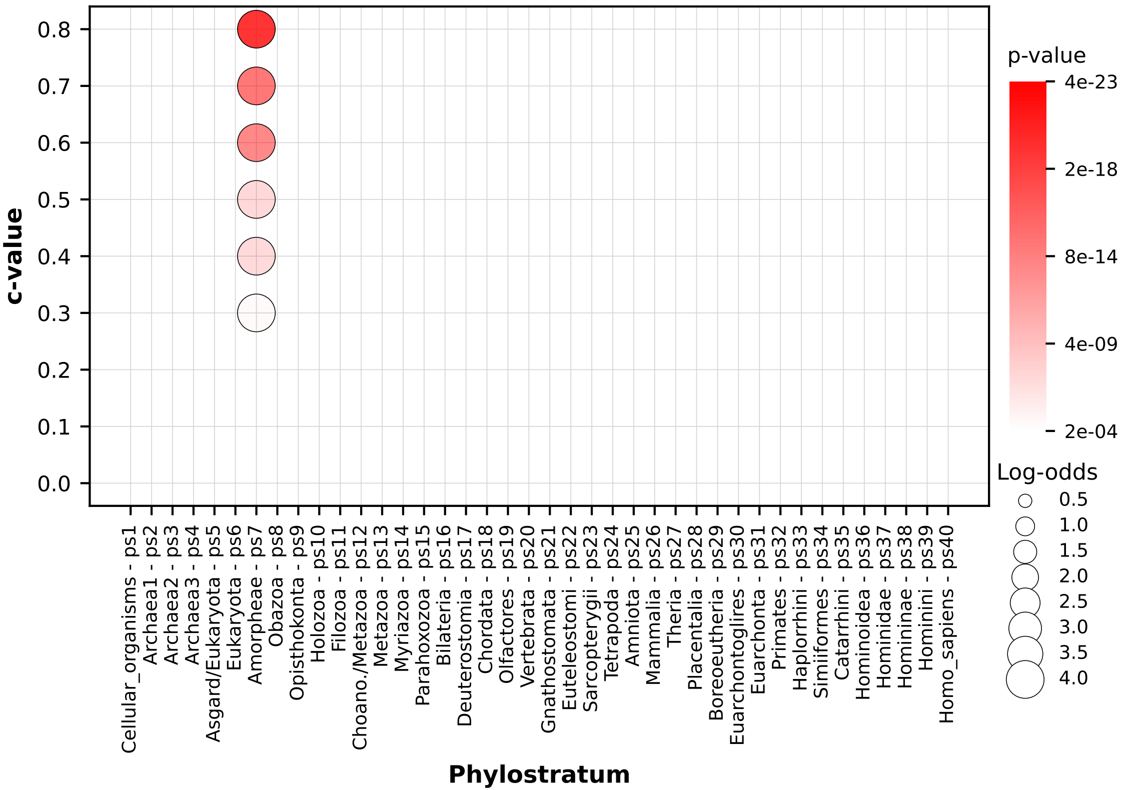

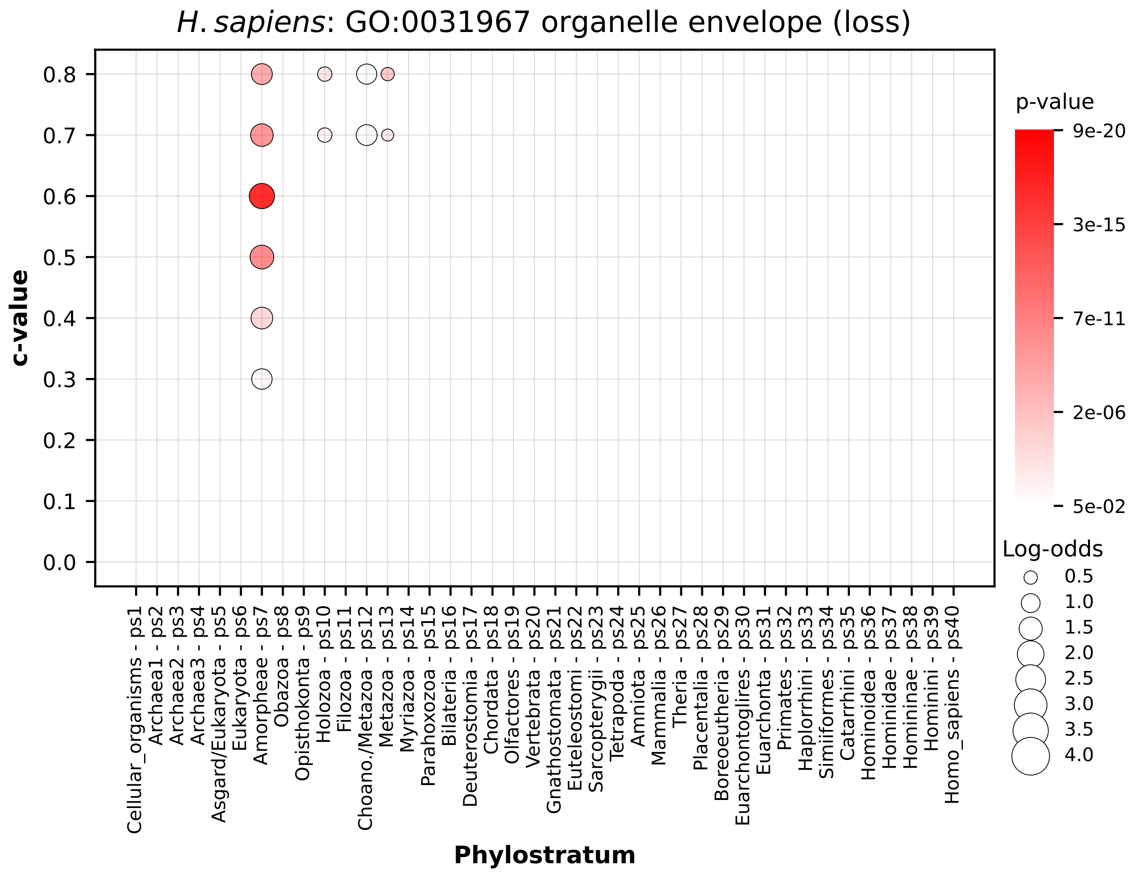

H. sapiens: GO:0031967 organelle envelope (loss)
c-value
0.8
0.7
0.6
0.5
0.4
0.3
0.2
0.1
0.0
p-value
9e-20
3e-15
7e-11
2e-06
5e-02
Log-odds
0.5
1.0
1.5
2.0
2.5
3.0
3.5
4.0
Cellular_organisms - ps1
Archaea1 - ps2
Archaea2 - ps3
Archaea3 - ps4
Asgard/Eukaryota - ps5
Eukaryota - ps6
Amorpheae - ps7
Obazoa - ps8
Opisthokonta - ps9
Holozoa - ps10
Filozoa - ps11
Choano./Metazoa - ps12
Metazoa - ps13
Myriazoa - ps14
Parahoxozoa - ps15
Bilateria - ps16
Deuterostomia - ps17
Chordata - ps18
Olfactores - ps19
Vertebrata - ps20
Gnathostomata - ps21
Euteleostomi - ps22
Sarcopterygii - ps23
Tetrapoda - ps24
Amniota - ps25
Mammalia - ps26
Theria - ps27
Placentalia - ps28
Boreoeutheria - ps29
Euarchontoglires - ps30
Euarchonta - ps31
Primates - ps32
Haplorrhini - ps33
Simiiformes - ps34
Catarrhini - ps35
Hominoidea - ps36
Hominidae - ps37
Homininae - ps38
Hominini - ps39
Homo_sapiens - ps40
Phylostratum

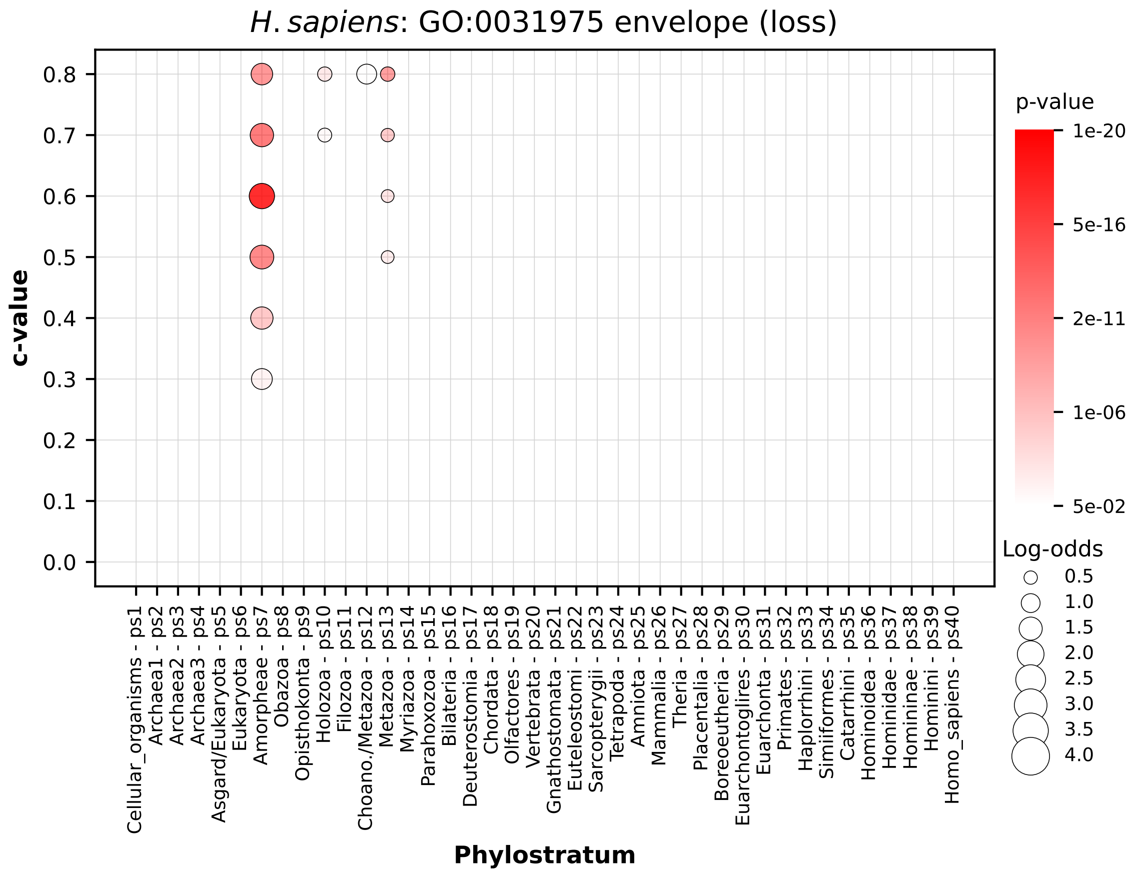

H. sapiens: GO:0031975 envelope (loss)
c-value
0.8
0.7
0.6
0.5
0.4
0.3
0.2
0.1
0.0
p-value
1e-20
5e-16
2e-11
1e-06
5e-02
Log-odds
0.5
1.0
1.5
2.0
2.5
3.0
3.5
4.0
Cellular_organisms - ps1
Archaea1 - ps2
Archaea2 - ps3
Archaea3 - ps4
Asgard/Eukaryota - ps5
Eukaryota - ps6
Amorpheae - ps7
Obazoa - ps8
Opisthokonta - ps9
Holozoa - ps10
Filozoa - ps11
Choano./Metazoa - ps12
Metazoa - ps13
Myriazoa - ps14
Parahoxozoa - ps15
Bilateria - ps16
Deuterostomia - ps17
Chordata - ps18
Olfactores - ps19
Vertebrata - ps20
Gnathostomata - ps21
Euteleostomi - ps22
Sarcopterygii - ps23
Tetrapoda - ps24
Amniota - ps25
Mammalia - ps26
Theria - ps27
Placentalia - ps28
Boreoeutheria - ps29
Euarchontoglires - ps30
Euarchonta - ps31
Primates - ps32
Haplorrhini - ps33
Simiiformes - ps34
Catarrhini - ps35
Hominoidea - ps36
Hominidae - ps37
Homininae - ps38
Hominini - ps39
Homo_sapiens - ps40
Phylostratum

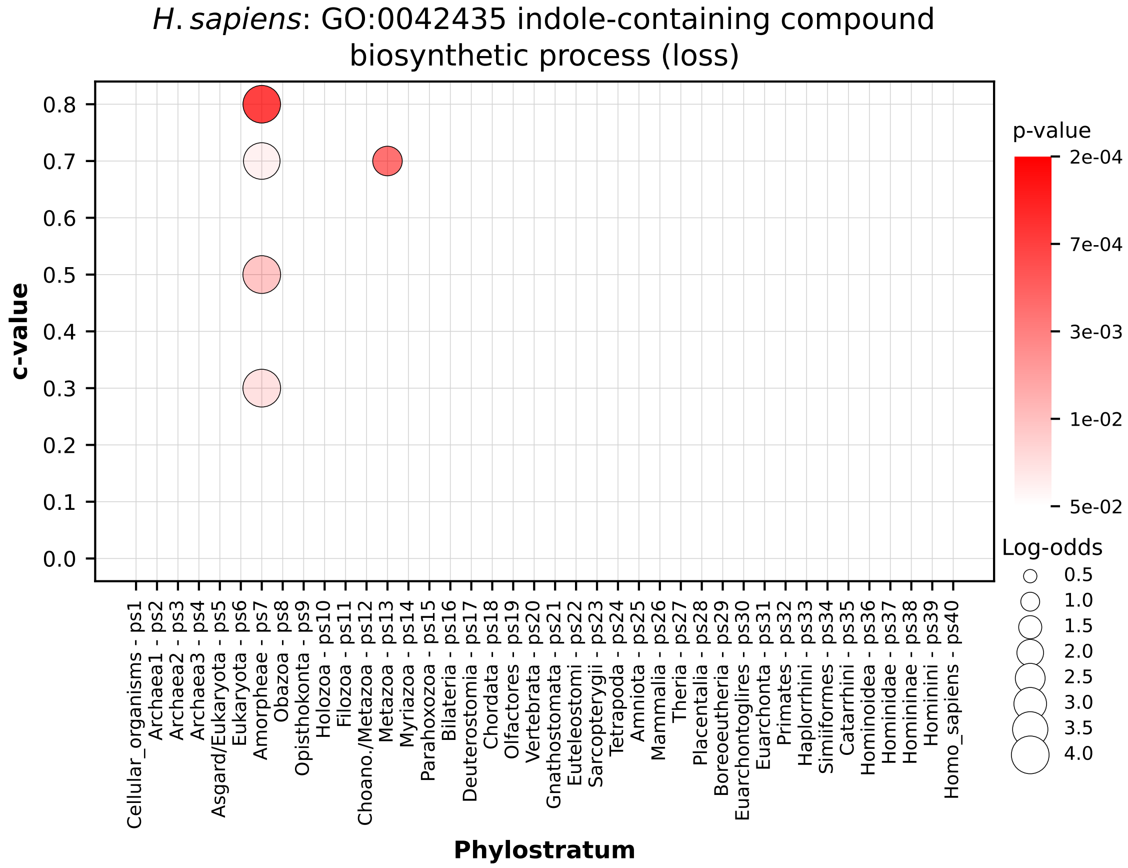

H. sapiens: GO:0042435 indole-containing compound biosynthetic process (loss)
c-value
0.0
0.1
0.2
0.3
0.4
0.5
0.6
0.7
0.8
p-value
2e-04
7e-04
3e-03
1e-02
5e-02
Log-odds
0.5
1.0
1.5
2.0
2.5
3.0
3.5
4.0
Cellular_organisms - ps1
Archaea1 - ps2
Archaea2 - ps3
Archaea3 - ps4
Asgard/Eukaryota - ps5
Eukaryota - ps6
Amorpheae - ps7
Obazoa - ps8
Opisthokonta - ps9
Holozoa - ps10
Filozoa - ps11
Choano./Metazoa - ps12
Metazoa - ps13
Myriazoa - ps14
Parahoxozoa - ps15
Bilateria - ps16
Deuterostomia - ps17
Chordata - ps18
Olfactores - ps19
Vertebrata - ps20
Gnathostomata - ps21
Euteleostomi - ps22
Sarcopterygii - ps23
Tetrapoda - ps24
Amniota - ps25
Mammalia - ps26
Theria - ps27
Placentalia - ps28
Boreoeutheria - ps29
Euarchontoglires - ps30
Euarchonta - ps31
Primates - ps32
Haplorrhini - ps33
Simiiformes - ps34
Catarrhini - ps35
Hominoidea - ps36
Hominidae - ps37
Homininae - ps38
Hominini - ps39
Homo_sapiens - ps40
Phylostratum

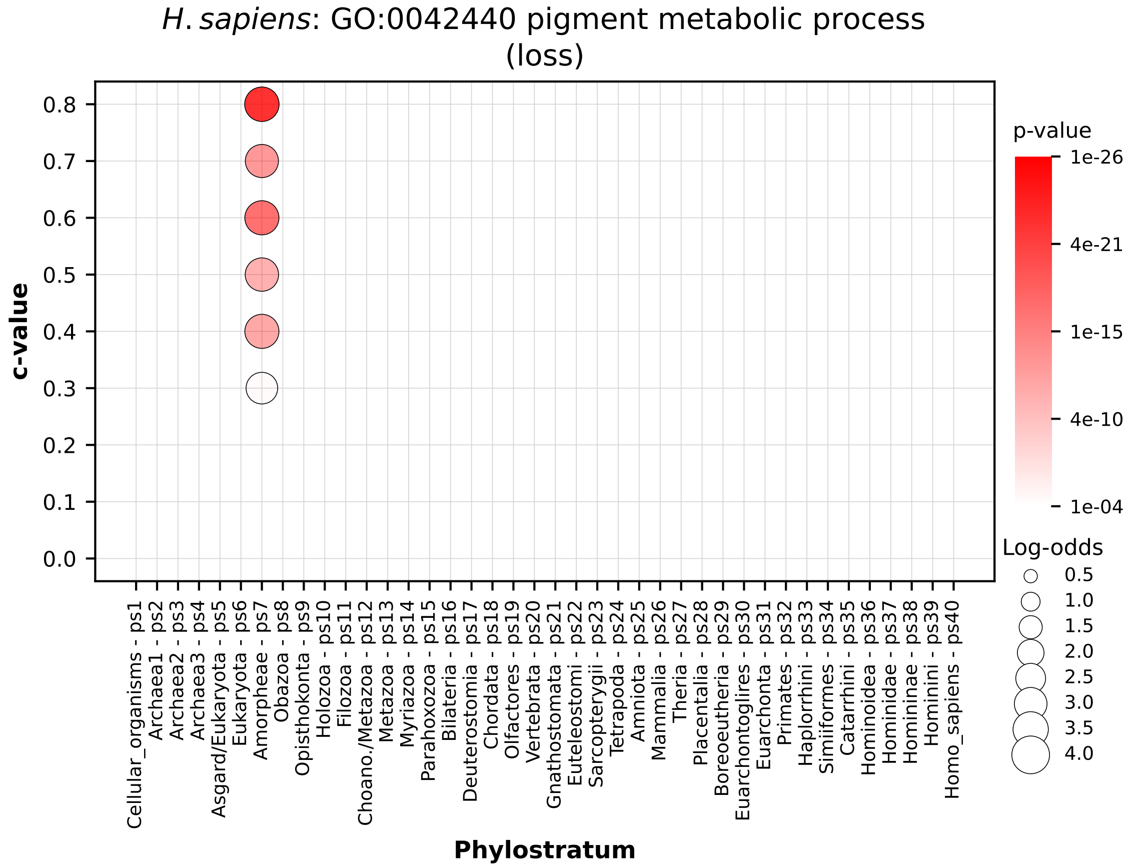

H. sapiens: GO:0042440 pigment metabolic process
(loss)
c-value
0.0
0.1
0.2
0.3
0.4
0.5
0.6
0.7
0.8
p-value
1e-26
4e-21
1e-15
4e-10
1e-04
Log-odds
0.5
1.0
1.5
2.0
2.5
3.0
3.5
4.0
Cellular_organisms - ps1
Archaea1 - ps2
Archaea2 - ps3
Archaea3 - ps4
Asgard/Eukaryota - ps5
Eukaryota - ps6
Amorpheae - ps7
Obazoa - ps8
Opisthokonta - ps9
Holozoa - ps10
Filozoa - ps11
Choano./Metazoa - ps12
Metazoa - ps13
Myriazoa - ps14
Parahoxozoa - ps15
Bilateria - ps16
Deuterostomia - ps17
Chordata - ps18
Olfactores - ps19
Vertebrata - ps20
Gnathostomata - ps21
Euteleostomi - ps22
Sarcopterygii - ps23
Tetrapoda - ps24
Amniota - ps25
Mammalia - ps26
Theria - ps27
Placentalia - ps28
Boreoeutheria - ps29
Euarchontoglires - ps30
Euarchonta - ps31
Primates - ps32
Haplorrhini - ps33
Simiiformes - ps34
Catarrhini - ps35
Hominoidea - ps36
Hominidae - ps37
Homininae - ps38
Hominini - ps39
Homo_sapiens - ps40
Phylostratum

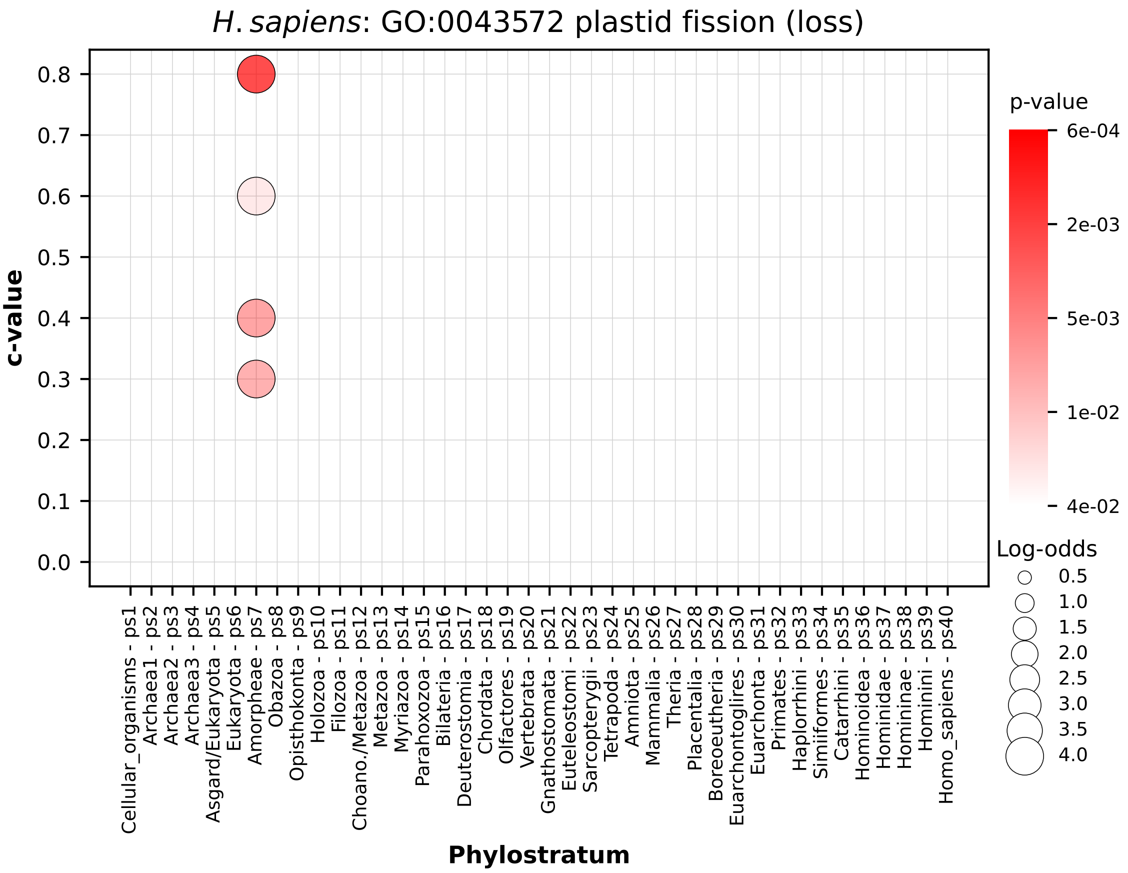

H. sapiens: GO:0043572 plastid fission (loss)
c-value
0.0
0.1
0.2
0.3
0.4
0.5
0.6
0.7
0.8
Phylostratum
Cellular_organisms - ps1
Archaea1 - ps2
Archaea2 - ps3
Archaea3 - ps4
Asgard/Eukaryota - ps5
Eukaryota - ps6
Amorpheae - ps7
Obazoa - ps8
Opisthokonta - ps9
Holozoa - ps10
Filozoa - ps11
Choano./Metazoa - ps12
Metazoa - ps13
Myriazoa - ps14
Parahoxozoa - ps15
Bilateria - ps16
Deuterostomia - ps17
Chordata - ps18
Olfactores - ps19
Vertebrata - ps20
Gnathostomata - ps21
Euteleostomi - ps22
Sarcopterygii - ps23
Tetrapoda - ps24
Amniota - ps25
Mammalia - ps26
Theria - ps27
Placentalia - ps28
Boreoeutheria - ps29
Euarchontoglires - ps30
Euarchonta - ps31
Primates - ps32
Haplorrhini - ps33
Simiiformes - ps34
Catarrhini - ps35
Hominoidea - ps36
Hominidae - ps37
Homininae - ps38
Hominini - ps39
Homo_sapiens - ps40
p-value
6e-04
2e-03
5e-03
1e-02
4e-02
Log-odds
0.5
1.0
1.5
2.0
2.5
3.0
3.5
4.0

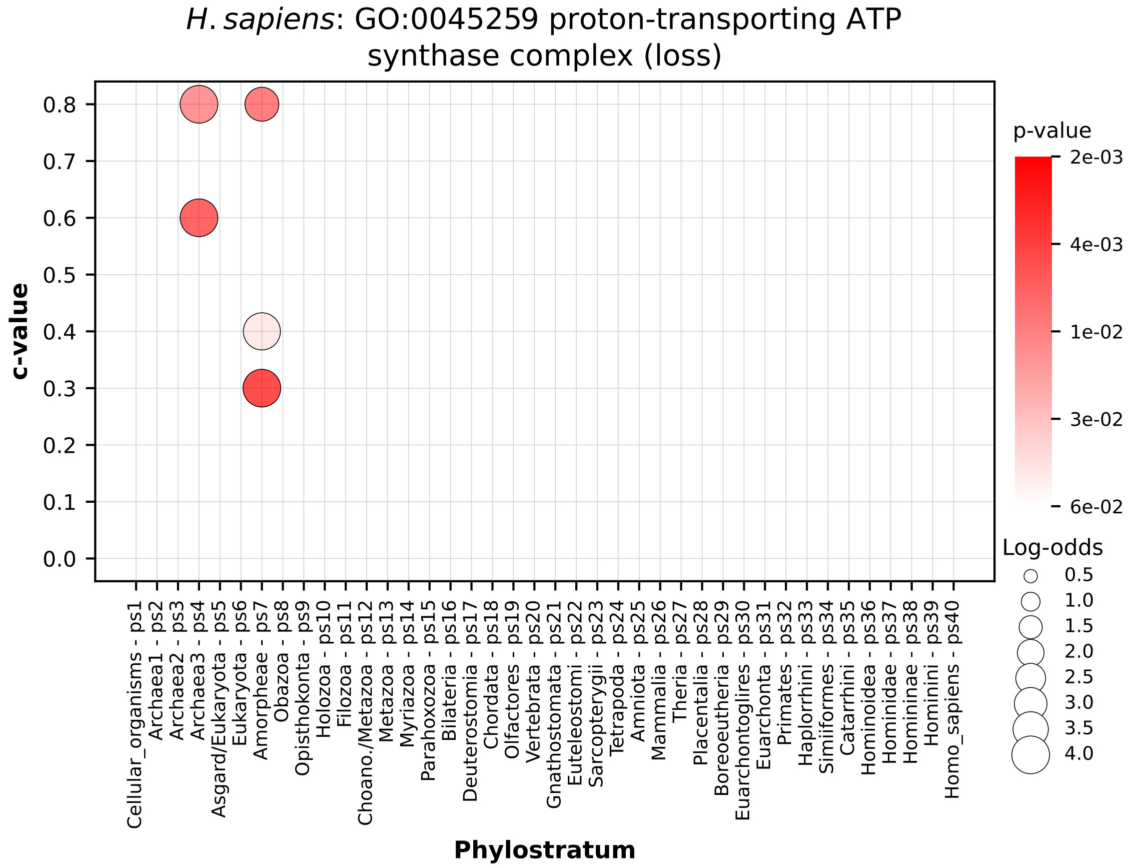

H. sapiens: GO:0045259 proton-transporting ATP synthase complex (loss)
c-value
0.8
0.7
0.6
0.5
0.4
0.3
0.2
0.1
0.0
Phylostratum
Cellular_organisms - ps1
Archaea1 - ps2
Archaea2 - ps3
Archaea3 - ps4
Asgard/Eukaryota - ps5
Eukaryota - ps6
Amorpheae - ps7
Obazoa - ps8
Opisthokonta - ps9
Holozoa - ps10
Filozoa - ps11
Choano./Metazoa - ps12
Metazoa - ps13
Myriazoa - ps14
Parahoxozoa - ps15
Bilateria - ps16
Deuterostomia - ps17
Chordata - ps18
Olfactores - ps19
Vertebrata - ps20
Gnathostomata - ps21
Euteleostomi - ps22
Sarcopterygii - ps23
Tetrapoda - ps24
Amniota - ps25
Mammalia - ps26
Theria - ps27
Placentalia - ps28
Boreoeutheria - ps29
Euarchontoglires - ps30
Euarchonta - ps31
Primates - ps32
Haplorrhini - ps33
Simiiformes - ps34
Catarrhini - ps35
Hominoidea - ps36
Hominidae - ps37
Homininae - ps38
Hominini - ps39
Homo_sapiens - ps40
p-value
2e-03
4e-03
1e-02
3e-02
6e-02
Log-odds
0.5
1.0
1.5
2.0
2.5
3.0
3.5
4.0

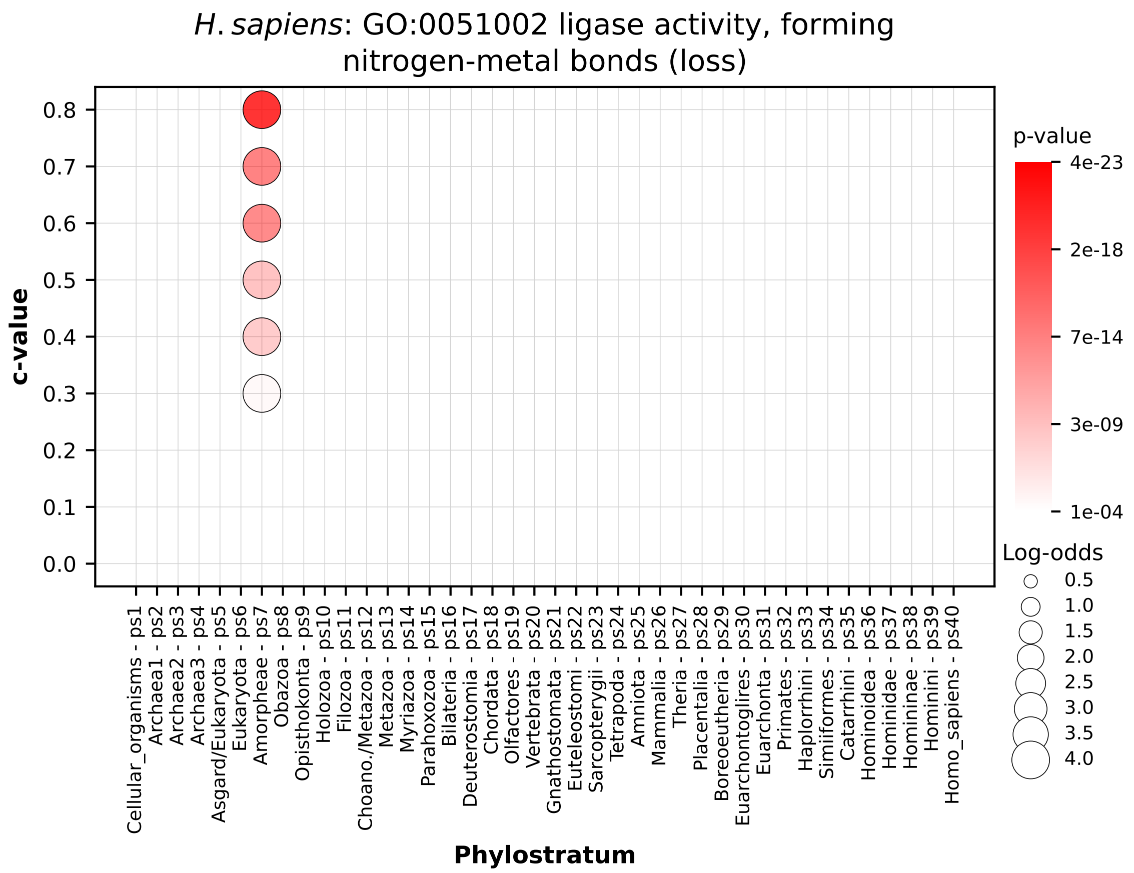
H. sapiens: GO:0051002 ligase activity, forming nitrogen-metal bonds (loss)
c-value
0.8
0.7
0.6
0.5
0.4
0.3
0.2
0.1
0.0
p-value
4e-23
2e-18
7e-14
3e-09
1e-04
Log-odds
0.5
1.0
1.5
2.0
2.5
3.0
3.5
4.0
Cellular_organisms - ps1
Archaea1 - ps2
Archaea2 - ps3
Archaea3 - ps4
Asgard/Eukaryota - ps5
Eukaryota - ps6
Amorpheae - ps7
Obazoa - ps8
Opisthokonta - ps9
Holozoa - ps10
Filozoa - ps11
Choano./Metazoa - ps12
Metazoa - ps13
Myriazoa - ps14
Parahoxozoa - ps15
Bilateria - ps16
Deuterostomia - ps17
Chordata - ps18
Olfactores - ps19
Vertebrata - ps20
Gnathostomata - ps21
Euteleostomi - ps22
Sarcopterygii - ps23
Tetrapoda - ps24
Amniota - ps25
Mammalia - ps26
Theria - ps27
Placentalia - ps28
Boreoeutheria - ps29
Euarchontoglires - ps30
Euarchonta - ps31
Primates - ps32
Haplorrhini - ps33
Simiiformes - ps34
Catarrhini - ps35
Hominoidea - ps36
Hominidae - ps37
Homininae - ps38
Hominini - ps39
Homo_sapiens - ps40
Phylostratum

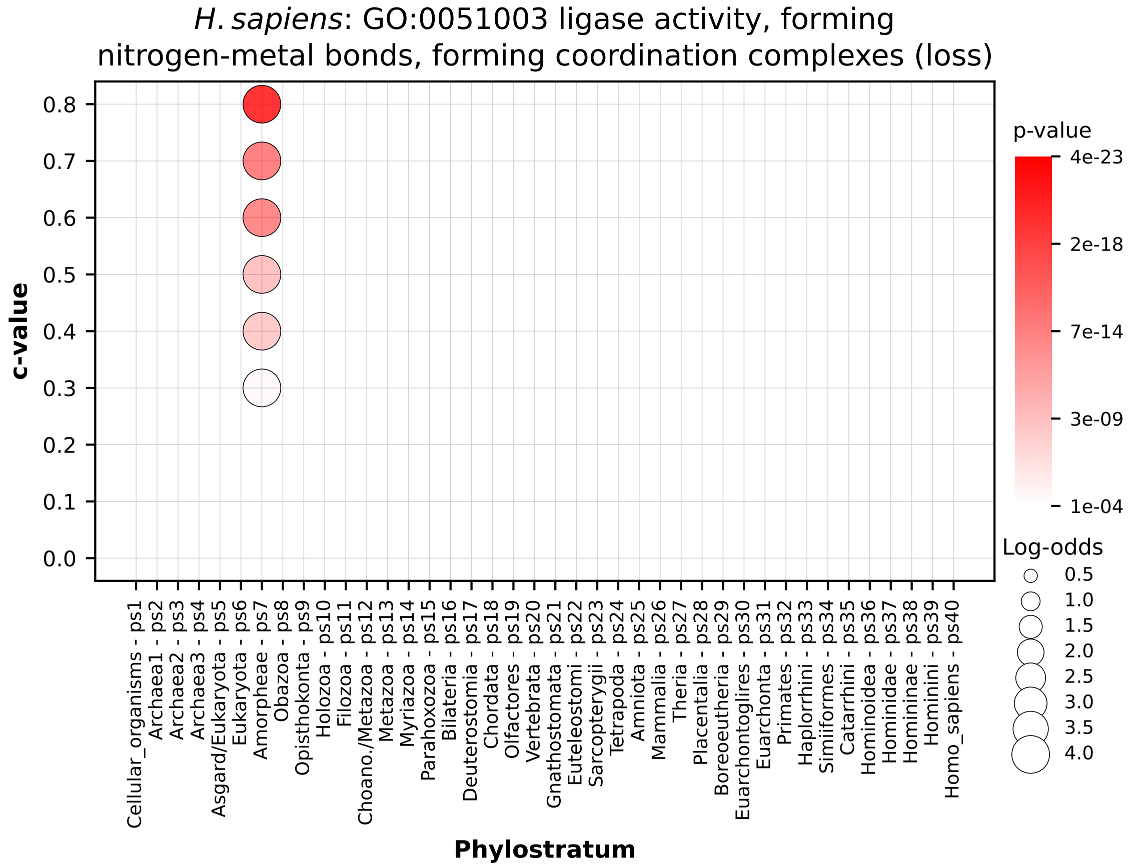

H. sapiens: GO:0051003 ligase activity, forming nitrogen-metal bonds, forming coordination complexes (loss)
c-value
0.8
0.7
0.6
0.5
0.4
0.3
0.2
0.1
0.0
p-value
4e-23
2e-18
7e-14
3e-09
1e-04
Log-odds
0.5
1.0
1.5
2.0
2.5
3.0
3.5
4.0
Cellular_organisms - ps1
Archaea1 - ps2
Archaea2 - ps3
Archaea3 - ps4
Asgard/Eukaryota - ps5
Eukaryota - ps6
Amorpheae - ps7
Obazoa - ps8
Opisthokonta - ps9
Holozoa - ps10
Filozoa - ps11
Choano./Metazoa - ps12
Metazoa - ps13
Myriazoa - ps14
Parahoxozoa - ps15
Bilateria - ps16
Deuterostomia - ps17
Chordata - ps18
Olfactores - ps19
Vertebrata - ps20
Gnathostomata - ps21
Euteleostomi - ps22
Sarcopterygii - ps23
Tetrapoda - ps24
Amniota - ps25
Mammalia - ps26
Theria - ps27
Placentalia - ps28
Boreoeutheria - ps29
Euarchontoglires - ps30
Euarchonta - ps31
Primates - ps32
Haplorrhini - ps33
Simiiformes - ps34
Catarrhini - ps35
Hominoidea - ps36
Hominidae - ps37
Homininae - ps38
Hominini - ps39
Homo_sapiens - ps40
Phylostratum

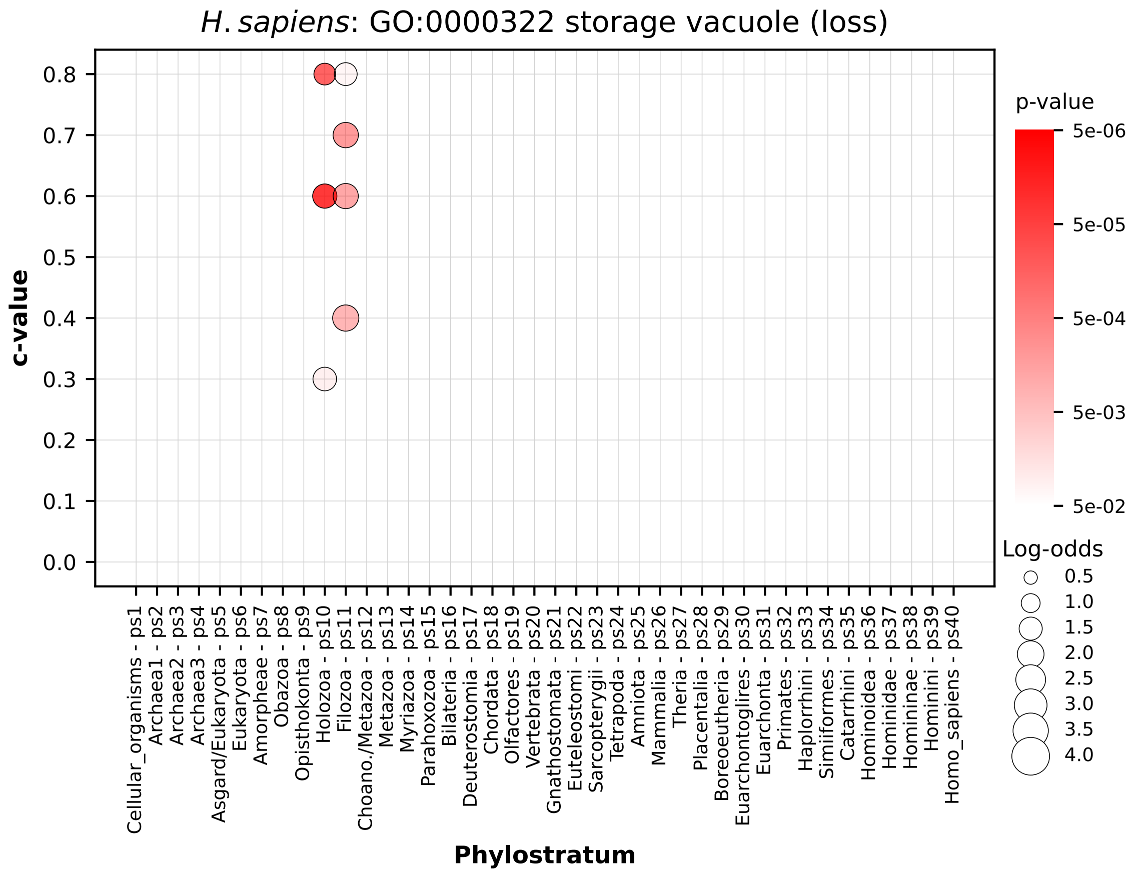

H. sapiens: GO:0000322 storage vacuole (loss)
c-value
0.8
0.7
0.6
0.5
0.4
0.3
0.2
0.1
0.0
Phylostratum
Cellular_organisms - ps1
Archaea1 - ps2
Archaea2 - ps3
Archaea3 - ps4
Asgard/Eukaryota - ps5
Eukaryota - ps6
Amorpheae - ps7
Obazoa - ps8
Opisthokonta - ps9
Holozoa - ps10
Filozoa - ps11
Choano./Metazoa - ps12
Metazoa - ps13
Myriazoa - ps14
Parahoxozoa - ps15
Bilateria - ps16
Deuterostomia - ps17
Chordata - ps18
Olfactores - ps19
Vertebrata - ps20
Gnathostomata - ps21
Euteleostomi - ps22
Sarcopterygii - ps23
Tetrapoda - ps24
Amniota - ps25
Mammalia - ps26
Theria - ps27
Placentalia - ps28
Boreoeutheria - ps29
Euarchontoglires - ps30
Euarchonta - ps31
Primates - ps32
Haplorrhini - ps33
Simiiformes - ps34
Catarrhini - ps35
Hominoidea - ps36
Hominidae - ps37
Homininae - ps38
Hominini - ps39
Homo_sapiens - ps40
p-value
5e-06
5e-05
5e-04
5e-03
5e-02
Log-odds
0.5
1.0
1.5
2.0
2.5
3.0
3.5
4.0

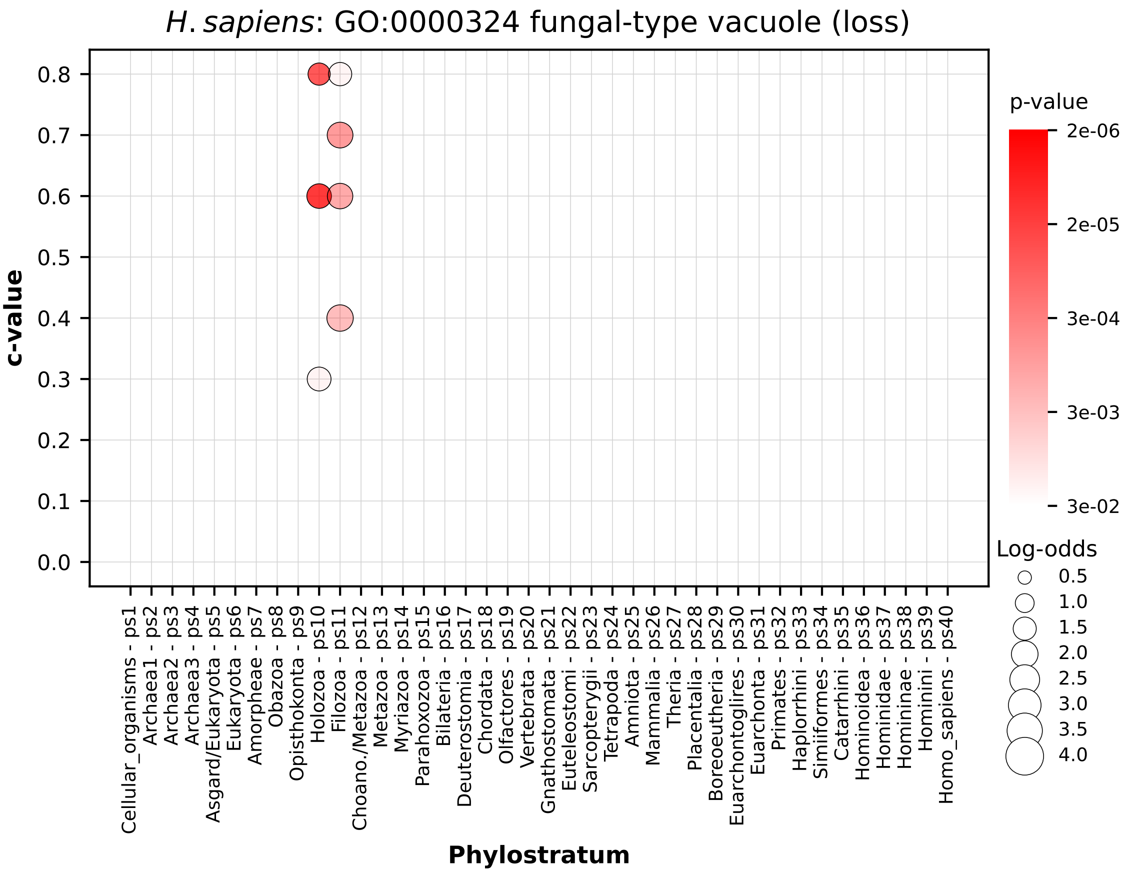

H. sapiens: GO:0000324 fungal-type vacuole (loss)
c-value
0.8
0.7
0.6
0.5
0.4
0.3
0.2
0.1
0.0
p-value
2e-06
2e-05
3e-04
3e-03
3e-02
Log-odds
0.5
1.0
1.5
2.0
2.5
3.0
3.5
4.0
Cellular_organisms - ps1
Archaea1 - ps2
Archaea2 - ps3
Archaea3 - ps4
Asgard/Eukaryota - ps5
Eukaryota - ps6
Amorpheae - ps7
Obazoa - ps8
Opisthokonta - ps9
Holozoa - ps10
Filozoa - ps11
Choano./Metazoa - ps12
Metazoa - ps13
Myriazoa - ps14
Parahoxozoa - ps15
Bilateria - ps16
Deuterostomia - ps17
Chordata - ps18
Olfactores - ps19
Vertebrata - ps20
Gnathostomata - ps21
Euteleostomi - ps22
Sarcopterygii - ps23
Tetrapoda - ps24
Amniota - ps25
Mammalia - ps26
Theria - ps27
Placentalia - ps28
Boreoeutheria - ps29
Euarchontoglires - ps30
Euarchonta - ps31
Primates - ps32
Haplorrhini - ps33
Simiiformes - ps34
Catarrhini - ps35
Hominoidea - ps36
Hominidae - ps37
Homininae - ps38
Hominini - ps39
Homo_sapiens - ps40
Phylostratum

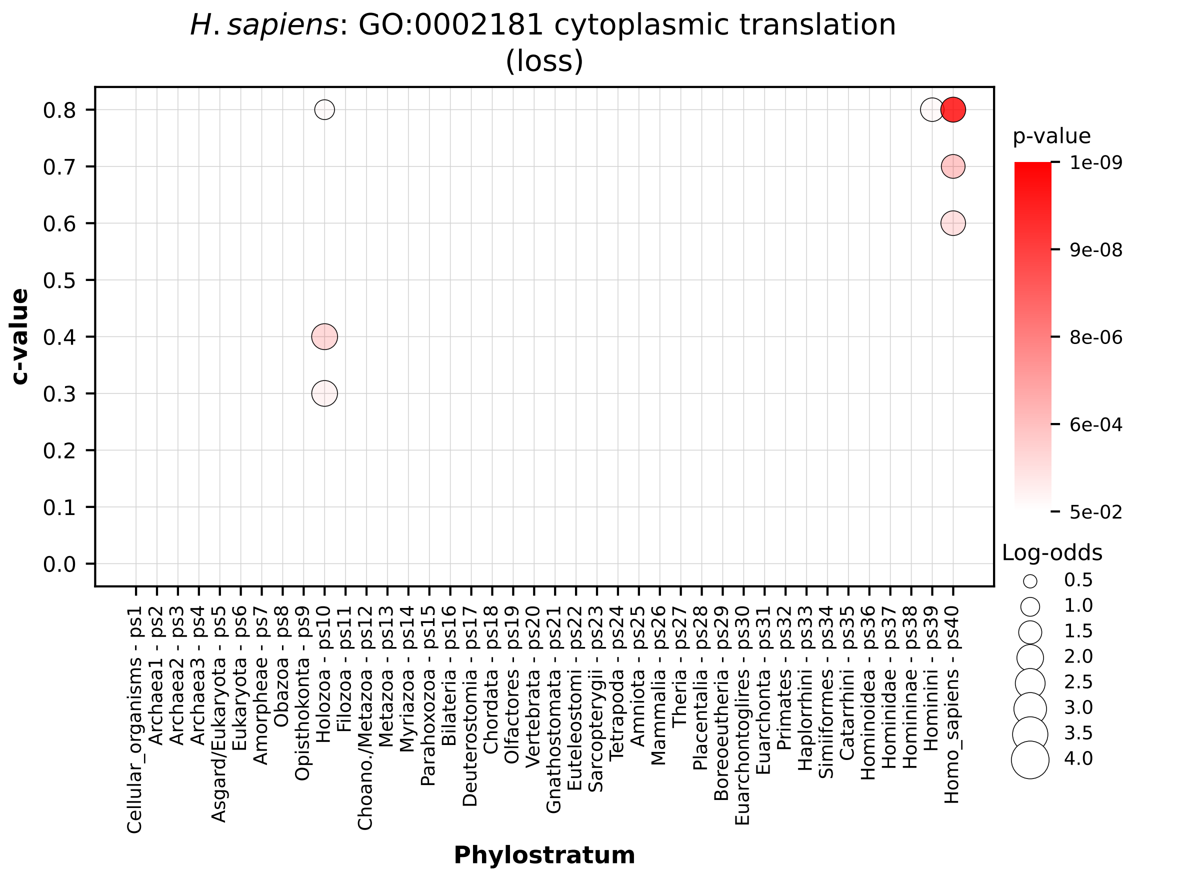

H. sapiens: GO:0002181 cytoplasmic translation
(loss)
c-value
0.8
0.7
0.6
0.5
0.4
0.3
0.2
0.1
0.0
Phylostratum
Cellular_organisms - ps1
Archaea1 - ps2
Archaea2 - ps3
Archaea3 - ps4
Asgard/Eukaryota - ps5
Eukaryota - ps6
Amorpheae - ps7
Obazoa - ps8
Opisthokonta - ps9
Holozoa - ps10
Filozoa - ps11
Choano./Metazoa - ps12
Metazoa - ps13
Myriazoa - ps14
Parahoxozoa - ps15
Bilateria - ps16
Deuterostomia - ps17
Chordata - ps18
Olfactores - ps19
Vertebrata - ps20
Gnathostomata - ps21
Euteleostomi - ps22
Sarcopterygii - ps23
Tetrapoda - ps24
Amniota - ps25
Mammalia - ps26
Theria - ps27
Placentalia - ps28
Boreoeutheria - ps29
Euarchontoglires - ps30
Euarchonta - ps31
Primates - ps32
Haplorrhini - ps33
Simiiformes - ps34
Catarrhini - ps35
Hominoidea - ps36
Hominidae - ps37
Homininae - ps38
Hominini - ps39
Homo_sapiens - ps40
p-value
1e-09
9e-08
8e-06
6e-04
5e-02
Log-odds
0.5
1.0
1.5
2.0
2.5
3.0
3.5
4.0

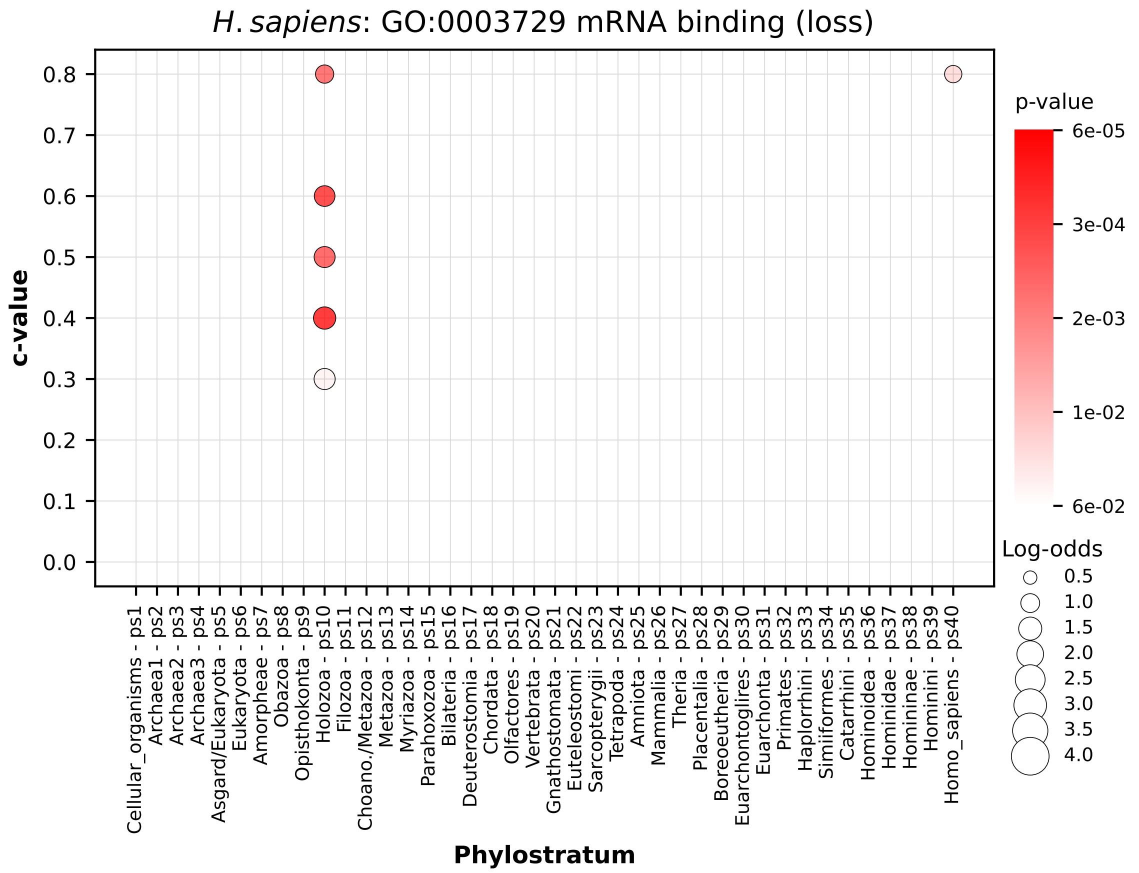

H. sapiens: GO:0003729 mRNA binding (loss)
c-value
0.0
0.1
0.2
0.3
0.4
0.5
0.6
0.7
0.8
Phylostratum
Cellular_organisms - ps1
Archaea1 - ps2
Archaea2 - ps3
Archaea3 - ps4
Asgard/Eukaryota - ps5
Eukaryota - ps6
Amorpheae - ps7
Obazoa - ps8
Opisthokonta - ps9
Holozoa - ps10
Filozoa - ps11
Choano./Metazoa - ps12
Metazoa - ps13
Myriazoa - ps14
Parahoxozoa - ps15
Bilateria - ps16
Deuterostomia - ps17
Chordata - ps18
Olfactores - ps19
Vertebrata - ps20
Gnathostomata - ps21
Euteleostomi - ps22
Sarcopterygii - ps23
Tetrapoda - ps24
Amniota - ps25
Mammalia - ps26
Theria - ps27
Placentalia - ps28
Boreoeutheria - ps29
Euarchontoglires - ps30
Euarchonta - ps31
Primates - ps32
Haplorrhini - ps33
Simiiformes - ps34
Catarrhini - ps35
Hominoidea - ps36
Hominidae - ps37
Homininae - ps38
Hominini - ps39
Homo_sapiens - ps40
p-value
6e-05
3e-04
2e-03
1e-02
6e-02
Log-odds
0.5
1.0
1.5
2.0
2.5
3.0
3.5
4.0

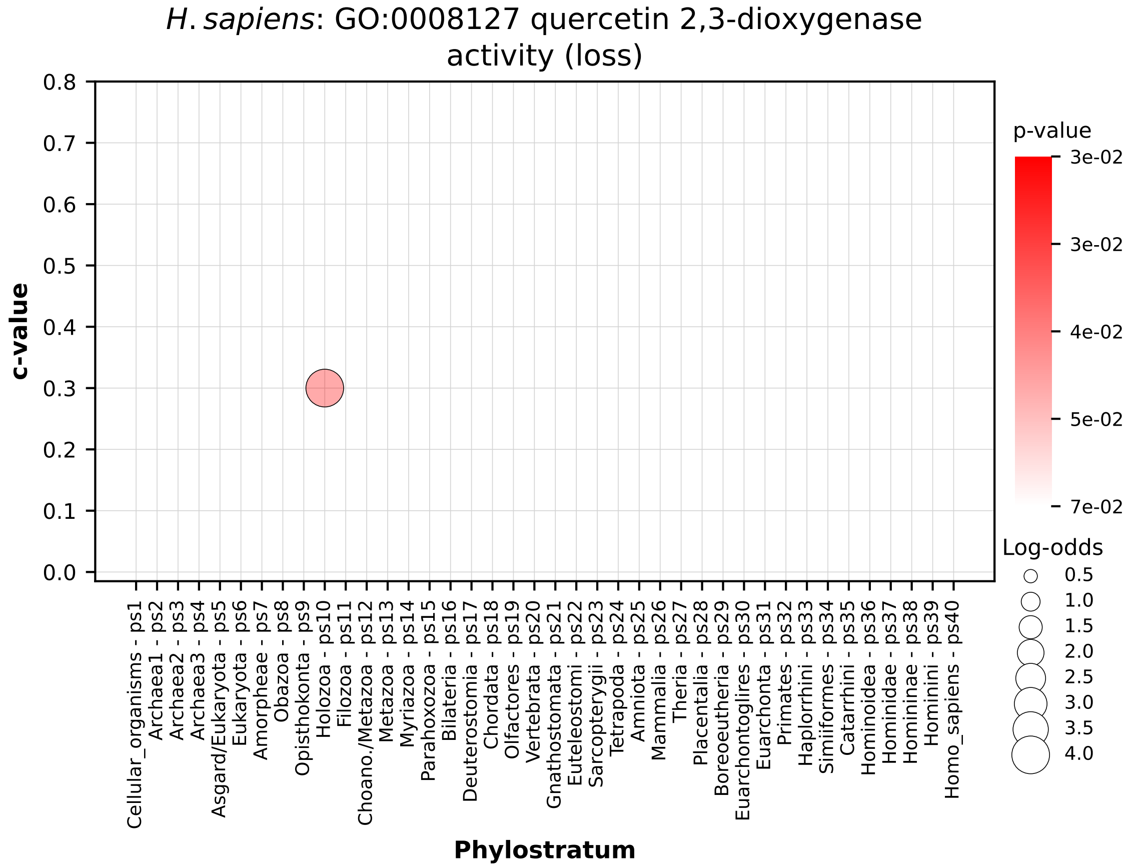

H. sapiens: GO:0008127 quercetin 2,3-dioxygenase activity (loss)
c-value
0.0
0.1
0.2
0.3
0.4
0.5
0.6
0.7
0.8
Phylostratum
Cellular_organisms - ps1
Archaea1 - ps2
Archaea2 - ps3
Archaea3 - ps4
Asgard/Eukaryota - ps5
Eukaryota - ps6
Amorpheae - ps7
Obazoa - ps8
Opisthokonta - ps9
Holozoa - ps10
Filozoa - ps11
Choano./Metazoa - ps12
Metazoa - ps13
Myriazoa - ps14
Parahoxozoa - ps15
Bilateria - ps16
Deuterostomia - ps17
Chordata - ps18
Olfactores - ps19
Vertebrata - ps20
Gnathostomata - ps21
Euteleostomi - ps22
Sarcopterygii - ps23
Tetrapoda - ps24
Amniota - ps25
Mammalia - ps26
Theria - ps27
Placentalia - ps28
Boreoeutheria - ps29
Euarchontoglires - ps30
Euarchonta - ps31
Primates - ps32
Haplorrhini - ps33
Simiiformes - ps34
Catarrhini - ps35
Hominoidea - ps36
Hominidae - ps37
Homininae - ps38
Hominini - ps39
Homo_sapiens - ps40
p-value
3e-02
3e-02
4e-02
5e-02
7e-02
Log-odds
0.5
1.0
1.5
2.0
2.5
3.0
3.5
4.0

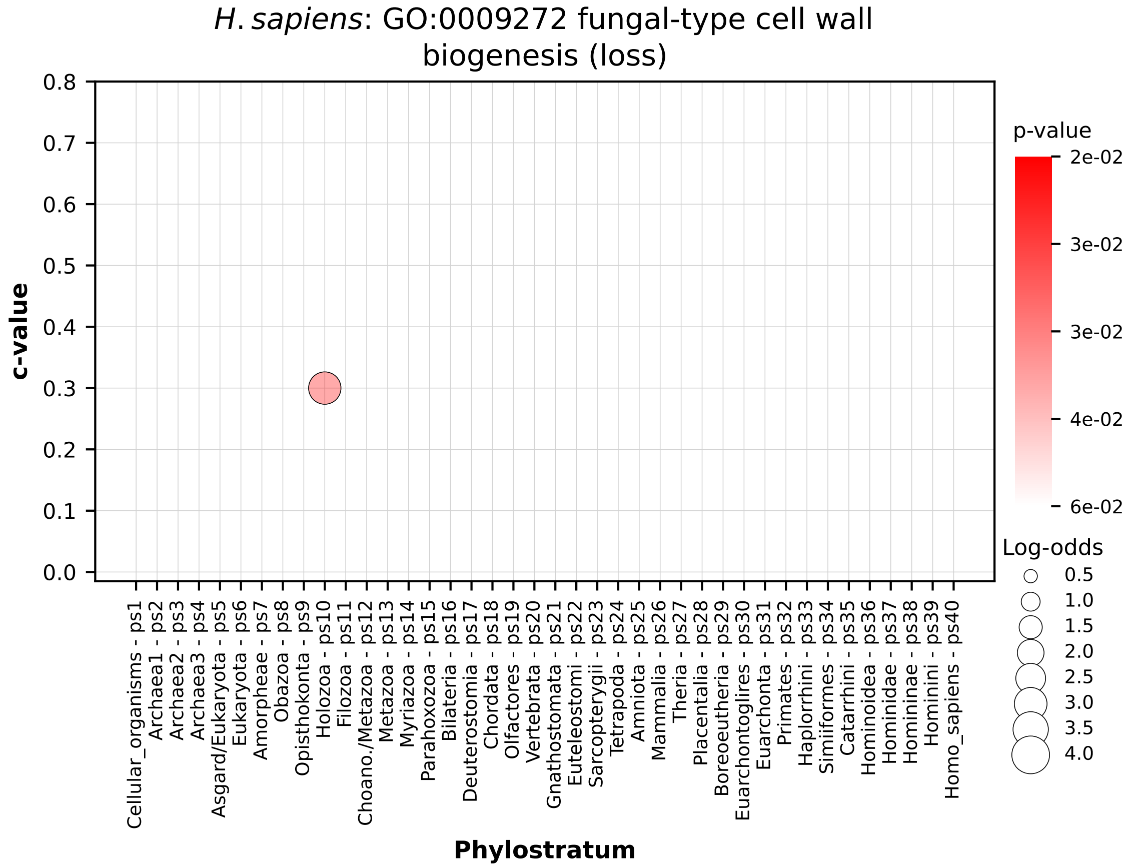

H. sapiens: GO:0009272 fungal-type cell wall biogenesis (loss)
c-value
0.8
0.7
0.6
0.5
0.4
0.3
0.2
0.1
0.0
Phylostratum
Cellular_organisms - ps1
Archaea1 - ps2
Archaea2 - ps3
Archaea3 - ps4
Asgard/Eukaryota - ps5
Eukaryota - ps6
Amorpheae - ps7
Obazoa - ps8
Opisthokonta - ps9
Holozoa - ps10
Filozoa - ps11
Choano./Metazoa - ps12
Metazoa - ps13
Myriazoa - ps14
Parahoxozoa - ps15
Bilateria - ps16
Deuterostomia - ps17
Chordata - ps18
Olfactores - ps19
Vertebrata - ps20
Gnathostomata - ps21
Euteleostomi - ps22
Sarcopterygii - ps23
Tetrapoda - ps24
Amniota - ps25
Mammalia - ps26
Theria - ps27
Placentalia - ps28
Boreoeutheria - ps29
Euarchontoglires - ps30
Euarchonta - ps31
Primates - ps32
Haplorrhini - ps33
Simiiformes - ps34
Catarrhini - ps35
Hominoidea - ps36
Hominidae - ps37
Homininae - ps38
Hominini - ps39
Homo_sapiens - ps40
p-value
2e-02
3e-02
3e-02
4e-02
6e-02
Log-odds
0.5
1.0
1.5
2.0
2.5
3.0
3.5
4.0

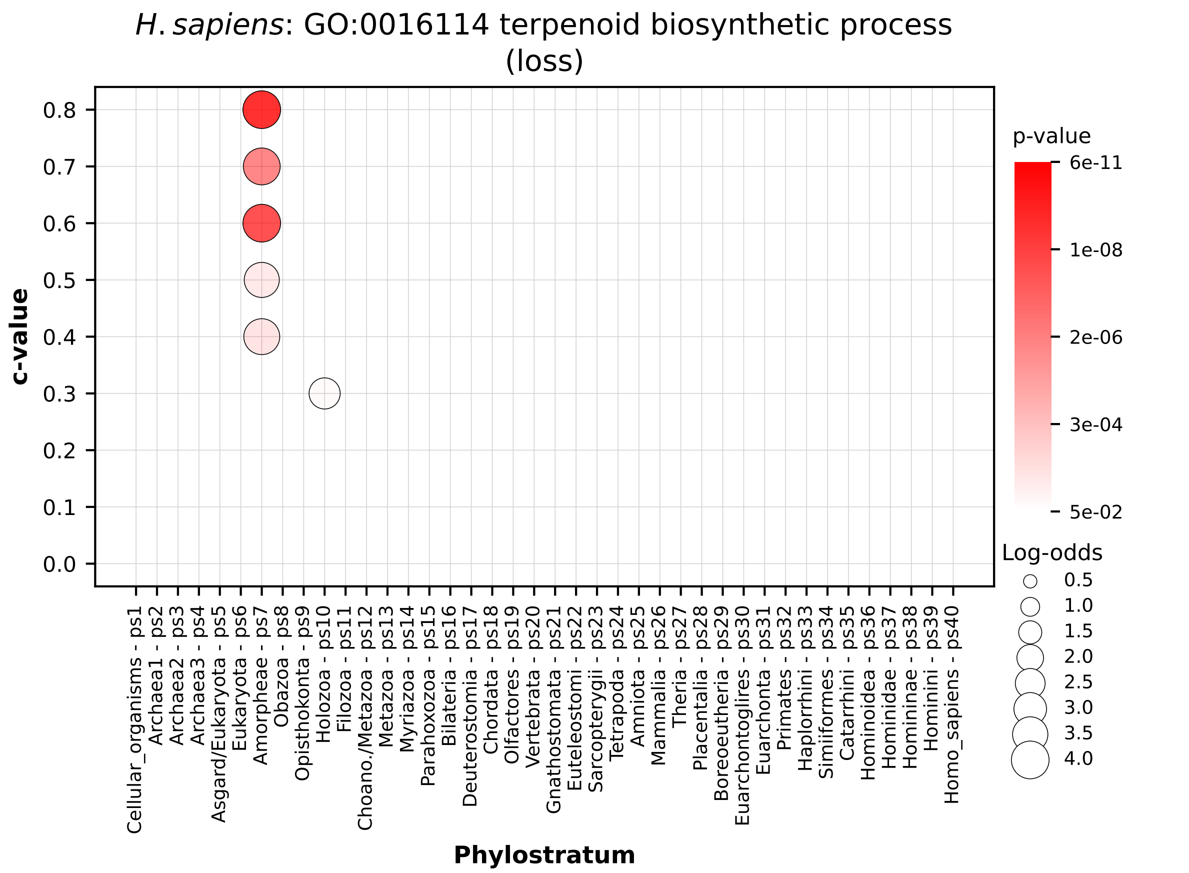

H. sapiens: GO:0016114 terpenoid biosynthetic process
(loss)
c-value
0.0
0.1
0.2
0.3
0.4
0.5
0.6
0.7
0.8
Phylostratum
Cellular_organisms - ps1
Archaea1 - ps2
Archaea2 - ps3
Archaea3 - ps4
Asgard/Eukaryota - ps5
Eukaryota - ps6
Amorpheae - ps7
Obazoa - ps8
Opisthokonta - ps9
Holozoa - ps10
Filozoa - ps11
Choano./Metazoa - ps12
Metazoa - ps13
Myriazoa - ps14
Parahoxozoa - ps15
Bilateria - ps16
Deuterostomia - ps17
Chordata - ps18
Olfactores - ps19
Vertebrata - ps20
Gnathostomata - ps21
Euteleostomi - ps22
Sarcopterygii - ps23
Tetrapoda - ps24
Amniota - ps25
Mammalia - ps26
Theria - ps27
Placentalia - ps28
Boreoeutheria - ps29
Euarchontoglires - ps30
Euarchonta - ps31
Primates - ps32
Haplorrhini - ps33
Simiiformes - ps34
Catarrhini - ps35
Hominoidea - ps36
Hominidae - ps37
Homininae - ps38
Hominini - ps39
Homo_sapiens - ps40
p-value
6e-11
1e-08
2e-06
3e-04
5e-02
Log-odds
0.5
1.0
1.5
2.0
2.5
3.0
3.5
4.0

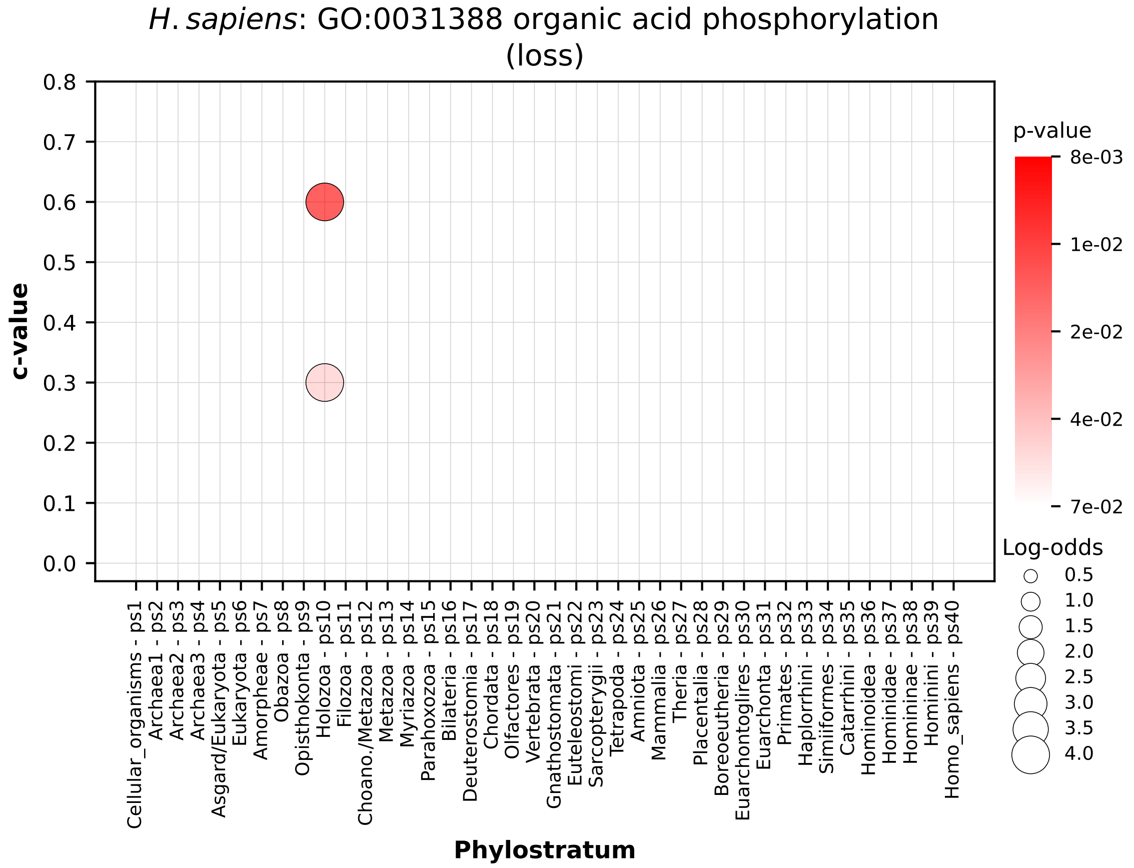

H. sapiens: GO:0031388 organic acid phosphorylation
(loss)
c-value
0.8
0.7
0.6
0.5
0.4
0.3
0.2
0.1
0.0
p-value
8e-03
1e-02
2e-02
4e-02
7e-02
Log-odds
0.5
1.0
1.5
2.0
2.5
3.0
3.5
4.0
Cellular_organisms - ps1
Archaea1 - ps2
Archaea2 - ps3
Archaea3 - ps4
Asgard/Eukaryota - ps5
Eukaryota - ps6
Amorpheae - ps7
Obazoa - ps8
Opisthokonta - ps9
Holozoa - ps10
Filozoa - ps11
Choano./Metazoa - ps12
Metazoa - ps13
Myriazoa - ps14
Parahoxozoa - ps15
Bilateria - ps16
Deuterostomia - ps17
Chordata - ps18
Olfactores - ps19
Vertebrata - ps20
Gnathostomata - ps21
Euteleostomi - ps22
Sarcopterygii - ps23
Tetrapoda - ps24
Amniota - ps25
Mammalia - ps26
Theria - ps27
Placentalia - ps28
Boreoeutheria - ps29
Euarchontoglires - ps30
Euarchonta - ps31
Primates - ps32
Haplorrhini - ps33
Simiiformes - ps34
Catarrhini - ps35
Hominoidea - ps36
Hominidae - ps37
Homininae - ps38
Hominini - ps39
Homo_sapiens - ps40
Phylostratum

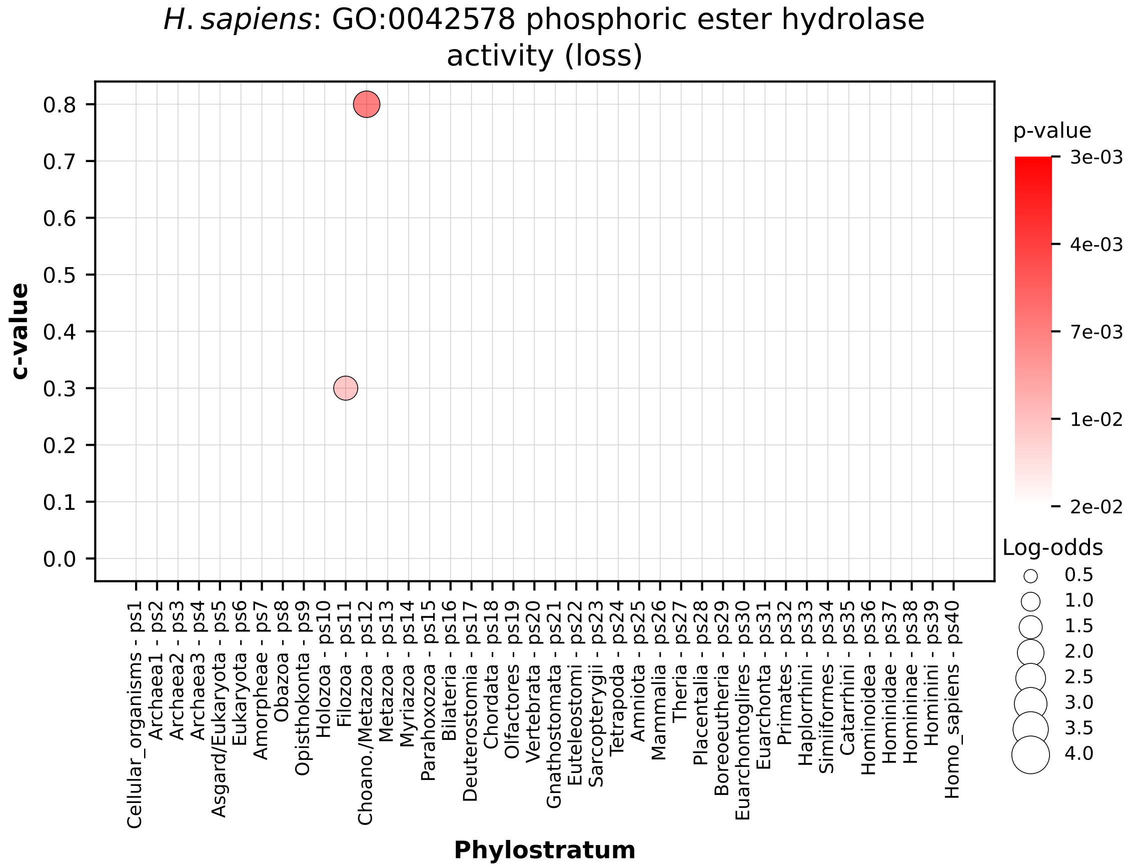
H. sapiens: GO:0042578 phosphoric ester hydrolase activity (loss)
c-value
0.0
0.1
0.2
0.3
0.4
0.5
0.6
0.7
0.8
Phylostratum
Cellular_organisms - ps1
Archaea1 - ps2
Archaea2 - ps3
Archaea3 - ps4
Asgard/Eukaryota - ps5
Eukaryota - ps6
Amorpheae - ps7
Obazoa - ps8
Opisthokonta - ps9
Holozoa - ps10
Filozoa - ps11
Choano./Metazoa - ps12
Metazoa - ps13
Myriazoa - ps14
Parahoxozoa - ps15
Bilateria - ps16
Deuterostomia - ps17
Chordata - ps18
Olfactores - ps19
Vertebrata - ps20
Gnathostomata - ps21
Euteleostomi - ps22
Sarcopterygii - ps23
Tetrapoda - ps24
Amniota - ps25
Mammalia - ps26
Theria - ps27
Placentalia - ps28
Boreoeutheria - ps29
Euarchontoglires - ps30
Euarchonta - ps31
Primates - ps32
Haplorrhini - ps33
Simiiformes - ps34
Catarrhini - ps35
Hominoidea - ps36
Hominidae - ps37
Homininae - ps38
Hominini - ps39
Homo_sapiens - ps40
p-value
3e-03
4e-03
7e-03
1e-02
2e-02
Log-odds
0.5
1.0
1.5
2.0
2.5
3.0
3.5
4.0

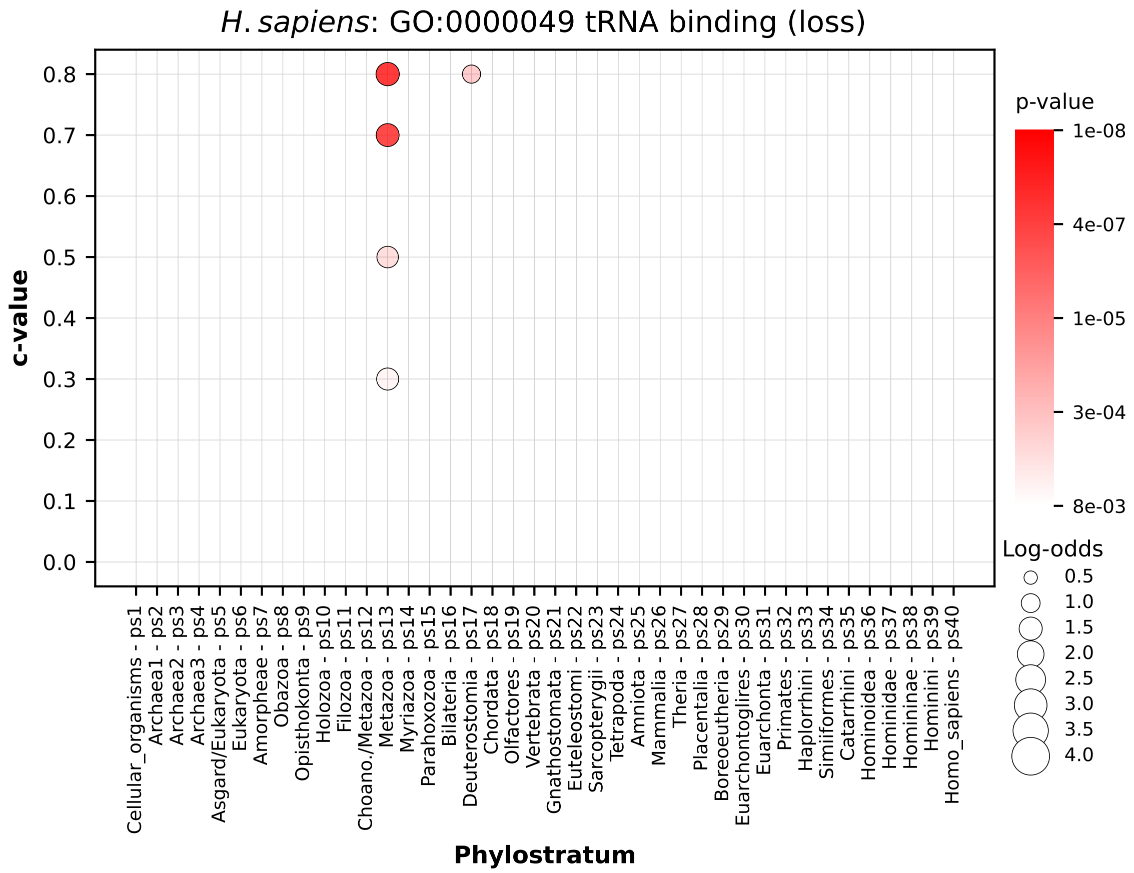

H. sapiens: GO:0000049 tRNA binding (loss)
c-value
0.0
0.1
0.2
0.3
0.4
0.5
0.6
0.7
0.8
p-value
1e-08
4e-07
1e-05
3e-04
8e-03
Log-odds
0.5
1.0
1.5
2.0
2.5
3.0
3.5
4.0
Cellular_organisms - ps1
Archaea1 - ps2
Archaea2 - ps3
Archaea3 - ps4
Asgard/Eukaryota - ps5
Eukaryota - ps6
Amorpheae - ps7
Obazoa - ps8
Opisthokonta - ps9
Holozoa - ps10
Filozoa - ps11
Choano./Metazoa - ps12
Metazoa - ps13
Myriazoa - ps14
Parahoxozoa - ps15
Bilateria - ps16
Deuterostomia - ps17
Chordata - ps18
Olfactores - ps19
Vertebrata - ps20
Gnathostomata - ps21
Euteleostomi - ps22
Sarcopterygii - ps23
Tetrapoda - ps24
Amniota - ps25
Mammalia - ps26
Theria - ps27
Placentalia - ps28
Boreoeutheria - ps29
Euarchontoglires - ps30
Euarchonta - ps31
Primates - ps32
Haplorrhini - ps33
Simiiformes - ps34
Catarrhini - ps35
Hominoidea - ps36
Hominidae - ps37
Homininae - ps38
Hominini - ps39
Homo_sapiens - ps40
Phylostratum

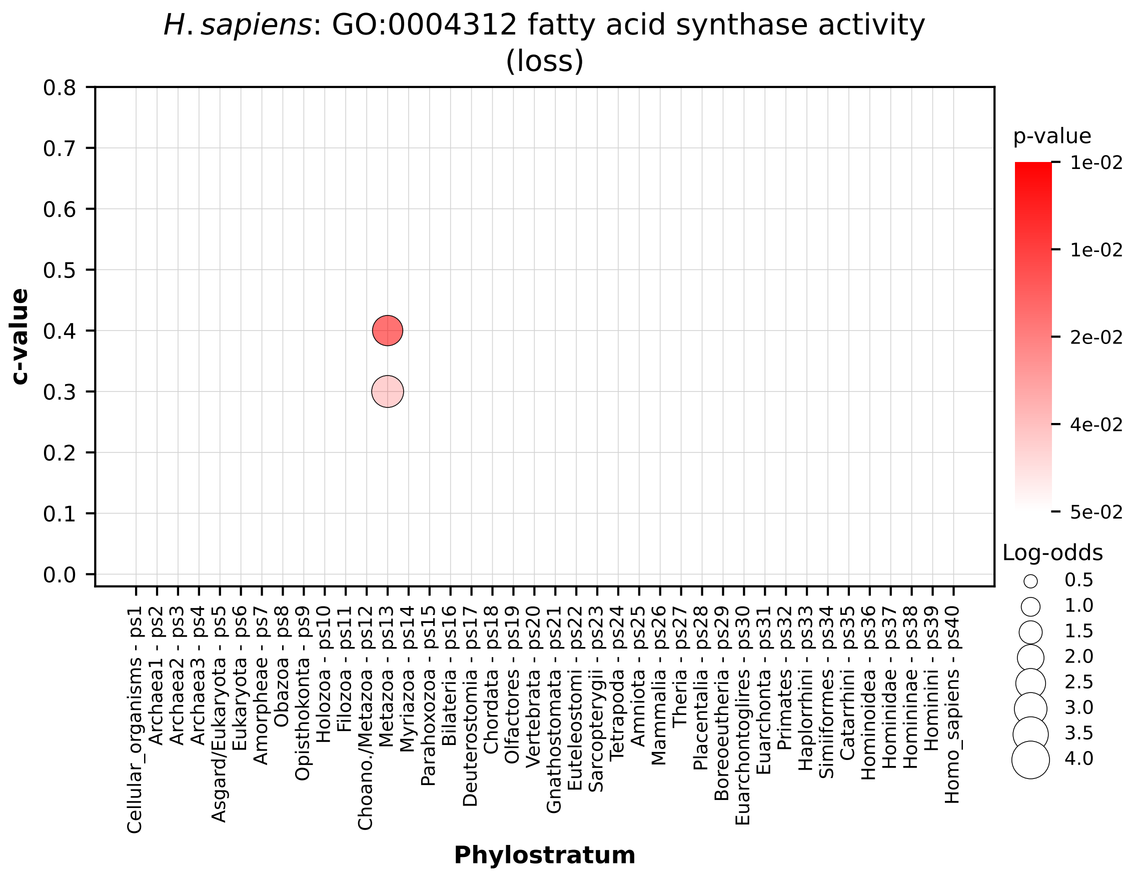

H. sapiens: GO:0004312 fatty acid synthase activity
(loss)
c-value
0.0
0.1
0.2
0.3
0.4
0.5
0.6
0.7
0.8
p-value
1e-02
1e-02
2e-02
4e-02
5e-02
Log-odds
0.5
1.0
1.5
2.0
2.5
3.0
3.5
4.0
Cellular_organisms - ps1
Archaea1 - ps2
Archaea2 - ps3
Archaea3 - ps4
Asgard/Eukaryota - ps5
Eukaryota - ps6
Amorpheae - ps7
Obazoa - ps8
Opisthokonta - ps9
Holozoa - ps10
Filozoa - ps11
Choano./Metazoa - ps12
Metazoa - ps13
Myriazoa - ps14
Parahoxozoa - ps15
Bilateria - ps16
Deuterostomia - ps17
Chordata - ps18
Olfactores - ps19
Vertebrata - ps20
Gnathostomata - ps21
Euteleostomi - ps22
Sarcopterygii - ps23
Tetrapoda - ps24
Amniota - ps25
Mammalia - ps26
Theria - ps27
Placentalia - ps28
Boreoeutheria - ps29
Euarchontoglires - ps30
Euarchonta - ps31
Primates - ps32
Haplorrhini - ps33
Simiiformes - ps34
Catarrhini - ps35
Hominoidea - ps36
Hominidae - ps37
Homininae - ps38
Hominini - ps39
Homo_sapiens - ps40
Phylostratum

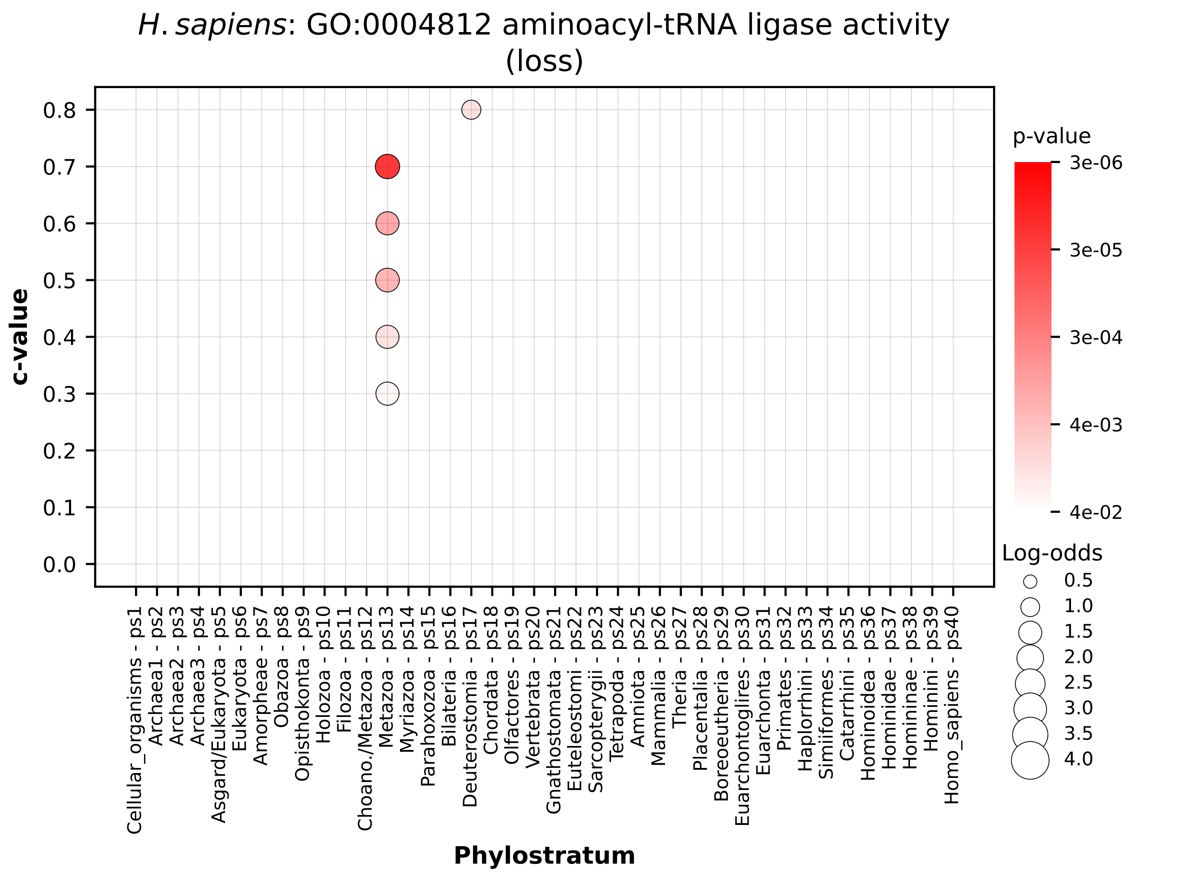

H. sapiens: GO:0004812 aminoacyl-tRNA ligase activity
(loss)
c-value
0.8
0.7
0.6
0.5
0.4
0.3
0.2
0.1
0.0
p-value
3e-06
3e-05
3e-04
4e-03
4e-02
Log-odds
0.5
1.0
1.5
2.0
2.5
3.0
3.5
4.0
Cellular_organisms - ps1
Archaea1 - ps2
Archaea2 - ps3
Archaea3 - ps4
Asgard/Eukaryota - ps5
Eukaryota - ps6
Amorpheae - ps7
Obazoa - ps8
Opisthokonta - ps9
Holozoa - ps10
Filozoa - ps11
Choano./Metazoa - ps12
Metazoa - ps13
Myriazoa - ps14
Parahoxozoa - ps15
Bilateria - ps16
Deuterostomia - ps17
Chordata - ps18
Olfactores - ps19
Vertebrata - ps20
Gnathostomata - ps21
Euteleostomi - ps22
Sarcopterygii - ps23
Tetrapoda - ps24
Amniota - ps25
Mammalia - ps26
Theria - ps27
Placentalia - ps28
Boreoeutheria - ps29
Euarchontoglires - ps30
Euarchonta - ps31
Primates - ps32
Haplorrhini - ps33
Simiiformes - ps34
Catarrhini - ps35
Hominoidea - ps36
Hominidae - ps37
Homininae - ps38
Hominini - ps39
Homo_sapiens - ps40
Phylostratum

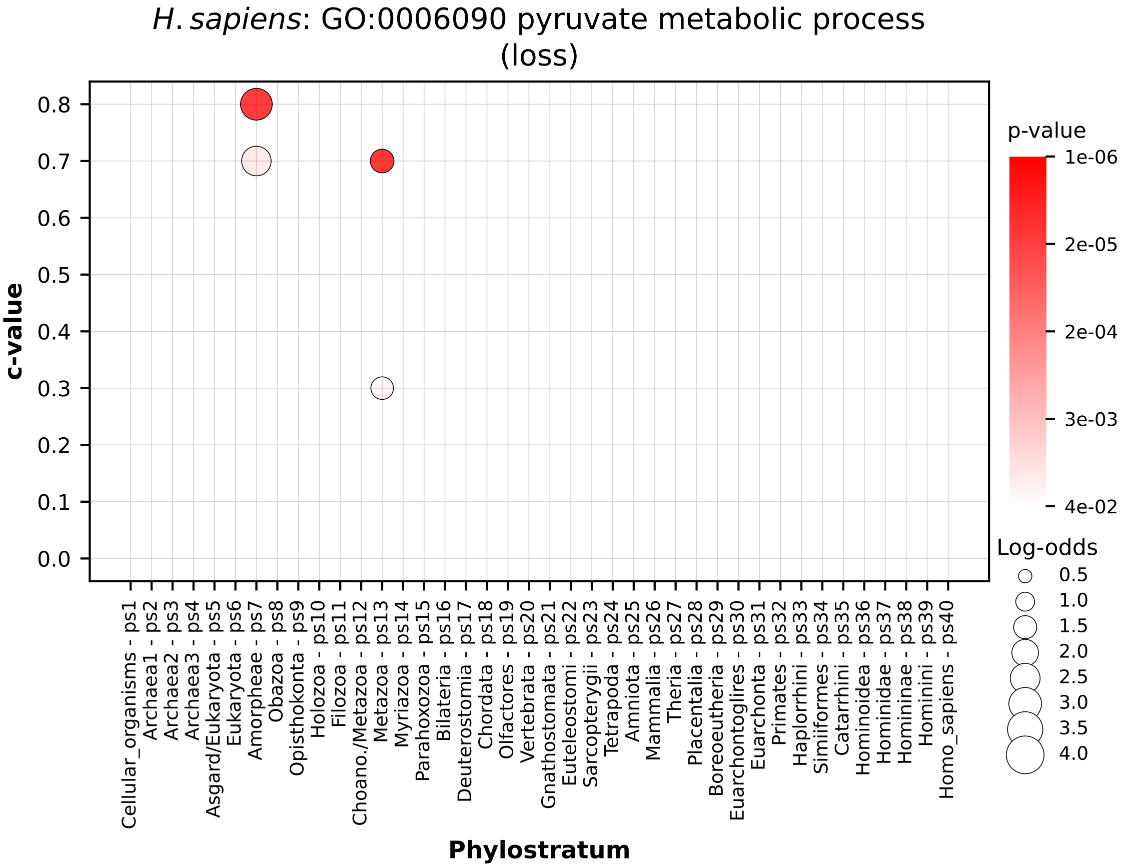

H. sapiens: GO:0006090 pyruvate metabolic process
(loss)
c-value
0.0
0.1
0.2
0.3
0.4
0.5
0.6
0.7
0.8
Phylostratum
Cellular_organisms - ps1
Archaea1 - ps2
Archaea2 - ps3
Archaea3 - ps4
Asgard/Eukaryota - ps5
Eukaryota - ps6
Amorpheae - ps7
Obazoa - ps8
Opisthokonta - ps9
Holozoa - ps10
Filozoa - ps11
Choano./Metazoa - ps12
Metazoa - ps13
Myriazoa - ps14
Parahoxozoa - ps15
Bilateria - ps16
Deuterostomia - ps17
Chordata - ps18
Olfactores - ps19
Vertebrata - ps20
Gnathostomata - ps21
Euteleostomi - ps22
Sarcopterygii - ps23
Tetrapoda - ps24
Amniota - ps25
Mammalia - ps26
Theria - ps27
Placentalia - ps28
Boreoeutheria - ps29
Euarchontoglires - ps30
Euarchonta - ps31
Primates - ps32
Haplorrhini - ps33
Simiiformes - ps34
Catarrhini - ps35
Hominoidea - ps36
Hominidae - ps37
Homininae - ps38
Hominini - ps39
Homo_sapiens - ps40
p-value
1e-06
2e-05
2e-04
3e-03
4e-02
Log-odds
0.5
1.0
1.5
2.0
2.5
3.0
3.5
4.0

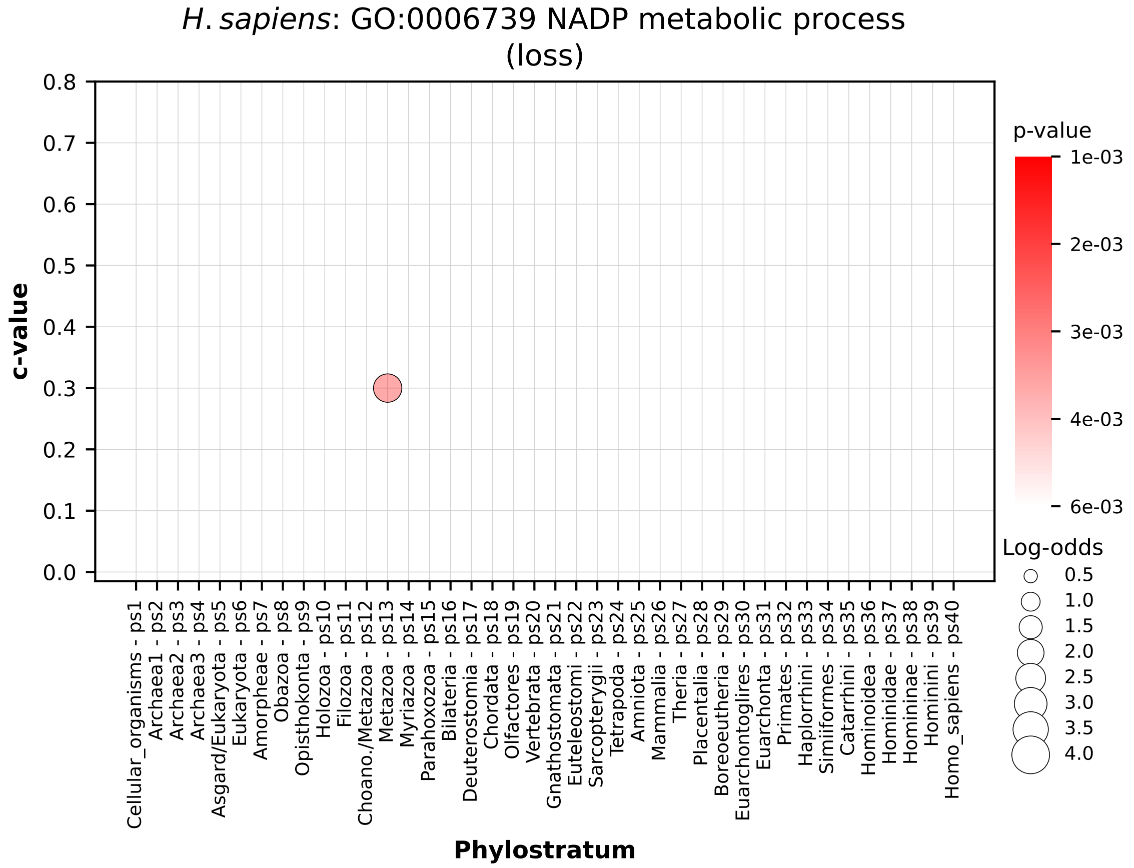
H. sapiens: GO:0006739 NADP metabolic process
(loss)
c-value
0.0
0.1
0.2
0.3
0.4
0.5
0.6
0.7
0.8
Phylostratum
Cellular_organisms - ps1
Archaea1 - ps2
Archaea2 - ps3
Archaea3 - ps4
Asgard/Eukaryota - ps5
Eukaryota - ps6
Amorpheae - ps7
Obazoa - ps8
Opisthokonta - ps9
Holozoa - ps10
Filozoa - ps11
Choano./Metazoa - ps12
Metazoa - ps13
Myriazoa - ps14
Parahoxozoa - ps15
Bilateria - ps16
Deuterostomia - ps17
Chordata - ps18
Olfactores - ps19
Vertebrata - ps20
Gnathostomata - ps21
Euteleostomi - ps22
Sarcopterygii - ps23
Tetrapoda - ps24
Amniota - ps25
Mammalia - ps26
Theria - ps27
Placentalia - ps28
Boreoeutheria - ps29
Euarchontoglires - ps30
Euarchonta - ps31
Primates - ps32
Haplorrhini - ps33
Simiiformes - ps34
Catarrhini - ps35
Hominoidea - ps36
Hominidae - ps37
Homininae - ps38
Hominini - ps39
Homo_sapiens - ps40
p-value
1e-03
2e-03
3e-03
4e-03
6e-03
Log-odds
0.5
1.0
1.5
2.0
2.5
3.0
3.5
4.0

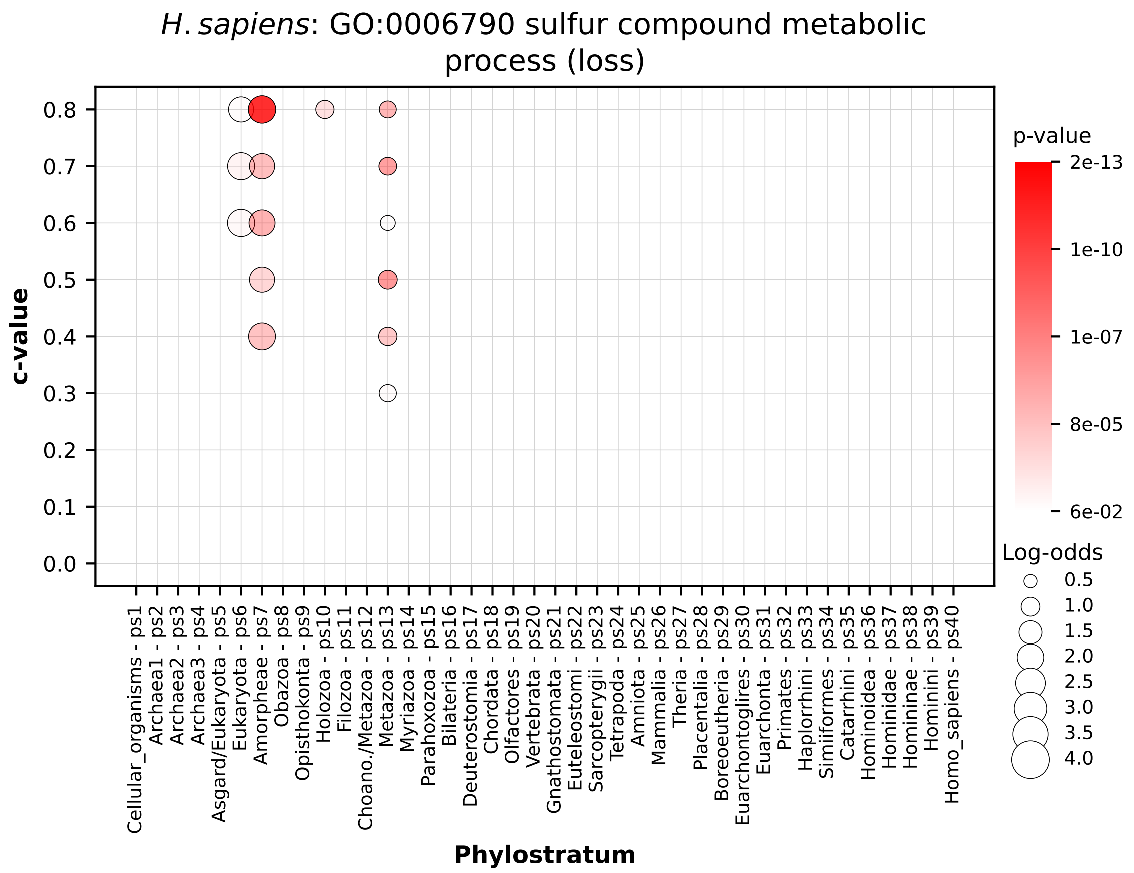

H. sapiens: GO:0006790 sulfur compound metabolic process (loss)
c-value
0.0
0.1
0.2
0.3
0.4
0.5
0.6
0.7
0.8
Phylostratum
Cellular_organisms - ps1
Archaea1 - ps2
Archaea2 - ps3
Archaea3 - ps4
Asgard/Eukaryota - ps5
Eukaryota - ps6
Amorpheae - ps7
Obazoa - ps8
Opisthokonta - ps9
Holozoa - ps10
Filozoa - ps11
Choano./Metazoa - ps12
Metazoa - ps13
Myriazoa - ps14
Parahoxozoa - ps15
Bilateria - ps16
Deuterostomia - ps17
Chordata - ps18
Olfactores - ps19
Vertebrata - ps20
Gnathostomata - ps21
Euteleostomi - ps22
Sarcopterygii - ps23
Tetrapoda - ps24
Amniota - ps25
Mammalia - ps26
Theria - ps27
Placentalia - ps28
Boreoeutheria - ps29
Euarchontoglires - ps30
Euarchonta - ps31
Primates - ps32
Haplorrhini - ps33
Simiiformes - ps34
Catarrhini - ps35
Hominoidea - ps36
Hominidae - ps37
Homininae - ps38
Hominini - ps39
Homo_sapiens - ps40
p-value
2e-13
1e-10
1e-07
8e-05
6e-02
Log-odds
0.5
1.0
1.5
2.0
2.5
3.0
3.5
4.0

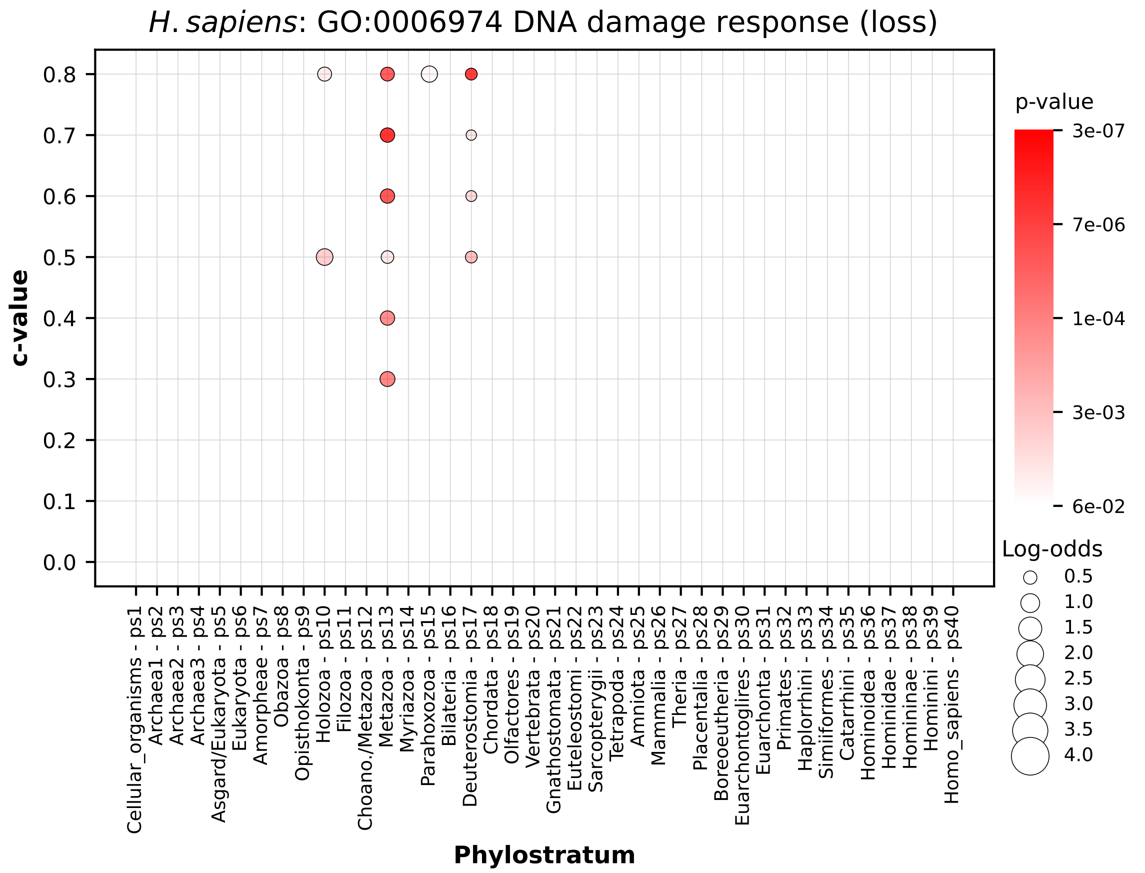

H. sapiens: GO:0006974 DNA damage response (loss)
c-value
0.8
0.7
0.6
0.5
0.4
0.3
0.2
0.1
0.0
p-value
3e-07
7e-06
1e-04
3e-03
6e-02
Log-odds
0.5
1.0
1.5
2.0
2.5
3.0
3.5
4.0
Cellular_organisms - ps1
Archaea1 - ps2
Archaea2 - ps3
Archaea3 - ps4
Asgard/Eukaryota - ps5
Eukaryota - ps6
Amorpheae - ps7
Obazoa - ps8
Opisthokonta - ps9
Holozoa - ps10
Filozoa - ps11
Choano./Metazoa - ps12
Metazoa - ps13
Myriazoa - ps14
Parahoxozoa - ps15
Bilateria - ps16
Deuterostomia - ps17
Chordata - ps18
Olfactores - ps19
Vertebrata - ps20
Gnathostomata - ps21
Euteleostomi - ps22
Sarcopterygii - ps23
Tetrapoda - ps24
Amniota - ps25
Mammalia - ps26
Theria - ps27
Placentalia - ps28
Boreoeutheria - ps29
Euarchontoglires - ps30
Euarchonta - ps31
Primates - ps32
Haplorrhini - ps33
Simiiformes - ps34
Catarrhini - ps35
Hominoidea - ps36
Hominidae - ps37
Homininae - ps38
Hominini - ps39
Homo_sapiens - ps40
Phylostratum

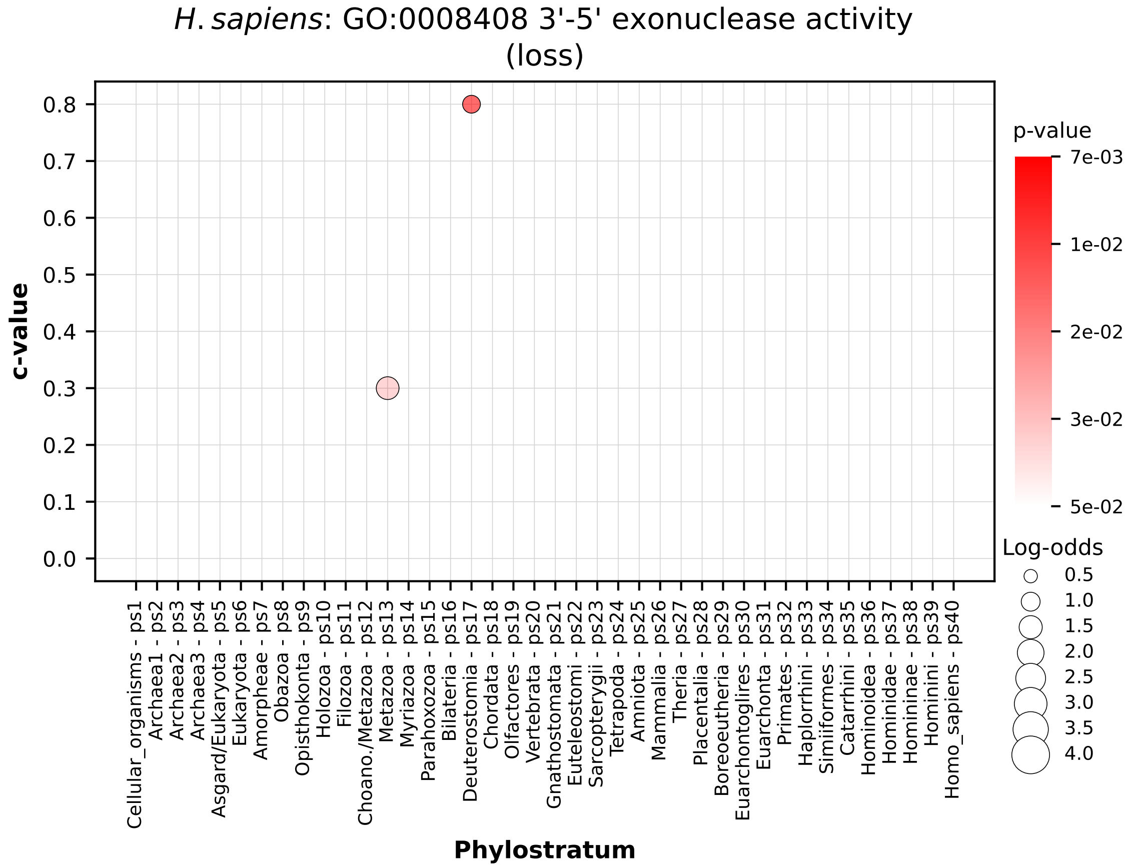

H. sapiens: GO:0008408 3'-5' exonuclease activity (loss)
c-value
0.0
0.1
0.2
0.3
0.4
0.5
0.6
0.7
0.8
p-value
7e-03
1e-02
2e-02
3e-02
5e-02
Log-odds
0.5
1.0
1.5
2.0
2.5
3.0
3.5
4.0
Cellular_organisms - ps1
Archaea1 - ps2
Archaea2 - ps3
Archaea3 - ps4
Asgard/Eukaryota - ps5
Eukaryota - ps6
Amorpheae - ps7
Obazoa - ps8
Opisthokonta - ps9
Holozoa - ps10
Filozoa - ps11
Choano./Metazoa - ps12
Metazoa - ps13
Myriazoa - ps14
Parahoxozoa - ps15
Bilateria - ps16
Deuterostomia - ps17
Chordata - ps18
Olfactores - ps19
Vertebrata - ps20
Gnathostomata - ps21
Euteleostomi - ps22
Sarcopterygii - ps23
Tetrapoda - ps24
Amniota - ps25
Mammalia - ps26
Theria - ps27
Placentalia - ps28
Boreoeutheria - ps29
Euarchontoglires - ps30
Euarchonta - ps31
Primates - ps32
Haplorrhini - ps33
Simiiformes - ps34
Catarrhini - ps35
Hominoidea - ps36
Hominidae - ps37
Homininae - ps38
Hominini - ps39
Homo_sapiens - ps40
Phylostratum

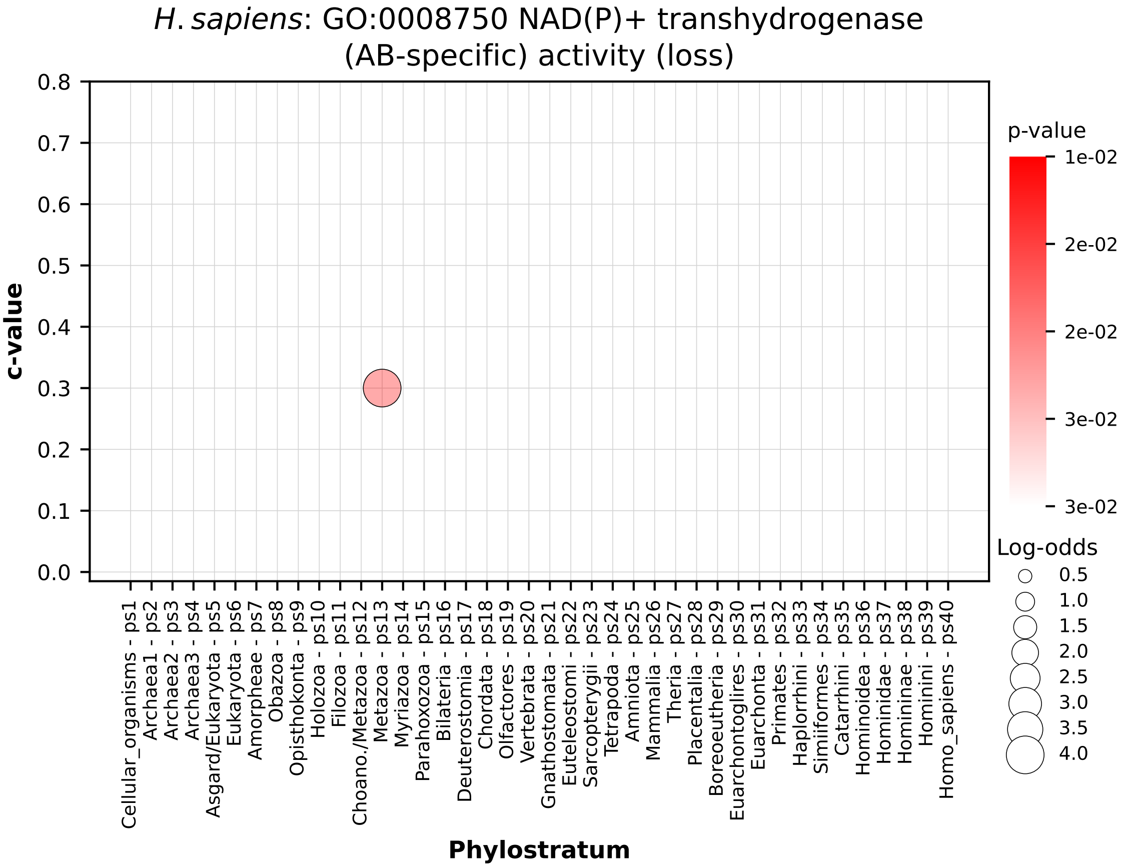
H. sapiens: GO:0008750 NAD(P)+ transhydrogenase (AB-specific) activity (loss)
c-value
0.0
0.1
0.2
0.3
0.4
0.5
0.6
0.7
0.8
Phylostratum
Cellular_organisms - ps1
Archaea1 - ps2
Archaea2 - ps3
Archaea3 - ps4
Asgard/Eukaryota - ps5
Eukaryota - ps6
Amorpheae - ps7
Obazoa - ps8
Opisthokonta - ps9
Holozoa - ps10
Filozoa - ps11
Choano./Metazoa - ps12
Metazoa - ps13
Myriazoa - ps14
Parahoxozoa - ps15
Bilateria - ps16
Deuterostomia - ps17
Chordata - ps18
Olfactores - ps19
Vertebrata - ps20
Gnathostomata - ps21
Euteleostomi - ps22
Sarcopterygii - ps23
Tetrapoda - ps24
Amniota - ps25
Mammalia - ps26
Theria - ps27
Placentalia - ps28
Boreoeutheria - ps29
Euarchontoglires - ps30
Euarchonta - ps31
Primates - ps32
Haplorrhini - ps33
Simiiformes - ps34
Catarrhini - ps35
Hominoidea - ps36
Hominidae - ps37
Homininae - ps38
Hominini - ps39
Homo_sapiens - ps40
p-value
1e-02
2e-02
2e-02
3e-02
3e-02
Log-odds
0.5
1.0
1.5
2.0
2.5
3.0
3.5
4.0

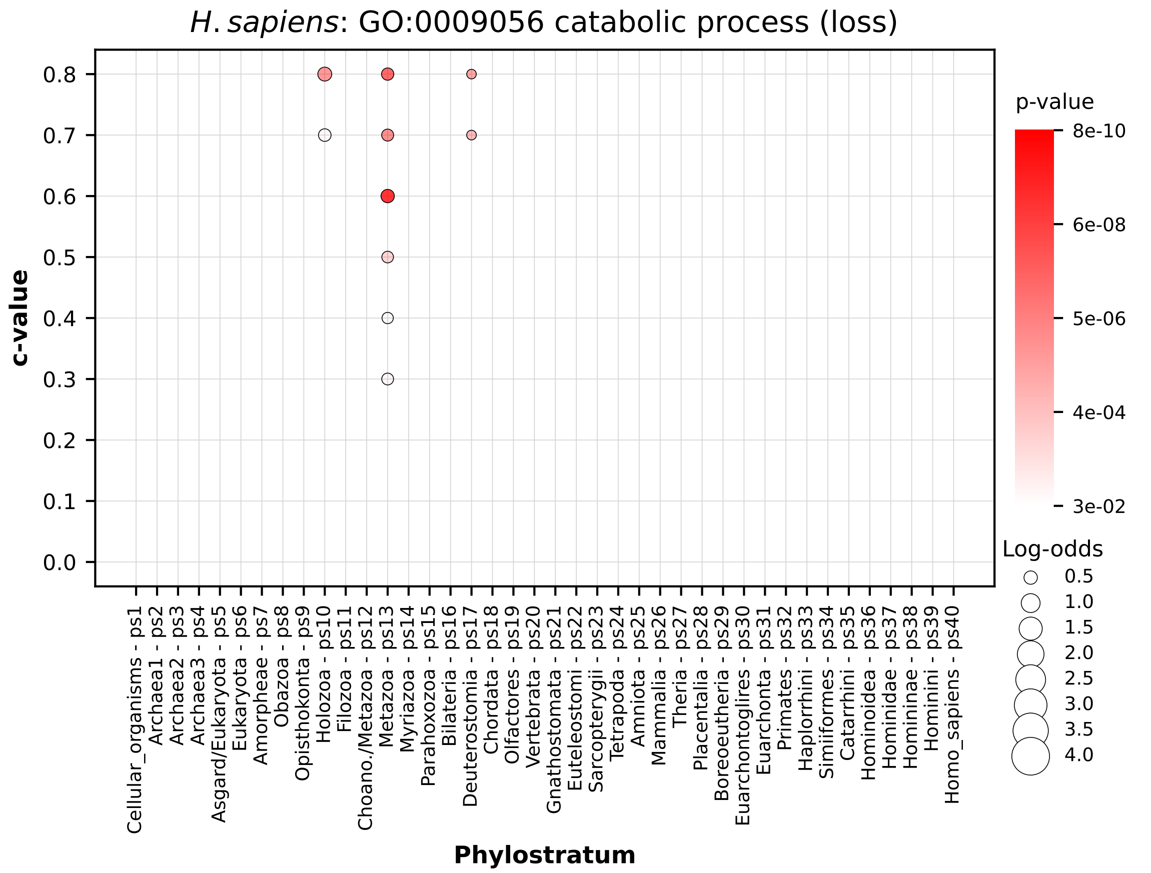

H. sapiens: GO:0009056 catabolic process (loss)
c-value
0.0
0.1
0.2
0.3
0.4
0.5
0.6
0.7
0.8
Phylostratum
Cellular_organisms - ps1
Archaea1 - ps2
Archaea2 - ps3
Archaea3 - ps4
Asgard/Eukaryota - ps5
Eukaryota - ps6
Amorpheae - ps7
Obazoa - ps8
Opisthokonta - ps9
Holozoa - ps10
Filozoa - ps11
Choano./Metazoa - ps12
Metazoa - ps13
Myriazoa - ps14
Parahoxozoa - ps15
Bilateria - ps16
Deuterostomia - ps17
Chordata - ps18
Olfactores - ps19
Vertebrata - ps20
Gnathostomata - ps21
Euteleostomi - ps22
Sarcopterygii - ps23
Tetrapoda - ps24
Amniota - ps25
Mammalia - ps26
Theria - ps27
Placentalia - ps28
Boreoeutheria - ps29
Euarchontoglires - ps30
Euarchonta - ps31
Primates - ps32
Haplorrhini - ps33
Simiiformes - ps34
Catarrhini - ps35
Hominoidea - ps36
Hominidae - ps37
Homininae - ps38
Hominini - ps39
Homo_sapiens - ps40
p-value
8e-10
6e-08
5e-06
4e-04
3e-02
Log-odds
0.5
1.0
1.5
2.0
2.5
3.0
3.5
4.0

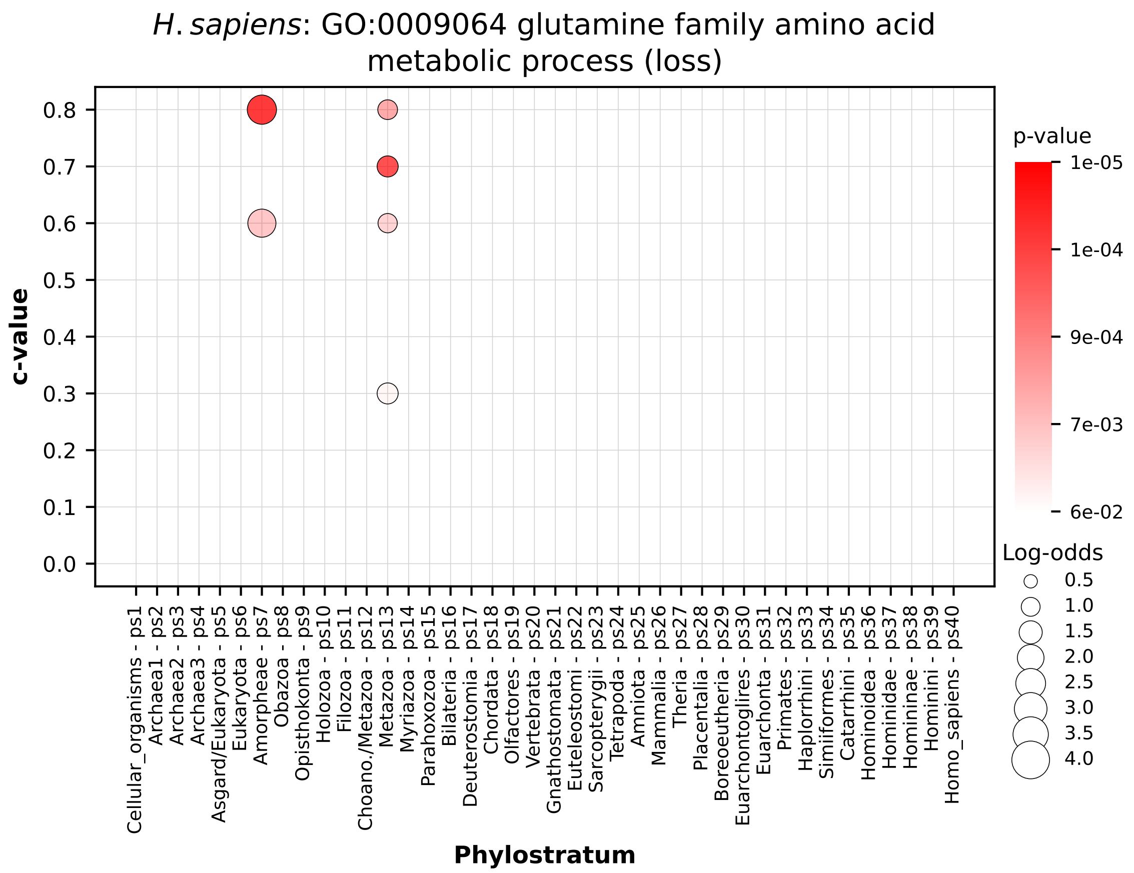

H. sapiens: GO:0009064 glutamine family amino acid metabolic process (loss)
c-value
0.8
0.7
0.6
0.5
0.4
0.3
0.2
0.1
0.0
Phylostratum
Cellular_organisms - ps1
Archaea1 - ps2
Archaea2 - ps3
Archaea3 - ps4
Asgard/Eukaryota - ps5
Eukaryota - ps6
Amorpheae - ps7
Obazoa - ps8
Opisthokonta - ps9
Holozoa - ps10
Filozoa - ps11
Choano./Metazoa - ps12
Metazoa - ps13
Myriazoa - ps14
Parahoxozoa - ps15
Bilateria - ps16
Deuterostomia - ps17
Chordata - ps18
Olfactores - ps19
Vertebrata - ps20
Gnathostomata - ps21
Euteleostomi - ps22
Sarcopterygii - ps23
Tetrapoda - ps24
Amniota - ps25
Mammalia - ps26
Theria - ps27
Placentalia - ps28
Boreoeutheria - ps29
Euarchontoglires - ps30
Euarchonta - ps31
Primates - ps32
Haplorrhini - ps33
Simiiformes - ps34
Catarrhini - ps35
Hominoidea - ps36
Hominidae - ps37
Homininae - ps38
Hominini - ps39
Homo_sapiens - ps40
p-value
1e-05
1e-04
9e-04
7e-03
6e-02
Log-odds
0.5
1.0
1.5
2.0
2.5
3.0
3.5
4.0

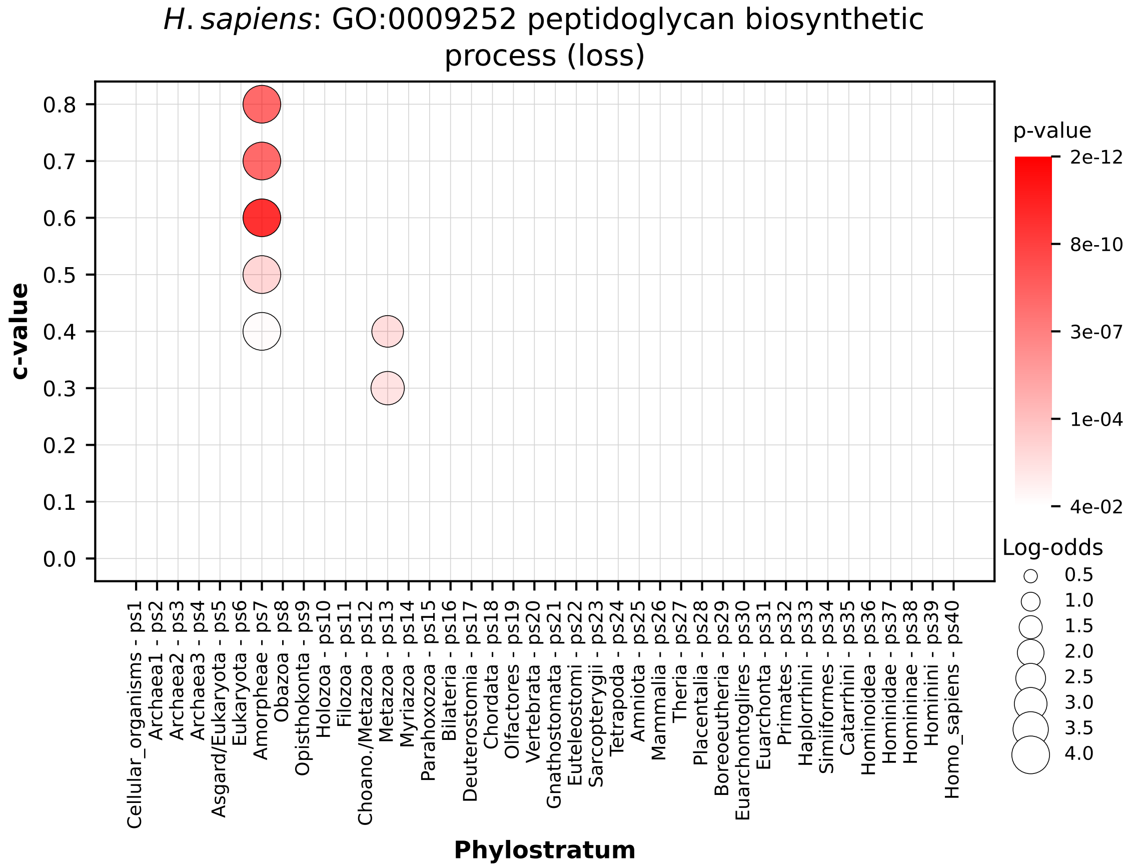

H. sapiens: GO:0009252 peptidoglycan biosynthetic process (loss)
c-value
0.8
0.7
0.6
0.5
0.4
0.3
0.2
0.1
0.0
p-value
2e-12
8e-10
3e-07
1e-04
4e-02
Log-odds
0.5
1.0
1.5
2.0
2.5
3.0
3.5
4.0
Cellular_organisms - ps1
Archaea1 - ps2
Archaea2 - ps3
Archaea3 - ps4
Asgard/Eukaryota - ps5
Eukaryota - ps6
Amorpheae - ps7
Obazoa - ps8
Opisthokonta - ps9
Holozoa - ps10
Filozoa - ps11
Choano./Metazoa - ps12
Metazoa - ps13
Myriazoa - ps14
Parahoxozoa - ps15
Bilateria - ps16
Deuterostomia - ps17
Chordata - ps18
Olfactores - ps19
Vertebrata - ps20
Gnathostomata - ps21
Euteleostomi - ps22
Sarcopterygii - ps23
Tetrapoda - ps24
Amniota - ps25
Mammalia - ps26
Theria - ps27
Placentalia - ps28
Boreoeutheria - ps29
Euarchontoglires - ps30
Euarchonta - ps31
Primates - ps32
Haplorrhini - ps33
Simiiformes - ps34
Catarrhini - ps35
Hominoidea - ps36
Hominidae - ps37
Homininae - ps38
Hominini - ps39
Homo_sapiens - ps40
Phylostratum

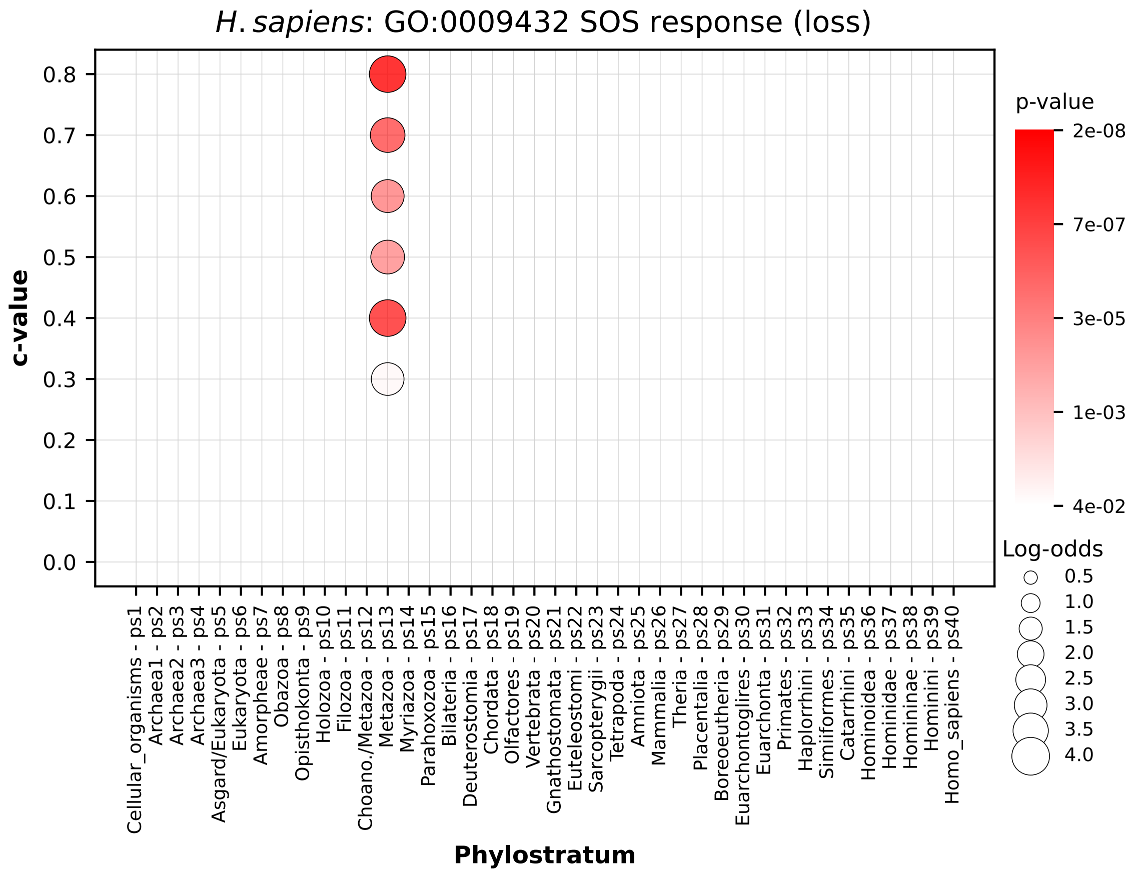

H. sapiens: GO:0009432 SOS response (loss)
c-value
0.8
0.7
0.6
0.5
0.4
0.3
0.2
0.1
0.0
p-value
2e-08
7e-07
3e-05
1e-03
4e-02
Log-odds
0.5
1.0
1.5
2.0
2.5
3.0
3.5
4.0
Cellular_organisms - ps1
Archaea1 - ps2
Archaea2 - ps3
Archaea3 - ps4
Asgard/Eukaryota - ps5
Eukaryota - ps6
Amorpheae - ps7
Obazoa - ps8
Opisthokonta - ps9
Holozoa - ps10
Filozoa - ps11
Choano./Metazoa - ps12
Metazoa - ps13
Myriazoa - ps14
Parahoxozoa - ps15
Bilateria - ps16
Deuterostomia - ps17
Chordata - ps18
Olfactores - ps19
Vertebrata - ps20
Gnathostomata - ps21
Euteleostomi - ps22
Sarcopterygii - ps23
Tetrapoda - ps24
Amniota - ps25
Mammalia - ps26
Theria - ps27
Placentalia - ps28
Boreoeutheria - ps29
Euarchontoglires - ps30
Euarchonta - ps31
Primates - ps32
Haplorrhini - ps33
Simiiformes - ps34
Catarrhini - ps35
Hominoidea - ps36
Hominidae - ps37
Homininae - ps38
Hominini - ps39
Homo_sapiens - ps40
Phylostratum

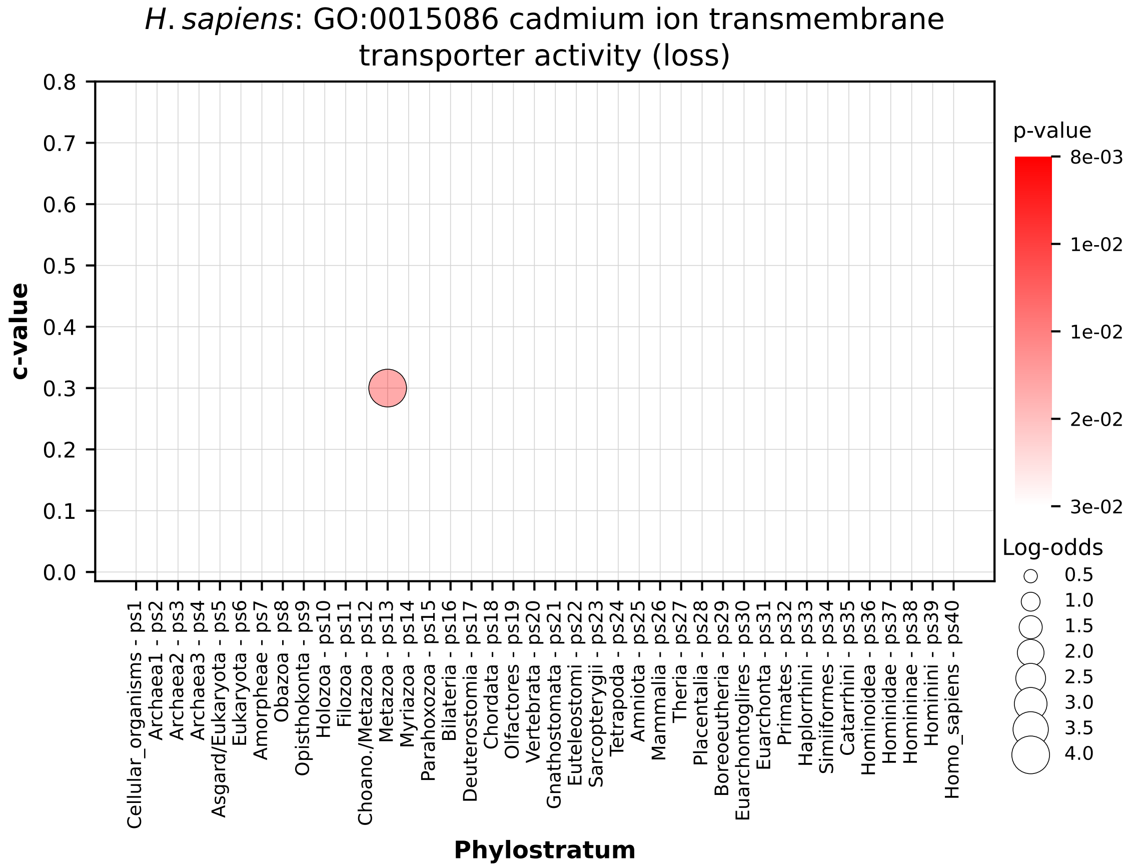

H. sapiens: GO:0015086 cadmium ion transmembrane transporter activity (loss)
c-value
0.0
0.1
0.2
0.3
0.4
0.5
0.6
0.7
0.8
Phylostratum
Cellular_organisms - ps1
Archaea1 - ps2
Archaea2 - ps3
Archaea3 - ps4
Asgard/Eukaryota - ps5
Eukaryota - ps6
Amorpheae - ps7
Obazoa - ps8
Opisthokonta - ps9
Holozoa - ps10
Filozoa - ps11
Choano./Metazoa - ps12
Metazoa - ps13
Myriazoa - ps14
Parahoxozoa - ps15
Bilateria - ps16
Deuterostomia - ps17
Chordata - ps18
Olfactores - ps19
Vertebrata - ps20
Gnathostomata - ps21
Euteleostomi - ps22
Sarcopterygii - ps23
Tetrapoda - ps24
Amniota - ps25
Mammalia - ps26
Theria - ps27
Placentalia - ps28
Boreoeutheria - ps29
Euarchontoglires - ps30
Euarchonta - ps31
Primates - ps32
Haplorrhini - ps33
Simiiformes - ps34
Catarrhini - ps35
Hominoidea - ps36
Hominidae - ps37
Homininae - ps38
Hominini - ps39
Homo_sapiens - ps40
p-value
8e-03
1e-02
1e-02
2e-02
3e-02
Log-odds
0.5
1.0
1.5
2.0
2.5
3.0
3.5
4.0

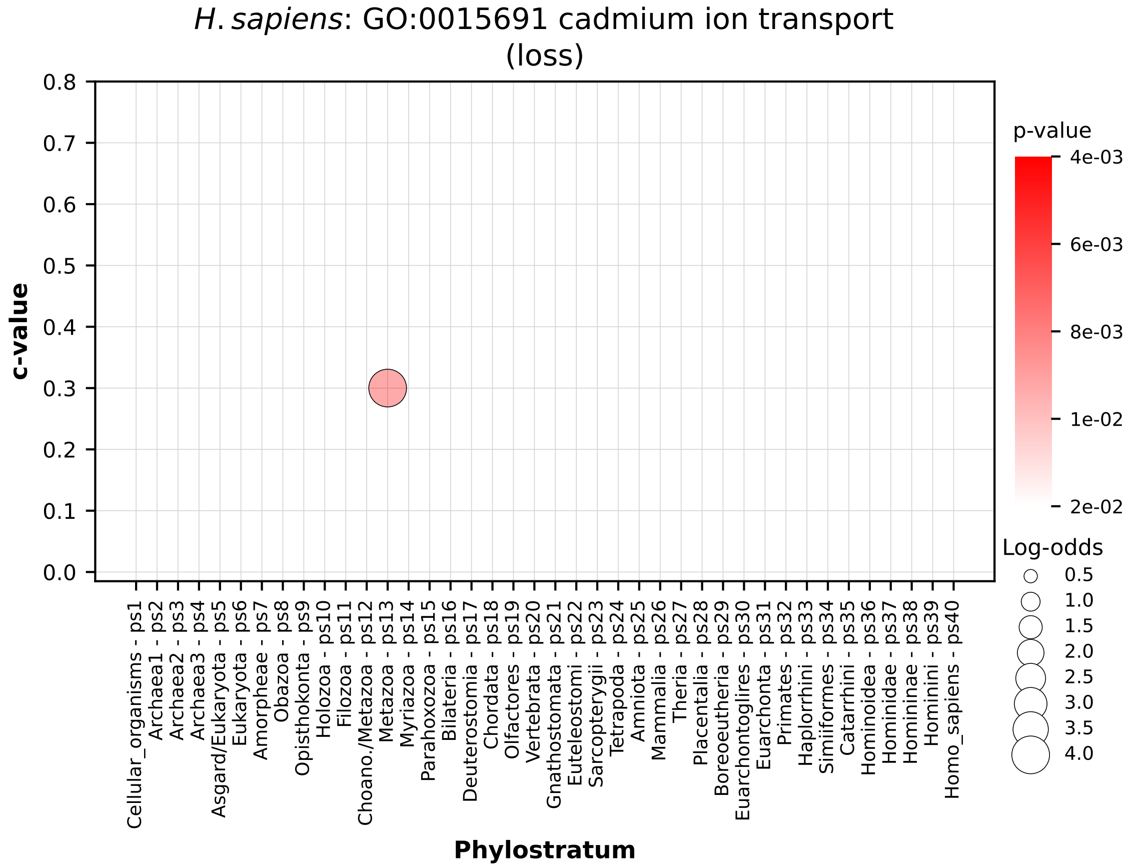
H. sapiens: GO:0015691 cadmium ion transport
(loss)
c-value
0.8
0.7
0.6
0.5
0.4
0.3
0.2
0.1
0.0
Phylostratum
Cellular_organisms - ps1
Archaea1 - ps2
Archaea2 - ps3
Archaea3 - ps4
Asgard/Eukaryota - ps5
Eukaryota - ps6
Amorpheae - ps7
Obazoa - ps8
Opisthokonta - ps9
Holozoa - ps10
Filozoa - ps11
Choano./Metazoa - ps12
Metazoa - ps13
Myriazoa - ps14
Parahoxozoa - ps15
Bilateria - ps16
Deuterostomia - ps17
Chordata - ps18
Olfactores - ps19
Vertebrata - ps20
Gnathostomata - ps21
Euteleostomi - ps22
Sarcopterygii - ps23
Tetrapoda - ps24
Amniota - ps25
Mammalia - ps26
Theria - ps27
Placentalia - ps28
Boreoeutheria - ps29
Euarchontoglires - ps30
Euarchonta - ps31
Primates - ps32
Haplorrhini - ps33
Simiiformes - ps34
Catarrhini - ps35
Hominoidea - ps36
Hominidae - ps37
Homininae - ps38
Hominini - ps39
Homo_sapiens - ps40
p-value
4e-03
6e-03
8e-03
1e-02
2e-02
Log-odds
0.5
1.0
1.5
2.0
2.5
3.0
3.5
4.0

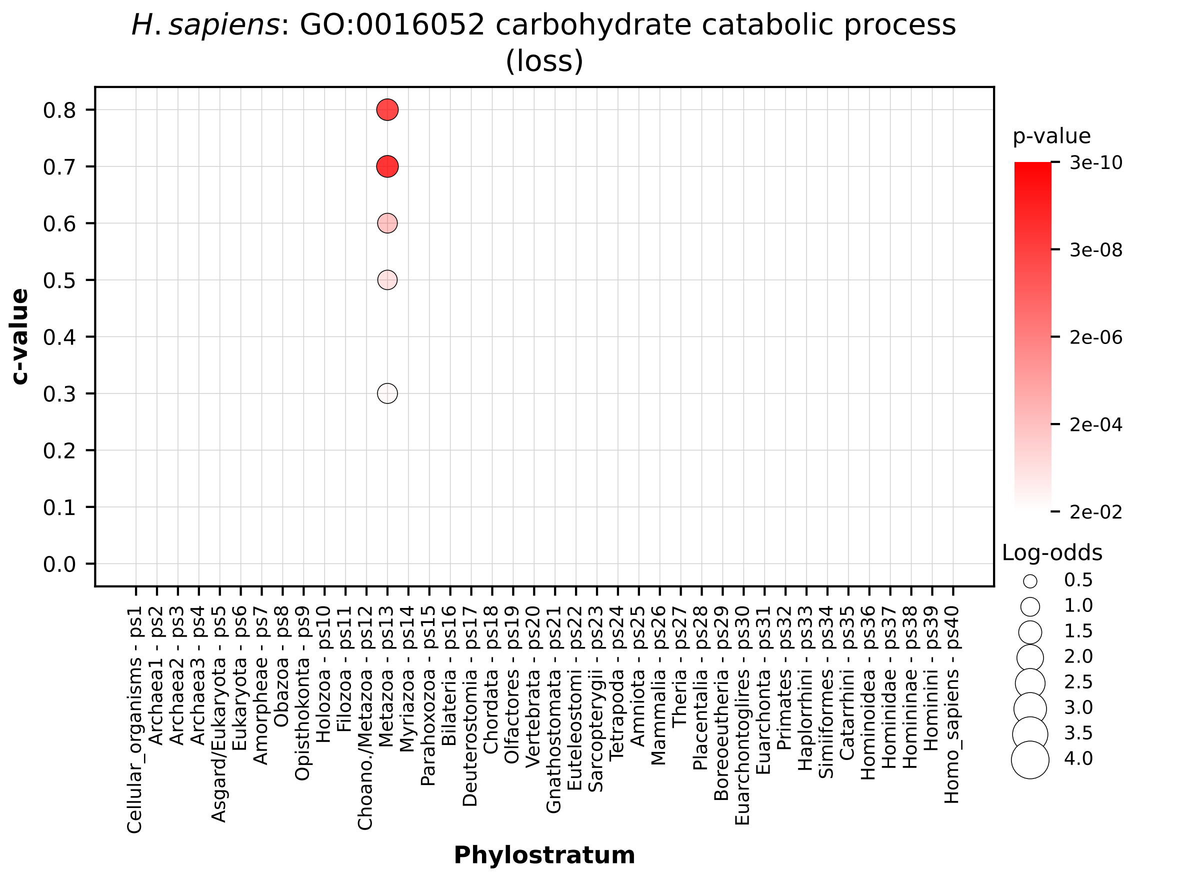

H. sapiens: GO:0016052 carbohydrate catabolic process
(loss)
c-value
0.8
0.7
0.6
0.5
0.4
0.3
0.2
0.1
0.0
p-value
3e-10
3e-08
2e-06
2e-04
2e-02
Log-odds
0.5
1.0
1.5
2.0
2.5
3.0
3.5
4.0
Cellular_organisms - ps1
Archaea1 - ps2
Archaea2 - ps3
Archaea3 - ps4
Asgard/Eukaryota - ps5
Eukaryota - ps6
Amorpheae - ps7
Obazoa - ps8
Opisthokonta - ps9
Holozoa - ps10
Filozoa - ps11
Choano./Metazoa - ps12
Metazoa - ps13
Myriazoa - ps14
Parahoxozoa - ps15
Bilateria - ps16
Deuterostomia - ps17
Chordata - ps18
Olfactores - ps19
Vertebrata - ps20
Gnathostomata - ps21
Euteleostomi - ps22
Sarcopterygii - ps23
Tetrapoda - ps24
Amniota - ps25
Mammalia - ps26
Theria - ps27
Placentalia - ps28
Boreoeutheria - ps29
Euarchontoglires - ps30
Euarchonta - ps31
Primates - ps32
Haplorrhini - ps33
Simiiformes - ps34
Catarrhini - ps35
Hominoidea - ps36
Hominidae - ps37
Homininae - ps38
Hominini - ps39
Homo_sapiens - ps40
Phylostratum

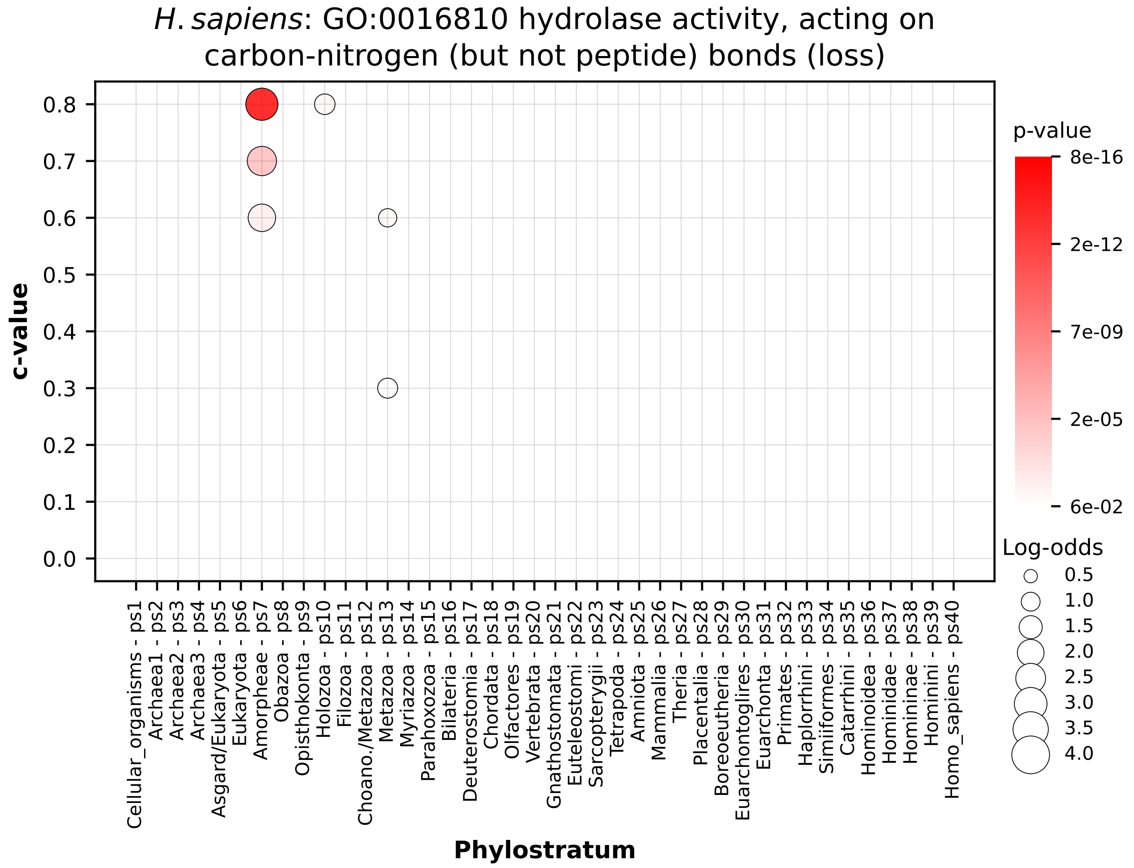

H. sapiens: GO:0016810 hydrolase activity, acting on carbon-nitrogen (but not peptide) bonds (loss)
c-value
0.0
0.1
0.2
0.3
0.4
0.5
0.6
0.7
0.8
p-value
8e-16
2e-12
7e-09
2e-05
6e-02
Log-odds
0.5
1.0
1.5
2.0
2.5
3.0
3.5
4.0
Cellular_organisms - ps1
Archaea1 - ps2
Archaea2 - ps3
Archaea3 - ps4
Asgard/Eukaryota - ps5
Eukaryota - ps6
Amorpheae - ps7
Obazoa - ps8
Opisthokonta - ps9
Holozoa - ps10
Filozoa - ps11
Choano./Metazoa - ps12
Metazoa - ps13
Myriazoa - ps14
Parahoxozoa - ps15
Bilateria - ps16
Deuterostomia - ps17
Chordata - ps18
Olfactores - ps19
Vertebrata - ps20
Gnathostomata - ps21
Euteleostomi - ps22
Sarcopterygii - ps23
Tetrapoda - ps24
Amniota - ps25
Mammalia - ps26
Theria - ps27
Placentalia - ps28
Boreoeutheria - ps29
Euarchontoglires - ps30
Euarchonta - ps31
Primates - ps32
Haplorrhini - ps33
Simiiformes - ps34
Catarrhini - ps35
Hominoidea - ps36
Hominidae - ps37
Homininae - ps38
Hominini - ps39
Homo_sapiens - ps40
Phylostratum

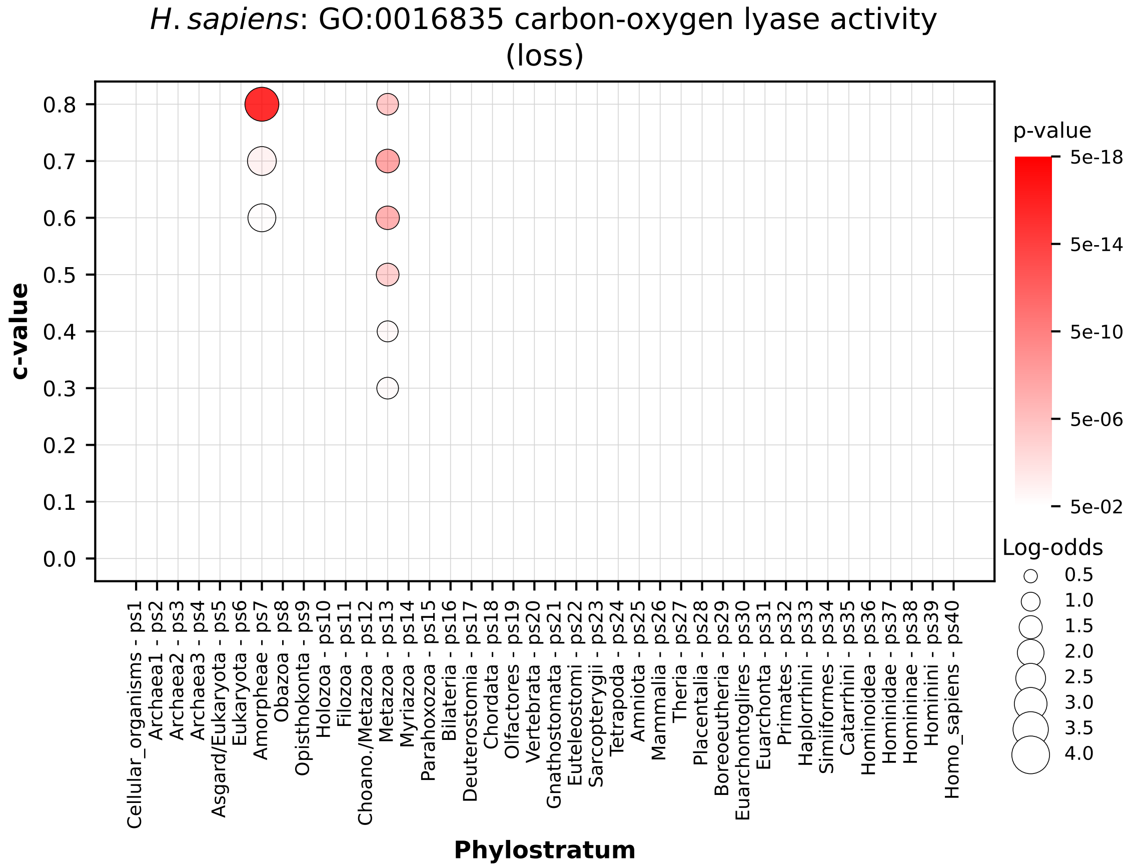

H. sapiens: GO:0016835 carbon-oxygen lyase activity (loss)
c-value
0.0
0.1
0.2
0.3
0.4
0.5
0.6
0.7
0.8
p-value
5e-18
5e-14
5e-10
5e-06
5e-02
Log-odds
0.5
1.0
1.5
2.0
2.5
3.0
3.5
4.0
Cellular_organisms - ps1
Archaea1 - ps2
Archaea2 - ps3
Archaea3 - ps4
Asgard/Eukaryota - ps5
Eukaryota - ps6
Amorpheae - ps7
Obazoa - ps8
Opisthokonta - ps9
Holozoa - ps10
Filozoa - ps11
Choano./Metazoa - ps12
Metazoa - ps13
Myriazoa - ps14
Parahoxozoa - ps15
Bilateria - ps16
Deuterostomia - ps17
Chordata - ps18
Olfactores - ps19
Vertebrata - ps20
Gnathostomata - ps21
Euteleostomi - ps22
Sarcopterygii - ps23
Tetrapoda - ps24
Amniota - ps25
Mammalia - ps26
Theria - ps27
Placentalia - ps28
Boreoeutheria - ps29
Euarchontoglires - ps30
Euarchonta - ps31
Primates - ps32
Haplorrhini - ps33
Simiiformes - ps34
Catarrhini - ps35
Hominoidea - ps36
Hominidae - ps37
Homininae - ps38
Hominini - ps39
Homo_sapiens - ps40
Phylostratum

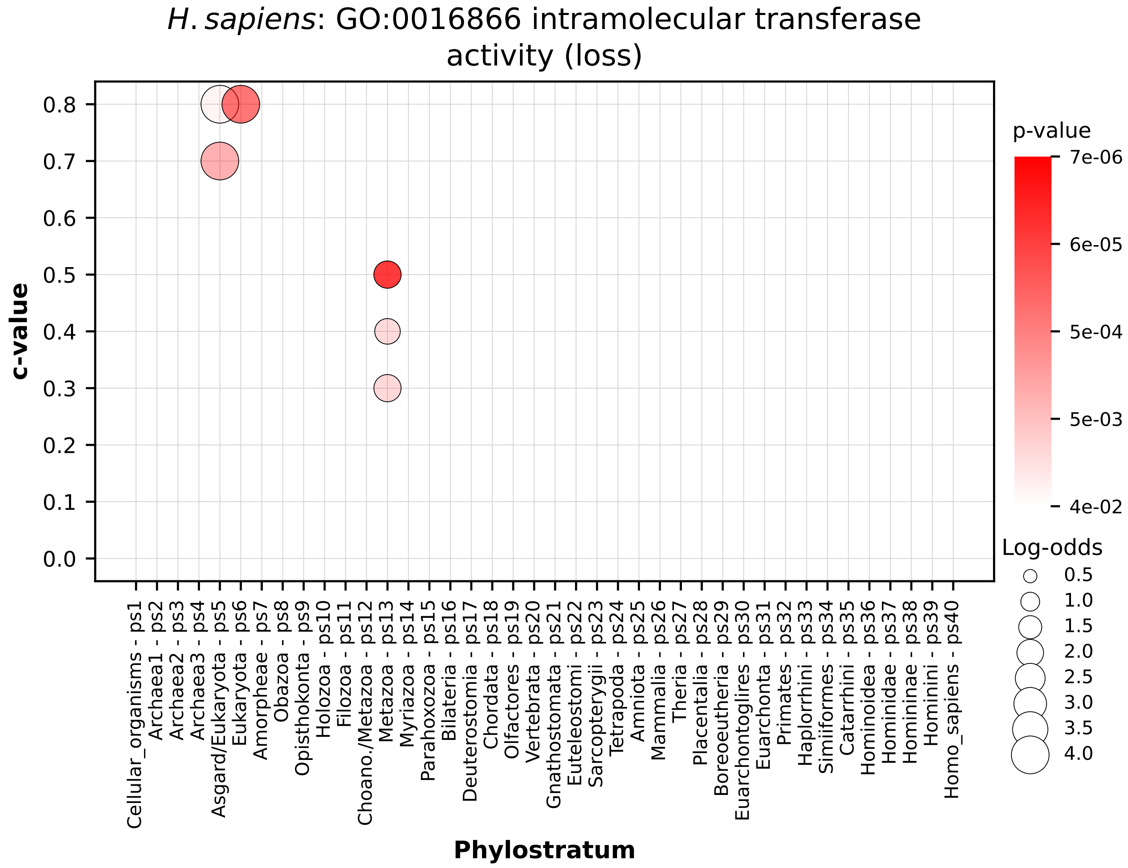

H. sapiens: GO:0016866 intramolecular transferase activity (loss)
c-value
0.8
0.7
0.6
0.5
0.4
0.3
0.2
0.1
0.0
Phylostratum
Cellular_organisms - ps1
Archaea1 - ps2
Archaea2 - ps3
Archaea3 - ps4
Asgard/Eukaryota - ps5
Eukaryota - ps6
Amorpheae - ps7
Obazoa - ps8
Opisthokonta - ps9
Holozoa - ps10
Filozoa - ps11
Choano./Metazoa - ps12
Metazoa - ps13
Myriazoa - ps14
Parahoxozoa - ps15
Bilateria - ps16
Deuterostomia - ps17
Chordata - ps18
Olfactores - ps19
Vertebrata - ps20
Gnathostomata - ps21
Euteleostomi - ps22
Sarcopterygii - ps23
Tetrapoda - ps24
Amniota - ps25
Mammalia - ps26
Theria - ps27
Placentalia - ps28
Boreoeutheria - ps29
Euarchontoglires - ps30
Euarchonta - ps31
Primates - ps32
Haplorrhini - ps33
Simiiformes - ps34
Catarrhini - ps35
Hominoidea - ps36
Hominidae - ps37
Homininae - ps38
Hominini - ps39
Homo_sapiens - ps40
p-value
7e-06
6e-05
5e-04
5e-03
4e-02
Log-odds
0.5
1.0
1.5
2.0
2.5
3.0
3.5
4.0

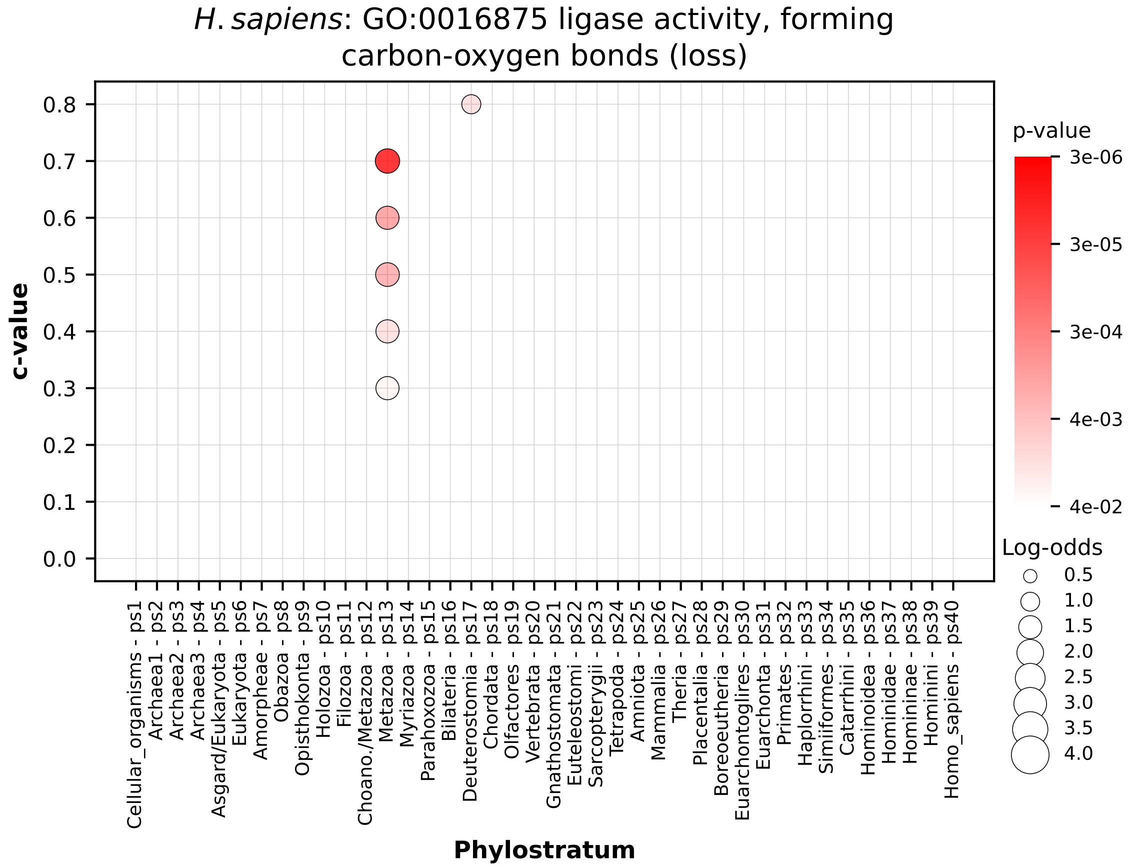
H. sapiens: GO:0016875 ligase activity, forming carbon-oxygen bonds (loss)
c-value
0.0
0.1
0.2
0.3
0.4
0.5
0.6
0.7
0.8
Phylostratum
Cellular_organisms - ps1
Archaea1 - ps2
Archaea2 - ps3
Archaea3 - ps4
Asgard/Eukaryota - ps5
Eukaryota - ps6
Amorpheae - ps7
Obazoa - ps8
Opisthokonta - ps9
Holozoa - ps10
Filozoa - ps11
Choano./Metazoa - ps12
Metazoa - ps13
Myriazoa - ps14
Parahoxozoa - ps15
Bilateria - ps16
Deuterostomia - ps17
Chordata - ps18
Olfactores - ps19
Vertebrata - ps20
Gnathostomata - ps21
Euteleostomi - ps22
Sarcopterygii - ps23
Tetrapoda - ps24
Amniota - ps25
Mammalia - ps26
Theria - ps27
Placentalia - ps28
Boreoeutheria - ps29
Euarchontoglires - ps30
Euarchonta - ps31
Primates - ps32
Haplorrhini - ps33
Simiiformes - ps34
Catarrhini - ps35
Hominoidea - ps36
Hominidae - ps37
Homininae - ps38
Hominini - ps39
Homo_sapiens - ps40
p-value
3e-06
3e-05
3e-04
4e-03
4e-02
Log-odds
0.5
1.0
1.5
2.0
2.5
3.0
3.5
4.0

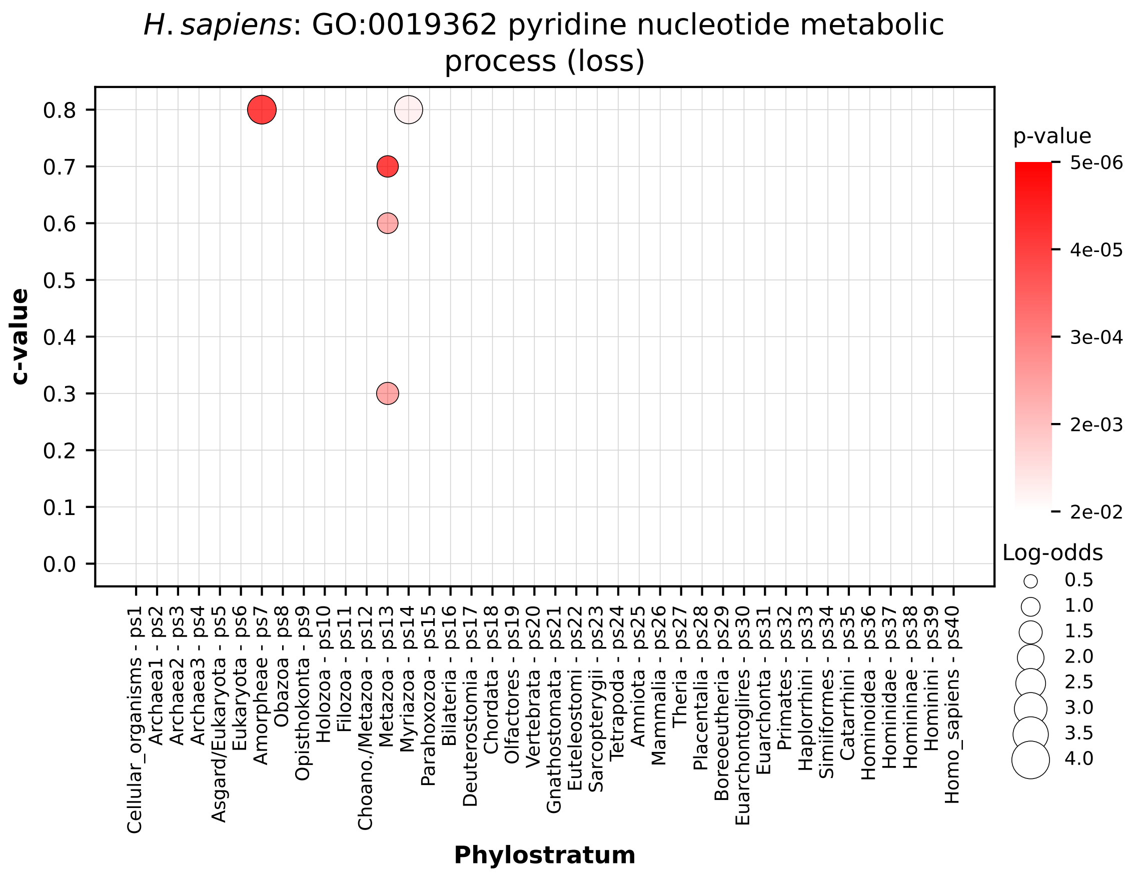

H. sapiens: GO:0019362 pyridine nucleotide metabolic process (loss)
c-value
0.8
0.7
0.6
0.5
0.4
0.3
0.2
0.1
0.0
p-value
5e-06
4e-05
3e-04
2e-03
2e-02
Log-odds
0.5
1.0
1.5
2.0
2.5
3.0
3.5
4.0
Cellular_organisms - ps1
Archaea1 - ps2
Archaea2 - ps3
Archaea3 - ps4
Asgard/Eukaryota - ps5
Eukaryota - ps6
Amorpheae - ps7
Obazoa - ps8
Opisthokonta - ps9
Holozoa - ps10
Filozoa - ps11
Choano./Metazoa - ps12
Metazoa - ps13
Myriazoa - ps14
Parahoxozoa - ps15
Bilateria - ps16
Deuterostomia - ps17
Chordata - ps18
Olfactores - ps19
Vertebrata - ps20
Gnathostomata - ps21
Euteleostomi - ps22
Sarcopterygii - ps23
Tetrapoda - ps24
Amniota - ps25
Mammalia - ps26
Theria - ps27
Placentalia - ps28
Boreoeutheria - ps29
Euarchontoglires - ps30
Euarchonta - ps31
Primates - ps32
Haplorrhini - ps33
Simiiformes - ps34
Catarrhini - ps35
Hominoidea - ps36
Hominidae - ps37
Homininae - ps38
Hominini - ps39
Homo_sapiens - ps40
Phylostratum

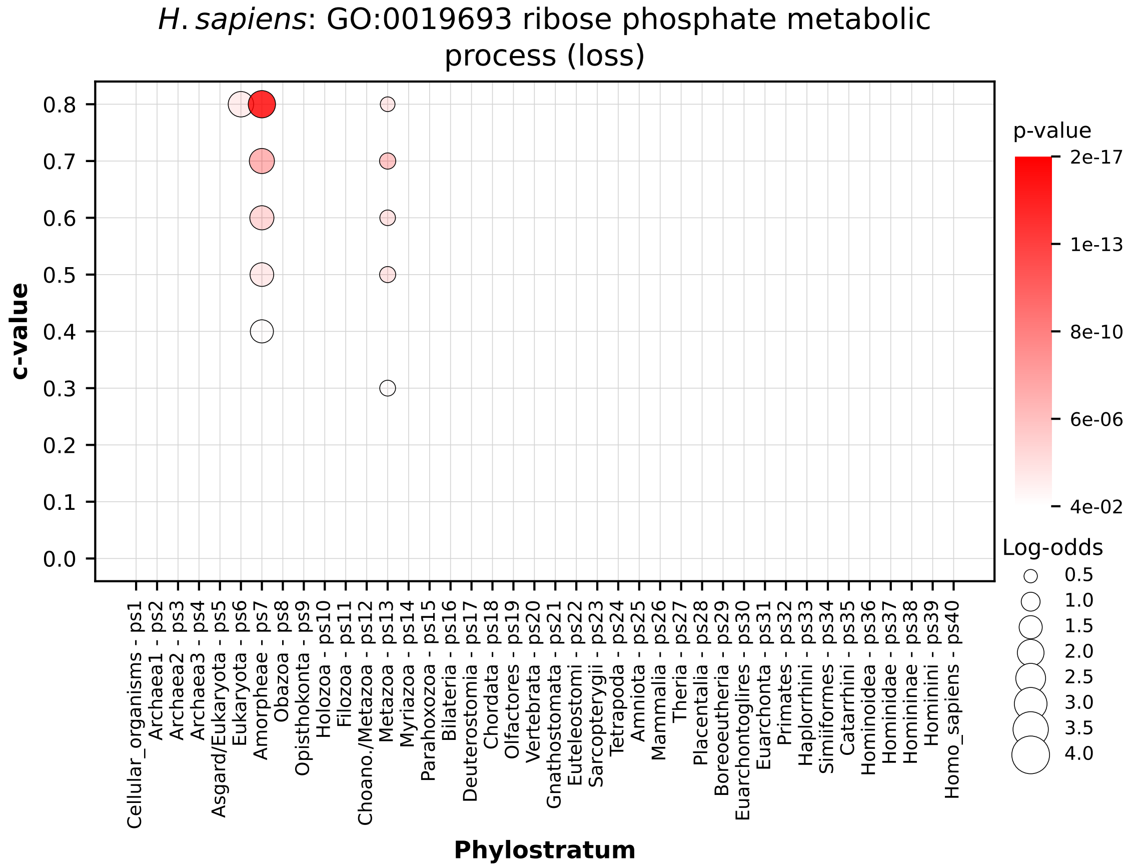
H. sapiens: GO:0019693 ribose phosphate metabolic process (loss)
c-value
0.0
0.1
0.2
0.3
0.4
0.5
0.6
0.7
0.8
Phylostratum
Cellular_organisms - ps1
Archaea1 - ps2
Archaea2 - ps3
Archaea3 - ps4
Asgard/Eukaryota - ps5
Eukaryota - ps6
Amorpheae - ps7
Obazoa - ps8
Opisthokonta - ps9
Holozoa - ps10
Filozoa - ps11
Choano./Metazoa - ps12
Metazoa - ps13
Myriazoa - ps14
Parahoxozoa - ps15
Bilateria - ps16
Deuterostomia - ps17
Chordata - ps18
Olfactores - ps19
Vertebrata - ps20
Gnathostomata - ps21
Euteleostomi - ps22
Sarcopterygii - ps23
Tetrapoda - ps24
Amniota - ps25
Mammalia - ps26
Theria - ps27
Placentalia - ps28
Boreoeutheria - ps29
Euarchontoglires - ps30
Euarchonta - ps31
Primates - ps32
Haplorrhini - ps33
Simiiformes - ps34
Catarrhini - ps35
Hominoidea - ps36
Hominidae - ps37
Homininae - ps38
Hominini - ps39
Homo_sapiens - ps40
p-value
2e-17
1e-13
8e-10
6e-06
4e-02
Log-odds
0.5
1.0
1.5
2.0
2.5
3.0
3.5
4.0

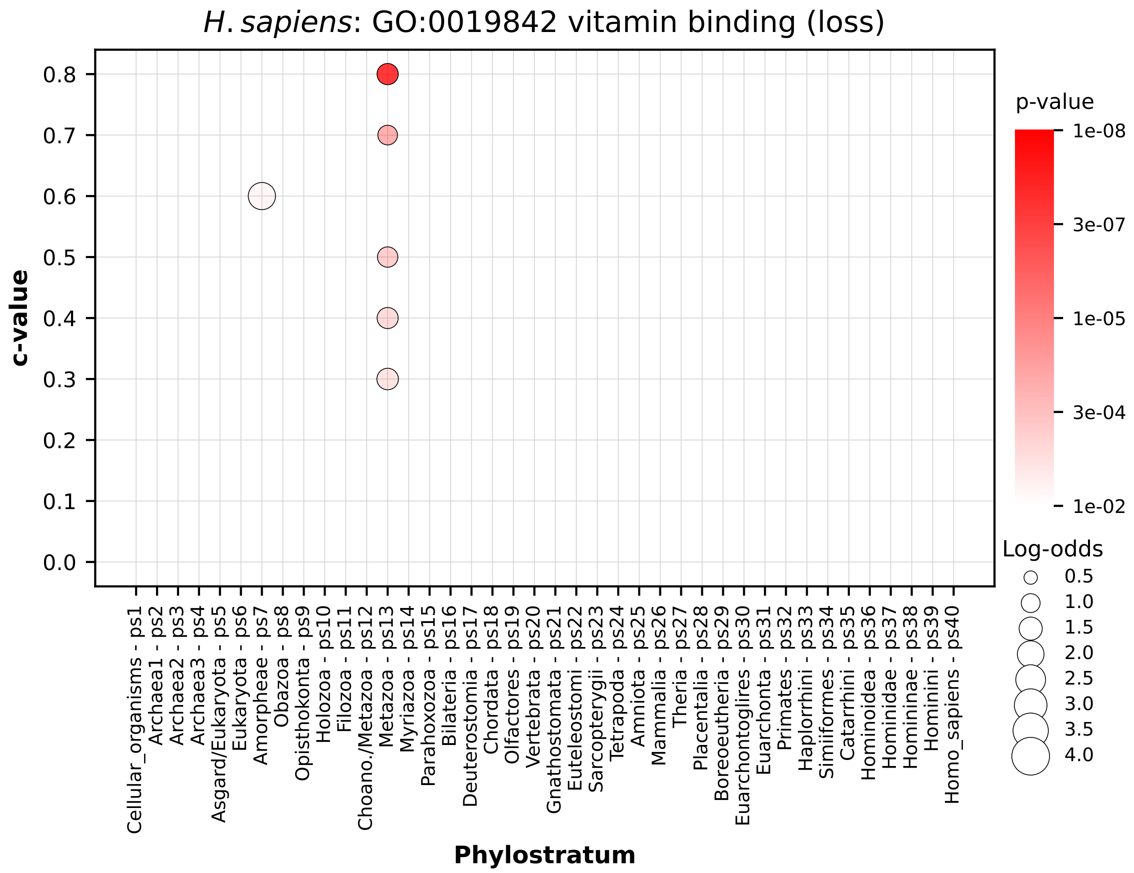

H. sapiens: GO:0019842 vitamin binding (loss)
c-value
0.8
0.7
0.6
0.5
0.4
0.3
0.2
0.1
0.0
Phylostratum
Cellular_organisms - ps1
Archaea1 - ps2
Archaea2 - ps3
Archaea3 - ps4
Asgard/Eukaryota - ps5
Eukaryota - ps6
Amorpheae - ps7
Obazoa - ps8
Opisthokonta - ps9
Holozoa - ps10
Filozoa - ps11
Choano./Metazoa - ps12
Metazoa - ps13
Myriazoa - ps14
Parahoxozoa - ps15
Bilateria - ps16
Deuterostomia - ps17
Chordata - ps18
Olfactores - ps19
Vertebrata - ps20
Gnathostomata - ps21
Euteleostomi - ps22
Sarcopterygii - ps23
Tetrapoda - ps24
Amniota - ps25
Mammalia - ps26
Theria - ps27
Placentalia - ps28
Boreoeutheria - ps29
Euarchontoglires - ps30
Euarchonta - ps31
Primates - ps32
Haplorrhini - ps33
Simiiformes - ps34
Catarrhini - ps35
Hominoidea - ps36
Hominidae - ps37
Homininae - ps38
Hominini - ps39
Homo_sapiens - ps40
p-value
1e-08
3e-07
1e-05
3e-04
1e-02
Log-odds
0.5
1.0
1.5
2.0
2.5
3.0
3.5
4.0

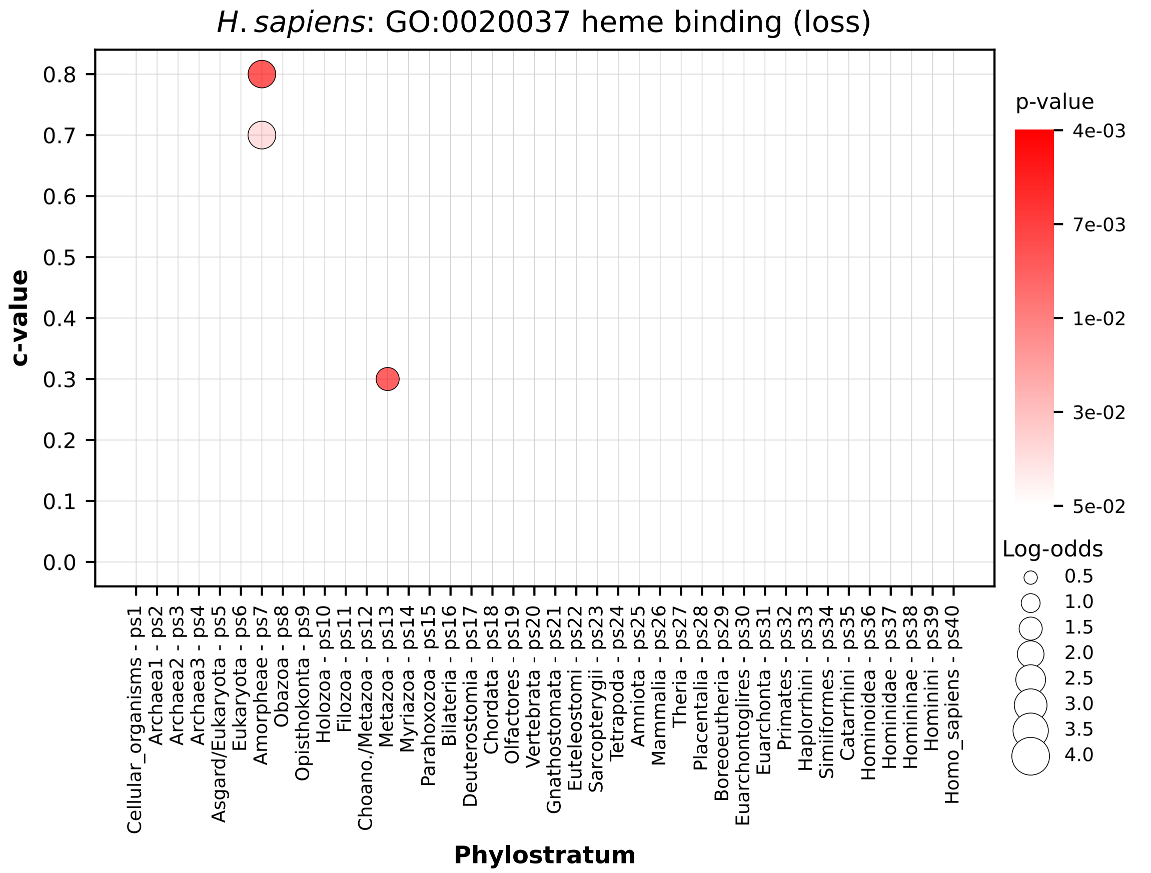

H. sapiens: GO:0020037 heme binding (loss)
c-value
0.0
0.1
0.2
0.3
0.4
0.5
0.6
0.7
0.8
Phylostratum
Cellular_organisms - ps1
Archaea1 - ps2
Archaea2 - ps3
Archaea3 - ps4
Asgard/Eukaryota - ps5
Eukaryota - ps6
Amorpheae - ps7
Obazoa - ps8
Opisthokonta - ps9
Holozoa - ps10
Filozoa - ps11
Choano./Metazoa - ps12
Metazoa - ps13
Myriazoa - ps14
Parahoxozoa - ps15
Bilateria - ps16
Deuterostomia - ps17
Chordata - ps18
Olfactores - ps19
Vertebrata - ps20
Gnathostomata - ps21
Euteleostomi - ps22
Sarcopterygii - ps23
Tetrapoda - ps24
Amniota - ps25
Mammalia - ps26
Theria - ps27
Placentalia - ps28
Boreoeutheria - ps29
Euarchontoglires - ps30
Euarchonta - ps31
Primates - ps32
Haplorrhini - ps33
Simiiformes - ps34
Catarrhini - ps35
Hominoidea - ps36
Hominidae - ps37
Homininae - ps38
Hominini - ps39
Homo_sapiens - ps40
p-value
4e-03
7e-03
1e-02
3e-02
5e-02
Log-odds
0.5
1.0
1.5
2.0
2.5
3.0
3.5
4.0

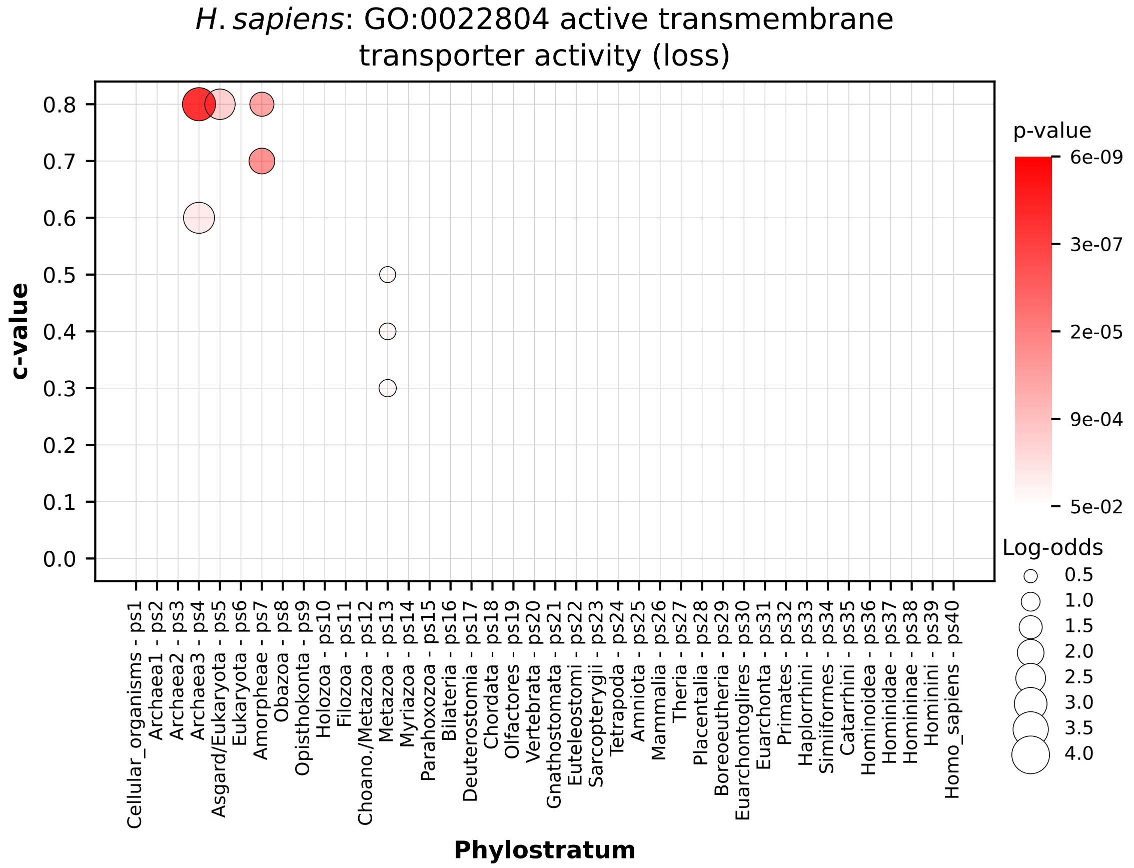

H. sapiens: GO:0022804 active transmembrane transporter activity (loss)
c-value
0.8
0.7
0.6
0.5
0.4
0.3
0.2
0.1
0.0
p-value
6e-09
3e-07
2e-05
9e-04
5e-02
Log-odds
0.5
1.0
1.5
2.0
2.5
3.0
3.5
4.0
Cellular_organisms - ps1
Archaea1 - ps2
Archaea2 - ps3
Archaea3 - ps4
Asgard/Eukaryota - ps5
Eukaryota - ps6
Amorpheae - ps7
Obazoa - ps8
Opisthokonta - ps9
Holozoa - ps10
Filozoa - ps11
Choano./Metazoa - ps12
Metazoa - ps13
Myriazoa - ps14
Parahoxozoa - ps15
Bilateria - ps16
Deuterostomia - ps17
Chordata - ps18
Olfactores - ps19
Vertebrata - ps20
Gnathostomata - ps21
Euteleostomi - ps22
Sarcopterygii - ps23
Tetrapoda - ps24
Amniota - ps25
Mammalia - ps26
Theria - ps27
Placentalia - ps28
Boreoeutheria - ps29
Euarchontoglires - ps30
Euarchonta - ps31
Primates - ps32
Haplorrhini - ps33
Simiiformes - ps34
Catarrhini - ps35
Hominoidea - ps36
Hominidae - ps37
Homininae - ps38
Hominini - ps39
Homo_sapiens - ps40
Phylostratum

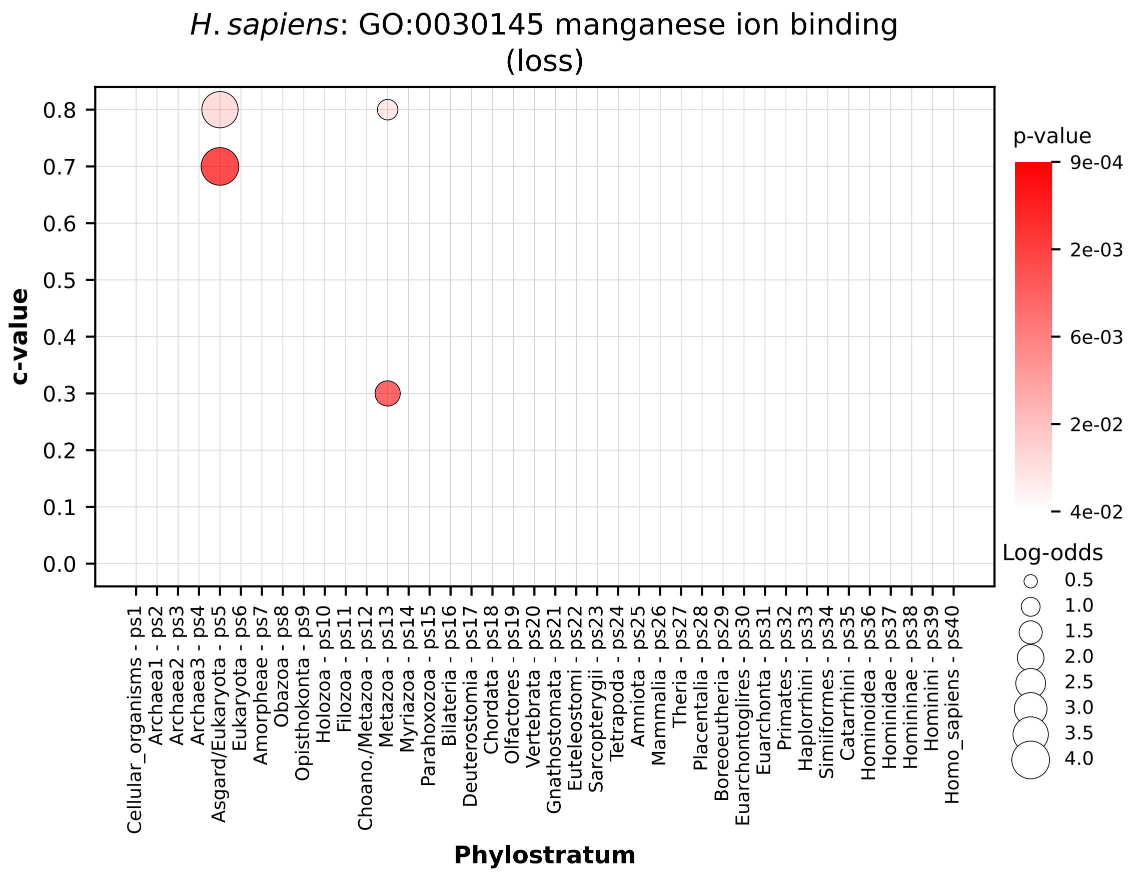
H. sapiens: GO:0030145 manganese ion binding
(loss)
c-value
0.8
0.7
0.6
0.5
0.4
0.3
0.2
0.1
0.0
p-value
9e-04
2e-03
6e-03
2e-02
4e-02
Log-odds
0.5
1.0
1.5
2.0
2.5
3.0
3.5
4.0
Cellular_organisms - ps1
Archaea1 - ps2
Archaea2 - ps3
Archaea3 - ps4
Asgard/Eukaryota - ps5
Eukaryota - ps6
Amorpheae - ps7
Obazoa - ps8
Opisthokonta - ps9
Holozoa - ps10
Filozoa - ps11
Choano./Metazoa - ps12
Metazoa - ps13
Myriazoa - ps14
Parahoxozoa - ps15
Bilateria - ps16
Deuterostomia - ps17
Chordata - ps18
Olfactores - ps19
Vertebrata - ps20
Gnathostomata - ps21
Euteleostomi - ps22
Sarcopterygii - ps23
Tetrapoda - ps24
Amniota - ps25
Mammalia - ps26
Theria - ps27
Placentalia - ps28
Boreoeutheria - ps29
Euarchontoglires - ps30
Euarchonta - ps31
Primates - ps32
Haplorrhini - ps33
Simiiformes - ps34
Catarrhini - ps35
Hominoidea - ps36
Hominidae - ps37
Homininae - ps38
Hominini - ps39
Homo_sapiens - ps40
Phylostratum

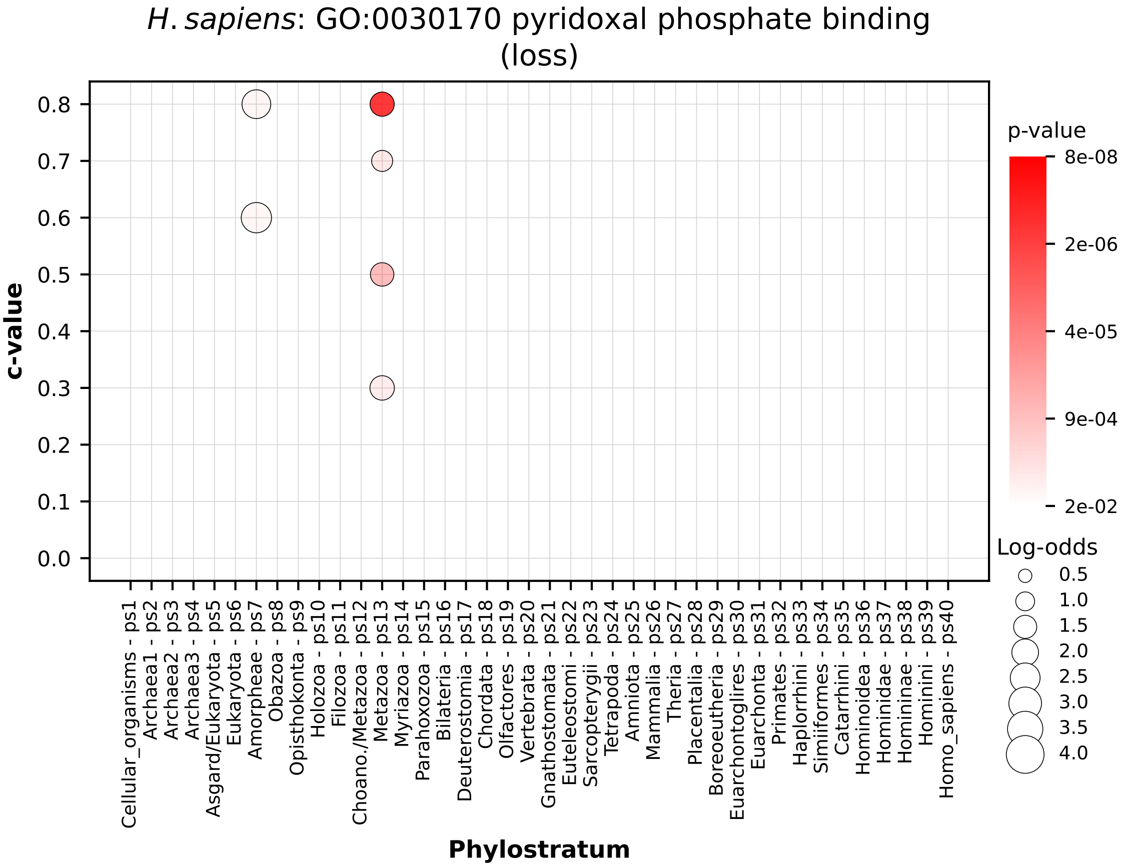
H. sapiens: GO:0030170 pyridoxal phosphate binding (loss)
c-value
0.0
0.1
0.2
0.3
0.4
0.5
0.6
0.7
0.8
Phylostratum
Cellular_organisms - ps1
Archaea1 - ps2
Archaea2 - ps3
Archaea3 - ps4
Asgard/Eukaryota - ps5
Eukaryota - ps6
Amorpheae - ps7
Obazoa - ps8
Opisthokonta - ps9
Holozoa - ps10
Filozoa - ps11
Choano./Metazoa - ps12
Metazoa - ps13
Myriazoa - ps14
Parahoxozoa - ps15
Bilateria - ps16
Deuterostomia - ps17
Chordata - ps18
Olfactores - ps19
Vertebrata - ps20
Gnathostomata - ps21
Euteleostomi - ps22
Sarcopterygii - ps23
Tetrapoda - ps24
Amniota - ps25
Mammalia - ps26
Theria - ps27
Placentalia - ps28
Boreoeutheria - ps29
Euarchontoglires - ps30
Euarchonta - ps31
Primates - ps32
Haplorrhini - ps33
Simiiformes - ps34
Catarrhini - ps35
Hominoidea - ps36
Hominidae - ps37
Homininae - ps38
Hominini - ps39
Homo_sapiens - ps40
p-value
8e-08
2e-06
4e-05
9e-04
2e-02
Log-odds
0.5
1.0
1.5
2.0
2.5
3.0
3.5
4.0

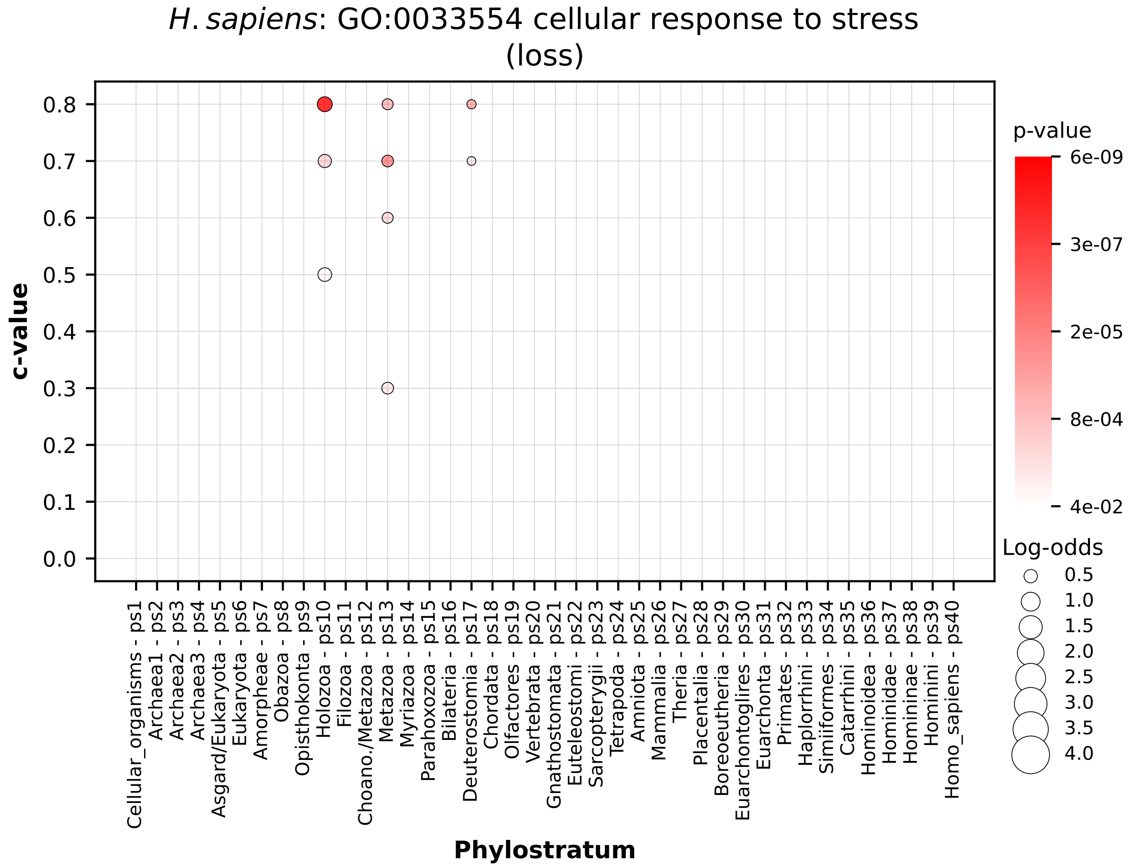

H. sapiens: GO:0033554 cellular response to stress (loss)
c-value
0.0
0.1
0.2
0.3
0.4
0.5
0.6
0.7
0.8
p-value
6e-09
3e-07
2e-05
8e-04
4e-02
Log-odds
0.5
1.0
1.5
2.0
2.5
3.0
3.5
4.0
Cellular_organisms - ps1
Archaea1 - ps2
Archaea2 - ps3
Archaea3 - ps4
Asgard/Eukaryota - ps5
Eukaryota - ps6
Amorpheae - ps7
Obazoa - ps8
Opisthokonta - ps9
Holozoa - ps10
Filozoa - ps11
Choano./Metazoa - ps12
Metazoa - ps13
Myriazoa - ps14
Parahoxozoa - ps15
Bilateria - ps16
Deuterostomia - ps17
Chordata - ps18
Olfactores - ps19
Vertebrata - ps20
Gnathostomata - ps21
Euteleostomi - ps22
Sarcopterygii - ps23
Tetrapoda - ps24
Amniota - ps25
Mammalia - ps26
Theria - ps27
Placentalia - ps28
Boreoeutheria - ps29
Euarchontoglires - ps30
Euarchonta - ps31
Primates - ps32
Haplorrhini - ps33
Simiiformes - ps34
Catarrhini - ps35
Hominoidea - ps36
Hominidae - ps37
Homininae - ps38
Hominini - ps39
Homo_sapiens - ps40
Phylostratum

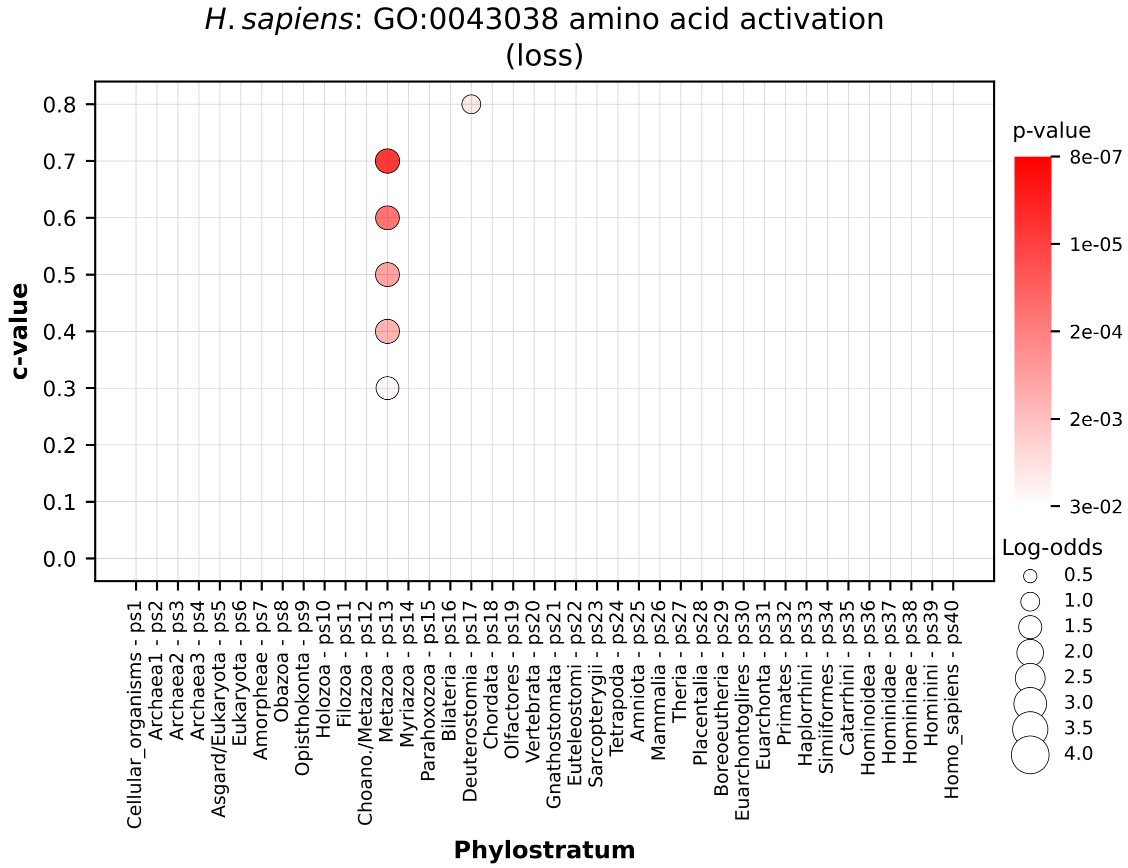

H. sapiens: GO:0043038 amino acid activation
(loss)
c-value
0.8
0.7
0.6
0.5
0.4
0.3
0.2
0.1
0.0
p-value
8e-07
1e-05
2e-04
2e-03
3e-02
Log-odds
0.5
1.0
1.5
2.0
2.5
3.0
3.5
4.0
Cellular_organisms - ps1
Archaea1 - ps2
Archaea2 - ps3
Archaea3 - ps4
Asgard/Eukaryota - ps5
Eukaryota - ps6
Amorpheae - ps7
Obazoa - ps8
Opisthokonta - ps9
Holozoa - ps10
Filozoa - ps11
Choano./Metazoa - ps12
Metazoa - ps13
Myriazoa - ps14
Parahoxozoa - ps15
Bilateria - ps16
Deuterostomia - ps17
Chordata - ps18
Olfactores - ps19
Vertebrata - ps20
Gnathostomata - ps21
Euteleostomi - ps22
Sarcopterygii - ps23
Tetrapoda - ps24
Amniota - ps25
Mammalia - ps26
Theria - ps27
Placentalia - ps28
Boreoeutheria - ps29
Euarchontoglires - ps30
Euarchonta - ps31
Primates - ps32
Haplorrhini - ps33
Simiiformes - ps34
Catarrhini - ps35
Hominoidea - ps36
Hominidae - ps37
Homininae - ps38
Hominini - ps39
Homo_sapiens - ps40
Phylostratum

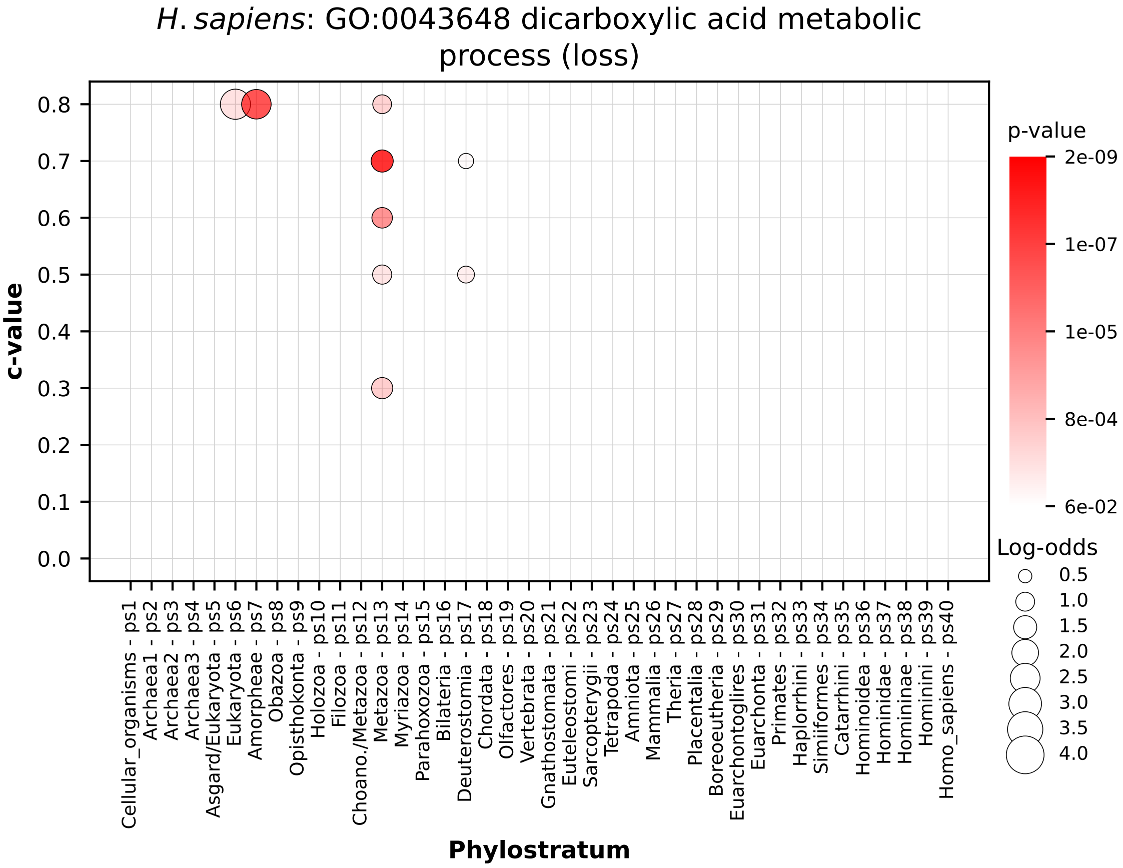

H. sapiens: GO:0043648 dicarboxylic acid metabolic process (loss)
c-value
0.0
0.1
0.2
0.3
0.4
0.5
0.6
0.7
0.8
Phylostratum
Cellular_organisms - ps1
Archaea1 - ps2
Archaea2 - ps3
Archaea3 - ps4
Asgard/Eukaryota - ps5
Eukaryota - ps6
Amorpheae - ps7
Obazoa - ps8
Opisthokonta - ps9
Holozoa - ps10
Filozoa - ps11
Choano./Metazoa - ps12
Metazoa - ps13
Myriazoa - ps14
Parahoxozoa - ps15
Bilateria - ps16
Deuterostomia - ps17
Chordata - ps18
Olfactores - ps19
Vertebrata - ps20
Gnathostomata - ps21
Euteleostomi - ps22
Sarcopterygii - ps23
Tetrapoda - ps24
Amniota - ps25
Mammalia - ps26
Theria - ps27
Placentalia - ps28
Boreoeutheria - ps29
Euarchontoglires - ps30
Euarchonta - ps31
Primates - ps32
Haplorrhini - ps33
Simiiformes - ps34
Catarrhini - ps35
Hominoidea - ps36
Hominidae - ps37
Homininae - ps38
Hominini - ps39
Homo_sapiens - ps40
p-value
2e-09
1e-07
1e-05
8e-04
6e-02
Log-odds
0.5
1.0
1.5
2.0
2.5
3.0
3.5
4.0

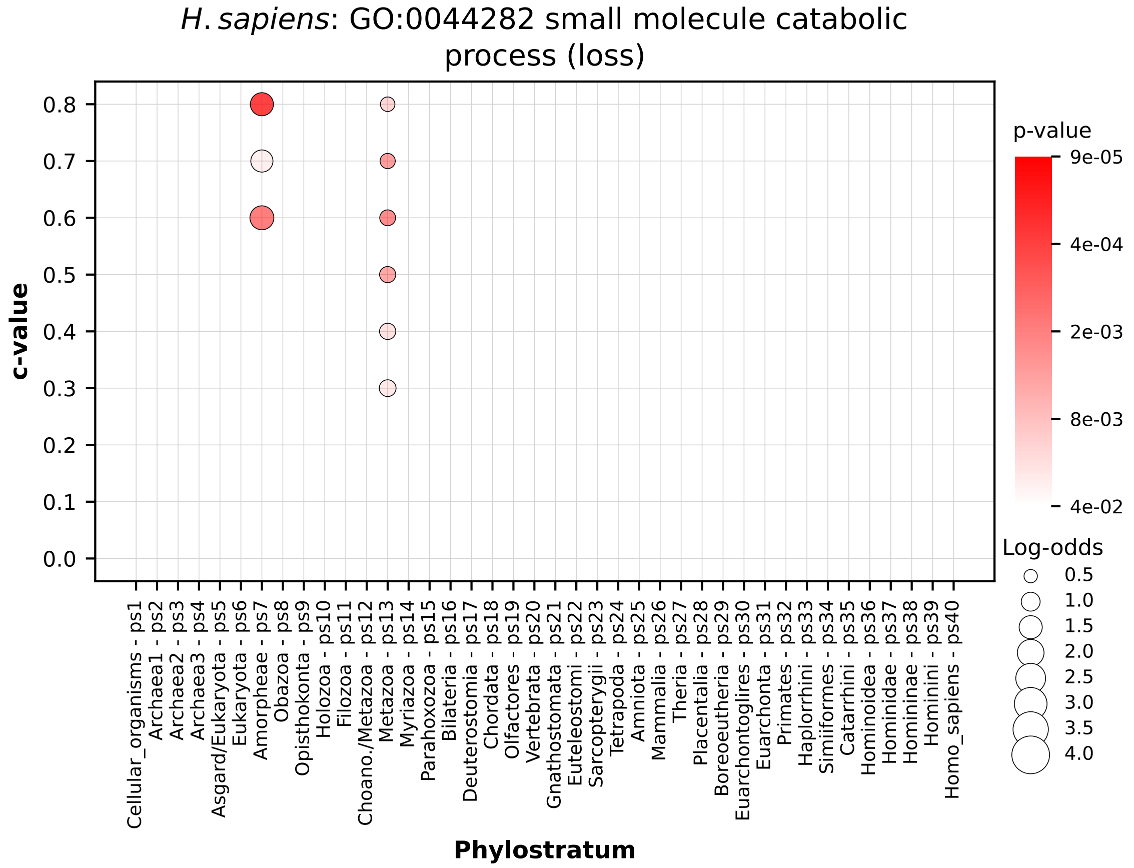

H. sapiens: GO:0044282 small molecule catabolic process (loss)
c-value
0.0
0.1
0.2
0.3
0.4
0.5
0.6
0.7
0.8
Phylostratum
Cellular_organisms - ps1
Archaea1 - ps2
Archaea2 - ps3
Archaea3 - ps4
Asgard/Eukaryota - ps5
Eukaryota - ps6
Amorpheae - ps7
Obazoa - ps8
Opisthokonta - ps9
Holozoa - ps10
Filozoa - ps11
Choano./Metazoa - ps12
Metazoa - ps13
Myriazoa - ps14
Parahoxozoa - ps15
Bilateria - ps16
Deuterostomia - ps17
Chordata - ps18
Olfactores - ps19
Vertebrata - ps20
Gnathostomata - ps21
Euteleostomi - ps22
Sarcopterygii - ps23
Tetrapoda - ps24
Amniota - ps25
Mammalia - ps26
Theria - ps27
Placentalia - ps28
Boreoeutheria - ps29
Euarchontoglires - ps30
Euarchonta - ps31
Primates - ps32
Haplorrhini - ps33
Simiiformes - ps34
Catarrhini - ps35
Hominoidea - ps36
Hominidae - ps37
Homininae - ps38
Hominini - ps39
Homo_sapiens - ps40
p-value
9e-05
4e-04
2e-03
8e-03
4e-02
Log-odds
0.5
1.0
1.5
2.0
2.5
3.0
3.5
4.0

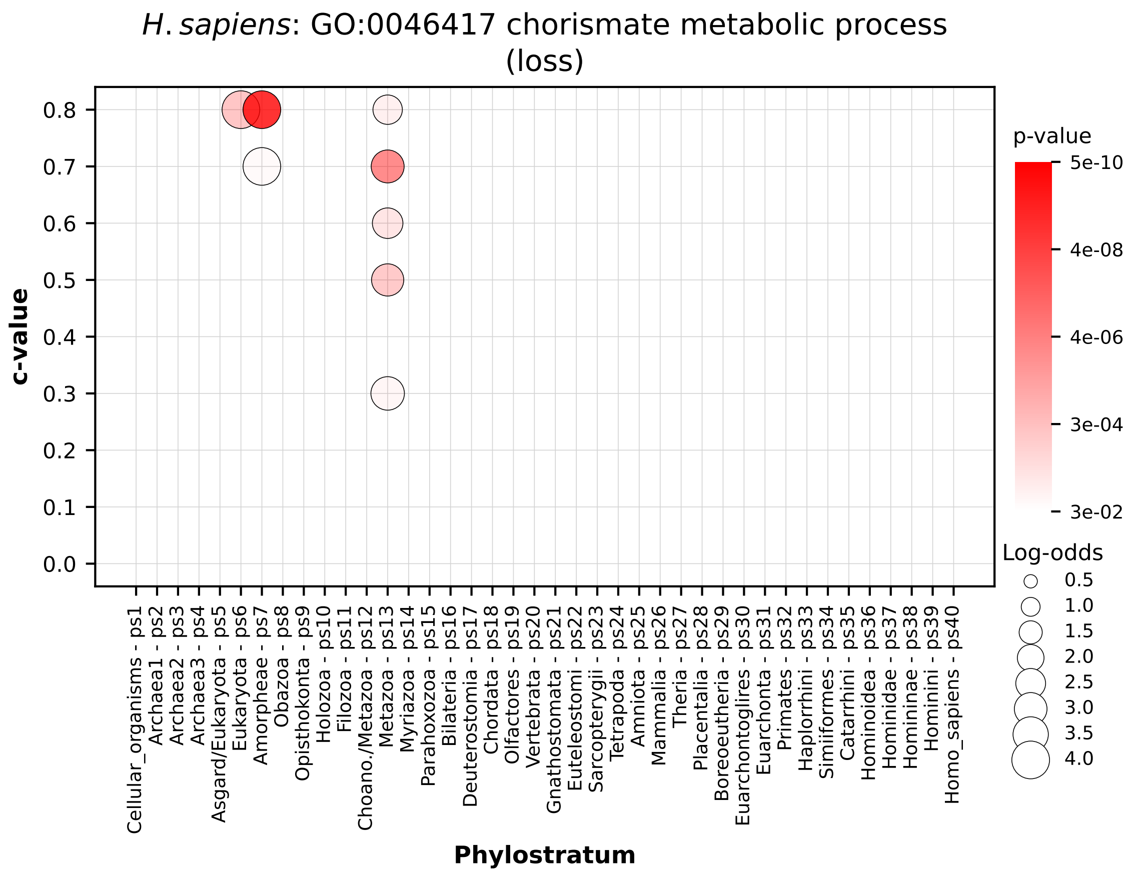
H. sapiens: GO:0046417 chorismate metabolic process
(loss)
c-value
0.8
0.7
0.6
0.5
0.4
0.3
0.2
0.1
0.0
p-value
5e-10
4e-08
4e-06
3e-04
3e-02
Log-odds
0.5
1.0
1.5
2.0
2.5
3.0
3.5
4.0
Cellular_organisms - ps1
Archaea1 - ps2
Archaea2 - ps3
Archaea3 - ps4
Asgard/Eukaryota - ps5
Eukaryota - ps6
Amorpheae - ps7
Obazoa - ps8
Opisthokonta - ps9
Holozoa - ps10
Filozoa - ps11
Choano./Metazoa - ps12
Metazoa - ps13
Myriazoa - ps14
Parahoxozoa - ps15
Bilateria - ps16
Deuterostomia - ps17
Chordata - ps18
Olfactores - ps19
Vertebrata - ps20
Gnathostomata - ps21
Euteleostomi - ps22
Sarcopterygii - ps23
Tetrapoda - ps24
Amniota - ps25
Mammalia - ps26
Theria - ps27
Placentalia - ps28
Boreoeutheria - ps29
Euarchontoglires - ps30
Euarchonta - ps31
Primates - ps32
Haplorrhini - ps33
Simiiformes - ps34
Catarrhini - ps35
Hominoidea - ps36
Hominidae - ps37
Homininae - ps38
Hominini - ps39
Homo_sapiens - ps40
Phylostratum

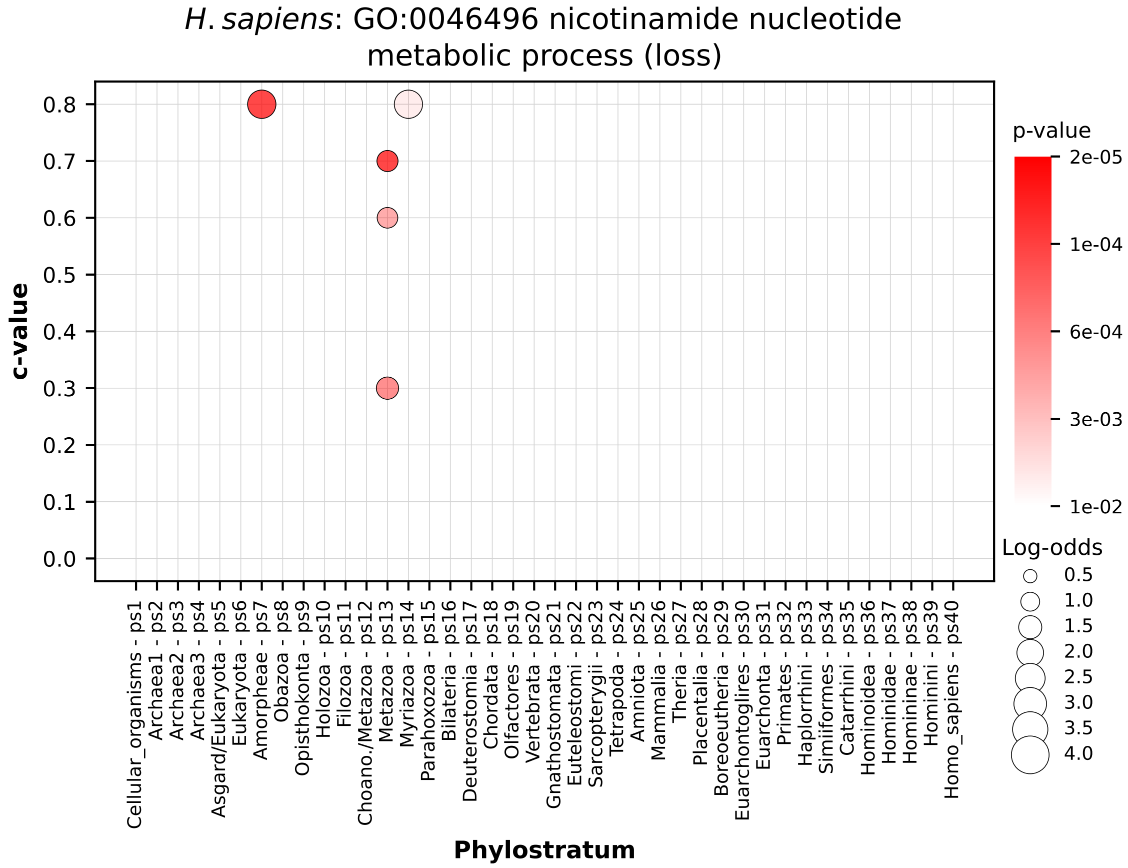

H. sapiens: GO:0046496 nicotinamide nucleotide metabolic process (loss)
c-value
0.8
0.7
0.6
0.5
0.4
0.3
0.2
0.1
0.0
p-value
2e-05
1e-04
6e-04
3e-03
1e-02
Log-odds
0.5
1.0
1.5
2.0
2.5
3.0
3.5
4.0
Cellular_organisms - ps1
Archaea1 - ps2
Archaea2 - ps3
Archaea3 - ps4
Asgard/Eukaryota - ps5
Eukaryota - ps6
Amorpheae - ps7
Obazoa - ps8
Opisthokonta - ps9
Holozoa - ps10
Filozoa - ps11
Choano./Metazoa - ps12
Metazoa - ps13
Myriazoa - ps14
Parahoxozoa - ps15
Bilateria - ps16
Deuterostomia - ps17
Chordata - ps18
Olfactores - ps19
Vertebrata - ps20
Gnathostomata - ps21
Euteleostomi - ps22
Sarcopterygii - ps23
Tetrapoda - ps24
Amniota - ps25
Mammalia - ps26
Theria - ps27
Placentalia - ps28
Boreoeutheria - ps29
Euarchontoglires - ps30
Euarchonta - ps31
Primates - ps32
Haplorrhini - ps33
Simiiformes - ps34
Catarrhini - ps35
Hominoidea - ps36
Hominidae - ps37
Homininae - ps38
Hominini - ps39
Homo_sapiens - ps40
Phylostratum

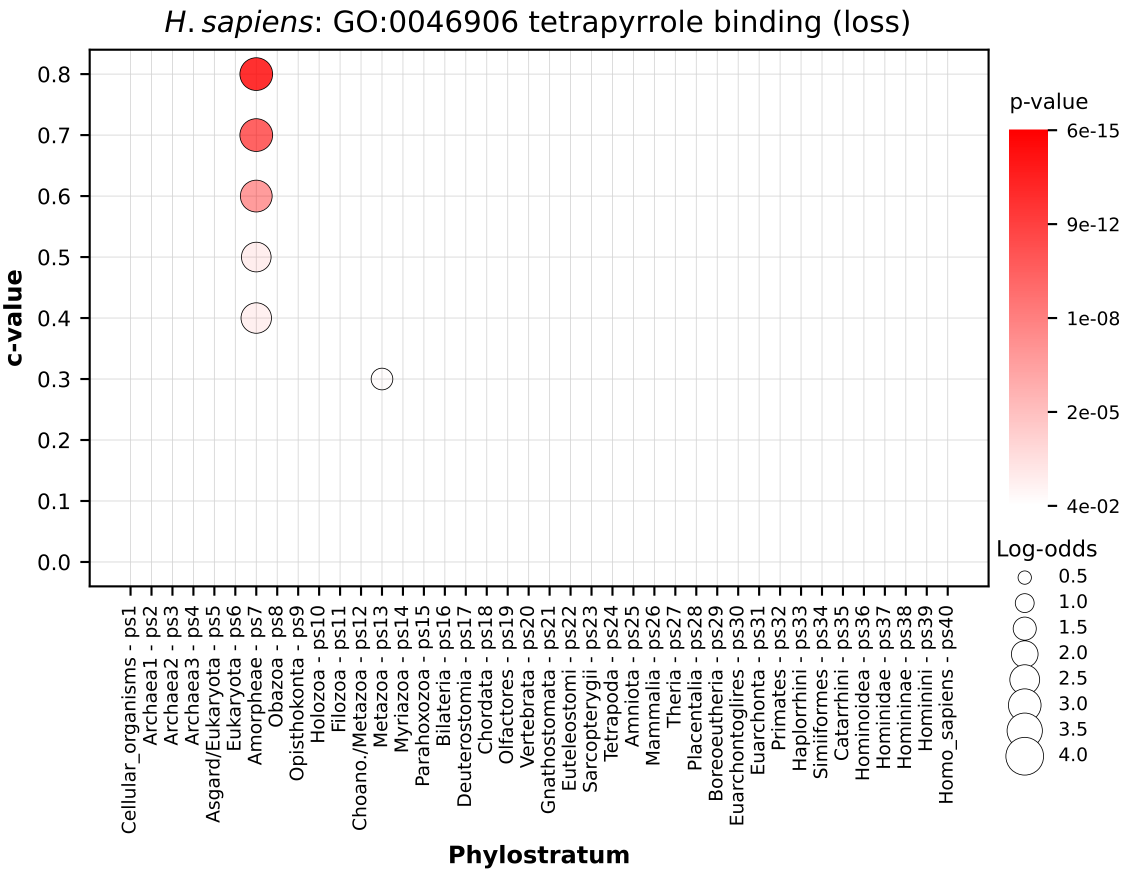

H. sapiens: GO:0046906 tetrapyrrole binding (loss)
c-value
0.8
0.7
0.6
0.5
0.4
0.3
0.2
0.1
0.0
Phylostratum
Cellular_organisms - ps1
Archaea1 - ps2
Archaea2 - ps3
Archaea3 - ps4
Asgard/Eukaryota - ps5
Eukaryota - ps6
Amorpheae - ps7
Obazoa - ps8
Opisthokonta - ps9
Holozoa - ps10
Filozoa - ps11
Choano./Metazoa - ps12
Metazoa - ps13
Myriazoa - ps14
Parahoxozoa - ps15
Bilateria - ps16
Deuterostomia - ps17
Chordata - ps18
Olfactores - ps19
Vertebrata - ps20
Gnathostomata - ps21
Euteleostomi - ps22
Sarcopterygii - ps23
Tetrapoda - ps24
Amniota - ps25
Mammalia - ps26
Theria - ps27
Placentalia - ps28
Boreoeutheria - ps29
Euarchontoglires - ps30
Euarchonta - ps31
Primates - ps32
Haplorrhini - ps33
Simiiformes - ps34
Catarrhini - ps35
Hominoidea - ps36
Hominidae - ps37
Homininae - ps38
Hominini - ps39
Homo_sapiens - ps40
p-value
6e-15
9e-12
1e-08
2e-05
4e-02
Log-odds
0.5
1.0
1.5
2.0
2.5
3.0
3.5
4.0

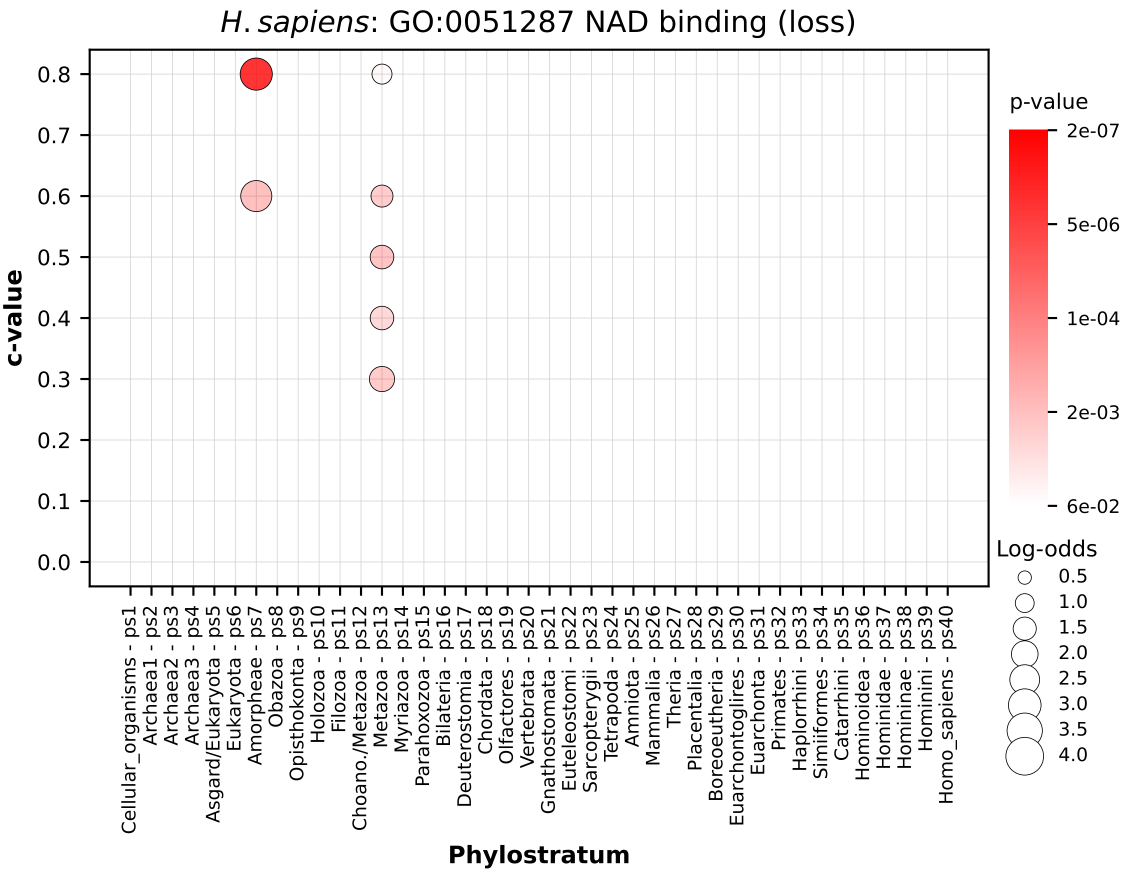

H. sapiens: GO:0051287 NAD binding (loss)
c-value
0.0
0.1
0.2
0.3
0.4
0.5
0.6
0.7
0.8
Phylostratum
Cellular_organisms - ps1
Archaea1 - ps2
Archaea2 - ps3
Archaea3 - ps4
Asgard/Eukaryota - ps5
Eukaryota - ps6
Amorpheae - ps7
Obazoa - ps8
Opisthokonta - ps9
Holozoa - ps10
Filozoa - ps11
Choano./Metazoa - ps12
Metazoa - ps13
Myriazoa - ps14
Parahoxozoa - ps15
Bilateria - ps16
Deuterostomia - ps17
Chordata - ps18
Olfactores - ps19
Vertebrata - ps20
Gnathostomata - ps21
Euteleostomi - ps22
Sarcopterygii - ps23
Tetrapoda - ps24
Amniota - ps25
Mammalia - ps26
Theria - ps27
Placentalia - ps28
Boreoeutheria - ps29
Euarchontoglires - ps30
Euarchonta - ps31
Primates - ps32
Haplorrhini - ps33
Simiiformes - ps34
Catarrhini - ps35
Hominoidea - ps36
Hominidae - ps37
Homininae - ps38
Hominini - ps39
Homo_sapiens - ps40
p-value
2e-07
5e-06
1e-04
2e-03
6e-02
Log-odds
0.5
1.0
1.5
2.0
2.5
3.0
3.5
4.0

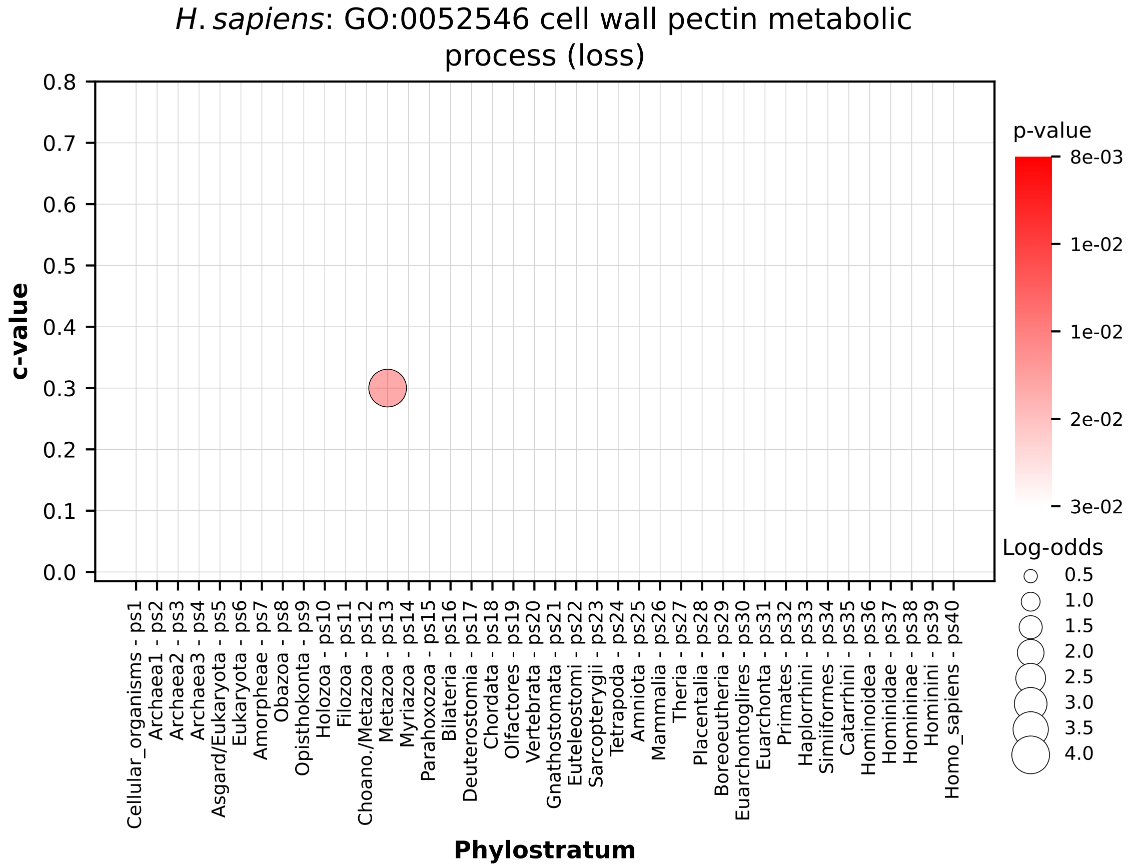

H. sapiens: GO:0052546 cell wall pectin metabolic process (loss)
c-value
0.8
0.7
0.6
0.5
0.4
0.3
0.2
0.1
0.0
p-value
8e-03
1e-02
1e-02
2e-02
3e-02
Log-odds
0.5
1.0
1.5
2.0
2.5
3.0
3.5
4.0
Cellular_organisms - ps1
Archaea1 - ps2
Archaea2 - ps3
Archaea3 - ps4
Asgard/Eukaryota - ps5
Eukaryota - ps6
Amorpheae - ps7
Obazoa - ps8
Opisthokonta - ps9
Holozoa - ps10
Filozoa - ps11
Choano./Metazoa - ps12
Metazoa - ps13
Myriazoa - ps14
Parahoxozoa - ps15
Bilateria - ps16
Deuterostomia - ps17
Chordata - ps18
Olfactores - ps19
Vertebrata - ps20
Gnathostomata - ps21
Euteleostomi - ps22
Sarcopterygii - ps23
Tetrapoda - ps24
Amniota - ps25
Mammalia - ps26
Theria - ps27
Placentalia - ps28
Boreoeutheria - ps29
Euarchontoglires - ps30
Euarchonta - ps31
Primates - ps32
Haplorrhini - ps33
Simiiformes - ps34
Catarrhini - ps35
Hominoidea - ps36
Hominidae - ps37
Homininae - ps38
Hominini - ps39
Homo_sapiens - ps40
Phylostratum

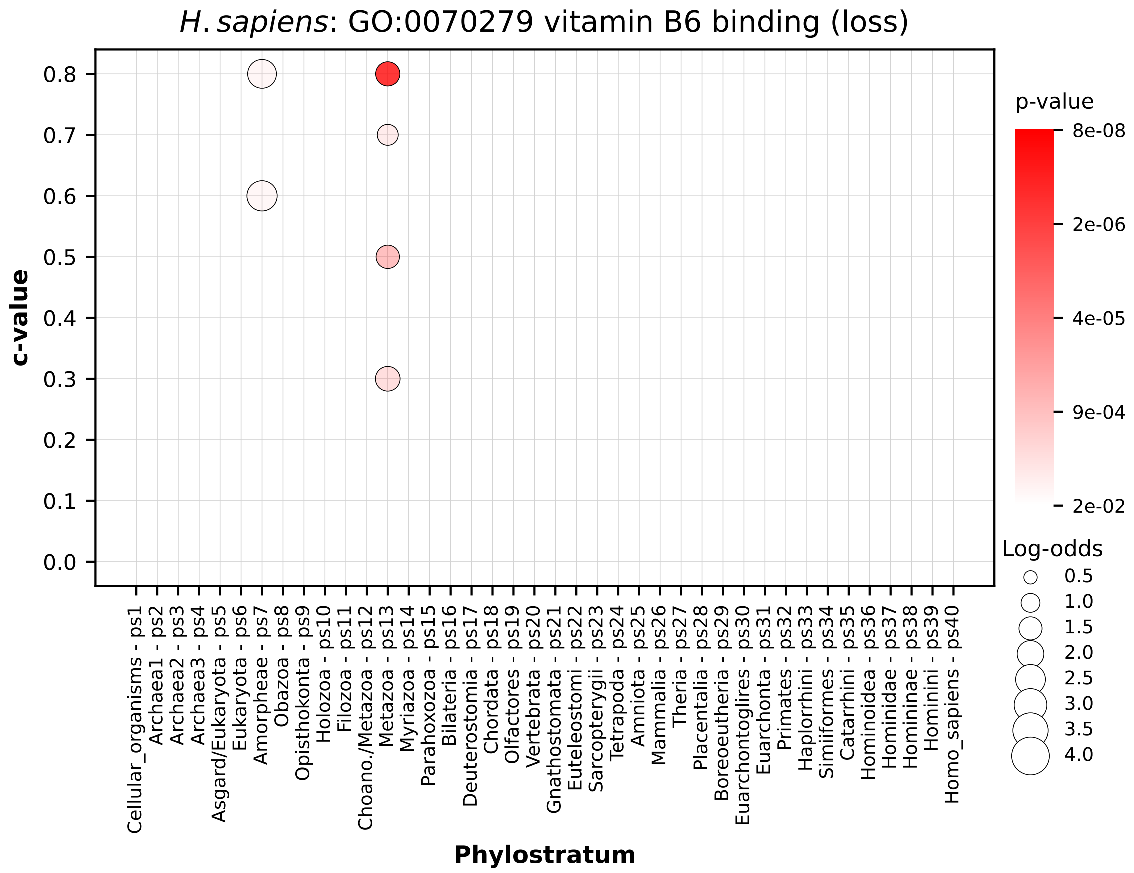

H. sapiens: GO:0070279 vitamin B6 binding (loss)
c-value
0.8
0.7
0.6
0.5
0.4
0.3
0.2
0.1
0.0
p-value
8e-08
2e-06
4e-05
9e-04
2e-02
Log-odds
0.5
1.0
1.5
2.0
2.5
3.0
3.5
4.0
Cellular_organisms - ps1
Archaea1 - ps2
Archaea2 - ps3
Archaea3 - ps4
Asgard/Eukaryota - ps5
Eukaryota - ps6
Amorpheae - ps7
Obazoa - ps8
Opisthokonta - ps9
Holozoa - ps10
Filozoa - ps11
Choano./Metazoa - ps12
Metazoa - ps13
Myriazoa - ps14
Parahoxozoa - ps15
Bilateria - ps16
Deuterostomia - ps17
Chordata - ps18
Olfactores - ps19
Vertebrata - ps20
Gnathostomata - ps21
Euteleostomi - ps22
Sarcopterygii - ps23
Tetrapoda - ps24
Amniota - ps25
Mammalia - ps26
Theria - ps27
Placentalia - ps28
Boreoeutheria - ps29
Euarchontoglires - ps30
Euarchonta - ps31
Primates - ps32
Haplorrhini - ps33
Simiiformes - ps34
Catarrhini - ps35
Hominoidea - ps36
Hominidae - ps37
Homininae - ps38
Hominini - ps39
Homo_sapiens - ps40
Phylostratum

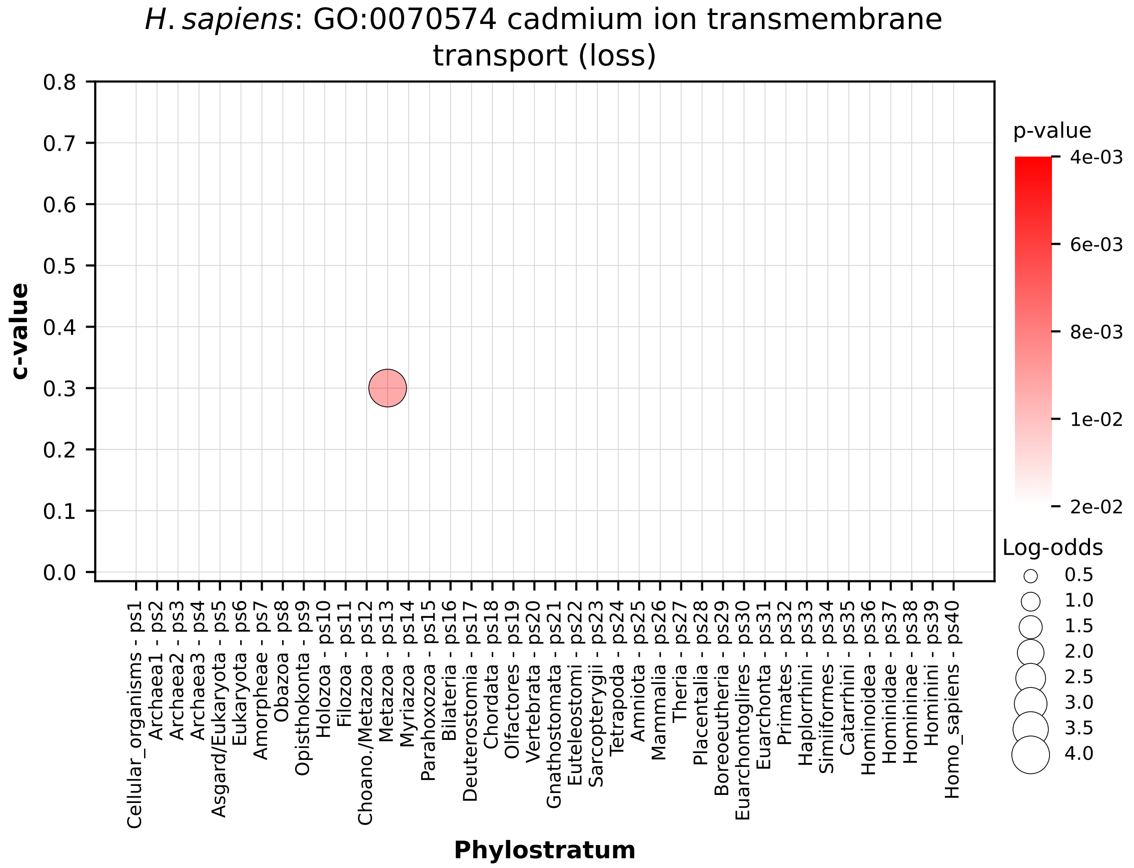
H. sapiens: GO:0070574 cadmium ion transmembrane transport (loss)
c-value
0.0
0.1
0.2
0.3
0.4
0.5
0.6
0.7
0.8
Phylostratum
Cellular_organisms - ps1
Archaea1 - ps2
Archaea2 - ps3
Archaea3 - ps4
Asgard/Eukaryota - ps5
Eukaryota - ps6
Amorpheae - ps7
Obazoa - ps8
Opisthokonta - ps9
Holozoa - ps10
Filozoa - ps11
Choano./Metazoa - ps12
Metazoa - ps13
Myriazoa - ps14
Parahoxozoa - ps15
Bilateria - ps16
Deuterostomia - ps17
Chordata - ps18
Olfactores - ps19
Vertebrata - ps20
Gnathostomata - ps21
Euteleostomi - ps22
Sarcopterygii - ps23
Tetrapoda - ps24
Amniota - ps25
Mammalia - ps26
Theria - ps27
Placentalia - ps28
Boreoeutheria - ps29
Euarchontoglires - ps30
Euarchonta - ps31
Primates - ps32
Haplorrhini - ps33
Simiiformes - ps34
Catarrhini - ps35
Hominoidea - ps36
Hominidae - ps37
Homininae - ps38
Hominini - ps39
Homo_sapiens - ps40
p-value
4e-03
6e-03
8e-03
1e-02
2e-02
Log-odds
0.5
1.0
1.5
2.0
2.5
3.0
3.5
4.0

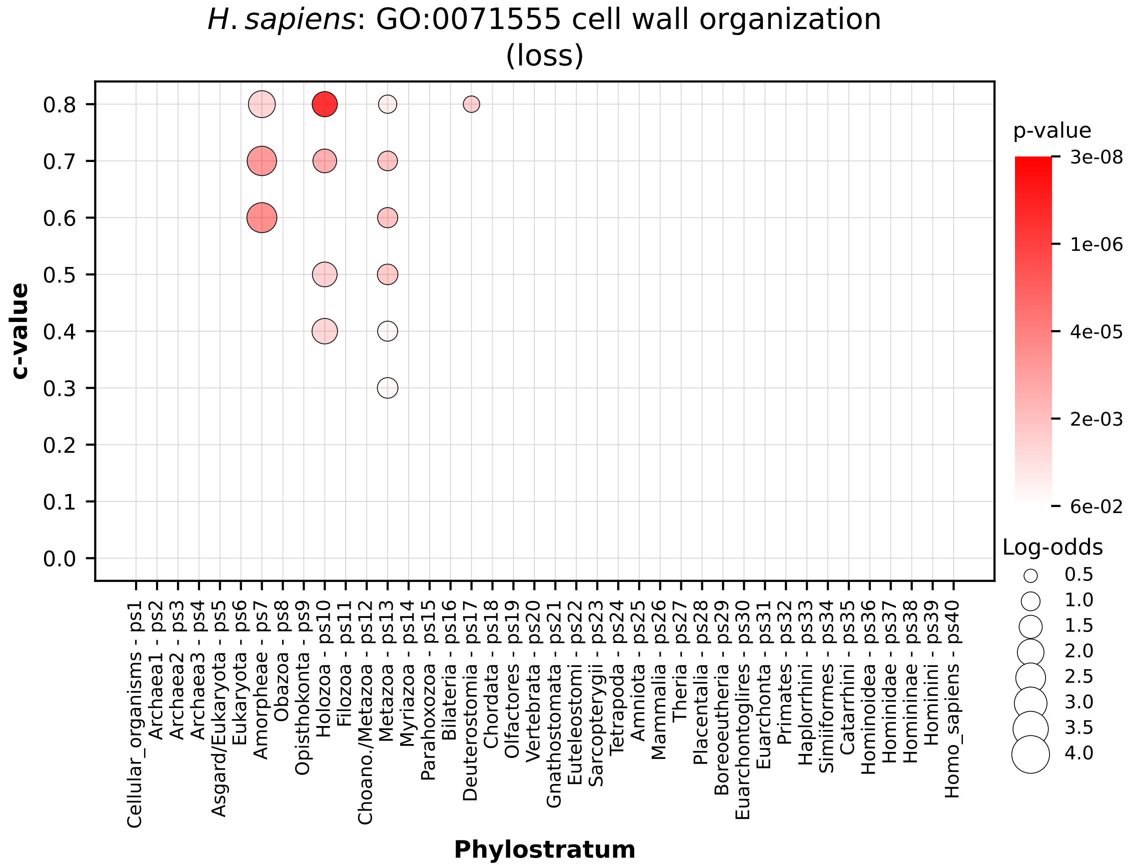

H. sapiens: GO:0071555 cell wall organization
(loss)
c-value
0.8
0.7
0.6
0.5
0.4
0.3
0.2
0.1
0.0
p-value
3e-08
1e-06
4e-05
2e-03
6e-02
Log-odds
0.5
1.0
1.5
2.0
2.5
3.0
3.5
4.0
Cellular_organisms - ps1
Archaea1 - ps2
Archaea2 - ps3
Archaea3 - ps4
Asgard/Eukaryota - ps5
Eukaryota - ps6
Amorpheae - ps7
Obazoa - ps8
Opisthokonta - ps9
Holozoa - ps10
Filozoa - ps11
Choano./Metazoa - ps12
Metazoa - ps13
Myriazoa - ps14
Parahoxozoa - ps15
Bilateria - ps16
Deuterostomia - ps17
Chordata - ps18
Olfactores - ps19
Vertebrata - ps20
Gnathostomata - ps21
Euteleostomi - ps22
Sarcopterygii - ps23
Tetrapoda - ps24
Amniota - ps25
Mammalia - ps26
Theria - ps27
Placentalia - ps28
Boreoeutheria - ps29
Euarchontoglires - ps30
Euarchonta - ps31
Primates - ps32
Haplorrhini - ps33
Simiiformes - ps34
Catarrhini - ps35
Hominoidea - ps36
Hominidae - ps37
Homininae - ps38
Hominini - ps39
Homo_sapiens - ps40
Phylostratum

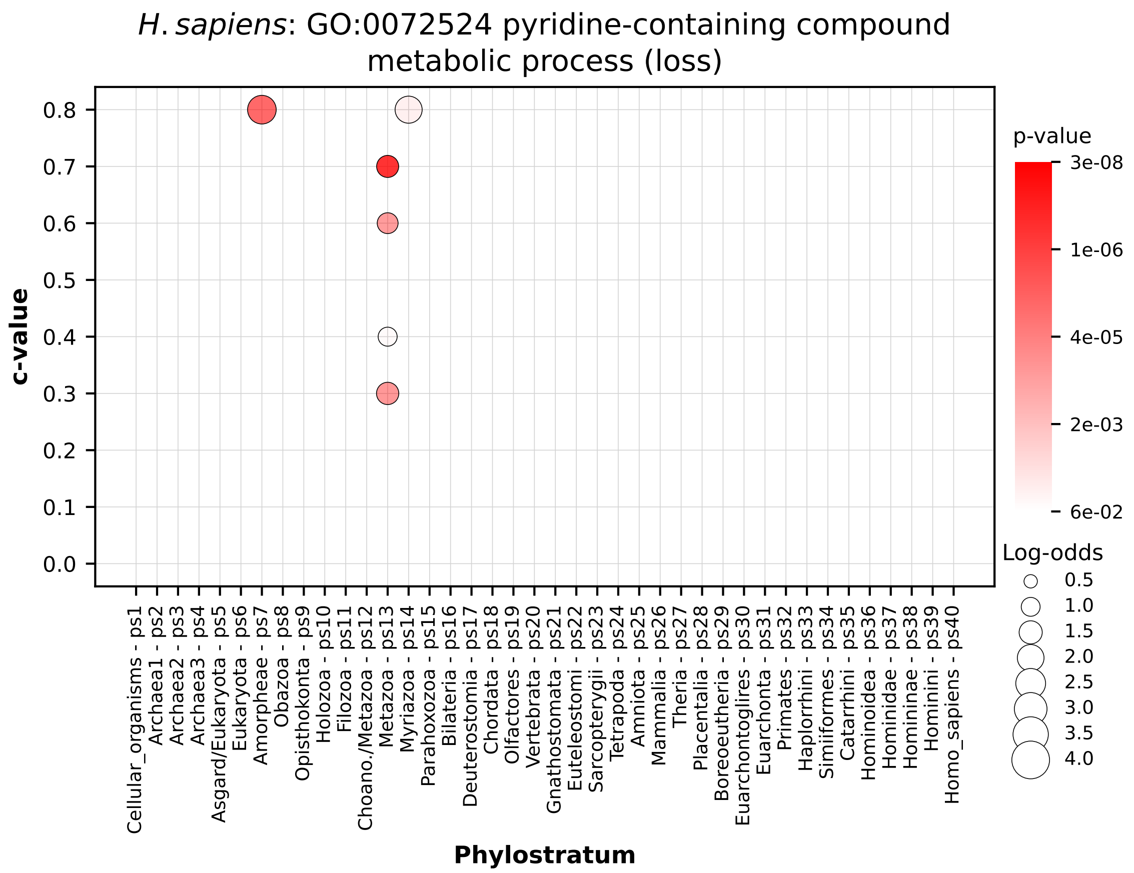
H. sapiens: GO:0072524 pyridine-containing compound metabolic process (loss)
c-value
0.8
0.7
0.6
0.5
0.4
0.3
0.2
0.1
0.0
p-value
3e-08
1e-06
4e-05
2e-03
6e-02
Log-odds
0.5
1.0
1.5
2.0
2.5
3.0
3.5
4.0
Cellular_organisms - ps1
Archaea1 - ps2
Archaea2 - ps3
Archaea3 - ps4
Asgard/Eukaryota - ps5
Eukaryota - ps6
Amorpheae - ps7
Obazoa - ps8
Opisthokonta - ps9
Holozoa - ps10
Filozoa - ps11
Choano./Metazoa - ps12
Metazoa - ps13
Myriazoa - ps14
Parahoxozoa - ps15
Bilateria - ps16
Deuterostomia - ps17
Chordata - ps18
Olfactores - ps19
Vertebrata - ps20
Gnathostomata - ps21
Euteleostomi - ps22
Sarcopterygii - ps23
Tetrapoda - ps24
Amniota - ps25
Mammalia - ps26
Theria - ps27
Placentalia - ps28
Boreoeutheria - ps29
Euarchontoglires - ps30
Euarchonta - ps31
Primates - ps32
Haplorrhini - ps33
Simiiformes - ps34
Catarrhini - ps35
Hominoidea - ps36
Hominidae - ps37
Homininae - ps38
Hominini - ps39
Homo_sapiens - ps40
Phylostratum

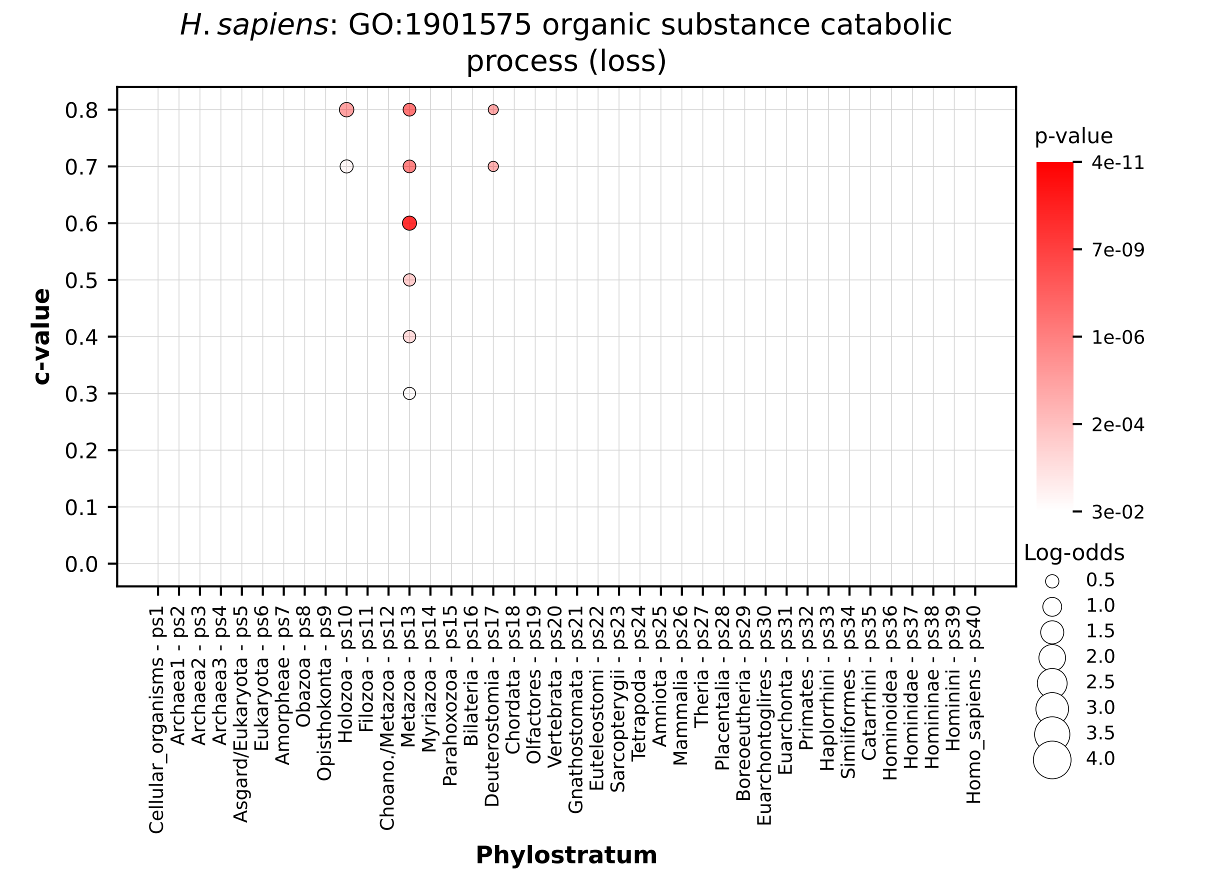

H. sapiens: GO:1901575 organic substance catabolic process (loss)
c-value
0.8
0.7
0.6
0.5
0.4
0.3
0.2
0.1
0.0
p-value
4e-11
7e-09
1e-06
2e-04
3e-02
Log-odds
0.5
1.0
1.5
2.0
2.5
3.0
3.5
4.0
Cellular_organisms - ps1
Archaea1 - ps2
Archaea2 - ps3
Archaea3 - ps4
Asgard/Eukaryota - ps5
Eukaryota - ps6
Amorpheae - ps7
Obazoa - ps8
Opisthokonta - ps9
Holozoa - ps10
Filozoa - ps11
Choano./Metazoa - ps12
Metazoa - ps13
Myriazoa - ps14
Parahoxozoa - ps15
Bilateria - ps16
Deuterostomia - ps17
Chordata - ps18
Olfactores - ps19
Vertebrata - ps20
Gnathostomata - ps21
Euteleostomi - ps22
Sarcopterygii - ps23
Tetrapoda - ps24
Amniota - ps25
Mammalia - ps26
Theria - ps27
Placentalia - ps28
Boreoeutheria - ps29
Euarchontoglires - ps30
Euarchonta - ps31
Primates - ps32
Haplorrhini - ps33
Simiiformes - ps34
Catarrhini - ps35
Hominoidea - ps36
Hominidae - ps37
Homininae - ps38
Hominini - ps39
Homo_sapiens - ps40
Phylostratum

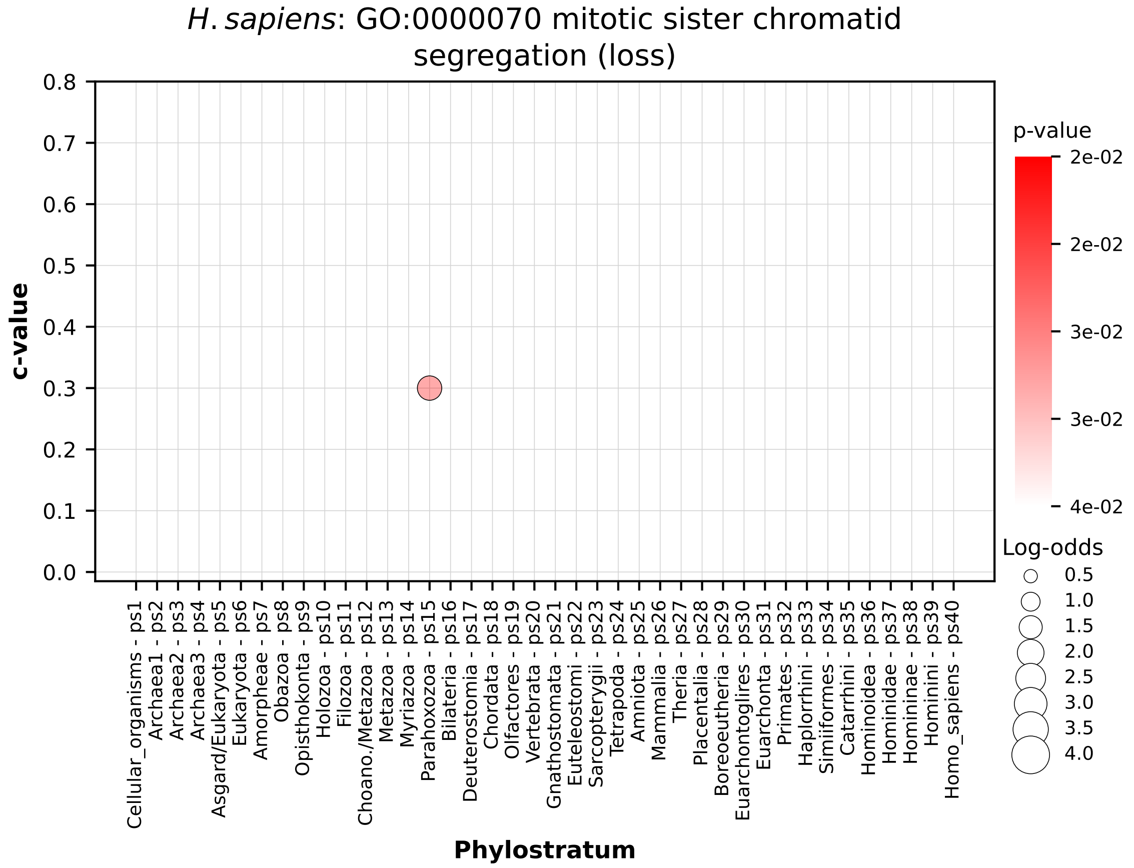

H. sapiens: GO:0000070 mitotic sister chromatid segregation (loss)
c-value
0.8
0.7
0.6
0.5
0.4
0.3
0.2
0.1
0.0
Phylostratum
Cellular_organisms - ps1
Archaea1 - ps2
Archaea2 - ps3
Archaea3 - ps4
Asgard/Eukaryota - ps5
Eukaryota - ps6
Amorpheae - ps7
Obazoa - ps8
Opisthokonta - ps9
Holozoa - ps10
Filozoa - ps11
Choano./Metazoa - ps12
Metazoa - ps13
Myriazoa - ps14
Parahoxozoa - ps15
Bilateria - ps16
Deuterostomia - ps17
Chordata - ps18
Olfactores - ps19
Vertebrata - ps20
Gnathostomata - ps21
Euteleostomi - ps22
Sarcopterygii - ps23
Tetrapoda - ps24
Amniota - ps25
Mammalia - ps26
Theria - ps27
Placentalia - ps28
Boreoeutheria - ps29
Euarchontoglires - ps30
Euarchonta - ps31
Primates - ps32
Haplorrhini - ps33
Simiiformes - ps34
Catarrhini - ps35
Hominoidea - ps36
Hominidae - ps37
Homininae - ps38
Hominini - ps39
Homo_sapiens - ps40
p-value
2e-02
2e-02
3e-02
3e-02
4e-02
Log-odds
0.5
1.0
1.5
2.0
2.5
3.0
3.5
4.0

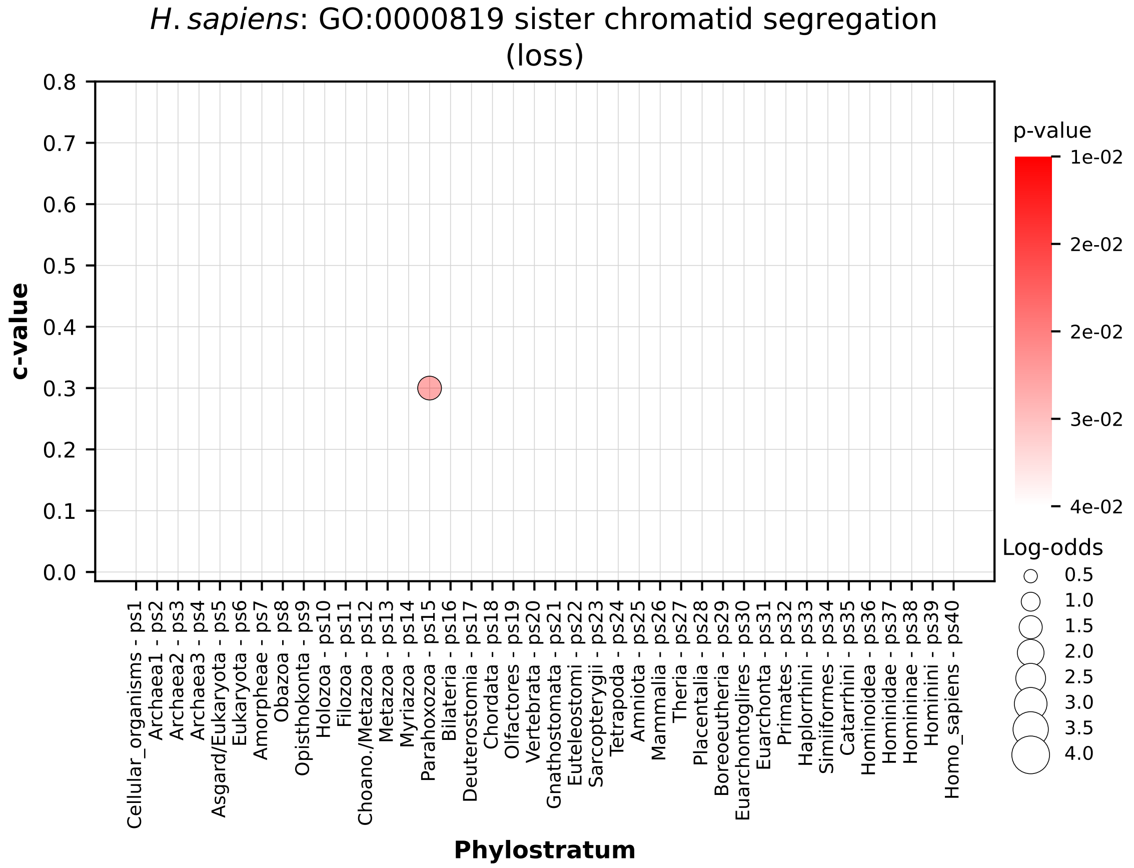

H. sapiens: GO:0000819 sister chromatid segregation (loss)
c-value
0.8
0.7
0.6
0.5
0.4
0.3
0.2
0.1
0.0
Phylostratum
Cellular_organisms - ps1
Archaea1 - ps2
Archaea2 - ps3
Archaea3 - ps4
Asgard/Eukaryota - ps5
Eukaryota - ps6
Amorpheae - ps7
Obazoa - ps8
Opisthokonta - ps9
Holozoa - ps10
Filozoa - ps11
Choano./Metazoa - ps12
Metazoa - ps13
Myriazoa - ps14
Parahoxozoa - ps15
Bilateria - ps16
Deuterostomia - ps17
Chordata - ps18
Olfactores - ps19
Vertebrata - ps20
Gnathostomata - ps21
Euteleostomi - ps22
Sarcopterygii - ps23
Tetrapoda - ps24
Amniota - ps25
Mammalia - ps26
Theria - ps27
Placentalia - ps28
Boreoeutheria - ps29
Euarchontoglires - ps30
Euarchonta - ps31
Primates - ps32
Haplorrhini - ps33
Simiiformes - ps34
Catarrhini - ps35
Hominoidea - ps36
Hominidae - ps37
Homininae - ps38
Hominini - ps39
Homo_sapiens - ps40
p-value
1e-02
2e-02
2e-02
3e-02
4e-02
Log-odds
0.5
1.0
1.5
2.0
2.5
3.0
3.5
4.0

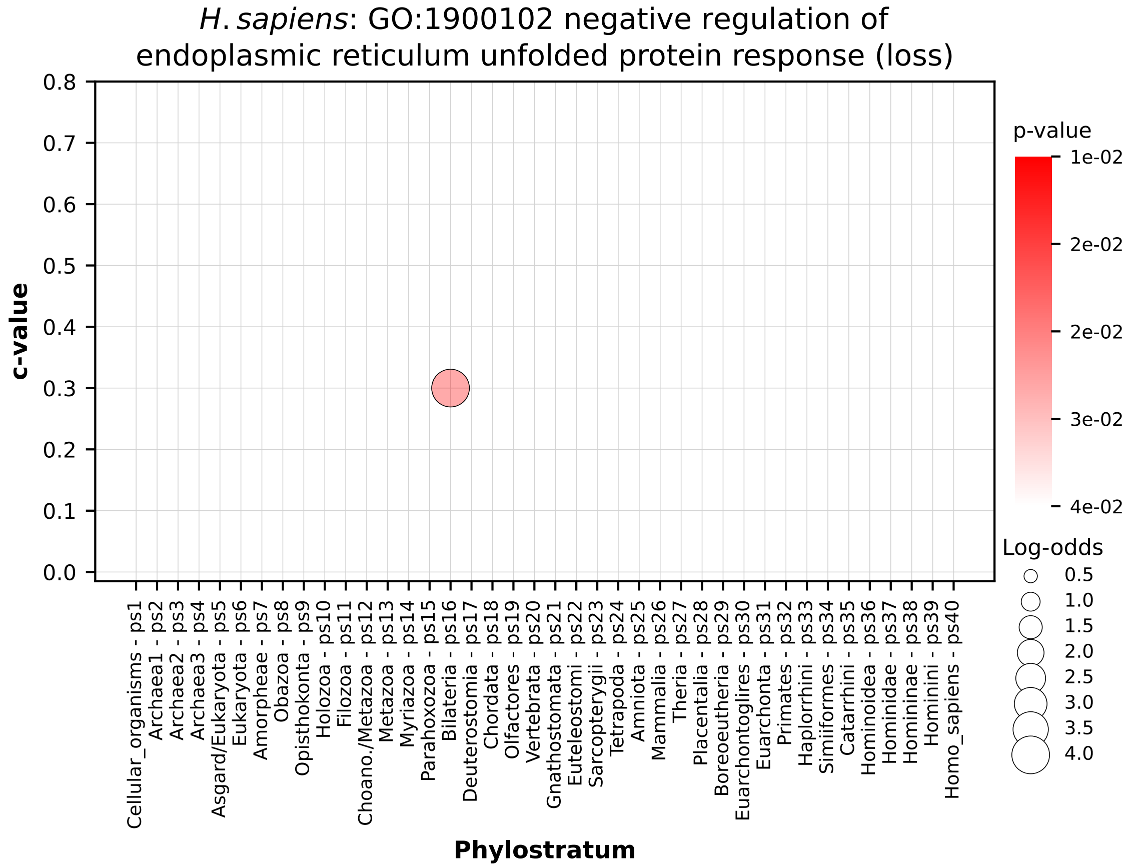

H. sapiens: GO:1900102 negative regulation of endoplasmic reticulum unfolded protein response (loss)
c-value
0.0
0.1
0.2
0.3
0.4
0.5
0.6
0.7
0.8
Phylostratum
Cellular_organisms - ps1
Archaea1 - ps2
Archaea2 - ps3
Archaea3 - ps4
Asgard/Eukaryota - ps5
Eukaryota - ps6
Amorpheae - ps7
Obazoa - ps8
Opisthokonta - ps9
Holozoa - ps10
Filozoa - ps11
Choano./Metazoa - ps12
Metazoa - ps13
Myriazoa - ps14
Parahoxozoa - ps15
Bilateria - ps16
Deuterostomia - ps17
Chordata - ps18
Olfactores - ps19
Vertebrata - ps20
Gnathostomata - ps21
Euteleostomi - ps22
Sarcopterygii - ps23
Tetrapoda - ps24
Amniota - ps25
Mammalia - ps26
Theria - ps27
Placentalia - ps28
Boreoeutheria - ps29
Euarchontoglires - ps30
Euarchonta - ps31
Primates - ps32
Haplorrhini - ps33
Simiiformes - ps34
Catarrhini - ps35
Hominoidea - ps36
Hominidae - ps37
Homininae - ps38
Hominini - ps39
Homo_sapiens - ps40
p-value
1e-02
2e-02
2e-02
3e-02
4e-02
Log-odds
0.5
1.0
1.5
2.0
2.5
3.0
3.5
4.0

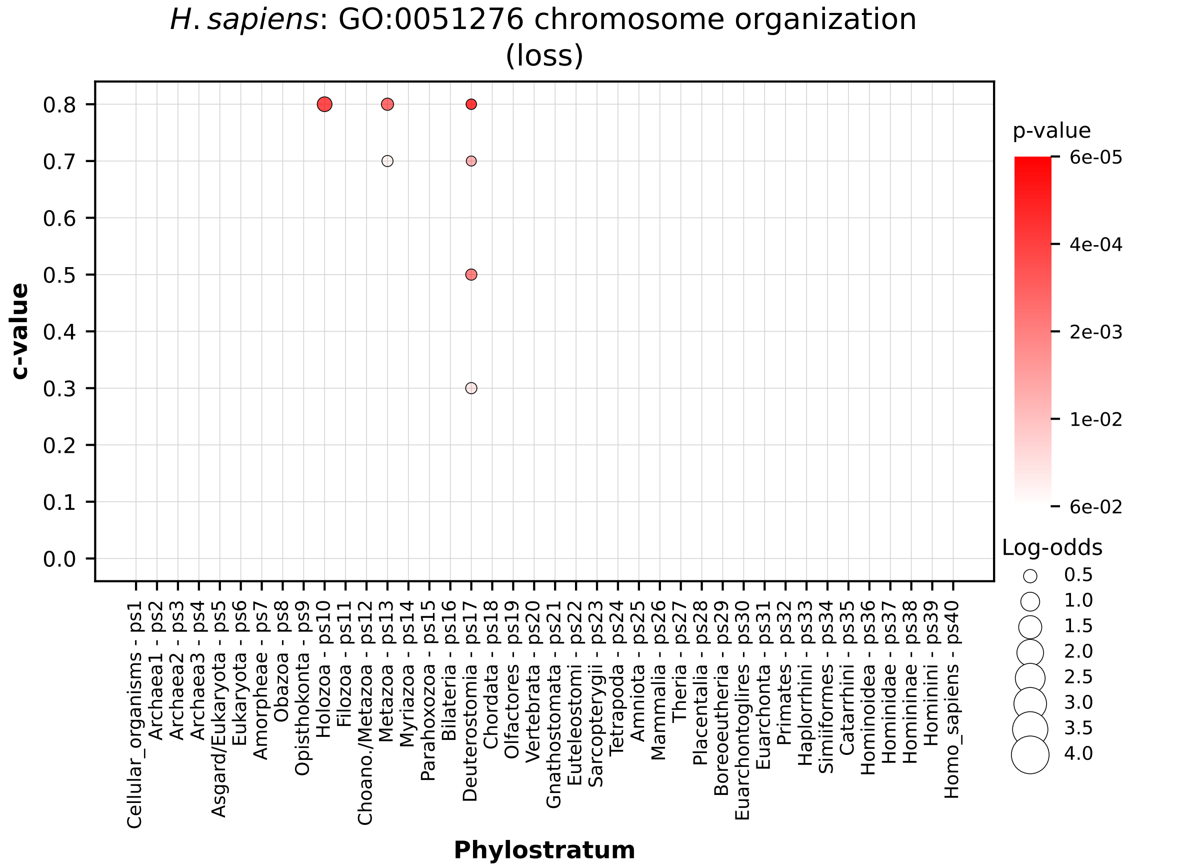

H. sapiens: GO:0051276 chromosome organization
(loss)
c-value
0.8
0.7
0.6
0.5
0.4
0.3
0.2
0.1
0.0
p-value
6e-05
4e-04
2e-03
1e-02
6e-02
Log-odds
0.5
1.0
1.5
2.0
2.5
3.0
3.5
4.0
Cellular_organisms - ps1
Archaea1 - ps2
Archaea2 - ps3
Archaea3 - ps4
Asgard/Eukaryota - ps5
Eukaryota - ps6
Amorpheae - ps7
Obazoa - ps8
Opisthokonta - ps9
Holozoa - ps10
Filozoa - ps11
Choano./Metazoa - ps12
Metazoa - ps13
Myriazoa - ps14
Parahoxozoa - ps15
Bilateria - ps16
Deuterostomia - ps17
Chordata - ps18
Olfactores - ps19
Vertebrata - ps20
Gnathostomata - ps21
Euteleostomi - ps22
Sarcopterygii - ps23
Tetrapoda - ps24
Amniota - ps25
Mammalia - ps26
Theria - ps27
Placentalia - ps28
Boreoeutheria - ps29
Euarchontoglires - ps30
Euarchonta - ps31
Primates - ps32
Haplorrhini - ps33
Simiiformes - ps34
Catarrhini - ps35
Hominoidea - ps36
Hominidae - ps37
Homininae - ps38
Hominini - ps39
Homo_sapiens - ps40
Phylostratum

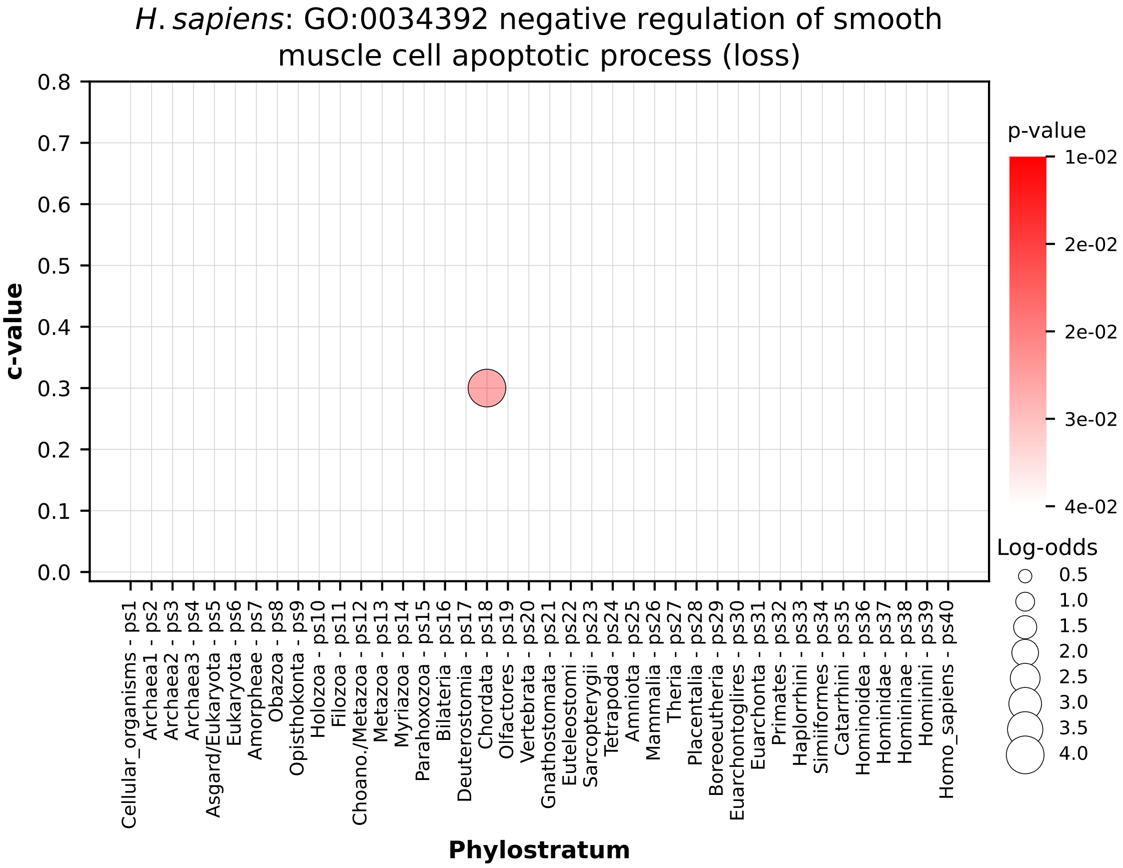
H. sapiens: GO:0034392 negative regulation of smooth muscle cell apoptotic process (loss)
c-value
0.8
0.7
0.6
0.5
0.4
0.3
0.2
0.1
0.0
Phylostratum
Cellular_organisms - ps1
Archaea1 - ps2
Archaea2 - ps3
Archaea3 - ps4
Asgard/Eukaryota - ps5
Eukaryota - ps6
Amorpheae - ps7
Obazoa - ps8
Opisthokonta - ps9
Holozoa - ps10
Filozoa - ps11
Choano./Metazoa - ps12
Metazoa - ps13
Myriazoa - ps14
Parahoxozoa - ps15
Bilateria - ps16
Deuterostomia - ps17
Chordata - ps18
Olfactores - ps19
Vertebrata - ps20
Gnathostomata - ps21
Euteleostomi - ps22
Sarcopterygii - ps23
Tetrapoda - ps24
Amniota - ps25
Mammalia - ps26
Theria - ps27
Placentalia - ps28
Boreoeutheria - ps29
Euarchontoglires - ps30
Euarchonta - ps31
Primates - ps32
Haplorrhini - ps33
Simiiformes - ps34
Catarrhini - ps35
Hominoidea - ps36
Hominidae - ps37
Homininae - ps38
Hominini - ps39
Homo_sapiens - ps40
p-value
1e-02
2e-02
2e-02
3e-02
4e-02
Log-odds
0.5
1.0
1.5
2.0
2.5
3.0
3.5
4.0

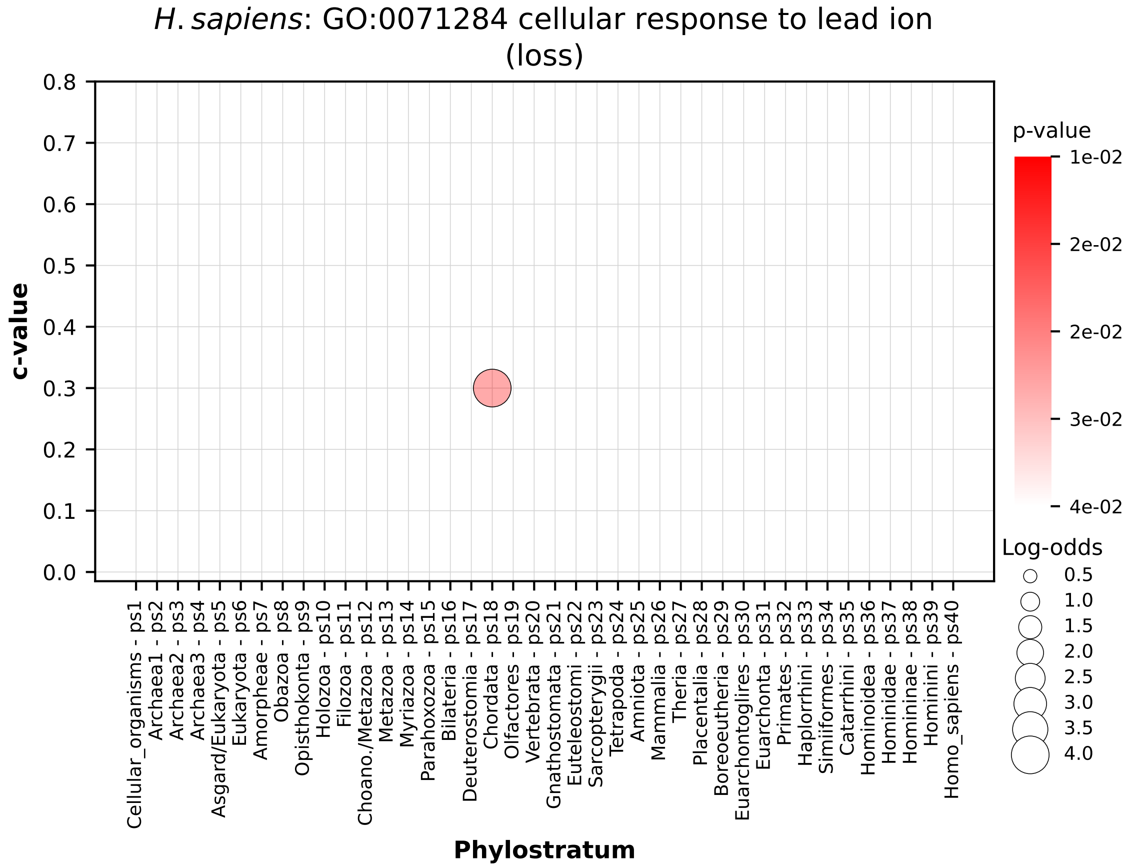

H. sapiens: GO:0071284 cellular response to lead ion
(loss)
c-value
0.8
0.7
0.6
0.5
0.4
0.3
0.2
0.1
0.0
Phylostratum
Cellular_organisms - ps1
Archaea1 - ps2
Archaea2 - ps3
Archaea3 - ps4
Asgard/Eukaryota - ps5
Eukaryota - ps6
Amorpheae - ps7
Obazoa - ps8
Opisthokonta - ps9
Holozoa - ps10
Filozoa - ps11
Choano./Metazoa - ps12
Metazoa - ps13
Myriazoa - ps14
Parahoxozoa - ps15
Bilateria - ps16
Deuterostomia - ps17
Chordata - ps18
Olfactores - ps19
Vertebrata - ps20
Gnathostomata - ps21
Euteleostomi - ps22
Sarcopterygii - ps23
Tetrapoda - ps24
Amniota - ps25
Mammalia - ps26
Theria - ps27
Placentalia - ps28
Boreoeutheria - ps29
Euarchontoglires - ps30
Euarchonta - ps31
Primates - ps32
Haplorrhini - ps33
Simiiformes - ps34
Catarrhini - ps35
Hominoidea - ps36
Hominidae - ps37
Homininae - ps38
Hominini - ps39
Homo_sapiens - ps40
p-value
1e-02
2e-02
2e-02
3e-02
4e-02
Log-odds
0.5
1.0
1.5
2.0
2.5
3.0
3.5
4.0

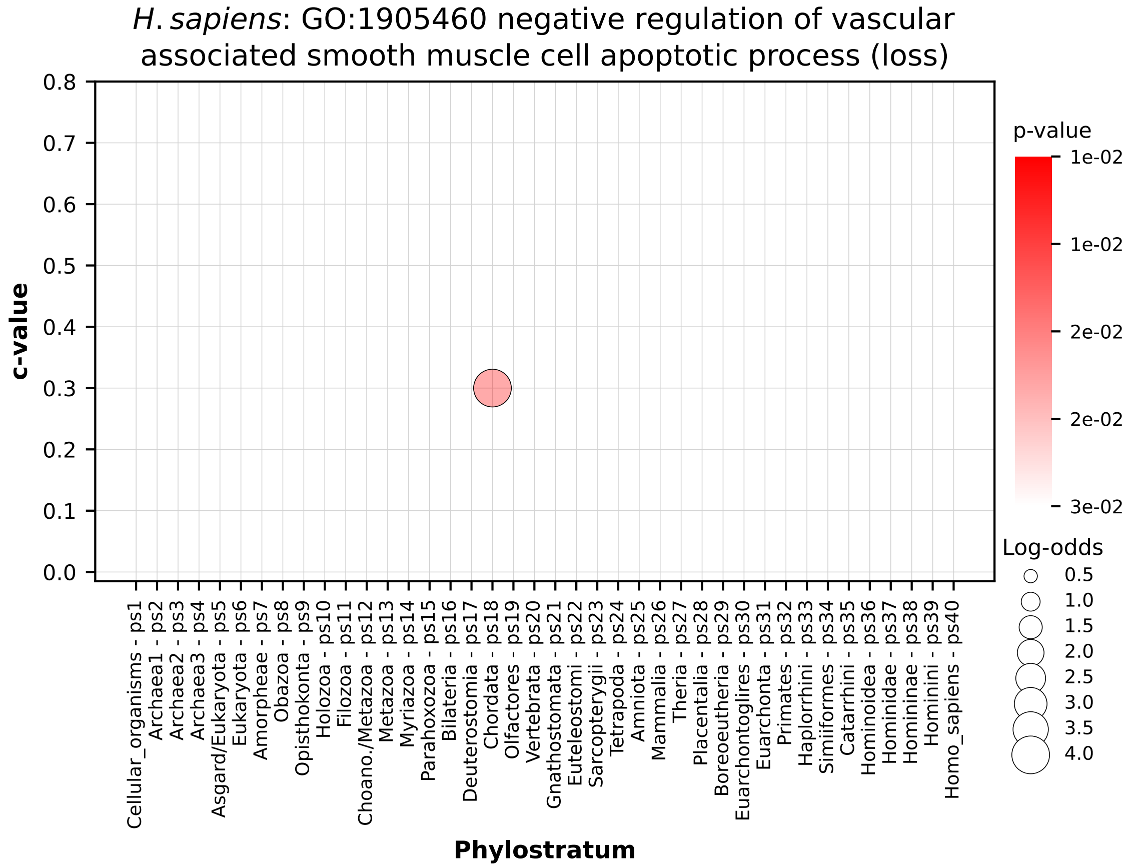

H. sapiens: GO:1905460 negative regulation of vascular associated smooth muscle cell apoptotic process (loss)
c-value
0.8
0.7
0.6
0.5
0.4
0.3
0.2
0.1
0.0
Phylostratum
Cellular_organisms - ps1
Archaea1 - ps2
Archaea2 - ps3
Archaea3 - ps4
Asgard/Eukaryota - ps5
Eukaryota - ps6
Amorpheae - ps7
Obazoa - ps8
Opisthokonta - ps9
Holozoa - ps10
Filozoa - ps11
Choano./Metazoa - ps12
Metazoa - ps13
Myriazoa - ps14
Parahoxozoa - ps15
Bilateria - ps16
Deuterostomia - ps17
Chordata - ps18
Olfactores - ps19
Vertebrata - ps20
Gnathostomata - ps21
Euteleostomi - ps22
Sarcopterygii - ps23
Tetrapoda - ps24
Amniota - ps25
Mammalia - ps26
Theria - ps27
Placentalia - ps28
Boreoeutheria - ps29
Euarchontoglires - ps30
Euarchonta - ps31
Primates - ps32
Haplorrhini - ps33
Simiiformes - ps34
Catarrhini - ps35
Hominoidea - ps36
Hominidae - ps37
Homininae - ps38
Hominini - ps39
Homo_sapiens - ps40
p-value
1e-02
1e-02
2e-02
2e-02
3e-02
Log-odds
0.5
1.0
1.5
2.0
2.5
3.0
3.5
4.0

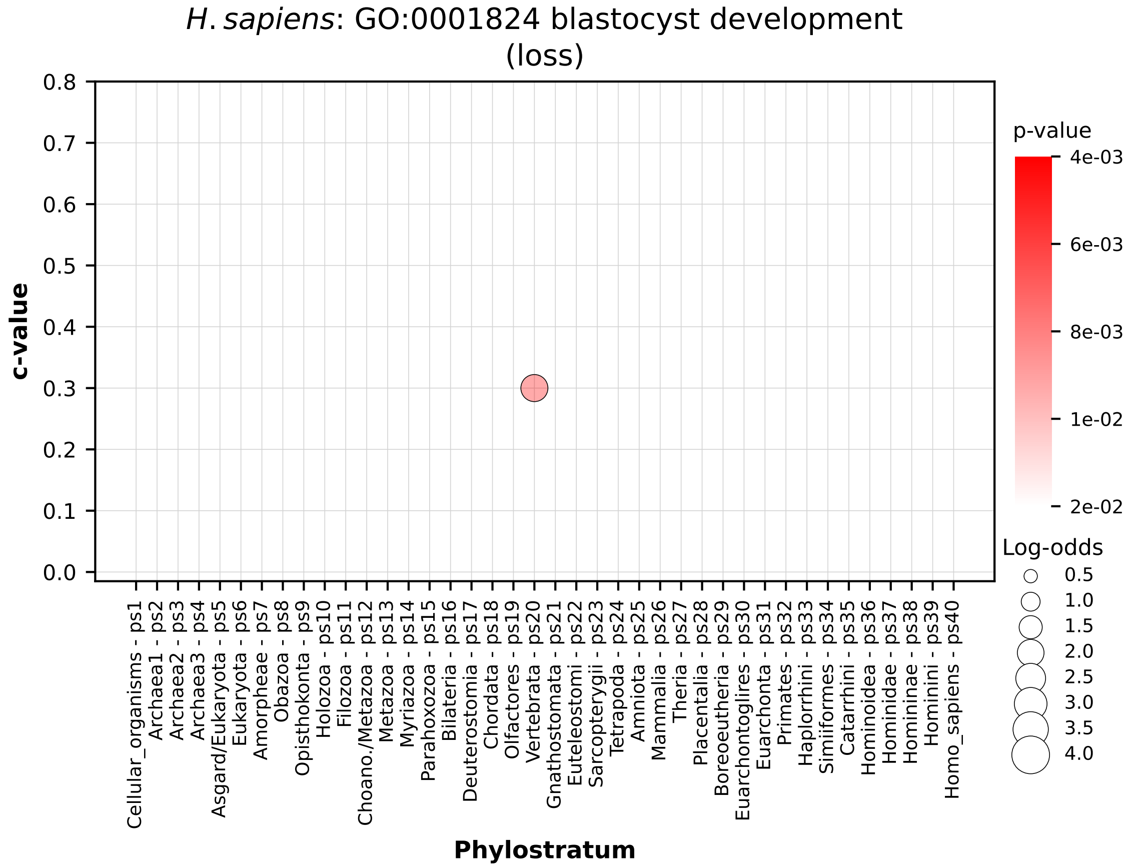
H. sapiens: GO:0001824 blastocyst development (loss)
c-value
0.8
0.7
0.6
0.5
0.4
0.3
0.2
0.1
0.0
p-value
4e-03
6e-03
8e-03
1e-02
2e-02
Log-odds
0.5
1.0
1.5
2.0
2.5
3.0
3.5
4.0
Cellular_organisms - ps1
Archaea1 - ps2
Archaea2 - ps3
Archaea3 - ps4
Asgard/Eukaryota - ps5
Eukaryota - ps6
Amorpheae - ps7
Obazoa - ps8
Opisthokonta - ps9
Holozoa - ps10
Filozoa - ps11
Choano./Metazoa - ps12
Metazoa - ps13
Myriazoa - ps14
Parahoxozoa - ps15
Bilateria - ps16
Deuterostomia - ps17
Chordata - ps18
Olfactores - ps19
Vertebrata - ps20
Gnathostomata - ps21
Euteleostomi - ps22
Sarcopterygii - ps23
Tetrapoda - ps24
Amniota - ps25
Mammalia - ps26
Theria - ps27
Placentalia - ps28
Boreoeutheria - ps29
Euarchontoglires - ps30
Euarchonta - ps31
Primates - ps32
Haplorrhini - ps33
Simiiformes - ps34
Catarrhini - ps35
Hominoidea - ps36
Hominidae - ps37
Homininae - ps38
Hominini - ps39
Homo_sapiens - ps40
Phylostratum

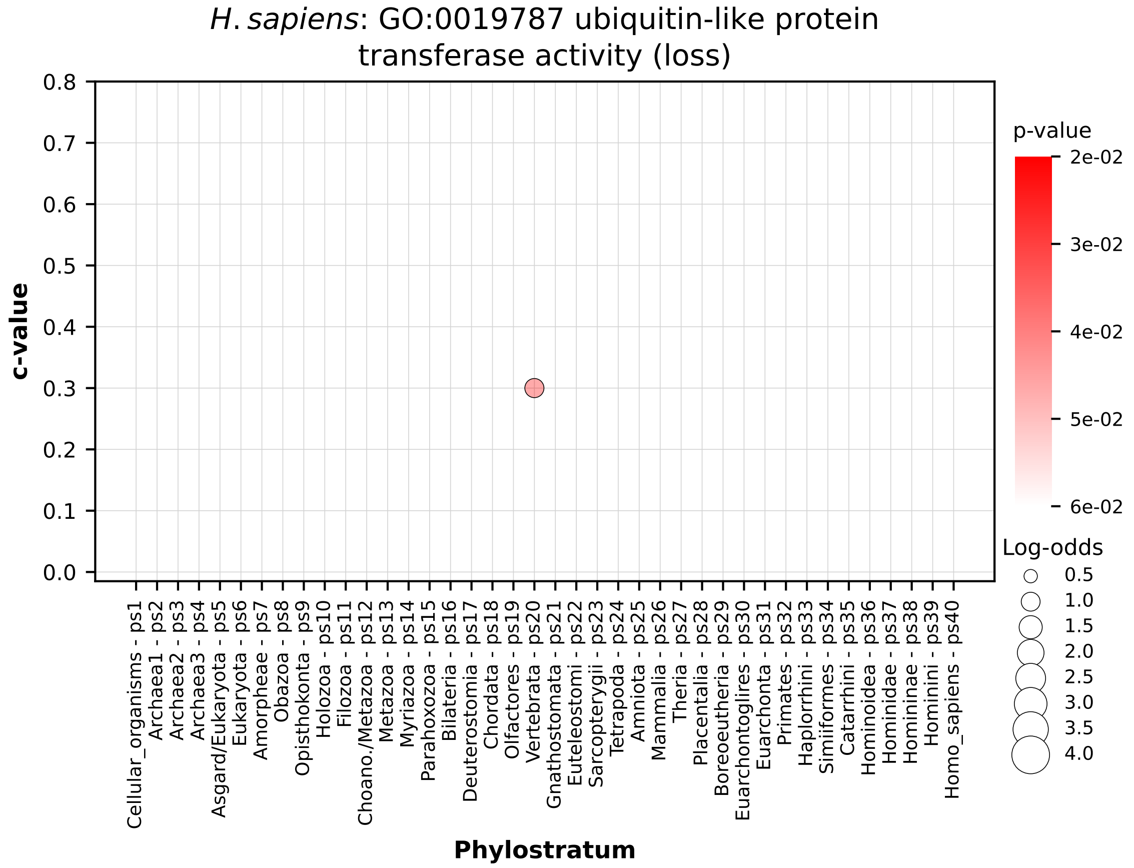

H. sapiens: GO:0019787 ubiquitin-like protein transferase activity (loss)
c-value
0.8
0.7
0.6
0.5
0.4
0.3
0.2
0.1
0.0
Phylostratum
Cellular_organisms - ps1
Archaea1 - ps2
Archaea2 - ps3
Archaea3 - ps4
Asgard/Eukaryota - ps5
Eukaryota - ps6
Amorpheae - ps7
Obazoa - ps8
Opisthokonta - ps9
Holozoa - ps10
Filozoa - ps11
Choano./Metazoa - ps12
Metazoa - ps13
Myriazoa - ps14
Parahoxozoa - ps15
Bilateria - ps16
Deuterostomia - ps17
Chordata - ps18
Olfactores - ps19
Vertebrata - ps20
Gnathostomata - ps21
Euteleostomi - ps22
Sarcopterygii - ps23
Tetrapoda - ps24
Amniota - ps25
Mammalia - ps26
Theria - ps27
Placentalia - ps28
Boreoeutheria - ps29
Euarchontoglires - ps30
Euarchonta - ps31
Primates - ps32
Haplorrhini - ps33
Simiiformes - ps34
Catarrhini - ps35
Hominoidea - ps36
Hominidae - ps37
Homininae - ps38
Hominini - ps39
Homo_sapiens - ps40
p-value
2e-02
3e-02
4e-02
5e-02
6e-02
Log-odds
0.5
1.0
1.5
2.0
2.5
3.0
3.5
4.0

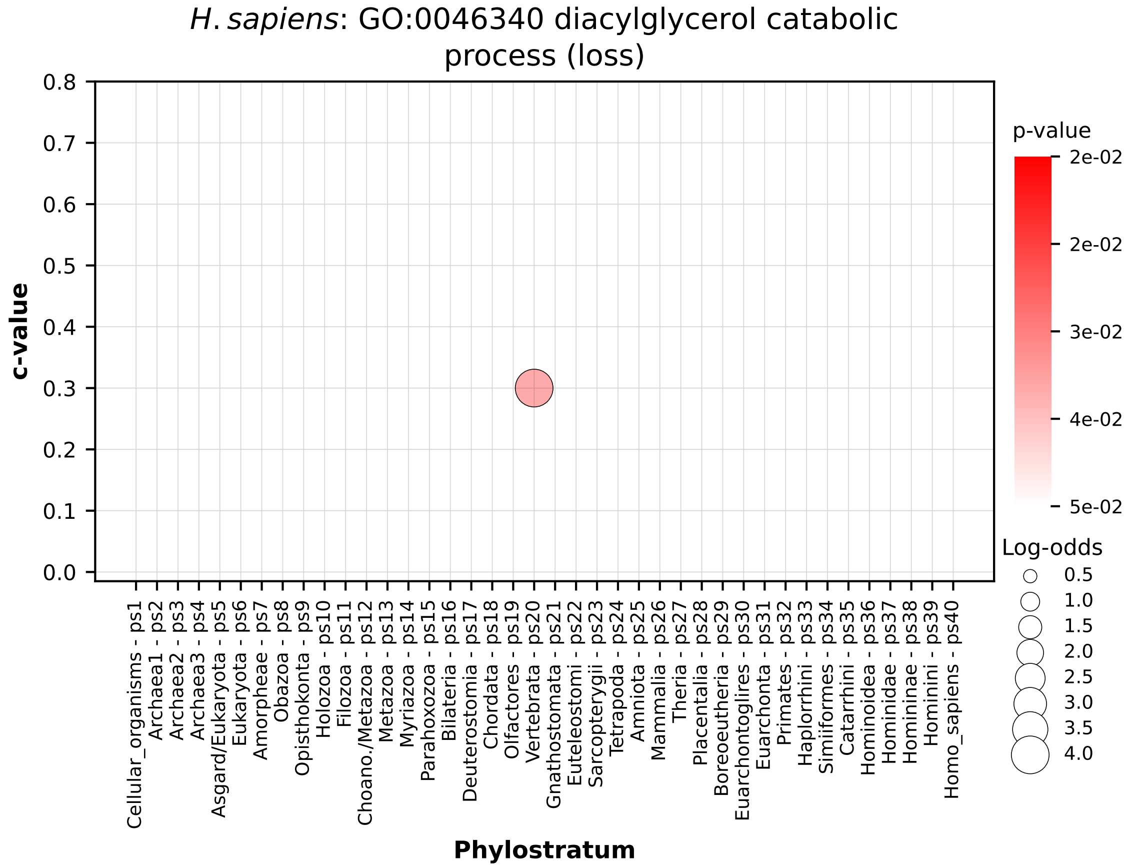

H. sapiens: GO:0046340 diacylglycerol catabolic process (loss)
c-value
0.8
0.7
0.6
0.5
0.4
0.3
0.2
0.1
0.0
Phylostratum
Cellular_organisms - ps1
Archaea1 - ps2
Archaea2 - ps3
Archaea3 - ps4
Asgard/Eukaryota - ps5
Eukaryota - ps6
Amorpheae - ps7
Obazoa - ps8
Opisthokonta - ps9
Holozoa - ps10
Filozoa - ps11
Choano./Metazoa - ps12
Metazoa - ps13
Myriazoa - ps14
Parahoxozoa - ps15
Bilateria - ps16
Deuterostomia - ps17
Chordata - ps18
Olfactores - ps19
Vertebrata - ps20
Gnathostomata - ps21
Euteleostomi - ps22
Sarcopterygii - ps23
Tetrapoda - ps24
Amniota - ps25
Mammalia - ps26
Theria - ps27
Placentalia - ps28
Boreoeutheria - ps29
Euarchontoglires - ps30
Euarchonta - ps31
Primates - ps32
Haplorrhini - ps33
Simiiformes - ps34
Catarrhini - ps35
Hominoidea - ps36
Hominidae - ps37
Homininae - ps38
Hominini - ps39
Homo_sapiens - ps40
p-value
2e-02
2e-02
3e-02
4e-02
5e-02
Log-odds
0.5
1.0
1.5
2.0
2.5
3.0
3.5
4.0

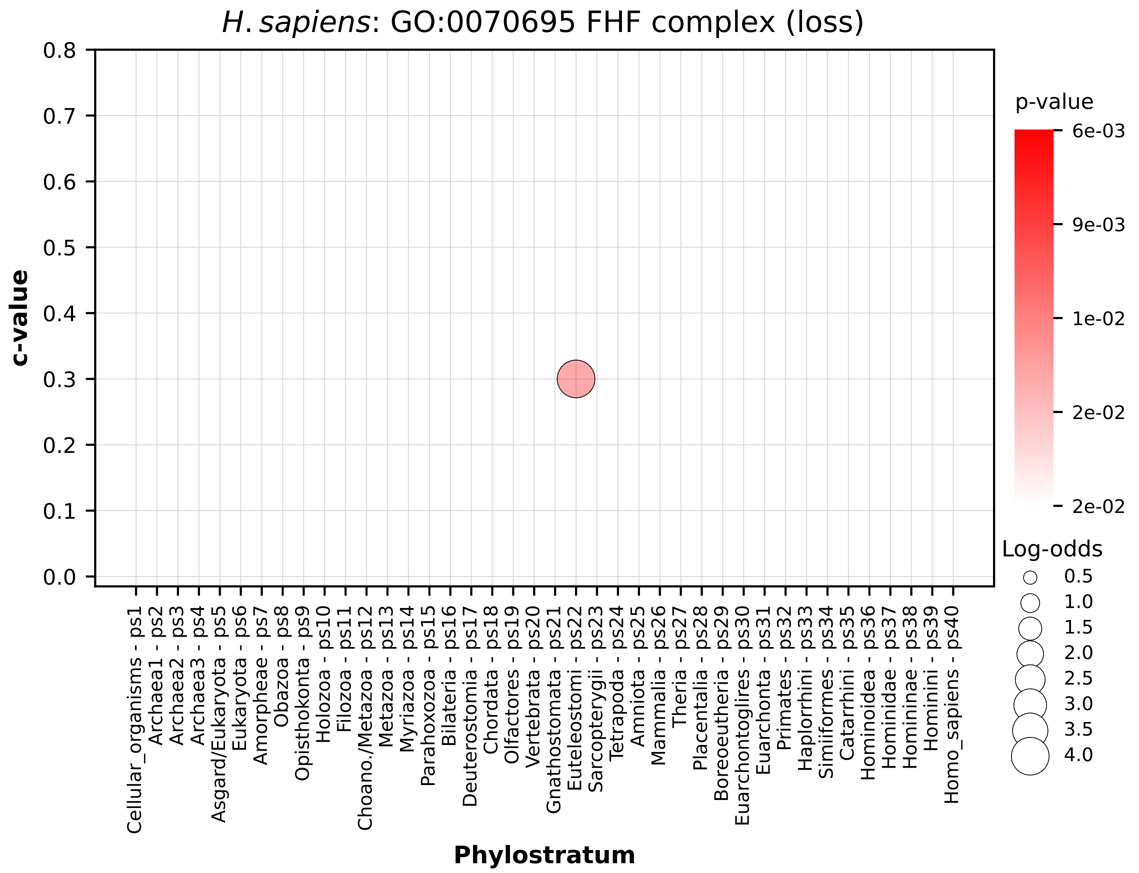

H. sapiens: GO:0070695 FHF complex (loss)
c-value
0.0
0.1
0.2
0.3
0.4
0.5
0.6
0.7
0.8
Phylostratum
Cellular_organisms - ps1
Archaea1 - ps2
Archaea2 - ps3
Archaea3 - ps4
Asgard/Eukaryota - ps5
Eukaryota - ps6
Amorpheae - ps7
Obazoa - ps8
Opisthokonta - ps9
Holozoa - ps10
Filozoa - ps11
Choano./Metazoa - ps12
Metazoa - ps13
Myriazoa - ps14
Parahoxozoa - ps15
Bilateria - ps16
Deuterostomia - ps17
Chordata - ps18
Olfactores - ps19
Vertebrata - ps20
Gnathostomata - ps21
Euteleostomi - ps22
Sarcopterygii - ps23
Tetrapoda - ps24
Amniota - ps25
Mammalia - ps26
Theria - ps27
Placentalia - ps28
Boreoeutheria - ps29
Euarchontoglires - ps30
Euarchonta - ps31
Primates - ps32
Haplorrhini - ps33
Simiiformes - ps34
Catarrhini - ps35
Hominoidea - ps36
Hominidae - ps37
Homininae - ps38
Hominini - ps39
Homo_sapiens - ps40
p-value
6e-03
9e-03
1e-02
2e-02
2e-02
Log-odds
0.5
1.0
1.5
2.0
2.5
3.0
3.5
4.0

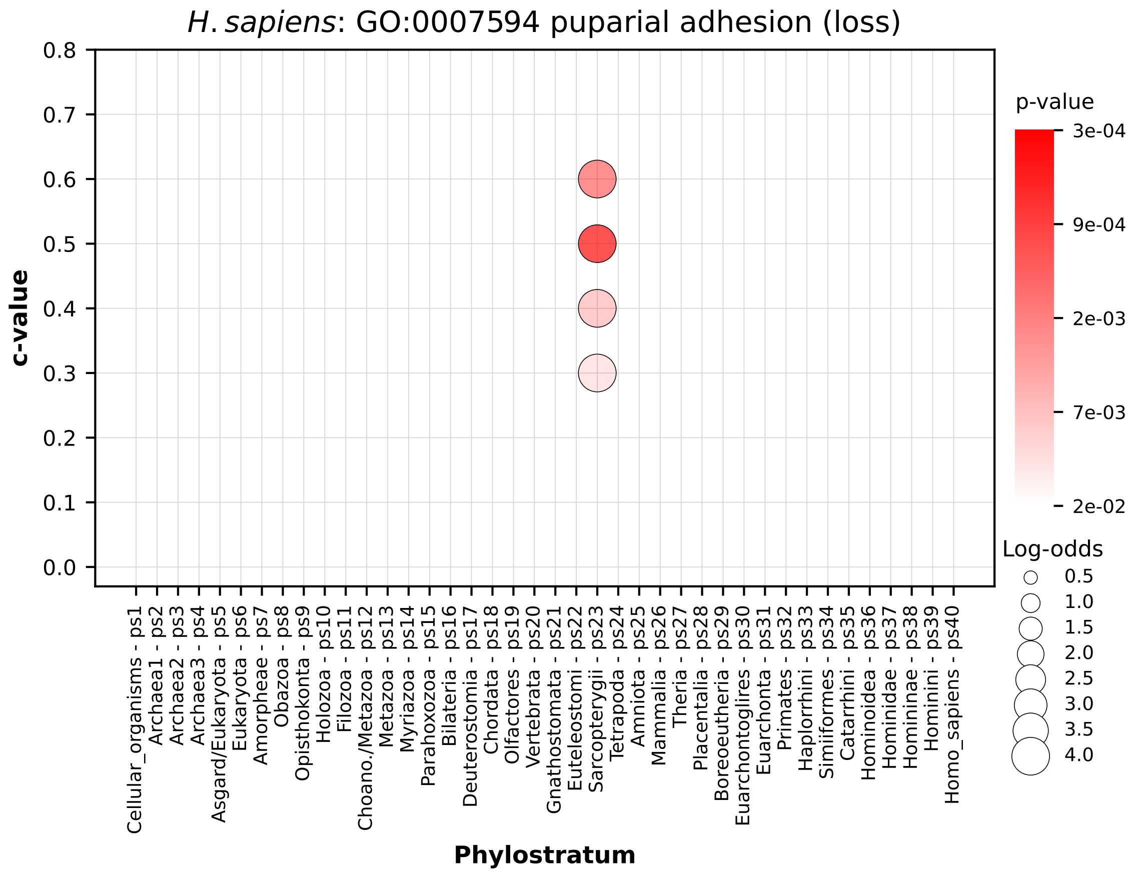

H. sapiens: GO:0007594 puparial adhesion (loss)
c-value
0.8
0.7
0.6
0.5
0.4
0.3
0.2
0.1
0.0
p-value
3e-04
9e-04
2e-03
7e-03
2e-02
Log-odds
0.5
1.0
1.5
2.0
2.5
3.0
3.5
4.0
Cellular_organisms - ps1
Archaea1 - ps2
Archaea2 - ps3
Archaea3 - ps4
Asgard/Eukaryota - ps5
Eukaryota - ps6
Amorpheae - ps7
Obazoa - ps8
Opisthokonta - ps9
Holozoa - ps10
Filozoa - ps11
Choano./Metazoa - ps12
Metazoa - ps13
Myriazoa - ps14
Parahoxozoa - ps15
Bilateria - ps16
Deuterostomia - ps17
Chordata - ps18
Olfactores - ps19
Vertebrata - ps20
Gnathostomata - ps21
Euteleostomi - ps22
Sarcopterygii - ps23
Tetrapoda - ps24
Amniota - ps25
Mammalia - ps26
Theria - ps27
Placentalia - ps28
Boreoeutheria - ps29
Euarchontoglires - ps30
Euarchonta - ps31
Primates - ps32
Haplorrhini - ps33
Simiiformes - ps34
Catarrhini - ps35
Hominoidea - ps36
Hominidae - ps37
Homininae - ps38
Hominini - ps39
Homo_sapiens - ps40
Phylostratum

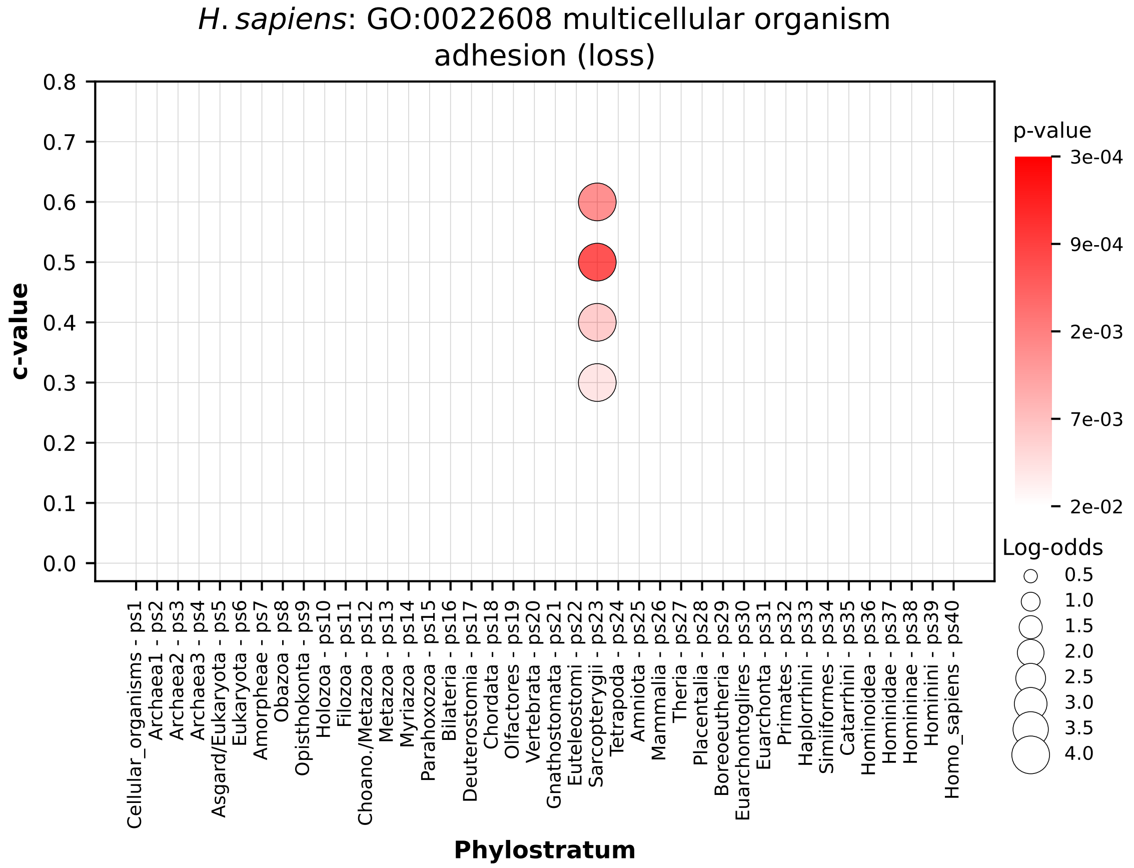

H. sapiens: GO:0022608 multicellular organism adhesion (loss)
c-value
0.8
0.7
0.6
0.5
0.4
0.3
0.2
0.1
0.0
p-value
3e-04
9e-04
2e-03
7e-03
2e-02
Log-odds
0.5
1.0
1.5
2.0
2.5
3.0
3.5
4.0
Cellular_organisms - ps1
Archaea1 - ps2
Archaea2 - ps3
Archaea3 - ps4
Asgard/Eukaryota - ps5
Eukaryota - ps6
Amorpheae - ps7
Obazoa - ps8
Opisthokonta - ps9
Holozoa - ps10
Filozoa - ps11
Choano./Metazoa - ps12
Metazoa - ps13
Myriazoa - ps14
Parahoxozoa - ps15
Bilateria - ps16
Deuterostomia - ps17
Chordata - ps18
Olfactores - ps19
Vertebrata - ps20
Gnathostomata - ps21
Euteleostomi - ps22
Sarcopterygii - ps23
Tetrapoda - ps24
Amniota - ps25
Mammalia - ps26
Theria - ps27
Placentalia - ps28
Boreoeutheria - ps29
Euarchontoglires - ps30
Euarchonta - ps31
Primates - ps32
Haplorrhini - ps33
Simiiformes - ps34
Catarrhini - ps35
Hominoidea - ps36
Hominidae - ps37
Homininae - ps38
Hominini - ps39
Homo_sapiens - ps40
Phylostratum

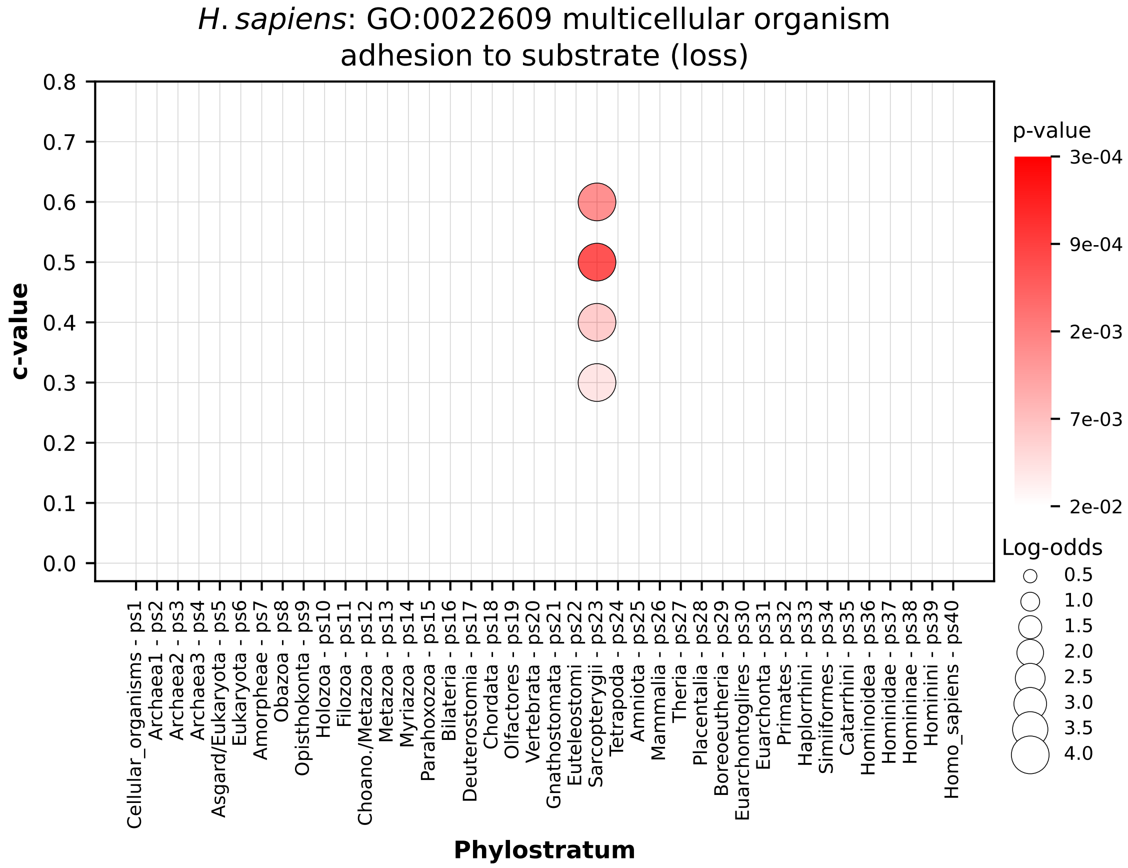

H. sapiens: GO:0022609 multicellular organism adhesion to substrate (loss)
c-value
0.8
0.7
0.6
0.5
0.4
0.3
0.2
0.1
0.0
p-value
3e-04
9e-04
2e-03
7e-03
2e-02
Log-odds
0.5
1.0
1.5
2.0
2.5
3.0
3.5
4.0
Cellular_organisms - ps1
Archaea1 - ps2
Archaea2 - ps3
Archaea3 - ps4
Asgard/Eukaryota - ps5
Eukaryota - ps6
Amorpheae - ps7
Obazoa - ps8
Opisthokonta - ps9
Holozoa - ps10
Filozoa - ps11
Choano./Metazoa - ps12
Metazoa - ps13
Myriazoa - ps14
Parahoxozoa - ps15
Bilateria - ps16
Deuterostomia - ps17
Chordata - ps18
Olfactores - ps19
Vertebrata - ps20
Gnathostomata - ps21
Euteleostomi - ps22
Sarcopterygii - ps23
Tetrapoda - ps24
Amniota - ps25
Mammalia - ps26
Theria - ps27
Placentalia - ps28
Boreoeutheria - ps29
Euarchontoglires - ps30
Euarchonta - ps31
Primates - ps32
Haplorrhini - ps33
Simiiformes - ps34
Catarrhini - ps35
Hominoidea - ps36
Hominidae - ps37
Homininae - ps38
Hominini - ps39
Homo_sapiens - ps40
Phylostratum

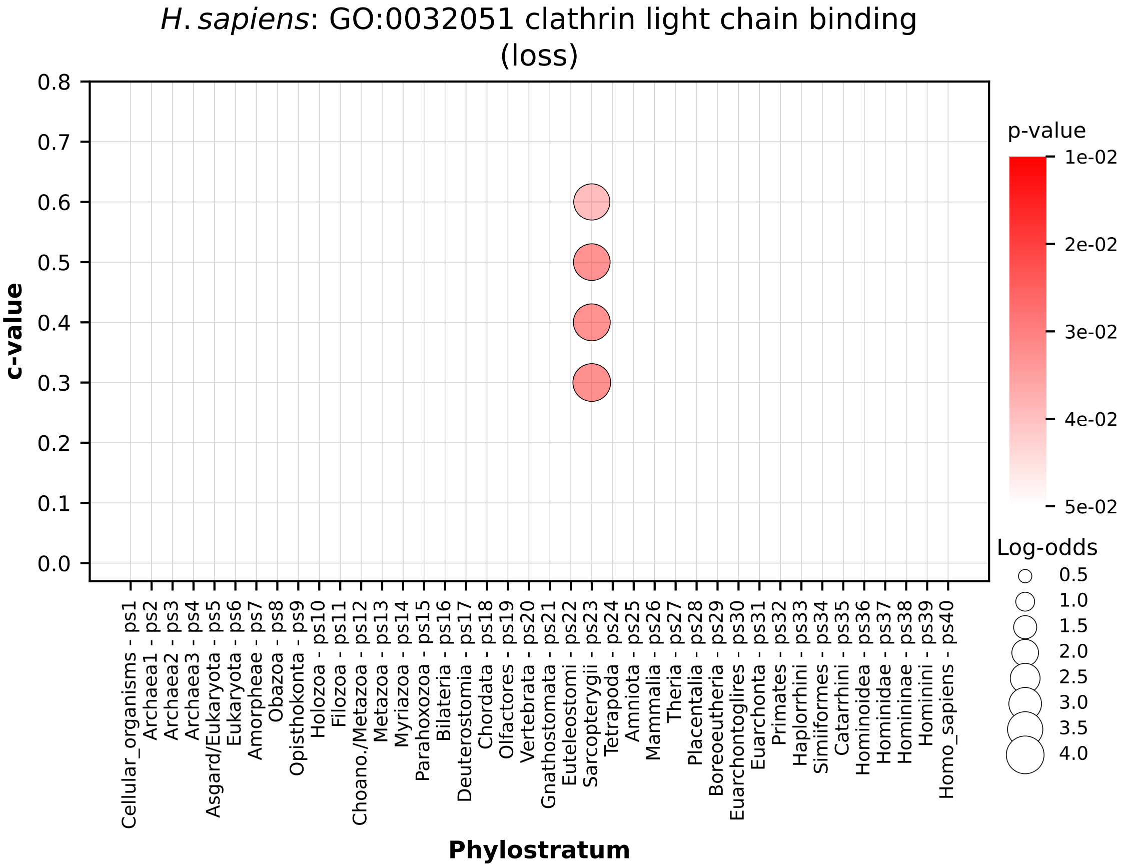

H. sapiens: GO:0032051 clathrin light chain binding
(loss)
c-value
0.8
0.7
0.6
0.5
0.4
0.3
0.2
0.1
0.0
p-value
1e-02
2e-02
3e-02
4e-02
5e-02
Log-odds
0.5
1.0
1.5
2.0
2.5
3.0
3.5
4.0
Cellular_organisms - ps1
Archaea1 - ps2
Archaea2 - ps3
Archaea3 - ps4
Asgard/Eukaryota - ps5
Eukaryota - ps6
Amorpheae - ps7
Obazoa - ps8
Opisthokonta - ps9
Holozoa - ps10
Filozoa - ps11
Choano./Metazoa - ps12
Metazoa - ps13
Myriazoa - ps14
Parahoxozoa - ps15
Bilateria - ps16
Deuterostomia - ps17
Chordata - ps18
Olfactores - ps19
Vertebrata - ps20
Gnathostomata - ps21
Euteleostomi - ps22
Sarcopterygii - ps23
Tetrapoda - ps24
Amniota - ps25
Mammalia - ps26
Theria - ps27
Placentalia - ps28
Boreoeutheria - ps29
Euarchontoglires - ps30
Euarchonta - ps31
Primates - ps32
Haplorrhini - ps33
Simiiformes - ps34
Catarrhini - ps35
Hominoidea - ps36
Hominidae - ps37
Homininae - ps38
Hominini - ps39
Homo_sapiens - ps40
Phylostratum

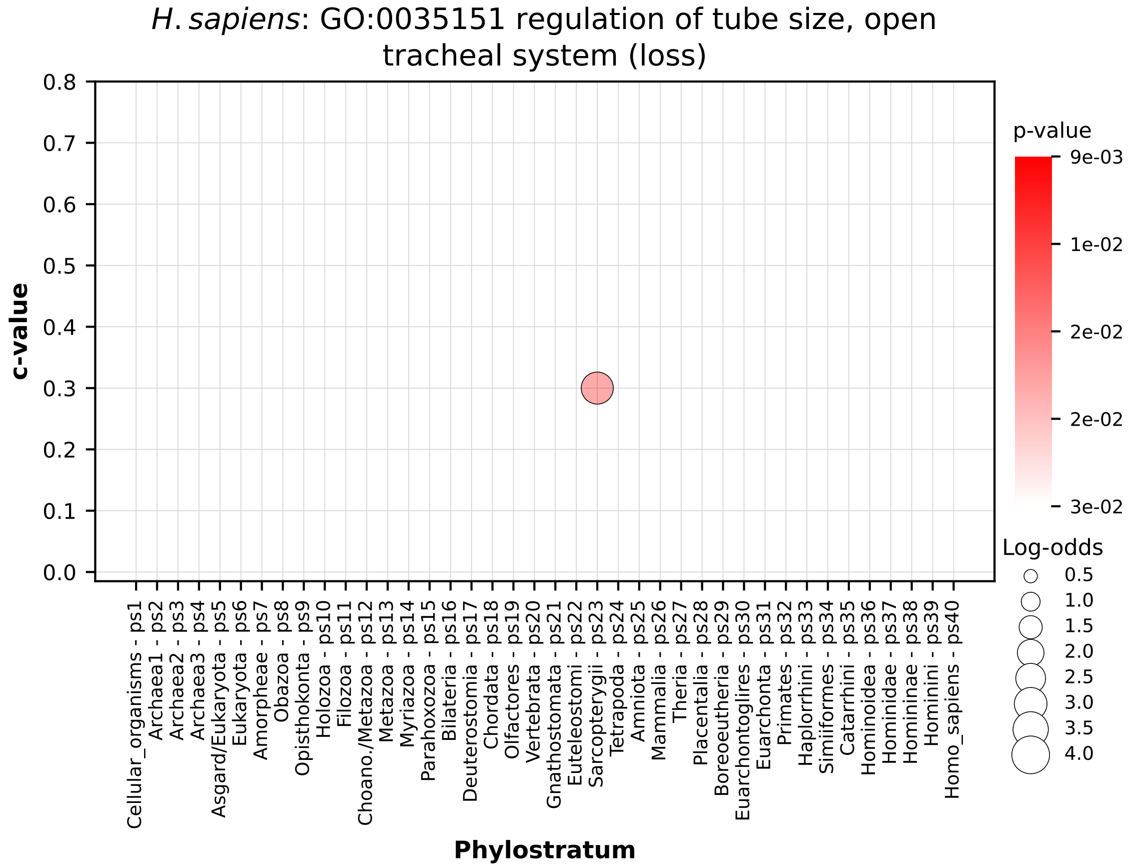

H. sapiens: GO:0035151 regulation of tube size, open tracheal system (loss)
c-value
0.8
0.7
0.6
0.5
0.4
0.3
0.2
0.1
0.0
Phylostratum
Cellular_organisms - ps1
Archaea1 - ps2
Archaea2 - ps3
Archaea3 - ps4
Asgard/Eukaryota - ps5
Eukaryota - ps6
Amorpheae - ps7
Obazoa - ps8
Opisthokonta - ps9
Holozoa - ps10
Filozoa - ps11
Choano./Metazoa - ps12
Metazoa - ps13
Myriazoa - ps14
Parahoxozoa - ps15
Bilateria - ps16
Deuterostomia - ps17
Chordata - ps18
Olfactores - ps19
Vertebrata - ps20
Gnathostomata - ps21
Euteleostomi - ps22
Sarcopterygii - ps23
Tetrapoda - ps24
Amniota - ps25
Mammalia - ps26
Theria - ps27
Placentalia - ps28
Boreoeutheria - ps29
Euarchontoglires - ps30
Euarchonta - ps31
Primates - ps32
Haplorrhini - ps33
Simiiformes - ps34
Catarrhini - ps35
Hominoidea - ps36
Hominidae - ps37
Homininae - ps38
Hominini - ps39
Homo_sapiens - ps40
p-value
9e-03
1e-02
2e-02
2e-02
3e-02
Log-odds
0.5
1.0
1.5
2.0
2.5
3.0
3.5
4.0

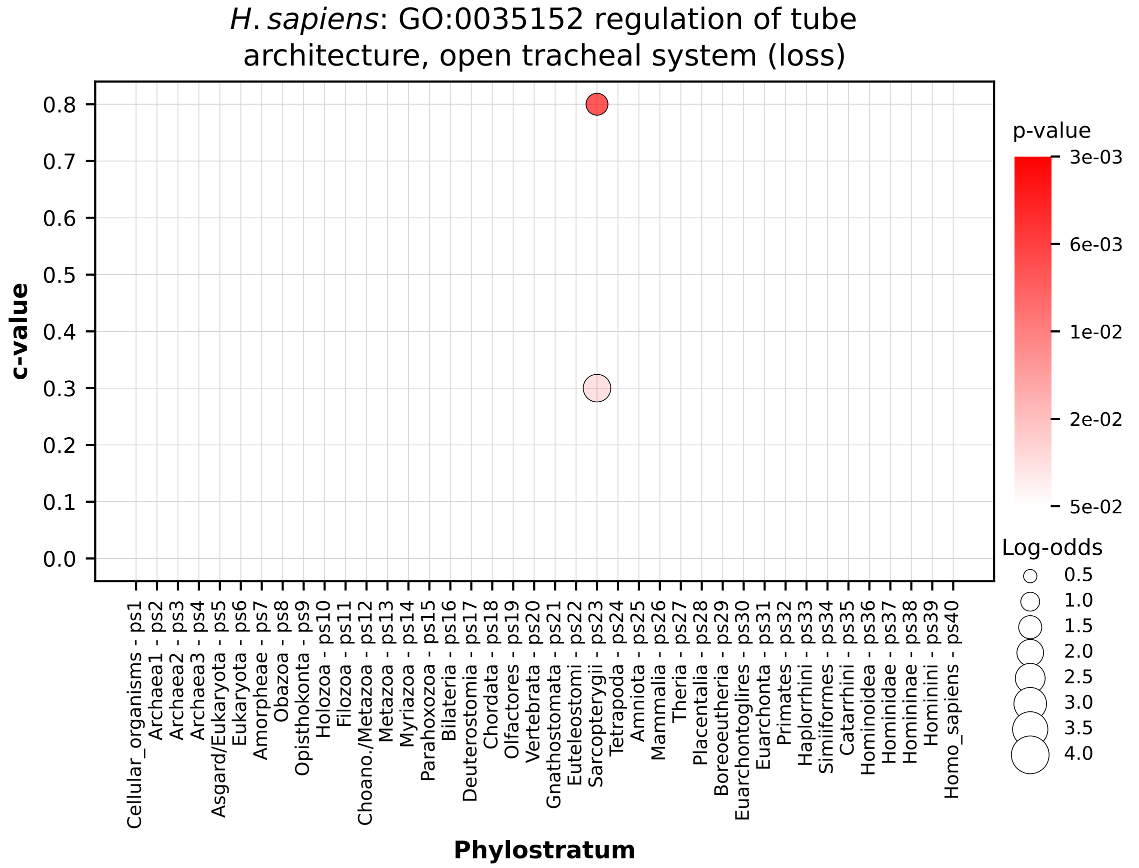

H. sapiens: GO:0035152 regulation of tube architecture, open tracheal system (loss)
c-value
0.0
0.1
0.2
0.3
0.4
0.5
0.6
0.7
0.8
Phylostratum
Cellular_organisms - ps1
Archaea1 - ps2
Archaea2 - ps3
Archaea3 - ps4
Asgard/Eukaryota - ps5
Eukaryota - ps6
Amorpheae - ps7
Obazoa - ps8
Opisthokonta - ps9
Holozoa - ps10
Filozoa - ps11
Choano./Metazoa - ps12
Metazoa - ps13
Myriazoa - ps14
Parahoxozoa - ps15
Bilateria - ps16
Deuterostomia - ps17
Chordata - ps18
Olfactores - ps19
Vertebrata - ps20
Gnathostomata - ps21
Euteleostomi - ps22
Sarcopterygii - ps23
Tetrapoda - ps24
Amniota - ps25
Mammalia - ps26
Theria - ps27
Placentalia - ps28
Boreoeutheria - ps29
Euarchontoglires - ps30
Euarchonta - ps31
Primates - ps32
Haplorrhini - ps33
Simiiformes - ps34
Catarrhini - ps35
Hominoidea - ps36
Hominidae - ps37
Homininae - ps38
Hominini - ps39
Homo_sapiens - ps40
p-value
3e-03
6e-03
1e-02
2e-02
5e-02
Log-odds
0.5
1.0
1.5
2.0
2.5
3.0
3.5
4.0

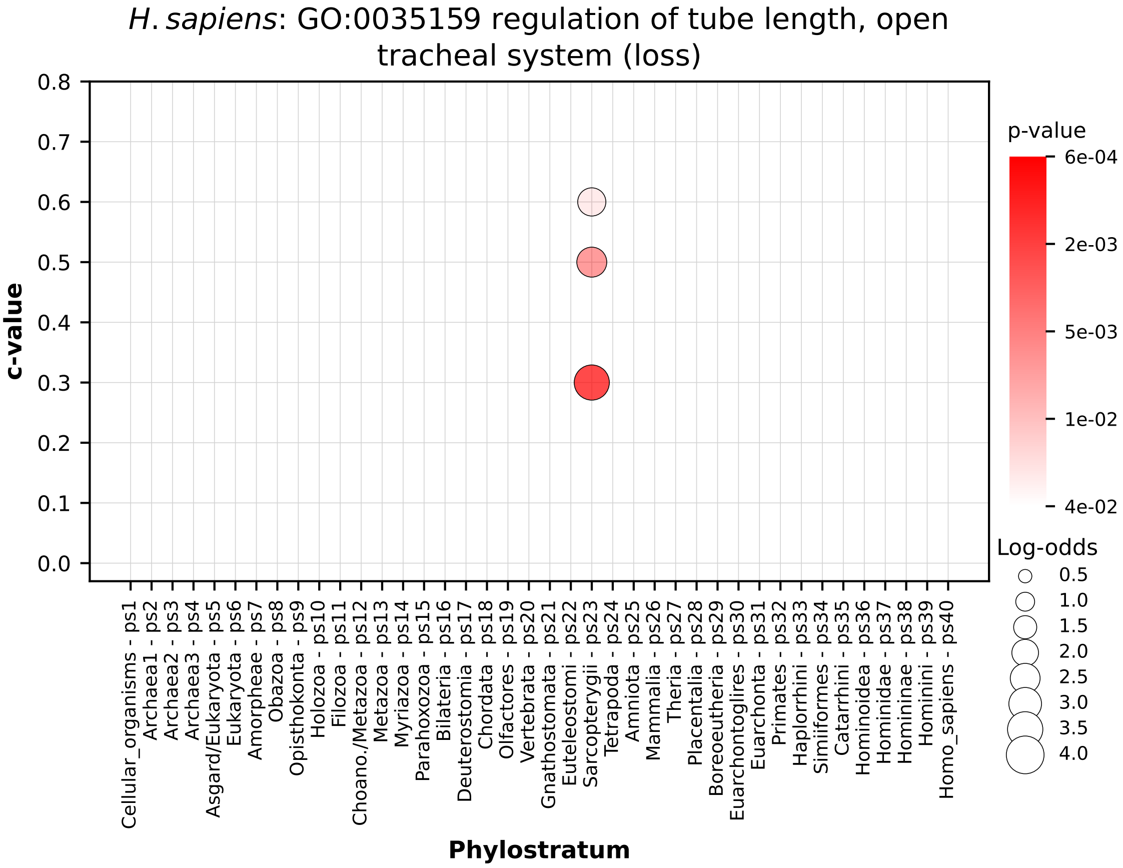

H. sapiens: GO:0035159 regulation of tube length, open tracheal system (loss)
c-value
0.0
0.1
0.2
0.3
0.4
0.5
0.6
0.7
0.8
Phylostratum
Cellular_organisms - ps1
Archaea1 - ps2
Archaea2 - ps3
Archaea3 - ps4
Asgard/Eukaryota - ps5
Eukaryota - ps6
Amorpheae - ps7
Obazoa - ps8
Opisthokonta - ps9
Holozoa - ps10
Filozoa - ps11
Choano./Metazoa - ps12
Metazoa - ps13
Myriazoa - ps14
Parahoxozoa - ps15
Bilateria - ps16
Deuterostomia - ps17
Chordata - ps18
Olfactores - ps19
Vertebrata - ps20
Gnathostomata - ps21
Euteleostomi - ps22
Sarcopterygii - ps23
Tetrapoda - ps24
Amniota - ps25
Mammalia - ps26
Theria - ps27
Placentalia - ps28
Boreoeutheria - ps29
Euarchontoglires - ps30
Euarchonta - ps31
Primates - ps32
Haplorrhini - ps33
Simiiformes - ps34
Catarrhini - ps35
Hominoidea - ps36
Hominidae - ps37
Homininae - ps38
Hominini - ps39
Homo_sapiens - ps40
p-value
6e-04
2e-03
5e-03
1e-02
4e-02
Log-odds
0.5
1.0
1.5
2.0
2.5
3.0
3.5
4.0

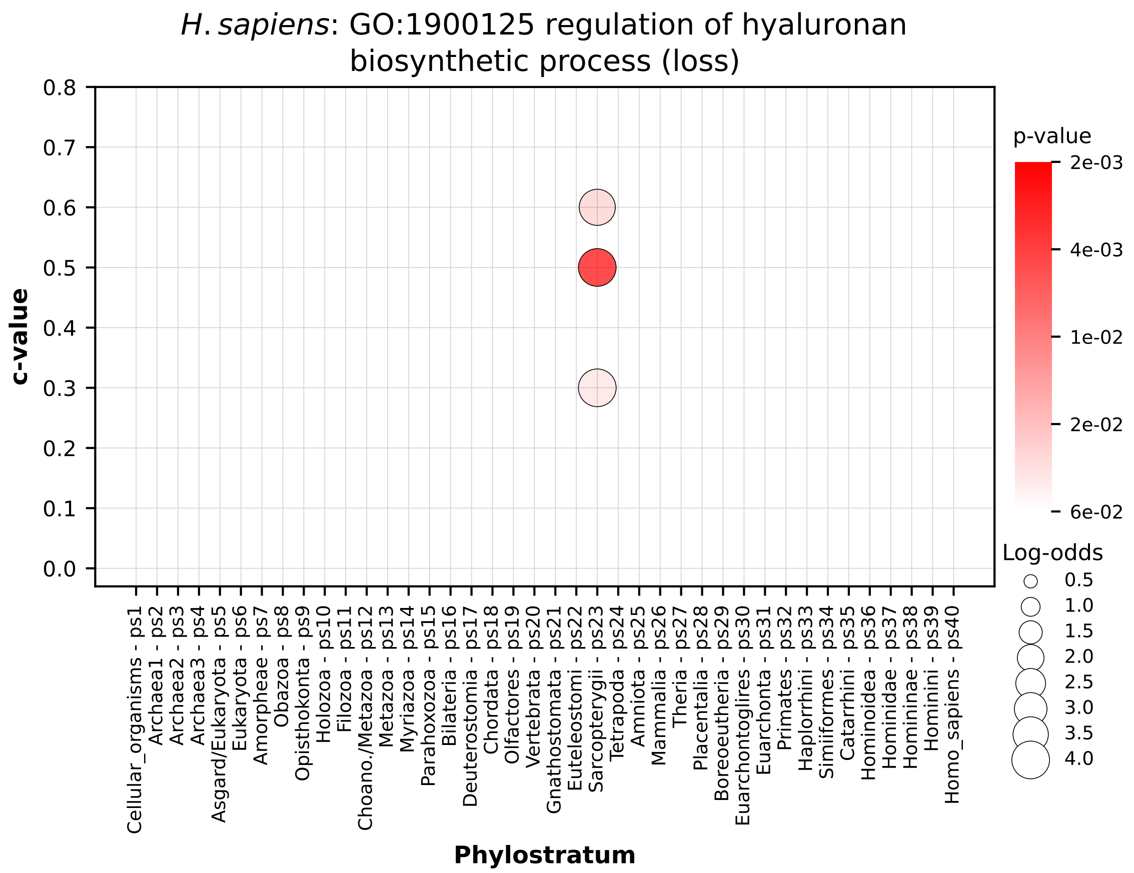
H. sapiens: GO:1900125 regulation of hyaluronan biosynthetic process (loss)
c-value
Phylostratum
p-value
2e-03
4e-03
1e-02
2e-02
6e-02
Log-odds
0.5
1.0
1.5
2.0
2.5
3.0
3.5
4.0
Cellular_organisms - ps1
Archaea1 - ps2
Archaea2 - ps3
Archaea3 - ps4
Asgard/Eukaryota - ps5
Eukaryota - ps6
Amorpheae - ps7
Obazoa - ps8
Opisthokonta - ps9
Holozoa - ps10
Filozoa - ps11
Choano./Metazoa - ps12
Metazoa - ps13
Myriazoa - ps14
Parahoxozoa - ps15
Bilateria - ps16
Deuterostomia - ps17
Chordata - ps18
Olfactores - ps19
Vertebrata - ps20
Gnathostomata - ps21
Euteleostomi - ps22
Sarcopterygii - ps23
Tetrapoda - ps24
Amniota - ps25
Mammalia - ps26
Theria - ps27
Placentalia - ps28
Boreoeutheria - ps29
Euarchontoglires - ps30
Euarchonta - ps31
Primates - ps32
Haplorrhini - ps33
Simiiformes - ps34
Catarrhini - ps35
Hominoidea - ps36
Hominidae - ps37
Homininae - ps38
Hominini - ps39
Homo_sapiens - ps40

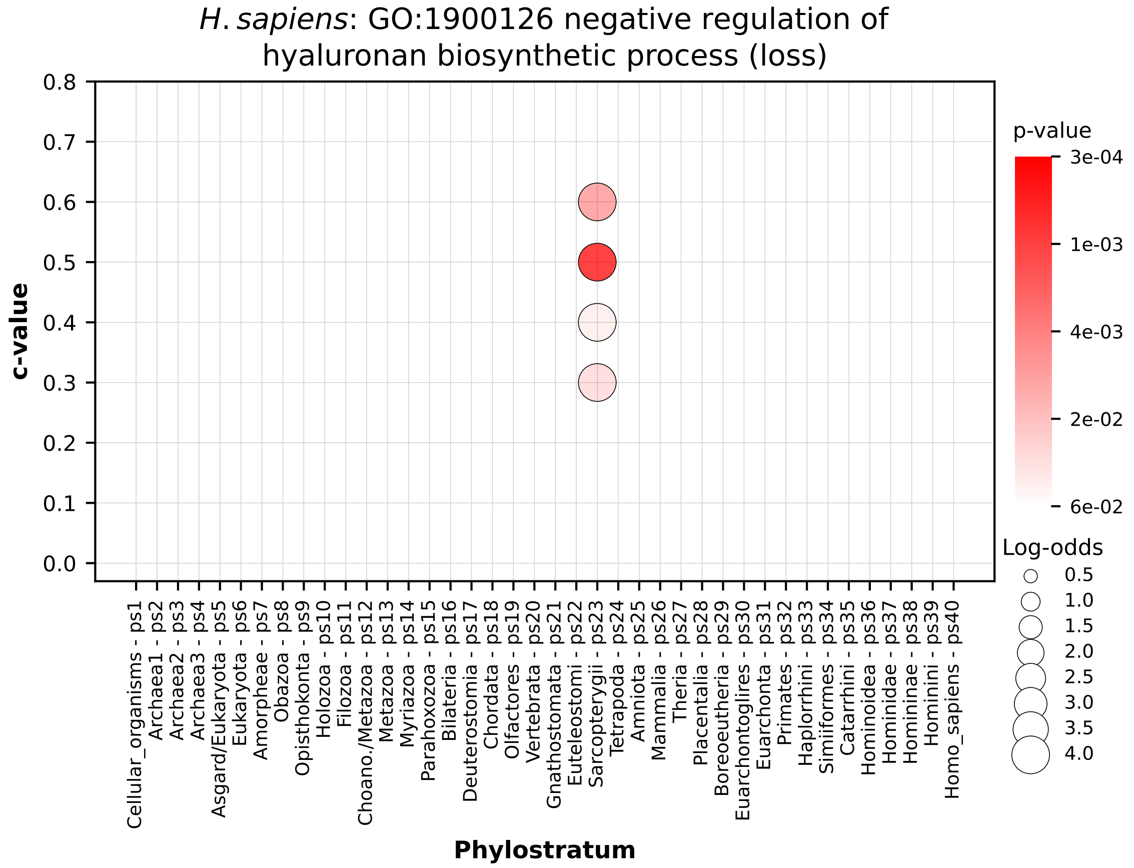

H. sapiens: GO:1900126 negative regulation of hyaluronan biosynthetic process (loss)
c-value
0.0
0.1
0.2
0.3
0.4
0.5
0.6
0.7
0.8
Phylostratum
Cellular_organisms - ps1
Archaea1 - ps2
Archaea2 - ps3
Archaea3 - ps4
Asgard/Eukaryota - ps5
Eukaryota - ps6
Amorpheae - ps7
Obazoa - ps8
Opisthokonta - ps9
Holozoa - ps10
Filozoa - ps11
Choano./Metazoa - ps12
Metazoa - ps13
Myriazoa - ps14
Parahoxozoa - ps15
Bilateria - ps16
Deuterostomia - ps17
Chordata - ps18
Olfactores - ps19
Vertebrata - ps20
Gnathostomata - ps21
Euteleostomi - ps22
Sarcopterygii - ps23
Tetrapoda - ps24
Amniota - ps25
Mammalia - ps26
Theria - ps27
Placentalia - ps28
Boreoeutheria - ps29
Euarchontoglires - ps30
Euarchonta - ps31
Primates - ps32
Haplorrhini - ps33
Simiiformes - ps34
Catarrhini - ps35
Hominoidea - ps36
Hominidae - ps37
Homininae - ps38
Hominini - ps39
Homo_sapiens - ps40
p-value
3e-04
1e-03
4e-03
2e-02
6e-02
Log-odds
0.5
1.0
1.5
2.0
2.5
3.0
3.5
4.0

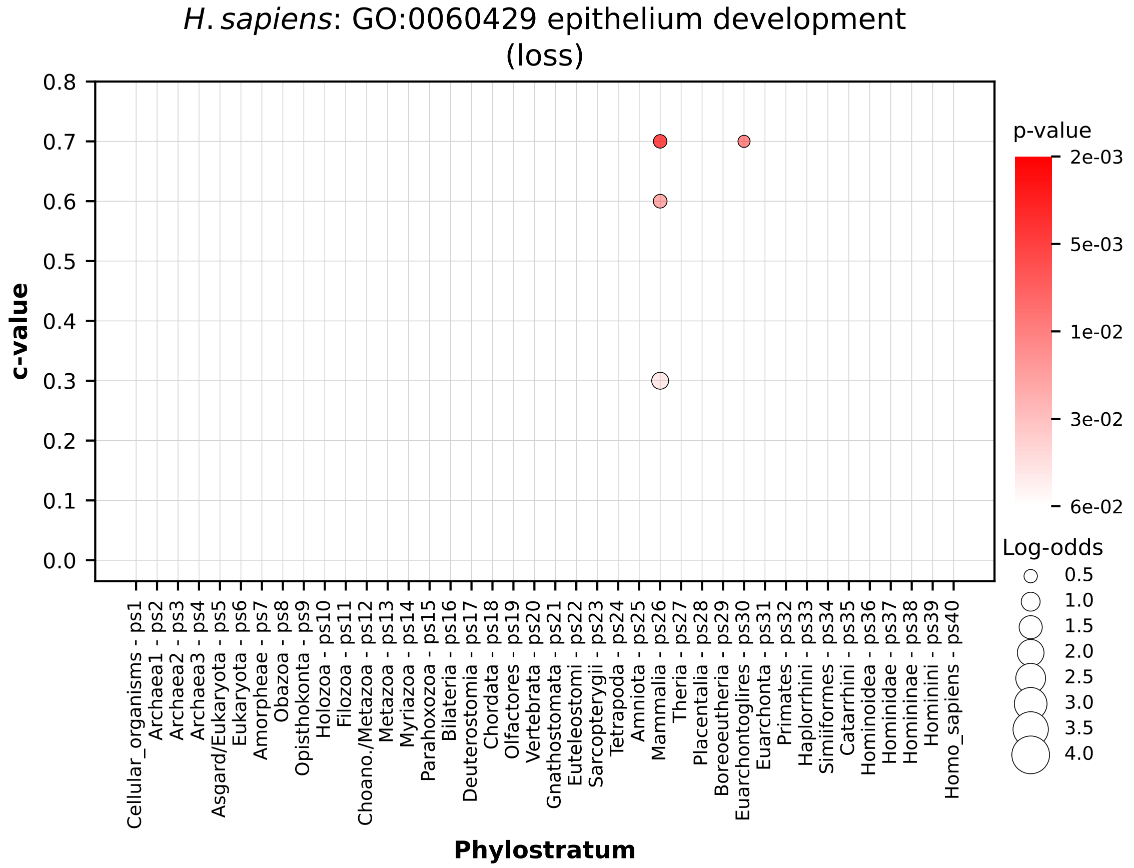

H. sapiens: GO:0060429 epithelium development
(loss)
c-value
0.8
0.7
0.6
0.5
0.4
0.3
0.2
0.1
0.0
p-value
2e-03
5e-03
1e-02
3e-02
6e-02
Log-odds
0.5
1.0
1.5
2.0
2.5
3.0
3.5
4.0
Cellular_organisms - ps1
Archaea1 - ps2
Archaea2 - ps3
Archaea3 - ps4
Asgard/Eukaryota - ps5
Eukaryota - ps6
Amorpheae - ps7
Obazoa - ps8
Opisthokonta - ps9
Holozoa - ps10
Filozoa - ps11
Choano./Metazoa - ps12
Metazoa - ps13
Myriazoa - ps14
Parahoxozoa - ps15
Bilateria - ps16
Deuterostomia - ps17
Chordata - ps18
Olfactores - ps19
Vertebrata - ps20
Gnathostomata - ps21
Euteleostomi - ps22
Sarcopterygii - ps23
Tetrapoda - ps24
Amniota - ps25
Mammalia - ps26
Theria - ps27
Placentalia - ps28
Boreoeutheria - ps29
Euarchontoglires - ps30
Euarchonta - ps31
Primates - ps32
Haplorrhini - ps33
Simiiformes - ps34
Catarrhini - ps35
Hominoidea - ps36
Hominidae - ps37
Homininae - ps38
Hominini - ps39
Homo_sapiens - ps40
Phylostratum

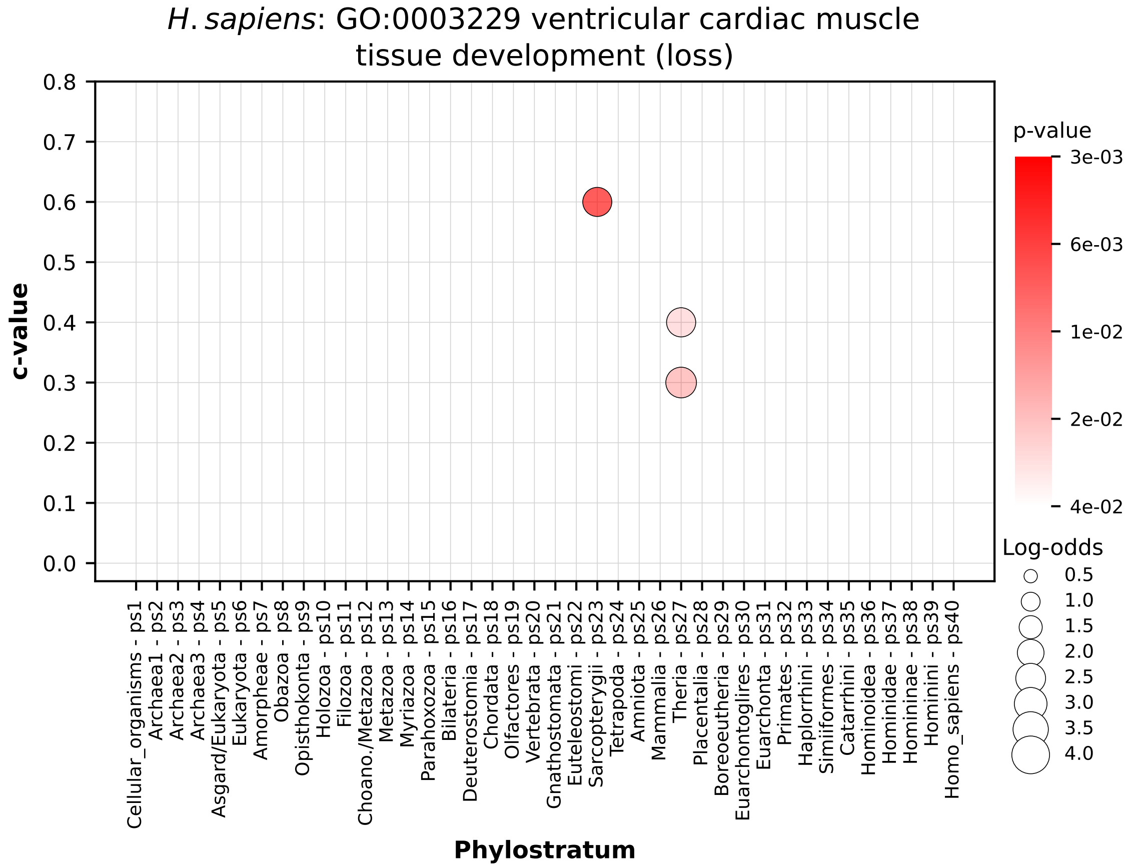

H. sapiens: GO:0003229 ventricular cardiac muscle tissue development (loss)
c-value
0.0
0.1
0.2
0.3
0.4
0.5
0.6
0.7
0.8
Phylostratum
Cellular_organisms - ps1
Archaea1 - ps2
Archaea2 - ps3
Archaea3 - ps4
Asgard/Eukaryota - ps5
Eukaryota - ps6
Amorpheae - ps7
Obazoa - ps8
Opisthokonta - ps9
Holozoa - ps10
Filozoa - ps11
Choano./Metazoa - ps12
Metazoa - ps13
Myriazoa - ps14
Parahoxozoa - ps15
Bilateria - ps16
Deuterostomia - ps17
Chordata - ps18
Olfactores - ps19
Vertebrata - ps20
Gnathostomata - ps21
Euteleostomi - ps22
Sarcopterygii - ps23
Tetrapoda - ps24
Amniota - ps25
Mammalia - ps26
Theria - ps27
Placentalia - ps28
Boreoeutheria - ps29
Euarchontoglires - ps30
Euarchonta - ps31
Primates - ps32
Haplorrhini - ps33
Simiiformes - ps34
Catarrhini - ps35
Hominoidea - ps36
Hominidae - ps37
Homininae - ps38
Hominini - ps39
Homo_sapiens - ps40
p-value
3e-03
6e-03
1e-02
2e-02
4e-02
Log-odds
0.5
1.0
1.5
2.0
2.5
3.0
3.5
4.0

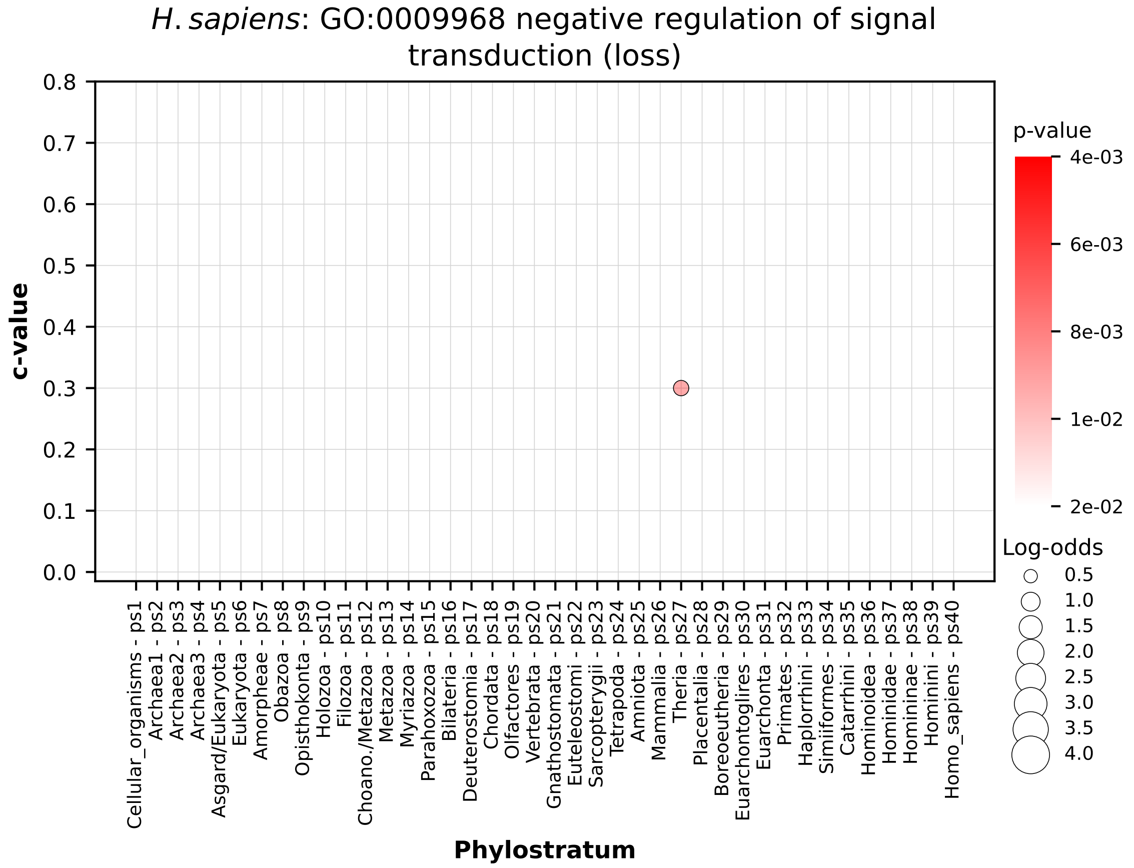
H. sapiens: GO:0009968 negative regulation of signal transduction (loss)
c-value
0.0
0.1
0.2
0.3
0.4
0.5
0.6
0.7
0.8
p-value
4e-03
6e-03
8e-03
1e-02
2e-02
Log-odds
0.5
1.0
1.5
2.0
2.5
3.0
3.5
4.0
Cellular_organisms - ps1
Archaea1 - ps2
Archaea2 - ps3
Archaea3 - ps4
Asgard/Eukaryota - ps5
Eukaryota - ps6
Amorpheae - ps7
Obazoa - ps8
Opisthokonta - ps9
Holozoa - ps10
Filozoa - ps11
Choano./Metazoa - ps12
Metazoa - ps13
Myriazoa - ps14
Parahoxozoa - ps15
Bilateria - ps16
Deuterostomia - ps17
Chordata - ps18
Olfactores - ps19
Vertebrata - ps20
Gnathostomata - ps21
Euteleostomi - ps22
Sarcopterygii - ps23
Tetrapoda - ps24
Amniota - ps25
Mammalia - ps26
Theria - ps27
Placentalia - ps28
Boreoeutheria - ps29
Euarchontoglires - ps30
Euarchonta - ps31
Primates - ps32
Haplorrhini - ps33
Simiiformes - ps34
Catarrhini - ps35
Hominoidea - ps36
Hominidae - ps37
Homininae - ps38
Hominini - ps39
Homo_sapiens - ps40
Phylostratum

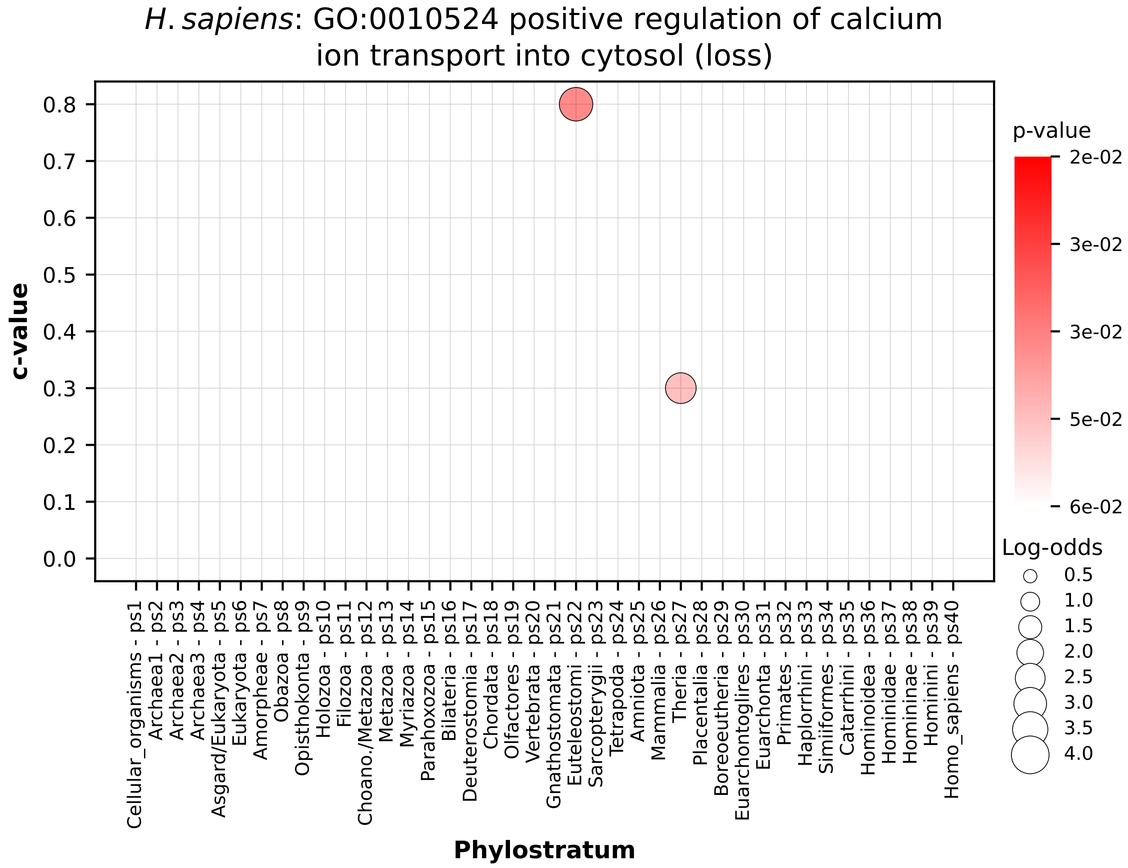

H. sapiens: GO:0010524 positive regulation of calcium ion transport into cytosol (loss)
c-value
0.0
0.1
0.2
0.3
0.4
0.5
0.6
0.7
0.8
Phylostratum
Cellular_organisms - ps1
Archaea1 - ps2
Archaea2 - ps3
Archaea3 - ps4
Asgard/Eukaryota - ps5
Eukaryota - ps6
Amorpheae - ps7
Obazoa - ps8
Opisthokonta - ps9
Holozoa - ps10
Filozoa - ps11
Choano./Metazoa - ps12
Metazoa - ps13
Myriazoa - ps14
Parahoxozoa - ps15
Bilateria - ps16
Deuterostomia - ps17
Chordata - ps18
Olfactores - ps19
Vertebrata - ps20
Gnathostomata - ps21
Euteleostomi - ps22
Sarcopterygii - ps23
Tetrapoda - ps24
Amniota - ps25
Mammalia - ps26
Theria - ps27
Placentalia - ps28
Boreoeutheria - ps29
Euarchontoglires - ps30
Euarchonta - ps31
Primates - ps32
Haplorrhini - ps33
Simiiformes - ps34
Catarrhini - ps35
Hominoidea - ps36
Hominidae - ps37
Homininae - ps38
Hominini - ps39
Homo_sapiens - ps40
p-value
2e-02
3e-02
3e-02
5e-02
6e-02
Log-odds
0.5
1.0
1.5
2.0
2.5
3.0
3.5
4.0

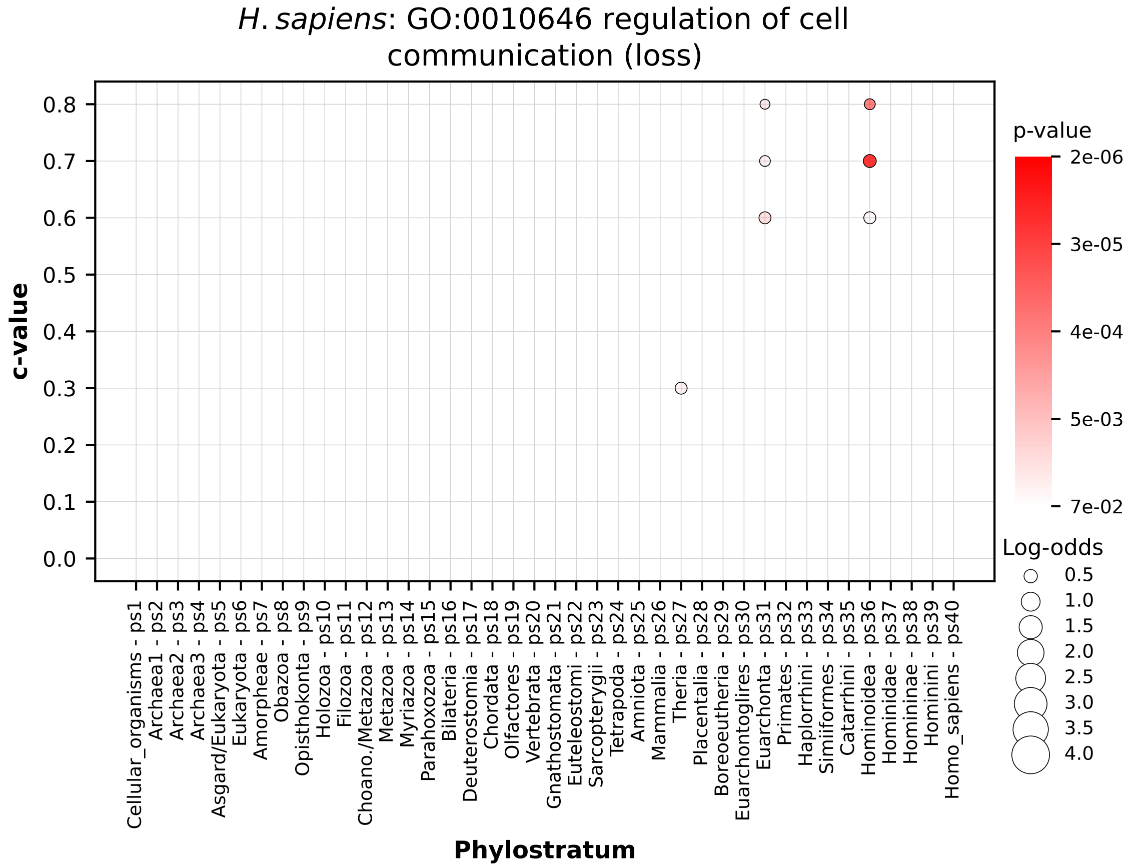

H. sapiens: GO:0010646 regulation of cell communication (loss)
c-value
0.0
0.1
0.2
0.3
0.4
0.5
0.6
0.7
0.8
Phylostratum
Cellular_organisms - ps1
Archaea1 - ps2
Archaea2 - ps3
Archaea3 - ps4
Asgard/Eukaryota - ps5
Eukaryota - ps6
Amorpheae - ps7
Obazoa - ps8
Opisthokonta - ps9
Holozoa - ps10
Filozoa - ps11
Choano./Metazoa - ps12
Metazoa - ps13
Myriazoa - ps14
Parahoxozoa - ps15
Bilateria - ps16
Deuterostomia - ps17
Chordata - ps18
Olfactores - ps19
Vertebrata - ps20
Gnathostomata - ps21
Euteleostomi - ps22
Sarcopterygii - ps23
Tetrapoda - ps24
Amniota - ps25
Mammalia - ps26
Theria - ps27
Placentalia - ps28
Boreoeutheria - ps29
Euarchontoglires - ps30
Euarchonta - ps31
Primates - ps32
Haplorrhini - ps33
Simiiformes - ps34
Catarrhini - ps35
Hominoidea - ps36
Hominidae - ps37
Homininae - ps38
Hominini - ps39
Homo_sapiens - ps40
p-value
2e-06
3e-05
4e-04
5e-03
7e-02
Log-odds
0.5
1.0
1.5
2.0
2.5
3.0
3.5
4.0

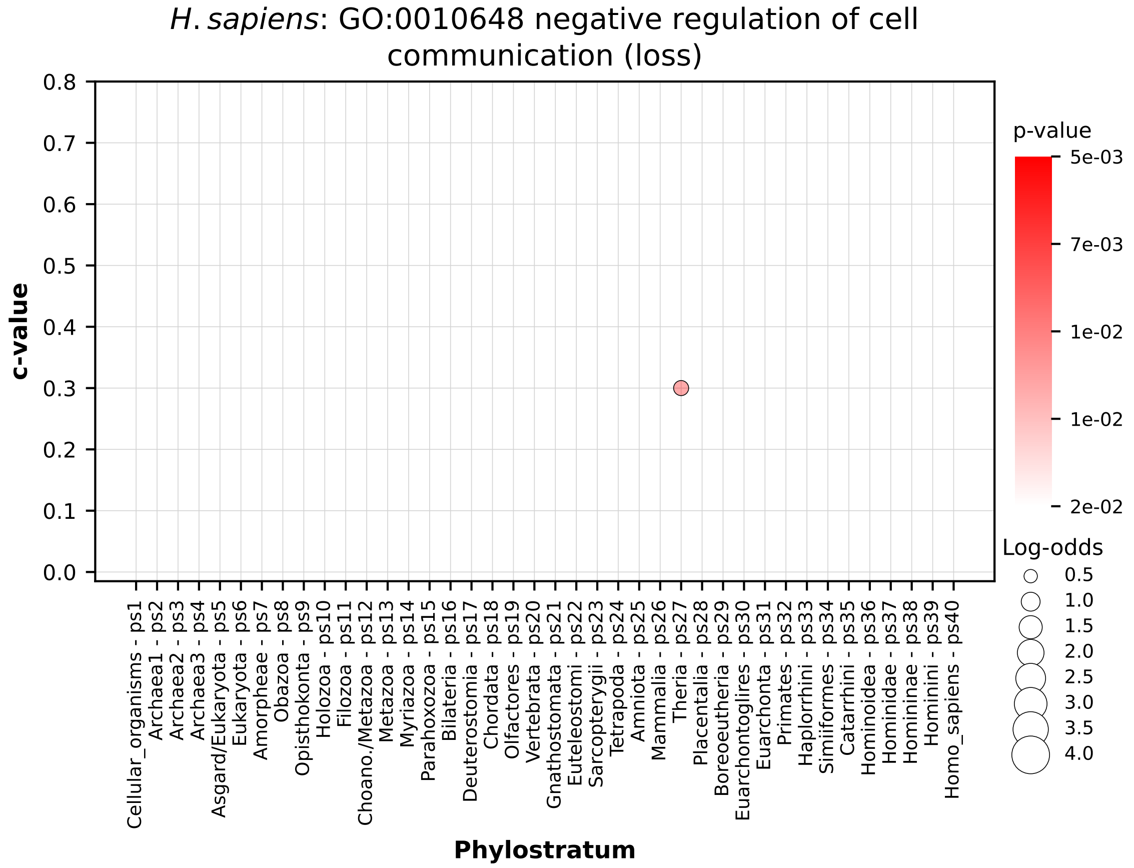

H. sapiens: GO:0010648 negative regulation of cell communication (loss)
c-value
0.0
0.1
0.2
0.3
0.4
0.5
0.6
0.7
0.8
Phylostratum
Cellular_organisms - ps1
Archaea1 - ps2
Archaea2 - ps3
Archaea3 - ps4
Asgard/Eukaryota - ps5
Eukaryota - ps6
Amorpheae - ps7
Obazoa - ps8
Opisthokonta - ps9
Holozoa - ps10
Filozoa - ps11
Choano./Metazoa - ps12
Metazoa - ps13
Myriazoa - ps14
Parahoxozoa - ps15
Bilateria - ps16
Deuterostomia - ps17
Chordata - ps18
Olfactores - ps19
Vertebrata - ps20
Gnathostomata - ps21
Euteleostomi - ps22
Sarcopterygii - ps23
Tetrapoda - ps24
Amniota - ps25
Mammalia - ps26
Theria - ps27
Placentalia - ps28
Boreoeutheria - ps29
Euarchontoglires - ps30
Euarchonta - ps31
Primates - ps32
Haplorrhini - ps33
Simiiformes - ps34
Catarrhini - ps35
Hominoidea - ps36
Hominidae - ps37
Homininae - ps38
Hominini - ps39
Homo_sapiens - ps40
p-value
5e-03
7e-03
1e-02
1e-02
2e-02
Log-odds
0.5
1.0
1.5
2.0
2.5
3.0
3.5
4.0

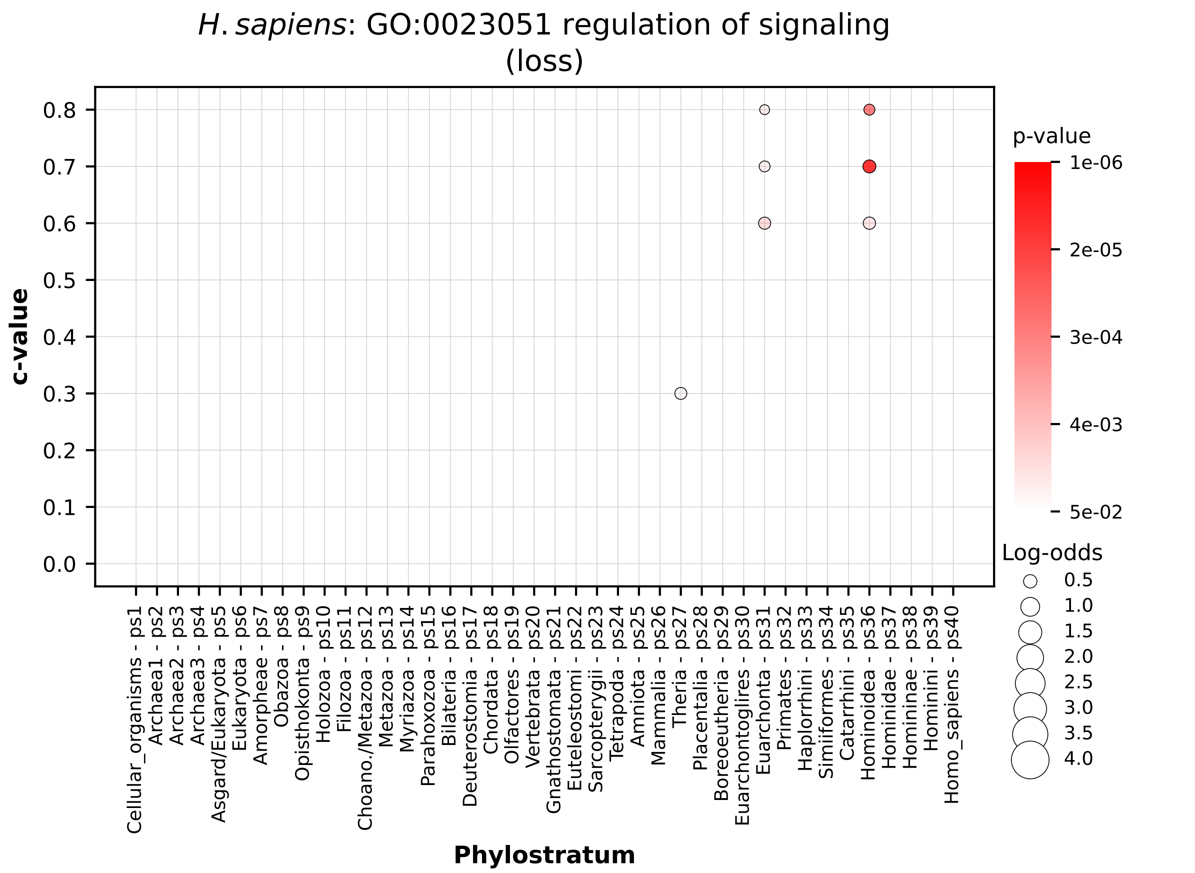

H. sapiens: GO:0023051 regulation of signaling
(loss)
c-value
0.0
0.1
0.2
0.3
0.4
0.5
0.6
0.7
0.8
Phylostratum
Cellular_organisms - ps1
Archaea1 - ps2
Archaea2 - ps3
Archaea3 - ps4
Asgard/Eukaryota - ps5
Eukaryota - ps6
Amorpheae - ps7
Obazoa - ps8
Opisthokonta - ps9
Holozoa - ps10
Filozoa - ps11
Choano./Metazoa - ps12
Metazoa - ps13
Myriazoa - ps14
Parahoxozoa - ps15
Bilateria - ps16
Deuterostomia - ps17
Chordata - ps18
Olfactores - ps19
Vertebrata - ps20
Gnathostomata - ps21
Euteleostomi - ps22
Sarcopterygii - ps23
Tetrapoda - ps24
Amniota - ps25
Mammalia - ps26
Theria - ps27
Placentalia - ps28
Boreoeutheria - ps29
Euarchontoglires - ps30
Euarchonta - ps31
Primates - ps32
Haplorrhini - ps33
Simiiformes - ps34
Catarrhini - ps35
Hominoidea - ps36
Hominidae - ps37
Homininae - ps38
Hominini - ps39
Homo_sapiens - ps40
p-value
1e-06
2e-05
3e-04
4e-03
5e-02
Log-odds
0.5
1.0
1.5
2.0
2.5
3.0
3.5
4.0

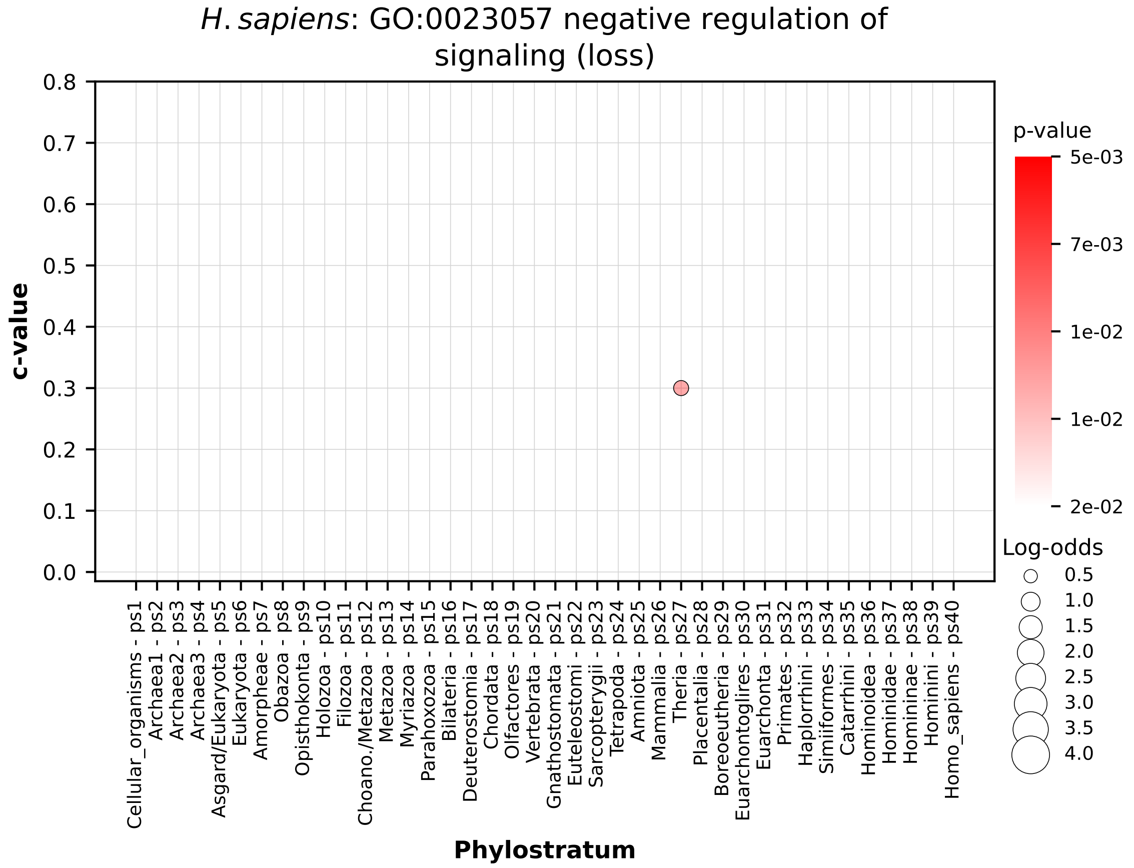

H. sapiens: GO:0023057 negative regulation of signaling (loss)
c-value
0.8
0.7
0.6
0.5
0.4
0.3
0.2
0.1
0.0
p-value
5e-03
7e-03
1e-02
1e-02
2e-02
Log-odds
0.5
1.0
1.5
2.0
2.5
3.0
3.5
4.0
Cellular_organisms - ps1
Archaea1 - ps2
Archaea2 - ps3
Archaea3 - ps4
Asgard/Eukaryota - ps5
Eukaryota - ps6
Amorpheae - ps7
Obazoa - ps8
Opisthokonta - ps9
Holozoa - ps10
Filozoa - ps11
Choano./Metazoa - ps12
Metazoa - ps13
Myriazoa - ps14
Parahoxozoa - ps15
Bilateria - ps16
Deuterostomia - ps17
Chordata - ps18
Olfactores - ps19
Vertebrata - ps20
Gnathostomata - ps21
Euteleostomi - ps22
Sarcopterygii - ps23
Tetrapoda - ps24
Amniota - ps25
Mammalia - ps26
Theria - ps27
Placentalia - ps28
Boreoeutheria - ps29
Euarchontoglires - ps30
Euarchonta - ps31
Primates - ps32
Haplorrhini - ps33
Simiiformes - ps34
Catarrhini - ps35
Hominoidea - ps36
Hominidae - ps37
Homininae - ps38
Hominini - ps39
Homo_sapiens - ps40
Phylostratum

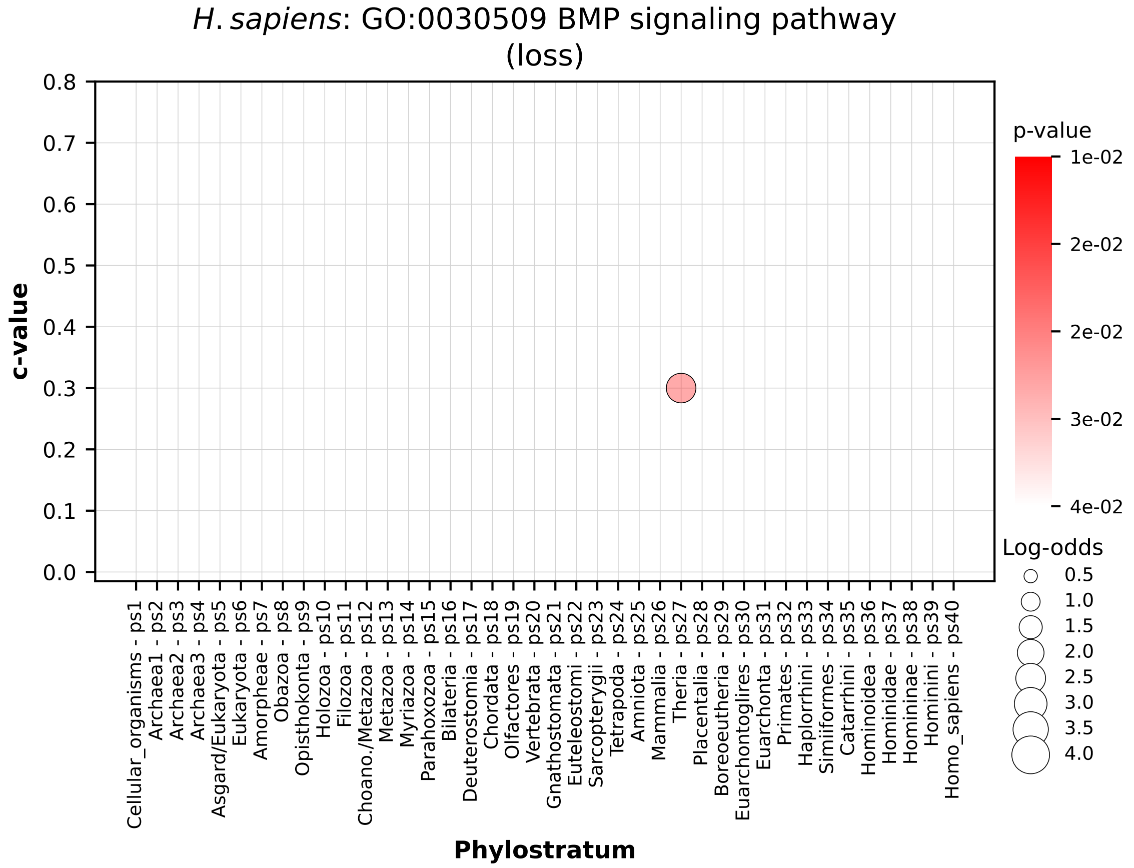

H. sapiens: GO:0030509 BMP signaling pathway
(loss)
c-value
Phylostratum
p-value
1e-02
2e-02
2e-02
3e-02
4e-02
Log-odds
0.5
1.0
1.5
2.0
2.5
3.0
3.5
4.0
Cellular_organisms - ps1
Archaea1 - ps2
Archaea2 - ps3
Archaea3 - ps4
Asgard/Eukaryota - ps5
Eukaryota - ps6
Amorpheae - ps7
Obazoa - ps8
Opisthokonta - ps9
Holozoa - ps10
Filozoa - ps11
Choano./Metazoa - ps12
Metazoa - ps13
Myriazoa - ps14
Parahoxozoa - ps15
Bilateria - ps16
Deuterostomia - ps17
Chordata - ps18
Olfactores - ps19
Vertebrata - ps20
Gnathostomata - ps21
Euteleostomi - ps22
Sarcopterygii - ps23
Tetrapoda - ps24
Amniota - ps25
Mammalia - ps26
Theria - ps27
Placentalia - ps28
Boreoeutheria - ps29
Euarchontoglires - ps30
Euarchonta - ps31
Primates - ps32
Haplorrhini - ps33
Simiiformes - ps34
Catarrhini - ps35
Hominoidea - ps36
Hominidae - ps37
Homininae - ps38
Hominini - ps39
Homo_sapiens - ps40

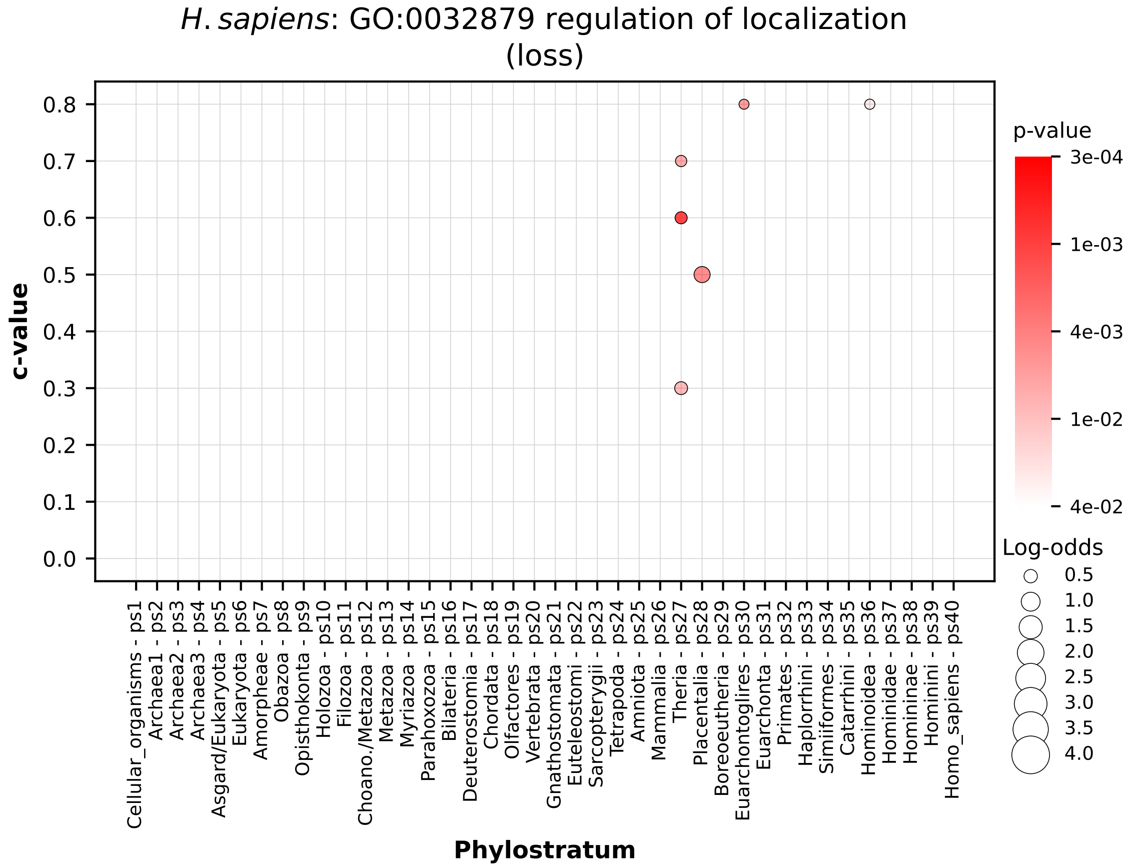

H. sapiens: GO:0032879 regulation of localization (loss)
c-value
0.0
0.1
0.2
0.3
0.4
0.5
0.6
0.7
0.8
Phylostratum
Cellular_organisms - ps1
Archaea1 - ps2
Archaea2 - ps3
Archaea3 - ps4
Asgard/Eukaryota - ps5
Eukaryota - ps6
Amorpheae - ps7
Obazoa - ps8
Opisthokonta - ps9
Holozoa - ps10
Filozoa - ps11
Choano./Metazoa - ps12
Metazoa - ps13
Myriazoa - ps14
Parahoxozoa - ps15
Bilateria - ps16
Deuterostomia - ps17
Chordata - ps18
Olfactores - ps19
Vertebrata - ps20
Gnathostomata - ps21
Euteleostomi - ps22
Sarcopterygii - ps23
Tetrapoda - ps24
Amniota - ps25
Mammalia - ps26
Theria - ps27
Placentalia - ps28
Boreoeutheria - ps29
Euarchontoglires - ps30
Euarchonta - ps31
Primates - ps32
Haplorrhini - ps33
Simiiformes - ps34
Catarrhini - ps35
Hominoidea - ps36
Hominidae - ps37
Homininae - ps38
Hominini - ps39
Homo_sapiens - ps40
p-value
3e-04
1e-03
4e-03
1e-02
4e-02
Log-odds
0.5
1.0
1.5
2.0
2.5
3.0
3.5
4.0

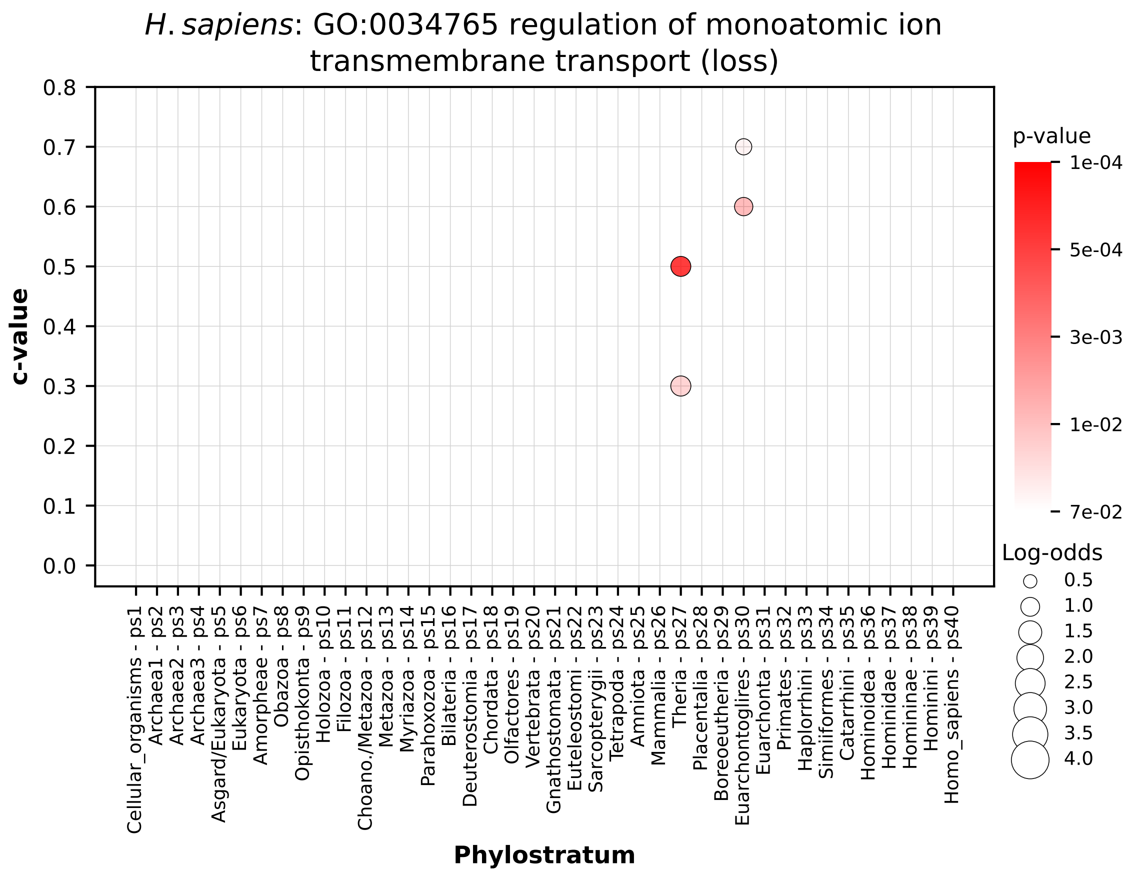

H. sapiens: GO:0034765 regulation of monoatomic ion transmembrane transport (loss)
c-value
0.8
0.7
0.6
0.5
0.4
0.3
0.2
0.1
0.0
p-value
1e-04
5e-04
3e-03
1e-02
7e-02
Log-odds
0.5
1.0
1.5
2.0
2.5
3.0
3.5
4.0
Cellular_organisms - ps1
Archaea1 - ps2
Archaea2 - ps3
Archaea3 - ps4
Asgard/Eukaryota - ps5
Eukaryota - ps6
Amorpheae - ps7
Obazoa - ps8
Opisthokonta - ps9
Holozoa - ps10
Filozoa - ps11
Choano./Metazoa - ps12
Metazoa - ps13
Myriazoa - ps14
Parahoxozoa - ps15
Bilateria - ps16
Deuterostomia - ps17
Chordata - ps18
Olfactores - ps19
Vertebrata - ps20
Gnathostomata - ps21
Euteleostomi - ps22
Sarcopterygii - ps23
Tetrapoda - ps24
Amniota - ps25
Mammalia - ps26
Theria - ps27
Placentalia - ps28
Boreoeutheria - ps29
Euarchontoglires - ps30
Euarchonta - ps31
Primates - ps32
Haplorrhini - ps33
Simiiformes - ps34
Catarrhini - ps35
Hominoidea - ps36
Hominidae - ps37
Homininae - ps38
Hominini - ps39
Homo_sapiens - ps40
Phylostratum

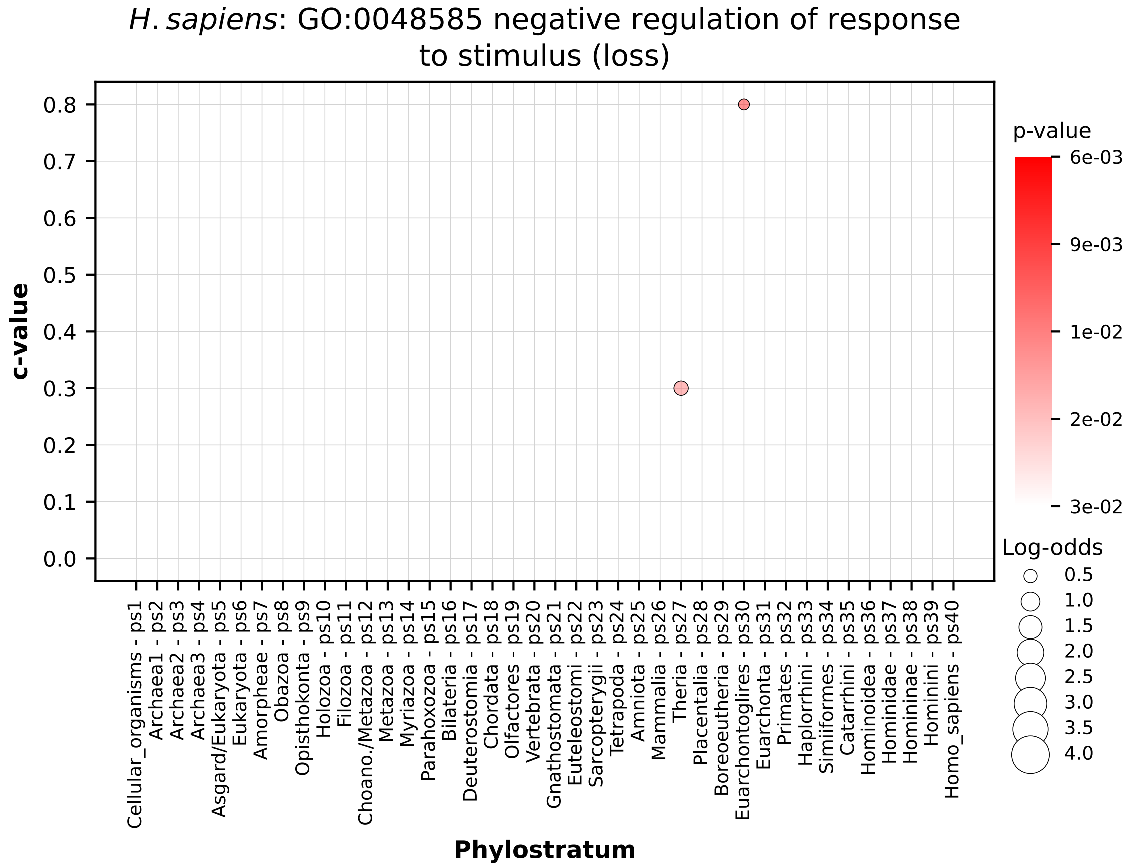

H. sapiens: GO:0048585 negative regulation of response to stimulus (loss)
c-value
0.0
0.1
0.2
0.3
0.4
0.5
0.6
0.7
0.8
Phylostratum
Cellular_organisms - ps1
Archaea1 - ps2
Archaea2 - ps3
Archaea3 - ps4
Asgard/Eukaryota - ps5
Eukaryota - ps6
Amorpheae - ps7
Obazoa - ps8
Opisthokonta - ps9
Holozoa - ps10
Filozoa - ps11
Choano./Metazoa - ps12
Metazoa - ps13
Myriazoa - ps14
Parahoxozoa - ps15
Bilateria - ps16
Deuterostomia - ps17
Chordata - ps18
Olfactores - ps19
Vertebrata - ps20
Gnathostomata - ps21
Euteleostomi - ps22
Sarcopterygii - ps23
Tetrapoda - ps24
Amniota - ps25
Mammalia - ps26
Theria - ps27
Placentalia - ps28
Boreoeutheria - ps29
Euarchontoglires - ps30
Euarchonta - ps31
Primates - ps32
Haplorrhini - ps33
Simiiformes - ps34
Catarrhini - ps35
Hominoidea - ps36
Hominidae - ps37
Homininae - ps38
Hominini - ps39
Homo_sapiens - ps40
p-value
6e-03
9e-03
1e-02
2e-02
3e-02
Log-odds
0.5
1.0
1.5
2.0
2.5
3.0
3.5
4.0

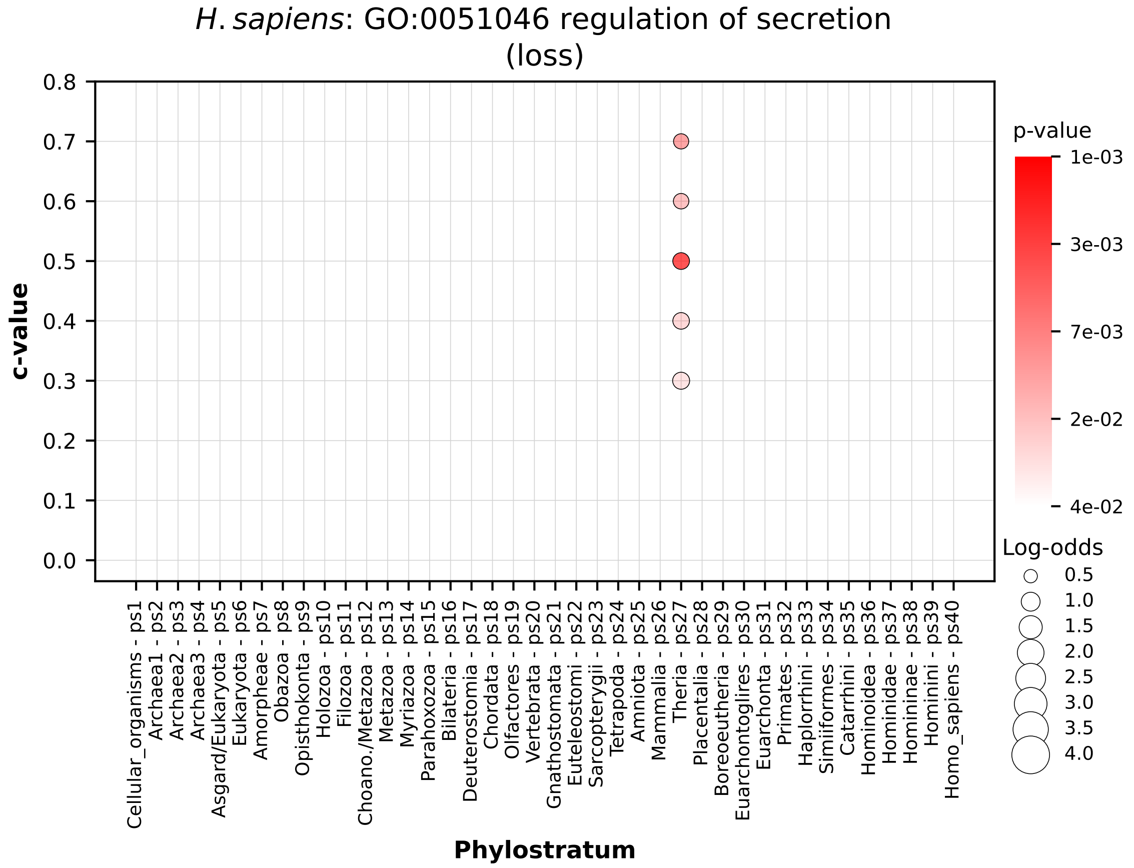
H. sapiens: GO:0051046 regulation of secretion
(loss)
c-value
0.0
0.1
0.2
0.3
0.4
0.5
0.6
0.7
0.8
p-value
1e-03
3e-03
7e-03
2e-02
4e-02
Log-odds
0.5
1.0
1.5
2.0
2.5
3.0
3.5
4.0
Cellular_organisms - ps1
Archaea1 - ps2
Archaea2 - ps3
Archaea3 - ps4
Asgard/Eukaryota - ps5
Eukaryota - ps6
Amorpheae - ps7
Obazoa - ps8
Opisthokonta - ps9
Holozoa - ps10
Filozoa - ps11
Choano./Metazoa - ps12
Metazoa - ps13
Myriazoa - ps14
Parahoxozoa - ps15
Bilateria - ps16
Deuterostomia - ps17
Chordata - ps18
Olfactores - ps19
Vertebrata - ps20
Gnathostomata - ps21
Euteleostomi - ps22
Sarcopterygii - ps23
Tetrapoda - ps24
Amniota - ps25
Mammalia - ps26
Theria - ps27
Placentalia - ps28
Boreoeutheria - ps29
Euarchontoglires - ps30
Euarchonta - ps31
Primates - ps32
Haplorrhini - ps33
Simiiformes - ps34
Catarrhini - ps35
Hominoidea - ps36
Hominidae - ps37
Homininae - ps38
Hominini - ps39
Homo_sapiens - ps40
Phylostratum

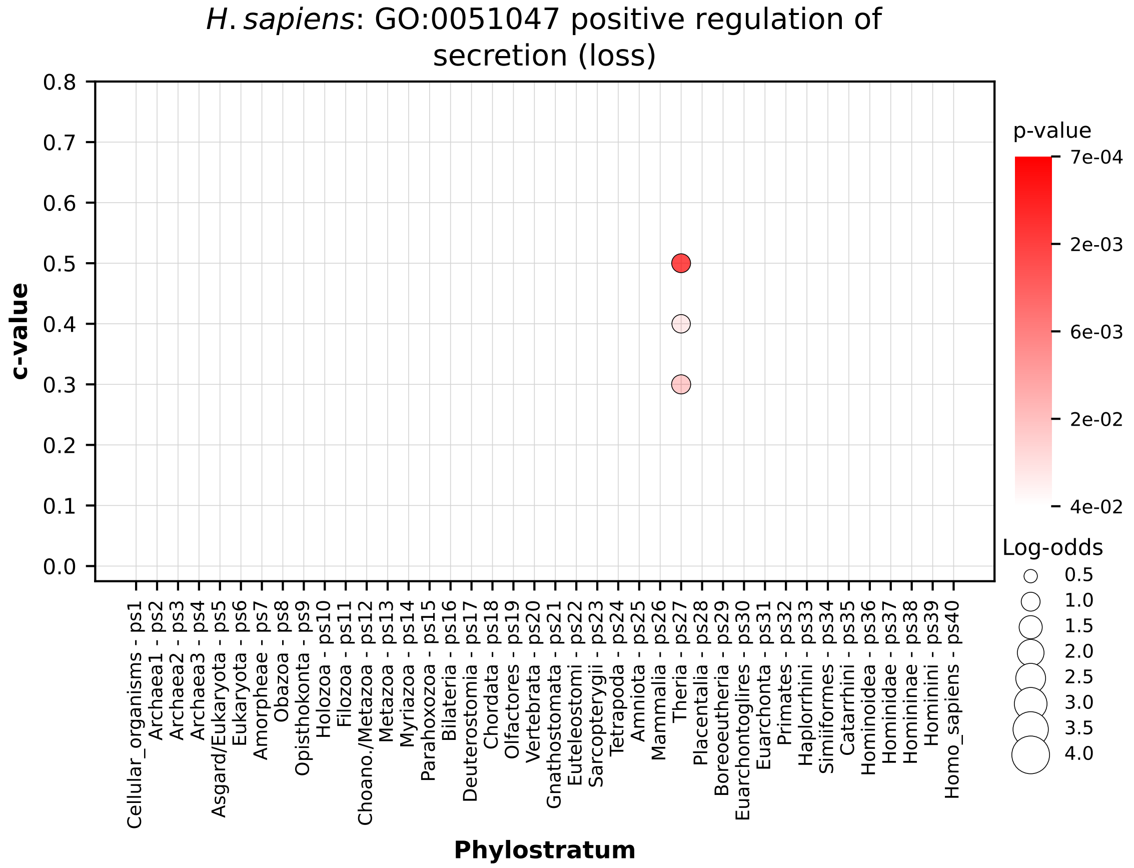

H. sapiens: GO:0051047 positive regulation of secretion (loss)
c-value
0.8
0.7
0.6
0.5
0.4
0.3
0.2
0.1
0.0
Phylostratum
Cellular_organisms - ps1
Archaea1 - ps2
Archaea2 - ps3
Archaea3 - ps4
Asgard/Eukaryota - ps5
Eukaryota - ps6
Amorpheae - ps7
Obazoa - ps8
Opisthokonta - ps9
Holozoa - ps10
Filozoa - ps11
Choano./Metazoa - ps12
Metazoa - ps13
Myriazoa - ps14
Parahoxozoa - ps15
Bilateria - ps16
Deuterostomia - ps17
Chordata - ps18
Olfactores - ps19
Vertebrata - ps20
Gnathostomata - ps21
Euteleostomi - ps22
Sarcopterygii - ps23
Tetrapoda - ps24
Amniota - ps25
Mammalia - ps26
Theria - ps27
Placentalia - ps28
Boreoeutheria - ps29
Euarchontoglires - ps30
Euarchonta - ps31
Primates - ps32
Haplorrhini - ps33
Simiiformes - ps34
Catarrhini - ps35
Hominoidea - ps36
Hominidae - ps37
Homininae - ps38
Hominini - ps39
Homo_sapiens - ps40
p-value
7e-04
2e-03
6e-03
2e-02
4e-02
Log-odds
0.5
1.0
1.5
2.0
2.5
3.0
3.5
4.0

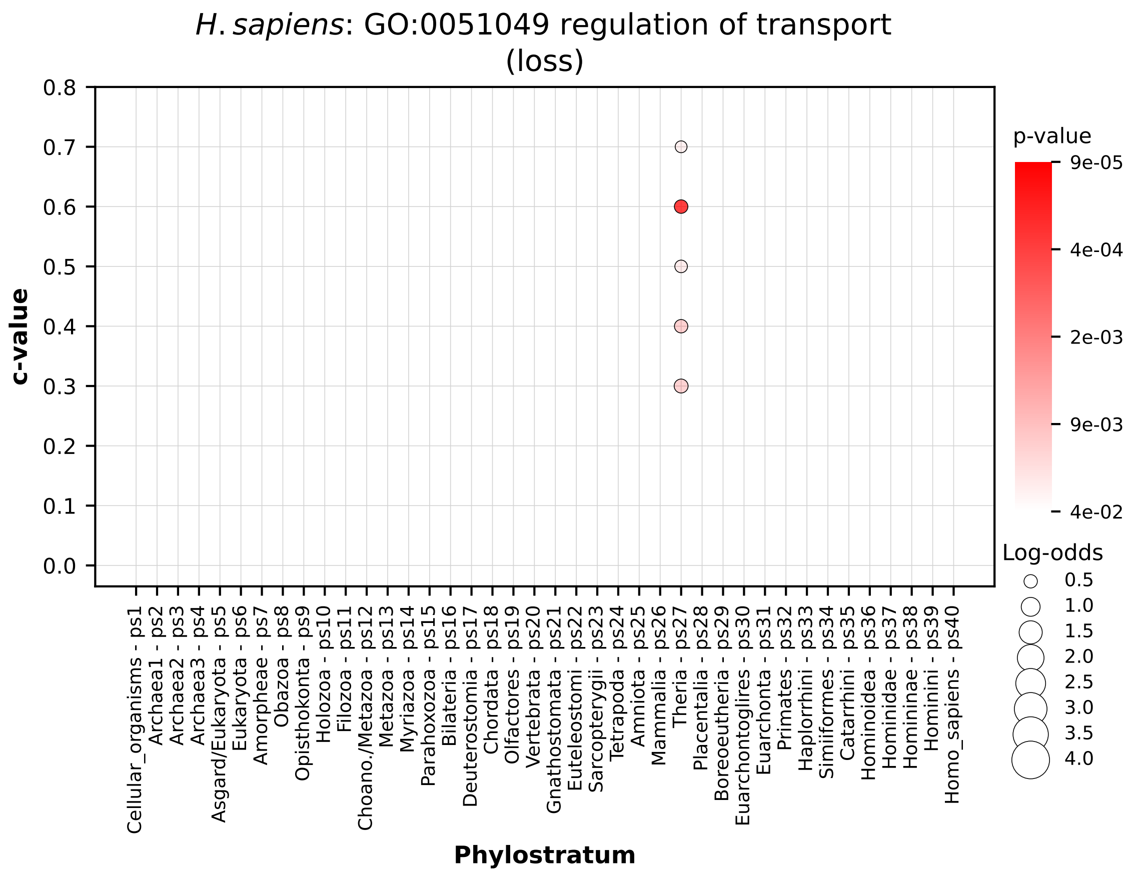

H. sapiens: GO:0051049 regulation of transport
(loss)
c-value
0.8
0.7
0.6
0.5
0.4
0.3
0.2
0.1
0.0
p-value
9e-05
4e-04
2e-03
9e-03
4e-02
Log-odds
0.5
1.0
1.5
2.0
2.5
3.0
3.5
4.0
Cellular_organisms - ps1
Archaea1 - ps2
Archaea2 - ps3
Archaea3 - ps4
Asgard/Eukaryota - ps5
Eukaryota - ps6
Amorpheae - ps7
Obazoa - ps8
Opisthokonta - ps9
Holozoa - ps10
Filozoa - ps11
Choano./Metazoa - ps12
Metazoa - ps13
Myriazoa - ps14
Parahoxozoa - ps15
Bilateria - ps16
Deuterostomia - ps17
Chordata - ps18
Olfactores - ps19
Vertebrata - ps20
Gnathostomata - ps21
Euteleostomi - ps22
Sarcopterygii - ps23
Tetrapoda - ps24
Amniota - ps25
Mammalia - ps26
Theria - ps27
Placentalia - ps28
Boreoeutheria - ps29
Euarchontoglires - ps30
Euarchonta - ps31
Primates - ps32
Haplorrhini - ps33
Simiiformes - ps34
Catarrhini - ps35
Hominoidea - ps36
Hominidae - ps37
Homininae - ps38
Hominini - ps39
Homo_sapiens - ps40
Phylostratum

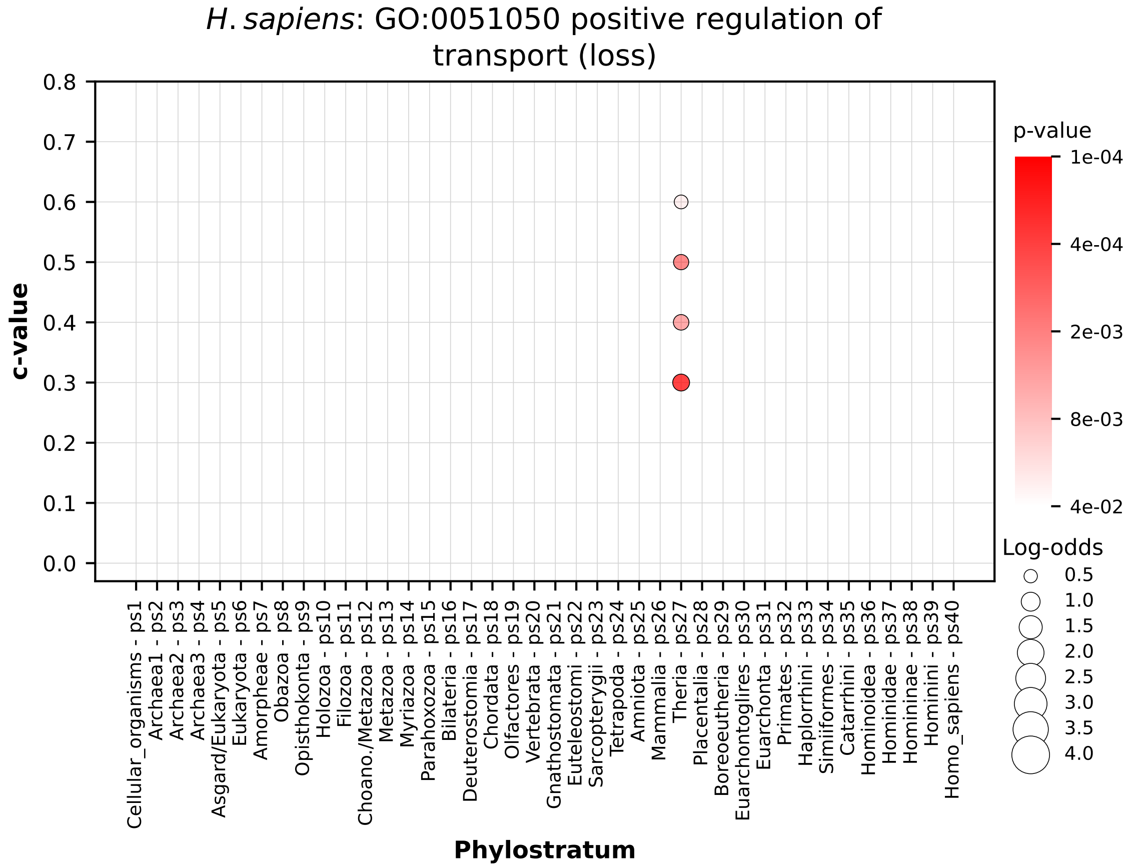

H. sapiens: GO:0051050 positive regulation of transport (loss)
c-value
0.0
0.1
0.2
0.3
0.4
0.5
0.6
0.7
0.8
Phylostratum
Cellular_organisms - ps1
Archaea1 - ps2
Archaea2 - ps3
Archaea3 - ps4
Asgard/Eukaryota - ps5
Eukaryota - ps6
Amorpheae - ps7
Obazoa - ps8
Opisthokonta - ps9
Holozoa - ps10
Filozoa - ps11
Choano./Metazoa - ps12
Metazoa - ps13
Myriazoa - ps14
Parahoxozoa - ps15
Bilateria - ps16
Deuterostomia - ps17
Chordata - ps18
Olfactores - ps19
Vertebrata - ps20
Gnathostomata - ps21
Euteleostomi - ps22
Sarcopterygii - ps23
Tetrapoda - ps24
Amniota - ps25
Mammalia - ps26
Theria - ps27
Placentalia - ps28
Boreoeutheria - ps29
Euarchontoglires - ps30
Euarchonta - ps31
Primates - ps32
Haplorrhini - ps33
Simiiformes - ps34
Catarrhini - ps35
Hominoidea - ps36
Hominidae - ps37
Homininae - ps38
Hominini - ps39
Homo_sapiens - ps40
p-value
1e-04
4e-04
2e-03
8e-03
4e-02
Log-odds
0.5
1.0
1.5
2.0
2.5
3.0
3.5
4.0

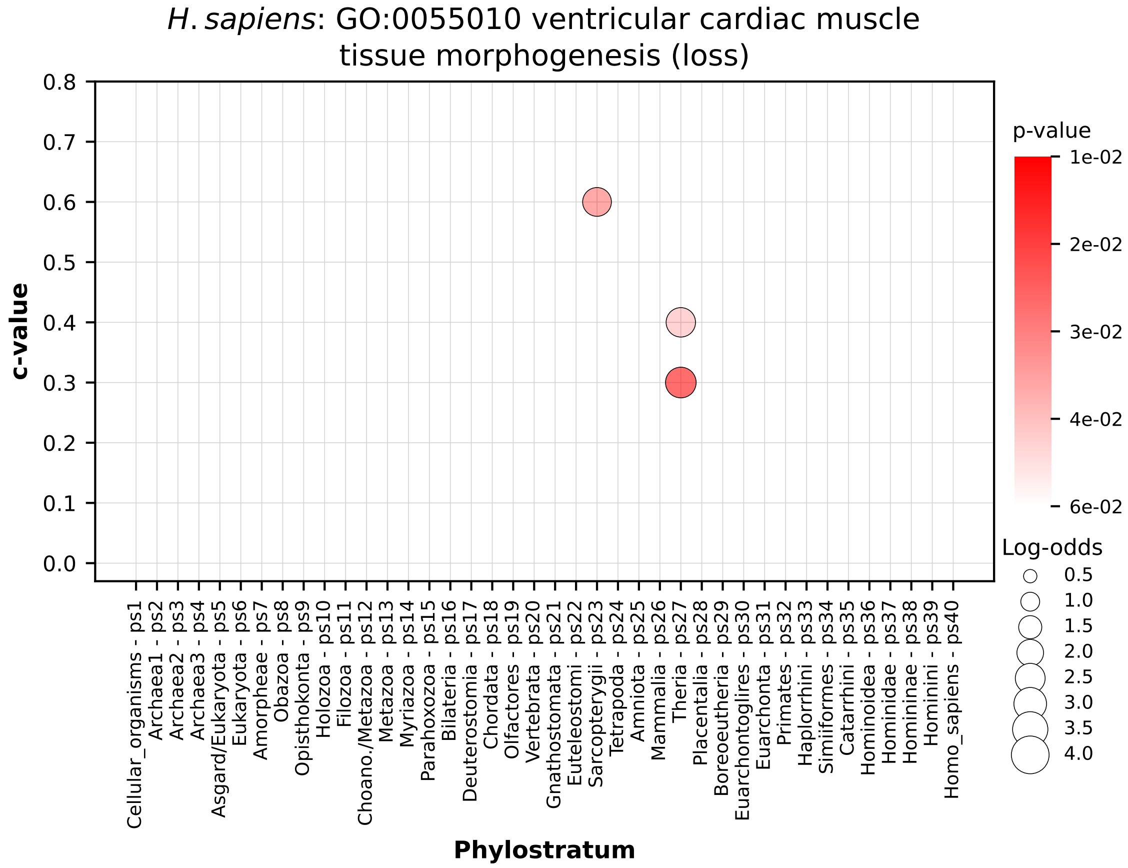

H. sapiens: GO:0055010 ventricular cardiac muscle tissue morphogenesis (loss)
c-value
0.8
0.7
0.6
0.5
0.4
0.3
0.2
0.1
0.0
p-value
1e-02
2e-02
3e-02
4e-02
6e-02
Log-odds
0.5
1.0
1.5
2.0
2.5
3.0
3.5
4.0
Cellular_organisms - ps1
Archaea1 - ps2
Archaea2 - ps3
Archaea3 - ps4
Asgard/Eukaryota - ps5
Eukaryota - ps6
Amorpheae - ps7
Obazoa - ps8
Opisthokonta - ps9
Holozoa - ps10
Filozoa - ps11
Choano./Metazoa - ps12
Metazoa - ps13
Myriazoa - ps14
Parahoxozoa - ps15
Bilateria - ps16
Deuterostomia - ps17
Chordata - ps18
Olfactores - ps19
Vertebrata - ps20
Gnathostomata - ps21
Euteleostomi - ps22
Sarcopterygii - ps23
Tetrapoda - ps24
Amniota - ps25
Mammalia - ps26
Theria - ps27
Placentalia - ps28
Boreoeutheria - ps29
Euarchontoglires - ps30
Euarchonta - ps31
Primates - ps32
Haplorrhini - ps33
Simiiformes - ps34
Catarrhini - ps35
Hominoidea - ps36
Hominidae - ps37
Homininae - ps38
Hominini - ps39
Homo_sapiens - ps40
Phylostratum

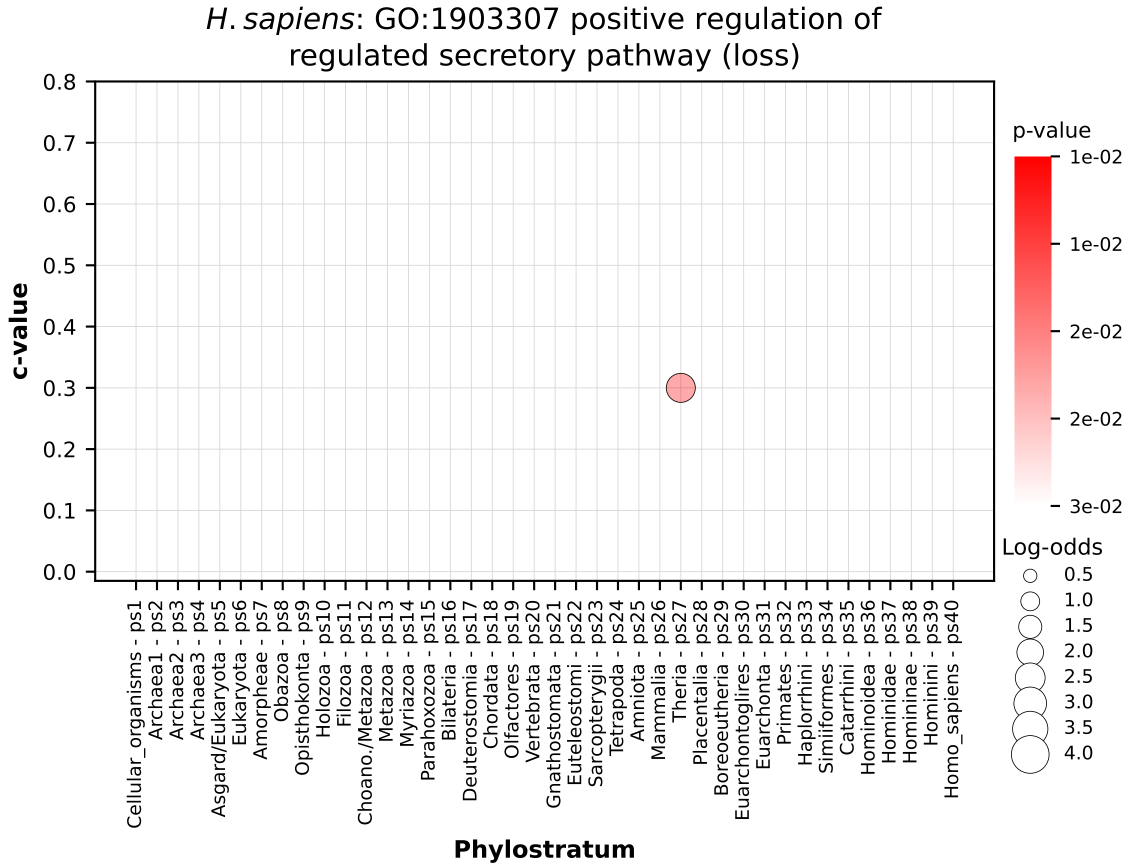

H. sapiens: GO:1903307 positive regulation of regulated secretory pathway (loss)
c-value
0.8
0.7
0.6
0.5
0.4
0.3
0.2
0.1
0.0
Phylostratum
Cellular_organisms - ps1
Archaea1 - ps2
Archaea2 - ps3
Archaea3 - ps4
Asgard/Eukaryota - ps5
Eukaryota - ps6
Amorpheae - ps7
Obazoa - ps8
Opisthokonta - ps9
Holozoa - ps10
Filozoa - ps11
Choano./Metazoa - ps12
Metazoa - ps13
Myriazoa - ps14
Parahoxozoa - ps15
Bilateria - ps16
Deuterostomia - ps17
Chordata - ps18
Olfactores - ps19
Vertebrata - ps20
Gnathostomata - ps21
Euteleostomi - ps22
Sarcopterygii - ps23
Tetrapoda - ps24
Amniota - ps25
Mammalia - ps26
Theria - ps27
Placentalia - ps28
Boreoeutheria - ps29
Euarchontoglires - ps30
Euarchonta - ps31
Primates - ps32
Haplorrhini - ps33
Simiiformes - ps34
Catarrhini - ps35
Hominoidea - ps36
Hominidae - ps37
Homininae - ps38
Hominini - ps39
Homo_sapiens - ps40
p-value
1e-02
1e-02
2e-02
2e-02
3e-02
Log-odds
0.5
1.0
1.5
2.0
2.5
3.0
3.5
4.0

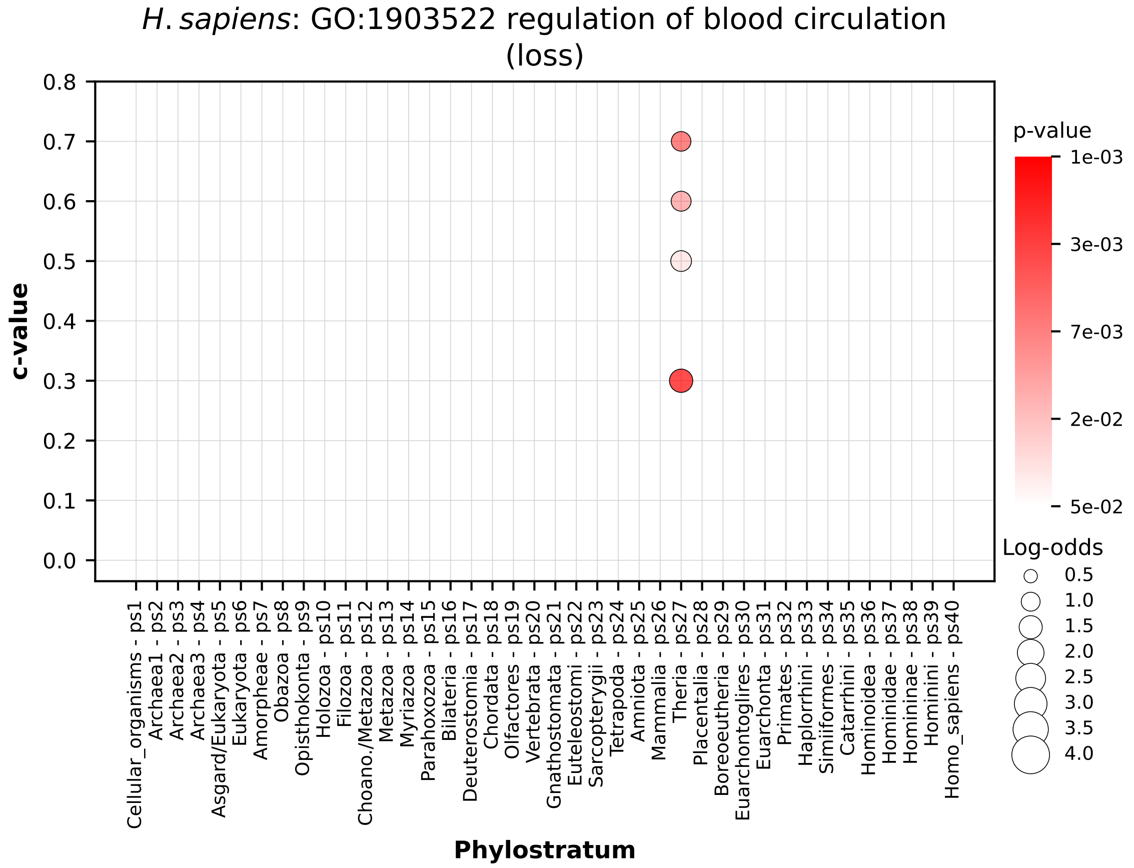
H. sapiens: GO:1903522 regulation of blood circulation
(loss)
c-value
0.8
0.7
0.6
0.5
0.4
0.3
0.2
0.1
0.0
p-value
1e-03
3e-03
7e-03
2e-02
5e-02
Log-odds
0.5
1.0
1.5
2.0
2.5
3.0
3.5
4.0
Cellular_organisms - ps1
Archaea1 - ps2
Archaea2 - ps3
Archaea3 - ps4
Asgard/Eukaryota - ps5
Eukaryota - ps6
Amorpheae - ps7
Obazoa - ps8
Opisthokonta - ps9
Holozoa - ps10
Filozoa - ps11
Choano./Metazoa - ps12
Metazoa - ps13
Myriazoa - ps14
Parahoxozoa - ps15
Bilateria - ps16
Deuterostomia - ps17
Chordata - ps18
Olfactores - ps19
Vertebrata - ps20
Gnathostomata - ps21
Euteleostomi - ps22
Sarcopterygii - ps23
Tetrapoda - ps24
Amniota - ps25
Mammalia - ps26
Theria - ps27
Placentalia - ps28
Boreoeutheria - ps29
Euarchontoglires - ps30
Euarchonta - ps31
Primates - ps32
Haplorrhini - ps33
Simiiformes - ps34
Catarrhini - ps35
Hominoidea - ps36
Hominidae - ps37
Homininae - ps38
Hominini - ps39
Homo_sapiens - ps40
Phylostratum

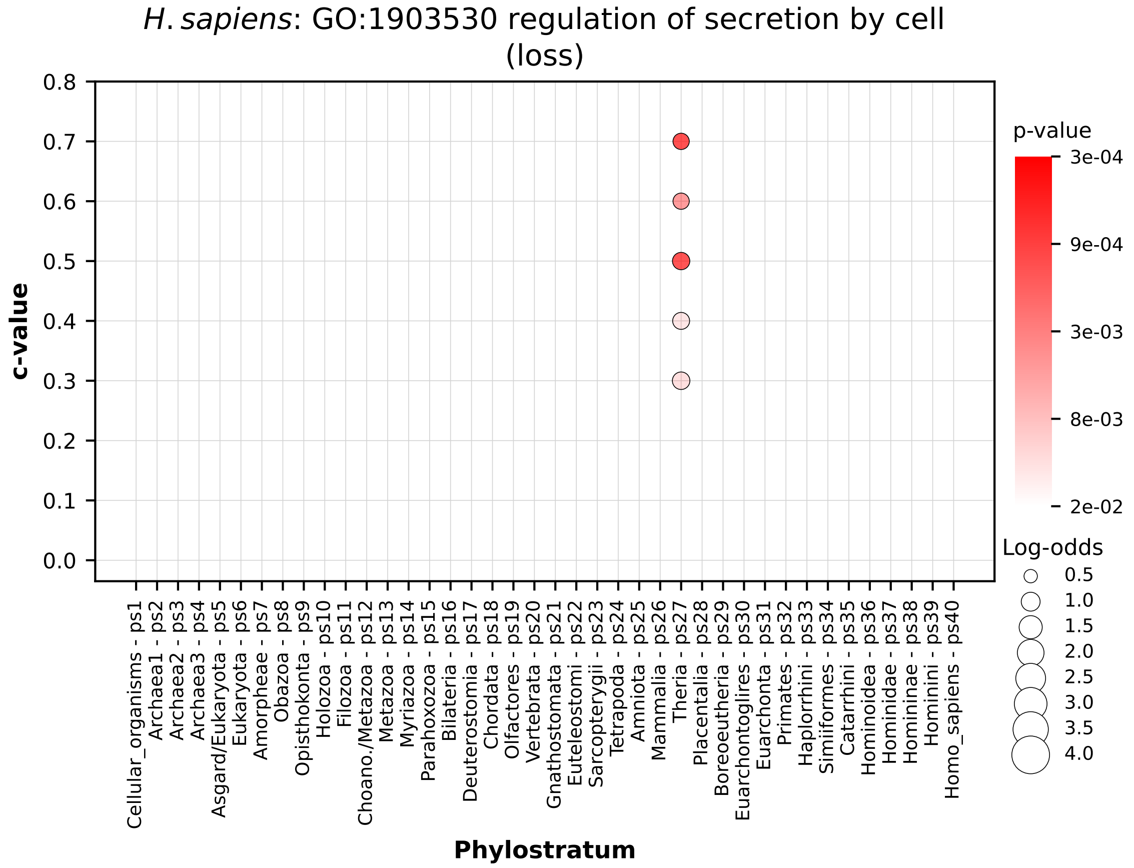

H. sapiens: GO:1903530 regulation of secretion by cell
(loss)
c-value
0.0
0.1
0.2
0.3
0.4
0.5
0.6
0.7
0.8
Phylostratum
Cellular_organisms - ps1
Archaea1 - ps2
Archaea2 - ps3
Archaea3 - ps4
Asgard/Eukaryota - ps5
Eukaryota - ps6
Amorpheae - ps7
Obazoa - ps8
Opisthokonta - ps9
Holozoa - ps10
Filozoa - ps11
Choano./Metazoa - ps12
Metazoa - ps13
Myriazoa - ps14
Parahoxozoa - ps15
Bilateria - ps16
Deuterostomia - ps17
Chordata - ps18
Olfactores - ps19
Vertebrata - ps20
Gnathostomata - ps21
Euteleostomi - ps22
Sarcopterygii - ps23
Tetrapoda - ps24
Amniota - ps25
Mammalia - ps26
Theria - ps27
Placentalia - ps28
Boreoeutheria - ps29
Euarchontoglires - ps30
Euarchonta - ps31
Primates - ps32
Haplorrhini - ps33
Simiiformes - ps34
Catarrhini - ps35
Hominoidea - ps36
Hominidae - ps37
Homininae - ps38
Hominini - ps39
Homo_sapiens - ps40
p-value
3e-04
9e-04
3e-03
8e-03
2e-02
Log-odds
0.5
1.0
1.5
2.0
2.5
3.0
3.5
4.0

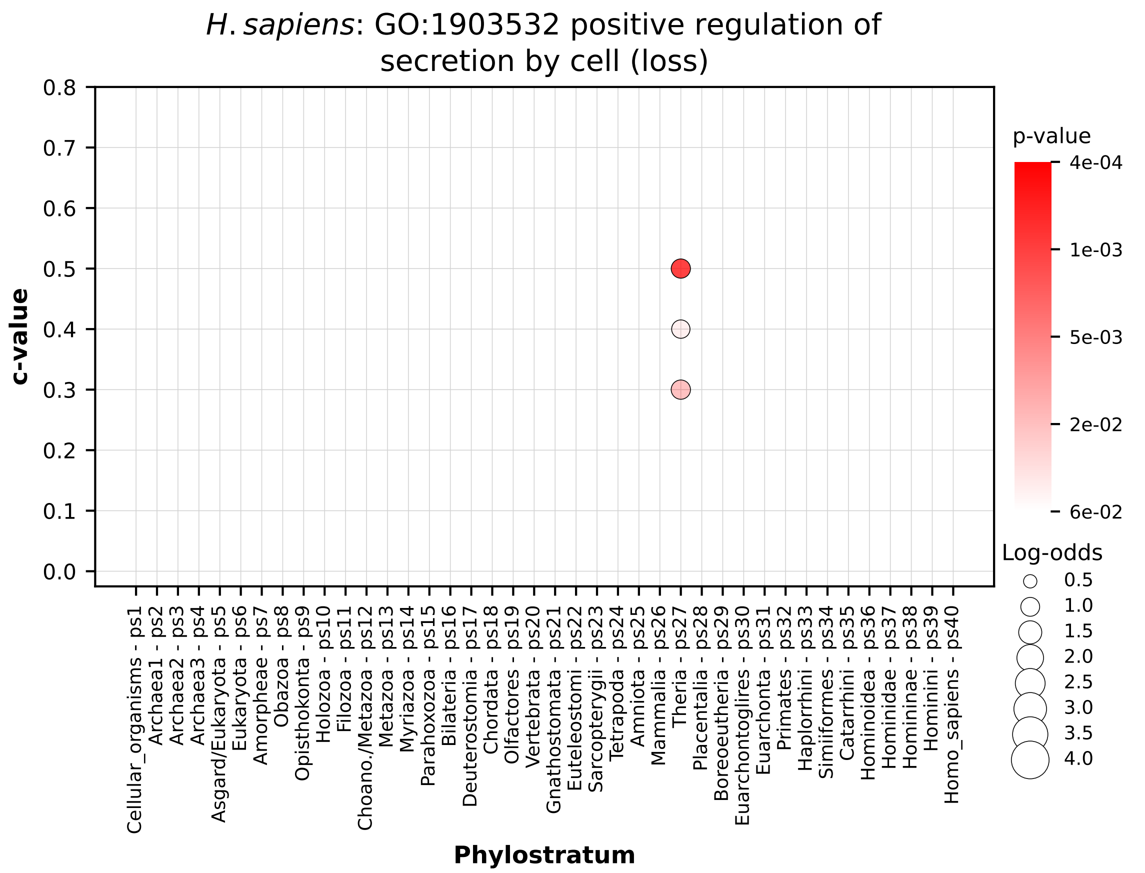

H. sapiens: GO:1903532 positive regulation of secretion by cell (loss)
c-value
0.8
0.7
0.6
0.5
0.4
0.3
0.2
0.1
0.0
Phylostratum
Cellular_organisms - ps1
Archaea1 - ps2
Archaea2 - ps3
Archaea3 - ps4
Asgard/Eukaryota - ps5
Eukaryota - ps6
Amorpheae - ps7
Obazoa - ps8
Opisthokonta - ps9
Holozoa - ps10
Filozoa - ps11
Choano./Metazoa - ps12
Metazoa - ps13
Myriazoa - ps14
Parahoxozoa - ps15
Bilateria - ps16
Deuterostomia - ps17
Chordata - ps18
Olfactores - ps19
Vertebrata - ps20
Gnathostomata - ps21
Euteleostomi - ps22
Sarcopterygii - ps23
Tetrapoda - ps24
Amniota - ps25
Mammalia - ps26
Theria - ps27
Placentalia - ps28
Boreoeutheria - ps29
Euarchontoglires - ps30
Euarchonta - ps31
Primates - ps32
Haplorrhini - ps33
Simiiformes - ps34
Catarrhini - ps35
Hominoidea - ps36
Hominidae - ps37
Homininae - ps38
Hominini - ps39
Homo_sapiens - ps40
p-value
4e-04
1e-03
5e-03
2e-02
6e-02
Log-odds
0.5
1.0
1.5
2.0
2.5
3.0
3.5
4.0

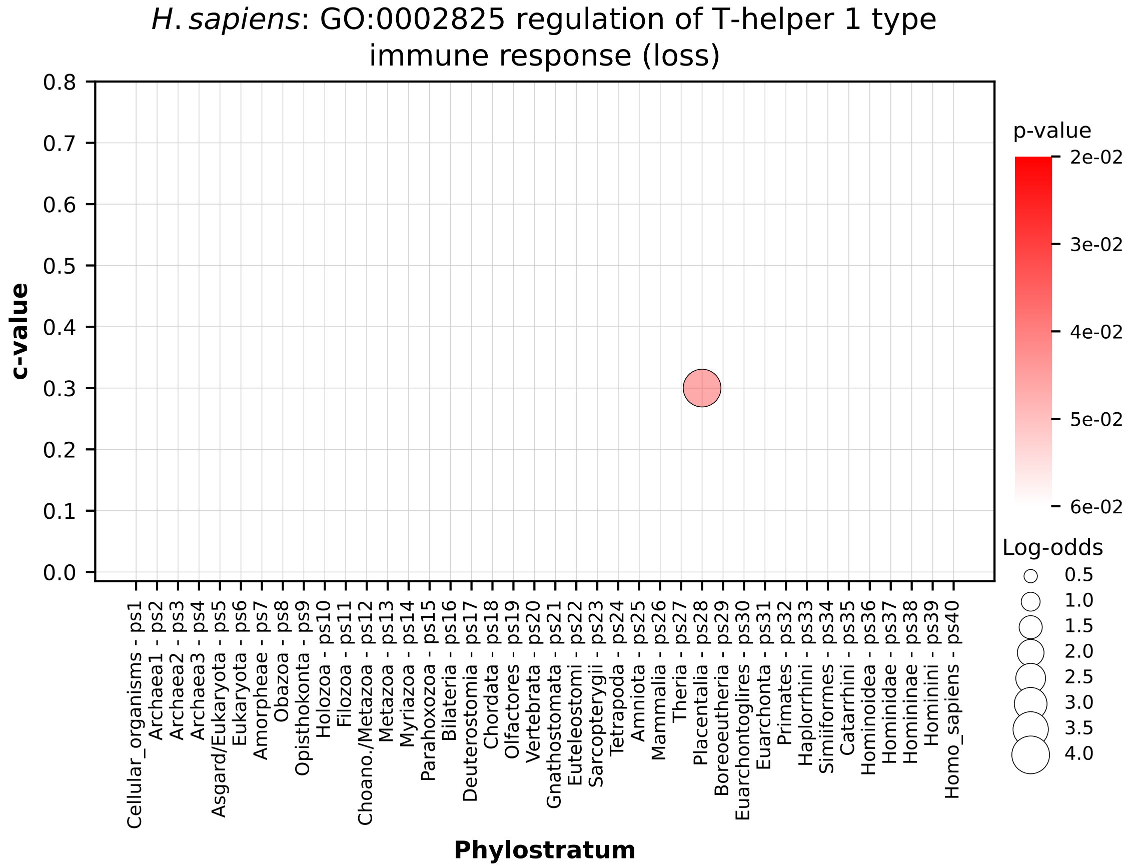
H. sapiens: GO:0002825 regulation of T-helper 1 type immune response (loss)
c-value
0.0
0.1
0.2
0.3
0.4
0.5
0.6
0.7
0.8
Phylostratum
Cellular_organisms - ps1
Archaea1 - ps2
Archaea2 - ps3
Archaea3 - ps4
Asgard/Eukaryota - ps5
Eukaryota - ps6
Amorpheae - ps7
Obazoa - ps8
Opisthokonta - ps9
Holozoa - ps10
Filozoa - ps11
Choano./Metazoa - ps12
Metazoa - ps13
Myriazoa - ps14
Parahoxozoa - ps15
Bilateria - ps16
Deuterostomia - ps17
Chordata - ps18
Olfactores - ps19
Vertebrata - ps20
Gnathostomata - ps21
Euteleostomi - ps22
Sarcopterygii - ps23
Tetrapoda - ps24
Amniota - ps25
Mammalia - ps26
Theria - ps27
Placentalia - ps28
Boreoeutheria - ps29
Euarchontoglires - ps30
Euarchonta - ps31
Primates - ps32
Haplorrhini - ps33
Simiiformes - ps34
Catarrhini - ps35
Hominoidea - ps36
Hominidae - ps37
Homininae - ps38
Hominini - ps39
Homo_sapiens - ps40
p-value
2e-02
3e-02
4e-02
5e-02
6e-02
Log-odds
0.5
1.0
1.5
2.0
2.5
3.0
3.5
4.0

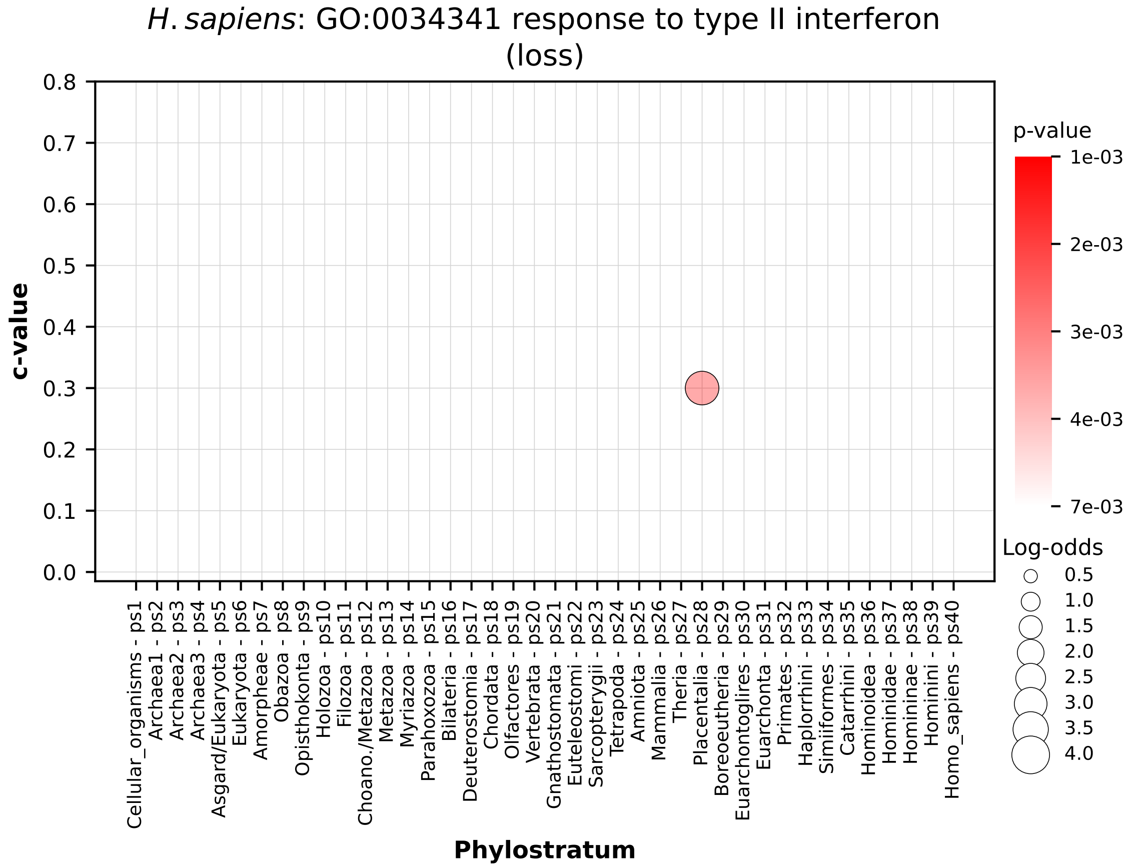

H. sapiens: GO:0034341 response to type II interferon (loss)
c-value
0.8
0.7
0.6
0.5
0.4
0.3
0.2
0.1
0.0
p-value
1e-03
2e-03
3e-03
4e-03
7e-03
Log-odds
0.5
1.0
1.5
2.0
2.5
3.0
3.5
4.0
Cellular_organisms - ps1
Archaea1 - ps2
Archaea2 - ps3
Archaea3 - ps4
Asgard/Eukaryota - ps5
Eukaryota - ps6
Amorpheae - ps7
Obazoa - ps8
Opisthokonta - ps9
Holozoa - ps10
Filozoa - ps11
Choano./Metazoa - ps12
Metazoa - ps13
Myriazoa - ps14
Parahoxozoa - ps15
Bilateria - ps16
Deuterostomia - ps17
Chordata - ps18
Olfactores - ps19
Vertebrata - ps20
Gnathostomata - ps21
Euteleostomi - ps22
Sarcopterygii - ps23
Tetrapoda - ps24
Amniota - ps25
Mammalia - ps26
Theria - ps27
Placentalia - ps28
Boreoeutheria - ps29
Euarchontoglires - ps30
Euarchonta - ps31
Primates - ps32
Haplorrhini - ps33
Simiiformes - ps34
Catarrhini - ps35
Hominoidea - ps36
Hominidae - ps37
Homininae - ps38
Hominini - ps39
Homo_sapiens - ps40
Phylostratum

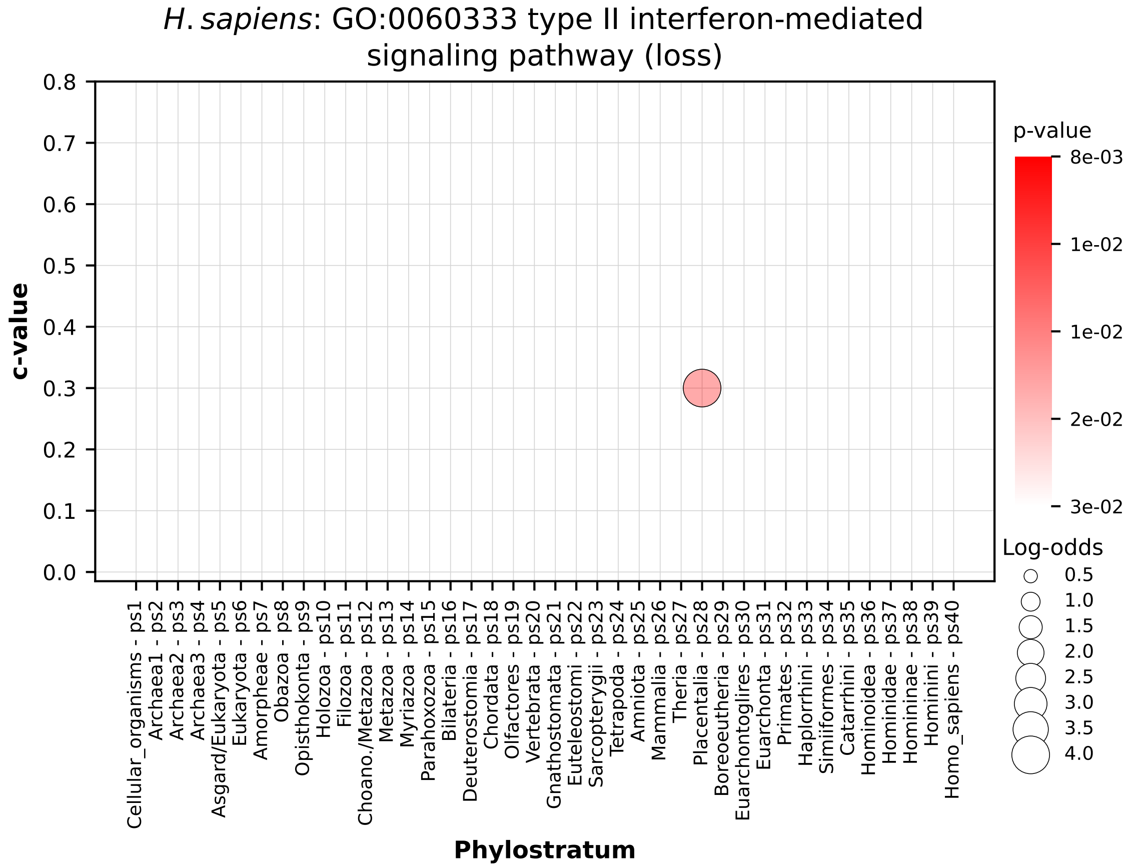

H. sapiens: GO:0060333 type II interferon-mediated signaling pathway (loss)
c-value
0.8
0.7
0.6
0.5
0.4
0.3
0.2
0.1
0.0
Phylostratum
Cellular_organisms - ps1
Archaea1 - ps2
Archaea2 - ps3
Archaea3 - ps4
Asgard/Eukaryota - ps5
Eukaryota - ps6
Amorpheae - ps7
Obazoa - ps8
Opisthokonta - ps9
Holozoa - ps10
Filozoa - ps11
Choano./Metazoa - ps12
Metazoa - ps13
Myriazoa - ps14
Parahoxozoa - ps15
Bilateria - ps16
Deuterostomia - ps17
Chordata - ps18
Olfactores - ps19
Vertebrata - ps20
Gnathostomata - ps21
Euteleostomi - ps22
Sarcopterygii - ps23
Tetrapoda - ps24
Amniota - ps25
Mammalia - ps26
Theria - ps27
Placentalia - ps28
Boreoeutheria - ps29
Euarchontoglires - ps30
Euarchonta - ps31
Primates - ps32
Haplorrhini - ps33
Simiiformes - ps34
Catarrhini - ps35
Hominoidea - ps36
Hominidae - ps37
Homininae - ps38
Hominini - ps39
Homo_sapiens - ps40
p-value
8e-03
1e-02
1e-02
2e-02
3e-02
Log-odds
0.5
1.0
1.5
2.0
2.5
3.0
3.5
4.0

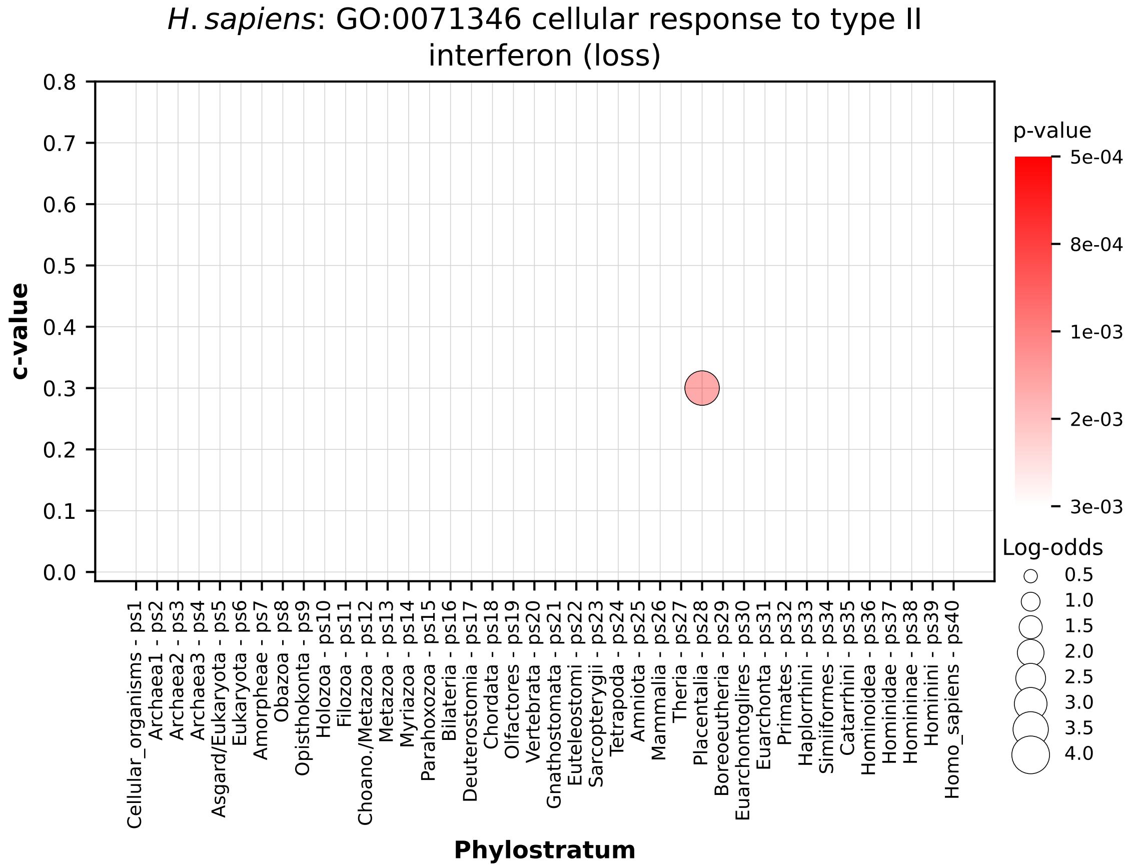

H. sapiens: GO:0071346 cellular response to type II interferon (loss)
c-value
0.0
0.1
0.2
0.3
0.4
0.5
0.6
0.7
0.8
Phylostratum
Cellular_organisms - ps1
Archaea1 - ps2
Archaea2 - ps3
Archaea3 - ps4
Asgard/Eukaryota - ps5
Eukaryota - ps6
Amorpheae - ps7
Obazoa - ps8
Opisthokonta - ps9
Holozoa - ps10
Filozoa - ps11
Choano./Metazoa - ps12
Metazoa - ps13
Myriazoa - ps14
Parahoxozoa - ps15
Bilateria - ps16
Deuterostomia - ps17
Chordata - ps18
Olfactores - ps19
Vertebrata - ps20
Gnathostomata - ps21
Euteleostomi - ps22
Sarcopterygii - ps23
Tetrapoda - ps24
Amniota - ps25
Mammalia - ps26
Theria - ps27
Placentalia - ps28
Boreoeutheria - ps29
Euarchontoglires - ps30
Euarchonta - ps31
Primates - ps32
Haplorrhini - ps33
Simiiformes - ps34
Catarrhini - ps35
Hominoidea - ps36
Hominidae - ps37
Homininae - ps38
Hominini - ps39
Homo_sapiens - ps40
p-value
5e-04
8e-04
1e-03
2e-03
3e-03
Log-odds
0.5
1.0
1.5
2.0
2.5
3.0
3.5
4.0

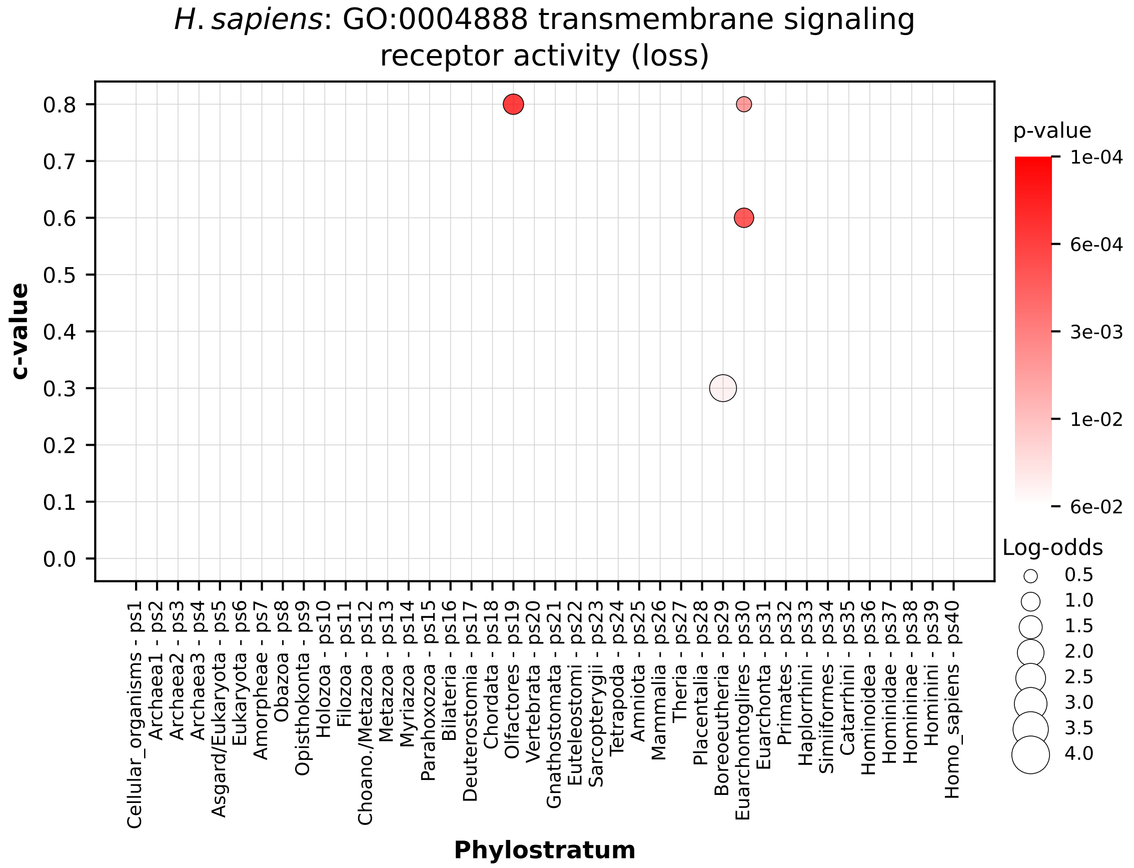
H. sapiens: GO:0004888 transmembrane signaling receptor activity (loss)
c-value
Phylostratum
p-value
1e-04
6e-04
3e-03
1e-02
6e-02
Log-odds
0.5
1.0
1.5
2.0
2.5
3.0
3.5
4.0
Cellular_organisms - ps1
Archaea1 - ps2
Archaea2 - ps3
Archaea3 - ps4
Asgard/Eukaryota - ps5
Eukaryota - ps6
Amorpheae - ps7
Obazoa - ps8
Opisthokonta - ps9
Holozoa - ps10
Filozoa - ps11
Choano./Metazoa - ps12
Metazoa - ps13
Myriazoa - ps14
Parahoxozoa - ps15
Bilateria - ps16
Deuterostomia - ps17
Chordata - ps18
Olfactores - ps19
Vertebrata - ps20
Gnathostomata - ps21
Euteleostomi - ps22
Sarcopterygii - ps23
Tetrapoda - ps24
Amniota - ps25
Mammalia - ps26
Theria - ps27
Placentalia - ps28
Boreoeutheria - ps29
Euarchontoglires - ps30
Euarchonta - ps31
Primates - ps32
Haplorrhini - ps33
Simiiformes - ps34
Catarrhini - ps35
Hominoidea - ps36
Hominidae - ps37
Homininae - ps38
Hominini - ps39
Homo_sapiens - ps40

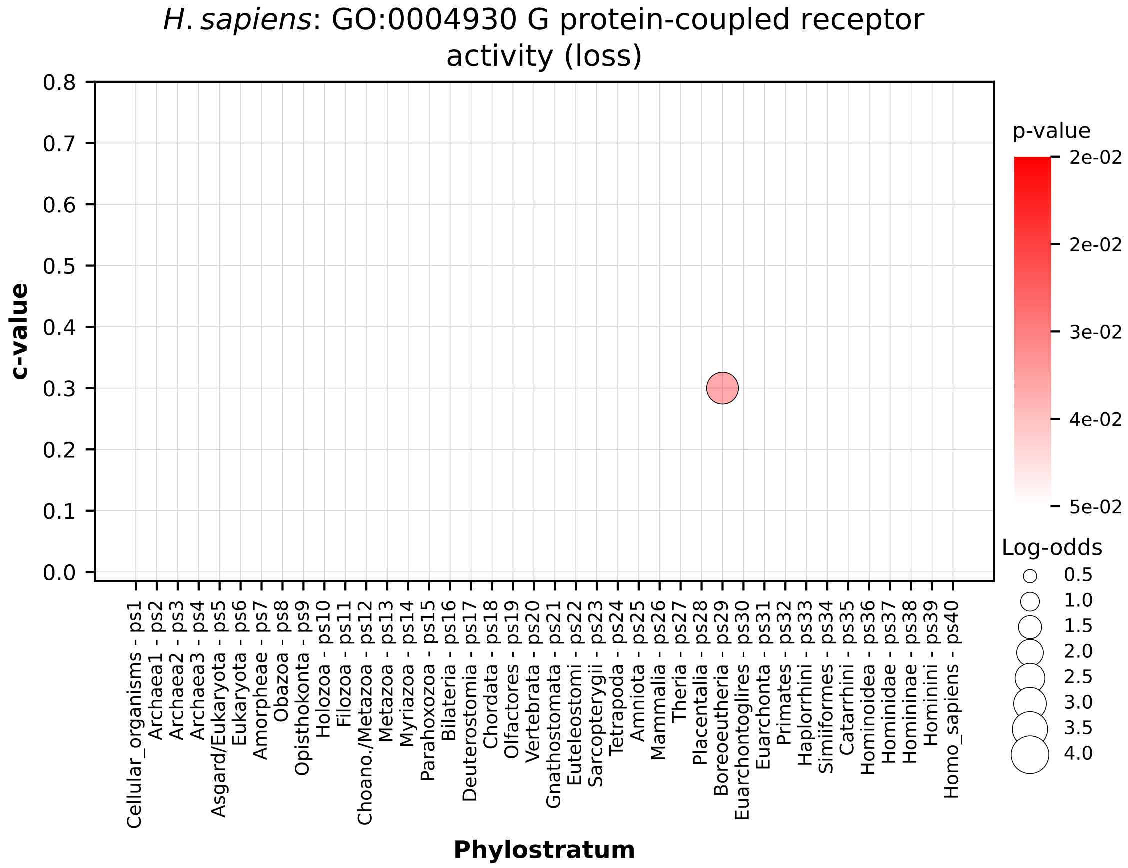

H. sapiens: GO:0004930 G protein-coupled receptor activity (loss)
c-value
0.8
0.7
0.6
0.5
0.4
0.3
0.2
0.1
0.0
Phylostratum
Cellular_organisms - ps1
Archaea1 - ps2
Archaea2 - ps3
Archaea3 - ps4
Asgard/Eukaryota - ps5
Eukaryota - ps6
Amorpheae - ps7
Obazoa - ps8
Opisthokonta - ps9
Holozoa - ps10
Filozoa - ps11
Choano./Metazoa - ps12
Metazoa - ps13
Myriazoa - ps14
Parahoxozoa - ps15
Bilateria - ps16
Deuterostomia - ps17
Chordata - ps18
Olfactores - ps19
Vertebrata - ps20
Gnathostomata - ps21
Euteleostomi - ps22
Sarcopterygii - ps23
Tetrapoda - ps24
Amniota - ps25
Mammalia - ps26
Theria - ps27
Placentalia - ps28
Boreoeutheria - ps29
Euarchontoglires - ps30
Euarchonta - ps31
Primates - ps32
Haplorrhini - ps33
Simiiformes - ps34
Catarrhini - ps35
Hominoidea - ps36
Hominidae - ps37
Homininae - ps38
Hominini - ps39
Homo_sapiens - ps40
p-value
2e-02
2e-02
3e-02
4e-02
5e-02
Log-odds
0.5
1.0
1.5
2.0
2.5
3.0
3.5
4.0

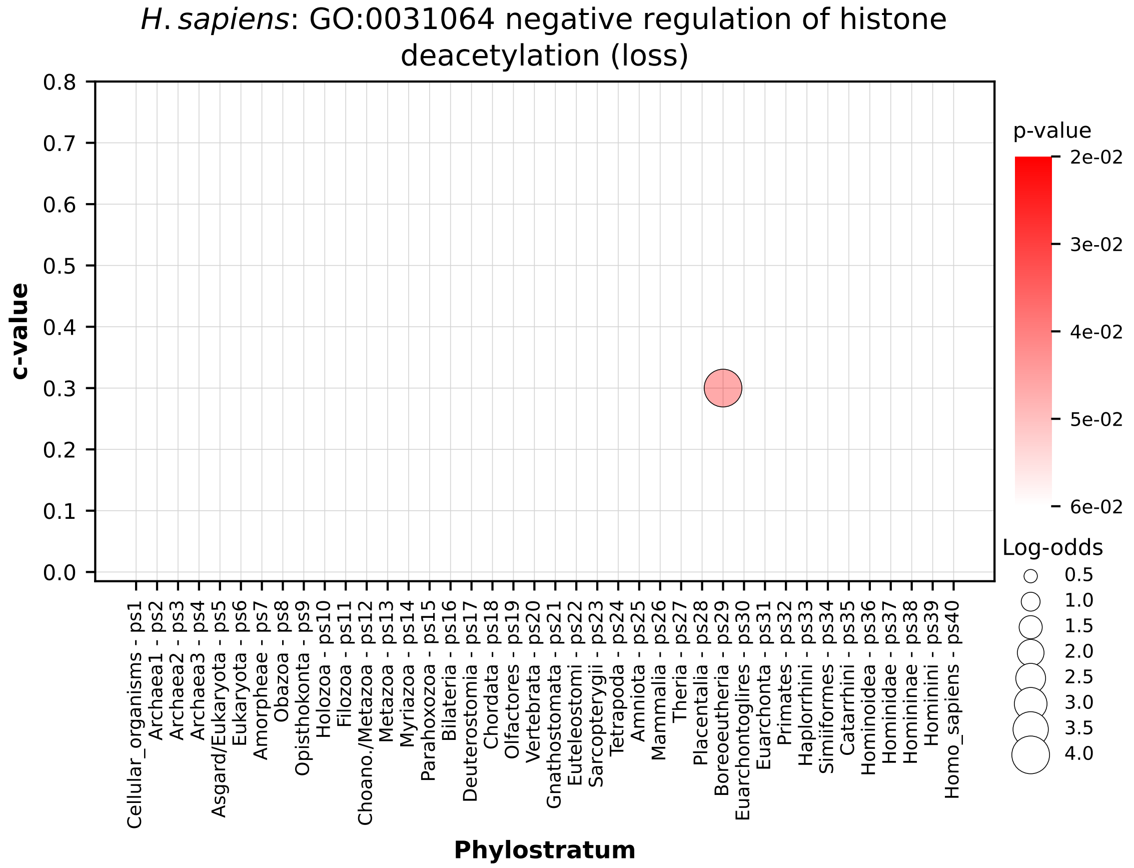

H. sapiens: GO:0031064 negative regulation of histone deacetylation (loss)
c-value
0.8
0.7
0.6
0.5
0.4
0.3
0.2
0.1
0.0
Phylostratum
Cellular_organisms - ps1
Archaea1 - ps2
Archaea2 - ps3
Archaea3 - ps4
Asgard/Eukaryota - ps5
Eukaryota - ps6
Amorpheae - ps7
Obazoa - ps8
Opisthokonta - ps9
Holozoa - ps10
Filozoa - ps11
Choano./Metazoa - ps12
Metazoa - ps13
Myriazoa - ps14
Parahoxozoa - ps15
Bilateria - ps16
Deuterostomia - ps17
Chordata - ps18
Olfactores - ps19
Vertebrata - ps20
Gnathostomata - ps21
Euteleostomi - ps22
Sarcopterygii - ps23
Tetrapoda - ps24
Amniota - ps25
Mammalia - ps26
Theria - ps27
Placentalia - ps28
Boreoeutheria - ps29
Euarchontoglires - ps30
Euarchonta - ps31
Primates - ps32
Haplorrhini - ps33
Simiiformes - ps34
Catarrhini - ps35
Hominoidea - ps36
Hominidae - ps37
Homininae - ps38
Hominini - ps39
Homo_sapiens - ps40
p-value
2e-02
3e-02
4e-02
5e-02
6e-02
Log-odds
0.5
1.0
1.5
2.0
2.5
3.0
3.5
4.0

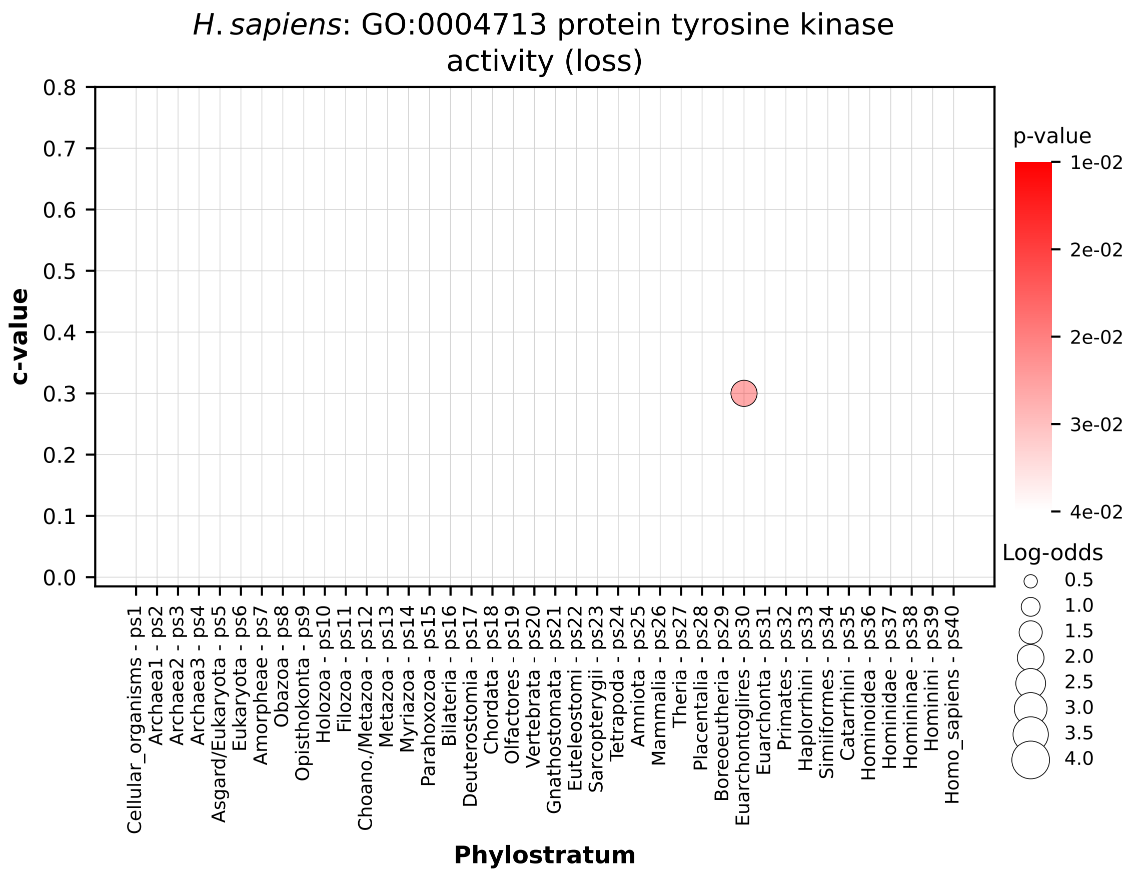

H. sapiens: GO:0004713 protein tyrosine kinase activity (loss)
c-value
0.0
0.1
0.2
0.3
0.4
0.5
0.6
0.7
0.8
Phylostratum
Cellular_organisms - ps1
Archaea1 - ps2
Archaea2 - ps3
Archaea3 - ps4
Asgard/Eukaryota - ps5
Eukaryota - ps6
Amorpheae - ps7
Obazoa - ps8
Opisthokonta - ps9
Holozoa - ps10
Filozoa - ps11
Choano./Metazoa - ps12
Metazoa - ps13
Myriazoa - ps14
Parahoxozoa - ps15
Bilateria - ps16
Deuterostomia - ps17
Chordata - ps18
Olfactores - ps19
Vertebrata - ps20
Gnathostomata - ps21
Euteleostomi - ps22
Sarcopterygii - ps23
Tetrapoda - ps24
Amniota - ps25
Mammalia - ps26
Theria - ps27
Placentalia - ps28
Boreoeutheria - ps29
Euarchontoglires - ps30
Euarchonta - ps31
Primates - ps32
Haplorrhini - ps33
Simiiformes - ps34
Catarrhini - ps35
Hominoidea - ps36
Hominidae - ps37
Homininae - ps38
Hominini - ps39
Homo_sapiens - ps40
p-value
1e-02
2e-02
2e-02
3e-02
4e-02
Log-odds
0.5
1.0
1.5
2.0
2.5
3.0
3.5
4.0

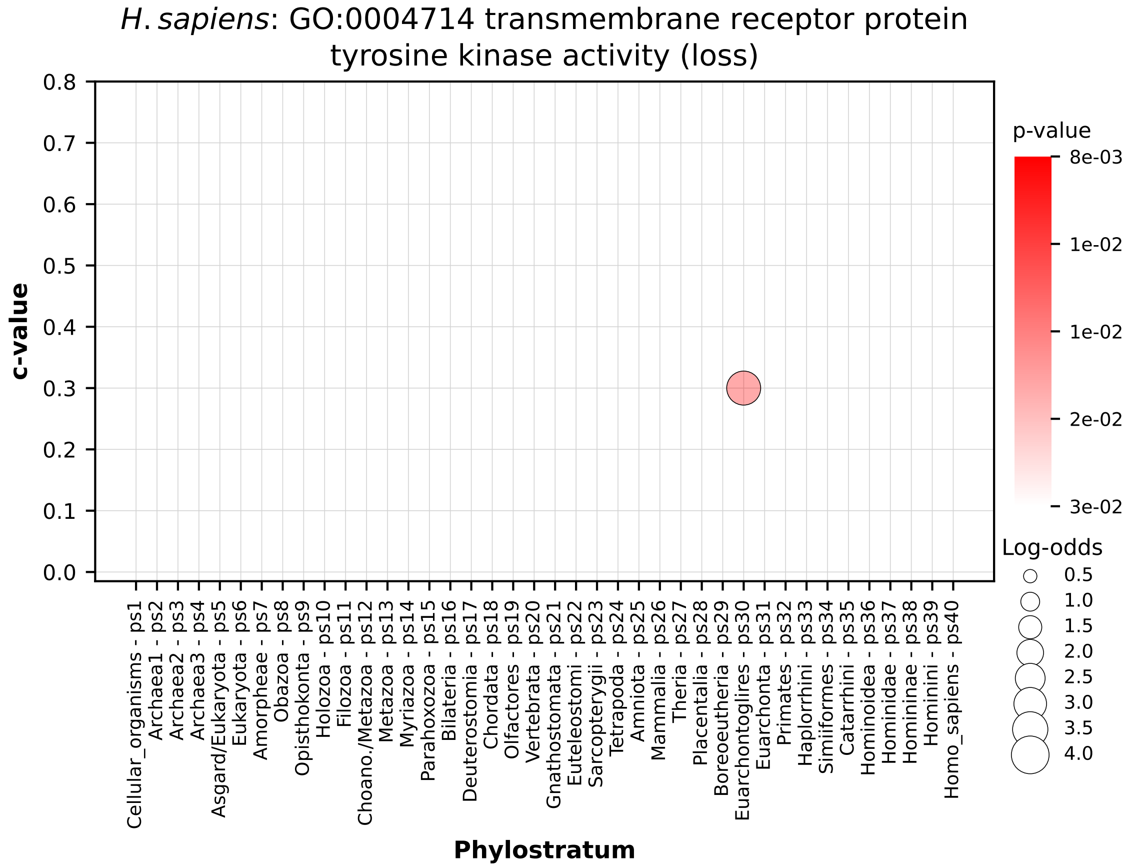
H. sapiens: GO:0004714 transmembrane receptor protein tyrosine kinase activity (loss)
c-value
0.8
0.7
0.6
0.5
0.4
0.3
0.2
0.1
0.0
Phylostratum
Cellular_organisms - ps1
Archaea1 - ps2
Archaea2 - ps3
Archaea3 - ps4
Asgard/Eukaryota - ps5
Eukaryota - ps6
Amorpheae - ps7
Obazoa - ps8
Opisthokonta - ps9
Holozoa - ps10
Filozoa - ps11
Choano./Metazoa - ps12
Metazoa - ps13
Myriazoa - ps14
Parahoxozoa - ps15
Bilateria - ps16
Deuterostomia - ps17
Chordata - ps18
Olfactores - ps19
Vertebrata - ps20
Gnathostomata - ps21
Euteleostomi - ps22
Sarcopterygii - ps23
Tetrapoda - ps24
Amniota - ps25
Mammalia - ps26
Theria - ps27
Placentalia - ps28
Boreoeutheria - ps29
Euarchontoglires - ps30
Euarchonta - ps31
Primates - ps32
Haplorrhini - ps33
Simiiformes - ps34
Catarrhini - ps35
Hominoidea - ps36
Hominidae - ps37
Homininae - ps38
Hominini - ps39
Homo_sapiens - ps40
p-value
8e-03
1e-02
1e-02
2e-02
3e-02
Log-odds
0.5
1.0
1.5
2.0
2.5
3.0
3.5
4.0

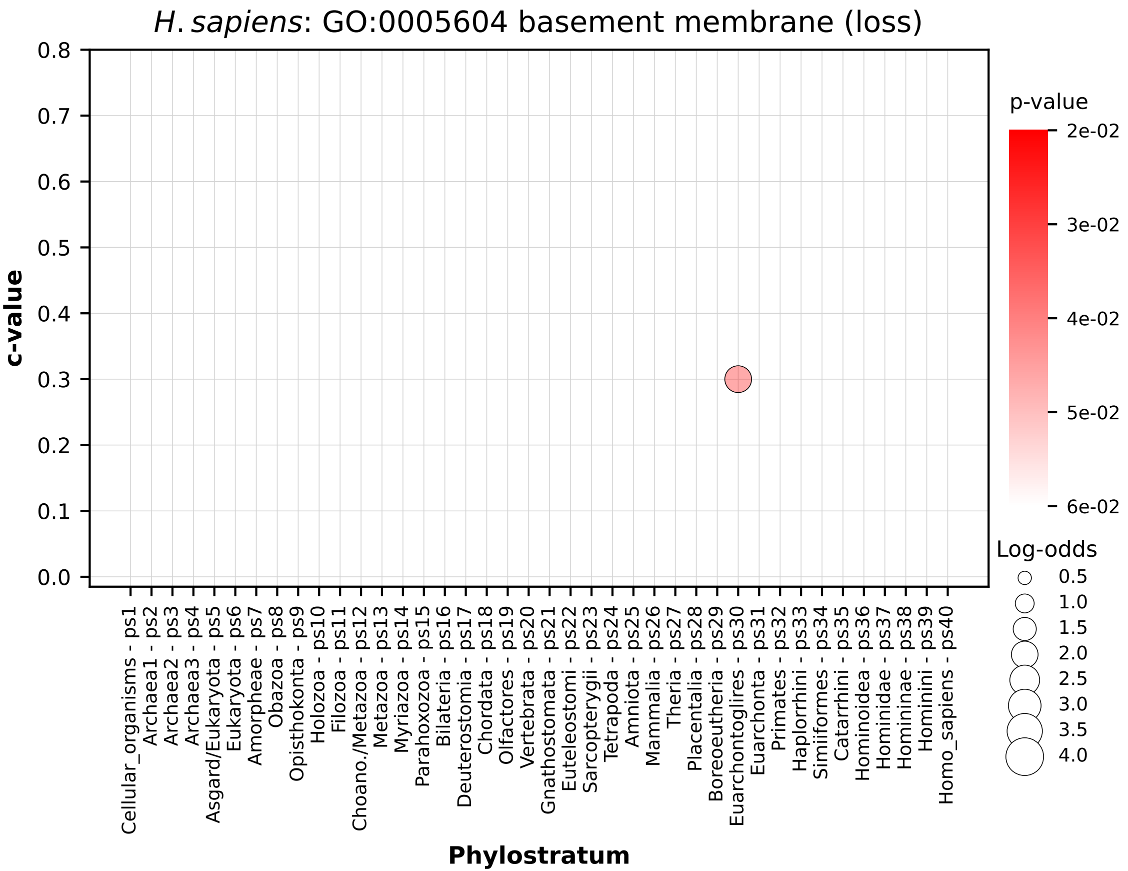

H. sapiens: GO:0005604 basement membrane (loss)
c-value
0.0
0.1
0.2
0.3
0.4
0.5
0.6
0.7
0.8
Phylostratum
Cellular_organisms - ps1
Archaea1 - ps2
Archaea2 - ps3
Archaea3 - ps4
Asgard/Eukaryota - ps5
Eukaryota - ps6
Amorpheae - ps7
Obazoa - ps8
Opisthokonta - ps9
Holozoa - ps10
Filozoa - ps11
Choano./Metazoa - ps12
Metazoa - ps13
Myriazoa - ps14
Parahoxozoa - ps15
Bilateria - ps16
Deuterostomia - ps17
Chordata - ps18
Olfactores - ps19
Vertebrata - ps20
Gnathostomata - ps21
Euteleostomi - ps22
Sarcopterygii - ps23
Tetrapoda - ps24
Amniota - ps25
Mammalia - ps26
Theria - ps27
Placentalia - ps28
Boreoeutheria - ps29
Euarchontoglires - ps30
Euarchonta - ps31
Primates - ps32
Haplorrhini - ps33
Simiiformes - ps34
Catarrhini - ps35
Hominoidea - ps36
Hominidae - ps37
Homininae - ps38
Hominini - ps39
Homo_sapiens - ps40
p-value
2e-02
3e-02
4e-02
5e-02
6e-02
Log-odds
0.5
1.0
1.5
2.0
2.5
3.0
3.5
4.0

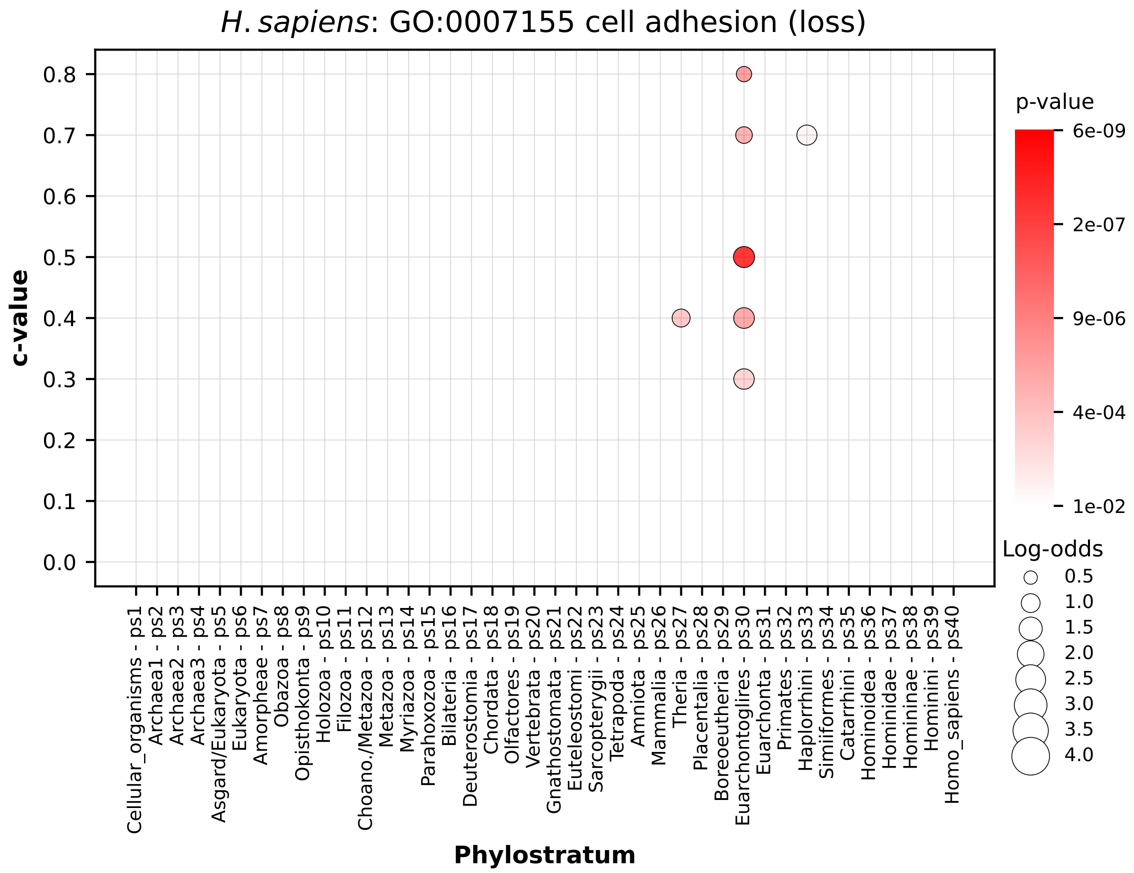

H. sapiens: GOː0007155 cell adhesion (loss)
c-value
0.0
0.1
0.2
0.3
0.4
0.5
0.6
0.7
0.8
Phylostratum
Cellular_organisms - ps1
Archaea1 - ps2
Archaea2 - ps3
Archaea3 - ps4
Asgard/Eukaryota - ps5
Eukaryota - ps6
Amorpheae - ps7
Obazoa - ps8
Opisthokonta - ps9
Holozoa - ps10
Filozoa - ps11
Choano./Metazoa - ps12
Metazoa - ps13
Myriazoa - ps14
Parahoxozoa - ps15
Bilateria - ps16
Deuterostomia - ps17
Chordata - ps18
Olfactores - ps19
Vertebrata - ps20
Gnathostomata - ps21
Euteleostomi - ps22
Sarcopterygii - ps23
Tetrapoda - ps24
Amniota - ps25
Mammalia - ps26
Theria - ps27
Placentalia - ps28
Boreoeutheria - ps29
Euarchontoglires - ps30
Euarchonta - ps31
Primates - ps32
Haplorrhini - ps33
Simiiformes - ps34
Catarrhini - ps35
Hominoidea - ps36
Hominidae - ps37
Homininae - ps38
Hominini - ps39
Homo_sapiens - ps40
p-value
6e-09
2e-07
9e-06
4e-04
1e-02
Log-odds
0.5
1.0
1.5
2.0
2.5
3.0
3.5
4.0

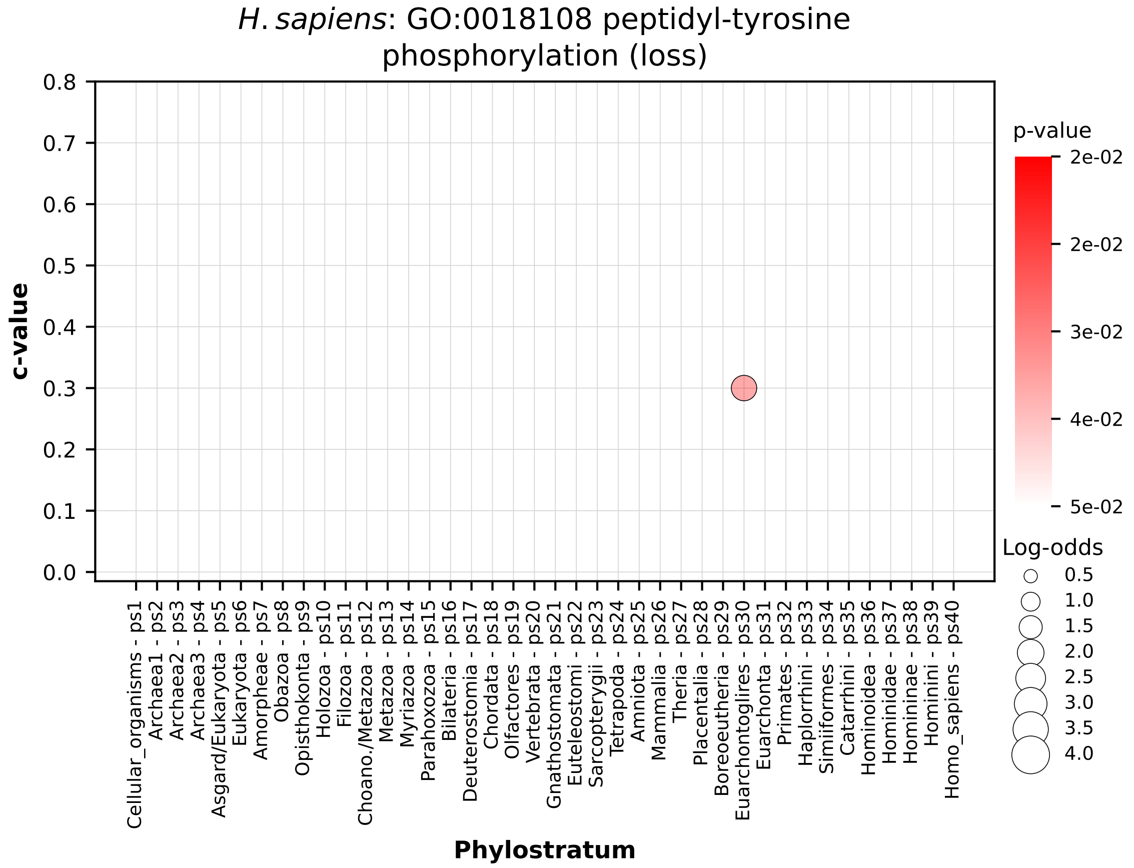

H. sapiens: GO:0018108 peptidyl-tyrosine phosphorylation (loss)
c-value
0.8
0.7
0.6
0.5
0.4
0.3
0.2
0.1
0.0
Phylostratum
Cellular_organisms - ps1
Archaea1 - ps2
Archaea2 - ps3
Archaea3 - ps4
Asgard/Eukaryota - ps5
Eukaryota - ps6
Amorpheae - ps7
Obazoa - ps8
Opisthokonta - ps9
Holozoa - ps10
Filozoa - ps11
Choano./Metazoa - ps12
Metazoa - ps13
Myriazoa - ps14
Parahoxozoa - ps15
Bilateria - ps16
Deuterostomia - ps17
Chordata - ps18
Olfactores - ps19
Vertebrata - ps20
Gnathostomata - ps21
Euteleostomi - ps22
Sarcopterygii - ps23
Tetrapoda - ps24
Amniota - ps25
Mammalia - ps26
Theria - ps27
Placentalia - ps28
Boreoeutheria - ps29
Euarchontoglires - ps30
Euarchonta - ps31
Primates - ps32
Haplorrhini - ps33
Simiiformes - ps34
Catarrhini - ps35
Hominoidea - ps36
Hominidae - ps37
Homininae - ps38
Hominini - ps39
Homo_sapiens - ps40
p-value
2e-02
2e-02
3e-02
4e-02
5e-02
Log-odds
0.5
1.0
1.5
2.0
2.5
3.0
3.5
4.0

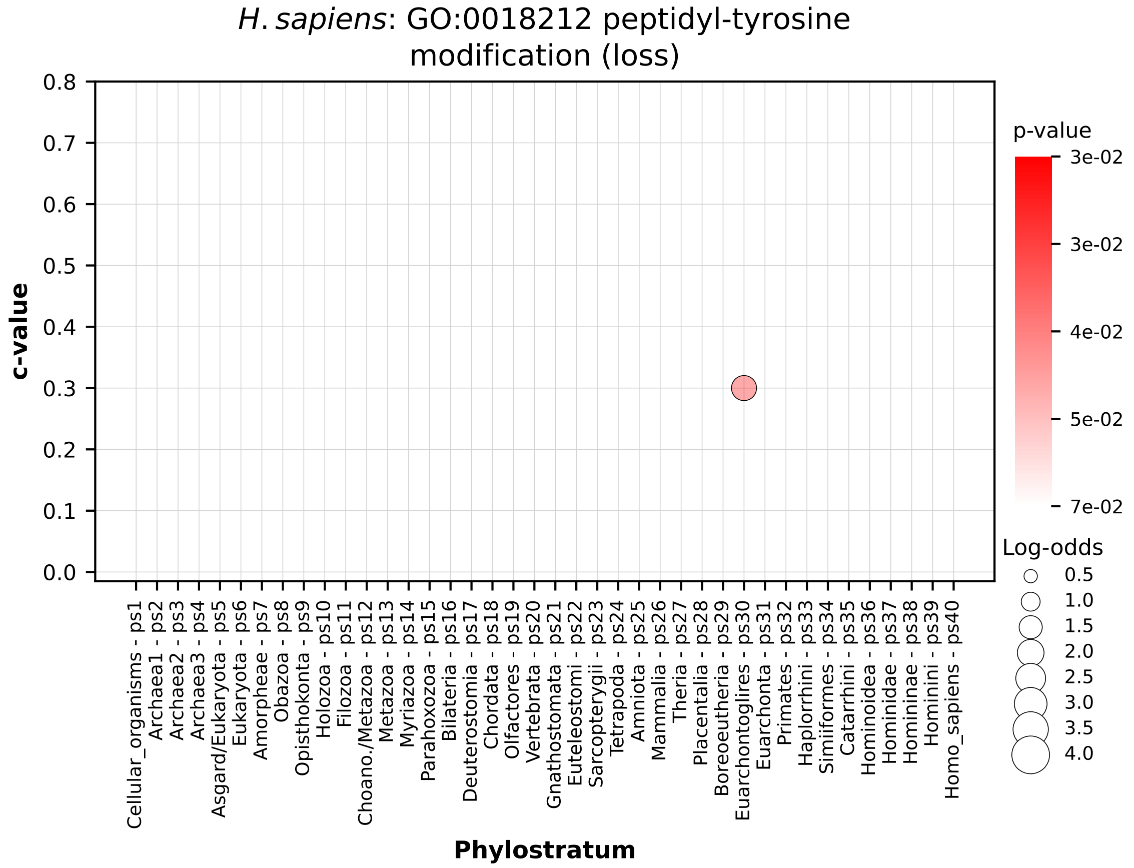
H. sapiens: GO:0018212 peptidyl-tyrosine modification (loss)
c-value
0.0
0.1
0.2
0.3
0.4
0.5
0.6
0.7
0.8
Phylostratum
Cellular_organisms - ps1
Archaea1 - ps2
Archaea2 - ps3
Archaea3 - ps4
Asgard/Eukaryota - ps5
Eukaryota - ps6
Amorpheae - ps7
Obazoa - ps8
Opisthokonta - ps9
Holozoa - ps10
Filozoa - ps11
Choano./Metazoa - ps12
Metazoa - ps13
Myriazoa - ps14
Parahoxozoa - ps15
Bilateria - ps16
Deuterostomia - ps17
Chordata - ps18
Olfactores - ps19
Vertebrata - ps20
Gnathostomata - ps21
Euteleostomi - ps22
Sarcopterygii - ps23
Tetrapoda - ps24
Amniota - ps25
Mammalia - ps26
Theria - ps27
Placentalia - ps28
Boreoeutheria - ps29
Euarchontoglires - ps30
Euarchonta - ps31
Primates - ps32
Haplorrhini - ps33
Simiiformes - ps34
Catarrhini - ps35
Hominoidea - ps36
Hominidae - ps37
Homininae - ps38
Hominini - ps39
Homo_sapiens - ps40
p-value
3e-02
3e-02
4e-02
5e-02
7e-02
Log-odds
0.5
1.0
1.5
2.0
2.5
3.0
3.5
4.0

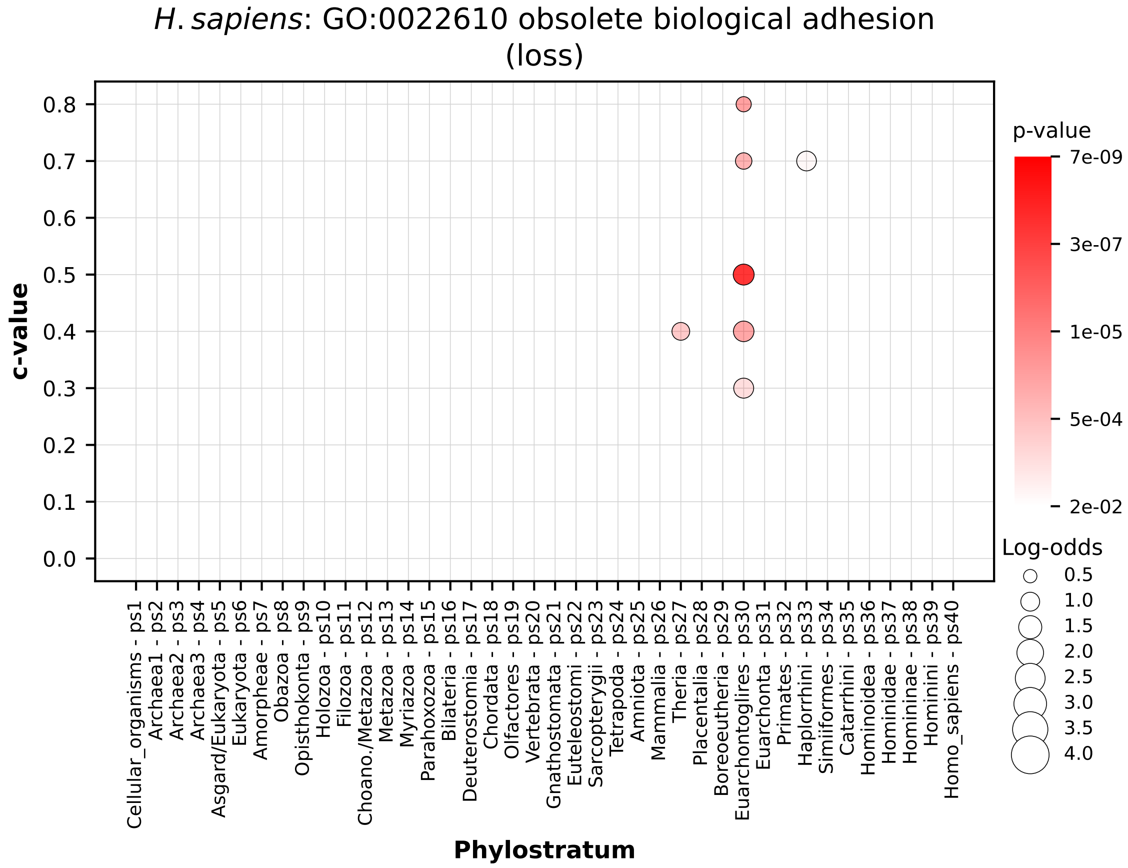
H. sapiens: GO:0022610 obsolete biological adhesion (loss)
c-value
0.0
0.1
0.2
0.3
0.4
0.5
0.6
0.7
0.8
Phylostratum
Cellular_organisms - ps1
Archaea1 - ps2
Archaea2 - ps3
Archaea3 - ps4
Asgard/Eukaryota - ps5
Eukaryota - ps6
Amorpheae - ps7
Obazoa - ps8
Opisthokonta - ps9
Holozoa - ps10
Filozoa - ps11
Choano./Metazoa - ps12
Metazoa - ps13
Myriazoa - ps14
Parahoxozoa - ps15
Bilateria - ps16
Deuterostomia - ps17
Chordata - ps18
Olfactores - ps19
Vertebrata - ps20
Gnathostomata - ps21
Euteleostomi - ps22
Sarcopterygii - ps23
Tetrapoda - ps24
Amniota - ps25
Mammalia - ps26
Theria - ps27
Placentalia - ps28
Boreoeutheria - ps29
Euarchontoglires - ps30
Euarchonta - ps31
Primates - ps32
Haplorrhini - ps33
Simiiformes - ps34
Catarrhini - ps35
Hominoidea - ps36
Hominidae - ps37
Homininae - ps38
Hominini - ps39
Homo_sapiens - ps40
p-value
7e-09
3e-07
1e-05
5e-04
2e-02
Log-odds
0.5
1.0
1.5
2.0
2.5
3.0
3.5
4.0

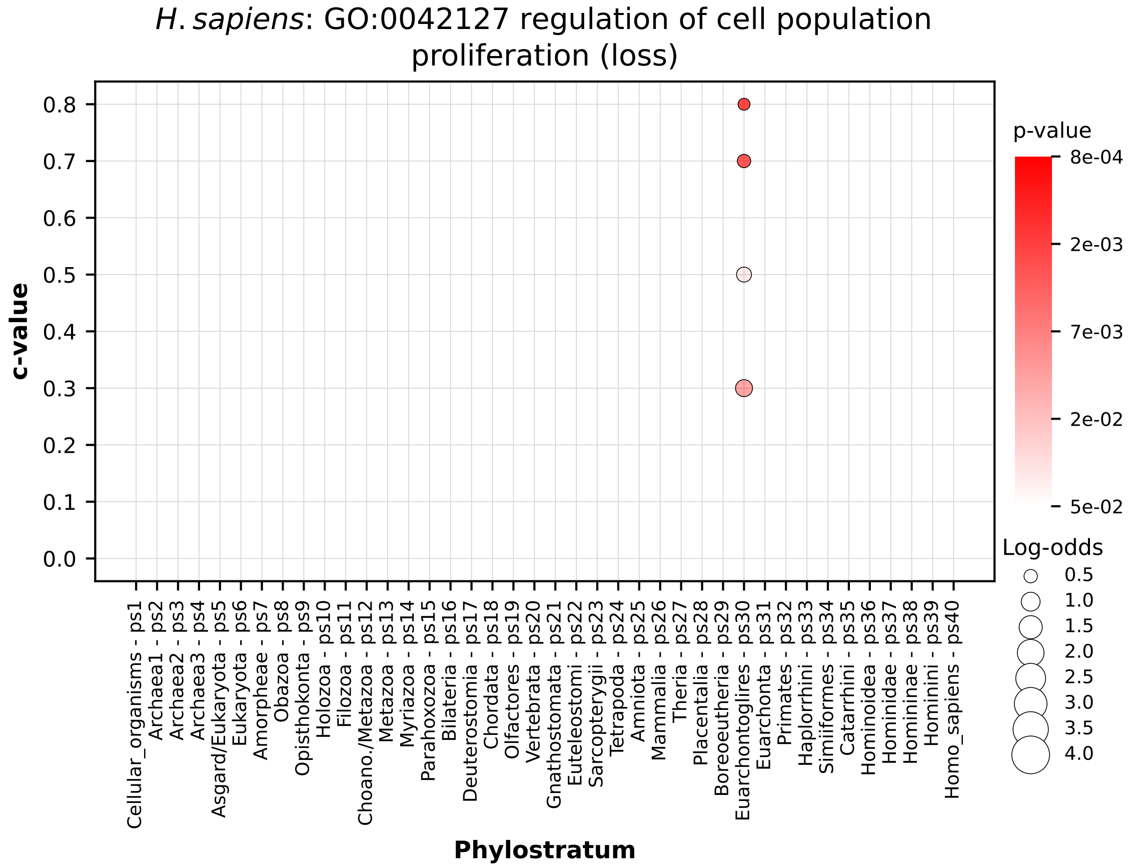

H. sapiens: GO:0042127 regulation of cell population proliferation (loss)
c-value
0.8
0.7
0.6
0.5
0.4
0.3
0.2
0.1
0.0
Phylostratum
Cellular_organisms - ps1
Archaea1 - ps2
Archaea2 - ps3
Archaea3 - ps4
Asgard/Eukaryota - ps5
Eukaryota - ps6
Amorpheae - ps7
Obazoa - ps8
Opisthokonta - ps9
Holozoa - ps10
Filozoa - ps11
Choano./Metazoa - ps12
Metazoa - ps13
Myriazoa - ps14
Parahoxozoa - ps15
Bilateria - ps16
Deuterostomia - ps17
Chordata - ps18
Olfactores - ps19
Vertebrata - ps20
Gnathostomata - ps21
Euteleostomi - ps22
Sarcopterygii - ps23
Tetrapoda - ps24
Amniota - ps25
Mammalia - ps26
Theria - ps27
Placentalia - ps28
Boreoeutheria - ps29
Euarchontoglires - ps30
Euarchonta - ps31
Primates - ps32
Haplorrhini - ps33
Simiiformes - ps34
Catarrhini - ps35
Hominoidea - ps36
Hominidae - ps37
Homininae - ps38
Hominini - ps39
Homo_sapiens - ps40
p-value
8e-04
2e-03
7e-03
2e-02
5e-02
Log-odds
0.5
1.0
1.5
2.0
2.5
3.0
3.5
4.0

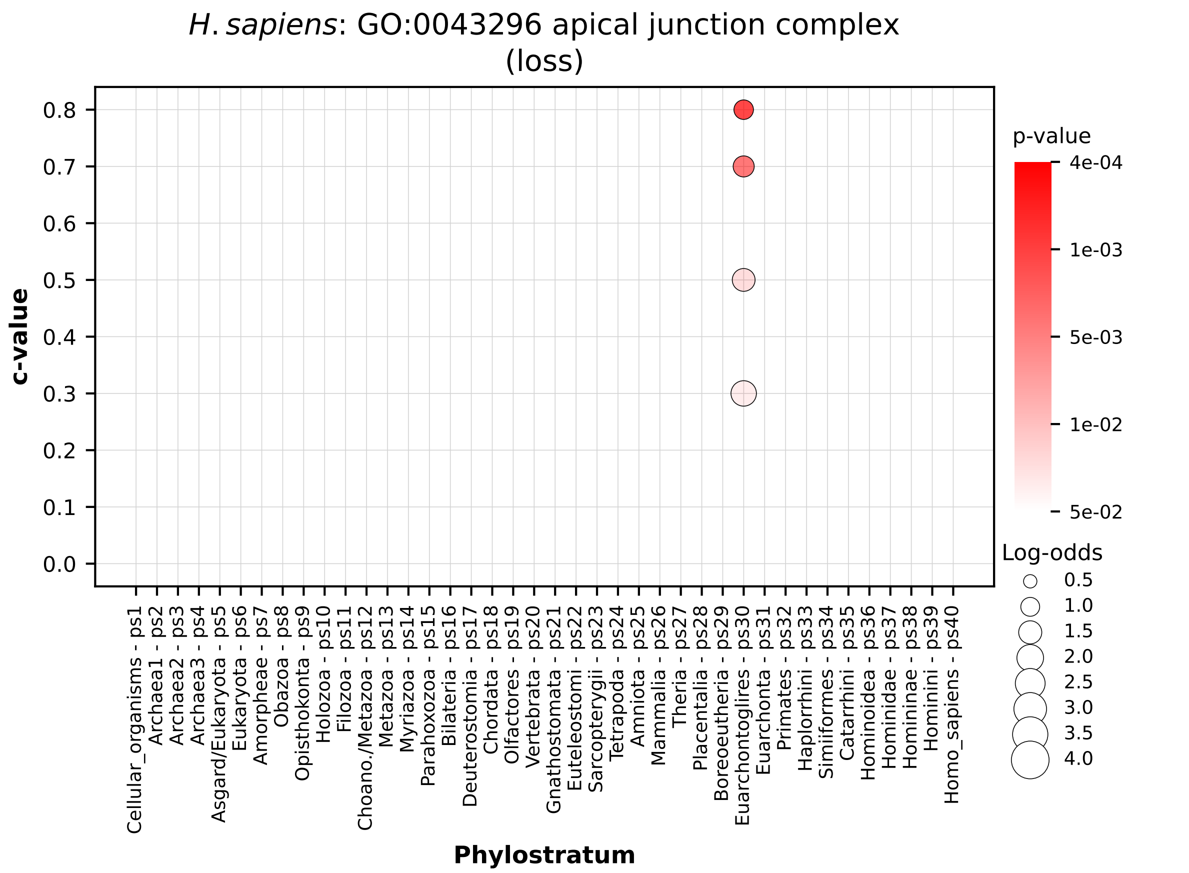
H. sapiens: GO:0043296 apical junction complex
(loss)
c-value
0.0
0.1
0.2
0.3
0.4
0.5
0.6
0.7
0.8
Phylostratum
Cellular_organisms - ps1
Archaea1 - ps2
Archaea2 - ps3
Archaea3 - ps4
Asgard/Eukaryota - ps5
Eukaryota - ps6
Amorpheae - ps7
Obazoa - ps8
Opisthokonta - ps9
Holozoa - ps10
Filozoa - ps11
Choano./Metazoa - ps12
Metazoa - ps13
Myriazoa - ps14
Parahoxozoa - ps15
Bilateria - ps16
Deuterostomia - ps17
Chordata - ps18
Olfactores - ps19
Vertebrata - ps20
Gnathostomata - ps21
Euteleostomi - ps22
Sarcopterygii - ps23
Tetrapoda - ps24
Amniota - ps25
Mammalia - ps26
Theria - ps27
Placentalia - ps28
Boreoeutheria - ps29
Euarchontoglires - ps30
Euarchonta - ps31
Primates - ps32
Haplorrhini - ps33
Simiiformes - ps34
Catarrhini - ps35
Hominoidea - ps36
Hominidae - ps37
Homininae - ps38
Hominini - ps39
Homo_sapiens - ps40
p-value
4e-04
1e-03
5e-03
1e-02
5e-02
Log-odds
0.5
1.0
1.5
2.0
2.5
3.0
3.5
4.0

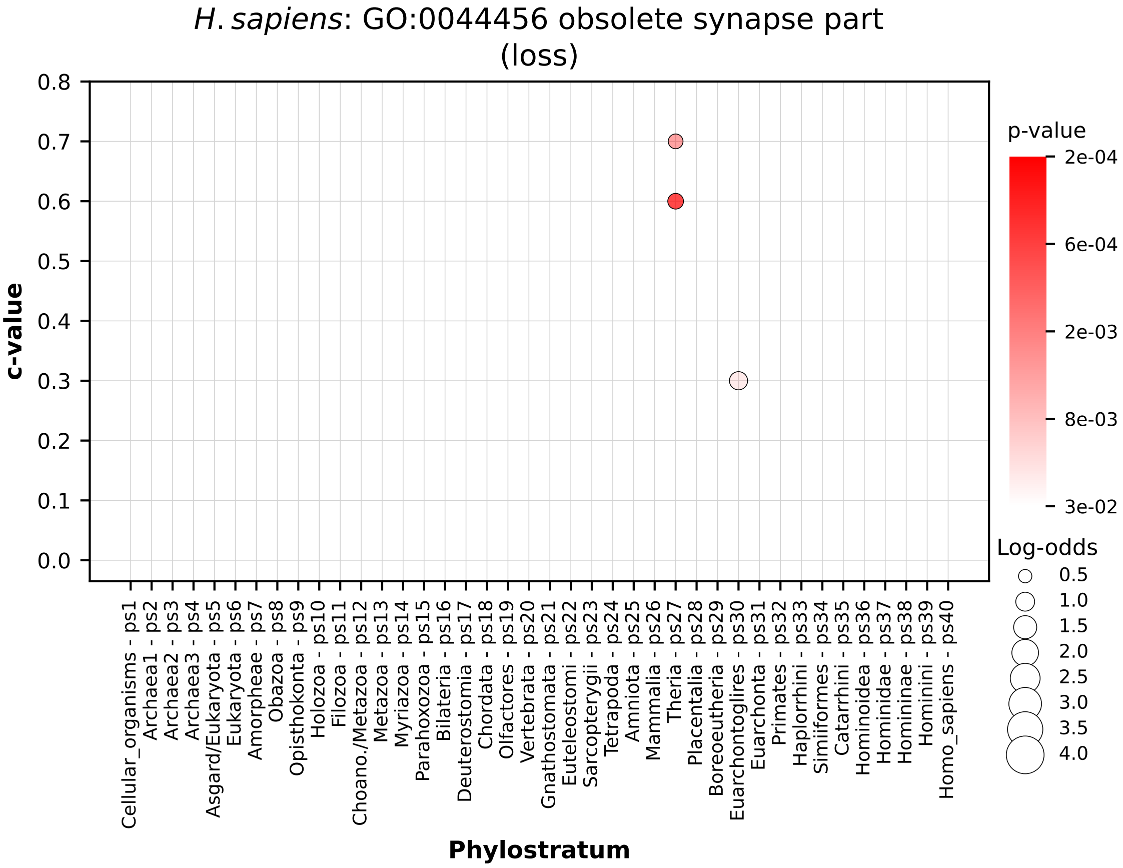

H. sapiens: GO:0044456 obsolete synapse part
(loss)
c-value
0.8
0.7
0.6
0.5
0.4
0.3
0.2
0.1
0.0
p-value
2e-04
6e-04
2e-03
8e-03
3e-02
Log-odds
0.5
1.0
1.5
2.0
2.5
3.0
3.5
4.0
Cellular_organisms - ps1
Archaea1 - ps2
Archaea2 - ps3
Archaea3 - ps4
Asgard/Eukaryota - ps5
Eukaryota - ps6
Amorpheae - ps7
Obazoa - ps8
Opisthokonta - ps9
Holozoa - ps10
Filozoa - ps11
Choano./Metazoa - ps12
Metazoa - ps13
Myriazoa - ps14
Parahoxozoa - ps15
Bilateria - ps16
Deuterostomia - ps17
Chordata - ps18
Olfactores - ps19
Vertebrata - ps20
Gnathostomata - ps21
Euteleostomi - ps22
Sarcopterygii - ps23
Tetrapoda - ps24
Amniota - ps25
Mammalia - ps26
Theria - ps27
Placentalia - ps28
Boreoeutheria - ps29
Euarchontoglires - ps30
Euarchonta - ps31
Primates - ps32
Haplorrhini - ps33
Simiiformes - ps34
Catarrhini - ps35
Hominoidea - ps36
Hominidae - ps37
Homininae - ps38
Hominini - ps39
Homo_sapiens - ps40
Phylostratum

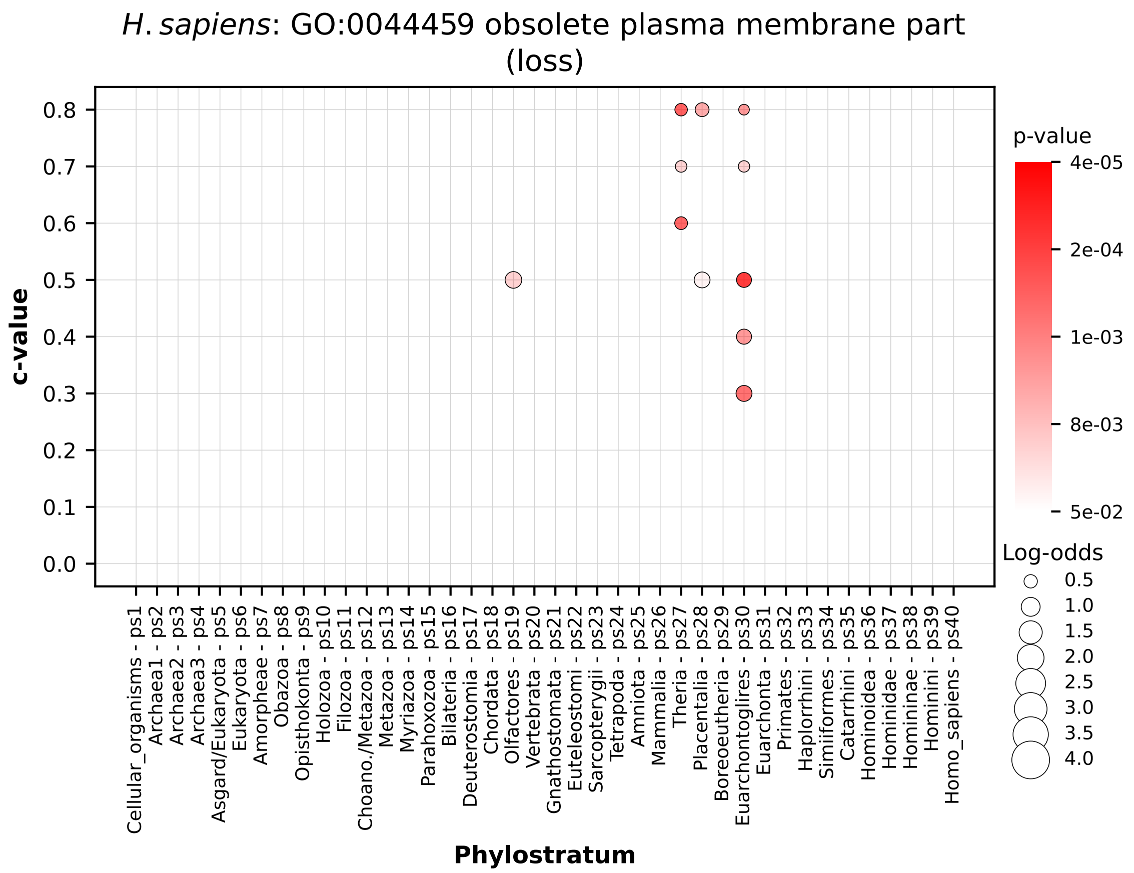

H. sapiens: GO:0044459 obsolete plasma membrane part (loss)
c-value
0.8
0.7
0.6
0.5
0.4
0.3
0.2
0.1
0.0
Phylostratum
Cellular_organisms - ps1
Archaea1 - ps2
Archaea2 - ps3
Archaea3 - ps4
Asgard/Eukaryota - ps5
Eukaryota - ps6
Amorpheae - ps7
Obazoa - ps8
Opisthokonta - ps9
Holozoa - ps10
Filozoa - ps11
Choano./Metazoa - ps12
Metazoa - ps13
Myriazoa - ps14
Parahoxozoa - ps15
Bilateria - ps16
Deuterostomia - ps17
Chordata - ps18
Olfactores - ps19
Vertebrata - ps20
Gnathostomata - ps21
Euteleostomi - ps22
Sarcopterygii - ps23
Tetrapoda - ps24
Amniota - ps25
Mammalia - ps26
Theria - ps27
Placentalia - ps28
Boreoeutheria - ps29
Euarchontoglires - ps30
Euarchonta - ps31
Primates - ps32
Haplorrhini - ps33
Simiiformes - ps34
Catarrhini - ps35
Hominoidea - ps36
Hominidae - ps37
Homininae - ps38
Hominini - ps39
Homo_sapiens - ps40
p-value
4e-05
2e-04
1e-03
8e-03
5e-02
Log-odds
0.5
1.0
1.5
2.0
2.5
3.0
3.5
4.0

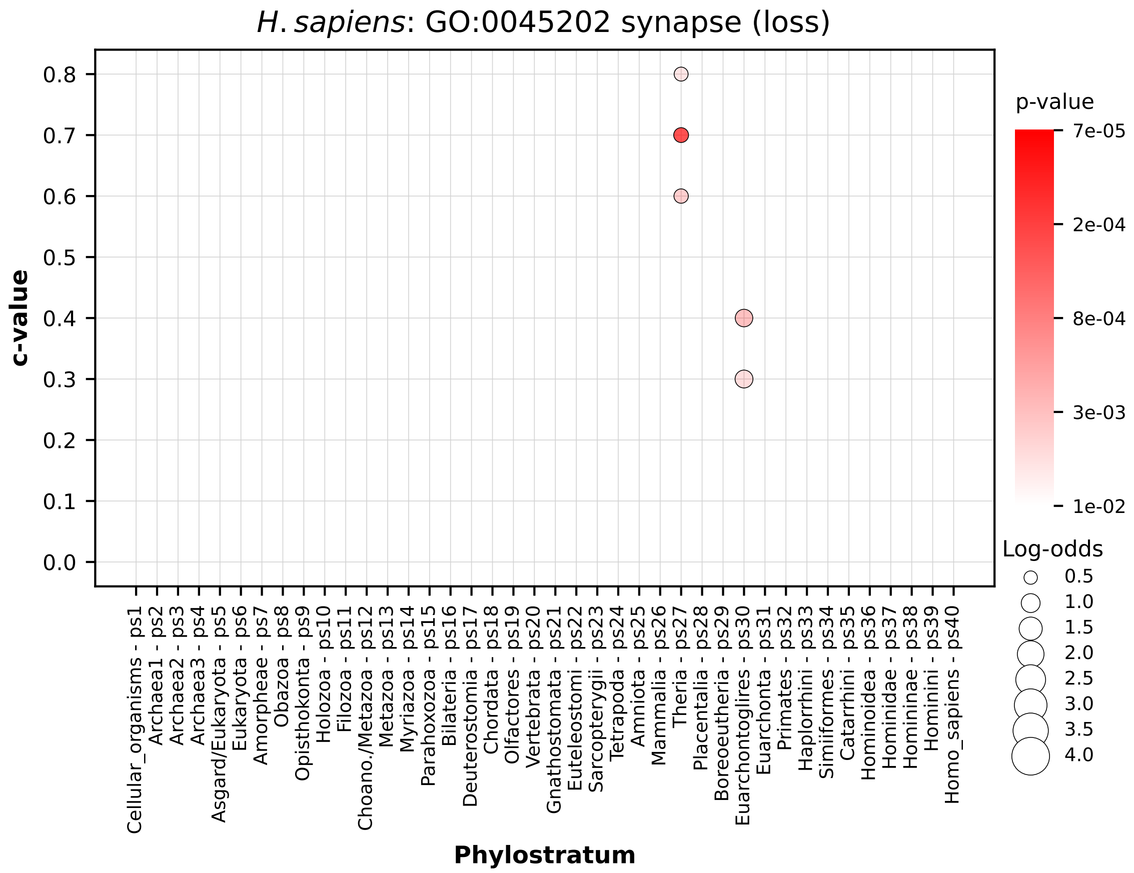

H. sapiens: GO:0045202 synapse (loss)
c-value
0.8
0.7
0.6
0.5
0.4
0.3
0.2
0.1
0.0
p-value
7e-05
2e-04
8e-04
3e-03
1e-02
Log-odds
0.5
1.0
1.5
2.0
2.5
3.0
3.5
4.0
Cellular_organisms - ps1
Archaea1 - ps2
Archaea2 - ps3
Archaea3 - ps4
Asgard/Eukaryota - ps5
Eukaryota - ps6
Amorpheae - ps7
Obazoa - ps8
Opisthokonta - ps9
Holozoa - ps10
Filozoa - ps11
Choano./Metazoa - ps12
Metazoa - ps13
Myriazoa - ps14
Parahoxozoa - ps15
Bilateria - ps16
Deuterostomia - ps17
Chordata - ps18
Olfactores - ps19
Vertebrata - ps20
Gnathostomata - ps21
Euteleostomi - ps22
Sarcopterygii - ps23
Tetrapoda - ps24
Amniota - ps25
Mammalia - ps26
Theria - ps27
Placentalia - ps28
Boreoeutheria - ps29
Euarchontoglires - ps30
Euarchonta - ps31
Primates - ps32
Haplorrhini - ps33
Simiiformes - ps34
Catarrhini - ps35
Hominoidea - ps36
Hominidae - ps37
Homininae - ps38
Hominini - ps39
Homo_sapiens - ps40
Phylostratum

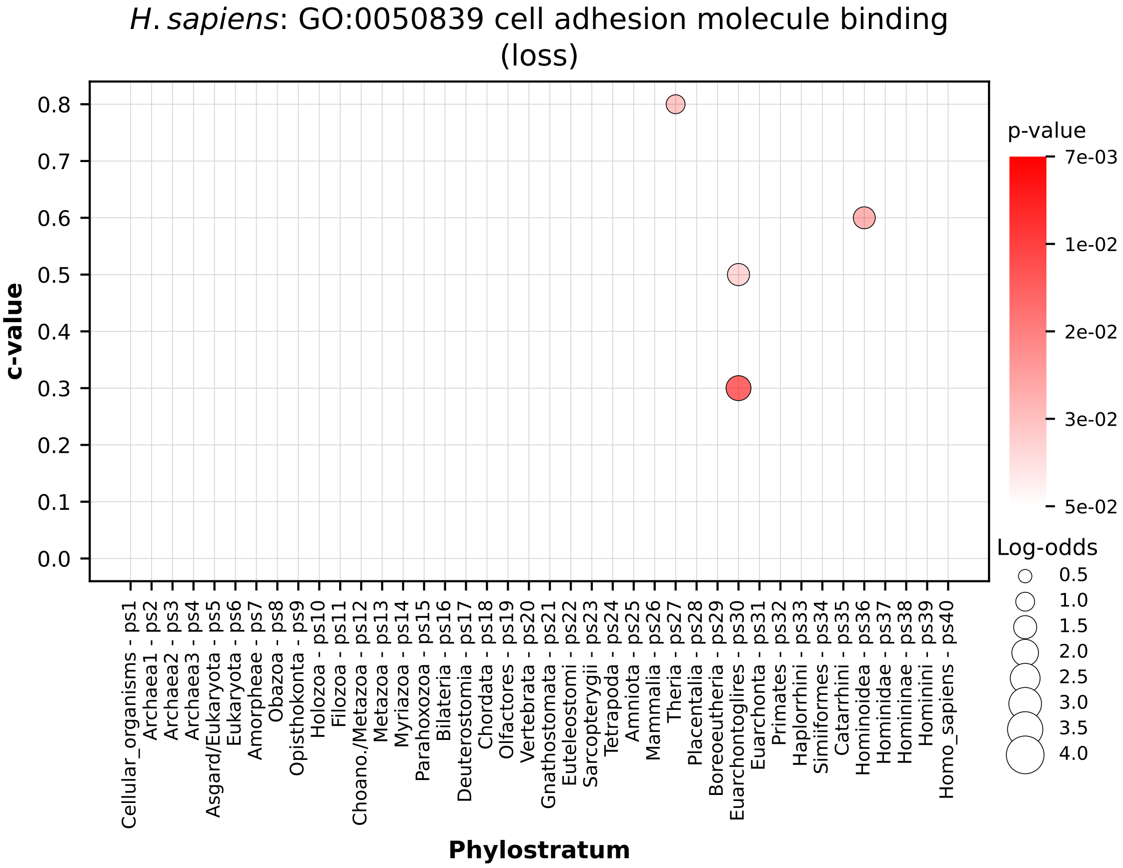

H. sapiens: GO:0050839 cell adhesion molecule binding
(loss)
c-value
0.8
0.7
0.6
0.5
0.4
0.3
0.2
0.1
0.0
p-value
7e-03
1e-02
2e-02
3e-02
5e-02
Log-odds
0.5
1.0
1.5
2.0
2.5
3.0
3.5
4.0
Cellular_organisms - ps1
Archaea1 - ps2
Archaea2 - ps3
Archaea3 - ps4
Asgard/Eukaryota - ps5
Eukaryota - ps6
Amorpheae - ps7
Obazoa - ps8
Opisthokonta - ps9
Holozoa - ps10
Filozoa - ps11
Choano./Metazoa - ps12
Metazoa - ps13
Myriazoa - ps14
Parahoxozoa - ps15
Bilateria - ps16
Deuterostomia - ps17
Chordata - ps18
Olfactores - ps19
Vertebrata - ps20
Gnathostomata - ps21
Euteleostomi - ps22
Sarcopterygii - ps23
Tetrapoda - ps24
Amniota - ps25
Mammalia - ps26
Theria - ps27
Placentalia - ps28
Boreoeutheria - ps29
Euarchontoglires - ps30
Euarchonta - ps31
Primates - ps32
Haplorrhini - ps33
Simiiformes - ps34
Catarrhini - ps35
Hominoidea - ps36
Hominidae - ps37
Homininae - ps38
Hominini - ps39
Homo_sapiens - ps40
Phylostratum

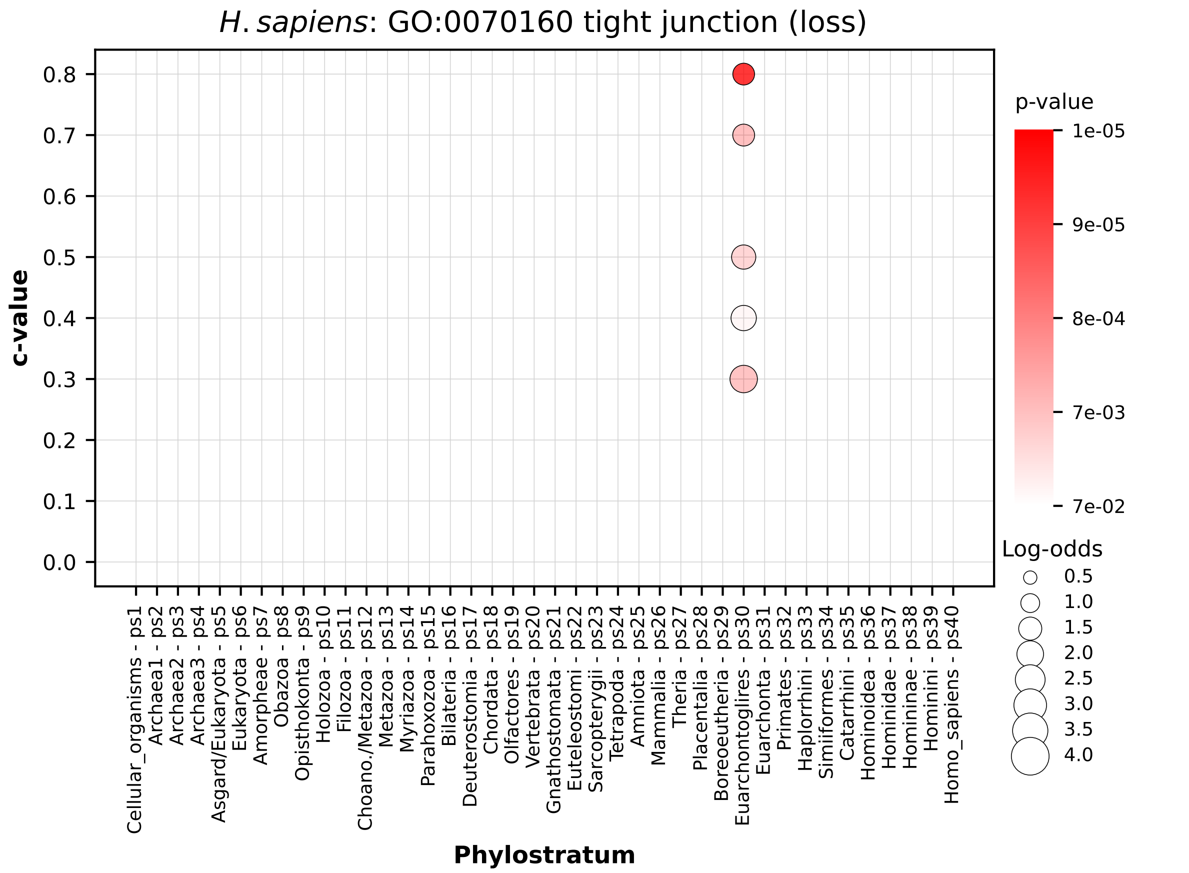

H. sapiens: GO:0070160 tight junction (loss)
c-value
0.0
0.1
0.2
0.3
0.4
0.5
0.6
0.7
0.8
Phylostratum
Cellular_organisms - ps1
Archaea1 - ps2
Archaea2 - ps3
Archaea3 - ps4
Asgard/Eukaryota - ps5
Eukaryota - ps6
Amorpheae - ps7
Obazoa - ps8
Opisthokonta - ps9
Holozoa - ps10
Filozoa - ps11
Choano./Metazoa - ps12
Metazoa - ps13
Myriazoa - ps14
Parahoxozoa - ps15
Bilateria - ps16
Deuterostomia - ps17
Chordata - ps18
Olfactores - ps19
Vertebrata - ps20
Gnathostomata - ps21
Euteleostomi - ps22
Sarcopterygii - ps23
Tetrapoda - ps24
Amniota - ps25
Mammalia - ps26
Theria - ps27
Placentalia - ps28
Boreoeutheria - ps29
Euarchontoglires - ps30
Euarchonta - ps31
Primates - ps32
Haplorrhini - ps33
Simiiformes - ps34
Catarrhini - ps35
Hominoidea - ps36
Hominidae - ps37
Homininae - ps38
Hominini - ps39
Homo_sapiens - ps40
p-value
1e-05
9e-05
8e-04
7e-03
7e-02
Log-odds
0.5
1.0
1.5
2.0
2.5
3.0
3.5
4.0

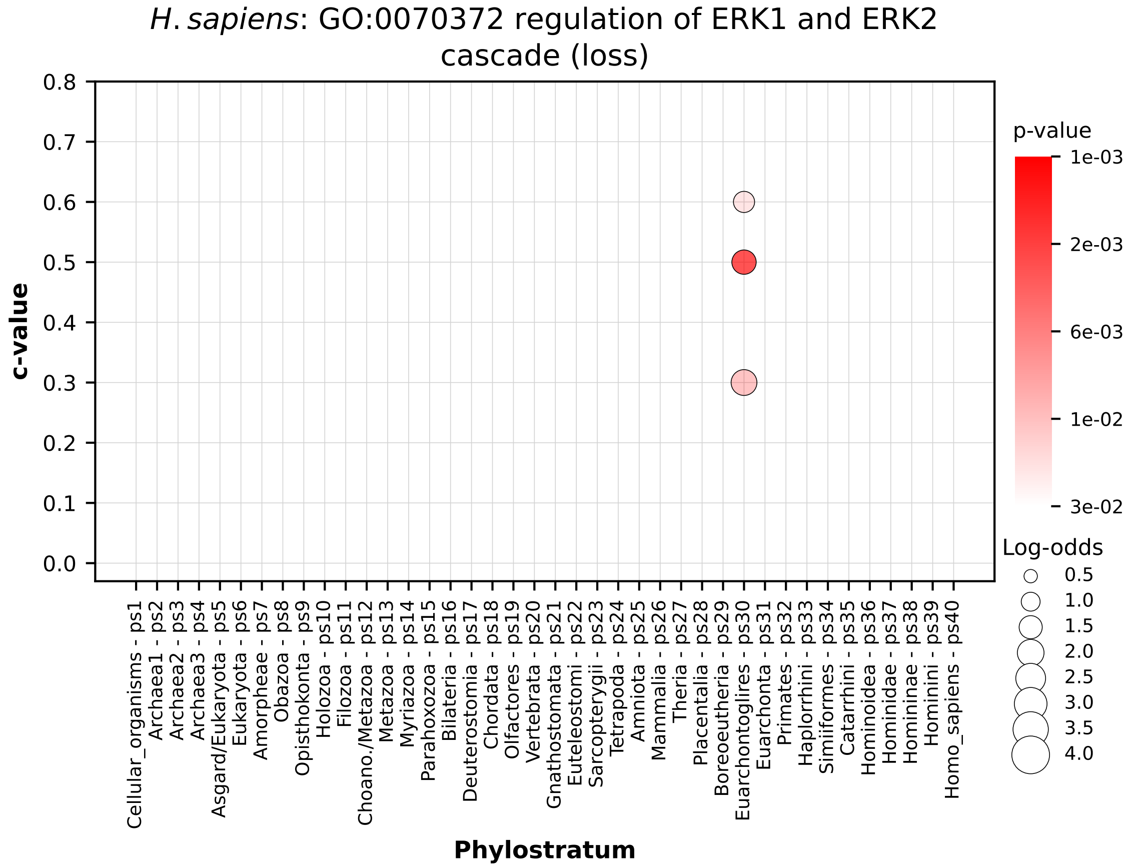

H. sapiens: GO:0070372 regulation of ERK1 and ERK2 cascade (loss)
c-value
0.8
0.7
0.6
0.5
0.4
0.3
0.2
0.1
0.0
p-value
1e-03
2e-03
6e-03
1e-02
3e-02
Log-odds
0.5
1.0
1.5
2.0
2.5
3.0
3.5
4.0
Cellular_organisms - ps1
Archaea1 - ps2
Archaea2 - ps3
Archaea3 - ps4
Asgard/Eukaryota - ps5
Eukaryota - ps6
Amorpheae - ps7
Obazoa - ps8
Opisthokonta - ps9
Holozoa - ps10
Filozoa - ps11
Choano./Metazoa - ps12
Metazoa - ps13
Myriazoa - ps14
Parahoxozoa - ps15
Bilateria - ps16
Deuterostomia - ps17
Chordata - ps18
Olfactores - ps19
Vertebrata - ps20
Gnathostomata - ps21
Euteleostomi - ps22
Sarcopterygii - ps23
Tetrapoda - ps24
Amniota - ps25
Mammalia - ps26
Theria - ps27
Placentalia - ps28
Boreoeutheria - ps29
Euarchontoglires - ps30
Euarchonta - ps31
Primates - ps32
Haplorrhini - ps33
Simiiformes - ps34
Catarrhini - ps35
Hominoidea - ps36
Hominidae - ps37
Homininae - ps38
Hominini - ps39
Homo_sapiens - ps40
Phylostratum

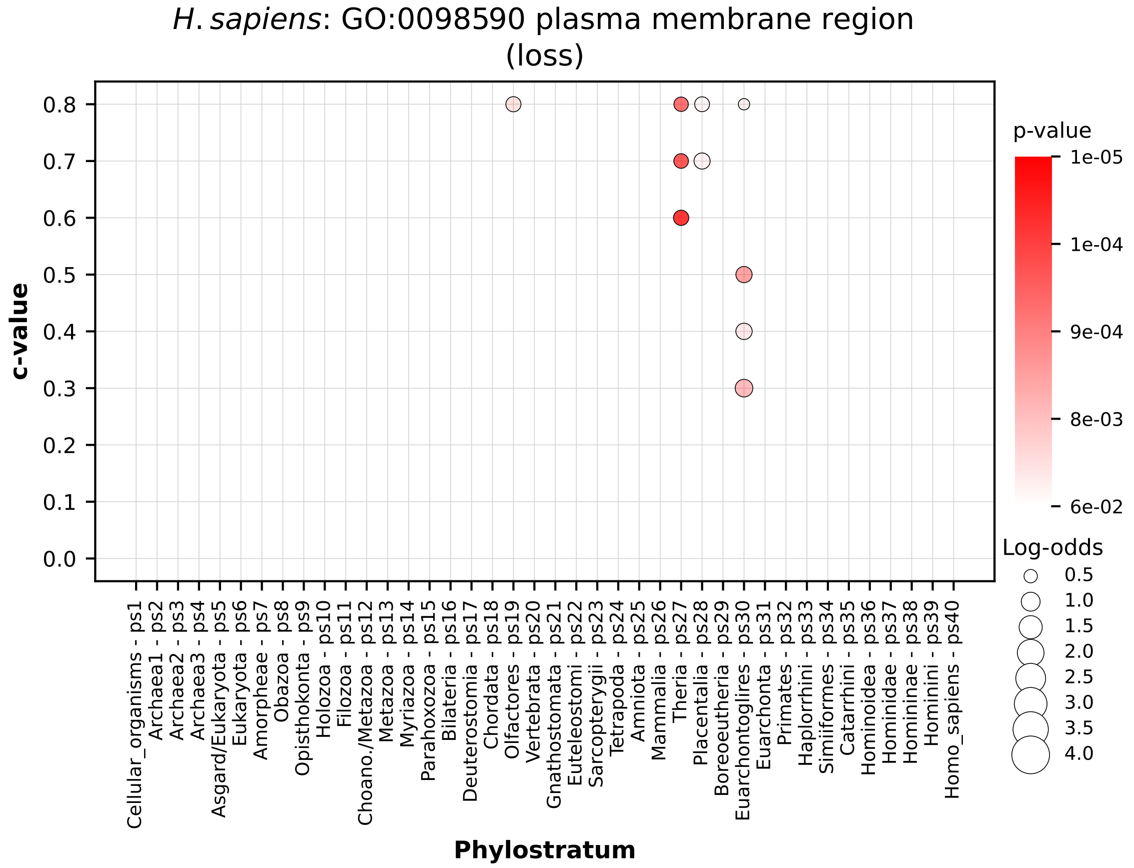
H. sapiens: GO:0098590 plasma membrane region
(loss)
c-value
0.0
0.1
0.2
0.3
0.4
0.5
0.6
0.7
0.8
Phylostratum
Cellular_organisms - ps1
Archaea1 - ps2
Archaea2 - ps3
Archaea3 - ps4
Asgard/Eukaryota - ps5
Eukaryota - ps6
Amorpheae - ps7
Obazoa - ps8
Opisthokonta - ps9
Holozoa - ps10
Filozoa - ps11
Choano./Metazoa - ps12
Metazoa - ps13
Myriazoa - ps14
Parahoxozoa - ps15
Bilateria - ps16
Deuterostomia - ps17
Chordata - ps18
Olfactores - ps19
Vertebrata - ps20
Gnathostomata - ps21
Euteleostomi - ps22
Sarcopterygii - ps23
Tetrapoda - ps24
Amniota - ps25
Mammalia - ps26
Theria - ps27
Placentalia - ps28
Boreoeutheria - ps29
Euarchontoglires - ps30
Euarchonta - ps31
Primates - ps32
Haplorrhini - ps33
Simiiformes - ps34
Catarrhini - ps35
Hominoidea - ps36
Hominidae - ps37
Homininae - ps38
Hominini - ps39
Homo_sapiens - ps40
p-value
1e-05
1e-04
9e-04
8e-03
6e-02
Log-odds
0.5
1.0
1.5
2.0
2.5
3.0
3.5
4.0

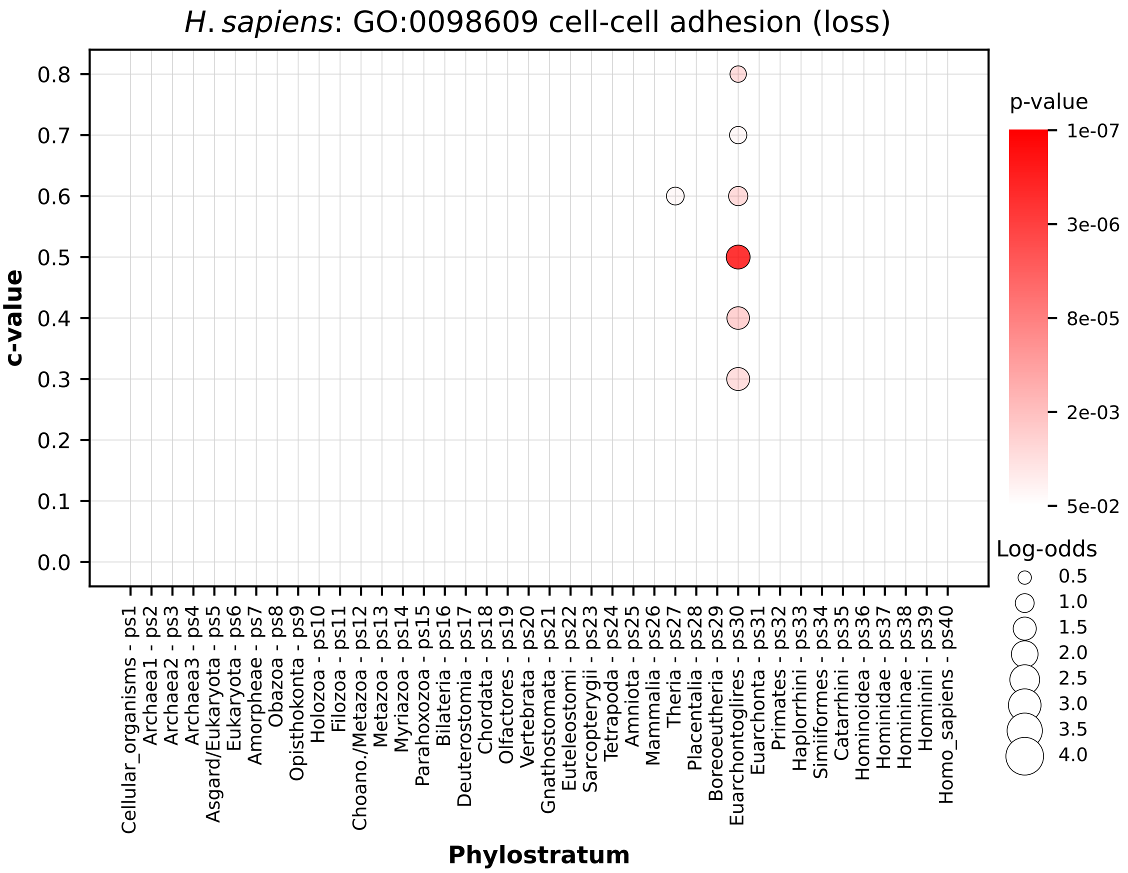
H. sapiens: GO:0098609 cell-cell adhesion (loss)
c-value
0.8
0.7
0.6
0.5
0.4
0.3
0.2
0.1
0.0
Phylostratum
Cellular_organisms - ps1
Archaea1 - ps2
Archaea2 - ps3
Archaea3 - ps4
Asgard/Eukaryota - ps5
Eukaryota - ps6
Amorpheae - ps7
Obazoa - ps8
Opisthokonta - ps9
Holozoa - ps10
Filozoa - ps11
Choano./Metazoa - ps12
Metazoa - ps13
Myriazoa - ps14
Parahoxozoa - ps15
Bilateria - ps16
Deuterostomia - ps17
Chordata - ps18
Olfactores - ps19
Vertebrata - ps20
Gnathostomata - ps21
Euteleostomi - ps22
Sarcopterygii - ps23
Tetrapoda - ps24
Amniota - ps25
Mammalia - ps26
Theria - ps27
Placentalia - ps28
Boreoeutheria - ps29
Euarchontoglires - ps30
Euarchonta - ps31
Primates - ps32
Haplorrhini - ps33
Simiiformes - ps34
Catarrhini - ps35
Hominoidea - ps36
Hominidae - ps37
Homininae - ps38
Hominini - ps39
Homo_sapiens - ps40
p-value
1e-07
3e-06
8e-05
2e-03
5e-02
Log-odds
0.5
1.0
1.5
2.0
2.5
3.0
3.5
4.0

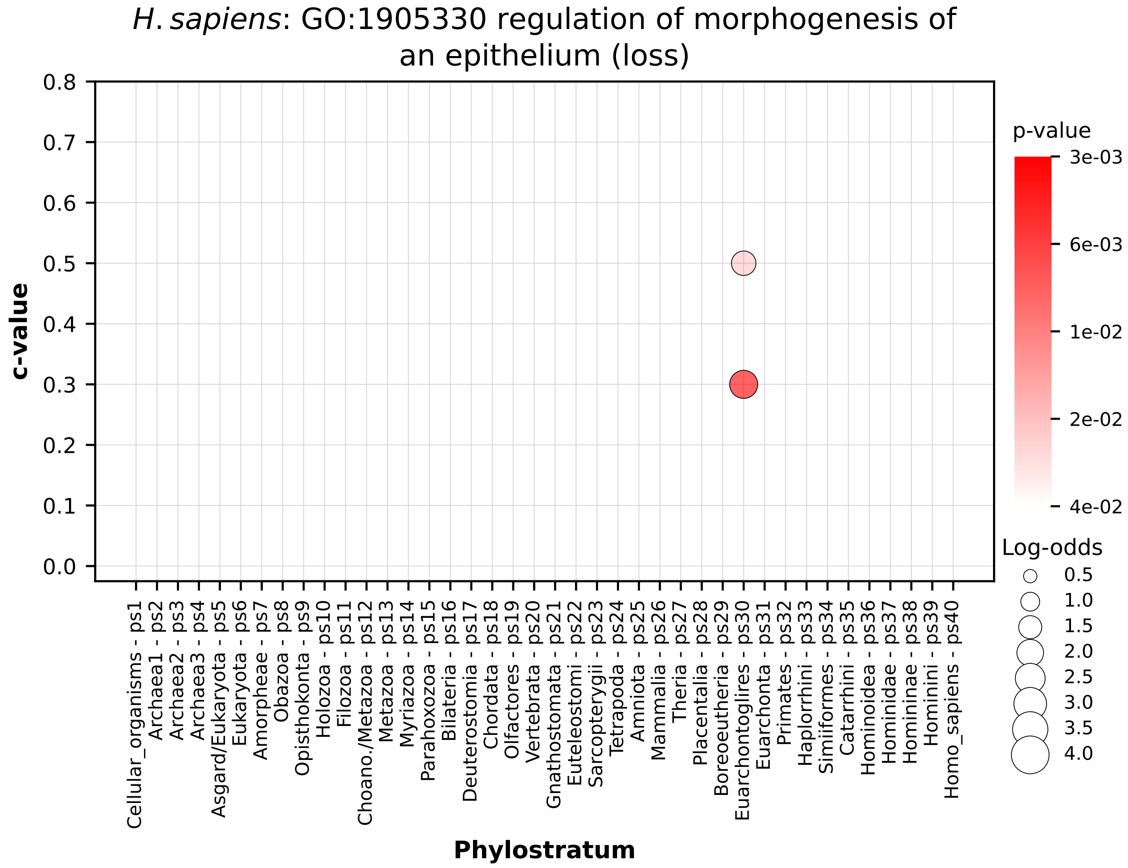

H. sapiens: GO:1905330 regulation of morphogenesis of an epithelium (loss)
c-value
0.0
0.1
0.2
0.3
0.4
0.5
0.6
0.7
0.8
p-value
3e-03
6e-03
1e-02
2e-02
4e-02
Log-odds
0.5
1.0
1.5
2.0
2.5
3.0
3.5
4.0
Cellular_organisms - ps1
Archaea1 - ps2
Archaea2 - ps3
Archaea3 - ps4
Asgard/Eukaryota - ps5
Eukaryota - ps6
Amorpheae - ps7
Obazoa - ps8
Opisthokonta - ps9
Holozoa - ps10
Filozoa - ps11
Choano./Metazoa - ps12
Metazoa - ps13
Myriazoa - ps14
Parahoxozoa - ps15
Bilateria - ps16
Deuterostomia - ps17
Chordata - ps18
Olfactores - ps19
Vertebrata - ps20
Gnathostomata - ps21
Euteleostomi - ps22
Sarcopterygii - ps23
Tetrapoda - ps24
Amniota - ps25
Mammalia - ps26
Theria - ps27
Placentalia - ps28
Boreoeutheria - ps29
Euarchontoglires - ps30
Euarchonta - ps31
Primates - ps32
Haplorrhini - ps33
Simiiformes - ps34
Catarrhini - ps35
Hominoidea - ps36
Hominidae - ps37
Homininae - ps38
Hominini - ps39
Homo_sapiens - ps40
Phylostratum

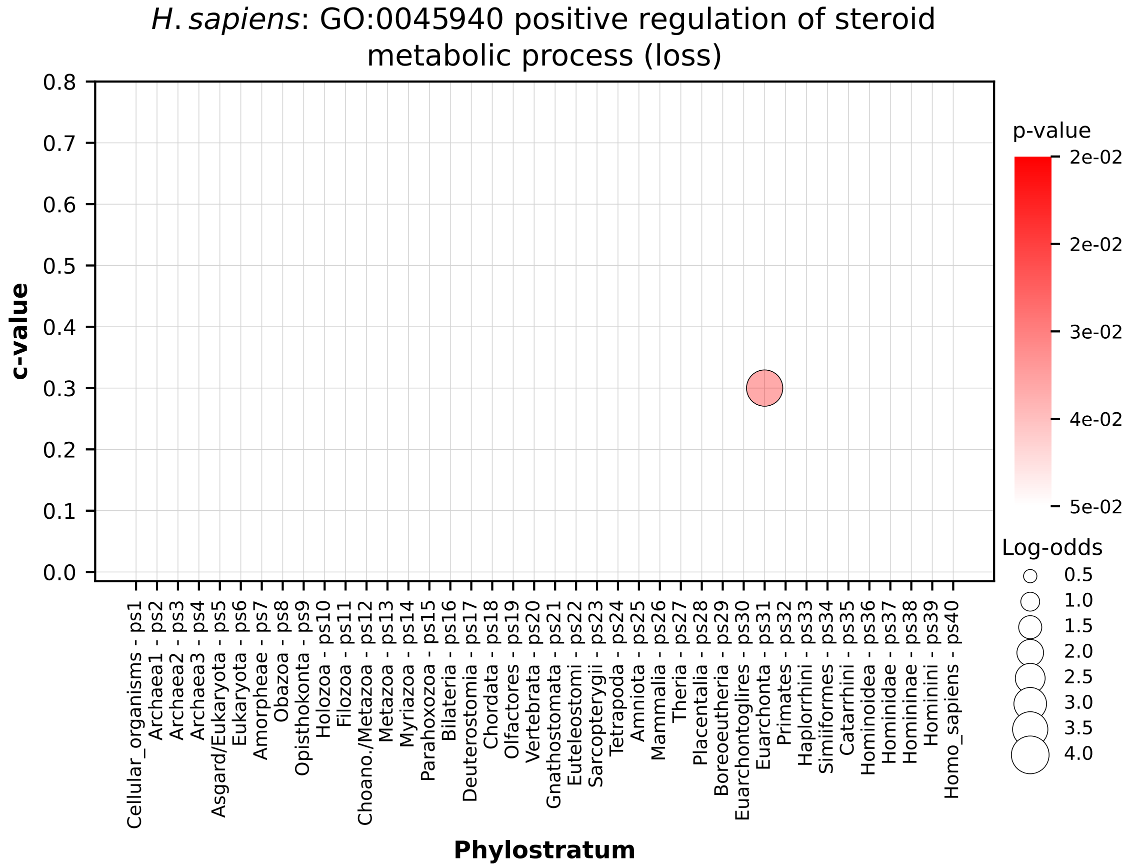

H. sapiens: GO:0045940 positive regulation of steroid metabolic process (loss)
c-value
0.8
0.7
0.6
0.5
0.4
0.3
0.2
0.1
0.0
Phylostratum
Cellular_organisms - ps1
Archaea1 - ps2
Archaea2 - ps3
Archaea3 - ps4
Asgard/Eukaryota - ps5
Eukaryota - ps6
Amorpheae - ps7
Obazoa - ps8
Opisthokonta - ps9
Holozoa - ps10
Filozoa - ps11
Choano./Metazoa - ps12
Metazoa - ps13
Myriazoa - ps14
Parahoxozoa - ps15
Bilateria - ps16
Deuterostomia - ps17
Chordata - ps18
Olfactores - ps19
Vertebrata - ps20
Gnathostomata - ps21
Euteleostomi - ps22
Sarcopterygii - ps23
Tetrapoda - ps24
Amniota - ps25
Mammalia - ps26
Theria - ps27
Placentalia - ps28
Boreoeutheria - ps29
Euarchontoglires - ps30
Euarchonta - ps31
Primates - ps32
Haplorrhini - ps33
Simiiformes - ps34
Catarrhini - ps35
Hominoidea - ps36
Hominidae - ps37
Homininae - ps38
Hominini - ps39
Homo_sapiens - ps40
p-value
2e-02
2e-02
3e-02
4e-02
5e-02
Log-odds
0.5
1.0
1.5
2.0
2.5
3.0
3.5
4.0

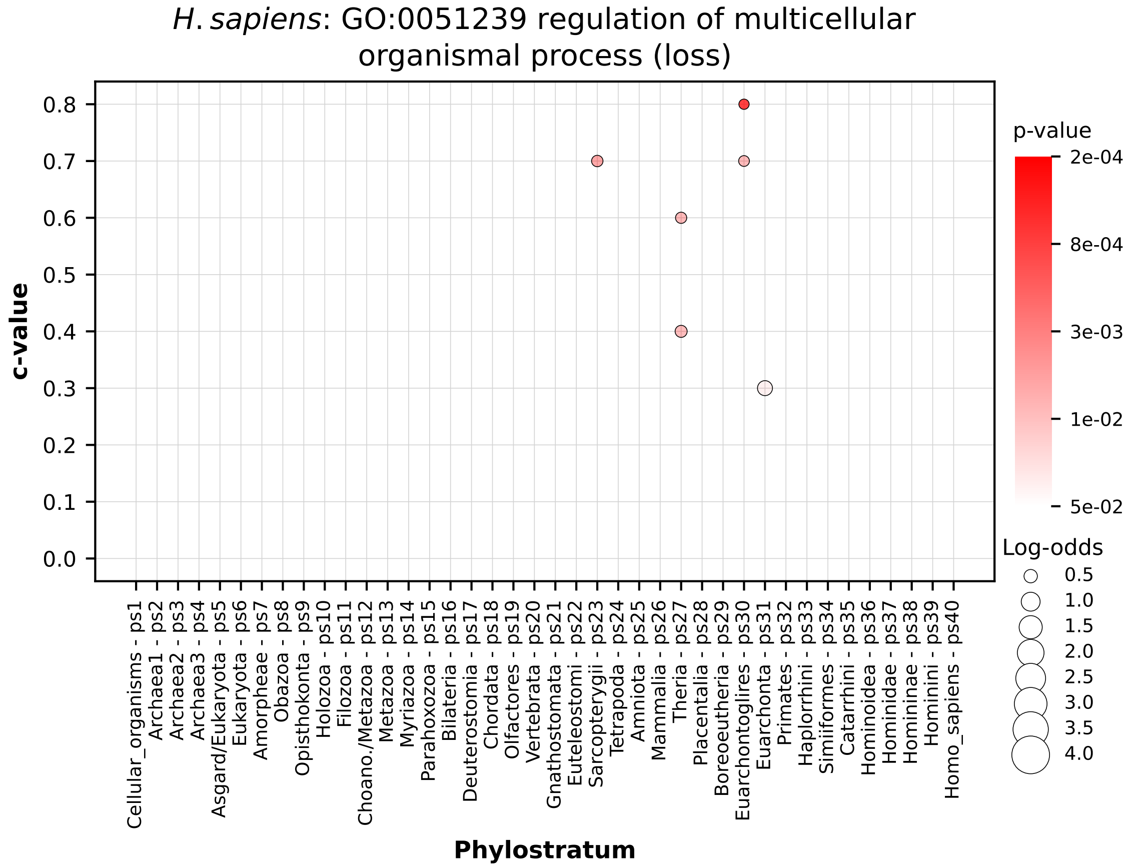

H. sapiens: GO:0051239 regulation of multicellular organismal process (loss)
c-value
0.0
0.1
0.2
0.3
0.4
0.5
0.6
0.7
0.8
Phylostratum
Cellular_organisms - ps1
Archaea1 - ps2
Archaea2 - ps3
Archaea3 - ps4
Asgard/Eukaryota - ps5
Eukaryota - ps6
Amorpheae - ps7
Obazoa - ps8
Opisthokonta - ps9
Holozoa - ps10
Filozoa - ps11
Choano./Metazoa - ps12
Metazoa - ps13
Myriazoa - ps14
Parahoxozoa - ps15
Bilateria - ps16
Deuterostomia - ps17
Chordata - ps18
Olfactores - ps19
Vertebrata - ps20
Gnathostomata - ps21
Euteleostomi - ps22
Sarcopterygii - ps23
Tetrapoda - ps24
Amniota - ps25
Mammalia - ps26
Theria - ps27
Placentalia - ps28
Boreoeutheria - ps29
Euarchontoglires - ps30
Euarchonta - ps31
Primates - ps32
Haplorrhini - ps33
Simiiformes - ps34
Catarrhini - ps35
Hominoidea - ps36
Hominidae - ps37
Homininae - ps38
Hominini - ps39
Homo_sapiens - ps40
p-value
2e-04
8e-04
3e-03
1e-02
5e-02
Log-odds
0.5
1.0
1.5
2.0
2.5
3.0
3.5
4.0

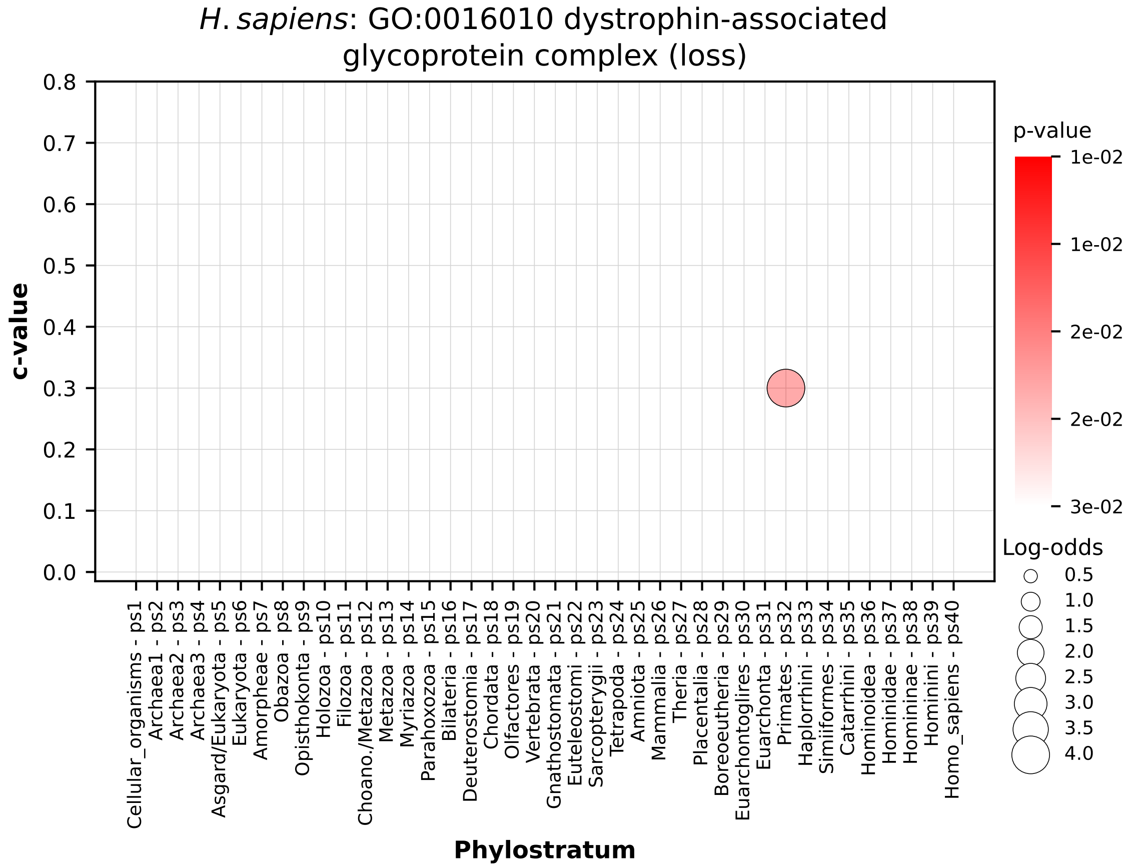

H. sapiens: GO:0016010 dystrophin-associated glycoprotein complex (loss)
c-value
0.8
0.7
0.6
0.5
0.4
0.3
0.2
0.1
0.0
Phylostratum
Cellular_organisms - ps1
Archaea1 - ps2
Archaea2 - ps3
Archaea3 - ps4
Asgard/Eukaryota - ps5
Eukaryota - ps6
Amorpheae - ps7
Obazoa - ps8
Opisthokonta - ps9
Holozoa - ps10
Filozoa - ps11
Choano./Metazoa - ps12
Metazoa - ps13
Myriazoa - ps14
Parahoxozoa - ps15
Bilateria - ps16
Deuterostomia - ps17
Chordata - ps18
Olfactores - ps19
Vertebrata - ps20
Gnathostomata - ps21
Euteleostomi - ps22
Sarcopterygii - ps23
Tetrapoda - ps24
Amniota - ps25
Mammalia - ps26
Theria - ps27
Placentalia - ps28
Boreoeutheria - ps29
Euarchontoglires - ps30
Euarchonta - ps31
Primates - ps32
Haplorrhini - ps33
Simiiformes - ps34
Catarrhini - ps35
Hominoidea - ps36
Hominidae - ps37
Homininae - ps38
Hominini - ps39
Homo_sapiens - ps40
p-value
1e-02
1e-02
2e-02
2e-02
3e-02
Log-odds
0.5
1.0
1.5
2.0
2.5
3.0
3.5
4.0

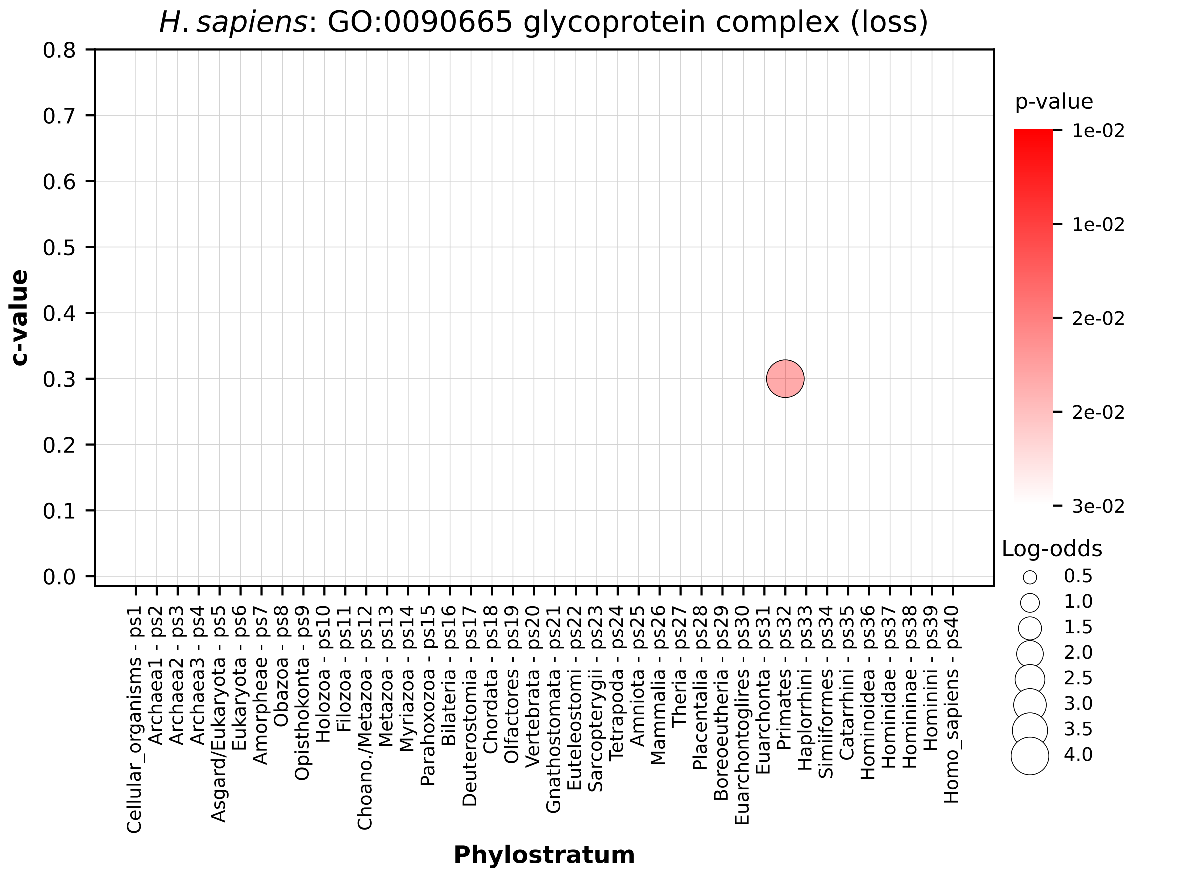

H. sapiens: GO:0090665 glycoprotein complex (loss)
c-value
0.8
0.7
0.6
0.5
0.4
0.3
0.2
0.1
0.0
Phylostratum
Cellular_organisms - ps1
Archaea1 - ps2
Archaea2 - ps3
Archaea3 - ps4
Asgard/Eukaryota - ps5
Eukaryota - ps6
Amorpheae - ps7
Obazoa - ps8
Opisthokonta - ps9
Holozoa - ps10
Filozoa - ps11
Choano./Metazoa - ps12
Metazoa - ps13
Myriazoa - ps14
Parahoxozoa - ps15
Bilateria - ps16
Deuterostomia - ps17
Chordata - ps18
Olfactores - ps19
Vertebrata - ps20
Gnathostomata - ps21
Euteleostomi - ps22
Sarcopterygii - ps23
Tetrapoda - ps24
Amniota - ps25
Mammalia - ps26
Theria - ps27
Placentalia - ps28
Boreoeutheria - ps29
Euarchontoglires - ps30
Euarchonta - ps31
Primates - ps32
Haplorrhini - ps33
Simiiformes - ps34
Catarrhini - ps35
Hominoidea - ps36
Hominidae - ps37
Homininae - ps38
Hominini - ps39
Homo_sapiens - ps40
p-value
1e-02
1e-02
2e-02
2e-02
3e-02
Log-odds
0.5
1.0
1.5
2.0
2.5
3.0
3.5
4.0

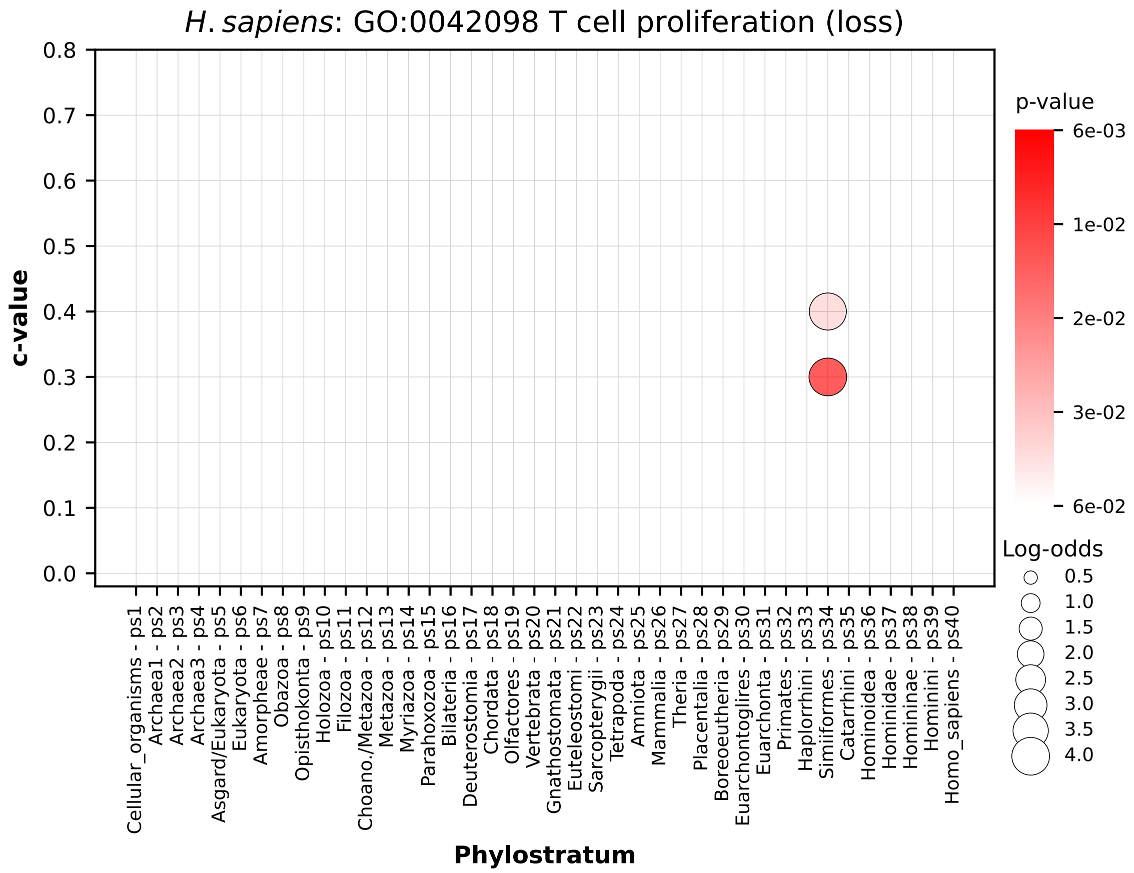
H. sapiens: GO:0042098 T cell proliferation (loss)
c-value
0.8
0.7
0.6
0.5
0.4
0.3
0.2
0.1
0.0
Phylostratum
Cellular_organisms - ps1
Archaea1 - ps2
Archaea2 - ps3
Archaea3 - ps4
Asgard/Eukaryota - ps5
Eukaryota - ps6
Amorpheae - ps7
Obazoa - ps8
Opisthokonta - ps9
Holozoa - ps10
Filozoa - ps11
Choano./Metazoa - ps12
Metazoa - ps13
Myriazoa - ps14
Parahoxozoa - ps15
Bilateria - ps16
Deuterostomia - ps17
Chordata - ps18
Olfactores - ps19
Vertebrata - ps20
Gnathostomata - ps21
Euteleostomi - ps22
Sarcopterygii - ps23
Tetrapoda - ps24
Amniota - ps25
Mammalia - ps26
Theria - ps27
Placentalia - ps28
Boreoeutheria - ps29
Euarchontoglires - ps30
Euarchonta - ps31
Primates - ps32
Haplorrhini - ps33
Simiiformes - ps34
Catarrhini - ps35
Hominoidea - ps36
Hominidae - ps37
Homininae - ps38
Hominini - ps39
Homo_sapiens - ps40
p-value
6e-03
1e-02
2e-02
3e-02
6e-02
Log-odds
0.5
1.0
1.5
2.0
2.5
3.0
3.5
4.0

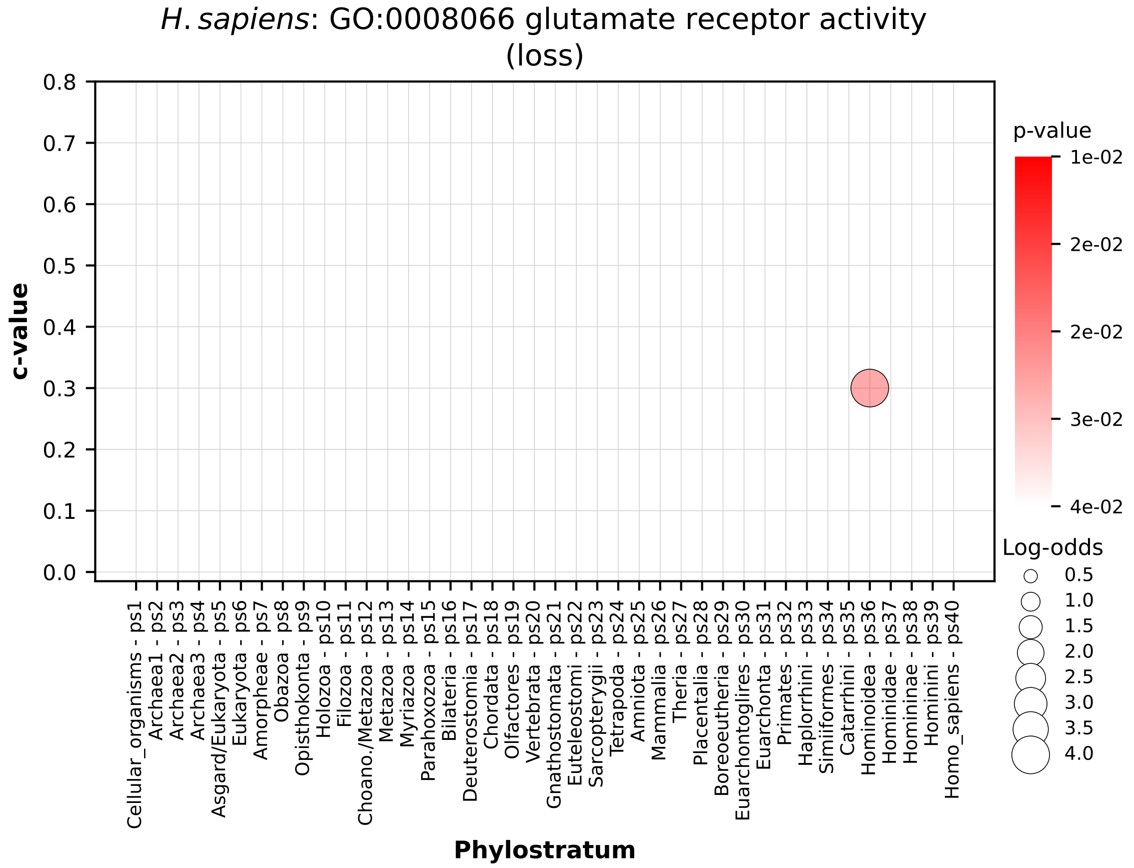

H. sapiens: GO:0008066 glutamate receptor activity
(loss)
c-value
0.8
0.7
0.6
0.5
0.4
0.3
0.2
0.1
0.0
Cellular_organisms - ps1
Archaea1 - ps2
Archaea2 - ps3
Archaea3 - ps4
Asgard/Eukaryota - ps5
Eukaryota - ps6
Amorpheae - ps7
Obazoa - ps8
Opisthokonta - ps9
Holozoa - ps10
Filozoa - ps11
Choano./Metazoa - ps12
Metazoa - ps13
Myriazoa - ps14
Parahoxozoa - ps15
Bilateria - ps16
Deuterostomia - ps17
Chordata - ps18
Olfactores - ps19
Vertebrata - ps20
Gnathostomata - ps21
Euteleostomi - ps22
Sarcopterygii - ps23
Tetrapoda - ps24
Amniota - ps25
Mammalia - ps26
Theria - ps27
Placentalia - ps28
Boreoeutheria - ps29
Euarchontoglires - ps30
Euarchonta - ps31
Primates - ps32
Haplorrhini - ps33
Simiiformes - ps34
Catarrhini - ps35
Hominoidea - ps36
Hominidae - ps37
Homininae - ps38
Hominini - ps39
Homo_sapiens - ps40
Phylostratum
p-value
1e-02
2e-02
2e-02
3e-02
4e-02
Log-odds
0.5
1.0
1.5
2.0
2.5
3.0
3.5
4.0

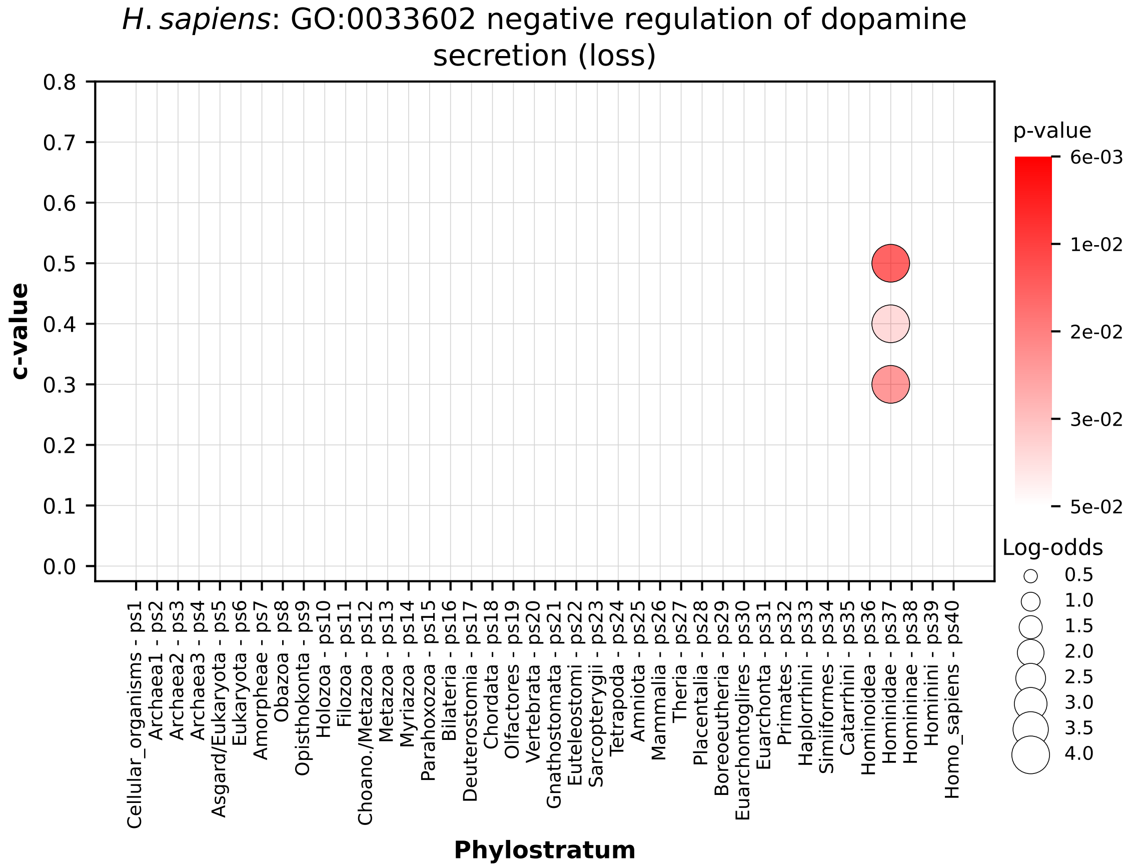

H. sapiens: GO:0033602 negative regulation of dopamine secretion (loss)
c-value
0.8
0.7
0.6
0.5
0.4
0.3
0.2
0.1
0.0
p-value
6e-03
1e-02
2e-02
3e-02
5e-02
Log-odds
0.5
1.0
1.5
2.0
2.5
3.0
3.5
4.0
Cellular_organisms - ps1
Archaea1 - ps2
Archaea2 - ps3
Archaea3 - ps4
Asgard/Eukaryota - ps5
Eukaryota - ps6
Amorpheae - ps7
Obazoa - ps8
Opisthokonta - ps9
Holozoa - ps10
Filozoa - ps11
Choano./Metazoa - ps12
Metazoa - ps13
Myriazoa - ps14
Parahoxozoa - ps15
Bilateria - ps16
Deuterostomia - ps17
Chordata - ps18
Olfactores - ps19
Vertebrata - ps20
Gnathostomata - ps21
Euteleostomi - ps22
Sarcopterygii - ps23
Tetrapoda - ps24
Amniota - ps25
Mammalia - ps26
Theria - ps27
Placentalia - ps28
Boreoeutheria - ps29
Euarchontoglires - ps30
Euarchonta - ps31
Primates - ps32
Haplorrhini - ps33
Simiiformes - ps34
Catarrhini - ps35
Hominoidea - ps36
Hominidae - ps37
Homininae - ps38
Hominini - ps39
Homo_sapiens - ps40
Phylostratum

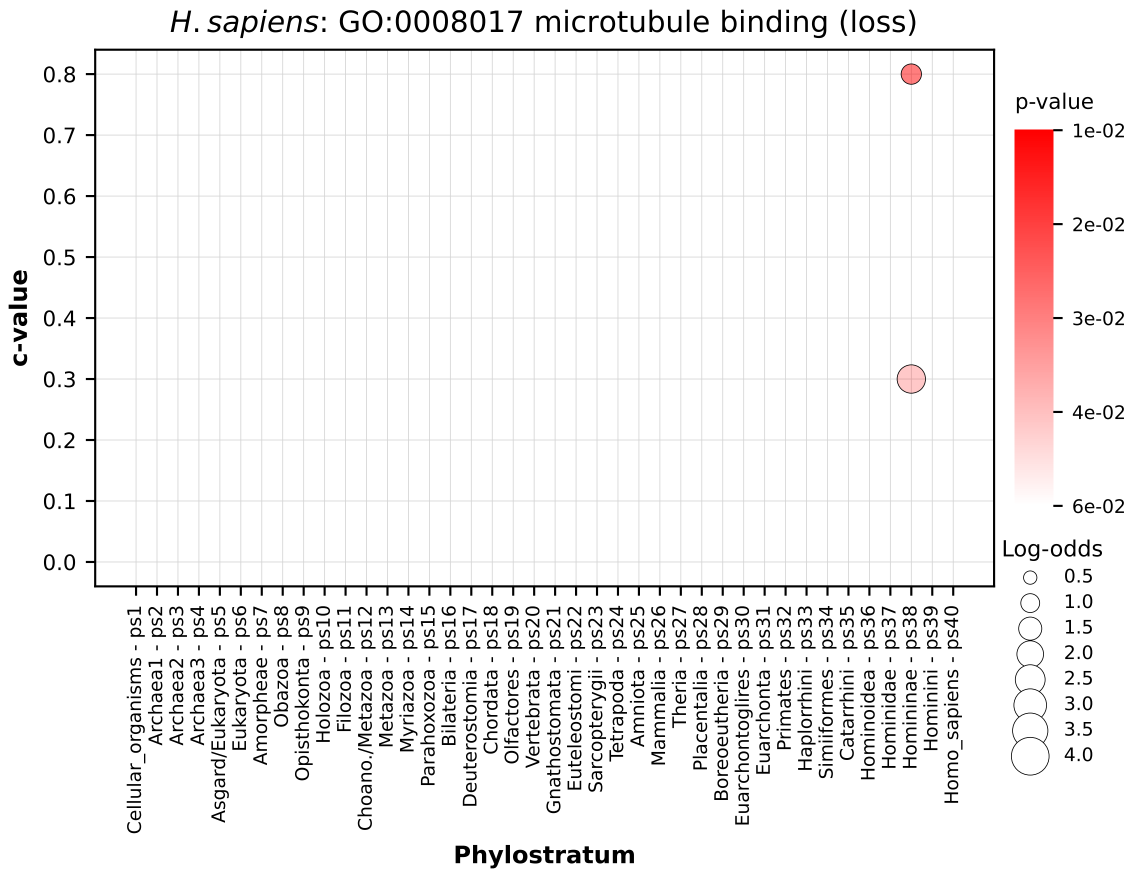

H. sapiens: GO:0008017 microtubule binding (loss)
c-value
0.8
0.7
0.6
0.5
0.4
0.3
0.2
0.1
0.0
p-value
1e-02
2e-02
3e-02
4e-02
6e-02
Log-odds
0.5
1.0
1.5
2.0
2.5
3.0
3.5
4.0
Cellular_organisms - ps1
Archaea1 - ps2
Archaea2 - ps3
Archaea3 - ps4
Asgard/Eukaryota - ps5
Eukaryota - ps6
Amorpheae - ps7
Obazoa - ps8
Opisthokonta - ps9
Holozoa - ps10
Filozoa - ps11
Choano./Metazoa - ps12
Metazoa - ps13
Myriazoa - ps14
Parahoxozoa - ps15
Bilateria - ps16
Deuterostomia - ps17
Chordata - ps18
Olfactores - ps19
Vertebrata - ps20
Gnathostomata - ps21
Euteleostomi - ps22
Sarcopterygii - ps23
Tetrapoda - ps24
Amniota - ps25
Mammalia - ps26
Theria - ps27
Placentalia - ps28
Boreoeutheria - ps29
Euarchontoglires - ps30
Euarchonta - ps31
Primates - ps32
Haplorrhini - ps33
Simiiformes - ps34
Catarrhini - ps35
Hominoidea - ps36
Hominidae - ps37
Homininae - ps38
Hominini - ps39
Homo_sapiens - ps40
Phylostratum

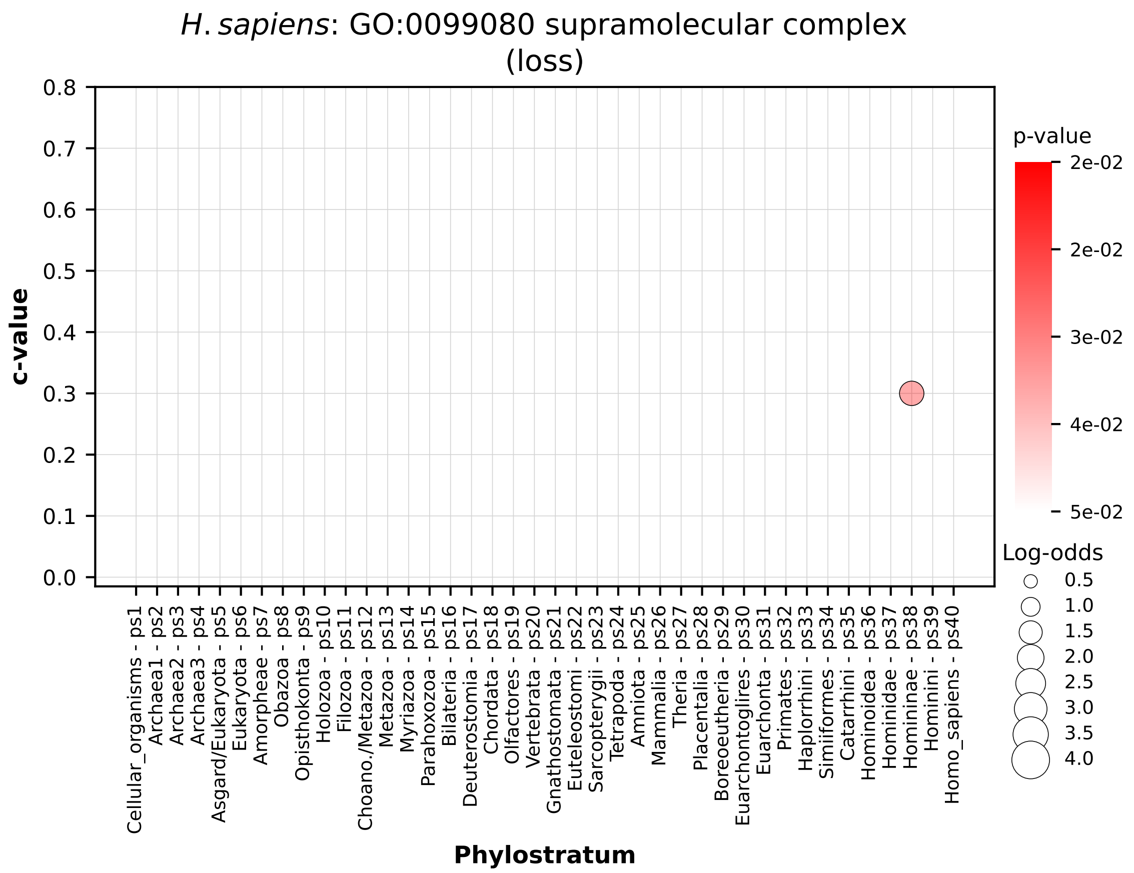

H. sapiens: GO:0099080 supramolecular complex
(loss)
c-value
0.0
0.1
0.2
0.3
0.4
0.5
0.6
0.7
0.8
Phylostratum
Cellular_organisms - ps1
Archaea1 - ps2
Archaea2 - ps3
Archaea3 - ps4
Asgard/Eukaryota - ps5
Eukaryota - ps6
Amorpheae - ps7
Obazoa - ps8
Opisthokonta - ps9
Holozoa - ps10
Filozoa - ps11
Choano./Metazoa - ps12
Metazoa - ps13
Myriazoa - ps14
Parahoxozoa - ps15
Bilateria - ps16
Deuterostomia - ps17
Chordata - ps18
Olfactores - ps19
Vertebrata - ps20
Gnathostomata - ps21
Euteleostomi - ps22
Sarcopterygii - ps23
Tetrapoda - ps24
Amniota - ps25
Mammalia - ps26
Theria - ps27
Placentalia - ps28
Boreoeutheria - ps29
Euarchontoglires - ps30
Euarchonta - ps31
Primates - ps32
Haplorrhini - ps33
Simiiformes - ps34
Catarrhini - ps35
Hominoidea - ps36
Hominidae - ps37
Homininae - ps38
Hominini - ps39
Homo_sapiens - ps40
p-value
2e-02
2e-02
3e-02
4e-02
5e-02
Log-odds
0.5
1.0
1.5
2.0
2.5
3.0
3.5
4.0

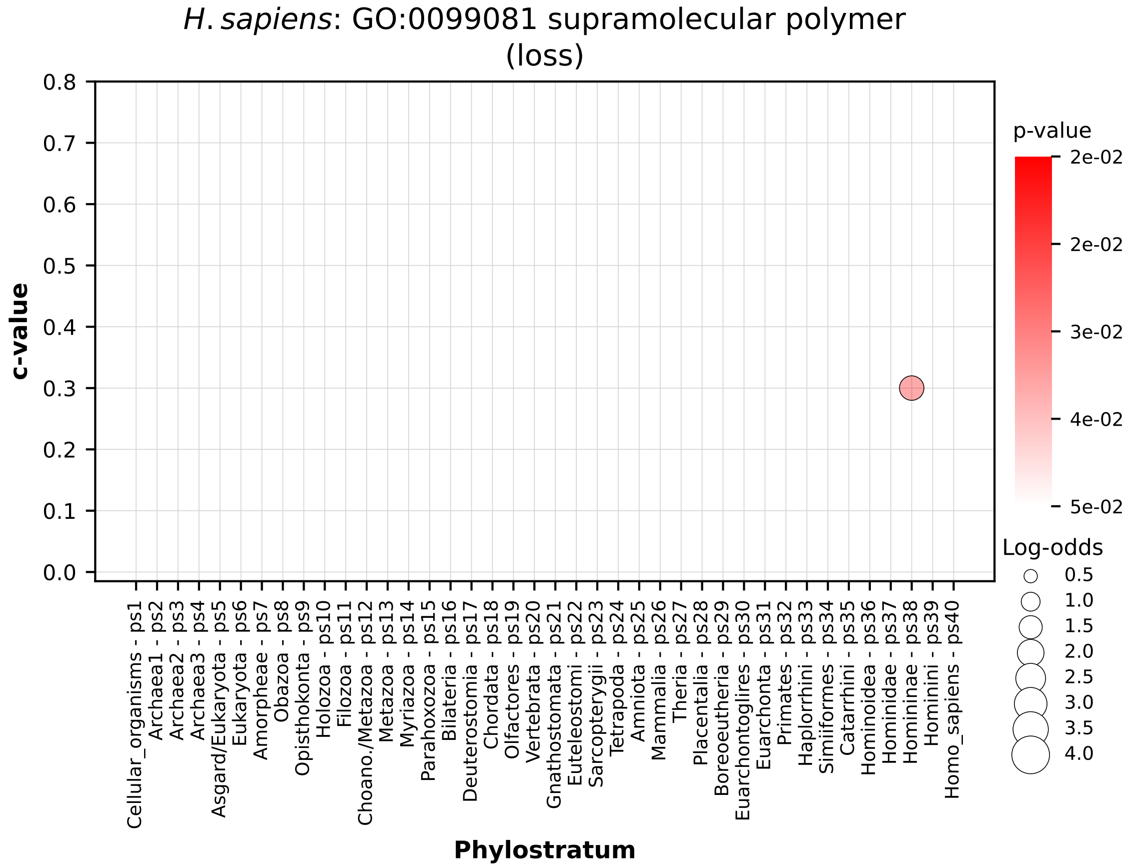
H. sapiens: GO:0099081 supramolecular polymer (loss)
c-value
Phylostratum
p-value
2e-02
2e-02
3e-02
4e-02
5e-02
Log-odds
0.5
1.0
1.5
2.0
2.5
3.0
3.5
4.0
Cellular_organisms - ps1
Archaea1 - ps2
Archaea2 - ps3
Archaea3 - ps4
Asgard/Eukaryota - ps5
Eukaryota - ps6
Amorpheae - ps7
Obazoa - ps8
Opisthokonta - ps9
Holozoa - ps10
Filozoa - ps11
Choano./Metazoa - ps12
Metazoa - ps13
Myriazoa - ps14
Parahoxozoa - ps15
Bilateria - ps16
Deuterostomia - ps17
Chordata - ps18
Olfactores - ps19
Vertebrata - ps20
Gnathostomata - ps21
Euteleostomi - ps22
Sarcopterygii - ps23
Tetrapoda - ps24
Amniota - ps25
Mammalia - ps26
Theria - ps27
Placentalia - ps28
Boreoeutheria - ps29
Euarchontoglires - ps30
Euarchonta - ps31
Primates - ps32
Haplorrhini - ps33
Simiiformes - ps34
Catarrhini - ps35
Hominoidea - ps36
Hominidae - ps37
Homininae - ps38
Hominini - ps39
Homo_sapiens - ps40

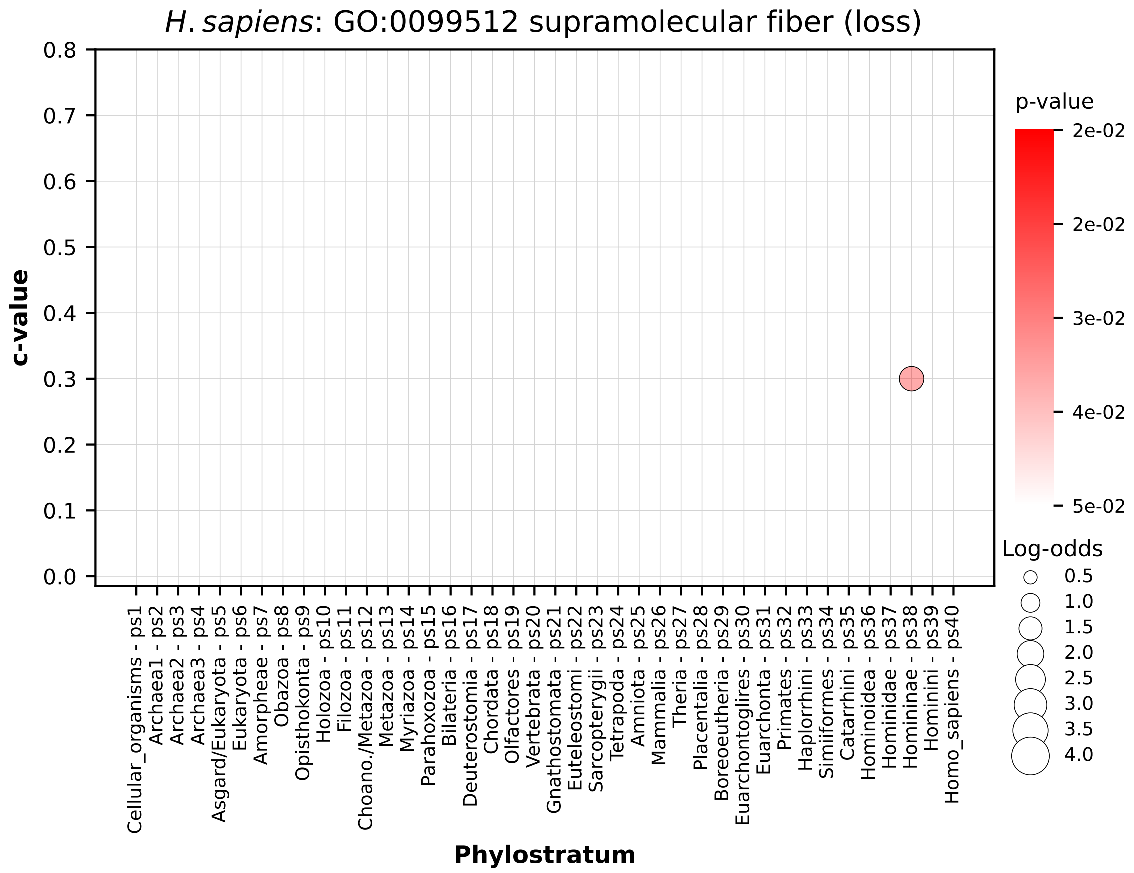
H. sapiens: GO:0099512 supramolecular fiber (loss)
c-value
0.8
0.7
0.6
0.5
0.4
0.3
0.2
0.1
0.0
Phylostratum
Cellular_organisms - ps1
Archaea1 - ps2
Archaea2 - ps3
Archaea3 - ps4
Asgard/Eukaryota - ps5
Eukaryota - ps6
Amorpheae - ps7
Obazoa - ps8
Opisthokonta - ps9
Holozoa - ps10
Filozoa - ps11
Choano./Metazoa - ps12
Metazoa - ps13
Myriazoa - ps14
Parahoxozoa - ps15
Bilateria - ps16
Deuterostomia - ps17
Chordata - ps18
Olfactores - ps19
Vertebrata - ps20
Gnathostomata - ps21
Euteleostomi - ps22
Sarcopterygii - ps23
Tetrapoda - ps24
Amniota - ps25
Mammalia - ps26
Theria - ps27
Placentalia - ps28
Boreoeutheria - ps29
Euarchontoglires - ps30
Euarchonta - ps31
Primates - ps32
Haplorrhini - ps33
Simiiformes - ps34
Catarrhini - ps35
Hominoidea - ps36
Hominidae - ps37
Homininae - ps38
Hominini - ps39
Homo_sapiens - ps40
p-value
2e-02
2e-02
3e-02
4e-02
5e-02
Log-odds
0.5
1.0
1.5
2.0
2.5
3.0
3.5
4.0

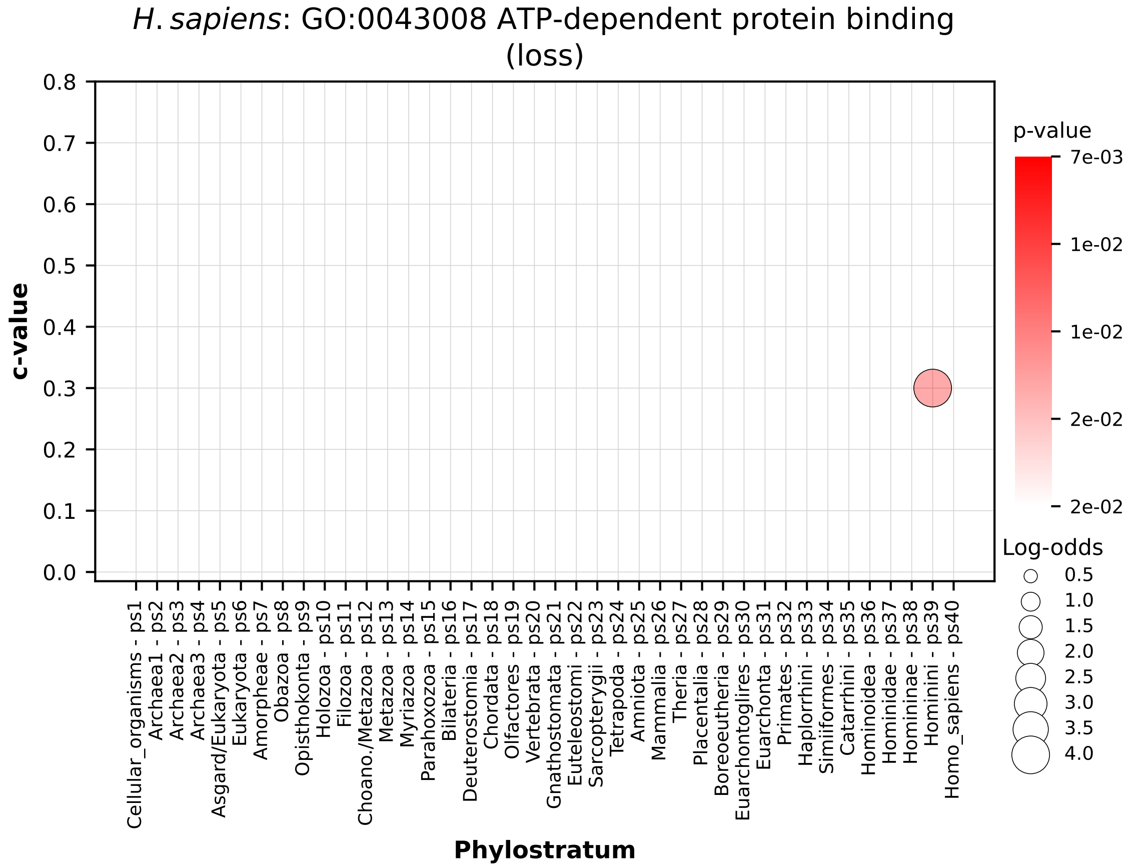

H. sapiens: GO:0043008 ATP-dependent protein binding (loss)
c-value
0.8
0.7
0.6
0.5
0.4
0.3
0.2
0.1
0.0
p-value
7e-03
1e-02
1e-02
2e-02
2e-02
Log-odds
0.5
1.0
1.5
2.0
2.5
3.0
3.5
4.0
Cellular_organisms - ps1
Archaea1 - ps2
Archaea2 - ps3
Archaea3 - ps4
Asgard/Eukaryota - ps5
Eukaryota - ps6
Amorpheae - ps7
Obazoa - ps8
Opisthokonta - ps9
Holozoa - ps10
Filozoa - ps11
Choano./Metazoa - ps12
Metazoa - ps13
Myriazoa - ps14
Parahoxozoa - ps15
Bilateria - ps16
Deuterostomia - ps17
Chordata - ps18
Olfactores - ps19
Vertebrata - ps20
Gnathostomata - ps21
Euteleostomi - ps22
Sarcopterygii - ps23
Tetrapoda - ps24
Amniota - ps25
Mammalia - ps26
Theria - ps27
Placentalia - ps28
Boreoeutheria - ps29
Euarchontoglires - ps30
Euarchonta - ps31
Primates - ps32
Haplorrhini - ps33
Simiiformes - ps34
Catarrhini - ps35
Hominoidea - ps36
Hominidae - ps37
Homininae - ps38
Hominini - ps39
Homo_sapiens - ps40
Phylostratum

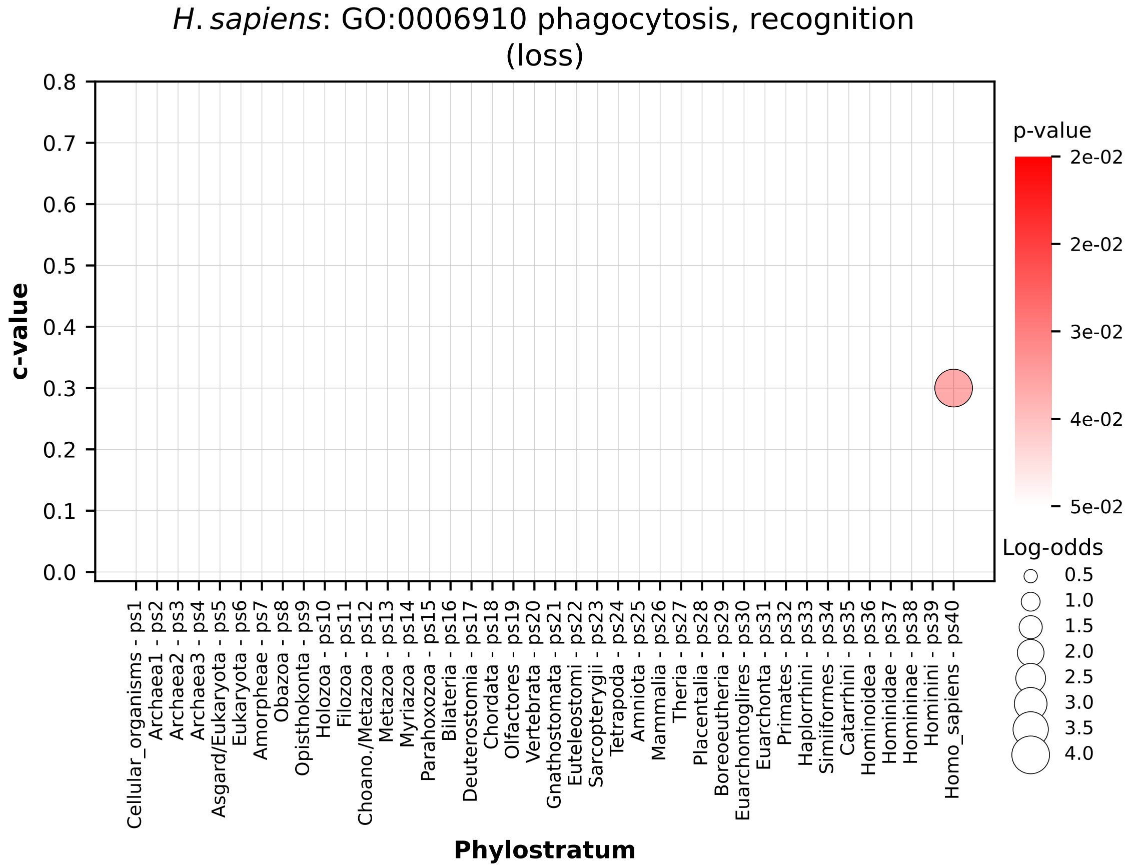
H. sapiens: GO:0006910 phagocytosis, recognition (loss)
c-value
0.8
0.7
0.6
0.5
0.4
0.3
0.2
0.1
0.0
Phylostratum
Cellular_organisms - ps1
Archaea1 - ps2
Archaea2 - ps3
Archaea3 - ps4
Asgard/Eukaryota - ps5
Eukaryota - ps6
Amorpheae - ps7
Obazoa - ps8
Opisthokonta - ps9
Holozoa - ps10
Filozoa - ps11
Choano./Metazoa - ps12
Metazoa - ps13
Myriazoa - ps14
Parahoxozoa - ps15
Bilateria - ps16
Deuterostomia - ps17
Chordata - ps18
Olfactores - ps19
Vertebrata - ps20
Gnathostomata - ps21
Euteleostomi - ps22
Sarcopterygii - ps23
Tetrapoda - ps24
Amniota - ps25
Mammalia - ps26
Theria - ps27
Placentalia - ps28
Boreoeutheria - ps29
Euarchontoglires - ps30
Euarchonta - ps31
Primates - ps32
Haplorrhini - ps33
Simiiformes - ps34
Catarrhini - ps35
Hominoidea - ps36
Hominidae - ps37
Homininae - ps38
Hominini - ps39
Homo_sapiens - ps40
p-value
2e-02
2e-02
3e-02
4e-02
5e-02
Log-odds
0.5
1.0
1.5
2.0
2.5
3.0
3.5
4.0

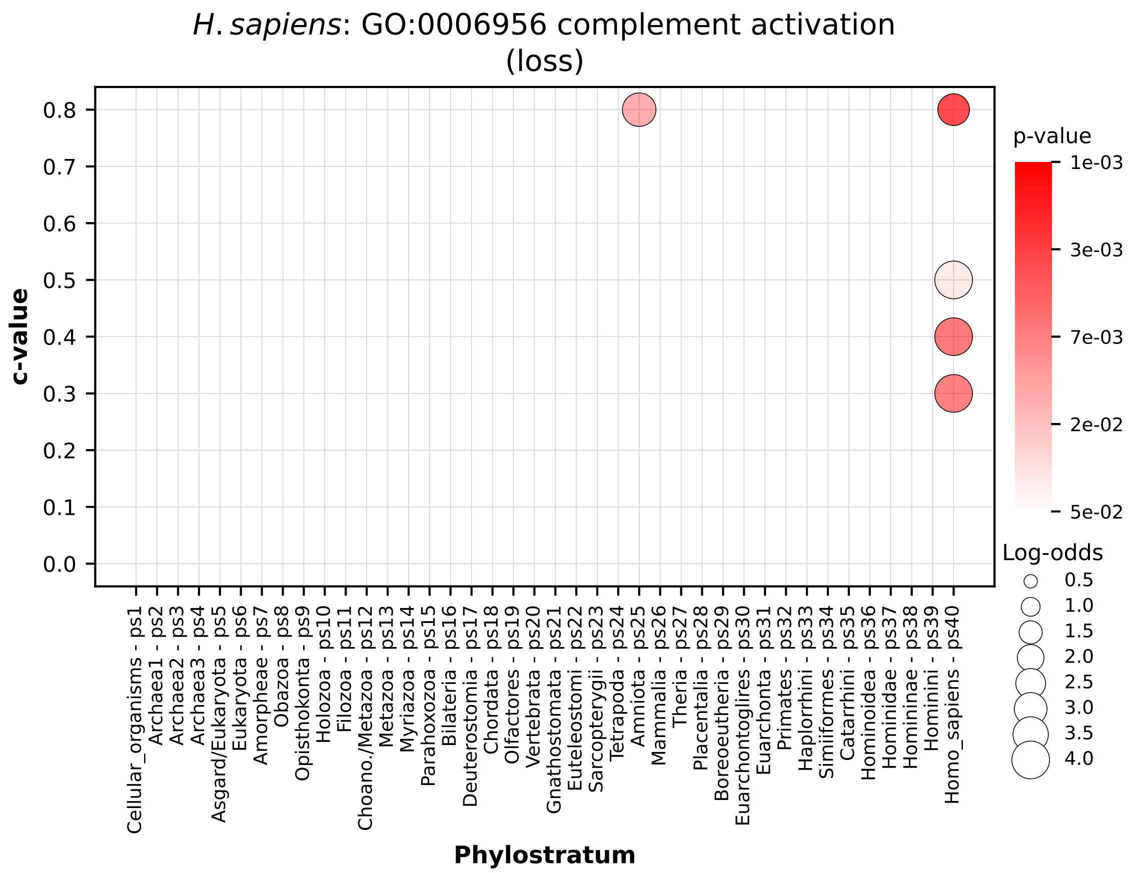

H. sapiens: GO:0006956 complement activation
(loss)
c-value
0.8
0.7
0.6
0.5
0.4
0.3
0.2
0.1
0.0
Phylostratum
Cellular_organisms - ps1
Archaea1 - ps2
Archaea2 - ps3
Archaea3 - ps4
Asgard/Eukaryota - ps5
Eukaryota - ps6
Amorpheae - ps7
Obazoa - ps8
Opisthokonta - ps9
Holozoa - ps10
Filozoa - ps11
Choano./Metazoa - ps12
Metazoa - ps13
Myriazoa - ps14
Parahoxozoa - ps15
Bilateria - ps16
Deuterostomia - ps17
Chordata - ps18
Olfactores - ps19
Vertebrata - ps20
Gnathostomata - ps21
Euteleostomi - ps22
Sarcopterygii - ps23
Tetrapoda - ps24
Amniota - ps25
Mammalia - ps26
Theria - ps27
Placentalia - ps28
Boreoeutheria - ps29
Euarchontoglires - ps30
Euarchonta - ps31
Primates - ps32
Haplorrhini - ps33
Simiiformes - ps34
Catarrhini - ps35
Hominoidea - ps36
Hominidae - ps37
Homininae - ps38
Hominini - ps39
Homo_sapiens - ps40
p-value
1e-03
3e-03
7e-03
2e-02
5e-02
Log-odds
0.5
1.0
1.5
2.0
2.5
3.0
3.5
4.0

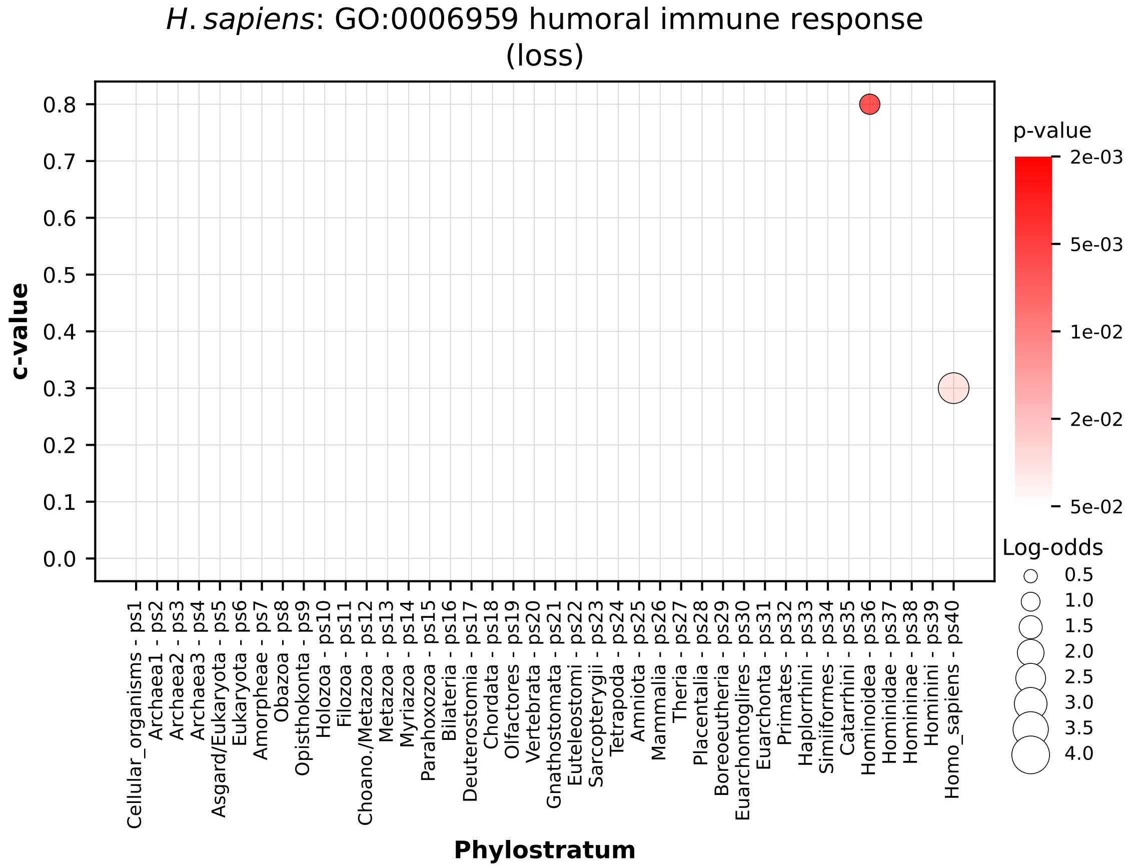

H. sapiens: GO:0006959 humoral immune response
(loss)
c-value
0.8
0.7
0.6
0.5
0.4
0.3
0.2
0.1
0.0
Phylostratum
Cellular_organisms - ps1
Archaea1 - ps2
Archaea2 - ps3
Archaea3 - ps4
Asgard/Eukaryota - ps5
Eukaryota - ps6
Amorpheae - ps7
Obazoa - ps8
Opisthokonta - ps9
Holozoa - ps10
Filozoa - ps11
Choano./Metazoa - ps12
Metazoa - ps13
Myriazoa - ps14
Parahoxozoa - ps15
Bilateria - ps16
Deuterostomia - ps17
Chordata - ps18
Olfactores - ps19
Vertebrata - ps20
Gnathostomata - ps21
Euteleostomi - ps22
Sarcopterygii - ps23
Tetrapoda - ps24
Amniota - ps25
Mammalia - ps26
Theria - ps27
Placentalia - ps28
Boreoeutheria - ps29
Euarchontoglires - ps30
Euarchonta - ps31
Primates - ps32
Haplorrhini - ps33
Simiiformes - ps34
Catarrhini - ps35
Hominoidea - ps36
Hominidae - ps37
Homininae - ps38
Hominini - ps39
Homo_sapiens - ps40
p-value
2e-03
5e-03
1e-02
2e-02
5e-02
Log-odds
0.5
1.0
1.5
2.0
2.5
3.0
3.5
4.0

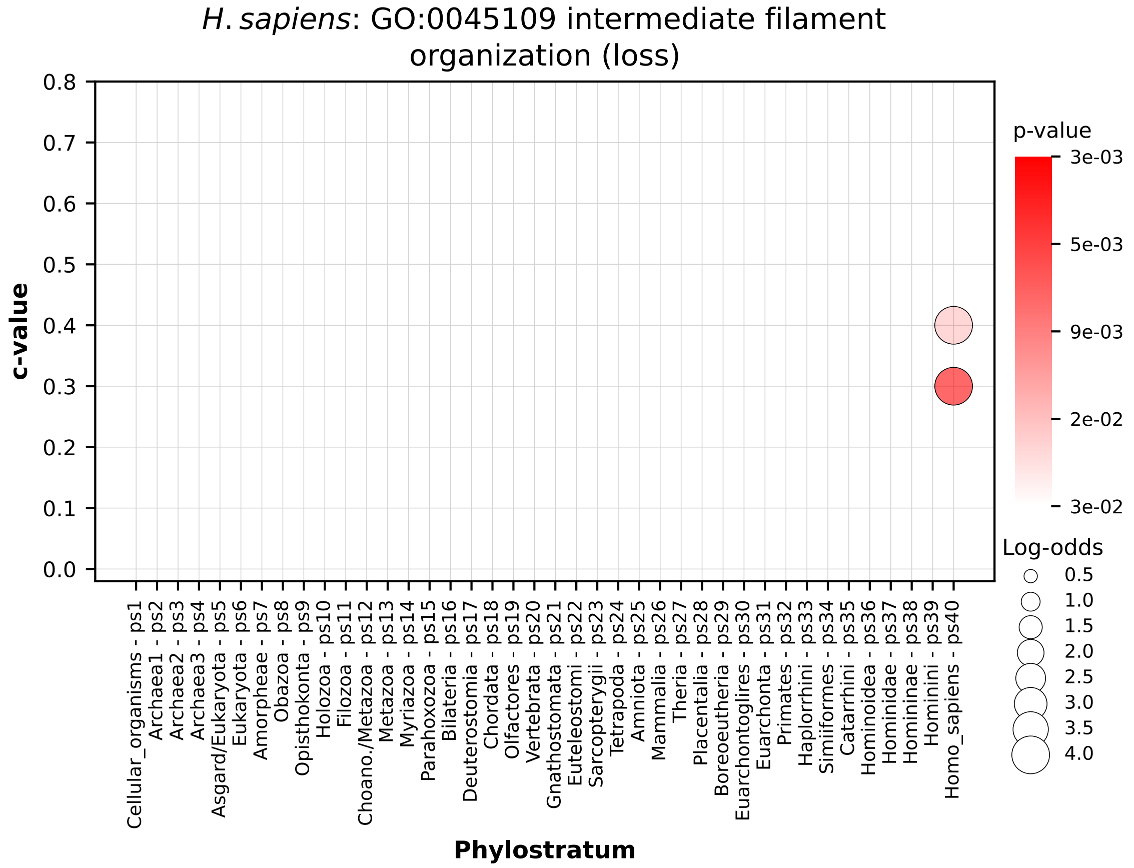
H. sapiens: GO:0045109 intermediate filament organization (loss)
c-value
Phylostratum
p-value
3e-03
5e-03
9e-03
2e-02
3e-02
Log-odds
0.5
1.0
1.5
2.0
2.5
3.0
3.5
4.0
Cellular_organisms - ps1
Archaea1 - ps2
Archaea2 - ps3
Archaea3 - ps4
Asgard/Eukaryota - ps5
Eukaryota - ps6
Amorpheae - ps7
Obazoa - ps8
Opisthokonta - ps9
Holozoa - ps10
Filozoa - ps11
Choano./Metazoa - ps12
Metazoa - ps13
Myriazoa - ps14
Parahoxozoa - ps15
Bilateria - ps16
Deuterostomia - ps17
Chordata - ps18
Olfactores - ps19
Vertebrata - ps20
Gnathostomata - ps21
Euteleostomi - ps22
Sarcopterygii - ps23
Tetrapoda - ps24
Amniota - ps25
Mammalia - ps26
Theria - ps27
Placentalia - ps28
Boreoeutheria - ps29
Euarchontoglires - ps30
Euarchonta - ps31
Primates - ps32
Haplorrhini - ps33
Simiiformes - ps34
Catarrhini - ps35
Hominoidea - ps36
Hominidae - ps37
Homininae - ps38
Hominini - ps39
Homo_sapiens - ps40

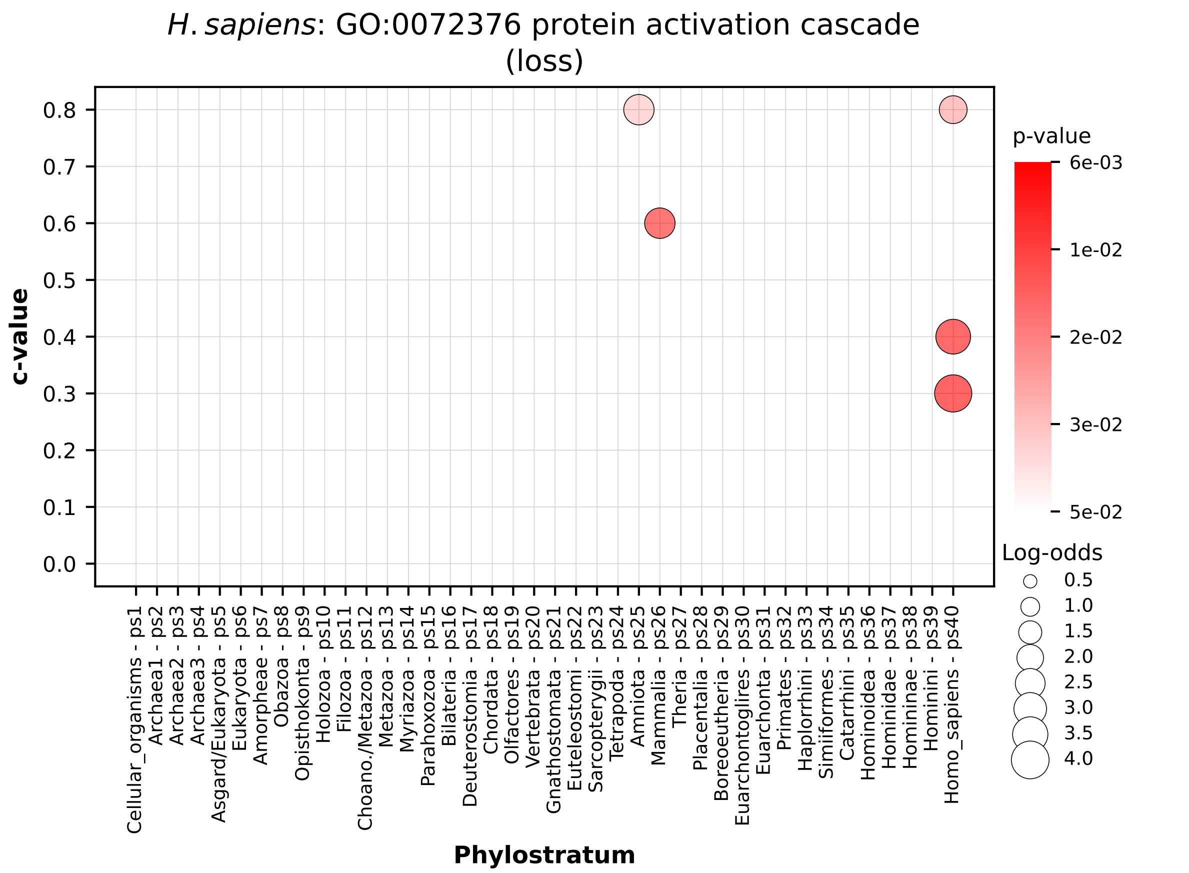

H. sapiens: GO:0072376 protein activation cascade
(loss)
c-value
0.8
0.7
0.6
0.5
0.4
0.3
0.2
0.1
0.0
p-value
6e-03
1e-02
2e-02
3e-02
5e-02
Log-odds
0.5
1.0
1.5
2.0
2.5
3.0
3.5
4.0
Cellular_organisms - ps1
Archaea1 - ps2
Archaea2 - ps3
Archaea3 - ps4
Asgard/Eukaryota - ps5
Eukaryota - ps6
Amorpheae - ps7
Obazoa - ps8
Opisthokonta - ps9
Holozoa - ps10
Filozoa - ps11
Choano./Metazoa - ps12
Metazoa - ps13
Myriazoa - ps14
Parahoxozoa - ps15
Bilateria - ps16
Deuterostomia - ps17
Chordata - ps18
Olfactores - ps19
Vertebrata - ps20
Gnathostomata - ps21
Euteleostomi - ps22
Sarcopterygii - ps23
Tetrapoda - ps24
Amniota - ps25
Mammalia - ps26
Theria - ps27
Placentalia - ps28
Boreoeutheria - ps29
Euarchontoglires - ps30
Euarchonta - ps31
Primates - ps32
Haplorrhini - ps33
Simiiformes - ps34
Catarrhini - ps35
Hominoidea - ps36
Hominidae - ps37
Homininae - ps38
Hominini - ps39
Homo_sapiens - ps40
Phylostratum

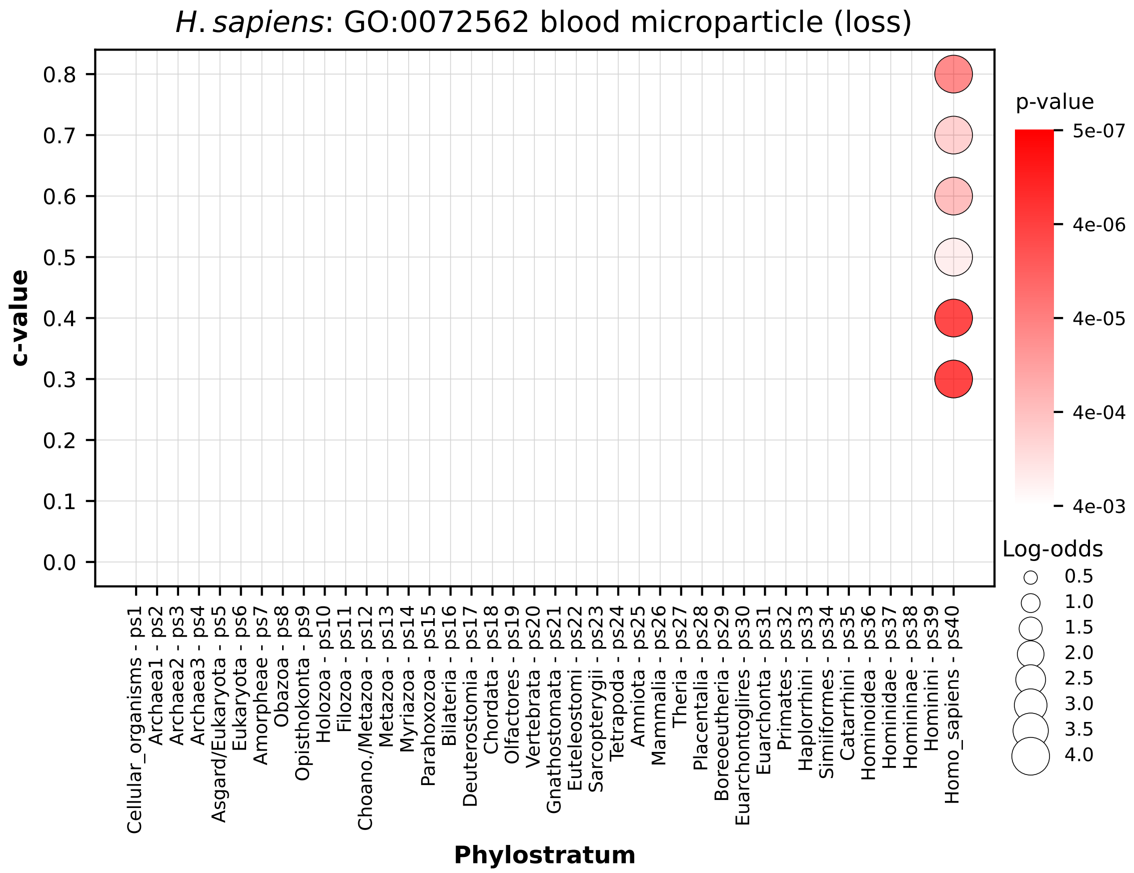

H. sapiens: GO:0072562 blood microparticle (loss)
c-value
0.8
0.7
0.6
0.5
0.4
0.3
0.2
0.1
0.0
p-value
5e-07
4e-06
4e-05
4e-04
4e-03
Log-odds
0.5
1.0
1.5
2.0
2.5
3.0
3.5
4.0
Cellular_organisms - ps1
Archaea1 - ps2
Archaea2 - ps3
Archaea3 - ps4
Asgard/Eukaryota - ps5
Eukaryota - ps6
Amorpheae - ps7
Obazoa - ps8
Opisthokonta - ps9
Holozoa - ps10
Filozoa - ps11
Choano./Metazoa - ps12
Metazoa - ps13
Myriazoa - ps14
Parahoxozoa - ps15
Bilateria - ps16
Deuterostomia - ps17
Chordata - ps18
Olfactores - ps19
Vertebrata - ps20
Gnathostomata - ps21
Euteleostomi - ps22
Sarcopterygii - ps23
Tetrapoda - ps24
Amniota - ps25
Mammalia - ps26
Theria - ps27
Placentalia - ps28
Boreoeutheria - ps29
Euarchontoglires - ps30
Euarchonta - ps31
Primates - ps32
Haplorrhini - ps33
Simiiformes - ps34
Catarrhini - ps35
Hominoidea - ps36
Hominidae - ps37
Homininae - ps38
Hominini - ps39
Homo_sapiens - ps40
Phylostratum

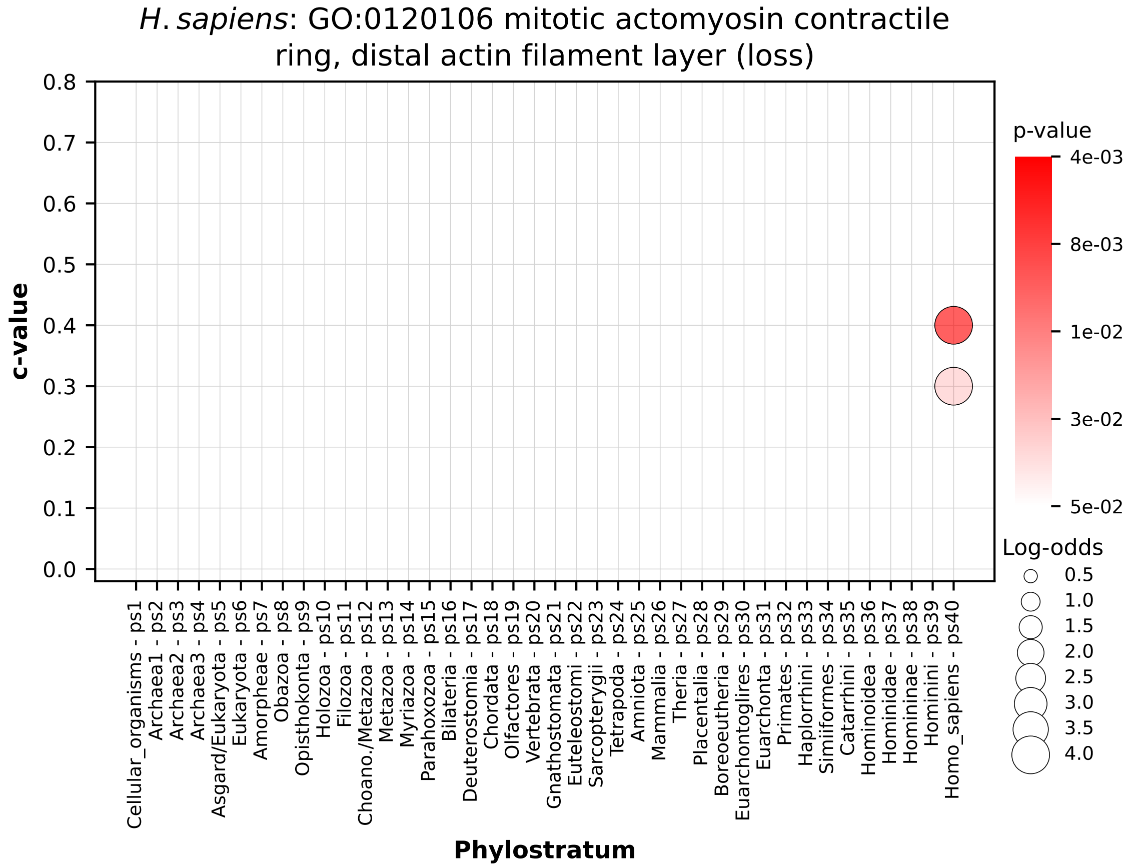

H. sapiens: GO:0120106 mitotic actomyosin contractile ring, distal actin filament layer (loss)
c-value
0.0
0.1
0.2
0.3
0.4
0.5
0.6
0.7
0.8
Phylostratum
Cellular_organisms - ps1
Archaea1 - ps2
Archaea2 - ps3
Archaea3 - ps4
Asgard/Eukaryota - ps5
Eukaryota - ps6
Amorpheae - ps7
Obazoa - ps8
Opisthokonta - ps9
Holozoa - ps10
Filozoa - ps11
Choano./Metazoa - ps12
Metazoa - ps13
Myriazoa - ps14
Parahoxozoa - ps15
Bilateria - ps16
Deuterostomia - ps17
Chordata - ps18
Olfactores - ps19
Vertebrata - ps20
Gnathostomata - ps21
Euteleostomi - ps22
Sarcopterygii - ps23
Tetrapoda - ps24
Amniota - ps25
Mammalia - ps26
Theria - ps27
Placentalia - ps28
Boreoeutheria - ps29
Euarchontoglires - ps30
Euarchonta - ps31
Primates - ps32
Haplorrhini - ps33
Simiiformes - ps34
Catarrhini - ps35
Hominoidea - ps36
Hominidae - ps37
Homininae - ps38
Hominini - ps39
Homo_sapiens - ps40
p-value
4e-03
8e-03
1e-02
3e-02
5e-02
Log-odds
0.5
1.0
1.5
2.0
2.5
3.0
3.5
4.0

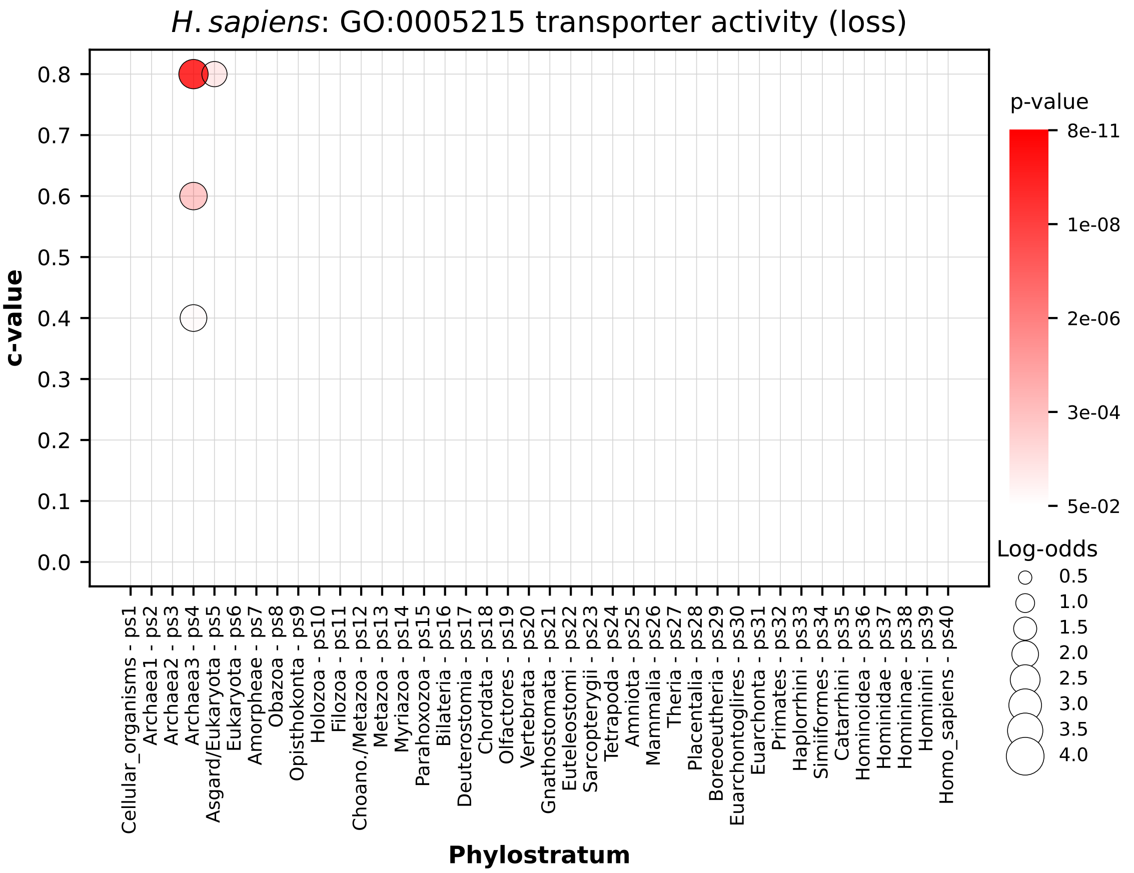

H. sapiens: GO:0005215 transporter activity (loss)
c-value
0.8
0.7
0.6
0.5
0.4
0.3
0.2
0.1
0.0
Phylostratum
Cellular_organisms - ps1
Archaea1 - ps2
Archaea2 - ps3
Archaea3 - ps4
Asgard/Eukaryota - ps5
Eukaryota - ps6
Amorpheae - ps7
Obazoa - ps8
Opisthokonta - ps9
Holozoa - ps10
Filozoa - ps11
Choano./Metazoa - ps12
Metazoa - ps13
Myriazoa - ps14
Parahoxozoa - ps15
Bilateria - ps16
Deuterostomia - ps17
Chordata - ps18
Olfactores - ps19
Vertebrata - ps20
Gnathostomata - ps21
Euteleostomi - ps22
Sarcopterygii - ps23
Tetrapoda - ps24
Amniota - ps25
Mammalia - ps26
Theria - ps27
Placentalia - ps28
Boreoeutheria - ps29
Euarchontoglires - ps30
Euarchonta - ps31
Primates - ps32
Haplorrhini - ps33
Simiiformes - ps34
Catarrhini - ps35
Hominoidea - ps36
Hominidae - ps37
Homininae - ps38
Hominini - ps39
Homo_sapiens - ps40
p-value
8e-11
1e-08
2e-06
3e-04
5e-02
Log-odds
0.5
1.0
1.5
2.0
2.5
3.0
3.5
4.0

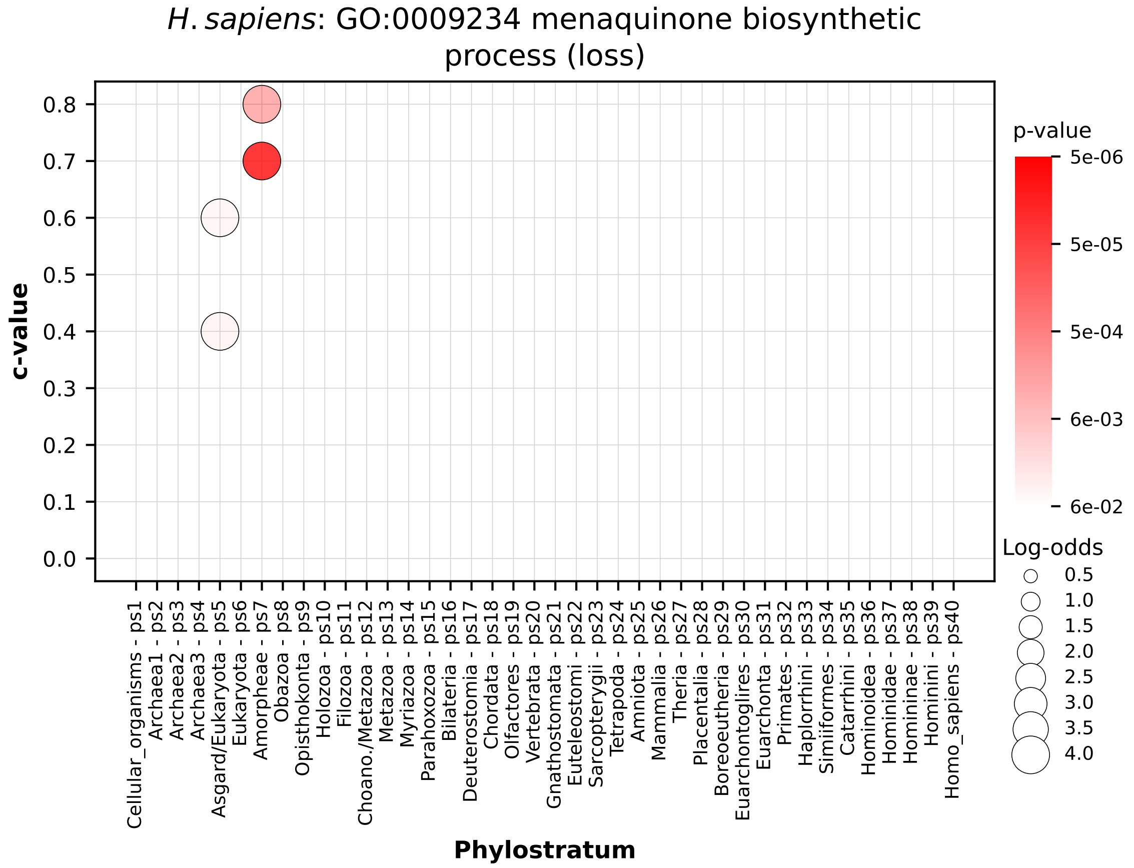

H. sapiens: GO:0009234 menaquinone biosynthetic process (loss)
c-value
0.8
0.7
0.6
0.5
0.4
0.3
0.2
0.1
0.0
Phylostratum
Cellular_organisms - ps1
Archaea1 - ps2
Archaea2 - ps3
Archaea3 - ps4
Asgard/Eukaryota - ps5
Eukaryota - ps6
Amorpheae - ps7
Obazoa - ps8
Opisthokonta - ps9
Holozoa - ps10
Filozoa - ps11
Choano./Metazoa - ps12
Metazoa - ps13
Myriazoa - ps14
Parahoxozoa - ps15
Bilateria - ps16
Deuterostomia - ps17
Chordata - ps18
Olfactores - ps19
Vertebrata - ps20
Gnathostomata - ps21
Euteleostomi - ps22
Sarcopterygii - ps23
Tetrapoda - ps24
Amniota - ps25
Mammalia - ps26
Theria - ps27
Placentalia - ps28
Boreoeutheria - ps29
Euarchontoglires - ps30
Euarchonta - ps31
Primates - ps32
Haplorrhini - ps33
Simiiformes - ps34
Catarrhini - ps35
Hominoidea - ps36
Hominidae - ps37
Homininae - ps38
Hominini - ps39
Homo_sapiens - ps40
p-value
5e-06
5e-05
5e-04
6e-03
6e-02
Log-odds
0.5
1.0
1.5
2.0
2.5
3.0
3.5
4.0

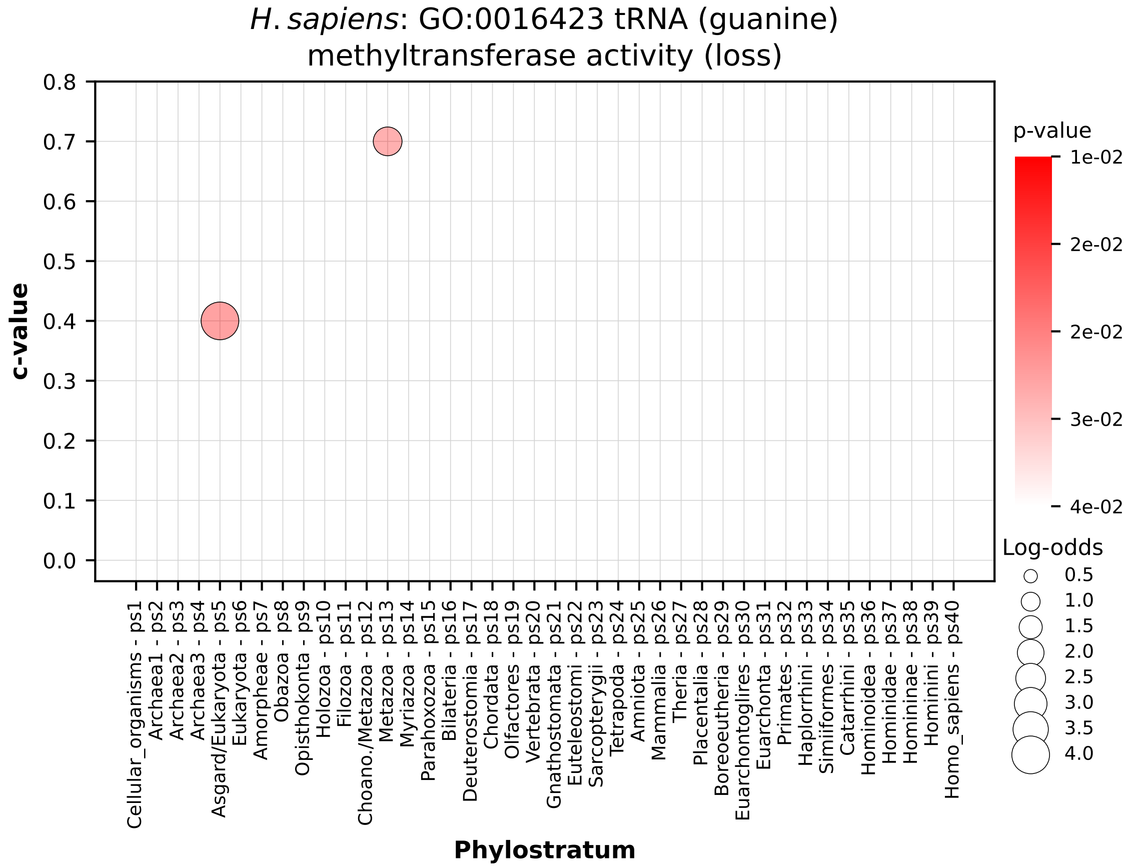

$H. sapiens$: GO:0016423 tRNA (guanine) methyltransferase activity (loss)
c-value
0.0
0.1
0.2
0.3
0.4
0.5
0.6
0.7
0.8
Phylostratum
Cellular_organisms - ps1
Archaea1 - ps2
Archaea2 - ps3
Archaea3 - ps4
Asgard/Eukaryota - ps5
Eukaryota - ps6
Amorpheae - ps7
Obazoa - ps8
Opisthokonta - ps9
Holozoa - ps10
Filozoa - ps11
Choano./Metazoa - ps12
Metazoa - ps13
Myriazoa - ps14
Parahoxozoa - ps15
Bilateria - ps16
Deuterostomia - ps17
Chordata - ps18
Olfactores - ps19
Vertebrata - ps20
Gnathostomata - ps21
Euteleostomi - ps22
Sarcopterygii - ps23
Tetrapoda - ps24
Amniota - ps25
Mammalia - ps26
Theria - ps27
Placentalia - ps28
Boreoeutheria - ps29
Euarchontoglires - ps30
Euarchonta - ps31
Primates - ps32
Haplorrhini - ps33
Simiiformes - ps34
Catarrhini - ps35
Hominoidea - ps36
Hominidae - ps37
Homininae - ps38
Hominini - ps39
Homo_sapiens - ps40
p-value
1e-02
2e-02
2e-02
3e-02
4e-02
Log-odds
0.5
1.0
1.5
2.0
2.5
3.0
3.5
4.0

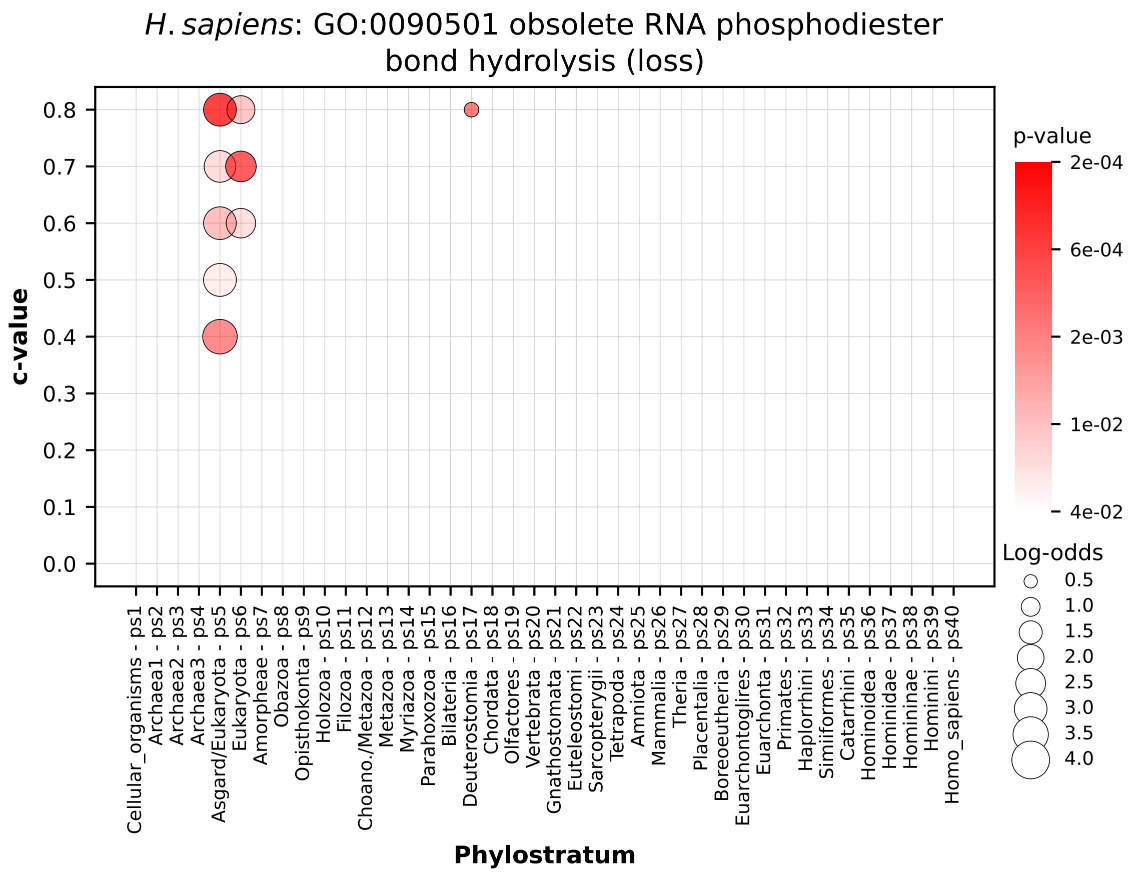

H. sapiens: GO:0090501 obsolete RNA phosphodiester bond hydrolysis (loss)
c-value
0.8
0.7
0.6
0.5
0.4
0.3
0.2
0.1
0.0
p-value
2e-04
6e-04
2e-03
1e-02
4e-02
Log-odds
0.5
1.0
1.5
2.0
2.5
3.0
3.5
4.0
Cellular_organisms - ps1
Archaea1 - ps2
Archaea2 - ps3
Archaea3 - ps4
Asgard/Eukaryota - ps5
Eukaryota - ps6
Amorpheae - ps7
Obazoa - ps8
Opisthokonta - ps9
Holozoa - ps10
Filozoa - ps11
Choano./Metazoa - ps12
Metazoa - ps13
Myriazoa - ps14
Parahoxozoa - ps15
Bilateria - ps16
Deuterostomia - ps17
Chordata - ps18
Olfactores - ps19
Vertebrata - ps20
Gnathostomata - ps21
Euteleostomi - ps22
Sarcopterygii - ps23
Tetrapoda - ps24
Amniota - ps25
Mammalia - ps26
Theria - ps27
Placentalia - ps28
Boreoeutheria - ps29
Euarchontoglires - ps30
Euarchonta - ps31
Primates - ps32
Haplorrhini - ps33
Simiiformes - ps34
Catarrhini - ps35
Hominoidea - ps36
Hominidae - ps37
Homininae - ps38
Hominini - ps39
Homo_sapiens - ps40
Phylostratum

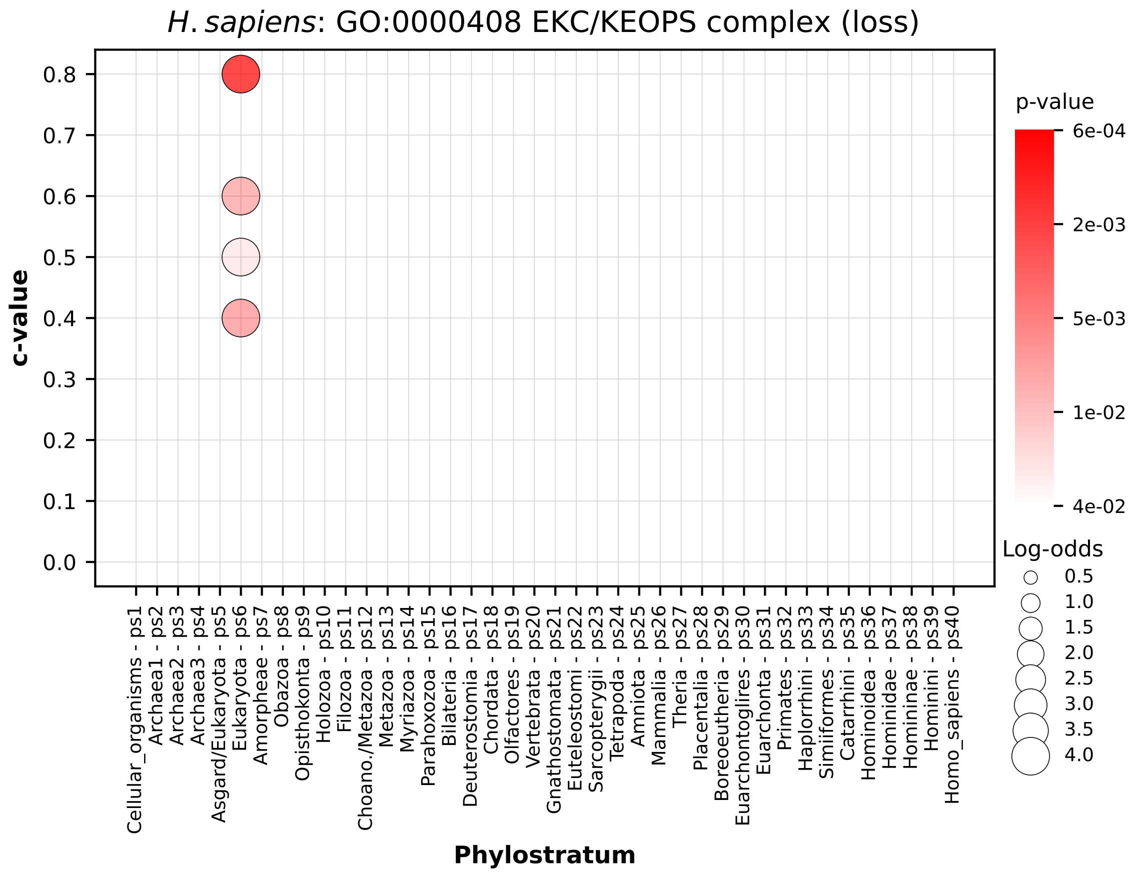

H. sapiens: GO:0000408 EKC/KEOPS complex (loss)
c-value
0.0
0.1
0.2
0.3
0.4
0.5
0.6
0.7
0.8
Phylostratum
Cellular_organisms - ps1
Archaea1 - ps2
Archaea2 - ps3
Archaea3 - ps4
Asgard/Eukaryota - ps5
Eukaryota - ps6
Amorpheae - ps7
Obazoa - ps8
Opisthokonta - ps9
Holozoa - ps10
Filozoa - ps11
Choano./Metazoa - ps12
Metazoa - ps13
Myriazoa - ps14
Parahoxozoa - ps15
Bilateria - ps16
Deuterostomia - ps17
Chordata - ps18
Olfactores - ps19
Vertebrata - ps20
Gnathostomata - ps21
Euteleostomi - ps22
Sarcopterygii - ps23
Tetrapoda - ps24
Amniota - ps25
Mammalia - ps26
Theria - ps27
Placentalia - ps28
Boreoeutheria - ps29
Euarchontoglires - ps30
Euarchonta - ps31
Primates - ps32
Haplorrhini - ps33
Simiiformes - ps34
Catarrhini - ps35
Hominoidea - ps36
Hominidae - ps37
Homininae - ps38
Hominini - ps39
Homo_sapiens - ps40
p-value
6e-04
2e-03
5e-03
1e-02
4e-02
Log-odds
0.5
1.0
1.5
2.0
2.5
3.0
3.5
4.0

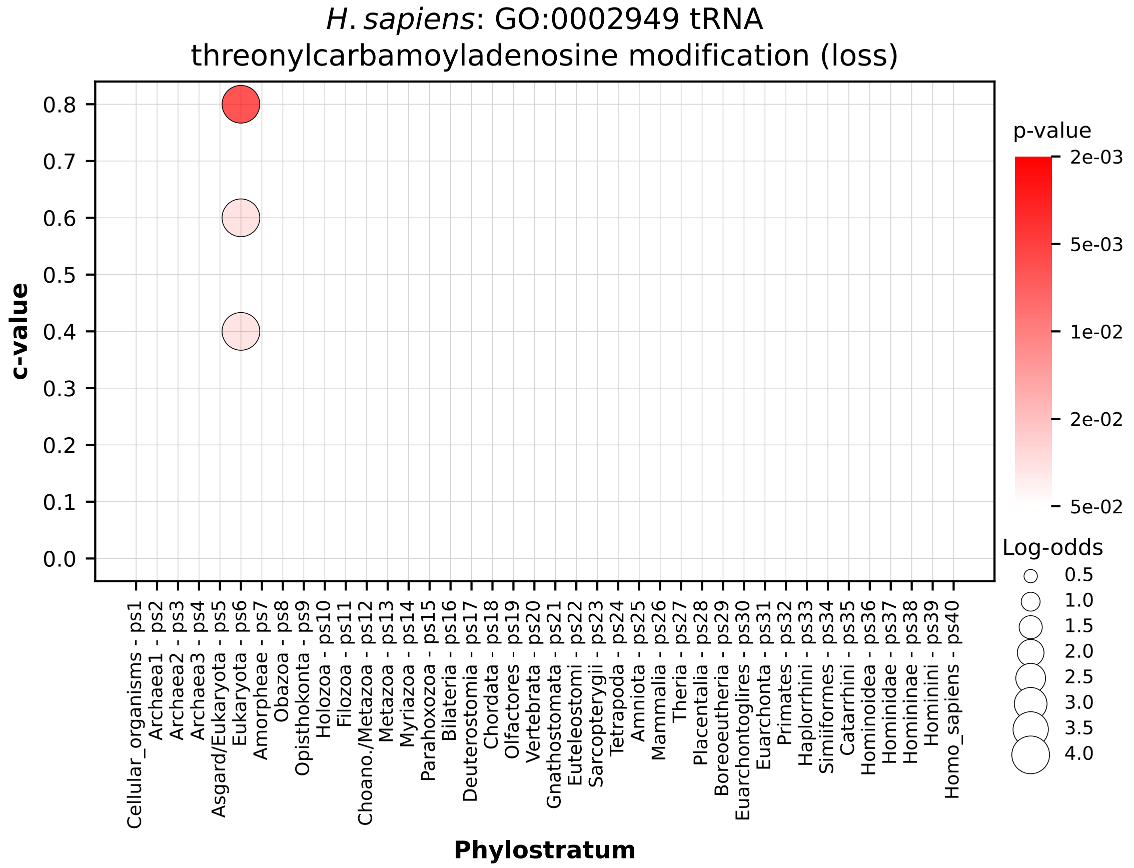

H. sapiens: GO:0002949 tRNA
threonylcarbamoyladenosine modification (loss)
c-value
0.8
0.7
0.6
0.5
0.4
0.3
0.2
0.1
0.0
p-value
2e-03
5e-03
1e-02
2e-02
5e-02
Log-odds
0.5
1.0
1.5
2.0
2.5
3.0
3.5
4.0
Cellular_organisms - ps1
Archaea1 - ps2
Archaea2 - ps3
Archaea3 - ps4
Asgard/Eukaryota - ps5
Eukaryota - ps6
Amorpheae - ps7
Obazoa - ps8
Opisthokonta - ps9
Holozoa - ps10
Filozoa - ps11
Choano./Metazoa - ps12
Metazoa - ps13
Myriazoa - ps14
Parahoxozoa - ps15
Bilateria - ps16
Deuterostomia - ps17
Chordata - ps18
Olfactores - ps19
Vertebrata - ps20
Gnathostomata - ps21
Euteleostomi - ps22
Sarcopterygii - ps23
Tetrapoda - ps24
Amniota - ps25
Mammalia - ps26
Theria - ps27
Placentalia - ps28
Boreoeutheria - ps29
Euarchontoglires - ps30
Euarchonta - ps31
Primates - ps32
Haplorrhini - ps33
Similformes - ps34
Catarrhini - ps35
Hominoidea - ps36
Hominidae - ps37
Homininae - ps38
Hominini - ps39
Homo_sapiens - ps40
Phylostratum

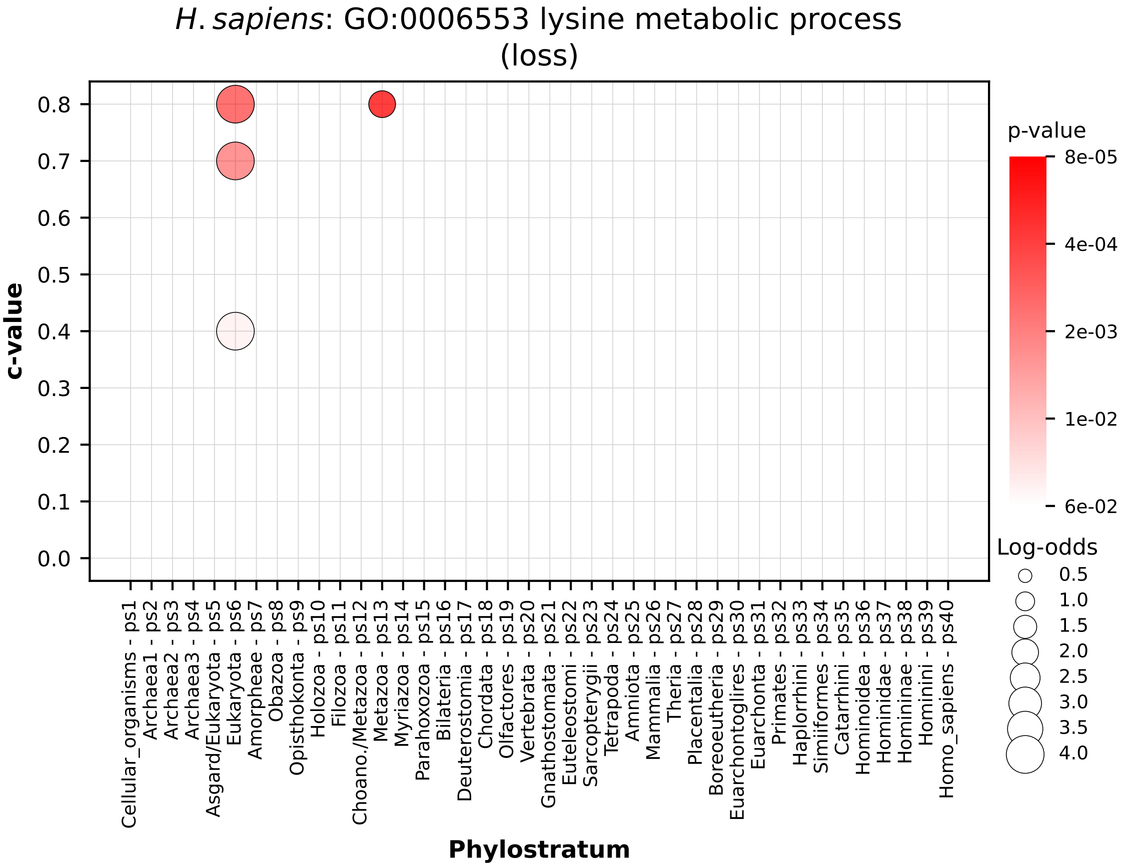

H. sapiens: GO:0006553 lysine metabolic process
(loss)
c-value
0.8
0.7
0.6
0.5
0.4
0.3
0.2
0.1
0.0
p-value
8e-05
4e-04
2e-03
1e-02
6e-02
Log-odds
0.5
1.0
1.5
2.0
2.5
3.0
3.5
4.0
Cellular_organisms - ps1
Archaea1 - ps2
Archaea2 - ps3
Archaea3 - ps4
Asgard/Eukaryota - ps5
Eukaryota - ps6
Amorpheae - ps7
Obazoa - ps8
Opisthokonta - ps9
Holozoa - ps10
Filozoa - ps11
Choano./Metazoa - ps12
Metazoa - ps13
Myriazoa - ps14
Parahoxozoa - ps15
Bilateria - ps16
Deuterostomia - ps17
Chordata - ps18
Olfactores - ps19
Vertebrata - ps20
Gnathostomata - ps21
Euteleostomi - ps22
Sarcopterygii - ps23
Tetrapoda - ps24
Amniota - ps25
Mammalia - ps26
Theria - ps27
Placentalia - ps28
Boreoeutheria - ps29
Euarchontoglires - ps30
Euarchonta - ps31
Primates - ps32
Haplorrhini - ps33
Simiiformes - ps34
Catarrhini - ps35
Hominoidea - ps36
Hominidae - ps37
Homininae - ps38
Hominini - ps39
Homo_sapiens - ps40
Phylostratum

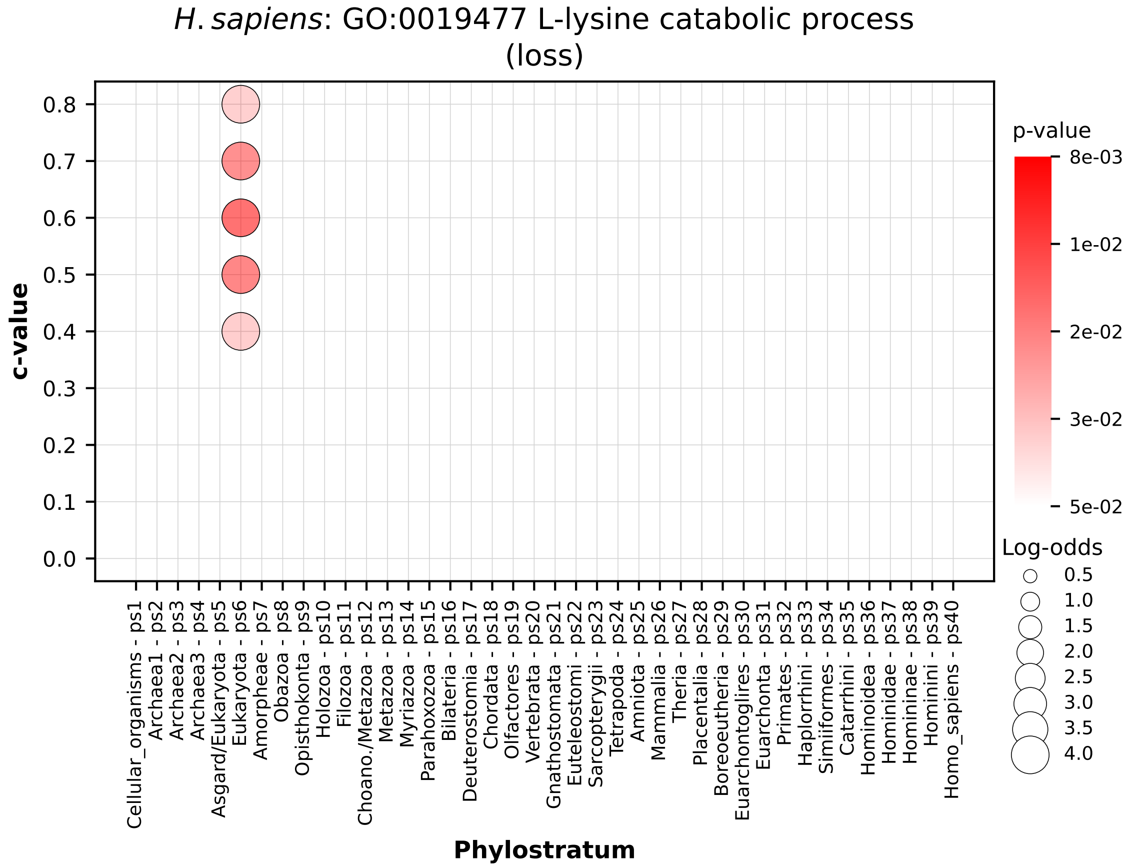
H. sapiens: GO:0019477 L-lysine catabolic process
(loss)
c-value
0.0
0.1
0.2
0.3
0.4
0.5
0.6
0.7
0.8
p-value
8e-03
1e-02
2e-02
3e-02
5e-02
Log-odds
0.5
1.0
1.5
2.0
2.5
3.0
3.5
4.0
Cellular_organisms - ps1
Archaea1 - ps2
Archaea2 - ps3
Archaea3 - ps4
Asgard/Eukaryota - ps5
Eukaryota - ps6
Amorpheae - ps7
Obazoa - ps8
Opisthokonta - ps9
Holozoa - ps10
Filozoa - ps11
Choano./Metazoa - ps12
Metazoa - ps13
Myriazoa - ps14
Parahoxozoa - ps15
Bilateria - ps16
Deuterostomia - ps17
Chordata - ps18
Olfactores - ps19
Vertebrata - ps20
Gnathostomata - ps21
Euteleostomi - ps22
Sarcopterygii - ps23
Tetrapoda - ps24
Amniota - ps25
Mammalia - ps26
Theria - ps27
Placentalia - ps28
Boreoeutheria - ps29
Euarchontoglires - ps30
Euarchonta - ps31
Primates - ps32
Haplorrhini - ps33
Simiiformes - ps34
Catarrhini - ps35
Hominoidea - ps36
Hominidae - ps37
Homininae - ps38
Hominini - ps39
Homo_sapiens - ps40
Phylostratum

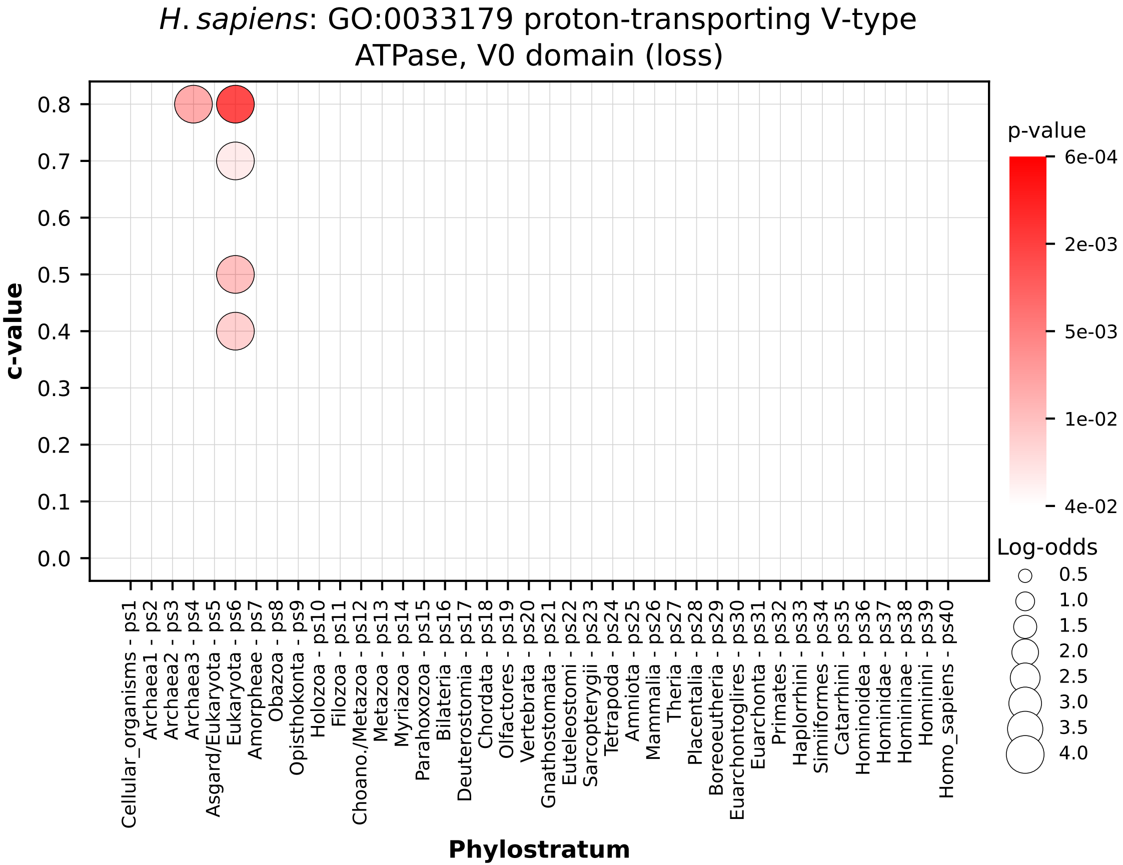

H. sapiens: GO:0033179 proton-transporting V-type ATPase, V0 domain (loss)
c-value
0.0
0.1
0.2
0.3
0.4
0.5
0.6
0.7
0.8
p-value
6e-04
2e-03
5e-03
1e-02
4e-02
Log-odds
0.5
1.0
1.5
2.0
2.5
3.0
3.5
4.0
Cellular_organisms - ps1
Archaea1 - ps2
Archaea2 - ps3
Archaea3 - ps4
Asgard/Eukaryota - ps5
Eukaryota - ps6
Amorpheae - ps7
Obazoa - ps8
Opisthokonta - ps9
Holozoa - ps10
Filozoa - ps11
Choano./Metazoa - ps12
Metazoa - ps13
Myriazoa - ps14
Parahoxozoa - ps15
Bilateria - ps16
Deuterostomia - ps17
Chordata - ps18
Olfactores - ps19
Vertebrata - ps20
Gnathostomata - ps21
Euteleostomi - ps22
Sarcopterygii - ps23
Tetrapoda - ps24
Amniota - ps25
Mammalia - ps26
Theria - ps27
Placentalia - ps28
Boreoeutheria - ps29
Euarchontoglires - ps30
Euarchonta - ps31
Primates - ps32
Haplorrhini - ps33
Simiiformes - ps34
Catarrhini - ps35
Hominoidea - ps36
Hominidae - ps37
Homininae - ps38
Hominini - ps39
Homo_sapiens - ps40
Phylostratum

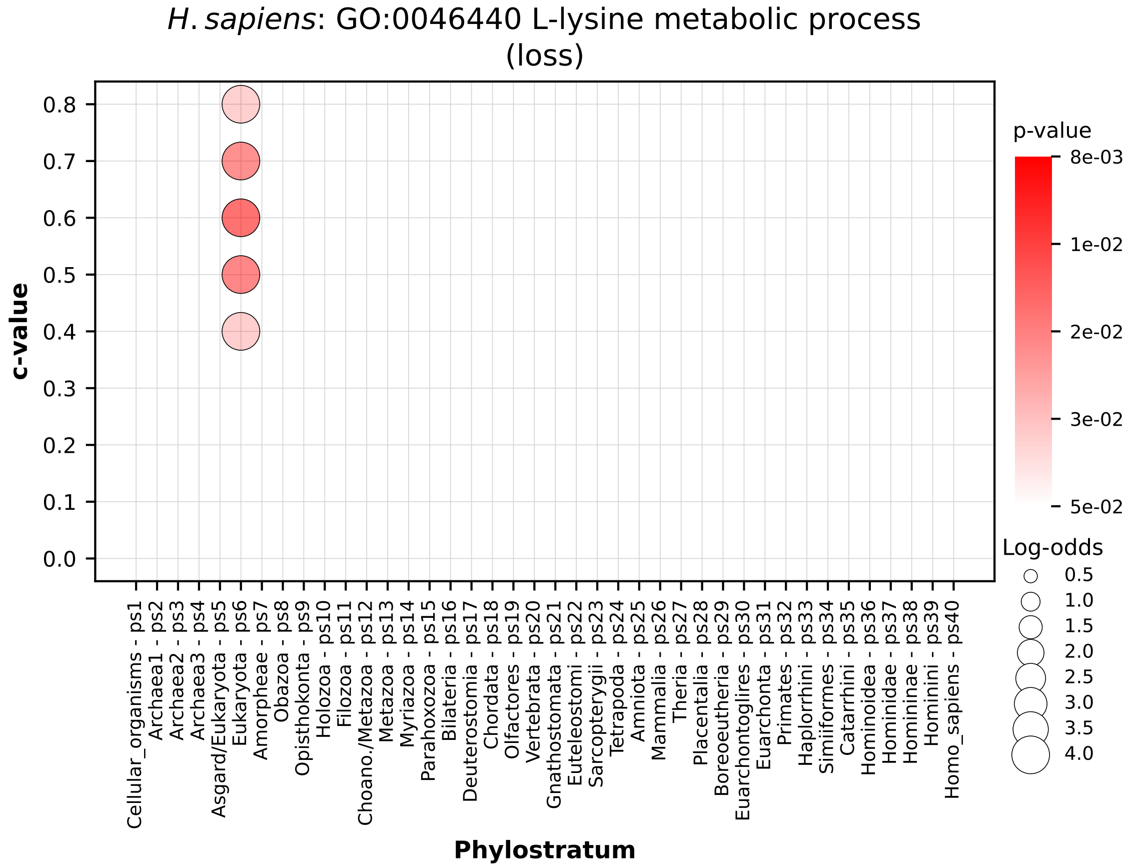
H. sapiens: GO:0046440 L-lysine metabolic process
(loss)
c-value
0.8
0.7
0.6
0.5
0.4
0.3
0.2
0.1
0.0
Phylostratum
Cellular_organisms - ps1
Archaea1 - ps2
Archaea2 - ps3
Archaea3 - ps4
Asgard/Eukaryota - ps5
Eukaryota - ps6
Amorpheae - ps7
Obazoa - ps8
Opisthokonta - ps9
Holozoa - ps10
Filozoa - ps11
Choano./Metazoa - ps12
Metazoa - ps13
Myriazoa - ps14
Parahoxozoa - ps15
Bilateria - ps16
Deuterostomia - ps17
Chordata - ps18
Olfactores - ps19
Vertebrata - ps20
Gnathostomata - ps21
Euteleostomi - ps22
Sarcopterygii - ps23
Tetrapoda - ps24
Amniota - ps25
Mammalia - ps26
Theria - ps27
Placentalia - ps28
Boreoeutheria - ps29
Euarchontoglires - ps30
Euarchonta - ps31
Primates - ps32
Haplorrhini - ps33
Simiiformes - ps34
Catarrhini - ps35
Hominoidea - ps36
Hominidae - ps37
Homininae - ps38
Hominini - ps39
Homo_sapiens - ps40
p-value
8e-03
1e-02
2e-02
3e-02
5e-02
Log-odds
0.5
1.0
1.5
2.0
2.5
3.0
3.5
4.0

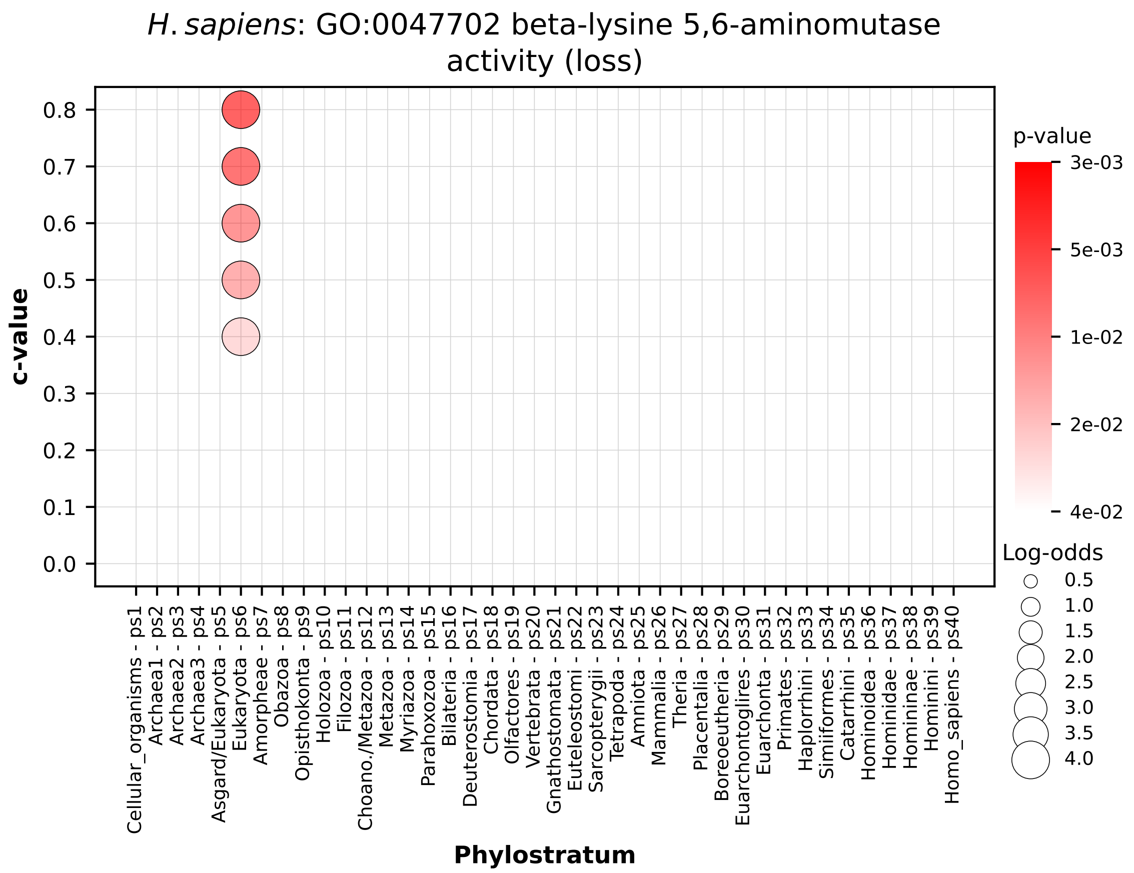
H. sapiens: GO:0047702 beta-lysine 5,6-aminomutase activity (loss)
c-value
0.0
0.1
0.2
0.3
0.4
0.5
0.6
0.7
0.8
Phylostratum
Cellular_organisms - ps1
Archaea1 - ps2
Archaea2 - ps3
Archaea3 - ps4
Asgard/Eukaryota - ps5
Eukaryota - ps6
Amorpheae - ps7
Obazoa - ps8
Opisthokonta - ps9
Holozoa - ps10
Filozoa - ps11
Choano./Metazoa - ps12
Metazoa - ps13
Myriazoa - ps14
Parahoxozoa - ps15
Bilateria - ps16
Deuterostomia - ps17
Chordata - ps18
Olfactores - ps19
Vertebrata - ps20
Gnathostomata - ps21
Euteleostomi - ps22
Sarcopterygii - ps23
Tetrapoda - ps24
Amniota - ps25
Mammalia - ps26
Theria - ps27
Placentalia - ps28
Boreoeutheria - ps29
Euarchontoglires - ps30
Euarchonta - ps31
Primates - ps32
Haplorrhini - ps33
Simiiformes - ps34
Catarrhini - ps35
Hominoidea - ps36
Hominidae - ps37
Homininae - ps38
Hominini - ps39
Homo_sapiens - ps40
p-value
3e-03
5e-03
1e-02
2e-02
4e-02
Log-odds
0.5
1.0
1.5
2.0
2.5
3.0
3.5
4.0

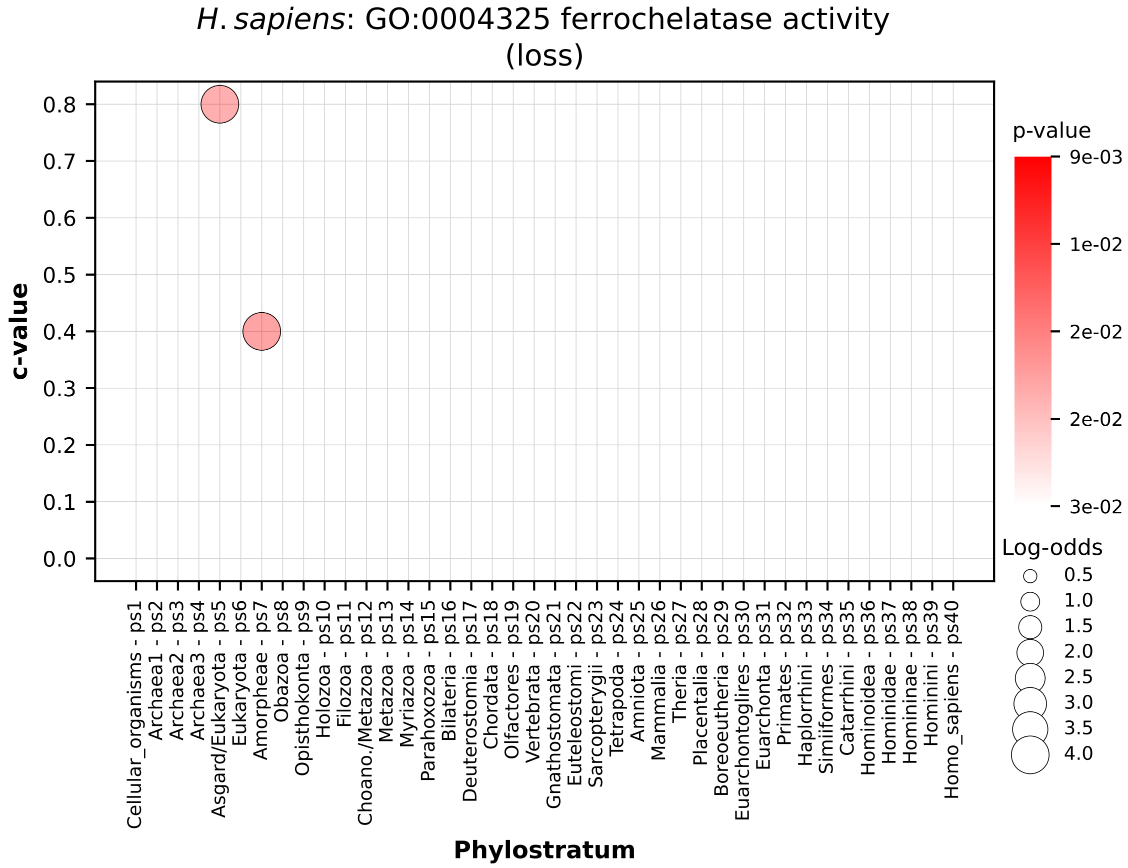

H. sapiens: GO:0004325 ferrochelatase activity
(loss)
c-value
0.8
0.7
0.6
0.5
0.4
0.3
0.2
0.1
0.0
p-value
9e-03
1e-02
2e-02
2e-02
3e-02
Log-odds
0.5
1.0
1.5
2.0
2.5
3.0
3.5
4.0
Cellular_organisms - ps1
Archaea1 - ps2
Archaea2 - ps3
Archaea3 - ps4
Asgard/Eukaryota - ps5
Eukaryota - ps6
Amorpheae - ps7
Obazoa - ps8
Opisthokonta - ps9
Holozoa - ps10
Filozoa - ps11
Choano./Metazoa - ps12
Metazoa - ps13
Myriazoa - ps14
Parahoxozoa - ps15
Bilateria - ps16
Deuterostomia - ps17
Chordata - ps18
Olfactores - ps19
Vertebrata - ps20
Gnathostomata - ps21
Euteleostomi - ps22
Sarcopterygii - ps23
Tetrapoda - ps24
Amniota - ps25
Mammalia - ps26
Theria - ps27
Placentalia - ps28
Boreoeutheria - ps29
Euarchontoglires - ps30
Euarchonta - ps31
Primates - ps32
Haplorrhini - ps33
Simiiformes - ps34
Catarrhini - ps35
Hominoidea - ps36
Hominidae - ps37
Homininae - ps38
Hominini - ps39
Homo_sapiens - ps40
Phylostratum

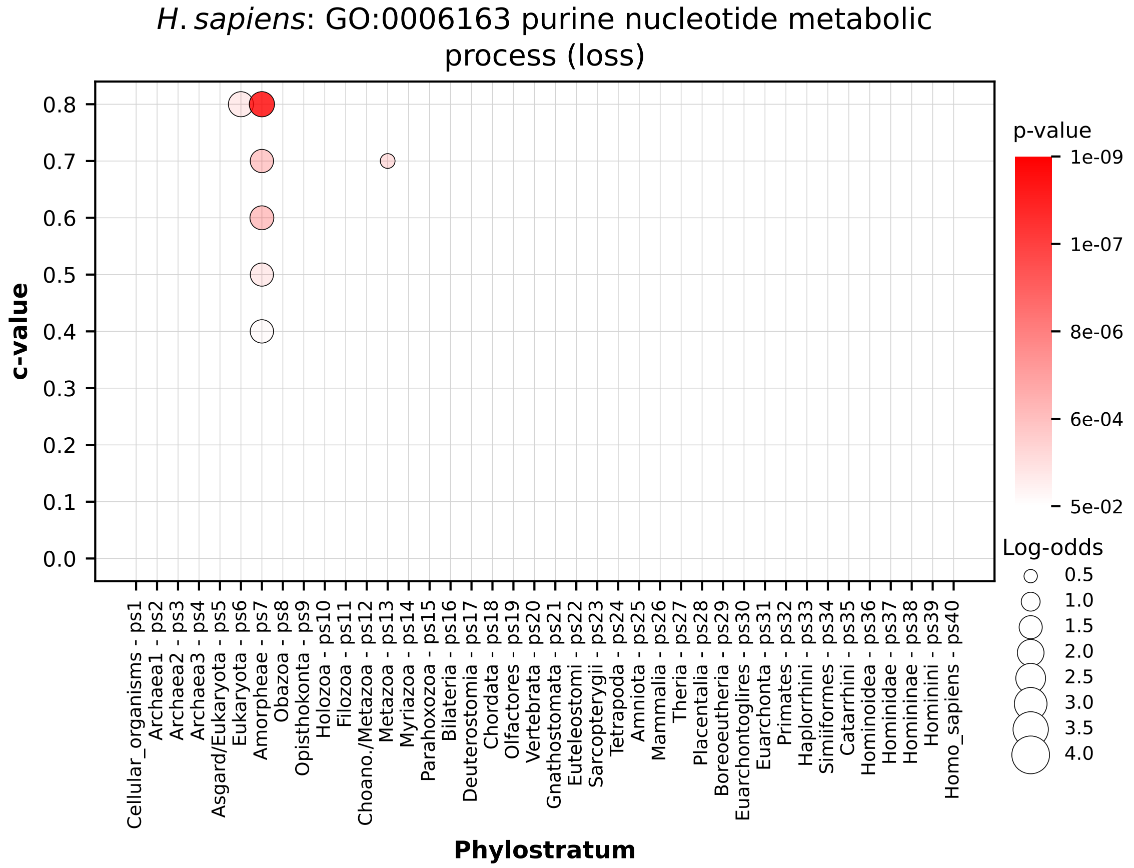

H. sapiens: GO:0006163 purine nucleotide metabolic process (loss)
c-value
0.8
0.7
0.6
0.5
0.4
0.3
0.2
0.1
0.0
Phylostratum
Cellular_organisms - ps1
Archaea1 - ps2
Archaea2 - ps3
Archaea3 - ps4
Asgard/Eukaryota - ps5
Eukaryota - ps6
Amorpheae - ps7
Obazoa - ps8
Opisthokonta - ps9
Holozoa - ps10
Filozoa - ps11
Choano./Metazoa - ps12
Metazoa - ps13
Myriazoa - ps14
Parahoxozoa - ps15
Bilateria - ps16
Deuterostomia - ps17
Chordata - ps18
Olfactores - ps19
Vertebrata - ps20
Gnathostomata - ps21
Euteleostomi - ps22
Sarcopterygii - ps23
Tetrapoda - ps24
Amniota - ps25
Mammalia - ps26
Theria - ps27
Placentalia - ps28
Boreoeutheria - ps29
Euarchontoglires - ps30
Euarchonta - ps31
Primates - ps32
Haplorrhini - ps33
Simiiformes - ps34
Catarrhini - ps35
Hominoidea - ps36
Hominidae - ps37
Homininae - ps38
Hominini - ps39
Homo_sapiens - ps40
p-value
1e-09
1e-07
8e-06
6e-04
5e-02
Log-odds
0.5
1.0
1.5
2.0
2.5
3.0
3.5
4.0

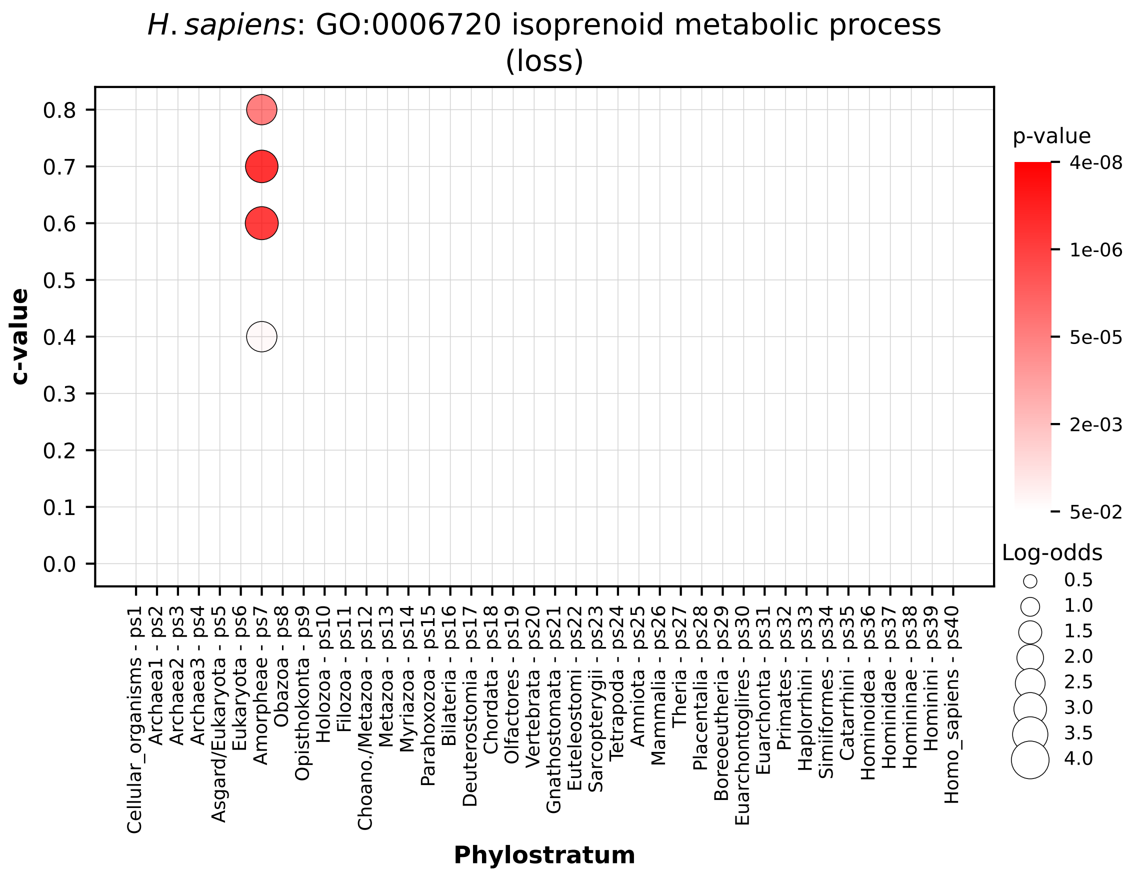

H. sapiens: GO:0006720 isoprenoid metabolic process
(loss)
c-value
0.0
0.1
0.2
0.3
0.4
0.5
0.6
0.7
0.8
p-value
4e-08
1e-06
5e-05
2e-03
5e-02
Log-odds
0.5
1.0
1.5
2.0
2.5
3.0
3.5
4.0
Cellular_organisms - ps1
Archaea1 - ps2
Archaea2 - ps3
Archaea3 - ps4
Asgard/Eukaryota - ps5
Eukaryota - ps6
Amorpheae - ps7
Obazoa - ps8
Opisthokonta - ps9
Holozoa - ps10
Filozoa - ps11
Choano./Metazoa - ps12
Metazoa - ps13
Myriazoa - ps14
Parahoxozoa - ps15
Bilateria - ps16
Deuterostomia - ps17
Chordata - ps18
Olfactores - ps19
Vertebrata - ps20
Gnathostomata - ps21
Euteleostomi - ps22
Sarcopterygii - ps23
Tetrapoda - ps24
Amniota - ps25
Mammalia - ps26
Theria - ps27
Placentalia - ps28
Boreoeutheria - ps29
Euarchontoglires - ps30
Euarchonta - ps31
Primates - ps32
Haplorrhini - ps33
Simiiformes - ps34
Catarrhini - ps35
Hominoidea - ps36
Hominidae - ps37
Homininae - ps38
Hominini - ps39
Homo_sapiens - ps40
Phylostratum

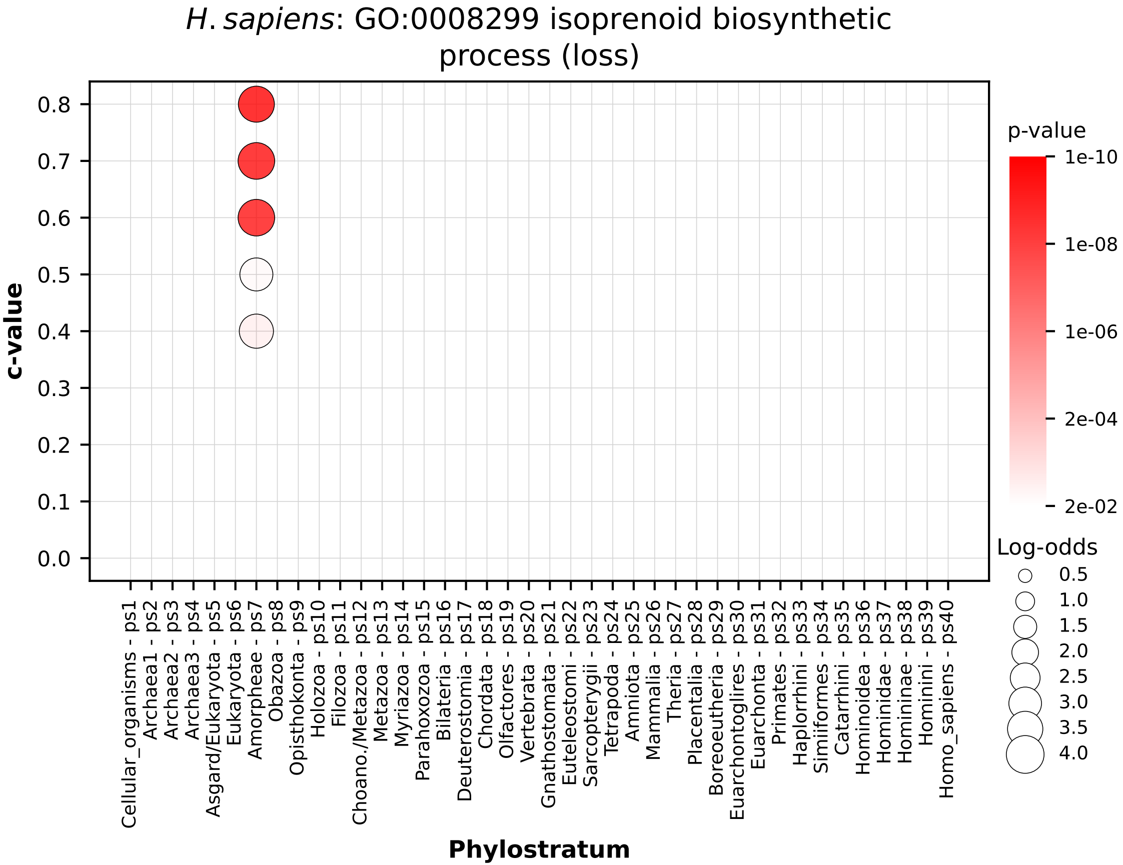
H. sapiens: GO:0008299 isoprenoid biosynthetic process (loss)
c-value
0.8
0.7
0.6
0.5
0.4
0.3
0.2
0.1
0.0
Phylostratum
Cellular_organisms - ps1
Archaea1 - ps2
Archaea2 - ps3
Archaea3 - ps4
Asgard/Eukaryota - ps5
Eukaryota - ps6
Amorpheae - ps7
Obazoa - ps8
Opisthokonta - ps9
Holozoa - ps10
Filozoa - ps11
Choano./Metazoa - ps12
Metazoa - ps13
Myriazoa - ps14
Parahoxozoa - ps15
Bilateria - ps16
Deuterostomia - ps17
Chordata - ps18
Olfactores - ps19
Vertebrata - ps20
Gnathostomata - ps21
Euteleostomi - ps22
Sarcopterygii - ps23
Tetrapoda - ps24
Amniota - ps25
Mammalia - ps26
Theria - ps27
Placentalia - ps28
Boreoeutheria - ps29
Euarchontoglires - ps30
Euarchonta - ps31
Primates - ps32
Haplorrhini - ps33
Simiiformes - ps34
Catarrhini - ps35
Hominoidea - ps36
Hominidae - ps37
Homininae - ps38
Hominini - ps39
Homo_sapiens - ps40
p-value
1e-10
1e-08
1e-06
2e-04
2e-02
Log-odds
0.5
1.0
1.5
2.0
2.5
3.0
3.5
4.0

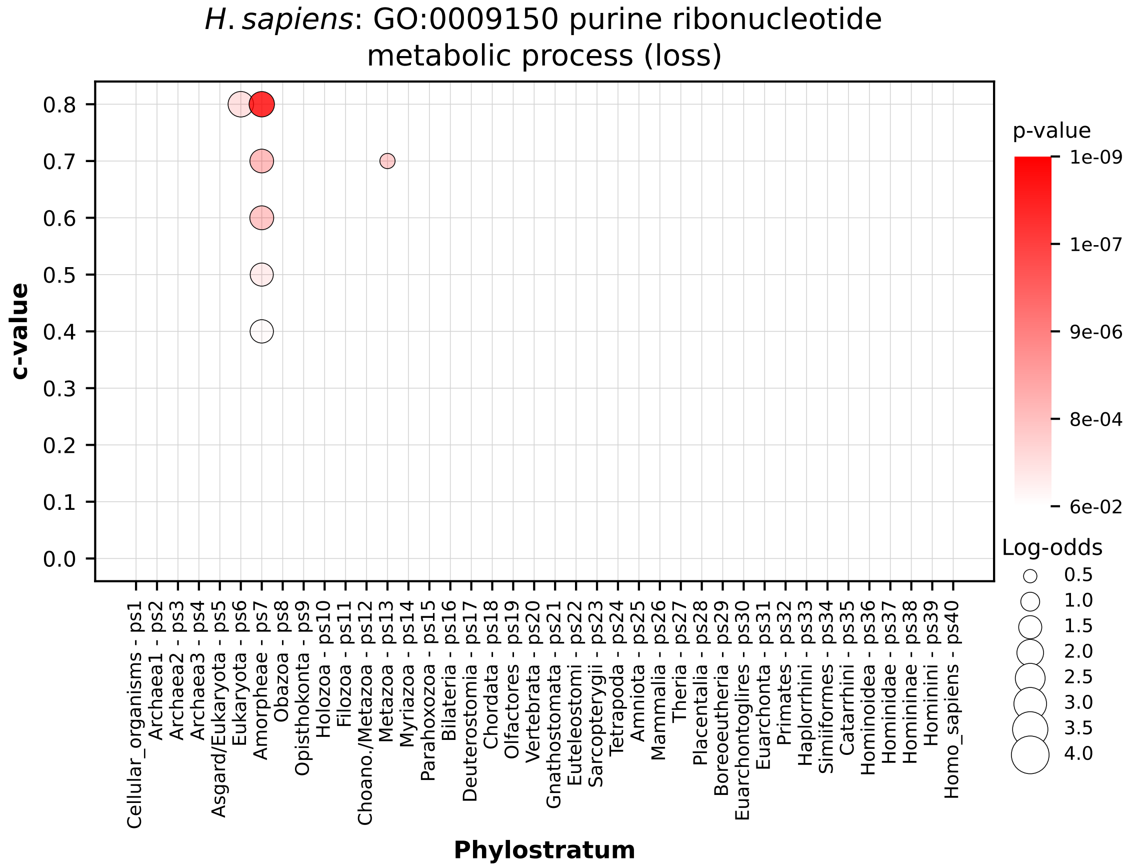

H. sapiens: GO:0009150 purine ribonucleotide metabolic process (loss)
c-value
0.8
0.7
0.6
0.5
0.4
0.3
0.2
0.1
0.0
p-value
1e-09
1e-07
9e-06
8e-04
6e-02
Log-odds
0.5
1.0
1.5
2.0
2.5
3.0
3.5
4.0
Cellular_organisms - ps1
Archaea1 - ps2
Archaea2 - ps3
Archaea3 - ps4
Asgard/Eukaryota - ps5
Eukaryota - ps6
Amorpheae - ps7
Obazoa - ps8
Opisthokonta - ps9
Holozoa - ps10
Filozoa - ps11
Choano./Metazoa - ps12
Metazoa - ps13
Myriazoa - ps14
Parahoxozoa - ps15
Bilateria - ps16
Deuterostomia - ps17
Chordata - ps18
Olfactores - ps19
Vertebrata - ps20
Gnathostomata - ps21
Euteleostomi - ps22
Sarcopterygii - ps23
Tetrapoda - ps24
Amniota - ps25
Mammalia - ps26
Theria - ps27
Placentalia - ps28
Boreoeutheria - ps29
Euarchontoglires - ps30
Euarchonta - ps31
Primates - ps32
Haplorrhini - ps33
Simiiformes - ps34
Catarrhini - ps35
Hominoidea - ps36
Hominidae - ps37
Homininae - ps38
Hominini - ps39
Homo_sapiens - ps40
Phylostratum

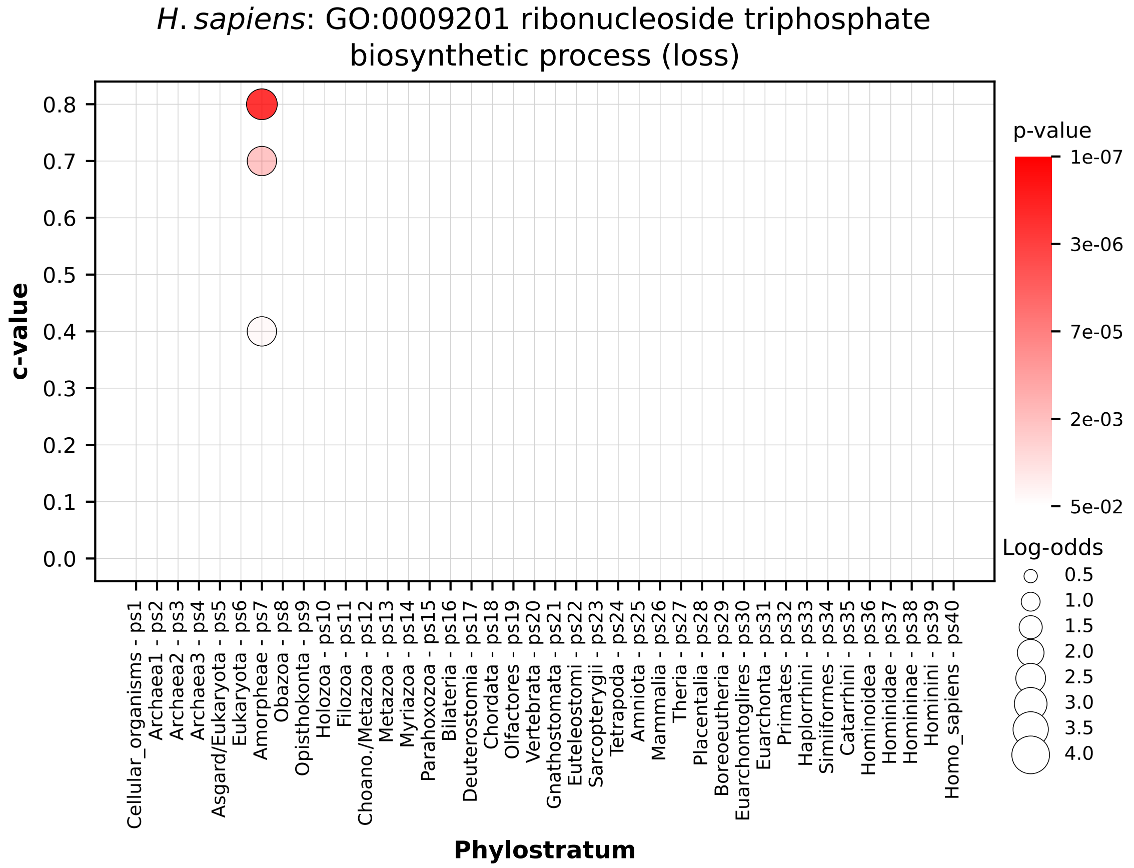

H. sapiens: GO:0009201 ribonucleoside triphosphate biosynthetic process (loss)
c-value
0.0
0.1
0.2
0.3
0.4
0.5
0.6
0.7
0.8
Phylostratum
Cellular_organisms - ps1
Archaea1 - ps2
Archaea2 - ps3
Archaea3 - ps4
Asgard/Eukaryota - ps5
Eukaryota - ps6
Amorpheae - ps7
Obazoa - ps8
Opisthokonta - ps9
Holozoa - ps10
Filozoa - ps11
Choano./Metazoa - ps12
Metazoa - ps13
Myriazoa - ps14
Parahoxozoa - ps15
Bilateria - ps16
Deuterostomia - ps17
Chordata - ps18
Olfactores - ps19
Vertebrata - ps20
Gnathostomata - ps21
Euteleostomi - ps22
Sarcopterygii - ps23
Tetrapoda - ps24
Amniota - ps25
Mammalia - ps26
Theria - ps27
Placentalia - ps28
Boreoeutheria - ps29
Euarchontoglires - ps30
Euarchonta - ps31
Primates - ps32
Haplorrhini - ps33
Simiiformes - ps34
Catarrhini - ps35
Hominoidea - ps36
Hominidae - ps37
Homininae - ps38
Hominini - ps39
Homo_sapiens - ps40
p-value
1e-07
3e-06
7e-05
2e-03
5e-02
Log-odds
0.5
1.0
1.5
2.0
2.5
3.0
3.5
4.0

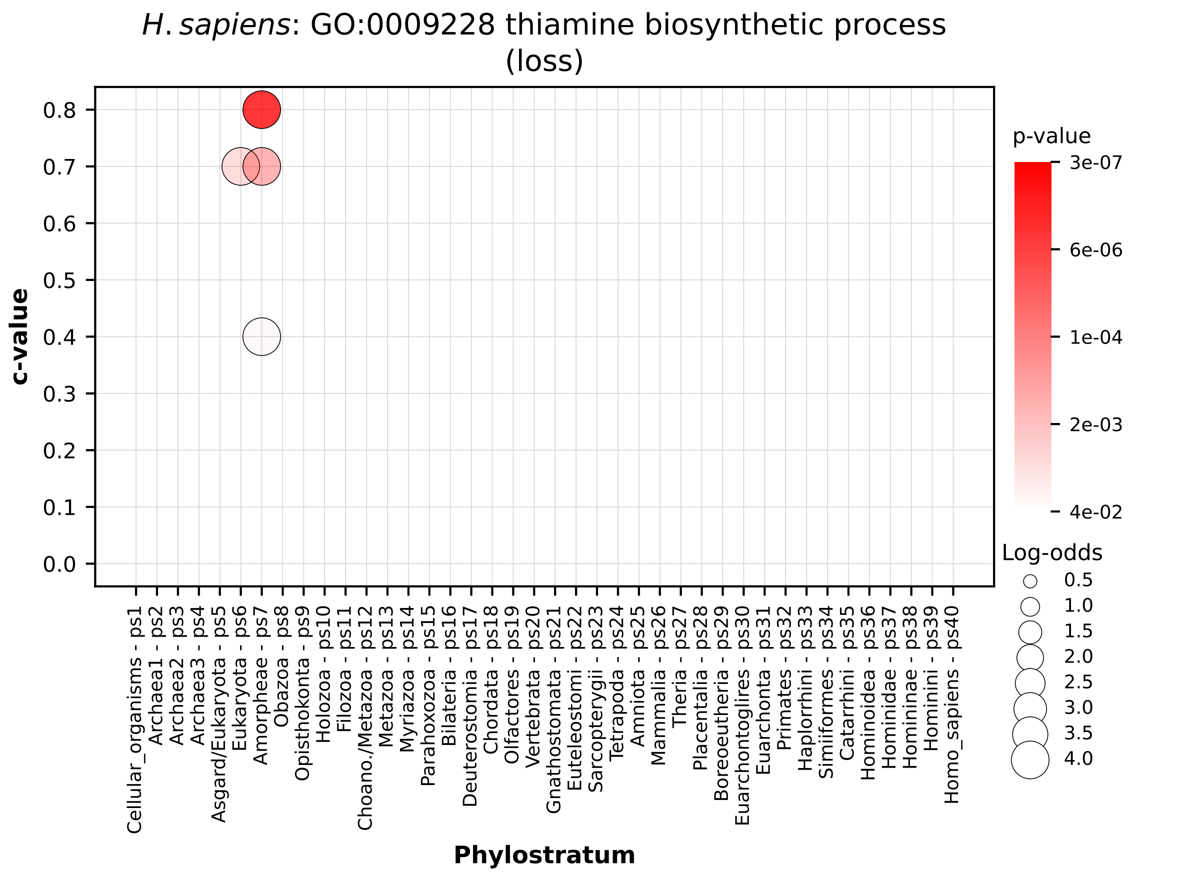

H. sapiens: GO:0009228 thiamine biosynthetic process
(loss)
c-value
0.0
0.1
0.2
0.3
0.4
0.5
0.6
0.7
0.8
Phylostratum
Cellular_organisms - ps1
Archaea1 - ps2
Archaea2 - ps3
Archaea3 - ps4
Asgard/Eukaryota - ps5
Eukaryota - ps6
Amorpheae - ps7
Obazoa - ps8
Opisthokonta - ps9
Holozoa - ps10
Filozoa - ps11
Choano./Metazoa - ps12
Metazoa - ps13
Myriazoa - ps14
Parahoxozoa - ps15
Bilateria - ps16
Deuterostomia - ps17
Chordata - ps18
Olfactores - ps19
Vertebrata - ps20
Gnathostomata - ps21
Euteleostomi - ps22
Sarcopterygii - ps23
Tetrapoda - ps24
Amniota - ps25
Mammalia - ps26
Theria - ps27
Placentalia - ps28
Boreoeutheria - ps29
Euarchontoglires - ps30
Euarchonta - ps31
Primates - ps32
Haplorrhini - ps33
Simiiformes - ps34
Catarrhini - ps35
Hominoidea - ps36
Hominidae - ps37
Homininae - ps38
Hominini - ps39
Homo_sapiens - ps40
p-value
3e-07
6e-06
1e-04
2e-03
4e-02
Log-odds
0.5
1.0
1.5
2.0
2.5
3.0
3.5
4.0

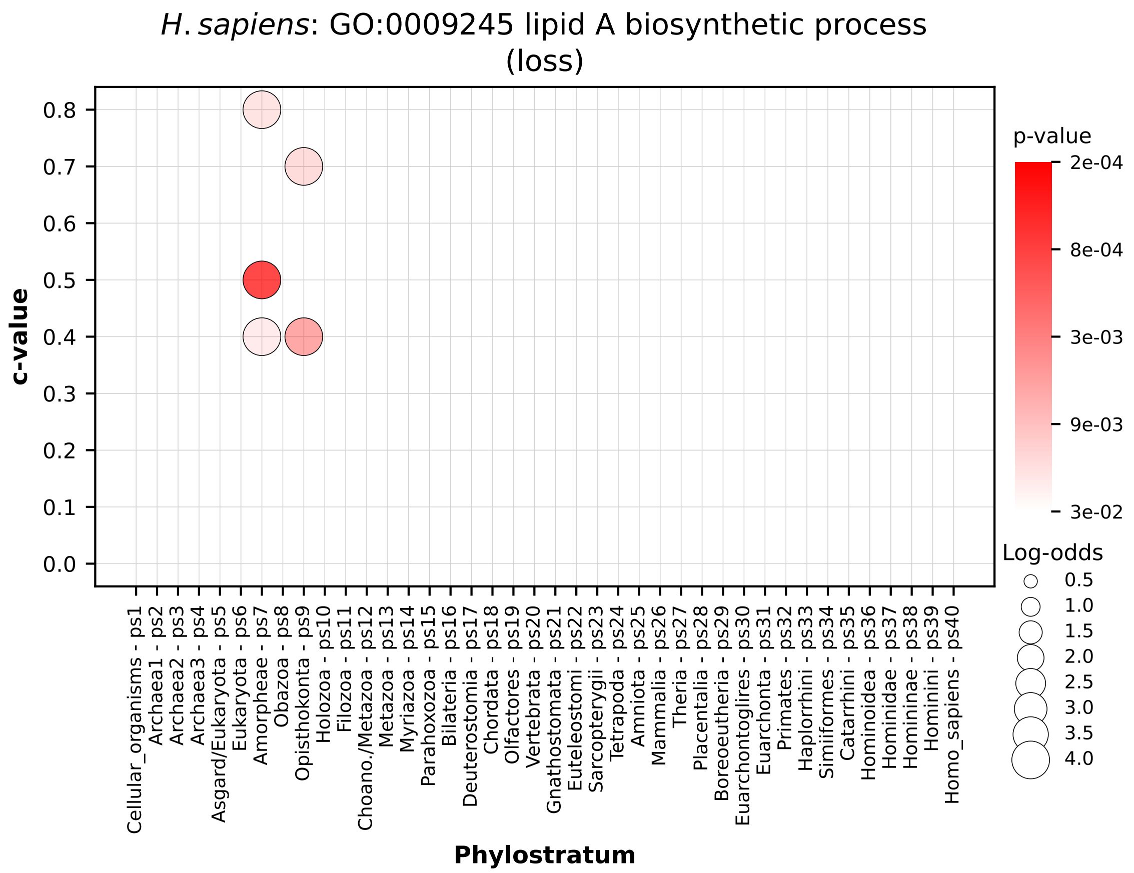
H. sapiens: GO:0009245 lipid A biosynthetic process
(loss)
c-value
0.8
0.7
0.6
0.5
0.4
0.3
0.2
0.1
0.0
p-value
2e-04
8e-04
3e-03
9e-03
3e-02
Log-odds
0.5
1.0
1.5
2.0
2.5
3.0
3.5
4.0
Cellular_organisms - ps1
Archaea1 - ps2
Archaea2 - ps3
Archaea3 - ps4
Asgard/Eukaryota - ps5
Eukaryota - ps6
Amorpheae - ps7
Obazoa - ps8
Opisthokonta - ps9
Holozoa - ps10
Filozoa - ps11
Choano./Metazoa - ps12
Metazoa - ps13
Myriazoa - ps14
Parahoxozoa - ps15
Bilateria - ps16
Deuterostomia - ps17
Chordata - ps18
Olfactores - ps19
Vertebrata - ps20
Gnathostomata - ps21
Euteleostomi - ps22
Sarcopterygii - ps23
Tetrapoda - ps24
Amniota - ps25
Mammalia - ps26
Theria - ps27
Placentalia - ps28
Boreoeutheria - ps29
Euarchontoglires - ps30
Euarchonta - ps31
Primates - ps32
Haplorrhini - ps33
Simiiformes - ps34
Catarrhini - ps35
Hominoidea - ps36
Hominidae - ps37
Homininae - ps38
Hominini - ps39
Homo_sapiens - ps40
Phylostratum

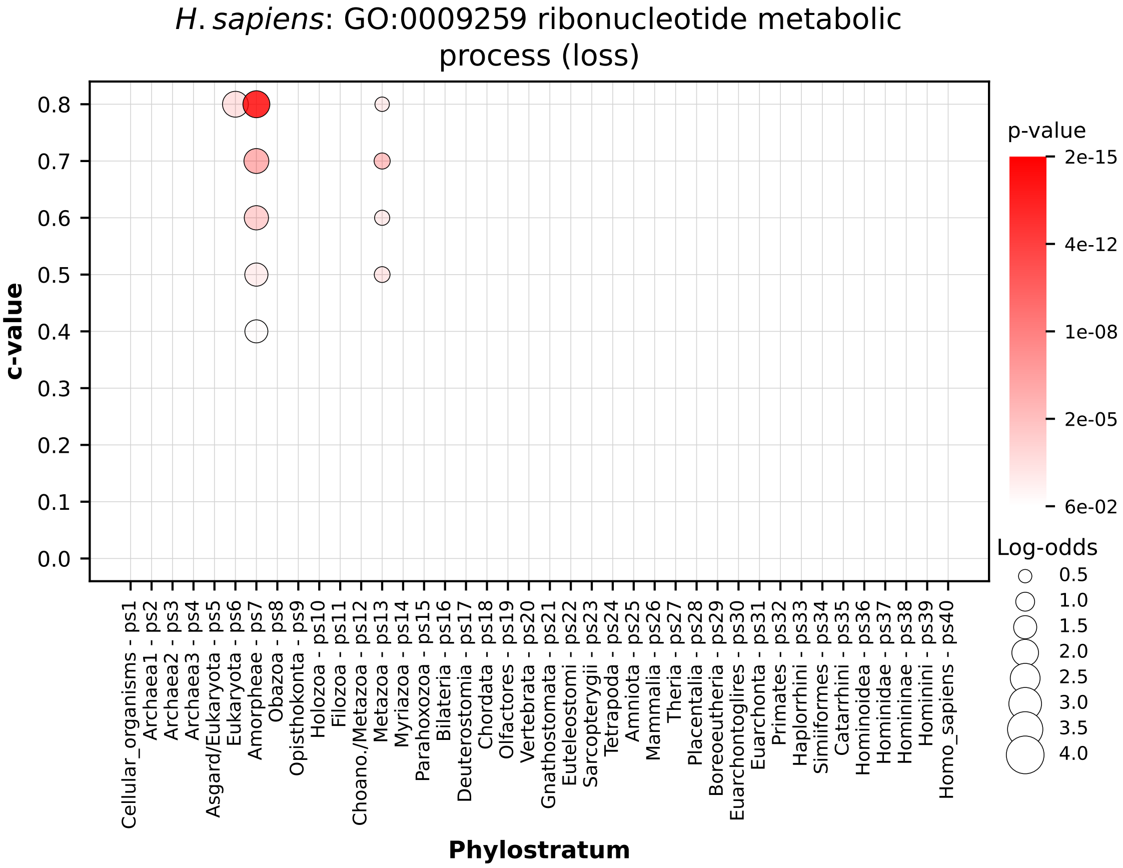

H. sapiens: GO:0009259 ribonucleotide metabolic process (loss)
c-value
0.0
0.1
0.2
0.3
0.4
0.5
0.6
0.7
0.8
Phylostratum
Cellular_organisms - ps1
Archaea1 - ps2
Archaea2 - ps3
Archaea3 - ps4
Asgard/Eukaryota - ps5
Eukaryota - ps6
Amorpheae - ps7
Obazoa - ps8
Opisthokonta - ps9
Holozoa - ps10
Filozoa - ps11
Choano./Metazoa - ps12
Metazoa - ps13
Myriazoa - ps14
Parahoxozoa - ps15
Bilateria - ps16
Deuterostomia - ps17
Chordata - ps18
Olfactores - ps19
Vertebrata - ps20
Gnathostomata - ps21
Euteleostomi - ps22
Sarcopterygii - ps23
Tetrapoda - ps24
Amniota - ps25
Mammalia - ps26
Theria - ps27
Placentalia - ps28
Boreoeutheria - ps29
Euarchontoglires - ps30
Euarchonta - ps31
Primates - ps32
Haplorrhini - ps33
Simiiformes - ps34
Catarrhini - ps35
Hominoidea - ps36
Hominidae - ps37
Homininae - ps38
Hominini - ps39
Homo_sapiens - ps40
p-value
2e-15
4e-12
1e-08
2e-05
6e-02
Log-odds
0.5
1.0
1.5
2.0
2.5
3.0
3.5
4.0

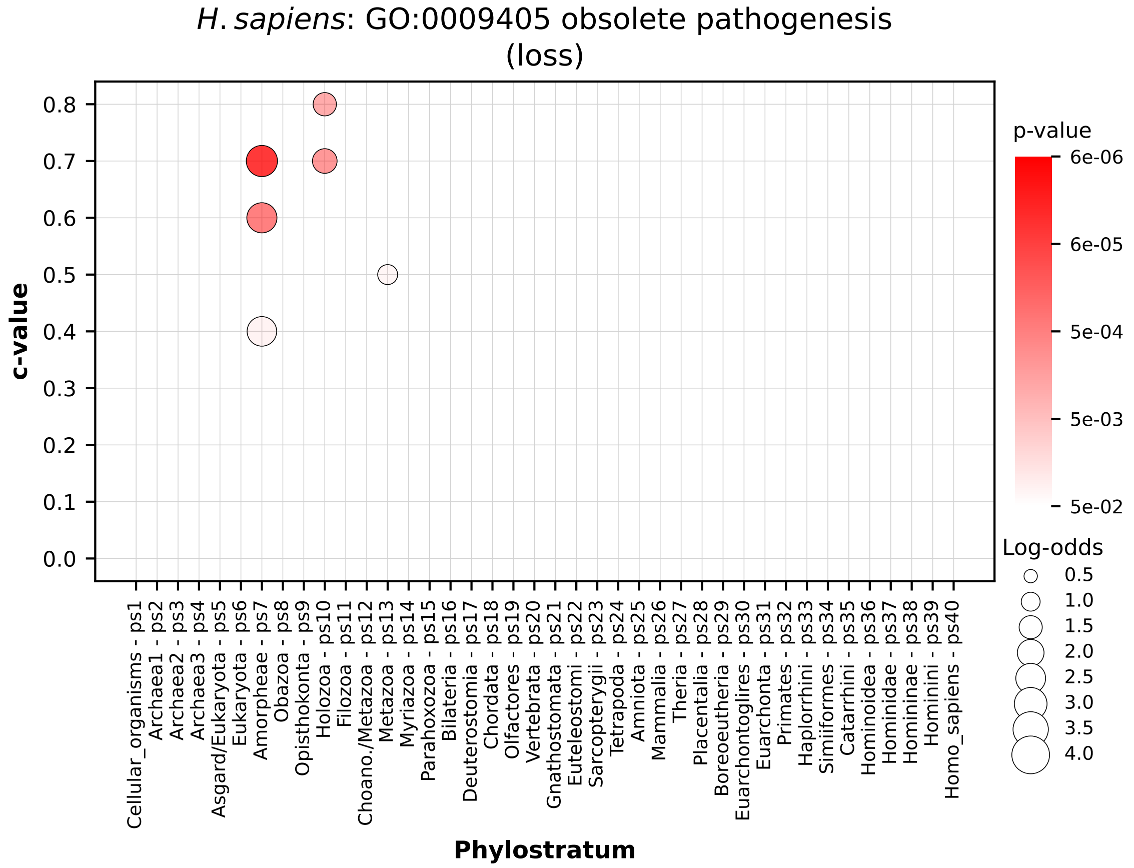

H. sapiens: GO:0009405 obsolete pathogenesis
(loss)
c-value
0.0
0.1
0.2
0.3
0.4
0.5
0.6
0.7
0.8
Phylostratum
Cellular_organisms - ps1
Archaea1 - ps2
Archaea2 - ps3
Archaea3 - ps4
Asgard/Eukaryota - ps5
Eukaryota - ps6
Amorpheae - ps7
Obazoa - ps8
Opisthokonta - ps9
Holozoa - ps10
Filozoa - ps11
Choano./Metazoa - ps12
Metazoa - ps13
Myriazoa - ps14
Parahoxozoa - ps15
Bilateria - ps16
Deuterostomia - ps17
Chordata - ps18
Olfactores - ps19
Vertebrata - ps20
Gnathostomata - ps21
Euteleostomi - ps22
Sarcopterygii - ps23
Tetrapoda - ps24
Amniota - ps25
Mammalia - ps26
Theria - ps27
Placentalia - ps28
Boreoeutheria - ps29
Euarchontoglires - ps30
Euarchonta - ps31
Primates - ps32
Haplorrhini - ps33
Simiiformes - ps34
Catarrhini - ps35
Hominoidea - ps36
Hominidae - ps37
Homininae - ps38
Hominini - ps39
Homo_sapiens - ps40
p-value
6e-06
6e-05
5e-04
5e-03
5e-02
Log-odds
0.5
1.0
1.5
2.0
2.5
3.0
3.5
4.0

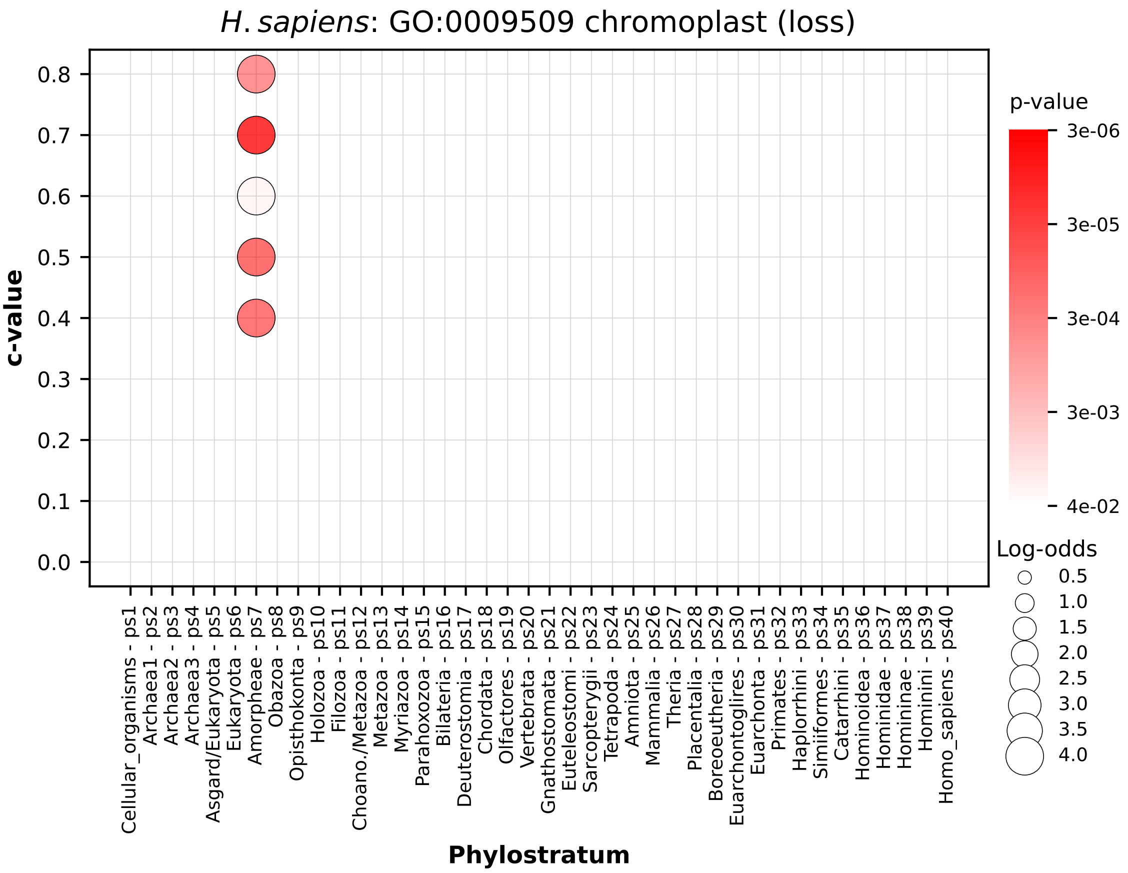

H. sapiens: GO:0009509 chromoplast (loss)
c-value
0.8
0.7
0.6
0.5
0.4
0.3
0.2
0.1
0.0
Phylostratum
Cellular_organisms - ps1
Archaea1 - ps2
Archaea2 - ps3
Archaea3 - ps4
Asgard/Eukaryota - ps5
Eukaryota - ps6
Amorpheae - ps7
Obazoa - ps8
Opisthokonta - ps9
Holozoa - ps10
Filozoa - ps11
Choano./Metazoa - ps12
Metazoa - ps13
Myriazoa - ps14
Parahoxozoa - ps15
Bilateria - ps16
Deuterostomia - ps17
Chordata - ps18
Olfactores - ps19
Vertebrata - ps20
Gnathostomata - ps21
Euteleostomi - ps22
Sarcopterygii - ps23
Tetrapoda - ps24
Amniota - ps25
Mammalia - ps26
Theria - ps27
Placentalia - ps28
Boreoeutheria - ps29
Euarchontoglires - ps30
Euarchonta - ps31
Primates - ps32
Haplorrhini - ps33
Simiiformes - ps34
Catarrhini - ps35
Hominoidea - ps36
Hominidae - ps37
Homininae - ps38
Hominini - ps39
Homo_sapiens - ps40
p-value
3e-06
3e-05
3e-04
3e-03
4e-02
Log-odds
0.5
1.0
1.5
2.0
2.5
3.0
3.5
4.0

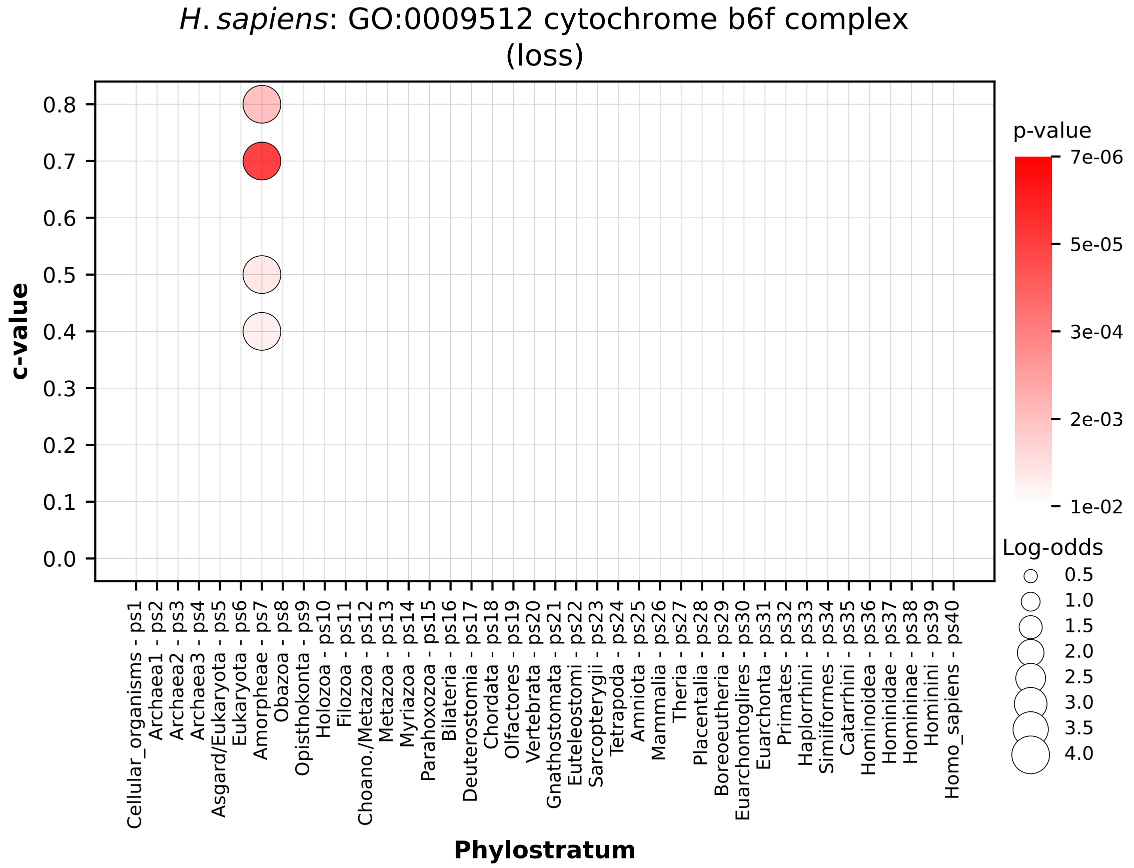
H. sapiens: GO:0009512 cytochrome b6f complex
(loss)
c-value
0.0
0.1
0.2
0.3
0.4
0.5
0.6
0.7
0.8
Phylostratum
Cellular_organisms - ps1
Archaea1 - ps2
Archaea2 - ps3
Archaea3 - ps4
Asgard/Eukaryota - ps5
Eukaryota - ps6
Amorpheae - ps7
Obazoa - ps8
Opisthokonta - ps9
Holozoa - ps10
Filozoa - ps11
Choano./Metazoa - ps12
Metazoa - ps13
Myriazoa - ps14
Parahoxozoa - ps15
Bilateria - ps16
Deuterostomia - ps17
Chordata - ps18
Olfactores - ps19
Vertebrata - ps20
Gnathostomata - ps21
Euteleostomi - ps22
Sarcopterygii - ps23
Tetrapoda - ps24
Amniota - ps25
Mammalia - ps26
Theria - ps27
Placentalia - ps28
Boreoeutheria - ps29
Euarchontoglires - ps30
Euarchonta - ps31
Primates - ps32
Haplorrhini - ps33
Simiiformes - ps34
Catarrhini - ps35
Hominoidea - ps36
Hominidae - ps37
Homininae - ps38
Hominini - ps39
Homo_sapiens - ps40
p-value
7e-06
5e-05
3e-04
2e-03
1e-02
Log-odds
0.5
1.0
1.5
2.0
2.5
3.0
3.5
4.0

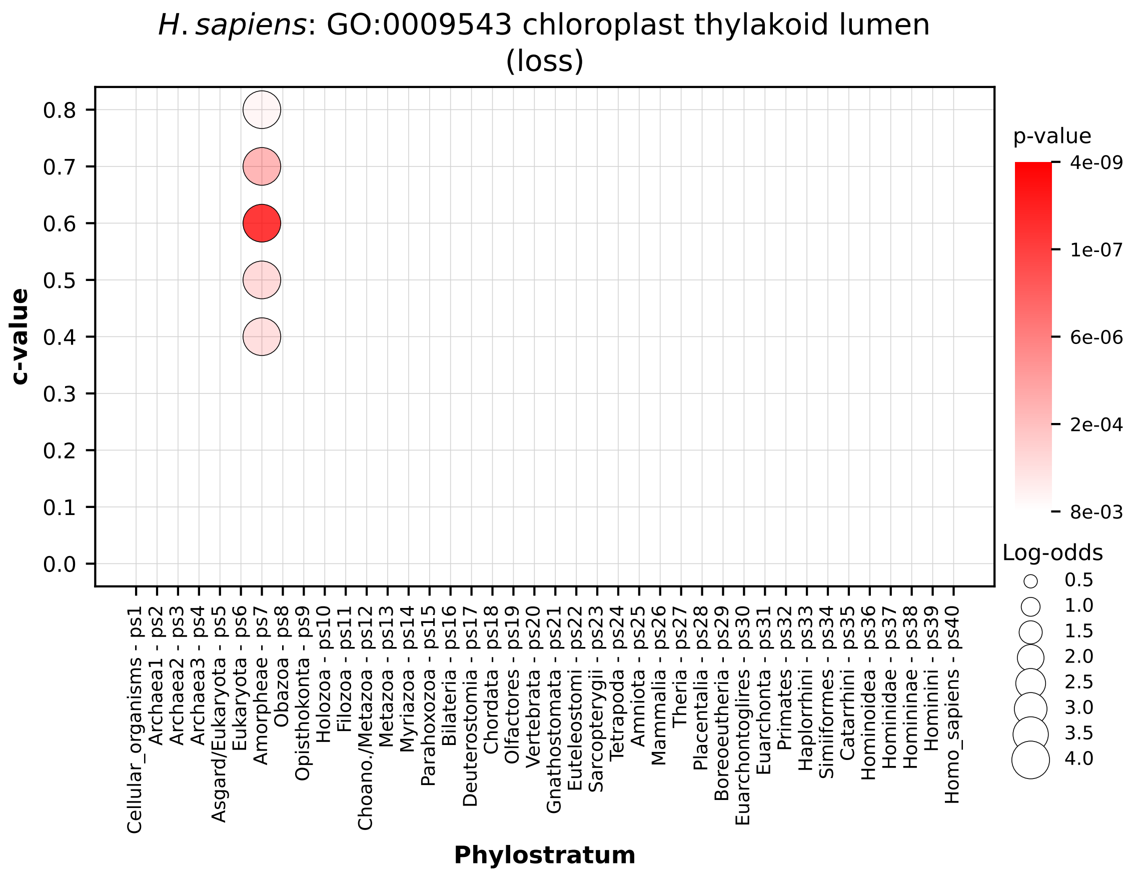

H. sapiens: GO:0009543 chloroplast thylakoid lumen
(loss)
c-value
0.0
0.1
0.2
0.3
0.4
0.5
0.6
0.7
0.8
p-value
4e-09
1e-07
6e-06
2e-04
8e-03
Log-odds
0.5
1.0
1.5
2.0
2.5
3.0
3.5
4.0
Phylostratum
Cellular_organisms - ps1
Archaea1 - ps2
Archaea2 - ps3
Archaea3 - ps4
Asgard/Eukaryota - ps5
Eukaryota - ps6
Amorpheae - ps7
Obazoa - ps8
Opisthokonta - ps9
Holozoa - ps10
Filozoa - ps11
Choano./Metazoa - ps12
Metazoa - ps13
Myriazoa - ps14
Parahoxozoa - ps15
Bilateria - ps16
Deuterostomia - ps17
Chordata - ps18
Olfactores - ps19
Vertebrata - ps20
Gnathostomata - ps21
Euteleostomi - ps22
Sarcopterygii - ps23
Tetrapoda - ps24
Amniota - ps25
Mammalia - ps26
Theria - ps27
Placentalia - ps28
Boreoeutheria - ps29
Euarchontoglires - ps30
Euarchonta - ps31
Primates - ps32
Haplorrhini - ps33
Simiiformes - ps34
Catarrhini - ps35
Hominoidea - ps36
Hominidae - ps37
Homininae - ps38
Hominini - ps39
Homo_sapiens - ps40

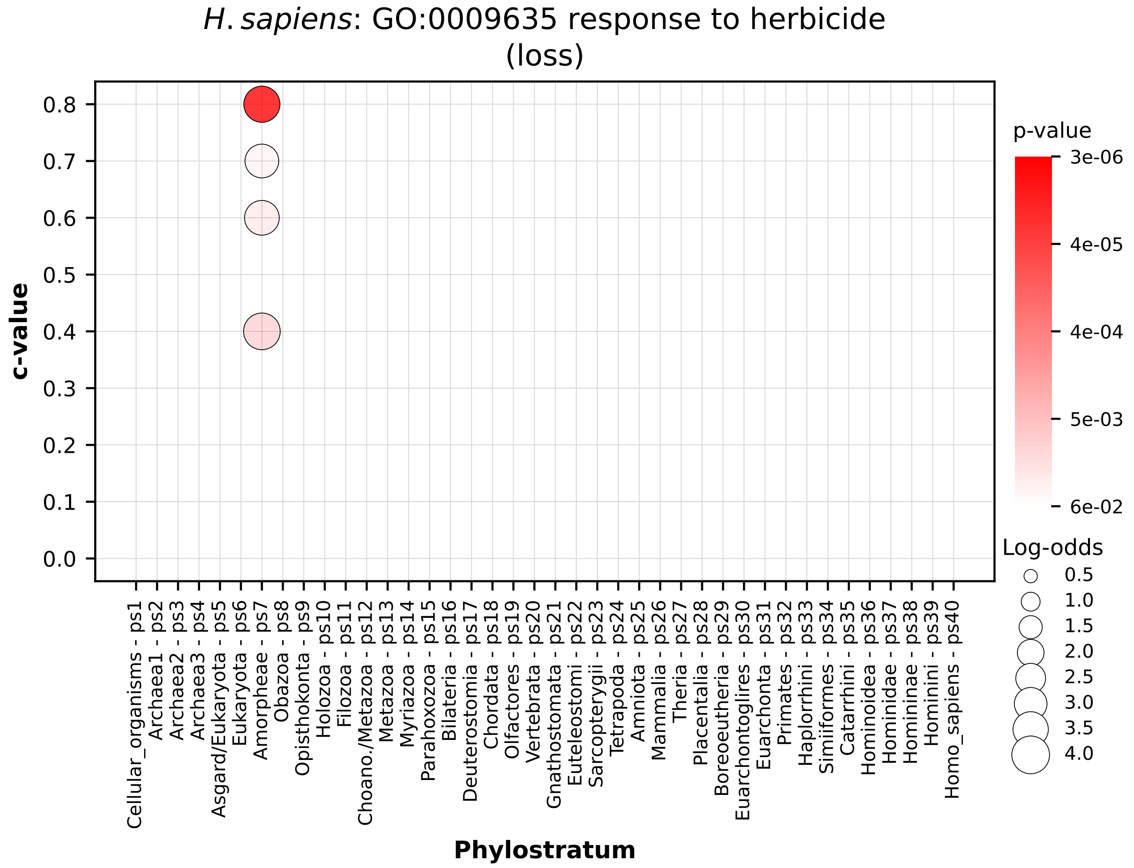

H. sapiens: GO:0009635 response to herbicide
(loss)
c-value
0.0
0.1
0.2
0.3
0.4
0.5
0.6
0.7
0.8
Phylostratum
Cellular_organisms - ps1
Archaea1 - ps2
Archaea2 - ps3
Archaea3 - ps4
Asgard/Eukaryota - ps5
Eukaryota - ps6
Amorpheae - ps7
Obazoa - ps8
Opisthokonta - ps9
Holozoa - ps10
Filozoa - ps11
Choano./Metazoa - ps12
Metazoa - ps13
Myriazoa - ps14
Parahoxozoa - ps15
Bilateria - ps16
Deuterostomia - ps17
Chordata - ps18
Olfactores - ps19
Vertebrata - ps20
Gnathostomata - ps21
Euteleostomi - ps22
Sarcopterygii - ps23
Tetrapoda - ps24
Amniota - ps25
Mammalia - ps26
Theria - ps27
Placentalia - ps28
Boreoeutheria - ps29
Euarchontoglires - ps30
Euarchonta - ps31
Primates - ps32
Haplorrhini - ps33
Simiiformes - ps34
Catarrhini - ps35
Hominoidea - ps36
Hominidae - ps37
Homininae - ps38
Hominini - ps39
Homo_sapiens - ps40
p-value
3e-06
4e-05
4e-04
5e-03
6e-02
Log-odds
0.5
1.0
1.5
2.0
2.5
3.0
3.5
4.0

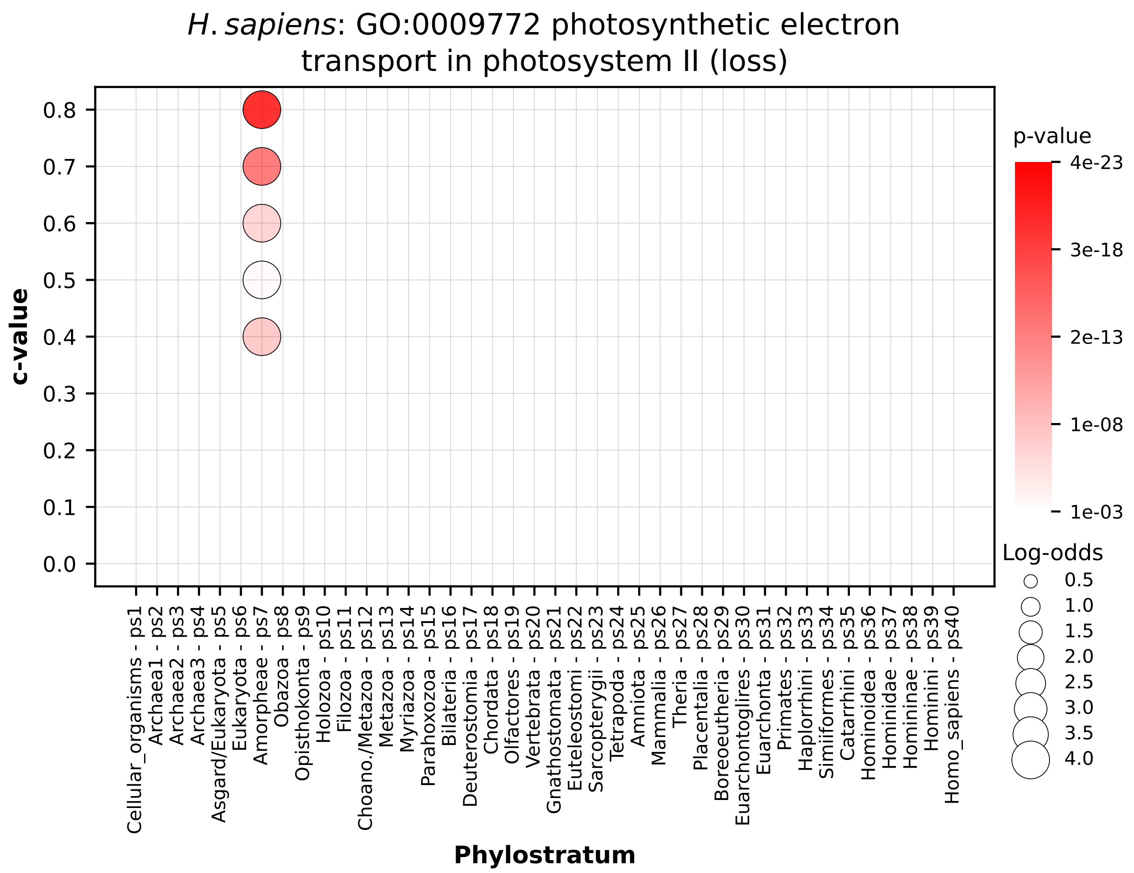

H. sapiens: GO:0009772 photosynthetic electron transport in photosystem II (loss)
c-value
0.8
0.7
0.6
0.5
0.4
0.3
0.2
0.1
0.0
p-value
4e-23
3e-18
2e-13
1e-08
1e-03
Log-odds
0.5
1.0
1.5
2.0
2.5
3.0
3.5
4.0
Cellular_organisms - ps1
Archaea1 - ps2
Archaea2 - ps3
Archaea3 - ps4
Asgard/Eukaryota - ps5
Eukaryota - ps6
Amorpheae - ps7
Obazoa - ps8
Opisthokonta - ps9
Holozoa - ps10
Filozoa - ps11
Choano./Metazoa - ps12
Metazoa - ps13
Myriazoa - ps14
Parahoxozoa - ps15
Bilateria - ps16
Deuterostomia - ps17
Chordata - ps18
Olfactores - ps19
Vertebrata - ps20
Gnathostomata - ps21
Euteleostomi - ps22
Sarcopterygii - ps23
Tetrapoda - ps24
Amniota - ps25
Mammalia - ps26
Theria - ps27
Placentalia - ps28
Boreoeutheria - ps29
Euarchontoglires - ps30
Euarchonta - ps31
Primates - ps32
Haplorrhini - ps33
Simiiformes - ps34
Catarrhini - ps35
Hominoidea - ps36
Hominidae - ps37
Homininae - ps38
Hominini - ps39
Homo_sapiens - ps40
Phylostratum

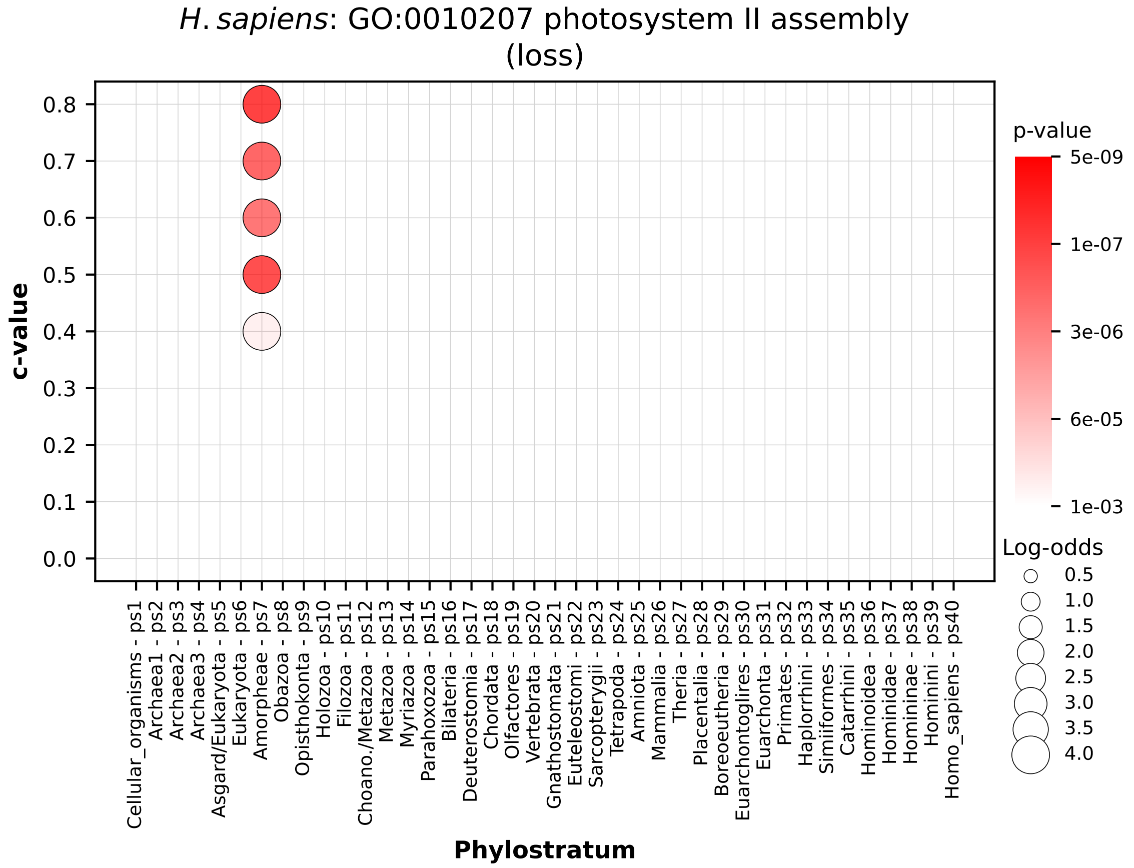

$H. sapiens$: GO:0010207 photosystem II assembly
(loss)
c-value
0.0
0.1
0.2
0.3
0.4
0.5
0.6
0.7
0.8
Phylostratum
Cellular_organisms - ps1
Archaea1 - ps2
Archaea2 - ps3
Archaea3 - ps4
Asgard/Eukaryota - ps5
Eukaryota - ps6
Amorpheae - ps7
Obazoa - ps8
Opisthokonta - ps9
Holozoa - ps10
Filozoa - ps11
Choano./Metazoa - ps12
Metazoa - ps13
Myriazoa - ps14
Parahoxozoa - ps15
Bilateria - ps16
Deuterostomia - ps17
Chordata - ps18
Olfactores - ps19
Vertebrata - ps20
Gnathostomata - ps21
Euteleostomi - ps22
Sarcopterygii - ps23
Tetrapoda - ps24
Amniota - ps25
Mammalia - ps26
Theria - ps27
Placentalia - ps28
Boreoeutheria - ps29
Euarchontoglires - ps30
Euarchonta - ps31
Primates - ps32
Haplorrhini - ps33
Simiiformes - ps34
Catarrhini - ps35
Hominoidea - ps36
Hominidae - ps37
Homininae - ps38
Hominini - ps39
Homo_sapiens - ps40
p-value
5e-09
1e-07
3e-06
6e-05
1e-03
Log-odds
0.5
1.0
1.5
2.0
2.5
3.0
3.5
4.0

H. sapiens: GO:0016108 tetraterpenoid metabolic process (loss)

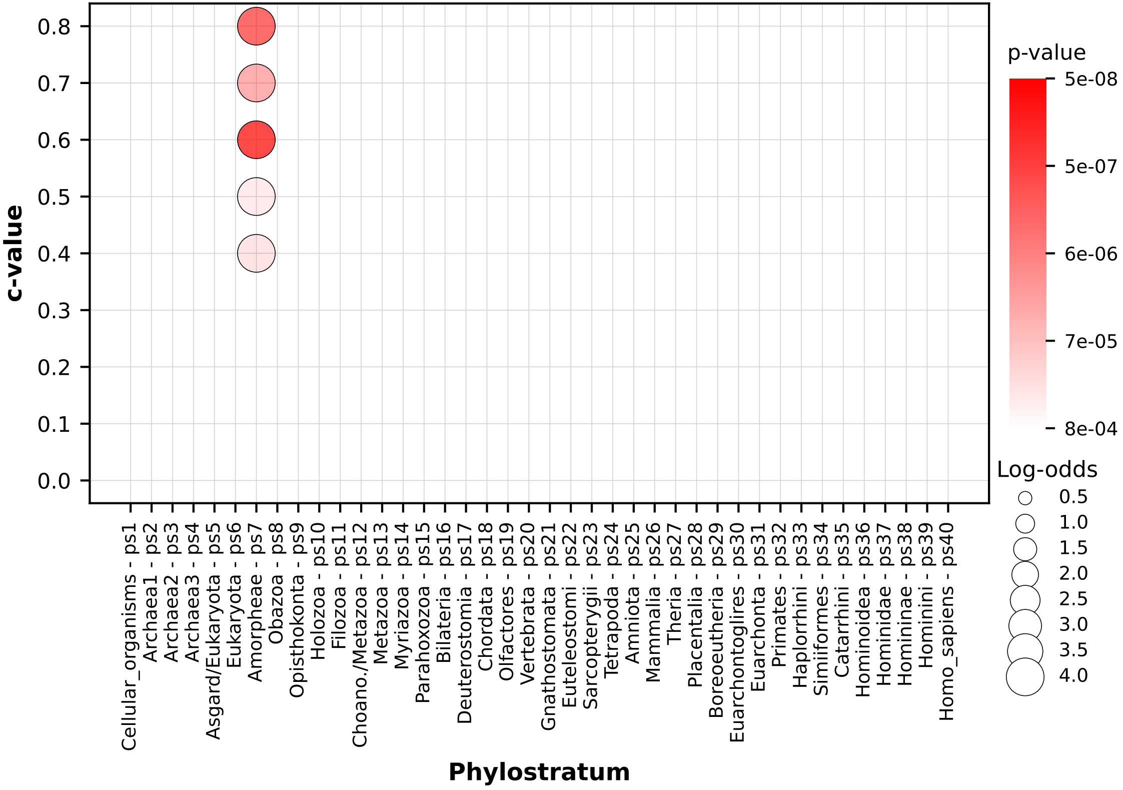

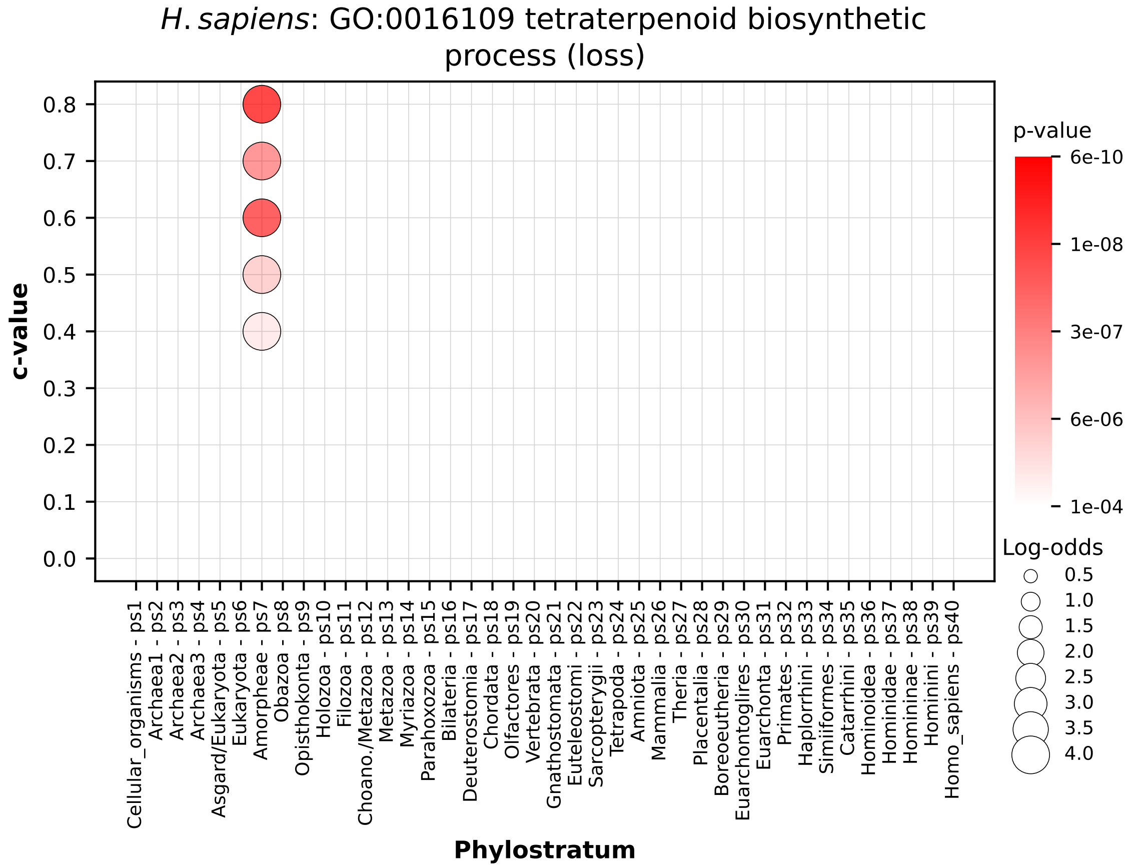

H. sapiens: GO:0016109 tetraterpenoid biosynthetic process (loss)
c-value
0.0
0.1
0.2
0.3
0.4
0.5
0.6
0.7
0.8
Phylostratum
Cellular_organisms - ps1
Archaea1 - ps2
Archaea2 - ps3
Archaea3 - ps4
Asgard/Eukaryota - ps5
Eukaryota - ps6
Amorpheae - ps7
Obazoa - ps8
Opisthokonta - ps9
Holozoa - ps10
Filozoa - ps11
Choano./Metazoa - ps12
Metazoa - ps13
Myriazoa - ps14
Parahoxozoa - ps15
Bilateria - ps16
Deuterostomia - ps17
Chordata - ps18
Olfactores - ps19
Vertebrata - ps20
Gnathostomata - ps21
Euteleostomi - ps22
Sarcopterygii - ps23
Tetrapoda - ps24
Amniota - ps25
Mammalia - ps26
Theria - ps27
Placentalia - ps28
Boreoeutheria - ps29
Euarchontoglires - ps30
Euarchonta - ps31
Primates - ps32
Haplorrhini - ps33
Simiiformes - ps34
Catarrhini - ps35
Hominoidea - ps36
Hominidae - ps37
Homininae - ps38
Hominini - ps39
Homo_sapiens - ps40
p-value
6e-10
1e-08
3e-07
6e-06
1e-04
Log-odds
0.5
1.0
1.5
2.0
2.5
3.0
3.5
4.0

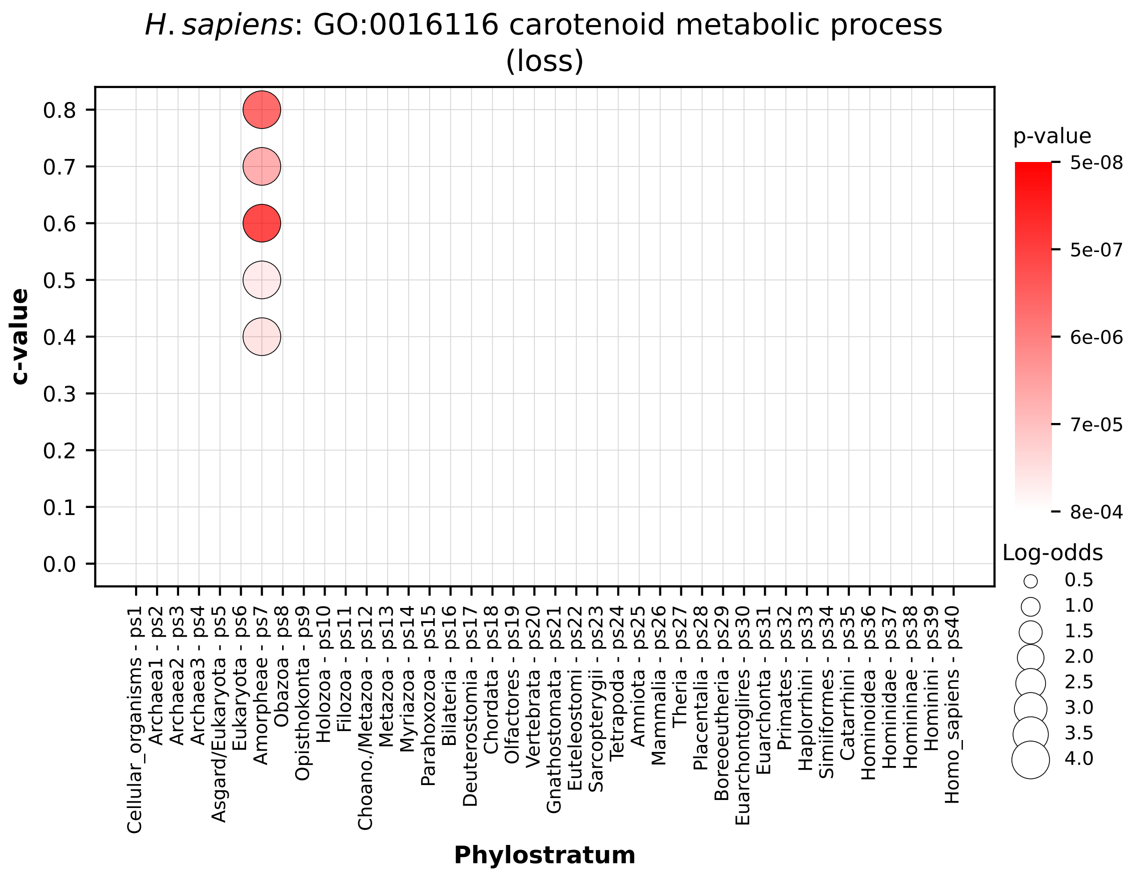

H. sapiens: GO:0016116 carotenoid metabolic process
(loss)
c-value
0.0
0.1
0.2
0.3
0.4
0.5
0.6
0.7
0.8
Phylostratum
Cellular_organisms - ps1
Archaea1 - ps2
Archaea2 - ps3
Archaea3 - ps4
Asgard/Eukaryota - ps5
Eukaryota - ps6
Amorpheae - ps7
Obazoa - ps8
Opisthokonta - ps9
Holozoa - ps10
Filozoa - ps11
Choano./Metazoa - ps12
Metazoa - ps13
Myriazoa - ps14
Parahoxozoa - ps15
Bilateria - ps16
Deuterostomia - ps17
Chordata - ps18
Olfactores - ps19
Vertebrata - ps20
Gnathostomata - ps21
Euteleostomi - ps22
Sarcopterygii - ps23
Tetrapoda - ps24
Amniota - ps25
Mammalia - ps26
Theria - ps27
Placentalia - ps28
Boreoeutheria - ps29
Euarchontoglires - ps30
Euarchonta - ps31
Primates - ps32
Haplorrhini - ps33
Simiiformes - ps34
Catarrhini - ps35
Hominoidea - ps36
Hominidae - ps37
Homininae - ps38
Hominini - ps39
Homo_sapiens - ps40
p-value
5e-08
5e-07
6e-06
7e-05
8e-04
Log-odds
0.5
1.0
1.5
2.0
2.5
3.0
3.5
4.0

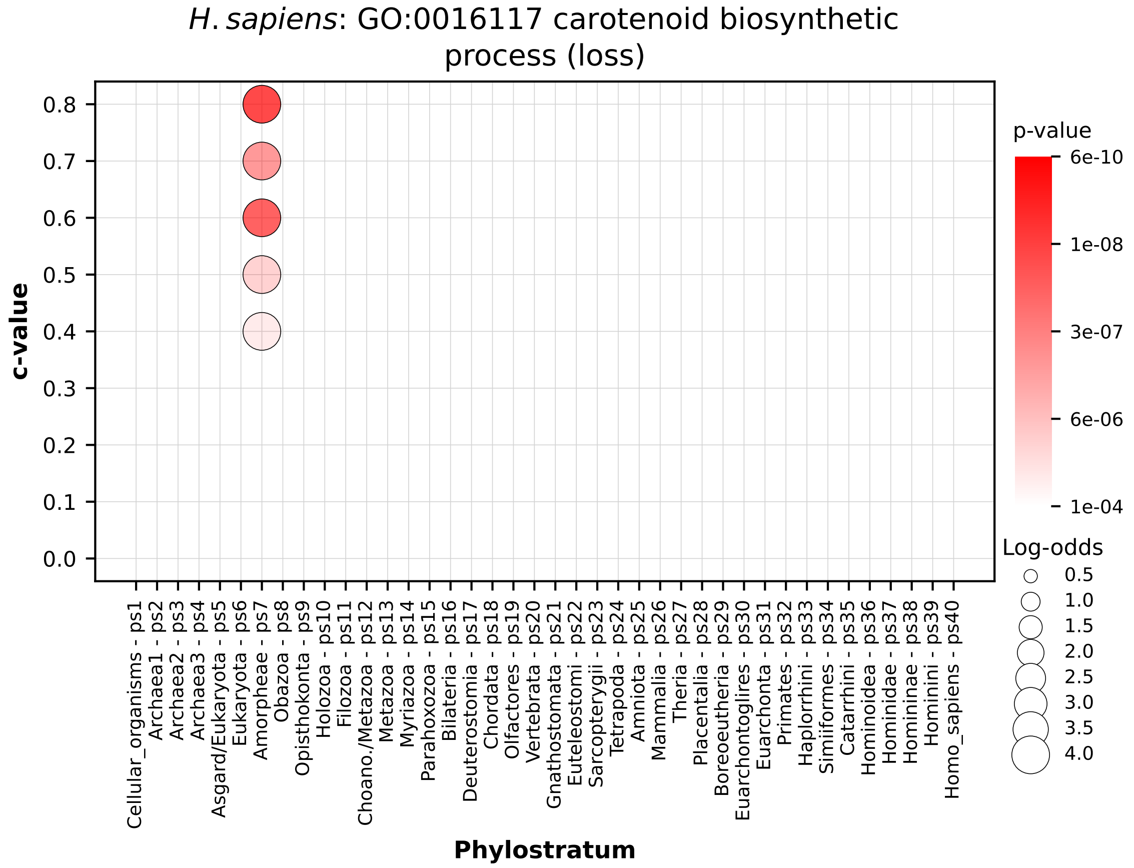

H. sapiens: GO:0016117 carotenoid biosynthetic process (loss)
c-value
0.0
0.1
0.2
0.3
0.4
0.5
0.6
0.7
0.8
Phylostratum
Cellular_organisms - ps1
Archaea1 - ps2
Archaea2 - ps3
Archaea3 - ps4
Asgard/Eukaryota - ps5
Eukaryota - ps6
Amorpheae - ps7
Obazoa - ps8
Opisthokonta - ps9
Holozoa - ps10
Filozoa - ps11
Choano./Metazoa - ps12
Metazoa - ps13
Myriazoa - ps14
Parahoxozoa - ps15
Bilateria - ps16
Deuterostomia - ps17
Chordata - ps18
Olfactores - ps19
Vertebrata - ps20
Gnathostomata - ps21
Euteleostomi - ps22
Sarcopterygii - ps23
Tetrapoda - ps24
Amniota - ps25
Mammalia - ps26
Theria - ps27
Placentalia - ps28
Boreoeutheria - ps29
Euarchontoglires - ps30
Euarchonta - ps31
Primates - ps32
Haplorrhini - ps33
Simiiformes - ps34
Catarrhini - ps35
Hominoidea - ps36
Hominidae - ps37
Homininae - ps38
Hominini - ps39
Homo_sapiens - ps40
p-value
6e-10
1e-08
3e-07
6e-06
1e-04
Log-odds
0.5
1.0
1.5
2.0
2.5
3.0
3.5
4.0

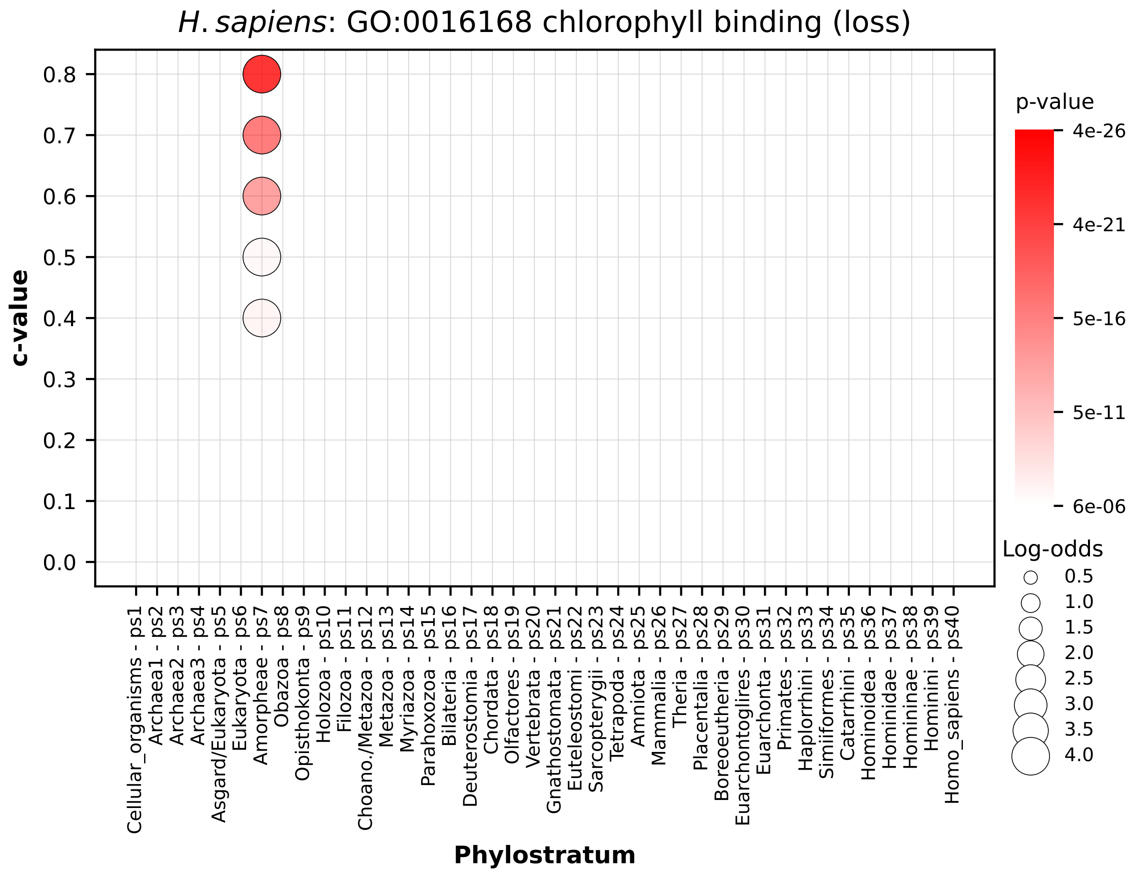

$H. sapiens$: GO:0016168 chlorophyll binding (loss)
c-value
0.8
0.7
0.6
0.5
0.4
0.3
0.2
0.1
0.0
p-value
4e-26
4e-21
5e-16
5e-11
6e-06
Log-odds
0.5
1.0
1.5
2.0
2.5
3.0
3.5
4.0
Cellular_organisms - ps1
Archaea1 - ps2
Archaea2 - ps3
Archaea3 - ps4
Asgard/Eukaryota - ps5
Eukaryota - ps6
Amorpheae - ps7
Obazoa - ps8
Opisthokonta - ps9
Holozoa - ps10
Filozoa - ps11
Choano./Metazoa - ps12
Metazoa - ps13
Myriazoa - ps14
Parahoxozoa - ps15
Bilateria - ps16
Deuterostomia - ps17
Chordata - ps18
Olfactores - ps19
Vertebrata - ps20
Gnathostomata - ps21
Euteleostomi - ps22
Sarcopterygii - ps23
Tetrapoda - ps24
Amniota - ps25
Mammalia - ps26
Theria - ps27
Placentalia - ps28
Boreoeutheria - ps29
Euarchontoglires - ps30
Euarchonta - ps31
Primates - ps32
Haplorrhini - ps33
Simiiformes - ps34
Catarrhini - ps35
Hominoidea - ps36
Hominidae - ps37
Homininae - ps38
Hominini - ps39
Homo_sapiens - ps40
Phylostratum

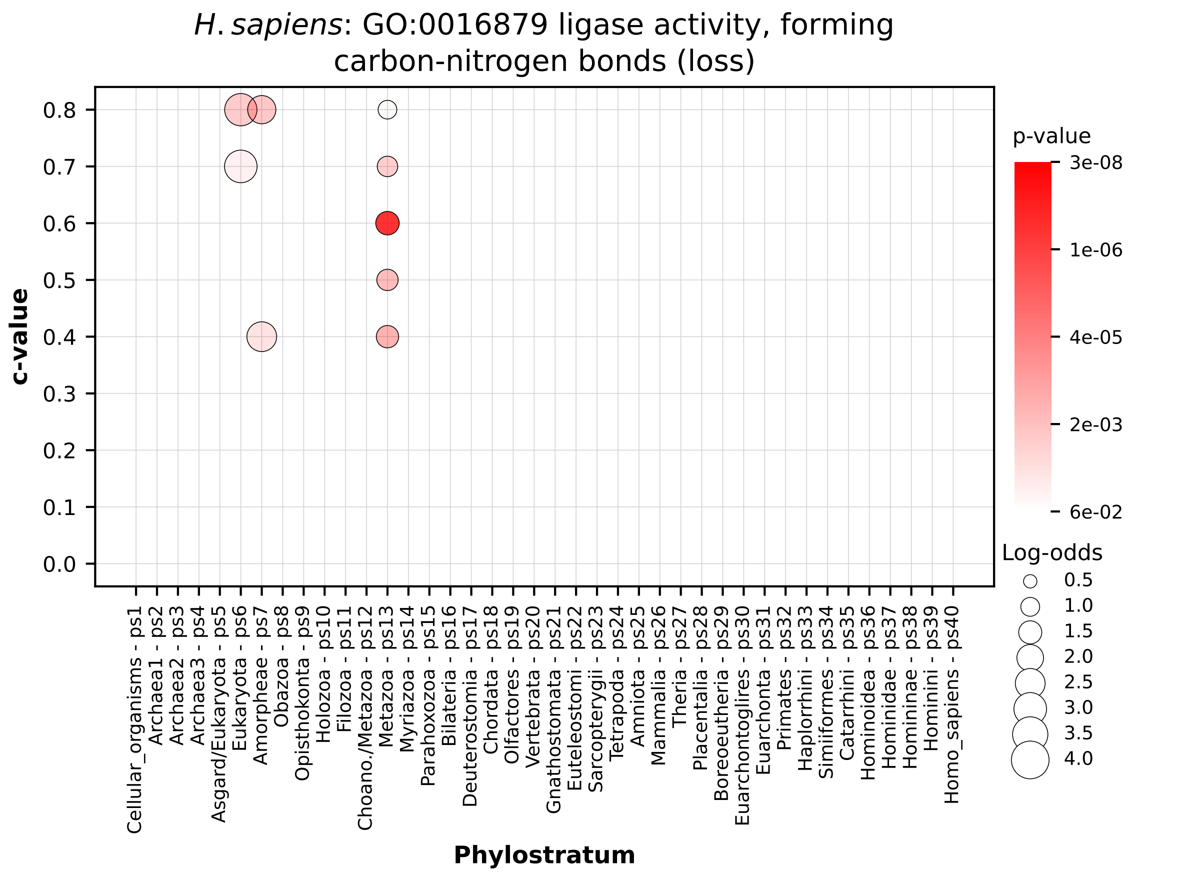
H. sapiens: GO:0016879 ligase activity, forming carbon-nitrogen bonds (loss)
c-value
0.8
0.7
0.6
0.5
0.4
0.3
0.2
0.1
0.0
Phylostratum
Cellular_organisms - ps1
Archaea1 - ps2
Archaea2 - ps3
Archaea3 - ps4
Asgard/Eukaryota - ps5
Eukaryota - ps6
Amorpheae - ps7
Obazoa - ps8
Opisthokonta - ps9
Holozoa - ps10
Filozoa - ps11
Choano./Metazoa - ps12
Metazoa - ps13
Myriazoa - ps14
Parahoxozoa - ps15
Bilateria - ps16
Deuterostomia - ps17
Chordata - ps18
Olfactores - ps19
Vertebrata - ps20
Gnathostomata - ps21
Euteleostomi - ps22
Sarcopterygii - ps23
Tetrapoda - ps24
Amniota - ps25
Mammalia - ps26
Theria - ps27
Placentalia - ps28
Boreoeutheria - ps29
Euarchontoglires - ps30
Euarchonta - ps31
Primates - ps32
Haplorrhini - ps33
Simiiformes - ps34
Catarrhini - ps35
Hominoidea - ps36
Hominidae - ps37
Homininae - ps38
Hominini - ps39
Homo_sapiens - ps40
p-value
3e-08
1e-06
4e-05
2e-03
6e-02
Log-odds
0.5
1.0
1.5
2.0
2.5
3.0
3.5
4.0

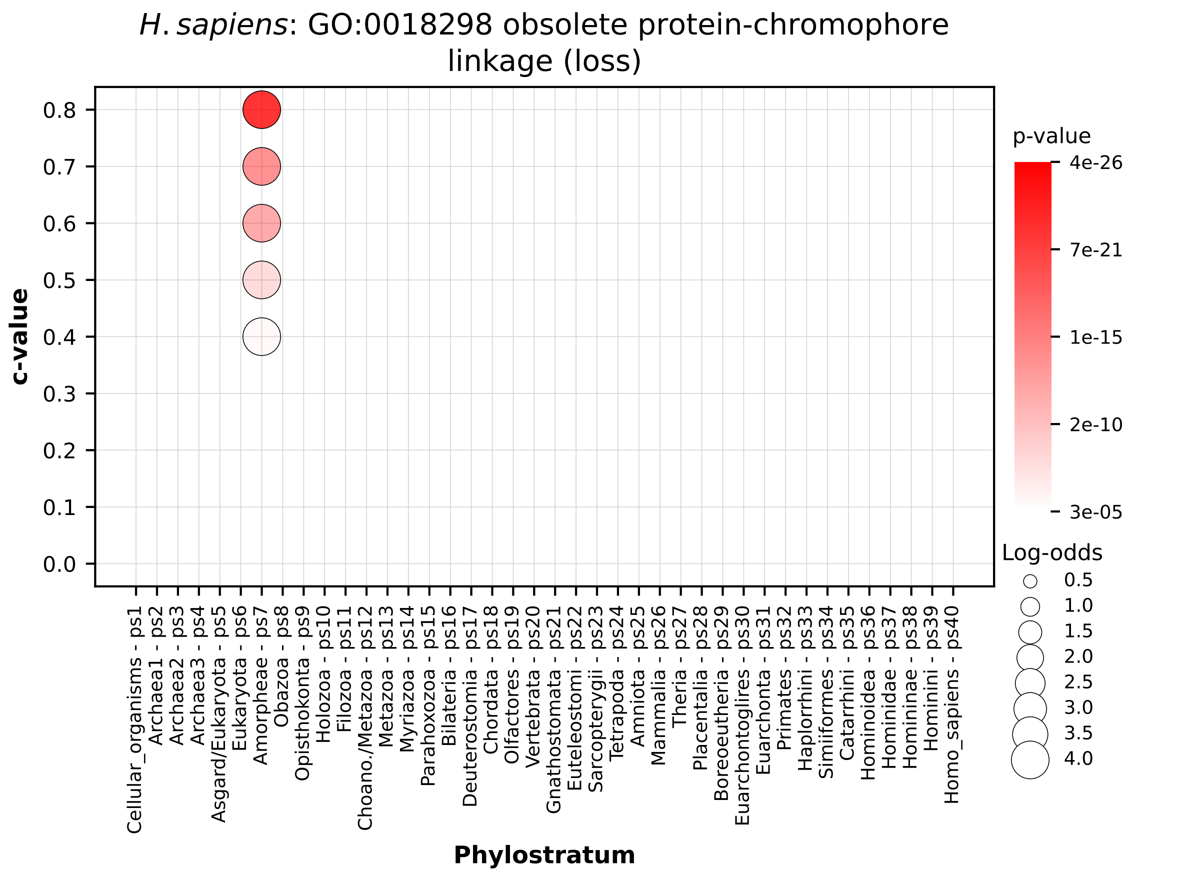

H. sapiens: GO:0018298 obsolete protein-chromophore linkage (loss)
c-value
0.8
0.7
0.6
0.5
0.4
0.3
0.2
0.1
0.0
p-value
4e-26
7e-21
1e-15
2e-10
3e-05
Log-odds
0.5
1.0
1.5
2.0
2.5
3.0
3.5
4.0
Cellular_organisms - ps1
Archaea1 - ps2
Archaea2 - ps3
Archaea3 - ps4
Asgard/Eukaryota - ps5
Eukaryota - ps6
Amorpheae - ps7
Obazoa - ps8
Opisthokonta - ps9
Holozoa - ps10
Filozoa - ps11
Choano./Metazoa - ps12
Metazoa - ps13
Myriazoa - ps14
Parahoxozoa - ps15
Bilateria - ps16
Deuterostomia - ps17
Chordata - ps18
Olfactores - ps19
Vertebrata - ps20
Gnathostomata - ps21
Euteleostomi - ps22
Sarcopterygii - ps23
Tetrapoda - ps24
Amniota - ps25
Mammalia - ps26
Theria - ps27
Placentalia - ps28
Boreoeutheria - ps29
Euarchontoglires - ps30
Euarchonta - ps31
Primates - ps32
Haplorrhini - ps33
Simiiformes - ps34
Catarrhini - ps35
Hominoidea - ps36
Hominidae - ps37
Homininae - ps38
Hominini - ps39
Homo_sapiens - ps40
Phylostratum

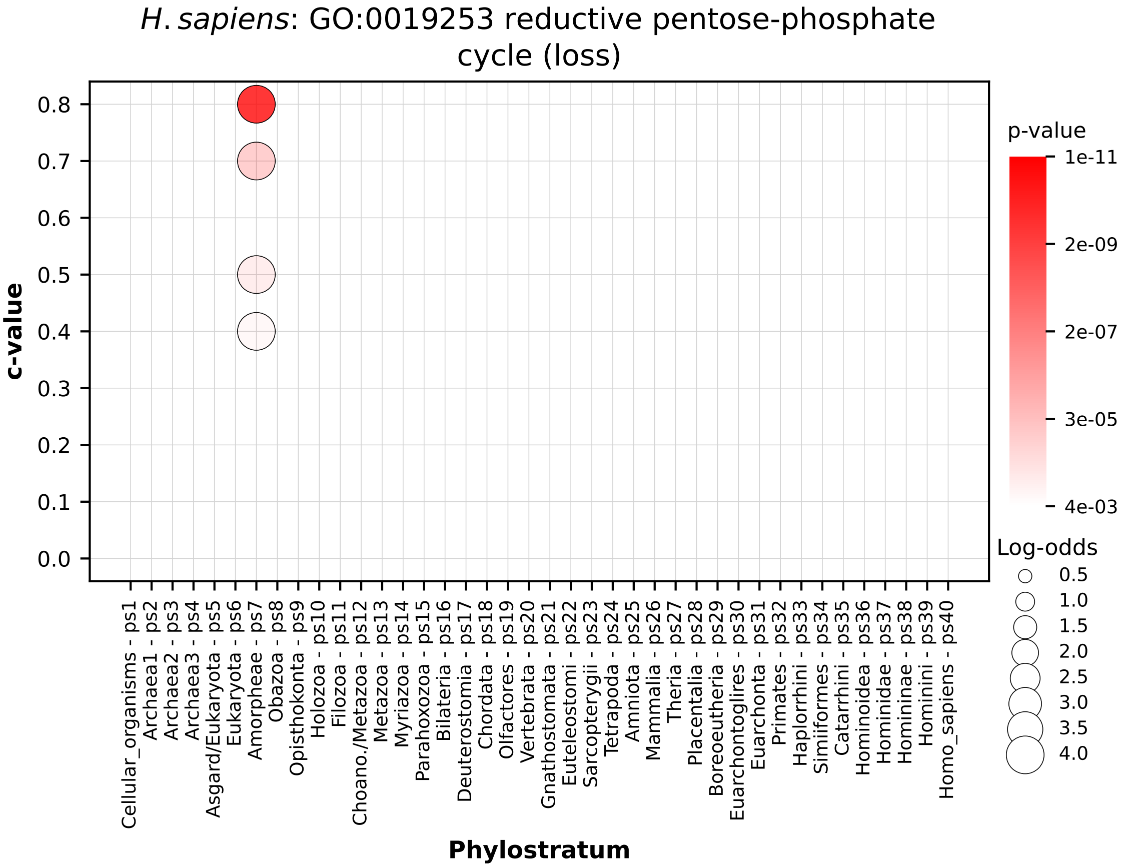

H. sapiens: GO:0019253 reductive pentose-phosphate cycle (loss)
c-value
0.8
0.7
0.6
0.5
0.4
0.3
0.2
0.1
0.0
p-value
1e-11
2e-09
2e-07
3e-05
4e-03
Log-odds
0.5
1.0
1.5
2.0
2.5
3.0
3.5
4.0
Cellular_organisms - ps1
Archaea1 - ps2
Archaea2 - ps3
Archaea3 - ps4
Asgard/Eukaryota - ps5
Eukaryota - ps6
Amorpheae - ps7
Obazoa - ps8
Opisthokonta - ps9
Holozoa - ps10
Filozoa - ps11
Choano./Metazoa - ps12
Metazoa - ps13
Myriazoa - ps14
Parahoxozoa - ps15
Bilateria - ps16
Deuterostomia - ps17
Chordata - ps18
Olfactores - ps19
Vertebrata - ps20
Gnathostomata - ps21
Euteleostomi - ps22
Sarcopterygii - ps23
Tetrapoda - ps24
Amniota - ps25
Mammalia - ps26
Theria - ps27
Placentalia - ps28
Boreoeutheria - ps29
Euarchontoglires - ps30
Euarchonta - ps31
Primates - ps32
Haplorrhini - ps33
Simiiformes - ps34
Catarrhini - ps35
Hominoidea - ps36
Hominidae - ps37
Homininae - ps38
Hominini - ps39
Homo_sapiens - ps40
Phylostratum

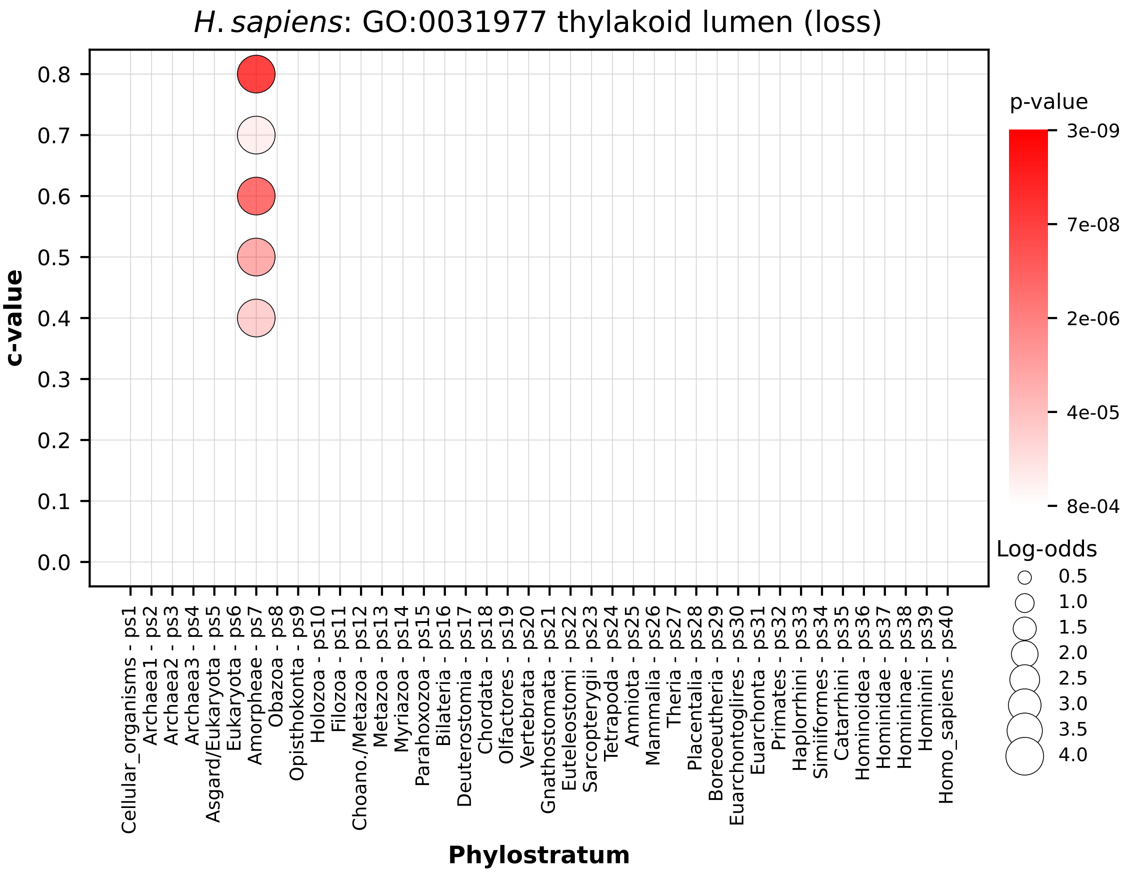
H. sapiens: GO:0031977 thylakoid lumen (loss)
c-value
0.8
0.7
0.6
0.5
0.4
0.3
0.2
0.1
0.0
Phylostratum
Cellular_organisms - ps1
Archaea1 - ps2
Archaea2 - ps3
Archaea3 - ps4
Asgard/Eukaryota - ps5
Eukaryota - ps6
Amorpheae - ps7
Obazoa - ps8
Opisthokonta - ps9
Holozoa - ps10
Filozoa - ps11
Choano./Metazoa - ps12
Metazoa - ps13
Myriazoa - ps14
Parahoxozoa - ps15
Bilateria - ps16
Deuterostomia - ps17
Chordata - ps18
Olfactores - ps19
Vertebrata - ps20
Gnathostomata - ps21
Euteleostomi - ps22
Sarcopterygii - ps23
Tetrapoda - ps24
Amniota - ps25
Mammalia - ps26
Theria - ps27
Placentalia - ps28
Boreoeutheria - ps29
Euarchontoglires - ps30
Euarchonta - ps31
Primates - ps32
Haplorrhini - ps33
Simiiformes - ps34
Catarrhini - ps35
Hominoidea - ps36
Hominidae - ps37
Homininae - ps38
Hominini - ps39
Homo_sapiens - ps40
p-value
3e-09
7e-08
2e-06
4e-05
8e-04
Log-odds
0.5
1.0
1.5
2.0
2.5
3.0
3.5
4.0

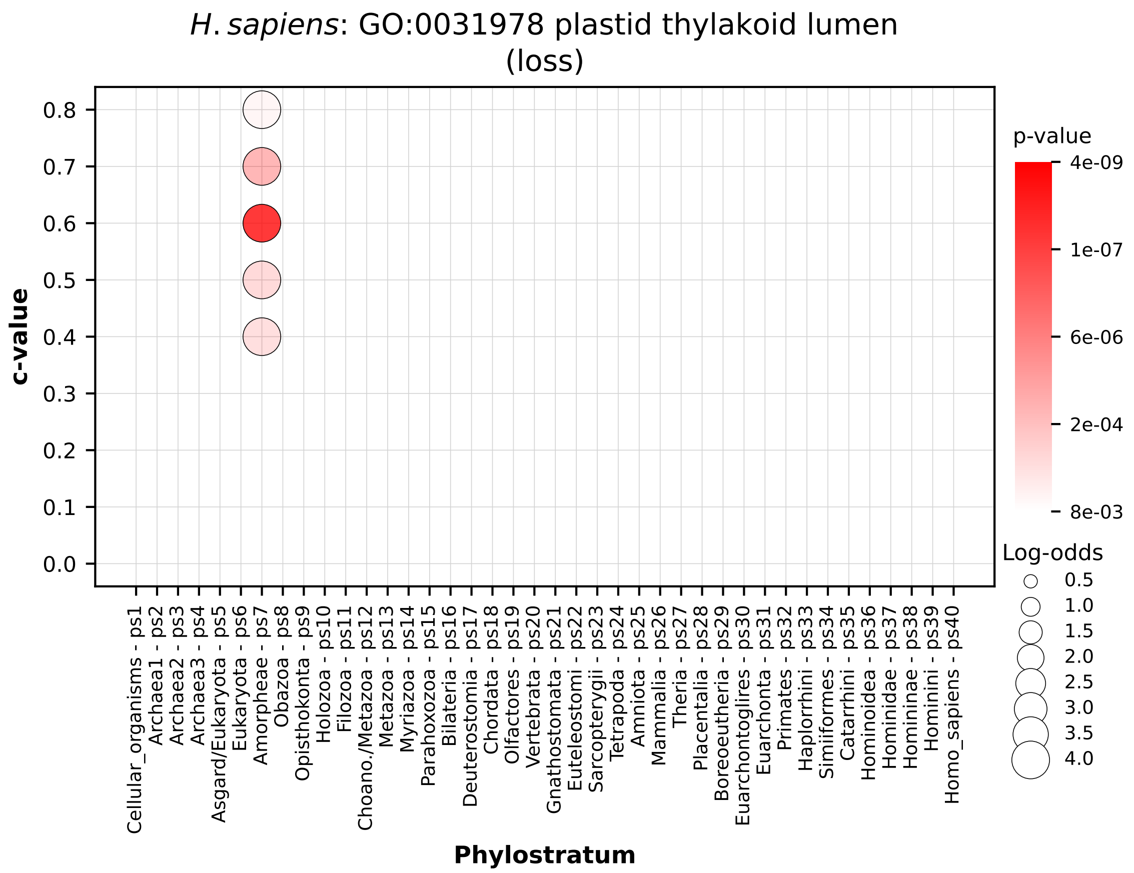

H. sapiens: GO:0031978 plastid thylakoid lumen
(loss)
c-value
0.8
0.7
0.6
0.5
0.4
0.3
0.2
0.1
0.0
p-value
4e-09
1e-07
6e-06
2e-04
8e-03
Log-odds
0.5
1.0
1.5
2.0
2.5
3.0
3.5
4.0
Cellular_organisms - ps1
Archaea1 - ps2
Archaea2 - ps3
Archaea3 - ps4
Asgard/Eukaryota - ps5
Eukaryota - ps6
Amorpheae - ps7
Obazoa - ps8
Opisthokonta - ps9
Holozoa - ps10
Filozoa - ps11
Choano./Metazoa - ps12
Metazoa - ps13
Myriazoa - ps14
Parahoxozoa - ps15
Bilateria - ps16
Deuterostomia - ps17
Chordata - ps18
Olfactores - ps19
Vertebrata - ps20
Gnathostomata - ps21
Euteleostomi - ps22
Sarcopterygii - ps23
Tetrapoda - ps24
Amniota - ps25
Mammalia - ps26
Theria - ps27
Placentalia - ps28
Boreoeutheria - ps29
Euarchontoglires - ps30
Euarchonta - ps31
Primates - ps32
Haplorrhini - ps33
Simiiformes - ps34
Catarrhini - ps35
Hominoidea - ps36
Hominidae - ps37
Homininae - ps38
Hominini - ps39
Homo_sapiens - ps40
Phylostratum

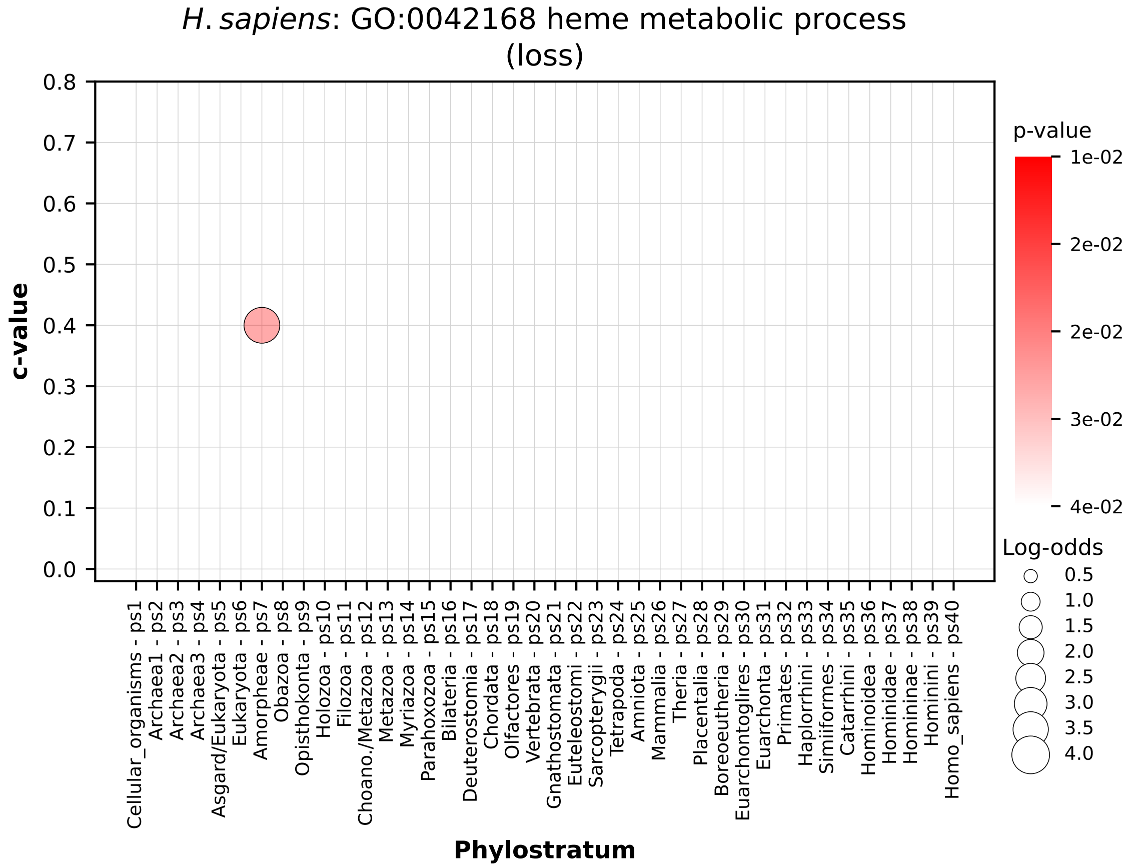

H. sapiens: GO:0042168 heme metabolic process
(loss)
c-value
0.0
0.1
0.2
0.3
0.4
0.5
0.6
0.7
0.8
Phylostratum
Cellular_organisms - ps1
Archaea1 - ps2
Archaea2 - ps3
Archaea3 - ps4
Asgard/Eukaryota - ps5
Eukaryota - ps6
Amorpheae - ps7
Obazoa - ps8
Opisthokonta - ps9
Holozoa - ps10
Filozoa - ps11
Choano./Metazoa - ps12
Metazoa - ps13
Myriazoa - ps14
Parahoxozoa - ps15
Bilateria - ps16
Deuterostomia - ps17
Chordata - ps18
Olfactores - ps19
Vertebrata - ps20
Gnathostomata - ps21
Euteleostomi - ps22
Sarcopterygii - ps23
Tetrapoda - ps24
Amniota - ps25
Mammalia - ps26
Theria - ps27
Placentalia - ps28
Boreoeutheria - ps29
Euarchontoglires - ps30
Euarchonta - ps31
Primates - ps32
Haplorrhini - ps33
Simiiformes - ps34
Catarrhini - ps35
Hominoidea - ps36
Hominidae - ps37
Homininae - ps38
Hominini - ps39
Homo_sapiens - ps40
p-value
1e-02
2e-02
2e-02
3e-02
4e-02
Log-odds
0.5
1.0
1.5
2.0
2.5
3.0
3.5
4.0

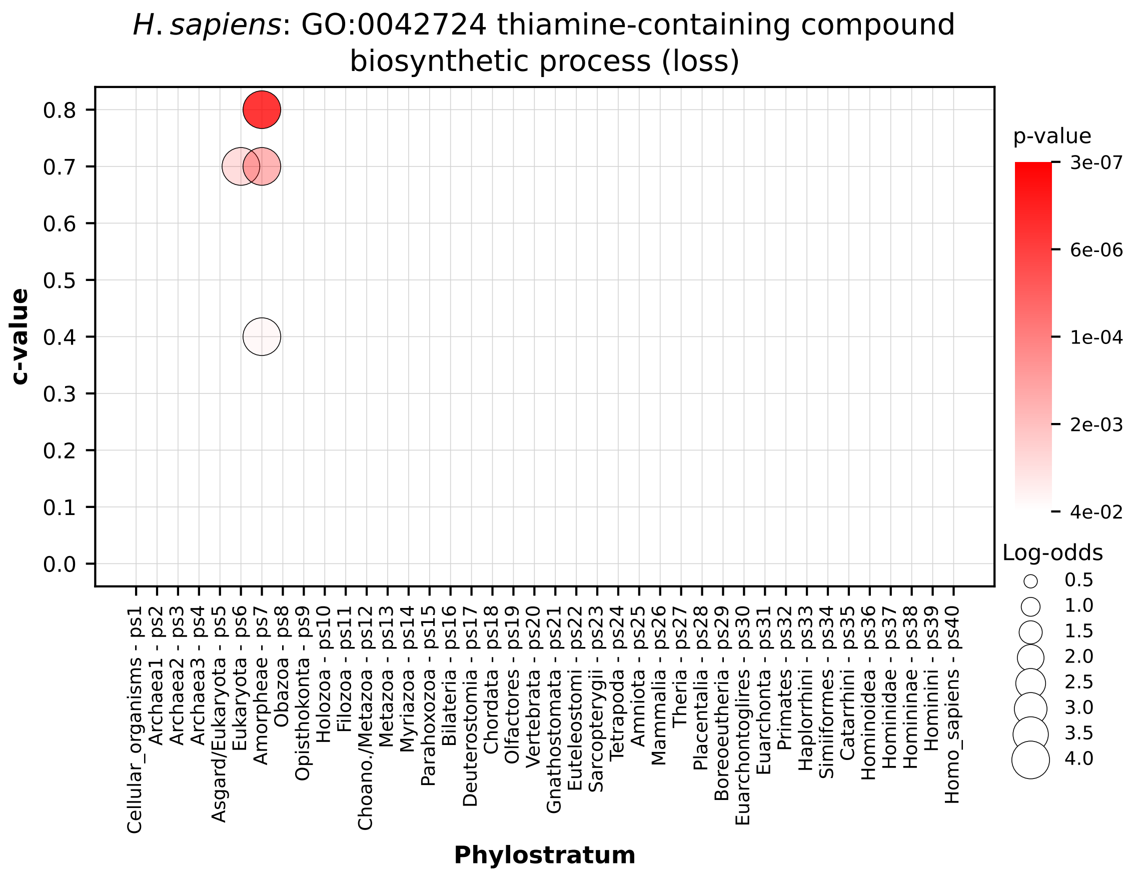

H. sapiens: GO:0042724 thiamine-containing compound biosynthetic process (loss)
c-value
0.8
0.7
0.6
0.5
0.4
0.3
0.2
0.1
0.0
p-value
3e-07
6e-06
1e-04
2e-03
4e-02
Log-odds
0.5
1.0
1.5
2.0
2.5
3.0
3.5
4.0
Cellular_organisms - ps1
Archaea1 - ps2
Archaea2 - ps3
Archaea3 - ps4
Asgard/Eukaryota - ps5
Eukaryota - ps6
Amorpheae - ps7
Obazoa - ps8
Opisthokonta - ps9
Holozoa - ps10
Filozoa - ps11
Choano./Metazoa - ps12
Metazoa - ps13
Myriazoa - ps14
Parahoxozoa - ps15
Bilateria - ps16
Deuterostomia - ps17
Chordata - ps18
Olfactores - ps19
Vertebrata - ps20
Gnathostomata - ps21
Euteleostomi - ps22
Sarcopterygii - ps23
Tetrapoda - ps24
Amniota - ps25
Mammalia - ps26
Theria - ps27
Placentalia - ps28
Boreoeutheria - ps29
Euarchontoglires - ps30
Euarchonta - ps31
Primates - ps32
Haplorrhini - ps33
Simiiformes - ps34
Catarrhini - ps35
Hominoidea - ps36
Hominidae - ps37
Homininae - ps38
Hominini - ps39
Homo_sapiens - ps40
Phylostratum

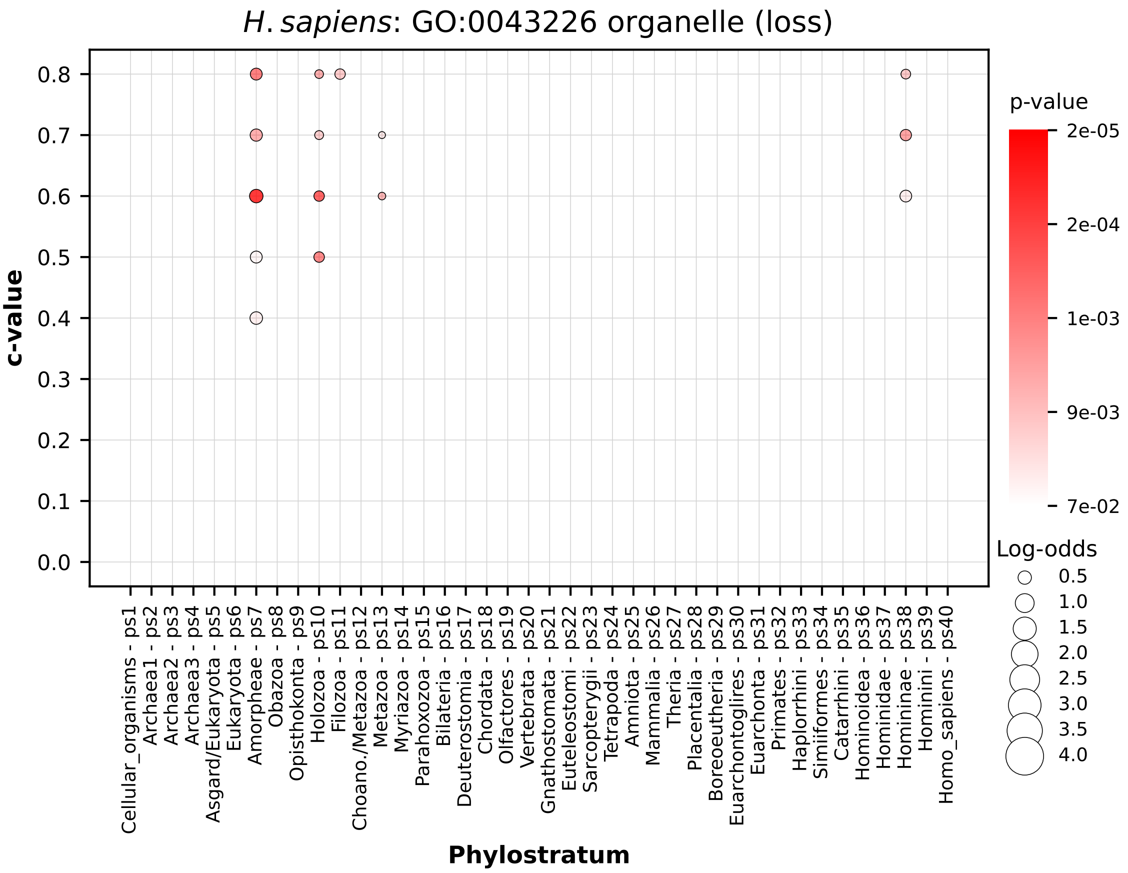

$H. sapiens$: GO:0043226 organelle (loss)
c-value
0.0
0.1
0.2
0.3
0.4
0.5
0.6
0.7
0.8
Phylostratum
Cellular_organisms - ps1
Archaea1 - ps2
Archaea2 - ps3
Archaea3 - ps4
Asgard/Eukaryota - ps5
Eukaryota - ps6
Amorpheae - ps7
Obazoa - ps8
Opisthokonta - ps9
Holozoa - ps10
Filozoa - ps11
Choano./Metazoa - ps12
Metazoa - ps13
Myriazoa - ps14
Parahoxozoa - ps15
Bilateria - ps16
Deuterostomia - ps17
Chordata - ps18
Olfactores - ps19
Vertebrata - ps20
Gnathostomata - ps21
Euteleostomi - ps22
Sarcopterygii - ps23
Tetrapoda - ps24
Amniota - ps25
Mammalia - ps26
Theria - ps27
Placentalia - ps28
Boreoeutheria - ps29
Euarchontoglires - ps30
Euarchonta - ps31
Primates - ps32
Haplorrhini - ps33
Simiiformes - ps34
Catarrhini - ps35
Hominoidea - ps36
Hominidae - ps37
Homininae - ps38
Hominini - ps39
Homo_sapiens - ps40
p-value
2e-05
2e-04
1e-03
9e-03
7e-02
Log-odds
0.5
1.0
1.5
2.0
2.5
3.0
3.5
4.0

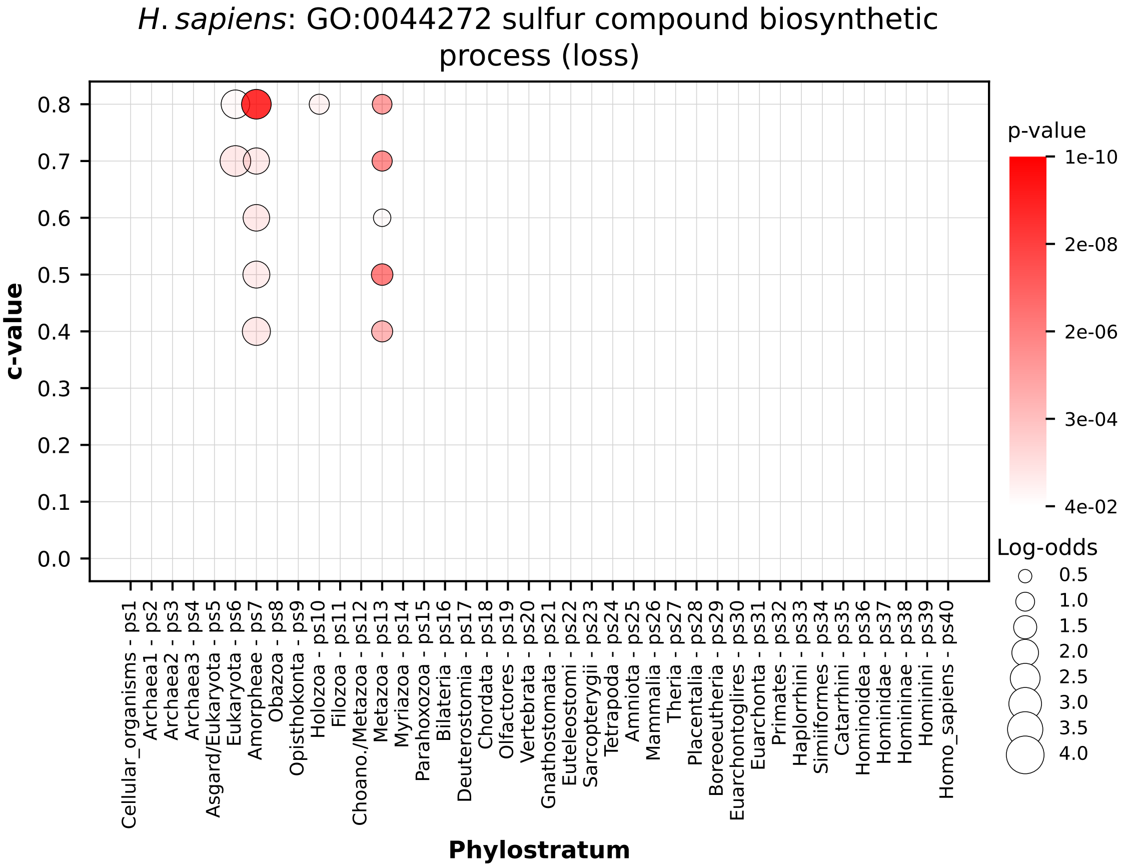
H. sapiens: GO:0044272 sulfur compound biosynthetic process (loss)
c-value
0.8
0.7
0.6
0.5
0.4
0.3
0.2
0.1
0.0
p-value
1e-10
2e-08
2e-06
3e-04
4e-02
Log-odds
0.5
1.0
1.5
2.0
2.5
3.0
3.5
4.0
Cellular_organisms - ps1
Archaea1 - ps2
Archaea2 - ps3
Archaea3 - ps4
Asgard/Eukaryota - ps5
Eukaryota - ps6
Amorpheae - ps7
Obazoa - ps8
Opisthokonta - ps9
Holozoa - ps10
Filozoa - ps11
Choano./Metazoa - ps12
Metazoa - ps13
Myriazoa - ps14
Parahoxozoa - ps15
Bilateria - ps16
Deuterostomia - ps17
Chordata - ps18
Olfactores - ps19
Vertebrata - ps20
Gnathostomata - ps21
Euteleostomi - ps22
Sarcopterygii - ps23
Tetrapoda - ps24
Amniota - ps25
Mammalia - ps26
Theria - ps27
Placentalia - ps28
Boreoeutheria - ps29
Euarchontoglires - ps30
Euarchonta - ps31
Primates - ps32
Haplorrhini - ps33
Simiiformes - ps34
Catarrhini - ps35
Hominoidea - ps36
Hominidae - ps37
Homininae - ps38
Hominini - ps39
Homo_sapiens - ps40
Phylostratum

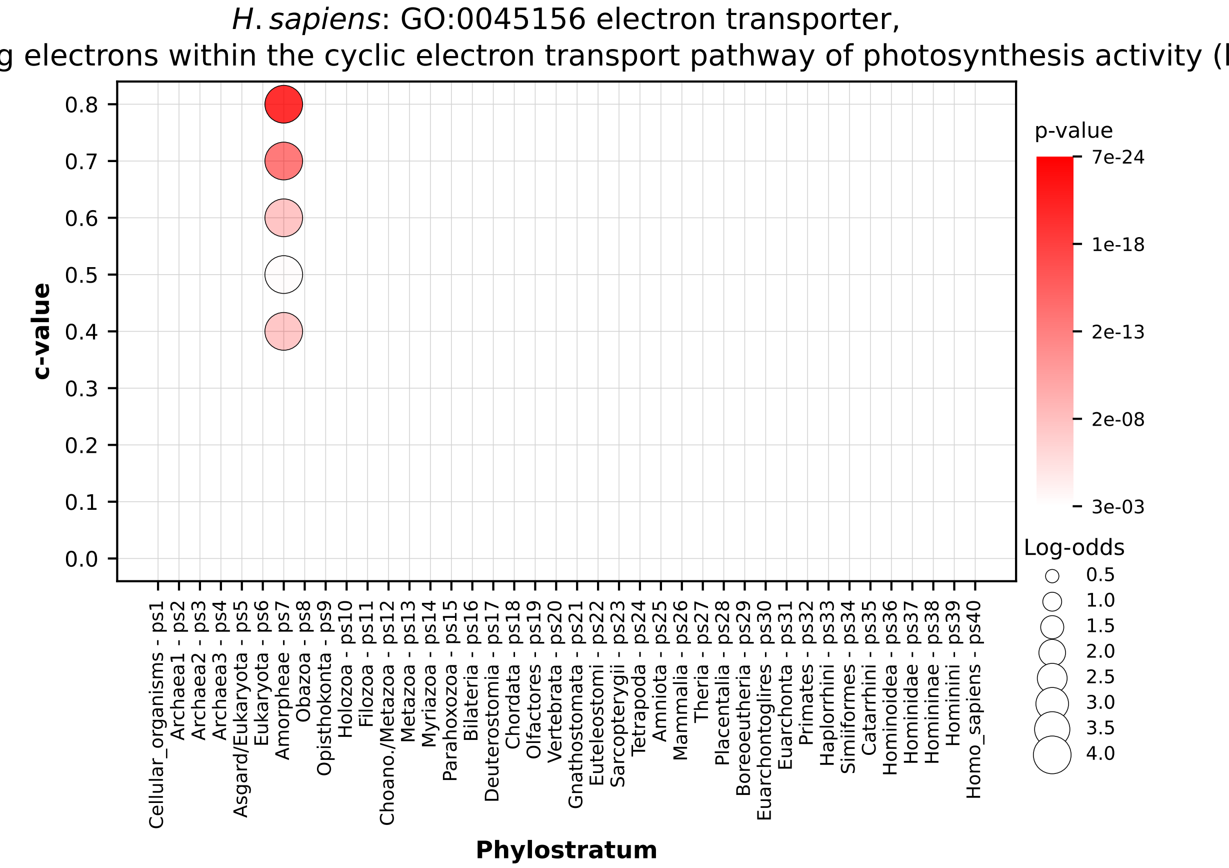

H. sapiens: GO:0045156 electron transporter,
g electrons within the cyclic electron transport pathway of photosynthesis activity (I
c-value
0.8
0.7
0.6
0.5
0.4
0.3
0.2
0.1
0.0
p-value
7e-24
1e-18
2e-13
2e-08
3e-03
Log-odds
0.5
1.0
1.5
2.0
2.5
3.0
3.5
4.0
Cellular_organisms - ps1
Archaea1 - ps2
Archaea2 - ps3
Archaea3 - ps4
Asgard/Eukaryota - ps5
Eukaryota - ps6
Amorpheae - ps7
Obazoa - ps8
Opisthokonta - ps9
Holozoa - ps10
Filozoa - ps11
Choano./Metazoa - ps12
Metazoa - ps13
Myriazoa - ps14
Parahoxozoa - ps15
Bilateria - ps16
Deuterostomia - ps17
Chordata - ps18
Olfactores - ps19
Vertebrata - ps20
Gnathostomata - ps21
Euteleostomi - ps22
Sarcopterygii - ps23
Tetrapoda - ps24
Amniota - ps25
Mammalia - ps26
Theria - ps27
Placentalia - ps28
Boreoeutheria - ps29
Euarchontoglires - ps30
Euarchonta - ps31
Primates - ps32
Haplorrhini - ps33
Simiiformes - ps34
Catarrhini - ps35
Hominoidea - ps36
Hominidae - ps37
Homininae - ps38
Hominini - ps39
Homo_sapiens - ps40
Phylostratum

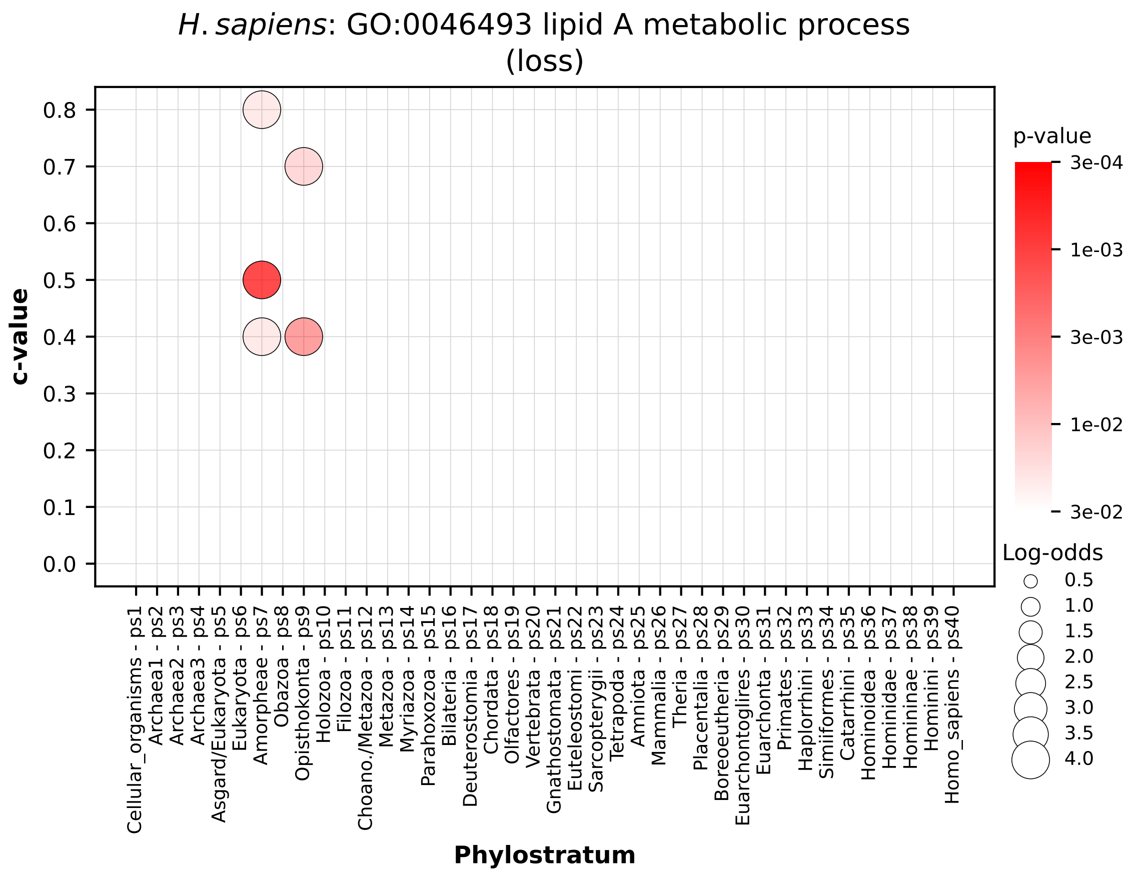

H. sapiens: GO:0046493 lipid A metabolic process
(loss)
c-value
0.8
0.7
0.6
0.5
0.4
0.3
0.2
0.1
0.0
p-value
3e-04
1e-03
3e-03
1e-02
3e-02
Log-odds
0.5
1.0
1.5
2.0
2.5
3.0
3.5
4.0
Cellular_organisms - ps1
Archaea1 - ps2
Archaea2 - ps3
Archaea3 - ps4
Asgard/Eukaryota - ps5
Eukaryota - ps6
Amorpheae - ps7
Obazoa - ps8
Opisthokonta - ps9
Holozoa - ps10
Filozoa - ps11
Choano./Metazoa - ps12
Metazoa - ps13
Myriazoa - ps14
Parahoxozoa - ps15
Bilateria - ps16
Deuterostomia - ps17
Chordata - ps18
Olfactores - ps19
Vertebrata - ps20
Gnathostomata - ps21
Euteleostomi - ps22
Sarcopterygii - ps23
Tetrapoda - ps24
Amniota - ps25
Mammalia - ps26
Theria - ps27
Placentalia - ps28
Boreoeutheria - ps29
Euarchontoglires - ps30
Euarchonta - ps31
Primates - ps32
Haplorrhini - ps33
Simiiformes - ps34
Catarrhini - ps35
Hominoidea - ps36
Hominidae - ps37
Homininae - ps38
Hominini - ps39
Homo_sapiens - ps40
Phylostratum

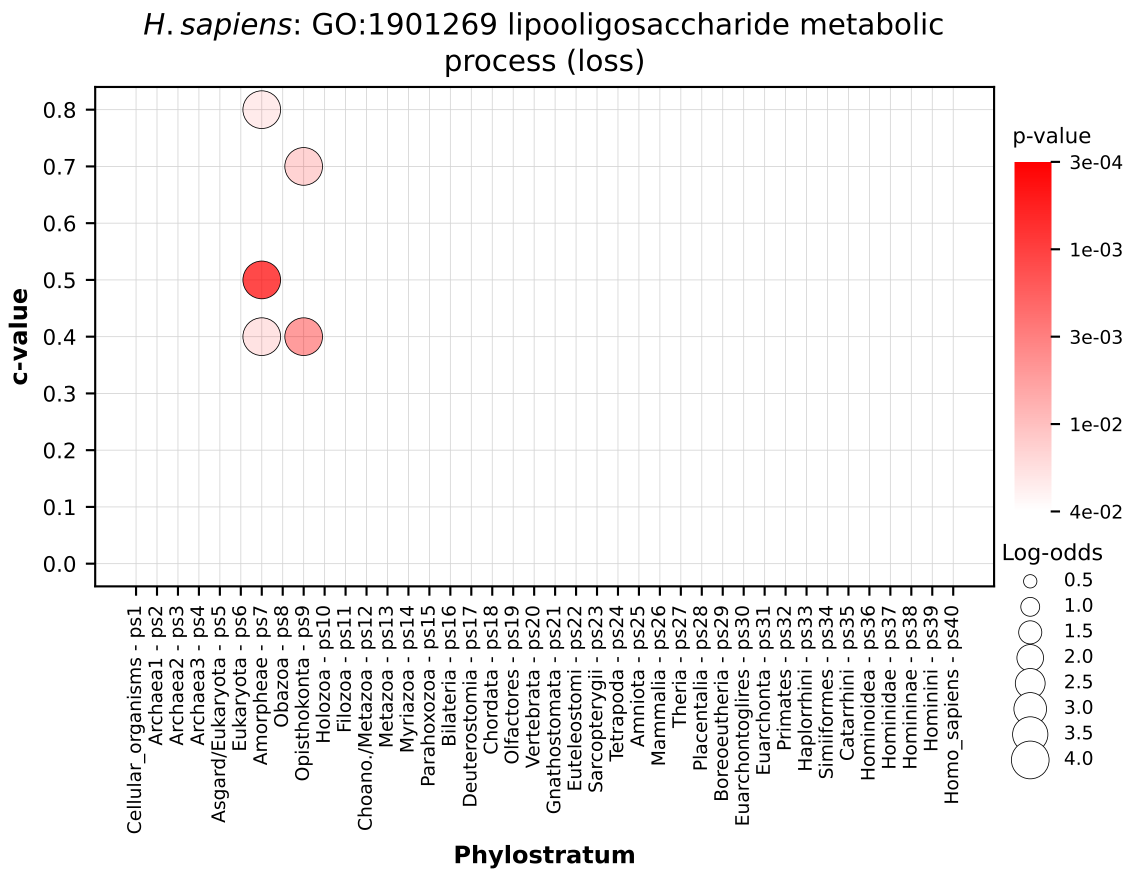
H. sapiens: GO:1901269 lipooligosaccharide metabolic process (loss)
c-value
0.0
0.1
0.2
0.3
0.4
0.5
0.6
0.7
0.8
p-value
3e-04
1e-03
3e-03
1e-02
4e-02
Log-odds
0.5
1.0
1.5
2.0
2.5
3.0
3.5
4.0
Cellular_organisms - ps1
Archaea1 - ps2
Archaea2 - ps3
Archaea3 - ps4
Asgard/Eukaryota - ps5
Eukaryota - ps6
Amorpheae - ps7
Obazoa - ps8
Opisthokonta - ps9
Holozoa - ps10
Filozoa - ps11
Choano./Metazoa - ps12
Metazoa - ps13
Myriazoa - ps14
Parahoxozoa - ps15
Bilateria - ps16
Deuterostomia - ps17
Chordata - ps18
Olfactores - ps19
Vertebrata - ps20
Gnathostomata - ps21
Euteleostomi - ps22
Sarcopterygii - ps23
Tetrapoda - ps24
Amniota - ps25
Mammalia - ps26
Theria - ps27
Placentalia - ps28
Boreoeutheria - ps29
Euarchontoglires - ps30
Euarchonta - ps31
Primates - ps32
Haplorrhini - ps33
Simiiformes - ps34
Catarrhini - ps35
Hominoidea - ps36
Hominidae - ps37
Homininae - ps38
Hominini - ps39
Homo_sapiens - ps40
Phylostratum

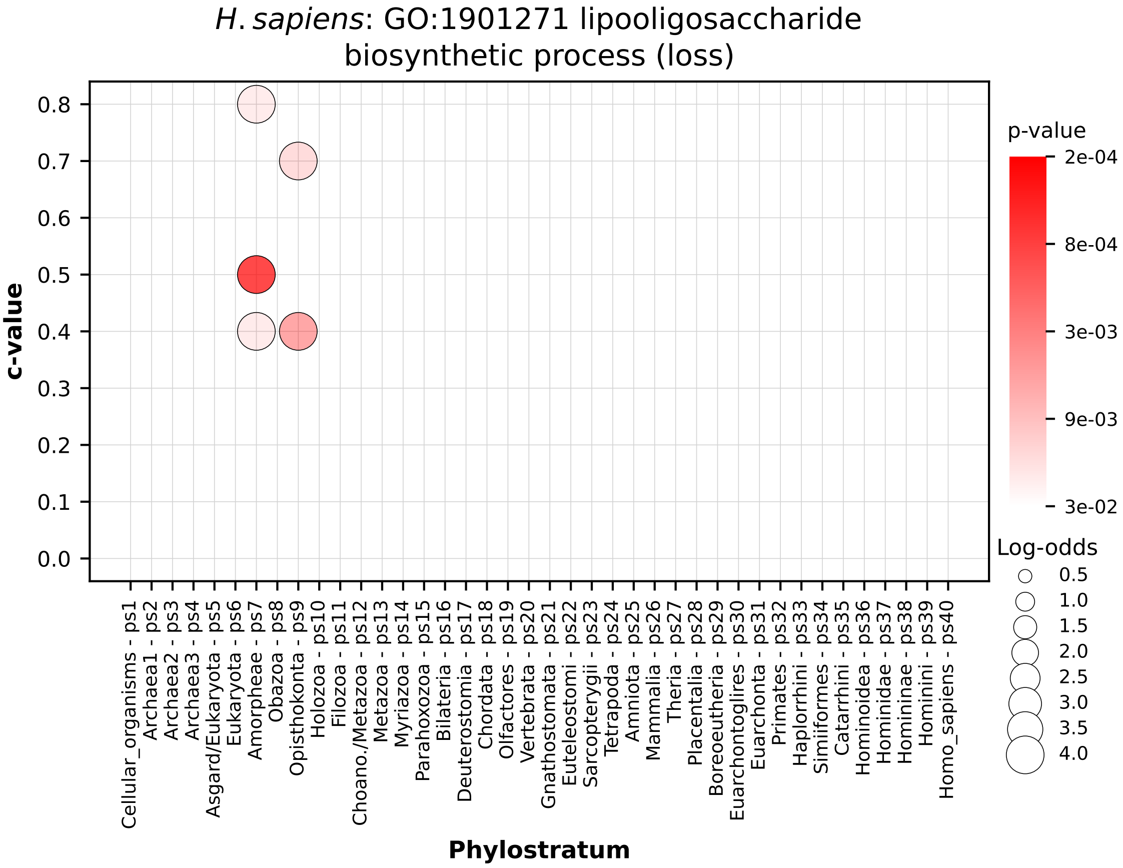

H. sapiens: GO:1901271 lipooligosaccharide biosynthetic process (loss)
c-value
0.0
0.1
0.2
0.3
0.4
0.5
0.6
0.7
0.8
Phylostratum
Cellular_organisms - ps1
Archaea1 - ps2
Archaea2 - ps3
Archaea3 - ps4
Asgard/Eukaryota - ps5
Eukaryota - ps6
Amorpheae - ps7
Obazoa - ps8
Opisthokonta - ps9
Holozoa - ps10
Filozoa - ps11
Choano./Metazoa - ps12
Metazoa - ps13
Myriazoa - ps14
Parahoxozoa - ps15
Bilateria - ps16
Deuterostomia - ps17
Chordata - ps18
Olfactores - ps19
Vertebrata - ps20
Gnathostomata - ps21
Euteleostomi - ps22
Sarcopterygii - ps23
Tetrapoda - ps24
Amniota - ps25
Mammalia - ps26
Theria - ps27
Placentalia - ps28
Boreoeutheria - ps29
Euarchontoglires - ps30
Euarchonta - ps31
Primates - ps32
Haplorrhini - ps33
Simiiformes - ps34
Catarrhini - ps35
Hominoidea - ps36
Hominidae - ps37
Homininae - ps38
Hominini - ps39
Homo_sapiens - ps40
p-value
2e-04
8e-04
3e-03
9e-03
3e-02
Log-odds
0.5
1.0
1.5
2.0
2.5
3.0
3.5
4.0

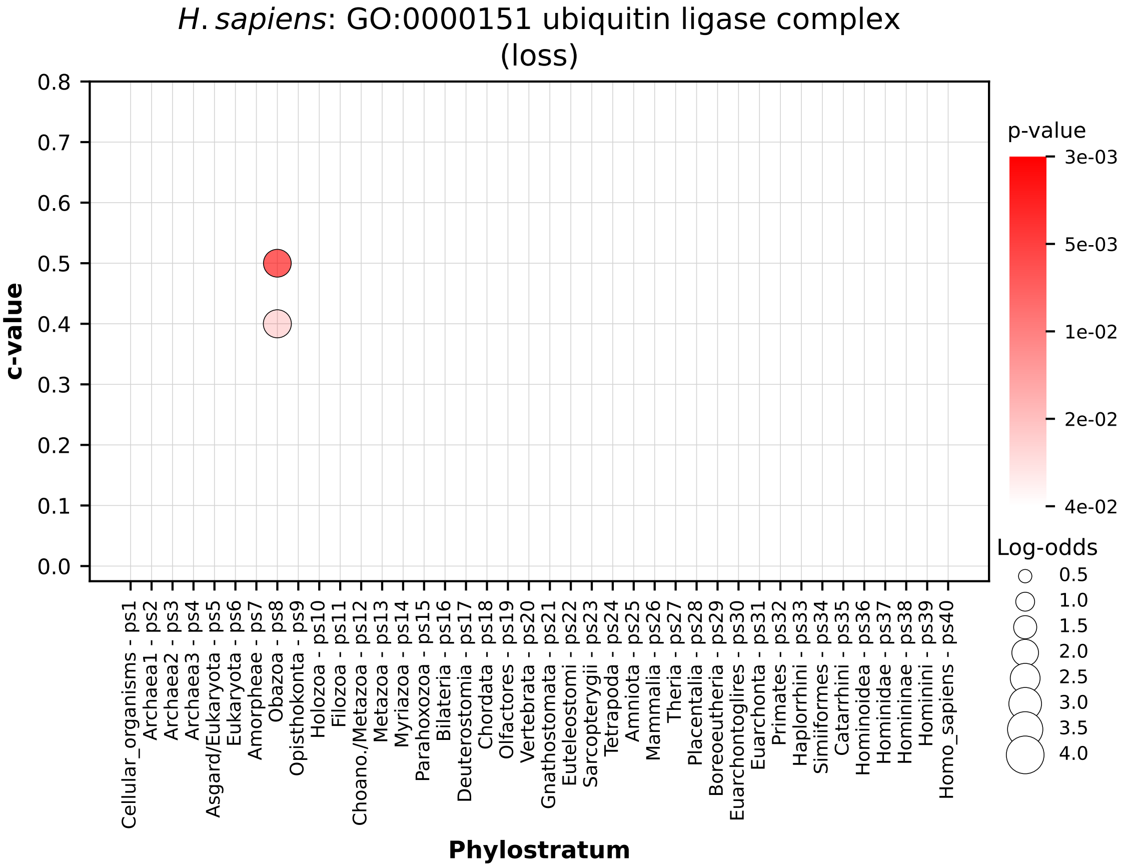

H. sapiens: GO:0000151 ubiquitin ligase complex
(loss)
c-value
0.8
0.7
0.6
0.5
0.4
0.3
0.2
0.1
0.0
Phylostratum
Cellular_organisms - ps1
Archaea1 - ps2
Archaea2 - ps3
Archaea3 - ps4
Asgard/Eukaryota - ps5
Eukaryota - ps6
Amorpheae - ps7
Obazoa - ps8
Opisthokonta - ps9
Holozoa - ps10
Filozoa - ps11
Choano./Metazoa - ps12
Metazoa - ps13
Myriazoa - ps14
Parahoxozoa - ps15
Bilateria - ps16
Deuterostomia - ps17
Chordata - ps18
Olfactores - ps19
Vertebrata - ps20
Gnathostomata - ps21
Euteleostomi - ps22
Sarcopterygii - ps23
Tetrapoda - ps24
Amniota - ps25
Mammalia - ps26
Theria - ps27
Placentalia - ps28
Boreoeutheria - ps29
Euarchontoglires - ps30
Euarchonta - ps31
Primates - ps32
Haplorrhini - ps33
Simiiformes - ps34
Catarrhini - ps35
Hominoidea - ps36
Hominidae - ps37
Homininae - ps38
Hominini - ps39
Homo_sapiens - ps40
p-value
3e-03
5e-03
1e-02
2e-02
4e-02
Log-odds
0.5
1.0
1.5
2.0
2.5
3.0
3.5
4.0

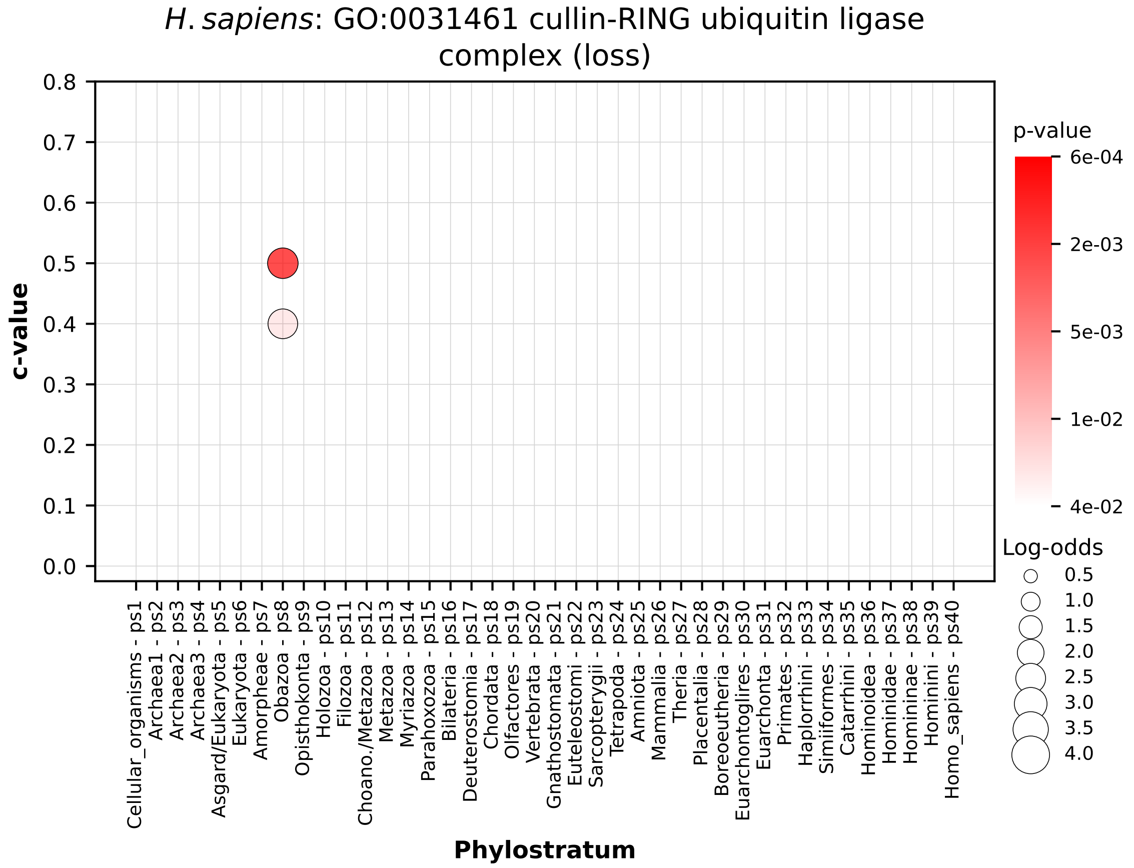

H. sapiens: GO:0031461 cullin-RING ubiquitin ligase complex (loss)
c-value
0.8
0.7
0.6
0.5
0.4
0.3
0.2
0.1
0.0
Phylostratum
Cellular_organisms - ps1
Archaea1 - ps2
Archaea2 - ps3
Archaea3 - ps4
Asgard/Eukaryota - ps5
Eukaryota - ps6
Amorpheae - ps7
Obazoa - ps8
Opisthokonta - ps9
Holozoa - ps10
Filozoa - ps11
Choano./Metazoa - ps12
Metazoa - ps13
Myriazoa - ps14
Parahoxozoa - ps15
Bilateria - ps16
Deuterostomia - ps17
Chordata - ps18
Olfactores - ps19
Vertebrata - ps20
Gnathostomata - ps21
Euteleostomi - ps22
Sarcopterygii - ps23
Tetrapoda - ps24
Amniota - ps25
Mammalia - ps26
Theria - ps27
Placentalia - ps28
Boreoeutheria - ps29
Euarchontoglires - ps30
Euarchonta - ps31
Primates - ps32
Haplorrhini - ps33
Simiiformes - ps34
Catarrhini - ps35
Hominoidea - ps36
Hominidae - ps37
Homininae - ps38
Hominini - ps39
Homo_sapiens - ps40
p-value
6e-04
2e-03
5e-03
1e-02
4e-02
Log-odds
0.5
1.0
1.5
2.0
2.5
3.0
3.5
4.0

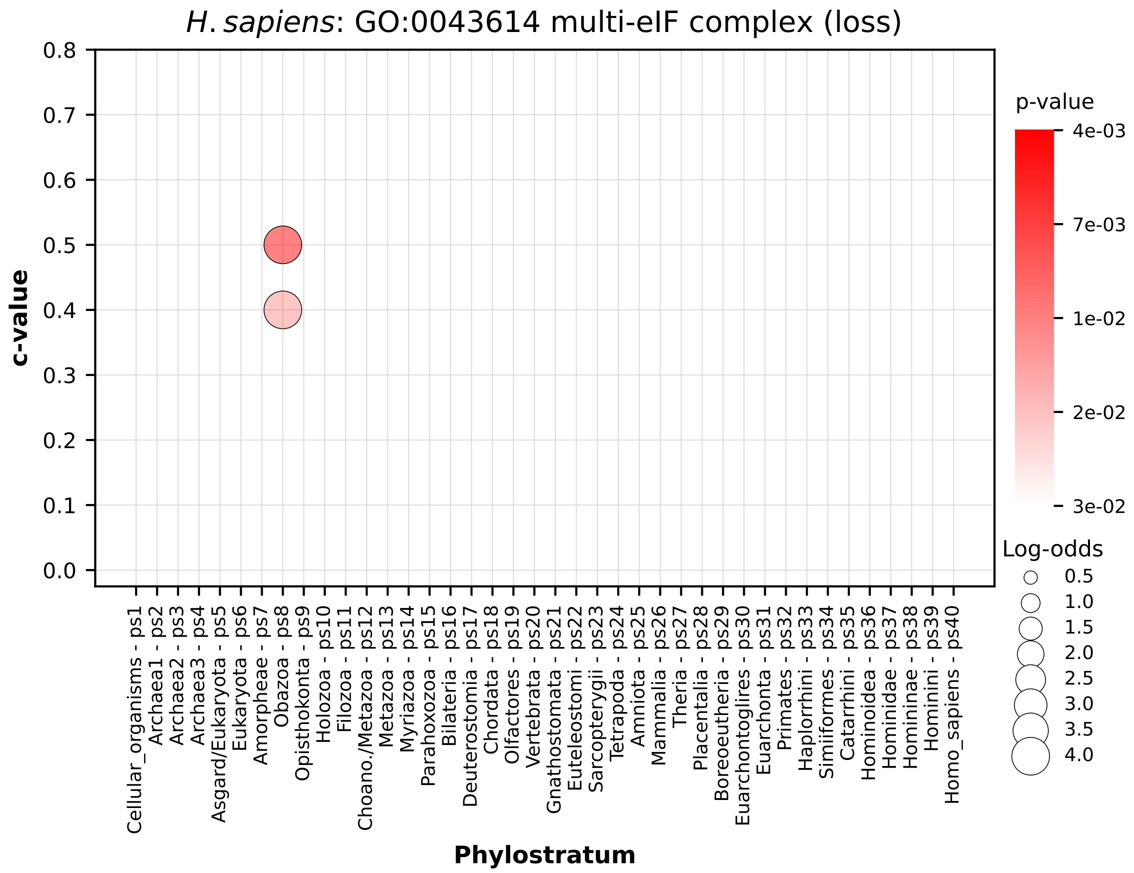

H. sapiens: GO:0043614 multi-eIF complex (loss)
c-value
0.8
0.7
0.6
0.5
0.4
0.3
0.2
0.1
0.0
p-value
4e-03
7e-03
1e-02
2e-02
3e-02
Log-odds
0.5
1.0
1.5
2.0
2.5
3.0
3.5
4.0
Cellular_organisms - ps1
Archaea1 - ps2
Archaea2 - ps3
Archaea3 - ps4
Asgard/Eukaryota - ps5
Eukaryota - ps6
Amorpheae - ps7
Obazoa - ps8
Opisthokonta - ps9
Holozoa - ps10
Filozoa - ps11
Choano./Metazoa - ps12
Metazoa - ps13
Myriazoa - ps14
Parahoxozoa - ps15
Bilateria - ps16
Deuterostomia - ps17
Chordata - ps18
Olfactores - ps19
Vertebrata - ps20
Gnathostomata - ps21
Euteleostomi - ps22
Sarcopterygii - ps23
Tetrapoda - ps24
Amniota - ps25
Mammalia - ps26
Theria - ps27
Placentalia - ps28
Boreoeutheria - ps29
Euarchontoglires - ps30
Euarchonta - ps31
Primates - ps32
Haplorrhini - ps33
Simiiformes - ps34
Catarrhini - ps35
Hominoidea - ps36
Hominidae - ps37
Homininae - ps38
Hominini - ps39
Homo_sapiens - ps40
Phylostratum

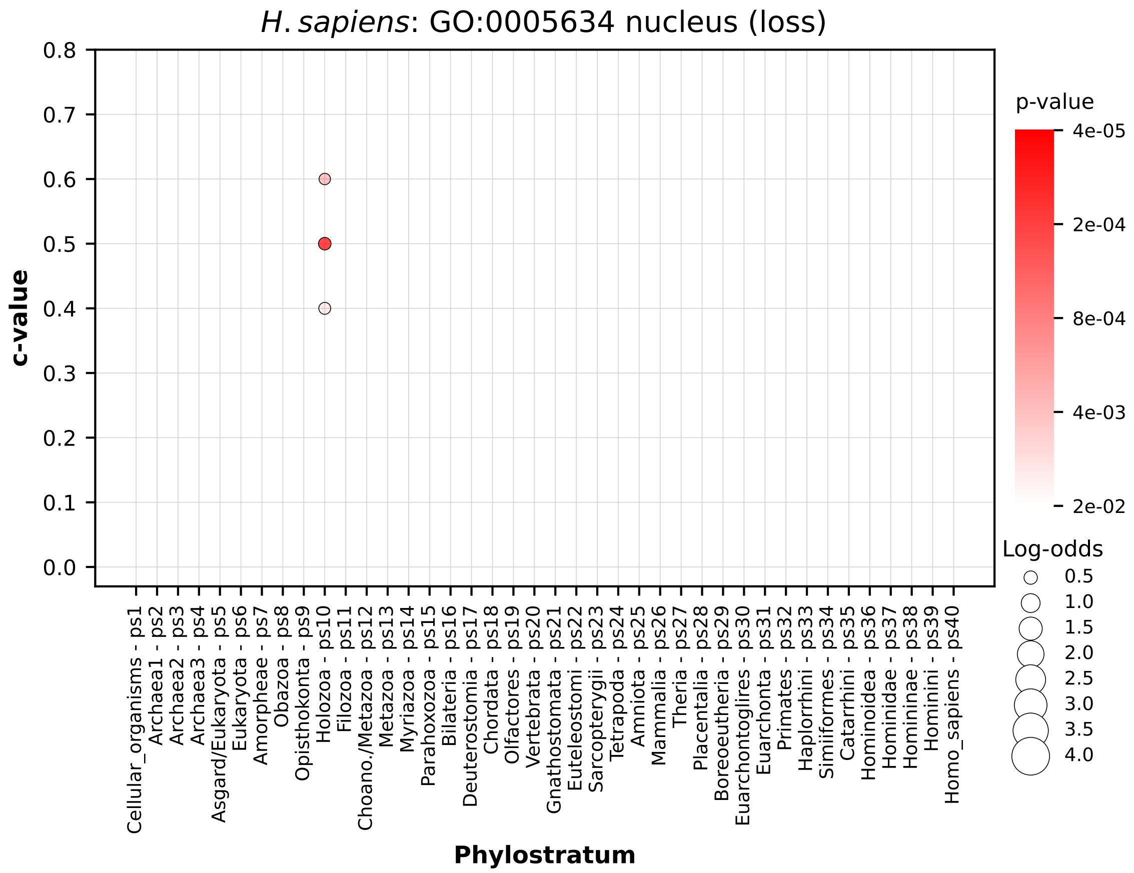

H. sapiens: GO:0005634 nucleus (loss)
c-value
0.8
0.7
0.6
0.5
0.4
0.3
0.2
0.1
0.0
p-value
4e-05
2e-04
8e-04
4e-03
2e-02
Log-odds
0.5
1.0
1.5
2.0
2.5
3.0
3.5
4.0
Cellular_organisms - ps1
Archaea1 - ps2
Archaea2 - ps3
Archaea3 - ps4
Asgard/Eukaryota - ps5
Eukaryota - ps6
Amorpheae - ps7
Obazoa - ps8
Opisthokonta - ps9
Holozoa - ps10
Filozoa - ps11
Choano./Metazoa - ps12
Metazoa - ps13
Myriazoa - ps14
Parahoxozoa - ps15
Bilateria - ps16
Deuterostomia - ps17
Chordata - ps18
Olfactores - ps19
Vertebrata - ps20
Gnathostomata - ps21
Euteleostomi - ps22
Sarcopterygii - ps23
Tetrapoda - ps24
Amniota - ps25
Mammalia - ps26
Theria - ps27
Placentalia - ps28
Boreoeutheria - ps29
Euarchontoglires - ps30
Euarchonta - ps31
Primates - ps32
Haplorrhini - ps33
Simiiformes - ps34
Catarrhini - ps35
Hominoidea - ps36
Hominidae - ps37
Homininae - ps38
Hominini - ps39
Homo_sapiens - ps40
Phylostratum

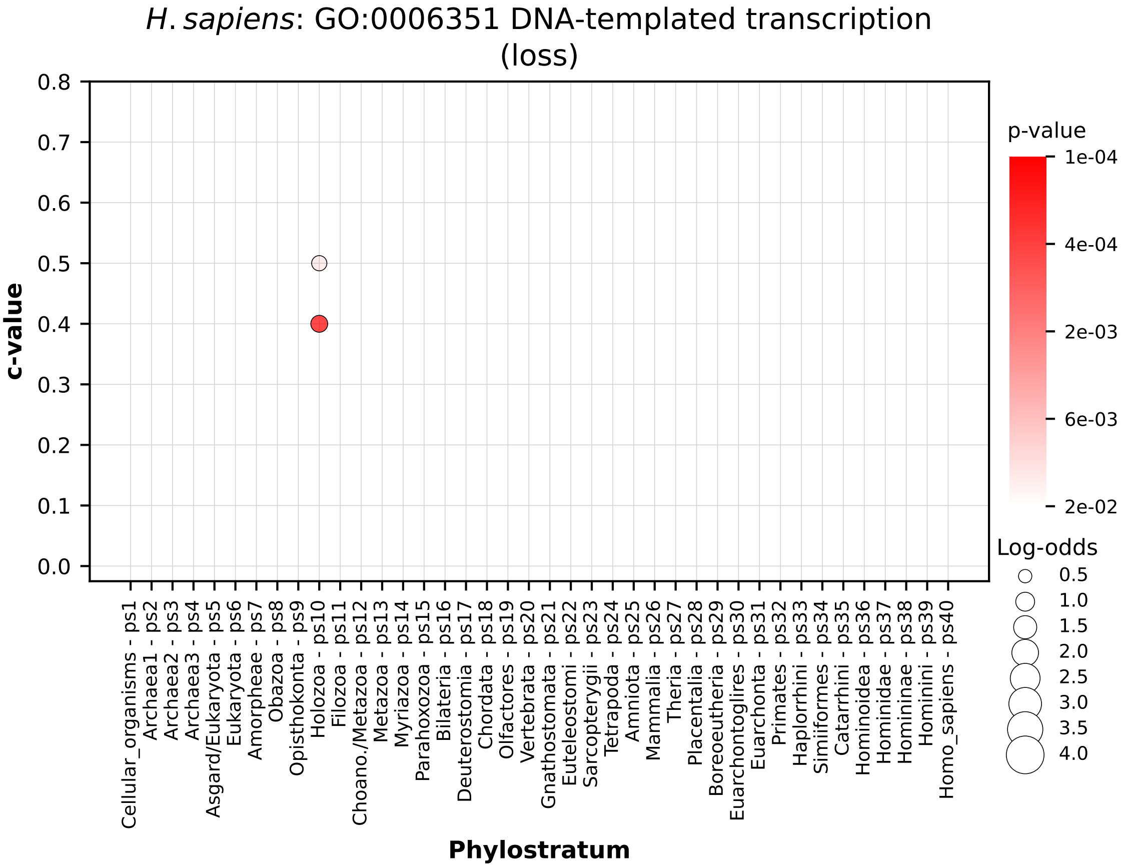
H. sapiens: GO:0006351 DNA-templated transcription
(loss)
c-value
0.8
0.7
0.6
0.5
0.4
0.3
0.2
0.1
0.0
Phylostratum
Cellular_organisms - ps1
Archaea1 - ps2
Archaea2 - ps3
Archaea3 - ps4
Asgard/Eukaryota - ps5
Eukaryota - ps6
Amorpheae - ps7
Obazoa - ps8
Opisthokonta - ps9
Holozoa - ps10
Filozoa - ps11
Choano./Metazoa - ps12
Metazoa - ps13
Myriazoa - ps14
Parahoxozoa - ps15
Bilateria - ps16
Deuterostomia - ps17
Chordata - ps18
Olfactores - ps19
Vertebrata - ps20
Gnathostomata - ps21
Euteleostomi - ps22
Sarcopterygii - ps23
Tetrapoda - ps24
Amniota - ps25
Mammalia - ps26
Theria - ps27
Placentalia - ps28
Boreoeutheria - ps29
Euarchontoglires - ps30
Euarchonta - ps31
Primates - ps32
Haplorrhini - ps33
Simiiformes - ps34
Catarrhini - ps35
Hominoidea - ps36
Hominidae - ps37
Homininae - ps38
Hominini - ps39
Homo_sapiens - ps40
p-value
1e-04
4e-04
2e-03
6e-03
2e-02
Log-odds
0.5
1.0
1.5
2.0
2.5
3.0
3.5
4.0

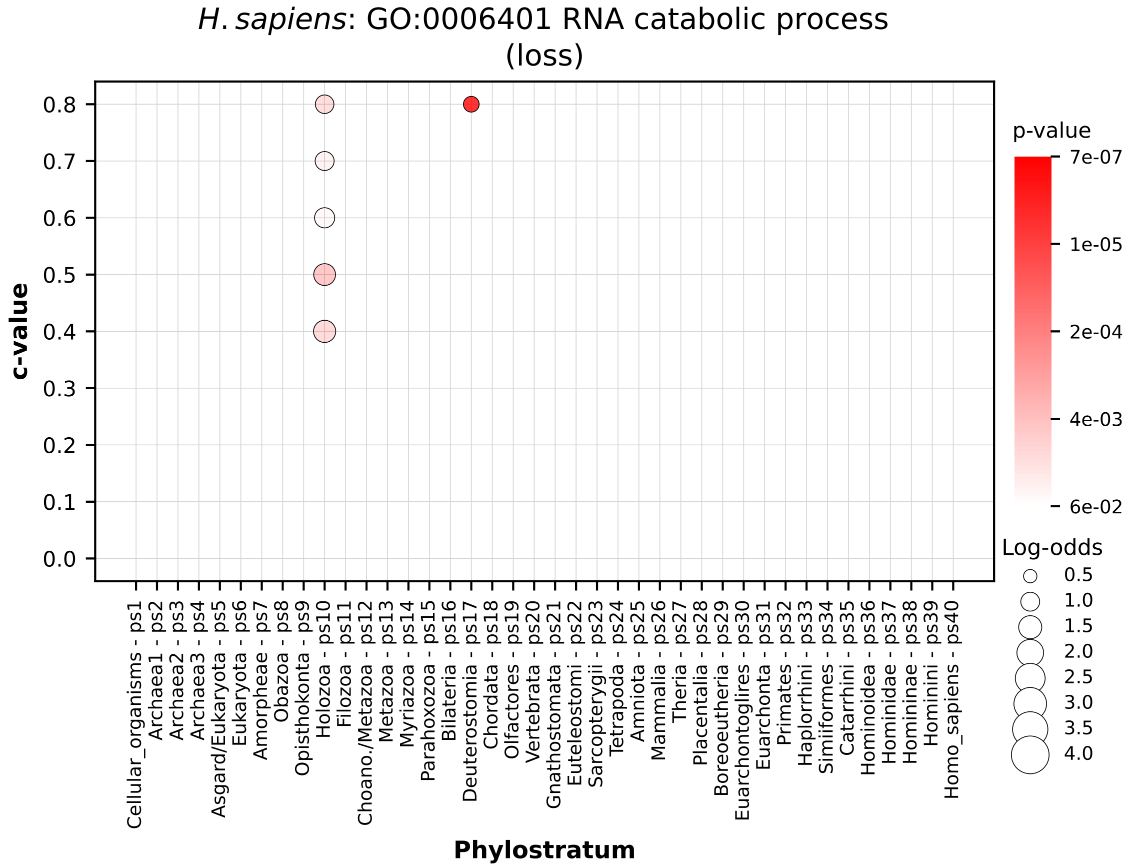

H. sapiens: GO:0006401 RNA catabolic process
(loss)
c-value
0.8
0.7
0.6
0.5
0.4
0.3
0.2
0.1
0.0
p-value
7e-07
1e-05
2e-04
4e-03
6e-02
Log-odds
0.5
1.0
1.5
2.0
2.5
3.0
3.5
4.0
Cellular_organisms - ps1
Archaea1 - ps2
Archaea2 - ps3
Archaea3 - ps4
Asgard/Eukaryota - ps5
Eukaryota - ps6
Amorpheae - ps7
Obazoa - ps8
Opisthokonta - ps9
Holozoa - ps10
Filozoa - ps11
Choano./Metazoa - ps12
Metazoa - ps13
Myriazoa - ps14
Parahoxozoa - ps15
Bilateria - ps16
Deuterostomia - ps17
Chordata - ps18
Olfactores - ps19
Vertebrata - ps20
Gnathostomata - ps21
Euteleostomi - ps22
Sarcopterygii - ps23
Tetrapoda - ps24
Amniota - ps25
Mammalia - ps26
Theria - ps27
Placentalia - ps28
Boreoeutheria - ps29
Euarchontoglires - ps30
Euarchonta - ps31
Primates - ps32
Haplorrhini - ps33
Simiiformes - ps34
Catarrhini - ps35
Hominoidea - ps36
Hominidae - ps37
Homininae - ps38
Hominini - ps39
Homo_sapiens - ps40
Phylostratum

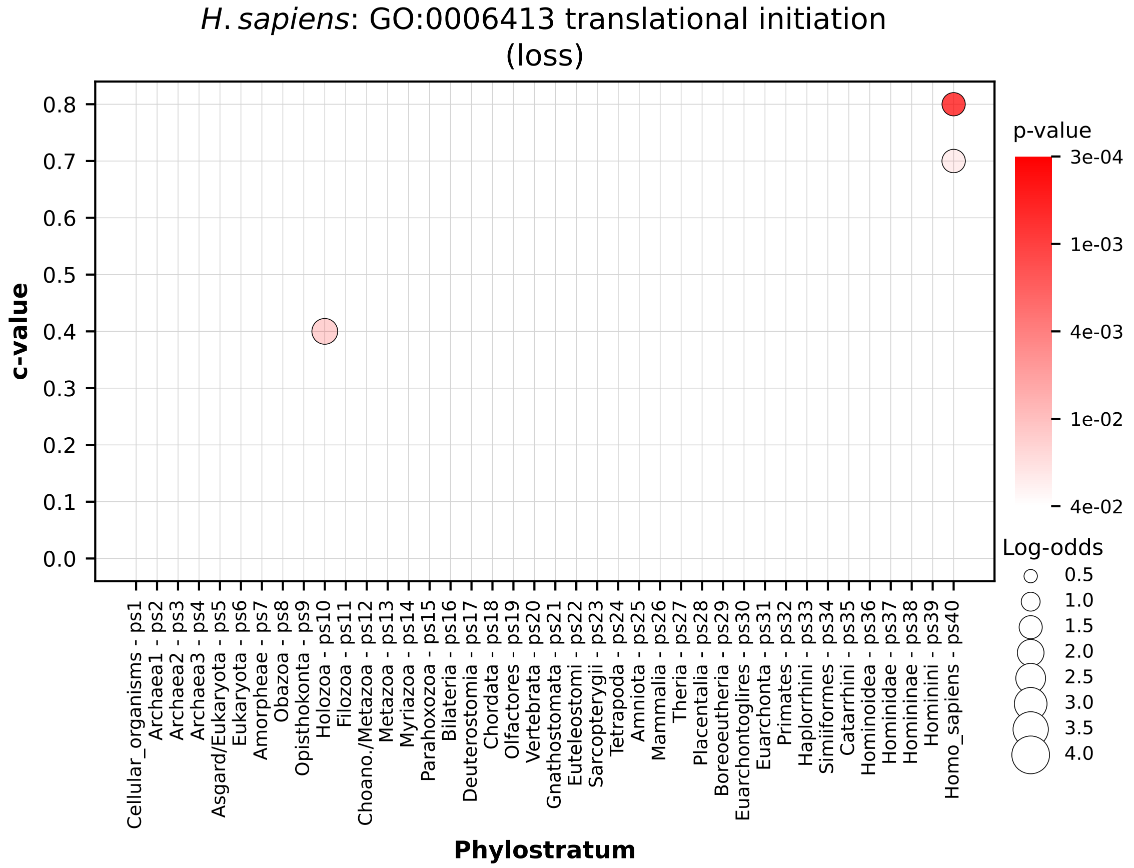
H. sapiens: GO:0006413 translational initiation
(loss)
c-value
0.0
0.1
0.2
0.3
0.4
0.5
0.6
0.7
0.8
Phylostratum
Cellular_organisms - ps1
Archaea1 - ps2
Archaea2 - ps3
Archaea3 - ps4
Asgard/Eukaryota - ps5
Eukaryota - ps6
Amorpheae - ps7
Obazoa - ps8
Opisthokonta - ps9
Holozoa - ps10
Filozoa - ps11
Choano./Metazoa - ps12
Metazoa - ps13
Myriazoa - ps14
Parahoxozoa - ps15
Bilateria - ps16
Deuterostomia - ps17
Chordata - ps18
Olfactores - ps19
Vertebrata - ps20
Gnathostomata - ps21
Euteleostomi - ps22
Sarcopterygii - ps23
Tetrapoda - ps24
Amniota - ps25
Mammalia - ps26
Theria - ps27
Placentalia - ps28
Boreoeutheria - ps29
Euarchontoglires - ps30
Euarchonta - ps31
Primates - ps32
Haplorrhini - ps33
Simiiformes - ps34
Catarrhini - ps35
Hominoidea - ps36
Hominidae - ps37
Homininae - ps38
Hominini - ps39
Homo_sapiens - ps40
p-value
3e-04
1e-03
4e-03
1e-02
4e-02
Log-odds
0.5
1.0
1.5
2.0
2.5
3.0
3.5
4.0

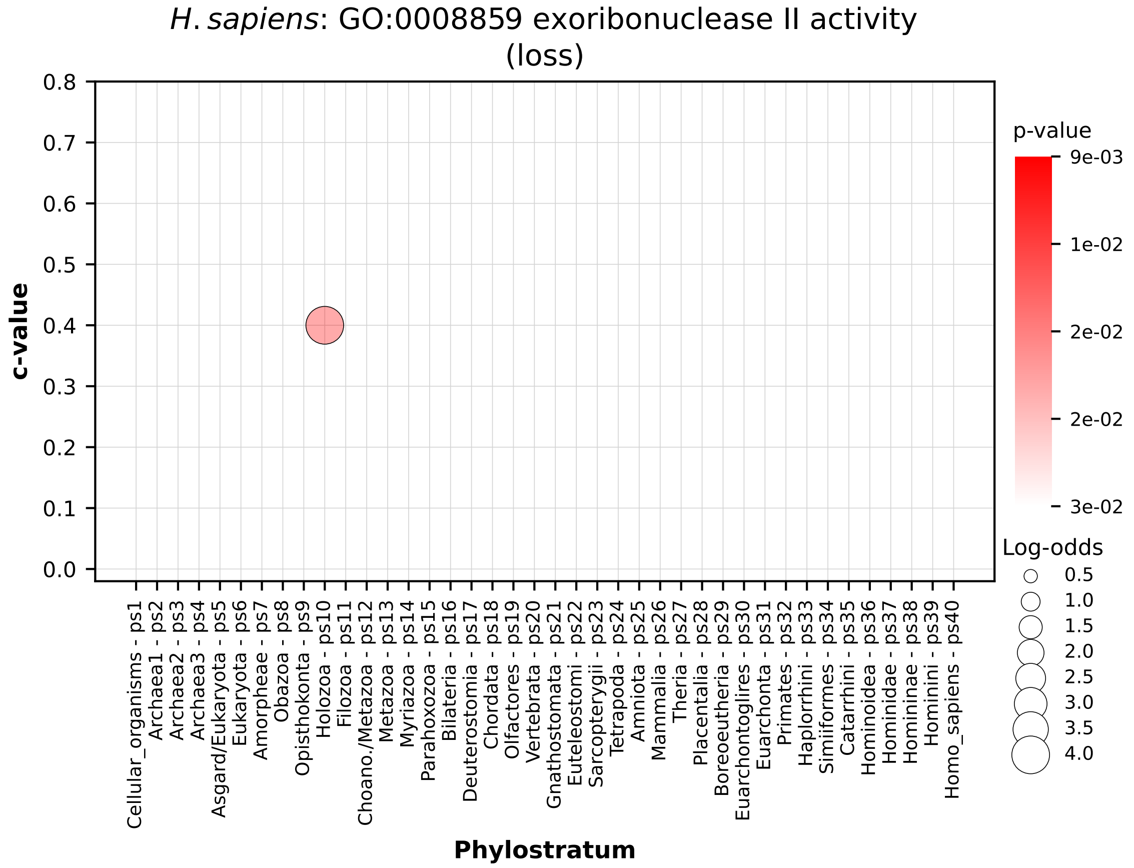
H. sapiens: GO:0008859 exoribonuclease II activity
(loss)
c-value
0.0
0.1
0.2
0.3
0.4
0.5
0.6
0.7
0.8
Phylostratum
Cellular_organisms - ps1
Archaea1 - ps2
Archaea2 - ps3
Archaea3 - ps4
Asgard/Eukaryota - ps5
Eukaryota - ps6
Amorpheae - ps7
Obazoa - ps8
Opisthokonta - ps9
Holozoa - ps10
Filozoa - ps11
Choano./Metazoa - ps12
Metazoa - ps13
Myriazoa - ps14
Parahoxozoa - ps15
Bilateria - ps16
Deuterostomia - ps17
Chordata - ps18
Olfactores - ps19
Vertebrata - ps20
Gnathostomata - ps21
Euteleostomi - ps22
Sarcopterygii - ps23
Tetrapoda - ps24
Amniota - ps25
Mammalia - ps26
Theria - ps27
Placentalia - ps28
Boreoeutheria - ps29
Euarchontoglires - ps30
Euarchonta - ps31
Primates - ps32
Haplorrhini - ps33
Simiiformes - ps34
Catarrhini - ps35
Hominoidea - ps36
Hominidae - ps37
Homininae - ps38
Hominini - ps39
Homo_sapiens - ps40
p-value
9e-03
1e-02
2e-02
2e-02
3e-02
Log-odds
0.5
1.0
1.5
2.0
2.5
3.0
3.5
4.0

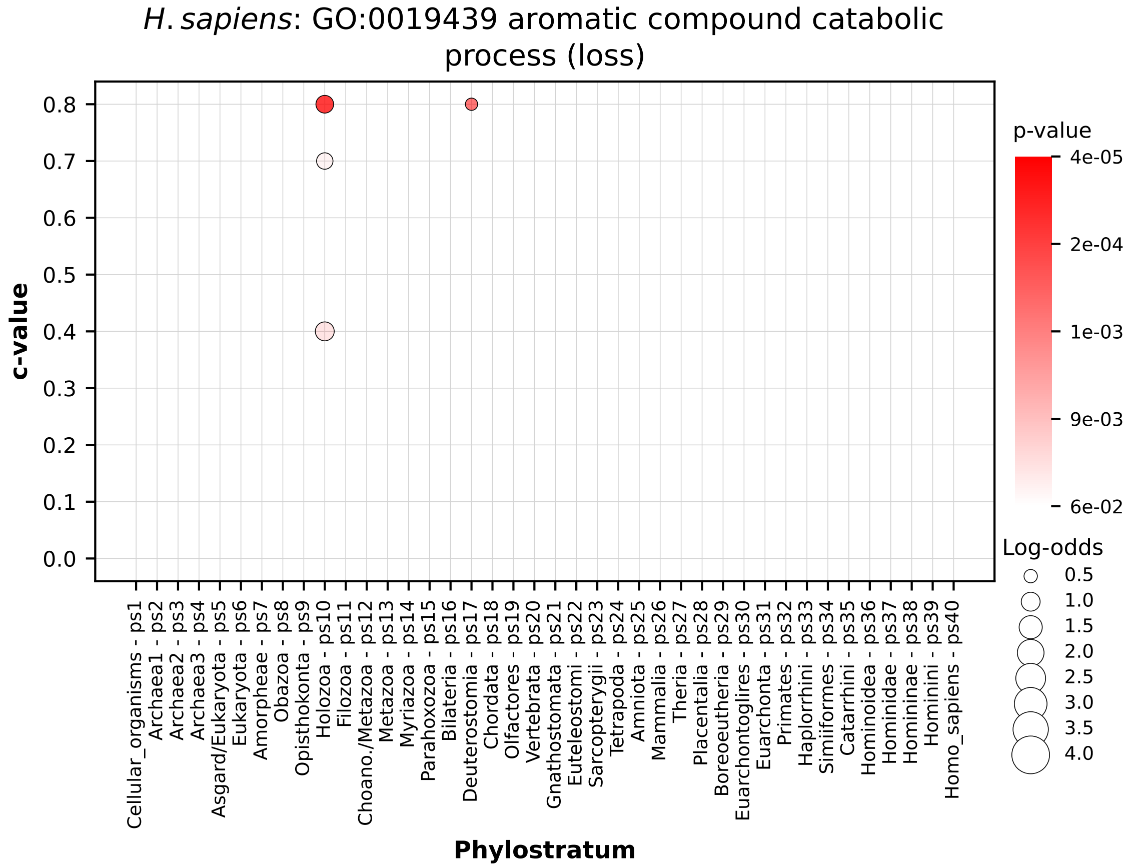
H. sapiens: GO:0019439 aromatic compound catabolic process (loss)
c-value
0.0
0.1
0.2
0.3
0.4
0.5
0.6
0.7
0.8
Phylostratum
Cellular_organisms - ps1
Archaea1 - ps2
Archaea2 - ps3
Archaea3 - ps4
Asgard/Eukaryota - ps5
Eukaryota - ps6
Amorpheae - ps7
Obazoa - ps8
Opisthokonta - ps9
Holozoa - ps10
Filozoa - ps11
Choano./Metazoa - ps12
Metazoa - ps13
Myriazoa - ps14
Parahoxozoa - ps15
Bilateria - ps16
Deuterostomia - ps17
Chordata - ps18
Olfactores - ps19
Vertebrata - ps20
Gnathostomata - ps21
Euteleostomi - ps22
Sarcopterygii - ps23
Tetrapoda - ps24
Amniota - ps25
Mammalia - ps26
Theria - ps27
Placentalia - ps28
Boreoeutheria - ps29
Euarchontoglires - ps30
Euarchonta - ps31
Primates - ps32
Haplorrhini - ps33
Simiiformes - ps34
Catarrhini - ps35
Hominoidea - ps36
Hominidae - ps37
Homininae - ps38
Hominini - ps39
Homo_sapiens - ps40
p-value
4e-05
2e-04
1e-03
9e-03
6e-02
Log-odds
0.5
1.0
1.5
2.0
2.5
3.0
3.5
4.0

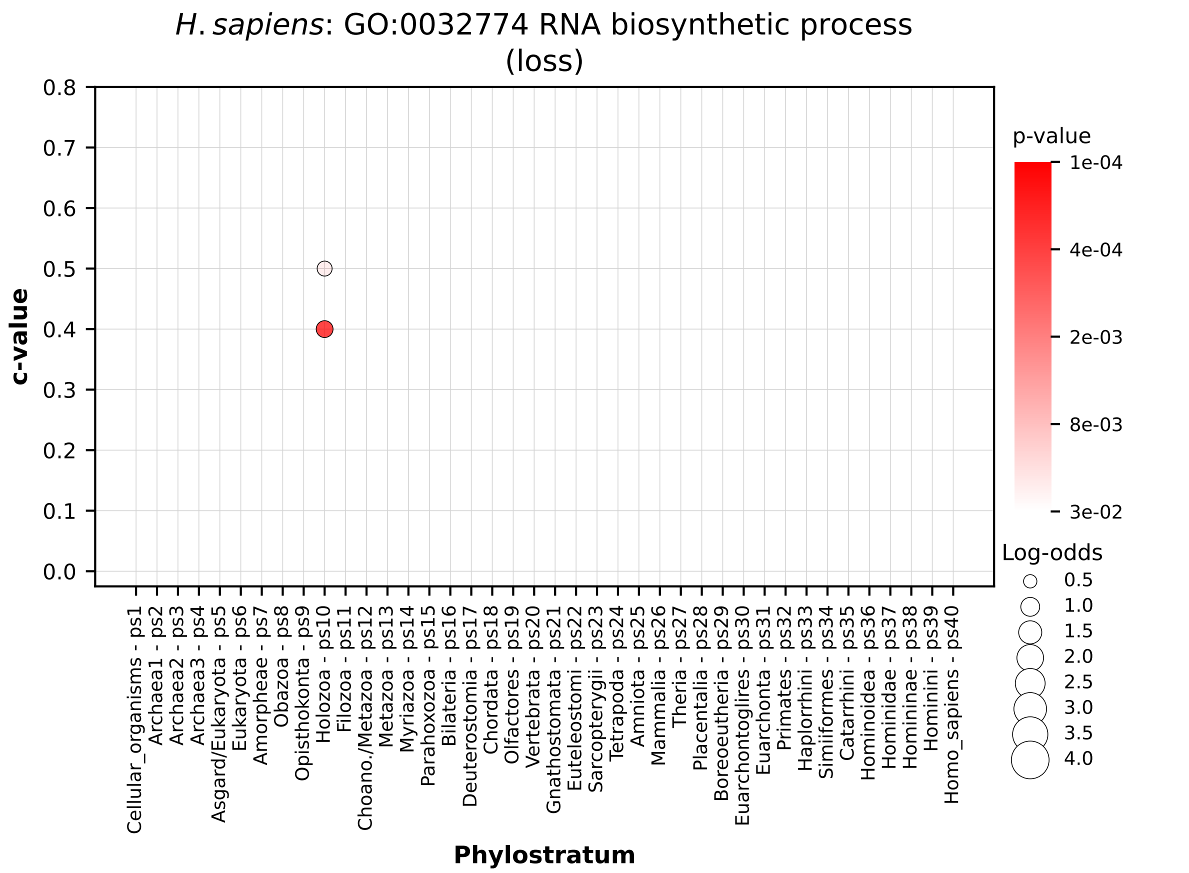

H. sapiens: GO:0032774 RNA biosynthetic process
(loss)
c-value
0.8
0.7
0.6
0.5
0.4
0.3
0.2
0.1
0.0
p-value
1e-04
4e-04
2e-03
8e-03
3e-02
Log-odds
0.5
1.0
1.5
2.0
2.5
3.0
3.5
4.0
Cellular_organisms - ps1
Archaea1 - ps2
Archaea2 - ps3
Archaea3 - ps4
Asgard/Eukaryota - ps5
Eukaryota - ps6
Amorpheae - ps7
Obazoa - ps8
Opisthokonta - ps9
Holozoa - ps10
Filozoa - ps11
Choano./Metazoa - ps12
Metazoa - ps13
Myriazoa - ps14
Parahoxozoa - ps15
Bilateria - ps16
Deuterostomia - ps17
Chordata - ps18
Olfactores - ps19
Vertebrata - ps20
Gnathostomata - ps21
Euteleostomi - ps22
Sarcopterygii - ps23
Tetrapoda - ps24
Amniota - ps25
Mammalia - ps26
Theria - ps27
Placentalia - ps28
Boreoeutheria - ps29
Euarchontoglires - ps30
Euarchonta - ps31
Primates - ps32
Haplorrhini - ps33
Simiiformes - ps34
Catarrhini - ps35
Hominoidea - ps36
Hominidae - ps37
Homininae - ps38
Hominini - ps39
Homo_sapiens - ps40
Phylostratum

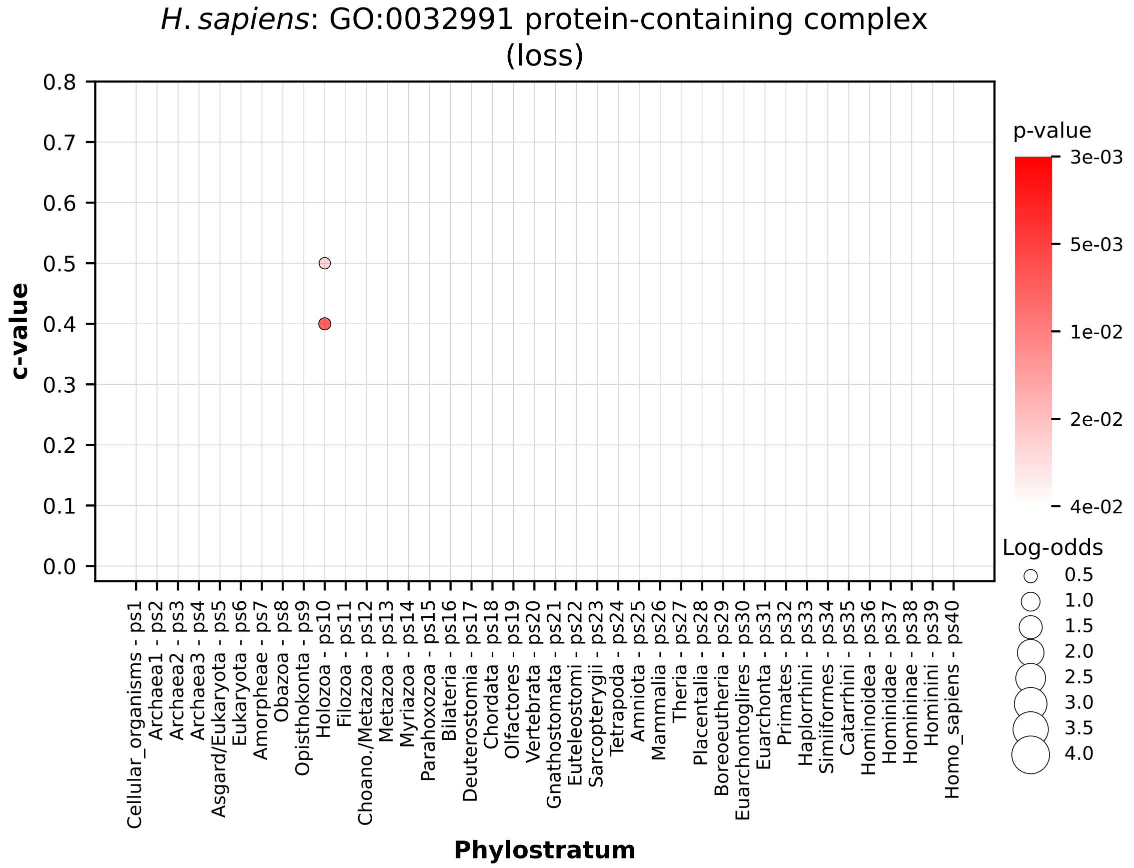
H. sapiens: GO:0032991 protein-containing complex
(loss)
c-value
0.8
0.7
0.6
0.5
0.4
0.3
0.2
0.1
0.0
p-value
3e-03
5e-03
1e-02
2e-02
4e-02
Log-odds
0.5
1.0
1.5
2.0
2.5
3.0
3.5
4.0
Cellular_organisms - ps1
Archaea1 - ps2
Archaea2 - ps3
Archaea3 - ps4
Asgard/Eukaryota - ps5
Eukaryota - ps6
Amorpheae - ps7
Obazoa - ps8
Opisthokonta - ps9
Holozoa - ps10
Filozoa - ps11
Choano./Metazoa - ps12
Metazoa - ps13
Myriazoa - ps14
Parahoxozoa - ps15
Bilateria - ps16
Deuterostomia - ps17
Chordata - ps18
Olfactores - ps19
Vertebrata - ps20
Gnathostomata - ps21
Euteleostomi - ps22
Sarcopterygii - ps23
Tetrapoda - ps24
Amniota - ps25
Mammalia - ps26
Theria - ps27
Placentalia - ps28
Boreoeutheria - ps29
Euarchontoglires - ps30
Euarchonta - ps31
Primates - ps32
Haplorrhini - ps33
Simiiformes - ps34
Catarrhini - ps35
Hominoidea - ps36
Hominidae - ps37
Homininae - ps38
Hominini - ps39
Homo_sapiens - ps40
Phylostratum

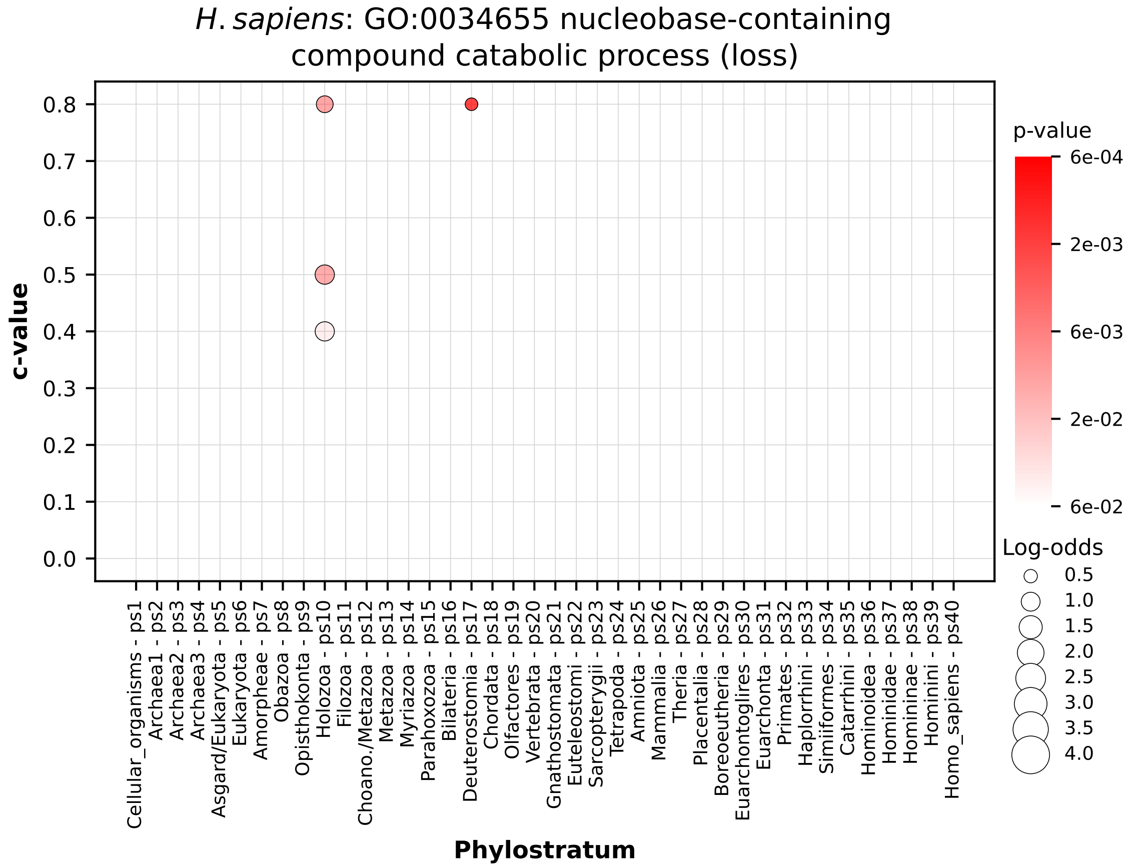

H. sapiens: GO:0034655 nucleobase-containing compound catabolic process (loss)
c-value
0.0
0.1
0.2
0.3
0.4
0.5
0.6
0.7
0.8
Phylostratum
Cellular_organisms - ps1
Archaea1 - ps2
Archaea2 - ps3
Archaea3 - ps4
Asgard/Eukaryota - ps5
Eukaryota - ps6
Amorpheae - ps7
Obazoa - ps8
Opisthokonta - ps9
Holozoa - ps10
Filozoa - ps11
Choano./Metazoa - ps12
Metazoa - ps13
Myriazoa - ps14
Parahoxozoa - ps15
Bilateria - ps16
Deuterostomia - ps17
Chordata - ps18
Olfactores - ps19
Vertebrata - ps20
Gnathostomata - ps21
Euteleostomi - ps22
Sarcopterygii - ps23
Tetrapoda - ps24
Amniota - ps25
Mammalia - ps26
Theria - ps27
Placentalia - ps28
Boreoeutheria - ps29
Euarchontoglires - ps30
Euarchonta - ps31
Primates - ps32
Haplorrhini - ps33
Simiiformes - ps34
Catarrhini - ps35
Hominoidea - ps36
Hominidae - ps37
Homininae - ps38
Hominini - ps39
Homo_sapiens - ps40
p-value
6e-04
2e-03
6e-03
2e-02
6e-02
Log-odds
0.5
1.0
1.5
2.0
2.5
3.0
3.5
4.0

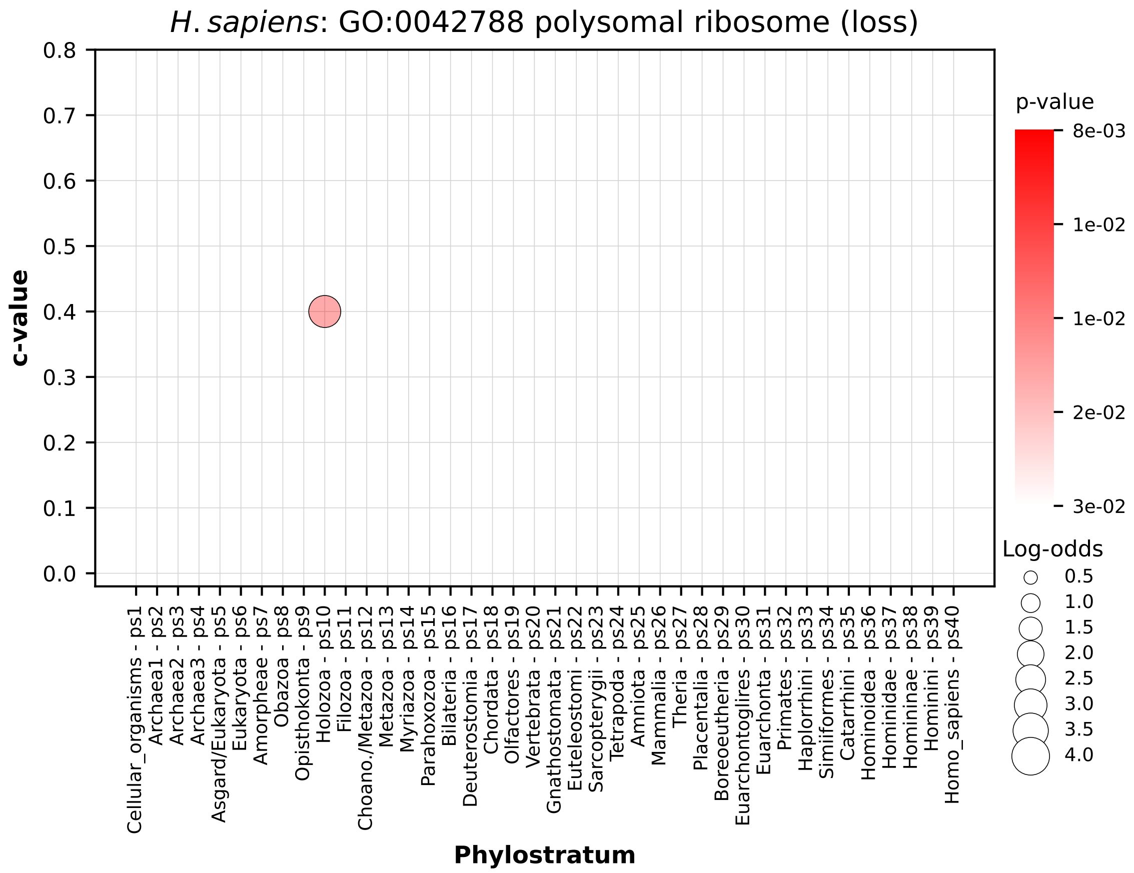

H. sapiens: GO:0042788 polysomal ribosome (loss)
c-value
0.8
0.7
0.6
0.5
0.4
0.3
0.2
0.1
0.0
Phylostratum
Cellular_organisms - ps1
Archaea1 - ps2
Archaea2 - ps3
Archaea3 - ps4
Asgard/Eukaryota - ps5
Eukaryota - ps6
Amorpheae - ps7
Obazoa - ps8
Opisthokonta - ps9
Holozoa - ps10
Filozoa - ps11
Choano./Metazoa - ps12
Metazoa - ps13
Myriazoa - ps14
Parahoxozoa - ps15
Bilateria - ps16
Deuterostomia - ps17
Chordata - ps18
Olfactores - ps19
Vertebrata - ps20
Gnathostomata - ps21
Euteleostomi - ps22
Sarcopterygii - ps23
Tetrapoda - ps24
Amniota - ps25
Mammalia - ps26
Theria - ps27
Placentalia - ps28
Boreoeutheria - ps29
Euarchontoglires - ps30
Euarchonta - ps31
Primates - ps32
Haplorrhini - ps33
Simiiformes - ps34
Catarrhini - ps35
Hominoidea - ps36
Hominidae - ps37
Homininae - ps38
Hominini - ps39
Homo_sapiens - ps40
p-value
8e-03
1e-02
1e-02
2e-02
3e-02
Log-odds
0.5
1.0
1.5
2.0
2.5
3.0
3.5
4.0

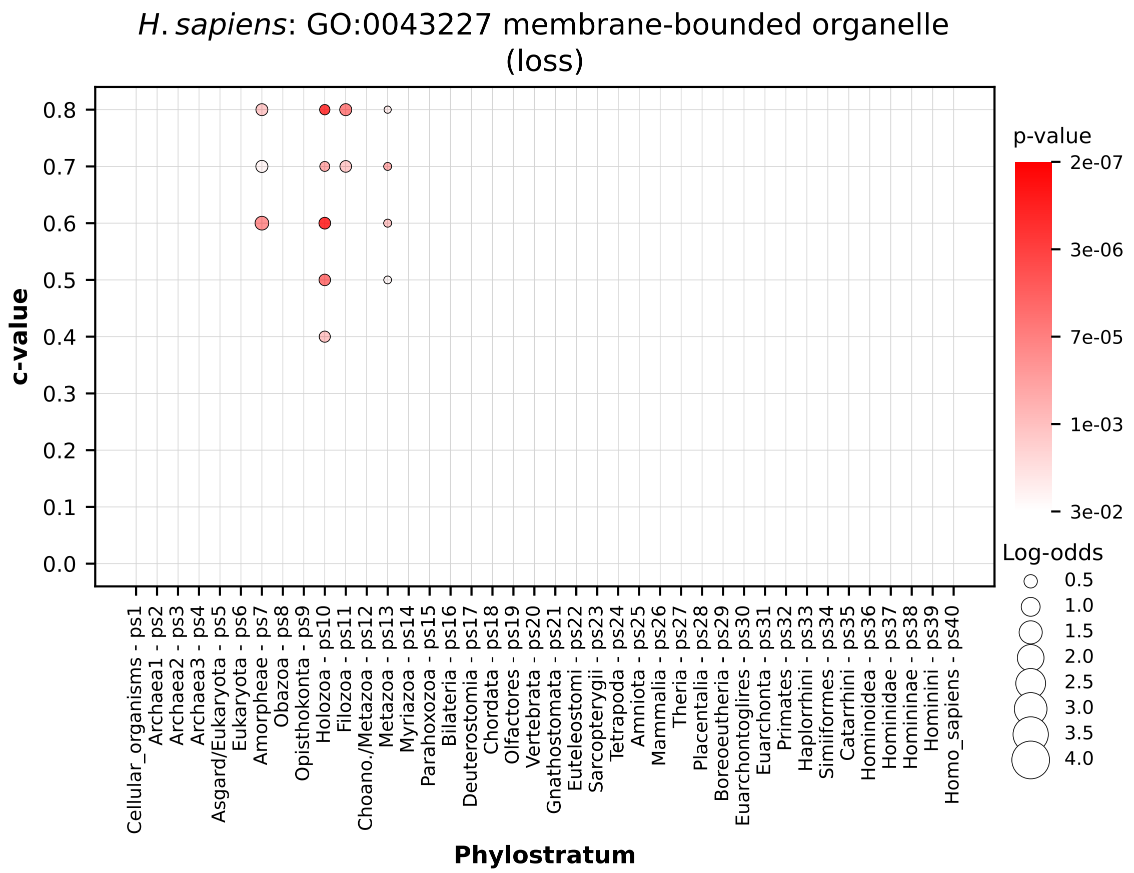

H. sapiens: GO:0043227 membrane-bounded organelle
(loss)
c-value
0.8
0.7
0.6
0.5
0.4
0.3
0.2
0.1
0.0
Phylostratum
Cellular_organisms - ps1
Archaea1 - ps2
Archaea2 - ps3
Archaea3 - ps4
Asgard/Eukaryota - ps5
Eukaryota - ps6
Amorpheae - ps7
Obazoa - ps8
Opisthokonta - ps9
Holozoa - ps10
Filozoa - ps11
Choano./Metazoa - ps12
Metazoa - ps13
Myriazoa - ps14
Parahoxozoa - ps15
Bilateria - ps16
Deuterostomia - ps17
Chordata - ps18
Olfactores - ps19
Vertebrata - ps20
Gnathostomata - ps21
Euteleostomi - ps22
Sarcopterygii - ps23
Tetrapoda - ps24
Amniota - ps25
Mammalia - ps26
Theria - ps27
Placentalia - ps28
Boreoeutheria - ps29
Euarchontoglires - ps30
Euarchonta - ps31
Primates - ps32
Haplorrhini - ps33
Simiiformes - ps34
Catarrhini - ps35
Hominoidea - ps36
Hominidae - ps37
Homininae - ps38
Hominini - ps39
Homo_sapiens - ps40
p-value
2e-07
3e-06
7e-05
1e-03
3e-02
Log-odds
0.5
1.0
1.5
2.0
2.5
3.0
3.5
4.0

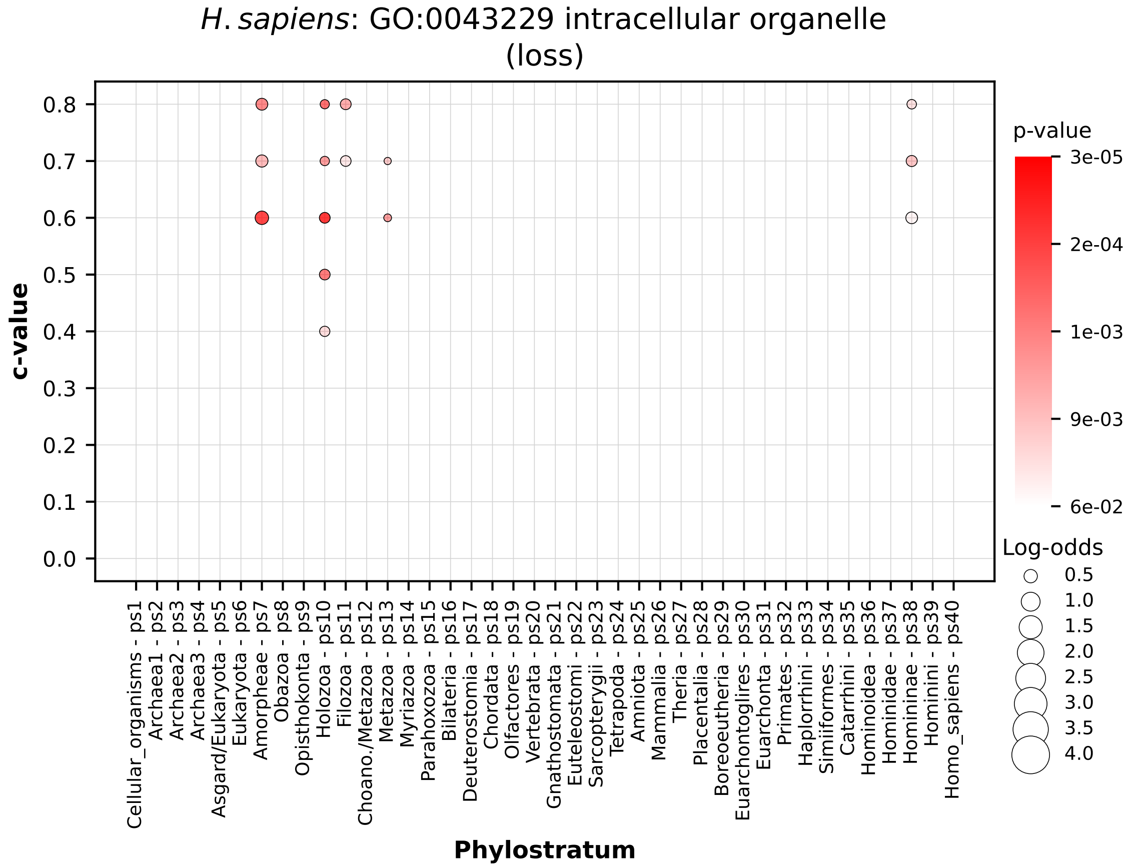

H. sapiens: GO:0043229 intracellular organelle (loss)
c-value
0.0
0.1
0.2
0.3
0.4
0.5
0.6
0.7
0.8
Phylostratum
Cellular_organisms - ps1
Archaea1 - ps2
Archaea2 - ps3
Archaea3 - ps4
Asgard/Eukaryota - ps5
Eukaryota - ps6
Amorpheae - ps7
Obazoa - ps8
Opisthokonta - ps9
Holozoa - ps10
Filozoa - ps11
Choano./Metazoa - ps12
Metazoa - ps13
Myriazoa - ps14
Parahoxozoa - ps15
Bilateria - ps16
Deuterostomia - ps17
Chordata - ps18
Olfactores - ps19
Vertebrata - ps20
Gnathostomata - ps21
Euteleostomi - ps22
Sarcopterygii - ps23
Tetrapoda - ps24
Amniota - ps25
Mammalia - ps26
Theria - ps27
Placentalia - ps28
Boreoeutheria - ps29
Euarchontoglires - ps30
Euarchonta - ps31
Primates - ps32
Haplorrhini - ps33
Simiiformes - ps34
Catarrhini - ps35
Hominoidea - ps36
Hominidae - ps37
Homininae - ps38
Hominini - ps39
Homo_sapiens - ps40
p-value
3e-05
2e-04
1e-03
9e-03
6e-02
Log-odds
0.5
1.0
1.5
2.0
2.5
3.0
3.5
4.0

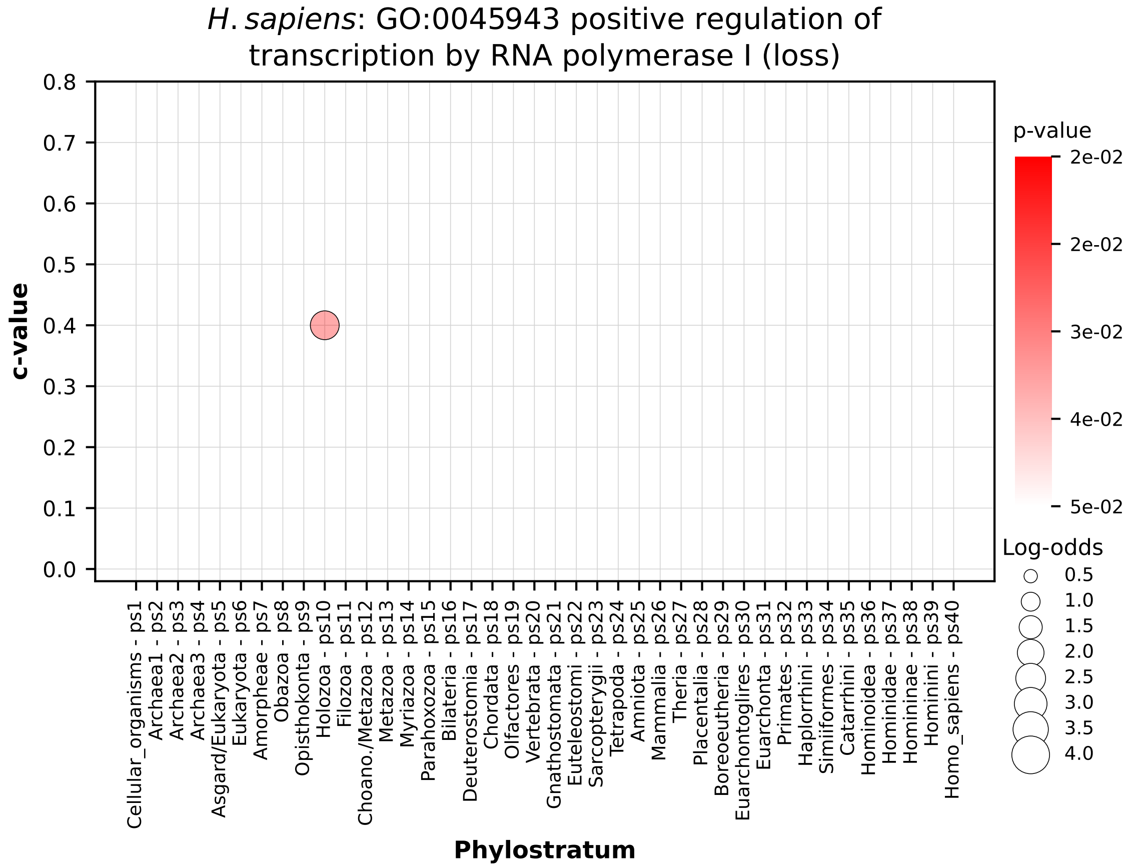

H. sapiens: GO:0045943 positive regulation of transcription by RNA polymerase I (loss)
c-value
0.8
0.7
0.6
0.5
0.4
0.3
0.2
0.1
0.0
Phylostratum
Cellular_organisms - ps1
Archaea1 - ps2
Archaea2 - ps3
Archaea3 - ps4
Asgard/Eukaryota - ps5
Eukaryota - ps6
Amorpheae - ps7
Obazoa - ps8
Opisthokonta - ps9
Holozoa - ps10
Filozoa - ps11
Choano./Metazoa - ps12
Metazoa - ps13
Myriazoa - ps14
Parahoxozoa - ps15
Bilateria - ps16
Deuterostomia - ps17
Chordata - ps18
Olfactores - ps19
Vertebrata - ps20
Gnathostomata - ps21
Euteleostomi - ps22
Sarcopterygii - ps23
Tetrapoda - ps24
Amniota - ps25
Mammalia - ps26
Theria - ps27
Placentalia - ps28
Boreoeutheria - ps29
Euarchontoglires - ps30
Euarchonta - ps31
Primates - ps32
Haplorrhini - ps33
Simiiformes - ps34
Catarrhini - ps35
Hominoidea - ps36
Hominidae - ps37
Homininae - ps38
Hominini - ps39
Homo_sapiens - ps40
p-value
2e-02
2e-02
3e-02
4e-02
5e-02
Log-odds
0.5
1.0
1.5
2.0
2.5
3.0
3.5
4.0

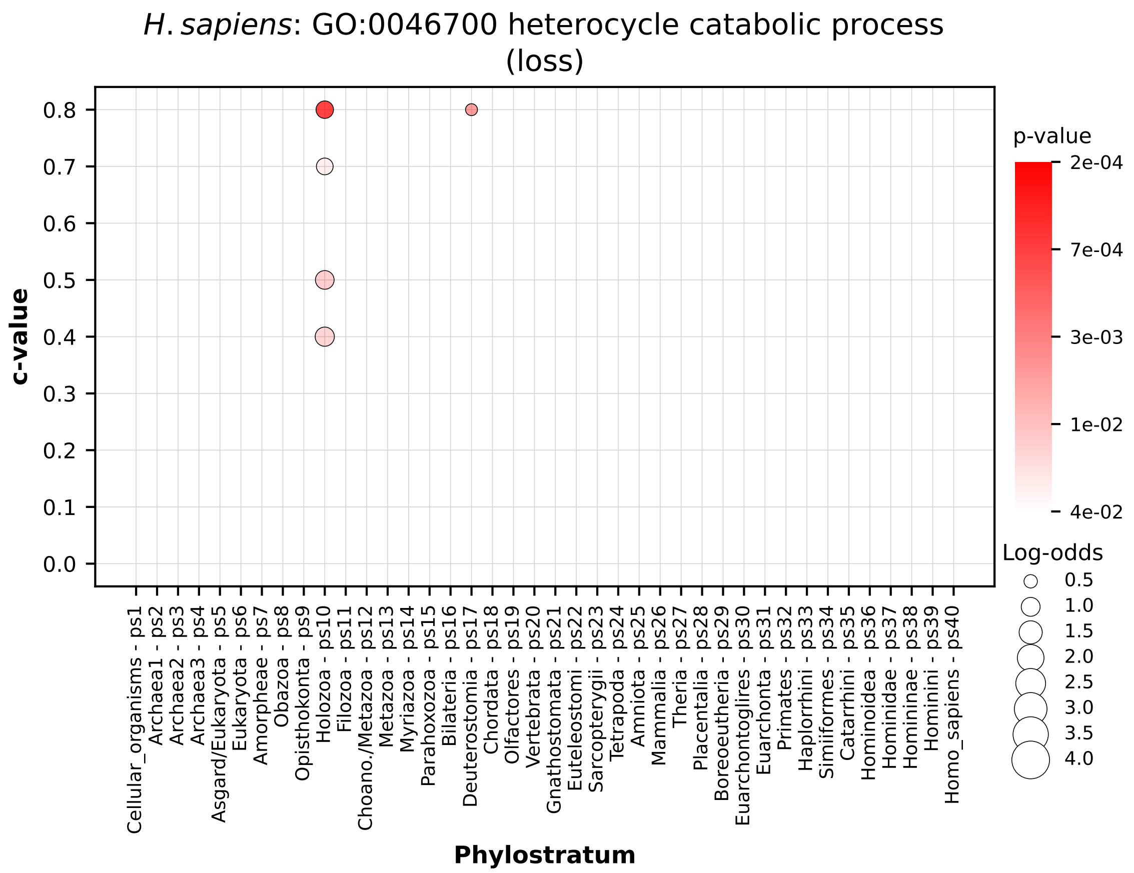
H. sapiens: GO:0046700 heterocycle catabolic process
(loss)
c-value
0.0
0.1
0.2
0.3
0.4
0.5
0.6
0.7
0.8
Phylostratum
Cellular_organisms - ps1
Archaea1 - ps2
Archaea2 - ps3
Archaea3 - ps4
Asgard/Eukaryota - ps5
Eukaryota - ps6
Amorpheae - ps7
Obazoa - ps8
Opisthokonta - ps9
Holozoa - ps10
Filozoa - ps11
Choano./Metazoa - ps12
Metazoa - ps13
Myriazoa - ps14
Parahoxozoa - ps15
Bilateria - ps16
Deuterostomia - ps17
Chordata - ps18
Olfactores - ps19
Vertebrata - ps20
Gnathostomata - ps21
Euteleostomi - ps22
Sarcopterygii - ps23
Tetrapoda - ps24
Amniota - ps25
Mammalia - ps26
Theria - ps27
Placentalia - ps28
Boreoeutheria - ps29
Euarchontoglires - ps30
Euarchonta - ps31
Primates - ps32
Haplorrhini - ps33
Simiiformes - ps34
Catarrhini - ps35
Hominoidea - ps36
Hominidae - ps37
Homininae - ps38
Hominini - ps39
Homo_sapiens - ps40
p-value
2e-04
7e-04
3e-03
1e-02
4e-02
Log-odds
0.5
1.0
1.5
2.0
2.5
3.0
3.5
4.0

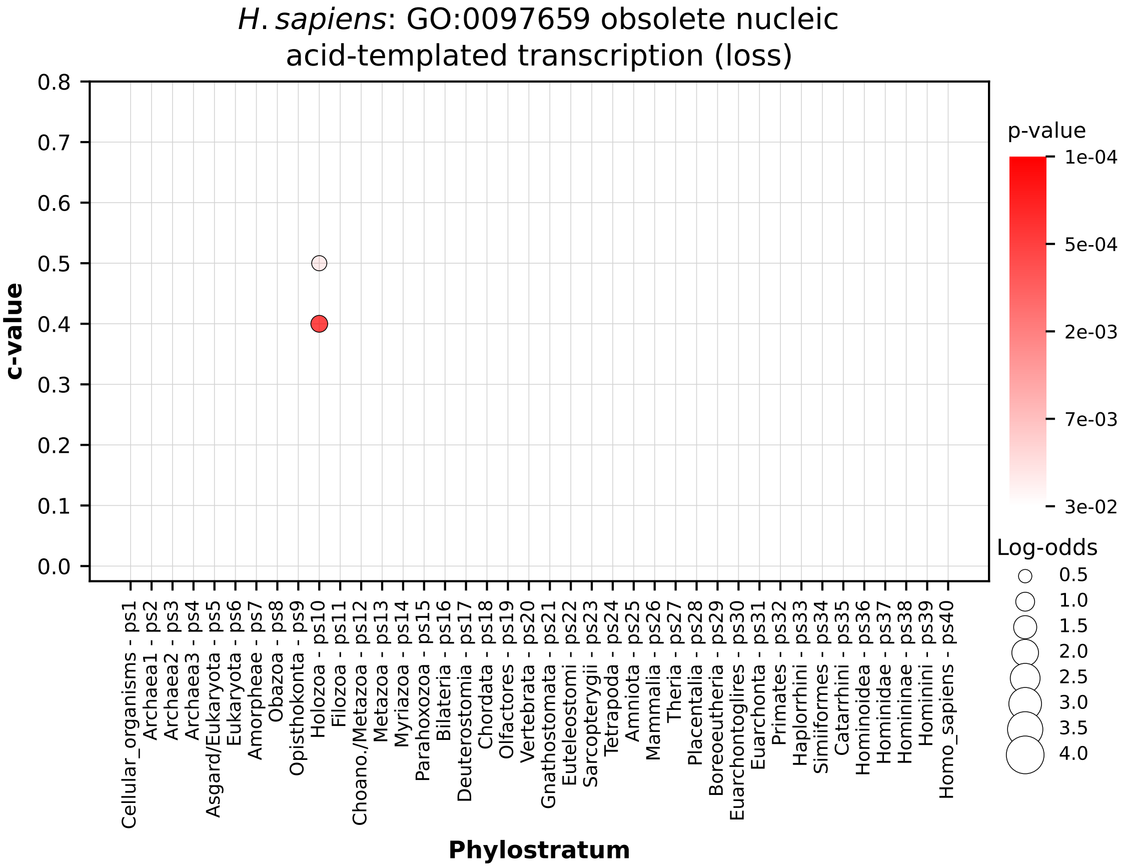

H. sapiens: GO:0097659 obsolete nucleic acid-templated transcription (loss)
c-value
0.8
0.7
0.6
0.5
0.4
0.3
0.2
0.1
0.0
Phylostratum
Cellular_organisms - ps1
Archaea1 - ps2
Archaea2 - ps3
Archaea3 - ps4
Asgard/Eukaryota - ps5
Eukaryota - ps6
Amorpheae - ps7
Obazoa - ps8
Opisthokonta - ps9
Holozoa - ps10
Filozoa - ps11
Choano./Metazoa - ps12
Metazoa - ps13
Myriazoa - ps14
Parahoxozoa - ps15
Bilateria - ps16
Deuterostomia - ps17
Chordata - ps18
Olfactores - ps19
Vertebrata - ps20
Gnathostomata - ps21
Euteleostomi - ps22
Sarcopterygii - ps23
Tetrapoda - ps24
Amniota - ps25
Mammalia - ps26
Theria - ps27
Placentalia - ps28
Boreoeutheria - ps29
Euarchontoglires - ps30
Euarchonta - ps31
Primates - ps32
Haplorrhini - ps33
Simiiformes - ps34
Catarrhini - ps35
Hominoidea - ps36
Hominidae - ps37
Homininae - ps38
Hominini - ps39
Homo_sapiens - ps40
p-value
1e-04
5e-04
2e-03
7e-03
3e-02
Log-odds
0.5
1.0
1.5
2.0
2.5
3.0
3.5
4.0

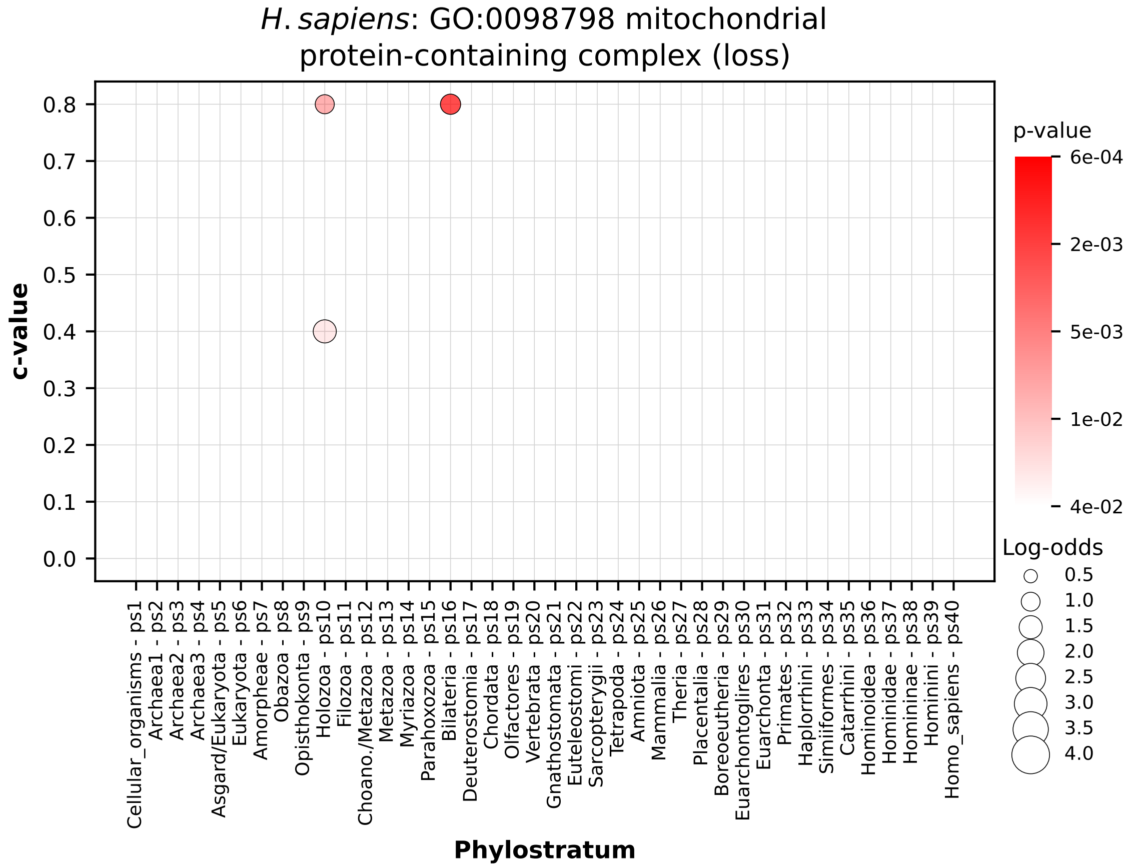
H. sapiens: GO:0098798 mitochondrial protein-containing complex (loss)
c-value
0.0
0.1
0.2
0.3
0.4
0.5
0.6
0.7
0.8
p-value
6e-04
2e-03
5e-03
1e-02
4e-02
Log-odds
0.5
1.0
1.5
2.0
2.5
3.0
3.5
4.0
Cellular_organisms - ps1
Archaea1 - ps2
Archaea2 - ps3
Archaea3 - ps4
Asgard/Eukaryota - ps5
Eukaryota - ps6
Amorpheae - ps7
Obazoa - ps8
Opisthokonta - ps9
Holozoa - ps10
Filozoa - ps11
Choano./Metazoa - ps12
Metazoa - ps13
Myriazoa - ps14
Parahoxozoa - ps15
Bilateria - ps16
Deuterostomia - ps17
Chordata - ps18
Olfactores - ps19
Vertebrata - ps20
Gnathostomata - ps21
Euteleostomi - ps22
Sarcopterygii - ps23
Tetrapoda - ps24
Amniota - ps25
Mammalia - ps26
Theria - ps27
Placentalia - ps28
Boreoeutheria - ps29
Euarchontoglires - ps30
Euarchonta - ps31
Primates - ps32
Haplorrhini - ps33
Simiiformes - ps34
Catarrhini - ps35
Hominoidea - ps36
Hominidae - ps37
Homininae - ps38
Hominini - ps39
Homo_sapiens - ps40
Phylostratum

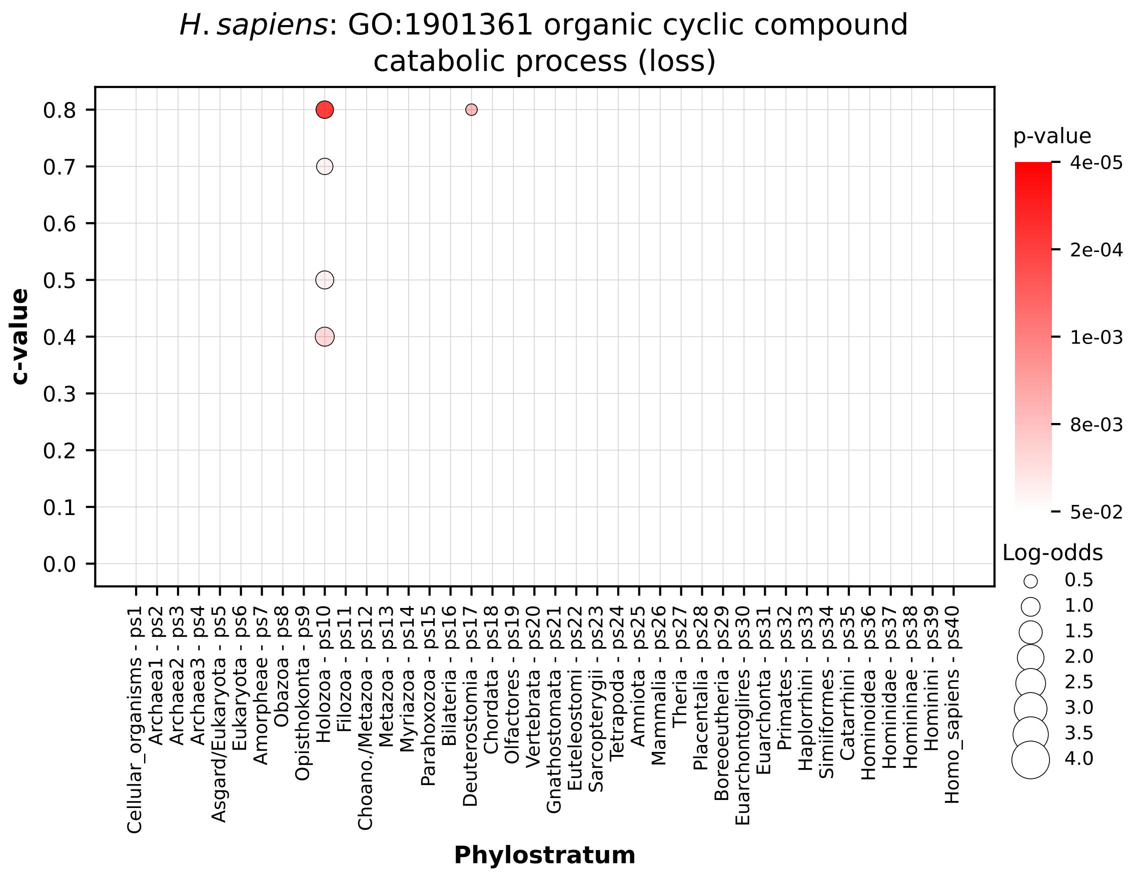
H. sapiens: GO:1901361 organic cyclic compound catabolic process (loss)
c-value
0.0
0.1
0.2
0.3
0.4
0.5
0.6
0.7
0.8
Phylostratum
Cellular_organisms - ps1
Archaea1 - ps2
Archaea2 - ps3
Archaea3 - ps4
Asgard/Eukaryota - ps5
Eukaryota - ps6
Amorpheae - ps7
Obazoa - ps8
Opisthokonta - ps9
Holozoa - ps10
Filozoa - ps11
Choano./Metazoa - ps12
Metazoa - ps13
Myriazoa - ps14
Parahoxozoa - ps15
Bilateria - ps16
Deuterostomia - ps17
Chordata - ps18
Olfactores - ps19
Vertebrata - ps20
Gnathostomata - ps21
Euteleostomi - ps22
Sarcopterygii - ps23
Tetrapoda - ps24
Amniota - ps25
Mammalia - ps26
Theria - ps27
Placentalia - ps28
Boreoeutheria - ps29
Euarchontoglires - ps30
Euarchonta - ps31
Primates - ps32
Haplorrhini - ps33
Simiiformes - ps34
Catarrhini - ps35
Hominoidea - ps36
Hominidae - ps37
Homininae - ps38
Hominini - ps39
Homo_sapiens - ps40
p-value
4e-05
2e-04
1e-03
8e-03
5e-02
Log-odds
0.5
1.0
1.5
2.0
2.5
3.0
3.5
4.0

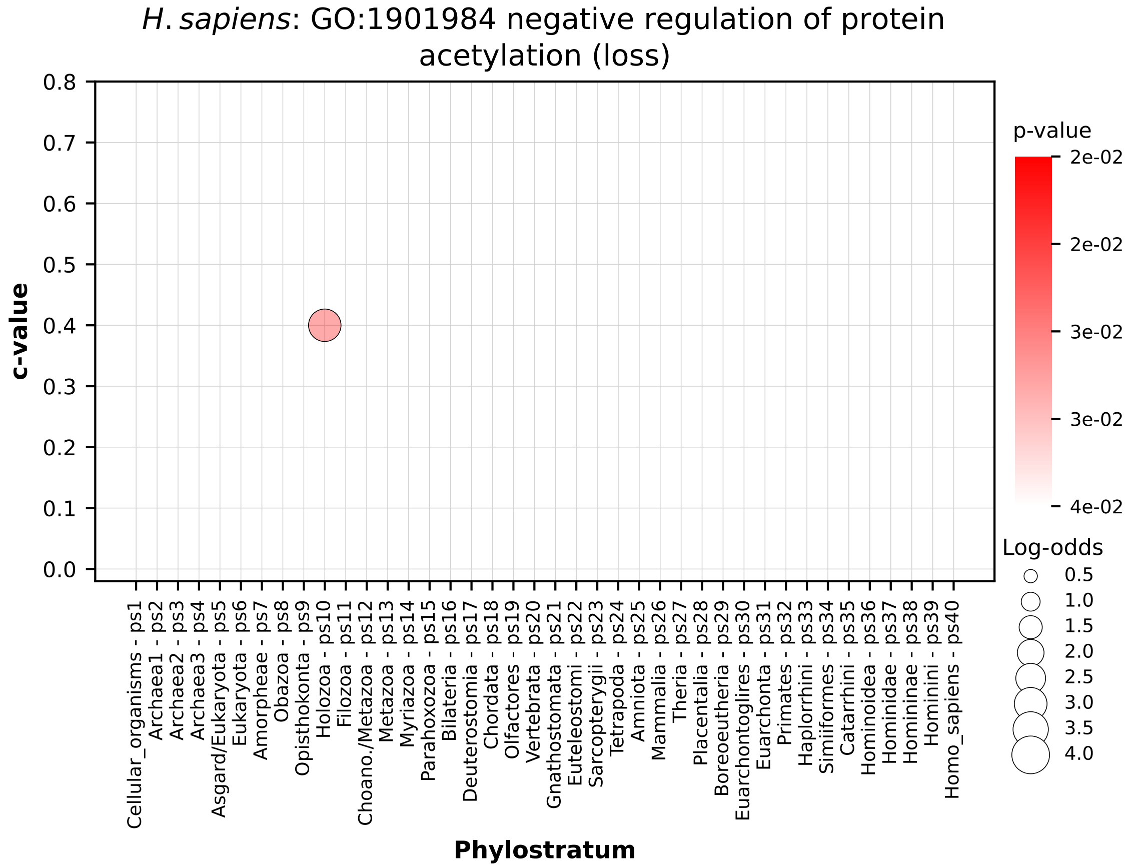
H. sapiens: GO:1901984 negative regulation of protein acetylation (loss)
c-value
0.8
0.7
0.6
0.5
0.4
0.3
0.2
0.1
0.0
p-value
2e-02
2e-02
3e-02
3e-02
4e-02
Log-odds
0.5
1.0
1.5
2.0
2.5
3.0
3.5
4.0
Cellular_organisms - ps1
Archaea1 - ps2
Archaea2 - ps3
Archaea3 - ps4
Asgard/Eukaryota - ps5
Eukaryota - ps6
Amorpheae - ps7
Obazoa - ps8
Opisthokonta - ps9
Holozoa - ps10
Filozoa - ps11
Choano./Metazoa - ps12
Metazoa - ps13
Myriazoa - ps14
Parahoxozoa - ps15
Bilateria - ps16
Deuterostomia - ps17
Chordata - ps18
Olfactores - ps19
Vertebrata - ps20
Gnathostomata - ps21
Euteleostomi - ps22
Sarcopterygii - ps23
Tetrapoda - ps24
Amniota - ps25
Mammalia - ps26
Theria - ps27
Placentalia - ps28
Boreoeutheria - ps29
Euarchontoglires - ps30
Euarchonta - ps31
Primates - ps32
Haplorrhini - ps33
Simiiformes - ps34
Catarrhini - ps35
Hominoidea - ps36
Hominidae - ps37
Homininae - ps38
Hominini - ps39
Homo_sapiens - ps40
Phylostratum

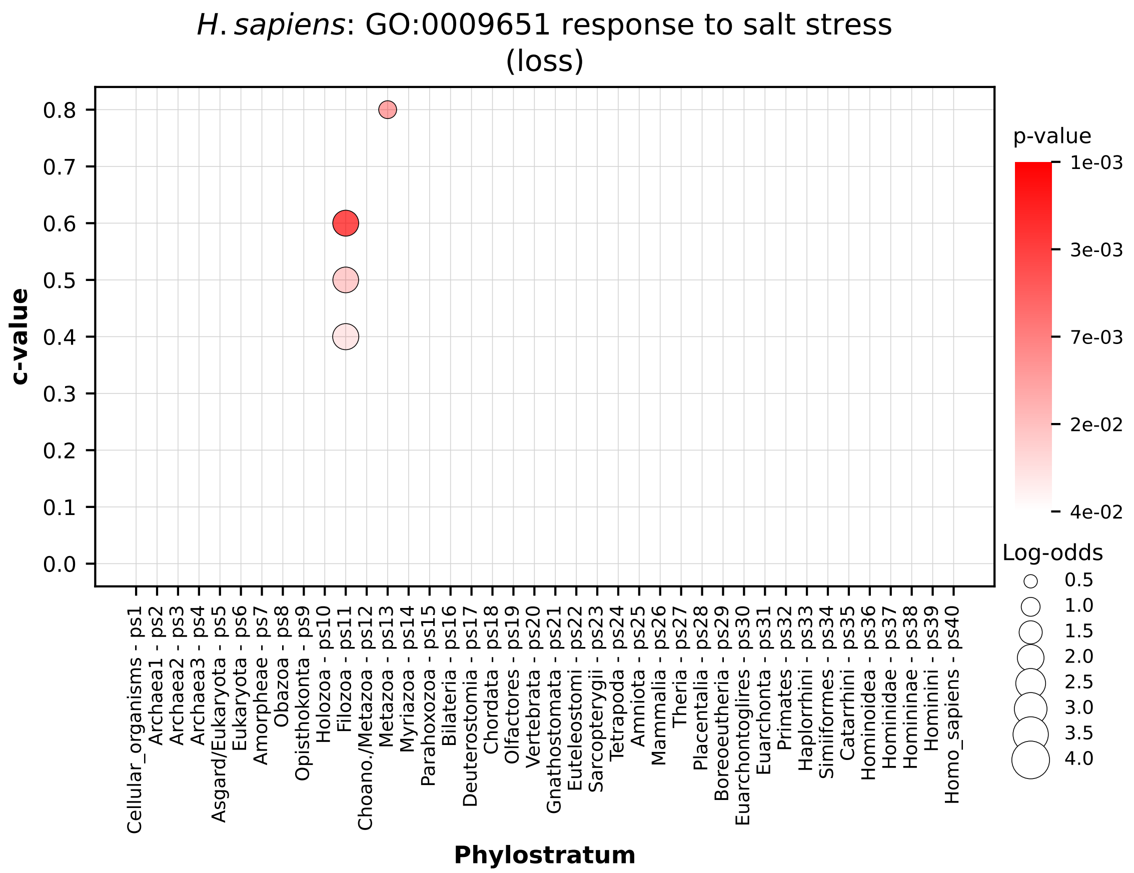
H. sapiens: GO:0009651 response to salt stress
(loss)
c-value
0.0
0.1
0.2
0.3
0.4
0.5
0.6
0.7
0.8
p-value
1e-03
3e-03
7e-03
2e-02
4e-02
Log-odds
0.5
1.0
1.5
2.0
2.5
3.0
3.5
4.0
Cellular_organisms - ps1
Archaea1 - ps2
Archaea2 - ps3
Archaea3 - ps4
Asgard/Eukaryota - ps5
Eukaryota - ps6
Amorpheae - ps7
Obazoa - ps8
Opisthokonta - ps9
Holozoa - ps10
Filozoa - ps11
Choano./Metazoa - ps12
Metazoa - ps13
Myriazoa - ps14
Parahoxozoa - ps15
Bilateria - ps16
Deuterostomia - ps17
Chordata - ps18
Olfactores - ps19
Vertebrata - ps20
Gnathostomata - ps21
Euteleostomi - ps22
Sarcopterygii - ps23
Tetrapoda - ps24
Amniota - ps25
Mammalia - ps26
Theria - ps27
Placentalia - ps28
Boreoeutheria - ps29
Euarchontoglires - ps30
Euarchonta - ps31
Primates - ps32
Haplorrhini - ps33
Simiiformes - ps34
Catarrhini - ps35
Hominoidea - ps36
Hominidae - ps37
Homininae - ps38
Hominini - ps39
Homo_sapiens - ps40
Phylostratum

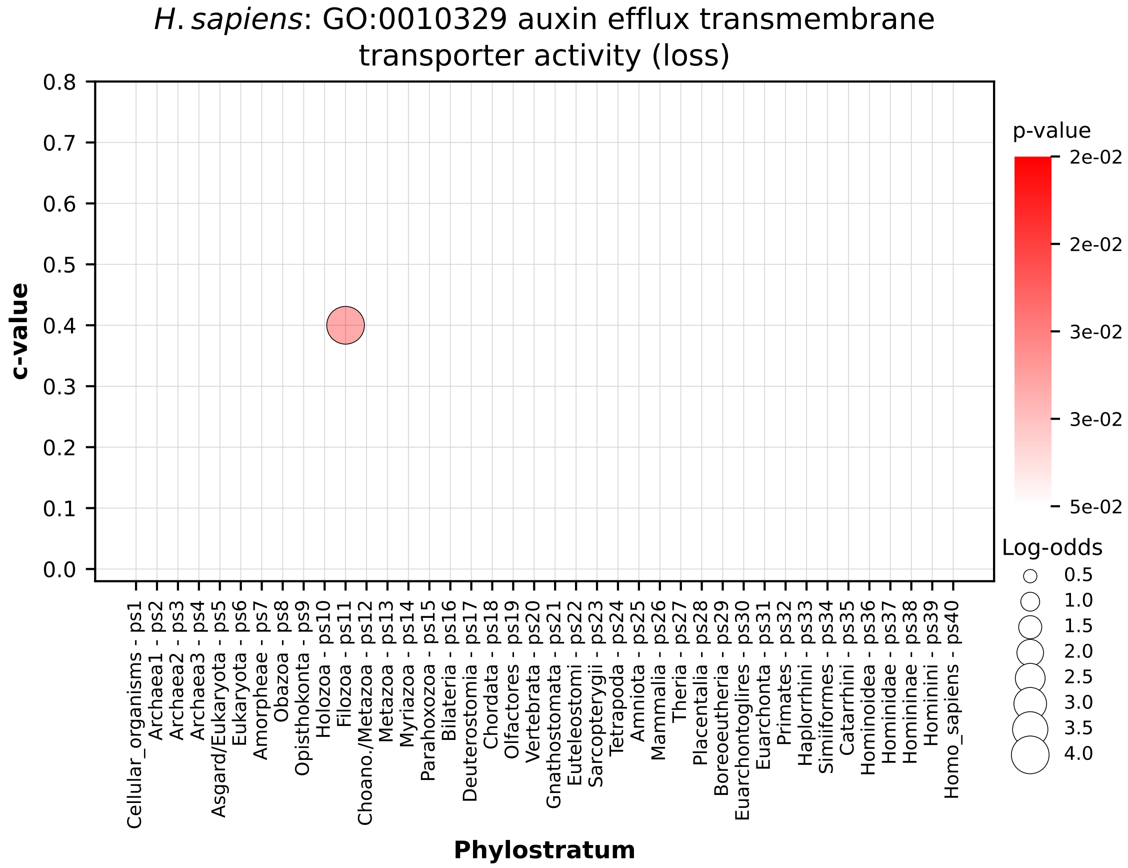

H. sapiens: GO:0010329 auxin efflux transmembrane transporter activity (loss)
c-value
0.8
0.7
0.6
0.5
0.4
0.3
0.2
0.1
0.0
Phylostratum
Cellular_organisms - ps1
Archaea1 - ps2
Archaea2 - ps3
Archaea3 - ps4
Asgard/Eukaryota - ps5
Eukaryota - ps6
Amorpheae - ps7
Obazoa - ps8
Opisthokonta - ps9
Holozoa - ps10
Filozoa - ps11
Choano./Metazoa - ps12
Metazoa - ps13
Myriazoa - ps14
Parahoxozoa - ps15
Bilateria - ps16
Deuterostomia - ps17
Chordata - ps18
Olfactores - ps19
Vertebrata - ps20
Gnathostomata - ps21
Euteleostomi - ps22
Sarcopterygii - ps23
Tetrapoda - ps24
Amniota - ps25
Mammalia - ps26
Theria - ps27
Placentalia - ps28
Boreoeutheria - ps29
Euarchontoglires - ps30
Euarchonta - ps31
Primates - ps32
Haplorrhini - ps33
Simiiformes - ps34
Catarrhini - ps35
Hominoidea - ps36
Hominidae - ps37
Homininae - ps38
Hominini - ps39
Homo_sapiens - ps40
p-value
2e-02
2e-02
3e-02
3e-02
5e-02
Log-odds
0.5
1.0
1.5
2.0
2.5
3.0
3.5
4.0

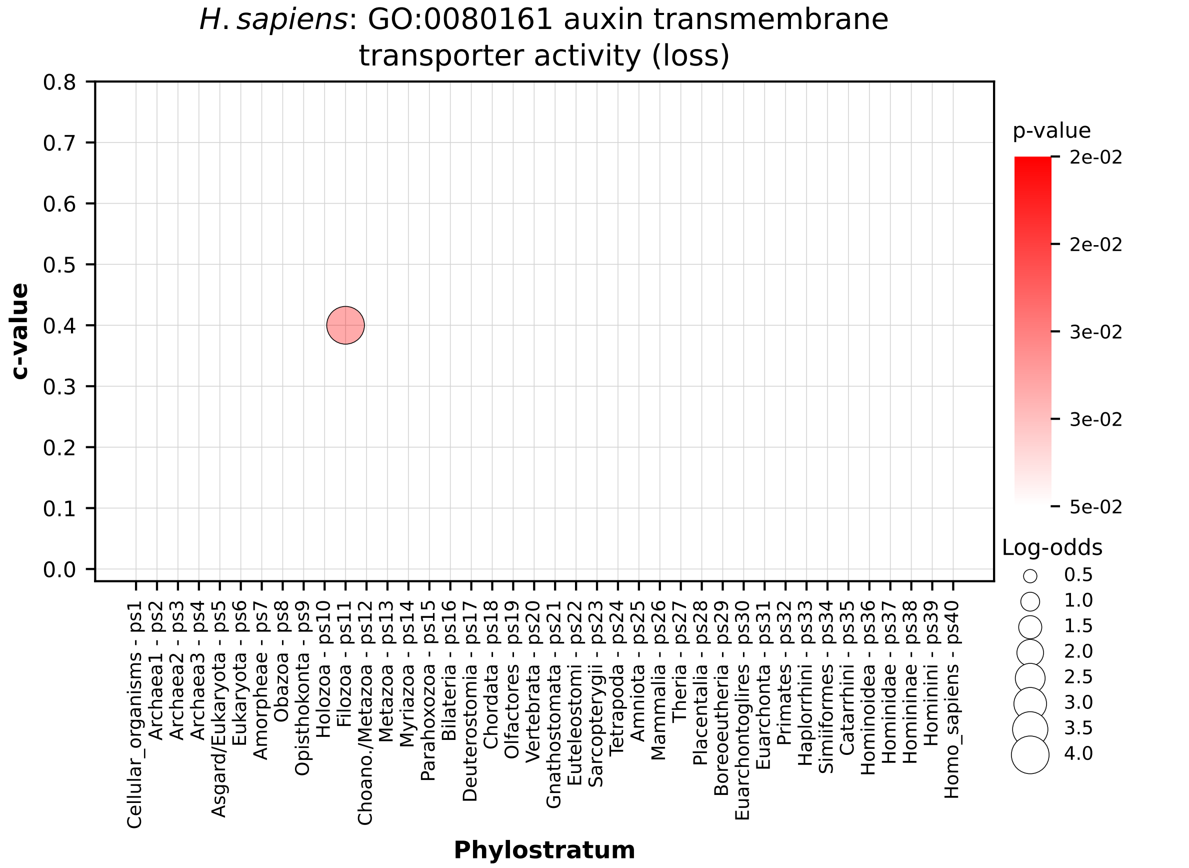

H. sapiens: GO:0080161 auxin transmembrane transporter activity (loss)
c-value
0.8
0.7
0.6
0.5
0.4
0.3
0.2
0.1
0.0
p-value
2e-02
2e-02
3e-02
3e-02
5e-02
Log-odds
0.5
1.0
1.5
2.0
2.5
3.0
3.5
4.0
Cellular_organisms - ps1
Archaea1 - ps2
Archaea2 - ps3
Archaea3 - ps4
Asgard/Eukaryota - ps5
Eukaryota - ps6
Amorpheae - ps7
Obazoa - ps8
Opisthokonta - ps9
Holozoa - ps10
Filozoa - ps11
Choano./Metazoa - ps12
Metazoa - ps13
Myriazoa - ps14
Parahoxozoa - ps15
Bilateria - ps16
Deuterostomia - ps17
Chordata - ps18
Olfactores - ps19
Vertebrata - ps20
Gnathostomata - ps21
Euteleostomi - ps22
Sarcopterygii - ps23
Tetrapoda - ps24
Amniota - ps25
Mammalia - ps26
Theria - ps27
Placentalia - ps28
Boreoeutheria - ps29
Euarchontoglires - ps30
Euarchonta - ps31
Primates - ps32
Haplorrhini - ps33
Simiiformes - ps34
Catarrhini - ps35
Hominoidea - ps36
Hominidae - ps37
Homininae - ps38
Hominini - ps39
Homo_sapiens - ps40
Phylostratum

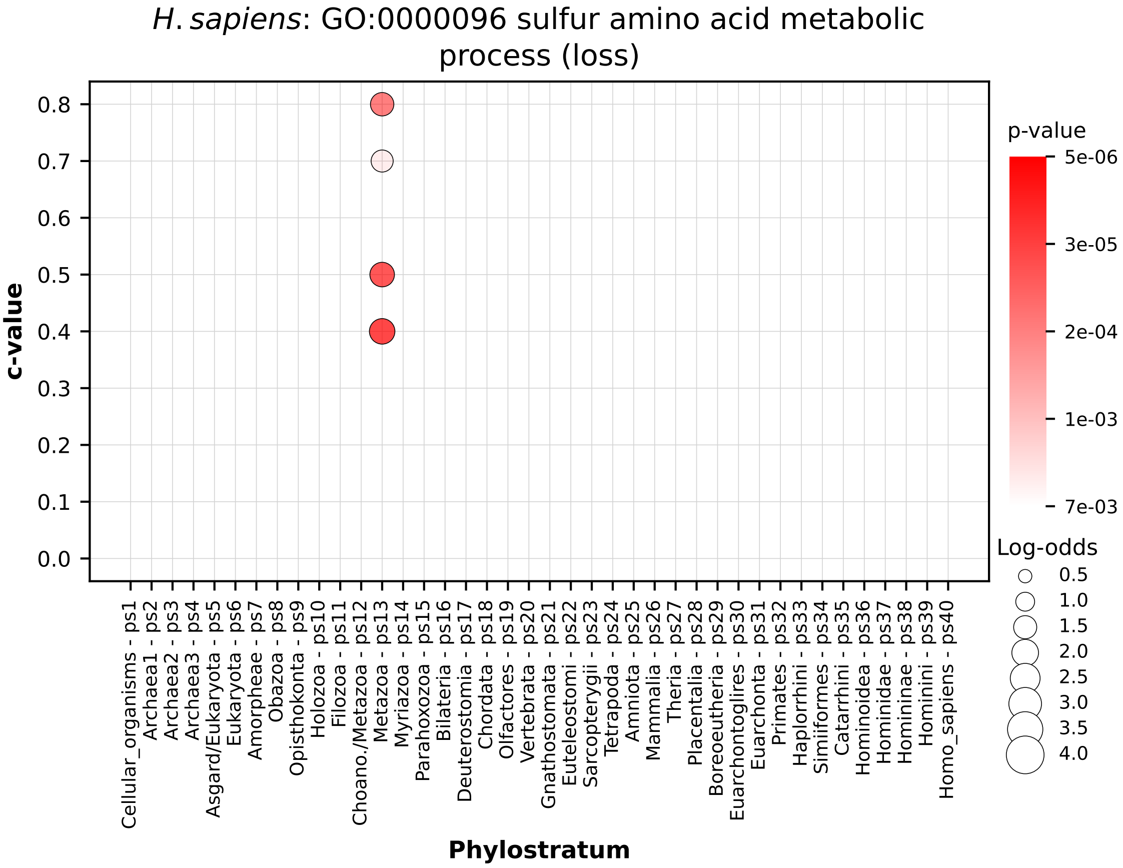

H. sapiens: GO:0000096 sulfur amino acid metabolic process (loss)
c-value
0.8
0.7
0.6
0.5
0.4
0.3
0.2
0.1
0.0
p-value
5e-06
3e-05
2e-04
1e-03
7e-03
Log-odds
0.5
1.0
1.5
2.0
2.5
3.0
3.5
4.0
Cellular_organisms - ps1
Archaea1 - ps2
Archaea2 - ps3
Archaea3 - ps4
Asgard/Eukaryota - ps5
Eukaryota - ps6
Amorpheae - ps7
Obazoa - ps8
Opisthokonta - ps9
Holozoa - ps10
Filozoa - ps11
Choano./Metazoa - ps12
Metazoa - ps13
Myriazoa - ps14
Parahoxozoa - ps15
Bilateria - ps16
Deuterostomia - ps17
Chordata - ps18
Olfactores - ps19
Vertebrata - ps20
Gnathostomata - ps21
Euteleostomi - ps22
Sarcopterygii - ps23
Tetrapoda - ps24
Amniota - ps25
Mammalia - ps26
Theria - ps27
Placentalia - ps28
Boreoeutheria - ps29
Euarchontoglires - ps30
Euarchonta - ps31
Primates - ps32
Haplorrhini - ps33
Simiiformes - ps34
Catarrhini - ps35
Hominoidea - ps36
Hominidae - ps37
Homininae - ps38
Hominini - ps39
Homo_sapiens - ps40
Phylostratum

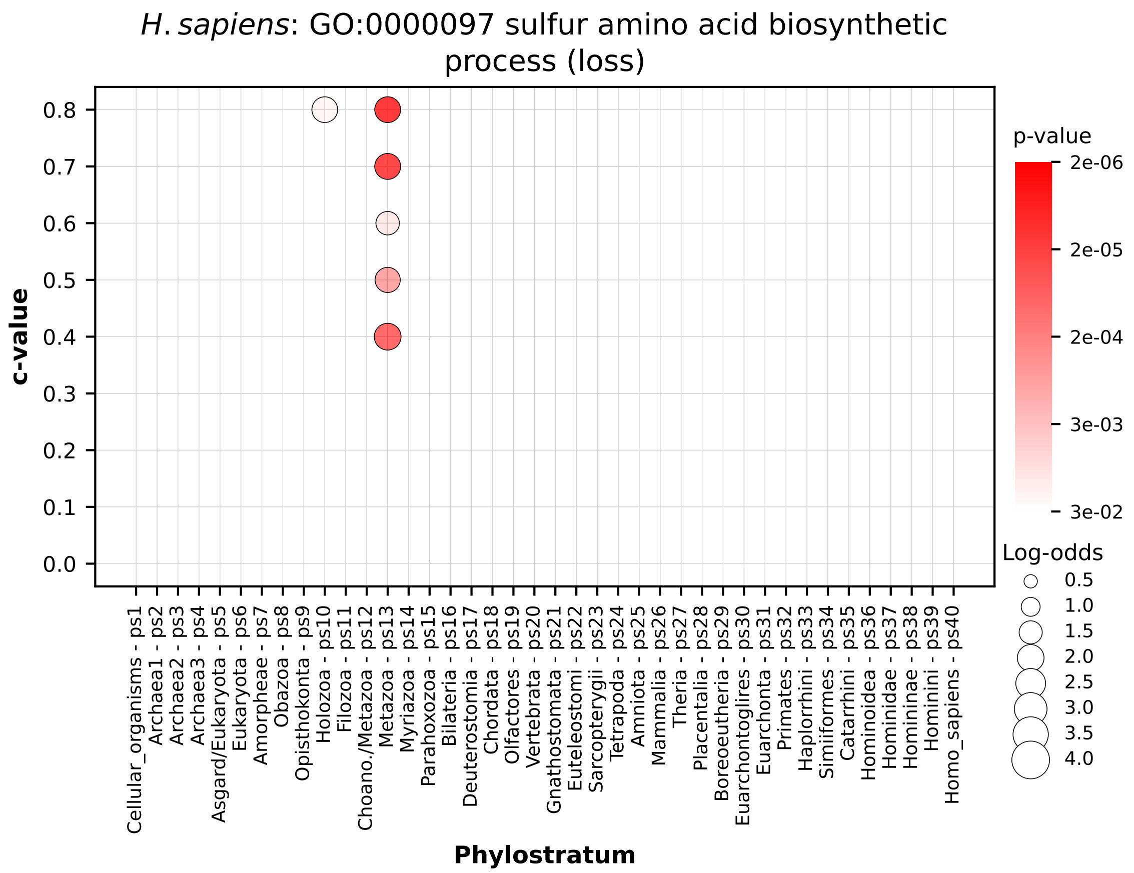

H. sapiens: GO:0000097 sulfur amino acid biosynthetic process (loss)
c-value
0.8
0.7
0.6
0.5
0.4
0.3
0.2
0.1
0.0
p-value
2e-06
2e-05
2e-04
3e-03
3e-02
Log-odds
0.5
1.0
1.5
2.0
2.5
3.0
3.5
4.0
Cellular_organisms - ps1
Archaea1 - ps2
Archaea2 - ps3
Archaea3 - ps4
Asgard/Eukaryota - ps5
Eukaryota - ps6
Amorpheae - ps7
Obazoa - ps8
Opisthokonta - ps9
Holozoa - ps10
Filozoa - ps11
Choano./Metazoa - ps12
Metazoa - ps13
Myriazoa - ps14
Parahoxozoa - ps15
Bilateria - ps16
Deuterostomia - ps17
Chordata - ps18
Olfactores - ps19
Vertebrata - ps20
Gnathostomata - ps21
Euteleostomi - ps22
Sarcopterygii - ps23
Tetrapoda - ps24
Amniota - ps25
Mammalia - ps26
Theria - ps27
Placentalia - ps28
Boreoeutheria - ps29
Euarchontoglires - ps30
Euarchonta - ps31
Primates - ps32
Haplorrhini - ps33
Simiiformes - ps34
Catarrhini - ps35
Hominoidea - ps36
Hominidae - ps37
Homininae - ps38
Hominini - ps39
Homo_sapiens - ps40
Phylostratum

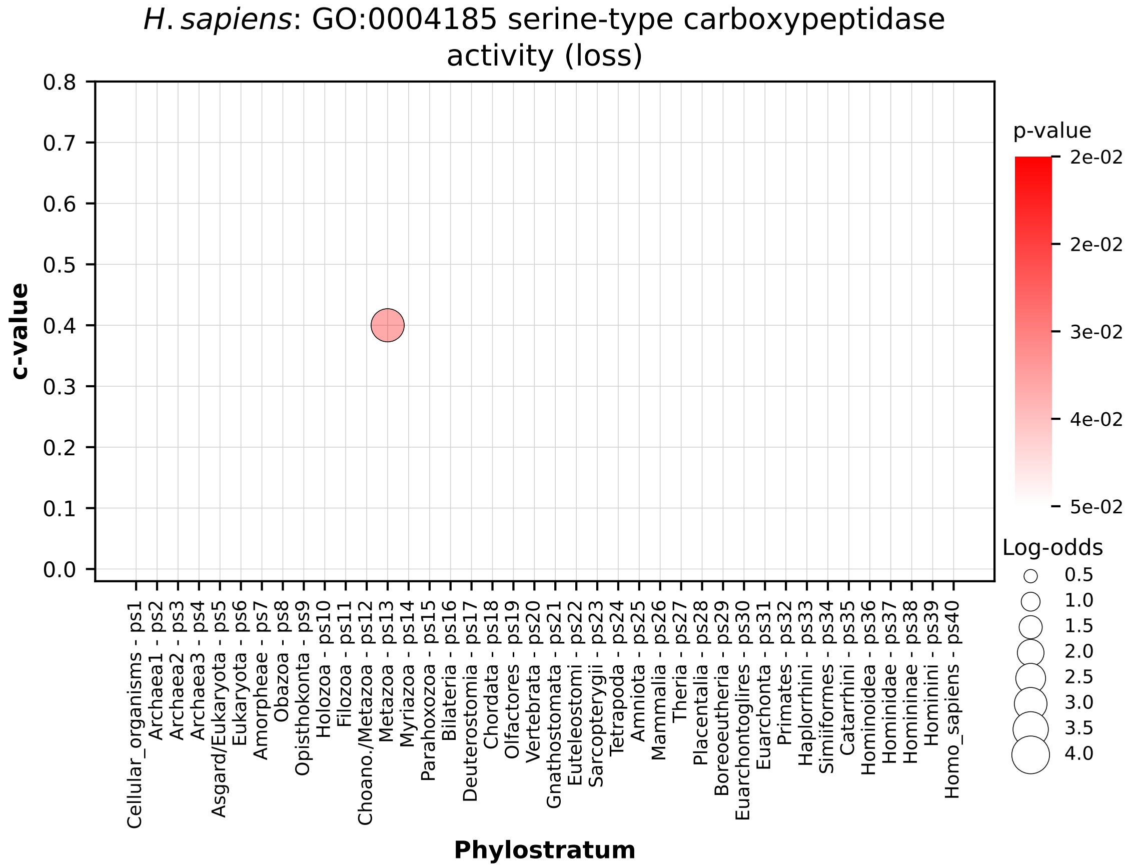

H. sapiens: GO:0004185 serine-type carboxypeptidase activity (loss)
c-value
0.0
0.1
0.2
0.3
0.4
0.5
0.6
0.7
0.8
Phylostratum
Cellular_organisms - ps1
Archaea1 - ps2
Archaea2 - ps3
Archaea3 - ps4
Asgard/Eukaryota - ps5
Eukaryota - ps6
Amorpheae - ps7
Obazoa - ps8
Opisthokonta - ps9
Holozoa - ps10
Filozoa - ps11
Choano./Metazoa - ps12
Metazoa - ps13
Myriazoa - ps14
Parahoxozoa - ps15
Bilateria - ps16
Deuterostomia - ps17
Chordata - ps18
Olfactores - ps19
Vertebrata - ps20
Gnathostomata - ps21
Euteleostomi - ps22
Sarcopterygii - ps23
Tetrapoda - ps24
Amniota - ps25
Mammalia - ps26
Theria - ps27
Placentalia - ps28
Boreoeutheria - ps29
Euarchontoglires - ps30
Euarchonta - ps31
Primates - ps32
Haplorrhini - ps33
Simiiformes - ps34
Catarrhini - ps35
Hominoidea - ps36
Hominidae - ps37
Homininae - ps38
Hominini - ps39
Homo_sapiens - ps40
p-value
2e-02
2e-02
3e-02
4e-02
5e-02
Log-odds
0.5
1.0
1.5
2.0
2.5
3.0
3.5
4.0

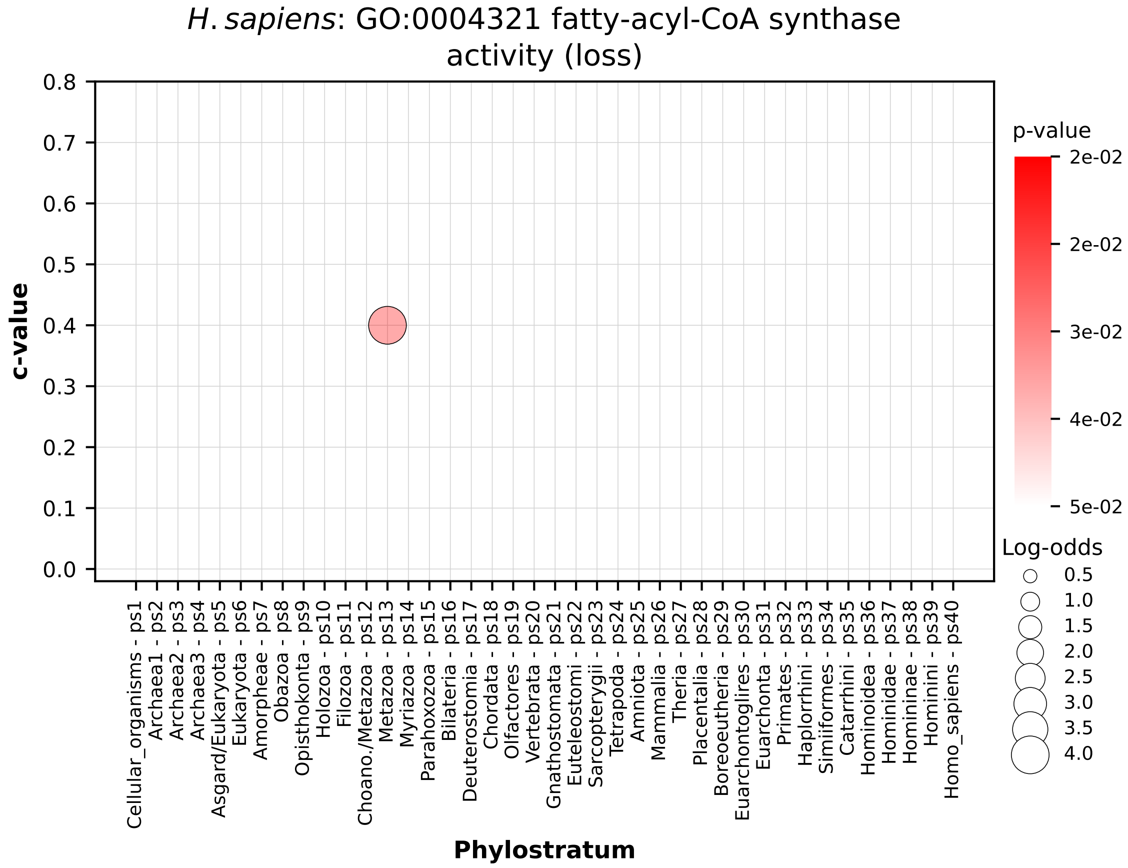

H. sapiens: GO:0004321 fatty-acyl-CoA synthase activity (loss)
c-value
0.8
0.7
0.6
0.5
0.4
0.3
0.2
0.1
0.0
Phylostratum
Cellular_organisms - ps1
Archaea1 - ps2
Archaea2 - ps3
Archaea3 - ps4
Asgard/Eukaryota - ps5
Eukaryota - ps6
Amorpheae - ps7
Obazoa - ps8
Opisthokonta - ps9
Holozoa - ps10
Filozoa - ps11
Choano./Metazoa - ps12
Metazoa - ps13
Myriazoa - ps14
Parahoxozoa - ps15
Bilateria - ps16
Deuterostomia - ps17
Chordata - ps18
Olfactores - ps19
Vertebrata - ps20
Gnathostomata - ps21
Euteleostomi - ps22
Sarcopterygii - ps23
Tetrapoda - ps24
Amniota - ps25
Mammalia - ps26
Theria - ps27
Placentalia - ps28
Boreoeutheria - ps29
Euarchontoglires - ps30
Euarchonta - ps31
Primates - ps32
Haplorrhini - ps33
Simiiformes - ps34
Catarrhini - ps35
Hominoidea - ps36
Hominidae - ps37
Homininae - ps38
Hominini - ps39
Homo_sapiens - ps40
p-value
2e-02
2e-02
3e-02
4e-02
5e-02
Log-odds
0.5
1.0
1.5
2.0
2.5
3.0
3.5
4.0

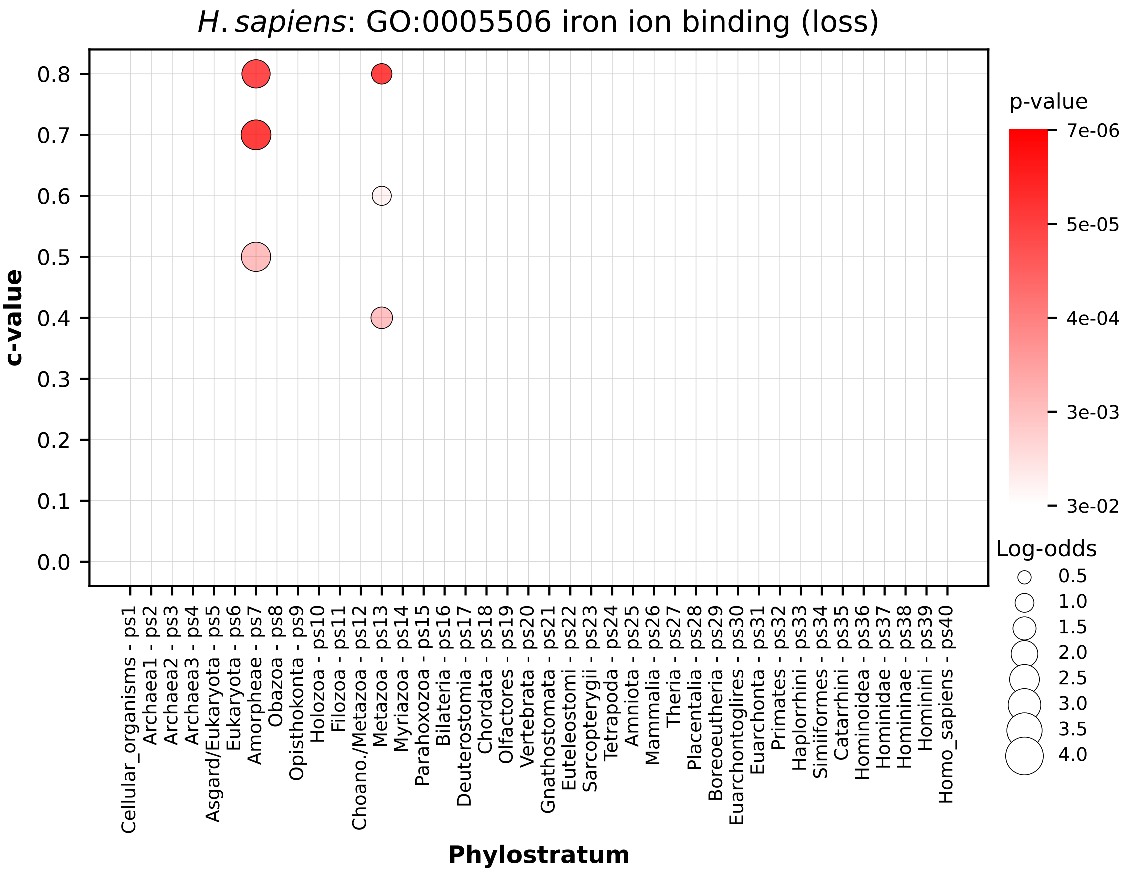
H. sapiens: GO:0005506 iron ion binding (loss)
c-value
0.8
0.7
0.6
0.5
0.4
0.3
0.2
0.1
0.0
p-value
7e-06
5e-05
4e-04
3e-03
3e-02
Log-odds
0.5
1.0
1.5
2.0
2.5
3.0
3.5
4.0
Cellular_organisms - ps1
Archaea1 - ps2
Archaea2 - ps3
Archaea3 - ps4
Asgard/Eukaryota - ps5
Eukaryota - ps6
Amorpheae - ps7
Obazoa - ps8
Opisthokonta - ps9
Holozoa - ps10
Filozoa - ps11
Choano./Metazoa - ps12
Metazoa - ps13
Myriazoa - ps14
Parahoxozoa - ps15
Bilateria - ps16
Deuterostomia - ps17
Chordata - ps18
Olfactores - ps19
Vertebrata - ps20
Gnathostomata - ps21
Euteleostomi - ps22
Sarcopterygii - ps23
Tetrapoda - ps24
Amniota - ps25
Mammalia - ps26
Theria - ps27
Placentalia - ps28
Boreoeutheria - ps29
Euarchontoglires - ps30
Euarchonta - ps31
Primates - ps32
Haplorrhini - ps33
Simiiformes - ps34
Catarrhini - ps35
Hominoidea - ps36
Hominidae - ps37
Homininae - ps38
Hominini - ps39
Homo_sapiens - ps40
Phylostratum

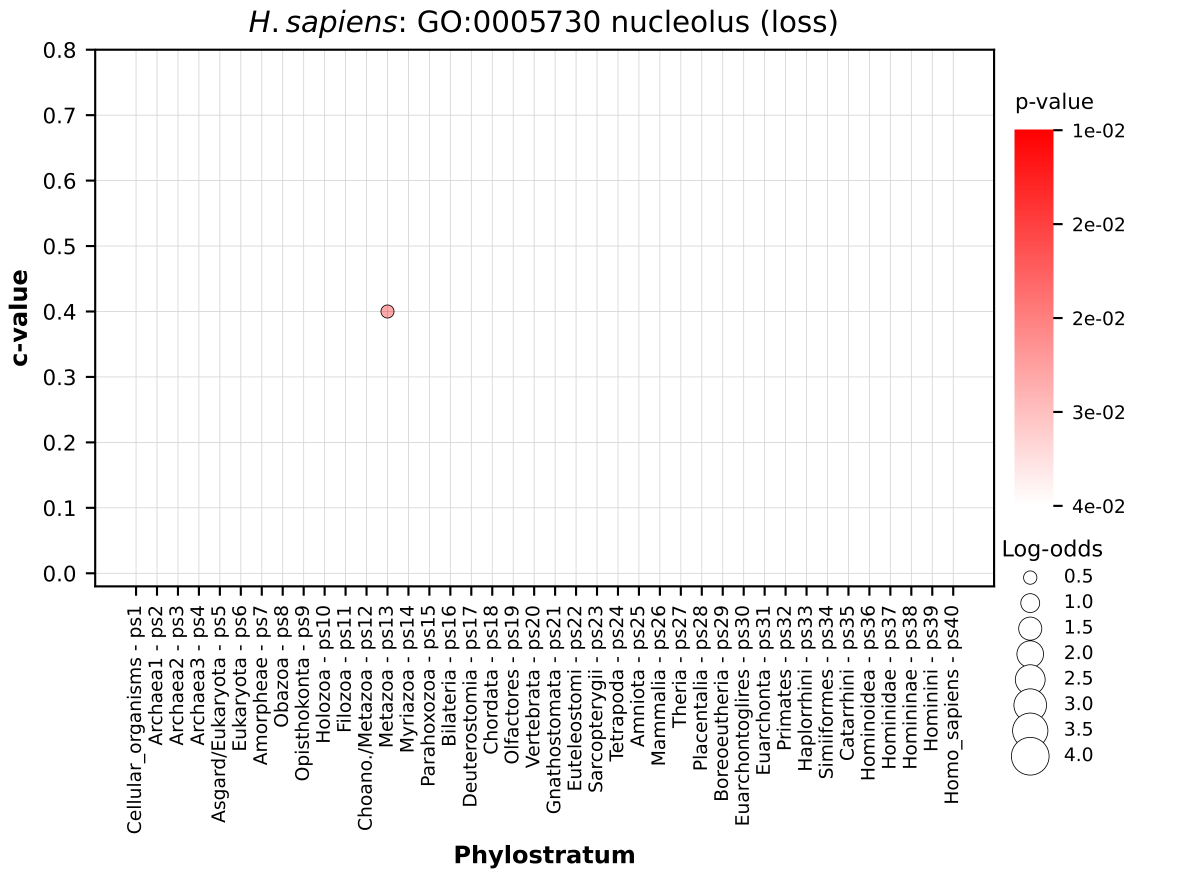

H. sapiens: GO:0005730 nucleolus (loss)
c-value
0.8
0.7
0.6
0.5
0.4
0.3
0.2
0.1
0.0
Phylostratum
Cellular_organisms - ps1
Archaea1 - ps2
Archaea2 - ps3
Archaea3 - ps4
Asgard/Eukaryota - ps5
Eukaryota - ps6
Amorpheae - ps7
Obazoa - ps8
Opisthokonta - ps9
Holozoa - ps10
Filozoa - ps11
Choano./Metazoa - ps12
Metazoa - ps13
Myriazoa - ps14
Parahoxozoa - ps15
Bilateria - ps16
Deuterostomia - ps17
Chordata - ps18
Olfactores - ps19
Vertebrata - ps20
Gnathostomata - ps21
Euteleostomi - ps22
Sarcopterygii - ps23
Tetrapoda - ps24
Amniota - ps25
Mammalia - ps26
Theria - ps27
Placentalia - ps28
Boreoeutheria - ps29
Euarchontoglires - ps30
Euarchonta - ps31
Primates - ps32
Haplorrhini - ps33
Simiiformes - ps34
Catarrhini - ps35
Hominoidea - ps36
Hominidae - ps37
Homininae - ps38
Hominini - ps39
Homo_sapiens - ps40
p-value
1e-02
2e-02
2e-02
3e-02
4e-02
Log-odds
0.5
1.0
1.5
2.0
2.5
3.0
3.5
4.0

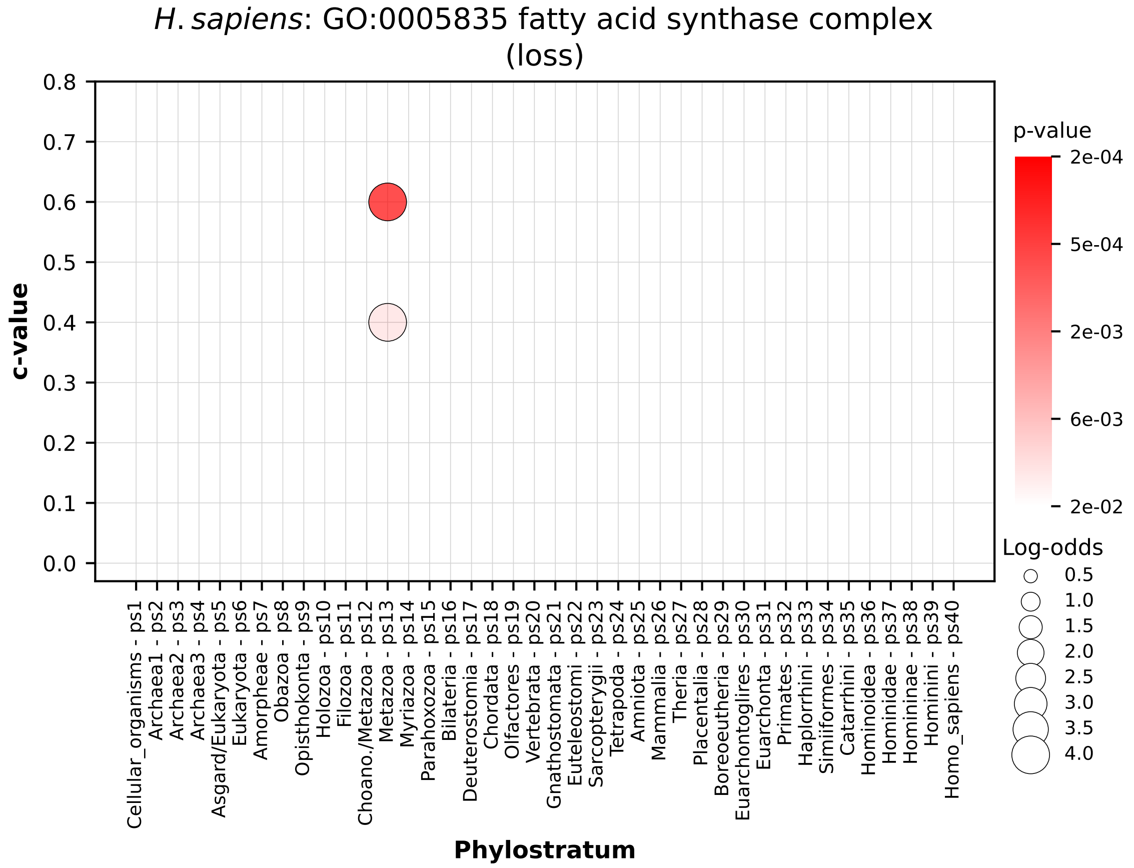

H. sapiens: GO:0005835 fatty acid synthase complex
(loss)
c-value
0.8
0.7
0.6
0.5
0.4
0.3
0.2
0.1
0.0
p-value
2e-04
5e-04
2e-03
6e-03
2e-02
Log-odds
0.5
1.0
1.5
2.0
2.5
3.0
3.5
4.0
Cellular_organisms - ps1
Archaea1 - ps2
Archaea2 - ps3
Archaea3 - ps4
Asgard/Eukaryota - ps5
Eukaryota - ps6
Amorpheae - ps7
Obazoa - ps8
Opisthokonta - ps9
Holozoa - ps10
Filozoa - ps11
Choano./Metazoa - ps12
Metazoa - ps13
Myriazoa - ps14
Parahoxozoa - ps15
Bilateria - ps16
Deuterostomia - ps17
Chordata - ps18
Olfactores - ps19
Vertebrata - ps20
Gnathostomata - ps21
Euteleostomi - ps22
Sarcopterygii - ps23
Tetrapoda - ps24
Amniota - ps25
Mammalia - ps26
Theria - ps27
Placentalia - ps28
Boreoeutheria - ps29
Euarchontoglires - ps30
Euarchonta - ps31
Primates - ps32
Haplorrhini - ps33
Simiiformes - ps34
Catarrhini - ps35
Hominoidea - ps36
Hominidae - ps37
Homininae - ps38
Hominini - ps39
Homo_sapiens - ps40
Phylostratum

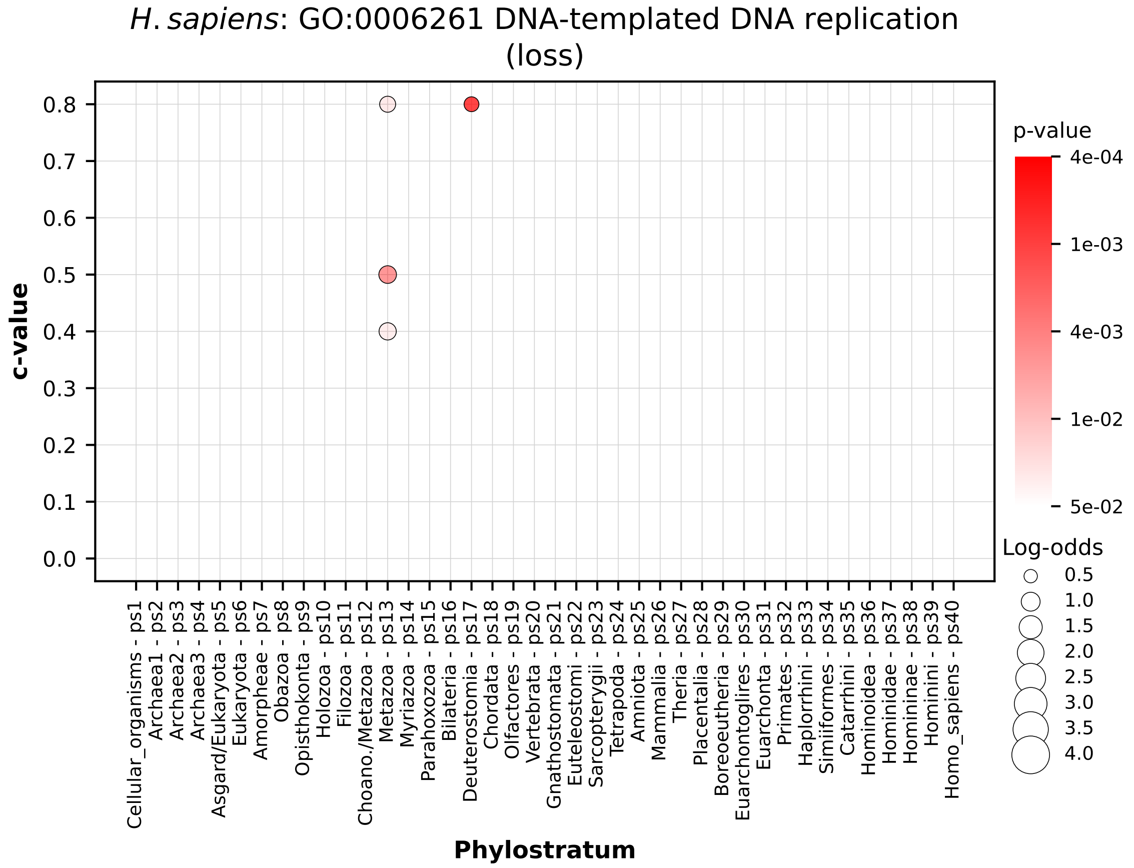

H. sapiens: GO:0006261 DNA-templated DNA replication
(loss)
c-value
0.8
0.7
0.6
0.5
0.4
0.3
0.2
0.1
0.0
p-value
4e-04
1e-03
4e-03
1e-02
5e-02
Log-odds
0.5
1.0
1.5
2.0
2.5
3.0
3.5
4.0
Cellular_organisms - ps1
Archaea1 - ps2
Archaea2 - ps3
Archaea3 - ps4
Asgard/Eukaryota - ps5
Eukaryota - ps6
Amorpheae - ps7
Obazoa - ps8
Opisthokonta - ps9
Holozoa - ps10
Filozoa - ps11
Choano./Metazoa - ps12
Metazoa - ps13
Myriazoa - ps14
Parahoxozoa - ps15
Bilateria - ps16
Deuterostomia - ps17
Chordata - ps18
Olfactores - ps19
Vertebrata - ps20
Gnathostomata - ps21
Euteleostomi - ps22
Sarcopterygii - ps23
Tetrapoda - ps24
Amniota - ps25
Mammalia - ps26
Theria - ps27
Placentalia - ps28
Boreoeutheria - ps29
Euarchontoglires - ps30
Euarchonta - ps31
Primates - ps32
Haplorrhini - ps33
Simiiformes - ps34
Catarrhini - ps35
Hominoidea - ps36
Hominidae - ps37
Homininae - ps38
Hominini - ps39
Homo_sapiens - ps40
Phylostratum

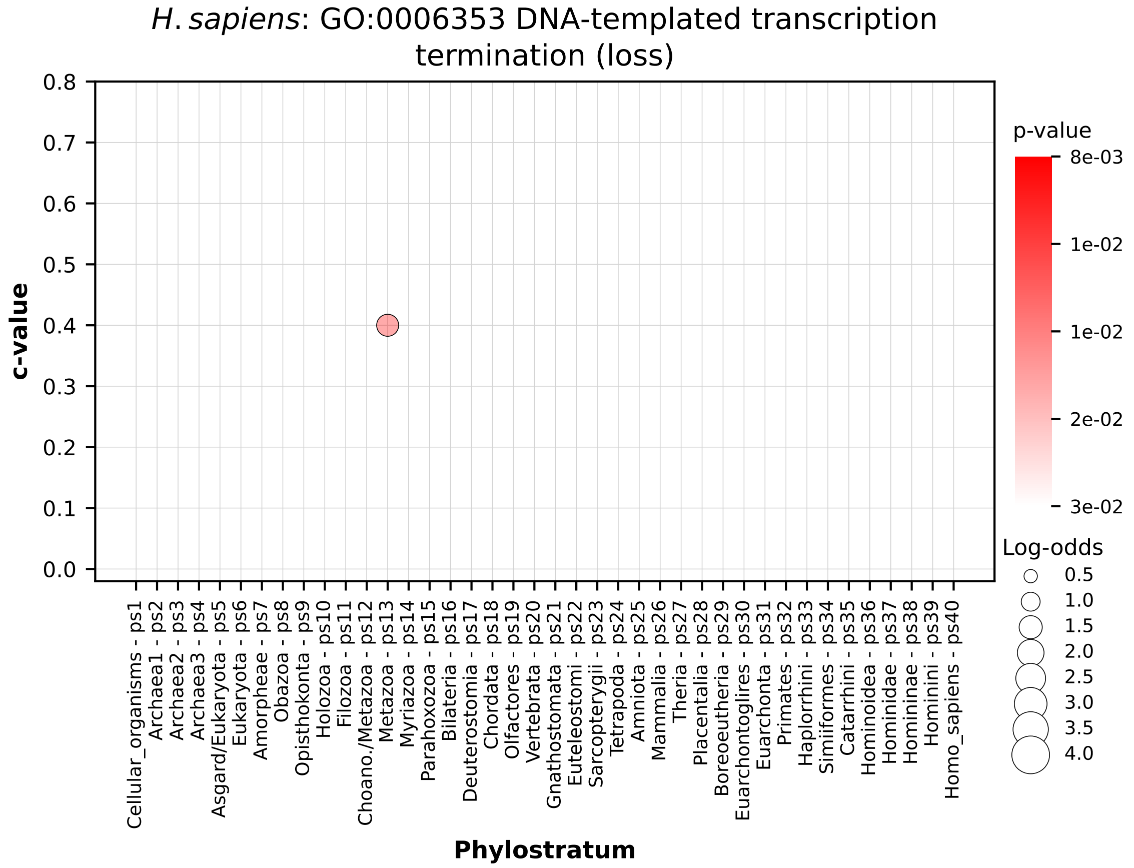

H. sapiens: GO:0006353 DNA-templated transcription termination (loss)
c-value
0.8
0.7
0.6
0.5
0.4
0.3
0.2
0.1
0.0
Phylostratum
Cellular_organisms - ps1
Archaea1 - ps2
Archaea2 - ps3
Archaea3 - ps4
Asgard/Eukaryota - ps5
Eukaryota - ps6
Amorpheae - ps7
Obazoa - ps8
Opisthokonta - ps9
Holozoa - ps10
Filozoa - ps11
Choano./Metazoa - ps12
Metazoa - ps13
Myriazoa - ps14
Parahoxozoa - ps15
Bilateria - ps16
Deuterostomia - ps17
Chordata - ps18
Olfactores - ps19
Vertebrata - ps20
Gnathostomata - ps21
Euteleostomi - ps22
Sarcopterygii - ps23
Tetrapoda - ps24
Amniota - ps25
Mammalia - ps26
Theria - ps27
Placentalia - ps28
Boreoeutheria - ps29
Euarchontoglires - ps30
Euarchonta - ps31
Primates - ps32
Haplorrhini - ps33
Simiiformes - ps34
Catarrhini - ps35
Hominoidea - ps36
Hominidae - ps37
Homininae - ps38
Hominini - ps39
Homo_sapiens - ps40
p-value
8e-03
1e-02
1e-02
2e-02
3e-02
Log-odds
0.5
1.0
1.5
2.0
2.5
3.0
3.5
4.0

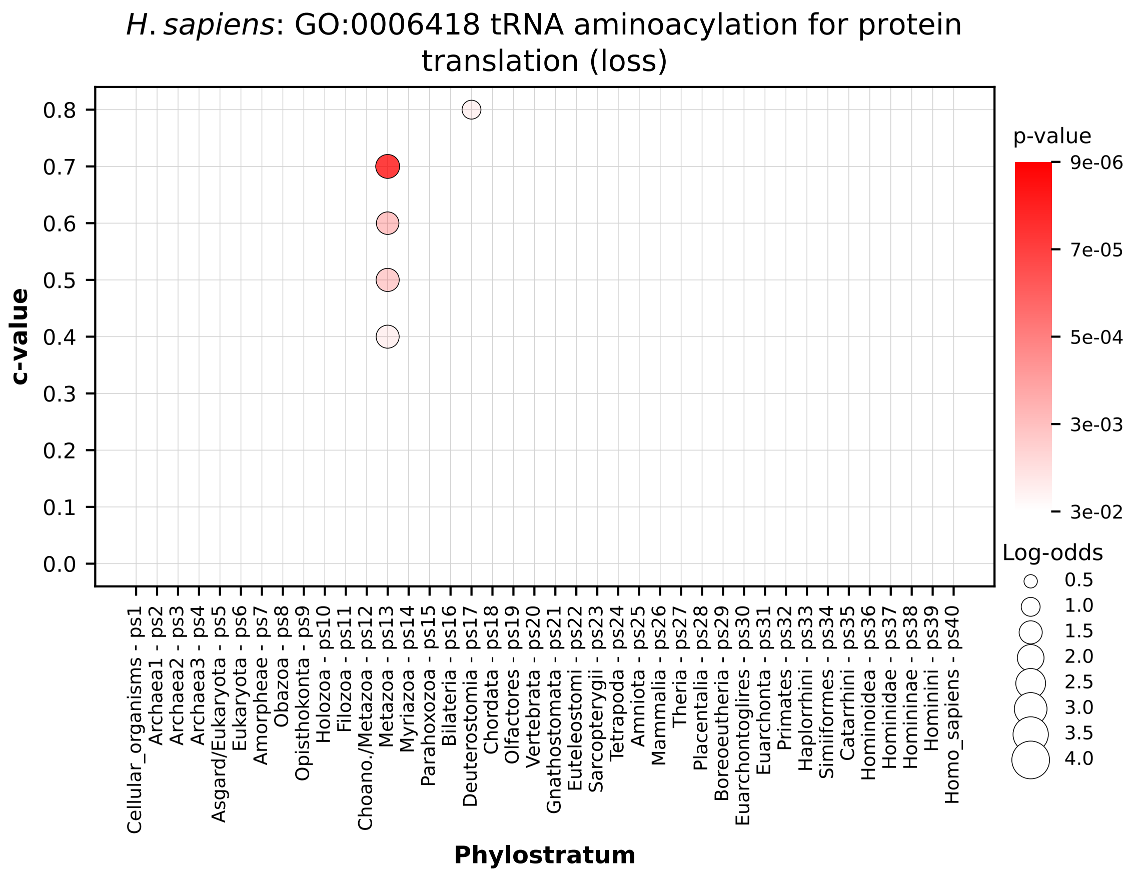

H. sapiens: GO:0006418 tRNA aminoacylation for protein translation (loss)
c-value
0.0
0.1
0.2
0.3
0.4
0.5
0.6
0.7
0.8
Phylostratum
Cellular_organisms - ps1
Archaea1 - ps2
Archaea2 - ps3
Archaea3 - ps4
Asgard/Eukaryota - ps5
Eukaryota - ps6
Amorpheae - ps7
Obazoa - ps8
Opisthokonta - ps9
Holozoa - ps10
Filozoa - ps11
Choano./Metazoa - ps12
Metazoa - ps13
Myriazoa - ps14
Parahoxozoa - ps15
Bilateria - ps16
Deuterostomia - ps17
Chordata - ps18
Olfactores - ps19
Vertebrata - ps20
Gnathostomata - ps21
Euteleostomi - ps22
Sarcopterygii - ps23
Tetrapoda - ps24
Amniota - ps25
Mammalia - ps26
Theria - ps27
Placentalia - ps28
Boreoeutheria - ps29
Euarchontoglires - ps30
Euarchonta - ps31
Primates - ps32
Haplorrhini - ps33
Simiiformes - ps34
Catarrhini - ps35
Hominoidea - ps36
Hominidae - ps37
Homininae - ps38
Hominini - ps39
Homo_sapiens - ps40
p-value
9e-06
7e-05
5e-04
3e-03
3e-02
Log-odds
0.5
1.0
1.5
2.0
2.5
3.0
3.5
4.0

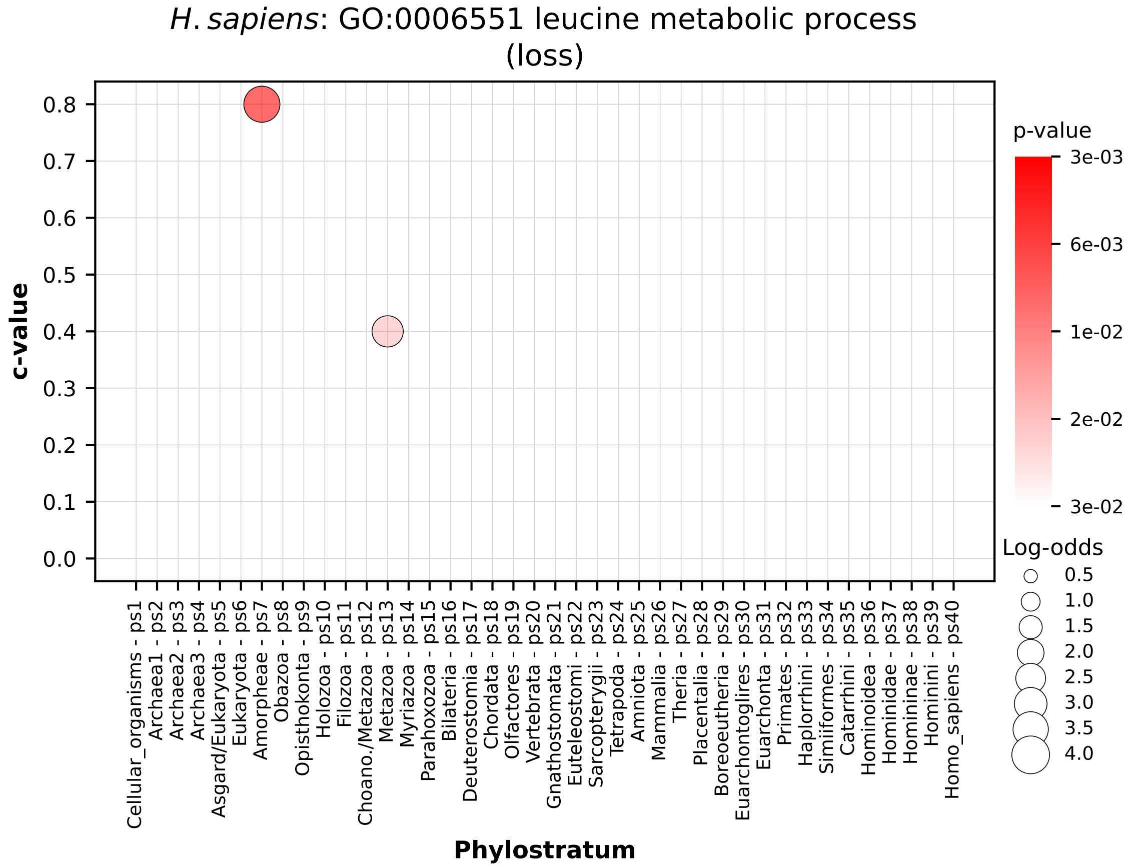

H. sapiens: GO:0006551 leucine metabolic process
(loss)
c-value
0.8
0.7
0.6
0.5
0.4
0.3
0.2
0.1
0.0
p-value
3e-03
6e-03
1e-02
2e-02
3e-02
Log-odds
0.5
1.0
1.5
2.0
2.5
3.0
3.5
4.0
Cellular_organisms - ps1
Archaea1 - ps2
Archaea2 - ps3
Archaea3 - ps4
Asgard/Eukaryota - ps5
Eukaryota - ps6
Amorpheae - ps7
Obazoa - ps8
Opisthokonta - ps9
Holozoa - ps10
Filozoa - ps11
Choano./Metazoa - ps12
Metazoa - ps13
Myriazoa - ps14
Parahoxozoa - ps15
Bilateria - ps16
Deuterostomia - ps17
Chordata - ps18
Olfactores - ps19
Vertebrata - ps20
Gnathostomata - ps21
Euteleostomi - ps22
Sarcopterygii - ps23
Tetrapoda - ps24
Amniota - ps25
Mammalia - ps26
Theria - ps27
Placentalia - ps28
Boreoeutheria - ps29
Euarchontoglires - ps30
Euarchonta - ps31
Primates - ps32
Haplorrhini - ps33
Simiiformes - ps34
Catarrhini - ps35
Hominoidea - ps36
Hominidae - ps37
Homininae - ps38
Hominini - ps39
Homo_sapiens - ps40
Phylostratum

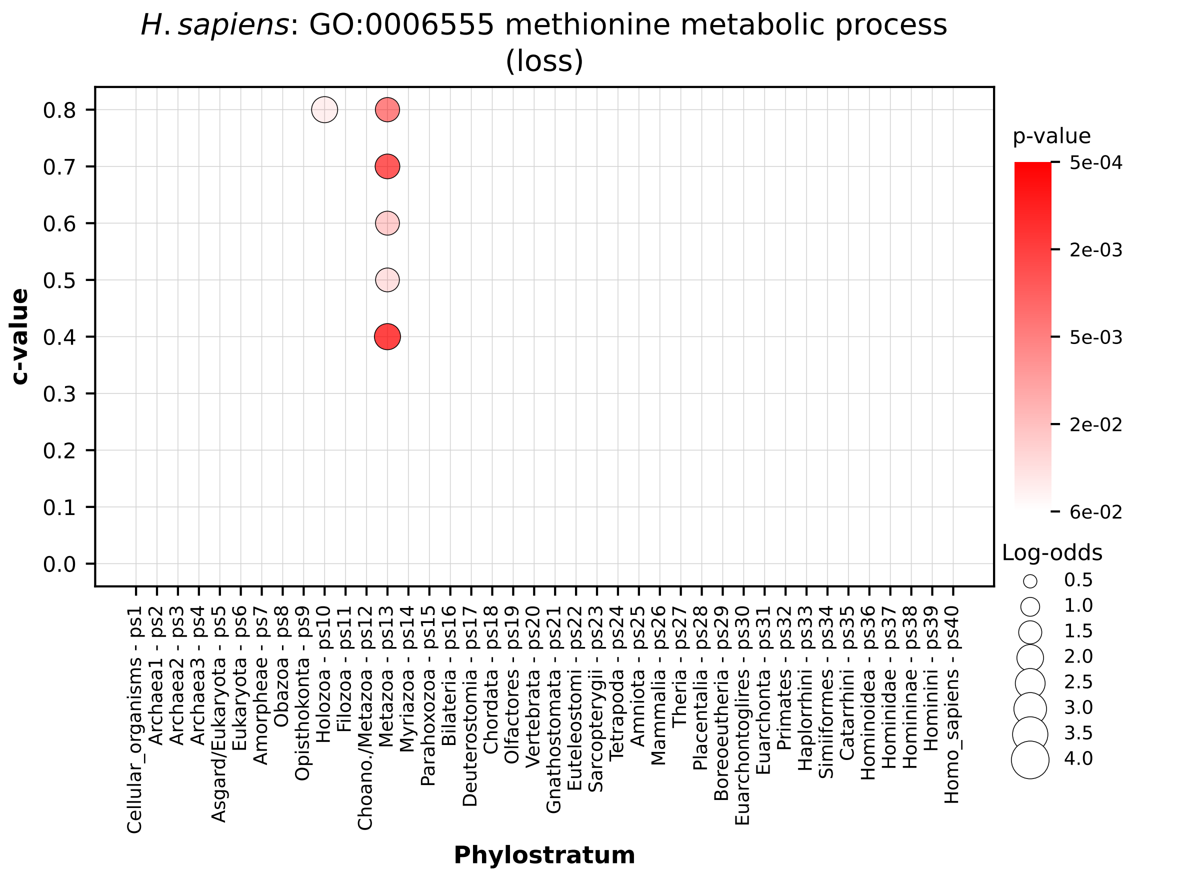

H. sapiens: GO:0006555 methionine metabolic process (loss)
c-value
0.8
0.7
0.6
0.5
0.4
0.3
0.2
0.1
0.0
p-value
5e-04
2e-03
5e-03
2e-02
6e-02
Log-odds
0.5
1.0
1.5
2.0
2.5
3.0
3.5
4.0
Cellular_organisms - ps1
Archaea1 - ps2
Archaea2 - ps3
Archaea3 - ps4
Asgard/Eukaryota - ps5
Eukaryota - ps6
Amorpheae - ps7
Obazoa - ps8
Opisthokonta - ps9
Holozoa - ps10
Filozoa - ps11
Choano./Metazoa - ps12
Metazoa - ps13
Myriazoa - ps14
Parahoxozoa - ps15
Bilateria - ps16
Deuterostomia - ps17
Chordata - ps18
Olfactores - ps19
Vertebrata - ps20
Gnathostomata - ps21
Euteleostomi - ps22
Sarcopterygii - ps23
Tetrapoda - ps24
Amniota - ps25
Mammalia - ps26
Theria - ps27
Placentalia - ps28
Boreoeutheria - ps29
Euarchontoglires - ps30
Euarchonta - ps31
Primates - ps32
Haplorrhini - ps33
Simiiformes - ps34
Catarrhini - ps35
Hominoidea - ps36
Hominidae - ps37
Homininae - ps38
Hominini - ps39
Homo_sapiens - ps40
Phylostratum

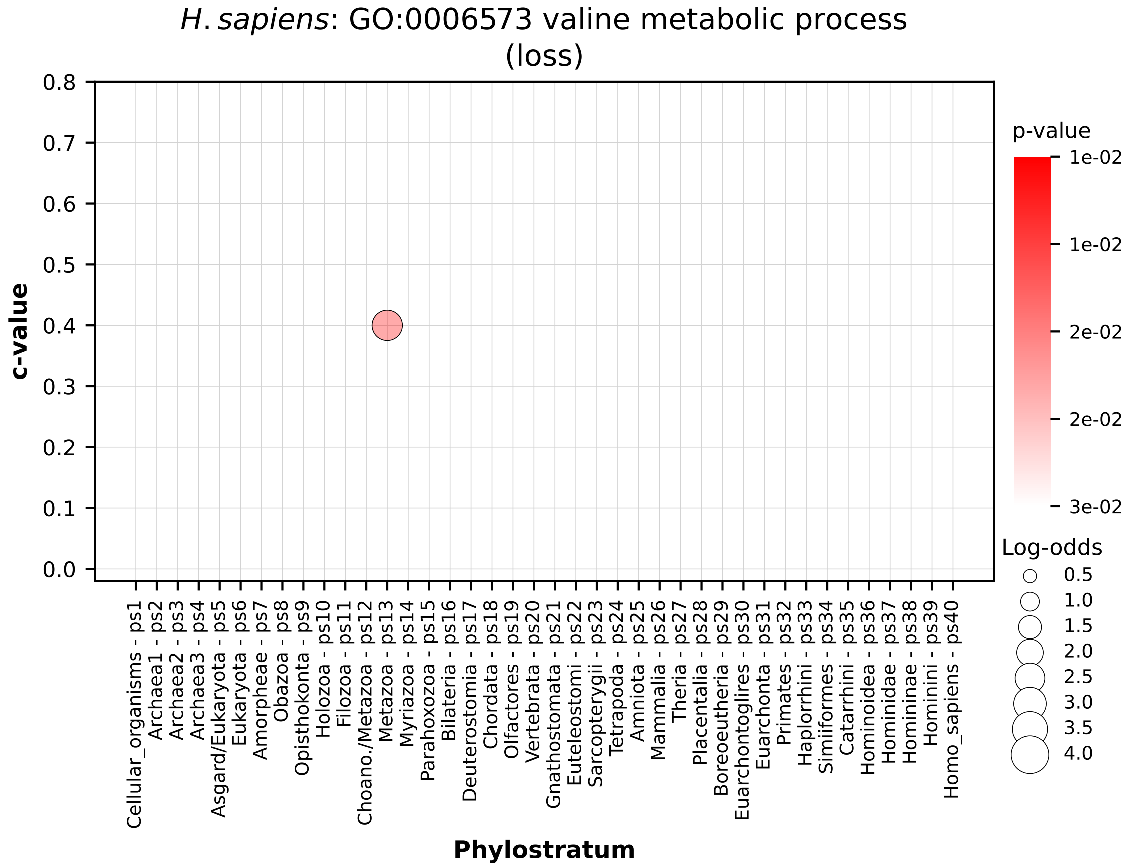
H. sapiens: GO:0006573 valine metabolic process
(loss)
c-value
0.0
0.1
0.2
0.3
0.4
0.5
0.6
0.7
0.8
Phylostratum
Cellular_organisms - ps1
Archaea1 - ps2
Archaea2 - ps3
Archaea3 - ps4
Asgard/Eukaryota - ps5
Eukaryota - ps6
Amorpheae - ps7
Obazoa - ps8
Opisthokonta - ps9
Holozoa - ps10
Filozoa - ps11
Choano./Metazoa - ps12
Metazoa - ps13
Myriazoa - ps14
Parahoxozoa - ps15
Bilateria - ps16
Deuterostomia - ps17
Chordata - ps18
Olfactores - ps19
Vertebrata - ps20
Gnathostomata - ps21
Euteleostomi - ps22
Sarcopterygii - ps23
Tetrapoda - ps24
Amniota - ps25
Mammalia - ps26
Theria - ps27
Placentalia - ps28
Boreoeutheria - ps29
Euarchontoglires - ps30
Euarchonta - ps31
Primates - ps32
Haplorrhini - ps33
Simiiformes - ps34
Catarrhini - ps35
Hominoidea - ps36
Hominidae - ps37
Homininae - ps38
Hominini - ps39
Homo_sapiens - ps40
p-value
1e-02
1e-02
2e-02
2e-02
3e-02
Log-odds
0.5
1.0
1.5
2.0
2.5
3.0
3.5
4.0

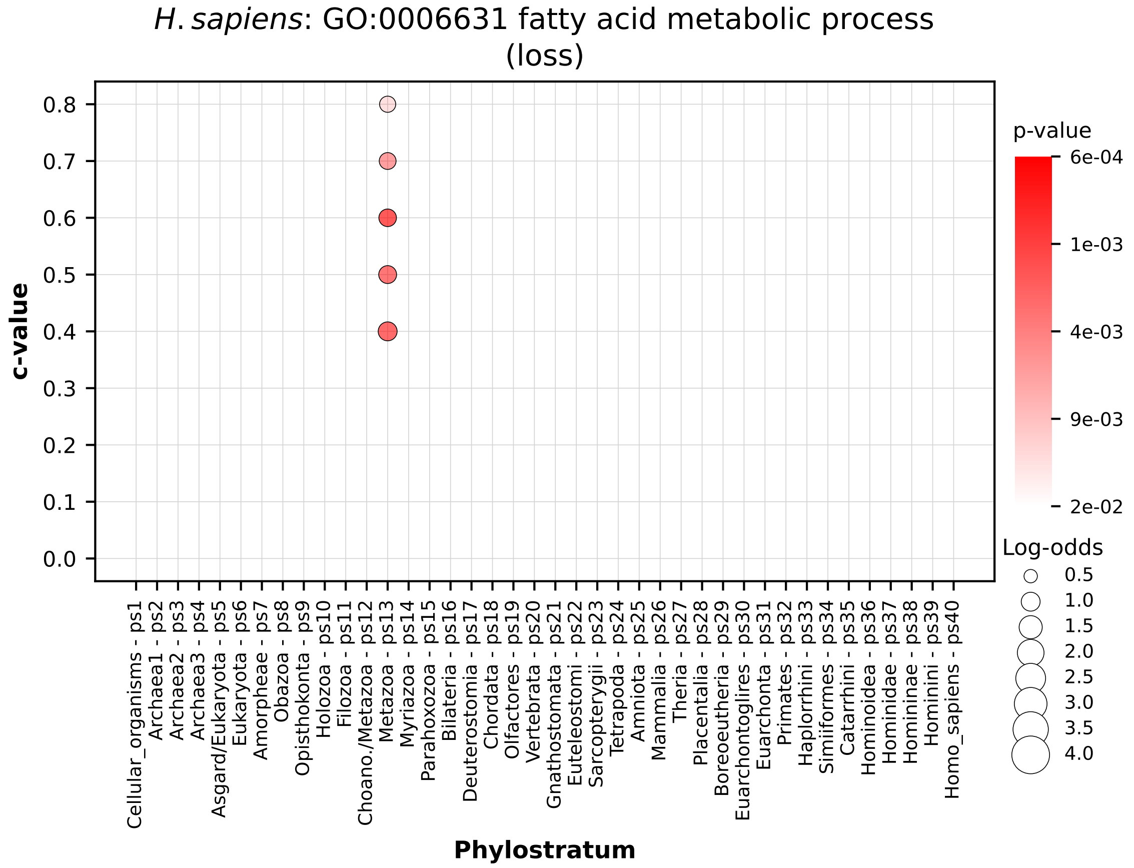

H. sapiens: GO:0006631 fatty acid metabolic process
(loss)
c-value
0.0
0.1
0.2
0.3
0.4
0.5
0.6
0.7
0.8
Phylostratum
Cellular_organisms - ps1
Archaea1 - ps2
Archaea2 - ps3
Archaea3 - ps4
Asgard/Eukaryota - ps5
Eukaryota - ps6
Amorpheae - ps7
Obazoa - ps8
Opisthokonta - ps9
Holozoa - ps10
Filozoa - ps11
Choano./Metazoa - ps12
Metazoa - ps13
Myriazoa - ps14
Parahoxozoa - ps15
Bilateria - ps16
Deuterostomia - ps17
Chordata - ps18
Olfactores - ps19
Vertebrata - ps20
Gnathostomata - ps21
Euteleostomi - ps22
Sarcopterygii - ps23
Tetrapoda - ps24
Amniota - ps25
Mammalia - ps26
Theria - ps27
Placentalia - ps28
Boreoeutheria - ps29
Euarchontoglires - ps30
Euarchonta - ps31
Primates - ps32
Haplorrhini - ps33
Simiiformes - ps34
Catarrhini - ps35
Hominoidea - ps36
Hominidae - ps37
Homininae - ps38
Hominini - ps39
Homo_sapiens - ps40
p-value
6e-04
1e-03
4e-03
9e-03
2e-02
Log-odds
0.5
1.0
1.5
2.0
2.5
3.0
3.5
4.0

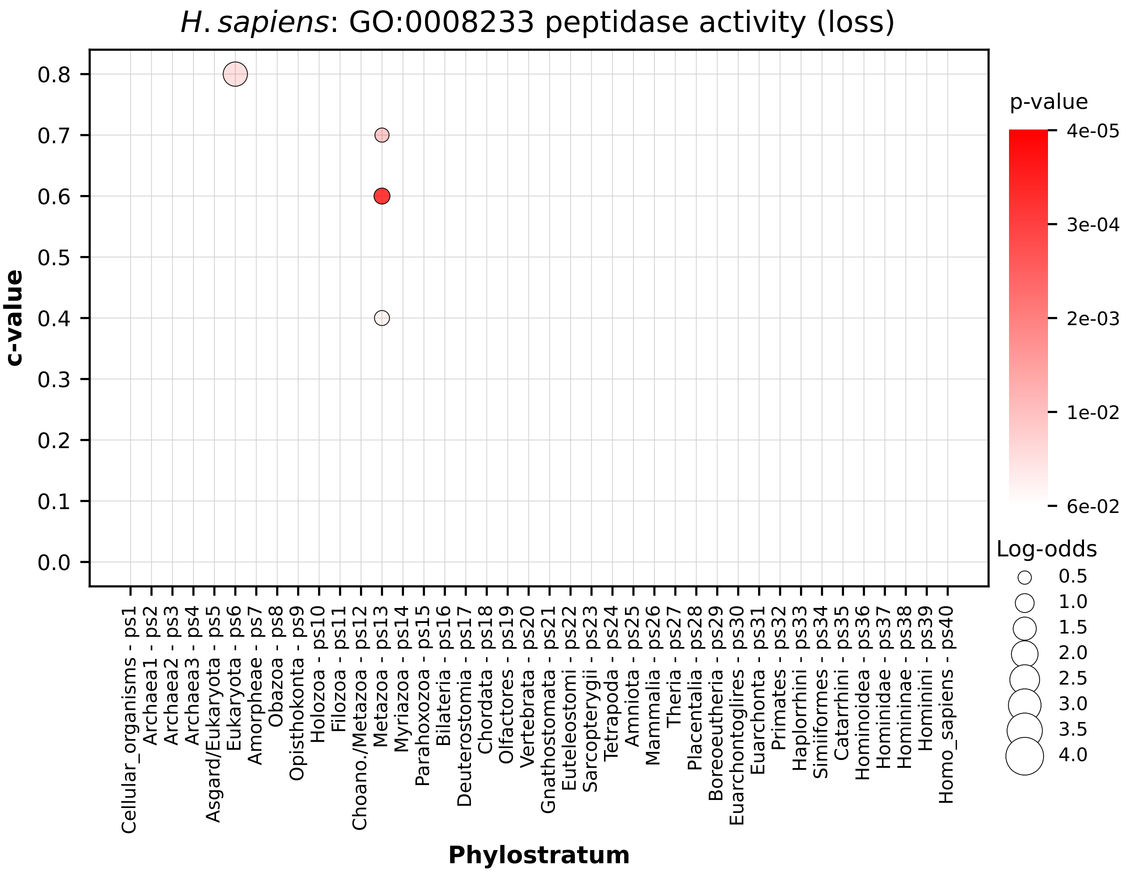
H. sapiens: GO:0008233 peptidase activity (loss)
c-value
0.8
0.7
0.6
0.5
0.4
0.3
0.2
0.1
0.0
Phylostratum
Cellular_organisms - ps1
Archaea1 - ps2
Archaea2 - ps3
Archaea3 - ps4
Asgard/Eukaryota - ps5
Eukaryota - ps6
Amorpheae - ps7
Obazoa - ps8
Opisthokonta - ps9
Holozoa - ps10
Filozoa - ps11
Choano./Metazoa - ps12
Metazoa - ps13
Myriazoa - ps14
Parahoxozoa - ps15
Bilateria - ps16
Deuterostomia - ps17
Chordata - ps18
Olfactores - ps19
Vertebrata - ps20
Gnathostomata - ps21
Euteleostomi - ps22
Sarcopterygii - ps23
Tetrapoda - ps24
Amniota - ps25
Mammalia - ps26
Theria - ps27
Placentalia - ps28
Boreoeutheria - ps29
Euarchontoglires - ps30
Euarchonta - ps31
Primates - ps32
Haplorrhini - ps33
Simiiformes - ps34
Catarrhini - ps35
Hominoidea - ps36
Hominidae - ps37
Homininae - ps38
Hominini - ps39
Homo_sapiens - ps40
p-value
4e-05
3e-04
2e-03
1e-02
6e-02
Log-odds
0.5
1.0
1.5
2.0
2.5
3.0
3.5
4.0

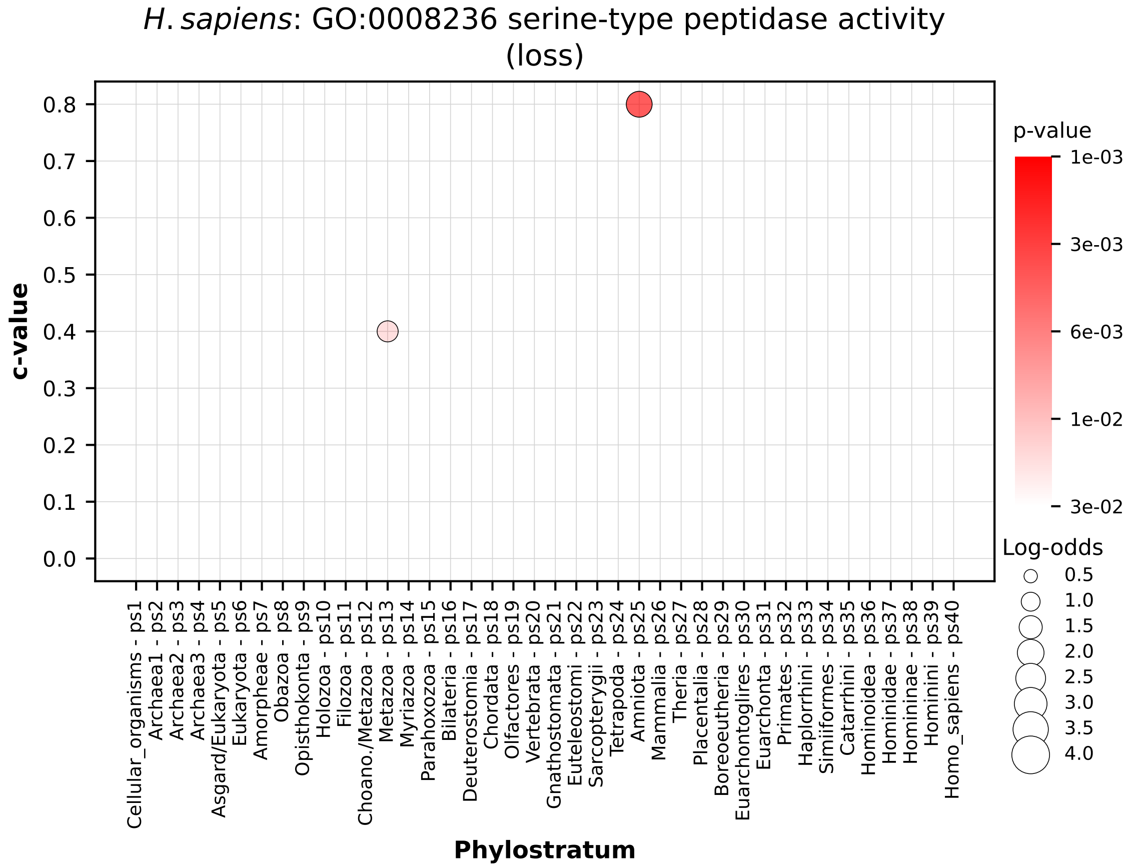

H. sapiens: GO:0008236 serine-type peptidase activity
(loss)
c-value
0.0
0.1
0.2
0.3
0.4
0.5
0.6
0.7
0.8
Phylostratum
Cellular_organisms - ps1
Archaea1 - ps2
Archaea2 - ps3
Archaea3 - ps4
Asgard/Eukaryota - ps5
Eukaryota - ps6
Amorpheae - ps7
Obazoa - ps8
Opisthokonta - ps9
Holozoa - ps10
Filozoa - ps11
Choano./Metazoa - ps12
Metazoa - ps13
Myriazoa - ps14
Parahoxozoa - ps15
Bilateria - ps16
Deuterostomia - ps17
Chordata - ps18
Olfactores - ps19
Vertebrata - ps20
Gnathostomata - ps21
Euteleostomi - ps22
Sarcopterygii - ps23
Tetrapoda - ps24
Amniota - ps25
Mammalia - ps26
Theria - ps27
Placentalia - ps28
Boreoeutheria - ps29
Euarchontoglires - ps30
Euarchonta - ps31
Primates - ps32
Haplorrhini - ps33
Simiiformes - ps34
Catarrhini - ps35
Hominoidea - ps36
Hominidae - ps37
Homininae - ps38
Hominini - ps39
Homo_sapiens - ps40
p-value
1e-03
3e-03
6e-03
1e-02
3e-02
Log-odds
0.5
1.0
1.5
2.0
2.5
3.0
3.5
4.0

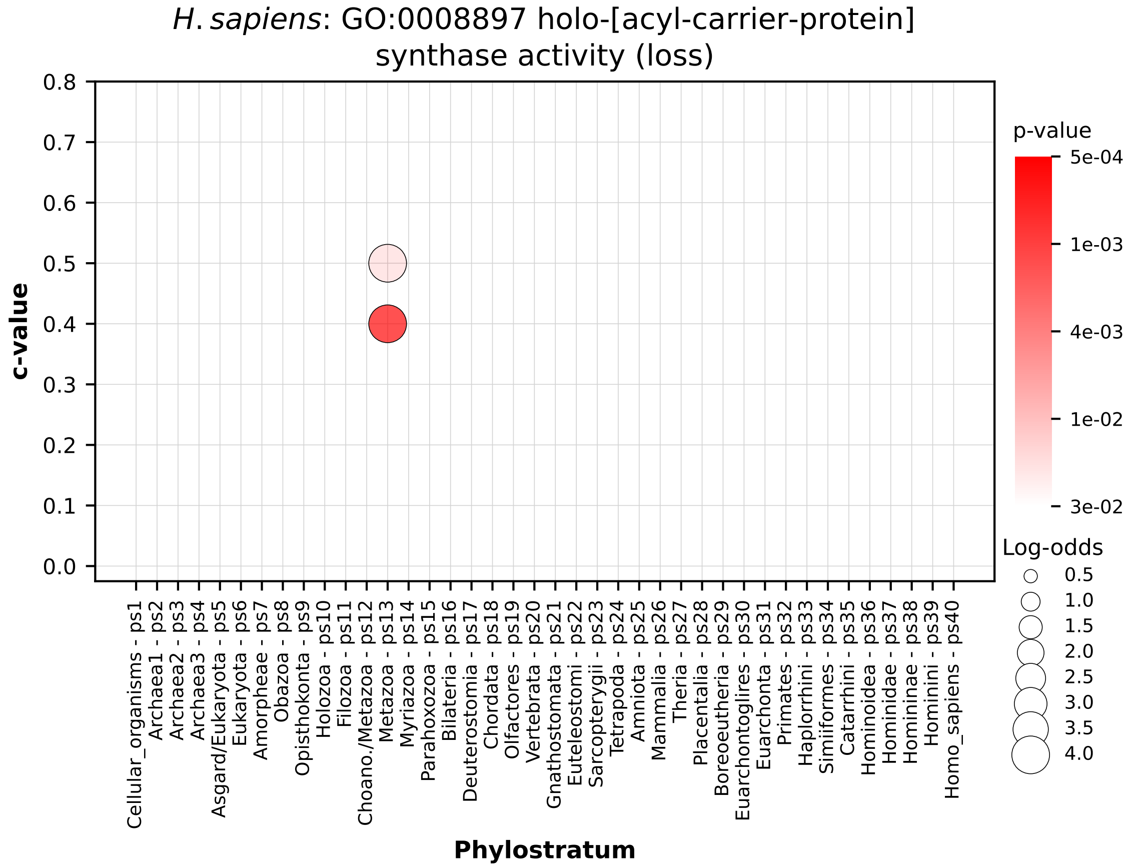

H. sapiens: GO:0008897 holo-[acyl-carrier-protein] synthase activity (loss)
c-value
0.8
0.7
0.6
0.5
0.4
0.3
0.2
0.1
0.0
Phylostratum
Cellular_organisms - ps1
Archaea1 - ps2
Archaea2 - ps3
Archaea3 - ps4
Asgard/Eukaryota - ps5
Eukaryota - ps6
Amorpheae - ps7
Obazoa - ps8
Opisthokonta - ps9
Holozoa - ps10
Filozoa - ps11
Choano./Metazoa - ps12
Metazoa - ps13
Myriazoa - ps14
Parahoxozoa - ps15
Bilateria - ps16
Deuterostomia - ps17
Chordata - ps18
Olfactores - ps19
Vertebrata - ps20
Gnathostomata - ps21
Euteleostomi - ps22
Sarcopterygii - ps23
Tetrapoda - ps24
Amniota - ps25
Mammalia - ps26
Theria - ps27
Placentalia - ps28
Boreoeutheria - ps29
Euarchontoglires - ps30
Euarchonta - ps31
Primates - ps32
Haplorrhini - ps33
Simiiformes - ps34
Catarrhini - ps35
Hominoidea - ps36
Hominidae - ps37
Homininae - ps38
Hominini - ps39
Homo_sapiens - ps40
p-value
5e-04
1e-03
4e-03
1e-02
3e-02
Log-odds
0.5
1.0
1.5
2.0
2.5
3.0
3.5
4.0

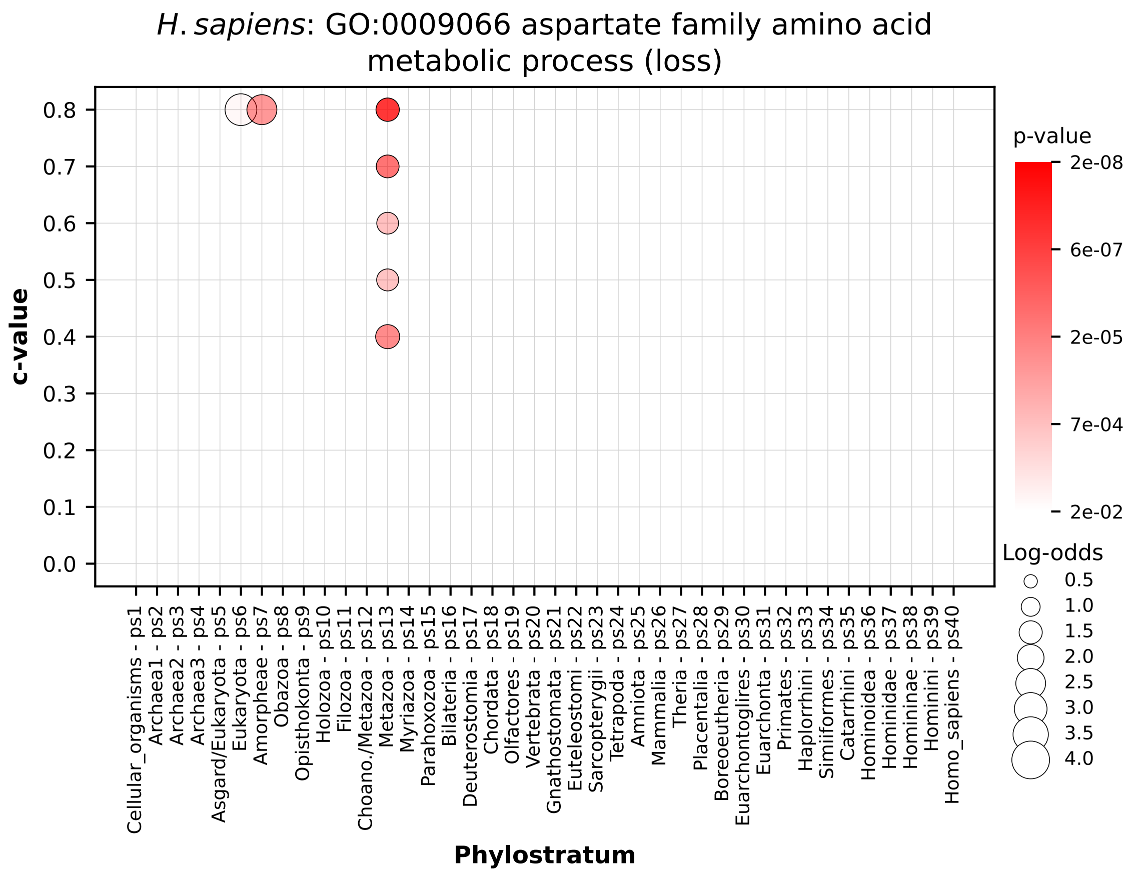

H. sapiens: GO:0009066 aspartate family amino acid metabolic process (loss)
c-value
0.0
0.1
0.2
0.3
0.4
0.5
0.6
0.7
0.8
p-value
2e-08
6e-07
2e-05
7e-04
2e-02
Log-odds
0.5
1.0
1.5
2.0
2.5
3.0
3.5
4.0
Cellular_organisms - ps1
Archaea1 - ps2
Archaea2 - ps3
Archaea3 - ps4
Asgard/Eukaryota - ps5
Eukaryota - ps6
Amorpheae - ps7
Obazoa - ps8
Opisthokonta - ps9
Holozoa - ps10
Filozoa - ps11
Choano./Metazoa - ps12
Metazoa - ps13
Myriazoa - ps14
Parahoxozoa - ps15
Bilateria - ps16
Deuterostomia - ps17
Chordata - ps18
Olfactores - ps19
Vertebrata - ps20
Gnathostomata - ps21
Euteleostomi - ps22
Sarcopterygii - ps23
Tetrapoda - ps24
Amniota - ps25
Mammalia - ps26
Theria - ps27
Placentalia - ps28
Boreoeutheria - ps29
Euarchontoglires - ps30
Euarchonta - ps31
Primates - ps32
Haplorrhini - ps33
Simiiformes - ps34
Catarrhini - ps35
Hominoidea - ps36
Hominidae - ps37
Homininae - ps38
Hominini - ps39
Homo_sapiens - ps40
Phylostratum

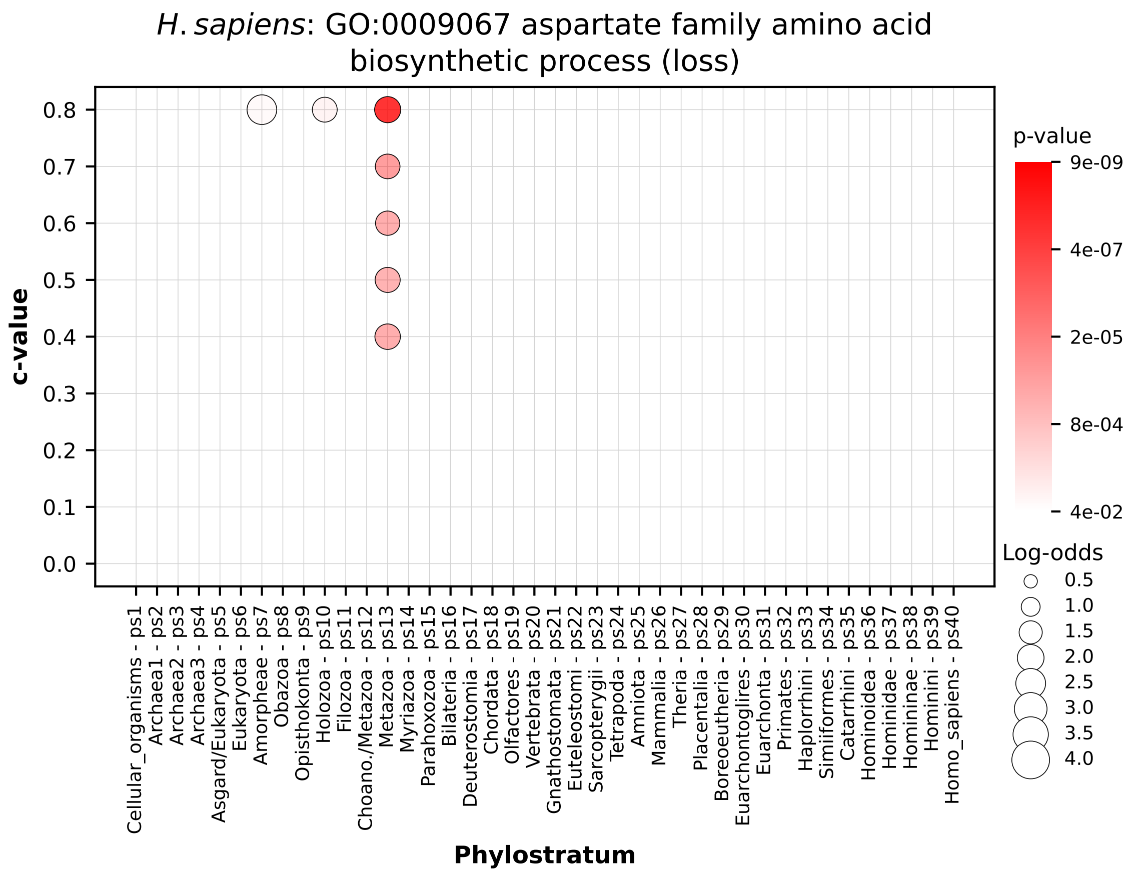

H. sapiens: GO:0009067 aspartate family amino acid biosynthetic process (loss)
c-value
0.8
0.7
0.6
0.5
0.4
0.3
0.2
0.1
0.0
p-value
9e-09
4e-07
2e-05
8e-04
4e-02
Log-odds
0.5
1.0
1.5
2.0
2.5
3.0
3.5
4.0
Cellular_organisms - ps1
Archaea1 - ps2
Archaea2 - ps3
Archaea3 - ps4
Asgard/Eukaryota - ps5
Eukaryota - ps6
Amorpheae - ps7
Obazoa - ps8
Opisthokonta - ps9
Holozoa - ps10
Filozoa - ps11
Choano./Metazoa - ps12
Metazoa - ps13
Myriazoa - ps14
Parahoxozoa - ps15
Bilateria - ps16
Deuterostomia - ps17
Chordata - ps18
Olfactores - ps19
Vertebrata - ps20
Gnathostomata - ps21
Euteleostomi - ps22
Sarcopterygii - ps23
Tetrapoda - ps24
Amniota - ps25
Mammalia - ps26
Theria - ps27
Placentalia - ps28
Boreoeutheria - ps29
Euarchontoglires - ps30
Euarchonta - ps31
Primates - ps32
Haplorrhini - ps33
Simiiformes - ps34
Catarrhini - ps35
Hominoidea - ps36
Hominidae - ps37
Homininae - ps38
Hominini - ps39
Homo_sapiens - ps40
Phylostratum

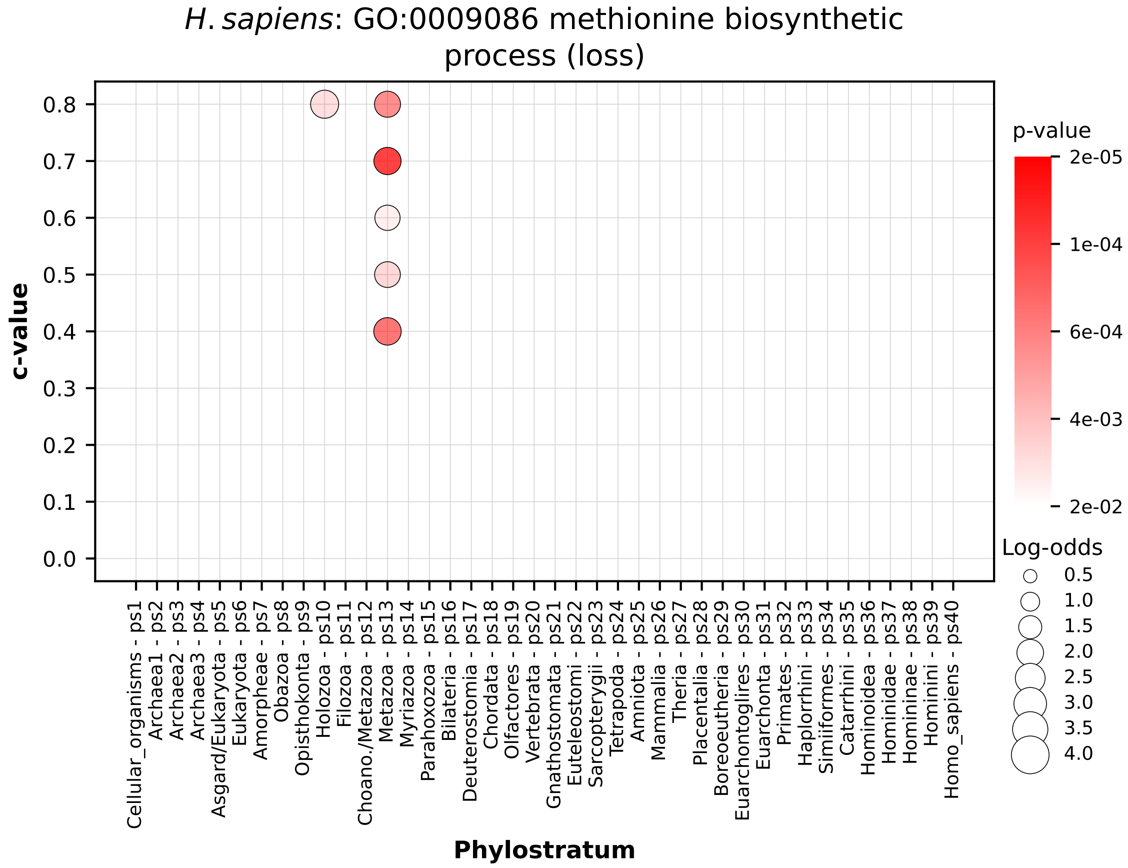
H. sapiens: GO:0009086 methionine biosynthetic process (loss)
c-value
0.0
0.1
0.2
0.3
0.4
0.5
0.6
0.7
0.8
Phylostratum
Cellular_organisms - ps1
Archaea1 - ps2
Archaea2 - ps3
Archaea3 - ps4
Asgard/Eukaryota - ps5
Eukaryota - ps6
Amorpheae - ps7
Obazoa - ps8
Opisthokonta - ps9
Holozoa - ps10
Filozoa - ps11
Choano./Metazoa - ps12
Metazoa - ps13
Myriazoa - ps14
Parahoxozoa - ps15
Bilateria - ps16
Deuterostomia - ps17
Chordata - ps18
Olfactores - ps19
Vertebrata - ps20
Gnathostomata - ps21
Euteleostomi - ps22
Sarcopterygii - ps23
Tetrapoda - ps24
Amniota - ps25
Mammalia - ps26
Theria - ps27
Placentalia - ps28
Boreoeutheria - ps29
Euarchontoglires - ps30
Euarchonta - ps31
Primates - ps32
Haplorrhini - ps33
Simiiformes - ps34
Catarrhini - ps35
Hominoidea - ps36
Hominidae - ps37
Homininae - ps38
Hominini - ps39
Homo_sapiens - ps40
p-value
2e-05
1e-04
6e-04
4e-03
2e-02
Log-odds
0.5
1.0
1.5
2.0
2.5
3.0
3.5
4.0

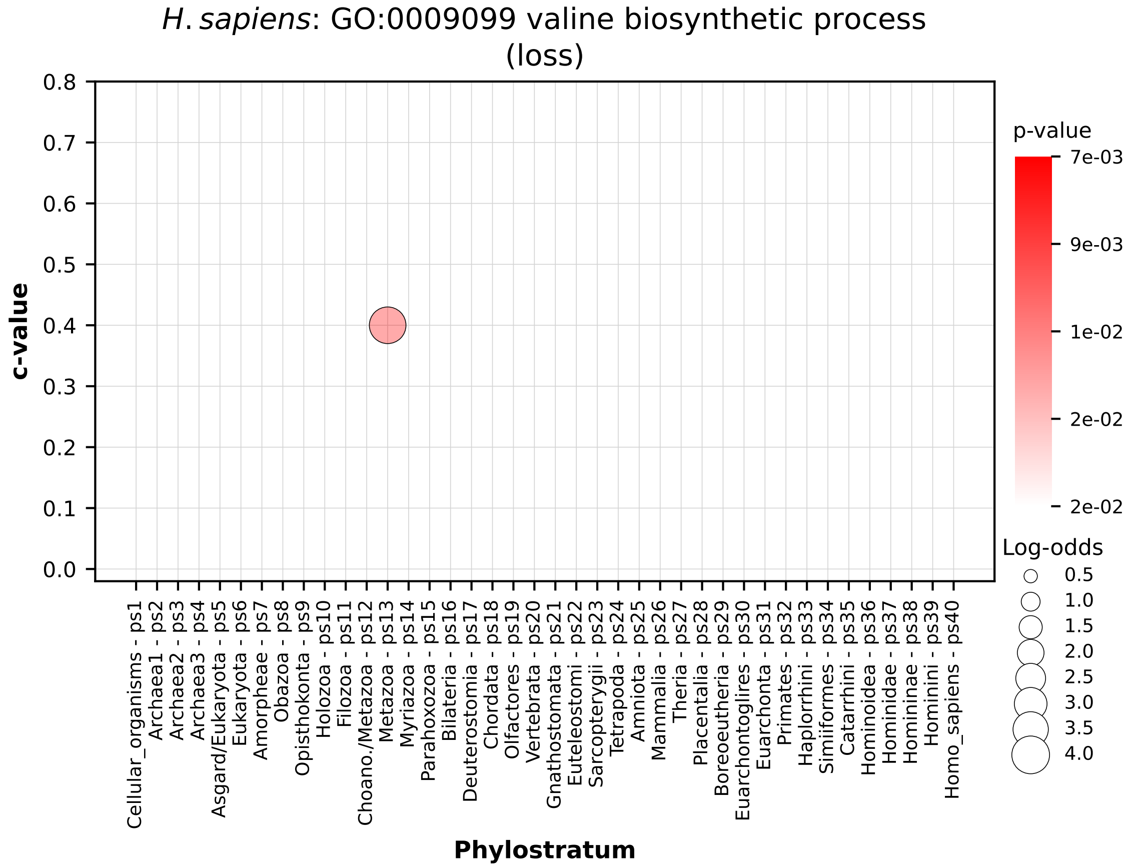

H. sapiens: GO:0009099 valine biosynthetic process
(loss)
c-value
0.0
0.1
0.2
0.3
0.4
0.5
0.6
0.7
0.8
Phylostratum
Cellular_organisms - ps1
Archaea1 - ps2
Archaea2 - ps3
Archaea3 - ps4
Asgard/Eukaryota - ps5
Eukaryota - ps6
Amorpheae - ps7
Obazoa - ps8
Opisthokonta - ps9
Holozoa - ps10
Filozoa - ps11
Choano./Metazoa - ps12
Metazoa - ps13
Myriazoa - ps14
Parahoxozoa - ps15
Bilateria - ps16
Deuterostomia - ps17
Chordata - ps18
Olfactores - ps19
Vertebrata - ps20
Gnathostomata - ps21
Euteleostomi - ps22
Sarcopterygii - ps23
Tetrapoda - ps24
Amniota - ps25
Mammalia - ps26
Theria - ps27
Placentalia - ps28
Boreoeutheria - ps29
Euarchontoglires - ps30
Euarchonta - ps31
Primates - ps32
Haplorrhini - ps33
Simiiformes - ps34
Catarrhini - ps35
Hominoidea - ps36
Hominidae - ps37
Homininae - ps38
Hominini - ps39
Homo_sapiens - ps40
p-value
7e-03
9e-03
1e-02
2e-02
2e-02
Log-odds
0.5
1.0
1.5
2.0
2.5
3.0
3.5
4.0

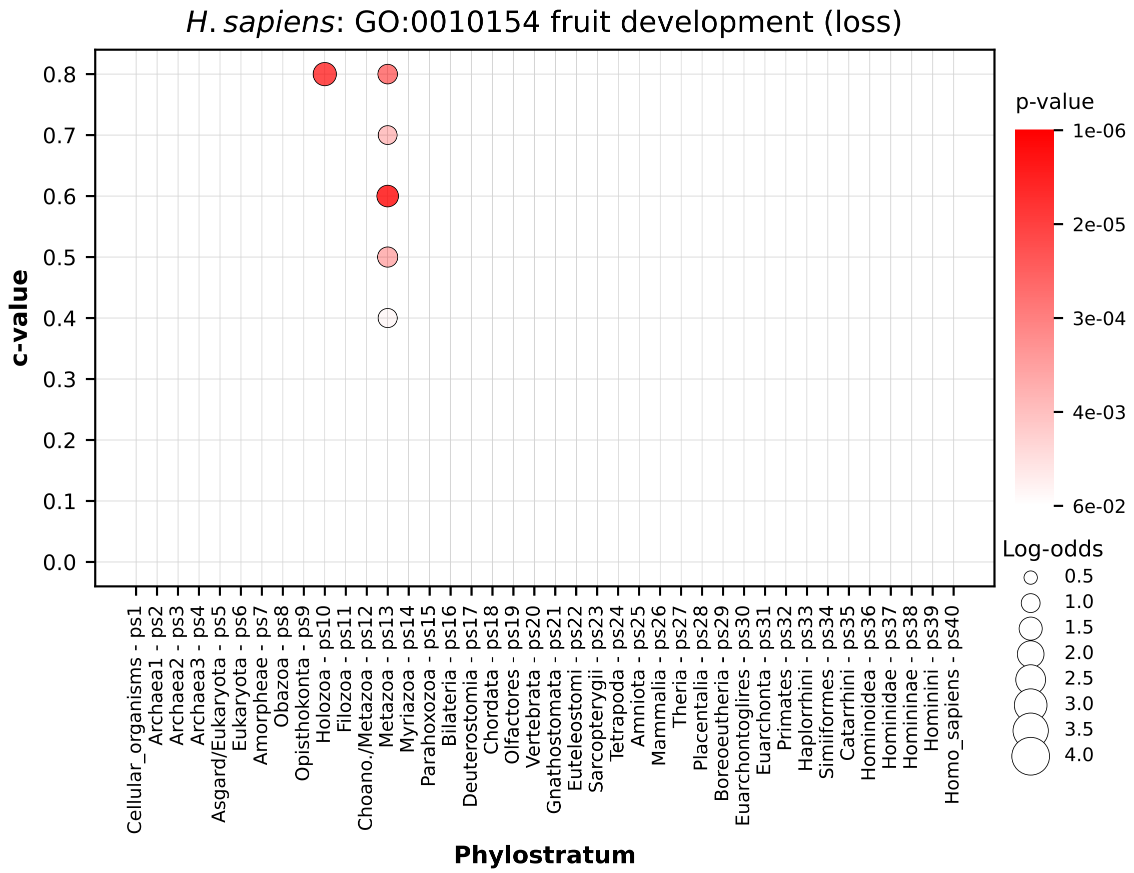

H. sapiens: GO:0010154 fruit development (loss)
c-value
0.0
0.1
0.2
0.3
0.4
0.5
0.6
0.7
0.8
Phylostratum
Cellular_organisms - ps1
Archaea1 - ps2
Archaea2 - ps3
Archaea3 - ps4
Asgard/Eukaryota - ps5
Eukaryota - ps6
Amorpheae - ps7
Obazoa - ps8
Opisthokonta - ps9
Holozoa - ps10
Filozoa - ps11
Choano./Metazoa - ps12
Metazoa - ps13
Myriazoa - ps14
Parahoxozoa - ps15
Bilateria - ps16
Deuterostomia - ps17
Chordata - ps18
Olfactores - ps19
Vertebrata - ps20
Gnathostomata - ps21
Euteleostomi - ps22
Sarcopterygii - ps23
Tetrapoda - ps24
Amniota - ps25
Mammalia - ps26
Theria - ps27
Placentalia - ps28
Boreoeutheria - ps29
Euarchontoglires - ps30
Euarchonta - ps31
Primates - ps32
Haplorrhini - ps33
Simiiformes - ps34
Catarrhini - ps35
Hominoidea - ps36
Hominidae - ps37
Homininae - ps38
Hominini - ps39
Homo_sapiens - ps40
p-value
1e-06
2e-05
3e-04
4e-03
6e-02
Log-odds
0.5
1.0
1.5
2.0
2.5
3.0
3.5
4.0

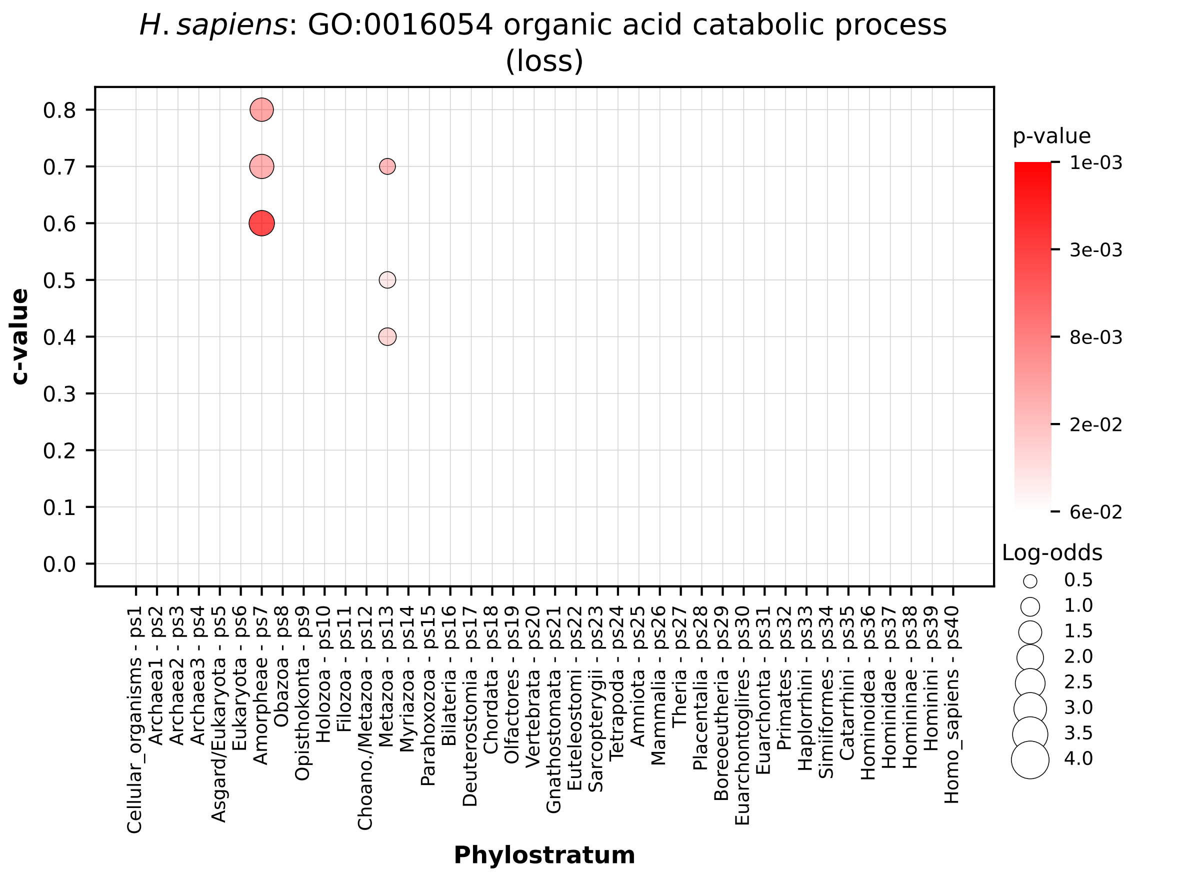

H. sapiens: GO:0016054 organic acid catabolic process (loss)
c-value
0.8
0.7
0.6
0.5
0.4
0.3
0.2
0.1
0.0
p-value
1e-03
3e-03
8e-03
2e-02
6e-02
Log-odds
0.5
1.0
1.5
2.0
2.5
3.0
3.5
4.0
Cellular_organisms - ps1
Archaea1 - ps2
Archaea2 - ps3
Archaea3 - ps4
Asgard/Eukaryota - ps5
Eukaryota - ps6
Amorpheae - ps7
Obazoa - ps8
Opisthokonta - ps9
Holozoa - ps10
Filozoa - ps11
Choano./Metazoa - ps12
Metazoa - ps13
Myriazoa - ps14
Parahoxozoa - ps15
Bilateria - ps16
Deuterostomia - ps17
Chordata - ps18
Olfactores - ps19
Vertebrata - ps20
Gnathostomata - ps21
Euteleostomi - ps22
Sarcopterygii - ps23
Tetrapoda - ps24
Amniota - ps25
Mammalia - ps26
Theria - ps27
Placentalia - ps28
Boreoeutheria - ps29
Euarchontoglires - ps30
Euarchonta - ps31
Primates - ps32
Haplorrhini - ps33
Simiiformes - ps34
Catarrhini - ps35
Hominoidea - ps36
Hominidae - ps37
Homininae - ps38
Hominini - ps39
Homo_sapiens - ps40
Phylostratum

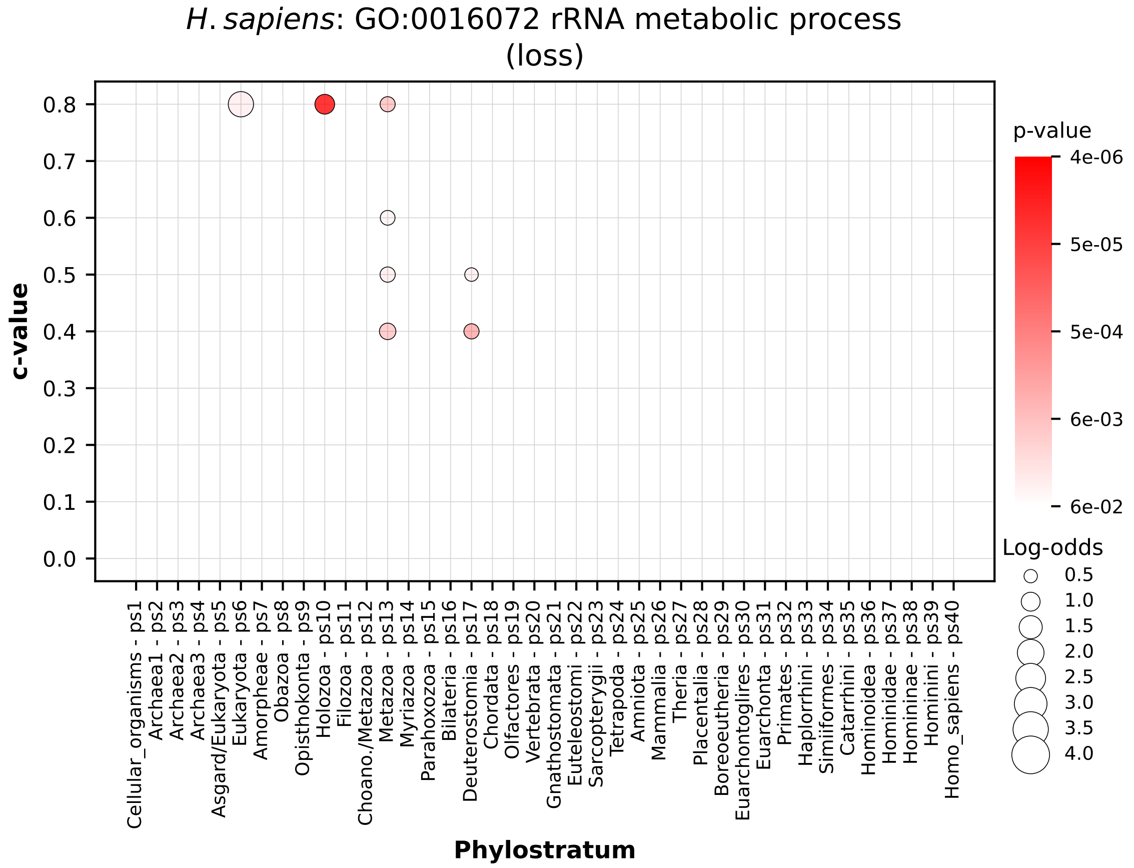
H. sapiens: GO:0016072 rRNA metabolic process
(loss)
c-value
0.8
0.7
0.6
0.5
0.4
0.3
0.2
0.1
0.0
p-value
4e-06
5e-05
5e-04
6e-03
6e-02
Log-odds
0.5
1.0
1.5
2.0
2.5
3.0
3.5
4.0
Cellular_organisms - ps1
Archaea1 - ps2
Archaea2 - ps3
Archaea3 - ps4
Asgard/Eukaryota - ps5
Eukaryota - ps6
Amorpheae - ps7
Obazoa - ps8
Opisthokonta - ps9
Holozoa - ps10
Filozoa - ps11
Choano./Metazoa - ps12
Metazoa - ps13
Myriazoa - ps14
Parahoxozoa - ps15
Bilateria - ps16
Deuterostomia - ps17
Chordata - ps18
Olfactores - ps19
Vertebrata - ps20
Gnathostomata - ps21
Euteleostomi - ps22
Sarcopterygii - ps23
Tetrapoda - ps24
Amniota - ps25
Mammalia - ps26
Theria - ps27
Placentalia - ps28
Boreoeutheria - ps29
Euarchontoglires - ps30
Euarchonta - ps31
Primates - ps32
Haplorrhini - ps33
Simiiformes - ps34
Catarrhini - ps35
Hominoidea - ps36
Hominidae - ps37
Homininae - ps38
Hominini - ps39
Homo_sapiens - ps40
Phylostratum

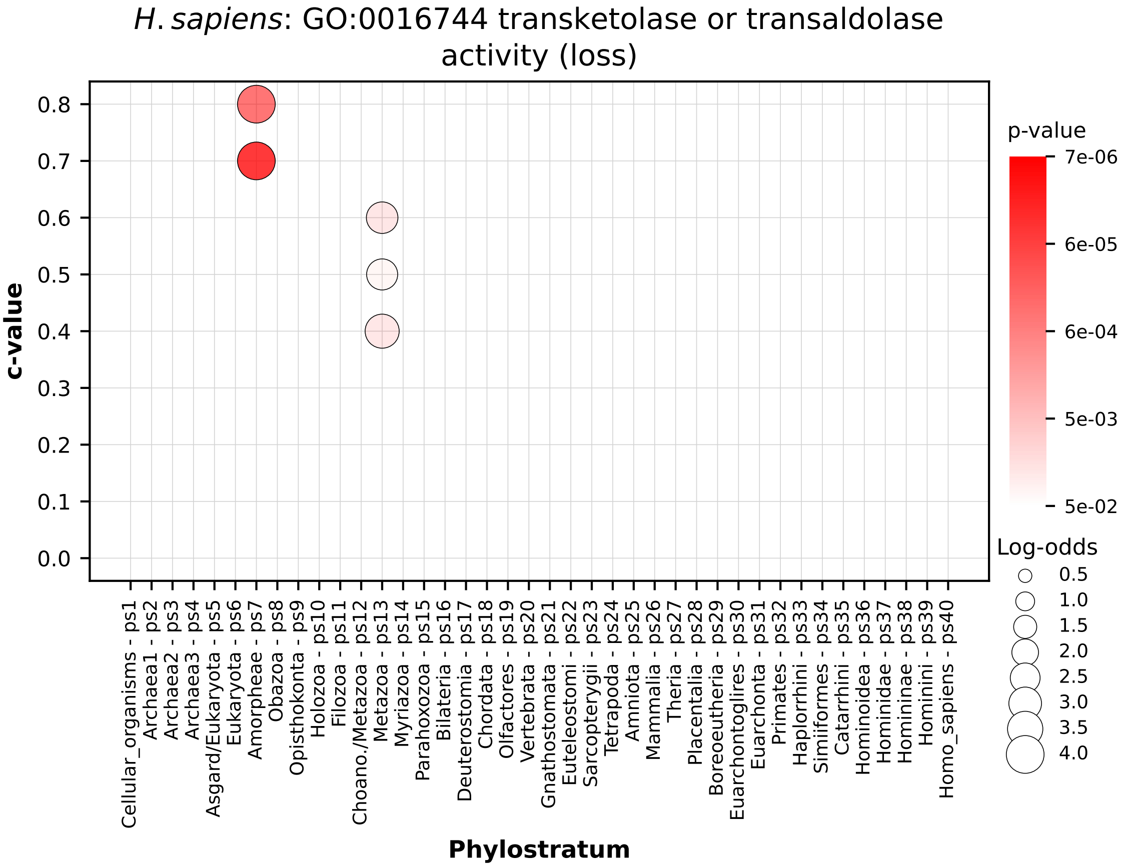

H. sapiens: GO:0016744 transketolase or transaldolase activity (loss)
c-value
0.0
0.1
0.2
0.3
0.4
0.5
0.6
0.7
0.8
Phylostratum
Cellular_organisms - ps1
Archaea1 - ps2
Archaea2 - ps3
Archaea3 - ps4
Asgard/Eukaryota - ps5
Eukaryota - ps6
Amorpheae - ps7
Obazoa - ps8
Opisthokonta - ps9
Holozoa - ps10
Filozoa - ps11
Choano./Metazoa - ps12
Metazoa - ps13
Myriazoa - ps14
Parahoxozoa - ps15
Bilateria - ps16
Deuterostomia - ps17
Chordata - ps18
Olfactores - ps19
Vertebrata - ps20
Gnathostomata - ps21
Euteleostomi - ps22
Sarcopterygii - ps23
Tetrapoda - ps24
Amniota - ps25
Mammalia - ps26
Theria - ps27
Placentalia - ps28
Boreoeutheria - ps29
Euarchontoglires - ps30
Euarchonta - ps31
Primates - ps32
Haplorrhini - ps33
Simiiformes - ps34
Catarrhini - ps35
Hominoidea - ps36
Hominidae - ps37
Homininae - ps38
Hominini - ps39
Homo_sapiens - ps40
p-value
7e-06
6e-05
6e-04
5e-03
5e-02
Log-odds
0.5
1.0
1.5
2.0
2.5
3.0
3.5
4.0

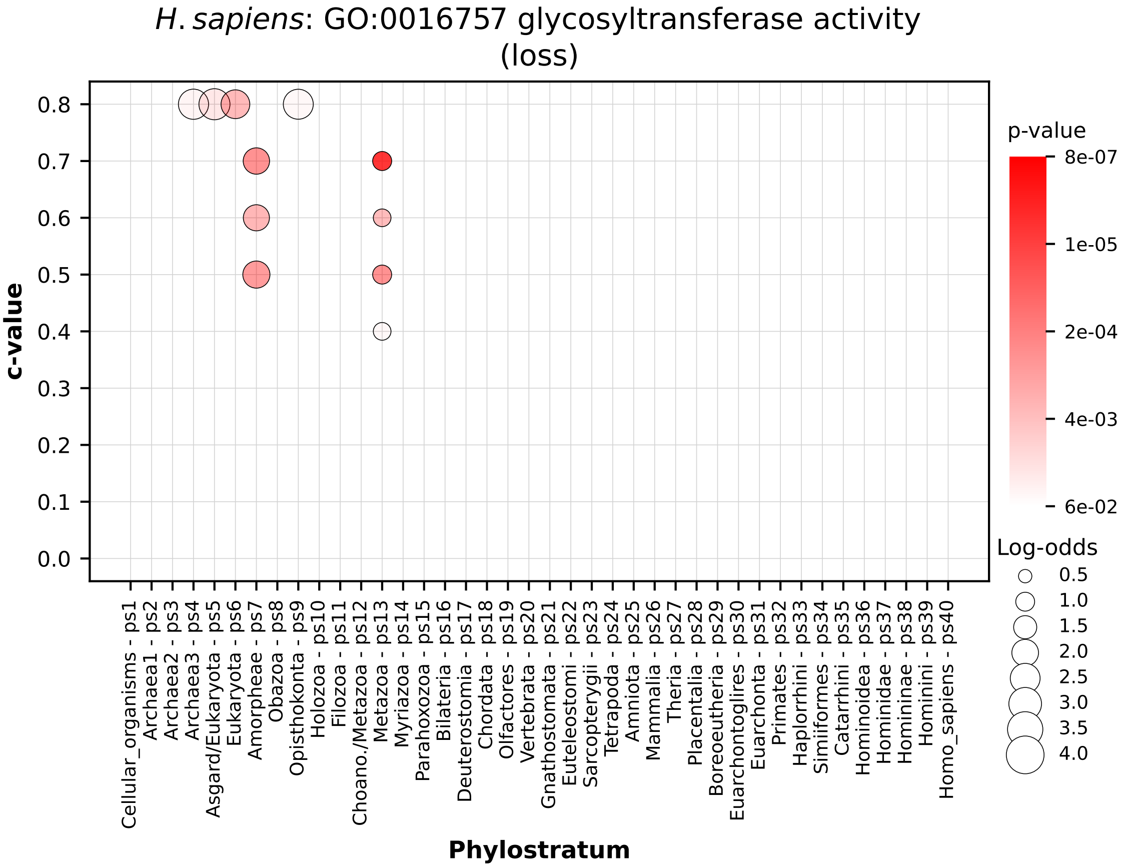

H. sapiens: GO:0016757 glycosyltransferase activity
(loss)
c-value
0.8
0.7
0.6
0.5
0.4
0.3
0.2
0.1
0.0
p-value
8e-07
1e-05
2e-04
4e-03
6e-02
Log-odds
0.5
1.0
1.5
2.0
2.5
3.0
3.5
4.0
Cellular_organisms - ps1
Archaea1 - ps2
Archaea2 - ps3
Archaea3 - ps4
Asgard/Eukaryota - ps5
Eukaryota - ps6
Amorpheae - ps7
Obazoa - ps8
Opisthokonta - ps9
Holozoa - ps10
Filozoa - ps11
Choano./Metazoa - ps12
Metazoa - ps13
Myriazoa - ps14
Parahoxozoa - ps15
Bilateria - ps16
Deuterostomia - ps17
Chordata - ps18
Olfactores - ps19
Vertebrata - ps20
Gnathostomata - ps21
Euteleostomi - ps22
Sarcopterygii - ps23
Tetrapoda - ps24
Amniota - ps25
Mammalia - ps26
Theria - ps27
Placentalia - ps28
Boreoeutheria - ps29
Euarchontoglires - ps30
Euarchonta - ps31
Primates - ps32
Haplorrhini - ps33
Simiiformes - ps34
Catarrhini - ps35
Hominoidea - ps36
Hominidae - ps37
Homininae - ps38
Hominini - ps39
Homo_sapiens - ps40
Phylostratum

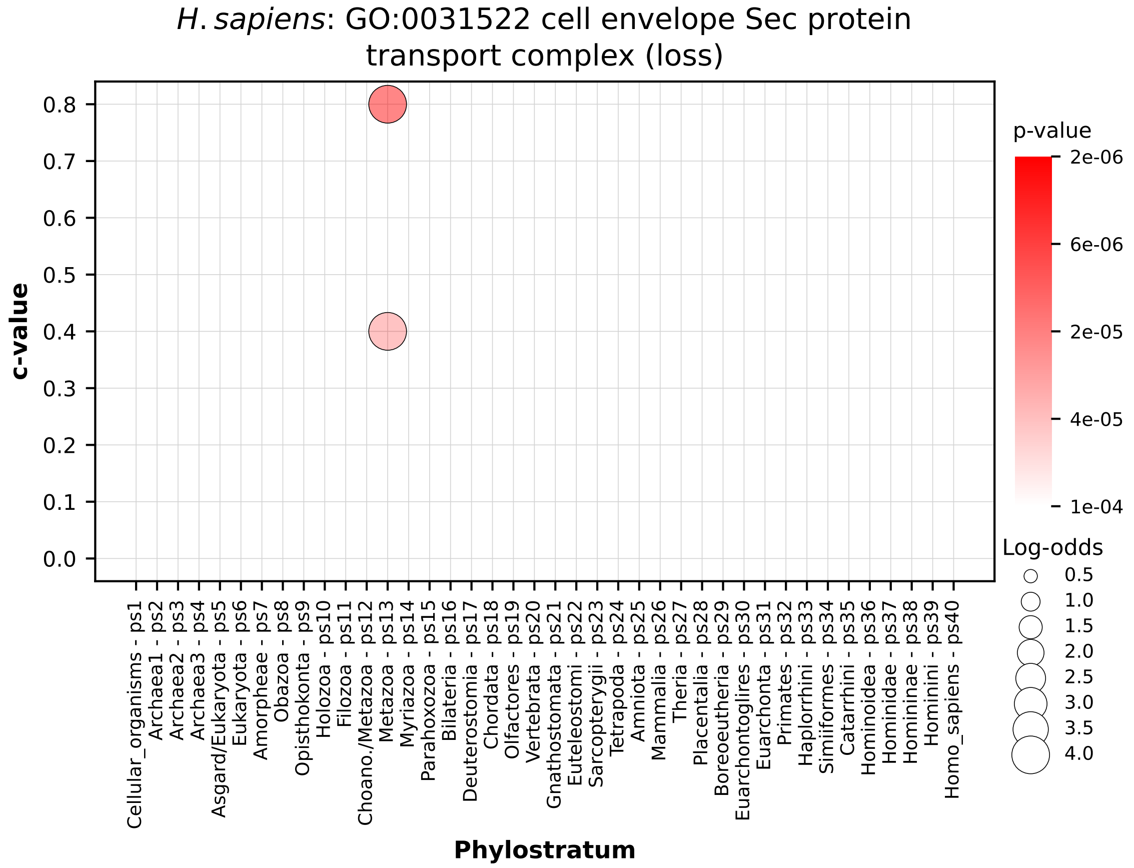

H. sapiens: GO:0031522 cell envelope Sec protein transport complex (loss)
c-value
0.0
0.1
0.2
0.3
0.4
0.5
0.6
0.7
0.8
Phylostratum
Cellular_organisms - ps1
Archaea1 - ps2
Archaea2 - ps3
Archaea3 - ps4
Asgard/Eukaryota - ps5
Eukaryota - ps6
Amorpheae - ps7
Obazoa - ps8
Opisthokonta - ps9
Holozoa - ps10
Filozoa - ps11
Choano./Metazoa - ps12
Metazoa - ps13
Myriazoa - ps14
Parahoxozoa - ps15
Bilateria - ps16
Deuterostomia - ps17
Chordata - ps18
Olfactores - ps19
Vertebrata - ps20
Gnathostomata - ps21
Euteleostomi - ps22
Sarcopterygii - ps23
Tetrapoda - ps24
Amniota - ps25
Mammalia - ps26
Theria - ps27
Placentalia - ps28
Boreoeutheria - ps29
Euarchontoglires - ps30
Euarchonta - ps31
Primates - ps32
Haplorrhini - ps33
Simiiformes - ps34
Catarrhini - ps35
Hominoidea - ps36
Hominidae - ps37
Homininae - ps38
Hominini - ps39
Homo_sapiens - ps40
p-value
2e-06
6e-06
2e-05
4e-05
1e-04
Log-odds
0.5
1.0
1.5
2.0
2.5
3.0
3.5
4.0

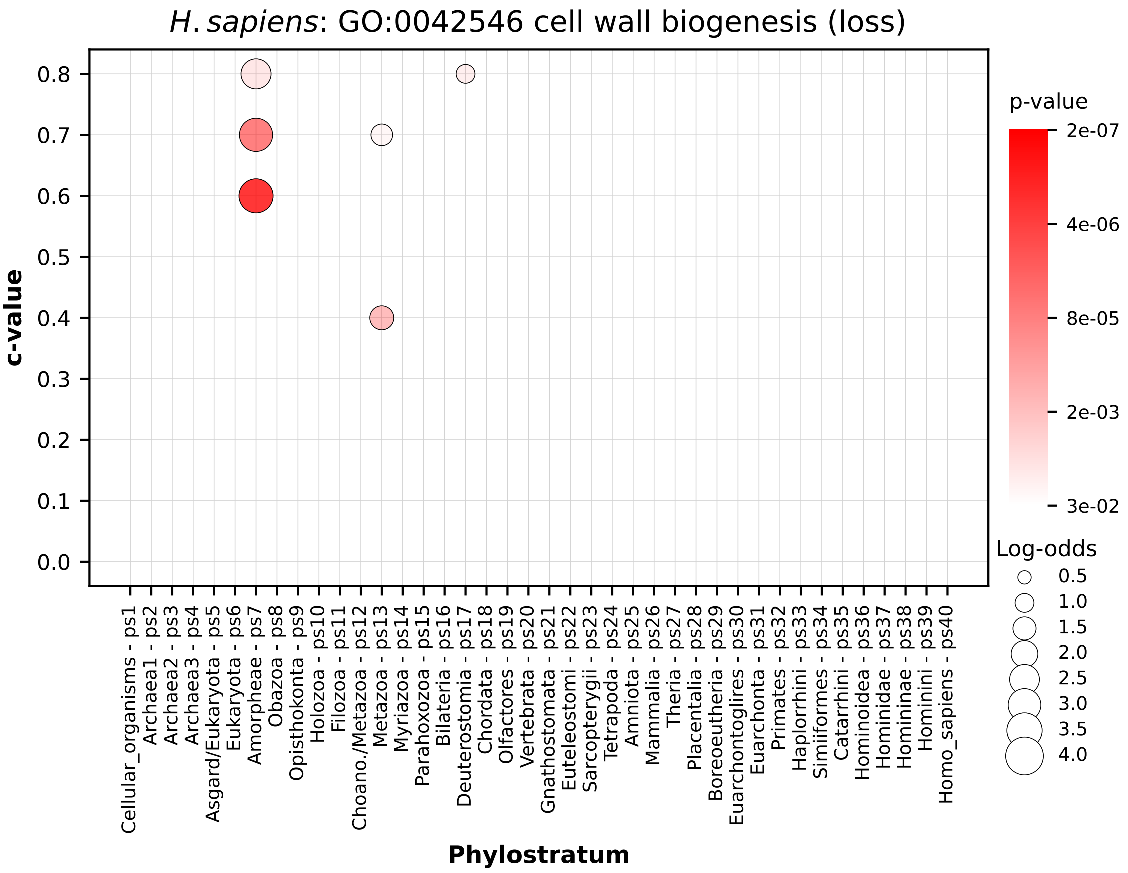

H. sapiens: GO:0042546 cell wall biogenesis (loss)
c-value
0.8
0.7
0.6
0.5
0.4
0.3
0.2
0.1
0.0
p-value
2e-07
4e-06
8e-05
2e-03
3e-02
Log-odds
0.5
1.0
1.5
2.0
2.5
3.0
3.5
4.0
Cellular_organisms - ps1
Archaea1 - ps2
Archaea2 - ps3
Archaea3 - ps4
Asgard/Eukaryota - ps5
Eukaryota - ps6
Amorpheae - ps7
Obazoa - ps8
Opisthokonta - ps9
Holozoa - ps10
Filozoa - ps11
Choano./Metazoa - ps12
Metazoa - ps13
Myriazoa - ps14
Parahoxozoa - ps15
Bilateria - ps16
Deuterostomia - ps17
Chordata - ps18
Olfactores - ps19
Vertebrata - ps20
Gnathostomata - ps21
Euteleostomi - ps22
Sarcopterygii - ps23
Tetrapoda - ps24
Amniota - ps25
Mammalia - ps26
Theria - ps27
Placentalia - ps28
Boreoeutheria - ps29
Euarchontoglires - ps30
Euarchonta - ps31
Primates - ps32
Haplorrhini - ps33
Simiiformes - ps34
Catarrhini - ps35
Hominoidea - ps36
Hominidae - ps37
Homininae - ps38
Hominini - ps39
Homo_sapiens - ps40
Phylostratum

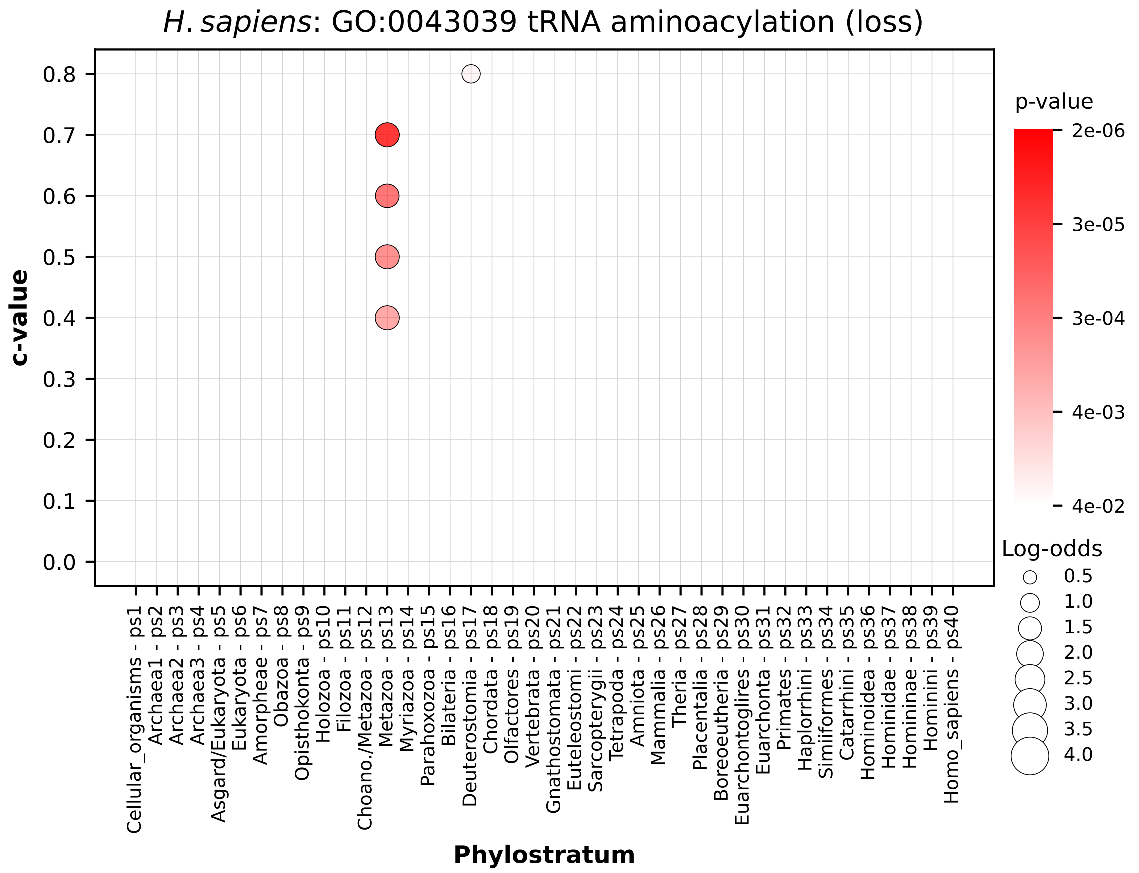
H. sapiens: GO:0043039 tRNA aminoacylation (loss)
c-value
0.8
0.7
0.6
0.5
0.4
0.3
0.2
0.1
0.0
p-value
2e-06
3e-05
3e-04
4e-03
4e-02
Log-odds
0.5
1.0
1.5
2.0
2.5
3.0
3.5
4.0
Cellular_organisms - ps1
Archaea1 - ps2
Archaea2 - ps3
Archaea3 - ps4
Asgard/Eukaryota - ps5
Eukaryota - ps6
Amorpheae - ps7
Obazoa - ps8
Opisthokonta - ps9
Holozoa - ps10
Filozoa - ps11
Choano./Metazoa - ps12
Metazoa - ps13
Myriazoa - ps14
Parahoxozoa - ps15
Bilateria - ps16
Deuterostomia - ps17
Chordata - ps18
Olfactores - ps19
Vertebrata - ps20
Gnathostomata - ps21
Euteleostomi - ps22
Sarcopterygii - ps23
Tetrapoda - ps24
Amniota - ps25
Mammalia - ps26
Theria - ps27
Placentalia - ps28
Boreoeutheria - ps29
Euarchontoglires - ps30
Euarchonta - ps31
Primates - ps32
Haplorrhini - ps33
Simiiformes - ps34
Catarrhini - ps35
Hominoidea - ps36
Hominidae - ps37
Homininae - ps38
Hominini - ps39
Homo_sapiens - ps40
Phylostratum

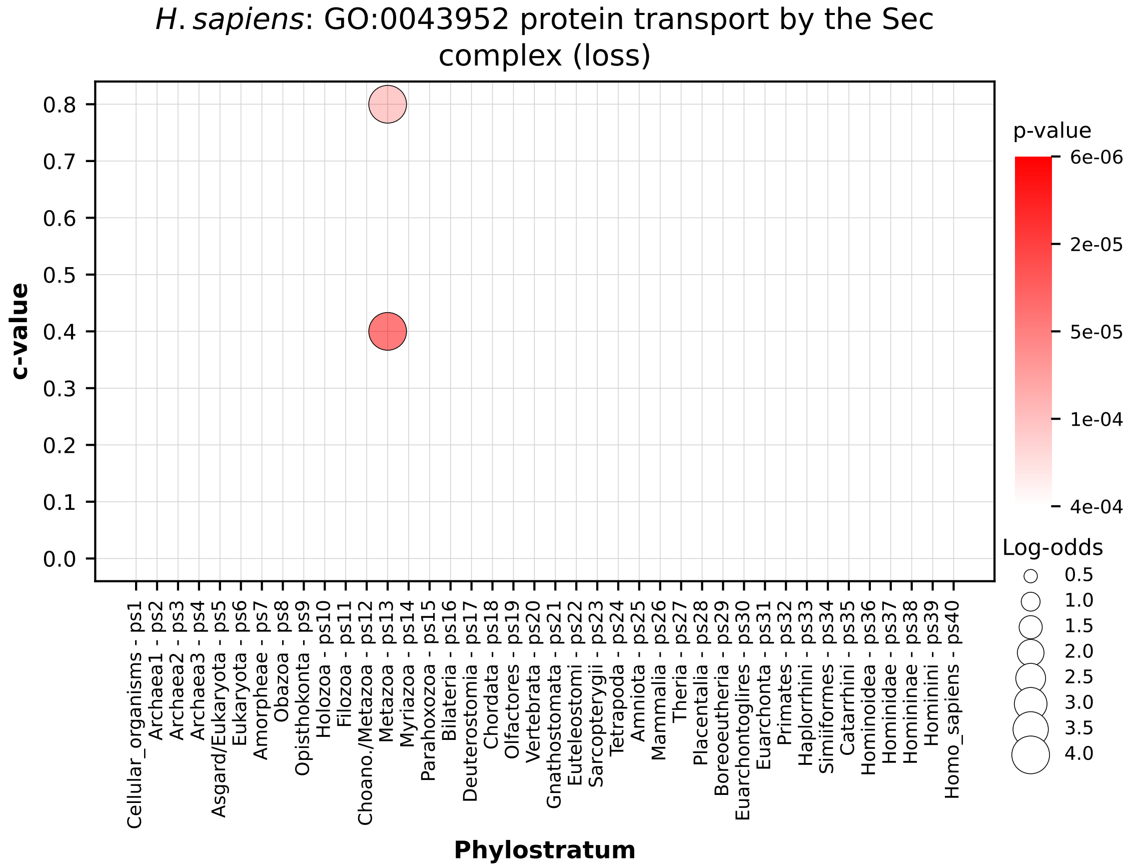

H. sapiens: GO:0043952 protein transport by the Sec complex (loss)
c-value
0.0
0.1
0.2
0.3
0.4
0.5
0.6
0.7
0.8
p-value
6e-06
2e-05
5e-05
1e-04
4e-04
Log-odds
0.5
1.0
1.5
2.0
2.5
3.0
3.5
4.0
Cellular_organisms - ps1
Archaea1 - ps2
Archaea2 - ps3
Archaea3 - ps4
Asgard/Eukaryota - ps5
Eukaryota - ps6
Amorpheae - ps7
Obazoa - ps8
Opisthokonta - ps9
Holozoa - ps10
Filozoa - ps11
Choano./Metazoa - ps12
Metazoa - ps13
Myriazoa - ps14
Parahoxozoa - ps15
Bilateria - ps16
Deuterostomia - ps17
Chordata - ps18
Olfactores - ps19
Vertebrata - ps20
Gnathostomata - ps21
Euteleostomi - ps22
Sarcopterygii - ps23
Tetrapoda - ps24
Amniota - ps25
Mammalia - ps26
Theria - ps27
Placentalia - ps28
Boreoeutheria - ps29
Euarchontoglires - ps30
Euarchonta - ps31
Primates - ps32
Haplorrhini - ps33
Simiiformes - ps34
Catarrhini - ps35
Hominoidea - ps36
Hominidae - ps37
Homininae - ps38
Hominini - ps39
Homo_sapiens - ps40
Phylostratum

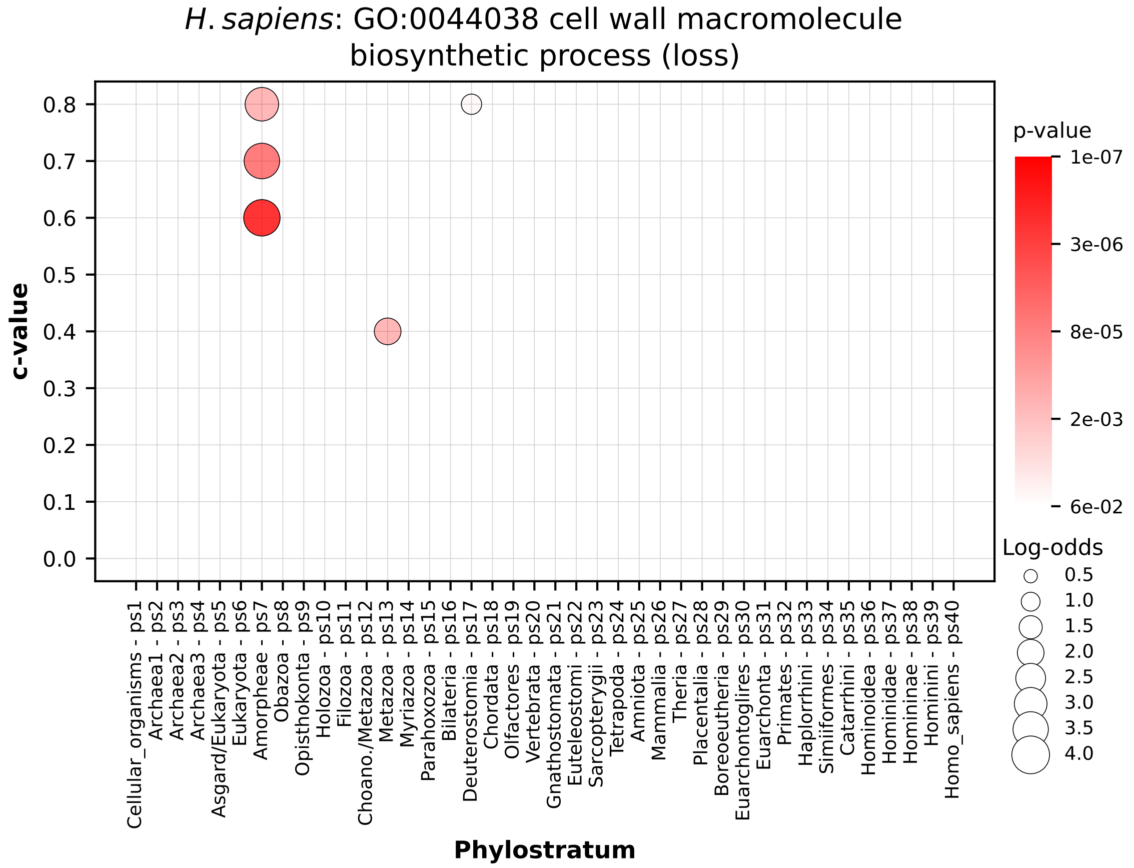

H. sapiens: GO:0044038 cell wall macromolecule biosynthetic process (loss)
c-value
0.8
0.7
0.6
0.5
0.4
0.3
0.2
0.1
0.0
p-value
1e-07
3e-06
8e-05
2e-03
6e-02
Log-odds
0.5
1.0
1.5
2.0
2.5
3.0
3.5
4.0
Cellular_organisms - ps1
Archaea1 - ps2
Archaea2 - ps3
Archaea3 - ps4
Asgard/Eukaryota - ps5
Eukaryota - ps6
Amorpheae - ps7
Obazoa - ps8
Opisthokonta - ps9
Holozoa - ps10
Filozoa - ps11
Choano./Metazoa - ps12
Metazoa - ps13
Myriazoa - ps14
Parahoxozoa - ps15
Bilateria - ps16
Deuterostomia - ps17
Chordata - ps18
Olfactores - ps19
Vertebrata - ps20
Gnathostomata - ps21
Euteleostomi - ps22
Sarcopterygii - ps23
Tetrapoda - ps24
Amniota - ps25
Mammalia - ps26
Theria - ps27
Placentalia - ps28
Boreoeutheria - ps29
Euarchontoglires - ps30
Euarchonta - ps31
Primates - ps32
Haplorrhini - ps33
Simiiformes - ps34
Catarrhini - ps35
Hominoidea - ps36
Hominidae - ps37
Homininae - ps38
Hominini - ps39
Homo_sapiens - ps40
Phylostratum

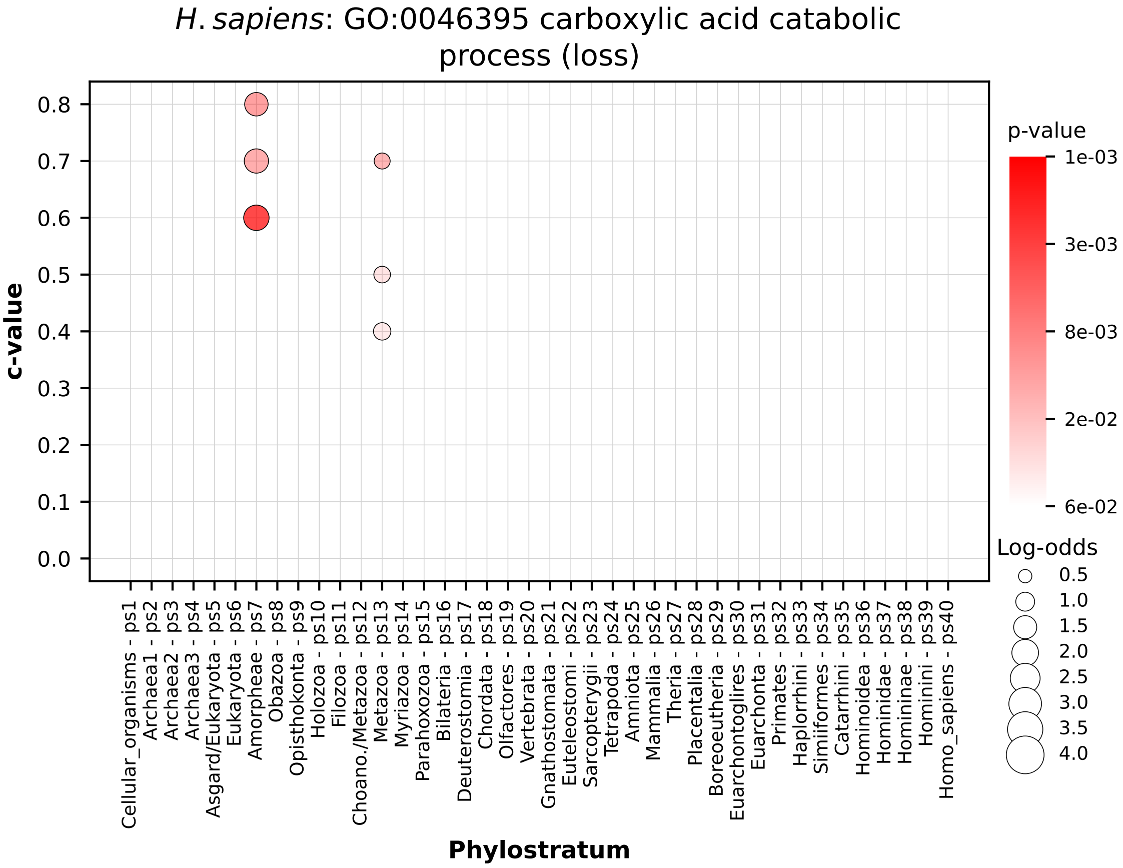
H. sapiens: GO:0046395 carboxylic acid catabolic process (loss)
c-value
0.0
0.1
0.2
0.3
0.4
0.5
0.6
0.7
0.8
Phylostratum
Cellular_organisms - ps1
Archaea1 - ps2
Archaea2 - ps3
Archaea3 - ps4
Asgard/Eukaryota - ps5
Eukaryota - ps6
Amorpheae - ps7
Obazoa - ps8
Opisthokonta - ps9
Holozoa - ps10
Filozoa - ps11
Choano./Metazoa - ps12
Metazoa - ps13
Myriazoa - ps14
Parahoxozoa - ps15
Bilateria - ps16
Deuterostomia - ps17
Chordata - ps18
Olfactores - ps19
Vertebrata - ps20
Gnathostomata - ps21
Euteleostomi - ps22
Sarcopterygii - ps23
Tetrapoda - ps24
Amniota - ps25
Mammalia - ps26
Theria - ps27
Placentalia - ps28
Boreoeutheria - ps29
Euarchontoglires - ps30
Euarchonta - ps31
Primates - ps32
Haplorrhini - ps33
Simiiformes - ps34
Catarrhini - ps35
Hominoidea - ps36
Hominidae - ps37
Homininae - ps38
Hominini - ps39
Homo_sapiens - ps40
p-value
1e-03
3e-03
8e-03
2e-02
6e-02
Log-odds
0.5
1.0
1.5
2.0
2.5
3.0
3.5
4.0

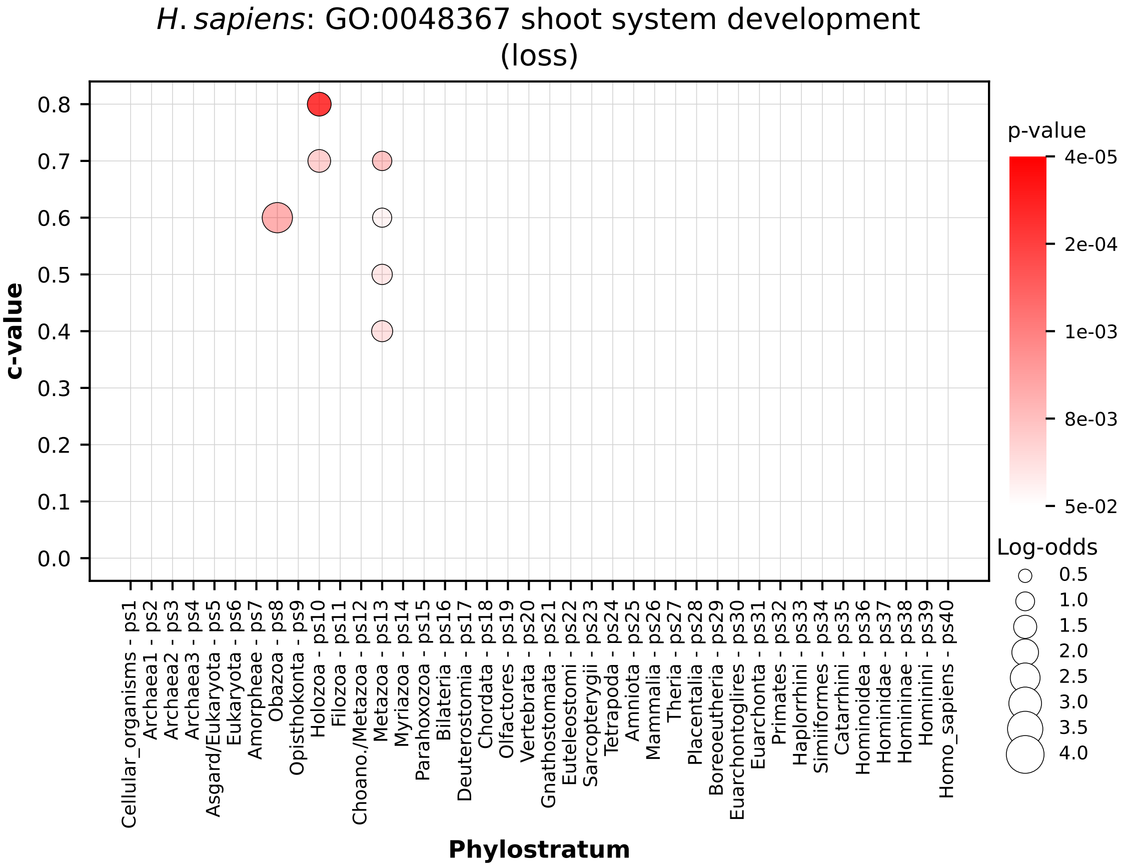

H. sapiens: GO:0048367 shoot system development (loss)
c-value
0.8
0.7
0.6
0.5
0.4
0.3
0.2
0.1
0.0
Phylostratum
Cellular_organisms - ps1
Archaea1 - ps2
Archaea2 - ps3
Archaea3 - ps4
Asgard/Eukaryota - ps5
Eukaryota - ps6
Amorpheae - ps7
Obazoa - ps8
Opisthokonta - ps9
Holozoa - ps10
Filozoa - ps11
Choano./Metazoa - ps12
Metazoa - ps13
Myriazoa - ps14
Parahoxozoa - ps15
Bilateria - ps16
Deuterostomia - ps17
Chordata - ps18
Olfactores - ps19
Vertebrata - ps20
Gnathostomata - ps21
Euteleostomi - ps22
Sarcopterygii - ps23
Tetrapoda - ps24
Amniota - ps25
Mammalia - ps26
Theria - ps27
Placentalia - ps28
Boreoeutheria - ps29
Euarchontoglires - ps30
Euarchonta - ps31
Primates - ps32
Haplorrhini - ps33
Simiiformes - ps34
Catarrhini - ps35
Hominoidea - ps36
Hominidae - ps37
Homininae - ps38
Hominini - ps39
Homo_sapiens - ps40
p-value
4e-05
2e-04
1e-03
8e-03
5e-02
Log-odds
0.5
1.0
1.5
2.0
2.5
3.0
3.5
4.0

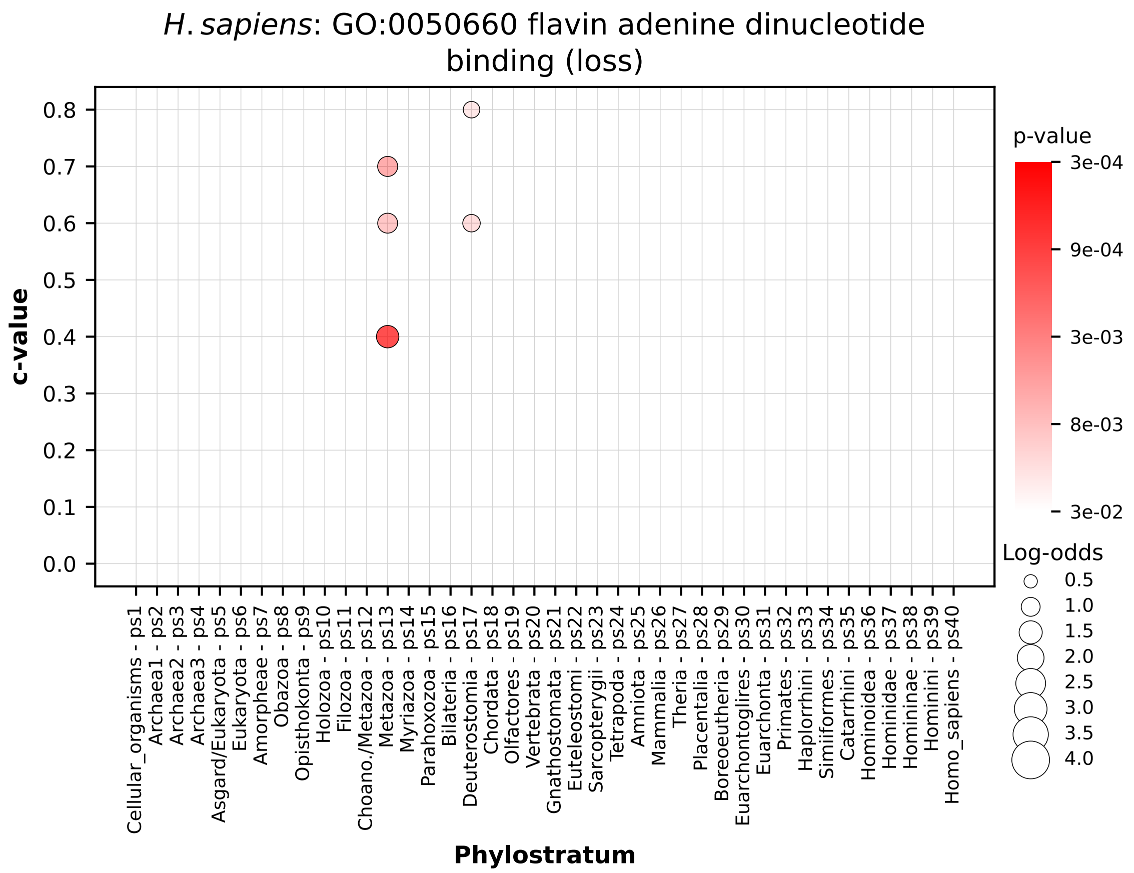

H. sapiens: GO:0050660 flavin adenine dinucleotide binding (loss)
c-value
0.0
0.1
0.2
0.3
0.4
0.5
0.6
0.7
0.8
p-value
3e-04
9e-04
3e-03
8e-03
3e-02
Log-odds
0.5
1.0
1.5
2.0
2.5
3.0
3.5
4.0
Cellular_organisms - ps1
Archaea1 - ps2
Archaea2 - ps3
Archaea3 - ps4
Asgard/Eukaryota - ps5
Eukaryota - ps6
Amorpheae - ps7
Obazoa - ps8
Opisthokonta - ps9
Holozoa - ps10
Filozoa - ps11
Choano./Metazoa - ps12
Metazoa - ps13
Myriazoa - ps14
Parahoxozoa - ps15
Bilateria - ps16
Deuterostomia - ps17
Chordata - ps18
Olfactores - ps19
Vertebrata - ps20
Gnathostomata - ps21
Euteleostomi - ps22
Sarcopterygii - ps23
Tetrapoda - ps24
Amniota - ps25
Mammalia - ps26
Theria - ps27
Placentalia - ps28
Boreoeutheria - ps29
Euarchontoglires - ps30
Euarchonta - ps31
Primates - ps32
Haplorrhini - ps33
Simiiformes - ps34
Catarrhini - ps35
Hominoidea - ps36
Hominidae - ps37
Homininae - ps38
Hominini - ps39
Homo_sapiens - ps40
Phylostratum

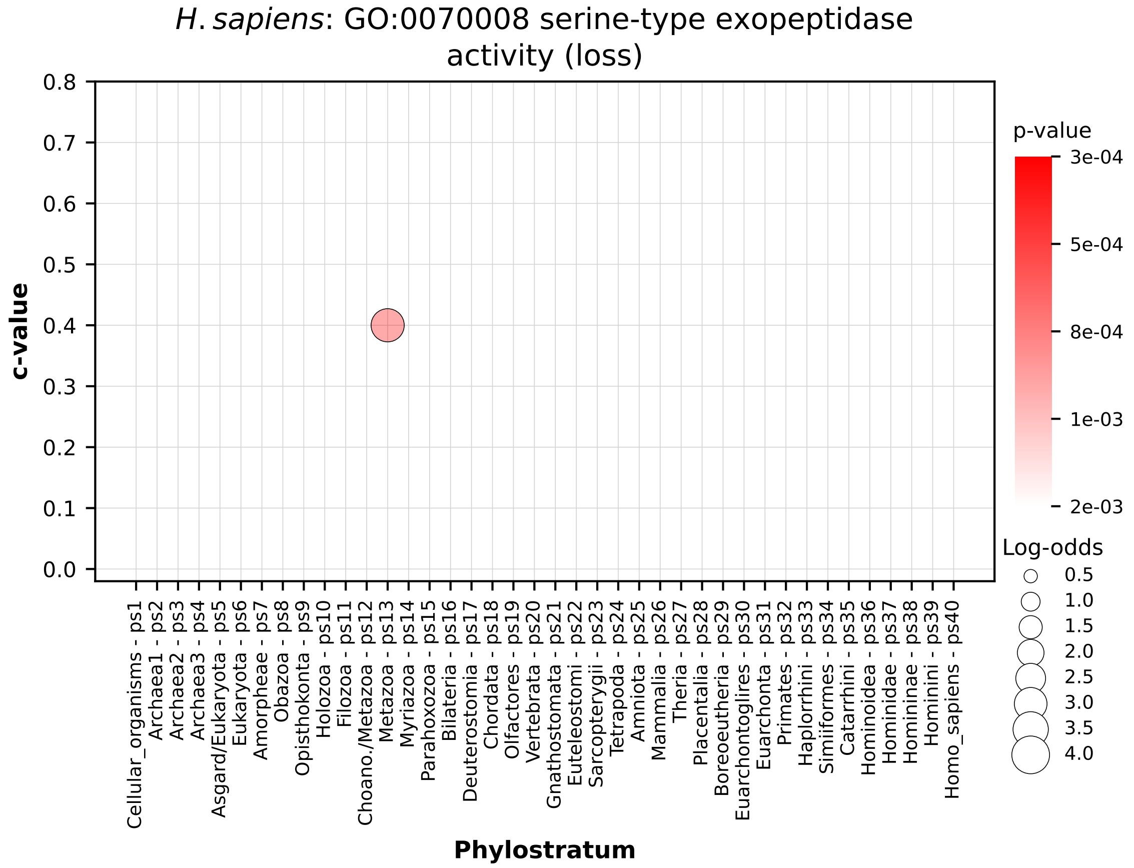

H. sapiens: GO:0070008 serine-type exopeptidase activity (loss)
c-value
0.0
0.1
0.2
0.3
0.4
0.5
0.6
0.7
0.8
Phylostratum
Cellular_organisms - ps1
Archaea1 - ps2
Archaea2 - ps3
Archaea3 - ps4
Asgard/Eukaryota - ps5
Eukaryota - ps6
Amorpheae - ps7
Obazoa - ps8
Opisthokonta - ps9
Holozoa - ps10
Filozoa - ps11
Choano./Metazoa - ps12
Metazoa - ps13
Myriazoa - ps14
Parahoxozoa - ps15
Bilateria - ps16
Deuterostomia - ps17
Chordata - ps18
Olfactores - ps19
Vertebrata - ps20
Gnathostomata - ps21
Euteleostomi - ps22
Sarcopterygii - ps23
Tetrapoda - ps24
Amniota - ps25
Mammalia - ps26
Theria - ps27
Placentalia - ps28
Boreoeutheria - ps29
Euarchontoglires - ps30
Euarchonta - ps31
Primates - ps32
Haplorrhini - ps33
Simiiformes - ps34
Catarrhini - ps35
Hominoidea - ps36
Hominidae - ps37
Homininae - ps38
Hominini - ps39
Homo_sapiens - ps40
p-value
3e-04
5e-04
8e-04
1e-03
2e-03
Log-odds
0.5
1.0
1.5
2.0
2.5
3.0
3.5
4.0

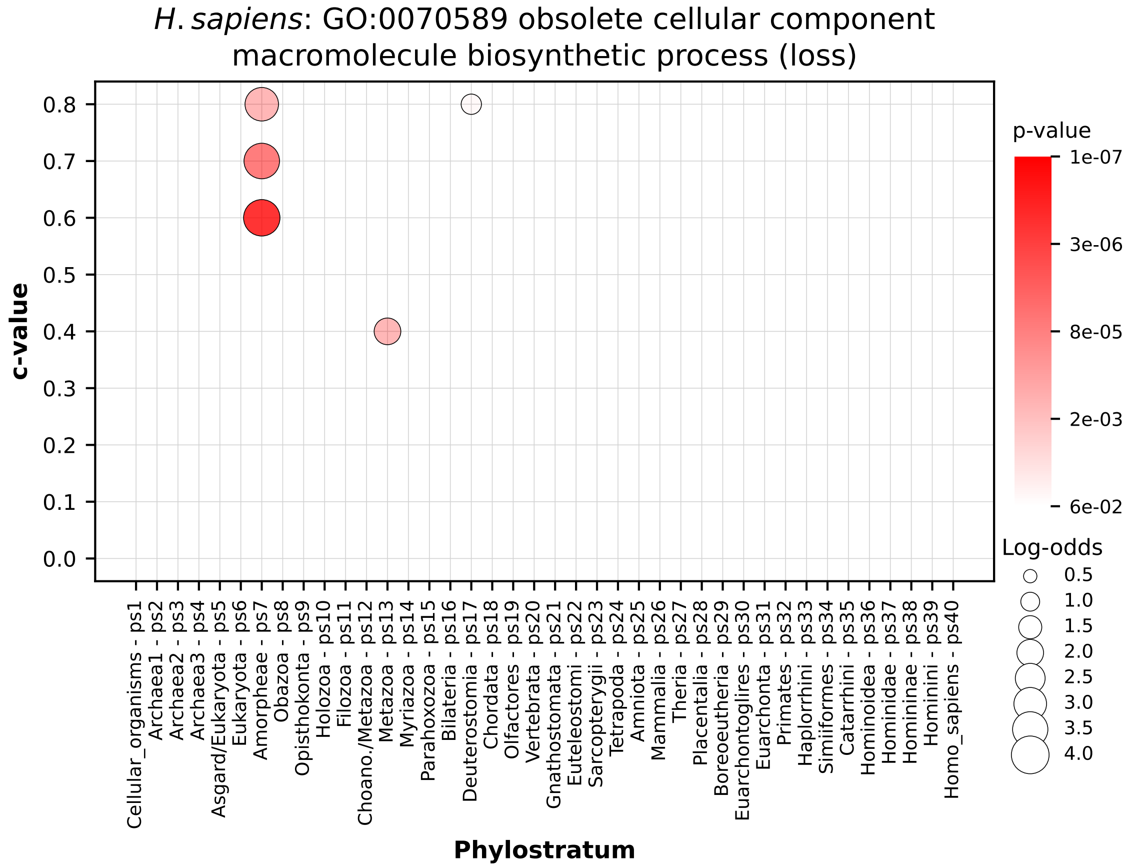

H. sapiens: GO:0070589 obsolete cellular component macromolecule biosynthetic process (loss)
c-value
0.0
0.1
0.2
0.3
0.4
0.5
0.6
0.7
0.8
p-value
1e-07
3e-06
8e-05
2e-03
6e-02
Log-odds
0.5
1.0
1.5
2.0
2.5
3.0
3.5
4.0
Cellular_organisms - ps1
Archaea1 - ps2
Archaea2 - ps3
Archaea3 - ps4
Asgard/Eukaryota - ps5
Eukaryota - ps6
Amorpheae - ps7
Obazoa - ps8
Opisthokonta - ps9
Holozoa - ps10
Filozoa - ps11
Choano./Metazoa - ps12
Metazoa - ps13
Myriazoa - ps14
Parahoxozoa - ps15
Bilateria - ps16
Deuterostomia - ps17
Chordata - ps18
Olfactores - ps19
Vertebrata - ps20
Gnathostomata - ps21
Euteleostomi - ps22
Sarcopterygii - ps23
Tetrapoda - ps24
Amniota - ps25
Mammalia - ps26
Theria - ps27
Placentalia - ps28
Boreoeutheria - ps29
Euarchontoglires - ps30
Euarchonta - ps31
Primates - ps32
Haplorrhini - ps33
Simiiformes - ps34
Catarrhini - ps35
Hominoidea - ps36
Hominidae - ps37
Homininae - ps38
Hominini - ps39
Homo_sapiens - ps40
Phylostratum

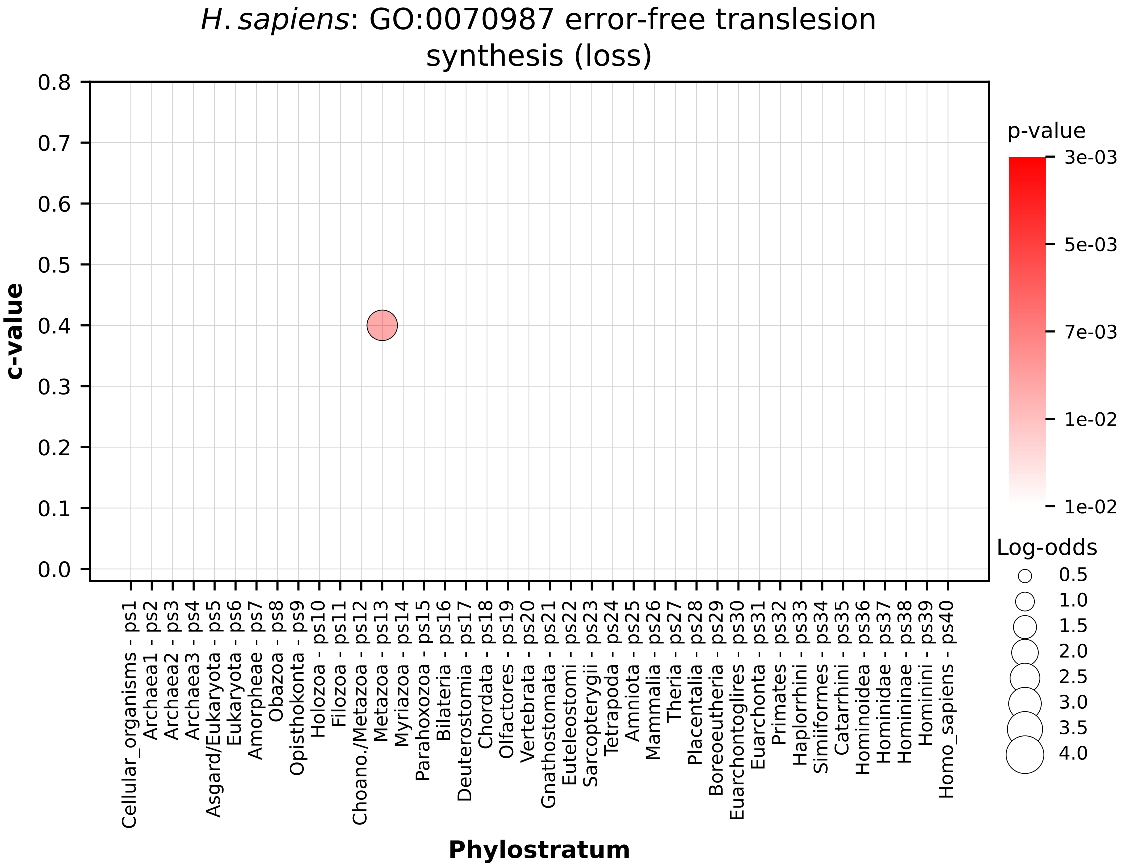

H. sapiens: GO:0070987 error-free translesion synthesis (loss)
c-value
0.8
0.7
0.6
0.5
0.4
0.3
0.2
0.1
0.0
p-value
3e-03
5e-03
7e-03
1e-02
1e-02
Log-odds
0.5
1.0
1.5
2.0
2.5
3.0
3.5
4.0
Cellular_organisms - ps1
Archaea1 - ps2
Archaea2 - ps3
Archaea3 - ps4
Asgard/Eukaryota - ps5
Eukaryota - ps6
Amorpheae - ps7
Obazoa - ps8
Opisthokonta - ps9
Holozoa - ps10
Filozoa - ps11
Choano./Metazoa - ps12
Metazoa - ps13
Myriazoa - ps14
Parahoxozoa - ps15
Bilateria - ps16
Deuterostomia - ps17
Chordata - ps18
Olfactores - ps19
Vertebrata - ps20
Gnathostomata - ps21
Euteleostomi - ps22
Sarcopterygii - ps23
Tetrapoda - ps24
Amniota - ps25
Mammalia - ps26
Theria - ps27
Placentalia - ps28
Boreoeutheria - ps29
Euarchontoglires - ps30
Euarchonta - ps31
Primates - ps32
Haplorrhini - ps33
Simiiformes - ps34
Catarrhini - ps35
Hominoidea - ps36
Hominidae - ps37
Homininae - ps38
Hominini - ps39
Homo_sapiens - ps40
Phylostratum

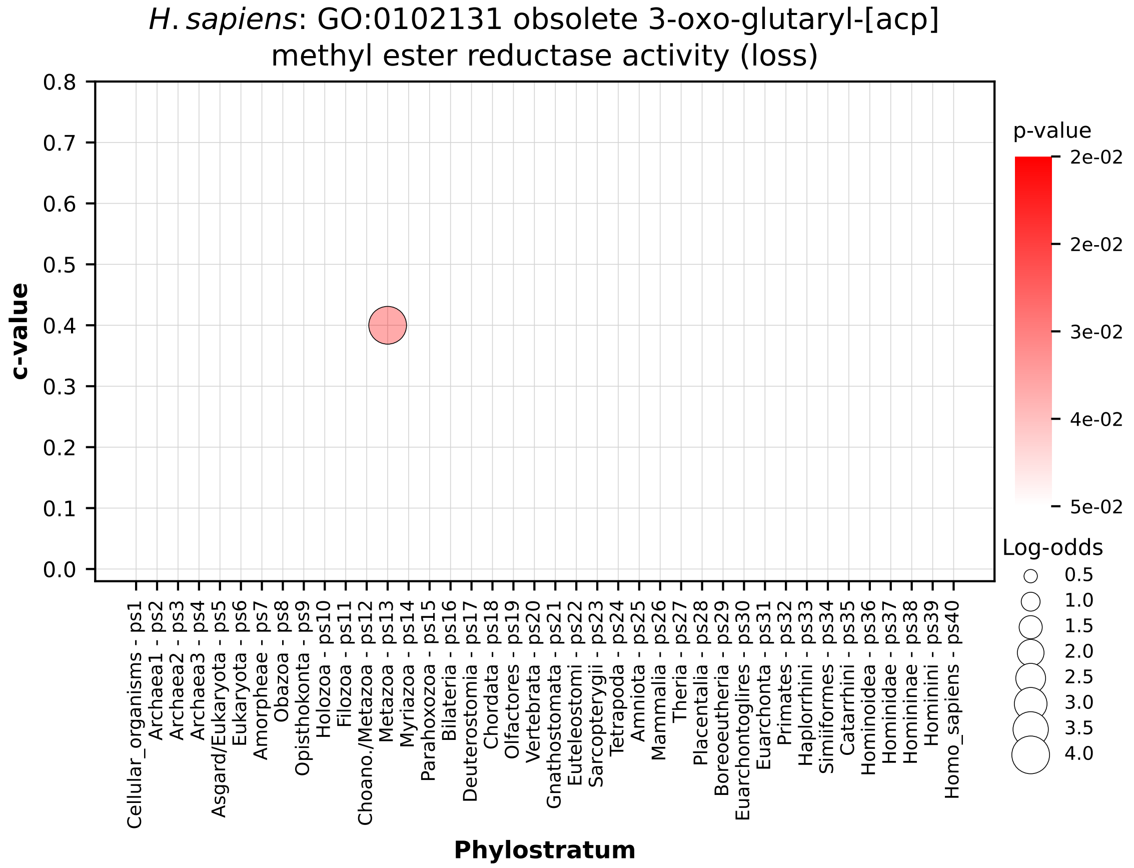

H. sapiens: GO:0102131 obsolete 3-oxo-glutaryl-[acp] methyl ester reductase activity (loss)
c-value
0.0
0.1
0.2
0.3
0.4
0.5
0.6
0.7
0.8
p-value
2e-02
2e-02
3e-02
4e-02
5e-02
Log-odds
0.5
1.0
1.5
2.0
2.5
3.0
3.5
4.0
Cellular_organisms - ps1
Archaea1 - ps2
Archaea2 - ps3
Archaea3 - ps4
Asgard/Eukaryota - ps5
Eukaryota - ps6
Amorpheae - ps7
Obazoa - ps8
Opisthokonta - ps9
Holozoa - ps10
Filozoa - ps11
Choano./Metazoa - ps12
Metazoa - ps13
Myriazoa - ps14
Parahoxozoa - ps15
Bilateria - ps16
Deuterostomia - ps17
Chordata - ps18
Olfactores - ps19
Vertebrata - ps20
Gnathostomata - ps21
Euteleostomi - ps22
Sarcopterygii - ps23
Tetrapoda - ps24
Amniota - ps25
Mammalia - ps26
Theria - ps27
Placentalia - ps28
Boreoeutheria - ps29
Euarchontoglires - ps30
Euarchonta - ps31
Primates - ps32
Haplorrhini - ps33
Simiiformes - ps34
Catarrhini - ps35
Hominoidea - ps36
Hominidae - ps37
Homininae - ps38
Hominini - ps39
Homo_sapiens - ps40
Phylostratum

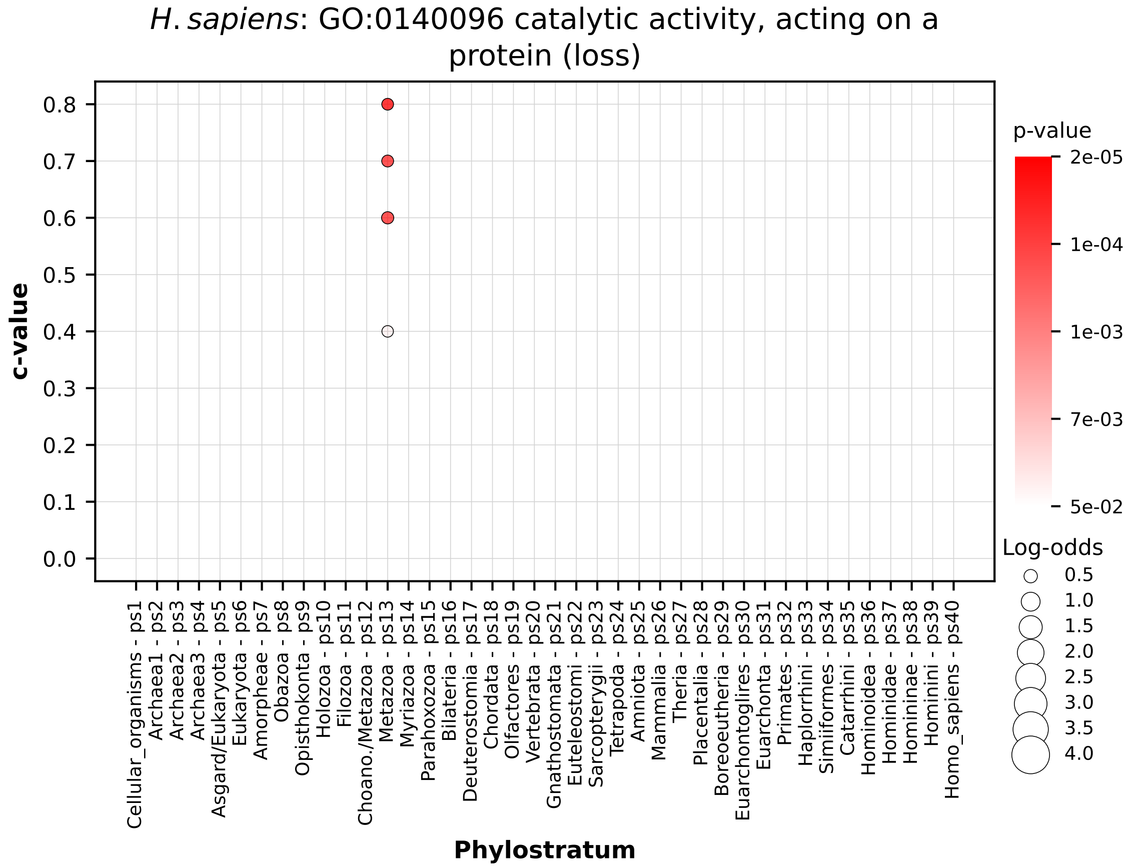

H. sapiens: GO:0140096 catalytic activity, acting on a protein (loss)
c-value
0.0
0.1
0.2
0.3
0.4
0.5
0.6
0.7
0.8
Phylostratum
Cellular_organisms - ps1
Archaea1 - ps2
Archaea2 - ps3
Archaea3 - ps4
Asgard/Eukaryota - ps5
Eukaryota - ps6
Amorpheae - ps7
Obazoa - ps8
Opisthokonta - ps9
Holozoa - ps10
Filozoa - ps11
Choano./Metazoa - ps12
Metazoa - ps13
Myriazoa - ps14
Parahoxozoa - ps15
Bilateria - ps16
Deuterostomia - ps17
Chordata - ps18
Olfactores - ps19
Vertebrata - ps20
Gnathostomata - ps21
Euteleostomi - ps22
Sarcopterygii - ps23
Tetrapoda - ps24
Amniota - ps25
Mammalia - ps26
Theria - ps27
Placentalia - ps28
Boreoeutheria - ps29
Euarchontoglires - ps30
Euarchonta - ps31
Primates - ps32
Haplorrhini - ps33
Simiiformes - ps34
Catarrhini - ps35
Hominoidea - ps36
Hominidae - ps37
Homininae - ps38
Hominini - ps39
Homo_sapiens - ps40
p-value
2e-05
1e-04
1e-03
7e-03
5e-02
Log-odds
0.5
1.0
1.5
2.0
2.5
3.0
3.5
4.0

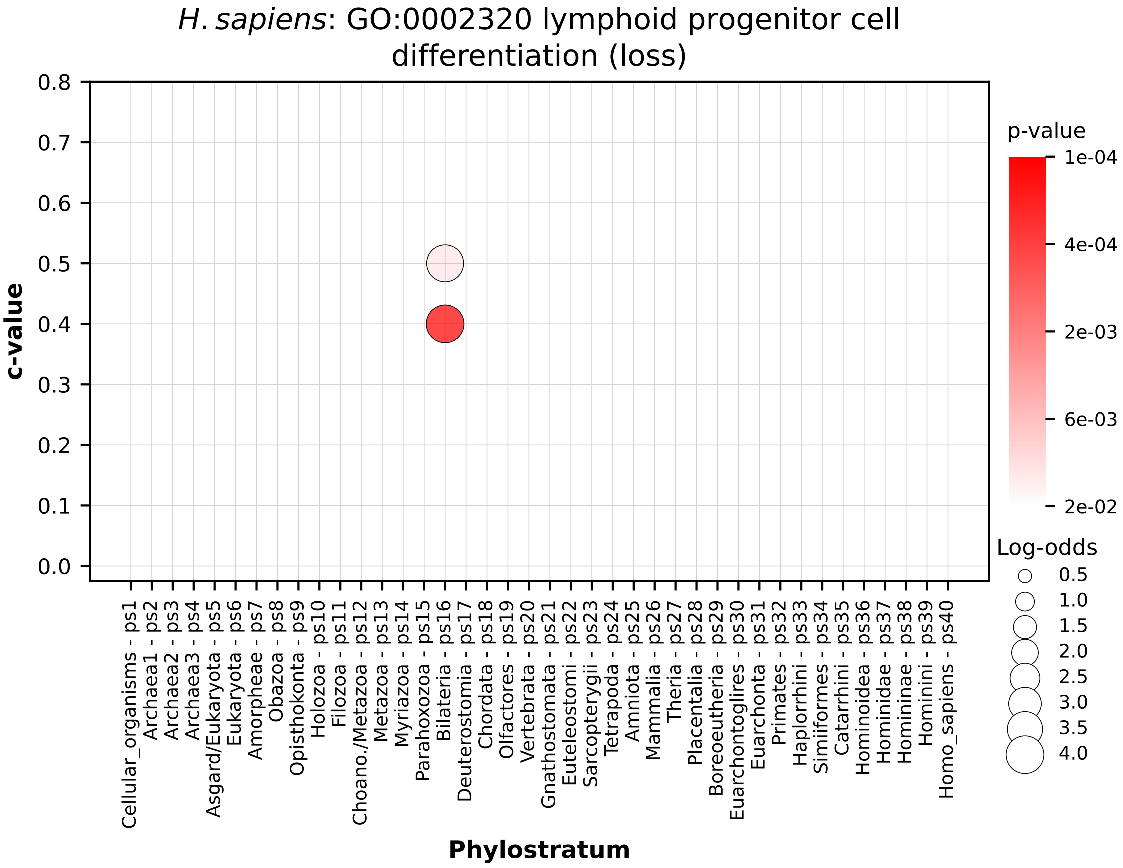

H. sapiens: GO:0002320 lymphoid progenitor cell differentiation (loss)
c-value
0.0
0.1
0.2
0.3
0.4
0.5
0.6
0.7
0.8
Phylostratum
Cellular_organisms - ps1
Archaea1 - ps2
Archaea2 - ps3
Archaea3 - ps4
Asgard/Eukaryota - ps5
Eukaryota - ps6
Amorpheae - ps7
Obazoa - ps8
Opisthokonta - ps9
Holozoa - ps10
Filozoa - ps11
Choano./Metazoa - ps12
Metazoa - ps13
Myriazoa - ps14
Parahoxozoa - ps15
Bilateria - ps16
Deuterostomia - ps17
Chordata - ps18
Olfactores - ps19
Vertebrata - ps20
Gnathostomata - ps21
Euteleostomi - ps22
Sarcopterygii - ps23
Tetrapoda - ps24
Amniota - ps25
Mammalia - ps26
Theria - ps27
Placentalia - ps28
Boreoeutheria - ps29
Euarchontoglires - ps30
Euarchonta - ps31
Primates - ps32
Haplorrhini - ps33
Simiiformes - ps34
Catarrhini - ps35
Hominoidea - ps36
Hominidae - ps37
Homininae - ps38
Hominini - ps39
Homo_sapiens - ps40
p-value
1e-04
4e-04
2e-03
6e-03
2e-02
Log-odds
0.5
1.0
1.5
2.0
2.5
3.0
3.5
4.0

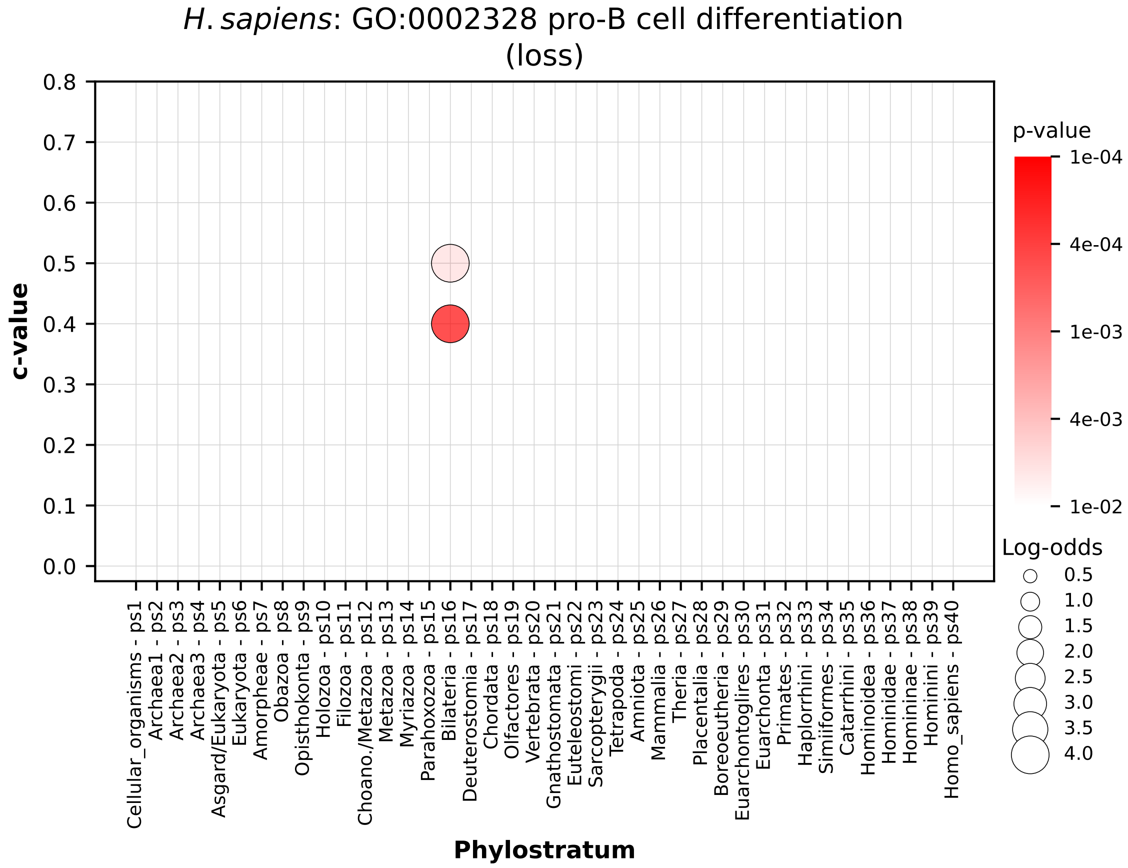

H. sapiens: GO:0002328 pro-B cell differentiation (loss)
c-value
0.0
0.1
0.2
0.3
0.4
0.5
0.6
0.7
0.8
Phylostratum
Cellular_organisms - ps1
Archaea1 - ps2
Archaea2 - ps3
Archaea3 - ps4
Asgard/Eukaryota - ps5
Eukaryota - ps6
Amorpheae - ps7
Obazoa - ps8
Opisthokonta - ps9
Holozoa - ps10
Filozoa - ps11
Choano./Metazoa - ps12
Metazoa - ps13
Myriazoa - ps14
Parahoxozoa - ps15
Bilateria - ps16
Deuterostomia - ps17
Chordata - ps18
Olfactores - ps19
Vertebrata - ps20
Gnathostomata - ps21
Euteleostomi - ps22
Sarcopterygii - ps23
Tetrapoda - ps24
Amniota - ps25
Mammalia - ps26
Theria - ps27
Placentalia - ps28
Boreoeutheria - ps29
Euarchontoglires - ps30
Euarchonta - ps31
Primates - ps32
Haplorrhini - ps33
Simiiformes - ps34
Catarrhini - ps35
Hominoidea - ps36
Hominidae - ps37
Homininae - ps38
Hominini - ps39
Homo_sapiens - ps40
p-value
1e-04
4e-04
1e-03
4e-03
1e-02
Log-odds
0.5
1.0
1.5
2.0
2.5
3.0
3.5
4.0

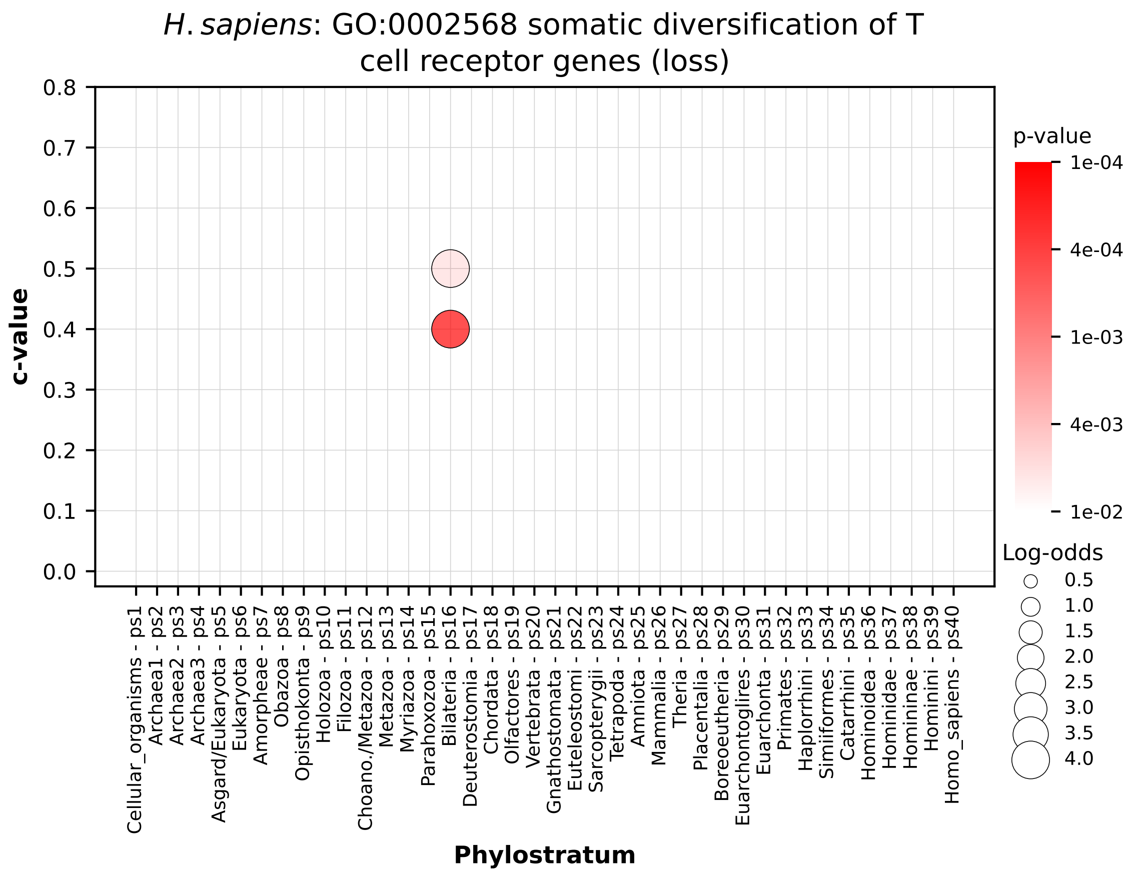

H. sapiens: GO:0002568 somatic diversification of T cell receptor genes (loss)
c-value
0.8
0.7
0.6
0.5
0.4
0.3
0.2
0.1
0.0
p-value
1e-04
4e-04
1e-03
4e-03
1e-02
Log-odds
0.5
1.0
1.5
2.0
2.5
3.0
3.5
4.0
Cellular_organisms - ps1
Archaea1 - ps2
Archaea2 - ps3
Archaea3 - ps4
Asgard/Eukaryota - ps5
Eukaryota - ps6
Amorpheae - ps7
Obazoa - ps8
Opisthokonta - ps9
Holozoa - ps10
Filozoa - ps11
Choano./Metazoa - ps12
Metazoa - ps13
Myriazoa - ps14
Parahoxozoa - ps15
Bilateria - ps16
Deuterostomia - ps17
Chordata - ps18
Olfactores - ps19
Vertebrata - ps20
Gnathostomata - ps21
Euteleostomi - ps22
Sarcopterygii - ps23
Tetrapoda - ps24
Amniota - ps25
Mammalia - ps26
Theria - ps27
Placentalia - ps28
Boreoeutheria - ps29
Euarchontoglires - ps30
Euarchonta - ps31
Primates - ps32
Haplorrhini - ps33
Simiiformes - ps34
Catarrhini - ps35
Hominoidea - ps36
Hominidae - ps37
Homininae - ps38
Hominini - ps39
Homo_sapiens - ps40
Phylostratum

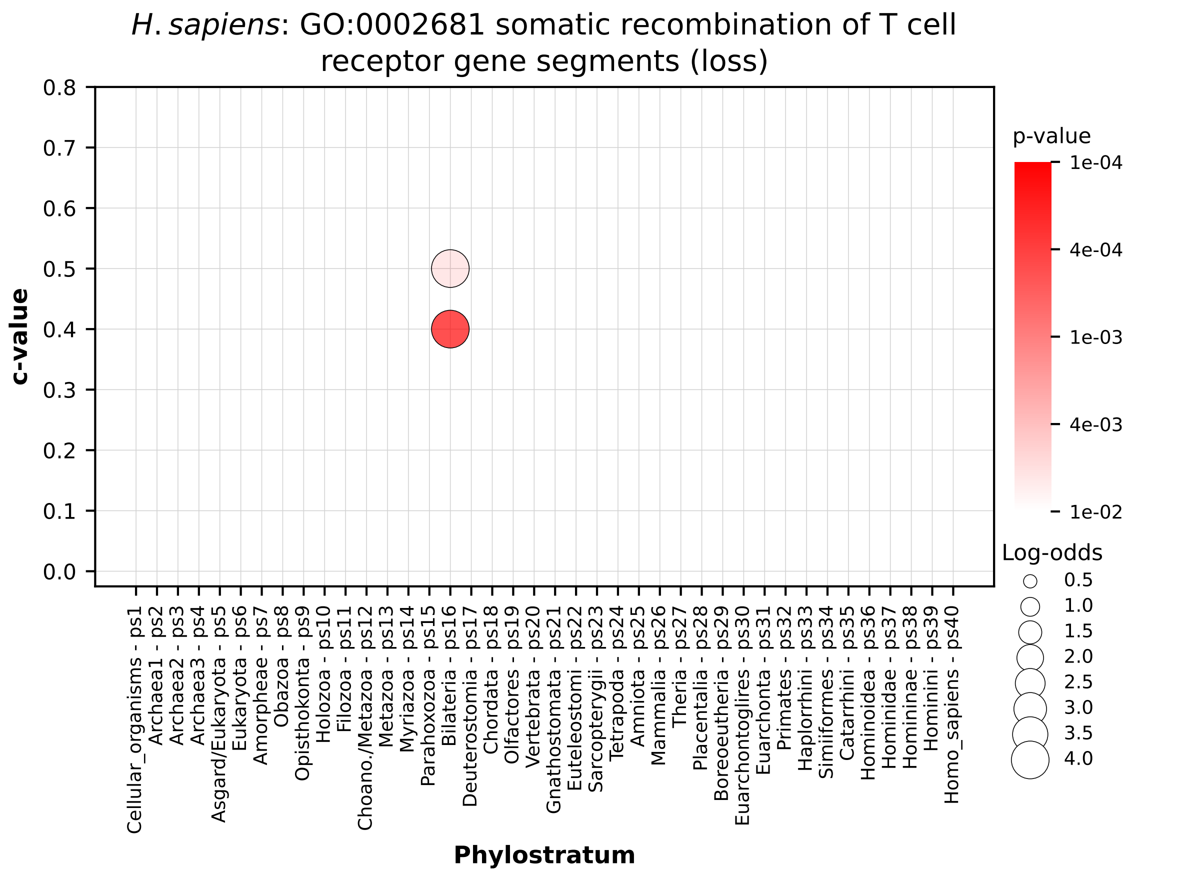

H. sapiens: GO:0002681 somatic recombination of T cell receptor gene segments (loss)
c-value
0.8
0.7
0.6
0.5
0.4
0.3
0.2
0.1
0.0
Phylostratum
Cellular_organisms - ps1
Archaea1 - ps2
Archaea2 - ps3
Archaea3 - ps4
Asgard/Eukaryota - ps5
Eukaryota - ps6
Amorpheae - ps7
Obazoa - ps8
Opisthokonta - ps9
Holozoa - ps10
Filozoa - ps11
Choano./Metazoa - ps12
Metazoa - ps13
Myriazoa - ps14
Parahoxozoa - ps15
Bilateria - ps16
Deuterostomia - ps17
Chordata - ps18
Olfactores - ps19
Vertebrata - ps20
Gnathostomata - ps21
Euteleostomi - ps22
Sarcopterygii - ps23
Tetrapoda - ps24
Amniota - ps25
Mammalia - ps26
Theria - ps27
Placentalia - ps28
Boreoeutheria - ps29
Euarchontoglires - ps30
Euarchonta - ps31
Primates - ps32
Haplorrhini - ps33
Simiiformes - ps34
Catarrhini - ps35
Hominoidea - ps36
Hominidae - ps37
Homininae - ps38
Hominini - ps39
Homo_sapiens - ps40
p-value
1e-04
4e-04
1e-03
4e-03
1e-02
Log-odds
0.5
1.0
1.5
2.0
2.5
3.0
3.5
4.0

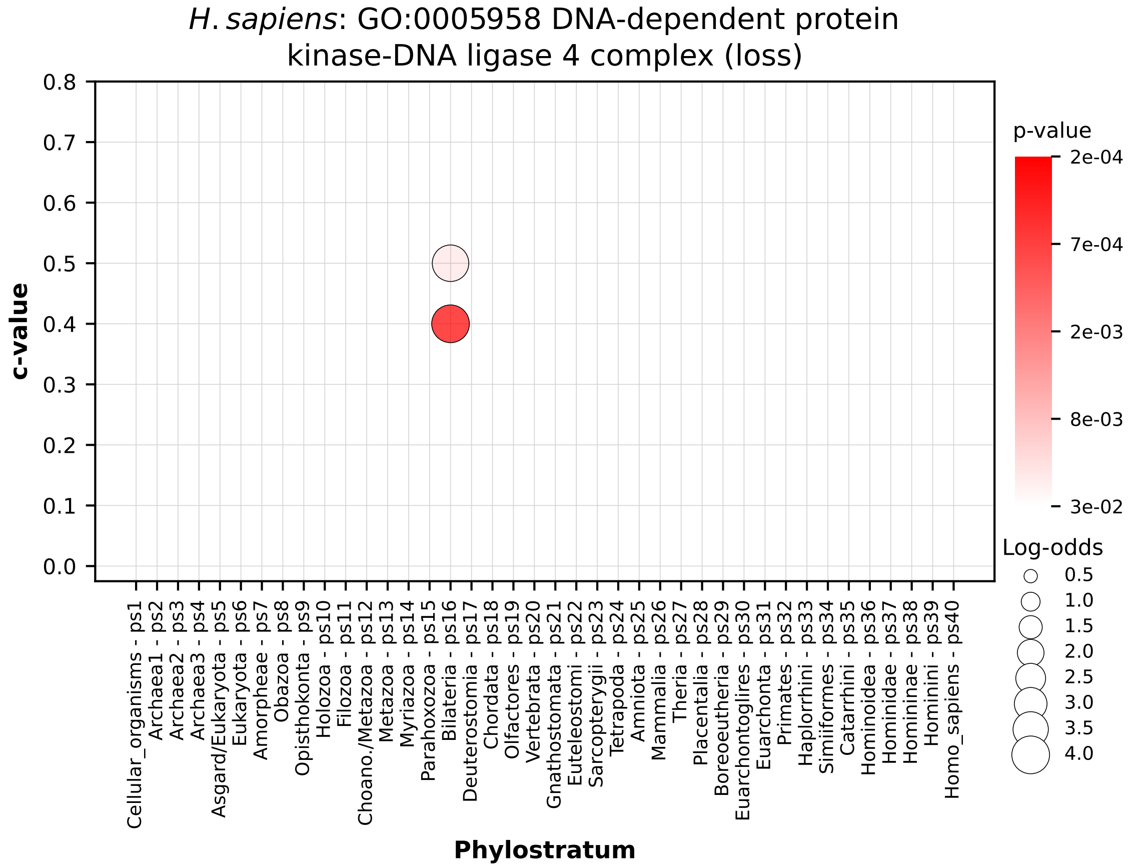

H. sapiens: GO:0005958 DNA-dependent protein kinase-DNA ligase 4 complex (loss)
c-value
0.8
0.7
0.6
0.5
0.4
0.3
0.2
0.1
0.0
Phylostratum
Cellular_organisms - ps1
Archaea1 - ps2
Archaea2 - ps3
Archaea3 - ps4
Asgard/Eukaryota - ps5
Eukaryota - ps6
Amorpheae - ps7
Obazoa - ps8
Opisthokonta - ps9
Holozoa - ps10
Filozoa - ps11
Choano./Metazoa - ps12
Metazoa - ps13
Myriazoa - ps14
Parahoxozoa - ps15
Bilateria - ps16
Deuterostomia - ps17
Chordata - ps18
Olfactores - ps19
Vertebrata - ps20
Gnathostomata - ps21
Euteleostomi - ps22
Sarcopterygii - ps23
Tetrapoda - ps24
Amniota - ps25
Mammalia - ps26
Theria - ps27
Placentalia - ps28
Boreoeutheria - ps29
Euarchontoglires - ps30
Euarchonta - ps31
Primates - ps32
Haplorrhini - ps33
Simiiformes - ps34
Catarrhini - ps35
Hominoidea - ps36
Hominidae - ps37
Homininae - ps38
Hominini - ps39
Homo_sapiens - ps40
p-value
2e-04
7e-04
2e-03
8e-03
3e-02
Log-odds
0.5
1.0
1.5
2.0
2.5
3.0
3.5
4.0

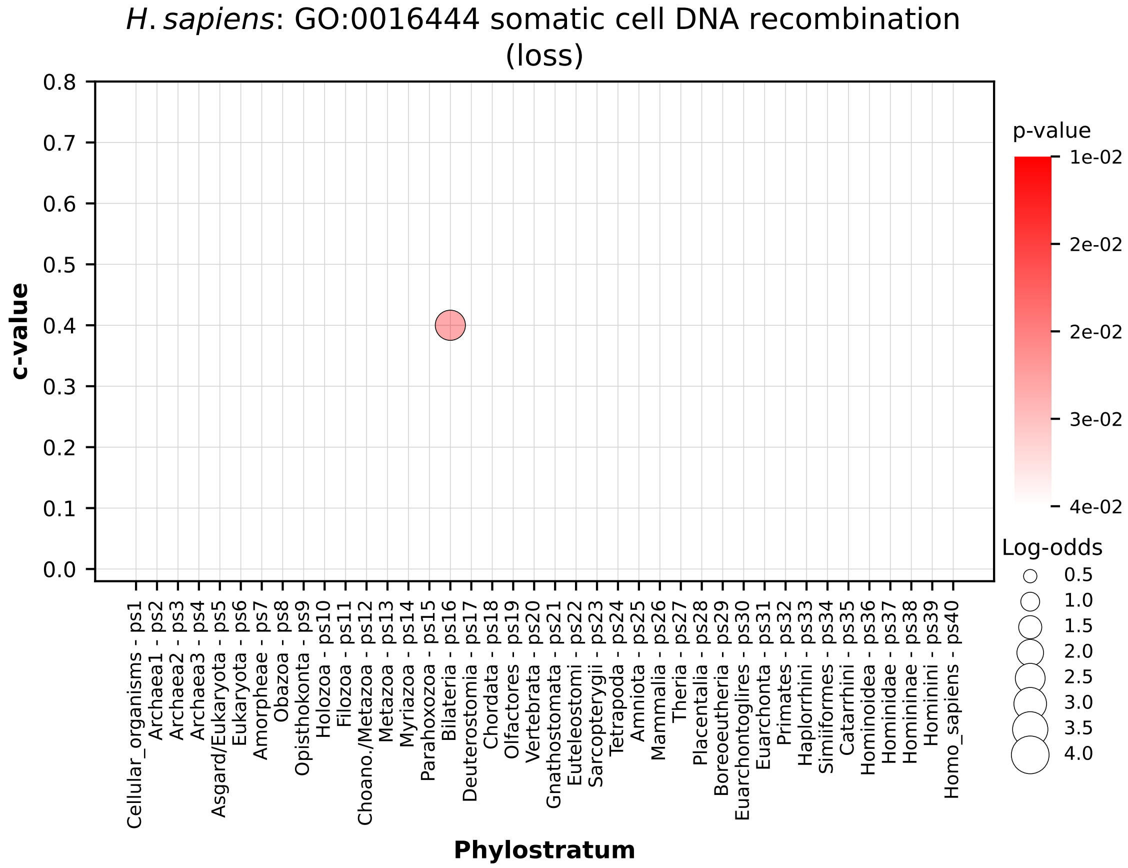

H. sapiens: GO:0016444 somatic cell DNA recombination (loss)
c-value
0.8
0.7
0.6
0.5
0.4
0.3
0.2
0.1
0.0
Phylostratum
Cellular_organisms - ps1
Archaea1 - ps2
Archaea2 - ps3
Archaea3 - ps4
Asgard/Eukaryota - ps5
Eukaryota - ps6
Amorpheae - ps7
Obazoa - ps8
Opisthokonta - ps9
Holozoa - ps10
Filozoa - ps11
Choano./Metazoa - ps12
Metazoa - ps13
Myriazoa - ps14
Parahoxozoa - ps15
Bilateria - ps16
Deuterostomia - ps17
Chordata - ps18
Olfactores - ps19
Vertebrata - ps20
Gnathostomata - ps21
Euteleostomi - ps22
Sarcopterygii - ps23
Tetrapoda - ps24
Amniota - ps25
Mammalia - ps26
Theria - ps27
Placentalia - ps28
Boreoeutheria - ps29
Euarchontoglires - ps30
Euarchonta - ps31
Primates - ps32
Haplorrhini - ps33
Simiiformes - ps34
Catarrhini - ps35
Hominoidea - ps36
Hominidae - ps37
Homininae - ps38
Hominini - ps39
Homo_sapiens - ps40
p-value
1e-02
2e-02
2e-02
3e-02
4e-02
Log-odds
0.5
1.0
1.5
2.0
2.5
3.0
3.5
4.0

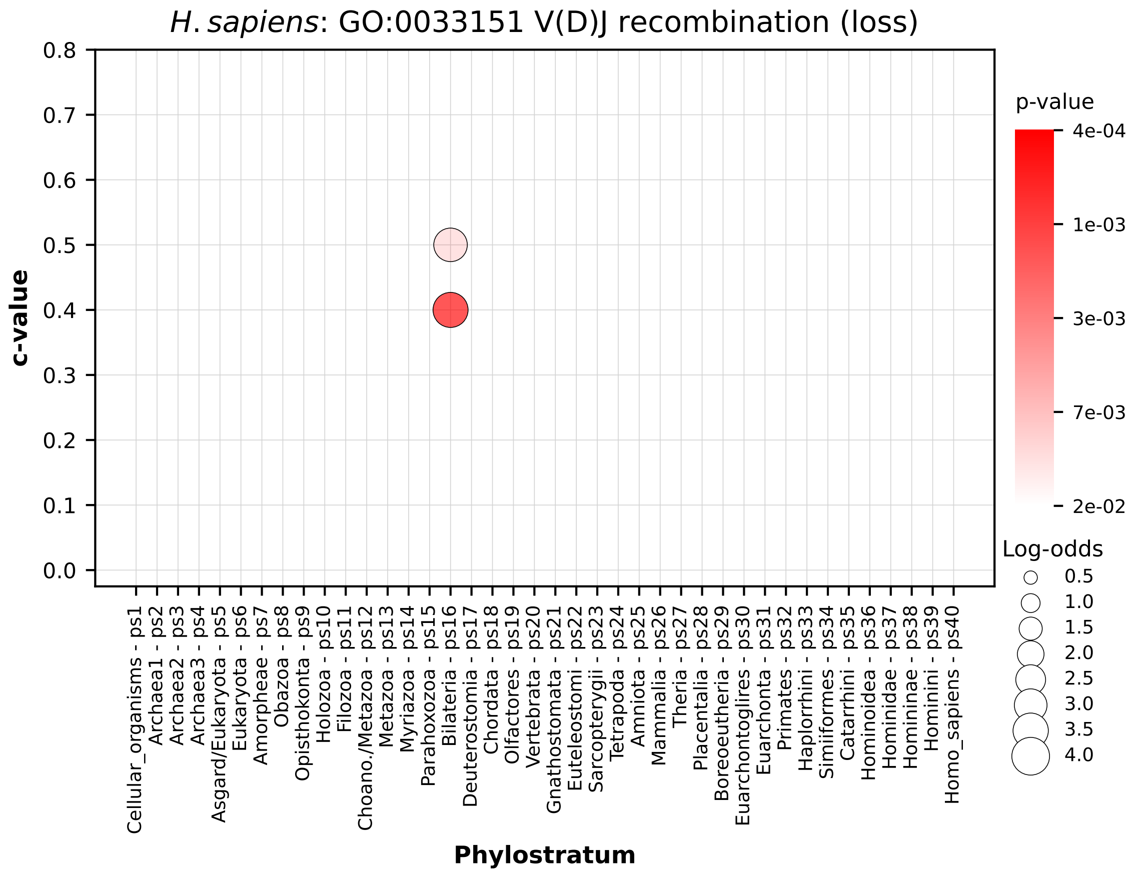
H. sapiens: GO:0033151 V(D)J recombination (loss)
c-value
0.0
0.1
0.2
0.3
0.4
0.5
0.6
0.7
0.8
Phylostratum
Cellular_organisms - ps1
Archaea1 - ps2
Archaea2 - ps3
Archaea3 - ps4
Asgard/Eukaryota - ps5
Eukaryota - ps6
Amorpheae - ps7
Obazoa - ps8
Opisthokonta - ps9
Holozoa - ps10
Filozoa - ps11
Choano./Metazoa - ps12
Metazoa - ps13
Myriazoa - ps14
Parahoxozoa - ps15
Bilateria - ps16
Deuterostomia - ps17
Chordata - ps18
Olfactores - ps19
Vertebrata - ps20
Gnathostomata - ps21
Euteleostomi - ps22
Sarcopterygii - ps23
Tetrapoda - ps24
Amniota - ps25
Mammalia - ps26
Theria - ps27
Placentalia - ps28
Boreoeutheria - ps29
Euarchontoglires - ps30
Euarchonta - ps31
Primates - ps32
Haplorrhini - ps33
Simiiformes - ps34
Catarrhini - ps35
Hominoidea - ps36
Hominidae - ps37
Homininae - ps38
Hominini - ps39
Homo_sapiens - ps40
p-value
4e-04
1e-03
3e-03
7e-03
2e-02
Log-odds
0.5
1.0
1.5
2.0
2.5
3.0
3.5
4.0

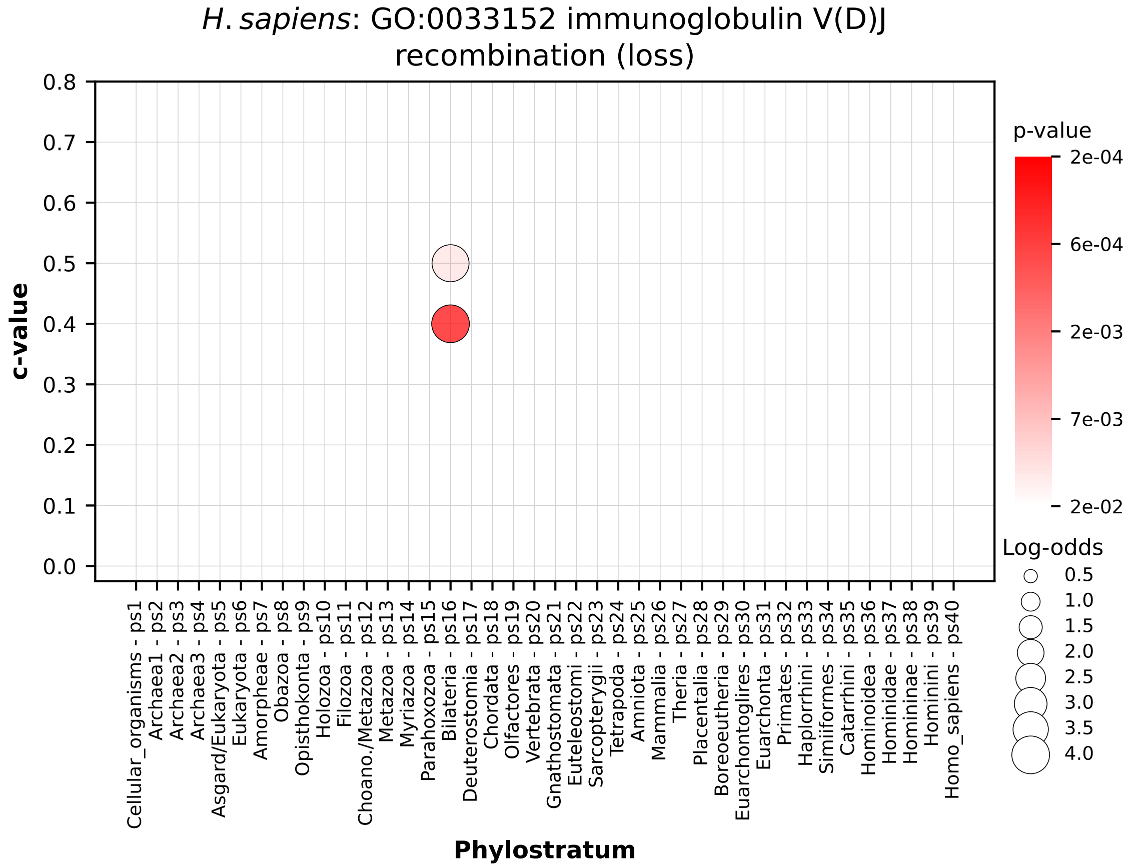

H. sapiens: GO:0033152 immunoglobulin V(D)J recombination (loss)
c-value
0.0
0.1
0.2
0.3
0.4
0.5
0.6
0.7
0.8
Phylostratum
Cellular_organisms - ps1
Archaea1 - ps2
Archaea2 - ps3
Archaea3 - ps4
Asgard/Eukaryota - ps5
Eukaryota - ps6
Amorpheae - ps7
Obazoa - ps8
Opisthokonta - ps9
Holozoa - ps10
Filozoa - ps11
Choano./Metazoa - ps12
Metazoa - ps13
Myriazoa - ps14
Parahoxozoa - ps15
Bilateria - ps16
Deuterostomia - ps17
Chordata - ps18
Olfactores - ps19
Vertebrata - ps20
Gnathostomata - ps21
Euteleostomi - ps22
Sarcopterygii - ps23
Tetrapoda - ps24
Amniota - ps25
Mammalia - ps26
Theria - ps27
Placentalia - ps28
Boreoeutheria - ps29
Euarchontoglires - ps30
Euarchonta - ps31
Primates - ps32
Haplorrhini - ps33
Simiiformes - ps34
Catarrhini - ps35
Hominoidea - ps36
Hominidae - ps37
Homininae - ps38
Hominini - ps39
Homo_sapiens - ps40
p-value
2e-04
6e-04
2e-03
7e-03
2e-02
Log-odds
0.5
1.0
1.5
2.0
2.5
3.0
3.5
4.0

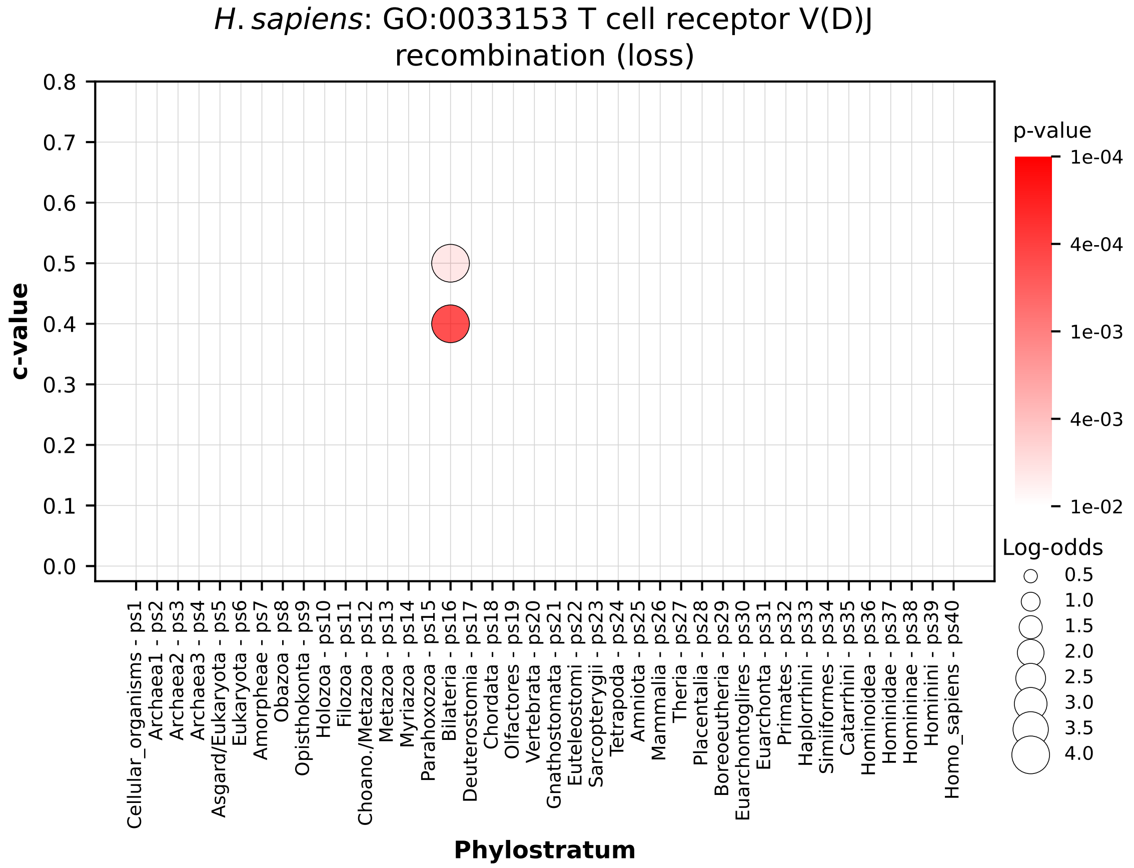

H. sapiens: GO:0033153 T cell receptor V(D)J recombination (loss)
c-value
0.0
0.1
0.2
0.3
0.4
0.5
0.6
0.7
0.8
Phylostratum
Cellular_organisms - ps1
Archaea1 - ps2
Archaea2 - ps3
Archaea3 - ps4
Asgard/Eukaryota - ps5
Eukaryota - ps6
Amorpheae - ps7
Obazoa - ps8
Opisthokonta - ps9
Holozoa - ps10
Filozoa - ps11
Choano./Metazoa - ps12
Metazoa - ps13
Myriazoa - ps14
Parahoxozoa - ps15
Bilateria - ps16
Deuterostomia - ps17
Chordata - ps18
Olfactores - ps19
Vertebrata - ps20
Gnathostomata - ps21
Euteleostomi - ps22
Sarcopterygii - ps23
Tetrapoda - ps24
Amniota - ps25
Mammalia - ps26
Theria - ps27
Placentalia - ps28
Boreoeutheria - ps29
Euarchontoglires - ps30
Euarchonta - ps31
Primates - ps32
Haplorrhini - ps33
Simiiformes - ps34
Catarrhini - ps35
Hominoidea - ps36
Hominidae - ps37
Homininae - ps38
Hominini - ps39
Homo_sapiens - ps40
p-value
1e-04
4e-04
1e-03
4e-03
1e-02
Log-odds
0.5
1.0
1.5
2.0
2.5
3.0
3.5
4.0

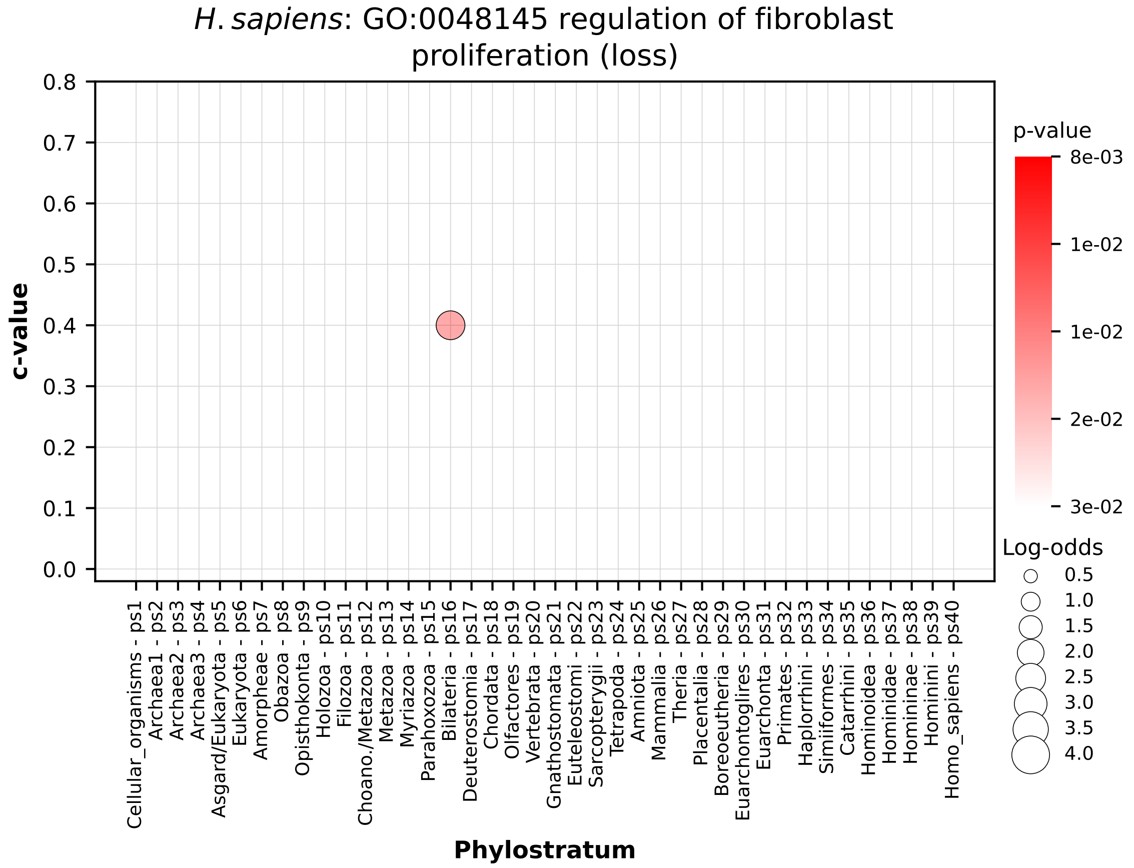

H. sapiens: GO:0048145 regulation of fibroblast proliferation (loss)
c-value
0.8
0.7
0.6
0.5
0.4
0.3
0.2
0.1
0.0
Phylostratum
Cellular_organisms - ps1
Archaea1 - ps2
Archaea2 - ps3
Archaea3 - ps4
Asgard/Eukaryota - ps5
Eukaryota - ps6
Amorpheae - ps7
Obazoa - ps8
Opisthokonta - ps9
Holozoa - ps10
Filozoa - ps11
Choano./Metazoa - ps12
Metazoa - ps13
Myriazoa - ps14
Parahoxozoa - ps15
Bilateria - ps16
Deuterostomia - ps17
Chordata - ps18
Olfactores - ps19
Vertebrata - ps20
Gnathostomata - ps21
Euteleostomi - ps22
Sarcopterygii - ps23
Tetrapoda - ps24
Amniota - ps25
Mammalia - ps26
Theria - ps27
Placentalia - ps28
Boreoeutheria - ps29
Euarchontoglires - ps30
Euarchonta - ps31
Primates - ps32
Haplorrhini - ps33
Simiiformes - ps34
Catarrhini - ps35
Hominoidea - ps36
Hominidae - ps37
Homininae - ps38
Hominini - ps39
Homo_sapiens - ps40
p-value
8e-03
1e-02
1e-02
2e-02
3e-02
Log-odds
0.5
1.0
1.5
2.0
2.5
3.0
3.5
4.0

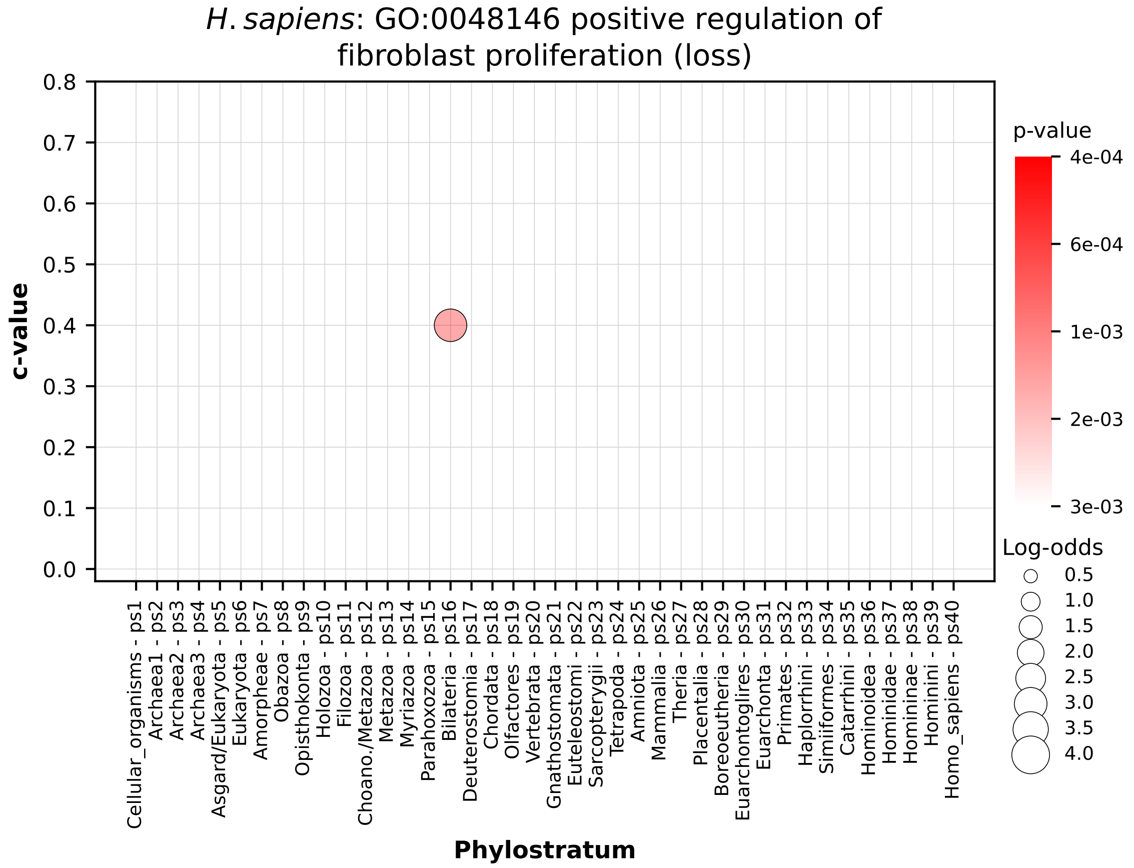

H. sapiens: GO:0048146 positive regulation of fibroblast proliferation (loss)
c-value
0.8
0.7
0.6
0.5
0.4
0.3
0.2
0.1
0.0
Phylostratum
Cellular_organisms - ps1
Archaea1 - ps2
Archaea2 - ps3
Archaea3 - ps4
Asgard/Eukaryota - ps5
Eukaryota - ps6
Amorpheae - ps7
Obazoa - ps8
Opisthokonta - ps9
Holozoa - ps10
Filozoa - ps11
Choano./Metazoa - ps12
Metazoa - ps13
Myriazoa - ps14
Parahoxozoa - ps15
Bilateria - ps16
Deuterostomia - ps17
Chordata - ps18
Olfactores - ps19
Vertebrata - ps20
Gnathostomata - ps21
Euteleostomi - ps22
Sarcopterygii - ps23
Tetrapoda - ps24
Amniota - ps25
Mammalia - ps26
Theria - ps27
Placentalia - ps28
Boreoeutheria - ps29
Euarchontoglires - ps30
Euarchonta - ps31
Primates - ps32
Haplorrhini - ps33
Simiiformes - ps34
Catarrhini - ps35
Hominoidea - ps36
Hominidae - ps37
Homininae - ps38
Hominini - ps39
Homo_sapiens - ps40
p-value
4e-04
6e-04
1e-03
2e-03
3e-03
Log-odds
0.5
1.0
1.5
2.0
2.5
3.0
3.5
4.0

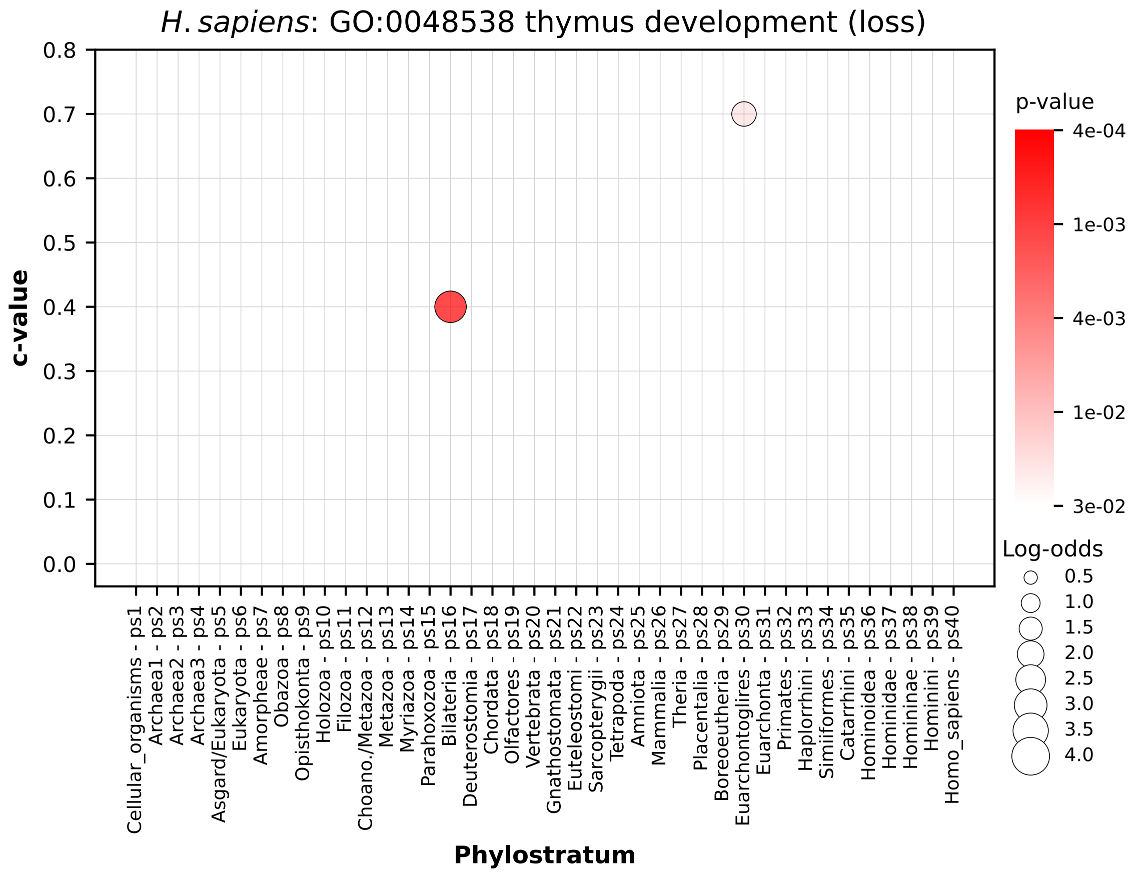

H. sapiens: GO:0048538 thymus development (loss)
c-value
0.8
0.7
0.6
0.5
0.4
0.3
0.2
0.1
0.0
p-value
4e-04
1e-03
4e-03
1e-02
3e-02
Log-odds
0.5
1.0
1.5
2.0
2.5
3.0
3.5
4.0
Cellular_organisms - ps1
Archaea1 - ps2
Archaea2 - ps3
Archaea3 - ps4
Asgard/Eukaryota - ps5
Eukaryota - ps6
Amorpheae - ps7
Obazoa - ps8
Opisthokonta - ps9
Holozoa - ps10
Filozoa - ps11
Choano./Metazoa - ps12
Metazoa - ps13
Myriazoa - ps14
Parahoxozoa - ps15
Bilateria - ps16
Deuterostomia - ps17
Chordata - ps18
Olfactores - ps19
Vertebrata - ps20
Gnathostomata - ps21
Euteleostomi - ps22
Sarcopterygii - ps23
Tetrapoda - ps24
Amniota - ps25
Mammalia - ps26
Theria - ps27
Placentalia - ps28
Boreoeutheria - ps29
Euarchontoglires - ps30
Euarchonta - ps31
Primates - ps32
Haplorrhini - ps33
Simiiformes - ps34
Catarrhini - ps35
Hominoidea - ps36
Hominidae - ps37
Homininae - ps38
Hominini - ps39
Homo_sapiens - ps40
Phylostratum

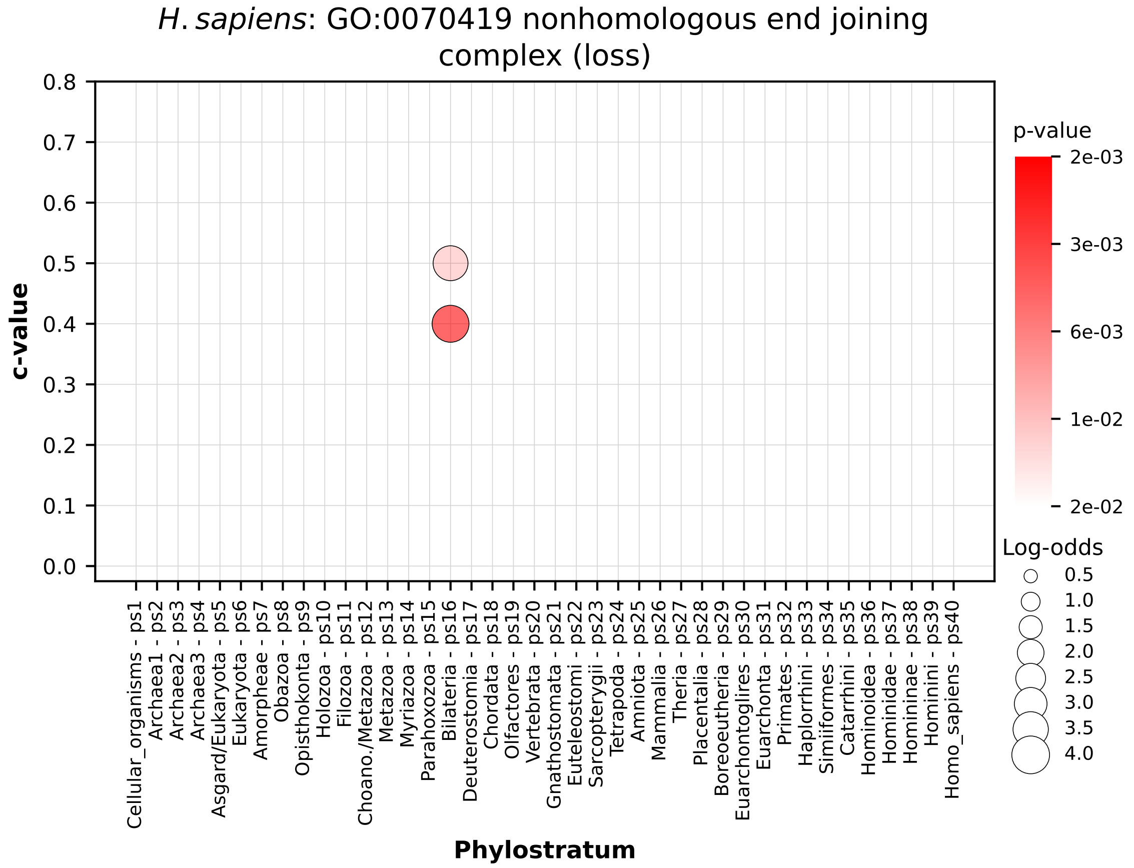

H. sapiens: GO:0070419 nonhomologous end joining complex (loss)
c-value
0.8
0.7
0.6
0.5
0.4
0.3
0.2
0.1
0.0
Phylostratum
Cellular_organisms - ps1
Archaea1 - ps2
Archaea2 - ps3
Archaea3 - ps4
Asgard/Eukaryota - ps5
Eukaryota - ps6
Amorpheae - ps7
Obazoa - ps8
Opisthokonta - ps9
Holozoa - ps10
Filozoa - ps11
Choano./Metazoa - ps12
Metazoa - ps13
Myriazoa - ps14
Parahoxozoa - ps15
Bilateria - ps16
Deuterostomia - ps17
Chordata - ps18
Olfactores - ps19
Vertebrata - ps20
Gnathostomata - ps21
Euteleostomi - ps22
Sarcopterygii - ps23
Tetrapoda - ps24
Amniota - ps25
Mammalia - ps26
Theria - ps27
Placentalia - ps28
Boreoeutheria - ps29
Euarchontoglires - ps30
Euarchonta - ps31
Primates - ps32
Haplorrhini - ps33
Simiiformes - ps34
Catarrhini - ps35
Hominoidea - ps36
Hominidae - ps37
Homininae - ps38
Hominini - ps39
Homo_sapiens - ps40
p-value
2e-03
3e-03
6e-03
1e-02
2e-02
Log-odds
0.5
1.0
1.5
2.0
2.5
3.0
3.5
4.0

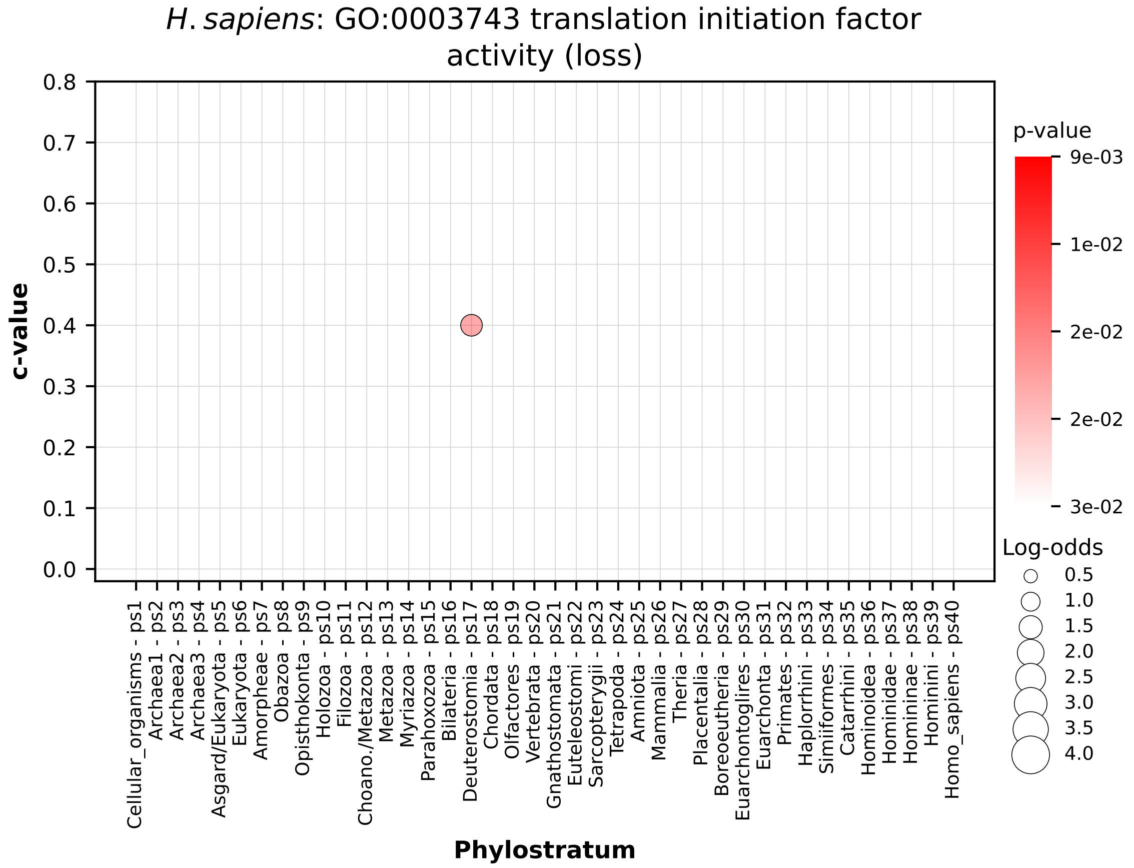
H. sapiens: GO:0003743 translation initiation factor activity (loss)
c-value
0.8
0.7
0.6
0.5
0.4
0.3
0.2
0.1
0.0
p-value
9e-03
1e-02
2e-02
2e-02
3e-02
Log-odds
0.5
1.0
1.5
2.0
2.5
3.0
3.5
4.0
Cellular_organisms - ps1
Archaea1 - ps2
Archaea2 - ps3
Archaea3 - ps4
Asgard/Eukaryota - ps5
Eukaryota - ps6
Amorpheae - ps7
Obazoa - ps8
Opisthokonta - ps9
Holozoa - ps10
Filozoa - ps11
Choano./Metazoa - ps12
Metazoa - ps13
Myriazoa - ps14
Parahoxozoa - ps15
Bilateria - ps16
Deuterostomia - ps17
Chordata - ps18
Olfactores - ps19
Vertebrata - ps20
Gnathostomata - ps21
Euteleostomi - ps22
Sarcopterygii - ps23
Tetrapoda - ps24
Amniota - ps25
Mammalia - ps26
Theria - ps27
Placentalia - ps28
Boreoeutheria - ps29
Euarchontoglires - ps30
Euarchonta - ps31
Primates - ps32
Haplorrhini - ps33
Simiiformes - ps34
Catarrhini - ps35
Hominoidea - ps36
Hominidae - ps37
Homininae - ps38
Hominini - ps39
Homo_sapiens - ps40
Phylostratum

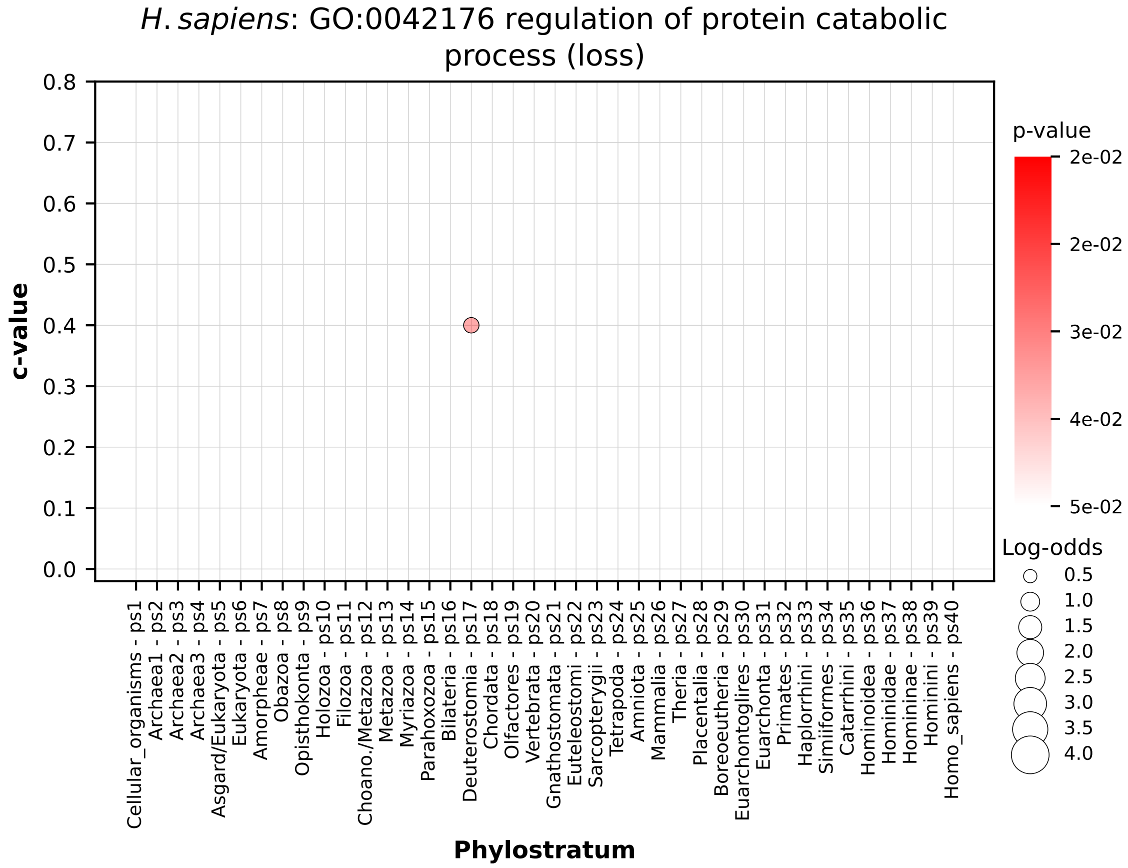

H. sapiens: GO:0042176 regulation of protein catabolic process (loss)
c-value
0.0
0.1
0.2
0.3
0.4
0.5
0.6
0.7
0.8
Phylostratum
Cellular_organisms - ps1
Archaea1 - ps2
Archaea2 - ps3
Archaea3 - ps4
Asgard/Eukaryota - ps5
Eukaryota - ps6
Amorpheae - ps7
Obazoa - ps8
Opisthokonta - ps9
Holozoa - ps10
Filozoa - ps11
Choano./Metazoa - ps12
Metazoa - ps13
Myriazoa - ps14
Parahoxozoa - ps15
Bilateria - ps16
Deuterostomia - ps17
Chordata - ps18
Olfactores - ps19
Vertebrata - ps20
Gnathostomata - ps21
Euteleostomi - ps22
Sarcopterygii - ps23
Tetrapoda - ps24
Amniota - ps25
Mammalia - ps26
Theria - ps27
Placentalia - ps28
Boreoeutheria - ps29
Euarchontoglires - ps30
Euarchonta - ps31
Primates - ps32
Haplorrhini - ps33
Simiiformes - ps34
Catarrhini - ps35
Hominoidea - ps36
Hominidae - ps37
Homininae - ps38
Hominini - ps39
Homo_sapiens - ps40
p-value
2e-02
2e-02
3e-02
4e-02
5e-02
Log-odds
0.5
1.0
1.5
2.0
2.5
3.0
3.5
4.0

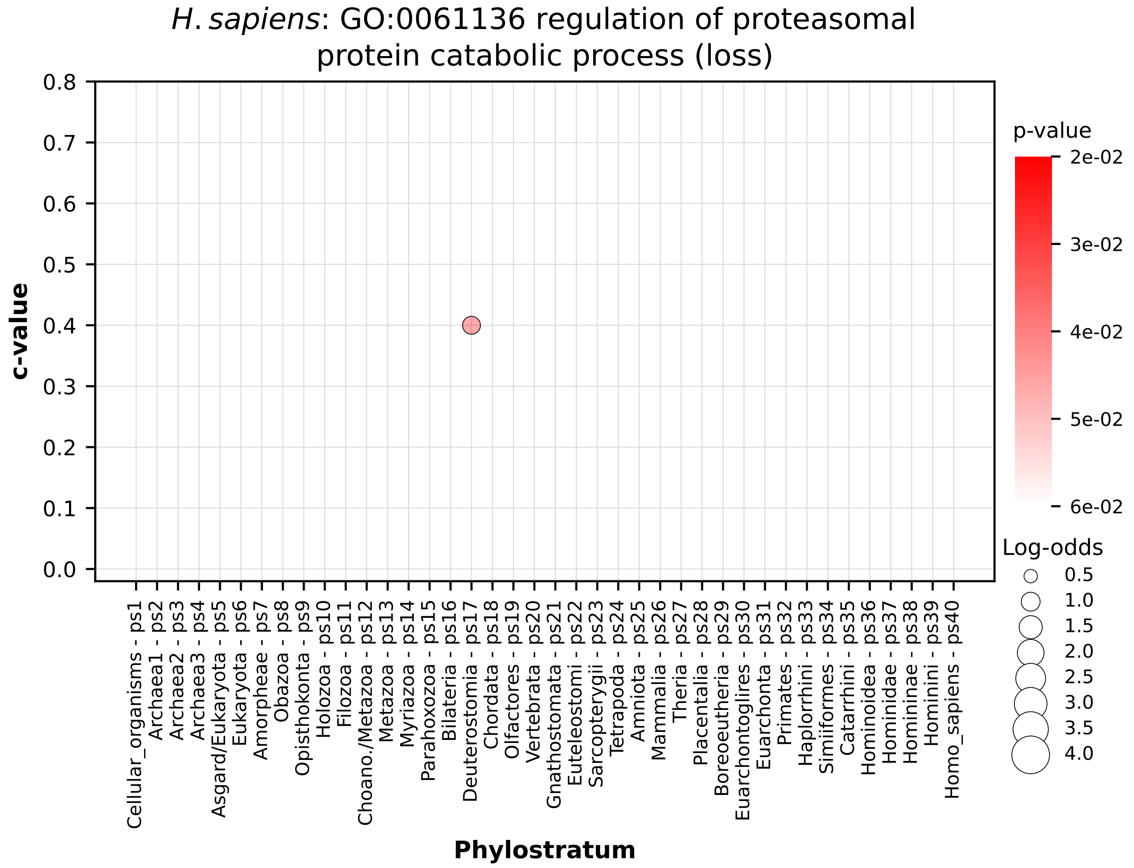

H. sapiens: GO:0061136 regulation of proteasomal protein catabolic process (loss)
c-value
0.0
0.1
0.2
0.3
0.4
0.5
0.6
0.7
0.8
Phylostratum
Cellular_organisms - ps1
Archaea1 - ps2
Archaea2 - ps3
Archaea3 - ps4
Asgard/Eukaryota - ps5
Eukaryota - ps6
Amorpheae - ps7
Obazoa - ps8
Opisthokonta - ps9
Holozoa - ps10
Filozoa - ps11
Choano./Metazoa - ps12
Metazoa - ps13
Myriazoa - ps14
Parahoxozoa - ps15
Bilateria - ps16
Deuterostomia - ps17
Chordata - ps18
Olfactores - ps19
Vertebrata - ps20
Gnathostomata - ps21
Euteleostomi - ps22
Sarcopterygii - ps23
Tetrapoda - ps24
Amniota - ps25
Mammalia - ps26
Theria - ps27
Placentalia - ps28
Boreoeutheria - ps29
Euarchontoglires - ps30
Euarchonta - ps31
Primates - ps32
Haplorrhini - ps33
Simiiformes - ps34
Catarrhini - ps35
Hominoidea - ps36
Hominidae - ps37
Homininae - ps38
Hominini - ps39
Homo_sapiens - ps40
p-value
2e-02
3e-02
4e-02
5e-02
6e-02
Log-odds
0.5
1.0
1.5
2.0
2.5
3.0
3.5
4.0

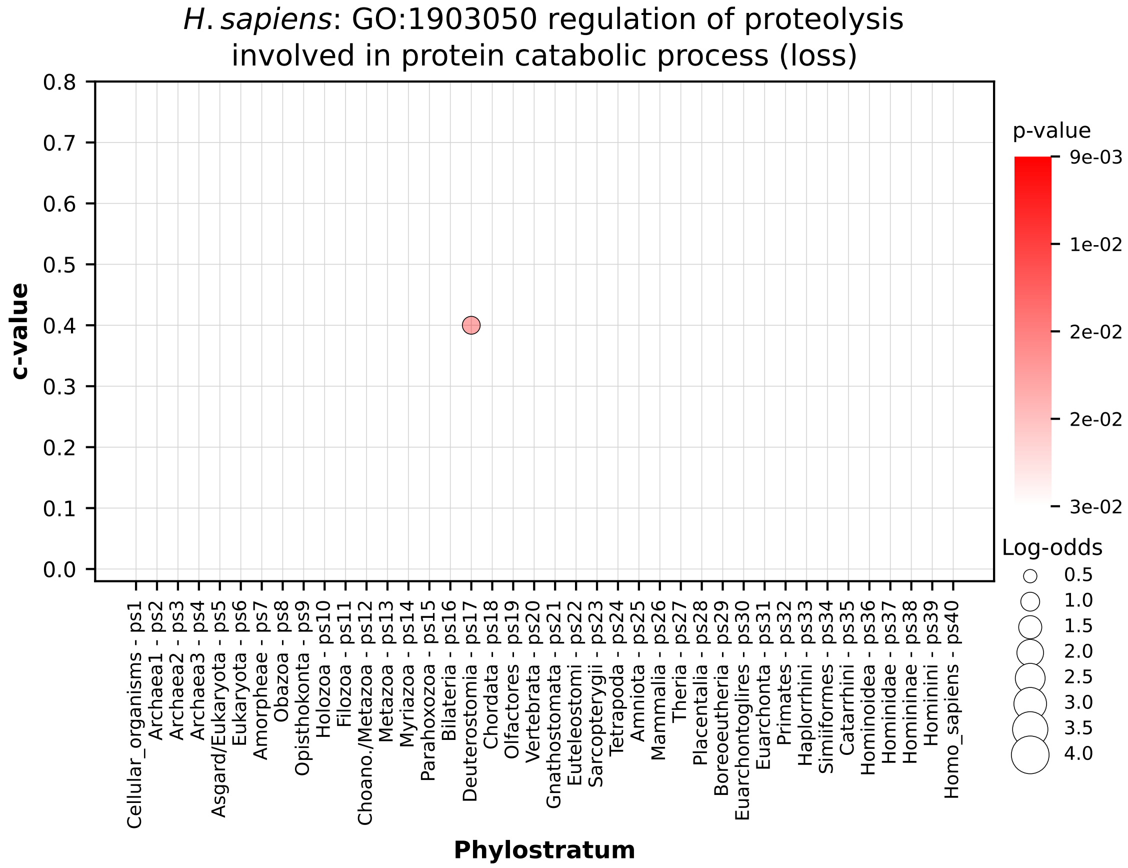

H. sapiens: GO:1903050 regulation of proteolysis involved in protein catabolic process (loss)
c-value
0.0
0.1
0.2
0.3
0.4
0.5
0.6
0.7
0.8
Phylostratum
Cellular_organisms - ps1
Archaea1 - ps2
Archaea2 - ps3
Archaea3 - ps4
Asgard/Eukaryota - ps5
Eukaryota - ps6
Amorpheae - ps7
Obazoa - ps8
Opisthokonta - ps9
Holozoa - ps10
Filozoa - ps11
Choano./Metazoa - ps12
Metazoa - ps13
Myriazoa - ps14
Parahoxozoa - ps15
Bilateria - ps16
Deuterostomia - ps17
Chordata - ps18
Olfactores - ps19
Vertebrata - ps20
Gnathostomata - ps21
Euteleostomi - ps22
Sarcopterygii - ps23
Tetrapoda - ps24
Amniota - ps25
Mammalia - ps26
Theria - ps27
Placentalia - ps28
Boreoeutheria - ps29
Euarchontoglires - ps30
Euarchonta - ps31
Primates - ps32
Haplorrhini - ps33
Simiiformes - ps34
Catarrhini - ps35
Hominoidea - ps36
Hominidae - ps37
Homininae - ps38
Hominini - ps39
Homo_sapiens - ps40
p-value
9e-03
1e-02
2e-02
2e-02
3e-02
Log-odds
0.5
1.0
1.5
2.0
2.5
3.0
3.5
4.0

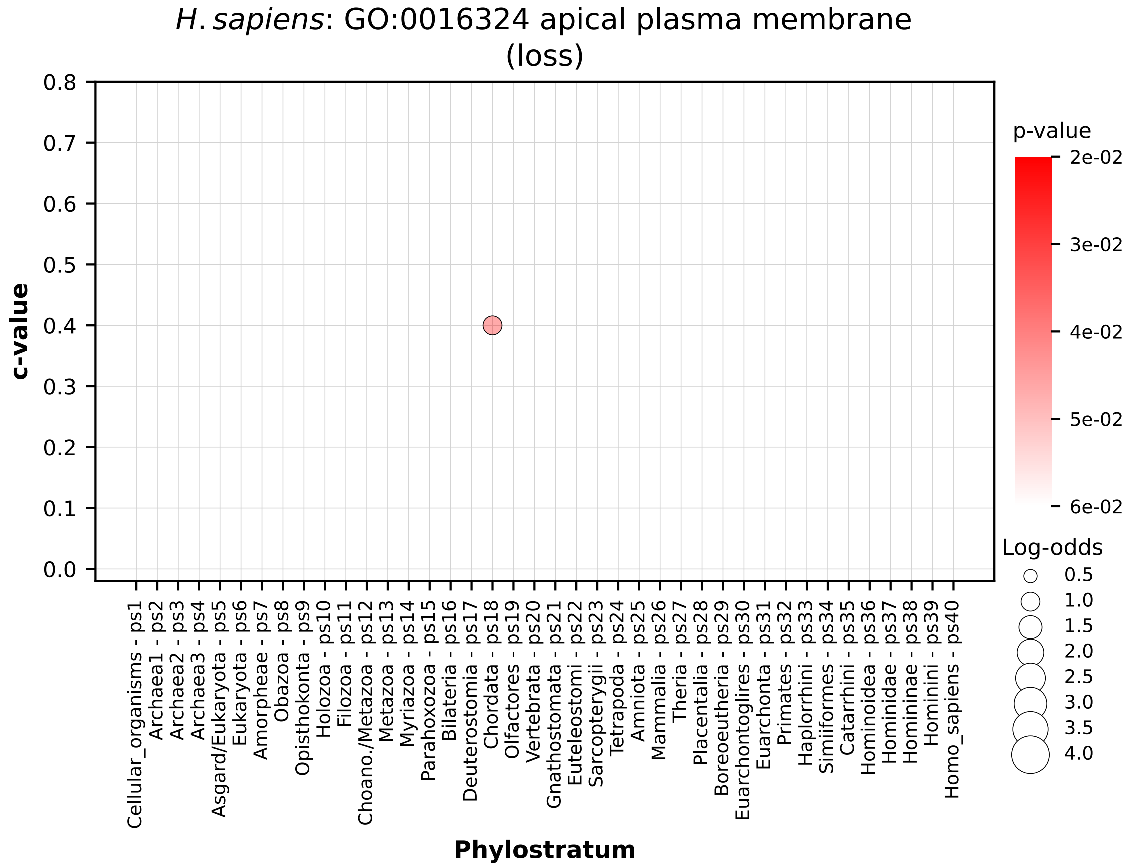

H. sapiens: GO:0016324 apical plasma membrane
(loss)
c-value
0.8
0.7
0.6
0.5
0.4
0.3
0.2
0.1
0.0
Phylostratum
Cellular_organisms - ps1
Archaea1 - ps2
Archaea2 - ps3
Archaea3 - ps4
Asgard/Eukaryota - ps5
Eukaryota - ps6
Amorpheae - ps7
Obazoa - ps8
Opisthokonta - ps9
Holozoa - ps10
Filozoa - ps11
Choano./Metazoa - ps12
Metazoa - ps13
Myriazoa - ps14
Parahoxozoa - ps15
Bilateria - ps16
Deuterostomia - ps17
Chordata - ps18
Olfactores - ps19
Vertebrata - ps20
Gnathostomata - ps21
Euteleostomi - ps22
Sarcopterygii - ps23
Tetrapoda - ps24
Amniota - ps25
Mammalia - ps26
Theria - ps27
Placentalia - ps28
Boreoeutheria - ps29
Euarchontoglires - ps30
Euarchonta - ps31
Primates - ps32
Haplorrhini - ps33
Simiiformes - ps34
Catarrhini - ps35
Hominoidea - ps36
Hominidae - ps37
Homininae - ps38
Hominini - ps39
Homo_sapiens - ps40
p-value
2e-02
3e-02
4e-02
5e-02
6e-02
Log-odds
0.5
1.0
1.5
2.0
2.5
3.0
3.5
4.0

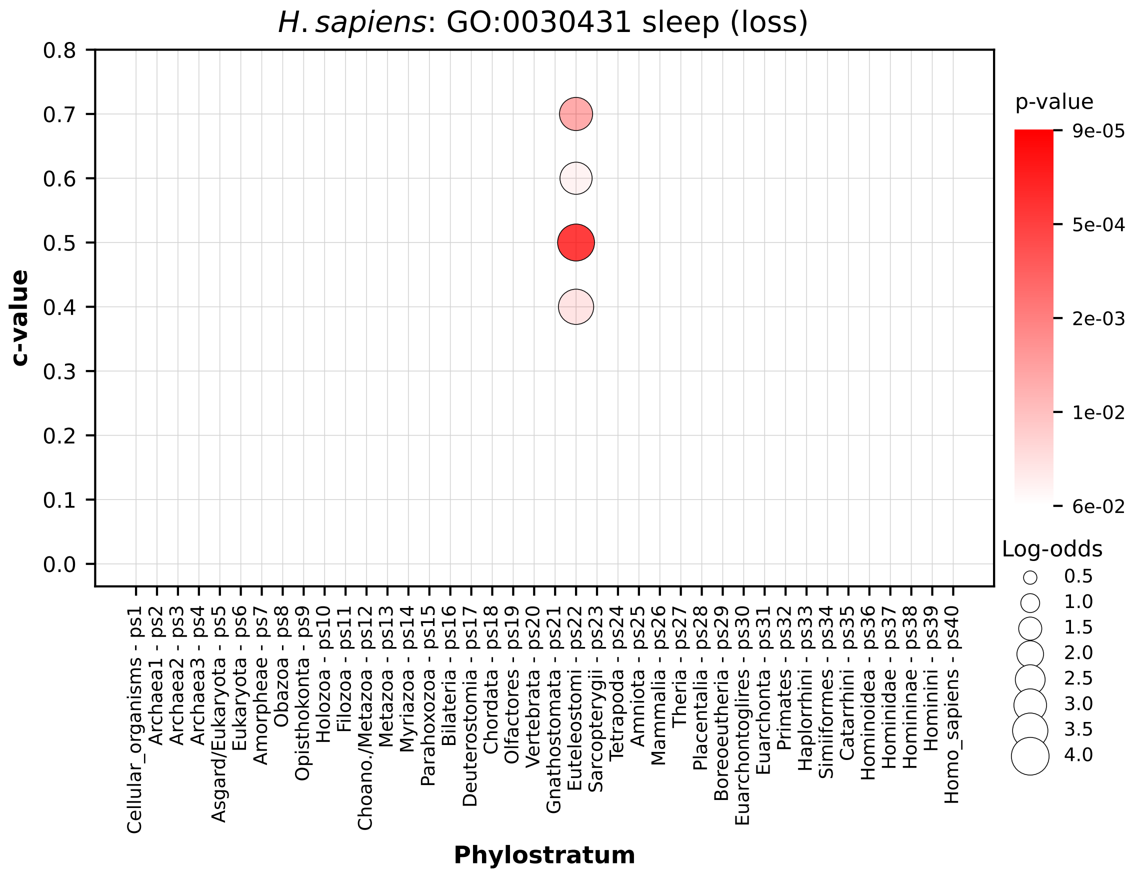

H. sapiens: GO:0030431 sleep (loss)
c-value
0.0
0.1
0.2
0.3
0.4
0.5
0.6
0.7
0.8
Phylostratum
Cellular_organisms - ps1
Archaea1 - ps2
Archaea2 - ps3
Archaea3 - ps4
Asgard/Eukaryota - ps5
Eukaryota - ps6
Amorpheae - ps7
Obazoa - ps8
Opisthokonta - ps9
Holozoa - ps10
Filozoa - ps11
Choano./Metazoa - ps12
Metazoa - ps13
Myriazoa - ps14
Parahoxozoa - ps15
Bilateria - ps16
Deuterostomia - ps17
Chordata - ps18
Olfactores - ps19
Vertebrata - ps20
Gnathostomata - ps21
Euteleostomi - ps22
Sarcopterygii - ps23
Tetrapoda - ps24
Amniota - ps25
Mammalia - ps26
Theria - ps27
Placentalia - ps28
Boreoeutheria - ps29
Euarchontoglires - ps30
Euarchonta - ps31
Primates - ps32
Haplorrhini - ps33
Simiiformes - ps34
Catarrhini - ps35
Hominoidea - ps36
Hominidae - ps37
Homininae - ps38
Hominini - ps39
Homo_sapiens - ps40
p-value
9e-05
5e-04
2e-03
1e-02
6e-02
Log-odds
0.5
1.0
1.5
2.0
2.5
3.0
3.5
4.0

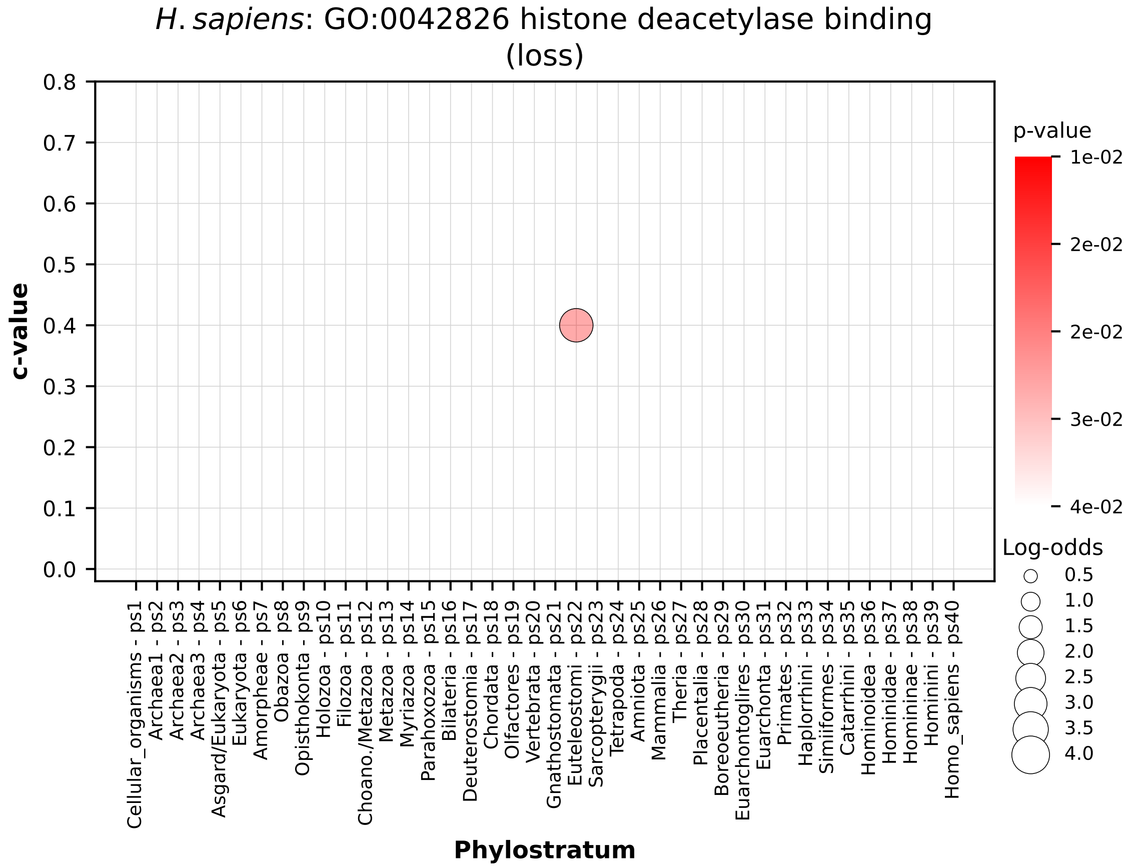

H. sapiens: GO:0042826 histone deacetylase binding (loss)
c-value
0.0
0.1
0.2
0.3
0.4
0.5
0.6
0.7
0.8
Phylostratum
Cellular_organisms - ps1
Archaea1 - ps2
Archaea2 - ps3
Archaea3 - ps4
Asgard/Eukaryota - ps5
Eukaryota - ps6
Amorpheae - ps7
Obazoa - ps8
Opisthokonta - ps9
Holozoa - ps10
Filozoa - ps11
Choano./Metazoa - ps12
Metazoa - ps13
Myriazoa - ps14
Parahoxozoa - ps15
Bilateria - ps16
Deuterostomia - ps17
Chordata - ps18
Olfactores - ps19
Vertebrata - ps20
Gnathostomata - ps21
Euteleostomi - ps22
Sarcopterygii - ps23
Tetrapoda - ps24
Amniota - ps25
Mammalia - ps26
Theria - ps27
Placentalia - ps28
Boreoeutheria - ps29
Euarchontoglires - ps30
Euarchonta - ps31
Primates - ps32
Haplorrhini - ps33
Simiiformes - ps34
Catarrhini - ps35
Hominoidea - ps36
Hominidae - ps37
Homininae - ps38
Hominini - ps39
Homo_sapiens - ps40
p-value
1e-02
2e-02
2e-02
3e-02
4e-02
Log-odds
0.5
1.0
1.5
2.0
2.5
3.0
3.5
4.0

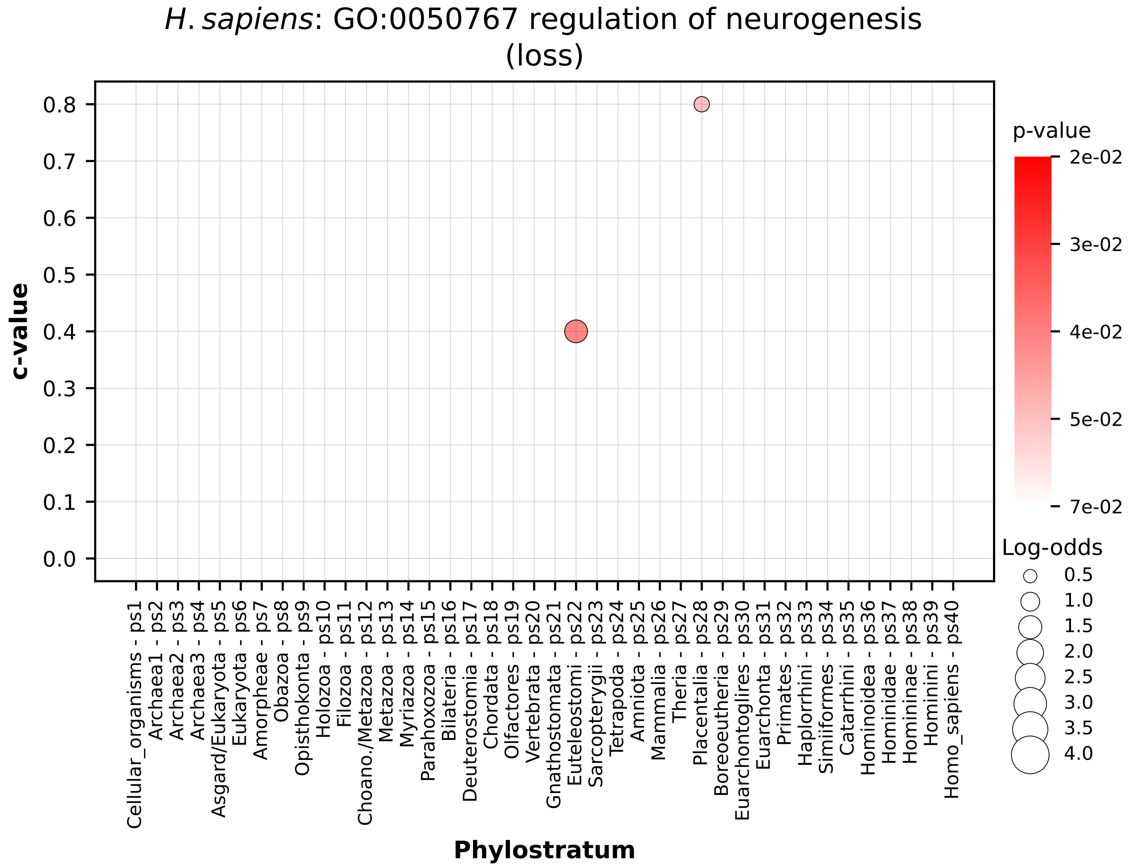

H. sapiens: GO:0050767 regulation of neurogenesis (loss)
c-value
0.0
0.1
0.2
0.3
0.4
0.5
0.6
0.7
0.8
Phylostratum
Cellular_organisms - ps1
Archaea1 - ps2
Archaea2 - ps3
Archaea3 - ps4
Asgard/Eukaryota - ps5
Eukaryota - ps6
Amorpheae - ps7
Obazoa - ps8
Opisthokonta - ps9
Holozoa - ps10
Filozoa - ps11
Choano./Metazoa - ps12
Metazoa - ps13
Myriazoa - ps14
Parahoxozoa - ps15
Bilateria - ps16
Deuterostomia - ps17
Chordata - ps18
Olfactores - ps19
Vertebrata - ps20
Gnathostomata - ps21
Euteleostomi - ps22
Sarcopterygii - ps23
Tetrapoda - ps24
Amniota - ps25
Mammalia - ps26
Theria - ps27
Placentalia - ps28
Boreoeutheria - ps29
Euarchontoglires - ps30
Euarchonta - ps31
Primates - ps32
Haplorrhini - ps33
Simiiformes - ps34
Catarrhini - ps35
Hominoidea - ps36
Hominidae - ps37
Homininae - ps38
Hominini - ps39
Homo_sapiens - ps40
p-value
2e-02
3e-02
4e-02
5e-02
7e-02
Log-odds
0.5
1.0
1.5
2.0
2.5
3.0
3.5
4.0

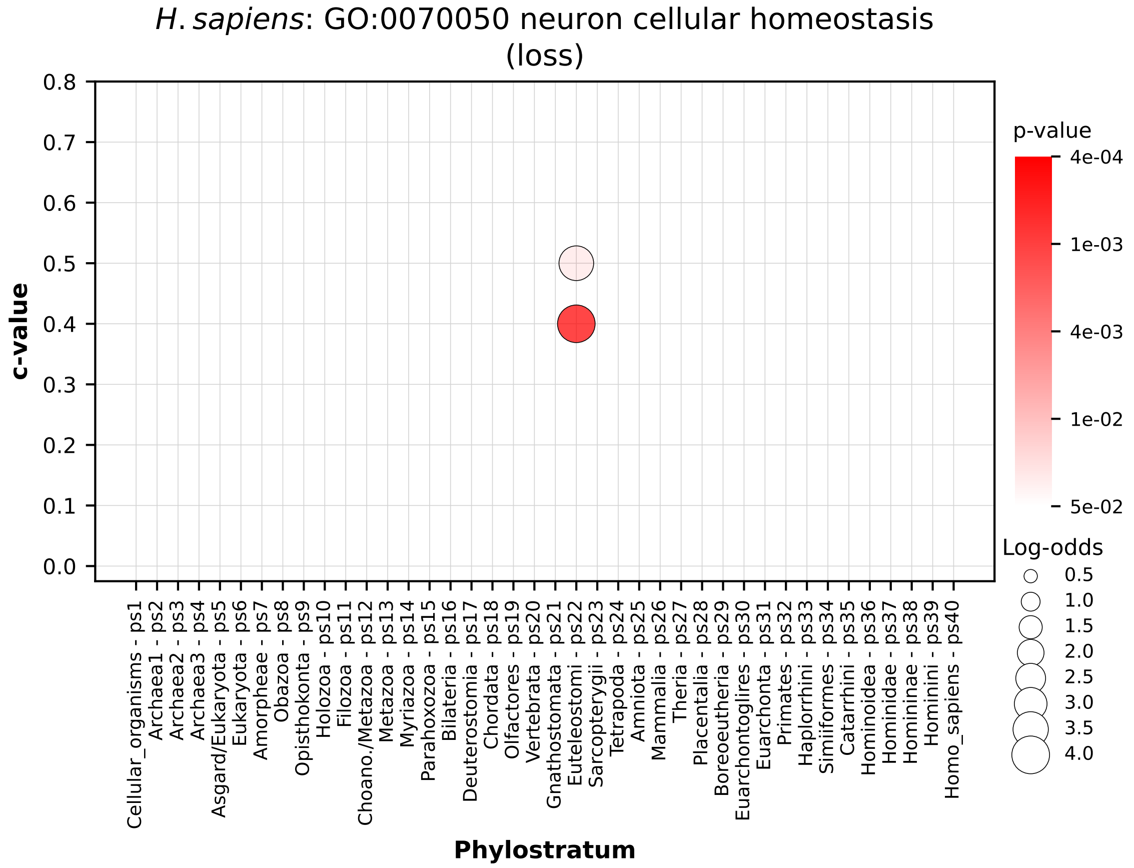

H. sapiens: GO:0070050 neuron cellular homeostasis (loss)
c-value
0.8
0.7
0.6
0.5
0.4
0.3
0.2
0.1
0.0
p-value
4e-04
1e-03
4e-03
1e-02
5e-02
Log-odds
0.5
1.0
1.5
2.0
2.5
3.0
3.5
4.0
Cellular_organisms - ps1
Archaea1 - ps2
Archaea2 - ps3
Archaea3 - ps4
Asgard/Eukaryota - ps5
Eukaryota - ps6
Amorpheae - ps7
Obazoa - ps8
Opisthokonta - ps9
Holozoa - ps10
Filozoa - ps11
Choano./Metazoa - ps12
Metazoa - ps13
Myriazoa - ps14
Parahoxozoa - ps15
Bilateria - ps16
Deuterostomia - ps17
Chordata - ps18
Olfactores - ps19
Vertebrata - ps20
Gnathostomata - ps21
Euteleostomi - ps22
Sarcopterygii - ps23
Tetrapoda - ps24
Amniota - ps25
Mammalia - ps26
Theria - ps27
Placentalia - ps28
Boreoeutheria - ps29
Euarchontoglires - ps30
Euarchonta - ps31
Primates - ps32
Haplorrhini - ps33
Simiiformes - ps34
Catarrhini - ps35
Hominoidea - ps36
Hominidae - ps37
Homininae - ps38
Hominini - ps39
Homo_sapiens - ps40
Phylostratum

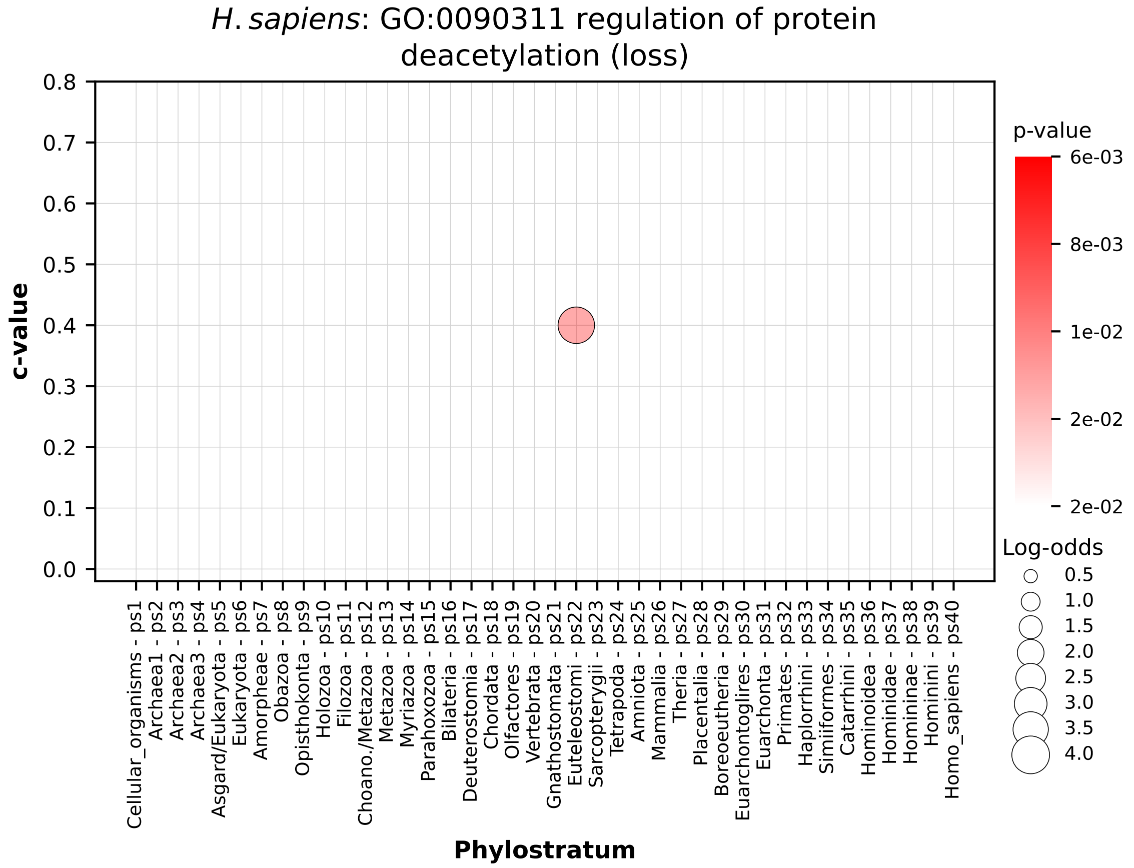

H. sapiens: GO:0090311 regulation of protein deacetylation (loss)
c-value
0.8
0.7
0.6
0.5
0.4
0.3
0.2
0.1
0.0
Phylostratum
Cellular_organisms - ps1
Archaea1 - ps2
Archaea2 - ps3
Archaea3 - ps4
Asgard/Eukaryota - ps5
Eukaryota - ps6
Amorpheae - ps7
Obazoa - ps8
Opisthokonta - ps9
Holozoa - ps10
Filozoa - ps11
Choano./Metazoa - ps12
Metazoa - ps13
Myriazoa - ps14
Parahoxozoa - ps15
Bilateria - ps16
Deuterostomia - ps17
Chordata - ps18
Olfactores - ps19
Vertebrata - ps20
Gnathostomata - ps21
Euteleostomi - ps22
Sarcopterygii - ps23
Tetrapoda - ps24
Amniota - ps25
Mammalia - ps26
Theria - ps27
Placentalia - ps28
Boreoeutheria - ps29
Euarchontoglires - ps30
Euarchonta - ps31
Primates - ps32
Haplorrhini - ps33
Simiiformes - ps34
Catarrhini - ps35
Hominoidea - ps36
Hominidae - ps37
Homininae - ps38
Hominini - ps39
Homo_sapiens - ps40
p-value
6e-03
8e-03
1e-02
2e-02
2e-02
Log-odds
0.5
1.0
1.5
2.0
2.5
3.0
3.5
4.0

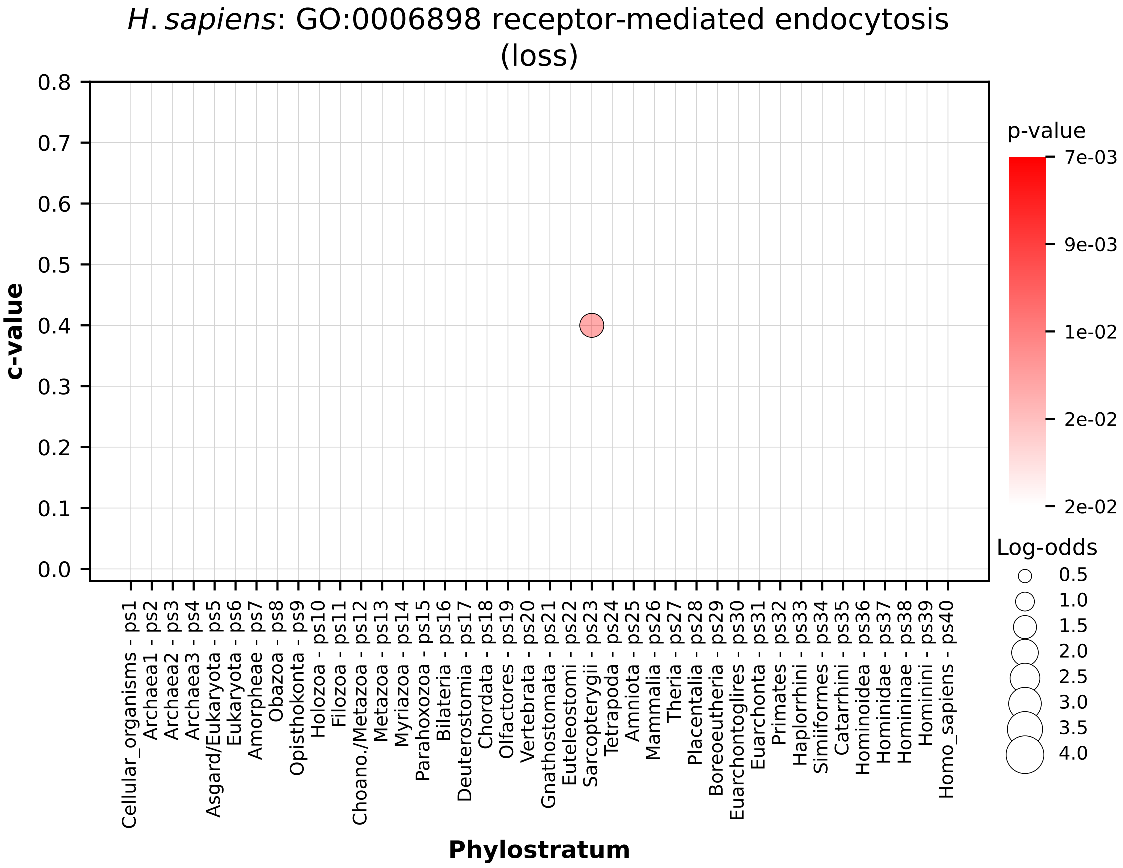

H. sapiens: GO:0006898 receptor-mediated endocytosis
(loss)
c-value
0.8
0.7
0.6
0.5
0.4
0.3
0.2
0.1
0.0
p-value
7e-03
9e-03
1e-02
2e-02
2e-02
Log-odds
0.5
1.0
1.5
2.0
2.5
3.0
3.5
4.0
Cellular_organisms - ps1
Archaea1 - ps2
Archaea2 - ps3
Archaea3 - ps4
Asgard/Eukaryota - ps5
Eukaryota - ps6
Amorpheae - ps7
Obazoa - ps8
Opisthokonta - ps9
Holozoa - ps10
Filozoa - ps11
Choano./Metazoa - ps12
Metazoa - ps13
Myriazoa - ps14
Parahoxozoa - ps15
Bilateria - ps16
Deuterostomia - ps17
Chordata - ps18
Olfactores - ps19
Vertebrata - ps20
Gnathostomata - ps21
Euteleostomi - ps22
Sarcopterygii - ps23
Tetrapoda - ps24
Amniota - ps25
Mammalia - ps26
Theria - ps27
Placentalia - ps28
Boreoeutheria - ps29
Euarchontoglires - ps30
Euarchonta - ps31
Primates - ps32
Haplorrhini - ps33
Simiiformes - ps34
Catarrhini - ps35
Hominoidea - ps36
Hominidae - ps37
Homininae - ps38
Hominini - ps39
Homo_sapiens - ps40
Phylostratum

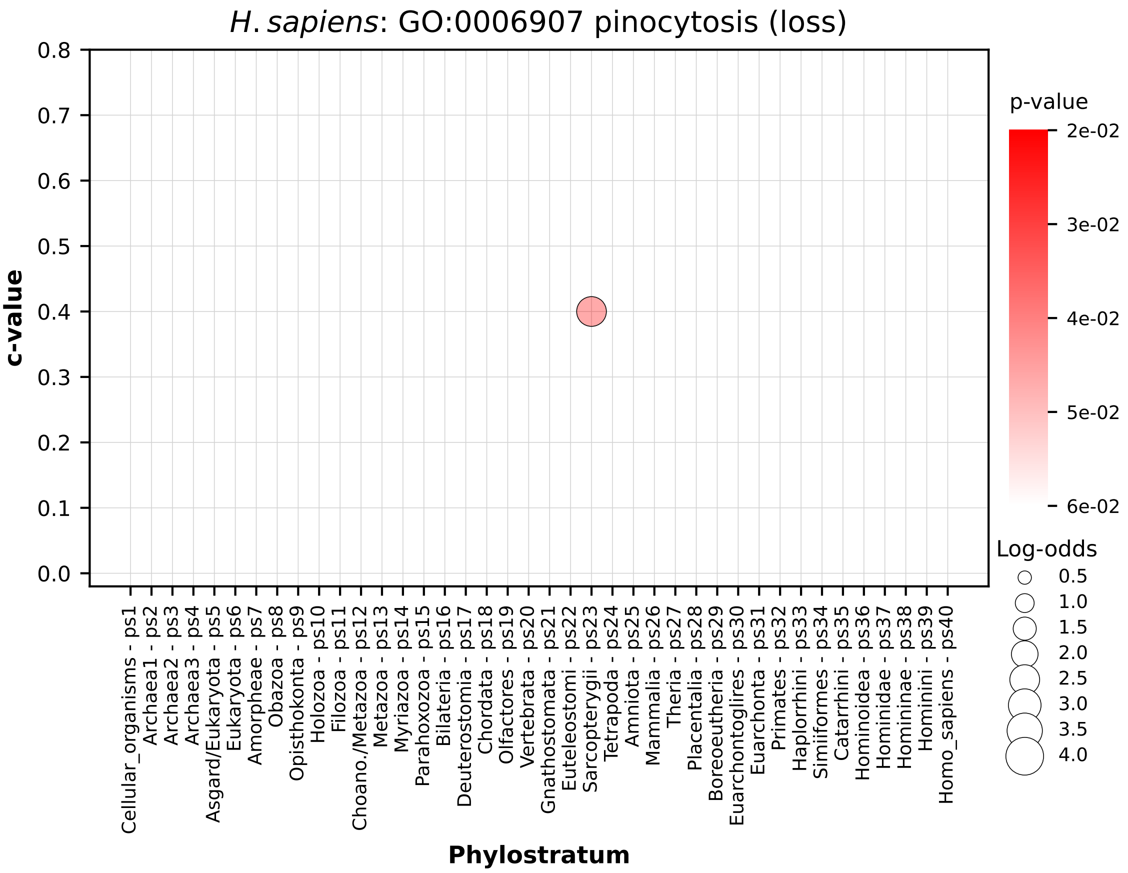

H. sapiens: GO:0006907 pinocytosis (loss)
c-value
0.0
0.1
0.2
0.3
0.4
0.5
0.6
0.7
0.8
Phylostratum
Cellular_organisms - ps1
Archaea1 - ps2
Archaea2 - ps3
Archaea3 - ps4
Asgard/Eukaryota - ps5
Eukaryota - ps6
Amorpheae - ps7
Obazoa - ps8
Opisthokonta - ps9
Holozoa - ps10
Filozoa - ps11
Choano./Metazoa - ps12
Metazoa - ps13
Myriazoa - ps14
Parahoxozoa - ps15
Bilateria - ps16
Deuterostomia - ps17
Chordata - ps18
Olfactores - ps19
Vertebrata - ps20
Gnathostomata - ps21
Euteleostomi - ps22
Sarcopterygii - ps23
Tetrapoda - ps24
Amniota - ps25
Mammalia - ps26
Theria - ps27
Placentalia - ps28
Boreoeutheria - ps29
Euarchontoglires - ps30
Euarchonta - ps31
Primates - ps32
Haplorrhini - ps33
Simiiformes - ps34
Catarrhini - ps35
Hominoidea - ps36
Hominidae - ps37
Homininae - ps38
Hominini - ps39
Homo_sapiens - ps40
p-value
2e-02
3e-02
4e-02
5e-02
6e-02
Log-odds
0.5
1.0
1.5
2.0
2.5
3.0
3.5
4.0

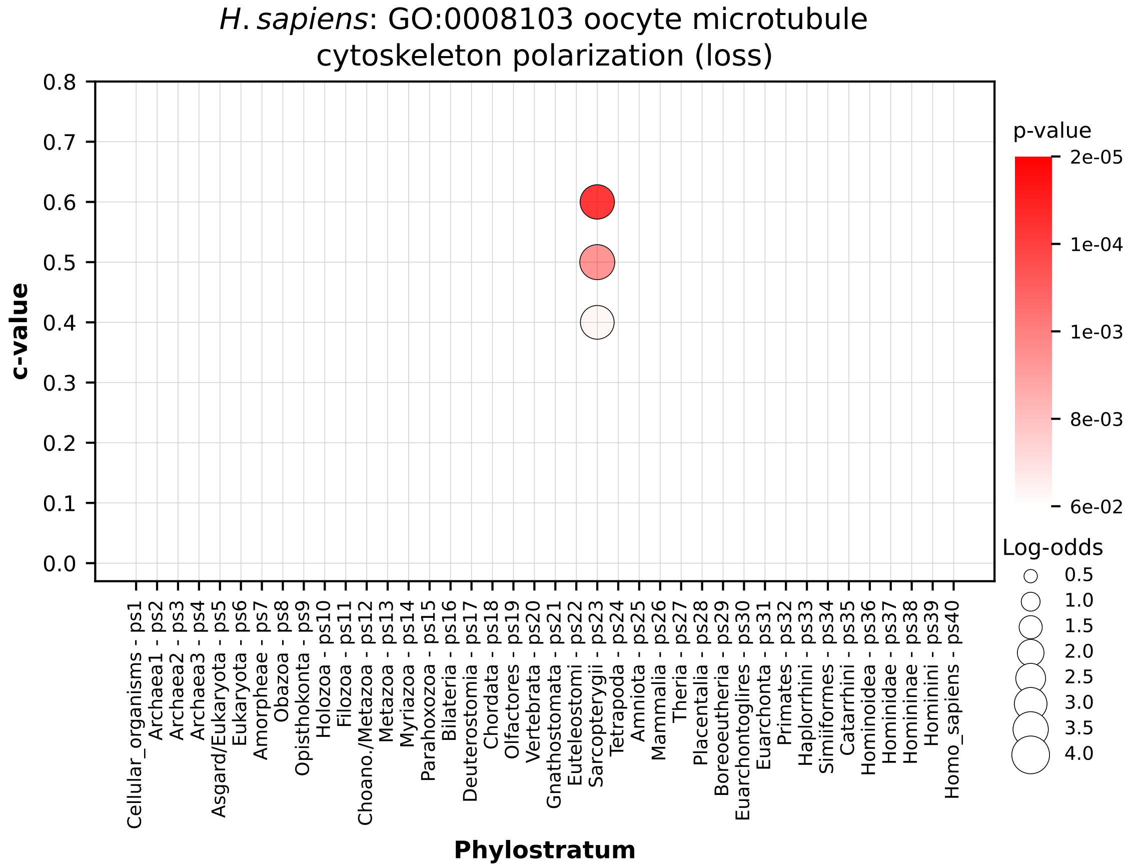

H. sapiens: GO:0008103 oocyte microtubule cytoskeleton polarization (loss)
c-value
0.8
0.7
0.6
0.5
0.4
0.3
0.2
0.1
0.0
Phylostratum
Cellular_organisms - ps1
Archaea1 - ps2
Archaea2 - ps3
Archaea3 - ps4
Asgard/Eukaryota - ps5
Eukaryota - ps6
Amorpheae - ps7
Obazoa - ps8
Opisthokonta - ps9
Holozoa - ps10
Filozoa - ps11
Choano./Metazoa - ps12
Metazoa - ps13
Myriazoa - ps14
Parahoxozoa - ps15
Bilateria - ps16
Deuterostomia - ps17
Chordata - ps18
Olfactores - ps19
Vertebrata - ps20
Gnathostomata - ps21
Euteleostomi - ps22
Sarcopterygii - ps23
Tetrapoda - ps24
Amniota - ps25
Mammalia - ps26
Theria - ps27
Placentalia - ps28
Boreoeutheria - ps29
Euarchontoglires - ps30
Euarchonta - ps31
Primates - ps32
Haplorrhini - ps33
Simiiformes - ps34
Catarrhini - ps35
Hominoidea - ps36
Hominidae - ps37
Homininae - ps38
Hominini - ps39
Homo_sapiens - ps40
p-value
2e-05
1e-04
1e-03
8e-03
6e-02
Log-odds
0.5
1.0
1.5
2.0
2.5
3.0
3.5
4.0

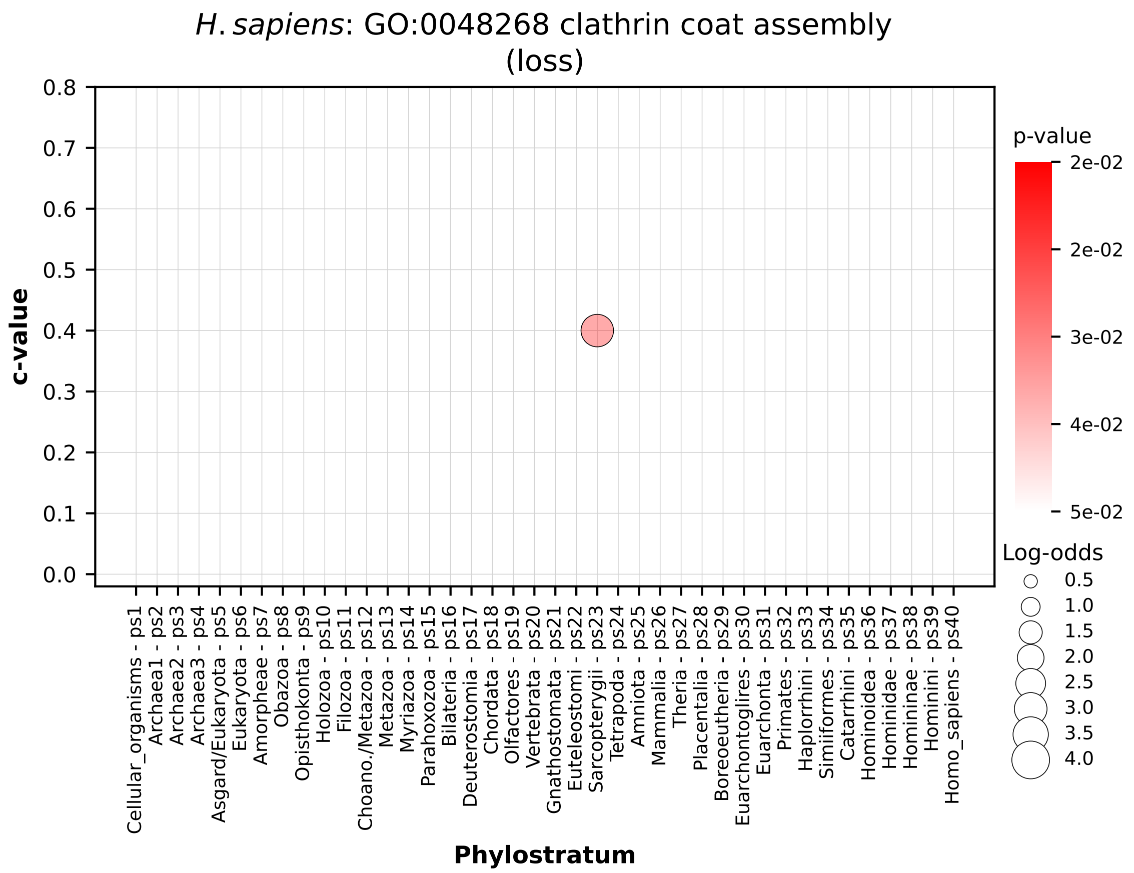

H. sapiens: GO:0048268 clathrin coat assembly
(loss)
c-value
0.0
0.1
0.2
0.3
0.4
0.5
0.6
0.7
0.8
Phylostratum
Cellular_organisms - ps1
Archaea1 - ps2
Archaea2 - ps3
Archaea3 - ps4
Asgard/Eukaryota - ps5
Eukaryota - ps6
Amorpheae - ps7
Obazoa - ps8
Opisthokonta - ps9
Holozoa - ps10
Filozoa - ps11
Choano./Metazoa - ps12
Metazoa - ps13
Myriazoa - ps14
Parahoxozoa - ps15
Bilateria - ps16
Deuterostomia - ps17
Chordata - ps18
Olfactores - ps19
Vertebrata - ps20
Gnathostomata - ps21
Euteleostomi - ps22
Sarcopterygii - ps23
Tetrapoda - ps24
Amniota - ps25
Mammalia - ps26
Theria - ps27
Placentalia - ps28
Boreoeutheria - ps29
Euarchontoglires - ps30
Euarchonta - ps31
Primates - ps32
Haplorrhini - ps33
Simiiformes - ps34
Catarrhini - ps35
Hominoidea - ps36
Hominidae - ps37
Homininae - ps38
Hominini - ps39
Homo_sapiens - ps40
p-value
2e-02
2e-02
3e-02
4e-02
5e-02
Log-odds
0.5
1.0
1.5
2.0
2.5
3.0
3.5
4.0

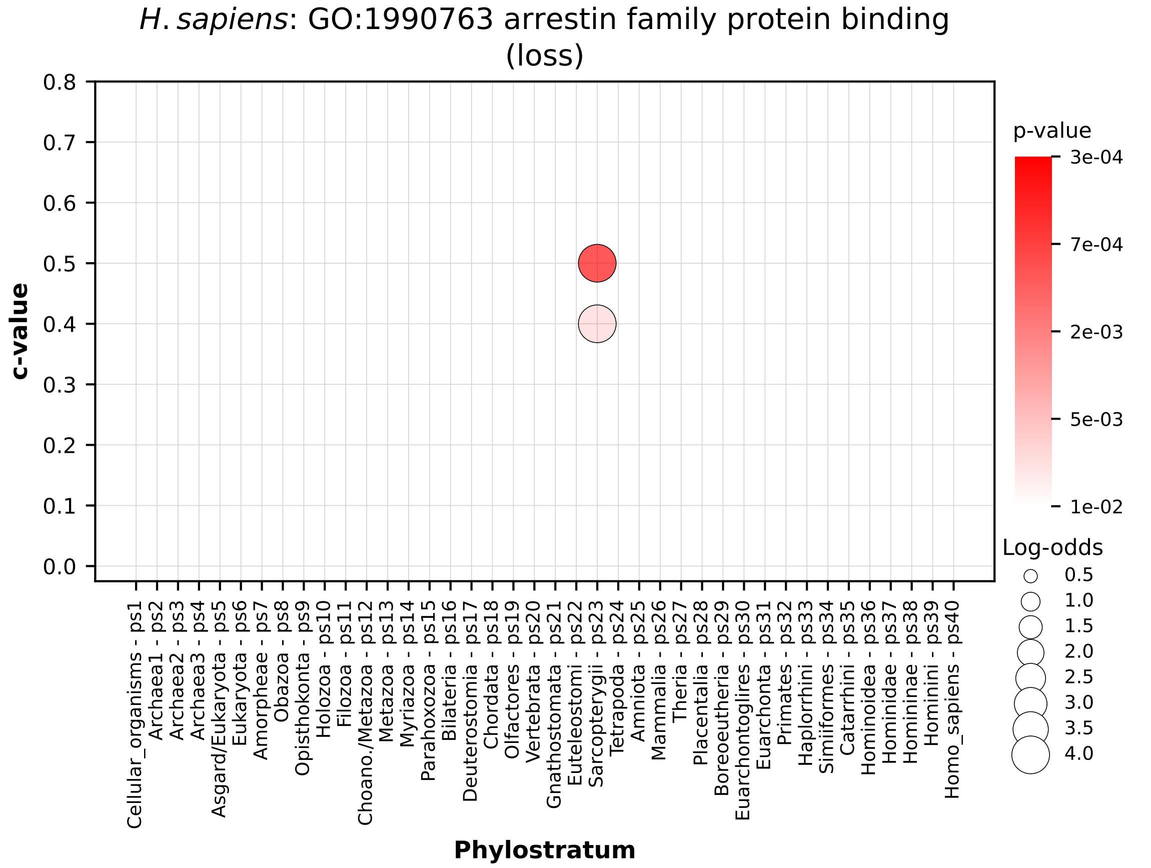

H. sapiens: GO:1990763 arrestin family protein binding (loss)
c-value
0.8
0.7
0.6
0.5
0.4
0.3
0.2
0.1
0.0
Cellular_organisms - ps1
Archaea1 - ps2
Archaea2 - ps3
Archaea3 - ps4
Asgard/Eukaryota - ps5
Eukaryota - ps6
Amorpheae - ps7
Obazoa - ps8
Opisthokonta - ps9
Holozoa - ps10
Filozoa - ps11
Choano./Metazoa - ps12
Metazoa - ps13
Myriazoa - ps14
Parahoxozoa - ps15
Bilateria - ps16
Deuterostomia - ps17
Chordata - ps18
Olfactores - ps19
Vertebrata - ps20
Gnathostomata - ps21
Euteleostomi - ps22
Sarcopterygii - ps23
Tetrapoda - ps24
Amniota - ps25
Mammalia - ps26
Theria - ps27
Placentalia - ps28
Boreoeutheria - ps29
Euarchontoglires - ps30
Euarchonta - ps31
Primates - ps32
Haplorrhini - ps33
Simiiformes - ps34
Catarrhini - ps35
Hominoidea - ps36
Hominidae - ps37
Homininae - ps38
Hominini - ps39
Homo_sapiens - ps40
Phylostratum
p-value
3e-04
7e-04
2e-03
5e-03
1e-02
Log-odds
0.5
1.0
1.5
2.0
2.5
3.0
3.5
4.0

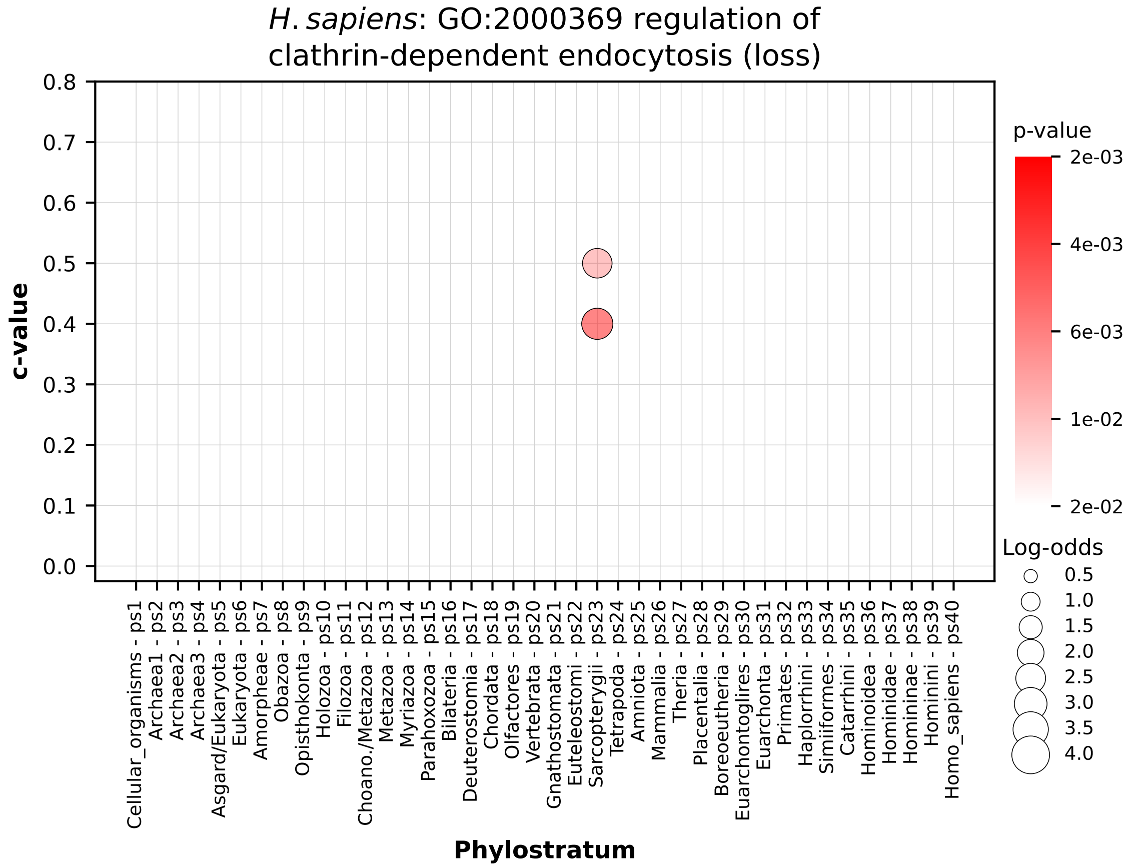

H. sapiens: GO:2000369 regulation of clathrin-dependent endocytosis (loss)
c-value
0.8
0.7
0.6
0.5
0.4
0.3
0.2
0.1
0.0
p-value
2e-03
4e-03
6e-03
1e-02
2e-02
Log-odds
0.5
1.0
1.5
2.0
2.5
3.0
3.5
4.0
Cellular_organisms - ps1
Archaea1 - ps2
Archaea2 - ps3
Archaea3 - ps4
Asgard/Eukaryota - ps5
Eukaryota - ps6
Amorpheae - ps7
Obazoa - ps8
Opisthokonta - ps9
Holozoa - ps10
Filozoa - ps11
Choano./Metazoa - ps12
Metazoa - ps13
Myriazoa - ps14
Parahoxozoa - ps15
Bilateria - ps16
Deuterostomia - ps17
Chordata - ps18
Olfactores - ps19
Vertebrata - ps20
Gnathostomata - ps21
Euteleostomi - ps22
Sarcopterygii - ps23
Tetrapoda - ps24
Amniota - ps25
Mammalia - ps26
Theria - ps27
Placentalia - ps28
Boreoeutheria - ps29
Euarchontoglires - ps30
Euarchonta - ps31
Primates - ps32
Haplorrhini - ps33
Simiiformes - ps34
Catarrhini - ps35
Hominoidea - ps36
Hominidae - ps37
Homininae - ps38
Hominini - ps39
Homo_sapiens - ps40
Phylostratum

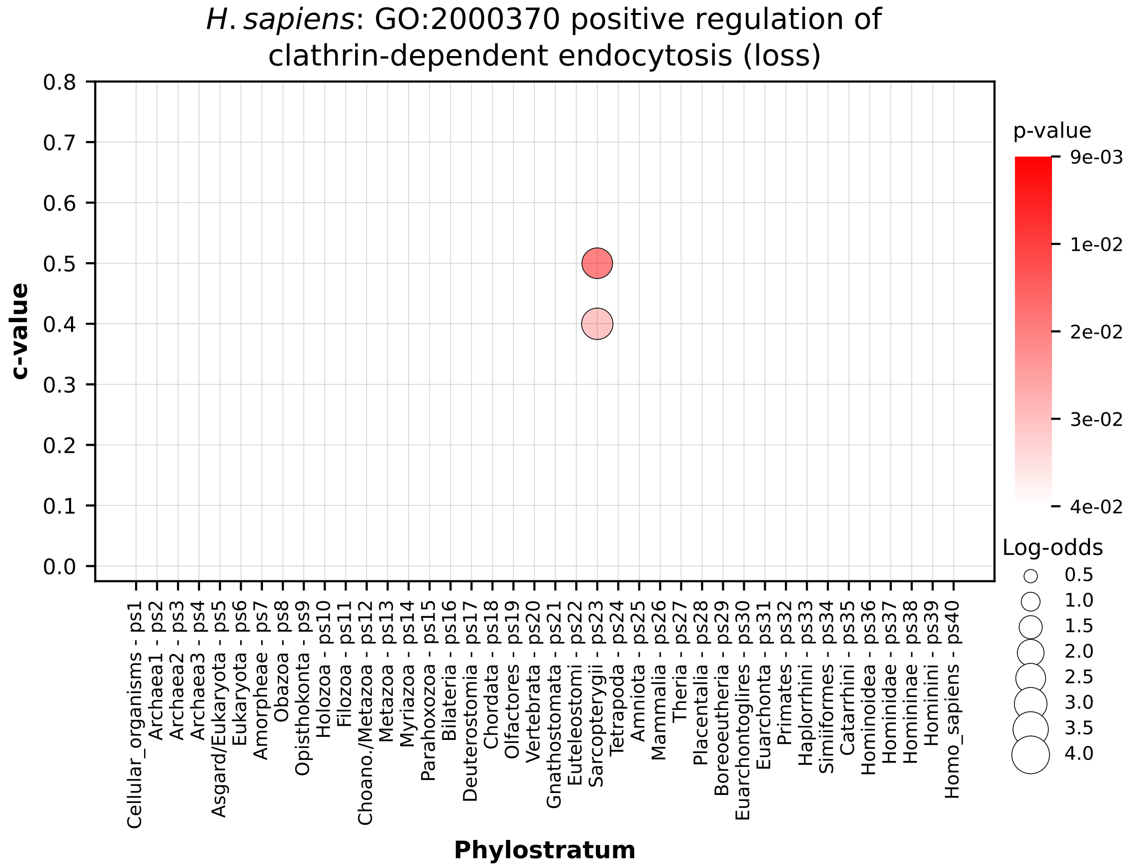
H. sapiens: GO:2000370 positive regulation of clathrin-dependent endocytosis (loss)
c-value
0.8
0.7
0.6
0.5
0.4
0.3
0.2
0.1
0.0
Phylostratum
Cellular_organisms - ps1
Archaea1 - ps2
Archaea2 - ps3
Archaea3 - ps4
Asgard/Eukaryota - ps5
Eukaryota - ps6
Amorpheae - ps7
Obazoa - ps8
Opisthokonta - ps9
Holozoa - ps10
Filozoa - ps11
Choano./Metazoa - ps12
Metazoa - ps13
Myriazoa - ps14
Parahoxozoa - ps15
Bilateria - ps16
Deuterostomia - ps17
Chordata - ps18
Olfactores - ps19
Vertebrata - ps20
Gnathostomata - ps21
Euteleostomi - ps22
Sarcopterygii - ps23
Tetrapoda - ps24
Amniota - ps25
Mammalia - ps26
Theria - ps27
Placentalia - ps28
Boreoeutheria - ps29
Euarchontoglires - ps30
Euarchonta - ps31
Primates - ps32
Haplorrhini - ps33
Simiiformes - ps34
Catarrhini - ps35
Hominoidea - ps36
Hominidae - ps37
Homininae - ps38
Hominini - ps39
Homo_sapiens - ps40
p-value
9e-03
1e-02
2e-02
3e-02
4e-02
Log-odds
0.5
1.0
1.5
2.0
2.5
3.0
3.5
4.0

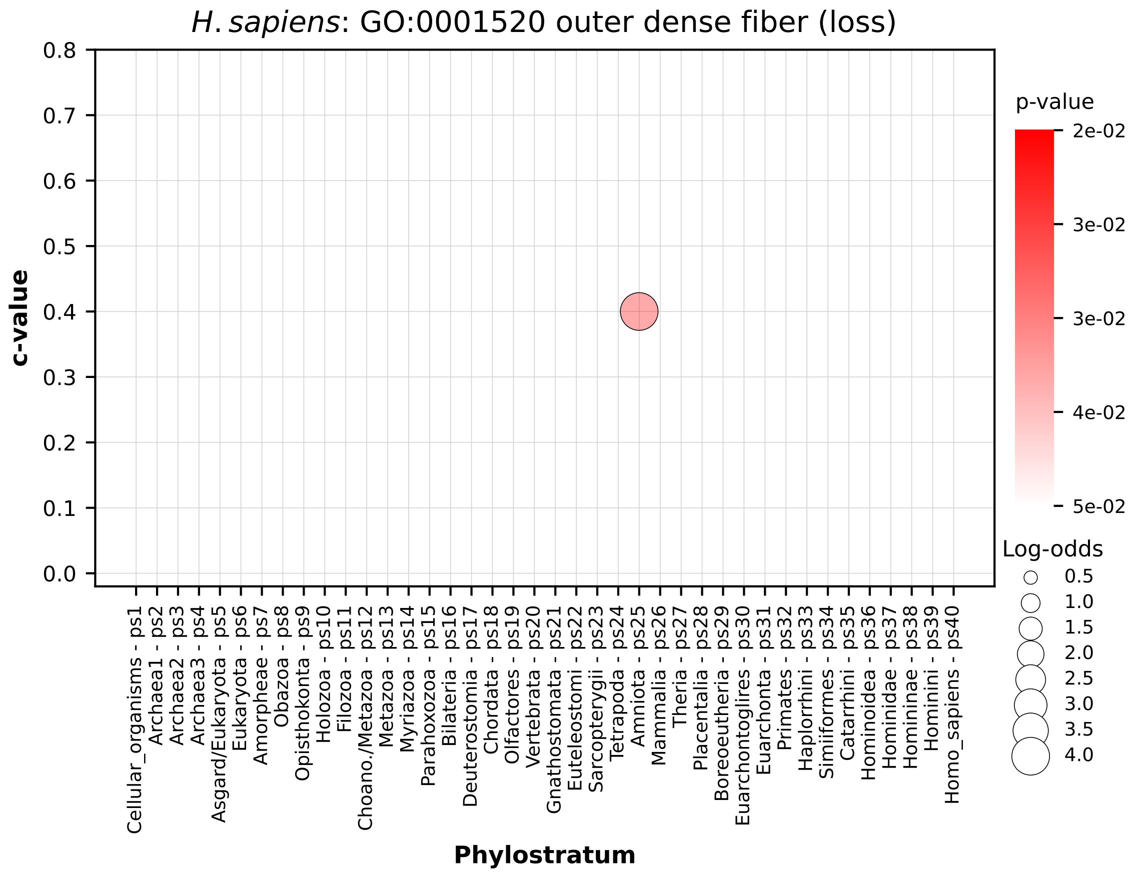

H. sapiens: GO:0001520 outer dense fiber (loss)
c-value
0.0
0.1
0.2
0.3
0.4
0.5
0.6
0.7
0.8
Phylostratum
Cellular_organisms - ps1
Archaea1 - ps2
Archaea2 - ps3
Archaea3 - ps4
Asgard/Eukaryota - ps5
Eukaryota - ps6
Amorpheae - ps7
Obazoa - ps8
Opisthokonta - ps9
Holozoa - ps10
Filozoa - ps11
Choano./Metazoa - ps12
Metazoa - ps13
Myriazoa - ps14
Parahoxozoa - ps15
Bilateria - ps16
Deuterostomia - ps17
Chordata - ps18
Olfactores - ps19
Vertebrata - ps20
Gnathostomata - ps21
Euteleostomi - ps22
Sarcopterygii - ps23
Tetrapoda - ps24
Amniota - ps25
Mammalia - ps26
Theria - ps27
Placentalia - ps28
Boreoeutheria - ps29
Euarchontoglires - ps30
Euarchonta - ps31
Primates - ps32
Haplorrhini - ps33
Simiiformes - ps34
Catarrhini - ps35
Hominoidea - ps36
Hominidae - ps37
Homininae - ps38
Hominini - ps39
Homo_sapiens - ps40
p-value
2e-02
3e-02
3e-02
4e-02
5e-02
Log-odds
0.5
1.0
1.5
2.0
2.5
3.0
3.5
4.0

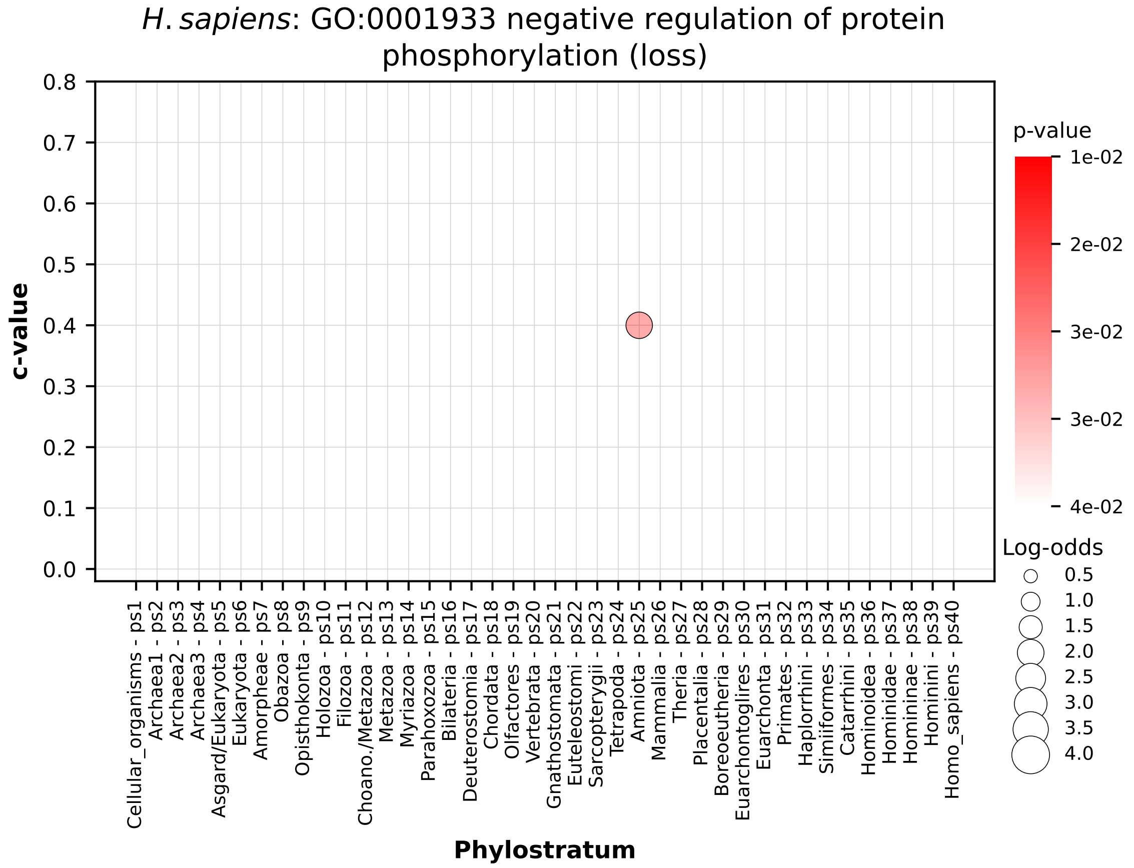

H. sapiens: GO:0001933 negative regulation of protein phosphorylation (loss)
c-value
0.8
0.7
0.6
0.5
0.4
0.3
0.2
0.1
0.0
Phylostratum
Cellular_organisms - ps1
Archaea1 - ps2
Archaea2 - ps3
Archaea3 - ps4
Asgard/Eukaryota - ps5
Eukaryota - ps6
Amorpheae - ps7
Obazoa - ps8
Opisthokonta - ps9
Holozoa - ps10
Filozoa - ps11
Choano./Metazoa - ps12
Metazoa - ps13
Myriazoa - ps14
Parahoxozoa - ps15
Bilateria - ps16
Deuterostomia - ps17
Chordata - ps18
Olfactores - ps19
Vertebrata - ps20
Gnathostomata - ps21
Euteleostomi - ps22
Sarcopterygii - ps23
Tetrapoda - ps24
Amniota - ps25
Mammalia - ps26
Theria - ps27
Placentalia - ps28
Boreoeutheria - ps29
Euarchontoglires - ps30
Euarchonta - ps31
Primates - ps32
Haplorrhini - ps33
Simiiformes - ps34
Catarrhini - ps35
Hominoidea - ps36
Hominidae - ps37
Homininae - ps38
Hominini - ps39
Homo_sapiens - ps40
p-value
1e-02
2e-02
3e-02
3e-02
4e-02
Log-odds
0.5
1.0
1.5
2.0
2.5
3.0
3.5
4.0

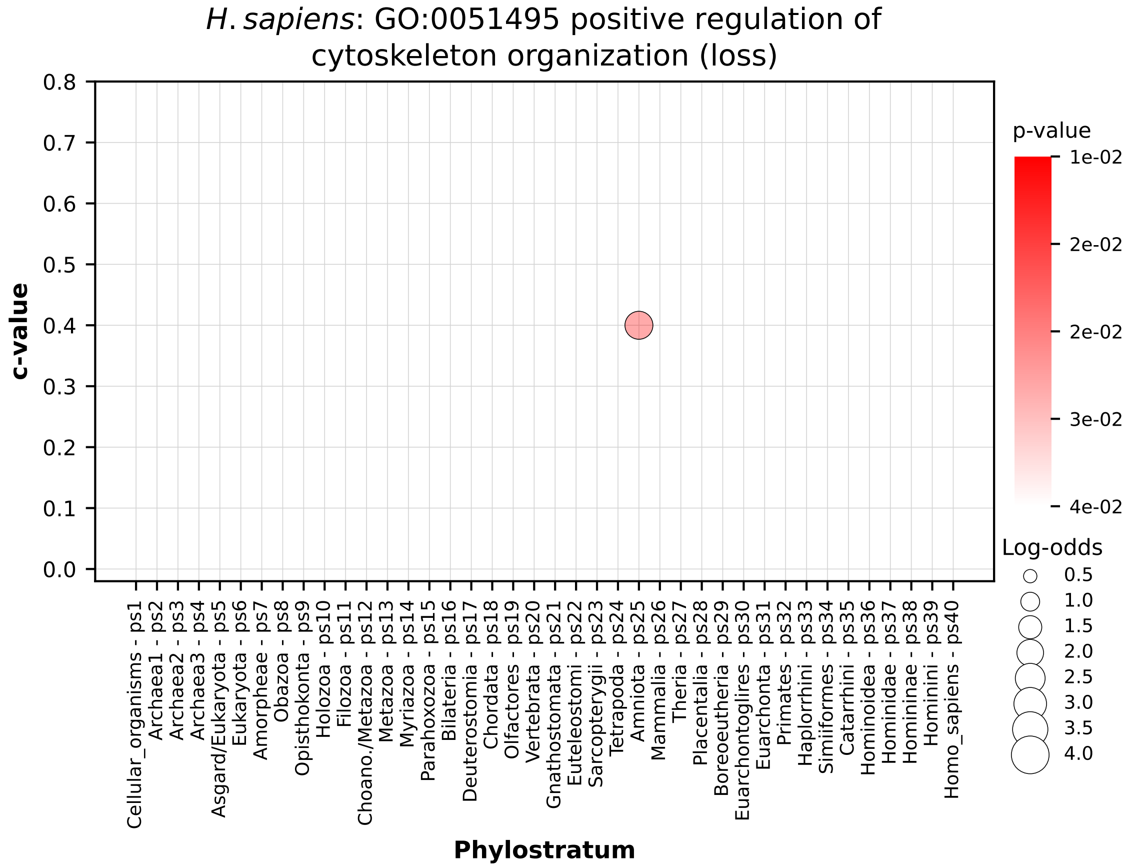

H. sapiens: GO:0051495 positive regulation of cytoskeleton organization (loss)
c-value
0.8
0.7
0.6
0.5
0.4
0.3
0.2
0.1
0.0
p-value
1e-02
2e-02
2e-02
3e-02
4e-02
Log-odds
0.5
1.0
1.5
2.0
2.5
3.0
3.5
4.0
Cellular_organisms - ps1
Archaea1 - ps2
Archaea2 - ps3
Archaea3 - ps4
Asgard/Eukaryota - ps5
Eukaryota - ps6
Amorpheae - ps7
Obazoa - ps8
Opisthokonta - ps9
Holozoa - ps10
Filozoa - ps11
Choano./Metazoa - ps12
Metazoa - ps13
Myriazoa - ps14
Parahoxozoa - ps15
Bilateria - ps16
Deuterostomia - ps17
Chordata - ps18
Olfactores - ps19
Vertebrata - ps20
Gnathostomata - ps21
Euteleostomi - ps22
Sarcopterygii - ps23
Tetrapoda - ps24
Amniota - ps25
Mammalia - ps26
Theria - ps27
Placentalia - ps28
Boreoeutheria - ps29
Euarchontoglires - ps30
Euarchonta - ps31
Primates - ps32
Haplorrhini - ps33
Simiiformes - ps34
Catarrhini - ps35
Hominoidea - ps36
Hominidae - ps37
Homininae - ps38
Hominini - ps39
Homo_sapiens - ps40
Phylostratum

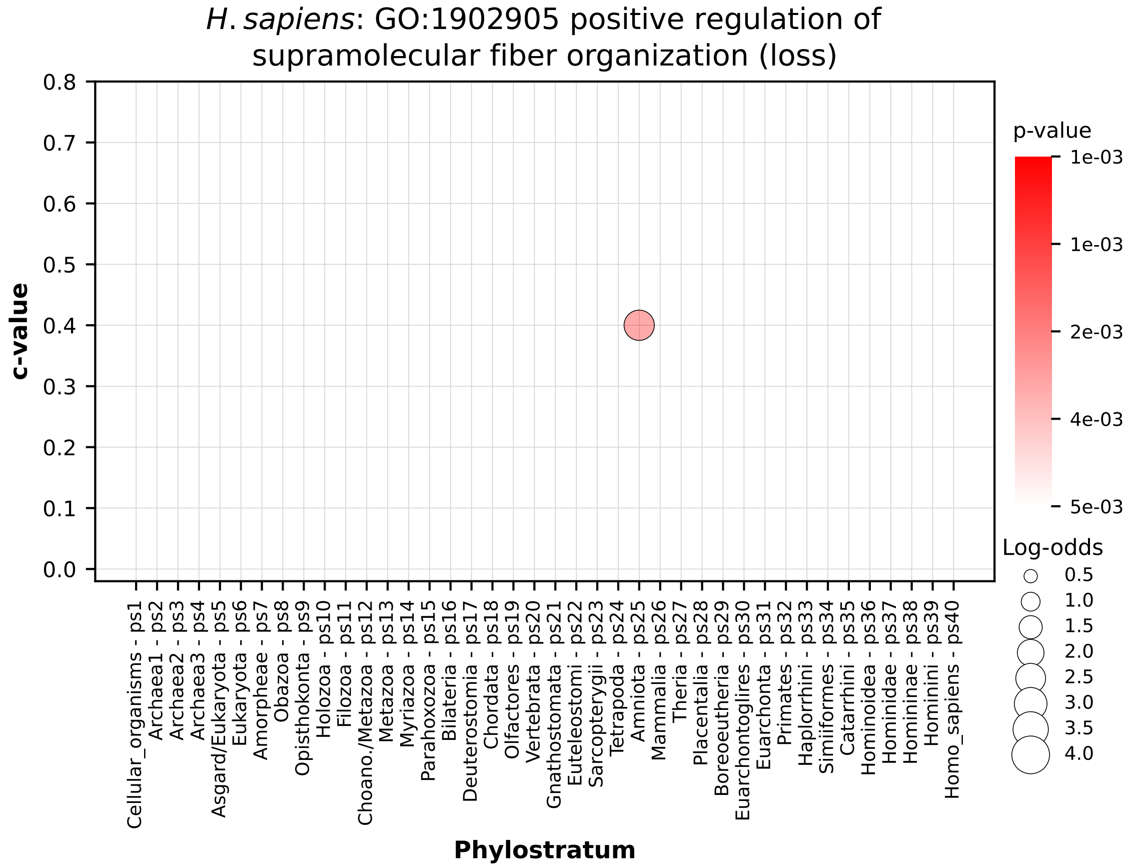

H. sapiens: GO:1902905 positive regulation of supramolecular fiber organization (loss)
c-value
0.0
0.1
0.2
0.3
0.4
0.5
0.6
0.7
0.8
Phylostratum
Cellular_organisms - ps1
Archaea1 - ps2
Archaea2 - ps3
Archaea3 - ps4
Asgard/Eukaryota - ps5
Eukaryota - ps6
Amorpheae - ps7
Obazoa - ps8
Opisthokonta - ps9
Holozoa - ps10
Filozoa - ps11
Choano./Metazoa - ps12
Metazoa - ps13
Myriazoa - ps14
Parahoxozoa - ps15
Bilateria - ps16
Deuterostomia - ps17
Chordata - ps18
Olfactores - ps19
Vertebrata - ps20
Gnathostomata - ps21
Euteleostomi - ps22
Sarcopterygii - ps23
Tetrapoda - ps24
Amniota - ps25
Mammalia - ps26
Theria - ps27
Placentalia - ps28
Boreoeutheria - ps29
Euarchontoglires - ps30
Euarchonta - ps31
Primates - ps32
Haplorrhini - ps33
Simiiformes - ps34
Catarrhini - ps35
Hominoidea - ps36
Hominidae - ps37
Homininae - ps38
Hominini - ps39
Homo_sapiens - ps40
p-value
1e-03
1e-03
2e-03
4e-03
5e-03
Log-odds
0.5
1.0
1.5
2.0
2.5
3.0
3.5
4.0

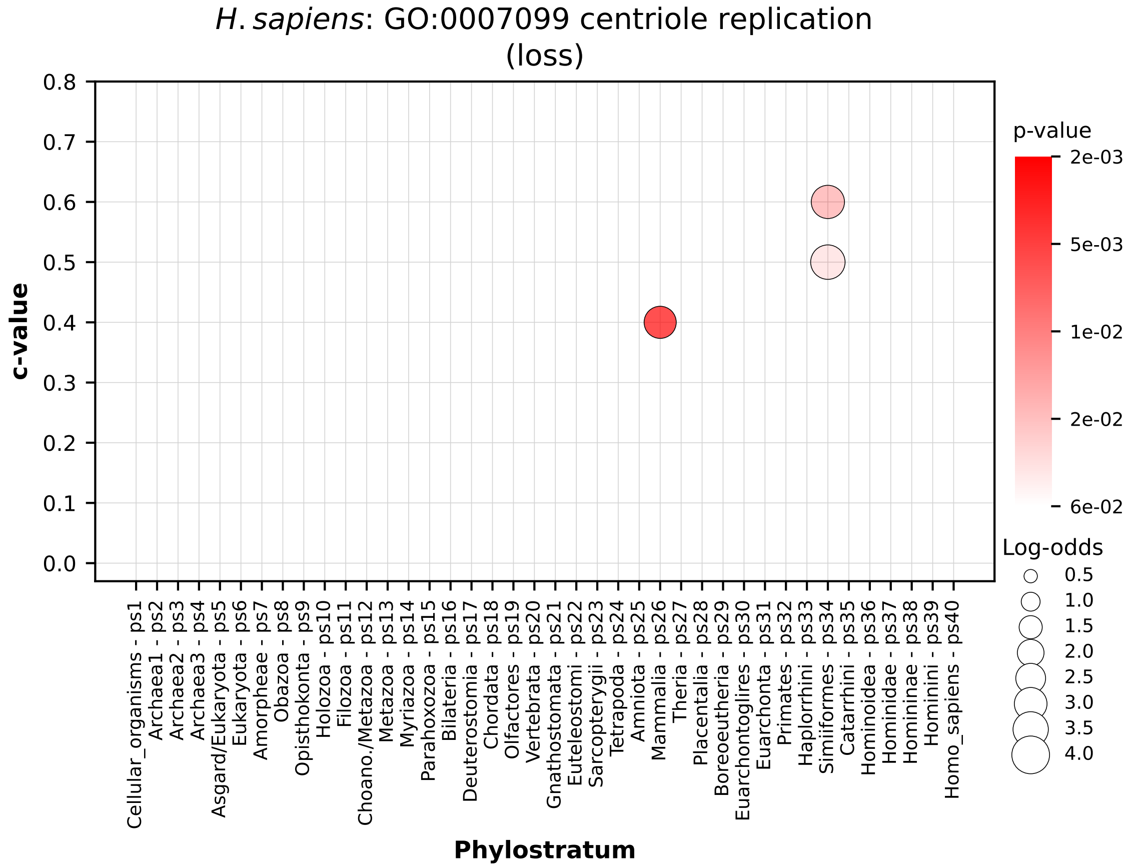

H. sapiens: GO:0007099 centriole replication
(loss)
c-value
0.0
0.1
0.2
0.3
0.4
0.5
0.6
0.7
0.8
p-value
2e-03
5e-03
1e-02
2e-02
6e-02
Log-odds
0.5
1.0
1.5
2.0
2.5
3.0
3.5
4.0
Cellular_organisms - ps1
Archaea1 - ps2
Archaea2 - ps3
Archaea3 - ps4
Asgard/Eukaryota - ps5
Eukaryota - ps6
Amorpheae - ps7
Obazoa - ps8
Opisthokonta - ps9
Holozoa - ps10
Filozoa - ps11
Choano./Metazoa - ps12
Metazoa - ps13
Myriazoa - ps14
Parahoxozoa - ps15
Bilateria - ps16
Deuterostomia - ps17
Chordata - ps18
Olfactores - ps19
Vertebrata - ps20
Gnathostomata - ps21
Euteleostomi - ps22
Sarcopterygii - ps23
Tetrapoda - ps24
Amniota - ps25
Mammalia - ps26
Theria - ps27
Placentalia - ps28
Boreoeutheria - ps29
Euarchontoglires - ps30
Euarchonta - ps31
Primates - ps32
Haplorrhini - ps33
Simiiformes - ps34
Catarrhini - ps35
Hominoidea - ps36
Hominidae - ps37
Homininae - ps38
Hominini - ps39
Homo_sapiens - ps40
Phylostratum

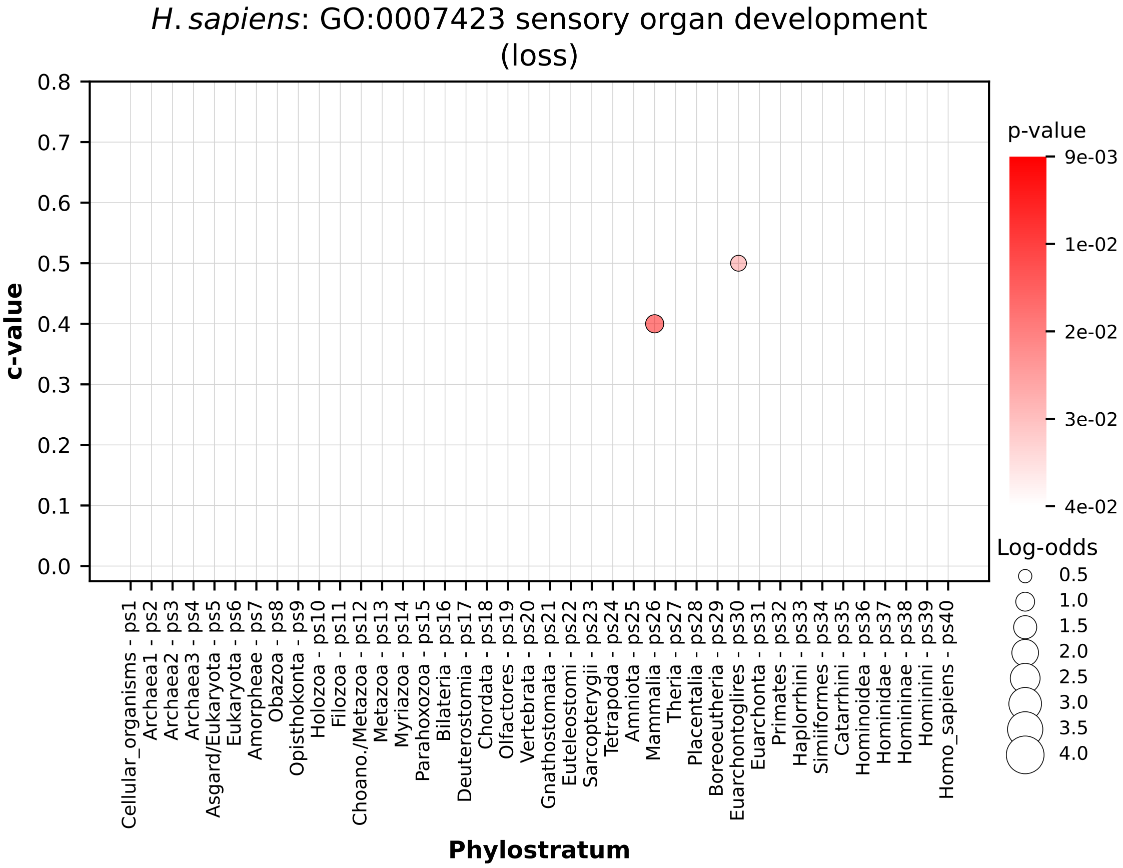

H. sapiens: GO:0007423 sensory organ development (loss)
c-value
0.8
0.7
0.6
0.5
0.4
0.3
0.2
0.1
0.0
Cellular_organisms - ps1
Archaea1 - ps2
Archaea2 - ps3
Archaea3 - ps4
Asgard/Eukaryota - ps5
Eukaryota - ps6
Amorpheae - ps7
Obazoa - ps8
Opisthokonta - ps9
Holozoa - ps10
Filozoa - ps11
Choano./Metazoa - ps12
Metazoa - ps13
Myriazoa - ps14
Parahoxozoa - ps15
Bilateria - ps16
Deuterostomia - ps17
Chordata - ps18
Olfactores - ps19
Vertebrata - ps20
Gnathostomata - ps21
Euteleostomi - ps22
Sarcopterygii - ps23
Tetrapoda - ps24
Amniota - ps25
Mammalia - ps26
Theria - ps27
Placentalia - ps28
Boreoeutheria - ps29
Euarchontoglires - ps30
Euarchonta - ps31
Primates - ps32
Haplorrhini - ps33
Simiiformes - ps34
Catarrhini - ps35
Hominoidea - ps36
Hominidae - ps37
Homininae - ps38
Hominini - ps39
Homo_sapiens - ps40
Phylostratum
p-value
9e-03
1e-02
2e-02
3e-02
4e-02
Log-odds
0.5
1.0
1.5
2.0
2.5
3.0
3.5
4.0

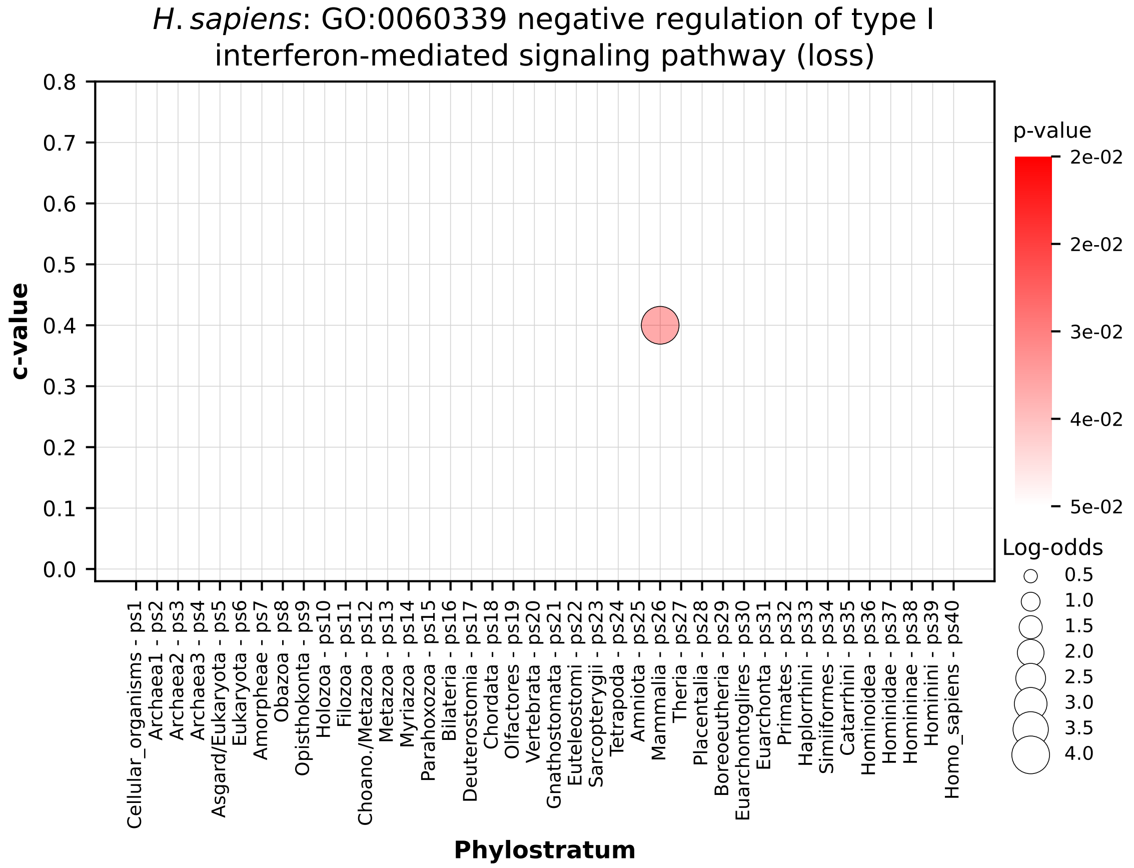

H. sapiens: GO:0060339 negative regulation of type I interferon-mediated signaling pathway (loss)
c-value
0.0
0.1
0.2
0.3
0.4
0.5
0.6
0.7
0.8
Phylostratum
Cellular_organisms - ps1
Archaea1 - ps2
Archaea2 - ps3
Archaea3 - ps4
Asgard/Eukaryota - ps5
Eukaryota - ps6
Amorpheae - ps7
Obazoa - ps8
Opisthokonta - ps9
Holozoa - ps10
Filozoa - ps11
Choano./Metazoa - ps12
Metazoa - ps13
Myriazoa - ps14
Parahoxozoa - ps15
Bilateria - ps16
Deuterostomia - ps17
Chordata - ps18
Olfactores - ps19
Vertebrata - ps20
Gnathostomata - ps21
Euteleostomi - ps22
Sarcopterygii - ps23
Tetrapoda - ps24
Amniota - ps25
Mammalia - ps26
Theria - ps27
Placentalia - ps28
Boreoeutheria - ps29
Euarchontoglires - ps30
Euarchonta - ps31
Primates - ps32
Haplorrhini - ps33
Simiiformes - ps34
Catarrhini - ps35
Hominoidea - ps36
Hominidae - ps37
Homininae - ps38
Hominini - ps39
Homo_sapiens - ps40
p-value
2e-02
2e-02
3e-02
4e-02
5e-02
Log-odds
0.5
1.0
1.5
2.0
2.5
3.0
3.5
4.0

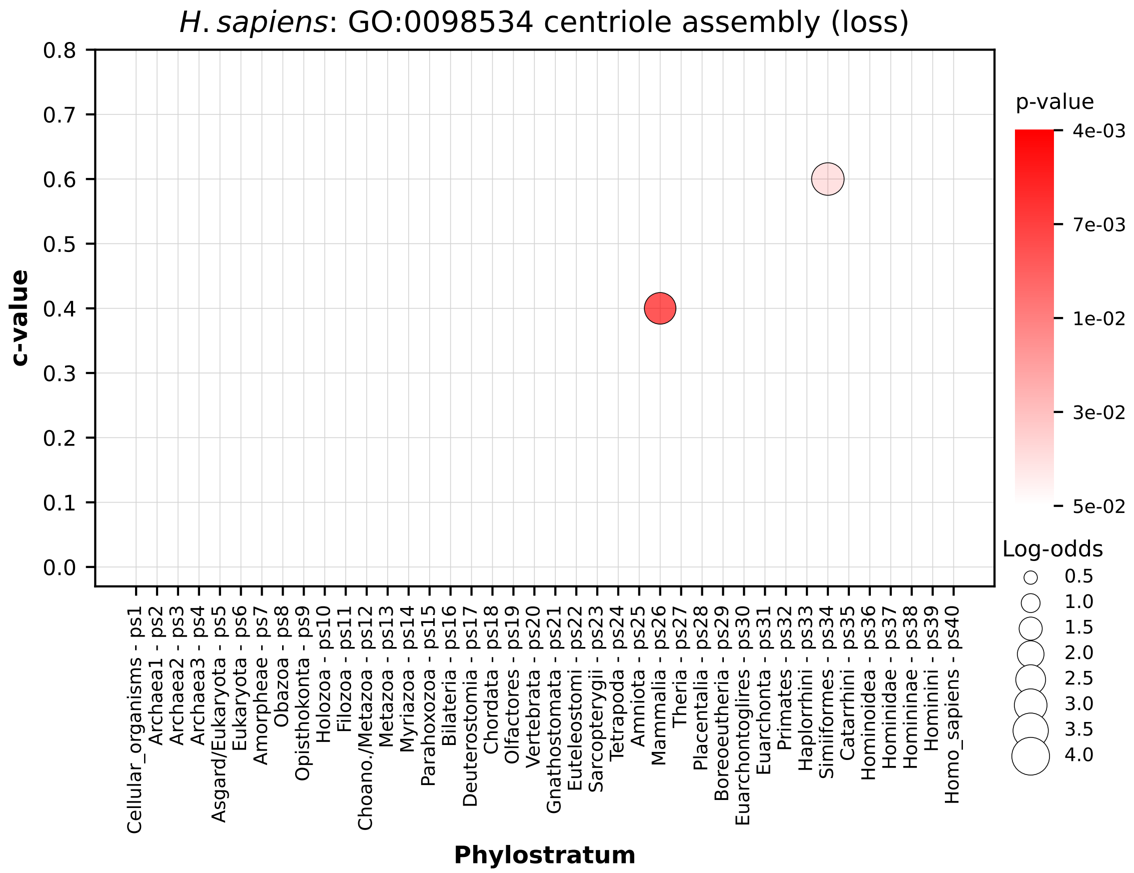

H. sapiens: GO:0098534 centriole assembly (loss)
c-value
0.0
0.1
0.2
0.3
0.4
0.5
0.6
0.7
0.8
Phylostratum
Cellular_organisms - ps1
Archaea1 - ps2
Archaea2 - ps3
Archaea3 - ps4
Asgard/Eukaryota - ps5
Eukaryota - ps6
Amorpheae - ps7
Obazoa - ps8
Opisthokonta - ps9
Holozoa - ps10
Filozoa - ps11
Choano./Metazoa - ps12
Metazoa - ps13
Myriazoa - ps14
Parahoxozoa - ps15
Bilateria - ps16
Deuterostomia - ps17
Chordata - ps18
Olfactores - ps19
Vertebrata - ps20
Gnathostomata - ps21
Euteleostomi - ps22
Sarcopterygii - ps23
Tetrapoda - ps24
Amniota - ps25
Mammalia - ps26
Theria - ps27
Placentalia - ps28
Boreoeutheria - ps29
Euarchontoglires - ps30
Euarchonta - ps31
Primates - ps32
Haplorrhini - ps33
Simiiformes - ps34
Catarrhini - ps35
Hominoidea - ps36
Hominidae - ps37
Homininae - ps38
Hominini - ps39
Homo_sapiens - ps40
p-value
4e-03
7e-03
1e-02
3e-02
5e-02
Log-odds
0.5
1.0
1.5
2.0
2.5
3.0
3.5
4.0

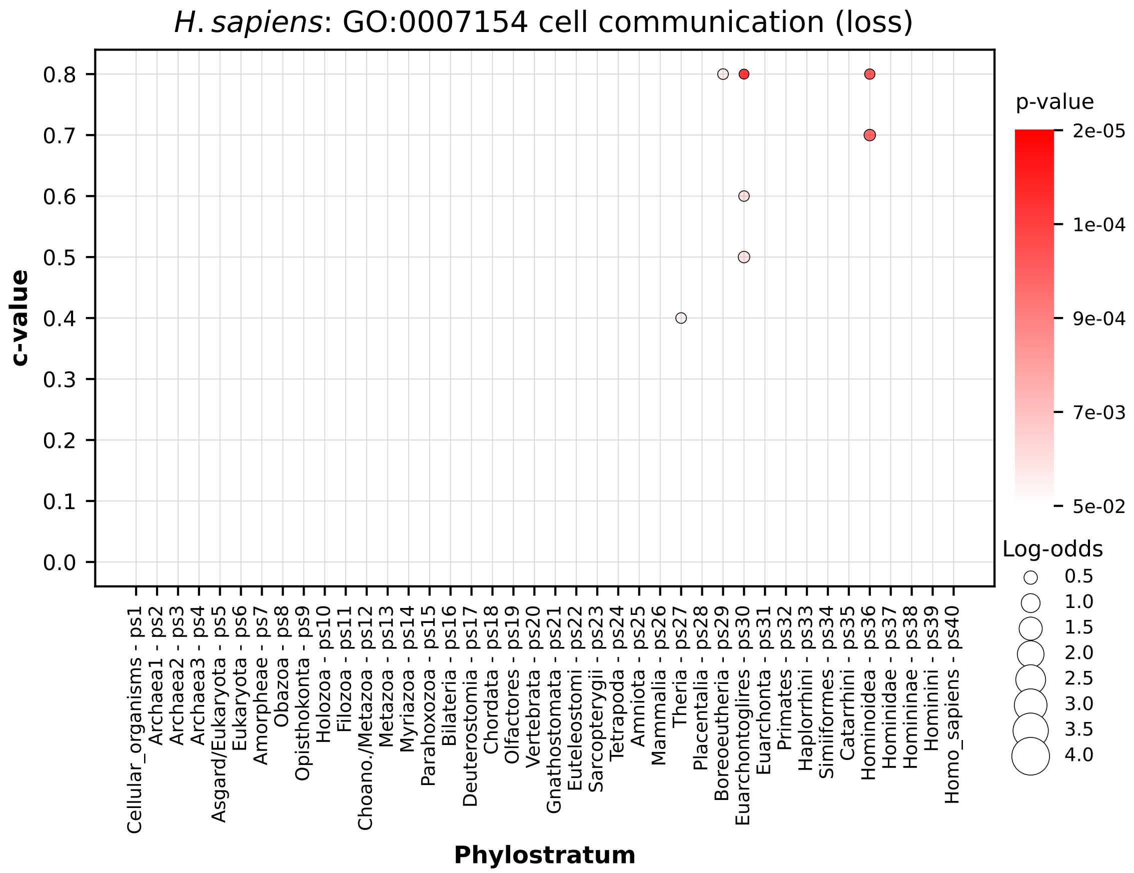

H. sapiens: GO:0007154 cell communication (loss)
c-value
0.0
0.1
0.2
0.3
0.4
0.5
0.6
0.7
0.8
Phylostratum
Cellular_organisms - ps1
Archaea1 - ps2
Archaea2 - ps3
Archaea3 - ps4
Asgard/Eukaryota - ps5
Eukaryota - ps6
Amorpheae - ps7
Obazoa - ps8
Opisthokonta - ps9
Holozoa - ps10
Filozoa - ps11
Choano./Metazoa - ps12
Metazoa - ps13
Myriazoa - ps14
Parahoxozoa - ps15
Bilateria - ps16
Deuterostomia - ps17
Chordata - ps18
Olfactores - ps19
Vertebrata - ps20
Gnathostomata - ps21
Euteleostomi - ps22
Sarcopterygii - ps23
Tetrapoda - ps24
Amniota - ps25
Mammalia - ps26
Theria - ps27
Placentalia - ps28
Boreoeutheria - ps29
Euarchontoglires - ps30
Euarchonta - ps31
Primates - ps32
Haplorrhini - ps33
Simiiformes - ps34
Catarrhini - ps35
Hominoidea - ps36
Hominidae - ps37
Homininae - ps38
Hominini - ps39
Homo_sapiens - ps40
p-value
2e-05
1e-04
9e-04
7e-03
5e-02
Log-odds
0.5
1.0
1.5
2.0
2.5
3.0
3.5
4.0

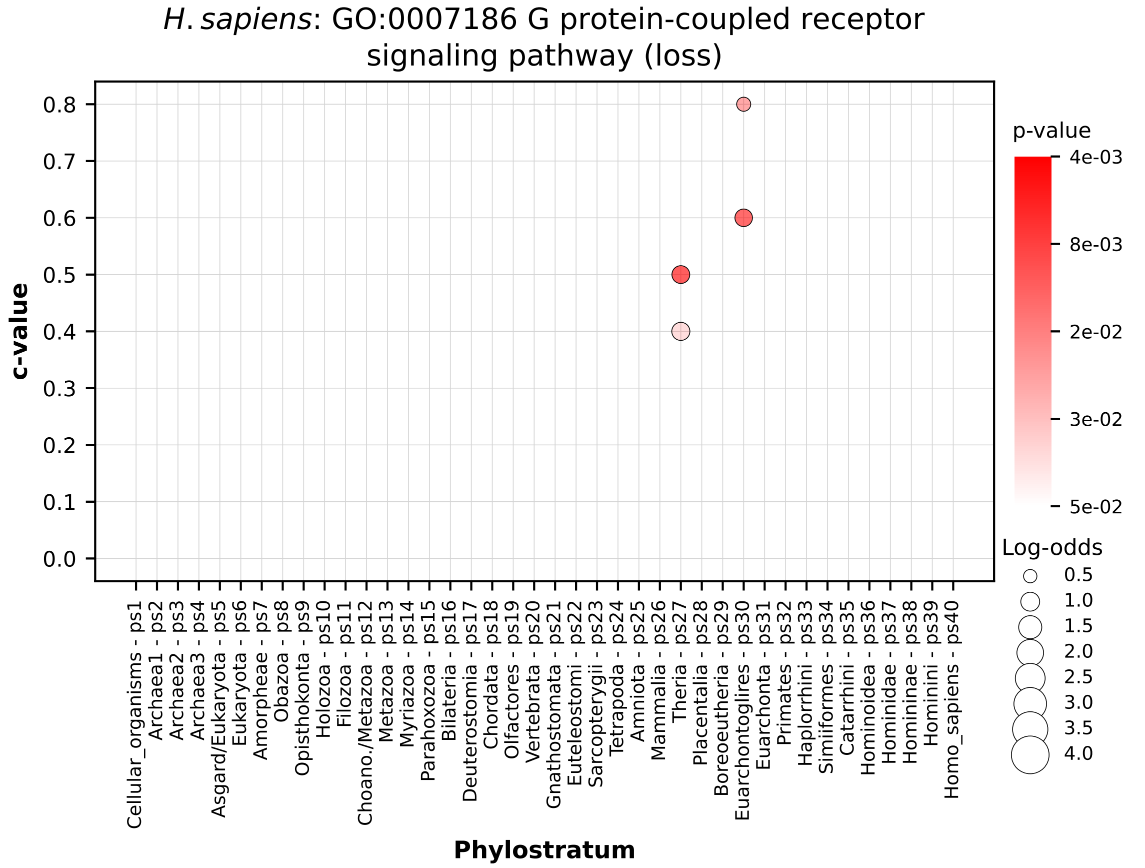

H. sapiens: GO:0007186 G protein-coupled receptor signaling pathway (loss)
c-value
0.8
0.7
0.6
0.5
0.4
0.3
0.2
0.1
0.0
p-value
4e-03
8e-03
2e-02
3e-02
5e-02
Log-odds
0.5
1.0
1.5
2.0
2.5
3.0
3.5
4.0
Cellular_organisms - ps1
Archaea1 - ps2
Archaea2 - ps3
Archaea3 - ps4
Asgard/Eukaryota - ps5
Eukaryota - ps6
Amorpheae - ps7
Obazoa - ps8
Opisthokonta - ps9
Holozoa - ps10
Filozoa - ps11
Choano./Metazoa - ps12
Metazoa - ps13
Myriazoa - ps14
Parahoxozoa - ps15
Bilateria - ps16
Deuterostomia - ps17
Chordata - ps18
Olfactores - ps19
Vertebrata - ps20
Gnathostomata - ps21
Euteleostomi - ps22
Sarcopterygii - ps23
Tetrapoda - ps24
Amniota - ps25
Mammalia - ps26
Theria - ps27
Placentalia - ps28
Boreoeutheria - ps29
Euarchontoglires - ps30
Euarchonta - ps31
Primates - ps32
Haplorrhini - ps33
Simiiformes - ps34
Catarrhini - ps35
Hominoidea - ps36
Hominidae - ps37
Homininae - ps38
Hominini - ps39
Homo_sapiens - ps40
Phylostratum

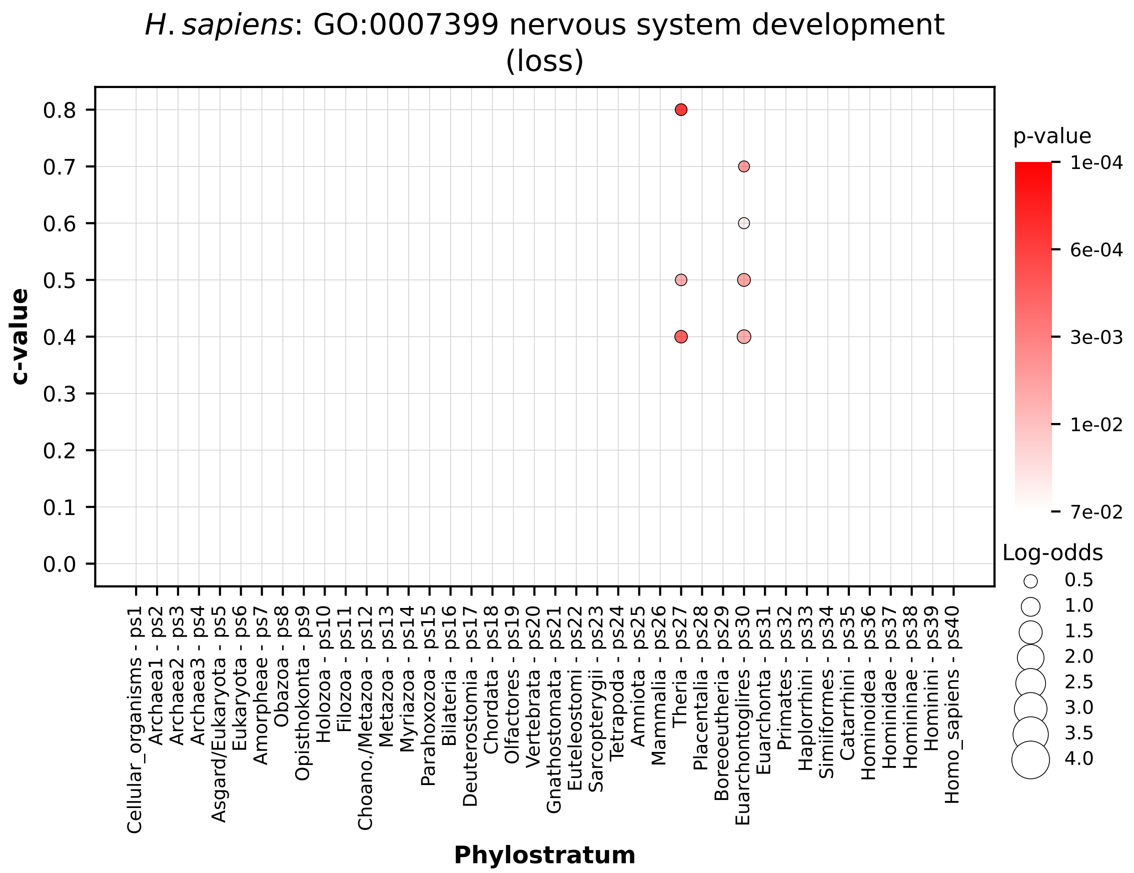

H. sapiens: GO:0007399 nervous system development
(loss)
c-value
0.8
0.7
0.6
0.5
0.4
0.3
0.2
0.1
0.0
p-value
1e-04
6e-04
3e-03
1e-02
7e-02
Log-odds
0.5
1.0
1.5
2.0
2.5
3.0
3.5
4.0
Cellular_organisms - ps1
Archaea1 - ps2
Archaea2 - ps3
Archaea3 - ps4
Asgard/Eukaryota - ps5
Eukaryota - ps6
Amorpheae - ps7
Obazoa - ps8
Opisthokonta - ps9
Holozoa - ps10
Filozoa - ps11
Choano./Metazoa - ps12
Metazoa - ps13
Myriazoa - ps14
Parahoxozoa - ps15
Bilateria - ps16
Deuterostomia - ps17
Chordata - ps18
Olfactores - ps19
Vertebrata - ps20
Gnathostomata - ps21
Euteleostomi - ps22
Sarcopterygii - ps23
Tetrapoda - ps24
Amniota - ps25
Mammalia - ps26
Theria - ps27
Placentalia - ps28
Boreoeutheria - ps29
Euarchontoglires - ps30
Euarchonta - ps31
Primates - ps32
Haplorrhini - ps33
Simiiformes - ps34
Catarrhini - ps35
Hominoidea - ps36
Hominidae - ps37
Homininae - ps38
Hominini - ps39
Homo_sapiens - ps40
Phylostratum

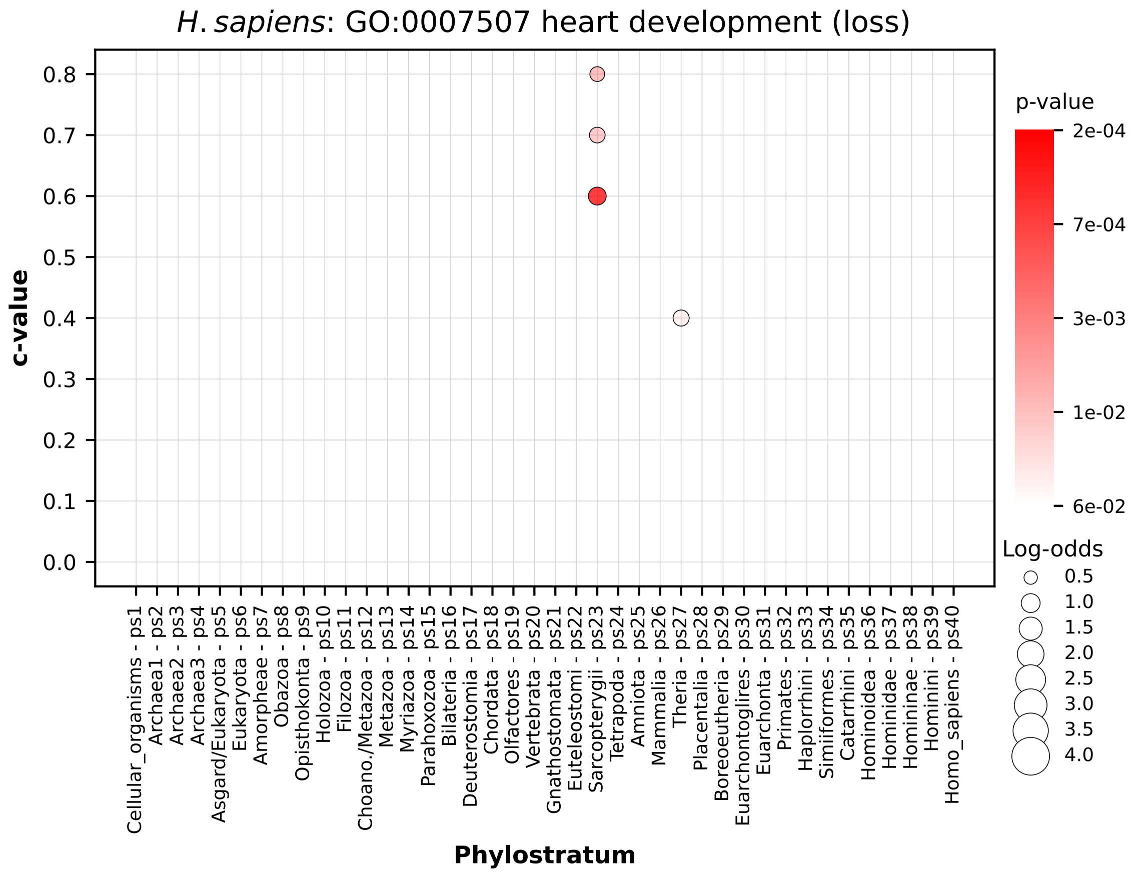
H. sapiens: GO:0007507 heart development (loss)
c-value
0.0
0.1
0.2
0.3
0.4
0.5
0.6
0.7
0.8
Phylostratum
Cellular_organisms - ps1
Archaea1 - ps2
Archaea2 - ps3
Archaea3 - ps4
Asgard/Eukaryota - ps5
Eukaryota - ps6
Amorpheae - ps7
Obazoa - ps8
Opisthokonta - ps9
Holozoa - ps10
Filozoa - ps11
Choano./Metazoa - ps12
Metazoa - ps13
Myriazoa - ps14
Parahoxozoa - ps15
Bilateria - ps16
Deuterostomia - ps17
Chordata - ps18
Olfactores - ps19
Vertebrata - ps20
Gnathostomata - ps21
Euteleostomi - ps22
Sarcopterygii - ps23
Tetrapoda - ps24
Amniota - ps25
Mammalia - ps26
Theria - ps27
Placentalia - ps28
Boreoeutheria - ps29
Euarchontoglires - ps30
Euarchonta - ps31
Primates - ps32
Haplorrhini - ps33
Simiiformes - ps34
Catarrhini - ps35
Hominoidea - ps36
Hominidae - ps37
Homininae - ps38
Hominini - ps39
Homo_sapiens - ps40
p-value
2e-04
7e-04
3e-03
1e-02
6e-02
Log-odds
0.5
1.0
1.5
2.0
2.5
3.0
3.5
4.0

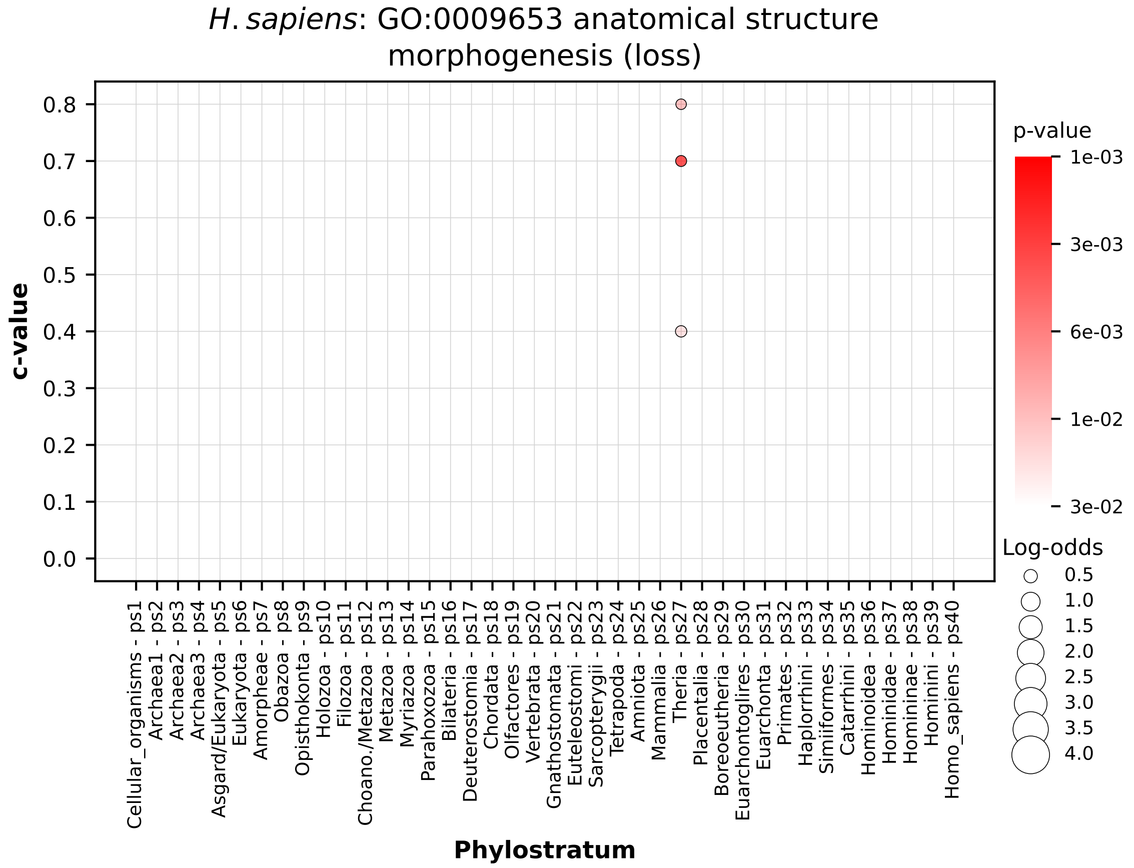

H. sapiens: GO:0009653 anatomical structure morphogenesis (loss)
c-value
0.8
0.7
0.6
0.5
0.4
0.3
0.2
0.1
0.0
p-value
1e-03
3e-03
6e-03
1e-02
3e-02
Log-odds
0.5
1.0
1.5
2.0
2.5
3.0
3.5
4.0
Cellular_organisms - ps1
Archaea1 - ps2
Archaea2 - ps3
Archaea3 - ps4
Asgard/Eukaryota - ps5
Eukaryota - ps6
Amorpheae - ps7
Obazoa - ps8
Opisthokonta - ps9
Holozoa - ps10
Filozoa - ps11
Choano./Metazoa - ps12
Metazoa - ps13
Myriazoa - ps14
Parahoxozoa - ps15
Bilateria - ps16
Deuterostomia - ps17
Chordata - ps18
Olfactores - ps19
Vertebrata - ps20
Gnathostomata - ps21
Euteleostomi - ps22
Sarcopterygii - ps23
Tetrapoda - ps24
Amniota - ps25
Mammalia - ps26
Theria - ps27
Placentalia - ps28
Boreoeutheria - ps29
Euarchontoglires - ps30
Euarchonta - ps31
Primates - ps32
Haplorrhini - ps33
Simiiformes - ps34
Catarrhini - ps35
Hominoidea - ps36
Hominidae - ps37
Homininae - ps38
Hominini - ps39
Homo_sapiens - ps40
Phylostratum

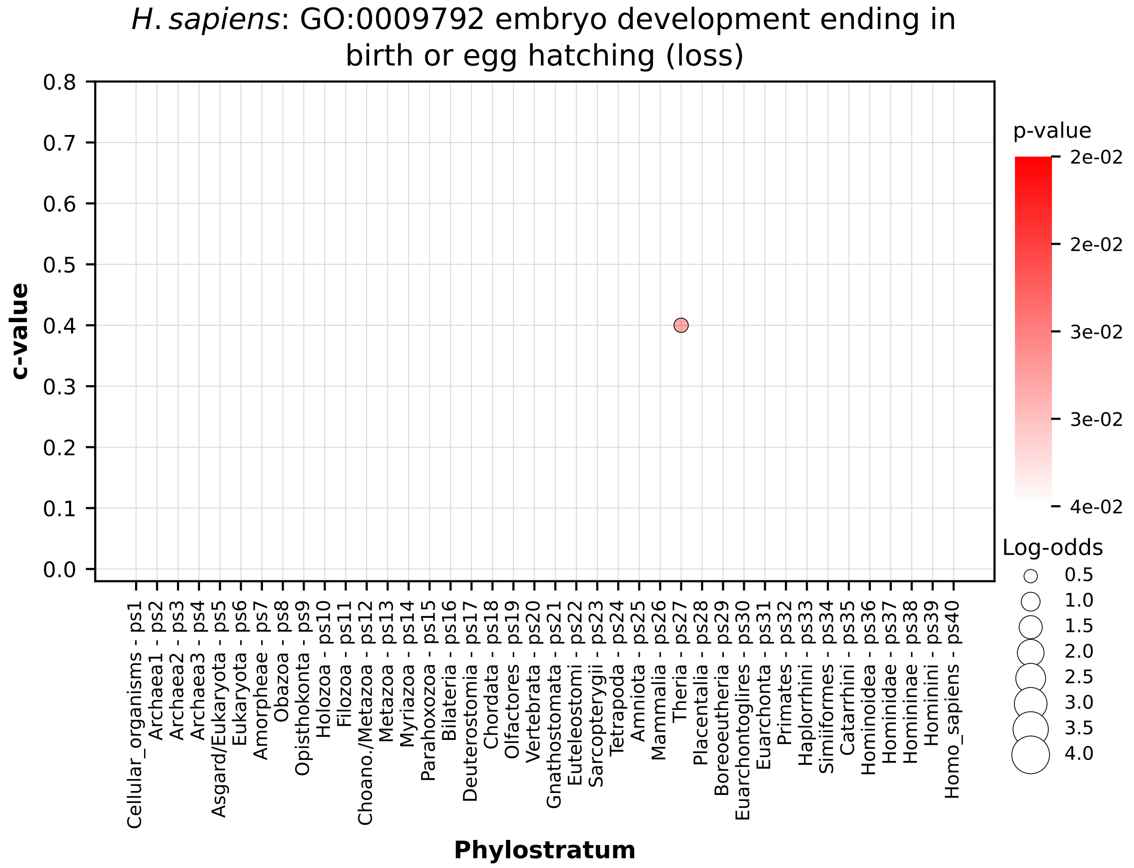
H. sapiens: GO:0009792 embryo development ending in birth or egg hatching (loss)
c-value
0.8
0.7
0.6
0.5
0.4
0.3
0.2
0.1
0.0
Phylostratum
Cellular_organisms - ps1
Archaea1 - ps2
Archaea2 - ps3
Archaea3 - ps4
Asgard/Eukaryota - ps5
Eukaryota - ps6
Amorpheae - ps7
Obazoa - ps8
Opisthokonta - ps9
Holozoa - ps10
Filozoa - ps11
Choano./Metazoa - ps12
Metazoa - ps13
Myriazoa - ps14
Parahoxozoa - ps15
Bilateria - ps16
Deuterostomia - ps17
Chordata - ps18
Olfactores - ps19
Vertebrata - ps20
Gnathostomata - ps21
Euteleostomi - ps22
Sarcopterygii - ps23
Tetrapoda - ps24
Amniota - ps25
Mammalia - ps26
Theria - ps27
Placentalia - ps28
Boreoeutheria - ps29
Euarchontoglires - ps30
Euarchonta - ps31
Primates - ps32
Haplorrhini - ps33
Simiiformes - ps34
Catarrhini - ps35
Hominoidea - ps36
Hominidae - ps37
Homininae - ps38
Hominini - ps39
Homo_sapiens - ps40
p-value
2e-02
2e-02
3e-02
3e-02
4e-02
Log-odds
0.5
1.0
1.5
2.0
2.5
3.0
3.5
4.0

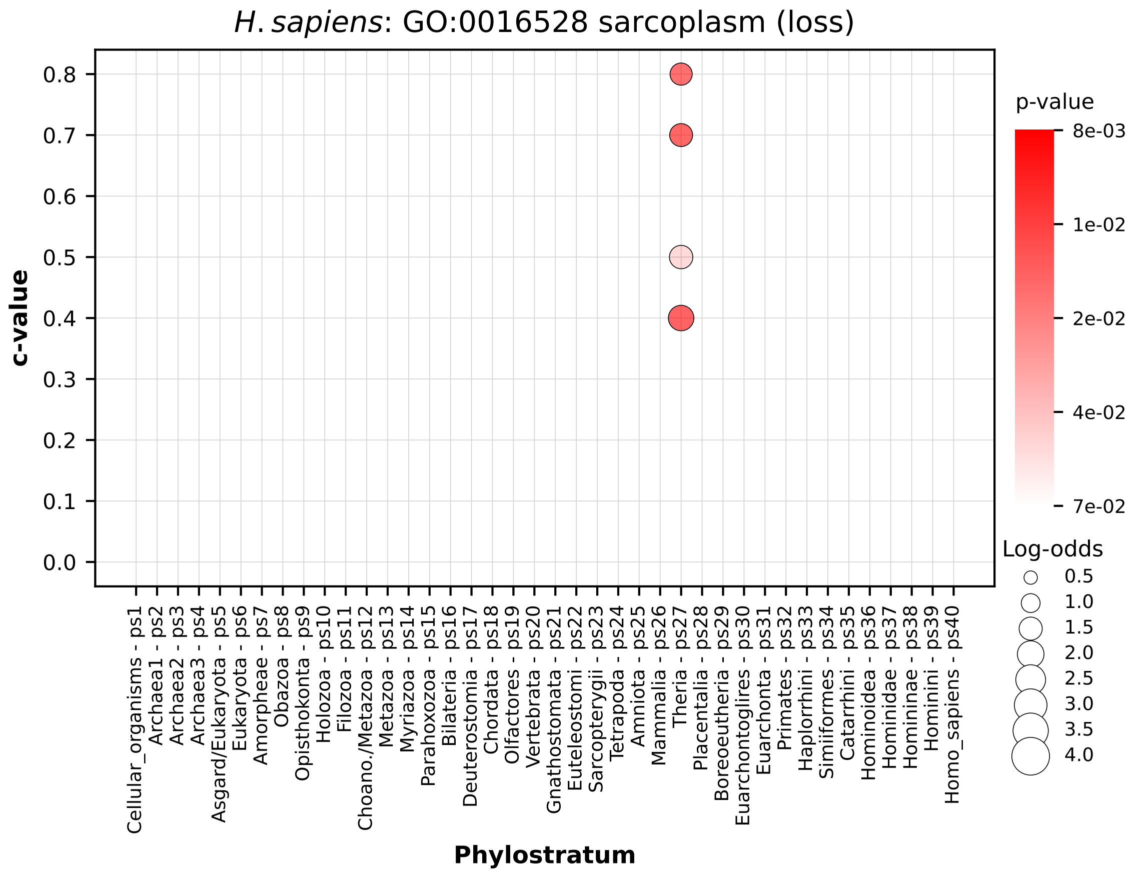
H. sapiens: GO:0016528 sarcoplasm (loss)
c-value
0.8
0.7
0.6
0.5
0.4
0.3
0.2
0.1
0.0
p-value
8e-03
1e-02
2e-02
4e-02
7e-02
Log-odds
0.5
1.0
1.5
2.0
2.5
3.0
3.5
4.0
Cellular_organisms - ps1
Archaea1 - ps2
Archaea2 - ps3
Archaea3 - ps4
Asgard/Eukaryota - ps5
Eukaryota - ps6
Amorpheae - ps7
Obazoa - ps8
Opisthokonta - ps9
Holozoa - ps10
Filozoa - ps11
Choano./Metazoa - ps12
Metazoa - ps13
Myriazoa - ps14
Parahoxozoa - ps15
Bilateria - ps16
Deuterostomia - ps17
Chordata - ps18
Olfactores - ps19
Vertebrata - ps20
Gnathostomata - ps21
Euteleostomi - ps22
Sarcopterygii - ps23
Tetrapoda - ps24
Amniota - ps25
Mammalia - ps26
Theria - ps27
Placentalia - ps28
Boreoeutheria - ps29
Euarchontoglires - ps30
Euarchonta - ps31
Primates - ps32
Haplorrhini - ps33
Simiiformes - ps34
Catarrhini - ps35
Hominoidea - ps36
Hominidae - ps37
Homininae - ps38
Hominini - ps39
Homo_sapiens - ps40
Phylostratum

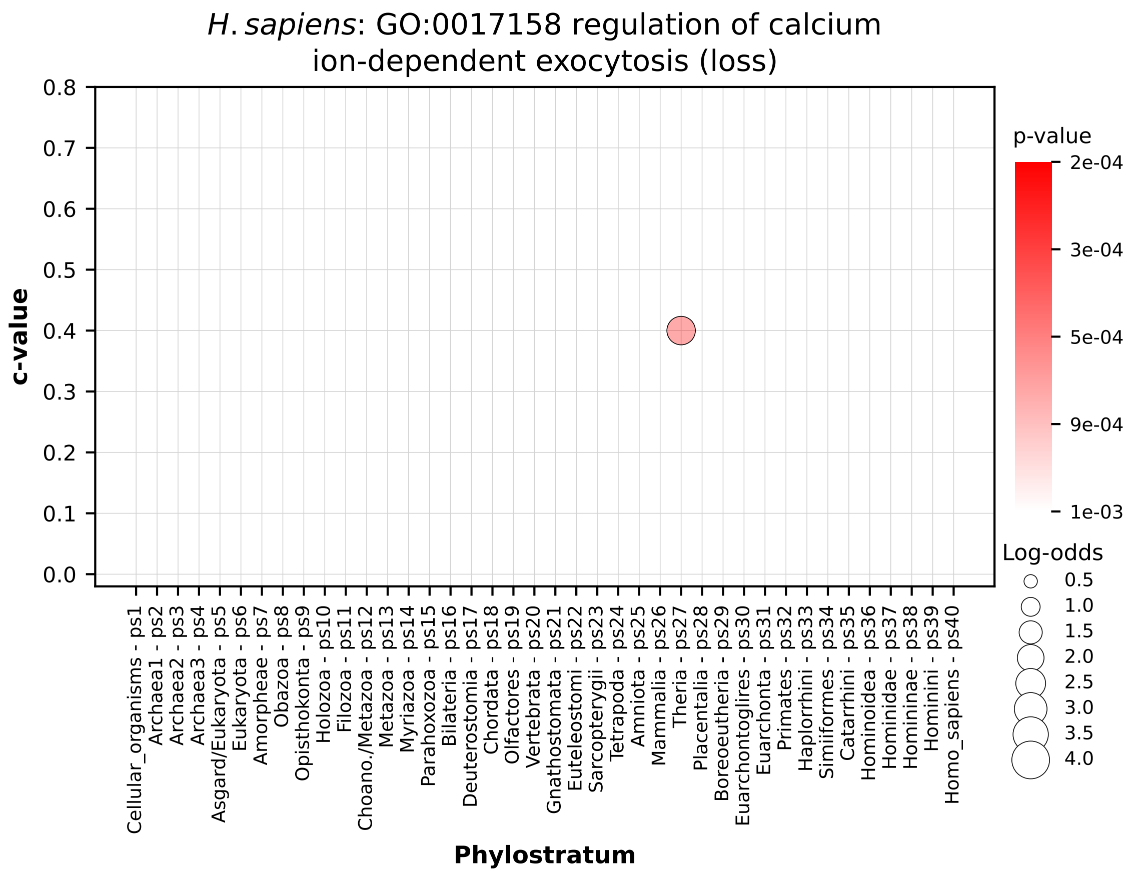
H. sapiens: GO:0017158 regulation of calcium ion-dependent exocytosis (loss)
c-value
0.0
0.1
0.2
0.3
0.4
0.5
0.6
0.7
0.8
Phylostratum
Cellular_organisms - ps1
Archaea1 - ps2
Archaea2 - ps3
Archaea3 - ps4
Asgard/Eukaryota - ps5
Eukaryota - ps6
Amorpheae - ps7
Obazoa - ps8
Opisthokonta - ps9
Holozoa - ps10
Filozoa - ps11
Choano./Metazoa - ps12
Metazoa - ps13
Myriazoa - ps14
Parahoxozoa - ps15
Bilateria - ps16
Deuterostomia - ps17
Chordata - ps18
Olfactores - ps19
Vertebrata - ps20
Gnathostomata - ps21
Euteleostomi - ps22
Sarcopterygii - ps23
Tetrapoda - ps24
Amniota - ps25
Mammalia - ps26
Theria - ps27
Placentalia - ps28
Boreoeutheria - ps29
Euarchontoglires - ps30
Euarchonta - ps31
Primates - ps32
Haplorrhini - ps33
Simiiformes - ps34
Catarrhini - ps35
Hominoidea - ps36
Hominidae - ps37
Homininae - ps38
Hominini - ps39
Homo_sapiens - ps40
p-value
2e-04
3e-04
5e-04
9e-04
1e-03
Log-odds
0.5
1.0
1.5
2.0
2.5
3.0
3.5
4.0

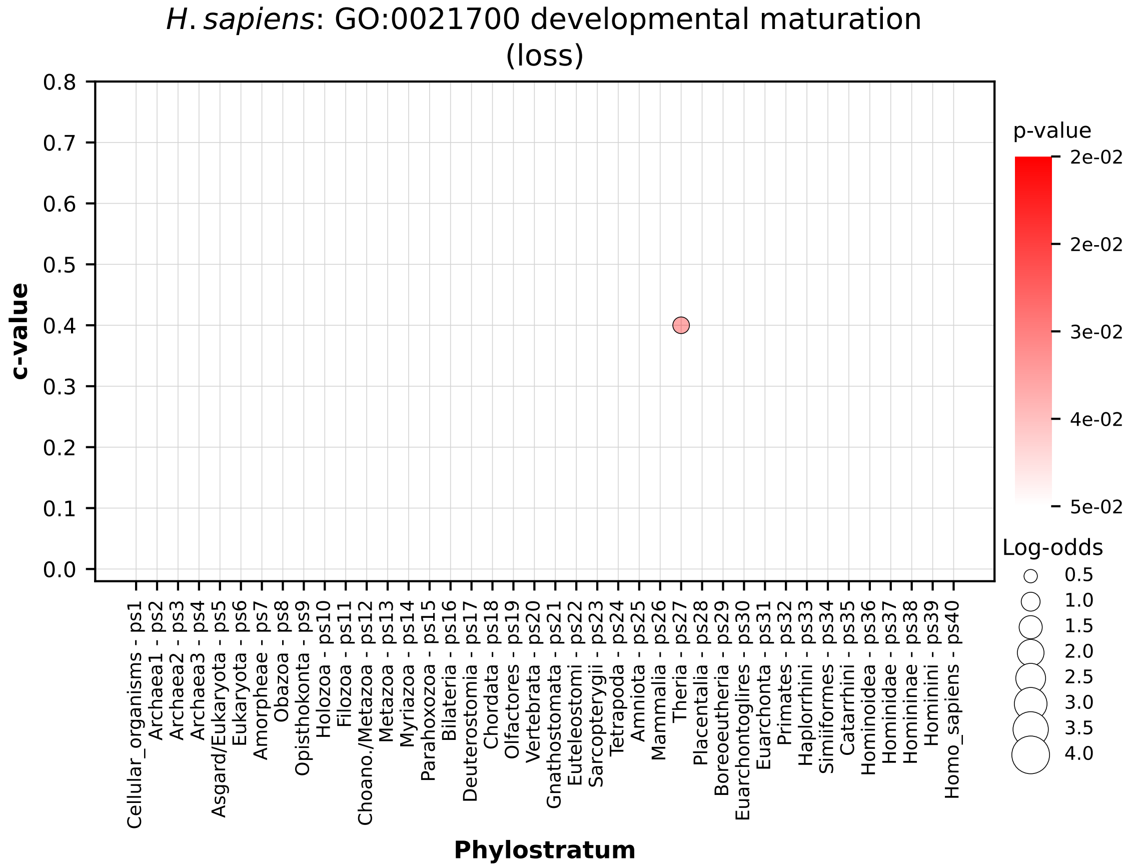

H. sapiens: GO:0021700 developmental maturation
(loss)
c-value
0.0
0.1
0.2
0.3
0.4
0.5
0.6
0.7
0.8
Phylostratum
Cellular_organisms - ps1
Archaea1 - ps2
Archaea2 - ps3
Archaea3 - ps4
Asgard/Eukaryota - ps5
Eukaryota - ps6
Amorpheae - ps7
Obazoa - ps8
Opisthokonta - ps9
Holozoa - ps10
Filozoa - ps11
Choano./Metazoa - ps12
Metazoa - ps13
Myriazoa - ps14
Parahoxozoa - ps15
Bilateria - ps16
Deuterostomia - ps17
Chordata - ps18
Olfactores - ps19
Vertebrata - ps20
Gnathostomata - ps21
Euteleostomi - ps22
Sarcopterygii - ps23
Tetrapoda - ps24
Amniota - ps25
Mammalia - ps26
Theria - ps27
Placentalia - ps28
Boreoeutheria - ps29
Euarchontoglires - ps30
Euarchonta - ps31
Primates - ps32
Haplorrhini - ps33
Simiiformes - ps34
Catarrhini - ps35
Hominoidea - ps36
Hominidae - ps37
Homininae - ps38
Hominini - ps39
Homo_sapiens - ps40
p-value
2e-02
2e-02
3e-02
4e-02
5e-02
Log-odds
0.5
1.0
1.5
2.0
2.5
3.0
3.5
4.0

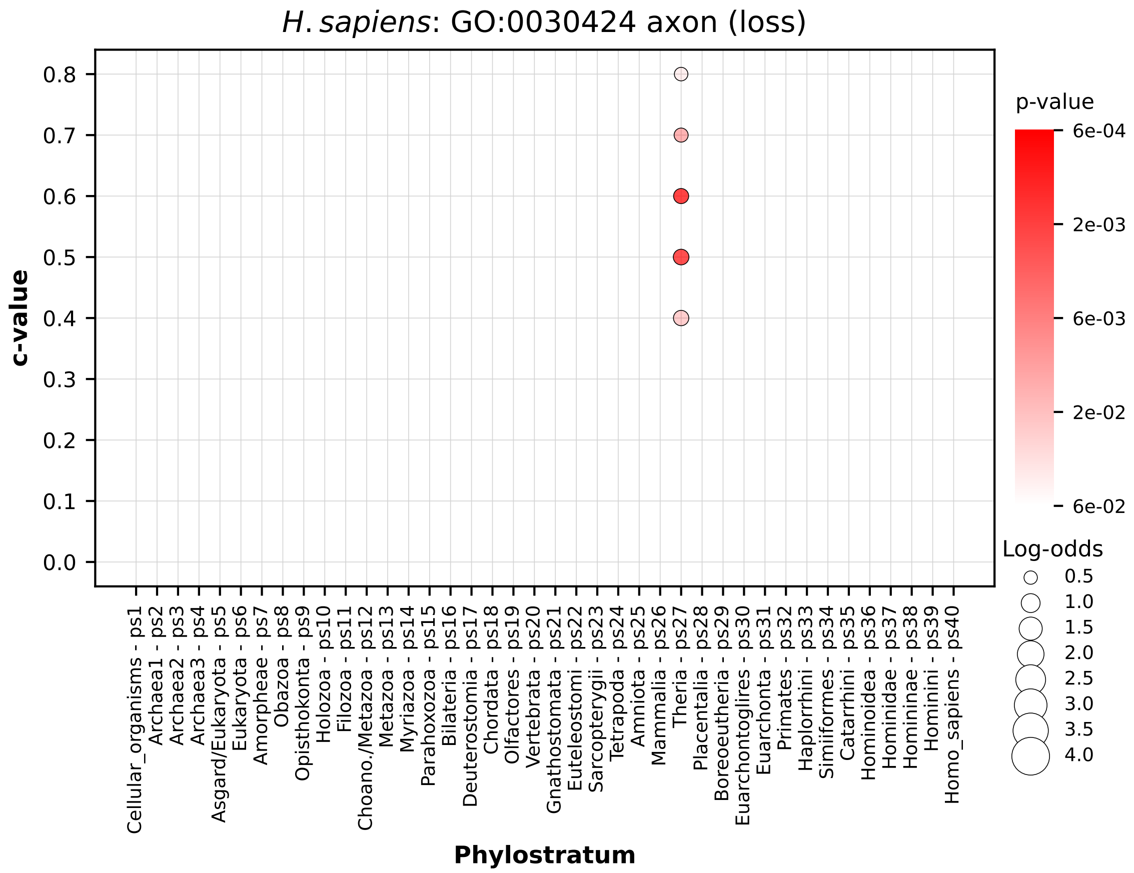
H. sapiens: GO:0030424 axon (loss)
c-value
0.0
0.1
0.2
0.3
0.4
0.5
0.6
0.7
0.8
Phylostratum
Cellular_organisms - ps1
Archaea1 - ps2
Archaea2 - ps3
Archaea3 - ps4
Asgard/Eukaryota - ps5
Eukaryota - ps6
Amorpheae - ps7
Obazoa - ps8
Opisthokonta - ps9
Holozoa - ps10
Filozoa - ps11
Choano./Metazoa - ps12
Metazoa - ps13
Myriazoa - ps14
Parahoxozoa - ps15
Bilateria - ps16
Deuterostomia - ps17
Chordata - ps18
Olfactores - ps19
Vertebrata - ps20
Gnathostomata - ps21
Euteleostomi - ps22
Sarcopterygii - ps23
Tetrapoda - ps24
Amniota - ps25
Mammalia - ps26
Theria - ps27
Placentalia - ps28
Boreoeutheria - ps29
Euarchontoglires - ps30
Euarchonta - ps31
Primates - ps32
Haplorrhini - ps33
Simiiformes - ps34
Catarrhini - ps35
Hominoidea - ps36
Hominidae - ps37
Homininae - ps38
Hominini - ps39
Homo_sapiens - ps40
p-value
6e-04
2e-03
6e-03
2e-02
6e-02
Log-odds
0.5
1.0
1.5
2.0
2.5
3.0
3.5
4.0

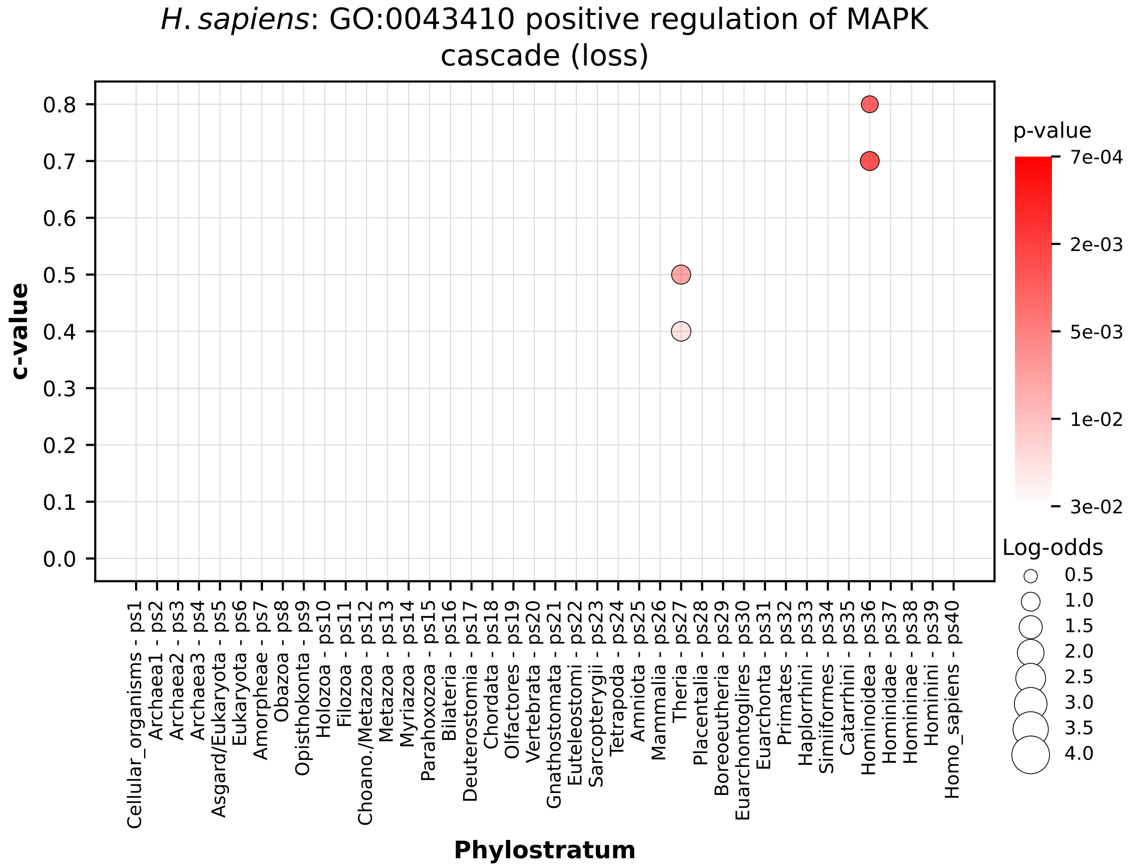

H. sapiens: GO:0043410 positive regulation of MAPK cascade (loss)
c-value
0.0
0.1
0.2
0.3
0.4
0.5
0.6
0.7
0.8
Phylostratum
Cellular_organisms - ps1
Archaea1 - ps2
Archaea2 - ps3
Archaea3 - ps4
Asgard/Eukaryota - ps5
Eukaryota - ps6
Amorpheae - ps7
Obazoa - ps8
Opisthokonta - ps9
Holozoa - ps10
Filozoa - ps11
Choano./Metazoa - ps12
Metazoa - ps13
Myriazoa - ps14
Parahoxozoa - ps15
Bilateria - ps16
Deuterostomia - ps17
Chordata - ps18
Olfactores - ps19
Vertebrata - ps20
Gnathostomata - ps21
Euteleostomi - ps22
Sarcopterygii - ps23
Tetrapoda - ps24
Amniota - ps25
Mammalia - ps26
Theria - ps27
Placentalia - ps28
Boreoeutheria - ps29
Euarchontoglires - ps30
Euarchonta - ps31
Primates - ps32
Haplorrhini - ps33
Simiiformes - ps34
Catarrhini - ps35
Hominoidea - ps36
Hominidae - ps37
Homininae - ps38
Hominini - ps39
Homo_sapiens - ps40
p-value
7e-04
2e-03
5e-03
1e-02
3e-02
Log-odds
0.5
1.0
1.5
2.0
2.5
3.0
3.5
4.0

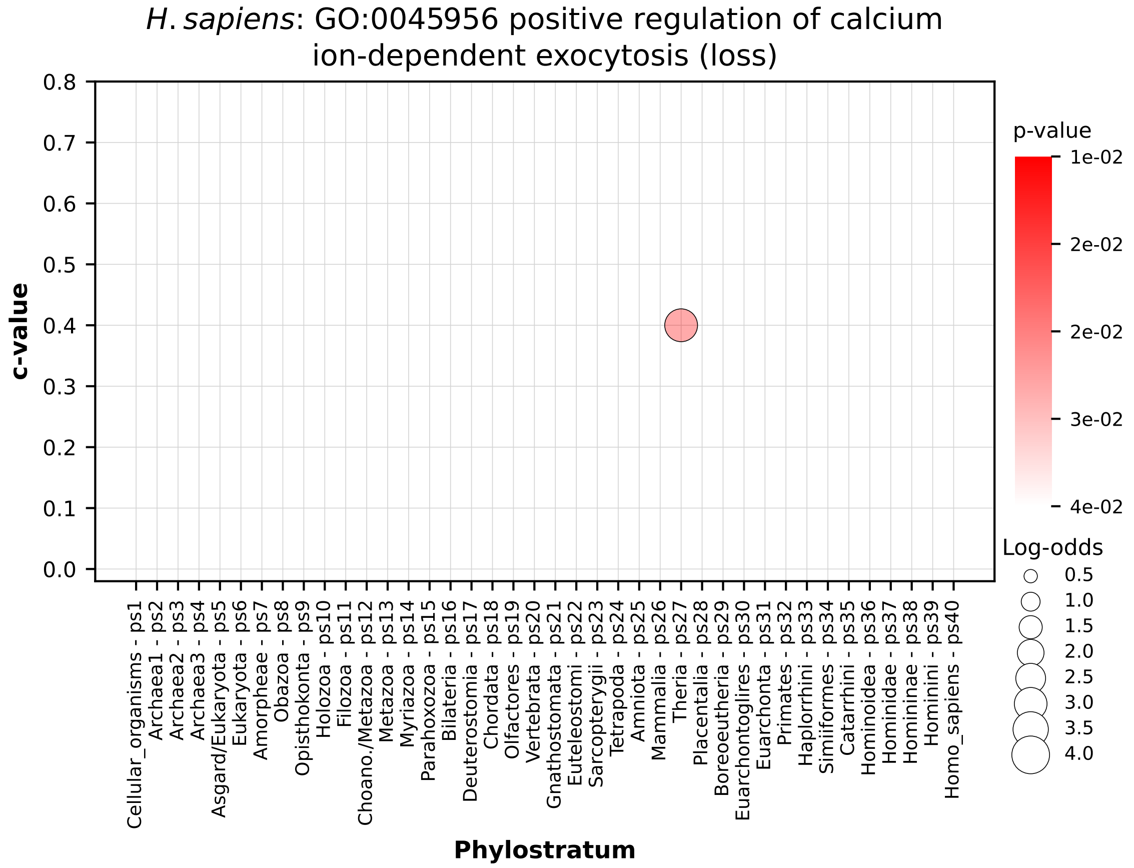

H. sapiens: GO:0045956 positive regulation of calcium ion-dependent exocytosis (loss)
c-value
0.8
0.7
0.6
0.5
0.4
0.3
0.2
0.1
0.0
Phylostratum
Cellular_organisms - ps1
Archaea1 - ps2
Archaea2 - ps3
Archaea3 - ps4
Asgard/Eukaryota - ps5
Eukaryota - ps6
Amorpheae - ps7
Obazoa - ps8
Opisthokonta - ps9
Holozoa - ps10
Filozoa - ps11
Choano./Metazoa - ps12
Metazoa - ps13
Myriazoa - ps14
Parahoxozoa - ps15
Bilateria - ps16
Deuterostomia - ps17
Chordata - ps18
Olfactores - ps19
Vertebrata - ps20
Gnathostomata - ps21
Euteleostomi - ps22
Sarcopterygii - ps23
Tetrapoda - ps24
Amniota - ps25
Mammalia - ps26
Theria - ps27
Placentalia - ps28
Boreoeutheria - ps29
Euarchontoglires - ps30
Euarchonta - ps31
Primates - ps32
Haplorrhini - ps33
Simiiformes - ps34
Catarrhini - ps35
Hominoidea - ps36
Hominidae - ps37
Homininae - ps38
Hominini - ps39
Homo_sapiens - ps40
p-value
1e-02
2e-02
2e-02
3e-02
4e-02
Log-odds
0.5
1.0
1.5
2.0
2.5
3.0
3.5
4.0

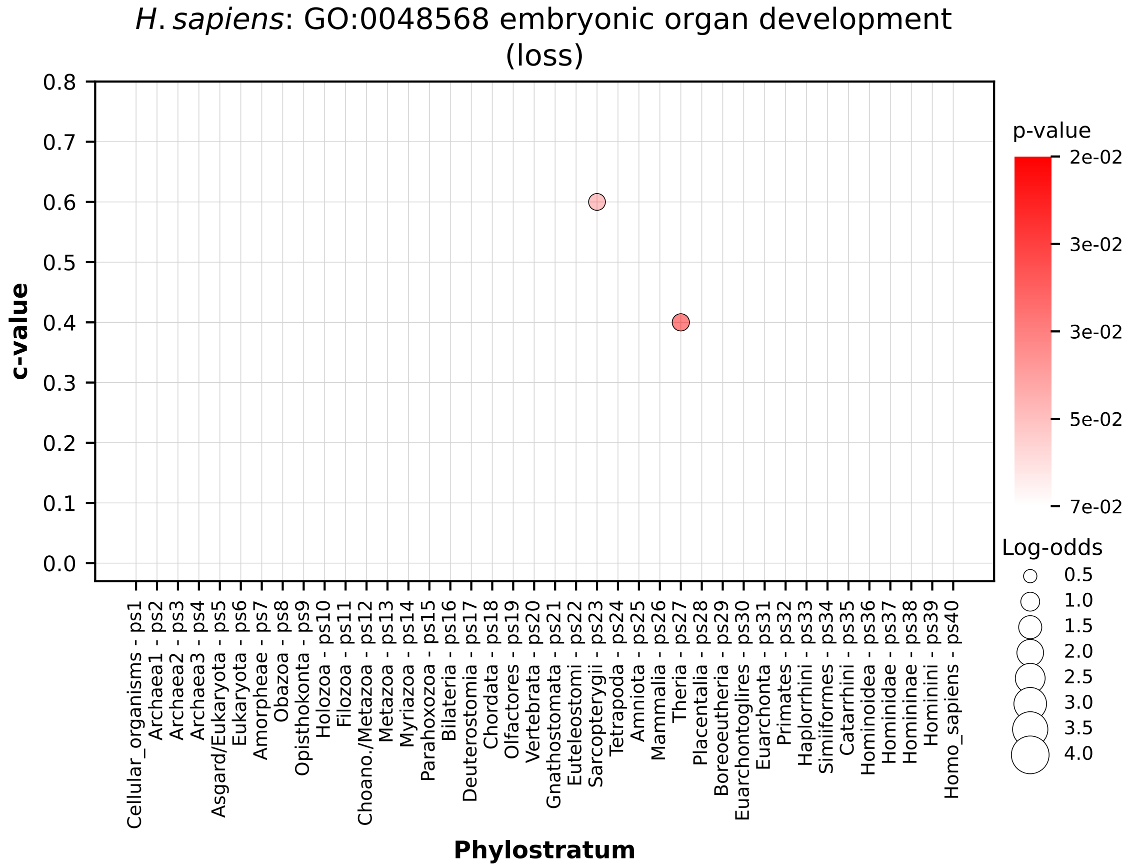

$H. sapiens$: GO:0048568 embryonic organ development (loss)
c-value
0.0
0.1
0.2
0.3
0.4
0.5
0.6
0.7
0.8
Phylostratum
Cellular_organisms - ps1
Archaea1 - ps2
Archaea2 - ps3
Archaea3 - ps4
Asgard/Eukaryota - ps5
Eukaryota - ps6
Amorpheae - ps7
Obazoa - ps8
Opisthokonta - ps9
Holozoa - ps10
Filozoa - ps11
Choano./Metazoa - ps12
Metazoa - ps13
Myriazoa - ps14
Parahoxozoa - ps15
Bilateria - ps16
Deuterostomia - ps17
Chordata - ps18
Olfactores - ps19
Vertebrata - ps20
Gnathostomata - ps21
Euteleostomi - ps22
Sarcopterygii - ps23
Tetrapoda - ps24
Amniota - ps25
Mammalia - ps26
Theria - ps27
Placentalia - ps28
Boreoeutheria - ps29
Euarchontoglires - ps30
Euarchonta - ps31
Primates - ps32
Haplorrhini - ps33
Simiiformes - ps34
Catarrhini - ps35
Hominoidea - ps36
Hominidae - ps37
Homininae - ps38
Hominini - ps39
Homo_sapiens - ps40
p-value
2e-02
3e-02
3e-02
5e-02
7e-02
Log-odds
0.5
1.0
1.5
2.0
2.5
3.0
3.5
4.0

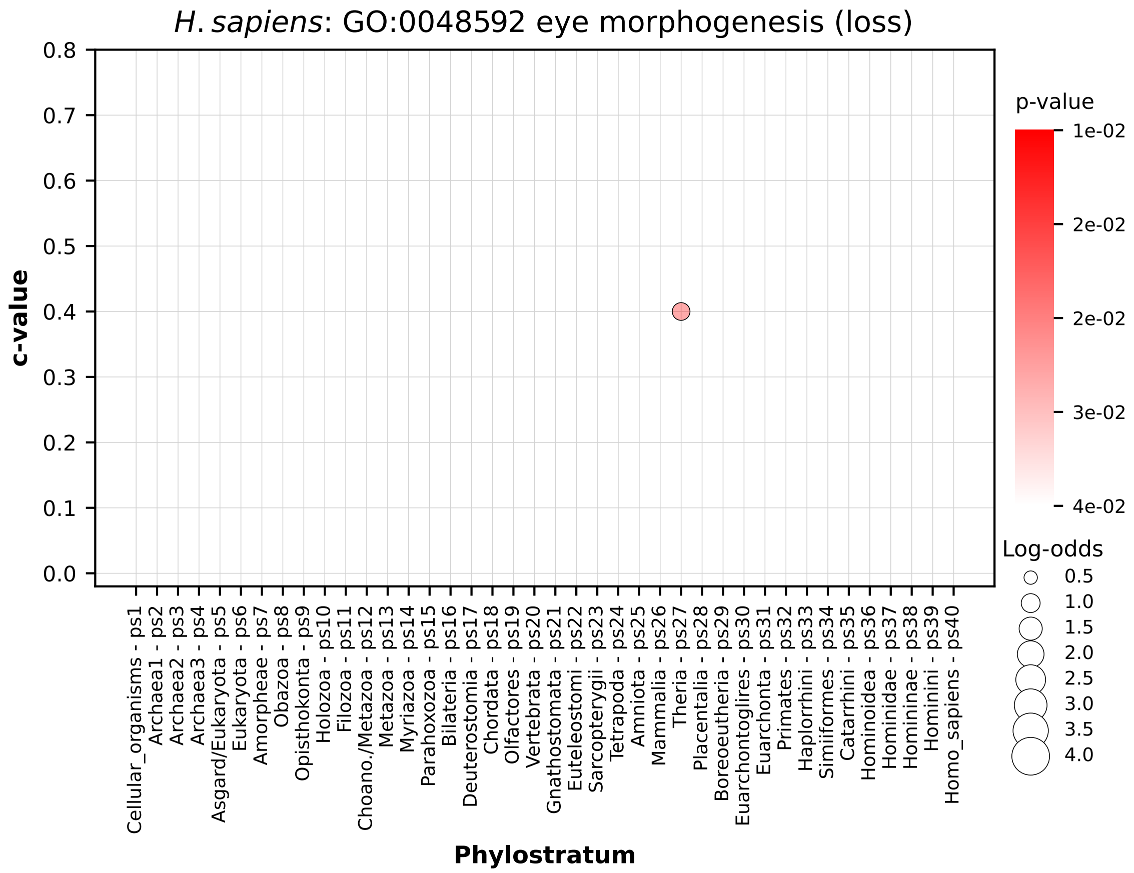

H. sapiens: GO:0048592 eye morphogenesis (loss)
c-value
0.8
0.7
0.6
0.5
0.4
0.3
0.2
0.1
0.0
Phylostratum
Cellular_organisms - ps1
Archaea1 - ps2
Archaea2 - ps3
Archaea3 - ps4
Asgard/Eukaryota - ps5
Eukaryota - ps6
Amorpheae - ps7
Obazoa - ps8
Opisthokonta - ps9
Holozoa - ps10
Filozoa - ps11
Choano./Metazoa - ps12
Metazoa - ps13
Myriazoa - ps14
Parahoxozoa - ps15
Bilateria - ps16
Deuterostomia - ps17
Chordata - ps18
Olfactores - ps19
Vertebrata - ps20
Gnathostomata - ps21
Euteleostomi - ps22
Sarcopterygii - ps23
Tetrapoda - ps24
Amniota - ps25
Mammalia - ps26
Theria - ps27
Placentalia - ps28
Boreoeutheria - ps29
Euarchontoglires - ps30
Euarchonta - ps31
Primates - ps32
Haplorrhini - ps33
Simiiformes - ps34
Catarrhini - ps35
Hominoidea - ps36
Hominidae - ps37
Homininae - ps38
Hominini - ps39
Homo_sapiens - ps40
p-value
1e-02
2e-02
2e-02
3e-02
4e-02
Log-odds
0.5
1.0
1.5
2.0
2.5
3.0
3.5
4.0

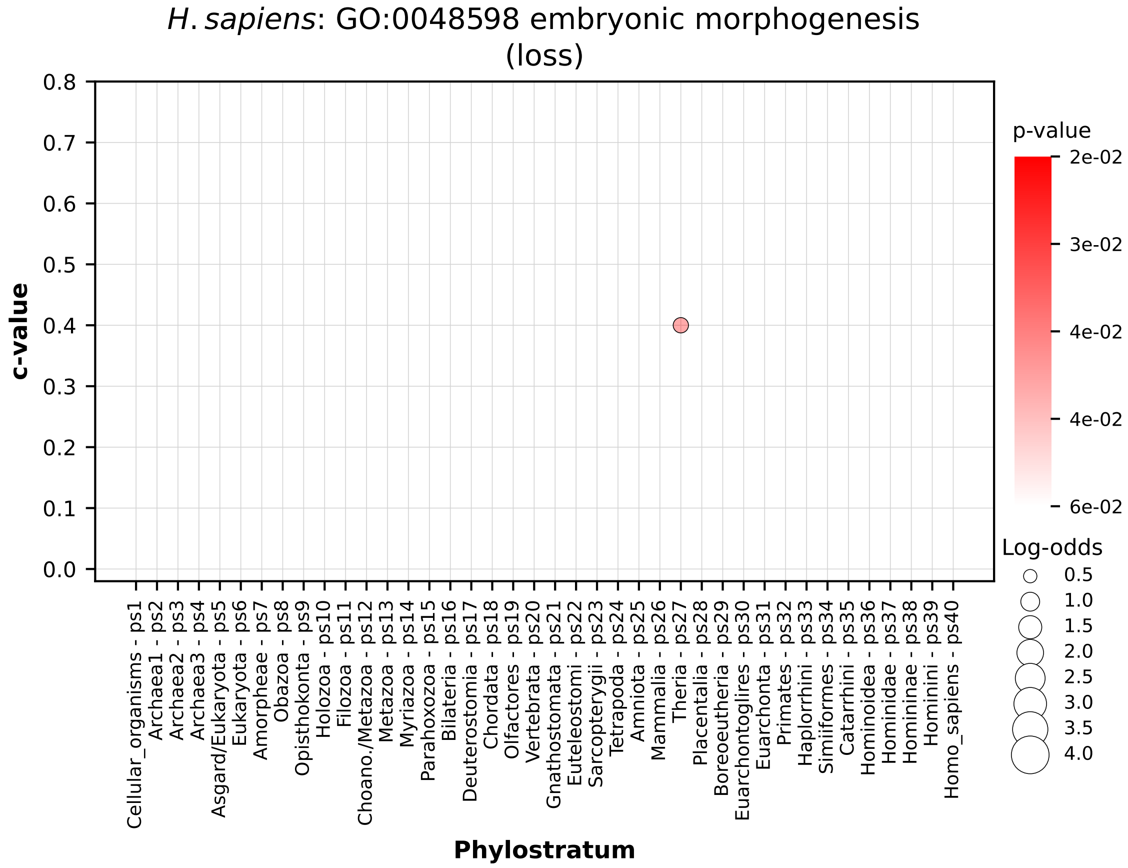
H. sapiens: GO:0048598 embryonic morphogenesis
(loss)
c-value
0.0
0.1
0.2
0.3
0.4
0.5
0.6
0.7
0.8
Phylostratum
Cellular_organisms - ps1
Archaea1 - ps2
Archaea2 - ps3
Archaea3 - ps4
Asgard/Eukaryota - ps5
Eukaryota - ps6
Amorpheae - ps7
Obazoa - ps8
Opisthokonta - ps9
Holozoa - ps10
Filozoa - ps11
Choano./Metazoa - ps12
Metazoa - ps13
Myriazoa - ps14
Parahoxozoa - ps15
Bilateria - ps16
Deuterostomia - ps17
Chordata - ps18
Olfactores - ps19
Vertebrata - ps20
Gnathostomata - ps21
Euteleostomi - ps22
Sarcopterygii - ps23
Tetrapoda - ps24
Amniota - ps25
Mammalia - ps26
Theria - ps27
Placentalia - ps28
Boreoeutheria - ps29
Euarchontoglires - ps30
Euarchonta - ps31
Primates - ps32
Haplorrhini - ps33
Simiiformes - ps34
Catarrhini - ps35
Hominoidea - ps36
Hominidae - ps37
Homininae - ps38
Hominini - ps39
Homo_sapiens - ps40
p-value
2e-02
3e-02
4e-02
4e-02
6e-02
Log-odds
0.5
1.0
1.5
2.0
2.5
3.0
3.5
4.0

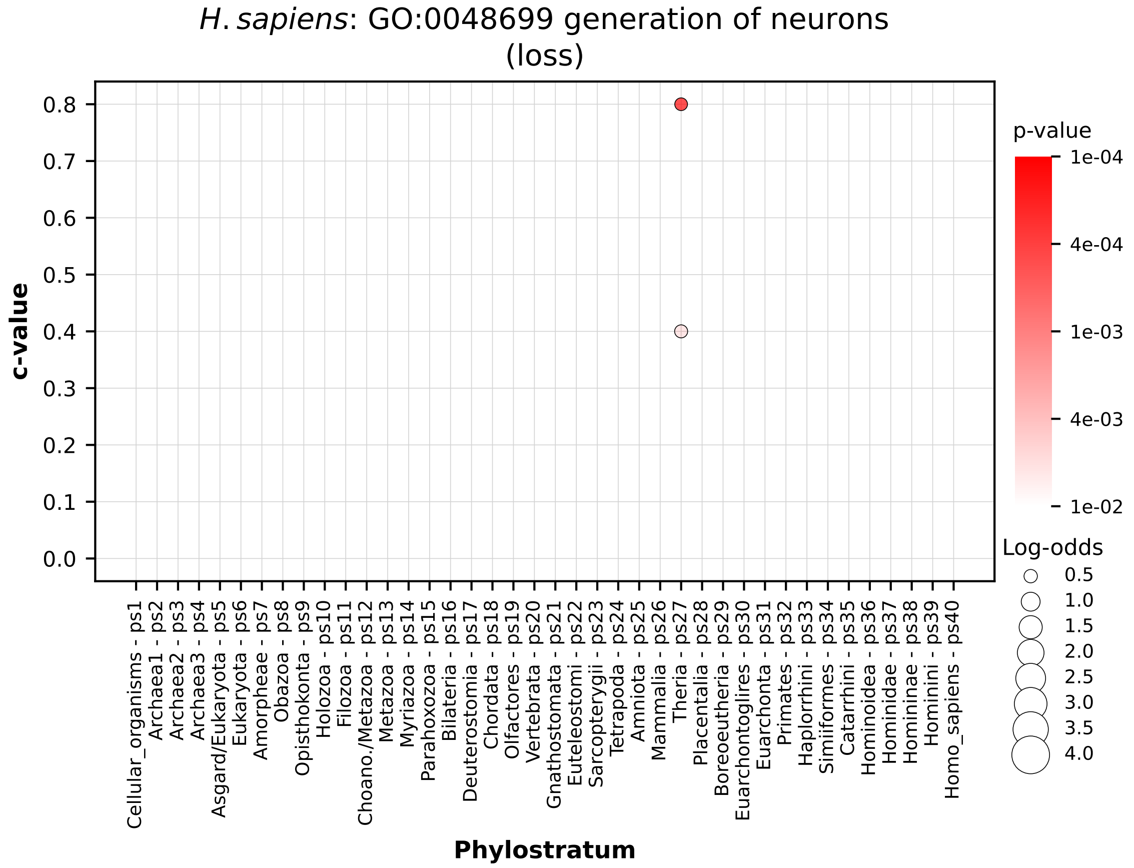

$H. sapiens$: GO:0048699 generation of neurons
(loss)
c-value
0.0
0.1
0.2
0.3
0.4
0.5
0.6
0.7
0.8
p-value
1e-04
4e-04
1e-03
4e-03
1e-02
Log-odds
0.5
1.0
1.5
2.0
2.5
3.0
3.5
4.0
Cellular_organisms - ps1
Archaea1 - ps2
Archaea2 - ps3
Archaea3 - ps4
Asgard/Eukaryota - ps5
Eukaryota - ps6
Amorpheae - ps7
Obazoa - ps8
Opisthokonta - ps9
Holozoa - ps10
Filozoa - ps11
Choano./Metazoa - ps12
Metazoa - ps13
Myriazoa - ps14
Parahoxozoa - ps15
Bilateria - ps16
Deuterostomia - ps17
Chordata - ps18
Olfactores - ps19
Vertebrata - ps20
Gnathostomata - ps21
Euteleostomi - ps22
Sarcopterygii - ps23
Tetrapoda - ps24
Amniota - ps25
Mammalia - ps26
Theria - ps27
Placentalia - ps28
Boreoeutheria - ps29
Euarchontoglires - ps30
Euarchonta - ps31
Primates - ps32
Haplorrhini - ps33
Simiiformes - ps34
Catarrhini - ps35
Hominoidea - ps36
Hominidae - ps37
Homininae - ps38
Hominini - ps39
Homo_sapiens - ps40
Phylostratum

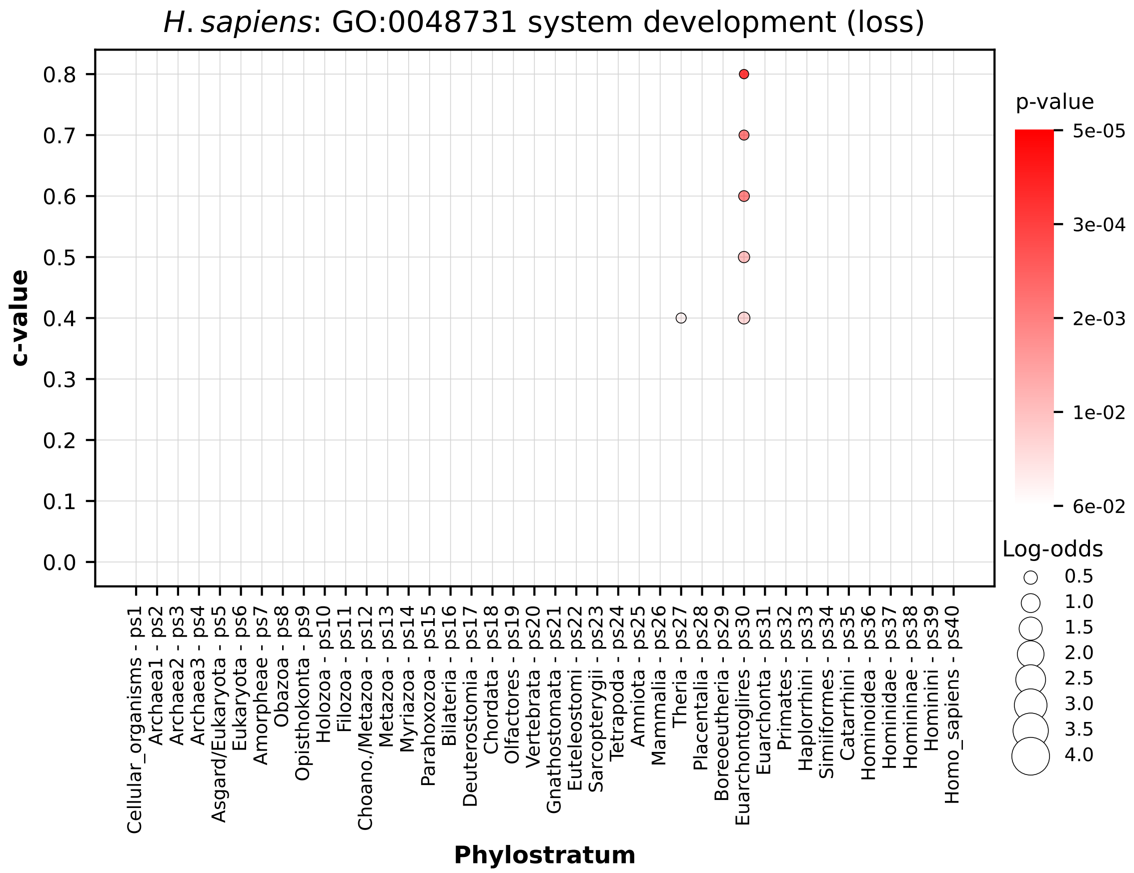

H. sapiens: GO:0048731 system development (loss)
c-value
0.0
0.1
0.2
0.3
0.4
0.5
0.6
0.7
0.8
p-value
5e-05
3e-04
2e-03
1e-02
6e-02
Log-odds
0.5
1.0
1.5
2.0
2.5
3.0
3.5
4.0
Cellular_organisms - ps1
Archaea1 - ps2
Archaea2 - ps3
Archaea3 - ps4
Asgard/Eukaryota - ps5
Eukaryota - ps6
Amorpheae - ps7
Obazoa - ps8
Opisthokonta - ps9
Holozoa - ps10
Filozoa - ps11
Choano./Metazoa - ps12
Metazoa - ps13
Myriazoa - ps14
Parahoxozoa - ps15
Bilateria - ps16
Deuterostomia - ps17
Chordata - ps18
Olfactores - ps19
Vertebrata - ps20
Gnathostomata - ps21
Euteleostomi - ps22
Sarcopterygii - ps23
Tetrapoda - ps24
Amniota - ps25
Mammalia - ps26
Theria - ps27
Placentalia - ps28
Boreoeutheria - ps29
Euarchontoglires - ps30
Euarchonta - ps31
Primates - ps32
Haplorrhini - ps33
Simiiformes - ps34
Catarrhini - ps35
Hominoidea - ps36
Hominidae - ps37
Homininae - ps38
Hominini - ps39
Homo_sapiens - ps40
Phylostratum

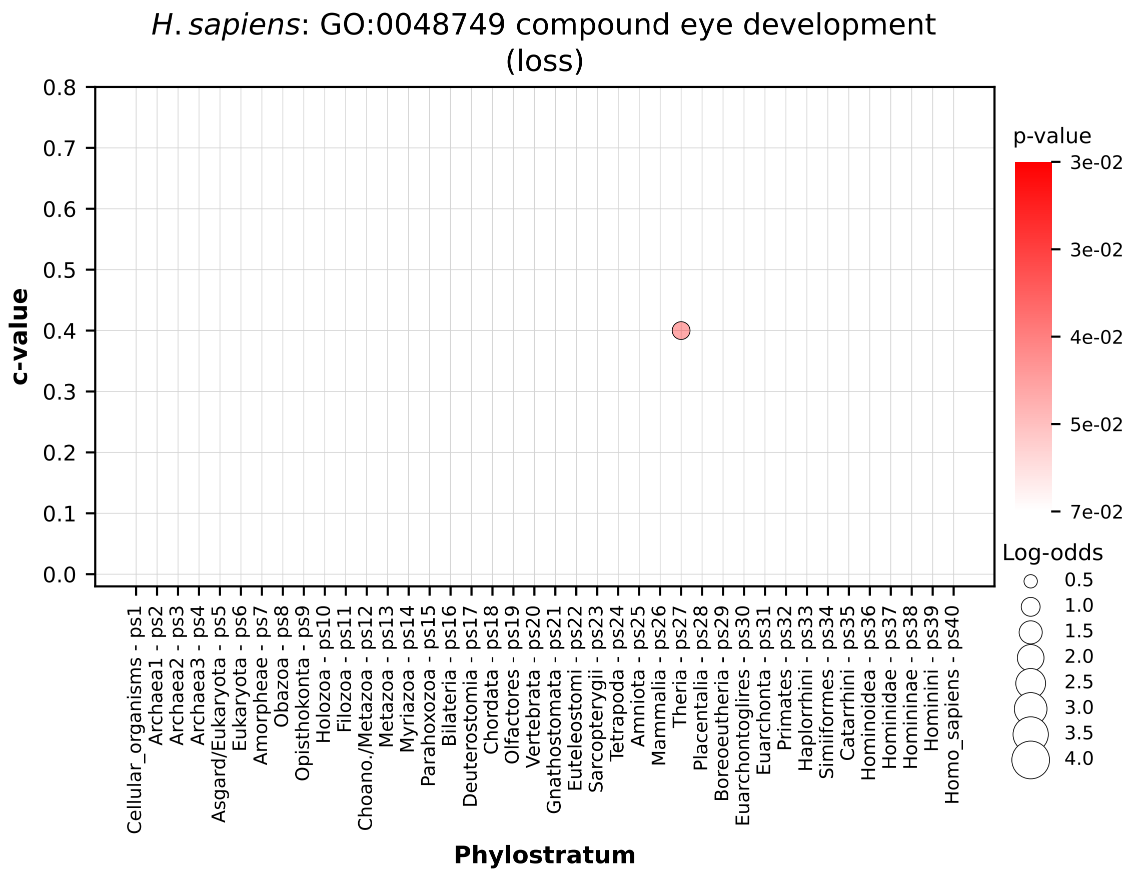

H. sapiens: GO:0048749 compound eye development
(loss)
c-value
0.8
0.7
0.6
0.5
0.4
0.3
0.2
0.1
0.0
Phylostratum
Cellular_organisms - ps1
Archaea1 - ps2
Archaea2 - ps3
Archaea3 - ps4
Asgard/Eukaryota - ps5
Eukaryota - ps6
Amorpheae - ps7
Obazoa - ps8
Opisthokonta - ps9
Holozoa - ps10
Filozoa - ps11
Choano./Metazoa - ps12
Metazoa - ps13
Myriazoa - ps14
Parahoxozoa - ps15
Bilateria - ps16
Deuterostomia - ps17
Chordata - ps18
Olfactores - ps19
Vertebrata - ps20
Gnathostomata - ps21
Euteleostomi - ps22
Sarcopterygii - ps23
Tetrapoda - ps24
Amniota - ps25
Mammalia - ps26
Theria - ps27
Placentalia - ps28
Boreoeutheria - ps29
Euarchontoglires - ps30
Euarchonta - ps31
Primates - ps32
Haplorrhini - ps33
Simiiformes - ps34
Catarrhini - ps35
Hominoidea - ps36
Hominidae - ps37
Homininae - ps38
Hominini - ps39
Homo_sapiens - ps40
p-value
3e-02
3e-02
4e-02
5e-02
7e-02
Log-odds
0.5
1.0
1.5
2.0
2.5
3.0
3.5
4.0

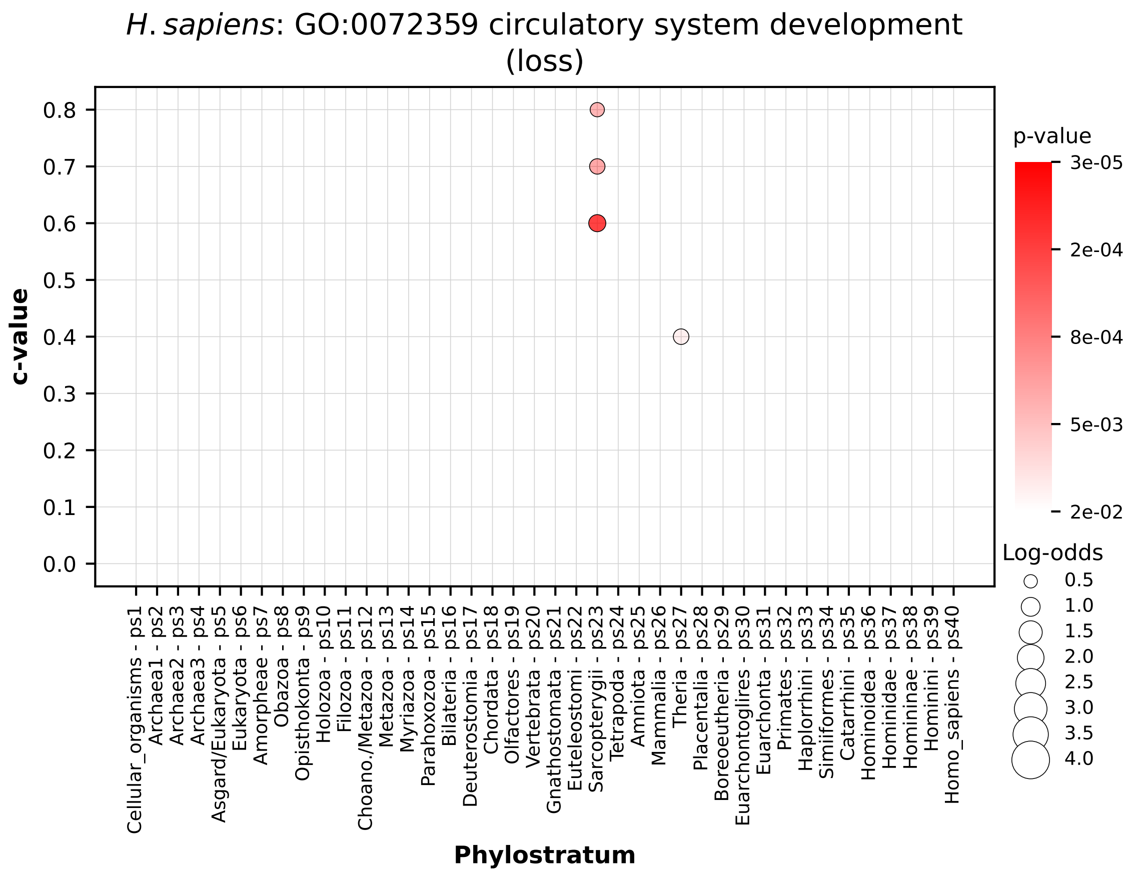

H. sapiens: GO:0072359 circulatory system development
(loss)
c-value
0.8
0.7
0.6
0.5
0.4
0.3
0.2
0.1
0.0
p-value
3e-05
2e-04
8e-04
5e-03
2e-02
Log-odds
0.5
1.0
1.5
2.0
2.5
3.0
3.5
4.0
Cellular_organisms - ps1
Archaea1 - ps2
Archaea2 - ps3
Archaea3 - ps4
Asgard/Eukaryota - ps5
Eukaryota - ps6
Amorpheae - ps7
Obazoa - ps8
Opisthokonta - ps9
Holozoa - ps10
Filozoa - ps11
Choano./Metazoa - ps12
Metazoa - ps13
Myriazoa - ps14
Parahoxozoa - ps15
Bilateria - ps16
Deuterostomia - ps17
Chordata - ps18
Olfactores - ps19
Vertebrata - ps20
Gnathostomata - ps21
Euteleostomi - ps22
Sarcopterygii - ps23
Tetrapoda - ps24
Amniota - ps25
Mammalia - ps26
Theria - ps27
Placentalia - ps28
Boreoeutheria - ps29
Euarchontoglires - ps30
Euarchonta - ps31
Primates - ps32
Haplorrhini - ps33
Simiiformes - ps34
Catarrhini - ps35
Hominoidea - ps36
Hominidae - ps37
Homininae - ps38
Hominini - ps39
Homo_sapiens - ps40
Phylostratum

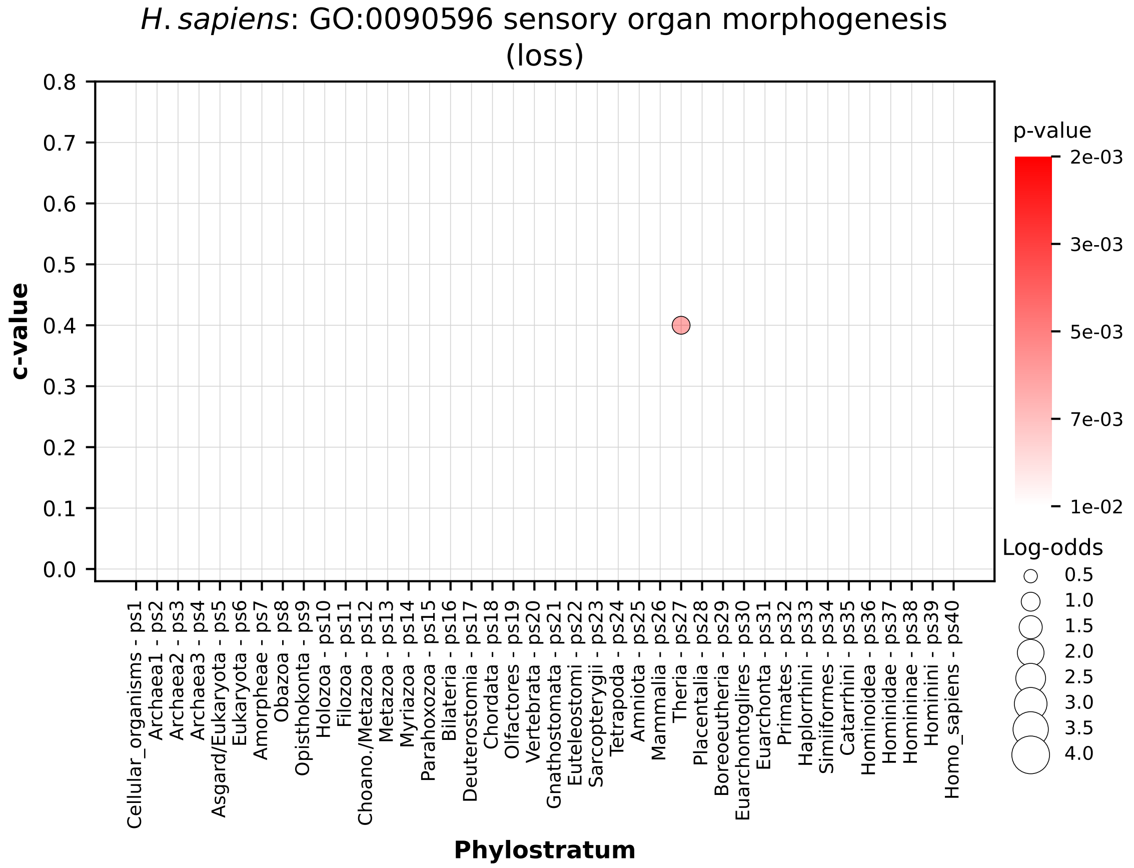
H. sapiens: GO:0090596 sensory organ morphogenesis
(loss)
c-value
0.0
0.1
0.2
0.3
0.4
0.5
0.6
0.7
0.8
Phylostratum
Cellular_organisms - ps1
Archaea1 - ps2
Archaea2 - ps3
Archaea3 - ps4
Asgard/Eukaryota - ps5
Eukaryota - ps6
Amorpheae - ps7
Obazoa - ps8
Opisthokonta - ps9
Holozoa - ps10
Filozoa - ps11
Choano./Metazoa - ps12
Metazoa - ps13
Myriazoa - ps14
Parahoxozoa - ps15
Bilateria - ps16
Deuterostomia - ps17
Chordata - ps18
Olfactores - ps19
Vertebrata - ps20
Gnathostomata - ps21
Euteleostomi - ps22
Sarcopterygii - ps23
Tetrapoda - ps24
Amniota - ps25
Mammalia - ps26
Theria - ps27
Placentalia - ps28
Boreoeutheria - ps29
Euarchontoglires - ps30
Euarchonta - ps31
Primates - ps32
Haplorrhini - ps33
Simiiformes - ps34
Catarrhini - ps35
Hominoidea - ps36
Hominidae - ps37
Homininae - ps38
Hominini - ps39
Homo_sapiens - ps40
p-value
2e-03
3e-03
5e-03
7e-03
1e-02
Log-odds
0.5
1.0
1.5
2.0
2.5
3.0
3.5
4.0

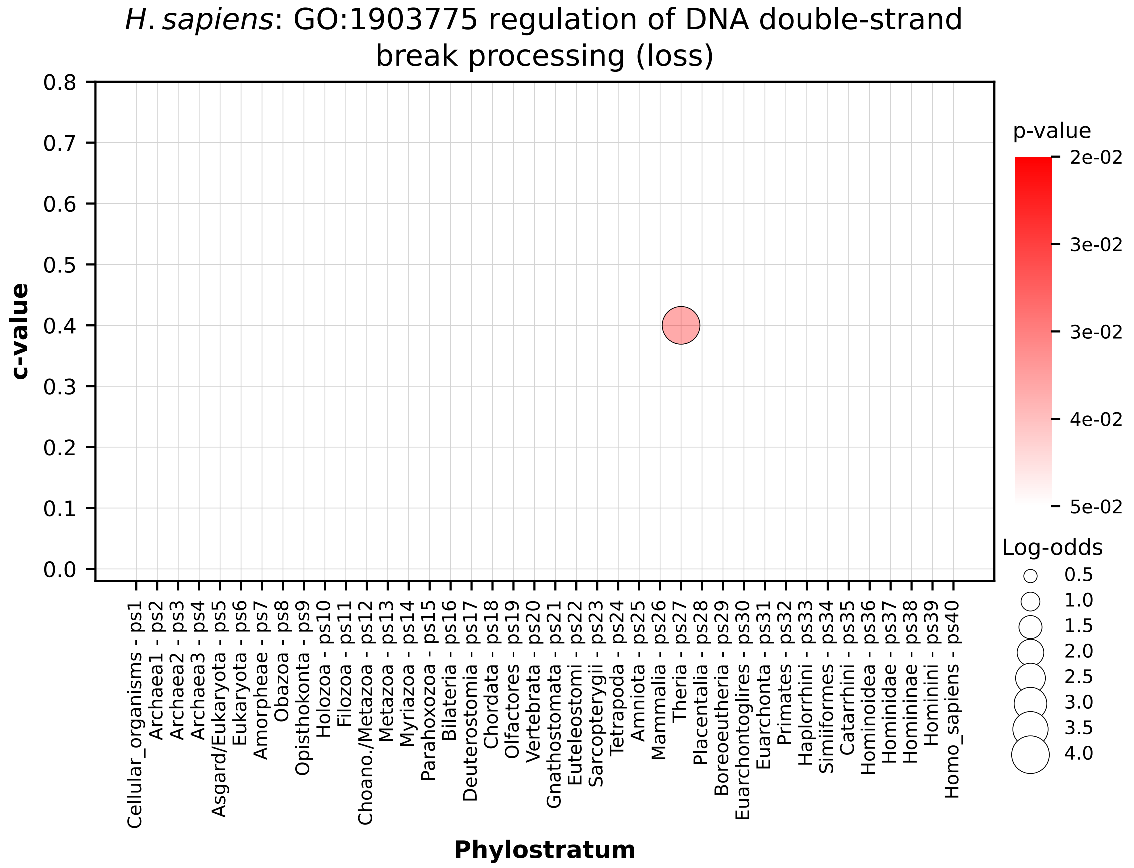
H. sapiens: GO:1903775 regulation of DNA double-strand break processing (loss)
c-value
0.0
0.1
0.2
0.3
0.4
0.5
0.6
0.7
0.8
Phylostratum
Cellular_organisms - ps1
Archaea1 - ps2
Archaea2 - ps3
Archaea3 - ps4
Asgard/Eukaryota - ps5
Eukaryota - ps6
Amorpheae - ps7
Obazoa - ps8
Opisthokonta - ps9
Holozoa - ps10
Filozoa - ps11
Choano./Metazoa - ps12
Metazoa - ps13
Myriazoa - ps14
Parahoxozoa - ps15
Bilateria - ps16
Deuterostomia - ps17
Chordata - ps18
Olfactores - ps19
Vertebrata - ps20
Gnathostomata - ps21
Euteleostomi - ps22
Sarcopterygii - ps23
Tetrapoda - ps24
Amniota - ps25
Mammalia - ps26
Theria - ps27
Placentalia - ps28
Boreoeutheria - ps29
Euarchontoglires - ps30
Euarchonta - ps31
Primates - ps32
Haplorrhini - ps33
Simiiformes - ps34
Catarrhini - ps35
Hominoidea - ps36
Hominidae - ps37
Homininae - ps38
Hominini - ps39
Homo_sapiens - ps40
p-value
2e-02
3e-02
3e-02
4e-02
5e-02
Log-odds
0.5
1.0
1.5
2.0
2.5
3.0
3.5
4.0

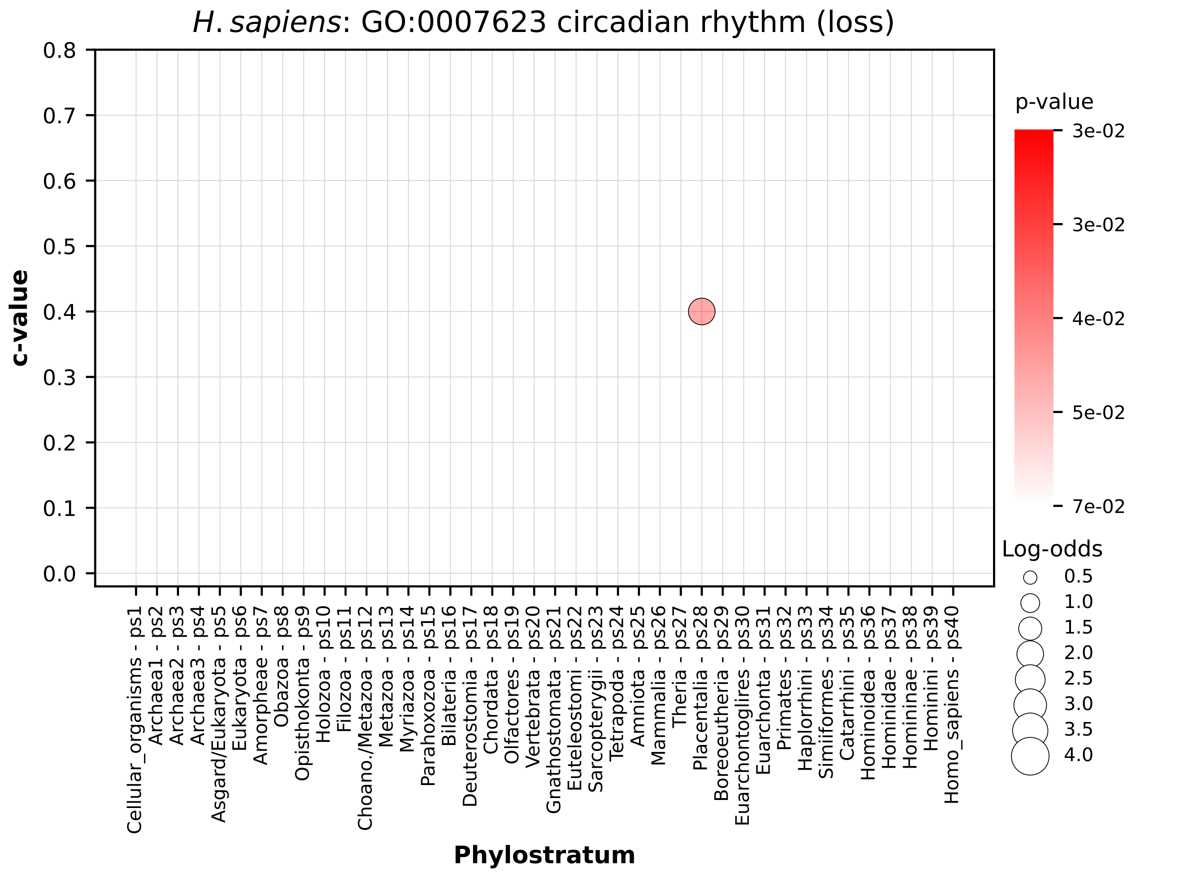

H. sapiens: GO:0007623 circadian rhythm (loss)
c-value
0.0
0.1
0.2
0.3
0.4
0.5
0.6
0.7
0.8
Phylostratum
Cellular_organisms - ps1
Archaea1 - ps2
Archaea2 - ps3
Archaea3 - ps4
Asgard/Eukaryota - ps5
Eukaryota - ps6
Amorpheae - ps7
Obazoa - ps8
Opisthokonta - ps9
Holozoa - ps10
Filozoa - ps11
Choano./Metazoa - ps12
Metazoa - ps13
Myriazoa - ps14
Parahoxozoa - ps15
Bilateria - ps16
Deuterostomia - ps17
Chordata - ps18
Olfactores - ps19
Vertebrata - ps20
Gnathostomata - ps21
Euteleostomi - ps22
Sarcopterygii - ps23
Tetrapoda - ps24
Amniota - ps25
Mammalia - ps26
Theria - ps27
Placentalia - ps28
Boreoeutheria - ps29
Euarchontoglires - ps30
Euarchonta - ps31
Primates - ps32
Haplorrhini - ps33
Simiiformes - ps34
Catarrhini - ps35
Hominoidea - ps36
Hominidae - ps37
Homininae - ps38
Hominini - ps39
Homo_sapiens - ps40
p-value
3e-02
3e-02
4e-02
5e-02
7e-02
Log-odds
0.5
1.0
1.5
2.0
2.5
3.0
3.5
4.0

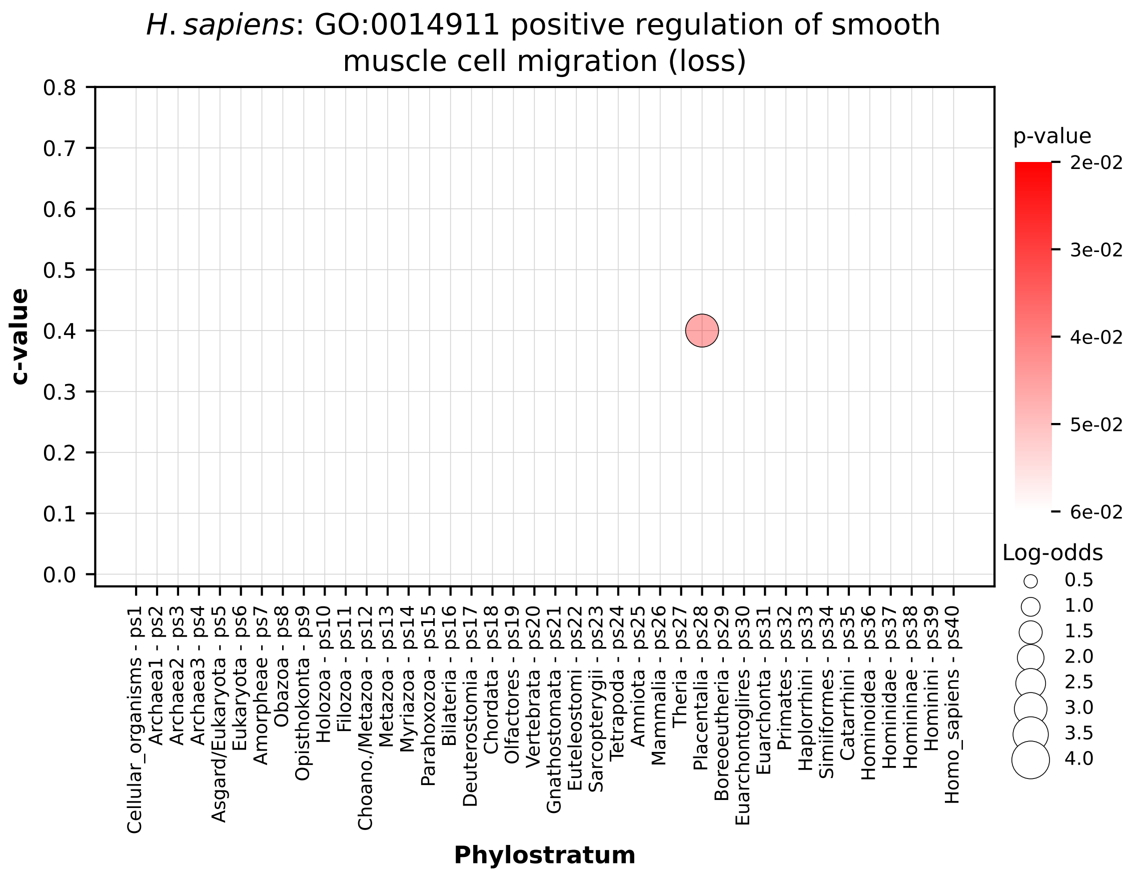
H. sapiens: GO:0014911 positive regulation of smooth muscle cell migration (loss)
c-value
0.0
0.1
0.2
0.3
0.4
0.5
0.6
0.7
0.8
Phylostratum
Cellular_organisms - ps1
Archaea1 - ps2
Archaea2 - ps3
Archaea3 - ps4
Asgard/Eukaryota - ps5
Eukaryota - ps6
Amorpheae - ps7
Obazoa - ps8
Opisthokonta - ps9
Holozoa - ps10
Filozoa - ps11
Choano./Metazoa - ps12
Metazoa - ps13
Myriazoa - ps14
Parahoxozoa - ps15
Bilateria - ps16
Deuterostomia - ps17
Chordata - ps18
Olfactores - ps19
Vertebrata - ps20
Gnathostomata - ps21
Euteleostomi - ps22
Sarcopterygii - ps23
Tetrapoda - ps24
Amniota - ps25
Mammalia - ps26
Theria - ps27
Placentalia - ps28
Boreoeutheria - ps29
Euarchontoglires - ps30
Euarchonta - ps31
Primates - ps32
Haplorrhini - ps33
Simiiformes - ps34
Catarrhini - ps35
Hominoidea - ps36
Hominidae - ps37
Homininae - ps38
Hominini - ps39
Homo_sapiens - ps40
p-value
2e-02
3e-02
4e-02
5e-02
6e-02
Log-odds
0.5
1.0
1.5
2.0
2.5
3.0
3.5
4.0

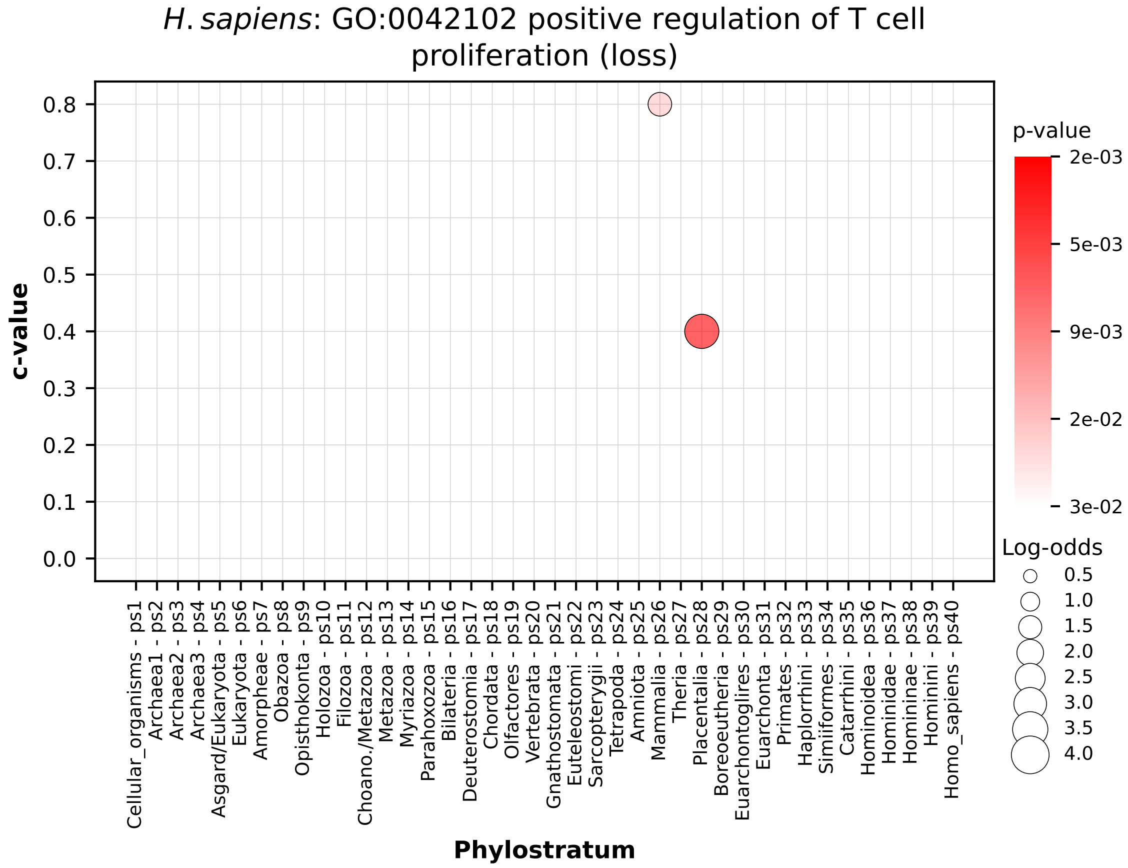

H. sapiens: GO:0042102 positive regulation of T cell proliferation (loss)
c-value
Phylostratum
p-value
2e-03
5e-03
9e-03
2e-02
3e-02
Log-odds
0.5
1.0
1.5
2.0
2.5
3.0
3.5
4.0
Cellular_organisms - ps1
Archaea1 - ps2
Archaea2 - ps3
Archaea3 - ps4
Asgard/Eukaryota - ps5
Eukaryota - ps6
Amorpheae - ps7
Obazoa - ps8
Opisthokonta - ps9
Holozoa - ps10
Filozoa - ps11
Choano./Metazoa - ps12
Metazoa - ps13
Myriazoa - ps14
Parahoxozoa - ps15
Bilateria - ps16
Deuterostomia - ps17
Chordata - ps18
Olfactores - ps19
Vertebrata - ps20
Gnathostomata - ps21
Euteleostomi - ps22
Sarcopterygii - ps23
Tetrapoda - ps24
Amniota - ps25
Mammalia - ps26
Theria - ps27
Placentalia - ps28
Boreoeutheria - ps29
Euarchontoglires - ps30
Euarchonta - ps31
Primates - ps32
Haplorrhini - ps33
Simiiformes - ps34
Catarrhini - ps35
Hominoidea - ps36
Hominidae - ps37
Homininae - ps38
Hominini - ps39
Homo_sapiens - ps40

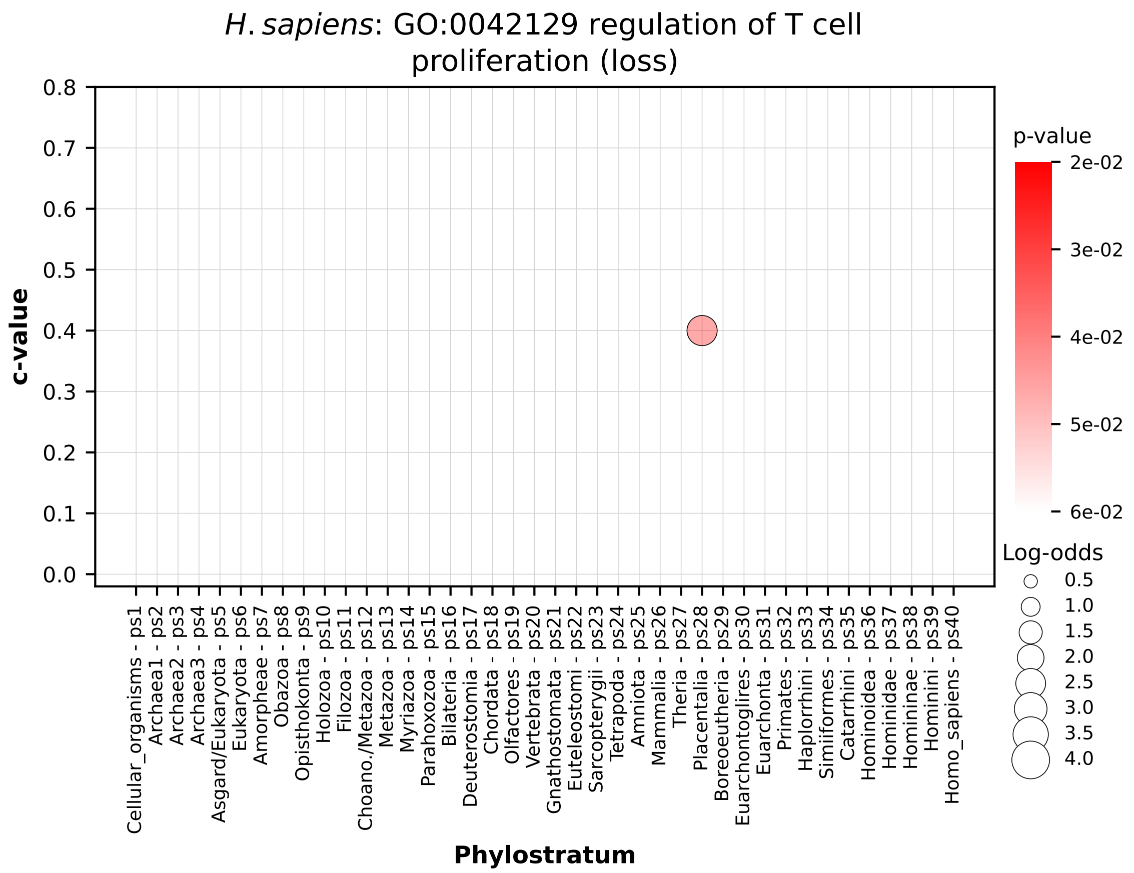

H. sapiens: GO:0042129 regulation of T cell proliferation (loss)
c-value
0.0
0.1
0.2
0.3
0.4
0.5
0.6
0.7
0.8
Phylostratum
Cellular_organisms - ps1
Archaea1 - ps2
Archaea2 - ps3
Archaea3 - ps4
Asgard/Eukaryota - ps5
Eukaryota - ps6
Amorpheae - ps7
Obazoa - ps8
Opisthokonta - ps9
Holozoa - ps10
Filozoa - ps11
Choano./Metazoa - ps12
Metazoa - ps13
Myriazoa - ps14
Parahoxozoa - ps15
Bilateria - ps16
Deuterostomia - ps17
Chordata - ps18
Olfactores - ps19
Vertebrata - ps20
Gnathostomata - ps21
Euteleostomi - ps22
Sarcopterygii - ps23
Tetrapoda - ps24
Amniota - ps25
Mammalia - ps26
Theria - ps27
Placentalia - ps28
Boreoeutheria - ps29
Euarchontoglires - ps30
Euarchonta - ps31
Primates - ps32
Haplorrhini - ps33
Simiiformes - ps34
Catarrhini - ps35
Hominoidea - ps36
Hominidae - ps37
Homininae - ps38
Hominini - ps39
Homo_sapiens - ps40
p-value
2e-02
3e-02
4e-02
5e-02
6e-02
Log-odds
0.5
1.0
1.5
2.0
2.5
3.0
3.5
4.0

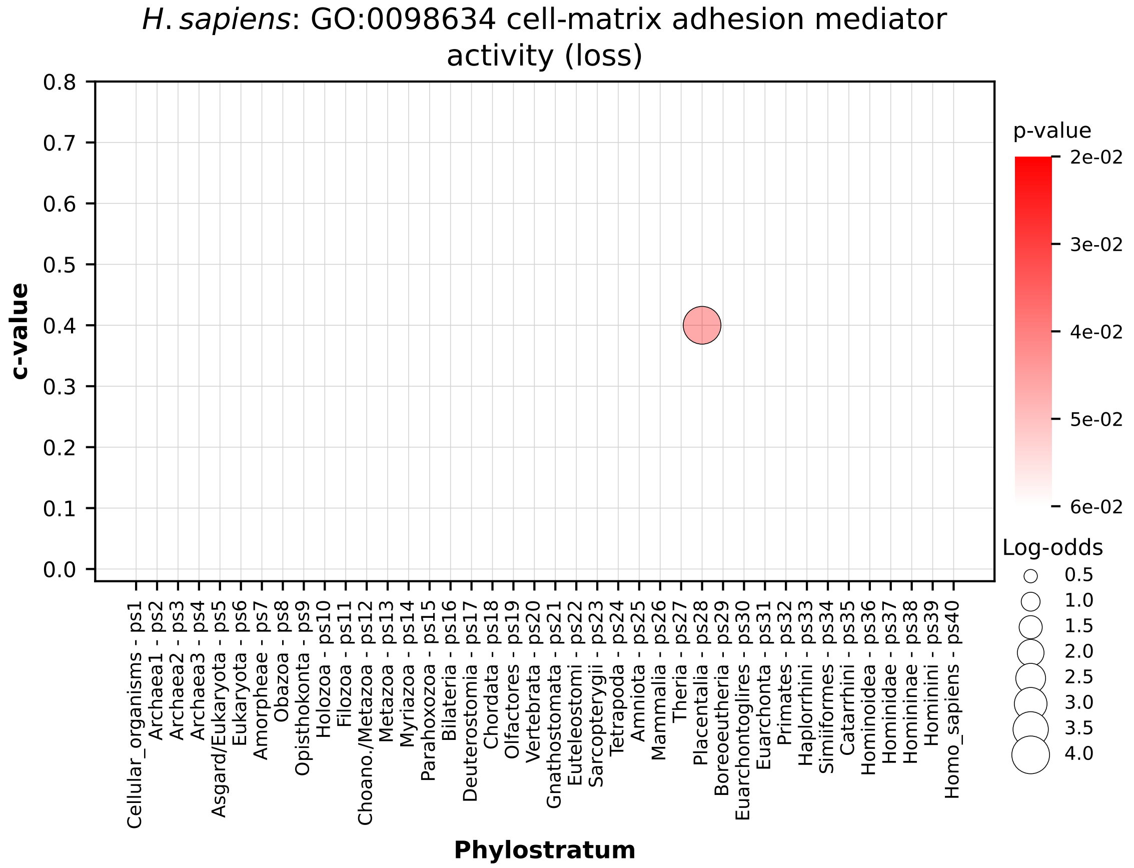
H. sapiens: GO:0098634 cell-matrix adhesion mediator activity (loss)
c-value
0.0
0.1
0.2
0.3
0.4
0.5
0.6
0.7
0.8
Phylostratum
Cellular_organisms - ps1
Archaea1 - ps2
Archaea2 - ps3
Archaea3 - ps4
Asgard/Eukaryota - ps5
Eukaryota - ps6
Amorpheae - ps7
Obazoa - ps8
Opisthokonta - ps9
Holozoa - ps10
Filozoa - ps11
Choano./Metazoa - ps12
Metazoa - ps13
Myriazoa - ps14
Parahoxozoa - ps15
Bilateria - ps16
Deuterostomia - ps17
Chordata - ps18
Olfactores - ps19
Vertebrata - ps20
Gnathostomata - ps21
Euteleostomi - ps22
Sarcopterygii - ps23
Tetrapoda - ps24
Amniota - ps25
Mammalia - ps26
Theria - ps27
Placentalia - ps28
Boreoeutheria - ps29
Euarchontoglires - ps30
Euarchonta - ps31
Primates - ps32
Haplorrhini - ps33
Simiiformes - ps34
Catarrhini - ps35
Hominoidea - ps36
Hominidae - ps37
Homininae - ps38
Hominini - ps39
Homo_sapiens - ps40
p-value
2e-02
3e-02
4e-02
5e-02
6e-02
Log-odds
0.5
1.0
1.5
2.0
2.5
3.0
3.5
4.0

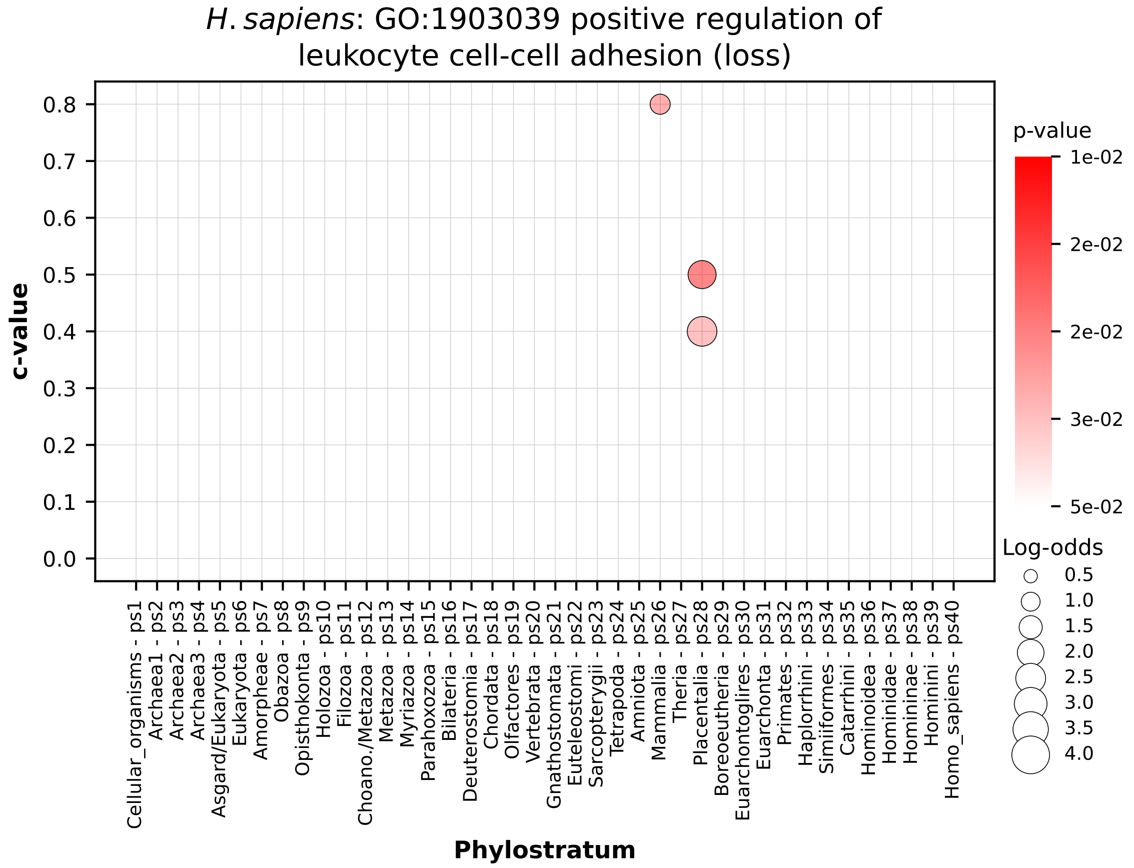

H. sapiens: GO:1903039 positive regulation of leukocyte cell-cell adhesion (loss)
c-value
0.0
0.1
0.2
0.3
0.4
0.5
0.6
0.7
0.8
Phylostratum
Cellular_organisms - ps1
Archaea1 - ps2
Archaea2 - ps3
Archaea3 - ps4
Asgard/Eukaryota - ps5
Eukaryota - ps6
Amorpheae - ps7
Obazoa - ps8
Opisthokonta - ps9
Holozoa - ps10
Filozoa - ps11
Choano./Metazoa - ps12
Metazoa - ps13
Myriazoa - ps14
Parahoxozoa - ps15
Bilateria - ps16
Deuterostomia - ps17
Chordata - ps18
Olfactores - ps19
Vertebrata - ps20
Gnathostomata - ps21
Euteleostomi - ps22
Sarcopterygii - ps23
Tetrapoda - ps24
Amniota - ps25
Mammalia - ps26
Theria - ps27
Placentalia - ps28
Boreoeutheria - ps29
Euarchontoglires - ps30
Euarchonta - ps31
Primates - ps32
Haplorrhini - ps33
Simiiformes - ps34
Catarrhini - ps35
Hominoidea - ps36
Hominidae - ps37
Homininae - ps38
Hominini - ps39
Homo_sapiens - ps40
p-value
1e-02
2e-02
2e-02
3e-02
5e-02
Log-odds
0.5
1.0
1.5
2.0
2.5
3.0
3.5
4.0

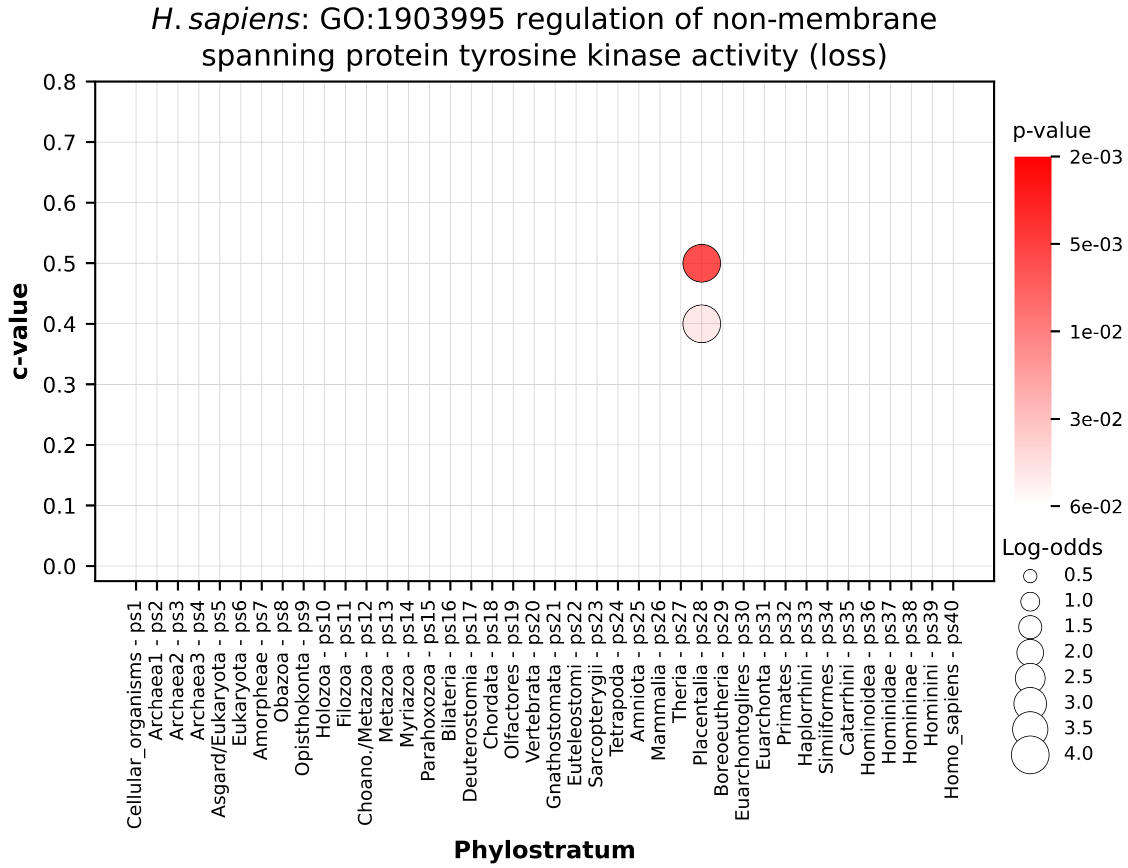

H. sapiens: GO:1903995 regulation of non-membrane spanning protein tyrosine kinase activity (loss)
c-value
0.8
0.7
0.6
0.5
0.4
0.3
0.2
0.1
0.0
Phylostratum
Cellular_organisms - ps1
Archaea1 - ps2
Archaea2 - ps3
Archaea3 - ps4
Asgard/Eukaryota - ps5
Eukaryota - ps6
Amorpheae - ps7
Obazoa - ps8
Opisthokonta - ps9
Holozoa - ps10
Filozoa - ps11
Choano./Metazoa - ps12
Metazoa - ps13
Myriazoa - ps14
Parahoxozoa - ps15
Bilateria - ps16
Deuterostomia - ps17
Chordata - ps18
Olfactores - ps19
Vertebrata - ps20
Gnathostomata - ps21
Euteleostomi - ps22
Sarcopterygii - ps23
Tetrapoda - ps24
Amniota - ps25
Mammalia - ps26
Theria - ps27
Placentalia - ps28
Boreoeutheria - ps29
Euarchontoglires - ps30
Euarchonta - ps31
Primates - ps32
Haplorrhini - ps33
Simiiformes - ps34
Catarrhini - ps35
Hominoidea - ps36
Hominidae - ps37
Homininae - ps38
Hominini - ps39
Homo_sapiens - ps40
p-value
2e-03
5e-03
1e-02
3e-02
6e-02
Log-odds
0.5
1.0
1.5
2.0
2.5
3.0
3.5
4.0

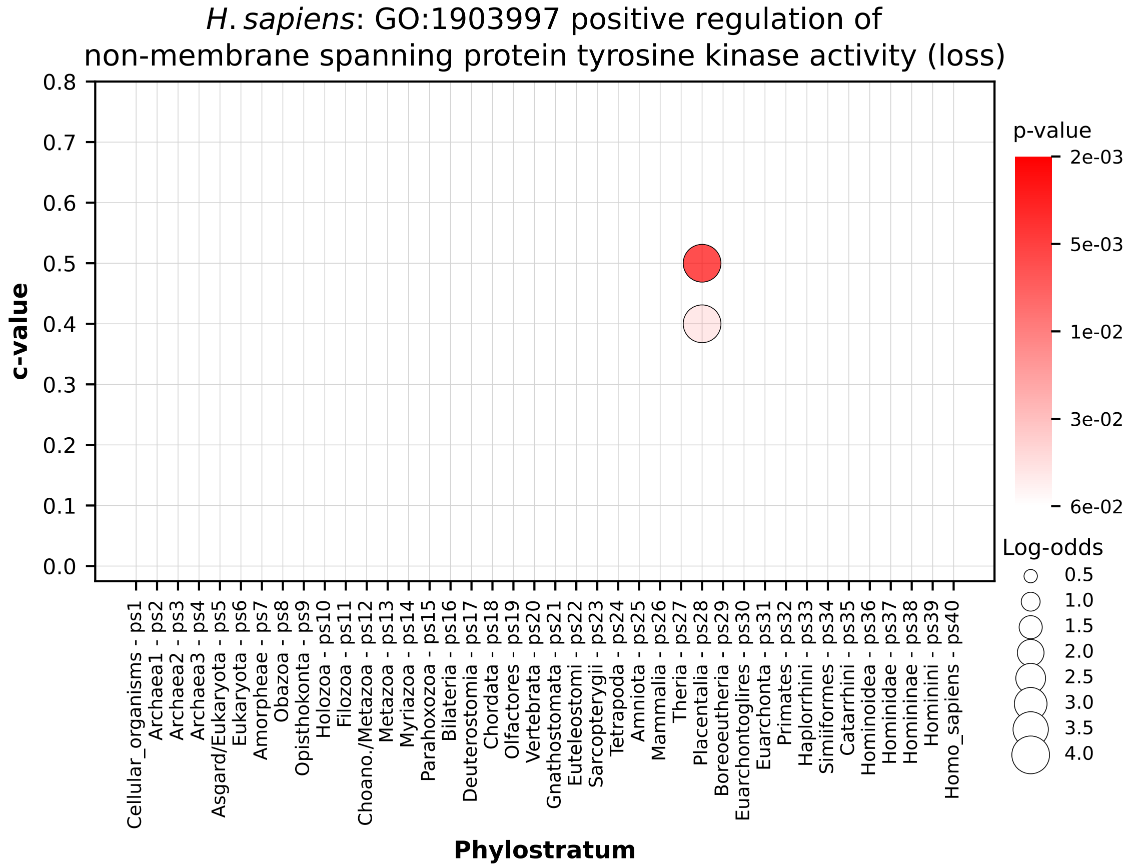
H. sapiens: GO:1903997 positive regulation of non-membrane spanning protein tyrosine kinase activity (loss)
c-value
0.0
0.1
0.2
0.3
0.4
0.5
0.6
0.7
0.8
Phylostratum
Cellular_organisms - ps1
Archaea1 - ps2
Archaea2 - ps3
Archaea3 - ps4
Asgard/Eukaryota - ps5
Eukaryota - ps6
Amorpheae - ps7
Obazoa - ps8
Opisthokonta - ps9
Holozoa - ps10
Filozoa - ps11
Choano./Metazoa - ps12
Metazoa - ps13
Myriazoa - ps14
Parahoxozoa - ps15
Bilateria - ps16
Deuterostomia - ps17
Chordata - ps18
Olfactores - ps19
Vertebrata - ps20
Gnathostomata - ps21
Euteleostomi - ps22
Sarcopterygii - ps23
Tetrapoda - ps24
Amniota - ps25
Mammalia - ps26
Theria - ps27
Placentalia - ps28
Boreoeutheria - ps29
Euarchontoglires - ps30
Euarchonta - ps31
Primates - ps32
Haplorrhini - ps33
Simiiformes - ps34
Catarrhini - ps35
Hominoidea - ps36
Hominidae - ps37
Homininae - ps38
Hominini - ps39
Homo_sapiens - ps40
p-value
2e-03
5e-03
1e-02
3e-02
6e-02
Log-odds
0.5
1.0
1.5
2.0
2.5
3.0
3.5
4.0

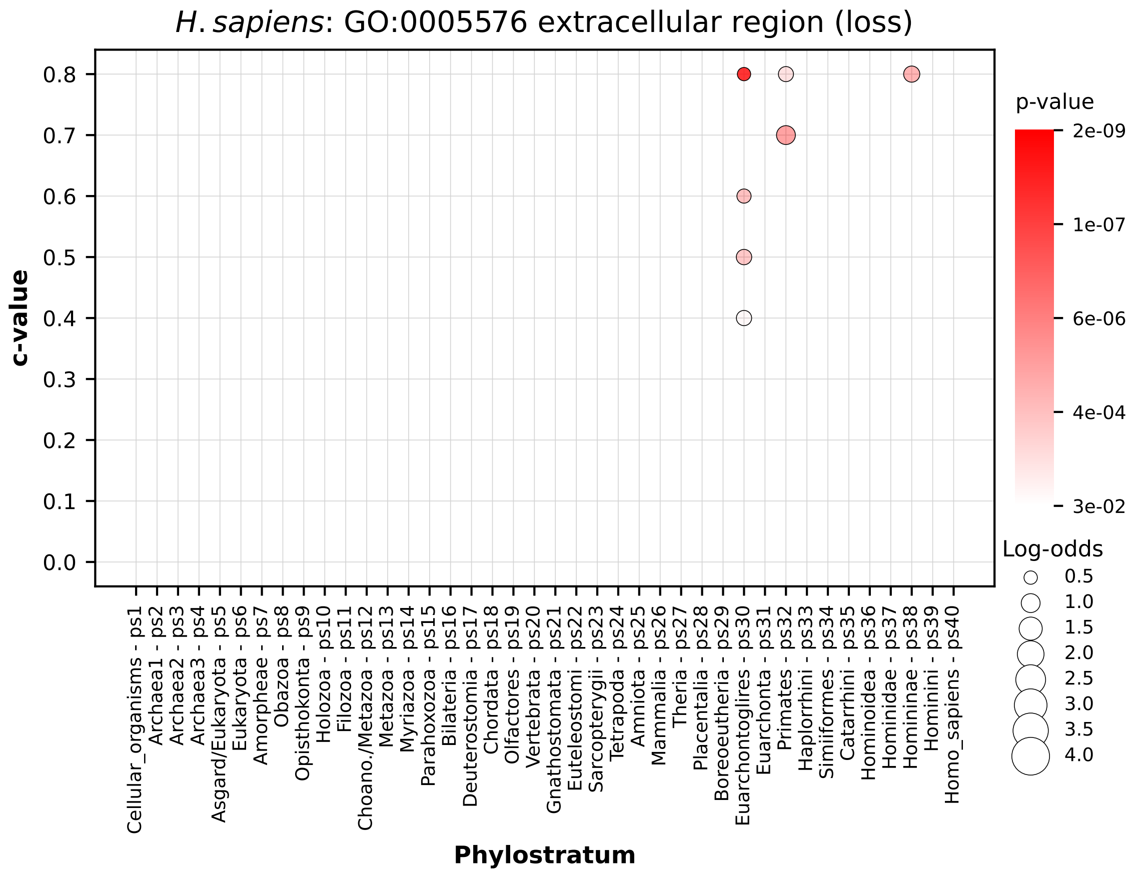

H. sapiens: GO:0005576 extracellular region (loss)
c-value
0.8
0.7
0.6
0.5
0.4
0.3
0.2
0.1
0.0
Phylostratum
Cellular_organisms - ps1
Archaea1 - ps2
Archaea2 - ps3
Archaea3 - ps4
Asgard/Eukaryota - ps5
Eukaryota - ps6
Amorpheae - ps7
Obazoa - ps8
Opisthokonta - ps9
Holozoa - ps10
Filozoa - ps11
Choano./Metazoa - ps12
Metazoa - ps13
Myriazoa - ps14
Parahoxozoa - ps15
Bilateria - ps16
Deuterostomia - ps17
Chordata - ps18
Olfactores - ps19
Vertebrata - ps20
Gnathostomata - ps21
Euteleostomi - ps22
Sarcopterygii - ps23
Tetrapoda - ps24
Amniota - ps25
Mammalia - ps26
Theria - ps27
Placentalia - ps28
Boreoeutheria - ps29
Euarchontoglires - ps30
Euarchonta - ps31
Primates - ps32
Haplorrhini - ps33
Simiiformes - ps34
Catarrhini - ps35
Hominoidea - ps36
Hominidae - ps37
Homininae - ps38
Hominini - ps39
Homo_sapiens - ps40
p-value
2e-09
1e-07
6e-06
4e-04
3e-02
Log-odds
0.5
1.0
1.5
2.0
2.5
3.0
3.5
4.0

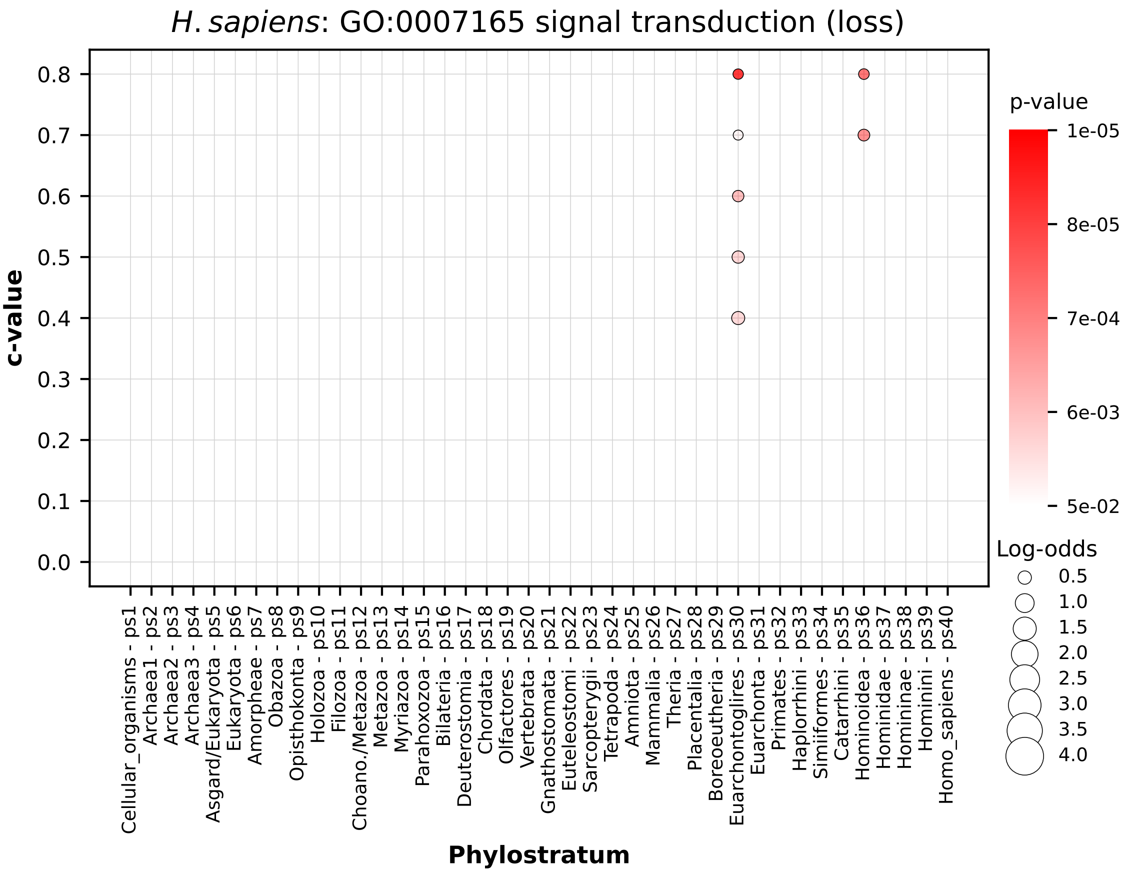

H. sapiens: GO:0007165 signal transduction (loss)
c-value
0.0
0.1
0.2
0.3
0.4
0.5
0.6
0.7
0.8
Phylostratum
Cellular_organisms - ps1
Archaea1 - ps2
Archaea2 - ps3
Archaea3 - ps4
Asgard/Eukaryota - ps5
Eukaryota - ps6
Amorpheae - ps7
Obazoa - ps8
Opisthokonta - ps9
Holozoa - ps10
Filozoa - ps11
Choano./Metazoa - ps12
Metazoa - ps13
Myriazoa - ps14
Parahoxozoa - ps15
Bilateria - ps16
Deuterostomia - ps17
Chordata - ps18
Olfactores - ps19
Vertebrata - ps20
Gnathostomata - ps21
Euteleostomi - ps22
Sarcopterygii - ps23
Tetrapoda - ps24
Amniota - ps25
Mammalia - ps26
Theria - ps27
Placentalia - ps28
Boreoeutheria - ps29
Euarchontoglires - ps30
Euarchonta - ps31
Primates - ps32
Haplorrhini - ps33
Simiiformes - ps34
Catarrhini - ps35
Hominoidea - ps36
Hominidae - ps37
Homininae - ps38
Hominini - ps39
Homo_sapiens - ps40
p-value
1e-05
8e-05
7e-04
6e-03
5e-02
Log-odds
0.5
1.0
1.5
2.0
2.5
3.0
3.5
4.0

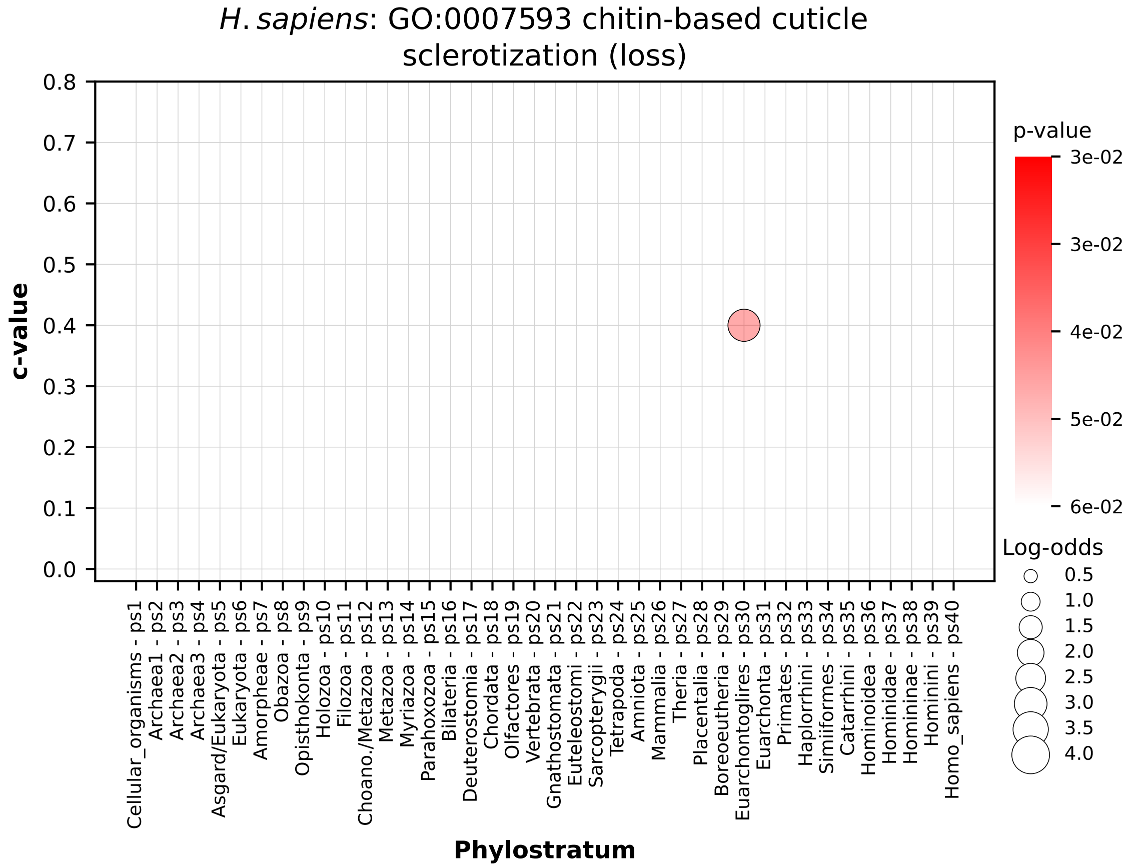

H. sapiens: GO:0007593 chitin-based cuticle sclerotization (loss)
c-value
0.8
0.7
0.6
0.5
0.4
0.3
0.2
0.1
0.0
p-value
3e-02
3e-02
4e-02
5e-02
6e-02
Log-odds
0.5
1.0
1.5
2.0
2.5
3.0
3.5
4.0
Cellular_organisms - ps1
Archaea1 - ps2
Archaea2 - ps3
Archaea3 - ps4
Asgard/Eukaryota - ps5
Eukaryota - ps6
Amorpheae - ps7
Obazoa - ps8
Opisthokonta - ps9
Holozoa - ps10
Filozoa - ps11
Choano./Metazoa - ps12
Metazoa - ps13
Myriazoa - ps14
Parahoxozoa - ps15
Bilateria - ps16
Deuterostomia - ps17
Chordata - ps18
Olfactores - ps19
Vertebrata - ps20
Gnathostomata - ps21
Euteleostomi - ps22
Sarcopterygii - ps23
Tetrapoda - ps24
Amniota - ps25
Mammalia - ps26
Theria - ps27
Placentalia - ps28
Boreoeutheria - ps29
Euarchontoglires - ps30
Euarchonta - ps31
Primates - ps32
Haplorrhini - ps33
Simiiformes - ps34
Catarrhini - ps35
Hominoidea - ps36
Hominidae - ps37
Homininae - ps38
Hominini - ps39
Homo_sapiens - ps40
Phylostratum

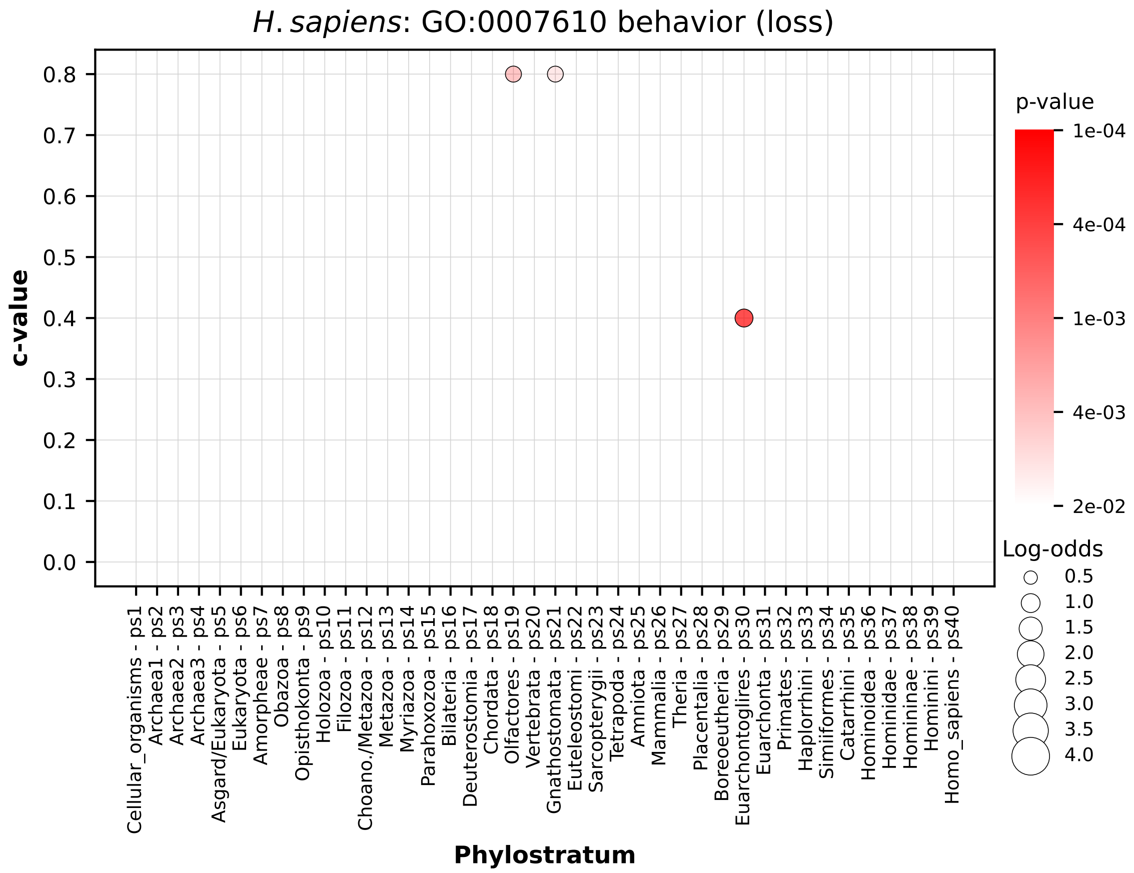

H. sapiens: GO:0007610 behavior (loss)
c-value
0.0
0.1
0.2
0.3
0.4
0.5
0.6
0.7
0.8
Phylostratum
Cellular_organisms - ps1
Archaea1 - ps2
Archaea2 - ps3
Archaea3 - ps4
Asgard/Eukaryota - ps5
Eukaryota - ps6
Amorpheae - ps7
Obazoa - ps8
Opisthokonta - ps9
Holozoa - ps10
Filozoa - ps11
Choano./Metazoa - ps12
Metazoa - ps13
Myriazoa - ps14
Parahoxozoa - ps15
Bilateria - ps16
Deuterostomia - ps17
Chordata - ps18
Olfactores - ps19
Vertebrata - ps20
Gnathostomata - ps21
Euteleostomi - ps22
Sarcopterygii - ps23
Tetrapoda - ps24
Amniota - ps25
Mammalia - ps26
Theria - ps27
Placentalia - ps28
Boreoeutheria - ps29
Euarchontoglires - ps30
Euarchonta - ps31
Primates - ps32
Haplorrhini - ps33
Simiiformes - ps34
Catarrhini - ps35
Hominoidea - ps36
Hominidae - ps37
Homininae - ps38
Hominini - ps39
Homo_sapiens - ps40
p-value
1e-04
4e-04
1e-03
4e-03
2e-02
Log-odds
0.5
1.0
1.5
2.0
2.5
3.0
3.5
4.0

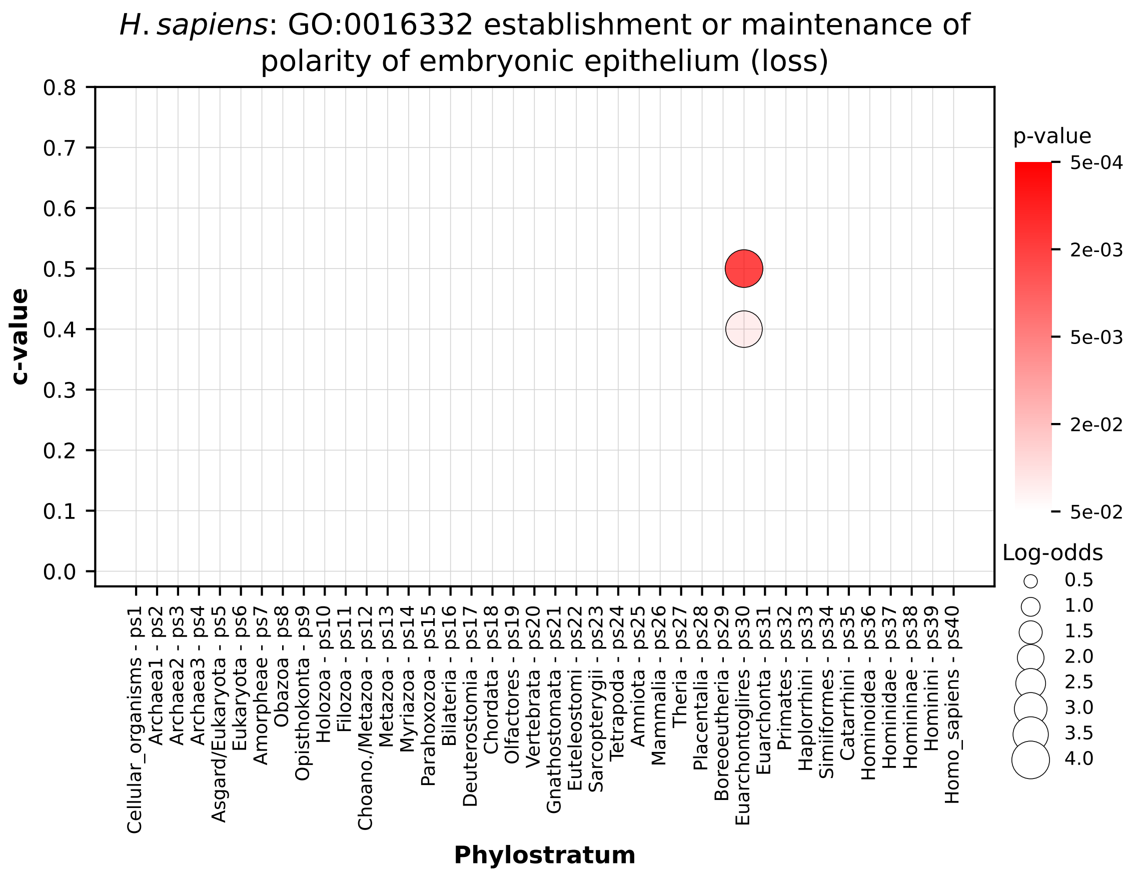

H. sapiens: GO:0016332 establishment or maintenance of polarity of embryonic epithelium (loss)
c-value
0.8
0.7
0.6
0.5
0.4
0.3
0.2
0.1
0.0
Phylostratum
Cellular_organisms - ps1
Archaea1 - ps2
Archaea2 - ps3
Archaea3 - ps4
Asgard/Eukaryota - ps5
Eukaryota - ps6
Amorpheae - ps7
Obazoa - ps8
Opisthokonta - ps9
Holozoa - ps10
Filozoa - ps11
Choano./Metazoa - ps12
Metazoa - ps13
Myriazoa - ps14
Parahoxozoa - ps15
Bilateria - ps16
Deuterostomia - ps17
Chordata - ps18
Olfactores - ps19
Vertebrata - ps20
Gnathostomata - ps21
Euteleostomi - ps22
Sarcopterygii - ps23
Tetrapoda - ps24
Amniota - ps25
Mammalia - ps26
Theria - ps27
Placentalia - ps28
Boreoeutheria - ps29
Euarchontoglires - ps30
Euarchonta - ps31
Primates - ps32
Haplorrhini - ps33
Simiiformes - ps34
Catarrhini - ps35
Hominoidea - ps36
Hominidae - ps37
Homininae - ps38
Hominini - ps39
Homo_sapiens - ps40
p-value
5e-04
2e-03
5e-03
2e-02
5e-02
Log-odds
0.5
1.0
1.5
2.0
2.5
3.0
3.5
4.0

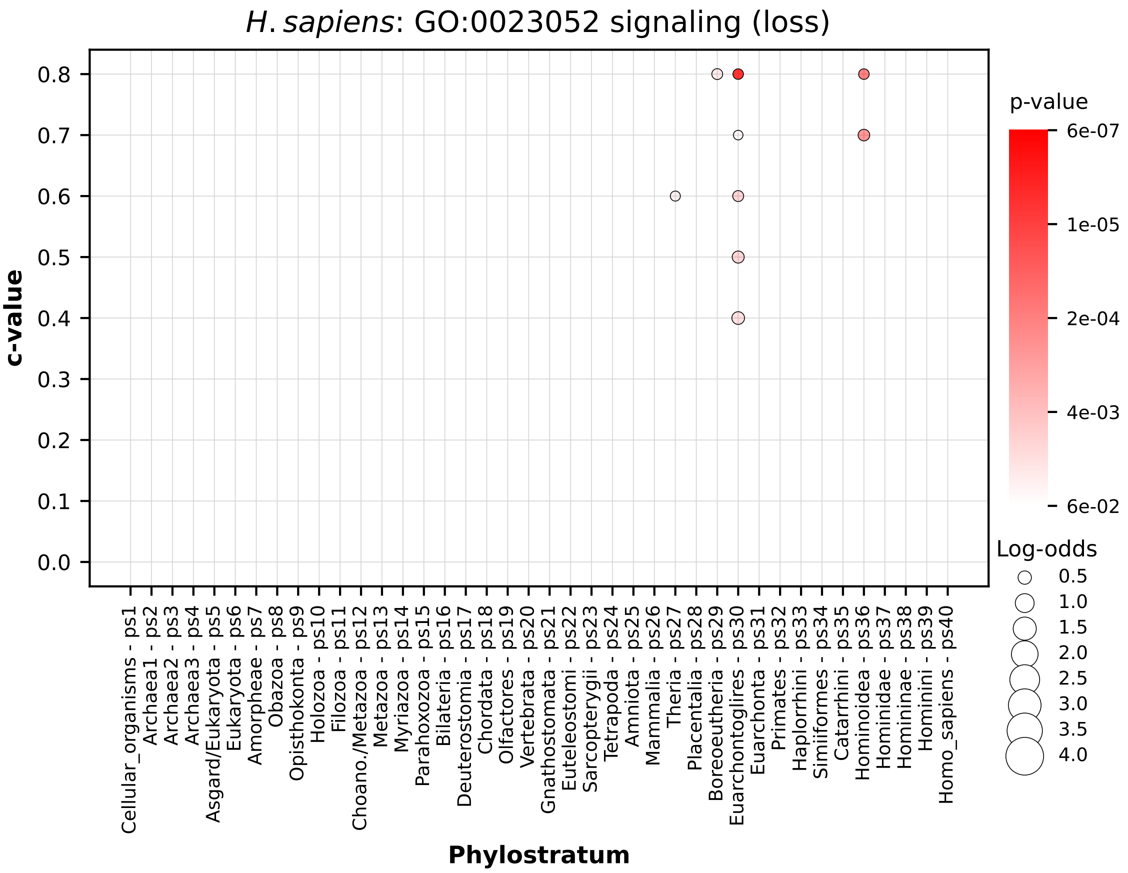

H. sapiens: GO:0023052 signaling (loss)
c-value
0.0
0.1
0.2
0.3
0.4
0.5
0.6
0.7
0.8
p-value
6e-07
1e-05
2e-04
4e-03
6e-02
Log-odds
0.5
1.0
1.5
2.0
2.5
3.0
3.5
4.0
Cellular_organisms - ps1
Archaea1 - ps2
Archaea2 - ps3
Archaea3 - ps4
Asgard/Eukaryota - ps5
Eukaryota - ps6
Amorpheae - ps7
Obazoa - ps8
Opisthokonta - ps9
Holozoa - ps10
Filozoa - ps11
Choano./Metazoa - ps12
Metazoa - ps13
Myriazoa - ps14
Parahoxozoa - ps15
Bilateria - ps16
Deuterostomia - ps17
Chordata - ps18
Olfactores - ps19
Vertebrata - ps20
Gnathostomata - ps21
Euteleostomi - ps22
Sarcopterygii - ps23
Tetrapoda - ps24
Amniota - ps25
Mammalia - ps26
Theria - ps27
Placentalia - ps28
Boreoeutheria - ps29
Euarchontoglires - ps30
Euarchonta - ps31
Primates - ps32
Haplorrhini - ps33
Simiiformes - ps34
Catarrhini - ps35
Hominoidea - ps36
Hominidae - ps37
Homininae - ps38
Hominini - ps39
Homo_sapiens - ps40
Phylostratum

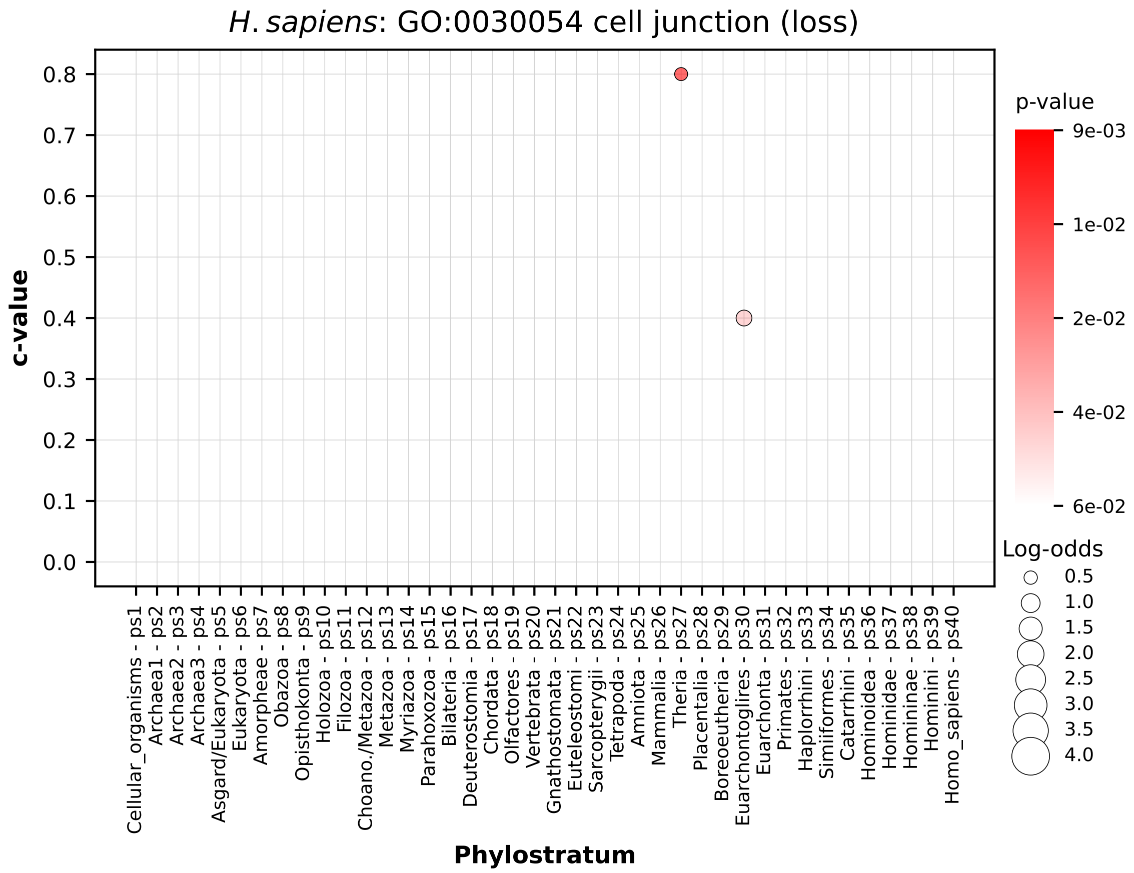

H. sapiens: GO:0030054 cell junction (loss)
c-value
0.8
0.7
0.6
0.5
0.4
0.3
0.2
0.1
0.0
p-value
9e-03
1e-02
2e-02
4e-02
6e-02
Log-odds
0.5
1.0
1.5
2.0
2.5
3.0
3.5
4.0
Cellular_organisms - ps1
Archaea1 - ps2
Archaea2 - ps3
Archaea3 - ps4
Asgard/Eukaryota - ps5
Eukaryota - ps6
Amorpheae - ps7
Obazoa - ps8
Opisthokonta - ps9
Holozoa - ps10
Filozoa - ps11
Choano./Metazoa - ps12
Metazoa - ps13
Myriazoa - ps14
Parahoxozoa - ps15
Bilateria - ps16
Deuterostomia - ps17
Chordata - ps18
Olfactores - ps19
Vertebrata - ps20
Gnathostomata - ps21
Euteleostomi - ps22
Sarcopterygii - ps23
Tetrapoda - ps24
Amniota - ps25
Mammalia - ps26
Theria - ps27
Placentalia - ps28
Boreoeutheria - ps29
Euarchontoglires - ps30
Euarchonta - ps31
Primates - ps32
Haplorrhini - ps33
Similformes - ps34
Catarrhini - ps35
Hominoidea - ps36
Hominidae - ps37
Homininae - ps38
Hominini - ps39
Homo_sapiens - ps40
Phylostratum

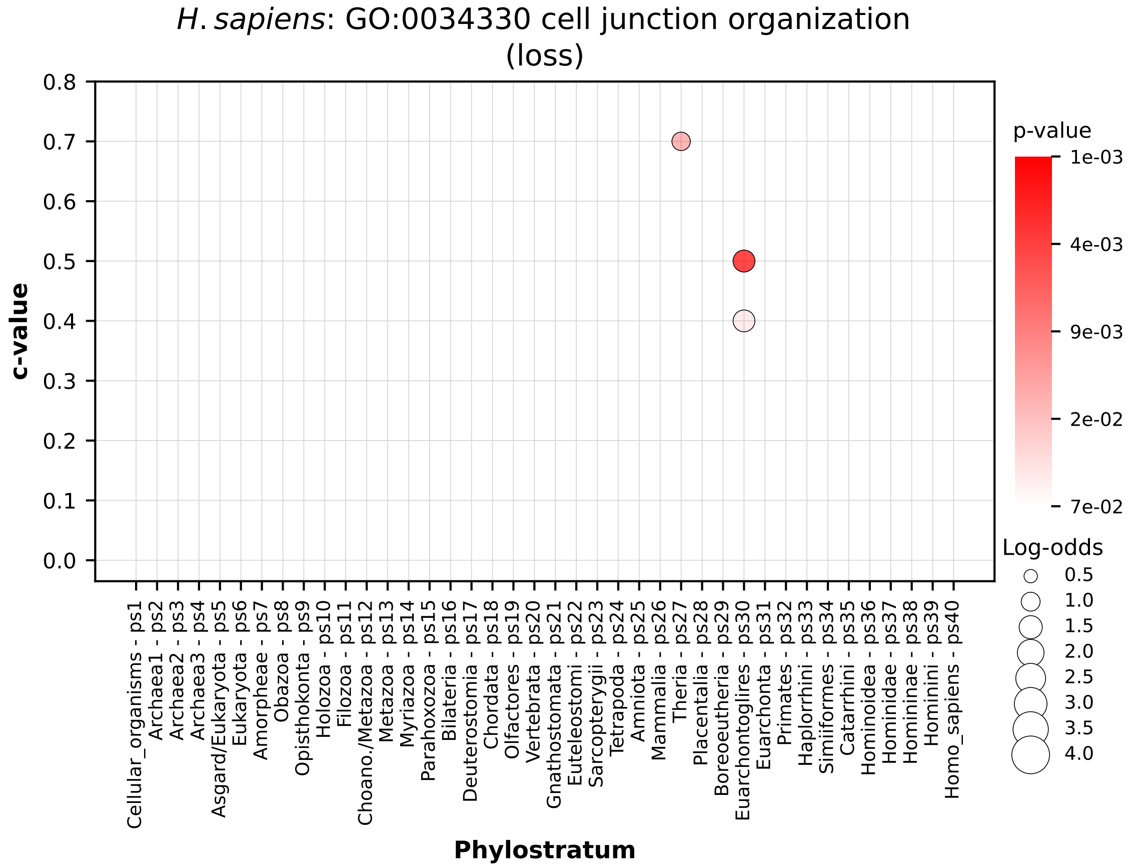

H. sapiens: GO:0034330 cell junction organization
(loss)
c-value
0.8
0.7
0.6
0.5
0.4
0.3
0.2
0.1
0.0
p-value
1e-03
4e-03
9e-03
2e-02
7e-02
Log-odds
0.5
1.0
1.5
2.0
2.5
3.0
3.5
4.0
Cellular_organisms - ps1
Archaea1 - ps2
Archaea2 - ps3
Archaea3 - ps4
Asgard/Eukaryota - ps5
Eukaryota - ps6
Amorpheae - ps7
Obazoa - ps8
Opisthokonta - ps9
Holozoa - ps10
Filozoa - ps11
Choano./Metazoa - ps12
Metazoa - ps13
Myriazoa - ps14
Parahoxozoa - ps15
Bilateria - ps16
Deuterostomia - ps17
Chordata - ps18
Olfactores - ps19
Vertebrata - ps20
Gnathostomata - ps21
Euteleostomi - ps22
Sarcopterygii - ps23
Tetrapoda - ps24
Amniota - ps25
Mammalia - ps26
Theria - ps27
Placentalia - ps28
Boreoeutheria - ps29
Euarchontoglires - ps30
Euarchonta - ps31
Primates - ps32
Haplorrhini - ps33
Simiiformes - ps34
Catarrhini - ps35
Hominoidea - ps36
Hominidae - ps37
Homininae - ps38
Hominini - ps39
Homo_sapiens - ps40
Phylostratum

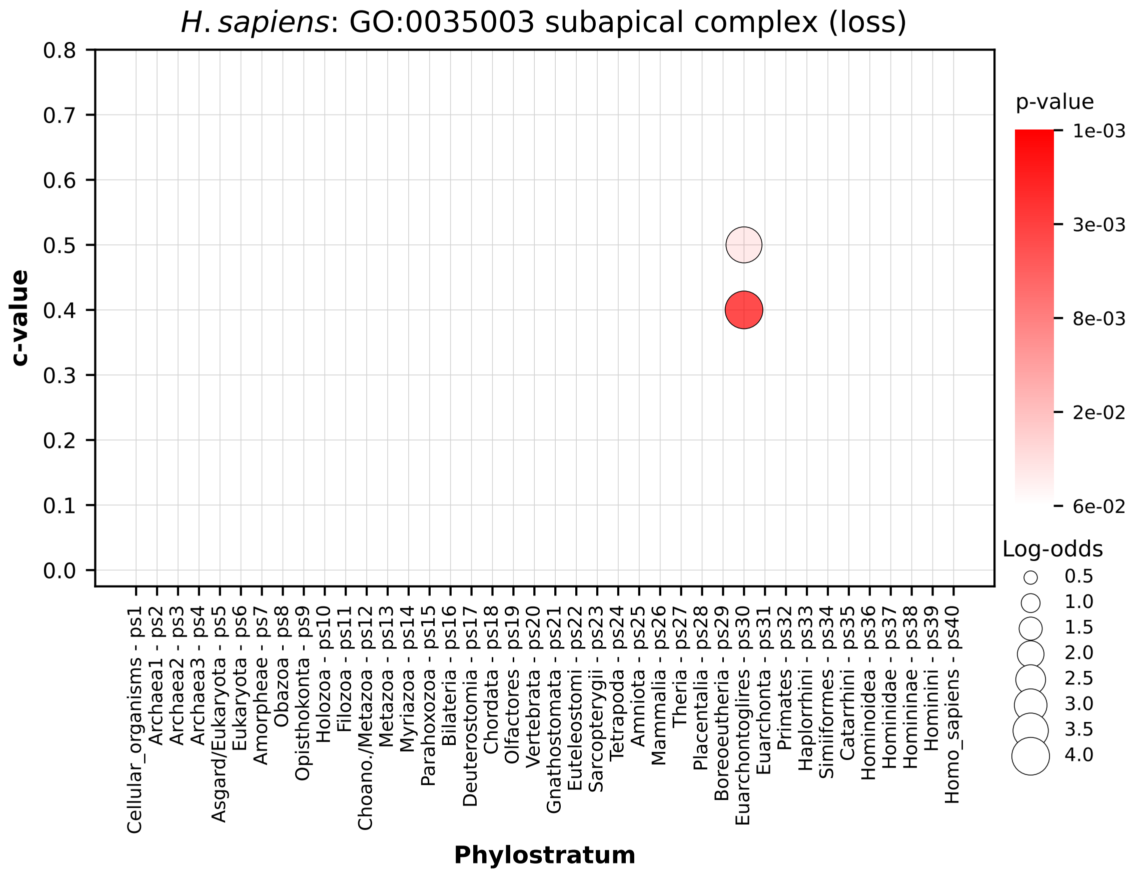

H. sapiens: GO:0035003 subapical complex (loss)
c-value
0.8
0.7
0.6
0.5
0.4
0.3
0.2
0.1
0.0
p-value
1e-03
3e-03
8e-03
2e-02
6e-02
Log-odds
0.5
1.0
1.5
2.0
2.5
3.0
3.5
4.0
Cellular_organisms - ps1
Archaea1 - ps2
Archaea2 - ps3
Archaea3 - ps4
Asgard/Eukaryota - ps5
Eukaryota - ps6
Amorpheae - ps7
Obazoa - ps8
Opisthokonta - ps9
Holozoa - ps10
Filozoa - ps11
Choano./Metazoa - ps12
Metazoa - ps13
Myriazoa - ps14
Parahoxozoa - ps15
Bilateria - ps16
Deuterostomia - ps17
Chordata - ps18
Olfactores - ps19
Vertebrata - ps20
Gnathostomata - ps21
Euteleostomi - ps22
Sarcopterygii - ps23
Tetrapoda - ps24
Amniota - ps25
Mammalia - ps26
Theria - ps27
Placentalia - ps28
Boreoeutheria - ps29
Euarchontoglires - ps30
Euarchonta - ps31
Primates - ps32
Haplorrhini - ps33
Simiiformes - ps34
Catarrhini - ps35
Hominoidea - ps36
Hominidae - ps37
Homininae - ps38
Hominini - ps39
Homo_sapiens - ps40
Phylostratum

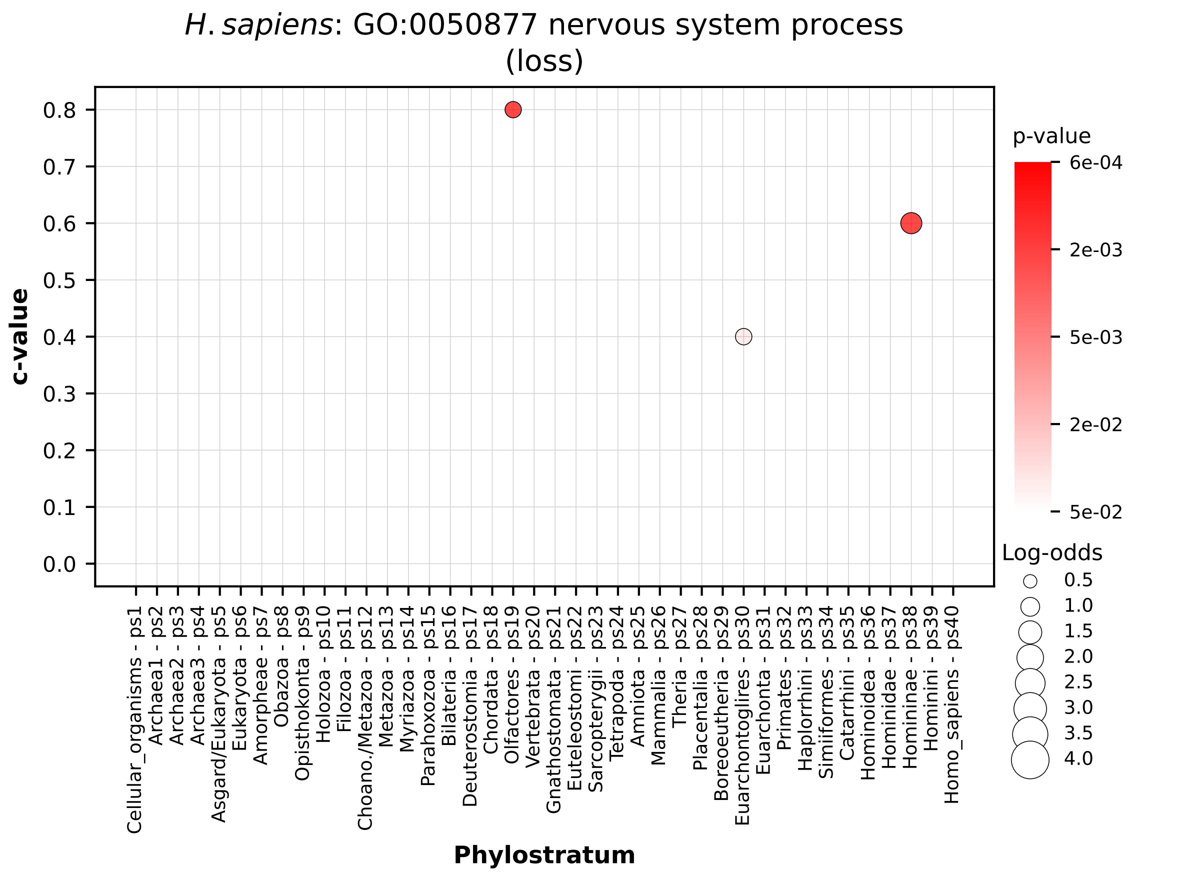
H. sapiens: GO:0050877 nervous system process
(loss)
c-value
0.0
0.1
0.2
0.3
0.4
0.5
0.6
0.7
0.8
p-value
6e-04
2e-03
5e-03
2e-02
5e-02
Log-odds
0.5
1.0
1.5
2.0
2.5
3.0
3.5
4.0
Cellular_organisms - ps1
Archaea1 - ps2
Archaea2 - ps3
Archaea3 - ps4
Asgard/Eukaryota - ps5
Eukaryota - ps6
Amorpheae - ps7
Obazoa - ps8
Opisthokonta - ps9
Holozoa - ps10
Filozoa - ps11
Choano./Metazoa - ps12
Metazoa - ps13
Myriazoa - ps14
Parahoxozoa - ps15
Bilateria - ps16
Deuterostomia - ps17
Chordata - ps18
Olfactores - ps19
Vertebrata - ps20
Gnathostomata - ps21
Euteleostomi - ps22
Sarcopterygii - ps23
Tetrapoda - ps24
Amniota - ps25
Mammalia - ps26
Theria - ps27
Placentalia - ps28
Boreoeutheria - ps29
Euarchontoglires - ps30
Euarchonta - ps31
Primates - ps32
Haplorrhini - ps33
Simiiformes - ps34
Catarrhini - ps35
Hominoidea - ps36
Hominidae - ps37
Homininae - ps38
Hominini - ps39
Homo_sapiens - ps40
Phylostratum

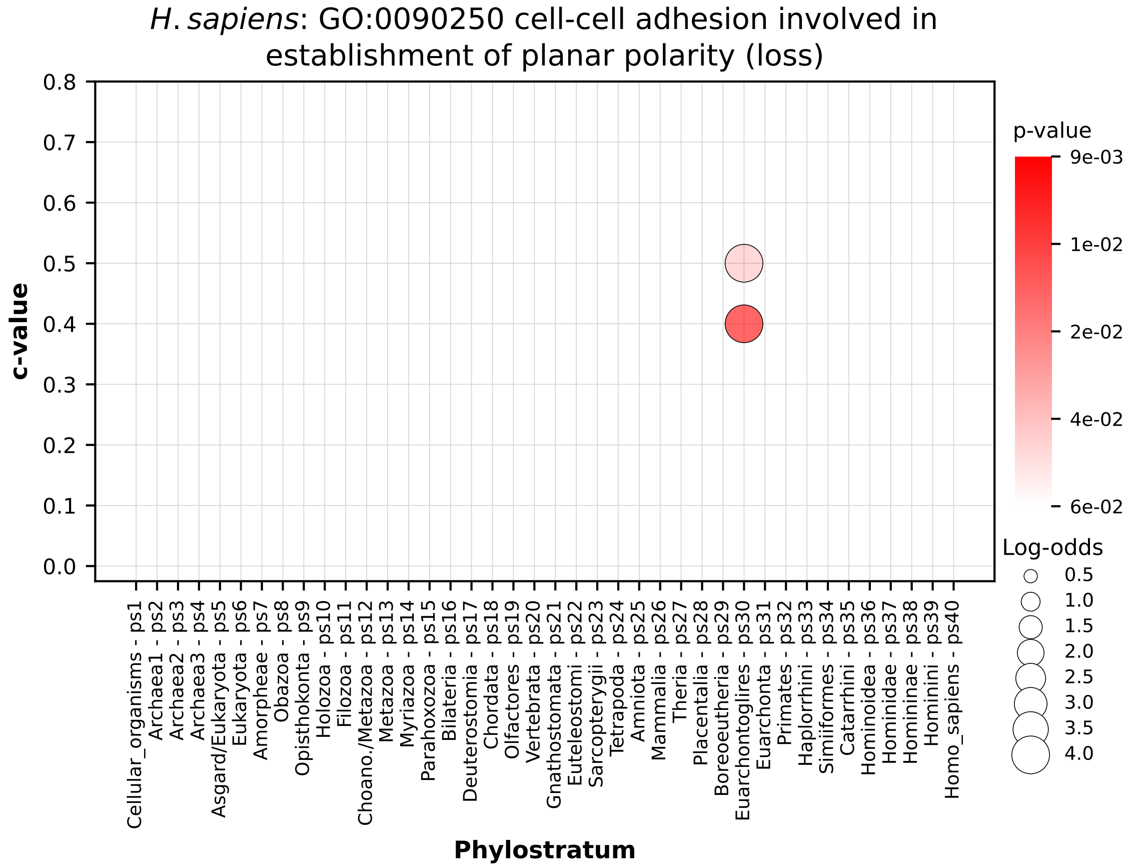

H. sapiens: GO:0090250 cell-cell adhesion involved in establishment of planar polarity (loss)
c-value
0.0
0.1
0.2
0.3
0.4
0.5
0.6
0.7
0.8
Phylostratum
Cellular_organisms - ps1
Archaea1 - ps2
Archaea2 - ps3
Archaea3 - ps4
Asgard/Eukaryota - ps5
Eukaryota - ps6
Amorpheae - ps7
Obazoa - ps8
Opisthokonta - ps9
Holozoa - ps10
Filozoa - ps11
Choano./Metazoa - ps12
Metazoa - ps13
Myriazoa - ps14
Parahoxozoa - ps15
Bilateria - ps16
Deuterostomia - ps17
Chordata - ps18
Olfactores - ps19
Vertebrata - ps20
Gnathostomata - ps21
Euteleostomi - ps22
Sarcopterygii - ps23
Tetrapoda - ps24
Amniota - ps25
Mammalia - ps26
Theria - ps27
Placentalia - ps28
Boreoeutheria - ps29
Euarchontoglires - ps30
Euarchonta - ps31
Primates - ps32
Haplorrhini - ps33
Simiiformes - ps34
Catarrhini - ps35
Hominoidea - ps36
Hominidae - ps37
Homininae - ps38
Hominini - ps39
Homo_sapiens - ps40
p-value
9e-03
1e-02
2e-02
4e-02
6e-02
Log-odds
0.5
1.0
1.5
2.0
2.5
3.0
3.5
4.0

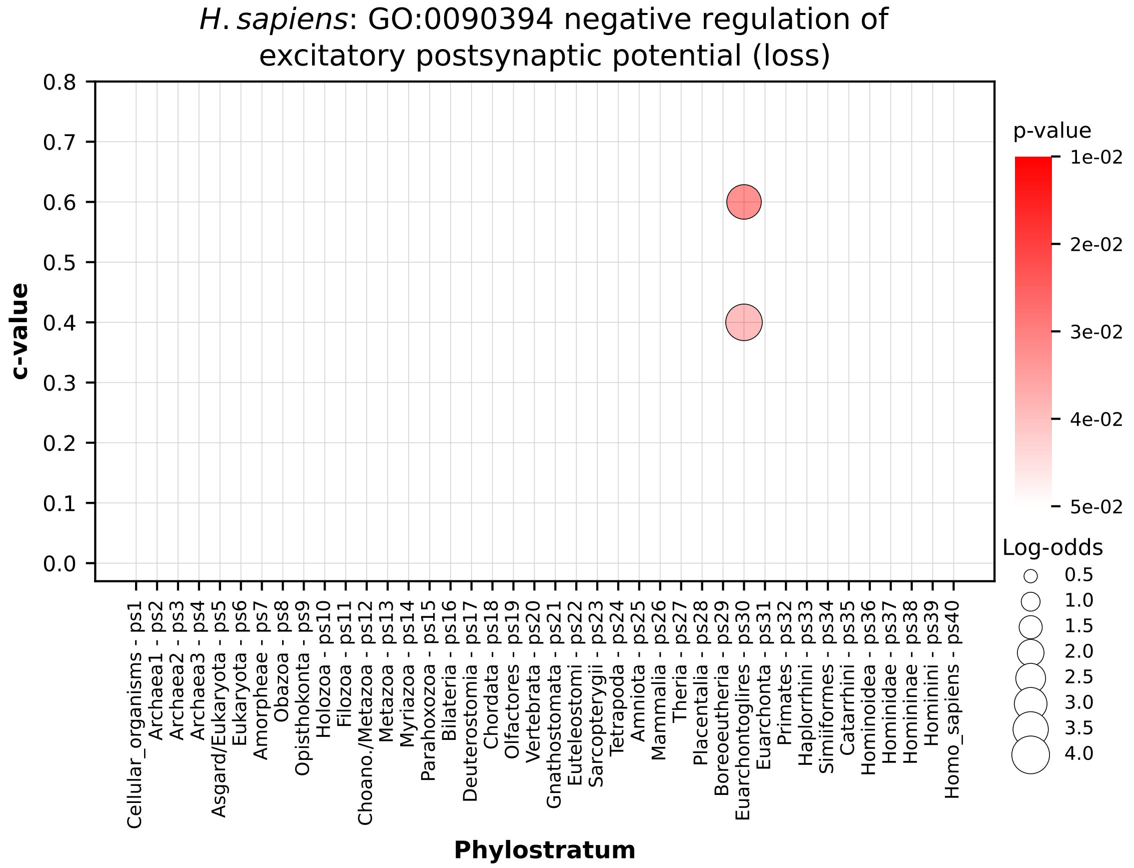

H. sapiens: GO:0090394 negative regulation of excitatory postsynaptic potential (loss)
c-value
0.8
0.7
0.6
0.5
0.4
0.3
0.2
0.1
0.0
Phylostratum
Cellular_organisms - ps1
Archaea1 - ps2
Archaea2 - ps3
Archaea3 - ps4
Asgard/Eukaryota - ps5
Eukaryota - ps6
Amorpheae - ps7
Obazoa - ps8
Opisthokonta - ps9
Holozoa - ps10
Filozoa - ps11
Choano./Metazoa - ps12
Metazoa - ps13
Myriazoa - ps14
Parahoxozoa - ps15
Bilateria - ps16
Deuterostomia - ps17
Chordata - ps18
Olfactores - ps19
Vertebrata - ps20
Gnathostomata - ps21
Euteleostomi - ps22
Sarcopterygii - ps23
Tetrapoda - ps24
Amniota - ps25
Mammalia - ps26
Theria - ps27
Placentalia - ps28
Boreoeutheria - ps29
Euarchontoglires - ps30
Euarchonta - ps31
Primates - ps32
Haplorrhini - ps33
Simiiformes - ps34
Catarrhini - ps35
Hominoidea - ps36
Hominidae - ps37
Homininae - ps38
Hominini - ps39
Homo_sapiens - ps40
p-value
1e-02
2e-02
3e-02
4e-02
5e-02
Log-odds
0.5
1.0
1.5
2.0
2.5
3.0
3.5
4.0

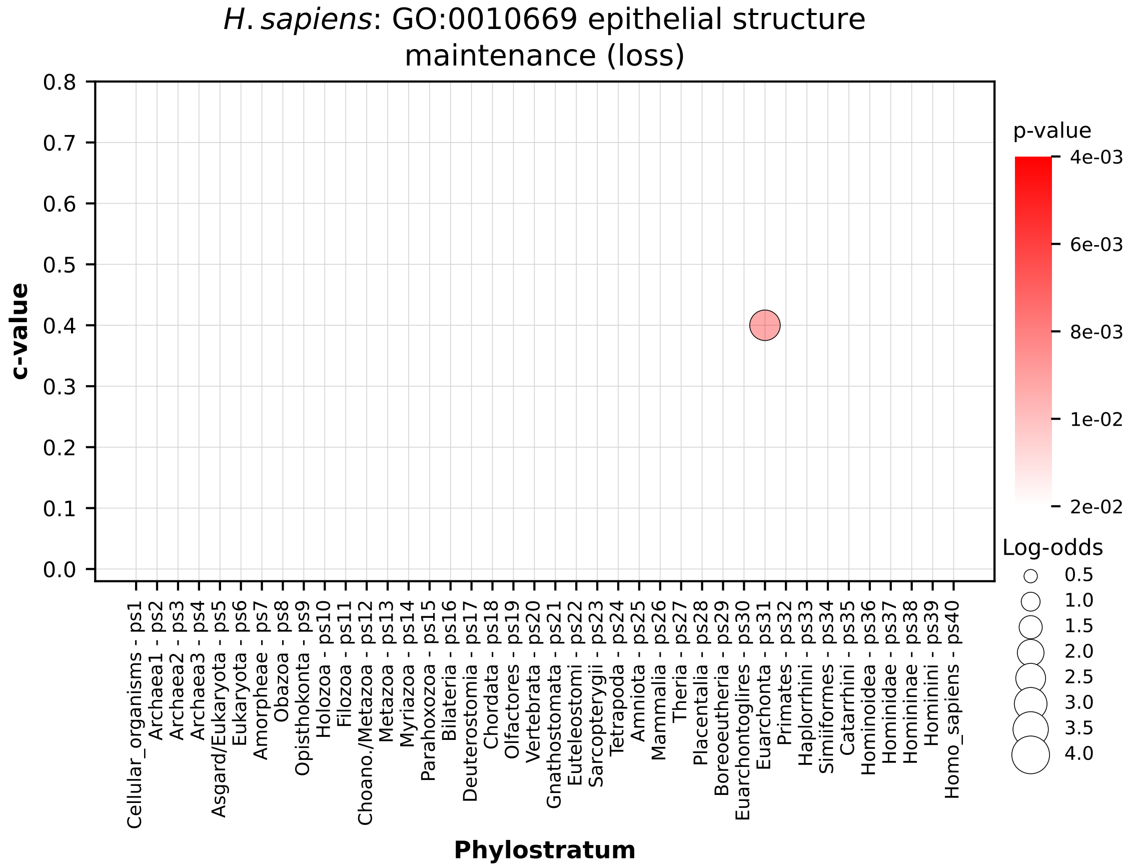

H. sapiens: GO:0010669 epithelial structure maintenance (loss)
c-value
0.8
0.7
0.6
0.5
0.4
0.3
0.2
0.1
0.0
Phylostratum
Cellular_organisms - ps1
Archaea1 - ps2
Archaea2 - ps3
Archaea3 - ps4
Asgard/Eukaryota - ps5
Eukaryota - ps6
Amorpheae - ps7
Obazoa - ps8
Opisthokonta - ps9
Holozoa - ps10
Filozoa - ps11
Choano./Metazoa - ps12
Metazoa - ps13
Myriazoa - ps14
Parahoxozoa - ps15
Bilateria - ps16
Deuterostomia - ps17
Chordata - ps18
Olfactores - ps19
Vertebrata - ps20
Gnathostomata - ps21
Euteleostomi - ps22
Sarcopterygii - ps23
Tetrapoda - ps24
Amniota - ps25
Mammalia - ps26
Theria - ps27
Placentalia - ps28
Boreoeutheria - ps29
Euarchontoglires - ps30
Euarchonta - ps31
Primates - ps32
Haplorrhini - ps33
Simiiformes - ps34
Catarrhini - ps35
Hominoidea - ps36
Hominidae - ps37
Homininae - ps38
Hominini - ps39
Homo_sapiens - ps40
p-value
4e-03
6e-03
8e-03
1e-02
2e-02
Log-odds
0.5
1.0
1.5
2.0
2.5
3.0
3.5
4.0

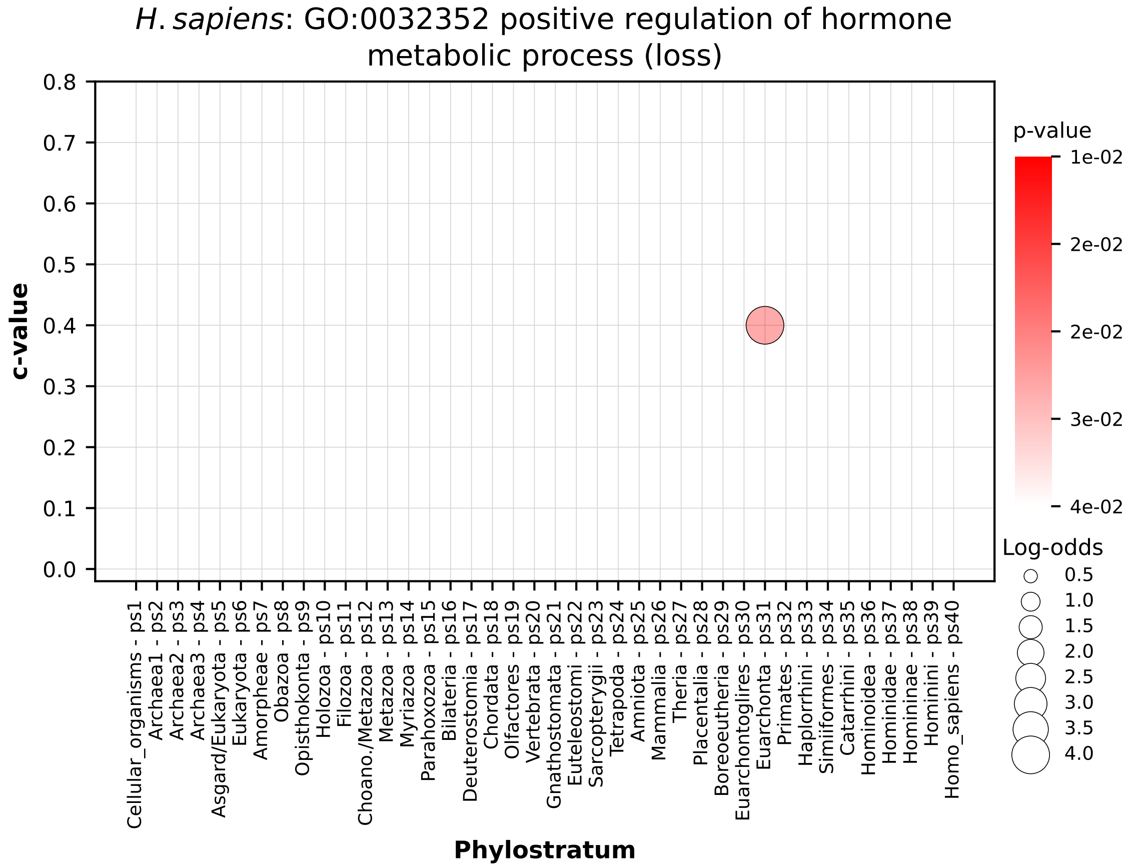

H. sapiens: GO:0032352 positive regulation of hormone metabolic process (loss)
c-value
0.8
0.7
0.6
0.5
0.4
0.3
0.2
0.1
0.0
Phylostratum
Cellular_organisms - ps1
Archaea1 - ps2
Archaea2 - ps3
Archaea3 - ps4
Asgard/Eukaryota - ps5
Eukaryota - ps6
Amorpheae - ps7
Obazoa - ps8
Opisthokonta - ps9
Holozoa - ps10
Filozoa - ps11
Choano./Metazoa - ps12
Metazoa - ps13
Myriazoa - ps14
Parahoxozoa - ps15
Bilateria - ps16
Deuterostomia - ps17
Chordata - ps18
Olfactores - ps19
Vertebrata - ps20
Gnathostomata - ps21
Euteleostomi - ps22
Sarcopterygii - ps23
Tetrapoda - ps24
Amniota - ps25
Mammalia - ps26
Theria - ps27
Placentalia - ps28
Boreoeutheria - ps29
Euarchontoglires - ps30
Euarchonta - ps31
Primates - ps32
Haplorrhini - ps33
Simiiformes - ps34
Catarrhini - ps35
Hominoidea - ps36
Hominidae - ps37
Homininae - ps38
Hominini - ps39
Homo_sapiens - ps40
p-value
1e-02
2e-02
2e-02
3e-02
4e-02
Log-odds
0.5
1.0
1.5
2.0
2.5
3.0
3.5
4.0

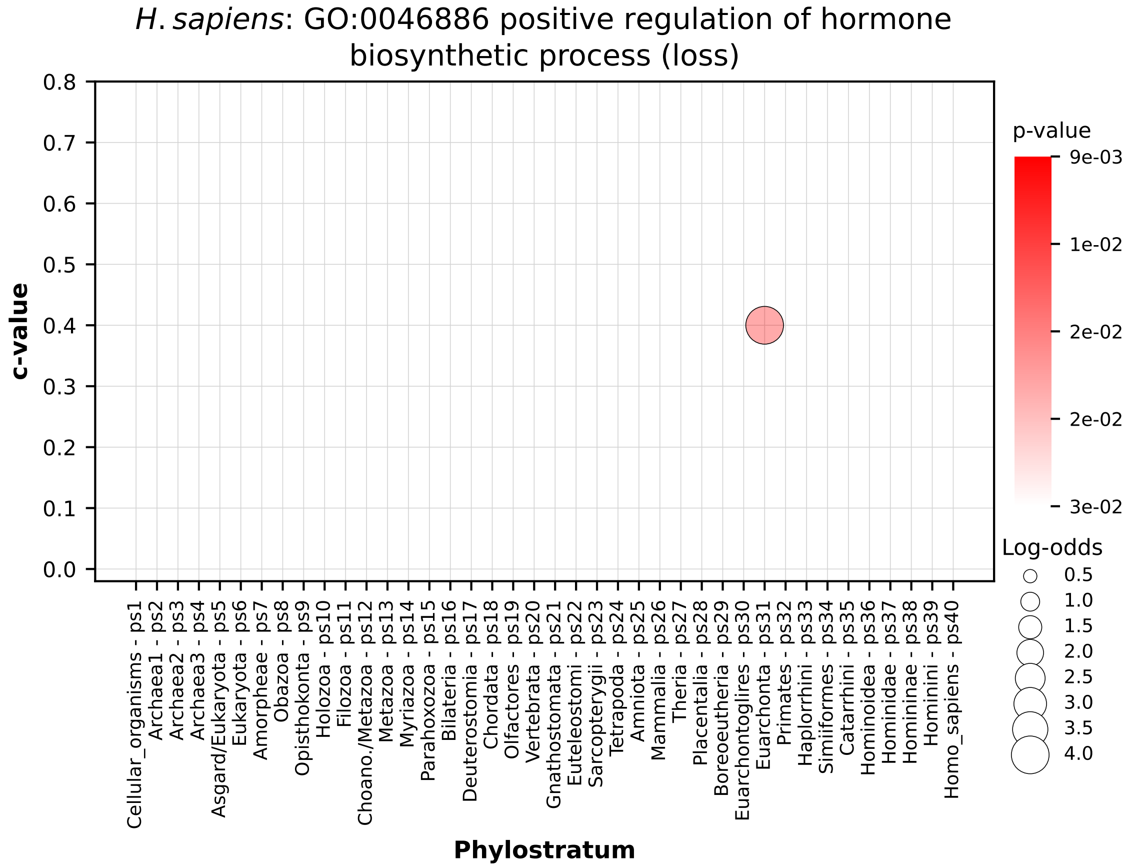

H. sapiens: GO:0046886 positive regulation of hormone biosynthetic process (loss)
c-value
0.0
0.1
0.2
0.3
0.4
0.5
0.6
0.7
0.8
Phylostratum
Cellular_organisms - ps1
Archaea1 - ps2
Archaea2 - ps3
Archaea3 - ps4
Asgard/Eukaryota - ps5
Eukaryota - ps6
Amorpheae - ps7
Obazoa - ps8
Opisthokonta - ps9
Holozoa - ps10
Filozoa - ps11
Choano./Metazoa - ps12
Metazoa - ps13
Myriazoa - ps14
Parahoxozoa - ps15
Bilateria - ps16
Deuterostomia - ps17
Chordata - ps18
Olfactores - ps19
Vertebrata - ps20
Gnathostomata - ps21
Euteleostomi - ps22
Sarcopterygii - ps23
Tetrapoda - ps24
Amniota - ps25
Mammalia - ps26
Theria - ps27
Placentalia - ps28
Boreoeutheria - ps29
Euarchontoglires - ps30
Euarchonta - ps31
Primates - ps32
Haplorrhini - ps33
Simiiformes - ps34
Catarrhini - ps35
Hominoidea - ps36
Hominidae - ps37
Homininae - ps38
Hominini - ps39
Homo_sapiens - ps40
p-value
9e-03
1e-02
2e-02
2e-02
3e-02
Log-odds
0.5
1.0
1.5
2.0
2.5
3.0
3.5
4.0

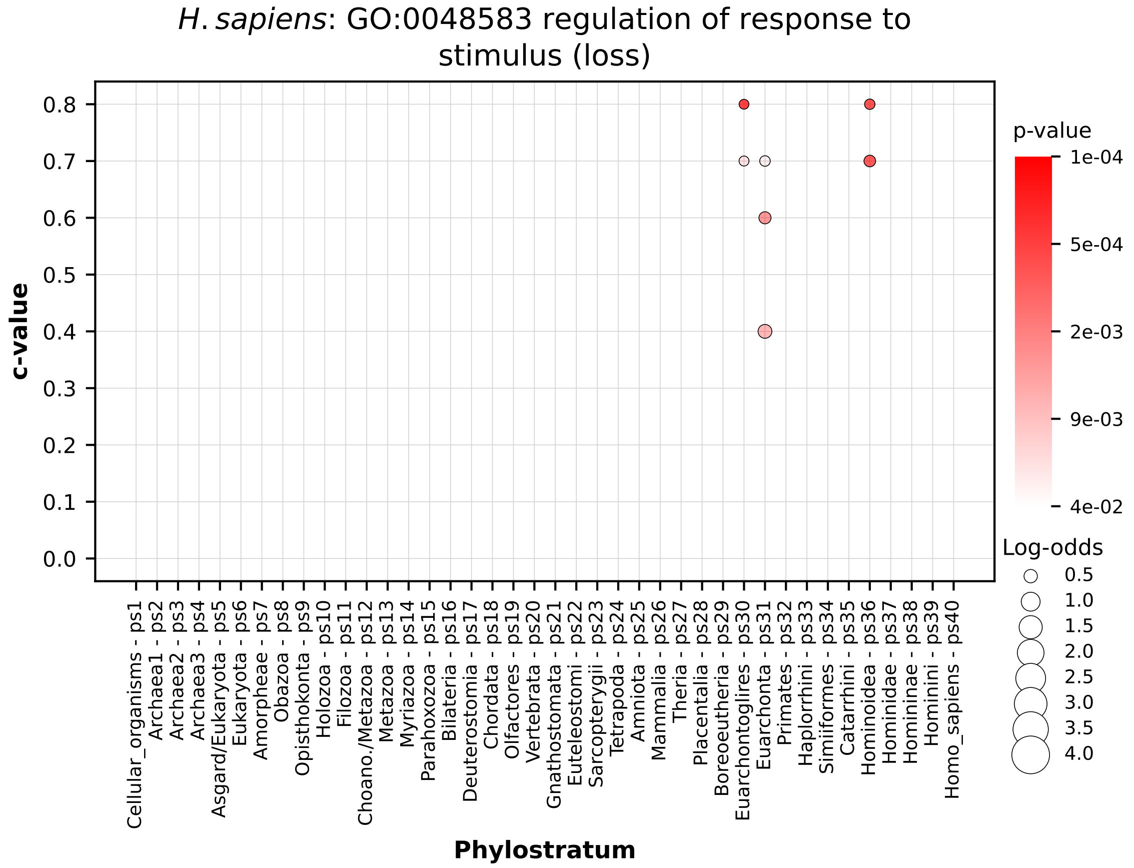

H. sapiens: GO:0048583 regulation of response to stimulus (loss)
c-value
0.0
0.1
0.2
0.3
0.4
0.5
0.6
0.7
0.8
Phylostratum
Cellular_organisms - ps1
Archaea1 - ps2
Archaea2 - ps3
Archaea3 - ps4
Asgard/Eukaryota - ps5
Eukaryota - ps6
Amorpheae - ps7
Obazoa - ps8
Opisthokonta - ps9
Holozoa - ps10
Filozoa - ps11
Choano./Metazoa - ps12
Metazoa - ps13
Myriazoa - ps14
Parahoxozoa - ps15
Bilateria - ps16
Deuterostomia - ps17
Chordata - ps18
Olfactores - ps19
Vertebrata - ps20
Gnathostomata - ps21
Euteleostomi - ps22
Sarcopterygii - ps23
Tetrapoda - ps24
Amniota - ps25
Mammalia - ps26
Theria - ps27
Placentalia - ps28
Boreoeutheria - ps29
Euarchontoglires - ps30
Euarchonta - ps31
Primates - ps32
Haplorrhini - ps33
Simiiformes - ps34
Catarrhini - ps35
Hominoidea - ps36
Hominidae - ps37
Homininae - ps38
Hominini - ps39
Homo_sapiens - ps40
p-value
1e-04
5e-04
2e-03
9e-03
4e-02
Log-odds
0.5
1.0
1.5
2.0
2.5
3.0
3.5
4.0

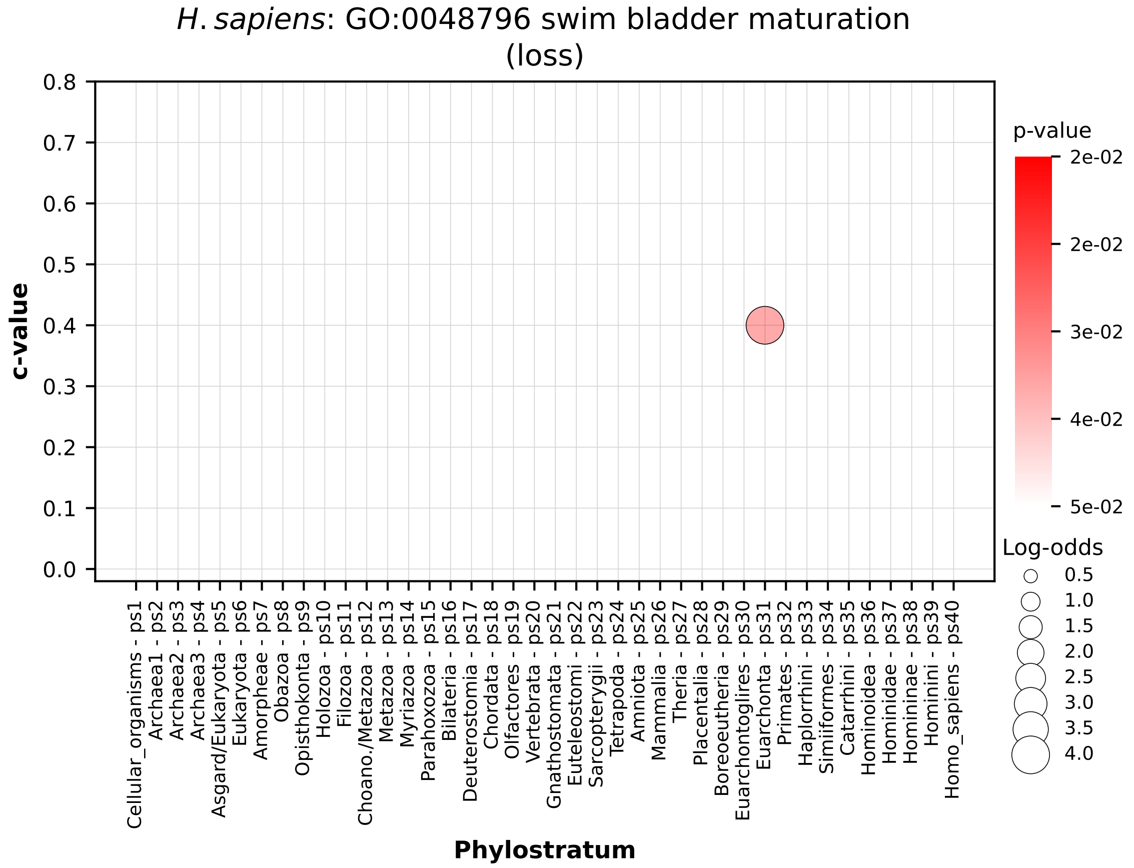
H. sapiens: GO:0048796 swim bladder maturation (loss)
c-value
0.0
0.1
0.2
0.3
0.4
0.5
0.6
0.7
0.8
Phylostratum
Cellular_organisms - ps1
Archaea1 - ps2
Archaea2 - ps3
Archaea3 - ps4
Asgard/Eukaryota - ps5
Eukaryota - ps6
Amorpheae - ps7
Obazoa - ps8
Opisthokonta - ps9
Holozoa - ps10
Filozoa - ps11
Choano./Metazoa - ps12
Metazoa - ps13
Myriazoa - ps14
Parahoxozoa - ps15
Bilateria - ps16
Deuterostomia - ps17
Chordata - ps18
Olfactores - ps19
Vertebrata - ps20
Gnathostomata - ps21
Euteleostomi - ps22
Sarcopterygii - ps23
Tetrapoda - ps24
Amniota - ps25
Mammalia - ps26
Theria - ps27
Placentalia - ps28
Boreoeutheria - ps29
Euarchontoglires - ps30
Euarchonta - ps31
Primates - ps32
Haplorrhini - ps33
Simiiformes - ps34
Catarrhini - ps35
Hominoidea - ps36
Hominidae - ps37
Homininae - ps38
Hominini - ps39
Homo_sapiens - ps40
p-value
2e-02
2e-02
3e-02
4e-02
5e-02
Log-odds
0.5
1.0
1.5
2.0
2.5
3.0
3.5
4.0

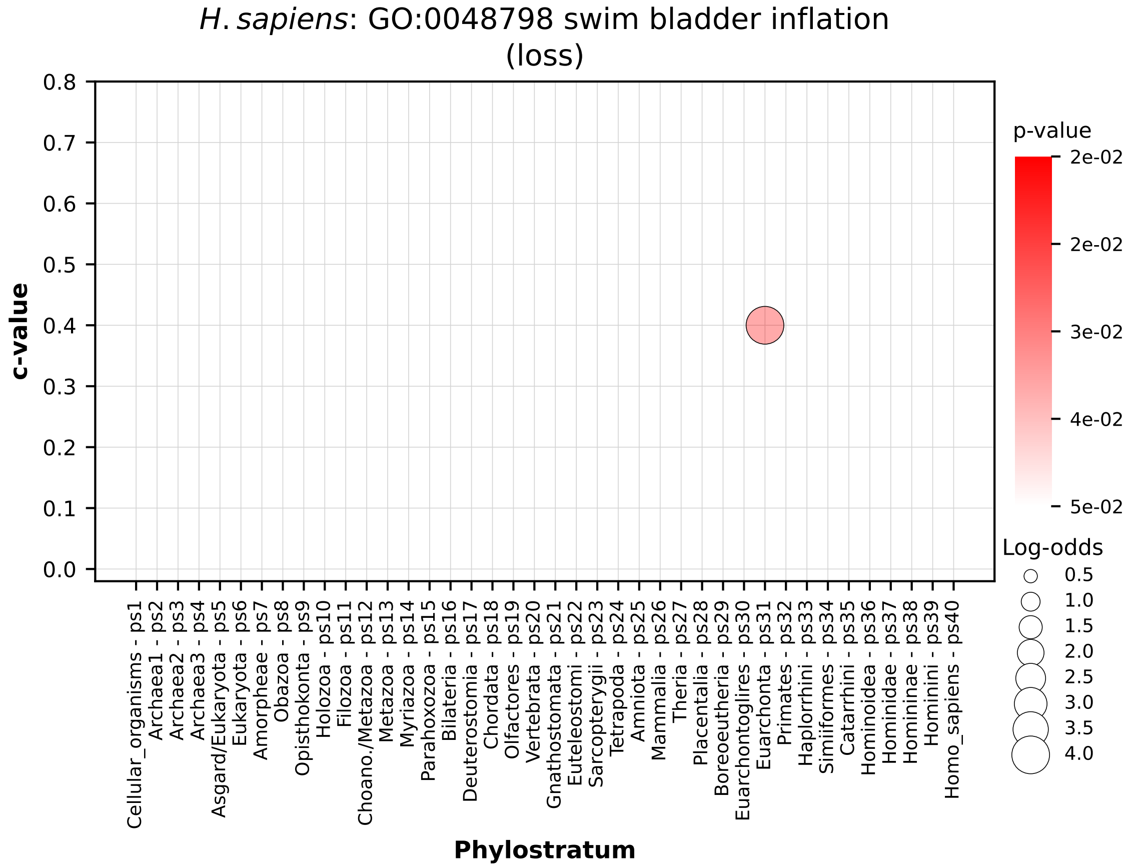

H. sapiens: GO:0048798 swim bladder inflation
(loss)
c-value
0.8
0.7
0.6
0.5
0.4
0.3
0.2
0.1
0.0
Phylostratum
Cellular_organisms - ps1
Archaea1 - ps2
Archaea2 - ps3
Archaea3 - ps4
Asgard/Eukaryota - ps5
Eukaryota - ps6
Amorpheae - ps7
Obazoa - ps8
Opisthokonta - ps9
Holozoa - ps10
Filozoa - ps11
Choano./Metazoa - ps12
Metazoa - ps13
Myriazoa - ps14
Parahoxozoa - ps15
Bilateria - ps16
Deuterostomia - ps17
Chordata - ps18
Olfactores - ps19
Vertebrata - ps20
Gnathostomata - ps21
Euteleostomi - ps22
Sarcopterygii - ps23
Tetrapoda - ps24
Amniota - ps25
Mammalia - ps26
Theria - ps27
Placentalia - ps28
Boreoeutheria - ps29
Euarchontoglires - ps30
Euarchonta - ps31
Primates - ps32
Haplorrhini - ps33
Simiiformes - ps34
Catarrhini - ps35
Hominoidea - ps36
Hominidae - ps37
Homininae - ps38
Hominini - ps39
Homo_sapiens - ps40
p-value
2e-02
2e-02
3e-02
4e-02
5e-02
Log-odds
0.5
1.0
1.5
2.0
2.5
3.0
3.5
4.0

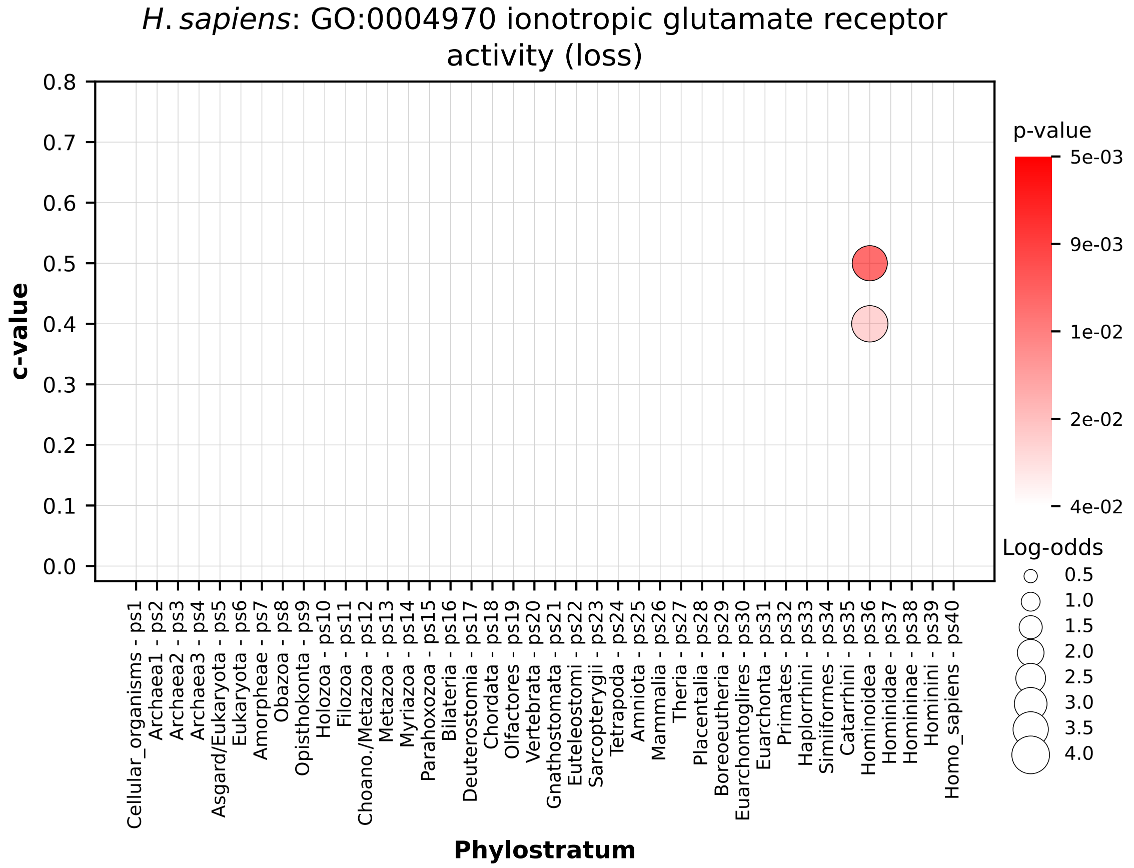

H. sapiens: GO:0004970 ionotropic glutamate receptor activity (loss)
c-value
0.0
0.1
0.2
0.3
0.4
0.5
0.6
0.7
0.8
Phylostratum
Cellular_organisms - ps1
Archaea1 - ps2
Archaea2 - ps3
Archaea3 - ps4
Asgard/Eukaryota - ps5
Eukaryota - ps6
Amorpheae - ps7
Obazoa - ps8
Opisthokonta - ps9
Holozoa - ps10
Filozoa - ps11
Choano./Metazoa - ps12
Metazoa - ps13
Myriazoa - ps14
Parahoxozoa - ps15
Bilateria - ps16
Deuterostomia - ps17
Chordata - ps18
Olfactores - ps19
Vertebrata - ps20
Gnathostomata - ps21
Euteleostomi - ps22
Sarcopterygii - ps23
Tetrapoda - ps24
Amniota - ps25
Mammalia - ps26
Theria - ps27
Placentalia - ps28
Boreoeutheria - ps29
Euarchontoglires - ps30
Euarchonta - ps31
Primates - ps32
Haplorrhini - ps33
Simiiformes - ps34
Catarrhini - ps35
Hominoidea - ps36
Hominidae - ps37
Homininae - ps38
Hominini - ps39
Homo_sapiens - ps40
p-value
5e-03
9e-03
1e-02
2e-02
4e-02
Log-odds
0.5
1.0
1.5
2.0
2.5
3.0
3.5
4.0

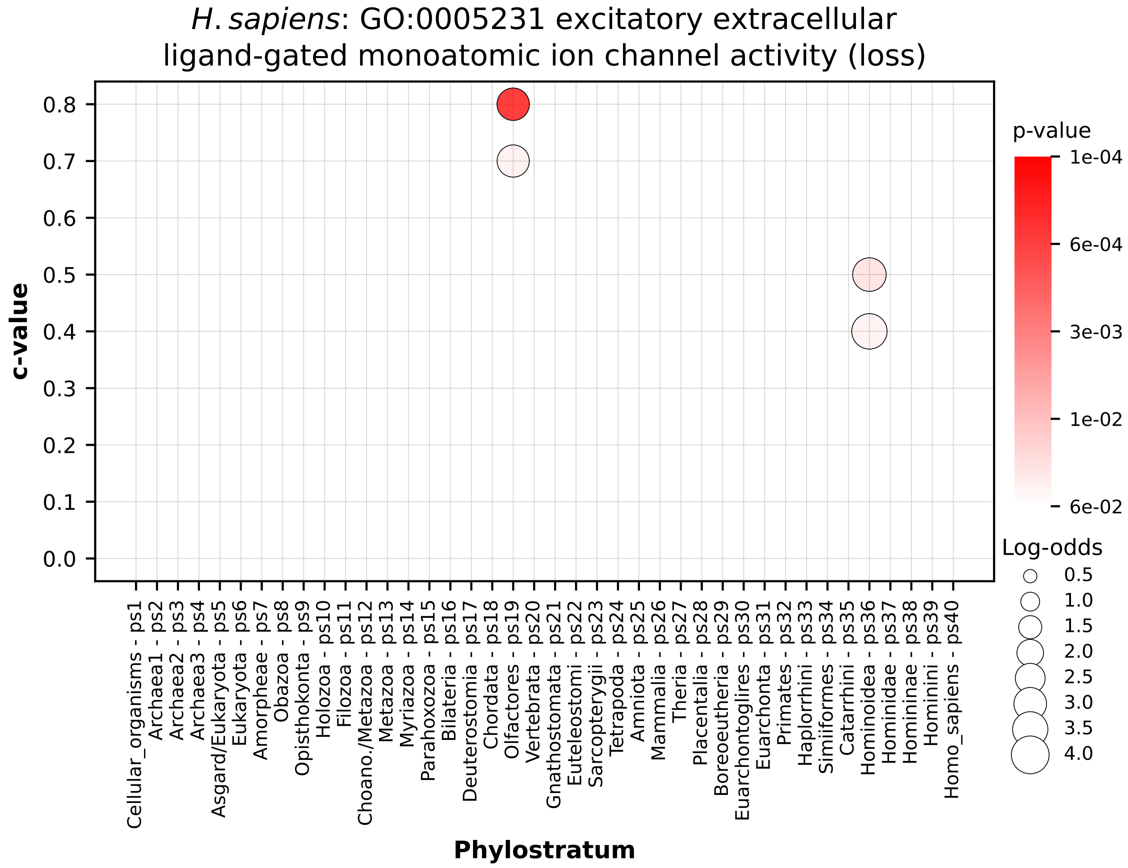

H. sapiens: GO:0005231 excitatory extracellular ligand-gated monoatomic ion channel activity (loss)
c-value
0.8
0.7
0.6
0.5
0.4
0.3
0.2
0.1
0.0
Phylostratum
Cellular_organisms - ps1
Archaea1 - ps2
Archaea2 - ps3
Archaea3 - ps4
Asgard/Eukaryota - ps5
Eukaryota - ps6
Amorpheae - ps7
Obazoa - ps8
Opisthokonta - ps9
Holozoa - ps10
Filozoa - ps11
Choano./Metazoa - ps12
Metazoa - ps13
Myriazoa - ps14
Parahoxozoa - ps15
Bilateria - ps16
Deuterostomia - ps17
Chordata - ps18
Olfactores - ps19
Vertebrata - ps20
Gnathostomata - ps21
Euteleostomi - ps22
Sarcopterygii - ps23
Tetrapoda - ps24
Amniota - ps25
Mammalia - ps26
Theria - ps27
Placentalia - ps28
Boreoeutheria - ps29
Euarchontoglires - ps30
Euarchonta - ps31
Primates - ps32
Haplorrhini - ps33
Simiiformes - ps34
Catarrhini - ps35
Hominoidea - ps36
Hominidae - ps37
Homininae - ps38
Hominini - ps39
Homo_sapiens - ps40
p-value
1e-04
6e-04
3e-03
1e-02
6e-02
Log-odds
0.5
1.0
1.5
2.0
2.5
3.0
3.5
4.0

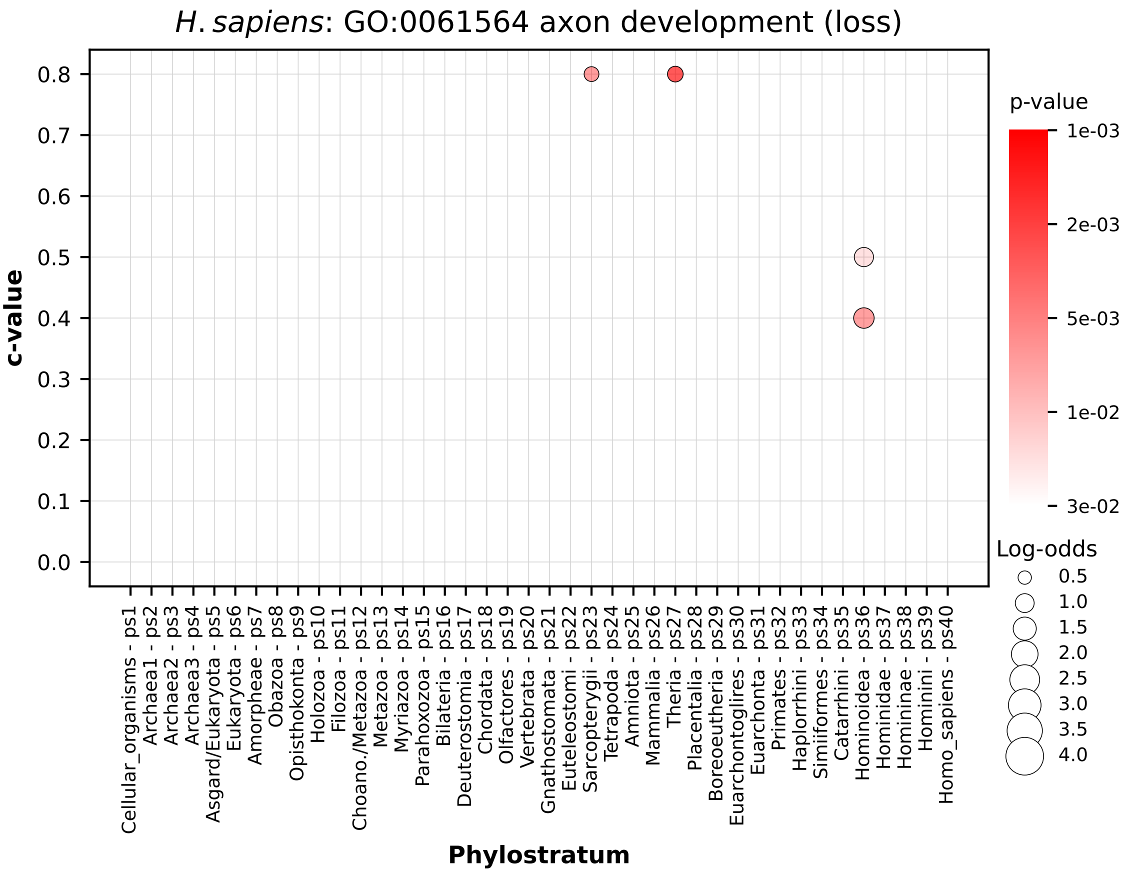

H. sapiens: GO:0061564 axon development (loss)
c-value
0.0
0.1
0.2
0.3
0.4
0.5
0.6
0.7
0.8
Phylostratum
Cellular_organisms - ps1
Archaea1 - ps2
Archaea2 - ps3
Archaea3 - ps4
Asgard/Eukaryota - ps5
Eukaryota - ps6
Amorpheae - ps7
Obazoa - ps8
Opisthokonta - ps9
Holozoa - ps10
Filozoa - ps11
Choano./Metazoa - ps12
Metazoa - ps13
Myriazoa - ps14
Parahoxozoa - ps15
Bilateria - ps16
Deuterostomia - ps17
Chordata - ps18
Olfactores - ps19
Vertebrata - ps20
Gnathostomata - ps21
Euteleostomi - ps22
Sarcopterygii - ps23
Tetrapoda - ps24
Amniota - ps25
Mammalia - ps26
Theria - ps27
Placentalia - ps28
Boreoeutheria - ps29
Euarchontoglires - ps30
Euarchonta - ps31
Primates - ps32
Haplorrhini - ps33
Simiiformes - ps34
Catarrhini - ps35
Hominoidea - ps36
Hominidae - ps37
Homininae - ps38
Hominini - ps39
Homo_sapiens - ps40
p-value
1e-03
2e-03
5e-03
1e-02
3e-02
Log-odds
0.5
1.0
1.5
2.0
2.5
3.0
3.5
4.0

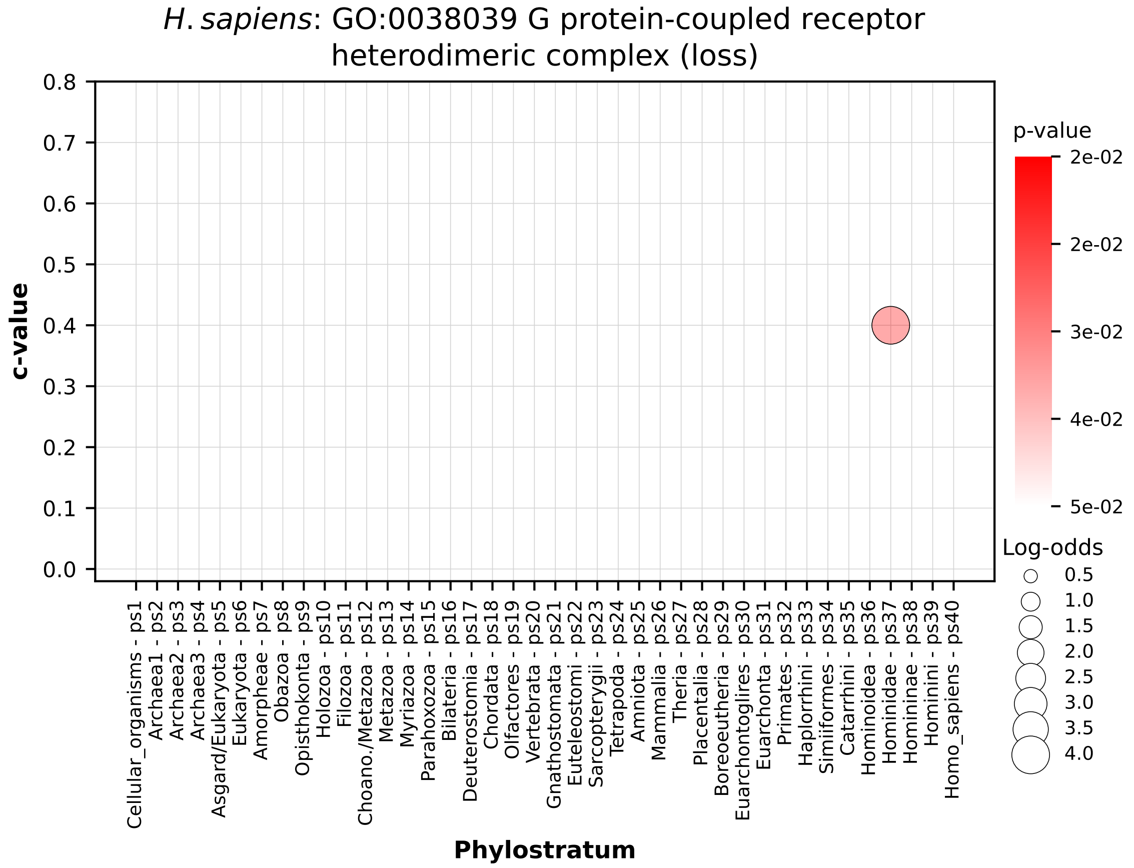

H. sapiens: GO:0038039 G protein-coupled receptor heterodimeric complex (loss)
c-value
0.0
0.1
0.2
0.3
0.4
0.5
0.6
0.7
0.8
Phylostratum
Cellular_organisms - ps1
Archaea1 - ps2
Archaea2 - ps3
Archaea3 - ps4
Asgard/Eukaryota - ps5
Eukaryota - ps6
Amorpheae - ps7
Obazoa - ps8
Opisthokonta - ps9
Holozoa - ps10
Filozoa - ps11
Choano./Metazoa - ps12
Metazoa - ps13
Myriazoa - ps14
Parahoxozoa - ps15
Bilateria - ps16
Deuterostomia - ps17
Chordata - ps18
Olfactores - ps19
Vertebrata - ps20
Gnathostomata - ps21
Euteleostomi - ps22
Sarcopterygii - ps23
Tetrapoda - ps24
Amniota - ps25
Mammalia - ps26
Theria - ps27
Placentalia - ps28
Boreoeutheria - ps29
Euarchontoglires - ps30
Euarchonta - ps31
Primates - ps32
Haplorrhini - ps33
Simiiformes - ps34
Catarrhini - ps35
Hominoidea - ps36
Hominidae - ps37
Homininae - ps38
Hominini - ps39
Homo_sapiens - ps40
p-value
2e-02
2e-02
3e-02
4e-02
5e-02
Log-odds
0.5
1.0
1.5
2.0
2.5
3.0
3.5
4.0

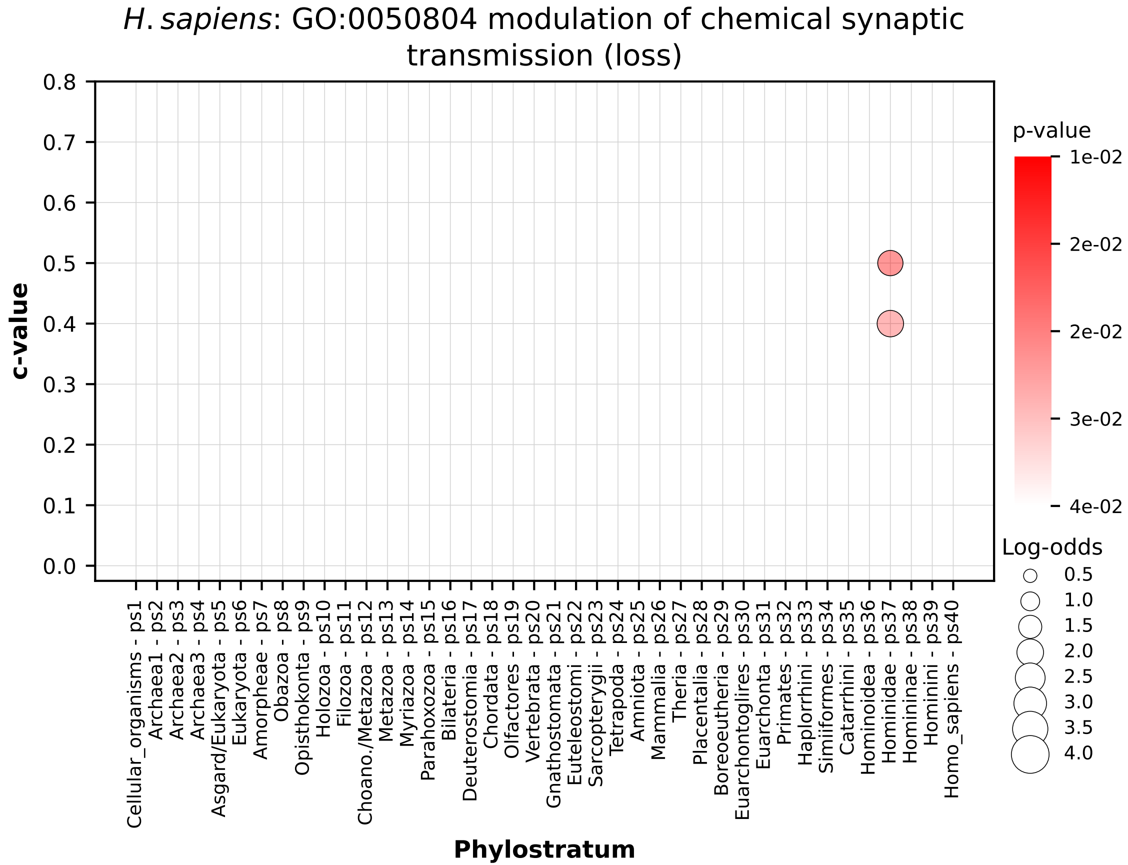

H. sapiens: GO:0050804 modulation of chemical synaptic transmission (loss)
c-value
0.8
0.7
0.6
0.5
0.4
0.3
0.2
0.1
0.0
Phylostratum
Cellular_organisms - ps1
Archaea1 - ps2
Archaea2 - ps3
Archaea3 - ps4
Asgard/Eukaryota - ps5
Eukaryota - ps6
Amorpheae - ps7
Obazoa - ps8
Opisthokonta - ps9
Holozoa - ps10
Filozoa - ps11
Choano./Metazoa - ps12
Metazoa - ps13
Myriazoa - ps14
Parahoxozoa - ps15
Bilateria - ps16
Deuterostomia - ps17
Chordata - ps18
Olfactores - ps19
Vertebrata - ps20
Gnathostomata - ps21
Euteleostomi - ps22
Sarcopterygii - ps23
Tetrapoda - ps24
Amniota - ps25
Mammalia - ps26
Theria - ps27
Placentalia - ps28
Boreoeutheria - ps29
Euarchontoglires - ps30
Euarchonta - ps31
Primates - ps32
Haplorrhini - ps33
Simiiformes - ps34
Catarrhini - ps35
Hominoidea - ps36
Hominidae - ps37
Homininae - ps38
Hominini - ps39
Homo_sapiens - ps40
p-value
1e-02
2e-02
2e-02
3e-02
4e-02
Log-odds
0.5
1.0
1.5
2.0
2.5
3.0
3.5
4.0

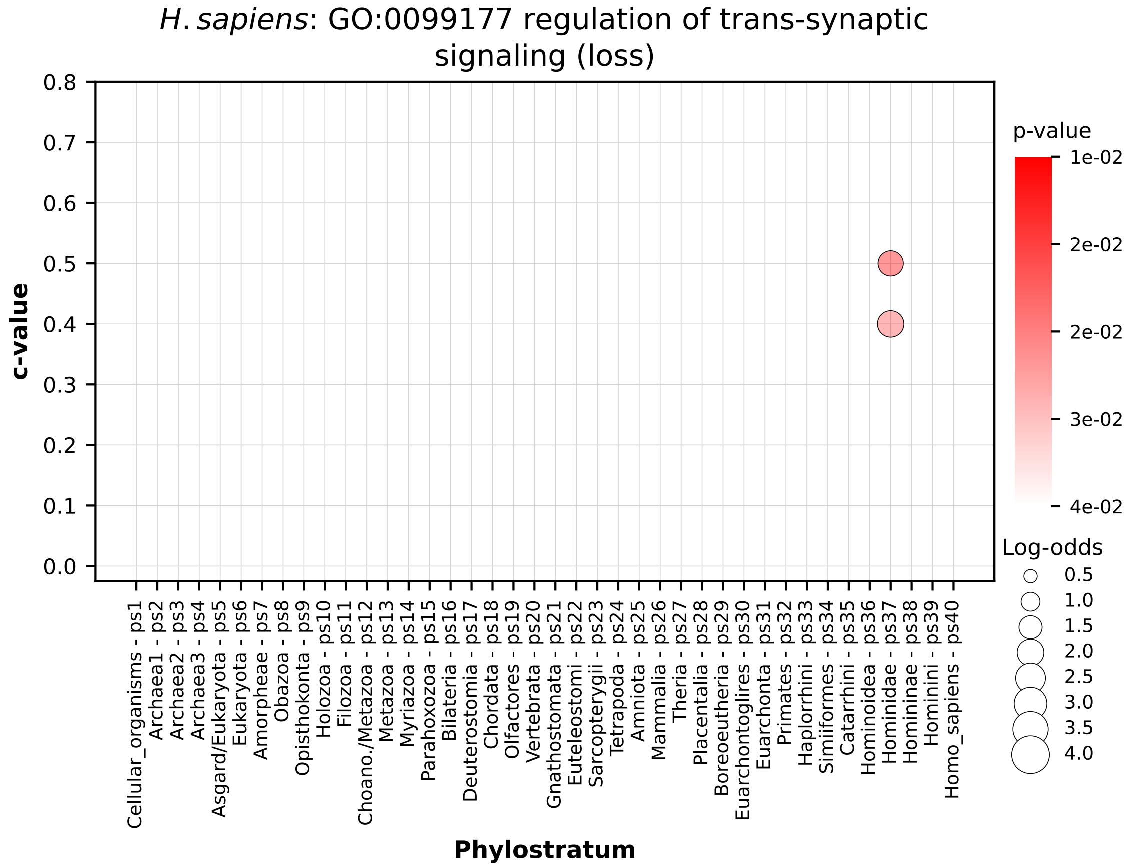

H. sapiens: GO:0099177 regulation of trans-synaptic signaling (loss)
c-value
0.0
0.1
0.2
0.3
0.4
0.5
0.6
0.7
0.8
Phylostratum
Cellular_organisms - ps1
Archaea1 - ps2
Archaea2 - ps3
Archaea3 - ps4
Asgard/Eukaryota - ps5
Eukaryota - ps6
Amorpheae - ps7
Obazoa - ps8
Opisthokonta - ps9
Holozoa - ps10
Filozoa - ps11
Choano./Metazoa - ps12
Metazoa - ps13
Myriazoa - ps14
Parahoxozoa - ps15
Bilateria - ps16
Deuterostomia - ps17
Chordata - ps18
Olfactores - ps19
Vertebrata - ps20
Gnathostomata - ps21
Euteleostomi - ps22
Sarcopterygii - ps23
Tetrapoda - ps24
Amniota - ps25
Mammalia - ps26
Theria - ps27
Placentalia - ps28
Boreoeutheria - ps29
Euarchontoglires - ps30
Euarchonta - ps31
Primates - ps32
Haplorrhini - ps33
Simiiformes - ps34
Catarrhini - ps35
Hominoidea - ps36
Hominidae - ps37
Homininae - ps38
Hominini - ps39
Homo_sapiens - ps40
p-value
1e-02
2e-02
2e-02
3e-02
4e-02
Log-odds
0.5
1.0
1.5
2.0
2.5
3.0
3.5
4.0

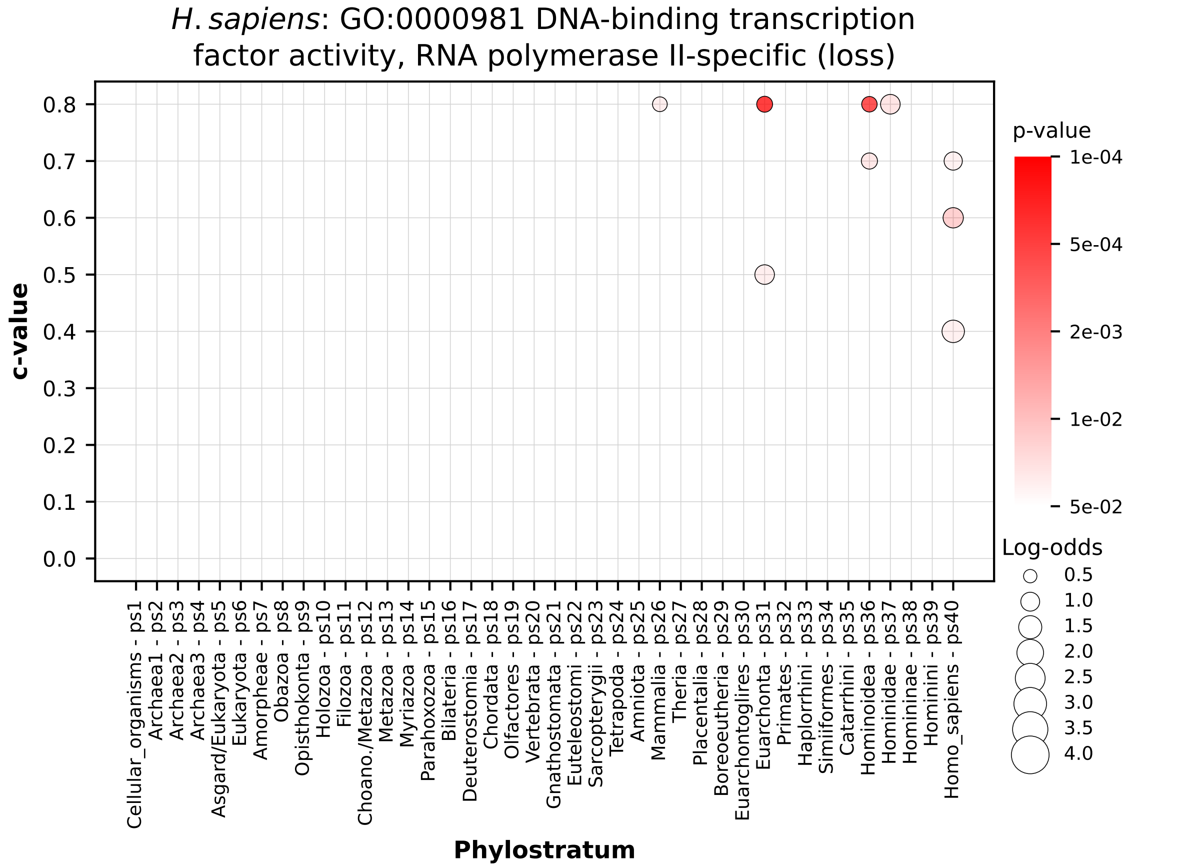

H. sapiens: GO:0000981 DNA-binding transcription factor activity, RNA polymerase II-specific (loss)
c-value
0.0
0.1
0.2
0.3
0.4
0.5
0.6
0.7
0.8
Phylostratum
Cellular_organisms - ps1
Archaea1 - ps2
Archaea2 - ps3
Archaea3 - ps4
Asgard/Eukaryota - ps5
Eukaryota - ps6
Amorpheae - ps7
Obazoa - ps8
Opisthokonta - ps9
Holozoa - ps10
Filozoa - ps11
Choano./Metazoa - ps12
Metazoa - ps13
Myriazoa - ps14
Parahoxozoa - ps15
Bilateria - ps16
Deuterostomia - ps17
Chordata - ps18
Olfactores - ps19
Vertebrata - ps20
Gnathostomata - ps21
Euteleostomi - ps22
Sarcopterygii - ps23
Tetrapoda - ps24
Amniota - ps25
Mammalia - ps26
Theria - ps27
Placentalia - ps28
Boreoeutheria - ps29
Euarchontoglires - ps30
Euarchonta - ps31
Primates - ps32
Haplorrhini - ps33
Simiiformes - ps34
Catarrhini - ps35
Hominoidea - ps36
Hominidae - ps37
Homininae - ps38
Hominini - ps39
Homo_sapiens - ps40
p-value
1e-04
5e-04
2e-03
1e-02
5e-02
Log-odds
0.5
1.0
1.5
2.0
2.5
3.0
3.5
4.0

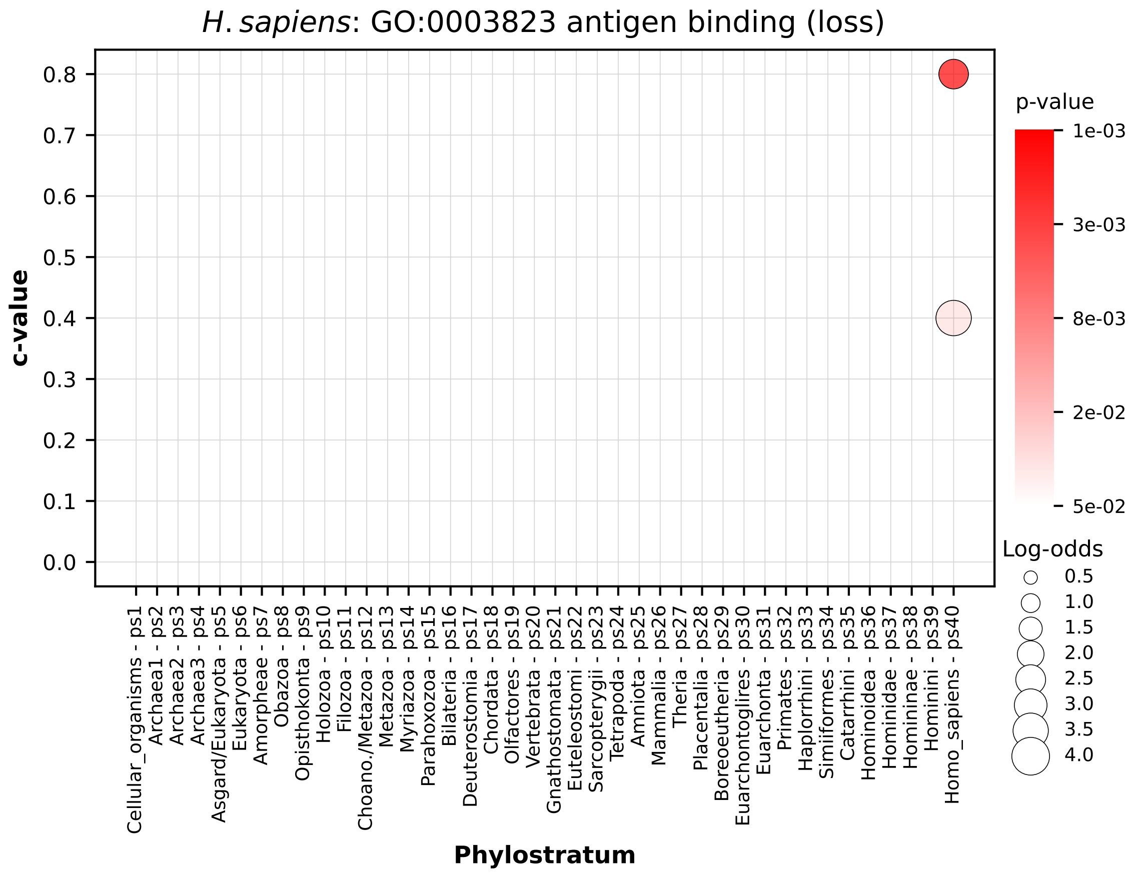
H. sapiens: GO:0003823 antigen binding (loss)
c-value
0.0
0.1
0.2
0.3
0.4
0.5
0.6
0.7
0.8
p-value
1e-03
3e-03
8e-03
2e-02
5e-02
Log-odds
0.5
1.0
1.5
2.0
2.5
3.0
3.5
4.0
Cellular_organisms - ps1
Archaea1 - ps2
Archaea2 - ps3
Archaea3 - ps4
Asgard/Eukaryota - ps5
Eukaryota - ps6
Amorpheae - ps7
Obazoa - ps8
Opisthokonta - ps9
Holozoa - ps10
Filozoa - ps11
Choano./Metazoa - ps12
Metazoa - ps13
Myriazoa - ps14
Parahoxozoa - ps15
Bilateria - ps16
Deuterostomia - ps17
Chordata - ps18
Olfactores - ps19
Vertebrata - ps20
Gnathostomata - ps21
Euteleostomi - ps22
Sarcopterygii - ps23
Tetrapoda - ps24
Amniota - ps25
Mammalia - ps26
Theria - ps27
Placentalia - ps28
Boreoeutheria - ps29
Euarchontoglires - ps30
Euarchonta - ps31
Primates - ps32
Haplorrhini - ps33
Simiiformes - ps34
Catarrhini - ps35
Hominoidea - ps36
Hominidae - ps37
Homininae - ps38
Hominini - ps39
Homo_sapiens - ps40
Phylostratum

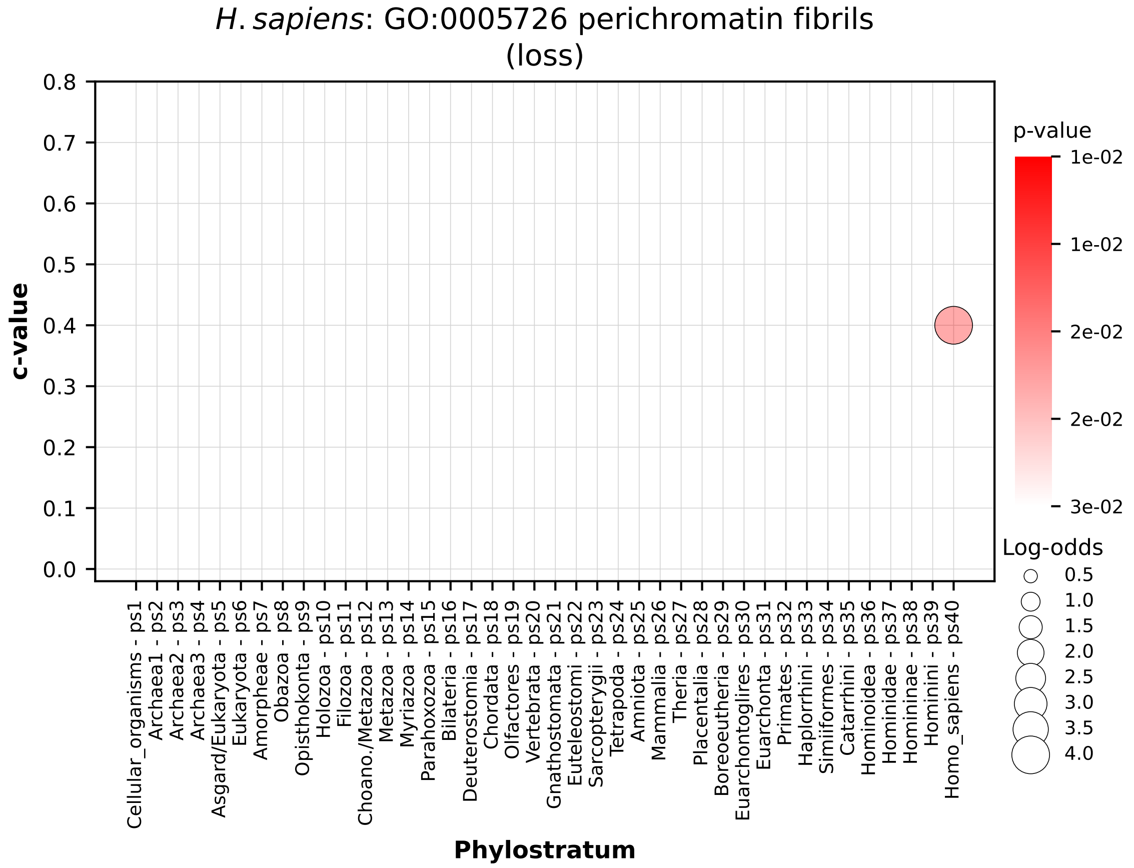

H. sapiens: GO:0005726 perichromatin fibrils
(loss)
c-value
0.8
0.7
0.6
0.5
0.4
0.3
0.2
0.1
0.0
p-value
1e-02
1e-02
2e-02
2e-02
3e-02
Log-odds
0.5
1.0
1.5
2.0
2.5
3.0
3.5
4.0
Cellular_organisms - ps1
Archaea1 - ps2
Archaea2 - ps3
Archaea3 - ps4
Asgard/Eukaryota - ps5
Eukaryota - ps6
Amorpheae - ps7
Obazoa - ps8
Opisthokonta - ps9
Holozoa - ps10
Filozoa - ps11
Choano./Metazoa - ps12
Metazoa - ps13
Myriazoa - ps14
Parahoxozoa - ps15
Bilateria - ps16
Deuterostomia - ps17
Chordata - ps18
Olfactores - ps19
Vertebrata - ps20
Gnathostomata - ps21
Euteleostomi - ps22
Sarcopterygii - ps23
Tetrapoda - ps24
Amniota - ps25
Mammalia - ps26
Theria - ps27
Placentalia - ps28
Boreoeutheria - ps29
Euarchontoglires - ps30
Euarchonta - ps31
Primates - ps32
Haplorrhini - ps33
Simiiformes - ps34
Catarrhini - ps35
Hominoidea - ps36
Hominidae - ps37
Homininae - ps38
Hominini - ps39
Homo_sapiens - ps40
Phylostratum

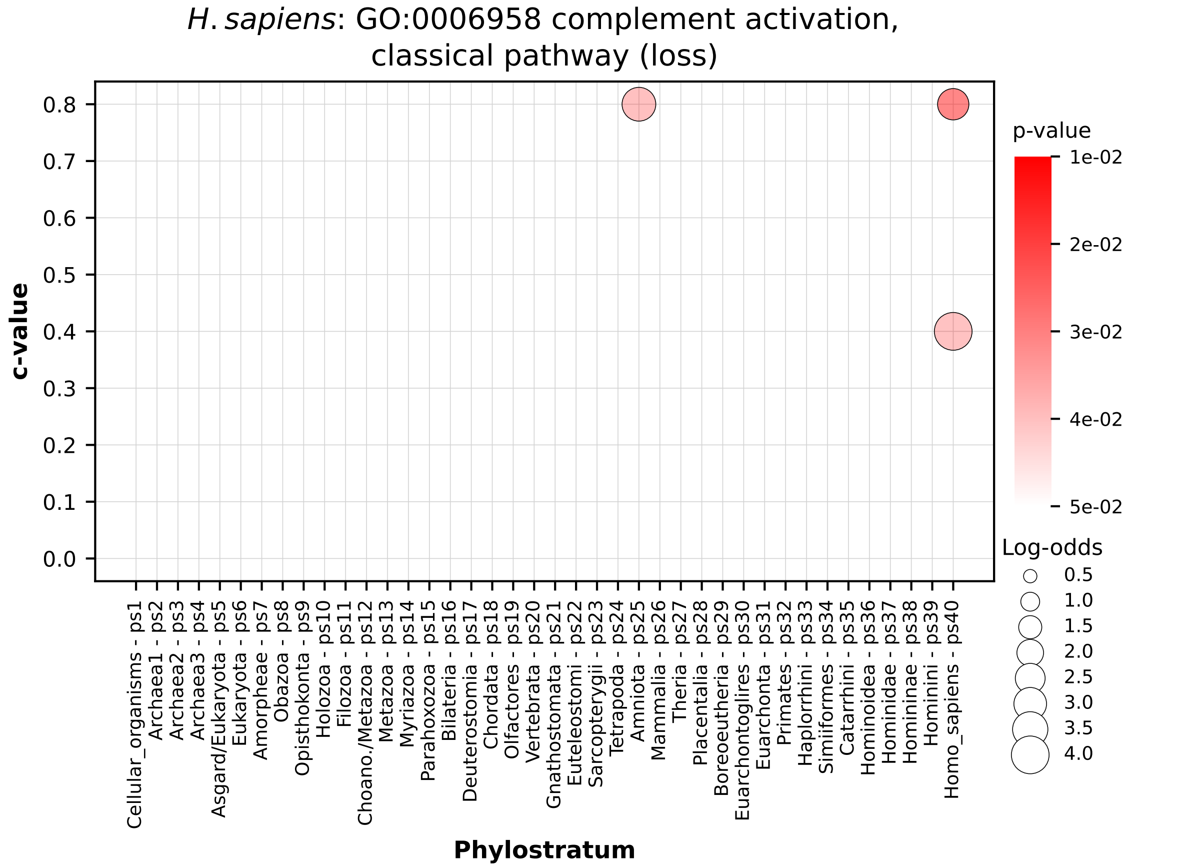

H. sapiens: GO:0006958 complement activation, classical pathway (loss)
c-value
0.0
0.1
0.2
0.3
0.4
0.5
0.6
0.7
0.8
Phylostratum
Cellular_organisms - ps1
Archaea1 - ps2
Archaea2 - ps3
Archaea3 - ps4
Asgard/Eukaryota - ps5
Eukaryota - ps6
Amorpheae - ps7
Obazoa - ps8
Opisthokonta - ps9
Holozoa - ps10
Filozoa - ps11
Choano./Metazoa - ps12
Metazoa - ps13
Myriazoa - ps14
Parahoxozoa - ps15
Bilateria - ps16
Deuterostomia - ps17
Chordata - ps18
Olfactores - ps19
Vertebrata - ps20
Gnathostomata - ps21
Euteleostomi - ps22
Sarcopterygii - ps23
Tetrapoda - ps24
Amniota - ps25
Mammalia - ps26
Theria - ps27
Placentalia - ps28
Boreoeutheria - ps29
Euarchontoglires - ps30
Euarchonta - ps31
Primates - ps32
Haplorrhini - ps33
Simiiformes - ps34
Catarrhini - ps35
Hominoidea - ps36
Hominidae - ps37
Homininae - ps38
Hominini - ps39
Homo_sapiens - ps40
p-value
1e-02
2e-02
3e-02
4e-02
5e-02
Log-odds
0.5
1.0
1.5
2.0
2.5
3.0
3.5
4.0

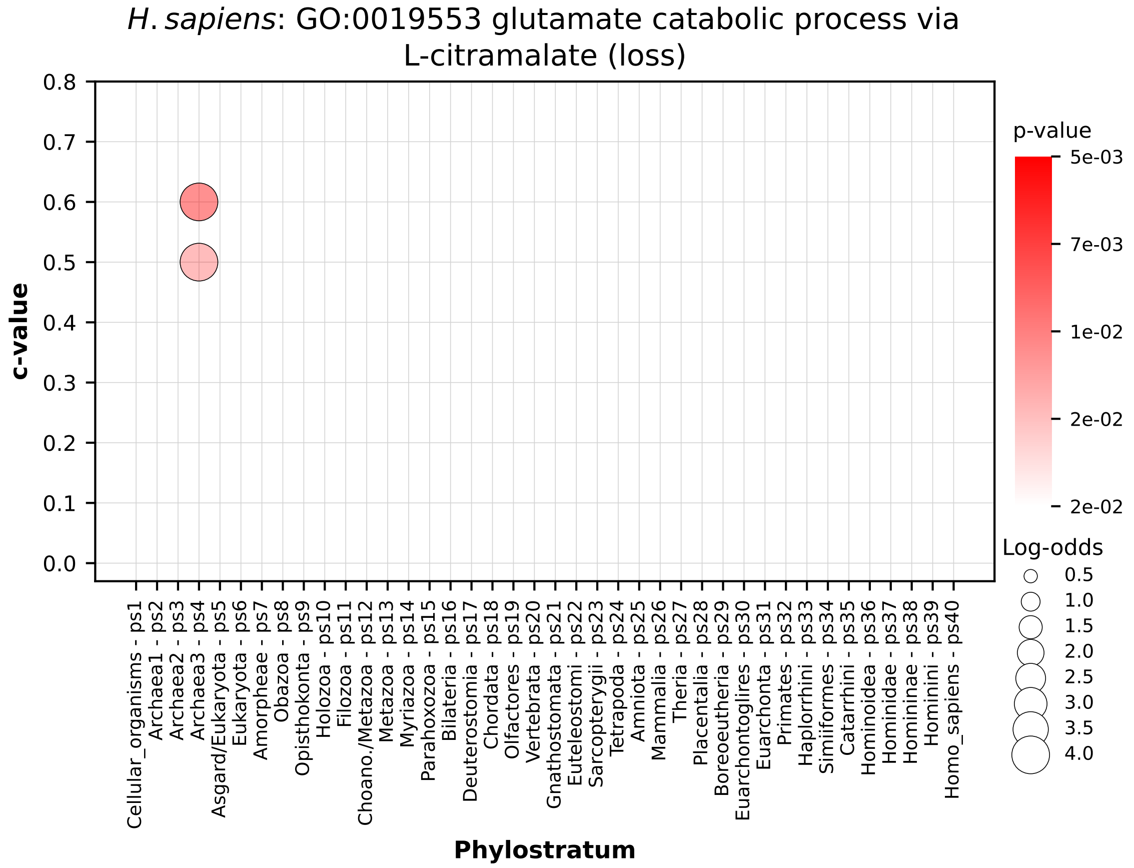

H. sapiens: GO:0019553 glutamate catabolic process via L-citramalate (loss)
c-value
0.8
0.7
0.6
0.5
0.4
0.3
0.2
0.1
0.0
p-value
5e-03
7e-03
1e-02
2e-02
2e-02
Log-odds
0.5
1.0
1.5
2.0
2.5
3.0
3.5
4.0
Cellular_organisms - ps1
Archaea1 - ps2
Archaea2 - ps3
Archaea3 - ps4
Asgard/Eukaryota - ps5
Eukaryota - ps6
Amorpheae - ps7
Obazoa - ps8
Opisthokonta - ps9
Holozoa - ps10
Filozoa - ps11
Choano./Metazoa - ps12
Metazoa - ps13
Myriazoa - ps14
Parahoxozoa - ps15
Bilateria - ps16
Deuterostomia - ps17
Chordata - ps18
Olfactores - ps19
Vertebrata - ps20
Gnathostomata - ps21
Euteleostomi - ps22
Sarcopterygii - ps23
Tetrapoda - ps24
Amniota - ps25
Mammalia - ps26
Theria - ps27
Placentalia - ps28
Boreoeutheria - ps29
Euarchontoglires - ps30
Euarchonta - ps31
Primates - ps32
Haplorrhini - ps33
Simiiformes - ps34
Catarrhini - ps35
Hominoidea - ps36
Hominidae - ps37
Homininae - ps38
Hominini - ps39
Homo_sapiens - ps40
Phylostratum

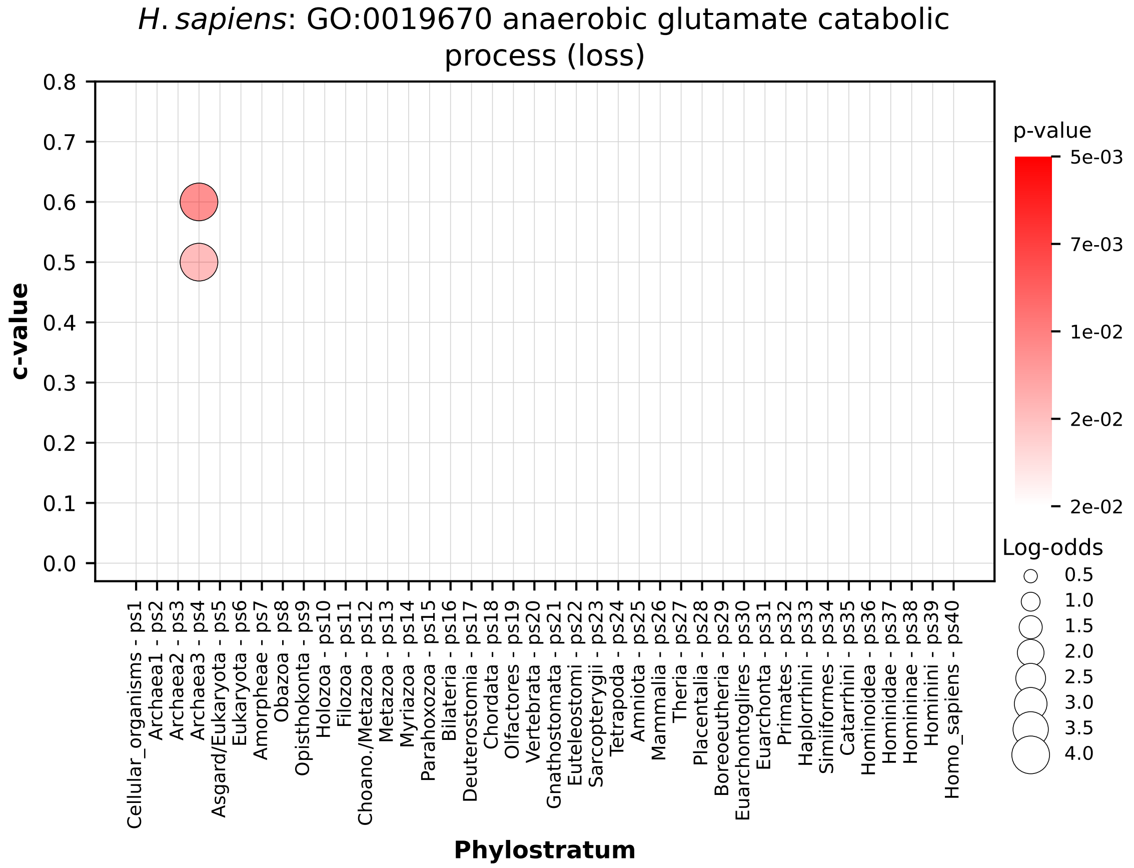

H. sapiens: GO:0019670 anaerobic glutamate catabolic process (loss)
c-value
0.0
0.1
0.2
0.3
0.4
0.5
0.6
0.7
0.8
Phylostratum
Cellular_organisms - ps1
Archaea1 - ps2
Archaea2 - ps3
Archaea3 - ps4
Asgard/Eukaryota - ps5
Eukaryota - ps6
Amorpheae - ps7
Obazoa - ps8
Opisthokonta - ps9
Holozoa - ps10
Filozoa - ps11
Choano./Metazoa - ps12
Metazoa - ps13
Myriazoa - ps14
Parahoxozoa - ps15
Bilateria - ps16
Deuterostomia - ps17
Chordata - ps18
Olfactores - ps19
Vertebrata - ps20
Gnathostomata - ps21
Euteleostomi - ps22
Sarcopterygii - ps23
Tetrapoda - ps24
Amniota - ps25
Mammalia - ps26
Theria - ps27
Placentalia - ps28
Boreoeutheria - ps29
Euarchontoglires - ps30
Euarchonta - ps31
Primates - ps32
Haplorrhini - ps33
Simiiformes - ps34
Catarrhini - ps35
Hominoidea - ps36
Hominidae - ps37
Homininae - ps38
Hominini - ps39
Homo_sapiens - ps40
p-value
5e-03
7e-03
1e-02
2e-02
2e-02
Log-odds
0.5
1.0
1.5
2.0
2.5
3.0
3.5
4.0

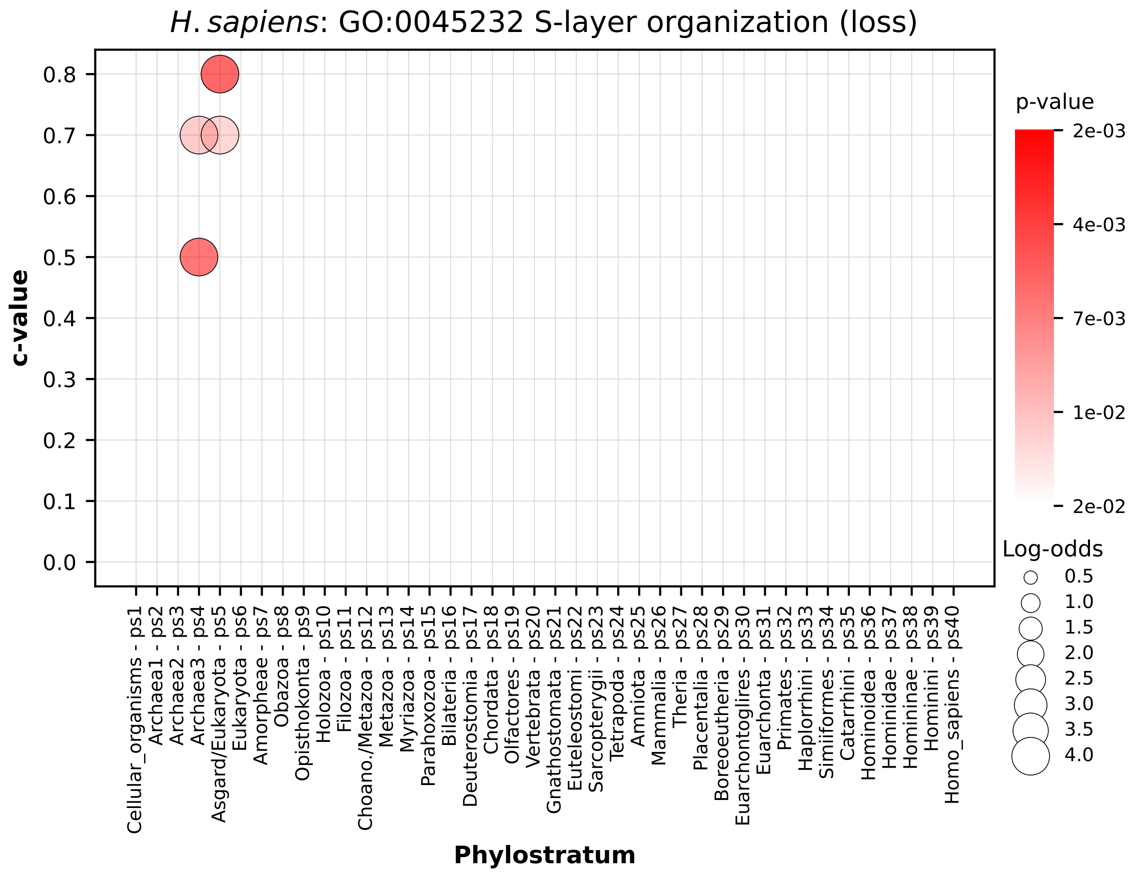

H. sapiens: GO:0045232 S-layer organization (loss)
c-value
0.0
0.1
0.2
0.3
0.4
0.5
0.6
0.7
0.8
Phylostratum
Cellular_organisms - ps1
Archaea1 - ps2
Archaea2 - ps3
Archaea3 - ps4
Asgard/Eukaryota - ps5
Eukaryota - ps6
Amorpheae - ps7
Obazoa - ps8
Opisthokonta - ps9
Holozoa - ps10
Filozoa - ps11
Choano./Metazoa - ps12
Metazoa - ps13
Myriazoa - ps14
Parahoxozoa - ps15
Bilateria - ps16
Deuterostomia - ps17
Chordata - ps18
Olfactores - ps19
Vertebrata - ps20
Gnathostomata - ps21
Euteleostomi - ps22
Sarcopterygii - ps23
Tetrapoda - ps24
Amniota - ps25
Mammalia - ps26
Theria - ps27
Placentalia - ps28
Boreoeutheria - ps29
Euarchontoglires - ps30
Euarchonta - ps31
Primates - ps32
Haplorrhini - ps33
Simiiformes - ps34
Catarrhini - ps35
Hominoidea - ps36
Hominidae - ps37
Homininae - ps38
Hominini - ps39
Homo_sapiens - ps40
p-value
2e-03
4e-03
7e-03
1e-02
2e-02
Log-odds
0.5
1.0
1.5
2.0
2.5
3.0
3.5
4.0

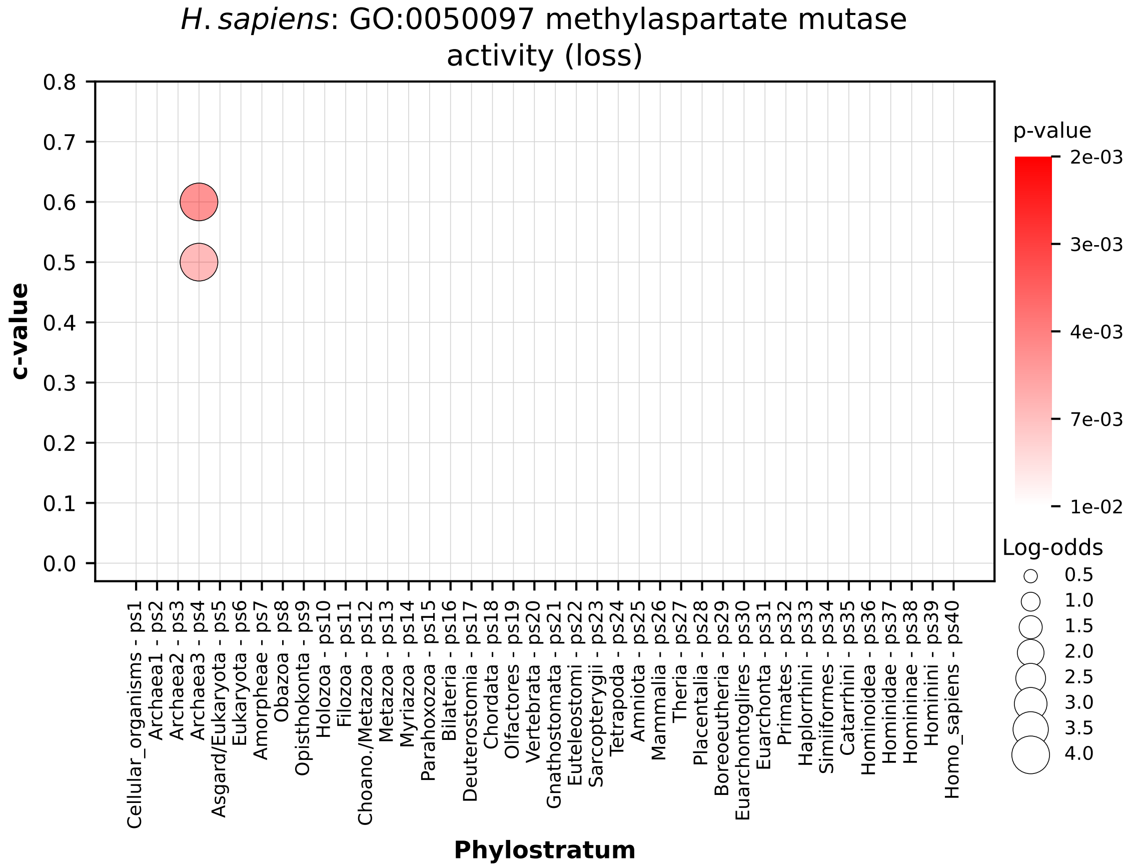

H. sapiens: GO:0050097 methylaspartate mutase activity (loss)
c-value
0.8
0.7
0.6
0.5
0.4
0.3
0.2
0.1
0.0
p-value
2e-03
3e-03
4e-03
7e-03
1e-02
Log-odds
0.5
1.0
1.5
2.0
2.5
3.0
3.5
4.0
Cellular_organisms - ps1
Archaea1 - ps2
Archaea2 - ps3
Archaea3 - ps4
Asgard/Eukaryota - ps5
Eukaryota - ps6
Amorpheae - ps7
Obazoa - ps8
Opisthokonta - ps9
Holozoa - ps10
Filozoa - ps11
Choano./Metazoa - ps12
Metazoa - ps13
Myriazoa - ps14
Parahoxozoa - ps15
Bilateria - ps16
Deuterostomia - ps17
Chordata - ps18
Olfactores - ps19
Vertebrata - ps20
Gnathostomata - ps21
Euteleostomi - ps22
Sarcopterygii - ps23
Tetrapoda - ps24
Amniota - ps25
Mammalia - ps26
Theria - ps27
Placentalia - ps28
Boreoeutheria - ps29
Euarchontoglires - ps30
Euarchonta - ps31
Primates - ps32
Haplorrhini - ps33
Simiiformes - ps34
Catarrhini - ps35
Hominoidea - ps36
Hominidae - ps37
Homininae - ps38
Hominini - ps39
Homo_sapiens - ps40
Phylostratum

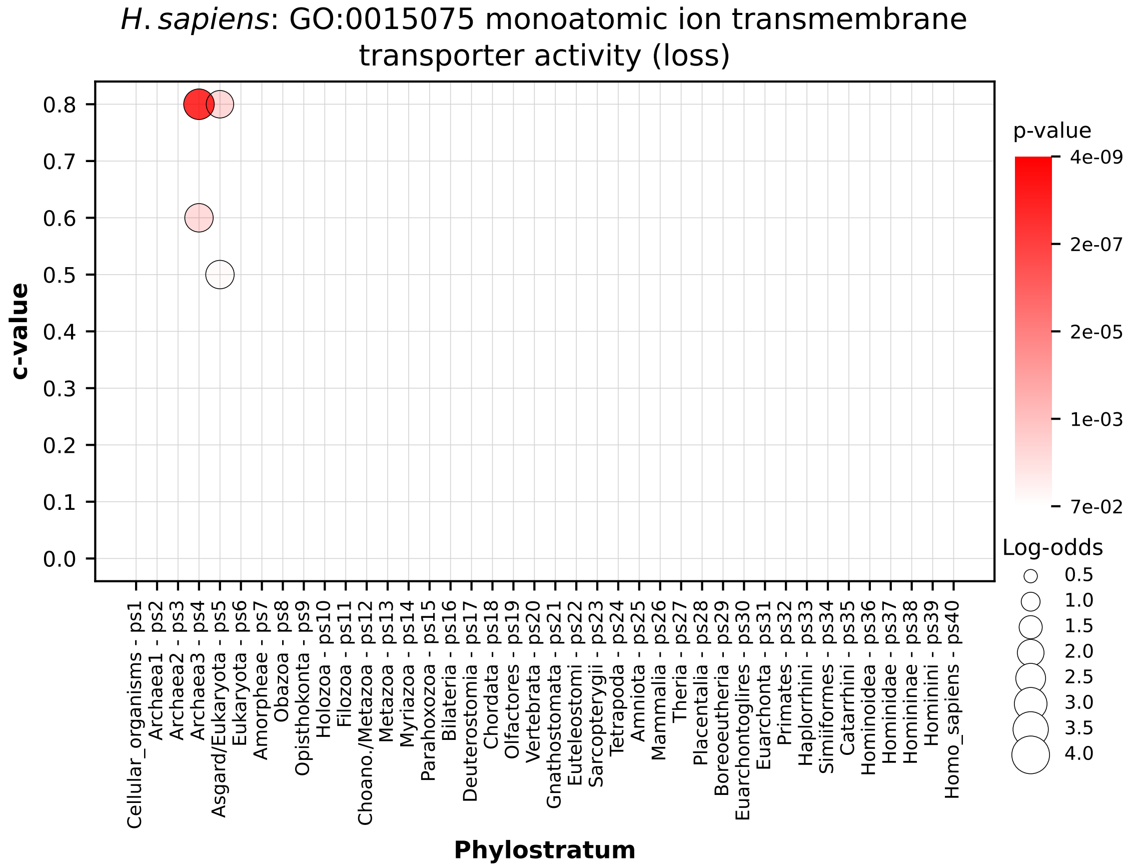
H. sapiens: GO:0015075 monoatomic ion transmembrane transporter activity (loss)
c-value
0.0
0.1
0.2
0.3
0.4
0.5
0.6
0.7
0.8
p-value
4e-09
2e-07
2e-05
1e-03
7e-02
Log-odds
0.5
1.0
1.5
2.0
2.5
3.0
3.5
4.0
Cellular_organisms - ps1
Archaea1 - ps2
Archaea2 - ps3
Archaea3 - ps4
Asgard/Eukaryota - ps5
Eukaryota - ps6
Amorpheae - ps7
Obazoa - ps8
Opisthokonta - ps9
Holozoa - ps10
Filozoa - ps11
Choano./Metazoa - ps12
Metazoa - ps13
Myriazoa - ps14
Parahoxozoa - ps15
Bilateria - ps16
Deuterostomia - ps17
Chordata - ps18
Olfactores - ps19
Vertebrata - ps20
Gnathostomata - ps21
Euteleostomi - ps22
Sarcopterygii - ps23
Tetrapoda - ps24
Amniota - ps25
Mammalia - ps26
Theria - ps27
Placentalia - ps28
Boreoeutheria - ps29
Euarchontoglires - ps30
Euarchonta - ps31
Primates - ps32
Haplorrhini - ps33
Simiiformes - ps34
Catarrhini - ps35
Hominoidea - ps36
Hominidae - ps37
Homininae - ps38
Hominini - ps39
Homo_sapiens - ps40
Phylostratum

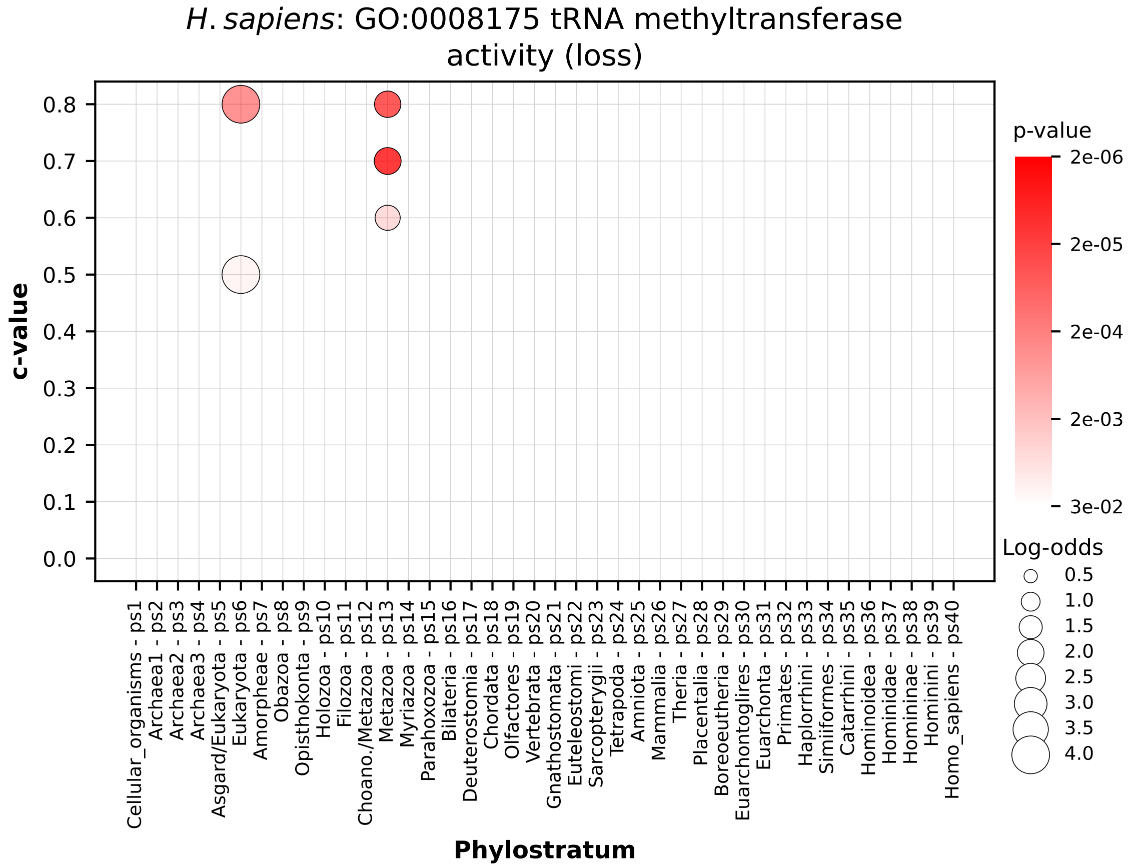

H. sapiens: GO:0008175 tRNA methyltransferase activity (loss)
c-value
0.0
0.1
0.2
0.3
0.4
0.5
0.6
0.7
0.8
Phylostratum
Cellular_organisms - ps1
Archaea1 - ps2
Archaea2 - ps3
Archaea3 - ps4
Asgard/Eukaryota - ps5
Eukaryota - ps6
Amorpheae - ps7
Obazoa - ps8
Opisthokonta - ps9
Holozoa - ps10
Filozoa - ps11
Choano./Metazoa - ps12
Metazoa - ps13
Myriazoa - ps14
Parahoxozoa - ps15
Bilateria - ps16
Deuterostomia - ps17
Chordata - ps18
Olfactores - ps19
Vertebrata - ps20
Gnathostomata - ps21
Euteleostomi - ps22
Sarcopterygii - ps23
Tetrapoda - ps24
Amniota - ps25
Mammalia - ps26
Theria - ps27
Placentalia - ps28
Boreoeutheria - ps29
Euarchontoglires - ps30
Euarchonta - ps31
Primates - ps32
Haplorrhini - ps33
Simiiformes - ps34
Catarrhini - ps35
Hominoidea - ps36
Hominidae - ps37
Homininae - ps38
Hominini - ps39
Homo_sapiens - ps40
p-value
2e-06
2e-05
2e-04
2e-03
3e-02
Log-odds
0.5
1.0
1.5
2.0
2.5
3.0
3.5
4.0

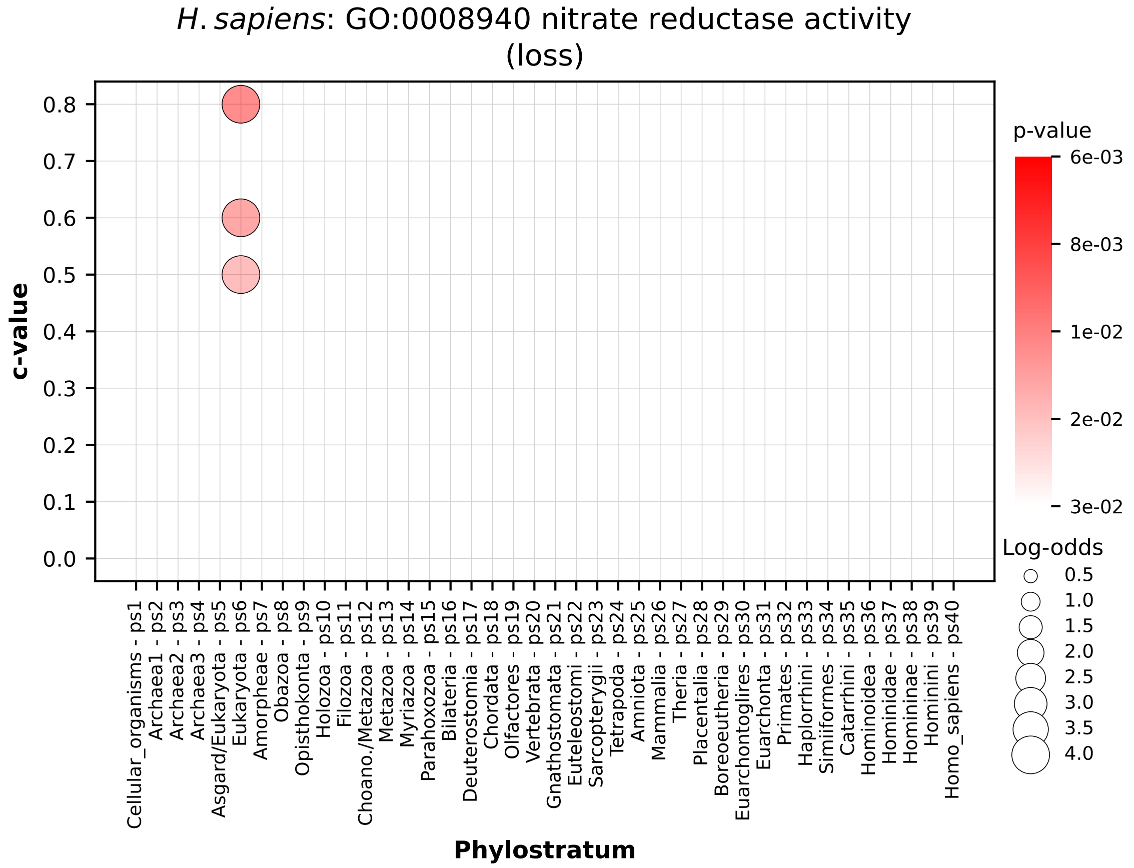

H. sapiens: GO:0008940 nitrate reductase activity
(loss)
c-value
0.8
0.7
0.6
0.5
0.4
0.3
0.2
0.1
0.0
p-value
6e-03
8e-03
1e-02
2e-02
3e-02
Log-odds
0.5
1.0
1.5
2.0
2.5
3.0
3.5
4.0
Cellular_organisms - ps1
Archaea1 - ps2
Archaea2 - ps3
Archaea3 - ps4
Asgard/Eukaryota - ps5
Eukaryota - ps6
Amorpheae - ps7
Obazoa - ps8
Opisthokonta - ps9
Holozoa - ps10
Filozoa - ps11
Choano./Metazoa - ps12
Metazoa - ps13
Myriazoa - ps14
Parahoxozoa - ps15
Bilateria - ps16
Deuterostomia - ps17
Chordata - ps18
Olfactores - ps19
Vertebrata - ps20
Gnathostomata - ps21
Euteleostomi - ps22
Sarcopterygii - ps23
Tetrapoda - ps24
Amniota - ps25
Mammalia - ps26
Theria - ps27
Placentalia - ps28
Boreoeutheria - ps29
Euarchontoglires - ps30
Euarchonta - ps31
Primates - ps32
Haplorrhini - ps33
Simiiformes - ps34
Catarrhini - ps35
Hominoidea - ps36
Hominidae - ps37
Homininae - ps38
Hominini - ps39
Homo_sapiens - ps40
Phylostratum

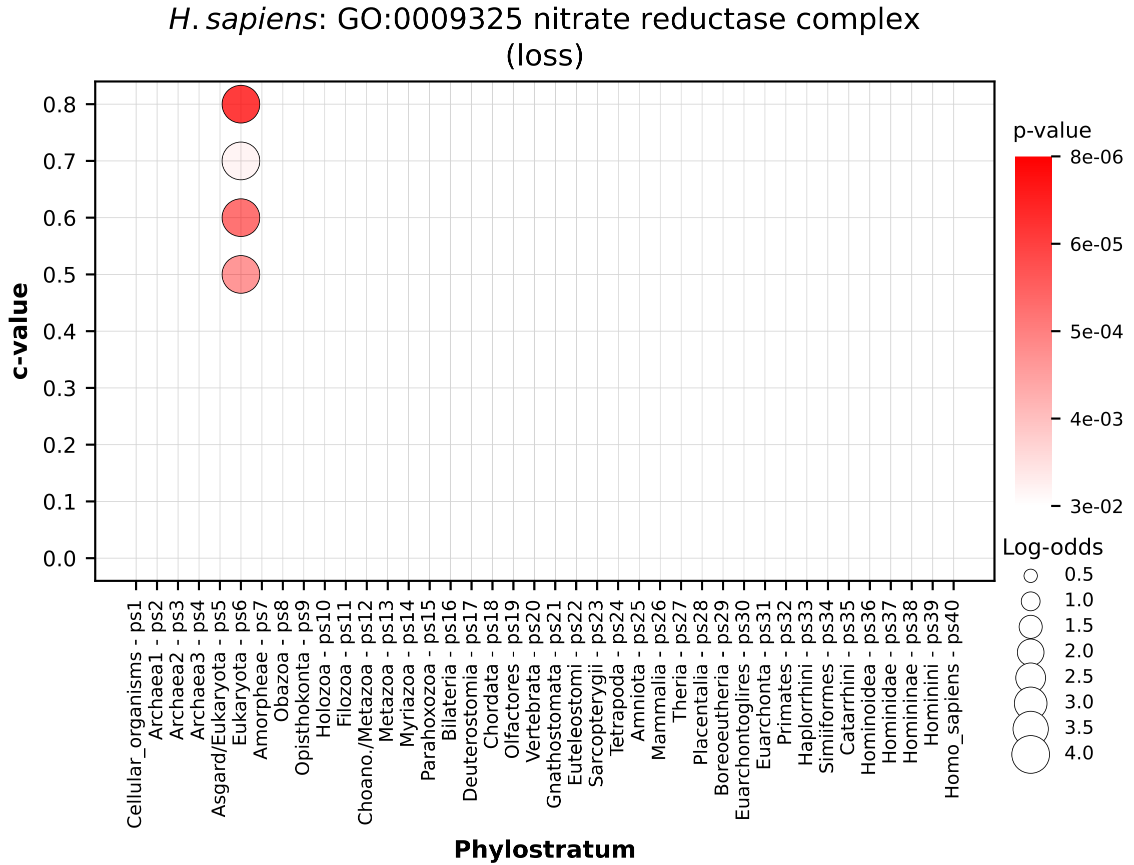

$H. sapiens$: GO:0009325 nitrate reductase complex
(loss)
c-value
0.8
0.7
0.6
0.5
0.4
0.3
0.2
0.1
0.0
p-value
8e-06
6e-05
5e-04
4e-03
3e-02
Log-odds
0.5
1.0
1.5
2.0
2.5
3.0
3.5
4.0
Cellular_organisms - ps1
Archaea1 - ps2
Archaea2 - ps3
Archaea3 - ps4
Asgard/Eukaryota - ps5
Eukaryota - ps6
Amorpheae - ps7
Obazoa - ps8
Opisthokonta - ps9
Holozoa - ps10
Filozoa - ps11
Choano./Metazoa - ps12
Metazoa - ps13
Myriazoa - ps14
Parahoxozoa - ps15
Bilateria - ps16
Deuterostomia - ps17
Chordata - ps18
Olfactores - ps19
Vertebrata - ps20
Gnathostomata - ps21
Euteleostomi - ps22
Sarcopterygii - ps23
Tetrapoda - ps24
Amniota - ps25
Mammalia - ps26
Theria - ps27
Placentalia - ps28
Boreoeutheria - ps29
Euarchontoglires - ps30
Euarchonta - ps31
Primates - ps32
Haplorrhini - ps33
Simiiformes - ps34
Catarrhini - ps35
Hominoidea - ps36
Hominidae - ps37
Homininae - ps38
Hominini - ps39
Homo_sapiens - ps40
Phylostratum

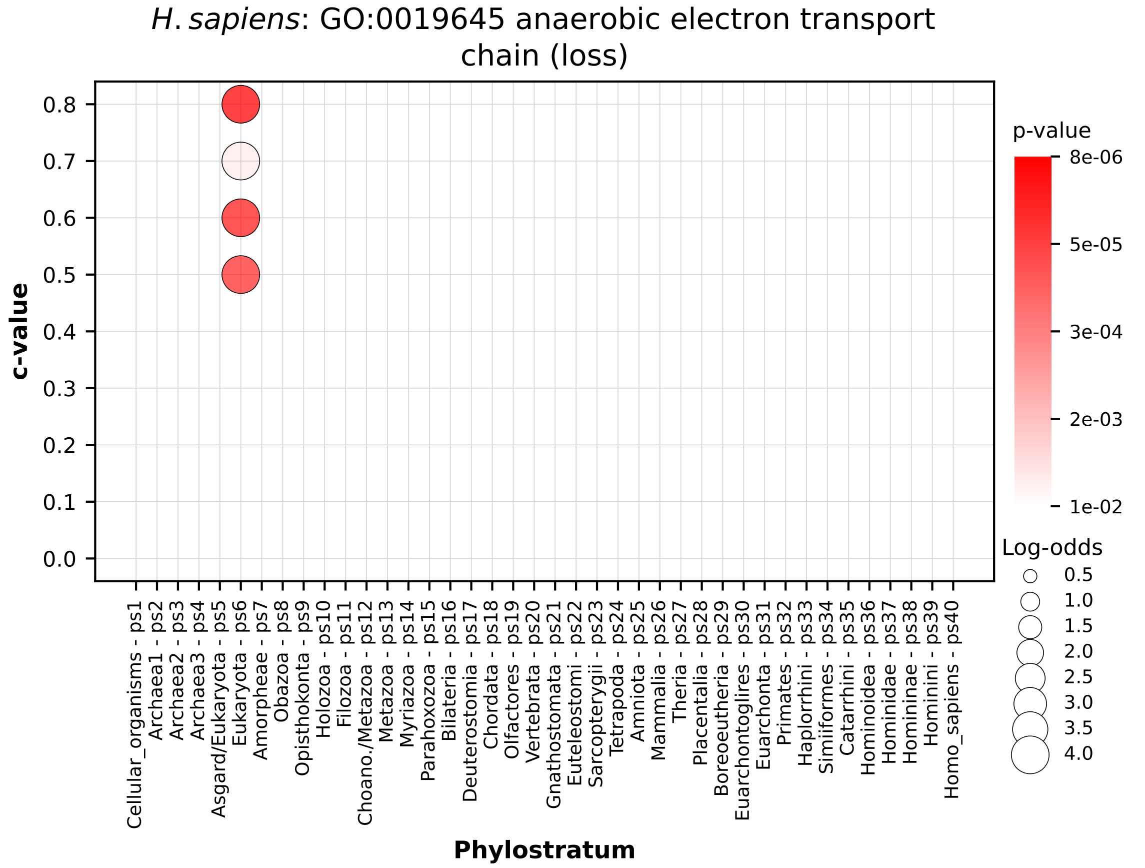

H. sapiens: GO:0019645 anaerobic electron transport chain (loss)
c-value
0.8
0.7
0.6
0.5
0.4
0.3
0.2
0.1
0.0
p-value
8e-06
5e-05
3e-04
2e-03
1e-02
Log-odds
0.5
1.0
1.5
2.0
2.5
3.0
3.5
4.0
Cellular_organisms - ps1
Archaea1 - ps2
Archaea2 - ps3
Archaea3 - ps4
Asgard/Eukaryota - ps5
Eukaryota - ps6
Amorpheae - ps7
Obazoa - ps8
Opisthokonta - ps9
Holozoa - ps10
Filozoa - ps11
Choano./Metazoa - ps12
Metazoa - ps13
Myriazoa - ps14
Parahoxozoa - ps15
Bilateria - ps16
Deuterostomia - ps17
Chordata - ps18
Olfactores - ps19
Vertebrata - ps20
Gnathostomata - ps21
Euteleostomi - ps22
Sarcopterygii - ps23
Tetrapoda - ps24
Amniota - ps25
Mammalia - ps26
Theria - ps27
Placentalia - ps28
Boreoeutheria - ps29
Euarchontoglires - ps30
Euarchonta - ps31
Primates - ps32
Haplorrhini - ps33
Simiiformes - ps34
Catarrhini - ps35
Hominoidea - ps36
Hominidae - ps37
Homininae - ps38
Hominini - ps39
Homo_sapiens - ps40
Phylostratum

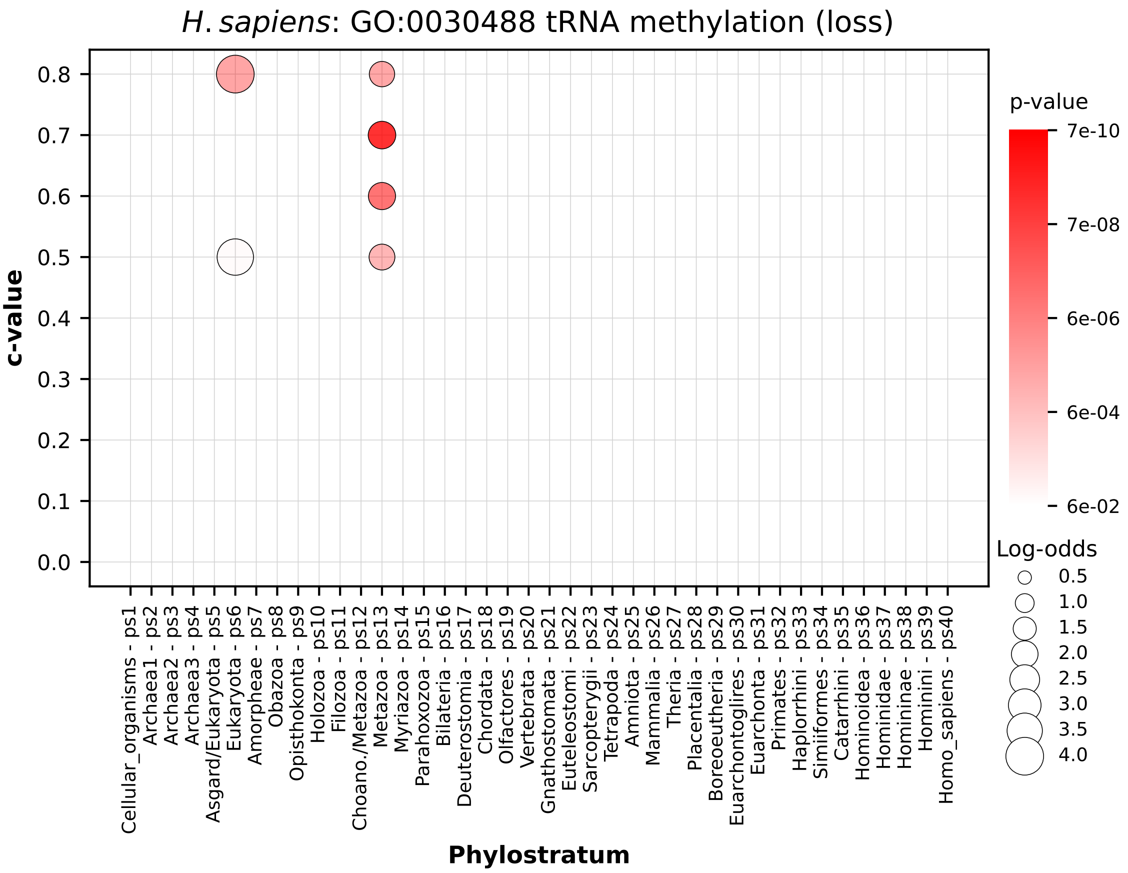

H. sapiens: GO:0030488 tRNA methylation (loss)
c-value
0.0
0.1
0.2
0.3
0.4
0.5
0.6
0.7
0.8
Phylostratum
Cellular_organisms - ps1
Archaea1 - ps2
Archaea2 - ps3
Archaea3 - ps4
Asgard/Eukaryota - ps5
Eukaryota - ps6
Amorpheae - ps7
Obazoa - ps8
Opisthokonta - ps9
Holozoa - ps10
Filozoa - ps11
Choano./Metazoa - ps12
Metazoa - ps13
Myriazoa - ps14
Parahoxozoa - ps15
Bilateria - ps16
Deuterostomia - ps17
Chordata - ps18
Olfactores - ps19
Vertebrata - ps20
Gnathostomata - ps21
Euteleostomi - ps22
Sarcopterygii - ps23
Tetrapoda - ps24
Amniota - ps25
Mammalia - ps26
Theria - ps27
Placentalia - ps28
Boreoeutheria - ps29
Euarchontoglires - ps30
Euarchonta - ps31
Primates - ps32
Haplorrhini - ps33
Simiiformes - ps34
Catarrhini - ps35
Hominoidea - ps36
Hominidae - ps37
Homininae - ps38
Hominini - ps39
Homo_sapiens - ps40
p-value
7e-10
7e-08
6e-06
6e-04
6e-02
Log-odds
0.5
1.0
1.5
2.0
2.5
3.0
3.5
4.0

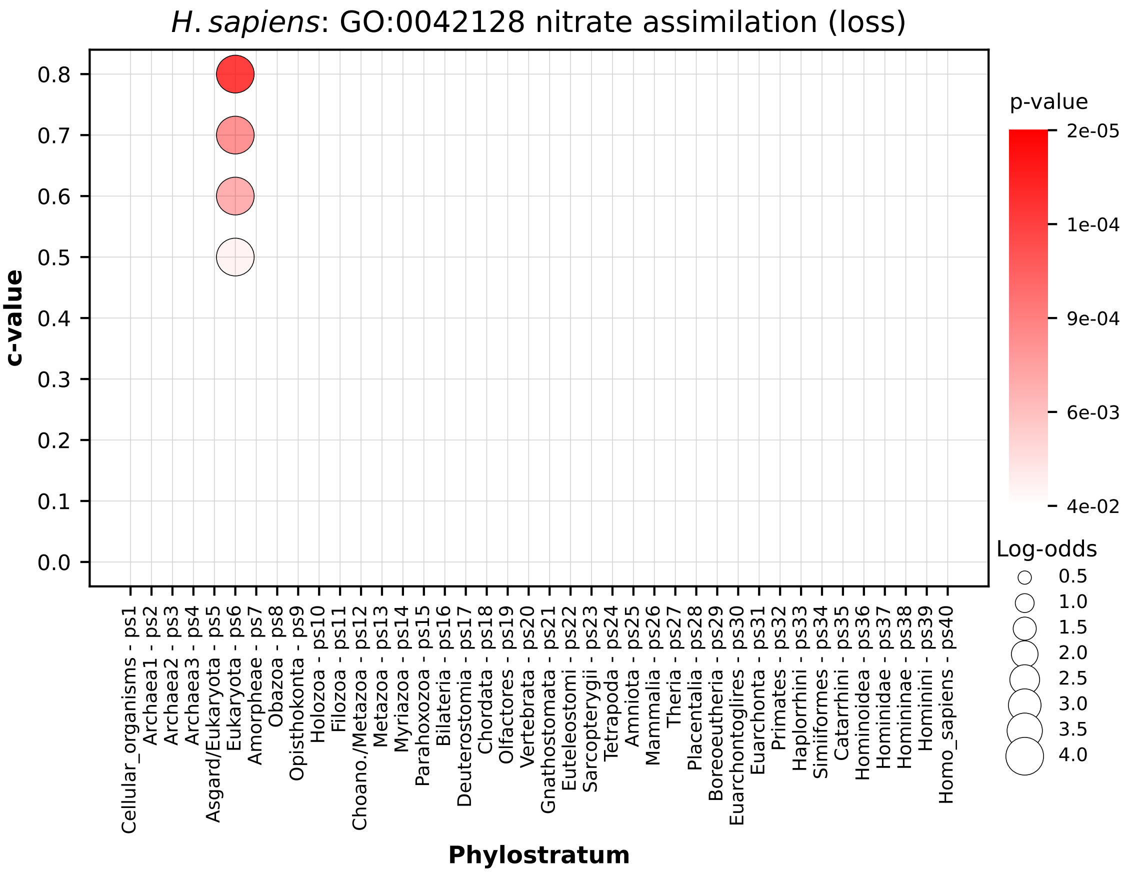
H. sapiens: GO:0042128 nitrate assimilation (loss)
c-value
0.8
0.7
0.6
0.5
0.4
0.3
0.2
0.1
0.0
Phylostratum
Cellular_organisms - ps1
Archaea1 - ps2
Archaea2 - ps3
Archaea3 - ps4
Asgard/Eukaryota - ps5
Eukaryota - ps6
Amorpheae - ps7
Obazoa - ps8
Opisthokonta - ps9
Holozoa - ps10
Filozoa - ps11
Choano./Metazoa - ps12
Metazoa - ps13
Myriazoa - ps14
Parahoxozoa - ps15
Bilateria - ps16
Deuterostomia - ps17
Chordata - ps18
Olfactores - ps19
Vertebrata - ps20
Gnathostomata - ps21
Euteleostomi - ps22
Sarcopterygii - ps23
Tetrapoda - ps24
Amniota - ps25
Mammalia - ps26
Theria - ps27
Placentalia - ps28
Boreoeutheria - ps29
Euarchontoglires - ps30
Euarchonta - ps31
Primates - ps32
Haplorrhini - ps33
Simiiformes - ps34
Catarrhini - ps35
Hominoidea - ps36
Hominidae - ps37
Homininae - ps38
Hominini - ps39
Homo_sapiens - ps40
p-value
2e-05
1e-04
9e-04
6e-03
4e-02
Log-odds
0.5
1.0
1.5
2.0
2.5
3.0
3.5
4.0

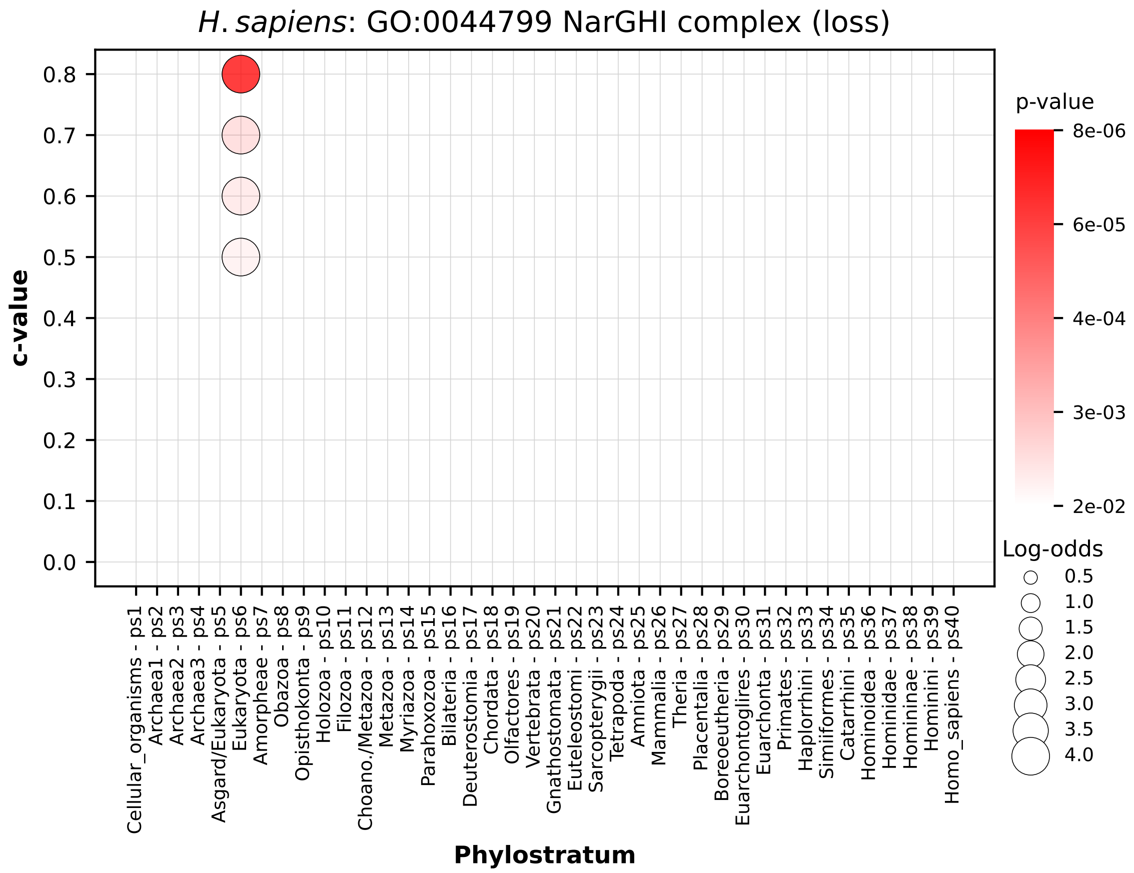

H. sapiens: GO:0044799 NarGHI complex (loss)
c-value
0.8
0.7
0.6
0.5
0.4
0.3
0.2
0.1
0.0
p-value
8e-06
6e-05
4e-04
3e-03
2e-02
Log-odds
0.5
1.0
1.5
2.0
2.5
3.0
3.5
4.0
Cellular_organisms - ps1
Archaea1 - ps2
Archaea2 - ps3
Archaea3 - ps4
Asgard/Eukaryota - ps5
Eukaryota - ps6
Amorpheae - ps7
Obazoa - ps8
Opisthokonta - ps9
Holozoa - ps10
Filozoa - ps11
Choano./Metazoa - ps12
Metazoa - ps13
Myriazoa - ps14
Parahoxozoa - ps15
Bilateria - ps16
Deuterostomia - ps17
Chordata - ps18
Olfactores - ps19
Vertebrata - ps20
Gnathostomata - ps21
Euteleostomi - ps22
Sarcopterygii - ps23
Tetrapoda - ps24
Amniota - ps25
Mammalia - ps26
Theria - ps27
Placentalia - ps28
Boreoeutheria - ps29
Euarchontoglires - ps30
Euarchonta - ps31
Primates - ps32
Haplorrhini - ps33
Simiiformes - ps34
Catarrhini - ps35
Hominoidea - ps36
Hominidae - ps37
Homininae - ps38
Hominini - ps39
Homo_sapiens - ps40
Phylostratum

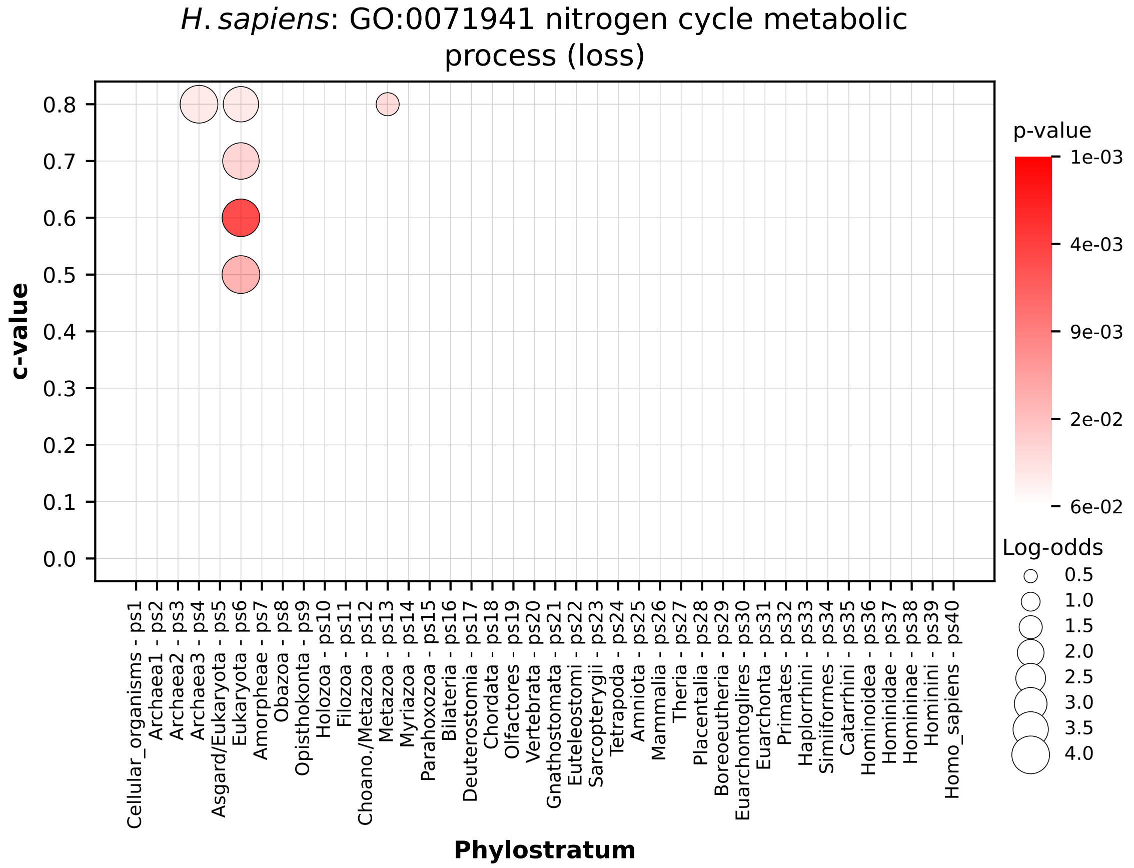
H. sapiens: GO:0071941 nitrogen cycle metabolic process (loss)
c-value
0.0
0.1
0.2
0.3
0.4
0.5
0.6
0.7
0.8
Phylostratum
Cellular_organisms - ps1
Archaea1 - ps2
Archaea2 - ps3
Archaea3 - ps4
Asgard/Eukaryota - ps5
Eukaryota - ps6
Amorpheae - ps7
Obazoa - ps8
Opisthokonta - ps9
Holozoa - ps10
Filozoa - ps11
Choano./Metazoa - ps12
Metazoa - ps13
Myriazoa - ps14
Parahoxozoa - ps15
Bilateria - ps16
Deuterostomia - ps17
Chordata - ps18
Olfactores - ps19
Vertebrata - ps20
Gnathostomata - ps21
Euteleostomi - ps22
Sarcopterygii - ps23
Tetrapoda - ps24
Amniota - ps25
Mammalia - ps26
Theria - ps27
Placentalia - ps28
Boreoeutheria - ps29
Euarchontoglires - ps30
Euarchonta - ps31
Primates - ps32
Haplorrhini - ps33
Simiiformes - ps34
Catarrhini - ps35
Hominoidea - ps36
Hominidae - ps37
Homininae - ps38
Hominini - ps39
Homo_sapiens - ps40
p-value
1e-03
4e-03
9e-03
2e-02
6e-02
Log-odds
0.5
1.0
1.5
2.0
2.5
3.0
3.5
4.0

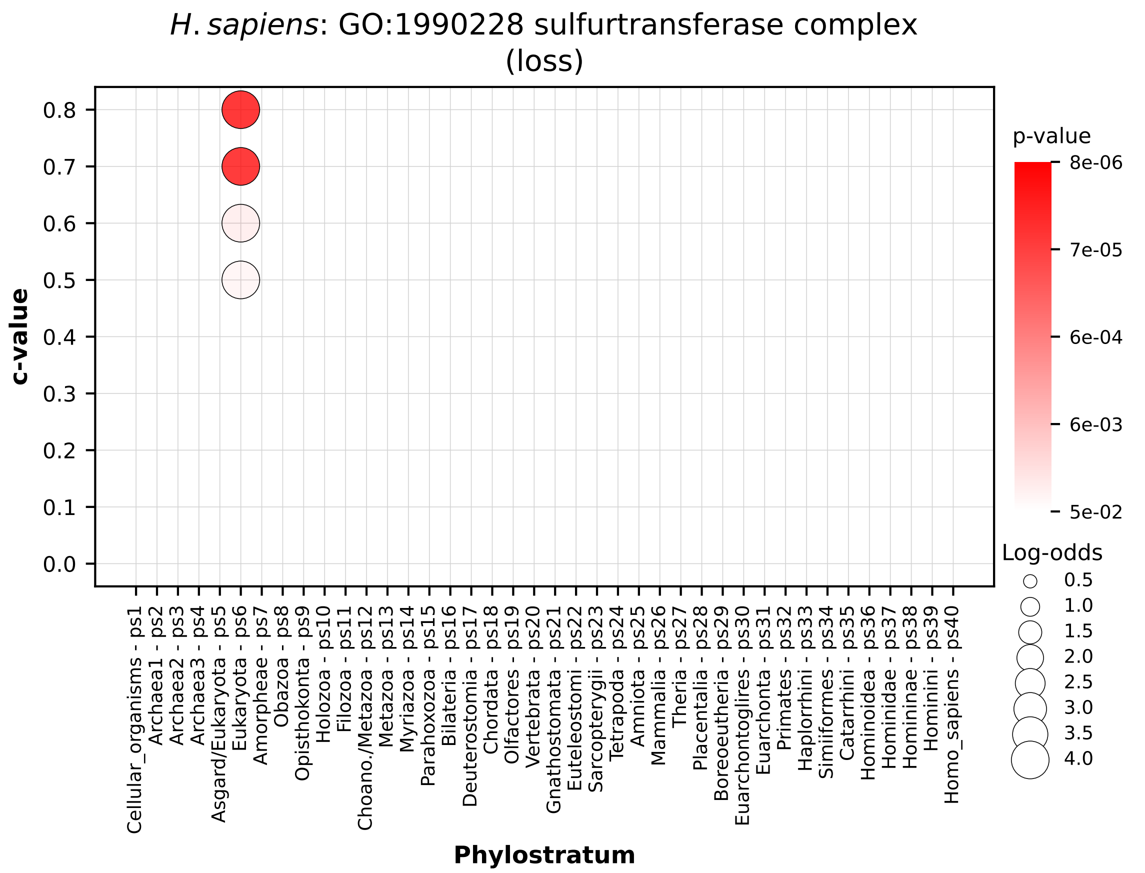

H. sapiens: GO:1990228 sulfurtransferase complex
(loss)
c-value
0.0
0.1
0.2
0.3
0.4
0.5
0.6
0.7
0.8
Phylostratum
Cellular_organisms - ps1
Archaea1 - ps2
Archaea2 - ps3
Archaea3 - ps4
Asgard/Eukaryota - ps5
Eukaryota - ps6
Amorpheae - ps7
Obazoa - ps8
Opisthokonta - ps9
Holozoa - ps10
Filozoa - ps11
Choano./Metazoa - ps12
Metazoa - ps13
Myriazoa - ps14
Parahoxozoa - ps15
Bilateria - ps16
Deuterostomia - ps17
Chordata - ps18
Olfactores - ps19
Vertebrata - ps20
Gnathostomata - ps21
Euteleostomi - ps22
Sarcopterygii - ps23
Tetrapoda - ps24
Amniota - ps25
Mammalia - ps26
Theria - ps27
Placentalia - ps28
Boreoeutheria - ps29
Euarchontoglires - ps30
Euarchonta - ps31
Primates - ps32
Haplorrhini - ps33
Simiiformes - ps34
Catarrhini - ps35
Hominoidea - ps36
Hominidae - ps37
Homininae - ps38
Hominini - ps39
Homo_sapiens - ps40
p-value
8e-06
7e-05
6e-04
6e-03
5e-02
Log-odds
0.5
1.0
1.5
2.0
2.5
3.0
3.5
4.0

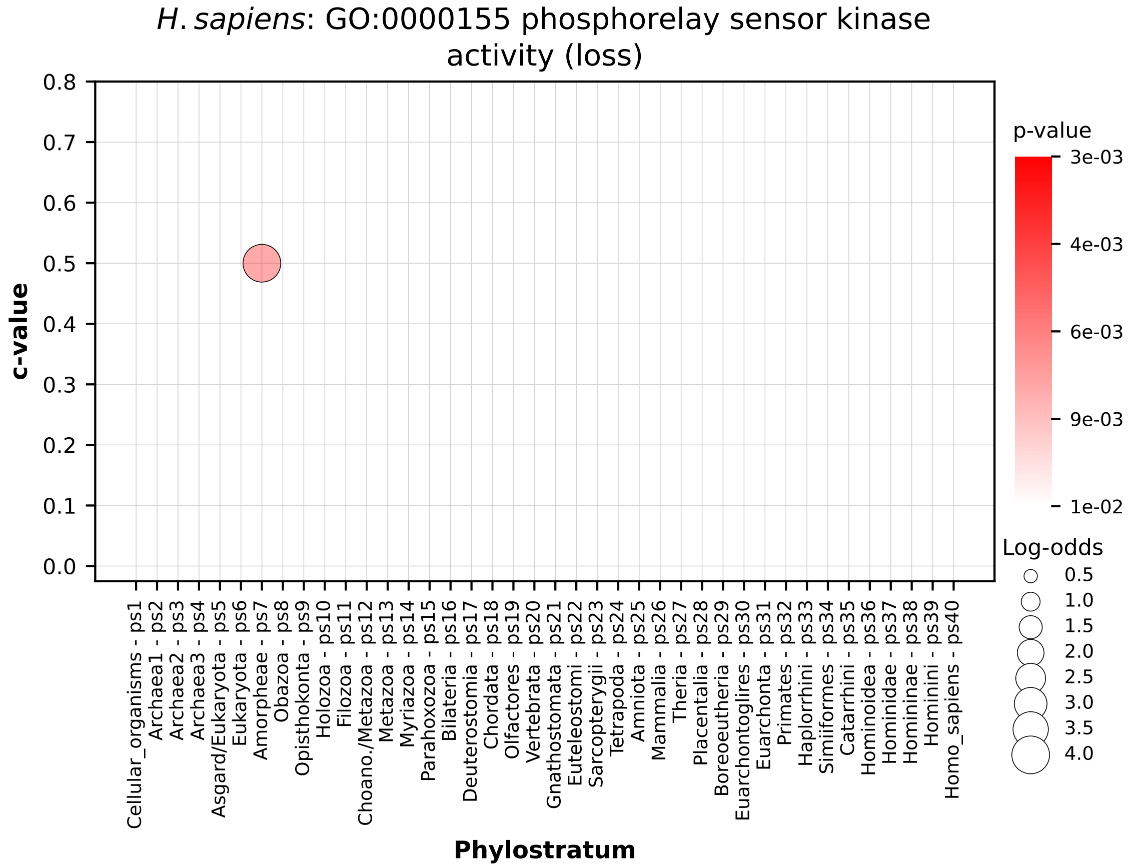
H. sapiens: GO:0000155 phosphorelay sensor kinase activity (loss)
c-value
0.0
0.1
0.2
0.3
0.4
0.5
0.6
0.7
0.8
Phylostratum
Cellular_organisms - ps1
Archaea1 - ps2
Archaea2 - ps3
Archaea3 - ps4
Asgard/Eukaryota - ps5
Eukaryota - ps6
Amorpheae - ps7
Obazoa - ps8
Opisthokonta - ps9
Holozoa - ps10
Filozoa - ps11
Choano./Metazoa - ps12
Metazoa - ps13
Myriazoa - ps14
Parahoxozoa - ps15
Bilateria - ps16
Deuterostomia - ps17
Chordata - ps18
Olfactores - ps19
Vertebrata - ps20
Gnathostomata - ps21
Euteleostomi - ps22
Sarcopterygii - ps23
Tetrapoda - ps24
Amniota - ps25
Mammalia - ps26
Theria - ps27
Placentalia - ps28
Boreoeutheria - ps29
Euarchontoglires - ps30
Euarchonta - ps31
Primates - ps32
Haplorrhini - ps33
Simiiformes - ps34
Catarrhini - ps35
Hominoidea - ps36
Hominidae - ps37
Homininae - ps38
Hominini - ps39
Homo_sapiens - ps40
p-value
3e-03
4e-03
6e-03
9e-03
1e-02
Log-odds
0.5
1.0
1.5
2.0
2.5
3.0
3.5
4.0

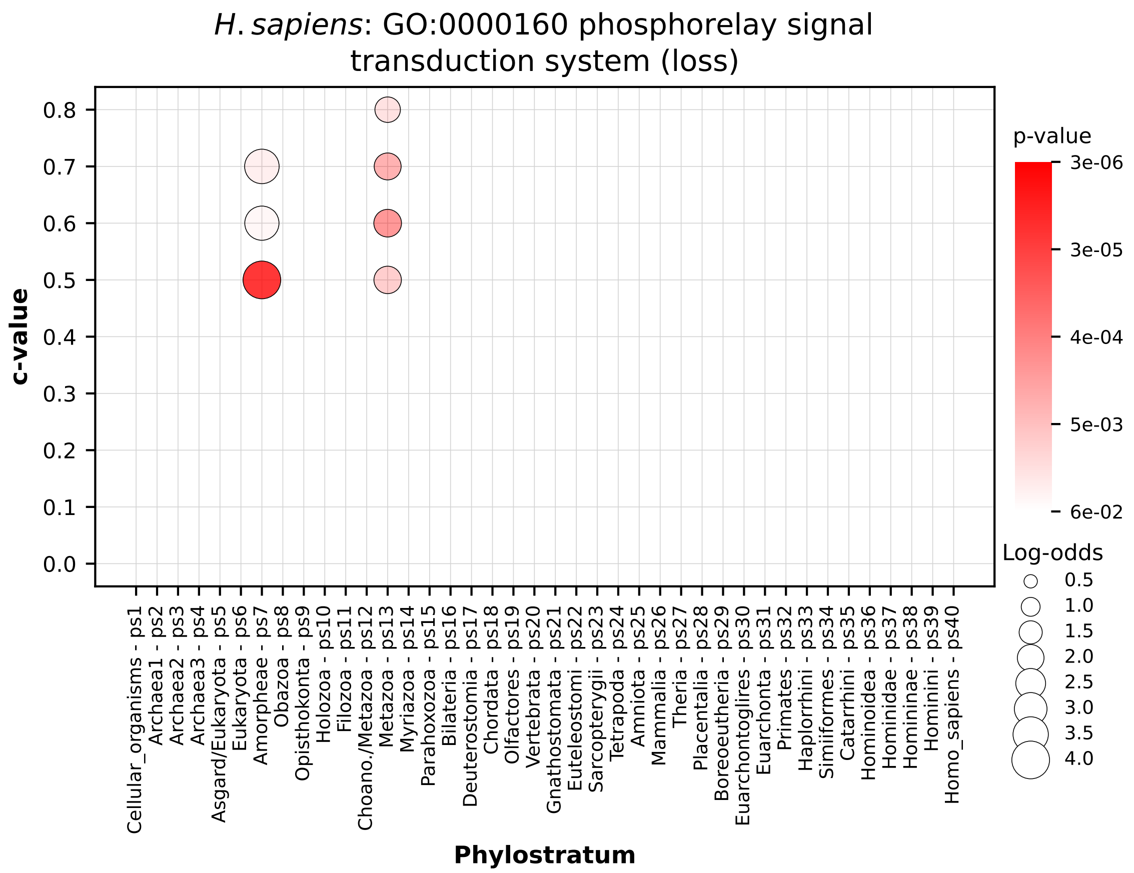
H. sapiens: GO:0000160 phosphorelay signal transduction system (loss)
c-value
0.0
0.1
0.2
0.3
0.4
0.5
0.6
0.7
0.8
Phylostratum
Cellular_organisms - ps1
Archaea1 - ps2
Archaea2 - ps3
Archaea3 - ps4
Asgard/Eukaryota - ps5
Eukaryota - ps6
Amorpheae - ps7
Obazoa - ps8
Opisthokonta - ps9
Holozoa - ps10
Filozoa - ps11
Choano./Metazoa - ps12
Metazoa - ps13
Myriazoa - ps14
Parahoxozoa - ps15
Bilateria - ps16
Deuterostomia - ps17
Chordata - ps18
Olfactores - ps19
Vertebrata - ps20
Gnathostomata - ps21
Euteleostomi - ps22
Sarcopterygii - ps23
Tetrapoda - ps24
Amniota - ps25
Mammalia - ps26
Theria - ps27
Placentalia - ps28
Boreoeutheria - ps29
Euarchontoglires - ps30
Euarchonta - ps31
Primates - ps32
Haplorrhini - ps33
Simiiformes - ps34
Catarrhini - ps35
Hominoidea - ps36
Hominidae - ps37
Homininae - ps38
Hominini - ps39
Homo_sapiens - ps40
p-value
3e-06
3e-05
4e-04
5e-03
6e-02
Log-odds
0.5
1.0
1.5
2.0
2.5
3.0
3.5
4.0

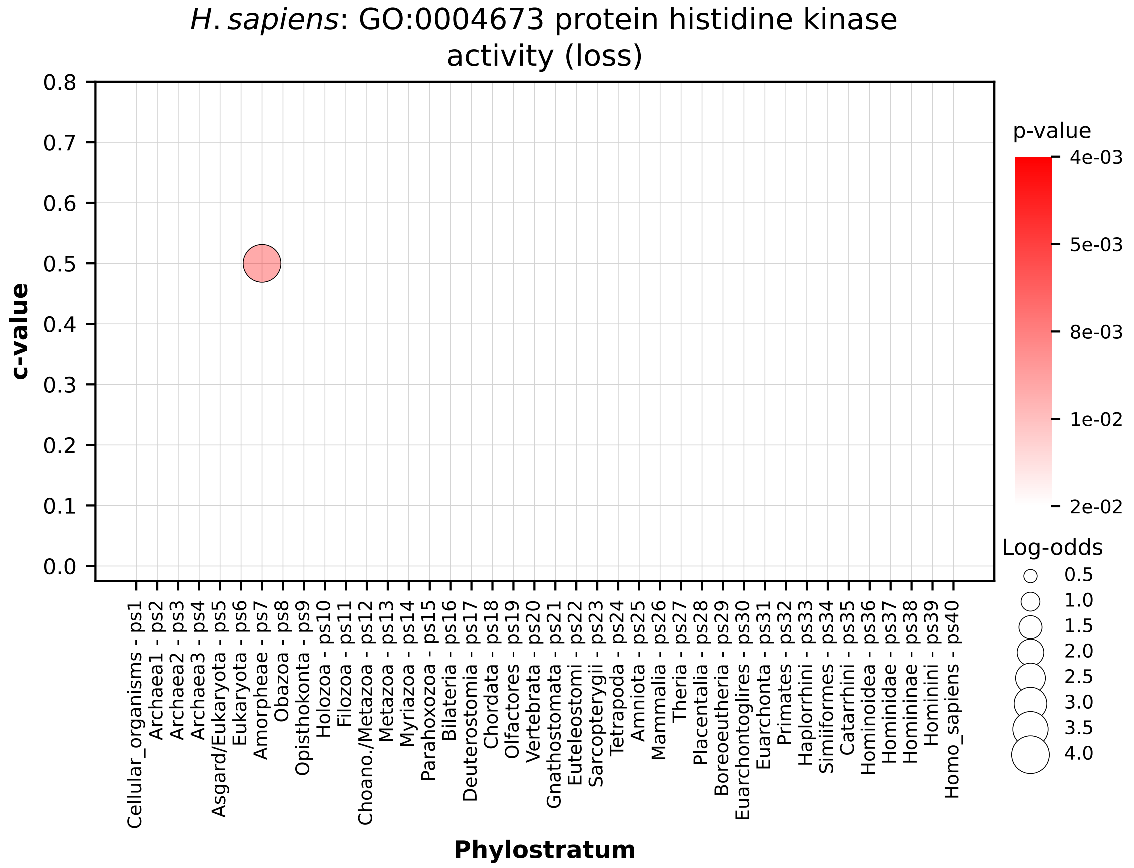

H. sapiens: GO:0004673 protein histidine kinase activity (loss)
c-value
0.0
0.1
0.2
0.3
0.4
0.5
0.6
0.7
0.8
Phylostratum
Cellular_organisms - ps1
Archaea1 - ps2
Archaea2 - ps3
Archaea3 - ps4
Asgard/Eukaryota - ps5
Eukaryota - ps6
Amorpheae - ps7
Obazoa - ps8
Opisthokonta - ps9
Holozoa - ps10
Filozoa - ps11
Choano./Metazoa - ps12
Metazoa - ps13
Myriazoa - ps14
Parahoxozoa - ps15
Bilateria - ps16
Deuterostomia - ps17
Chordata - ps18
Olfactores - ps19
Vertebrata - ps20
Gnathostomata - ps21
Euteleostomi - ps22
Sarcopterygii - ps23
Tetrapoda - ps24
Amniota - ps25
Mammalia - ps26
Theria - ps27
Placentalia - ps28
Boreoeutheria - ps29
Euarchontoglires - ps30
Euarchonta - ps31
Primates - ps32
Haplorrhini - ps33
Simiiformes - ps34
Catarrhini - ps35
Hominoidea - ps36
Hominidae - ps37
Homininae - ps38
Hominini - ps39
Homo_sapiens - ps40
p-value
4e-03
5e-03
8e-03
1e-02
2e-02
Log-odds
0.5
1.0
1.5
2.0
2.5
3.0
3.5
4.0

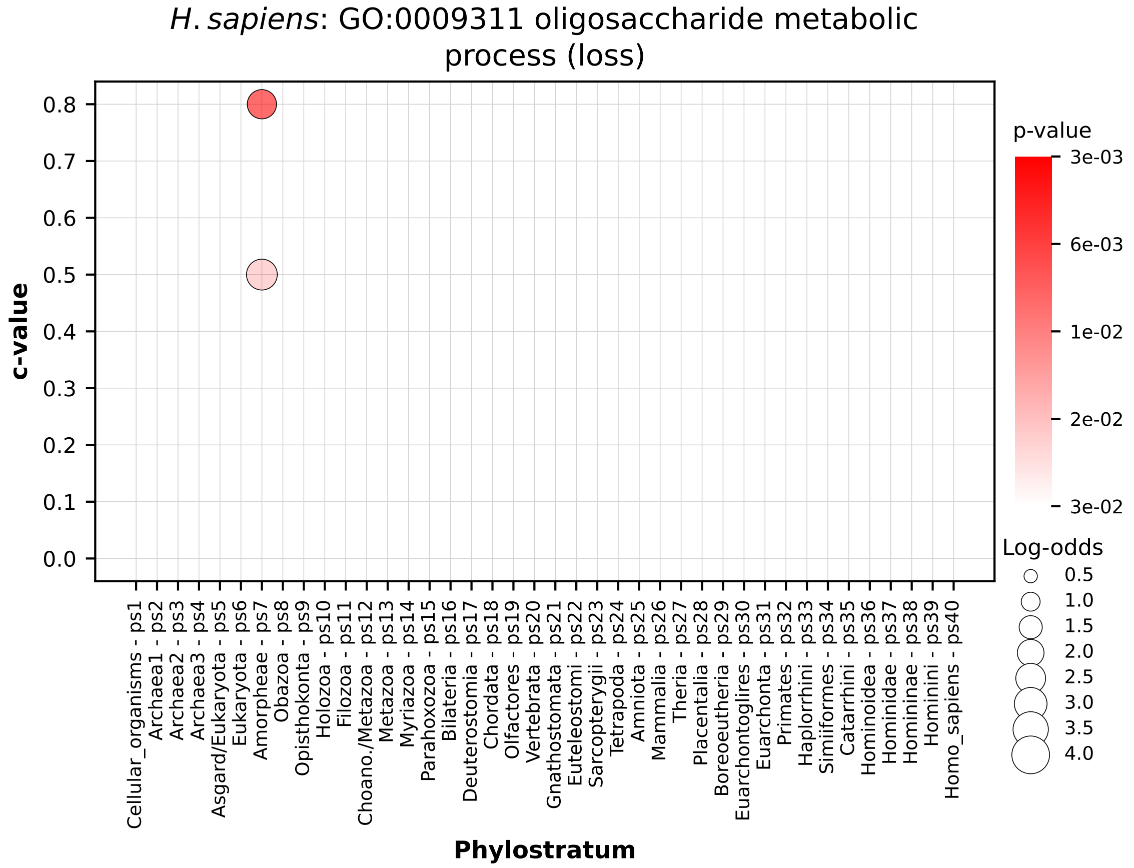

H. sapiens: GO:0009311 oligosaccharide metabolic process (loss)
c-value
0.8
0.7
0.6
0.5
0.4
0.3
0.2
0.1
0.0
p-value
3e-03
6e-03
1e-02
2e-02
3e-02
Log-odds
0.5
1.0
1.5
2.0
2.5
3.0
3.5
4.0
Cellular_organisms - ps1
Archaea1 - ps2
Archaea2 - ps3
Archaea3 - ps4
Asgard/Eukaryota - ps5
Eukaryota - ps6
Amorpheae - ps7
Obazoa - ps8
Opisthokonta - ps9
Holozoa - ps10
Filozoa - ps11
Choano./Metazoa - ps12
Metazoa - ps13
Myriazoa - ps14
Parahoxozoa - ps15
Bilateria - ps16
Deuterostomia - ps17
Chordata - ps18
Olfactores - ps19
Vertebrata - ps20
Gnathostomata - ps21
Euteleostomi - ps22
Sarcopterygii - ps23
Tetrapoda - ps24
Amniota - ps25
Mammalia - ps26
Theria - ps27
Placentalia - ps28
Boreoeutheria - ps29
Euarchontoglires - ps30
Euarchonta - ps31
Primates - ps32
Haplorrhini - ps33
Simiiformes - ps34
Catarrhini - ps35
Hominoidea - ps36
Hominidae - ps37
Homininae - ps38
Hominini - ps39
Homo_sapiens - ps40
Phylostratum

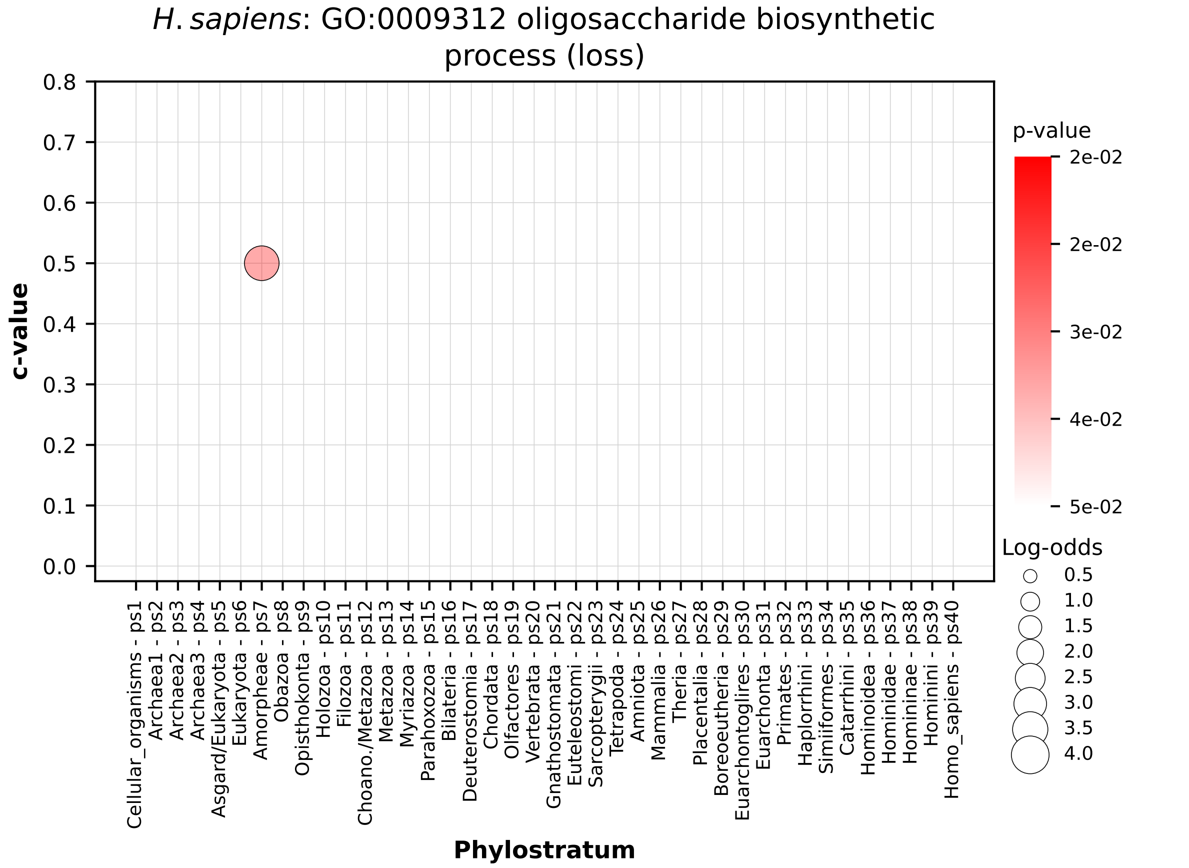

$H. sapiens$: GO:0009312 oligosaccharide biosynthetic process (loss)
c-value
0.0
0.1
0.2
0.3
0.4
0.5
0.6
0.7
0.8
Phylostratum
Cellular_organisms - ps1
Archaea1 - ps2
Archaea2 - ps3
Archaea3 - ps4
Asgard/Eukaryota - ps5
Eukaryota - ps6
Amorpheae - ps7
Obazoa - ps8
Opisthokonta - ps9
Holozoa - ps10
Filozoa - ps11
Choano./Metazoa - ps12
Metazoa - ps13
Myriazoa - ps14
Parahoxozoa - ps15
Bilateria - ps16
Deuterostomia - ps17
Chordata - ps18
Olfactores - ps19
Vertebrata - ps20
Gnathostomata - ps21
Euteleostomi - ps22
Sarcopterygii - ps23
Tetrapoda - ps24
Amniota - ps25
Mammalia - ps26
Theria - ps27
Placentalia - ps28
Boreoeutheria - ps29
Euarchontoglires - ps30
Euarchonta - ps31
Primates - ps32
Haplorrhini - ps33
Simiiformes - ps34
Catarrhini - ps35
Hominoidea - ps36
Hominidae - ps37
Homininae - ps38
Hominini - ps39
Homo_sapiens - ps40
p-value
2e-02
2e-02
3e-02
4e-02
5e-02
Log-odds
0.5
1.0
1.5
2.0
2.5
3.0
3.5
4.0

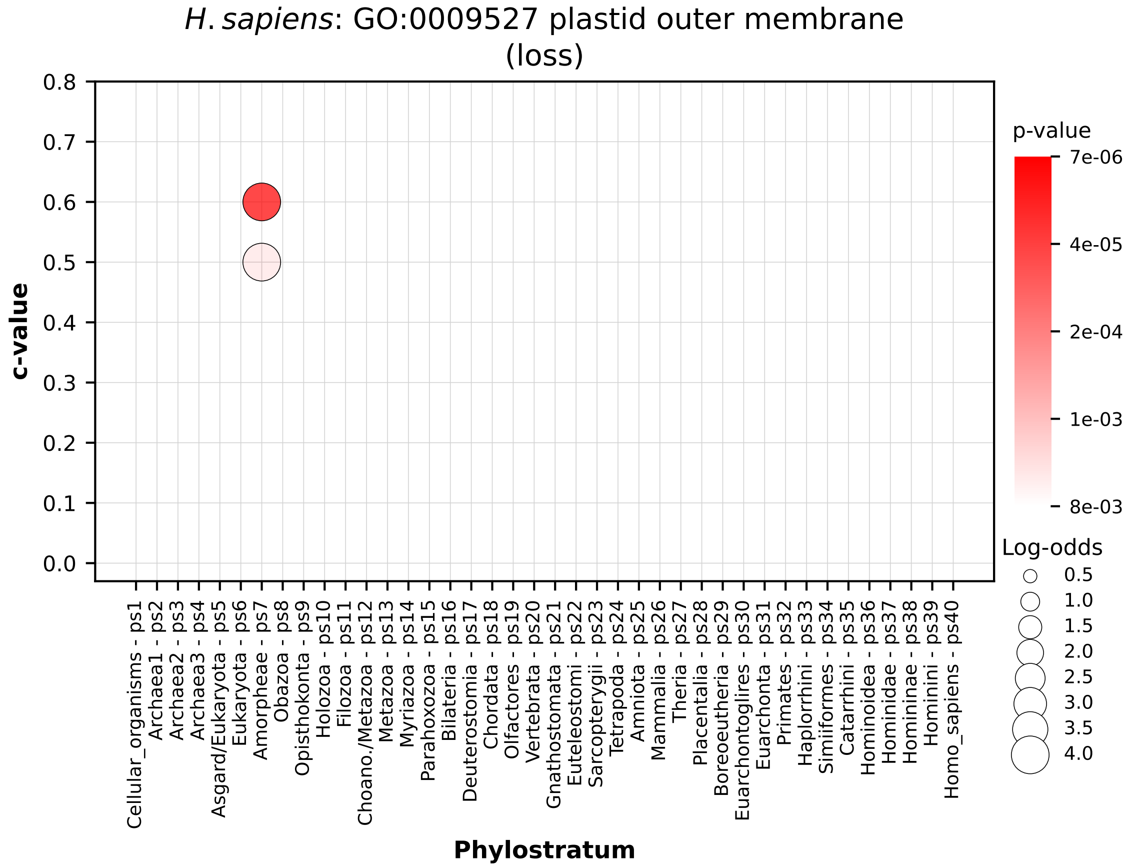

H. sapiens: GO:0009527 plastid outer membrane
(loss)
c-value
0.0
0.1
0.2
0.3
0.4
0.5
0.6
0.7
0.8
Phylostratum
Cellular_organisms - ps1
Archaea1 - ps2
Archaea2 - ps3
Archaea3 - ps4
Asgard/Eukaryota - ps5
Eukaryota - ps6
Amorpheae - ps7
Obazoa - ps8
Opisthokonta - ps9
Holozoa - ps10
Filozoa - ps11
Choano./Metazoa - ps12
Metazoa - ps13
Myriazoa - ps14
Parahoxozoa - ps15
Bilateria - ps16
Deuterostomia - ps17
Chordata - ps18
Olfactores - ps19
Vertebrata - ps20
Gnathostomata - ps21
Euteleostomi - ps22
Sarcopterygii - ps23
Tetrapoda - ps24
Amniota - ps25
Mammalia - ps26
Theria - ps27
Placentalia - ps28
Boreoeutheria - ps29
Euarchontoglires - ps30
Euarchonta - ps31
Primates - ps32
Haplorrhini - ps33
Simiiformes - ps34
Catarrhini - ps35
Hominoidea - ps36
Hominidae - ps37
Homininae - ps38
Hominini - ps39
Homo_sapiens - ps40
p-value
7e-06
4e-05
2e-04
1e-03
8e-03
Log-odds
0.5
1.0
1.5
2.0
2.5
3.0
3.5
4.0

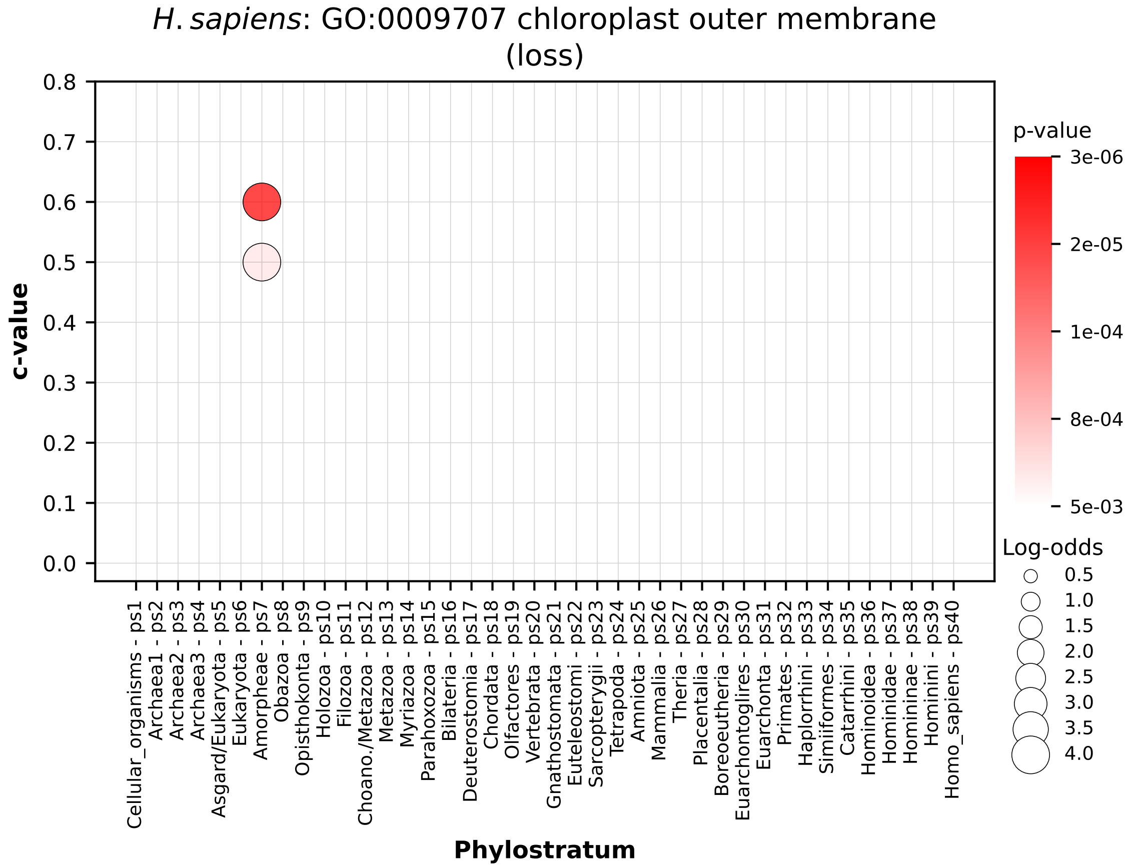

H. sapiens: GO:0009707 chloroplast outer membrane
(loss)
c-value
0.8
0.7
0.6
0.5
0.4
0.3
0.2
0.1
0.0
Phylostratum
Cellular_organisms - ps1
Archaea1 - ps2
Archaea2 - ps3
Archaea3 - ps4
Asgard/Eukaryota - ps5
Eukaryota - ps6
Amorpheae - ps7
Obazoa - ps8
Opisthokonta - ps9
Holozoa - ps10
Filozoa - ps11
Choano./Metazoa - ps12
Metazoa - ps13
Myriazoa - ps14
Parahoxozoa - ps15
Bilateria - ps16
Deuterostomia - ps17
Chordata - ps18
Olfactores - ps19
Vertebrata - ps20
Gnathostomata - ps21
Euteleostomi - ps22
Sarcopterygii - ps23
Tetrapoda - ps24
Amniota - ps25
Mammalia - ps26
Theria - ps27
Placentalia - ps28
Boreoeutheria - ps29
Euarchontoglires - ps30
Euarchonta - ps31
Primates - ps32
Haplorrhini - ps33
Simiiformes - ps34
Catarrhini - ps35
Hominoidea - ps36
Hominidae - ps37
Homininae - ps38
Hominini - ps39
Homo_sapiens - ps40
p-value
3e-06
2e-05
1e-04
8e-04
5e-03
Log-odds
0.5
1.0
1.5
2.0
2.5
3.0
3.5
4.0

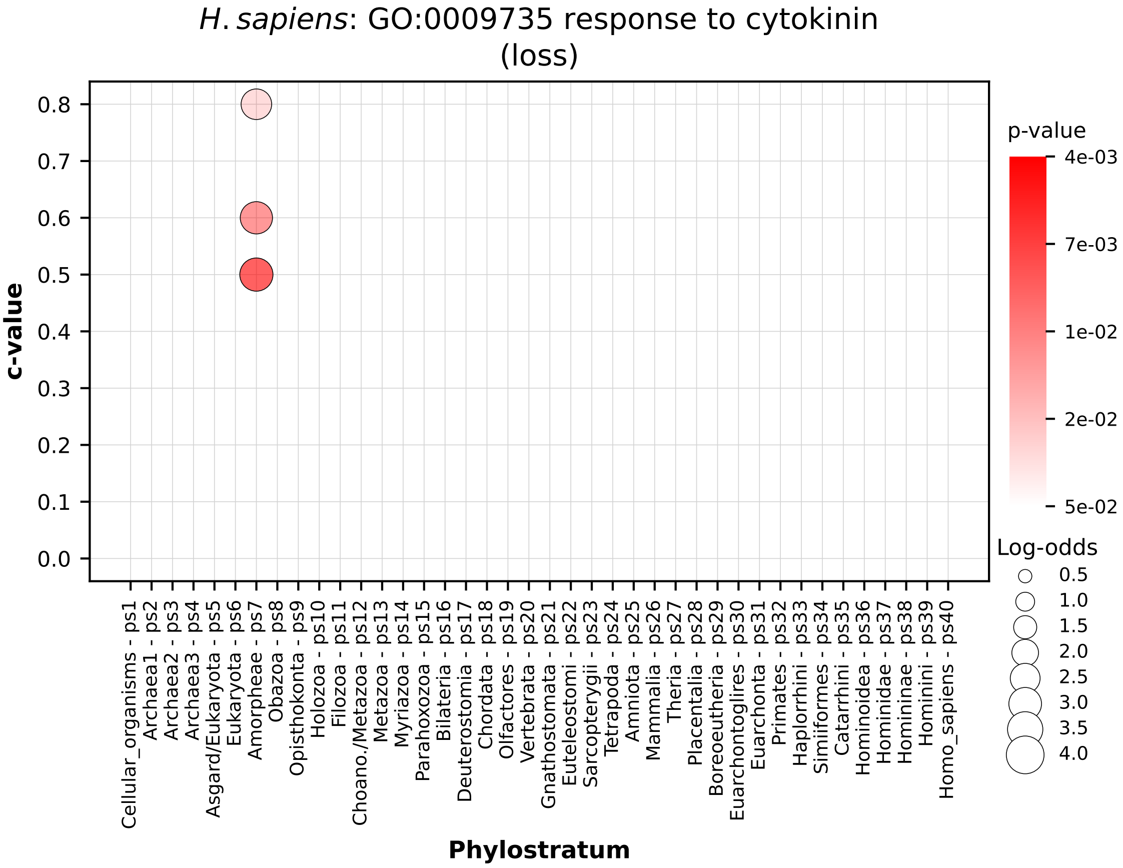
H. sapiens: GO:0009735 response to cytokinin
(loss)
c-value
0.8
0.7
0.6
0.5
0.4
0.3
0.2
0.1
0.0
p-value
4e-03
7e-03
1e-02
2e-02
5e-02
Log-odds
0.5
1.0
1.5
2.0
2.5
3.0
3.5
4.0
Cellular_organisms - ps1
Archaea1 - ps2
Archaea2 - ps3
Archaea3 - ps4
Asgard/Eukaryota - ps5
Eukaryota - ps6
Amorpheae - ps7
Obazoa - ps8
Opisthokonta - ps9
Holozoa - ps10
Filozoa - ps11
Choano./Metazoa - ps12
Metazoa - ps13
Myriazoa - ps14
Parahoxozoa - ps15
Bilateria - ps16
Deuterostomia - ps17
Chordata - ps18
Olfactores - ps19
Vertebrata - ps20
Gnathostomata - ps21
Euteleostomi - ps22
Sarcopterygii - ps23
Tetrapoda - ps24
Amniota - ps25
Mammalia - ps26
Theria - ps27
Placentalia - ps28
Boreoeutheria - ps29
Euarchontoglires - ps30
Euarchonta - ps31
Primates - ps32
Haplorrhini - ps33
Simiiformes - ps34
Catarrhini - ps35
Hominoidea - ps36
Hominidae - ps37
Homininae - ps38
Hominini - ps39
Homo_sapiens - ps40
Phylostratum

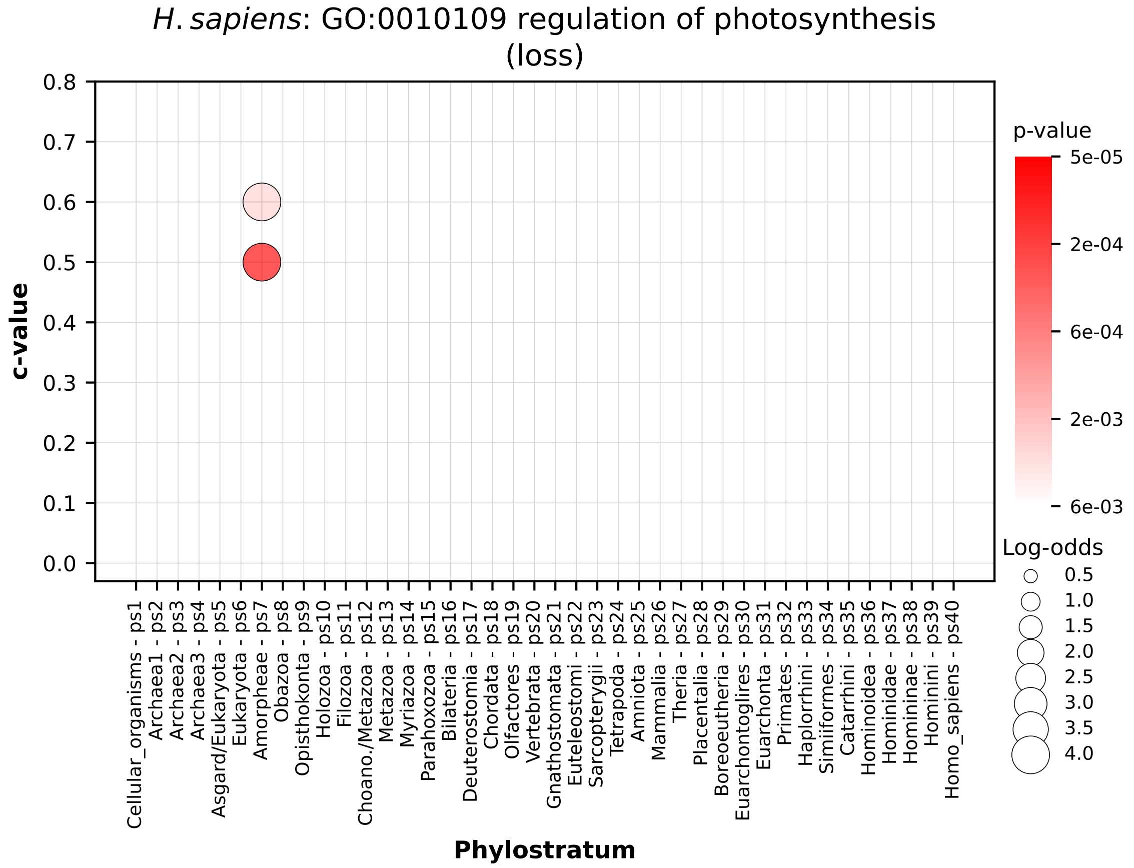

H. sapiens: GO:0010109 regulation of photosynthesis
(loss)
c-value
0.8
0.7
0.6
0.5
0.4
0.3
0.2
0.1
0.0
Phylostratum
Cellular_organisms - ps1
Archaea1 - ps2
Archaea2 - ps3
Archaea3 - ps4
Asgard/Eukaryota - ps5
Eukaryota - ps6
Amorpheae - ps7
Obazoa - ps8
Opisthokonta - ps9
Holozoa - ps10
Filozoa - ps11
Choano./Metazoa - ps12
Metazoa - ps13
Myriazoa - ps14
Parahoxozoa - ps15
Bilateria - ps16
Deuterostomia - ps17
Chordata - ps18
Olfactores - ps19
Vertebrata - ps20
Gnathostomata - ps21
Euteleostomi - ps22
Sarcopterygii - ps23
Tetrapoda - ps24
Amniota - ps25
Mammalia - ps26
Theria - ps27
Placentalia - ps28
Boreoeutheria - ps29
Euarchontoglires - ps30
Euarchonta - ps31
Primates - ps32
Haplorrhini - ps33
Simiiformes - ps34
Catarrhini - ps35
Hominoidea - ps36
Hominidae - ps37
Homininae - ps38
Hominini - ps39
Homo_sapiens - ps40
p-value
5e-05
2e-04
6e-04
2e-03
6e-03
Log-odds
0.5
1.0
1.5
2.0
2.5
3.0
3.5
4.0

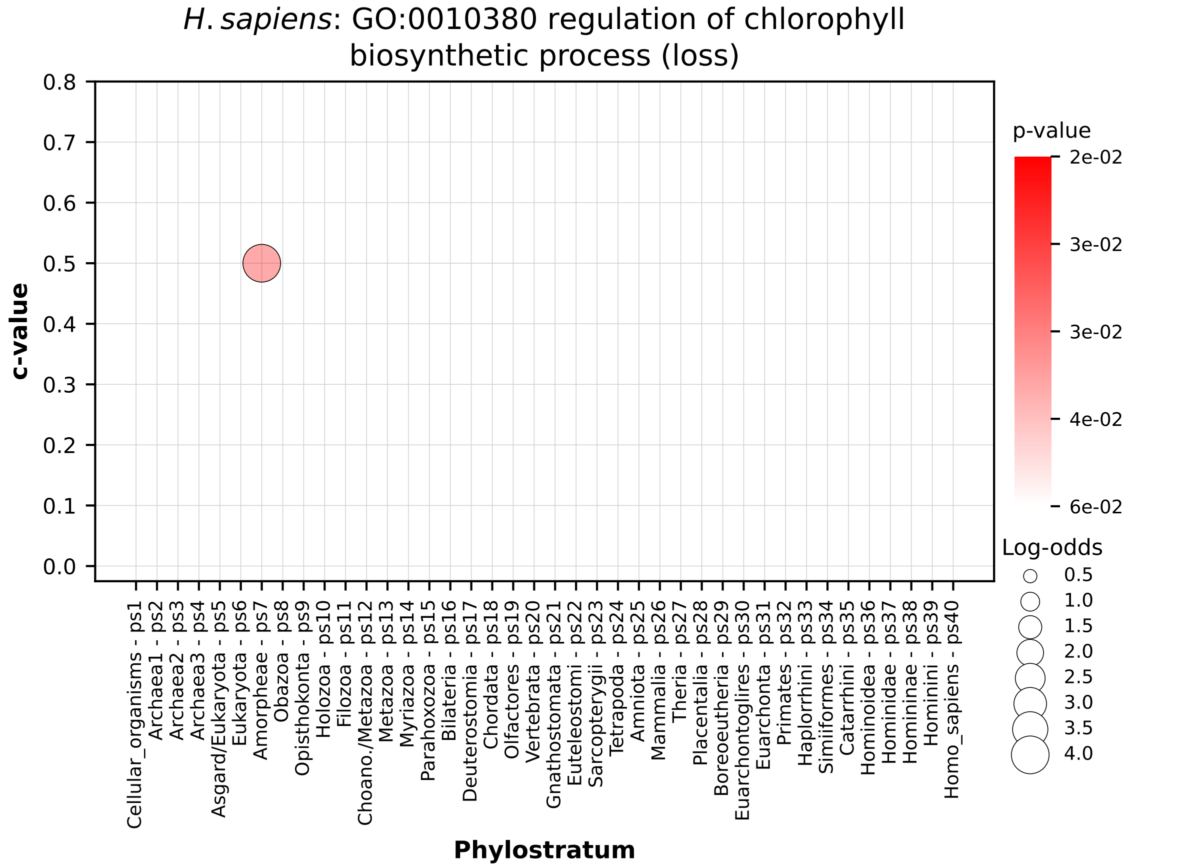

H. sapiens: GO:0010380 regulation of chlorophyll biosynthetic process (loss)
c-value
0.0
0.1
0.2
0.3
0.4
0.5
0.6
0.7
0.8
Phylostratum
Cellular_organisms - ps1
Archaea1 - ps2
Archaea2 - ps3
Archaea3 - ps4
Asgard/Eukaryota - ps5
Eukaryota - ps6
Amorpheae - ps7
Obazoa - ps8
Opisthokonta - ps9
Holozoa - ps10
Filozoa - ps11
Choano./Metazoa - ps12
Metazoa - ps13
Myriazoa - ps14
Parahoxozoa - ps15
Bilateria - ps16
Deuterostomia - ps17
Chordata - ps18
Olfactores - ps19
Vertebrata - ps20
Gnathostomata - ps21
Euteleostomi - ps22
Sarcopterygii - ps23
Tetrapoda - ps24
Amniota - ps25
Mammalia - ps26
Theria - ps27
Placentalia - ps28
Boreoeutheria - ps29
Euarchontoglires - ps30
Euarchonta - ps31
Primates - ps32
Haplorrhini - ps33
Simiiformes - ps34
Catarrhini - ps35
Hominoidea - ps36
Hominidae - ps37
Homininae - ps38
Hominini - ps39
Homo_sapiens - ps40
p-value
2e-02
3e-02
3e-02
4e-02
6e-02
Log-odds
0.5
1.0
1.5
2.0
2.5
3.0
3.5
4.0

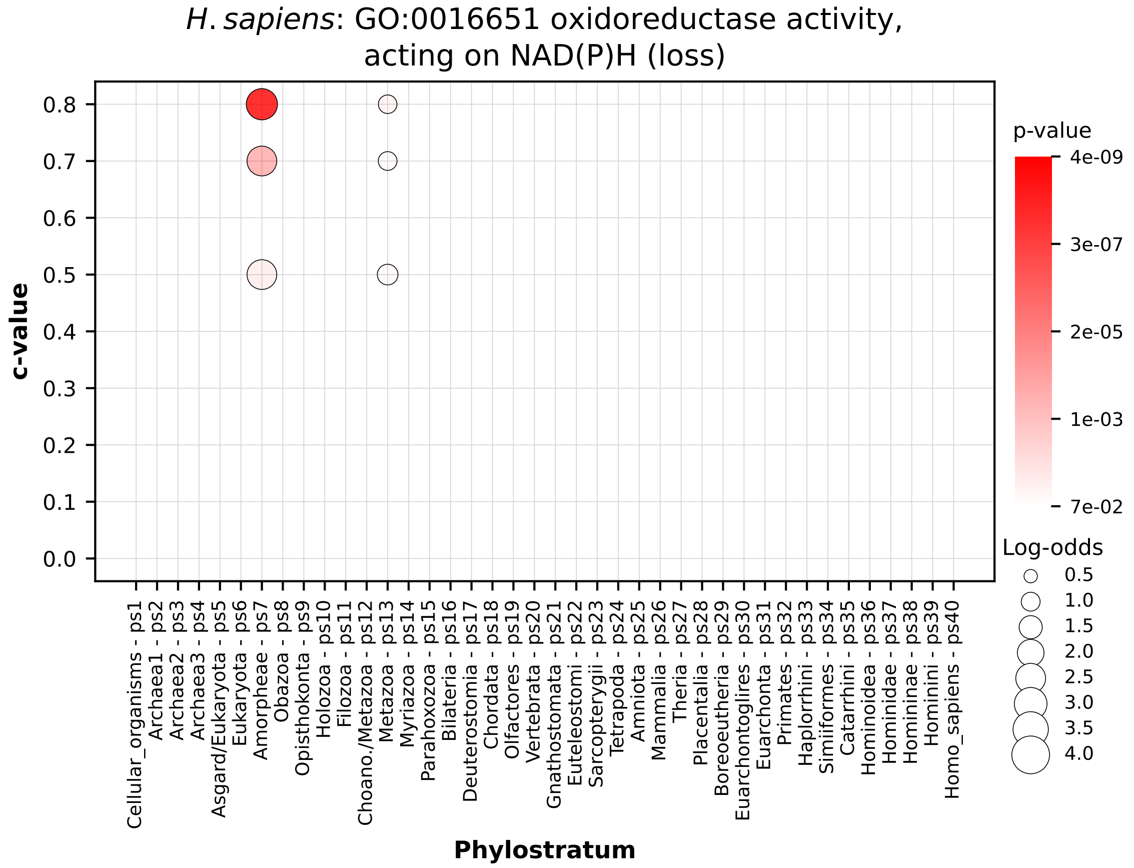

H. sapiens: GO:0016651 oxidoreductase activity, acting on NAD(P)H (loss)
c-value
0.8
0.7
0.6
0.5
0.4
0.3
0.2
0.1
0.0
p-value
4e-09
3e-07
2e-05
1e-03
7e-02
Log-odds
0.5
1.0
1.5
2.0
2.5
3.0
3.5
4.0
Cellular_organisms - ps1
Archaea1 - ps2
Archaea2 - ps3
Archaea3 - ps4
Asgard/Eukaryota - ps5
Eukaryota - ps6
Amorpheae - ps7
Obazoa - ps8
Opisthokonta - ps9
Holozoa - ps10
Filozoa - ps11
Choano./Metazoa - ps12
Metazoa - ps13
Myriazoa - ps14
Parahoxozoa - ps15
Bilateria - ps16
Deuterostomia - ps17
Chordata - ps18
Olfactores - ps19
Vertebrata - ps20
Gnathostomata - ps21
Euteleostomi - ps22
Sarcopterygii - ps23
Tetrapoda - ps24
Amniota - ps25
Mammalia - ps26
Theria - ps27
Placentalia - ps28
Boreoeutheria - ps29
Euarchontoglires - ps30
Euarchonta - ps31
Primates - ps32
Haplorrhini - ps33
Simiiformes - ps34
Catarrhini - ps35
Hominoidea - ps36
Hominidae - ps37
Homininae - ps38
Hominini - ps39
Homo_sapiens - ps40
Phylostratum

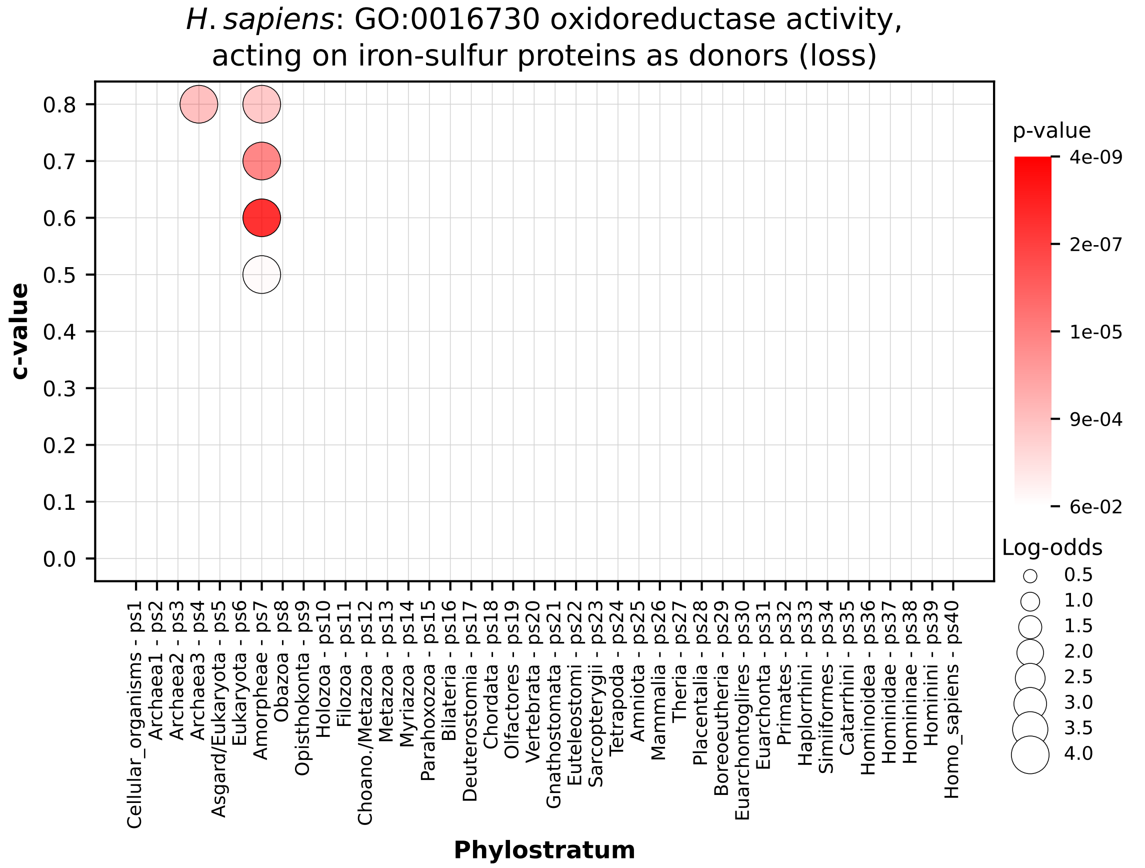

H. sapiens: GO:0016730 oxidoreductase activity,
acting on iron-sulfur proteins as donors (loss)
c-value
0.0
0.1
0.2
0.3
0.4
0.5
0.6
0.7
0.8
Phylostratum
Cellular_organisms - ps1
Archaea1 - ps2
Archaea2 - ps3
Archaea3 - ps4
Asgard/Eukaryota - ps5
Eukaryota - ps6
Amorpheae - ps7
Obazoa - ps8
Opisthokonta - ps9
Holozoa - ps10
Filozoa - ps11
Choano./Metazoa - ps12
Metazoa - ps13
Myriazoa - ps14
Parahoxozoa - ps15
Bilateria - ps16
Deuterostomia - ps17
Chordata - ps18
Olfactores - ps19
Vertebrata - ps20
Gnathostomata - ps21
Euteleostomi - ps22
Sarcopterygii - ps23
Tetrapoda - ps24
Amniota - ps25
Mammalia - ps26
Theria - ps27
Placentalia - ps28
Boreoeutheria - ps29
Euarchontoglires - ps30
Euarchonta - ps31
Primates - ps32
Haplorrhini - ps33
Simiiformes - ps34
Catarrhini - ps35
Hominoidea - ps36
Hominidae - ps37
Homininae - ps38
Hominini - ps39
Homo_sapiens - ps40
p-value
4e-09
2e-07
1e-05
9e-04
6e-02
Log-odds
0.5
1.0
1.5
2.0
2.5
3.0
3.5
4.0

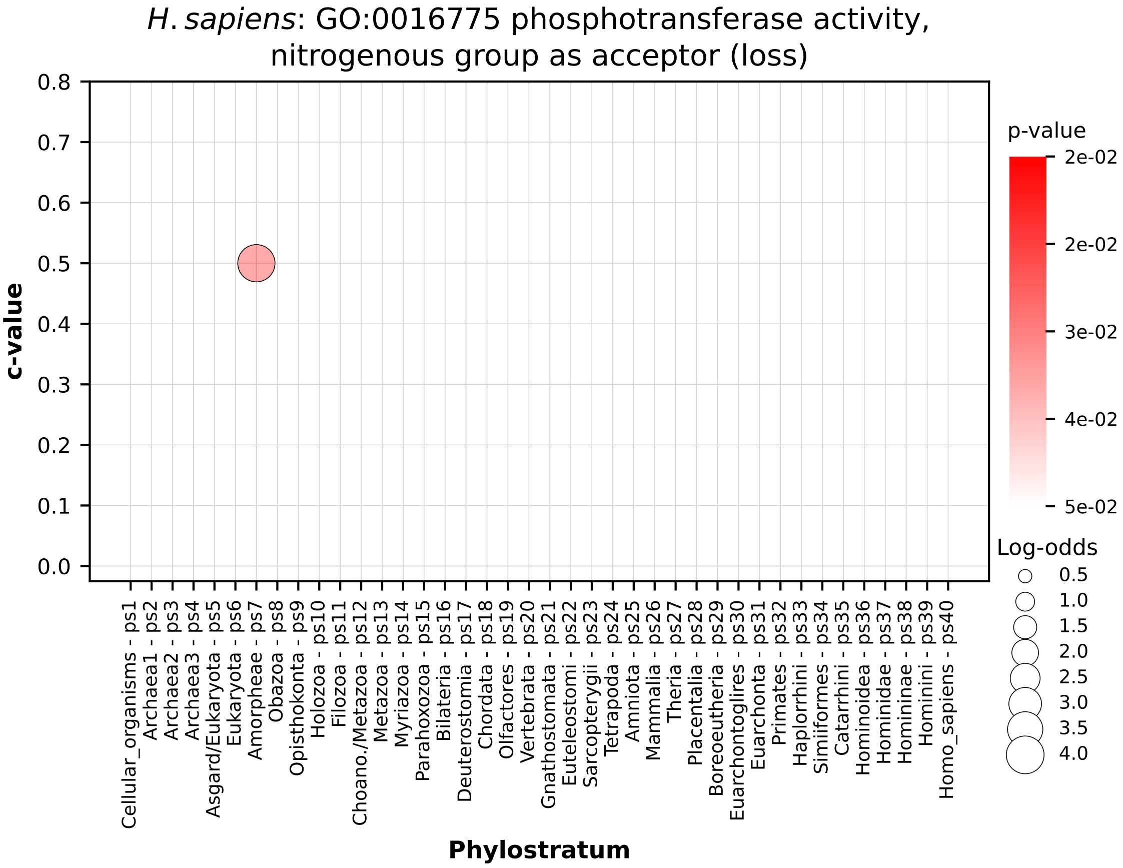

H. sapiens: GO:0016775 phosphotransferase activity, nitrogenous group as acceptor (loss)
c-value
0.8
0.7
0.6
0.5
0.4
0.3
0.2
0.1
0.0
Phylostratum
Cellular_organisms - ps1
Archaea1 - ps2
Archaea2 - ps3
Archaea3 - ps4
Asgard/Eukaryota - ps5
Eukaryota - ps6
Amorpheae - ps7
Obazoa - ps8
Opisthokonta - ps9
Holozoa - ps10
Filozoa - ps11
Choano./Metazoa - ps12
Metazoa - ps13
Myriazoa - ps14
Parahoxozoa - ps15
Bilateria - ps16
Deuterostomia - ps17
Chordata - ps18
Olfactores - ps19
Vertebrata - ps20
Gnathostomata - ps21
Euteleostomi - ps22
Sarcopterygii - ps23
Tetrapoda - ps24
Amniota - ps25
Mammalia - ps26
Theria - ps27
Placentalia - ps28
Boreoeutheria - ps29
Euarchontoglires - ps30
Euarchonta - ps31
Primates - ps32
Haplorrhini - ps33
Simiiformes - ps34
Catarrhini - ps35
Hominoidea - ps36
Hominidae - ps37
Homininae - ps38
Hominini - ps39
Homo_sapiens - ps40
p-value
2e-02
2e-02
3e-02
4e-02
5e-02
Log-odds
0.5
1.0
1.5
2.0
2.5
3.0
3.5
4.0

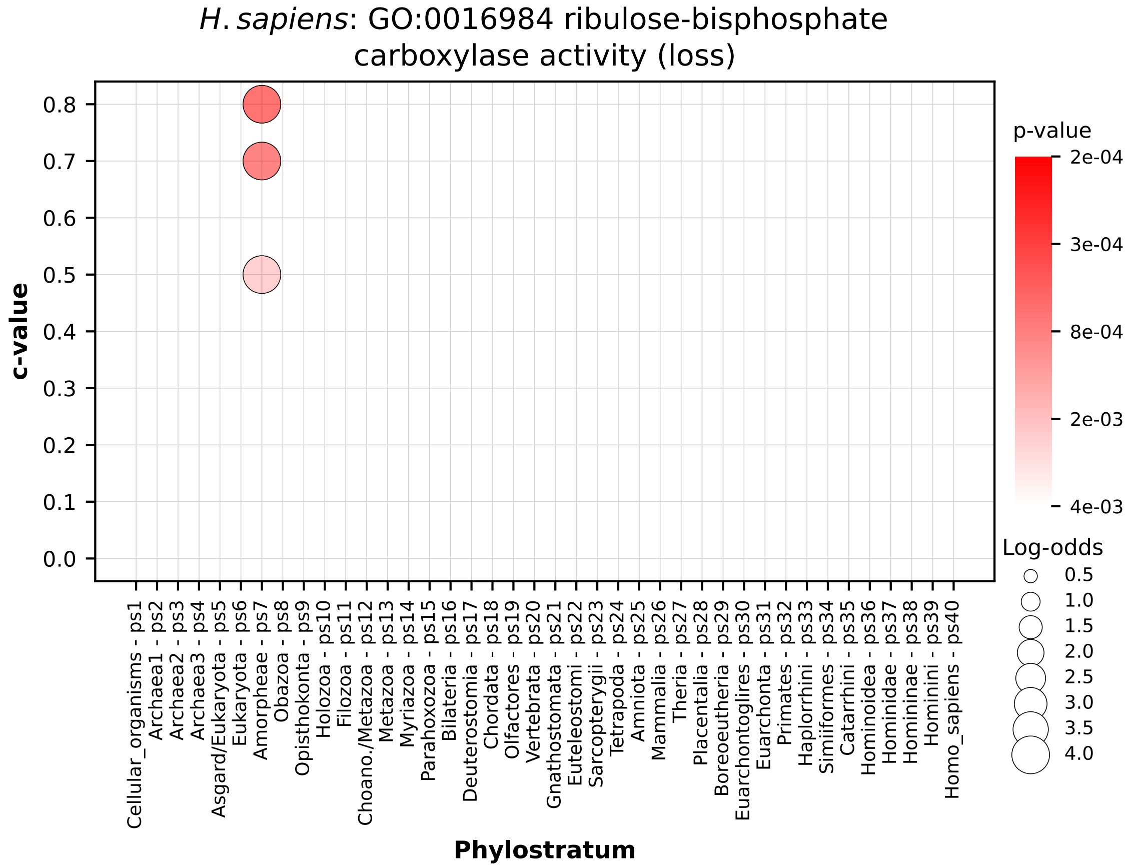
H. sapiens: GO:0016984 ribulose-bisphosphate carboxylase activity (loss)
c-value
0.8
0.7
0.6
0.5
0.4
0.3
0.2
0.1
0.0
Phylostratum
Cellular_organisms - ps1
Archaea1 - ps2
Archaea2 - ps3
Archaea3 - ps4
Asgard/Eukaryota - ps5
Eukaryota - ps6
Amorpheae - ps7
Obazoa - ps8
Opisthokonta - ps9
Holozoa - ps10
Filozoa - ps11
Choano./Metazoa - ps12
Metazoa - ps13
Myriazoa - ps14
Parahoxozoa - ps15
Bilateria - ps16
Deuterostomia - ps17
Chordata - ps18
Olfactores - ps19
Vertebrata - ps20
Gnathostomata - ps21
Euteleostomi - ps22
Sarcopterygii - ps23
Tetrapoda - ps24
Amniota - ps25
Mammalia - ps26
Theria - ps27
Placentalia - ps28
Boreoeutheria - ps29
Euarchontoglires - ps30
Euarchonta - ps31
Primates - ps32
Haplorrhini - ps33
Simiiformes - ps34
Catarrhini - ps35
Hominoidea - ps36
Hominidae - ps37
Homininae - ps38
Hominini - ps39
Homo_sapiens - ps40
p-value
2e-04
3e-04
8e-04
2e-03
4e-03
Log-odds
0.5
1.0
1.5
2.0
2.5
3.0
3.5
4.0

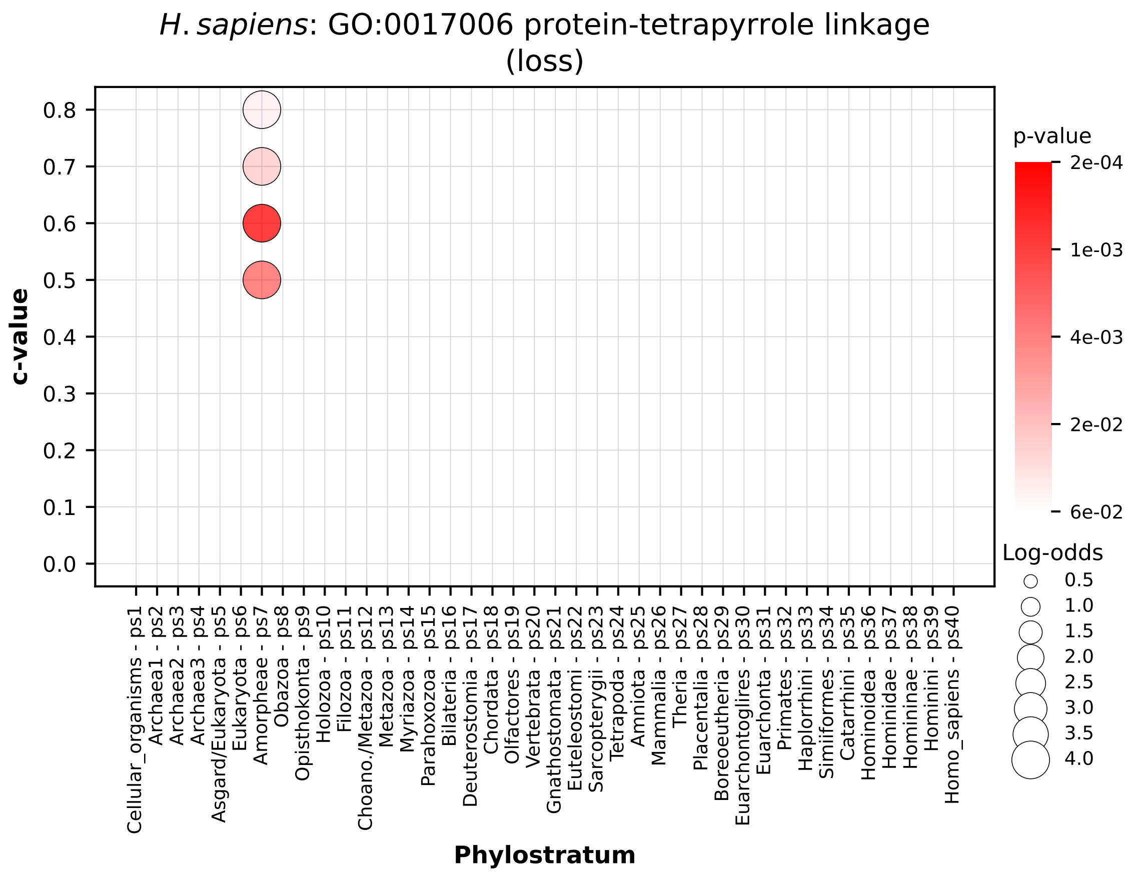

H. sapiens: GO:0017006 protein-tetrapyrrole linkage
(loss)
c-value
0.0
0.1
0.2
0.3
0.4
0.5
0.6
0.7
0.8
Phylostratum
Cellular_organisms - ps1
Archaea1 - ps2
Archaea2 - ps3
Archaea3 - ps4
Asgard/Eukaryota - ps5
Eukaryota - ps6
Amorpheae - ps7
Obazoa - ps8
Opisthokonta - ps9
Holozoa - ps10
Filozoa - ps11
Choano./Metazoa - ps12
Metazoa - ps13
Myriazoa - ps14
Parahoxozoa - ps15
Bilateria - ps16
Deuterostomia - ps17
Chordata - ps18
Olfactores - ps19
Vertebrata - ps20
Gnathostomata - ps21
Euteleostomi - ps22
Sarcopterygii - ps23
Tetrapoda - ps24
Amniota - ps25
Mammalia - ps26
Theria - ps27
Placentalia - ps28
Boreoeutheria - ps29
Euarchontoglires - ps30
Euarchonta - ps31
Primates - ps32
Haplorrhini - ps33
Simiiformes - ps34
Catarrhini - ps35
Hominoidea - ps36
Hominidae - ps37
Homininae - ps38
Hominini - ps39
Homo_sapiens - ps40
p-value
2e-04
1e-03
4e-03
2e-02
6e-02
Log-odds
0.5
1.0
1.5
2.0
2.5
3.0
3.5
4.0

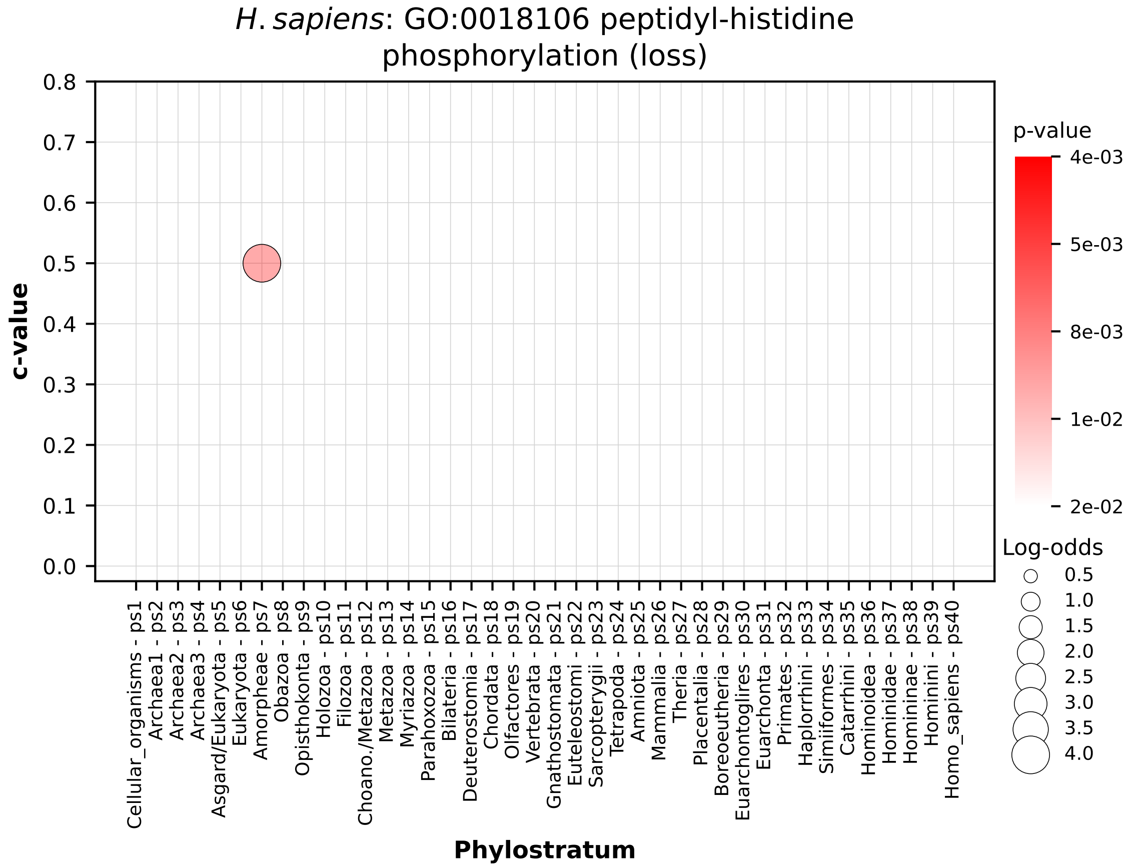

H. sapiens: GO:0018106 peptidyl-histidine phosphorylation (loss)
c-value
0.8
0.7
0.6
0.5
0.4
0.3
0.2
0.1
0.0
Phylostratum
Cellular_organisms - ps1
Archaea1 - ps2
Archaea2 - ps3
Archaea3 - ps4
Asgard/Eukaryota - ps5
Eukaryota - ps6
Amorpheae - ps7
Obazoa - ps8
Opisthokonta - ps9
Holozoa - ps10
Filozoa - ps11
Choano./Metazoa - ps12
Metazoa - ps13
Myriazoa - ps14
Parahoxozoa - ps15
Bilateria - ps16
Deuterostomia - ps17
Chordata - ps18
Olfactores - ps19
Vertebrata - ps20
Gnathostomata - ps21
Euteleostomi - ps22
Sarcopterygii - ps23
Tetrapoda - ps24
Amniota - ps25
Mammalia - ps26
Theria - ps27
Placentalia - ps28
Boreoeutheria - ps29
Euarchontoglires - ps30
Euarchonta - ps31
Primates - ps32
Haplorrhini - ps33
Simiiformes - ps34
Catarrhini - ps35
Hominoidea - ps36
Hominidae - ps37
Homininae - ps38
Hominini - ps39
Homo_sapiens - ps40
p-value
4e-03
5e-03
8e-03
1e-02
2e-02
Log-odds
0.5
1.0
1.5
2.0
2.5
3.0
3.5
4.0

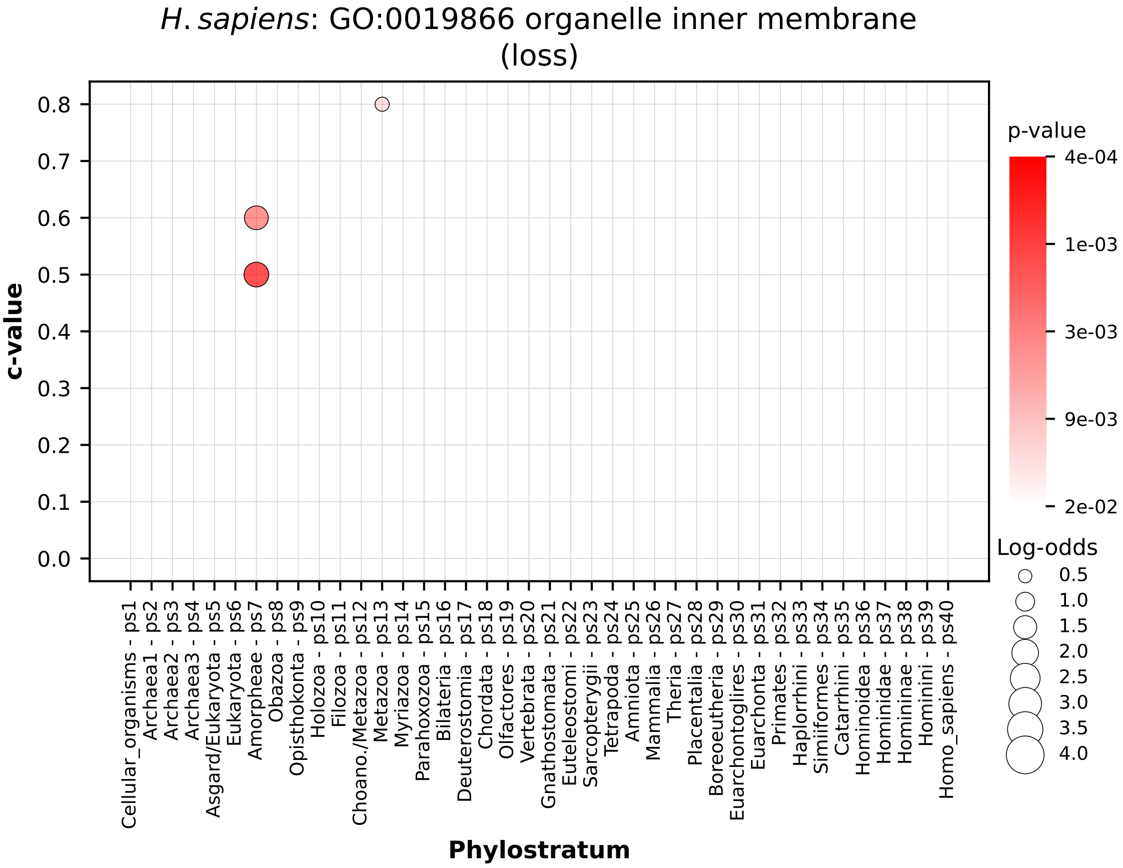

H. sapiens: GO:0019866 organelle inner membrane
(loss)
c-value
0.8
0.7
0.6
0.5
0.4
0.3
0.2
0.1
0.0
p-value
4e-04
1e-03
3e-03
9e-03
2e-02
Log-odds
0.5
1.0
1.5
2.0
2.5
3.0
3.5
4.0
Cellular_organisms - ps1
Archaea1 - ps2
Archaea2 - ps3
Archaea3 - ps4
Asgard/Eukaryota - ps5
Eukaryota - ps6
Amorpheae - ps7
Obazoa - ps8
Opisthokonta - ps9
Holozoa - ps10
Filozoa - ps11
Choano./Metazoa - ps12
Metazoa - ps13
Myriazoa - ps14
Parahoxozoa - ps15
Bilateria - ps16
Deuterostomia - ps17
Chordata - ps18
Olfactores - ps19
Vertebrata - ps20
Gnathostomata - ps21
Euteleostomi - ps22
Sarcopterygii - ps23
Tetrapoda - ps24
Amniota - ps25
Mammalia - ps26
Theria - ps27
Placentalia - ps28
Boreoeutheria - ps29
Euarchontoglires - ps30
Euarchonta - ps31
Primates - ps32
Haplorrhini - ps33
Simiiformes - ps34
Catarrhini - ps35
Hominoidea - ps36
Hominidae - ps37
Homininae - ps38
Hominini - ps39
Homo_sapiens - ps40
Phylostratum

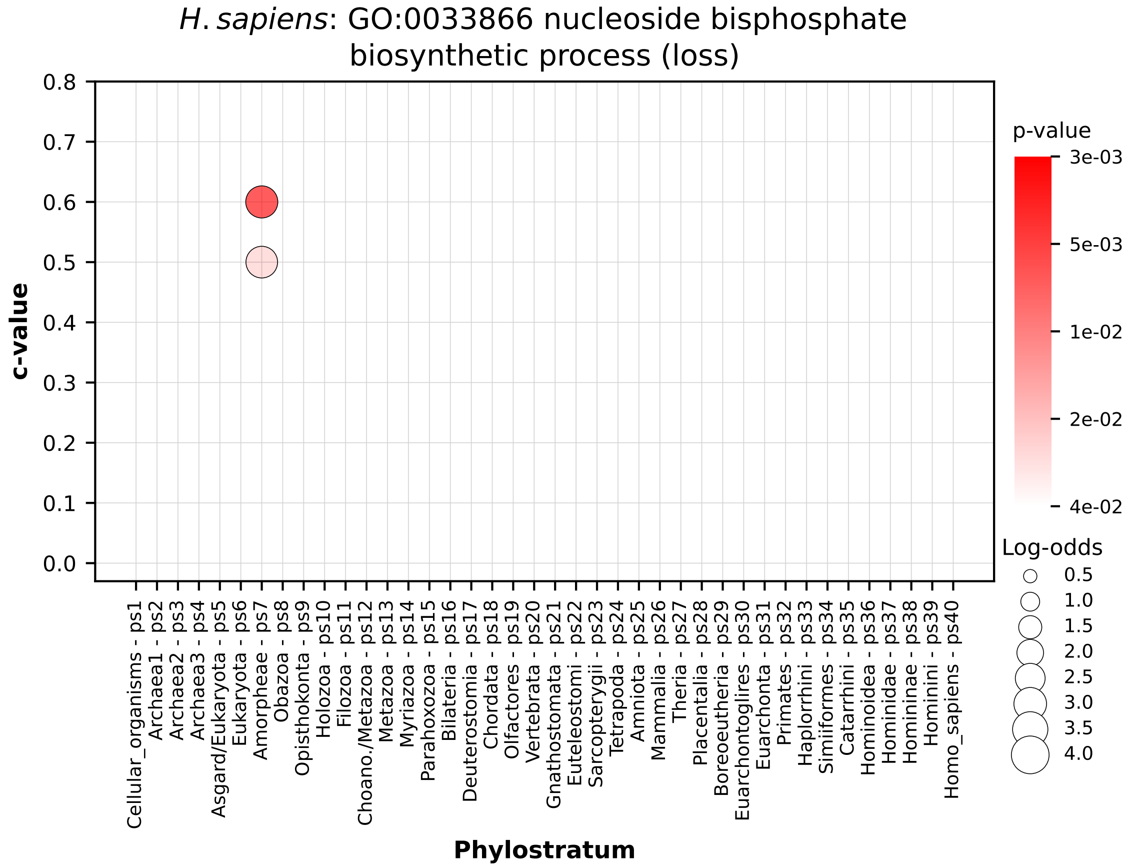

H. sapiens: GO:0033866 nucleoside bisphosphate biosynthetic process (loss)
c-value
0.8
0.7
0.6
0.5
0.4
0.3
0.2
0.1
0.0
p-value
3e-03
5e-03
1e-02
2e-02
4e-02
Log-odds
0.5
1.0
1.5
2.0
2.5
3.0
3.5
4.0
Cellular_organisms - ps1
Archaea1 - ps2
Archaea2 - ps3
Archaea3 - ps4
Asgard/Eukaryota - ps5
Eukaryota - ps6
Amorpheae - ps7
Obazoa - ps8
Opisthokonta - ps9
Holozoa - ps10
Filozoa - ps11
Choano./Metazoa - ps12
Metazoa - ps13
Myriazoa - ps14
Parahoxozoa - ps15
Bilateria - ps16
Deuterostomia - ps17
Chordata - ps18
Olfactores - ps19
Vertebrata - ps20
Gnathostomata - ps21
Euteleostomi - ps22
Sarcopterygii - ps23
Tetrapoda - ps24
Amniota - ps25
Mammalia - ps26
Theria - ps27
Placentalia - ps28
Boreoeutheria - ps29
Euarchontoglires - ps30
Euarchonta - ps31
Primates - ps32
Haplorrhini - ps33
Simiiformes - ps34
Catarrhini - ps35
Hominoidea - ps36
Hominidae - ps37
Homininae - ps38
Hominini - ps39
Homo_sapiens - ps40
Phylostratum

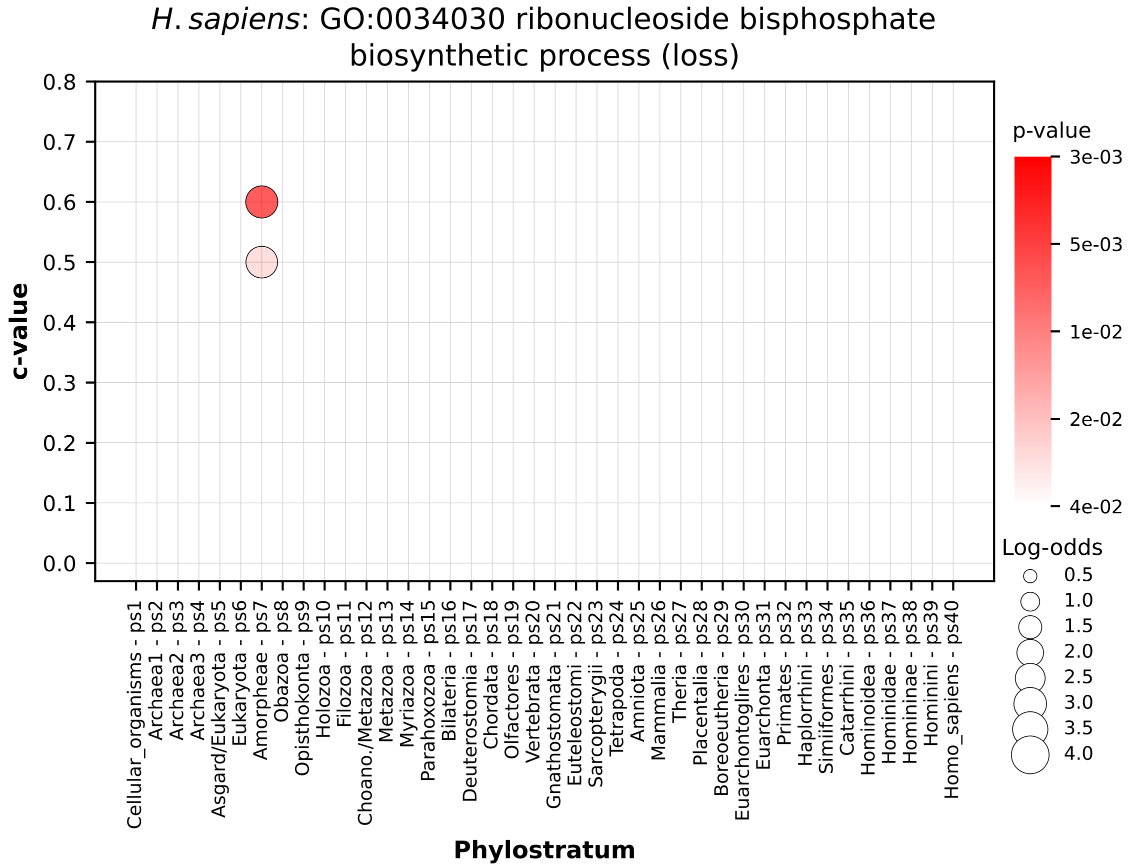

H. sapiens: GO:0034030 ribonucleoside bisphosphate biosynthetic process (loss)
c-value
0.8
0.7
0.6
0.5
0.4
0.3
0.2
0.1
0.0
Phylostratum
Cellular_organisms - ps1
Archaea1 - ps2
Archaea2 - ps3
Archaea3 - ps4
Asgard/Eukaryota - ps5
Eukaryota - ps6
Amorpheae - ps7
Obazoa - ps8
Opisthokonta - ps9
Holozoa - ps10
Filozoa - ps11
Choano./Metazoa - ps12
Metazoa - ps13
Myriazoa - ps14
Parahoxozoa - ps15
Bilateria - ps16
Deuterostomia - ps17
Chordata - ps18
Olfactores - ps19
Vertebrata - ps20
Gnathostomata - ps21
Euteleostomi - ps22
Sarcopterygii - ps23
Tetrapoda - ps24
Amniota - ps25
Mammalia - ps26
Theria - ps27
Placentalia - ps28
Boreoeutheria - ps29
Euarchontoglires - ps30
Euarchonta - ps31
Primates - ps32
Haplorrhini - ps33
Simiiformes - ps34
Catarrhini - ps35
Hominoidea - ps36
Hominidae - ps37
Homininae - ps38
Hominini - ps39
Homo_sapiens - ps40
p-value
3e-03
5e-03
1e-02
2e-02
4e-02
Log-odds
0.5
1.0
1.5
2.0
2.5
3.0
3.5
4.0

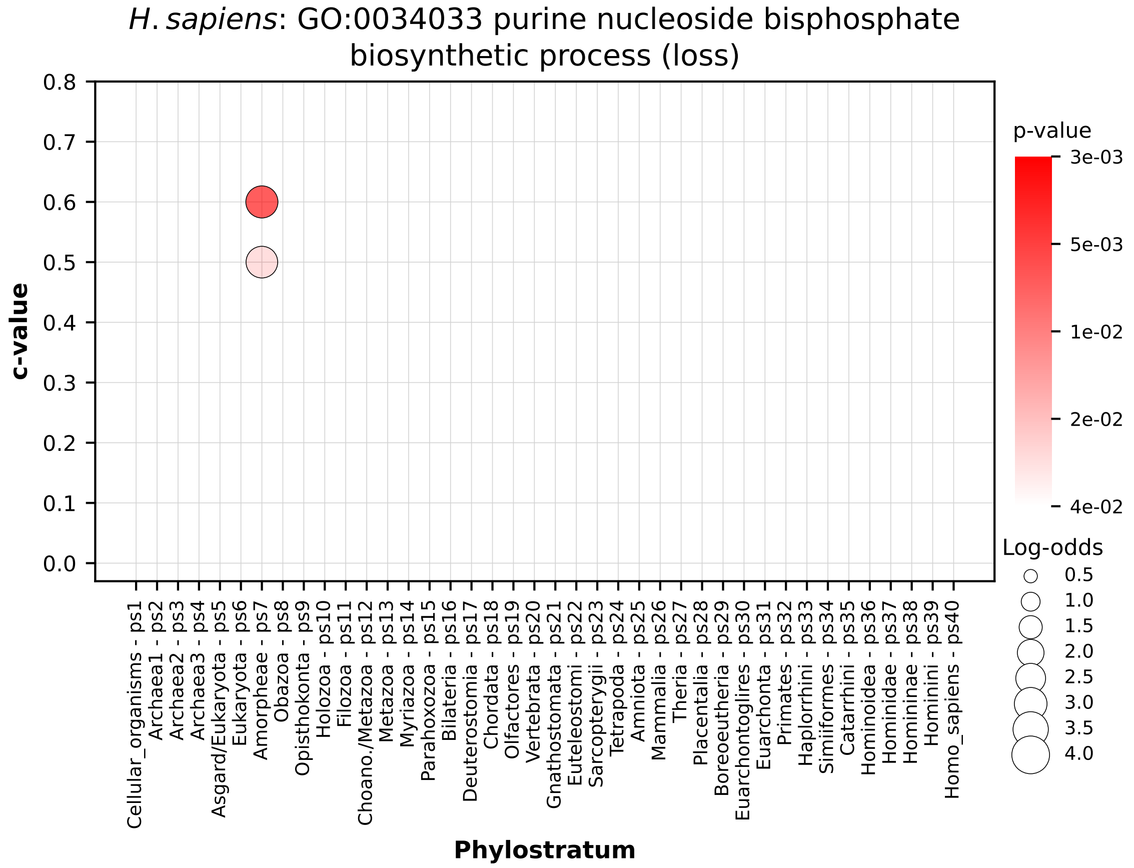

H. sapiens: GO:0034033 purine nucleoside bisphosphate biosynthetic process (loss)
c-value
0.8
0.7
0.6
0.5
0.4
0.3
0.2
0.1
0.0
p-value
3e-03
5e-03
1e-02
2e-02
4e-02
Log-odds
0.5
1.0
1.5
2.0
2.5
3.0
3.5
4.0
Cellular_organisms - ps1
Archaea1 - ps2
Archaea2 - ps3
Archaea3 - ps4
Asgard/Eukaryota - ps5
Eukaryota - ps6
Amorpheae - ps7
Obazoa - ps8
Opisthokonta - ps9
Holozoa - ps10
Filozoa - ps11
Choano./Metazoa - ps12
Metazoa - ps13
Myriazoa - ps14
Parahoxozoa - ps15
Bilateria - ps16
Deuterostomia - ps17
Chordata - ps18
Olfactores - ps19
Vertebrata - ps20
Gnathostomata - ps21
Euteleostomi - ps22
Sarcopterygii - ps23
Tetrapoda - ps24
Amniota - ps25
Mammalia - ps26
Theria - ps27
Placentalia - ps28
Boreoeutheria - ps29
Euarchontoglires - ps30
Euarchonta - ps31
Primates - ps32
Haplorrhini - ps33
Simiiformes - ps34
Catarrhini - ps35
Hominoidea - ps36
Hominidae - ps37
Homininae - ps38
Hominini - ps39
Homo_sapiens - ps40
Phylostratum

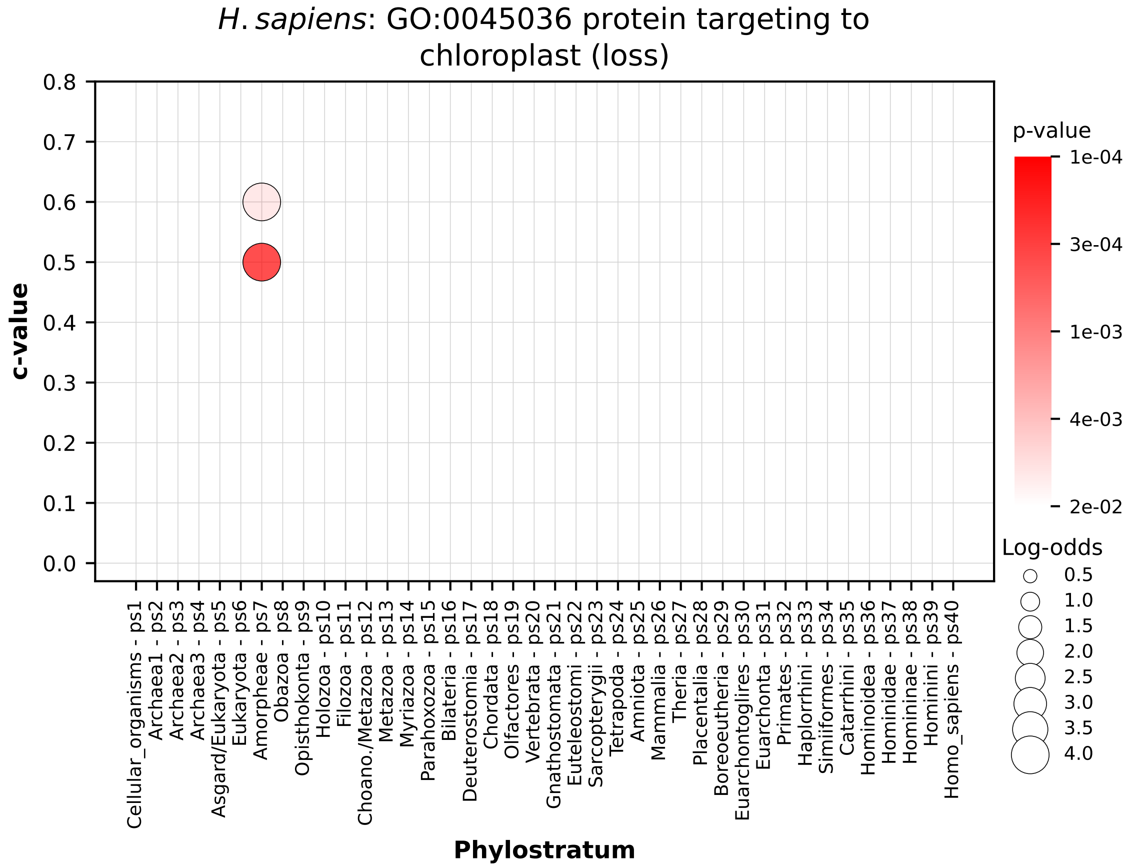

H. sapiens: GO:0045036 protein targeting to chloroplast (loss)
c-value
0.0
0.1
0.2
0.3
0.4
0.5
0.6
0.7
0.8
Phylostratum
Cellular_organisms - ps1
Archaea1 - ps2
Archaea2 - ps3
Archaea3 - ps4
Asgard/Eukaryota - ps5
Eukaryota - ps6
Amorpheae - ps7
Obazoa - ps8
Opisthokonta - ps9
Holozoa - ps10
Filozoa - ps11
Choano./Metazoa - ps12
Metazoa - ps13
Myriazoa - ps14
Parahoxozoa - ps15
Bilateria - ps16
Deuterostomia - ps17
Chordata - ps18
Olfactores - ps19
Vertebrata - ps20
Gnathostomata - ps21
Euteleostomi - ps22
Sarcopterygii - ps23
Tetrapoda - ps24
Amniota - ps25
Mammalia - ps26
Theria - ps27
Placentalia - ps28
Boreoeutheria - ps29
Euarchontoglires - ps30
Euarchonta - ps31
Primates - ps32
Haplorrhini - ps33
Simiiformes - ps34
Catarrhini - ps35
Hominoidea - ps36
Hominidae - ps37
Homininae - ps38
Hominini - ps39
Homo_sapiens - ps40
p-value
1e-04
3e-04
1e-03
4e-03
2e-02
Log-odds
0.5
1.0
1.5
2.0
2.5
3.0
3.5
4.0

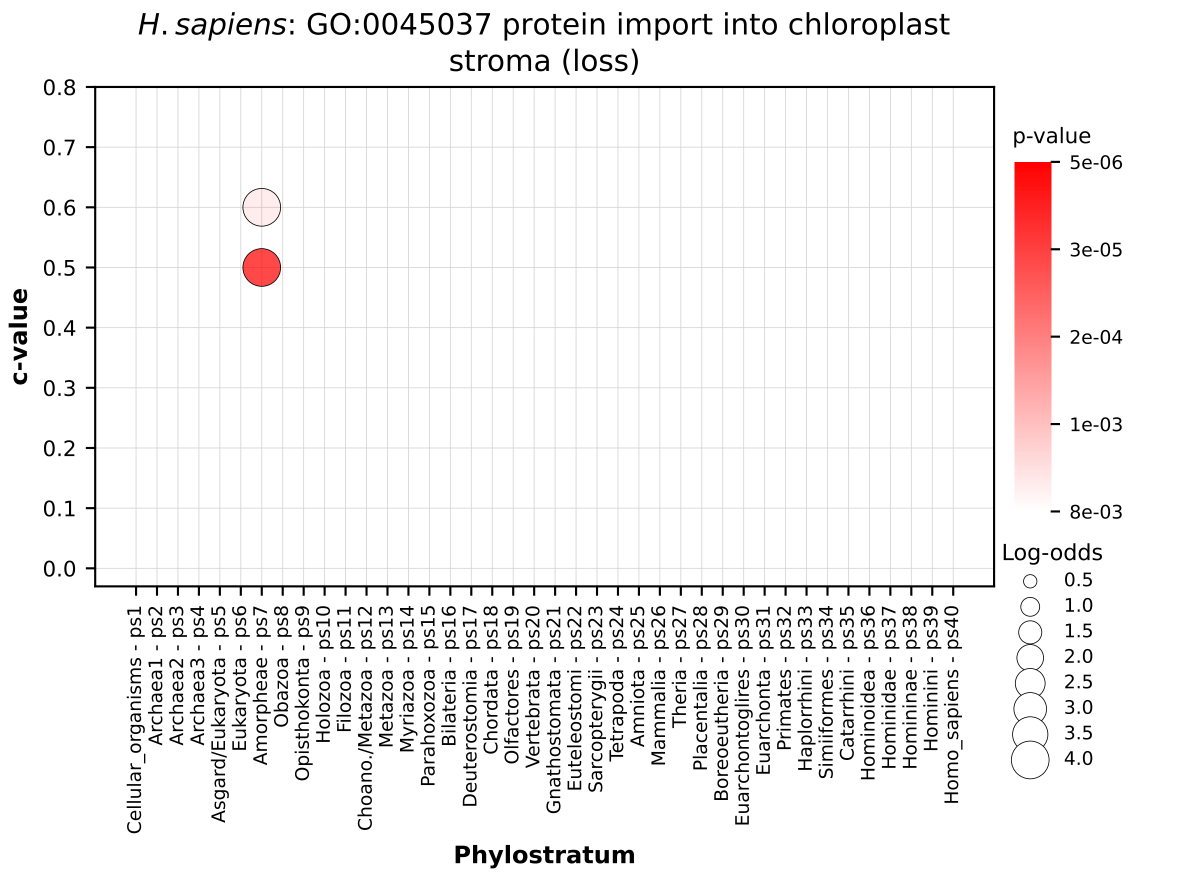

H. sapiens: GO:0045037 protein import into chloroplast stroma (loss)
c-value
0.0
0.1
0.2
0.3
0.4
0.5
0.6
0.7
0.8
Phylostratum
Cellular_organisms - ps1
Archaea1 - ps2
Archaea2 - ps3
Archaea3 - ps4
Asgard/Eukaryota - ps5
Eukaryota - ps6
Amorpheae - ps7
Obazoa - ps8
Opisthokonta - ps9
Holozoa - ps10
Filozoa - ps11
Choano./Metazoa - ps12
Metazoa - ps13
Myriazoa - ps14
Parahoxozoa - ps15
Bilateria - ps16
Deuterostomia - ps17
Chordata - ps18
Olfactores - ps19
Vertebrata - ps20
Gnathostomata - ps21
Euteleostomi - ps22
Sarcopterygii - ps23
Tetrapoda - ps24
Amniota - ps25
Mammalia - ps26
Theria - ps27
Placentalia - ps28
Boreoeutheria - ps29
Euarchontoglires - ps30
Euarchonta - ps31
Primates - ps32
Haplorrhini - ps33
Simiiformes - ps34
Catarrhini - ps35
Hominoidea - ps36
Hominidae - ps37
Homininae - ps38
Hominini - ps39
Homo_sapiens - ps40
p-value
5e-06
3e-05
2e-04
1e-03
8e-03
Log-odds
0.5
1.0
1.5
2.0
2.5
3.0
3.5
4.0

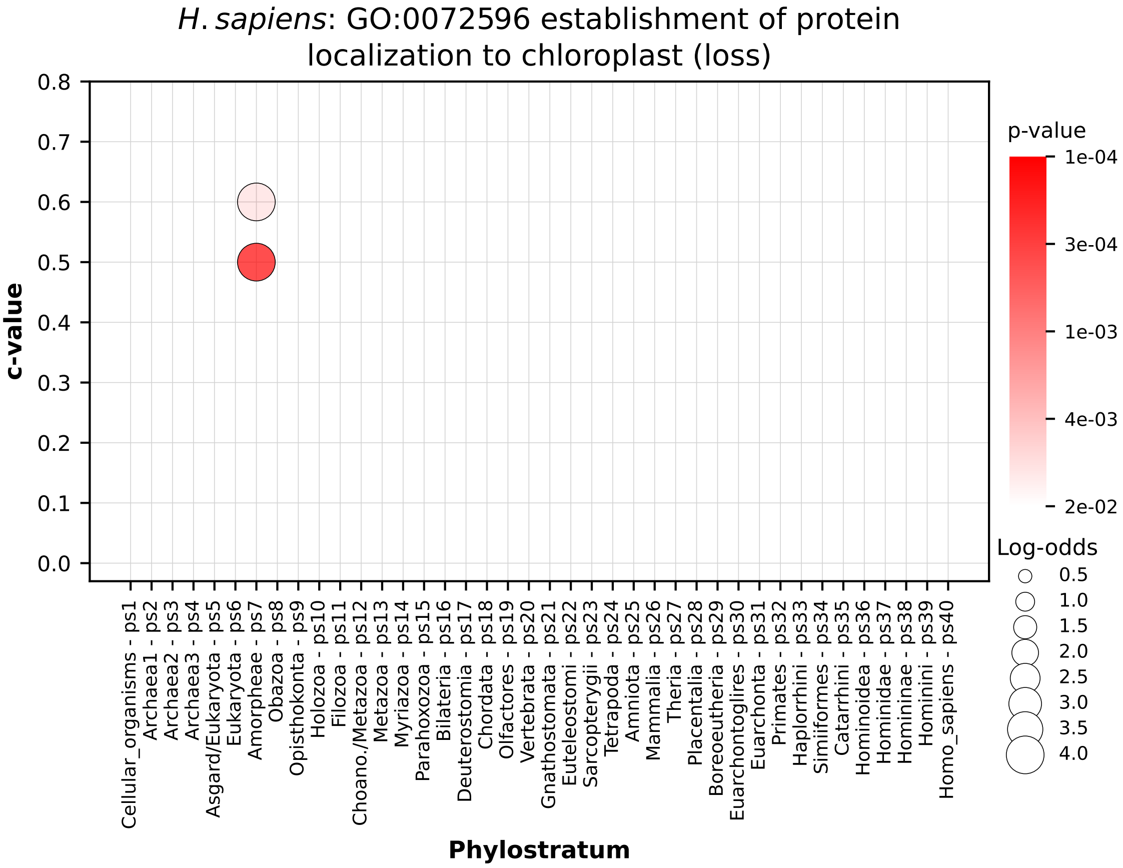

H. sapiens: GO:0072596 establishment of protein localization to chloroplast (loss)
c-value
0.0
0.1
0.2
0.3
0.4
0.5
0.6
0.7
0.8
Phylostratum
Cellular_organisms - ps1
Archaea1 - ps2
Archaea2 - ps3
Archaea3 - ps4
Asgard/Eukaryota - ps5
Eukaryota - ps6
Amorpheae - ps7
Obazoa - ps8
Opisthokonta - ps9
Holozoa - ps10
Filozoa - ps11
Choano./Metazoa - ps12
Metazoa - ps13
Myriazoa - ps14
Parahoxozoa - ps15
Bilateria - ps16
Deuterostomia - ps17
Chordata - ps18
Olfactores - ps19
Vertebrata - ps20
Gnathostomata - ps21
Euteleostomi - ps22
Sarcopterygii - ps23
Tetrapoda - ps24
Amniota - ps25
Mammalia - ps26
Theria - ps27
Placentalia - ps28
Boreoeutheria - ps29
Euarchontoglires - ps30
Euarchonta - ps31
Primates - ps32
Haplorrhini - ps33
Simiiformes - ps34
Catarrhini - ps35
Hominoidea - ps36
Hominidae - ps37
Homininae - ps38
Hominini - ps39
Homo_sapiens - ps40
p-value
1e-04
3e-04
1e-03
4e-03
2e-02
Log-odds
0.5
1.0
1.5
2.0
2.5
3.0
3.5
4.0

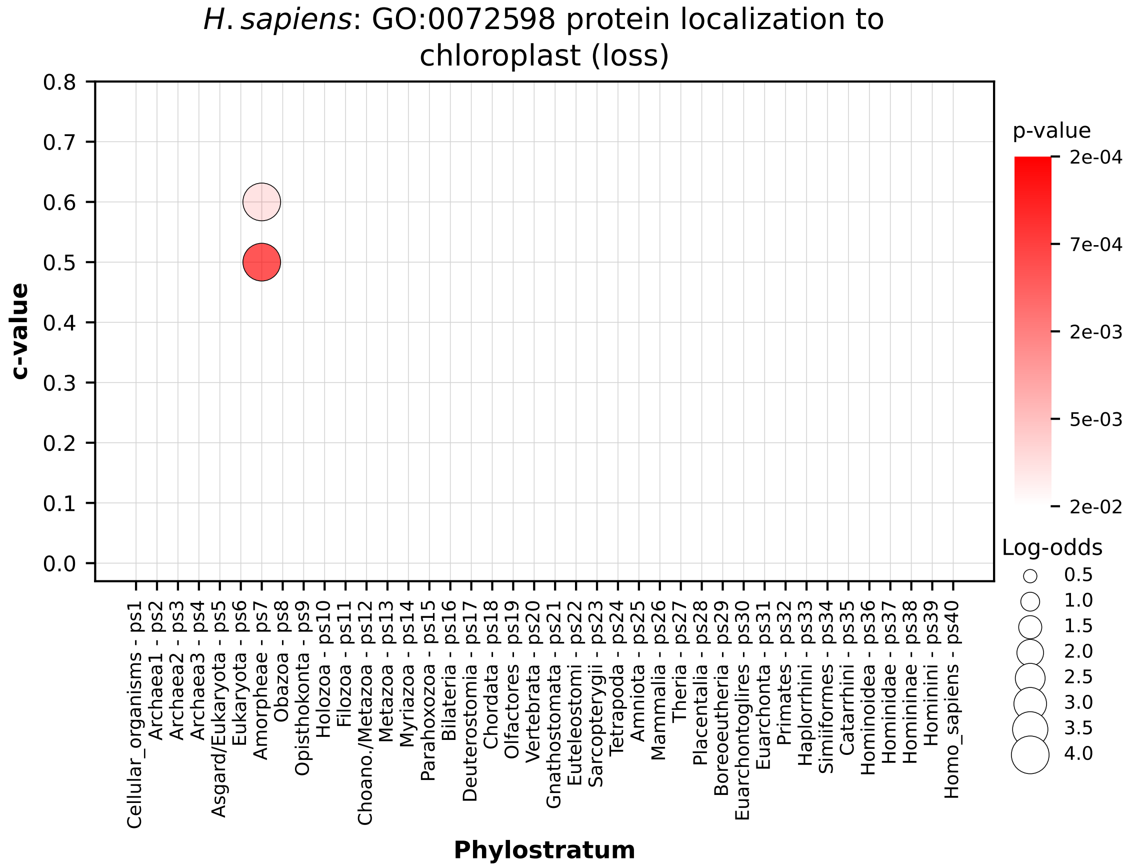

H. sapiens: GO:0072598 protein localization to chloroplast (loss)
c-value
0.0
0.1
0.2
0.3
0.4
0.5
0.6
0.7
0.8
Phylostratum
Cellular_organisms - ps1
Archaea1 - ps2
Archaea2 - ps3
Archaea3 - ps4
Asgard/Eukaryota - ps5
Eukaryota - ps6
Amorpheae - ps7
Obazoa - ps8
Opisthokonta - ps9
Holozoa - ps10
Filozoa - ps11
Choano./Metazoa - ps12
Metazoa - ps13
Myriazoa - ps14
Parahoxozoa - ps15
Bilateria - ps16
Deuterostomia - ps17
Chordata - ps18
Olfactores - ps19
Vertebrata - ps20
Gnathostomata - ps21
Euteleostomi - ps22
Sarcopterygii - ps23
Tetrapoda - ps24
Amniota - ps25
Mammalia - ps26
Theria - ps27
Placentalia - ps28
Boreoeutheria - ps29
Euarchontoglires - ps30
Euarchonta - ps31
Primates - ps32
Haplorrhini - ps33
Simiiformes - ps34
Catarrhini - ps35
Hominoidea - ps36
Hominidae - ps37
Homininae - ps38
Hominini - ps39
Homo_sapiens - ps40
p-value
2e-04
7e-04
2e-03
5e-03
2e-02
Log-odds
0.5
1.0
1.5
2.0
2.5
3.0
3.5
4.0

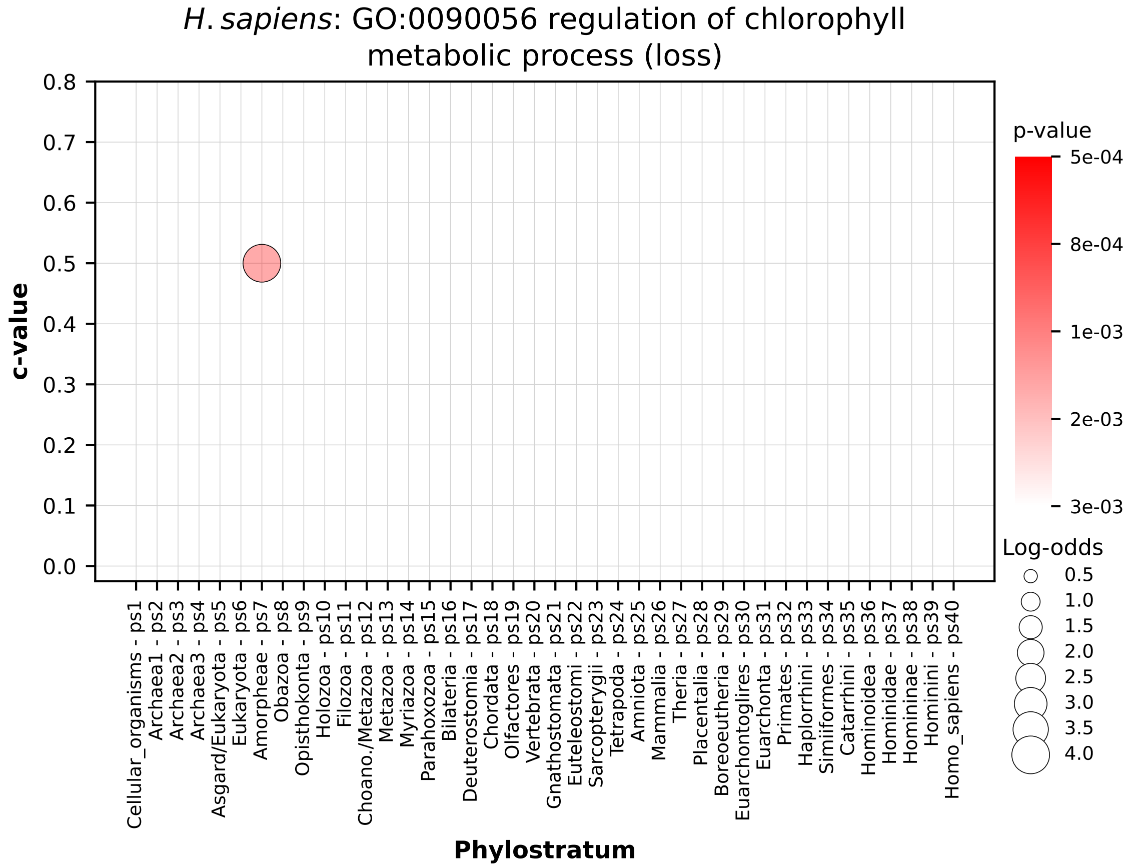

H. sapiens: GO:0090056 regulation of chlorophyll metabolic process (loss)
c-value
0.0
0.1
0.2
0.3
0.4
0.5
0.6
0.7
0.8
Phylostratum
Cellular_organisms - ps1
Archaea1 - ps2
Archaea2 - ps3
Archaea3 - ps4
Asgard/Eukaryota - ps5
Eukaryota - ps6
Amorpheae - ps7
Obazoa - ps8
Opisthokonta - ps9
Holozoa - ps10
Filozoa - ps11
Choano./Metazoa - ps12
Metazoa - ps13
Myriazoa - ps14
Parahoxozoa - ps15
Bilateria - ps16
Deuterostomia - ps17
Chordata - ps18
Olfactores - ps19
Vertebrata - ps20
Gnathostomata - ps21
Euteleostomi - ps22
Sarcopterygii - ps23
Tetrapoda - ps24
Amniota - ps25
Mammalia - ps26
Theria - ps27
Placentalia - ps28
Boreoeutheria - ps29
Euarchontoglires - ps30
Euarchonta - ps31
Primates - ps32
Haplorrhini - ps33
Simiiformes - ps34
Catarrhini - ps35
Hominoidea - ps36
Hominidae - ps37
Homininae - ps38
Hominini - ps39
Homo_sapiens - ps40
p-value
5e-04
8e-04
1e-03
2e-03
3e-03
Log-odds
0.5
1.0
1.5
2.0
2.5
3.0
3.5
4.0

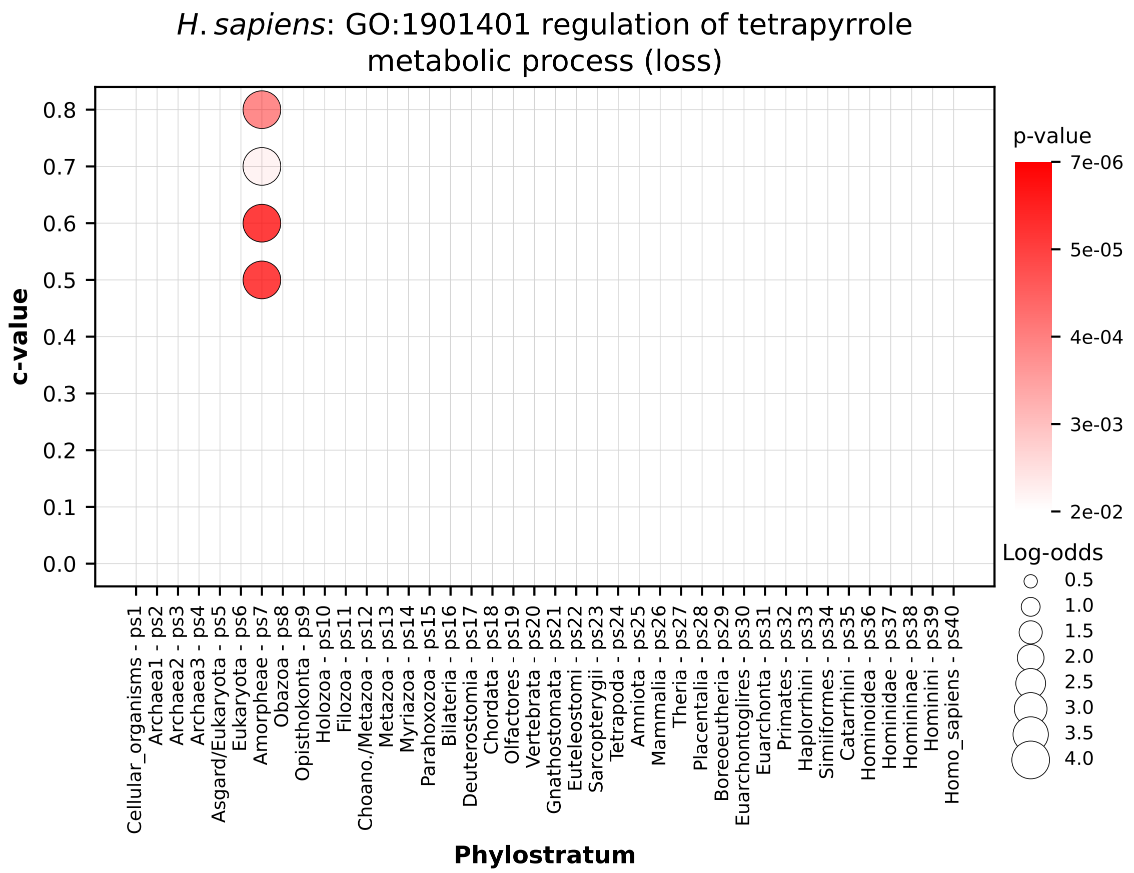
H. sapiens: GO:1901401 regulation of tetrapyrrole metabolic process (loss)
c-value
0.0
0.1
0.2
0.3
0.4
0.5
0.6
0.7
0.8
Phylostratum
Cellular_organisms - ps1
Archaea1 - ps2
Archaea2 - ps3
Archaea3 - ps4
Asgard/Eukaryota - ps5
Eukaryota - ps6
Amorpheae - ps7
Obazoa - ps8
Opisthokonta - ps9
Holozoa - ps10
Filozoa - ps11
Choano./Metazoa - ps12
Metazoa - ps13
Myriazoa - ps14
Parahoxozoa - ps15
Bilateria - ps16
Deuterostomia - ps17
Chordata - ps18
Olfactores - ps19
Vertebrata - ps20
Gnathostomata - ps21
Euteleostomi - ps22
Sarcopterygii - ps23
Tetrapoda - ps24
Amniota - ps25
Mammalia - ps26
Theria - ps27
Placentalia - ps28
Boreoeutheria - ps29
Euarchontoglires - ps30
Euarchonta - ps31
Primates - ps32
Haplorrhini - ps33
Simiiformes - ps34
Catarrhini - ps35
Hominoidea - ps36
Hominidae - ps37
Homininae - ps38
Hominini - ps39
Homo_sapiens - ps40
p-value
7e-06
5e-05
4e-04
3e-03
2e-02
Log-odds
0.5
1.0
1.5
2.0
2.5
3.0
3.5
4.0

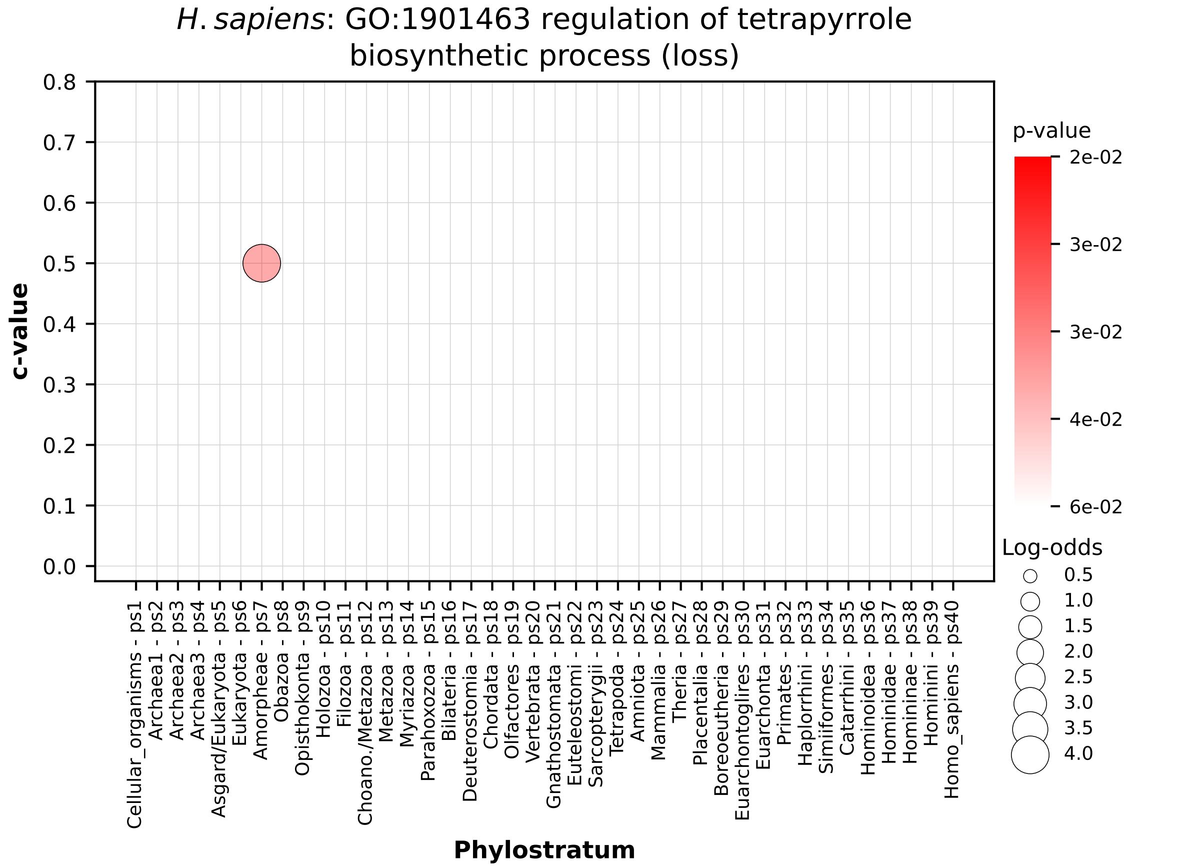

H. sapiens: GO:1901463 regulation of tetrapyrrole biosynthetic process (loss)
c-value
0.8
0.7
0.6
0.5
0.4
0.3
0.2
0.1
0.0
p-value
2e-02
3e-02
3e-02
4e-02
6e-02
Log-odds
0.5
1.0
1.5
2.0
2.5
3.0
3.5
4.0
Cellular_organisms - ps1
Archaea1 - ps2
Archaea2 - ps3
Archaea3 - ps4
Asgard/Eukaryota - ps5
Eukaryota - ps6
Amorpheae - ps7
Obazoa - ps8
Opisthokonta - ps9
Holozoa - ps10
Filozoa - ps11
Choano./Metazoa - ps12
Metazoa - ps13
Myriazoa - ps14
Parahoxozoa - ps15
Bilateria - ps16
Deuterostomia - ps17
Chordata - ps18
Olfactores - ps19
Vertebrata - ps20
Gnathostomata - ps21
Euteleostomi - ps22
Sarcopterygii - ps23
Tetrapoda - ps24
Amniota - ps25
Mammalia - ps26
Theria - ps27
Placentalia - ps28
Boreoeutheria - ps29
Euarchontoglires - ps30
Euarchonta - ps31
Primates - ps32
Haplorrhini - ps33
Simiiformes - ps34
Catarrhini - ps35
Hominoidea - ps36
Hominidae - ps37
Homininae - ps38
Hominini - ps39
Homo_sapiens - ps40
Phylostratum

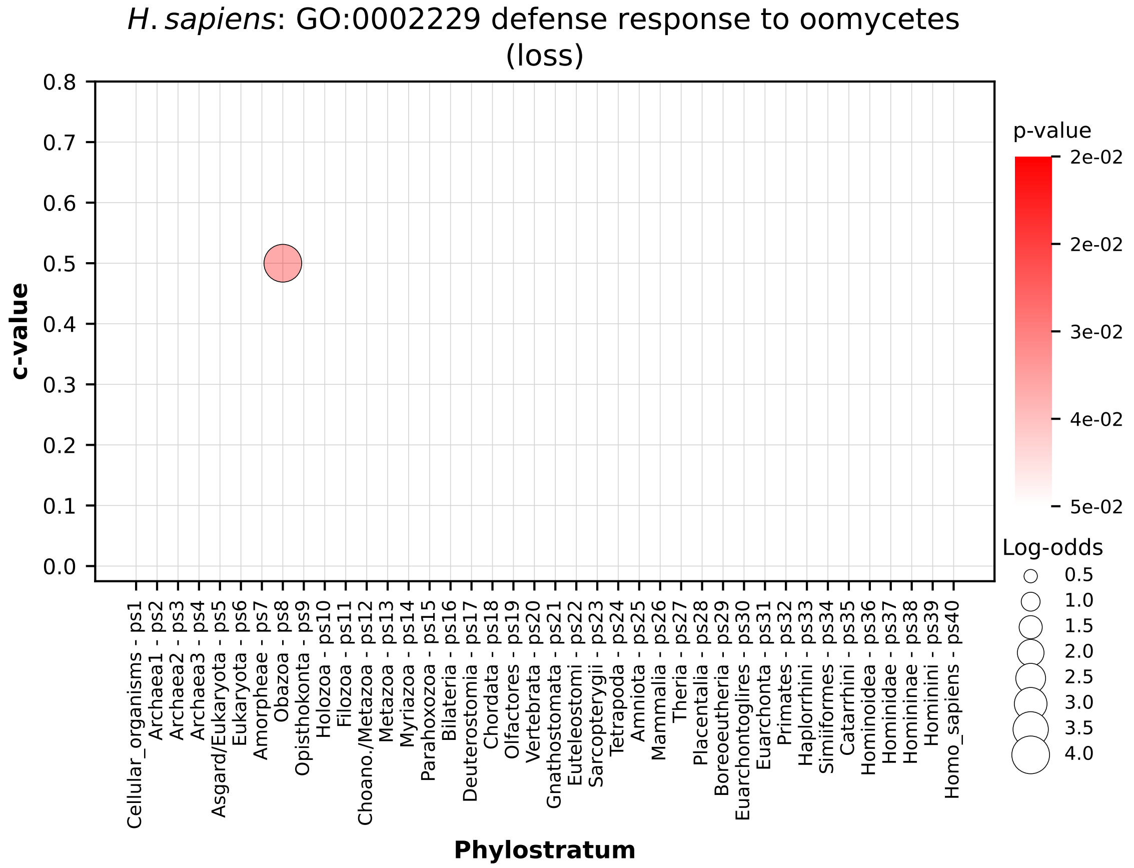

H. sapiens: GO:0002229 defense response to oomycetes
(loss)
c-value
0.8
0.7
0.6
0.5
0.4
0.3
0.2
0.1
0.0
Phylostratum
Cellular_organisms - ps1
Archaea1 - ps2
Archaea2 - ps3
Archaea3 - ps4
Asgard/Eukaryota - ps5
Eukaryota - ps6
Amorpheae - ps7
Obazoa - ps8
Opisthokonta - ps9
Holozoa - ps10
Filozoa - ps11
Choano./Metazoa - ps12
Metazoa - ps13
Myriazoa - ps14
Parahoxozoa - ps15
Bilateria - ps16
Deuterostomia - ps17
Chordata - ps18
Olfactores - ps19
Vertebrata - ps20
Gnathostomata - ps21
Euteleostomi - ps22
Sarcopterygii - ps23
Tetrapoda - ps24
Amniota - ps25
Mammalia - ps26
Theria - ps27
Placentalia - ps28
Boreoeutheria - ps29
Euarchontoglires - ps30
Euarchonta - ps31
Primates - ps32
Haplorrhini - ps33
Simiiformes - ps34
Catarrhini - ps35
Hominoidea - ps36
Hominidae - ps37
Homininae - ps38
Hominini - ps39
Homo_sapiens - ps40
p-value
2e-02
2e-02
3e-02
4e-02
5e-02
Log-odds
0.5
1.0
1.5
2.0
2.5
3.0
3.5
4.0

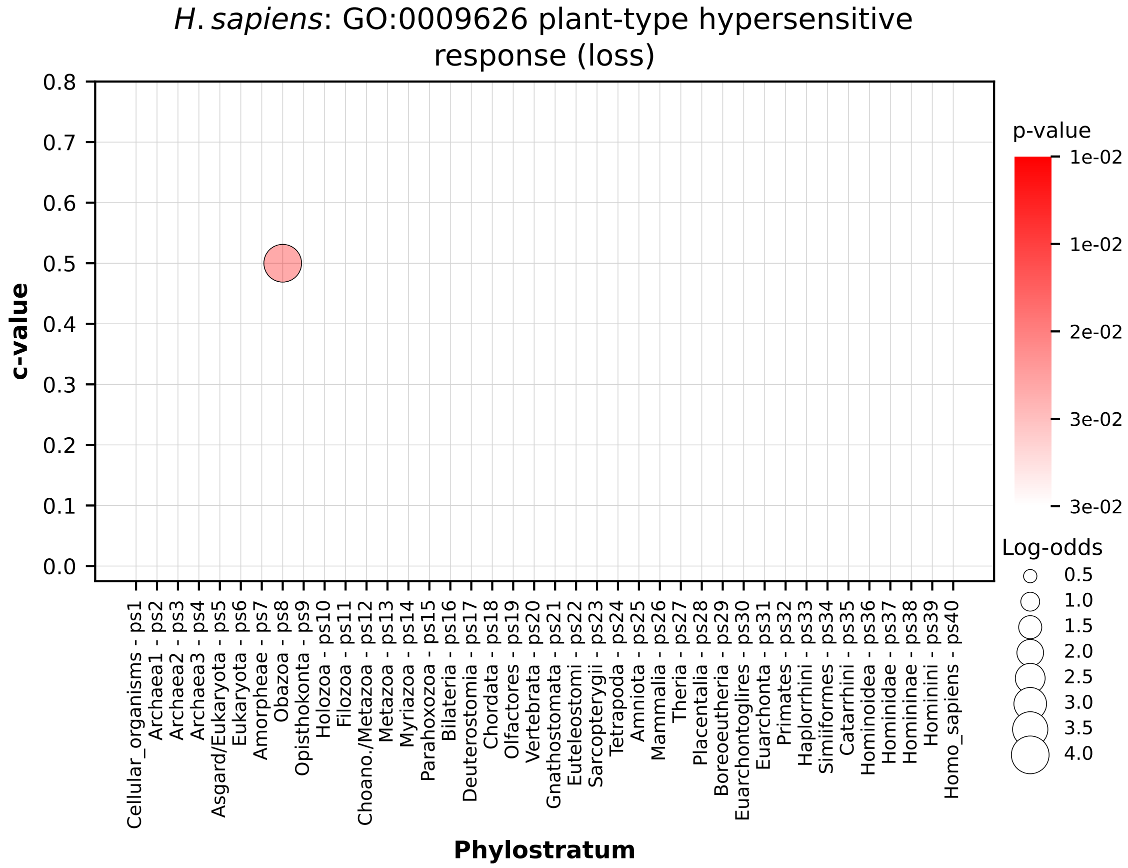

H. sapiens: GO:0009626 plant-type hypersensitive response (loss)
c-value
0.8
0.7
0.6
0.5
0.4
0.3
0.2
0.1
0.0
p-value
1e-02
1e-02
2e-02
3e-02
3e-02
Log-odds
0.5
1.0
1.5
2.0
2.5
3.0
3.5
4.0
Cellular_organisms - ps1
Archaea1 - ps2
Archaea2 - ps3
Archaea3 - ps4
Asgard/Eukaryota - ps5
Eukaryota - ps6
Amorpheae - ps7
Obazoa - ps8
Opisthokonta - ps9
Holozoa - ps10
Filozoa - ps11
Choano./Metazoa - ps12
Metazoa - ps13
Myriazoa - ps14
Parahoxozoa - ps15
Bilateria - ps16
Deuterostomia - ps17
Chordata - ps18
Olfactores - ps19
Vertebrata - ps20
Gnathostomata - ps21
Euteleostomi - ps22
Sarcopterygii - ps23
Tetrapoda - ps24
Amniota - ps25
Mammalia - ps26
Theria - ps27
Placentalia - ps28
Boreoeutheria - ps29
Euarchontoglires - ps30
Euarchonta - ps31
Primates - ps32
Haplorrhini - ps33
Simiiformes - ps34
Catarrhini - ps35
Hominoidea - ps36
Hominidae - ps37
Homininae - ps38
Hominini - ps39
Homo_sapiens - ps40
Phylostratum

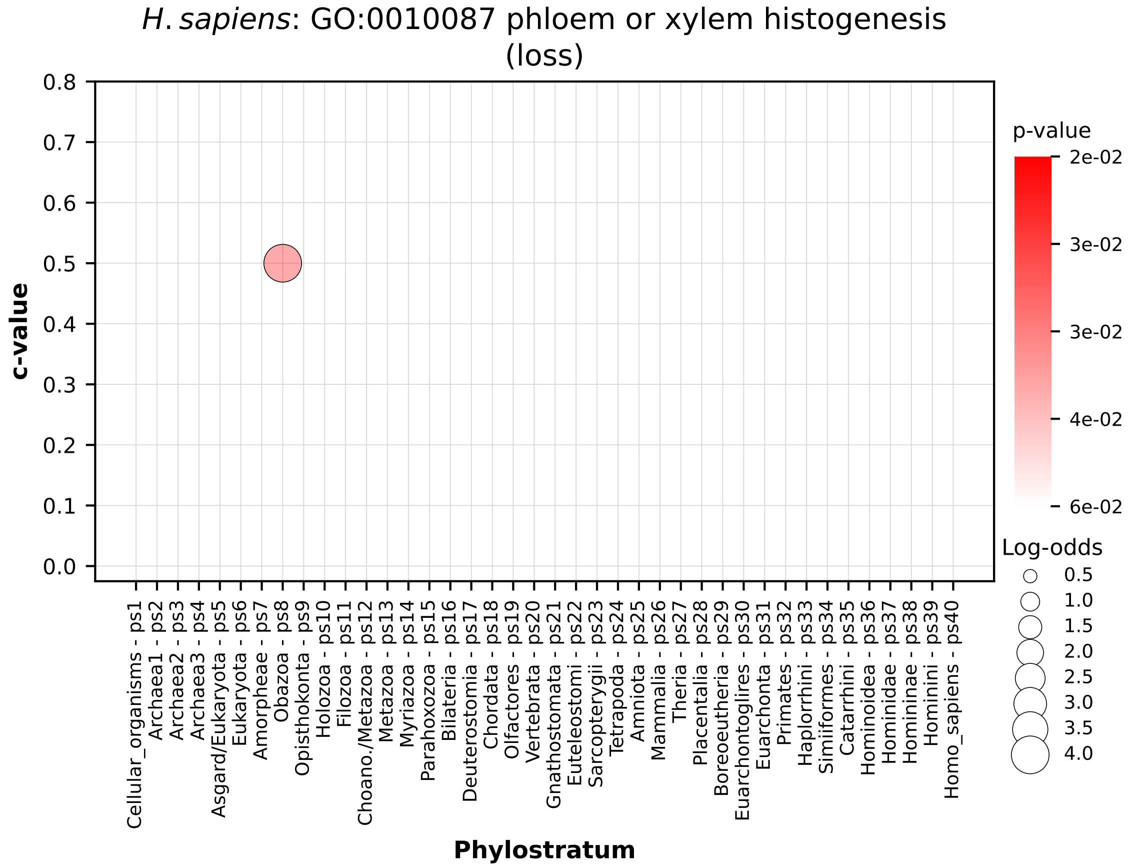
H. sapiens: GO:0010087 phloem or xylem histogenesis
(loss)
c-value
0.8
0.7
0.6
0.5
0.4
0.3
0.2
0.1
0.0
p-value
2e-02
3e-02
3e-02
4e-02
6e-02
Log-odds
0.5
1.0
1.5
2.0
2.5
3.0
3.5
4.0
Cellular_organisms - ps1
Archaea1 - ps2
Archaea2 - ps3
Archaea3 - ps4
Asgard/Eukaryota - ps5
Eukaryota - ps6
Amorpheae - ps7
Obazoa - ps8
Opisthokonta - ps9
Holozoa - ps10
Filozoa - ps11
Choano./Metazoa - ps12
Metazoa - ps13
Myriazoa - ps14
Parahoxozoa - ps15
Bilateria - ps16
Deuterostomia - ps17
Chordata - ps18
Olfactores - ps19
Vertebrata - ps20
Gnathostomata - ps21
Euteleostomi - ps22
Sarcopterygii - ps23
Tetrapoda - ps24
Amniota - ps25
Mammalia - ps26
Theria - ps27
Placentalia - ps28
Boreoeutheria - ps29
Euarchontoglires - ps30
Euarchonta - ps31
Primates - ps32
Haplorrhini - ps33
Simiiformes - ps34
Catarrhini - ps35
Hominoidea - ps36
Hominidae - ps37
Homininae - ps38
Hominini - ps39
Homo_sapiens - ps40
Phylostratum

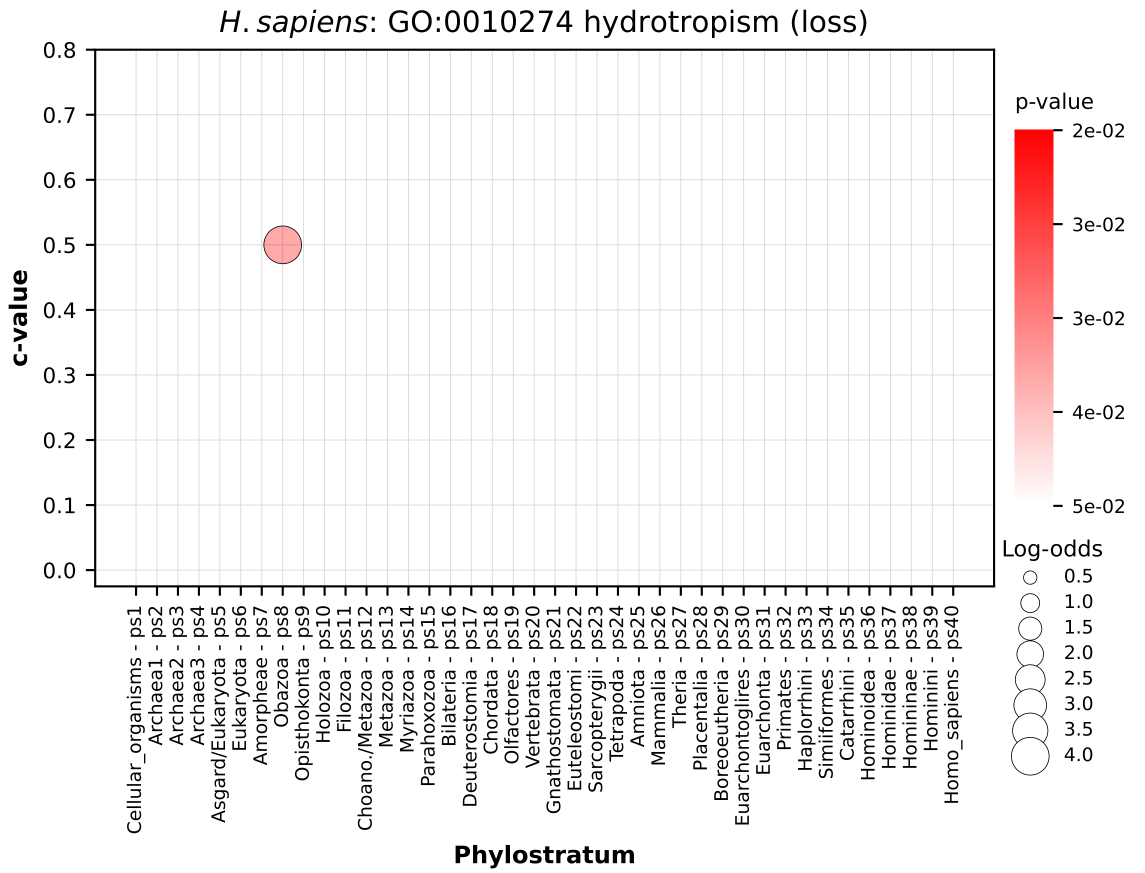

H. sapiens: GO:0010274 hydrotropism (loss)
c-value
0.8
0.7
0.6
0.5
0.4
0.3
0.2
0.1
0.0
p-value
2e-02
3e-02
3e-02
4e-02
5e-02
Log-odds
0.5
1.0
1.5
2.0
2.5
3.0
3.5
4.0
Cellular_organisms - ps1
Archaea1 - ps2
Archaea2 - ps3
Archaea3 - ps4
Asgard/Eukaryota - ps5
Eukaryota - ps6
Amorpheae - ps7
Obazoa - ps8
Opisthokonta - ps9
Holozoa - ps10
Filozoa - ps11
Choano./Metazoa - ps12
Metazoa - ps13
Myriazoa - ps14
Parahoxozoa - ps15
Bilateria - ps16
Deuterostomia - ps17
Chordata - ps18
Olfactores - ps19
Vertebrata - ps20
Gnathostomata - ps21
Euteleostomi - ps22
Sarcopterygii - ps23
Tetrapoda - ps24
Amniota - ps25
Mammalia - ps26
Theria - ps27
Placentalia - ps28
Boreoeutheria - ps29
Euarchontoglires - ps30
Euarchonta - ps31
Primates - ps32
Haplorrhini - ps33
Simiiformes - ps34
Catarrhini - ps35
Hominoidea - ps36
Hominidae - ps37
Homininae - ps38
Hominini - ps39
Homo_sapiens - ps40
Phylostratum

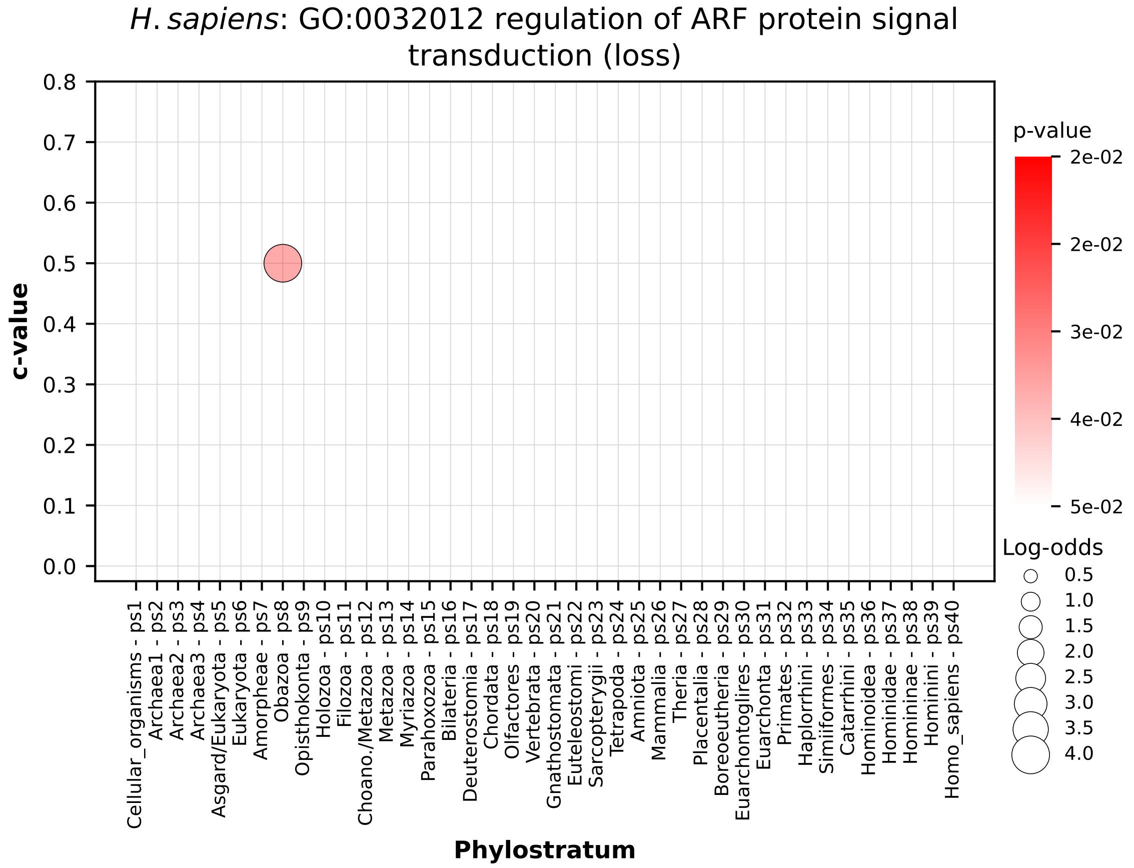

H. sapiens: GO:0032012 regulation of ARF protein signal transduction (loss)
c-value
0.8
0.7
0.6
0.5
0.4
0.3
0.2
0.1
0.0
p-value
2e-02
2e-02
3e-02
4e-02
5e-02
Log-odds
0.5
1.0
1.5
2.0
2.5
3.0
3.5
4.0
Cellular_organisms - ps1
Archaea1 - ps2
Archaea2 - ps3
Archaea3 - ps4
Asgard/Eukaryota - ps5
Eukaryota - ps6
Amorpheae - ps7
Obazoa - ps8
Opisthokonta - ps9
Holozoa - ps10
Filozoa - ps11
Choano./Metazoa - ps12
Metazoa - ps13
Myriazoa - ps14
Parahoxozoa - ps15
Bilateria - ps16
Deuterostomia - ps17
Chordata - ps18
Olfactores - ps19
Vertebrata - ps20
Gnathostomata - ps21
Euteleostomi - ps22
Sarcopterygii - ps23
Tetrapoda - ps24
Amniota - ps25
Mammalia - ps26
Theria - ps27
Placentalia - ps28
Boreoeutheria - ps29
Euarchontoglires - ps30
Euarchonta - ps31
Primates - ps32
Haplorrhini - ps33
Simiiformes - ps34
Catarrhini - ps35
Hominoidea - ps36
Hominidae - ps37
Homininae - ps38
Hominini - ps39
Homo_sapiens - ps40
Phylostratum

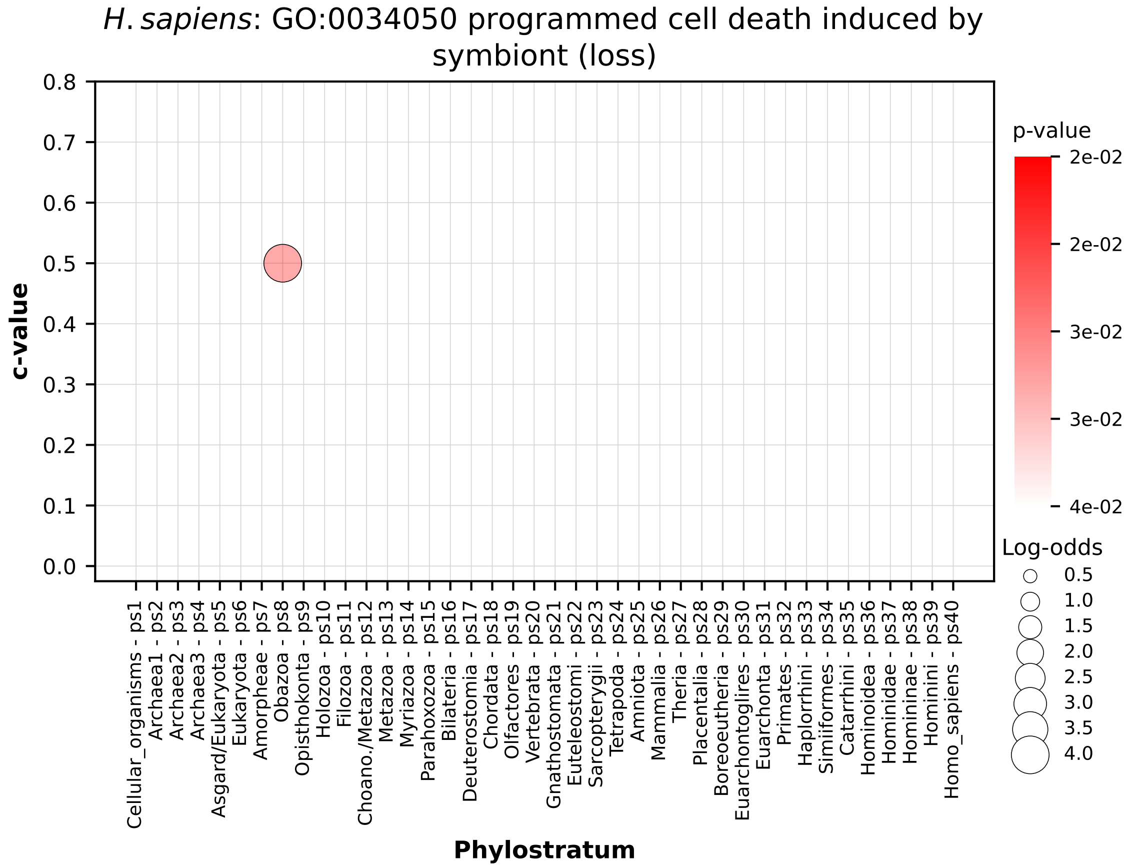

H. sapiens: GO:0034050 programmed cell death induced by symbiont (loss)
c-value
0.8
0.7
0.6
0.5
0.4
0.3
0.2
0.1
0.0
p-value
2e-02
2e-02
3e-02
3e-02
4e-02
Log-odds
0.5
1.0
1.5
2.0
2.5
3.0
3.5
4.0
Cellular_organisms - ps1
Archaea1 - ps2
Archaea2 - ps3
Archaea3 - ps4
Asgard/Eukaryota - ps5
Eukaryota - ps6
Amorpheae - ps7
Obazoa - ps8
Opisthokonta - ps9
Holozoa - ps10
Filozoa - ps11
Choano./Metazoa - ps12
Metazoa - ps13
Myriazoa - ps14
Parahoxozoa - ps15
Bilateria - ps16
Deuterostomia - ps17
Chordata - ps18
Olfactores - ps19
Vertebrata - ps20
Gnathostomata - ps21
Euteleostomi - ps22
Sarcopterygii - ps23
Tetrapoda - ps24
Amniota - ps25
Mammalia - ps26
Theria - ps27
Placentalia - ps28
Boreoeutheria - ps29
Euarchontoglires - ps30
Euarchonta - ps31
Primates - ps32
Haplorrhini - ps33
Simiiformes - ps34
Catarrhini - ps35
Hominoidea - ps36
Hominidae - ps37
Homininae - ps38
Hominini - ps39
Homo_sapiens - ps40
Phylostratum

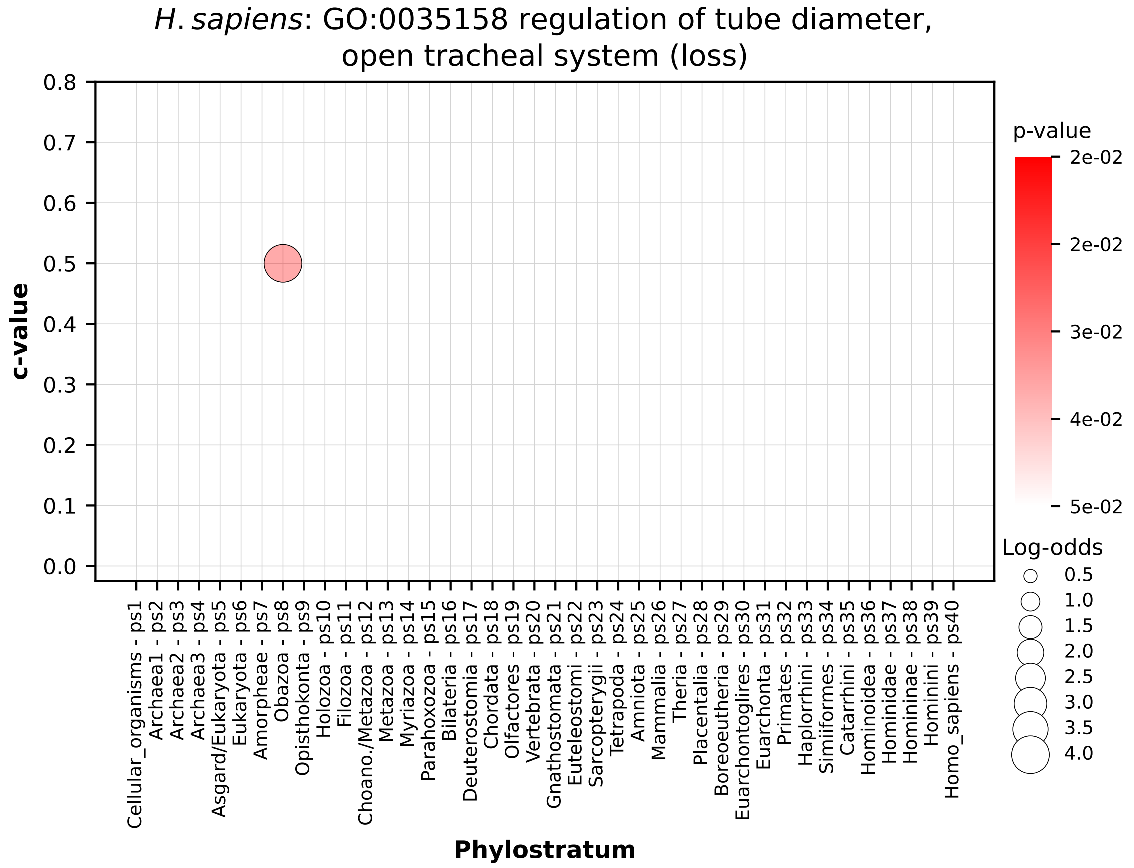

H. sapiens: GO:0035158 regulation of tube diameter, open tracheal system (loss)
c-value
0.8
0.7
0.6
0.5
0.4
0.3
0.2
0.1
0.0
Phylostratum
Cellular_organisms - ps1
Archaea1 - ps2
Archaea2 - ps3
Archaea3 - ps4
Asgard/Eukaryota - ps5
Eukaryota - ps6
Amorpheae - ps7
Obazoa - ps8
Opisthokonta - ps9
Holozoa - ps10
Filozoa - ps11
Choano./Metazoa - ps12
Metazoa - ps13
Myriazoa - ps14
Parahoxozoa - ps15
Bilateria - ps16
Deuterostomia - ps17
Chordata - ps18
Olfactores - ps19
Vertebrata - ps20
Gnathostomata - ps21
Euteleostomi - ps22
Sarcopterygii - ps23
Tetrapoda - ps24
Amniota - ps25
Mammalia - ps26
Theria - ps27
Placentalia - ps28
Boreoeutheria - ps29
Euarchontoglires - ps30
Euarchonta - ps31
Primates - ps32
Haplorrhini - ps33
Simiiformes - ps34
Catarrhini - ps35
Hominoidea - ps36
Hominidae - ps37
Homininae - ps38
Hominini - ps39
Homo_sapiens - ps40
p-value
2e-02
2e-02
3e-02
4e-02
5e-02
Log-odds
0.5
1.0
1.5
2.0
2.5
3.0
3.5
4.0

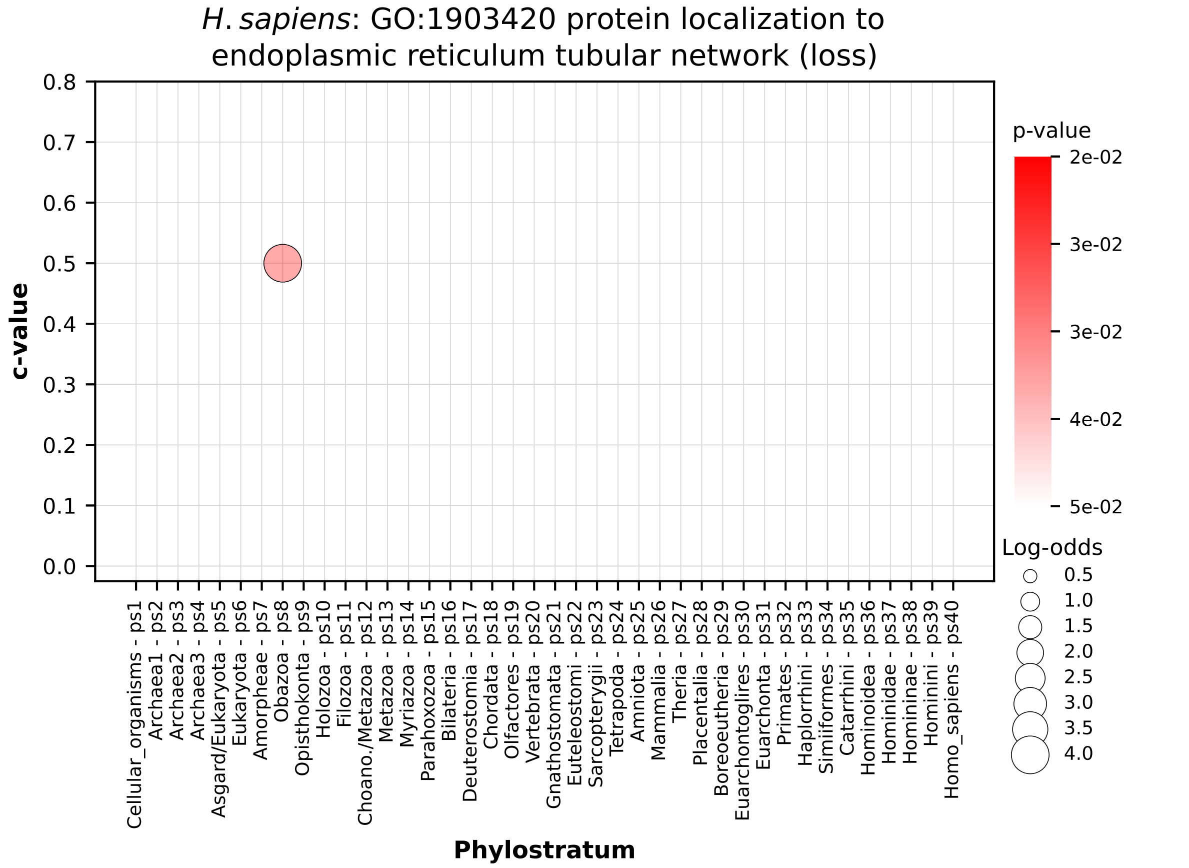

H. sapiens: GO:1903420 protein localization to endoplasmic reticulum tubular network (loss)
c-value
0.0
0.1
0.2
0.3
0.4
0.5
0.6
0.7
0.8
Phylostratum
Cellular_organisms - ps1
Archaea1 - ps2
Archaea2 - ps3
Archaea3 - ps4
Asgard/Eukaryota - ps5
Eukaryota - ps6
Amorpheae - ps7
Obazoa - ps8
Opisthokonta - ps9
Holozoa - ps10
Filozoa - ps11
Choano./Metazoa - ps12
Metazoa - ps13
Myriazoa - ps14
Parahoxozoa - ps15
Bilateria - ps16
Deuterostomia - ps17
Chordata - ps18
Olfactores - ps19
Vertebrata - ps20
Gnathostomata - ps21
Euteleostomi - ps22
Sarcopterygii - ps23
Tetrapoda - ps24
Amniota - ps25
Mammalia - ps26
Theria - ps27
Placentalia - ps28
Boreoeutheria - ps29
Euarchontoglires - ps30
Euarchonta - ps31
Primates - ps32
Haplorrhini - ps33
Simiiformes - ps34
Catarrhini - ps35
Hominoidea - ps36
Hominidae - ps37
Homininae - ps38
Hominini - ps39
Homo_sapiens - ps40
p-value
2e-02
3e-02
3e-02
4e-02
5e-02
Log-odds
0.5
1.0
1.5
2.0
2.5
3.0
3.5
4.0

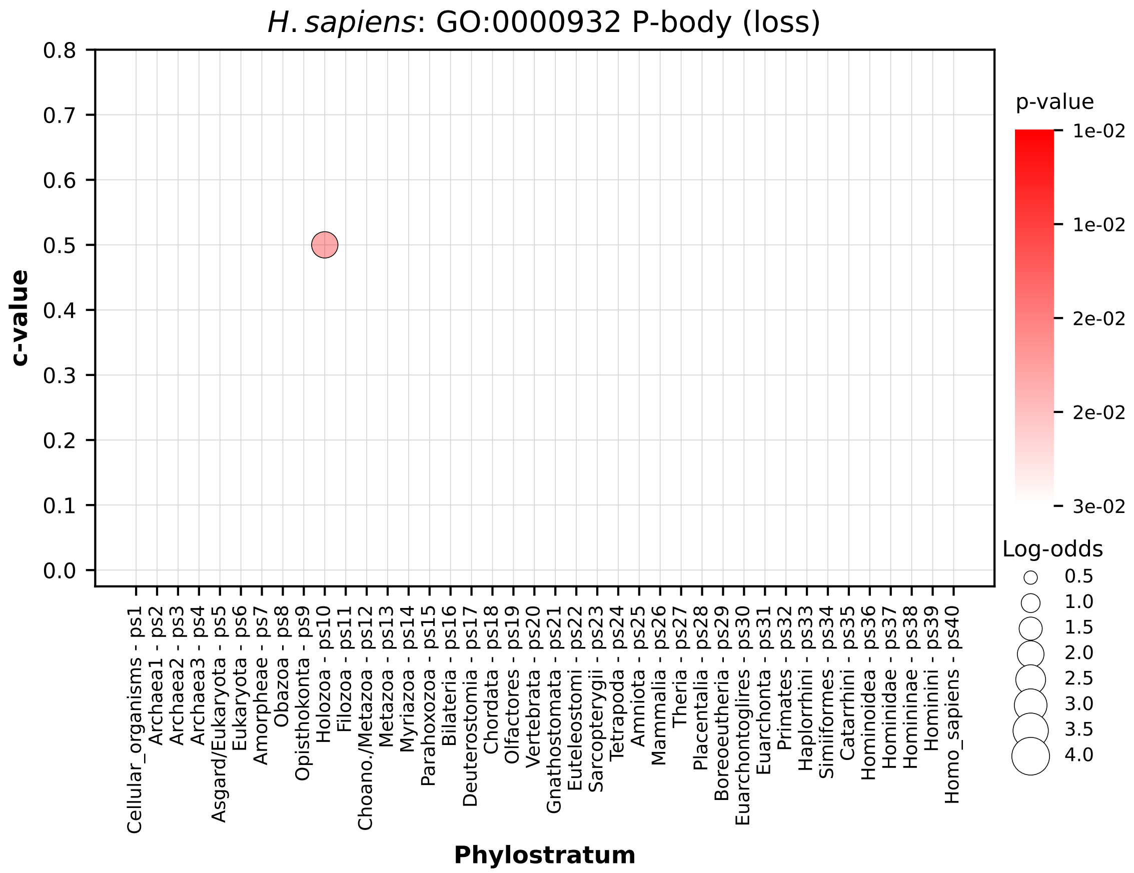

H. sapiens: GO:0000932 P-body (loss)
c-value
0.8
0.7
0.6
0.5
0.4
0.3
0.2
0.1
0.0
Phylostratum
Cellular_organisms - ps1
Archaea1 - ps2
Archaea2 - ps3
Archaea3 - ps4
Asgard/Eukaryota - ps5
Eukaryota - ps6
Amorpheae - ps7
Obazoa - ps8
Opisthokonta - ps9
Holozoa - ps10
Filozoa - ps11
Choano./Metazoa - ps12
Metazoa - ps13
Myriazoa - ps14
Parahoxozoa - ps15
Bilateria - ps16
Deuterostomia - ps17
Chordata - ps18
Olfactores - ps19
Vertebrata - ps20
Gnathostomata - ps21
Euteleostomi - ps22
Sarcopterygii - ps23
Tetrapoda - ps24
Amniota - ps25
Mammalia - ps26
Theria - ps27
Placentalia - ps28
Boreoeutheria - ps29
Euarchontoglires - ps30
Euarchonta - ps31
Primates - ps32
Haplorrhini - ps33
Simiiformes - ps34
Catarrhini - ps35
Hominoidea - ps36
Hominidae - ps37
Homininae - ps38
Hominini - ps39
Homo_sapiens - ps40
p-value
1e-02
1e-02
2e-02
2e-02
3e-02
Log-odds
0.5
1.0
1.5
2.0
2.5
3.0
3.5
4.0

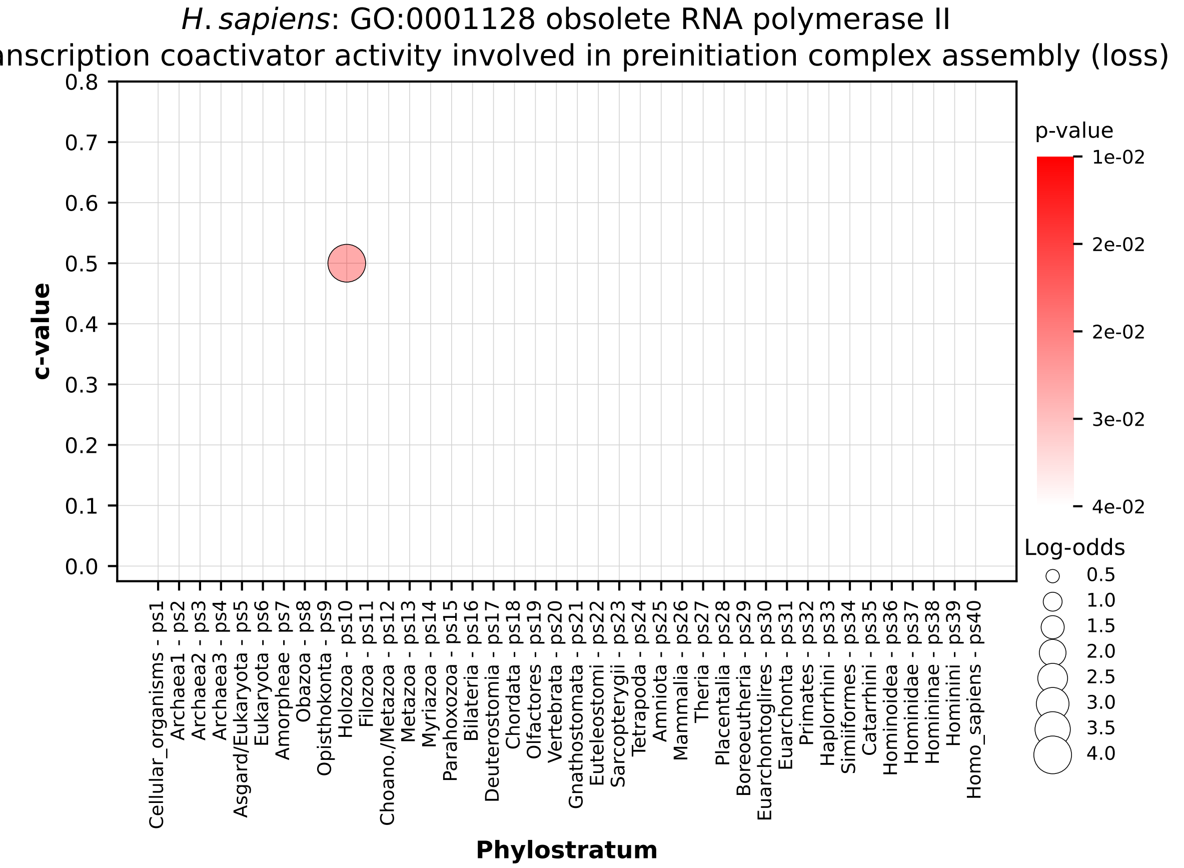

H. sapiens: GO:0001128 obsolete RNA polymerase II
anscription coactivator activity involved in preinitiation complex assembly (loss)
c-value
0.8
0.7
0.6
0.5
0.4
0.3
0.2
0.1
0.0
Phylostratum
Cellular_organisms - ps1
Archaea1 - ps2
Archaea2 - ps3
Archaea3 - ps4
Asgard/Eukaryota - ps5
Eukaryota - ps6
Amorpheae - ps7
Obazoa - ps8
Opisthokonta - ps9
Holozoa - ps10
Filozoa - ps11
Choano./Metazoa - ps12
Metazoa - ps13
Myriazoa - ps14
Parahoxozoa - ps15
Bilateria - ps16
Deuterostomia - ps17
Chordata - ps18
Olfactores - ps19
Vertebrata - ps20
Gnathostomata - ps21
Euteleostomi - ps22
Sarcopterygii - ps23
Tetrapoda - ps24
Amniota - ps25
Mammalia - ps26
Theria - ps27
Placentalia - ps28
Boreoeutheria - ps29
Euarchontoglires - ps30
Euarchonta - ps31
Primates - ps32
Haplorrhini - ps33
Simiiformes - ps34
Catarrhini - ps35
Hominoidea - ps36
Hominidae - ps37
Homininae - ps38
Hominini - ps39
Homo_sapiens - ps40
p-value
1e-02
2e-02
2e-02
3e-02
4e-02
Log-odds
0.5
1.0
1.5
2.0
2.5
3.0
3.5
4.0

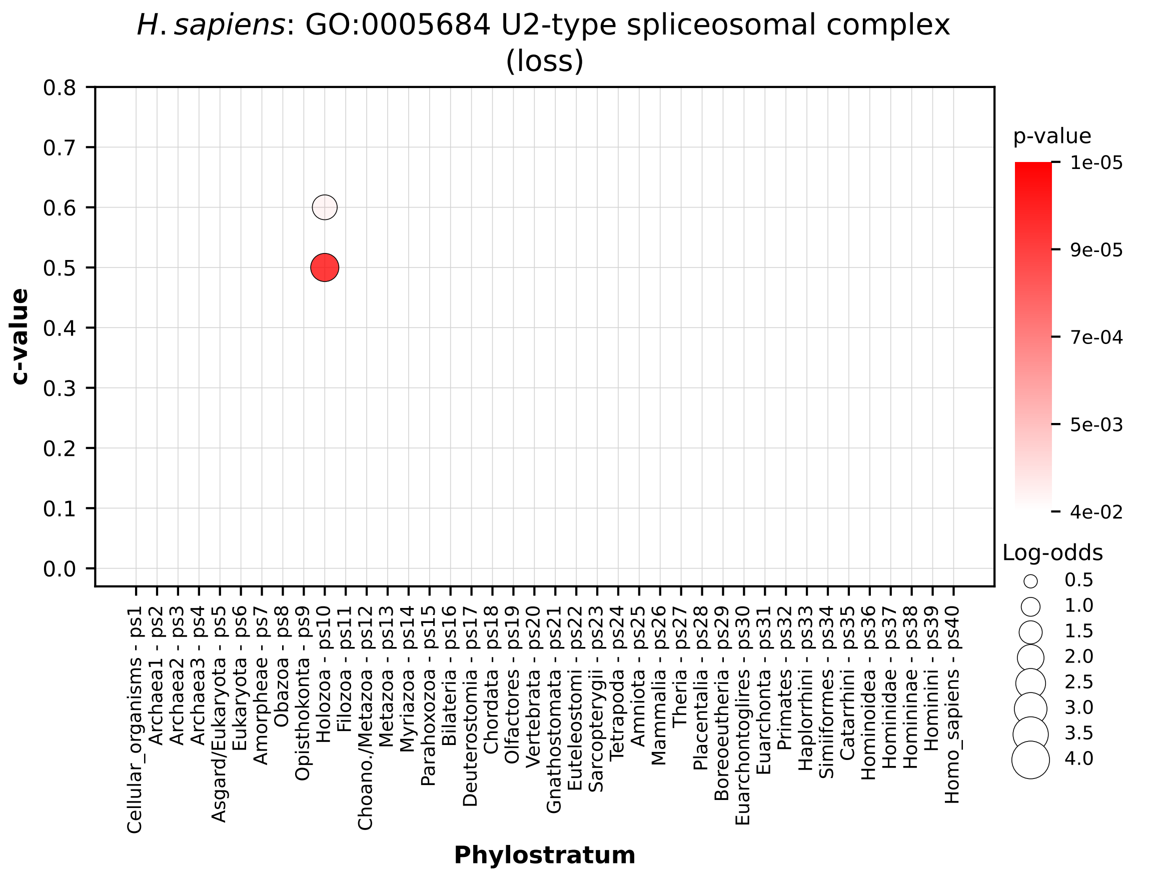

H. sapiens: GO:0005684 U2-type spliceosomal complex
(loss)
c-value
0.8
0.7
0.6
0.5
0.4
0.3
0.2
0.1
0.0
p-value
1e-05
9e-05
7e-04
5e-03
4e-02
Log-odds
0.5
1.0
1.5
2.0
2.5
3.0
3.5
4.0
Cellular_organisms - ps1
Archaea1 - ps2
Archaea2 - ps3
Archaea3 - ps4
Asgard/Eukaryota - ps5
Eukaryota - ps6
Amorpheae - ps7
Obazoa - ps8
Opisthokonta - ps9
Holozoa - ps10
Filozoa - ps11
Choano./Metazoa - ps12
Metazoa - ps13
Myriazoa - ps14
Parahoxozoa - ps15
Bilateria - ps16
Deuterostomia - ps17
Chordata - ps18
Olfactores - ps19
Vertebrata - ps20
Gnathostomata - ps21
Euteleostomi - ps22
Sarcopterygii - ps23
Tetrapoda - ps24
Amniota - ps25
Mammalia - ps26
Theria - ps27
Placentalia - ps28
Boreoeutheria - ps29
Euarchontoglires - ps30
Euarchonta - ps31
Primates - ps32
Haplorrhini - ps33
Simiiformes - ps34
Catarrhini - ps35
Hominoidea - ps36
Hominidae - ps37
Homininae - ps38
Hominini - ps39
Homo_sapiens - ps40
Phylostratum

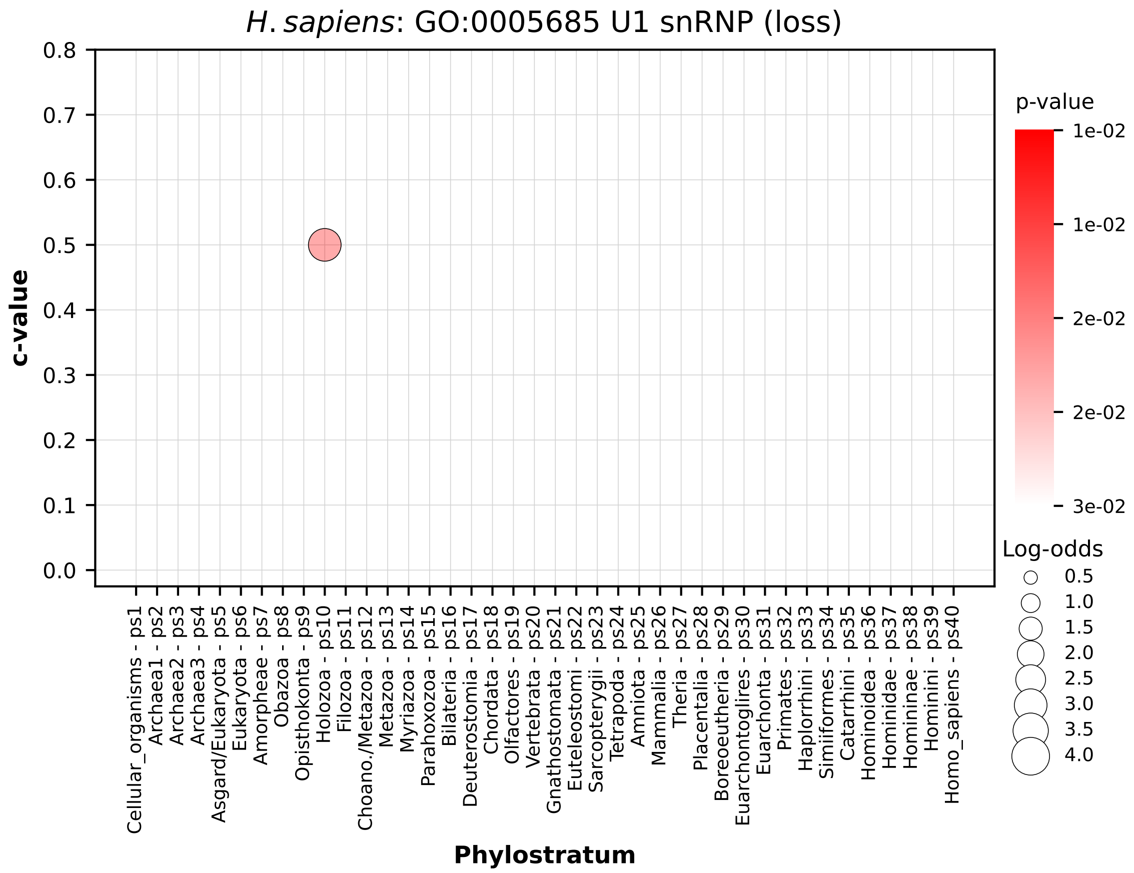

H. sapiens: GO:0005685 U1 snRNP (loss)
c-value
0.0
0.1
0.2
0.3
0.4
0.5
0.6
0.7
0.8
p-value
1e-02
1e-02
2e-02
2e-02
3e-02
Log-odds
0.5
1.0
1.5
2.0
2.5
3.0
3.5
4.0
Cellular_organisms - ps1
Archaea1 - ps2
Archaea2 - ps3
Archaea3 - ps4
Asgard/Eukaryota - ps5
Eukaryota - ps6
Amorpheae - ps7
Obazoa - ps8
Opisthokonta - ps9
Holozoa - ps10
Filozoa - ps11
Choano./Metazoa - ps12
Metazoa - ps13
Myriazoa - ps14
Parahoxozoa - ps15
Bilateria - ps16
Deuterostomia - ps17
Chordata - ps18
Olfactores - ps19
Vertebrata - ps20
Gnathostomata - ps21
Euteleostomi - ps22
Sarcopterygii - ps23
Tetrapoda - ps24
Amniota - ps25
Mammalia - ps26
Theria - ps27
Placentalia - ps28
Boreoeutheria - ps29
Euarchontoglires - ps30
Euarchonta - ps31
Primates - ps32
Haplorrhini - ps33
Simiiformes - ps34
Catarrhini - ps35
Hominoidea - ps36
Hominidae - ps37
Homininae - ps38
Hominini - ps39
Homo_sapiens - ps40
Phylostratum

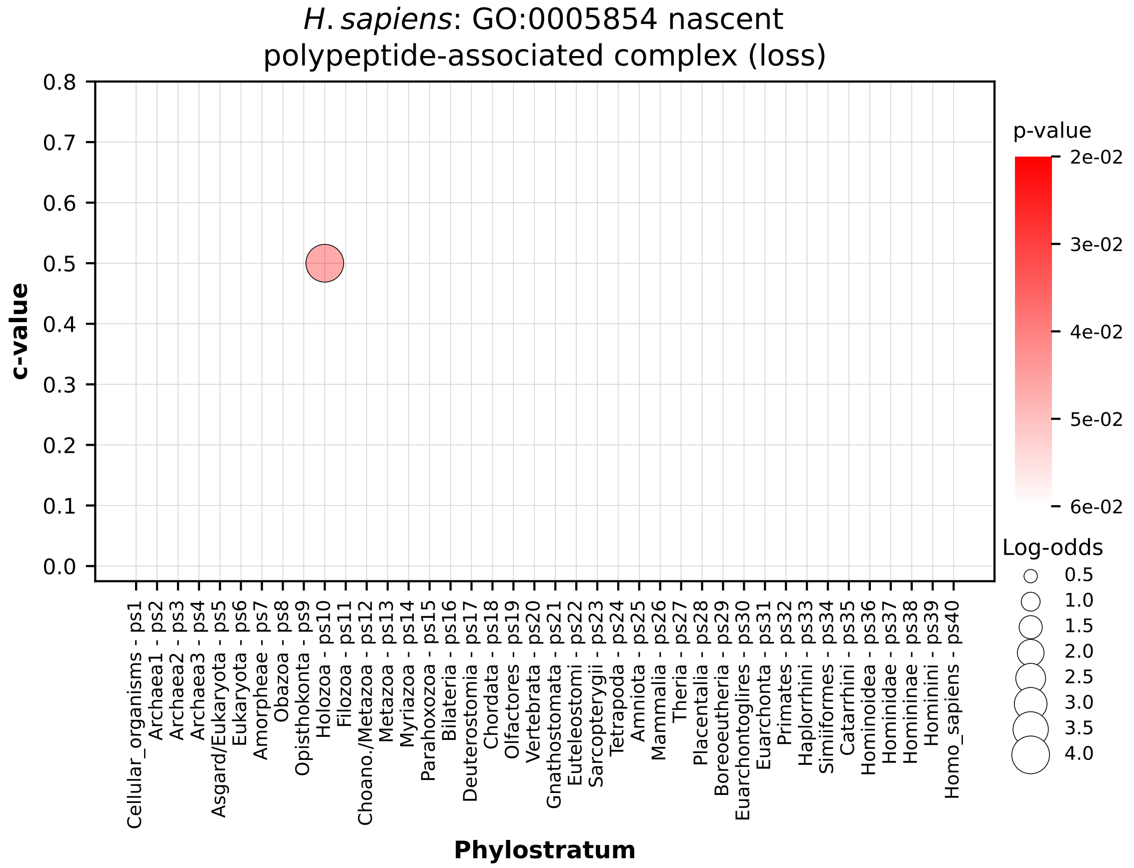
H. sapiens: GO:0005854 nascent polypeptide-associated complex (loss)
c-value
0.0
0.1
0.2
0.3
0.4
0.5
0.6
0.7
0.8
Phylostratum
Cellular_organisms - ps1
Archaea1 - ps2
Archaea2 - ps3
Archaea3 - ps4
Asgard/Eukaryota - ps5
Eukaryota - ps6
Amorpheae - ps7
Obazoa - ps8
Opisthokonta - ps9
Holozoa - ps10
Filozoa - ps11
Choano./Metazoa - ps12
Metazoa - ps13
Myriazoa - ps14
Parahoxozoa - ps15
Bilateria - ps16
Deuterostomia - ps17
Chordata - ps18
Olfactores - ps19
Vertebrata - ps20
Gnathostomata - ps21
Euteleostomi - ps22
Sarcopterygii - ps23
Tetrapoda - ps24
Amniota - ps25
Mammalia - ps26
Theria - ps27
Placentalia - ps28
Boreoeutheria - ps29
Euarchontoglires - ps30
Euarchonta - ps31
Primates - ps32
Haplorrhini - ps33
Simiiformes - ps34
Catarrhini - ps35
Hominoidea - ps36
Hominidae - ps37
Homininae - ps38
Hominini - ps39
Homo_sapiens - ps40
p-value
2e-02
3e-02
4e-02
5e-02
6e-02
Log-odds
0.5
1.0
1.5
2.0
2.5
3.0
3.5
4.0

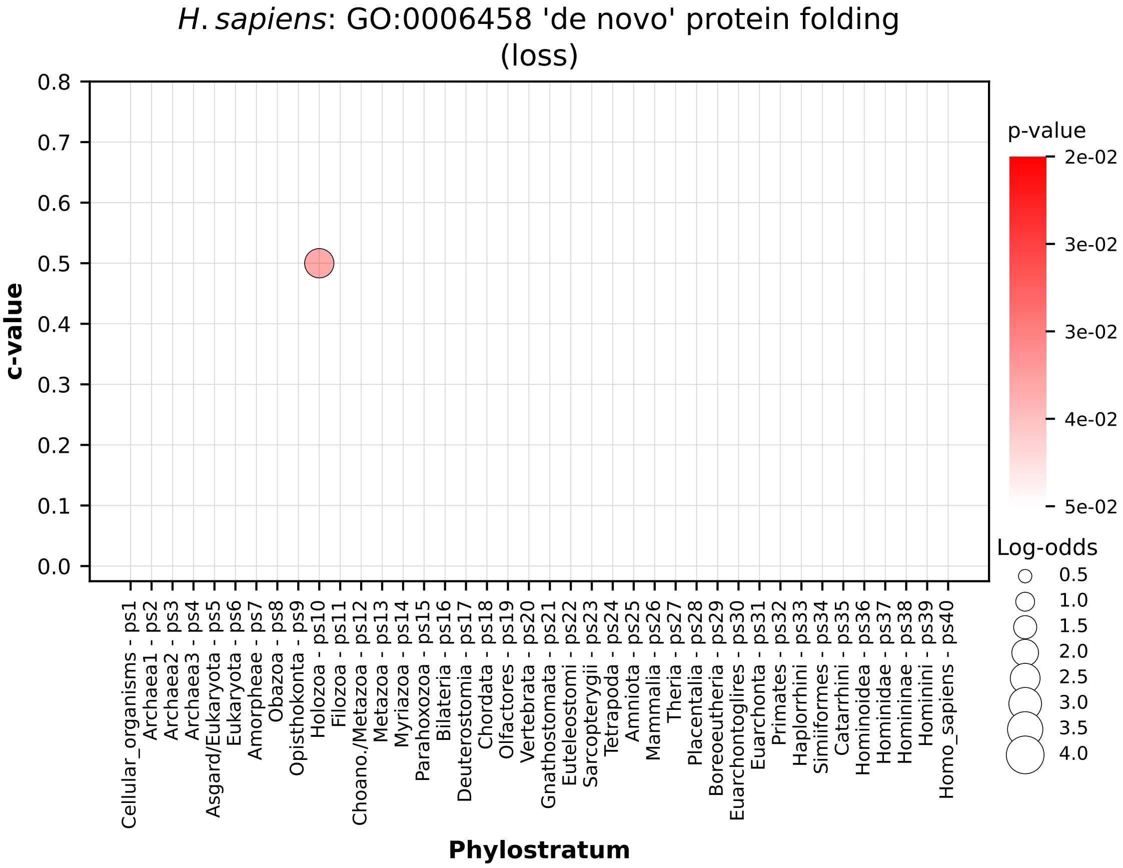
H. sapiens: GO:0006458 'de novo' protein folding
(loss)
c-value
0.8
0.7
0.6
0.5
0.4
0.3
0.2
0.1
0.0
p-value
2e-02
3e-02
3e-02
4e-02
5e-02
Log-odds
0.5
1.0
1.5
2.0
2.5
3.0
3.5
4.0
Cellular_organisms - ps1
Archaea1 - ps2
Archaea2 - ps3
Archaea3 - ps4
Asgard/Eukaryota - ps5
Eukaryota - ps6
Amorpheae - ps7
Obazoa - ps8
Opisthokonta - ps9
Holozoa - ps10
Filozoa - ps11
Choano./Metazoa - ps12
Metazoa - ps13
Myriazoa - ps14
Parahoxozoa - ps15
Bilateria - ps16
Deuterostomia - ps17
Chordata - ps18
Olfactores - ps19
Vertebrata - ps20
Gnathostomata - ps21
Euteleostomi - ps22
Sarcopterygii - ps23
Tetrapoda - ps24
Amniota - ps25
Mammalia - ps26
Theria - ps27
Placentalia - ps28
Boreoeutheria - ps29
Euarchontoglires - ps30
Euarchonta - ps31
Primates - ps32
Haplorrhini - ps33
Simiiformes - ps34
Catarrhini - ps35
Hominoidea - ps36
Hominidae - ps37
Homininae - ps38
Hominini - ps39
Homo_sapiens - ps40
Phylostratum

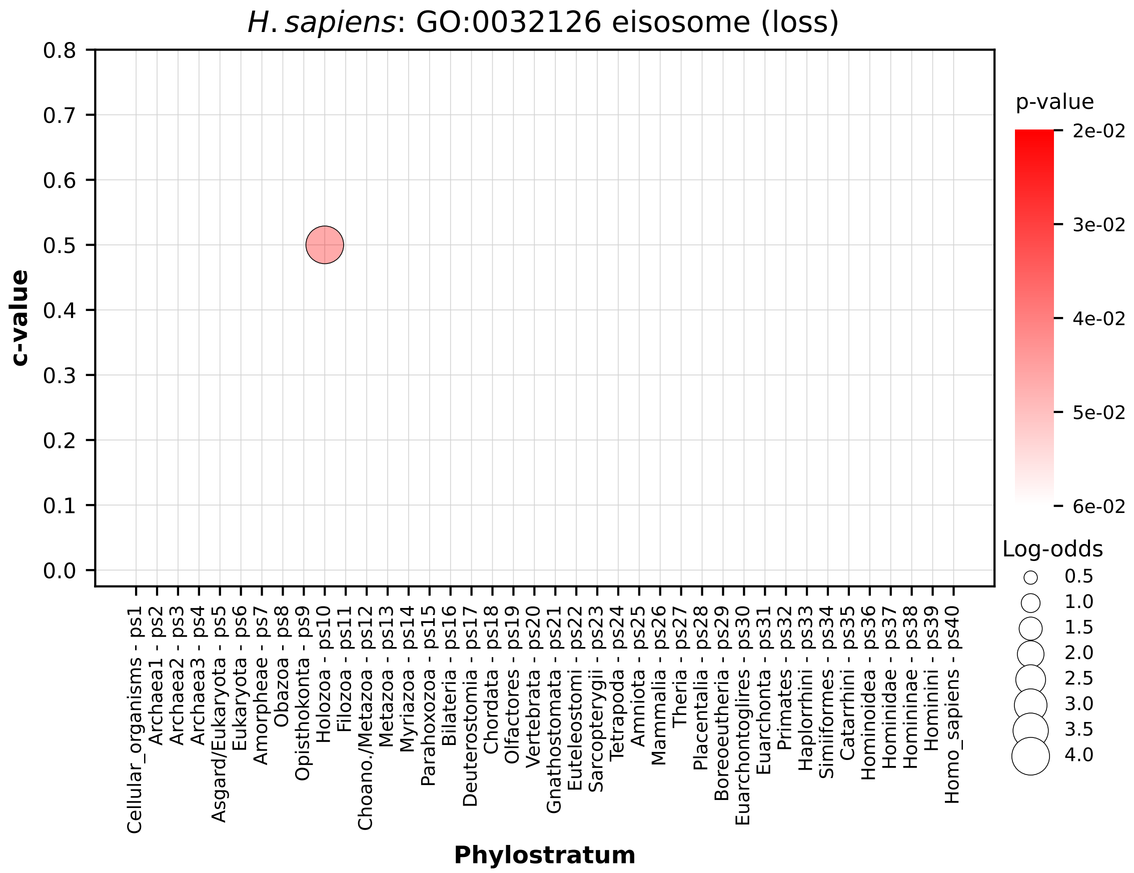
H. sapiens: GO:0032126 eisosome (loss)
c-value
0.8
0.7
0.6
0.5
0.4
0.3
0.2
0.1
0.0
Phylostratum
Cellular_organisms - ps1
Archaea1 - ps2
Archaea2 - ps3
Archaea3 - ps4
Asgard/Eukaryota - ps5
Eukaryota - ps6
Amorpheae - ps7
Obazoa - ps8
Opisthokonta - ps9
Holozoa - ps10
Filozoa - ps11
Choano./Metazoa - ps12
Metazoa - ps13
Myriazoa - ps14
Parahoxozoa - ps15
Bilateria - ps16
Deuterostomia - ps17
Chordata - ps18
Olfactores - ps19
Vertebrata - ps20
Gnathostomata - ps21
Euteleostomi - ps22
Sarcopterygii - ps23
Tetrapoda - ps24
Amniota - ps25
Mammalia - ps26
Theria - ps27
Placentalia - ps28
Boreoeutheria - ps29
Euarchontoglires - ps30
Euarchonta - ps31
Primates - ps32
Haplorrhini - ps33
Simiiformes - ps34
Catarrhini - ps35
Hominoidea - ps36
Hominidae - ps37
Homininae - ps38
Hominini - ps39
Homo_sapiens - ps40
p-value
2e-02
3e-02
4e-02
5e-02
6e-02
Log-odds
0.5
1.0
1.5
2.0
2.5
3.0
3.5
4.0

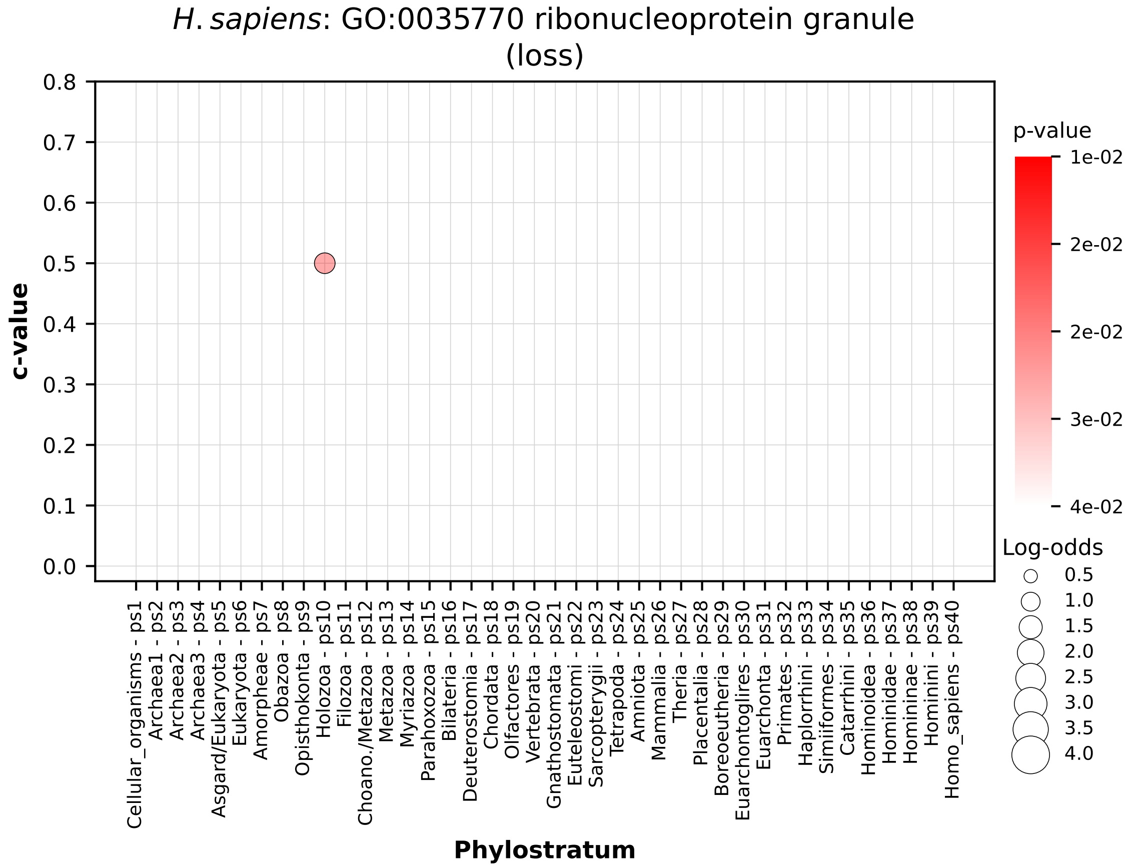

H. sapiens: GO:0035770 ribonucleoprotein granule
(loss)
c-value
0.8
0.7
0.6
0.5
0.4
0.3
0.2
0.1
0.0
p-value
1e-02
2e-02
2e-02
3e-02
4e-02
Log-odds
0.5
1.0
1.5
2.0
2.5
3.0
3.5
4.0
Cellular_organisms - ps1
Archaea1 - ps2
Archaea2 - ps3
Archaea3 - ps4
Asgard/Eukaryota - ps5
Eukaryota - ps6
Amorpheae - ps7
Obazoa - ps8
Opisthokonta - ps9
Holozoa - ps10
Filozoa - ps11
Choano./Metazoa - ps12
Metazoa - ps13
Myriazoa - ps14
Parahoxozoa - ps15
Bilateria - ps16
Deuterostomia - ps17
Chordata - ps18
Olfactores - ps19
Vertebrata - ps20
Gnathostomata - ps21
Euteleostomi - ps22
Sarcopterygii - ps23
Tetrapoda - ps24
Amniota - ps25
Mammalia - ps26
Theria - ps27
Placentalia - ps28
Boreoeutheria - ps29
Euarchontoglires - ps30
Euarchonta - ps31
Primates - ps32
Haplorrhini - ps33
Simiiformes - ps34
Catarrhini - ps35
Hominoidea - ps36
Hominidae - ps37
Homininae - ps38
Hominini - ps39
Homo_sapiens - ps40
Phylostratum

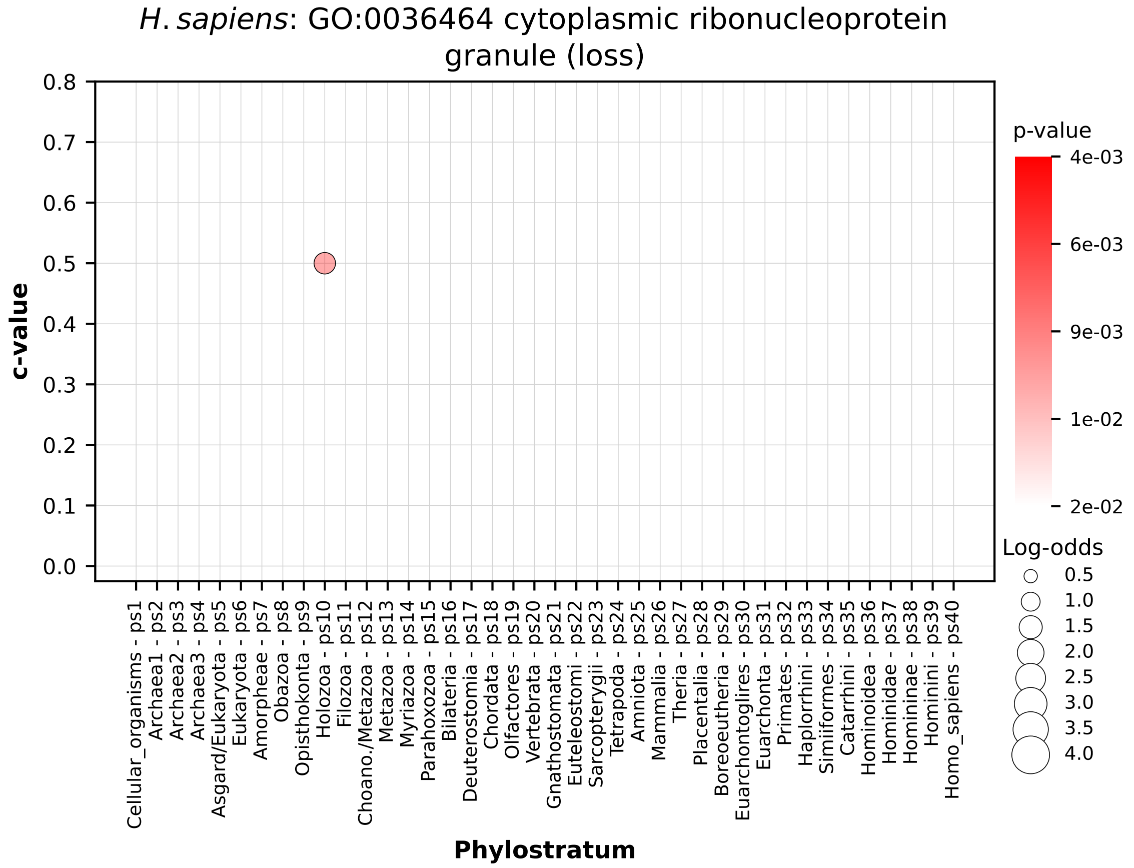
H. sapiens: GO:0036464 cytoplasmic ribonucleoprotein granule (loss)
c-value
0.8
0.7
0.6
0.5
0.4
0.3
0.2
0.1
0.0
p-value
4e-03
6e-03
9e-03
1e-02
2e-02
Log-odds
0.5
1.0
1.5
2.0
2.5
3.0
3.5
4.0
Cellular_organisms - ps1
Archaea1 - ps2
Archaea2 - ps3
Archaea3 - ps4
Asgard/Eukaryota - ps5
Eukaryota - ps6
Amorpheae - ps7
Obazoa - ps8
Opisthokonta - ps9
Holozoa - ps10
Filozoa - ps11
Choano./Metazoa - ps12
Metazoa - ps13
Myriazoa - ps14
Parahoxozoa - ps15
Bilateria - ps16
Deuterostomia - ps17
Chordata - ps18
Olfactores - ps19
Vertebrata - ps20
Gnathostomata - ps21
Euteleostomi - ps22
Sarcopterygii - ps23
Tetrapoda - ps24
Amniota - ps25
Mammalia - ps26
Theria - ps27
Placentalia - ps28
Boreoeutheria - ps29
Euarchontoglires - ps30
Euarchonta - ps31
Primates - ps32
Haplorrhini - ps33
Simiiformes - ps34
Catarrhini - ps35
Hominoidea - ps36
Hominidae - ps37
Homininae - ps38
Hominini - ps39
Homo_sapiens - ps40
Phylostratum

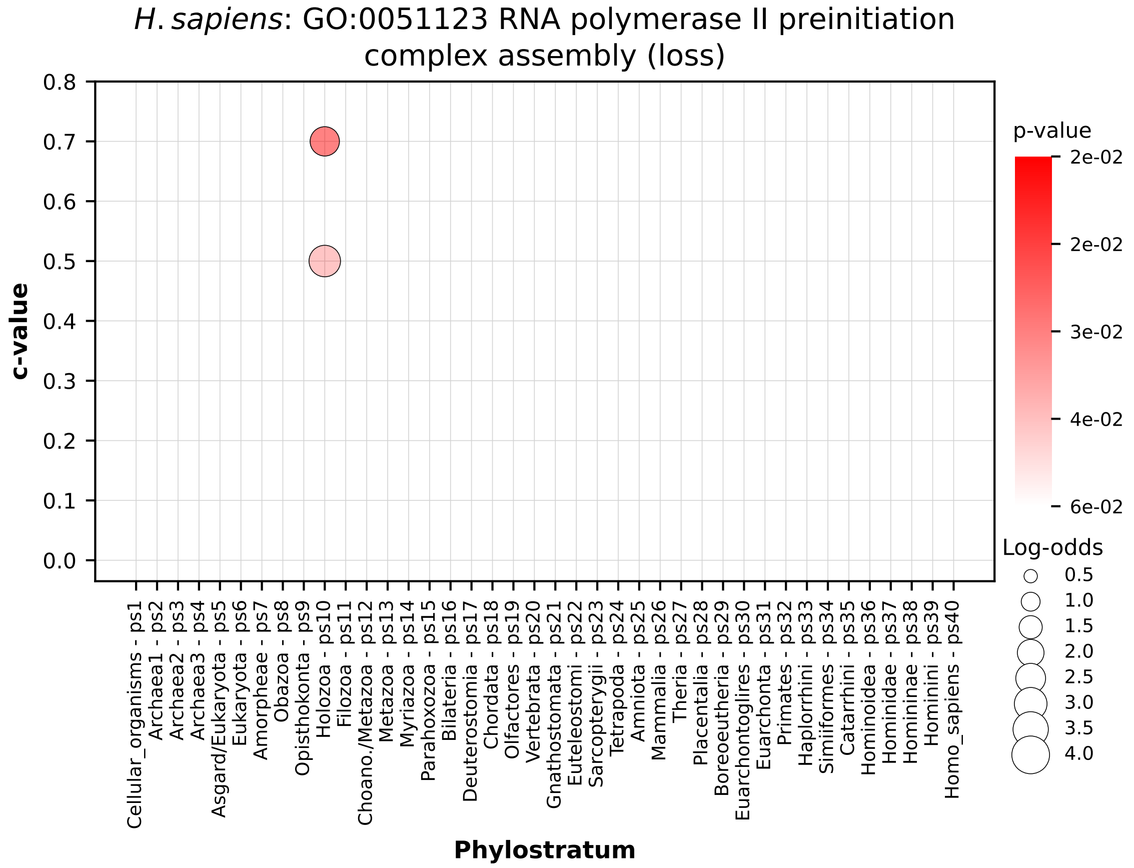

H. sapiens: GO:0051123 RNA polymerase II preinitiation complex assembly (loss)
c-value
0.8
0.7
0.6
0.5
0.4
0.3
0.2
0.1
0.0
p-value
2e-02
2e-02
3e-02
4e-02
6e-02
Log-odds
0.5
1.0
1.5
2.0
2.5
3.0
3.5
4.0
Cellular_organisms - ps1
Archaea1 - ps2
Archaea2 - ps3
Archaea3 - ps4
Asgard/Eukaryota - ps5
Eukaryota - ps6
Amorpheae - ps7
Obazoa - ps8
Opisthokonta - ps9
Holozoa - ps10
Filozoa - ps11
Choano./Metazoa - ps12
Metazoa - ps13
Myriazoa - ps14
Parahoxozoa - ps15
Bilateria - ps16
Deuterostomia - ps17
Chordata - ps18
Olfactores - ps19
Vertebrata - ps20
Gnathostomata - ps21
Euteleostomi - ps22
Sarcopterygii - ps23
Tetrapoda - ps24
Amniota - ps25
Mammalia - ps26
Theria - ps27
Placentalia - ps28
Boreoeutheria - ps29
Euarchontoglires - ps30
Euarchonta - ps31
Primates - ps32
Haplorrhini - ps33
Simiiformes - ps34
Catarrhini - ps35
Hominoidea - ps36
Hominidae - ps37
Homininae - ps38
Hominini - ps39
Homo_sapiens - ps40
Phylostratum

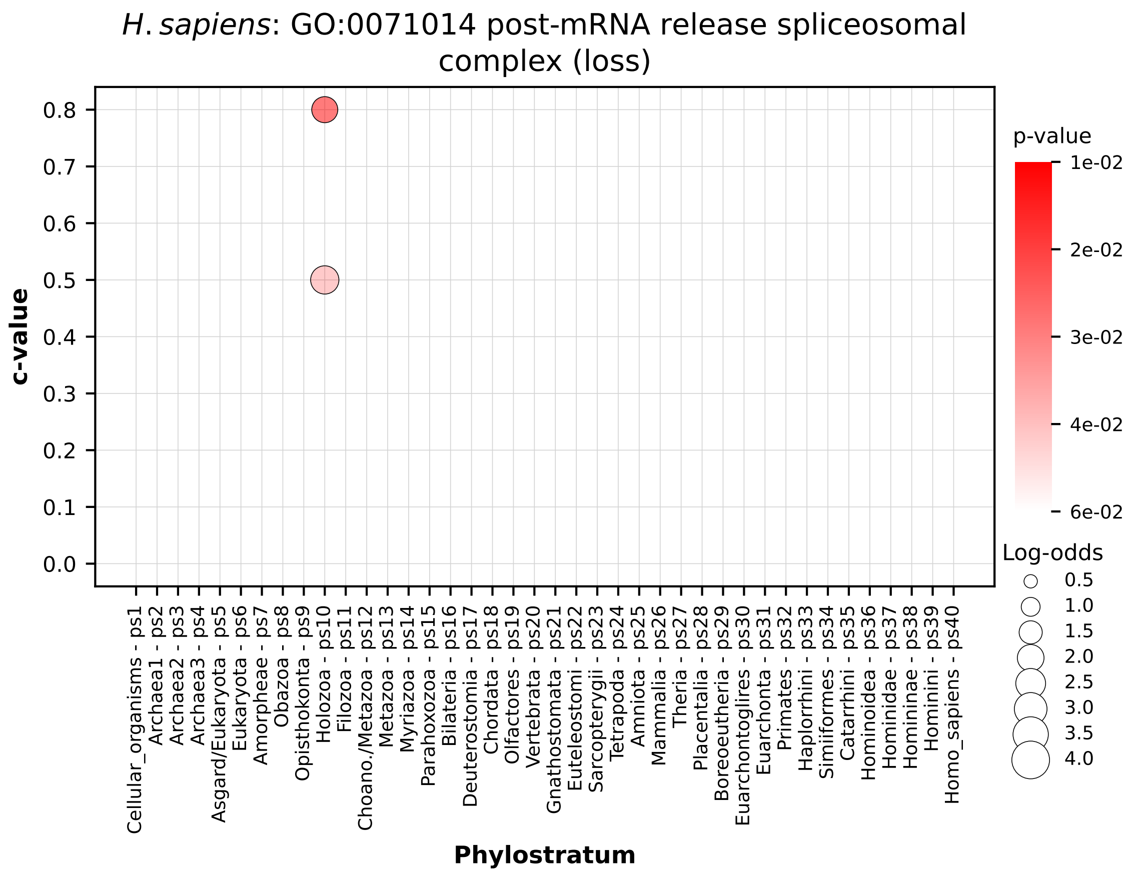

H. sapiens: GO:0071014 post-mRNA release spliceosomal complex (loss)
c-value
0.0
0.1
0.2
0.3
0.4
0.5
0.6
0.7
0.8
p-value
1e-02
2e-02
3e-02
4e-02
6e-02
Log-odds
0.5
1.0
1.5
2.0
2.5
3.0
3.5
4.0
Cellular_organisms - ps1
Archaea1 - ps2
Archaea2 - ps3
Archaea3 - ps4
Asgard/Eukaryota - ps5
Eukaryota - ps6
Amorpheae - ps7
Obazoa - ps8
Opisthokonta - ps9
Holozoa - ps10
Filozoa - ps11
Choano./Metazoa - ps12
Metazoa - ps13
Myriazoa - ps14
Parahoxozoa - ps15
Bilateria - ps16
Deuterostomia - ps17
Chordata - ps18
Olfactores - ps19
Vertebrata - ps20
Gnathostomata - ps21
Euteleostomi - ps22
Sarcopterygii - ps23
Tetrapoda - ps24
Amniota - ps25
Mammalia - ps26
Theria - ps27
Placentalia - ps28
Boreoeutheria - ps29
Euarchontoglires - ps30
Euarchonta - ps31
Primates - ps32
Haplorrhini - ps33
Simiiformes - ps34
Catarrhini - ps35
Hominoidea - ps36
Hominidae - ps37
Homininae - ps38
Hominini - ps39
Homo_sapiens - ps40
Phylostratum

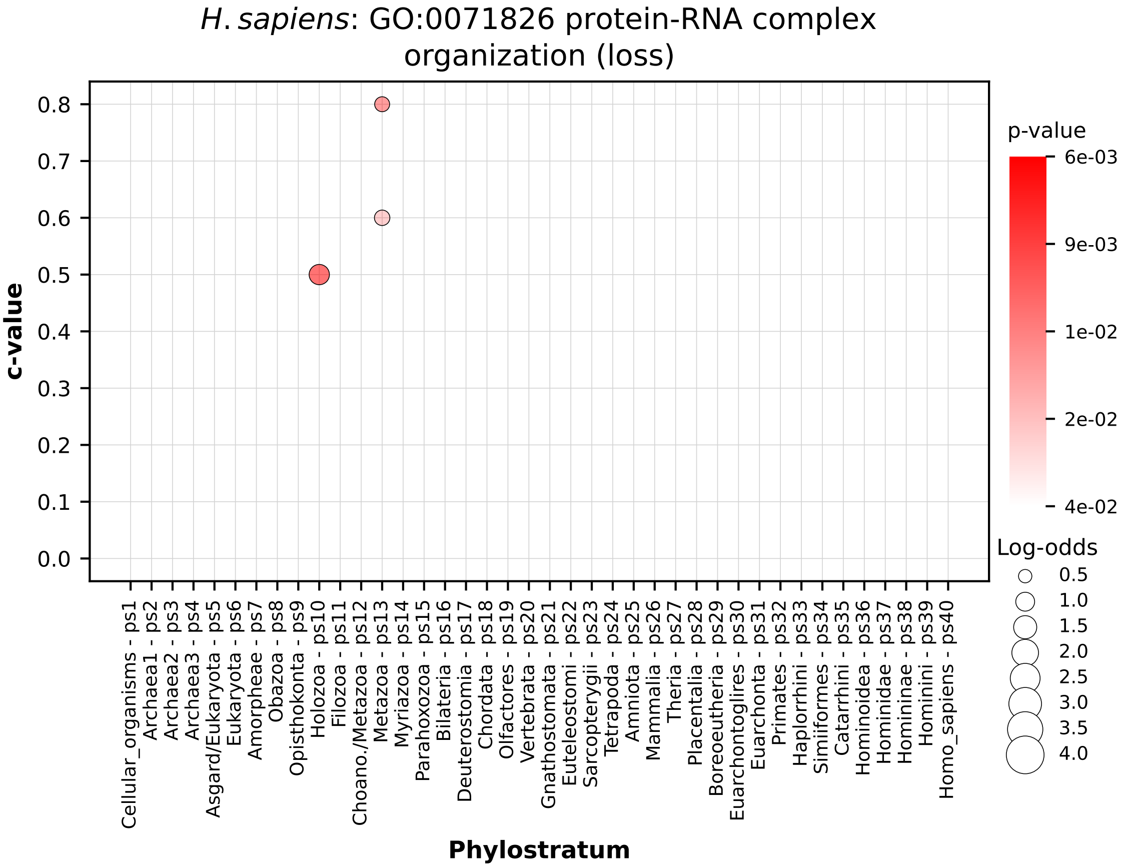

H. sapiens: GO:0071826 protein-RNA complex organization (loss)
c-value
0.0
0.1
0.2
0.3
0.4
0.5
0.6
0.7
0.8
Phylostratum
Cellular_organisms - ps1
Archaea1 - ps2
Archaea2 - ps3
Archaea3 - ps4
Asgard/Eukaryota - ps5
Eukaryota - ps6
Amorpheae - ps7
Obazoa - ps8
Opisthokonta - ps9
Holozoa - ps10
Filozoa - ps11
Choano./Metazoa - ps12
Metazoa - ps13
Myriazoa - ps14
Parahoxozoa - ps15
Bilateria - ps16
Deuterostomia - ps17
Chordata - ps18
Olfactores - ps19
Vertebrata - ps20
Gnathostomata - ps21
Euteleostomi - ps22
Sarcopterygii - ps23
Tetrapoda - ps24
Amniota - ps25
Mammalia - ps26
Theria - ps27
Placentalia - ps28
Boreoeutheria - ps29
Euarchontoglires - ps30
Euarchonta - ps31
Primates - ps32
Haplorrhini - ps33
Simiiformes - ps34
Catarrhini - ps35
Hominoidea - ps36
Hominidae - ps37
Homininae - ps38
Hominini - ps39
Homo_sapiens - ps40
p-value
6e-03
9e-03
1e-02
2e-02
4e-02
Log-odds
0.5
1.0
1.5
2.0
2.5
3.0
3.5
4.0

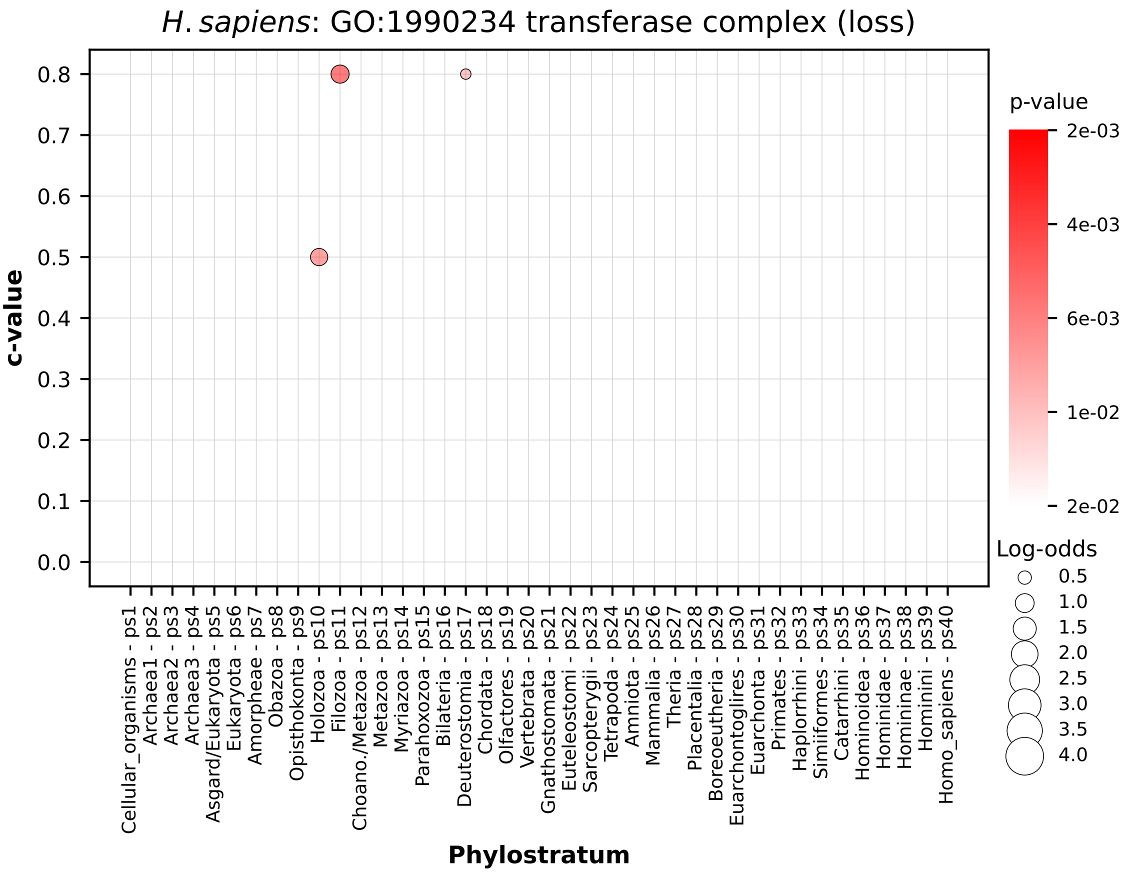

H. sapiens: GO:1990234 transferase complex (loss)
c-value
0.8
0.7
0.6
0.5
0.4
0.3
0.2
0.1
0.0
p-value
2e-03
4e-03
6e-03
1e-02
2e-02
Log-odds
0.5
1.0
1.5
2.0
2.5
3.0
3.5
4.0
Cellular_organisms - ps1
Archaea1 - ps2
Archaea2 - ps3
Archaea3 - ps4
Asgard/Eukaryota - ps5
Eukaryota - ps6
Amorpheae - ps7
Obazoa - ps8
Opisthokonta - ps9
Holozoa - ps10
Filozoa - ps11
Choano./Metazoa - ps12
Metazoa - ps13
Myriazoa - ps14
Parahoxozoa - ps15
Bilateria - ps16
Deuterostomia - ps17
Chordata - ps18
Olfactores - ps19
Vertebrata - ps20
Gnathostomata - ps21
Euteleostomi - ps22
Sarcopterygii - ps23
Tetrapoda - ps24
Amniota - ps25
Mammalia - ps26
Theria - ps27
Placentalia - ps28
Boreoeutheria - ps29
Euarchontoglires - ps30
Euarchonta - ps31
Primates - ps32
Haplorrhini - ps33
Simiiformes - ps34
Catarrhini - ps35
Hominoidea - ps36
Hominidae - ps37
Homininae - ps38
Hominini - ps39
Homo_sapiens - ps40
Phylostratum

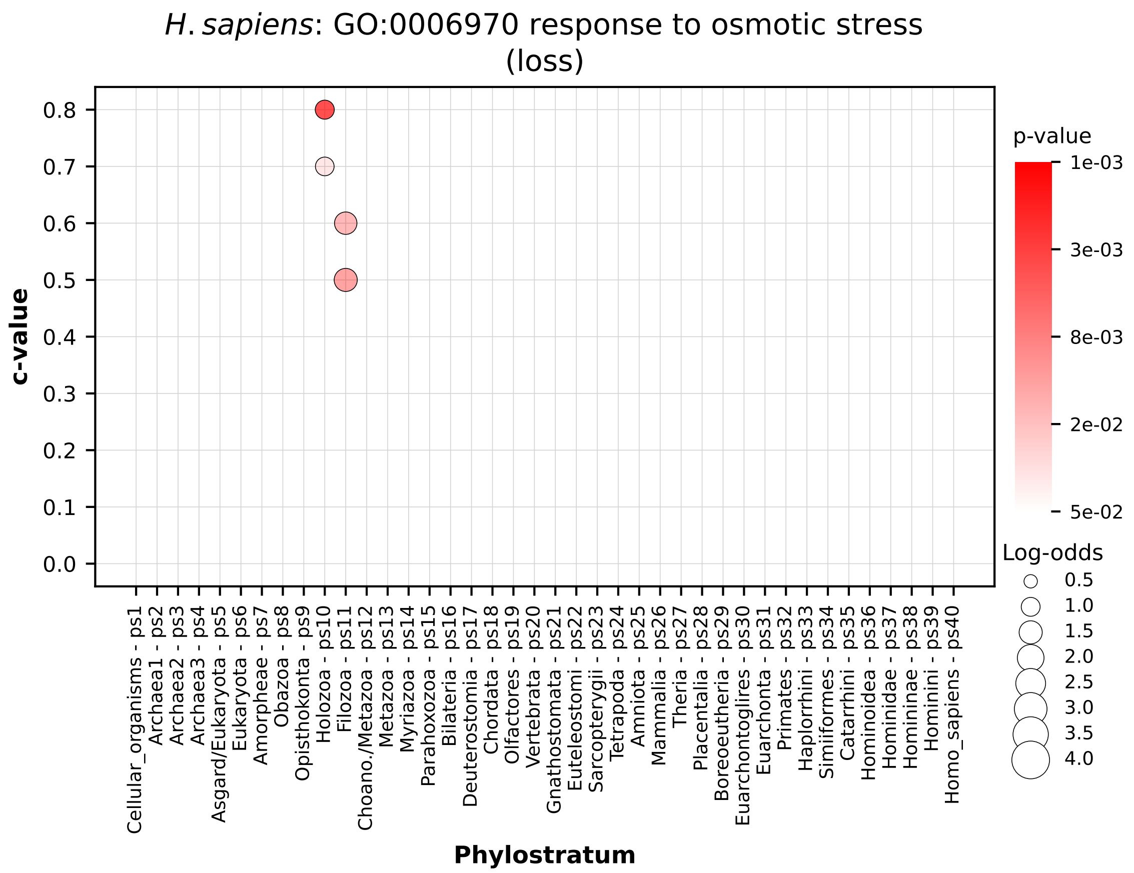

H. sapiens: GO:0006970 response to osmotic stress
(loss)
c-value
0.0
0.1
0.2
0.3
0.4
0.5
0.6
0.7
0.8
Phylostratum
Cellular_organisms - ps1
Archaea1 - ps2
Archaea2 - ps3
Archaea3 - ps4
Asgard/Eukaryota - ps5
Eukaryota - ps6
Amorpheae - ps7
Obazoa - ps8
Opisthokonta - ps9
Holozoa - ps10
Filozoa - ps11
Choano./Metazoa - ps12
Metazoa - ps13
Myriazoa - ps14
Parahoxozoa - ps15
Bilateria - ps16
Deuterostomia - ps17
Chordata - ps18
Olfactores - ps19
Vertebrata - ps20
Gnathostomata - ps21
Euteleostomi - ps22
Sarcopterygii - ps23
Tetrapoda - ps24
Amniota - ps25
Mammalia - ps26
Theria - ps27
Placentalia - ps28
Boreoeutheria - ps29
Euarchontoglires - ps30
Euarchonta - ps31
Primates - ps32
Haplorrhini - ps33
Simiiformes - ps34
Catarrhini - ps35
Hominoidea - ps36
Hominidae - ps37
Homininae - ps38
Hominini - ps39
Homo_sapiens - ps40
p-value
1e-03
3e-03
8e-03
2e-02
5e-02
Log-odds
0.5
1.0
1.5
2.0
2.5
3.0
3.5
4.0

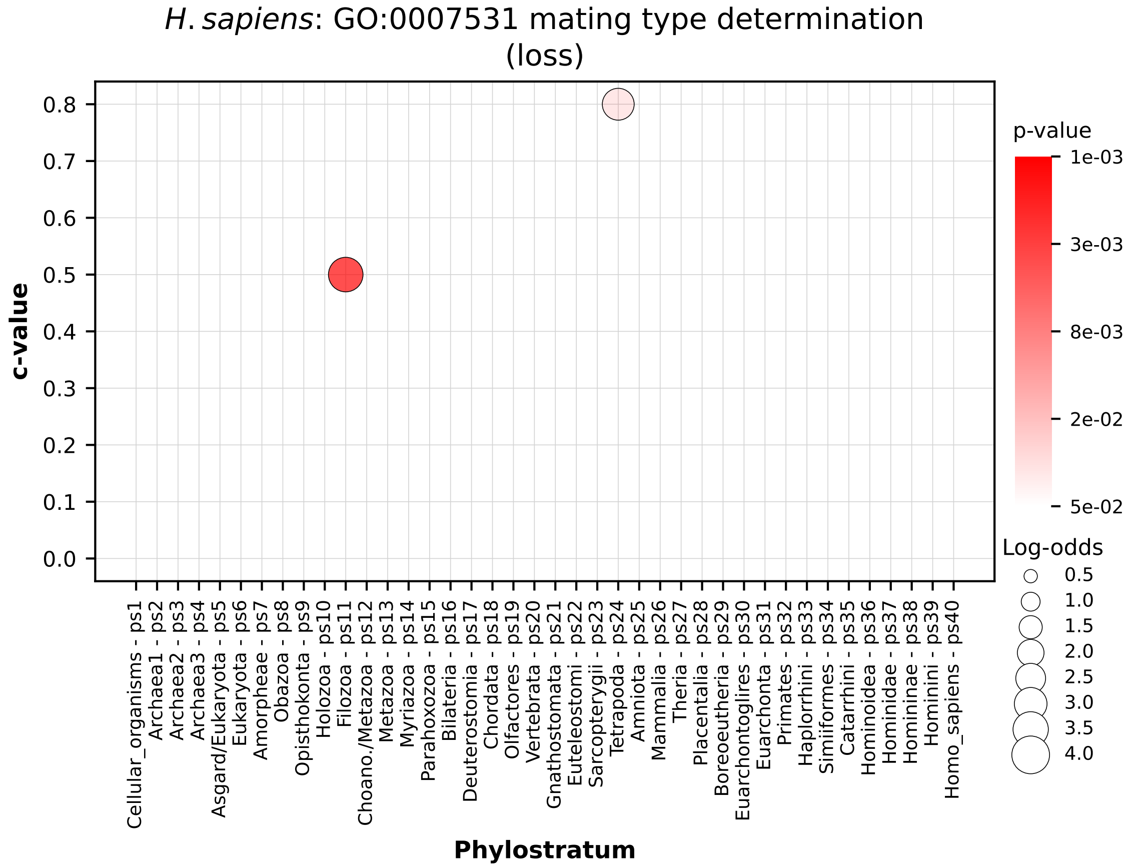

H. sapiens: GO:0007531 mating type determination (loss)
c-value
0.0
0.1
0.2
0.3
0.4
0.5
0.6
0.7
0.8
p-value
1e-03
3e-03
8e-03
2e-02
5e-02
Log-odds
0.5
1.0
1.5
2.0
2.5
3.0
3.5
4.0
Cellular_organisms - ps1
Archaea1 - ps2
Archaea2 - ps3
Archaea3 - ps4
Asgard/Eukaryota - ps5
Eukaryota - ps6
Amorpheae - ps7
Obazoa - ps8
Opisthokonta - ps9
Holozoa - ps10
Filozoa - ps11
Choano./Metazoa - ps12
Metazoa - ps13
Myriazoa - ps14
Parahoxozoa - ps15
Bilateria - ps16
Deuterostomia - ps17
Chordata - ps18
Olfactores - ps19
Vertebrata - ps20
Gnathostomata - ps21
Euteleostomi - ps22
Sarcopterygii - ps23
Tetrapoda - ps24
Amniota - ps25
Mammalia - ps26
Theria - ps27
Placentalia - ps28
Boreoeutheria - ps29
Euarchontoglires - ps30
Euarchonta - ps31
Primates - ps32
Haplorrhini - ps33
Simiiformes - ps34
Catarrhini - ps35
Hominoidea - ps36
Hominidae - ps37
Homininae - ps38
Hominini - ps39
Homo_sapiens - ps40
Phylostratum

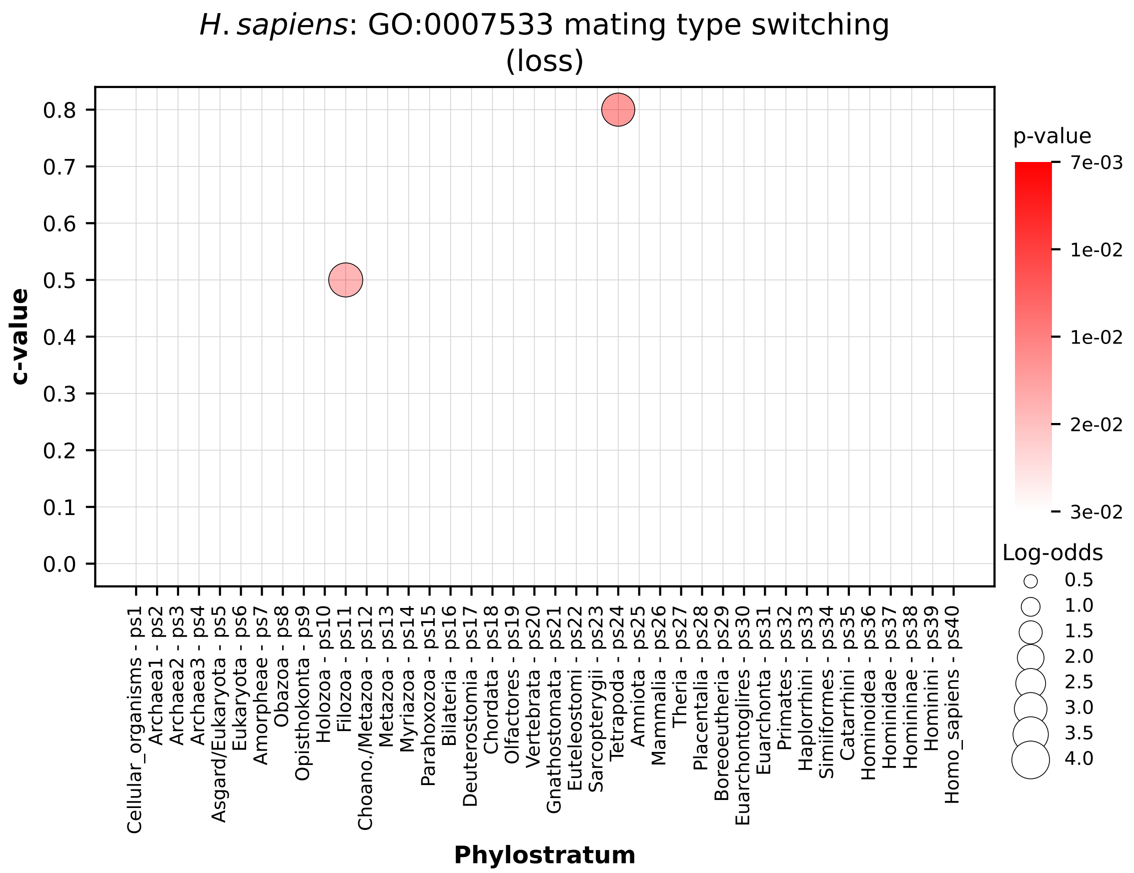

H. sapiens: GO:0007533 mating type switching
(loss)
c-value
0.0
0.1
0.2
0.3
0.4
0.5
0.6
0.7
0.8
p-value
7e-03
1e-02
1e-02
2e-02
3e-02
Log-odds
0.5
1.0
1.5
2.0
2.5
3.0
3.5
4.0
Cellular_organisms - ps1
Archaea1 - ps2
Archaea2 - ps3
Archaea3 - ps4
Asgard/Eukaryota - ps5
Eukaryota - ps6
Amorpheae - ps7
Obazoa - ps8
Opisthokonta - ps9
Holozoa - ps10
Filozoa - ps11
Choano./Metazoa - ps12
Metazoa - ps13
Myriazoa - ps14
Parahoxozoa - ps15
Bilateria - ps16
Deuterostomia - ps17
Chordata - ps18
Olfactores - ps19
Vertebrata - ps20
Gnathostomata - ps21
Euteleostomi - ps22
Sarcopterygii - ps23
Tetrapoda - ps24
Amniota - ps25
Mammalia - ps26
Theria - ps27
Placentalia - ps28
Boreoeutheria - ps29
Euarchontoglires - ps30
Euarchonta - ps31
Primates - ps32
Haplorrhini - ps33
Simiiformes - ps34
Catarrhini - ps35
Hominoidea - ps36
Hominidae - ps37
Homininae - ps38
Hominini - ps39
Homo_sapiens - ps40
Phylostratum

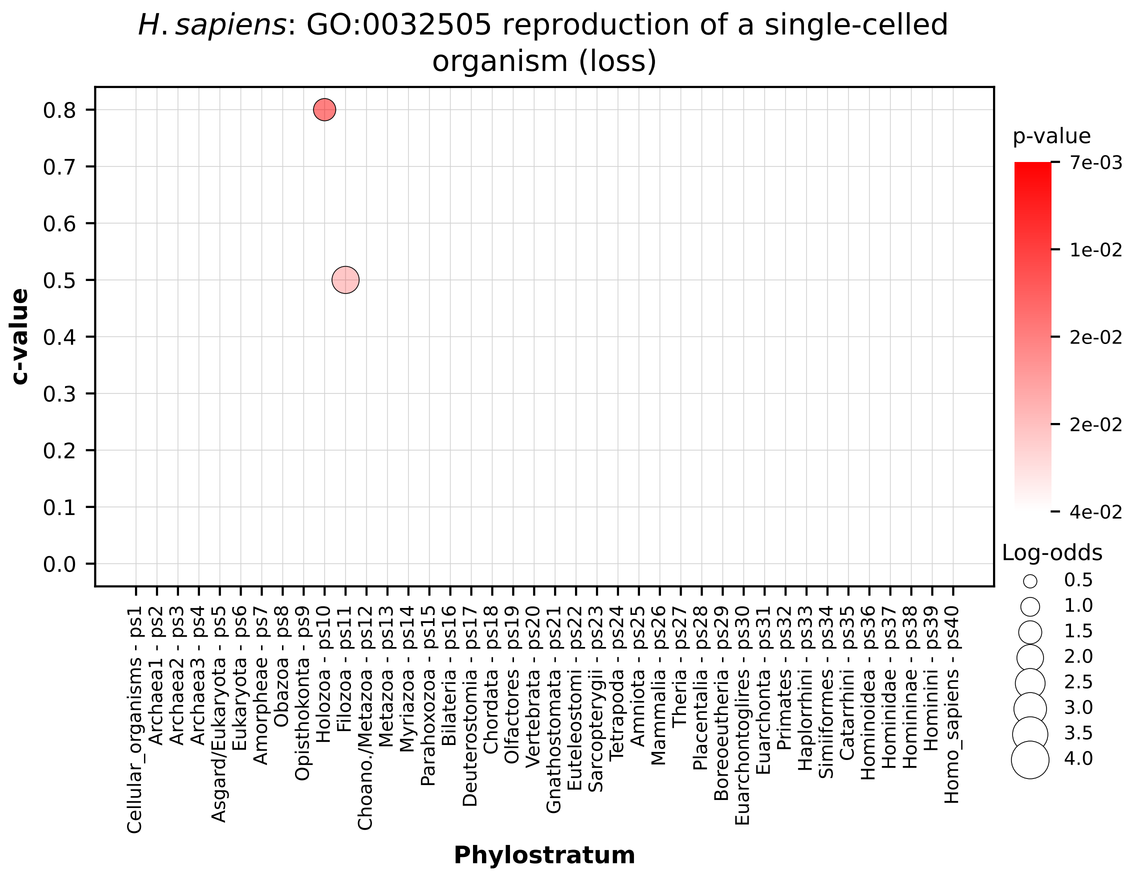

H. sapiens: GO:0032505 reproduction of a single-celled organism (loss)
c-value
0.0
0.1
0.2
0.3
0.4
0.5
0.6
0.7
0.8
p-value
7e-03
1e-02
2e-02
2e-02
4e-02
Log-odds
0.5
1.0
1.5
2.0
2.5
3.0
3.5
4.0
Cellular_organisms - ps1
Archaea1 - ps2
Archaea2 - ps3
Archaea3 - ps4
Asgard/Eukaryota - ps5
Eukaryota - ps6
Amorpheae - ps7
Obazoa - ps8
Opisthokonta - ps9
Holozoa - ps10
Filozoa - ps11
Choano./Metazoa - ps12
Metazoa - ps13
Myriazoa - ps14
Parahoxozoa - ps15
Bilateria - ps16
Deuterostomia - ps17
Chordata - ps18
Olfactores - ps19
Vertebrata - ps20
Gnathostomata - ps21
Euteleostomi - ps22
Sarcopterygii - ps23
Tetrapoda - ps24
Amniota - ps25
Mammalia - ps26
Theria - ps27
Placentalia - ps28
Boreoeutheria - ps29
Euarchontoglires - ps30
Euarchonta - ps31
Primates - ps32
Haplorrhini - ps33
Simiiformes - ps34
Catarrhini - ps35
Hominoidea - ps36
Hominidae - ps37
Homininae - ps38
Hominini - ps39
Homo_sapiens - ps40
Phylostratum

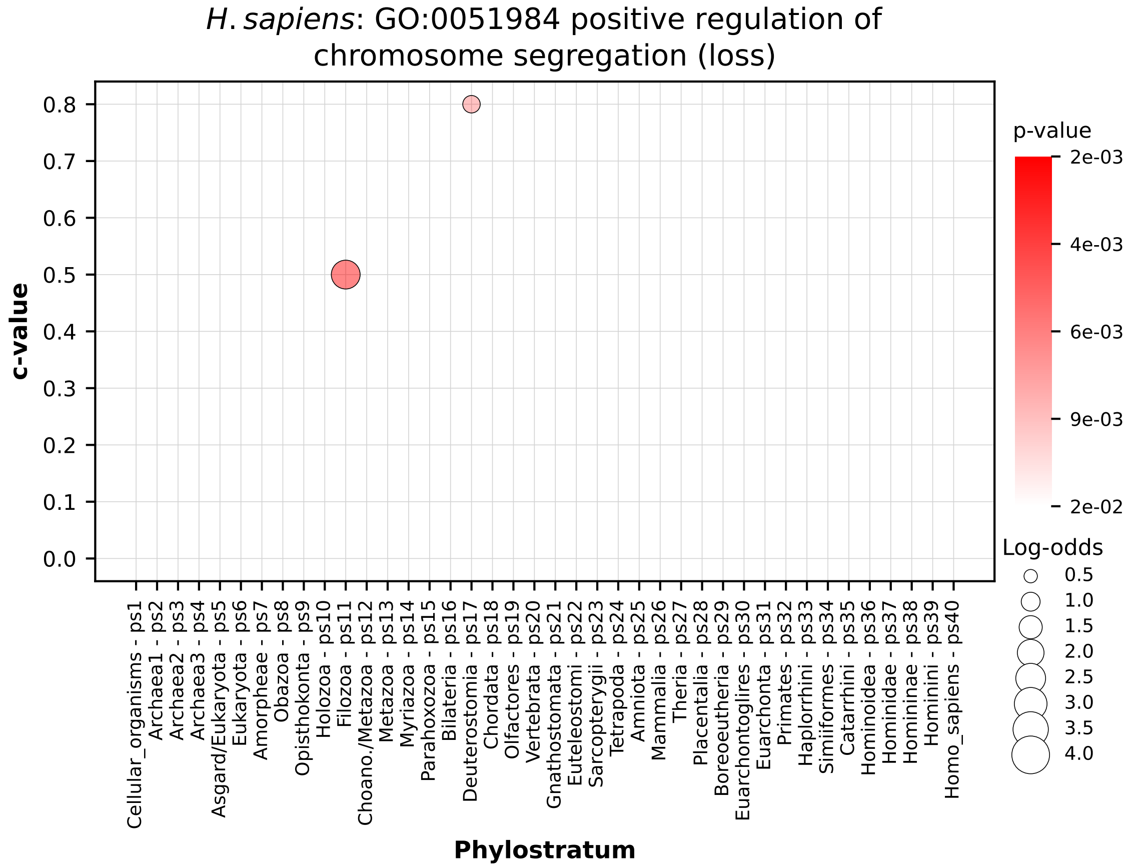

H. sapiens: GO:0051984 positive regulation of chromosome segregation (loss)
c-value
0.0
0.1
0.2
0.3
0.4
0.5
0.6
0.7
0.8
Phylostratum
Cellular_organisms - ps1
Archaea1 - ps2
Archaea2 - ps3
Archaea3 - ps4
Asgard/Eukaryota - ps5
Eukaryota - ps6
Amorpheae - ps7
Obazoa - ps8
Opisthokonta - ps9
Holozoa - ps10
Filozoa - ps11
Choano./Metazoa - ps12
Metazoa - ps13
Myriazoa - ps14
Parahoxozoa - ps15
Bilateria - ps16
Deuterostomia - ps17
Chordata - ps18
Olfactores - ps19
Vertebrata - ps20
Gnathostomata - ps21
Euteleostomi - ps22
Sarcopterygii - ps23
Tetrapoda - ps24
Amniota - ps25
Mammalia - ps26
Theria - ps27
Placentalia - ps28
Boreoeutheria - ps29
Euarchontoglires - ps30
Euarchonta - ps31
Primates - ps32
Haplorrhini - ps33
Simiiformes - ps34
Catarrhini - ps35
Hominoidea - ps36
Hominidae - ps37
Homininae - ps38
Hominini - ps39
Homo_sapiens - ps40
p-value
2e-03
4e-03
6e-03
9e-03
2e-02
Log-odds
0.5
1.0
1.5
2.0
2.5
3.0
3.5
4.0

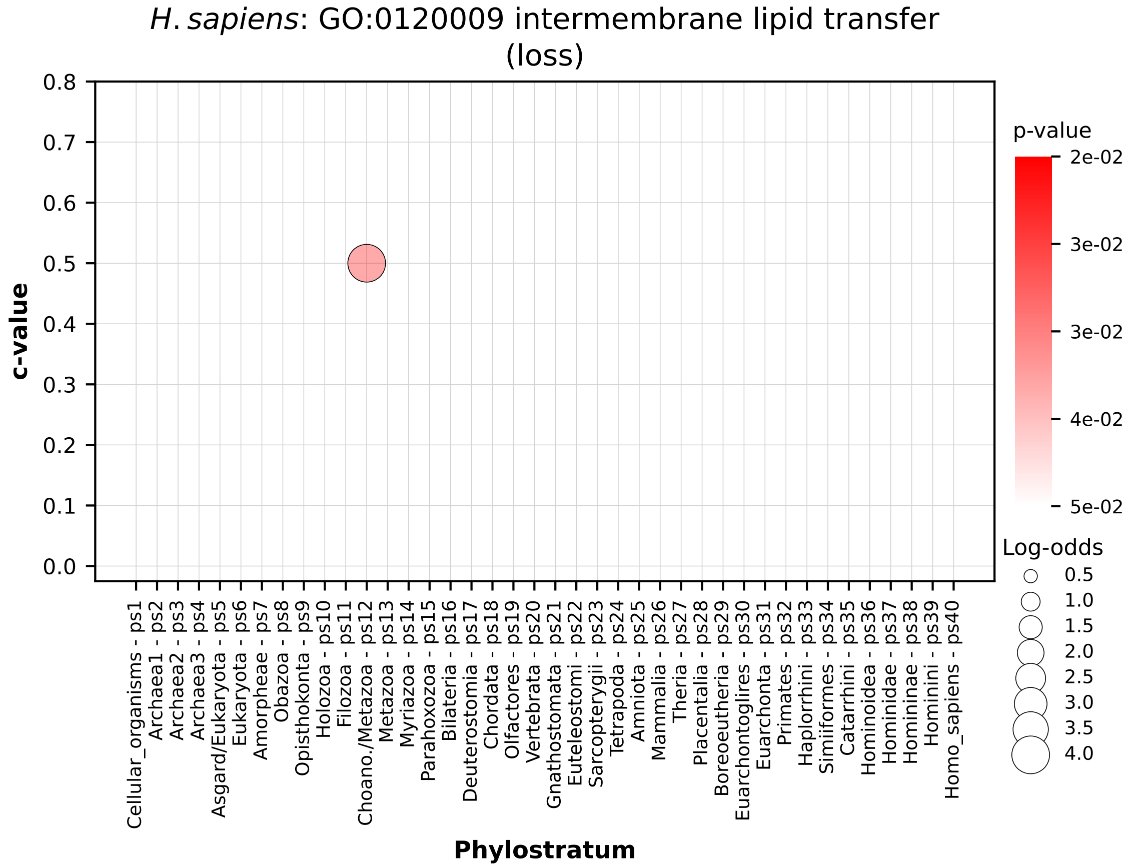

H. sapiens: GO:0120009 intermembrane lipid transfer
(loss)
c-value
0.0
0.1
0.2
0.3
0.4
0.5
0.6
0.7
0.8
p-value
2e-02
3e-02
3e-02
4e-02
5e-02
Log-odds
0.5
1.0
1.5
2.0
2.5
3.0
3.5
4.0
Cellular_organisms - ps1
Archaea1 - ps2
Archaea2 - ps3
Archaea3 - ps4
Asgard/Eukaryota - ps5
Eukaryota - ps6
Amorpheae - ps7
Obazoa - ps8
Opisthokonta - ps9
Holozoa - ps10
Filozoa - ps11
Choano./Metazoa - ps12
Metazoa - ps13
Myriazoa - ps14
Parahoxozoa - ps15
Bilateria - ps16
Deuterostomia - ps17
Chordata - ps18
Olfactores - ps19
Vertebrata - ps20
Gnathostomata - ps21
Euteleostomi - ps22
Sarcopterygii - ps23
Tetrapoda - ps24
Amniota - ps25
Mammalia - ps26
Theria - ps27
Placentalia - ps28
Boreoeutheria - ps29
Euarchontoglires - ps30
Euarchonta - ps31
Primates - ps32
Haplorrhini - ps33
Simiiformes - ps34
Catarrhini - ps35
Hominoidea - ps36
Hominidae - ps37
Homininae - ps38
Hominini - ps39
Homo_sapiens - ps40
Phylostratum

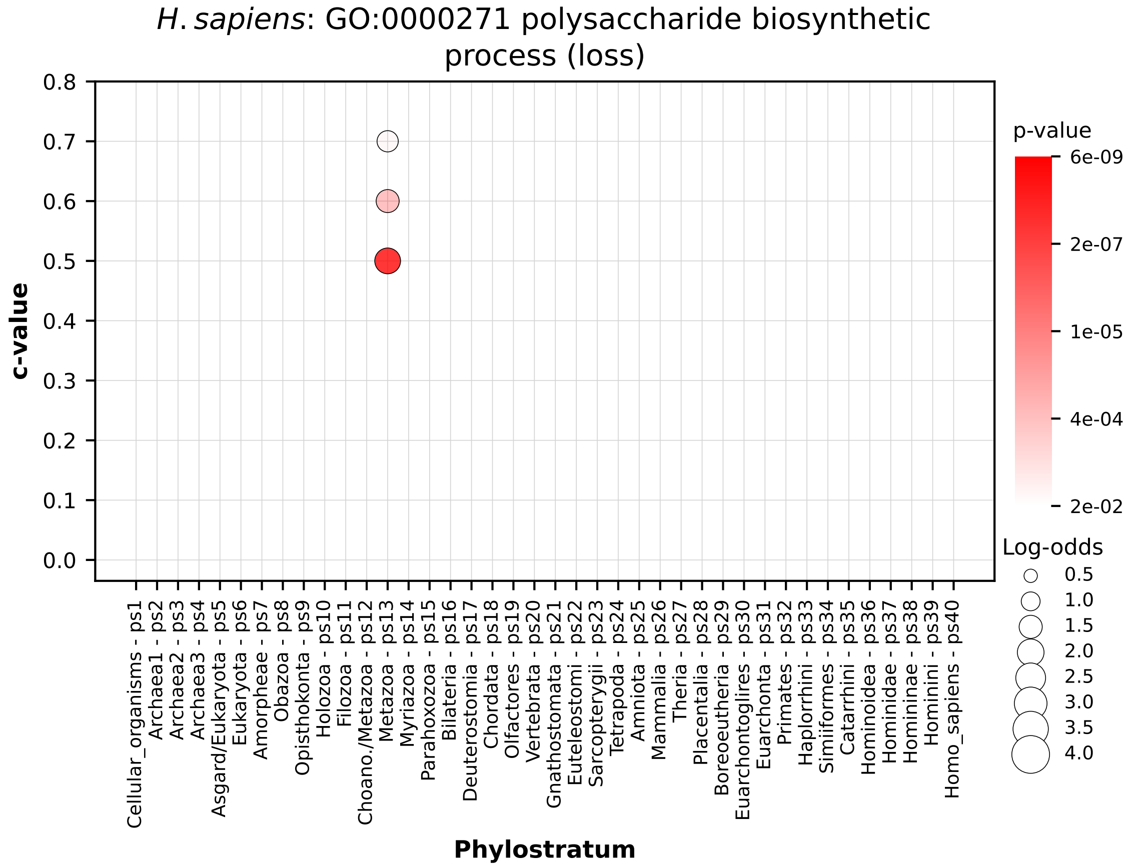

H. sapiens: GO:0000271 polysaccharide biosynthetic process (loss)
c-value
0.8
0.7
0.6
0.5
0.4
0.3
0.2
0.1
0.0
p-value
6e-09
2e-07
1e-05
4e-04
2e-02
Log-odds
0.5
1.0
1.5
2.0
2.5
3.0
3.5
4.0
Cellular_organisms - ps1
Archaea1 - ps2
Archaea2 - ps3
Archaea3 - ps4
Asgard/Eukaryota - ps5
Eukaryota - ps6
Amorpheae - ps7
Obazoa - ps8
Opisthokonta - ps9
Holozoa - ps10
Filozoa - ps11
Choano./Metazoa - ps12
Metazoa - ps13
Myriazoa - ps14
Parahoxozoa - ps15
Bilateria - ps16
Deuterostomia - ps17
Chordata - ps18
Olfactores - ps19
Vertebrata - ps20
Gnathostomata - ps21
Euteleostomi - ps22
Sarcopterygii - ps23
Tetrapoda - ps24
Amniota - ps25
Mammalia - ps26
Theria - ps27
Placentalia - ps28
Boreoeutheria - ps29
Euarchontoglires - ps30
Euarchonta - ps31
Primates - ps32
Haplorrhini - ps33
Simiiformes - ps34
Catarrhini - ps35
Hominoidea - ps36
Hominidae - ps37
Homininae - ps38
Hominini - ps39
Homo_sapiens - ps40
Phylostratum

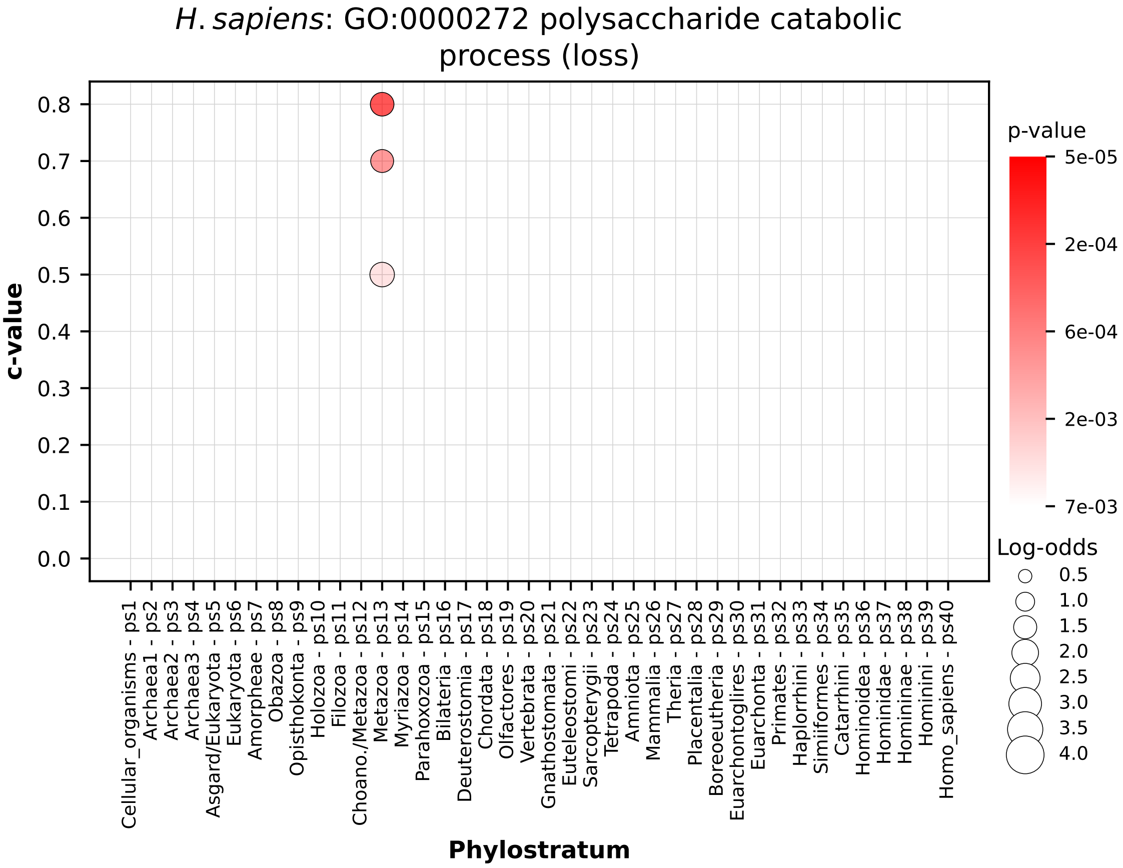

$H. sapiens$: GO:0000272 polysaccharide catabolic process (loss)
c-value
0.0
0.1
0.2
0.3
0.4
0.5
0.6
0.7
0.8
Phylostratum
Cellular_organisms - ps1
Archaea1 - ps2
Archaea2 - ps3
Archaea3 - ps4
Asgard/Eukaryota - ps5
Eukaryota - ps6
Amorpheae - ps7
Obazoa - ps8
Opisthokonta - ps9
Holozoa - ps10
Filozoa - ps11
Choano./Metazoa - ps12
Metazoa - ps13
Myriazoa - ps14
Parahoxozoa - ps15
Bilateria - ps16
Deuterostomia - ps17
Chordata - ps18
Olfactores - ps19
Vertebrata - ps20
Gnathostomata - ps21
Euteleostomi - ps22
Sarcopterygii - ps23
Tetrapoda - ps24
Amniota - ps25
Mammalia - ps26
Theria - ps27
Placentalia - ps28
Boreoeutheria - ps29
Euarchontoglires - ps30
Euarchonta - ps31
Primates - ps32
Haplorrhini - ps33
Simiiformes - ps34
Catarrhini - ps35
Hominoidea - ps36
Hominidae - ps37
Homininae - ps38
Hominini - ps39
Homo_sapiens - ps40
p-value
5e-05
2e-04
6e-04
2e-03
7e-03
Log-odds
0.5
1.0
1.5
2.0
2.5
3.0
3.5
4.0

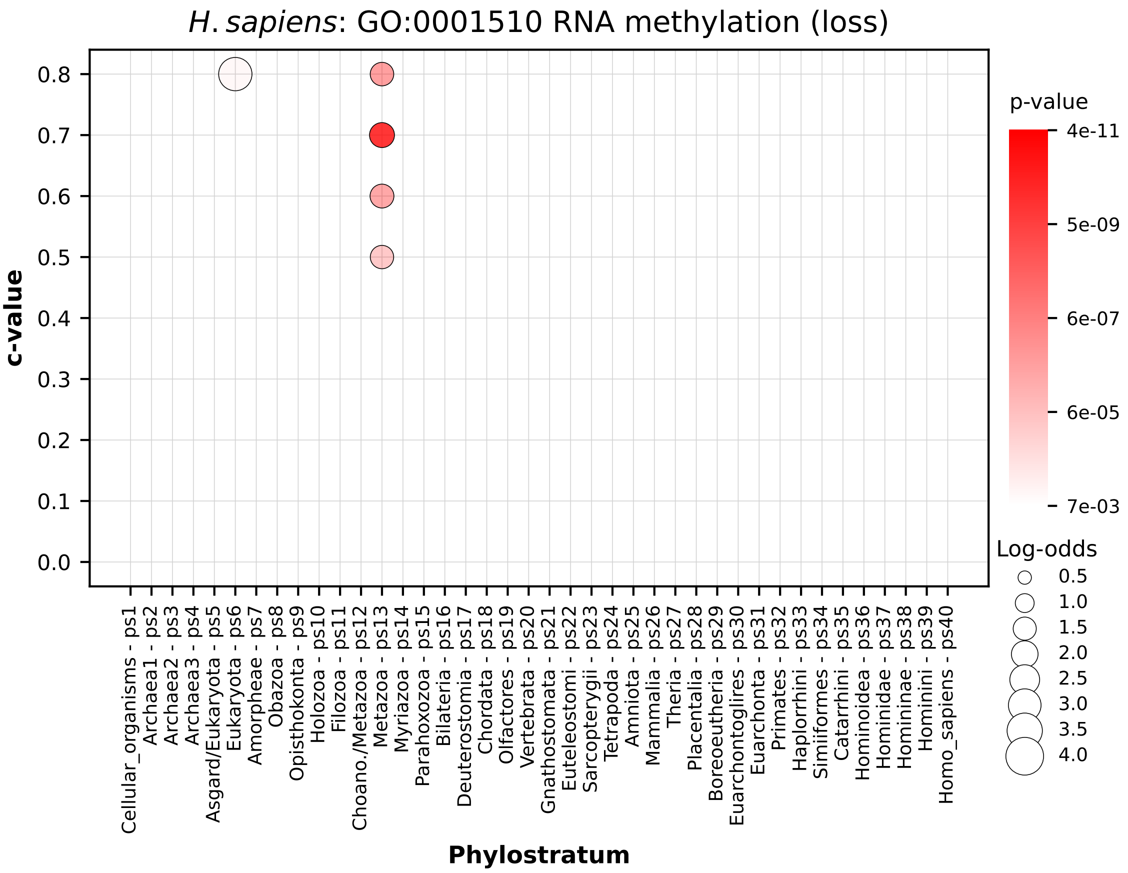
H. sapiens: GO:0001510 RNA methylation (loss)
c-value
0.0
0.1
0.2
0.3
0.4
0.5
0.6
0.7
0.8
Phylostratum
Cellular_organisms - ps1
Archaea1 - ps2
Archaea2 - ps3
Archaea3 - ps4
Asgard/Eukaryota - ps5
Eukaryota - ps6
Amorpheae - ps7
Obazoa - ps8
Opisthokonta - ps9
Holozoa - ps10
Filozoa - ps11
Choano./Metazoa - ps12
Metazoa - ps13
Myriazoa - ps14
Parahoxozoa - ps15
Bilateria - ps16
Deuterostomia - ps17
Chordata - ps18
Olfactores - ps19
Vertebrata - ps20
Gnathostomata - ps21
Euteleostomi - ps22
Sarcopterygii - ps23
Tetrapoda - ps24
Amniota - ps25
Mammalia - ps26
Theria - ps27
Placentalia - ps28
Boreoeutheria - ps29
Euarchontoglires - ps30
Euarchonta - ps31
Primates - ps32
Haplorrhini - ps33
Simiiformes - ps34
Catarrhini - ps35
Hominoidea - ps36
Hominidae - ps37
Homininae - ps38
Hominini - ps39
Homo_sapiens - ps40
p-value
4e-11
5e-09
6e-07
6e-05
7e-03
Log-odds
0.5
1.0
1.5
2.0
2.5
3.0
3.5
4.0

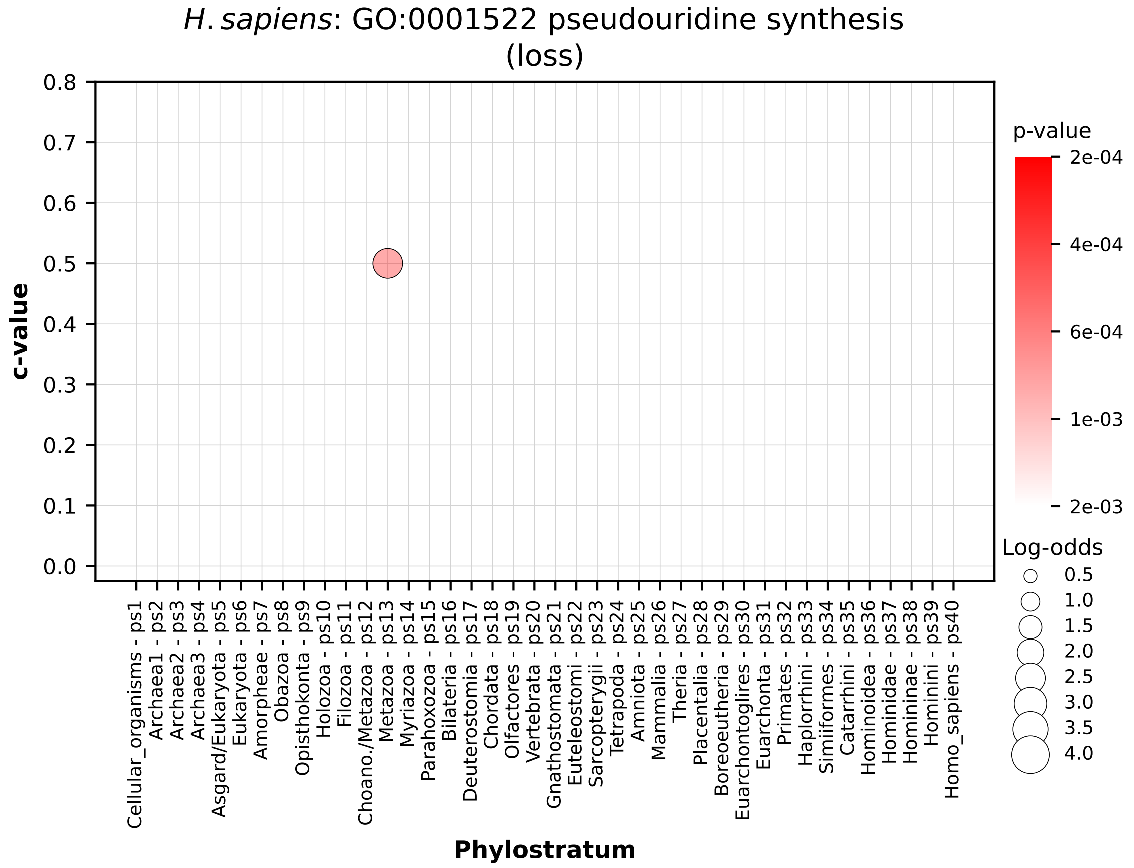

H. sapiens: GO:0001522 pseudouridine synthesis
(loss)
c-value
0.8
0.7
0.6
0.5
0.4
0.3
0.2
0.1
0.0
p-value
2e-04
4e-04
6e-04
1e-03
2e-03
Log-odds
0.5
1.0
1.5
2.0
2.5
3.0
3.5
4.0
Cellular_organisms - ps1
Archaea1 - ps2
Archaea2 - ps3
Archaea3 - ps4
Asgard/Eukaryota - ps5
Eukaryota - ps6
Amorpheae - ps7
Obazoa - ps8
Opisthokonta - ps9
Holozoa - ps10
Filozoa - ps11
Choano./Metazoa - ps12
Metazoa - ps13
Myriazoa - ps14
Parahoxozoa - ps15
Bilateria - ps16
Deuterostomia - ps17
Chordata - ps18
Olfactores - ps19
Vertebrata - ps20
Gnathostomata - ps21
Euteleostomi - ps22
Sarcopterygii - ps23
Tetrapoda - ps24
Amniota - ps25
Mammalia - ps26
Theria - ps27
Placentalia - ps28
Boreoeutheria - ps29
Euarchontoglires - ps30
Euarchonta - ps31
Primates - ps32
Haplorrhini - ps33
Simiiformes - ps34
Catarrhini - ps35
Hominoidea - ps36
Hominidae - ps37
Homininae - ps38
Hominini - ps39
Homo_sapiens - ps40
Phylostratum

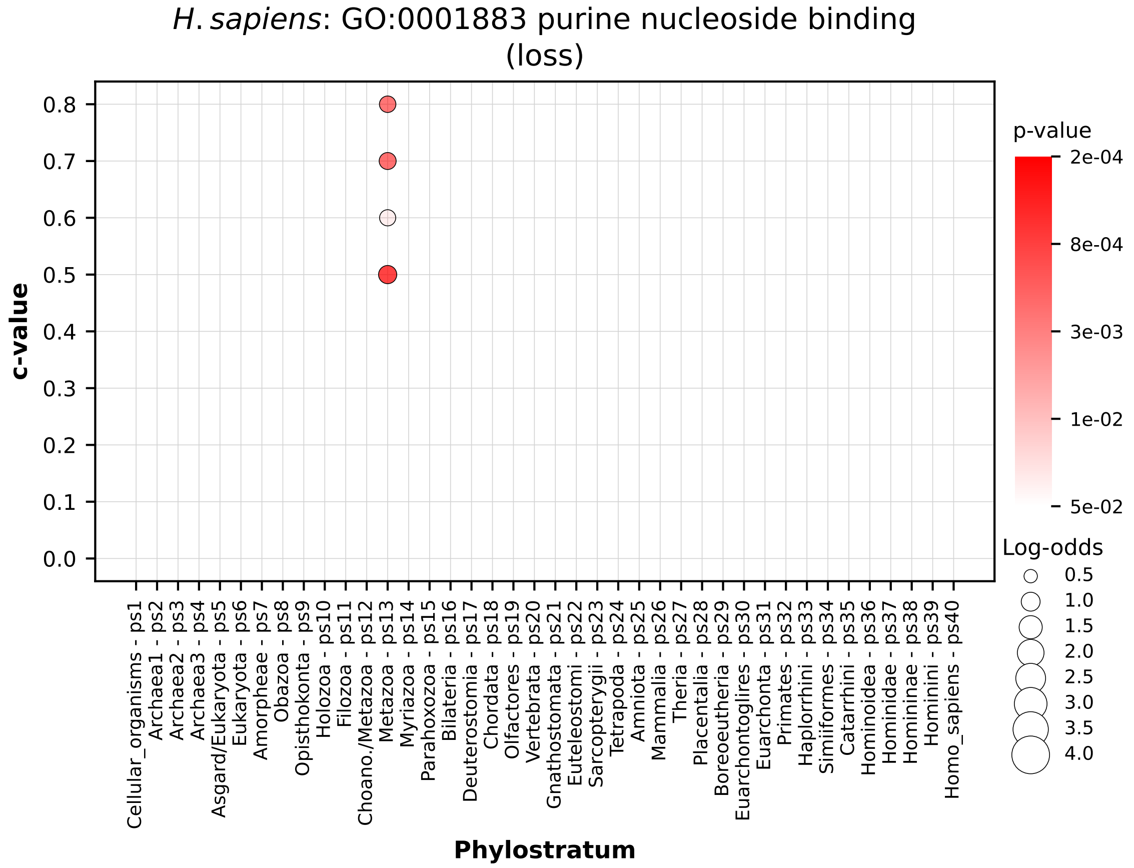

H. sapiens: GO:0001883 purine nucleoside binding
(loss)
c-value
0.0
0.1
0.2
0.3
0.4
0.5
0.6
0.7
0.8
p-value
2e-04
8e-04
3e-03
1e-02
5e-02
Log-odds
0.5
1.0
1.5
2.0
2.5
3.0
3.5
4.0
Cellular_organisms - ps1
Archaea1 - ps2
Archaea2 - ps3
Archaea3 - ps4
Asgard/Eukaryota - ps5
Eukaryota - ps6
Amorpheae - ps7
Obazoa - ps8
Opisthokonta - ps9
Holozoa - ps10
Filozoa - ps11
Choano./Metazoa - ps12
Metazoa - ps13
Myriazoa - ps14
Parahoxozoa - ps15
Bilateria - ps16
Deuterostomia - ps17
Chordata - ps18
Olfactores - ps19
Vertebrata - ps20
Gnathostomata - ps21
Euteleostomi - ps22
Sarcopterygii - ps23
Tetrapoda - ps24
Amniota - ps25
Mammalia - ps26
Theria - ps27
Placentalia - ps28
Boreoeutheria - ps29
Euarchontoglires - ps30
Euarchonta - ps31
Primates - ps32
Haplorrhini - ps33
Simiiformes - ps34
Catarrhini - ps35
Hominoidea - ps36
Hominidae - ps37
Homininae - ps38
Hominini - ps39
Homo_sapiens - ps40
Phylostratum

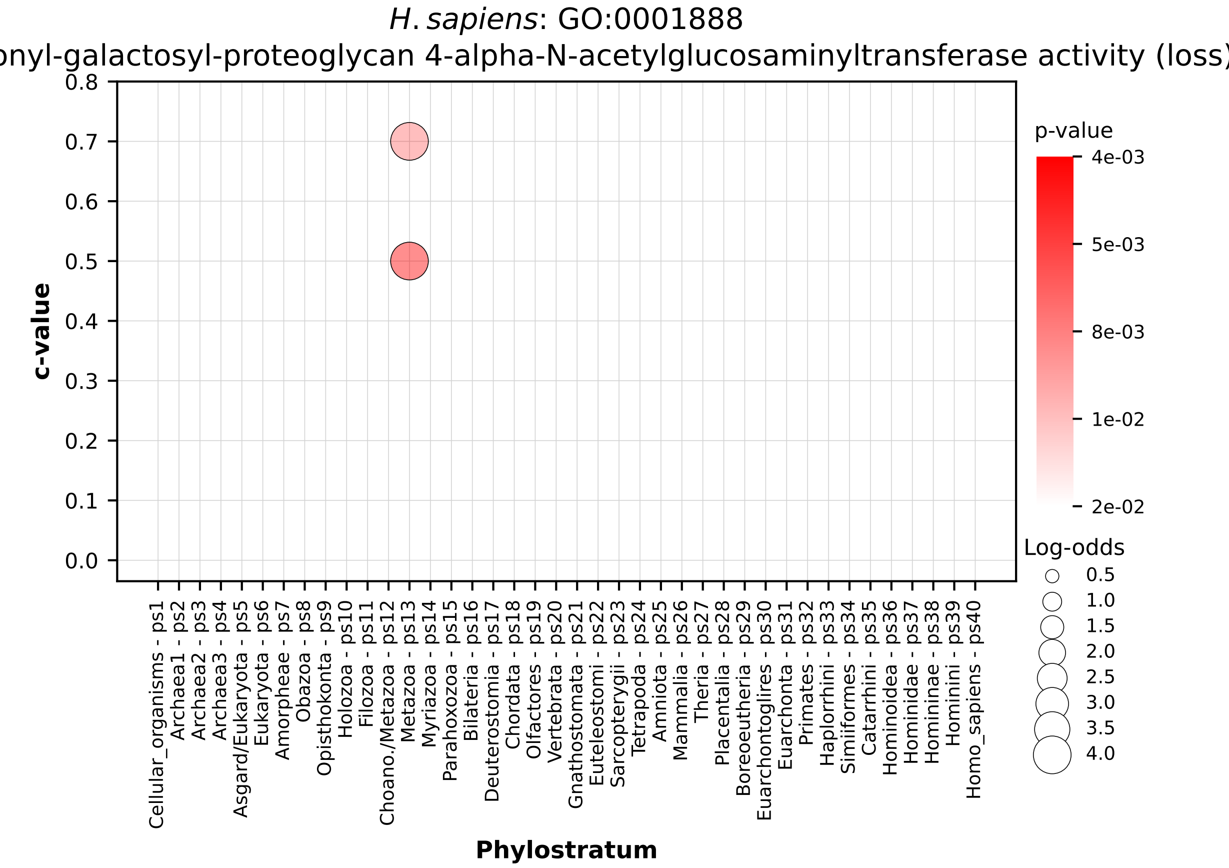
H. sapiens: GO:0001888
onyl-galactosyl-proteoglycan 4-alpha-N-acetylglucosaminyltransferase activity (loss)
c-value
0.8
0.7
0.6
0.5
0.4
0.3
0.2
0.1
0.0
p-value
4e-03
5e-03
8e-03
1e-02
2e-02
Log-odds
0.5
1.0
1.5
2.0
2.5
3.0
3.5
4.0
Cellular_organisms - ps1
Archaea1 - ps2
Archaea2 - ps3
Archaea3 - ps4
Asgard/Eukaryota - ps5
Eukaryota - ps6
Amorpheae - ps7
Obazoa - ps8
Opisthokonta - ps9
Holozoa - ps10
Filozoa - ps11
Choano./Metazoa - ps12
Metazoa - ps13
Myriazoa - ps14
Parahoxozoa - ps15
Bilateria - ps16
Deuterostomia - ps17
Chordata - ps18
Olfactores - ps19
Vertebrata - ps20
Gnathostomata - ps21
Euteleostomi - ps22
Sarcopterygii - ps23
Tetrapoda - ps24
Amniota - ps25
Mammalia - ps26
Theria - ps27
Placentalia - ps28
Boreoeutheria - ps29
Euarchontoglires - ps30
Euarchonta - ps31
Primates - ps32
Haplorrhini - ps33
Simiiformes - ps34
Catarrhini - ps35
Hominoidea - ps36
Hominidae - ps37
Homininae - ps38
Hominini - ps39
Homo_sapiens - ps40
Phylostratum

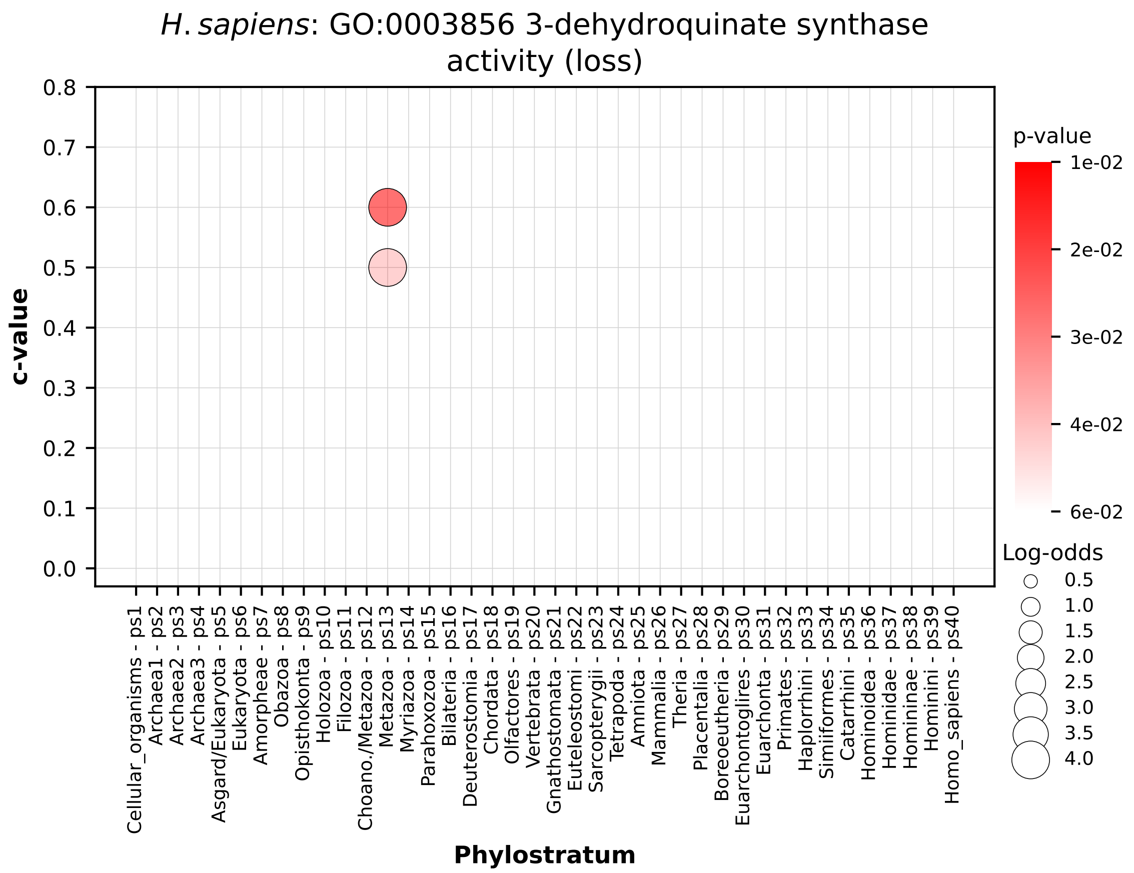

H. sapiens: GO:0003856 3-dehydroquinate synthase activity (loss)
c-value
0.8
0.7
0.6
0.5
0.4
0.3
0.2
0.1
0.0
p-value
1e-02
2e-02
3e-02
4e-02
6e-02
Log-odds
0.5
1.0
1.5
2.0
2.5
3.0
3.5
4.0
Cellular_organisms - ps1
Archaea1 - ps2
Archaea2 - ps3
Archaea3 - ps4
Asgard/Eukaryota - ps5
Eukaryota - ps6
Amorpheae - ps7
Obazoa - ps8
Opisthokonta - ps9
Holozoa - ps10
Filozoa - ps11
Choano./Metazoa - ps12
Metazoa - ps13
Myriazoa - ps14
Parahoxozoa - ps15
Bilateria - ps16
Deuterostomia - ps17
Chordata - ps18
Olfactores - ps19
Vertebrata - ps20
Gnathostomata - ps21
Euteleostomi - ps22
Sarcopterygii - ps23
Tetrapoda - ps24
Amniota - ps25
Mammalia - ps26
Theria - ps27
Placentalia - ps28
Boreoeutheria - ps29
Euarchontoglires - ps30
Euarchonta - ps31
Primates - ps32
Haplorrhini - ps33
Simiiformes - ps34
Catarrhini - ps35
Hominoidea - ps36
Hominidae - ps37
Homininae - ps38
Hominini - ps39
Homo_sapiens - ps40
Phylostratum

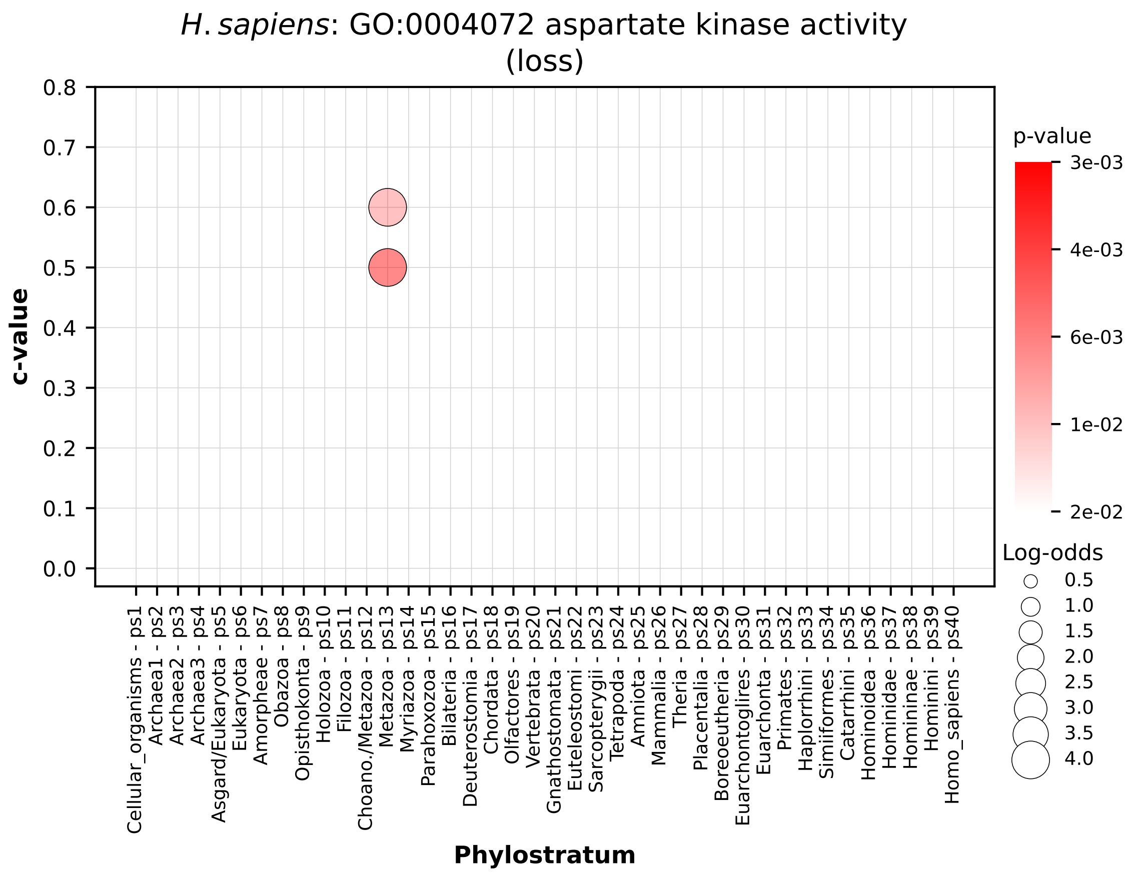

H. sapiens: GO:0004072 aspartate kinase activity
(loss)
c-value
0.0
0.1
0.2
0.3
0.4
0.5
0.6
0.7
0.8
Phylostratum
Cellular_organisms - ps1
Archaea1 - ps2
Archaea2 - ps3
Archaea3 - ps4
Asgard/Eukaryota - ps5
Eukaryota - ps6
Amorpheae - ps7
Obazoa - ps8
Opisthokonta - ps9
Holozoa - ps10
Filozoa - ps11
Choano./Metazoa - ps12
Metazoa - ps13
Myriazoa - ps14
Parahoxozoa - ps15
Bilateria - ps16
Deuterostomia - ps17
Chordata - ps18
Olfactores - ps19
Vertebrata - ps20
Gnathostomata - ps21
Euteleostomi - ps22
Sarcopterygii - ps23
Tetrapoda - ps24
Amniota - ps25
Mammalia - ps26
Theria - ps27
Placentalia - ps28
Boreoeutheria - ps29
Euarchontoglires - ps30
Euarchonta - ps31
Primates - ps32
Haplorrhini - ps33
Simiiformes - ps34
Catarrhini - ps35
Hominoidea - ps36
Hominidae - ps37
Homininae - ps38
Hominini - ps39
Homo_sapiens - ps40
p-value
3e-03
4e-03
6e-03
1e-02
2e-02
Log-odds
0.5
1.0
1.5
2.0
2.5
3.0
3.5
4.0

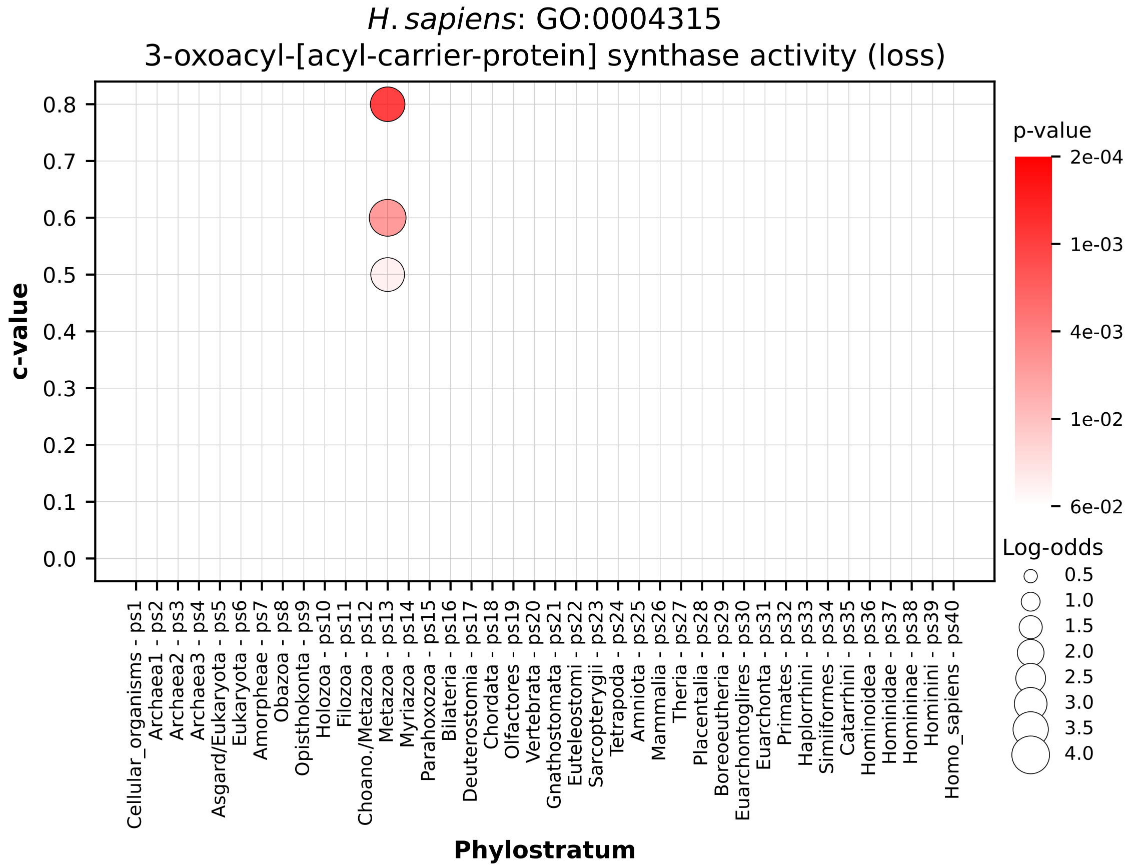

H. sapiens: GO:0004315
3-oxoacyl-[acyl-carrier-protein] synthase activity (loss)
c-value
0.8
0.7
0.6
0.5
0.4
0.3
0.2
0.1
0.0
Phylostratum
Cellular_organisms - ps1
Archaea1 - ps2
Archaea2 - ps3
Archaea3 - ps4
Asgard/Eukaryota - ps5
Eukaryota - ps6
Amorpheae - ps7
Obazoa - ps8
Opisthokonta - ps9
Holozoa - ps10
Filozoa - ps11
Choano./Metazoa - ps12
Metazoa - ps13
Myriazoa - ps14
Parahoxozoa - ps15
Bilateria - ps16
Deuterostomia - ps17
Chordata - ps18
Olfactores - ps19
Vertebrata - ps20
Gnathostomata - ps21
Euteleostomi - ps22
Sarcopterygii - ps23
Tetrapoda - ps24
Amniota - ps25
Mammalia - ps26
Theria - ps27
Placentalia - ps28
Boreoeutheria - ps29
Euarchontoglires - ps30
Euarchonta - ps31
Primates - ps32
Haplorrhini - ps33
Simiiformes - ps34
Catarrhini - ps35
Hominoidea - ps36
Hominidae - ps37
Homininae - ps38
Hominini - ps39
Homo_sapiens - ps40
p-value
2e-04
1e-03
4e-03
1e-02
6e-02
Log-odds
0.5
1.0
1.5
2.0
2.5
3.0
3.5
4.0

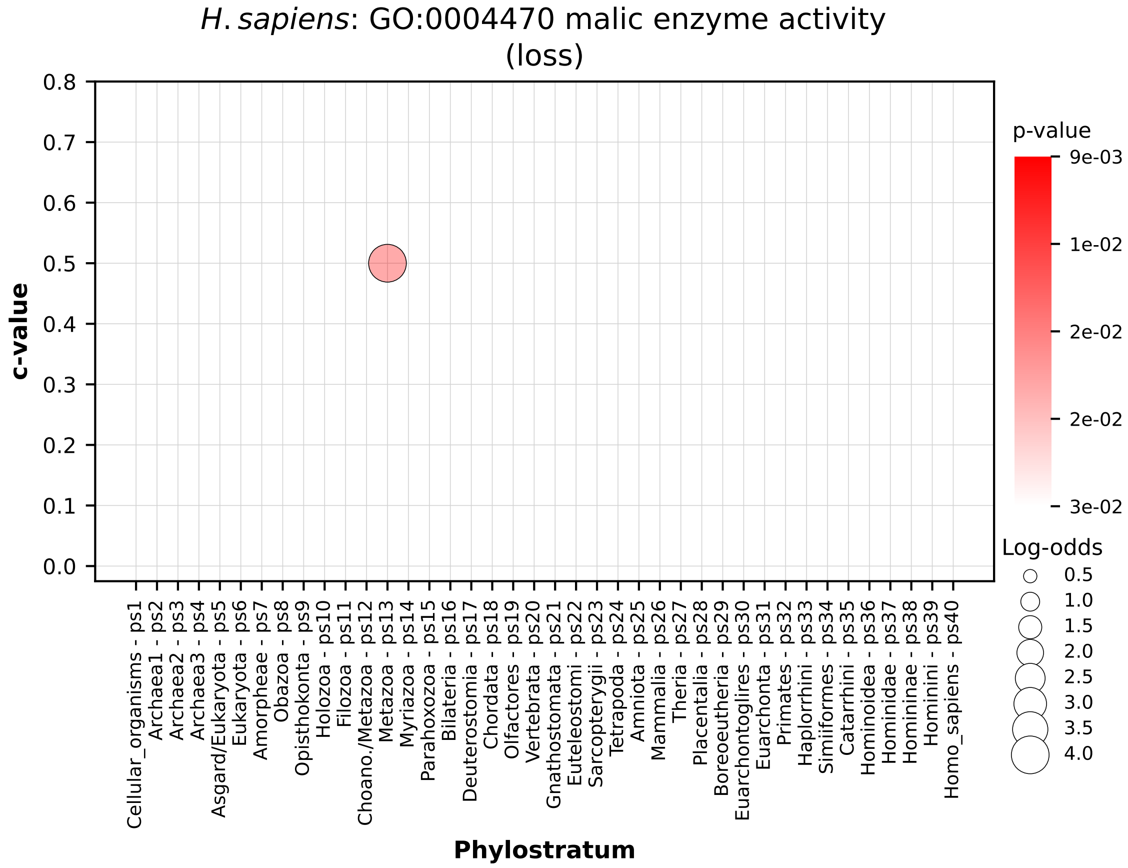

H. sapiens: GO:0004470 malic enzyme activity
(loss)
c-value
0.8
0.7
0.6
0.5
0.4
0.3
0.2
0.1
0.0
p-value
9e-03
1e-02
2e-02
2e-02
3e-02
Log-odds
0.5
1.0
1.5
2.0
2.5
3.0
3.5
4.0
Cellular_organisms - ps1
Archaea1 - ps2
Archaea2 - ps3
Archaea3 - ps4
Asgard/Eukaryota - ps5
Eukaryota - ps6
Amorpheae - ps7
Obazoa - ps8
Opisthokonta - ps9
Holozoa - ps10
Filozoa - ps11
Choano./Metazoa - ps12
Metazoa - ps13
Myriazoa - ps14
Parahoxozoa - ps15
Bilateria - ps16
Deuterostomia - ps17
Chordata - ps18
Olfactores - ps19
Vertebrata - ps20
Gnathostomata - ps21
Euteleostomi - ps22
Sarcopterygii - ps23
Tetrapoda - ps24
Amniota - ps25
Mammalia - ps26
Theria - ps27
Placentalia - ps28
Boreoeutheria - ps29
Euarchontoglires - ps30
Euarchonta - ps31
Primates - ps32
Haplorrhini - ps33
Simiiformes - ps34
Catarrhini - ps35
Hominoidea - ps36
Hominidae - ps37
Homininae - ps38
Hominini - ps39
Homo_sapiens - ps40
Phylostratum

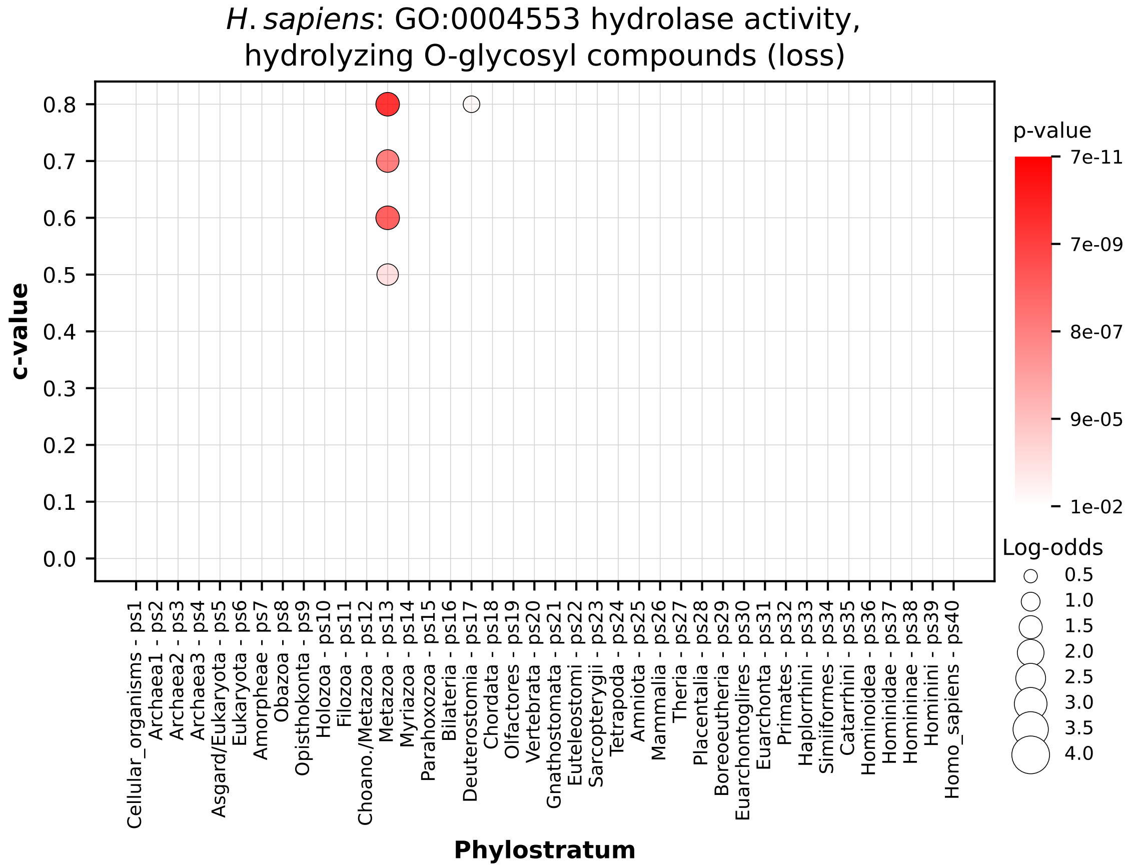
H. sapiens: GO:0004553 hydrolase activity, hydrolyzing O-glycosyl compounds (loss)
c-value
0.0
0.1
0.2
0.3
0.4
0.5
0.6
0.7
0.8
Phylostratum
Cellular_organisms - ps1
Archaea1 - ps2
Archaea2 - ps3
Archaea3 - ps4
Asgard/Eukaryota - ps5
Eukaryota - ps6
Amorpheae - ps7
Obazoa - ps8
Opisthokonta - ps9
Holozoa - ps10
Filozoa - ps11
Choano./Metazoa - ps12
Metazoa - ps13
Myriazoa - ps14
Parahoxozoa - ps15
Bilateria - ps16
Deuterostomia - ps17
Chordata - ps18
Olfactores - ps19
Vertebrata - ps20
Gnathostomata - ps21
Euteleostomi - ps22
Sarcopterygii - ps23
Tetrapoda - ps24
Amniota - ps25
Mammalia - ps26
Theria - ps27
Placentalia - ps28
Boreoeutheria - ps29
Euarchontoglires - ps30
Euarchonta - ps31
Primates - ps32
Haplorrhini - ps33
Simiiformes - ps34
Catarrhini - ps35
Hominoidea - ps36
Hominidae - ps37
Homininae - ps38
Hominini - ps39
Homo_sapiens - ps40
p-value
7e-11
7e-09
8e-07
9e-05
1e-02
Log-odds
0.5
1.0
1.5
2.0
2.5
3.0
3.5
4.0

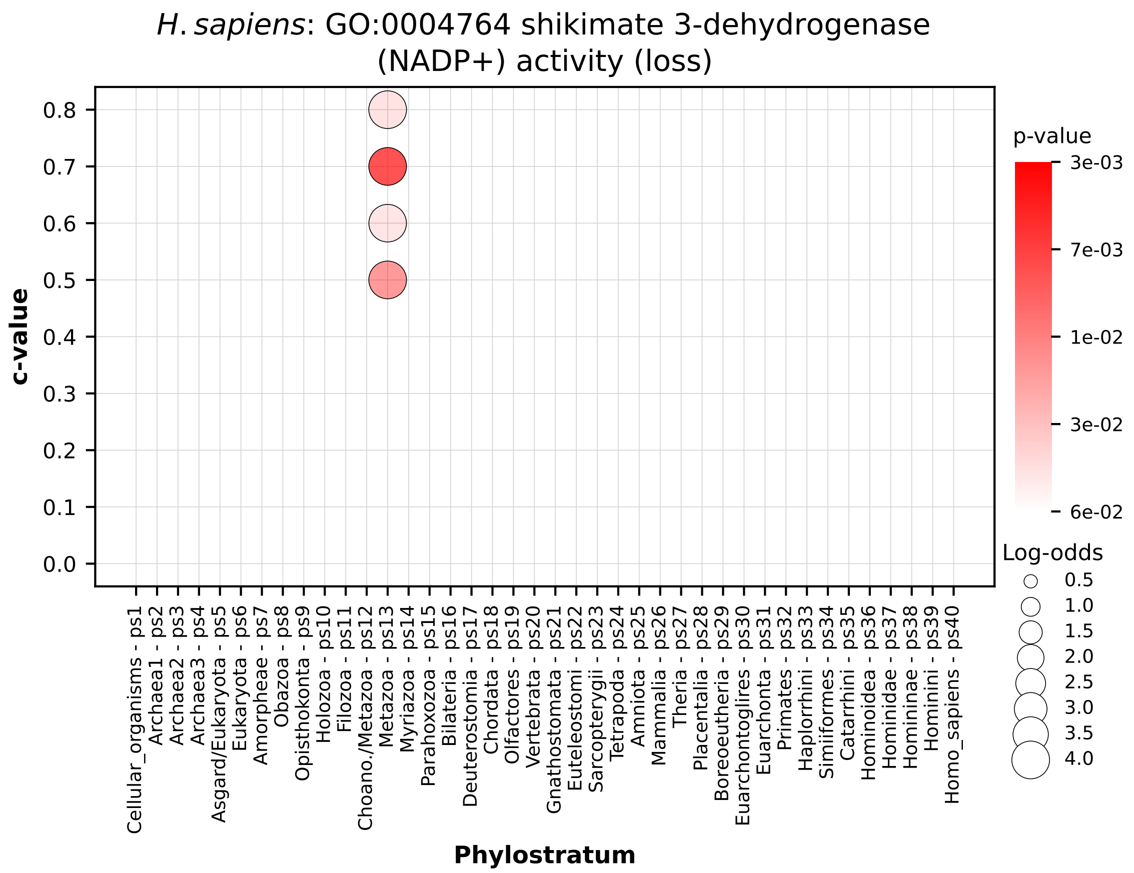
H. sapiens: GO:0004764 shikimate 3-dehydrogenase (NADP+) activity (loss)
c-value
0.0
0.1
0.2
0.3
0.4
0.5
0.6
0.7
0.8
Phylostratum
Cellular_organisms - ps1
Archaea1 - ps2
Archaea2 - ps3
Archaea3 - ps4
Asgard/Eukaryota - ps5
Eukaryota - ps6
Amorpheae - ps7
Obazoa - ps8
Opisthokonta - ps9
Holozoa - ps10
Filozoa - ps11
Choano./Metazoa - ps12
Metazoa - ps13
Myriazoa - ps14
Parahoxozoa - ps15
Bilateria - ps16
Deuterostomia - ps17
Chordata - ps18
Olfactores - ps19
Vertebrata - ps20
Gnathostomata - ps21
Euteleostomi - ps22
Sarcopterygii - ps23
Tetrapoda - ps24
Amniota - ps25
Mammalia - ps26
Theria - ps27
Placentalia - ps28
Boreoeutheria - ps29
Euarchontoglires - ps30
Euarchonta - ps31
Primates - ps32
Haplorrhini - ps33
Simiiformes - ps34
Catarrhini - ps35
Hominoidea - ps36
Hominidae - ps37
Homininae - ps38
Hominini - ps39
Homo_sapiens - ps40
p-value
3e-03
7e-03
1e-02
3e-02
6e-02
Log-odds
0.5
1.0
1.5
2.0
2.5
3.0
3.5
4.0

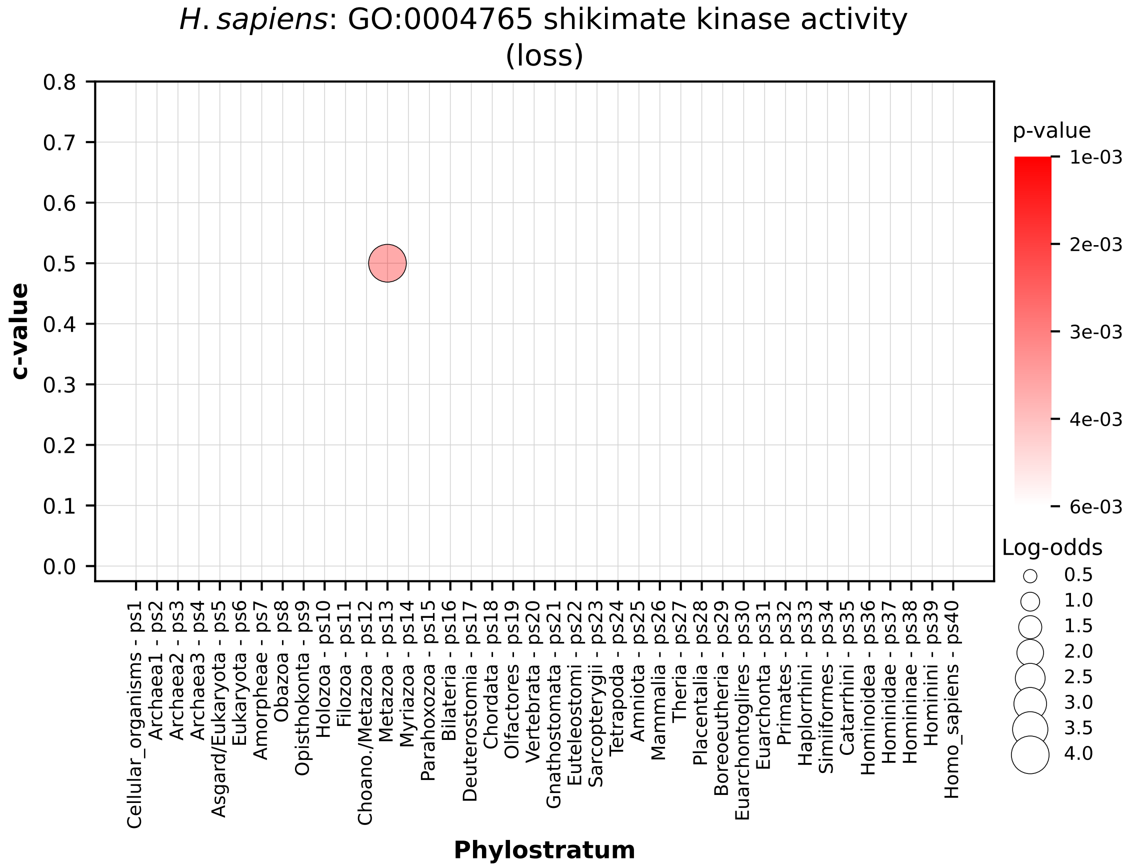

H. sapiens: GO:0004765 shikimate kinase activity
(loss)
c-value
0.0
0.1
0.2
0.3
0.4
0.5
0.6
0.7
0.8
p-value
1e-03
2e-03
3e-03
4e-03
6e-03
Log-odds
0.5
1.0
1.5
2.0
2.5
3.0
3.5
4.0
Cellular_organisms - ps1
Archaea1 - ps2
Archaea2 - ps3
Archaea3 - ps4
Asgard/Eukaryota - ps5
Eukaryota - ps6
Amorpheae - ps7
Obazoa - ps8
Opisthokonta - ps9
Holozoa - ps10
Filozoa - ps11
Choano./Metazoa - ps12
Metazoa - ps13
Myriazoa - ps14
Parahoxozoa - ps15
Bilateria - ps16
Deuterostomia - ps17
Chordata - ps18
Olfactores - ps19
Vertebrata - ps20
Gnathostomata - ps21
Euteleostomi - ps22
Sarcopterygii - ps23
Tetrapoda - ps24
Amniota - ps25
Mammalia - ps26
Theria - ps27
Placentalia - ps28
Boreoeutheria - ps29
Euarchontoglires - ps30
Euarchonta - ps31
Primates - ps32
Haplorrhini - ps33
Simiiformes - ps34
Catarrhini - ps35
Hominoidea - ps36
Hominidae - ps37
Homininae - ps38
Hominini - ps39
Homo_sapiens - ps40
Phylostratum

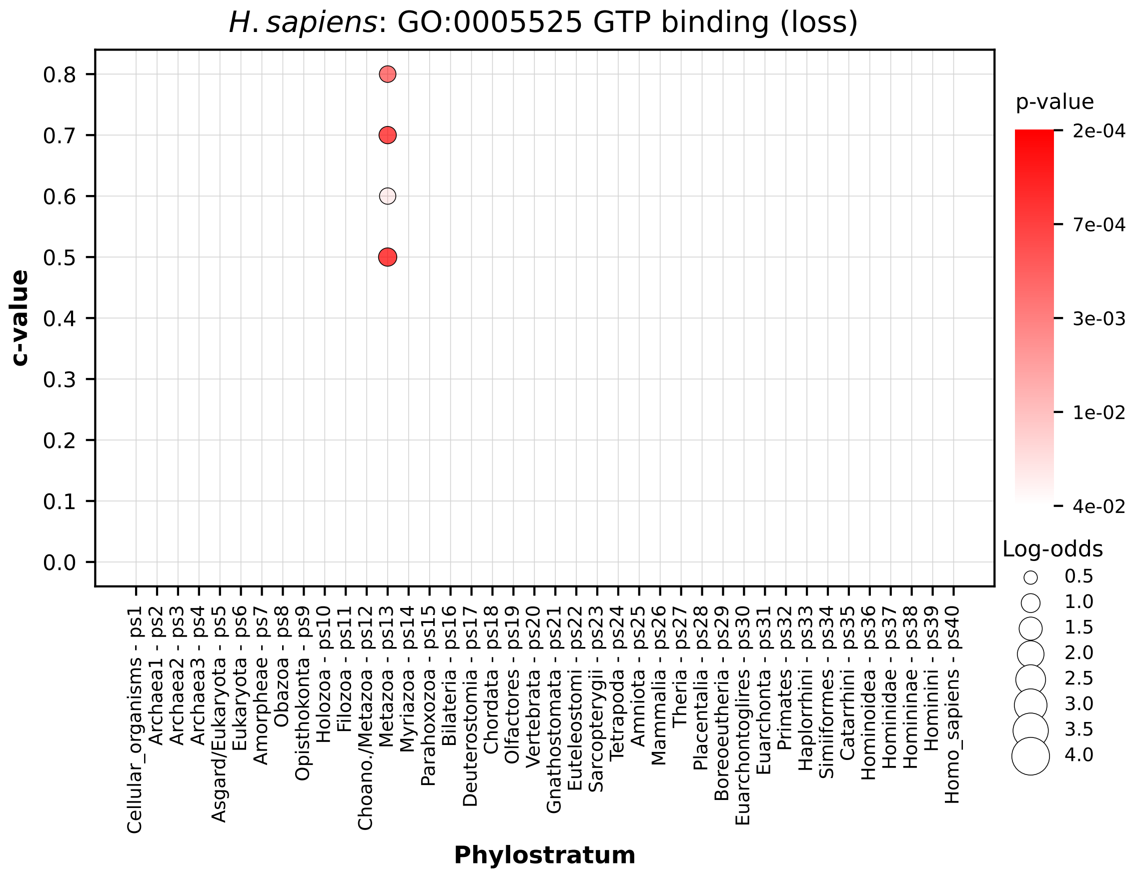

H. sapiens: GO:0005525 GTP binding (loss)
c-value
0.0
0.1
0.2
0.3
0.4
0.5
0.6
0.7
0.8
Phylostratum
Cellular_organisms - ps1
Archaea1 - ps2
Archaea2 - ps3
Archaea3 - ps4
Asgard/Eukaryota - ps5
Eukaryota - ps6
Amorpheae - ps7
Obazoa - ps8
Opisthokonta - ps9
Holozoa - ps10
Filozoa - ps11
Choano./Metazoa - ps12
Metazoa - ps13
Myriazoa - ps14
Parahoxozoa - ps15
Bilateria - ps16
Deuterostomia - ps17
Chordata - ps18
Olfactores - ps19
Vertebrata - ps20
Gnathostomata - ps21
Euteleostomi - ps22
Sarcopterygii - ps23
Tetrapoda - ps24
Amniota - ps25
Mammalia - ps26
Theria - ps27
Placentalia - ps28
Boreoeutheria - ps29
Euarchontoglires - ps30
Euarchonta - ps31
Primates - ps32
Haplorrhini - ps33
Simiiformes - ps34
Catarrhini - ps35
Hominoidea - ps36
Hominidae - ps37
Homininae - ps38
Hominini - ps39
Homo_sapiens - ps40
p-value
2e-04
7e-04
3e-03
1e-02
4e-02
Log-odds
0.5
1.0
1.5
2.0
2.5
3.0
3.5
4.0

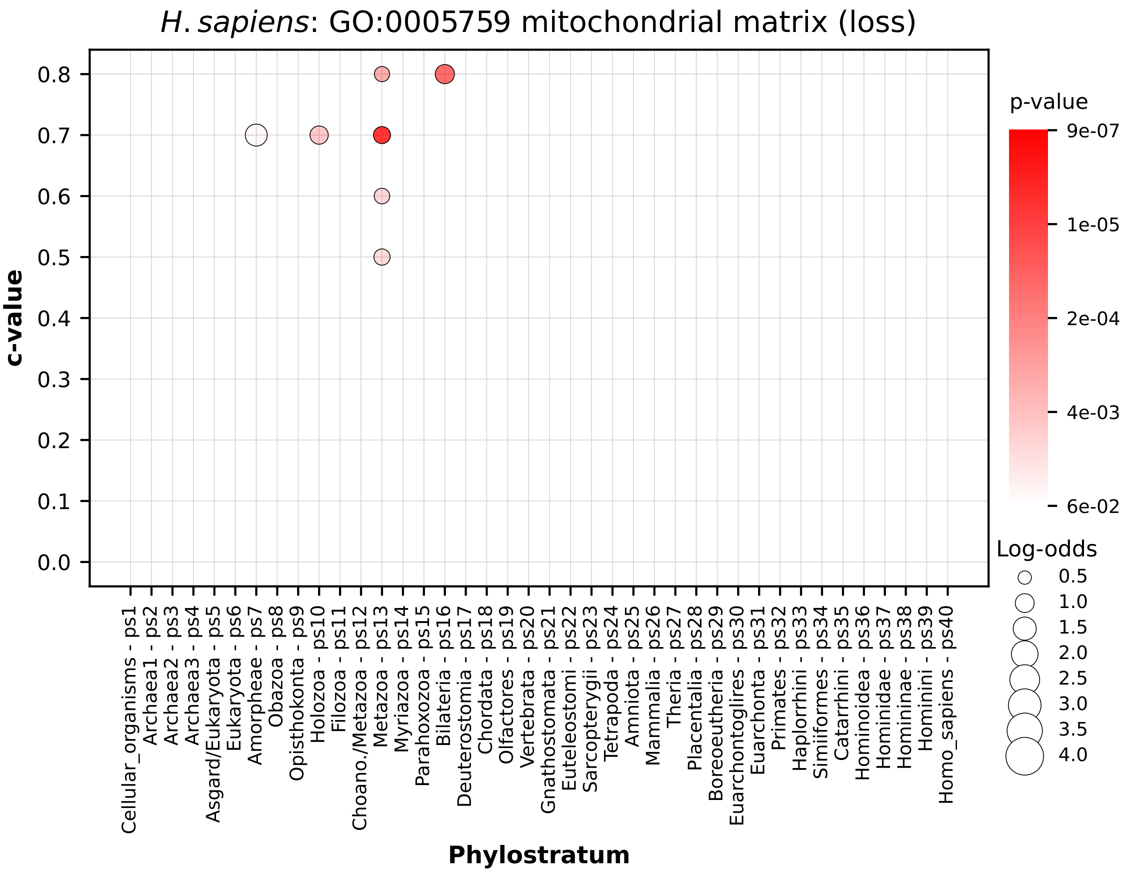

H. sapiens: GO:0005759 mitochondrial matrix (loss)
c-value
0.8
0.7
0.6
0.5
0.4
0.3
0.2
0.1
0.0
Phylostratum
Cellular_organisms - ps1
Archaea1 - ps2
Archaea2 - ps3
Archaea3 - ps4
Asgard/Eukaryota - ps5
Eukaryota - ps6
Amorpheae - ps7
Obazoa - ps8
Opisthokonta - ps9
Holozoa - ps10
Filozoa - ps11
Choano./Metazoa - ps12
Metazoa - ps13
Myriazoa - ps14
Parahoxozoa - ps15
Bilateria - ps16
Deuterostomia - ps17
Chordata - ps18
Olfactores - ps19
Vertebrata - ps20
Gnathostomata - ps21
Euteleostomi - ps22
Sarcopterygii - ps23
Tetrapoda - ps24
Amniota - ps25
Mammalia - ps26
Theria - ps27
Placentalia - ps28
Boreoeutheria - ps29
Euarchontoglires - ps30
Euarchonta - ps31
Primates - ps32
Haplorrhini - ps33
Simiiformes - ps34
Catarrhini - ps35
Hominoidea - ps36
Hominidae - ps37
Homininae - ps38
Hominini - ps39
Homo_sapiens - ps40
p-value
9e-07
1e-05
2e-04
4e-03
6e-02
Log-odds
0.5
1.0
1.5
2.0
2.5
3.0
3.5
4.0

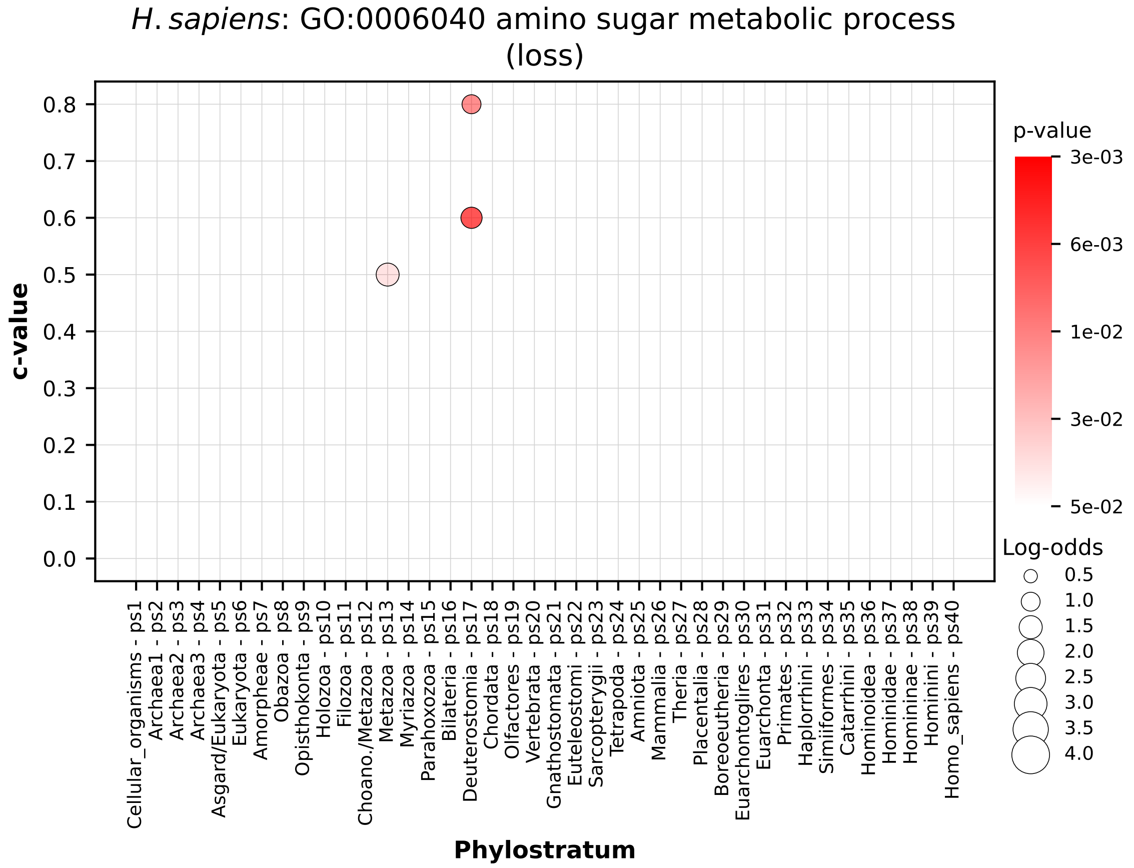

H. sapiens: GO:0006040 amino sugar metabolic process
(loss)
c-value
0.0
0.1
0.2
0.3
0.4
0.5
0.6
0.7
0.8
p-value
3e-03
6e-03
1e-02
3e-02
5e-02
Log-odds
0.5
1.0
1.5
2.0
2.5
3.0
3.5
4.0
Cellular_organisms - ps1
Archaea1 - ps2
Archaea2 - ps3
Archaea3 - ps4
Asgard/Eukaryota - ps5
Eukaryota - ps6
Amorpheae - ps7
Obazoa - ps8
Opisthokonta - ps9
Holozoa - ps10
Filozoa - ps11
Choano./Metazoa - ps12
Metazoa - ps13
Myriazoa - ps14
Parahoxozoa - ps15
Bilateria - ps16
Deuterostomia - ps17
Chordata - ps18
Olfactores - ps19
Vertebrata - ps20
Gnathostomata - ps21
Euteleostomi - ps22
Sarcopterygii - ps23
Tetrapoda - ps24
Amniota - ps25
Mammalia - ps26
Theria - ps27
Placentalia - ps28
Boreoeutheria - ps29
Euarchontoglires - ps30
Euarchonta - ps31
Primates - ps32
Haplorrhini - ps33
Simiiformes - ps34
Catarrhini - ps35
Hominoidea - ps36
Hominidae - ps37
Homininae - ps38
Hominini - ps39
Homo_sapiens - ps40
Phylostratum

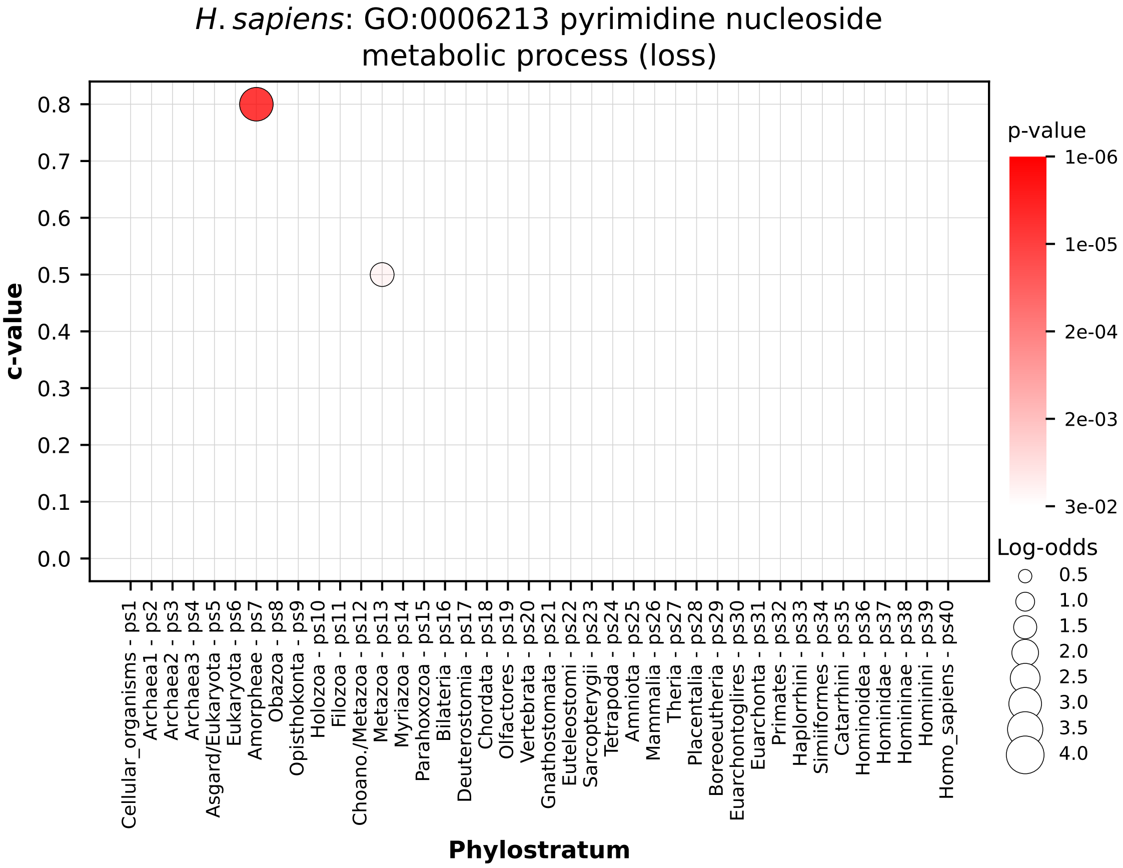
H. sapiens: GO:0006213 pyrimidine nucleoside metabolic process (loss)
c-value
0.8
0.7
0.6
0.5
0.4
0.3
0.2
0.1
0.0
p-value
1e-06
1e-05
2e-04
2e-03
3e-02
Log-odds
0.5
1.0
1.5
2.0
2.5
3.0
3.5
4.0
Cellular_organisms - ps1
Archaea1 - ps2
Archaea2 - ps3
Archaea3 - ps4
Asgard/Eukaryota - ps5
Eukaryota - ps6
Amorpheae - ps7
Obazoa - ps8
Opisthokonta - ps9
Holozoa - ps10
Filozoa - ps11
Choano./Metazoa - ps12
Metazoa - ps13
Myriazoa - ps14
Parahoxozoa - ps15
Bilateria - ps16
Deuterostomia - ps17
Chordata - ps18
Olfactores - ps19
Vertebrata - ps20
Gnathostomata - ps21
Euteleostomi - ps22
Sarcopterygii - ps23
Tetrapoda - ps24
Amniota - ps25
Mammalia - ps26
Theria - ps27
Placentalia - ps28
Boreoeutheria - ps29
Euarchontoglires - ps30
Euarchonta - ps31
Primates - ps32
Haplorrhini - ps33
Simiiformes - ps34
Catarrhini - ps35
Hominoidea - ps36
Hominidae - ps37
Homininae - ps38
Hominini - ps39
Homo_sapiens - ps40
Phylostratum

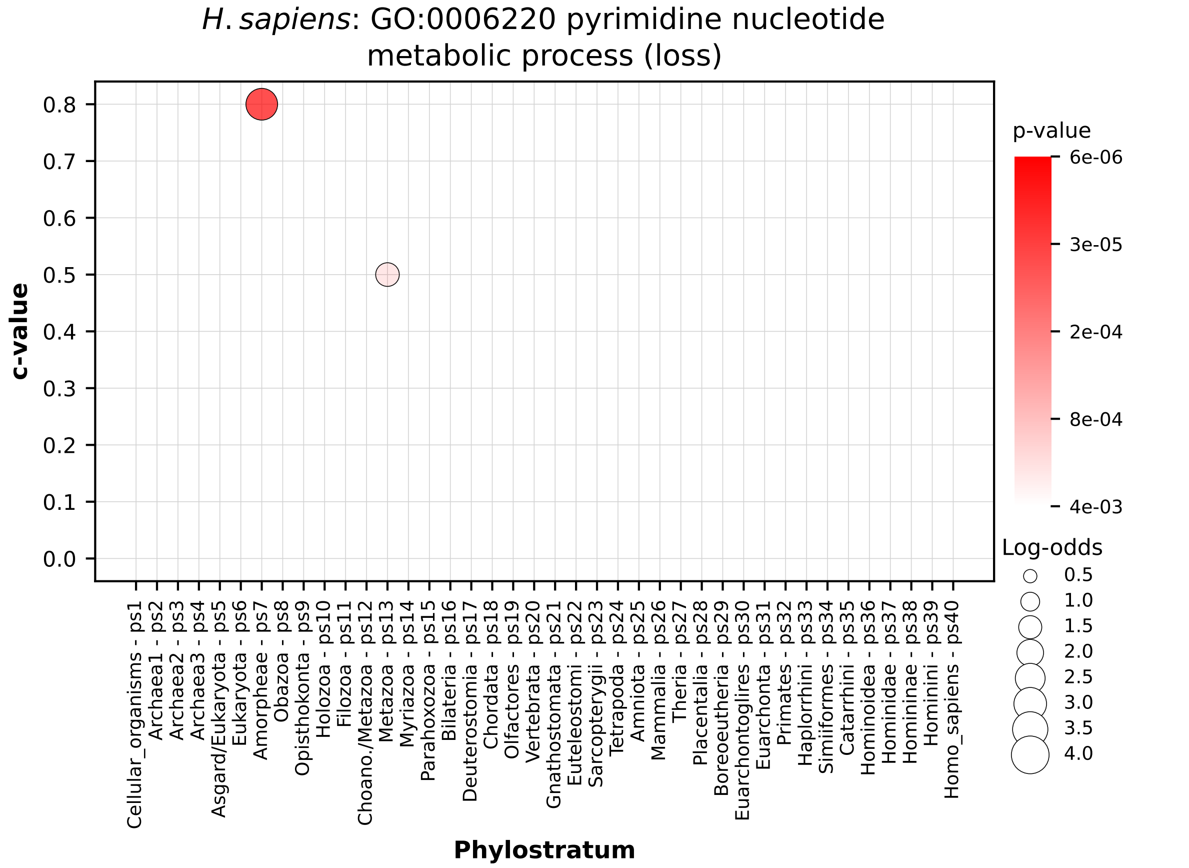
H. sapiens: GO:0006220 pyrimidine nucleotide metabolic process (loss)
c-value
0.8
0.7
0.6
0.5
0.4
0.3
0.2
0.1
0.0
Phylostratum
Cellular_organisms - ps1
Archaea1 - ps2
Archaea2 - ps3
Archaea3 - ps4
Asgard/Eukaryota - ps5
Eukaryota - ps6
Amorpheae - ps7
Obazoa - ps8
Opisthokonta - ps9
Holozoa - ps10
Filozoa - ps11
Choano./Metazoa - ps12
Metazoa - ps13
Myriazoa - ps14
Parahoxozoa - ps15
Bilateria - ps16
Deuterostomia - ps17
Chordata - ps18
Olfactores - ps19
Vertebrata - ps20
Gnathostomata - ps21
Euteleostomi - ps22
Sarcopterygii - ps23
Tetrapoda - ps24
Amniota - ps25
Mammalia - ps26
Theria - ps27
Placentalia - ps28
Boreoeutheria - ps29
Euarchontoglires - ps30
Euarchonta - ps31
Primates - ps32
Haplorrhini - ps33
Simiiformes - ps34
Catarrhini - ps35
Hominoidea - ps36
Hominidae - ps37
Homininae - ps38
Hominini - ps39
Homo_sapiens - ps40
p-value
6e-06
3e-05
2e-04
8e-04
4e-03
Log-odds
0.5
1.0
1.5
2.0
2.5
3.0
3.5
4.0

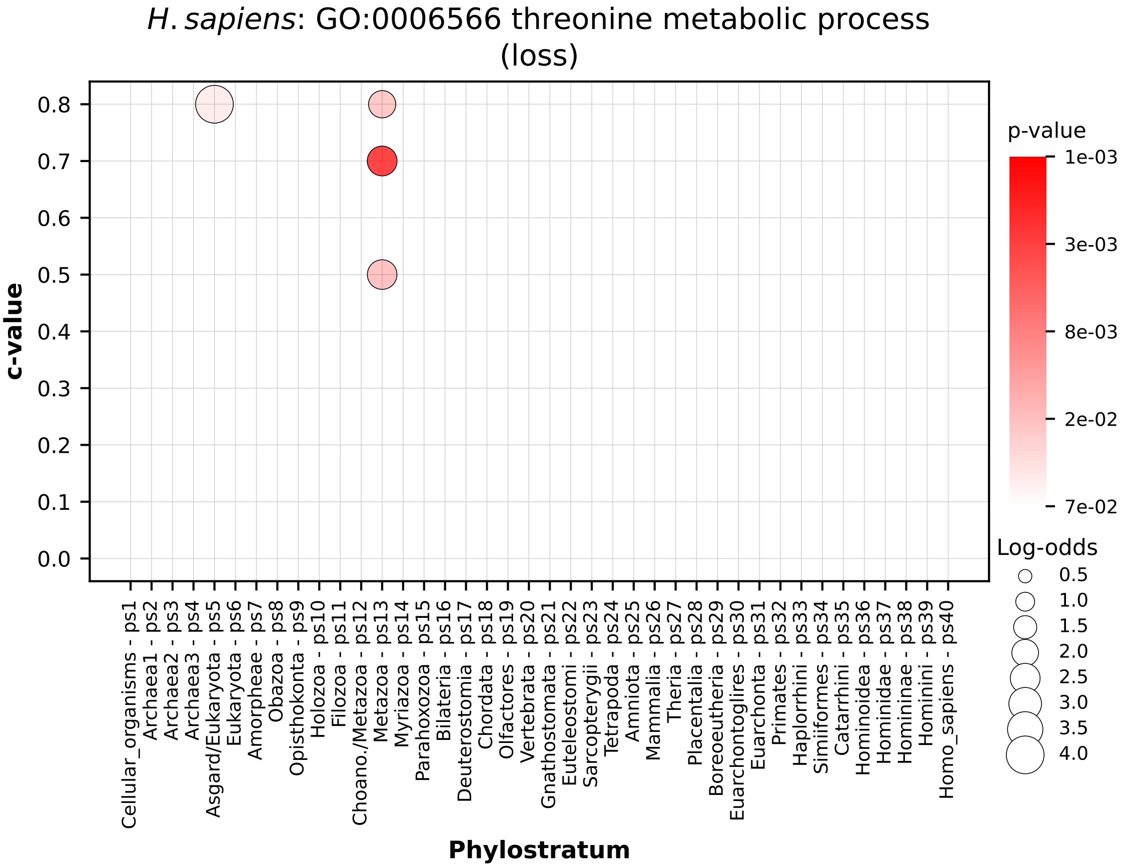

H. sapiens: GO:0006566 threonine metabolic process
(loss)
c-value
0.0
0.1
0.2
0.3
0.4
0.5
0.6
0.7
0.8
Phylostratum
Cellular_organisms - ps1
Archaea1 - ps2
Archaea2 - ps3
Archaea3 - ps4
Asgard/Eukaryota - ps5
Eukaryota - ps6
Amorpheae - ps7
Obazoa - ps8
Opisthokonta - ps9
Holozoa - ps10
Filozoa - ps11
Choano./Metazoa - ps12
Metazoa - ps13
Myriazoa - ps14
Parahoxozoa - ps15
Bilateria - ps16
Deuterostomia - ps17
Chordata - ps18
Olfactores - ps19
Vertebrata - ps20
Gnathostomata - ps21
Euteleostomi - ps22
Sarcopterygii - ps23
Tetrapoda - ps24
Amniota - ps25
Mammalia - ps26
Theria - ps27
Placentalia - ps28
Boreoeutheria - ps29
Euarchontoglires - ps30
Euarchonta - ps31
Primates - ps32
Haplorrhini - ps33
Simiiformes - ps34
Catarrhini - ps35
Hominoidea - ps36
Hominidae - ps37
Homininae - ps38
Hominini - ps39
Homo_sapiens - ps40
p-value
1e-03
3e-03
8e-03
2e-02
7e-02
Log-odds
0.5
1.0
1.5
2.0
2.5
3.0
3.5
4.0

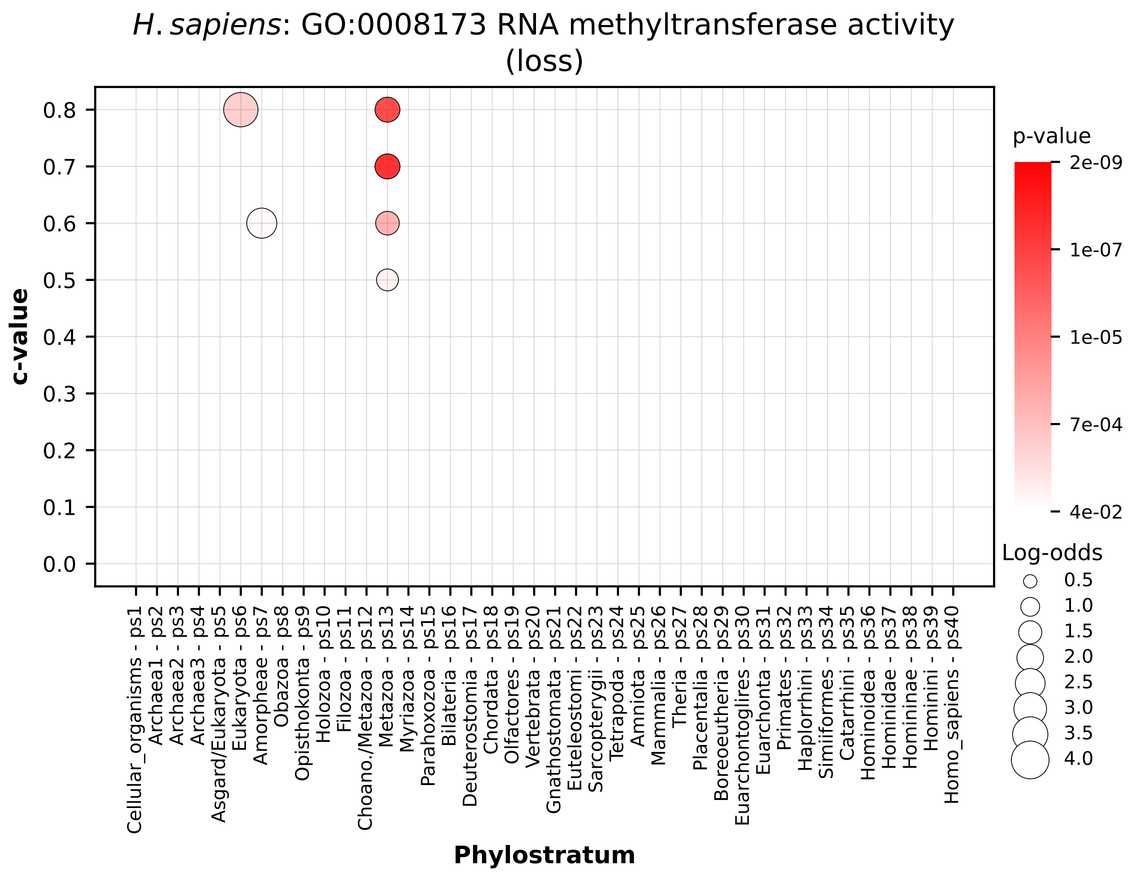

H. sapiens: GO:0008173 RNA methyltransferase activity
(loss)
c-value
0.8
0.7
0.6
0.5
0.4
0.3
0.2
0.1
0.0
p-value
2e-09
1e-07
1e-05
7e-04
4e-02
Log-odds
0.5
1.0
1.5
2.0
2.5
3.0
3.5
4.0
Cellular_organisms - ps1
Archaea1 - ps2
Archaea2 - ps3
Archaea3 - ps4
Asgard/Eukaryota - ps5
Eukaryota - ps6
Amorpheae - ps7
Obazoa - ps8
Opisthokonta - ps9
Holozoa - ps10
Filozoa - ps11
Choano./Metazoa - ps12
Metazoa - ps13
Myriazoa - ps14
Parahoxozoa - ps15
Bilateria - ps16
Deuterostomia - ps17
Chordata - ps18
Olfactores - ps19
Vertebrata - ps20
Gnathostomata - ps21
Euteleostomi - ps22
Sarcopterygii - ps23
Tetrapoda - ps24
Amniota - ps25
Mammalia - ps26
Theria - ps27
Placentalia - ps28
Boreoeutheria - ps29
Euarchontoglires - ps30
Euarchonta - ps31
Primates - ps32
Haplorrhini - ps33
Simiiformes - ps34
Catarrhini - ps35
Hominoidea - ps36
Hominidae - ps37
Homininae - ps38
Hominini - ps39
Homo_sapiens - ps40
Phylostratum

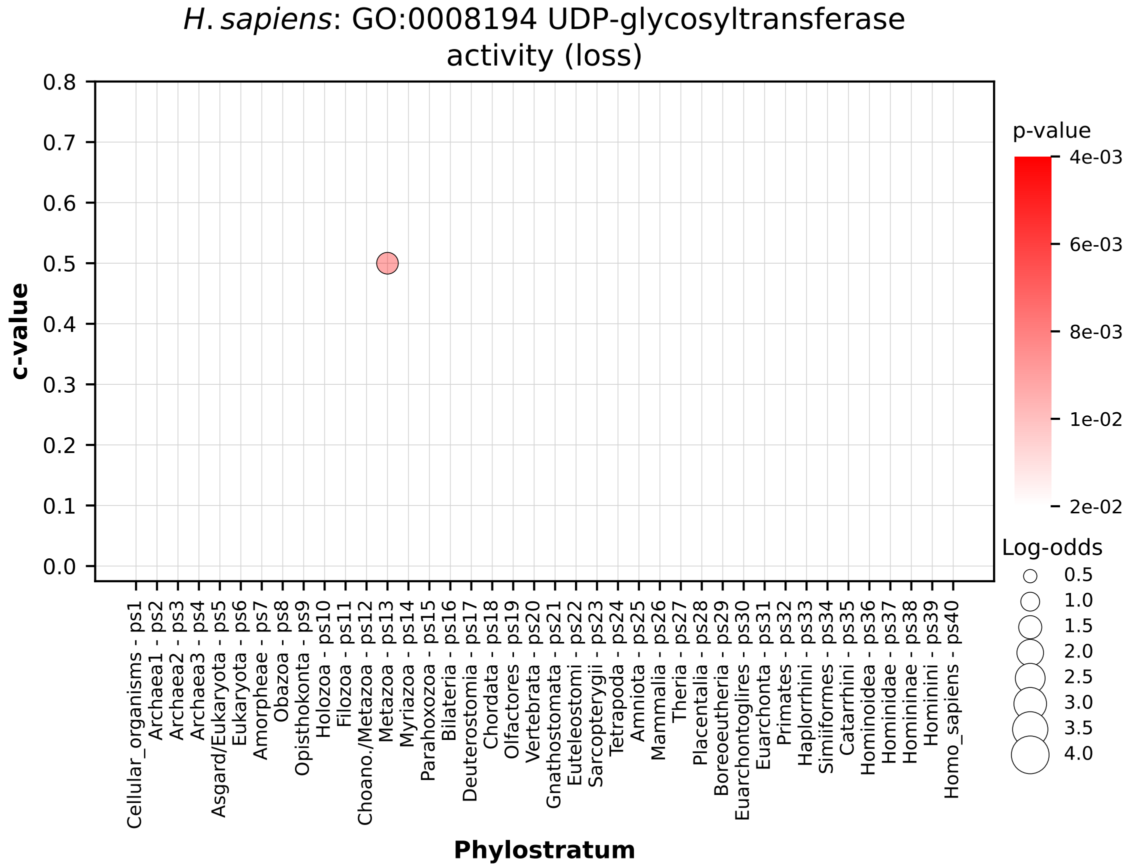

H. sapiens: GO:0008194 UDP-glycosyltransferase activity (loss)
c-value
0.8
0.7
0.6
0.5
0.4
0.3
0.2
0.1
0.0
Phylostratum
Cellular_organisms - ps1
Archaea1 - ps2
Archaea2 - ps3
Archaea3 - ps4
Asgard/Eukaryota - ps5
Eukaryota - ps6
Amorpheae - ps7
Obazoa - ps8
Opisthokonta - ps9
Holozoa - ps10
Filozoa - ps11
Choano./Metazoa - ps12
Metazoa - ps13
Myriazoa - ps14
Parahoxozoa - ps15
Bilateria - ps16
Deuterostomia - ps17
Chordata - ps18
Olfactores - ps19
Vertebrata - ps20
Gnathostomata - ps21
Euteleostomi - ps22
Sarcopterygii - ps23
Tetrapoda - ps24
Amniota - ps25
Mammalia - ps26
Theria - ps27
Placentalia - ps28
Boreoeutheria - ps29
Euarchontoglires - ps30
Euarchonta - ps31
Primates - ps32
Haplorrhini - ps33
Simiiformes - ps34
Catarrhini - ps35
Hominoidea - ps36
Hominidae - ps37
Homininae - ps38
Hominini - ps39
Homo_sapiens - ps40
p-value
4e-03
6e-03
8e-03
1e-02
2e-02
Log-odds
0.5
1.0
1.5
2.0
2.5
3.0
3.5
4.0

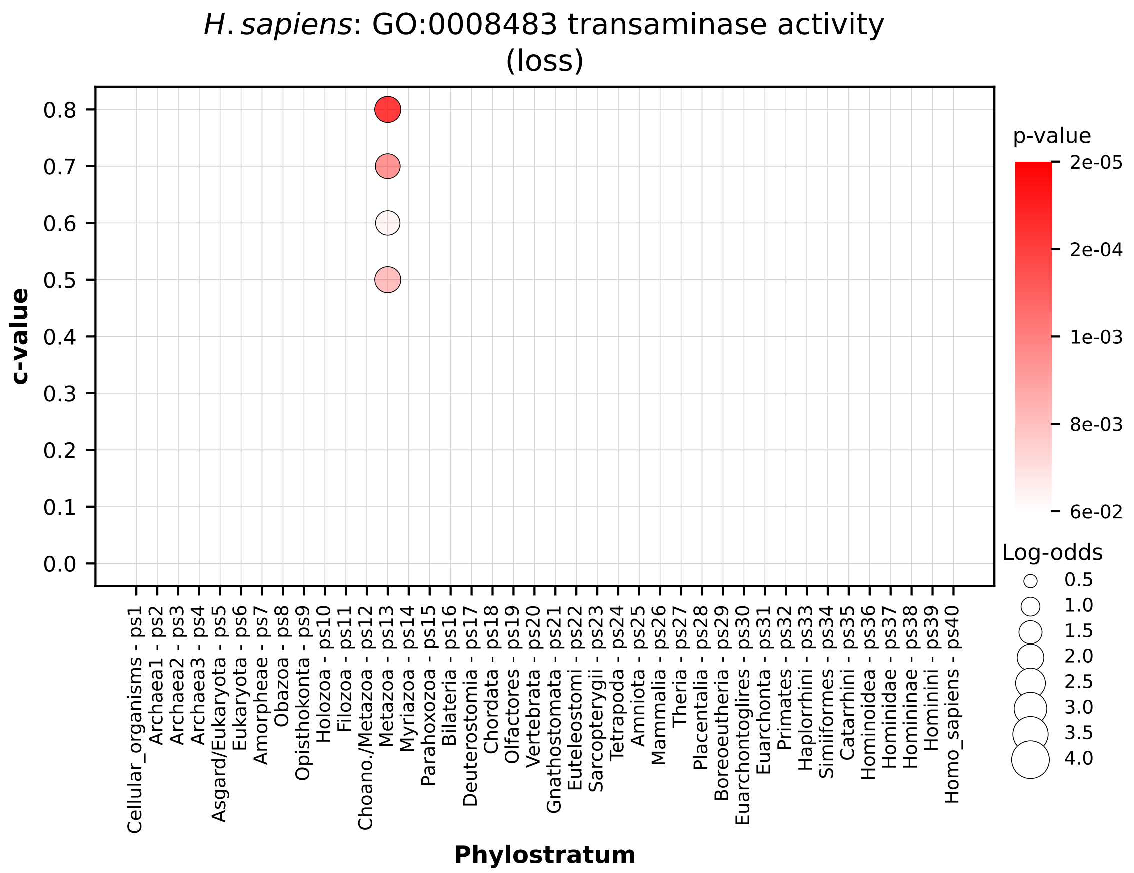

H. sapiens: GO:0008483 transaminase activity
(loss)
c-value
0.8
0.7
0.6
0.5
0.4
0.3
0.2
0.1
0.0
p-value
2e-05
2e-04
1e-03
8e-03
6e-02
Log-odds
0.5
1.0
1.5
2.0
2.5
3.0
3.5
4.0
Cellular_organisms - ps1
Archaea1 - ps2
Archaea2 - ps3
Archaea3 - ps4
Asgard/Eukaryota - ps5
Eukaryota - ps6
Amorpheae - ps7
Obazoa - ps8
Opisthokonta - ps9
Holozoa - ps10
Filozoa - ps11
Choano./Metazoa - ps12
Metazoa - ps13
Myriazoa - ps14
Parahoxozoa - ps15
Bilateria - ps16
Deuterostomia - ps17
Chordata - ps18
Olfactores - ps19
Vertebrata - ps20
Gnathostomata - ps21
Euteleostomi - ps22
Sarcopterygii - ps23
Tetrapoda - ps24
Amniota - ps25
Mammalia - ps26
Theria - ps27
Placentalia - ps28
Boreoeutheria - ps29
Euarchontoglires - ps30
Euarchonta - ps31
Primates - ps32
Haplorrhini - ps33
Simiiformes - ps34
Catarrhini - ps35
Hominoidea - ps36
Hominidae - ps37
Homininae - ps38
Hominini - ps39
Homo_sapiens - ps40
Phylostratum

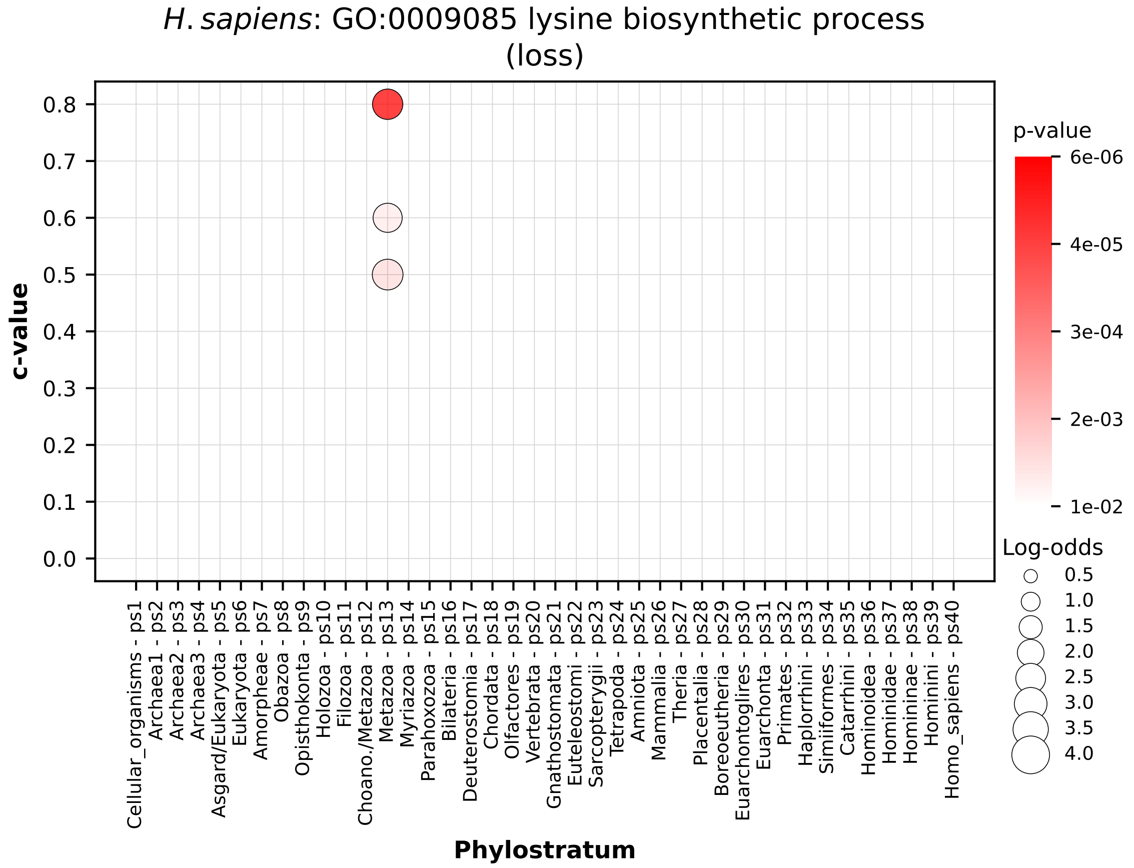

H. sapiens: GO:0009085 lysine biosynthetic process
(loss)
c-value
0.0
0.1
0.2
0.3
0.4
0.5
0.6
0.7
0.8
Phylostratum
Cellular_organisms - ps1
Archaea1 - ps2
Archaea2 - ps3
Archaea3 - ps4
Asgard/Eukaryota - ps5
Eukaryota - ps6
Amorpheae - ps7
Obazoa - ps8
Opisthokonta - ps9
Holozoa - ps10
Filozoa - ps11
Choano./Metazoa - ps12
Metazoa - ps13
Myriazoa - ps14
Parahoxozoa - ps15
Bilateria - ps16
Deuterostomia - ps17
Chordata - ps18
Olfactores - ps19
Vertebrata - ps20
Gnathostomata - ps21
Euteleostomi - ps22
Sarcopterygii - ps23
Tetrapoda - ps24
Amniota - ps25
Mammalia - ps26
Theria - ps27
Placentalia - ps28
Boreoeutheria - ps29
Euarchontoglires - ps30
Euarchonta - ps31
Primates - ps32
Haplorrhini - ps33
Simiiformes - ps34
Catarrhini - ps35
Hominoidea - ps36
Hominidae - ps37
Homininae - ps38
Hominini - ps39
Homo_sapiens - ps40
p-value
6e-06
4e-05
3e-04
2e-03
1e-02
Log-odds
0.5
1.0
1.5
2.0
2.5
3.0
3.5
4.0

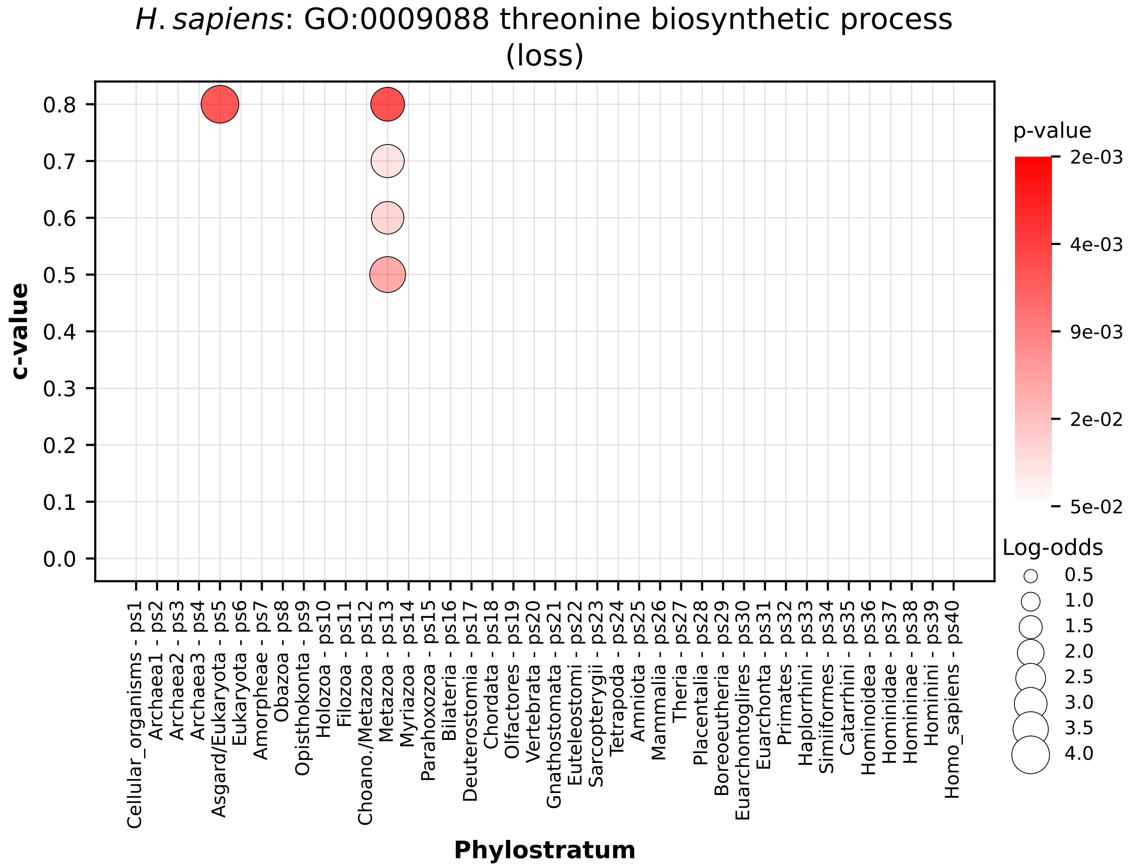

H. sapiens: GO:0009088 threonine biosynthetic process
(loss)
c-value
0.0
0.1
0.2
0.3
0.4
0.5
0.6
0.7
0.8
p-value
2e-03
4e-03
9e-03
2e-02
5e-02
Log-odds
0.5
1.0
1.5
2.0
2.5
3.0
3.5
4.0
Cellular_organisms - ps1
Archaea1 - ps2
Archaea2 - ps3
Archaea3 - ps4
Asgard/Eukaryota - ps5
Eukaryota - ps6
Amorpheae - ps7
Obazoa - ps8
Opisthokonta - ps9
Holozoa - ps10
Filozoa - ps11
Choano./Metazoa - ps12
Metazoa - ps13
Myriazoa - ps14
Parahoxozoa - ps15
Bilateria - ps16
Deuterostomia - ps17
Chordata - ps18
Olfactores - ps19
Vertebrata - ps20
Gnathostomata - ps21
Euteleostomi - ps22
Sarcopterygii - ps23
Tetrapoda - ps24
Amniota - ps25
Mammalia - ps26
Theria - ps27
Placentalia - ps28
Boreoeutheria - ps29
Euarchontoglires - ps30
Euarchonta - ps31
Primates - ps32
Haplorrhini - ps33
Simiiformes - ps34
Catarrhini - ps35
Hominoidea - ps36
Hominidae - ps37
Homininae - ps38
Hominini - ps39
Homo_sapiens - ps40
Phylostratum

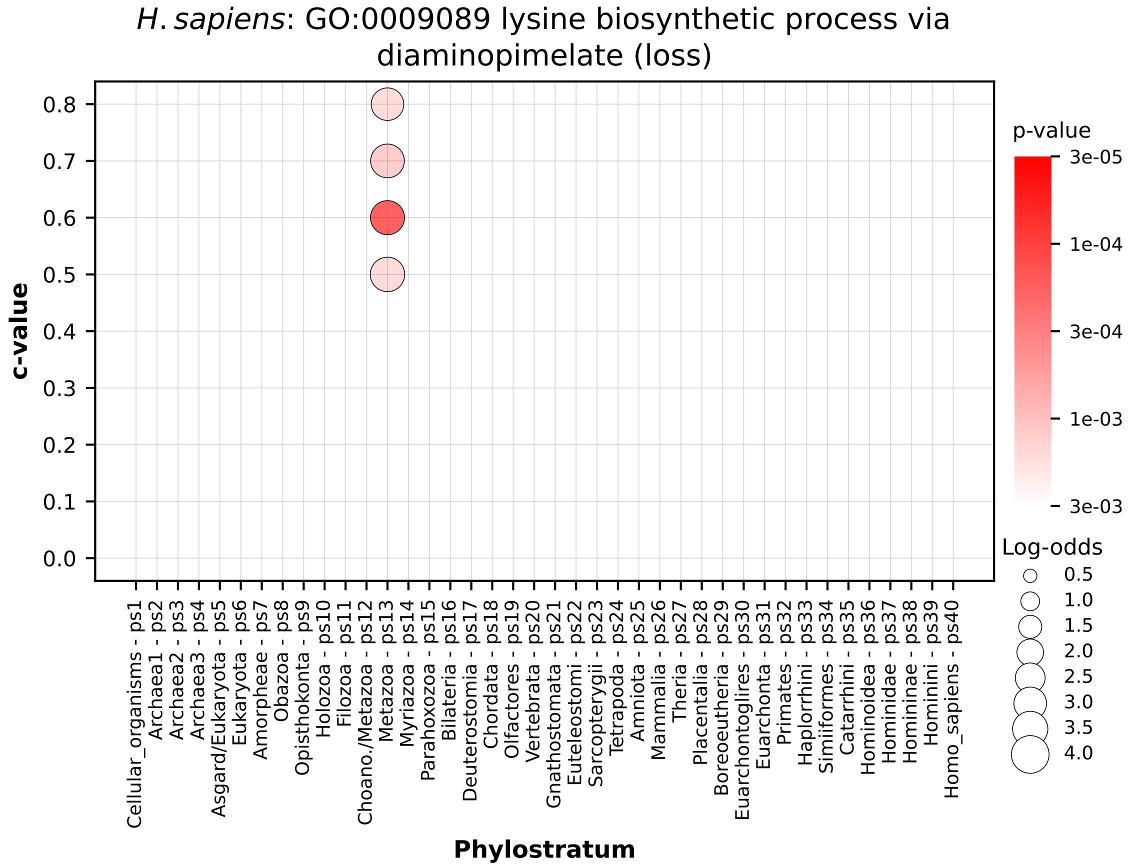

H. sapiens: GO:0009089 lysine biosynthetic process via diaminopimelate (loss)
c-value
0.8
0.7
0.6
0.5
0.4
0.3
0.2
0.1
0.0
p-value
3e-05
1e-04
3e-04
1e-03
3e-03
Log-odds
0.5
1.0
1.5
2.0
2.5
3.0
3.5
4.0
Cellular_organisms - ps1
Archaea1 - ps2
Archaea2 - ps3
Archaea3 - ps4
Asgard/Eukaryota - ps5
Eukaryota - ps6
Amorpheae - ps7
Obazoa - ps8
Opisthokonta - ps9
Holozoa - ps10
Filozoa - ps11
Choano./Metazoa - ps12
Metazoa - ps13
Myriazoa - ps14
Parahoxozoa - ps15
Bilateria - ps16
Deuterostomia - ps17
Chordata - ps18
Olfactores - ps19
Vertebrata - ps20
Gnathostomata - ps21
Euteleostomi - ps22
Sarcopterygii - ps23
Tetrapoda - ps24
Amniota - ps25
Mammalia - ps26
Theria - ps27
Placentalia - ps28
Boreoeutheria - ps29
Euarchontoglires - ps30
Euarchonta - ps31
Primates - ps32
Haplorrhini - ps33
Simiiformes - ps34
Catarrhini - ps35
Hominoidea - ps36
Hominidae - ps37
Homininae - ps38
Hominini - ps39
Homo_sapiens - ps40
Phylostratum

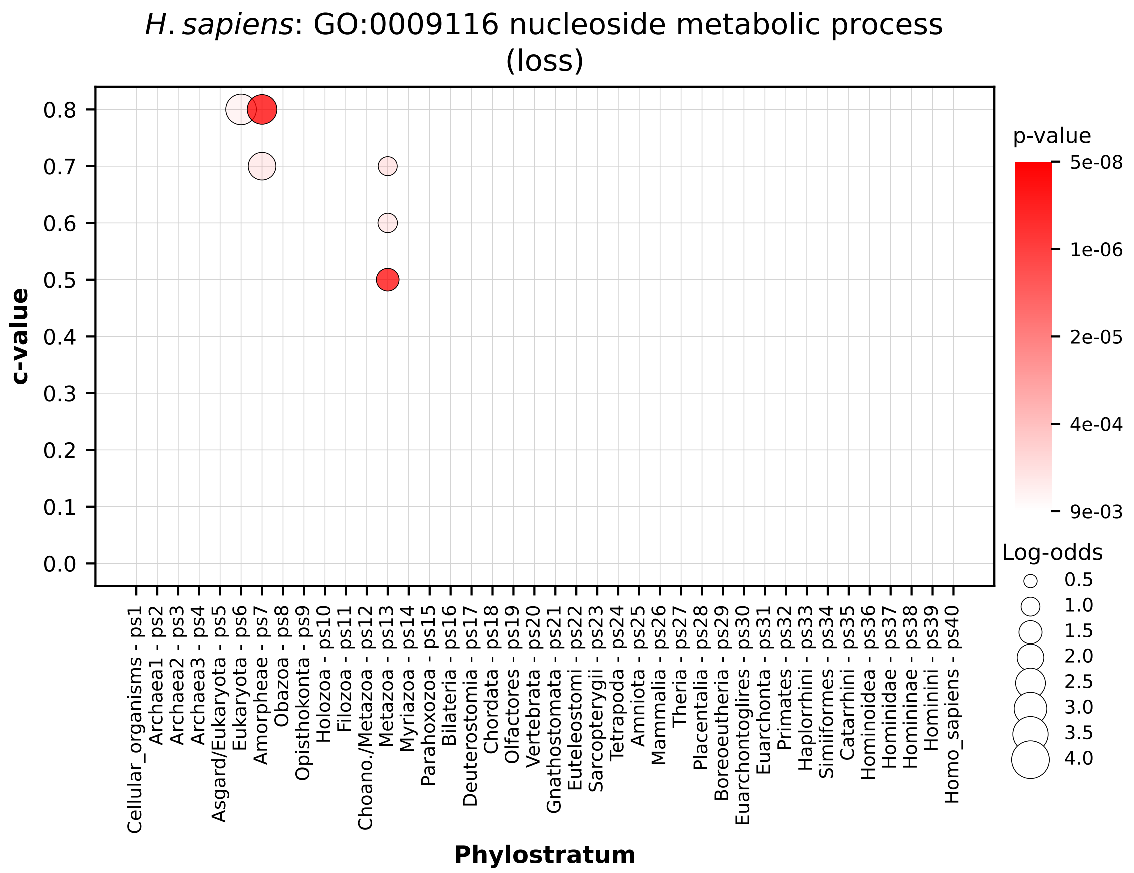

H. sapiens: GO:0009116 nucleoside metabolic process
(loss)
c-value
0.0
0.1
0.2
0.3
0.4
0.5
0.6
0.7
0.8
Phylostratum
Cellular_organisms - ps1
Archaea1 - ps2
Archaea2 - ps3
Archaea3 - ps4
Asgard/Eukaryota - ps5
Eukaryota - ps6
Amorpheae - ps7
Obazoa - ps8
Opisthokonta - ps9
Holozoa - ps10
Filozoa - ps11
Choano./Metazoa - ps12
Metazoa - ps13
Myriazoa - ps14
Parahoxozoa - ps15
Bilateria - ps16
Deuterostomia - ps17
Chordata - ps18
Olfactores - ps19
Vertebrata - ps20
Gnathostomata - ps21
Euteleostomi - ps22
Sarcopterygii - ps23
Tetrapoda - ps24
Amniota - ps25
Mammalia - ps26
Theria - ps27
Placentalia - ps28
Boreoeutheria - ps29
Euarchontoglires - ps30
Euarchonta - ps31
Primates - ps32
Haplorrhini - ps33
Simiiformes - ps34
Catarrhini - ps35
Hominoidea - ps36
Hominidae - ps37
Homininae - ps38
Hominini - ps39
Homo_sapiens - ps40
p-value
5e-08
1e-06
2e-05
4e-04
9e-03
Log-odds
0.5
1.0
1.5
2.0
2.5
3.0
3.5
4.0

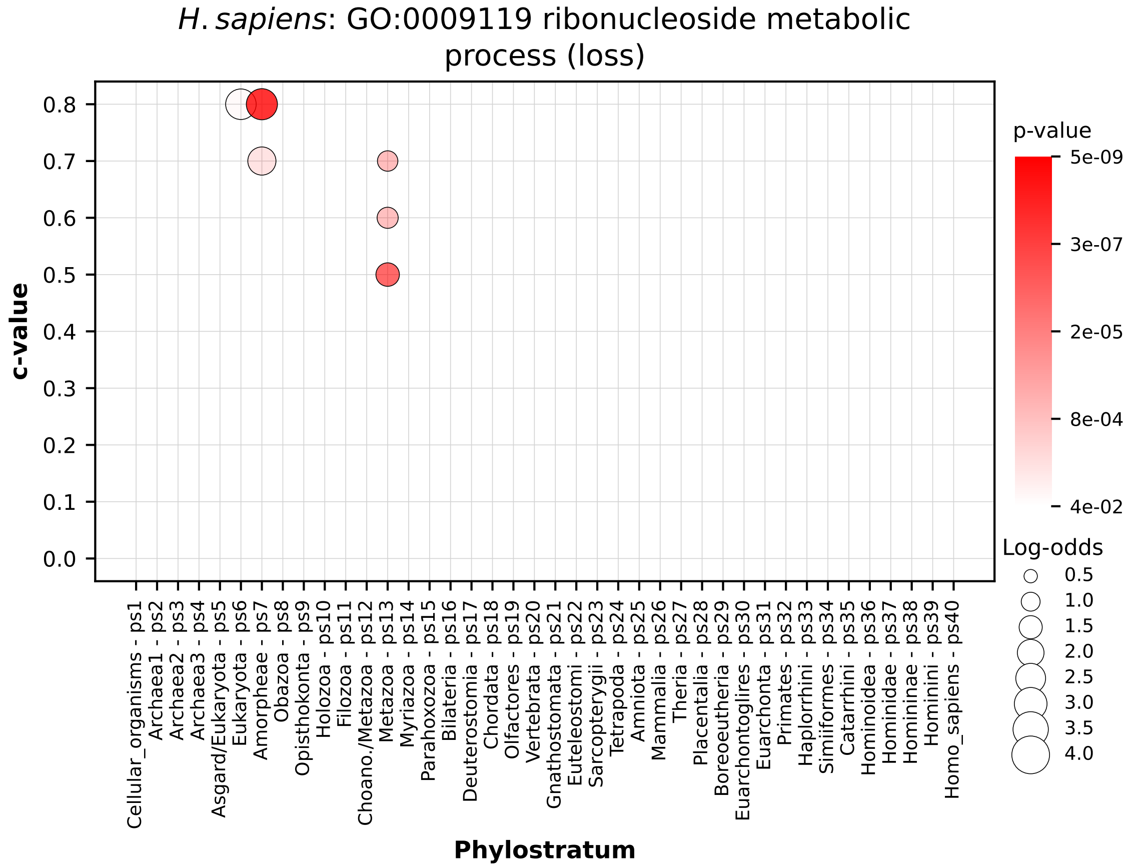

H. sapiens: GO:0009119 ribonucleoside metabolic process (loss)
c-value
0.8
0.7
0.6
0.5
0.4
0.3
0.2
0.1
0.0
p-value
5e-09
3e-07
2e-05
8e-04
4e-02
Log-odds
0.5
1.0
1.5
2.0
2.5
3.0
3.5
4.0
Cellular_organisms - ps1
Archaea1 - ps2
Archaea2 - ps3
Archaea3 - ps4
Asgard/Eukaryota - ps5
Eukaryota - ps6
Amorpheae - ps7
Obazoa - ps8
Opisthokonta - ps9
Holozoa - ps10
Filozoa - ps11
Choano./Metazoa - ps12
Metazoa - ps13
Myriazoa - ps14
Parahoxozoa - ps15
Bilateria - ps16
Deuterostomia - ps17
Chordata - ps18
Olfactores - ps19
Vertebrata - ps20
Gnathostomata - ps21
Euteleostomi - ps22
Sarcopterygii - ps23
Tetrapoda - ps24
Amniota - ps25
Mammalia - ps26
Theria - ps27
Placentalia - ps28
Boreoeutheria - ps29
Euarchontoglires - ps30
Euarchonta - ps31
Primates - ps32
Haplorrhini - ps33
Simiiformes - ps34
Catarrhini - ps35
Hominoidea - ps36
Hominidae - ps37
Homininae - ps38
Hominini - ps39
Homo_sapiens - ps40
Phylostratum

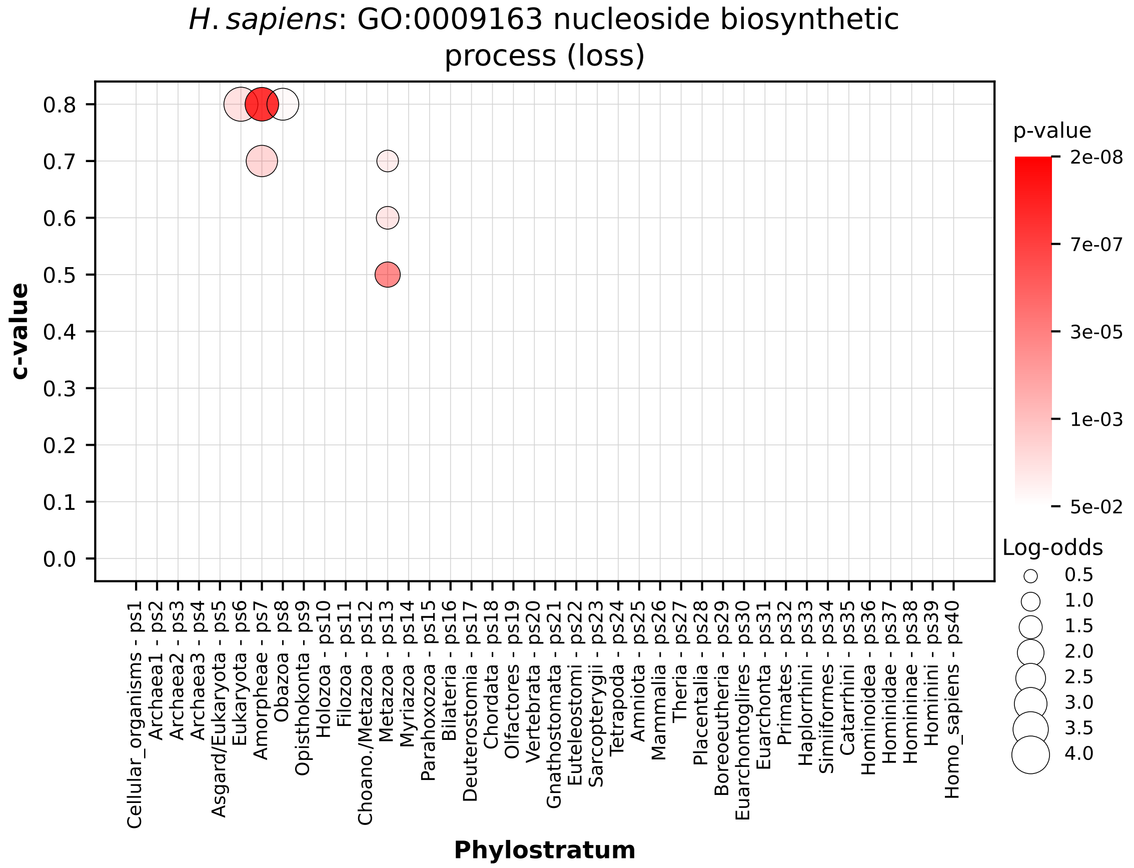

H. sapiens: GO:0009163 nucleoside biosynthetic process (loss)
c-value
0.8
0.7
0.6
0.5
0.4
0.3
0.2
0.1
0.0
p-value
2e-08
7e-07
3e-05
1e-03
5e-02
Log-odds
0.5
1.0
1.5
2.0
2.5
3.0
3.5
4.0
Cellular_organisms - ps1
Archaea1 - ps2
Archaea2 - ps3
Archaea3 - ps4
Asgard/Eukaryota - ps5
Eukaryota - ps6
Amorpheae - ps7
Obazoa - ps8
Opisthokonta - ps9
Holozoa - ps10
Filozoa - ps11
Choano./Metazoa - ps12
Metazoa - ps13
Myriazoa - ps14
Parahoxozoa - ps15
Bilateria - ps16
Deuterostomia - ps17
Chordata - ps18
Olfactores - ps19
Vertebrata - ps20
Gnathostomata - ps21
Euteleostomi - ps22
Sarcopterygii - ps23
Tetrapoda - ps24
Amniota - ps25
Mammalia - ps26
Theria - ps27
Placentalia - ps28
Boreoeutheria - ps29
Euarchontoglires - ps30
Euarchonta - ps31
Primates - ps32
Haplorrhini - ps33
Simiiformes - ps34
Catarrhini - ps35
Hominoidea - ps36
Hominidae - ps37
Homininae - ps38
Hominini - ps39
Homo_sapiens - ps40
Phylostratum

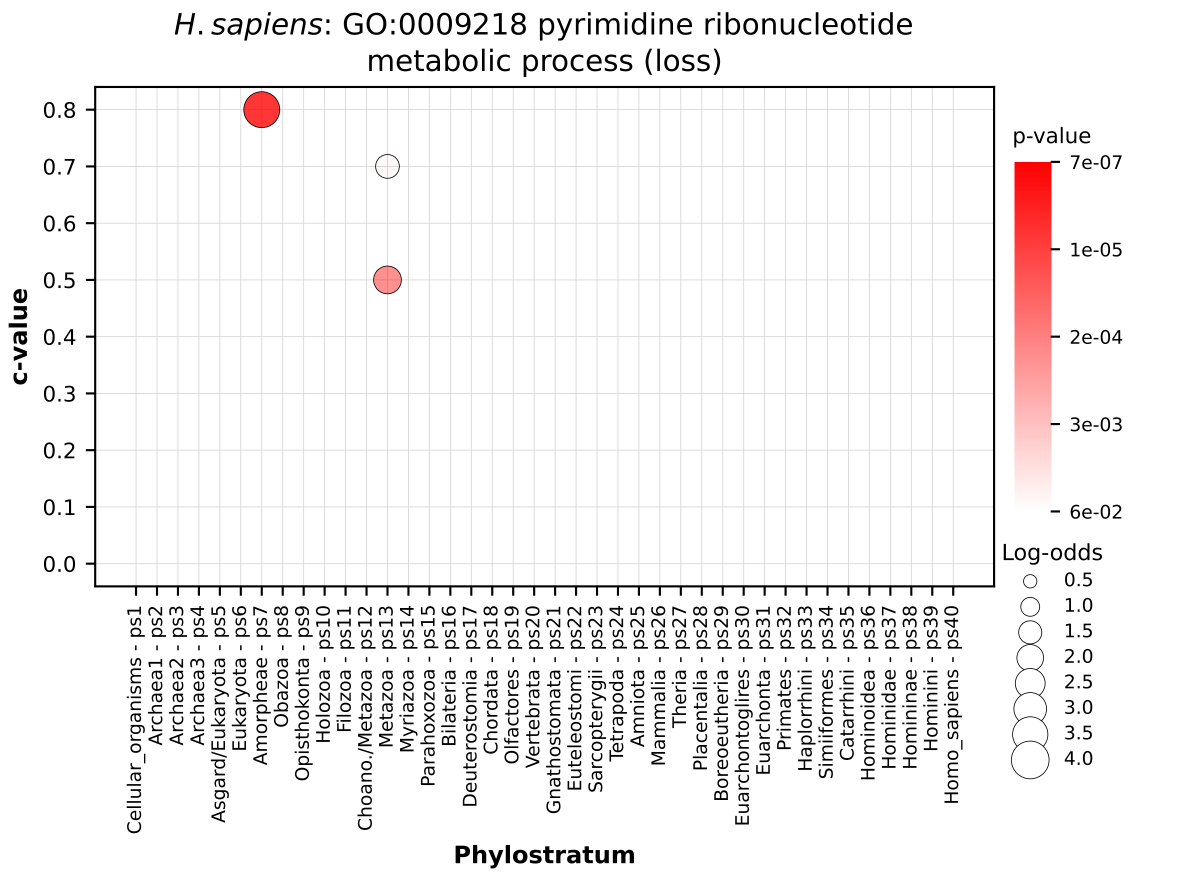

H. sapiens: GO:0009218 pyrimidine ribonucleotide metabolic process (loss)
c-value
0.0
0.1
0.2
0.3
0.4
0.5
0.6
0.7
0.8
Phylostratum
Cellular_organisms - ps1
Archaea1 - ps2
Archaea2 - ps3
Archaea3 - ps4
Asgard/Eukaryota - ps5
Eukaryota - ps6
Amorpheae - ps7
Obazoa - ps8
Opisthokonta - ps9
Holozoa - ps10
Filozoa - ps11
Choano./Metazoa - ps12
Metazoa - ps13
Myriazoa - ps14
Parahoxozoa - ps15
Bilateria - ps16
Deuterostomia - ps17
Chordata - ps18
Olfactores - ps19
Vertebrata - ps20
Gnathostomata - ps21
Euteleostomi - ps22
Sarcopterygii - ps23
Tetrapoda - ps24
Amniota - ps25
Mammalia - ps26
Theria - ps27
Placentalia - ps28
Boreoeutheria - ps29
Euarchontoglires - ps30
Euarchonta - ps31
Primates - ps32
Haplorrhini - ps33
Simiiformes - ps34
Catarrhini - ps35
Hominoidea - ps36
Hominidae - ps37
Homininae - ps38
Hominini - ps39
Homo_sapiens - ps40
p-value
7e-07
1e-05
2e-04
3e-03
6e-02
Log-odds
0.5
1.0
1.5
2.0
2.5
3.0
3.5
4.0

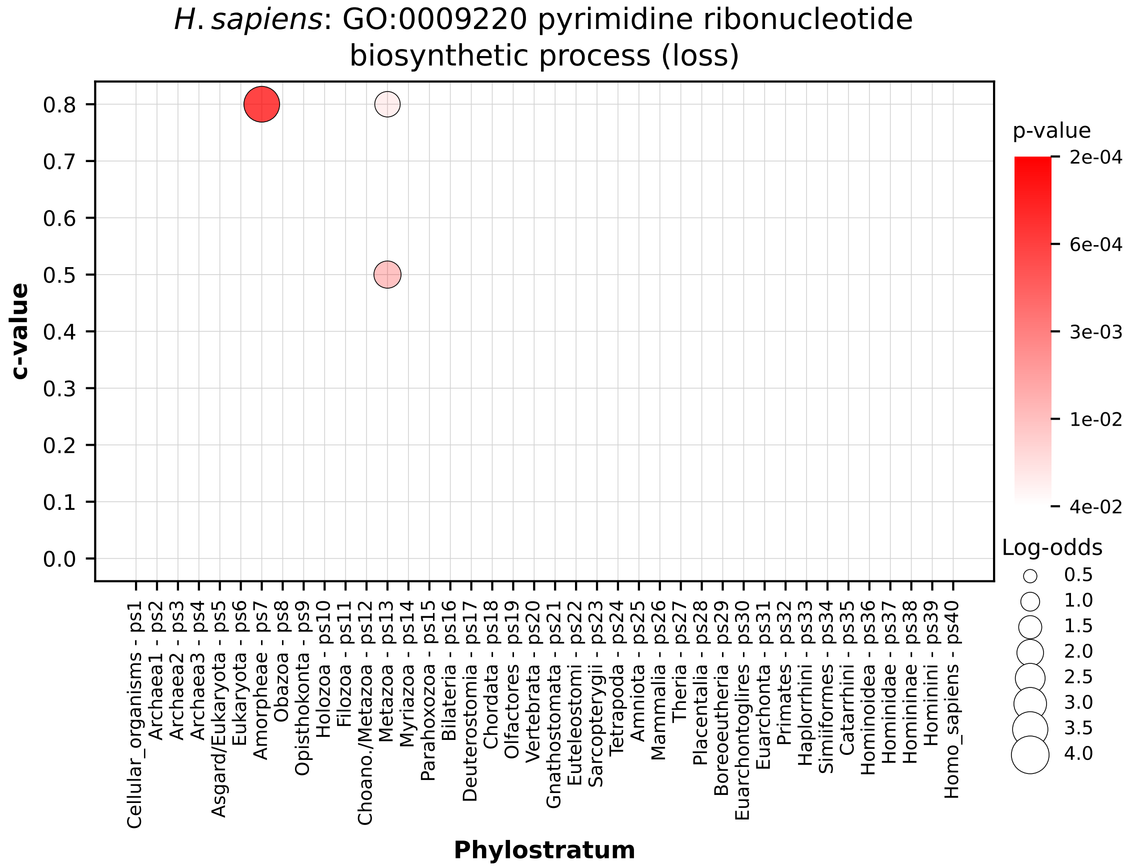

H. sapiens: GO:0009220 pyrimidine ribonucleotide biosynthetic process (loss)
c-value
0.0
0.1
0.2
0.3
0.4
0.5
0.6
0.7
0.8
Phylostratum
Cellular_organisms - ps1
Archaea1 - ps2
Archaea2 - ps3
Archaea3 - ps4
Asgard/Eukaryota - ps5
Eukaryota - ps6
Amorpheae - ps7
Obazoa - ps8
Opisthokonta - ps9
Holozoa - ps10
Filozoa - ps11
Choano./Metazoa - ps12
Metazoa - ps13
Myriazoa - ps14
Parahoxozoa - ps15
Bilateria - ps16
Deuterostomia - ps17
Chordata - ps18
Olfactores - ps19
Vertebrata - ps20
Gnathostomata - ps21
Euteleostomi - ps22
Sarcopterygii - ps23
Tetrapoda - ps24
Amniota - ps25
Mammalia - ps26
Theria - ps27
Placentalia - ps28
Boreoeutheria - ps29
Euarchontoglires - ps30
Euarchonta - ps31
Primates - ps32
Haplorrhini - ps33
Simiiformes - ps34
Catarrhini - ps35
Hominoidea - ps36
Hominidae - ps37
Homininae - ps38
Hominini - ps39
Homo_sapiens - ps40
p-value
2e-04
6e-04
3e-03
1e-02
4e-02
Log-odds
0.5
1.0
1.5
2.0
2.5
3.0
3.5
4.0

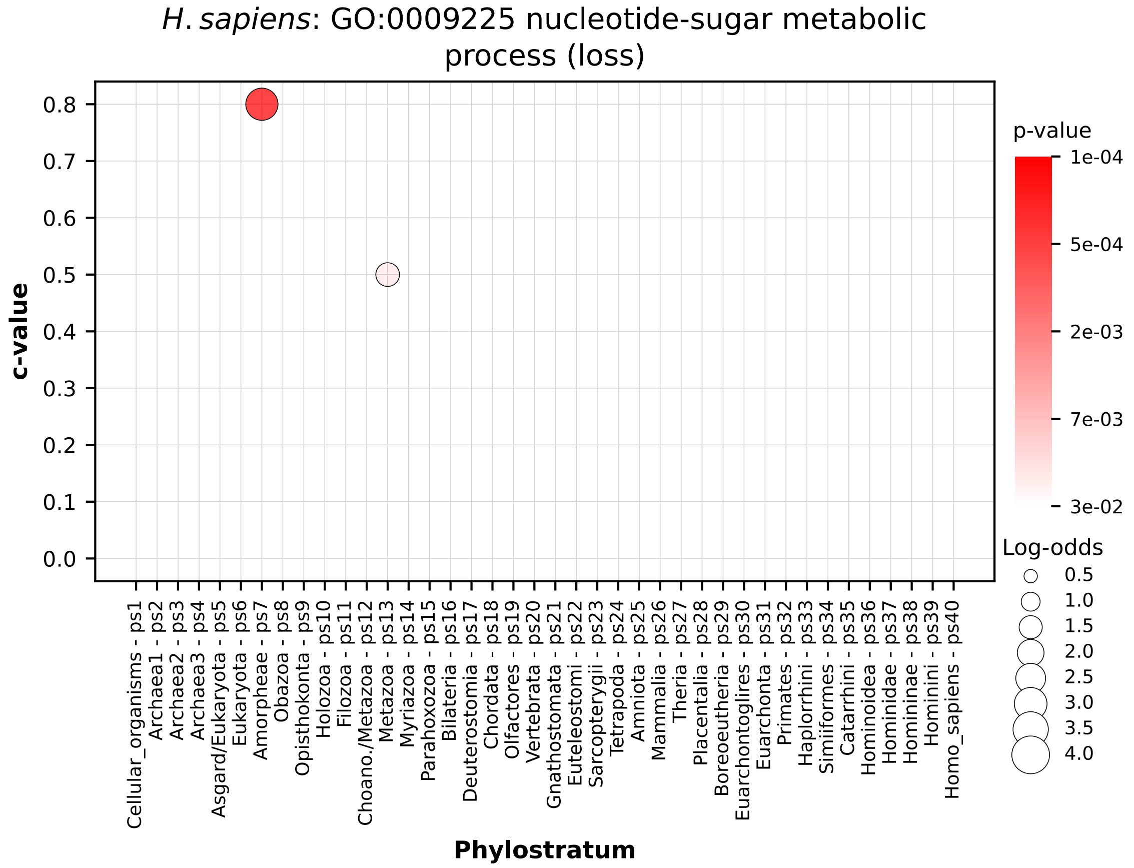

H. sapiens: GO:0009225 nucleotide-sugar metabolic process (loss)
c-value
0.8
0.7
0.6
0.5
0.4
0.3
0.2
0.1
0.0
Phylostratum
Cellular_organisms - ps1
Archaea1 - ps2
Archaea2 - ps3
Archaea3 - ps4
Asgard/Eukaryota - ps5
Eukaryota - ps6
Amorpheae - ps7
Obazoa - ps8
Opisthokonta - ps9
Holozoa - ps10
Filozoa - ps11
Choano./Metazoa - ps12
Metazoa - ps13
Myriazoa - ps14
Parahoxozoa - ps15
Bilateria - ps16
Deuterostomia - ps17
Chordata - ps18
Olfactores - ps19
Vertebrata - ps20
Gnathostomata - ps21
Euteleostomi - ps22
Sarcopterygii - ps23
Tetrapoda - ps24
Amniota - ps25
Mammalia - ps26
Theria - ps27
Placentalia - ps28
Boreoeutheria - ps29
Euarchontoglires - ps30
Euarchonta - ps31
Primates - ps32
Haplorrhini - ps33
Simiiformes - ps34
Catarrhini - ps35
Hominoidea - ps36
Hominidae - ps37
Homininae - ps38
Hominini - ps39
Homo_sapiens - ps40
p-value
1e-04
5e-04
2e-03
7e-03
3e-02
Log-odds
0.5
1.0
1.5
2.0
2.5
3.0
3.5
4.0

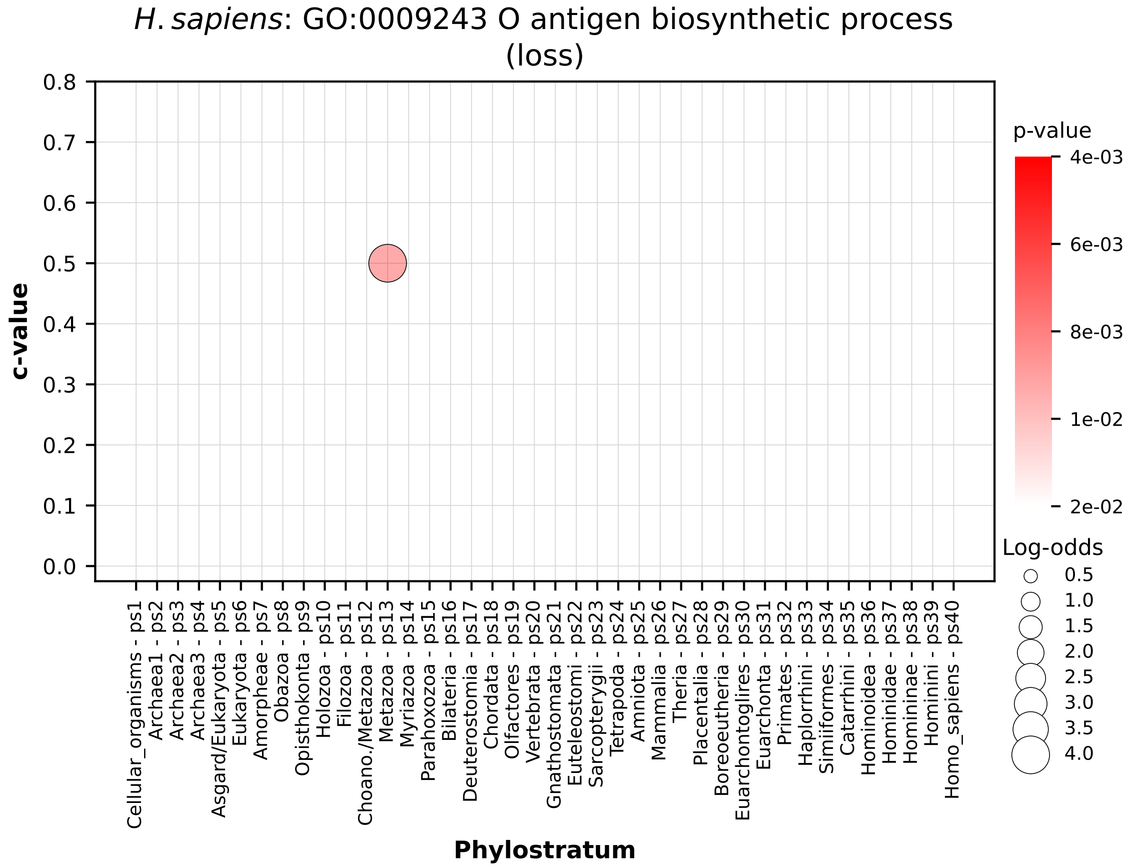

H. sapiens: GO:0009243 O antigen biosynthetic process
(loss)
c-value
0.8
0.7
0.6
0.5
0.4
0.3
0.2
0.1
0.0
p-value
4e-03
6e-03
8e-03
1e-02
2e-02
Log-odds
0.5
1.0
1.5
2.0
2.5
3.0
3.5
4.0
Cellular_organisms - ps1
Archaea1 - ps2
Archaea2 - ps3
Archaea3 - ps4
Asgard/Eukaryota - ps5
Eukaryota - ps6
Amorpheae - ps7
Obazoa - ps8
Opisthokonta - ps9
Holozoa - ps10
Filozoa - ps11
Choano./Metazoa - ps12
Metazoa - ps13
Myriazoa - ps14
Parahoxozoa - ps15
Bilateria - ps16
Deuterostomia - ps17
Chordata - ps18
Olfactores - ps19
Vertebrata - ps20
Gnathostomata - ps21
Euteleostomi - ps22
Sarcopterygii - ps23
Tetrapoda - ps24
Amniota - ps25
Mammalia - ps26
Theria - ps27
Placentalia - ps28
Boreoeutheria - ps29
Euarchontoglires - ps30
Euarchonta - ps31
Primates - ps32
Haplorrhini - ps33
Simiiformes - ps34
Catarrhini - ps35
Hominoidea - ps36
Hominidae - ps37
Homininae - ps38
Hominini - ps39
Homo_sapiens - ps40
Phylostratum

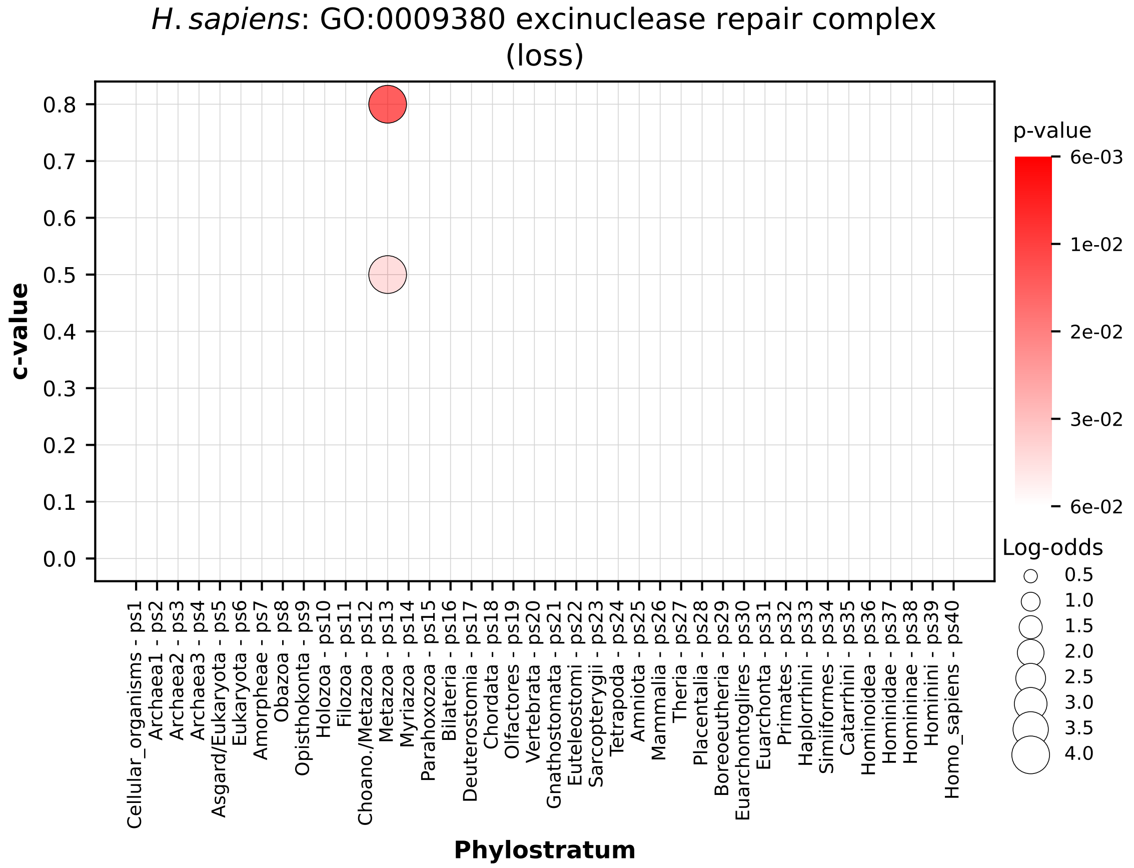

H. sapiens: GO:0009380 excinuclease repair complex
(loss)
c-value
0.8
0.7
0.6
0.5
0.4
0.3
0.2
0.1
0.0
p-value
6e-03
1e-02
2e-02
3e-02
6e-02
Log-odds
0.5
1.0
1.5
2.0
2.5
3.0
3.5
4.0
Cellular_organisms - ps1
Archaea1 - ps2
Archaea2 - ps3
Archaea3 - ps4
Asgard/Eukaryota - ps5
Eukaryota - ps6
Amorpheae - ps7
Obazoa - ps8
Opisthokonta - ps9
Holozoa - ps10
Filozoa - ps11
Choano./Metazoa - ps12
Metazoa - ps13
Myriazoa - ps14
Parahoxozoa - ps15
Bilateria - ps16
Deuterostomia - ps17
Chordata - ps18
Olfactores - ps19
Vertebrata - ps20
Gnathostomata - ps21
Euteleostomi - ps22
Sarcopterygii - ps23
Tetrapoda - ps24
Amniota - ps25
Mammalia - ps26
Theria - ps27
Placentalia - ps28
Boreoeutheria - ps29
Euarchontoglires - ps30
Euarchonta - ps31
Primates - ps32
Haplorrhini - ps33
Simiiformes - ps34
Catarrhini - ps35
Hominoidea - ps36
Hominidae - ps37
Homininae - ps38
Hominini - ps39
Homo_sapiens - ps40
Phylostratum

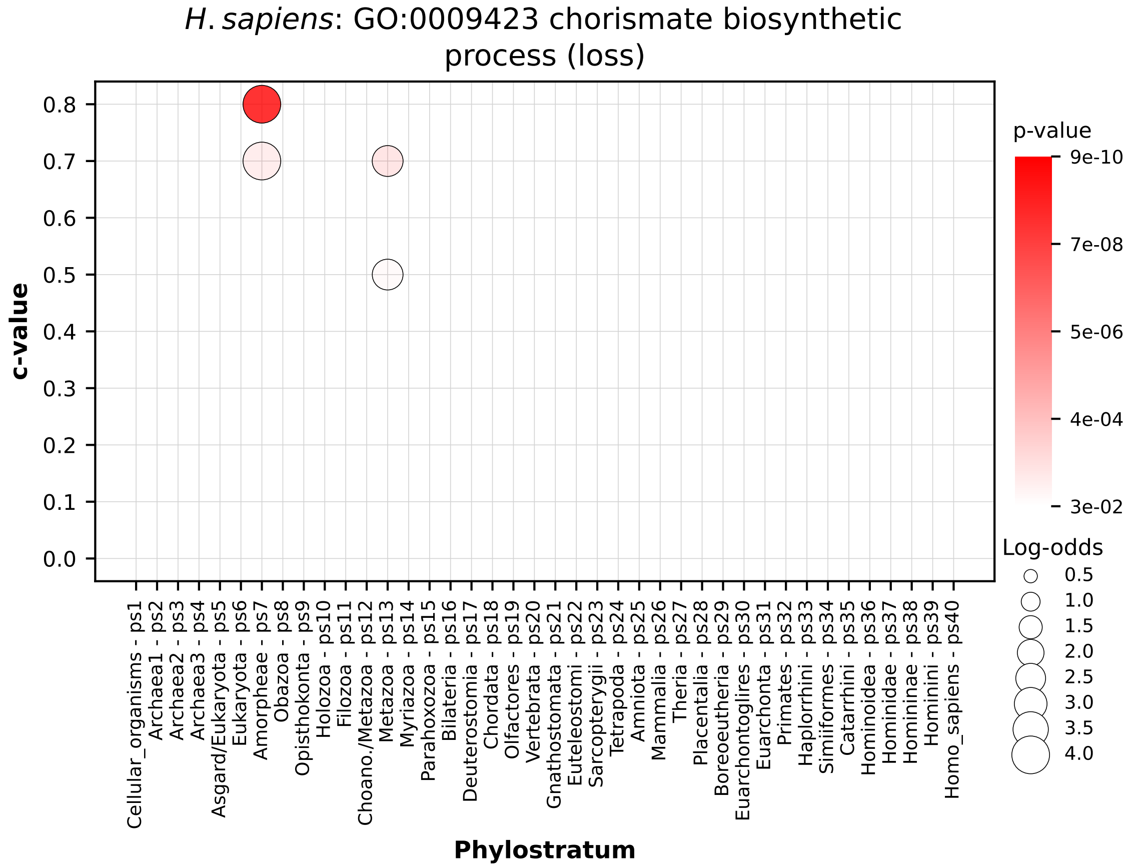

H. sapiens: GO:0009423 chorismate biosynthetic process (loss)
c-value
0.8
0.7
0.6
0.5
0.4
0.3
0.2
0.1
0.0
p-value
9e-10
7e-08
5e-06
4e-04
3e-02
Log-odds
0.5
1.0
1.5
2.0
2.5
3.0
3.5
4.0
Cellular_organisms - ps1
Archaea1 - ps2
Archaea2 - ps3
Archaea3 - ps4
Asgard/Eukaryota - ps5
Eukaryota - ps6
Amorpheae - ps7
Obazoa - ps8
Opisthokonta - ps9
Holozoa - ps10
Filozoa - ps11
Choano./Metazoa - ps12
Metazoa - ps13
Myriazoa - ps14
Parahoxozoa - ps15
Bilateria - ps16
Deuterostomia - ps17
Chordata - ps18
Olfactores - ps19
Vertebrata - ps20
Gnathostomata - ps21
Euteleostomi - ps22
Sarcopterygii - ps23
Tetrapoda - ps24
Amniota - ps25
Mammalia - ps26
Theria - ps27
Placentalia - ps28
Boreoeutheria - ps29
Euarchontoglires - ps30
Euarchonta - ps31
Primates - ps32
Haplorrhini - ps33
Simiiformes - ps34
Catarrhini - ps35
Hominoidea - ps36
Hominidae - ps37
Homininae - ps38
Hominini - ps39
Homo_sapiens - ps40
Phylostratum

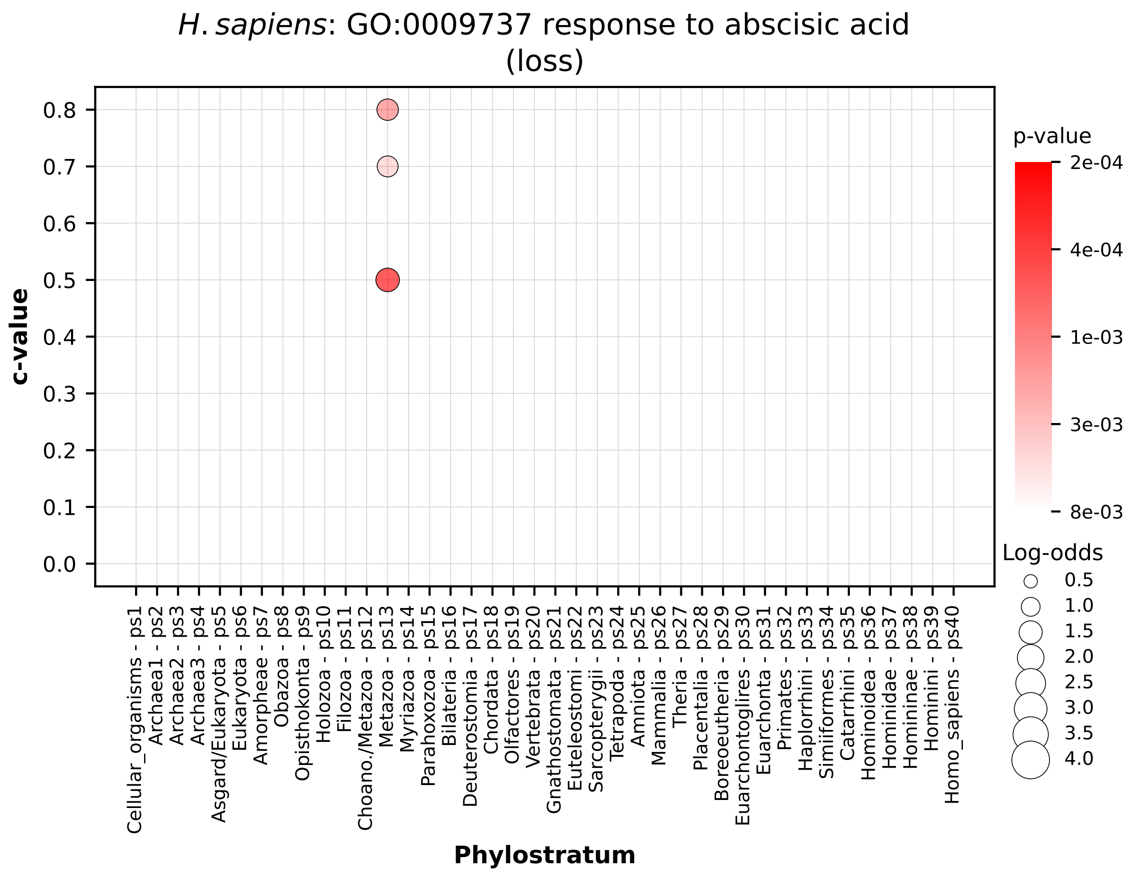
H. sapiens: GO:0009737 response to abscisic acid
(loss)
c-value
0.8
0.7
0.6
0.5
0.4
0.3
0.2
0.1
0.0
p-value
2e-04
4e-04
1e-03
3e-03
8e-03
Log-odds
0.5
1.0
1.5
2.0
2.5
3.0
3.5
4.0
Cellular_organisms - ps1
Archaea1 - ps2
Archaea2 - ps3
Archaea3 - ps4
Asgard/Eukaryota - ps5
Eukaryota - ps6
Amorpheae - ps7
Obazoa - ps8
Opisthokonta - ps9
Holozoa - ps10
Filozoa - ps11
Choano./Metazoa - ps12
Metazoa - ps13
Myriazoa - ps14
Parahoxozoa - ps15
Bilateria - ps16
Deuterostomia - ps17
Chordata - ps18
Olfactores - ps19
Vertebrata - ps20
Gnathostomata - ps21
Euteleostomi - ps22
Sarcopterygii - ps23
Tetrapoda - ps24
Amniota - ps25
Mammalia - ps26
Theria - ps27
Placentalia - ps28
Boreoeutheria - ps29
Euarchontoglires - ps30
Euarchonta - ps31
Primates - ps32
Haplorrhini - ps33
Simiiformes - ps34
Catarrhini - ps35
Hominoidea - ps36
Hominidae - ps37
Homininae - ps38
Hominini - ps39
Homo_sapiens - ps40
Phylostratum

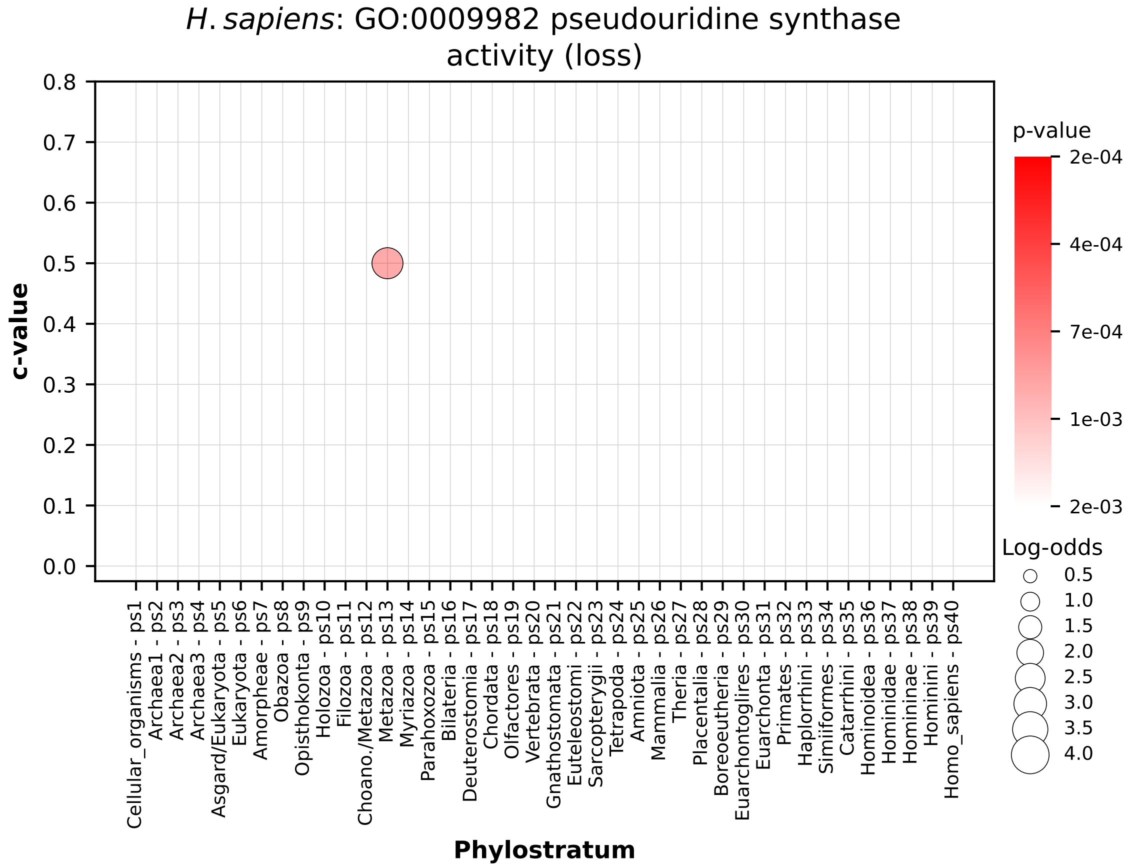

H. sapiens: GO:0009982 pseudouridine synthase activity (loss)
c-value
0.0
0.1
0.2
0.3
0.4
0.5
0.6
0.7
0.8
Phylostratum
Cellular_organisms - ps1
Archaea1 - ps2
Archaea2 - ps3
Archaea3 - ps4
Asgard/Eukaryota - ps5
Eukaryota - ps6
Amorpheae - ps7
Obazoa - ps8
Opisthokonta - ps9
Holozoa - ps10
Filozoa - ps11
Choano./Metazoa - ps12
Metazoa - ps13
Myriazoa - ps14
Parahoxozoa - ps15
Bilateria - ps16
Deuterostomia - ps17
Chordata - ps18
Olfactores - ps19
Vertebrata - ps20
Gnathostomata - ps21
Euteleostomi - ps22
Sarcopterygii - ps23
Tetrapoda - ps24
Amniota - ps25
Mammalia - ps26
Theria - ps27
Placentalia - ps28
Boreoeutheria - ps29
Euarchontoglires - ps30
Euarchonta - ps31
Primates - ps32
Haplorrhini - ps33
Simiiformes - ps34
Catarrhini - ps35
Hominoidea - ps36
Hominidae - ps37
Homininae - ps38
Hominini - ps39
Homo_sapiens - ps40
p-value
2e-04
4e-04
7e-04
1e-03
2e-03
Log-odds
0.5
1.0
1.5
2.0
2.5
3.0
3.5
4.0

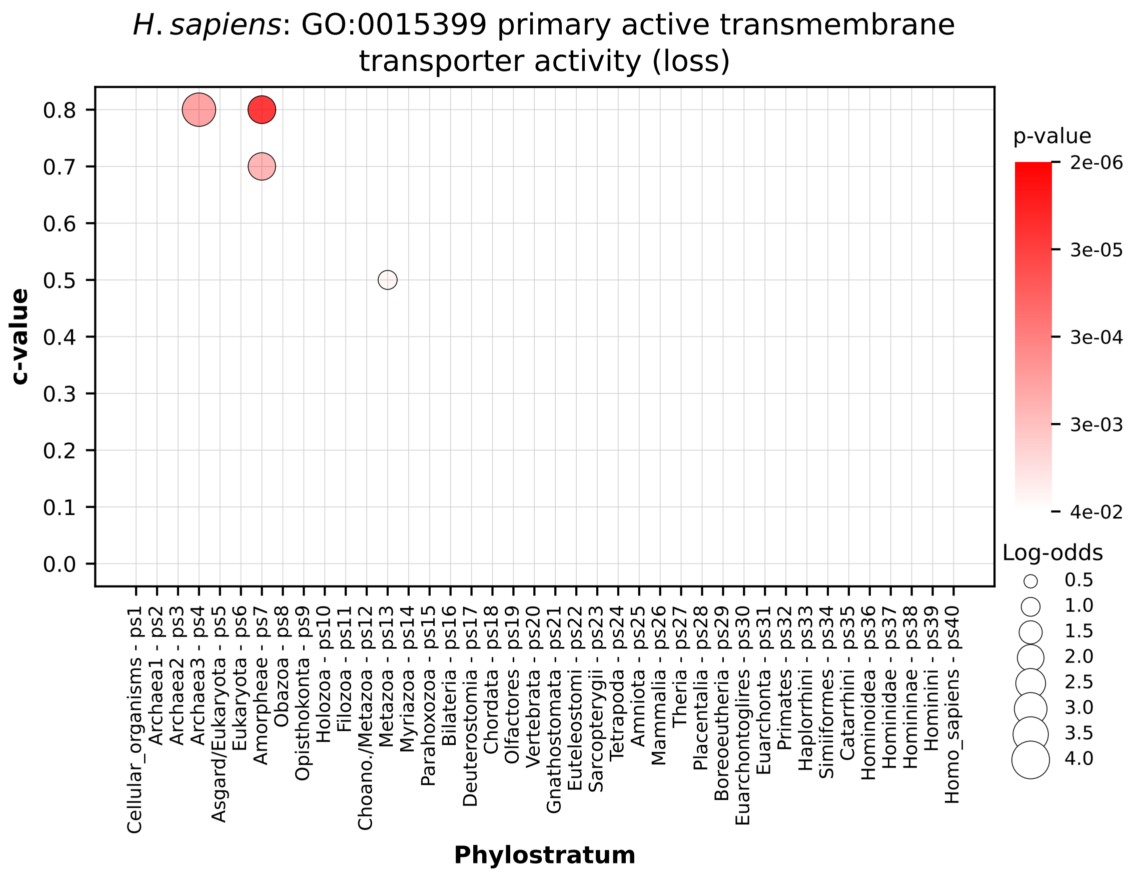
H. sapiens: GO:0015399 primary active transmembrane transporter activity (loss)
c-value
0.0
0.1
0.2
0.3
0.4
0.5
0.6
0.7
0.8
p-value
2e-06
3e-05
3e-04
3e-03
4e-02
Log-odds
0.5
1.0
1.5
2.0
2.5
3.0
3.5
4.0
Cellular_organisms - ps1
Archaea1 - ps2
Archaea2 - ps3
Archaea3 - ps4
Asgard/Eukaryota - ps5
Eukaryota - ps6
Amorpheae - ps7
Obazoa - ps8
Opisthokonta - ps9
Holozoa - ps10
Filozoa - ps11
Choano./Metazoa - ps12
Metazoa - ps13
Myriazoa - ps14
Parahoxozoa - ps15
Bilateria - ps16
Deuterostomia - ps17
Chordata - ps18
Olfactores - ps19
Vertebrata - ps20
Gnathostomata - ps21
Euteleostomi - ps22
Sarcopterygii - ps23
Tetrapoda - ps24
Amniota - ps25
Mammalia - ps26
Theria - ps27
Placentalia - ps28
Boreoeutheria - ps29
Euarchontoglires - ps30
Euarchonta - ps31
Primates - ps32
Haplorrhini - ps33
Simiiformes - ps34
Catarrhini - ps35
Hominoidea - ps36
Hominidae - ps37
Homininae - ps38
Hominini - ps39
Homo_sapiens - ps40
Phylostratum

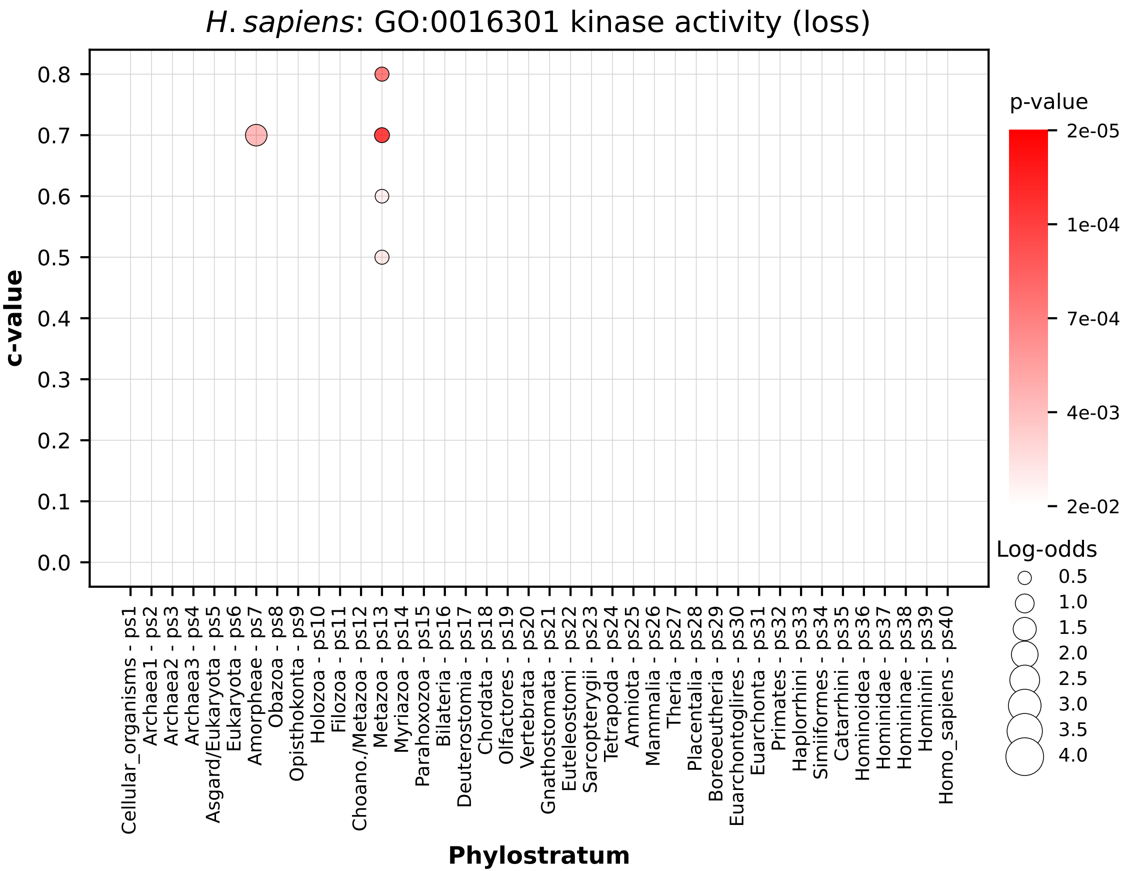

H. sapiens: GO:0016301 kinase activity (loss)
c-value
0.8
0.7
0.6
0.5
0.4
0.3
0.2
0.1
0.0
p-value
2e-05
1e-04
7e-04
4e-03
2e-02
Log-odds
0.5
1.0
1.5
2.0
2.5
3.0
3.5
4.0
Cellular_organisms - ps1
Archaea1 - ps2
Archaea2 - ps3
Archaea3 - ps4
Asgard/Eukaryota - ps5
Eukaryota - ps6
Amorpheae - ps7
Obazoa - ps8
Opisthokonta - ps9
Holozoa - ps10
Filozoa - ps11
Choano./Metazoa - ps12
Metazoa - ps13
Myriazoa - ps14
Parahoxozoa - ps15
Bilateria - ps16
Deuterostomia - ps17
Chordata - ps18
Olfactores - ps19
Vertebrata - ps20
Gnathostomata - ps21
Euteleostomi - ps22
Sarcopterygii - ps23
Tetrapoda - ps24
Amniota - ps25
Mammalia - ps26
Theria - ps27
Placentalia - ps28
Boreoeutheria - ps29
Euarchontoglires - ps30
Euarchonta - ps31
Primates - ps32
Haplorrhini - ps33
Simiiformes - ps34
Catarrhini - ps35
Hominoidea - ps36
Hominidae - ps37
Homininae - ps38
Hominini - ps39
Homo_sapiens - ps40
Phylostratum

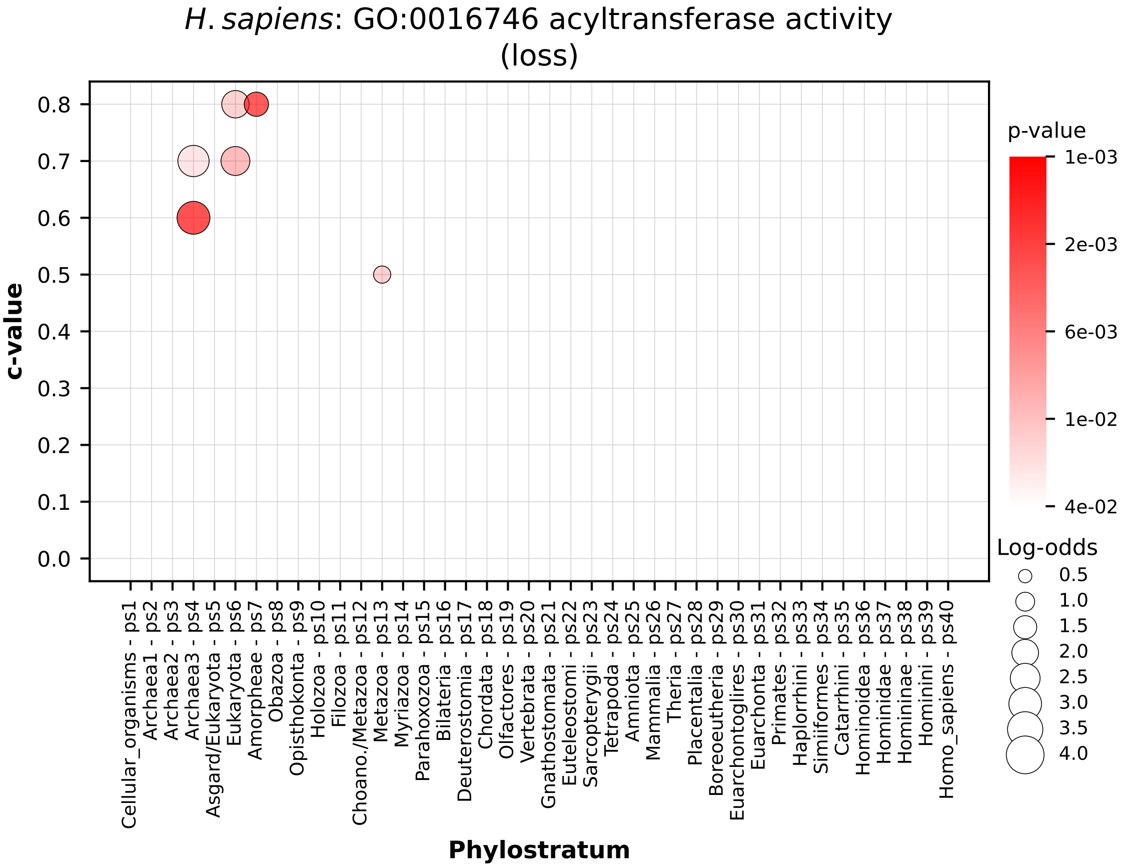
H. sapiens: GO:0016746 acyltransferase activity
(loss)
c-value
0.8
0.7
0.6
0.5
0.4
0.3
0.2
0.1
0.0
p-value
1e-03
2e-03
6e-03
1e-02
4e-02
Log-odds
0.5
1.0
1.5
2.0
2.5
3.0
3.5
4.0
Cellular_organisms - ps1
Archaea1 - ps2
Archaea2 - ps3
Archaea3 - ps4
Asgard/Eukaryota - ps5
Eukaryota - ps6
Amorpheae - ps7
Obazoa - ps8
Opisthokonta - ps9
Holozoa - ps10
Filozoa - ps11
Choano./Metazoa - ps12
Metazoa - ps13
Myriazoa - ps14
Parahoxozoa - ps15
Bilateria - ps16
Deuterostomia - ps17
Chordata - ps18
Olfactores - ps19
Vertebrata - ps20
Gnathostomata - ps21
Euteleostomi - ps22
Sarcopterygii - ps23
Tetrapoda - ps24
Amniota - ps25
Mammalia - ps26
Theria - ps27
Placentalia - ps28
Boreoeutheria - ps29
Euarchontoglires - ps30
Euarchonta - ps31
Primates - ps32
Haplorrhini - ps33
Simiiformes - ps34
Catarrhini - ps35
Hominoidea - ps36
Hominidae - ps37
Homininae - ps38
Hominini - ps39
Homo_sapiens - ps40
Phylostratum

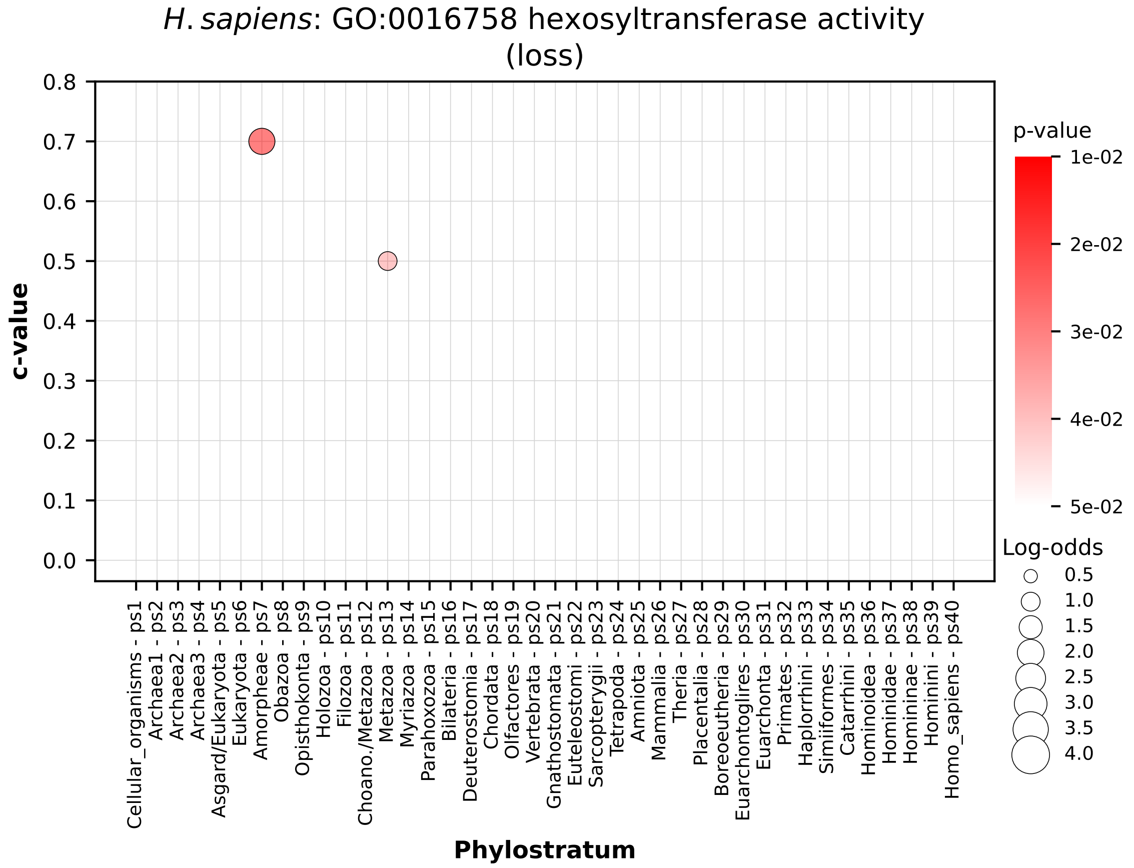

H. sapiens: GO:0016758 hexosyltransferase activity (loss)
c-value
0.0
0.1
0.2
0.3
0.4
0.5
0.6
0.7
0.8
Phylostratum
Cellular_organisms - ps1
Archaea1 - ps2
Archaea2 - ps3
Archaea3 - ps4
Asgard/Eukaryota - ps5
Eukaryota - ps6
Amorpheae - ps7
Obazoa - ps8
Opisthokonta - ps9
Holozoa - ps10
Filozoa - ps11
Choano./Metazoa - ps12
Metazoa - ps13
Myriazoa - ps14
Parahoxozoa - ps15
Bilateria - ps16
Deuterostomia - ps17
Chordata - ps18
Olfactores - ps19
Vertebrata - ps20
Gnathostomata - ps21
Euteleostomi - ps22
Sarcopterygii - ps23
Tetrapoda - ps24
Amniota - ps25
Mammalia - ps26
Theria - ps27
Placentalia - ps28
Boreoeutheria - ps29
Euarchontoglires - ps30
Euarchonta - ps31
Primates - ps32
Haplorrhini - ps33
Simiiformes - ps34
Catarrhini - ps35
Hominoidea - ps36
Hominidae - ps37
Homininae - ps38
Hominini - ps39
Homo_sapiens - ps40
p-value
1e-02
2e-02
3e-02
4e-02
5e-02
Log-odds
0.5
1.0
1.5
2.0
2.5
3.0
3.5
4.0

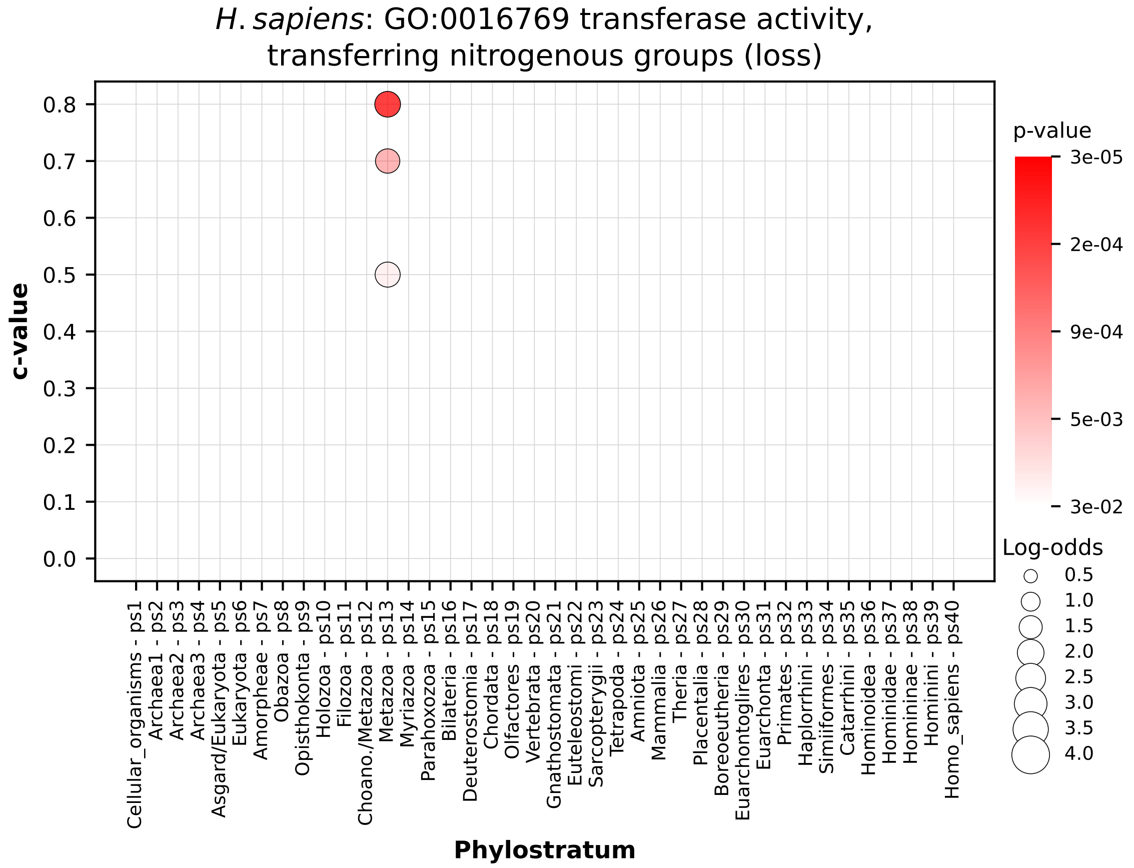

H. sapiens: GO:0016769 transferase activity, transferring nitrogenous groups (loss)
c-value
0.8
0.7
0.6
0.5
0.4
0.3
0.2
0.1
0.0
p-value
3e-05
2e-04
9e-04
5e-03
3e-02
Log-odds
0.5
1.0
1.5
2.0
2.5
3.0
3.5
4.0
Cellular_organisms - ps1
Archaea1 - ps2
Archaea2 - ps3
Archaea3 - ps4
Asgard/Eukaryota - ps5
Eukaryota - ps6
Amorpheae - ps7
Obazoa - ps8
Opisthokonta - ps9
Holozoa - ps10
Filozoa - ps11
Choano./Metazoa - ps12
Metazoa - ps13
Myriazoa - ps14
Parahoxozoa - ps15
Bilateria - ps16
Deuterostomia - ps17
Chordata - ps18
Olfactores - ps19
Vertebrata - ps20
Gnathostomata - ps21
Euteleostomi - ps22
Sarcopterygii - ps23
Tetrapoda - ps24
Amniota - ps25
Mammalia - ps26
Theria - ps27
Placentalia - ps28
Boreoeutheria - ps29
Euarchontoglires - ps30
Euarchonta - ps31
Primates - ps32
Haplorrhini - ps33
Simiiformes - ps34
Catarrhini - ps35
Hominoidea - ps36
Hominidae - ps37
Homininae - ps38
Hominini - ps39
Homo_sapiens - ps40
Phylostratum

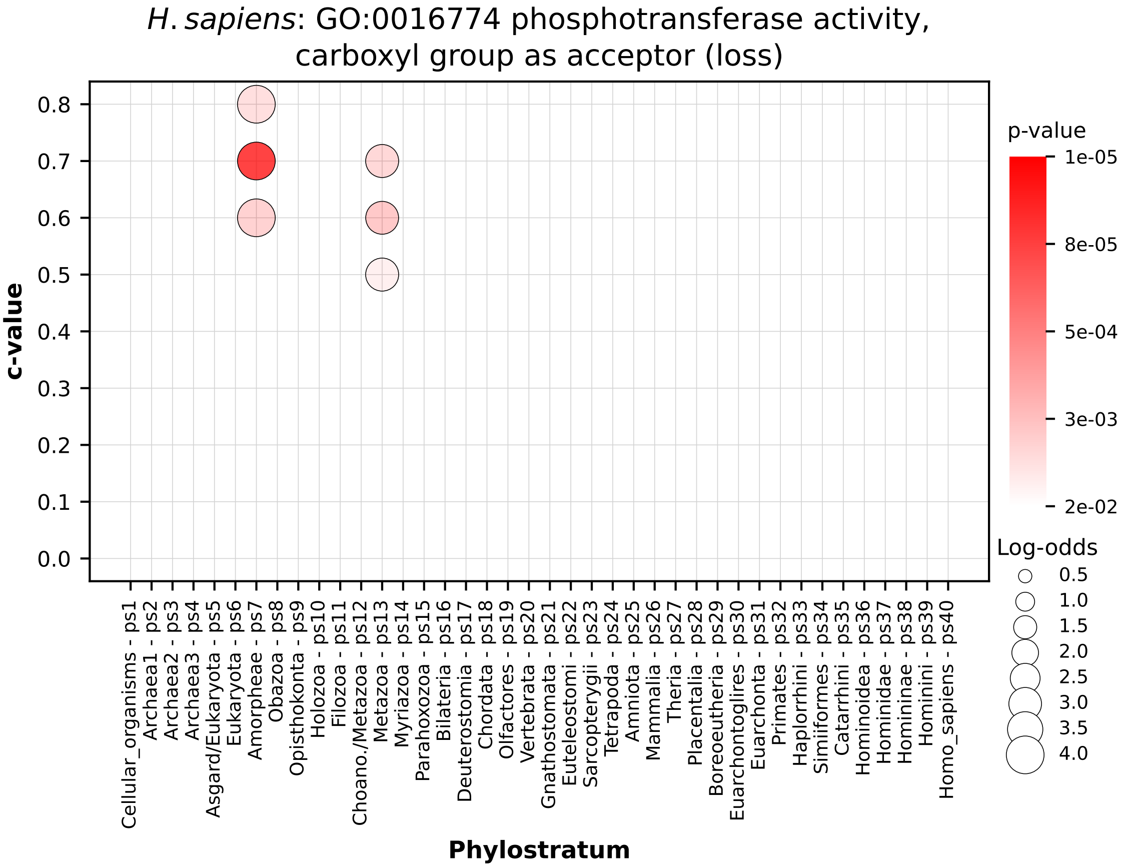

H. sapiens: GO:0016774 phosphotransferase activity, carboxyl group as acceptor (loss)
c-value
0.0
0.1
0.2
0.3
0.4
0.5
0.6
0.7
0.8
Phylostratum
Cellular_organisms - ps1
Archaea1 - ps2
Archaea2 - ps3
Archaea3 - ps4
Asgard/Eukaryota - ps5
Eukaryota - ps6
Amorpheae - ps7
Obazoa - ps8
Opisthokonta - ps9
Holozoa - ps10
Filozoa - ps11
Choano./Metazoa - ps12
Metazoa - ps13
Myriazoa - ps14
Parahoxozoa - ps15
Bilateria - ps16
Deuterostomia - ps17
Chordata - ps18
Olfactores - ps19
Vertebrata - ps20
Gnathostomata - ps21
Euteleostomi - ps22
Sarcopterygii - ps23
Tetrapoda - ps24
Amniota - ps25
Mammalia - ps26
Theria - ps27
Placentalia - ps28
Boreoeutheria - ps29
Euarchontoglires - ps30
Euarchonta - ps31
Primates - ps32
Haplorrhini - ps33
Simiiformes - ps34
Catarrhini - ps35
Hominoidea - ps36
Hominidae - ps37
Homininae - ps38
Hominini - ps39
Homo_sapiens - ps40
p-value
1e-05
8e-05
5e-04
3e-03
2e-02
Log-odds
0.5
1.0
1.5
2.0
2.5
3.0
3.5
4.0

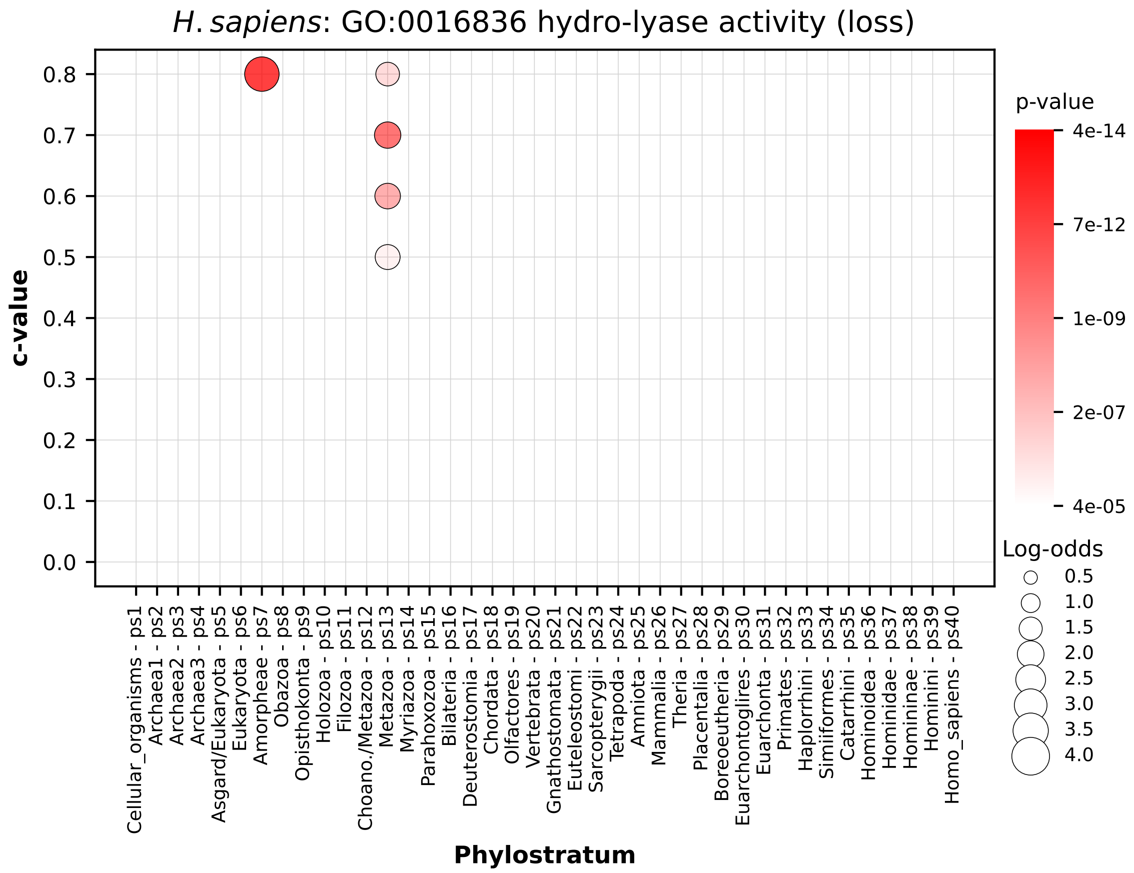

H. sapiens: GO:0016836 hydro-lyase activity (loss)
c-value
0.8
0.7
0.6
0.5
0.4
0.3
0.2
0.1
0.0
p-value
4e-14
7e-12
1e-09
2e-07
4e-05
Log-odds
0.5
1.0
1.5
2.0
2.5
3.0
3.5
4.0
Cellular_organisms - ps1
Archaea1 - ps2
Archaea2 - ps3
Archaea3 - ps4
Asgard/Eukaryota - ps5
Eukaryota - ps6
Amorpheae - ps7
Obazoa - ps8
Opisthokonta - ps9
Holozoa - ps10
Filozoa - ps11
Choano./Metazoa - ps12
Metazoa - ps13
Myriazoa - ps14
Parahoxozoa - ps15
Bilateria - ps16
Deuterostomia - ps17
Chordata - ps18
Olfactores - ps19
Vertebrata - ps20
Gnathostomata - ps21
Euteleostomi - ps22
Sarcopterygii - ps23
Tetrapoda - ps24
Amniota - ps25
Mammalia - ps26
Theria - ps27
Placentalia - ps28
Boreoeutheria - ps29
Euarchontoglires - ps30
Euarchonta - ps31
Primates - ps32
Haplorrhini - ps33
Simiiformes - ps34
Catarrhini - ps35
Hominoidea - ps36
Hominidae - ps37
Homininae - ps38
Hominini - ps39
Homo_sapiens - ps40
Phylostratum

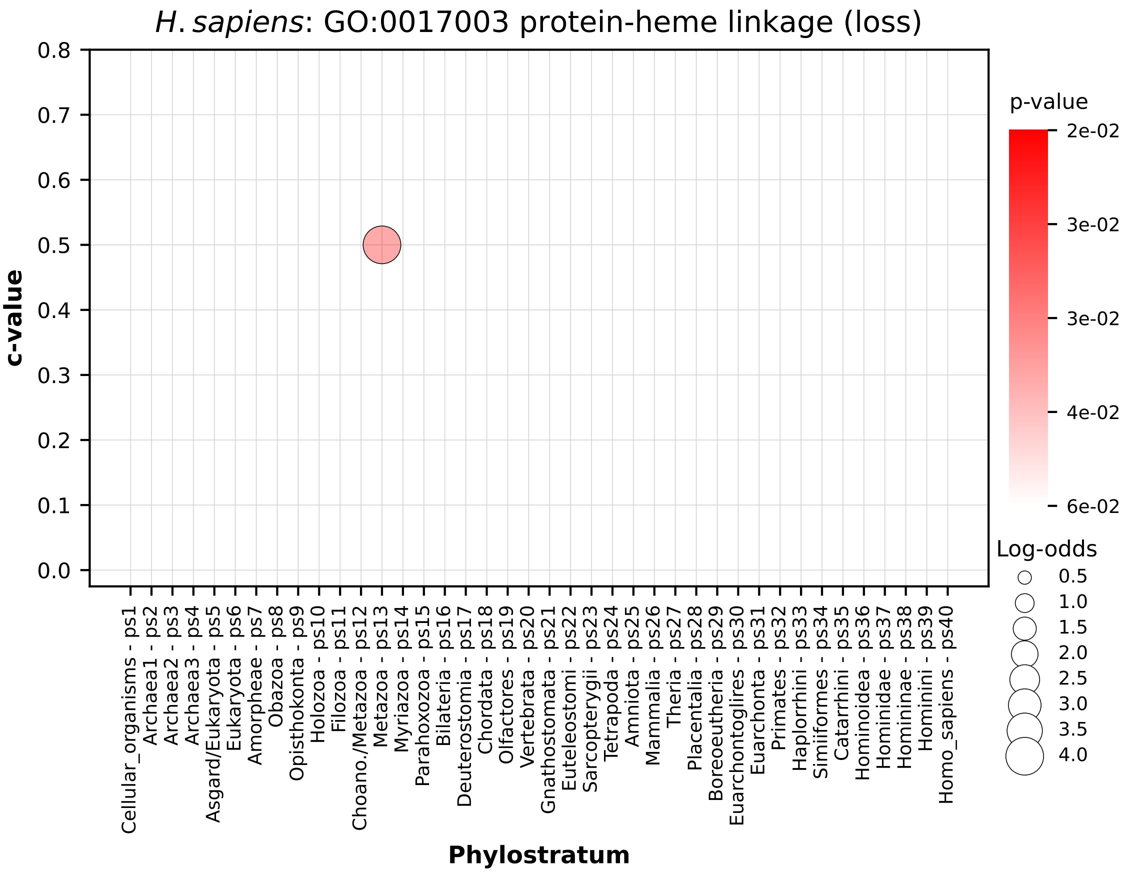

H. sapiens: GO:0017003 protein-heme linkage (loss)
c-value
0.8
0.7
0.6
0.5
0.4
0.3
0.2
0.1
0.0
p-value
2e-02
3e-02
3e-02
4e-02
6e-02
Log-odds
0.5
1.0
1.5
2.0
2.5
3.0
3.5
4.0
Cellular_organisms - ps1
Archaea1 - ps2
Archaea2 - ps3
Archaea3 - ps4
Asgard/Eukaryota - ps5
Eukaryota - ps6
Amorpheae - ps7
Obazoa - ps8
Opisthokonta - ps9
Holozoa - ps10
Filozoa - ps11
Choano./Metazoa - ps12
Metazoa - ps13
Myriazoa - ps14
Parahoxozoa - ps15
Bilateria - ps16
Deuterostomia - ps17
Chordata - ps18
Olfactores - ps19
Vertebrata - ps20
Gnathostomata - ps21
Euteleostomi - ps22
Sarcopterygii - ps23
Tetrapoda - ps24
Amniota - ps25
Mammalia - ps26
Theria - ps27
Placentalia - ps28
Boreoeutheria - ps29
Euarchontoglires - ps30
Euarchonta - ps31
Primates - ps32
Haplorrhini - ps33
Simiiformes - ps34
Catarrhini - ps35
Hominoidea - ps36
Hominidae - ps37
Homininae - ps38
Hominini - ps39
Homo_sapiens - ps40
Phylostratum

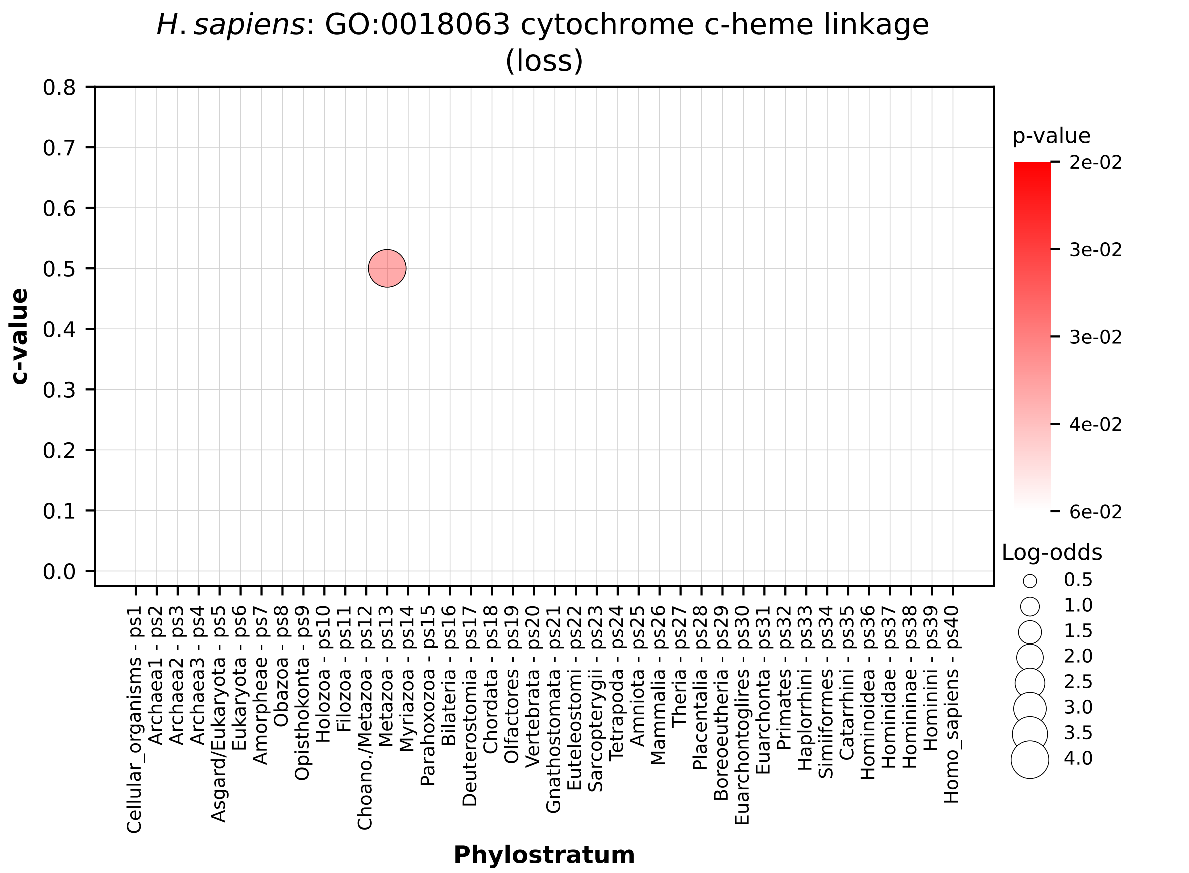

H. sapiens: GO:0018063 cytochrome c-heme linkage
(loss)
c-value
0.0
0.1
0.2
0.3
0.4
0.5
0.6
0.7
0.8
p-value
2e-02
3e-02
3e-02
4e-02
6e-02
Log-odds
0.5
1.0
1.5
2.0
2.5
3.0
3.5
4.0
Cellular_organisms - ps1
Archaea1 - ps2
Archaea2 - ps3
Archaea3 - ps4
Asgard/Eukaryota - ps5
Eukaryota - ps6
Amorpheae - ps7
Obazoa - ps8
Opisthokonta - ps9
Holozoa - ps10
Filozoa - ps11
Choano./Metazoa - ps12
Metazoa - ps13
Myriazoa - ps14
Parahoxozoa - ps15
Bilateria - ps16
Deuterostomia - ps17
Chordata - ps18
Olfactores - ps19
Vertebrata - ps20
Gnathostomata - ps21
Euteleostomi - ps22
Sarcopterygii - ps23
Tetrapoda - ps24
Amniota - ps25
Mammalia - ps26
Theria - ps27
Placentalia - ps28
Boreoeutheria - ps29
Euarchontoglires - ps30
Euarchonta - ps31
Primates - ps32
Haplorrhini - ps33
Simiiformes - ps34
Catarrhini - ps35
Hominoidea - ps36
Hominidae - ps37
Homininae - ps38
Hominini - ps39
Homo_sapiens - ps40
Phylostratum

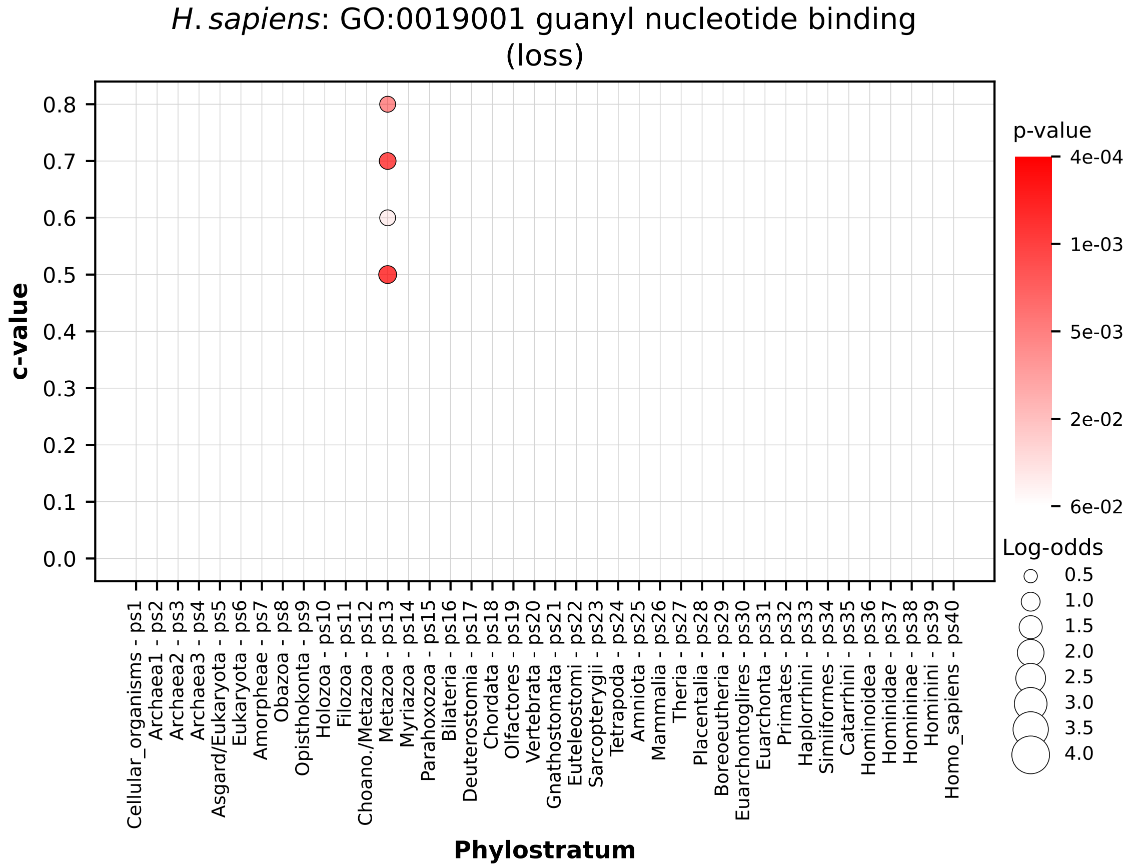

H. sapiens: GO:0019001 guanyl nucleotide binding (loss)
c-value
0.0
0.1
0.2
0.3
0.4
0.5
0.6
0.7
0.8
p-value
4e-04
1e-03
5e-03
2e-02
6e-02
Log-odds
0.5
1.0
1.5
2.0
2.5
3.0
3.5
4.0
Cellular_organisms - ps1
Archaea1 - ps2
Archaea2 - ps3
Archaea3 - ps4
Asgard/Eukaryota - ps5
Eukaryota - ps6
Amorpheae - ps7
Obazoa - ps8
Opisthokonta - ps9
Holozoa - ps10
Filozoa - ps11
Choano./Metazoa - ps12
Metazoa - ps13
Myriazoa - ps14
Parahoxozoa - ps15
Bilateria - ps16
Deuterostomia - ps17
Chordata - ps18
Olfactores - ps19
Vertebrata - ps20
Gnathostomata - ps21
Euteleostomi - ps22
Sarcopterygii - ps23
Tetrapoda - ps24
Amniota - ps25
Mammalia - ps26
Theria - ps27
Placentalia - ps28
Boreoeutheria - ps29
Euarchontoglires - ps30
Euarchonta - ps31
Primates - ps32
Haplorrhini - ps33
Simiiformes - ps34
Catarrhini - ps35
Hominoidea - ps36
Hominidae - ps37
Homininae - ps38
Hominini - ps39
Homo_sapiens - ps40
Phylostratum

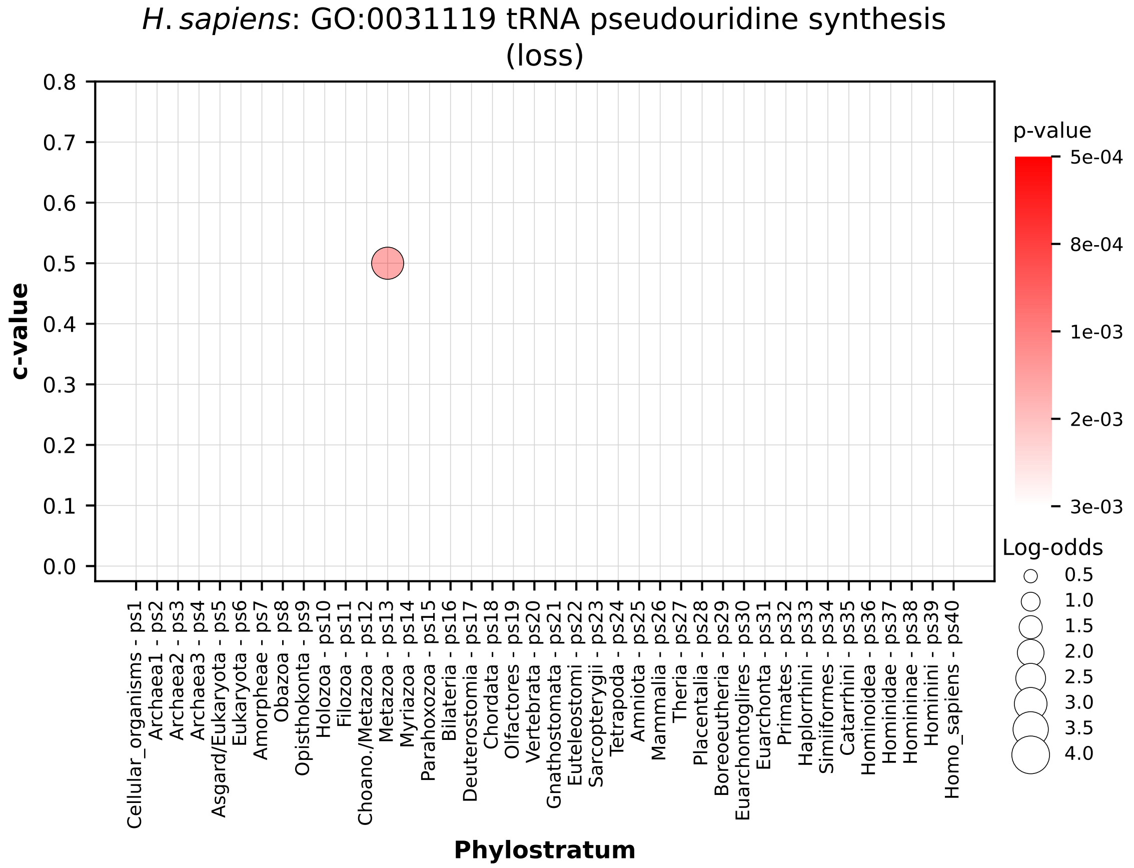
H. sapiens: GO:0031119 tRNA pseudouridine synthesis
(loss)
c-value
0.8
0.7
0.6
0.5
0.4
0.3
0.2
0.1
0.0
Phylostratum
Cellular_organisms - ps1
Archaea1 - ps2
Archaea2 - ps3
Archaea3 - ps4
Asgard/Eukaryota - ps5
Eukaryota - ps6
Amorpheae - ps7
Obazoa - ps8
Opisthokonta - ps9
Holozoa - ps10
Filozoa - ps11
Choano./Metazoa - ps12
Metazoa - ps13
Myriazoa - ps14
Parahoxozoa - ps15
Bilateria - ps16
Deuterostomia - ps17
Chordata - ps18
Olfactores - ps19
Vertebrata - ps20
Gnathostomata - ps21
Euteleostomi - ps22
Sarcopterygii - ps23
Tetrapoda - ps24
Amniota - ps25
Mammalia - ps26
Theria - ps27
Placentalia - ps28
Boreoeutheria - ps29
Euarchontoglires - ps30
Euarchonta - ps31
Primates - ps32
Haplorrhini - ps33
Simiiformes - ps34
Catarrhini - ps35
Hominoidea - ps36
Hominidae - ps37
Homininae - ps38
Hominini - ps39
Homo_sapiens - ps40
p-value
5e-04
8e-04
1e-03
2e-03
3e-03
Log-odds
0.5
1.0
1.5
2.0
2.5
3.0
3.5
4.0

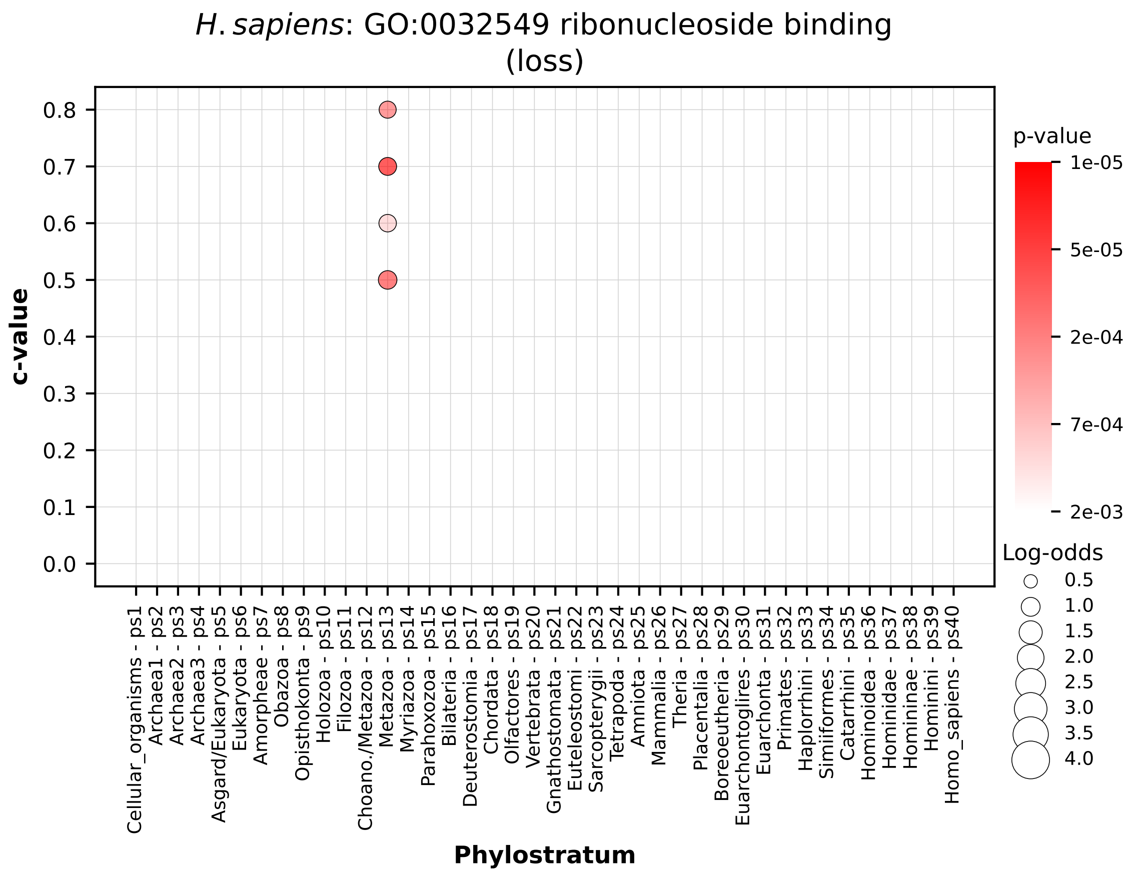
H. sapiens: GO:0032549 ribonucleoside binding
(loss)
c-value
0.8
0.7
0.6
0.5
0.4
0.3
0.2
0.1
0.0
p-value
1e-05
5e-05
2e-04
7e-04
2e-03
Log-odds
0.5
1.0
1.5
2.0
2.5
3.0
3.5
4.0
Cellular_organisms - ps1
Archaea1 - ps2
Archaea2 - ps3
Archaea3 - ps4
Asgard/Eukaryota - ps5
Eukaryota - ps6
Amorpheae - ps7
Obazoa - ps8
Opisthokonta - ps9
Holozoa - ps10
Filozoa - ps11
Choano./Metazoa - ps12
Metazoa - ps13
Myriazoa - ps14
Parahoxozoa - ps15
Bilateria - ps16
Deuterostomia - ps17
Chordata - ps18
Olfactores - ps19
Vertebrata - ps20
Gnathostomata - ps21
Euteleostomi - ps22
Sarcopterygii - ps23
Tetrapoda - ps24
Amniota - ps25
Mammalia - ps26
Theria - ps27
Placentalia - ps28
Boreoeutheria - ps29
Euarchontoglires - ps30
Euarchonta - ps31
Primates - ps32
Haplorrhini - ps33
Similformes - ps34
Catarrhini - ps35
Hominoidea - ps36
Hominidae - ps37
Homininae - ps38
Hominini - ps39
Homo_sapiens - ps40
Phylostratum

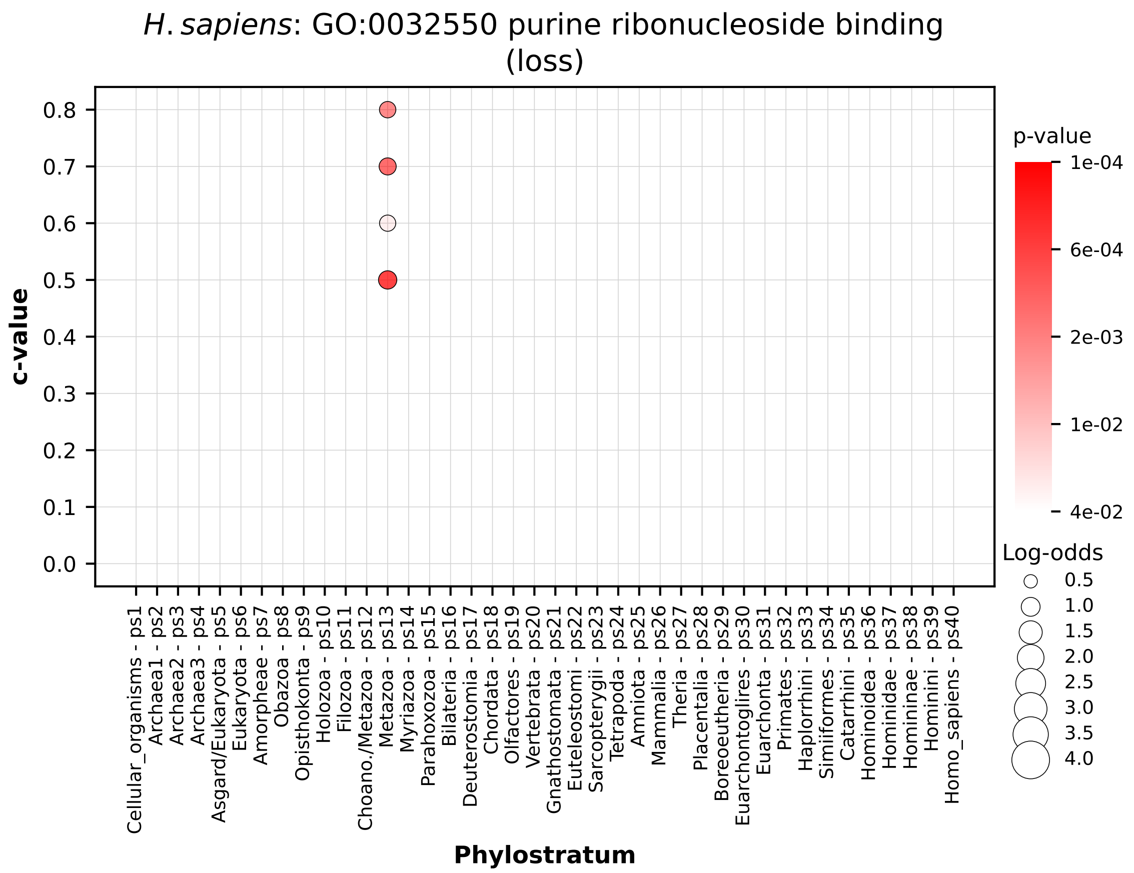
H. sapiens: GO:0032550 purine ribonucleoside binding (loss)
c-value
0.8
0.7
0.6
0.5
0.4
0.3
0.2
0.1
0.0
Phylostratum
Cellular_organisms - ps1
Archaea1 - ps2
Archaea2 - ps3
Archaea3 - ps4
Asgard/Eukaryota - ps5
Eukaryota - ps6
Amorpheae - ps7
Obazoa - ps8
Opisthokonta - ps9
Holozoa - ps10
Filozoa - ps11
Choano./Metazoa - ps12
Metazoa - ps13
Myriazoa - ps14
Parahoxozoa - ps15
Bilateria - ps16
Deuterostomia - ps17
Chordata - ps18
Olfactores - ps19
Vertebrata - ps20
Gnathostomata - ps21
Euteleostomi - ps22
Sarcopterygii - ps23
Tetrapoda - ps24
Amniota - ps25
Mammalia - ps26
Theria - ps27
Placentalia - ps28
Boreoeutheria - ps29
Euarchontoglires - ps30
Euarchonta - ps31
Primates - ps32
Haplorrhini - ps33
Simiiformes - ps34
Catarrhini - ps35
Hominoidea - ps36
Hominidae - ps37
Homininae - ps38
Hominini - ps39
Homo_sapiens - ps40
p-value
1e-04
6e-04
2e-03
1e-02
4e-02
Log-odds
0.5
1.0
1.5
2.0
2.5
3.0
3.5
4.0

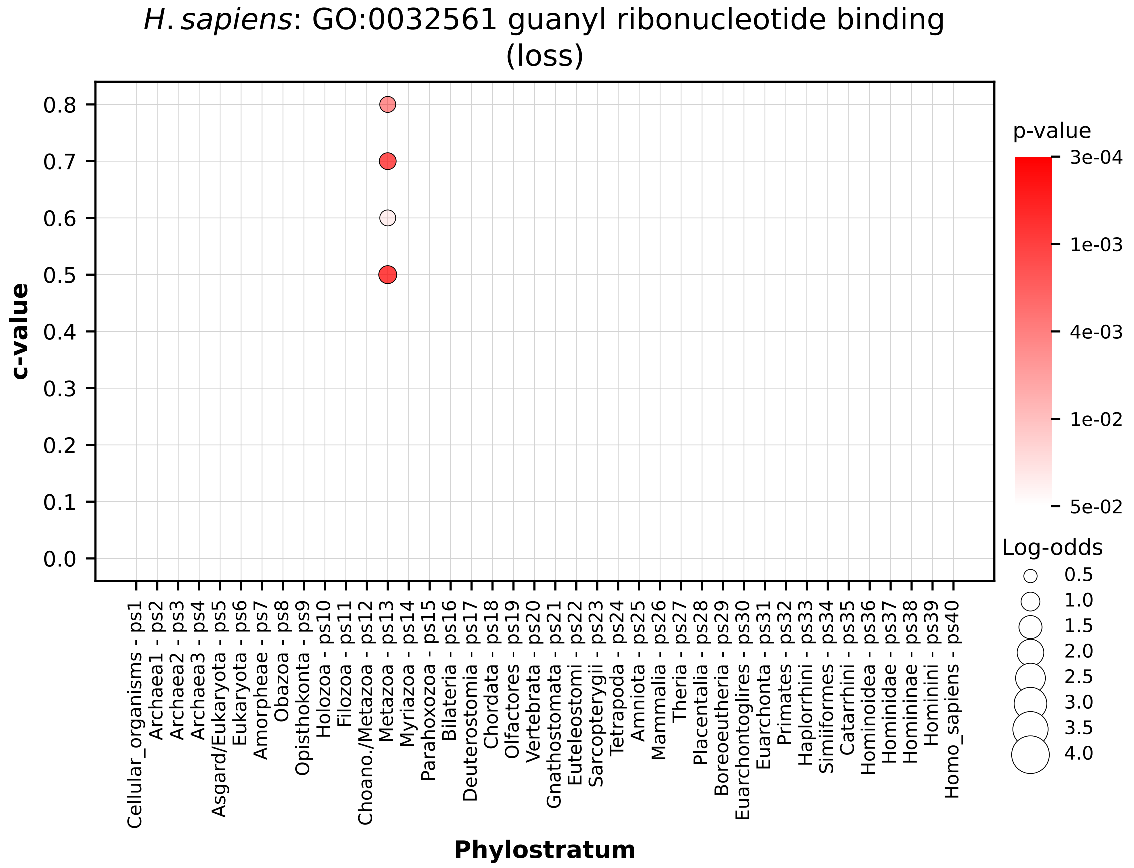

H. sapiens: GO:0032561 guanyl ribonucleotide binding
(loss)
c-value
0.0
0.1
0.2
0.3
0.4
0.5
0.6
0.7
0.8
Phylostratum
Cellular_organisms - ps1
Archaea1 - ps2
Archaea2 - ps3
Archaea3 - ps4
Asgard/Eukaryota - ps5
Eukaryota - ps6
Amorpheae - ps7
Obazoa - ps8
Opisthokonta - ps9
Holozoa - ps10
Filozoa - ps11
Choano./Metazoa - ps12
Metazoa - ps13
Myriazoa - ps14
Parahoxozoa - ps15
Bilateria - ps16
Deuterostomia - ps17
Chordata - ps18
Olfactores - ps19
Vertebrata - ps20
Gnathostomata - ps21
Euteleostomi - ps22
Sarcopterygii - ps23
Tetrapoda - ps24
Amniota - ps25
Mammalia - ps26
Theria - ps27
Placentalia - ps28
Boreoeutheria - ps29
Euarchontoglires - ps30
Euarchonta - ps31
Primates - ps32
Haplorrhini - ps33
Simiiformes - ps34
Catarrhini - ps35
Hominoidea - ps36
Hominidae - ps37
Homininae - ps38
Hominini - ps39
Homo_sapiens - ps40
p-value
3e-04
1e-03
4e-03
1e-02
5e-02
Log-odds
0.5
1.0
1.5
2.0
2.5
3.0
3.5
4.0

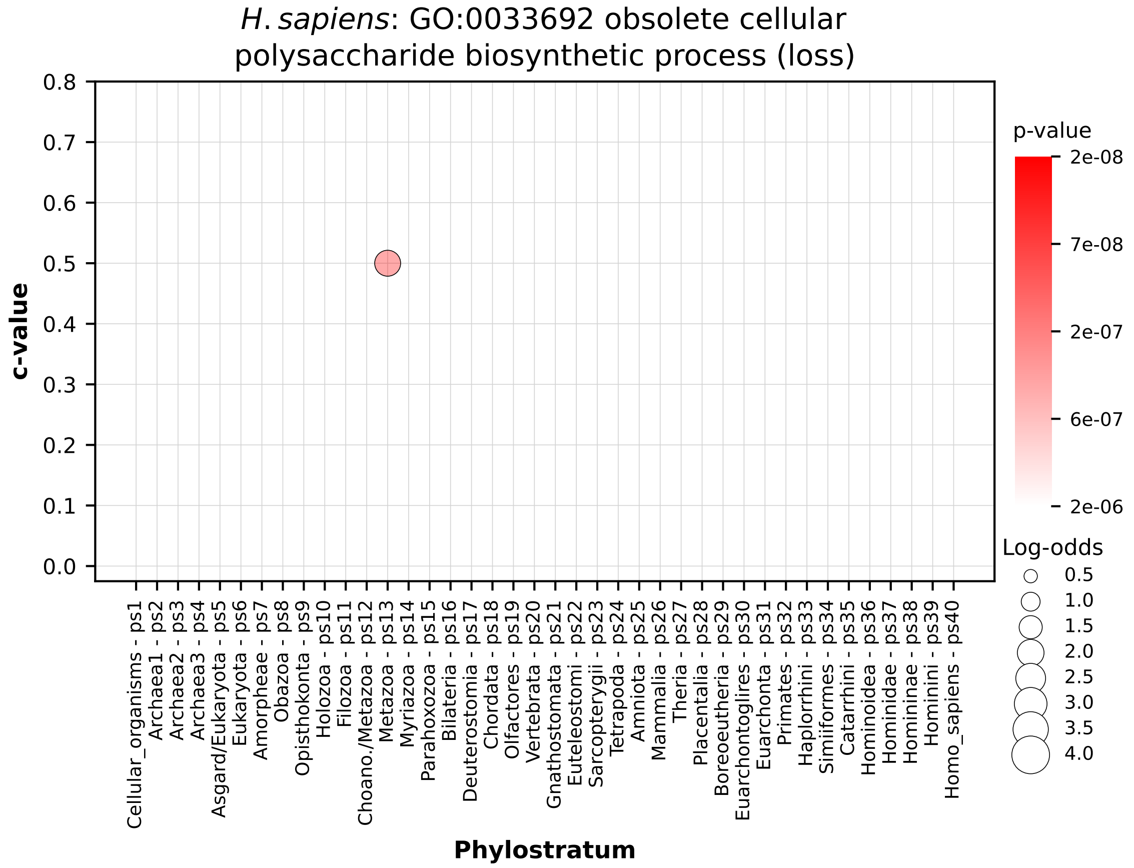
H. sapiens: GO:0033692 obsolete cellular polysaccharide biosynthetic process (loss)
c-value
0.0
0.1
0.2
0.3
0.4
0.5
0.6
0.7
0.8
Phylostratum
Cellular_organisms - ps1
Archaea1 - ps2
Archaea2 - ps3
Archaea3 - ps4
Asgard/Eukaryota - ps5
Eukaryota - ps6
Amorpheae - ps7
Obazoa - ps8
Opisthokonta - ps9
Holozoa - ps10
Filozoa - ps11
Choano./Metazoa - ps12
Metazoa - ps13
Myriazoa - ps14
Parahoxozoa - ps15
Bilateria - ps16
Deuterostomia - ps17
Chordata - ps18
Olfactores - ps19
Vertebrata - ps20
Gnathostomata - ps21
Euteleostomi - ps22
Sarcopterygii - ps23
Tetrapoda - ps24
Amniota - ps25
Mammalia - ps26
Theria - ps27
Placentalia - ps28
Boreoeutheria - ps29
Euarchontoglires - ps30
Euarchonta - ps31
Primates - ps32
Haplorrhini - ps33
Simiiformes - ps34
Catarrhini - ps35
Hominoidea - ps36
Hominidae - ps37
Homininae - ps38
Hominini - ps39
Homo_sapiens - ps40
p-value
2e-08
7e-08
2e-07
6e-07
2e-06
Log-odds
0.5
1.0
1.5
2.0
2.5
3.0
3.5
4.0

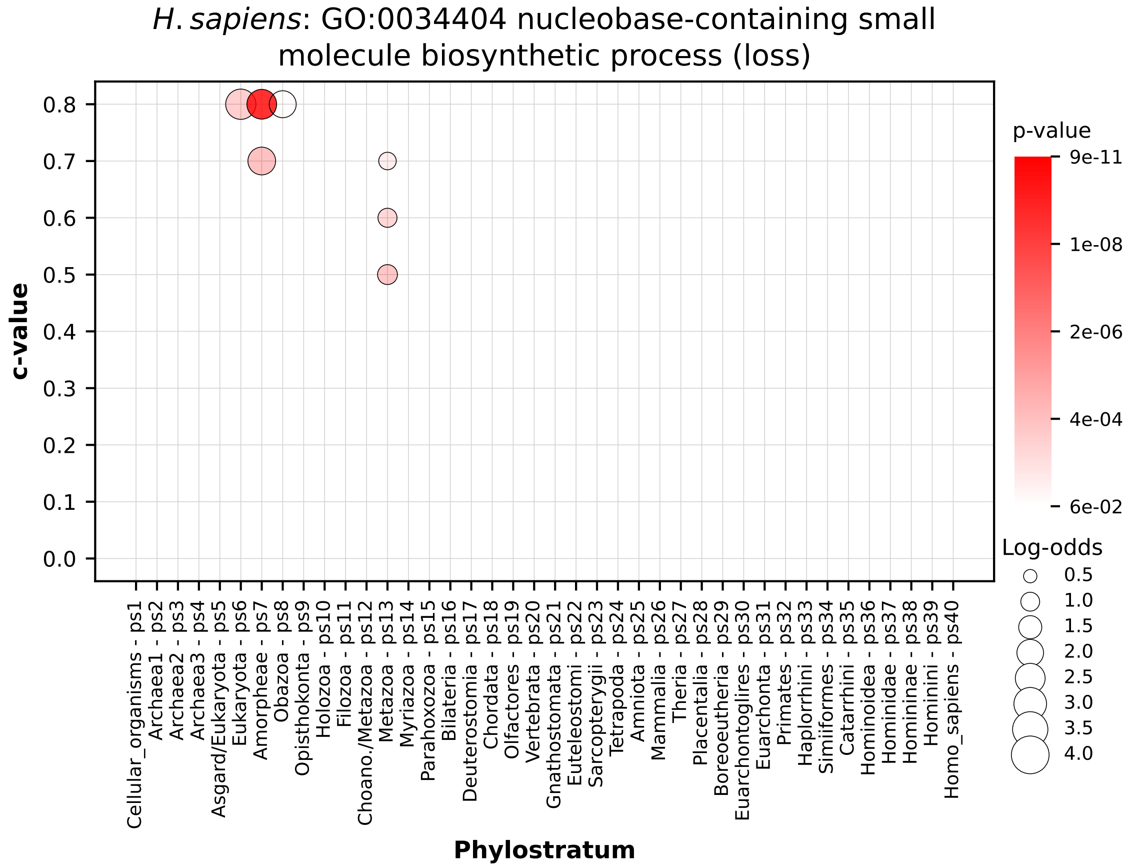

H. sapiens: GO:0034404 nucleobase-containing small molecule biosynthetic process (loss)
c-value
0.8
0.7
0.6
0.5
0.4
0.3
0.2
0.1
0.0
Phylostratum
Cellular_organisms - ps1
Archaea1 - ps2
Archaea2 - ps3
Archaea3 - ps4
Asgard/Eukaryota - ps5
Eukaryota - ps6
Amorpheae - ps7
Obazoa - ps8
Opisthokonta - ps9
Holozoa - ps10
Filozoa - ps11
Choano./Metazoa - ps12
Metazoa - ps13
Myriazoa - ps14
Parahoxozoa - ps15
Bilateria - ps16
Deuterostomia - ps17
Chordata - ps18
Olfactores - ps19
Vertebrata - ps20
Gnathostomata - ps21
Euteleostomi - ps22
Sarcopterygii - ps23
Tetrapoda - ps24
Amniota - ps25
Mammalia - ps26
Theria - ps27
Placentalia - ps28
Boreoeutheria - ps29
Euarchontoglires - ps30
Euarchonta - ps31
Primates - ps32
Haplorrhini - ps33
Simiiformes - ps34
Catarrhini - ps35
Hominoidea - ps36
Hominidae - ps37
Homininae - ps38
Hominini - ps39
Homo_sapiens - ps40
p-value
9e-11
1e-08
2e-06
4e-04
6e-02
Log-odds
0.5
1.0
1.5
2.0
2.5
3.0
3.5
4.0

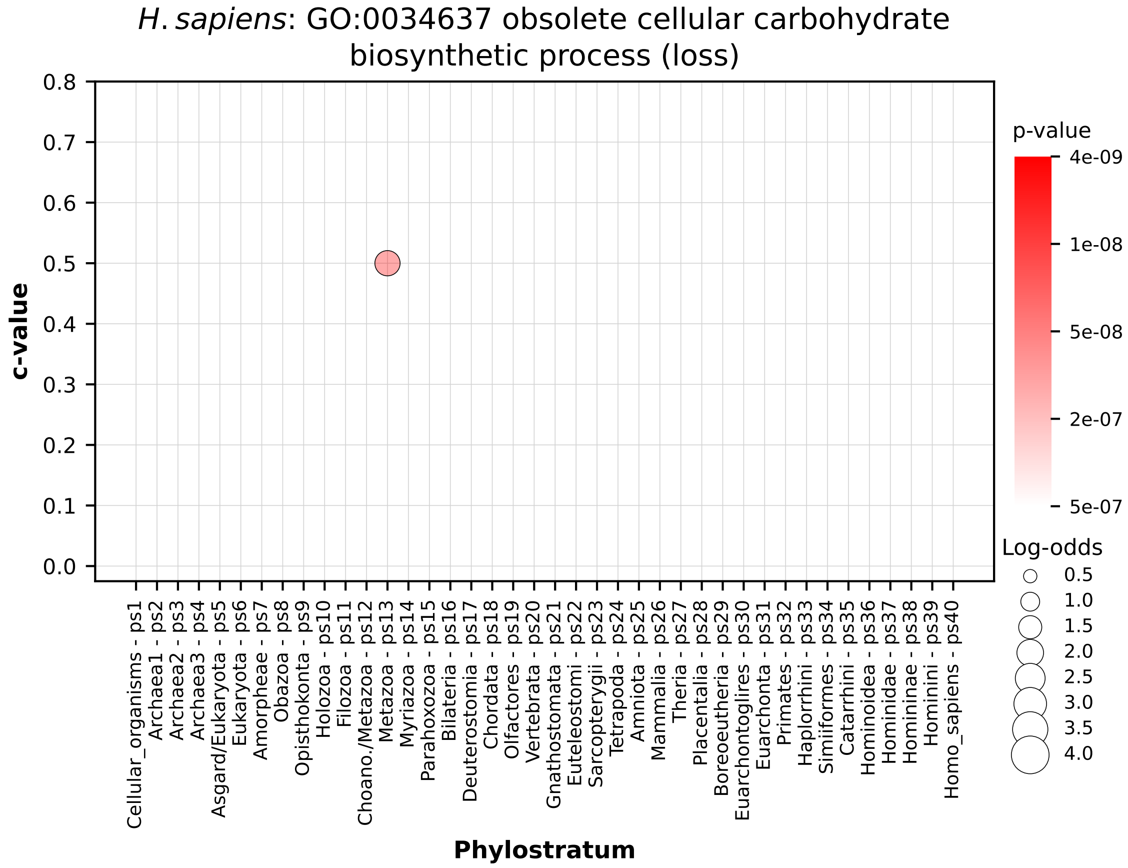
H. sapiens: GO:0034637 obsolete cellular carbohydrate biosynthetic process (loss)
c-value
0.0
0.1
0.2
0.3
0.4
0.5
0.6
0.7
0.8
Phylostratum
Cellular_organisms - ps1
Archaea1 - ps2
Archaea2 - ps3
Archaea3 - ps4
Asgard/Eukaryota - ps5
Eukaryota - ps6
Amorpheae - ps7
Obazoa - ps8
Opisthokonta - ps9
Holozoa - ps10
Filozoa - ps11
Choano./Metazoa - ps12
Metazoa - ps13
Myriazoa - ps14
Parahoxozoa - ps15
Bilateria - ps16
Deuterostomia - ps17
Chordata - ps18
Olfactores - ps19
Vertebrata - ps20
Gnathostomata - ps21
Euteleostomi - ps22
Sarcopterygii - ps23
Tetrapoda - ps24
Amniota - ps25
Mammalia - ps26
Theria - ps27
Placentalia - ps28
Boreoeutheria - ps29
Euarchontoglires - ps30
Euarchonta - ps31
Primates - ps32
Haplorrhini - ps33
Simiiformes - ps34
Catarrhini - ps35
Hominoidea - ps36
Hominidae - ps37
Homininae - ps38
Hominini - ps39
Homo_sapiens - ps40
p-value
4e-09
1e-08
5e-08
2e-07
5e-07
Log-odds
0.5
1.0
1.5
2.0
2.5
3.0
3.5
4.0

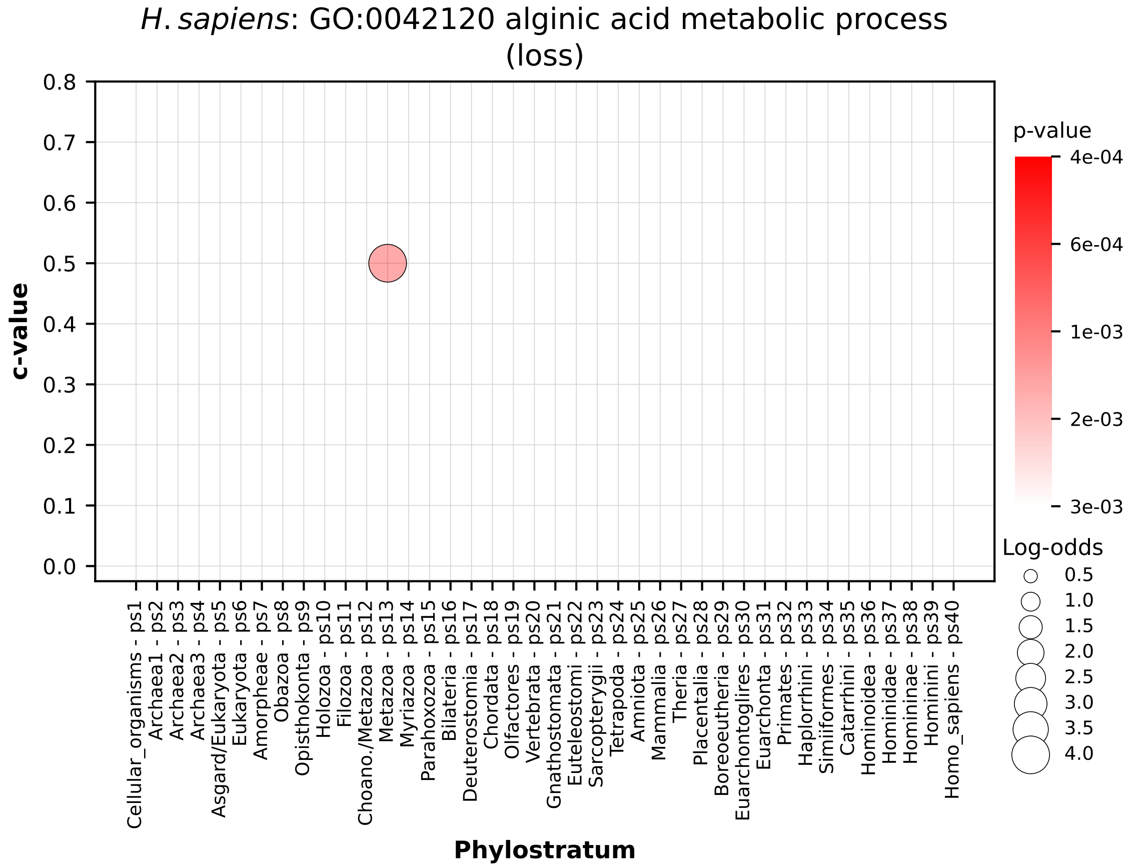

H. sapiens: GO:0042120 alginic acid metabolic process (loss)
c-value
0.0
0.1
0.2
0.3
0.4
0.5
0.6
0.7
0.8
Phylostratum
Cellular_organisms - ps1
Archaea1 - ps2
Archaea2 - ps3
Archaea3 - ps4
Asgard/Eukaryota - ps5
Eukaryota - ps6
Amorpheae - ps7
Obazoa - ps8
Opisthokonta - ps9
Holozoa - ps10
Filozoa - ps11
Choano./Metazoa - ps12
Metazoa - ps13
Myriazoa - ps14
Parahoxozoa - ps15
Bilateria - ps16
Deuterostomia - ps17
Chordata - ps18
Olfactores - ps19
Vertebrata - ps20
Gnathostomata - ps21
Euteleostomi - ps22
Sarcopterygii - ps23
Tetrapoda - ps24
Amniota - ps25
Mammalia - ps26
Theria - ps27
Placentalia - ps28
Boreoeutheria - ps29
Euarchontoglires - ps30
Euarchonta - ps31
Primates - ps32
Haplorrhini - ps33
Simiiformes - ps34
Catarrhini - ps35
Hominoidea - ps36
Hominidae - ps37
Homininae - ps38
Hominini - ps39
Homo_sapiens - ps40
p-value
4e-04
6e-04
1e-03
2e-03
3e-03
Log-odds
0.5
1.0
1.5
2.0
2.5
3.0
3.5
4.0

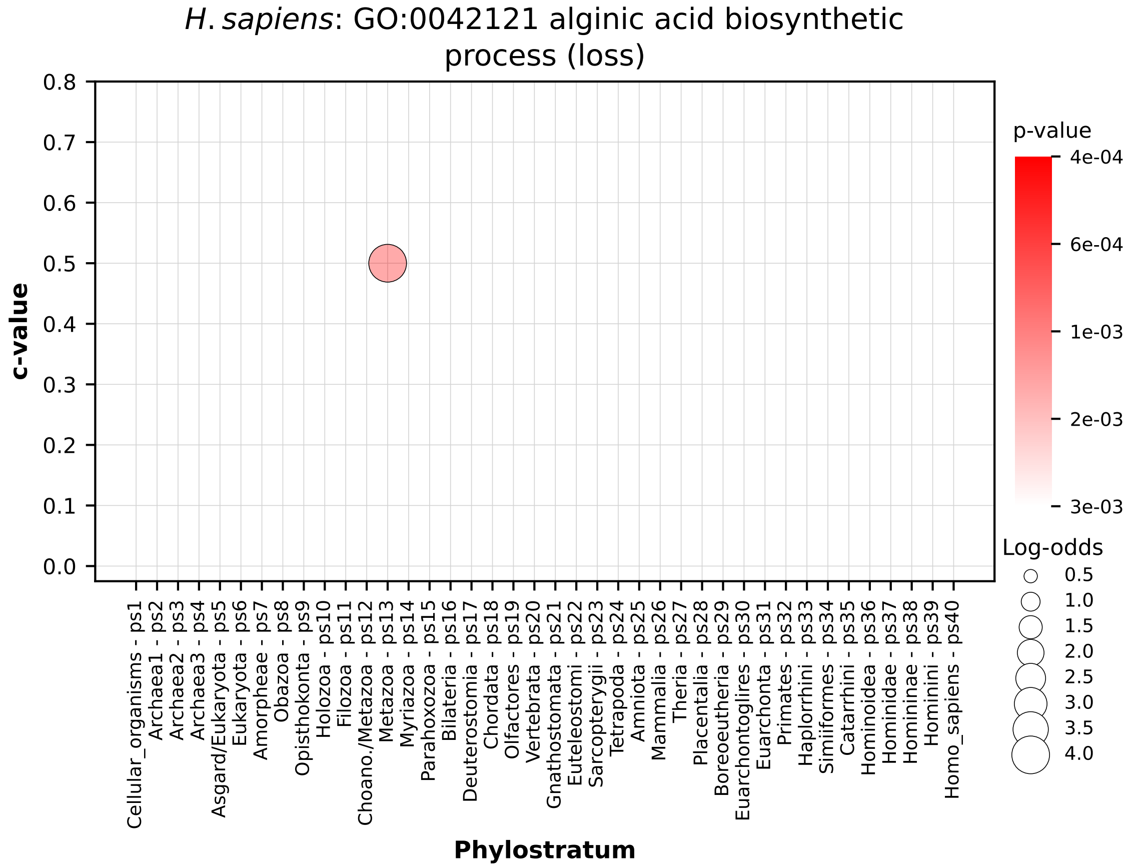
H. sapiens: GO:0042121 alginic acid biosynthetic process (loss)
c-value
Phylostratum
p-value
4e-04
6e-04
1e-03
2e-03
3e-03
Log-odds
0.5
1.0
1.5
2.0
2.5
3.0
3.5
4.0
Cellular_organisms - ps1
Archaea1 - ps2
Archaea2 - ps3
Archaea3 - ps4
Asgard/Eukaryota - ps5
Eukaryota - ps6
Amorpheae - ps7
Obazoa - ps8
Opisthokonta - ps9
Holozoa - ps10
Filozoa - ps11
Choano./Metazoa - ps12
Metazoa - ps13
Myriazoa - ps14
Parahoxozoa - ps15
Bilateria - ps16
Deuterostomia - ps17
Chordata - ps18
Olfactores - ps19
Vertebrata - ps20
Gnathostomata - ps21
Euteleostomi - ps22
Sarcopterygii - ps23
Tetrapoda - ps24
Amniota - ps25
Mammalia - ps26
Theria - ps27
Placentalia - ps28
Boreoeutheria - ps29
Euarchontoglires - ps30
Euarchonta - ps31
Primates - ps32
Haplorrhini - ps33
Simiiformes - ps34
Catarrhini - ps35
Hominoidea - ps36
Hominidae - ps37
Homininae - ps38
Hominini - ps39
Homo_sapiens - ps40

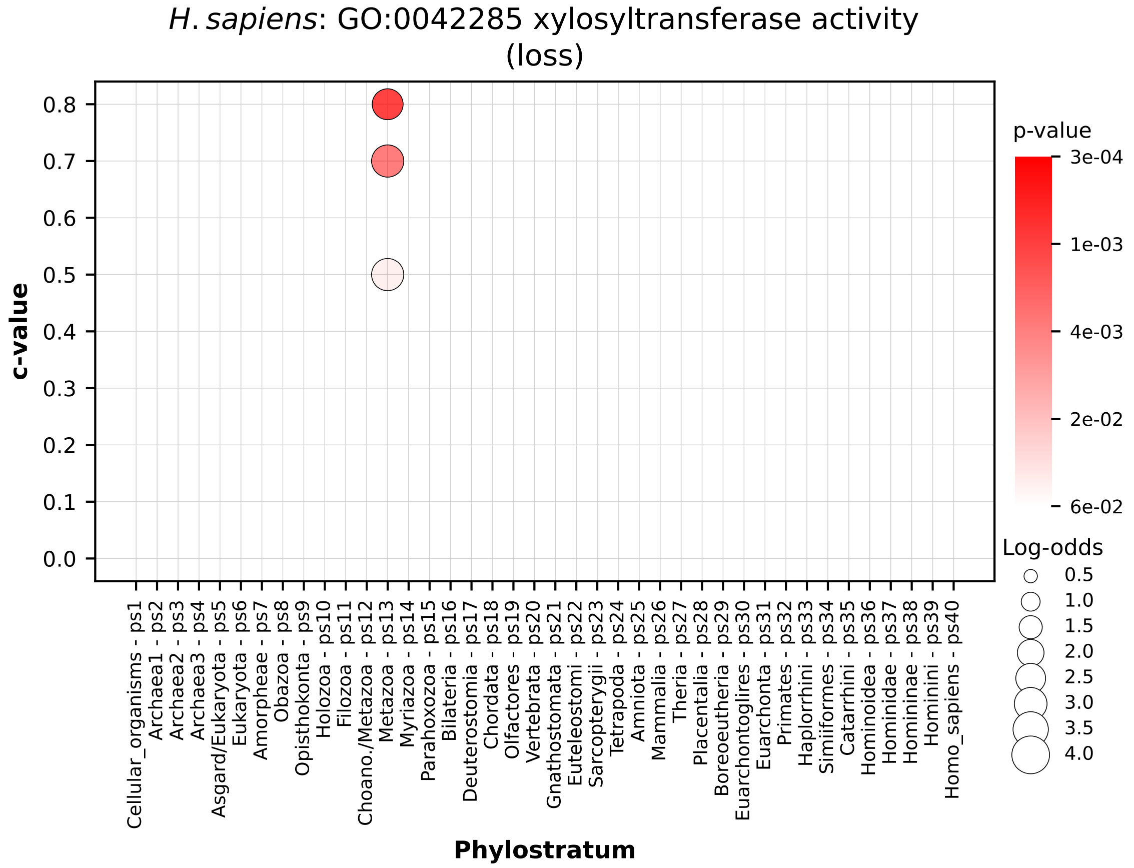

H. sapiens: GO:0042285 xylosyltransferase activity (loss)
c-value
0.8
0.7
0.6
0.5
0.4
0.3
0.2
0.1
0.0
p-value
3e-04
1e-03
4e-03
2e-02
6e-02
Log-odds
0.5
1.0
1.5
2.0
2.5
3.0
3.5
4.0
Cellular_organisms - ps1
Archaea1 - ps2
Archaea2 - ps3
Archaea3 - ps4
Asgard/Eukaryota - ps5
Eukaryota - ps6
Amorpheae - ps7
Obazoa - ps8
Opisthokonta - ps9
Holozoa - ps10
Filozoa - ps11
Choano./Metazoa - ps12
Metazoa - ps13
Myriazoa - ps14
Parahoxozoa - ps15
Bilateria - ps16
Deuterostomia - ps17
Chordata - ps18
Olfactores - ps19
Vertebrata - ps20
Gnathostomata - ps21
Euteleostomi - ps22
Sarcopterygii - ps23
Tetrapoda - ps24
Amniota - ps25
Mammalia - ps26
Theria - ps27
Placentalia - ps28
Boreoeutheria - ps29
Euarchontoglires - ps30
Euarchonta - ps31
Primates - ps32
Haplorrhini - ps33
Simiiformes - ps34
Catarrhini - ps35
Hominoidea - ps36
Hominidae - ps37
Homininae - ps38
Hominini - ps39
Homo_sapiens - ps40
Phylostratum

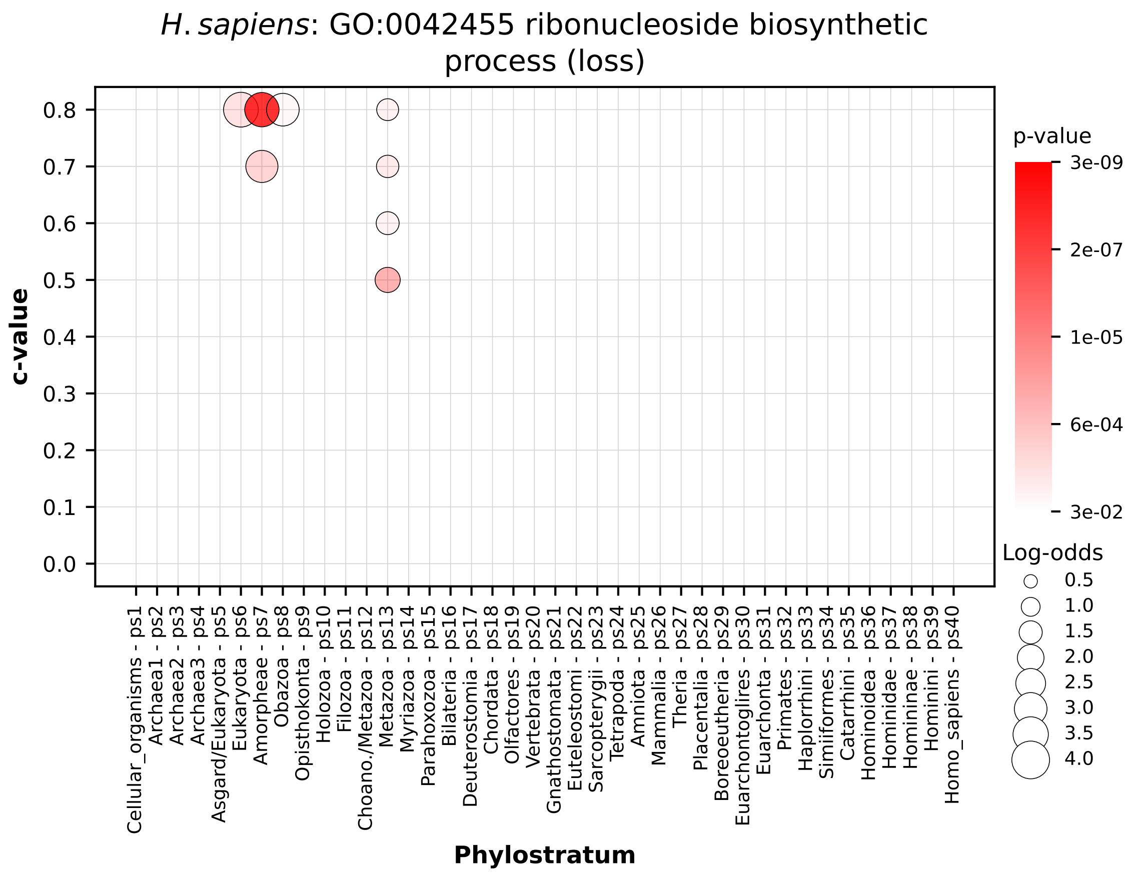

H. sapiens: GO:0042455 ribonucleoside biosynthetic process (loss)
c-value
0.0
0.1
0.2
0.3
0.4
0.5
0.6
0.7
0.8
Phylostratum
Cellular_organisms - ps1
Archaea1 - ps2
Archaea2 - ps3
Archaea3 - ps4
Asgard/Eukaryota - ps5
Eukaryota - ps6
Amorpheae - ps7
Obazoa - ps8
Opisthokonta - ps9
Holozoa - ps10
Filozoa - ps11
Choano./Metazoa - ps12
Metazoa - ps13
Myriazoa - ps14
Parahoxozoa - ps15
Bilateria - ps16
Deuterostomia - ps17
Chordata - ps18
Olfactores - ps19
Vertebrata - ps20
Gnathostomata - ps21
Euteleostomi - ps22
Sarcopterygii - ps23
Tetrapoda - ps24
Amniota - ps25
Mammalia - ps26
Theria - ps27
Placentalia - ps28
Boreoeutheria - ps29
Euarchontoglires - ps30
Euarchonta - ps31
Primates - ps32
Haplorrhini - ps33
Simiiformes - ps34
Catarrhini - ps35
Hominoidea - ps36
Hominidae - ps37
Homininae - ps38
Hominini - ps39
Homo_sapiens - ps40
p-value
3e-09
2e-07
1e-05
6e-04
3e-02
Log-odds
0.5
1.0
1.5
2.0
2.5
3.0
3.5
4.0

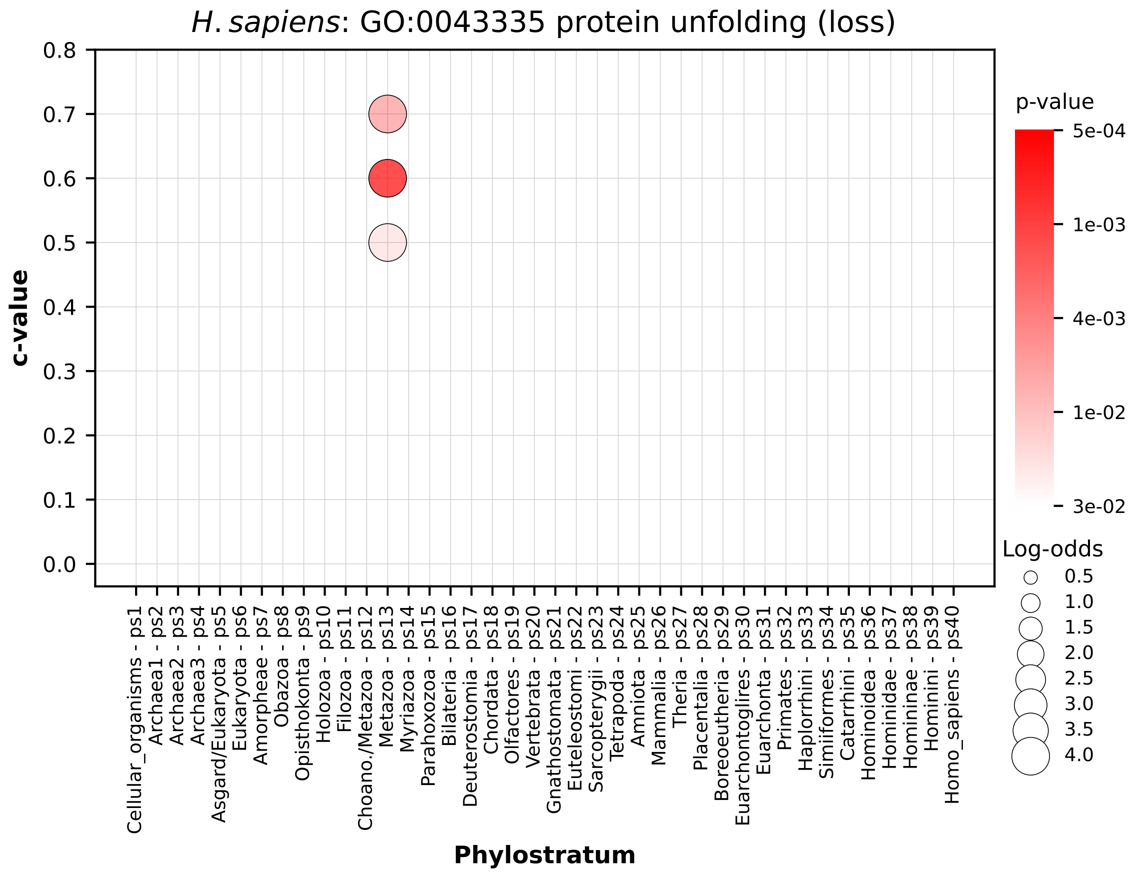

H. sapiens: GO:0043335 protein unfolding (loss)
c-value
0.0
0.1
0.2
0.3
0.4
0.5
0.6
0.7
0.8
Phylostratum
Cellular_organisms - ps1
Archaea1 - ps2
Archaea2 - ps3
Archaea3 - ps4
Asgard/Eukaryota - ps5
Eukaryota - ps6
Amorpheae - ps7
Obazoa - ps8
Opisthokonta - ps9
Holozoa - ps10
Filozoa - ps11
Choano./Metazoa - ps12
Metazoa - ps13
Myriazoa - ps14
Parahoxozoa - ps15
Bilateria - ps16
Deuterostomia - ps17
Chordata - ps18
Olfactores - ps19
Vertebrata - ps20
Gnathostomata - ps21
Euteleostomi - ps22
Sarcopterygii - ps23
Tetrapoda - ps24
Amniota - ps25
Mammalia - ps26
Theria - ps27
Placentalia - ps28
Boreoeutheria - ps29
Euarchontoglires - ps30
Euarchonta - ps31
Primates - ps32
Haplorrhini - ps33
Simiiformes - ps34
Catarrhini - ps35
Hominoidea - ps36
Hominidae - ps37
Homininae - ps38
Hominini - ps39
Homo_sapiens - ps40
p-value
5e-04
1e-03
4e-03
1e-02
3e-02
Log-odds
0.5
1.0
1.5
2.0
2.5
3.0
3.5
4.0

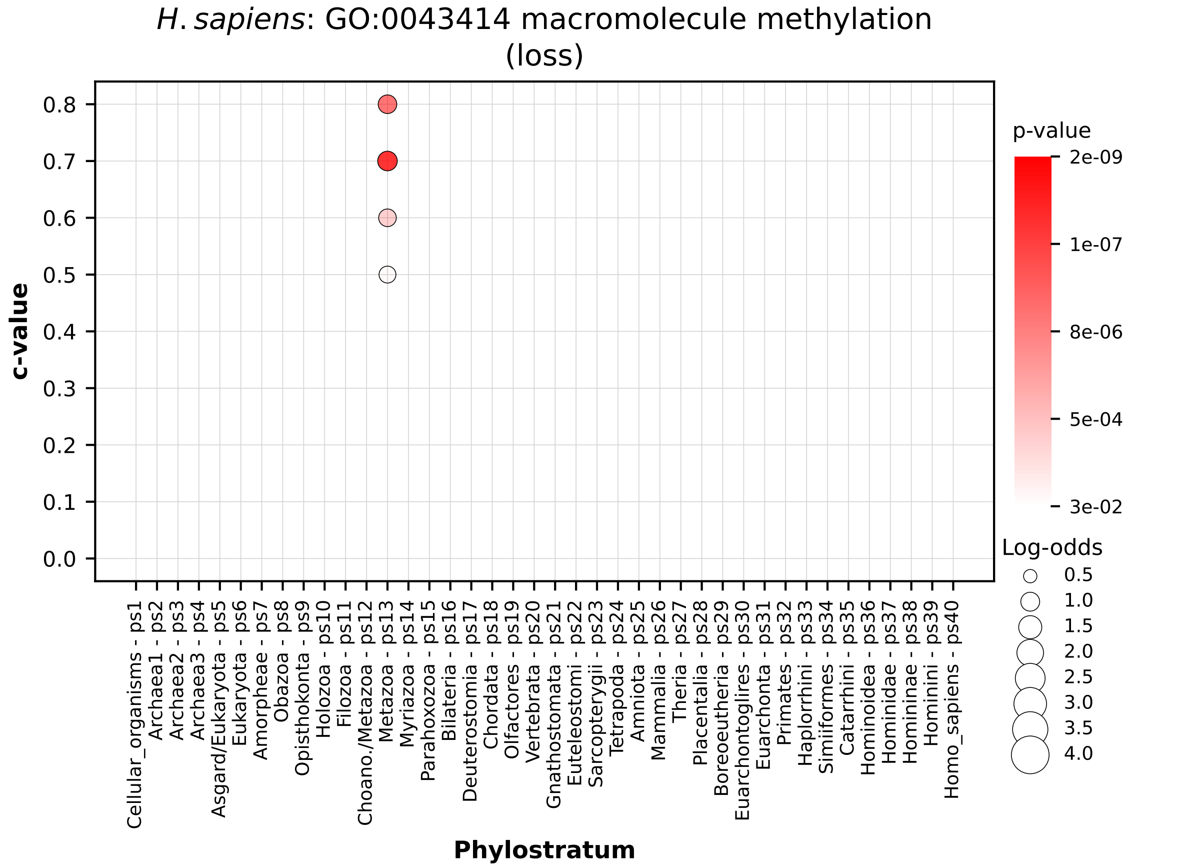

H. sapiens: GO:0043414 macromolecule methylation
(loss)
c-value
0.8
0.7
0.6
0.5
0.4
0.3
0.2
0.1
0.0
p-value
2e-09
1e-07
8e-06
5e-04
3e-02
Log-odds
0.5
1.0
1.5
2.0
2.5
3.0
3.5
4.0
Cellular_organisms - ps1
Archaea1 - ps2
Archaea2 - ps3
Archaea3 - ps4
Asgard/Eukaryota - ps5
Eukaryota - ps6
Amorpheae - ps7
Obazoa - ps8
Opisthokonta - ps9
Holozoa - ps10
Filozoa - ps11
Choano./Metazoa - ps12
Metazoa - ps13
Myriazoa - ps14
Parahoxozoa - ps15
Bilateria - ps16
Deuterostomia - ps17
Chordata - ps18
Olfactores - ps19
Vertebrata - ps20
Gnathostomata - ps21
Euteleostomi - ps22
Sarcopterygii - ps23
Tetrapoda - ps24
Amniota - ps25
Mammalia - ps26
Theria - ps27
Placentalia - ps28
Boreoeutheria - ps29
Euarchontoglires - ps30
Euarchonta - ps31
Primates - ps32
Haplorrhini - ps33
Similformes - ps34
Catarrhini - ps35
Hominoidea - ps36
Hominidae - ps37
Homininae - ps38
Hominini - ps39
Homo_sapiens - ps40
Phylostratum

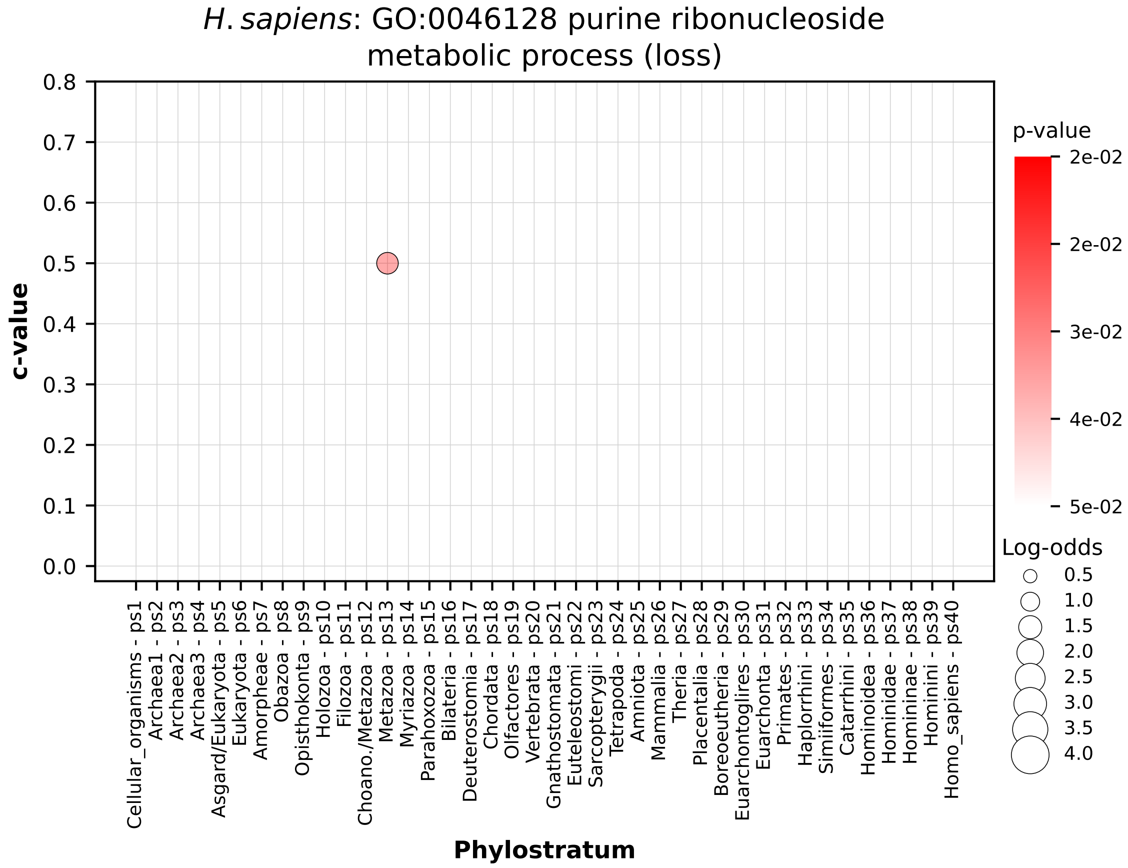

H. sapiens: GO:0046128 purine ribonucleoside metabolic process (loss)
c-value
0.8
0.7
0.6
0.5
0.4
0.3
0.2
0.1
0.0
p-value
2e-02
2e-02
3e-02
4e-02
5e-02
Log-odds
0.5
1.0
1.5
2.0
2.5
3.0
3.5
4.0
Cellular_organisms - ps1
Archaea1 - ps2
Archaea2 - ps3
Archaea3 - ps4
Asgard/Eukaryota - ps5
Eukaryota - ps6
Amorpheae - ps7
Obazoa - ps8
Opisthokonta - ps9
Holozoa - ps10
Filozoa - ps11
Choano./Metazoa - ps12
Metazoa - ps13
Myriazoa - ps14
Parahoxozoa - ps15
Bilateria - ps16
Deuterostomia - ps17
Chordata - ps18
Olfactores - ps19
Vertebrata - ps20
Gnathostomata - ps21
Euteleostomi - ps22
Sarcopterygii - ps23
Tetrapoda - ps24
Amniota - ps25
Mammalia - ps26
Theria - ps27
Placentalia - ps28
Boreoeutheria - ps29
Euarchontoglires - ps30
Euarchonta - ps31
Primates - ps32
Haplorrhini - ps33
Simiiformes - ps34
Catarrhini - ps35
Hominoidea - ps36
Hominidae - ps37
Homininae - ps38
Hominini - ps39
Homo_sapiens - ps40
Phylostratum

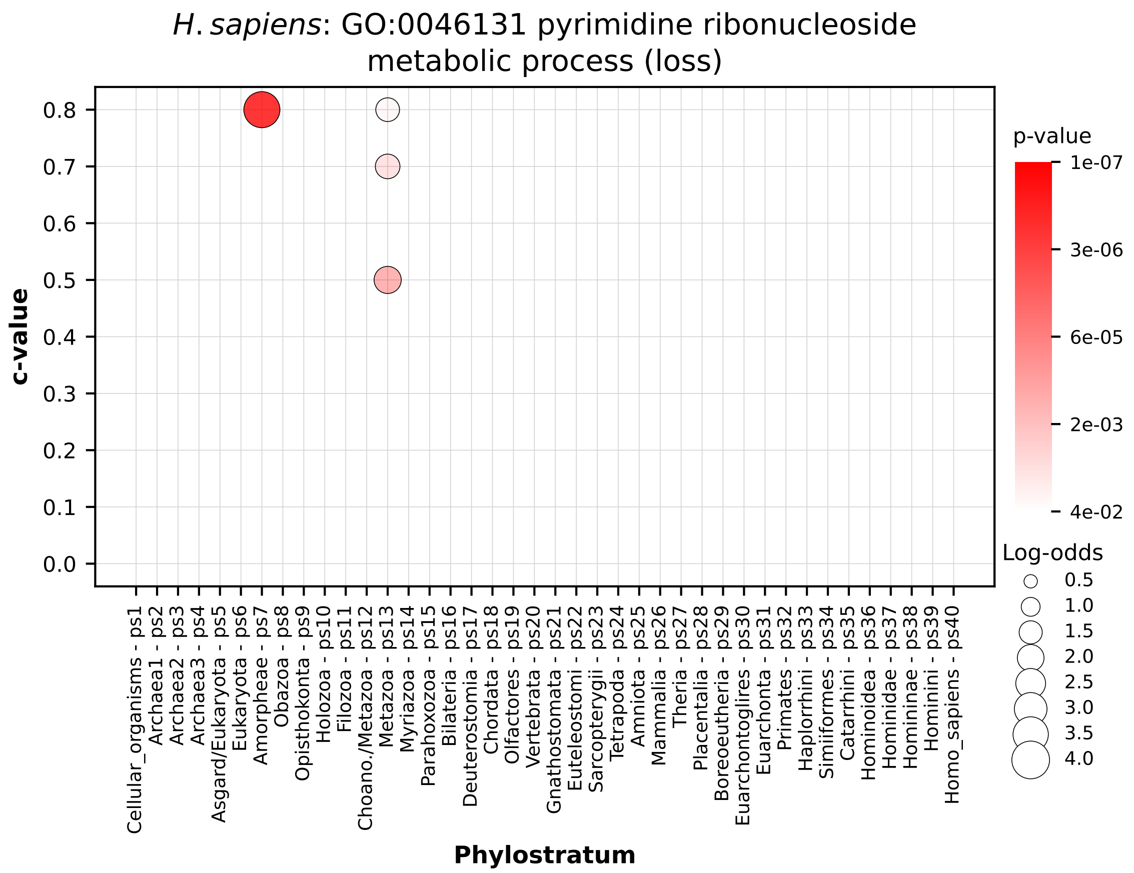
H. sapiens: GO:0046131 pyrimidine ribonucleoside metabolic process (loss)
c-value
0.0
0.1
0.2
0.3
0.4
0.5
0.6
0.7
0.8
Phylostratum
Cellular_organisms - ps1
Archaea1 - ps2
Archaea2 - ps3
Archaea3 - ps4
Asgard/Eukaryota - ps5
Eukaryota - ps6
Amorpheae - ps7
Obazoa - ps8
Opisthokonta - ps9
Holozoa - ps10
Filozoa - ps11
Choano./Metazoa - ps12
Metazoa - ps13
Myriazoa - ps14
Parahoxozoa - ps15
Bilateria - ps16
Deuterostomia - ps17
Chordata - ps18
Olfactores - ps19
Vertebrata - ps20
Gnathostomata - ps21
Euteleostomi - ps22
Sarcopterygii - ps23
Tetrapoda - ps24
Amniota - ps25
Mammalia - ps26
Theria - ps27
Placentalia - ps28
Boreoeutheria - ps29
Euarchontoglires - ps30
Euarchonta - ps31
Primates - ps32
Haplorrhini - ps33
Simiiformes - ps34
Catarrhini - ps35
Hominoidea - ps36
Hominidae - ps37
Homininae - ps38
Hominini - ps39
Homo_sapiens - ps40
p-value
1e-07
3e-06
6e-05
2e-03
4e-02
Log-odds
0.5
1.0
1.5
2.0
2.5
3.0
3.5
4.0

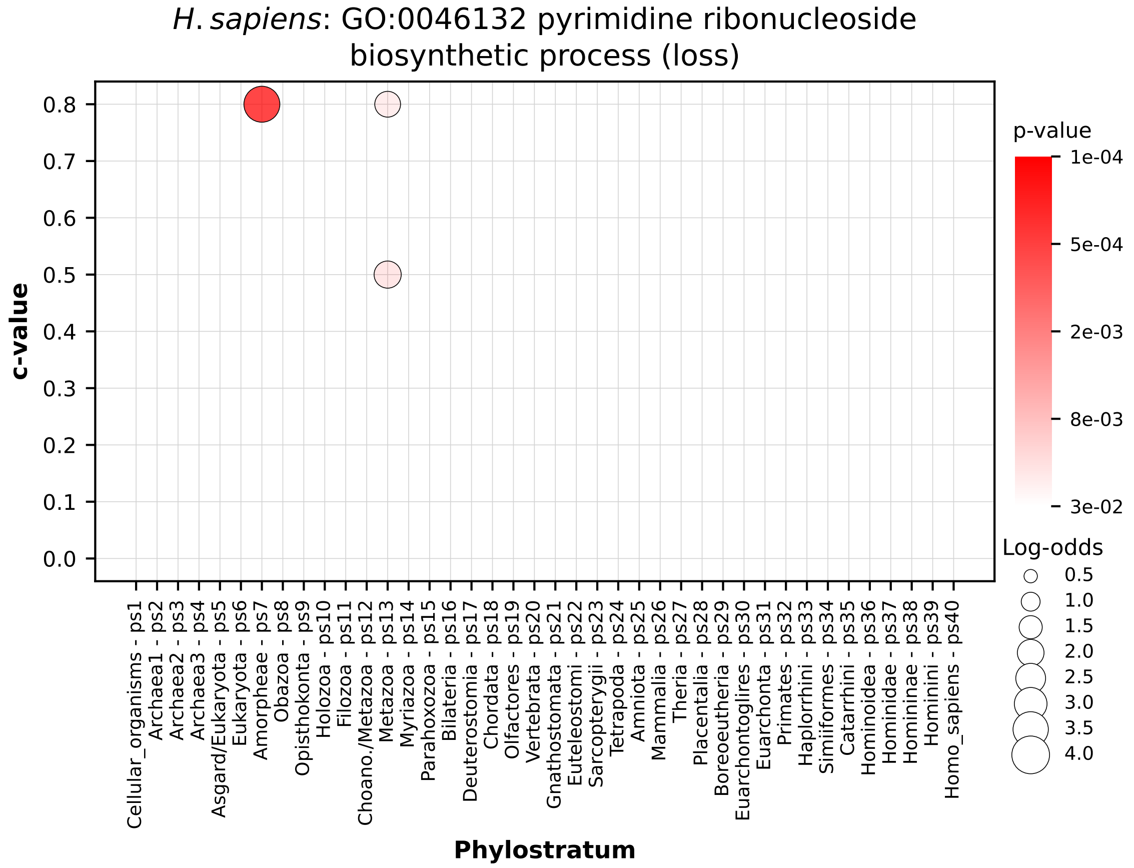
H. sapiens: GO:0046132 pyrimidine ribonucleoside biosynthetic process (loss)
c-value
0.0
0.1
0.2
0.3
0.4
0.5
0.6
0.7
0.8
p-value
1e-04
5e-04
2e-03
8e-03
3e-02
Log-odds
0.5
1.0
1.5
2.0
2.5
3.0
3.5
4.0
Cellular_organisms - ps1
Archaea1 - ps2
Archaea2 - ps3
Archaea3 - ps4
Asgard/Eukaryota - ps5
Eukaryota - ps6
Amorpheae - ps7
Obazoa - ps8
Opisthokonta - ps9
Holozoa - ps10
Filozoa - ps11
Choano./Metazoa - ps12
Metazoa - ps13
Myriazoa - ps14
Parahoxozoa - ps15
Bilateria - ps16
Deuterostomia - ps17
Chordata - ps18
Olfactores - ps19
Vertebrata - ps20
Gnathostomata - ps21
Euteleostomi - ps22
Sarcopterygii - ps23
Tetrapoda - ps24
Amniota - ps25
Mammalia - ps26
Theria - ps27
Placentalia - ps28
Boreoeutheria - ps29
Euarchontoglires - ps30
Euarchonta - ps31
Primates - ps32
Haplorrhini - ps33
Simiiformes - ps34
Catarrhini - ps35
Hominoidea - ps36
Hominidae - ps37
Homininae - ps38
Hominini - ps39
Homo_sapiens - ps40
Phylostratum

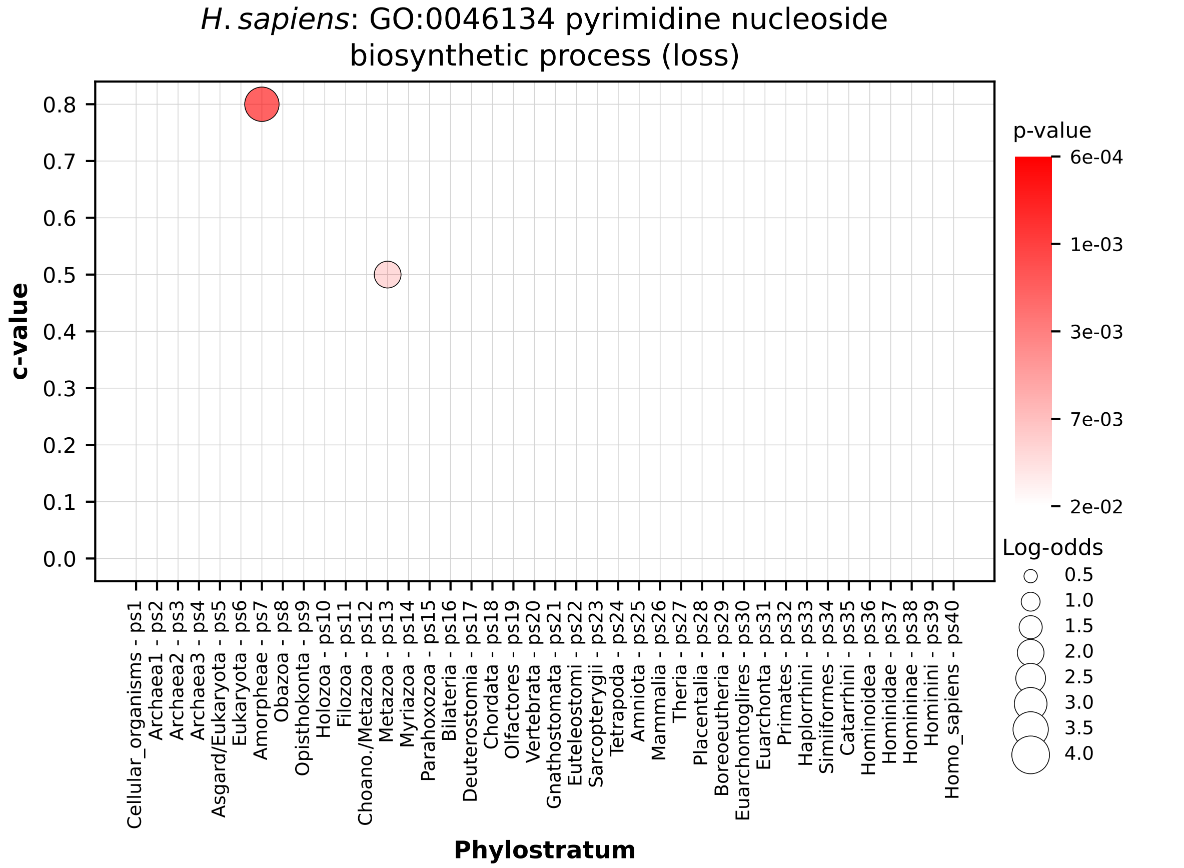

H. sapiens: GO:0046134 pyrimidine nucleoside biosynthetic process (loss)
c-value
0.0
0.1
0.2
0.3
0.4
0.5
0.6
0.7
0.8
Phylostratum
Cellular_organisms - ps1
Archaea1 - ps2
Archaea2 - ps3
Archaea3 - ps4
Asgard/Eukaryota - ps5
Eukaryota - ps6
Amorpheae - ps7
Obazoa - ps8
Opisthokonta - ps9
Holozoa - ps10
Filozoa - ps11
Choano./Metazoa - ps12
Metazoa - ps13
Myriazoa - ps14
Parahoxozoa - ps15
Bilateria - ps16
Deuterostomia - ps17
Chordata - ps18
Olfactores - ps19
Vertebrata - ps20
Gnathostomata - ps21
Euteleostomi - ps22
Sarcopterygii - ps23
Tetrapoda - ps24
Amniota - ps25
Mammalia - ps26
Theria - ps27
Placentalia - ps28
Boreoeutheria - ps29
Euarchontoglires - ps30
Euarchonta - ps31
Primates - ps32
Haplorrhini - ps33
Simiiformes - ps34
Catarrhini - ps35
Hominoidea - ps36
Hominidae - ps37
Homininae - ps38
Hominini - ps39
Homo_sapiens - ps40
p-value
6e-04
1e-03
3e-03
7e-03
2e-02
Log-odds
0.5
1.0
1.5
2.0
2.5
3.0
3.5
4.0

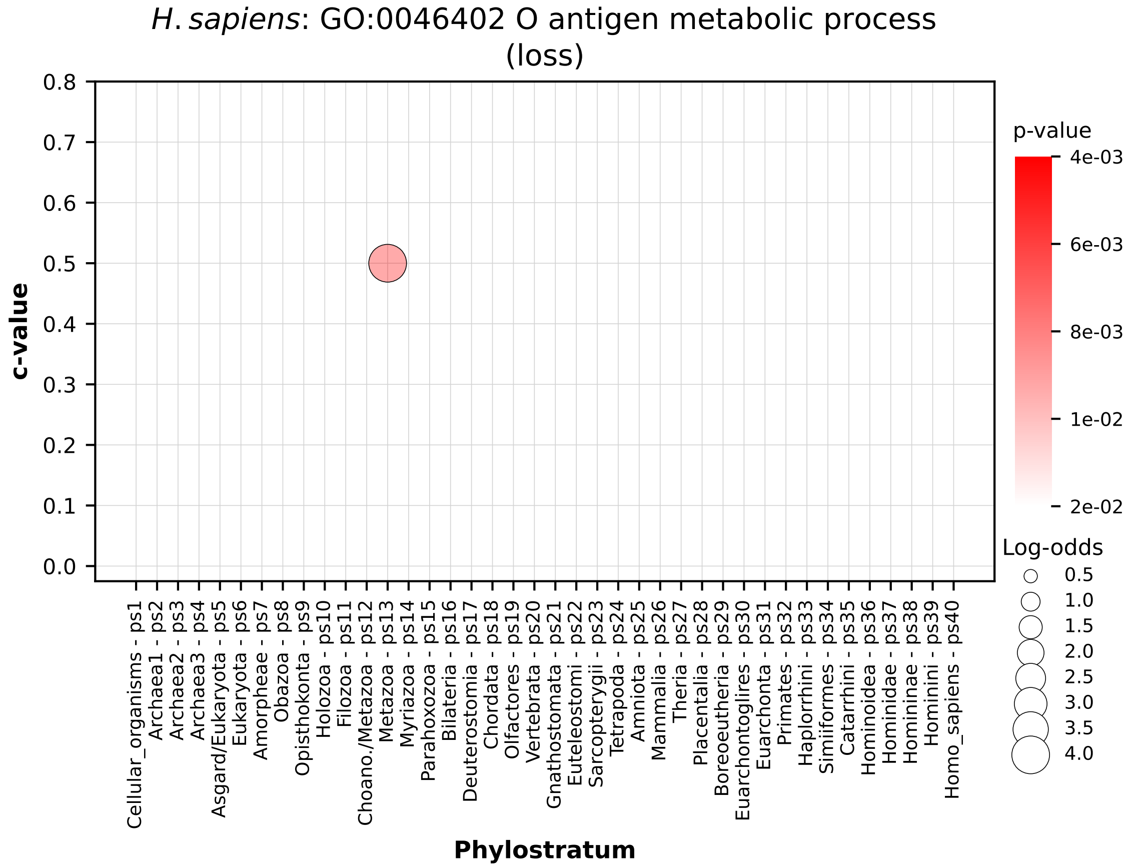

H. sapiens: GO:0046402 O antigen metabolic process
(loss)
c-value
0.8
0.7
0.6
0.5
0.4
0.3
0.2
0.1
0.0
p-value
4e-03
6e-03
8e-03
1e-02
2e-02
Log-odds
0.5
1.0
1.5
2.0
2.5
3.0
3.5
4.0
Cellular_organisms - ps1
Archaea1 - ps2
Archaea2 - ps3
Archaea3 - ps4
Asgard/Eukaryota - ps5
Eukaryota - ps6
Amorpheae - ps7
Obazoa - ps8
Opisthokonta - ps9
Holozoa - ps10
Filozoa - ps11
Choano./Metazoa - ps12
Metazoa - ps13
Myriazoa - ps14
Parahoxozoa - ps15
Bilateria - ps16
Deuterostomia - ps17
Chordata - ps18
Olfactores - ps19
Vertebrata - ps20
Gnathostomata - ps21
Euteleostomi - ps22
Sarcopterygii - ps23
Tetrapoda - ps24
Amniota - ps25
Mammalia - ps26
Theria - ps27
Placentalia - ps28
Boreoeutheria - ps29
Euarchontoglires - ps30
Euarchonta - ps31
Primates - ps32
Haplorrhini - ps33
Simiiformes - ps34
Catarrhini - ps35
Hominoidea - ps36
Hominidae - ps37
Homininae - ps38
Hominini - ps39
Homo_sapiens - ps40
Phylostratum

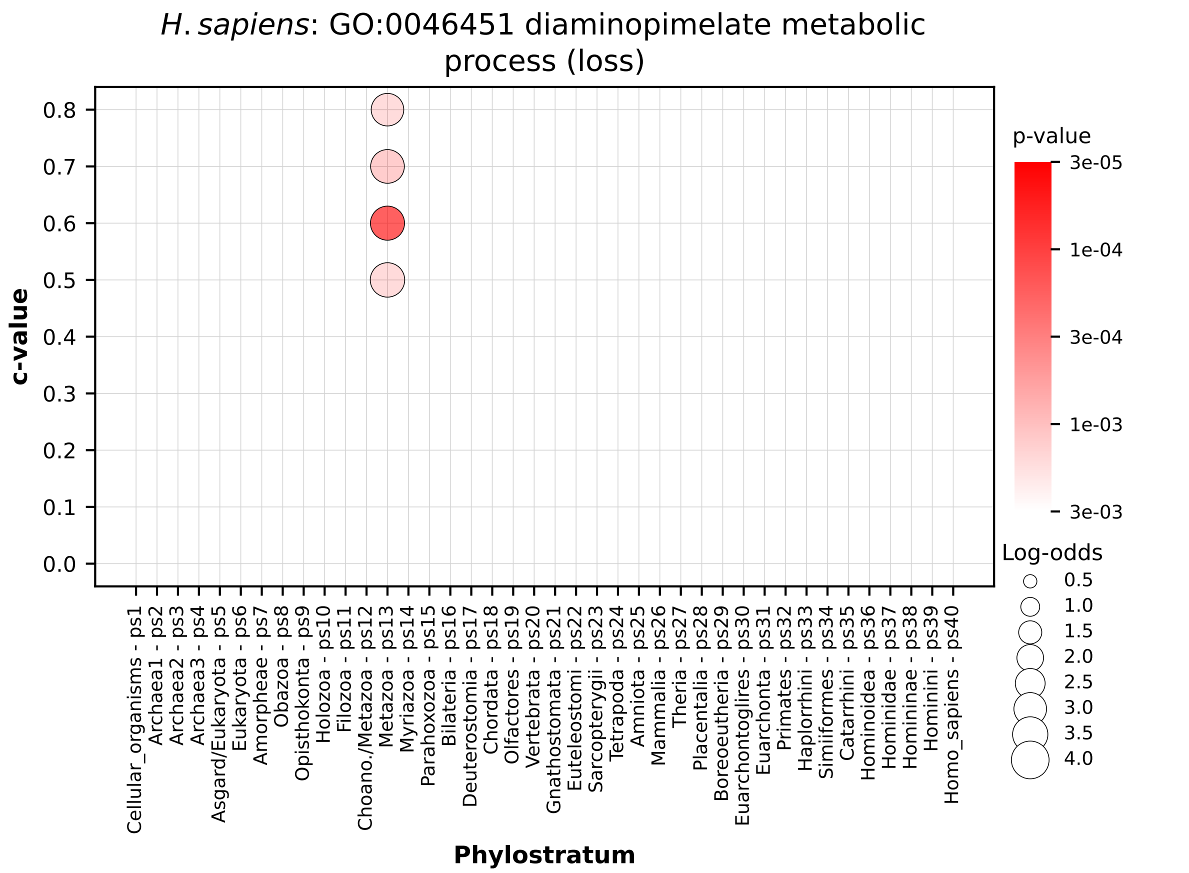

H. sapiens: GO:0046451 diaminopimelate metabolic process (loss)
c-value
0.0
0.1
0.2
0.3
0.4
0.5
0.6
0.7
0.8
Phylostratum
Cellular_organisms - ps1
Archaea1 - ps2
Archaea2 - ps3
Archaea3 - ps4
Asgard/Eukaryota - ps5
Eukaryota - ps6
Amorpheae - ps7
Obazoa - ps8
Opisthokonta - ps9
Holozoa - ps10
Filozoa - ps11
Choano./Metazoa - ps12
Metazoa - ps13
Myriazoa - ps14
Parahoxozoa - ps15
Bilateria - ps16
Deuterostomia - ps17
Chordata - ps18
Olfactores - ps19
Vertebrata - ps20
Gnathostomata - ps21
Euteleostomi - ps22
Sarcopterygii - ps23
Tetrapoda - ps24
Amniota - ps25
Mammalia - ps26
Theria - ps27
Placentalia - ps28
Boreoeutheria - ps29
Euarchontoglires - ps30
Euarchonta - ps31
Primates - ps32
Haplorrhini - ps33
Simiiformes - ps34
Catarrhini - ps35
Hominoidea - ps36
Hominidae - ps37
Homininae - ps38
Hominini - ps39
Homo_sapiens - ps40
p-value
3e-05
1e-04
3e-04
1e-03
3e-03
Log-odds
0.5
1.0
1.5
2.0
2.5
3.0
3.5
4.0

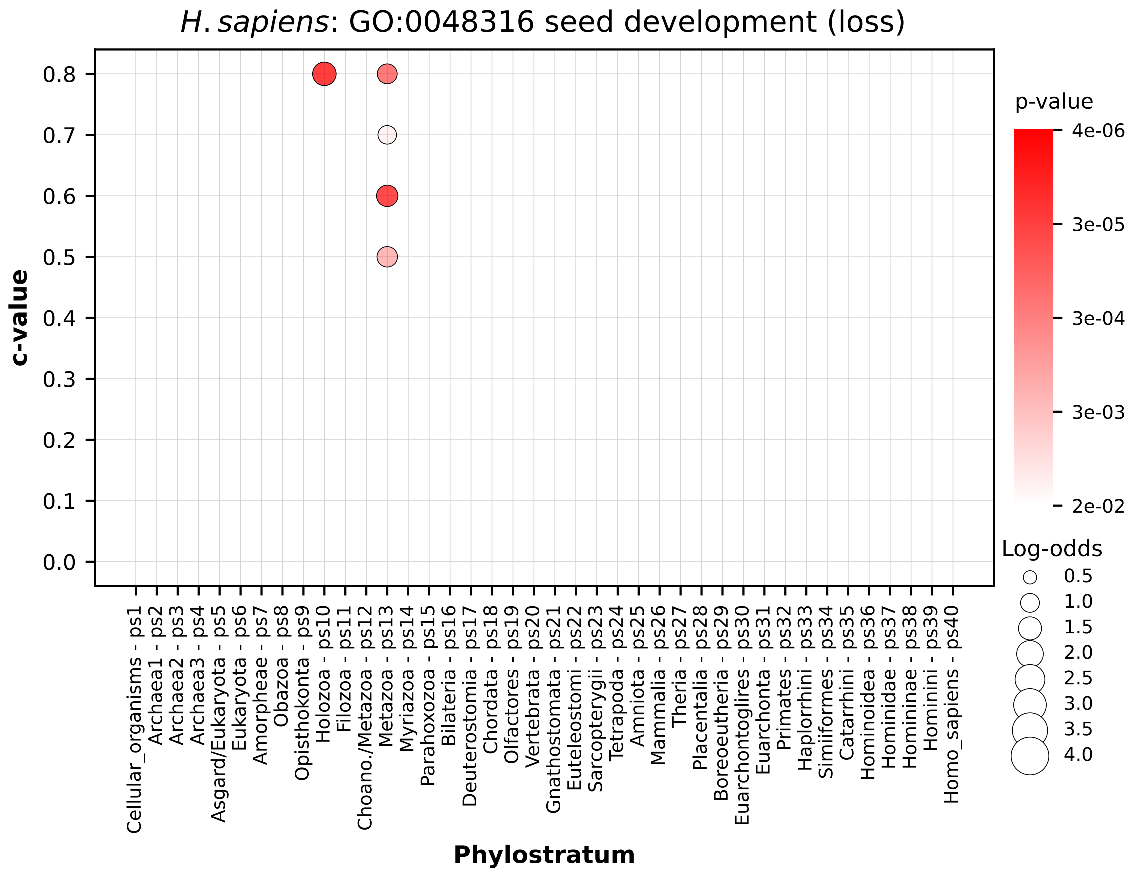
H. sapiens: GO:0048316 seed development (loss)
c-value
0.0
0.1
0.2
0.3
0.4
0.5
0.6
0.7
0.8
Phylostratum
Cellular_organisms - ps1
Archaea1 - ps2
Archaea2 - ps3
Archaea3 - ps4
Asgard/Eukaryota - ps5
Eukaryota - ps6
Amorpheae - ps7
Obazoa - ps8
Opisthokonta - ps9
Holozoa - ps10
Filozoa - ps11
Choano./Metazoa - ps12
Metazoa - ps13
Myriazoa - ps14
Parahoxozoa - ps15
Bilateria - ps16
Deuterostomia - ps17
Chordata - ps18
Olfactores - ps19
Vertebrata - ps20
Gnathostomata - ps21
Euteleostomi - ps22
Sarcopterygii - ps23
Tetrapoda - ps24
Amniota - ps25
Mammalia - ps26
Theria - ps27
Placentalia - ps28
Boreoeutheria - ps29
Euarchontoglires - ps30
Euarchonta - ps31
Primates - ps32
Haplorrhini - ps33
Simiiformes - ps34
Catarrhini - ps35
Hominoidea - ps36
Hominidae - ps37
Homininae - ps38
Hominini - ps39
Homo_sapiens - ps40
p-value
4e-06
3e-05
3e-04
3e-03
2e-02
Log-odds
0.5
1.0
1.5
2.0
2.5
3.0
3.5
4.0

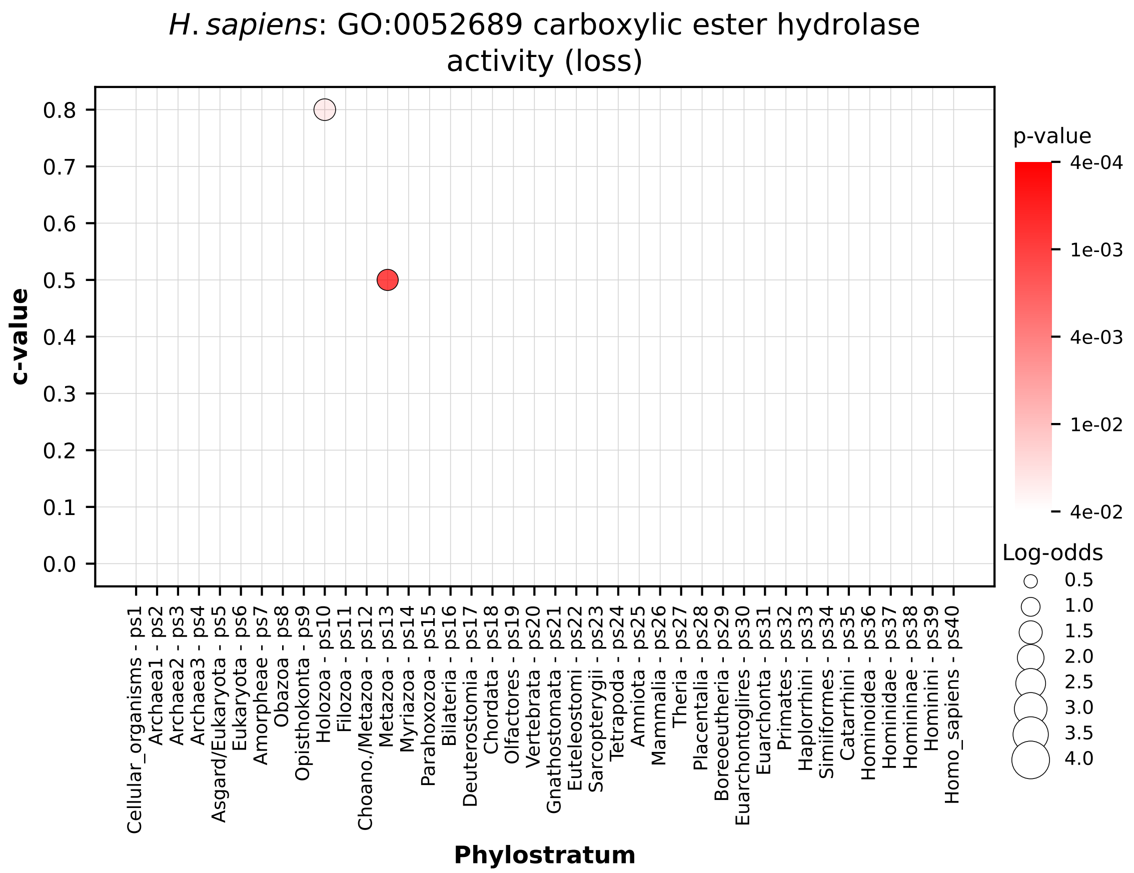
H. sapiens: GO:0052689 carboxylic ester hydrolase activity (loss)
c-value
0.0
0.1
0.2
0.3
0.4
0.5
0.6
0.7
0.8
Phylostratum
Cellular_organisms - ps1
Archaea1 - ps2
Archaea2 - ps3
Archaea3 - ps4
Asgard/Eukaryota - ps5
Eukaryota - ps6
Amorpheae - ps7
Obazoa - ps8
Opisthokonta - ps9
Holozoa - ps10
Filozoa - ps11
Choano./Metazoa - ps12
Metazoa - ps13
Myriazoa - ps14
Parahoxozoa - ps15
Bilateria - ps16
Deuterostomia - ps17
Chordata - ps18
Olfactores - ps19
Vertebrata - ps20
Gnathostomata - ps21
Euteleostomi - ps22
Sarcopterygii - ps23
Tetrapoda - ps24
Amniota - ps25
Mammalia - ps26
Theria - ps27
Placentalia - ps28
Boreoeutheria - ps29
Euarchontoglires - ps30
Euarchonta - ps31
Primates - ps32
Haplorrhini - ps33
Simiiformes - ps34
Catarrhini - ps35
Hominoidea - ps36
Hominidae - ps37
Homininae - ps38
Hominini - ps39
Homo_sapiens - ps40
p-value
4e-04
1e-03
4e-03
1e-02
4e-02
Log-odds
0.5
1.0
1.5
2.0
2.5
3.0
3.5
4.0

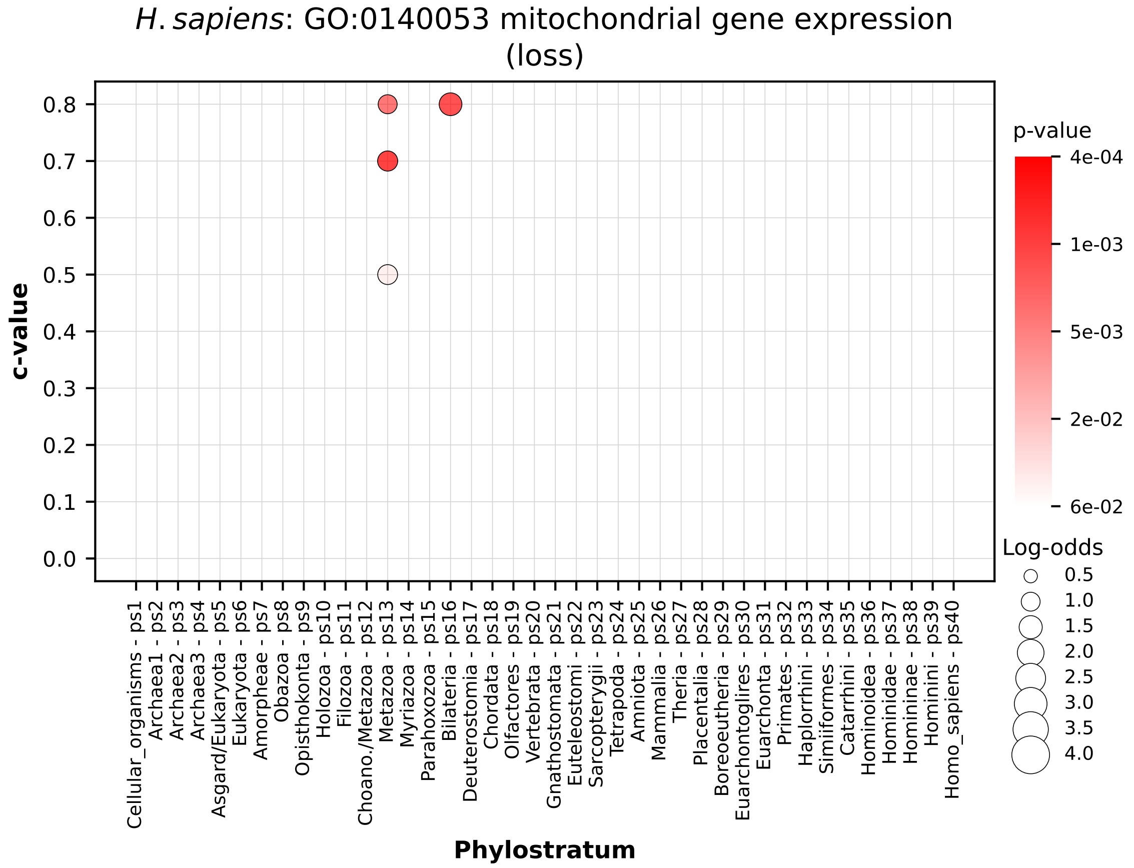
H. sapiens: GO:0140053 mitochondrial gene expression
(loss)
c-value
0.8
0.7
0.6
0.5
0.4
0.3
0.2
0.1
0.0
p-value
4e-04
1e-03
5e-03
2e-02
6e-02
Log-odds
0.5
1.0
1.5
2.0
2.5
3.0
3.5
4.0
Cellular_organisms - ps1
Archaea1 - ps2
Archaea2 - ps3
Archaea3 - ps4
Asgard/Eukaryota - ps5
Eukaryota - ps6
Amorpheae - ps7
Obazoa - ps8
Opisthokonta - ps9
Holozoa - ps10
Filozoa - ps11
Choano./Metazoa - ps12
Metazoa - ps13
Myriazoa - ps14
Parahoxozoa - ps15
Bilateria - ps16
Deuterostomia - ps17
Chordata - ps18
Olfactores - ps19
Vertebrata - ps20
Gnathostomata - ps21
Euteleostomi - ps22
Sarcopterygii - ps23
Tetrapoda - ps24
Amniota - ps25
Mammalia - ps26
Theria - ps27
Placentalia - ps28
Boreoeutheria - ps29
Euarchontoglires - ps30
Euarchonta - ps31
Primates - ps32
Haplorrhini - ps33
Simiiformes - ps34
Catarrhini - ps35
Hominoidea - ps36
Hominidae - ps37
Homininae - ps38
Hominini - ps39
Homo_sapiens - ps40
Phylostratum

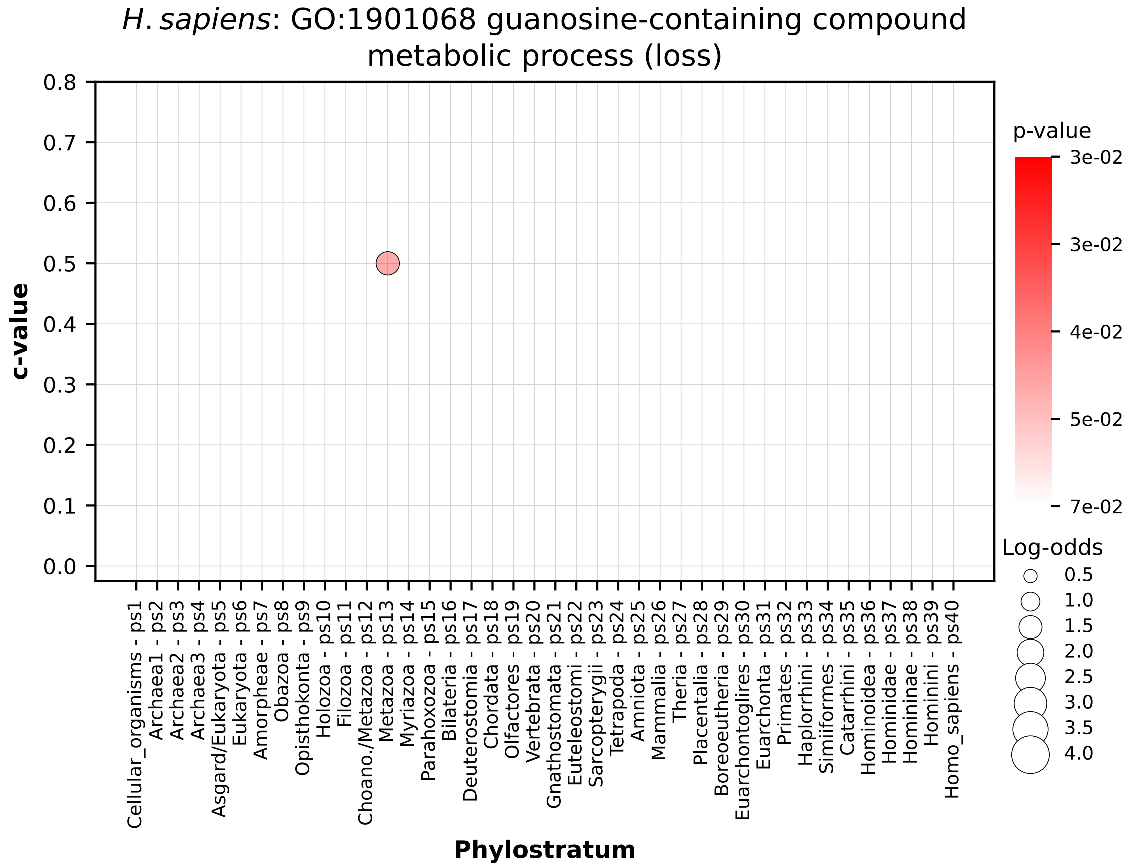
H. sapiens: GO:1901068 guanosine-containing compound metabolic process (loss)
c-value
0.0
0.1
0.2
0.3
0.4
0.5
0.6
0.7
0.8
Phylostratum
Cellular_organisms - ps1
Archaea1 - ps2
Archaea2 - ps3
Archaea3 - ps4
Asgard/Eukaryota - ps5
Eukaryota - ps6
Amorpheae - ps7
Obazoa - ps8
Opisthokonta - ps9
Holozoa - ps10
Filozoa - ps11
Choano./Metazoa - ps12
Metazoa - ps13
Myriazoa - ps14
Parahoxozoa - ps15
Bilateria - ps16
Deuterostomia - ps17
Chordata - ps18
Olfactores - ps19
Vertebrata - ps20
Gnathostomata - ps21
Euteleostomi - ps22
Sarcopterygii - ps23
Tetrapoda - ps24
Amniota - ps25
Mammalia - ps26
Theria - ps27
Placentalia - ps28
Boreoeutheria - ps29
Euarchontoglires - ps30
Euarchonta - ps31
Primates - ps32
Haplorrhini - ps33
Simiiformes - ps34
Catarrhini - ps35
Hominoidea - ps36
Hominidae - ps37
Homininae - ps38
Hominini - ps39
Homo_sapiens - ps40
p-value
3e-02
3e-02
4e-02
5e-02
7e-02
Log-odds
0.5
1.0
1.5
2.0
2.5
3.0
3.5
4.0

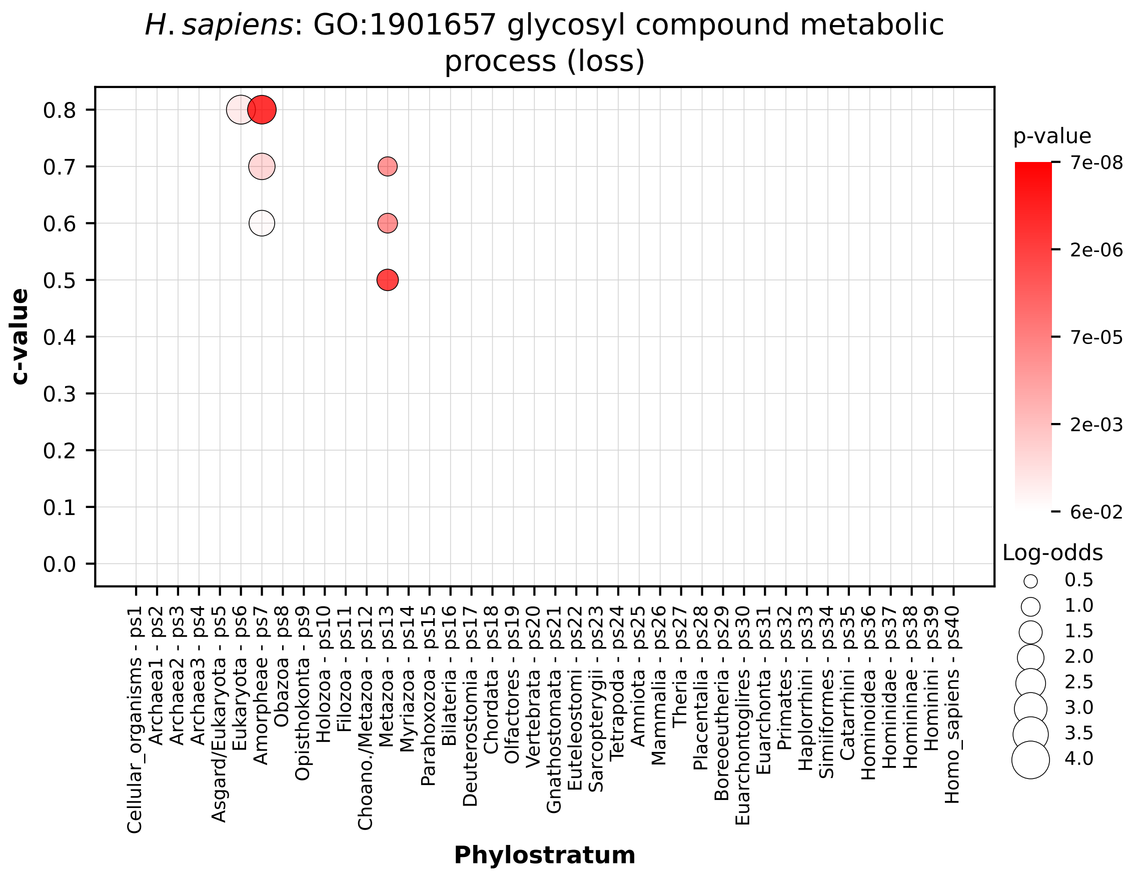

H. sapiens: GO:1901657 glycosyl compound metabolic process (loss)
c-value
0.0
0.1
0.2
0.3
0.4
0.5
0.6
0.7
0.8
Phylostratum
Cellular_organisms - ps1
Archaea1 - ps2
Archaea2 - ps3
Archaea3 - ps4
Asgard/Eukaryota - ps5
Eukaryota - ps6
Amorpheae - ps7
Obazoa - ps8
Opisthokonta - ps9
Holozoa - ps10
Filozoa - ps11
Choano./Metazoa - ps12
Metazoa - ps13
Myriazoa - ps14
Parahoxozoa - ps15
Bilateria - ps16
Deuterostomia - ps17
Chordata - ps18
Olfactores - ps19
Vertebrata - ps20
Gnathostomata - ps21
Euteleostomi - ps22
Sarcopterygii - ps23
Tetrapoda - ps24
Amniota - ps25
Mammalia - ps26
Theria - ps27
Placentalia - ps28
Boreoeutheria - ps29
Euarchontoglires - ps30
Euarchonta - ps31
Primates - ps32
Haplorrhini - ps33
Simiiformes - ps34
Catarrhini - ps35
Hominoidea - ps36
Hominidae - ps37
Homininae - ps38
Hominini - ps39
Homo_sapiens - ps40
p-value
7e-08
2e-06
7e-05
2e-03
6e-02
Log-odds
0.5
1.0
1.5
2.0
2.5
3.0
3.5
4.0

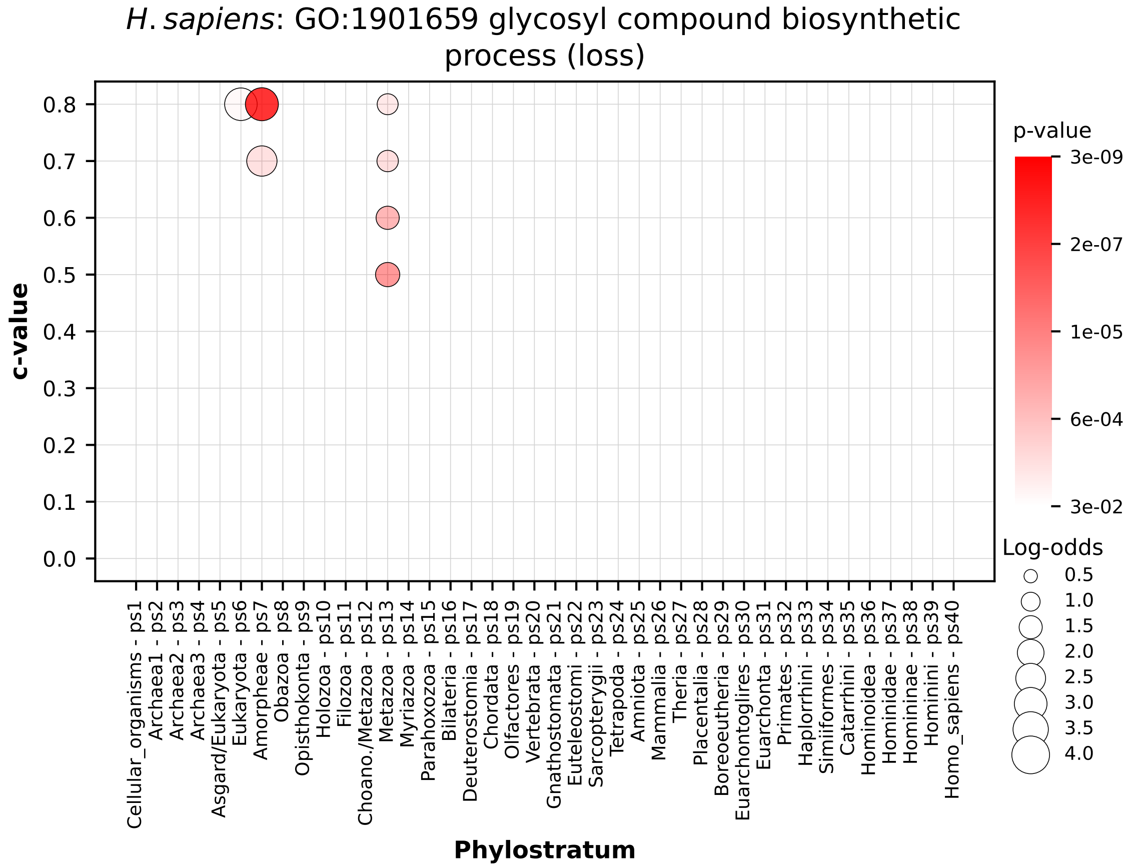

H. sapiens: GO:1901659 glycosyl compound biosynthetic process (loss)
c-value
0.8
0.7
0.6
0.5
0.4
0.3
0.2
0.1
0.0
Phylostratum
Cellular_organisms - ps1
Archaea1 - ps2
Archaea2 - ps3
Archaea3 - ps4
Asgard/Eukaryota - ps5
Eukaryota - ps6
Amorpheae - ps7
Obazoa - ps8
Opisthokonta - ps9
Holozoa - ps10
Filozoa - ps11
Choano./Metazoa - ps12
Metazoa - ps13
Myriazoa - ps14
Parahoxozoa - ps15
Bilateria - ps16
Deuterostomia - ps17
Chordata - ps18
Olfactores - ps19
Vertebrata - ps20
Gnathostomata - ps21
Euteleostomi - ps22
Sarcopterygii - ps23
Tetrapoda - ps24
Amniota - ps25
Mammalia - ps26
Theria - ps27
Placentalia - ps28
Boreoeutheria - ps29
Euarchontoglires - ps30
Euarchonta - ps31
Primates - ps32
Haplorrhini - ps33
Simiiformes - ps34
Catarrhini - ps35
Hominoidea - ps36
Hominidae - ps37
Homininae - ps38
Hominini - ps39
Homo_sapiens - ps40
p-value
3e-09
2e-07
1e-05
6e-04
3e-02
Log-odds
0.5
1.0
1.5
2.0
2.5
3.0
3.5
4.0

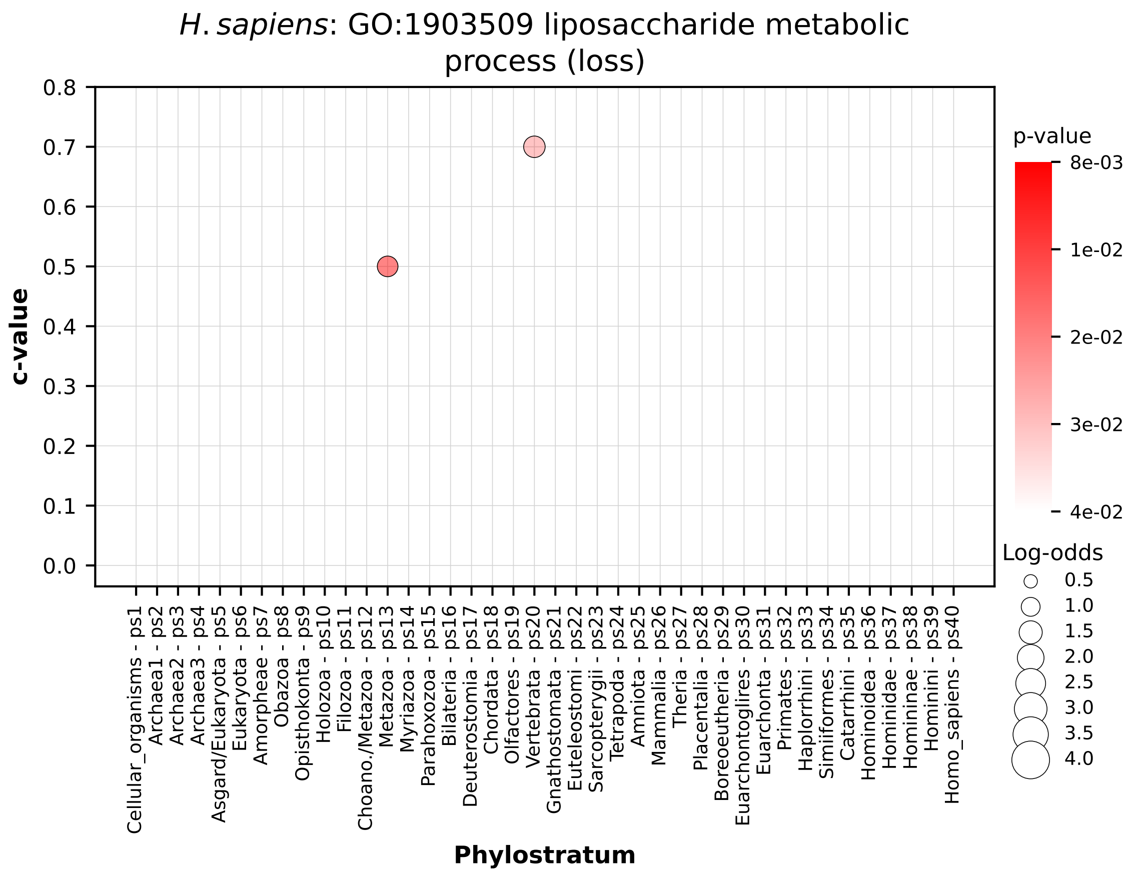
H. sapiens: GO:1903509 liposaccharide metabolic process (loss)
c-value
0.8
0.7
0.6
0.5
0.4
0.3
0.2
0.1
0.0
Phylostratum
Cellular_organisms - ps1
Archaea1 - ps2
Archaea2 - ps3
Archaea3 - ps4
Asgard/Eukaryota - ps5
Eukaryota - ps6
Amorpheae - ps7
Obazoa - ps8
Opisthokonta - ps9
Holozoa - ps10
Filozoa - ps11
Choano./Metazoa - ps12
Metazoa - ps13
Myriazoa - ps14
Parahoxozoa - ps15
Bilateria - ps16
Deuterostomia - ps17
Chordata - ps18
Olfactores - ps19
Vertebrata - ps20
Gnathostomata - ps21
Euteleostomi - ps22
Sarcopterygii - ps23
Tetrapoda - ps24
Amniota - ps25
Mammalia - ps26
Theria - ps27
Placentalia - ps28
Boreoeutheria - ps29
Euarchontoglires - ps30
Euarchonta - ps31
Primates - ps32
Haplorrhini - ps33
Simiiformes - ps34
Catarrhini - ps35
Hominoidea - ps36
Hominidae - ps37
Homininae - ps38
Hominini - ps39
Homo_sapiens - ps40
p-value
8e-03
1e-02
2e-02
3e-02
4e-02
Log-odds
0.5
1.0
1.5
2.0
2.5
3.0
3.5
4.0

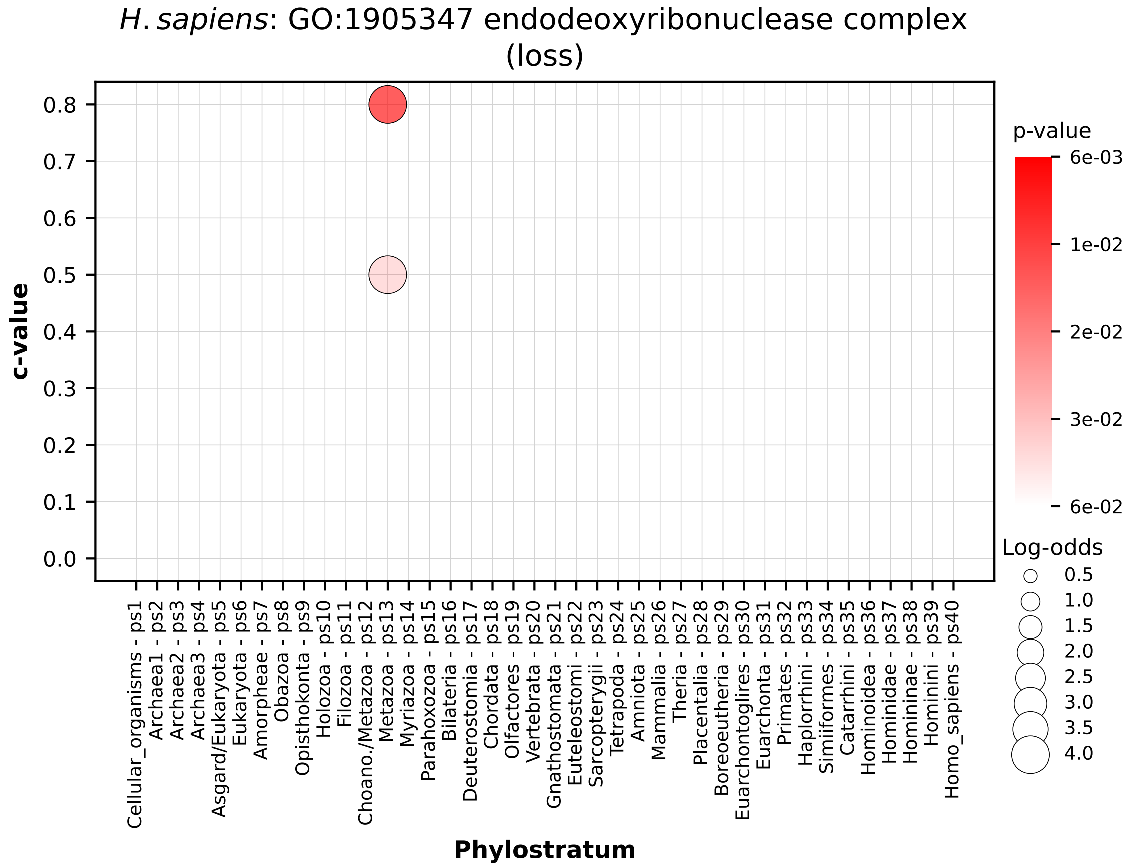

H. sapiens: GO:1905347 endodeoxyribonuclease complex (loss)
c-value
0.0
0.1
0.2
0.3
0.4
0.5
0.6
0.7
0.8
Phylostratum
Cellular_organisms - ps1
Archaea1 - ps2
Archaea2 - ps3
Archaea3 - ps4
Asgard/Eukaryota - ps5
Eukaryota - ps6
Amorpheae - ps7
Obazoa - ps8
Opisthokonta - ps9
Holozoa - ps10
Filozoa - ps11
Choano./Metazoa - ps12
Metazoa - ps13
Myriazoa - ps14
Parahoxozoa - ps15
Bilateria - ps16
Deuterostomia - ps17
Chordata - ps18
Olfactores - ps19
Vertebrata - ps20
Gnathostomata - ps21
Euteleostomi - ps22
Sarcopterygii - ps23
Tetrapoda - ps24
Amniota - ps25
Mammalia - ps26
Theria - ps27
Placentalia - ps28
Boreoeutheria - ps29
Euarchontoglires - ps30
Euarchonta - ps31
Primates - ps32
Haplorrhini - ps33
Simiiformes - ps34
Catarrhini - ps35
Hominoidea - ps36
Hominidae - ps37
Homininae - ps38
Hominini - ps39
Homo_sapiens - ps40
p-value
6e-03
1e-02
2e-02
3e-02
6e-02
Log-odds
0.5
1.0
1.5
2.0
2.5
3.0
3.5
4.0

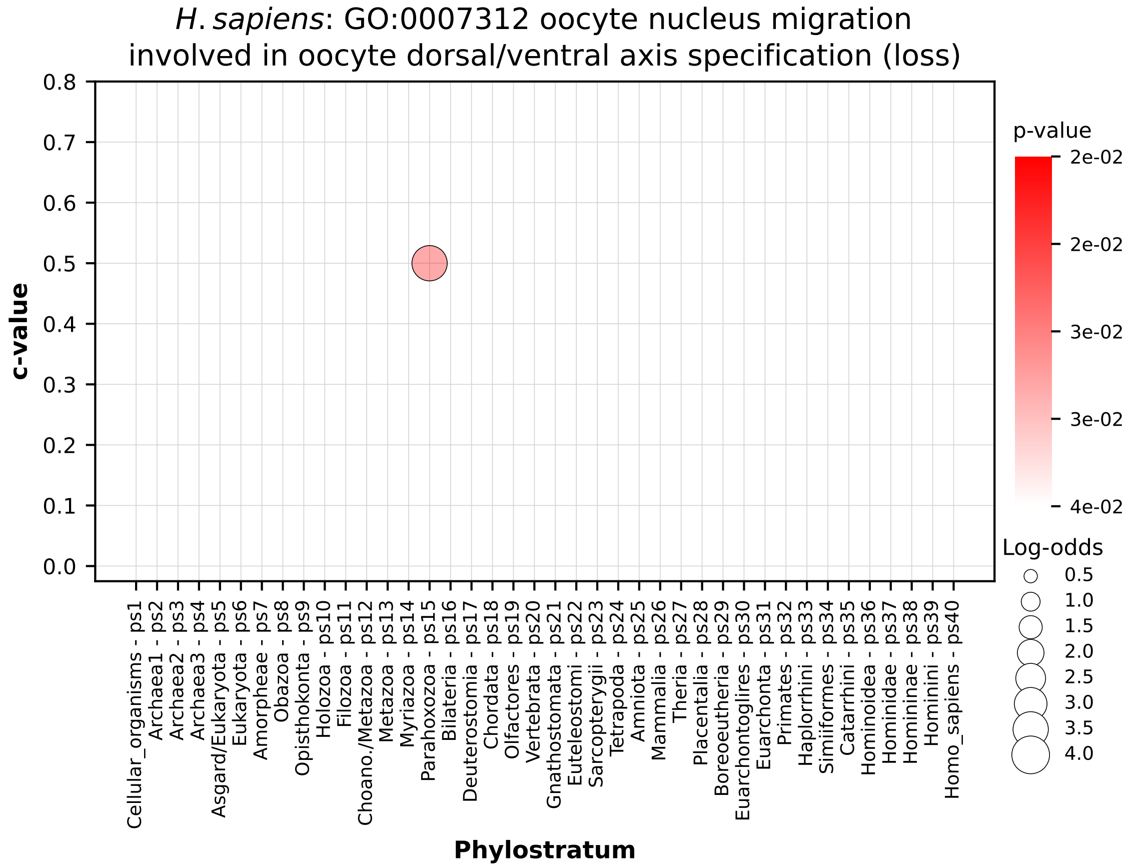
H. sapiens: GO:0007312 oocyte nucleus migration involved in oocyte dorsal/ventral axis specification (loss)
c-value
0.8
0.7
0.6
0.5
0.4
0.3
0.2
0.1
0.0
Phylostratum
Cellular_organisms - ps1
Archaea1 - ps2
Archaea2 - ps3
Archaea3 - ps4
Asgard/Eukaryota - ps5
Eukaryota - ps6
Amorpheae - ps7
Obazoa - ps8
Opisthokonta - ps9
Holozoa - ps10
Filozoa - ps11
Choano./Metazoa - ps12
Metazoa - ps13
Myriazoa - ps14
Parahoxozoa - ps15
Bilateria - ps16
Deuterostomia - ps17
Chordata - ps18
Olfactores - ps19
Vertebrata - ps20
Gnathostomata - ps21
Euteleostomi - ps22
Sarcopterygii - ps23
Tetrapoda - ps24
Amniota - ps25
Mammalia - ps26
Theria - ps27
Placentalia - ps28
Boreoeutheria - ps29
Euarchontoglires - ps30
Euarchonta - ps31
Primates - ps32
Haplorrhini - ps33
Simiiformes - ps34
Catarrhini - ps35
Hominoidea - ps36
Hominidae - ps37
Homininae - ps38
Hominini - ps39
Homo_sapiens - ps40
p-value
2e-02
2e-02
3e-02
3e-02
4e-02
Log-odds
0.5
1.0
1.5
2.0
2.5
3.0
3.5
4.0

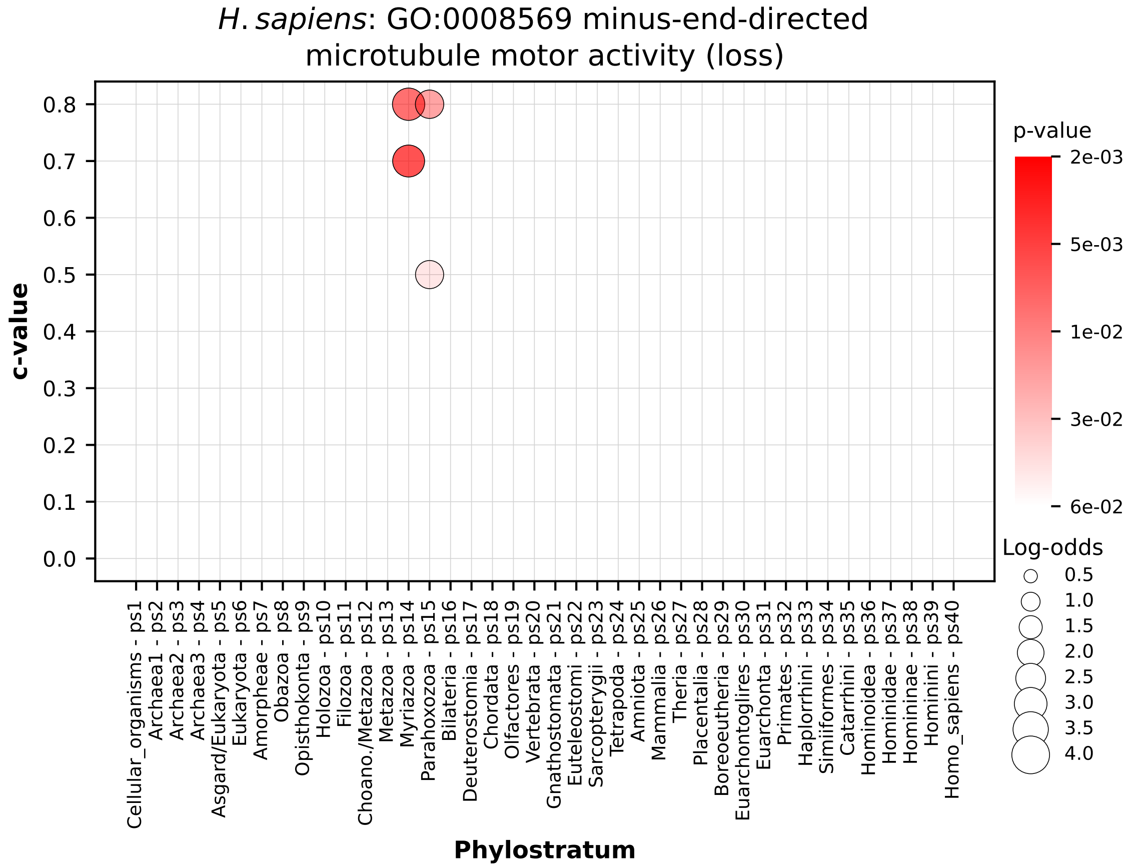

H. sapiens: GO:0008569 minus-end-directed microtubule motor activity (loss)
c-value
0.0
0.1
0.2
0.3
0.4
0.5
0.6
0.7
0.8
Phylostratum
Cellular_organisms - ps1
Archaea1 - ps2
Archaea2 - ps3
Archaea3 - ps4
Asgard/Eukaryota - ps5
Eukaryota - ps6
Amorpheae - ps7
Obazoa - ps8
Opisthokonta - ps9
Holozoa - ps10
Filozoa - ps11
Choano./Metazoa - ps12
Metazoa - ps13
Myriazoa - ps14
Parahoxozoa - ps15
Bilateria - ps16
Deuterostomia - ps17
Chordata - ps18
Olfactores - ps19
Vertebrata - ps20
Gnathostomata - ps21
Euteleostomi - ps22
Sarcopterygii - ps23
Tetrapoda - ps24
Amniota - ps25
Mammalia - ps26
Theria - ps27
Placentalia - ps28
Boreoeutheria - ps29
Euarchontoglires - ps30
Euarchonta - ps31
Primates - ps32
Haplorrhini - ps33
Simiiformes - ps34
Catarrhini - ps35
Hominoidea - ps36
Hominidae - ps37
Homininae - ps38
Hominini - ps39
Homo_sapiens - ps40
p-value
2e-03
5e-03
1e-02
3e-02
6e-02
Log-odds
0.5
1.0
1.5
2.0
2.5
3.0
3.5
4.0

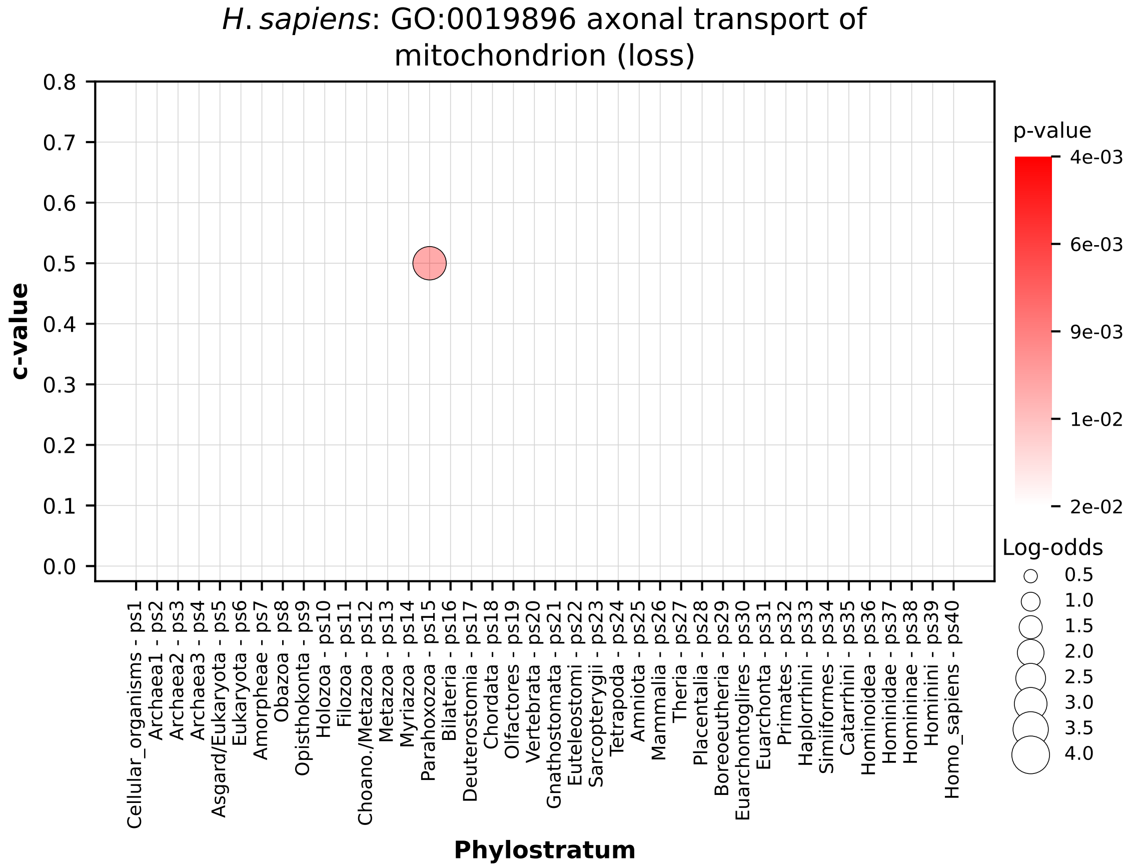

H. sapiens: GO:0019896 axonal transport of mitochondrion (loss)
c-value
0.8
0.7
0.6
0.5
0.4
0.3
0.2
0.1
0.0
Phylostratum
Cellular_organisms - ps1
Archaea1 - ps2
Archaea2 - ps3
Archaea3 - ps4
Asgard/Eukaryota - ps5
Eukaryota - ps6
Amorpheae - ps7
Obazoa - ps8
Opisthokonta - ps9
Holozoa - ps10
Filozoa - ps11
Choano./Metazoa - ps12
Metazoa - ps13
Myriazoa - ps14
Parahoxozoa - ps15
Bilateria - ps16
Deuterostomia - ps17
Chordata - ps18
Olfactores - ps19
Vertebrata - ps20
Gnathostomata - ps21
Euteleostomi - ps22
Sarcopterygii - ps23
Tetrapoda - ps24
Amniota - ps25
Mammalia - ps26
Theria - ps27
Placentalia - ps28
Boreoeutheria - ps29
Euarchontoglires - ps30
Euarchonta - ps31
Primates - ps32
Haplorrhini - ps33
Simiiformes - ps34
Catarrhini - ps35
Hominoidea - ps36
Hominidae - ps37
Homininae - ps38
Hominini - ps39
Homo_sapiens - ps40
p-value
4e-03
6e-03
9e-03
1e-02
2e-02
Log-odds
0.5
1.0
1.5
2.0
2.5
3.0
3.5
4.0

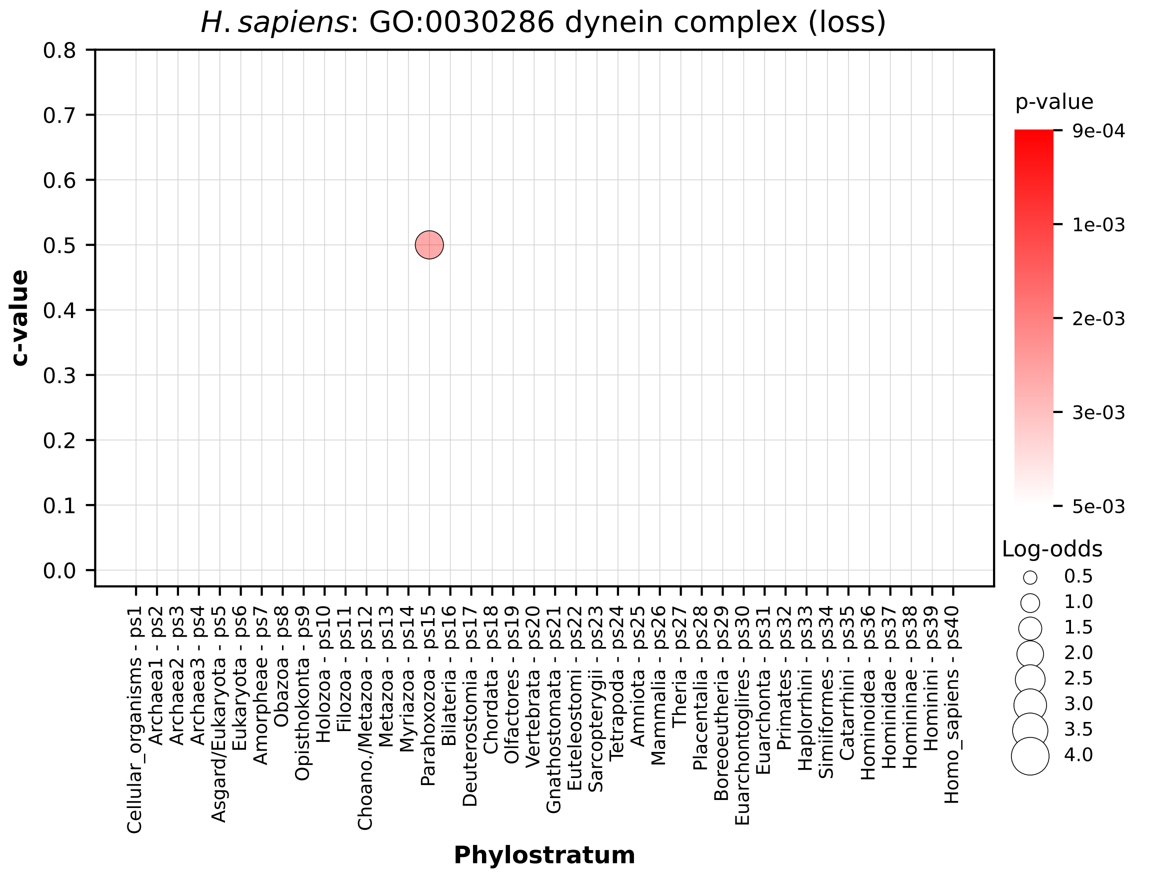
H. sapiens: GO:0030286 dynein complex (loss)
c-value
0.0
0.1
0.2
0.3
0.4
0.5
0.6
0.7
0.8
Phylostratum
Cellular_organisms - ps1
Archaea1 - ps2
Archaea2 - ps3
Archaea3 - ps4
Asgard/Eukaryota - ps5
Eukaryota - ps6
Amorpheae - ps7
Obazoa - ps8
Opisthokonta - ps9
Holozoa - ps10
Filozoa - ps11
Choano./Metazoa - ps12
Metazoa - ps13
Myriazoa - ps14
Parahoxozoa - ps15
Bilateria - ps16
Deuterostomia - ps17
Chordata - ps18
Olfactores - ps19
Vertebrata - ps20
Gnathostomata - ps21
Euteleostomi - ps22
Sarcopterygii - ps23
Tetrapoda - ps24
Amniota - ps25
Mammalia - ps26
Theria - ps27
Placentalia - ps28
Boreoeutheria - ps29
Euarchontoglires - ps30
Euarchonta - ps31
Primates - ps32
Haplorrhini - ps33
Simiiformes - ps34
Catarrhini - ps35
Hominoidea - ps36
Hominidae - ps37
Homininae - ps38
Hominini - ps39
Homo_sapiens - ps40
p-value
9e-04
1e-03
2e-03
3e-03
5e-03
Log-odds
0.5
1.0
1.5
2.0
2.5
3.0
3.5
4.0

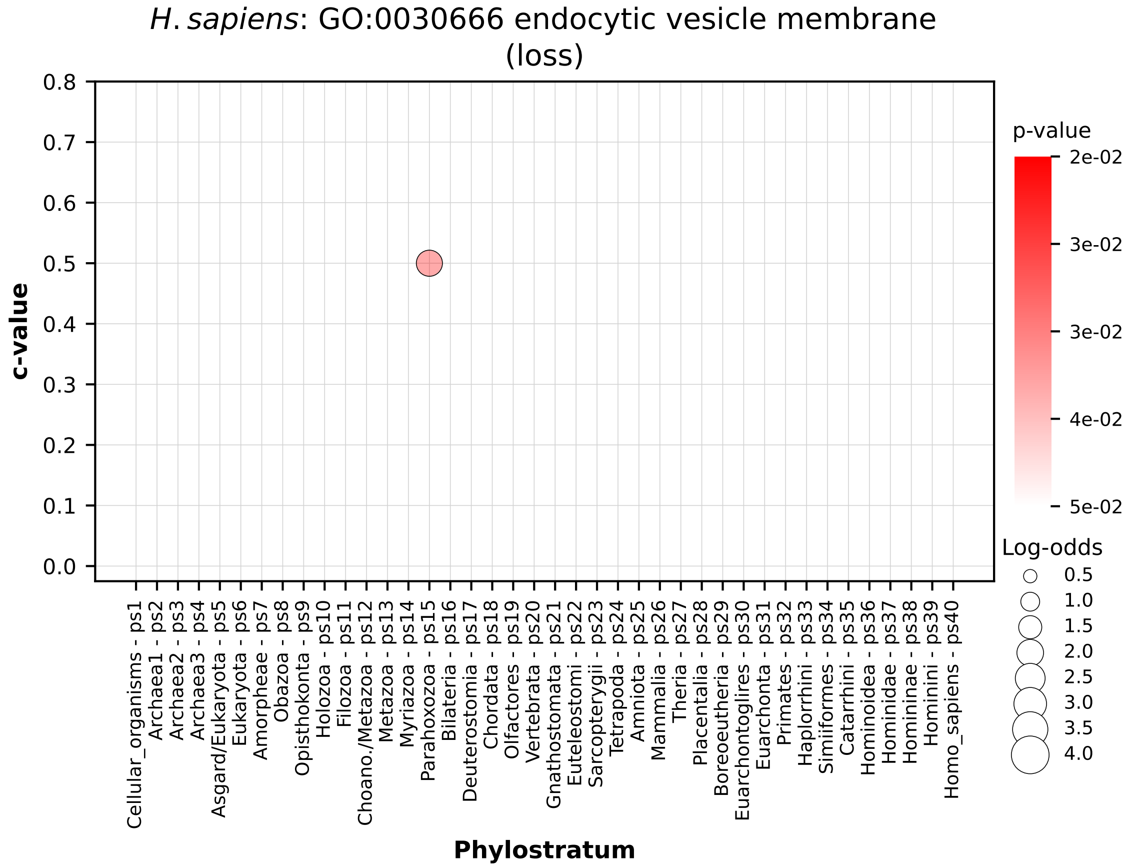

H. sapiens: GO:0030666 endocytic vesicle membrane
(loss)
c-value
0.8
0.7
0.6
0.5
0.4
0.3
0.2
0.1
0.0
Phylostratum
Cellular_organisms - ps1
Archaea1 - ps2
Archaea2 - ps3
Archaea3 - ps4
Asgard/Eukaryota - ps5
Eukaryota - ps6
Amorpheae - ps7
Obazoa - ps8
Opisthokonta - ps9
Holozoa - ps10
Filozoa - ps11
Choano./Metazoa - ps12
Metazoa - ps13
Myriazoa - ps14
Parahoxozoa - ps15
Bilateria - ps16
Deuterostomia - ps17
Chordata - ps18
Olfactores - ps19
Vertebrata - ps20
Gnathostomata - ps21
Euteleostomi - ps22
Sarcopterygii - ps23
Tetrapoda - ps24
Amniota - ps25
Mammalia - ps26
Theria - ps27
Placentalia - ps28
Boreoeutheria - ps29
Euarchontoglires - ps30
Euarchonta - ps31
Primates - ps32
Haplorrhini - ps33
Simiiformes - ps34
Catarrhini - ps35
Hominoidea - ps36
Hominidae - ps37
Homininae - ps38
Hominini - ps39
Homo_sapiens - ps40
p-value
2e-02
3e-02
3e-02
4e-02
5e-02
Log-odds
0.5
1.0
1.5
2.0
2.5
3.0
3.5
4.0

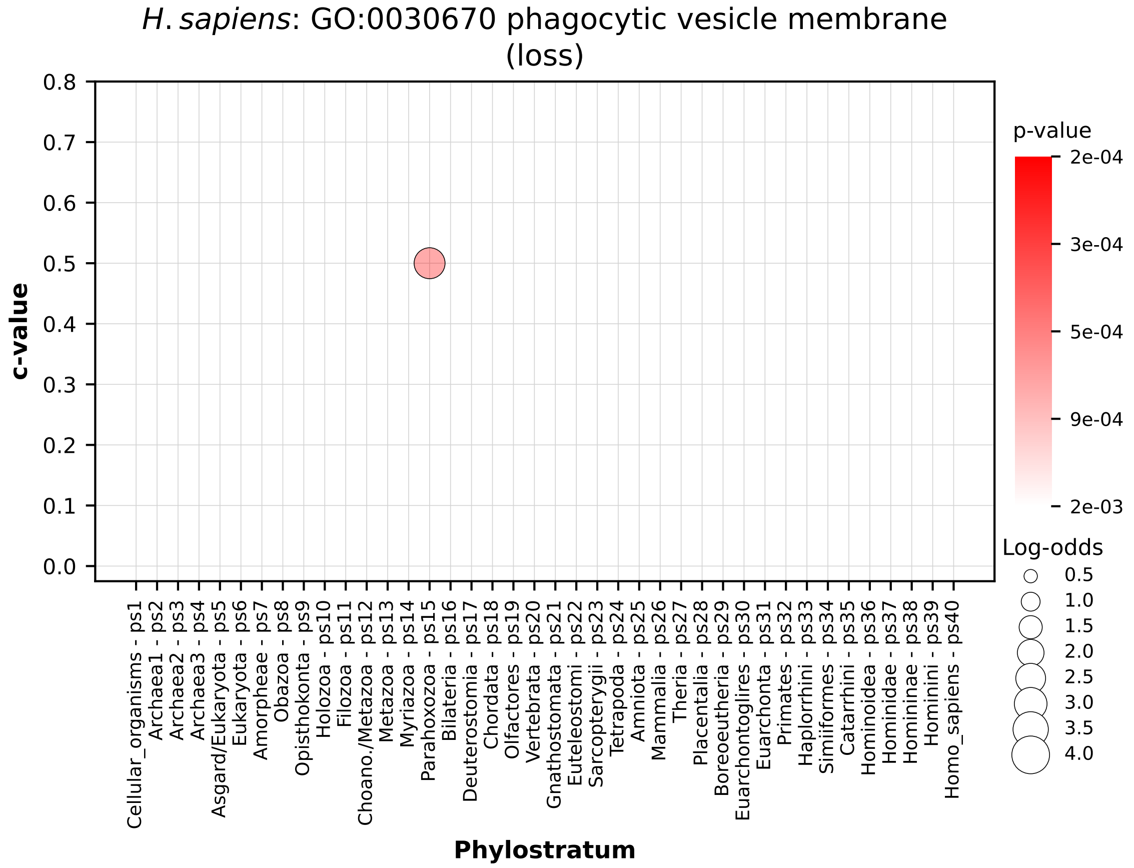
H. sapiens: GO:0030670 phagocytic vesicle membrane
(loss)
c-value
0.0
0.1
0.2
0.3
0.4
0.5
0.6
0.7
0.8
Phylostratum
Cellular_organisms - ps1
Archaea1 - ps2
Archaea2 - ps3
Archaea3 - ps4
Asgard/Eukaryota - ps5
Eukaryota - ps6
Amorpheae - ps7
Obazoa - ps8
Opisthokonta - ps9
Holozoa - ps10
Filozoa - ps11
Choano./Metazoa - ps12
Metazoa - ps13
Myriazoa - ps14
Parahoxozoa - ps15
Bilateria - ps16
Deuterostomia - ps17
Chordata - ps18
Olfactores - ps19
Vertebrata - ps20
Gnathostomata - ps21
Euteleostomi - ps22
Sarcopterygii - ps23
Tetrapoda - ps24
Amniota - ps25
Mammalia - ps26
Theria - ps27
Placentalia - ps28
Boreoeutheria - ps29
Euarchontoglires - ps30
Euarchonta - ps31
Primates - ps32
Haplorrhini - ps33
Simiiformes - ps34
Catarrhini - ps35
Hominoidea - ps36
Hominidae - ps37
Homininae - ps38
Hominini - ps39
Homo_sapiens - ps40
p-value
2e-04
3e-04
5e-04
9e-04
2e-03
Log-odds
0.5
1.0
1.5
2.0
2.5
3.0
3.5
4.0

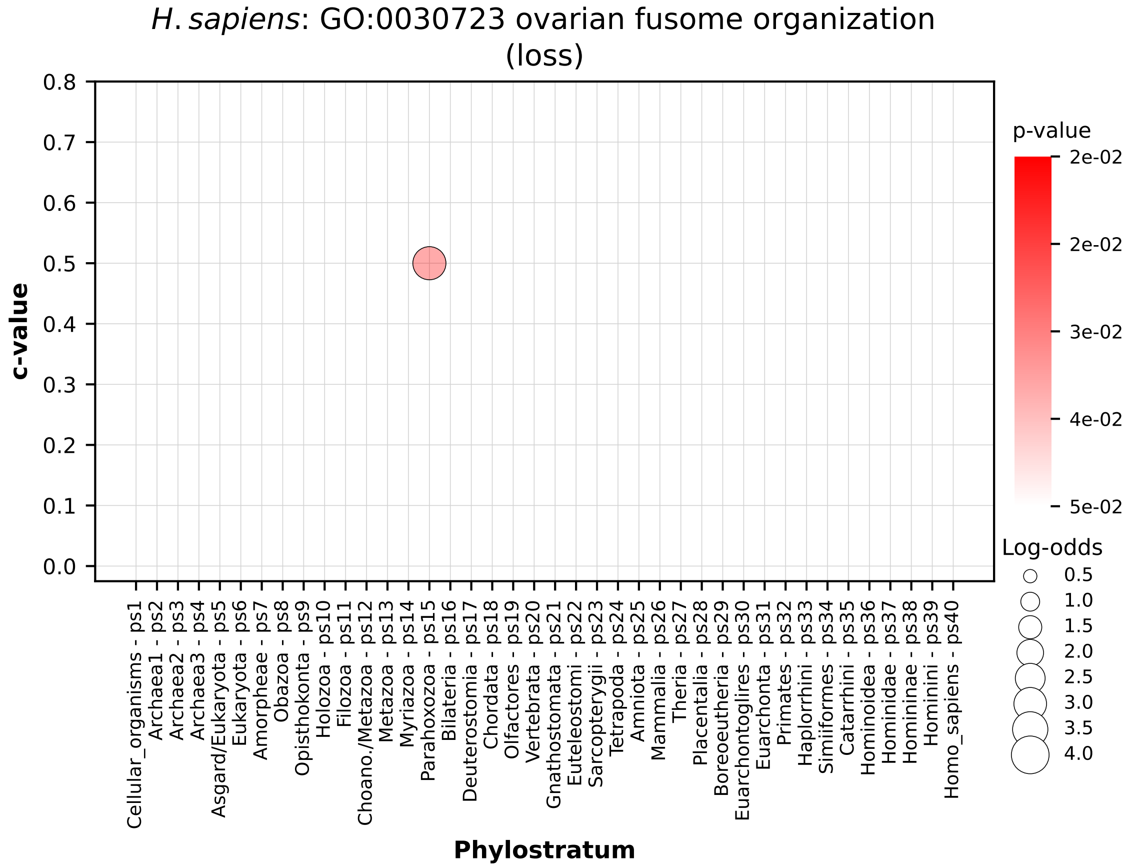

H. sapiens: GO:0030723 ovarian fusome organization (loss)
c-value
0.8
0.7
0.6
0.5
0.4
0.3
0.2
0.1
0.0
p-value
2e-02
2e-02
3e-02
4e-02
5e-02
Log-odds
0.5
1.0
1.5
2.0
2.5
3.0
3.5
4.0
Cellular_organisms - ps1
Archaea1 - ps2
Archaea2 - ps3
Archaea3 - ps4
Asgard/Eukaryota - ps5
Eukaryota - ps6
Amorpheae - ps7
Obazoa - ps8
Opisthokonta - ps9
Holozoa - ps10
Filozoa - ps11
Choano./Metazoa - ps12
Metazoa - ps13
Myriazoa - ps14
Parahoxozoa - ps15
Bilateria - ps16
Deuterostomia - ps17
Chordata - ps18
Olfactores - ps19
Vertebrata - ps20
Gnathostomata - ps21
Euteleostomi - ps22
Sarcopterygii - ps23
Tetrapoda - ps24
Amniota - ps25
Mammalia - ps26
Theria - ps27
Placentalia - ps28
Boreoeutheria - ps29
Euarchontoglires - ps30
Euarchonta - ps31
Primates - ps32
Haplorrhini - ps33
Simiiformes - ps34
Catarrhini - ps35
Hominoidea - ps36
Hominidae - ps37
Homininae - ps38
Hominini - ps39
Homo_sapiens - ps40
Phylostratum

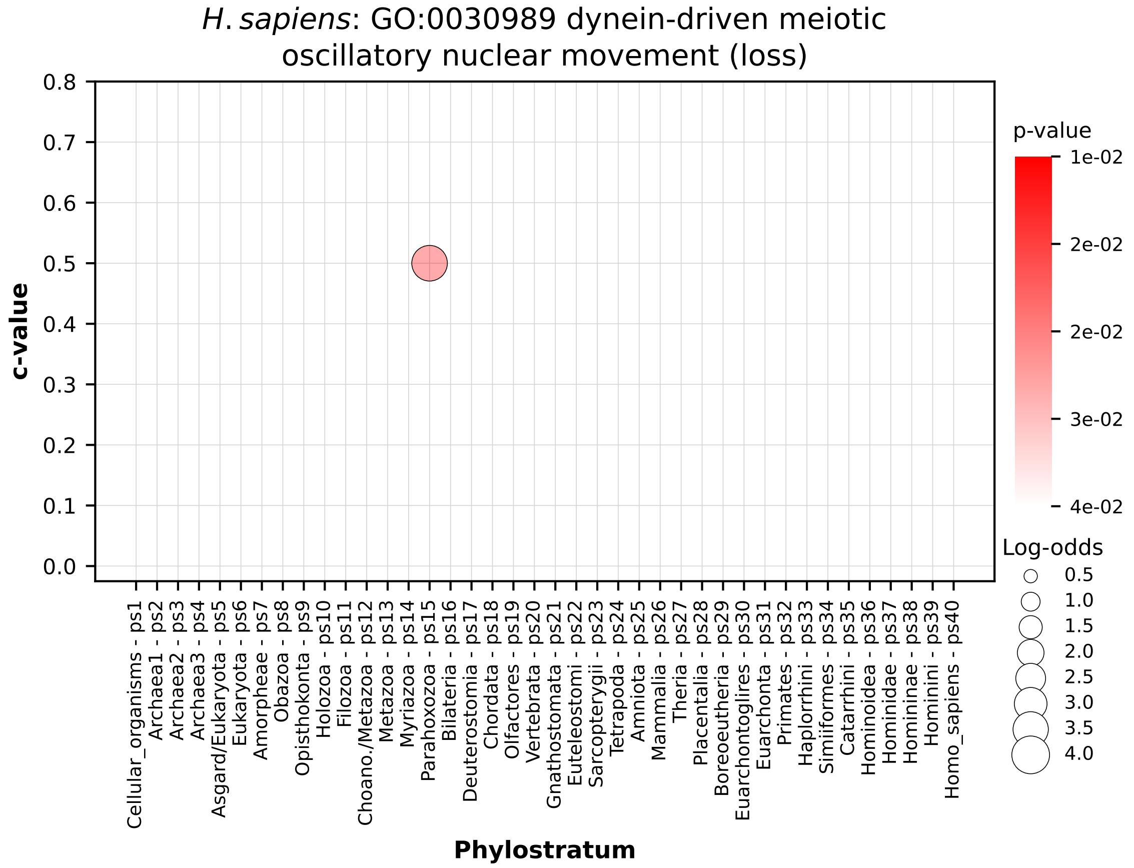

H. sapiens: GO:0030989 dynein-driven meiotic oscillatory nuclear movement (loss)
c-value
0.0
0.1
0.2
0.3
0.4
0.5
0.6
0.7
0.8
p-value
1e-02
2e-02
2e-02
3e-02
4e-02
Log-odds
0.5
1.0
1.5
2.0
2.5
3.0
3.5
4.0
Cellular_organisms - ps1
Archaea1 - ps2
Archaea2 - ps3
Archaea3 - ps4
Asgard/Eukaryota - ps5
Eukaryota - ps6
Amorpheae - ps7
Obazoa - ps8
Opisthokonta - ps9
Holozoa - ps10
Filozoa - ps11
Choano./Metazoa - ps12
Metazoa - ps13
Myriazoa - ps14
Parahoxozoa - ps15
Bilateria - ps16
Deuterostomia - ps17
Chordata - ps18
Olfactores - ps19
Vertebrata - ps20
Gnathostomata - ps21
Euteleostomi - ps22
Sarcopterygii - ps23
Tetrapoda - ps24
Amniota - ps25
Mammalia - ps26
Theria - ps27
Placentalia - ps28
Boreoeutheria - ps29
Euarchontoglires - ps30
Euarchonta - ps31
Primates - ps32
Haplorrhini - ps33
Simiiformes - ps34
Catarrhini - ps35
Hominoidea - ps36
Hominidae - ps37
Homininae - ps38
Hominini - ps39
Homo_sapiens - ps40
Phylostratum

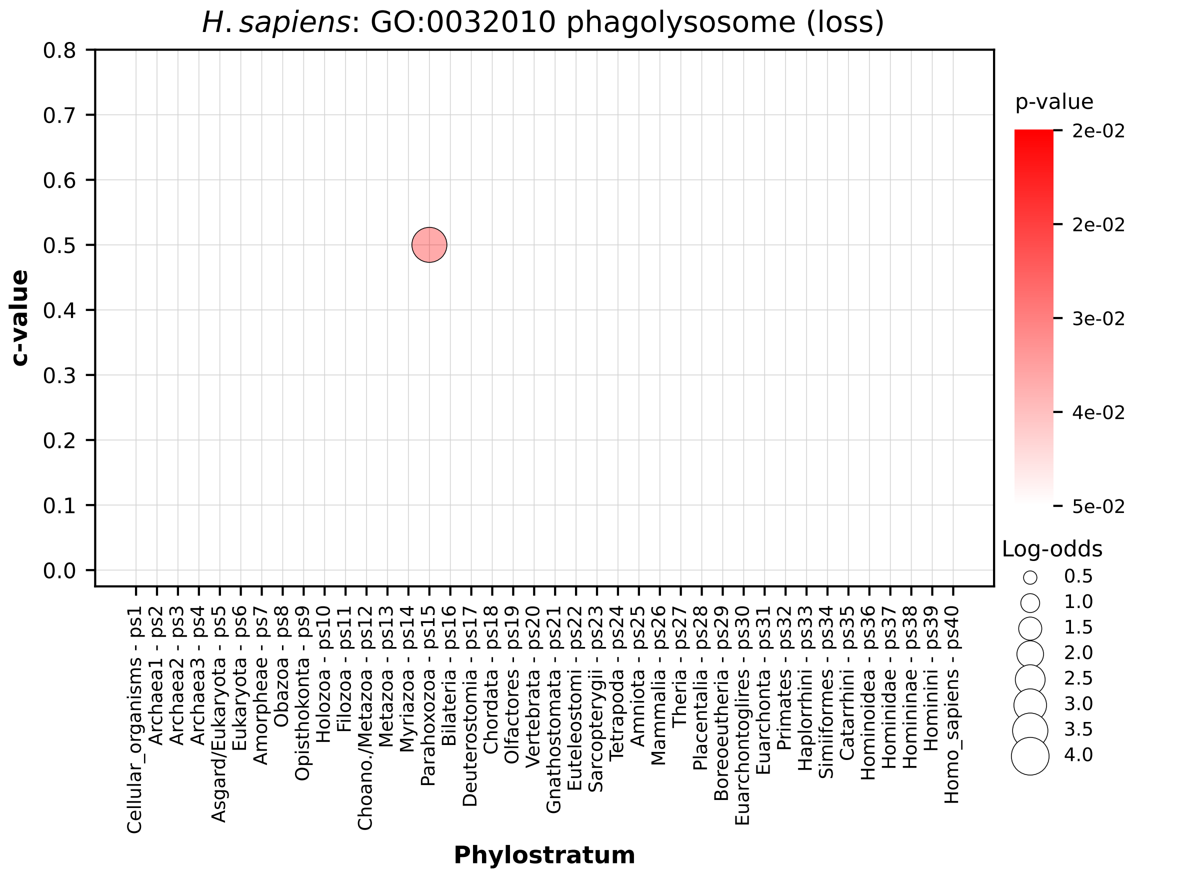
H. sapiens: GO:0032010 phagolysosome (loss)
c-value
0.8
0.7
0.6
0.5
0.4
0.3
0.2
0.1
0.0
p-value
2e-02
2e-02
3e-02
4e-02
5e-02
Log-odds
0.5
1.0
1.5
2.0
2.5
3.0
3.5
4.0
Cellular_organisms - ps1
Archaea1 - ps2
Archaea2 - ps3
Archaea3 - ps4
Asgard/Eukaryota - ps5
Eukaryota - ps6
Amorpheae - ps7
Obazoa - ps8
Opisthokonta - ps9
Holozoa - ps10
Filozoa - ps11
Choano./Metazoa - ps12
Metazoa - ps13
Myriazoa - ps14
Parahoxozoa - ps15
Bilateria - ps16
Deuterostomia - ps17
Chordata - ps18
Olfactores - ps19
Vertebrata - ps20
Gnathostomata - ps21
Euteleostomi - ps22
Sarcopterygii - ps23
Tetrapoda - ps24
Amniota - ps25
Mammalia - ps26
Theria - ps27
Placentalia - ps28
Boreoeutheria - ps29
Euarchontoglires - ps30
Euarchonta - ps31
Primates - ps32
Haplorrhini - ps33
Simiiformes - ps34
Catarrhini - ps35
Hominoidea - ps36
Hominidae - ps37
Homininae - ps38
Hominini - ps39
Homo_sapiens - ps40
Phylostratum

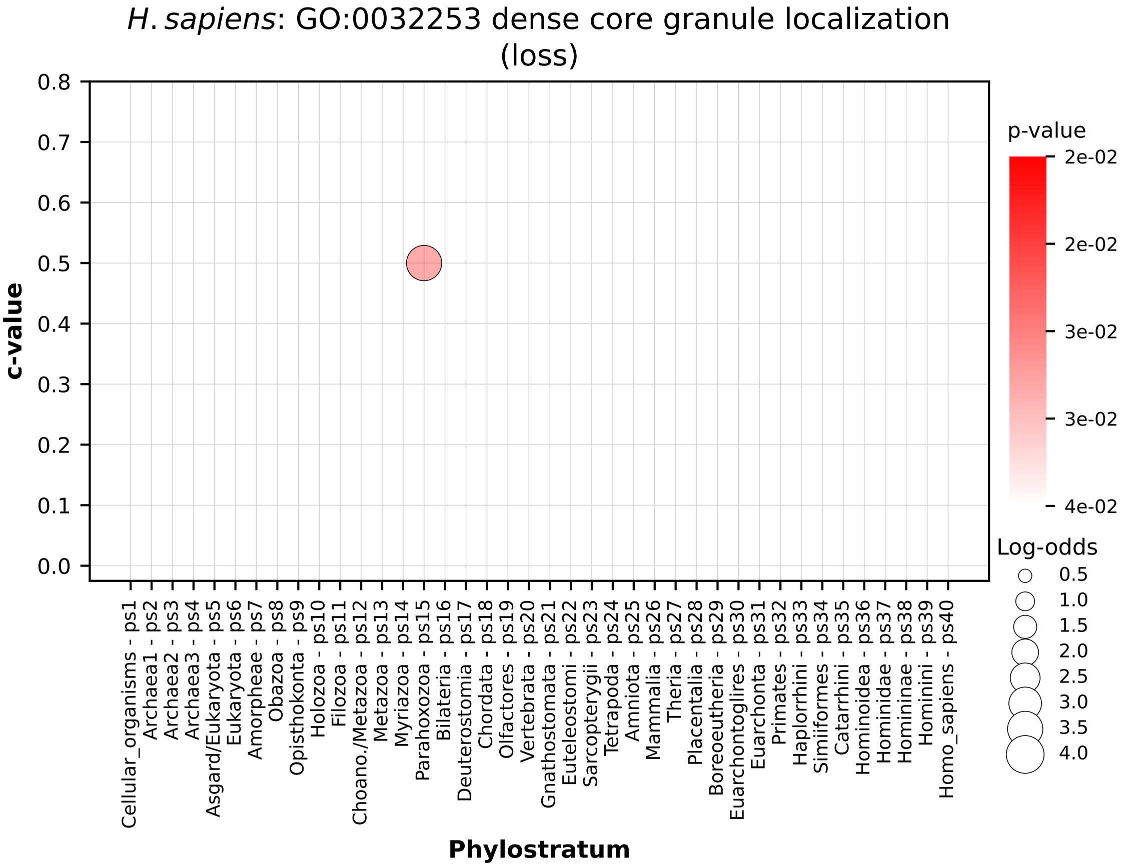

H. sapiens: GO:0032253 dense core granule localization (loss)
c-value
0.8
0.7
0.6
0.5
0.4
0.3
0.2
0.1
0.0
p-value
2e-02
2e-02
3e-02
3e-02
4e-02
Log-odds
0.5
1.0
1.5
2.0
2.5
3.0
3.5
4.0
Cellular_organisms - ps1
Archaea1 - ps2
Archaea2 - ps3
Archaea3 - ps4
Asgard/Eukaryota - ps5
Eukaryota - ps6
Amorpheae - ps7
Obazoa - ps8
Opisthokonta - ps9
Holozoa - ps10
Filozoa - ps11
Choano./Metazoa - ps12
Metazoa - ps13
Myriazoa - ps14
Parahoxozoa - ps15
Bilateria - ps16
Deuterostomia - ps17
Chordata - ps18
Olfactores - ps19
Vertebrata - ps20
Gnathostomata - ps21
Euteleostomi - ps22
Sarcopterygii - ps23
Tetrapoda - ps24
Amniota - ps25
Mammalia - ps26
Theria - ps27
Placentalia - ps28
Boreoeutheria - ps29
Euarchontoglires - ps30
Euarchonta - ps31
Primates - ps32
Haplorrhini - ps33
Simiiformes - ps34
Catarrhini - ps35
Hominoidea - ps36
Hominidae - ps37
Homininae - ps38
Hominini - ps39
Homo_sapiens - ps40
Phylostratum

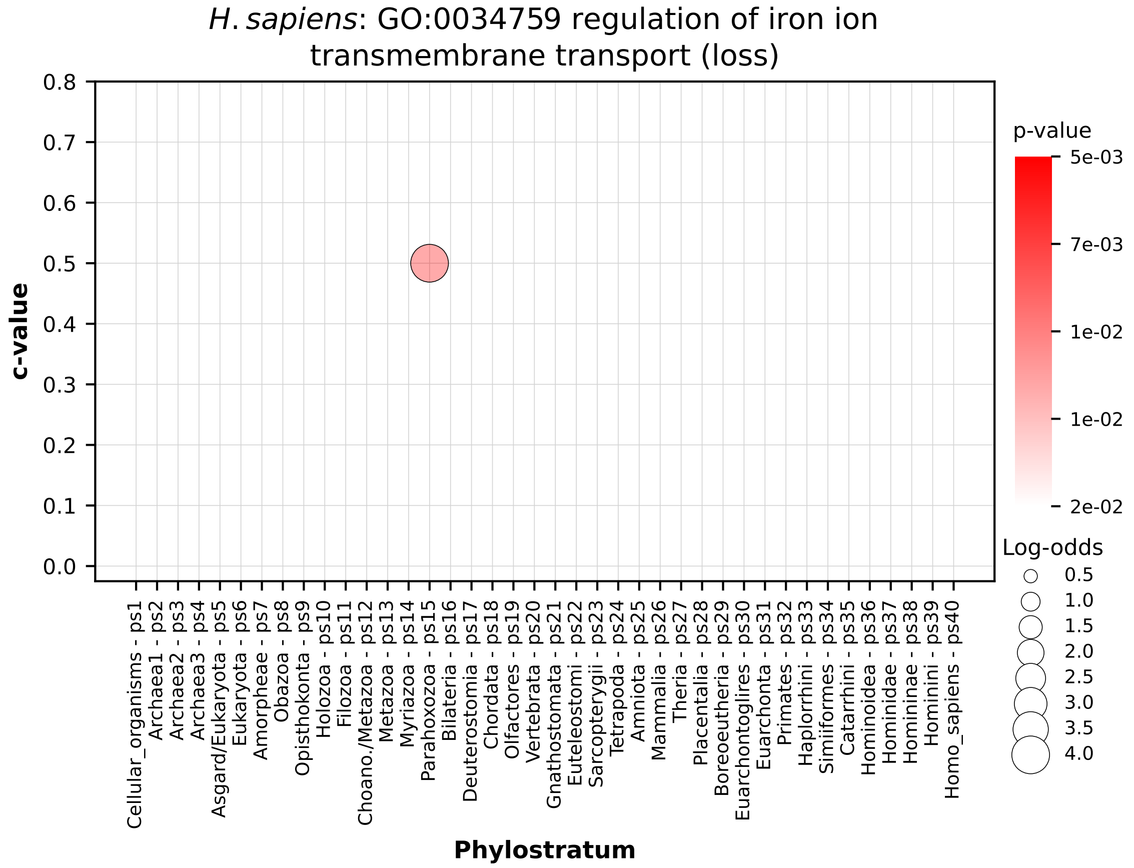

H. sapiens: GO:0034759 regulation of iron ion transmembrane transport (loss)
c-value
0.8
0.7
0.6
0.5
0.4
0.3
0.2
0.1
0.0
Phylostratum
Cellular_organisms - ps1
Archaea1 - ps2
Archaea2 - ps3
Archaea3 - ps4
Asgard/Eukaryota - ps5
Eukaryota - ps6
Amorpheae - ps7
Obazoa - ps8
Opisthokonta - ps9
Holozoa - ps10
Filozoa - ps11
Choano./Metazoa - ps12
Metazoa - ps13
Myriazoa - ps14
Parahoxozoa - ps15
Bilateria - ps16
Deuterostomia - ps17
Chordata - ps18
Olfactores - ps19
Vertebrata - ps20
Gnathostomata - ps21
Euteleostomi - ps22
Sarcopterygii - ps23
Tetrapoda - ps24
Amniota - ps25
Mammalia - ps26
Theria - ps27
Placentalia - ps28
Boreoeutheria - ps29
Euarchontoglires - ps30
Euarchonta - ps31
Primates - ps32
Haplorrhini - ps33
Simiiformes - ps34
Catarrhini - ps35
Hominoidea - ps36
Hominidae - ps37
Homininae - ps38
Hominini - ps39
Homo_sapiens - ps40
p-value
5e-03
7e-03
1e-02
1e-02
2e-02
Log-odds
0.5
1.0
1.5
2.0
2.5
3.0
3.5
4.0

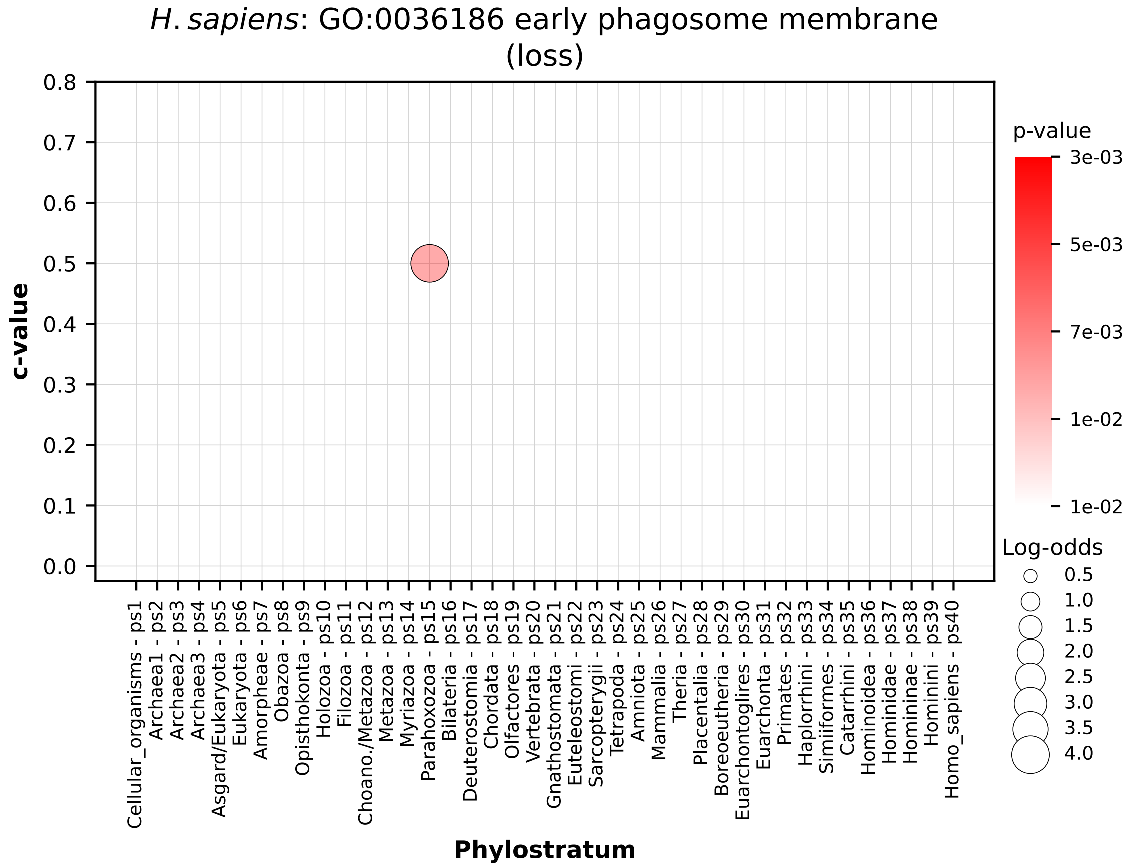

H. sapiens: GO:0036186 early phagosome membrane (loss)
c-value
0.8
0.7
0.6
0.5
0.4
0.3
0.2
0.1
0.0
p-value
3e-03
5e-03
7e-03
1e-02
1e-02
Log-odds
0.5
1.0
1.5
2.0
2.5
3.0
3.5
4.0
Cellular_organisms - ps1
Archaea1 - ps2
Archaea2 - ps3
Archaea3 - ps4
Asgard/Eukaryota - ps5
Eukaryota - ps6
Amorpheae - ps7
Obazoa - ps8
Opisthokonta - ps9
Holozoa - ps10
Filozoa - ps11
Choano./Metazoa - ps12
Metazoa - ps13
Myriazoa - ps14
Parahoxozoa - ps15
Bilateria - ps16
Deuterostomia - ps17
Chordata - ps18
Olfactores - ps19
Vertebrata - ps20
Gnathostomata - ps21
Euteleostomi - ps22
Sarcopterygii - ps23
Tetrapoda - ps24
Amniota - ps25
Mammalia - ps26
Theria - ps27
Placentalia - ps28
Boreoeutheria - ps29
Euarchontoglires - ps30
Euarchonta - ps31
Primates - ps32
Haplorrhini - ps33
Simiiformes - ps34
Catarrhini - ps35
Hominoidea - ps36
Hominidae - ps37
Homininae - ps38
Hominini - ps39
Homo_sapiens - ps40
Phylostratum

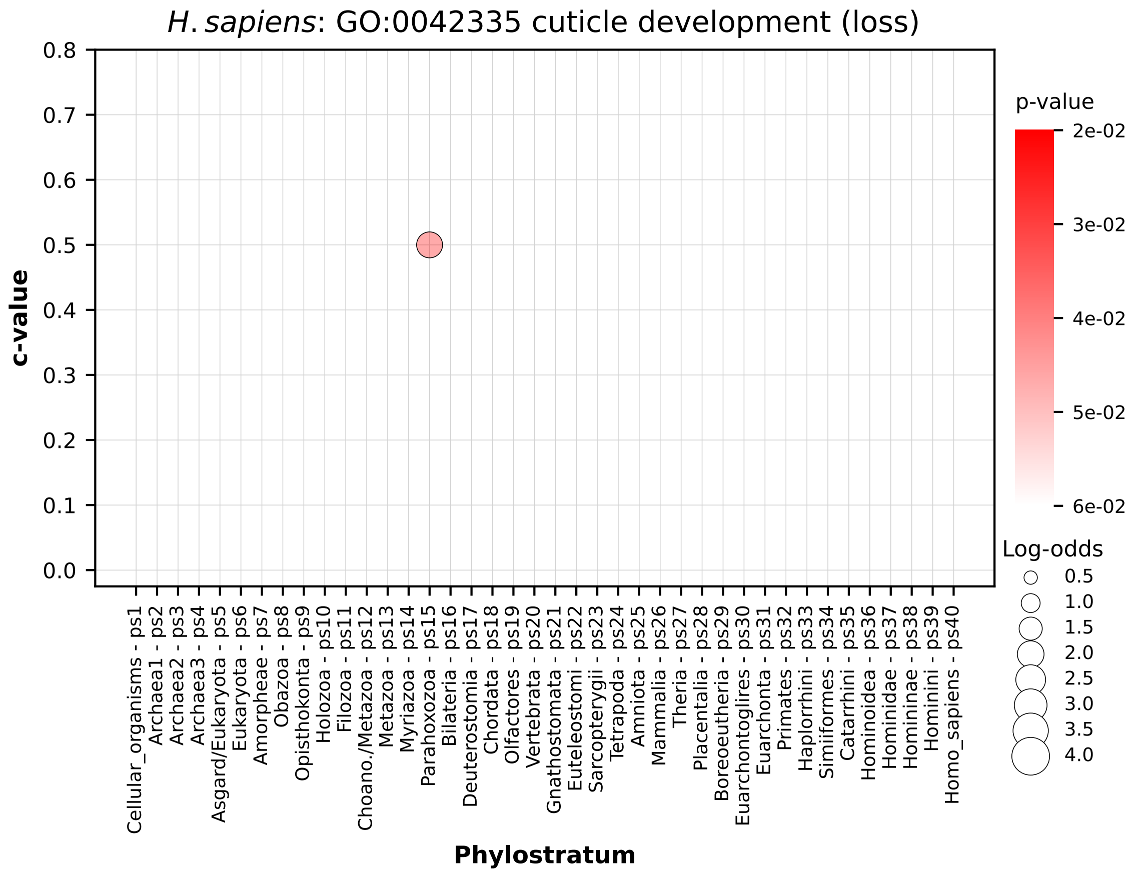

H. sapiens: GO:0042335 cuticle development (loss)
c-value
0.8
0.7
0.6
0.5
0.4
0.3
0.2
0.1
0.0
Phylostratum
Cellular_organisms - ps1
Archaea1 - ps2
Archaea2 - ps3
Archaea3 - ps4
Asgard/Eukaryota - ps5
Eukaryota - ps6
Amorpheae - ps7
Obazoa - ps8
Opisthokonta - ps9
Holozoa - ps10
Filozoa - ps11
Choano./Metazoa - ps12
Metazoa - ps13
Myriazoa - ps14
Parahoxozoa - ps15
Bilateria - ps16
Deuterostomia - ps17
Chordata - ps18
Olfactores - ps19
Vertebrata - ps20
Gnathostomata - ps21
Euteleostomi - ps22
Sarcopterygii - ps23
Tetrapoda - ps24
Amniota - ps25
Mammalia - ps26
Theria - ps27
Placentalia - ps28
Boreoeutheria - ps29
Euarchontoglires - ps30
Euarchonta - ps31
Primates - ps32
Haplorrhini - ps33
Simiiformes - ps34
Catarrhini - ps35
Hominoidea - ps36
Hominidae - ps37
Homininae - ps38
Hominini - ps39
Homo_sapiens - ps40
p-value
2e-02
3e-02
4e-02
5e-02
6e-02
Log-odds
0.5
1.0
1.5
2.0
2.5
3.0
3.5
4.0

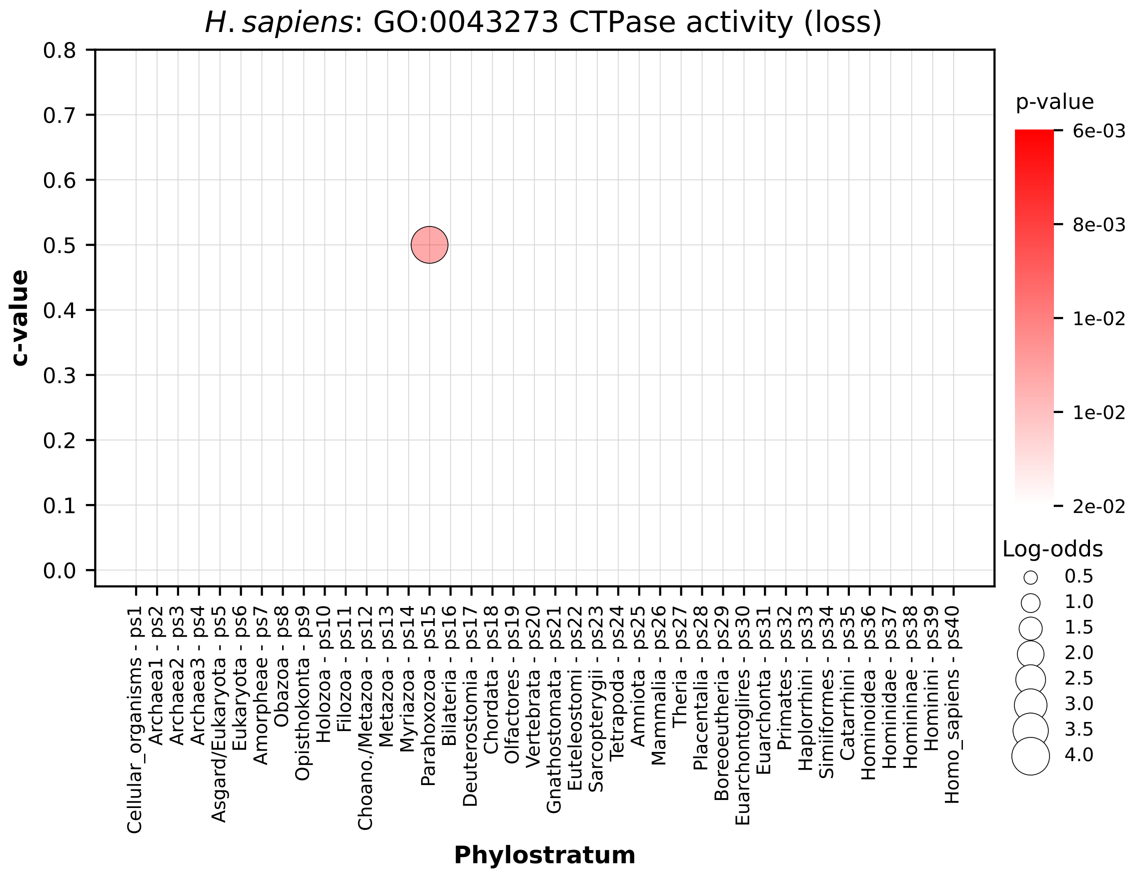
H. sapiens: GO:0043273 CTPase activity (loss)
c-value
0.0
0.1
0.2
0.3
0.4
0.5
0.6
0.7
0.8
Phylostratum
Cellular_organisms - ps1
Archaea1 - ps2
Archaea2 - ps3
Archaea3 - ps4
Asgard/Eukaryota - ps5
Eukaryota - ps6
Amorpheae - ps7
Obazoa - ps8
Opisthokonta - ps9
Holozoa - ps10
Filozoa - ps11
Choano./Metazoa - ps12
Metazoa - ps13
Myriazoa - ps14
Parahoxozoa - ps15
Bilateria - ps16
Deuterostomia - ps17
Chordata - ps18
Olfactores - ps19
Vertebrata - ps20
Gnathostomata - ps21
Euteleostomi - ps22
Sarcopterygii - ps23
Tetrapoda - ps24
Amniota - ps25
Mammalia - ps26
Theria - ps27
Placentalia - ps28
Boreoeutheria - ps29
Euarchontoglires - ps30
Euarchonta - ps31
Primates - ps32
Haplorrhini - ps33
Simiiformes - ps34
Catarrhini - ps35
Hominoidea - ps36
Hominidae - ps37
Homininae - ps38
Hominini - ps39
Homo_sapiens - ps40
p-value
6e-03
8e-03
1e-02
1e-02
2e-02
Log-odds
0.5
1.0
1.5
2.0
2.5
3.0
3.5
4.0

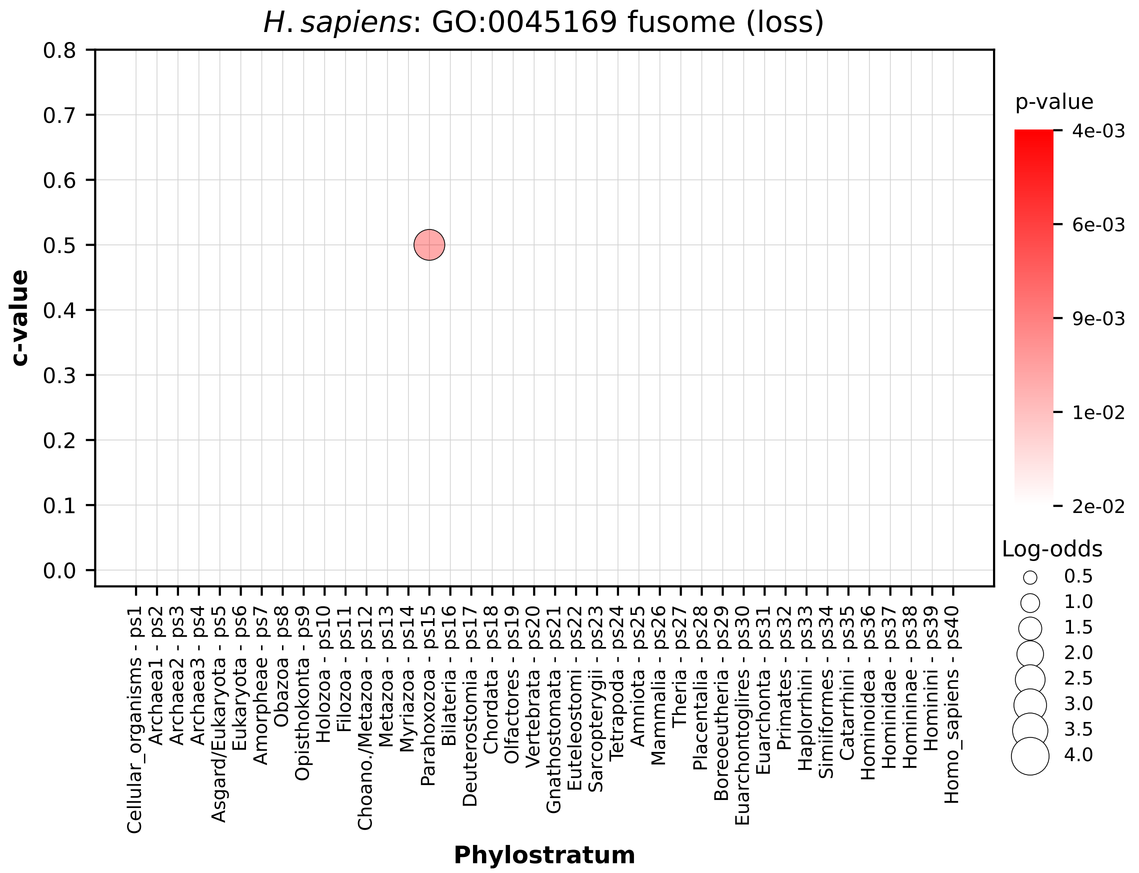
H. sapiens: GO:0045169 fusome (loss)
c-value
0.8
0.7
0.6
0.5
0.4
0.3
0.2
0.1
0.0
p-value
4e-03
6e-03
9e-03
1e-02
2e-02
Log-odds
0.5
1.0
1.5
2.0
2.5
3.0
3.5
4.0
Cellular_organisms - ps1
Archaea1 - ps2
Archaea2 - ps3
Archaea3 - ps4
Asgard/Eukaryota - ps5
Eukaryota - ps6
Amorpheae - ps7
Obazoa - ps8
Opisthokonta - ps9
Holozoa - ps10
Filozoa - ps11
Choano./Metazoa - ps12
Metazoa - ps13
Myriazoa - ps14
Parahoxozoa - ps15
Bilateria - ps16
Deuterostomia - ps17
Chordata - ps18
Olfactores - ps19
Vertebrata - ps20
Gnathostomata - ps21
Euteleostomi - ps22
Sarcopterygii - ps23
Tetrapoda - ps24
Amniota - ps25
Mammalia - ps26
Theria - ps27
Placentalia - ps28
Boreoeutheria - ps29
Euarchontoglires - ps30
Euarchonta - ps31
Primates - ps32
Haplorrhini - ps33
Simiiformes - ps34
Catarrhini - ps35
Hominoidea - ps36
Hominidae - ps37
Homininae - ps38
Hominini - ps39
Homo_sapiens - ps40
Phylostratum

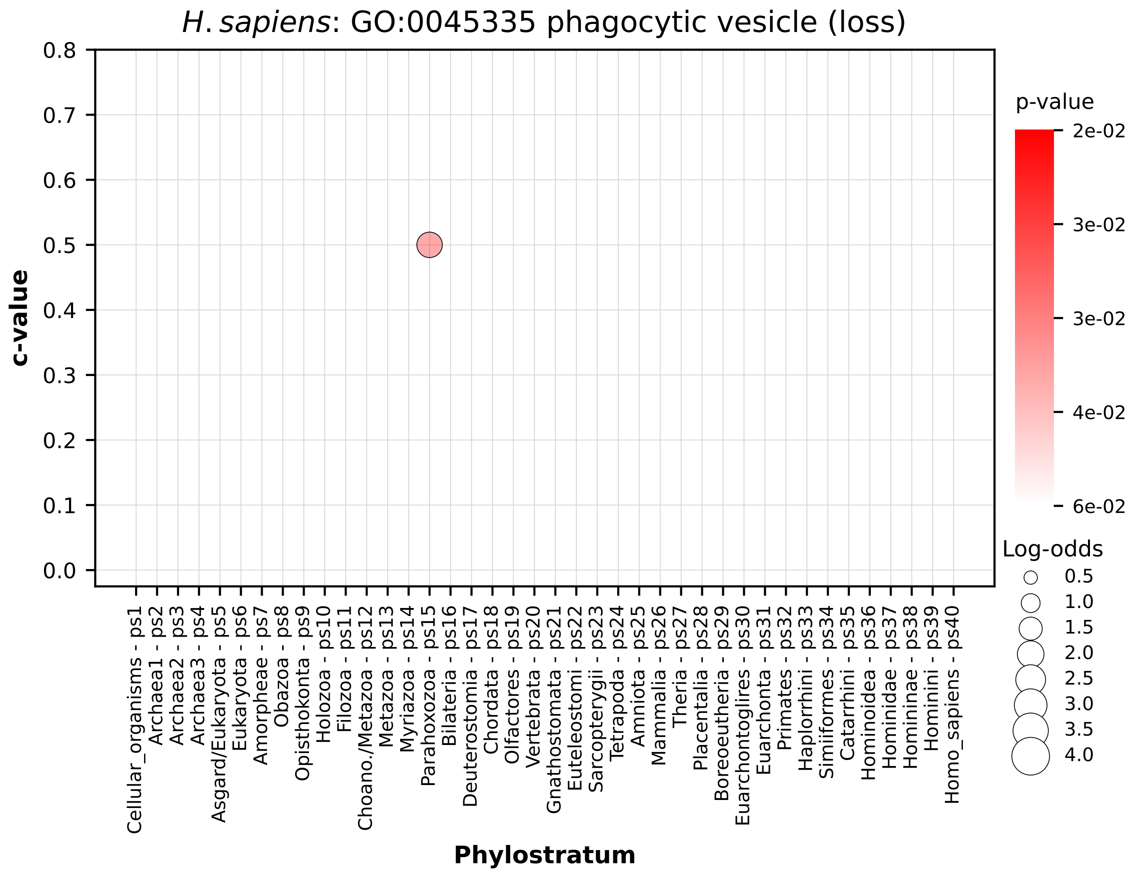

H. sapiens: GO:0045335 phagocytic vesicle (loss)
c-value
0.0
0.1
0.2
0.3
0.4
0.5
0.6
0.7
0.8
Phylostratum
Cellular_organisms - ps1
Archaea1 - ps2
Archaea2 - ps3
Archaea3 - ps4
Asgard/Eukaryota - ps5
Eukaryota - ps6
Amorpheae - ps7
Obazoa - ps8
Opisthokonta - ps9
Holozoa - ps10
Filozoa - ps11
Choano./Metazoa - ps12
Metazoa - ps13
Myriazoa - ps14
Parahoxozoa - ps15
Bilateria - ps16
Deuterostomia - ps17
Chordata - ps18
Olfactores - ps19
Vertebrata - ps20
Gnathostomata - ps21
Euteleostomi - ps22
Sarcopterygii - ps23
Tetrapoda - ps24
Amniota - ps25
Mammalia - ps26
Theria - ps27
Placentalia - ps28
Boreoeutheria - ps29
Euarchontoglires - ps30
Euarchonta - ps31
Primates - ps32
Haplorrhini - ps33
Simiiformes - ps34
Catarrhini - ps35
Hominoidea - ps36
Hominidae - ps37
Homininae - ps38
Hominini - ps39
Homo_sapiens - ps40
p-value
2e-02
3e-02
3e-02
4e-02
6e-02
Log-odds
0.5
1.0
1.5
2.0
2.5
3.0
3.5
4.0

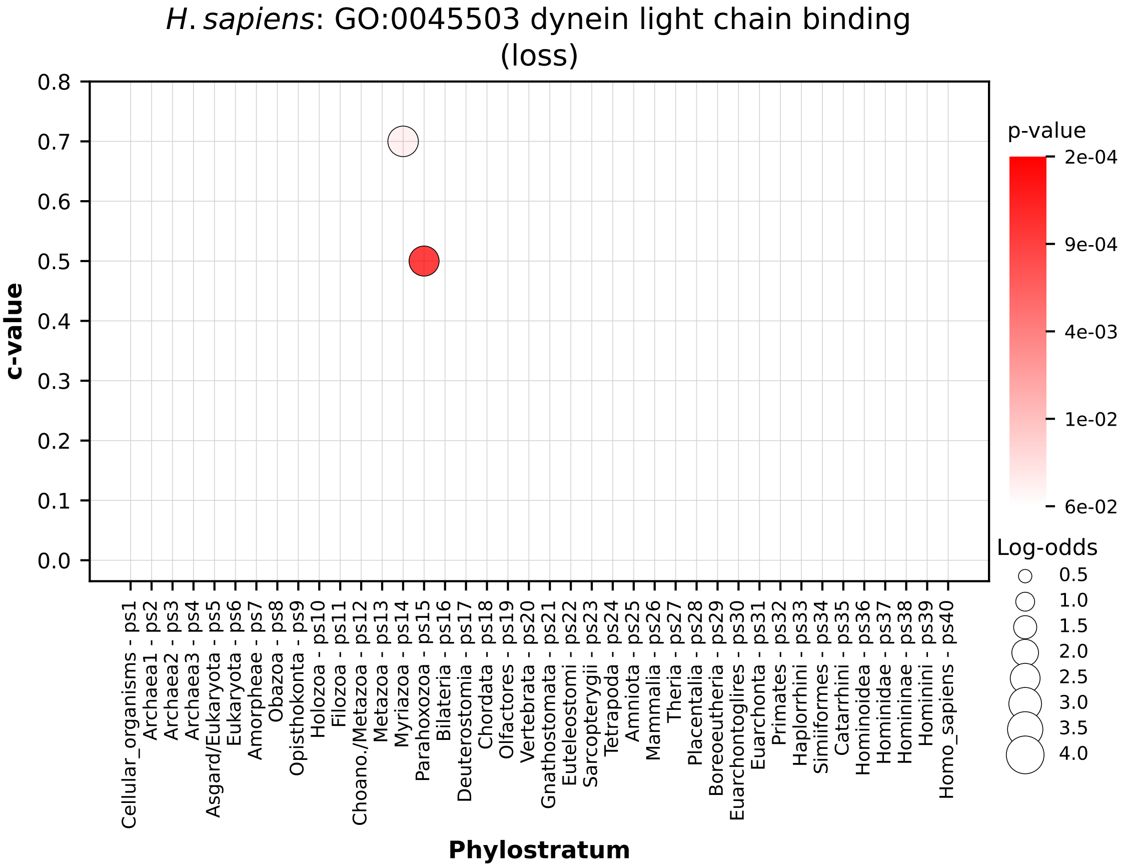
H. sapiens: GO:0045503 dynein light chain binding
(loss)
c-value
0.0
0.1
0.2
0.3
0.4
0.5
0.6
0.7
0.8
Phylostratum
Cellular_organisms - ps1
Archaea1 - ps2
Archaea2 - ps3
Archaea3 - ps4
Asgard/Eukaryota - ps5
Eukaryota - ps6
Amorpheae - ps7
Obazoa - ps8
Opisthokonta - ps9
Holozoa - ps10
Filozoa - ps11
Choano./Metazoa - ps12
Metazoa - ps13
Myriazoa - ps14
Parahoxozoa - ps15
Bilateria - ps16
Deuterostomia - ps17
Chordata - ps18
Olfactores - ps19
Vertebrata - ps20
Gnathostomata - ps21
Euteleostomi - ps22
Sarcopterygii - ps23
Tetrapoda - ps24
Amniota - ps25
Mammalia - ps26
Theria - ps27
Placentalia - ps28
Boreoeutheria - ps29
Euarchontoglires - ps30
Euarchonta - ps31
Primates - ps32
Haplorrhini - ps33
Simiiformes - ps34
Catarrhini - ps35
Hominoidea - ps36
Hominidae - ps37
Homininae - ps38
Hominini - ps39
Homo_sapiens - ps40
p-value
2e-04
9e-04
4e-03
1e-02
6e-02
Log-odds
0.5
1.0
1.5
2.0
2.5
3.0
3.5
4.0

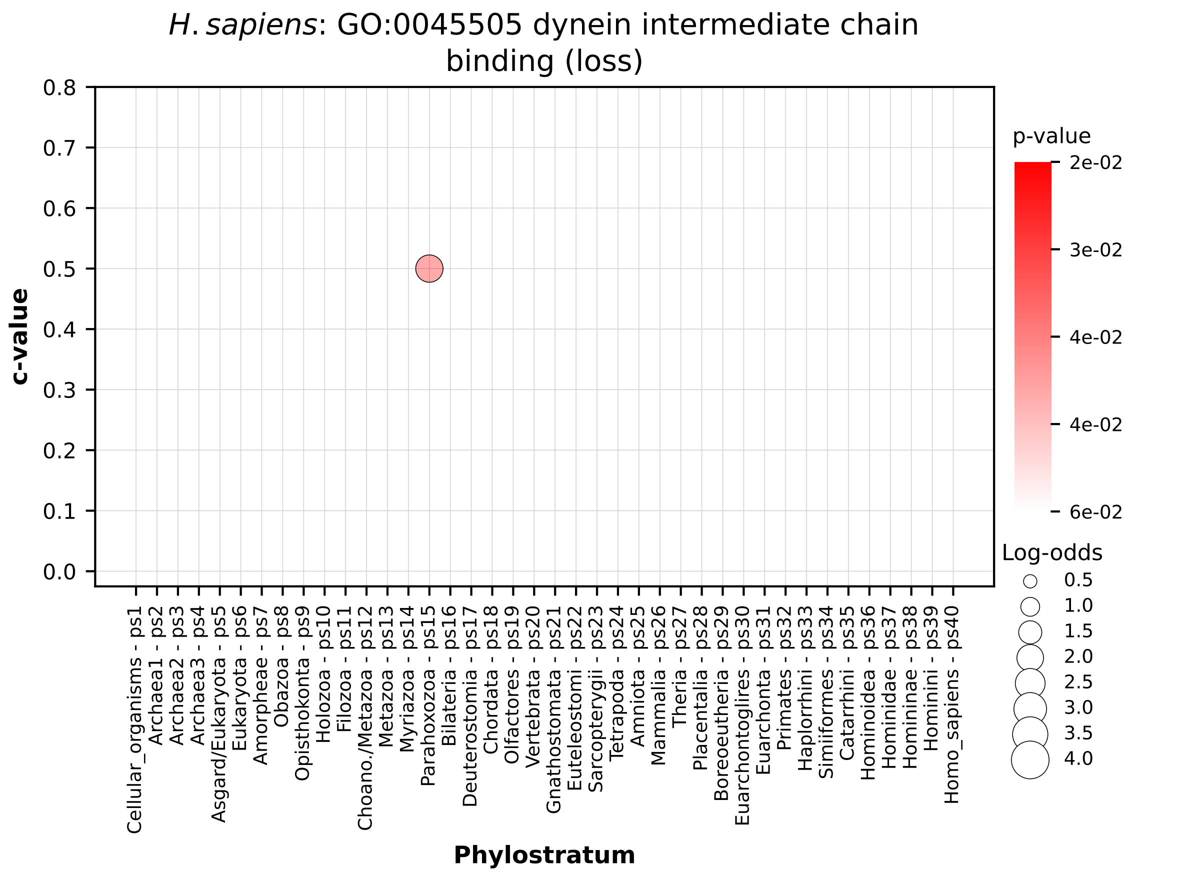

H. sapiens: GO:0045505 dynein intermediate chain binding (loss)
c-value
0.0
0.1
0.2
0.3
0.4
0.5
0.6
0.7
0.8
Phylostratum
Cellular_organisms - ps1
Archaea1 - ps2
Archaea2 - ps3
Archaea3 - ps4
Asgard/Eukaryota - ps5
Eukaryota - ps6
Amorpheae - ps7
Obazoa - ps8
Opisthokonta - ps9
Holozoa - ps10
Filozoa - ps11
Choano./Metazoa - ps12
Metazoa - ps13
Myriazoa - ps14
Parahoxozoa - ps15
Bilateria - ps16
Deuterostomia - ps17
Chordata - ps18
Olfactores - ps19
Vertebrata - ps20
Gnathostomata - ps21
Euteleostomi - ps22
Sarcopterygii - ps23
Tetrapoda - ps24
Amniota - ps25
Mammalia - ps26
Theria - ps27
Placentalia - ps28
Boreoeutheria - ps29
Euarchontoglires - ps30
Euarchonta - ps31
Primates - ps32
Haplorrhini - ps33
Simiiformes - ps34
Catarrhini - ps35
Hominoidea - ps36
Hominidae - ps37
Homininae - ps38
Hominini - ps39
Homo_sapiens - ps40
p-value
2e-02
3e-02
4e-02
4e-02
6e-02
Log-odds
0.5
1.0
1.5
2.0
2.5
3.0
3.5
4.0

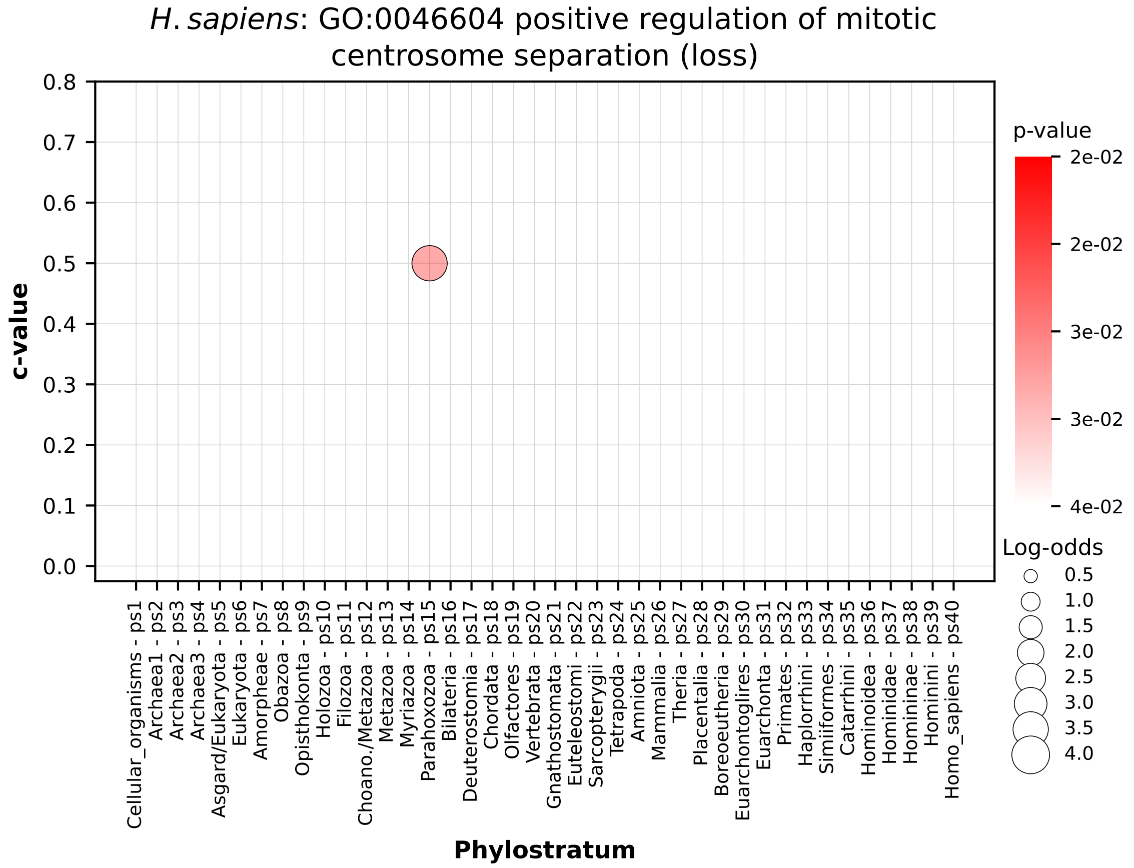

H. sapiens: GO:0046604 positive regulation of mitotic centrosome separation (loss)
c-value
0.8
0.7
0.6
0.5
0.4
0.3
0.2
0.1
0.0
p-value
2e-02
2e-02
3e-02
3e-02
4e-02
Log-odds
0.5
1.0
1.5
2.0
2.5
3.0
3.5
4.0
Cellular_organisms - ps1
Archaea1 - ps2
Archaea2 - ps3
Archaea3 - ps4
Asgard/Eukaryota - ps5
Eukaryota - ps6
Amorpheae - ps7
Obazoa - ps8
Opisthokonta - ps9
Holozoa - ps10
Filozoa - ps11
Choano./Metazoa - ps12
Metazoa - ps13
Myriazoa - ps14
Parahoxozoa - ps15
Bilateria - ps16
Deuterostomia - ps17
Chordata - ps18
Olfactores - ps19
Vertebrata - ps20
Gnathostomata - ps21
Euteleostomi - ps22
Sarcopterygii - ps23
Tetrapoda - ps24
Amniota - ps25
Mammalia - ps26
Theria - ps27
Placentalia - ps28
Boreoeutheria - ps29
Euarchontoglires - ps30
Euarchonta - ps31
Primates - ps32
Haplorrhini - ps33
Simiiformes - ps34
Catarrhini - ps35
Hominoidea - ps36
Hominidae - ps37
Homininae - ps38
Hominini - ps39
Homo_sapiens - ps40
Phylostratum

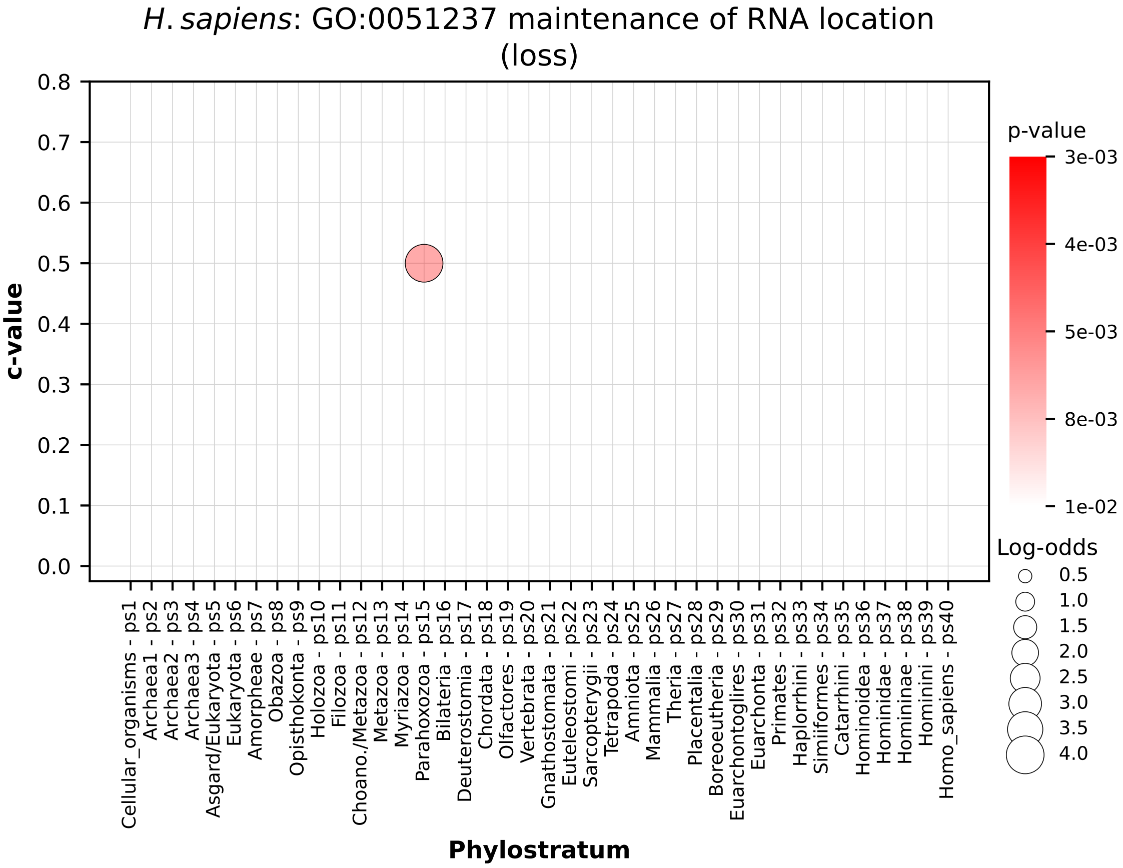

H. sapiens: GO:0051237 maintenance of RNA location
(loss)
c-value
0.8
0.7
0.6
0.5
0.4
0.3
0.2
0.1
0.0
p-value
3e-03
4e-03
5e-03
8e-03
1e-02
Log-odds
0.5
1.0
1.5
2.0
2.5
3.0
3.5
4.0
Cellular_organisms - ps1
Archaea1 - ps2
Archaea2 - ps3
Archaea3 - ps4
Asgard/Eukaryota - ps5
Eukaryota - ps6
Amorpheae - ps7
Obazoa - ps8
Opisthokonta - ps9
Holozoa - ps10
Filozoa - ps11
Choano./Metazoa - ps12
Metazoa - ps13
Myriazoa - ps14
Parahoxozoa - ps15
Bilateria - ps16
Deuterostomia - ps17
Chordata - ps18
Olfactores - ps19
Vertebrata - ps20
Gnathostomata - ps21
Euteleostomi - ps22
Sarcopterygii - ps23
Tetrapoda - ps24
Amniota - ps25
Mammalia - ps26
Theria - ps27
Placentalia - ps28
Boreoeutheria - ps29
Euarchontoglires - ps30
Euarchonta - ps31
Primates - ps32
Haplorrhini - ps33
Simiiformes - ps34
Catarrhini - ps35
Hominoidea - ps36
Hominidae - ps37
Homininae - ps38
Hominini - ps39
Homo_sapiens - ps40
Phylostratum

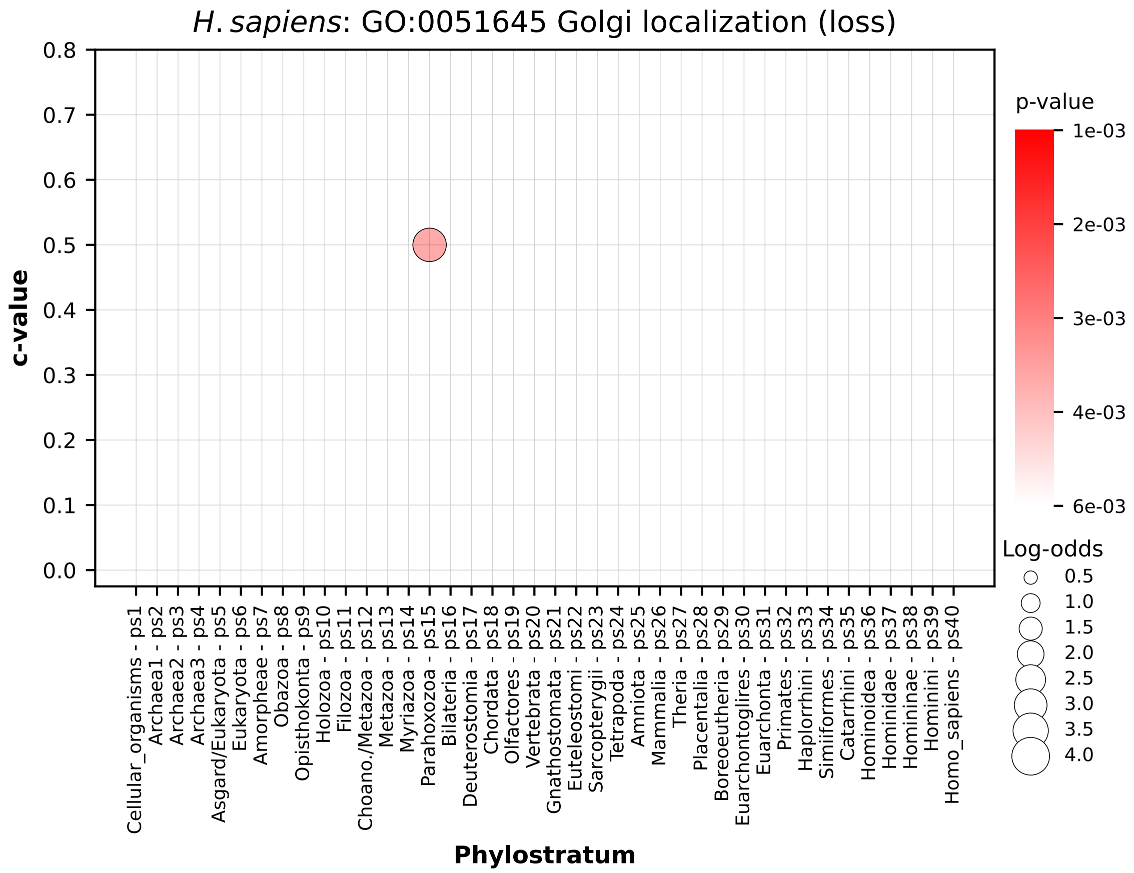
H. sapiens: GO:0051645 Golgi localization (loss)
c-value
0.8
0.7
0.6
0.5
0.4
0.3
0.2
0.1
0.0
Phylostratum
Cellular_organisms - ps1
Archaea1 - ps2
Archaea2 - ps3
Archaea3 - ps4
Asgard/Eukaryota - ps5
Eukaryota - ps6
Amorpheae - ps7
Obazoa - ps8
Opisthokonta - ps9
Holozoa - ps10
Filozoa - ps11
Choano./Metazoa - ps12
Metazoa - ps13
Myriazoa - ps14
Parahoxozoa - ps15
Bilateria - ps16
Deuterostomia - ps17
Chordata - ps18
Olfactores - ps19
Vertebrata - ps20
Gnathostomata - ps21
Euteleostomi - ps22
Sarcopterygii - ps23
Tetrapoda - ps24
Amniota - ps25
Mammalia - ps26
Theria - ps27
Placentalia - ps28
Boreoeutheria - ps29
Euarchontoglires - ps30
Euarchonta - ps31
Primates - ps32
Haplorrhini - ps33
Simiiformes - ps34
Catarrhini - ps35
Hominoidea - ps36
Hominidae - ps37
Homininae - ps38
Hominini - ps39
Homo_sapiens - ps40
p-value
1e-03
2e-03
3e-03
4e-03
6e-03
Log-odds
0.5
1.0
1.5
2.0
2.5
3.0
3.5
4.0

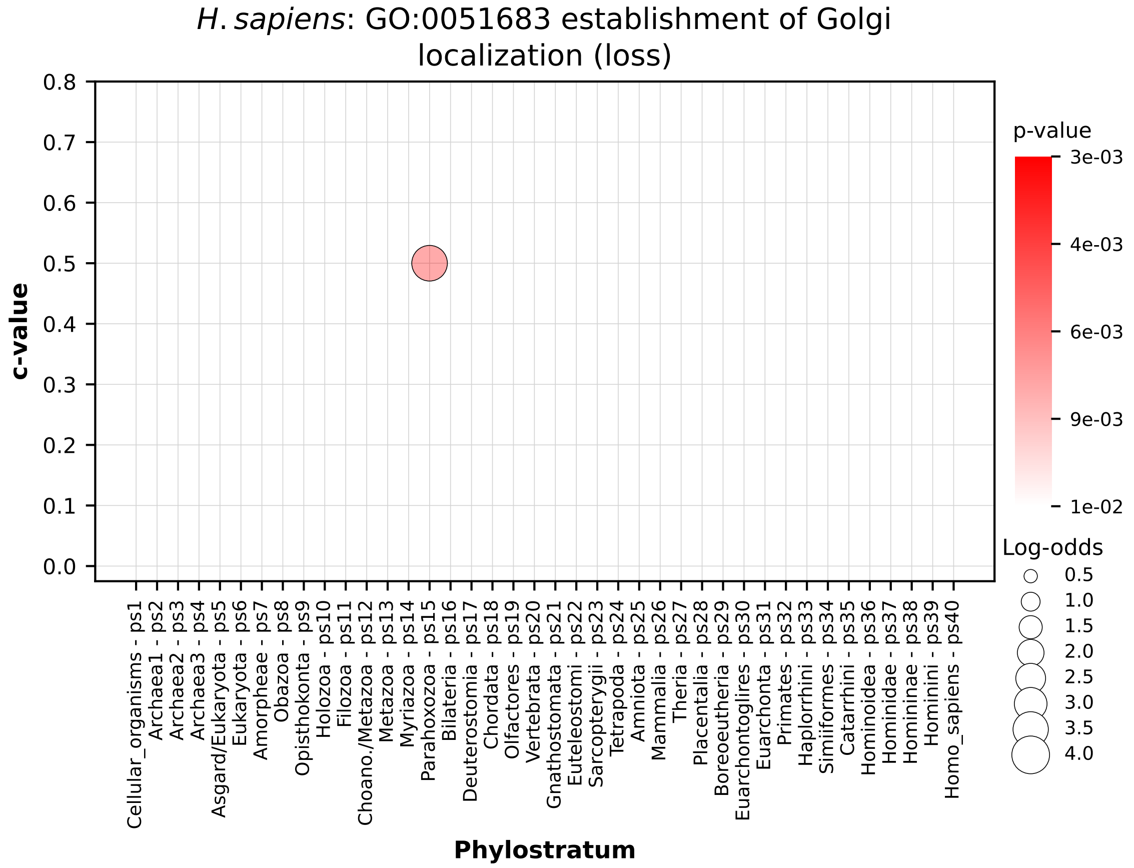

H. sapiens: GO:0051683 establishment of Golgi localization (loss)
c-value
0.8
0.7
0.6
0.5
0.4
0.3
0.2
0.1
0.0
p-value
3e-03
4e-03
6e-03
9e-03
1e-02
Log-odds
0.5
1.0
1.5
2.0
2.5
3.0
3.5
4.0
Cellular_organisms - ps1
Archaea1 - ps2
Archaea2 - ps3
Archaea3 - ps4
Asgard/Eukaryota - ps5
Eukaryota - ps6
Amorpheae - ps7
Obazoa - ps8
Opisthokonta - ps9
Holozoa - ps10
Filozoa - ps11
Choano./Metazoa - ps12
Metazoa - ps13
Myriazoa - ps14
Parahoxozoa - ps15
Bilateria - ps16
Deuterostomia - ps17
Chordata - ps18
Olfactores - ps19
Vertebrata - ps20
Gnathostomata - ps21
Euteleostomi - ps22
Sarcopterygii - ps23
Tetrapoda - ps24
Amniota - ps25
Mammalia - ps26
Theria - ps27
Placentalia - ps28
Boreoeutheria - ps29
Euarchontoglires - ps30
Euarchonta - ps31
Primates - ps32
Haplorrhini - ps33
Simiiformes - ps34
Catarrhini - ps35
Hominoidea - ps36
Hominidae - ps37
Homininae - ps38
Hominini - ps39
Homo_sapiens - ps40
Phylostratum

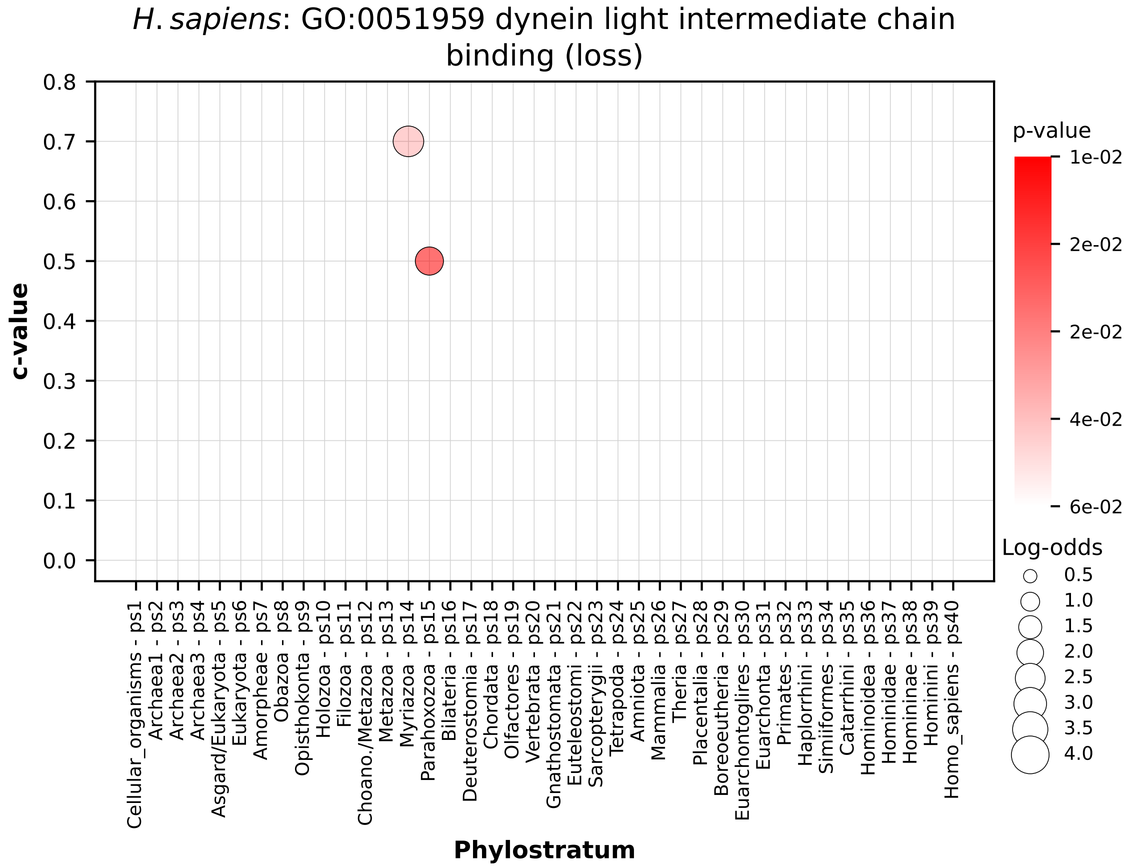

H. sapiens: GO:0051959 dynein light intermediate chain binding (loss)
c-value
0.8
0.7
0.6
0.5
0.4
0.3
0.2
0.1
0.0
Phylostratum
Cellular_organisms - ps1
Archaea1 - ps2
Archaea2 - ps3
Archaea3 - ps4
Asgard/Eukaryota - ps5
Eukaryota - ps6
Amorpheae - ps7
Obazoa - ps8
Opisthokonta - ps9
Holozoa - ps10
Filozoa - ps11
Choano./Metazoa - ps12
Metazoa - ps13
Myriazoa - ps14
Parahoxozoa - ps15
Bilateria - ps16
Deuterostomia - ps17
Chordata - ps18
Olfactores - ps19
Vertebrata - ps20
Gnathostomata - ps21
Euteleostomi - ps22
Sarcopterygii - ps23
Tetrapoda - ps24
Amniota - ps25
Mammalia - ps26
Theria - ps27
Placentalia - ps28
Boreoeutheria - ps29
Euarchontoglires - ps30
Euarchonta - ps31
Primates - ps32
Haplorrhini - ps33
Simiiformes - ps34
Catarrhini - ps35
Hominoidea - ps36
Hominidae - ps37
Homininae - ps38
Hominini - ps39
Homo_sapiens - ps40
p-value
1e-02
2e-02
2e-02
4e-02
6e-02
Log-odds
0.5
1.0
1.5
2.0
2.5
3.0
3.5
4.0

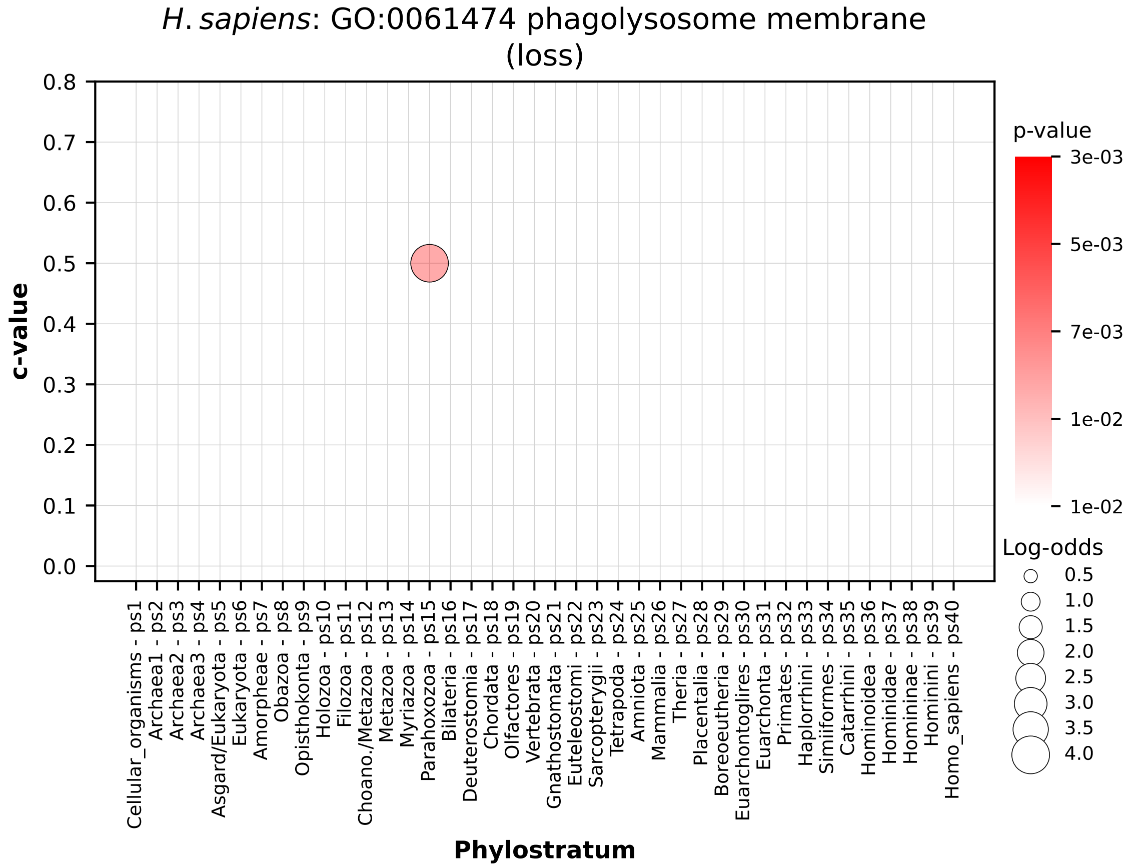

H. sapiens: GO:0061474 phagolysosome membrane
(loss)
c-value
0.8
0.7
0.6
0.5
0.4
0.3
0.2
0.1
0.0
p-value
3e-03
5e-03
7e-03
1e-02
1e-02
Log-odds
0.5
1.0
1.5
2.0
2.5
3.0
3.5
4.0
Cellular_organisms - ps1
Archaea1 - ps2
Archaea2 - ps3
Archaea3 - ps4
Asgard/Eukaryota - ps5
Eukaryota - ps6
Amorpheae - ps7
Obazoa - ps8
Opisthokonta - ps9
Holozoa - ps10
Filozoa - ps11
Choano./Metazoa - ps12
Metazoa - ps13
Myriazoa - ps14
Parahoxozoa - ps15
Bilateria - ps16
Deuterostomia - ps17
Chordata - ps18
Olfactores - ps19
Vertebrata - ps20
Gnathostomata - ps21
Euteleostomi - ps22
Sarcopterygii - ps23
Tetrapoda - ps24
Amniota - ps25
Mammalia - ps26
Theria - ps27
Placentalia - ps28
Boreoeutheria - ps29
Euarchontoglires - ps30
Euarchonta - ps31
Primates - ps32
Haplorrhini - ps33
Simiiformes - ps34
Catarrhini - ps35
Hominoidea - ps36
Hominidae - ps37
Homininae - ps38
Hominini - ps39
Homo_sapiens - ps40
Phylostratum

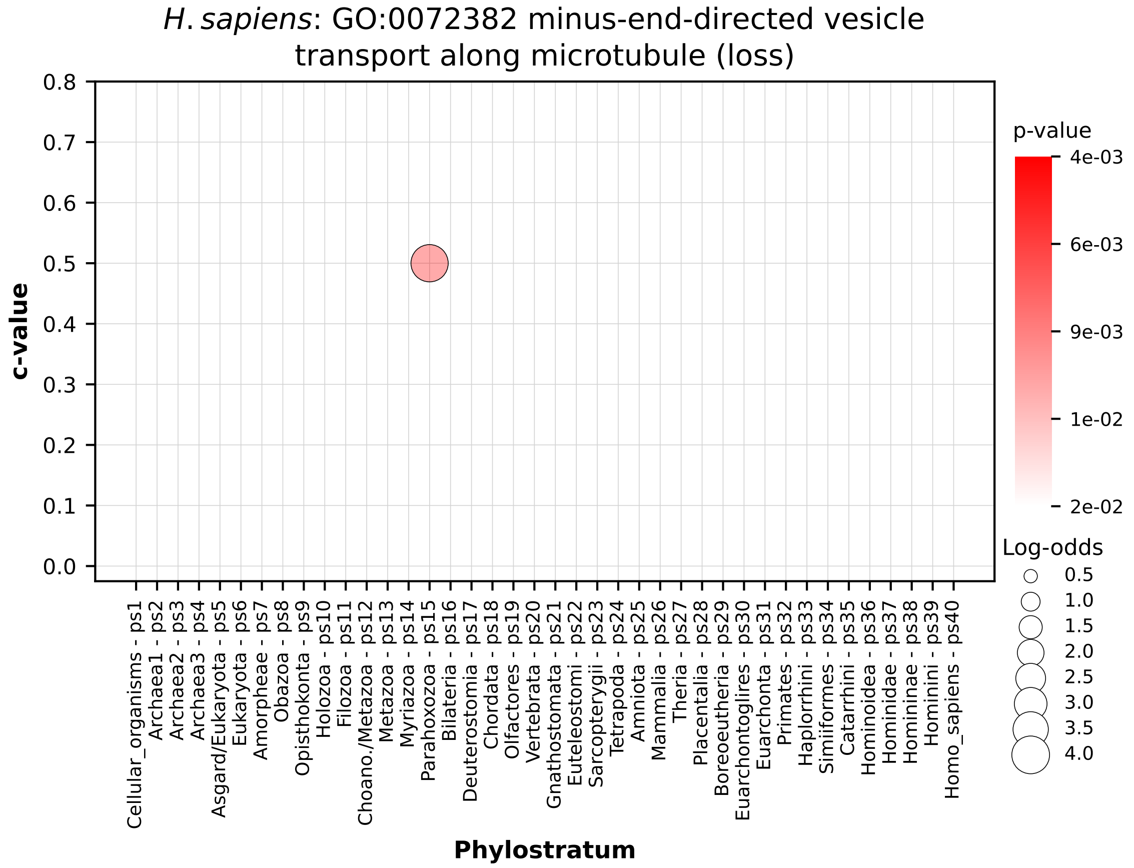

H. sapiens: GO:0072382 minus-end-directed vesicle transport along microtubule (loss)
c-value
0.8
0.7
0.6
0.5
0.4
0.3
0.2
0.1
0.0
p-value
4e-03
6e-03
9e-03
1e-02
2e-02
Log-odds
0.5
1.0
1.5
2.0
2.5
3.0
3.5
4.0
Cellular_organisms - ps1
Archaea1 - ps2
Archaea2 - ps3
Archaea3 - ps4
Asgard/Eukaryota - ps5
Eukaryota - ps6
Amorpheae - ps7
Obazoa - ps8
Opisthokonta - ps9
Holozoa - ps10
Filozoa - ps11
Choano./Metazoa - ps12
Metazoa - ps13
Myriazoa - ps14
Parahoxozoa - ps15
Bilateria - ps16
Deuterostomia - ps17
Chordata - ps18
Olfactores - ps19
Vertebrata - ps20
Gnathostomata - ps21
Euteleostomi - ps22
Sarcopterygii - ps23
Tetrapoda - ps24
Amniota - ps25
Mammalia - ps26
Theria - ps27
Placentalia - ps28
Boreoeutheria - ps29
Euarchontoglires - ps30
Euarchonta - ps31
Primates - ps32
Haplorrhini - ps33
Simiiformes - ps34
Catarrhini - ps35
Hominoidea - ps36
Hominidae - ps37
Homininae - ps38
Hominini - ps39
Homo_sapiens - ps40
Phylostratum

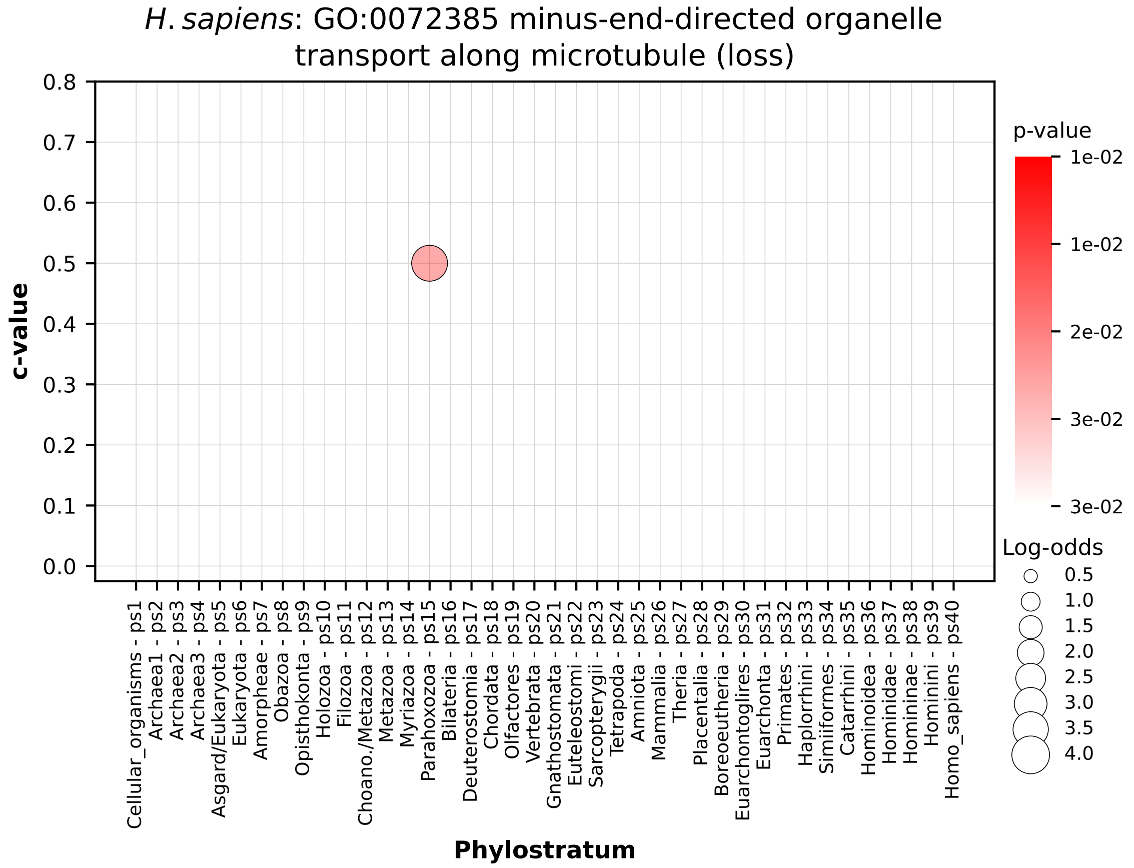

H. sapiens: GO:0072385 minus-end-directed organelle transport along microtubule (loss)
c-value
0.0
0.1
0.2
0.3
0.4
0.5
0.6
0.7
0.8
Phylostratum
Cellular_organisms - ps1
Archaea1 - ps2
Archaea2 - ps3
Archaea3 - ps4
Asgard/Eukaryota - ps5
Eukaryota - ps6
Amorpheae - ps7
Obazoa - ps8
Opisthokonta - ps9
Holozoa - ps10
Filozoa - ps11
Choano./Metazoa - ps12
Metazoa - ps13
Myriazoa - ps14
Parahoxozoa - ps15
Bilateria - ps16
Deuterostomia - ps17
Chordata - ps18
Olfactores - ps19
Vertebrata - ps20
Gnathostomata - ps21
Euteleostomi - ps22
Sarcopterygii - ps23
Tetrapoda - ps24
Amniota - ps25
Mammalia - ps26
Theria - ps27
Placentalia - ps28
Boreoeutheria - ps29
Euarchontoglires - ps30
Euarchonta - ps31
Primates - ps32
Haplorrhini - ps33
Simiiformes - ps34
Catarrhini - ps35
Hominoidea - ps36
Hominidae - ps37
Homininae - ps38
Hominini - ps39
Homo_sapiens - ps40
p-value
1e-02
1e-02
2e-02
3e-02
3e-02
Log-odds
0.5
1.0
1.5
2.0
2.5
3.0
3.5
4.0

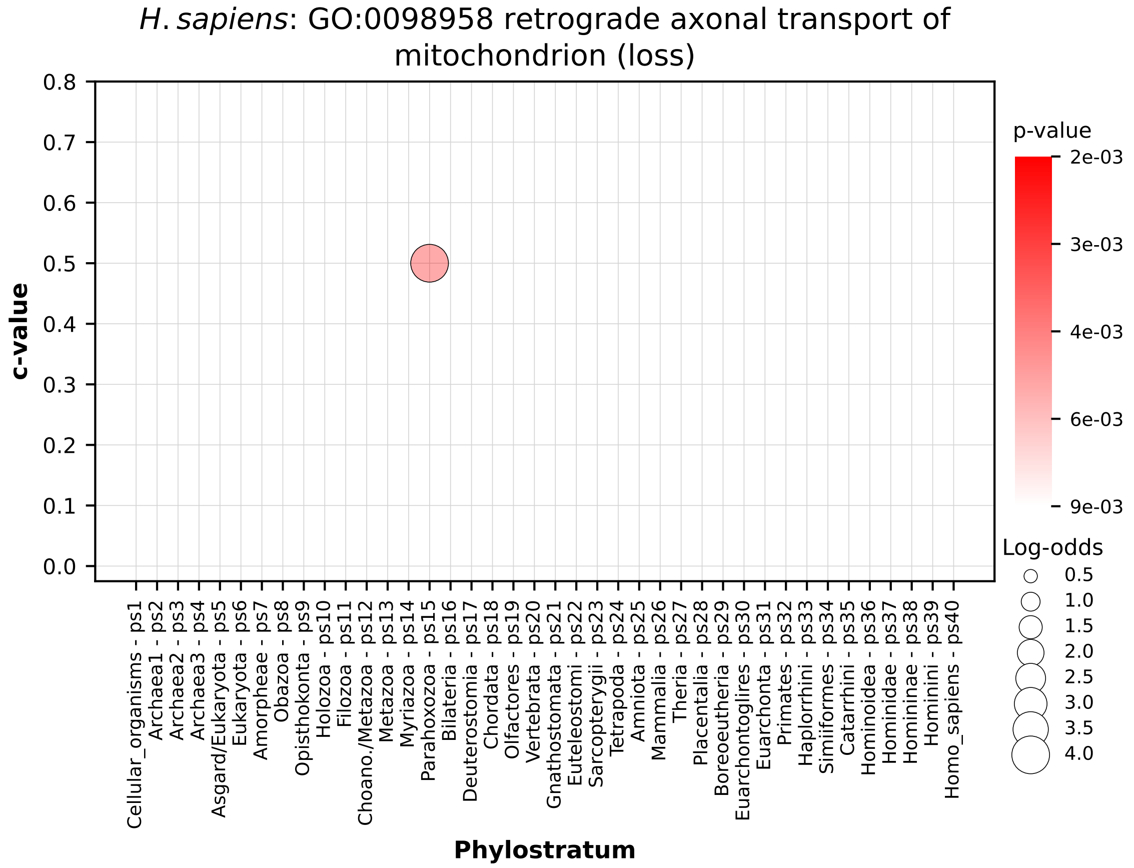
H. sapiens: GO:0098958 retrograde axonal transport of mitochondrion (loss)
c-value
0.8
0.7
0.6
0.5
0.4
0.3
0.2
0.1
0.0
p-value
2e-03
3e-03
4e-03
6e-03
9e-03
Log-odds
0.5
1.0
1.5
2.0
2.5
3.0
3.5
4.0
Cellular_organisms - ps1
Archaea1 - ps2
Archaea2 - ps3
Archaea3 - ps4
Asgard/Eukaryota - ps5
Eukaryota - ps6
Amorpheae - ps7
Obazoa - ps8
Opisthokonta - ps9
Holozoa - ps10
Filozoa - ps11
Choano./Metazoa - ps12
Metazoa - ps13
Myriazoa - ps14
Parahoxozoa - ps15
Bilateria - ps16
Deuterostomia - ps17
Chordata - ps18
Olfactores - ps19
Vertebrata - ps20
Gnathostomata - ps21
Euteleostomi - ps22
Sarcopterygii - ps23
Tetrapoda - ps24
Amniota - ps25
Mammalia - ps26
Theria - ps27
Placentalia - ps28
Boreoeutheria - ps29
Euarchontoglires - ps30
Euarchonta - ps31
Primates - ps32
Haplorrhini - ps33
Simiiformes - ps34
Catarrhini - ps35
Hominoidea - ps36
Hominidae - ps37
Homininae - ps38
Hominini - ps39
Homo_sapiens - ps40
Phylostratum

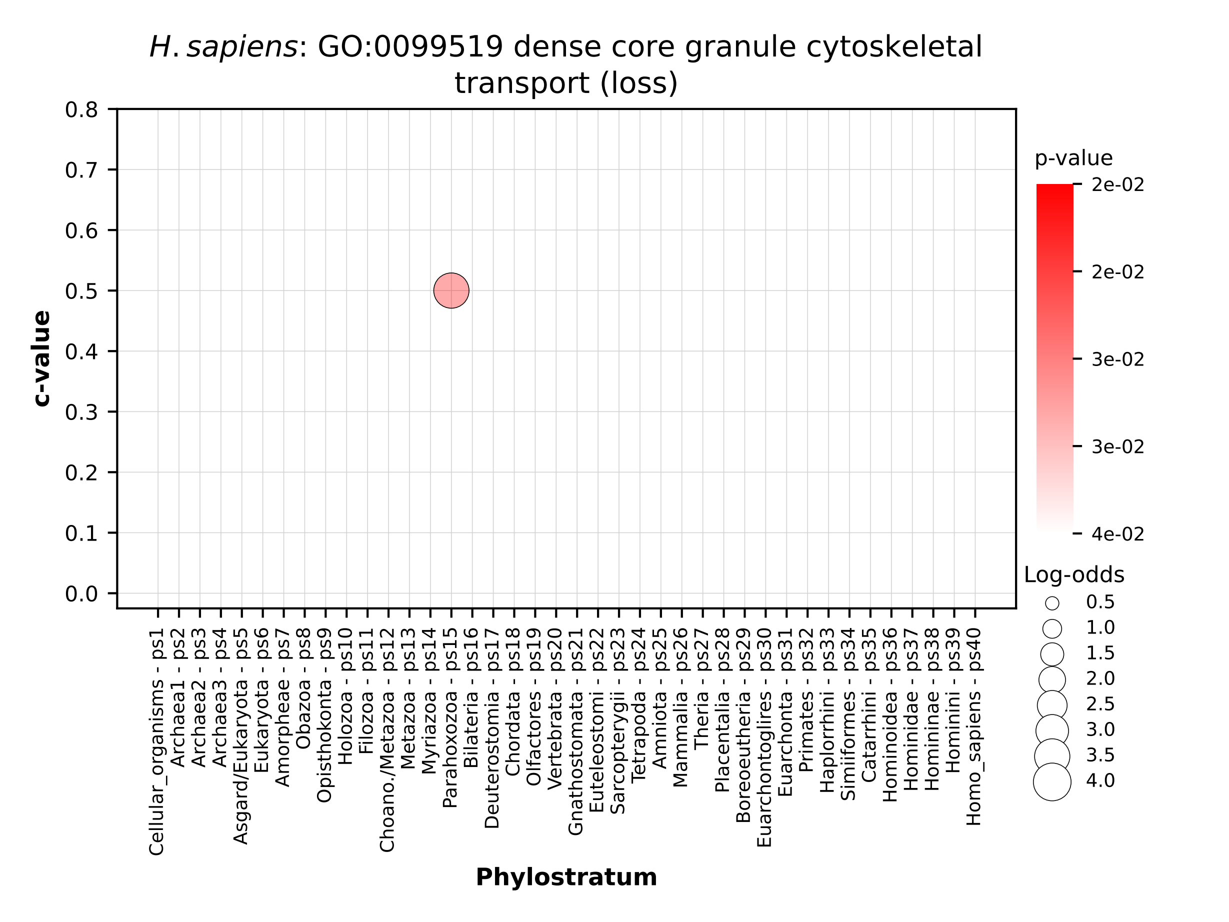

H. sapiens: GO:0099519 dense core granule cytoskeletal transport (loss)
c-value
0.0
0.1
0.2
0.3
0.4
0.5
0.6
0.7
0.8
Phylostratum
Cellular_organisms - ps1
Archaea1 - ps2
Archaea2 - ps3
Archaea3 - ps4
Asgard/Eukaryota - ps5
Eukaryota - ps6
Amorpheae - ps7
Obazoa - ps8
Opisthokonta - ps9
Holozoa - ps10
Filozoa - ps11
Choano./Metazoa - ps12
Metazoa - ps13
Myriazoa - ps14
Parahoxozoa - ps15
Bilateria - ps16
Deuterostomia - ps17
Chordata - ps18
Olfactores - ps19
Vertebrata - ps20
Gnathostomata - ps21
Euteleostomi - ps22
Sarcopterygii - ps23
Tetrapoda - ps24
Amniota - ps25
Mammalia - ps26
Theria - ps27
Placentalia - ps28
Boreoeutheria - ps29
Euarchontoglires - ps30
Euarchonta - ps31
Primates - ps32
Haplorrhini - ps33
Simiiformes - ps34
Catarrhini - ps35
Hominoidea - ps36
Hominidae - ps37
Homininae - ps38
Hominini - ps39
Homo_sapiens - ps40
p-value
2e-02
2e-02
3e-02
3e-02
4e-02
Log-odds
0.5
1.0
1.5
2.0
2.5
3.0
3.5
4.0

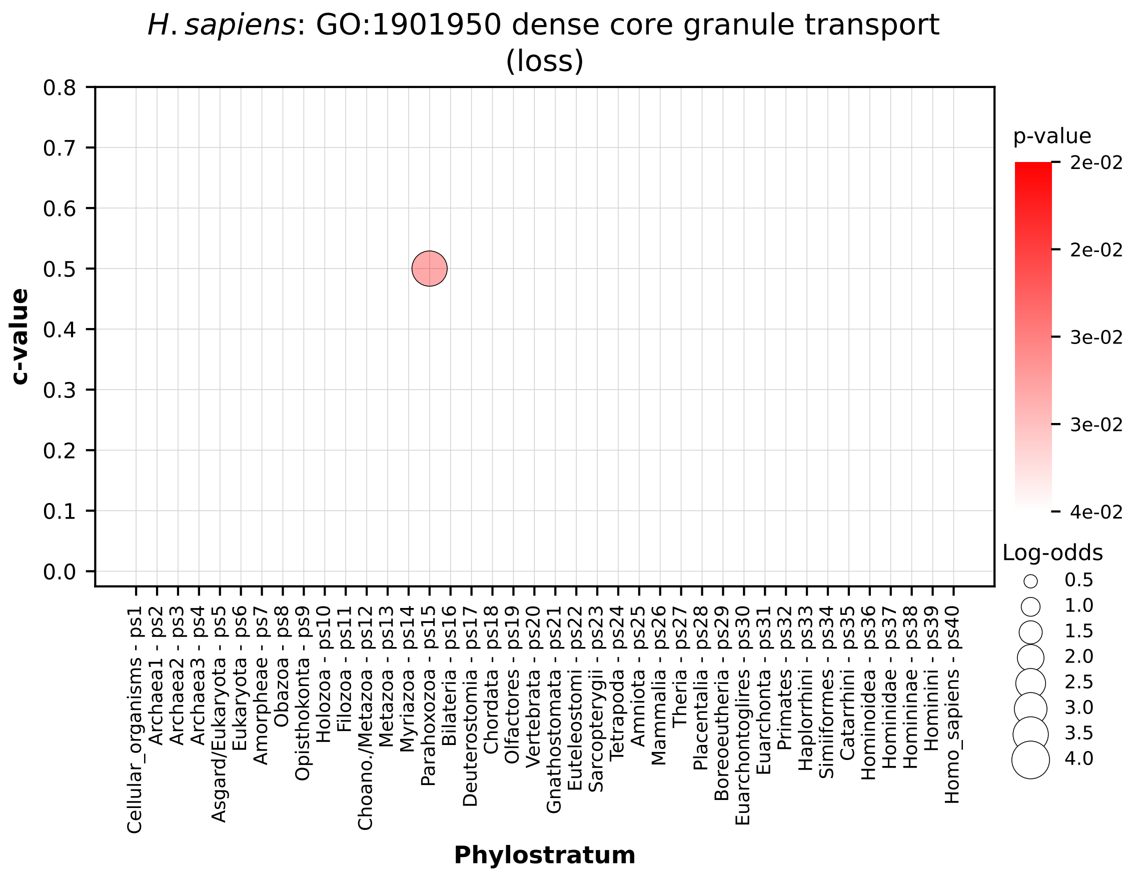

H. sapiens: GO:1901950 dense core granule transport
(loss)
c-value
0.8
0.7
0.6
0.5
0.4
0.3
0.2
0.1
0.0
p-value
2e-02
2e-02
3e-02
3e-02
4e-02
Log-odds
0.5
1.0
1.5
2.0
2.5
3.0
3.5
4.0
Cellular_organisms - ps1
Archaea1 - ps2
Archaea2 - ps3
Archaea3 - ps4
Asgard/Eukaryota - ps5
Eukaryota - ps6
Amorpheae - ps7
Obazoa - ps8
Opisthokonta - ps9
Holozoa - ps10
Filozoa - ps11
Choano./Metazoa - ps12
Metazoa - ps13
Myriazoa - ps14
Parahoxozoa - ps15
Bilateria - ps16
Deuterostomia - ps17
Chordata - ps18
Olfactores - ps19
Vertebrata - ps20
Gnathostomata - ps21
Euteleostomi - ps22
Sarcopterygii - ps23
Tetrapoda - ps24
Amniota - ps25
Mammalia - ps26
Theria - ps27
Placentalia - ps28
Boreoeutheria - ps29
Euarchontoglires - ps30
Euarchonta - ps31
Primates - ps32
Haplorrhini - ps33
Simiiformes - ps34
Catarrhini - ps35
Hominoidea - ps36
Hominidae - ps37
Homininae - ps38
Hominini - ps39
Homo_sapiens - ps40
Phylostratum

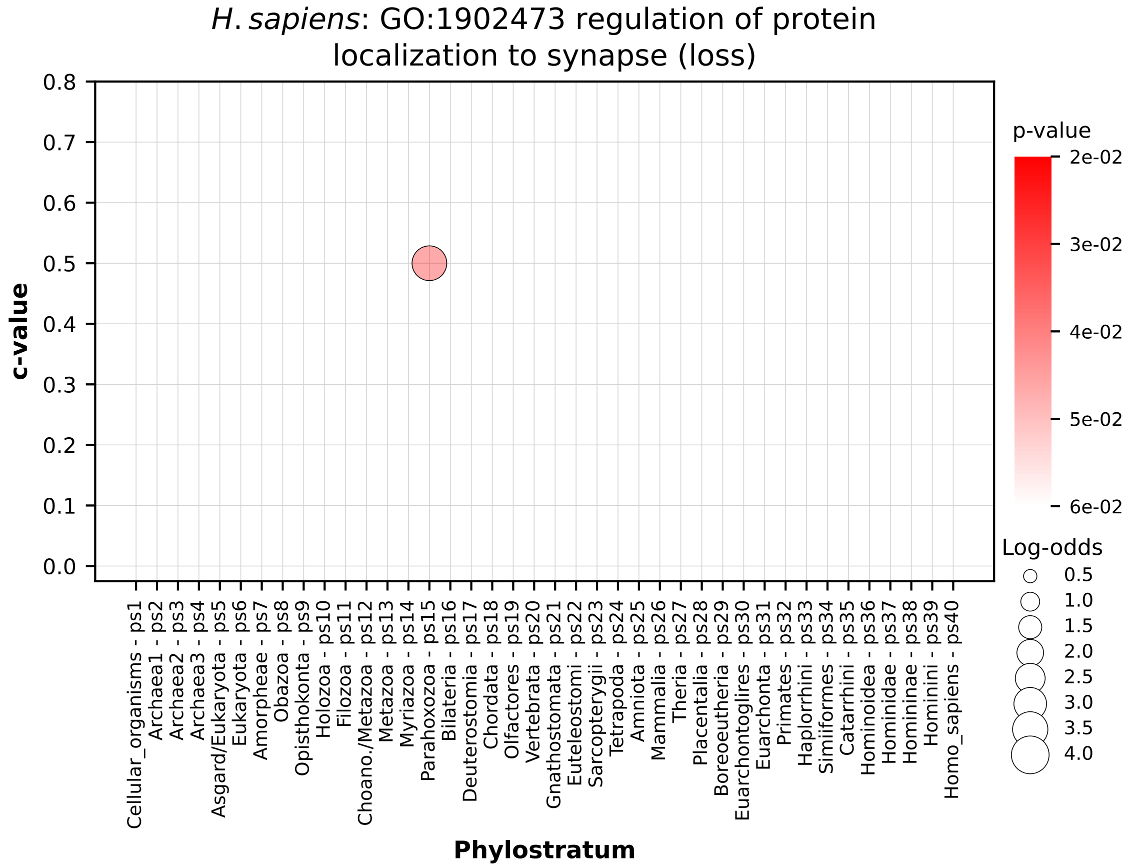

H. sapiens: GO:1902473 regulation of protein localization to synapse (loss)
c-value
0.8
0.7
0.6
0.5
0.4
0.3
0.2
0.1
0.0
Phylostratum
Cellular_organisms - ps1
Archaea1 - ps2
Archaea2 - ps3
Archaea3 - ps4
Asgard/Eukaryota - ps5
Eukaryota - ps6
Amorpheae - ps7
Obazoa - ps8
Opisthokonta - ps9
Holozoa - ps10
Filozoa - ps11
Choano./Metazoa - ps12
Metazoa - ps13
Myriazoa - ps14
Parahoxozoa - ps15
Bilateria - ps16
Deuterostomia - ps17
Chordata - ps18
Olfactores - ps19
Vertebrata - ps20
Gnathostomata - ps21
Euteleostomi - ps22
Sarcopterygii - ps23
Tetrapoda - ps24
Amniota - ps25
Mammalia - ps26
Theria - ps27
Placentalia - ps28
Boreoeutheria - ps29
Euarchontoglires - ps30
Euarchonta - ps31
Primates - ps32
Haplorrhini - ps33
Simiiformes - ps34
Catarrhini - ps35
Hominoidea - ps36
Hominidae - ps37
Homininae - ps38
Hominini - ps39
Homo_sapiens - ps40
p-value
2e-02
3e-02
4e-02
5e-02
6e-02
Log-odds
0.5
1.0
1.5
2.0
2.5
3.0
3.5
4.0

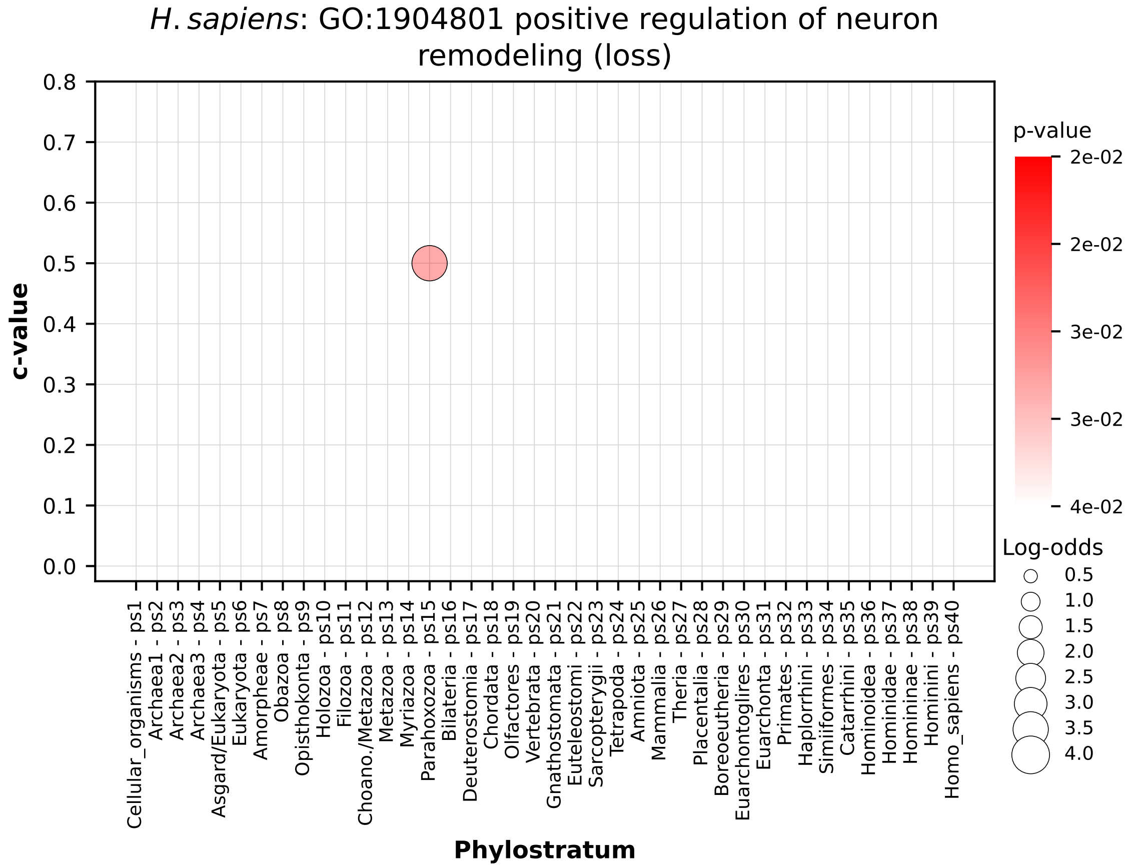
H. sapiens: GO:1904801 positive regulation of neuron remodeling (loss)
c-value
Phylostratum
p-value
2e-02
2e-02
3e-02
3e-02
4e-02
Log-odds
0.5
1.0
1.5
2.0
2.5
3.0
3.5
4.0
0.0
0.1
0.2
0.3
0.4
0.5
0.6
0.7
0.8
Cellular_organisms - ps1
Archaea1 - ps2
Archaea2 - ps3
Archaea3 - ps4
Asgard/Eukaryota - ps5
Eukaryota - ps6
Amorpheae - ps7
Obazoa - ps8
Opisthokonta - ps9
Holozoa - ps10
Filozoa - ps11
Choano./Metazoa - ps12
Metazoa - ps13
Myriazoa - ps14
Parahoxozoa - ps15
Bilateria - ps16
Deuterostomia - ps17
Chordata - ps18
Olfactores - ps19
Vertebrata - ps20
Gnathostomata - ps21
Euteleostomi - ps22
Sarcopterygii - ps23
Tetrapoda - ps24
Amniota - ps25
Mammalia - ps26
Theria - ps27
Placentalia - ps28
Boreoeutheria - ps29
Euarchontoglires - ps30
Euarchonta - ps31
Primates - ps32
Haplorrhini - ps33
Simiiformes - ps34
Catarrhini - ps35
Hominoidea - ps36
Hominidae - ps37
Homininae - ps38
Hominini - ps39
Homo_sapiens - ps40

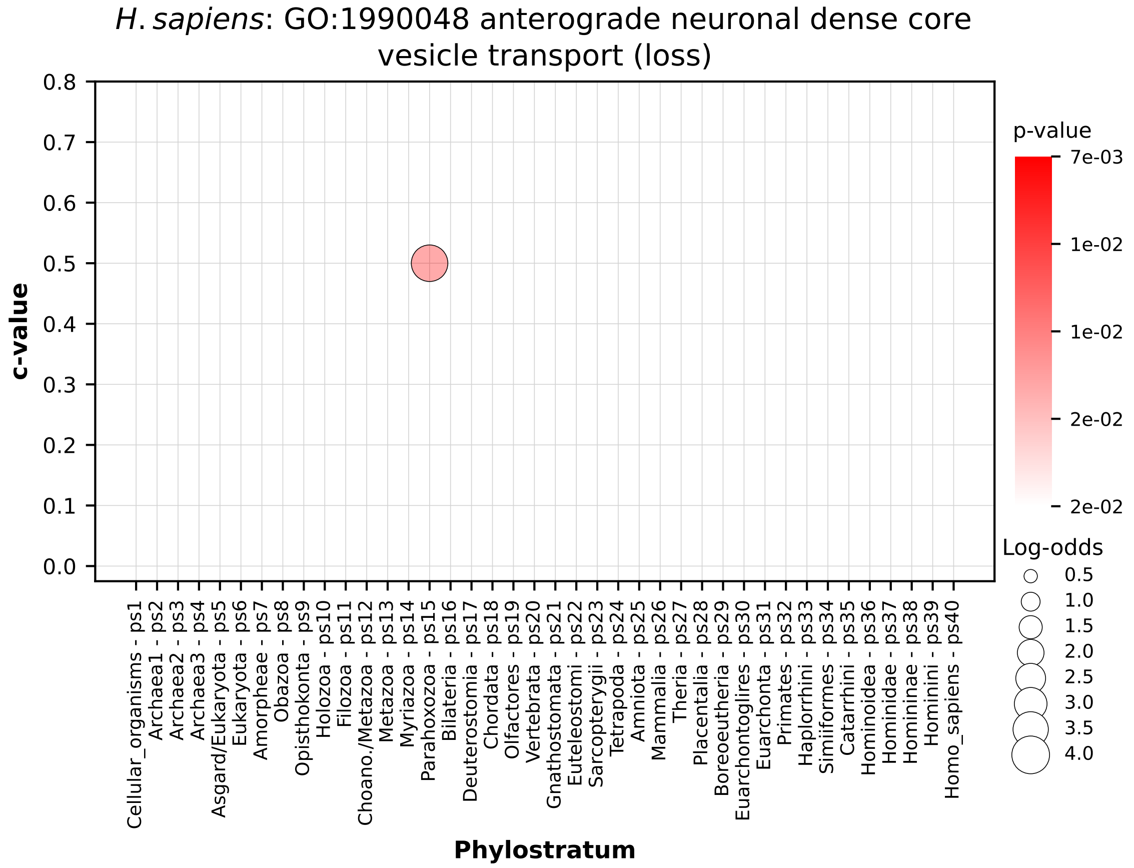

H. sapiens: GO:1990048 anterograde neuronal dense core vesicle transport (loss)
c-value
0.8
0.7
0.6
0.5
0.4
0.3
0.2
0.1
0.0
p-value
7e-03
1e-02
1e-02
2e-02
2e-02
Log-odds
0.5
1.0
1.5
2.0
2.5
3.0
3.5
4.0
Cellular_organisms - ps1
Archaea1 - ps2
Archaea2 - ps3
Archaea3 - ps4
Asgard/Eukaryota - ps5
Eukaryota - ps6
Amorpheae - ps7
Obazoa - ps8
Opisthokonta - ps9
Holozoa - ps10
Filozoa - ps11
Choano./Metazoa - ps12
Metazoa - ps13
Myriazoa - ps14
Parahoxozoa - ps15
Bilateria - ps16
Deuterostomia - ps17
Chordata - ps18
Olfactores - ps19
Vertebrata - ps20
Gnathostomata - ps21
Euteleostomi - ps22
Sarcopterygii - ps23
Tetrapoda - ps24
Amniota - ps25
Mammalia - ps26
Theria - ps27
Placentalia - ps28
Boreoeutheria - ps29
Euarchontoglires - ps30
Euarchonta - ps31
Primates - ps32
Haplorrhini - ps33
Simiiformes - ps34
Catarrhini - ps35
Hominoidea - ps36
Hominidae - ps37
Homininae - ps38
Hominini - ps39
Homo_sapiens - ps40
Phylostratum

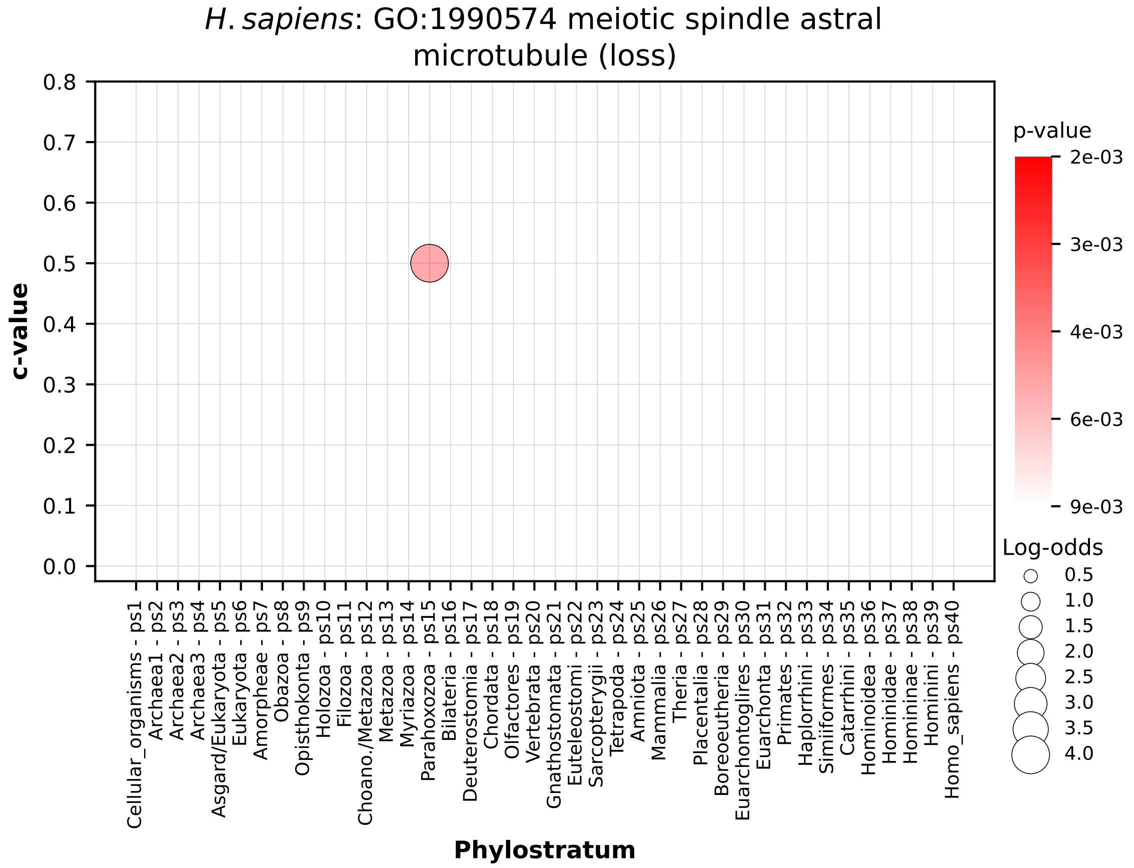

H. sapiens: GO:1990574 meiotic spindle astral microtubule (loss)
c-value
0.0
0.1
0.2
0.3
0.4
0.5
0.6
0.7
0.8
Phylostratum
Cellular_organisms - ps1
Archaea1 - ps2
Archaea2 - ps3
Archaea3 - ps4
Asgard/Eukaryota - ps5
Eukaryota - ps6
Amorpheae - ps7
Obazoa - ps8
Opisthokonta - ps9
Holozoa - ps10
Filozoa - ps11
Choano./Metazoa - ps12
Metazoa - ps13
Myriazoa - ps14
Parahoxozoa - ps15
Bilateria - ps16
Deuterostomia - ps17
Chordata - ps18
Olfactores - ps19
Vertebrata - ps20
Gnathostomata - ps21
Euteleostomi - ps22
Sarcopterygii - ps23
Tetrapoda - ps24
Amniota - ps25
Mammalia - ps26
Theria - ps27
Placentalia - ps28
Boreoeutheria - ps29
Euarchontoglires - ps30
Euarchonta - ps31
Primates - ps32
Haplorrhini - ps33
Simiiformes - ps34
Catarrhini - ps35
Hominoidea - ps36
Hominidae - ps37
Homininae - ps38
Hominini - ps39
Homo_sapiens - ps40
p-value
2e-03
3e-03
4e-03
6e-03
9e-03
Log-odds
0.5
1.0
1.5
2.0
2.5
3.0
3.5
4.0

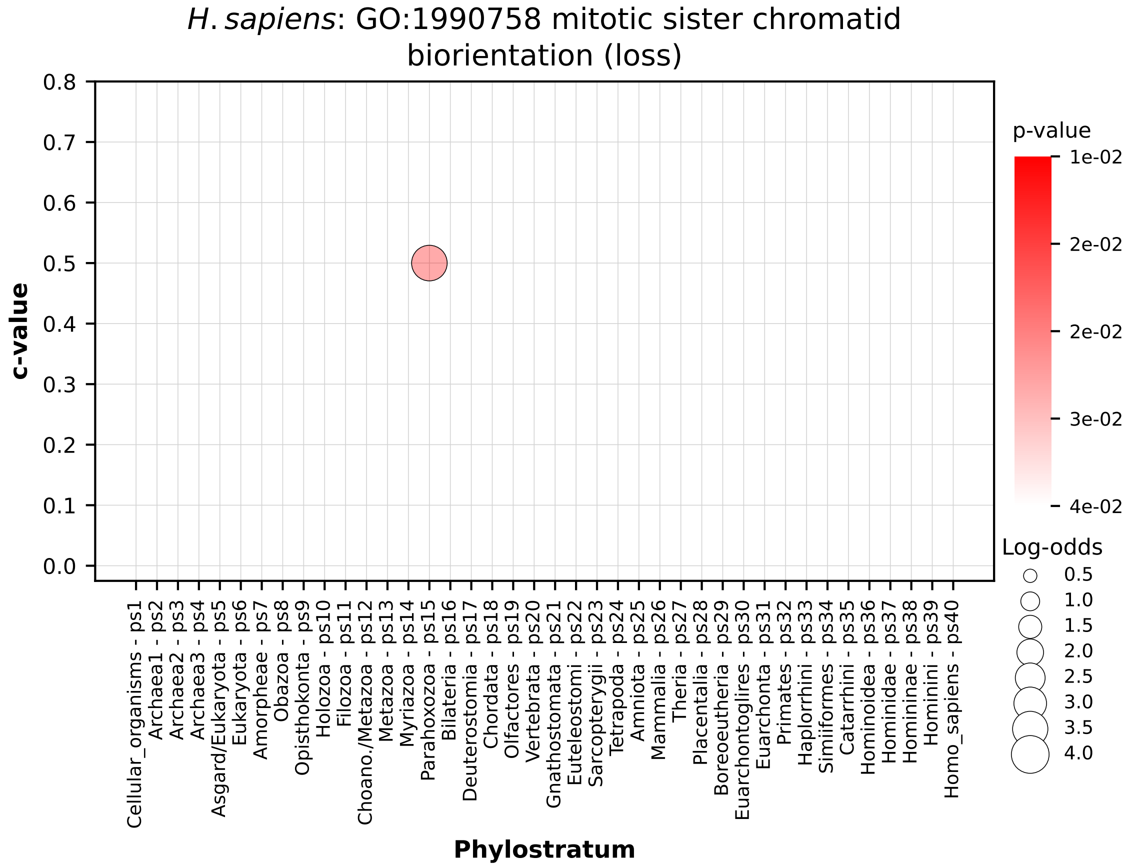

H. sapiens: GO:1990758 mitotic sister chromatid biorientation (loss)
c-value
0.0
0.1
0.2
0.3
0.4
0.5
0.6
0.7
0.8
Phylostratum
Cellular_organisms - ps1
Archaea1 - ps2
Archaea2 - ps3
Archaea3 - ps4
Asgard/Eukaryota - ps5
Eukaryota - ps6
Amorpheae - ps7
Obazoa - ps8
Opisthokonta - ps9
Holozoa - ps10
Filozoa - ps11
Choano./Metazoa - ps12
Metazoa - ps13
Myriazoa - ps14
Parahoxozoa - ps15
Bilateria - ps16
Deuterostomia - ps17
Chordata - ps18
Olfactores - ps19
Vertebrata - ps20
Gnathostomata - ps21
Euteleostomi - ps22
Sarcopterygii - ps23
Tetrapoda - ps24
Amniota - ps25
Mammalia - ps26
Theria - ps27
Placentalia - ps28
Boreoeutheria - ps29
Euarchontoglires - ps30
Euarchonta - ps31
Primates - ps32
Haplorrhini - ps33
Simiiformes - ps34
Catarrhini - ps35
Hominoidea - ps36
Hominidae - ps37
Homininae - ps38
Hominini - ps39
Homo_sapiens - ps40
p-value
1e-02
2e-02
2e-02
3e-02
4e-02
Log-odds
0.5
1.0
1.5
2.0
2.5
3.0
3.5
4.0

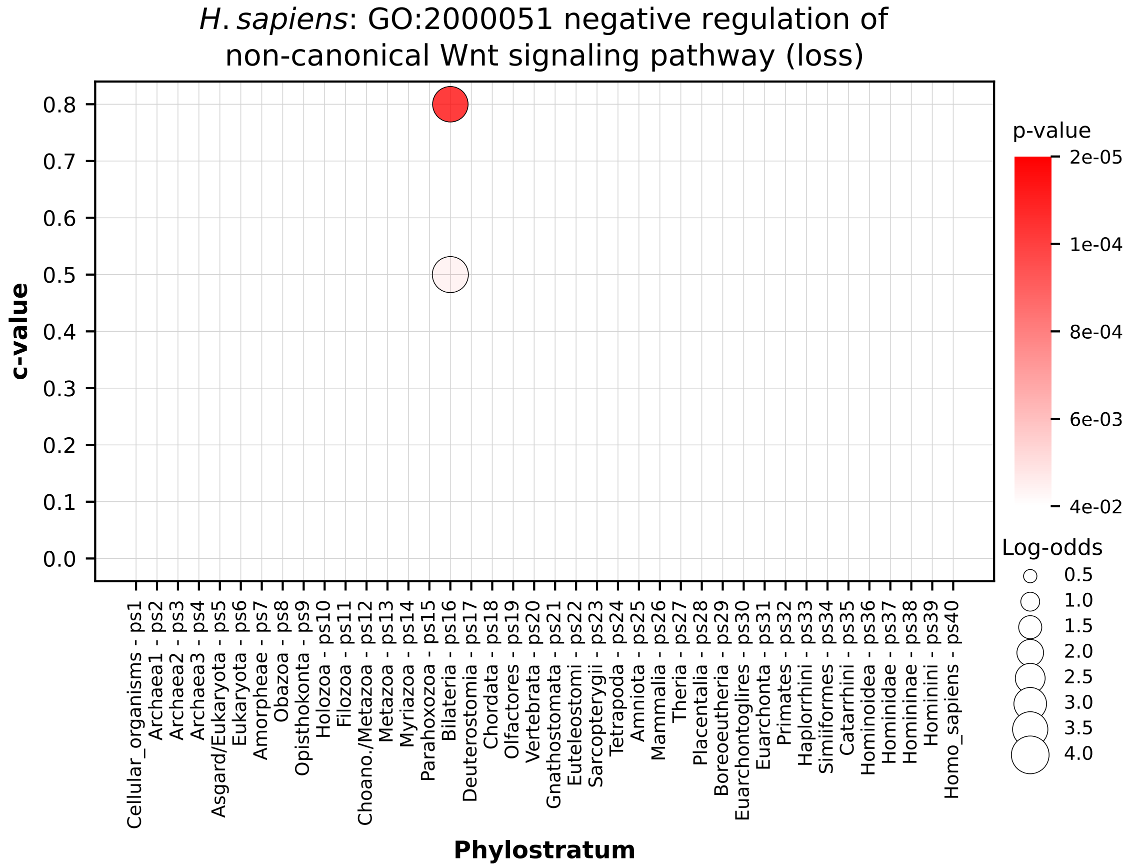
H. sapiens: GO:2000051 negative regulation of non-canonical Wnt signaling pathway (loss)
c-value
0.0
0.1
0.2
0.3
0.4
0.5
0.6
0.7
0.8
Phylostratum
Cellular_organisms - ps1
Archaea1 - ps2
Archaea2 - ps3
Archaea3 - ps4
Asgard/Eukaryota - ps5
Eukaryota - ps6
Amorpheae - ps7
Obazoa - ps8
Opisthokonta - ps9
Holozoa - ps10
Filozoa - ps11
Choano./Metazoa - ps12
Metazoa - ps13
Myriazoa - ps14
Parahoxozoa - ps15
Bilateria - ps16
Deuterostomia - ps17
Chordata - ps18
Olfactores - ps19
Vertebrata - ps20
Gnathostomata - ps21
Euteleostomi - ps22
Sarcopterygii - ps23
Tetrapoda - ps24
Amniota - ps25
Mammalia - ps26
Theria - ps27
Placentalia - ps28
Boreoeutheria - ps29
Euarchontoglires - ps30
Euarchonta - ps31
Primates - ps32
Haplorrhini - ps33
Simiiformes - ps34
Catarrhini - ps35
Hominoidea - ps36
Hominidae - ps37
Homininae - ps38
Hominini - ps39
Homo_sapiens - ps40
p-value
2e-05
1e-04
8e-04
6e-03
4e-02
Log-odds
0.5
1.0
1.5
2.0
2.5
3.0
3.5
4.0

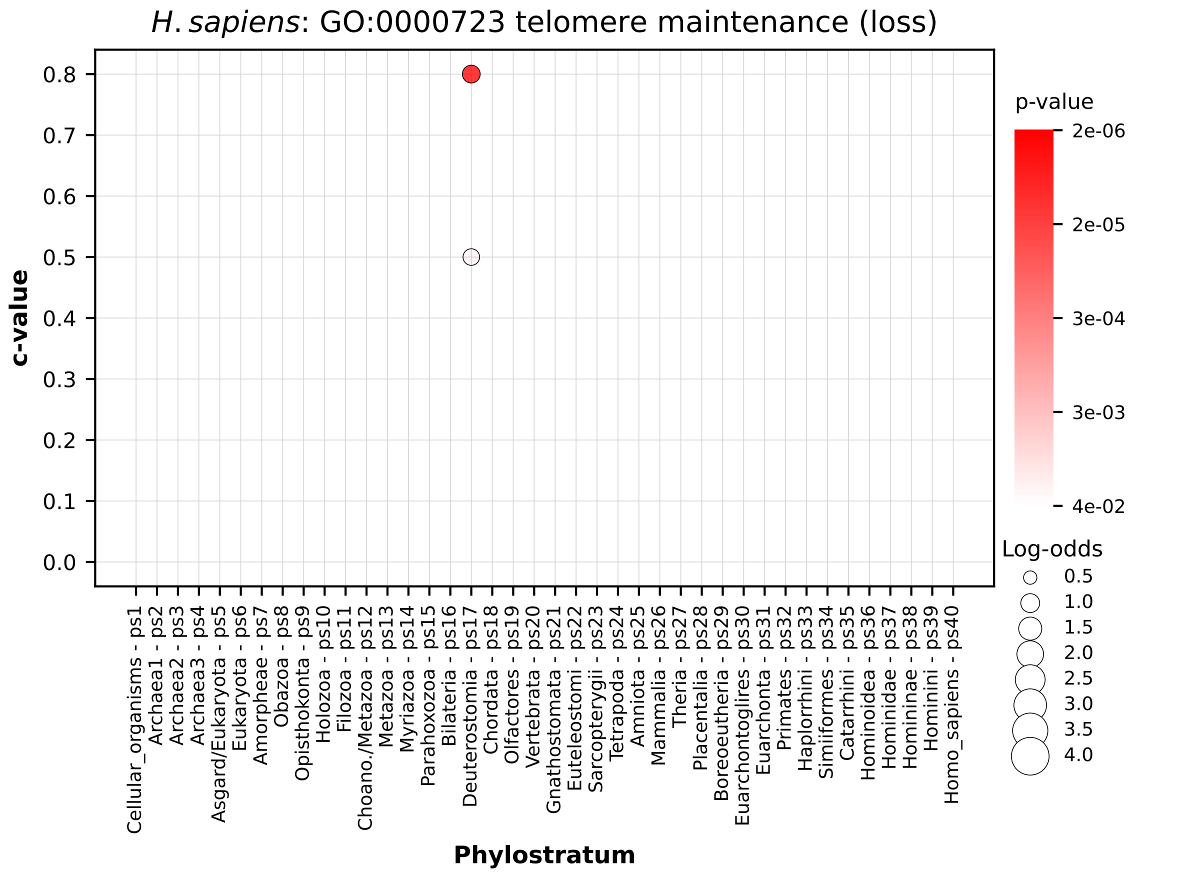

H. sapiens: GO:0000723 telomere maintenance (loss)
c-value
0.0
0.1
0.2
0.3
0.4
0.5
0.6
0.7
0.8
Phylostratum
Cellular_organisms - ps1
Archaea1 - ps2
Archaea2 - ps3
Archaea3 - ps4
Asgard/Eukaryota - ps5
Eukaryota - ps6
Amorpheae - ps7
Obazoa - ps8
Opisthokonta - ps9
Holozoa - ps10
Filozoa - ps11
Choano./Metazoa - ps12
Metazoa - ps13
Myriazoa - ps14
Parahoxozoa - ps15
Bilateria - ps16
Deuterostomia - ps17
Chordata - ps18
Olfactores - ps19
Vertebrata - ps20
Gnathostomata - ps21
Euteleostomi - ps22
Sarcopterygii - ps23
Tetrapoda - ps24
Amniota - ps25
Mammalia - ps26
Theria - ps27
Placentalia - ps28
Boreoeutheria - ps29
Euarchontoglires - ps30
Euarchonta - ps31
Primates - ps32
Haplorrhini - ps33
Simiiformes - ps34
Catarrhini - ps35
Hominoidea - ps36
Hominidae - ps37
Homininae - ps38
Hominini - ps39
Homo_sapiens - ps40
p-value
2e-06
2e-05
3e-04
3e-03
4e-02
Log-odds
0.5
1.0
1.5
2.0
2.5
3.0
3.5
4.0

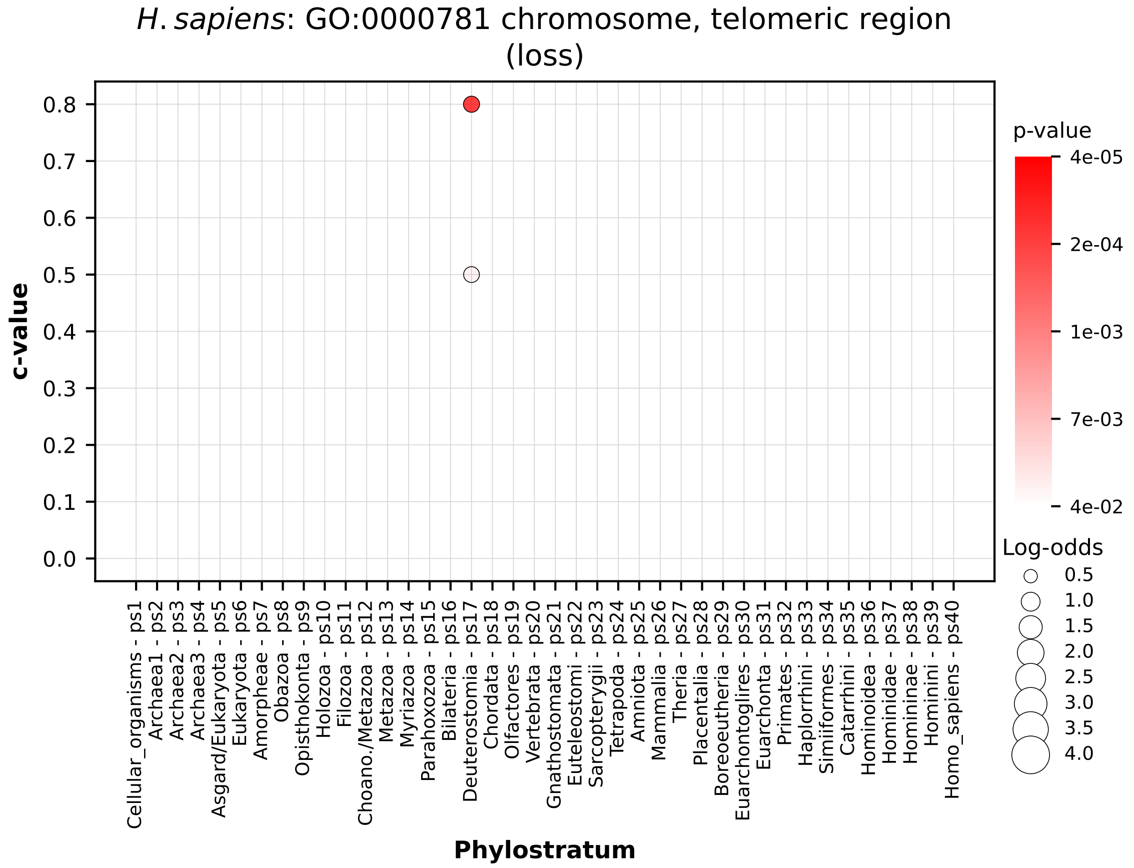

H. sapiens: GO:0000781 chromosome, telomeric region
(loss)
c-value
0.0
0.1
0.2
0.3
0.4
0.5
0.6
0.7
0.8
p-value
4e-05
2e-04
1e-03
7e-03
4e-02
Log-odds
0.5
1.0
1.5
2.0
2.5
3.0
3.5
4.0
Cellular_organisms - ps1
Archaea1 - ps2
Archaea2 - ps3
Archaea3 - ps4
Asgard/Eukaryota - ps5
Eukaryota - ps6
Amorpheae - ps7
Obazoa - ps8
Opisthokonta - ps9
Holozoa - ps10
Filozoa - ps11
Choano./Metazoa - ps12
Metazoa - ps13
Myriazoa - ps14
Parahoxozoa - ps15
Bilateria - ps16
Deuterostomia - ps17
Chordata - ps18
Olfactores - ps19
Vertebrata - ps20
Gnathostomata - ps21
Euteleostomi - ps22
Sarcopterygii - ps23
Tetrapoda - ps24
Amniota - ps25
Mammalia - ps26
Theria - ps27
Placentalia - ps28
Boreoeutheria - ps29
Euarchontoglires - ps30
Euarchonta - ps31
Primates - ps32
Haplorrhini - ps33
Simiiformes - ps34
Catarrhini - ps35
Hominoidea - ps36
Hominidae - ps37
Homininae - ps38
Hominini - ps39
Homo_sapiens - ps40
Phylostratum

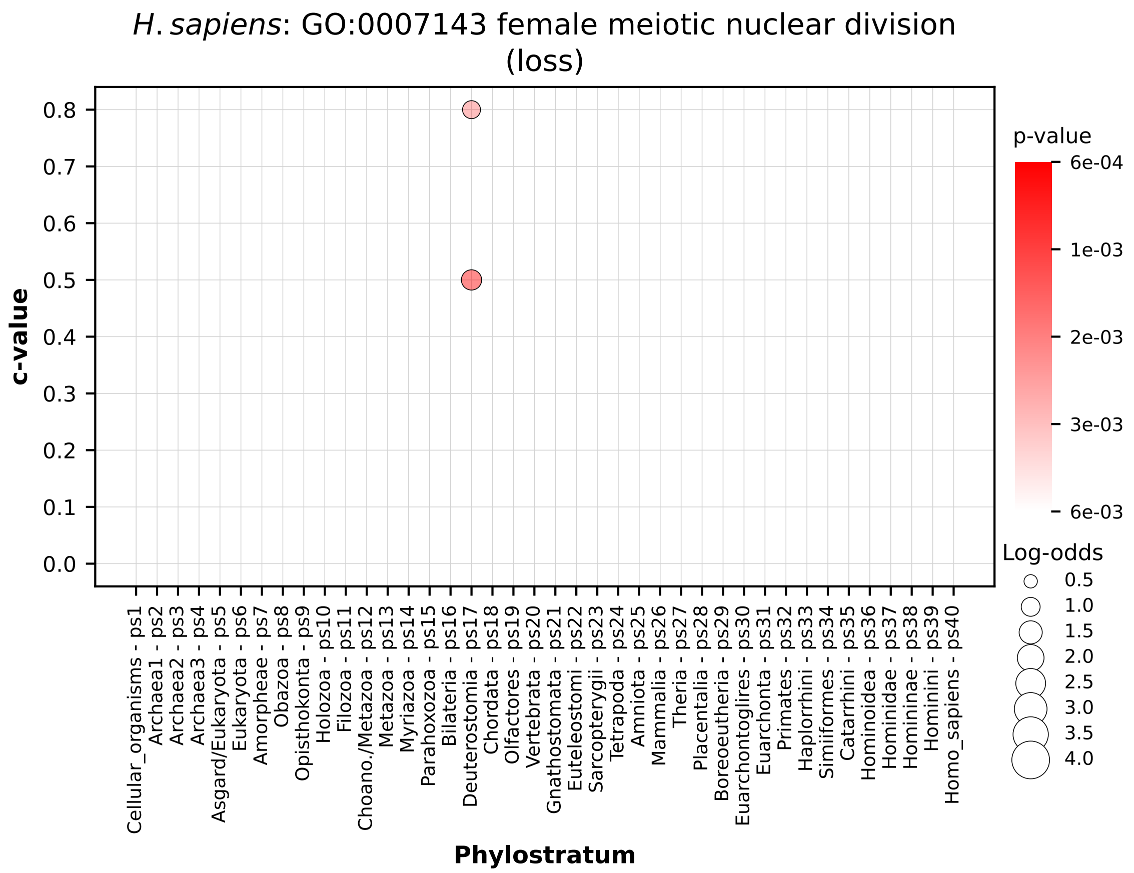

H. sapiens: GO:0007143 female meiotic nuclear division (loss)
c-value
0.0
0.1
0.2
0.3
0.4
0.5
0.6
0.7
0.8
p-value
6e-04
1e-03
2e-03
3e-03
6e-03
Log-odds
0.5
1.0
1.5
2.0
2.5
3.0
3.5
4.0
Cellular_organisms - ps1
Archaea1 - ps2
Archaea2 - ps3
Archaea3 - ps4
Asgard/Eukaryota - ps5
Eukaryota - ps6
Amorpheae - ps7
Obazoa - ps8
Opisthokonta - ps9
Holozoa - ps10
Filozoa - ps11
Choano./Metazoa - ps12
Metazoa - ps13
Myriazoa - ps14
Parahoxozoa - ps15
Bilateria - ps16
Deuterostomia - ps17
Chordata - ps18
Olfactores - ps19
Vertebrata - ps20
Gnathostomata - ps21
Euteleostomi - ps22
Sarcopterygii - ps23
Tetrapoda - ps24
Amniota - ps25
Mammalia - ps26
Theria - ps27
Placentalia - ps28
Boreoeutheria - ps29
Euarchontoglires - ps30
Euarchonta - ps31
Primates - ps32
Haplorrhini - ps33
Simiiformes - ps34
Catarrhini - ps35
Hominoidea - ps36
Hominidae - ps37
Homininae - ps38
Hominini - ps39
Homo_sapiens - ps40
Phylostratum

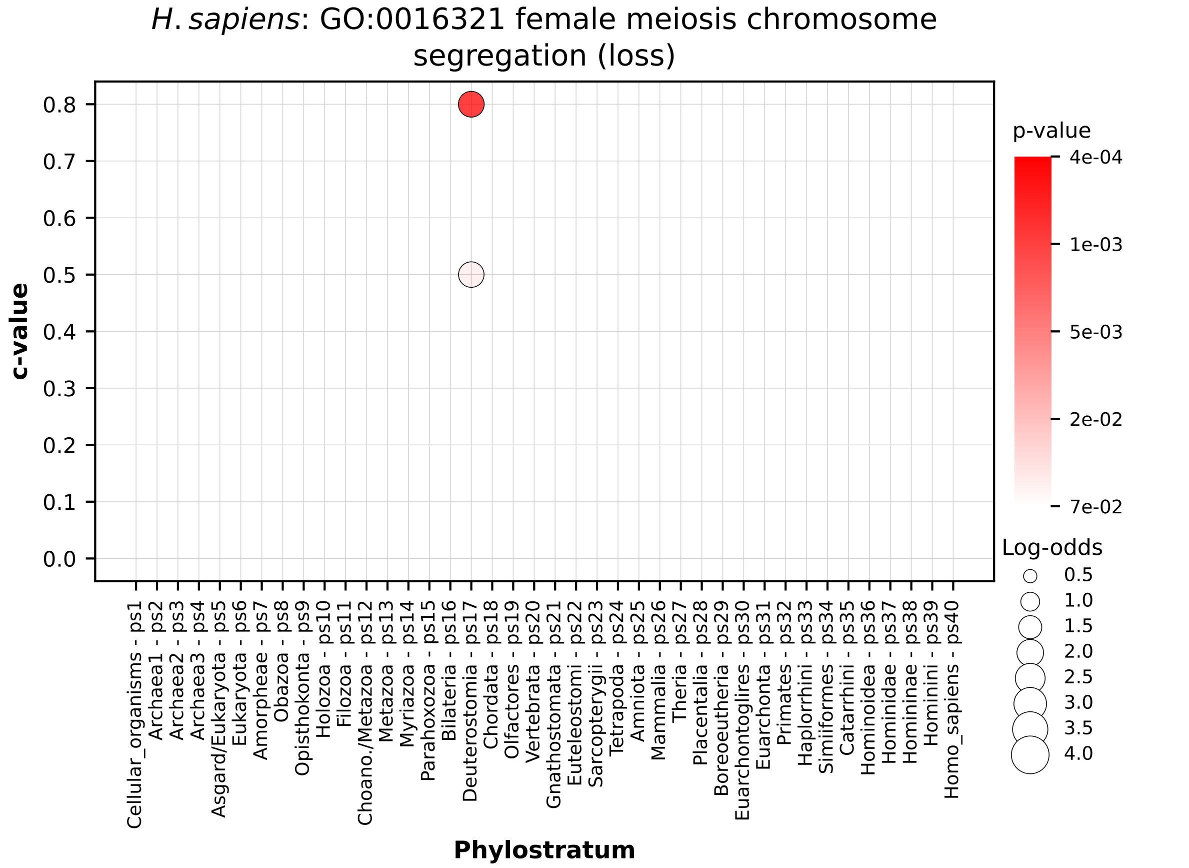

H. sapiens: GO:0016321 female meiosis chromosome segregation (loss)
c-value
0.8
0.7
0.6
0.5
0.4
0.3
0.2
0.1
0.0
p-value
4e-04
1e-03
5e-03
2e-02
7e-02
Log-odds
0.5
1.0
1.5
2.0
2.5
3.0
3.5
4.0
Cellular_organisms - ps1
Archaea1 - ps2
Archaea2 - ps3
Archaea3 - ps4
Asgard/Eukaryota - ps5
Eukaryota - ps6
Amorpheae - ps7
Obazoa - ps8
Opisthokonta - ps9
Holozoa - ps10
Filozoa - ps11
Choano./Metazoa - ps12
Metazoa - ps13
Myriazoa - ps14
Parahoxozoa - ps15
Bilateria - ps16
Deuterostomia - ps17
Chordata - ps18
Olfactores - ps19
Vertebrata - ps20
Gnathostomata - ps21
Euteleostomi - ps22
Sarcopterygii - ps23
Tetrapoda - ps24
Amniota - ps25
Mammalia - ps26
Theria - ps27
Placentalia - ps28
Boreoeutheria - ps29
Euarchontoglires - ps30
Euarchonta - ps31
Primates - ps32
Haplorrhini - ps33
Simiiformes - ps34
Catarrhini - ps35
Hominoidea - ps36
Hominidae - ps37
Homininae - ps38
Hominini - ps39
Homo_sapiens - ps40
Phylostratum

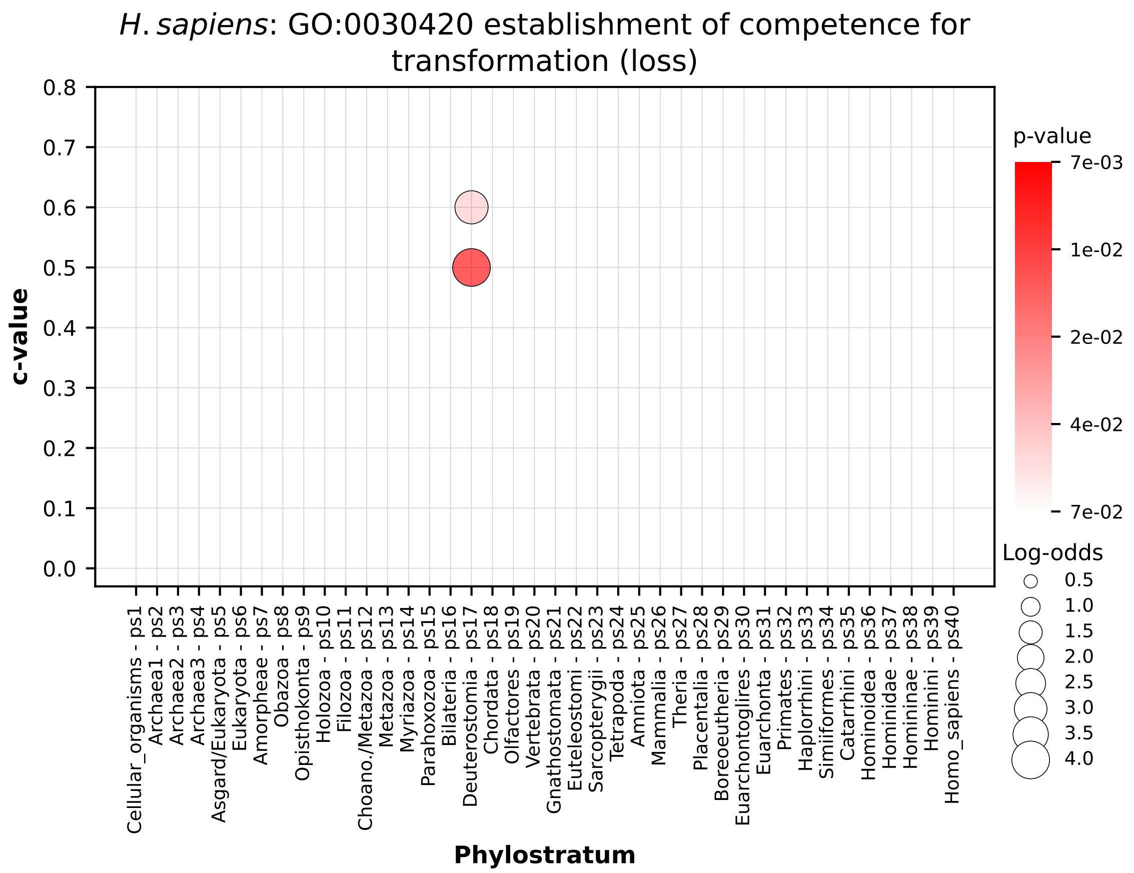

H. sapiens: GO:0030420 establishment of competence for transformation (loss)
c-value
0.8
0.7
0.6
0.5
0.4
0.3
0.2
0.1
0.0
Phylostratum
Cellular_organisms - ps1
Archaea1 - ps2
Archaea2 - ps3
Archaea3 - ps4
Asgard/Eukaryota - ps5
Eukaryota - ps6
Amorpheae - ps7
Obazoa - ps8
Opisthokonta - ps9
Holozoa - ps10
Filozoa - ps11
Choano./Metazoa - ps12
Metazoa - ps13
Myriazoa - ps14
Parahoxozoa - ps15
Bilateria - ps16
Deuterostomia - ps17
Chordata - ps18
Olfactores - ps19
Vertebrata - ps20
Gnathostomata - ps21
Euteleostomi - ps22
Sarcopterygii - ps23
Tetrapoda - ps24
Amniota - ps25
Mammalia - ps26
Theria - ps27
Placentalia - ps28
Boreoeutheria - ps29
Euarchontoglires - ps30
Euarchonta - ps31
Primates - ps32
Haplorrhini - ps33
Simiiformes - ps34
Catarrhini - ps35
Hominoidea - ps36
Hominidae - ps37
Homininae - ps38
Hominini - ps39
Homo_sapiens - ps40
p-value
7e-03
1e-02
2e-02
4e-02
7e-02
Log-odds
0.5
1.0
1.5
2.0
2.5
3.0
3.5
4.0

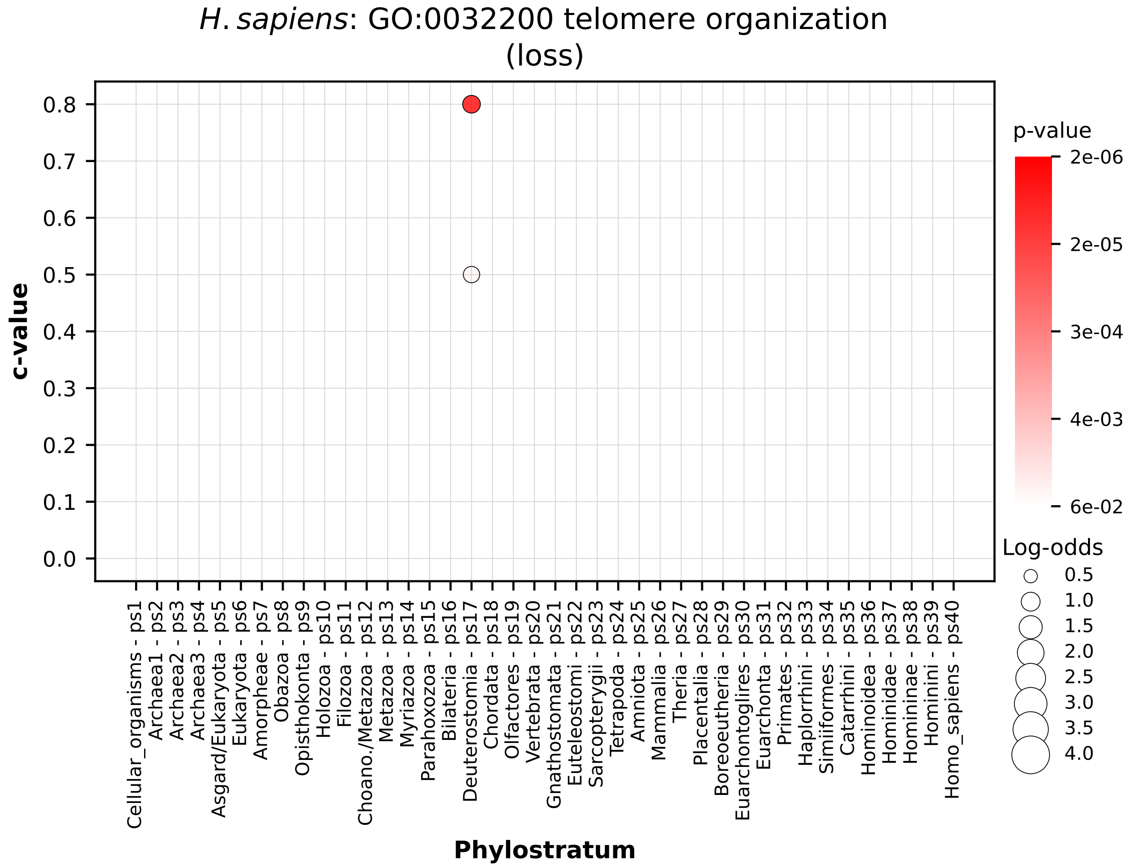

H. sapiens: GO:0032200 telomere organization
(loss)
c-value
0.0
0.1
0.2
0.3
0.4
0.5
0.6
0.7
0.8
Phylostratum
Cellular_organisms - ps1
Archaea1 - ps2
Archaea2 - ps3
Archaea3 - ps4
Asgard/Eukaryota - ps5
Eukaryota - ps6
Amorpheae - ps7
Obazoa - ps8
Opisthokonta - ps9
Holozoa - ps10
Filozoa - ps11
Choano./Metazoa - ps12
Metazoa - ps13
Myriazoa - ps14
Parahoxozoa - ps15
Bilateria - ps16
Deuterostomia - ps17
Chordata - ps18
Olfactores - ps19
Vertebrata - ps20
Gnathostomata - ps21
Euteleostomi - ps22
Sarcopterygii - ps23
Tetrapoda - ps24
Amniota - ps25
Mammalia - ps26
Theria - ps27
Placentalia - ps28
Boreoeutheria - ps29
Euarchontoglires - ps30
Euarchonta - ps31
Primates - ps32
Haplorrhini - ps33
Simiiformes - ps34
Catarrhini - ps35
Hominoidea - ps36
Hominidae - ps37
Homininae - ps38
Hominini - ps39
Homo_sapiens - ps40
p-value
2e-06
2e-05
3e-04
4e-03
6e-02
Log-odds
0.5
1.0
1.5
2.0
2.5
3.0
3.5
4.0

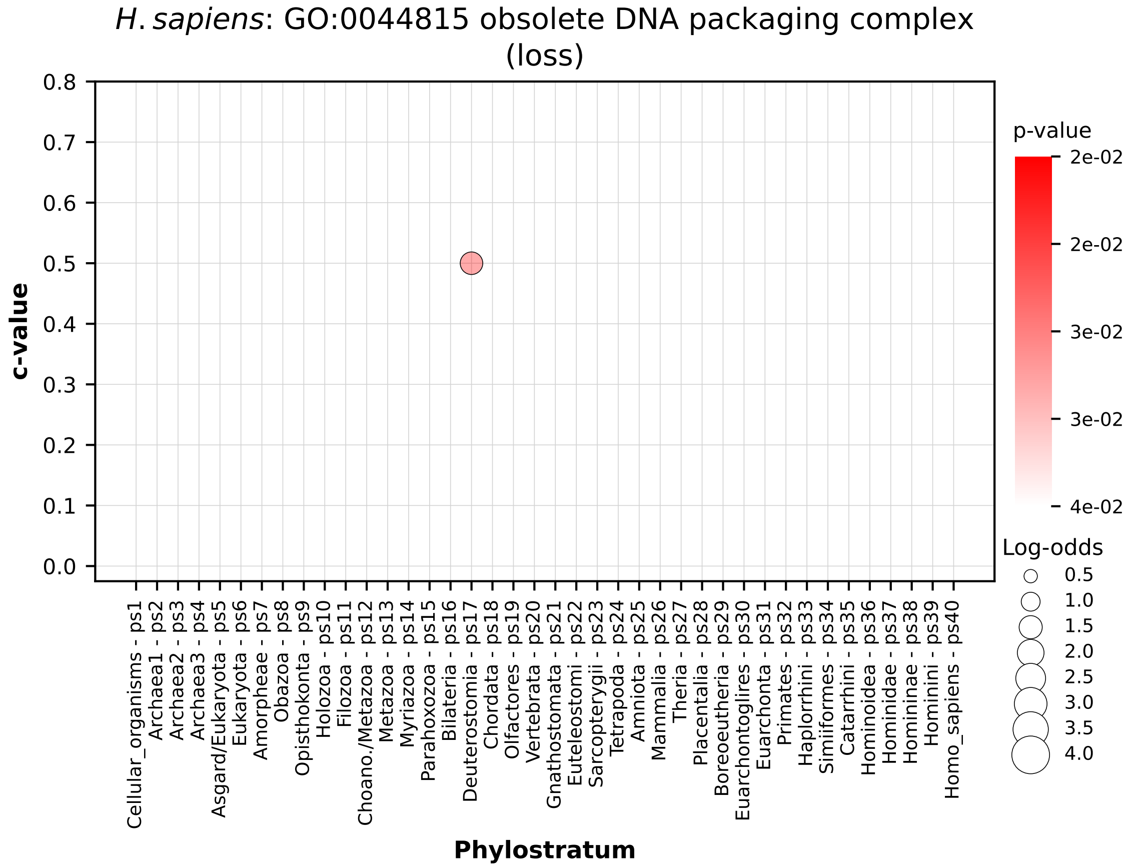

H. sapiens: GO:0044815 obsolete DNA packaging complex (loss)
c-value
0.8
0.7
0.6
0.5
0.4
0.3
0.2
0.1
0.0
Phylostratum
Cellular_organisms - ps1
Archaea1 - ps2
Archaea2 - ps3
Archaea3 - ps4
Asgard/Eukaryota - ps5
Eukaryota - ps6
Amorpheae - ps7
Obazoa - ps8
Opisthokonta - ps9
Holozoa - ps10
Filozoa - ps11
Choano./Metazoa - ps12
Metazoa - ps13
Myriazoa - ps14
Parahoxozoa - ps15
Bilateria - ps16
Deuterostomia - ps17
Chordata - ps18
Olfactores - ps19
Vertebrata - ps20
Gnathostomata - ps21
Euteleostomi - ps22
Sarcopterygii - ps23
Tetrapoda - ps24
Amniota - ps25
Mammalia - ps26
Theria - ps27
Placentalia - ps28
Boreoeutheria - ps29
Euarchontoglires - ps30
Euarchonta - ps31
Primates - ps32
Haplorrhini - ps33
Simiiformes - ps34
Catarrhini - ps35
Hominoidea - ps36
Hominidae - ps37
Homininae - ps38
Hominini - ps39
Homo_sapiens - ps40
p-value
2e-02
2e-02
3e-02
3e-02
4e-02
Log-odds
0.5
1.0
1.5
2.0
2.5
3.0
3.5
4.0

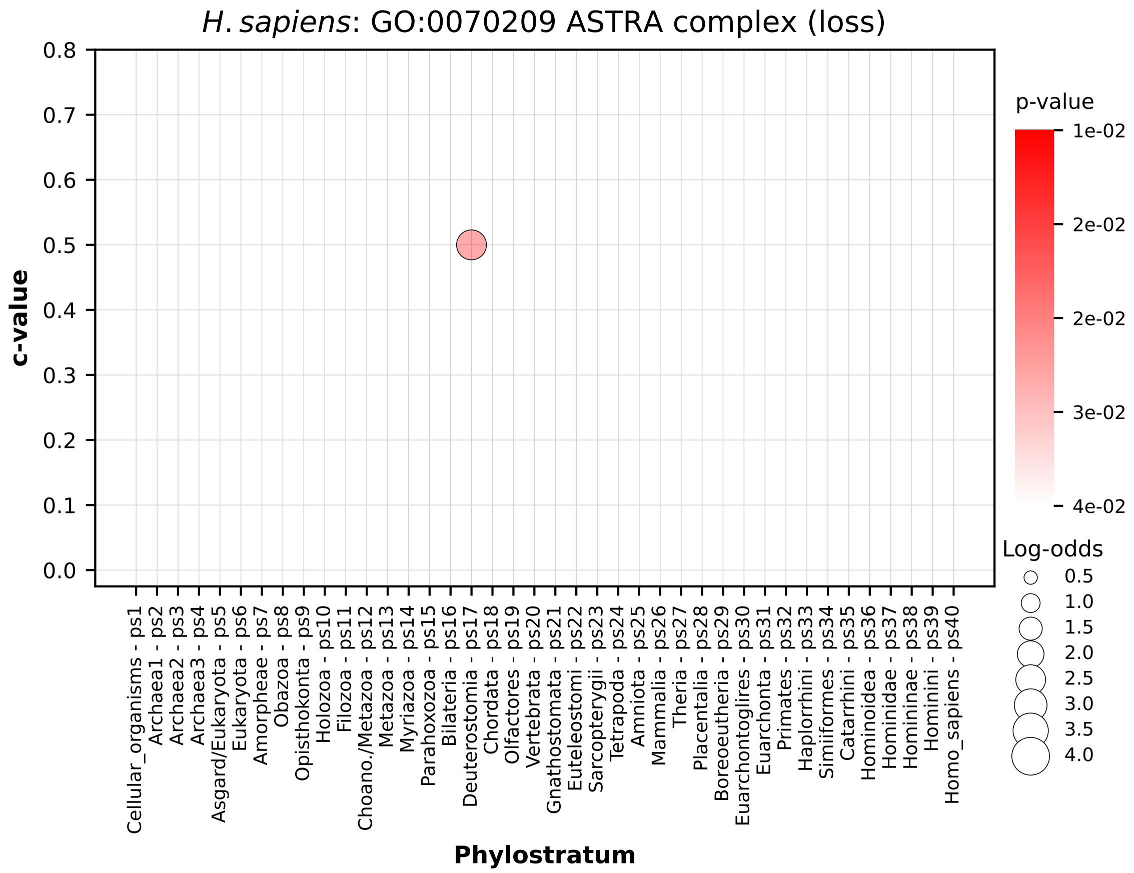
H. sapiens: GO:0070209 ASTRA complex (loss)
c-value
0.8
0.7
0.6
0.5
0.4
0.3
0.2
0.1
0.0
Phylostratum
Cellular_organisms - ps1
Archaea1 - ps2
Archaea2 - ps3
Archaea3 - ps4
Asgard/Eukaryota - ps5
Eukaryota - ps6
Amorpheae - ps7
Obazoa - ps8
Opisthokonta - ps9
Holozoa - ps10
Filozoa - ps11
Choano./Metazoa - ps12
Metazoa - ps13
Myriazoa - ps14
Parahoxozoa - ps15
Bilateria - ps16
Deuterostomia - ps17
Chordata - ps18
Olfactores - ps19
Vertebrata - ps20
Gnathostomata - ps21
Euteleostomi - ps22
Sarcopterygii - ps23
Tetrapoda - ps24
Amniota - ps25
Mammalia - ps26
Theria - ps27
Placentalia - ps28
Boreoeutheria - ps29
Euarchontoglires - ps30
Euarchonta - ps31
Primates - ps32
Haplorrhini - ps33
Simiiformes - ps34
Catarrhini - ps35
Hominoidea - ps36
Hominidae - ps37
Homininae - ps38
Hominini - ps39
Homo_sapiens - ps40
p-value
1e-02
2e-02
2e-02
3e-02
4e-02
Log-odds
0.5
1.0
1.5
2.0
2.5
3.0
3.5
4.0

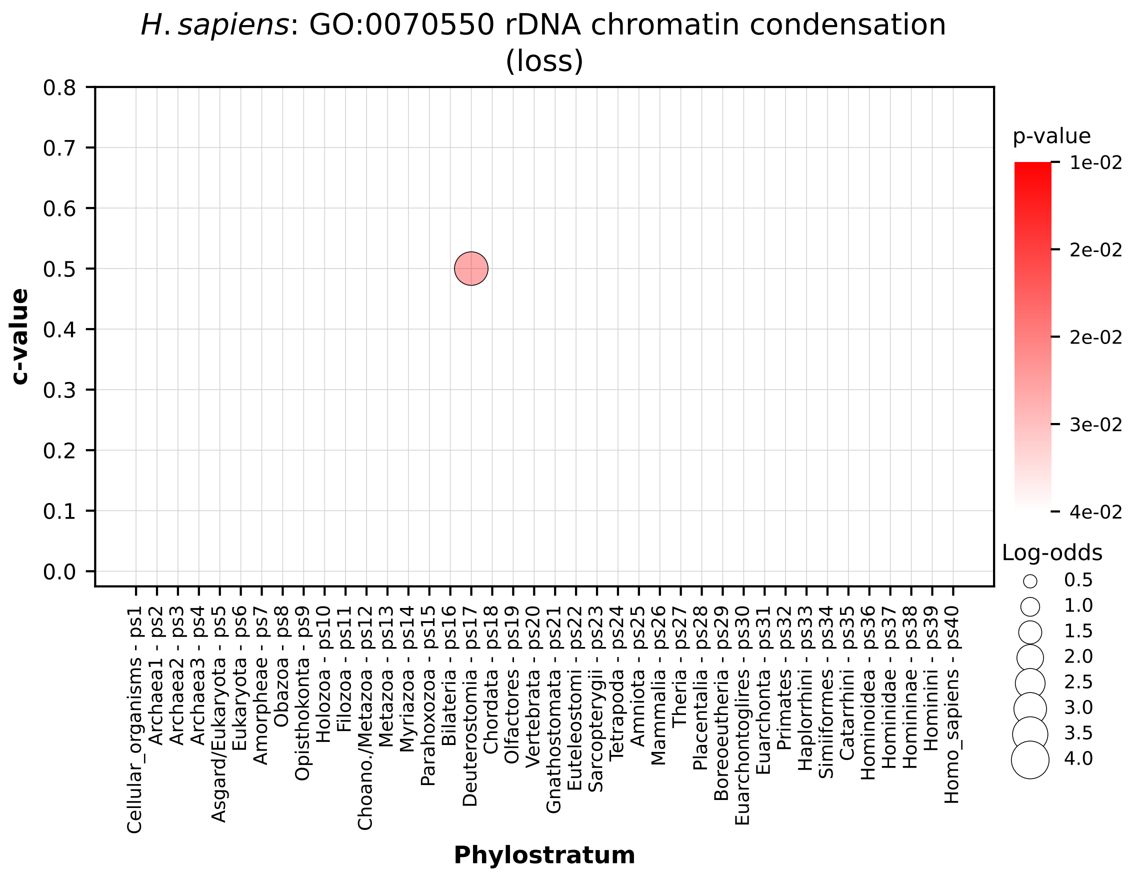

H. sapiens: GO:0070550 rDNA chromatin condensation
(loss)
c-value
0.8
0.7
0.6
0.5
0.4
0.3
0.2
0.1
0.0
p-value
1e-02
2e-02
2e-02
3e-02
4e-02
Log-odds
0.5
1.0
1.5
2.0
2.5
3.0
3.5
4.0
Cellular_organisms - ps1
Archaea1 - ps2
Archaea2 - ps3
Archaea3 - ps4
Asgard/Eukaryota - ps5
Eukaryota - ps6
Amorpheae - ps7
Obazoa - ps8
Opisthokonta - ps9
Holozoa - ps10
Filozoa - ps11
Choano./Metazoa - ps12
Metazoa - ps13
Myriazoa - ps14
Parahoxozoa - ps15
Bilateria - ps16
Deuterostomia - ps17
Chordata - ps18
Olfactores - ps19
Vertebrata - ps20
Gnathostomata - ps21
Euteleostomi - ps22
Sarcopterygii - ps23
Tetrapoda - ps24
Amniota - ps25
Mammalia - ps26
Theria - ps27
Placentalia - ps28
Boreoeutheria - ps29
Euarchontoglires - ps30
Euarchonta - ps31
Primates - ps32
Haplorrhini - ps33
Simiiformes - ps34
Catarrhini - ps35
Hominoidea - ps36
Hominidae - ps37
Homininae - ps38
Hominini - ps39
Homo_sapiens - ps40
Phylostratum

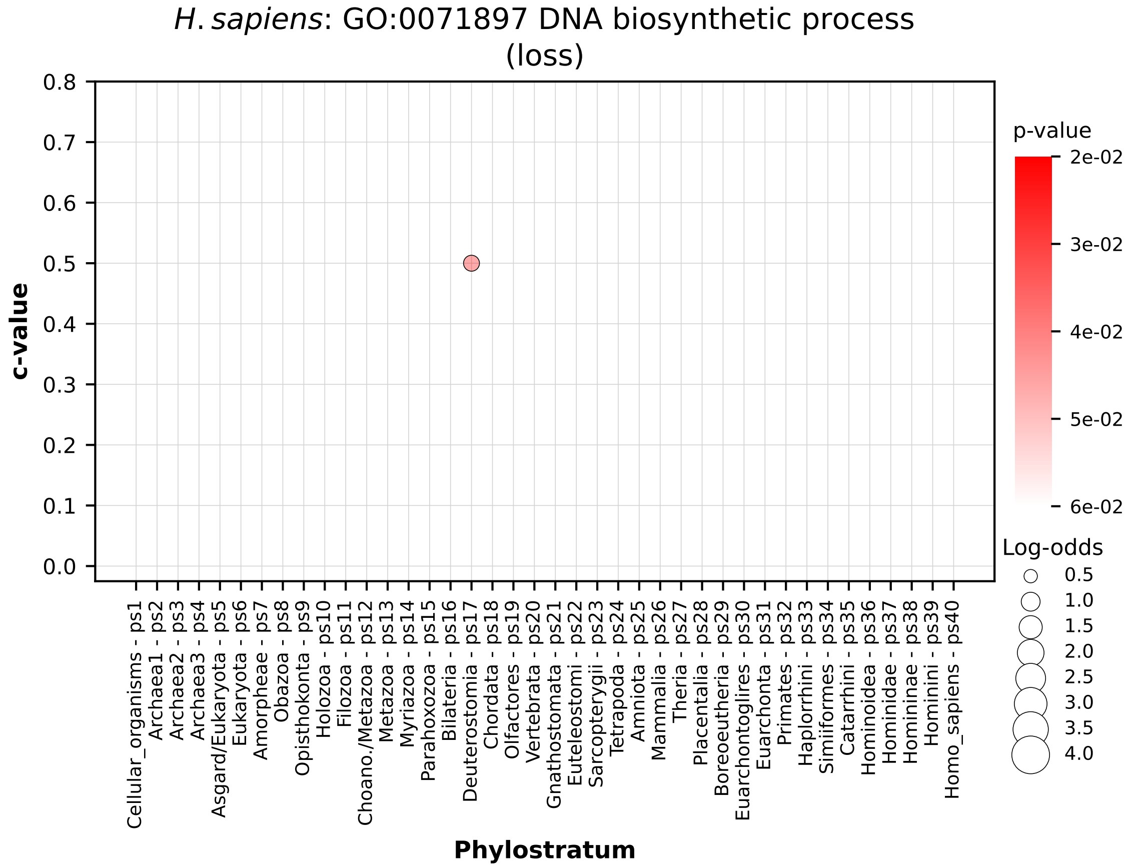

H. sapiens: GO:0071897 DNA biosynthetic process
(loss)
c-value
0.8
0.7
0.6
0.5
0.4
0.3
0.2
0.1
0.0
p-value
2e-02
3e-02
4e-02
5e-02
6e-02
Log-odds
0.5
1.0
1.5
2.0
2.5
3.0
3.5
4.0
Cellular_organisms - ps1
Archaea1 - ps2
Archaea2 - ps3
Archaea3 - ps4
Asgard/Eukaryota - ps5
Eukaryota - ps6
Amorpheae - ps7
Obazoa - ps8
Opisthokonta - ps9
Holozoa - ps10
Filozoa - ps11
Choano./Metazoa - ps12
Metazoa - ps13
Myriazoa - ps14
Parahoxozoa - ps15
Bilateria - ps16
Deuterostomia - ps17
Chordata - ps18
Olfactores - ps19
Vertebrata - ps20
Gnathostomata - ps21
Euteleostomi - ps22
Sarcopterygii - ps23
Tetrapoda - ps24
Amniota - ps25
Mammalia - ps26
Theria - ps27
Placentalia - ps28
Boreoeutheria - ps29
Euarchontoglires - ps30
Euarchonta - ps31
Primates - ps32
Haplorrhini - ps33
Simiiformes - ps34
Catarrhini - ps35
Hominoidea - ps36
Hominidae - ps37
Homininae - ps38
Hominini - ps39
Homo_sapiens - ps40
Phylostratum

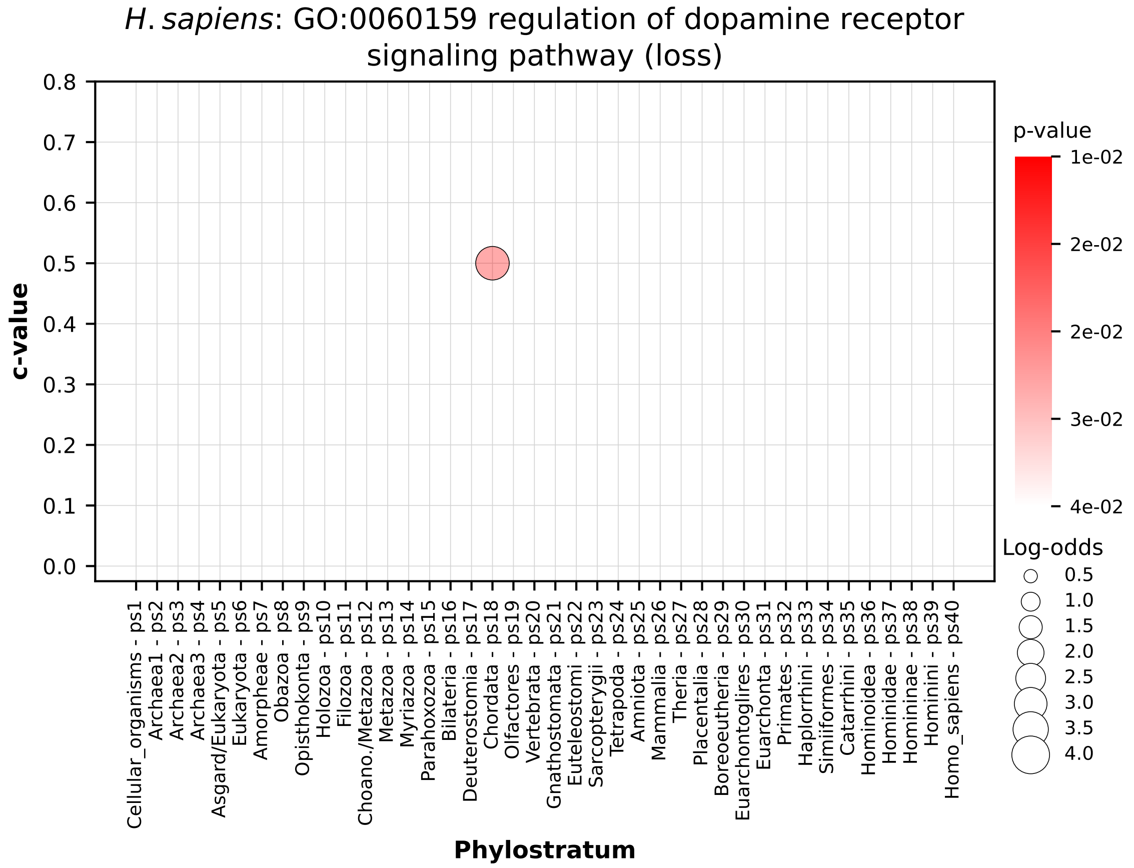
H. sapiens: GO:0060159 regulation of dopamine receptor signaling pathway (loss)
c-value
0.0
0.1
0.2
0.3
0.4
0.5
0.6
0.7
0.8
p-value
1e-02
2e-02
2e-02
3e-02
4e-02
Log-odds
0.5
1.0
1.5
2.0
2.5
3.0
3.5
4.0
Cellular_organisms - ps1
Archaea1 - ps2
Archaea2 - ps3
Archaea3 - ps4
Asgard/Eukaryota - ps5
Eukaryota - ps6
Amorpheae - ps7
Obazoa - ps8
Opisthokonta - ps9
Holozoa - ps10
Filozoa - ps11
Choano./Metazoa - ps12
Metazoa - ps13
Myriazoa - ps14
Parahoxozoa - ps15
Bilateria - ps16
Deuterostomia - ps17
Chordata - ps18
Olfactores - ps19
Vertebrata - ps20
Gnathostomata - ps21
Euteleostomi - ps22
Sarcopterygii - ps23
Tetrapoda - ps24
Amniota - ps25
Mammalia - ps26
Theria - ps27
Placentalia - ps28
Boreoeutheria - ps29
Euarchontoglires - ps30
Euarchonta - ps31
Primates - ps32
Haplorrhini - ps33
Simiiformes - ps34
Catarrhini - ps35
Hominoidea - ps36
Hominidae - ps37
Homininae - ps38
Hominini - ps39
Homo_sapiens - ps40
Phylostratum

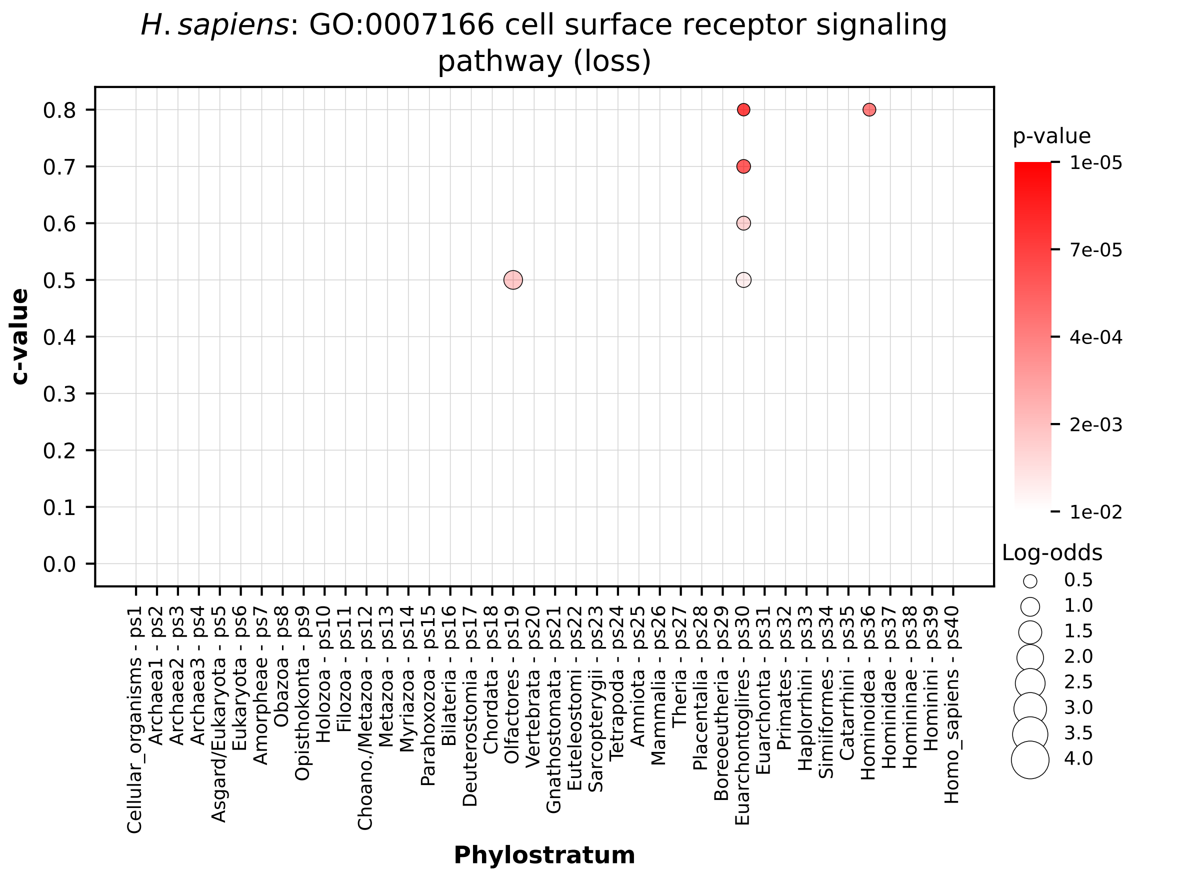

H. sapiens: GO:0007166 cell surface receptor signaling pathway (loss)
c-value
0.8
0.7
0.6
0.5
0.4
0.3
0.2
0.1
0.0
p-value
1e-05
7e-05
4e-04
2e-03
1e-02
Log-odds
0.5
1.0
1.5
2.0
2.5
3.0
3.5
4.0
Cellular_organisms - ps1
Archaea1 - ps2
Archaea2 - ps3
Archaea3 - ps4
Asgard/Eukaryota - ps5
Eukaryota - ps6
Amorpheae - ps7
Obazoa - ps8
Opisthokonta - ps9
Holozoa - ps10
Filozoa - ps11
Choano./Metazoa - ps12
Metazoa - ps13
Myriazoa - ps14
Parahoxozoa - ps15
Bilateria - ps16
Deuterostomia - ps17
Chordata - ps18
Olfactores - ps19
Vertebrata - ps20
Gnathostomata - ps21
Euteleostomi - ps22
Sarcopterygii - ps23
Tetrapoda - ps24
Amniota - ps25
Mammalia - ps26
Theria - ps27
Placentalia - ps28
Boreoeutheria - ps29
Euarchontoglires - ps30
Euarchonta - ps31
Primates - ps32
Haplorrhini - ps33
Simiiformes - ps34
Catarrhini - ps35
Hominoidea - ps36
Hominidae - ps37
Homininae - ps38
Hominini - ps39
Homo_sapiens - ps40
Phylostratum

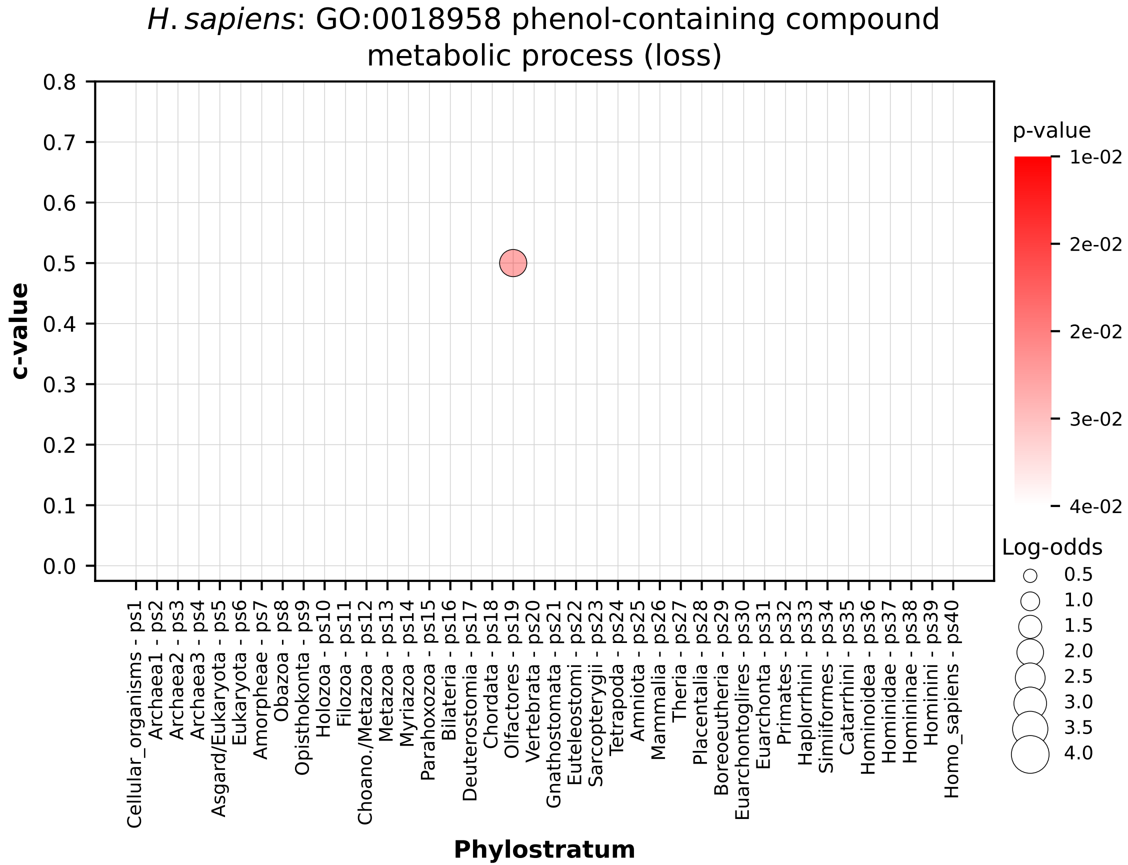

H. sapiens: GO:0018958 phenol-containing compound metabolic process (loss)
c-value
0.8
0.7
0.6
0.5
0.4
0.3
0.2
0.1
0.0
p-value
1e-02
2e-02
2e-02
3e-02
4e-02
Log-odds
0.5
1.0
1.5
2.0
2.5
3.0
3.5
4.0
Cellular_organisms - ps1
Archaea1 - ps2
Archaea2 - ps3
Archaea3 - ps4
Asgard/Eukaryota - ps5
Eukaryota - ps6
Amorpheae - ps7
Obazoa - ps8
Opisthokonta - ps9
Holozoa - ps10
Filozoa - ps11
Choano./Metazoa - ps12
Metazoa - ps13
Myriazoa - ps14
Parahoxozoa - ps15
Bilateria - ps16
Deuterostomia - ps17
Chordata - ps18
Olfactores - ps19
Vertebrata - ps20
Gnathostomata - ps21
Euteleostomi - ps22
Sarcopterygii - ps23
Tetrapoda - ps24
Amniota - ps25
Mammalia - ps26
Theria - ps27
Placentalia - ps28
Boreoeutheria - ps29
Euarchontoglires - ps30
Euarchonta - ps31
Primates - ps32
Haplorrhini - ps33
Simiiformes - ps34
Catarrhini - ps35
Hominoidea - ps36
Hominidae - ps37
Homininae - ps38
Hominini - ps39
Homo_sapiens - ps40
Phylostratum

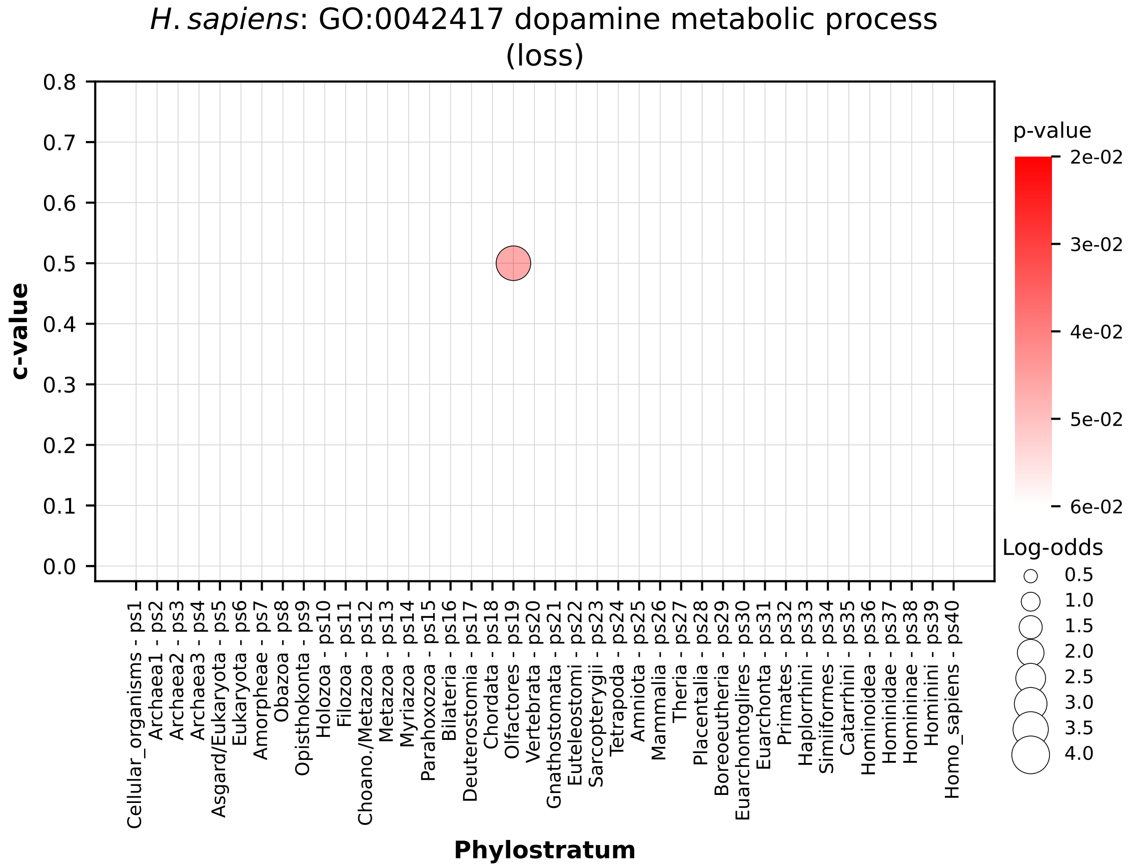

H. sapiens: GO:0042417 dopamine metabolic process (loss)
c-value
0.8
0.7
0.6
0.5
0.4
0.3
0.2
0.1
0.0
Phylostratum
Cellular_organisms - ps1
Archaea1 - ps2
Archaea2 - ps3
Archaea3 - ps4
Asgard/Eukaryota - ps5
Eukaryota - ps6
Amorpheae - ps7
Obazoa - ps8
Opisthokonta - ps9
Holozoa - ps10
Filozoa - ps11
Choano./Metazoa - ps12
Metazoa - ps13
Myriazoa - ps14
Parahoxozoa - ps15
Bilateria - ps16
Deuterostomia - ps17
Chordata - ps18
Olfactores - ps19
Vertebrata - ps20
Gnathostomata - ps21
Euteleostomi - ps22
Sarcopterygii - ps23
Tetrapoda - ps24
Amniota - ps25
Mammalia - ps26
Theria - ps27
Placentalia - ps28
Boreoeutheria - ps29
Euarchontoglires - ps30
Euarchonta - ps31
Primates - ps32
Haplorrhini - ps33
Simiiformes - ps34
Catarrhini - ps35
Hominoidea - ps36
Hominidae - ps37
Homininae - ps38
Hominini - ps39
Homo_sapiens - ps40
p-value
2e-02
3e-02
4e-02
5e-02
6e-02
Log-odds
0.5
1.0
1.5
2.0
2.5
3.0
3.5
4.0

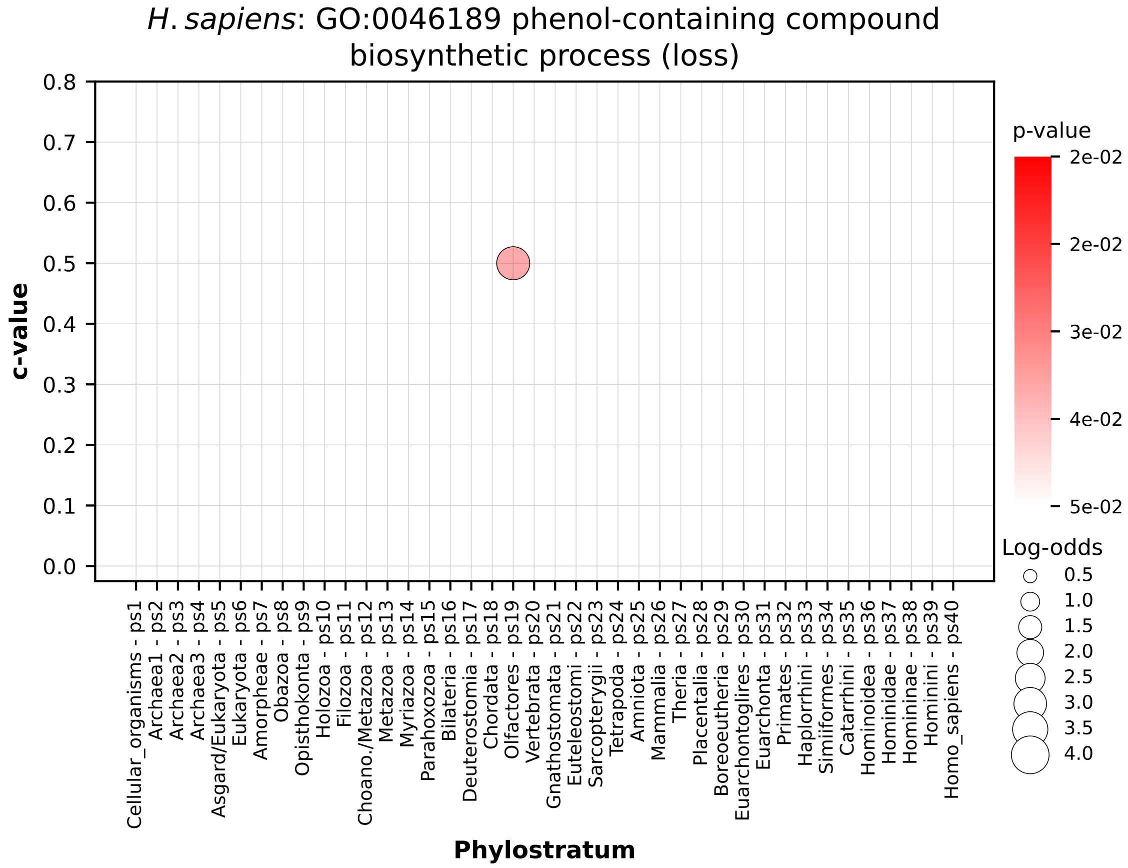

H. sapiens: GO:0046189 phenol-containing compound biosynthetic process (loss)
c-value
0.8
0.7
0.6
0.5
0.4
0.3
0.2
0.1
0.0
p-value
2e-02
2e-02
3e-02
4e-02
5e-02
Log-odds
0.5
1.0
1.5
2.0
2.5
3.0
3.5
4.0
Cellular_organisms - ps1
Archaea1 - ps2
Archaea2 - ps3
Archaea3 - ps4
Asgard/Eukaryota - ps5
Eukaryota - ps6
Amorpheae - ps7
Obazoa - ps8
Opisthokonta - ps9
Holozoa - ps10
Filozoa - ps11
Choano./Metazoa - ps12
Metazoa - ps13
Myriazoa - ps14
Parahoxozoa - ps15
Bilateria - ps16
Deuterostomia - ps17
Chordata - ps18
Olfactores - ps19
Vertebrata - ps20
Gnathostomata - ps21
Euteleostomi - ps22
Sarcopterygii - ps23
Tetrapoda - ps24
Amniota - ps25
Mammalia - ps26
Theria - ps27
Placentalia - ps28
Boreoeutheria - ps29
Euarchontoglires - ps30
Euarchonta - ps31
Primates - ps32
Haplorrhini - ps33
Simiiformes - ps34
Catarrhini - ps35
Hominoidea - ps36
Hominidae - ps37
Homininae - ps38
Hominini - ps39
Homo_sapiens - ps40
Phylostratum

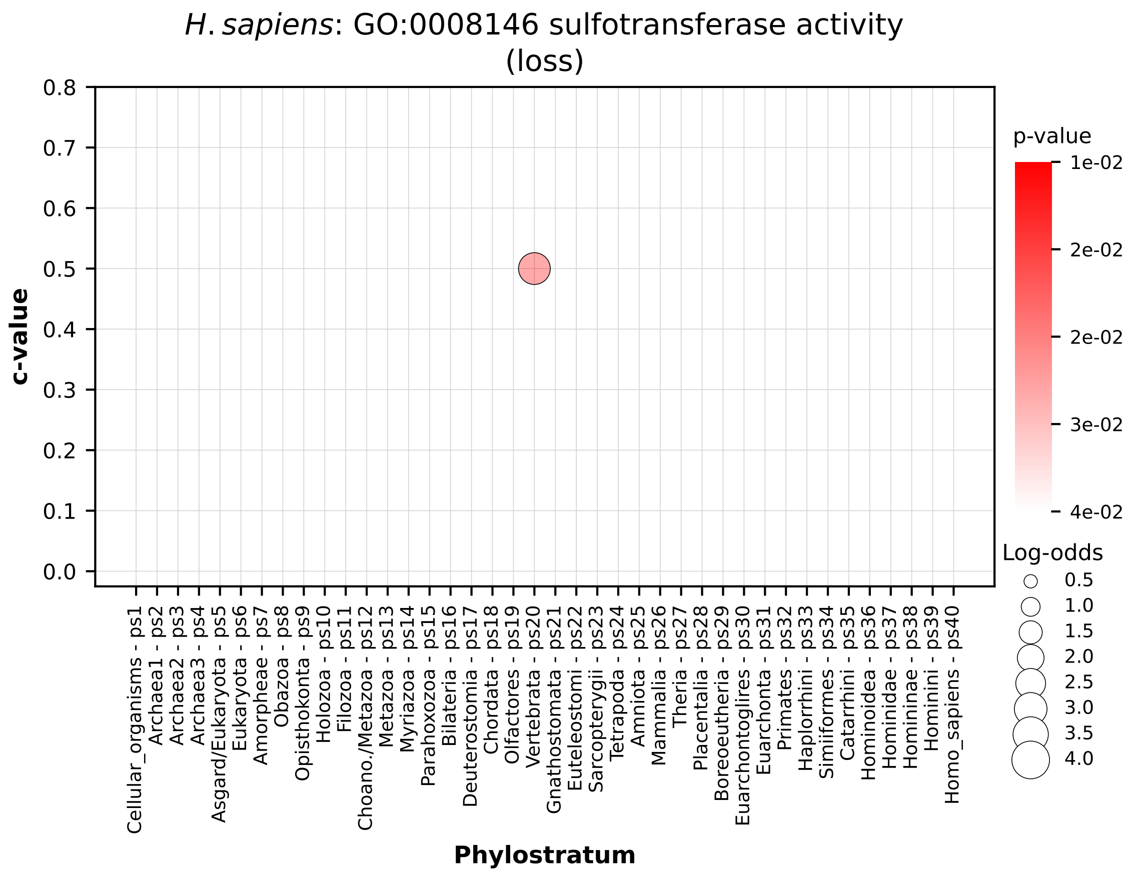

H. sapiens: GO:0008146 sulfotransferase activity (loss)
c-value
0.8
0.7
0.6
0.5
0.4
0.3
0.2
0.1
0.0
Phylostratum
Cellular_organisms - ps1
Archaea1 - ps2
Archaea2 - ps3
Archaea3 - ps4
Asgard/Eukaryota - ps5
Eukaryota - ps6
Amorpheae - ps7
Obazoa - ps8
Opisthokonta - ps9
Holozoa - ps10
Filozoa - ps11
Choano./Metazoa - ps12
Metazoa - ps13
Myriazoa - ps14
Parahoxozoa - ps15
Bilateria - ps16
Deuterostomia - ps17
Chordata - ps18
Olfactores - ps19
Vertebrata - ps20
Gnathostomata - ps21
Euteleostomi - ps22
Sarcopterygii - ps23
Tetrapoda - ps24
Amniota - ps25
Mammalia - ps26
Theria - ps27
Placentalia - ps28
Boreoeutheria - ps29
Euarchontoglires - ps30
Euarchonta - ps31
Primates - ps32
Haplorrhini - ps33
Simiiformes - ps34
Catarrhini - ps35
Hominoidea - ps36
Hominidae - ps37
Homininae - ps38
Hominini - ps39
Homo_sapiens - ps40
p-value
1e-02
2e-02
2e-02
3e-02
4e-02
Log-odds
0.5
1.0
1.5
2.0
2.5
3.0
3.5
4.0

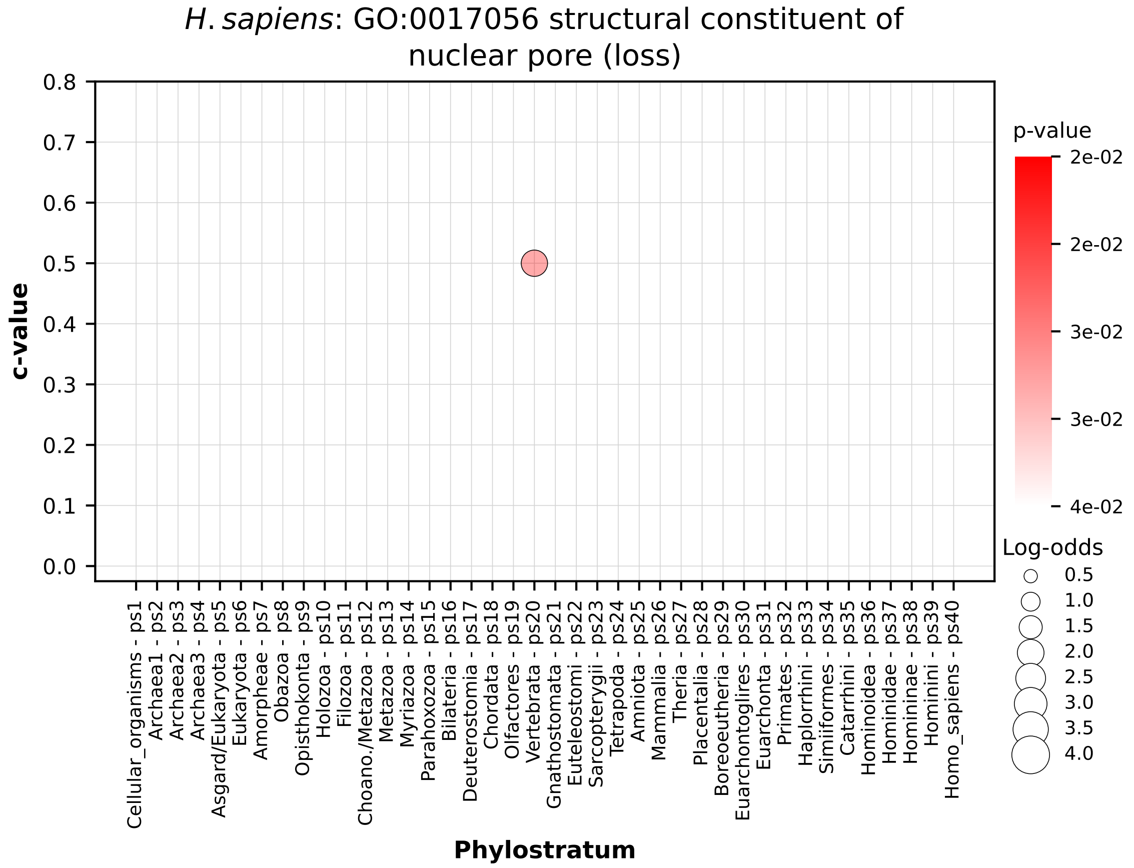

H. sapiens: GO:0017056 structural constituent of nuclear pore (loss)
c-value
0.0
0.1
0.2
0.3
0.4
0.5
0.6
0.7
0.8
Phylostratum
Cellular_organisms - ps1
Archaea1 - ps2
Archaea2 - ps3
Archaea3 - ps4
Asgard/Eukaryota - ps5
Eukaryota - ps6
Amorpheae - ps7
Obazoa - ps8
Opisthokonta - ps9
Holozoa - ps10
Filozoa - ps11
Choano./Metazoa - ps12
Metazoa - ps13
Myriazoa - ps14
Parahoxozoa - ps15
Bilateria - ps16
Deuterostomia - ps17
Chordata - ps18
Olfactores - ps19
Vertebrata - ps20
Gnathostomata - ps21
Euteleostomi - ps22
Sarcopterygii - ps23
Tetrapoda - ps24
Amniota - ps25
Mammalia - ps26
Theria - ps27
Placentalia - ps28
Boreoeutheria - ps29
Euarchontoglires - ps30
Euarchonta - ps31
Primates - ps32
Haplorrhini - ps33
Simiiformes - ps34
Catarrhini - ps35
Hominoidea - ps36
Hominidae - ps37
Homininae - ps38
Hominini - ps39
Homo_sapiens - ps40
p-value
2e-02
2e-02
3e-02
3e-02
4e-02
Log-odds
0.5
1.0
1.5
2.0
2.5
3.0
3.5
4.0

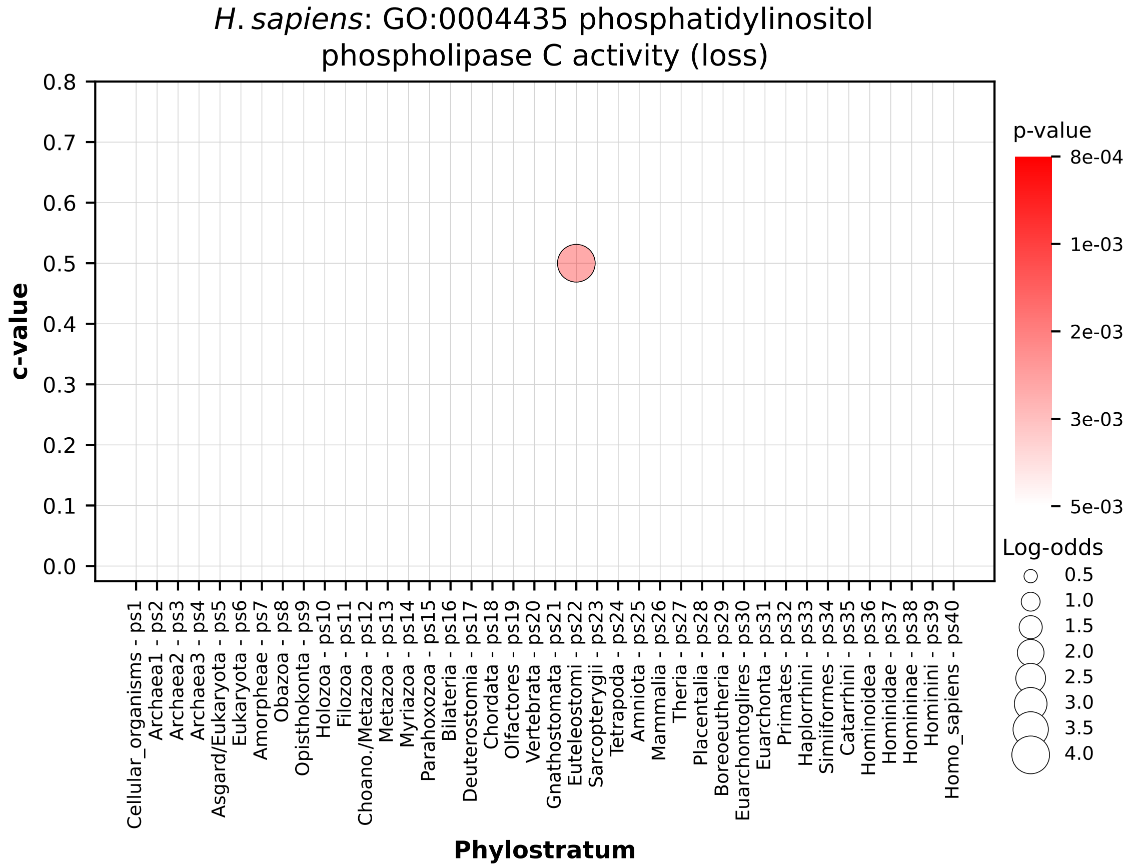

H. sapiens: GO:0004435 phosphatidylinositol phospholipase C activity (loss)
c-value
0.8
0.7
0.6
0.5
0.4
0.3
0.2
0.1
0.0
p-value
8e-04
1e-03
2e-03
3e-03
5e-03
Log-odds
0.5
1.0
1.5
2.0
2.5
3.0
3.5
4.0
Cellular_organisms - ps1
Archaea1 - ps2
Archaea2 - ps3
Archaea3 - ps4
Asgard/Eukaryota - ps5
Eukaryota - ps6
Amorpheae - ps7
Obazoa - ps8
Opisthokonta - ps9
Holozoa - ps10
Filozoa - ps11
Choano./Metazoa - ps12
Metazoa - ps13
Myriazoa - ps14
Parahoxozoa - ps15
Bilateria - ps16
Deuterostomia - ps17
Chordata - ps18
Olfactores - ps19
Vertebrata - ps20
Gnathostomata - ps21
Euteleostomi - ps22
Sarcopterygii - ps23
Tetrapoda - ps24
Amniota - ps25
Mammalia - ps26
Theria - ps27
Placentalia - ps28
Boreoeutheria - ps29
Euarchontoglires - ps30
Euarchonta - ps31
Primates - ps32
Haplorrhini - ps33
Simiiformes - ps34
Catarrhini - ps35
Hominoidea - ps36
Hominidae - ps37
Homininae - ps38
Hominini - ps39
Homo_sapiens - ps40
Phylostratum

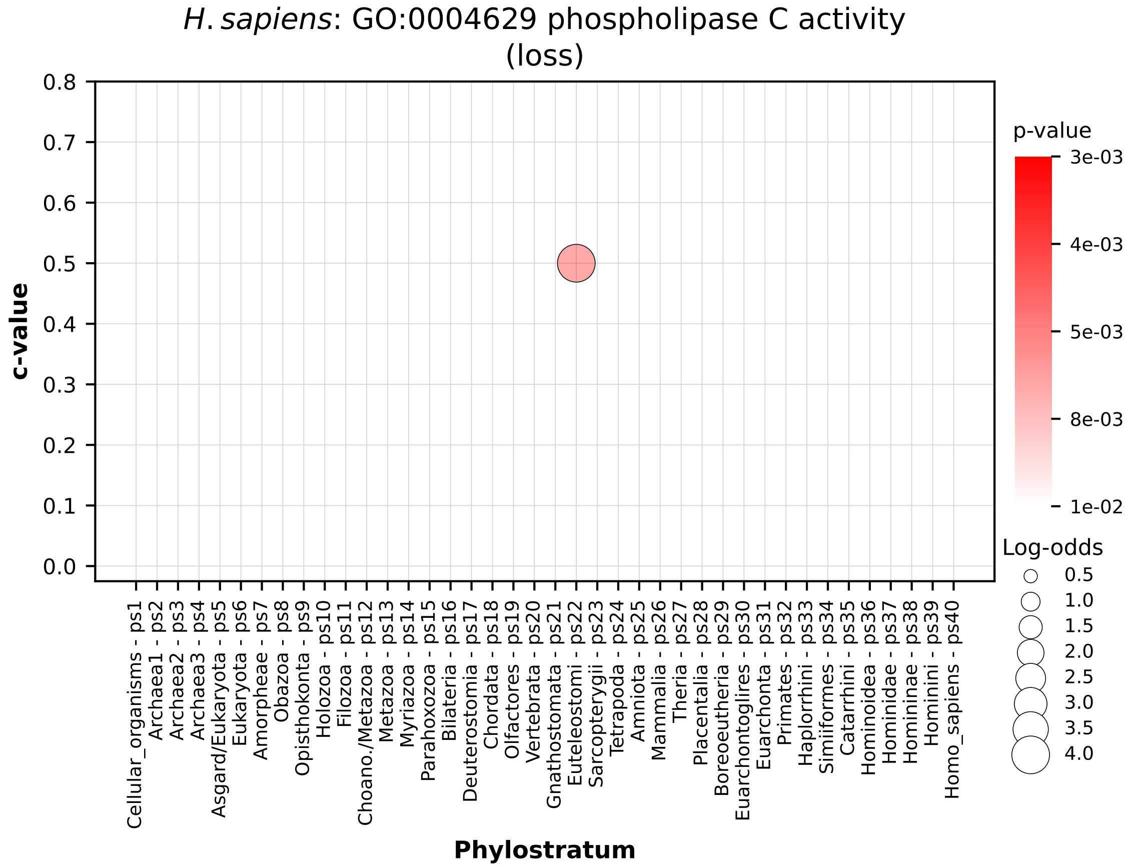

H. sapiens: GO:0004629 phospholipase C activity
(loss)
c-value
0.0
0.1
0.2
0.3
0.4
0.5
0.6
0.7
0.8
p-value
3e-03
4e-03
5e-03
8e-03
1e-02
Log-odds
0.5
1.0
1.5
2.0
2.5
3.0
3.5
4.0
Cellular_organisms - ps1
Archaea1 - ps2
Archaea2 - ps3
Archaea3 - ps4
Asgard/Eukaryota - ps5
Eukaryota - ps6
Amorpheae - ps7
Obazoa - ps8
Opisthokonta - ps9
Holozoa - ps10
Filozoa - ps11
Choano./Metazoa - ps12
Metazoa - ps13
Myriazoa - ps14
Parahoxozoa - ps15
Bilateria - ps16
Deuterostomia - ps17
Chordata - ps18
Olfactores - ps19
Vertebrata - ps20
Gnathostomata - ps21
Euteleostomi - ps22
Sarcopterygii - ps23
Tetrapoda - ps24
Amniota - ps25
Mammalia - ps26
Theria - ps27
Placentalia - ps28
Boreoeutheria - ps29
Euarchontoglires - ps30
Euarchonta - ps31
Primates - ps32
Haplorrhini - ps33
Simiiformes - ps34
Catarrhini - ps35
Hominoidea - ps36
Hominidae - ps37
Homininae - ps38
Hominini - ps39
Homo_sapiens - ps40
Phylostratum

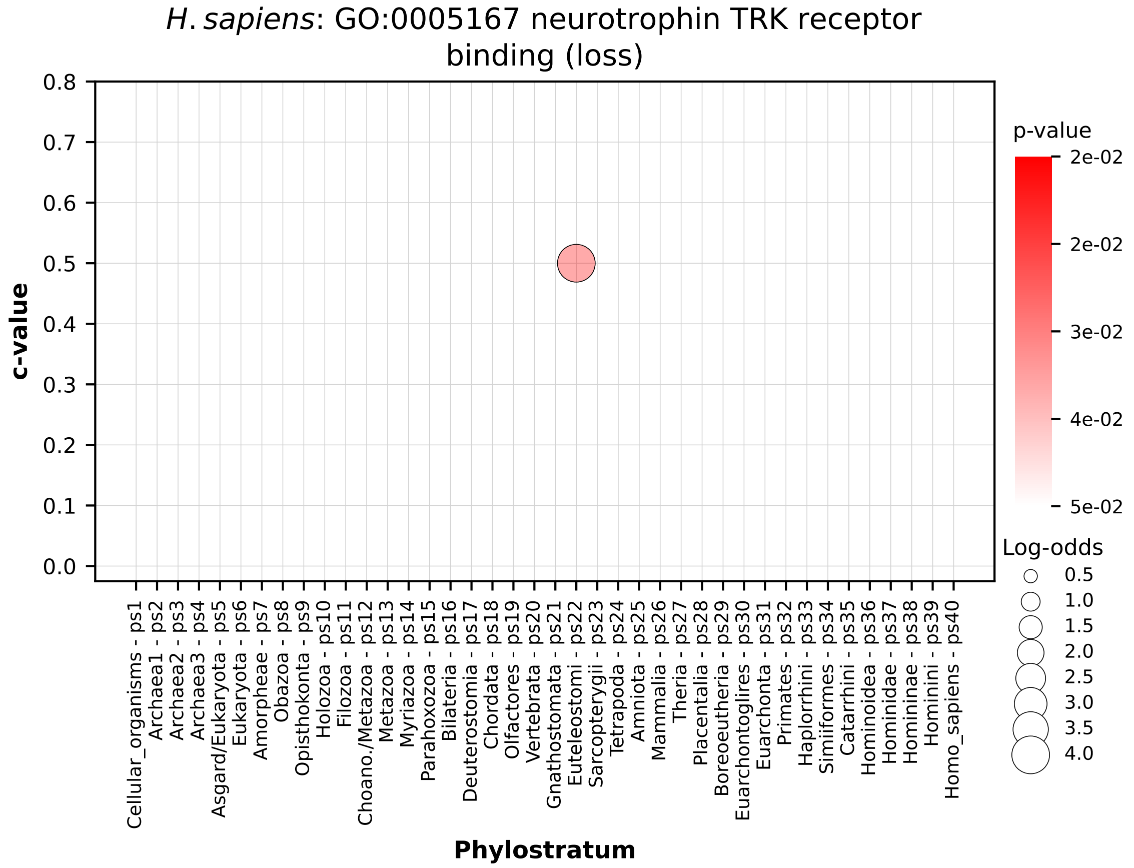

H. sapiens: GO:0005167 neurotrophin TRK receptor binding (loss)
c-value
0.8
0.7
0.6
0.5
0.4
0.3
0.2
0.1
0.0
p-value
2e-02
2e-02
3e-02
4e-02
5e-02
Log-odds
0.5
1.0
1.5
2.0
2.5
3.0
3.5
4.0
Cellular_organisms - ps1
Archaea1 - ps2
Archaea2 - ps3
Archaea3 - ps4
Asgard/Eukaryota - ps5
Eukaryota - ps6
Amorpheae - ps7
Obazoa - ps8
Opisthokonta - ps9
Holozoa - ps10
Filozoa - ps11
Choano./Metazoa - ps12
Metazoa - ps13
Myriazoa - ps14
Parahoxozoa - ps15
Bilateria - ps16
Deuterostomia - ps17
Chordata - ps18
Olfactores - ps19
Vertebrata - ps20
Gnathostomata - ps21
Euteleostomi - ps22
Sarcopterygii - ps23
Tetrapoda - ps24
Amniota - ps25
Mammalia - ps26
Theria - ps27
Placentalia - ps28
Boreoeutheria - ps29
Euarchontoglires - ps30
Euarchonta - ps31
Primates - ps32
Haplorrhini - ps33
Simiiformes - ps34
Catarrhini - ps35
Hominoidea - ps36
Hominidae - ps37
Homininae - ps38
Hominini - ps39
Homo_sapiens - ps40
Phylostratum

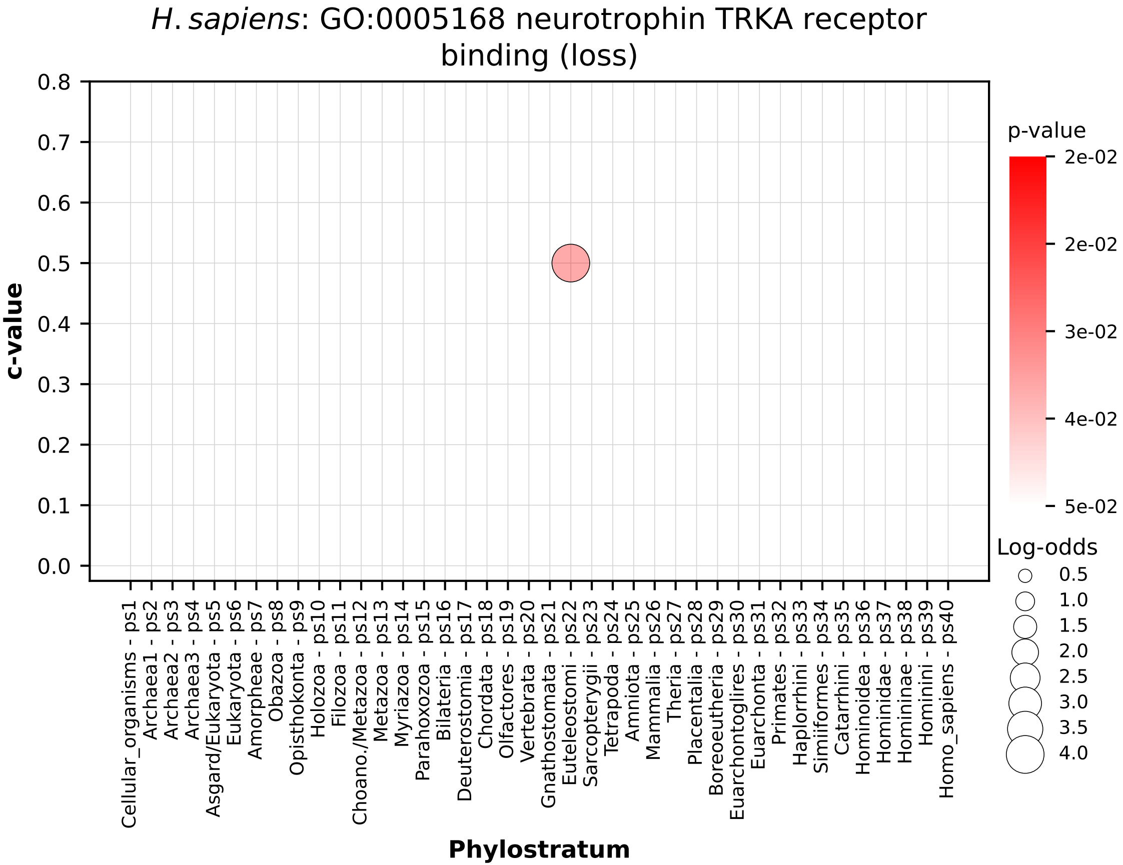

H. sapiens: GO:0005168 neurotrophin TRKA receptor binding (loss)
c-value
0.0
0.1
0.2
0.3
0.4
0.5
0.6
0.7
0.8
Phylostratum
Cellular_organisms - ps1
Archaea1 - ps2
Archaea2 - ps3
Archaea3 - ps4
Asgard/Eukaryota - ps5
Eukaryota - ps6
Amorpheae - ps7
Obazoa - ps8
Opisthokonta - ps9
Holozoa - ps10
Filozoa - ps11
Choano./Metazoa - ps12
Metazoa - ps13
Myriazoa - ps14
Parahoxozoa - ps15
Bilateria - ps16
Deuterostomia - ps17
Chordata - ps18
Olfactores - ps19
Vertebrata - ps20
Gnathostomata - ps21
Euteleostomi - ps22
Sarcopterygii - ps23
Tetrapoda - ps24
Amniota - ps25
Mammalia - ps26
Theria - ps27
Placentalia - ps28
Boreoeutheria - ps29
Euarchontoglires - ps30
Euarchonta - ps31
Primates - ps32
Haplorrhini - ps33
Simiiformes - ps34
Catarrhini - ps35
Hominoidea - ps36
Hominidae - ps37
Homininae - ps38
Hominini - ps39
Homo_sapiens - ps40
p-value
2e-02
2e-02
3e-02
4e-02
5e-02
Log-odds
0.5
1.0
1.5
2.0
2.5
3.0
3.5
4.0

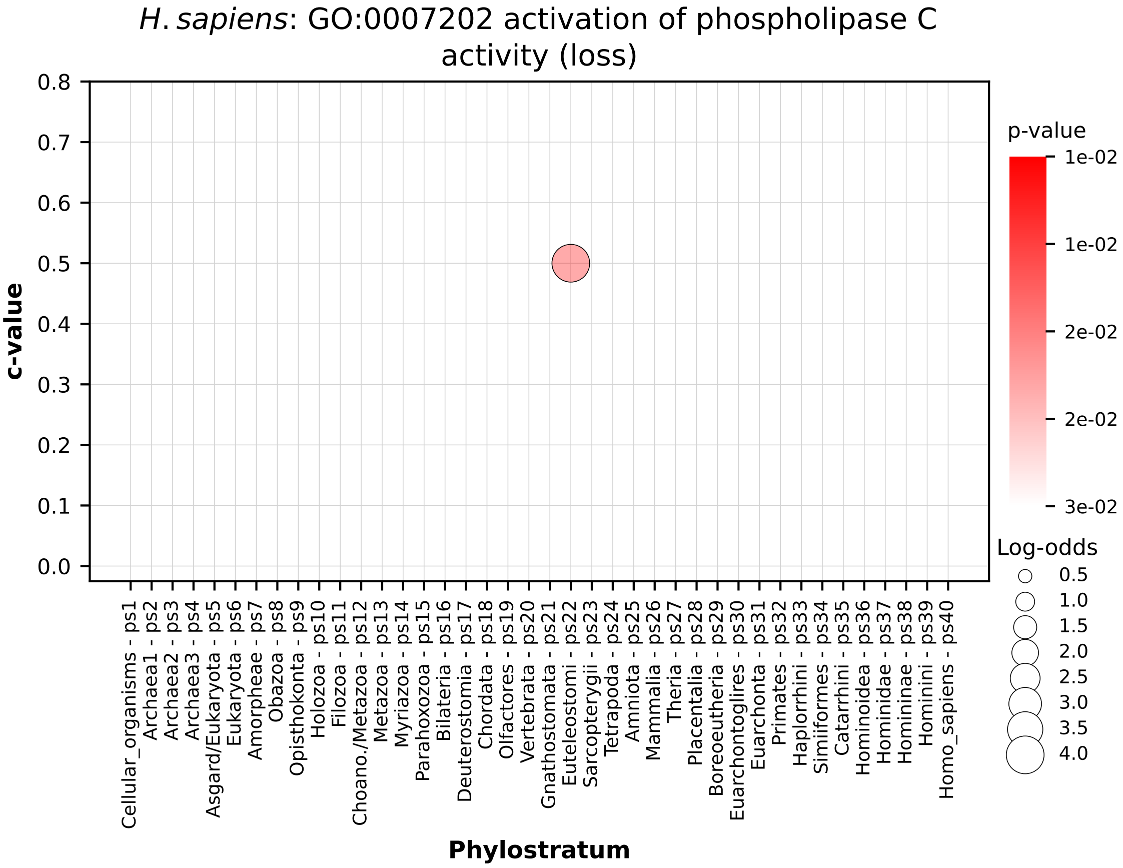

H. sapiens: GO:0007202 activation of phospholipase C activity (loss)
c-value
0.8
0.7
0.6
0.5
0.4
0.3
0.2
0.1
0.0
p-value
1e-02
1e-02
2e-02
2e-02
3e-02
Log-odds
0.5
1.0
1.5
2.0
2.5
3.0
3.5
4.0
Cellular_organisms - ps1
Archaea1 - ps2
Archaea2 - ps3
Archaea3 - ps4
Asgard/Eukaryota - ps5
Eukaryota - ps6
Amorpheae - ps7
Obazoa - ps8
Opisthokonta - ps9
Holozoa - ps10
Filozoa - ps11
Choano./Metazoa - ps12
Metazoa - ps13
Myriazoa - ps14
Parahoxozoa - ps15
Bilateria - ps16
Deuterostomia - ps17
Chordata - ps18
Olfactores - ps19
Vertebrata - ps20
Gnathostomata - ps21
Euteleostomi - ps22
Sarcopterygii - ps23
Tetrapoda - ps24
Amniota - ps25
Mammalia - ps26
Theria - ps27
Placentalia - ps28
Boreoeutheria - ps29
Euarchontoglires - ps30
Euarchonta - ps31
Primates - ps32
Haplorrhini - ps33
Simiiformes - ps34
Catarrhini - ps35
Hominoidea - ps36
Hominidae - ps37
Homininae - ps38
Hominini - ps39
Homo_sapiens - ps40
Phylostratum

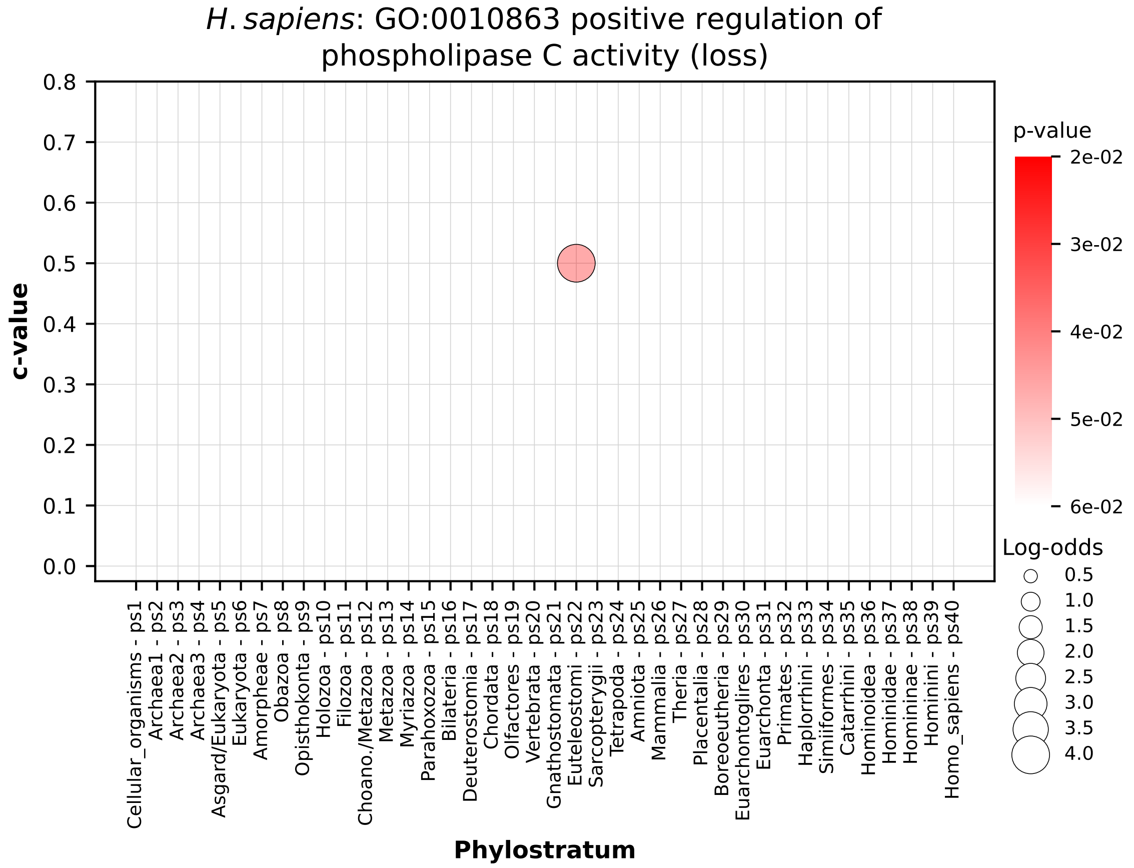

H. sapiens: GO:0010863 positive regulation of phospholipase C activity (loss)
c-value
0.8
0.7
0.6
0.5
0.4
0.3
0.2
0.1
0.0
Phylostratum
Cellular_organisms - ps1
Archaea1 - ps2
Archaea2 - ps3
Archaea3 - ps4
Asgard/Eukaryota - ps5
Eukaryota - ps6
Amorpheae - ps7
Obazoa - ps8
Opisthokonta - ps9
Holozoa - ps10
Filozoa - ps11
Choano./Metazoa - ps12
Metazoa - ps13
Myriazoa - ps14
Parahoxozoa - ps15
Bilateria - ps16
Deuterostomia - ps17
Chordata - ps18
Olfactores - ps19
Vertebrata - ps20
Gnathostomata - ps21
Euteleostomi - ps22
Sarcopterygii - ps23
Tetrapoda - ps24
Amniota - ps25
Mammalia - ps26
Theria - ps27
Placentalia - ps28
Boreoeutheria - ps29
Euarchontoglires - ps30
Euarchonta - ps31
Primates - ps32
Haplorrhini - ps33
Simiiformes - ps34
Catarrhini - ps35
Hominoidea - ps36
Hominidae - ps37
Homininae - ps38
Hominini - ps39
Homo_sapiens - ps40
p-value
2e-02
3e-02
4e-02
5e-02
6e-02
Log-odds
0.5
1.0
1.5
2.0
2.5
3.0
3.5
4.0

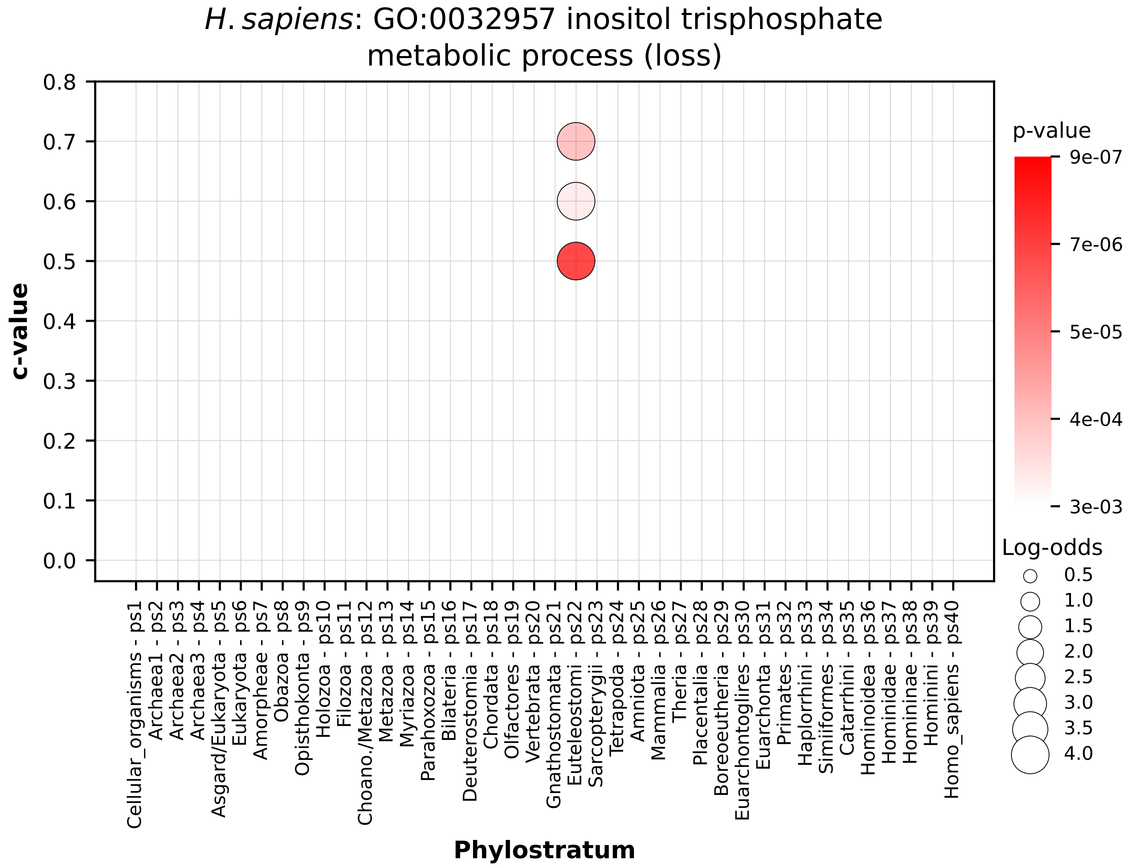

H. sapiens: GO:0032957 inositol trisphosphate metabolic process (loss)
c-value
0.8
0.7
0.6
0.5
0.4
0.3
0.2
0.1
0.0
p-value
9e-07
7e-06
5e-05
4e-04
3e-03
Log-odds
0.5
1.0
1.5
2.0
2.5
3.0
3.5
4.0
Cellular_organisms - ps1
Archaea1 - ps2
Archaea2 - ps3
Archaea3 - ps4
Asgard/Eukaryota - ps5
Eukaryota - ps6
Amorpheae - ps7
Obazoa - ps8
Opisthokonta - ps9
Holozoa - ps10
Filozoa - ps11
Choano./Metazoa - ps12
Metazoa - ps13
Myriazoa - ps14
Parahoxozoa - ps15
Bilateria - ps16
Deuterostomia - ps17
Chordata - ps18
Olfactores - ps19
Vertebrata - ps20
Gnathostomata - ps21
Euteleostomi - ps22
Sarcopterygii - ps23
Tetrapoda - ps24
Amniota - ps25
Mammalia - ps26
Theria - ps27
Placentalia - ps28
Boreoeutheria - ps29
Euarchontoglires - ps30
Euarchonta - ps31
Primates - ps32
Haplorrhini - ps33
Simiiformes - ps34
Catarrhini - ps35
Hominoidea - ps36
Hominidae - ps37
Homininae - ps38
Hominini - ps39
Homo_sapiens - ps40
Phylostratum

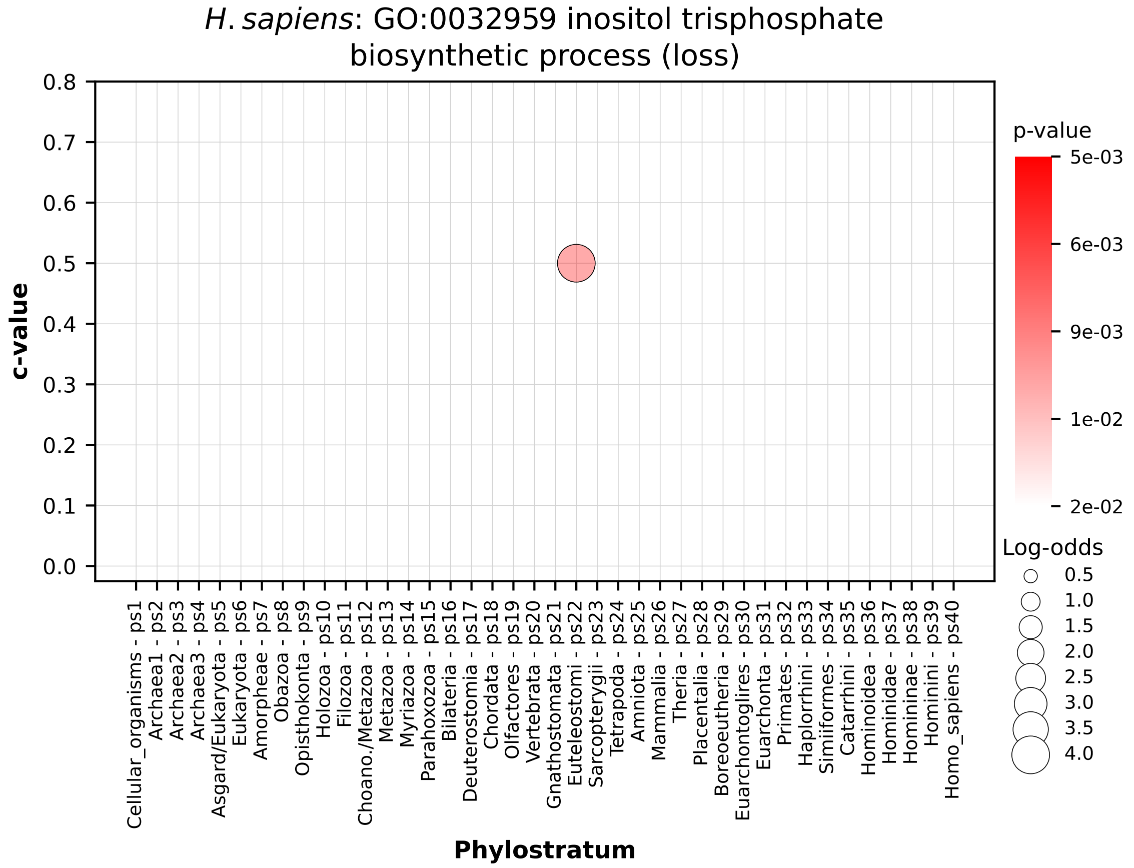

H. sapiens: GO:0032959 inositol trisphosphate biosynthetic process (loss)
c-value
0.0
0.1
0.2
0.3
0.4
0.5
0.6
0.7
0.8
Phylostratum
Cellular_organisms - ps1
Archaea1 - ps2
Archaea2 - ps3
Archaea3 - ps4
Asgard/Eukaryota - ps5
Eukaryota - ps6
Amorpheae - ps7
Obazoa - ps8
Opisthokonta - ps9
Holozoa - ps10
Filozoa - ps11
Choano./Metazoa - ps12
Metazoa - ps13
Myriazoa - ps14
Parahoxozoa - ps15
Bilateria - ps16
Deuterostomia - ps17
Chordata - ps18
Olfactores - ps19
Vertebrata - ps20
Gnathostomata - ps21
Euteleostomi - ps22
Sarcopterygii - ps23
Tetrapoda - ps24
Amniota - ps25
Mammalia - ps26
Theria - ps27
Placentalia - ps28
Boreoeutheria - ps29
Euarchontoglires - ps30
Euarchonta - ps31
Primates - ps32
Haplorrhini - ps33
Simiiformes - ps34
Catarrhini - ps35
Hominoidea - ps36
Hominidae - ps37
Homininae - ps38
Hominini - ps39
Homo_sapiens - ps40
p-value
5e-03
6e-03
9e-03
1e-02
2e-02
Log-odds
0.5
1.0
1.5
2.0
2.5
3.0
3.5
4.0

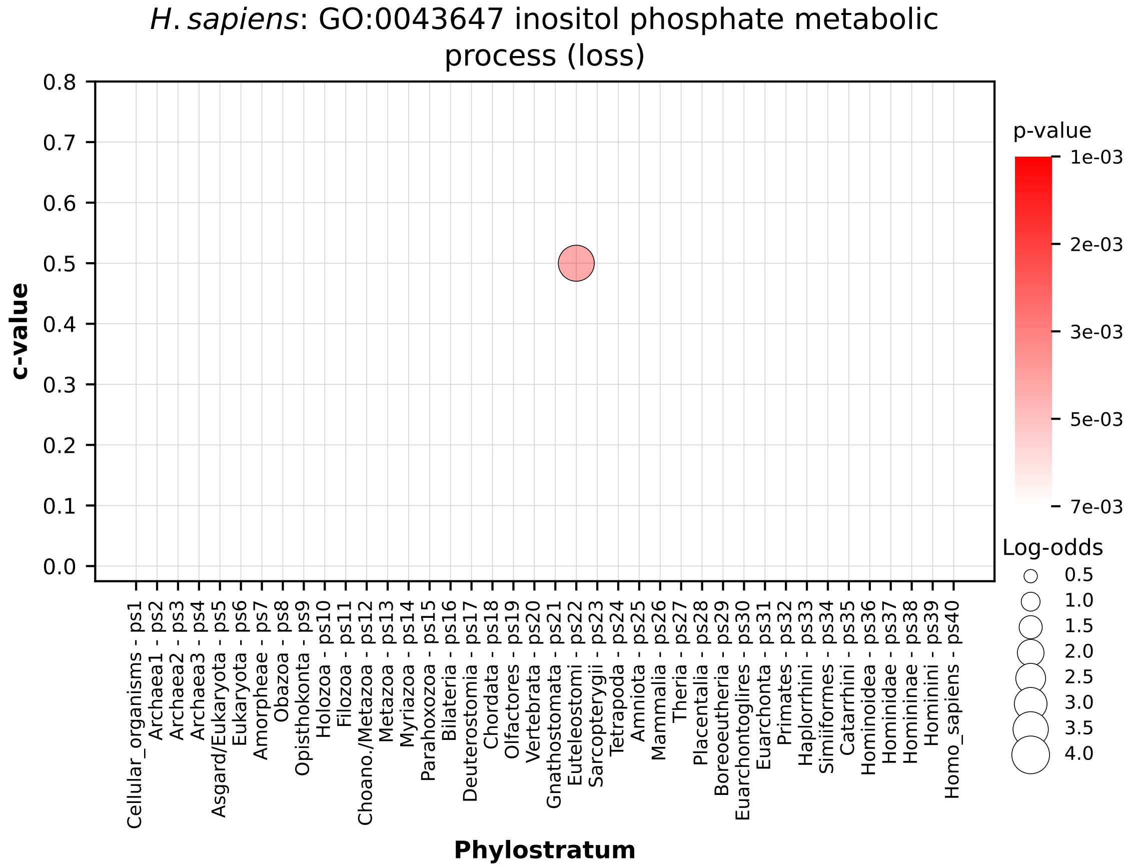

H. sapiens: GO:0043647 inositol phosphate metabolic process (loss)
c-value
0.0
0.1
0.2
0.3
0.4
0.5
0.6
0.7
0.8
Phylostratum
Cellular_organisms - ps1
Archaea1 - ps2
Archaea2 - ps3
Archaea3 - ps4
Asgard/Eukaryota - ps5
Eukaryota - ps6
Amorpheae - ps7
Obazoa - ps8
Opisthokonta - ps9
Holozoa - ps10
Filozoa - ps11
Choano./Metazoa - ps12
Metazoa - ps13
Myriazoa - ps14
Parahoxozoa - ps15
Bilateria - ps16
Deuterostomia - ps17
Chordata - ps18
Olfactores - ps19
Vertebrata - ps20
Gnathostomata - ps21
Euteleostomi - ps22
Sarcopterygii - ps23
Tetrapoda - ps24
Amniota - ps25
Mammalia - ps26
Theria - ps27
Placentalia - ps28
Boreoeutheria - ps29
Euarchontoglires - ps30
Euarchonta - ps31
Primates - ps32
Haplorrhini - ps33
Simiiformes - ps34
Catarrhini - ps35
Hominoidea - ps36
Hominidae - ps37
Homininae - ps38
Hominini - ps39
Homo_sapiens - ps40
p-value
1e-03
2e-03
3e-03
5e-03
7e-03
Log-odds
0.5
1.0
1.5
2.0
2.5
3.0
3.5
4.0

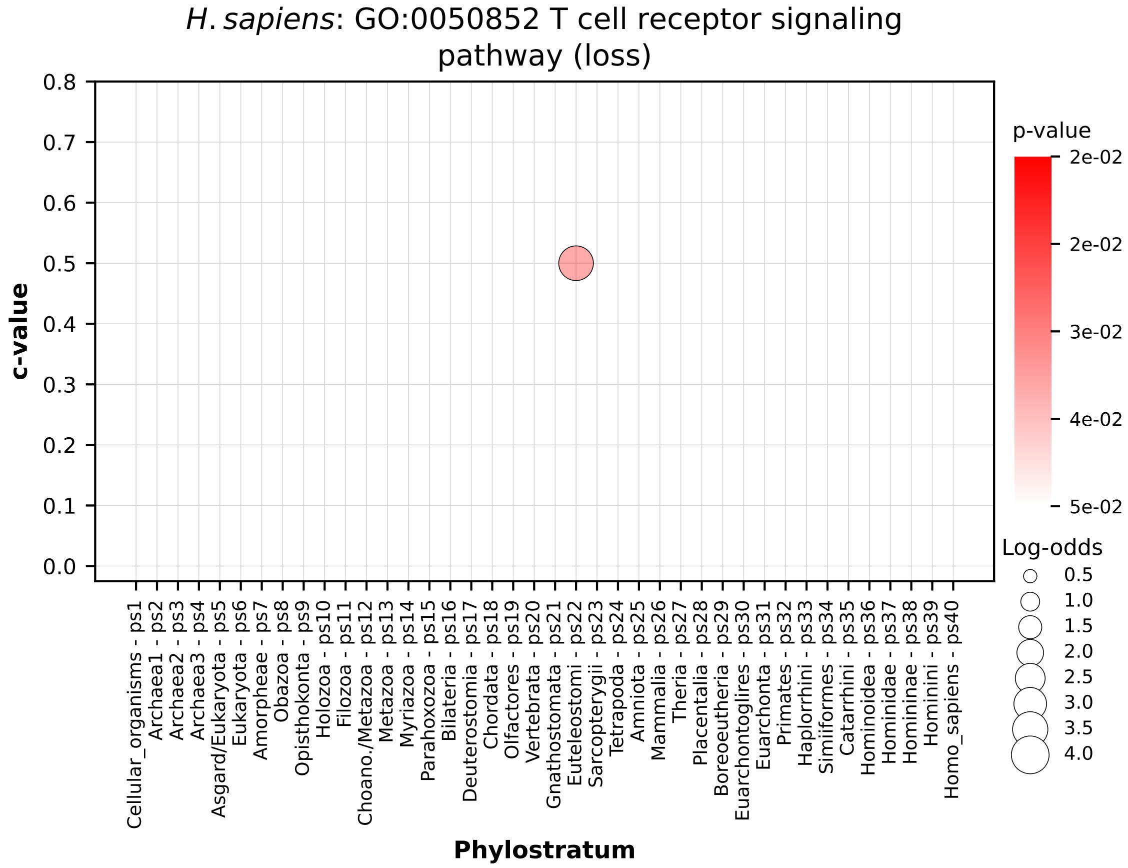

H. sapiens: GO:0050852 T cell receptor signaling pathway (loss)
c-value
0.8
0.7
0.6
0.5
0.4
0.3
0.2
0.1
0.0
p-value
2e-02
2e-02
3e-02
4e-02
5e-02
Log-odds
0.5
1.0
1.5
2.0
2.5
3.0
3.5
4.0
Cellular_organisms - ps1
Archaea1 - ps2
Archaea2 - ps3
Archaea3 - ps4
Asgard/Eukaryota - ps5
Eukaryota - ps6
Amorpheae - ps7
Obazoa - ps8
Opisthokonta - ps9
Holozoa - ps10
Filozoa - ps11
Choano./Metazoa - ps12
Metazoa - ps13
Myriazoa - ps14
Parahoxozoa - ps15
Bilateria - ps16
Deuterostomia - ps17
Chordata - ps18
Olfactores - ps19
Vertebrata - ps20
Gnathostomata - ps21
Euteleostomi - ps22
Sarcopterygii - ps23
Tetrapoda - ps24
Amniota - ps25
Mammalia - ps26
Theria - ps27
Placentalia - ps28
Boreoeutheria - ps29
Euarchontoglires - ps30
Euarchonta - ps31
Primates - ps32
Haplorrhini - ps33
Simiiformes - ps34
Catarrhini - ps35
Hominoidea - ps36
Hominidae - ps37
Homininae - ps38
Hominini - ps39
Homo_sapiens - ps40
Phylostratum

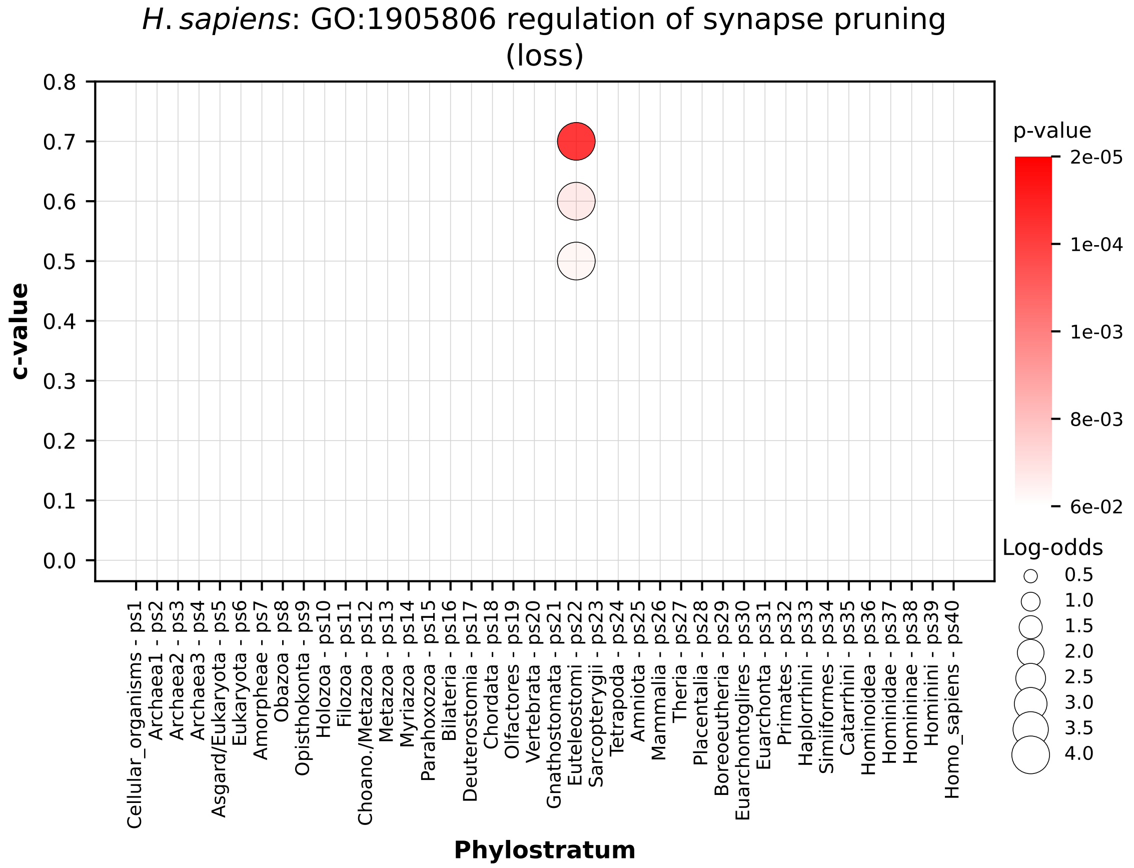

H. sapiens: GO:1905806 regulation of synapse pruning (loss)
c-value
0.8
0.7
0.6
0.5
0.4
0.3
0.2
0.1
0.0
Phylostratum
Cellular_organisms - ps1
Archaea1 - ps2
Archaea2 - ps3
Archaea3 - ps4
Asgard/Eukaryota - ps5
Eukaryota - ps6
Amorpheae - ps7
Obazoa - ps8
Opisthokonta - ps9
Holozoa - ps10
Filozoa - ps11
Choano./Metazoa - ps12
Metazoa - ps13
Myriazoa - ps14
Parahoxozoa - ps15
Bilateria - ps16
Deuterostomia - ps17
Chordata - ps18
Olfactores - ps19
Vertebrata - ps20
Gnathostomata - ps21
Euteleostomi - ps22
Sarcopterygii - ps23
Tetrapoda - ps24
Amniota - ps25
Mammalia - ps26
Theria - ps27
Placentalia - ps28
Boreoeutheria - ps29
Euarchontoglires - ps30
Euarchonta - ps31
Primates - ps32
Haplorrhini - ps33
Simiiformes - ps34
Catarrhini - ps35
Hominoidea - ps36
Hominidae - ps37
Homininae - ps38
Hominini - ps39
Homo_sapiens - ps40
p-value
2e-05
1e-04
1e-03
8e-03
6e-02
Log-odds
0.5
1.0
1.5
2.0
2.5
3.0
3.5
4.0

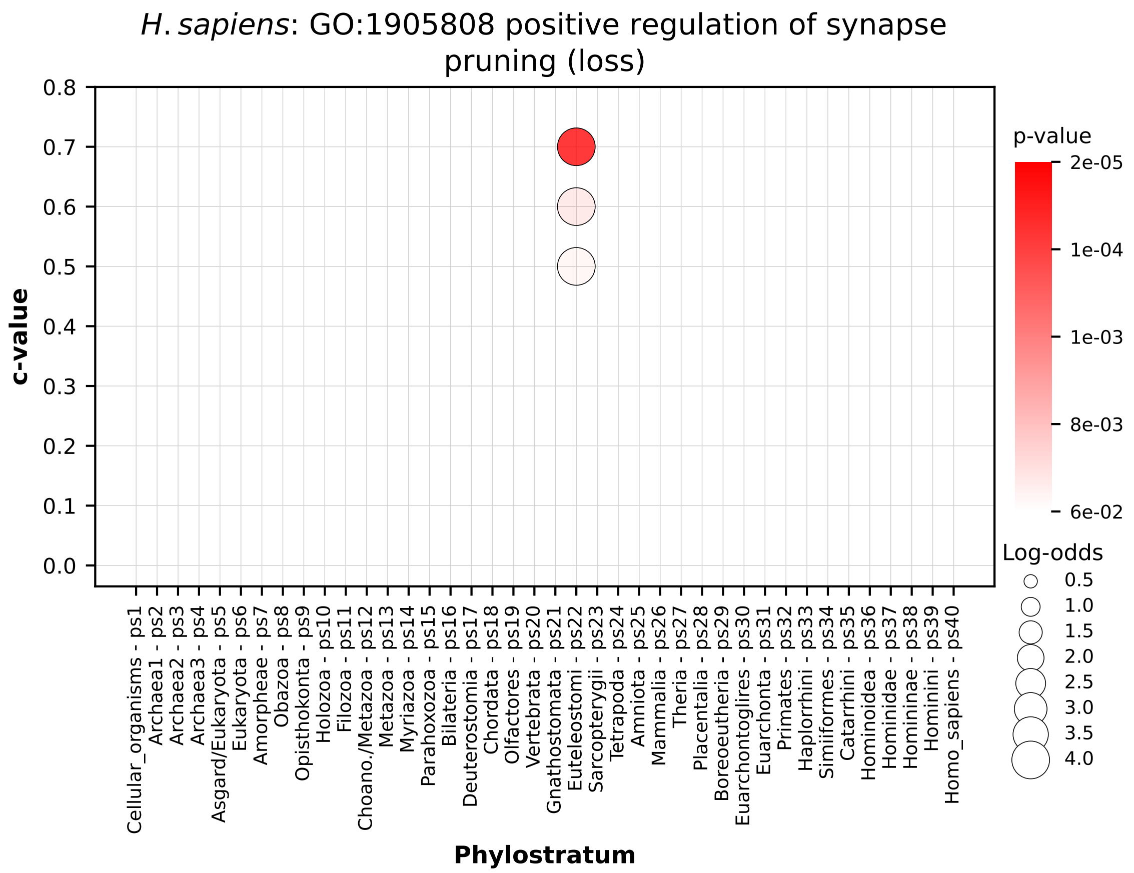

H. sapiens: GO:1905808 positive regulation of synapse pruning (loss)
c-value
0.8
0.7
0.6
0.5
0.4
0.3
0.2
0.1
0.0
p-value
2e-05
1e-04
1e-03
8e-03
6e-02
Log-odds
0.5
1.0
1.5
2.0
2.5
3.0
3.5
4.0
Cellular_organisms - ps1
Archaea1 - ps2
Archaea2 - ps3
Archaea3 - ps4
Asgard/Eukaryota - ps5
Eukaryota - ps6
Amorpheae - ps7
Obazoa - ps8
Opisthokonta - ps9
Holozoa - ps10
Filozoa - ps11
Choano./Metazoa - ps12
Metazoa - ps13
Myriazoa - ps14
Parahoxozoa - ps15
Bilateria - ps16
Deuterostomia - ps17
Chordata - ps18
Olfactores - ps19
Vertebrata - ps20
Gnathostomata - ps21
Euteleostomi - ps22
Sarcopterygii - ps23
Tetrapoda - ps24
Amniota - ps25
Mammalia - ps26
Theria - ps27
Placentalia - ps28
Boreoeutheria - ps29
Euarchontoglires - ps30
Euarchonta - ps31
Primates - ps32
Haplorrhini - ps33
Simiiformes - ps34
Catarrhini - ps35
Hominoidea - ps36
Hominidae - ps37
Homininae - ps38
Hominini - ps39
Homo_sapiens - ps40
Phylostratum

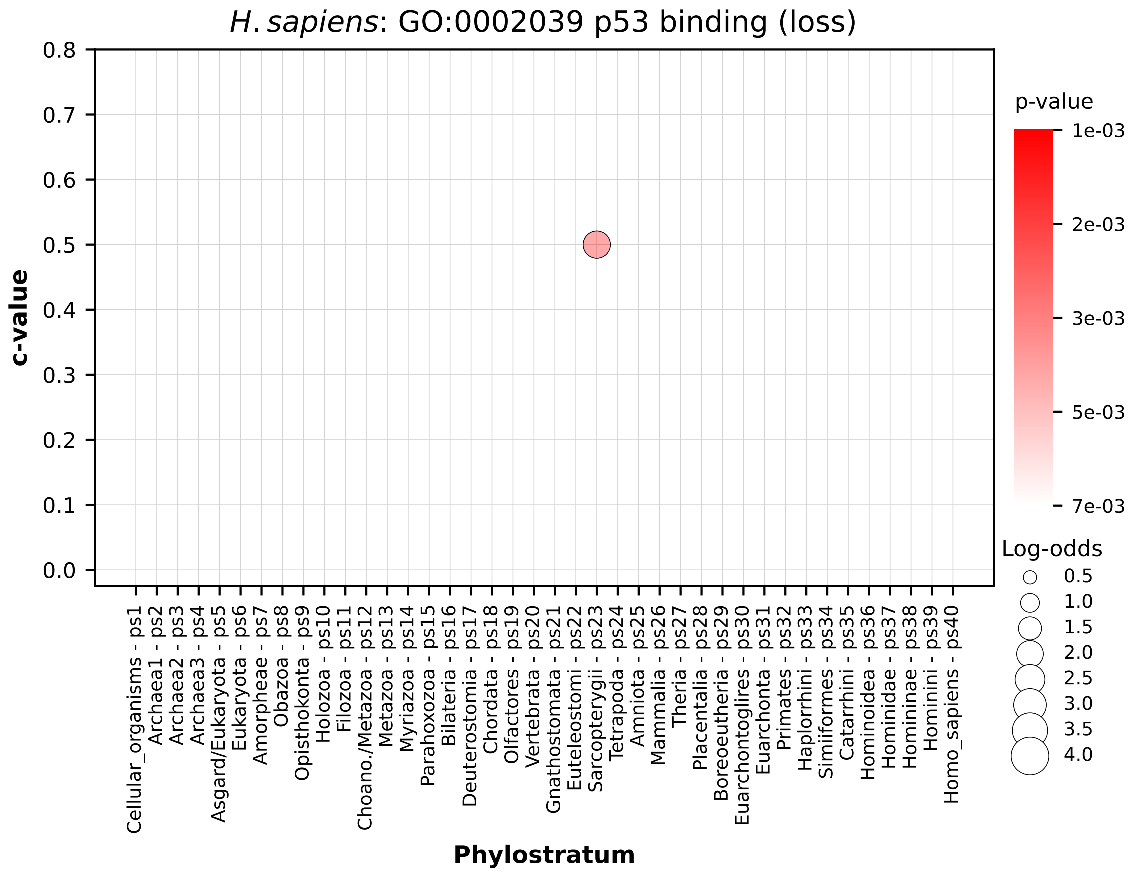

H. sapiens: GO:0002039 p53 binding (loss)
c-value
0.0
0.1
0.2
0.3
0.4
0.5
0.6
0.7
0.8
Phylostratum
Cellular_organisms - ps1
Archaea1 - ps2
Archaea2 - ps3
Archaea3 - ps4
Asgard/Eukaryota - ps5
Eukaryota - ps6
Amorpheae - ps7
Obazoa - ps8
Opisthokonta - ps9
Holozoa - ps10
Filozoa - ps11
Choano./Metazoa - ps12
Metazoa - ps13
Myriazoa - ps14
Parahoxozoa - ps15
Bilateria - ps16
Deuterostomia - ps17
Chordata - ps18
Olfactores - ps19
Vertebrata - ps20
Gnathostomata - ps21
Euteleostomi - ps22
Sarcopterygii - ps23
Tetrapoda - ps24
Amniota - ps25
Mammalia - ps26
Theria - ps27
Placentalia - ps28
Boreoeutheria - ps29
Euarchontoglires - ps30
Euarchonta - ps31
Primates - ps32
Haplorrhini - ps33
Simiiformes - ps34
Catarrhini - ps35
Hominoidea - ps36
Hominidae - ps37
Homininae - ps38
Hominini - ps39
Homo_sapiens - ps40
p-value
1e-03
2e-03
3e-03
5e-03
7e-03
Log-odds
0.5
1.0
1.5
2.0
2.5
3.0
3.5
4.0

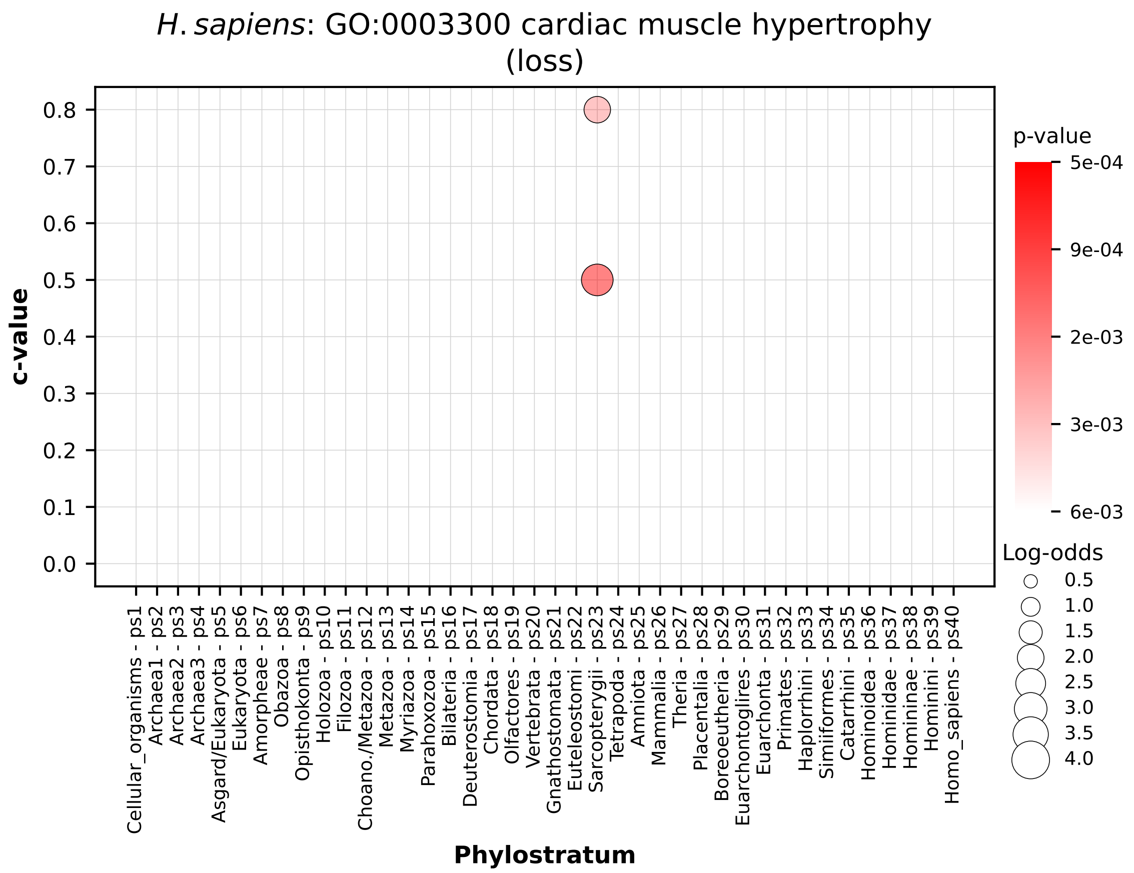
H. sapiens: GO:0003300 cardiac muscle hypertrophy
(loss)
c-value
0.0
0.1
0.2
0.3
0.4
0.5
0.6
0.7
0.8
p-value
5e-04
9e-04
2e-03
3e-03
6e-03
Log-odds
0.5
1.0
1.5
2.0
2.5
3.0
3.5
4.0
Cellular_organisms - ps1
Archaea1 - ps2
Archaea2 - ps3
Archaea3 - ps4
Asgard/Eukaryota - ps5
Eukaryota - ps6
Amorpheae - ps7
Obazoa - ps8
Opisthokonta - ps9
Holozoa - ps10
Filozoa - ps11
Choano./Metazoa - ps12
Metazoa - ps13
Myriazoa - ps14
Parahoxozoa - ps15
Bilateria - ps16
Deuterostomia - ps17
Chordata - ps18
Olfactores - ps19
Vertebrata - ps20
Gnathostomata - ps21
Euteleostomi - ps22
Sarcopterygii - ps23
Tetrapoda - ps24
Amniota - ps25
Mammalia - ps26
Theria - ps27
Placentalia - ps28
Boreoeutheria - ps29
Euarchontoglires - ps30
Euarchonta - ps31
Primates - ps32
Haplorrhini - ps33
Simiiformes - ps34
Catarrhini - ps35
Hominoidea - ps36
Hominidae - ps37
Homininae - ps38
Hominini - ps39
Homo_sapiens - ps40
Phylostratum

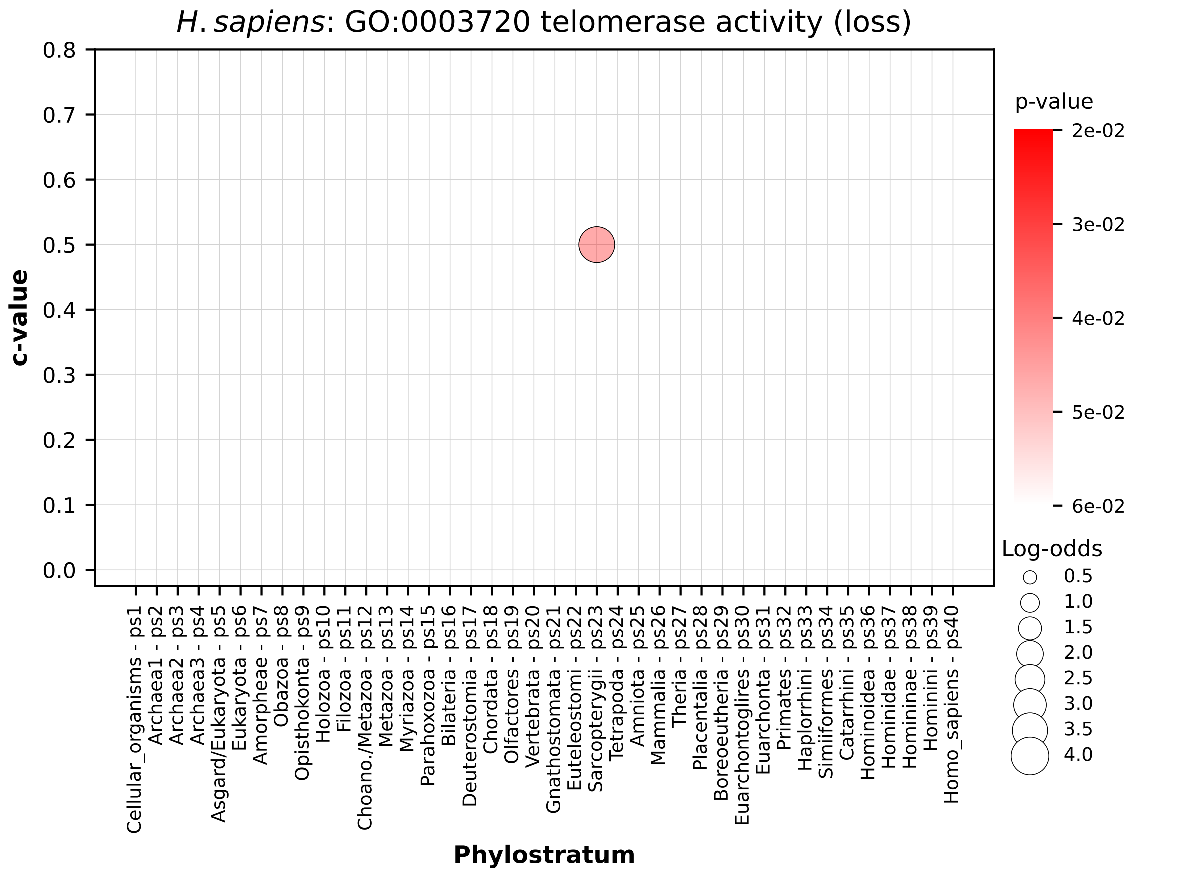

H. sapiens: GO:0003720 telomerase activity (loss)
c-value
0.0
0.1
0.2
0.3
0.4
0.5
0.6
0.7
0.8
Phylostratum
Cellular_organisms - ps1
Archaea1 - ps2
Archaea2 - ps3
Archaea3 - ps4
Asgard/Eukaryota - ps5
Eukaryota - ps6
Amorpheae - ps7
Obazoa - ps8
Opisthokonta - ps9
Holozoa - ps10
Filozoa - ps11
Choano./Metazoa - ps12
Metazoa - ps13
Myriazoa - ps14
Parahoxozoa - ps15
Bilateria - ps16
Deuterostomia - ps17
Chordata - ps18
Olfactores - ps19
Vertebrata - ps20
Gnathostomata - ps21
Euteleostomi - ps22
Sarcopterygii - ps23
Tetrapoda - ps24
Amniota - ps25
Mammalia - ps26
Theria - ps27
Placentalia - ps28
Boreoeutheria - ps29
Euarchontoglires - ps30
Euarchonta - ps31
Primates - ps32
Haplorrhini - ps33
Simiiformes - ps34
Catarrhini - ps35
Hominoidea - ps36
Hominidae - ps37
Homininae - ps38
Hominini - ps39
Homo_sapiens - ps40
p-value
2e-02
3e-02
4e-02
5e-02
6e-02
Log-odds
0.5
1.0
1.5
2.0
2.5
3.0
3.5
4.0

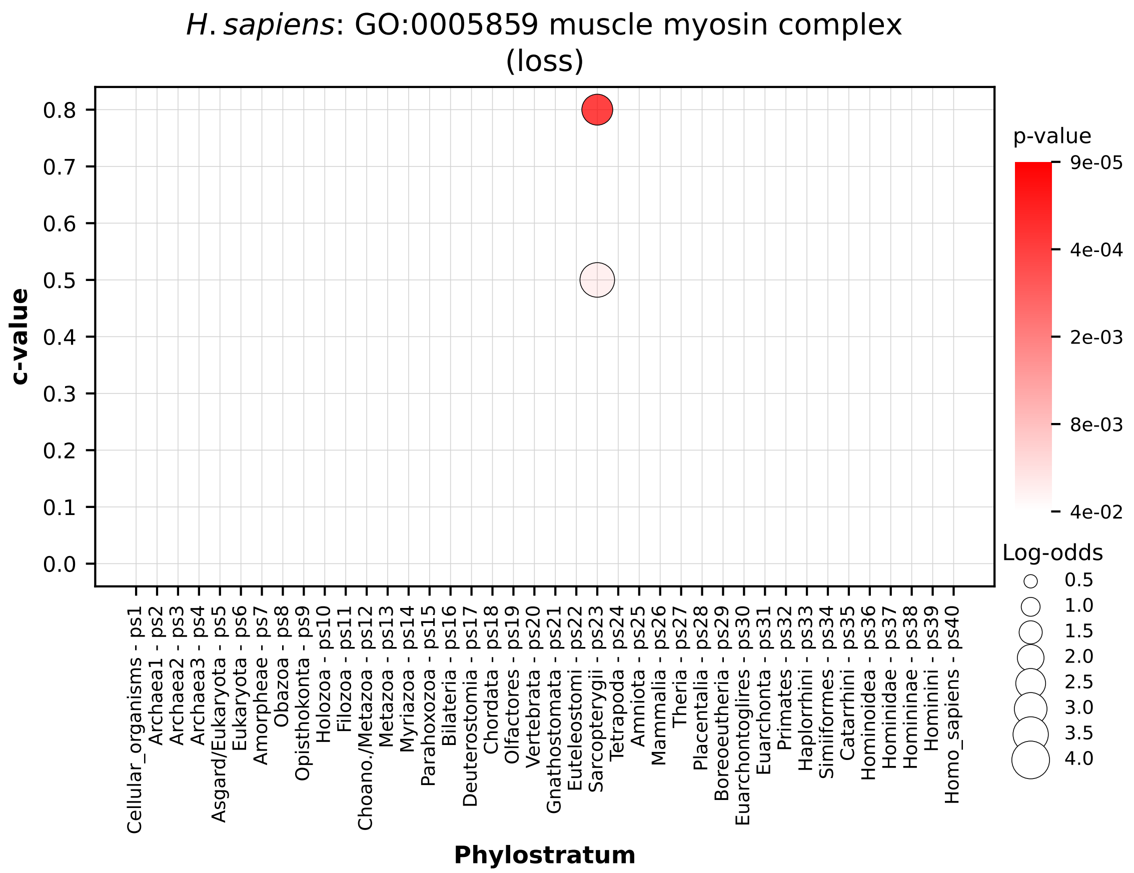

H. sapiens: GO:0005859 muscle myosin complex
(loss)
c-value
0.8
0.7
0.6
0.5
0.4
0.3
0.2
0.1
0.0
p-value
9e-05
4e-04
2e-03
8e-03
4e-02
Log-odds
0.5
1.0
1.5
2.0
2.5
3.0
3.5
4.0
Cellular_organisms - ps1
Archaea1 - ps2
Archaea2 - ps3
Archaea3 - ps4
Asgard/Eukaryota - ps5
Eukaryota - ps6
Amorpheae - ps7
Obazoa - ps8
Opisthokonta - ps9
Holozoa - ps10
Filozoa - ps11
Choano./Metazoa - ps12
Metazoa - ps13
Myriazoa - ps14
Parahoxozoa - ps15
Bilateria - ps16
Deuterostomia - ps17
Chordata - ps18
Olfactores - ps19
Vertebrata - ps20
Gnathostomata - ps21
Euteleostomi - ps22
Sarcopterygii - ps23
Tetrapoda - ps24
Amniota - ps25
Mammalia - ps26
Theria - ps27
Placentalia - ps28
Boreoeutheria - ps29
Euarchontoglires - ps30
Euarchonta - ps31
Primates - ps32
Haplorrhini - ps33
Simiiformes - ps34
Catarrhini - ps35
Hominoidea - ps36
Hominidae - ps37
Homininae - ps38
Hominini - ps39
Homo_sapiens - ps40
Phylostratum

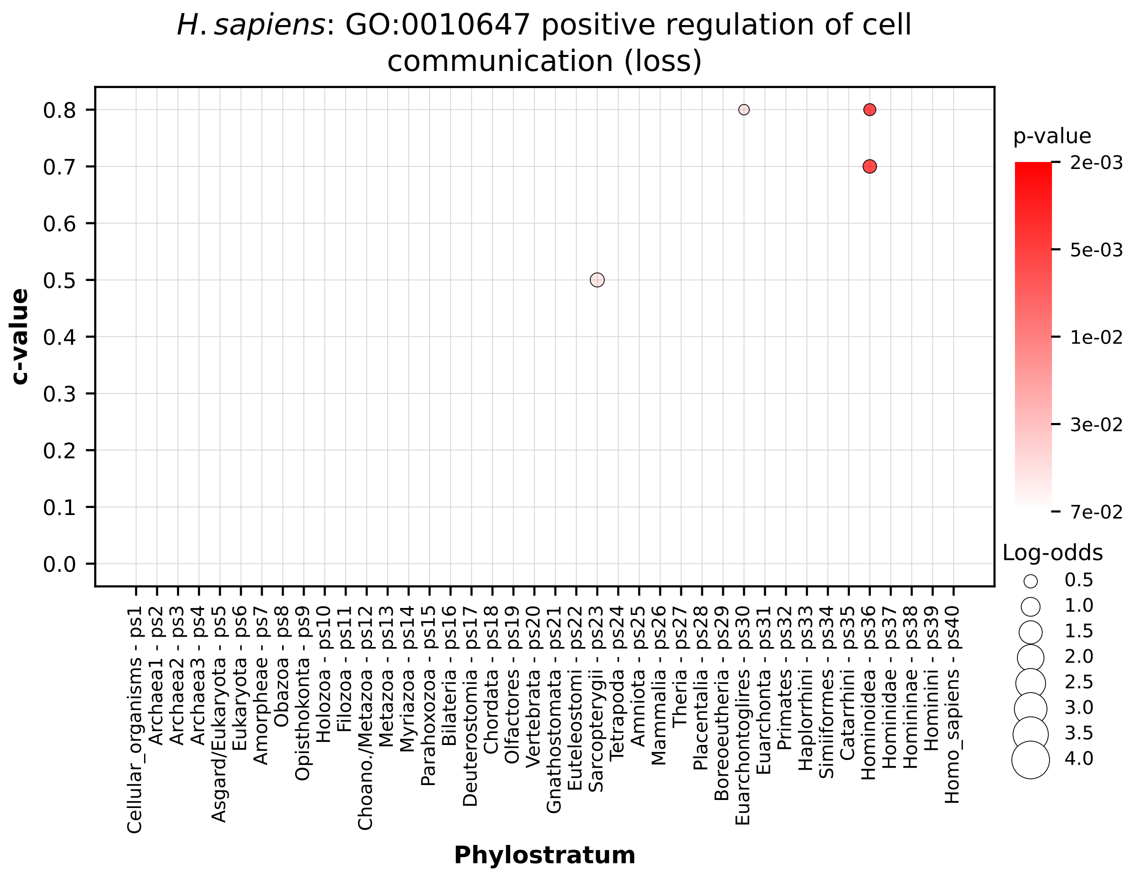

H. sapiens: GO:0010647 positive regulation of cell communication (loss)
c-value
0.0
0.1
0.2
0.3
0.4
0.5
0.6
0.7
0.8
Phylostratum
Cellular_organisms - ps1
Archaea1 - ps2
Archaea2 - ps3
Archaea3 - ps4
Asgard/Eukaryota - ps5
Eukaryota - ps6
Amorpheae - ps7
Obazoa - ps8
Opisthokonta - ps9
Holozoa - ps10
Filozoa - ps11
Choano./Metazoa - ps12
Metazoa - ps13
Myriazoa - ps14
Parahoxozoa - ps15
Bilateria - ps16
Deuterostomia - ps17
Chordata - ps18
Olfactores - ps19
Vertebrata - ps20
Gnathostomata - ps21
Euteleostomi - ps22
Sarcopterygii - ps23
Tetrapoda - ps24
Amniota - ps25
Mammalia - ps26
Theria - ps27
Placentalia - ps28
Boreoeutheria - ps29
Euarchontoglires - ps30
Euarchonta - ps31
Primates - ps32
Haplorrhini - ps33
Simiiformes - ps34
Catarrhini - ps35
Hominoidea - ps36
Hominidae - ps37
Homininae - ps38
Hominini - ps39
Homo_sapiens - ps40
p-value
2e-03
5e-03
1e-02
3e-02
7e-02
Log-odds
0.5
1.0
1.5
2.0
2.5
3.0
3.5
4.0

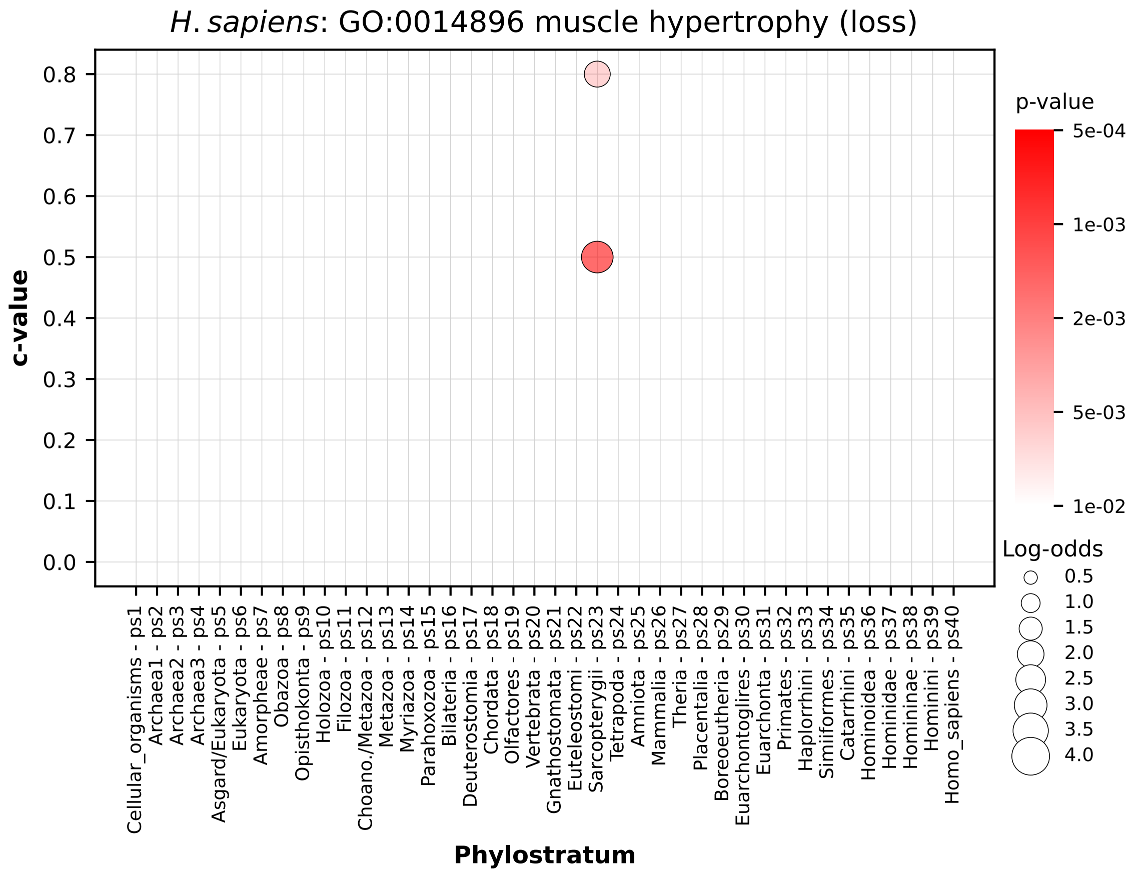

H. sapiens: GO:0014896 muscle hypertrophy (loss)
c-value
0.8
0.7
0.6
0.5
0.4
0.3
0.2
0.1
0.0
Phylostratum
Cellular_organisms - ps1
Archaea1 - ps2
Archaea2 - ps3
Archaea3 - ps4
Asgard/Eukaryota - ps5
Eukaryota - ps6
Amorpheae - ps7
Obazoa - ps8
Opisthokonta - ps9
Holozoa - ps10
Filozoa - ps11
Choano./Metazoa - ps12
Metazoa - ps13
Myriazoa - ps14
Parahoxozoa - ps15
Bilateria - ps16
Deuterostomia - ps17
Chordata - ps18
Olfactores - ps19
Vertebrata - ps20
Gnathostomata - ps21
Euteleostomi - ps22
Sarcopterygii - ps23
Tetrapoda - ps24
Amniota - ps25
Mammalia - ps26
Theria - ps27
Placentalia - ps28
Boreoeutheria - ps29
Euarchontoglires - ps30
Euarchonta - ps31
Primates - ps32
Haplorrhini - ps33
Simiiformes - ps34
Catarrhini - ps35
Hominoidea - ps36
Hominidae - ps37
Homininae - ps38
Hominini - ps39
Homo_sapiens - ps40
p-value
5e-04
1e-03
2e-03
5e-03
1e-02
Log-odds
0.5
1.0
1.5
2.0
2.5
3.0
3.5
4.0

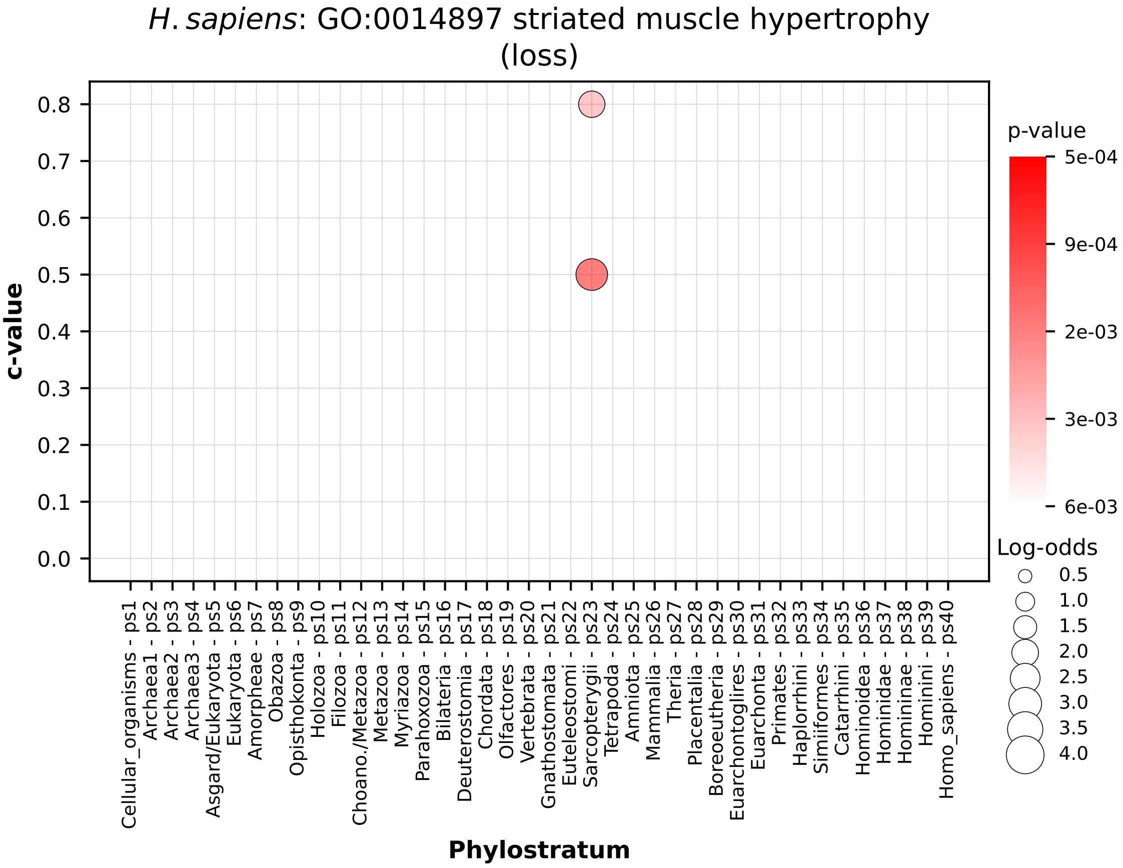

H. sapiens: GO:0014897 striated muscle hypertrophy
(loss)
c-value
0.8
0.7
0.6
0.5
0.4
0.3
0.2
0.1
0.0
p-value
5e-04
9e-04
2e-03
3e-03
6e-03
Log-odds
0.5
1.0
1.5
2.0
2.5
3.0
3.5
4.0
Cellular_organisms - ps1
Archaea1 - ps2
Archaea2 - ps3
Archaea3 - ps4
Asgard/Eukaryota - ps5
Eukaryota - ps6
Amorpheae - ps7
Obazoa - ps8
Opisthokonta - ps9
Holozoa - ps10
Filozoa - ps11
Choano./Metazoa - ps12
Metazoa - ps13
Myriazoa - ps14
Parahoxozoa - ps15
Bilateria - ps16
Deuterostomia - ps17
Chordata - ps18
Olfactores - ps19
Vertebrata - ps20
Gnathostomata - ps21
Euteleostomi - ps22
Sarcopterygii - ps23
Tetrapoda - ps24
Amniota - ps25
Mammalia - ps26
Theria - ps27
Placentalia - ps28
Boreoeutheria - ps29
Euarchontoglires - ps30
Euarchonta - ps31
Primates - ps32
Haplorrhini - ps33
Simiiformes - ps34
Catarrhini - ps35
Hominoidea - ps36
Hominidae - ps37
Homininae - ps38
Hominini - ps39
Homo_sapiens - ps40
Phylostratum

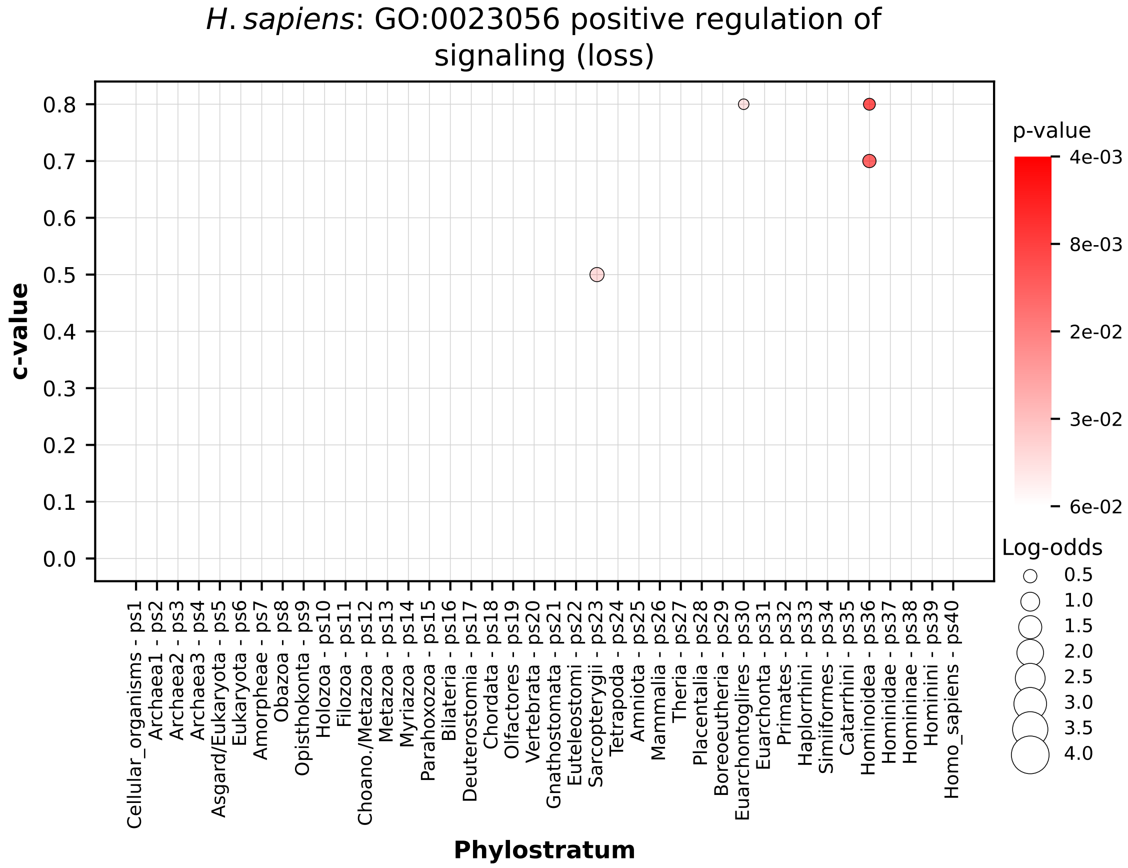

H. sapiens: GO:0023056 positive regulation of signaling (loss)
c-value
0.0
0.1
0.2
0.3
0.4
0.5
0.6
0.7
0.8
Phylostratum
Cellular_organisms - ps1
Archaea1 - ps2
Archaea2 - ps3
Archaea3 - ps4
Asgard/Eukaryota - ps5
Eukaryota - ps6
Amorpheae - ps7
Obazoa - ps8
Opisthokonta - ps9
Holozoa - ps10
Filozoa - ps11
Choano./Metazoa - ps12
Metazoa - ps13
Myriazoa - ps14
Parahoxozoa - ps15
Bilateria - ps16
Deuterostomia - ps17
Chordata - ps18
Olfactores - ps19
Vertebrata - ps20
Gnathostomata - ps21
Euteleostomi - ps22
Sarcopterygii - ps23
Tetrapoda - ps24
Amniota - ps25
Mammalia - ps26
Theria - ps27
Placentalia - ps28
Boreoeutheria - ps29
Euarchontoglires - ps30
Euarchonta - ps31
Primates - ps32
Haplorrhini - ps33
Simiiformes - ps34
Catarrhini - ps35
Hominoidea - ps36
Hominidae - ps37
Homininae - ps38
Hominini - ps39
Homo_sapiens - ps40
p-value
4e-03
8e-03
2e-02
3e-02
6e-02
Log-odds
0.5
1.0
1.5
2.0
2.5
3.0
3.5
4.0

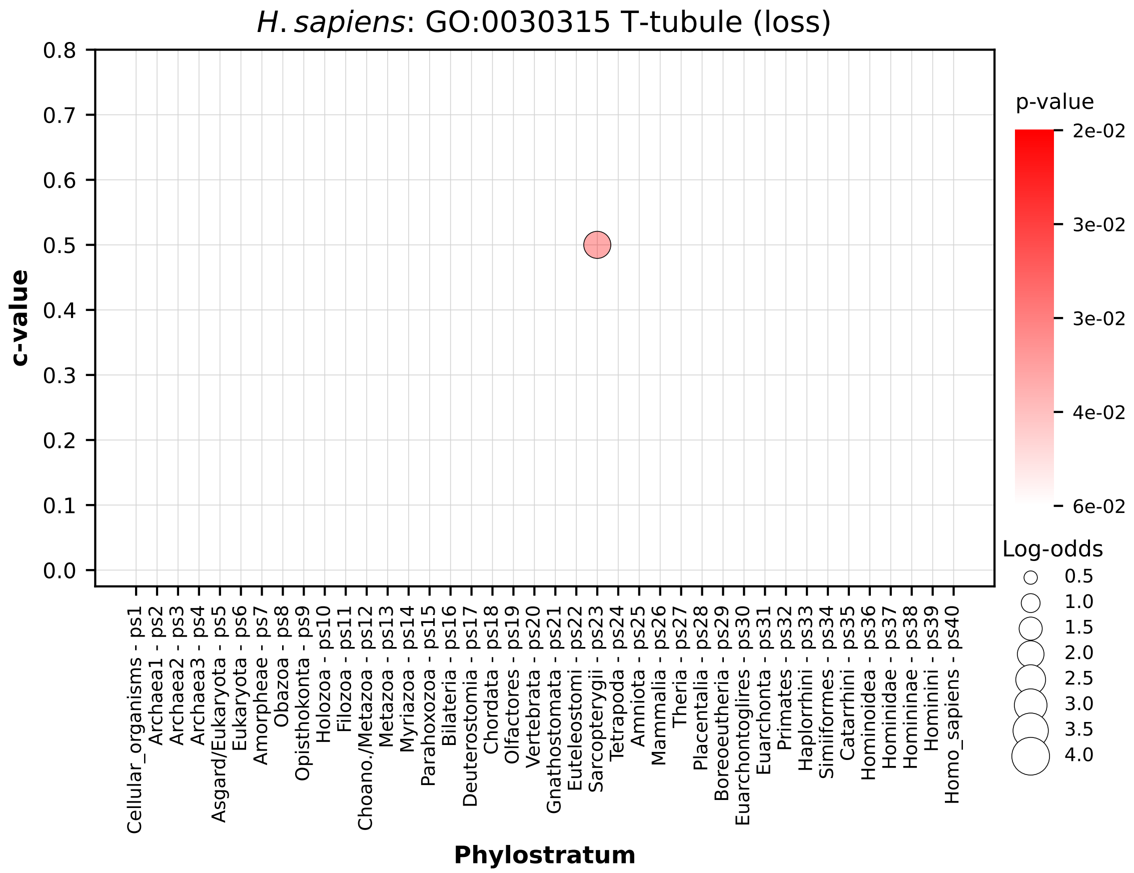

H. sapiens: GO:0030315 T-tubule (loss)
c-value
0.8
0.7
0.6
0.5
0.4
0.3
0.2
0.1
0.0
p-value
2e-02
3e-02
3e-02
4e-02
6e-02
Log-odds
0.5
1.0
1.5
2.0
2.5
3.0
3.5
4.0
Cellular_organisms - ps1
Archaea1 - ps2
Archaea2 - ps3
Archaea3 - ps4
Asgard/Eukaryota - ps5
Eukaryota - ps6
Amorpheae - ps7
Obazoa - ps8
Opisthokonta - ps9
Holozoa - ps10
Filozoa - ps11
Choano./Metazoa - ps12
Metazoa - ps13
Myriazoa - ps14
Parahoxozoa - ps15
Bilateria - ps16
Deuterostomia - ps17
Chordata - ps18
Olfactores - ps19
Vertebrata - ps20
Gnathostomata - ps21
Euteleostomi - ps22
Sarcopterygii - ps23
Tetrapoda - ps24
Amniota - ps25
Mammalia - ps26
Theria - ps27
Placentalia - ps28
Boreoeutheria - ps29
Euarchontoglires - ps30
Euarchonta - ps31
Primates - ps32
Haplorrhini - ps33
Simiiformes - ps34
Catarrhini - ps35
Hominoidea - ps36
Hominidae - ps37
Homininae - ps38
Hominini - ps39
Homo_sapiens - ps40
Phylostratum

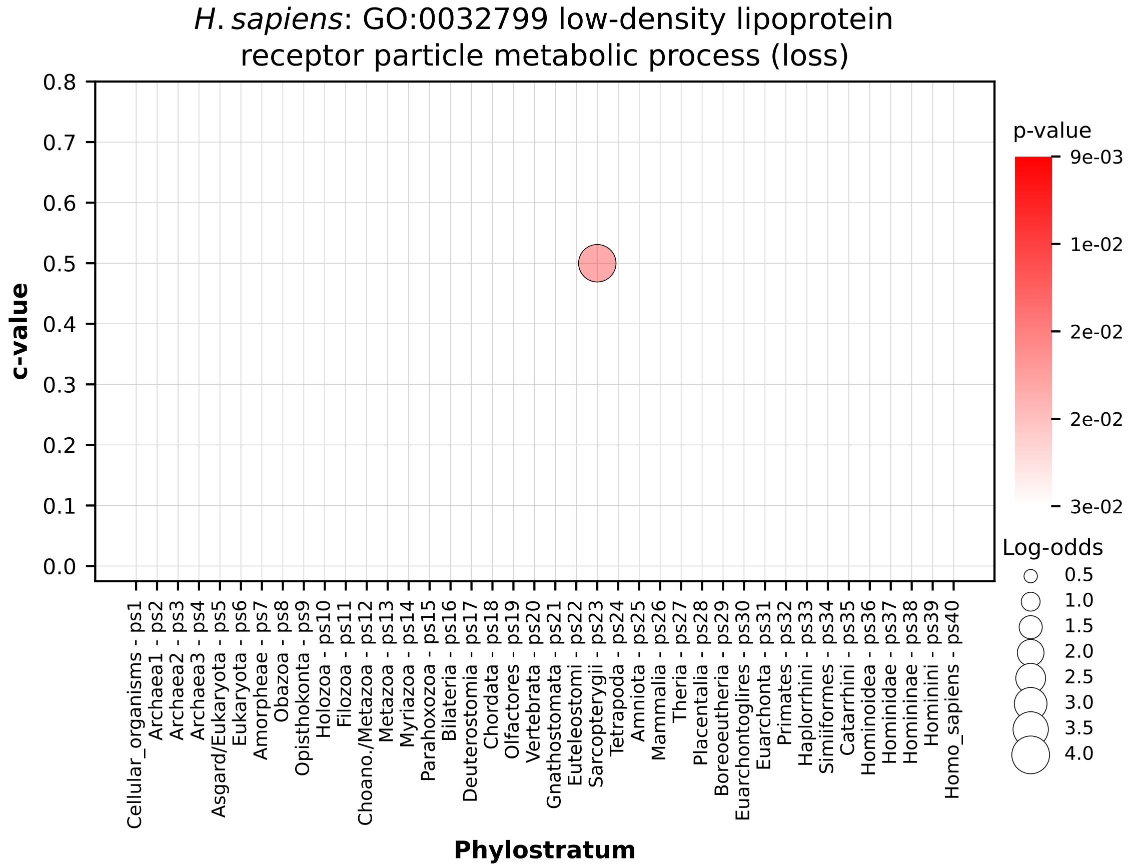

H. sapiens: GO:0032799 low-density lipoprotein receptor particle metabolic process (loss)
c-value
0.0
0.1
0.2
0.3
0.4
0.5
0.6
0.7
0.8
Phylostratum
Cellular_organisms - ps1
Archaea1 - ps2
Archaea2 - ps3
Archaea3 - ps4
Asgard/Eukaryota - ps5
Eukaryota - ps6
Amorpheae - ps7
Obazoa - ps8
Opisthokonta - ps9
Holozoa - ps10
Filozoa - ps11
Choano./Metazoa - ps12
Metazoa - ps13
Myriazoa - ps14
Parahoxozoa - ps15
Bilateria - ps16
Deuterostomia - ps17
Chordata - ps18
Olfactores - ps19
Vertebrata - ps20
Gnathostomata - ps21
Euteleostomi - ps22
Sarcopterygii - ps23
Tetrapoda - ps24
Amniota - ps25
Mammalia - ps26
Theria - ps27
Placentalia - ps28
Boreoeutheria - ps29
Euarchontoglires - ps30
Euarchonta - ps31
Primates - ps32
Haplorrhini - ps33
Simiiformes - ps34
Catarrhini - ps35
Hominoidea - ps36
Hominidae - ps37
Homininae - ps38
Hominini - ps39
Homo_sapiens - ps40
p-value
9e-03
1e-02
2e-02
2e-02
3e-02
Log-odds
0.5
1.0
1.5
2.0
2.5
3.0
3.5
4.0

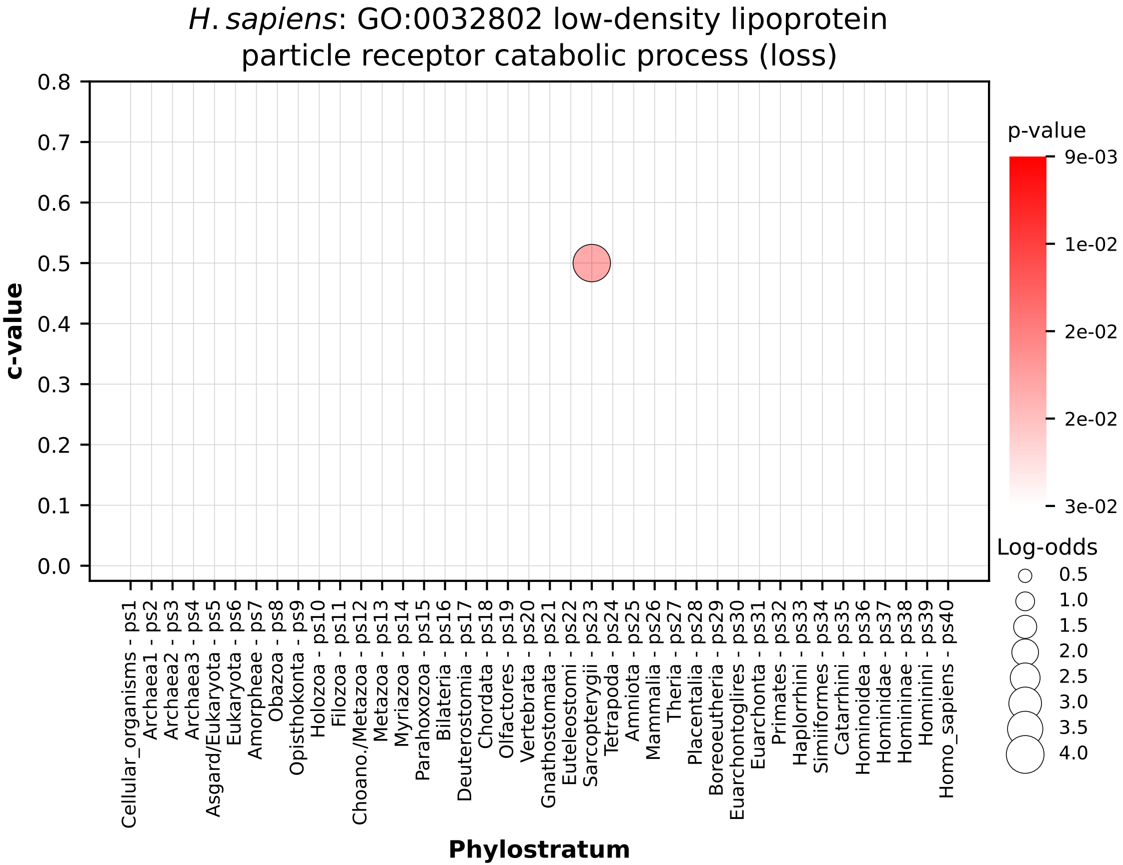

H. sapiens: GO:0032802 low-density lipoprotein particle receptor catabolic process (loss)
c-value
0.0
0.1
0.2
0.3
0.4
0.5
0.6
0.7
0.8
Phylostratum
Cellular_organisms - ps1
Archaea1 - ps2
Archaea2 - ps3
Archaea3 - ps4
Asgard/Eukaryota - ps5
Eukaryota - ps6
Amorpheae - ps7
Obazoa - ps8
Opisthokonta - ps9
Holozoa - ps10
Filozoa - ps11
Choano./Metazoa - ps12
Metazoa - ps13
Myriazoa - ps14
Parahoxozoa - ps15
Bilateria - ps16
Deuterostomia - ps17
Chordata - ps18
Olfactores - ps19
Vertebrata - ps20
Gnathostomata - ps21
Euteleostomi - ps22
Sarcopterygii - ps23
Tetrapoda - ps24
Amniota - ps25
Mammalia - ps26
Theria - ps27
Placentalia - ps28
Boreoeutheria - ps29
Euarchontoglires - ps30
Euarchonta - ps31
Primates - ps32
Haplorrhini - ps33
Simiiformes - ps34
Catarrhini - ps35
Hominoidea - ps36
Hominidae - ps37
Homininae - ps38
Hominini - ps39
Homo_sapiens - ps40
p-value
9e-03
1e-02
2e-02
2e-02
3e-02
Log-odds
0.5
1.0
1.5
2.0
2.5
3.0
3.5
4.0

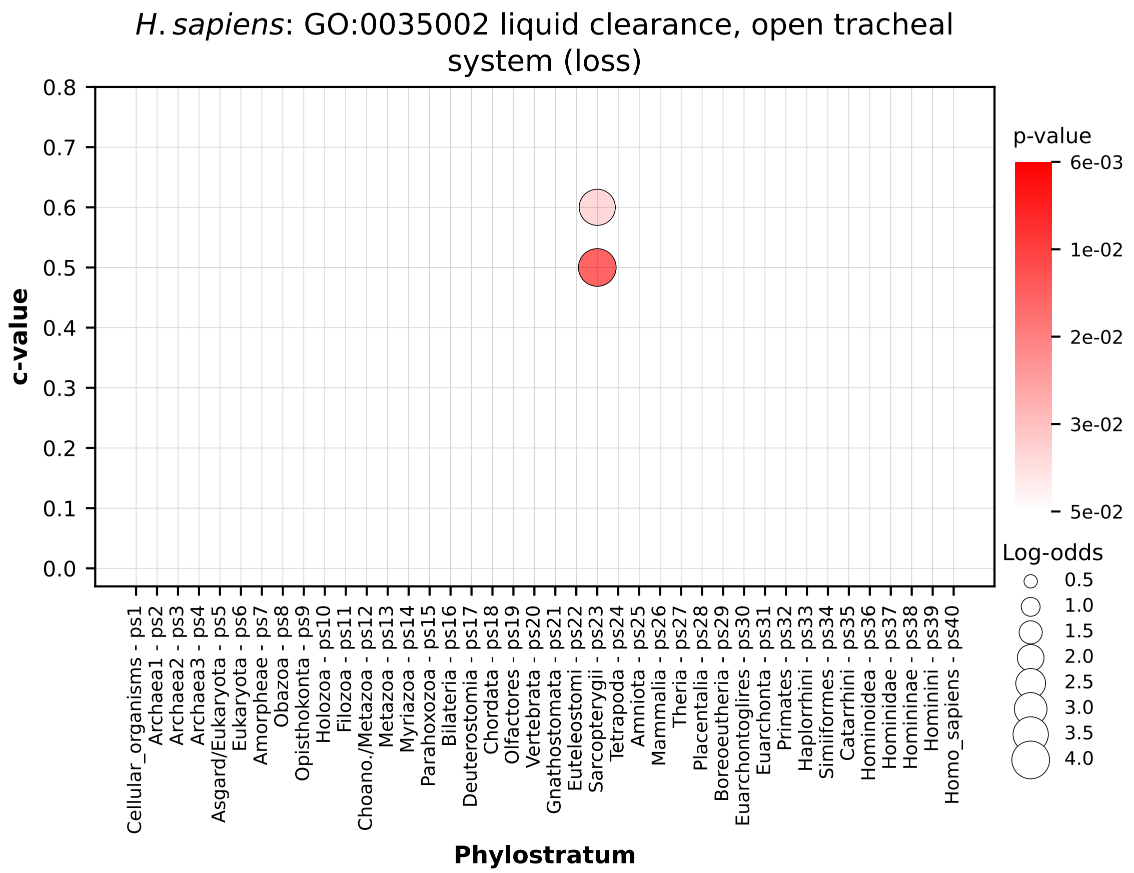

H. sapiens: GO:0035002 liquid clearance, open tracheal system (loss)
c-value
0.8
0.7
0.6
0.5
0.4
0.3
0.2
0.1
0.0
Phylostratum
Cellular_organisms - ps1
Archaea1 - ps2
Archaea2 - ps3
Archaea3 - ps4
Asgard/Eukaryota - ps5
Eukaryota - ps6
Amorpheae - ps7
Obazoa - ps8
Opisthokonta - ps9
Holozoa - ps10
Filozoa - ps11
Choano./Metazoa - ps12
Metazoa - ps13
Myriazoa - ps14
Parahoxozoa - ps15
Bilateria - ps16
Deuterostomia - ps17
Chordata - ps18
Olfactores - ps19
Vertebrata - ps20
Gnathostomata - ps21
Euteleostomi - ps22
Sarcopterygii - ps23
Tetrapoda - ps24
Amniota - ps25
Mammalia - ps26
Theria - ps27
Placentalia - ps28
Boreoeutheria - ps29
Euarchontoglires - ps30
Euarchonta - ps31
Primates - ps32
Haplorrhini - ps33
Simiiformes - ps34
Catarrhini - ps35
Hominoidea - ps36
Hominidae - ps37
Homininae - ps38
Hominini - ps39
Homo_sapiens - ps40
p-value
6e-03
1e-02
2e-02
3e-02
5e-02
Log-odds
0.5
1.0
1.5
2.0
2.5
3.0
3.5
4.0

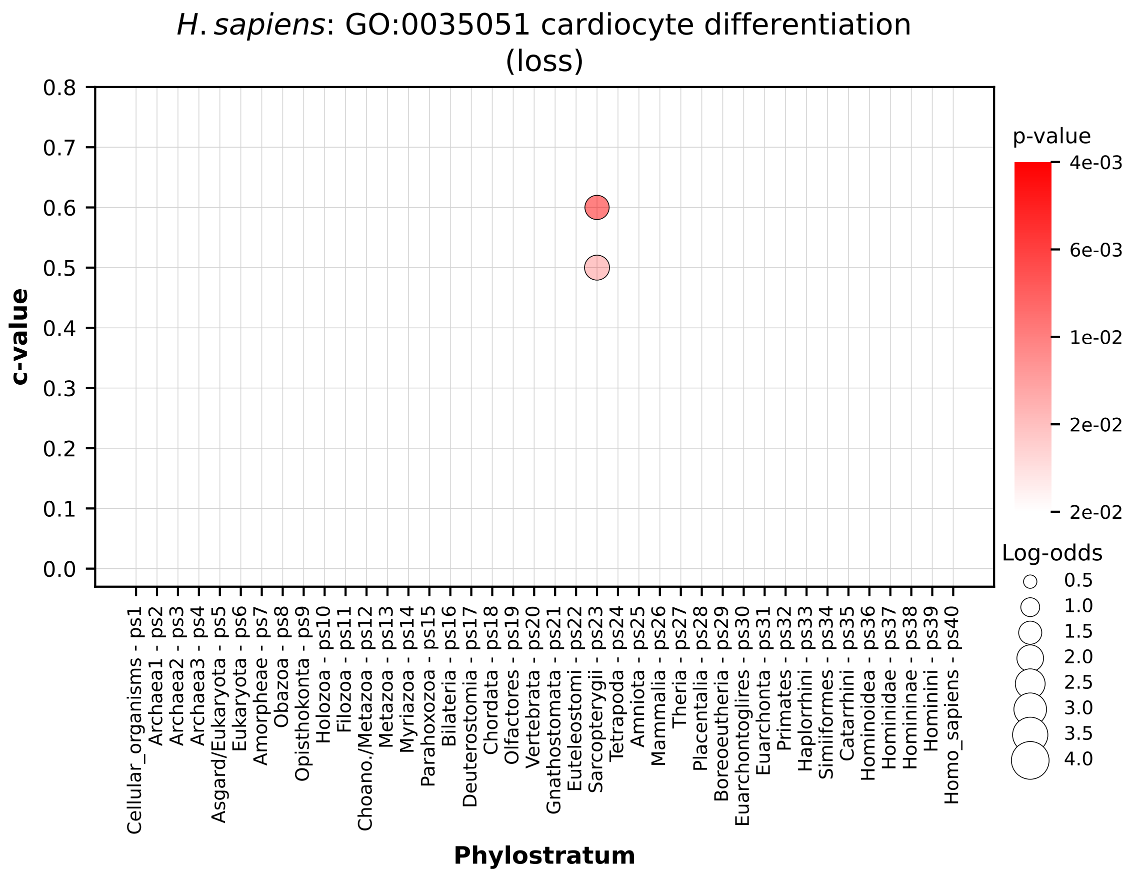
H. sapiens: GO:0035051 cardiocyte differentiation (loss)
c-value
0.8
0.7
0.6
0.5
0.4
0.3
0.2
0.1
0.0
p-value
4e-03
6e-03
1e-02
2e-02
2e-02
Log-odds
0.5
1.0
1.5
2.0
2.5
3.0
3.5
4.0
Cellular_organisms - ps1
Archaea1 - ps2
Archaea2 - ps3
Archaea3 - ps4
Asgard/Eukaryota - ps5
Eukaryota - ps6
Amorpheae - ps7
Obazoa - ps8
Opisthokonta - ps9
Holozoa - ps10
Filozoa - ps11
Choano./Metazoa - ps12
Metazoa - ps13
Myriazoa - ps14
Parahoxozoa - ps15
Bilateria - ps16
Deuterostomia - ps17
Chordata - ps18
Olfactores - ps19
Vertebrata - ps20
Gnathostomata - ps21
Euteleostomi - ps22
Sarcopterygii - ps23
Tetrapoda - ps24
Amniota - ps25
Mammalia - ps26
Theria - ps27
Placentalia - ps28
Boreoeutheria - ps29
Euarchontoglires - ps30
Euarchonta - ps31
Primates - ps32
Haplorrhini - ps33
Simiiformes - ps34
Catarrhini - ps35
Hominoidea - ps36
Hominidae - ps37
Homininae - ps38
Hominini - ps39
Homo_sapiens - ps40
Phylostratum

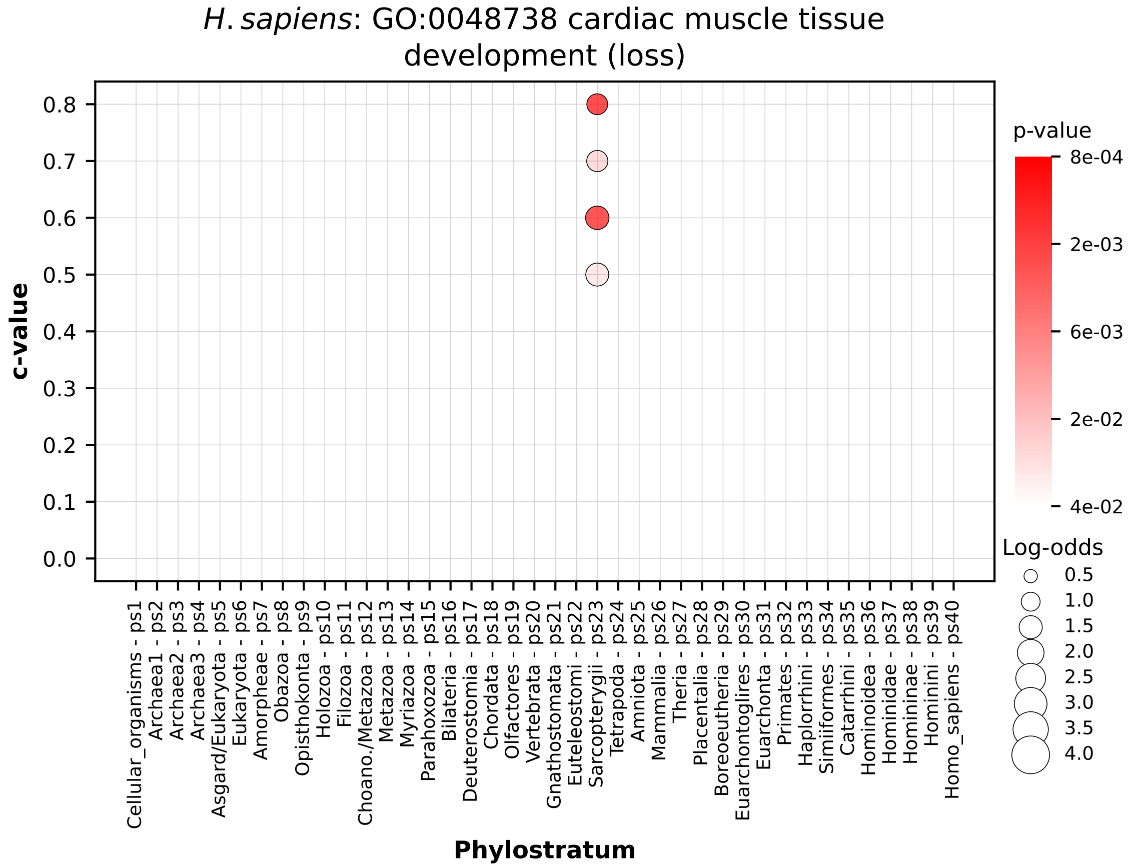

H. sapiens: GO:0048738 cardiac muscle tissue development (loss)
c-value
0.0
0.1
0.2
0.3
0.4
0.5
0.6
0.7
0.8
Phylostratum
Cellular_organisms - ps1
Archaea1 - ps2
Archaea2 - ps3
Archaea3 - ps4
Asgard/Eukaryota - ps5
Eukaryota - ps6
Amorpheae - ps7
Obazoa - ps8
Opisthokonta - ps9
Holozoa - ps10
Filozoa - ps11
Choano./Metazoa - ps12
Metazoa - ps13
Myriazoa - ps14
Parahoxozoa - ps15
Bilateria - ps16
Deuterostomia - ps17
Chordata - ps18
Olfactores - ps19
Vertebrata - ps20
Gnathostomata - ps21
Euteleostomi - ps22
Sarcopterygii - ps23
Tetrapoda - ps24
Amniota - ps25
Mammalia - ps26
Theria - ps27
Placentalia - ps28
Boreoeutheria - ps29
Euarchontoglires - ps30
Euarchonta - ps31
Primates - ps32
Haplorrhini - ps33
Simiiformes - ps34
Catarrhini - ps35
Hominoidea - ps36
Hominidae - ps37
Homininae - ps38
Hominini - ps39
Homo_sapiens - ps40
p-value
8e-04
2e-03
6e-03
2e-02
4e-02
Log-odds
0.5
1.0
1.5
2.0
2.5
3.0
3.5
4.0

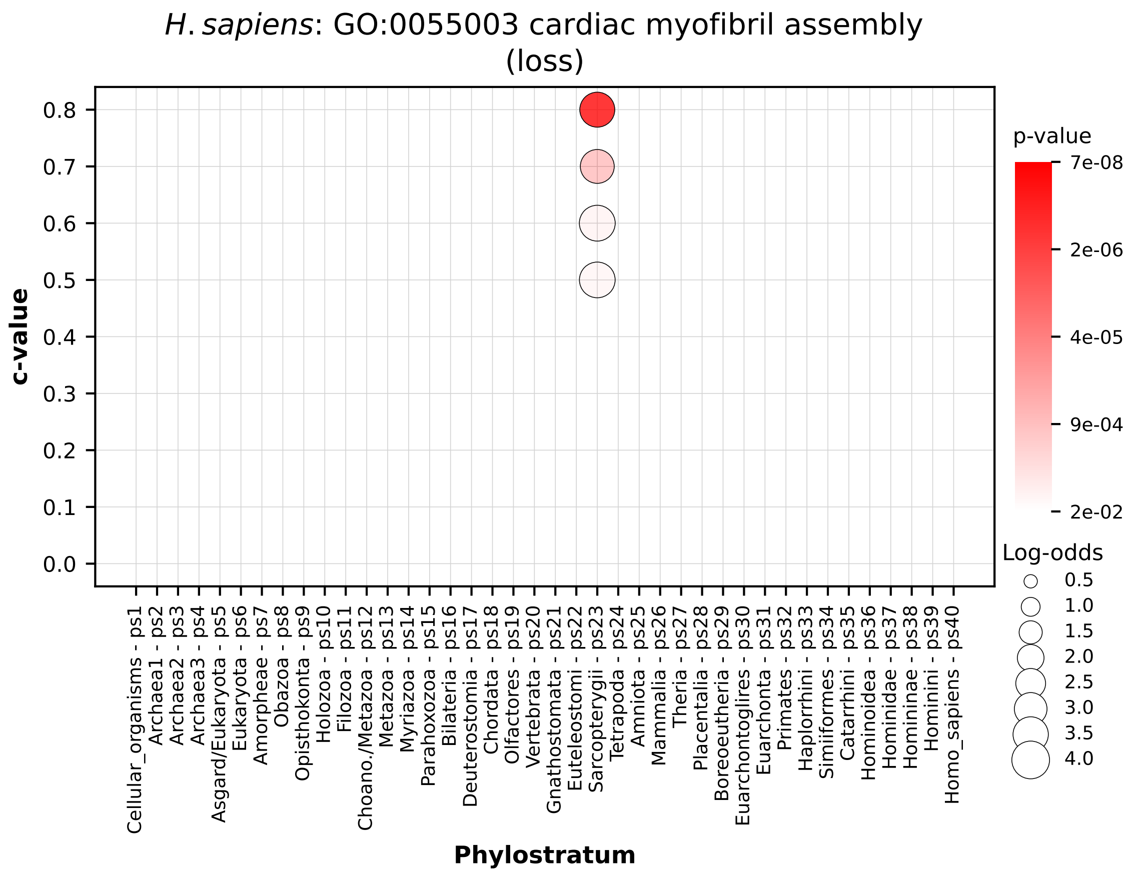

H. sapiens: GO:0055003 cardiac myofibril assembly
(loss)
c-value
0.8
0.7
0.6
0.5
0.4
0.3
0.2
0.1
0.0
p-value
7e-08
2e-06
4e-05
9e-04
2e-02
Log-odds
0.5
1.0
1.5
2.0
2.5
3.0
3.5
4.0
Cellular_organisms - ps1
Archaea1 - ps2
Archaea2 - ps3
Archaea3 - ps4
Asgard/Eukaryota - ps5
Eukaryota - ps6
Amorpheae - ps7
Obazoa - ps8
Opisthokonta - ps9
Holozoa - ps10
Filozoa - ps11
Choano./Metazoa - ps12
Metazoa - ps13
Myriazoa - ps14
Parahoxozoa - ps15
Bilateria - ps16
Deuterostomia - ps17
Chordata - ps18
Olfactores - ps19
Vertebrata - ps20
Gnathostomata - ps21
Euteleostomi - ps22
Sarcopterygii - ps23
Tetrapoda - ps24
Amniota - ps25
Mammalia - ps26
Theria - ps27
Placentalia - ps28
Boreoeutheria - ps29
Euarchontoglires - ps30
Euarchonta - ps31
Primates - ps32
Haplorrhini - ps33
Simiiformes - ps34
Catarrhini - ps35
Hominoidea - ps36
Hominidae - ps37
Homininae - ps38
Hominini - ps39
Homo_sapiens - ps40
Phylostratum

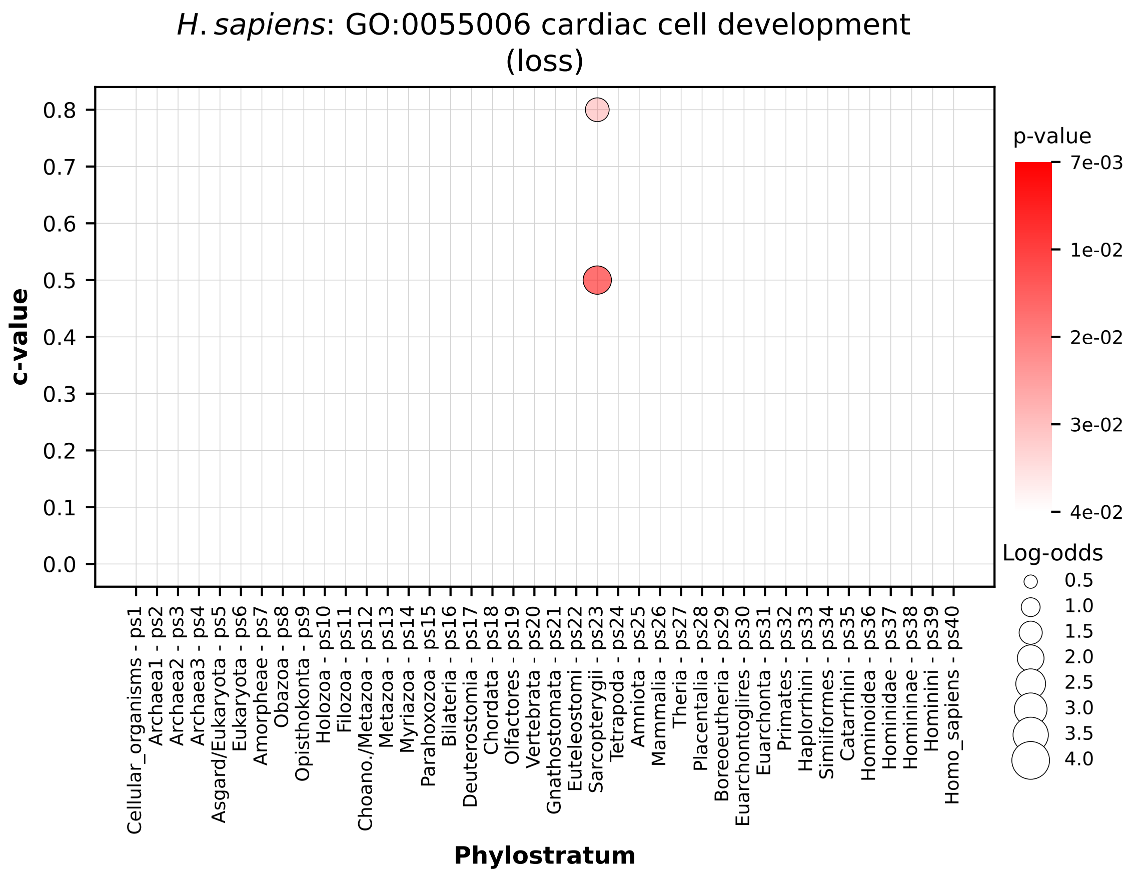
H. sapiens: GO:0055006 cardiac cell development
(loss)
c-value
0.8
0.7
0.6
0.5
0.4
0.3
0.2
0.1
0.0
Phylostratum
Cellular_organisms - ps1
Archaea1 - ps2
Archaea2 - ps3
Archaea3 - ps4
Asgard/Eukaryota - ps5
Eukaryota - ps6
Amorpheae - ps7
Obazoa - ps8
Opisthokonta - ps9
Holozoa - ps10
Filozoa - ps11
Choano./Metazoa - ps12
Metazoa - ps13
Myriazoa - ps14
Parahoxozoa - ps15
Bilateria - ps16
Deuterostomia - ps17
Chordata - ps18
Olfactores - ps19
Vertebrata - ps20
Gnathostomata - ps21
Euteleostomi - ps22
Sarcopterygii - ps23
Tetrapoda - ps24
Amniota - ps25
Mammalia - ps26
Theria - ps27
Placentalia - ps28
Boreoeutheria - ps29
Euarchontoglires - ps30
Euarchonta - ps31
Primates - ps32
Haplorrhini - ps33
Simiiformes - ps34
Catarrhini - ps35
Hominoidea - ps36
Hominidae - ps37
Homininae - ps38
Hominini - ps39
Homo_sapiens - ps40
p-value
7e-03
1e-02
2e-02
3e-02
4e-02
Log-odds
0.5
1.0
1.5
2.0
2.5
3.0
3.5
4.0

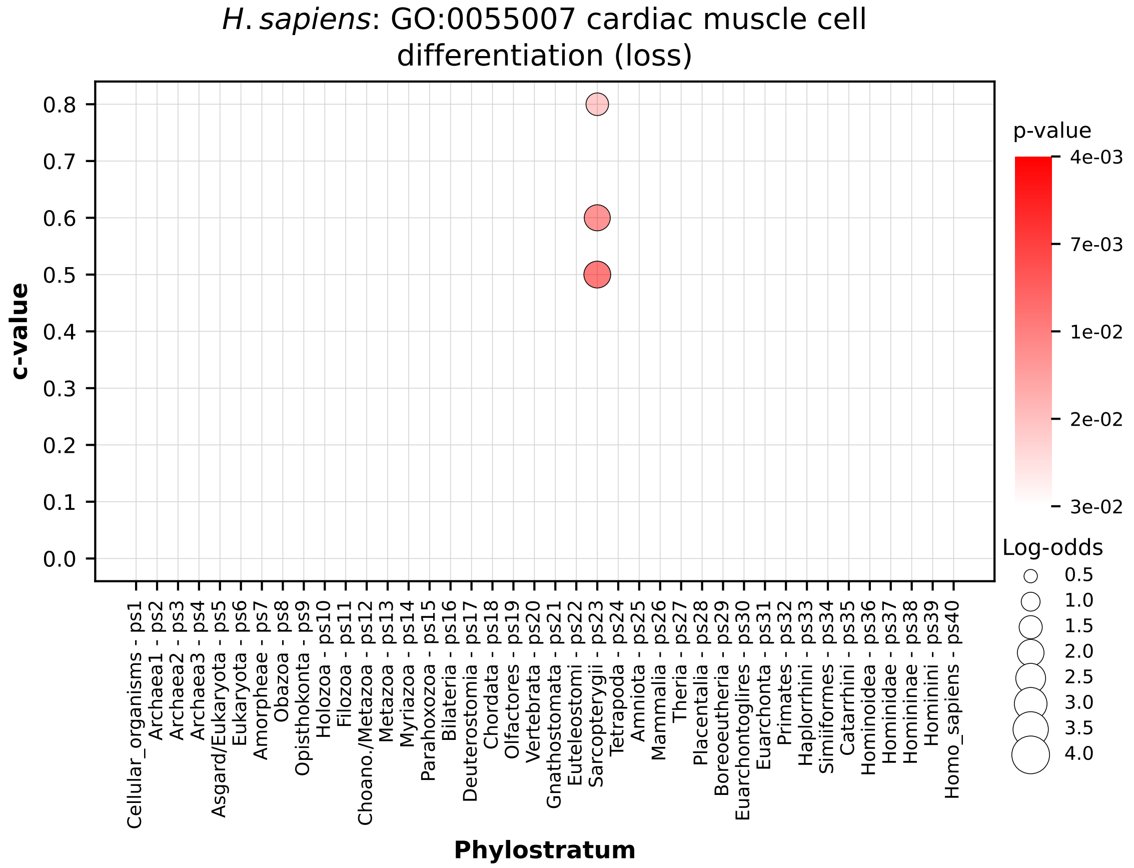
H. sapiens: GO:0055007 cardiac muscle cell differentiation (loss)
c-value
0.8
0.7
0.6
0.5
0.4
0.3
0.2
0.1
0.0
Phylostratum
Cellular_organisms - ps1
Archaea1 - ps2
Archaea2 - ps3
Archaea3 - ps4
Asgard/Eukaryota - ps5
Eukaryota - ps6
Amorpheae - ps7
Obazoa - ps8
Opisthokonta - ps9
Holozoa - ps10
Filozoa - ps11
Choano./Metazoa - ps12
Metazoa - ps13
Myriazoa - ps14
Parahoxozoa - ps15
Bilateria - ps16
Deuterostomia - ps17
Chordata - ps18
Olfactores - ps19
Vertebrata - ps20
Gnathostomata - ps21
Euteleostomi - ps22
Sarcopterygii - ps23
Tetrapoda - ps24
Amniota - ps25
Mammalia - ps26
Theria - ps27
Placentalia - ps28
Boreoeutheria - ps29
Euarchontoglires - ps30
Euarchonta - ps31
Primates - ps32
Haplorrhini - ps33
Simiiformes - ps34
Catarrhini - ps35
Hominoidea - ps36
Hominidae - ps37
Homininae - ps38
Hominini - ps39
Homo_sapiens - ps40
p-value
4e-03
7e-03
1e-02
2e-02
3e-02
Log-odds
0.5
1.0
1.5
2.0
2.5
3.0
3.5
4.0

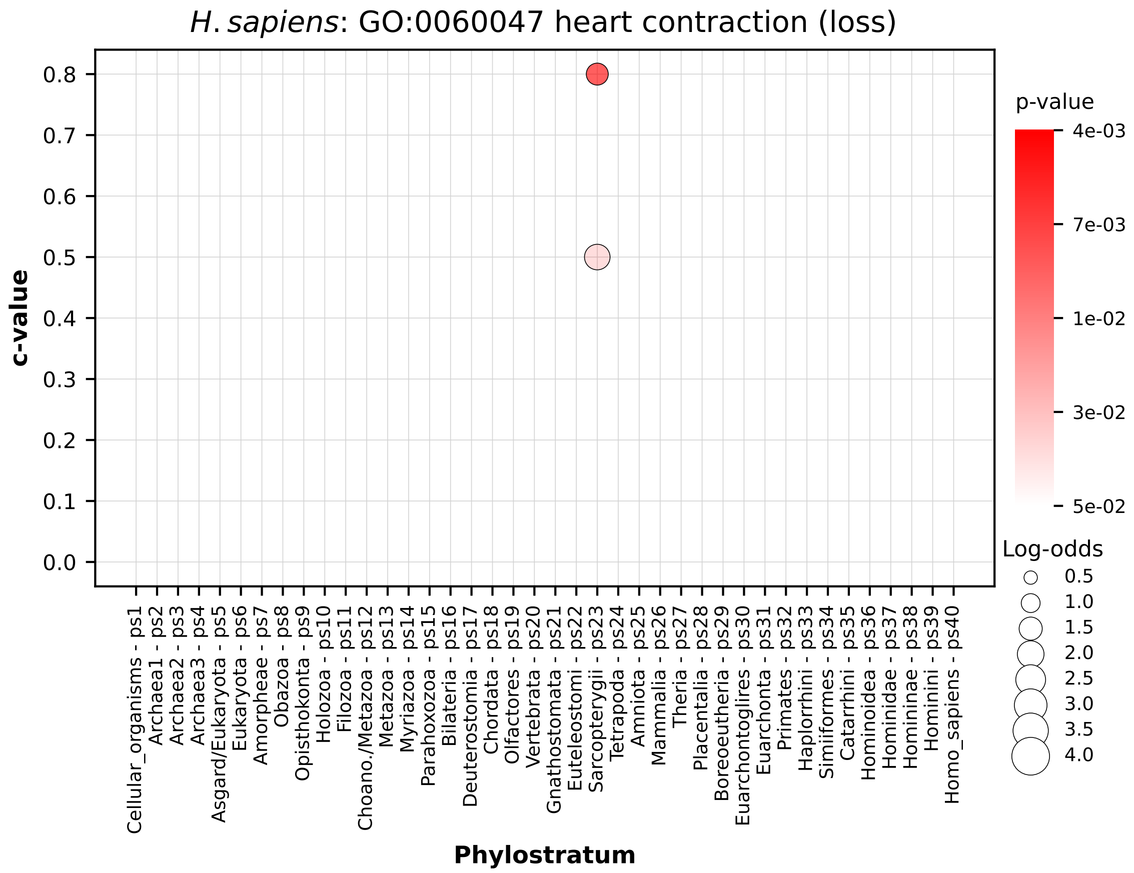

H. sapiens: GO:0060047 heart contraction (loss)
c-value
0.8
0.7
0.6
0.5
0.4
0.3
0.2
0.1
0.0
p-value
4e-03
7e-03
1e-02
3e-02
5e-02
Log-odds
0.5
1.0
1.5
2.0
2.5
3.0
3.5
4.0
Cellular_organisms - ps1
Archaea1 - ps2
Archaea2 - ps3
Archaea3 - ps4
Asgard/Eukaryota - ps5
Eukaryota - ps6
Amorpheae - ps7
Obazoa - ps8
Opisthokonta - ps9
Holozoa - ps10
Filozoa - ps11
Choano./Metazoa - ps12
Metazoa - ps13
Myriazoa - ps14
Parahoxozoa - ps15
Bilateria - ps16
Deuterostomia - ps17
Chordata - ps18
Olfactores - ps19
Vertebrata - ps20
Gnathostomata - ps21
Euteleostomi - ps22
Sarcopterygii - ps23
Tetrapoda - ps24
Amniota - ps25
Mammalia - ps26
Theria - ps27
Placentalia - ps28
Boreoeutheria - ps29
Euarchontoglires - ps30
Euarchonta - ps31
Primates - ps32
Haplorrhini - ps33
Simiiformes - ps34
Catarrhini - ps35
Hominoidea - ps36
Hominidae - ps37
Homininae - ps38
Hominini - ps39
Homo_sapiens - ps40
Phylostratum

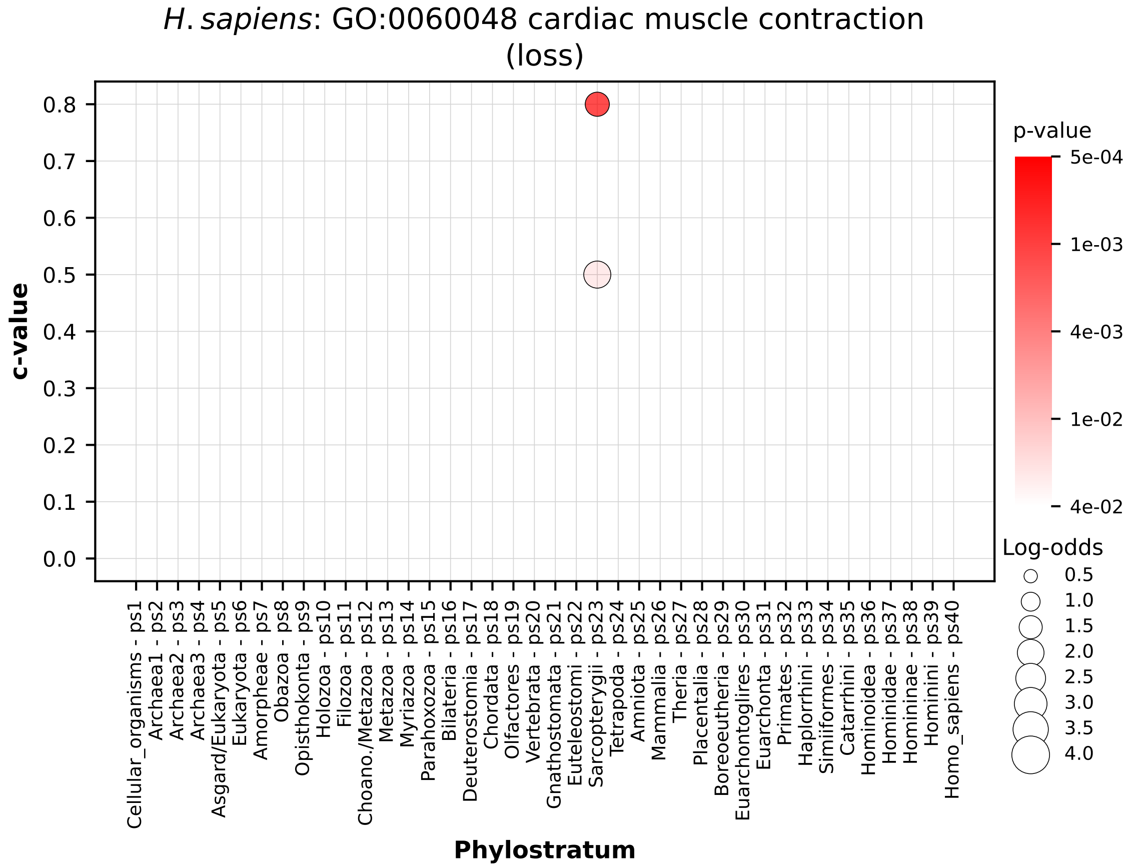
H. sapiens: GO:0060048 cardiac muscle contraction
(loss)
c-value
0.0
0.1
0.2
0.3
0.4
0.5
0.6
0.7
0.8
Phylostratum
Cellular_organisms - ps1
Archaea1 - ps2
Archaea2 - ps3
Archaea3 - ps4
Asgard/Eukaryota - ps5
Eukaryota - ps6
Amorpheae - ps7
Obazoa - ps8
Opisthokonta - ps9
Holozoa - ps10
Filozoa - ps11
Choano./Metazoa - ps12
Metazoa - ps13
Myriazoa - ps14
Parahoxozoa - ps15
Bilateria - ps16
Deuterostomia - ps17
Chordata - ps18
Olfactores - ps19
Vertebrata - ps20
Gnathostomata - ps21
Euteleostomi - ps22
Sarcopterygii - ps23
Tetrapoda - ps24
Amniota - ps25
Mammalia - ps26
Theria - ps27
Placentalia - ps28
Boreoeutheria - ps29
Euarchontoglires - ps30
Euarchonta - ps31
Primates - ps32
Haplorrhini - ps33
Simiiformes - ps34
Catarrhini - ps35
Hominoidea - ps36
Hominidae - ps37
Homininae - ps38
Hominini - ps39
Homo_sapiens - ps40
p-value
5e-04
1e-03
4e-03
1e-02
4e-02
Log-odds
0.5
1.0
1.5
2.0
2.5
3.0
3.5
4.0

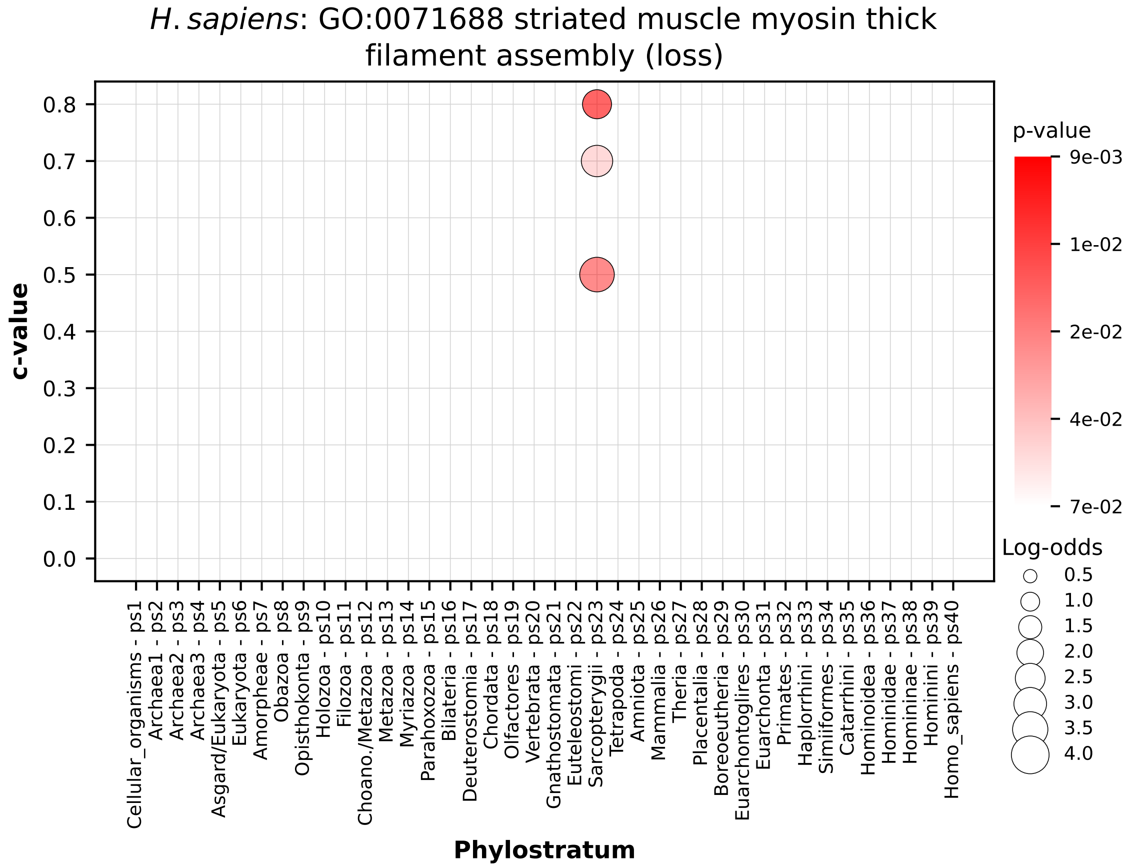

H. sapiens: GO:0071688 striated muscle myosin thick filament assembly (loss)
c-value
0.8
0.7
0.6
0.5
0.4
0.3
0.2
0.1
0.0
p-value
9e-03
1e-02
2e-02
4e-02
7e-02
Log-odds
0.5
1.0
1.5
2.0
2.5
3.0
3.5
4.0
Cellular_organisms - ps1
Archaea1 - ps2
Archaea2 - ps3
Archaea3 - ps4
Asgard/Eukaryota - ps5
Eukaryota - ps6
Amorpheae - ps7
Obazoa - ps8
Opisthokonta - ps9
Holozoa - ps10
Filozoa - ps11
Choano./Metazoa - ps12
Metazoa - ps13
Myriazoa - ps14
Parahoxozoa - ps15
Bilateria - ps16
Deuterostomia - ps17
Chordata - ps18
Olfactores - ps19
Vertebrata - ps20
Gnathostomata - ps21
Euteleostomi - ps22
Sarcopterygii - ps23
Tetrapoda - ps24
Amniota - ps25
Mammalia - ps26
Theria - ps27
Placentalia - ps28
Boreoeutheria - ps29
Euarchontoglires - ps30
Euarchonta - ps31
Primates - ps32
Haplorrhini - ps33
Simiiformes - ps34
Catarrhini - ps35
Hominoidea - ps36
Hominidae - ps37
Homininae - ps38
Hominini - ps39
Homo_sapiens - ps40
Phylostratum

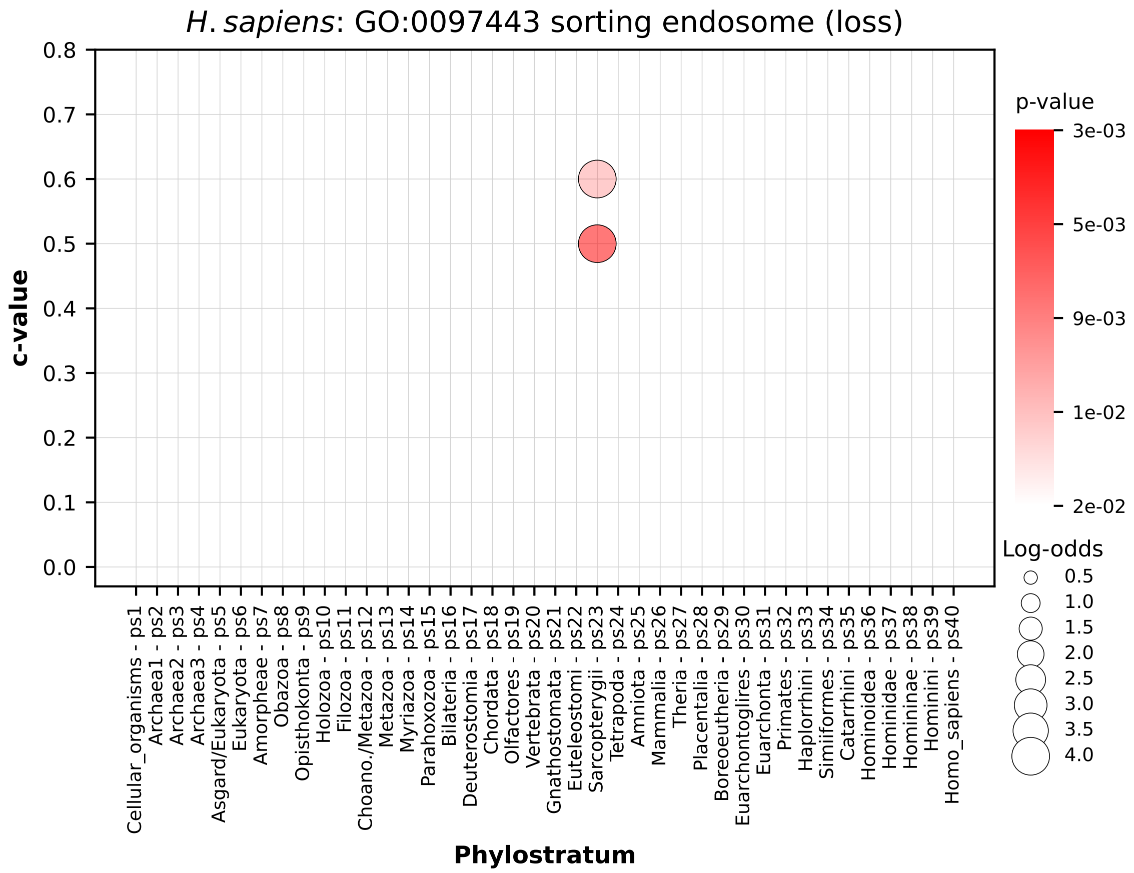
H. sapiens: GO:0097443 sorting endosome (loss)
c-value
0.0
0.1
0.2
0.3
0.4
0.5
0.6
0.7
0.8
Phylostratum
Cellular_organisms - ps1
Archaea1 - ps2
Archaea2 - ps3
Archaea3 - ps4
Asgard/Eukaryota - ps5
Eukaryota - ps6
Amorpheae - ps7
Obazoa - ps8
Opisthokonta - ps9
Holozoa - ps10
Filozoa - ps11
Choano./Metazoa - ps12
Metazoa - ps13
Myriazoa - ps14
Parahoxozoa - ps15
Bilateria - ps16
Deuterostomia - ps17
Chordata - ps18
Olfactores - ps19
Vertebrata - ps20
Gnathostomata - ps21
Euteleostomi - ps22
Sarcopterygii - ps23
Tetrapoda - ps24
Amniota - ps25
Mammalia - ps26
Theria - ps27
Placentalia - ps28
Boreoeutheria - ps29
Euarchontoglires - ps30
Euarchonta - ps31
Primates - ps32
Haplorrhini - ps33
Simiiformes - ps34
Catarrhini - ps35
Hominoidea - ps36
Hominidae - ps37
Homininae - ps38
Hominini - ps39
Homo_sapiens - ps40
p-value
3e-03
5e-03
9e-03
1e-02
2e-02
Log-odds
0.5
1.0
1.5
2.0
2.5
3.0
3.5
4.0

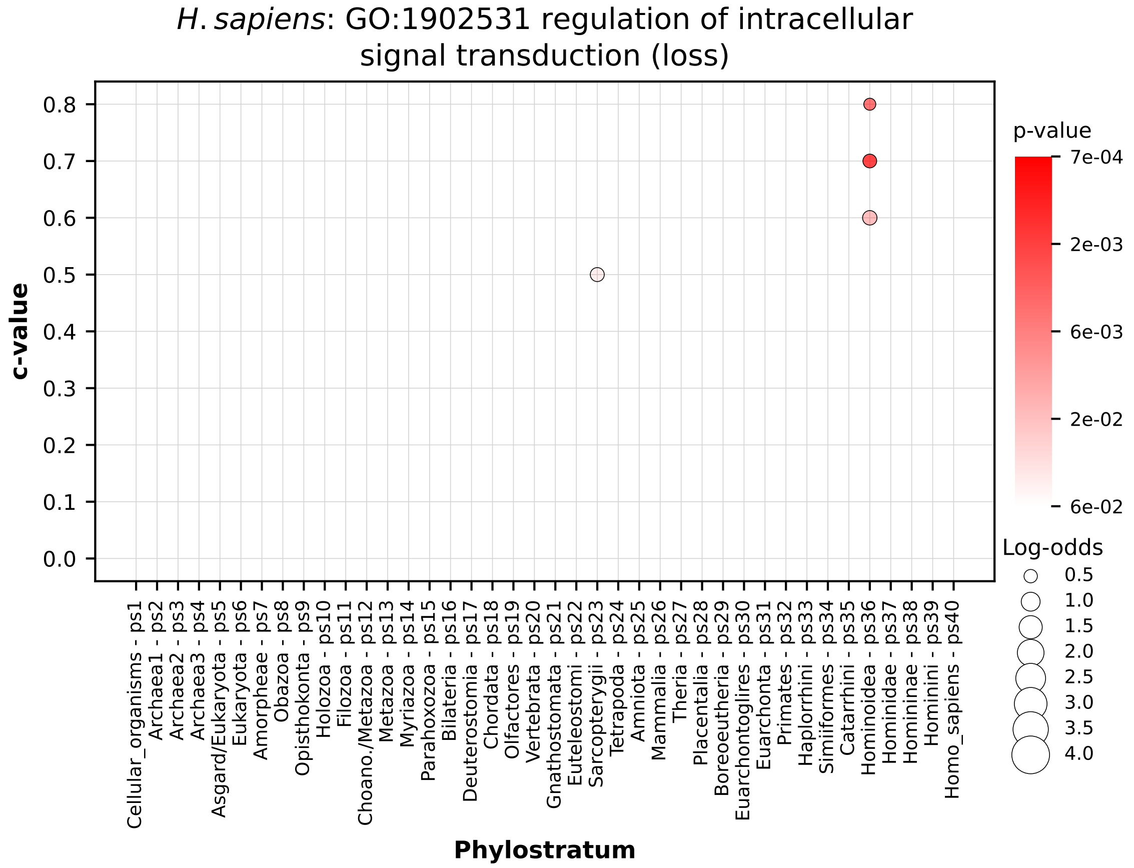

H. sapiens: GO:1902531 regulation of intracellular signal transduction (loss)
c-value
0.0
0.1
0.2
0.3
0.4
0.5
0.6
0.7
0.8
p-value
7e-04
2e-03
6e-03
2e-02
6e-02
Log-odds
0.5
1.0
1.5
2.0
2.5
3.0
3.5
4.0
Cellular_organisms - ps1
Archaea1 - ps2
Archaea2 - ps3
Archaea3 - ps4
Asgard/Eukaryota - ps5
Eukaryota - ps6
Amorpheae - ps7
Obazoa - ps8
Opisthokonta - ps9
Holozoa - ps10
Filozoa - ps11
Choano./Metazoa - ps12
Metazoa - ps13
Myriazoa - ps14
Parahoxozoa - ps15
Bilateria - ps16
Deuterostomia - ps17
Chordata - ps18
Olfactores - ps19
Vertebrata - ps20
Gnathostomata - ps21
Euteleostomi - ps22
Sarcopterygii - ps23
Tetrapoda - ps24
Amniota - ps25
Mammalia - ps26
Theria - ps27
Placentalia - ps28
Boreoeutheria - ps29
Euarchontoglires - ps30
Euarchonta - ps31
Primates - ps32
Haplorrhini - ps33
Simiiformes - ps34
Catarrhini - ps35
Hominoidea - ps36
Hominidae - ps37
Homininae - ps38
Hominini - ps39
Homo_sapiens - ps40
Phylostratum

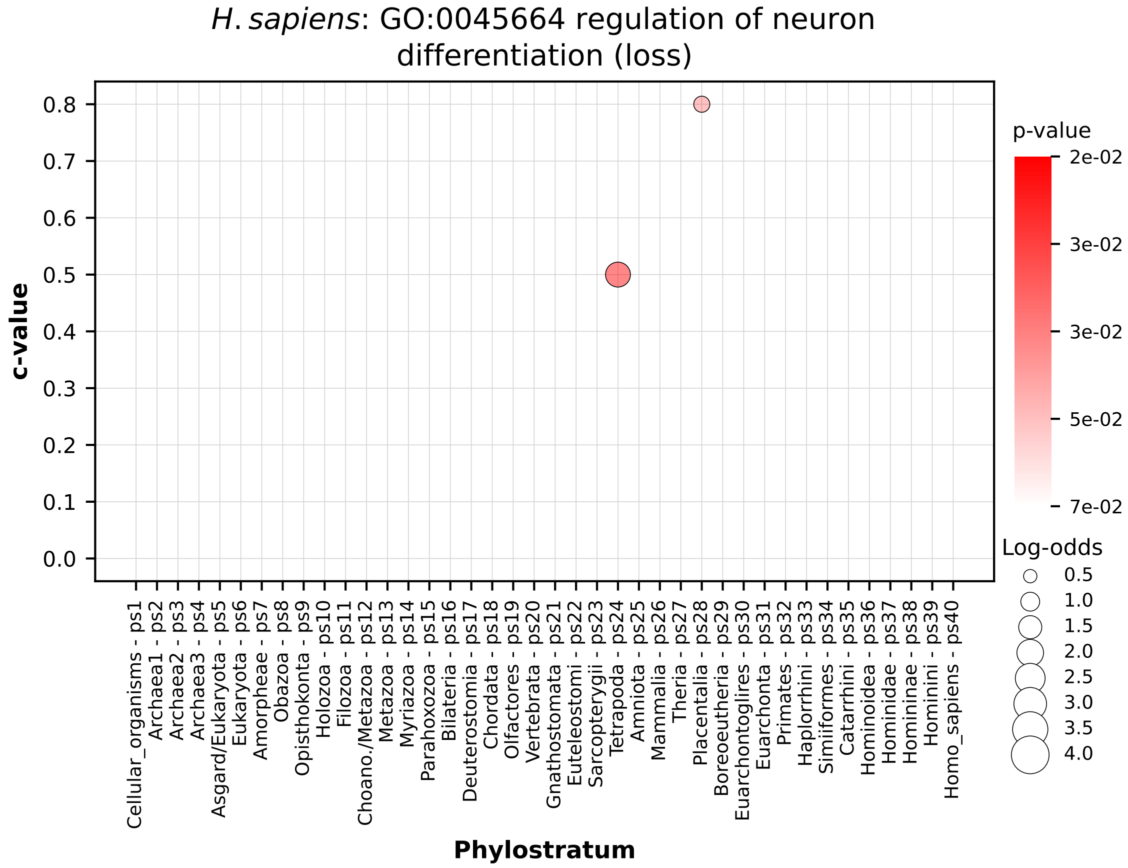

H. sapiens: GO:0045664 regulation of neuron differentiation (loss)
c-value
0.0
0.1
0.2
0.3
0.4
0.5
0.6
0.7
0.8
p-value
2e-02
3e-02
3e-02
5e-02
7e-02
Log-odds
0.5
1.0
1.5
2.0
2.5
3.0
3.5
4.0
Cellular_organisms - ps1
Archaea1 - ps2
Archaea2 - ps3
Archaea3 - ps4
Asgard/Eukaryota - ps5
Eukaryota - ps6
Amorpheae - ps7
Obazoa - ps8
Opisthokonta - ps9
Holozoa - ps10
Filozoa - ps11
Choano./Metazoa - ps12
Metazoa - ps13
Myriazoa - ps14
Parahoxozoa - ps15
Bilateria - ps16
Deuterostomia - ps17
Chordata - ps18
Olfactores - ps19
Vertebrata - ps20
Gnathostomata - ps21
Euteleostomi - ps22
Sarcopterygii - ps23
Tetrapoda - ps24
Amniota - ps25
Mammalia - ps26
Theria - ps27
Placentalia - ps28
Boreoeutheria - ps29
Euarchontoglires - ps30
Euarchonta - ps31
Primates - ps32
Haplorrhini - ps33
Simiiformes - ps34
Catarrhini - ps35
Hominoidea - ps36
Hominidae - ps37
Homininae - ps38
Hominini - ps39
Homo_sapiens - ps40
Phylostratum

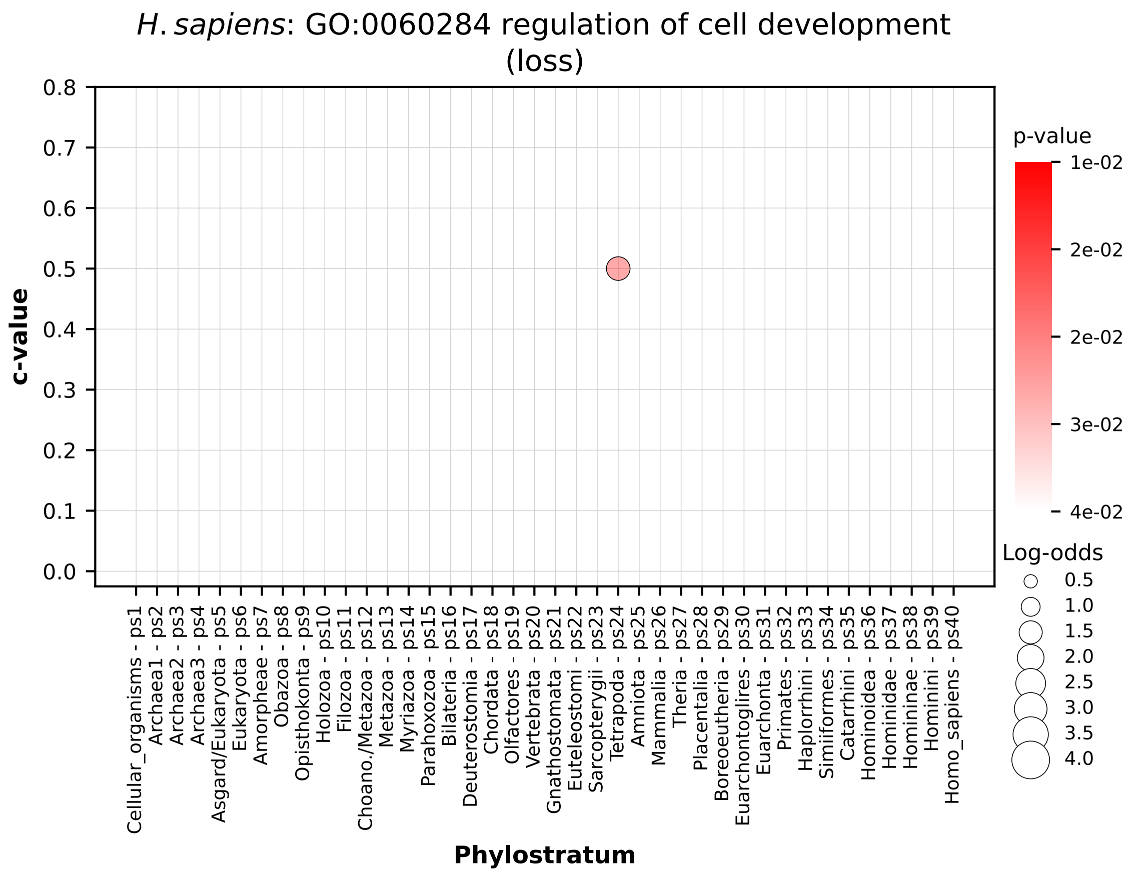

H. sapiens: GO:0060284 regulation of cell development
(loss)
c-value
0.8
0.7
0.6
0.5
0.4
0.3
0.2
0.1
0.0
p-value
1e-02
2e-02
2e-02
3e-02
4e-02
Log-odds
0.5
1.0
1.5
2.0
2.5
3.0
3.5
4.0
Cellular_organisms - ps1
Archaea1 - ps2
Archaea2 - ps3
Archaea3 - ps4
Asgard/Eukaryota - ps5
Eukaryota - ps6
Amorpheae - ps7
Obazoa - ps8
Opisthokonta - ps9
Holozoa - ps10
Filozoa - ps11
Choano./Metazoa - ps12
Metazoa - ps13
Myriazoa - ps14
Parahoxozoa - ps15
Bilateria - ps16
Deuterostomia - ps17
Chordata - ps18
Olfactores - ps19
Vertebrata - ps20
Gnathostomata - ps21
Euteleostomi - ps22
Sarcopterygii - ps23
Tetrapoda - ps24
Amniota - ps25
Mammalia - ps26
Theria - ps27
Placentalia - ps28
Boreoeutheria - ps29
Euarchontoglires - ps30
Euarchonta - ps31
Primates - ps32
Haplorrhini - ps33
Simiiformes - ps34
Catarrhini - ps35
Hominoidea - ps36
Hominidae - ps37
Homininae - ps38
Hominini - ps39
Homo_sapiens - ps40
Phylostratum

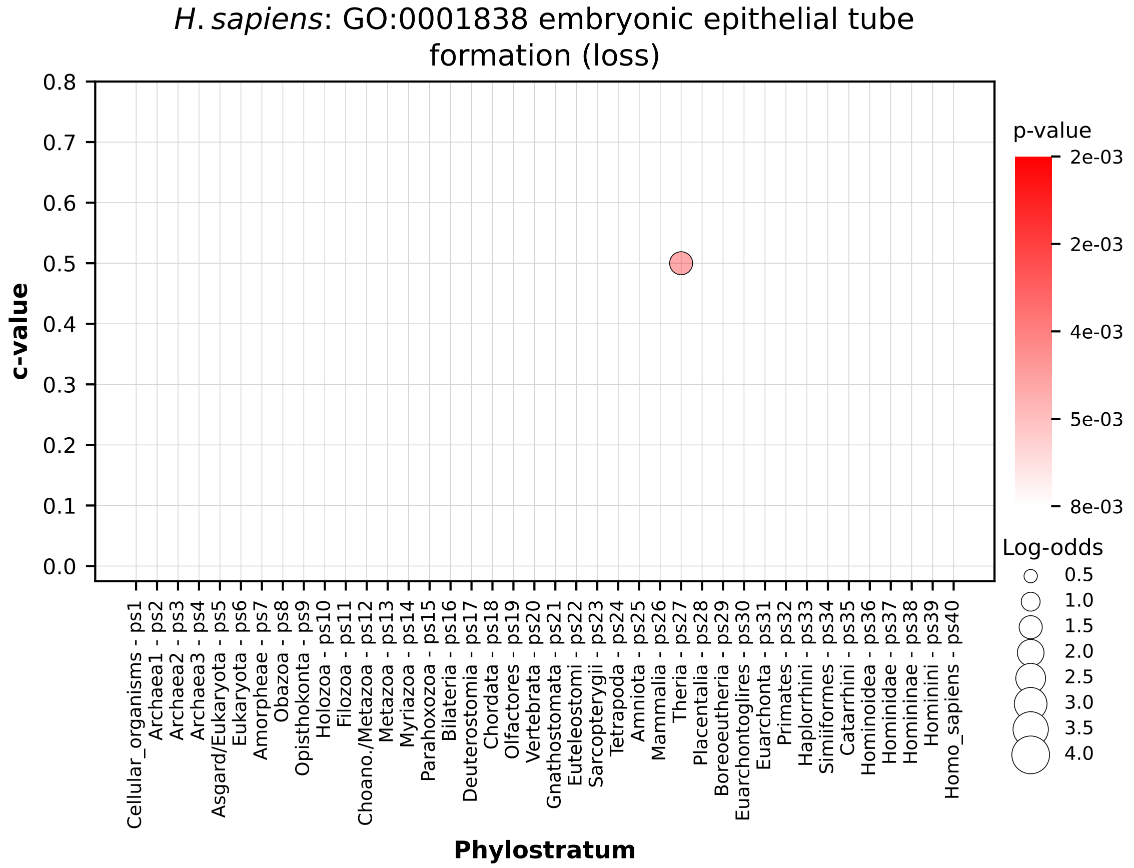

H. sapiens: GO:0001838 embryonic epithelial tube formation (loss)
c-value
0.0
0.1
0.2
0.3
0.4
0.5
0.6
0.7
0.8
Phylostratum
Cellular_organisms - ps1
Archaea1 - ps2
Archaea2 - ps3
Archaea3 - ps4
Asgard/Eukaryota - ps5
Eukaryota - ps6
Amorpheae - ps7
Obazoa - ps8
Opisthokonta - ps9
Holozoa - ps10
Filozoa - ps11
Choano./Metazoa - ps12
Metazoa - ps13
Myriazoa - ps14
Parahoxozoa - ps15
Bilateria - ps16
Deuterostomia - ps17
Chordata - ps18
Olfactores - ps19
Vertebrata - ps20
Gnathostomata - ps21
Euteleostomi - ps22
Sarcopterygii - ps23
Tetrapoda - ps24
Amniota - ps25
Mammalia - ps26
Theria - ps27
Placentalia - ps28
Boreoeutheria - ps29
Euarchontoglires - ps30
Euarchonta - ps31
Primates - ps32
Haplorrhini - ps33
Simiiformes - ps34
Catarrhini - ps35
Hominoidea - ps36
Hominidae - ps37
Homininae - ps38
Hominini - ps39
Homo_sapiens - ps40
p-value
2e-03
2e-03
4e-03
5e-03
8e-03
Log-odds
0.5
1.0
1.5
2.0
2.5
3.0
3.5
4.0

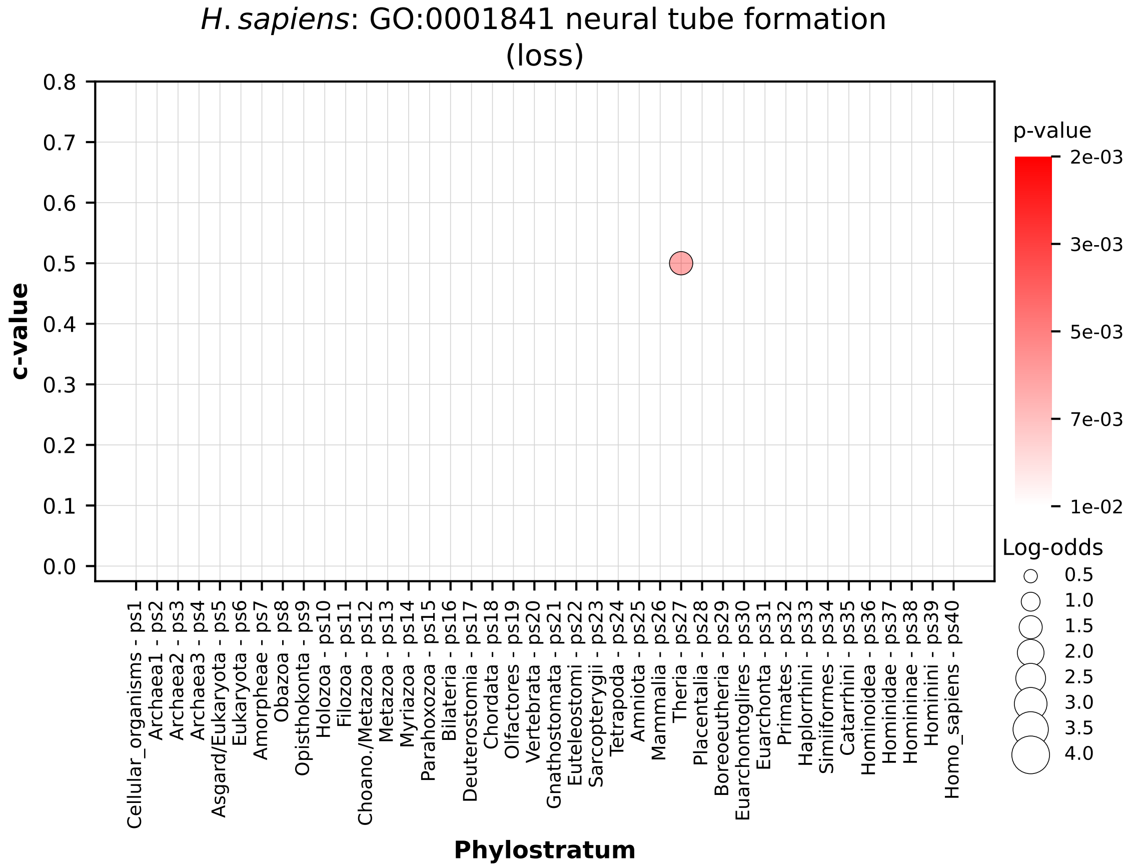

H. sapiens: GO:0001841 neural tube formation
(loss)
c-value
0.8
0.7
0.6
0.5
0.4
0.3
0.2
0.1
0.0
p-value
2e-03
3e-03
5e-03
7e-03
1e-02
Log-odds
0.5
1.0
1.5
2.0
2.5
3.0
3.5
4.0
Cellular_organisms - ps1
Archaea1 - ps2
Archaea2 - ps3
Archaea3 - ps4
Asgard/Eukaryota - ps5
Eukaryota - ps6
Amorpheae - ps7
Obazoa - ps8
Opisthokonta - ps9
Holozoa - ps10
Filozoa - ps11
Choano./Metazoa - ps12
Metazoa - ps13
Myriazoa - ps14
Parahoxozoa - ps15
Bilateria - ps16
Deuterostomia - ps17
Chordata - ps18
Olfactores - ps19
Vertebrata - ps20
Gnathostomata - ps21
Euteleostomi - ps22
Sarcopterygii - ps23
Tetrapoda - ps24
Amniota - ps25
Mammalia - ps26
Theria - ps27
Placentalia - ps28
Boreoeutheria - ps29
Euarchontoglires - ps30
Euarchonta - ps31
Primates - ps32
Haplorrhini - ps33
Simiiformes - ps34
Catarrhini - ps35
Hominoidea - ps36
Hominidae - ps37
Homininae - ps38
Hominini - ps39
Homo_sapiens - ps40
Phylostratum

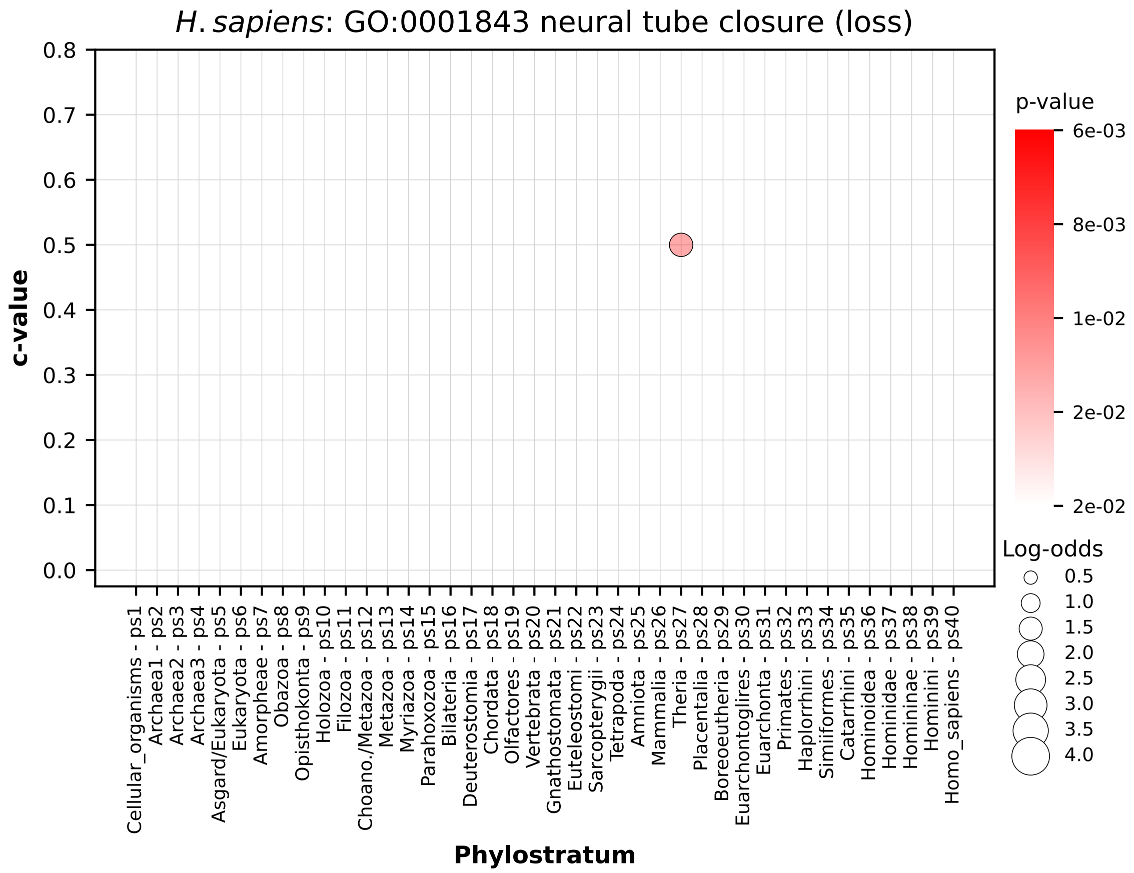

H. sapiens: GO:0001843 neural tube closure (loss)
c-value
0.8
0.7
0.6
0.5
0.4
0.3
0.2
0.1
0.0
Phylostratum
Cellular_organisms - ps1
Archaea1 - ps2
Archaea2 - ps3
Archaea3 - ps4
Asgard/Eukaryota - ps5
Eukaryota - ps6
Amorpheae - ps7
Obazoa - ps8
Opisthokonta - ps9
Holozoa - ps10
Filozoa - ps11
Choano./Metazoa - ps12
Metazoa - ps13
Myriazoa - ps14
Parahoxozoa - ps15
Bilateria - ps16
Deuterostomia - ps17
Chordata - ps18
Olfactores - ps19
Vertebrata - ps20
Gnathostomata - ps21
Euteleostomi - ps22
Sarcopterygii - ps23
Tetrapoda - ps24
Amniota - ps25
Mammalia - ps26
Theria - ps27
Placentalia - ps28
Boreoeutheria - ps29
Euarchontoglires - ps30
Euarchonta - ps31
Primates - ps32
Haplorrhini - ps33
Simiiformes - ps34
Catarrhini - ps35
Hominoidea - ps36
Hominidae - ps37
Homininae - ps38
Hominini - ps39
Homo_sapiens - ps40
p-value
6e-03
8e-03
1e-02
2e-02
2e-02
Log-odds
0.5
1.0
1.5
2.0
2.5
3.0
3.5
4.0

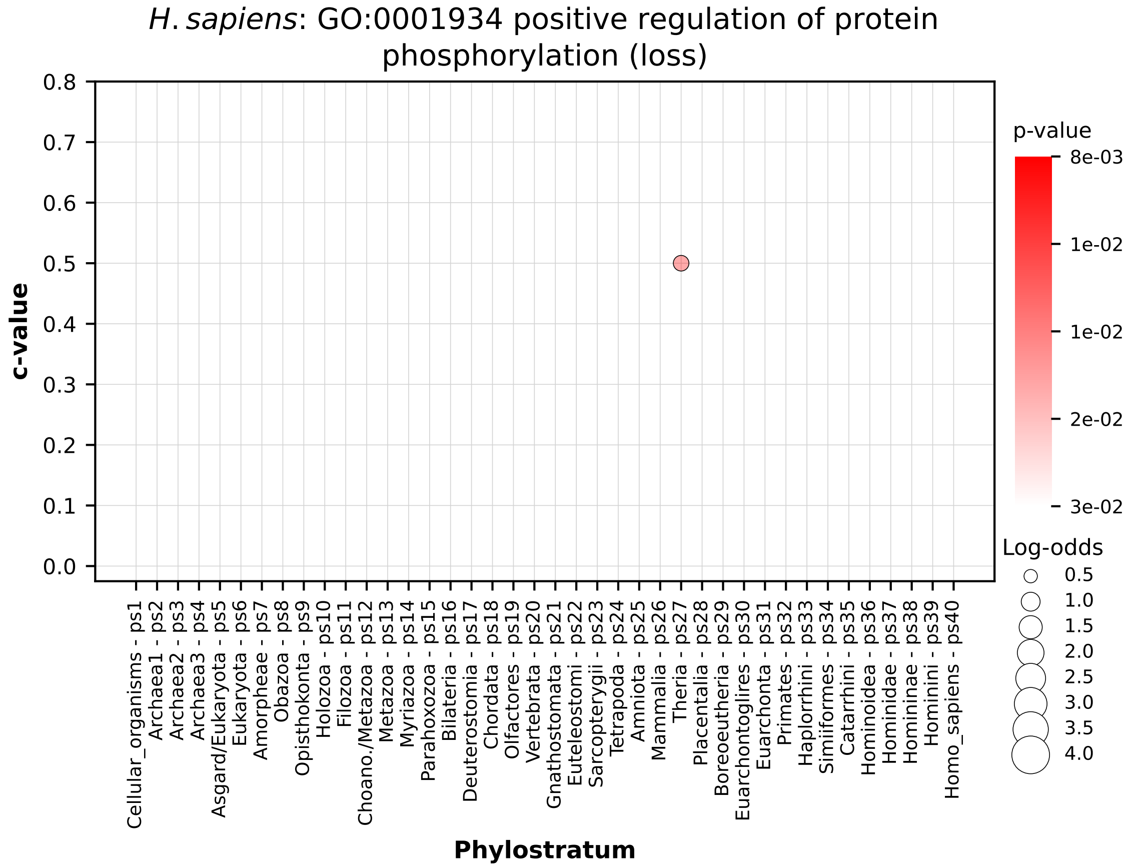

H. sapiens: GO:0001934 positive regulation of protein phosphorylation (loss)
c-value
0.8
0.7
0.6
0.5
0.4
0.3
0.2
0.1
0.0
Phylostratum
Cellular_organisms - ps1
Archaea1 - ps2
Archaea2 - ps3
Archaea3 - ps4
Asgard/Eukaryota - ps5
Eukaryota - ps6
Amorpheae - ps7
Obazoa - ps8
Opisthokonta - ps9
Holozoa - ps10
Filozoa - ps11
Choano./Metazoa - ps12
Metazoa - ps13
Myriazoa - ps14
Parahoxozoa - ps15
Bilateria - ps16
Deuterostomia - ps17
Chordata - ps18
Olfactores - ps19
Vertebrata - ps20
Gnathostomata - ps21
Euteleostomi - ps22
Sarcopterygii - ps23
Tetrapoda - ps24
Amniota - ps25
Mammalia - ps26
Theria - ps27
Placentalia - ps28
Boreoeutheria - ps29
Euarchontoglires - ps30
Euarchonta - ps31
Primates - ps32
Haplorrhini - ps33
Simiiformes - ps34
Catarrhini - ps35
Hominoidea - ps36
Hominidae - ps37
Homininae - ps38
Hominini - ps39
Homo_sapiens - ps40
p-value
8e-03
1e-02
1e-02
2e-02
3e-02
Log-odds
0.5
1.0
1.5
2.0
2.5
3.0
3.5
4.0

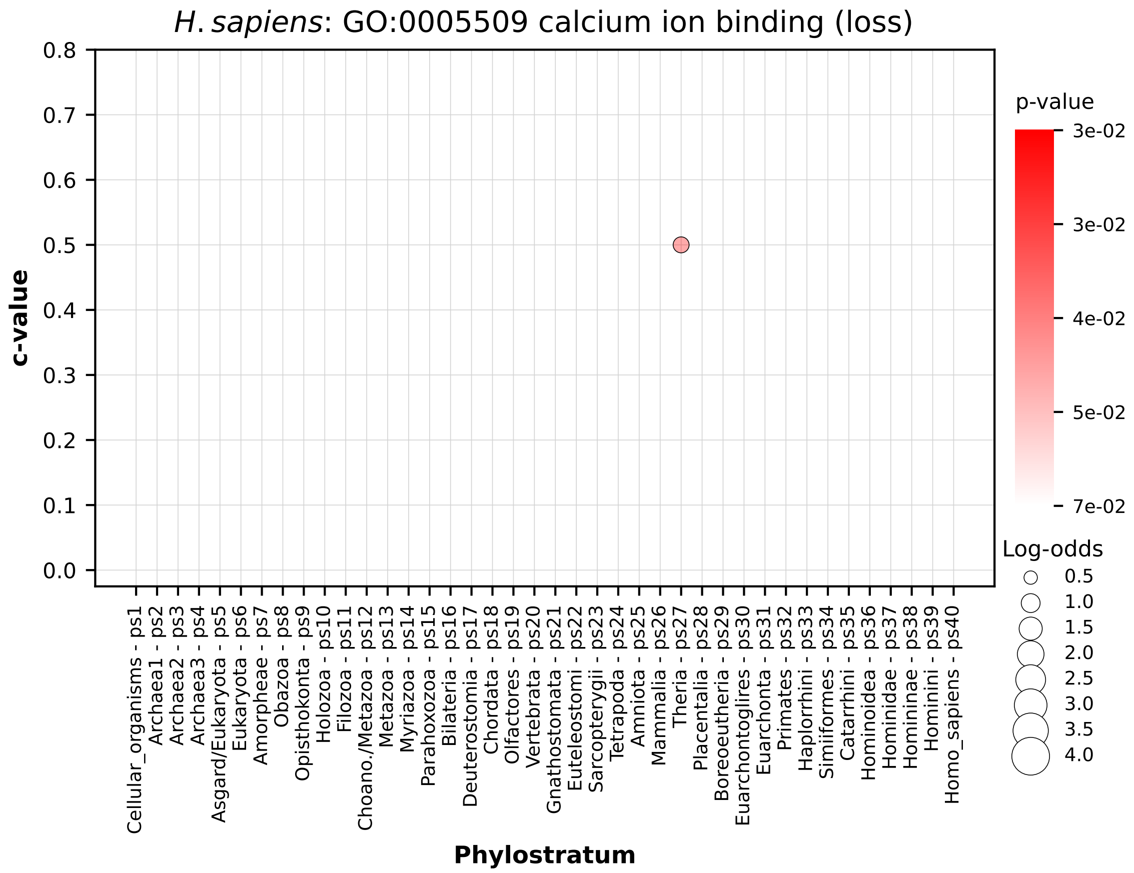

H. sapiens: GO:0005509 calcium ion binding (loss)
c-value
0.8
0.7
0.6
0.5
0.4
0.3
0.2
0.1
0.0
Phylostratum
Cellular_organisms - ps1
Archaea1 - ps2
Archaea2 - ps3
Archaea3 - ps4
Asgard/Eukaryota - ps5
Eukaryota - ps6
Amorpheae - ps7
Obazoa - ps8
Opisthokonta - ps9
Holozoa - ps10
Filozoa - ps11
Choano./Metazoa - ps12
Metazoa - ps13
Myriazoa - ps14
Parahoxozoa - ps15
Bilateria - ps16
Deuterostomia - ps17
Chordata - ps18
Olfactores - ps19
Vertebrata - ps20
Gnathostomata - ps21
Euteleostomi - ps22
Sarcopterygii - ps23
Tetrapoda - ps24
Amniota - ps25
Mammalia - ps26
Theria - ps27
Placentalia - ps28
Boreoeutheria - ps29
Euarchontoglires - ps30
Euarchonta - ps31
Primates - ps32
Haplorrhini - ps33
Simiiformes - ps34
Catarrhini - ps35
Hominoidea - ps36
Hominidae - ps37
Homininae - ps38
Hominini - ps39
Homo_sapiens - ps40
p-value
3e-02
3e-02
4e-02
5e-02
7e-02
Log-odds
0.5
1.0
1.5
2.0
2.5
3.0
3.5
4.0

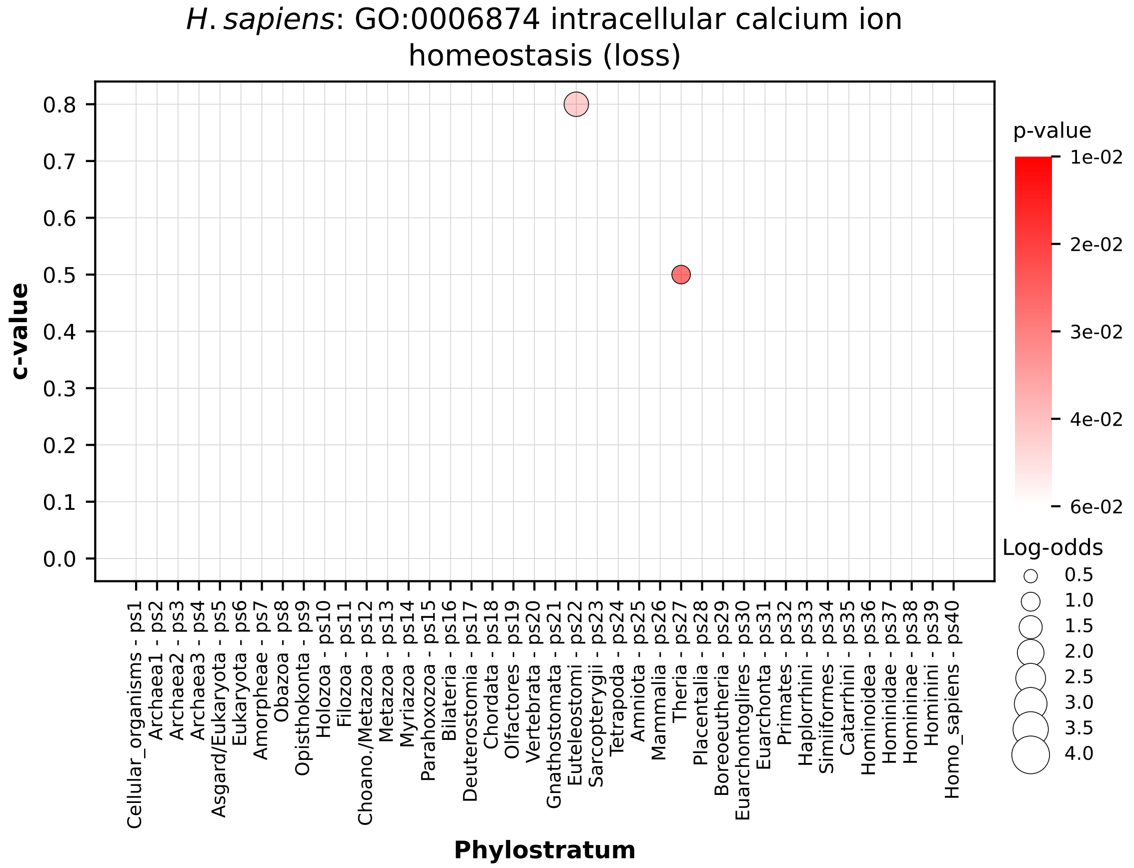

H. sapiens: GO:0006874 intracellular calcium ion homeostasis (loss)
c-value
0.0
0.1
0.2
0.3
0.4
0.5
0.6
0.7
0.8
Phylostratum
Cellular_organisms - ps1
Archaea1 - ps2
Archaea2 - ps3
Archaea3 - ps4
Asgard/Eukaryota - ps5
Eukaryota - ps6
Amorpheae - ps7
Obazoa - ps8
Opisthokonta - ps9
Holozoa - ps10
Filozoa - ps11
Choano./Metazoa - ps12
Metazoa - ps13
Myriazoa - ps14
Parahoxozoa - ps15
Bilateria - ps16
Deuterostomia - ps17
Chordata - ps18
Olfactores - ps19
Vertebrata - ps20
Gnathostomata - ps21
Euteleostomi - ps22
Sarcopterygii - ps23
Tetrapoda - ps24
Amniota - ps25
Mammalia - ps26
Theria - ps27
Placentalia - ps28
Boreoeutheria - ps29
Euarchontoglires - ps30
Euarchonta - ps31
Primates - ps32
Haplorrhini - ps33
Simiiformes - ps34
Catarrhini - ps35
Hominoidea - ps36
Hominidae - ps37
Homininae - ps38
Hominini - ps39
Homo_sapiens - ps40
p-value
1e-02
2e-02
3e-02
4e-02
6e-02
Log-odds
0.5
1.0
1.5
2.0
2.5
3.0
3.5
4.0

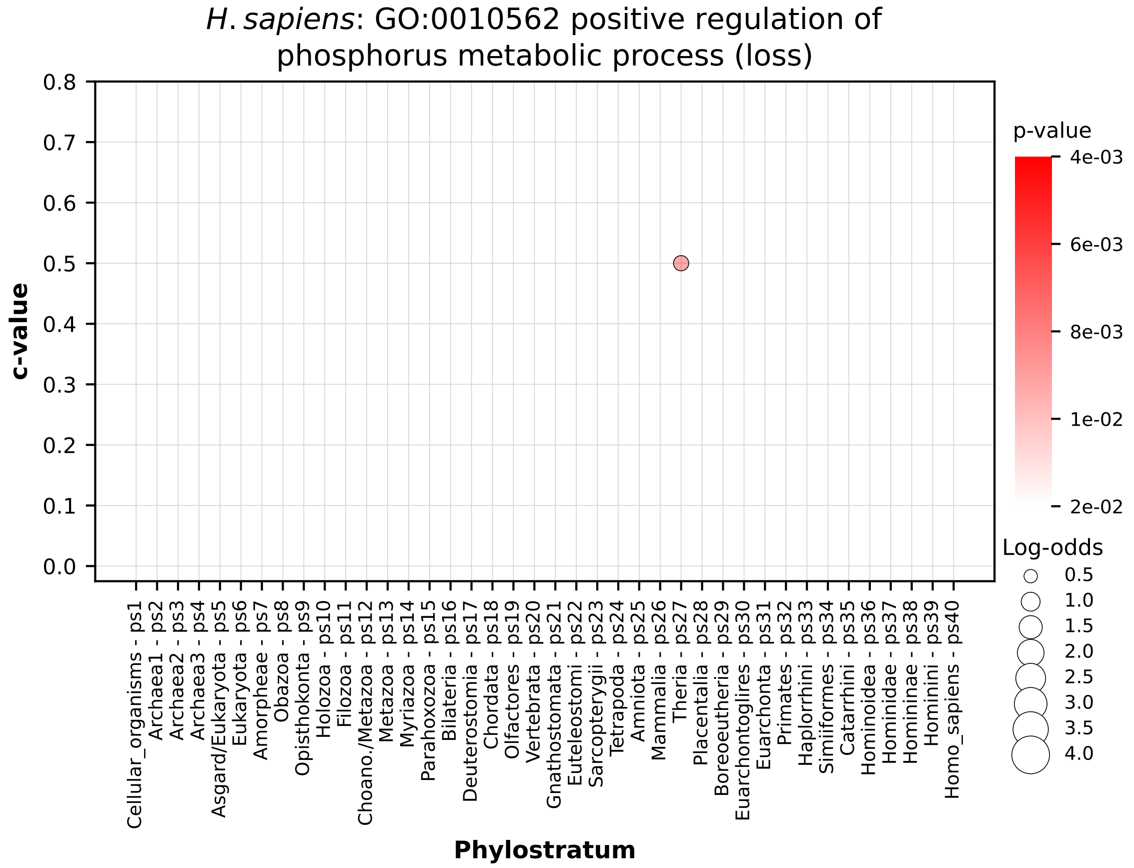
H. sapiens: GO:0010562 positive regulation of phosphorus metabolic process (loss)
c-value
0.8
0.7
0.6
0.5
0.4
0.3
0.2
0.1
0.0
Phylostratum
Cellular_organisms - ps1
Archaea1 - ps2
Archaea2 - ps3
Archaea3 - ps4
Asgard/Eukaryota - ps5
Eukaryota - ps6
Amorpheae - ps7
Obazoa - ps8
Opisthokonta - ps9
Holozoa - ps10
Filozoa - ps11
Choano./Metazoa - ps12
Metazoa - ps13
Myriazoa - ps14
Parahoxozoa - ps15
Bilateria - ps16
Deuterostomia - ps17
Chordata - ps18
Olfactores - ps19
Vertebrata - ps20
Gnathostomata - ps21
Euteleostomi - ps22
Sarcopterygii - ps23
Tetrapoda - ps24
Amniota - ps25
Mammalia - ps26
Theria - ps27
Placentalia - ps28
Boreoeutheria - ps29
Euarchontoglires - ps30
Euarchonta - ps31
Primates - ps32
Haplorrhini - ps33
Simiiformes - ps34
Catarrhini - ps35
Hominoidea - ps36
Hominidae - ps37
Homininae - ps38
Hominini - ps39
Homo_sapiens - ps40
p-value
4e-03
6e-03
8e-03
1e-02
2e-02
Log-odds
0.5
1.0
1.5
2.0
2.5
3.0
3.5
4.0

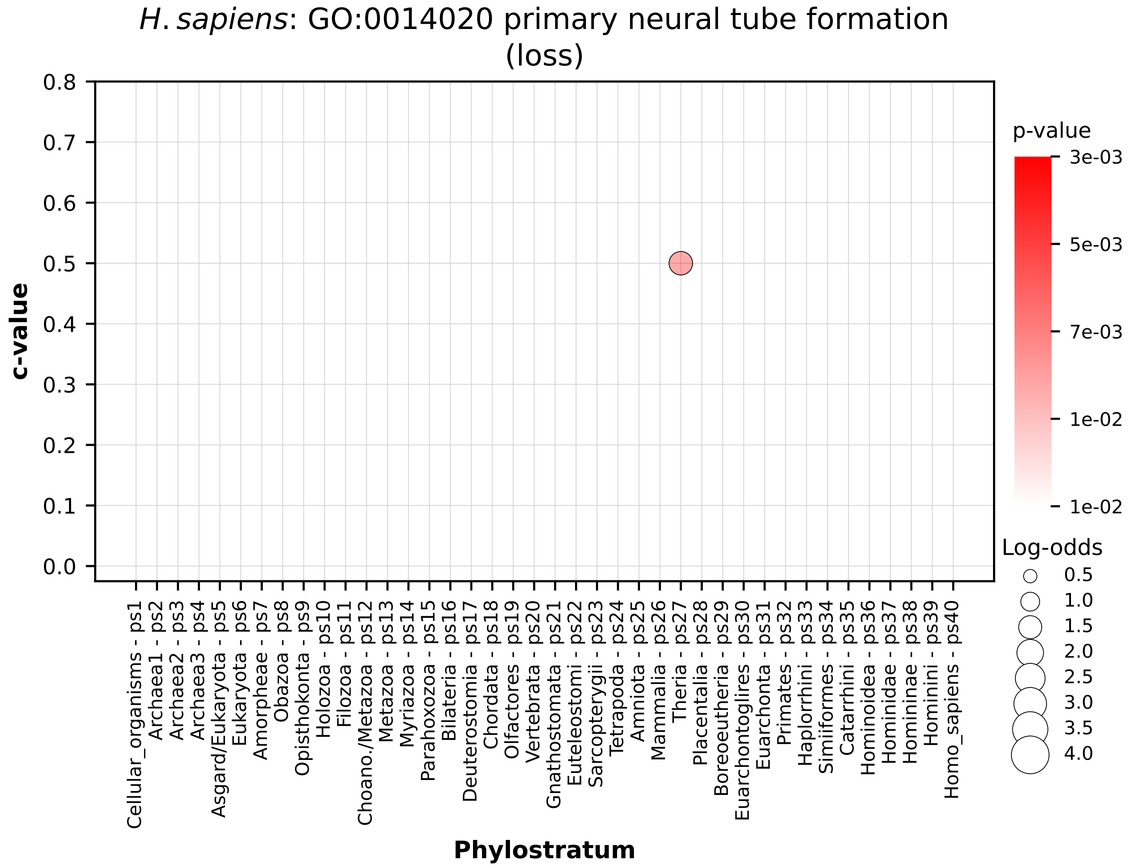
H. sapiens: GO:0014020 primary neural tube formation
(loss)
c-value
0.0
0.1
0.2
0.3
0.4
0.5
0.6
0.7
0.8
p-value
3e-03
5e-03
7e-03
1e-02
1e-02
Log-odds
0.5
1.0
1.5
2.0
2.5
3.0
3.5
4.0
Cellular_organisms - ps1
Archaea1 - ps2
Archaea2 - ps3
Archaea3 - ps4
Asgard/Eukaryota - ps5
Eukaryota - ps6
Amorpheae - ps7
Obazoa - ps8
Opisthokonta - ps9
Holozoa - ps10
Filozoa - ps11
Choano./Metazoa - ps12
Metazoa - ps13
Myriazoa - ps14
Parahoxozoa - ps15
Bilateria - ps16
Deuterostomia - ps17
Chordata - ps18
Olfactores - ps19
Vertebrata - ps20
Gnathostomata - ps21
Euteleostomi - ps22
Sarcopterygii - ps23
Tetrapoda - ps24
Amniota - ps25
Mammalia - ps26
Theria - ps27
Placentalia - ps28
Boreoeutheria - ps29
Euarchontoglires - ps30
Euarchonta - ps31
Primates - ps32
Haplorrhini - ps33
Simiiformes - ps34
Catarrhini - ps35
Hominoidea - ps36
Hominidae - ps37
Homininae - ps38
Hominini - ps39
Homo_sapiens - ps40
Phylostratum

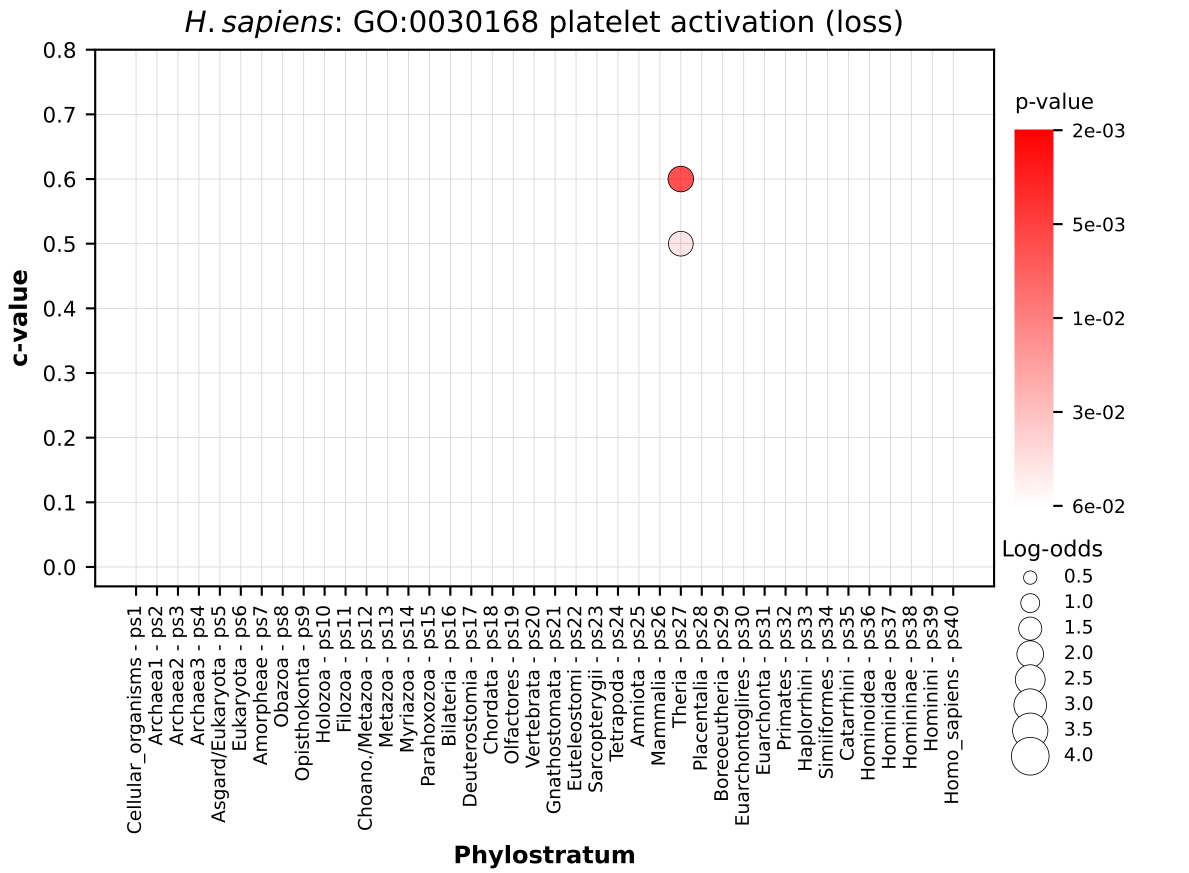

H. sapiens: GO:0030168 platelet activation (loss)
c-value
0.0
0.1
0.2
0.3
0.4
0.5
0.6
0.7
0.8
Phylostratum
Cellular_organisms - ps1
Archaea1 - ps2
Archaea2 - ps3
Archaea3 - ps4
Asgard/Eukaryota - ps5
Eukaryota - ps6
Amorpheae - ps7
Obazoa - ps8
Opisthokonta - ps9
Holozoa - ps10
Filozoa - ps11
Choano./Metazoa - ps12
Metazoa - ps13
Myriazoa - ps14
Parahoxozoa - ps15
Bilateria - ps16
Deuterostomia - ps17
Chordata - ps18
Olfactores - ps19
Vertebrata - ps20
Gnathostomata - ps21
Euteleostomi - ps22
Sarcopterygii - ps23
Tetrapoda - ps24
Amniota - ps25
Mammalia - ps26
Theria - ps27
Placentalia - ps28
Boreoeutheria - ps29
Euarchontoglires - ps30
Euarchonta - ps31
Primates - ps32
Haplorrhini - ps33
Simiiformes - ps34
Catarrhini - ps35
Hominoidea - ps36
Hominidae - ps37
Homininae - ps38
Hominini - ps39
Homo_sapiens - ps40
p-value
2e-03
5e-03
1e-02
3e-02
6e-02
Log-odds
0.5
1.0
1.5
2.0
2.5
3.0
3.5
4.0

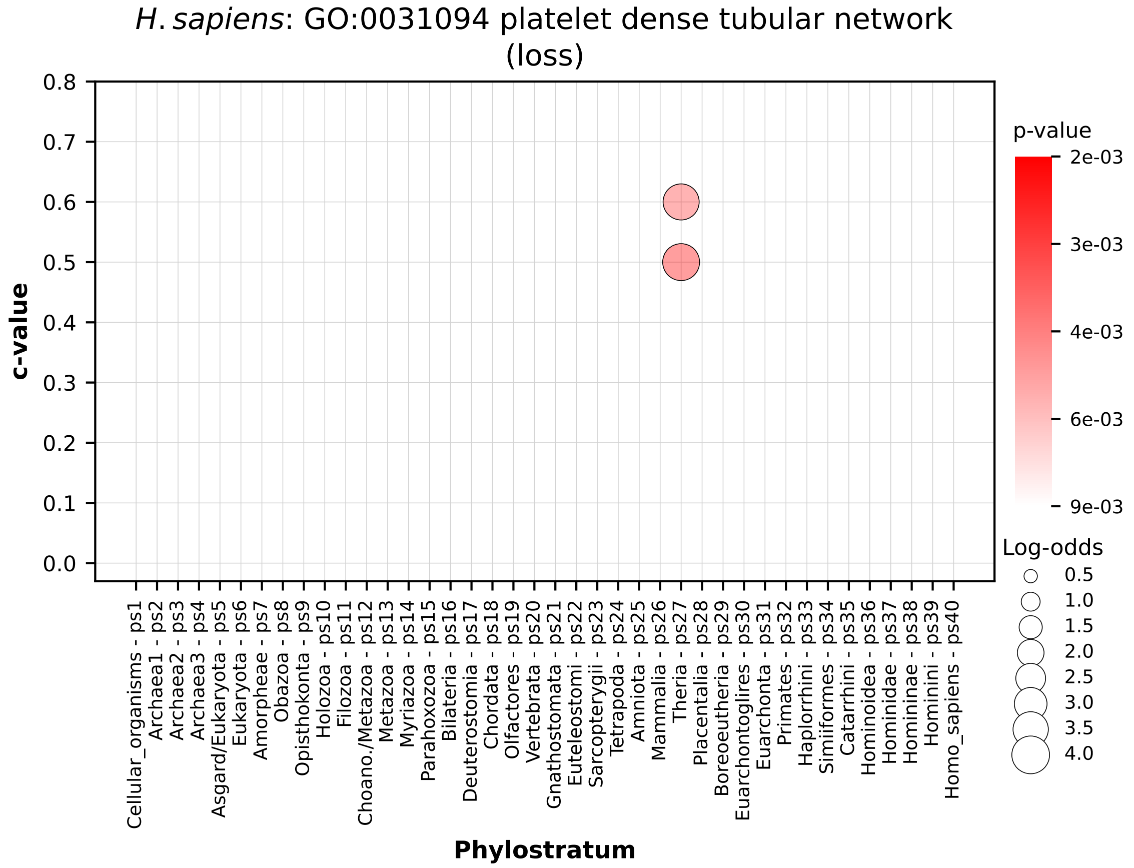

H. sapiens: GO:0031094 platelet dense tubular network
(loss)
c-value
0.8
0.7
0.6
0.5
0.4
0.3
0.2
0.1
0.0
Phylostratum
Cellular_organisms - ps1
Archaea1 - ps2
Archaea2 - ps3
Archaea3 - ps4
Asgard/Eukaryota - ps5
Eukaryota - ps6
Amorpheae - ps7
Obazoa - ps8
Opisthokonta - ps9
Holozoa - ps10
Filozoa - ps11
Choano./Metazoa - ps12
Metazoa - ps13
Myriazoa - ps14
Parahoxozoa - ps15
Bilateria - ps16
Deuterostomia - ps17
Chordata - ps18
Olfactores - ps19
Vertebrata - ps20
Gnathostomata - ps21
Euteleostomi - ps22
Sarcopterygii - ps23
Tetrapoda - ps24
Amniota - ps25
Mammalia - ps26
Theria - ps27
Placentalia - ps28
Boreoeutheria - ps29
Euarchontoglires - ps30
Euarchonta - ps31
Primates - ps32
Haplorrhini - ps33
Simiiformes - ps34
Catarrhini - ps35
Hominoidea - ps36
Hominidae - ps37
Homininae - ps38
Hominini - ps39
Homo_sapiens - ps40
p-value
2e-03
3e-03
4e-03
6e-03
9e-03
Log-odds
0.5
1.0
1.5
2.0
2.5
3.0
3.5
4.0

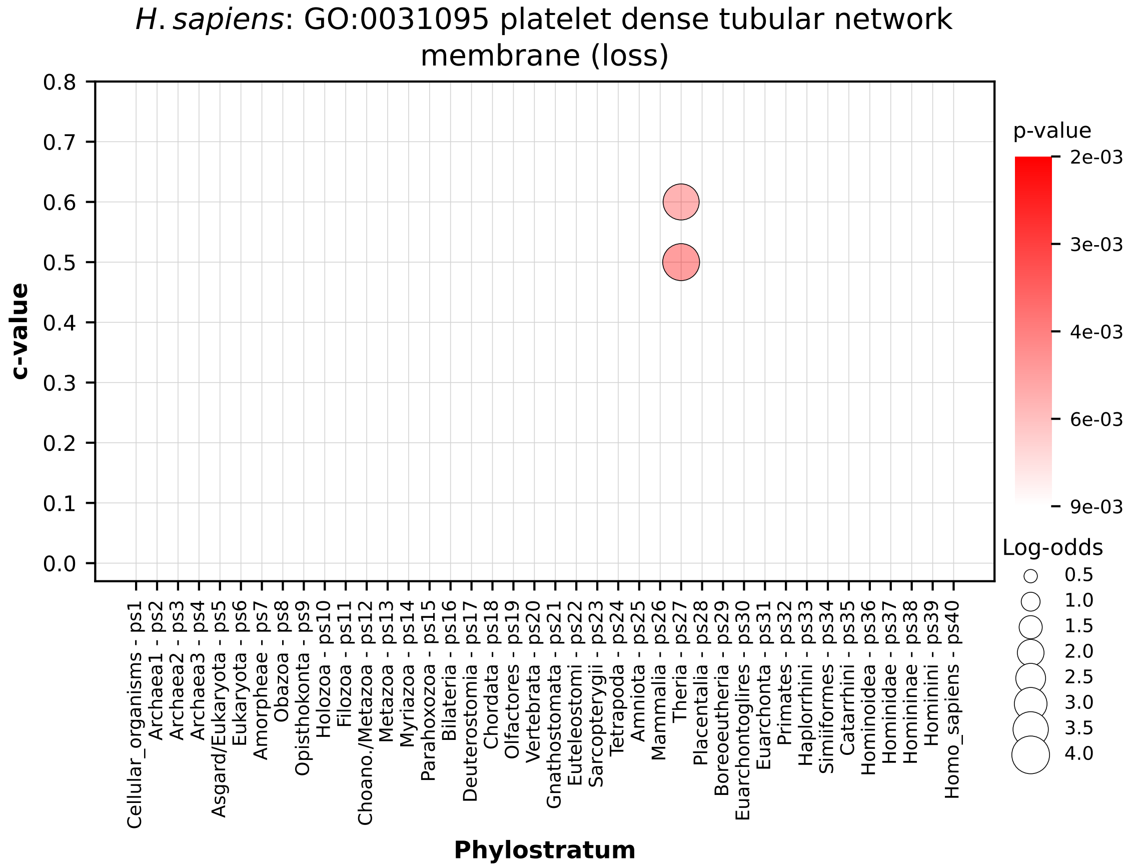

H. sapiens: GO:0031095 platelet dense tubular network membrane (loss)
c-value
0.8
0.7
0.6
0.5
0.4
0.3
0.2
0.1
0.0
Phylostratum
Cellular_organisms - ps1
Archaea1 - ps2
Archaea2 - ps3
Archaea3 - ps4
Asgard/Eukaryota - ps5
Eukaryota - ps6
Amorpheae - ps7
Obazoa - ps8
Opisthokonta - ps9
Holozoa - ps10
Filozoa - ps11
Choano./Metazoa - ps12
Metazoa - ps13
Myriazoa - ps14
Parahoxozoa - ps15
Bilateria - ps16
Deuterostomia - ps17
Chordata - ps18
Olfactores - ps19
Vertebrata - ps20
Gnathostomata - ps21
Euteleostomi - ps22
Sarcopterygii - ps23
Tetrapoda - ps24
Amniota - ps25
Mammalia - ps26
Theria - ps27
Placentalia - ps28
Boreoeutheria - ps29
Euarchontoglires - ps30
Euarchonta - ps31
Primates - ps32
Haplorrhini - ps33
Simiiformes - ps34
Catarrhini - ps35
Hominoidea - ps36
Hominidae - ps37
Homininae - ps38
Hominini - ps39
Homo_sapiens - ps40
p-value
2e-03
3e-03
4e-03
6e-03
9e-03
Log-odds
0.5
1.0
1.5
2.0
2.5
3.0
3.5
4.0

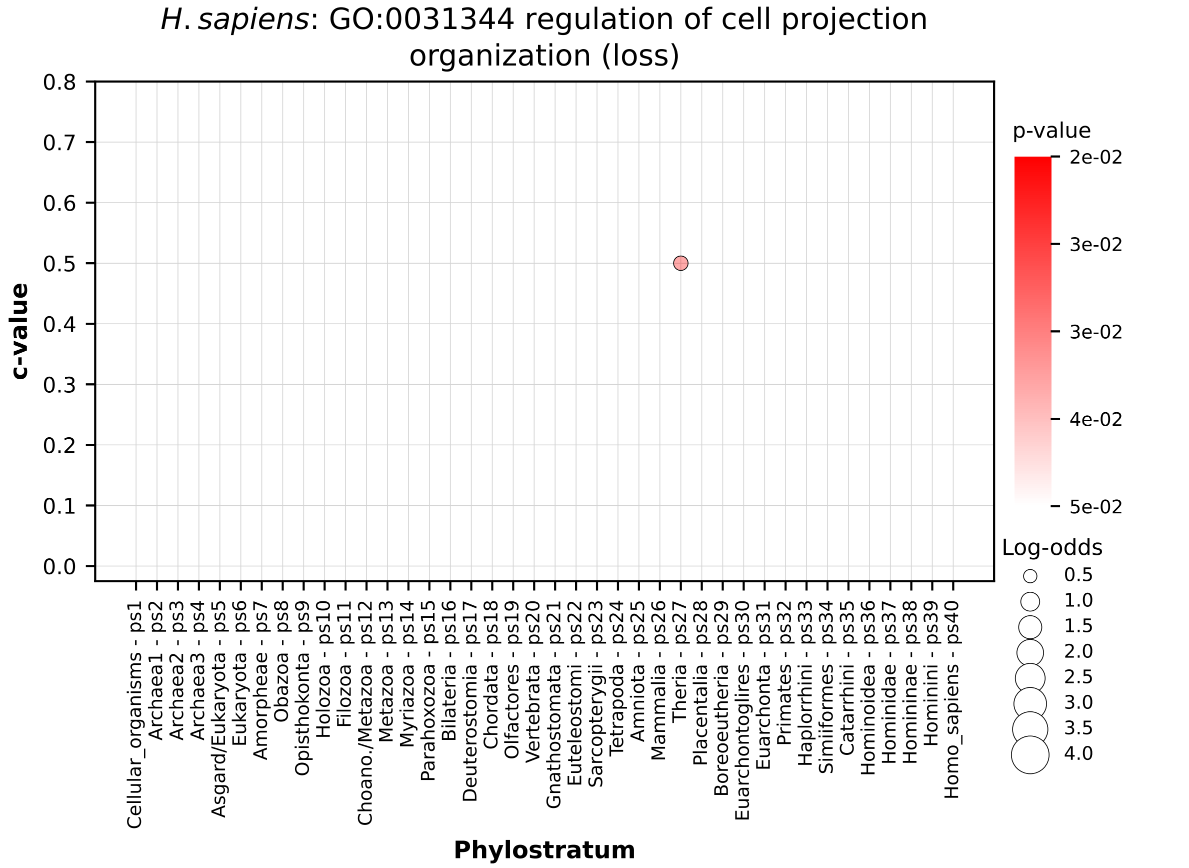
H. sapiens: GO:0031344 regulation of cell projection organization (loss)
c-value
0.8
0.7
0.6
0.5
0.4
0.3
0.2
0.1
0.0
p-value
2e-02
3e-02
3e-02
4e-02
5e-02
Log-odds
0.5
1.0
1.5
2.0
2.5
3.0
3.5
4.0
Cellular_organisms - ps1
Archaea1 - ps2
Archaea2 - ps3
Archaea3 - ps4
Asgard/Eukaryota - ps5
Eukaryota - ps6
Amorpheae - ps7
Obazoa - ps8
Opisthokonta - ps9
Holozoa - ps10
Filozoa - ps11
Choano./Metazoa - ps12
Metazoa - ps13
Myriazoa - ps14
Parahoxozoa - ps15
Bilateria - ps16
Deuterostomia - ps17
Chordata - ps18
Olfactores - ps19
Vertebrata - ps20
Gnathostomata - ps21
Euteleostomi - ps22
Sarcopterygii - ps23
Tetrapoda - ps24
Amniota - ps25
Mammalia - ps26
Theria - ps27
Placentalia - ps28
Boreoeutheria - ps29
Euarchontoglires - ps30
Euarchonta - ps31
Primates - ps32
Haplorrhini - ps33
Simiiformes - ps34
Catarrhini - ps35
Hominoidea - ps36
Hominidae - ps37
Homininae - ps38
Hominini - ps39
Homo_sapiens - ps40
Phylostratum

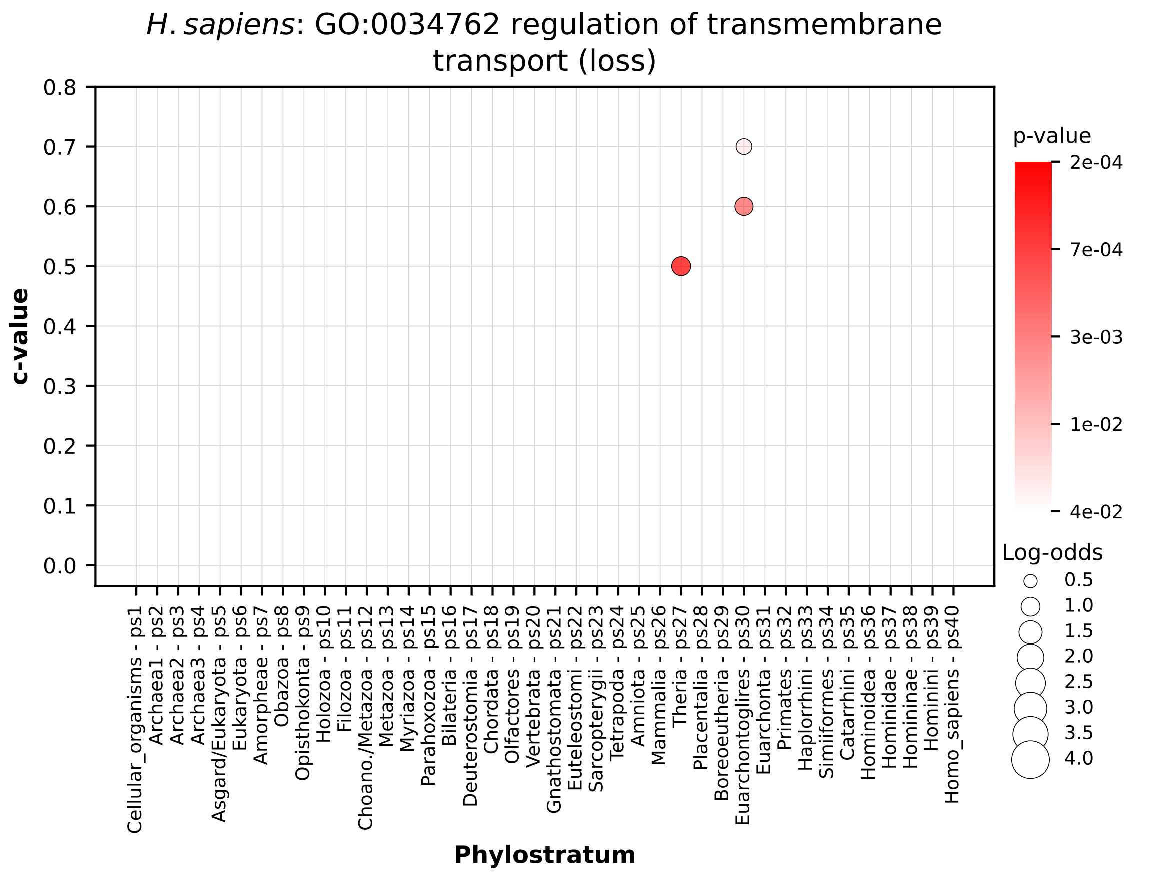

H. sapiens: GO:0034762 regulation of transmembrane transport (loss)
c-value
0.8
0.7
0.6
0.5
0.4
0.3
0.2
0.1
0.0
Phylostratum
Cellular_organisms - ps1
Archaea1 - ps2
Archaea2 - ps3
Archaea3 - ps4
Asgard/Eukaryota - ps5
Eukaryota - ps6
Amorpheae - ps7
Obazoa - ps8
Opisthokonta - ps9
Holozoa - ps10
Filozoa - ps11
Choano./Metazoa - ps12
Metazoa - ps13
Myriazoa - ps14
Parahoxozoa - ps15
Bilateria - ps16
Deuterostomia - ps17
Chordata - ps18
Olfactores - ps19
Vertebrata - ps20
Gnathostomata - ps21
Euteleostomi - ps22
Sarcopterygii - ps23
Tetrapoda - ps24
Amniota - ps25
Mammalia - ps26
Theria - ps27
Placentalia - ps28
Boreoeutheria - ps29
Euarchontoglires - ps30
Euarchonta - ps31
Primates - ps32
Haplorrhini - ps33
Simiiformes - ps34
Catarrhini - ps35
Hominoidea - ps36
Hominidae - ps37
Homininae - ps38
Hominini - ps39
Homo_sapiens - ps40
p-value
2e-04
7e-04
3e-03
1e-02
4e-02
Log-odds
0.5
1.0
1.5
2.0
2.5
3.0
3.5
4.0

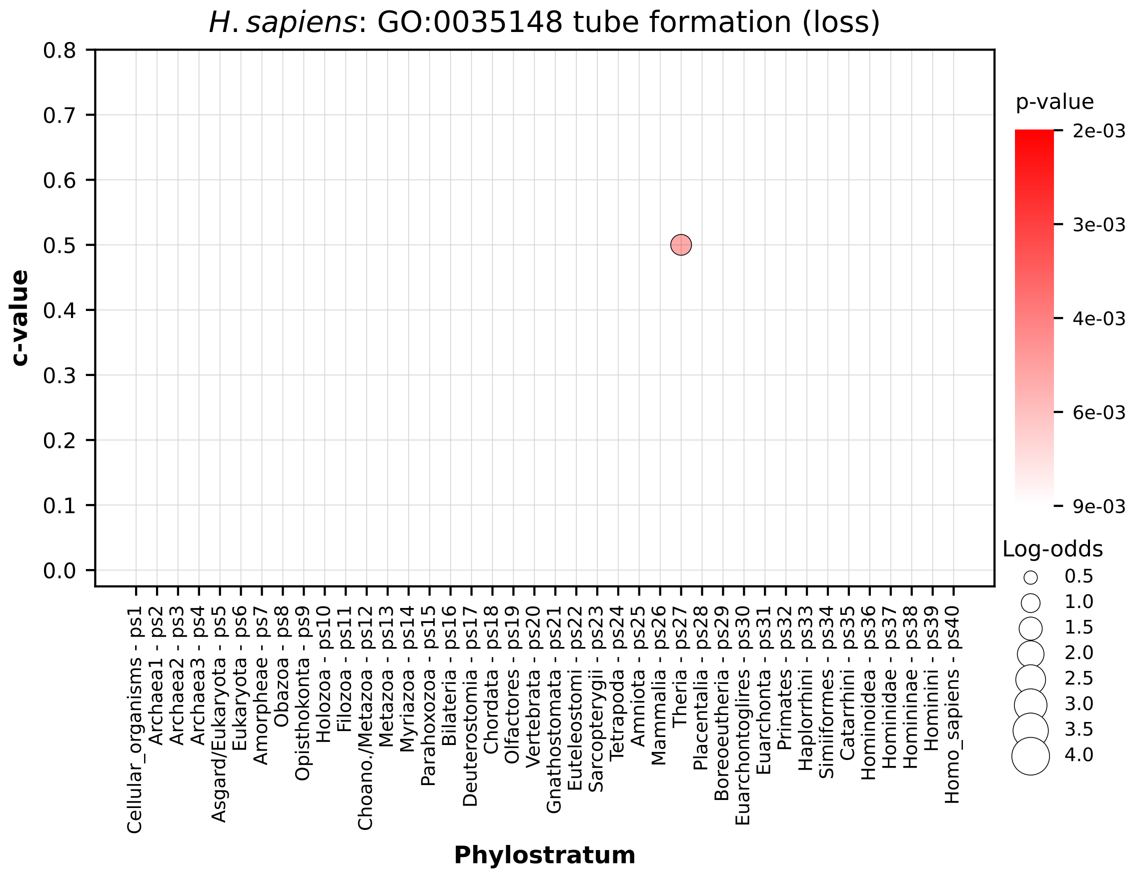

H. sapiens: GO:0035148 tube formation (loss)
c-value
0.0
0.1
0.2
0.3
0.4
0.5
0.6
0.7
0.8
Phylostratum
Cellular_organisms - ps1
Archaea1 - ps2
Archaea2 - ps3
Archaea3 - ps4
Asgard/Eukaryota - ps5
Eukaryota - ps6
Amorpheae - ps7
Obazoa - ps8
Opisthokonta - ps9
Holozoa - ps10
Filozoa - ps11
Choano./Metazoa - ps12
Metazoa - ps13
Myriazoa - ps14
Parahoxozoa - ps15
Bilateria - ps16
Deuterostomia - ps17
Chordata - ps18
Olfactores - ps19
Vertebrata - ps20
Gnathostomata - ps21
Euteleostomi - ps22
Sarcopterygii - ps23
Tetrapoda - ps24
Amniota - ps25
Mammalia - ps26
Theria - ps27
Placentalia - ps28
Boreoeutheria - ps29
Euarchontoglires - ps30
Euarchonta - ps31
Primates - ps32
Haplorrhini - ps33
Simiiformes - ps34
Catarrhini - ps35
Hominoidea - ps36
Hominidae - ps37
Homininae - ps38
Hominini - ps39
Homo_sapiens - ps40
p-value
2e-03
3e-03
4e-03
6e-03
9e-03
Log-odds
0.5
1.0
1.5
2.0
2.5
3.0
3.5
4.0

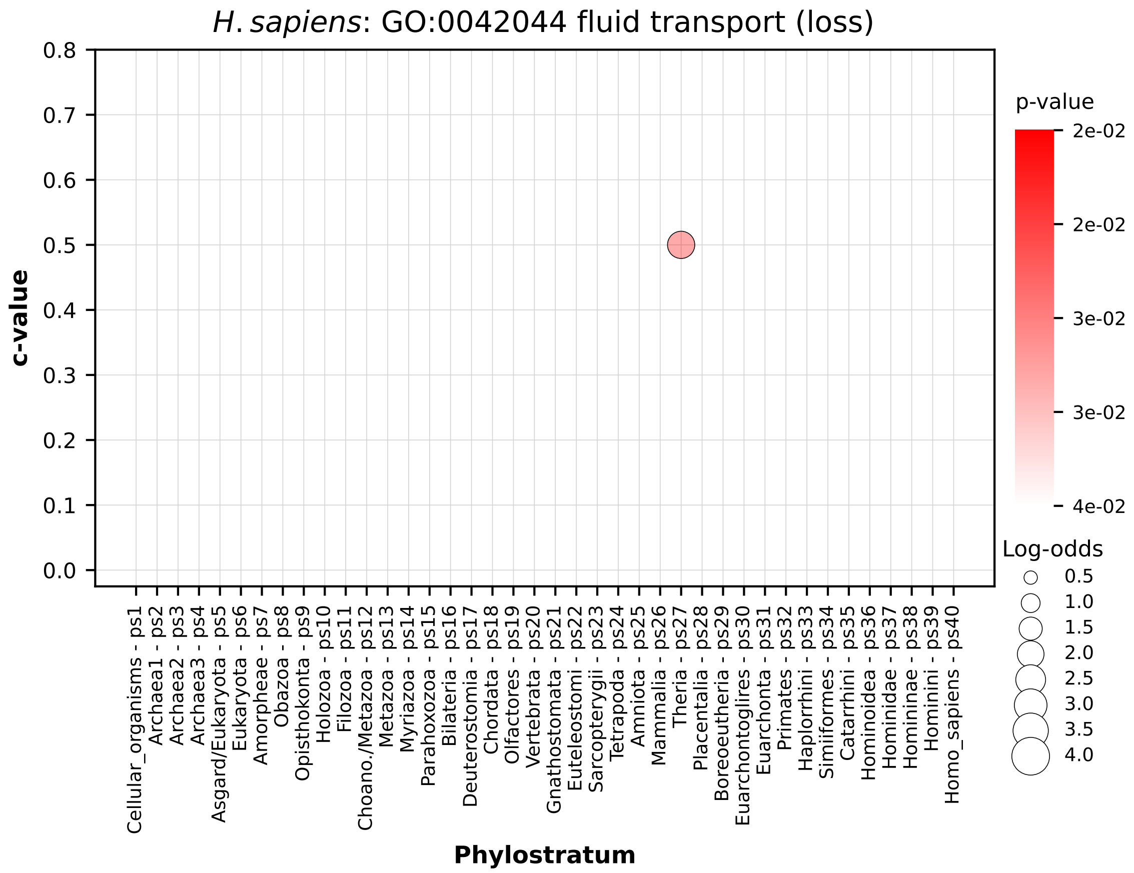

H. sapiens: GO:0042044 fluid transport (loss)
c-value
0.8
0.7
0.6
0.5
0.4
0.3
0.2
0.1
0.0
p-value
2e-02
2e-02
3e-02
3e-02
4e-02
Log-odds
0.5
1.0
1.5
2.0
2.5
3.0
3.5
4.0
Cellular_organisms - ps1
Archaea1 - ps2
Archaea2 - ps3
Archaea3 - ps4
Asgard/Eukaryota - ps5
Eukaryota - ps6
Amorpheae - ps7
Obazoa - ps8
Opisthokonta - ps9
Holozoa - ps10
Filozoa - ps11
Choano./Metazoa - ps12
Metazoa - ps13
Myriazoa - ps14
Parahoxozoa - ps15
Bilateria - ps16
Deuterostomia - ps17
Chordata - ps18
Olfactores - ps19
Vertebrata - ps20
Gnathostomata - ps21
Euteleostomi - ps22
Sarcopterygii - ps23
Tetrapoda - ps24
Amniota - ps25
Mammalia - ps26
Theria - ps27
Placentalia - ps28
Boreoeutheria - ps29
Euarchontoglires - ps30
Euarchonta - ps31
Primates - ps32
Haplorrhini - ps33
Simiiformes - ps34
Catarrhini - ps35
Hominoidea - ps36
Hominidae - ps37
Homininae - ps38
Hominini - ps39
Homo_sapiens - ps40
Phylostratum

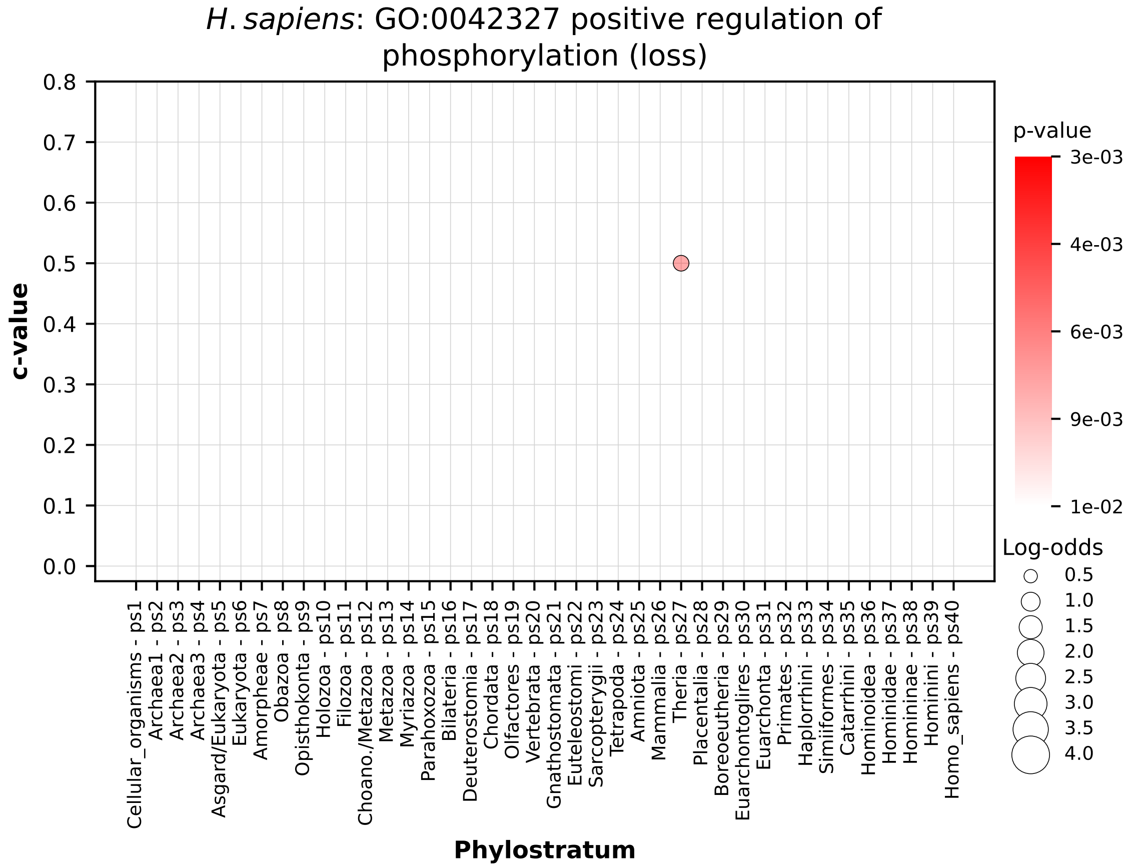

H. sapiens: GO:0042327 positive regulation of phosphorylation (loss)
c-value
0.8
0.7
0.6
0.5
0.4
0.3
0.2
0.1
0.0
p-value
3e-03
4e-03
6e-03
9e-03
1e-02
Log-odds
0.5
1.0
1.5
2.0
2.5
3.0
3.5
4.0
Cellular_organisms - ps1
Archaea1 - ps2
Archaea2 - ps3
Archaea3 - ps4
Asgard/Eukaryota - ps5
Eukaryota - ps6
Amorpheae - ps7
Obazoa - ps8
Opisthokonta - ps9
Holozoa - ps10
Filozoa - ps11
Choano./Metazoa - ps12
Metazoa - ps13
Myriazoa - ps14
Parahoxozoa - ps15
Bilateria - ps16
Deuterostomia - ps17
Chordata - ps18
Olfactores - ps19
Vertebrata - ps20
Gnathostomata - ps21
Euteleostomi - ps22
Sarcopterygii - ps23
Tetrapoda - ps24
Amniota - ps25
Mammalia - ps26
Theria - ps27
Placentalia - ps28
Boreoeutheria - ps29
Euarchontoglires - ps30
Euarchonta - ps31
Primates - ps32
Haplorrhini - ps33
Simiiformes - ps34
Catarrhini - ps35
Hominoidea - ps36
Hominidae - ps37
Homininae - ps38
Hominini - ps39
Homo_sapiens - ps40
Phylostratum

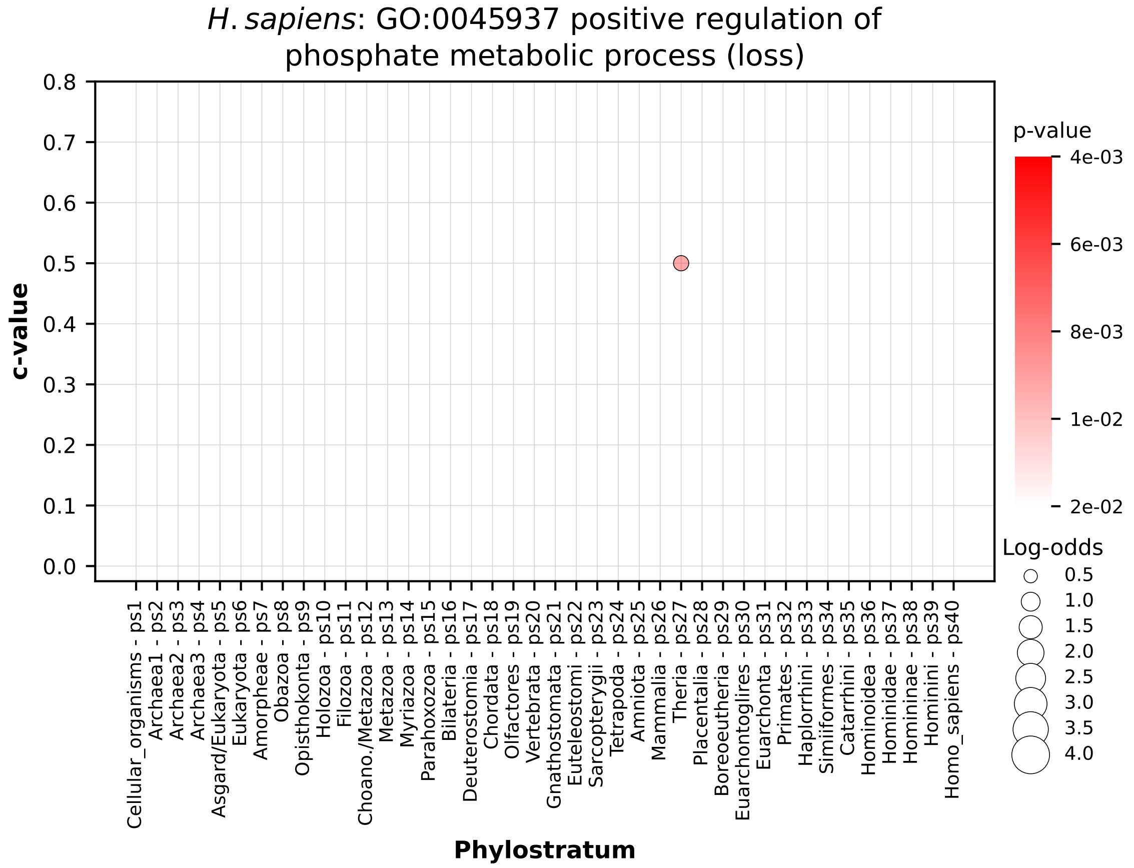
H. sapiens: GO:0045937 positive regulation of phosphate metabolic process (loss)
c-value
0.8
0.7
0.6
0.5
0.4
0.3
0.2
0.1
0.0
Phylostratum
Cellular_organisms - ps1
Archaea1 - ps2
Archaea2 - ps3
Archaea3 - ps4
Asgard/Eukaryota - ps5
Eukaryota - ps6
Amorpheae - ps7
Obazoa - ps8
Opisthokonta - ps9
Holozoa - ps10
Filozoa - ps11
Choano./Metazoa - ps12
Metazoa - ps13
Myriazoa - ps14
Parahoxozoa - ps15
Bilateria - ps16
Deuterostomia - ps17
Chordata - ps18
Olfactores - ps19
Vertebrata - ps20
Gnathostomata - ps21
Euteleostomi - ps22
Sarcopterygii - ps23
Tetrapoda - ps24
Amniota - ps25
Mammalia - ps26
Theria - ps27
Placentalia - ps28
Boreoeutheria - ps29
Euarchontoglires - ps30
Euarchonta - ps31
Primates - ps32
Haplorrhini - ps33
Simiiformes - ps34
Catarrhini - ps35
Hominoidea - ps36
Hominidae - ps37
Homininae - ps38
Hominini - ps39
Homo_sapiens - ps40
p-value
4e-03
6e-03
8e-03
1e-02
2e-02
Log-odds
0.5
1.0
1.5
2.0
2.5
3.0
3.5
4.0

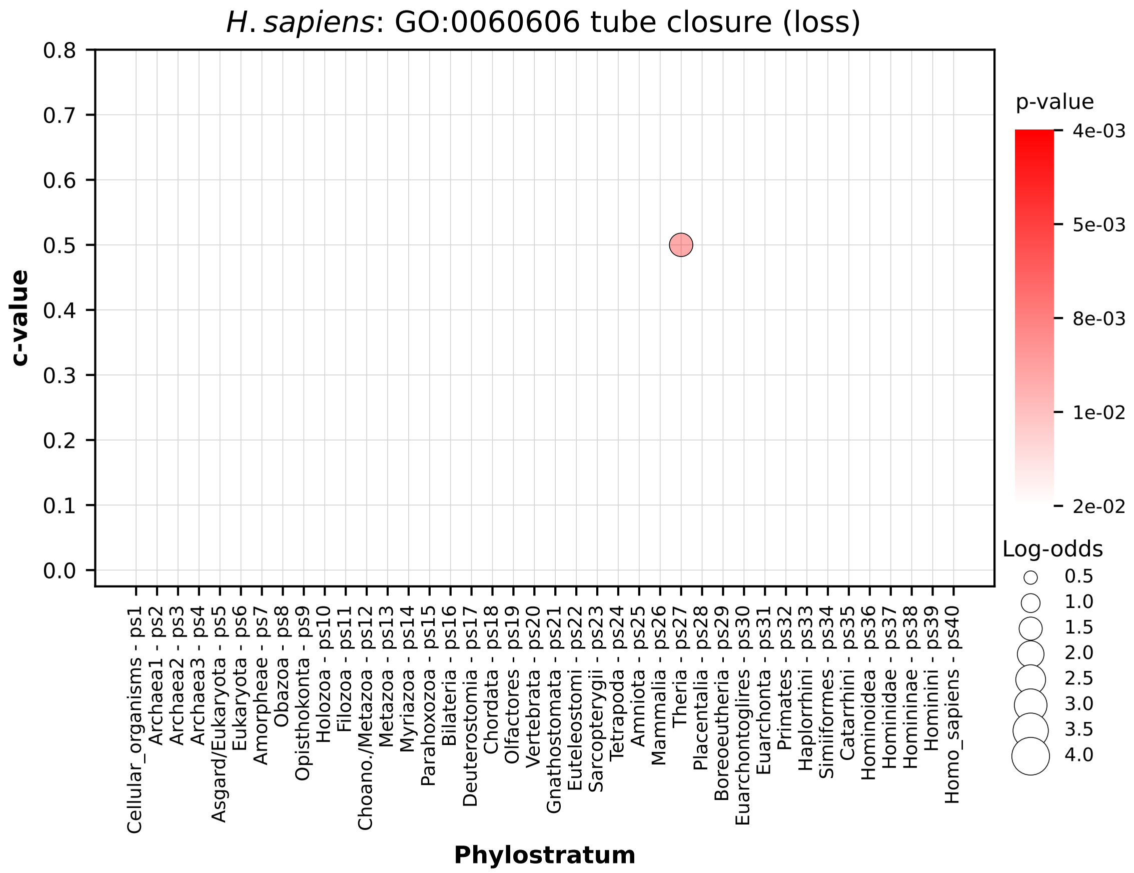

H. sapiens: GO:0060606 tube closure (loss)
c-value
0.8
0.7
0.6
0.5
0.4
0.3
0.2
0.1
0.0
Phylostratum
Cellular_organisms - ps1
Archaea1 - ps2
Archaea2 - ps3
Archaea3 - ps4
Asgard/Eukaryota - ps5
Eukaryota - ps6
Amorpheae - ps7
Obazoa - ps8
Opisthokonta - ps9
Holozoa - ps10
Filozoa - ps11
Choano./Metazoa - ps12
Metazoa - ps13
Myriazoa - ps14
Parahoxozoa - ps15
Bilateria - ps16
Deuterostomia - ps17
Chordata - ps18
Olfactores - ps19
Vertebrata - ps20
Gnathostomata - ps21
Euteleostomi - ps22
Sarcopterygii - ps23
Tetrapoda - ps24
Amniota - ps25
Mammalia - ps26
Theria - ps27
Placentalia - ps28
Boreoeutheria - ps29
Euarchontoglires - ps30
Euarchonta - ps31
Primates - ps32
Haplorrhini - ps33
Simiiformes - ps34
Catarrhini - ps35
Hominoidea - ps36
Hominidae - ps37
Homininae - ps38
Hominini - ps39
Homo_sapiens - ps40
p-value
4e-03
5e-03
8e-03
1e-02
2e-02
Log-odds
0.5
1.0
1.5
2.0
2.5
3.0
3.5
4.0

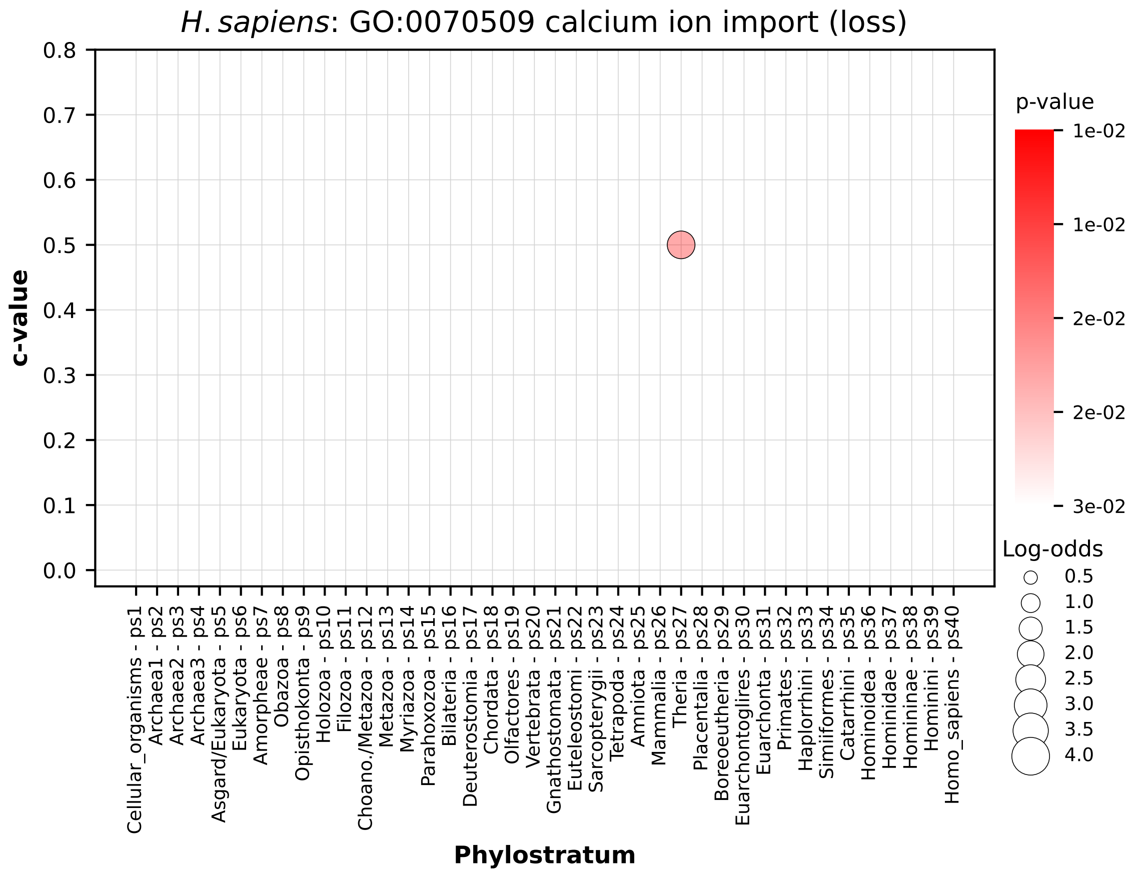

H. sapiens: GO:0070509 calcium ion import (loss)
c-value
0.8
0.7
0.6
0.5
0.4
0.3
0.2
0.1
0.0
p-value
1e-02
1e-02
2e-02
2e-02
3e-02
Log-odds
0.5
1.0
1.5
2.0
2.5
3.0
3.5
4.0
Cellular_organisms - ps1
Archaea1 - ps2
Archaea2 - ps3
Archaea3 - ps4
Asgard/Eukaryota - ps5
Eukaryota - ps6
Amorpheae - ps7
Obazoa - ps8
Opisthokonta - ps9
Holozoa - ps10
Filozoa - ps11
Choano./Metazoa - ps12
Metazoa - ps13
Myriazoa - ps14
Parahoxozoa - ps15
Bilateria - ps16
Deuterostomia - ps17
Chordata - ps18
Olfactores - ps19
Vertebrata - ps20
Gnathostomata - ps21
Euteleostomi - ps22
Sarcopterygii - ps23
Tetrapoda - ps24
Amniota - ps25
Mammalia - ps26
Theria - ps27
Placentalia - ps28
Boreoeutheria - ps29
Euarchontoglires - ps30
Euarchonta - ps31
Primates - ps32
Haplorrhini - ps33
Simiiformes - ps34
Catarrhini - ps35
Hominoidea - ps36
Hominidae - ps37
Homininae - ps38
Hominini - ps39
Homo_sapiens - ps40
Phylostratum

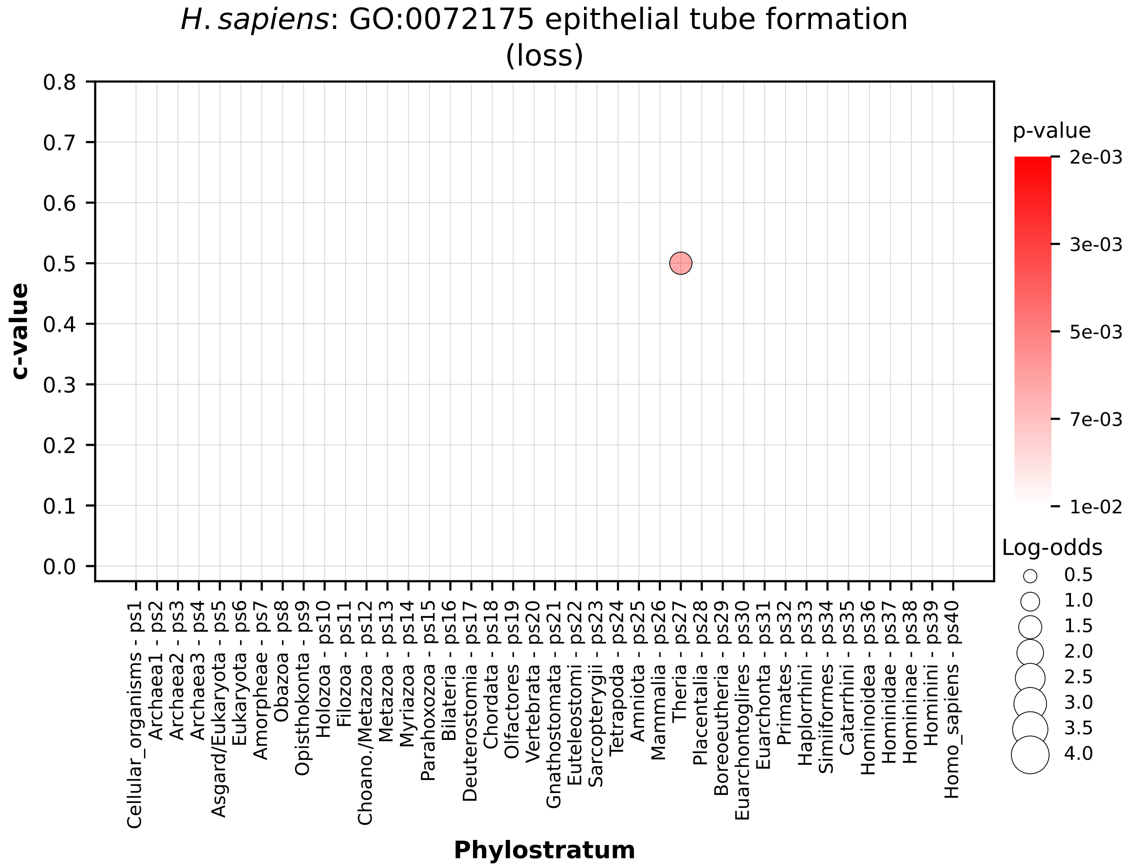

H. sapiens: GO:0072175 epithelial tube formation
(loss)
c-value
0.0
0.1
0.2
0.3
0.4
0.5
0.6
0.7
0.8
Phylostratum
Cellular_organisms - ps1
Archaea1 - ps2
Archaea2 - ps3
Archaea3 - ps4
Asgard/Eukaryota - ps5
Eukaryota - ps6
Amorpheae - ps7
Obazoa - ps8
Opisthokonta - ps9
Holozoa - ps10
Filozoa - ps11
Choano./Metazoa - ps12
Metazoa - ps13
Myriazoa - ps14
Parahoxozoa - ps15
Bilateria - ps16
Deuterostomia - ps17
Chordata - ps18
Olfactores - ps19
Vertebrata - ps20
Gnathostomata - ps21
Euteleostomi - ps22
Sarcopterygii - ps23
Tetrapoda - ps24
Amniota - ps25
Mammalia - ps26
Theria - ps27
Placentalia - ps28
Boreoeutheria - ps29
Euarchontoglires - ps30
Euarchonta - ps31
Primates - ps32
Haplorrhini - ps33
Simiiformes - ps34
Catarrhini - ps35
Hominoidea - ps36
Hominidae - ps37
Homininae - ps38
Hominini - ps39
Homo_sapiens - ps40
p-value
2e-03
3e-03
5e-03
7e-03
1e-02
Log-odds
0.5
1.0
1.5
2.0
2.5
3.0
3.5
4.0

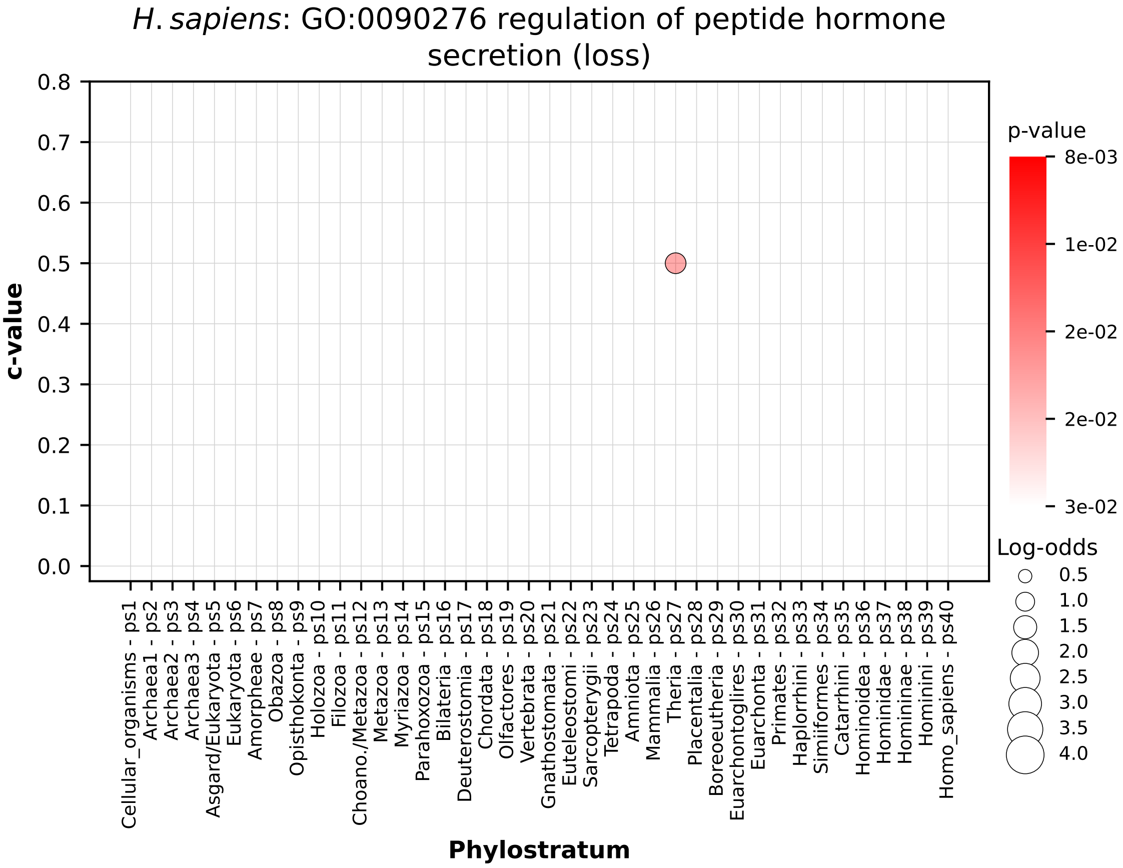

H. sapiens: GO:0090276 regulation of peptide hormone secretion (loss)
c-value
0.0
0.1
0.2
0.3
0.4
0.5
0.6
0.7
0.8
Phylostratum
Cellular_organisms - ps1
Archaea1 - ps2
Archaea2 - ps3
Archaea3 - ps4
Asgard/Eukaryota - ps5
Eukaryota - ps6
Amorpheae - ps7
Obazoa - ps8
Opisthokonta - ps9
Holozoa - ps10
Filozoa - ps11
Choano./Metazoa - ps12
Metazoa - ps13
Myriazoa - ps14
Parahoxozoa - ps15
Bilateria - ps16
Deuterostomia - ps17
Chordata - ps18
Olfactores - ps19
Vertebrata - ps20
Gnathostomata - ps21
Euteleostomi - ps22
Sarcopterygii - ps23
Tetrapoda - ps24
Amniota - ps25
Mammalia - ps26
Theria - ps27
Placentalia - ps28
Boreoeutheria - ps29
Euarchontoglires - ps30
Euarchonta - ps31
Primates - ps32
Haplorrhini - ps33
Simiiformes - ps34
Catarrhini - ps35
Hominoidea - ps36
Hominidae - ps37
Homininae - ps38
Hominini - ps39
Homo_sapiens - ps40
p-value
8e-03
1e-02
2e-02
2e-02
3e-02
Log-odds
0.5
1.0
1.5
2.0
2.5
3.0
3.5
4.0

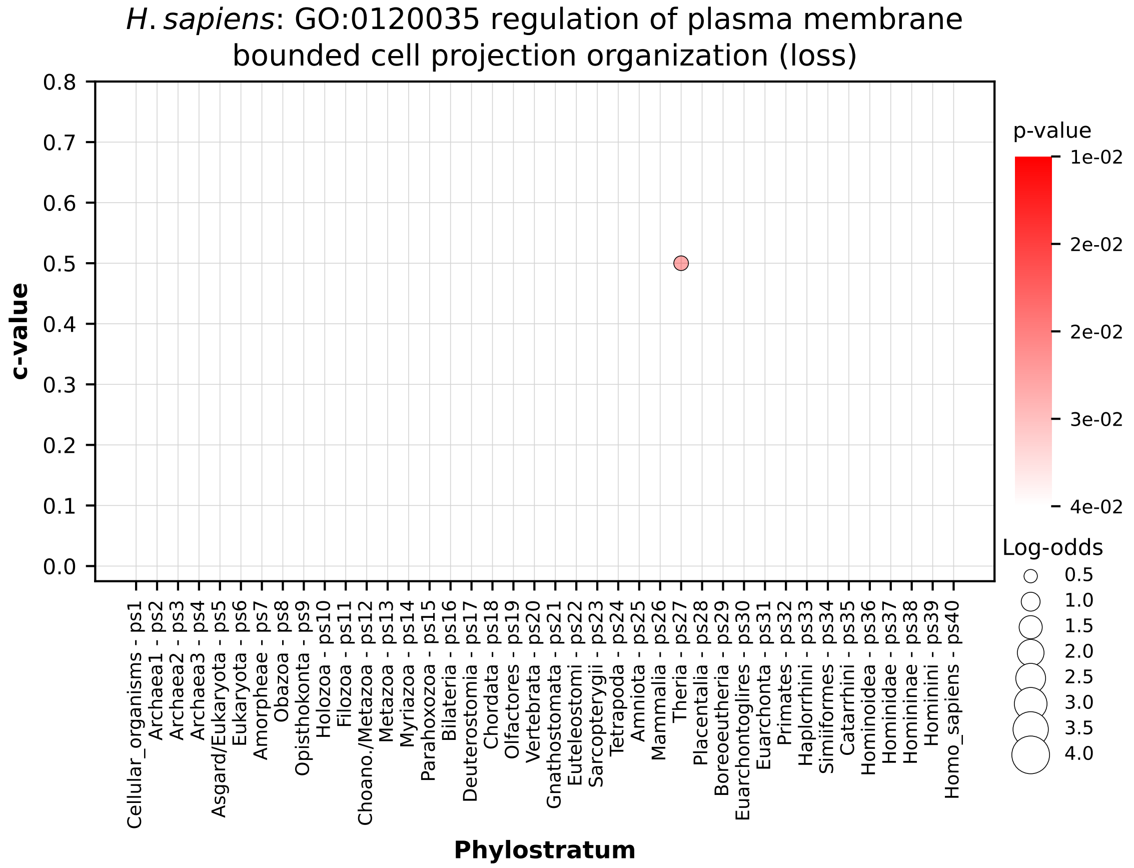

H. sapiens: GO:0120035 regulation of plasma membrane bounded cell projection organization (loss)
c-value
0.8
0.7
0.6
0.5
0.4
0.3
0.2
0.1
0.0
Phylostratum
Cellular_organisms - ps1
Archaea1 - ps2
Archaea2 - ps3
Archaea3 - ps4
Asgard/Eukaryota - ps5
Eukaryota - ps6
Amorpheae - ps7
Obazoa - ps8
Opisthokonta - ps9
Holozoa - ps10
Filozoa - ps11
Choano./Metazoa - ps12
Metazoa - ps13
Myriazoa - ps14
Parahoxozoa - ps15
Bilateria - ps16
Deuterostomia - ps17
Chordata - ps18
Olfactores - ps19
Vertebrata - ps20
Gnathostomata - ps21
Euteleostomi - ps22
Sarcopterygii - ps23
Tetrapoda - ps24
Amniota - ps25
Mammalia - ps26
Theria - ps27
Placentalia - ps28
Boreoeutheria - ps29
Euarchontoglires - ps30
Euarchonta - ps31
Primates - ps32
Haplorrhini - ps33
Simiiformes - ps34
Catarrhini - ps35
Hominoidea - ps36
Hominidae - ps37
Homininae - ps38
Hominini - ps39
Homo_sapiens - ps40
p-value
1e-02
2e-02
2e-02
3e-02
4e-02
Log-odds
0.5
1.0
1.5
2.0
2.5
3.0
3.5
4.0

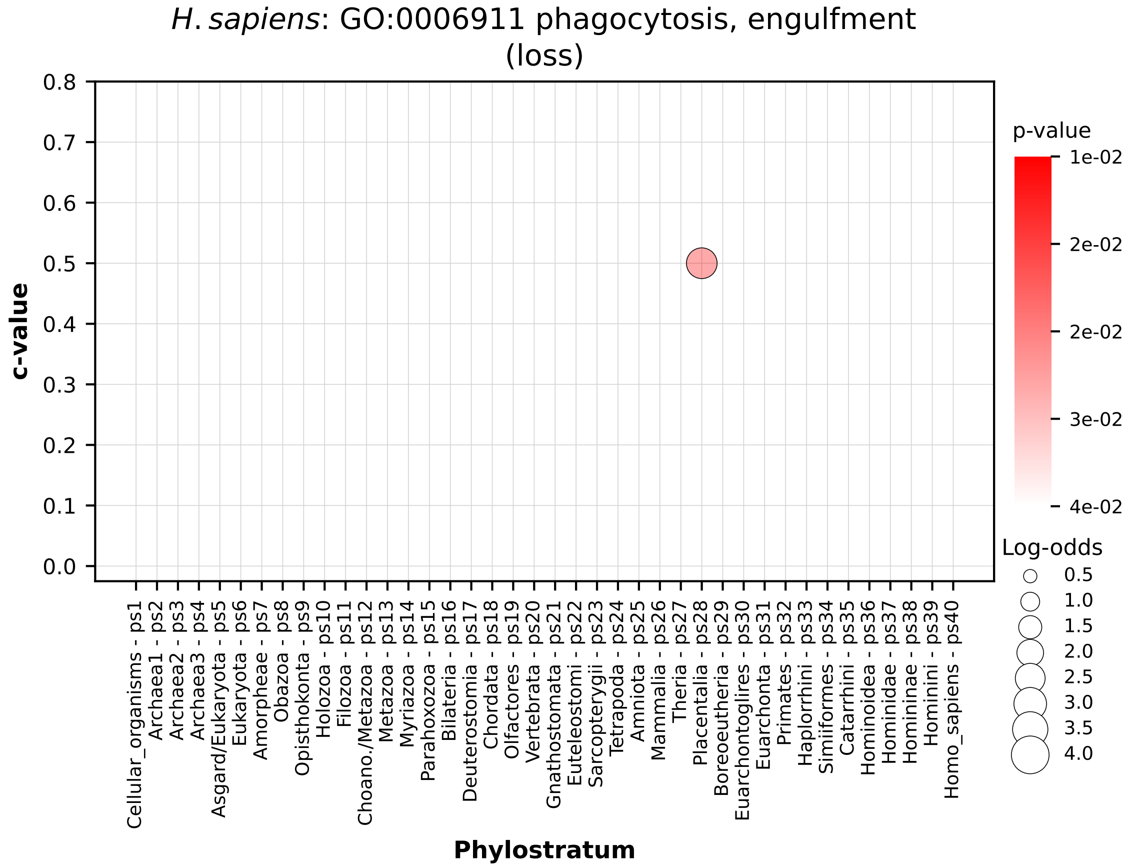

H. sapiens: GO:0006911 phagocytosis, engulfment (loss)
c-value
0.8
0.7
0.6
0.5
0.4
0.3
0.2
0.1
0.0
p-value
1e-02
2e-02
2e-02
3e-02
4e-02
Log-odds
0.5
1.0
1.5
2.0
2.5
3.0
3.5
4.0
Cellular_organisms - ps1
Archaea1 - ps2
Archaea2 - ps3
Archaea3 - ps4
Asgard/Eukaryota - ps5
Eukaryota - ps6
Amorpheae - ps7
Obazoa - ps8
Opisthokonta - ps9
Holozoa - ps10
Filozoa - ps11
Choano./Metazoa - ps12
Metazoa - ps13
Myriazoa - ps14
Parahoxozoa - ps15
Bilateria - ps16
Deuterostomia - ps17
Chordata - ps18
Olfactores - ps19
Vertebrata - ps20
Gnathostomata - ps21
Euteleostomi - ps22
Sarcopterygii - ps23
Tetrapoda - ps24
Amniota - ps25
Mammalia - ps26
Theria - ps27
Placentalia - ps28
Boreoeutheria - ps29
Euarchontoglires - ps30
Euarchonta - ps31
Primates - ps32
Haplorrhini - ps33
Simiiformes - ps34
Catarrhini - ps35
Hominoidea - ps36
Hominidae - ps37
Homininae - ps38
Hominini - ps39
Homo_sapiens - ps40
Phylostratum

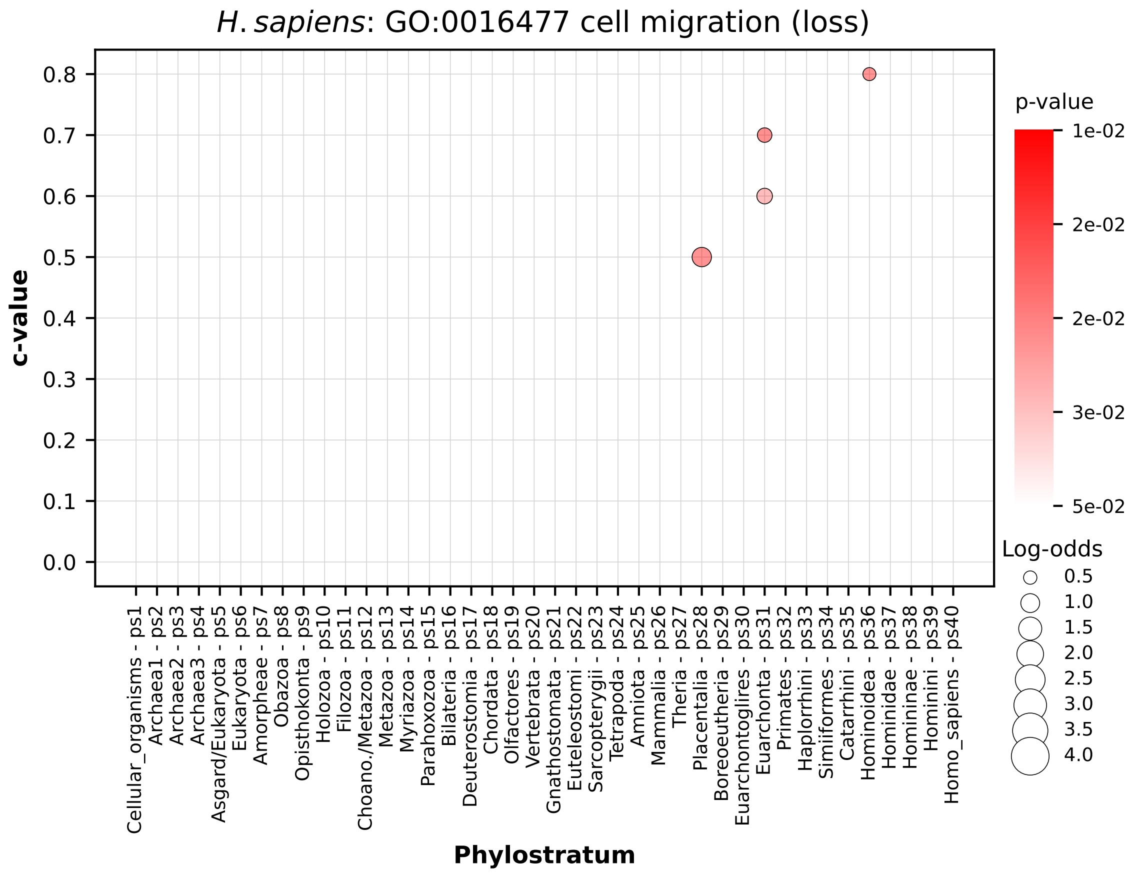

H. sapiens: GO:0016477 cell migration (loss)
c-value
0.8
0.7
0.6
0.5
0.4
0.3
0.2
0.1
0.0
p-value
1e-02
2e-02
2e-02
3e-02
5e-02
Log-odds
0.5
1.0
1.5
2.0
2.5
3.0
3.5
4.0
Cellular_organisms - ps1
Archaea1 - ps2
Archaea2 - ps3
Archaea3 - ps4
Asgard/Eukaryota - ps5
Eukaryota - ps6
Amorpheae - ps7
Obazoa - ps8
Opisthokonta - ps9
Holozoa - ps10
Filozoa - ps11
Choano./Metazoa - ps12
Metazoa - ps13
Myriazoa - ps14
Parahoxozoa - ps15
Bilateria - ps16
Deuterostomia - ps17
Chordata - ps18
Olfactores - ps19
Vertebrata - ps20
Gnathostomata - ps21
Euteleostomi - ps22
Sarcopterygii - ps23
Tetrapoda - ps24
Amniota - ps25
Mammalia - ps26
Theria - ps27
Placentalia - ps28
Boreoeutheria - ps29
Euarchontoglires - ps30
Euarchonta - ps31
Primates - ps32
Haplorrhini - ps33
Simiiformes - ps34
Catarrhini - ps35
Hominoidea - ps36
Hominidae - ps37
Homininae - ps38
Hominini - ps39
Homo_sapiens - ps40
Phylostratum

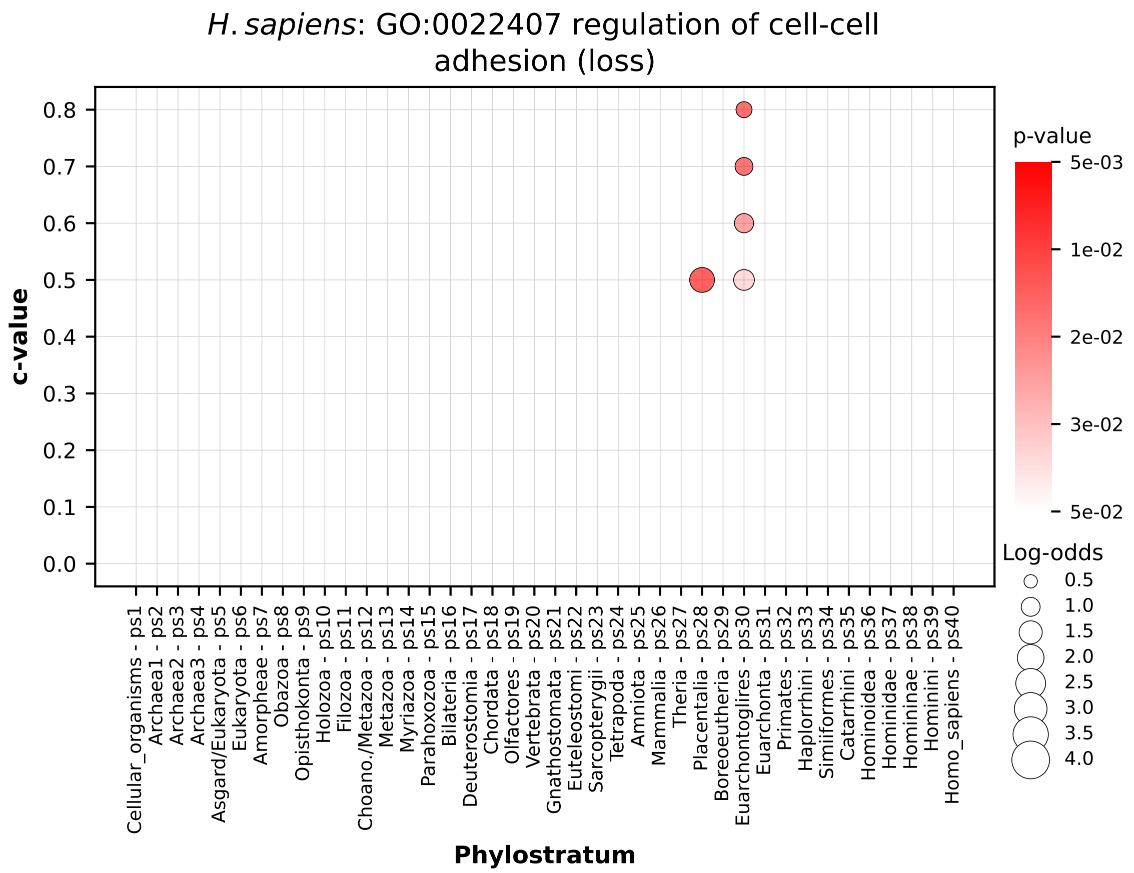

H. sapiens: GO:0022407 regulation of cell-cell adhesion (loss)
c-value
0.0
0.1
0.2
0.3
0.4
0.5
0.6
0.7
0.8
Phylostratum
Cellular_organisms - ps1
Archaea1 - ps2
Archaea2 - ps3
Archaea3 - ps4
Asgard/Eukaryota - ps5
Eukaryota - ps6
Amorpheae - ps7
Obazoa - ps8
Opisthokonta - ps9
Holozoa - ps10
Filozoa - ps11
Choano./Metazoa - ps12
Metazoa - ps13
Myriazoa - ps14
Parahoxozoa - ps15
Bilateria - ps16
Deuterostomia - ps17
Chordata - ps18
Olfactores - ps19
Vertebrata - ps20
Gnathostomata - ps21
Euteleostomi - ps22
Sarcopterygii - ps23
Tetrapoda - ps24
Amniota - ps25
Mammalia - ps26
Theria - ps27
Placentalia - ps28
Boreoeutheria - ps29
Euarchontoglires - ps30
Euarchonta - ps31
Primates - ps32
Haplorrhini - ps33
Simiiformes - ps34
Catarrhini - ps35
Hominoidea - ps36
Hominidae - ps37
Homininae - ps38
Hominini - ps39
Homo_sapiens - ps40
p-value
5e-03
1e-02
2e-02
3e-02
5e-02
Log-odds
0.5
1.0
1.5
2.0
2.5
3.0
3.5
4.0

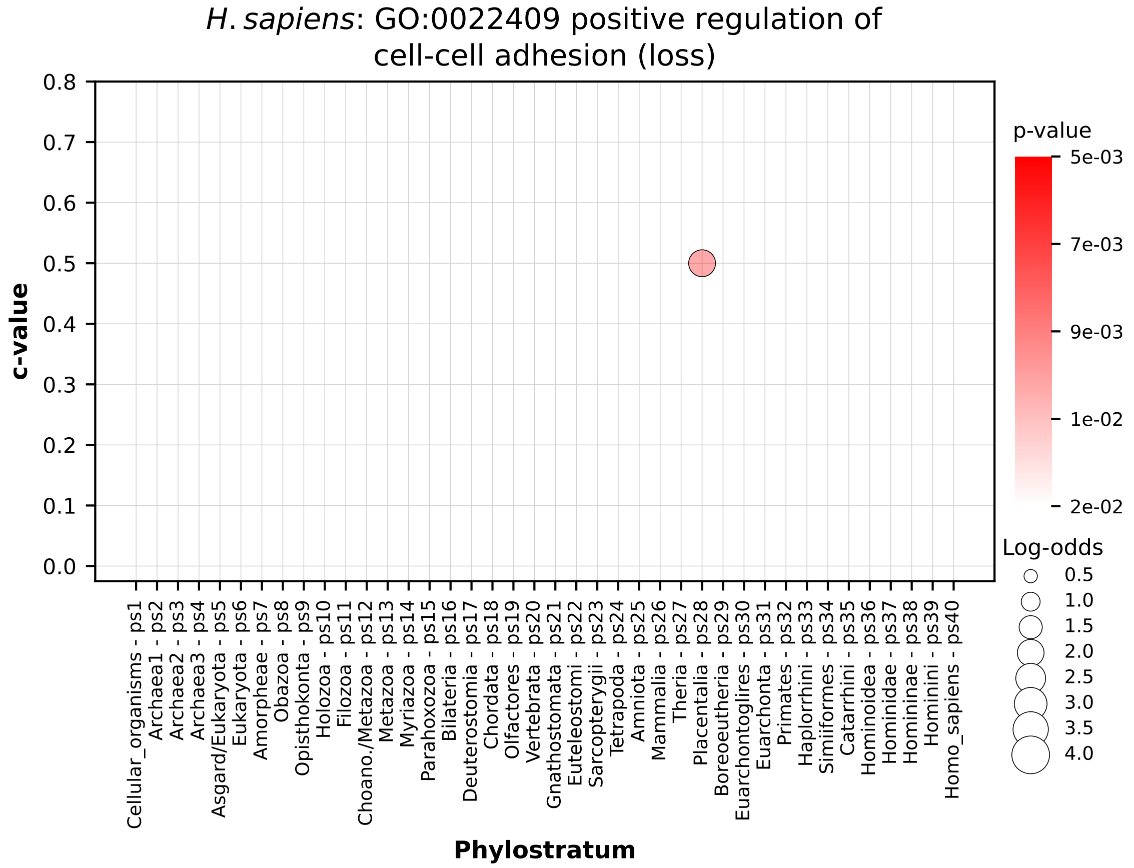

H. sapiens: GO:0022409 positive regulation of cell-cell adhesion (loss)
c-value
0.0
0.1
0.2
0.3
0.4
0.5
0.6
0.7
0.8
Phylostratum
Cellular_organisms - ps1
Archaea1 - ps2
Archaea2 - ps3
Archaea3 - ps4
Asgard/Eukaryota - ps5
Eukaryota - ps6
Amorpheae - ps7
Obazoa - ps8
Opisthokonta - ps9
Holozoa - ps10
Filozoa - ps11
Choano./Metazoa - ps12
Metazoa - ps13
Myriazoa - ps14
Parahoxozoa - ps15
Bilateria - ps16
Deuterostomia - ps17
Chordata - ps18
Olfactores - ps19
Vertebrata - ps20
Gnathostomata - ps21
Euteleostomi - ps22
Sarcopterygii - ps23
Tetrapoda - ps24
Amniota - ps25
Mammalia - ps26
Theria - ps27
Placentalia - ps28
Boreoeutheria - ps29
Euarchontoglires - ps30
Euarchonta - ps31
Primates - ps32
Haplorrhini - ps33
Simiiformes - ps34
Catarrhini - ps35
Hominoidea - ps36
Hominidae - ps37
Homininae - ps38
Hominini - ps39
Homo_sapiens - ps40
p-value
5e-03
7e-03
9e-03
1e-02
2e-02
Log-odds
0.5
1.0
1.5
2.0
2.5
3.0
3.5
4.0

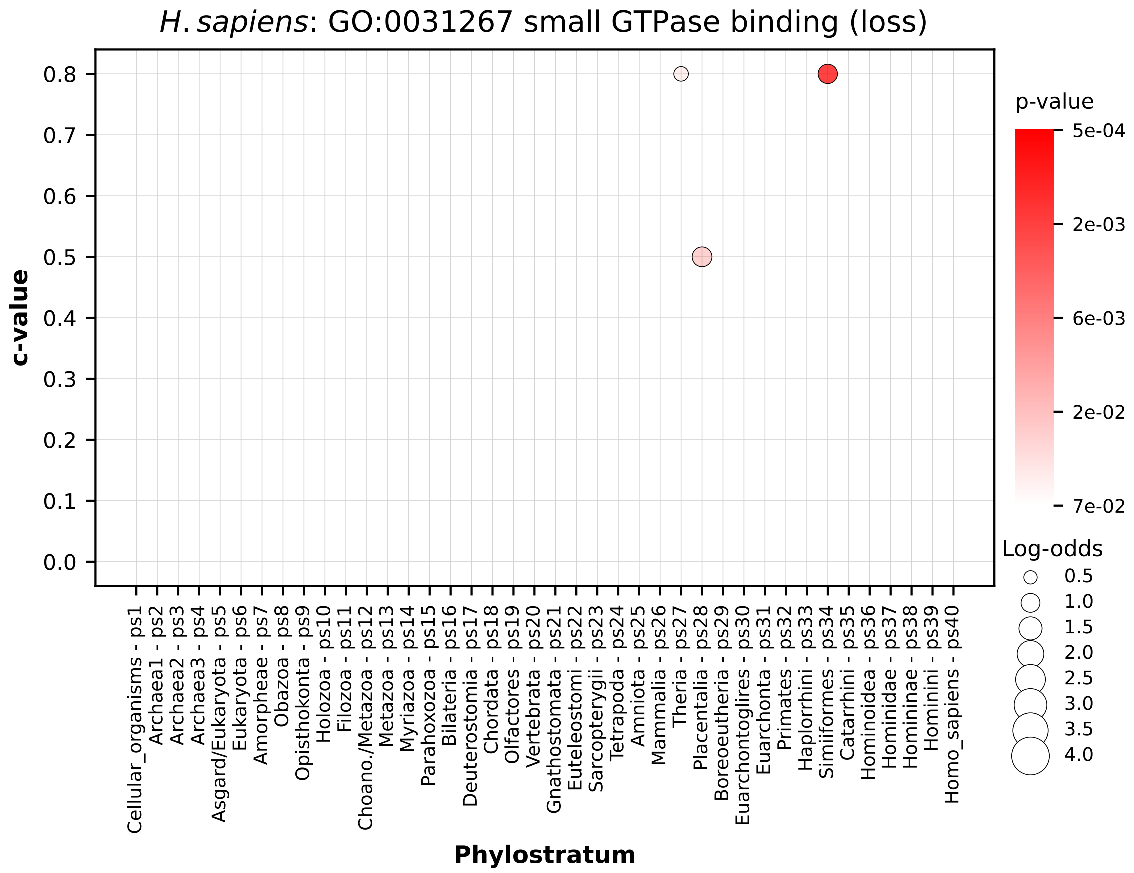

H. sapiens: GO:0031267 small GTPase binding (loss)
c-value
0.8
0.7
0.6
0.5
0.4
0.3
0.2
0.1
0.0
p-value
5e-04
2e-03
6e-03
2e-02
7e-02
Log-odds
0.5
1.0
1.5
2.0
2.5
3.0
3.5
4.0
Cellular_organisms - ps1
Archaea1 - ps2
Archaea2 - ps3
Archaea3 - ps4
Asgard/Eukaryota - ps5
Eukaryota - ps6
Amorpheae - ps7
Obazoa - ps8
Opisthokonta - ps9
Holozoa - ps10
Filozoa - ps11
Choano./Metazoa - ps12
Metazoa - ps13
Myriazoa - ps14
Parahoxozoa - ps15
Bilateria - ps16
Deuterostomia - ps17
Chordata - ps18
Olfactores - ps19
Vertebrata - ps20
Gnathostomata - ps21
Euteleostomi - ps22
Sarcopterygii - ps23
Tetrapoda - ps24
Amniota - ps25
Mammalia - ps26
Theria - ps27
Placentalia - ps28
Boreoeutheria - ps29
Euarchontoglires - ps30
Euarchonta - ps31
Primates - ps32
Haplorrhini - ps33
Simiiformes - ps34
Catarrhini - ps35
Hominoidea - ps36
Hominidae - ps37
Homininae - ps38
Hominini - ps39
Homo_sapiens - ps40
Phylostratum

H. sapiens: GO:0032045 guanyl-nucleotide exchange factor complex (loss)

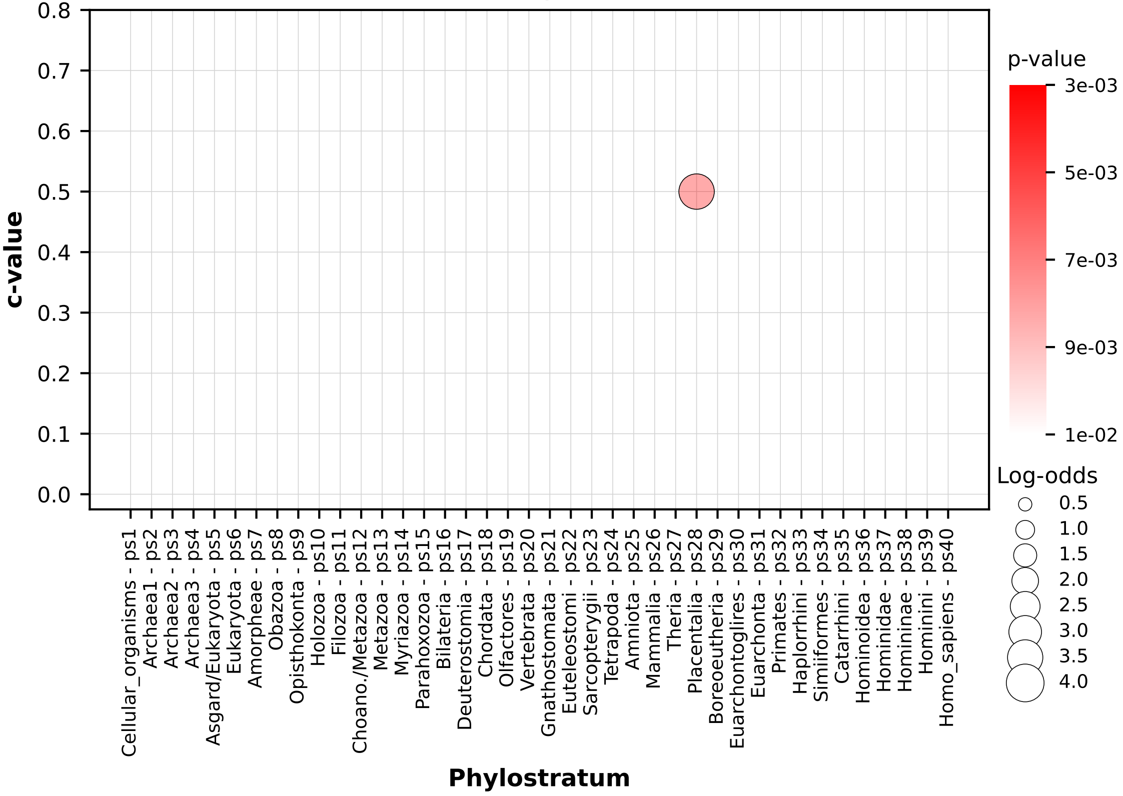

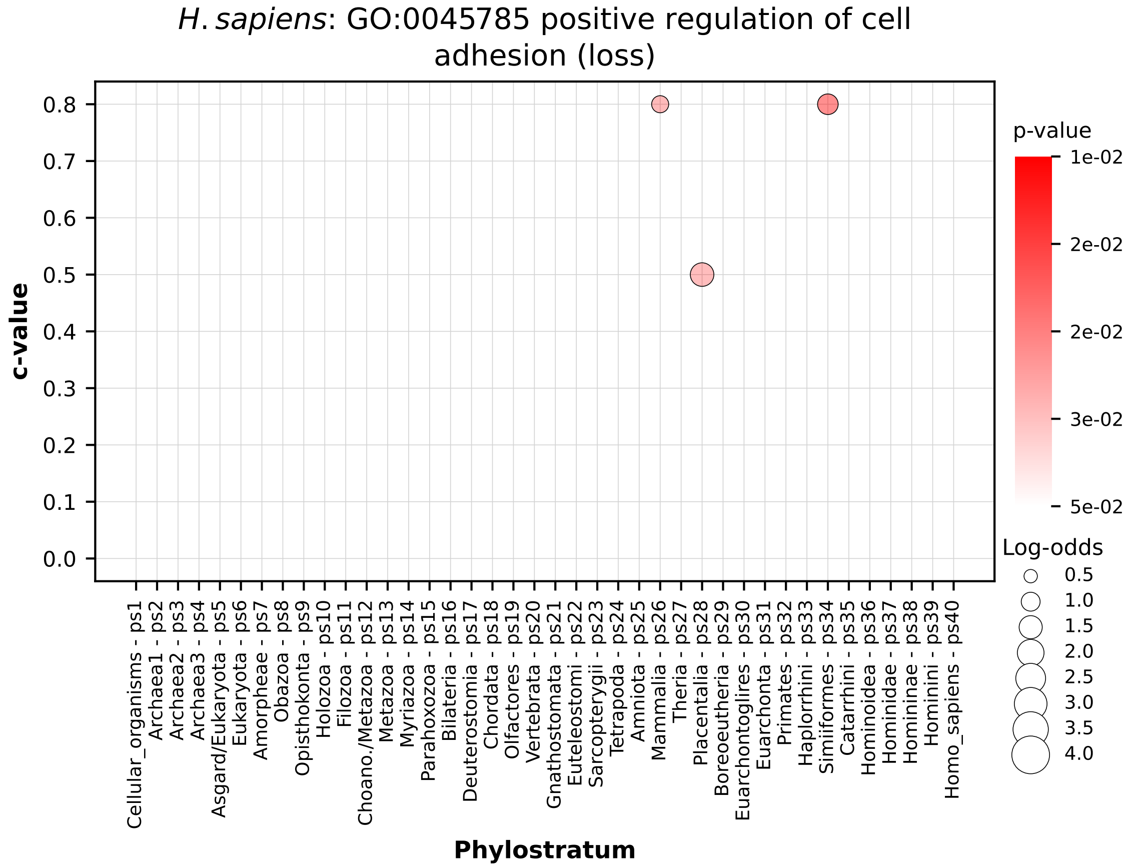

H. sapiens: GO:0045785 positive regulation of cell adhesion (loss)
c-value
0.8
0.7
0.6
0.5
0.4
0.3
0.2
0.1
0.0
p-value
1e-02
2e-02
2e-02
3e-02
5e-02
Log-odds
0.5
1.0
1.5
2.0
2.5
3.0
3.5
4.0
Cellular_organisms - ps1
Archaea1 - ps2
Archaea2 - ps3
Archaea3 - ps4
Asgard/Eukaryota - ps5
Eukaryota - ps6
Amorpheae - ps7
Obazoa - ps8
Opisthokonta - ps9
Holozoa - ps10
Filozoa - ps11
Choano./Metazoa - ps12
Metazoa - ps13
Myriazoa - ps14
Parahoxozoa - ps15
Bilateria - ps16
Deuterostomia - ps17
Chordata - ps18
Olfactores - ps19
Vertebrata - ps20
Gnathostomata - ps21
Euteleostomi - ps22
Sarcopterygii - ps23
Tetrapoda - ps24
Amniota - ps25
Mammalia - ps26
Theria - ps27
Placentalia - ps28
Boreoeutheria - ps29
Euarchontoglires - ps30
Euarchonta - ps31
Primates - ps32
Haplorrhini - ps33
Simiiformes - ps34
Catarrhini - ps35
Hominoidea - ps36
Hominidae - ps37
Homininae - ps38
Hominini - ps39
Homo_sapiens - ps40
Phylostratum

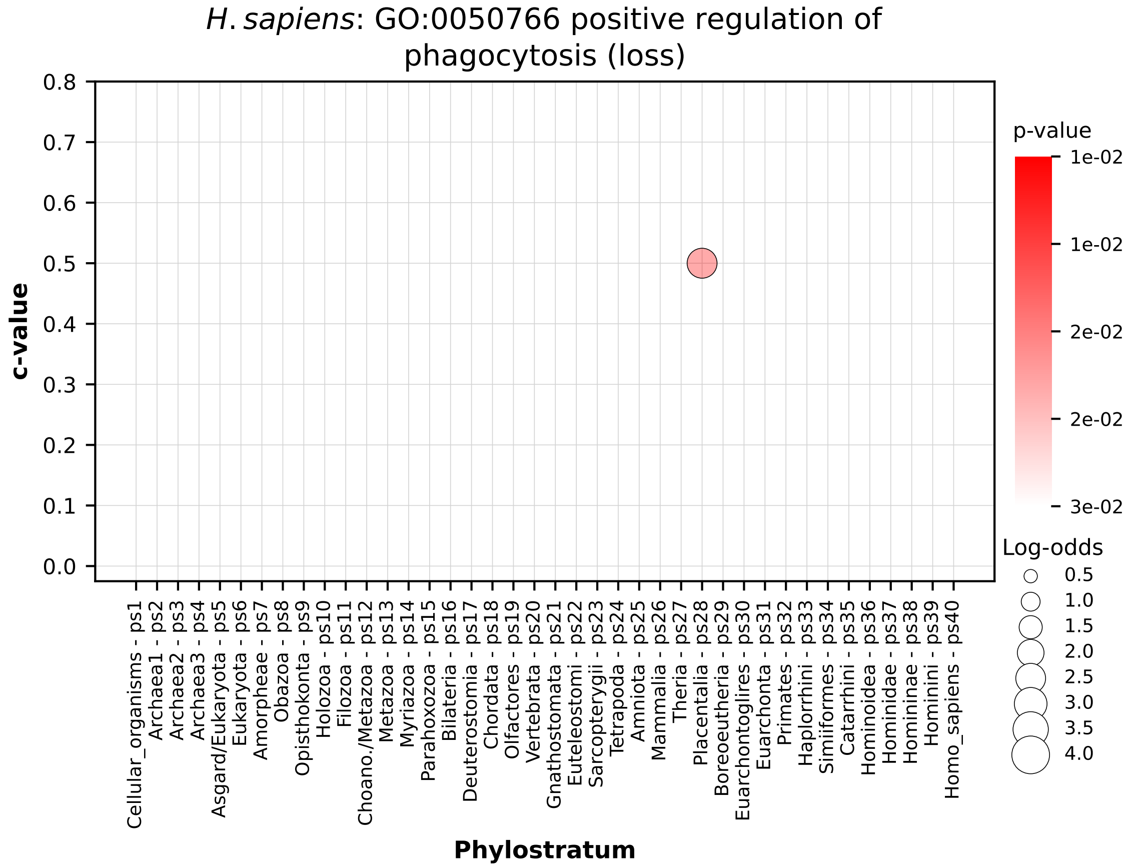
H. sapiens: GO:0050766 positive regulation of phagocytosis (loss)
c-value
0.8
0.7
0.6
0.5
0.4
0.3
0.2
0.1
0.0
Phylostratum
Cellular_organisms - ps1
Archaea1 - ps2
Archaea2 - ps3
Archaea3 - ps4
Asgard/Eukaryota - ps5
Eukaryota - ps6
Amorpheae - ps7
Obazoa - ps8
Opisthokonta - ps9
Holozoa - ps10
Filozoa - ps11
Choano./Metazoa - ps12
Metazoa - ps13
Myriazoa - ps14
Parahoxozoa - ps15
Bilateria - ps16
Deuterostomia - ps17
Chordata - ps18
Olfactores - ps19
Vertebrata - ps20
Gnathostomata - ps21
Euteleostomi - ps22
Sarcopterygii - ps23
Tetrapoda - ps24
Amniota - ps25
Mammalia - ps26
Theria - ps27
Placentalia - ps28
Boreoeutheria - ps29
Euarchontoglires - ps30
Euarchonta - ps31
Primates - ps32
Haplorrhini - ps33
Simiiformes - ps34
Catarrhini - ps35
Hominoidea - ps36
Hominidae - ps37
Homininae - ps38
Hominini - ps39
Homo_sapiens - ps40
p-value
1e-02
1e-02
2e-02
2e-02
3e-02
Log-odds
0.5
1.0
1.5
2.0
2.5
3.0
3.5
4.0

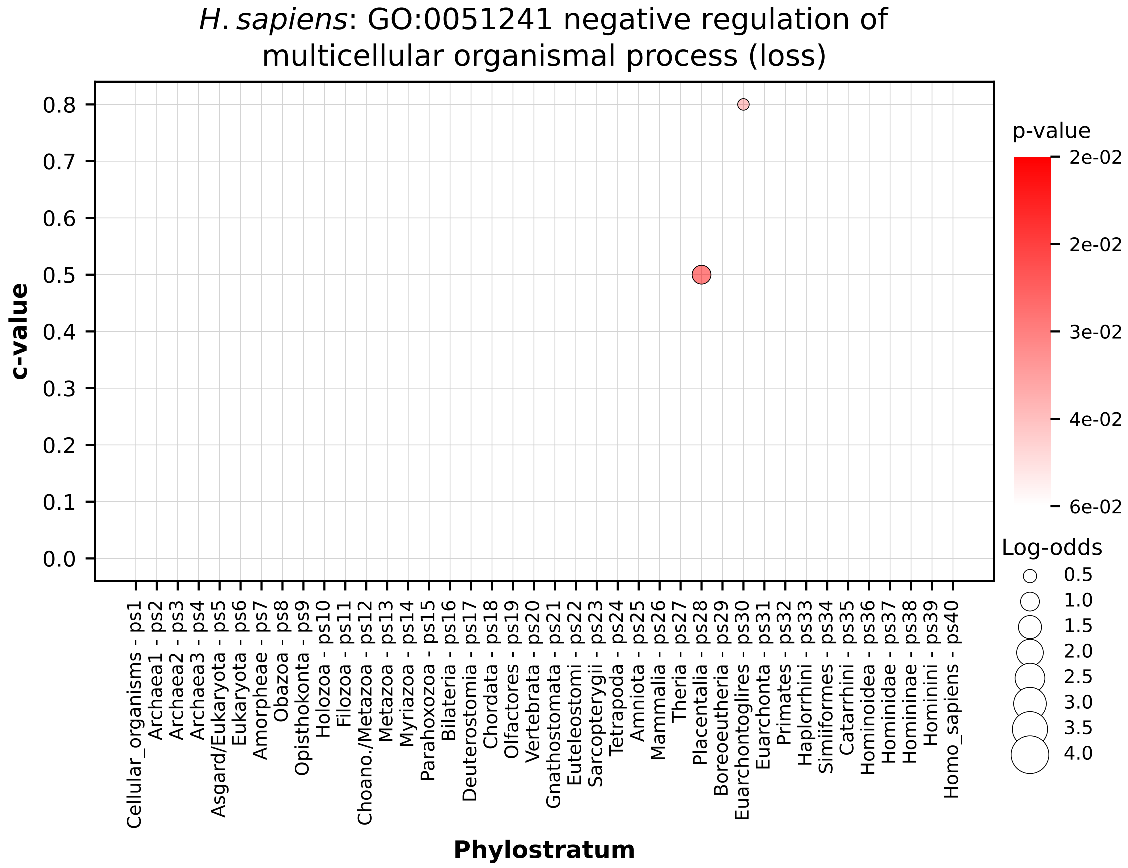

H. sapiens: GO:0051241 negative regulation of multicellular organismal process (loss)
c-value
0.8
0.7
0.6
0.5
0.4
0.3
0.2
0.1
0.0
Phylostratum
Cellular_organisms - ps1
Archaea1 - ps2
Archaea2 - ps3
Archaea3 - ps4
Asgard/Eukaryota - ps5
Eukaryota - ps6
Amorpheae - ps7
Obazoa - ps8
Opisthokonta - ps9
Holozoa - ps10
Filozoa - ps11
Choano./Metazoa - ps12
Metazoa - ps13
Myriazoa - ps14
Parahoxozoa - ps15
Bilateria - ps16
Deuterostomia - ps17
Chordata - ps18
Olfactores - ps19
Vertebrata - ps20
Gnathostomata - ps21
Euteleostomi - ps22
Sarcopterygii - ps23
Tetrapoda - ps24
Amniota - ps25
Mammalia - ps26
Theria - ps27
Placentalia - ps28
Boreoeutheria - ps29
Euarchontoglires - ps30
Euarchonta - ps31
Primates - ps32
Haplorrhini - ps33
Simiiformes - ps34
Catarrhini - ps35
Hominoidea - ps36
Hominidae - ps37
Homininae - ps38
Hominini - ps39
Homo_sapiens - ps40
p-value
2e-02
2e-02
3e-02
4e-02
6e-02
Log-odds
0.5
1.0
1.5
2.0
2.5
3.0
3.5
4.0

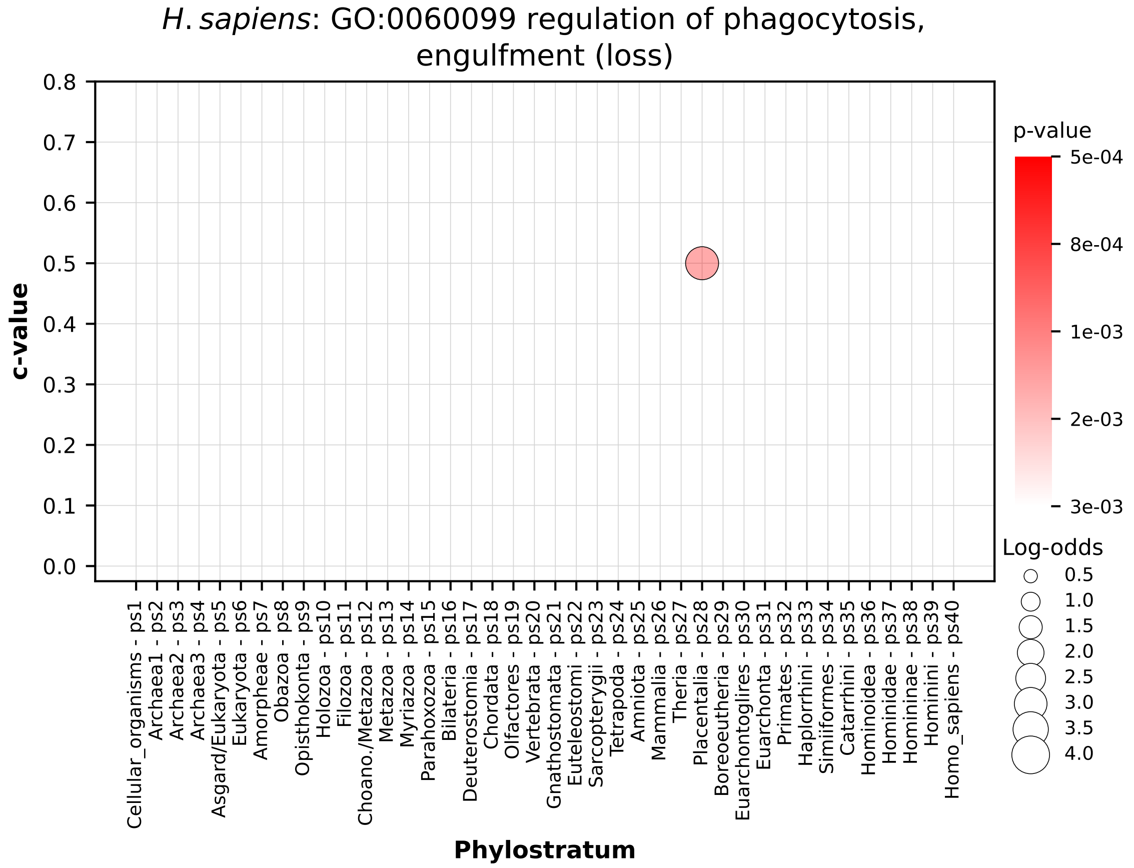

H. sapiens: GO:0060099 regulation of phagocytosis, engulfment (loss)
c-value
0.0
0.1
0.2
0.3
0.4
0.5
0.6
0.7
0.8
Phylostratum
Cellular_organisms - ps1
Archaea1 - ps2
Archaea2 - ps3
Archaea3 - ps4
Asgard/Eukaryota - ps5
Eukaryota - ps6
Amorpheae - ps7
Obazoa - ps8
Opisthokonta - ps9
Holozoa - ps10
Filozoa - ps11
Choano./Metazoa - ps12
Metazoa - ps13
Myriazoa - ps14
Parahoxozoa - ps15
Bilateria - ps16
Deuterostomia - ps17
Chordata - ps18
Olfactores - ps19
Vertebrata - ps20
Gnathostomata - ps21
Euteleostomi - ps22
Sarcopterygii - ps23
Tetrapoda - ps24
Amniota - ps25
Mammalia - ps26
Theria - ps27
Placentalia - ps28
Boreoeutheria - ps29
Euarchontoglires - ps30
Euarchonta - ps31
Primates - ps32
Haplorrhini - ps33
Simiiformes - ps34
Catarrhini - ps35
Hominoidea - ps36
Hominidae - ps37
Homininae - ps38
Hominini - ps39
Homo_sapiens - ps40
p-value
5e-04
8e-04
1e-03
2e-03
3e-03
Log-odds
0.5
1.0
1.5
2.0
2.5
3.0
3.5
4.0

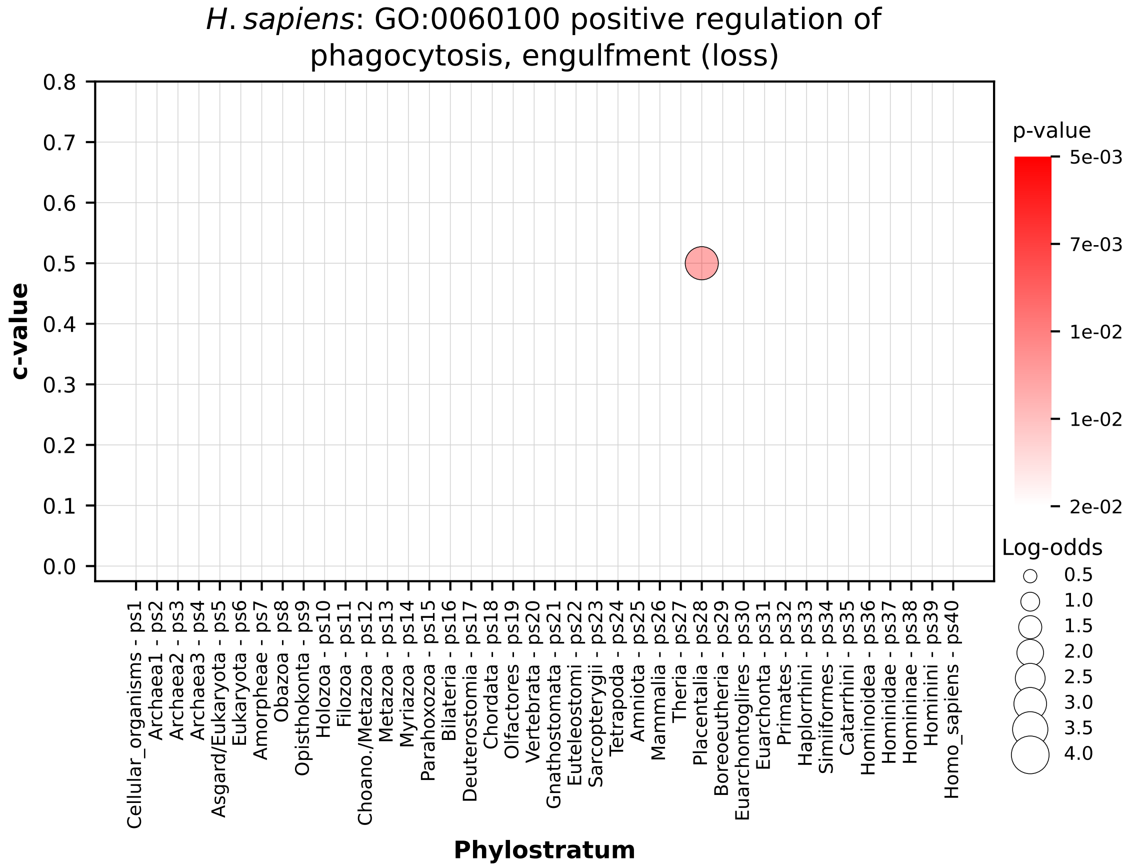

H. sapiens: GO:0060100 positive regulation of phagocytosis, engulfment (loss)
c-value
0.0
0.1
0.2
0.3
0.4
0.5
0.6
0.7
0.8
Phylostratum
Cellular_organisms - ps1
Archaea1 - ps2
Archaea2 - ps3
Archaea3 - ps4
Asgard/Eukaryota - ps5
Eukaryota - ps6
Amorpheae - ps7
Obazoa - ps8
Opisthokonta - ps9
Holozoa - ps10
Filozoa - ps11
Choano./Metazoa - ps12
Metazoa - ps13
Myriazoa - ps14
Parahoxozoa - ps15
Bilateria - ps16
Deuterostomia - ps17
Chordata - ps18
Olfactores - ps19
Vertebrata - ps20
Gnathostomata - ps21
Euteleostomi - ps22
Sarcopterygii - ps23
Tetrapoda - ps24
Amniota - ps25
Mammalia - ps26
Theria - ps27
Placentalia - ps28
Boreoeutheria - ps29
Euarchontoglires - ps30
Euarchonta - ps31
Primates - ps32
Haplorrhini - ps33
Simiiformes - ps34
Catarrhini - ps35
Hominoidea - ps36
Hominidae - ps37
Homininae - ps38
Hominini - ps39
Homo_sapiens - ps40
p-value
5e-03
7e-03
1e-02
1e-02
2e-02
Log-odds
0.5
1.0
1.5
2.0
2.5
3.0
3.5
4.0

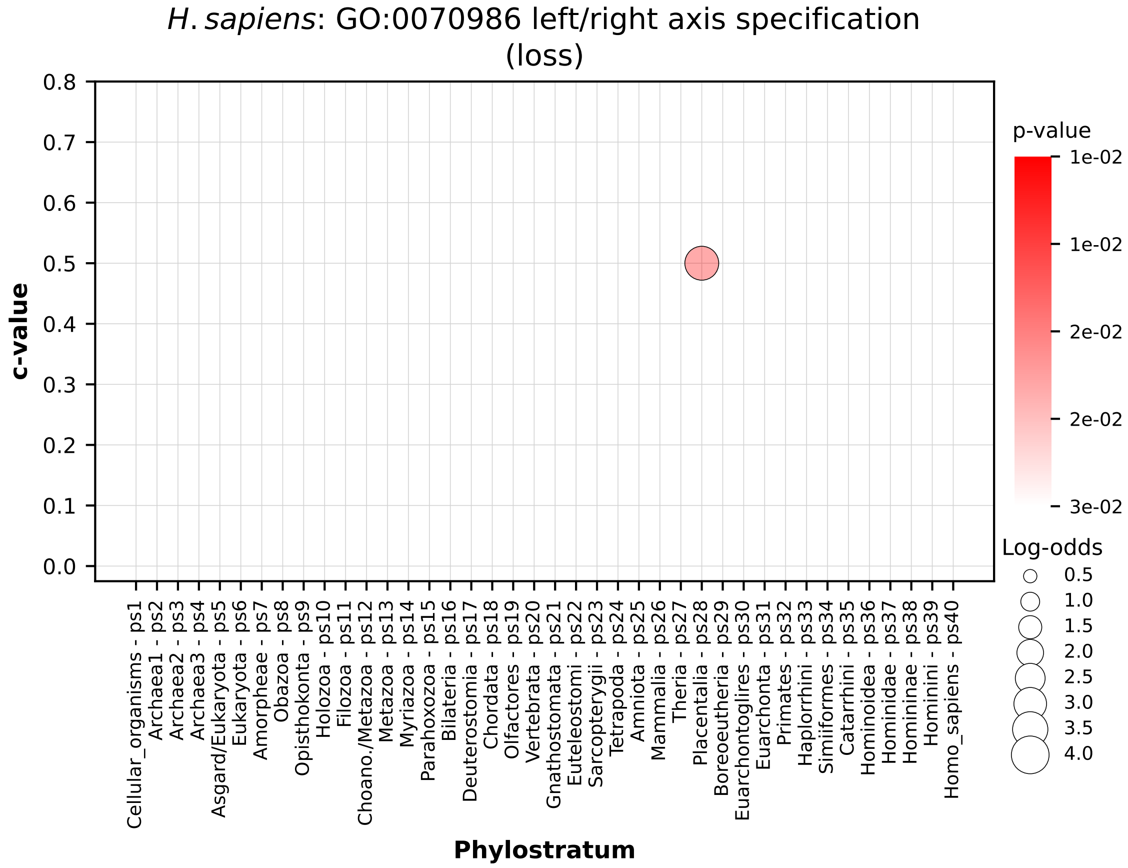

H. sapiens: GO:0070986 left/right axis specification
(loss)
c-value
0.8
0.7
0.6
0.5
0.4
0.3
0.2
0.1
0.0
p-value
1e-02
1e-02
2e-02
2e-02
3e-02
Log-odds
0.5
1.0
1.5
2.0
2.5
3.0
3.5
4.0
Cellular_organisms - ps1
Archaea1 - ps2
Archaea2 - ps3
Archaea3 - ps4
Asgard/Eukaryota - ps5
Eukaryota - ps6
Amorpheae - ps7
Obazoa - ps8
Opisthokonta - ps9
Holozoa - ps10
Filozoa - ps11
Choano./Metazoa - ps12
Metazoa - ps13
Myriazoa - ps14
Parahoxozoa - ps15
Bilateria - ps16
Deuterostomia - ps17
Chordata - ps18
Olfactores - ps19
Vertebrata - ps20
Gnathostomata - ps21
Euteleostomi - ps22
Sarcopterygii - ps23
Tetrapoda - ps24
Amniota - ps25
Mammalia - ps26
Theria - ps27
Placentalia - ps28
Boreoeutheria - ps29
Euarchontoglires - ps30
Euarchonta - ps31
Primates - ps32
Haplorrhini - ps33
Simiiformes - ps34
Catarrhini - ps35
Hominoidea - ps36
Hominidae - ps37
Homininae - ps38
Hominini - ps39
Homo_sapiens - ps40
Phylostratum

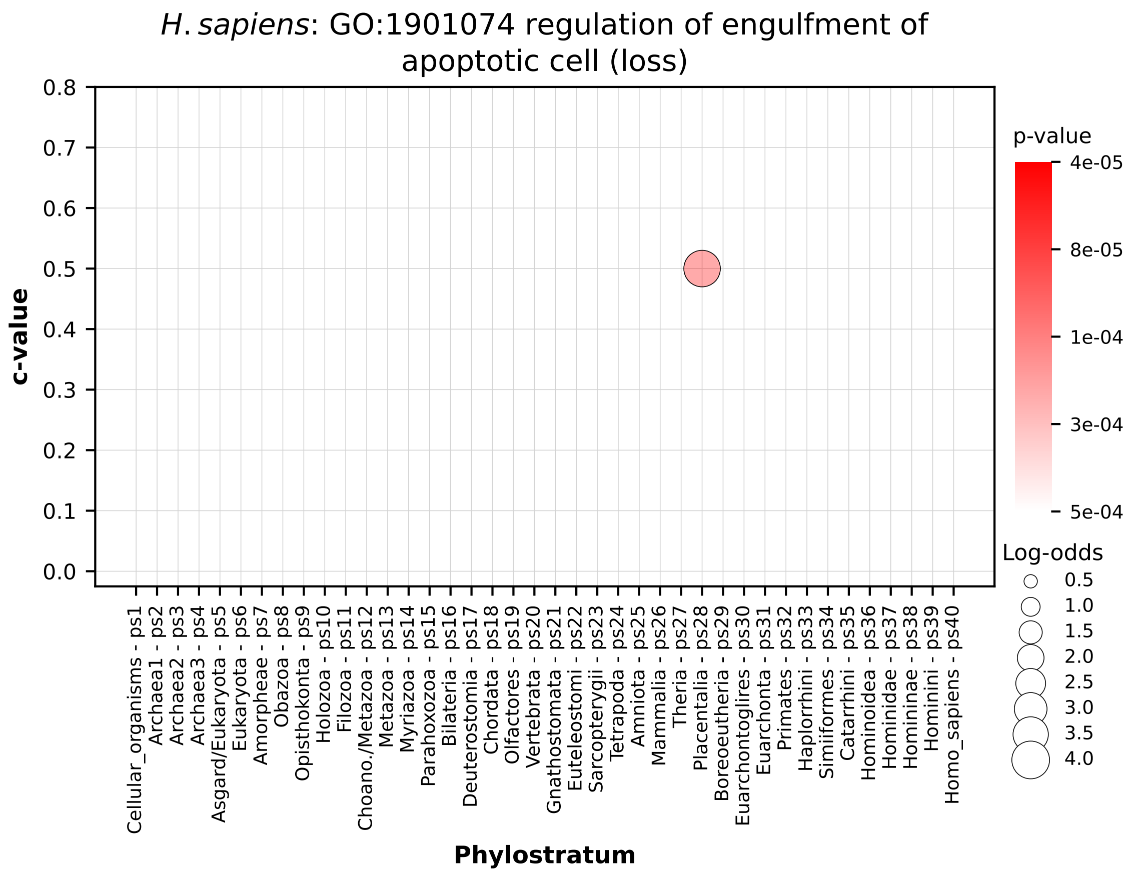

H. sapiens: GO:1901074 regulation of engulfment of apoptotic cell (loss)
c-value
0.0
0.1
0.2
0.3
0.4
0.5
0.6
0.7
0.8
Phylostratum
Cellular_organisms - ps1
Archaea1 - ps2
Archaea2 - ps3
Archaea3 - ps4
Asgard/Eukaryota - ps5
Eukaryota - ps6
Amorpheae - ps7
Obazoa - ps8
Opisthokonta - ps9
Holozoa - ps10
Filozoa - ps11
Choano./Metazoa - ps12
Metazoa - ps13
Myriazoa - ps14
Parahoxozoa - ps15
Bilateria - ps16
Deuterostomia - ps17
Chordata - ps18
Olfactores - ps19
Vertebrata - ps20
Gnathostomata - ps21
Euteleostomi - ps22
Sarcopterygii - ps23
Tetrapoda - ps24
Amniota - ps25
Mammalia - ps26
Theria - ps27
Placentalia - ps28
Boreoeutheria - ps29
Euarchontoglires - ps30
Euarchonta - ps31
Primates - ps32
Haplorrhini - ps33
Simiiformes - ps34
Catarrhini - ps35
Hominoidea - ps36
Hominidae - ps37
Homininae - ps38
Hominini - ps39
Homo_sapiens - ps40
p-value
4e-05
8e-05
1e-04
3e-04
5e-04
Log-odds
0.5
1.0
1.5
2.0
2.5
3.0
3.5
4.0

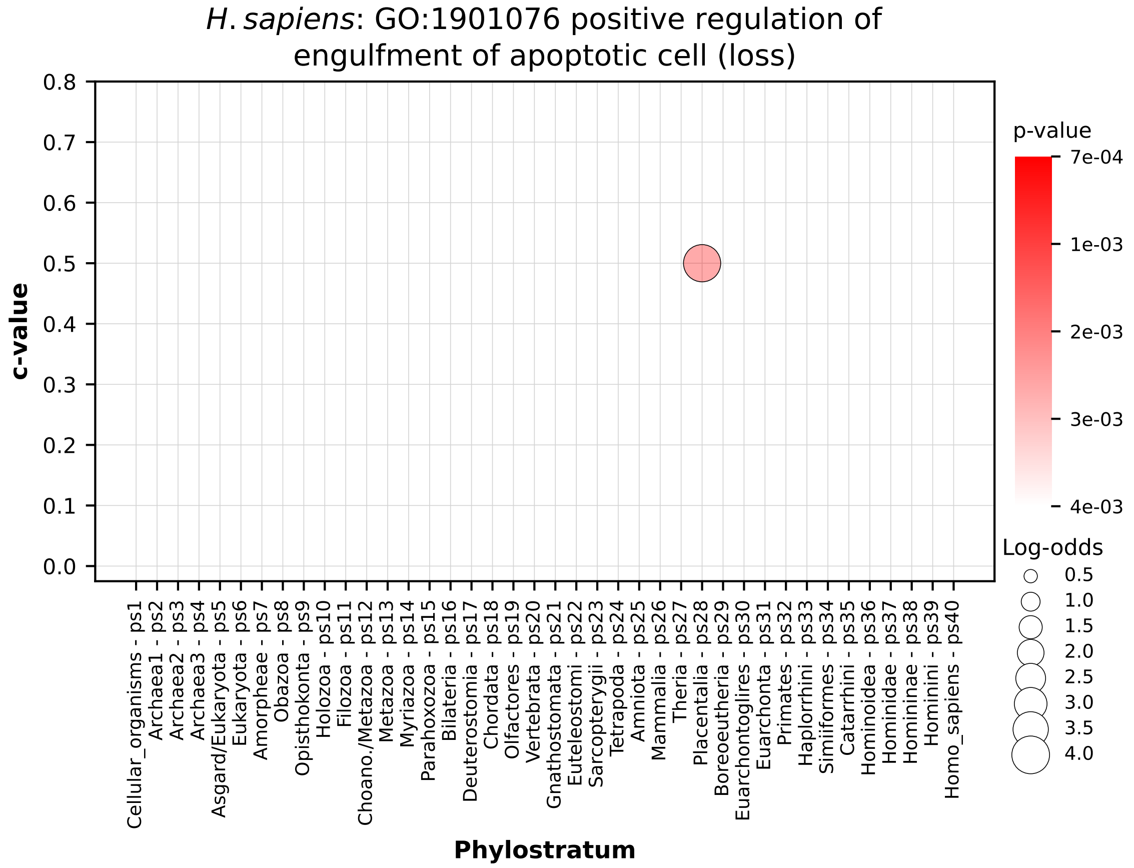

H. sapiens: GO:1901076 positive regulation of engulfment of apoptotic cell (loss)
c-value
0.8
0.7
0.6
0.5
0.4
0.3
0.2
0.1
0.0
p-value
7e-04
1e-03
2e-03
3e-03
4e-03
Log-odds
0.5
1.0
1.5
2.0
2.5
3.0
3.5
4.0
Cellular_organisms - ps1
Archaea1 - ps2
Archaea2 - ps3
Archaea3 - ps4
Asgard/Eukaryota - ps5
Eukaryota - ps6
Amorpheae - ps7
Obazoa - ps8
Opisthokonta - ps9
Holozoa - ps10
Filozoa - ps11
Choano./Metazoa - ps12
Metazoa - ps13
Myriazoa - ps14
Parahoxozoa - ps15
Bilateria - ps16
Deuterostomia - ps17
Chordata - ps18
Olfactores - ps19
Vertebrata - ps20
Gnathostomata - ps21
Euteleostomi - ps22
Sarcopterygii - ps23
Tetrapoda - ps24
Amniota - ps25
Mammalia - ps26
Theria - ps27
Placentalia - ps28
Boreoeutheria - ps29
Euarchontoglires - ps30
Euarchonta - ps31
Primates - ps32
Haplorrhini - ps33
Simiiformes - ps34
Catarrhini - ps35
Hominoidea - ps36
Hominidae - ps37
Homininae - ps38
Hominini - ps39
Homo_sapiens - ps40
Phylostratum

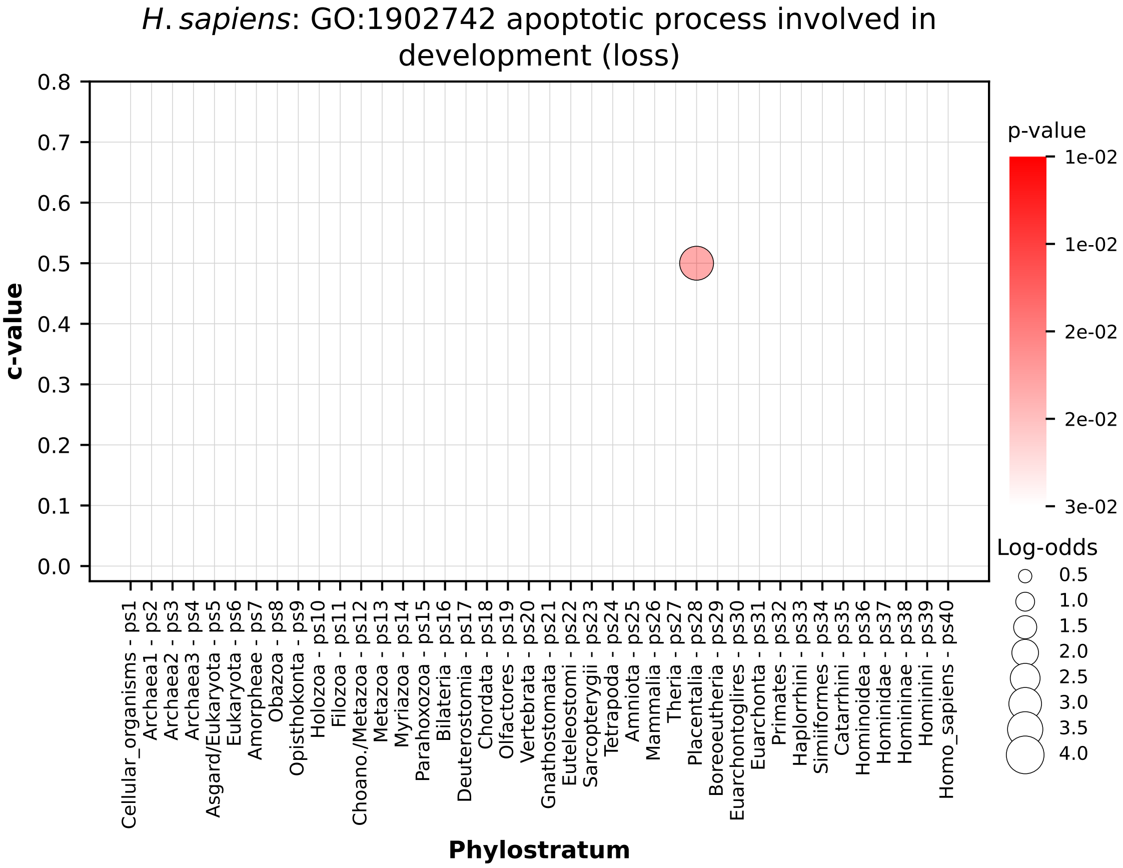

H. sapiens: GO:1902742 apoptotic process involved in development (loss)
c-value
0.8
0.7
0.6
0.5
0.4
0.3
0.2
0.1
0.0
p-value
1e-02
1e-02
2e-02
2e-02
3e-02
Log-odds
0.5
1.0
1.5
2.0
2.5
3.0
3.5
4.0
Cellular_organisms - ps1
Archaea1 - ps2
Archaea2 - ps3
Archaea3 - ps4
Asgard/Eukaryota - ps5
Eukaryota - ps6
Amorpheae - ps7
Obazoa - ps8
Opisthokonta - ps9
Holozoa - ps10
Filozoa - ps11
Choano./Metazoa - ps12
Metazoa - ps13
Myriazoa - ps14
Parahoxozoa - ps15
Bilateria - ps16
Deuterostomia - ps17
Chordata - ps18
Olfactores - ps19
Vertebrata - ps20
Gnathostomata - ps21
Euteleostomi - ps22
Sarcopterygii - ps23
Tetrapoda - ps24
Amniota - ps25
Mammalia - ps26
Theria - ps27
Placentalia - ps28
Boreoeutheria - ps29
Euarchontoglires - ps30
Euarchonta - ps31
Primates - ps32
Haplorrhini - ps33
Simiiformes - ps34
Catarrhini - ps35
Hominoidea - ps36
Hominidae - ps37
Homininae - ps38
Hominini - ps39
Homo_sapiens - ps40
Phylostratum

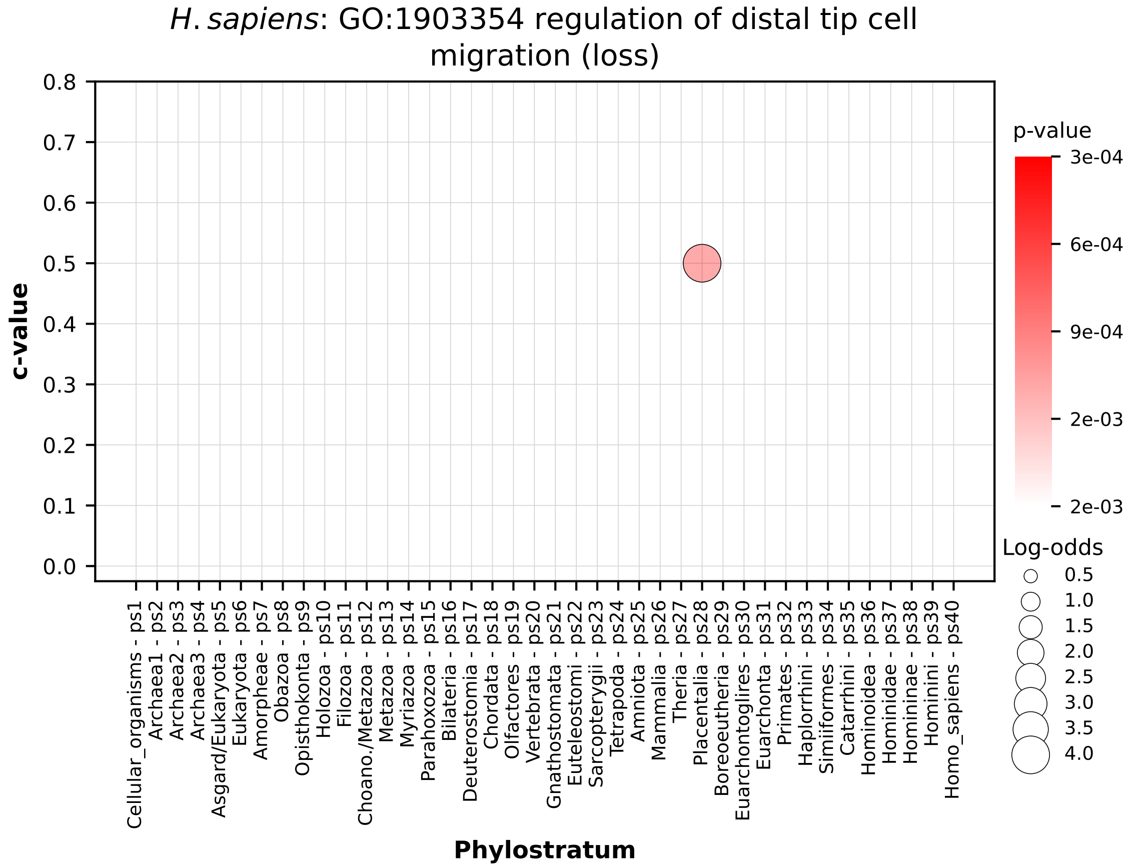
H. sapiens: GO:1903354 regulation of distal tip cell migration (loss)
c-value
0.8
0.7
0.6
0.5
0.4
0.3
0.2
0.1
0.0
Phylostratum
Cellular_organisms - ps1
Archaea1 - ps2
Archaea2 - ps3
Archaea3 - ps4
Asgard/Eukaryota - ps5
Eukaryota - ps6
Amorpheae - ps7
Obazoa - ps8
Opisthokonta - ps9
Holozoa - ps10
Filozoa - ps11
Choano./Metazoa - ps12
Metazoa - ps13
Myriazoa - ps14
Parahoxozoa - ps15
Bilateria - ps16
Deuterostomia - ps17
Chordata - ps18
Olfactores - ps19
Vertebrata - ps20
Gnathostomata - ps21
Euteleostomi - ps22
Sarcopterygii - ps23
Tetrapoda - ps24
Amniota - ps25
Mammalia - ps26
Theria - ps27
Placentalia - ps28
Boreoeutheria - ps29
Euarchontoglires - ps30
Euarchonta - ps31
Primates - ps32
Haplorrhini - ps33
Simiiformes - ps34
Catarrhini - ps35
Hominoidea - ps36
Hominidae - ps37
Homininae - ps38
Hominini - ps39
Homo_sapiens - ps40
p-value
3e-04
6e-04
9e-04
2e-03
2e-03
Log-odds
0.5
1.0
1.5
2.0
2.5
3.0
3.5
4.0

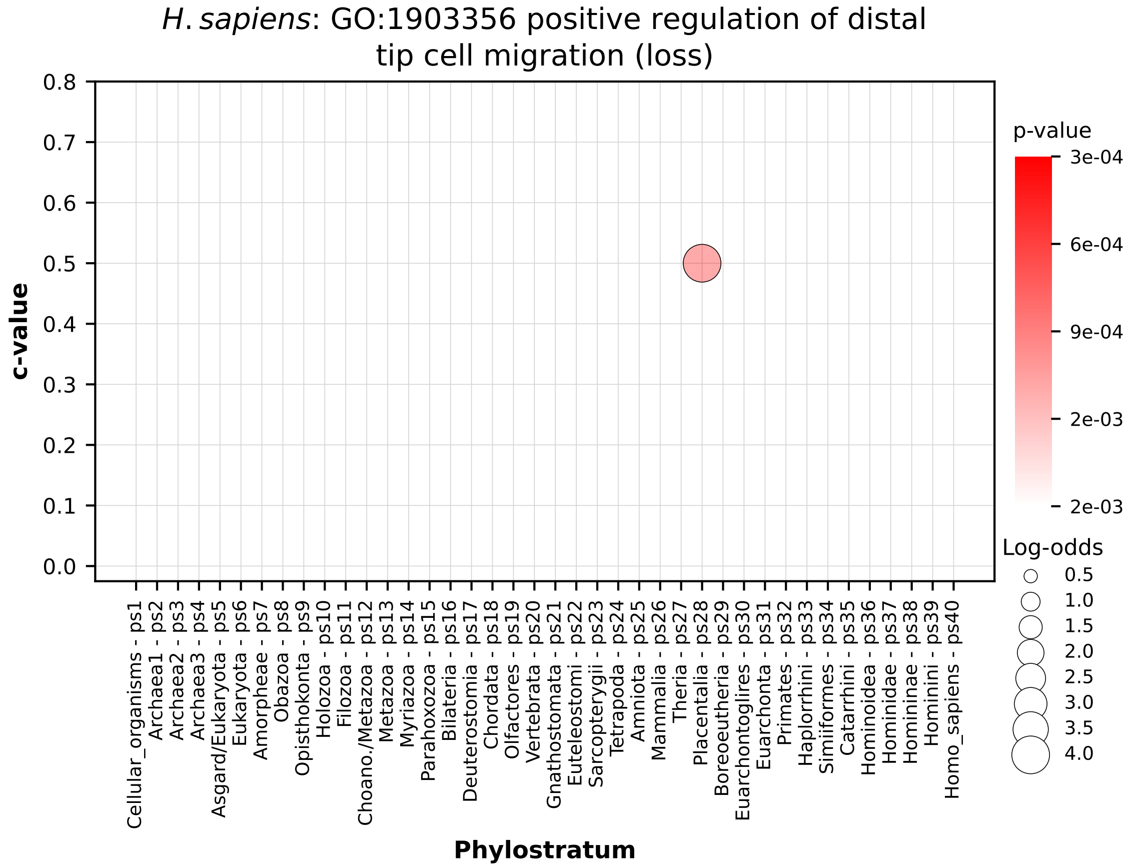

H. sapiens: GO:1903356 positive regulation of distal tip cell migration (loss)
c-value
0.8
0.7
0.6
0.5
0.4
0.3
0.2
0.1
0.0
Phylostratum
Cellular_organisms - ps1
Archaea1 - ps2
Archaea2 - ps3
Archaea3 - ps4
Asgard/Eukaryota - ps5
Eukaryota - ps6
Amorpheae - ps7
Obazoa - ps8
Opisthokonta - ps9
Holozoa - ps10
Filozoa - ps11
Choano./Metazoa - ps12
Metazoa - ps13
Myriazoa - ps14
Parahoxozoa - ps15
Bilateria - ps16
Deuterostomia - ps17
Chordata - ps18
Olfactores - ps19
Vertebrata - ps20
Gnathostomata - ps21
Euteleostomi - ps22
Sarcopterygii - ps23
Tetrapoda - ps24
Amniota - ps25
Mammalia - ps26
Theria - ps27
Placentalia - ps28
Boreoeutheria - ps29
Euarchontoglires - ps30
Euarchonta - ps31
Primates - ps32
Haplorrhini - ps33
Simiiformes - ps34
Catarrhini - ps35
Hominoidea - ps36
Hominidae - ps37
Homininae - ps38
Hominini - ps39
Homo_sapiens - ps40
p-value
3e-04
6e-04
9e-04
2e-03
2e-03
Log-odds
0.5
1.0
1.5
2.0
2.5
3.0
3.5
4.0

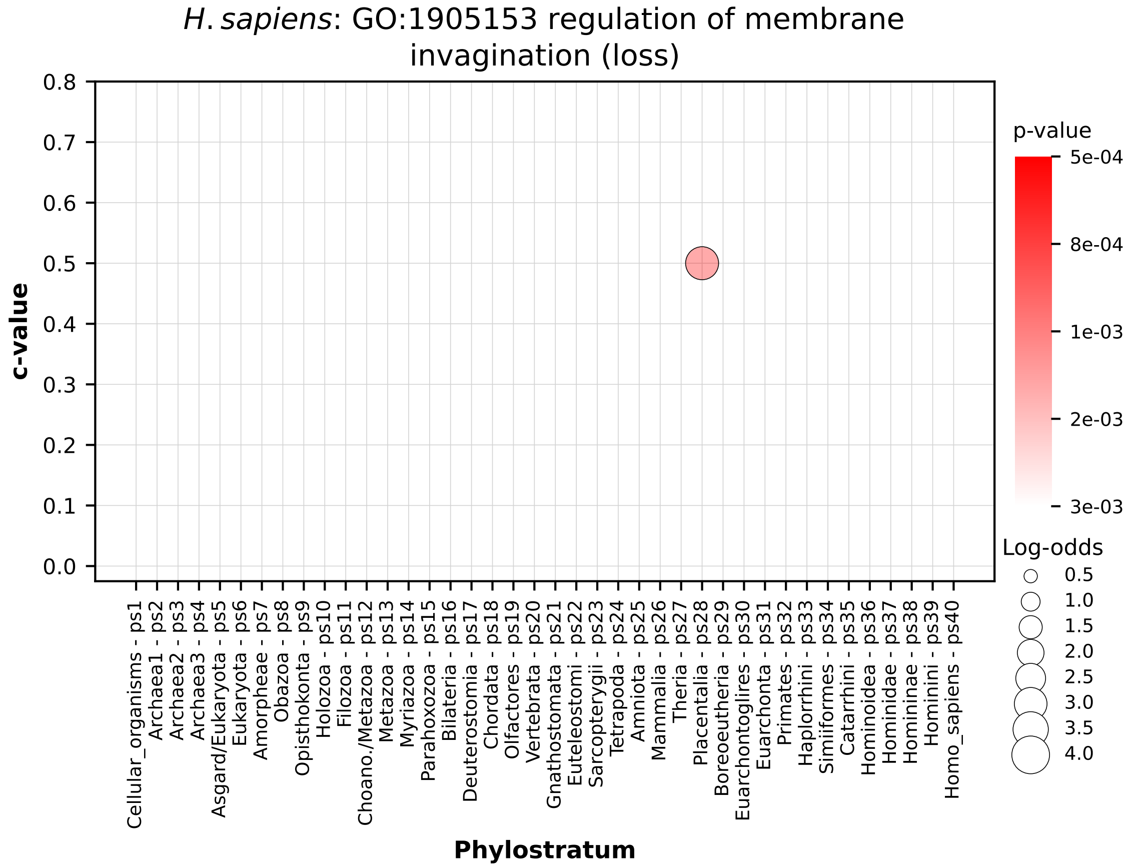

H. sapiens: GO:1905153 regulation of membrane invagination (loss)
c-value
0.0
0.1
0.2
0.3
0.4
0.5
0.6
0.7
0.8
Phylostratum
Cellular_organisms - ps1
Archaea1 - ps2
Archaea2 - ps3
Archaea3 - ps4
Asgard/Eukaryota - ps5
Eukaryota - ps6
Amorpheae - ps7
Obazoa - ps8
Opisthokonta - ps9
Holozoa - ps10
Filozoa - ps11
Choano./Metazoa - ps12
Metazoa - ps13
Myriazoa - ps14
Parahoxozoa - ps15
Bilateria - ps16
Deuterostomia - ps17
Chordata - ps18
Olfactores - ps19
Vertebrata - ps20
Gnathostomata - ps21
Euteleostomi - ps22
Sarcopterygii - ps23
Tetrapoda - ps24
Amniota - ps25
Mammalia - ps26
Theria - ps27
Placentalia - ps28
Boreoeutheria - ps29
Euarchontoglires - ps30
Euarchonta - ps31
Primates - ps32
Haplorrhini - ps33
Simiiformes - ps34
Catarrhini - ps35
Hominoidea - ps36
Hominidae - ps37
Homininae - ps38
Hominini - ps39
Homo_sapiens - ps40
p-value
5e-04
8e-04
1e-03
2e-03
3e-03
Log-odds
0.5
1.0
1.5
2.0
2.5
3.0
3.5
4.0

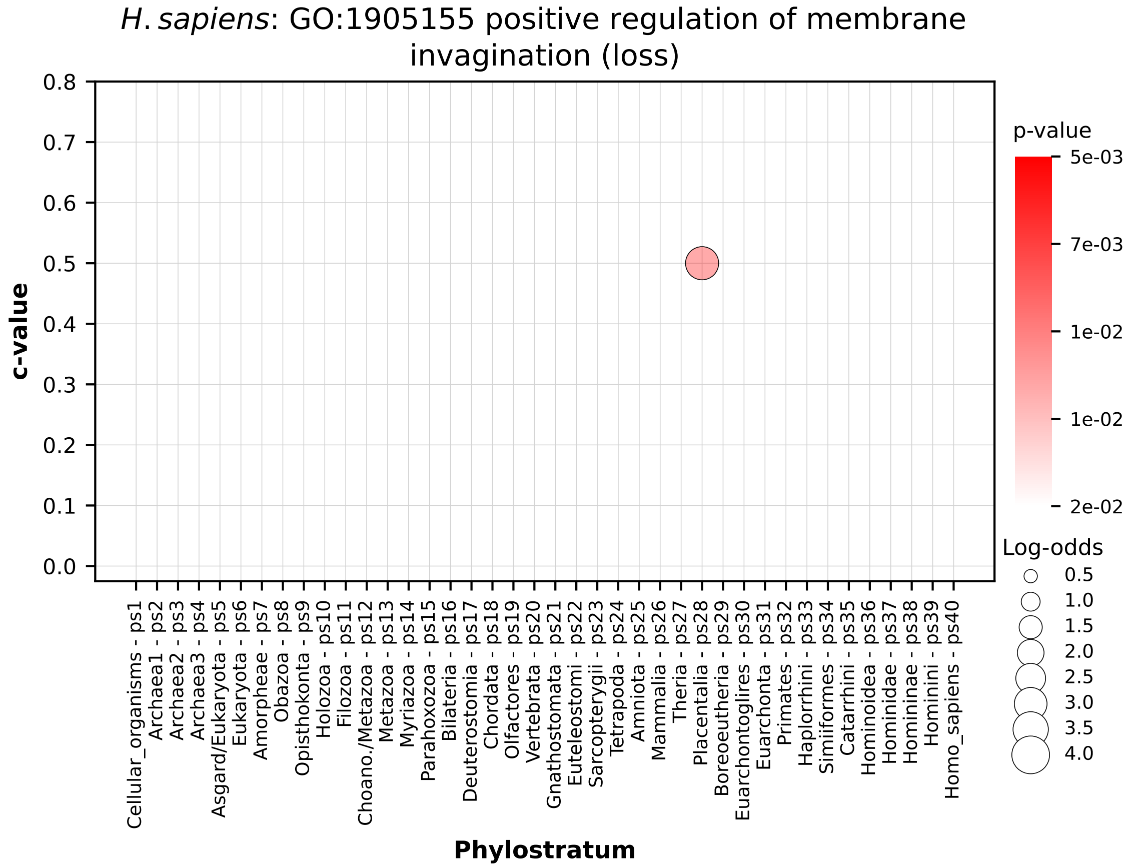

H. sapiens: GO:1905155 positive regulation of membrane invagination (loss)
c-value
0.8
0.7
0.6
0.5
0.4
0.3
0.2
0.1
0.0
p-value
5e-03
7e-03
1e-02
1e-02
2e-02
Log-odds
0.5
1.0
1.5
2.0
2.5
3.0
3.5
4.0
Cellular_organisms - ps1
Archaea1 - ps2
Archaea2 - ps3
Archaea3 - ps4
Asgard/Eukaryota - ps5
Eukaryota - ps6
Amorpheae - ps7
Obazoa - ps8
Opisthokonta - ps9
Holozoa - ps10
Filozoa - ps11
Choano./Metazoa - ps12
Metazoa - ps13
Myriazoa - ps14
Parahoxozoa - ps15
Bilateria - ps16
Deuterostomia - ps17
Chordata - ps18
Olfactores - ps19
Vertebrata - ps20
Gnathostomata - ps21
Euteleostomi - ps22
Sarcopterygii - ps23
Tetrapoda - ps24
Amniota - ps25
Mammalia - ps26
Theria - ps27
Placentalia - ps28
Boreoeutheria - ps29
Euarchontoglires - ps30
Euarchonta - ps31
Primates - ps32
Haplorrhini - ps33
Simiiformes - ps34
Catarrhini - ps35
Hominoidea - ps36
Hominidae - ps37
Homininae - ps38
Hominini - ps39
Homo_sapiens - ps40
Phylostratum

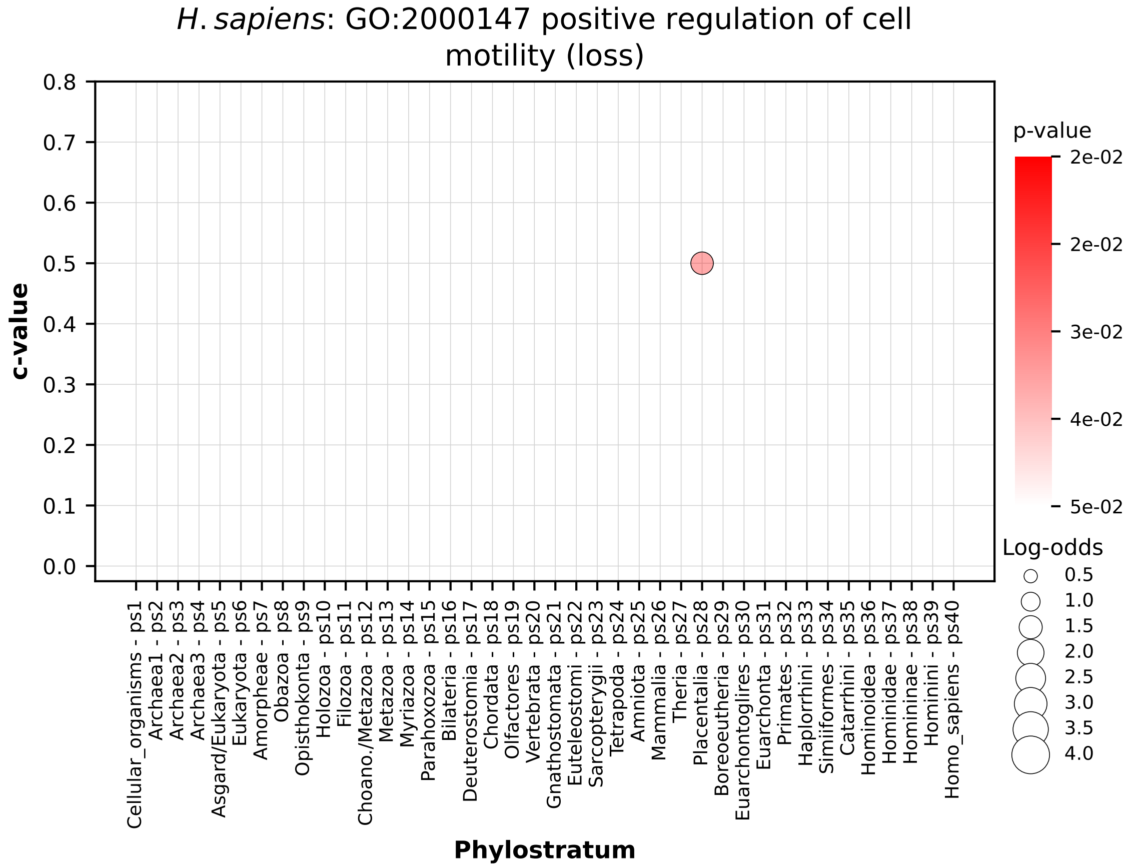

H. sapiens: GO:2000147 positive regulation of cell motility (loss)
c-value
0.8
0.7
0.6
0.5
0.4
0.3
0.2
0.1
0.0
Phylostratum
Cellular_organisms - ps1
Archaea1 - ps2
Archaea2 - ps3
Archaea3 - ps4
Asgard/Eukaryota - ps5
Eukaryota - ps6
Amorpheae - ps7
Obazoa - ps8
Opisthokonta - ps9
Holozoa - ps10
Filozoa - ps11
Choano./Metazoa - ps12
Metazoa - ps13
Myriazoa - ps14
Parahoxozoa - ps15
Bilateria - ps16
Deuterostomia - ps17
Chordata - ps18
Olfactores - ps19
Vertebrata - ps20
Gnathostomata - ps21
Euteleostomi - ps22
Sarcopterygii - ps23
Tetrapoda - ps24
Amniota - ps25
Mammalia - ps26
Theria - ps27
Placentalia - ps28
Boreoeutheria - ps29
Euarchontoglires - ps30
Euarchonta - ps31
Primates - ps32
Haplorrhini - ps33
Simiiformes - ps34
Catarrhini - ps35
Hominoidea - ps36
Hominidae - ps37
Homininae - ps38
Hominini - ps39
Homo_sapiens - ps40
p-value
2e-02
2e-02
3e-02
4e-02
5e-02
Log-odds
0.5
1.0
1.5
2.0
2.5
3.0
3.5
4.0

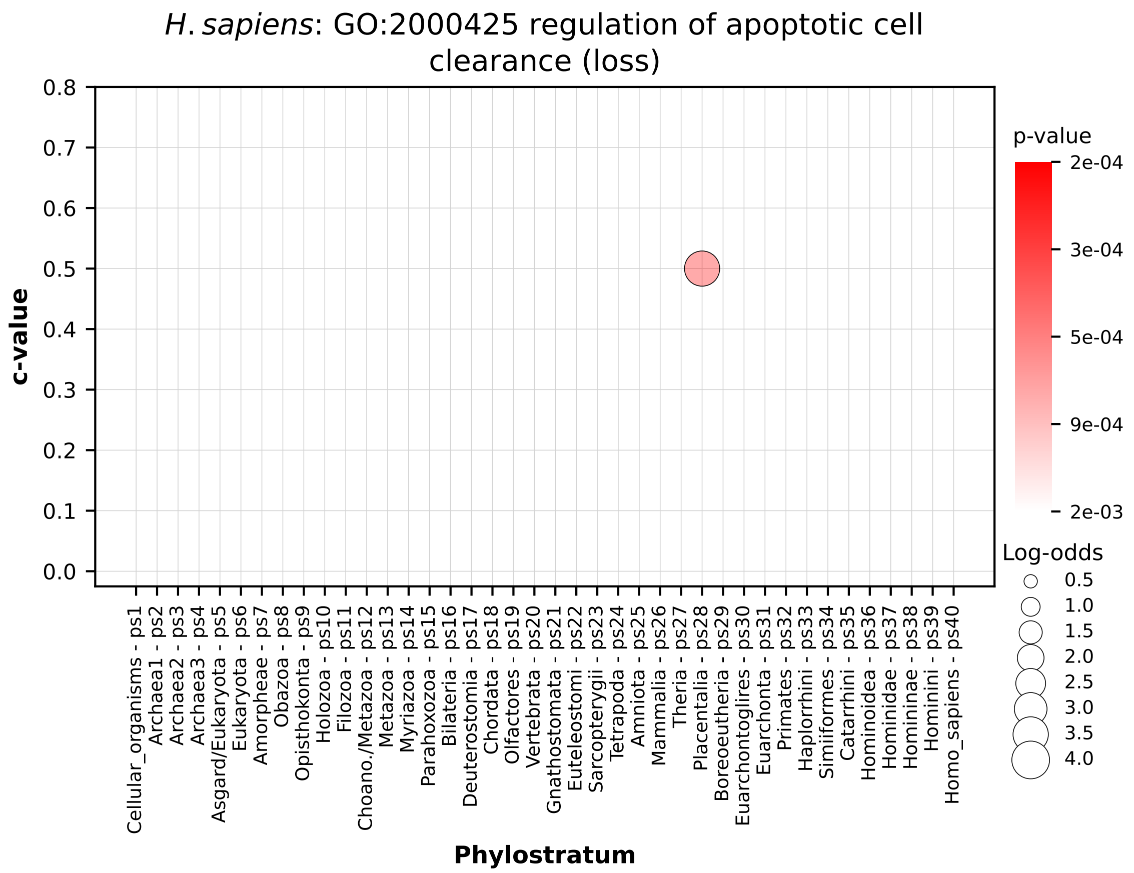

H. sapiens: GO:2000425 regulation of apoptotic cell clearance (loss)
c-value
0.0
0.1
0.2
0.3
0.4
0.5
0.6
0.7
0.8
Phylostratum
Cellular_organisms - ps1
Archaea1 - ps2
Archaea2 - ps3
Archaea3 - ps4
Asgard/Eukaryota - ps5
Eukaryota - ps6
Amorpheae - ps7
Obazoa - ps8
Opisthokonta - ps9
Holozoa - ps10
Filozoa - ps11
Choano./Metazoa - ps12
Metazoa - ps13
Myriazoa - ps14
Parahoxozoa - ps15
Bilateria - ps16
Deuterostomia - ps17
Chordata - ps18
Olfactores - ps19
Vertebrata - ps20
Gnathostomata - ps21
Euteleostomi - ps22
Sarcopterygii - ps23
Tetrapoda - ps24
Amniota - ps25
Mammalia - ps26
Theria - ps27
Placentalia - ps28
Boreoeutheria - ps29
Euarchontoglires - ps30
Euarchonta - ps31
Primates - ps32
Haplorrhini - ps33
Simiiformes - ps34
Catarrhini - ps35
Hominoidea - ps36
Hominidae - ps37
Homininae - ps38
Hominini - ps39
Homo_sapiens - ps40
p-value
2e-04
3e-04
5e-04
9e-04
2e-03
Log-odds
0.5
1.0
1.5
2.0
2.5
3.0
3.5
4.0

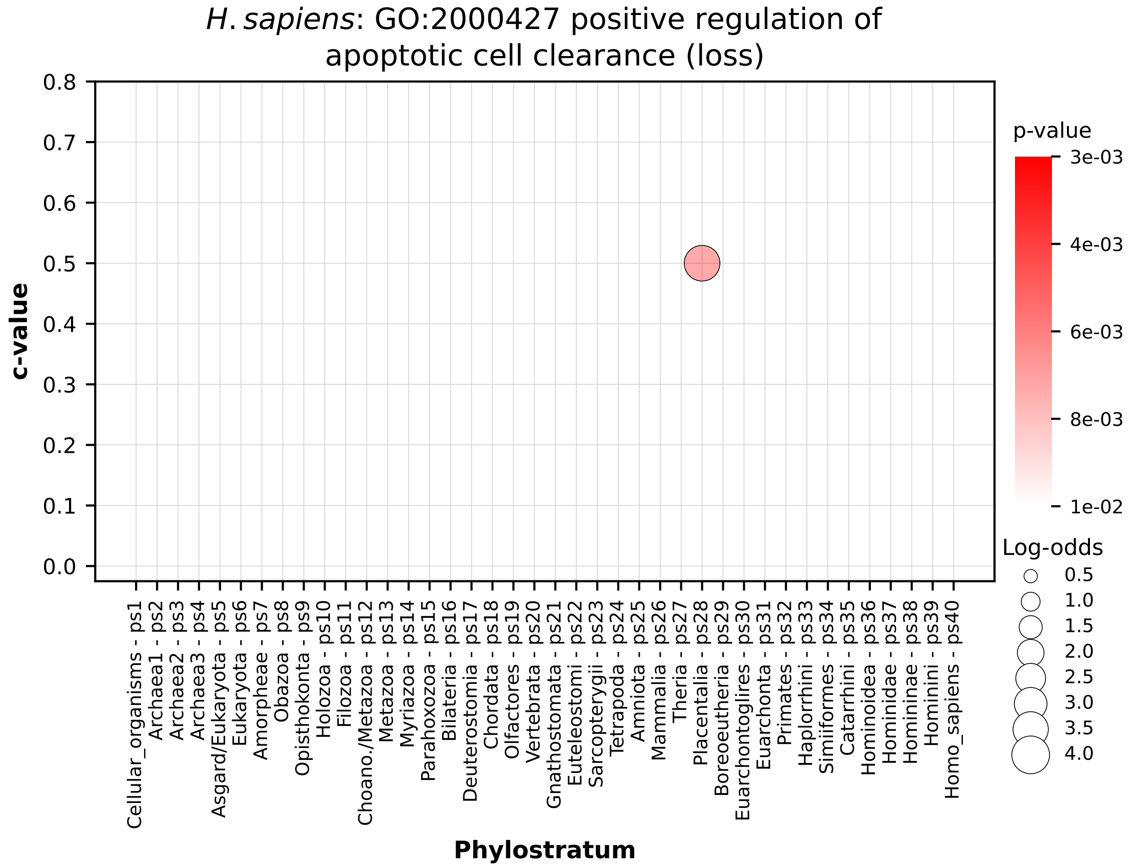

H. sapiens: GO:2000427 positive regulation of apoptotic cell clearance (loss)
c-value
0.0
0.1
0.2
0.3
0.4
0.5
0.6
0.7
0.8
Phylostratum
Cellular_organisms - ps1
Archaea1 - ps2
Archaea2 - ps3
Archaea3 - ps4
Asgard/Eukaryota - ps5
Eukaryota - ps6
Amorpheae - ps7
Obazoa - ps8
Opisthokonta - ps9
Holozoa - ps10
Filozoa - ps11
Choano./Metazoa - ps12
Metazoa - ps13
Myriazoa - ps14
Parahoxozoa - ps15
Bilateria - ps16
Deuterostomia - ps17
Chordata - ps18
Olfactores - ps19
Vertebrata - ps20
Gnathostomata - ps21
Euteleostomi - ps22
Sarcopterygii - ps23
Tetrapoda - ps24
Amniota - ps25
Mammalia - ps26
Theria - ps27
Placentalia - ps28
Boreoeutheria - ps29
Euarchontoglires - ps30
Euarchonta - ps31
Primates - ps32
Haplorrhini - ps33
Simiiformes - ps34
Catarrhini - ps35
Hominoidea - ps36
Hominidae - ps37
Homininae - ps38
Hominini - ps39
Homo_sapiens - ps40
p-value
3e-03
4e-03
6e-03
8e-03
1e-02
Log-odds
0.5
1.0
1.5
2.0
2.5
3.0
3.5
4.0

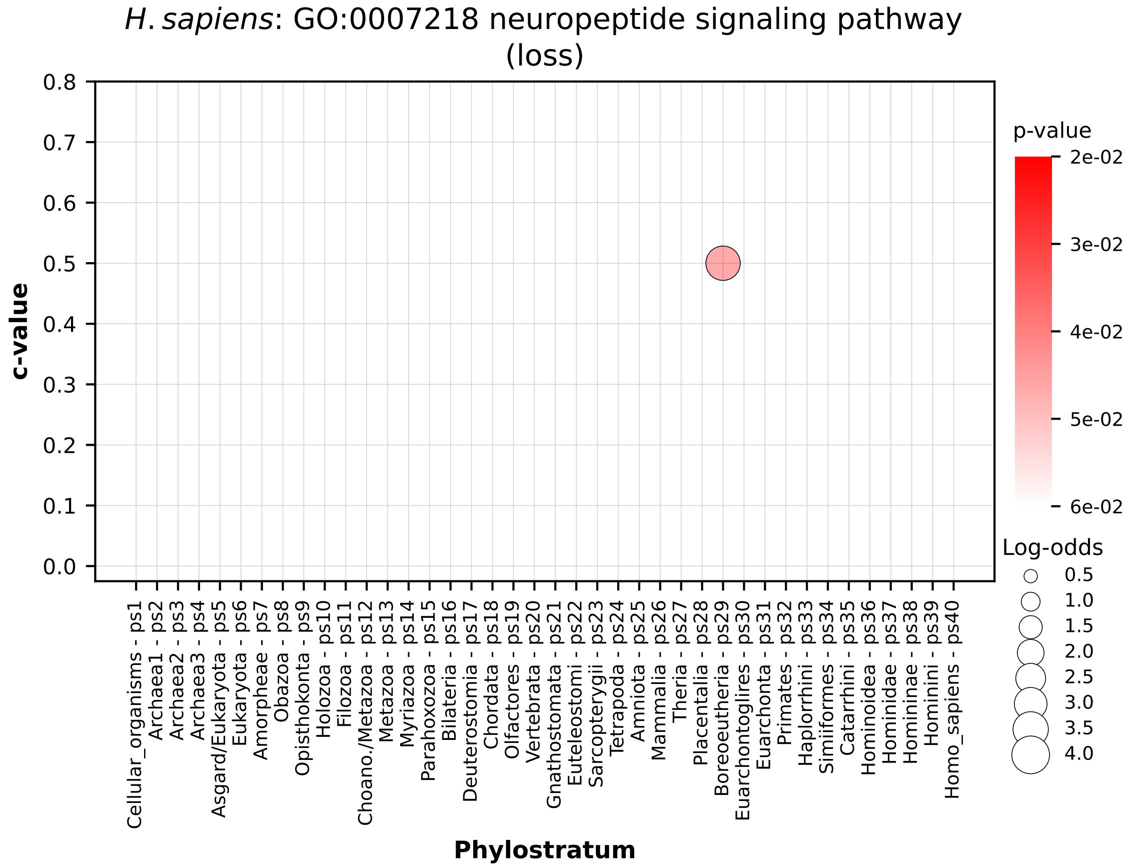

H. sapiens: GO:0007218 neuropeptide signaling pathway
(loss)
c-value
0.8
0.7
0.6
0.5
0.4
0.3
0.2
0.1
0.0
p-value
2e-02
3e-02
4e-02
5e-02
6e-02
Log-odds
0.5
1.0
1.5
2.0
2.5
3.0
3.5
4.0
Cellular_organisms - ps1
Archaea1 - ps2
Archaea2 - ps3
Archaea3 - ps4
Asgard/Eukaryota - ps5
Eukaryota - ps6
Amorpheae - ps7
Obazoa - ps8
Opisthokonta - ps9
Holozoa - ps10
Filozoa - ps11
Choano./Metazoa - ps12
Metazoa - ps13
Myriazoa - ps14
Parahoxozoa - ps15
Bilateria - ps16
Deuterostomia - ps17
Chordata - ps18
Olfactores - ps19
Vertebrata - ps20
Gnathostomata - ps21
Euteleostomi - ps22
Sarcopterygii - ps23
Tetrapoda - ps24
Amniota - ps25
Mammalia - ps26
Theria - ps27
Placentalia - ps28
Boreoeutheria - ps29
Euarchontoglires - ps30
Euarchonta - ps31
Primates - ps32
Haplorrhini - ps33
Simiiformes - ps34
Catarrhini - ps35
Hominoidea - ps36
Hominidae - ps37
Homininae - ps38
Hominini - ps39
Homo_sapiens - ps40
Phylostratum

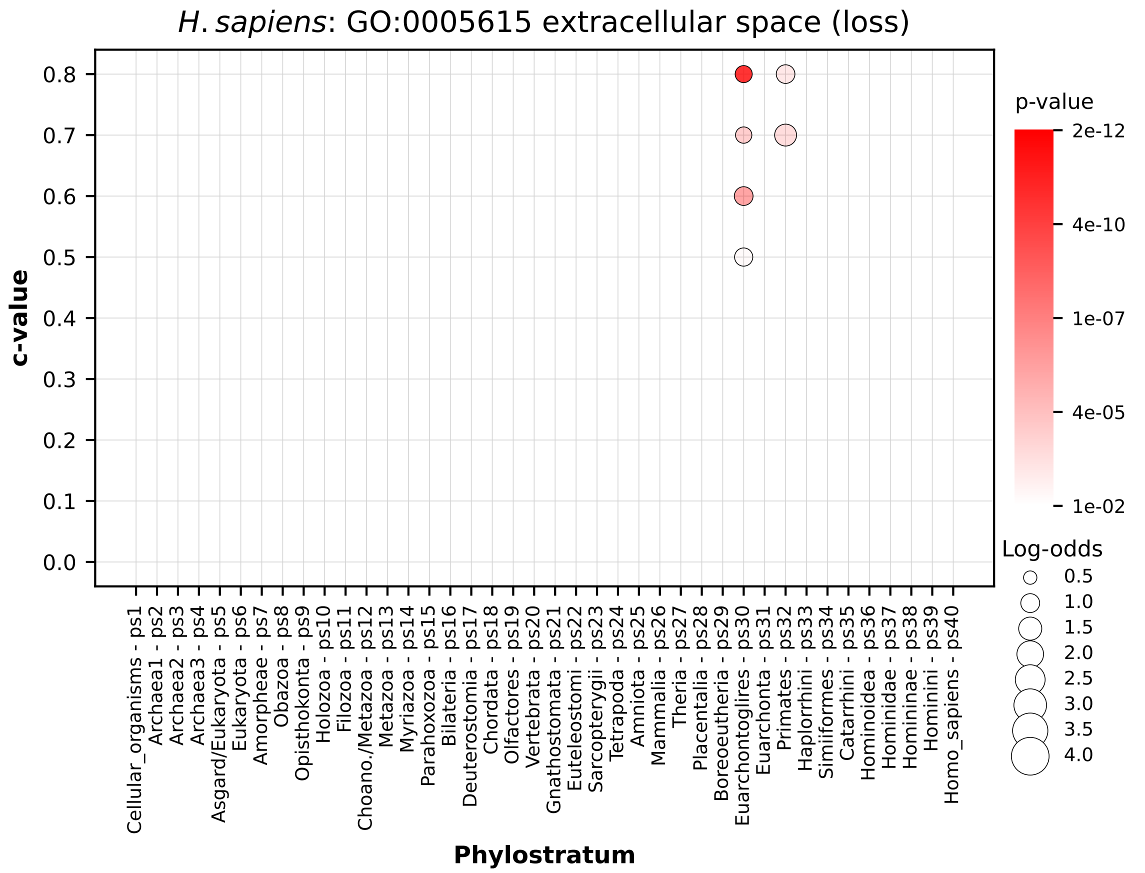

H. sapiens: GO:0005615 extracellular space (loss)
c-value
0.8
0.7
0.6
0.5
0.4
0.3
0.2
0.1
0.0
p-value
2e-12
4e-10
1e-07
4e-05
1e-02
Log-odds
0.5
1.0
1.5
2.0
2.5
3.0
3.5
4.0
Cellular_organisms - ps1
Archaea1 - ps2
Archaea2 - ps3
Archaea3 - ps4
Asgard/Eukaryota - ps5
Eukaryota - ps6
Amorpheae - ps7
Obazoa - ps8
Opisthokonta - ps9
Holozoa - ps10
Filozoa - ps11
Choano./Metazoa - ps12
Metazoa - ps13
Myriazoa - ps14
Parahoxozoa - ps15
Bilateria - ps16
Deuterostomia - ps17
Chordata - ps18
Olfactores - ps19
Vertebrata - ps20
Gnathostomata - ps21
Euteleostomi - ps22
Sarcopterygii - ps23
Tetrapoda - ps24
Amniota - ps25
Mammalia - ps26
Theria - ps27
Placentalia - ps28
Boreoeutheria - ps29
Euarchontoglires - ps30
Euarchonta - ps31
Primates - ps32
Haplorrhini - ps33
Simiiformes - ps34
Catarrhini - ps35
Hominoidea - ps36
Hominidae - ps37
Homininae - ps38
Hominini - ps39
Homo_sapiens - ps40
Phylostratum

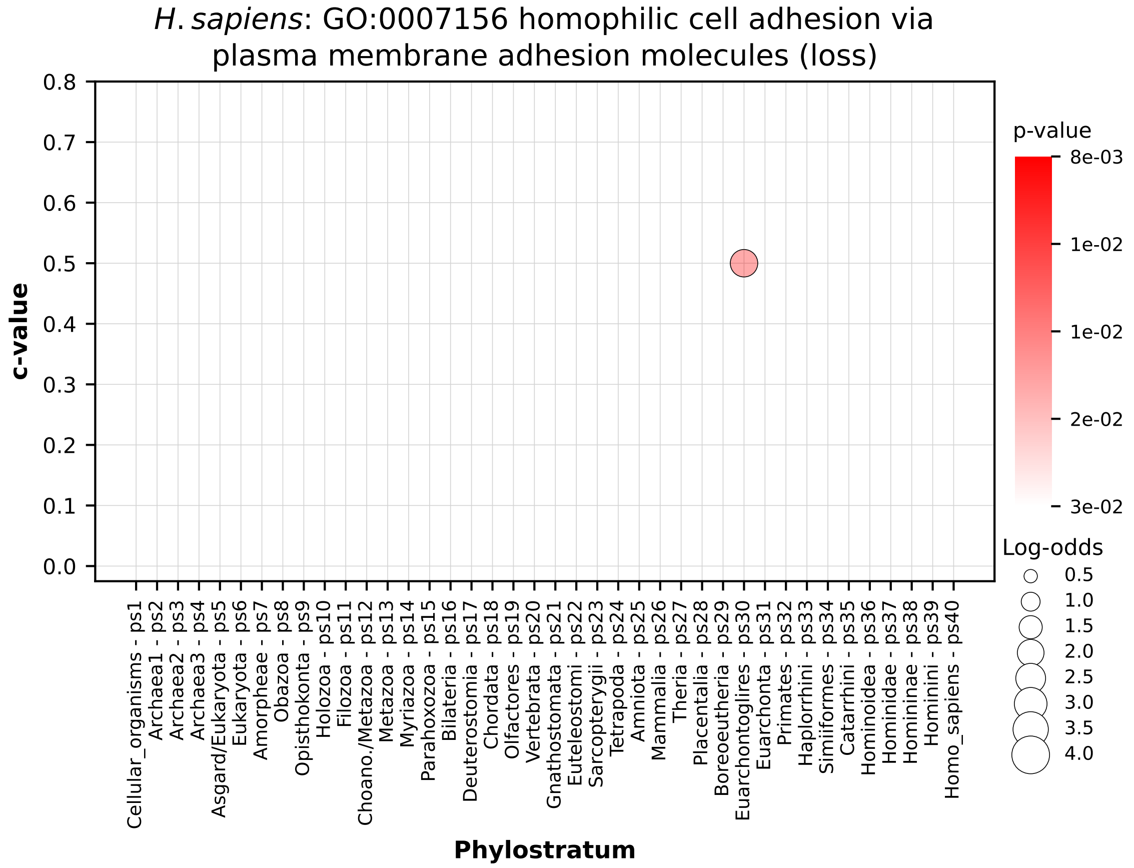

H. sapiens: GO:0007156 homophilic cell adhesion via plasma membrane adhesion molecules (loss)
c-value
0.8
0.7
0.6
0.5
0.4
0.3
0.2
0.1
0.0
p-value
8e-03
1e-02
1e-02
2e-02
3e-02
Log-odds
0.5
1.0
1.5
2.0
2.5
3.0
3.5
4.0
Cellular_organisms - ps1
Archaea1 - ps2
Archaea2 - ps3
Archaea3 - ps4
Asgard/Eukaryota - ps5
Eukaryota - ps6
Amorpheae - ps7
Obazoa - ps8
Opisthokonta - ps9
Holozoa - ps10
Filozoa - ps11
Choano./Metazoa - ps12
Metazoa - ps13
Myriazoa - ps14
Parahoxozoa - ps15
Bilateria - ps16
Deuterostomia - ps17
Chordata - ps18
Olfactores - ps19
Vertebrata - ps20
Gnathostomata - ps21
Euteleostomi - ps22
Sarcopterygii - ps23
Tetrapoda - ps24
Amniota - ps25
Mammalia - ps26
Theria - ps27
Placentalia - ps28
Boreoeutheria - ps29
Euarchontoglires - ps30
Euarchonta - ps31
Primates - ps32
Haplorrhini - ps33
Simiiformes - ps34
Catarrhini - ps35
Hominoidea - ps36
Hominidae - ps37
Homininae - ps38
Hominini - ps39
Homo_sapiens - ps40
Phylostratum

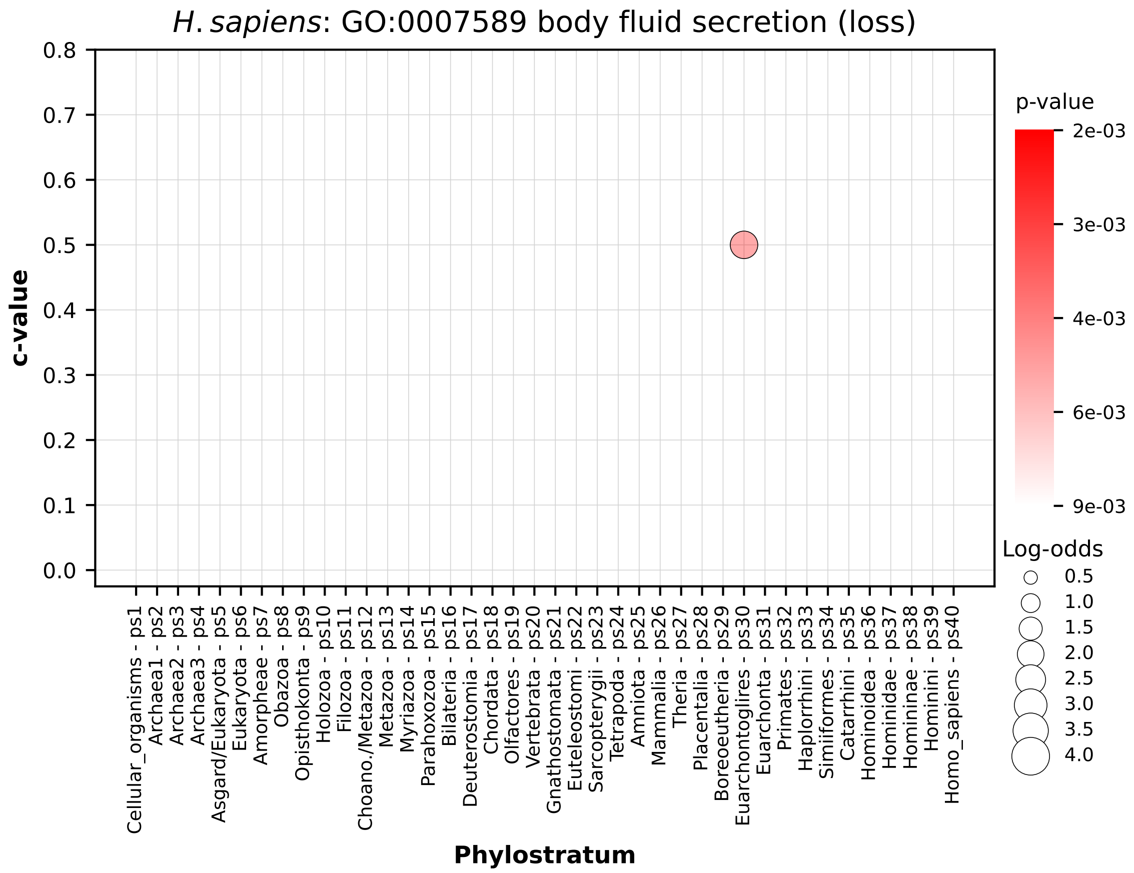
H. sapiens: GO:0007589 body fluid secretion (loss)
c-value
0.8
0.7
0.6
0.5
0.4
0.3
0.2
0.1
0.0
p-value
2e-03
3e-03
4e-03
6e-03
9e-03
Log-odds
0.5
1.0
1.5
2.0
2.5
3.0
3.5
4.0
Cellular_organisms - ps1
Archaea1 - ps2
Archaea2 - ps3
Archaea3 - ps4
Asgard/Eukaryota - ps5
Eukaryota - ps6
Amorpheae - ps7
Obazoa - ps8
Opisthokonta - ps9
Holozoa - ps10
Filozoa - ps11
Choano./Metazoa - ps12
Metazoa - ps13
Myriazoa - ps14
Parahoxozoa - ps15
Bilateria - ps16
Deuterostomia - ps17
Chordata - ps18
Olfactores - ps19
Vertebrata - ps20
Gnathostomata - ps21
Euteleostomi - ps22
Sarcopterygii - ps23
Tetrapoda - ps24
Amniota - ps25
Mammalia - ps26
Theria - ps27
Placentalia - ps28
Boreoeutheria - ps29
Euarchontoglires - ps30
Euarchonta - ps31
Primates - ps32
Haplorrhini - ps33
Simiiformes - ps34
Catarrhini - ps35
Hominoidea - ps36
Hominidae - ps37
Homininae - ps38
Hominini - ps39
Homo_sapiens - ps40
Phylostratum

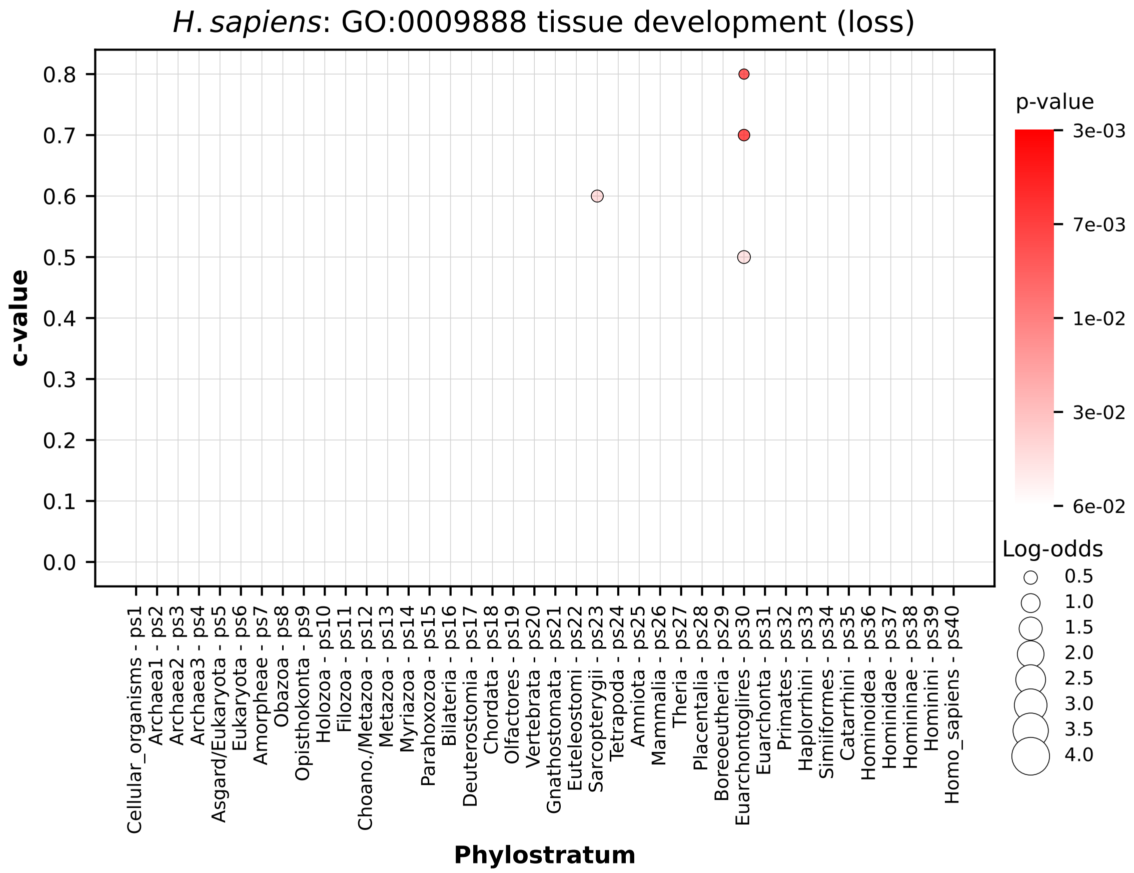

H. sapiens: GO:0009888 tissue development (loss)
c-value
0.0
0.1
0.2
0.3
0.4
0.5
0.6
0.7
0.8
p-value
3e-03
7e-03
1e-02
3e-02
6e-02
Log-odds
0.5
1.0
1.5
2.0
2.5
3.0
3.5
4.0
Cellular_organisms - ps1
Archaea1 - ps2
Archaea2 - ps3
Archaea3 - ps4
Asgard/Eukaryota - ps5
Eukaryota - ps6
Amorpheae - ps7
Obazoa - ps8
Opisthokonta - ps9
Holozoa - ps10
Filozoa - ps11
Choano./Metazoa - ps12
Metazoa - ps13
Myriazoa - ps14
Parahoxozoa - ps15
Bilateria - ps16
Deuterostomia - ps17
Chordata - ps18
Olfactores - ps19
Vertebrata - ps20
Gnathostomata - ps21
Euteleostomi - ps22
Sarcopterygii - ps23
Tetrapoda - ps24
Amniota - ps25
Mammalia - ps26
Theria - ps27
Placentalia - ps28
Boreoeutheria - ps29
Euarchontoglires - ps30
Euarchonta - ps31
Primates - ps32
Haplorrhini - ps33
Simiiformes - ps34
Catarrhini - ps35
Hominoidea - ps36
Hominidae - ps37
Homininae - ps38
Hominini - ps39
Homo_sapiens - ps40
Phylostratum

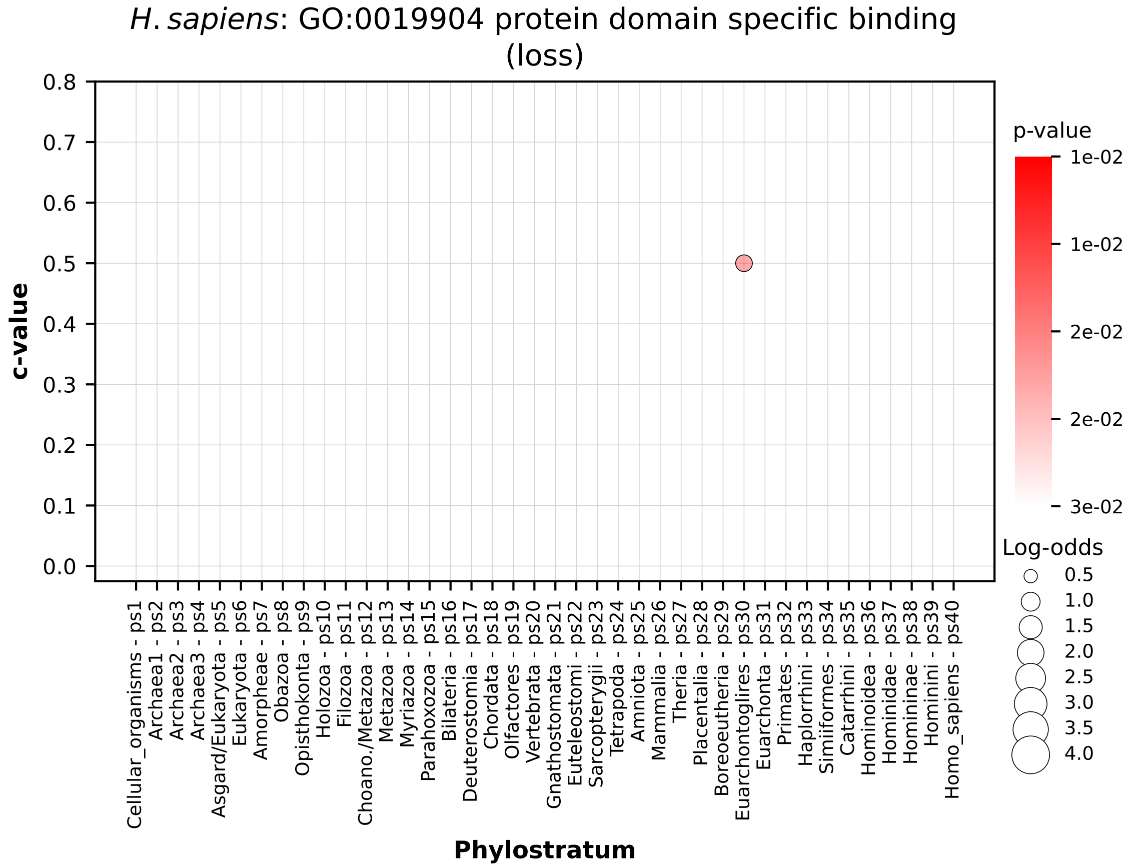

H. sapiens: GO:0019904 protein domain specific binding (loss)
c-value
0.8
0.7
0.6
0.5
0.4
0.3
0.2
0.1
0.0
p-value
1e-02
1e-02
2e-02
2e-02
3e-02
Log-odds
0.5
1.0
1.5
2.0
2.5
3.0
3.5
4.0
Cellular_organisms - ps1
Archaea1 - ps2
Archaea2 - ps3
Archaea3 - ps4
Asgard/Eukaryota - ps5
Eukaryota - ps6
Amorpheae - ps7
Obazoa - ps8
Opisthokonta - ps9
Holozoa - ps10
Filozoa - ps11
Choano./Metazoa - ps12
Metazoa - ps13
Myriazoa - ps14
Parahoxozoa - ps15
Bilateria - ps16
Deuterostomia - ps17
Chordata - ps18
Olfactores - ps19
Vertebrata - ps20
Gnathostomata - ps21
Euteleostomi - ps22
Sarcopterygii - ps23
Tetrapoda - ps24
Amniota - ps25
Mammalia - ps26
Theria - ps27
Placentalia - ps28
Boreoeutheria - ps29
Euarchontoglires - ps30
Euarchonta - ps31
Primates - ps32
Haplorrhini - ps33
Simiiformes - ps34
Catarrhini - ps35
Hominoidea - ps36
Hominidae - ps37
Homininae - ps38
Hominini - ps39
Homo_sapiens - ps40
Phylostratum

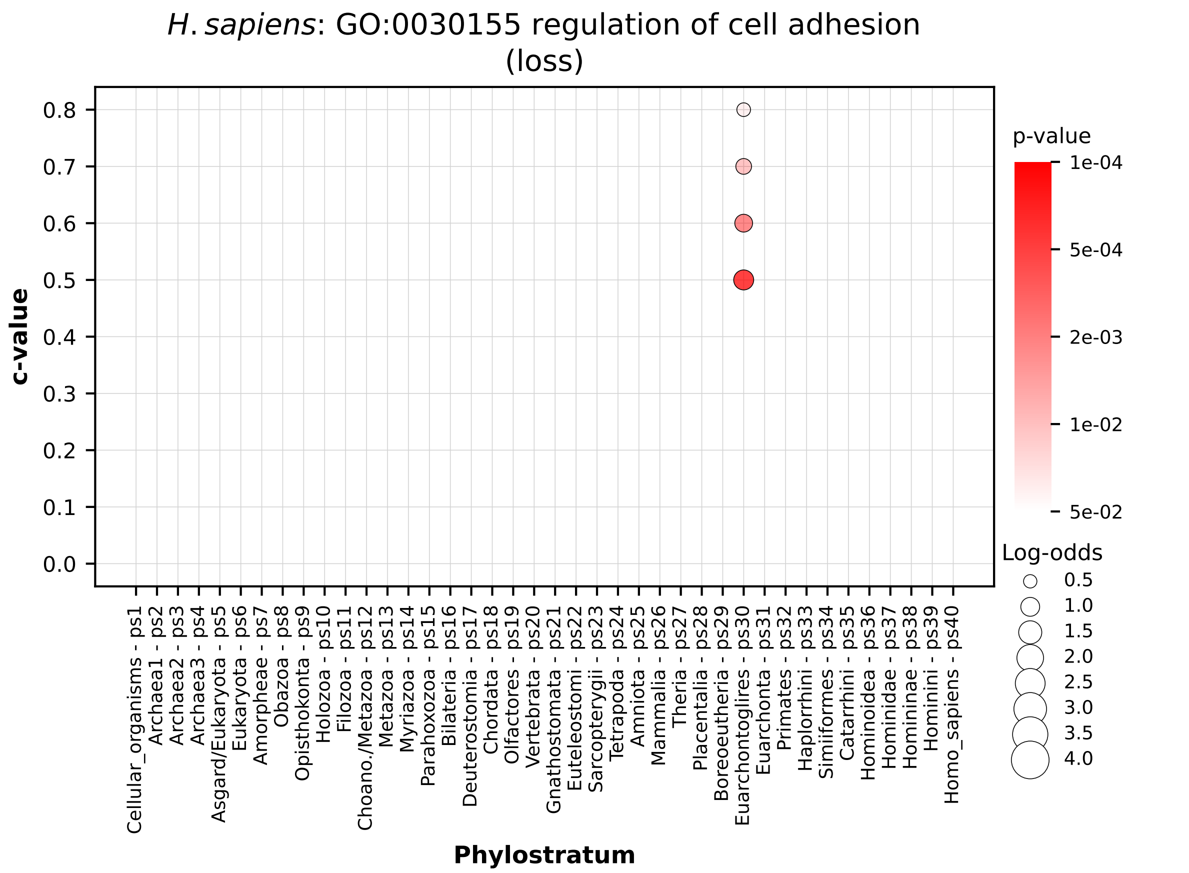

H. sapiens: GO:0030155 regulation of cell adhesion (loss)
c-value
0.0
0.1
0.2
0.3
0.4
0.5
0.6
0.7
0.8
p-value
1e-04
5e-04
2e-03
1e-02
5e-02
Log-odds
0.5
1.0
1.5
2.0
2.5
3.0
3.5
4.0
Cellular_organisms - ps1
Archaea1 - ps2
Archaea2 - ps3
Archaea3 - ps4
Asgard/Eukaryota - ps5
Eukaryota - ps6
Amorpheae - ps7
Obazoa - ps8
Opisthokonta - ps9
Holozoa - ps10
Filozoa - ps11
Choano./Metazoa - ps12
Metazoa - ps13
Myriazoa - ps14
Parahoxozoa - ps15
Bilateria - ps16
Deuterostomia - ps17
Chordata - ps18
Olfactores - ps19
Vertebrata - ps20
Gnathostomata - ps21
Euteleostomi - ps22
Sarcopterygii - ps23
Tetrapoda - ps24
Amniota - ps25
Mammalia - ps26
Theria - ps27
Placentalia - ps28
Boreoeutheria - ps29
Euarchontoglires - ps30
Euarchonta - ps31
Primates - ps32
Haplorrhini - ps33
Simiiformes - ps34
Catarrhini - ps35
Hominoidea - ps36
Hominidae - ps37
Homininae - ps38
Hominini - ps39
Homo_sapiens - ps40
Phylostratum

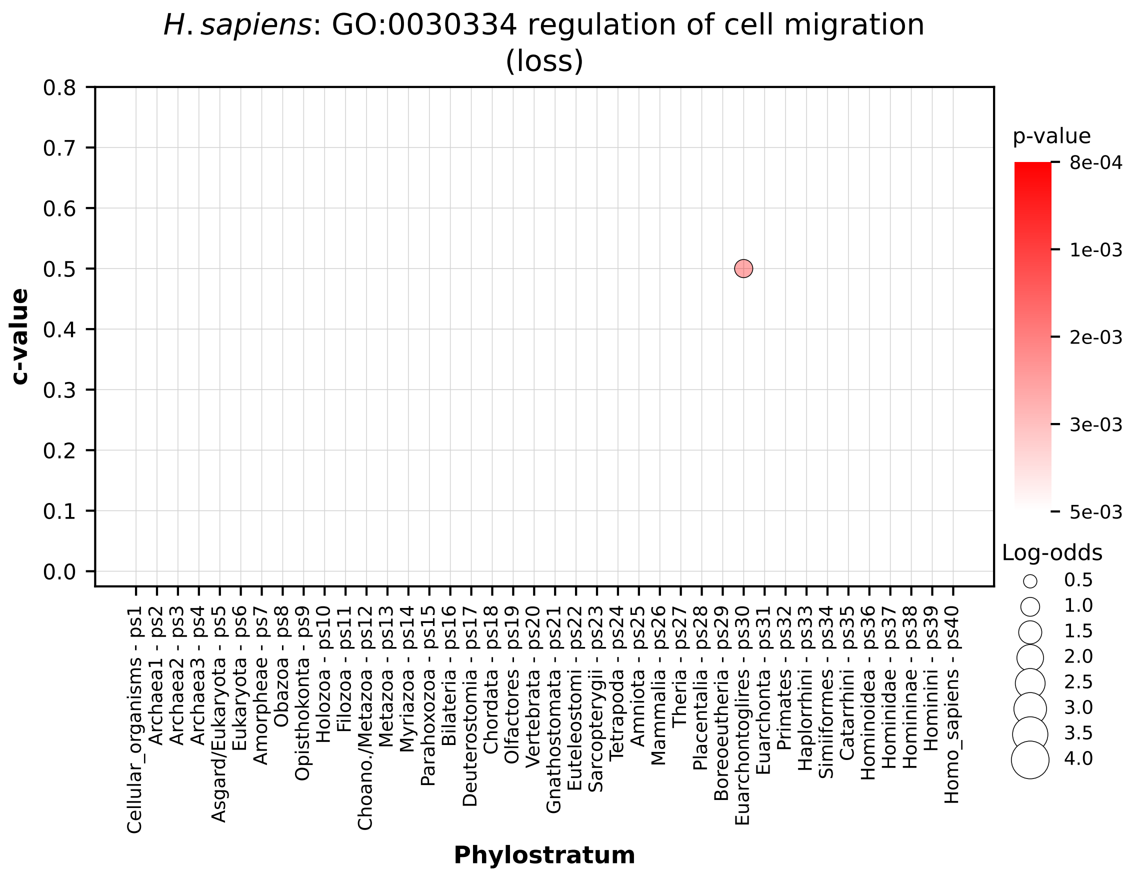

H. sapiens: GO:0030334 regulation of cell migration (loss)
c-value
0.0
0.1
0.2
0.3
0.4
0.5
0.6
0.7
0.8
Phylostratum
Cellular_organisms - ps1
Archaea1 - ps2
Archaea2 - ps3
Archaea3 - ps4
Asgard/Eukaryota - ps5
Eukaryota - ps6
Amorpheae - ps7
Obazoa - ps8
Opisthokonta - ps9
Holozoa - ps10
Filozoa - ps11
Choano./Metazoa - ps12
Metazoa - ps13
Myriazoa - ps14
Parahoxozoa - ps15
Bilateria - ps16
Deuterostomia - ps17
Chordata - ps18
Olfactores - ps19
Vertebrata - ps20
Gnathostomata - ps21
Euteleostomi - ps22
Sarcopterygii - ps23
Tetrapoda - ps24
Amniota - ps25
Mammalia - ps26
Theria - ps27
Placentalia - ps28
Boreoeutheria - ps29
Euarchontoglires - ps30
Euarchonta - ps31
Primates - ps32
Haplorrhini - ps33
Simiiformes - ps34
Catarrhini - ps35
Hominoidea - ps36
Hominidae - ps37
Homininae - ps38
Hominini - ps39
Homo_sapiens - ps40
p-value
8e-04
1e-03
2e-03
3e-03
5e-03
Log-odds
0.5
1.0
1.5
2.0
2.5
3.0
3.5
4.0

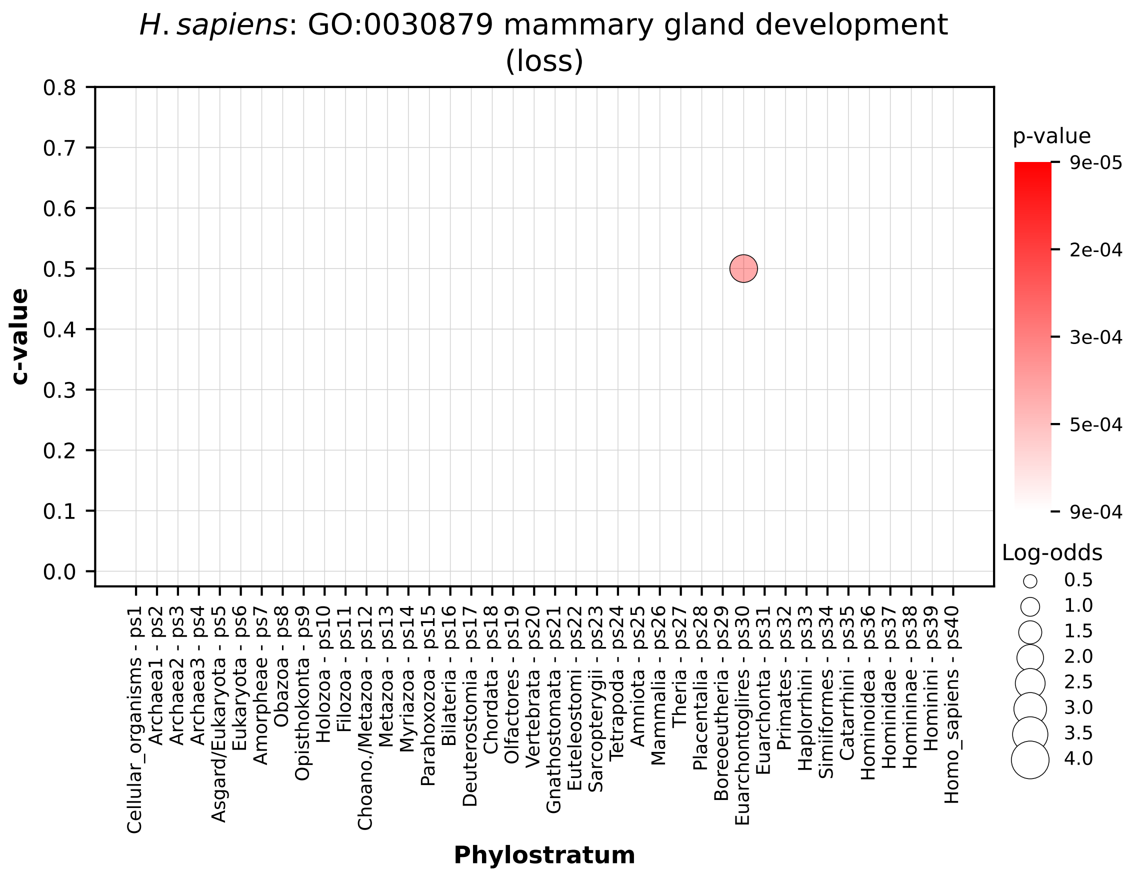

H. sapiens: GO:0030879 mammary gland development (loss)
c-value
0.0
0.1
0.2
0.3
0.4
0.5
0.6
0.7
0.8
Phylostratum
Cellular_organisms - ps1
Archaea1 - ps2
Archaea2 - ps3
Archaea3 - ps4
Asgard/Eukaryota - ps5
Eukaryota - ps6
Amorpheae - ps7
Obazoa - ps8
Opisthokonta - ps9
Holozoa - ps10
Filozoa - ps11
Choano./Metazoa - ps12
Metazoa - ps13
Myriazoa - ps14
Parahoxozoa - ps15
Bilateria - ps16
Deuterostomia - ps17
Chordata - ps18
Olfactores - ps19
Vertebrata - ps20
Gnathostomata - ps21
Euteleostomi - ps22
Sarcopterygii - ps23
Tetrapoda - ps24
Amniota - ps25
Mammalia - ps26
Theria - ps27
Placentalia - ps28
Boreoeutheria - ps29
Euarchontoglires - ps30
Euarchonta - ps31
Primates - ps32
Haplorrhini - ps33
Simiiformes - ps34
Catarrhini - ps35
Hominoidea - ps36
Hominidae - ps37
Homininae - ps38
Hominini - ps39
Homo_sapiens - ps40
p-value
9e-05
2e-04
3e-04
5e-04
9e-04
Log-odds
0.5
1.0
1.5
2.0
2.5
3.0
3.5
4.0

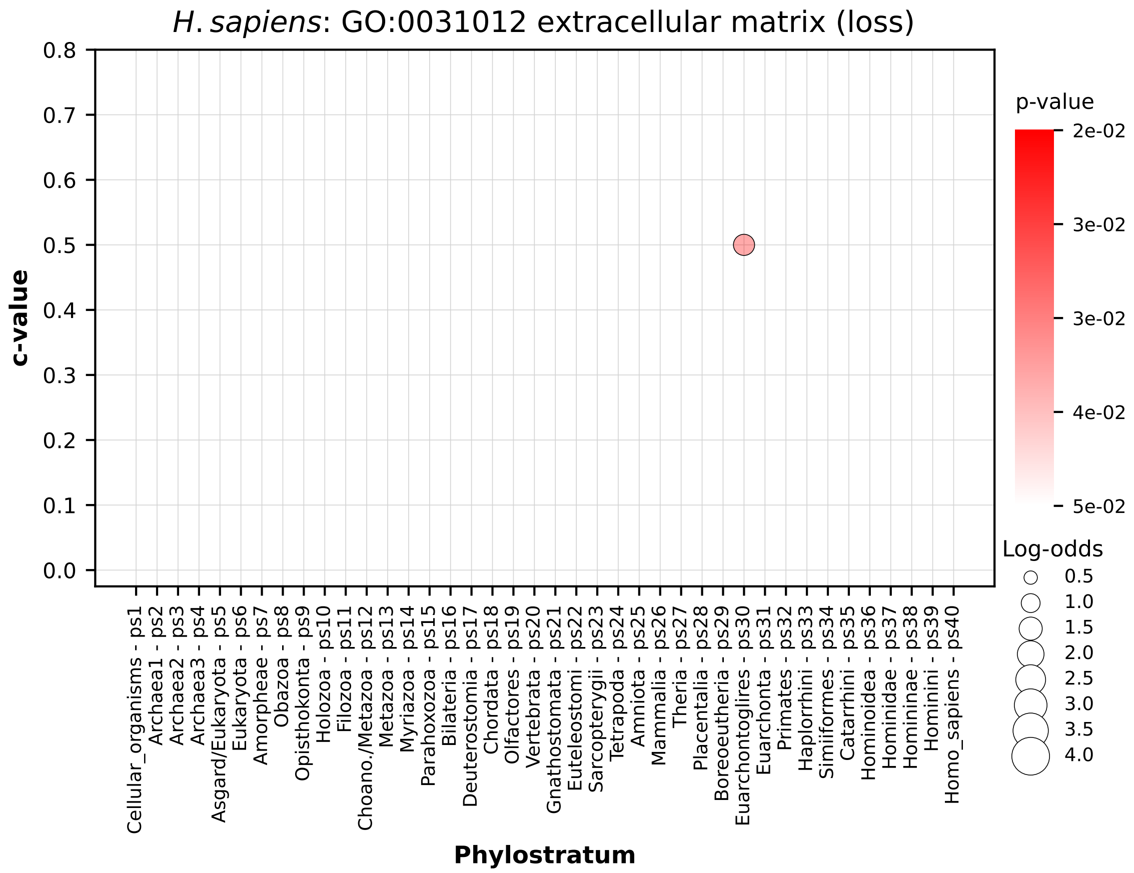
H. sapiens: GO:0031012 extracellular matrix (loss)
c-value
0.8
0.7
0.6
0.5
0.4
0.3
0.2
0.1
0.0
Phylostratum
Cellular_organisms - ps1
Archaea1 - ps2
Archaea2 - ps3
Archaea3 - ps4
Asgard/Eukaryota - ps5
Eukaryota - ps6
Amorpheae - ps7
Obazoa - ps8
Opisthokonta - ps9
Holozoa - ps10
Filozoa - ps11
Choano./Metazoa - ps12
Metazoa - ps13
Myriazoa - ps14
Parahoxozoa - ps15
Bilateria - ps16
Deuterostomia - ps17
Chordata - ps18
Olfactores - ps19
Vertebrata - ps20
Gnathostomata - ps21
Euteleostomi - ps22
Sarcopterygii - ps23
Tetrapoda - ps24
Amniota - ps25
Mammalia - ps26
Theria - ps27
Placentalia - ps28
Boreoeutheria - ps29
Euarchontoglires - ps30
Euarchonta - ps31
Primates - ps32
Haplorrhini - ps33
Simiiformes - ps34
Catarrhini - ps35
Hominoidea - ps36
Hominidae - ps37
Homininae - ps38
Hominini - ps39
Homo_sapiens - ps40
p-value
2e-02
3e-02
3e-02
4e-02
5e-02
Log-odds
0.5
1.0
1.5
2.0
2.5
3.0
3.5
4.0

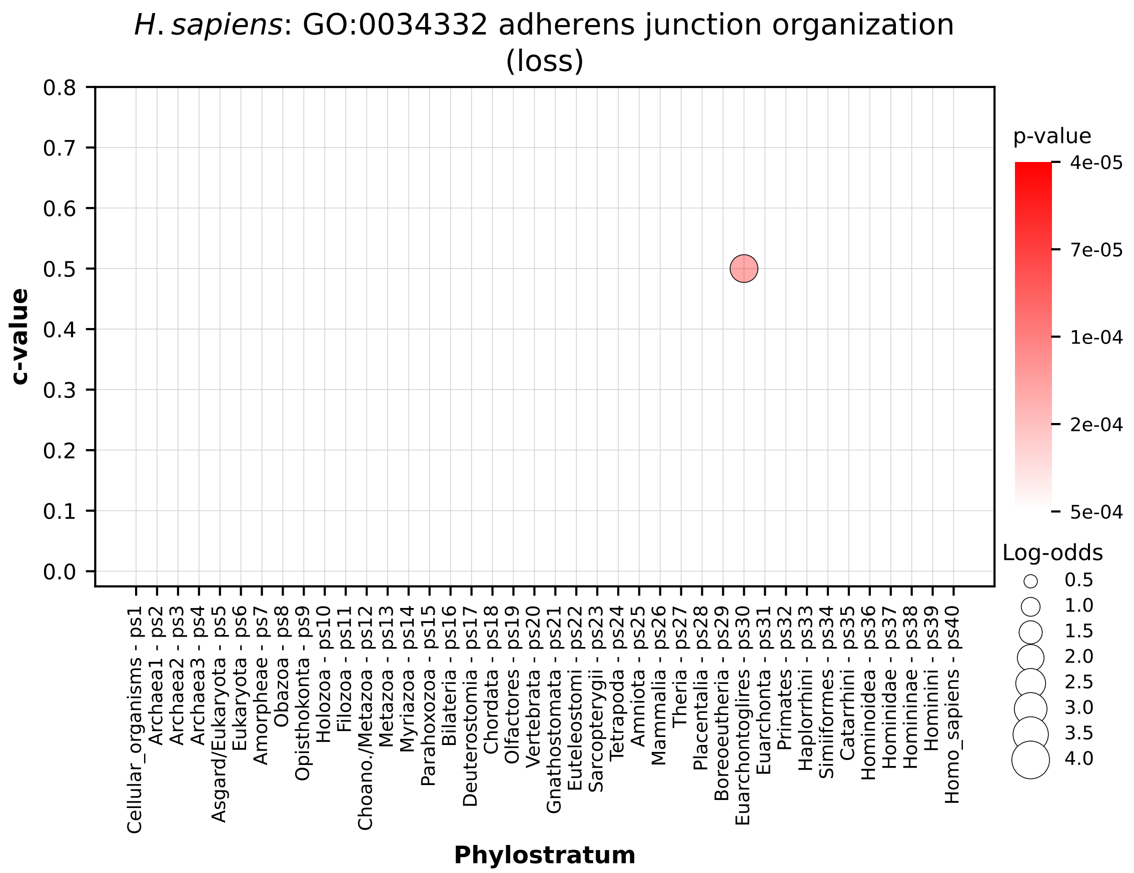
H. sapiens: GO:0034332 adherens junction organization
(loss)
c-value
0.8
0.7
0.6
0.5
0.4
0.3
0.2
0.1
0.0
Phylostratum
Cellular_organisms - ps1
Archaea1 - ps2
Archaea2 - ps3
Archaea3 - ps4
Asgard/Eukaryota - ps5
Eukaryota - ps6
Amorpheae - ps7
Obazoa - ps8
Opisthokonta - ps9
Holozoa - ps10
Filozoa - ps11
Choano./Metazoa - ps12
Metazoa - ps13
Myriazoa - ps14
Parahoxozoa - ps15
Bilateria - ps16
Deuterostomia - ps17
Chordata - ps18
Olfactores - ps19
Vertebrata - ps20
Gnathostomata - ps21
Euteleostomi - ps22
Sarcopterygii - ps23
Tetrapoda - ps24
Amniota - ps25
Mammalia - ps26
Theria - ps27
Placentalia - ps28
Boreoeutheria - ps29
Euarchontoglires - ps30
Euarchonta - ps31
Primates - ps32
Haplorrhini - ps33
Simiiformes - ps34
Catarrhini - ps35
Hominoidea - ps36
Hominidae - ps37
Homininae - ps38
Hominini - ps39
Homo_sapiens - ps40
p-value
4e-05
7e-05
1e-04
2e-04
5e-04
Log-odds
0.5
1.0
1.5
2.0
2.5
3.0
3.5
4.0

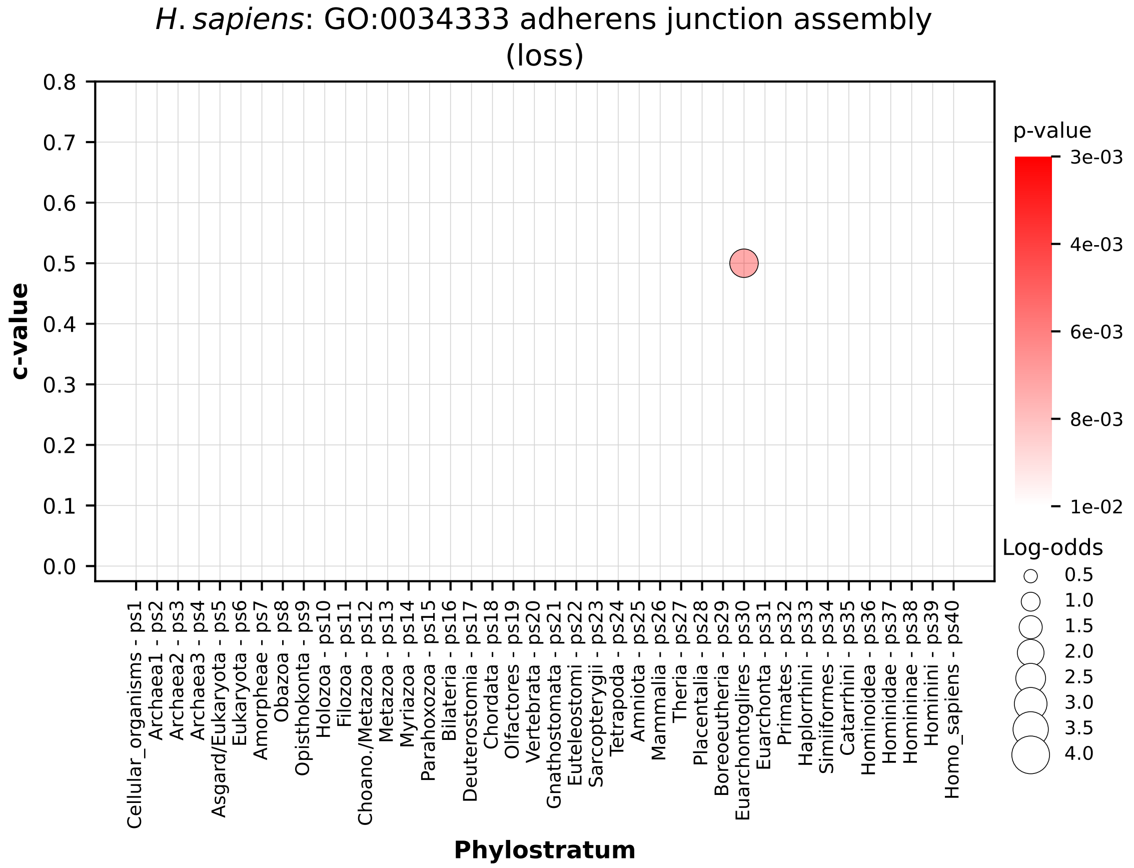

H. sapiens: GO:0034333 adherens junction assembly (loss)
c-value
0.8
0.7
0.6
0.5
0.4
0.3
0.2
0.1
0.0
Phylostratum
Cellular_organisms - ps1
Archaea1 - ps2
Archaea2 - ps3
Archaea3 - ps4
Asgard/Eukaryota - ps5
Eukaryota - ps6
Amorpheae - ps7
Obazoa - ps8
Opisthokonta - ps9
Holozoa - ps10
Filozoa - ps11
Choano./Metazoa - ps12
Metazoa - ps13
Myriazoa - ps14
Parahoxozoa - ps15
Bilateria - ps16
Deuterostomia - ps17
Chordata - ps18
Olfactores - ps19
Vertebrata - ps20
Gnathostomata - ps21
Euteleostomi - ps22
Sarcopterygii - ps23
Tetrapoda - ps24
Amniota - ps25
Mammalia - ps26
Theria - ps27
Placentalia - ps28
Boreoeutheria - ps29
Euarchontoglires - ps30
Euarchonta - ps31
Primates - ps32
Haplorrhini - ps33
Simiiformes - ps34
Catarrhini - ps35
Hominoidea - ps36
Hominidae - ps37
Homininae - ps38
Hominini - ps39
Homo_sapiens - ps40
p-value
3e-03
4e-03
6e-03
8e-03
1e-02
Log-odds
0.5
1.0
1.5
2.0
2.5
3.0
3.5
4.0

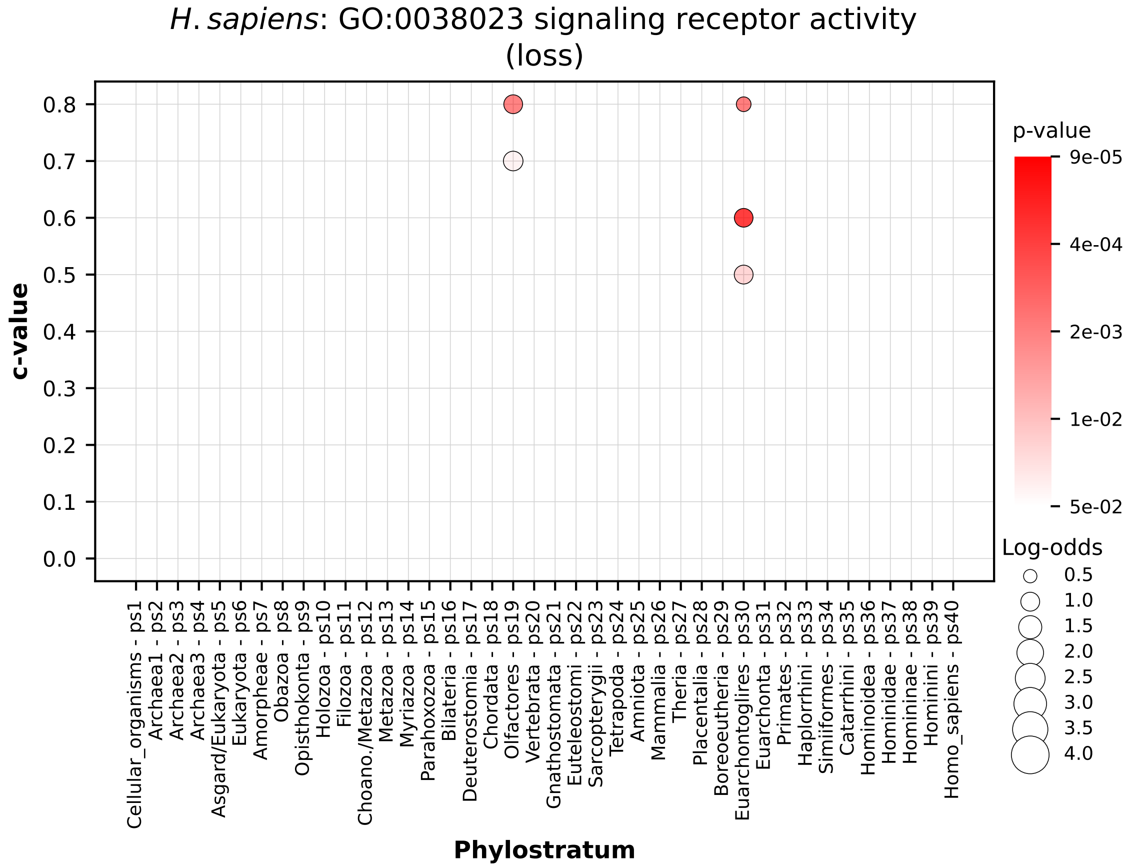

H. sapiens: GO:0038023 signaling receptor activity
(loss)
c-value
0.0
0.1
0.2
0.3
0.4
0.5
0.6
0.7
0.8
Phylostratum
Cellular_organisms - ps1
Archaea1 - ps2
Archaea2 - ps3
Archaea3 - ps4
Asgard/Eukaryota - ps5
Eukaryota - ps6
Amorpheae - ps7
Obazoa - ps8
Opisthokonta - ps9
Holozoa - ps10
Filozoa - ps11
Choano./Metazoa - ps12
Metazoa - ps13
Myriazoa - ps14
Parahoxozoa - ps15
Bilateria - ps16
Deuterostomia - ps17
Chordata - ps18
Olfactores - ps19
Vertebrata - ps20
Gnathostomata - ps21
Euteleostomi - ps22
Sarcopterygii - ps23
Tetrapoda - ps24
Amniota - ps25
Mammalia - ps26
Theria - ps27
Placentalia - ps28
Boreoeutheria - ps29
Euarchontoglires - ps30
Euarchonta - ps31
Primates - ps32
Haplorrhini - ps33
Simiiformes - ps34
Catarrhini - ps35
Hominoidea - ps36
Hominidae - ps37
Homininae - ps38
Hominini - ps39
Homo_sapiens - ps40
p-value
9e-05
4e-04
2e-03
1e-02
5e-02
Log-odds
0.5
1.0
1.5
2.0
2.5
3.0
3.5
4.0

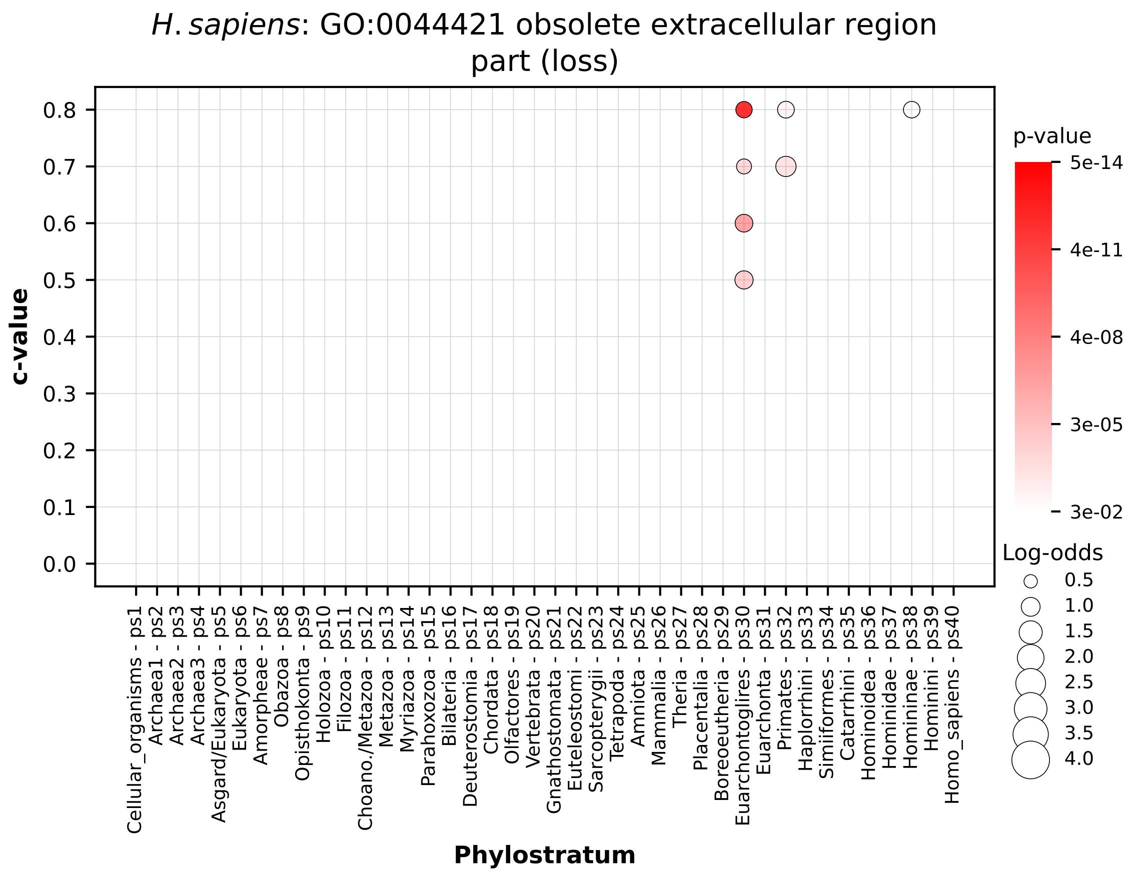

H. sapiens: GO:0044421 obsolete extracellular region part (loss)
c-value
0.0
0.1
0.2
0.3
0.4
0.5
0.6
0.7
0.8
Phylostratum
Cellular_organisms - ps1
Archaea1 - ps2
Archaea2 - ps3
Archaea3 - ps4
Asgard/Eukaryota - ps5
Eukaryota - ps6
Amorpheae - ps7
Obazoa - ps8
Opisthokonta - ps9
Holozoa - ps10
Filozoa - ps11
Choano./Metazoa - ps12
Metazoa - ps13
Myriazoa - ps14
Parahoxozoa - ps15
Bilateria - ps16
Deuterostomia - ps17
Chordata - ps18
Olfactores - ps19
Vertebrata - ps20
Gnathostomata - ps21
Euteleostomi - ps22
Sarcopterygii - ps23
Tetrapoda - ps24
Amniota - ps25
Mammalia - ps26
Theria - ps27
Placentalia - ps28
Boreoeutheria - ps29
Euarchontoglires - ps30
Euarchonta - ps31
Primates - ps32
Haplorrhini - ps33
Simiiformes - ps34
Catarrhini - ps35
Hominoidea - ps36
Hominidae - ps37
Homininae - ps38
Hominini - ps39
Homo_sapiens - ps40
p-value
5e-14
4e-11
4e-08
3e-05
3e-02
Log-odds
0.5
1.0
1.5
2.0
2.5
3.0
3.5
4.0

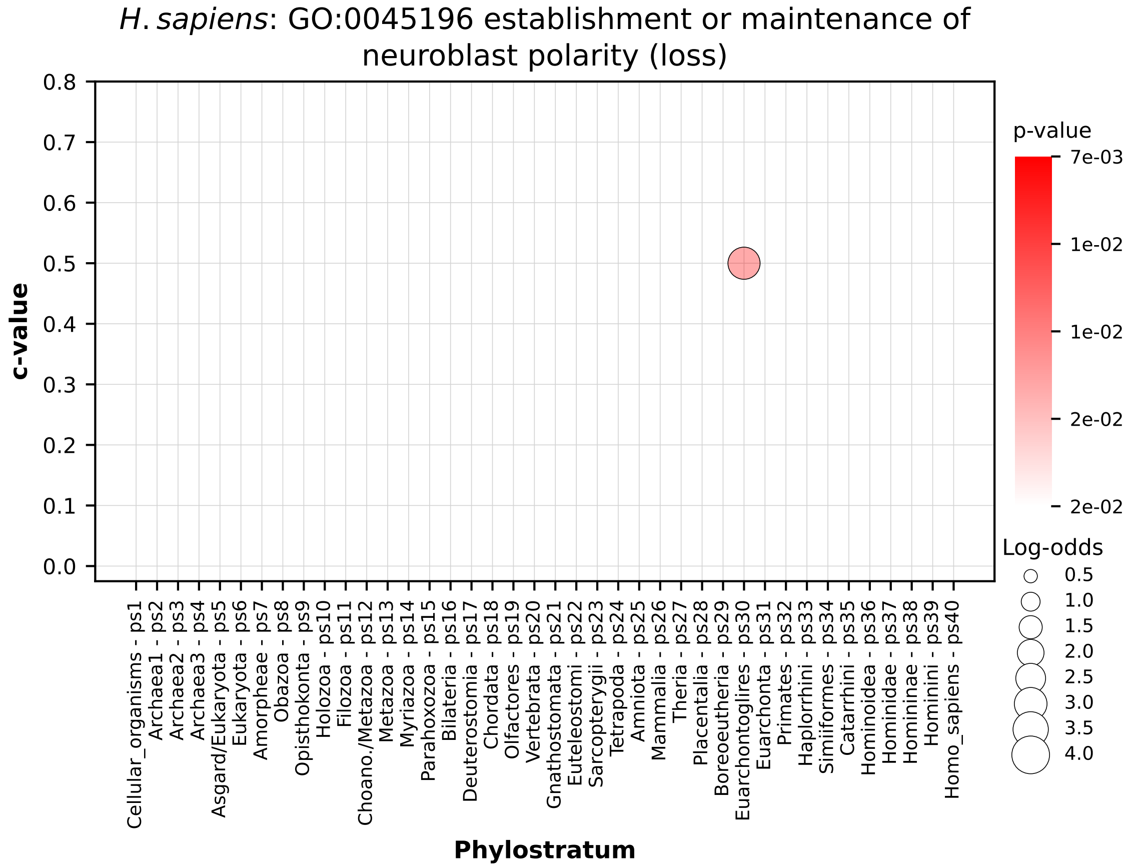
H. sapiens: GO:0045196 establishment or maintenance of neuroblast polarity (loss)
c-value
0.8
0.7
0.6
0.5
0.4
0.3
0.2
0.1
0.0
Phylostratum
Cellular_organisms - ps1
Archaea1 - ps2
Archaea2 - ps3
Archaea3 - ps4
Asgard/Eukaryota - ps5
Eukaryota - ps6
Amorpheae - ps7
Obazoa - ps8
Opisthokonta - ps9
Holozoa - ps10
Filozoa - ps11
Choano./Metazoa - ps12
Metazoa - ps13
Myriazoa - ps14
Parahoxozoa - ps15
Bilateria - ps16
Deuterostomia - ps17
Chordata - ps18
Olfactores - ps19
Vertebrata - ps20
Gnathostomata - ps21
Euteleostomi - ps22
Sarcopterygii - ps23
Tetrapoda - ps24
Amniota - ps25
Mammalia - ps26
Theria - ps27
Placentalia - ps28
Boreoeutheria - ps29
Euarchontoglires - ps30
Euarchonta - ps31
Primates - ps32
Haplorrhini - ps33
Simiiformes - ps34
Catarrhini - ps35
Hominoidea - ps36
Hominidae - ps37
Homininae - ps38
Hominini - ps39
Homo_sapiens - ps40
p-value
7e-03
1e-02
1e-02
2e-02
2e-02
Log-odds
0.5
1.0
1.5
2.0
2.5
3.0
3.5
4.0

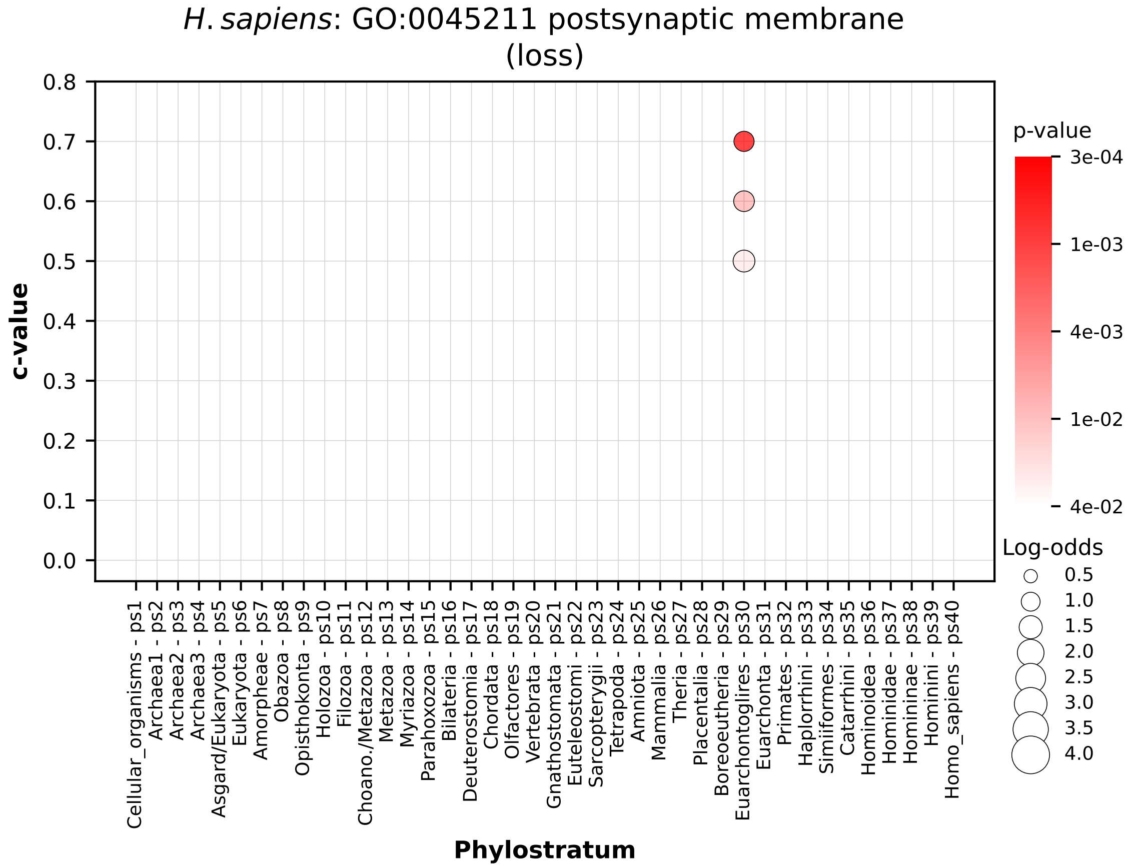
H. sapiens: GO:0045211 postsynaptic membrane
(loss)
c-value
0.8
0.7
0.6
0.5
0.4
0.3
0.2
0.1
0.0
Phylostratum
Cellular_organisms - ps1
Archaea1 - ps2
Archaea2 - ps3
Archaea3 - ps4
Asgard/Eukaryota - ps5
Eukaryota - ps6
Amorpheae - ps7
Obazoa - ps8
Opisthokonta - ps9
Holozoa - ps10
Filozoa - ps11
Choano./Metazoa - ps12
Metazoa - ps13
Myriazoa - ps14
Parahoxozoa - ps15
Bilateria - ps16
Deuterostomia - ps17
Chordata - ps18
Olfactores - ps19
Vertebrata - ps20
Gnathostomata - ps21
Euteleostomi - ps22
Sarcopterygii - ps23
Tetrapoda - ps24
Amniota - ps25
Mammalia - ps26
Theria - ps27
Placentalia - ps28
Boreoeutheria - ps29
Euarchontoglires - ps30
Euarchonta - ps31
Primates - ps32
Haplorrhini - ps33
Simiiformes - ps34
Catarrhini - ps35
Hominoidea - ps36
Hominidae - ps37
Homininae - ps38
Hominini - ps39
Homo_sapiens - ps40
p-value
3e-04
1e-03
4e-03
1e-02
4e-02
Log-odds
0.5
1.0
1.5
2.0
2.5
3.0
3.5
4.0

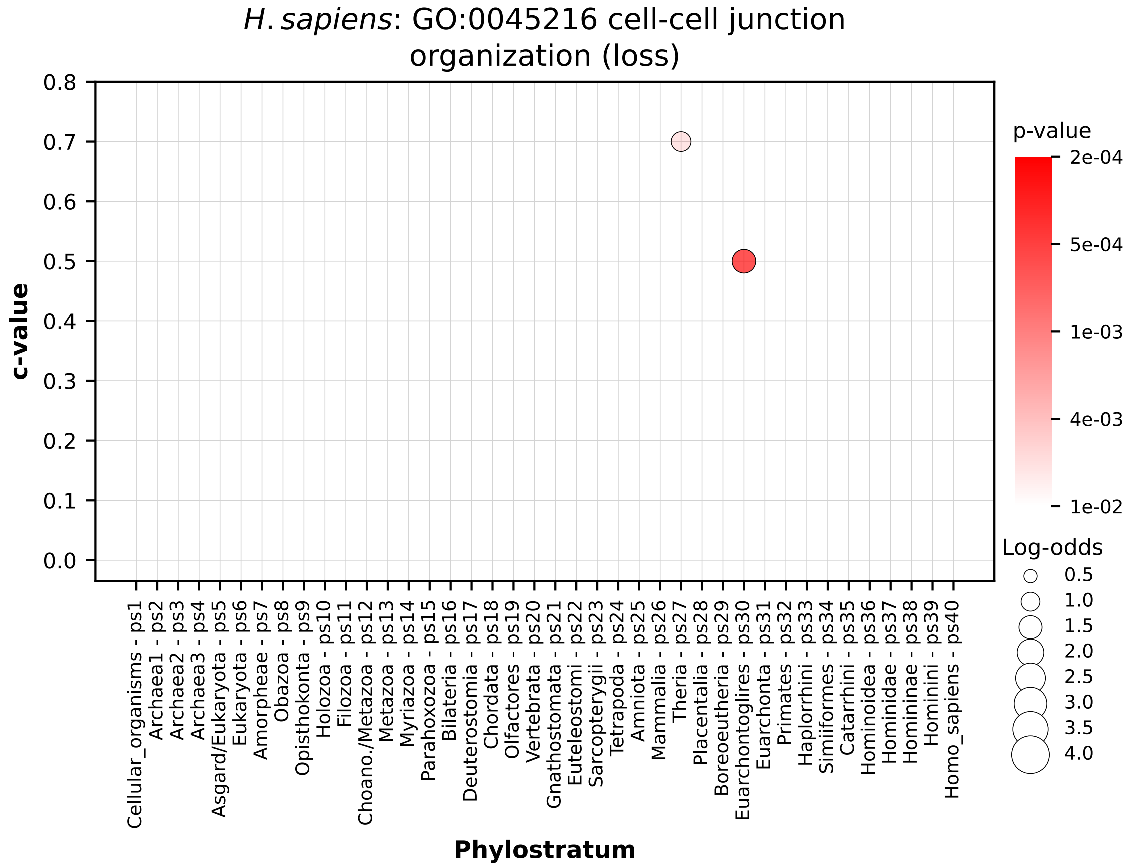
H. sapiens: GO:0045216 cell-cell junction organization (loss)
c-value
0.8
0.7
0.6
0.5
0.4
0.3
0.2
0.1
0.0
p-value
2e-04
5e-04
1e-03
4e-03
1e-02
Log-odds
0.5
1.0
1.5
2.0
2.5
3.0
3.5
4.0
Cellular_organisms - ps1
Archaea1 - ps2
Archaea2 - ps3
Archaea3 - ps4
Asgard/Eukaryota - ps5
Eukaryota - ps6
Amorpheae - ps7
Obazoa - ps8
Opisthokonta - ps9
Holozoa - ps10
Filozoa - ps11
Choano./Metazoa - ps12
Metazoa - ps13
Myriazoa - ps14
Parahoxozoa - ps15
Bilateria - ps16
Deuterostomia - ps17
Chordata - ps18
Olfactores - ps19
Vertebrata - ps20
Gnathostomata - ps21
Euteleostomi - ps22
Sarcopterygii - ps23
Tetrapoda - ps24
Amniota - ps25
Mammalia - ps26
Theria - ps27
Placentalia - ps28
Boreoeutheria - ps29
Euarchontoglires - ps30
Euarchonta - ps31
Primates - ps32
Haplorrhini - ps33
Simiiformes - ps34
Catarrhini - ps35
Hominoidea - ps36
Hominidae - ps37
Homininae - ps38
Hominini - ps39
Homo_sapiens - ps40
Phylostratum

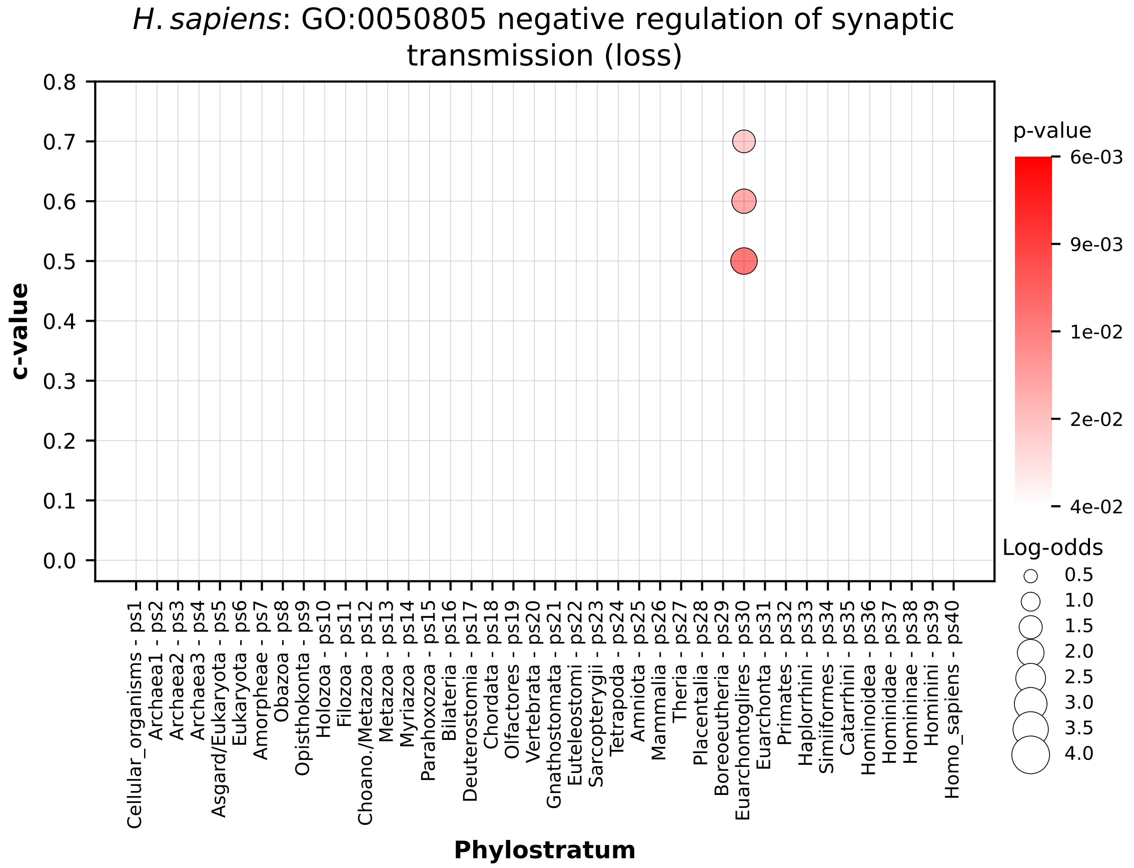

H. sapiens: GO:0050805 negative regulation of synaptic transmission (loss)
c-value
0.8
0.7
0.6
0.5
0.4
0.3
0.2
0.1
0.0
p-value
6e-03
9e-03
1e-02
2e-02
4e-02
Log-odds
0.5
1.0
1.5
2.0
2.5
3.0
3.5
4.0
Cellular_organisms - ps1
Archaea1 - ps2
Archaea2 - ps3
Archaea3 - ps4
Asgard/Eukaryota - ps5
Eukaryota - ps6
Amorpheae - ps7
Obazoa - ps8
Opisthokonta - ps9
Holozoa - ps10
Filozoa - ps11
Choano./Metazoa - ps12
Metazoa - ps13
Myriazoa - ps14
Parahoxozoa - ps15
Bilateria - ps16
Deuterostomia - ps17
Chordata - ps18
Olfactores - ps19
Vertebrata - ps20
Gnathostomata - ps21
Euteleostomi - ps22
Sarcopterygii - ps23
Tetrapoda - ps24
Amniota - ps25
Mammalia - ps26
Theria - ps27
Placentalia - ps28
Boreoeutheria - ps29
Euarchontoglires - ps30
Euarchonta - ps31
Primates - ps32
Haplorrhini - ps33
Simiiformes - ps34
Catarrhini - ps35
Hominoidea - ps36
Hominidae - ps37
Homininae - ps38
Hominini - ps39
Homo_sapiens - ps40
Phylostratum

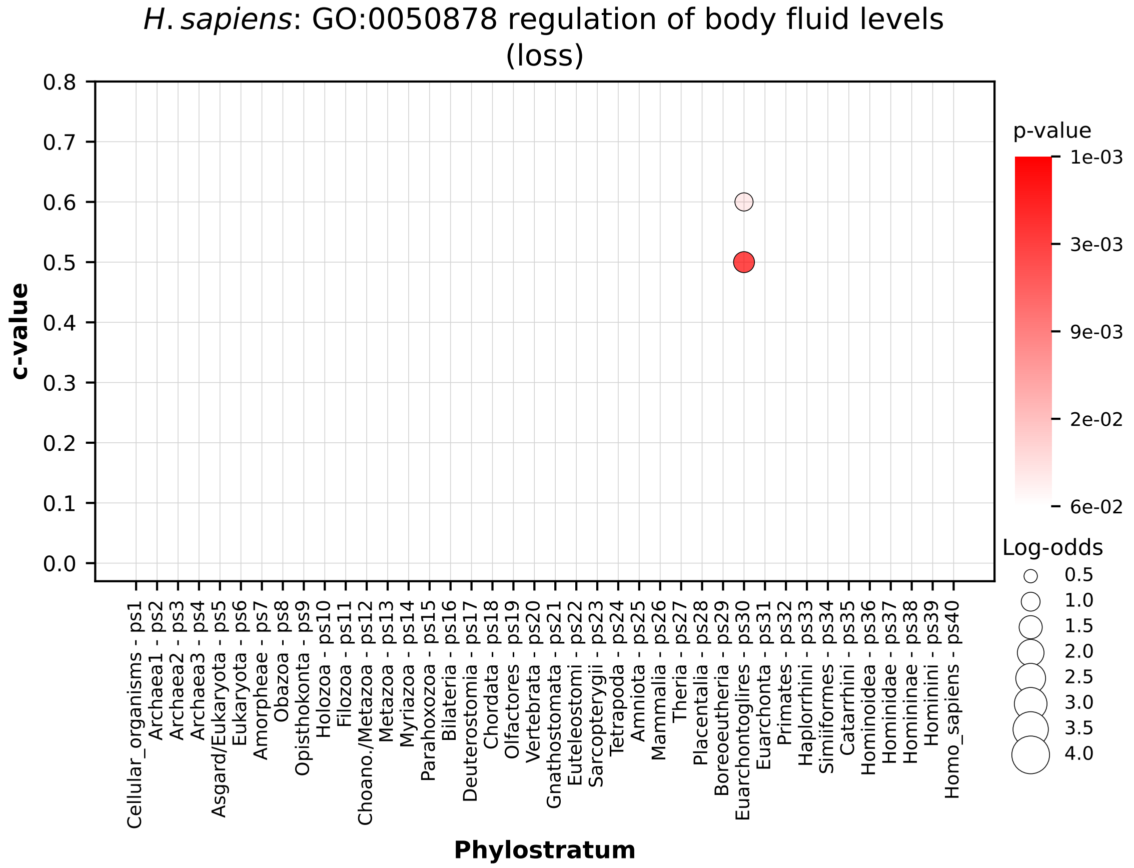

H. sapiens: GO:0050878 regulation of body fluid levels
(loss)
c-value
0.8
0.7
0.6
0.5
0.4
0.3
0.2
0.1
0.0
Phylostratum
Cellular_organisms - ps1
Archaea1 - ps2
Archaea2 - ps3
Archaea3 - ps4
Asgard/Eukaryota - ps5
Eukaryota - ps6
Amorpheae - ps7
Obazoa - ps8
Opisthokonta - ps9
Holozoa - ps10
Filozoa - ps11
Choano./Metazoa - ps12
Metazoa - ps13
Myriazoa - ps14
Parahoxozoa - ps15
Bilateria - ps16
Deuterostomia - ps17
Chordata - ps18
Olfactores - ps19
Vertebrata - ps20
Gnathostomata - ps21
Euteleostomi - ps22
Sarcopterygii - ps23
Tetrapoda - ps24
Amniota - ps25
Mammalia - ps26
Theria - ps27
Placentalia - ps28
Boreoeutheria - ps29
Euarchontoglires - ps30
Euarchonta - ps31
Primates - ps32
Haplorrhini - ps33
Simiiformes - ps34
Catarrhini - ps35
Hominoidea - ps36
Hominidae - ps37
Homininae - ps38
Hominini - ps39
Homo_sapiens - ps40
p-value
1e-03
3e-03
9e-03
2e-02
6e-02
Log-odds
0.5
1.0
1.5
2.0
2.5
3.0
3.5
4.0

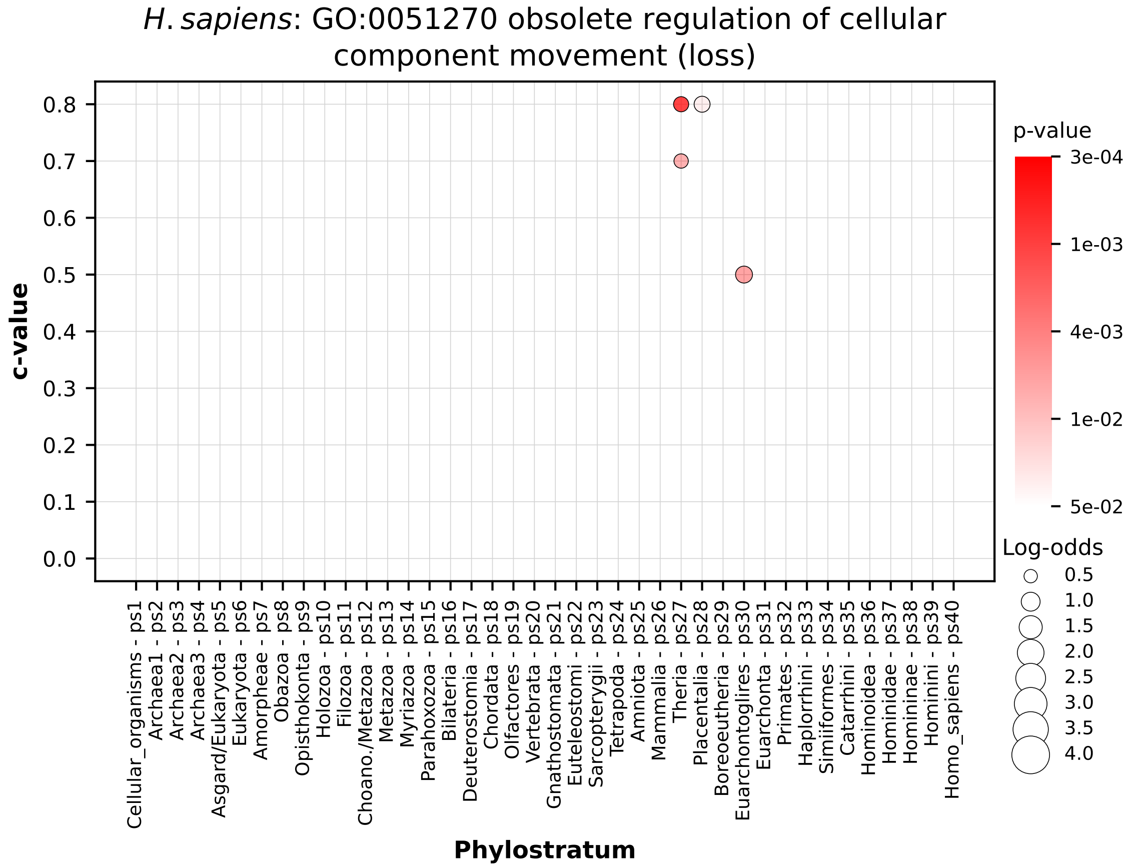
H. sapiens: GO:0051270 obsolete regulation of cellular component movement (loss)
c-value
0.0
0.1
0.2
0.3
0.4
0.5
0.6
0.7
0.8
p-value
3e-04
1e-03
4e-03
1e-02
5e-02
Log-odds
0.5
1.0
1.5
2.0
2.5
3.0
3.5
4.0
Cellular_organisms - ps1
Archaea1 - ps2
Archaea2 - ps3
Archaea3 - ps4
Asgard/Eukaryota - ps5
Eukaryota - ps6
Amorpheae - ps7
Obazoa - ps8
Opisthokonta - ps9
Holozoa - ps10
Filozoa - ps11
Choano./Metazoa - ps12
Metazoa - ps13
Myriazoa - ps14
Parahoxozoa - ps15
Bilateria - ps16
Deuterostomia - ps17
Chordata - ps18
Olfactores - ps19
Vertebrata - ps20
Gnathostomata - ps21
Euteleostomi - ps22
Sarcopterygii - ps23
Tetrapoda - ps24
Amniota - ps25
Mammalia - ps26
Theria - ps27
Placentalia - ps28
Boreoeutheria - ps29
Euarchontoglires - ps30
Euarchonta - ps31
Primates - ps32
Haplorrhini - ps33
Simiiformes - ps34
Catarrhini - ps35
Hominoidea - ps36
Hominidae - ps37
Homininae - ps38
Hominini - ps39
Homo_sapiens - ps40
Phylostratum

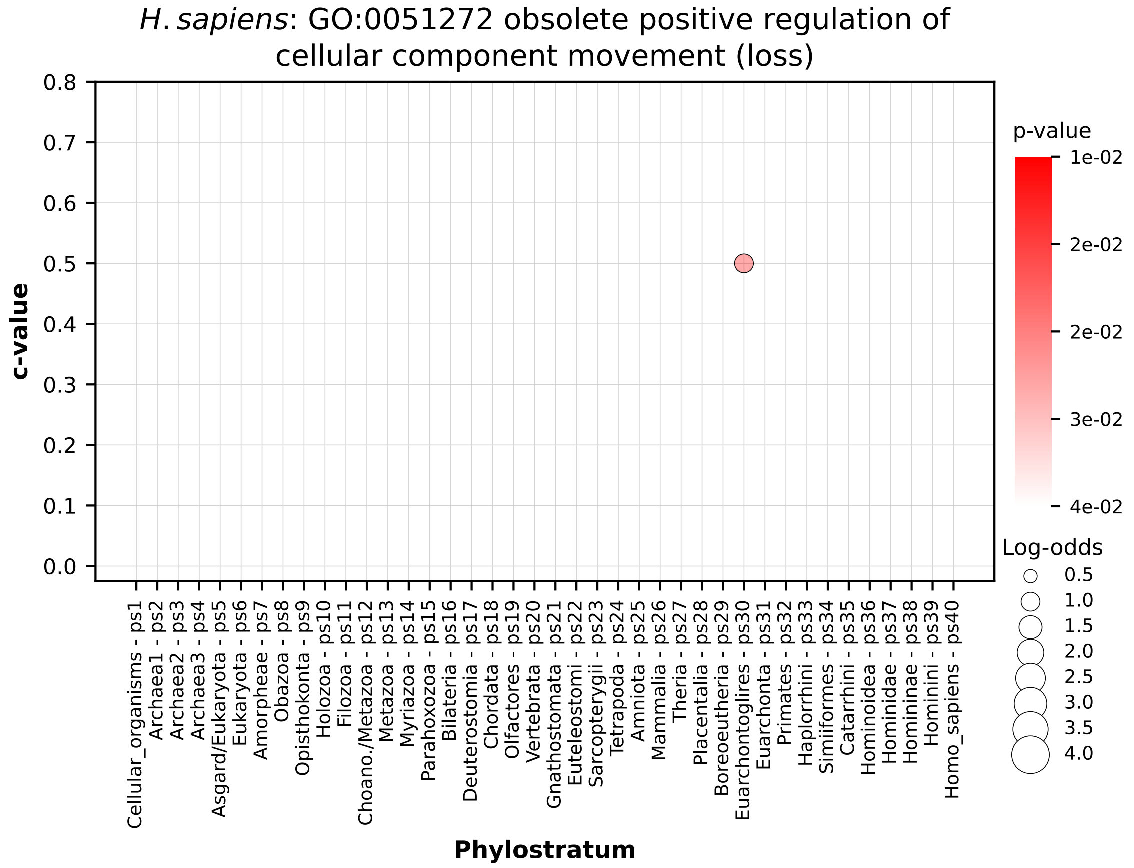

H. sapiens: GO:0051272 obsolete positive regulation of cellular component movement (loss)
c-value
0.0
0.1
0.2
0.3
0.4
0.5
0.6
0.7
0.8
Phylostratum
Cellular_organisms - ps1
Archaea1 - ps2
Archaea2 - ps3
Archaea3 - ps4
Asgard/Eukaryota - ps5
Eukaryota - ps6
Amorpheae - ps7
Obazoa - ps8
Opisthokonta - ps9
Holozoa - ps10
Filozoa - ps11
Choano./Metazoa - ps12
Metazoa - ps13
Myriazoa - ps14
Parahoxozoa - ps15
Bilateria - ps16
Deuterostomia - ps17
Chordata - ps18
Olfactores - ps19
Vertebrata - ps20
Gnathostomata - ps21
Euteleostomi - ps22
Sarcopterygii - ps23
Tetrapoda - ps24
Amniota - ps25
Mammalia - ps26
Theria - ps27
Placentalia - ps28
Boreoeutheria - ps29
Euarchontoglires - ps30
Euarchonta - ps31
Primates - ps32
Haplorrhini - ps33
Simiiformes - ps34
Catarrhini - ps35
Hominoidea - ps36
Hominidae - ps37
Homininae - ps38
Hominini - ps39
Homo_sapiens - ps40
p-value
1e-02
2e-02
2e-02
3e-02
4e-02
Log-odds
0.5
1.0
1.5
2.0
2.5
3.0
3.5
4.0

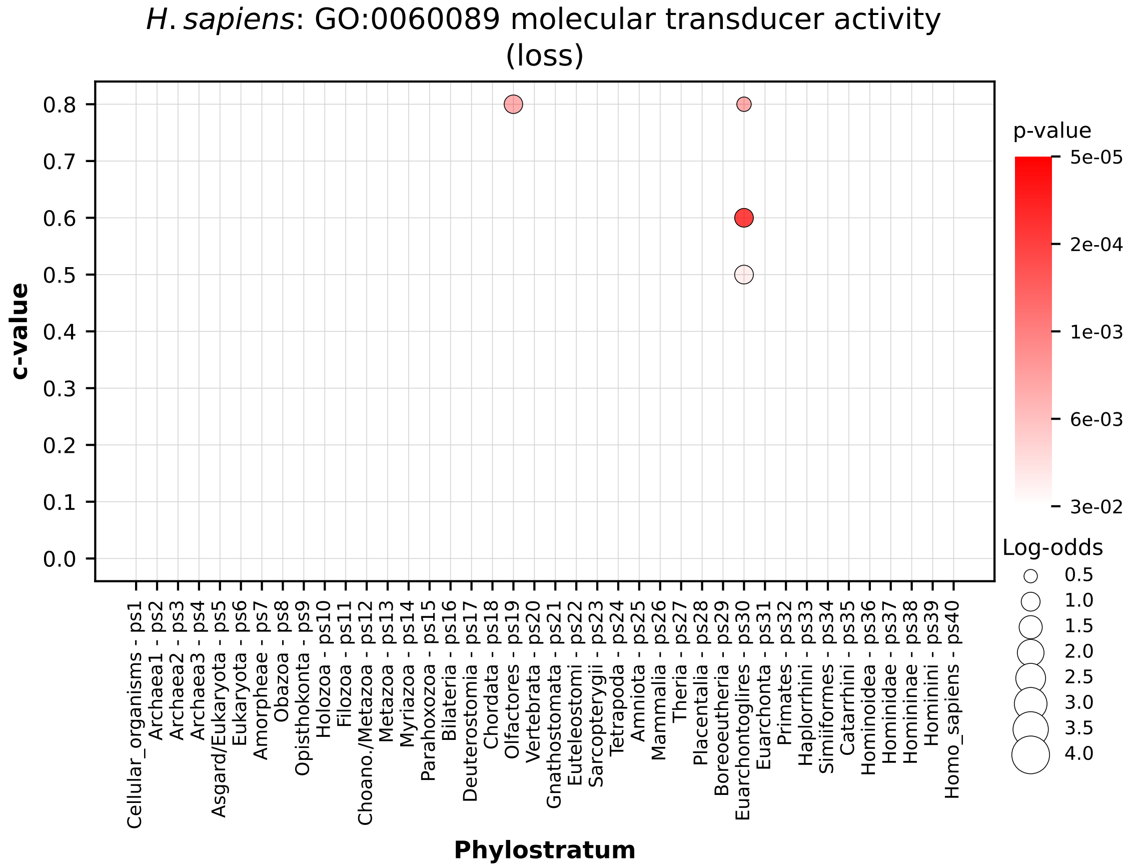

H. sapiens: GO:0060089 molecular transducer activity (loss)
c-value
0.0
0.1
0.2
0.3
0.4
0.5
0.6
0.7
0.8
Phylostratum
Cellular_organisms - ps1
Archaea1 - ps2
Archaea2 - ps3
Archaea3 - ps4
Asgard/Eukaryota - ps5
Eukaryota - ps6
Amorpheae - ps7
Obazoa - ps8
Opisthokonta - ps9
Holozoa - ps10
Filozoa - ps11
Choano./Metazoa - ps12
Metazoa - ps13
Myriazoa - ps14
Parahoxozoa - ps15
Bilateria - ps16
Deuterostomia - ps17
Chordata - ps18
Olfactores - ps19
Vertebrata - ps20
Gnathostomata - ps21
Euteleostomi - ps22
Sarcopterygii - ps23
Tetrapoda - ps24
Amniota - ps25
Mammalia - ps26
Theria - ps27
Placentalia - ps28
Boreoeutheria - ps29
Euarchontoglires - ps30
Euarchonta - ps31
Primates - ps32
Haplorrhini - ps33
Simiiformes - ps34
Catarrhini - ps35
Hominoidea - ps36
Hominidae - ps37
Homininae - ps38
Hominini - ps39
Homo_sapiens - ps40
p-value
5e-05
2e-04
1e-03
6e-03
3e-02
Log-odds
0.5
1.0
1.5
2.0
2.5
3.0
3.5
4.0

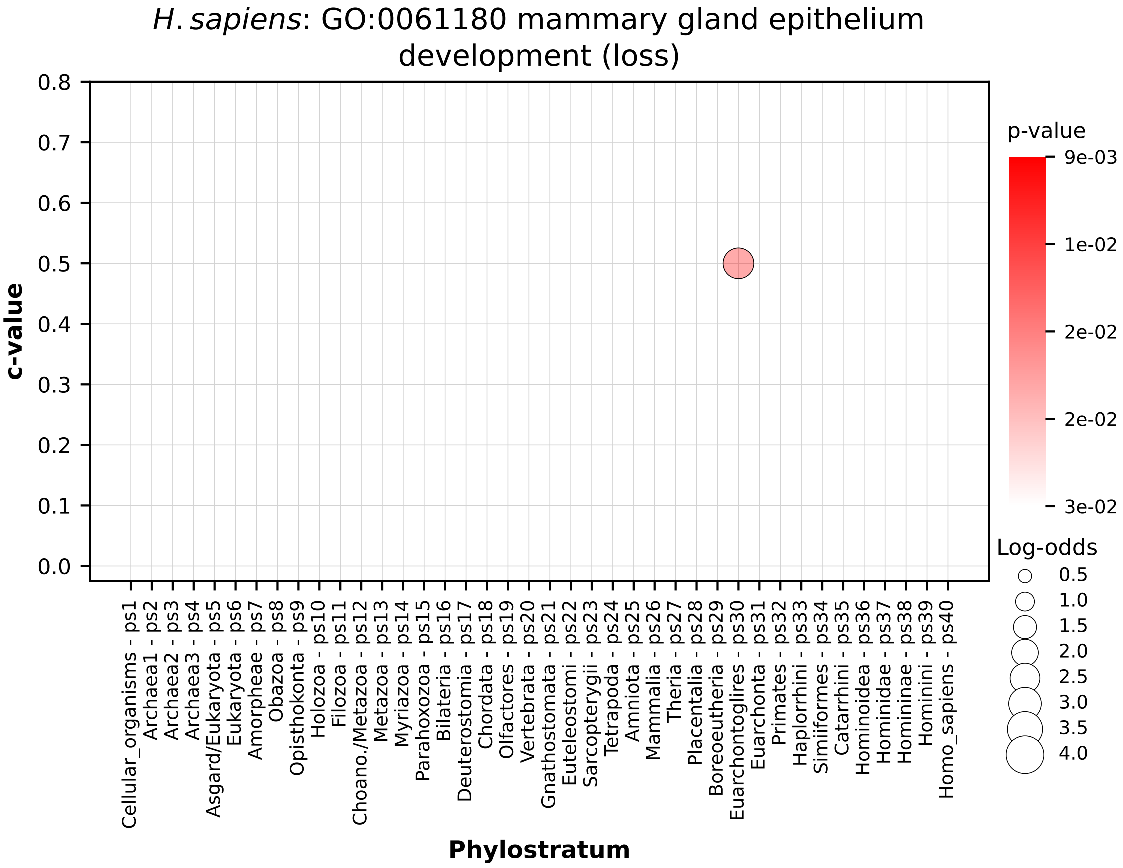
H. sapiens: GO:0061180 mammary gland epithelium development (loss)
c-value
0.0
0.1
0.2
0.3
0.4
0.5
0.6
0.7
0.8
Phylostratum
Cellular_organisms - ps1
Archaea1 - ps2
Archaea2 - ps3
Archaea3 - ps4
Asgard/Eukaryota - ps5
Eukaryota - ps6
Amorpheae - ps7
Obazoa - ps8
Opisthokonta - ps9
Holozoa - ps10
Filozoa - ps11
Choano./Metazoa - ps12
Metazoa - ps13
Myriazoa - ps14
Parahoxozoa - ps15
Bilateria - ps16
Deuterostomia - ps17
Chordata - ps18
Olfactores - ps19
Vertebrata - ps20
Gnathostomata - ps21
Euteleostomi - ps22
Sarcopterygii - ps23
Tetrapoda - ps24
Amniota - ps25
Mammalia - ps26
Theria - ps27
Placentalia - ps28
Boreoeutheria - ps29
Euarchontoglires - ps30
Euarchonta - ps31
Primates - ps32
Haplorrhini - ps33
Simiiformes - ps34
Catarrhini - ps35
Hominoidea - ps36
Hominidae - ps37
Homininae - ps38
Hominini - ps39
Homo_sapiens - ps40
p-value
9e-03
1e-02
2e-02
2e-02
3e-02
Log-odds
0.5
1.0
1.5
2.0
2.5
3.0
3.5
4.0

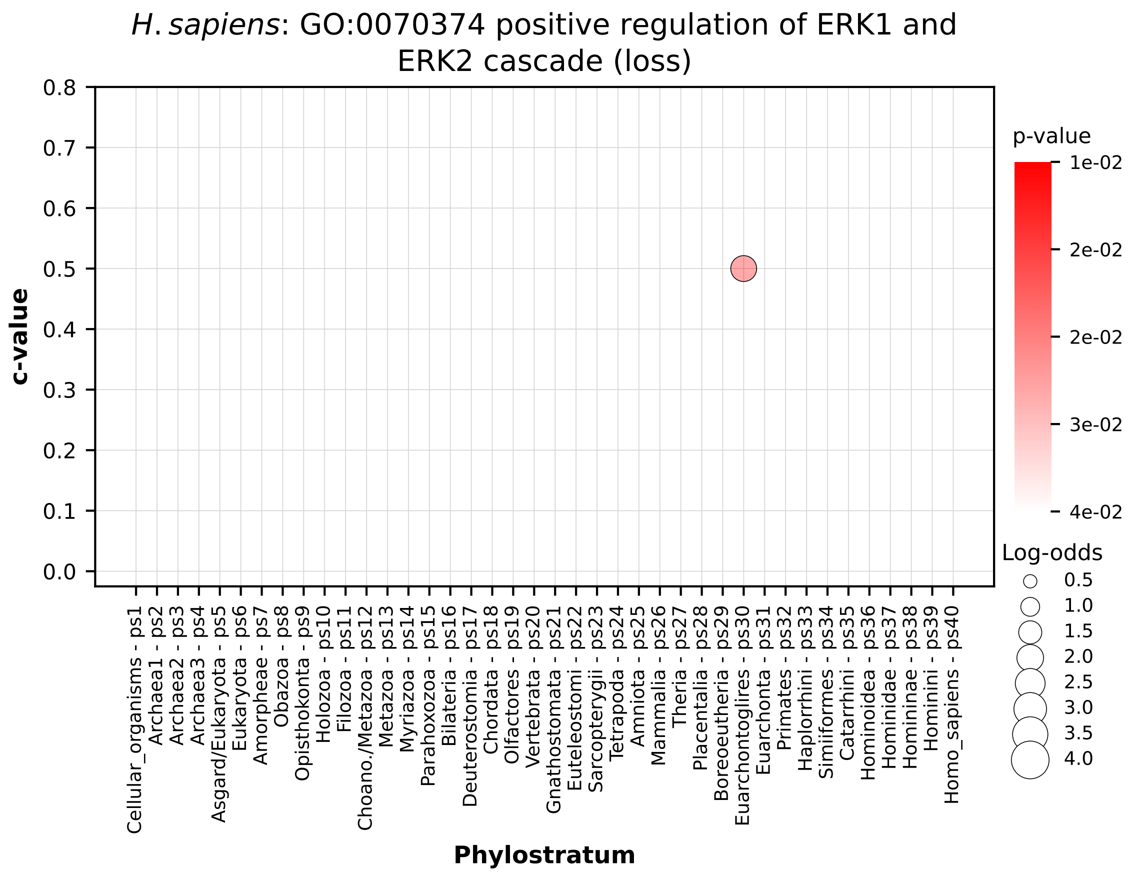

H. sapiens: GO:0070374 positive regulation of ERK1 and ERK2 cascade (loss)
c-value
0.0
0.1
0.2
0.3
0.4
0.5
0.6
0.7
0.8
Phylostratum
Cellular_organisms - ps1
Archaea1 - ps2
Archaea2 - ps3
Archaea3 - ps4
Asgard/Eukaryota - ps5
Eukaryota - ps6
Amorpheae - ps7
Obazoa - ps8
Opisthokonta - ps9
Holozoa - ps10
Filozoa - ps11
Choano./Metazoa - ps12
Metazoa - ps13
Myriazoa - ps14
Parahoxozoa - ps15
Bilateria - ps16
Deuterostomia - ps17
Chordata - ps18
Olfactores - ps19
Vertebrata - ps20
Gnathostomata - ps21
Euteleostomi - ps22
Sarcopterygii - ps23
Tetrapoda - ps24
Amniota - ps25
Mammalia - ps26
Theria - ps27
Placentalia - ps28
Boreoeutheria - ps29
Euarchontoglires - ps30
Euarchonta - ps31
Primates - ps32
Haplorrhini - ps33
Simiiformes - ps34
Catarrhini - ps35
Hominoidea - ps36
Hominidae - ps37
Homininae - ps38
Hominini - ps39
Homo_sapiens - ps40
p-value
1e-02
2e-02
2e-02
3e-02
4e-02
Log-odds
0.5
1.0
1.5
2.0
2.5
3.0
3.5
4.0

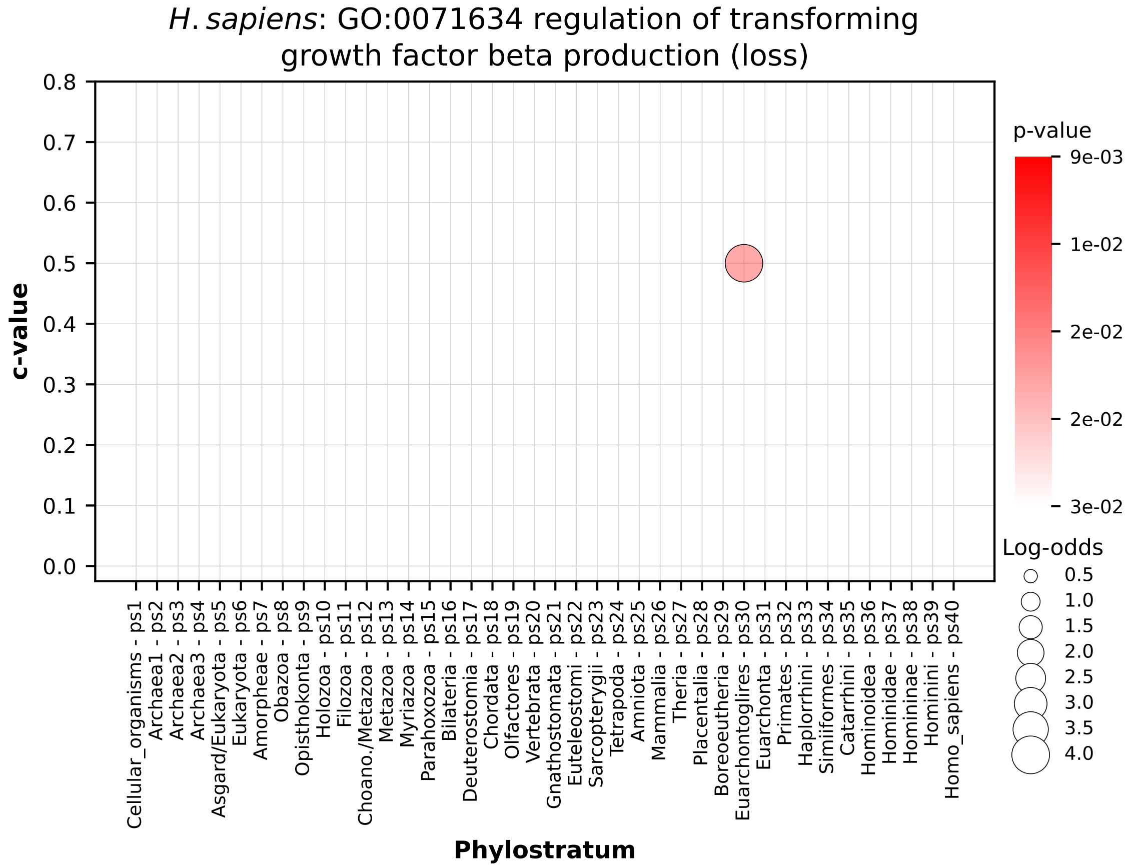
H. sapiens: GO:0071634 regulation of transforming growth factor beta production (loss)
c-value
0.0
0.1
0.2
0.3
0.4
0.5
0.6
0.7
0.8
Phylostratum
Cellular_organisms - ps1
Archaea1 - ps2
Archaea2 - ps3
Archaea3 - ps4
Asgard/Eukaryota - ps5
Eukaryota - ps6
Amorpheae - ps7
Obazoa - ps8
Opisthokonta - ps9
Holozoa - ps10
Filozoa - ps11
Choano./Metazoa - ps12
Metazoa - ps13
Myriazoa - ps14
Parahoxozoa - ps15
Bilateria - ps16
Deuterostomia - ps17
Chordata - ps18
Olfactores - ps19
Vertebrata - ps20
Gnathostomata - ps21
Euteleostomi - ps22
Sarcopterygii - ps23
Tetrapoda - ps24
Amniota - ps25
Mammalia - ps26
Theria - ps27
Placentalia - ps28
Boreoeutheria - ps29
Euarchontoglires - ps30
Euarchonta - ps31
Primates - ps32
Haplorrhini - ps33
Simiiformes - ps34
Catarrhini - ps35
Hominoidea - ps36
Hominidae - ps37
Homininae - ps38
Hominini - ps39
Homo_sapiens - ps40
p-value
9e-03
1e-02
2e-02
2e-02
3e-02
Log-odds
0.5
1.0
1.5
2.0
2.5
3.0
3.5
4.0

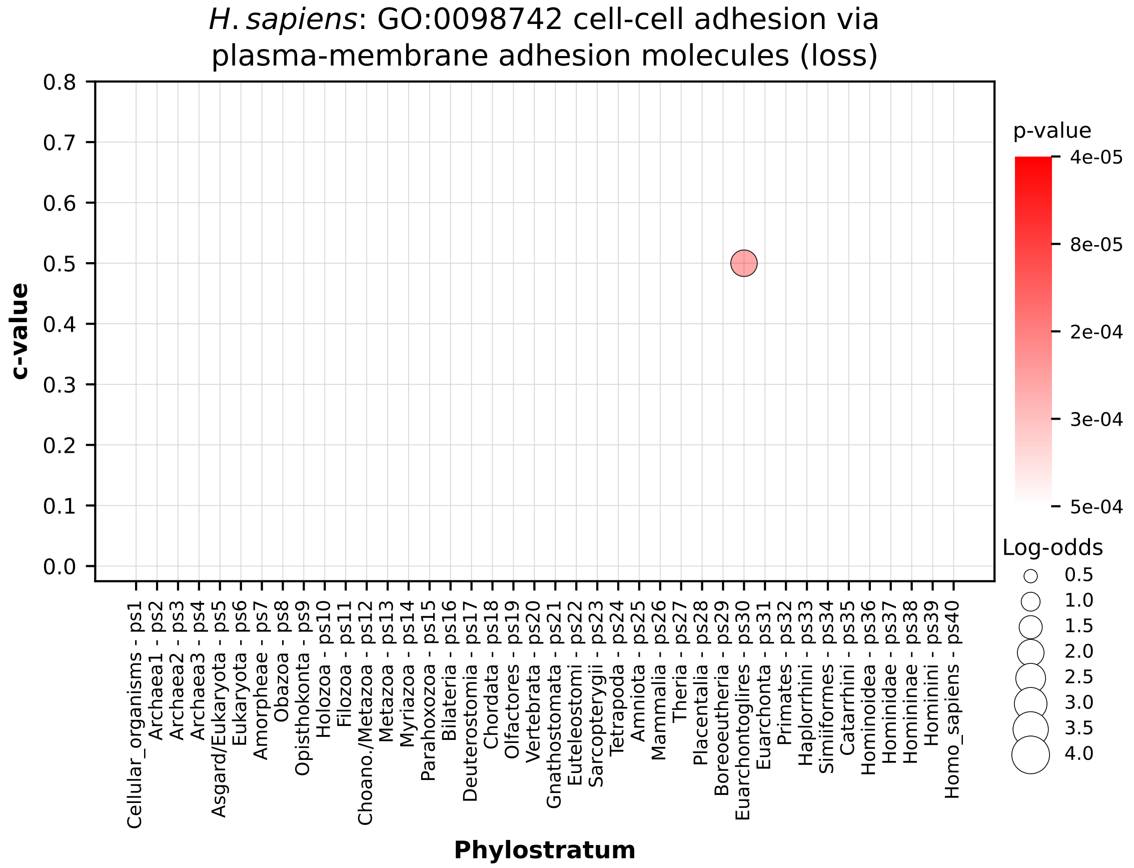

H. sapiens: GO:0098742 cell-cell adhesion via plasma-membrane adhesion molecules (loss)
c-value
0.8
0.7
0.6
0.5
0.4
0.3
0.2
0.1
0.0
Phylostratum
Cellular_organisms - ps1
Archaea1 - ps2
Archaea2 - ps3
Archaea3 - ps4
Asgard/Eukaryota - ps5
Eukaryota - ps6
Amorpheae - ps7
Obazoa - ps8
Opisthokonta - ps9
Holozoa - ps10
Filozoa - ps11
Choano./Metazoa - ps12
Metazoa - ps13
Myriazoa - ps14
Parahoxozoa - ps15
Bilateria - ps16
Deuterostomia - ps17
Chordata - ps18
Olfactores - ps19
Vertebrata - ps20
Gnathostomata - ps21
Euteleostomi - ps22
Sarcopterygii - ps23
Tetrapoda - ps24
Amniota - ps25
Mammalia - ps26
Theria - ps27
Placentalia - ps28
Boreoeutheria - ps29
Euarchontoglires - ps30
Euarchonta - ps31
Primates - ps32
Haplorrhini - ps33
Simiiformes - ps34
Catarrhini - ps35
Hominoidea - ps36
Hominidae - ps37
Homininae - ps38
Hominini - ps39
Homo_sapiens - ps40
p-value
4e-05
8e-05
2e-04
3e-04
5e-04
Log-odds
0.5
1.0
1.5
2.0
2.5
3.0
3.5
4.0

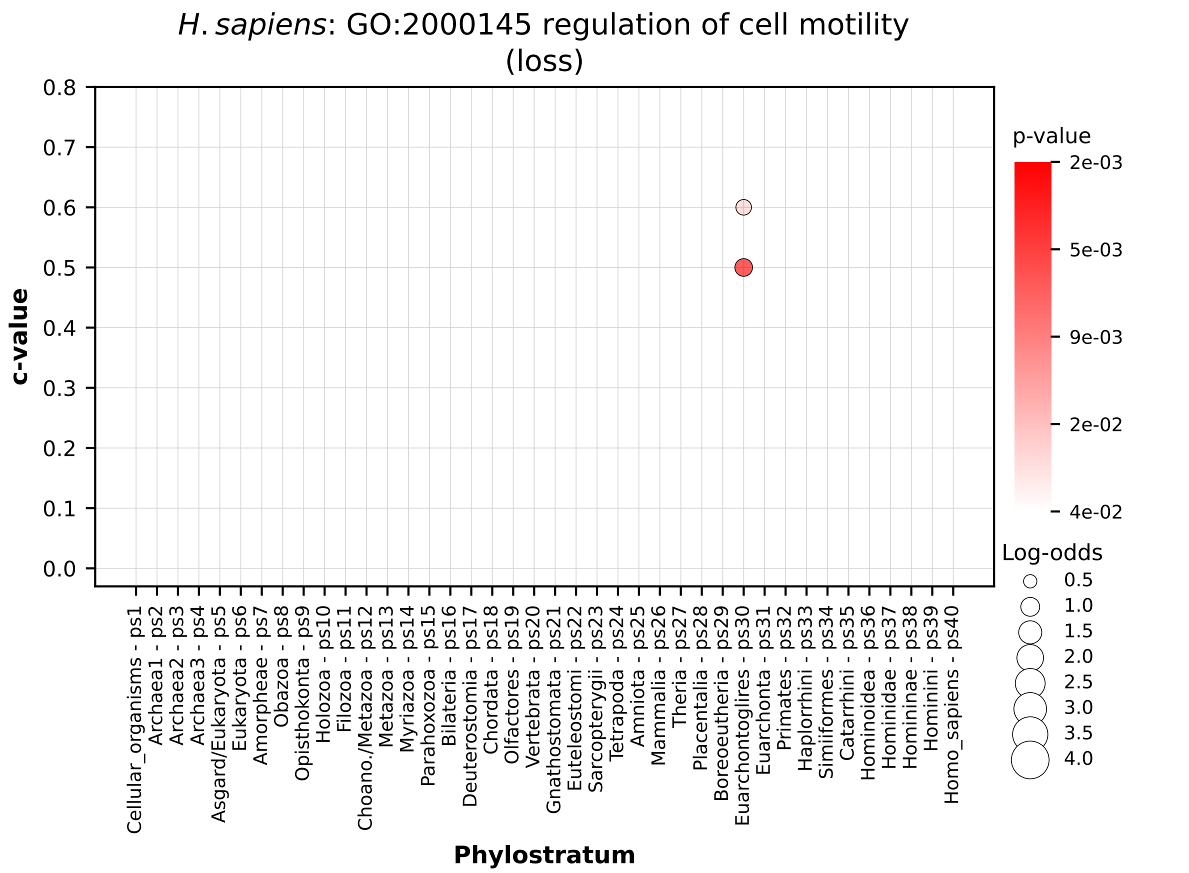

H. sapiens: GO:2000145 regulation of cell motility
(loss)
c-value
0.8
0.7
0.6
0.5
0.4
0.3
0.2
0.1
0.0
Phylostratum
Cellular_organisms - ps1
Archaea1 - ps2
Archaea2 - ps3
Archaea3 - ps4
Asgard/Eukaryota - ps5
Eukaryota - ps6
Amorpheae - ps7
Obazoa - ps8
Opisthokonta - ps9
Holozoa - ps10
Filozoa - ps11
Choano./Metazoa - ps12
Metazoa - ps13
Myriazoa - ps14
Parahoxozoa - ps15
Bilateria - ps16
Deuterostomia - ps17
Chordata - ps18
Olfactores - ps19
Vertebrata - ps20
Gnathostomata - ps21
Euteleostomi - ps22
Sarcopterygii - ps23
Tetrapoda - ps24
Amniota - ps25
Mammalia - ps26
Theria - ps27
Placentalia - ps28
Boreoeutheria - ps29
Euarchontoglires - ps30
Euarchonta - ps31
Primates - ps32
Haplorrhini - ps33
Simiiformes - ps34
Catarrhini - ps35
Hominoidea - ps36
Hominidae - ps37
Homininae - ps38
Hominini - ps39
Homo_sapiens - ps40
p-value
2e-03
5e-03
9e-03
2e-02
4e-02
Log-odds
0.5
1.0
1.5
2.0
2.5
3.0
3.5
4.0

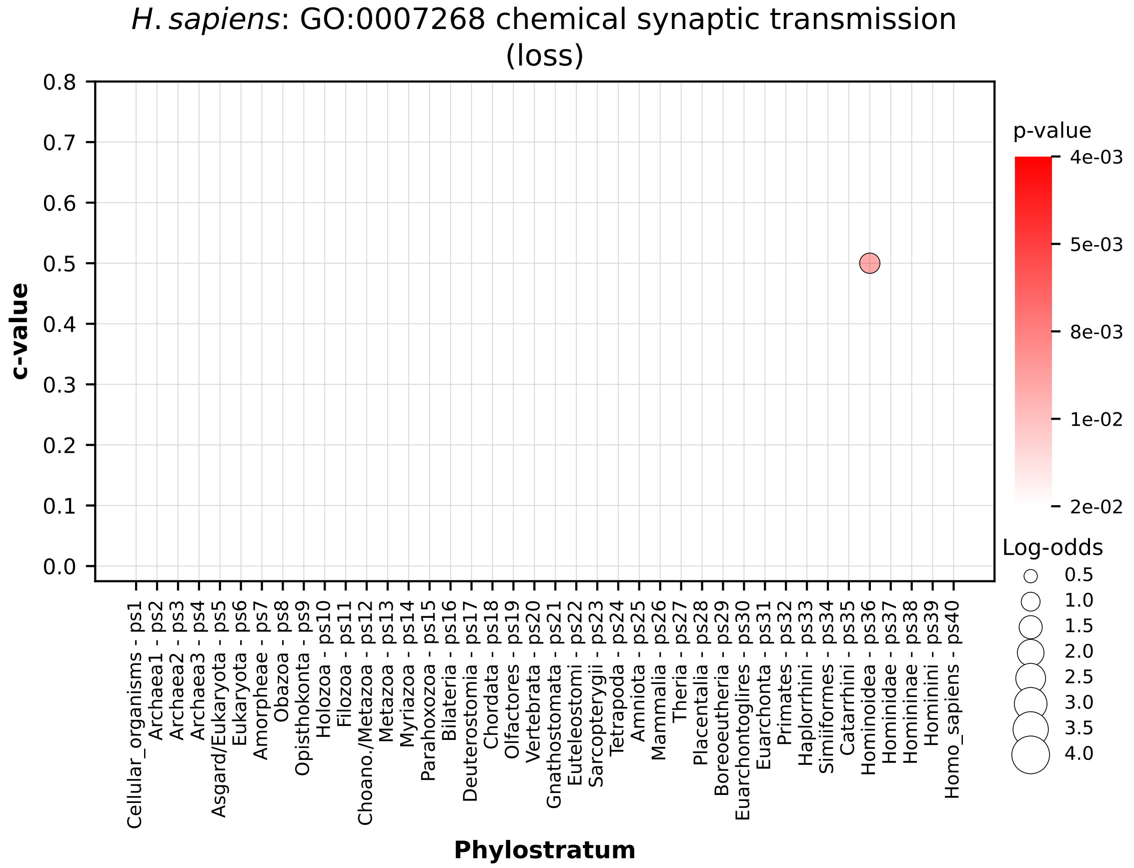
H. sapiens: GO:0007268 chemical synaptic transmission (loss)
c-value
0.0
0.1
0.2
0.3
0.4
0.5
0.6
0.7
0.8
Phylostratum
Cellular_organisms - ps1
Archaea1 - ps2
Archaea2 - ps3
Archaea3 - ps4
Asgard/Eukaryota - ps5
Eukaryota - ps6
Amorpheae - ps7
Obazoa - ps8
Opisthokonta - ps9
Holozoa - ps10
Filozoa - ps11
Choano./Metazoa - ps12
Metazoa - ps13
Myriazoa - ps14
Parahoxozoa - ps15
Bilateria - ps16
Deuterostomia - ps17
Chordata - ps18
Olfactores - ps19
Vertebrata - ps20
Gnathostomata - ps21
Euteleostomi - ps22
Sarcopterygii - ps23
Tetrapoda - ps24
Amniota - ps25
Mammalia - ps26
Theria - ps27
Placentalia - ps28
Boreoeutheria - ps29
Euarchontoglires - ps30
Euarchonta - ps31
Primates - ps32
Haplorrhini - ps33
Simiiformes - ps34
Catarrhini - ps35
Hominoidea - ps36
Hominidae - ps37
Homininae - ps38
Hominini - ps39
Homo_sapiens - ps40
p-value
4e-03
5e-03
8e-03
1e-02
2e-02
Log-odds
0.5
1.0
1.5
2.0
2.5
3.0
3.5
4.0

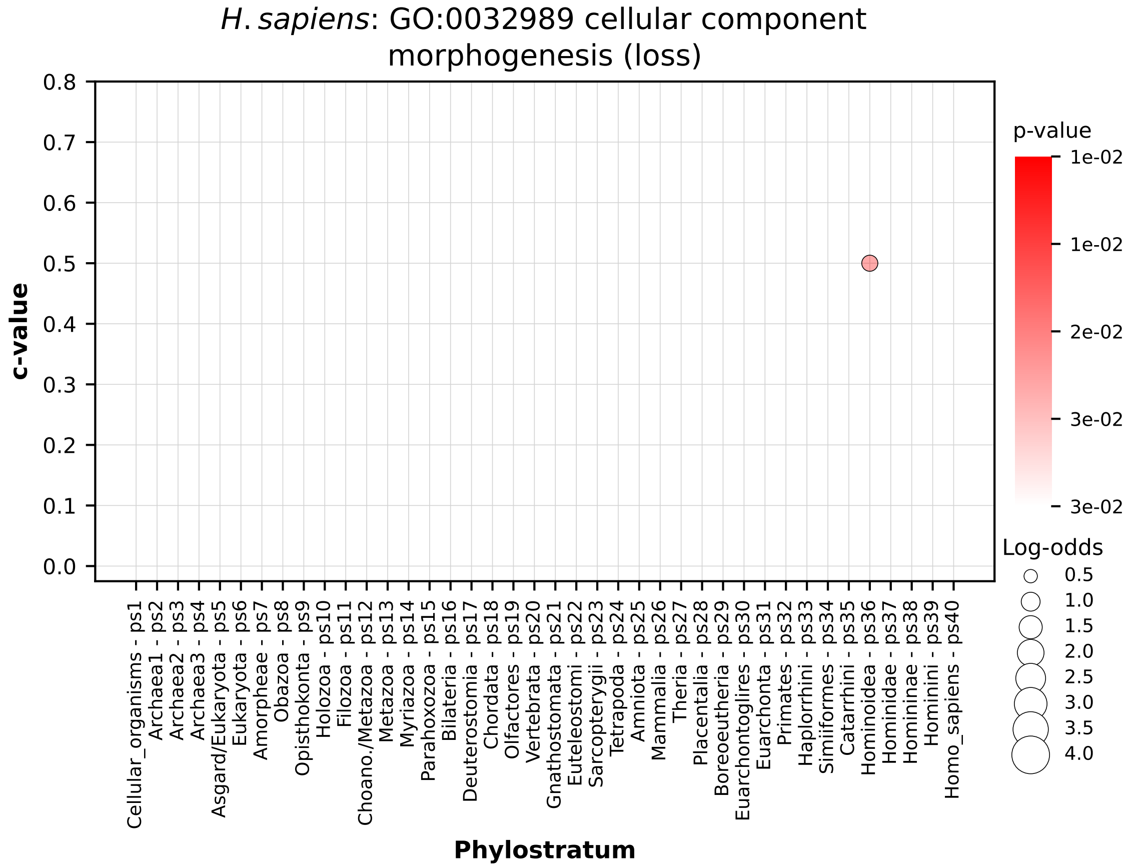

H. sapiens: GO:0032989 cellular component morphogenesis (loss)
c-value
0.0
0.1
0.2
0.3
0.4
0.5
0.6
0.7
0.8
Phylostratum
Cellular_organisms - ps1
Archaea1 - ps2
Archaea2 - ps3
Archaea3 - ps4
Asgard/Eukaryota - ps5
Eukaryota - ps6
Amorpheae - ps7
Obazoa - ps8
Opisthokonta - ps9
Holozoa - ps10
Filozoa - ps11
Choano./Metazoa - ps12
Metazoa - ps13
Myriazoa - ps14
Parahoxozoa - ps15
Bilateria - ps16
Deuterostomia - ps17
Chordata - ps18
Olfactores - ps19
Vertebrata - ps20
Gnathostomata - ps21
Euteleostomi - ps22
Sarcopterygii - ps23
Tetrapoda - ps24
Amniota - ps25
Mammalia - ps26
Theria - ps27
Placentalia - ps28
Boreoeutheria - ps29
Euarchontoglires - ps30
Euarchonta - ps31
Primates - ps32
Haplorrhini - ps33
Simiiformes - ps34
Catarrhini - ps35
Hominoidea - ps36
Hominidae - ps37
Homininae - ps38
Hominini - ps39
Homo_sapiens - ps40
p-value
1e-02
1e-02
2e-02
3e-02
3e-02
Log-odds
0.5
1.0
1.5
2.0
2.5
3.0
3.5
4.0

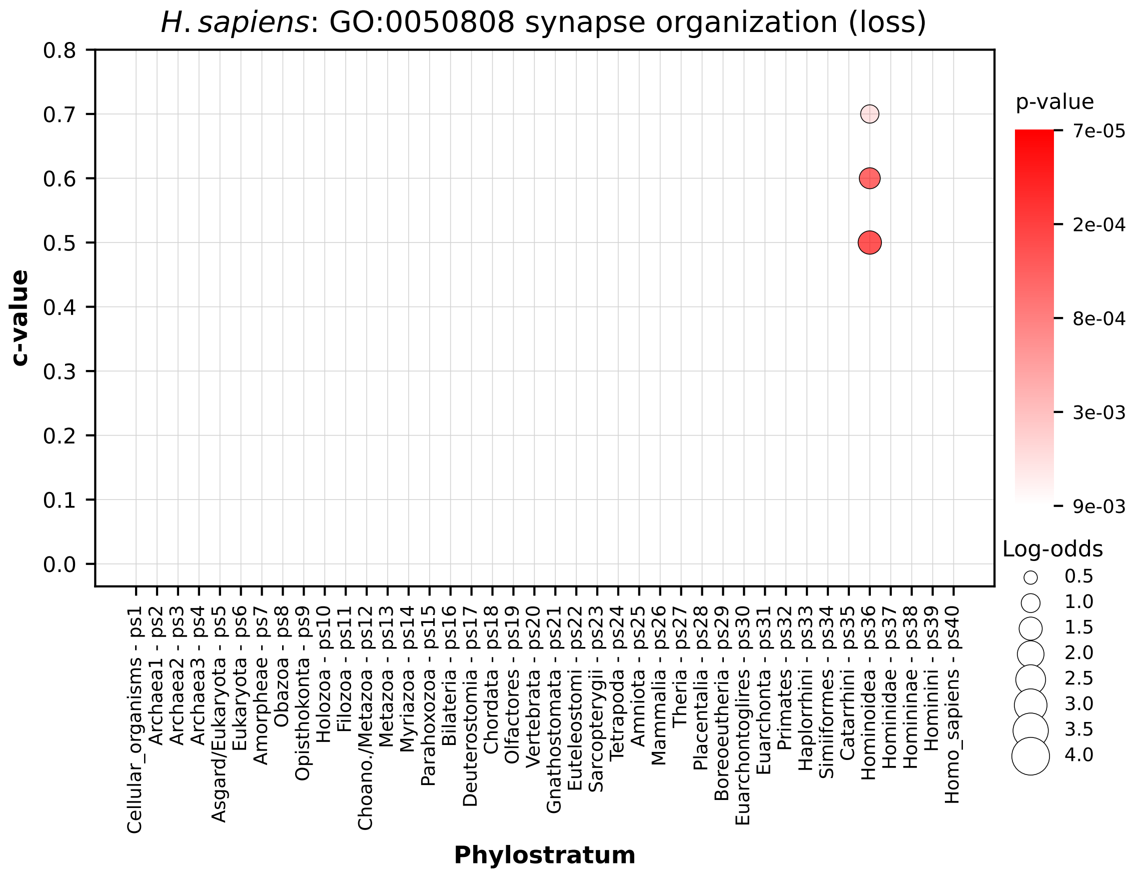

H. sapiens: GO:0050808 synapse organization (loss)
c-value
0.8
0.7
0.6
0.5
0.4
0.3
0.2
0.1
0.0
p-value
7e-05
2e-04
8e-04
3e-03
9e-03
Log-odds
0.5
1.0
1.5
2.0
2.5
3.0
3.5
4.0
Cellular_organisms - ps1
Archaea1 - ps2
Archaea2 - ps3
Archaea3 - ps4
Asgard/Eukaryota - ps5
Eukaryota - ps6
Amorpheae - ps7
Obazoa - ps8
Opisthokonta - ps9
Holozoa - ps10
Filozoa - ps11
Choano./Metazoa - ps12
Metazoa - ps13
Myriazoa - ps14
Parahoxozoa - ps15
Bilateria - ps16
Deuterostomia - ps17
Chordata - ps18
Olfactores - ps19
Vertebrata - ps20
Gnathostomata - ps21
Euteleostomi - ps22
Sarcopterygii - ps23
Tetrapoda - ps24
Amniota - ps25
Mammalia - ps26
Theria - ps27
Placentalia - ps28
Boreoeutheria - ps29
Euarchontoglires - ps30
Euarchonta - ps31
Primates - ps32
Haplorrhini - ps33
Simiiformes - ps34
Catarrhini - ps35
Hominoidea - ps36
Hominidae - ps37
Homininae - ps38
Hominini - ps39
Homo_sapiens - ps40
Phylostratum

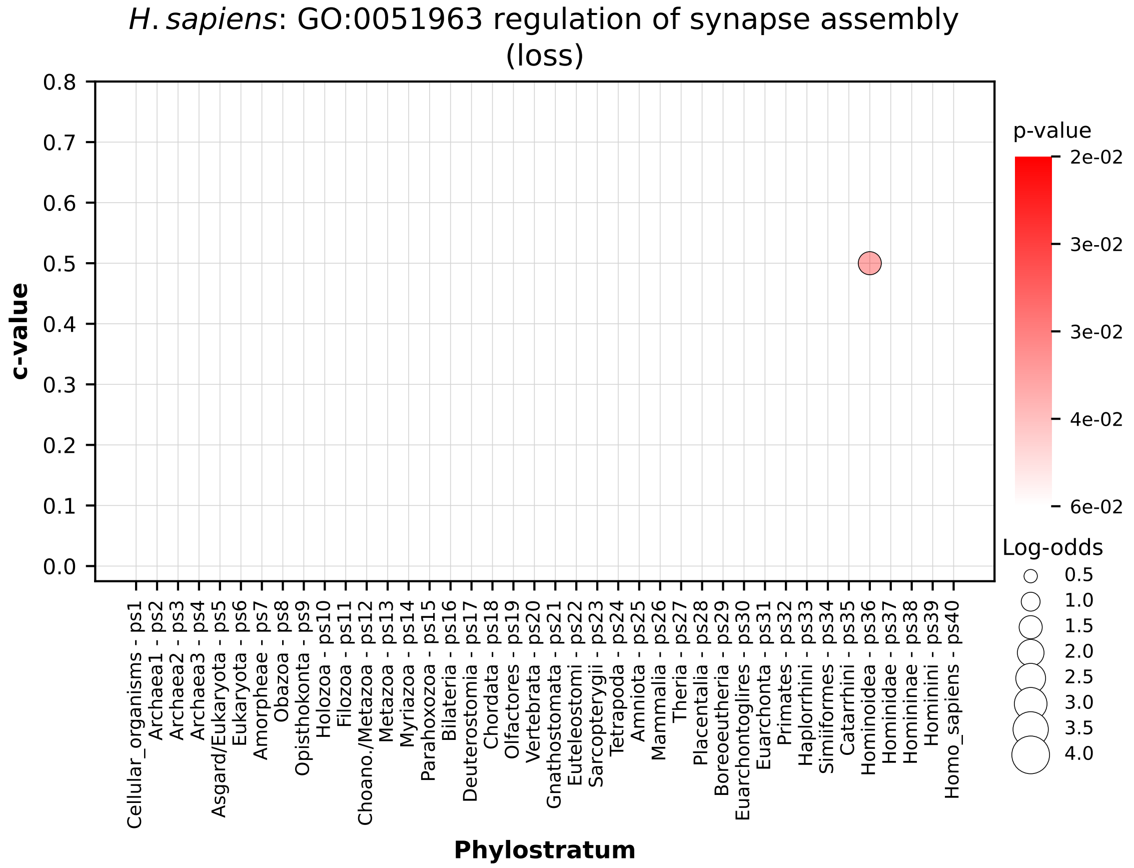
H. sapiens: GO:0051963 regulation of synapse assembly (loss)
c-value
0.8
0.7
0.6
0.5
0.4
0.3
0.2
0.1
0.0
p-value
2e-02
3e-02
3e-02
4e-02
6e-02
Log-odds
0.5
1.0
1.5
2.0
2.5
3.0
3.5
4.0
Cellular_organisms - ps1
Archaea1 - ps2
Archaea2 - ps3
Archaea3 - ps4
Asgard/Eukaryota - ps5
Eukaryota - ps6
Amorpheae - ps7
Obazoa - ps8
Opisthokonta - ps9
Holozoa - ps10
Filozoa - ps11
Choano./Metazoa - ps12
Metazoa - ps13
Myriazoa - ps14
Parahoxozoa - ps15
Bilateria - ps16
Deuterostomia - ps17
Chordata - ps18
Olfactores - ps19
Vertebrata - ps20
Gnathostomata - ps21
Euteleostomi - ps22
Sarcopterygii - ps23
Tetrapoda - ps24
Amniota - ps25
Mammalia - ps26
Theria - ps27
Placentalia - ps28
Boreoeutheria - ps29
Euarchontoglires - ps30
Euarchonta - ps31
Primates - ps32
Haplorrhini - ps33
Simiiformes - ps34
Catarrhini - ps35
Hominoidea - ps36
Hominidae - ps37
Homininae - ps38
Hominini - ps39
Homo_sapiens - ps40
Phylostratum

H. sapiens: GO:0098916 anterograde trans-synaptic signaling (loss)

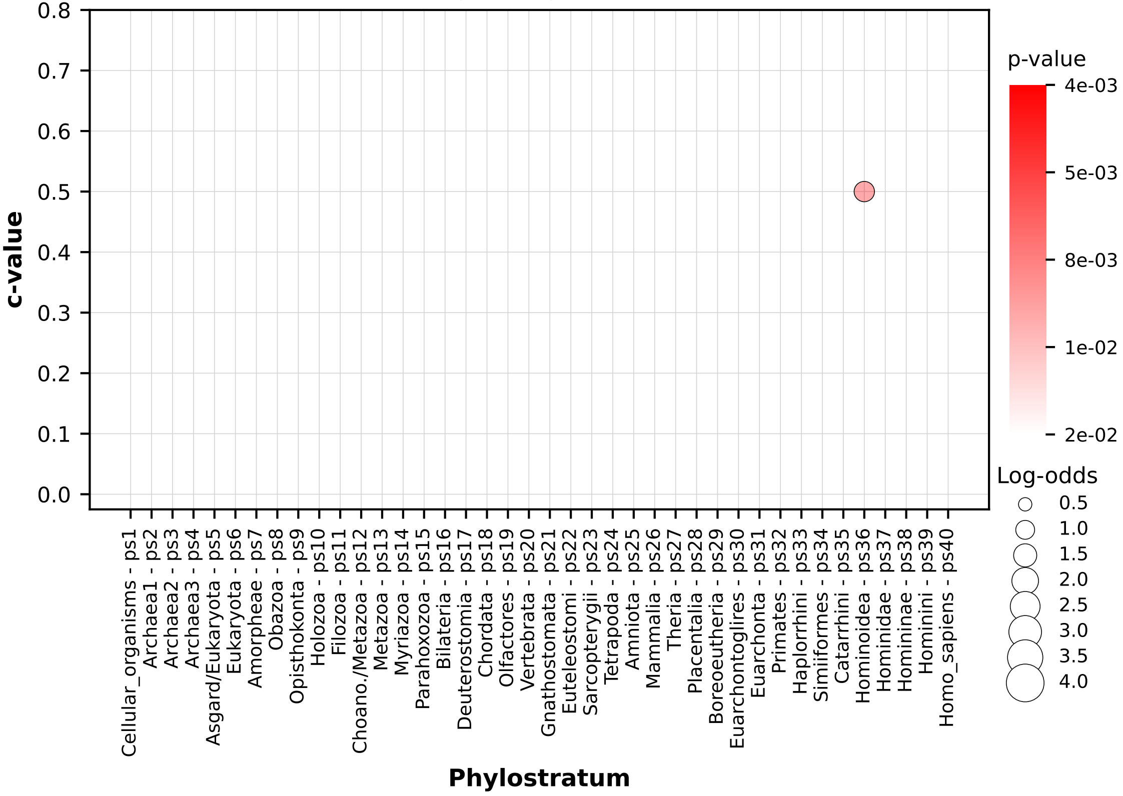

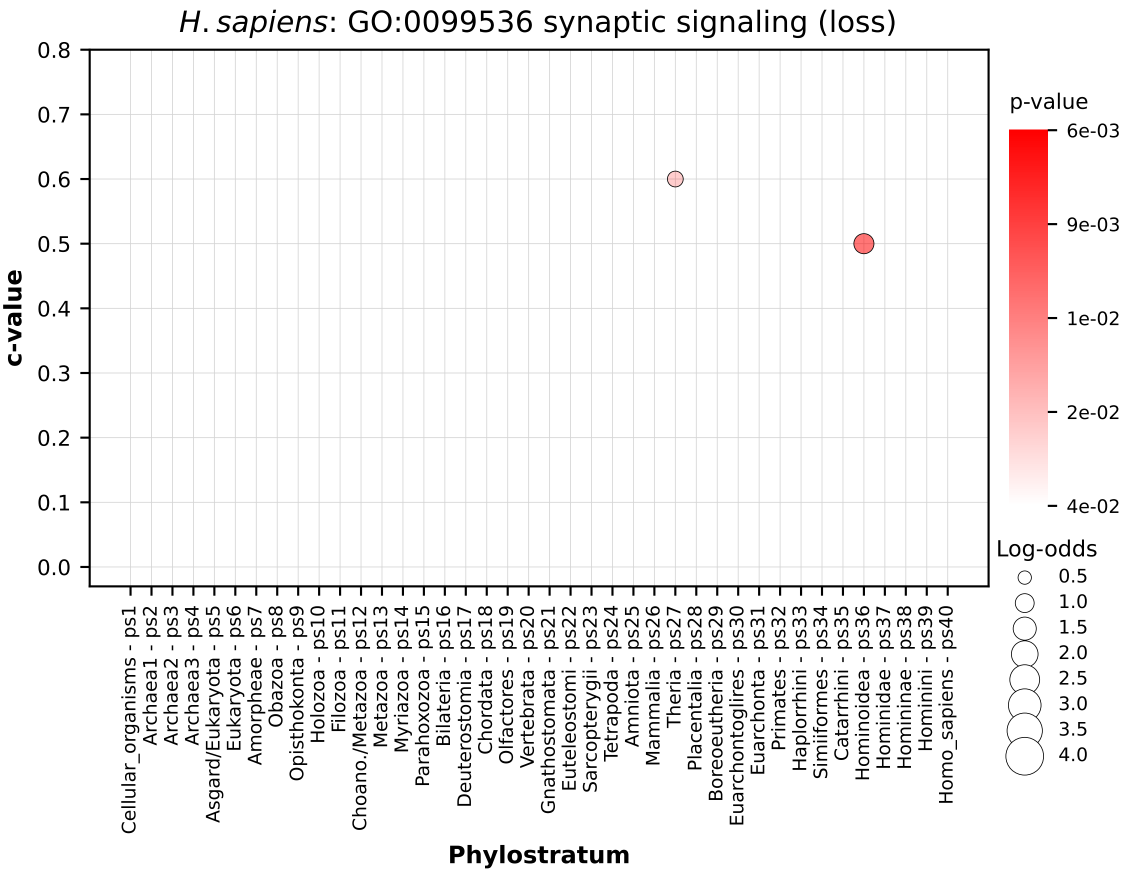

H. sapiens: GO:0099536 synaptic signaling (loss)
c-value
0.8
0.7
0.6
0.5
0.4
0.3
0.2
0.1
0.0
Phylostratum
Cellular_organisms - ps1
Archaea1 - ps2
Archaea2 - ps3
Archaea3 - ps4
Asgard/Eukaryota - ps5
Eukaryota - ps6
Amorpheae - ps7
Obazoa - ps8
Opisthokonta - ps9
Holozoa - ps10
Filozoa - ps11
Choano./Metazoa - ps12
Metazoa - ps13
Myriazoa - ps14
Parahoxozoa - ps15
Bilateria - ps16
Deuterostomia - ps17
Chordata - ps18
Olfactores - ps19
Vertebrata - ps20
Gnathostomata - ps21
Euteleostomi - ps22
Sarcopterygii - ps23
Tetrapoda - ps24
Amniota - ps25
Mammalia - ps26
Theria - ps27
Placentalia - ps28
Boreoeutheria - ps29
Euarchontoglires - ps30
Euarchonta - ps31
Primates - ps32
Haplorrhini - ps33
Simiiformes - ps34
Catarrhini - ps35
Hominoidea - ps36
Hominidae - ps37
Homininae - ps38
Hominini - ps39
Homo_sapiens - ps40
p-value
6e-03
9e-03
1e-02
2e-02
4e-02
Log-odds
0.5
1.0
1.5
2.0
2.5
3.0
3.5
4.0

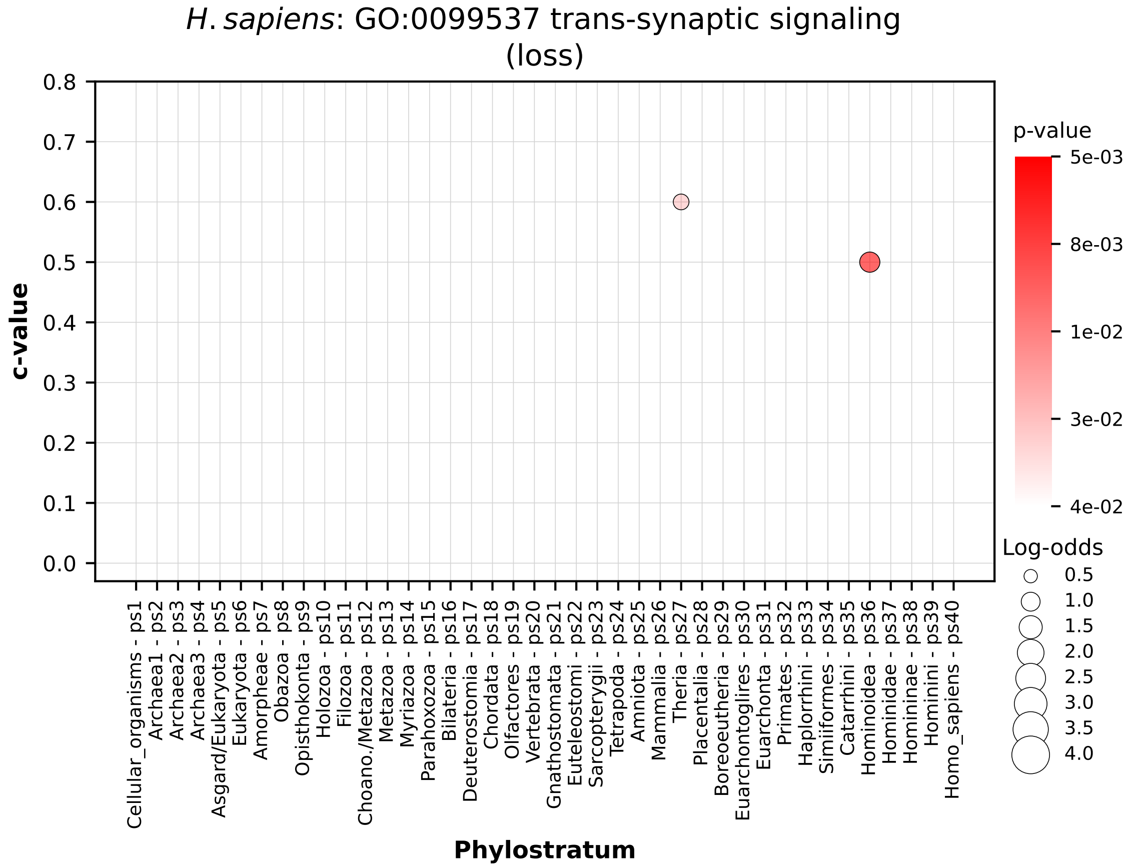

H. sapiens: GO:0099537 trans-synaptic signaling
(loss)
c-value
0.8
0.7
0.6
0.5
0.4
0.3
0.2
0.1
0.0
p-value
5e-03
8e-03
1e-02
3e-02
4e-02
Log-odds
0.5
1.0
1.5
2.0
2.5
3.0
3.5
4.0
Cellular_organisms - ps1
Archaea1 - ps2
Archaea2 - ps3
Archaea3 - ps4
Asgard/Eukaryota - ps5
Eukaryota - ps6
Amorpheae - ps7
Obazoa - ps8
Opisthokonta - ps9
Holozoa - ps10
Filozoa - ps11
Choano./Metazoa - ps12
Metazoa - ps13
Myriazoa - ps14
Parahoxozoa - ps15
Bilateria - ps16
Deuterostomia - ps17
Chordata - ps18
Olfactores - ps19
Vertebrata - ps20
Gnathostomata - ps21
Euteleostomi - ps22
Sarcopterygii - ps23
Tetrapoda - ps24
Amniota - ps25
Mammalia - ps26
Theria - ps27
Placentalia - ps28
Boreoeutheria - ps29
Euarchontoglires - ps30
Euarchonta - ps31
Primates - ps32
Haplorrhini - ps33
Simiiformes - ps34
Catarrhini - ps35
Hominoidea - ps36
Hominidae - ps37
Homininae - ps38
Hominini - ps39
Homo_sapiens - ps40
Phylostratum

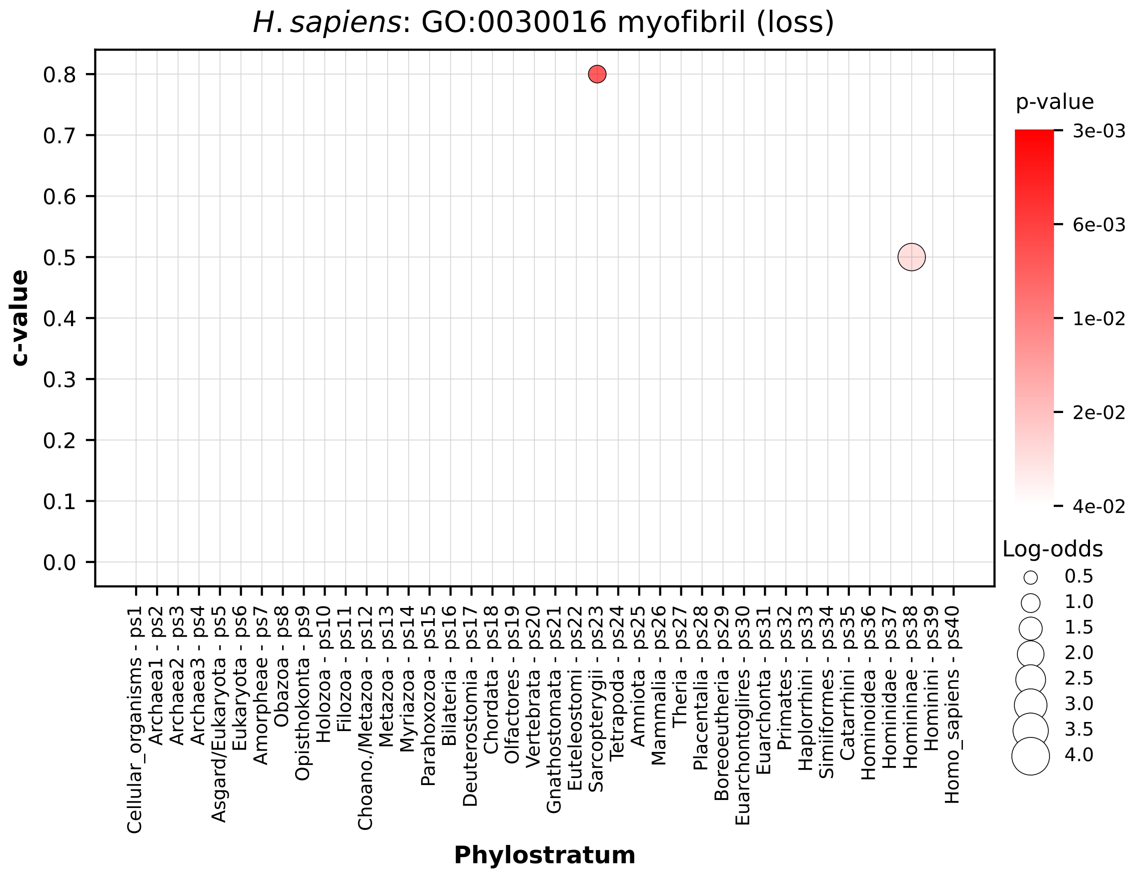

H. sapiens: GO:0030016 myofibril (loss)
c-value
0.8
0.7
0.6
0.5
0.4
0.3
0.2
0.1
0.0
p-value
3e-03
6e-03
1e-02
2e-02
4e-02
Log-odds
0.5
1.0
1.5
2.0
2.5
3.0
3.5
4.0
Cellular_organisms - ps1
Archaea1 - ps2
Archaea2 - ps3
Archaea3 - ps4
Asgard/Eukaryota - ps5
Eukaryota - ps6
Amorpheae - ps7
Obazoa - ps8
Opisthokonta - ps9
Holozoa - ps10
Filozoa - ps11
Choano./Metazoa - ps12
Metazoa - ps13
Myriazoa - ps14
Parahoxozoa - ps15
Bilateria - ps16
Deuterostomia - ps17
Chordata - ps18
Olfactores - ps19
Vertebrata - ps20
Gnathostomata - ps21
Euteleostomi - ps22
Sarcopterygii - ps23
Tetrapoda - ps24
Amniota - ps25
Mammalia - ps26
Theria - ps27
Placentalia - ps28
Boreoeutheria - ps29
Euarchontoglires - ps30
Euarchonta - ps31
Primates - ps32
Haplorrhini - ps33
Simiiformes - ps34
Catarrhini - ps35
Hominoidea - ps36
Hominidae - ps37
Homininae - ps38
Hominini - ps39
Homo_sapiens - ps40
Phylostratum

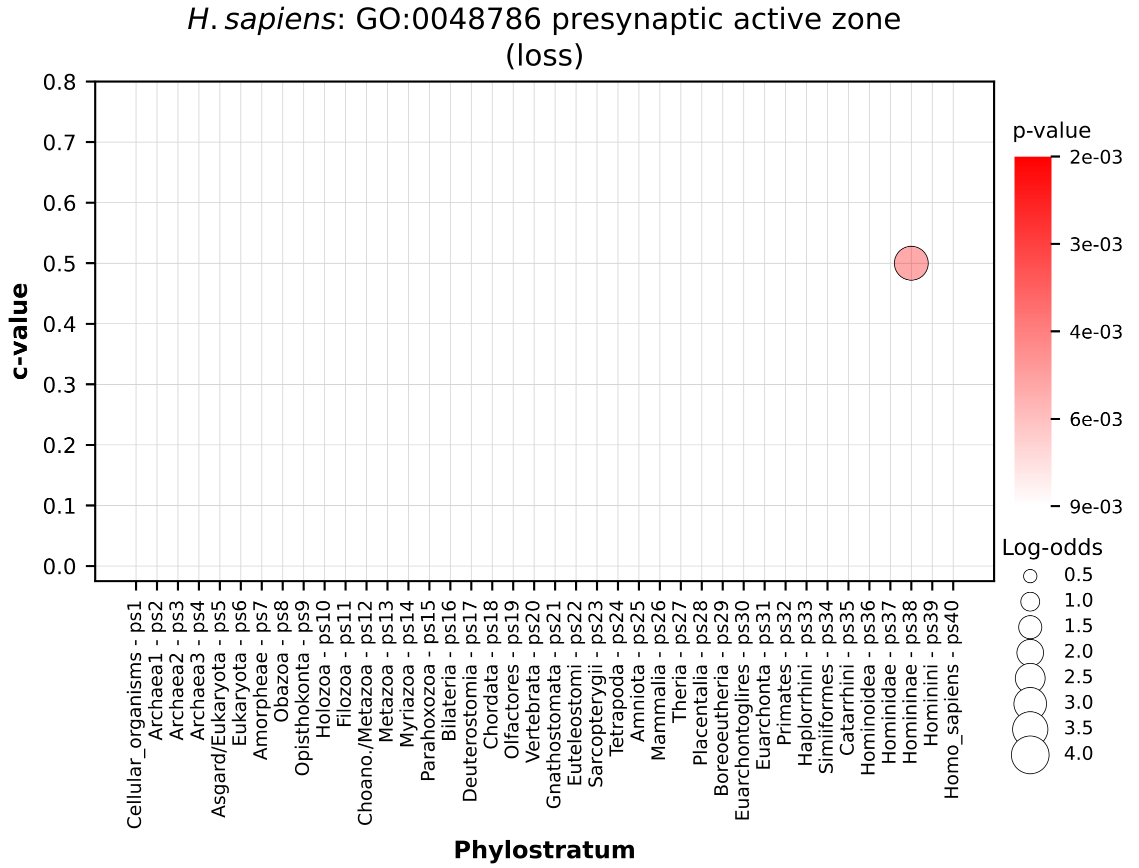

H. sapiens: GO:0048786 presynaptic active zone
(loss)
c-value
0.8
0.7
0.6
0.5
0.4
0.3
0.2
0.1
0.0
Phylostratum
Cellular_organisms - ps1
Archaea1 - ps2
Archaea2 - ps3
Archaea3 - ps4
Asgard/Eukaryota - ps5
Eukaryota - ps6
Amorpheae - ps7
Obazoa - ps8
Opisthokonta - ps9
Holozoa - ps10
Filozoa - ps11
Choano./Metazoa - ps12
Metazoa - ps13
Myriazoa - ps14
Parahoxozoa - ps15
Bilateria - ps16
Deuterostomia - ps17
Chordata - ps18
Olfactores - ps19
Vertebrata - ps20
Gnathostomata - ps21
Euteleostomi - ps22
Sarcopterygii - ps23
Tetrapoda - ps24
Amniota - ps25
Mammalia - ps26
Theria - ps27
Placentalia - ps28
Boreoeutheria - ps29
Euarchontoglires - ps30
Euarchonta - ps31
Primates - ps32
Haplorrhini - ps33
Simiiformes - ps34
Catarrhini - ps35
Hominoidea - ps36
Hominidae - ps37
Homininae - ps38
Hominini - ps39
Homo_sapiens - ps40
p-value
2e-03
3e-03
4e-03
6e-03
9e-03
Log-odds
0.5
1.0
1.5
2.0
2.5
3.0
3.5
4.0

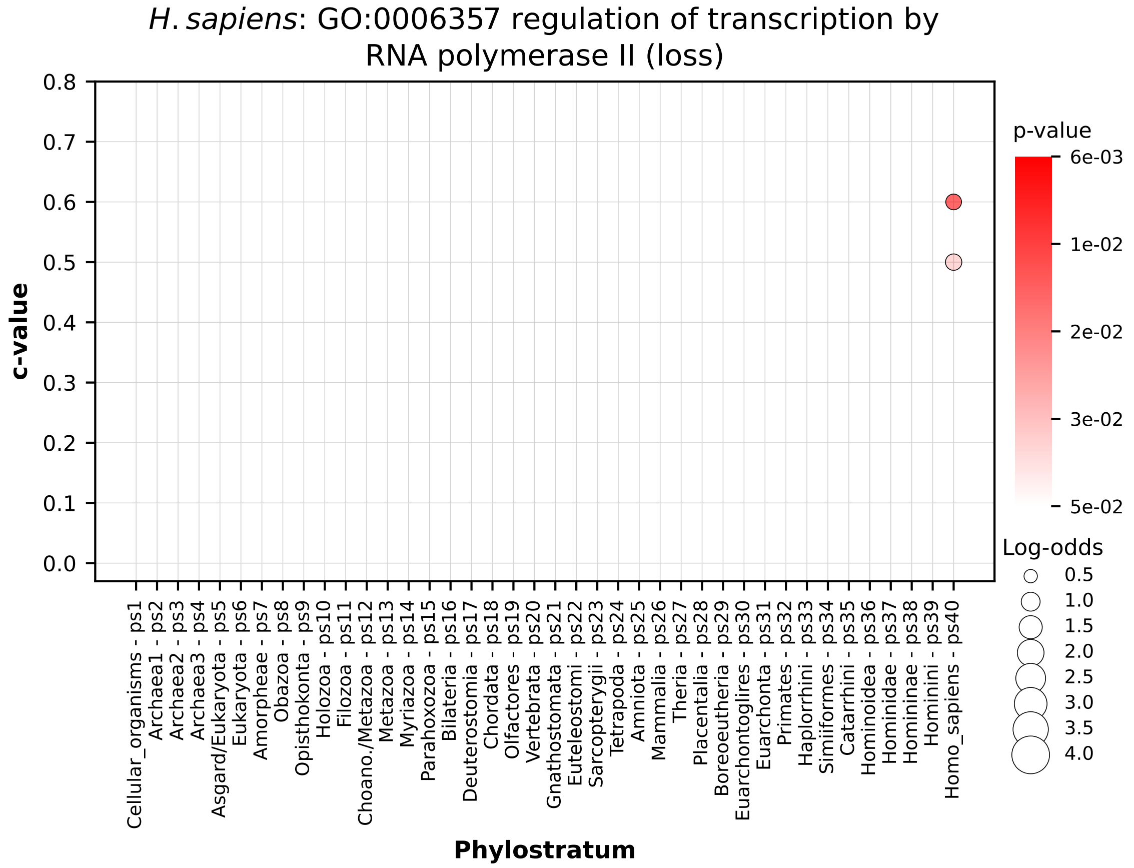
H. sapiens: GO:0006357 regulation of transcription by RNA polymerase II (loss)
c-value
0.8
0.7
0.6
0.5
0.4
0.3
0.2
0.1
0.0
p-value
6e-03
1e-02
2e-02
3e-02
5e-02
Log-odds
0.5
1.0
1.5
2.0
2.5
3.0
3.5
4.0
Cellular_organisms - ps1
Archaea1 - ps2
Archaea2 - ps3
Archaea3 - ps4
Asgard/Eukaryota - ps5
Eukaryota - ps6
Amorpheae - ps7
Obazoa - ps8
Opisthokonta - ps9
Holozoa - ps10
Filozoa - ps11
Choano./Metazoa - ps12
Metazoa - ps13
Myriazoa - ps14
Parahoxozoa - ps15
Bilateria - ps16
Deuterostomia - ps17
Chordata - ps18
Olfactores - ps19
Vertebrata - ps20
Gnathostomata - ps21
Euteleostomi - ps22
Sarcopterygii - ps23
Tetrapoda - ps24
Amniota - ps25
Mammalia - ps26
Theria - ps27
Placentalia - ps28
Boreoeutheria - ps29
Euarchontoglires - ps30
Euarchonta - ps31
Primates - ps32
Haplorrhini - ps33
Simiiformes - ps34
Catarrhini - ps35
Hominoidea - ps36
Hominidae - ps37
Homininae - ps38
Hominini - ps39
Homo_sapiens - ps40
Phylostratum

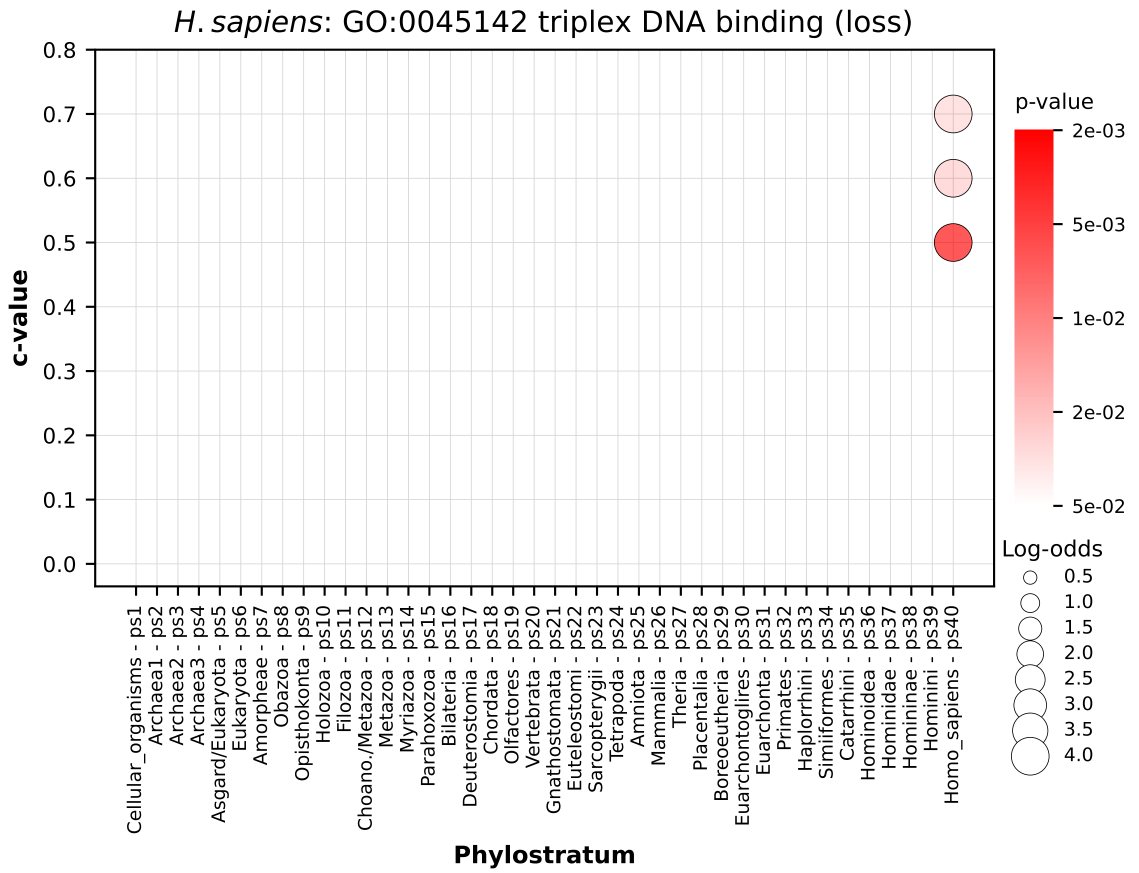

H. sapiens: GO:0045142 triplex DNA binding (loss)
c-value
0.8
0.7
0.6
0.5
0.4
0.3
0.2
0.1
0.0
p-value
2e-03
5e-03
1e-02
2e-02
5e-02
Log-odds
0.5
1.0
1.5
2.0
2.5
3.0
3.5
4.0
Cellular_organisms - ps1
Archaea1 - ps2
Archaea2 - ps3
Archaea3 - ps4
Asgard/Eukaryota - ps5
Eukaryota - ps6
Amorpheae - ps7
Obazoa - ps8
Opisthokonta - ps9
Holozoa - ps10
Filozoa - ps11
Choano./Metazoa - ps12
Metazoa - ps13
Myriazoa - ps14
Parahoxozoa - ps15
Bilateria - ps16
Deuterostomia - ps17
Chordata - ps18
Olfactores - ps19
Vertebrata - ps20
Gnathostomata - ps21
Euteleostomi - ps22
Sarcopterygii - ps23
Tetrapoda - ps24
Amniota - ps25
Mammalia - ps26
Theria - ps27
Placentalia - ps28
Boreoeutheria - ps29
Euarchontoglires - ps30
Euarchonta - ps31
Primates - ps32
Haplorrhini - ps33
Simiiformes - ps34
Catarrhini - ps35
Hominoidea - ps36
Hominidae - ps37
Homininae - ps38
Hominini - ps39
Homo_sapiens - ps40
Phylostratum

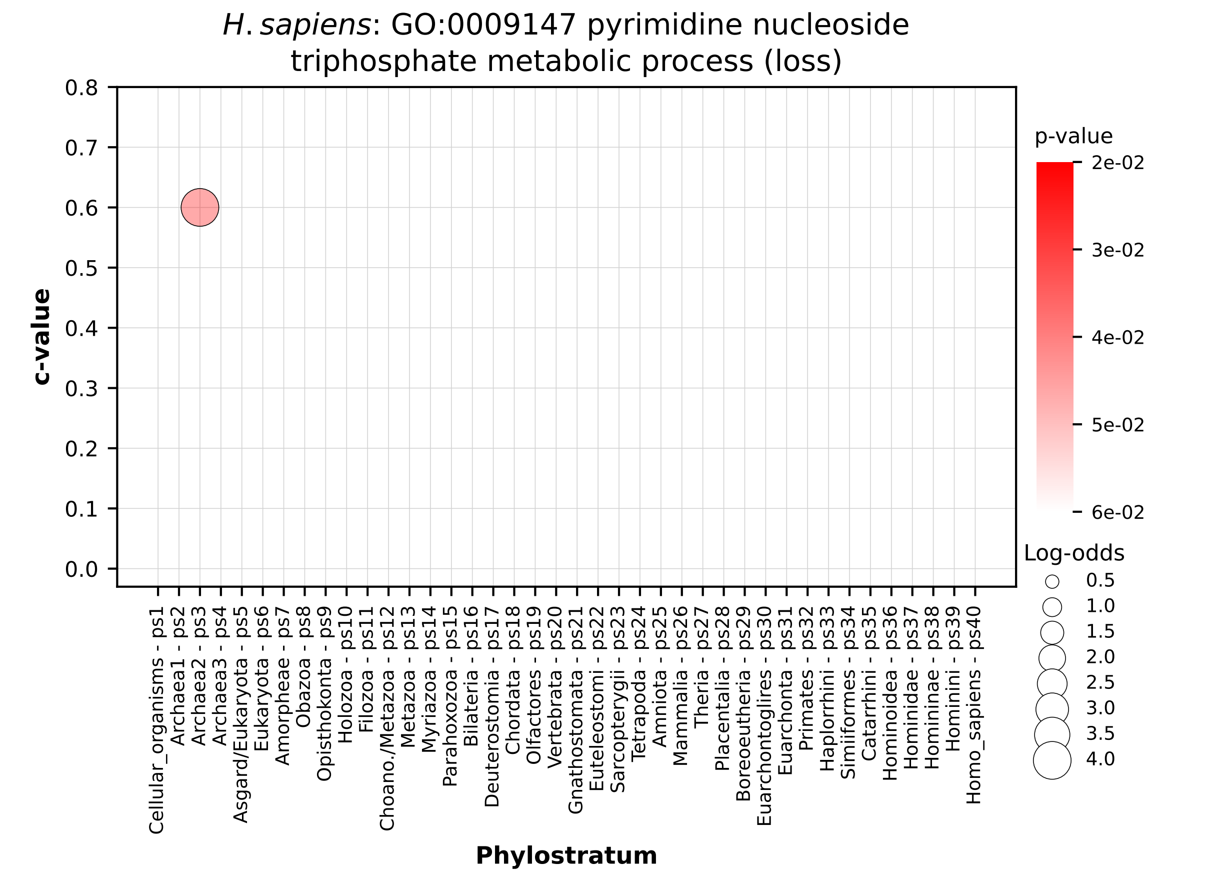

H. sapiens: GO:0009147 pyrimidine nucleoside triphosphate metabolic process (loss)
c-value
0.8
0.7
0.6
0.5
0.4
0.3
0.2
0.1
0.0
Phylostratum
Cellular_organisms - ps1
Archaea1 - ps2
Archaea2 - ps3
Archaea3 - ps4
Asgard/Eukaryota - ps5
Eukaryota - ps6
Amorpheae - ps7
Obazoa - ps8
Opisthokonta - ps9
Holozoa - ps10
Filozoa - ps11
Choano./Metazoa - ps12
Metazoa - ps13
Myriazoa - ps14
Parahoxozoa - ps15
Bilateria - ps16
Deuterostomia - ps17
Chordata - ps18
Olfactores - ps19
Vertebrata - ps20
Gnathostomata - ps21
Euteleostomi - ps22
Sarcopterygii - ps23
Tetrapoda - ps24
Amniota - ps25
Mammalia - ps26
Theria - ps27
Placentalia - ps28
Boreoeutheria - ps29
Euarchontoglires - ps30
Euarchonta - ps31
Primates - ps32
Haplorrhini - ps33
Simiiformes - ps34
Catarrhini - ps35
Hominoidea - ps36
Hominidae - ps37
Homininae - ps38
Hominini - ps39
Homo_sapiens - ps40
p-value
2e-02
3e-02
4e-02
5e-02
6e-02
Log-odds
0.5
1.0
1.5
2.0
2.5
3.0
3.5
4.0

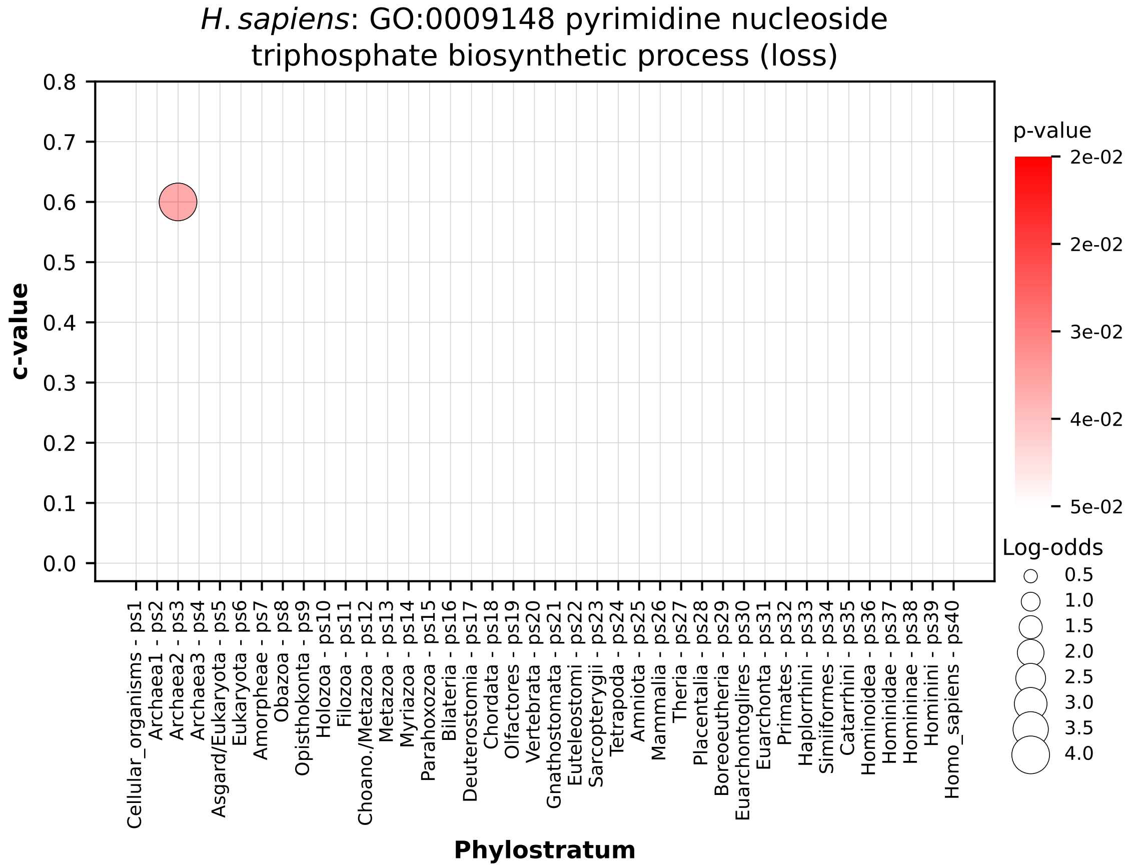

H. sapiens: GO:0009148 pyrimidine nucleoside triphosphate biosynthetic process (loss)
c-value
0.8
0.7
0.6
0.5
0.4
0.3
0.2
0.1
0.0
Phylostratum
Cellular_organisms - ps1
Archaea1 - ps2
Archaea2 - ps3
Archaea3 - ps4
Asgard/Eukaryota - ps5
Eukaryota - ps6
Amorpheae - ps7
Obazoa - ps8
Opisthokonta - ps9
Holozoa - ps10
Filozoa - ps11
Choano./Metazoa - ps12
Metazoa - ps13
Myriazoa - ps14
Parahoxozoa - ps15
Bilateria - ps16
Deuterostomia - ps17
Chordata - ps18
Olfactores - ps19
Vertebrata - ps20
Gnathostomata - ps21
Euteleostomi - ps22
Sarcopterygii - ps23
Tetrapoda - ps24
Amniota - ps25
Mammalia - ps26
Theria - ps27
Placentalia - ps28
Boreoeutheria - ps29
Euarchontoglires - ps30
Euarchonta - ps31
Primates - ps32
Haplorrhini - ps33
Simiiformes - ps34
Catarrhini - ps35
Hominoidea - ps36
Hominidae - ps37
Homininae - ps38
Hominini - ps39
Homo_sapiens - ps40
p-value
2e-02
2e-02
3e-02
4e-02
5e-02
Log-odds
0.5
1.0
1.5
2.0
2.5
3.0
3.5
4.0

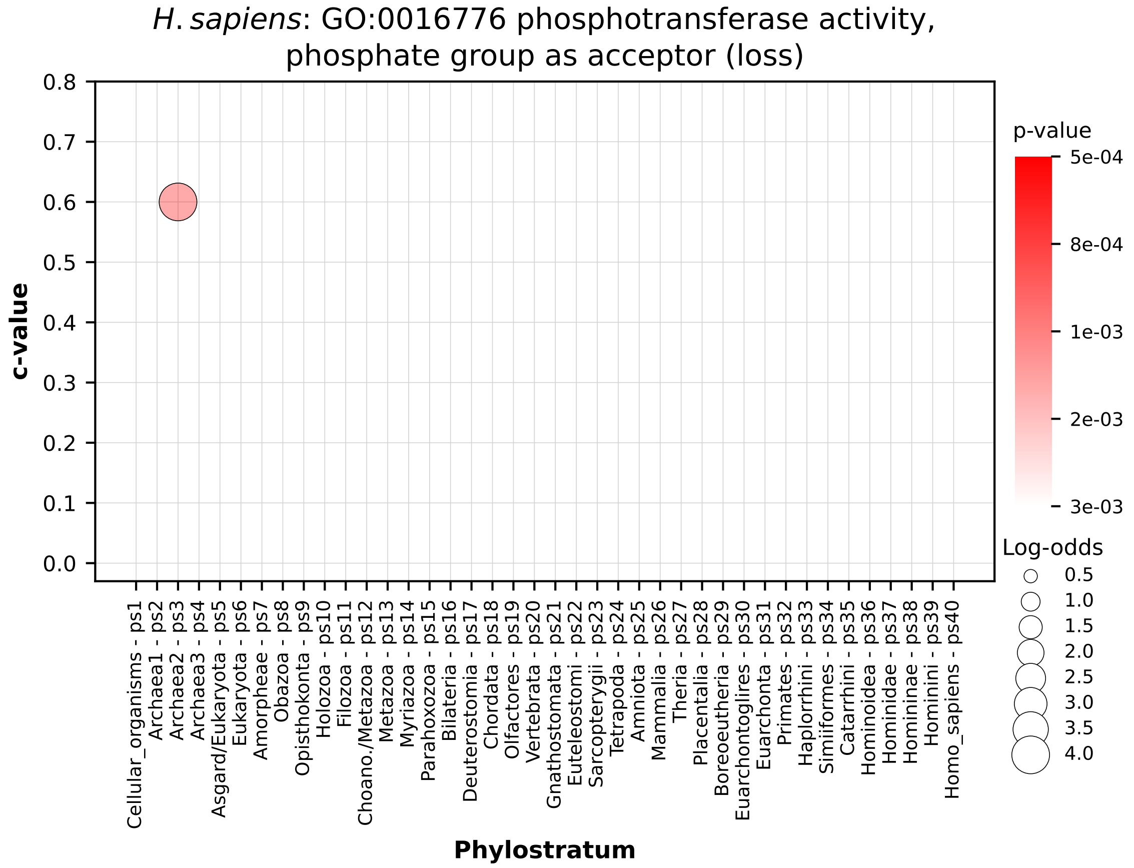

H. sapiens: GO:0016776 phosphotransferase activity, phosphate group as acceptor (loss)
c-value
0.8
0.7
0.6
0.5
0.4
0.3
0.2
0.1
0.0
p-value
5e-04
8e-04
1e-03
2e-03
3e-03
Log-odds
0.5
1.0
1.5
2.0
2.5
3.0
3.5
4.0
Cellular_organisms - ps1
Archaea1 - ps2
Archaea2 - ps3
Archaea3 - ps4
Asgard/Eukaryota - ps5
Eukaryota - ps6
Amorpheae - ps7
Obazoa - ps8
Opisthokonta - ps9
Holozoa - ps10
Filozoa - ps11
Choano./Metazoa - ps12
Metazoa - ps13
Myriazoa - ps14
Parahoxozoa - ps15
Bilateria - ps16
Deuterostomia - ps17
Chordata - ps18
Olfactores - ps19
Vertebrata - ps20
Gnathostomata - ps21
Euteleostomi - ps22
Sarcopterygii - ps23
Tetrapoda - ps24
Amniota - ps25
Mammalia - ps26
Theria - ps27
Placentalia - ps28
Boreoeutheria - ps29
Euarchontoglires - ps30
Euarchonta - ps31
Primates - ps32
Haplorrhini - ps33
Simiiformes - ps34
Catarrhini - ps35
Hominoidea - ps36
Hominidae - ps37
Homininae - ps38
Hominini - ps39
Homo_sapiens - ps40
Phylostratum

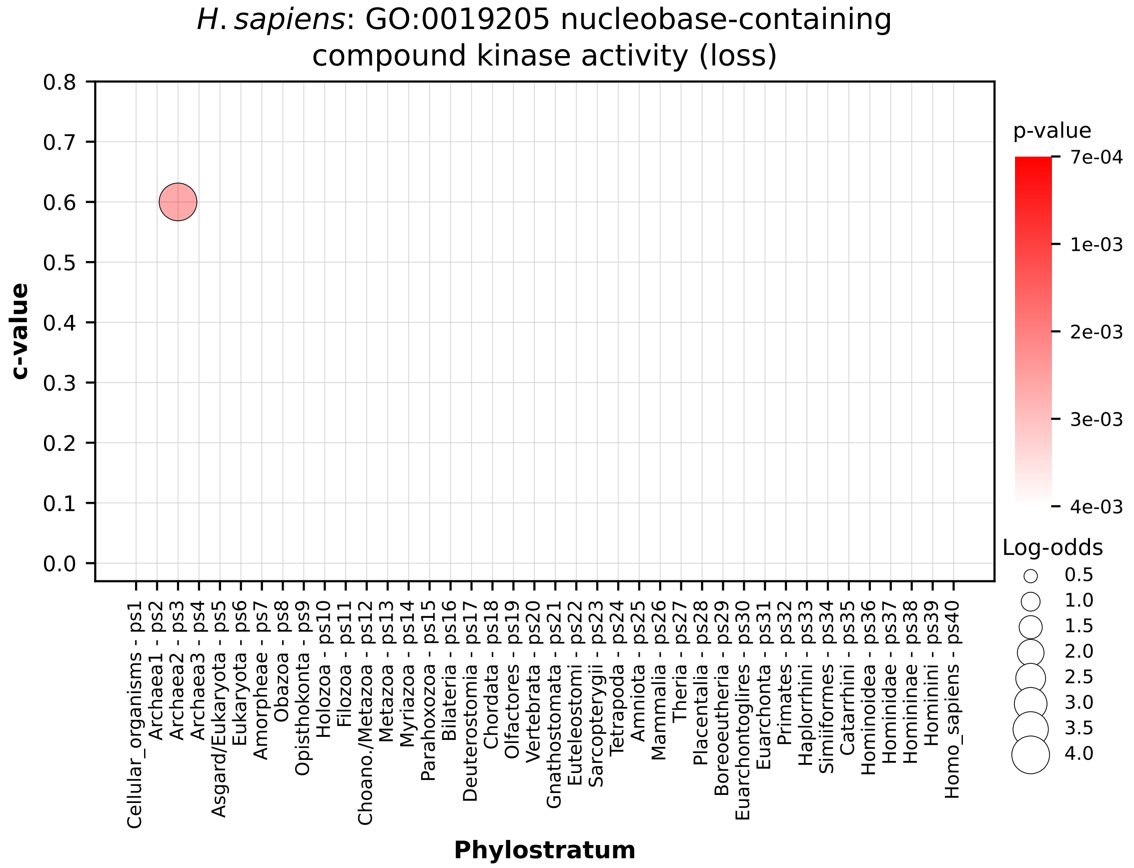

H. sapiens: GO:0019205 nucleobase-containing compound kinase activity (loss)
c-value
0.8
0.7
0.6
0.5
0.4
0.3
0.2
0.1
0.0
Phylostratum
Cellular_organisms - ps1
Archaea1 - ps2
Archaea2 - ps3
Archaea3 - ps4
Asgard/Eukaryota - ps5
Eukaryota - ps6
Amorpheae - ps7
Obazoa - ps8
Opisthokonta - ps9
Holozoa - ps10
Filozoa - ps11
Choano./Metazoa - ps12
Metazoa - ps13
Myriazoa - ps14
Parahoxozoa - ps15
Bilateria - ps16
Deuterostomia - ps17
Chordata - ps18
Olfactores - ps19
Vertebrata - ps20
Gnathostomata - ps21
Euteleostomi - ps22
Sarcopterygii - ps23
Tetrapoda - ps24
Amniota - ps25
Mammalia - ps26
Theria - ps27
Placentalia - ps28
Boreoeutheria - ps29
Euarchontoglires - ps30
Euarchonta - ps31
Primates - ps32
Haplorrhini - ps33
Simiiformes - ps34
Catarrhini - ps35
Hominoidea - ps36
Hominidae - ps37
Homininae - ps38
Hominini - ps39
Homo_sapiens - ps40
p-value
7e-04
1e-03
2e-03
3e-03
4e-03
Log-odds
0.5
1.0
1.5
2.0
2.5
3.0
3.5
4.0

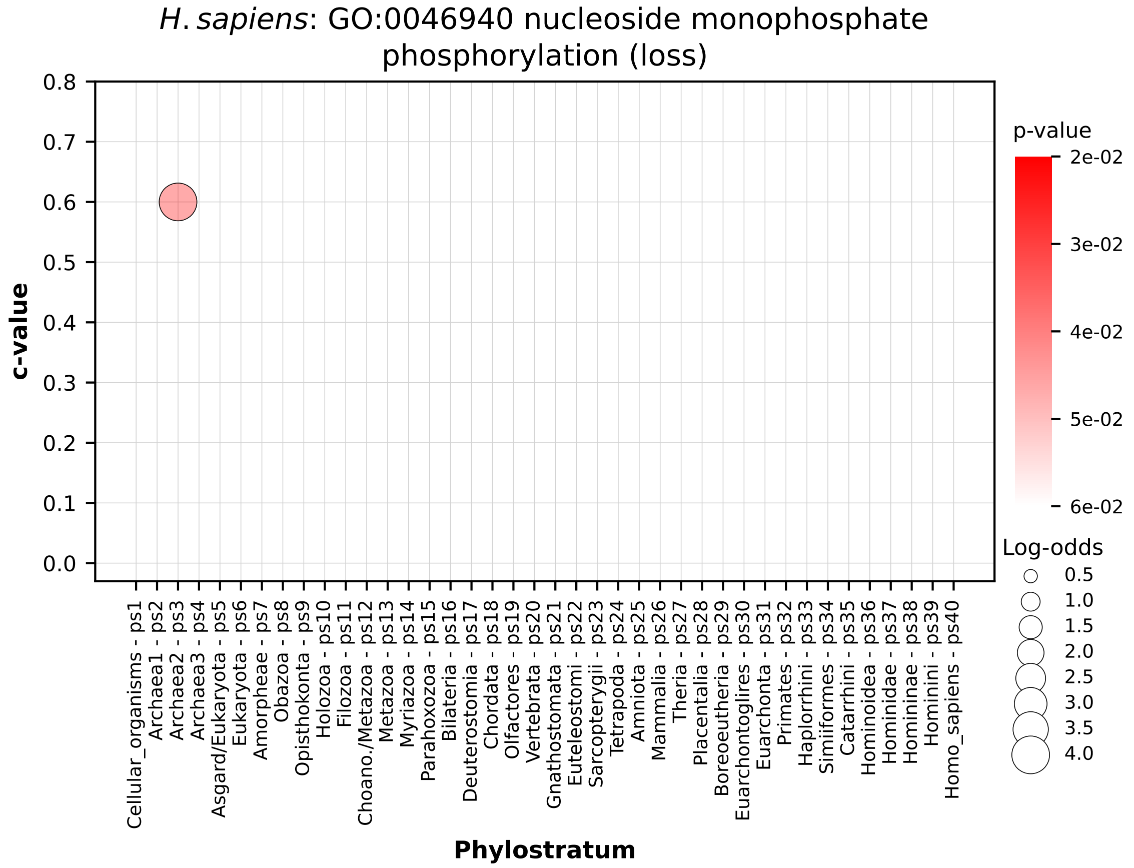
H. sapiens: GO:0046940 nucleoside monophosphate phosphorylation (loss)
c-value
0.8
0.7
0.6
0.5
0.4
0.3
0.2
0.1
0.0
Phylostratum
Cellular_organisms - ps1
Archaea1 - ps2
Archaea2 - ps3
Archaea3 - ps4
Asgard/Eukaryota - ps5
Eukaryota - ps6
Amorpheae - ps7
Obazoa - ps8
Opisthokonta - ps9
Holozoa - ps10
Filozoa - ps11
Choano./Metazoa - ps12
Metazoa - ps13
Myriazoa - ps14
Parahoxozoa - ps15
Bilateria - ps16
Deuterostomia - ps17
Chordata - ps18
Olfactores - ps19
Vertebrata - ps20
Gnathostomata - ps21
Euteleostomi - ps22
Sarcopterygii - ps23
Tetrapoda - ps24
Amniota - ps25
Mammalia - ps26
Theria - ps27
Placentalia - ps28
Boreoeutheria - ps29
Euarchontoglires - ps30
Euarchonta - ps31
Primates - ps32
Haplorrhini - ps33
Simiiformes - ps34
Catarrhini - ps35
Hominoidea - ps36
Hominidae - ps37
Homininae - ps38
Hominini - ps39
Homo_sapiens - ps40
p-value
2e-02
3e-02
4e-02
5e-02
6e-02
Log-odds
0.5
1.0
1.5
2.0
2.5
3.0
3.5
4.0

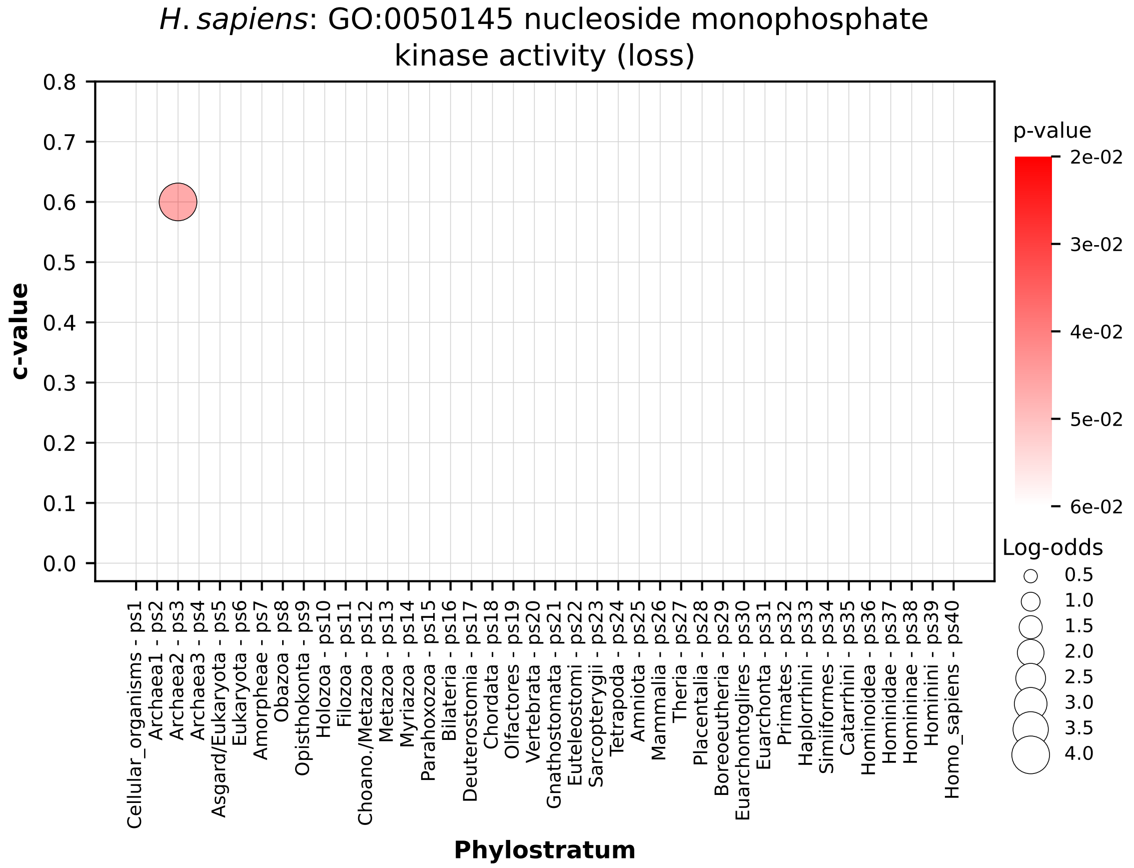

H. sapiens: GO:0050145 nucleoside monophosphate kinase activity (loss)
c-value
0.8
0.7
0.6
0.5
0.4
0.3
0.2
0.1
0.0
p-value
2e-02
3e-02
4e-02
5e-02
6e-02
Log-odds
0.5
1.0
1.5
2.0
2.5
3.0
3.5
4.0
Cellular_organisms - ps1
Archaea1 - ps2
Archaea2 - ps3
Archaea3 - ps4
Asgard/Eukaryota - ps5
Eukaryota - ps6
Amorpheae - ps7
Obazoa - ps8
Opisthokonta - ps9
Holozoa - ps10
Filozoa - ps11
Choano./Metazoa - ps12
Metazoa - ps13
Myriazoa - ps14
Parahoxozoa - ps15
Bilateria - ps16
Deuterostomia - ps17
Chordata - ps18
Olfactores - ps19
Vertebrata - ps20
Gnathostomata - ps21
Euteleostomi - ps22
Sarcopterygii - ps23
Tetrapoda - ps24
Amniota - ps25
Mammalia - ps26
Theria - ps27
Placentalia - ps28
Boreoeutheria - ps29
Euarchontoglires - ps30
Euarchonta - ps31
Primates - ps32
Haplorrhini - ps33
Simiiformes - ps34
Catarrhini - ps35
Hominoidea - ps36
Hominidae - ps37
Homininae - ps38
Hominini - ps39
Homo_sapiens - ps40
Phylostratum

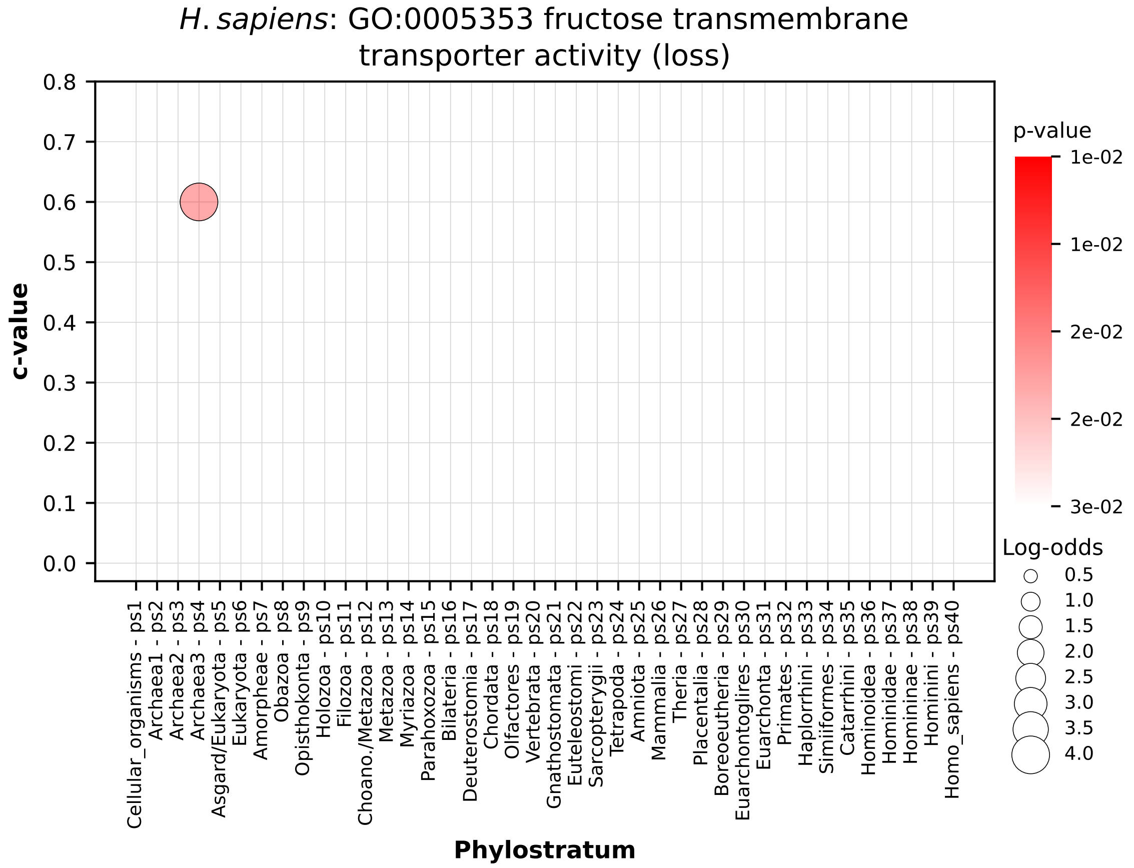

H. sapiens: GO:0005353 fructose transmembrane transporter activity (loss)
c-value
0.0
0.1
0.2
0.3
0.4
0.5
0.6
0.7
0.8
Phylostratum
Cellular_organisms - ps1
Archaea1 - ps2
Archaea2 - ps3
Archaea3 - ps4
Asgard/Eukaryota - ps5
Eukaryota - ps6
Amorpheae - ps7
Obazoa - ps8
Opisthokonta - ps9
Holozoa - ps10
Filozoa - ps11
Choano./Metazoa - ps12
Metazoa - ps13
Myriazoa - ps14
Parahoxozoa - ps15
Bilateria - ps16
Deuterostomia - ps17
Chordata - ps18
Olfactores - ps19
Vertebrata - ps20
Gnathostomata - ps21
Euteleostomi - ps22
Sarcopterygii - ps23
Tetrapoda - ps24
Amniota - ps25
Mammalia - ps26
Theria - ps27
Placentalia - ps28
Boreoeutheria - ps29
Euarchontoglires - ps30
Euarchonta - ps31
Primates - ps32
Haplorrhini - ps33
Simiiformes - ps34
Catarrhini - ps35
Hominoidea - ps36
Hominidae - ps37
Homininae - ps38
Hominini - ps39
Homo_sapiens - ps40
p-value
1e-02
1e-02
2e-02
2e-02
3e-02
Log-odds
0.5
1.0
1.5
2.0
2.5
3.0
3.5
4.0

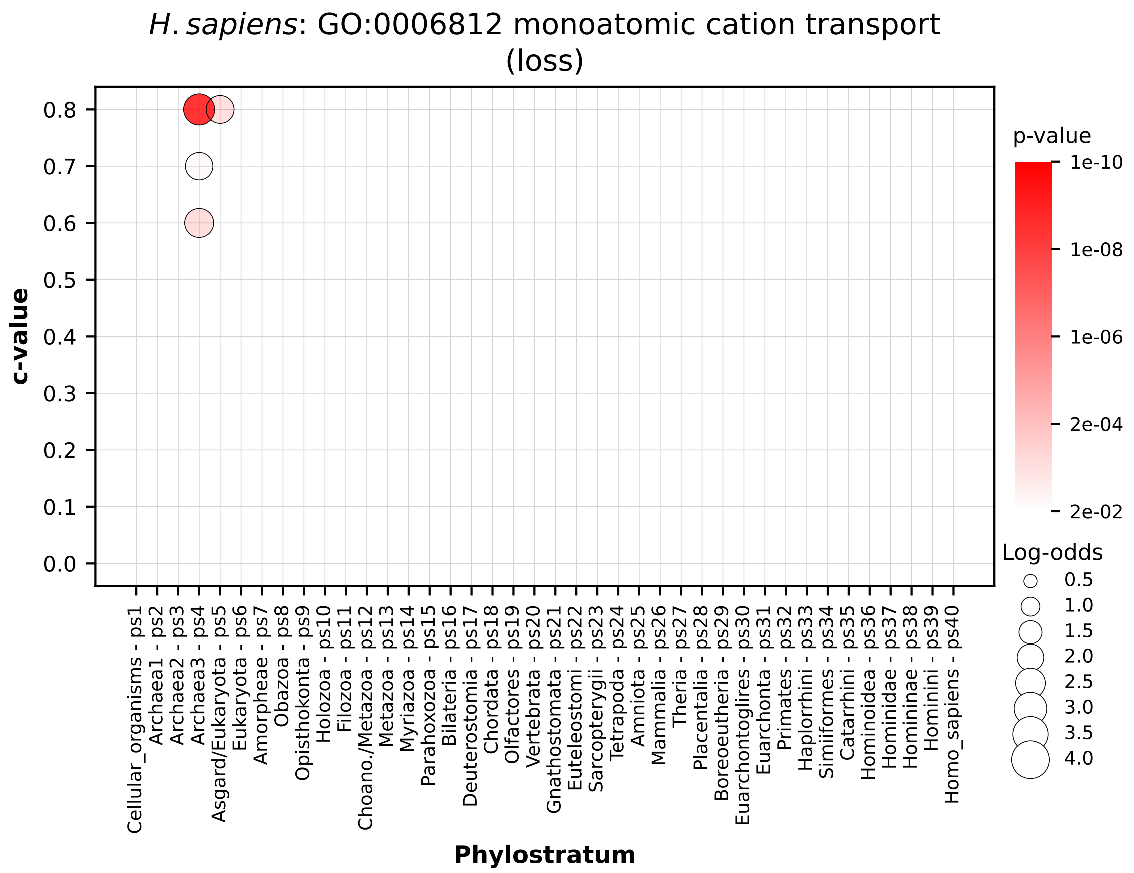

H. sapiens: GO:0006812 monoatomic cation transport
(loss)
c-value
0.8
0.7
0.6
0.5
0.4
0.3
0.2
0.1
0.0
p-value
1e-10
1e-08
1e-06
2e-04
2e-02
Log-odds
0.5
1.0
1.5
2.0
2.5
3.0
3.5
4.0
Cellular_organisms - ps1
Archaea1 - ps2
Archaea2 - ps3
Archaea3 - ps4
Asgard/Eukaryota - ps5
Eukaryota - ps6
Amorpheae - ps7
Obazoa - ps8
Opisthokonta - ps9
Holozoa - ps10
Filozoa - ps11
Choano./Metazoa - ps12
Metazoa - ps13
Myriazoa - ps14
Parahoxozoa - ps15
Bilateria - ps16
Deuterostomia - ps17
Chordata - ps18
Olfactores - ps19
Vertebrata - ps20
Gnathostomata - ps21
Euteleostomi - ps22
Sarcopterygii - ps23
Tetrapoda - ps24
Amniota - ps25
Mammalia - ps26
Theria - ps27
Placentalia - ps28
Boreoeutheria - ps29
Euarchontoglires - ps30
Euarchonta - ps31
Primates - ps32
Haplorrhini - ps33
Simiiformes - ps34
Catarrhini - ps35
Hominoidea - ps36
Hominidae - ps37
Homininae - ps38
Hominini - ps39
Homo_sapiens - ps40
Phylostratum

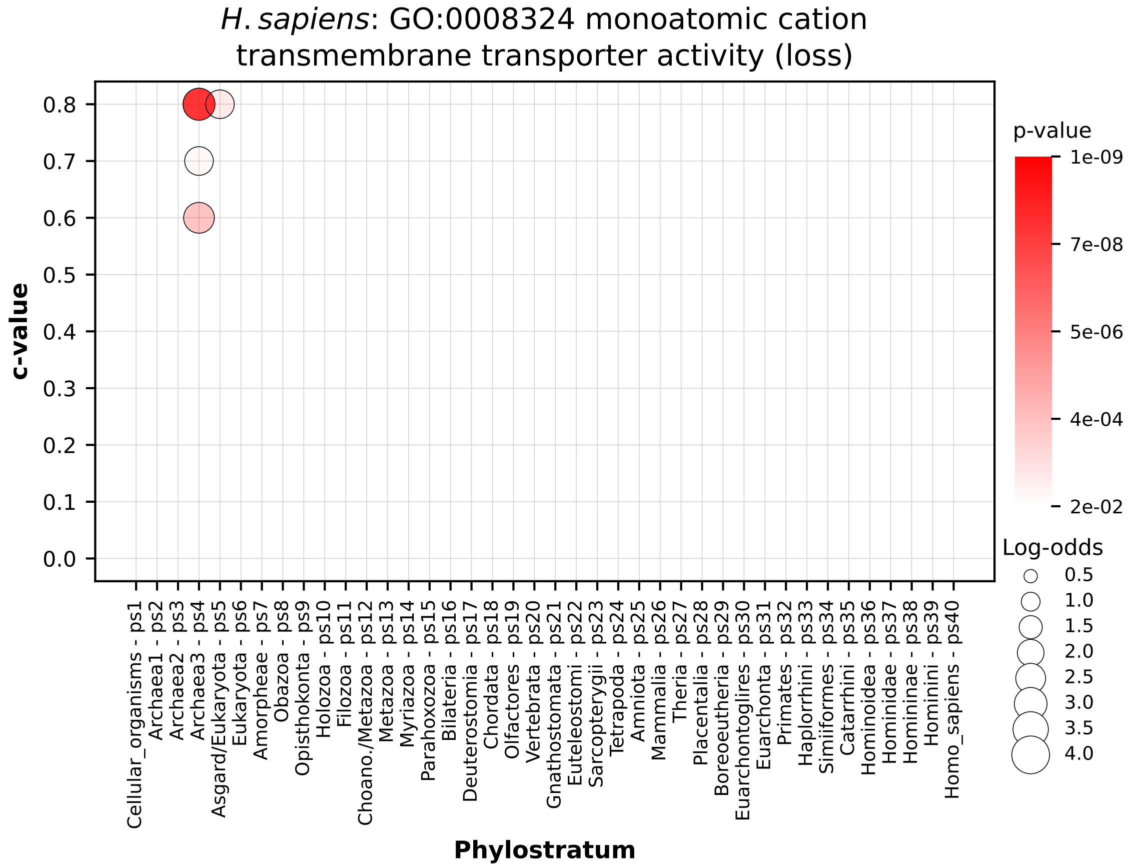

H. sapiens: GO:0008324 monoatomic cation transmembrane transporter activity (loss)
c-value
0.0
0.1
0.2
0.3
0.4
0.5
0.6
0.7
0.8
Phylostratum
Cellular_organisms - ps1
Archaea1 - ps2
Archaea2 - ps3
Archaea3 - ps4
Asgard/Eukaryota - ps5
Eukaryota - ps6
Amorpheae - ps7
Obazoa - ps8
Opisthokonta - ps9
Holozoa - ps10
Filozoa - ps11
Choano./Metazoa - ps12
Metazoa - ps13
Myriazoa - ps14
Parahoxozoa - ps15
Bilateria - ps16
Deuterostomia - ps17
Chordata - ps18
Olfactores - ps19
Vertebrata - ps20
Gnathostomata - ps21
Euteleostomi - ps22
Sarcopterygii - ps23
Tetrapoda - ps24
Amniota - ps25
Mammalia - ps26
Theria - ps27
Placentalia - ps28
Boreoeutheria - ps29
Euarchontoglires - ps30
Euarchonta - ps31
Primates - ps32
Haplorrhini - ps33
Simiiformes - ps34
Catarrhini - ps35
Hominoidea - ps36
Hominidae - ps37
Homininae - ps38
Hominini - ps39
Homo_sapiens - ps40
p-value
1e-09
7e-08
5e-06
4e-04
2e-02
Log-odds
0.5
1.0
1.5
2.0
2.5
3.0
3.5
4.0

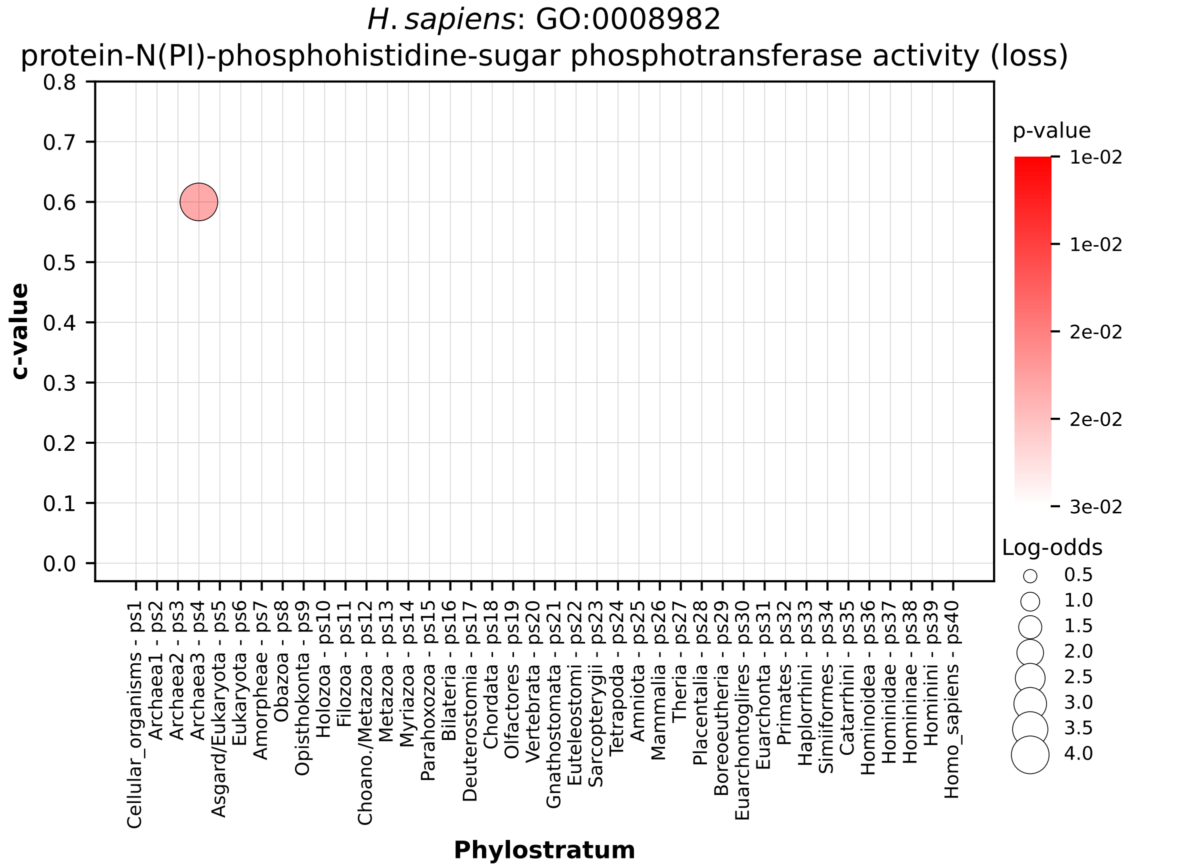

H. sapiens: GO:0008982
protein-N(PI)-phosphohistidine-sugar phosphotransferase activity (loss)
c-value
0.8
0.7
0.6
0.5
0.4
0.3
0.2
0.1
0.0
p-value
1e-02
1e-02
2e-02
2e-02
3e-02
Log-odds
0.5
1.0
1.5
2.0
2.5
3.0
3.5
4.0
Cellular_organisms - ps1
Archaea1 - ps2
Archaea2 - ps3
Archaea3 - ps4
Asgard/Eukaryota - ps5
Eukaryota - ps6
Amorpheae - ps7
Obazoa - ps8
Opisthokonta - ps9
Holozoa - ps10
Filozoa - ps11
Choano./Metazoa - ps12
Metazoa - ps13
Myriazoa - ps14
Parahoxozoa - ps15
Bilateria - ps16
Deuterostomia - ps17
Chordata - ps18
Olfactores - ps19
Vertebrata - ps20
Gnathostomata - ps21
Euteleostomi - ps22
Sarcopterygii - ps23
Tetrapoda - ps24
Amniota - ps25
Mammalia - ps26
Theria - ps27
Placentalia - ps28
Boreoeutheria - ps29
Euarchontoglires - ps30
Euarchonta - ps31
Primates - ps32
Haplorrhini - ps33
Simiiformes - ps34
Catarrhini - ps35
Hominoidea - ps36
Hominidae - ps37
Homininae - ps38
Hominini - ps39
Homo_sapiens - ps40
Phylostratum

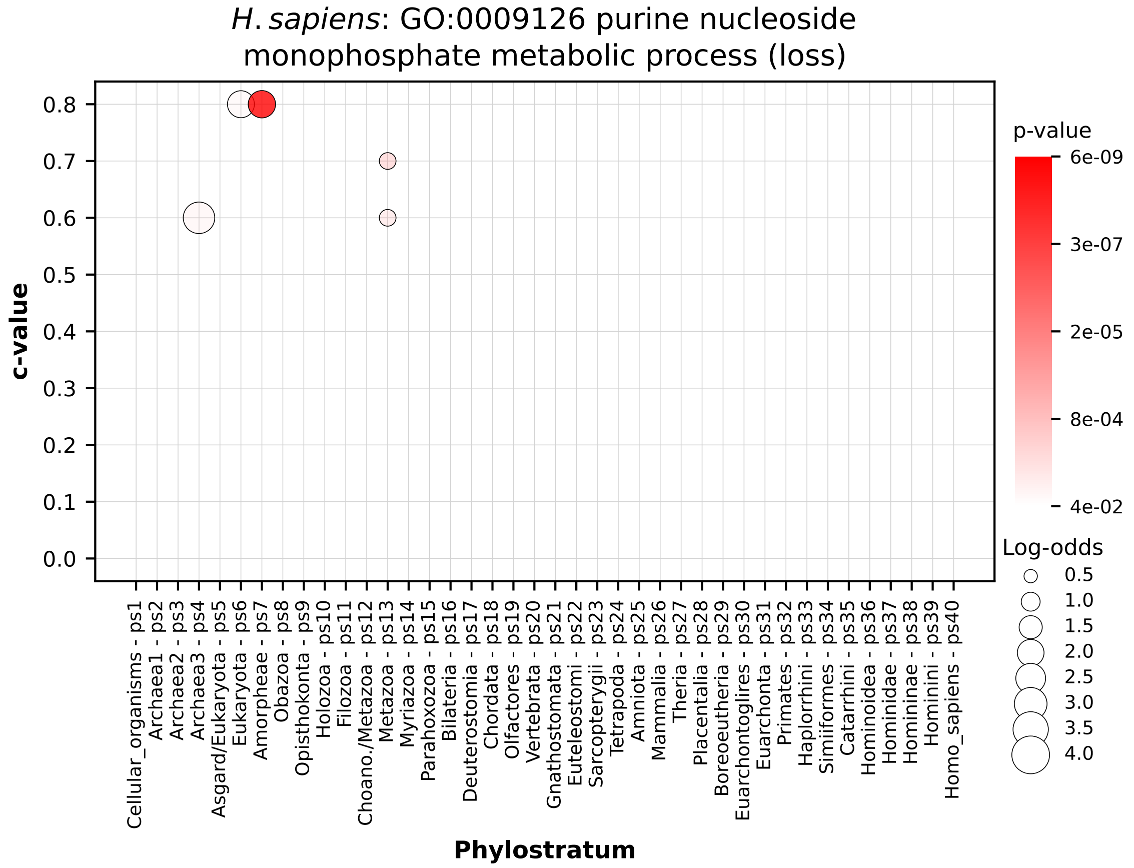

H. sapiens: GO:0009126 purine nucleoside monophosphate metabolic process (loss)
c-value
0.8
0.7
0.6
0.5
0.4
0.3
0.2
0.1
0.0
p-value
6e-09
3e-07
2e-05
8e-04
4e-02
Log-odds
0.5
1.0
1.5
2.0
2.5
3.0
3.5
4.0
Cellular_organisms - ps1
Archaea1 - ps2
Archaea2 - ps3
Archaea3 - ps4
Asgard/Eukaryota - ps5
Eukaryota - ps6
Amorpheae - ps7
Obazoa - ps8
Opisthokonta - ps9
Holozoa - ps10
Filozoa - ps11
Choano./Metazoa - ps12
Metazoa - ps13
Myriazoa - ps14
Parahoxozoa - ps15
Bilateria - ps16
Deuterostomia - ps17
Chordata - ps18
Olfactores - ps19
Vertebrata - ps20
Gnathostomata - ps21
Euteleostomi - ps22
Sarcopterygii - ps23
Tetrapoda - ps24
Amniota - ps25
Mammalia - ps26
Theria - ps27
Placentalia - ps28
Boreoeutheria - ps29
Euarchontoglires - ps30
Euarchonta - ps31
Primates - ps32
Haplorrhini - ps33
Simiiformes - ps34
Catarrhini - ps35
Hominoidea - ps36
Hominidae - ps37
Homininae - ps38
Hominini - ps39
Homo_sapiens - ps40
Phylostratum

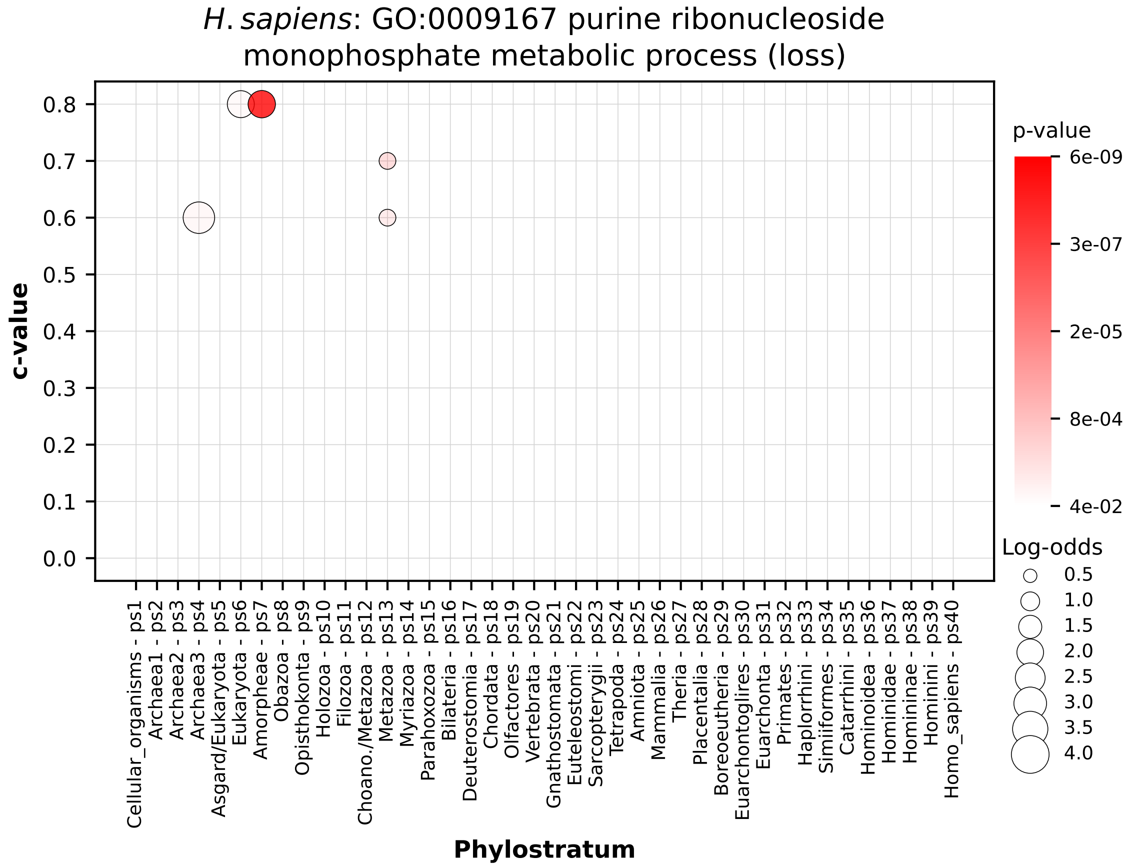
H. sapiens: GO:0009167 purine ribonucleoside monophosphate metabolic process (loss)
c-value
0.8
0.7
0.6
0.5
0.4
0.3
0.2
0.1
0.0
p-value
6e-09
3e-07
2e-05
8e-04
4e-02
Log-odds
0.5
1.0
1.5
2.0
2.5
3.0
3.5
4.0
Cellular_organisms - ps1
Archaea1 - ps2
Archaea2 - ps3
Archaea3 - ps4
Asgard/Eukaryota - ps5
Eukaryota - ps6
Amorpheae - ps7
Obazoa - ps8
Opisthokonta - ps9
Holozoa - ps10
Filozoa - ps11
Choano./Metazoa - ps12
Metazoa - ps13
Myriazoa - ps14
Parahoxozoa - ps15
Bilateria - ps16
Deuterostomia - ps17
Chordata - ps18
Olfactores - ps19
Vertebrata - ps20
Gnathostomata - ps21
Euteleostomi - ps22
Sarcopterygii - ps23
Tetrapoda - ps24
Amniota - ps25
Mammalia - ps26
Theria - ps27
Placentalia - ps28
Boreoeutheria - ps29
Euarchontoglires - ps30
Euarchonta - ps31
Primates - ps32
Haplorrhini - ps33
Simiiformes - ps34
Catarrhini - ps35
Hominoidea - ps36
Hominidae - ps37
Homininae - ps38
Hominini - ps39
Homo_sapiens - ps40
Phylostratum

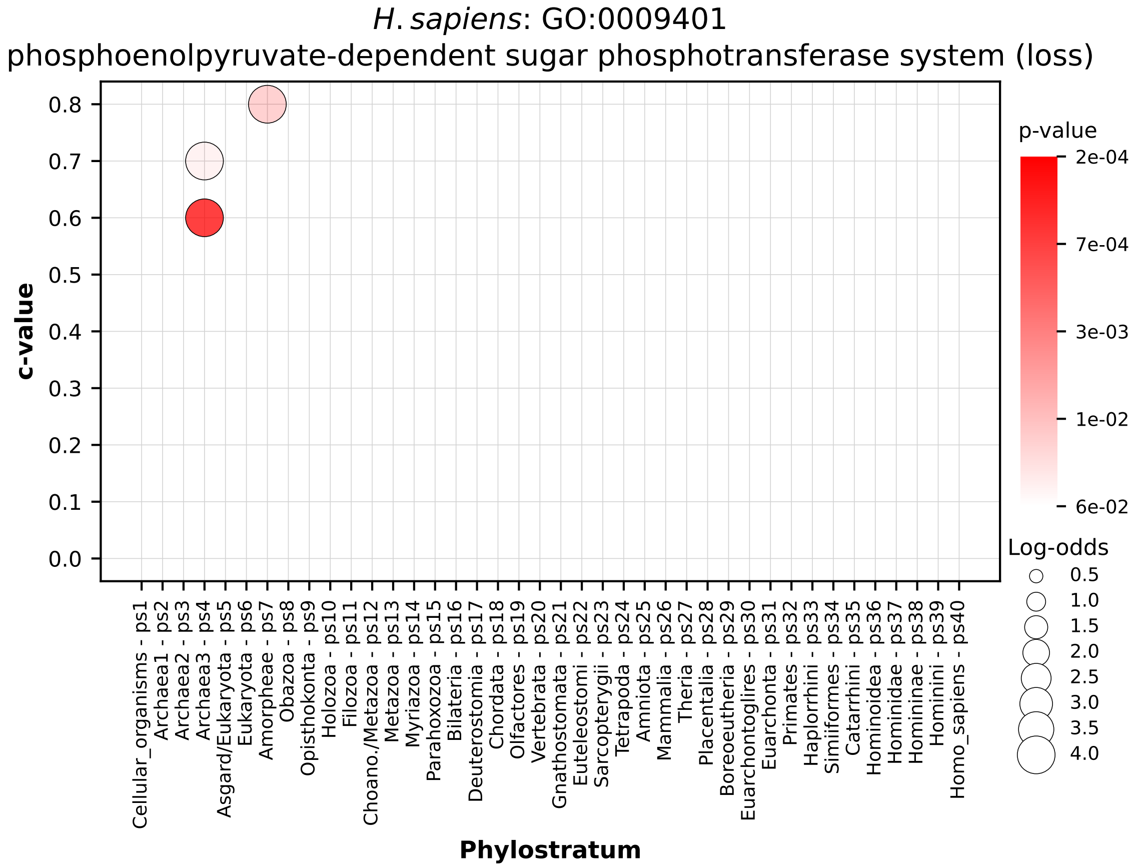
H. sapiens: GO:0009401
phosphoenolpyruvate-dependent sugar phosphotransferase system (loss)
c-value
0.0
0.1
0.2
0.3
0.4
0.5
0.6
0.7
0.8
p-value
2e-04
7e-04
3e-03
1e-02
6e-02
Log-odds
0.5
1.0
1.5
2.0
2.5
3.0
3.5
4.0
Cellular_organisms - ps1
Archaea1 - ps2
Archaea2 - ps3
Archaea3 - ps4
Asgard/Eukaryota - ps5
Eukaryota - ps6
Amorpheae - ps7
Obazoa - ps8
Opisthokonta - ps9
Holozoa - ps10
Filozoa - ps11
Choano./Metazoa - ps12
Metazoa - ps13
Myriazoa - ps14
Parahoxozoa - ps15
Bilateria - ps16
Deuterostomia - ps17
Chordata - ps18
Olfactores - ps19
Vertebrata - ps20
Gnathostomata - ps21
Euteleostomi - ps22
Sarcopterygii - ps23
Tetrapoda - ps24
Amniota - ps25
Mammalia - ps26
Theria - ps27
Placentalia - ps28
Boreoeutheria - ps29
Euarchontoglires - ps30
Euarchonta - ps31
Primates - ps32
Haplorrhini - ps33
Simiiformes - ps34
Catarrhini - ps35
Hominoidea - ps36
Hominidae - ps37
Homininae - ps38
Hominini - ps39
Homo_sapiens - ps40
Phylostratum

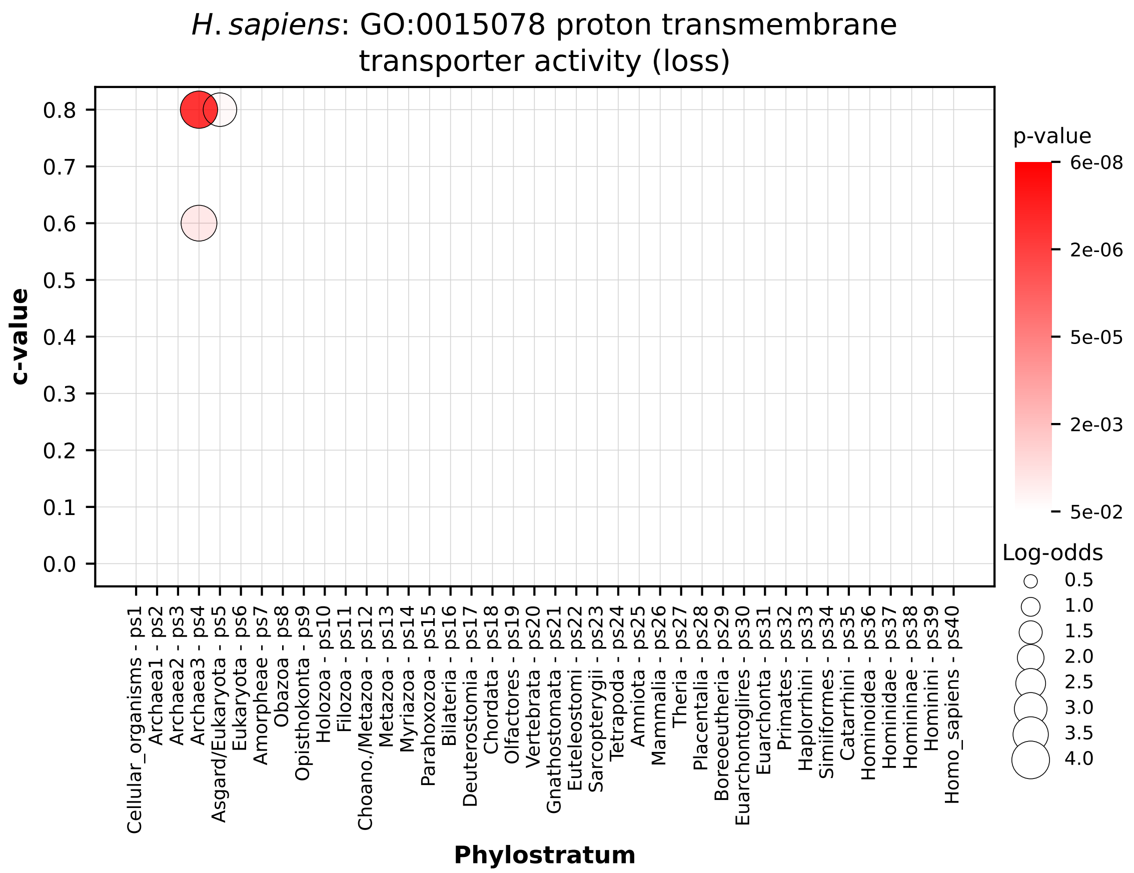

H. sapiens: GO:0015078 proton transmembrane transporter activity (loss)
c-value
0.8
0.7
0.6
0.5
0.4
0.3
0.2
0.1
0.0
p-value
6e-08
2e-06
5e-05
2e-03
5e-02
Log-odds
0.5
1.0
1.5
2.0
2.5
3.0
3.5
4.0
Cellular_organisms - ps1
Archaea1 - ps2
Archaea2 - ps3
Archaea3 - ps4
Asgard/Eukaryota - ps5
Eukaryota - ps6
Amorpheae - ps7
Obazoa - ps8
Opisthokonta - ps9
Holozoa - ps10
Filozoa - ps11
Choano./Metazoa - ps12
Metazoa - ps13
Myriazoa - ps14
Parahoxozoa - ps15
Bilateria - ps16
Deuterostomia - ps17
Chordata - ps18
Olfactores - ps19
Vertebrata - ps20
Gnathostomata - ps21
Euteleostomi - ps22
Sarcopterygii - ps23
Tetrapoda - ps24
Amniota - ps25
Mammalia - ps26
Theria - ps27
Placentalia - ps28
Boreoeutheria - ps29
Euarchontoglires - ps30
Euarchonta - ps31
Primates - ps32
Haplorrhini - ps33
Simiiformes - ps34
Catarrhini - ps35
Hominoidea - ps36
Hominidae - ps37
Homininae - ps38
Hominini - ps39
Homo_sapiens - ps40
Phylostratum

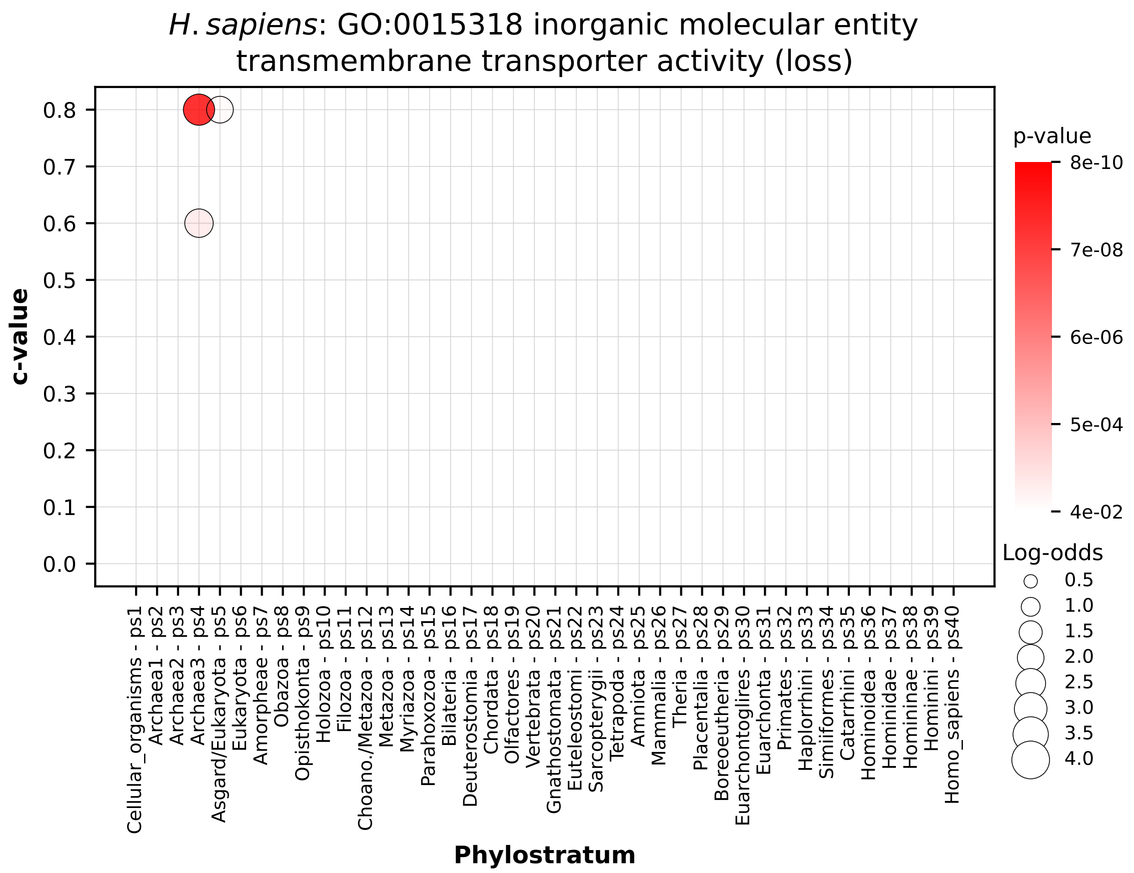

H. sapiens: GO:0015318 inorganic molecular entity transmembrane transporter activity (loss)
c-value
0.0
0.1
0.2
0.3
0.4
0.5
0.6
0.7
0.8
p-value
8e-10
7e-08
6e-06
5e-04
4e-02
Log-odds
0.5
1.0
1.5
2.0
2.5
3.0
3.5
4.0
Cellular_organisms - ps1
Archaea1 - ps2
Archaea2 - ps3
Archaea3 - ps4
Asgard/Eukaryota - ps5
Eukaryota - ps6
Amorpheae - ps7
Obazoa - ps8
Opisthokonta - ps9
Holozoa - ps10
Filozoa - ps11
Choano./Metazoa - ps12
Metazoa - ps13
Myriazoa - ps14
Parahoxozoa - ps15
Bilateria - ps16
Deuterostomia - ps17
Chordata - ps18
Olfactores - ps19
Vertebrata - ps20
Gnathostomata - ps21
Euteleostomi - ps22
Sarcopterygii - ps23
Tetrapoda - ps24
Amniota - ps25
Mammalia - ps26
Theria - ps27
Placentalia - ps28
Boreoeutheria - ps29
Euarchontoglires - ps30
Euarchonta - ps31
Primates - ps32
Haplorrhini - ps33
Simiiformes - ps34
Catarrhini - ps35
Hominoidea - ps36
Hominidae - ps37
Homininae - ps38
Hominini - ps39
Homo_sapiens - ps40
Phylostratum

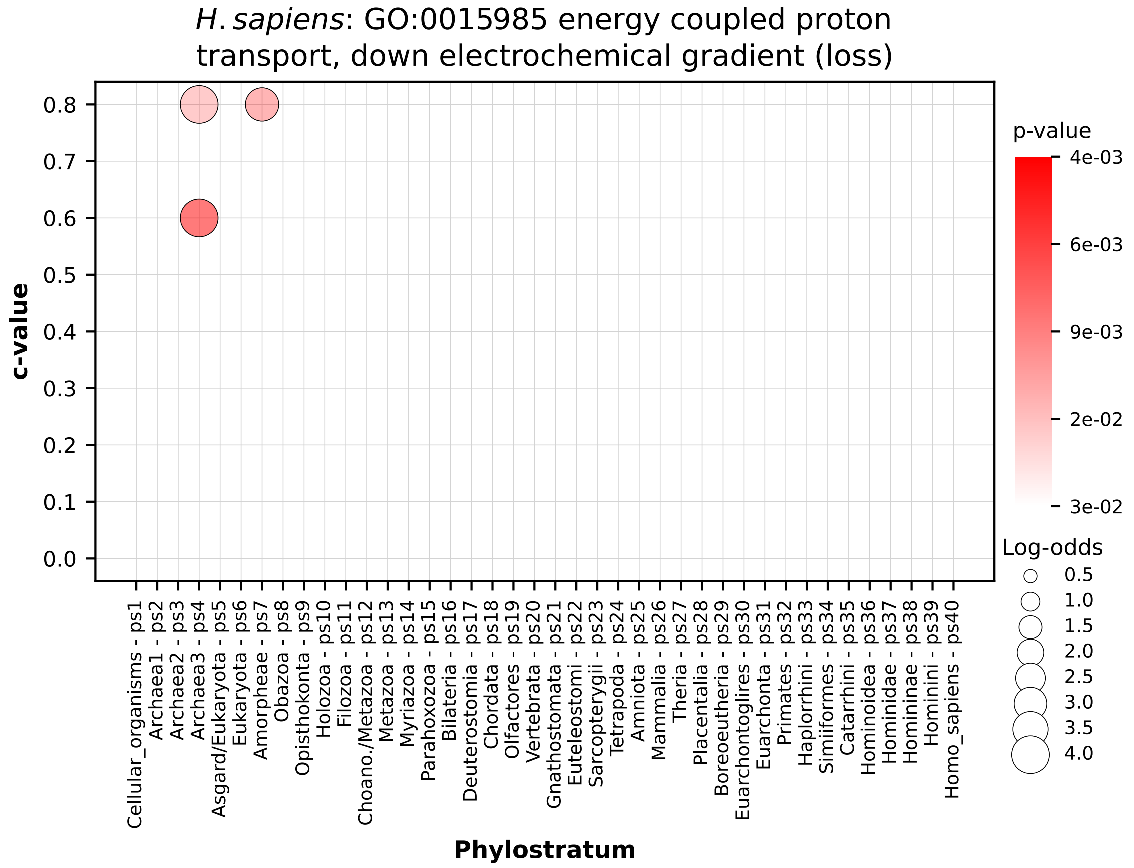

H. sapiens: GO:0015985 energy coupled proton transport, down electrochemical gradient (loss)
c-value
0.0
0.1
0.2
0.3
0.4
0.5
0.6
0.7
0.8
Phylostratum
Cellular_organisms - ps1
Archaea1 - ps2
Archaea2 - ps3
Archaea3 - ps4
Asgard/Eukaryota - ps5
Eukaryota - ps6
Amorpheae - ps7
Obazoa - ps8
Opisthokonta - ps9
Holozoa - ps10
Filozoa - ps11
Choano./Metazoa - ps12
Metazoa - ps13
Myriazoa - ps14
Parahoxozoa - ps15
Bilateria - ps16
Deuterostomia - ps17
Chordata - ps18
Olfactores - ps19
Vertebrata - ps20
Gnathostomata - ps21
Euteleostomi - ps22
Sarcopterygii - ps23
Tetrapoda - ps24
Amniota - ps25
Mammalia - ps26
Theria - ps27
Placentalia - ps28
Boreoeutheria - ps29
Euarchontoglires - ps30
Euarchonta - ps31
Primates - ps32
Haplorrhini - ps33
Simiiformes - ps34
Catarrhini - ps35
Hominoidea - ps36
Hominidae - ps37
Homininae - ps38
Hominini - ps39
Homo_sapiens - ps40
p-value
4e-03
6e-03
9e-03
2e-02
3e-02
Log-odds
0.5
1.0
1.5
2.0
2.5
3.0
3.5
4.0

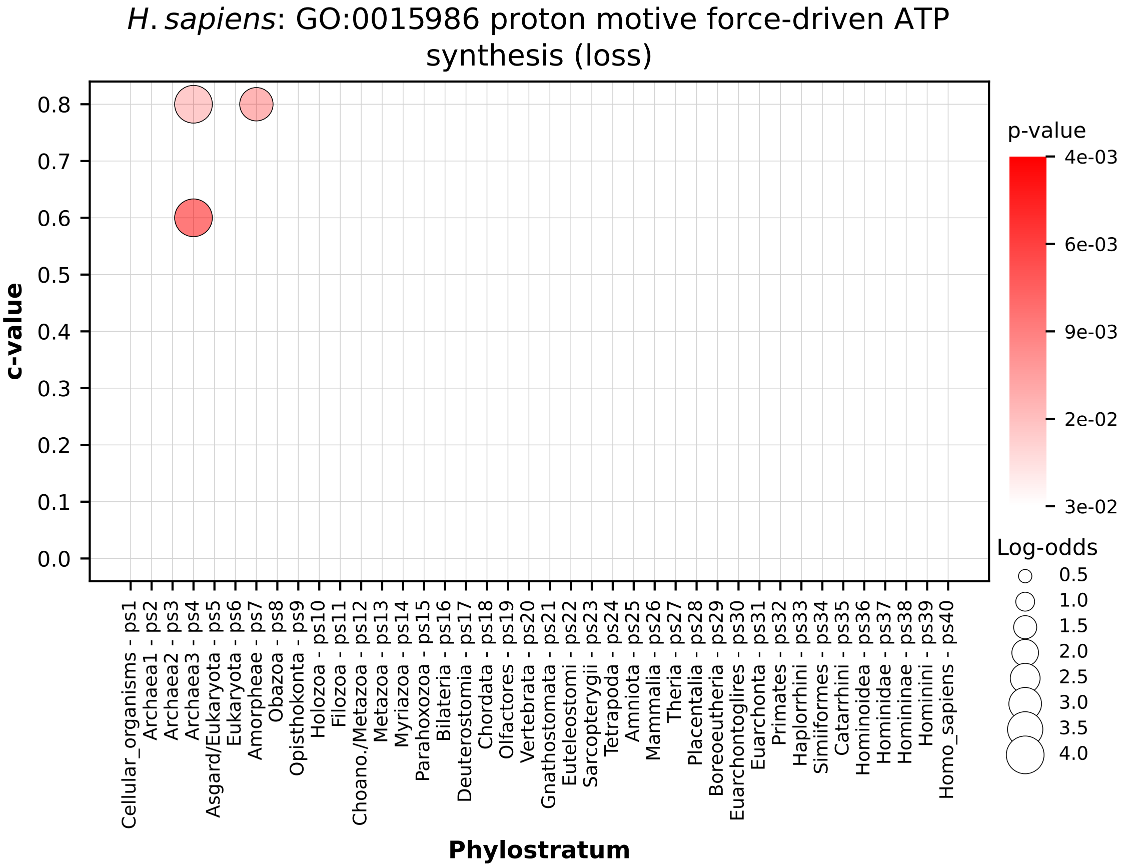
H. sapiens: GO:0015986 proton motive force-driven ATP synthesis (loss)
c-value
0.0
0.1
0.2
0.3
0.4
0.5
0.6
0.7
0.8
Phylostratum
Cellular_organisms - ps1
Archaea1 - ps2
Archaea2 - ps3
Archaea3 - ps4
Asgard/Eukaryota - ps5
Eukaryota - ps6
Amorpheae - ps7
Obazoa - ps8
Opisthokonta - ps9
Holozoa - ps10
Filozoa - ps11
Choano./Metazoa - ps12
Metazoa - ps13
Myriazoa - ps14
Parahoxozoa - ps15
Bilateria - ps16
Deuterostomia - ps17
Chordata - ps18
Olfactores - ps19
Vertebrata - ps20
Gnathostomata - ps21
Euteleostomi - ps22
Sarcopterygii - ps23
Tetrapoda - ps24
Amniota - ps25
Mammalia - ps26
Theria - ps27
Placentalia - ps28
Boreoeutheria - ps29
Euarchontoglires - ps30
Euarchonta - ps31
Primates - ps32
Haplorrhini - ps33
Simiiformes - ps34
Catarrhini - ps35
Hominoidea - ps36
Hominidae - ps37
Homininae - ps38
Hominini - ps39
Homo_sapiens - ps40
p-value
4e-03
6e-03
9e-03
2e-02
3e-02
Log-odds
0.5
1.0
1.5
2.0
2.5
3.0
3.5
4.0

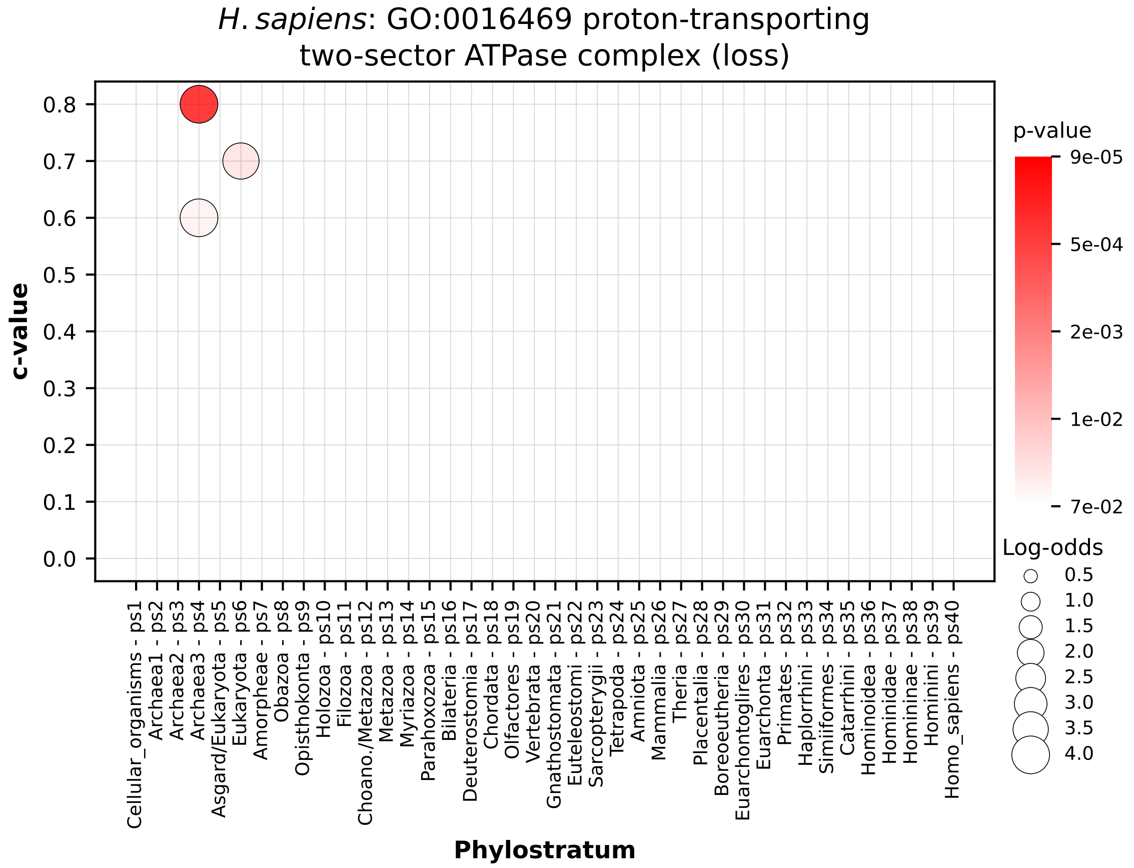

H. sapiens: GO:0016469 proton-transporting two-sector ATPase complex (loss)
c-value
0.0
0.1
0.2
0.3
0.4
0.5
0.6
0.7
0.8
Phylostratum
Cellular_organisms - ps1
Archaea1 - ps2
Archaea2 - ps3
Archaea3 - ps4
Asgard/Eukaryota - ps5
Eukaryota - ps6
Amorpheae - ps7
Obazoa - ps8
Opisthokonta - ps9
Holozoa - ps10
Filozoa - ps11
Choano./Metazoa - ps12
Metazoa - ps13
Myriazoa - ps14
Parahoxozoa - ps15
Bilateria - ps16
Deuterostomia - ps17
Chordata - ps18
Olfactores - ps19
Vertebrata - ps20
Gnathostomata - ps21
Euteleostomi - ps22
Sarcopterygii - ps23
Tetrapoda - ps24
Amniota - ps25
Mammalia - ps26
Theria - ps27
Placentalia - ps28
Boreoeutheria - ps29
Euarchontoglires - ps30
Euarchonta - ps31
Primates - ps32
Haplorrhini - ps33
Simiiformes - ps34
Catarrhini - ps35
Hominoidea - ps36
Hominidae - ps37
Homininae - ps38
Hominini - ps39
Homo_sapiens - ps40
p-value
9e-05
5e-04
2e-03
1e-02
7e-02
Log-odds
0.5
1.0
1.5
2.0
2.5
3.0
3.5
4.0

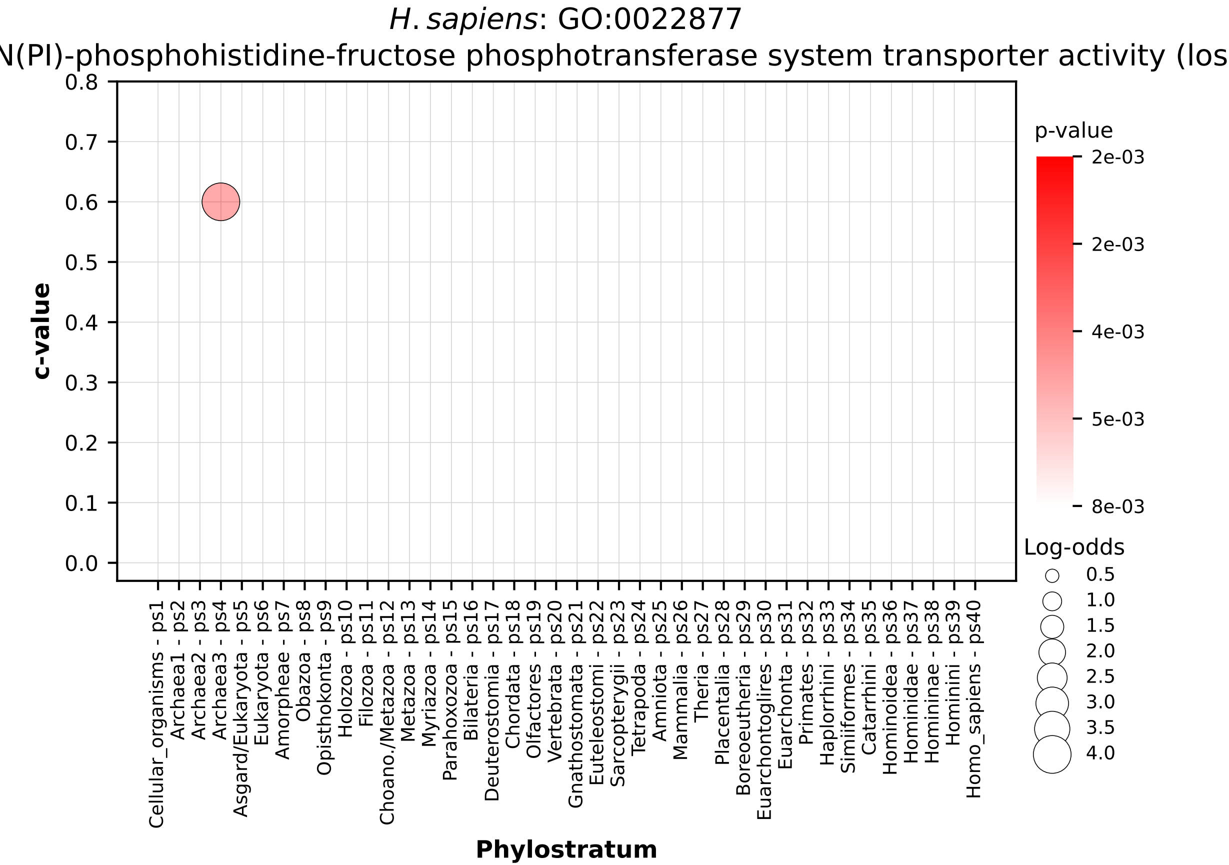

H. sapiens: GO:0022877
N(PI)-phosphohistidine-fructose phosphotransferase system transporter activity (los
c-value
0.8
0.7
0.6
0.5
0.4
0.3
0.2
0.1
0.0
p-value
2e-03
2e-03
4e-03
5e-03
8e-03
Log-odds
0.5
1.0
1.5
2.0
2.5
3.0
3.5
4.0
Cellular_organisms - ps1
Archaea1 - ps2
Archaea2 - ps3
Archaea3 - ps4
Asgard/Eukaryota - ps5
Eukaryota - ps6
Amorpheae - ps7
Obazoa - ps8
Opisthokonta - ps9
Holozoa - ps10
Filozoa - ps11
Choano./Metazoa - ps12
Metazoa - ps13
Myriazoa - ps14
Parahoxozoa - ps15
Bilateria - ps16
Deuterostomia - ps17
Chordata - ps18
Olfactores - ps19
Vertebrata - ps20
Gnathostomata - ps21
Euteleostomi - ps22
Sarcopterygii - ps23
Tetrapoda - ps24
Amniota - ps25
Mammalia - ps26
Theria - ps27
Placentalia - ps28
Boreoeutheria - ps29
Euarchontoglires - ps30
Euarchonta - ps31
Primates - ps32
Haplorrhini - ps33
Simiiformes - ps34
Catarrhini - ps35
Hominoidea - ps36
Hominidae - ps37
Homininae - ps38
Hominini - ps39
Homo_sapiens - ps40
Phylostratum

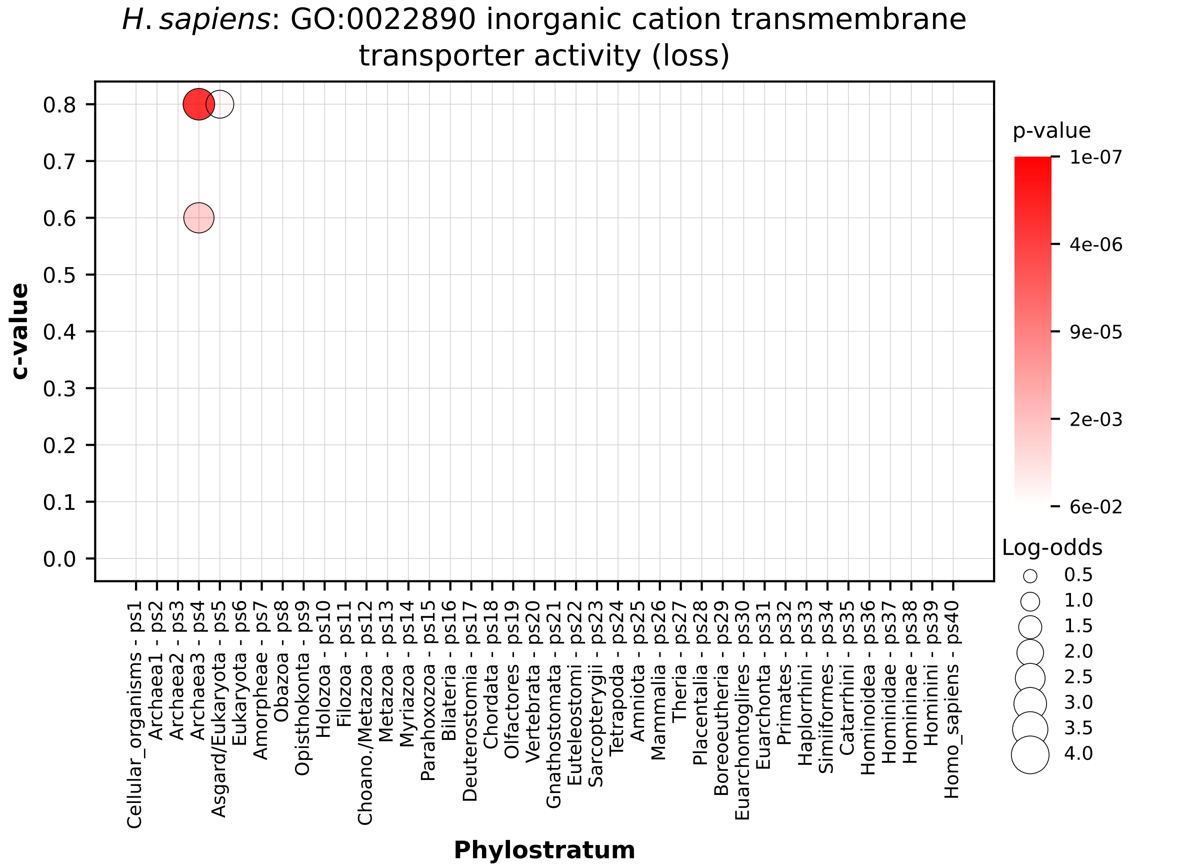
H. sapiens: GO:0022890 inorganic cation transmembrane transporter activity (loss)
c-value
0.0
0.1
0.2
0.3
0.4
0.5
0.6
0.7
0.8
Phylostratum
Cellular_organisms - ps1
Archaea1 - ps2
Archaea2 - ps3
Archaea3 - ps4
Asgard/Eukaryota - ps5
Eukaryota - ps6
Amorpheae - ps7
Obazoa - ps8
Opisthokonta - ps9
Holozoa - ps10
Filozoa - ps11
Choano./Metazoa - ps12
Metazoa - ps13
Myriazoa - ps14
Parahoxozoa - ps15
Bilateria - ps16
Deuterostomia - ps17
Chordata - ps18
Olfactores - ps19
Vertebrata - ps20
Gnathostomata - ps21
Euteleostomi - ps22
Sarcopterygii - ps23
Tetrapoda - ps24
Amniota - ps25
Mammalia - ps26
Theria - ps27
Placentalia - ps28
Boreoeutheria - ps29
Euarchontoglires - ps30
Euarchonta - ps31
Primates - ps32
Haplorrhini - ps33
Simiiformes - ps34
Catarrhini - ps35
Hominoidea - ps36
Hominidae - ps37
Homininae - ps38
Hominini - ps39
Homo_sapiens - ps40
p-value
1e-07
4e-06
9e-05
2e-03
6e-02
Log-odds
0.5
1.0
1.5
2.0
2.5
3.0
3.5
4.0

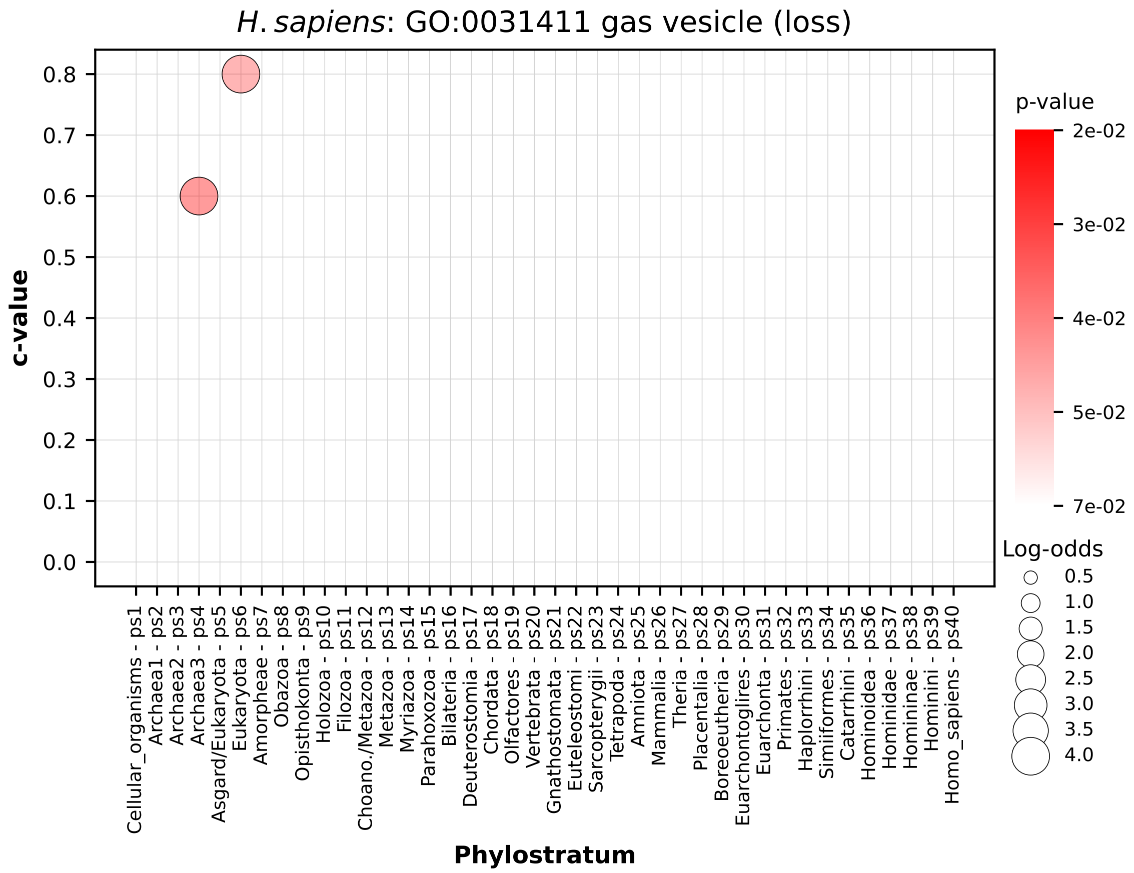

H. sapiens: GO:0031411 gas vesicle (loss)
c-value
Phylostratum
p-value
Log-odds
0.0
0.1
0.2
0.3
0.4
0.5
0.6
0.7
0.8
2e-02
3e-02
4e-02
5e-02
7e-02
0.5
1.0
1.5
2.0
2.5
3.0
3.5
4.0
Cellular_organisms - ps1
Archaea1 - ps2
Archaea2 - ps3
Archaea3 - ps4
Asgard/Eukaryota - ps5
Eukaryota - ps6
Amorpheae - ps7
Obazoa - ps8
Opisthokonta - ps9
Holozoa - ps10
Filozoa - ps11
Choano./Metazoa - ps12
Metazoa - ps13
Myriazoa - ps14
Parahoxozoa - ps15
Bilateria - ps16
Deuterostomia - ps17
Chordata - ps18
Olfactores - ps19
Vertebrata - ps20
Gnathostomata - ps21
Euteleostomi - ps22
Sarcopterygii - ps23
Tetrapoda - ps24
Amniota - ps25
Mammalia - ps26
Theria - ps27
Placentalia - ps28
Boreoeutheria - ps29
Euarchontoglires - ps30
Euarchonta - ps31
Primates - ps32
Haplorrhini - ps33
Simiiformes - ps34
Catarrhini - ps35
Hominoidea - ps36
Hominidae - ps37
Homininae - ps38
Hominini - ps39
Homo_sapiens - ps40

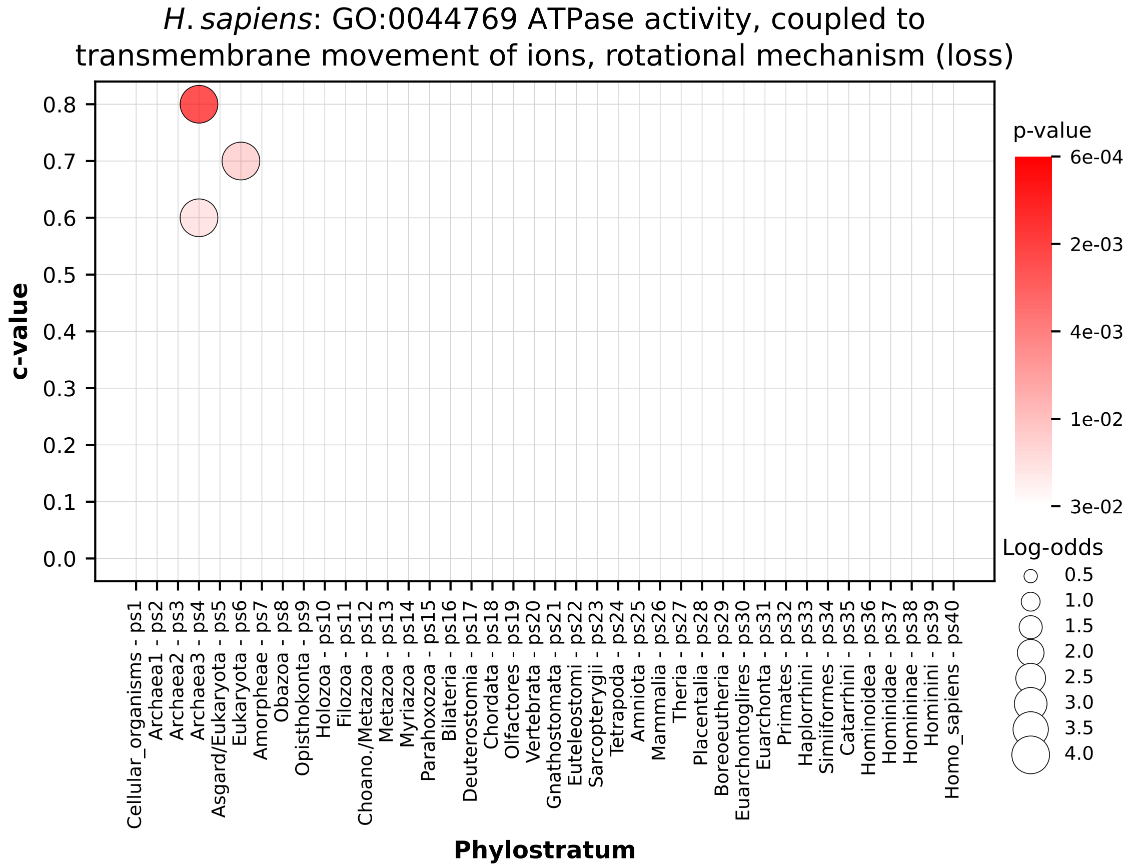

H. sapiens: GO:0044769 ATPase activity, coupled to transmembrane movement of ions, rotational mechanism (loss)
c-value
0.0
0.1
0.2
0.3
0.4
0.5
0.6
0.7
0.8
p-value
6e-04
2e-03
4e-03
1e-02
3e-02
Log-odds
0.5
1.0
1.5
2.0
2.5
3.0
3.5
4.0
Cellular_organisms - ps1
Archaea1 - ps2
Archaea2 - ps3
Archaea3 - ps4
Asgard/Eukaryota - ps5
Eukaryota - ps6
Amorpheae - ps7
Obazoa - ps8
Opisthokonta - ps9
Holozoa - ps10
Filozoa - ps11
Choano./Metazoa - ps12
Metazoa - ps13
Myriazoa - ps14
Parahoxozoa - ps15
Bilateria - ps16
Deuterostomia - ps17
Chordata - ps18
Olfactores - ps19
Vertebrata - ps20
Gnathostomata - ps21
Euteleostomi - ps22
Sarcopterygii - ps23
Tetrapoda - ps24
Amniota - ps25
Mammalia - ps26
Theria - ps27
Placentalia - ps28
Boreoeutheria - ps29
Euarchontoglires - ps30
Euarchonta - ps31
Primates - ps32
Haplorrhini - ps33
Simiiformes - ps34
Catarrhini - ps35
Hominoidea - ps36
Hominidae - ps37
Homininae - ps38
Hominini - ps39
Homo_sapiens - ps40
Phylostratum

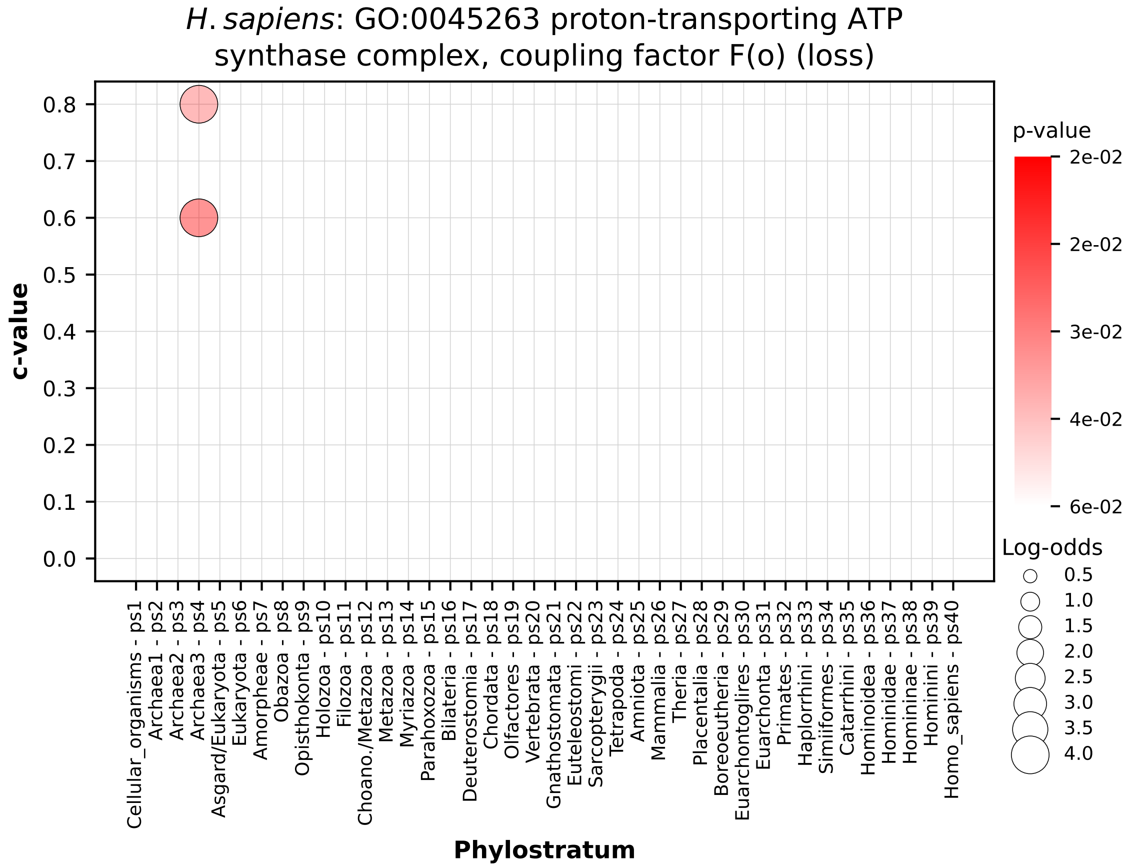

H. sapiens: GO:0045263 proton-transporting ATP synthase complex, coupling factor F(o) (loss)
c-value
0.8
0.7
0.6
0.5
0.4
0.3
0.2
0.1
0.0
p-value
2e-02
2e-02
3e-02
4e-02
6e-02
Log-odds
0.5
1.0
1.5
2.0
2.5
3.0
3.5
4.0
Cellular_organisms - ps1
Archaea1 - ps2
Archaea2 - ps3
Archaea3 - ps4
Asgard/Eukaryota - ps5
Eukaryota - ps6
Amorpheae - ps7
Obazoa - ps8
Opisthokonta - ps9
Holozoa - ps10
Filozoa - ps11
Choano./Metazoa - ps12
Metazoa - ps13
Myriazoa - ps14
Parahoxozoa - ps15
Bilateria - ps16
Deuterostomia - ps17
Chordata - ps18
Olfactores - ps19
Vertebrata - ps20
Gnathostomata - ps21
Euteleostomi - ps22
Sarcopterygii - ps23
Tetrapoda - ps24
Amniota - ps25
Mammalia - ps26
Theria - ps27
Placentalia - ps28
Boreoeutheria - ps29
Euarchontoglires - ps30
Euarchonta - ps31
Primates - ps32
Haplorrhini - ps33
Simiiformes - ps34
Catarrhini - ps35
Hominoidea - ps36
Hominidae - ps37
Homininae - ps38
Hominini - ps39
Homo_sapiens - ps40
Phylostratum

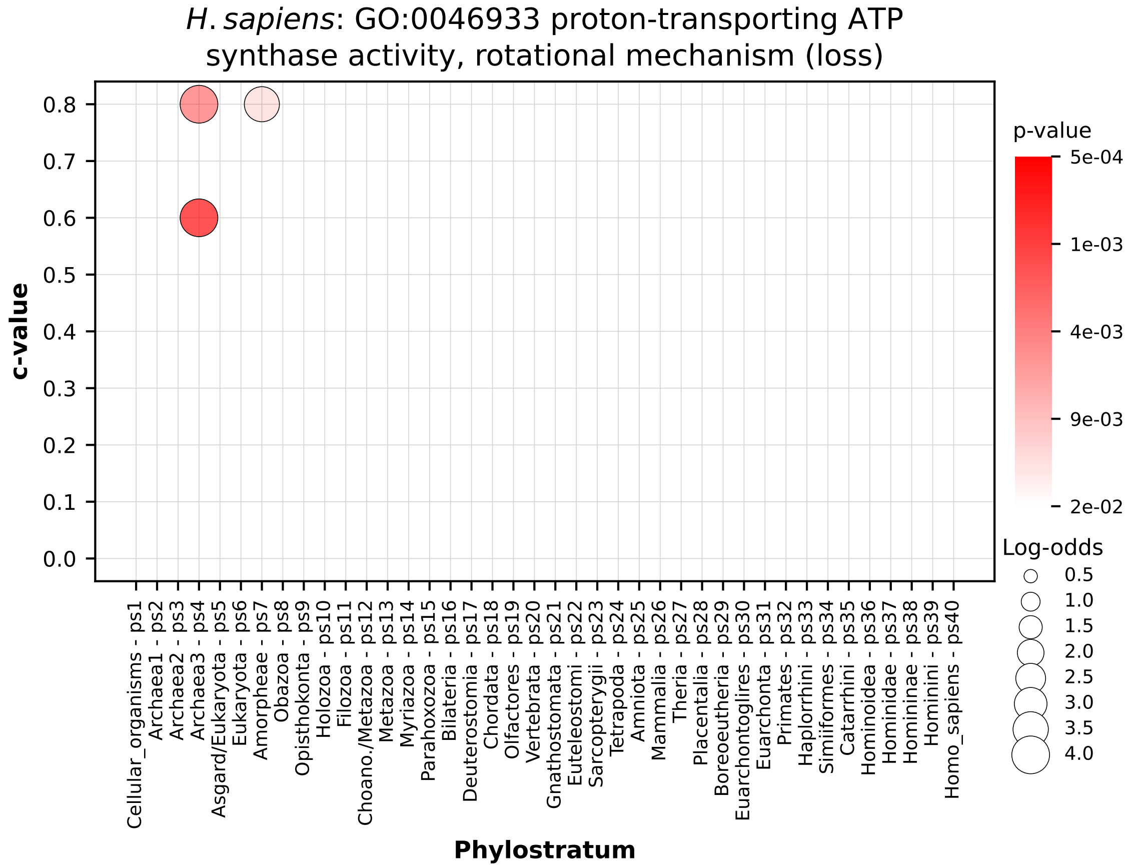

H. sapiens: GO:0046933 proton-transporting ATP synthase activity, rotational mechanism (loss)
c-value
0.0
0.1
0.2
0.3
0.4
0.5
0.6
0.7
0.8
p-value
5e-04
1e-03
4e-03
9e-03
2e-02
Log-odds
0.5
1.0
1.5
2.0
2.5
3.0
3.5
4.0
Cellular_organisms - ps1
Archaea1 - ps2
Archaea2 - ps3
Archaea3 - ps4
Asgard/Eukaryota - ps5
Eukaryota - ps6
Amorpheae - ps7
Obazoa - ps8
Opisthokonta - ps9
Holozoa - ps10
Filozoa - ps11
Choano./Metazoa - ps12
Metazoa - ps13
Myriazoa - ps14
Parahoxozoa - ps15
Bilateria - ps16
Deuterostomia - ps17
Chordata - ps18
Olfactores - ps19
Vertebrata - ps20
Gnathostomata - ps21
Euteleostomi - ps22
Sarcopterygii - ps23
Tetrapoda - ps24
Amniota - ps25
Mammalia - ps26
Theria - ps27
Placentalia - ps28
Boreoeutheria - ps29
Euarchontoglires - ps30
Euarchonta - ps31
Primates - ps32
Haplorrhini - ps33
Simiiformes - ps34
Catarrhini - ps35
Hominoidea - ps36
Hominidae - ps37
Homininae - ps38
Hominini - ps39
Homo_sapiens - ps40
Phylostratum

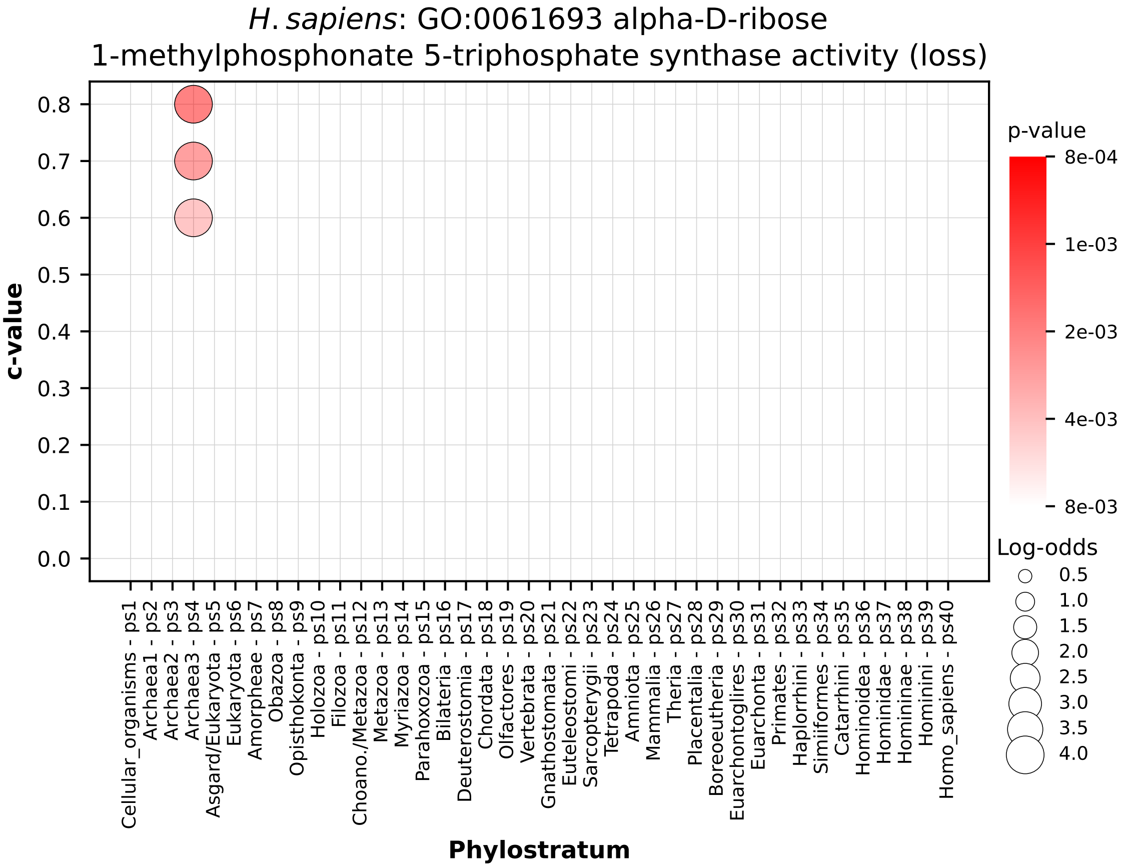

H. sapiens: GO:0061693 alpha-D-ribose
1-methylphosphonate 5-triphosphate synthase activity (loss)
c-value
0.8
0.7
0.6
0.5
0.4
0.3
0.2
0.1
0.0
Phylostratum
Cellular_organisms - ps1
Archaea1 - ps2
Archaea2 - ps3
Archaea3 - ps4
Asgard/Eukaryota - ps5
Eukaryota - ps6
Amorpheae - ps7
Obazoa - ps8
Opisthokonta - ps9
Holozoa - ps10
Filozoa - ps11
Choano./Metazoa - ps12
Metazoa - ps13
Myriazoa - ps14
Parahoxozoa - ps15
Bilateria - ps16
Deuterostomia - ps17
Chordata - ps18
Olfactores - ps19
Vertebrata - ps20
Gnathostomata - ps21
Euteleostomi - ps22
Sarcopterygii - ps23
Tetrapoda - ps24
Amniota - ps25
Mammalia - ps26
Theria - ps27
Placentalia - ps28
Boreoeutheria - ps29
Euarchontoglires - ps30
Euarchonta - ps31
Primates - ps32
Haplorrhini - ps33
Simiiformes - ps34
Catarrhini - ps35
Hominoidea - ps36
Hominidae - ps37
Homininae - ps38
Hominini - ps39
Homo_sapiens - ps40
p-value
8e-04
1e-03
2e-03
4e-03
8e-03
Log-odds
0.5
1.0
1.5
2.0
2.5
3.0
3.5
4.0

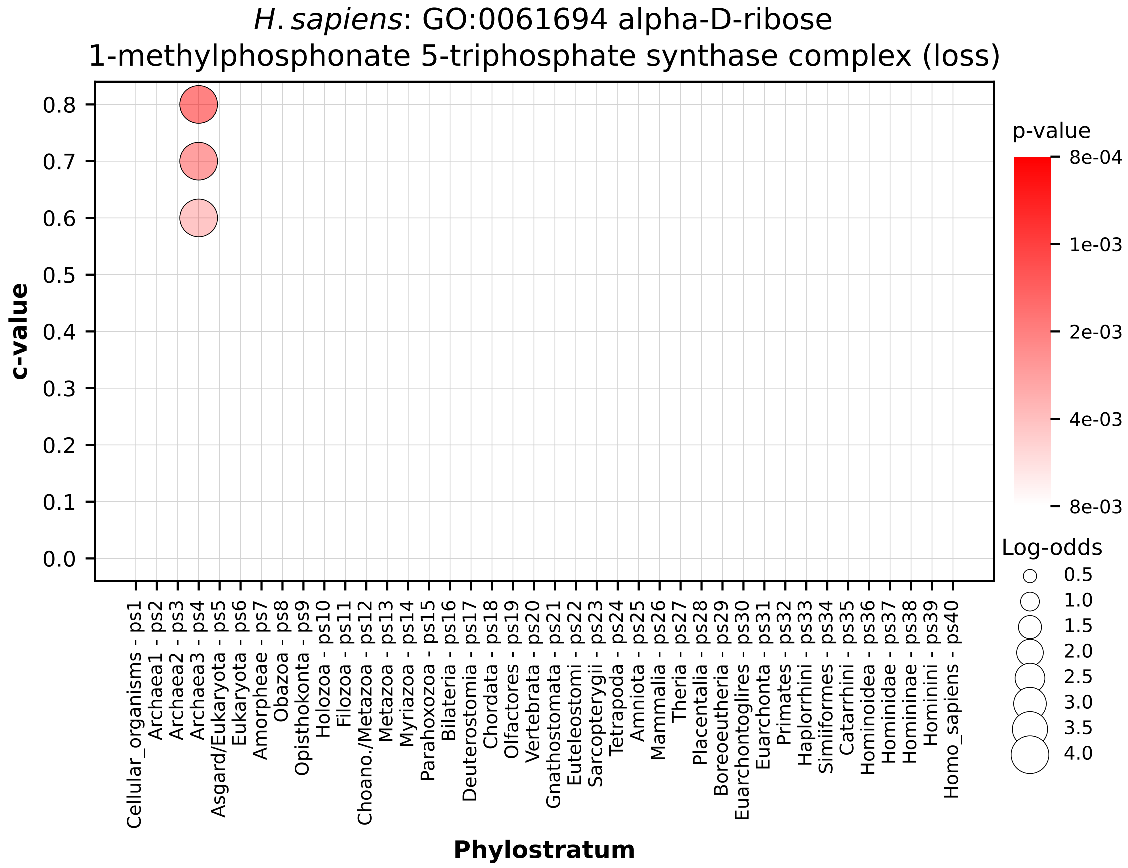

H. sapiens: GO:0061694 alpha-D-ribose
1-methylphosphonate 5-triphosphate synthase complex (loss)
c-value
0.0
0.1
0.2
0.3
0.4
0.5
0.6
0.7
0.8
Phylostratum
Cellular_organisms - ps1
Archaea1 - ps2
Archaea2 - ps3
Archaea3 - ps4
Asgard/Eukaryota - ps5
Eukaryota - ps6
Amorpheae - ps7
Obazoa - ps8
Opisthokonta - ps9
Holozoa - ps10
Filozoa - ps11
Choano./Metazoa - ps12
Metazoa - ps13
Myriazoa - ps14
Parahoxozoa - ps15
Bilateria - ps16
Deuterostomia - ps17
Chordata - ps18
Olfactores - ps19
Vertebrata - ps20
Gnathostomata - ps21
Euteleostomi - ps22
Sarcopterygii - ps23
Tetrapoda - ps24
Amniota - ps25
Mammalia - ps26
Theria - ps27
Placentalia - ps28
Boreoeutheria - ps29
Euarchontoglires - ps30
Euarchonta - ps31
Primates - ps32
Haplorrhini - ps33
Simiiformes - ps34
Catarrhini - ps35
Hominoidea - ps36
Hominidae - ps37
Homininae - ps38
Hominini - ps39
Homo_sapiens - ps40
p-value
8e-04
1e-03
2e-03
4e-03
8e-03
Log-odds
0.5
1.0
1.5
2.0
2.5
3.0
3.5
4.0

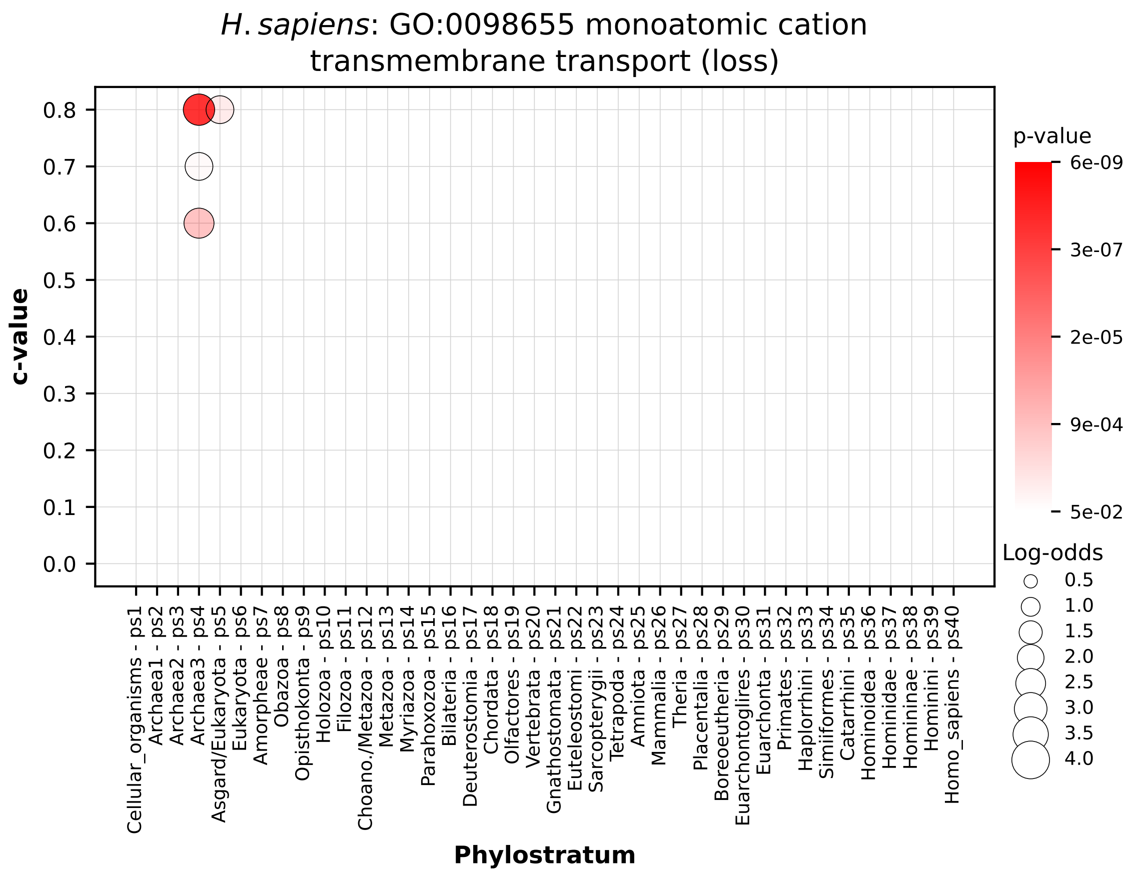

H. sapiens: GO:0098655 monoatomic cation transmembrane transport (loss)
c-value
0.0
0.1
0.2
0.3
0.4
0.5
0.6
0.7
0.8
Phylostratum
Cellular_organisms - ps1
Archaea1 - ps2
Archaea2 - ps3
Archaea3 - ps4
Asgard/Eukaryota - ps5
Eukaryota - ps6
Amorpheae - ps7
Obazoa - ps8
Opisthokonta - ps9
Holozoa - ps10
Filozoa - ps11
Choano./Metazoa - ps12
Metazoa - ps13
Myriazoa - ps14
Parahoxozoa - ps15
Bilateria - ps16
Deuterostomia - ps17
Chordata - ps18
Olfactores - ps19
Vertebrata - ps20
Gnathostomata - ps21
Euteleostomi - ps22
Sarcopterygii - ps23
Tetrapoda - ps24
Amniota - ps25
Mammalia - ps26
Theria - ps27
Placentalia - ps28
Boreoeutheria - ps29
Euarchontoglires - ps30
Euarchonta - ps31
Primates - ps32
Haplorrhini - ps33
Simiiformes - ps34
Catarrhini - ps35
Hominoidea - ps36
Hominidae - ps37
Homininae - ps38
Hominini - ps39
Homo_sapiens - ps40
p-value
6e-09
3e-07
2e-05
9e-04
5e-02
Log-odds
0.5
1.0
1.5
2.0
2.5
3.0
3.5
4.0

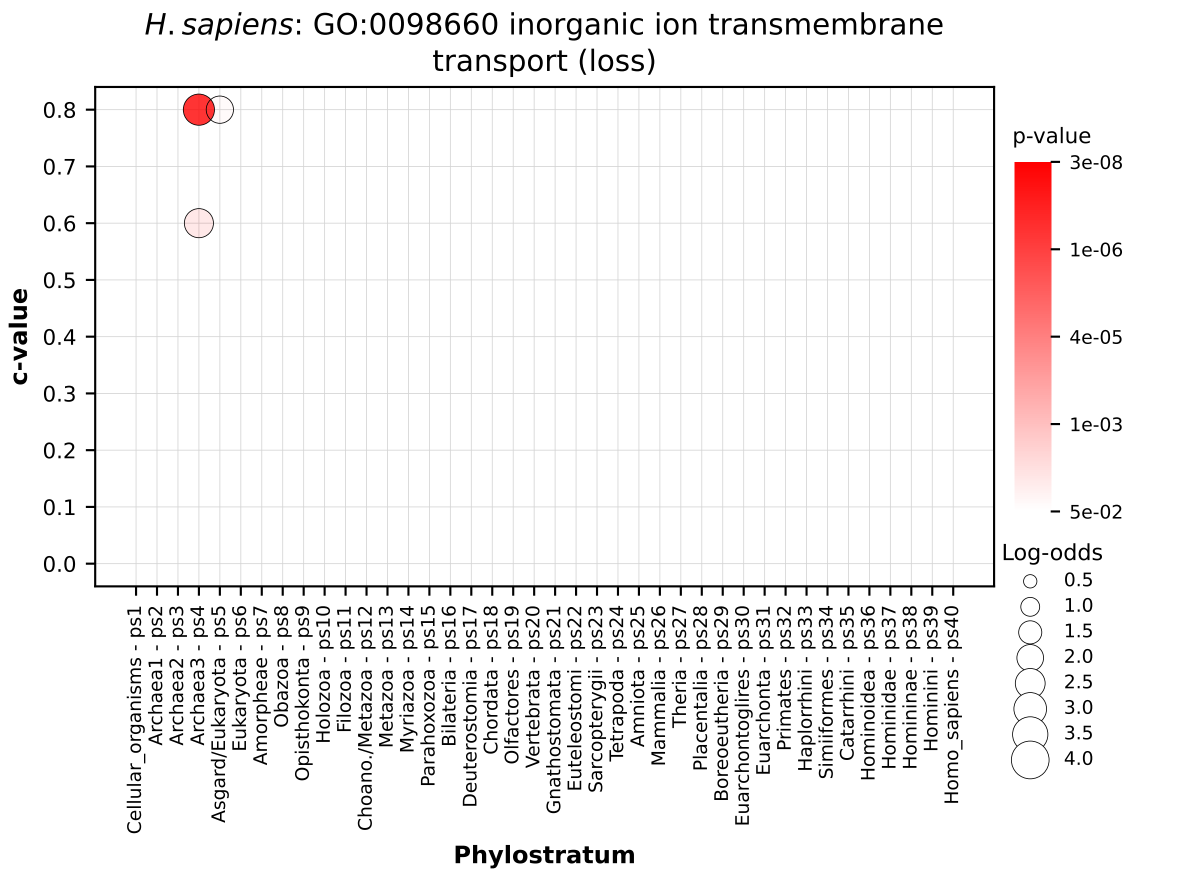

H. sapiens: GO:0098660 inorganic ion transmembrane transport (loss)
c-value
0.8
0.7
0.6
0.5
0.4
0.3
0.2
0.1
0.0
Phylostratum
Cellular_organisms - ps1
Archaea1 - ps2
Archaea2 - ps3
Archaea3 - ps4
Asgard/Eukaryota - ps5
Eukaryota - ps6
Amorpheae - ps7
Obazoa - ps8
Opisthokonta - ps9
Holozoa - ps10
Filozoa - ps11
Choano./Metazoa - ps12
Metazoa - ps13
Myriazoa - ps14
Parahoxozoa - ps15
Bilateria - ps16
Deuterostomia - ps17
Chordata - ps18
Olfactores - ps19
Vertebrata - ps20
Gnathostomata - ps21
Euteleostomi - ps22
Sarcopterygii - ps23
Tetrapoda - ps24
Amniota - ps25
Mammalia - ps26
Theria - ps27
Placentalia - ps28
Boreoeutheria - ps29
Euarchontoglires - ps30
Euarchonta - ps31
Primates - ps32
Haplorrhini - ps33
Simiiformes - ps34
Catarrhini - ps35
Hominoidea - ps36
Hominidae - ps37
Homininae - ps38
Hominini - ps39
Homo_sapiens - ps40
p-value
3e-08
1e-06
4e-05
1e-03
5e-02
Log-odds
0.5
1.0
1.5
2.0
2.5
3.0
3.5
4.0

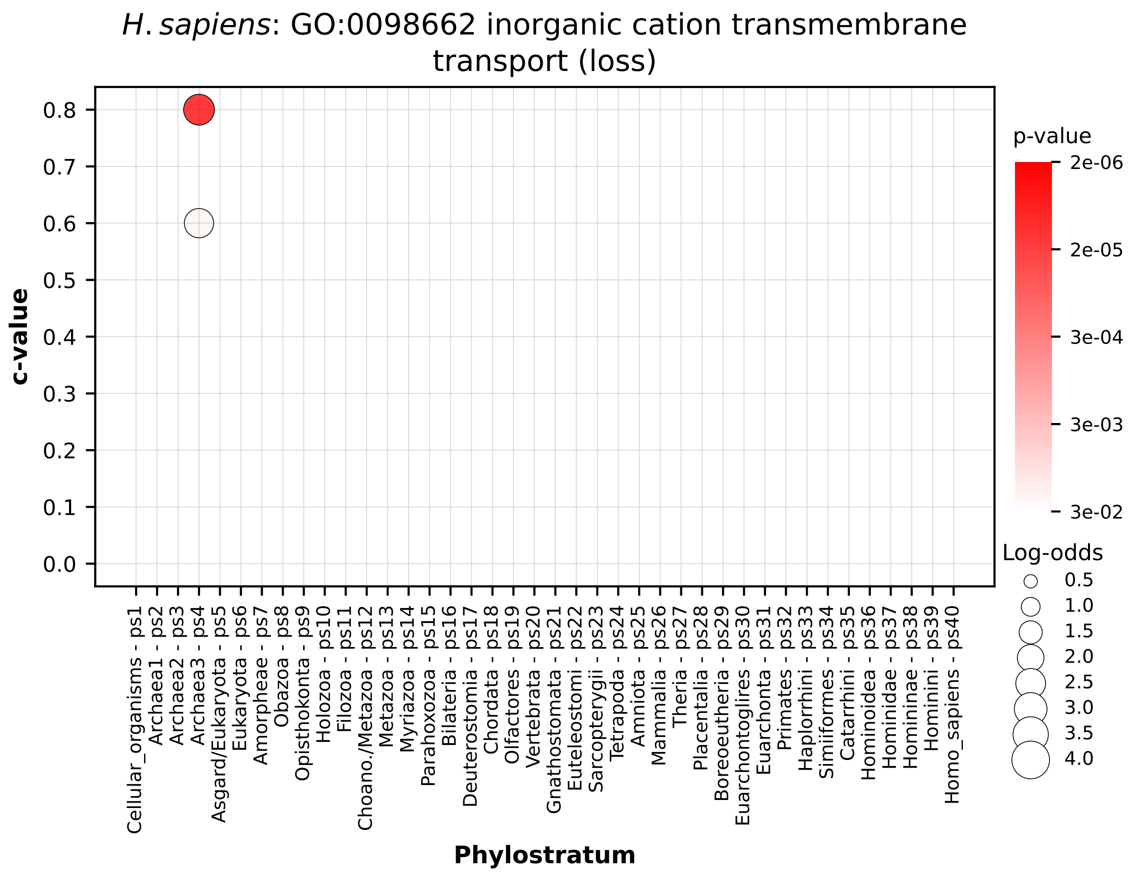

H. sapiens: GO:0098662 inorganic cation transmembrane transport (loss)
c-value
0.0
0.1
0.2
0.3
0.4
0.5
0.6
0.7
0.8
Phylostratum
Cellular_organisms - ps1
Archaea1 - ps2
Archaea2 - ps3
Archaea3 - ps4
Asgard/Eukaryota - ps5
Eukaryota - ps6
Amorpheae - ps7
Obazoa - ps8
Opisthokonta - ps9
Holozoa - ps10
Filozoa - ps11
Choano./Metazoa - ps12
Metazoa - ps13
Myriazoa - ps14
Parahoxozoa - ps15
Bilateria - ps16
Deuterostomia - ps17
Chordata - ps18
Olfactores - ps19
Vertebrata - ps20
Gnathostomata - ps21
Euteleostomi - ps22
Sarcopterygii - ps23
Tetrapoda - ps24
Amniota - ps25
Mammalia - ps26
Theria - ps27
Placentalia - ps28
Boreoeutheria - ps29
Euarchontoglires - ps30
Euarchonta - ps31
Primates - ps32
Haplorrhini - ps33
Simiiformes - ps34
Catarrhini - ps35
Hominoidea - ps36
Hominidae - ps37
Homininae - ps38
Hominini - ps39
Homo_sapiens - ps40
p-value
2e-06
2e-05
3e-04
3e-03
3e-02
Log-odds
0.5
1.0
1.5
2.0
2.5
3.0
3.5
4.0

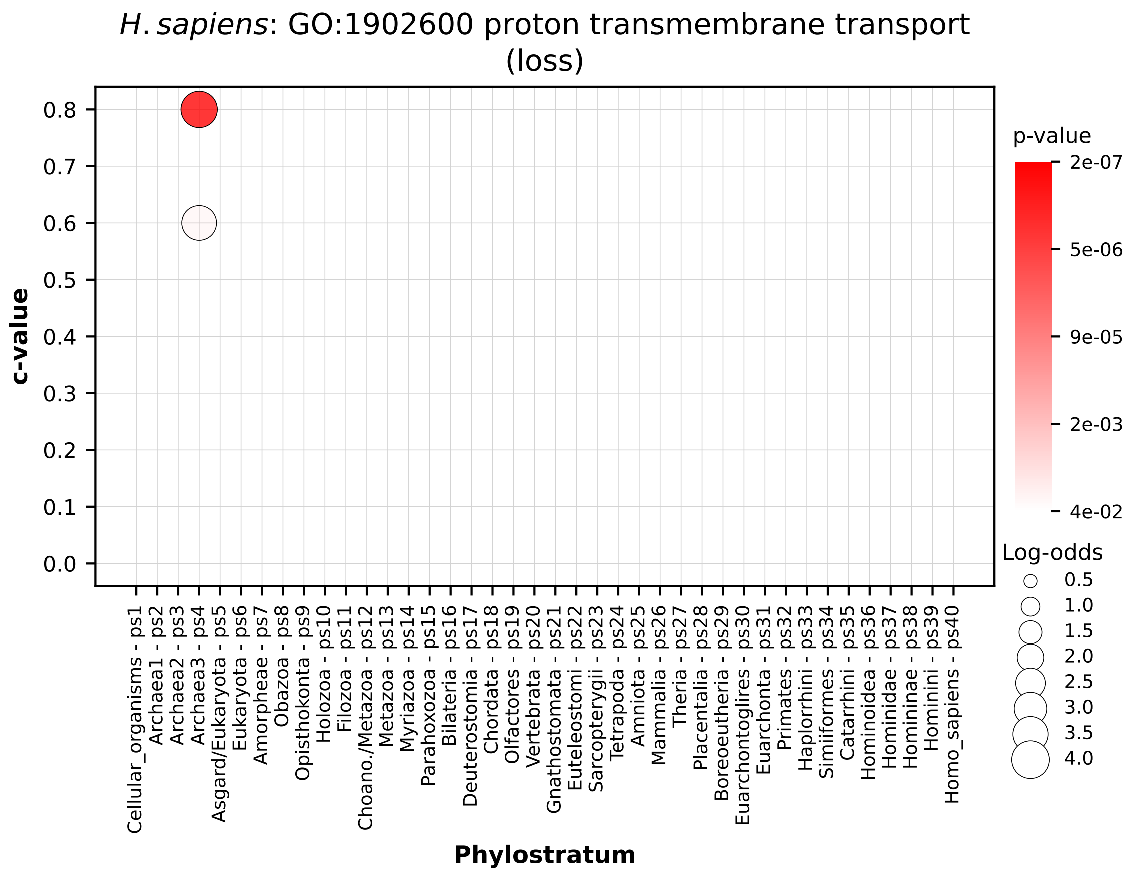

H. sapiens: GO:1902600 proton transmembrane transport
(loss)
c-value
0.8
0.7
0.6
0.5
0.4
0.3
0.2
0.1
0.0
p-value
2e-07
5e-06
9e-05
2e-03
4e-02
Log-odds
0.5
1.0
1.5
2.0
2.5
3.0
3.5
4.0
Cellular_organisms - ps1
Archaea1 - ps2
Archaea2 - ps3
Archaea3 - ps4
Asgard/Eukaryota - ps5
Eukaryota - ps6
Amorpheae - ps7
Obazoa - ps8
Opisthokonta - ps9
Holozoa - ps10
Filozoa - ps11
Choano./Metazoa - ps12
Metazoa - ps13
Myriazoa - ps14
Parahoxozoa - ps15
Bilateria - ps16
Deuterostomia - ps17
Chordata - ps18
Olfactores - ps19
Vertebrata - ps20
Gnathostomata - ps21
Euteleostomi - ps22
Sarcopterygii - ps23
Tetrapoda - ps24
Amniota - ps25
Mammalia - ps26
Theria - ps27
Placentalia - ps28
Boreoeutheria - ps29
Euarchontoglires - ps30
Euarchonta - ps31
Primates - ps32
Haplorrhini - ps33
Simiiformes - ps34
Catarrhini - ps35
Hominoidea - ps36
Hominidae - ps37
Homininae - ps38
Hominini - ps39
Homo_sapiens - ps40
Phylostratum

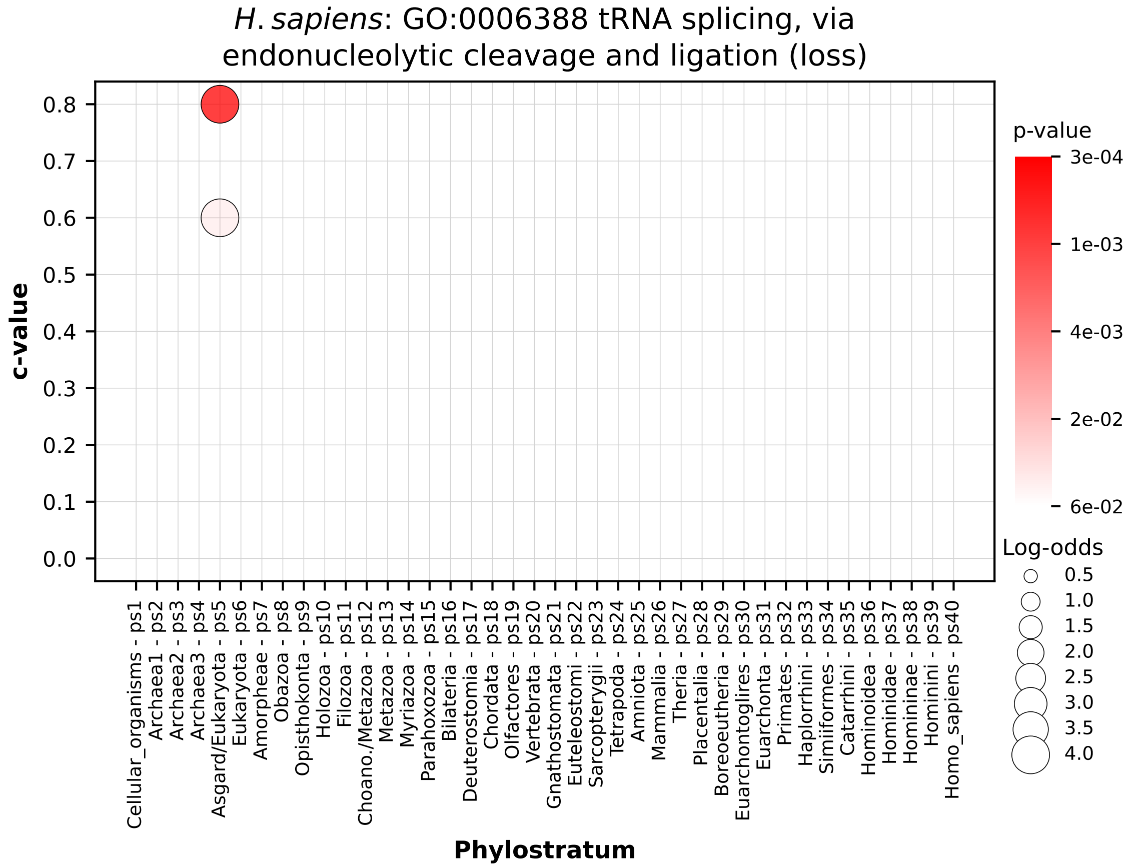

H. sapiens: GO:0006388 tRNA splicing, via endonucleolytic cleavage and ligation (loss)
c-value
0.0
0.1
0.2
0.3
0.4
0.5
0.6
0.7
0.8
p-value
3e-04
1e-03
4e-03
2e-02
6e-02
Log-odds
0.5
1.0
1.5
2.0
2.5
3.0
3.5
4.0
Cellular_organisms - ps1
Archaea1 - ps2
Archaea2 - ps3
Archaea3 - ps4
Asgard/Eukaryota - ps5
Eukaryota - ps6
Amorpheae - ps7
Obazoa - ps8
Opisthokonta - ps9
Holozoa - ps10
Filozoa - ps11
Choano./Metazoa - ps12
Metazoa - ps13
Myriazoa - ps14
Parahoxozoa - ps15
Bilateria - ps16
Deuterostomia - ps17
Chordata - ps18
Olfactores - ps19
Vertebrata - ps20
Gnathostomata - ps21
Euteleostomi - ps22
Sarcopterygii - ps23
Tetrapoda - ps24
Amniota - ps25
Mammalia - ps26
Theria - ps27
Placentalia - ps28
Boreoeutheria - ps29
Euarchontoglires - ps30
Euarchonta - ps31
Primates - ps32
Haplorrhini - ps33
Simiiformes - ps34
Catarrhini - ps35
Hominoidea - ps36
Hominidae - ps37
Homininae - ps38
Hominini - ps39
Homo_sapiens - ps40
Phylostratum

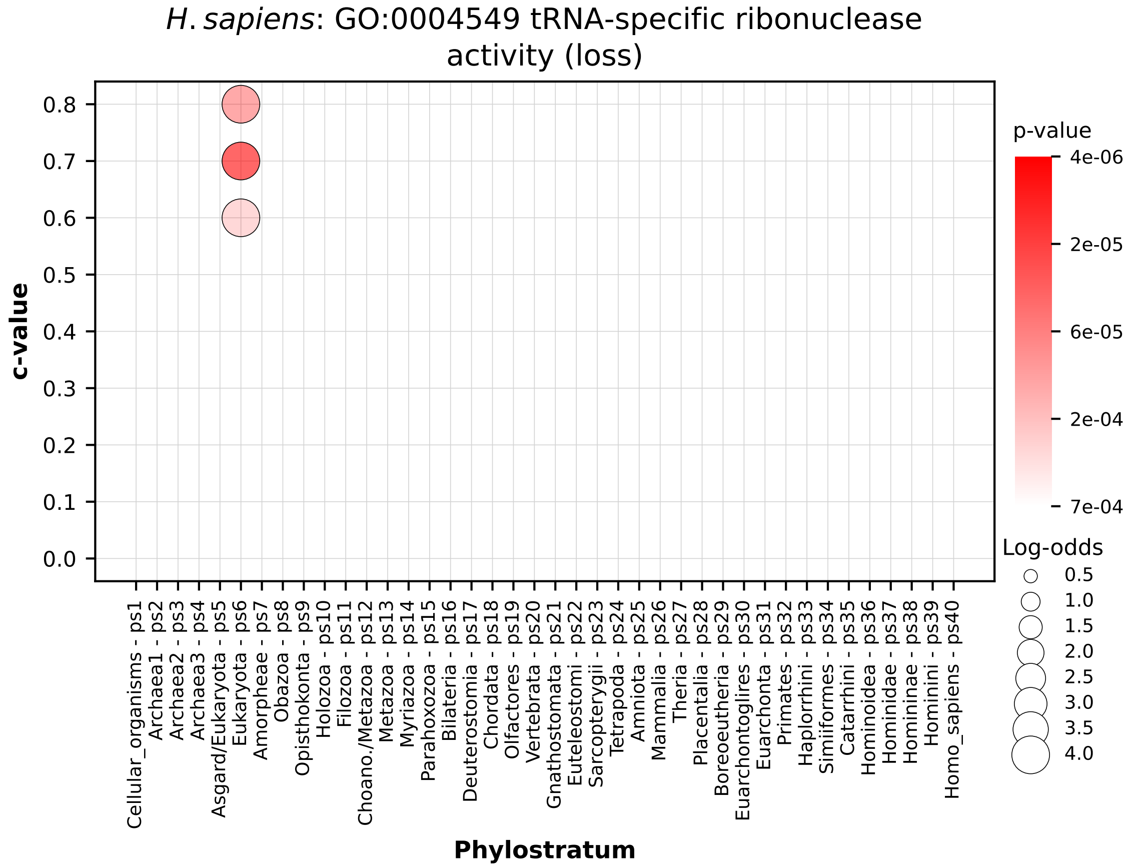

H. sapiens: GO:0004549 tRNA-specific ribonuclease activity (loss)
c-value
0.0
0.1
0.2
0.3
0.4
0.5
0.6
0.7
0.8
Phylostratum
Cellular_organisms - ps1
Archaea1 - ps2
Archaea2 - ps3
Archaea3 - ps4
Asgard/Eukaryota - ps5
Eukaryota - ps6
Amorpheae - ps7
Obazoa - ps8
Opisthokonta - ps9
Holozoa - ps10
Filozoa - ps11
Choano./Metazoa - ps12
Metazoa - ps13
Myriazoa - ps14
Parahoxozoa - ps15
Bilateria - ps16
Deuterostomia - ps17
Chordata - ps18
Olfactores - ps19
Vertebrata - ps20
Gnathostomata - ps21
Euteleostomi - ps22
Sarcopterygii - ps23
Tetrapoda - ps24
Amniota - ps25
Mammalia - ps26
Theria - ps27
Placentalia - ps28
Boreoeutheria - ps29
Euarchontoglires - ps30
Euarchonta - ps31
Primates - ps32
Haplorrhini - ps33
Simiiformes - ps34
Catarrhini - ps35
Hominoidea - ps36
Hominidae - ps37
Homininae - ps38
Hominini - ps39
Homo_sapiens - ps40
p-value
4e-06
2e-05
6e-05
2e-04
7e-04
Log-odds
0.5
1.0
1.5
2.0
2.5
3.0
3.5
4.0

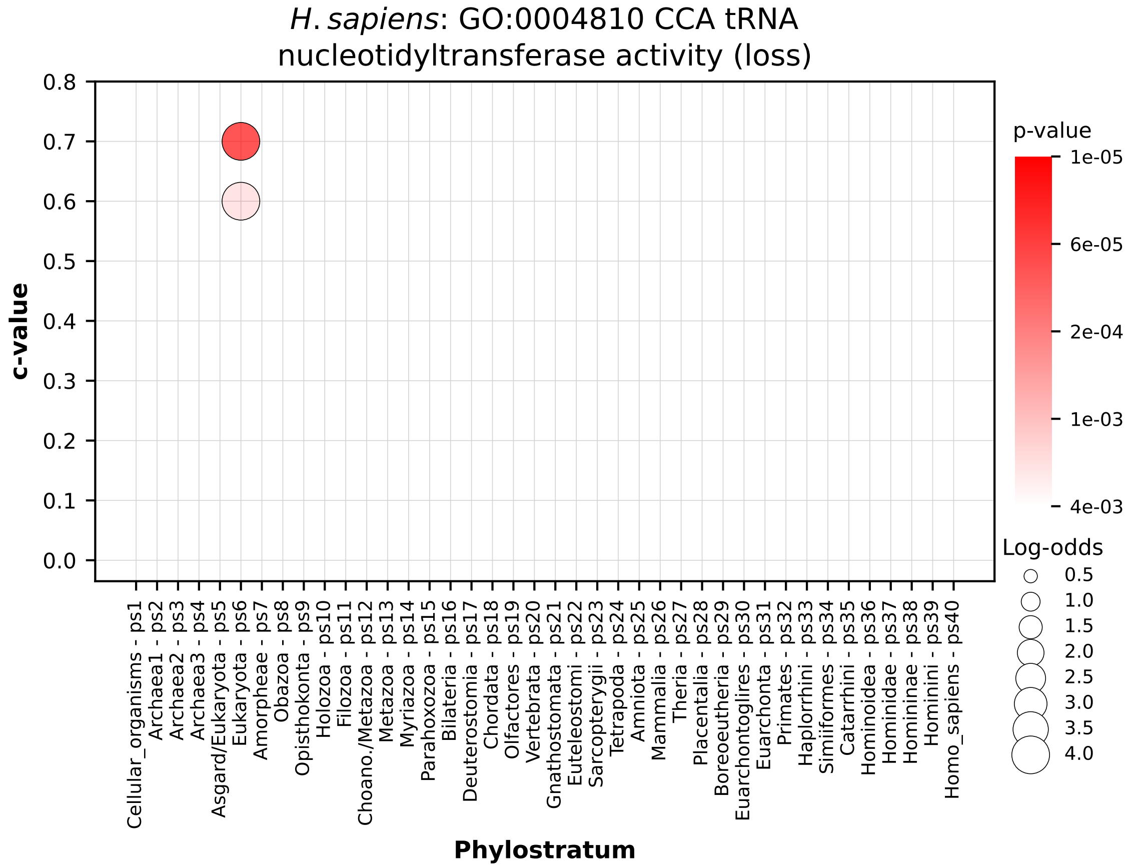

H. sapiens: GO:0004810 CCA tRNA nucleotidyltransferase activity (loss)
c-value
0.8
0.7
0.6
0.5
0.4
0.3
0.2
0.1
0.0
Phylostratum
Cellular_organisms - ps1
Archaea1 - ps2
Archaea2 - ps3
Archaea3 - ps4
Asgard/Eukaryota - ps5
Eukaryota - ps6
Amorpheae - ps7
Obazoa - ps8
Opisthokonta - ps9
Holozoa - ps10
Filozoa - ps11
Choano./Metazoa - ps12
Metazoa - ps13
Myriazoa - ps14
Parahoxozoa - ps15
Bilateria - ps16
Deuterostomia - ps17
Chordata - ps18
Olfactores - ps19
Vertebrata - ps20
Gnathostomata - ps21
Euteleostomi - ps22
Sarcopterygii - ps23
Tetrapoda - ps24
Amniota - ps25
Mammalia - ps26
Theria - ps27
Placentalia - ps28
Boreoeutheria - ps29
Euarchontoglires - ps30
Euarchonta - ps31
Primates - ps32
Haplorrhini - ps33
Simiiformes - ps34
Catarrhini - ps35
Hominoidea - ps36
Hominidae - ps37
Homininae - ps38
Hominini - ps39
Homo_sapiens - ps40
p-value
1e-05
6e-05
2e-04
1e-03
4e-03
Log-odds
0.5
1.0
1.5
2.0
2.5
3.0
3.5
4.0

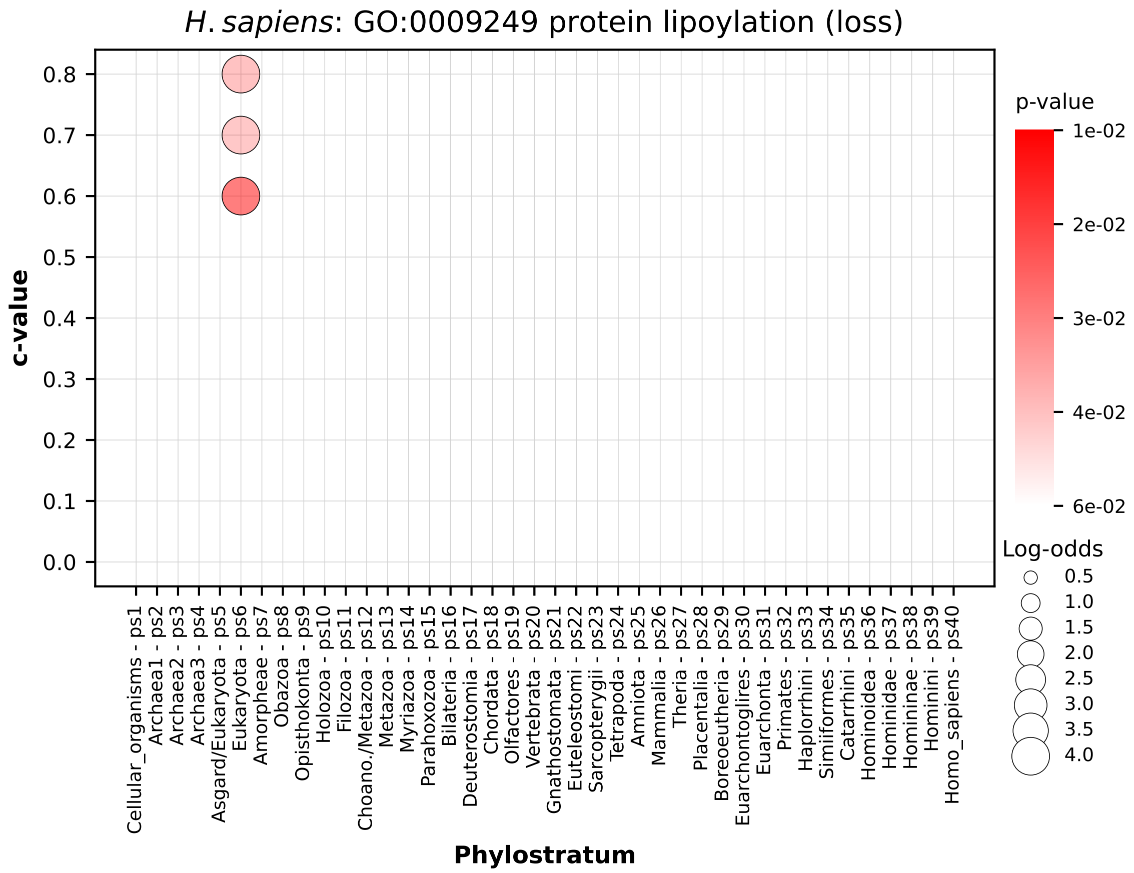

H. sapiens: GO:0009249 protein lipoylation (loss)
c-value
0.8
0.7
0.6
0.5
0.4
0.3
0.2
0.1
0.0
Phylostratum
Cellular_organisms - ps1
Archaea1 - ps2
Archaea2 - ps3
Archaea3 - ps4
Asgard/Eukaryota - ps5
Eukaryota - ps6
Amorpheae - ps7
Obazoa - ps8
Opisthokonta - ps9
Holozoa - ps10
Filozoa - ps11
Choano./Metazoa - ps12
Metazoa - ps13
Myriazoa - ps14
Parahoxozoa - ps15
Bilateria - ps16
Deuterostomia - ps17
Chordata - ps18
Olfactores - ps19
Vertebrata - ps20
Gnathostomata - ps21
Euteleostomi - ps22
Sarcopterygii - ps23
Tetrapoda - ps24
Amniota - ps25
Mammalia - ps26
Theria - ps27
Placentalia - ps28
Boreoeutheria - ps29
Euarchontoglires - ps30
Euarchonta - ps31
Primates - ps32
Haplorrhini - ps33
Simiiformes - ps34
Catarrhini - ps35
Hominoidea - ps36
Hominidae - ps37
Homininae - ps38
Hominini - ps39
Homo_sapiens - ps40
p-value
1e-02
2e-02
3e-02
4e-02
6e-02
Log-odds
0.5
1.0
1.5
2.0
2.5
3.0
3.5
4.0

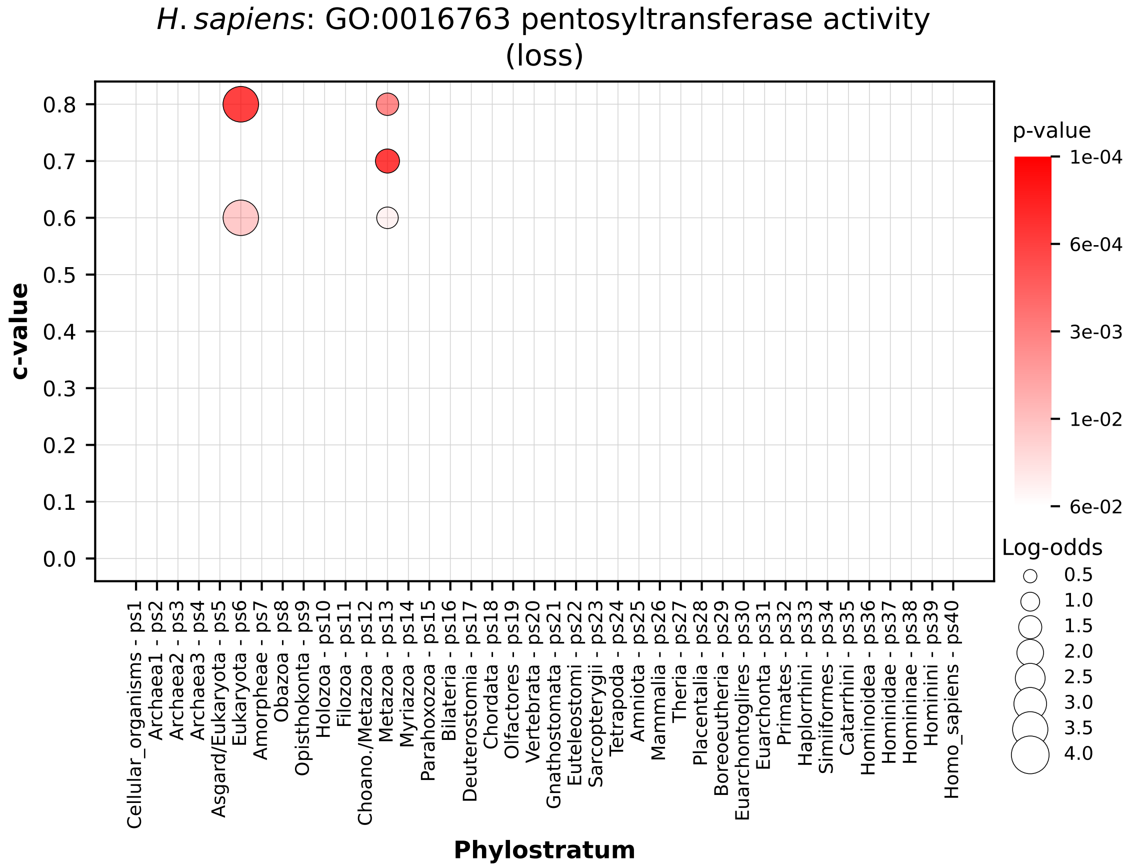

H. sapiens: GO:0016763 pentosyltransferase activity
(loss)
c-value
0.8
0.7
0.6
0.5
0.4
0.3
0.2
0.1
0.0
p-value
1e-04
6e-04
3e-03
1e-02
6e-02
Log-odds
0.5
1.0
1.5
2.0
2.5
3.0
3.5
4.0
Cellular_organisms - ps1
Archaea1 - ps2
Archaea2 - ps3
Archaea3 - ps4
Asgard/Eukaryota - ps5
Eukaryota - ps6
Amorpheae - ps7
Obazoa - ps8
Opisthokonta - ps9
Holozoa - ps10
Filozoa - ps11
Choano./Metazoa - ps12
Metazoa - ps13
Myriazoa - ps14
Parahoxozoa - ps15
Bilateria - ps16
Deuterostomia - ps17
Chordata - ps18
Olfactores - ps19
Vertebrata - ps20
Gnathostomata - ps21
Euteleostomi - ps22
Sarcopterygii - ps23
Tetrapoda - ps24
Amniota - ps25
Mammalia - ps26
Theria - ps27
Placentalia - ps28
Boreoeutheria - ps29
Euarchontoglires - ps30
Euarchonta - ps31
Primates - ps32
Haplorrhini - ps33
Simiiformes - ps34
Catarrhini - ps35
Hominoidea - ps36
Hominidae - ps37
Homininae - ps38
Hominini - ps39
Homo_sapiens - ps40
Phylostratum

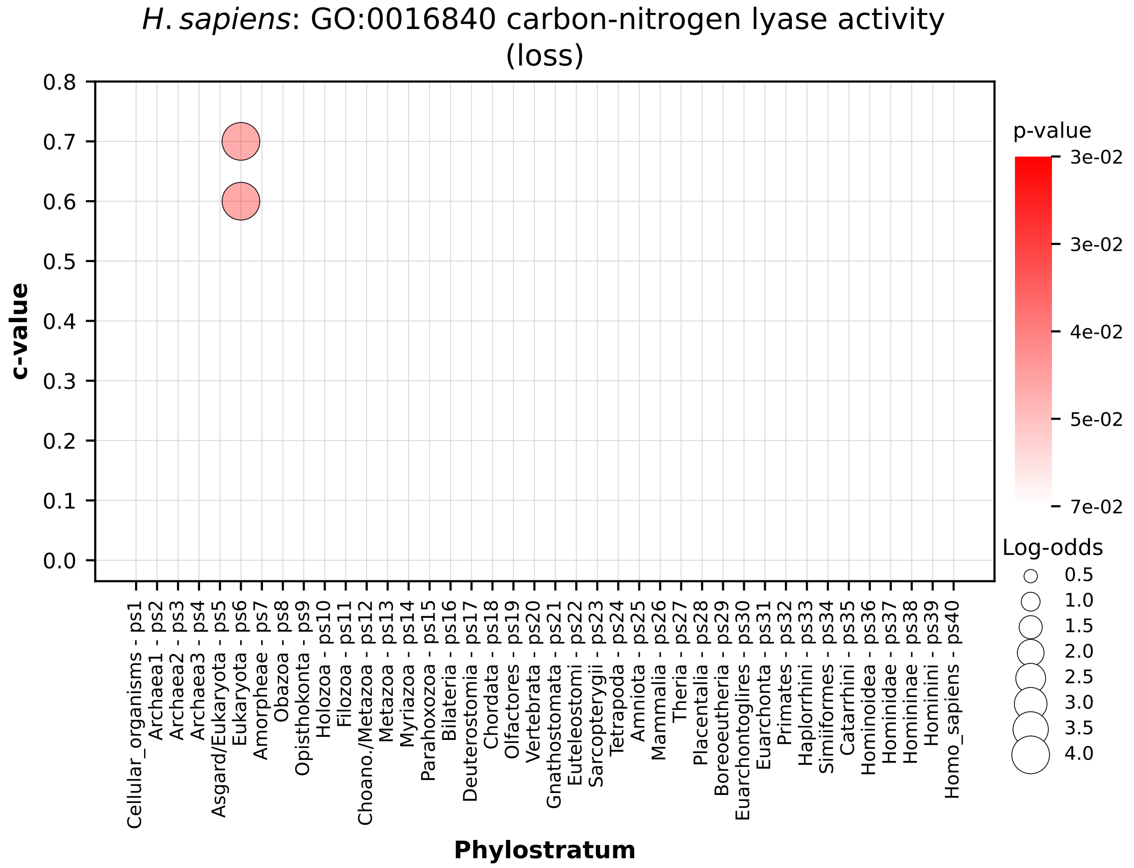
H. sapiens: GO:0016840 carbon-nitrogen lyase activity (loss)
c-value
0.0
0.1
0.2
0.3
0.4
0.5
0.6
0.7
0.8
Phylostratum
Cellular_organisms - ps1
Archaea1 - ps2
Archaea2 - ps3
Archaea3 - ps4
Asgard/Eukaryota - ps5
Eukaryota - ps6
Amorpheae - ps7
Obazoa - ps8
Opisthokonta - ps9
Holozoa - ps10
Filozoa - ps11
Choano./Metazoa - ps12
Metazoa - ps13
Myriazoa - ps14
Parahoxozoa - ps15
Bilateria - ps16
Deuterostomia - ps17
Chordata - ps18
Olfactores - ps19
Vertebrata - ps20
Gnathostomata - ps21
Euteleostomi - ps22
Sarcopterygii - ps23
Tetrapoda - ps24
Amniota - ps25
Mammalia - ps26
Theria - ps27
Placentalia - ps28
Boreoeutheria - ps29
Euarchontoglires - ps30
Euarchonta - ps31
Primates - ps32
Haplorrhini - ps33
Simiiformes - ps34
Catarrhini - ps35
Hominoidea - ps36
Hominidae - ps37
Homininae - ps38
Hominini - ps39
Homo_sapiens - ps40
p-value
3e-02
3e-02
4e-02
5e-02
7e-02
Log-odds
0.5
1.0
1.5
2.0
2.5
3.0
3.5
4.0

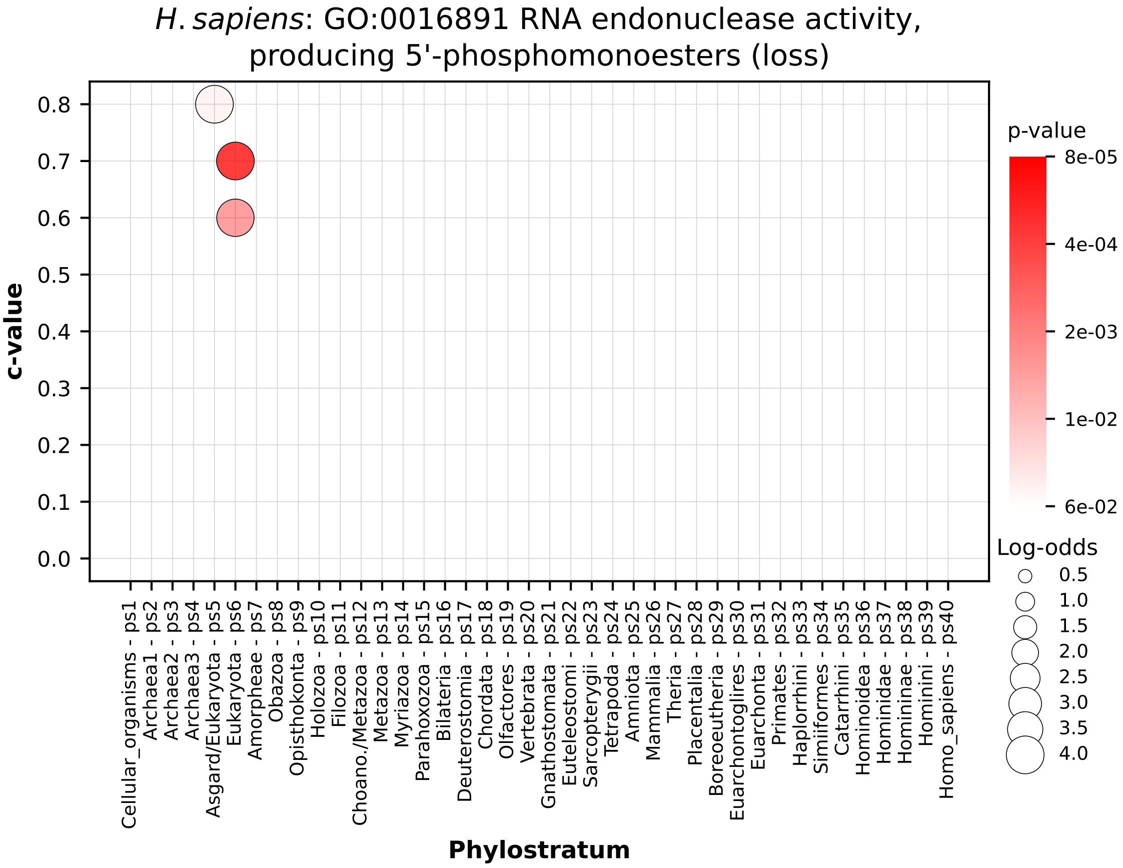

H. sapiens: GO:0016891 RNA endonuclease activity, producing 5'-phosphomonoesters (loss)
c-value
0.8
0.7
0.6
0.5
0.4
0.3
0.2
0.1
0.0
p-value
8e-05
4e-04
2e-03
1e-02
6e-02
Log-odds
0.5
1.0
1.5
2.0
2.5
3.0
3.5
4.0
Cellular_organisms - ps1
Archaea1 - ps2
Archaea2 - ps3
Archaea3 - ps4
Asgard/Eukaryota - ps5
Eukaryota - ps6
Amorpheae - ps7
Obazoa - ps8
Opisthokonta - ps9
Holozoa - ps10
Filozoa - ps11
Choano./Metazoa - ps12
Metazoa - ps13
Myriazoa - ps14
Parahoxozoa - ps15
Bilateria - ps16
Deuterostomia - ps17
Chordata - ps18
Olfactores - ps19
Vertebrata - ps20
Gnathostomata - ps21
Euteleostomi - ps22
Sarcopterygii - ps23
Tetrapoda - ps24
Amniota - ps25
Mammalia - ps26
Theria - ps27
Placentalia - ps28
Boreoeutheria - ps29
Euarchontoglires - ps30
Euarchonta - ps31
Primates - ps32
Haplorrhini - ps33
Simiiformes - ps34
Catarrhini - ps35
Hominoidea - ps36
Hominidae - ps37
Homininae - ps38
Hominini - ps39
Homo_sapiens - ps40
Phylostratum

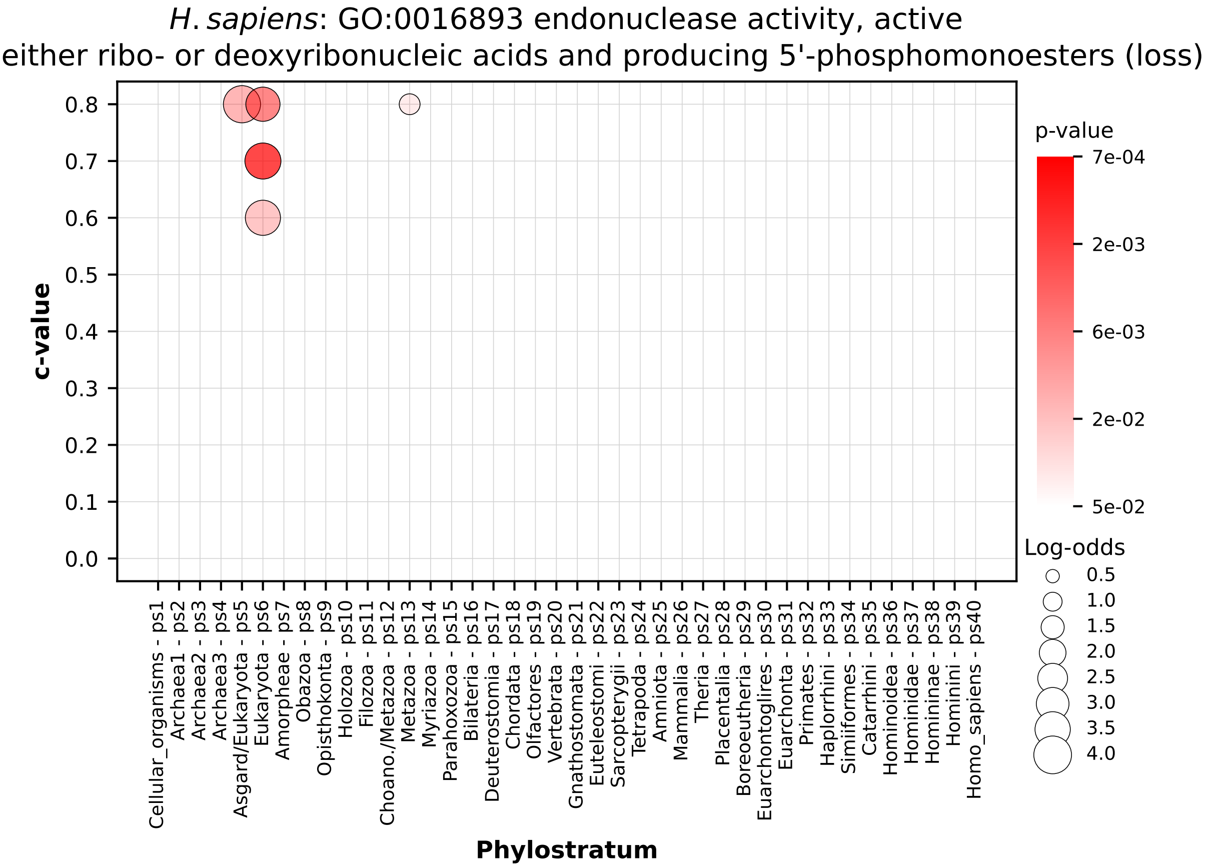

H. sapiens: GO:0016893 endonuclease activity, active
either ribo- or deoxyribonucleic acids and producing 5'-phosphomonoesters (loss)
c-value
0.8
0.7
0.6
0.5
0.4
0.3
0.2
0.1
0.0
p-value
7e-04
2e-03
6e-03
2e-02
5e-02
Log-odds
0.5
1.0
1.5
2.0
2.5
3.0
3.5
4.0
Cellular_organisms - ps1
Archaea1 - ps2
Archaea2 - ps3
Archaea3 - ps4
Asgard/Eukaryota - ps5
Eukaryota - ps6
Amorpheae - ps7
Obazoa - ps8
Opisthokonta - ps9
Holozoa - ps10
Filozoa - ps11
Choano./Metazoa - ps12
Metazoa - ps13
Myriazoa - ps14
Parahoxozoa - ps15
Bilateria - ps16
Deuterostomia - ps17
Chordata - ps18
Olfactores - ps19
Vertebrata - ps20
Gnathostomata - ps21
Euteleostomi - ps22
Sarcopterygii - ps23
Tetrapoda - ps24
Amniota - ps25
Mammalia - ps26
Theria - ps27
Placentalia - ps28
Boreoeutheria - ps29
Euarchontoglires - ps30
Euarchonta - ps31
Primates - ps32
Haplorrhini - ps33
Simiiformes - ps34
Catarrhini - ps35
Hominoidea - ps36
Hominidae - ps37
Homininae - ps38
Hominini - ps39
Homo_sapiens - ps40
Phylostratum

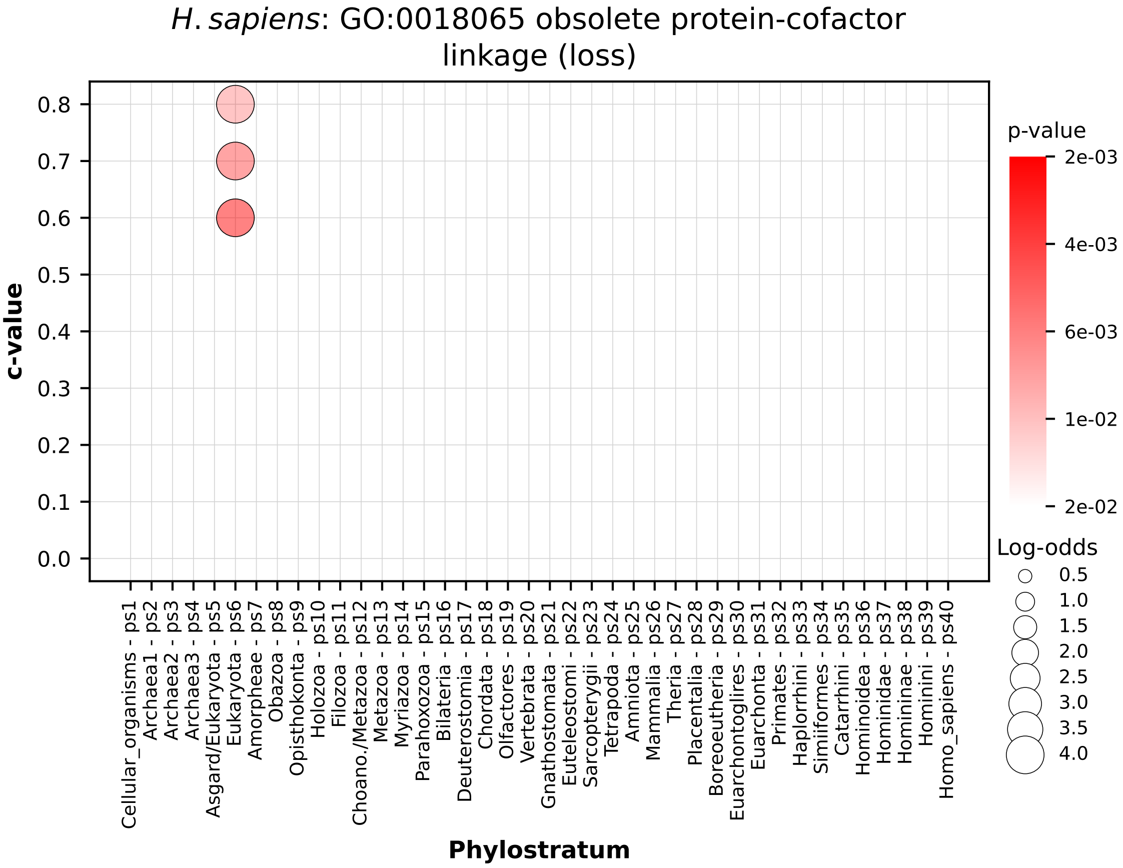
H. sapiens: GO:0018065 obsolete protein-cofactor linkage (loss)
c-value
0.8
0.7
0.6
0.5
0.4
0.3
0.2
0.1
0.0
p-value
2e-03
4e-03
6e-03
1e-02
2e-02
Log-odds
0.5
1.0
1.5
2.0
2.5
3.0
3.5
4.0
Cellular_organisms - ps1
Archaea1 - ps2
Archaea2 - ps3
Archaea3 - ps4
Asgard/Eukaryota - ps5
Eukaryota - ps6
Amorpheae - ps7
Obazoa - ps8
Opisthokonta - ps9
Holozoa - ps10
Filozoa - ps11
Choano./Metazoa - ps12
Metazoa - ps13
Myriazoa - ps14
Parahoxozoa - ps15
Bilateria - ps16
Deuterostomia - ps17
Chordata - ps18
Olfactores - ps19
Vertebrata - ps20
Gnathostomata - ps21
Euteleostomi - ps22
Sarcopterygii - ps23
Tetrapoda - ps24
Amniota - ps25
Mammalia - ps26
Theria - ps27
Placentalia - ps28
Boreoeutheria - ps29
Euarchontoglires - ps30
Euarchonta - ps31
Primates - ps32
Haplorrhini - ps33
Simiiformes - ps34
Catarrhini - ps35
Hominoidea - ps36
Hominidae - ps37
Homininae - ps38
Hominini - ps39
Homo_sapiens - ps40
Phylostratum

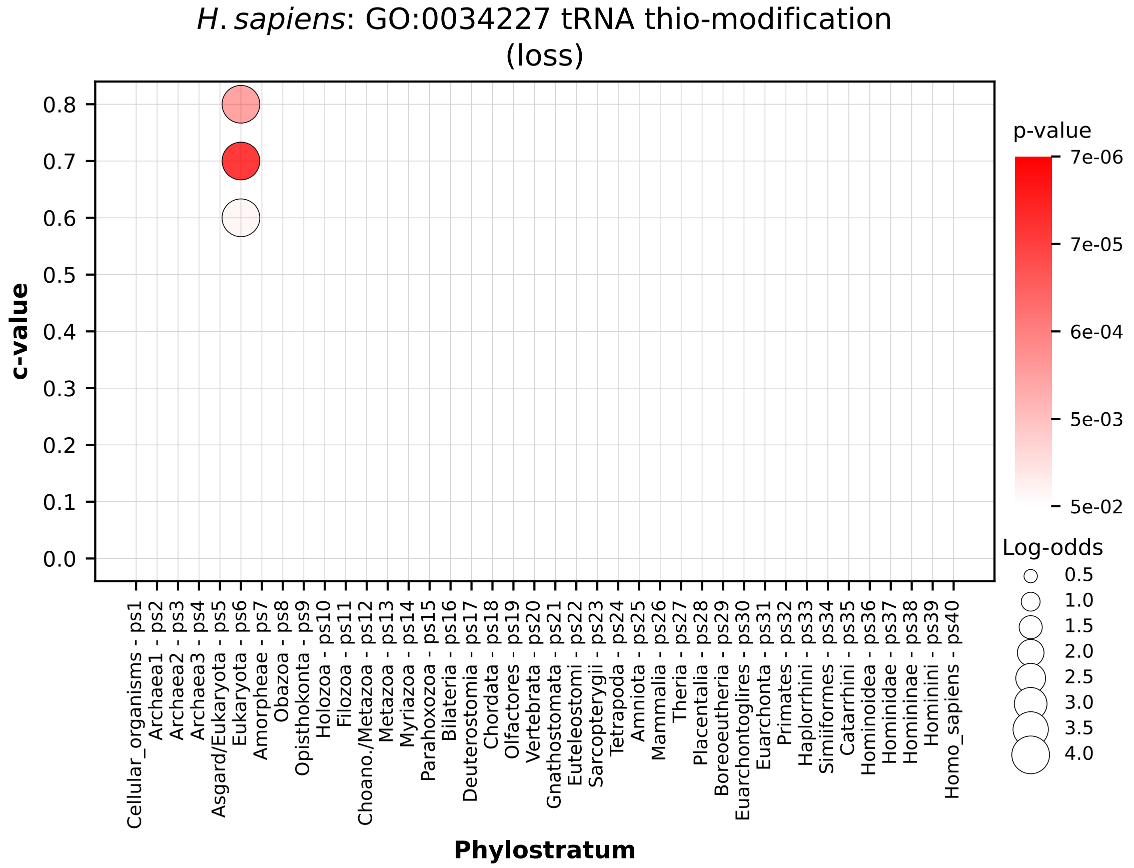

H. sapiens: GO:0034227 tRNA thio-modification
(loss)
c-value
0.0
0.1
0.2
0.3
0.4
0.5
0.6
0.7
0.8
p-value
7e-06
7e-05
6e-04
5e-03
5e-02
Log-odds
0.5
1.0
1.5
2.0
2.5
3.0
3.5
4.0
Cellular_organisms - ps1
Archaea1 - ps2
Archaea2 - ps3
Archaea3 - ps4
Asgard/Eukaryota - ps5
Eukaryota - ps6
Amorpheae - ps7
Obazoa - ps8
Opisthokonta - ps9
Holozoa - ps10
Filozoa - ps11
Choano./Metazoa - ps12
Metazoa - ps13
Myriazoa - ps14
Parahoxozoa - ps15
Bilateria - ps16
Deuterostomia - ps17
Chordata - ps18
Olfactores - ps19
Vertebrata - ps20
Gnathostomata - ps21
Euteleostomi - ps22
Sarcopterygii - ps23
Tetrapoda - ps24
Amniota - ps25
Mammalia - ps26
Theria - ps27
Placentalia - ps28
Boreoeutheria - ps29
Euarchontoglires - ps30
Euarchonta - ps31
Primates - ps32
Haplorrhini - ps33
Simiiformes - ps34
Catarrhini - ps35
Hominoidea - ps36
Hominidae - ps37
Homininae - ps38
Hominini - ps39
Homo_sapiens - ps40
Phylostratum

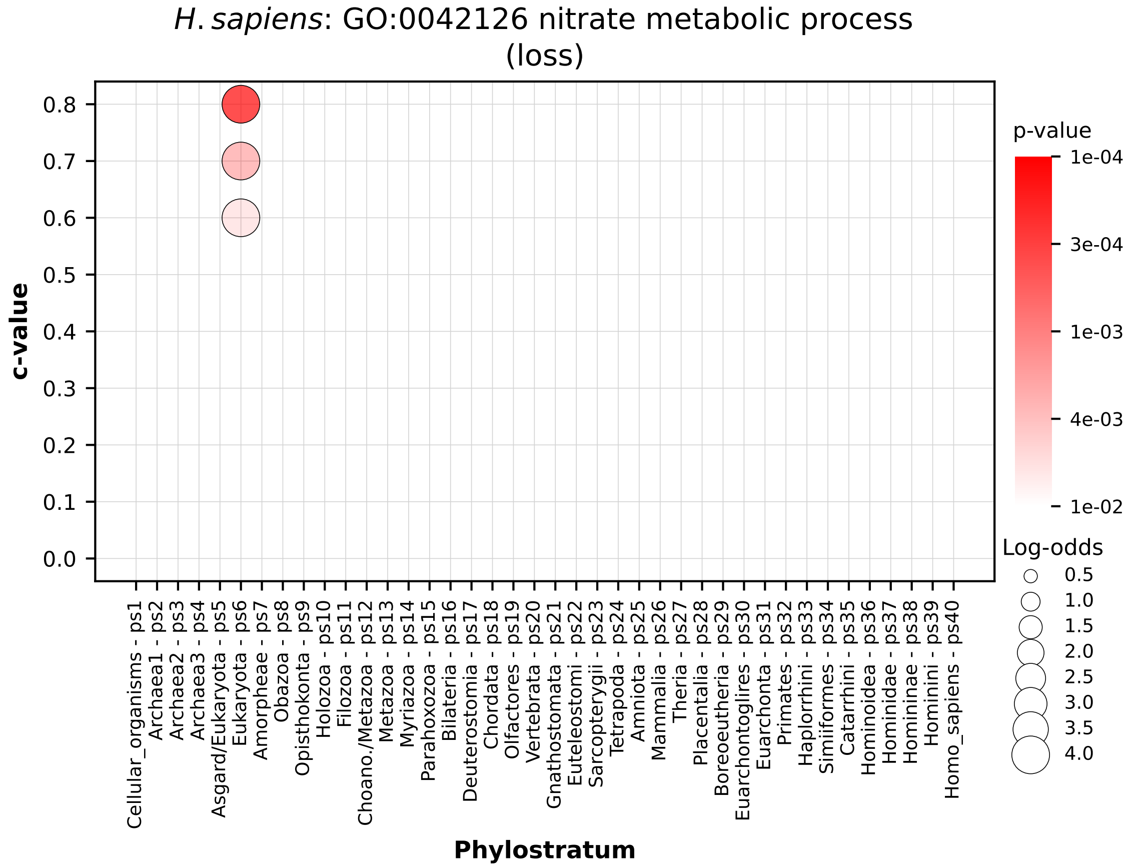

H. sapiens: GO:0042126 nitrate metabolic process
(loss)
c-value
0.0
0.1
0.2
0.3
0.4
0.5
0.6
0.7
0.8
p-value
1e-04
3e-04
1e-03
4e-03
1e-02
Log-odds
0.5
1.0
1.5
2.0
2.5
3.0
3.5
4.0
Cellular_organisms - ps1
Archaea1 - ps2
Archaea2 - ps3
Archaea3 - ps4
Asgard/Eukaryota - ps5
Eukaryota - ps6
Amorpheae - ps7
Obazoa - ps8
Opisthokonta - ps9
Holozoa - ps10
Filozoa - ps11
Choano./Metazoa - ps12
Metazoa - ps13
Myriazoa - ps14
Parahoxozoa - ps15
Bilateria - ps16
Deuterostomia - ps17
Chordata - ps18
Olfactores - ps19
Vertebrata - ps20
Gnathostomata - ps21
Euteleostomi - ps22
Sarcopterygii - ps23
Tetrapoda - ps24
Amniota - ps25
Mammalia - ps26
Theria - ps27
Placentalia - ps28
Boreoeutheria - ps29
Euarchontoglires - ps30
Euarchonta - ps31
Primates - ps32
Haplorrhini - ps33
Simiiformes - ps34
Catarrhini - ps35
Hominoidea - ps36
Hominidae - ps37
Homininae - ps38
Hominini - ps39
Homo_sapiens - ps40
Phylostratum

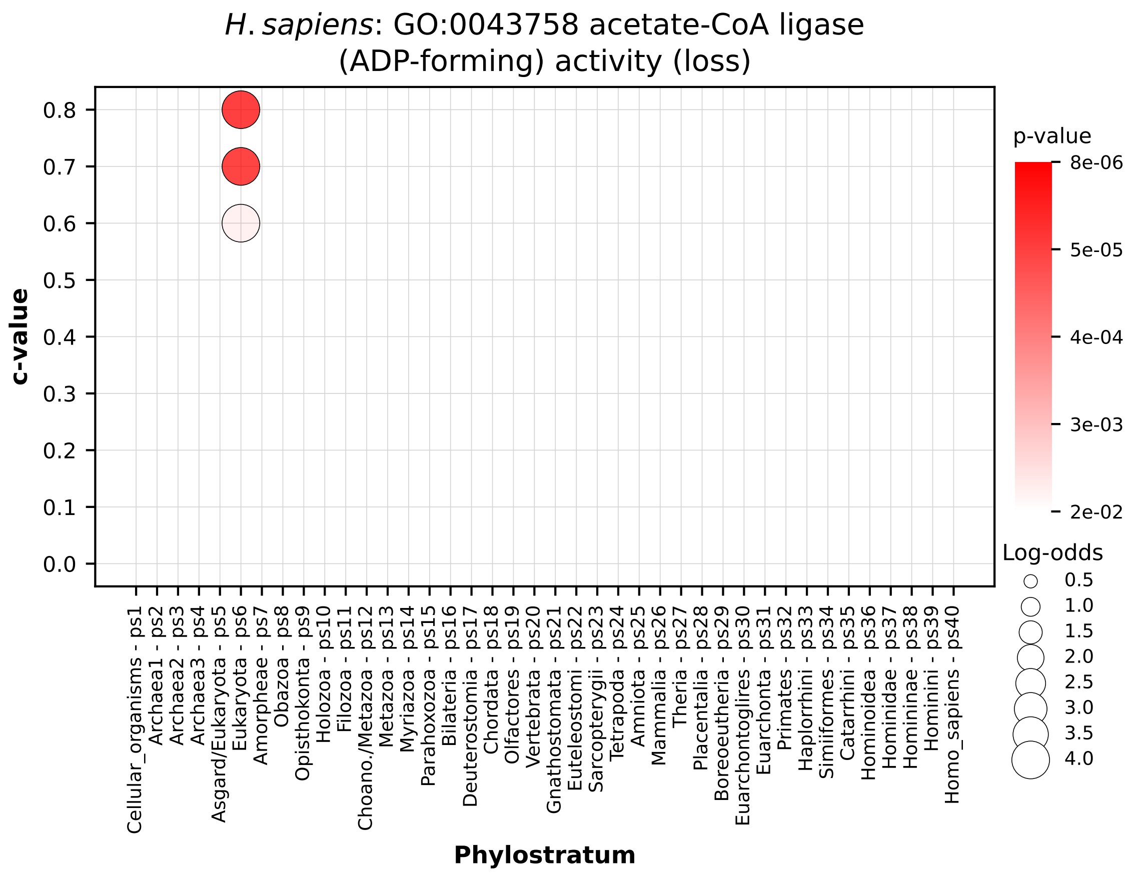

H. sapiens: GO:0043758 acetate-CoA ligase (ADP-forming) activity (loss)
c-value
0.0
0.1
0.2
0.3
0.4
0.5
0.6
0.7
0.8
p-value
8e-06
5e-05
4e-04
3e-03
2e-02
Log-odds
0.5
1.0
1.5
2.0
2.5
3.0
3.5
4.0
Cellular_organisms - ps1
Archaea1 - ps2
Archaea2 - ps3
Archaea3 - ps4
Asgard/Eukaryota - ps5
Eukaryota - ps6
Amorpheae - ps7
Obazoa - ps8
Opisthokonta - ps9
Holozoa - ps10
Filozoa - ps11
Choano./Metazoa - ps12
Metazoa - ps13
Myriazoa - ps14
Parahoxozoa - ps15
Bilateria - ps16
Deuterostomia - ps17
Chordata - ps18
Olfactores - ps19
Vertebrata - ps20
Gnathostomata - ps21
Euteleostomi - ps22
Sarcopterygii - ps23
Tetrapoda - ps24
Amniota - ps25
Mammalia - ps26
Theria - ps27
Placentalia - ps28
Boreoeutheria - ps29
Euarchontoglires - ps30
Euarchonta - ps31
Primates - ps32
Haplorrhini - ps33
Simiiformes - ps34
Catarrhini - ps35
Hominoidea - ps36
Hominidae - ps37
Homininae - ps38
Hominini - ps39
Homo_sapiens - ps40
Phylostratum

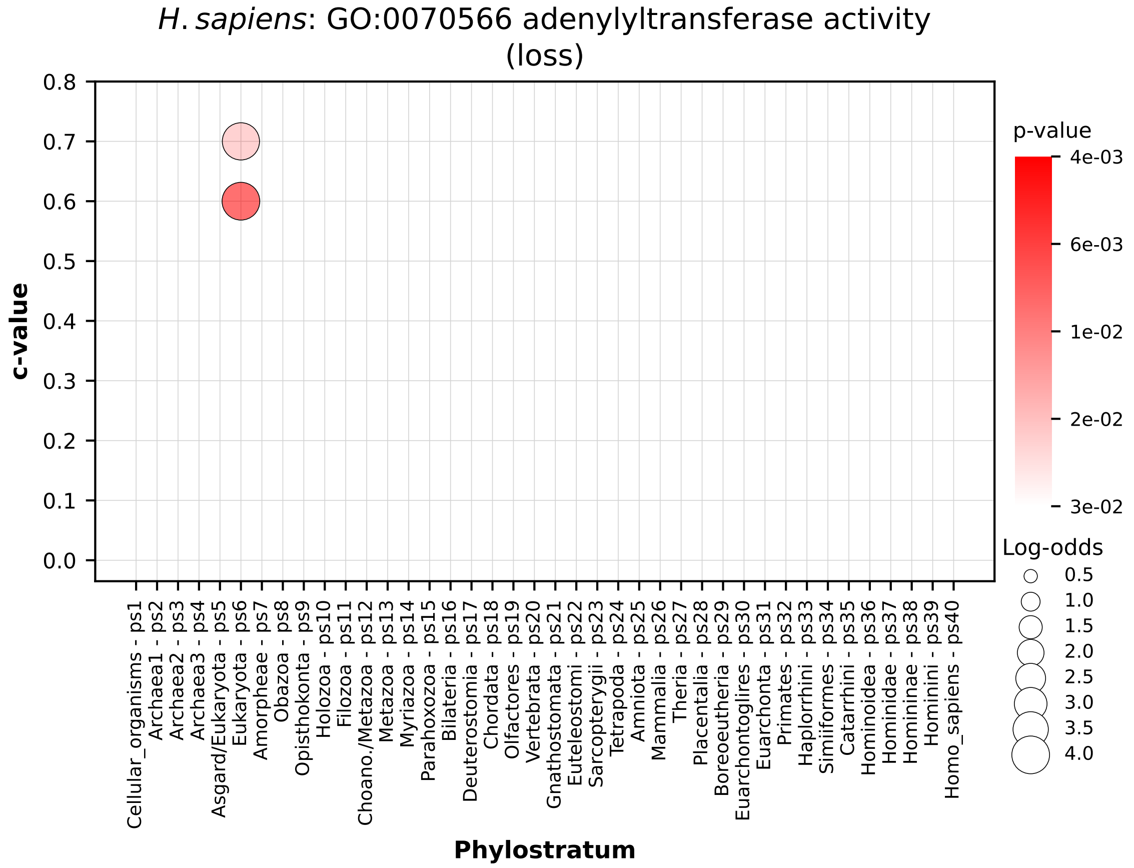
H. sapiens: GO:0070566 adenylyltransferase activity
(loss)
c-value
0.8
0.7
0.6
0.5
0.4
0.3
0.2
0.1
0.0
p-value
4e-03
6e-03
1e-02
2e-02
3e-02
Log-odds
0.5
1.0
1.5
2.0
2.5
3.0
3.5
4.0
Cellular_organisms - ps1
Archaea1 - ps2
Archaea2 - ps3
Archaea3 - ps4
Asgard/Eukaryota - ps5
Eukaryota - ps6
Amorpheae - ps7
Obazoa - ps8
Opisthokonta - ps9
Holozoa - ps10
Filozoa - ps11
Choano./Metazoa - ps12
Metazoa - ps13
Myriazoa - ps14
Parahoxozoa - ps15
Bilateria - ps16
Deuterostomia - ps17
Chordata - ps18
Olfactores - ps19
Vertebrata - ps20
Gnathostomata - ps21
Euteleostomi - ps22
Sarcopterygii - ps23
Tetrapoda - ps24
Amniota - ps25
Mammalia - ps26
Theria - ps27
Placentalia - ps28
Boreoeutheria - ps29
Euarchontoglires - ps30
Euarchonta - ps31
Primates - ps32
Haplorrhini - ps33
Simiiformes - ps34
Catarrhini - ps35
Hominoidea - ps36
Hominidae - ps37
Homininae - ps38
Hominini - ps39
Homo_sapiens - ps40
Phylostratum

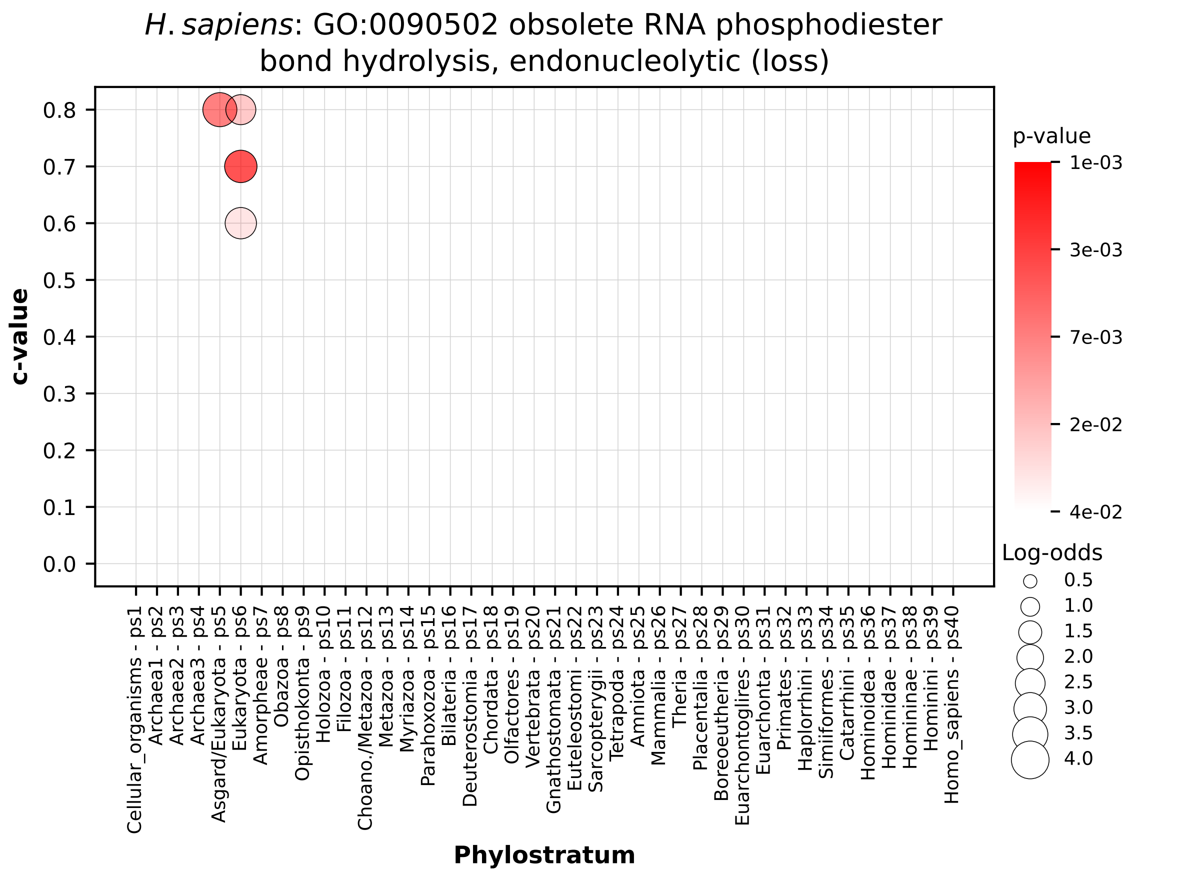

H. sapiens: GO:0090502 obsolete RNA phosphodiester bond hydrolysis, endonucleolytic (loss)
c-value
0.0
0.1
0.2
0.3
0.4
0.5
0.6
0.7
0.8
p-value
1e-03
3e-03
7e-03
2e-02
4e-02
Log-odds
0.5
1.0
1.5
2.0
2.5
3.0
3.5
4.0
Cellular_organisms - ps1
Archaea1 - ps2
Archaea2 - ps3
Archaea3 - ps4
Asgard/Eukaryota - ps5
Eukaryota - ps6
Amorpheae - ps7
Obazoa - ps8
Opisthokonta - ps9
Holozoa - ps10
Filozoa - ps11
Choano./Metazoa - ps12
Metazoa - ps13
Myriazoa - ps14
Parahoxozoa - ps15
Bilateria - ps16
Deuterostomia - ps17
Chordata - ps18
Olfactores - ps19
Vertebrata - ps20
Gnathostomata - ps21
Euteleostomi - ps22
Sarcopterygii - ps23
Tetrapoda - ps24
Amniota - ps25
Mammalia - ps26
Theria - ps27
Placentalia - ps28
Boreoeutheria - ps29
Euarchontoglires - ps30
Euarchonta - ps31
Primates - ps32
Haplorrhini - ps33
Simiiformes - ps34
Catarrhini - ps35
Hominoidea - ps36
Hominidae - ps37
Homininae - ps38
Hominini - ps39
Homo_sapiens - ps40
Phylostratum

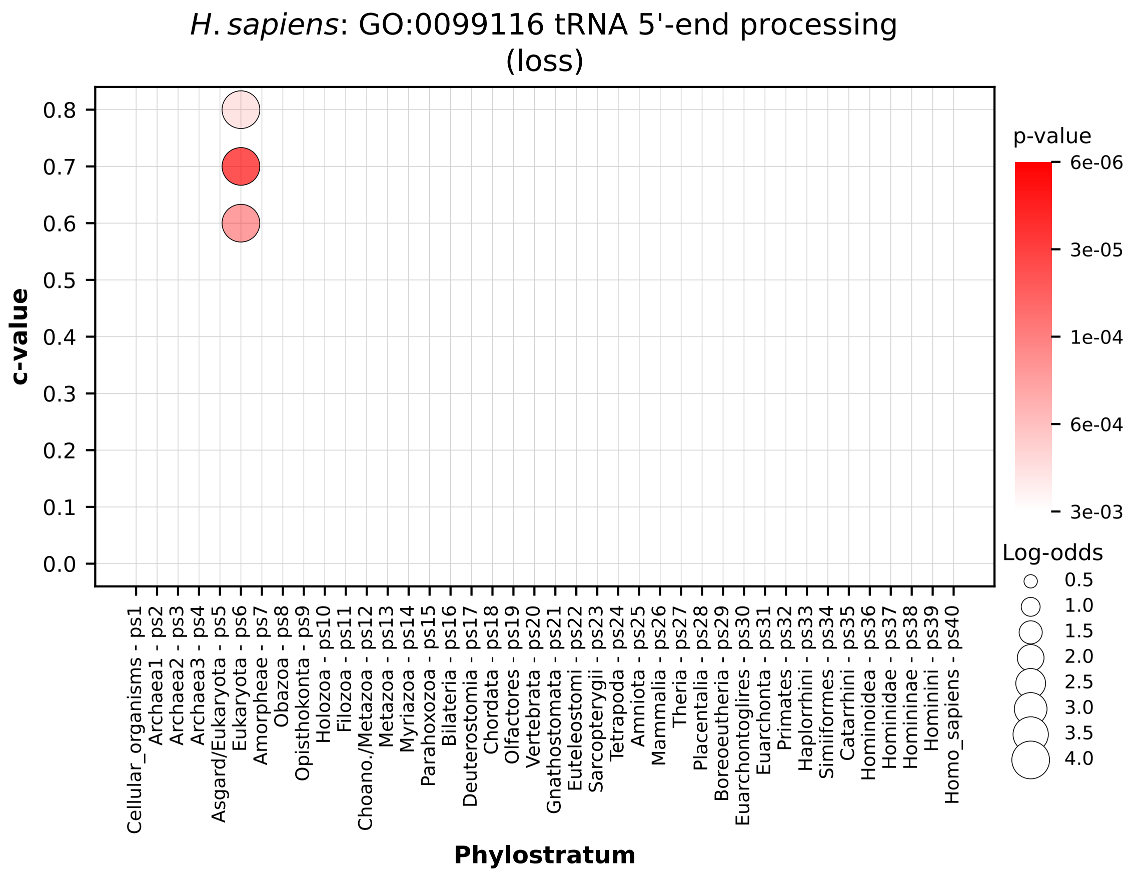
H. sapiens: GO:0099116 tRNA 5'-end processing
(loss)
c-value
0.8
0.7
0.6
0.5
0.4
0.3
0.2
0.1
0.0
p-value
6e-06
3e-05
1e-04
6e-04
3e-03
Log-odds
0.5
1.0
1.5
2.0
2.5
3.0
3.5
4.0
Cellular_organisms - ps1
Archaea1 - ps2
Archaea2 - ps3
Archaea3 - ps4
Asgard/Eukaryota - ps5
Eukaryota - ps6
Amorpheae - ps7
Obazoa - ps8
Opisthokonta - ps9
Holozoa - ps10
Filozoa - ps11
Choano./Metazoa - ps12
Metazoa - ps13
Myriazoa - ps14
Parahoxozoa - ps15
Bilateria - ps16
Deuterostomia - ps17
Chordata - ps18
Olfactores - ps19
Vertebrata - ps20
Gnathostomata - ps21
Euteleostomi - ps22
Sarcopterygii - ps23
Tetrapoda - ps24
Amniota - ps25
Mammalia - ps26
Theria - ps27
Placentalia - ps28
Boreoeutheria - ps29
Euarchontoglires - ps30
Euarchonta - ps31
Primates - ps32
Haplorrhini - ps33
Simiiformes - ps34
Catarrhini - ps35
Hominoidea - ps36
Hominidae - ps37
Homininae - ps38
Hominini - ps39
Homo_sapiens - ps40
Phylostratum

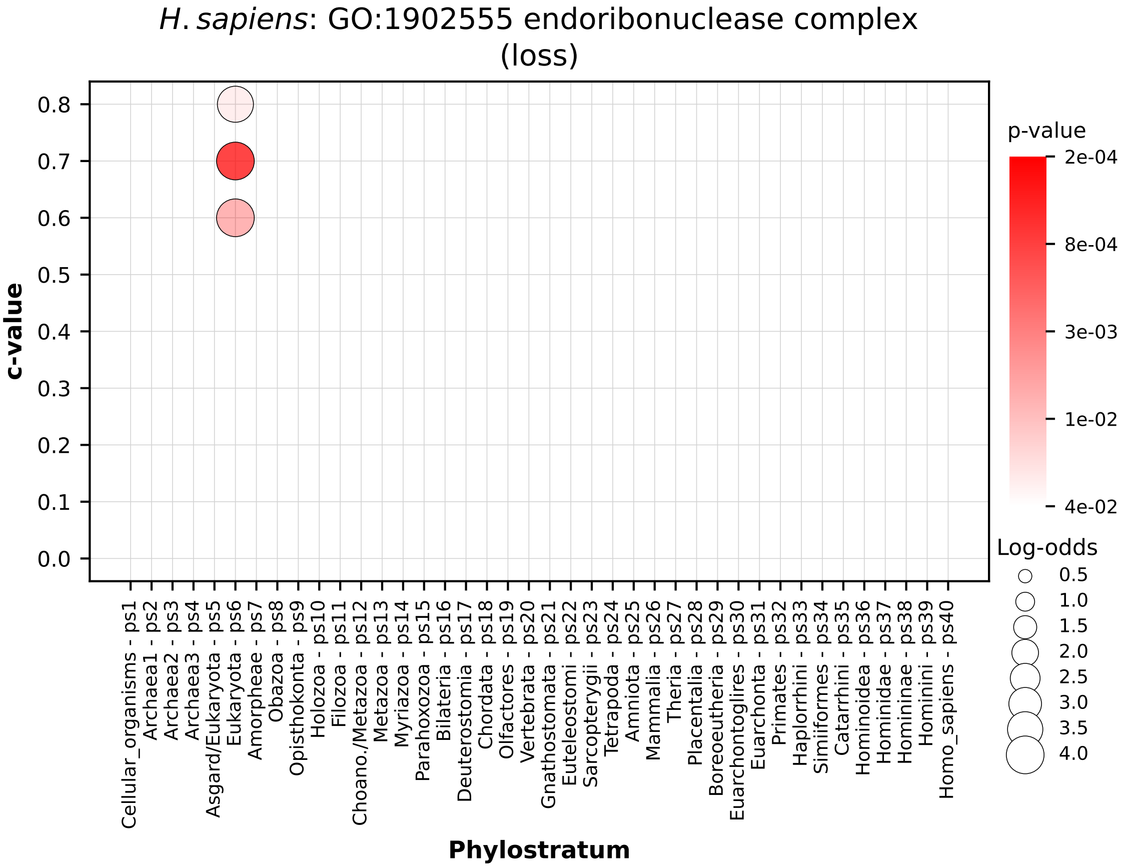
H. sapiens: GO:1902555 endoribonuclease complex
(loss)
c-value
0.0
0.1
0.2
0.3
0.4
0.5
0.6
0.7
0.8
Phylostratum
Cellular_organisms - ps1
Archaea1 - ps2
Archaea2 - ps3
Archaea3 - ps4
Asgard/Eukaryota - ps5
Eukaryota - ps6
Amorpheae - ps7
Obazoa - ps8
Opisthokonta - ps9
Holozoa - ps10
Filozoa - ps11
Choano./Metazoa - ps12
Metazoa - ps13
Myriazoa - ps14
Parahoxozoa - ps15
Bilateria - ps16
Deuterostomia - ps17
Chordata - ps18
Olfactores - ps19
Vertebrata - ps20
Gnathostomata - ps21
Euteleostomi - ps22
Sarcopterygii - ps23
Tetrapoda - ps24
Amniota - ps25
Mammalia - ps26
Theria - ps27
Placentalia - ps28
Boreoeutheria - ps29
Euarchontoglires - ps30
Euarchonta - ps31
Primates - ps32
Haplorrhini - ps33
Simiiformes - ps34
Catarrhini - ps35
Hominoidea - ps36
Hominidae - ps37
Homininae - ps38
Hominini - ps39
Homo_sapiens - ps40
p-value
2e-04
8e-04
3e-03
1e-02
4e-02
Log-odds
0.5
1.0
1.5
2.0
2.5
3.0
3.5
4.0

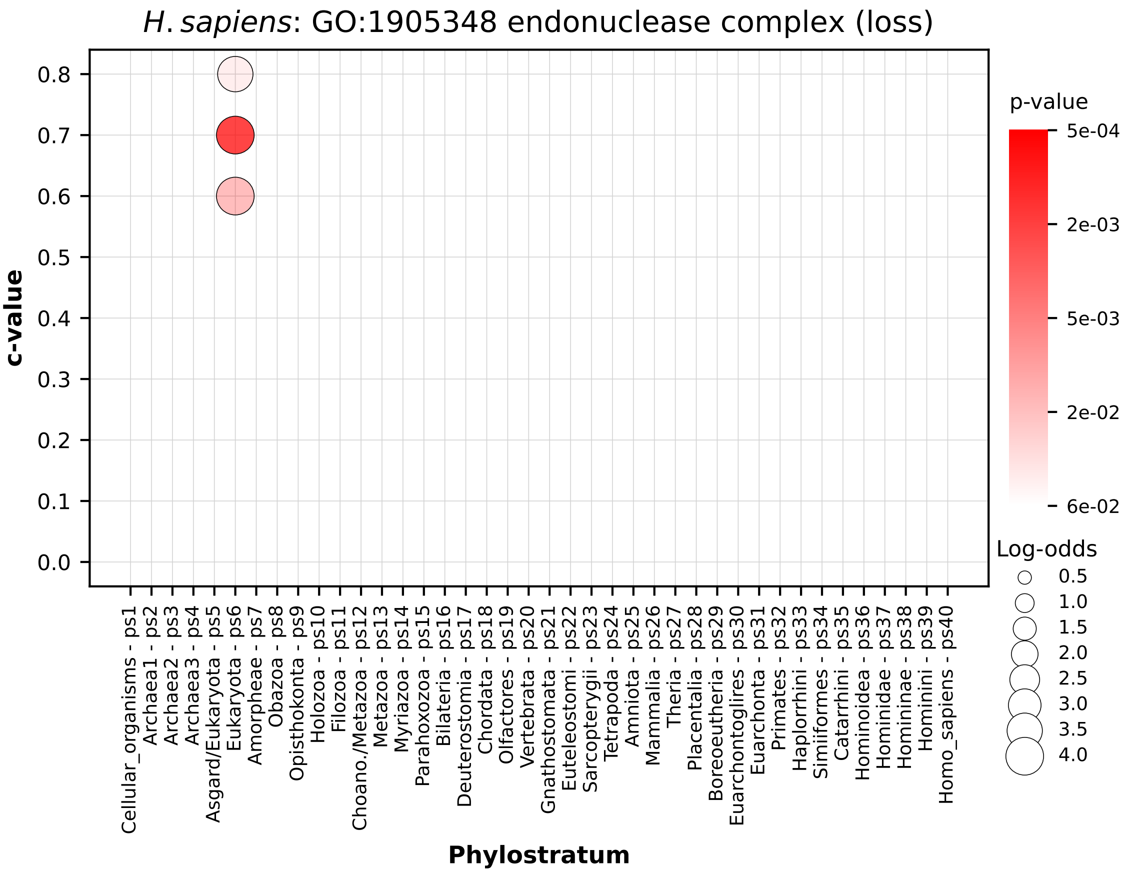

H. sapiens: GO:1905348 endonuclease complex (loss)
c-value
0.0
0.1
0.2
0.3
0.4
0.5
0.6
0.7
0.8
p-value
5e-04
2e-03
5e-03
2e-02
6e-02
Log-odds
0.5
1.0
1.5
2.0
2.5
3.0
3.5
4.0
Cellular_organisms - ps1
Archaea1 - ps2
Archaea2 - ps3
Archaea3 - ps4
Asgard/Eukaryota - ps5
Eukaryota - ps6
Amorpheae - ps7
Obazoa - ps8
Opisthokonta - ps9
Holozoa - ps10
Filozoa - ps11
Choano./Metazoa - ps12
Metazoa - ps13
Myriazoa - ps14
Parahoxozoa - ps15
Bilateria - ps16
Deuterostomia - ps17
Chordata - ps18
Olfactores - ps19
Vertebrata - ps20
Gnathostomata - ps21
Euteleostomi - ps22
Sarcopterygii - ps23
Tetrapoda - ps24
Amniota - ps25
Mammalia - ps26
Theria - ps27
Placentalia - ps28
Boreoeutheria - ps29
Euarchontoglires - ps30
Euarchonta - ps31
Primates - ps32
Haplorrhini - ps33
Simiiformes - ps34
Catarrhini - ps35
Hominoidea - ps36
Hominidae - ps37
Homininae - ps38
Hominini - ps39
Homo_sapiens - ps40
Phylostratum

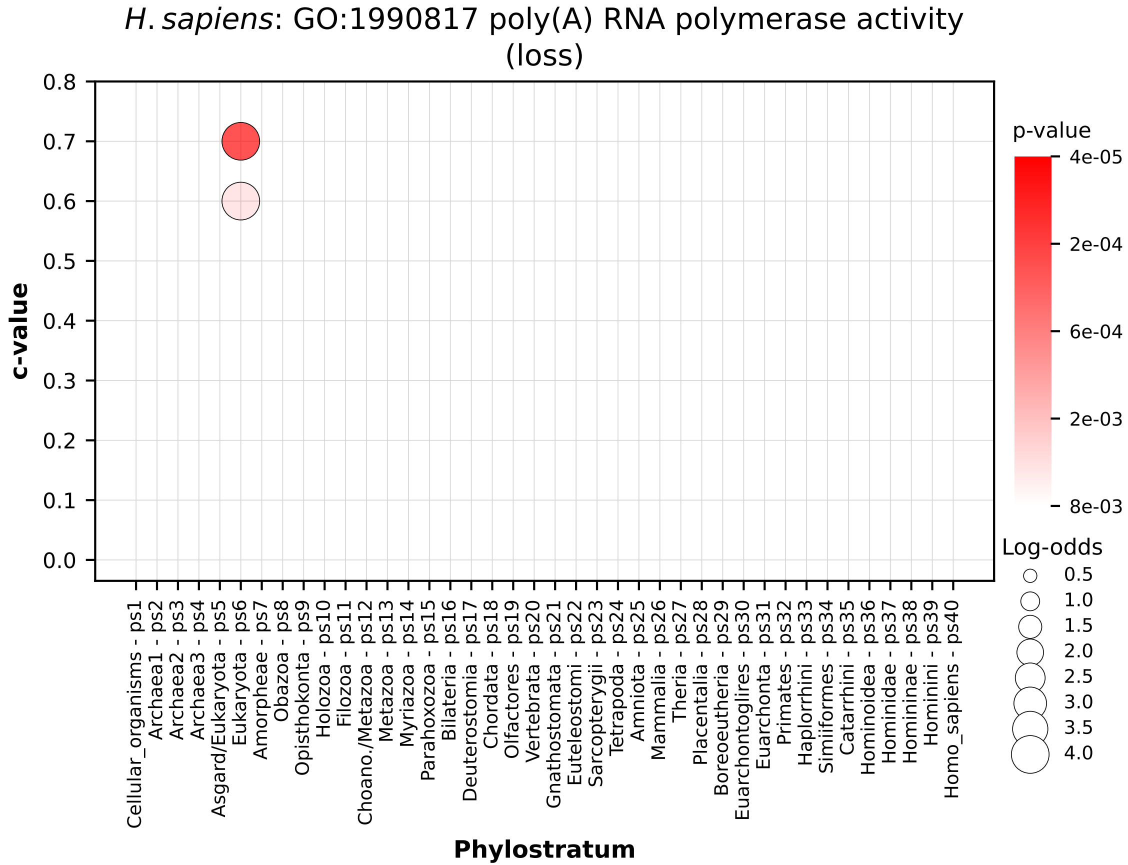

H. sapiens: GO:1990817 poly(A) RNA polymerase activity (loss)
c-value
0.8
0.7
0.6
0.5
0.4
0.3
0.2
0.1
0.0
p-value
4e-05
2e-04
6e-04
2e-03
8e-03
Log-odds
0.5
1.0
1.5
2.0
2.5
3.0
3.5
4.0
Cellular_organisms - ps1
Archaea1 - ps2
Archaea2 - ps3
Archaea3 - ps4
Asgard/Eukaryota - ps5
Eukaryota - ps6
Amorpheae - ps7
Obazoa - ps8
Opisthokonta - ps9
Holozoa - ps10
Filozoa - ps11
Choano./Metazoa - ps12
Metazoa - ps13
Myriazoa - ps14
Parahoxozoa - ps15
Bilateria - ps16
Deuterostomia - ps17
Chordata - ps18
Olfactores - ps19
Vertebrata - ps20
Gnathostomata - ps21
Euteleostomi - ps22
Sarcopterygii - ps23
Tetrapoda - ps24
Amniota - ps25
Mammalia - ps26
Theria - ps27
Placentalia - ps28
Boreoeutheria - ps29
Euarchontoglires - ps30
Euarchonta - ps31
Primates - ps32
Haplorrhini - ps33
Simiiformes - ps34
Catarrhini - ps35
Hominoidea - ps36
Hominidae - ps37
Homininae - ps38
Hominini - ps39
Homo_sapiens - ps40
Phylostratum

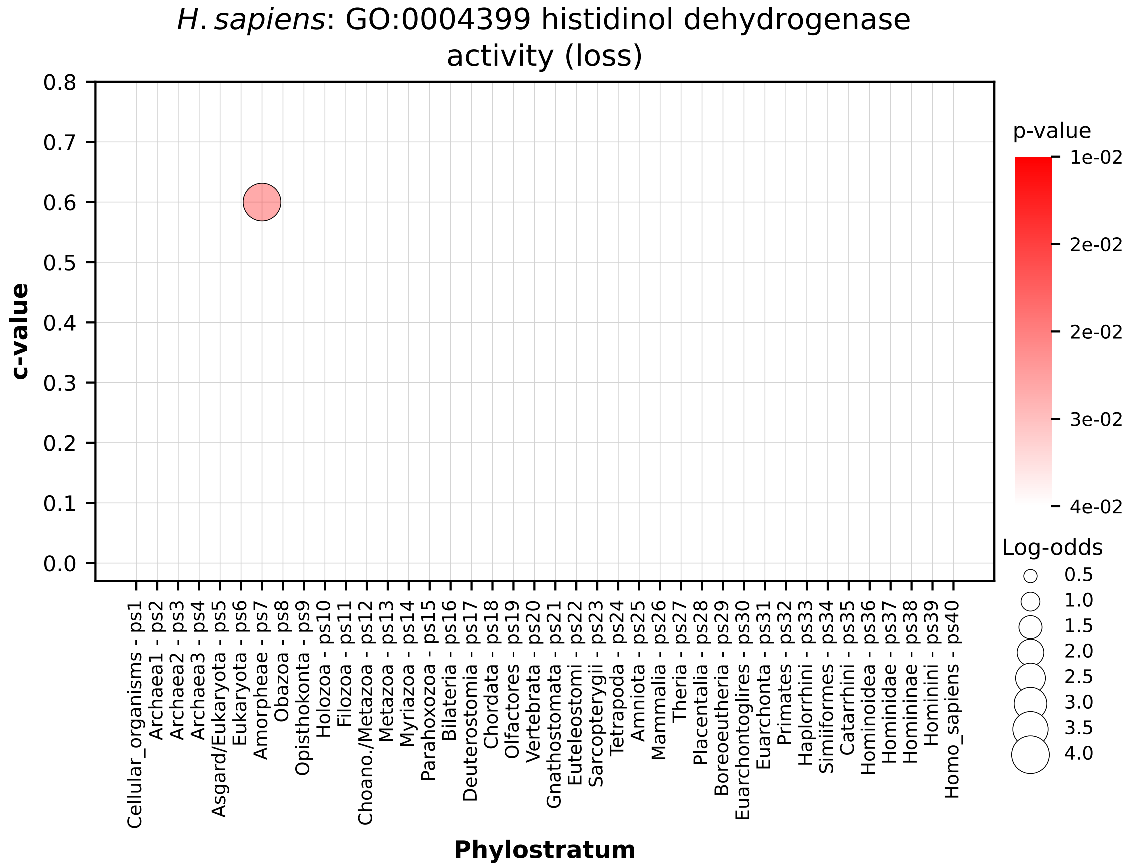
H. sapiens: GO:0004399 histidinol dehydrogenase activity (loss)
c-value
0.8
0.7
0.6
0.5
0.4
0.3
0.2
0.1
0.0
p-value
1e-02
2e-02
2e-02
3e-02
4e-02
Log-odds
0.5
1.0
1.5
2.0
2.5
3.0
3.5
4.0
Cellular_organisms - ps1
Archaea1 - ps2
Archaea2 - ps3
Archaea3 - ps4
Asgard/Eukaryota - ps5
Eukaryota - ps6
Amorpheae - ps7
Obazoa - ps8
Opisthokonta - ps9
Holozoa - ps10
Filozoa - ps11
Choano./Metazoa - ps12
Metazoa - ps13
Myriazoa - ps14
Parahoxozoa - ps15
Bilateria - ps16
Deuterostomia - ps17
Chordata - ps18
Olfactores - ps19
Vertebrata - ps20
Gnathostomata - ps21
Euteleostomi - ps22
Sarcopterygii - ps23
Tetrapoda - ps24
Amniota - ps25
Mammalia - ps26
Theria - ps27
Placentalia - ps28
Boreoeutheria - ps29
Euarchontoglires - ps30
Euarchonta - ps31
Primates - ps32
Haplorrhini - ps33
Simiiformes - ps34
Catarrhini - ps35
Hominoidea - ps36
Hominidae - ps37
Homininae - ps38
Hominini - ps39
Homo_sapiens - ps40
Phylostratum

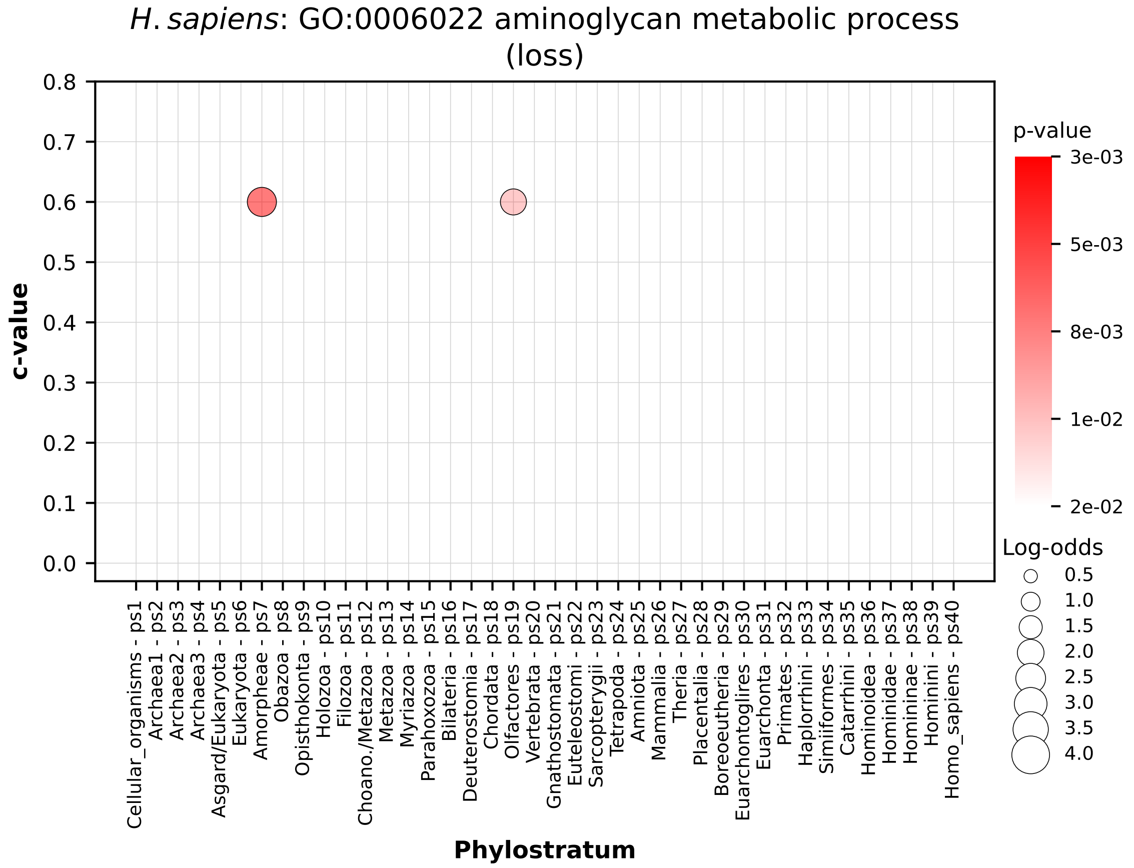

H. sapiens: GO:0006022 aminoglycan metabolic process
(loss)
c-value
0.8
0.7
0.6
0.5
0.4
0.3
0.2
0.1
0.0
p-value
3e-03
5e-03
8e-03
1e-02
2e-02
Log-odds
0.5
1.0
1.5
2.0
2.5
3.0
3.5
4.0
Cellular_organisms - ps1
Archaea1 - ps2
Archaea2 - ps3
Archaea3 - ps4
Asgard/Eukaryota - ps5
Eukaryota - ps6
Amorpheae - ps7
Obazoa - ps8
Opisthokonta - ps9
Holozoa - ps10
Filozoa - ps11
Choano./Metazoa - ps12
Metazoa - ps13
Myriazoa - ps14
Parahoxozoa - ps15
Bilateria - ps16
Deuterostomia - ps17
Chordata - ps18
Olfactores - ps19
Vertebrata - ps20
Gnathostomata - ps21
Euteleostomi - ps22
Sarcopterygii - ps23
Tetrapoda - ps24
Amniota - ps25
Mammalia - ps26
Theria - ps27
Placentalia - ps28
Boreoeutheria - ps29
Euarchontoglires - ps30
Euarchonta - ps31
Primates - ps32
Haplorrhini - ps33
Simiiformes - ps34
Catarrhini - ps35
Hominoidea - ps36
Hominidae - ps37
Homininae - ps38
Hominini - ps39
Homo_sapiens - ps40
Phylostratum

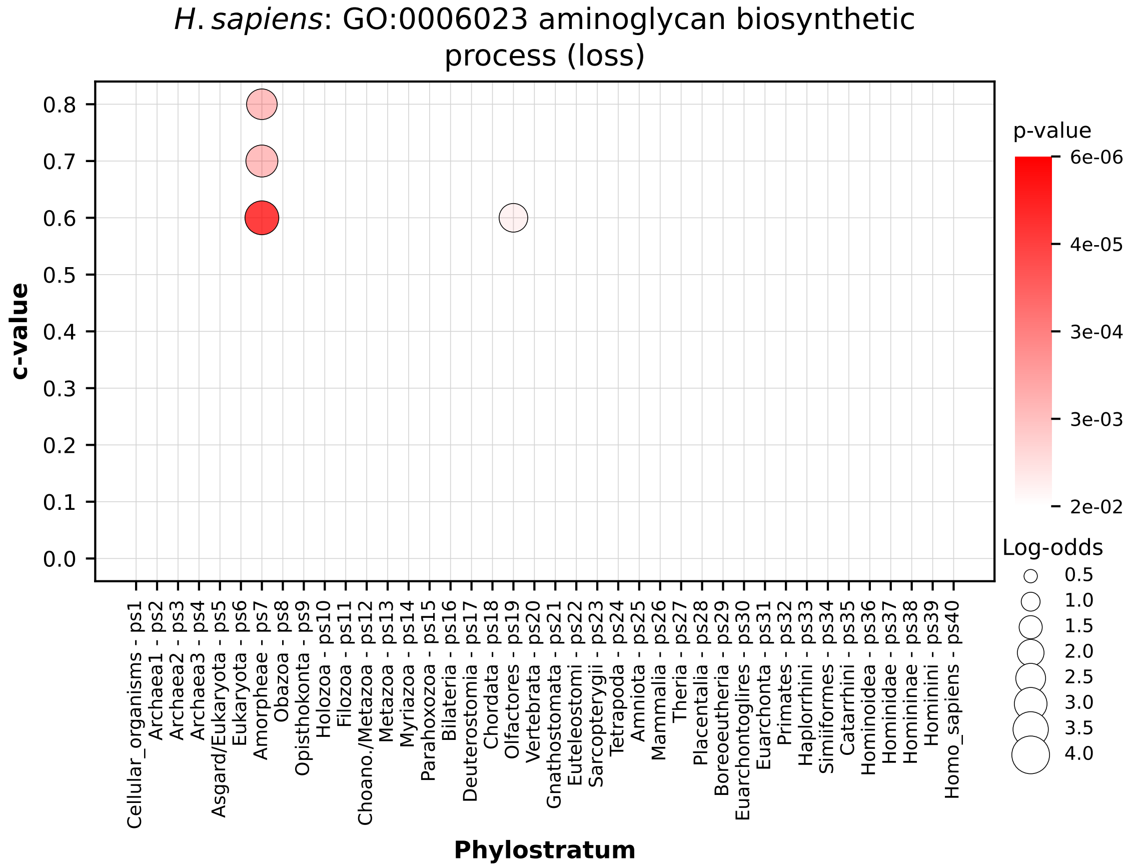

H. sapiens: GO:0006023 aminoglycan biosynthetic process (loss)
c-value
0.0
0.1
0.2
0.3
0.4
0.5
0.6
0.7
0.8
Phylostratum
Cellular_organisms - ps1
Archaea1 - ps2
Archaea2 - ps3
Archaea3 - ps4
Asgard/Eukaryota - ps5
Eukaryota - ps6
Amorpheae - ps7
Obazoa - ps8
Opisthokonta - ps9
Holozoa - ps10
Filozoa - ps11
Choano./Metazoa - ps12
Metazoa - ps13
Myriazoa - ps14
Parahoxozoa - ps15
Bilateria - ps16
Deuterostomia - ps17
Chordata - ps18
Olfactores - ps19
Vertebrata - ps20
Gnathostomata - ps21
Euteleostomi - ps22
Sarcopterygii - ps23
Tetrapoda - ps24
Amniota - ps25
Mammalia - ps26
Theria - ps27
Placentalia - ps28
Boreoeutheria - ps29
Euarchontoglires - ps30
Euarchonta - ps31
Primates - ps32
Haplorrhini - ps33
Simiiformes - ps34
Catarrhini - ps35
Hominoidea - ps36
Hominidae - ps37
Homininae - ps38
Hominini - ps39
Homo_sapiens - ps40
p-value
6e-06
4e-05
3e-04
3e-03
2e-02
Log-odds
0.5
1.0
1.5
2.0
2.5
3.0
3.5
4.0

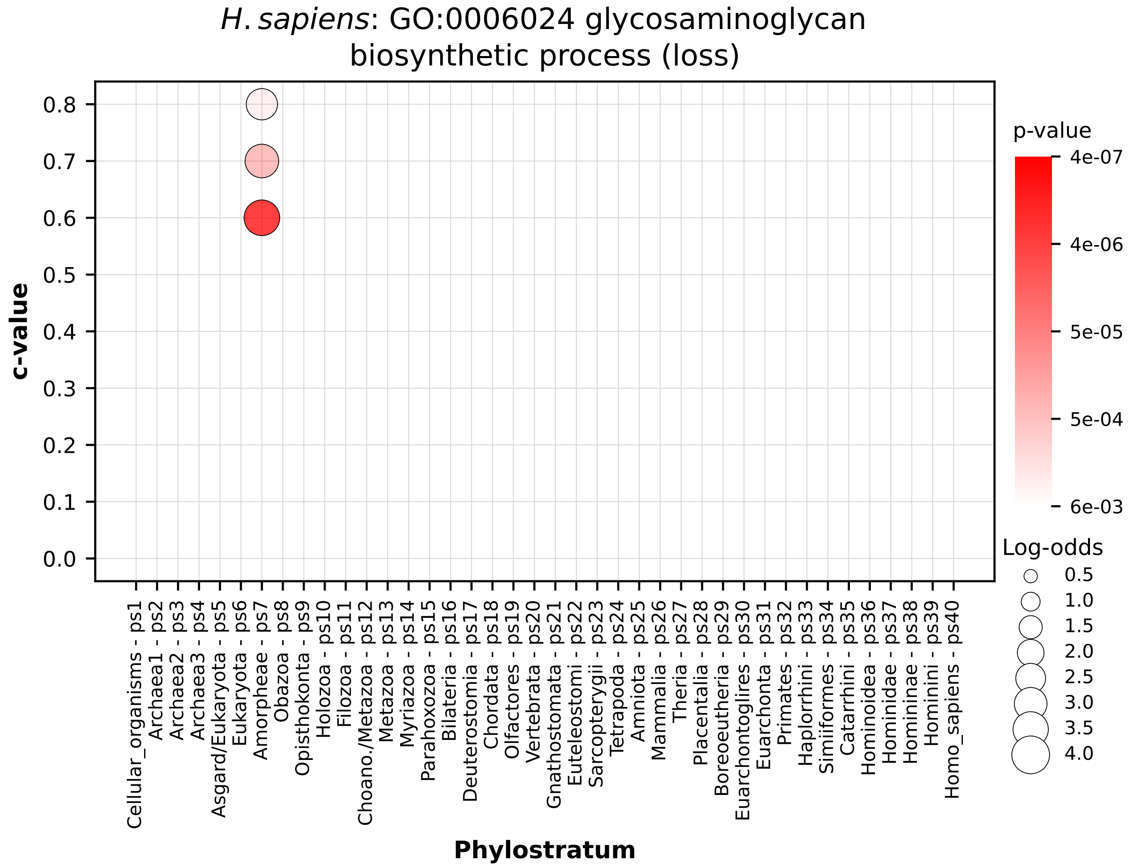

H. sapiens: GO:0006024 glycosaminoglycan biosynthetic process (loss)
c-value
0.8
0.7
0.6
0.5
0.4
0.3
0.2
0.1
0.0
p-value
4e-07
4e-06
5e-05
5e-04
6e-03
Log-odds
0.5
1.0
1.5
2.0
2.5
3.0
3.5
4.0
Cellular_organisms - ps1
Archaea1 - ps2
Archaea2 - ps3
Archaea3 - ps4
Asgard/Eukaryota - ps5
Eukaryota - ps6
Amorpheae - ps7
Obazoa - ps8
Opisthokonta - ps9
Holozoa - ps10
Filozoa - ps11
Choano./Metazoa - ps12
Metazoa - ps13
Myriazoa - ps14
Parahoxozoa - ps15
Bilateria - ps16
Deuterostomia - ps17
Chordata - ps18
Olfactores - ps19
Vertebrata - ps20
Gnathostomata - ps21
Euteleostomi - ps22
Sarcopterygii - ps23
Tetrapoda - ps24
Amniota - ps25
Mammalia - ps26
Theria - ps27
Placentalia - ps28
Boreoeutheria - ps29
Euarchontoglires - ps30
Euarchonta - ps31
Primates - ps32
Haplorrhini - ps33
Simiiformes - ps34
Catarrhini - ps35
Hominoidea - ps36
Hominidae - ps37
Homininae - ps38
Hominini - ps39
Homo_sapiens - ps40
Phylostratum

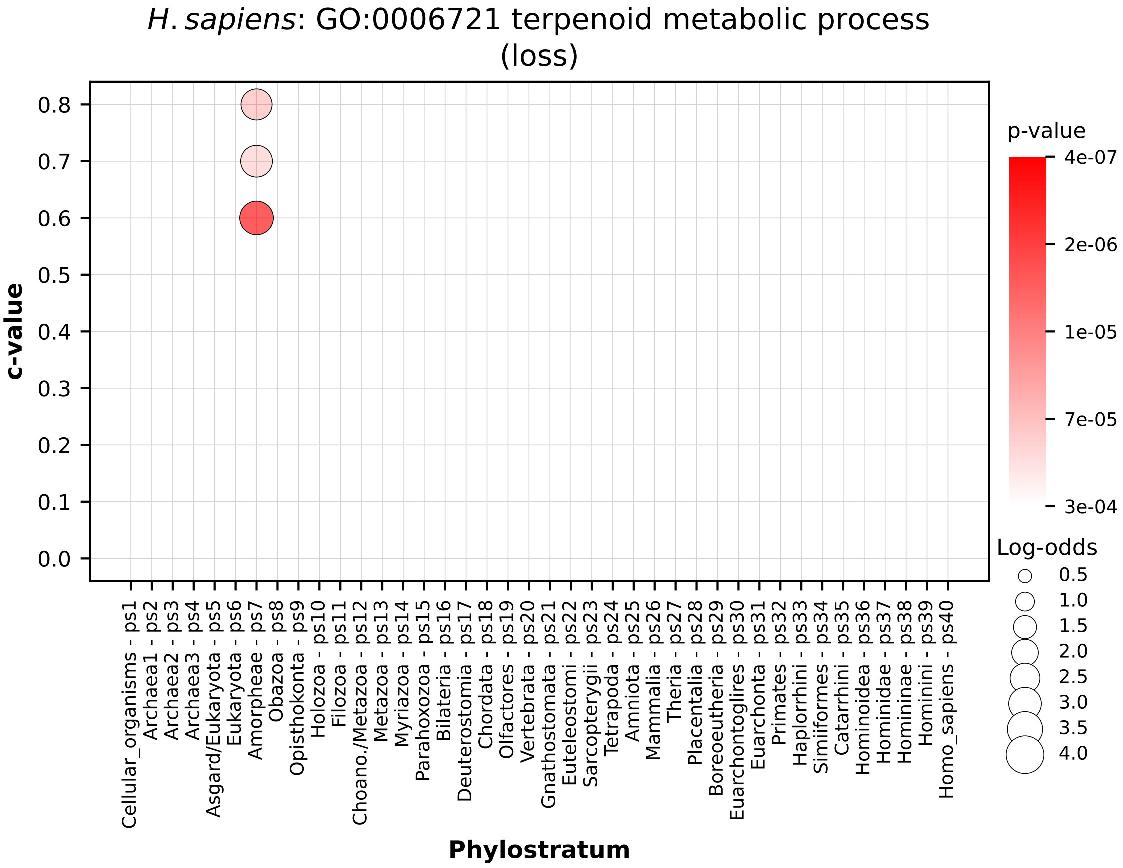

H. sapiens: GO:0006721 terpenoid metabolic process
(loss)
c-value
0.0
0.1
0.2
0.3
0.4
0.5
0.6
0.7
0.8
Phylostratum
Cellular_organisms - ps1
Archaea1 - ps2
Archaea2 - ps3
Archaea3 - ps4
Asgard/Eukaryota - ps5
Eukaryota - ps6
Amorpheae - ps7
Obazoa - ps8
Opisthokonta - ps9
Holozoa - ps10
Filozoa - ps11
Choano./Metazoa - ps12
Metazoa - ps13
Myriazoa - ps14
Parahoxozoa - ps15
Bilateria - ps16
Deuterostomia - ps17
Chordata - ps18
Olfactores - ps19
Vertebrata - ps20
Gnathostomata - ps21
Euteleostomi - ps22
Sarcopterygii - ps23
Tetrapoda - ps24
Amniota - ps25
Mammalia - ps26
Theria - ps27
Placentalia - ps28
Boreoeutheria - ps29
Euarchontoglires - ps30
Euarchonta - ps31
Primates - ps32
Haplorrhini - ps33
Simiiformes - ps34
Catarrhini - ps35
Hominoidea - ps36
Hominidae - ps37
Homininae - ps38
Hominini - ps39
Homo_sapiens - ps40
p-value
4e-07
2e-06
1e-05
7e-05
3e-04
Log-odds
0.5
1.0
1.5
2.0
2.5
3.0
3.5
4.0

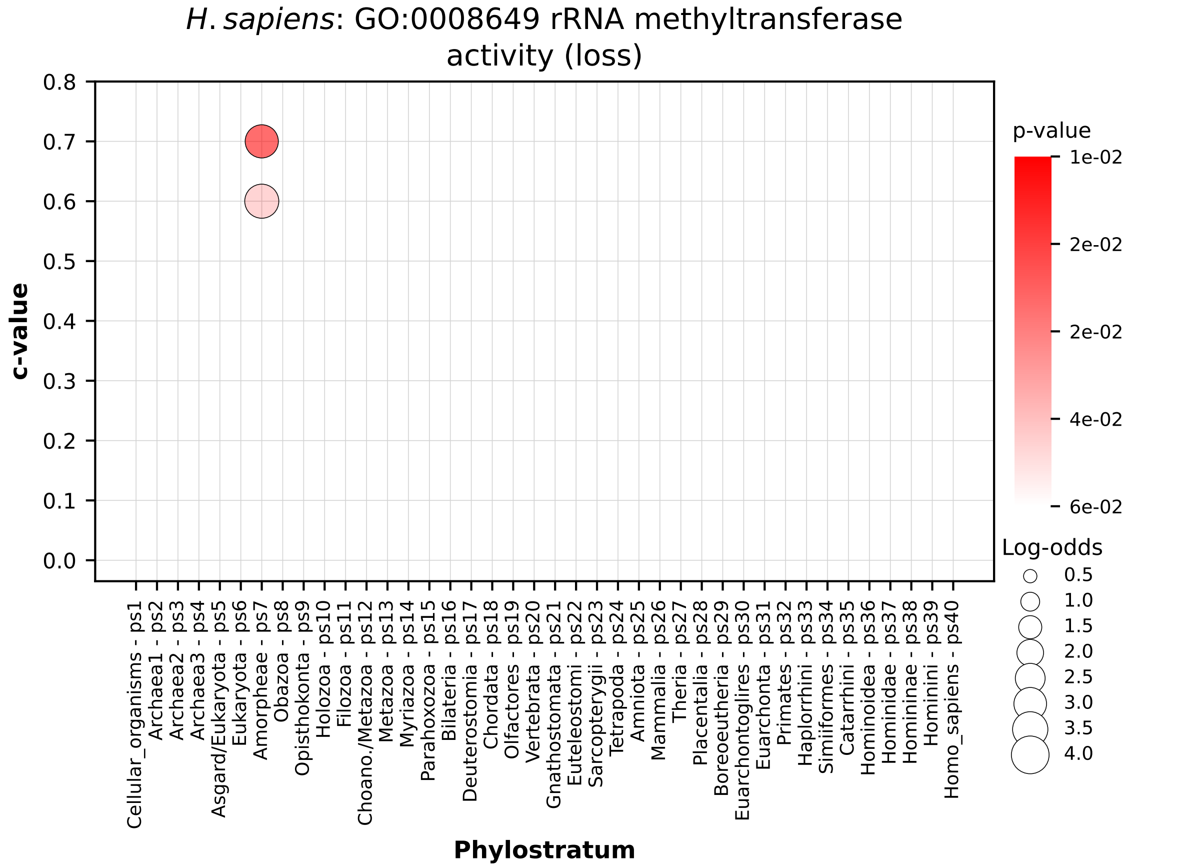
H. sapiens: GO:0008649 rRNA methyltransferase activity (loss)
c-value
0.0
0.1
0.2
0.3
0.4
0.5
0.6
0.7
0.8
Phylostratum
Cellular_organisms - ps1
Archaea1 - ps2
Archaea2 - ps3
Archaea3 - ps4
Asgard/Eukaryota - ps5
Eukaryota - ps6
Amorpheae - ps7
Obazoa - ps8
Opisthokonta - ps9
Holozoa - ps10
Filozoa - ps11
Choano./Metazoa - ps12
Metazoa - ps13
Myriazoa - ps14
Parahoxozoa - ps15
Bilateria - ps16
Deuterostomia - ps17
Chordata - ps18
Olfactores - ps19
Vertebrata - ps20
Gnathostomata - ps21
Euteleostomi - ps22
Sarcopterygii - ps23
Tetrapoda - ps24
Amniota - ps25
Mammalia - ps26
Theria - ps27
Placentalia - ps28
Boreoeutheria - ps29
Euarchontoglires - ps30
Euarchonta - ps31
Primates - ps32
Haplorrhini - ps33
Simiiformes - ps34
Catarrhini - ps35
Hominoidea - ps36
Hominidae - ps37
Homininae - ps38
Hominini - ps39
Homo_sapiens - ps40
p-value
1e-02
2e-02
2e-02
4e-02
6e-02
Log-odds
0.5
1.0
1.5
2.0
2.5
3.0
3.5
4.0

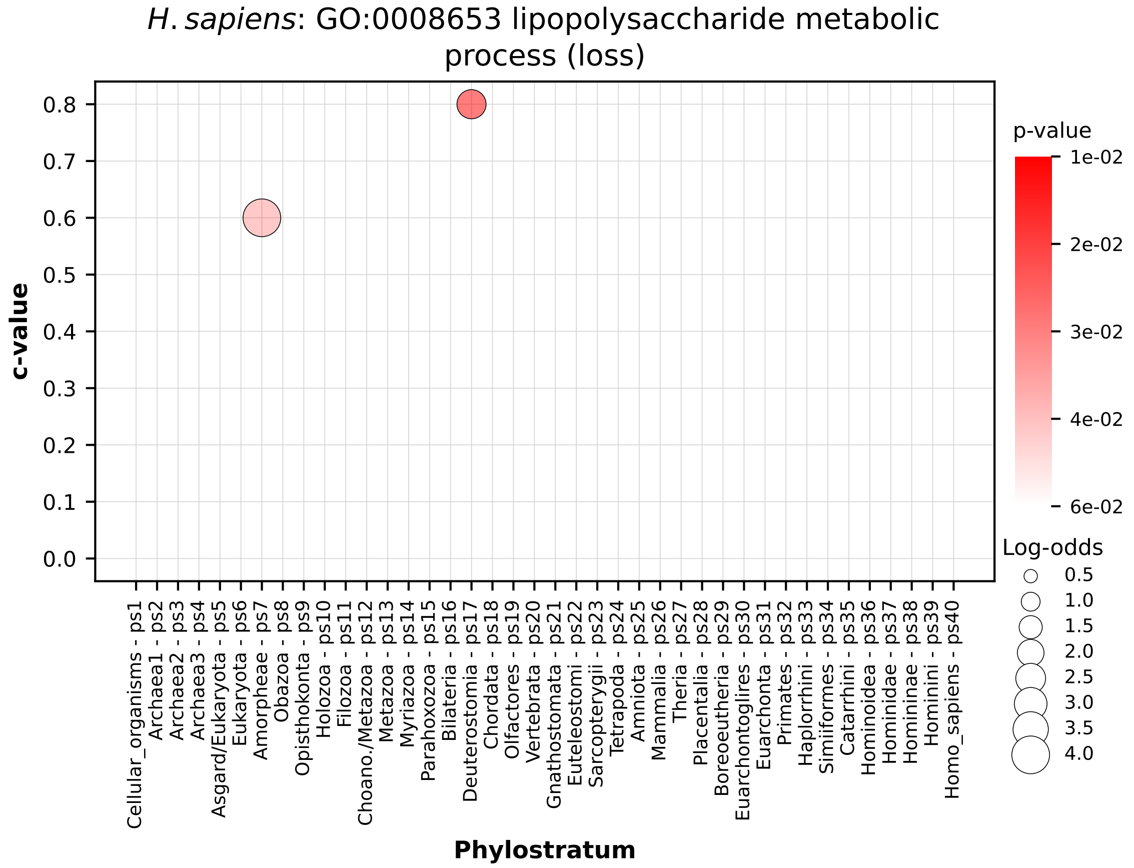

H. sapiens: GO:0008653 lipopolysaccharide metabolic process (loss)
c-value
0.8
0.7
0.6
0.5
0.4
0.3
0.2
0.1
0.0
Phylostratum
Cellular_organisms - ps1
Archaea1 - ps2
Archaea2 - ps3
Archaea3 - ps4
Asgard/Eukaryota - ps5
Eukaryota - ps6
Amorpheae - ps7
Obazoa - ps8
Opisthokonta - ps9
Holozoa - ps10
Filozoa - ps11
Choano./Metazoa - ps12
Metazoa - ps13
Myriazoa - ps14
Parahoxozoa - ps15
Bilateria - ps16
Deuterostomia - ps17
Chordata - ps18
Olfactores - ps19
Vertebrata - ps20
Gnathostomata - ps21
Euteleostomi - ps22
Sarcopterygii - ps23
Tetrapoda - ps24
Amniota - ps25
Mammalia - ps26
Theria - ps27
Placentalia - ps28
Boreoeutheria - ps29
Euarchontoglires - ps30
Euarchonta - ps31
Primates - ps32
Haplorrhini - ps33
Simiiformes - ps34
Catarrhini - ps35
Hominoidea - ps36
Hominidae - ps37
Homininae - ps38
Hominini - ps39
Homo_sapiens - ps40
p-value
1e-02
2e-02
3e-02
4e-02
6e-02
Log-odds
0.5
1.0
1.5
2.0
2.5
3.0
3.5
4.0

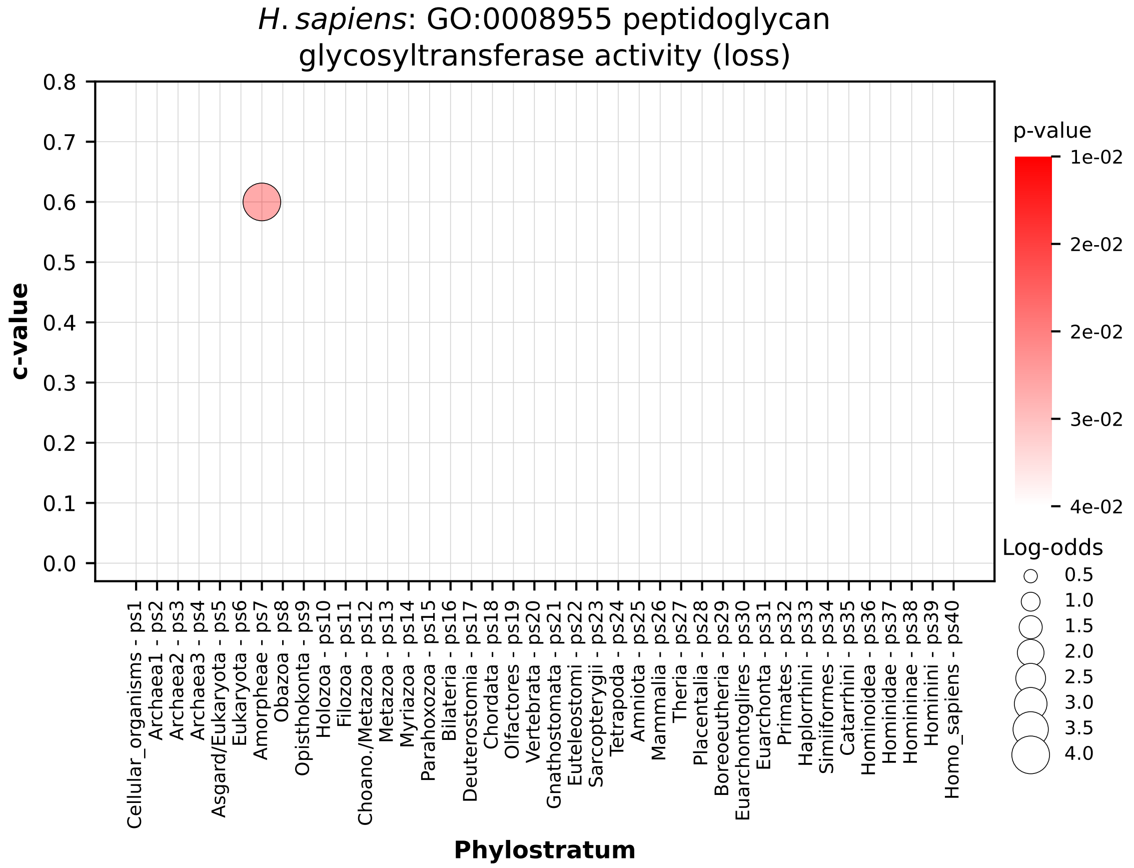

H. sapiens: GO:0008955 peptidoglycan glycosyltransferase activity (loss)
c-value
0.8
0.7
0.6
0.5
0.4
0.3
0.2
0.1
0.0
Phylostratum
Cellular_organisms - ps1
Archaea1 - ps2
Archaea2 - ps3
Archaea3 - ps4
Asgard/Eukaryota - ps5
Eukaryota - ps6
Amorpheae - ps7
Obazoa - ps8
Opisthokonta - ps9
Holozoa - ps10
Filozoa - ps11
Choano./Metazoa - ps12
Metazoa - ps13
Myriazoa - ps14
Parahoxozoa - ps15
Bilateria - ps16
Deuterostomia - ps17
Chordata - ps18
Olfactores - ps19
Vertebrata - ps20
Gnathostomata - ps21
Euteleostomi - ps22
Sarcopterygii - ps23
Tetrapoda - ps24
Amniota - ps25
Mammalia - ps26
Theria - ps27
Placentalia - ps28
Boreoeutheria - ps29
Euarchontoglires - ps30
Euarchonta - ps31
Primates - ps32
Haplorrhini - ps33
Simiiformes - ps34
Catarrhini - ps35
Hominoidea - ps36
Hominidae - ps37
Homininae - ps38
Hominini - ps39
Homo_sapiens - ps40
p-value
1e-02
2e-02
2e-02
3e-02
4e-02
Log-odds
0.5
1.0
1.5
2.0
2.5
3.0
3.5
4.0

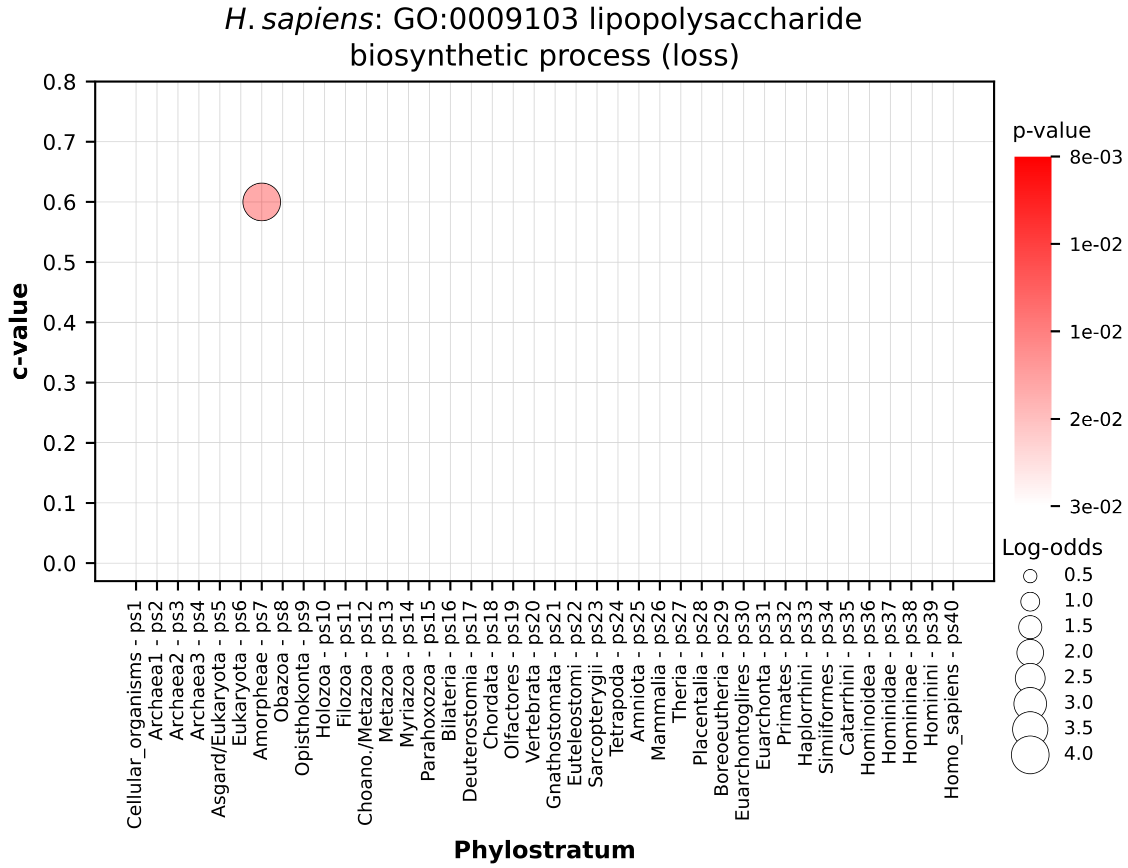

H. sapiens: GO:0009103 lipopolysaccharide biosynthetic process (loss)
c-value
0.0
0.1
0.2
0.3
0.4
0.5
0.6
0.7
0.8
Phylostratum
Cellular_organisms - ps1
Archaea1 - ps2
Archaea2 - ps3
Archaea3 - ps4
Asgard/Eukaryota - ps5
Eukaryota - ps6
Amorpheae - ps7
Obazoa - ps8
Opisthokonta - ps9
Holozoa - ps10
Filozoa - ps11
Choano./Metazoa - ps12
Metazoa - ps13
Myriazoa - ps14
Parahoxozoa - ps15
Bilateria - ps16
Deuterostomia - ps17
Chordata - ps18
Olfactores - ps19
Vertebrata - ps20
Gnathostomata - ps21
Euteleostomi - ps22
Sarcopterygii - ps23
Tetrapoda - ps24
Amniota - ps25
Mammalia - ps26
Theria - ps27
Placentalia - ps28
Boreoeutheria - ps29
Euarchontoglires - ps30
Euarchonta - ps31
Primates - ps32
Haplorrhini - ps33
Simiiformes - ps34
Catarrhini - ps35
Hominoidea - ps36
Hominidae - ps37
Homininae - ps38
Hominini - ps39
Homo_sapiens - ps40
p-value
8e-03
1e-02
1e-02
2e-02
3e-02
Log-odds
0.5
1.0
1.5
2.0
2.5
3.0
3.5
4.0

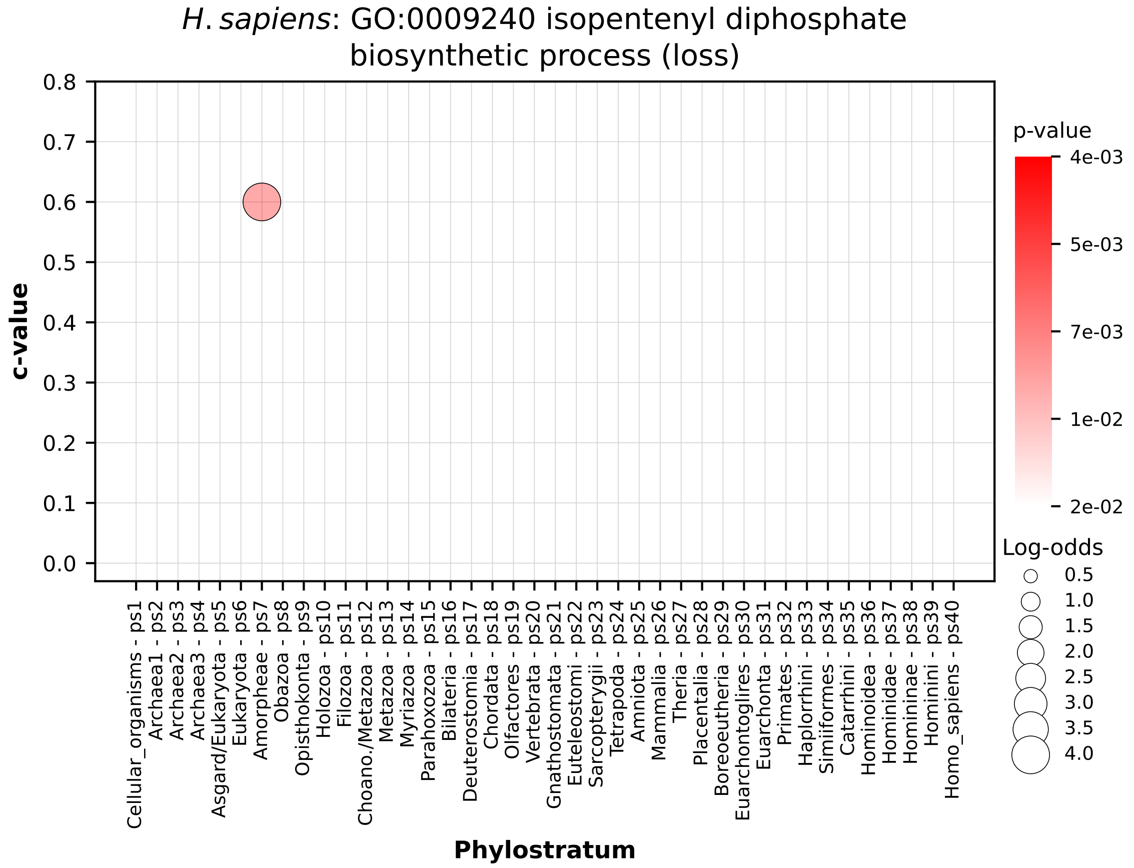

H. sapiens: GO:0009240 isopentenyl diphosphate biosynthetic process (loss)
c-value
0.8
0.7
0.6
0.5
0.4
0.3
0.2
0.1
0.0
Phylostratum
Cellular_organisms - ps1
Archaea1 - ps2
Archaea2 - ps3
Archaea3 - ps4
Asgard/Eukaryota - ps5
Eukaryota - ps6
Amorpheae - ps7
Obazoa - ps8
Opisthokonta - ps9
Holozoa - ps10
Filozoa - ps11
Choano./Metazoa - ps12
Metazoa - ps13
Myriazoa - ps14
Parahoxozoa - ps15
Bilateria - ps16
Deuterostomia - ps17
Chordata - ps18
Olfactores - ps19
Vertebrata - ps20
Gnathostomata - ps21
Euteleostomi - ps22
Sarcopterygii - ps23
Tetrapoda - ps24
Amniota - ps25
Mammalia - ps26
Theria - ps27
Placentalia - ps28
Boreoeutheria - ps29
Euarchontoglires - ps30
Euarchonta - ps31
Primates - ps32
Haplorrhini - ps33
Simiiformes - ps34
Catarrhini - ps35
Hominoidea - ps36
Hominidae - ps37
Homininae - ps38
Hominini - ps39
Homo_sapiens - ps40
p-value
4e-03
5e-03
7e-03
1e-02
2e-02
Log-odds
0.5
1.0
1.5
2.0
2.5
3.0
3.5
4.0

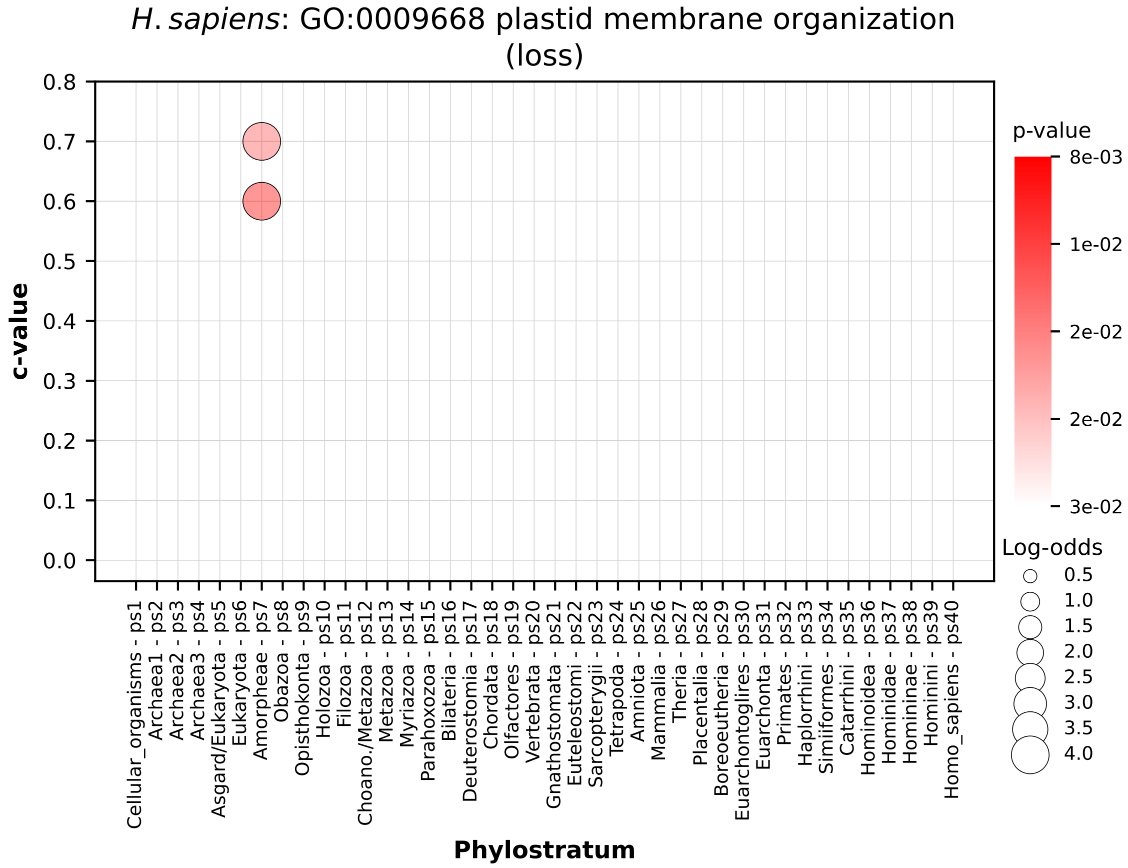

H. sapiens: GO:0009668 plastid membrane organization
(loss)
c-value
0.8
0.7
0.6
0.5
0.4
0.3
0.2
0.1
0.0
Phylostratum
Cellular_organisms - ps1
Archaea1 - ps2
Archaea2 - ps3
Archaea3 - ps4
Asgard/Eukaryota - ps5
Eukaryota - ps6
Amorpheae - ps7
Obazoa - ps8
Opisthokonta - ps9
Holozoa - ps10
Filozoa - ps11
Choano./Metazoa - ps12
Metazoa - ps13
Myriazoa - ps14
Parahoxozoa - ps15
Bilateria - ps16
Deuterostomia - ps17
Chordata - ps18
Olfactores - ps19
Vertebrata - ps20
Gnathostomata - ps21
Euteleostomi - ps22
Sarcopterygii - ps23
Tetrapoda - ps24
Amniota - ps25
Mammalia - ps26
Theria - ps27
Placentalia - ps28
Boreoeutheria - ps29
Euarchontoglires - ps30
Euarchonta - ps31
Primates - ps32
Haplorrhini - ps33
Simiiformes - ps34
Catarrhini - ps35
Hominoidea - ps36
Hominidae - ps37
Homininae - ps38
Hominini - ps39
Homo_sapiens - ps40
p-value
8e-03
1e-02
2e-02
2e-02
3e-02
Log-odds
0.5
1.0
1.5
2.0
2.5
3.0
3.5
4.0

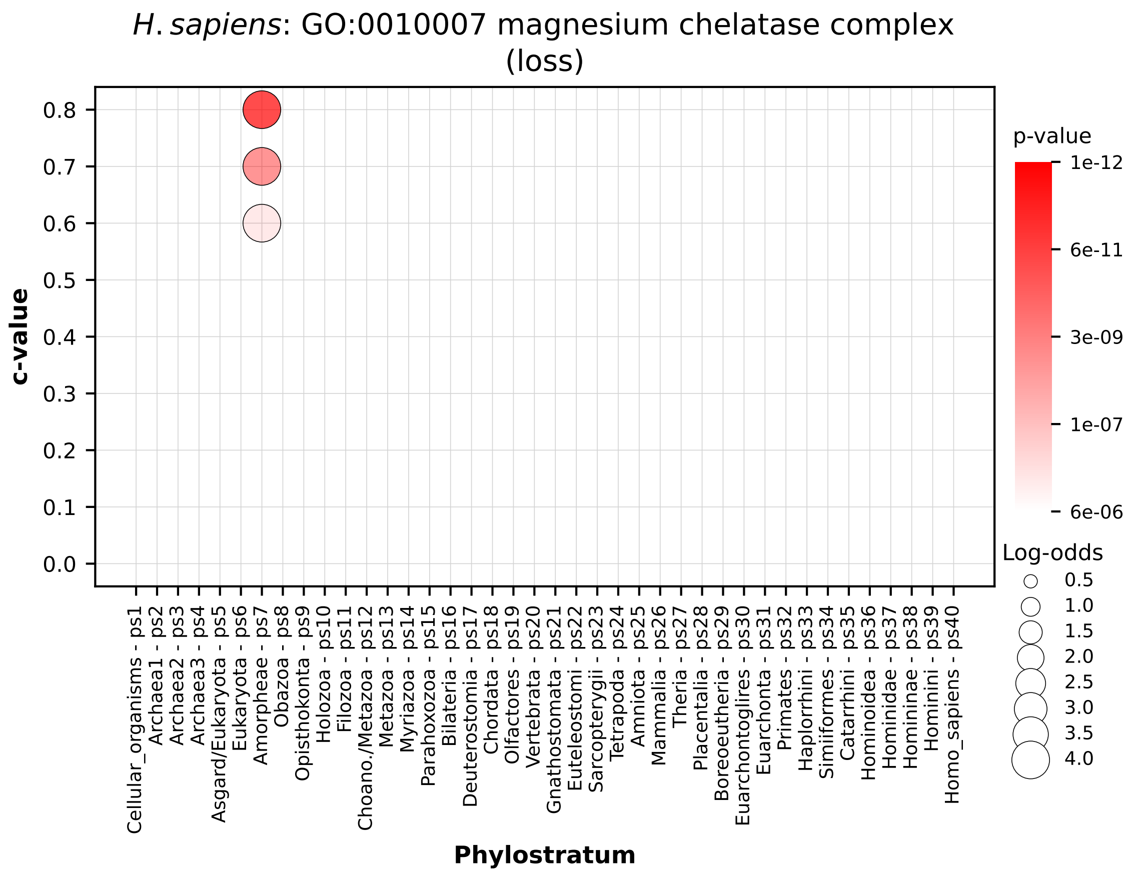

H. sapiens: GO:0010007 magnesium chelatase complex (loss)
c-value
0.0
0.1
0.2
0.3
0.4
0.5
0.6
0.7
0.8
Phylostratum
Cellular_organisms - ps1
Archaea1 - ps2
Archaea2 - ps3
Archaea3 - ps4
Asgard/Eukaryota - ps5
Eukaryota - ps6
Amorpheae - ps7
Obazoa - ps8
Opisthokonta - ps9
Holozoa - ps10
Filozoa - ps11
Choano./Metazoa - ps12
Metazoa - ps13
Myriazoa - ps14
Parahoxozoa - ps15
Bilateria - ps16
Deuterostomia - ps17
Chordata - ps18
Olfactores - ps19
Vertebrata - ps20
Gnathostomata - ps21
Euteleostomi - ps22
Sarcopterygii - ps23
Tetrapoda - ps24
Amniota - ps25
Mammalia - ps26
Theria - ps27
Placentalia - ps28
Boreoeutheria - ps29
Euarchontoglires - ps30
Euarchonta - ps31
Primates - ps32
Haplorrhini - ps33
Simiiformes - ps34
Catarrhini - ps35
Hominoidea - ps36
Hominidae - ps37
Homininae - ps38
Hominini - ps39
Homo_sapiens - ps40
p-value
1e-12
6e-11
3e-09
1e-07
6e-06
Log-odds
0.5
1.0
1.5
2.0
2.5
3.0
3.5
4.0

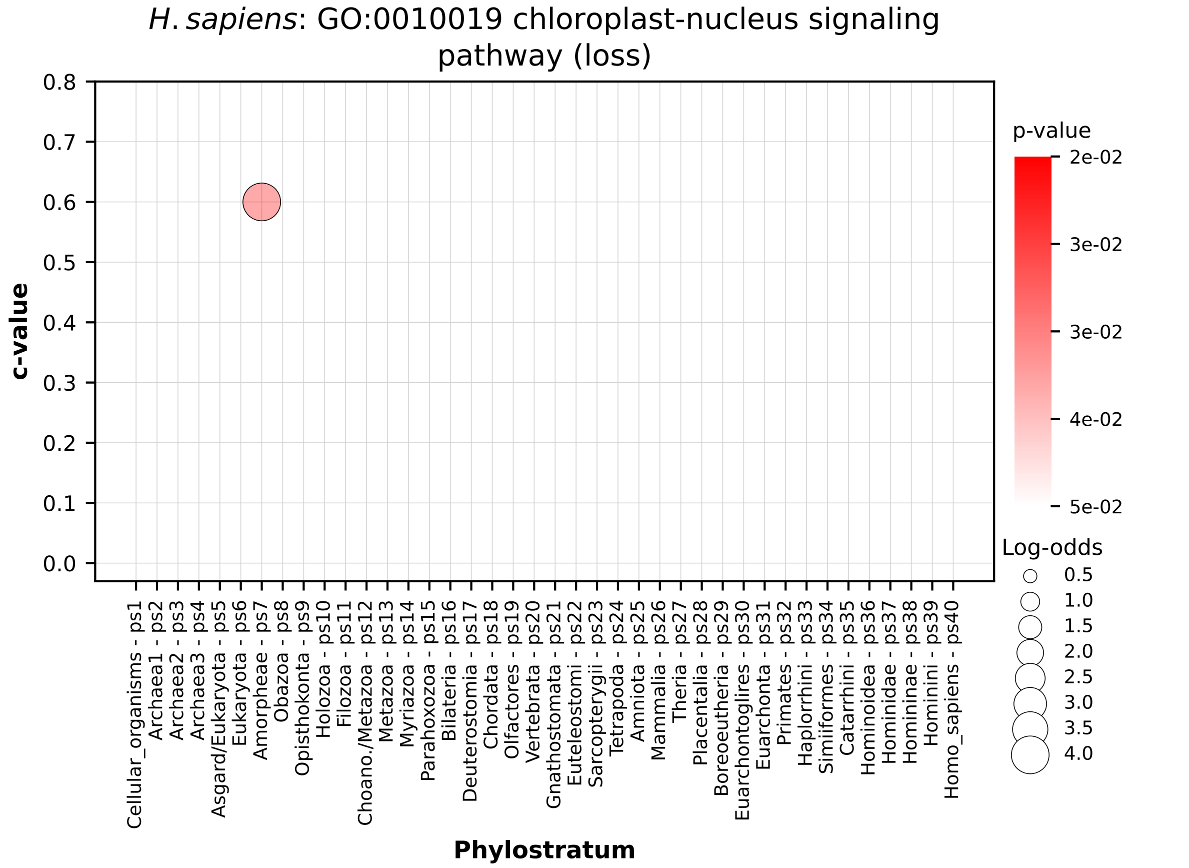

H. sapiens: GO:0010019 chloroplast-nucleus signaling pathway (loss)
c-value
0.8
0.7
0.6
0.5
0.4
0.3
0.2
0.1
0.0
Cellular_organisms - ps1
Archaea1 - ps2
Archaea2 - ps3
Archaea3 - ps4
Asgard/Eukaryota - ps5
Eukaryota - ps6
Amorpheae - ps7
Obazoa - ps8
Opisthokonta - ps9
Holozoa - ps10
Filozoa - ps11
Choano./Metazoa - ps12
Metazoa - ps13
Myriazoa - ps14
Parahoxozoa - ps15
Bilateria - ps16
Deuterostomia - ps17
Chordata - ps18
Olfactores - ps19
Vertebrata - ps20
Gnathostomata - ps21
Euteleostomi - ps22
Sarcopterygii - ps23
Tetrapoda - ps24
Amniota - ps25
Mammalia - ps26
Theria - ps27
Placentalia - ps28
Boreoeutheria - ps29
Euarchontoglires - ps30
Euarchonta - ps31
Primates - ps32
Haplorrhini - ps33
Simiiformes - ps34
Catarrhini - ps35
Hominoidea - ps36
Hominidae - ps37
Homininae - ps38
Hominini - ps39
Homo_sapiens - ps40
Phylostratum
p-value
2e-02
3e-02
3e-02
4e-02
5e-02
Log-odds
0.5
1.0
1.5
2.0
2.5
3.0
3.5
4.0

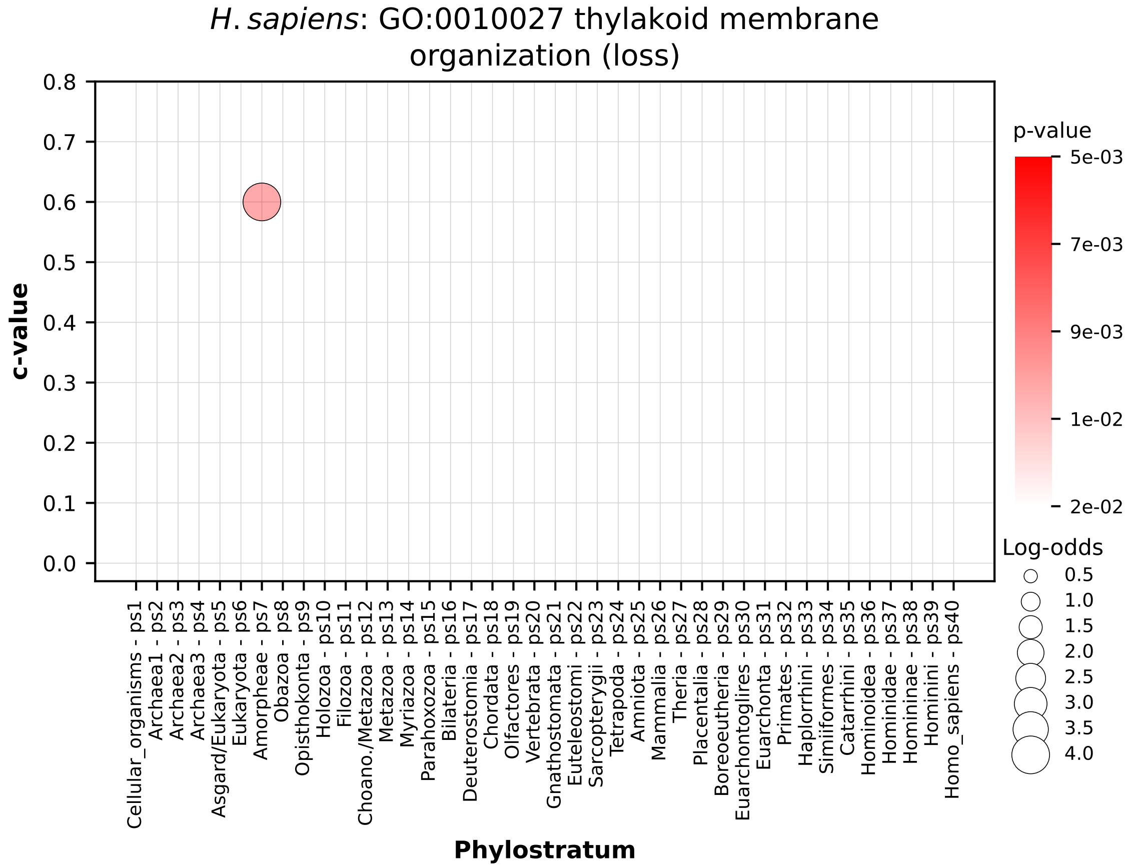

H. sapiens: GO:0010027 thylakoid membrane organization (loss)
c-value
0.8
0.7
0.6
0.5
0.4
0.3
0.2
0.1
0.0
p-value
5e-03
7e-03
9e-03
1e-02
2e-02
Log-odds
0.5
1.0
1.5
2.0
2.5
3.0
3.5
4.0
Cellular_organisms - ps1
Archaea1 - ps2
Archaea2 - ps3
Archaea3 - ps4
Asgard/Eukaryota - ps5
Eukaryota - ps6
Amorpheae - ps7
Obazoa - ps8
Opisthokonta - ps9
Holozoa - ps10
Filozoa - ps11
Choano./Metazoa - ps12
Metazoa - ps13
Myriazoa - ps14
Parahoxozoa - ps15
Bilateria - ps16
Deuterostomia - ps17
Chordata - ps18
Olfactores - ps19
Vertebrata - ps20
Gnathostomata - ps21
Euteleostomi - ps22
Sarcopterygii - ps23
Tetrapoda - ps24
Amniota - ps25
Mammalia - ps26
Theria - ps27
Placentalia - ps28
Boreoeutheria - ps29
Euarchontoglires - ps30
Euarchonta - ps31
Primates - ps32
Haplorrhini - ps33
Simiiformes - ps34
Catarrhini - ps35
Hominoidea - ps36
Hominidae - ps37
Homininae - ps38
Hominini - ps39
Homo_sapiens - ps40
Phylostratum

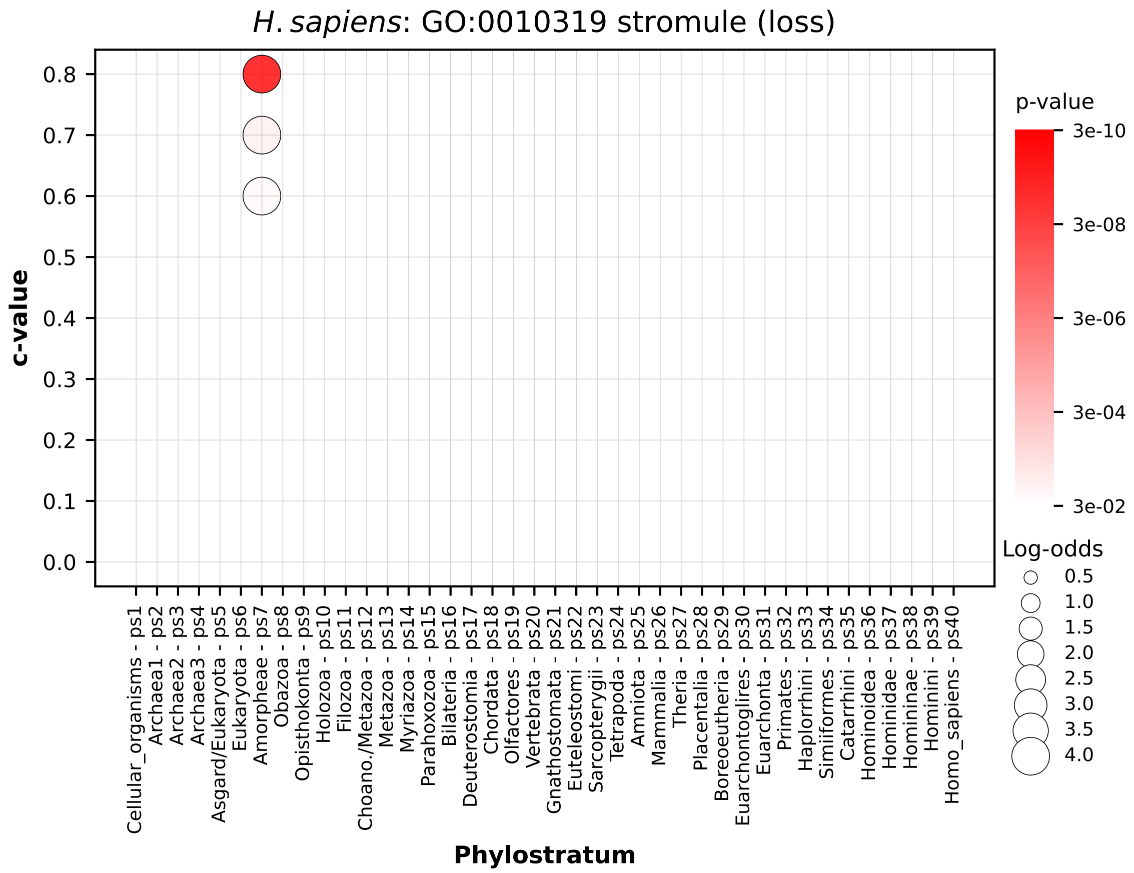

H. sapiens: GO:0010319 stromule (loss)
c-value
0.8
0.7
0.6
0.5
0.4
0.3
0.2
0.1
0.0
p-value
3e-10
3e-08
3e-06
3e-04
3e-02
Log-odds
0.5
1.0
1.5
2.0
2.5
3.0
3.5
4.0
Cellular_organisms - ps1
Archaea1 - ps2
Archaea2 - ps3
Archaea3 - ps4
Asgard/Eukaryota - ps5
Eukaryota - ps6
Amorpheae - ps7
Obazoa - ps8
Opisthokonta - ps9
Holozoa - ps10
Filozoa - ps11
Choano./Metazoa - ps12
Metazoa - ps13
Myriazoa - ps14
Parahoxozoa - ps15
Bilateria - ps16
Deuterostomia - ps17
Chordata - ps18
Olfactores - ps19
Vertebrata - ps20
Gnathostomata - ps21
Euteleostomi - ps22
Sarcopterygii - ps23
Tetrapoda - ps24
Amniota - ps25
Mammalia - ps26
Theria - ps27
Placentalia - ps28
Boreoeutheria - ps29
Euarchontoglires - ps30
Euarchonta - ps31
Primates - ps32
Haplorrhini - ps33
Simiiformes - ps34
Catarrhini - ps35
Hominoidea - ps36
Hominidae - ps37
Homininae - ps38
Hominini - ps39
Homo_sapiens - ps40
Phylostratum

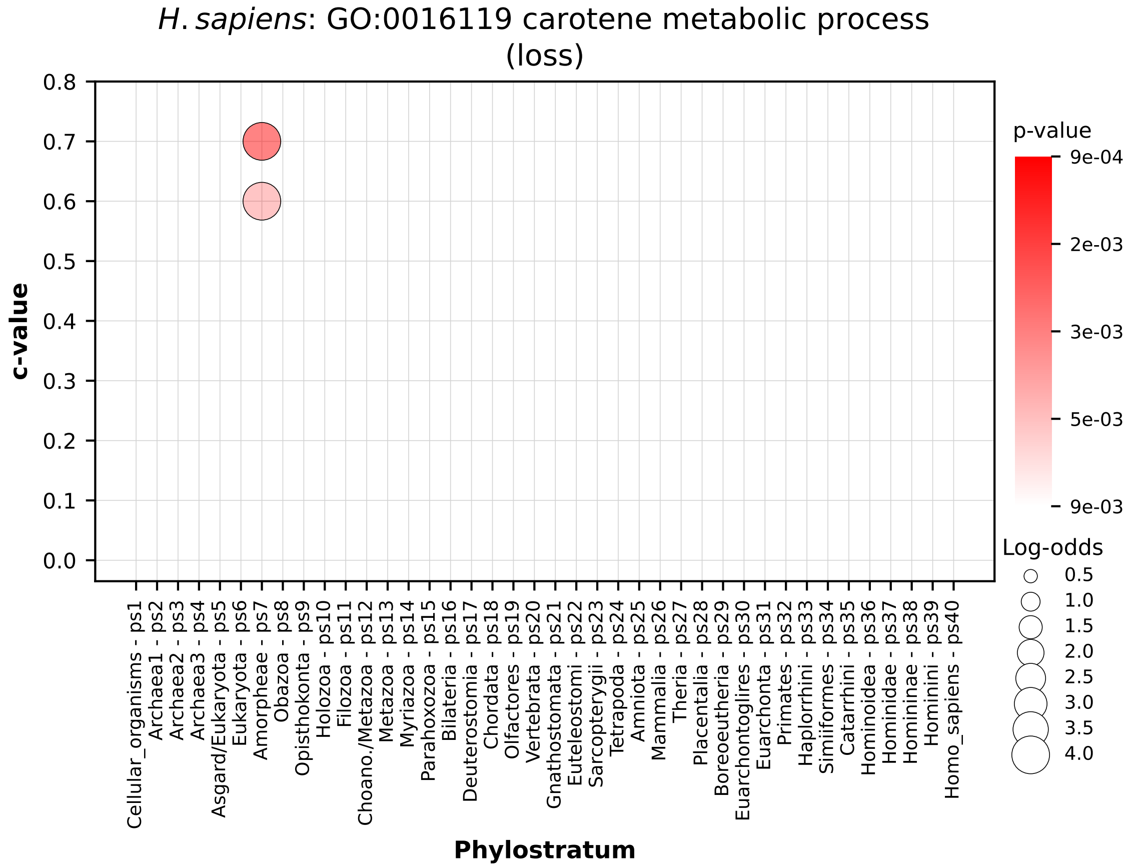

H. sapiens: GO:0016119 carotene metabolic process
(loss)
c-value
0.8
0.7
0.6
0.5
0.4
0.3
0.2
0.1
0.0
p-value
9e-04
2e-03
3e-03
5e-03
9e-03
Log-odds
0.5
1.0
1.5
2.0
2.5
3.0
3.5
4.0
Cellular_organisms - ps1
Archaea1 - ps2
Archaea2 - ps3
Archaea3 - ps4
Asgard/Eukaryota - ps5
Eukaryota - ps6
Amorpheae - ps7
Obazoa - ps8
Opisthokonta - ps9
Holozoa - ps10
Filozoa - ps11
Choano./Metazoa - ps12
Metazoa - ps13
Myriazoa - ps14
Parahoxozoa - ps15
Bilateria - ps16
Deuterostomia - ps17
Chordata - ps18
Olfactores - ps19
Vertebrata - ps20
Gnathostomata - ps21
Euteleostomi - ps22
Sarcopterygii - ps23
Tetrapoda - ps24
Amniota - ps25
Mammalia - ps26
Theria - ps27
Placentalia - ps28
Boreoeutheria - ps29
Euarchontoglires - ps30
Euarchonta - ps31
Primates - ps32
Haplorrhini - ps33
Simiiformes - ps34
Catarrhini - ps35
Hominoidea - ps36
Hominidae - ps37
Homininae - ps38
Hominini - ps39
Homo_sapiens - ps40
Phylostratum

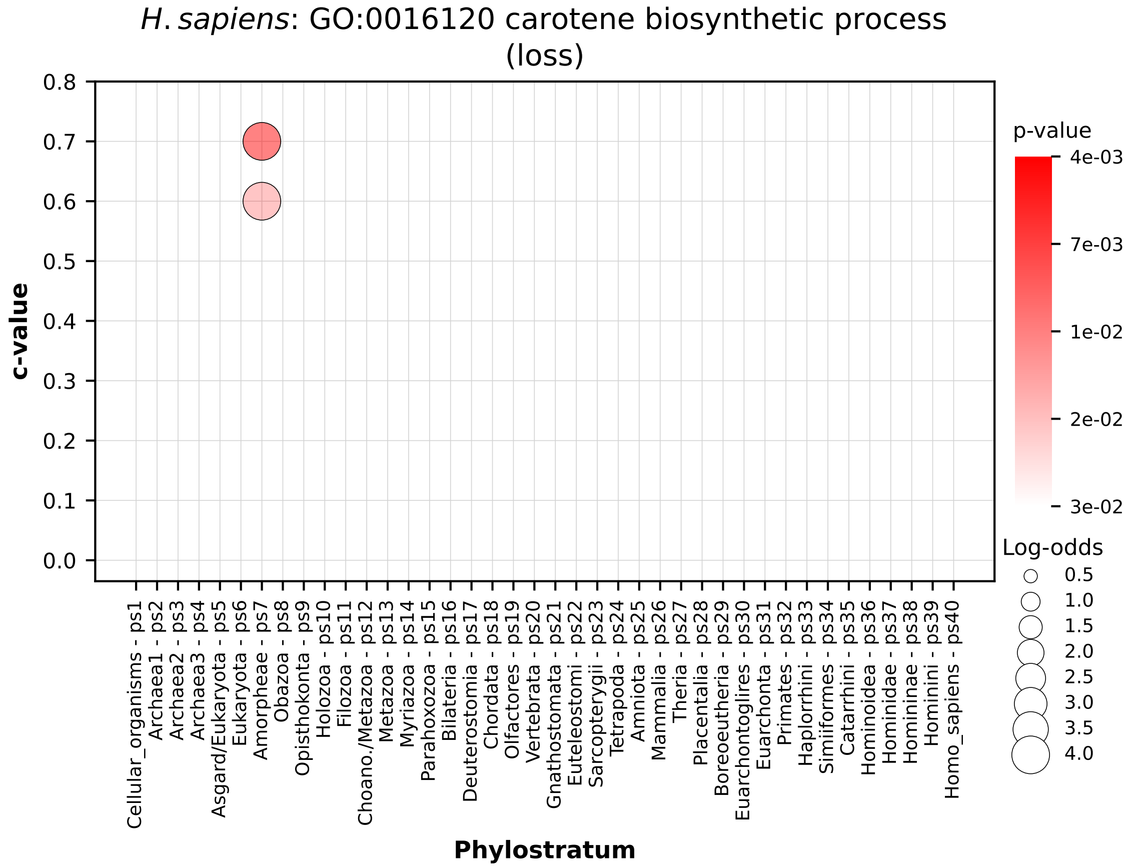

H. sapiens: GO:0016120 carotene biosynthetic process
(loss)
c-value
0.8
0.7
0.6
0.5
0.4
0.3
0.2
0.1
0.0
p-value
4e-03
7e-03
1e-02
2e-02
3e-02
Log-odds
0.5
1.0
1.5
2.0
2.5
3.0
3.5
4.0
Cellular_organisms - ps1
Archaea1 - ps2
Archaea2 - ps3
Archaea3 - ps4
Asgard/Eukaryota - ps5
Eukaryota - ps6
Amorpheae - ps7
Obazoa - ps8
Opisthokonta - ps9
Holozoa - ps10
Filozoa - ps11
Choano./Metazoa - ps12
Metazoa - ps13
Myriazoa - ps14
Parahoxozoa - ps15
Bilateria - ps16
Deuterostomia - ps17
Chordata - ps18
Olfactores - ps19
Vertebrata - ps20
Gnathostomata - ps21
Euteleostomi - ps22
Sarcopterygii - ps23
Tetrapoda - ps24
Amniota - ps25
Mammalia - ps26
Theria - ps27
Placentalia - ps28
Boreoeutheria - ps29
Euarchontoglires - ps30
Euarchonta - ps31
Primates - ps32
Haplorrhini - ps33
Simiiformes - ps34
Catarrhini - ps35
Hominoidea - ps36
Hominidae - ps37
Homininae - ps38
Hominini - ps39
Homo_sapiens - ps40
Phylostratum

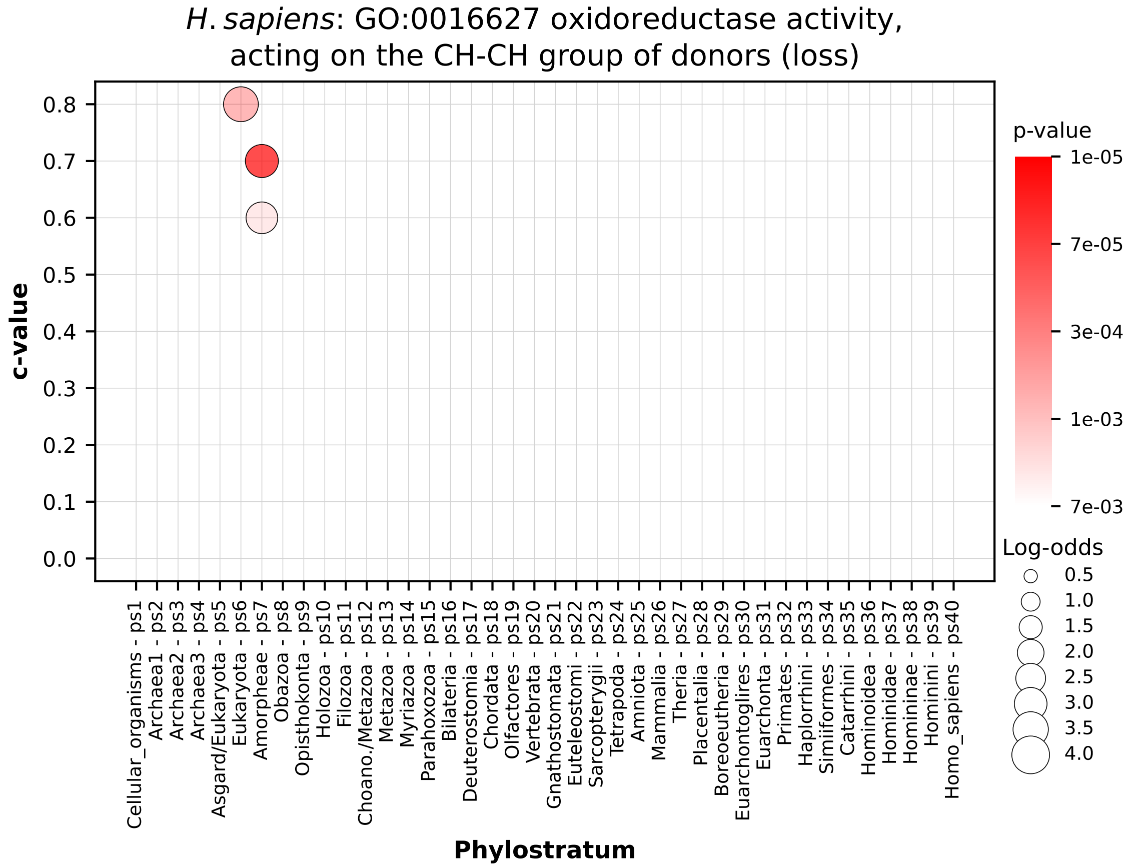

H. sapiens: GO:0016627 oxidoreductase activity, acting on the CH-CH group of donors (loss)
c-value
0.0
0.1
0.2
0.3
0.4
0.5
0.6
0.7
0.8
Phylostratum
Cellular_organisms - ps1
Archaea1 - ps2
Archaea2 - ps3
Archaea3 - ps4
Asgard/Eukaryota - ps5
Eukaryota - ps6
Amorpheae - ps7
Obazoa - ps8
Opisthokonta - ps9
Holozoa - ps10
Filozoa - ps11
Choano./Metazoa - ps12
Metazoa - ps13
Myriazoa - ps14
Parahoxozoa - ps15
Bilateria - ps16
Deuterostomia - ps17
Chordata - ps18
Olfactores - ps19
Vertebrata - ps20
Gnathostomata - ps21
Euteleostomi - ps22
Sarcopterygii - ps23
Tetrapoda - ps24
Amniota - ps25
Mammalia - ps26
Theria - ps27
Placentalia - ps28
Boreoeutheria - ps29
Euarchontoglires - ps30
Euarchonta - ps31
Primates - ps32
Haplorrhini - ps33
Simiiformes - ps34
Catarrhini - ps35
Hominoidea - ps36
Hominidae - ps37
Homininae - ps38
Hominini - ps39
Homo_sapiens - ps40
p-value
1e-05
7e-05
3e-04
1e-03
7e-03
Log-odds
0.5
1.0
1.5
2.0
2.5
3.0
3.5
4.0

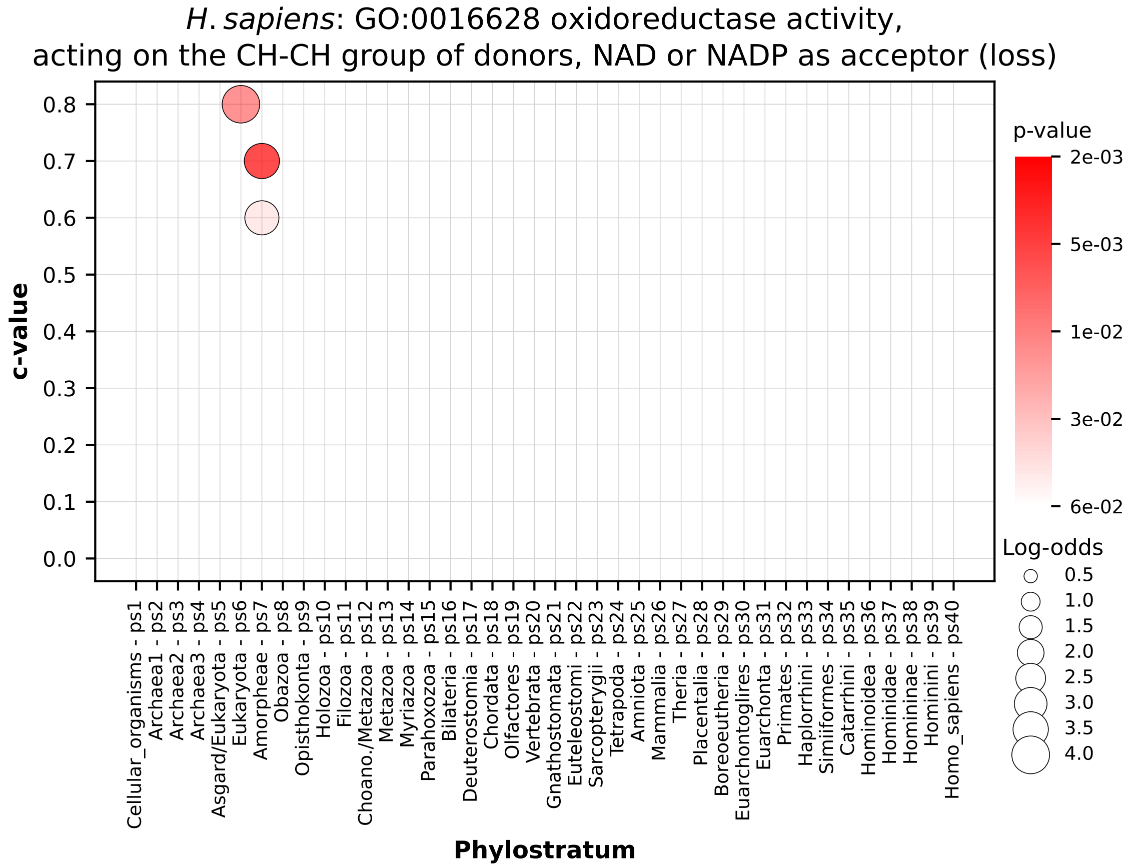

H. sapiens: GO:0016628 oxidoreductase activity,
acting on the CH-CH group of donors, NAD or NADP as acceptor (loss)
c-value
0.0
0.1
0.2
0.3
0.4
0.5
0.6
0.7
0.8
Phylostratum
Cellular_organisms - ps1
Archaea1 - ps2
Archaea2 - ps3
Archaea3 - ps4
Asgard/Eukaryota - ps5
Eukaryota - ps6
Amorpheae - ps7
Obazoa - ps8
Opisthokonta - ps9
Holozoa - ps10
Filozoa - ps11
Choano./Metazoa - ps12
Metazoa - ps13
Myriazoa - ps14
Parahoxozoa - ps15
Bilateria - ps16
Deuterostomia - ps17
Chordata - ps18
Olfactores - ps19
Vertebrata - ps20
Gnathostomata - ps21
Euteleostomi - ps22
Sarcopterygii - ps23
Tetrapoda - ps24
Amniota - ps25
Mammalia - ps26
Theria - ps27
Placentalia - ps28
Boreoeutheria - ps29
Euarchontoglires - ps30
Euarchonta - ps31
Primates - ps32
Haplorrhini - ps33
Simiiformes - ps34
Catarrhini - ps35
Hominoidea - ps36
Hominidae - ps37
Homininae - ps38
Hominini - ps39
Homo_sapiens - ps40
p-value
2e-03
5e-03
1e-02
3e-02
6e-02
Log-odds
0.5
1.0
1.5
2.0
2.5
3.0
3.5
4.0

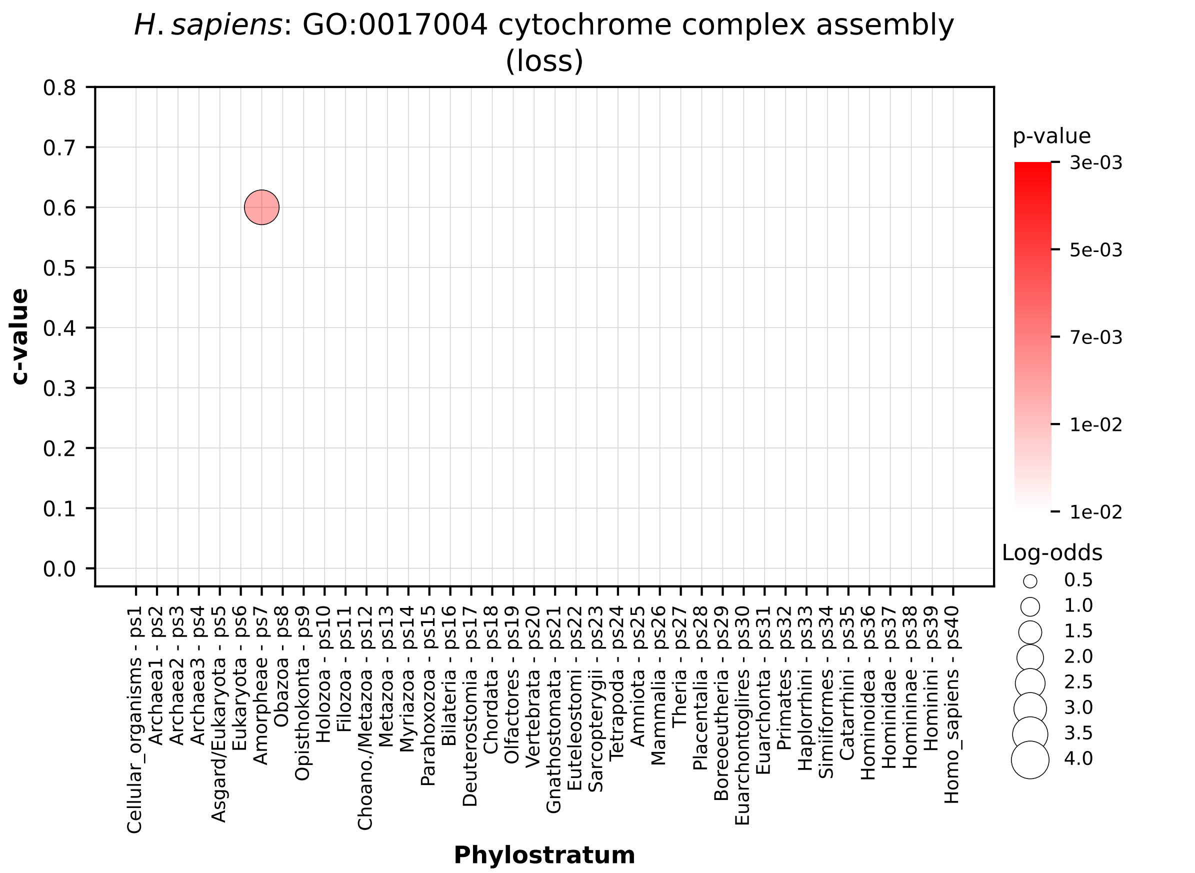
H. sapiens: GO:0017004 cytochrome complex assembly
(loss)
c-value
0.8
0.7
0.6
0.5
0.4
0.3
0.2
0.1
0.0
Phylostratum
Cellular_organisms - ps1
Archaea1 - ps2
Archaea2 - ps3
Archaea3 - ps4
Asgard/Eukaryota - ps5
Eukaryota - ps6
Amorpheae - ps7
Obazoa - ps8
Opisthokonta - ps9
Holozoa - ps10
Filozoa - ps11
Choano./Metazoa - ps12
Metazoa - ps13
Myriazoa - ps14
Parahoxozoa - ps15
Bilateria - ps16
Deuterostomia - ps17
Chordata - ps18
Olfactores - ps19
Vertebrata - ps20
Gnathostomata - ps21
Euteleostomi - ps22
Sarcopterygii - ps23
Tetrapoda - ps24
Amniota - ps25
Mammalia - ps26
Theria - ps27
Placentalia - ps28
Boreoeutheria - ps29
Euarchontoglires - ps30
Euarchonta - ps31
Primates - ps32
Haplorrhini - ps33
Simiiformes - ps34
Catarrhini - ps35
Hominoidea - ps36
Hominidae - ps37
Homininae - ps38
Hominini - ps39
Homo_sapiens - ps40
p-value
3e-03
5e-03
7e-03
1e-02
1e-02
Log-odds
0.5
1.0
1.5
2.0
2.5
3.0
3.5
4.0

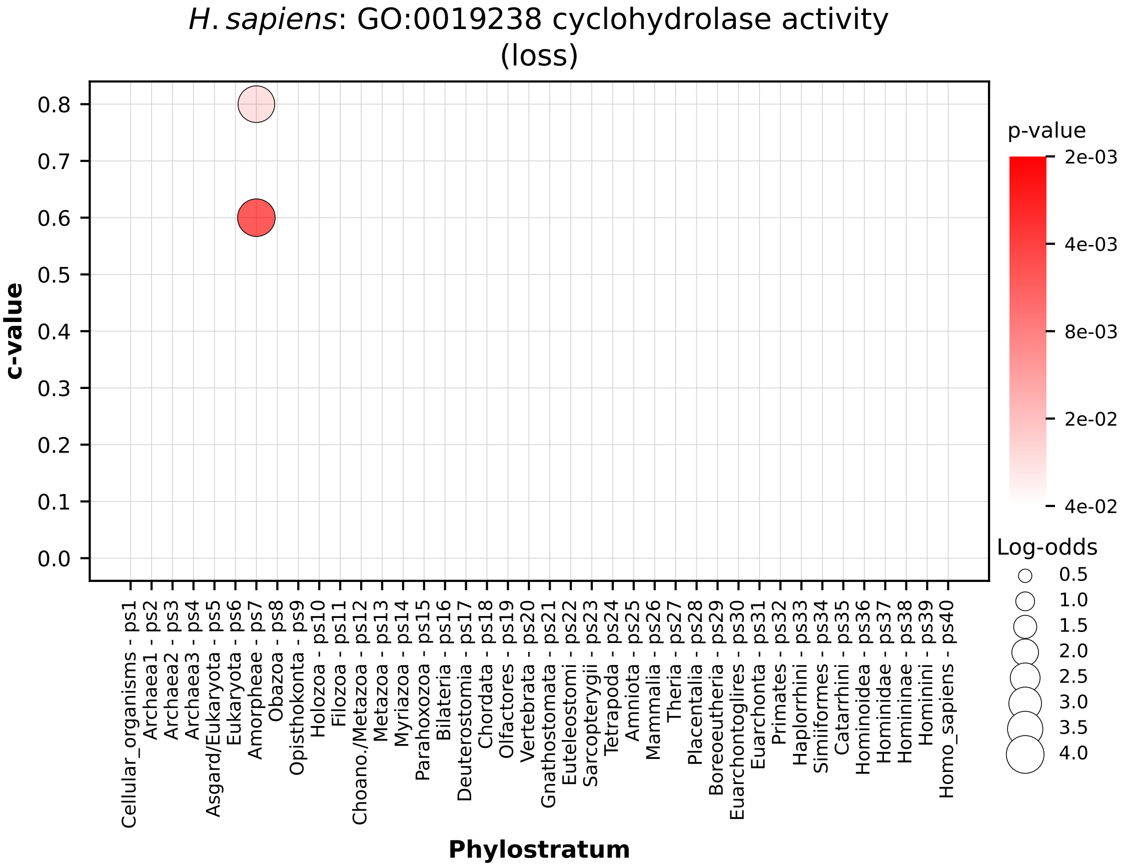
H. sapiens: GO:0019238 cyclohydrolase activity
(loss)
c-value
0.0
0.1
0.2
0.3
0.4
0.5
0.6
0.7
0.8
Phylostratum
Cellular_organisms - ps1
Archaea1 - ps2
Archaea2 - ps3
Archaea3 - ps4
Asgard/Eukaryota - ps5
Eukaryota - ps6
Amorpheae - ps7
Obazoa - ps8
Opisthokonta - ps9
Holozoa - ps10
Filozoa - ps11
Choano./Metazoa - ps12
Metazoa - ps13
Myriazoa - ps14
Parahoxozoa - ps15
Bilateria - ps16
Deuterostomia - ps17
Chordata - ps18
Olfactores - ps19
Vertebrata - ps20
Gnathostomata - ps21
Euteleostomi - ps22
Sarcopterygii - ps23
Tetrapoda - ps24
Amniota - ps25
Mammalia - ps26
Theria - ps27
Placentalia - ps28
Boreoeutheria - ps29
Euarchontoglires - ps30
Euarchonta - ps31
Primates - ps32
Haplorrhini - ps33
Simiiformes - ps34
Catarrhini - ps35
Hominoidea - ps36
Hominidae - ps37
Homininae - ps38
Hominini - ps39
Homo_sapiens - ps40
p-value
2e-03
4e-03
8e-03
2e-02
4e-02
Log-odds
0.5
1.0
1.5
2.0
2.5
3.0
3.5
4.0

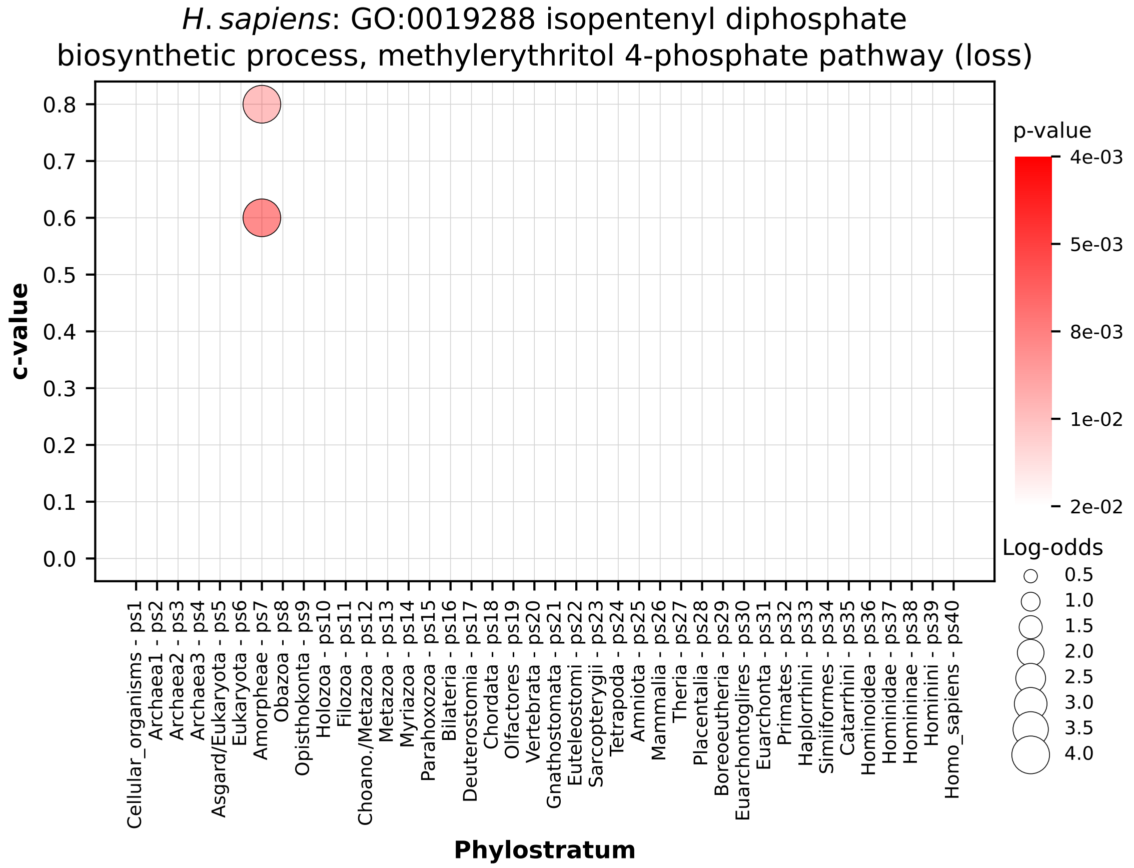

H. sapiens: GO:0019288 isopentenyl diphosphate biosynthetic process, methylerythritol 4-phosphate pathway (loss)
c-value
0.0
0.1
0.2
0.3
0.4
0.5
0.6
0.7
0.8
Phylostratum
Cellular_organisms - ps1
Archaea1 - ps2
Archaea2 - ps3
Archaea3 - ps4
Asgard/Eukaryota - ps5
Eukaryota - ps6
Amorpheae - ps7
Obazoa - ps8
Opisthokonta - ps9
Holozoa - ps10
Filozoa - ps11
Choano./Metazoa - ps12
Metazoa - ps13
Myriazoa - ps14
Parahoxozoa - ps15
Bilateria - ps16
Deuterostomia - ps17
Chordata - ps18
Olfactores - ps19
Vertebrata - ps20
Gnathostomata - ps21
Euteleostomi - ps22
Sarcopterygii - ps23
Tetrapoda - ps24
Amniota - ps25
Mammalia - ps26
Theria - ps27
Placentalia - ps28
Boreoeutheria - ps29
Euarchontoglires - ps30
Euarchonta - ps31
Primates - ps32
Haplorrhini - ps33
Simiiformes - ps34
Catarrhini - ps35
Hominoidea - ps36
Hominidae - ps37
Homininae - ps38
Hominini - ps39
Homo_sapiens - ps40
p-value
4e-03
5e-03
8e-03
1e-02
2e-02
Log-odds
0.5
1.0
1.5
2.0
2.5
3.0
3.5
4.0

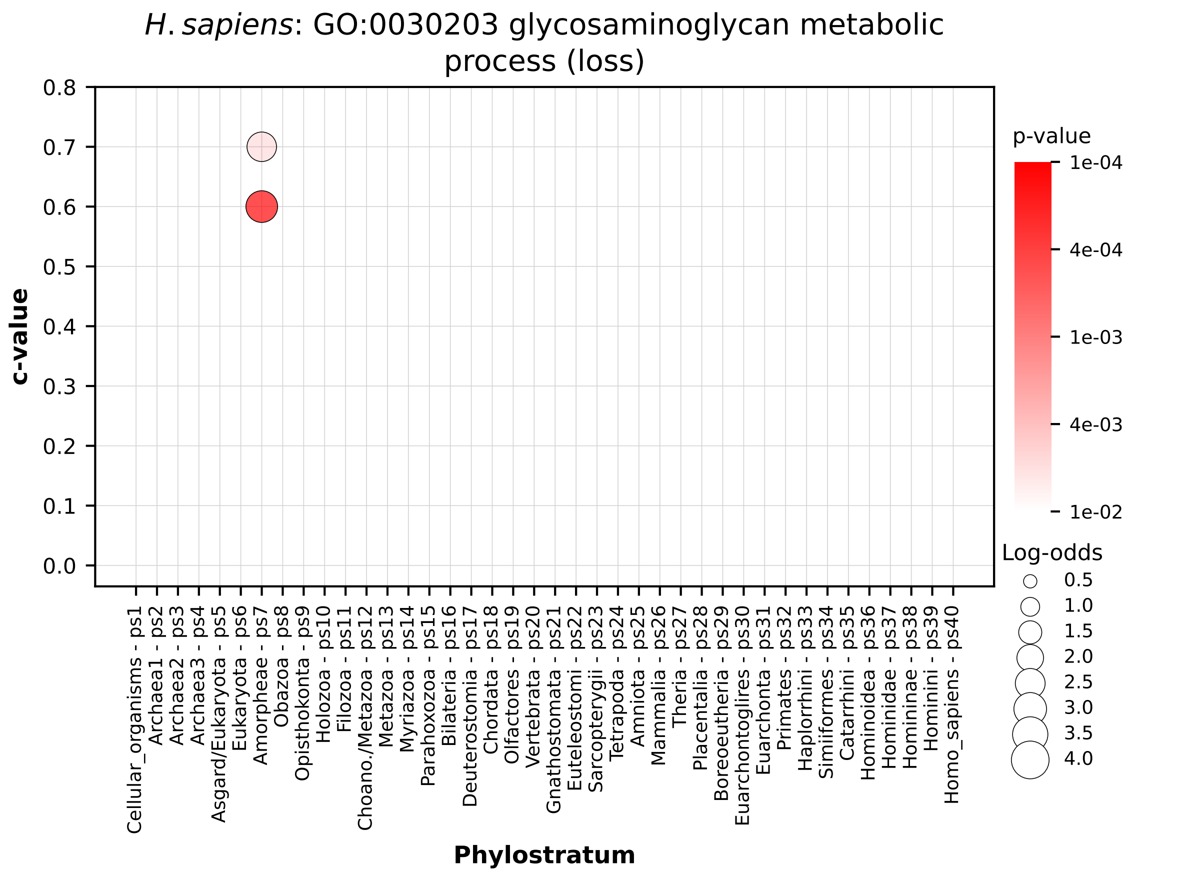

H. sapiens: GO:0030203 glycosaminoglycan metabolic process (loss)
c-value
0.8
0.7
0.6
0.5
0.4
0.3
0.2
0.1
0.0
Phylostratum
Cellular_organisms - ps1
Archaea1 - ps2
Archaea2 - ps3
Archaea3 - ps4
Asgard/Eukaryota - ps5
Eukaryota - ps6
Amorpheae - ps7
Obazoa - ps8
Opisthokonta - ps9
Holozoa - ps10
Filozoa - ps11
Choano./Metazoa - ps12
Metazoa - ps13
Myriazoa - ps14
Parahoxozoa - ps15
Bilateria - ps16
Deuterostomia - ps17
Chordata - ps18
Olfactores - ps19
Vertebrata - ps20
Gnathostomata - ps21
Euteleostomi - ps22
Sarcopterygii - ps23
Tetrapoda - ps24
Amniota - ps25
Mammalia - ps26
Theria - ps27
Placentalia - ps28
Boreoeutheria - ps29
Euarchontoglires - ps30
Euarchonta - ps31
Primates - ps32
Haplorrhini - ps33
Simiiformes - ps34
Catarrhini - ps35
Hominoidea - ps36
Hominidae - ps37
Homininae - ps38
Hominini - ps39
Homo_sapiens - ps40
p-value
1e-04
4e-04
1e-03
4e-03
1e-02
Log-odds
0.5
1.0
1.5
2.0
2.5
3.0
3.5
4.0

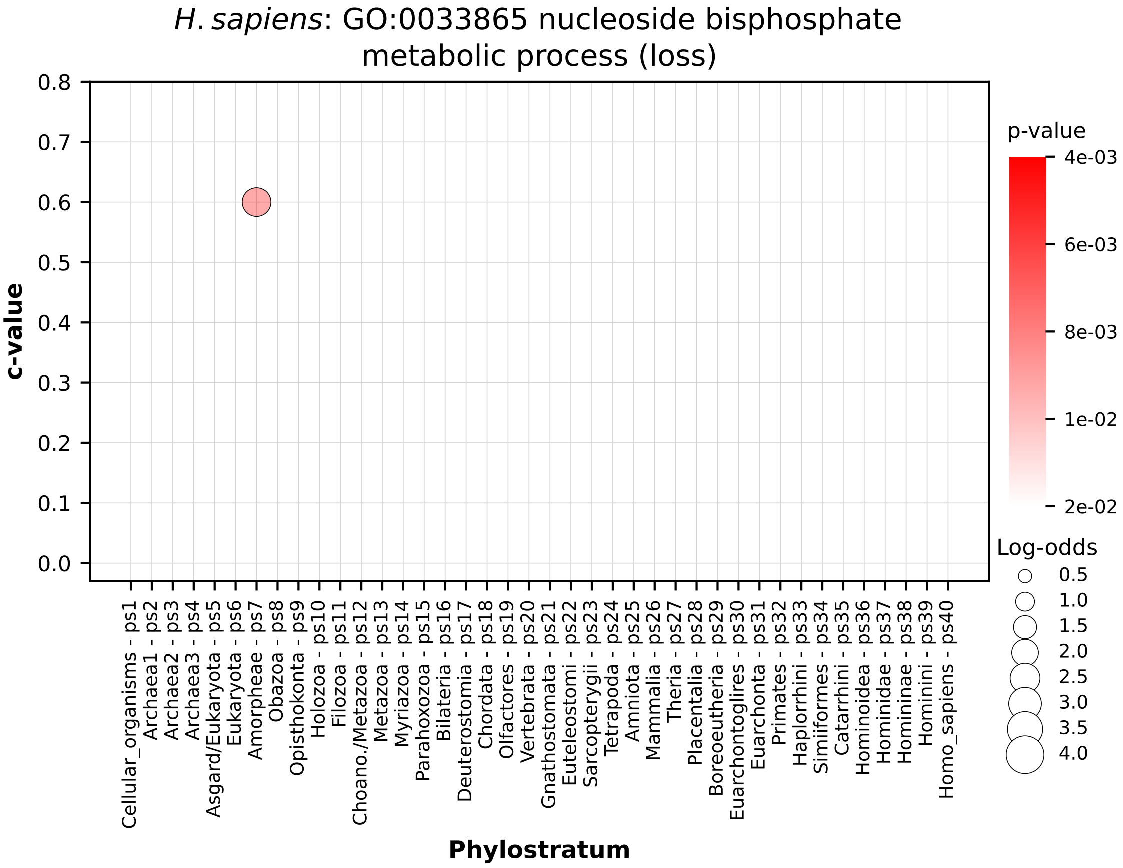

H. sapiens: GO:0033865 nucleoside bisphosphate metabolic process (loss)
c-value
0.0
0.1
0.2
0.3
0.4
0.5
0.6
0.7
0.8
Phylostratum
Cellular_organisms - ps1
Archaea1 - ps2
Archaea2 - ps3
Archaea3 - ps4
Asgard/Eukaryota - ps5
Eukaryota - ps6
Amorpheae - ps7
Obazoa - ps8
Opisthokonta - ps9
Holozoa - ps10
Filozoa - ps11
Choano./Metazoa - ps12
Metazoa - ps13
Myriazoa - ps14
Parahoxozoa - ps15
Bilateria - ps16
Deuterostomia - ps17
Chordata - ps18
Olfactores - ps19
Vertebrata - ps20
Gnathostomata - ps21
Euteleostomi - ps22
Sarcopterygii - ps23
Tetrapoda - ps24
Amniota - ps25
Mammalia - ps26
Theria - ps27
Placentalia - ps28
Boreoeutheria - ps29
Euarchontoglires - ps30
Euarchonta - ps31
Primates - ps32
Haplorrhini - ps33
Simiiformes - ps34
Catarrhini - ps35
Hominoidea - ps36
Hominidae - ps37
Homininae - ps38
Hominini - ps39
Homo_sapiens - ps40
p-value
4e-03
6e-03
8e-03
1e-02
2e-02
Log-odds
0.5
1.0
1.5
2.0
2.5
3.0
3.5
4.0

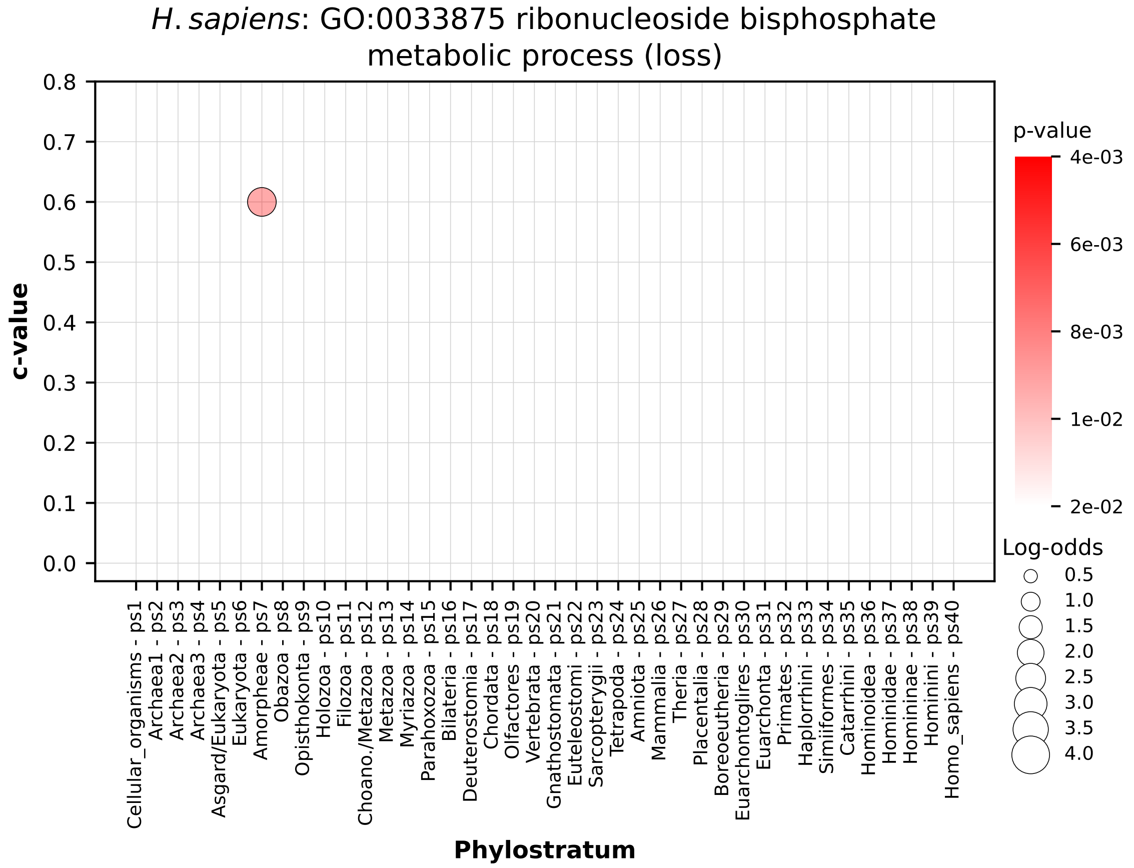

H. sapiens: GO:0033875 ribonucleoside bisphosphate metabolic process (loss)
c-value
0.8
0.7
0.6
0.5
0.4
0.3
0.2
0.1
0.0
Phylostratum
Cellular_organisms - ps1
Archaea1 - ps2
Archaea2 - ps3
Archaea3 - ps4
Asgard/Eukaryota - ps5
Eukaryota - ps6
Amorpheae - ps7
Obazoa - ps8
Opisthokonta - ps9
Holozoa - ps10
Filozoa - ps11
Choano./Metazoa - ps12
Metazoa - ps13
Myriazoa - ps14
Parahoxozoa - ps15
Bilateria - ps16
Deuterostomia - ps17
Chordata - ps18
Olfactores - ps19
Vertebrata - ps20
Gnathostomata - ps21
Euteleostomi - ps22
Sarcopterygii - ps23
Tetrapoda - ps24
Amniota - ps25
Mammalia - ps26
Theria - ps27
Placentalia - ps28
Boreoeutheria - ps29
Euarchontoglires - ps30
Euarchonta - ps31
Primates - ps32
Haplorrhini - ps33
Simiiformes - ps34
Catarrhini - ps35
Hominoidea - ps36
Hominidae - ps37
Homininae - ps38
Hominini - ps39
Homo_sapiens - ps40
p-value
4e-03
6e-03
8e-03
1e-02
2e-02
Log-odds
0.5
1.0
1.5
2.0
2.5
3.0
3.5
4.0

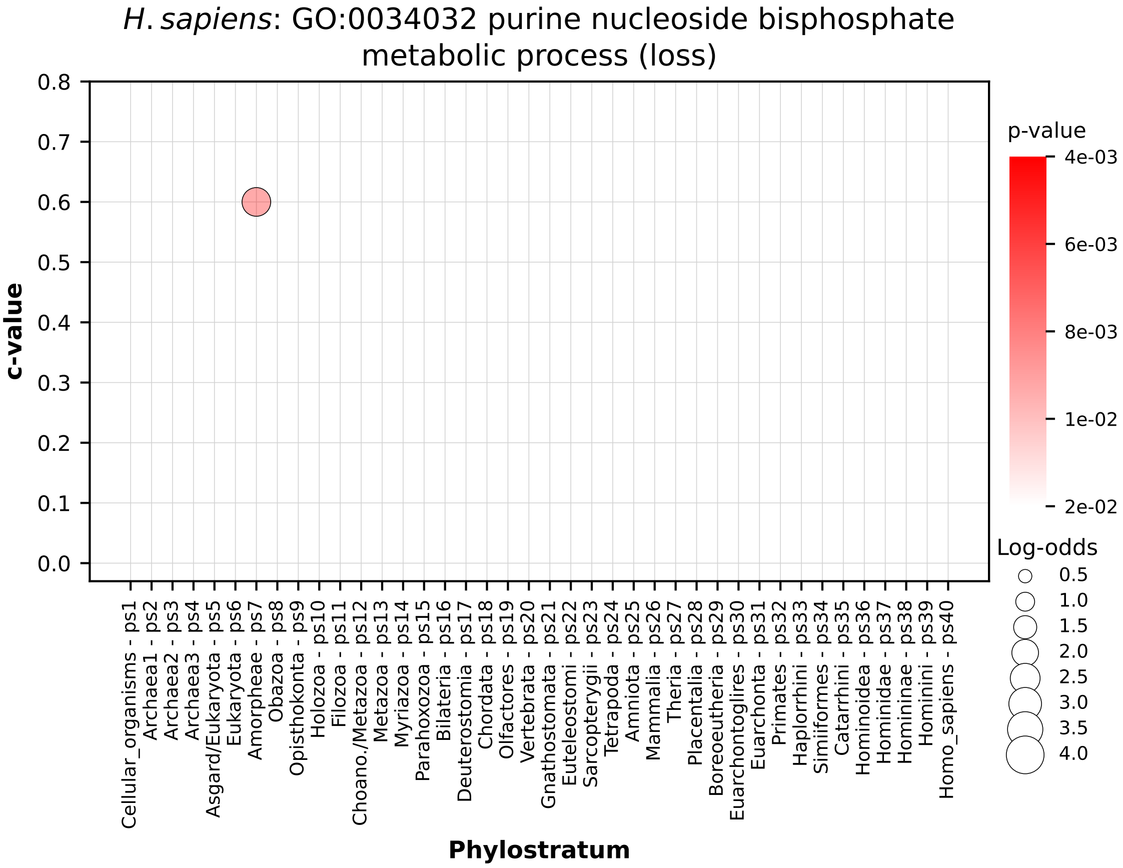
H. sapiens: GO:0034032 purine nucleoside bisphosphate metabolic process (loss)
c-value
0.8
0.7
0.6
0.5
0.4
0.3
0.2
0.1
0.0
Phylostratum
Cellular_organisms - ps1
Archaea1 - ps2
Archaea2 - ps3
Archaea3 - ps4
Asgard/Eukaryota - ps5
Eukaryota - ps6
Amorpheae - ps7
Obazoa - ps8
Opisthokonta - ps9
Holozoa - ps10
Filozoa - ps11
Choano./Metazoa - ps12
Metazoa - ps13
Myriazoa - ps14
Parahoxozoa - ps15
Bilateria - ps16
Deuterostomia - ps17
Chordata - ps18
Olfactores - ps19
Vertebrata - ps20
Gnathostomata - ps21
Euteleostomi - ps22
Sarcopterygii - ps23
Tetrapoda - ps24
Amniota - ps25
Mammalia - ps26
Theria - ps27
Placentalia - ps28
Boreoeutheria - ps29
Euarchontoglires - ps30
Euarchonta - ps31
Primates - ps32
Haplorrhini - ps33
Simiiformes - ps34
Catarrhini - ps35
Hominoidea - ps36
Hominidae - ps37
Homininae - ps38
Hominini - ps39
Homo_sapiens - ps40
p-value
4e-03
6e-03
8e-03
1e-02
2e-02
Log-odds
0.5
1.0
1.5
2.0
2.5
3.0
3.5
4.0

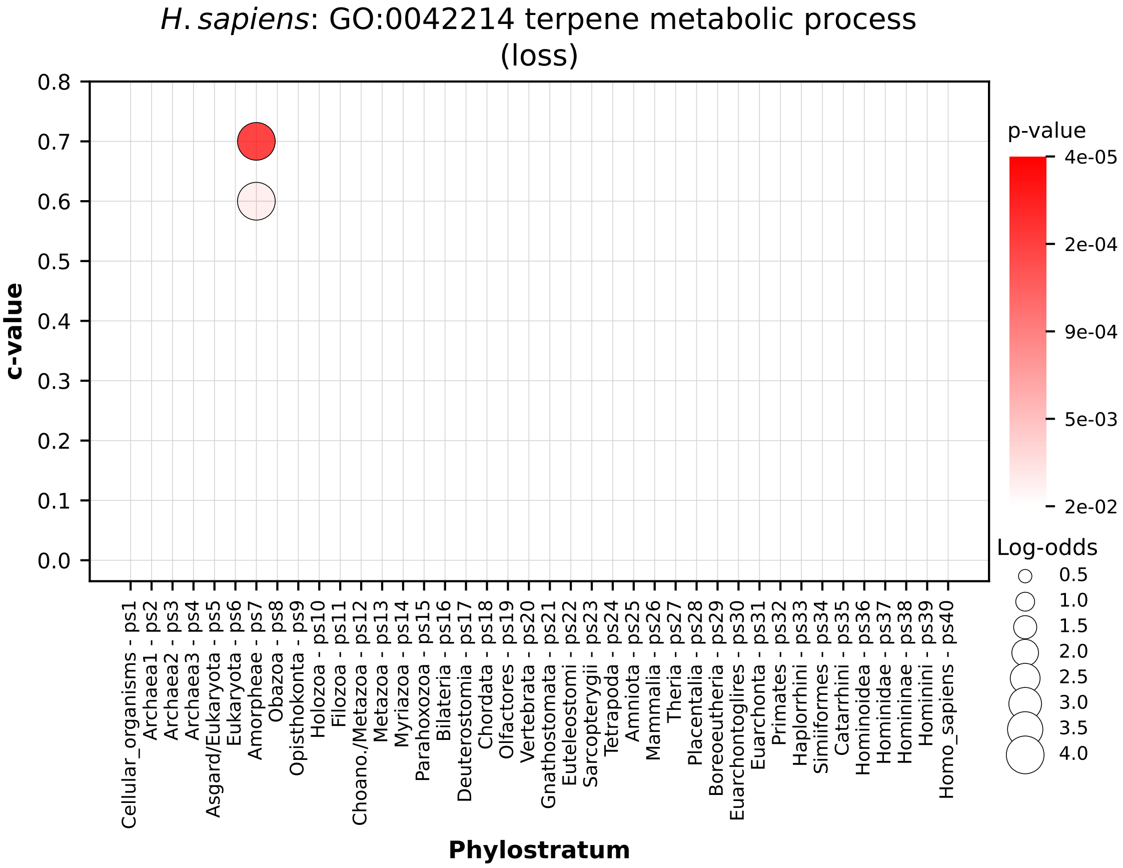
H. sapiens: GO:0042214 terpene metabolic process
(loss)
c-value
0.8
0.7
0.6
0.5
0.4
0.3
0.2
0.1
0.0
p-value
4e-05
2e-04
9e-04
5e-03
2e-02
Log-odds
0.5
1.0
1.5
2.0
2.5
3.0
3.5
4.0
Cellular_organisms - ps1
Archaea1 - ps2
Archaea2 - ps3
Archaea3 - ps4
Asgard/Eukaryota - ps5
Eukaryota - ps6
Amorpheae - ps7
Obazoa - ps8
Opisthokonta - ps9
Holozoa - ps10
Filozoa - ps11
Choano./Metazoa - ps12
Metazoa - ps13
Myriazoa - ps14
Parahoxozoa - ps15
Bilateria - ps16
Deuterostomia - ps17
Chordata - ps18
Olfactores - ps19
Vertebrata - ps20
Gnathostomata - ps21
Euteleostomi - ps22
Sarcopterygii - ps23
Tetrapoda - ps24
Amniota - ps25
Mammalia - ps26
Theria - ps27
Placentalia - ps28
Boreoeutheria - ps29
Euarchontoglires - ps30
Euarchonta - ps31
Primates - ps32
Haplorrhini - ps33
Simiiformes - ps34
Catarrhini - ps35
Hominoidea - ps36
Hominidae - ps37
Homininae - ps38
Hominini - ps39
Homo_sapiens - ps40
Phylostratum

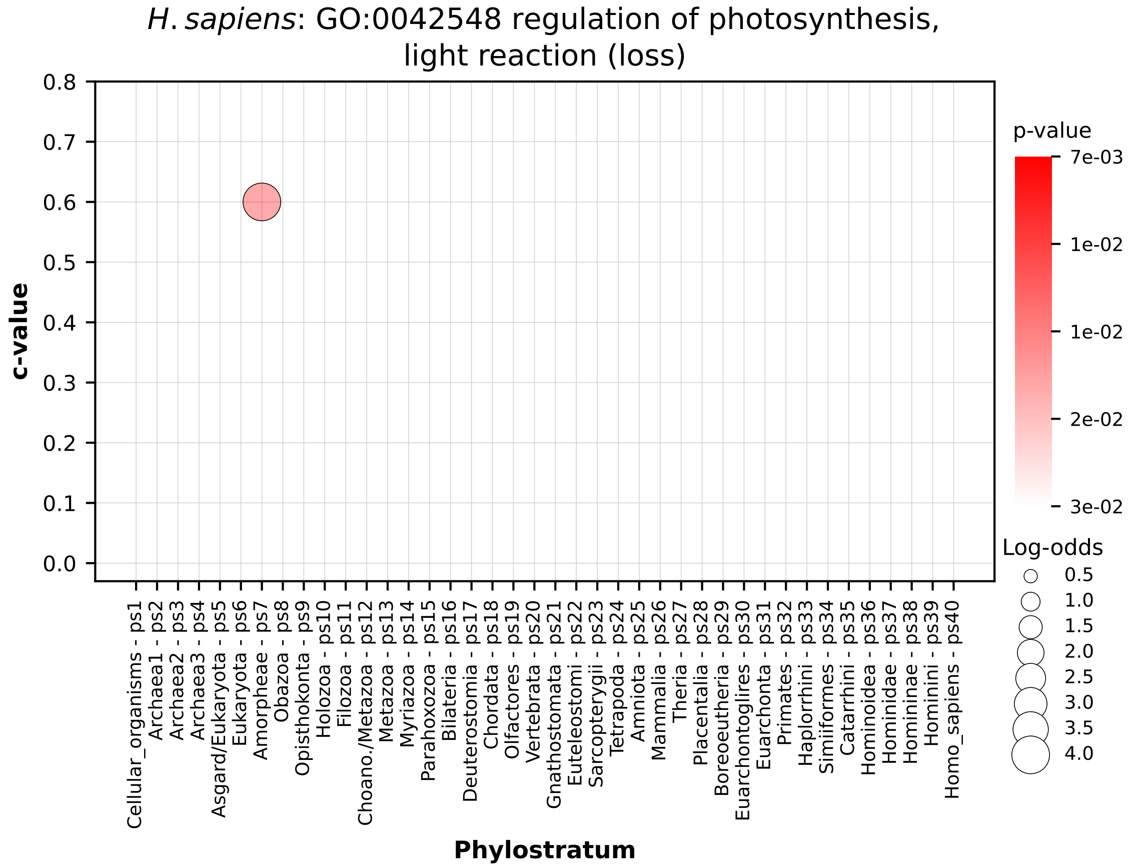

H. sapiens: GO:0042548 regulation of photosynthesis, light reaction (loss)
c-value
0.8
0.7
0.6
0.5
0.4
0.3
0.2
0.1
0.0
p-value
7e-03
1e-02
1e-02
2e-02
3e-02
Log-odds
0.5
1.0
1.5
2.0
2.5
3.0
3.5
4.0
Cellular_organisms - ps1
Archaea1 - ps2
Archaea2 - ps3
Archaea3 - ps4
Asgard/Eukaryota - ps5
Eukaryota - ps6
Amorpheae - ps7
Obazoa - ps8
Opisthokonta - ps9
Holozoa - ps10
Filozoa - ps11
Choano./Metazoa - ps12
Metazoa - ps13
Myriazoa - ps14
Parahoxozoa - ps15
Bilateria - ps16
Deuterostomia - ps17
Chordata - ps18
Olfactores - ps19
Vertebrata - ps20
Gnathostomata - ps21
Euteleostomi - ps22
Sarcopterygii - ps23
Tetrapoda - ps24
Amniota - ps25
Mammalia - ps26
Theria - ps27
Placentalia - ps28
Boreoeutheria - ps29
Euarchontoglires - ps30
Euarchonta - ps31
Primates - ps32
Haplorrhini - ps33
Simiiformes - ps34
Catarrhini - ps35
Hominoidea - ps36
Hominidae - ps37
Homininae - ps38
Hominini - ps39
Homo_sapiens - ps40
Phylostratum

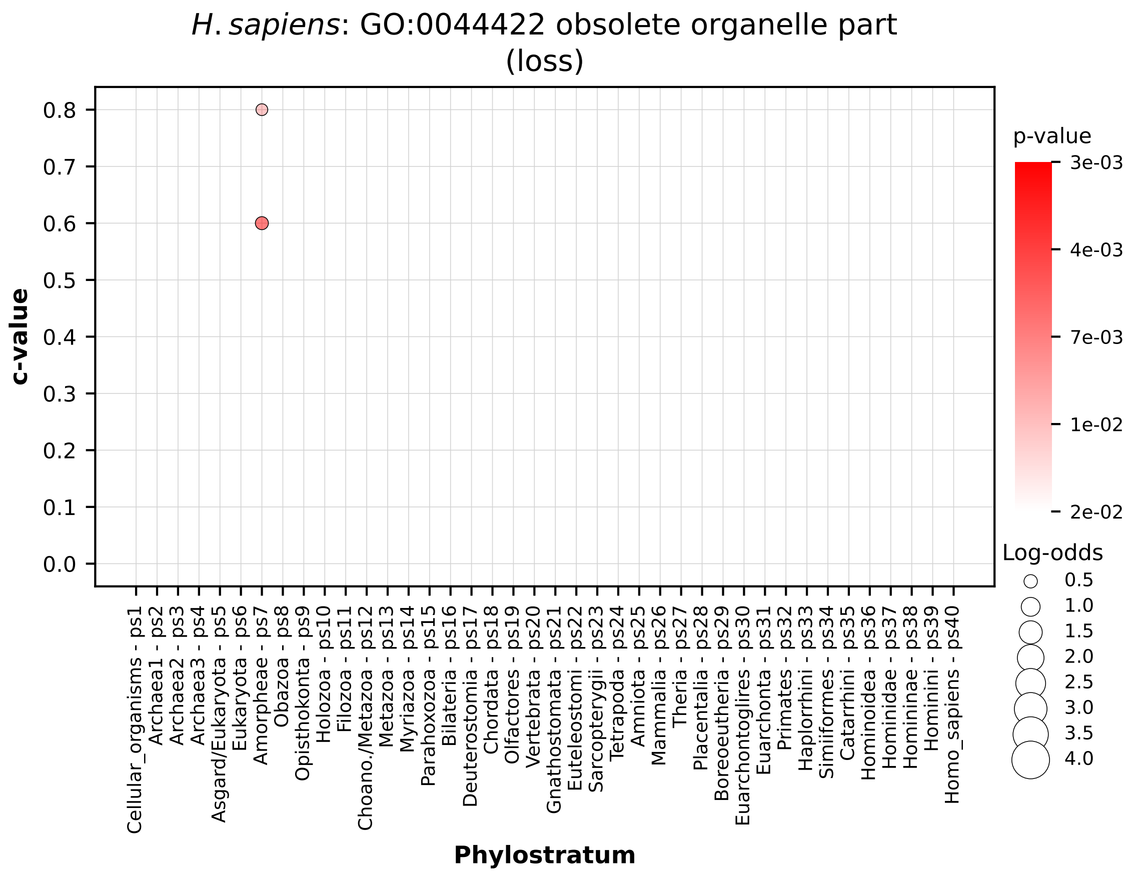

H. sapiens: GO:0044422 obsolete organelle part
(loss)
c-value
0.0
0.1
0.2
0.3
0.4
0.5
0.6
0.7
0.8
Phylostratum
Cellular_organisms - ps1
Archaea1 - ps2
Archaea2 - ps3
Archaea3 - ps4
Asgard/Eukaryota - ps5
Eukaryota - ps6
Amorpheae - ps7
Obazoa - ps8
Opisthokonta - ps9
Holozoa - ps10
Filozoa - ps11
Choano./Metazoa - ps12
Metazoa - ps13
Myriazoa - ps14
Parahoxozoa - ps15
Bilateria - ps16
Deuterostomia - ps17
Chordata - ps18
Olfactores - ps19
Vertebrata - ps20
Gnathostomata - ps21
Euteleostomi - ps22
Sarcopterygii - ps23
Tetrapoda - ps24
Amniota - ps25
Mammalia - ps26
Theria - ps27
Placentalia - ps28
Boreoeutheria - ps29
Euarchontoglires - ps30
Euarchonta - ps31
Primates - ps32
Haplorrhini - ps33
Simiiformes - ps34
Catarrhini - ps35
Hominoidea - ps36
Hominidae - ps37
Homininae - ps38
Hominini - ps39
Homo_sapiens - ps40
p-value
3e-03
4e-03
7e-03
1e-02
2e-02
Log-odds
0.5
1.0
1.5
2.0
2.5
3.0
3.5
4.0

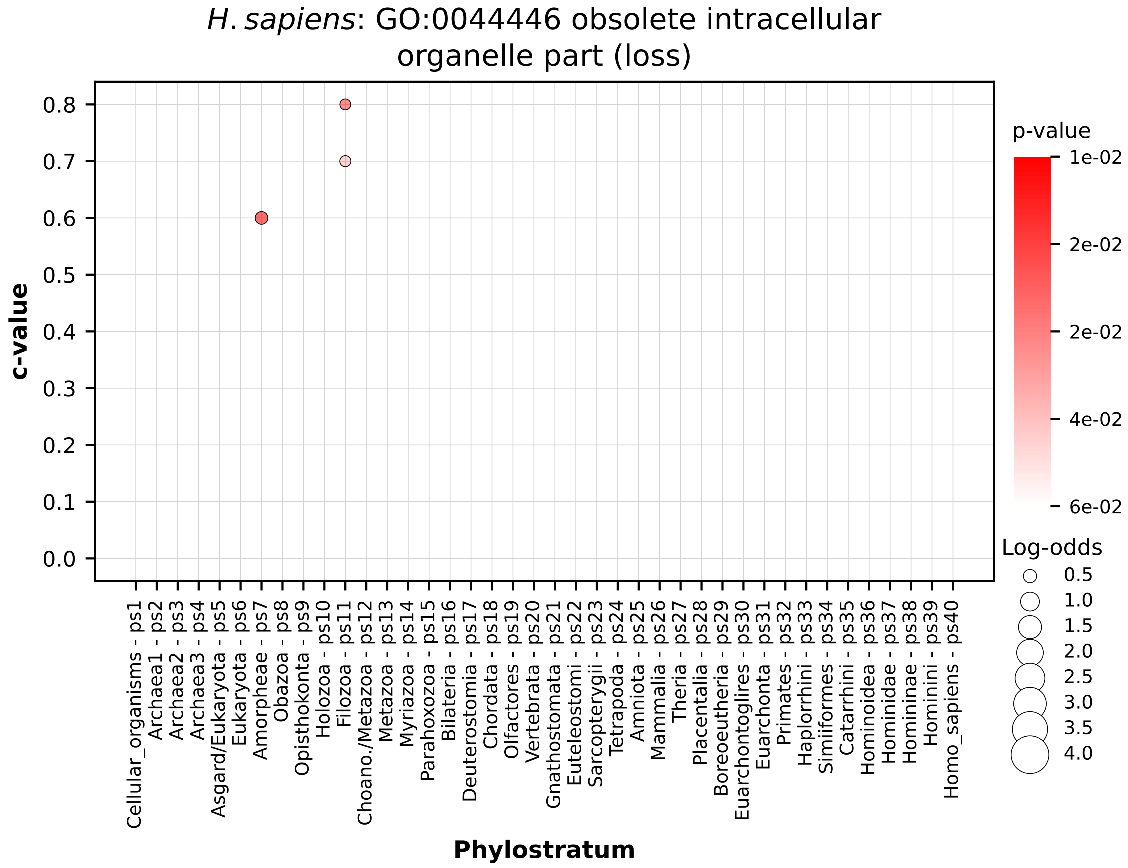

H. sapiens: GO:0044446 obsolete intracellular organelle part (loss)
c-value
0.8
0.7
0.6
0.5
0.4
0.3
0.2
0.1
0.0
p-value
1e-02
2e-02
2e-02
4e-02
6e-02
Log-odds
0.5
1.0
1.5
2.0
2.5
3.0
3.5
4.0
Cellular_organisms - ps1
Archaea1 - ps2
Archaea2 - ps3
Archaea3 - ps4
Asgard/Eukaryota - ps5
Eukaryota - ps6
Amorpheae - ps7
Obazoa - ps8
Opisthokonta - ps9
Holozoa - ps10
Filozoa - ps11
Choano./Metazoa - ps12
Metazoa - ps13
Myriazoa - ps14
Parahoxozoa - ps15
Bilateria - ps16
Deuterostomia - ps17
Chordata - ps18
Olfactores - ps19
Vertebrata - ps20
Gnathostomata - ps21
Euteleostomi - ps22
Sarcopterygii - ps23
Tetrapoda - ps24
Amniota - ps25
Mammalia - ps26
Theria - ps27
Placentalia - ps28
Boreoeutheria - ps29
Euarchontoglires - ps30
Euarchonta - ps31
Primates - ps32
Haplorrhini - ps33
Simiiformes - ps34
Catarrhini - ps35
Hominoidea - ps36
Hominidae - ps37
Homininae - ps38
Hominini - ps39
Homo_sapiens - ps40
Phylostratum

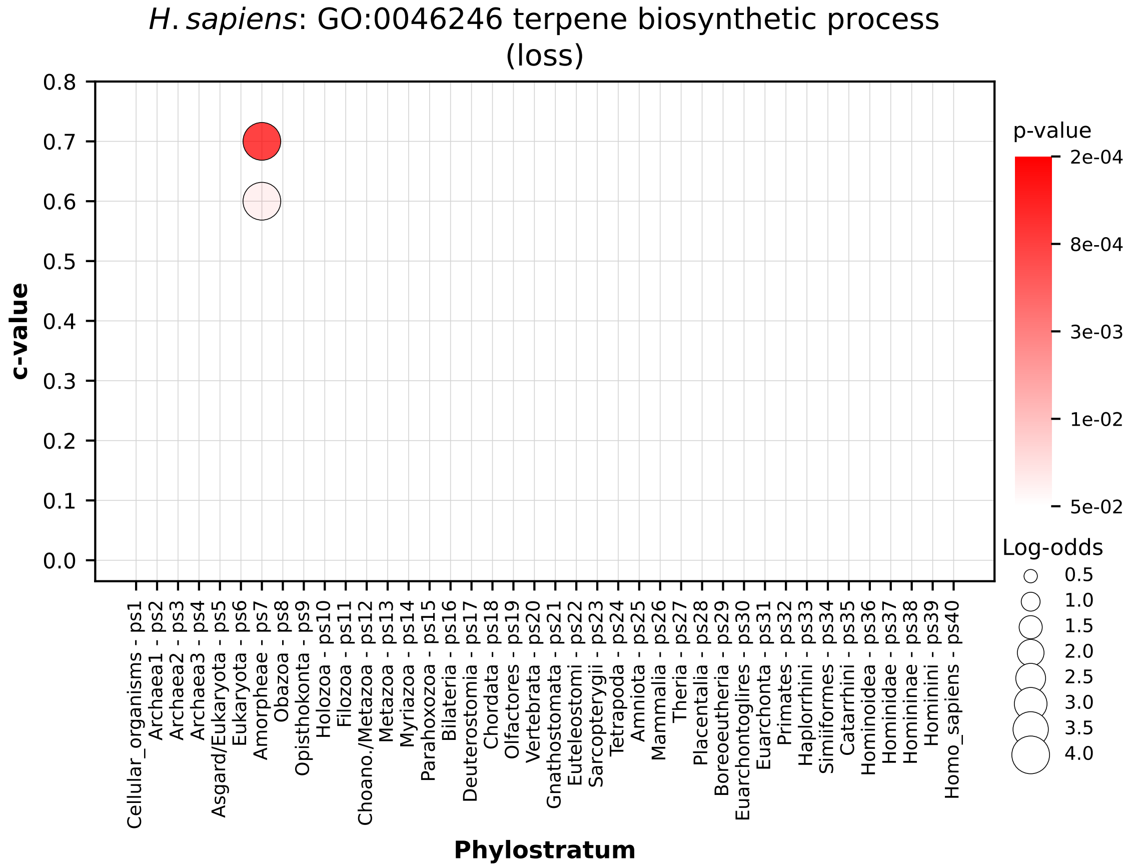
H. sapiens: GO:0046246 terpene biosynthetic process
(loss)
c-value
0.8
0.7
0.6
0.5
0.4
0.3
0.2
0.1
0.0
p-value
2e-04
8e-04
3e-03
1e-02
5e-02
Log-odds
0.5
1.0
1.5
2.0
2.5
3.0
3.5
4.0
Cellular_organisms - ps1
Archaea1 - ps2
Archaea2 - ps3
Archaea3 - ps4
Asgard/Eukaryota - ps5
Eukaryota - ps6
Amorpheae - ps7
Obazoa - ps8
Opisthokonta - ps9
Holozoa - ps10
Filozoa - ps11
Choano./Metazoa - ps12
Metazoa - ps13
Myriazoa - ps14
Parahoxozoa - ps15
Bilateria - ps16
Deuterostomia - ps17
Chordata - ps18
Olfactores - ps19
Vertebrata - ps20
Gnathostomata - ps21
Euteleostomi - ps22
Sarcopterygii - ps23
Tetrapoda - ps24
Amniota - ps25
Mammalia - ps26
Theria - ps27
Placentalia - ps28
Boreoeutheria - ps29
Euarchontoglires - ps30
Euarchonta - ps31
Primates - ps32
Haplorrhini - ps33
Simiiformes - ps34
Catarrhini - ps35
Hominoidea - ps36
Hominidae - ps37
Homininae - ps38
Hominini - ps39
Homo_sapiens - ps40
Phylostratum

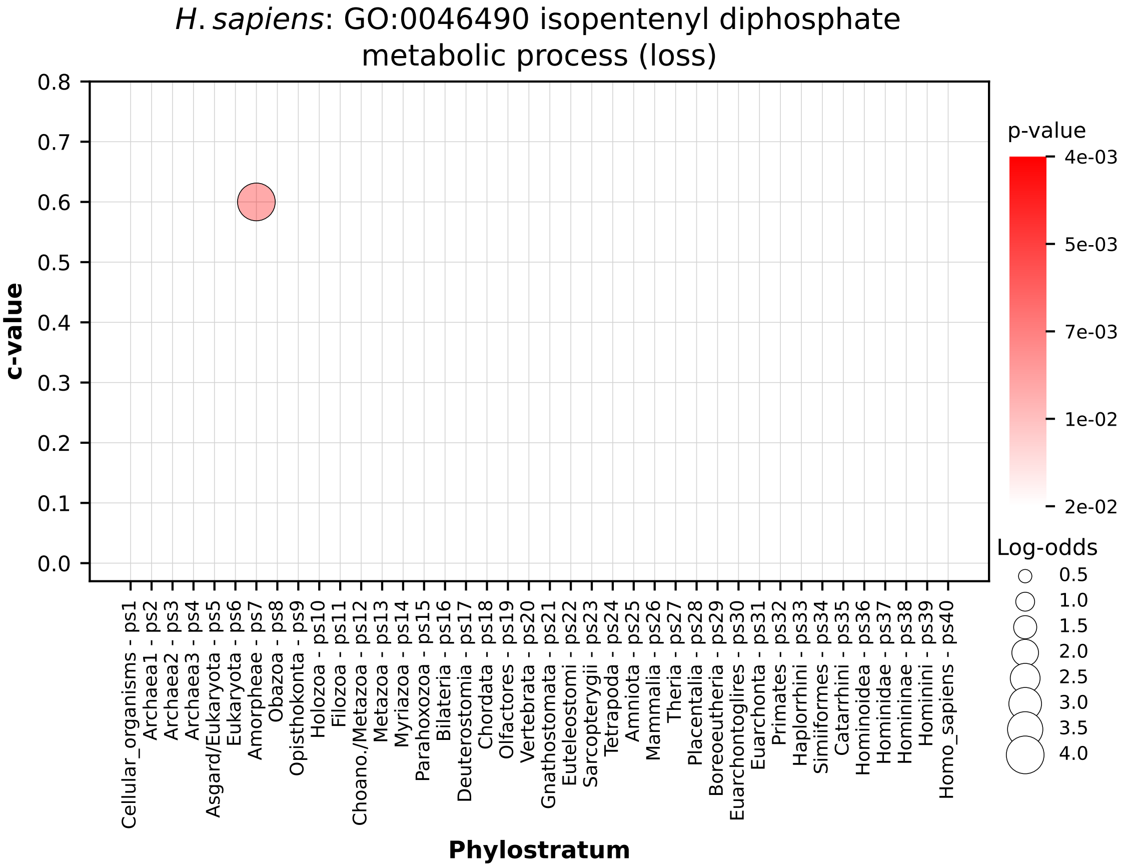

H. sapiens: GO:0046490 isopentenyl diphosphate metabolic process (loss)
c-value
0.8
0.7
0.6
0.5
0.4
0.3
0.2
0.1
0.0
p-value
4e-03
5e-03
7e-03
1e-02
2e-02
Log-odds
0.5
1.0
1.5
2.0
2.5
3.0
3.5
4.0
Cellular_organisms - ps1
Archaea1 - ps2
Archaea2 - ps3
Archaea3 - ps4
Asgard/Eukaryota - ps5
Eukaryota - ps6
Amorpheae - ps7
Obazoa - ps8
Opisthokonta - ps9
Holozoa - ps10
Filozoa - ps11
Choano./Metazoa - ps12
Metazoa - ps13
Myriazoa - ps14
Parahoxozoa - ps15
Bilateria - ps16
Deuterostomia - ps17
Chordata - ps18
Olfactores - ps19
Vertebrata - ps20
Gnathostomata - ps21
Euteleostomi - ps22
Sarcopterygii - ps23
Tetrapoda - ps24
Amniota - ps25
Mammalia - ps26
Theria - ps27
Placentalia - ps28
Boreoeutheria - ps29
Euarchontoglires - ps30
Euarchonta - ps31
Primates - ps32
Haplorrhini - ps33
Simiiformes - ps34
Catarrhini - ps35
Hominoidea - ps36
Hominidae - ps37
Homininae - ps38
Hominini - ps39
Homo_sapiens - ps40
Phylostratum

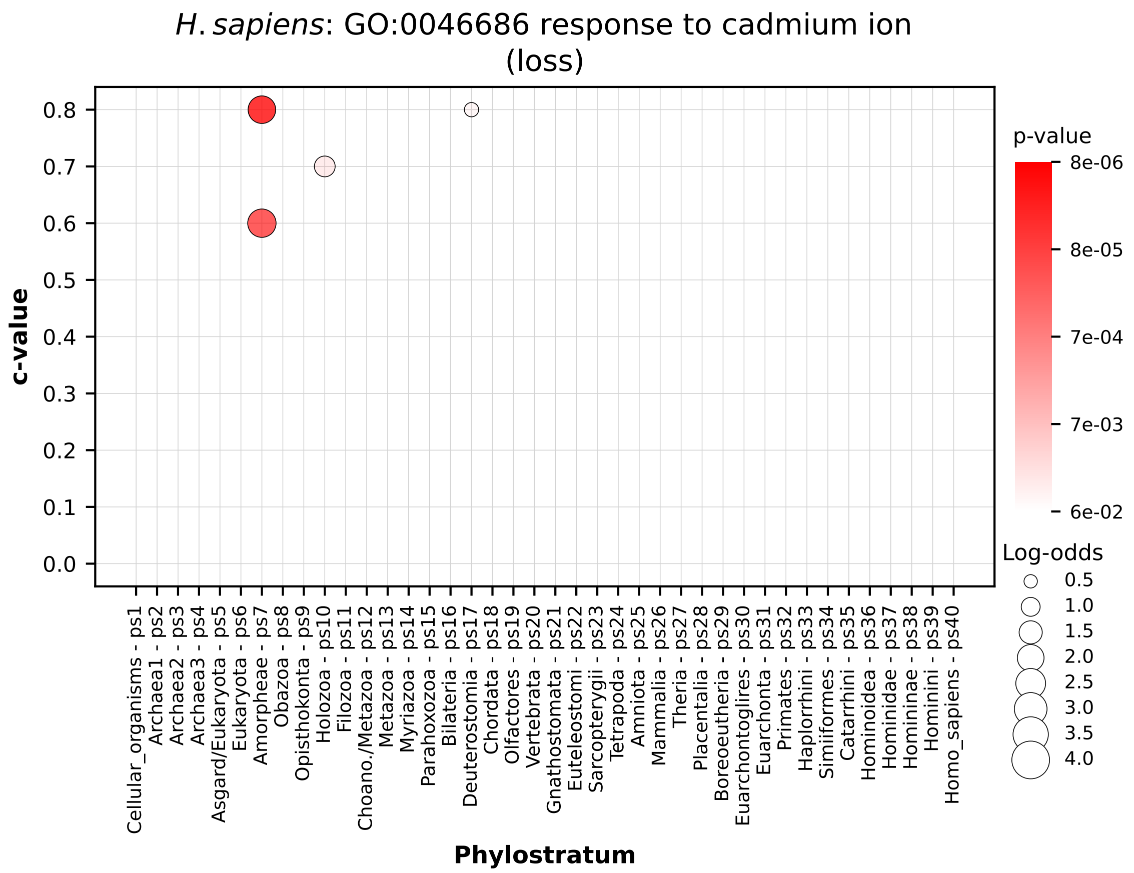
H. sapiens: GO:0046686 response to cadmium ion
(loss)
c-value
0.0
0.1
0.2
0.3
0.4
0.5
0.6
0.7
0.8
p-value
8e-06
8e-05
7e-04
7e-03
6e-02
Log-odds
0.5
1.0
1.5
2.0
2.5
3.0
3.5
4.0
Cellular_organisms - ps1
Archaea1 - ps2
Archaea2 - ps3
Archaea3 - ps4
Asgard/Eukaryota - ps5
Eukaryota - ps6
Amorpheae - ps7
Obazoa - ps8
Opisthokonta - ps9
Holozoa - ps10
Filozoa - ps11
Choano./Metazoa - ps12
Metazoa - ps13
Myriazoa - ps14
Parahoxozoa - ps15
Bilateria - ps16
Deuterostomia - ps17
Chordata - ps18
Olfactores - ps19
Vertebrata - ps20
Gnathostomata - ps21
Euteleostomi - ps22
Sarcopterygii - ps23
Tetrapoda - ps24
Amniota - ps25
Mammalia - ps26
Theria - ps27
Placentalia - ps28
Boreoeutheria - ps29
Euarchontoglires - ps30
Euarchonta - ps31
Primates - ps32
Haplorrhini - ps33
Simiiformes - ps34
Catarrhini - ps35
Hominoidea - ps36
Hominidae - ps37
Homininae - ps38
Hominini - ps39
Homo_sapiens - ps40
Phylostratum

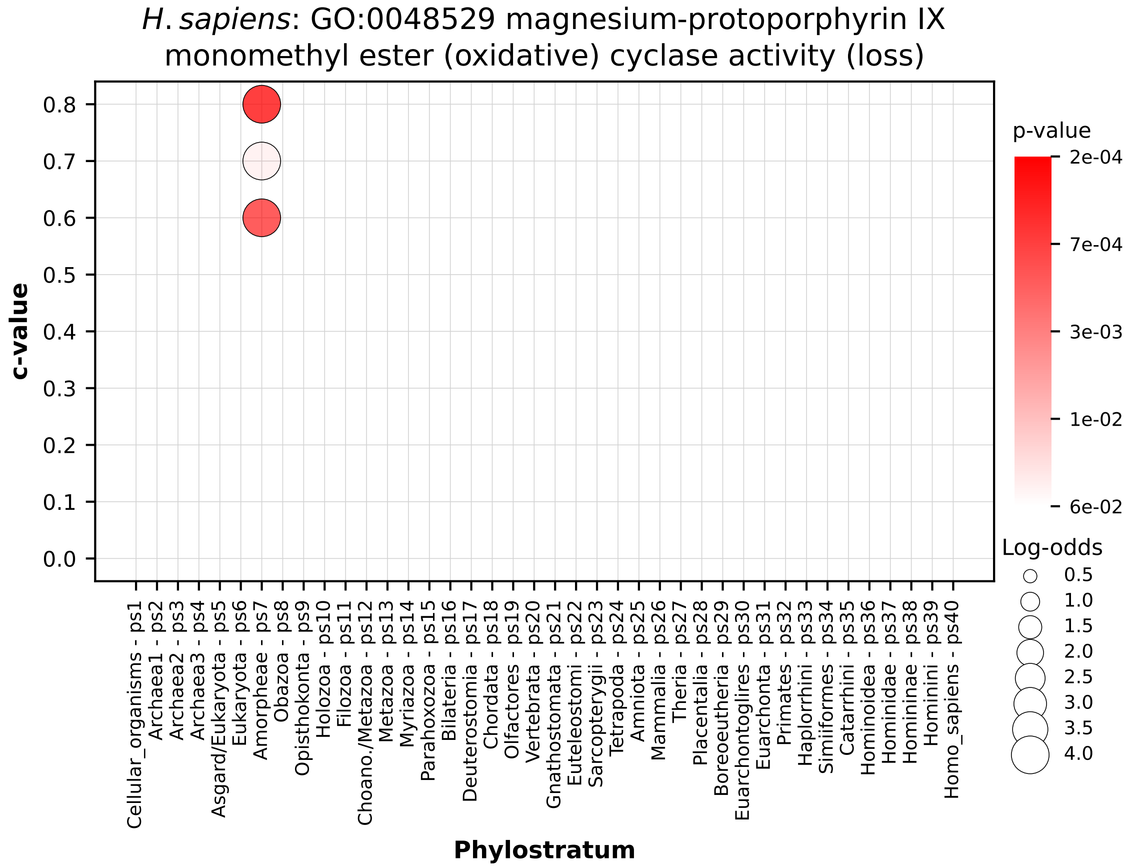

H. sapiens: GO:0048529 magnesium-protoporphyrin IX monomethyl ester (oxidative) cyclase activity (loss)
c-value
0.8
0.7
0.6
0.5
0.4
0.3
0.2
0.1
0.0
Phylostratum
Cellular_organisms - ps1
Archaea1 - ps2
Archaea2 - ps3
Archaea3 - ps4
Asgard/Eukaryota - ps5
Eukaryota - ps6
Amorpheae - ps7
Obazoa - ps8
Opisthokonta - ps9
Holozoa - ps10
Filozoa - ps11
Choano./Metazoa - ps12
Metazoa - ps13
Myriazoa - ps14
Parahoxozoa - ps15
Bilateria - ps16
Deuterostomia - ps17
Chordata - ps18
Olfactores - ps19
Vertebrata - ps20
Gnathostomata - ps21
Euteleostomi - ps22
Sarcopterygii - ps23
Tetrapoda - ps24
Amniota - ps25
Mammalia - ps26
Theria - ps27
Placentalia - ps28
Boreoeutheria - ps29
Euarchontoglires - ps30
Euarchonta - ps31
Primates - ps32
Haplorrhini - ps33
Simiiformes - ps34
Catarrhini - ps35
Hominoidea - ps36
Hominidae - ps37
Homininae - ps38
Hominini - ps39
Homo_sapiens - ps40
p-value
2e-04
7e-04
3e-03
1e-02
6e-02
Log-odds
0.5
1.0
1.5
2.0
2.5
3.0
3.5
4.0

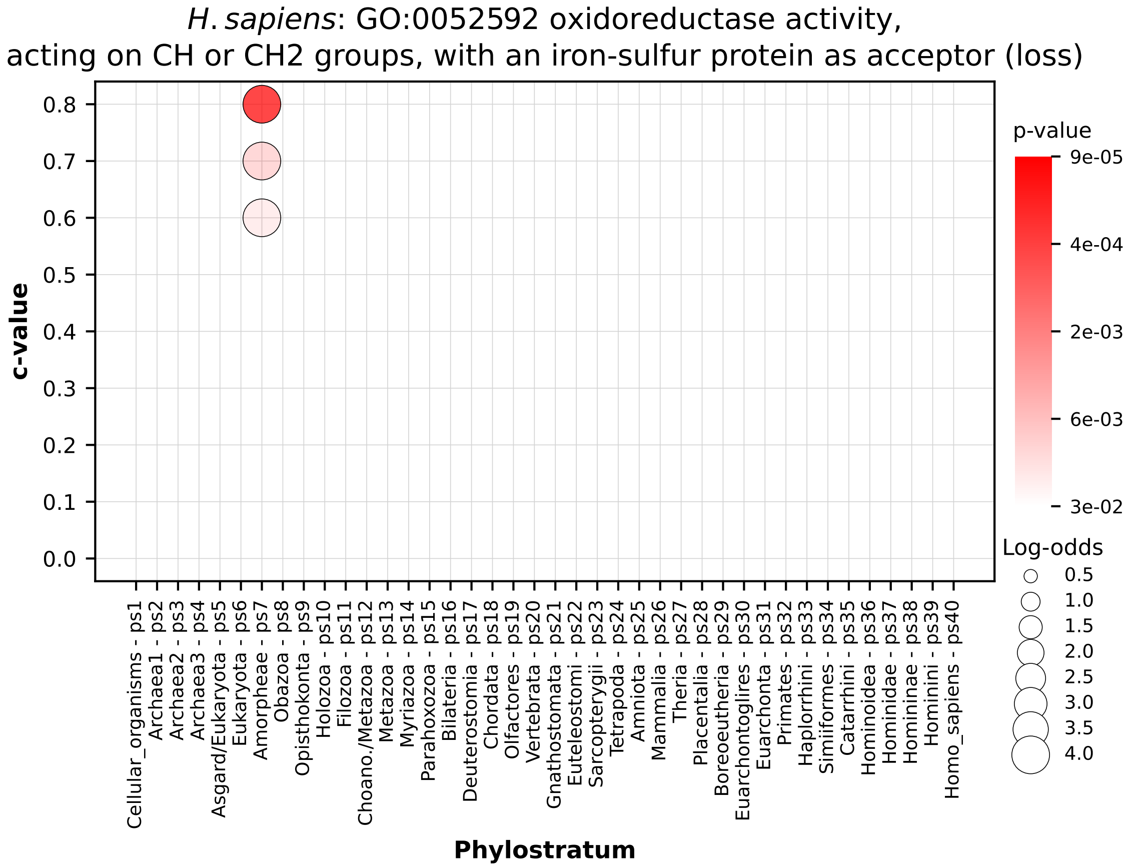

H. sapiens: GO:0052592 oxidoreductase activity,
acting on CH or CH2 groups, with an iron-sulfur protein as acceptor (loss)
c-value
0.0
0.1
0.2
0.3
0.4
0.5
0.6
0.7
0.8
p-value
9e-05
4e-04
2e-03
6e-03
3e-02
Log-odds
0.5
1.0
1.5
2.0
2.5
3.0
3.5
4.0
Cellular_organisms - ps1
Archaea1 - ps2
Archaea2 - ps3
Archaea3 - ps4
Asgard/Eukaryota - ps5
Eukaryota - ps6
Amorpheae - ps7
Obazoa - ps8
Opisthokonta - ps9
Holozoa - ps10
Filozoa - ps11
Choano./Metazoa - ps12
Metazoa - ps13
Myriazoa - ps14
Parahoxozoa - ps15
Bilateria - ps16
Deuterostomia - ps17
Chordata - ps18
Olfactores - ps19
Vertebrata - ps20
Gnathostomata - ps21
Euteleostomi - ps22
Sarcopterygii - ps23
Tetrapoda - ps24
Amniota - ps25
Mammalia - ps26
Theria - ps27
Placentalia - ps28
Boreoeutheria - ps29
Euarchontoglires - ps30
Euarchonta - ps31
Primates - ps32
Haplorrhini - ps33
Simiiformes - ps34
Catarrhini - ps35
Hominoidea - ps36
Hominidae - ps37
Homininae - ps38
Hominini - ps39
Homo_sapiens - ps40
Phylostratum

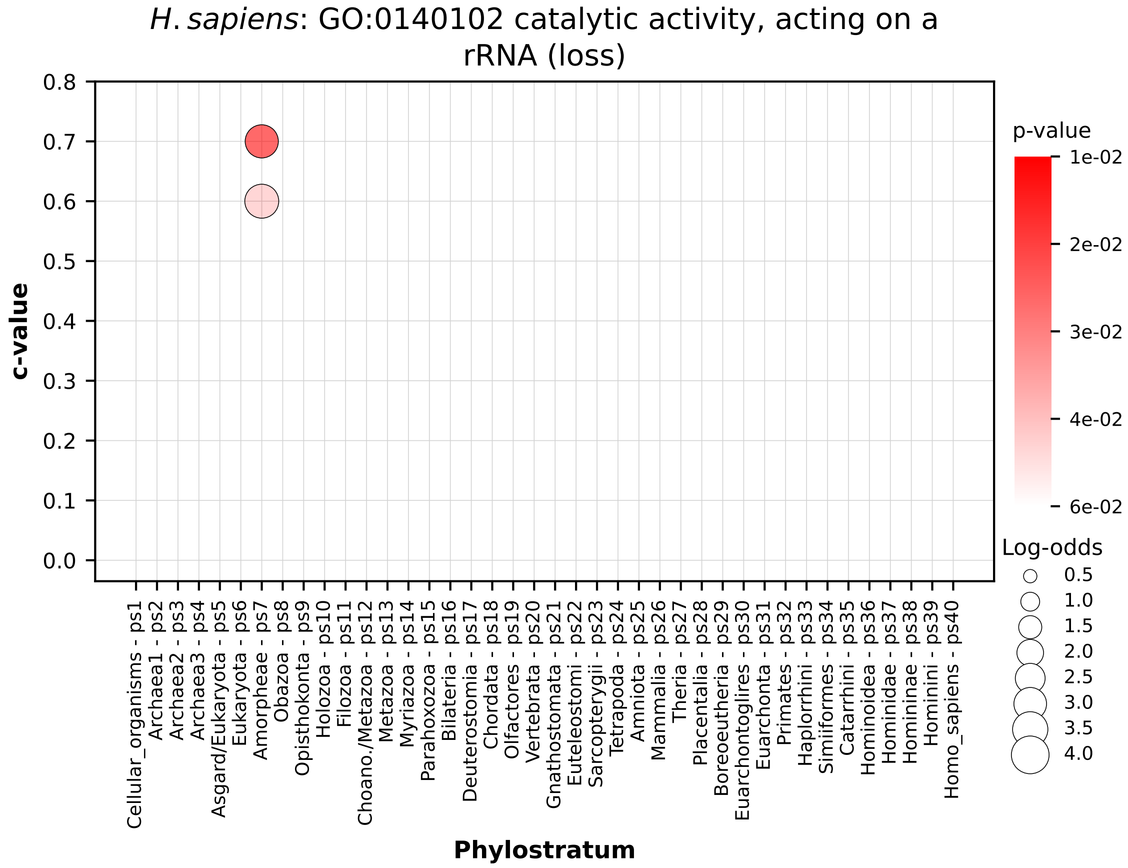

H. sapiens: GO:0140102 catalytic activity, acting on a rRNA (loss)
c-value
0.8
0.7
0.6
0.5
0.4
0.3
0.2
0.1
0.0
p-value
1e-02
2e-02
3e-02
4e-02
6e-02
Log-odds
0.5
1.0
1.5
2.0
2.5
3.0
3.5
4.0
Cellular_organisms - ps1
Archaea1 - ps2
Archaea2 - ps3
Archaea3 - ps4
Asgard/Eukaryota - ps5
Eukaryota - ps6
Amorpheae - ps7
Obazoa - ps8
Opisthokonta - ps9
Holozoa - ps10
Filozoa - ps11
Choano./Metazoa - ps12
Metazoa - ps13
Myriazoa - ps14
Parahoxozoa - ps15
Bilateria - ps16
Deuterostomia - ps17
Chordata - ps18
Olfactores - ps19
Vertebrata - ps20
Gnathostomata - ps21
Euteleostomi - ps22
Sarcopterygii - ps23
Tetrapoda - ps24
Amniota - ps25
Mammalia - ps26
Theria - ps27
Placentalia - ps28
Boreoeutheria - ps29
Euarchontoglires - ps30
Euarchonta - ps31
Primates - ps32
Haplorrhini - ps33
Simiiformes - ps34
Catarrhini - ps35
Hominoidea - ps36
Hominidae - ps37
Homininae - ps38
Hominini - ps39
Homo_sapiens - ps40
Phylostratum

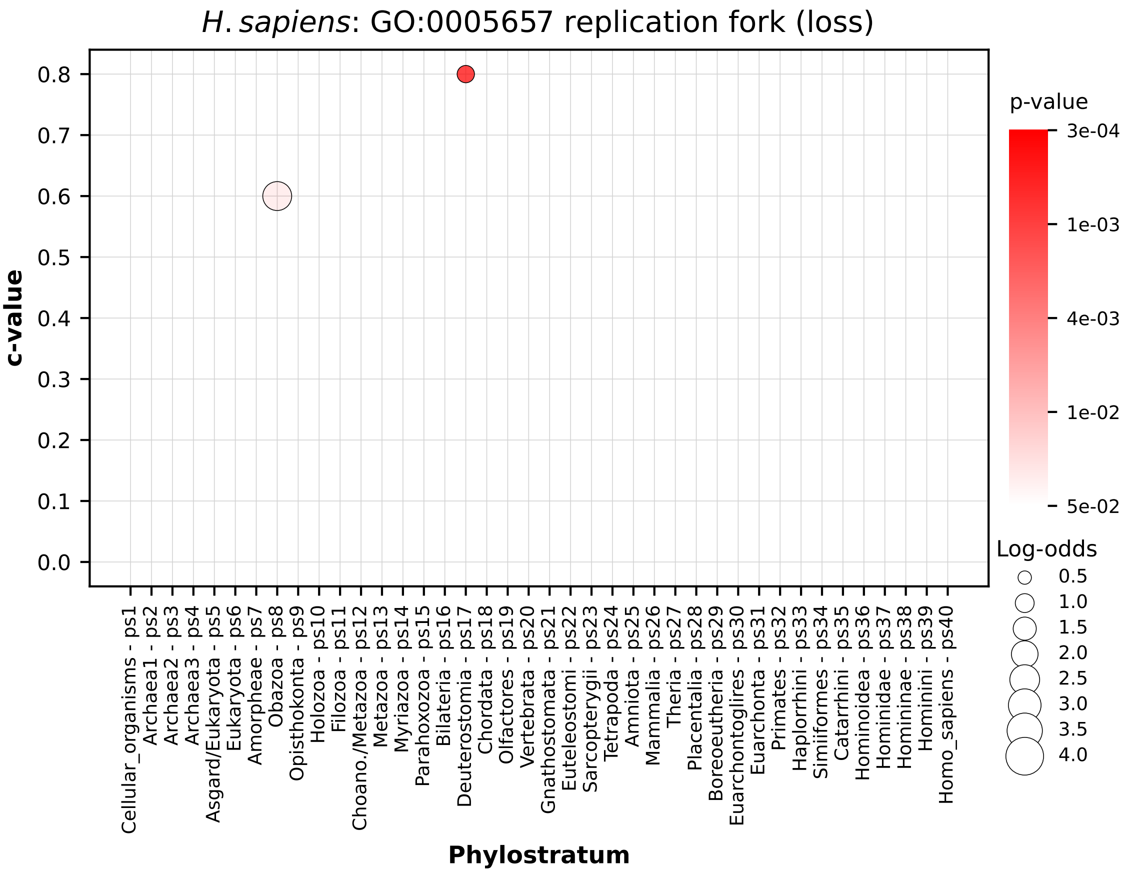

H. sapiens: GO:0005657 replication fork (loss)
c-value
0.0
0.1
0.2
0.3
0.4
0.5
0.6
0.7
0.8
Phylostratum
Cellular_organisms - ps1
Archaea1 - ps2
Archaea2 - ps3
Archaea3 - ps4
Asgard/Eukaryota - ps5
Eukaryota - ps6
Amorpheae - ps7
Obazoa - ps8
Opisthokonta - ps9
Holozoa - ps10
Filozoa - ps11
Choano./Metazoa - ps12
Metazoa - ps13
Myriazoa - ps14
Parahoxozoa - ps15
Bilateria - ps16
Deuterostomia - ps17
Chordata - ps18
Olfactores - ps19
Vertebrata - ps20
Gnathostomata - ps21
Euteleostomi - ps22
Sarcopterygii - ps23
Tetrapoda - ps24
Amniota - ps25
Mammalia - ps26
Theria - ps27
Placentalia - ps28
Boreoeutheria - ps29
Euarchontoglires - ps30
Euarchonta - ps31
Primates - ps32
Haplorrhini - ps33
Simiiformes - ps34
Catarrhini - ps35
Hominoidea - ps36
Hominidae - ps37
Homininae - ps38
Hominini - ps39
Homo_sapiens - ps40
p-value
3e-04
1e-03
4e-03
1e-02
5e-02
Log-odds
0.5
1.0
1.5
2.0
2.5
3.0
3.5
4.0

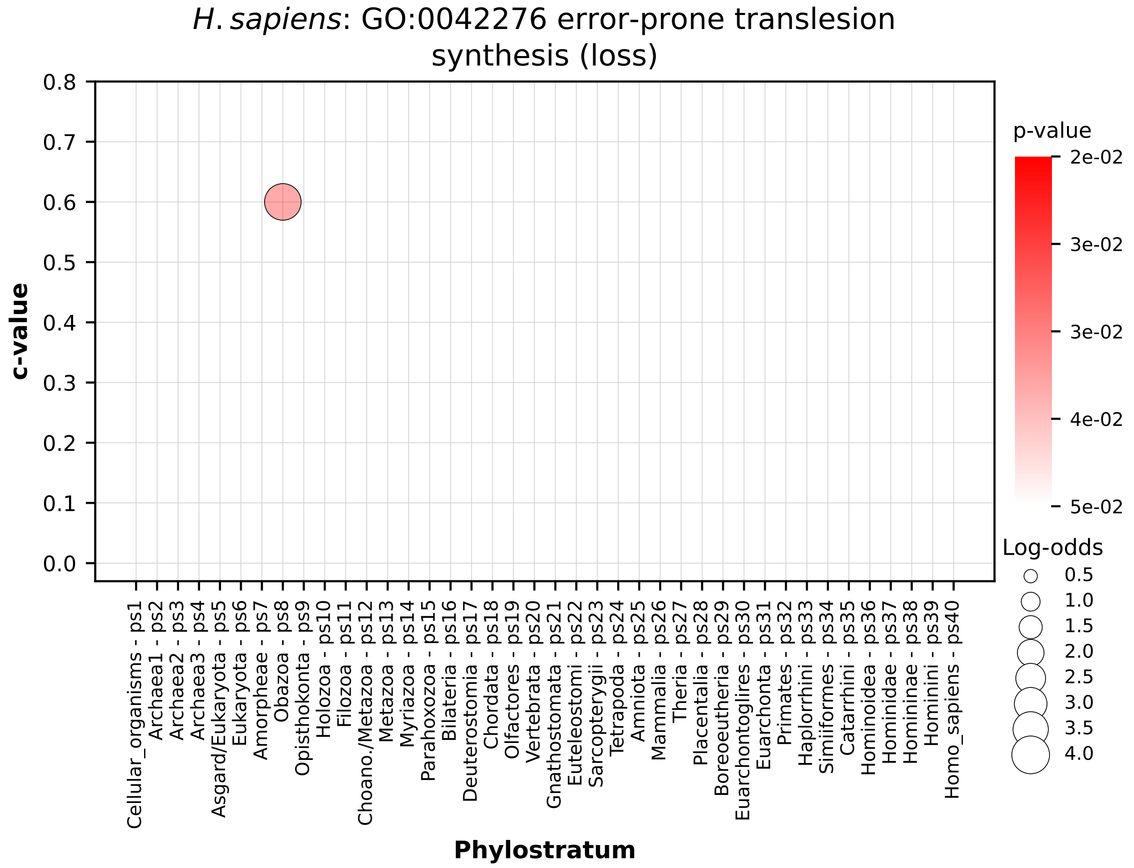

H. sapiens: GO:0042276 error-prone translesion synthesis (loss)
c-value
0.8
0.7
0.6
0.5
0.4
0.3
0.2
0.1
0.0
p-value
2e-02
3e-02
3e-02
4e-02
5e-02
Log-odds
0.5
1.0
1.5
2.0
2.5
3.0
3.5
4.0
Cellular_organisms - ps1
Archaea1 - ps2
Archaea2 - ps3
Archaea3 - ps4
Asgard/Eukaryota - ps5
Eukaryota - ps6
Amorpheae - ps7
Obazoa - ps8
Opisthokonta - ps9
Holozoa - ps10
Filozoa - ps11
Choano./Metazoa - ps12
Metazoa - ps13
Myriazoa - ps14
Parahoxozoa - ps15
Bilateria - ps16
Deuterostomia - ps17
Chordata - ps18
Olfactores - ps19
Vertebrata - ps20
Gnathostomata - ps21
Euteleostomi - ps22
Sarcopterygii - ps23
Tetrapoda - ps24
Amniota - ps25
Mammalia - ps26
Theria - ps27
Placentalia - ps28
Boreoeutheria - ps29
Euarchontoglires - ps30
Euarchonta - ps31
Primates - ps32
Haplorrhini - ps33
Simiiformes - ps34
Catarrhini - ps35
Hominoidea - ps36
Hominidae - ps37
Homininae - ps38
Hominini - ps39
Homo_sapiens - ps40
Phylostratum

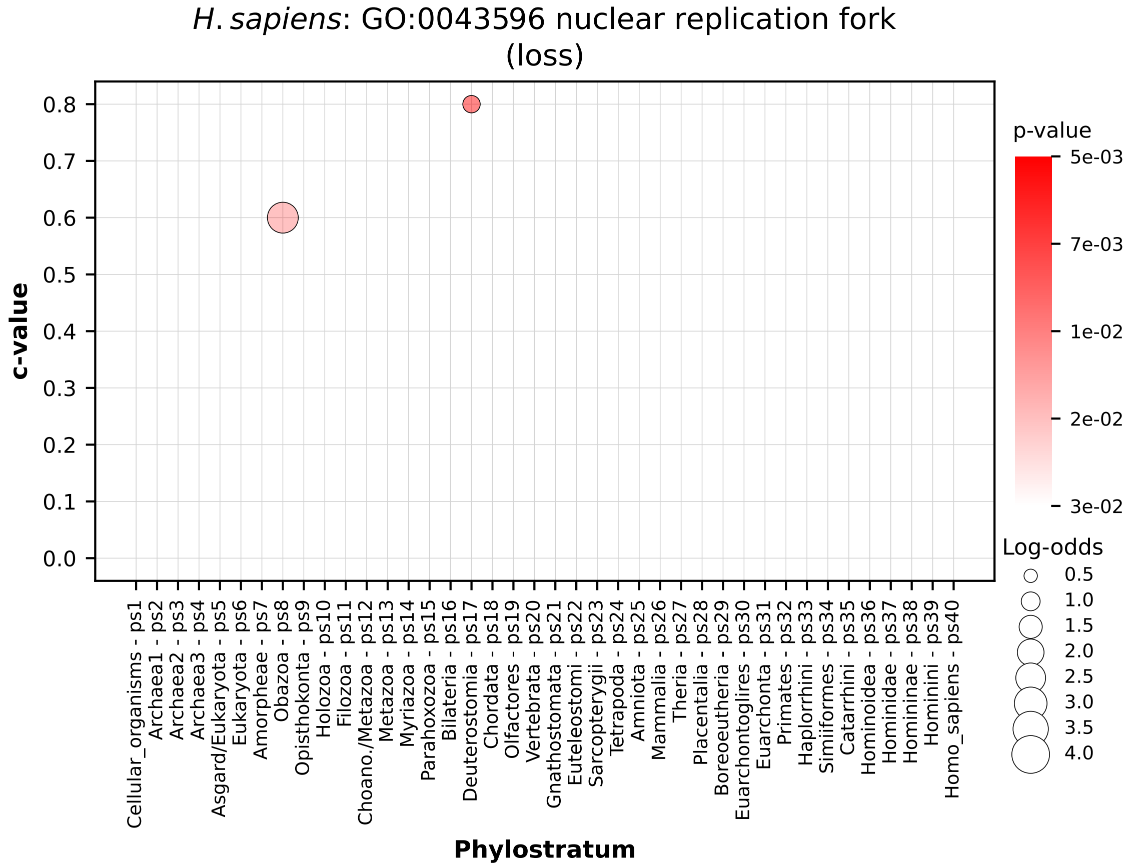
H. sapiens: GO:0043596 nuclear replication fork
(loss)
c-value
0.0
0.1
0.2
0.3
0.4
0.5
0.6
0.7
0.8
p-value
5e-03
7e-03
1e-02
2e-02
3e-02
Log-odds
0.5
1.0
1.5
2.0
2.5
3.0
3.5
4.0
Cellular_organisms - ps1
Archaea1 - ps2
Archaea2 - ps3
Archaea3 - ps4
Asgard/Eukaryota - ps5
Eukaryota - ps6
Amorpheae - ps7
Obazoa - ps8
Opisthokonta - ps9
Holozoa - ps10
Filozoa - ps11
Choano./Metazoa - ps12
Metazoa - ps13
Myriazoa - ps14
Parahoxozoa - ps15
Bilateria - ps16
Deuterostomia - ps17
Chordata - ps18
Olfactores - ps19
Vertebrata - ps20
Gnathostomata - ps21
Euteleostomi - ps22
Sarcopterygii - ps23
Tetrapoda - ps24
Amniota - ps25
Mammalia - ps26
Theria - ps27
Placentalia - ps28
Boreoeutheria - ps29
Euarchontoglires - ps30
Euarchonta - ps31
Primates - ps32
Haplorrhini - ps33
Simiiformes - ps34
Catarrhini - ps35
Hominoidea - ps36
Hominidae - ps37
Homininae - ps38
Hominini - ps39
Homo_sapiens - ps40
Phylostratum

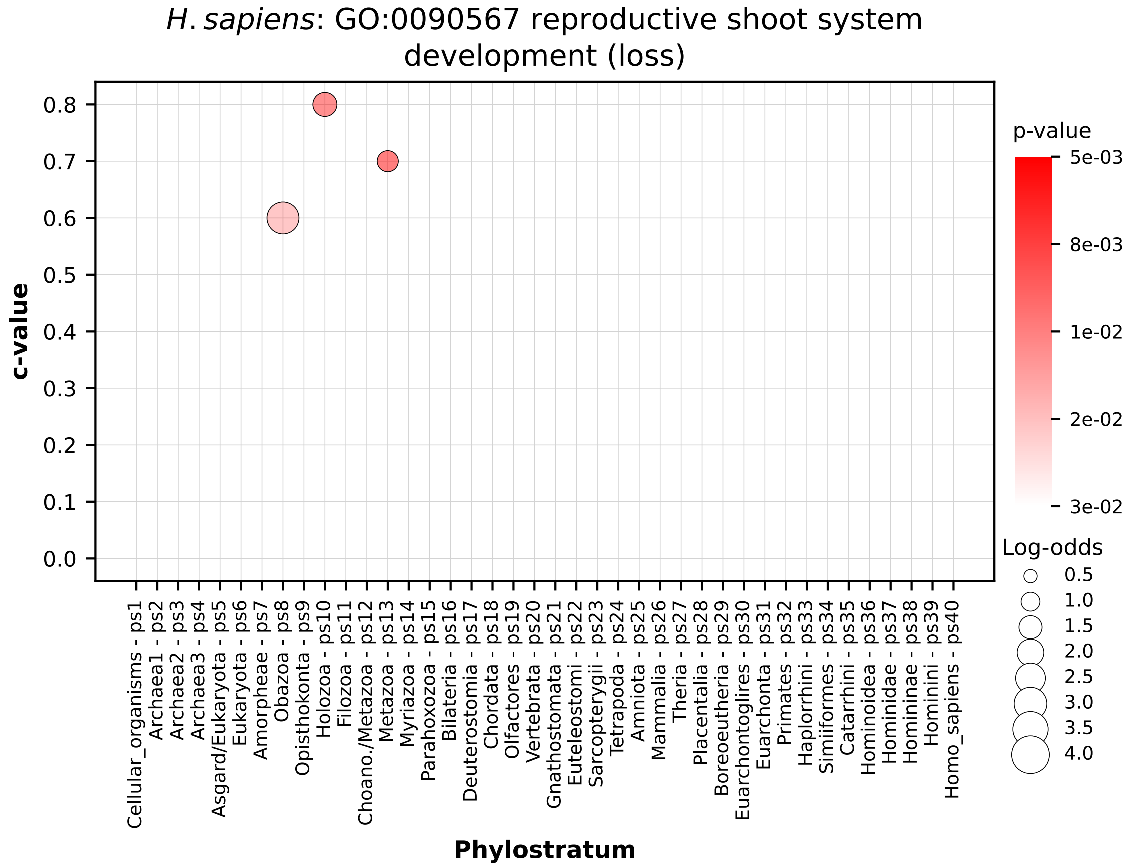

H. sapiens: GO:0090567 reproductive shoot system development (loss)
c-value
0.0
0.1
0.2
0.3
0.4
0.5
0.6
0.7
0.8
Phylostratum
Cellular_organisms - ps1
Archaea1 - ps2
Archaea2 - ps3
Archaea3 - ps4
Asgard/Eukaryota - ps5
Eukaryota - ps6
Amorpheae - ps7
Obazoa - ps8
Opisthokonta - ps9
Holozoa - ps10
Filozoa - ps11
Choano./Metazoa - ps12
Metazoa - ps13
Myriazoa - ps14
Parahoxozoa - ps15
Bilateria - ps16
Deuterostomia - ps17
Chordata - ps18
Olfactores - ps19
Vertebrata - ps20
Gnathostomata - ps21
Euteleostomi - ps22
Sarcopterygii - ps23
Tetrapoda - ps24
Amniota - ps25
Mammalia - ps26
Theria - ps27
Placentalia - ps28
Boreoeutheria - ps29
Euarchontoglires - ps30
Euarchonta - ps31
Primates - ps32
Haplorrhini - ps33
Simiiformes - ps34
Catarrhini - ps35
Hominoidea - ps36
Hominidae - ps37
Homininae - ps38
Hominini - ps39
Homo_sapiens - ps40
p-value
5e-03
8e-03
1e-02
2e-02
3e-02
Log-odds
0.5
1.0
1.5
2.0
2.5
3.0
3.5
4.0

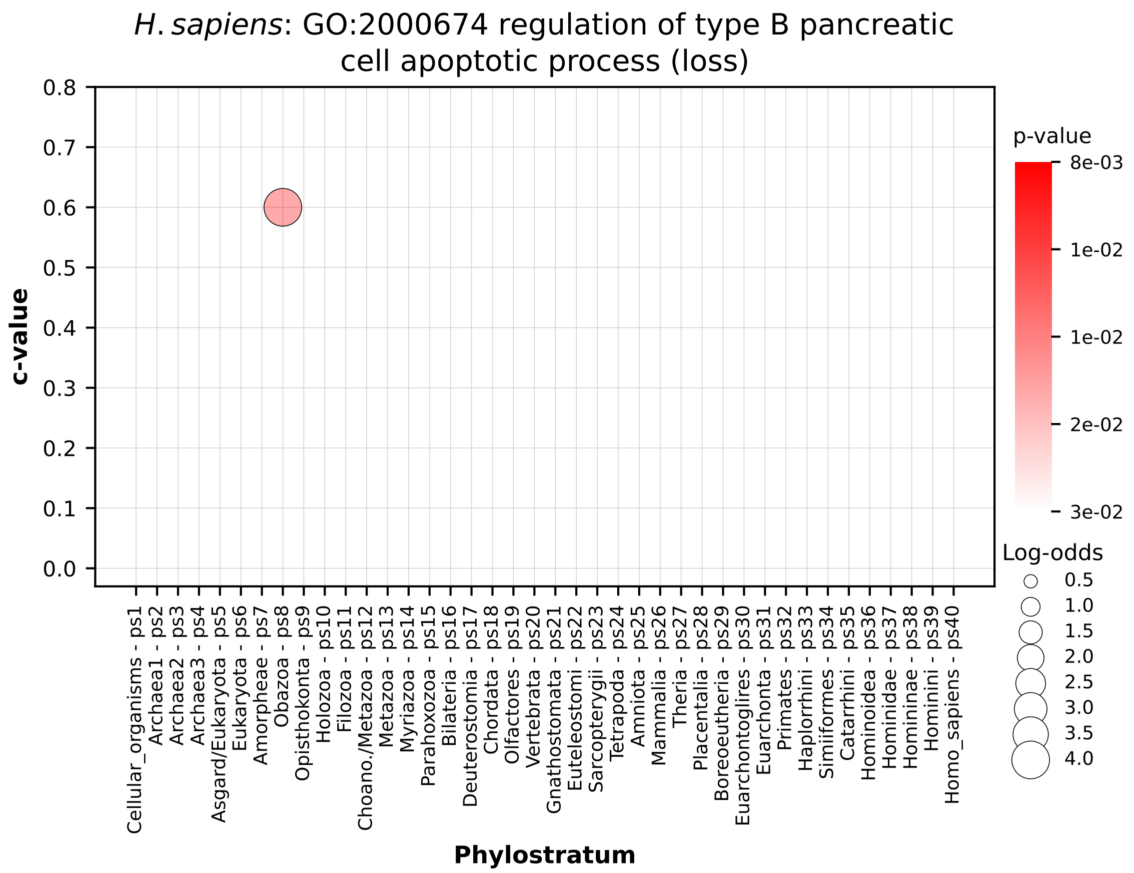

H. sapiens: GO:2000674 regulation of type B pancreatic cell apoptotic process (loss)
c-value
0.8
0.7
0.6
0.5
0.4
0.3
0.2
0.1
0.0
p-value
8e-03
1e-02
1e-02
2e-02
3e-02
Log-odds
0.5
1.0
1.5
2.0
2.5
3.0
3.5
4.0
Cellular_organisms - ps1
Archaea1 - ps2
Archaea2 - ps3
Archaea3 - ps4
Asgard/Eukaryota - ps5
Eukaryota - ps6
Amorpheae - ps7
Obazoa - ps8
Opisthokonta - ps9
Holozoa - ps10
Filozoa - ps11
Choano./Metazoa - ps12
Metazoa - ps13
Myriazoa - ps14
Parahoxozoa - ps15
Bilateria - ps16
Deuterostomia - ps17
Chordata - ps18
Olfactores - ps19
Vertebrata - ps20
Gnathostomata - ps21
Euteleostomi - ps22
Sarcopterygii - ps23
Tetrapoda - ps24
Amniota - ps25
Mammalia - ps26
Theria - ps27
Placentalia - ps28
Boreoeutheria - ps29
Euarchontoglires - ps30
Euarchonta - ps31
Primates - ps32
Haplorrhini - ps33
Simiiformes - ps34
Catarrhini - ps35
Hominoidea - ps36
Hominidae - ps37
Homininae - ps38
Hominini - ps39
Homo_sapiens - ps40
Phylostratum

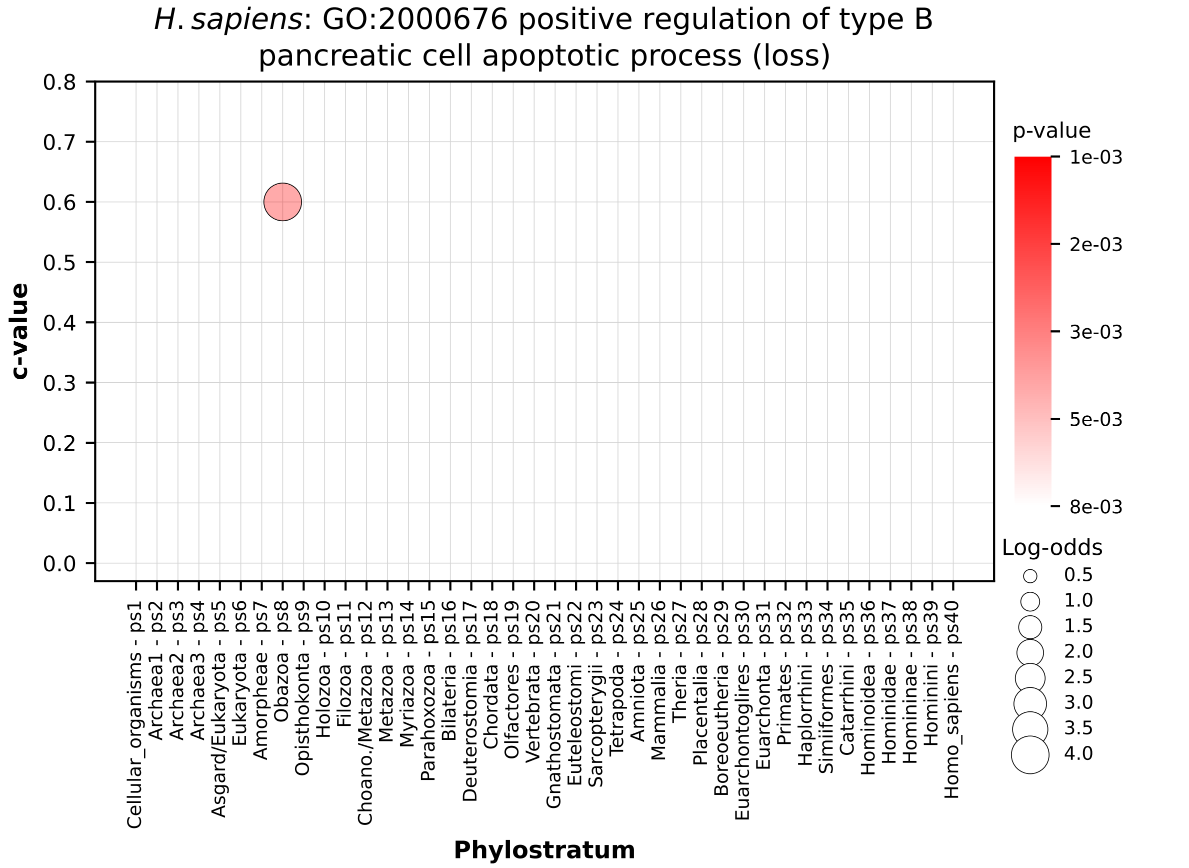

H. sapiens: GO:2000676 positive regulation of type B pancreatic cell apoptotic process (loss)
c-value
0.8
0.7
0.6
0.5
0.4
0.3
0.2
0.1
0.0
Phylostratum
Cellular_organisms - ps1
Archaea1 - ps2
Archaea2 - ps3
Archaea3 - ps4
Asgard/Eukaryota - ps5
Eukaryota - ps6
Amorpheae - ps7
Obazoa - ps8
Opisthokonta - ps9
Holozoa - ps10
Filozoa - ps11
Choano./Metazoa - ps12
Metazoa - ps13
Myriazoa - ps14
Parahoxozoa - ps15
Bilateria - ps16
Deuterostomia - ps17
Chordata - ps18
Olfactores - ps19
Vertebrata - ps20
Gnathostomata - ps21
Euteleostomi - ps22
Sarcopterygii - ps23
Tetrapoda - ps24
Amniota - ps25
Mammalia - ps26
Theria - ps27
Placentalia - ps28
Boreoeutheria - ps29
Euarchontoglires - ps30
Euarchonta - ps31
Primates - ps32
Haplorrhini - ps33
Simiiformes - ps34
Catarrhini - ps35
Hominoidea - ps36
Hominidae - ps37
Homininae - ps38
Hominini - ps39
Homo_sapiens - ps40
p-value
1e-03
2e-03
3e-03
5e-03
8e-03
Log-odds
0.5
1.0
1.5
2.0
2.5
3.0
3.5
4.0

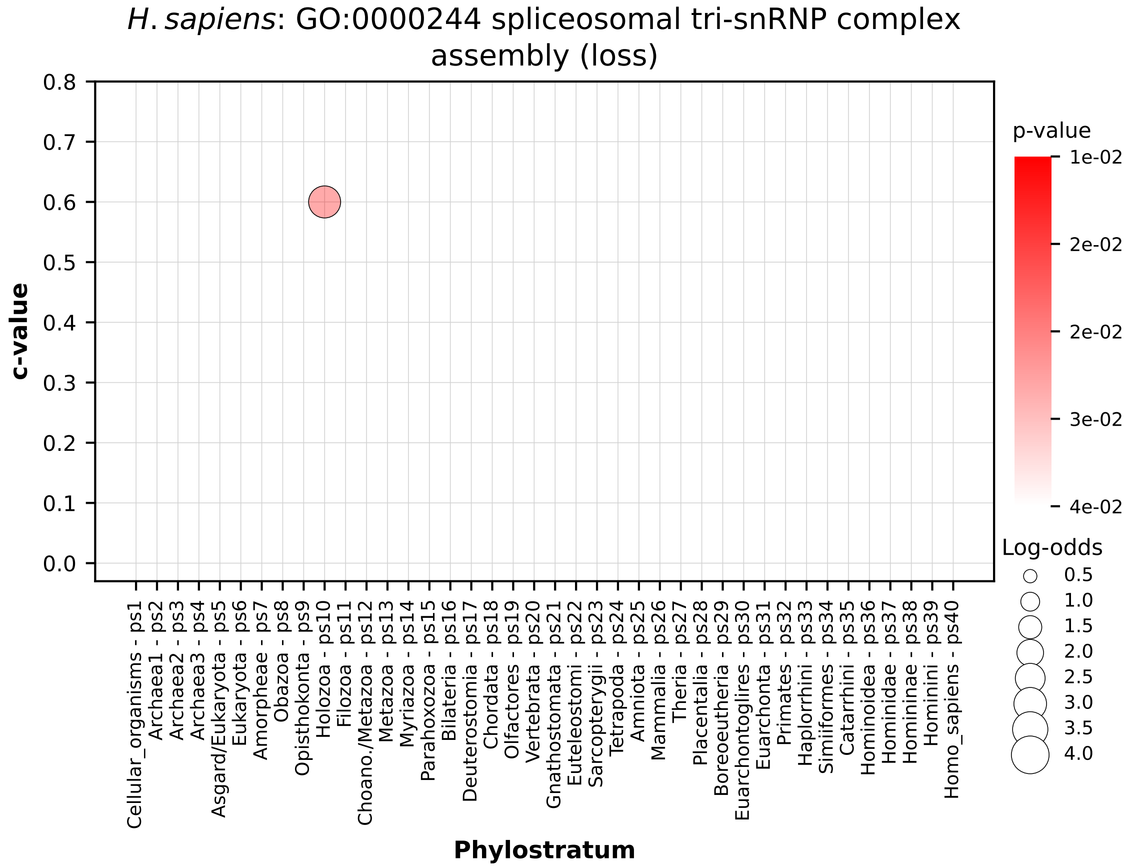

H. sapiens: GO:0000244 spliceosomal tri-snRNP complex assembly (loss)
c-value
0.8
0.7
0.6
0.5
0.4
0.3
0.2
0.1
0.0
Phylostratum
Cellular_organisms - ps1
Archaea1 - ps2
Archaea2 - ps3
Archaea3 - ps4
Asgard/Eukaryota - ps5
Eukaryota - ps6
Amorpheae - ps7
Obazoa - ps8
Opisthokonta - ps9
Holozoa - ps10
Filozoa - ps11
Choano./Metazoa - ps12
Metazoa - ps13
Myriazoa - ps14
Parahoxozoa - ps15
Bilateria - ps16
Deuterostomia - ps17
Chordata - ps18
Olfactores - ps19
Vertebrata - ps20
Gnathostomata - ps21
Euteleostomi - ps22
Sarcopterygii - ps23
Tetrapoda - ps24
Amniota - ps25
Mammalia - ps26
Theria - ps27
Placentalia - ps28
Boreoeutheria - ps29
Euarchontoglires - ps30
Euarchonta - ps31
Primates - ps32
Haplorrhini - ps33
Simiiformes - ps34
Catarrhini - ps35
Hominoidea - ps36
Hominidae - ps37
Homininae - ps38
Hominini - ps39
Homo_sapiens - ps40
p-value
1e-02
2e-02
2e-02
3e-02
4e-02
Log-odds
0.5
1.0
1.5
2.0
2.5
3.0
3.5
4.0

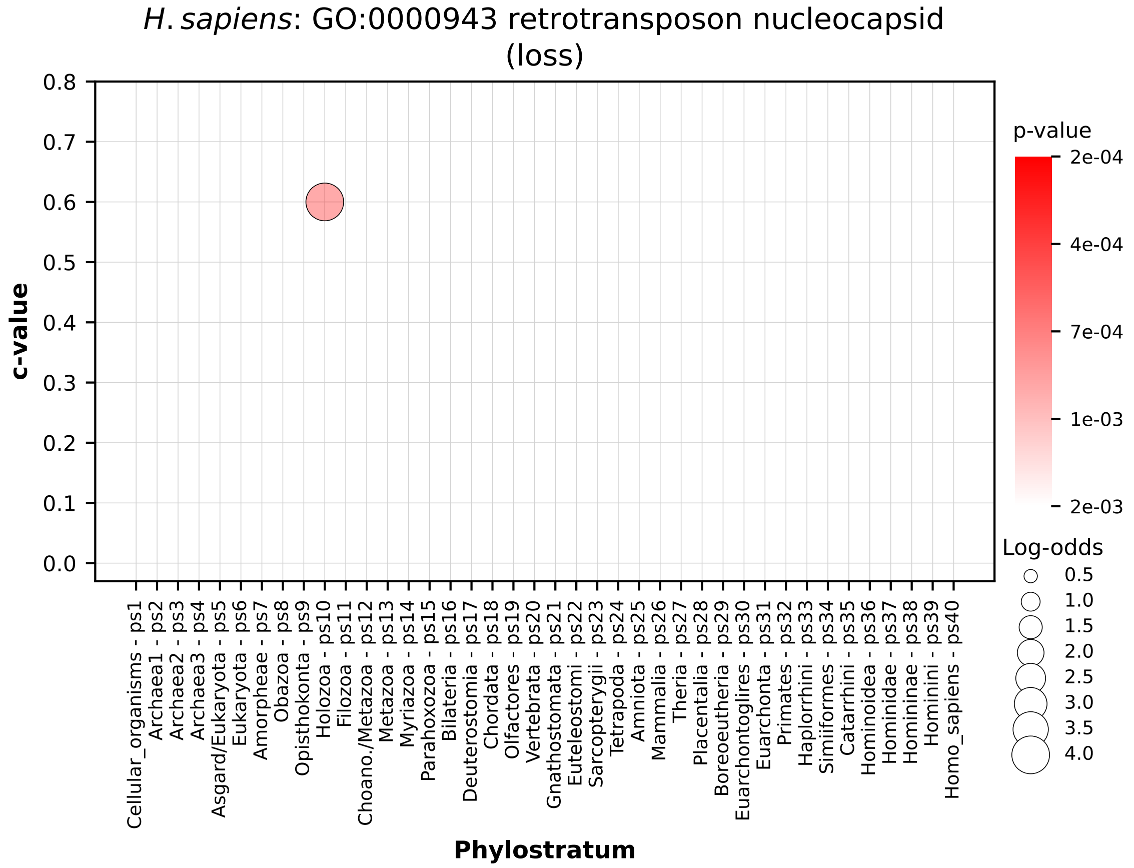
H. sapiens: GO:0000943 retrotransposon nucleocapsid
(loss)
c-value
0.8
0.7
0.6
0.5
0.4
0.3
0.2
0.1
0.0
p-value
2e-04
4e-04
7e-04
1e-03
2e-03
Log-odds
0.5
1.0
1.5
2.0
2.5
3.0
3.5
4.0
Cellular_organisms - ps1
Archaea1 - ps2
Archaea2 - ps3
Archaea3 - ps4
Asgard/Eukaryota - ps5
Eukaryota - ps6
Amorpheae - ps7
Obazoa - ps8
Opisthokonta - ps9
Holozoa - ps10
Filozoa - ps11
Choano./Metazoa - ps12
Metazoa - ps13
Myriazoa - ps14
Parahoxozoa - ps15
Bilateria - ps16
Deuterostomia - ps17
Chordata - ps18
Olfactores - ps19
Vertebrata - ps20
Gnathostomata - ps21
Euteleostomi - ps22
Sarcopterygii - ps23
Tetrapoda - ps24
Amniota - ps25
Mammalia - ps26
Theria - ps27
Placentalia - ps28
Boreoeutheria - ps29
Euarchontoglires - ps30
Euarchonta - ps31
Primates - ps32
Haplorrhini - ps33
Simiiformes - ps34
Catarrhini - ps35
Hominoidea - ps36
Hominidae - ps37
Homininae - ps38
Hominini - ps39
Homo_sapiens - ps40
Phylostratum

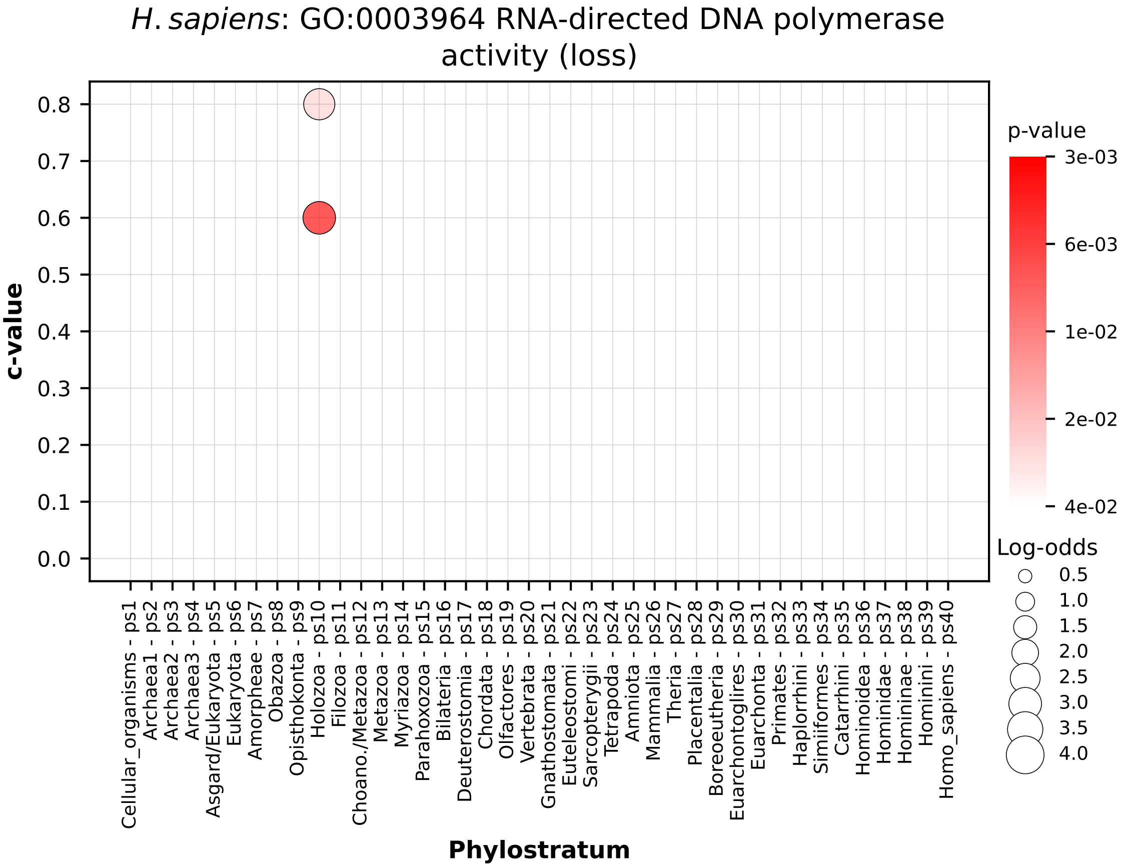

H. sapiens: GO:0003964 RNA-directed DNA polymerase activity (loss)
c-value
0.0
0.1
0.2
0.3
0.4
0.5
0.6
0.7
0.8
Phylostratum
Cellular_organisms - ps1
Archaea1 - ps2
Archaea2 - ps3
Archaea3 - ps4
Asgard/Eukaryota - ps5
Eukaryota - ps6
Amorpheae - ps7
Obazoa - ps8
Opisthokonta - ps9
Holozoa - ps10
Filozoa - ps11
Choano./Metazoa - ps12
Metazoa - ps13
Myriazoa - ps14
Parahoxozoa - ps15
Bilateria - ps16
Deuterostomia - ps17
Chordata - ps18
Olfactores - ps19
Vertebrata - ps20
Gnathostomata - ps21
Euteleostomi - ps22
Sarcopterygii - ps23
Tetrapoda - ps24
Amniota - ps25
Mammalia - ps26
Theria - ps27
Placentalia - ps28
Boreoeutheria - ps29
Euarchontoglires - ps30
Euarchonta - ps31
Primates - ps32
Haplorrhini - ps33
Simiiformes - ps34
Catarrhini - ps35
Hominoidea - ps36
Hominidae - ps37
Homininae - ps38
Hominini - ps39
Homo_sapiens - ps40
p-value
3e-03
6e-03
1e-02
2e-02
4e-02
Log-odds
0.5
1.0
1.5
2.0
2.5
3.0
3.5
4.0

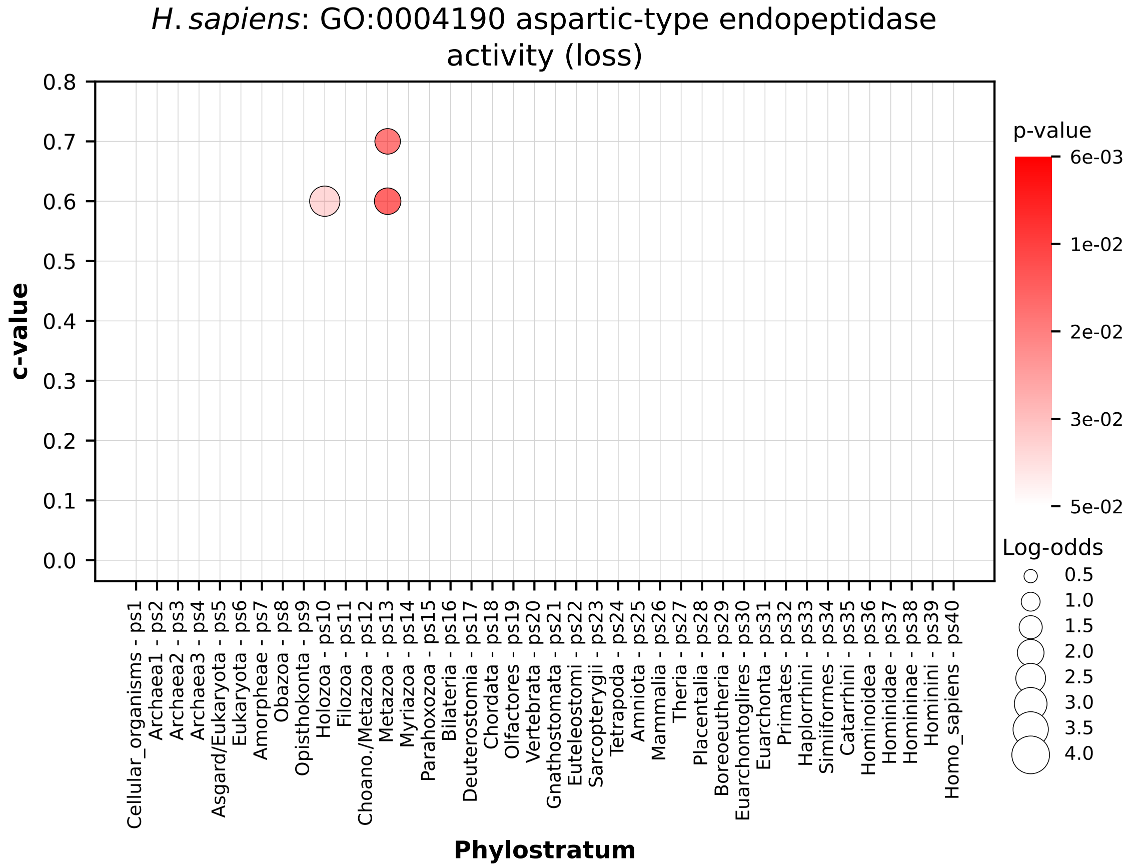

H. sapiens: GO:0004190 aspartic-type endopeptidase activity (loss)
c-value
0.8
0.7
0.6
0.5
0.4
0.3
0.2
0.1
0.0
Phylostratum
Cellular_organisms - ps1
Archaea1 - ps2
Archaea2 - ps3
Archaea3 - ps4
Asgard/Eukaryota - ps5
Eukaryota - ps6
Amorpheae - ps7
Obazoa - ps8
Opisthokonta - ps9
Holozoa - ps10
Filozoa - ps11
Choano./Metazoa - ps12
Metazoa - ps13
Myriazoa - ps14
Parahoxozoa - ps15
Bilateria - ps16
Deuterostomia - ps17
Chordata - ps18
Olfactores - ps19
Vertebrata - ps20
Gnathostomata - ps21
Euteleostomi - ps22
Sarcopterygii - ps23
Tetrapoda - ps24
Amniota - ps25
Mammalia - ps26
Theria - ps27
Placentalia - ps28
Boreoeutheria - ps29
Euarchontoglires - ps30
Euarchonta - ps31
Primates - ps32
Haplorrhini - ps33
Simiiformes - ps34
Catarrhini - ps35
Hominoidea - ps36
Hominidae - ps37
Homininae - ps38
Hominini - ps39
Homo_sapiens - ps40
p-value
6e-03
1e-02
2e-02
3e-02
5e-02
Log-odds
0.5
1.0
1.5
2.0
2.5
3.0
3.5
4.0

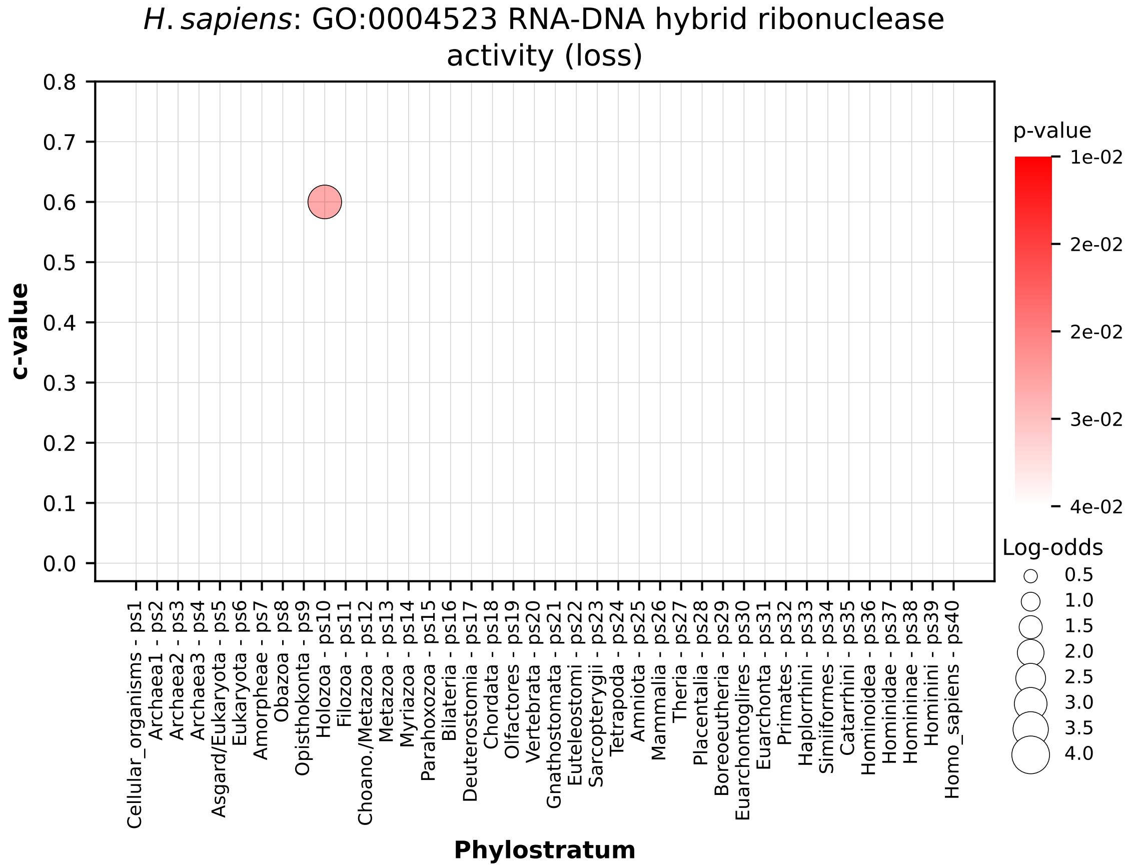

H. sapiens: GO:0004523 RNA-DNA hybrid ribonuclease activity (loss)
c-value
0.8
0.7
0.6
0.5
0.4
0.3
0.2
0.1
0.0
p-value
1e-02
2e-02
2e-02
3e-02
4e-02
Log-odds
0.5
1.0
1.5
2.0
2.5
3.0
3.5
4.0
Cellular_organisms - ps1
Archaea1 - ps2
Archaea2 - ps3
Archaea3 - ps4
Asgard/Eukaryota - ps5
Eukaryota - ps6
Amorpheae - ps7
Obazoa - ps8
Opisthokonta - ps9
Holozoa - ps10
Filozoa - ps11
Choano./Metazoa - ps12
Metazoa - ps13
Myriazoa - ps14
Parahoxozoa - ps15
Bilateria - ps16
Deuterostomia - ps17
Chordata - ps18
Olfactores - ps19
Vertebrata - ps20
Gnathostomata - ps21
Euteleostomi - ps22
Sarcopterygii - ps23
Tetrapoda - ps24
Amniota - ps25
Mammalia - ps26
Theria - ps27
Placentalia - ps28
Boreoeutheria - ps29
Euarchontoglires - ps30
Euarchonta - ps31
Primates - ps32
Haplorrhini - ps33
Simiiformes - ps34
Catarrhini - ps35
Hominoidea - ps36
Hominidae - ps37
Homininae - ps38
Hominini - ps39
Homo_sapiens - ps40
Phylostratum

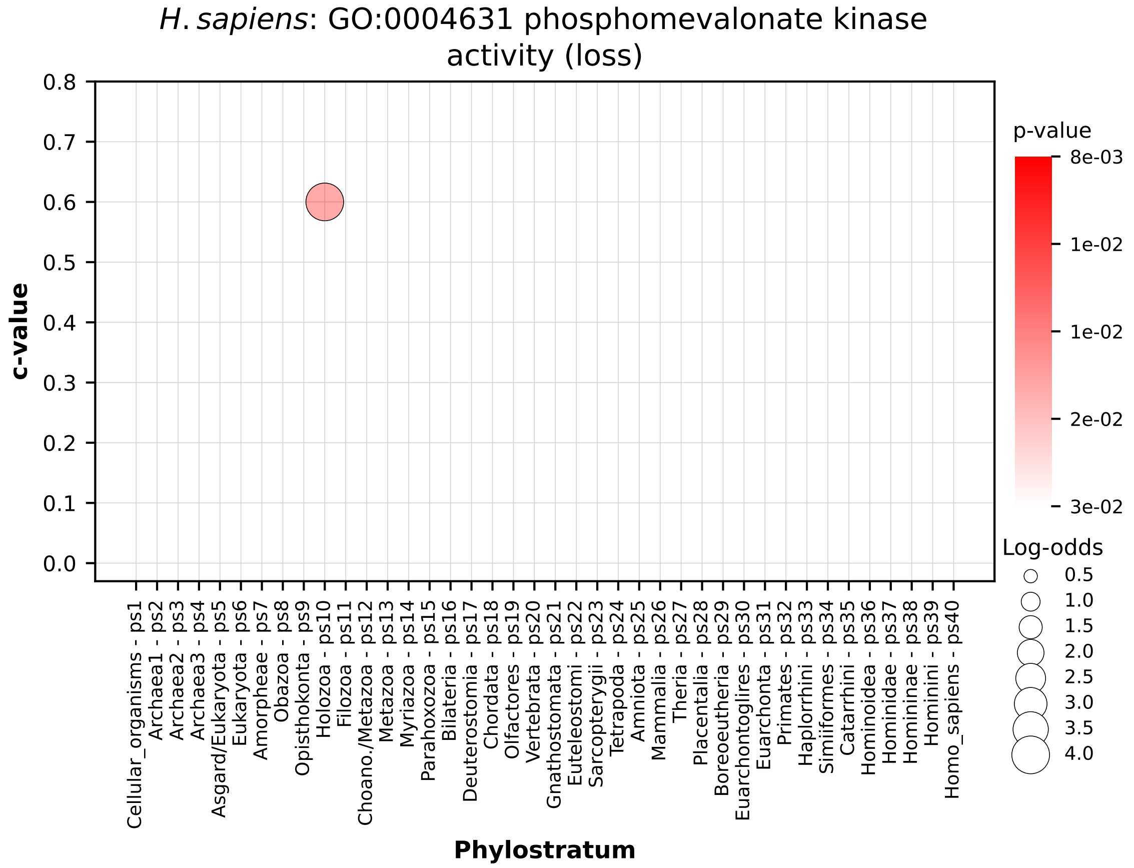
H. sapiens: GO:0004631 phosphomevalonate kinase activity (loss)
c-value
0.8
0.7
0.6
0.5
0.4
0.3
0.2
0.1
0.0
Phylostratum
Cellular_organisms - ps1
Archaea1 - ps2
Archaea2 - ps3
Archaea3 - ps4
Asgard/Eukaryota - ps5
Eukaryota - ps6
Amorpheae - ps7
Obazoa - ps8
Opisthokonta - ps9
Holozoa - ps10
Filozoa - ps11
Choano./Metazoa - ps12
Metazoa - ps13
Myriazoa - ps14
Parahoxozoa - ps15
Bilateria - ps16
Deuterostomia - ps17
Chordata - ps18
Olfactores - ps19
Vertebrata - ps20
Gnathostomata - ps21
Euteleostomi - ps22
Sarcopterygii - ps23
Tetrapoda - ps24
Amniota - ps25
Mammalia - ps26
Theria - ps27
Placentalia - ps28
Boreoeutheria - ps29
Euarchontoglires - ps30
Euarchonta - ps31
Primates - ps32
Haplorrhini - ps33
Simiiformes - ps34
Catarrhini - ps35
Hominoidea - ps36
Hominidae - ps37
Homininae - ps38
Hominini - ps39
Homo_sapiens - ps40
p-value
8e-03
1e-02
1e-02
2e-02
3e-02
Log-odds
0.5
1.0
1.5
2.0
2.5
3.0
3.5
4.0

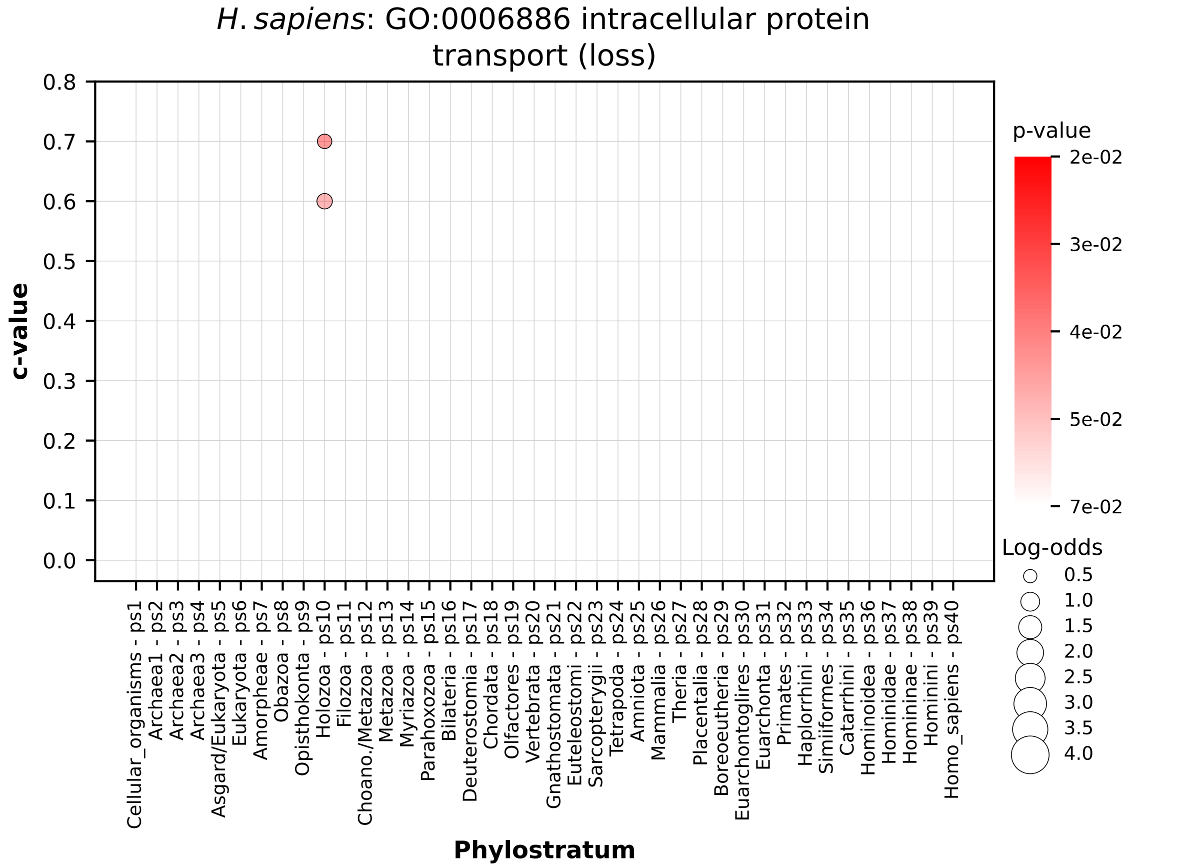

H. sapiens: GO:0006886 intracellular protein transport (loss)
c-value
0.8
0.7
0.6
0.5
0.4
0.3
0.2
0.1
0.0
p-value
2e-02
3e-02
4e-02
5e-02
7e-02
Log-odds
0.5
1.0
1.5
2.0
2.5
3.0
3.5
4.0
Cellular_organisms - ps1
Archaea1 - ps2
Archaea2 - ps3
Archaea3 - ps4
Asgard/Eukaryota - ps5
Eukaryota - ps6
Amorpheae - ps7
Obazoa - ps8
Opisthokonta - ps9
Holozoa - ps10
Filozoa - ps11
Choano./Metazoa - ps12
Metazoa - ps13
Myriazoa - ps14
Parahoxozoa - ps15
Bilateria - ps16
Deuterostomia - ps17
Chordata - ps18
Olfactores - ps19
Vertebrata - ps20
Gnathostomata - ps21
Euteleostomi - ps22
Sarcopterygii - ps23
Tetrapoda - ps24
Amniota - ps25
Mammalia - ps26
Theria - ps27
Placentalia - ps28
Boreoeutheria - ps29
Euarchontoglires - ps30
Euarchonta - ps31
Primates - ps32
Haplorrhini - ps33
Simiiformes - ps34
Catarrhini - ps35
Hominoidea - ps36
Hominidae - ps37
Homininae - ps38
Hominini - ps39
Homo_sapiens - ps40
Phylostratum

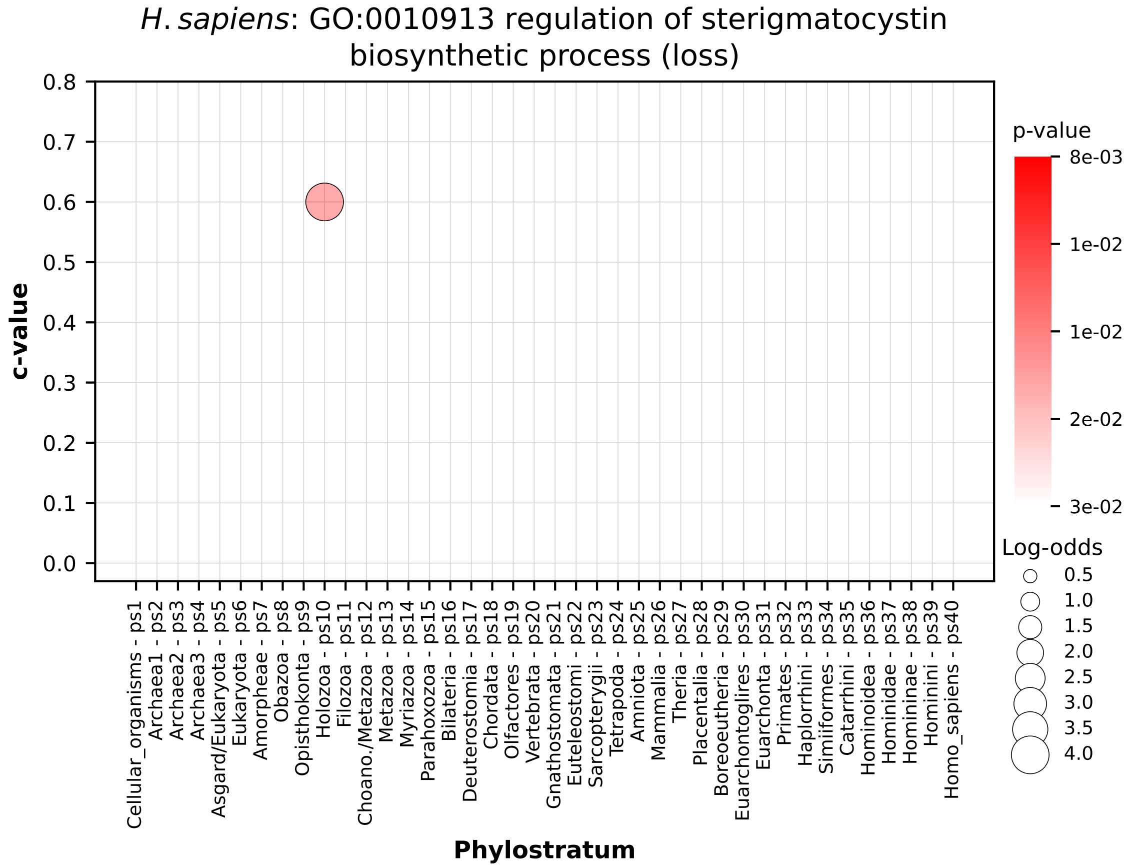

H. sapiens: GO:0010913 regulation of sterigmatocystin biosynthetic process (loss)
c-value
0.8
0.7
0.6
0.5
0.4
0.3
0.2
0.1
0.0
p-value
8e-03
1e-02
1e-02
2e-02
3e-02
Log-odds
0.5
1.0
1.5
2.0
2.5
3.0
3.5
4.0
Cellular_organisms - ps1
Archaea1 - ps2
Archaea2 - ps3
Archaea3 - ps4
Asgard/Eukaryota - ps5
Eukaryota - ps6
Amorpheae - ps7
Obazoa - ps8
Opisthokonta - ps9
Holozoa - ps10
Filozoa - ps11
Choano./Metazoa - ps12
Metazoa - ps13
Myriazoa - ps14
Parahoxozoa - ps15
Bilateria - ps16
Deuterostomia - ps17
Chordata - ps18
Olfactores - ps19
Vertebrata - ps20
Gnathostomata - ps21
Euteleostomi - ps22
Sarcopterygii - ps23
Tetrapoda - ps24
Amniota - ps25
Mammalia - ps26
Theria - ps27
Placentalia - ps28
Boreoeutheria - ps29
Euarchontoglires - ps30
Euarchonta - ps31
Primates - ps32
Haplorrhini - ps33
Simiiformes - ps34
Catarrhini - ps35
Hominoidea - ps36
Hominidae - ps37
Homininae - ps38
Hominini - ps39
Homo_sapiens - ps40
Phylostratum

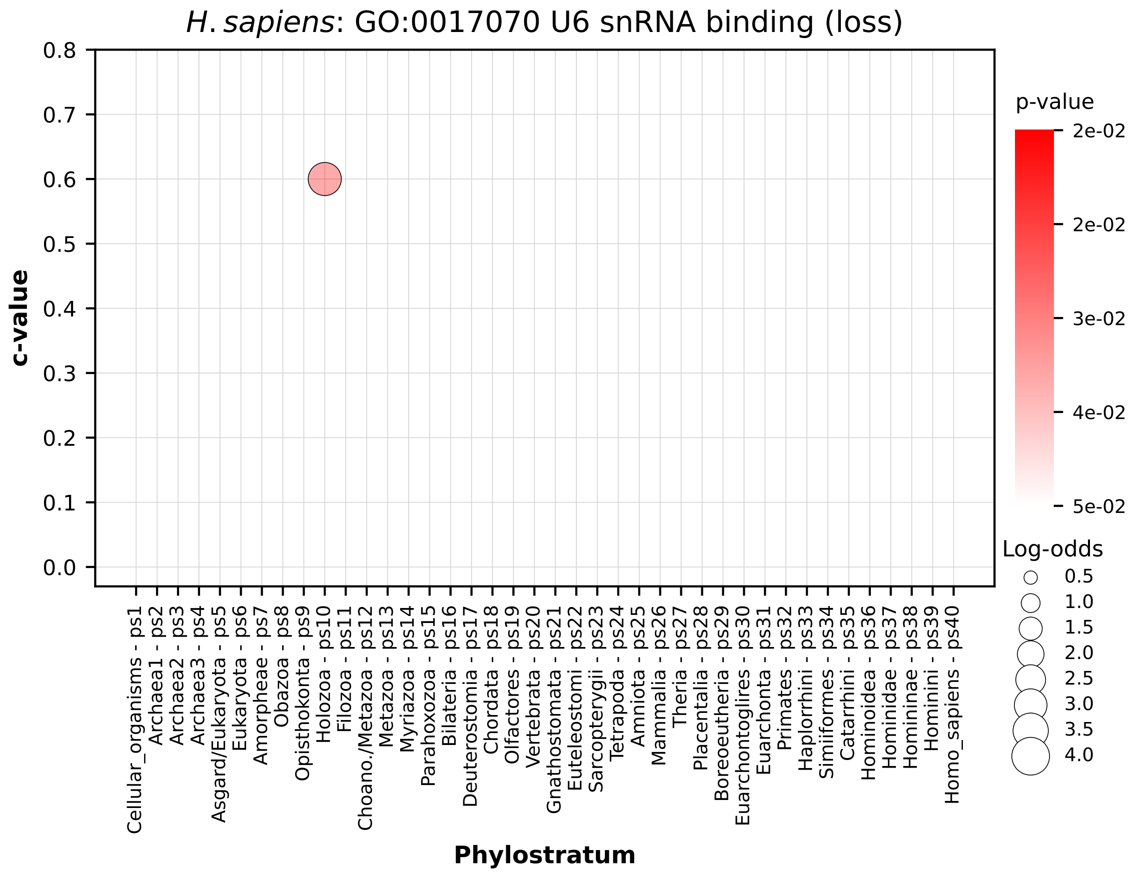

$H. sapiens$: GO:0017070 U6 snRNA binding (loss)
c-value
0.8
0.7
0.6
0.5
0.4
0.3
0.2
0.1
0.0
Phylostratum
Cellular_organisms - ps1
Archaea1 - ps2
Archaea2 - ps3
Archaea3 - ps4
Asgard/Eukaryota - ps5
Eukaryota - ps6
Amorpheae - ps7
Obazoa - ps8
Opisthokonta - ps9
Holozoa - ps10
Filozoa - ps11
Choano./Metazoa - ps12
Metazoa - ps13
Myriazoa - ps14
Parahoxozoa - ps15
Bilateria - ps16
Deuterostomia - ps17
Chordata - ps18
Olfactores - ps19
Vertebrata - ps20
Gnathostomata - ps21
Euteleostomi - ps22
Sarcopterygii - ps23
Tetrapoda - ps24
Amniota - ps25
Mammalia - ps26
Theria - ps27
Placentalia - ps28
Boreoeutheria - ps29
Euarchontoglires - ps30
Euarchonta - ps31
Primates - ps32
Haplorrhini - ps33
Simiiformes - ps34
Catarrhini - ps35
Hominoidea - ps36
Hominidae - ps37
Homininae - ps38
Hominini - ps39
Homo_sapiens - ps40
p-value
2e-02
2e-02
3e-02
4e-02
5e-02
Log-odds
0.5
1.0
1.5
2.0
2.5
3.0
3.5
4.0

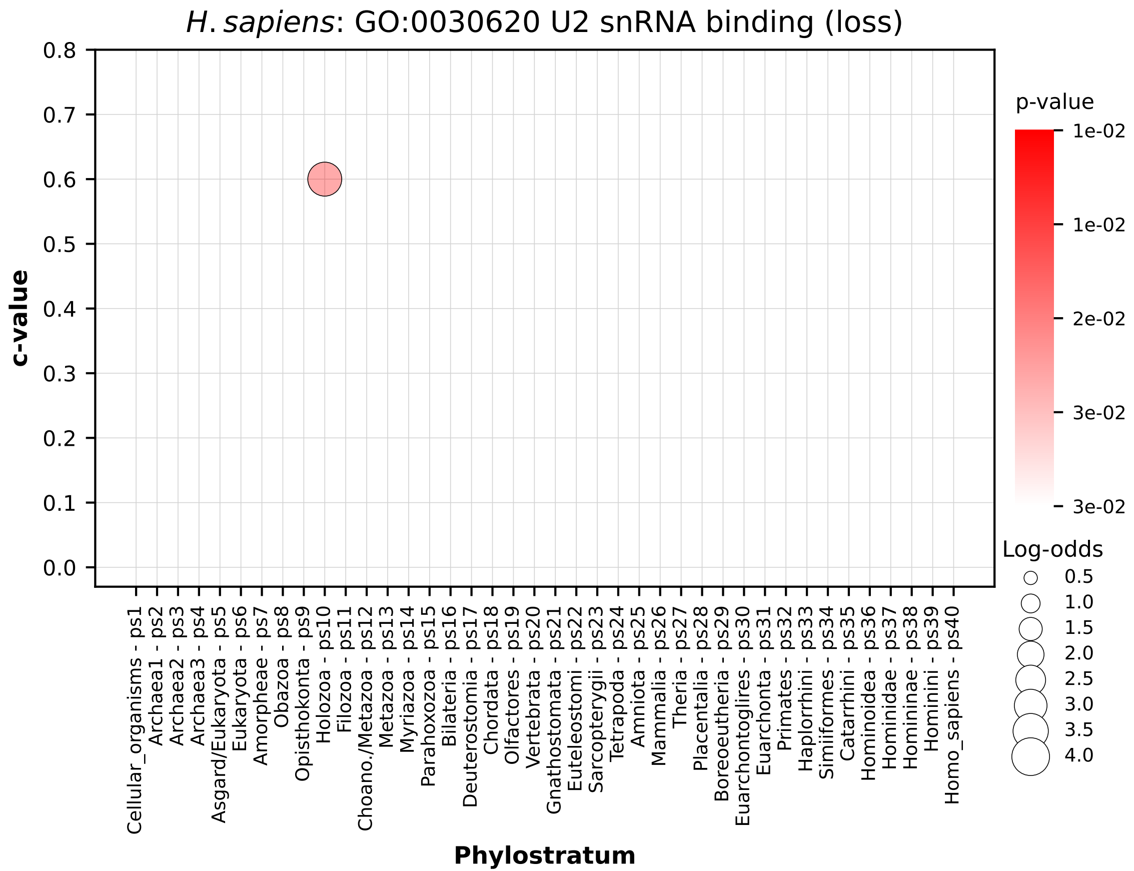

H. sapiens: GO:0030620 U2 snRNA binding (loss)
c-value
0.0
0.1
0.2
0.3
0.4
0.5
0.6
0.7
0.8
Phylostratum
Cellular_organisms - ps1
Archaea1 - ps2
Archaea2 - ps3
Archaea3 - ps4
Asgard/Eukaryota - ps5
Eukaryota - ps6
Amorpheae - ps7
Obazoa - ps8
Opisthokonta - ps9
Holozoa - ps10
Filozoa - ps11
Choano./Metazoa - ps12
Metazoa - ps13
Myriazoa - ps14
Parahoxozoa - ps15
Bilateria - ps16
Deuterostomia - ps17
Chordata - ps18
Olfactores - ps19
Vertebrata - ps20
Gnathostomata - ps21
Euteleostomi - ps22
Sarcopterygii - ps23
Tetrapoda - ps24
Amniota - ps25
Mammalia - ps26
Theria - ps27
Placentalia - ps28
Boreoeutheria - ps29
Euarchontoglires - ps30
Euarchonta - ps31
Primates - ps32
Haplorrhini - ps33
Simiiformes - ps34
Catarrhini - ps35
Hominoidea - ps36
Hominidae - ps37
Homininae - ps38
Hominini - ps39
Homo_sapiens - ps40
p-value
1e-02
1e-02
2e-02
3e-02
3e-02
Log-odds
0.5
1.0
1.5
2.0
2.5
3.0
3.5
4.0

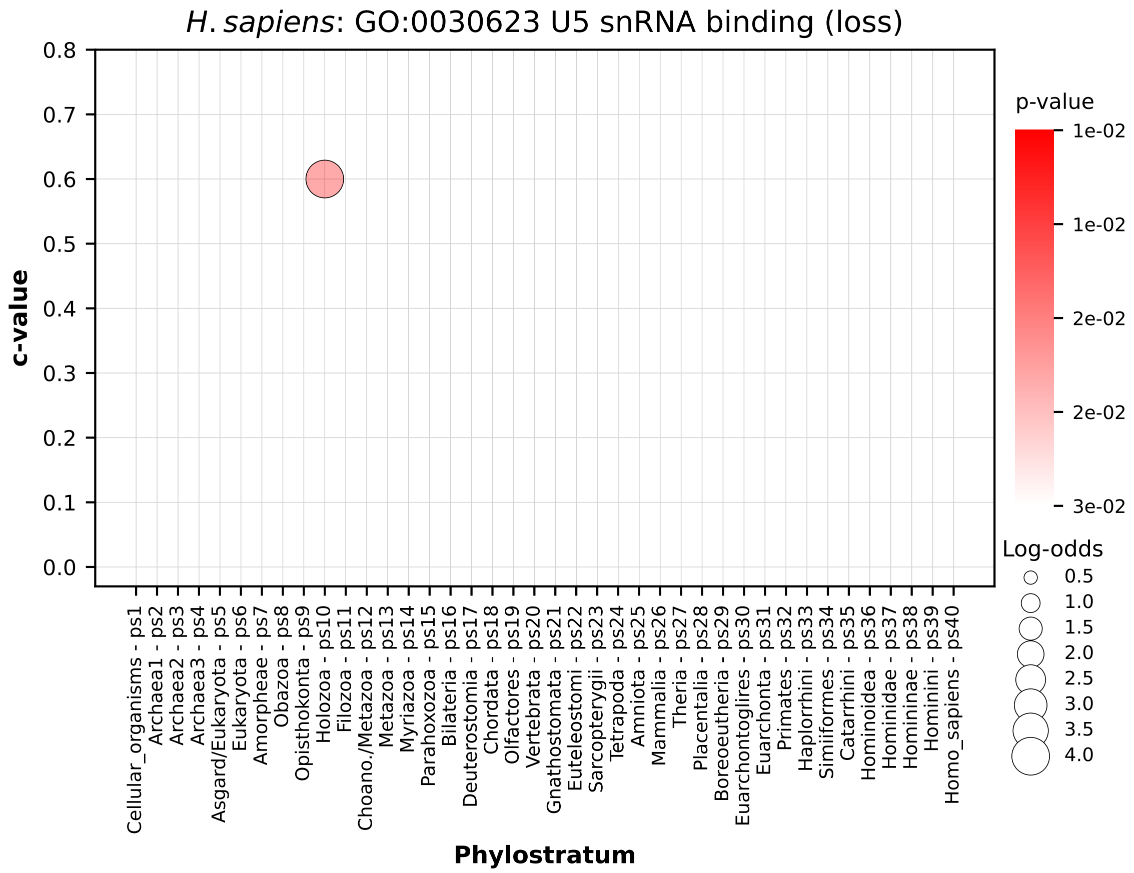

H. sapiens: GO:0030623 U5 snRNA binding (loss)
c-value
0.8
0.7
0.6
0.5
0.4
0.3
0.2
0.1
0.0
p-value
1e-02
1e-02
2e-02
2e-02
3e-02
Log-odds
0.5
1.0
1.5
2.0
2.5
3.0
3.5
4.0
Cellular_organisms - ps1
Archaea1 - ps2
Archaea2 - ps3
Archaea3 - ps4
Asgard/Eukaryota - ps5
Eukaryota - ps6
Amorpheae - ps7
Obazoa - ps8
Opisthokonta - ps9
Holozoa - ps10
Filozoa - ps11
Choano./Metazoa - ps12
Metazoa - ps13
Myriazoa - ps14
Parahoxozoa - ps15
Bilateria - ps16
Deuterostomia - ps17
Chordata - ps18
Olfactores - ps19
Vertebrata - ps20
Gnathostomata - ps21
Euteleostomi - ps22
Sarcopterygii - ps23
Tetrapoda - ps24
Amniota - ps25
Mammalia - ps26
Theria - ps27
Placentalia - ps28
Boreoeutheria - ps29
Euarchontoglires - ps30
Euarchonta - ps31
Primates - ps32
Haplorrhini - ps33
Simiiformes - ps34
Catarrhini - ps35
Hominoidea - ps36
Hominidae - ps37
Homininae - ps38
Hominini - ps39
Homo_sapiens - ps40
Phylostratum

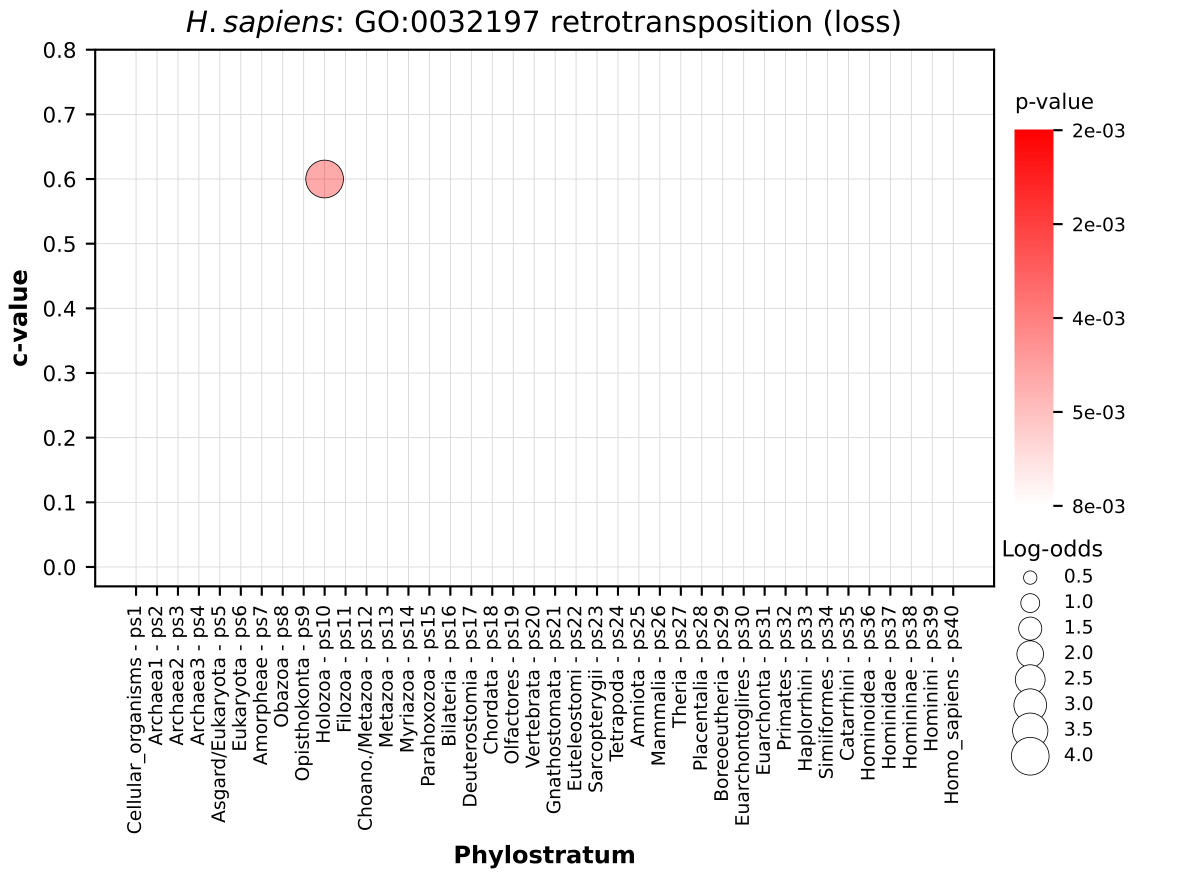

H. sapiens: GO:0032197 retrotransposition (loss)
c-value
0.8
0.7
0.6
0.5
0.4
0.3
0.2
0.1
0.0
p-value
2e-03
2e-03
4e-03
5e-03
8e-03
Log-odds
0.5
1.0
1.5
2.0
2.5
3.0
3.5
4.0
Cellular_organisms - ps1
Archaea1 - ps2
Archaea2 - ps3
Archaea3 - ps4
Asgard/Eukaryota - ps5
Eukaryota - ps6
Amorpheae - ps7
Obazoa - ps8
Opisthokonta - ps9
Holozoa - ps10
Filozoa - ps11
Choano./Metazoa - ps12
Metazoa - ps13
Myriazoa - ps14
Parahoxozoa - ps15
Bilateria - ps16
Deuterostomia - ps17
Chordata - ps18
Olfactores - ps19
Vertebrata - ps20
Gnathostomata - ps21
Euteleostomi - ps22
Sarcopterygii - ps23
Tetrapoda - ps24
Amniota - ps25
Mammalia - ps26
Theria - ps27
Placentalia - ps28
Boreoeutheria - ps29
Euarchontoglires - ps30
Euarchonta - ps31
Primates - ps32
Haplorrhini - ps33
Simiiformes - ps34
Catarrhini - ps35
Hominoidea - ps36
Hominidae - ps37
Homininae - ps38
Hominini - ps39
Homo_sapiens - ps40
Phylostratum

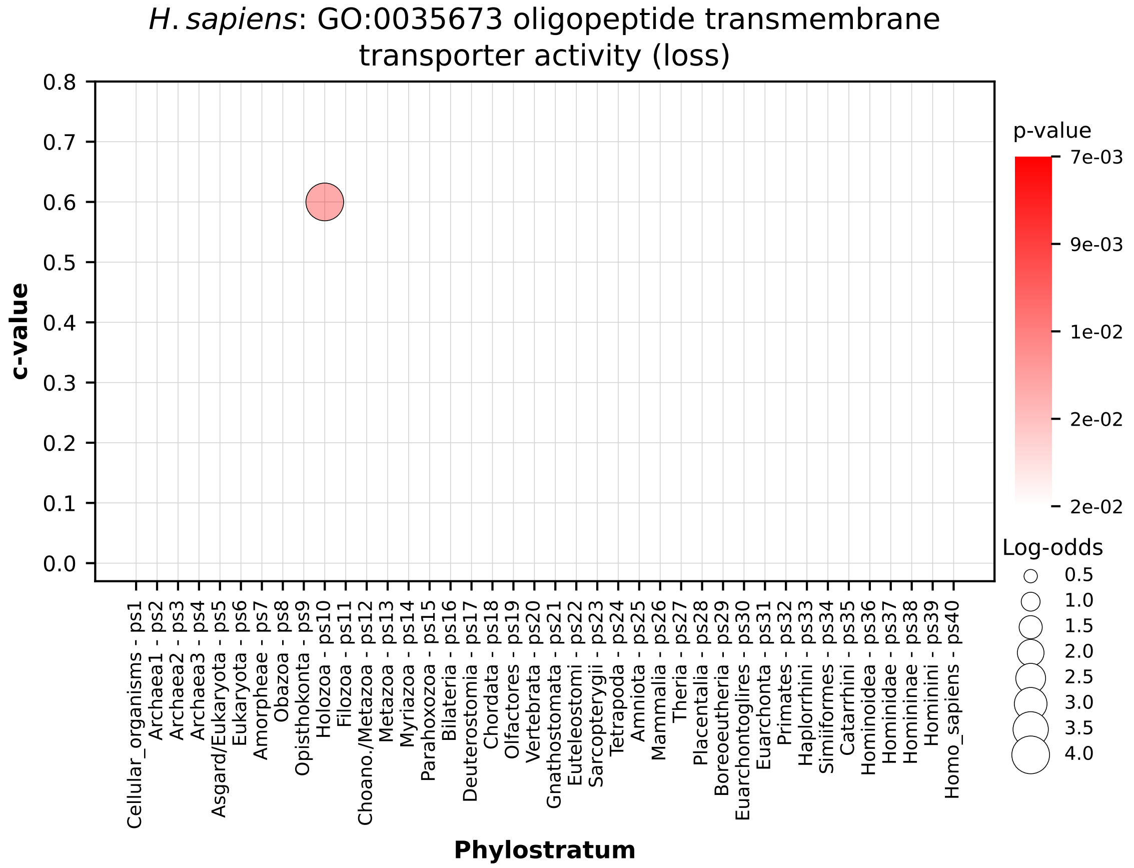

H. sapiens: GO:0035673 oligopeptide transmembrane transporter activity (loss)
c-value
0.0
0.1
0.2
0.3
0.4
0.5
0.6
0.7
0.8
Phylostratum
Cellular_organisms - ps1
Archaea1 - ps2
Archaea2 - ps3
Archaea3 - ps4
Asgard/Eukaryota - ps5
Eukaryota - ps6
Amorpheae - ps7
Obazoa - ps8
Opisthokonta - ps9
Holozoa - ps10
Filozoa - ps11
Choano./Metazoa - ps12
Metazoa - ps13
Myriazoa - ps14
Parahoxozoa - ps15
Bilateria - ps16
Deuterostomia - ps17
Chordata - ps18
Olfactores - ps19
Vertebrata - ps20
Gnathostomata - ps21
Euteleostomi - ps22
Sarcopterygii - ps23
Tetrapoda - ps24
Amniota - ps25
Mammalia - ps26
Theria - ps27
Placentalia - ps28
Boreoeutheria - ps29
Euarchontoglires - ps30
Euarchonta - ps31
Primates - ps32
Haplorrhini - ps33
Simiiformes - ps34
Catarrhini - ps35
Hominoidea - ps36
Hominidae - ps37
Homininae - ps38
Hominini - ps39
Homo_sapiens - ps40
p-value
7e-03
9e-03
1e-02
2e-02
2e-02
Log-odds
0.5
1.0
1.5
2.0
2.5
3.0
3.5
4.0

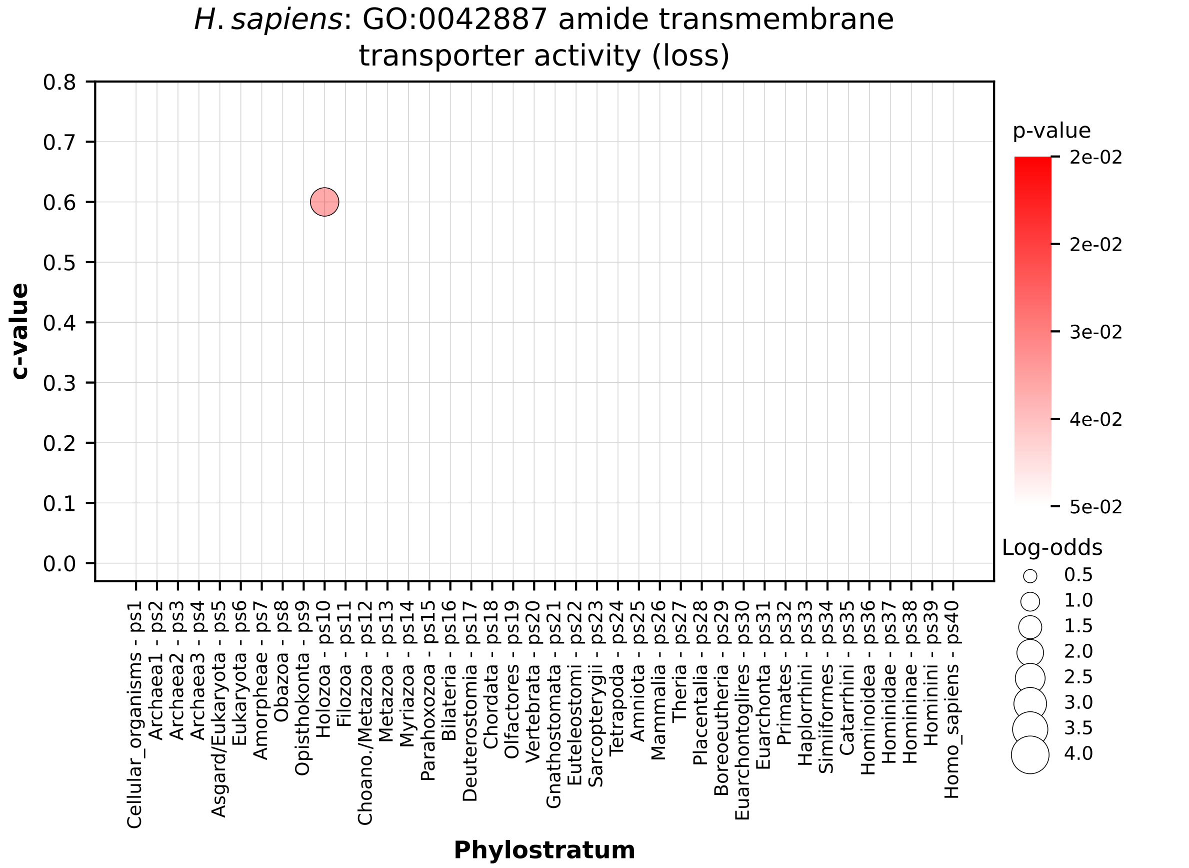
H. sapiens: GO:0042887 amide transmembrane transporter activity (loss)
c-value
Phylostratum
p-value
2e-02
2e-02
3e-02
4e-02
5e-02
Log-odds
0.5
1.0
1.5
2.0
2.5
3.0
3.5
4.0
0.0
0.1
0.2
0.3
0.4
0.5
0.6
0.7
0.8
Cellular_organisms - ps1
Archaea1 - ps2
Archaea2 - ps3
Archaea3 - ps4
Asgard/Eukaryota - ps5
Eukaryota - ps6
Amorpheae - ps7
Obazoa - ps8
Opisthokonta - ps9
Holozoa - ps10
Filozoa - ps11
Choano./Metazoa - ps12
Metazoa - ps13
Myriazoa - ps14
Parahoxozoa - ps15
Bilateria - ps16
Deuterostomia - ps17
Chordata - ps18
Olfactores - ps19
Vertebrata - ps20
Gnathostomata - ps21
Euteleostomi - ps22
Sarcopterygii - ps23
Tetrapoda - ps24
Amniota - ps25
Mammalia - ps26
Theria - ps27
Placentalia - ps28
Boreoeutheria - ps29
Euarchontoglires - ps30
Euarchonta - ps31
Primates - ps32
Haplorrhini - ps33
Simiiformes - ps34
Catarrhini - ps35
Hominoidea - ps36
Hominidae - ps37
Homininae - ps38
Hominini - ps39
Homo_sapiens - ps40

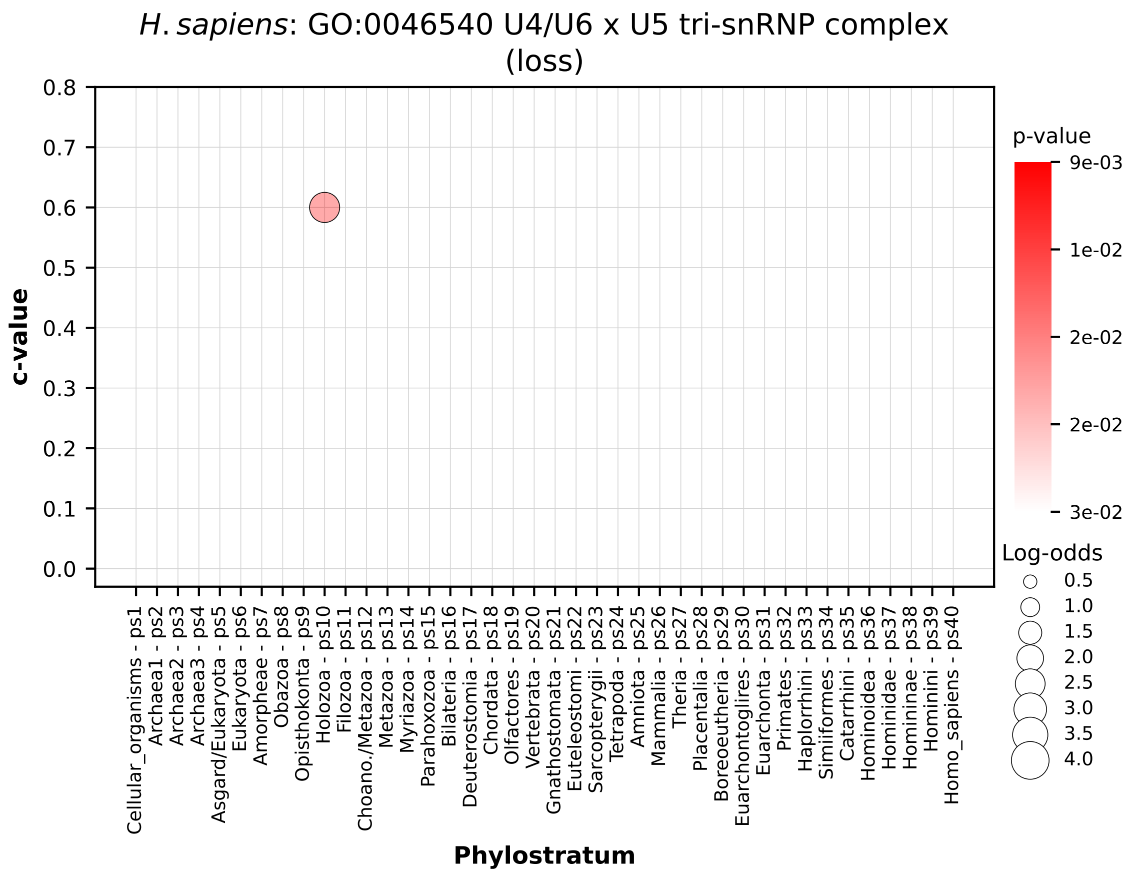
H. sapiens: GO:0046540 U4/U6 x U5 tri-snRNP complex (loss)
c-value
0.8
0.7
0.6
0.5
0.4
0.3
0.2
0.1
0.0
Phylostratum
Cellular_organisms - ps1
Archaea1 - ps2
Archaea2 - ps3
Archaea3 - ps4
Asgard/Eukaryota - ps5
Eukaryota - ps6
Amorpheae - ps7
Obazoa - ps8
Opisthokonta - ps9
Holozoa - ps10
Filozoa - ps11
Choano./Metazoa - ps12
Metazoa - ps13
Myriazoa - ps14
Parahoxozoa - ps15
Bilateria - ps16
Deuterostomia - ps17
Chordata - ps18
Olfactores - ps19
Vertebrata - ps20
Gnathostomata - ps21
Euteleostomi - ps22
Sarcopterygii - ps23
Tetrapoda - ps24
Amniota - ps25
Mammalia - ps26
Theria - ps27
Placentalia - ps28
Boreoeutheria - ps29
Euarchontoglires - ps30
Euarchonta - ps31
Primates - ps32
Haplorrhini - ps33
Simiiformes - ps34
Catarrhini - ps35
Hominoidea - ps36
Hominidae - ps37
Homininae - ps38
Hominini - ps39
Homo_sapiens - ps40
p-value
9e-03
1e-02
2e-02
2e-02
3e-02
Log-odds
0.5
1.0
1.5
2.0
2.5
3.0
3.5
4.0

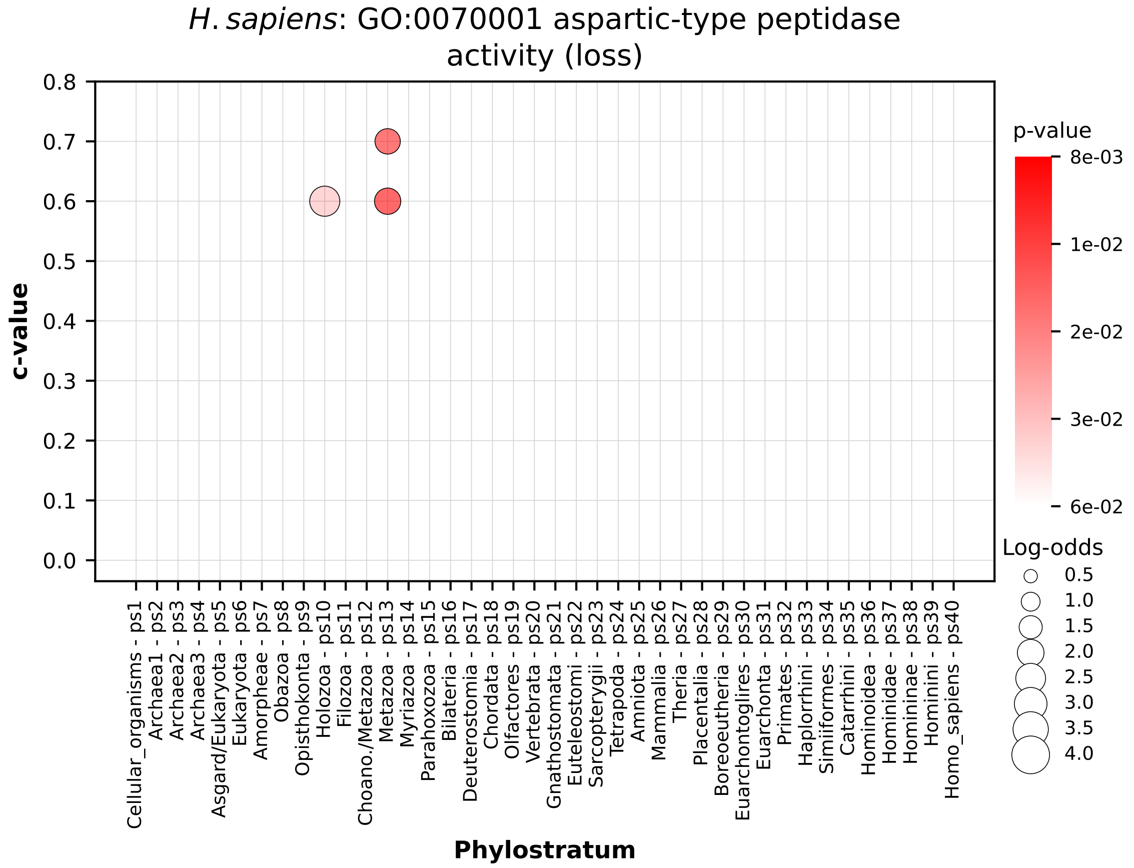

H. sapiens: GO:0070001 aspartic-type peptidase activity (loss)
c-value
0.8
0.7
0.6
0.5
0.4
0.3
0.2
0.1
0.0
p-value
8e-03
1e-02
2e-02
3e-02
6e-02
Log-odds
0.5
1.0
1.5
2.0
2.5
3.0
3.5
4.0
Cellular_organisms - ps1
Archaea1 - ps2
Archaea2 - ps3
Archaea3 - ps4
Asgard/Eukaryota - ps5
Eukaryota - ps6
Amorpheae - ps7
Obazoa - ps8
Opisthokonta - ps9
Holozoa - ps10
Filozoa - ps11
Choano./Metazoa - ps12
Metazoa - ps13
Myriazoa - ps14
Parahoxozoa - ps15
Bilateria - ps16
Deuterostomia - ps17
Chordata - ps18
Olfactores - ps19
Vertebrata - ps20
Gnathostomata - ps21
Euteleostomi - ps22
Sarcopterygii - ps23
Tetrapoda - ps24
Amniota - ps25
Mammalia - ps26
Theria - ps27
Placentalia - ps28
Boreoeutheria - ps29
Euarchontoglires - ps30
Euarchonta - ps31
Primates - ps32
Haplorrhini - ps33
Simiiformes - ps34
Catarrhini - ps35
Hominoidea - ps36
Hominidae - ps37
Homininae - ps38
Hominini - ps39
Homo_sapiens - ps40
Phylostratum

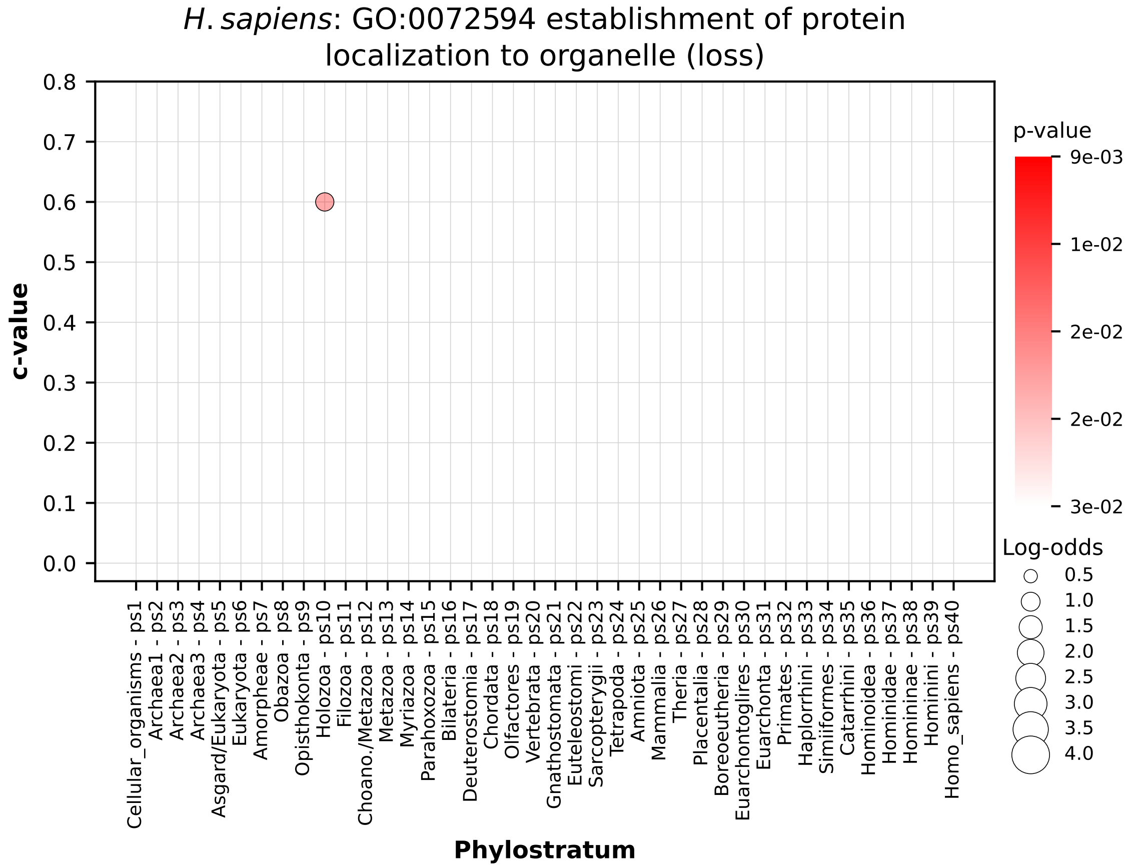

H. sapiens: GO:0072594 establishment of protein localization to organelle (loss)
c-value
0.0
0.1
0.2
0.3
0.4
0.5
0.6
0.7
0.8
Phylostratum
Cellular_organisms - ps1
Archaea1 - ps2
Archaea2 - ps3
Archaea3 - ps4
Asgard/Eukaryota - ps5
Eukaryota - ps6
Amorpheae - ps7
Obazoa - ps8
Opisthokonta - ps9
Holozoa - ps10
Filozoa - ps11
Choano./Metazoa - ps12
Metazoa - ps13
Myriazoa - ps14
Parahoxozoa - ps15
Bilateria - ps16
Deuterostomia - ps17
Chordata - ps18
Olfactores - ps19
Vertebrata - ps20
Gnathostomata - ps21
Euteleostomi - ps22
Sarcopterygii - ps23
Tetrapoda - ps24
Amniota - ps25
Mammalia - ps26
Theria - ps27
Placentalia - ps28
Boreoeutheria - ps29
Euarchontoglires - ps30
Euarchonta - ps31
Primates - ps32
Haplorrhini - ps33
Simiiformes - ps34
Catarrhini - ps35
Hominoidea - ps36
Hominidae - ps37
Homininae - ps38
Hominini - ps39
Homo_sapiens - ps40
p-value
9e-03
1e-02
2e-02
2e-02
3e-02
Log-odds
0.5
1.0
1.5
2.0
2.5
3.0
3.5
4.0

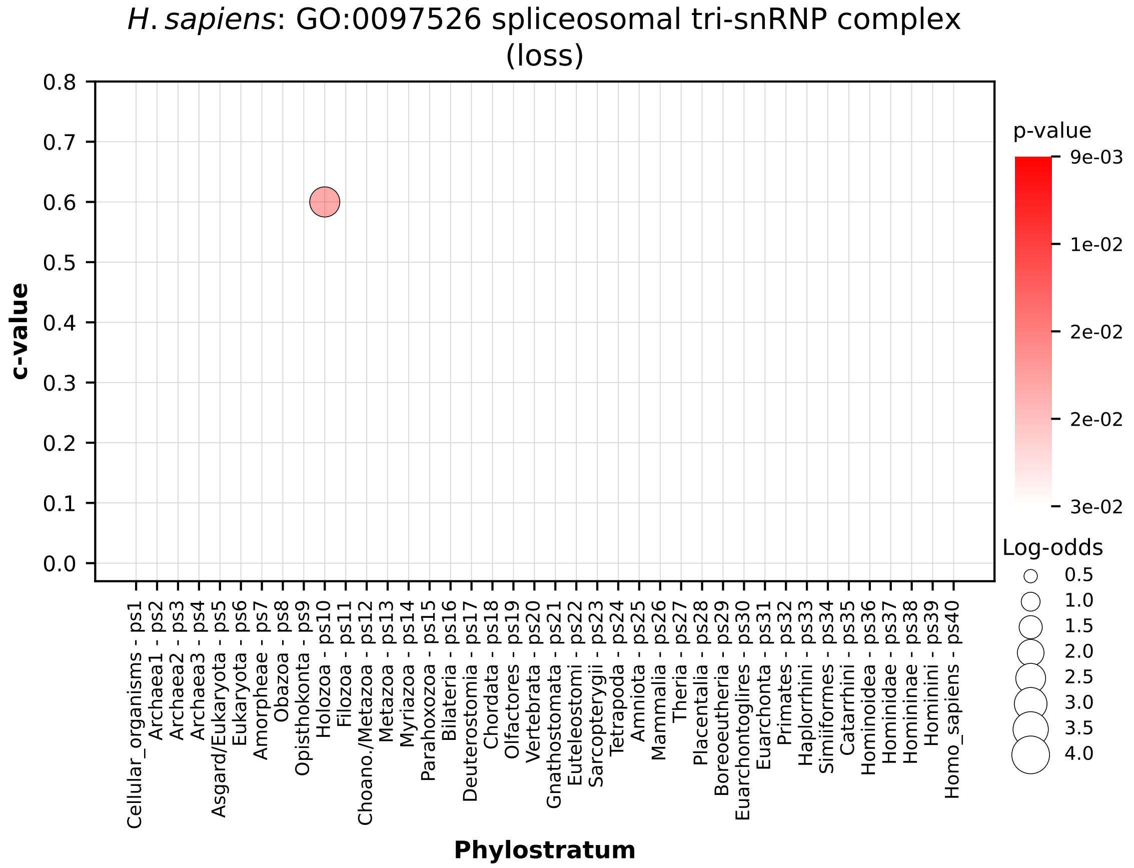
H. sapiens: GO:0097526 spliceosomal tri-snRNP complex (loss)
c-value
0.8
0.7
0.6
0.5
0.4
0.3
0.2
0.1
0.0
p-value
9e-03
1e-02
2e-02
2e-02
3e-02
Log-odds
0.5
1.0
1.5
2.0
2.5
3.0
3.5
4.0
Cellular_organisms - ps1
Archaea1 - ps2
Archaea2 - ps3
Archaea3 - ps4
Asgard/Eukaryota - ps5
Eukaryota - ps6
Amorpheae - ps7
Obazoa - ps8
Opisthokonta - ps9
Holozoa - ps10
Filozoa - ps11
Choano./Metazoa - ps12
Metazoa - ps13
Myriazoa - ps14
Parahoxozoa - ps15
Bilateria - ps16
Deuterostomia - ps17
Chordata - ps18
Olfactores - ps19
Vertebrata - ps20
Gnathostomata - ps21
Euteleostomi - ps22
Sarcopterygii - ps23
Tetrapoda - ps24
Amniota - ps25
Mammalia - ps26
Theria - ps27
Placentalia - ps28
Boreoeutheria - ps29
Euarchontoglires - ps30
Euarchonta - ps31
Primates - ps32
Haplorrhini - ps33
Simiiformes - ps34
Catarrhini - ps35
Hominoidea - ps36
Hominidae - ps37
Homininae - ps38
Hominini - ps39
Homo_sapiens - ps40
Phylostratum

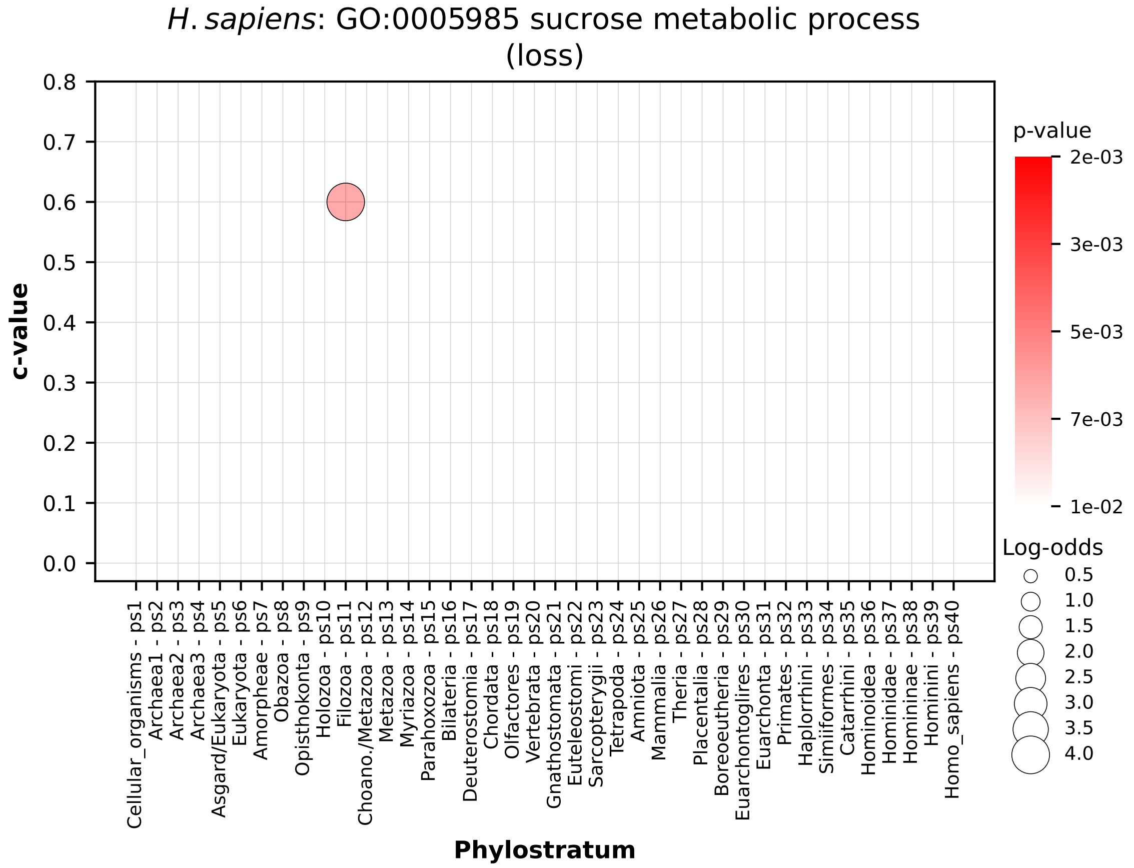

$H. sapiens$: GO:0005985 sucrose metabolic process
(loss)
c-value
0.8
0.7
0.6
0.5
0.4
0.3
0.2
0.1
0.0
p-value
2e-03
3e-03
5e-03
7e-03
1e-02
Log-odds
0.5
1.0
1.5
2.0
2.5
3.0
3.5
4.0
Cellular_organisms - ps1
Archaea1 - ps2
Archaea2 - ps3
Archaea3 - ps4
Asgard/Eukaryota - ps5
Eukaryota - ps6
Amorpheae - ps7
Obazoa - ps8
Opisthokonta - ps9
Holozoa - ps10
Filozoa - ps11
Choano./Metazoa - ps12
Metazoa - ps13
Myriazoa - ps14
Parahoxozoa - ps15
Bilateria - ps16
Deuterostomia - ps17
Chordata - ps18
Olfactores - ps19
Vertebrata - ps20
Gnathostomata - ps21
Euteleostomi - ps22
Sarcopterygii - ps23
Tetrapoda - ps24
Amniota - ps25
Mammalia - ps26
Theria - ps27
Placentalia - ps28
Boreoeutheria - ps29
Euarchontoglires - ps30
Euarchonta - ps31
Primates - ps32
Haplorrhini - ps33
Simiiformes - ps34
Catarrhini - ps35
Hominoidea - ps36
Hominidae - ps37
Homininae - ps38
Hominini - ps39
Homo_sapiens - ps40
Phylostratum

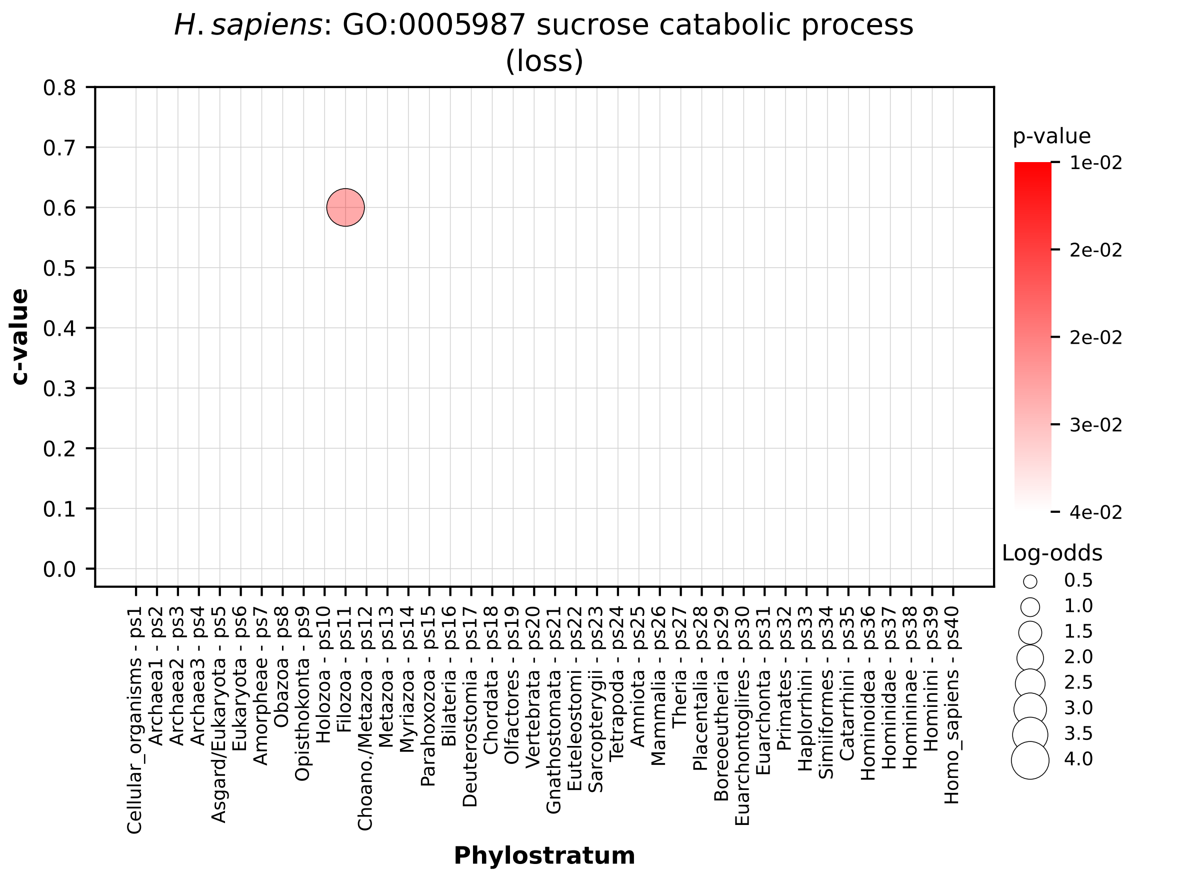

H. sapiens: GO:0005987 sucrose catabolic process
(loss)
c-value
0.8
0.7
0.6
0.5
0.4
0.3
0.2
0.1
0.0
p-value
1e-02
2e-02
2e-02
3e-02
4e-02
Log-odds
0.5
1.0
1.5
2.0
2.5
3.0
3.5
4.0
Cellular_organisms - ps1
Archaea1 - ps2
Archaea2 - ps3
Archaea3 - ps4
Asgard/Eukaryota - ps5
Eukaryota - ps6
Amorpheae - ps7
Obazoa - ps8
Opisthokonta - ps9
Holozoa - ps10
Filozoa - ps11
Choano./Metazoa - ps12
Metazoa - ps13
Myriazoa - ps14
Parahoxozoa - ps15
Bilateria - ps16
Deuterostomia - ps17
Chordata - ps18
Olfactores - ps19
Vertebrata - ps20
Gnathostomata - ps21
Euteleostomi - ps22
Sarcopterygii - ps23
Tetrapoda - ps24
Amniota - ps25
Mammalia - ps26
Theria - ps27
Placentalia - ps28
Boreoeutheria - ps29
Euarchontoglires - ps30
Euarchonta - ps31
Primates - ps32
Haplorrhini - ps33
Simiiformes - ps34
Catarrhini - ps35
Hominoidea - ps36
Hominidae - ps37
Homininae - ps38
Hominini - ps39
Homo_sapiens - ps40
Phylostratum

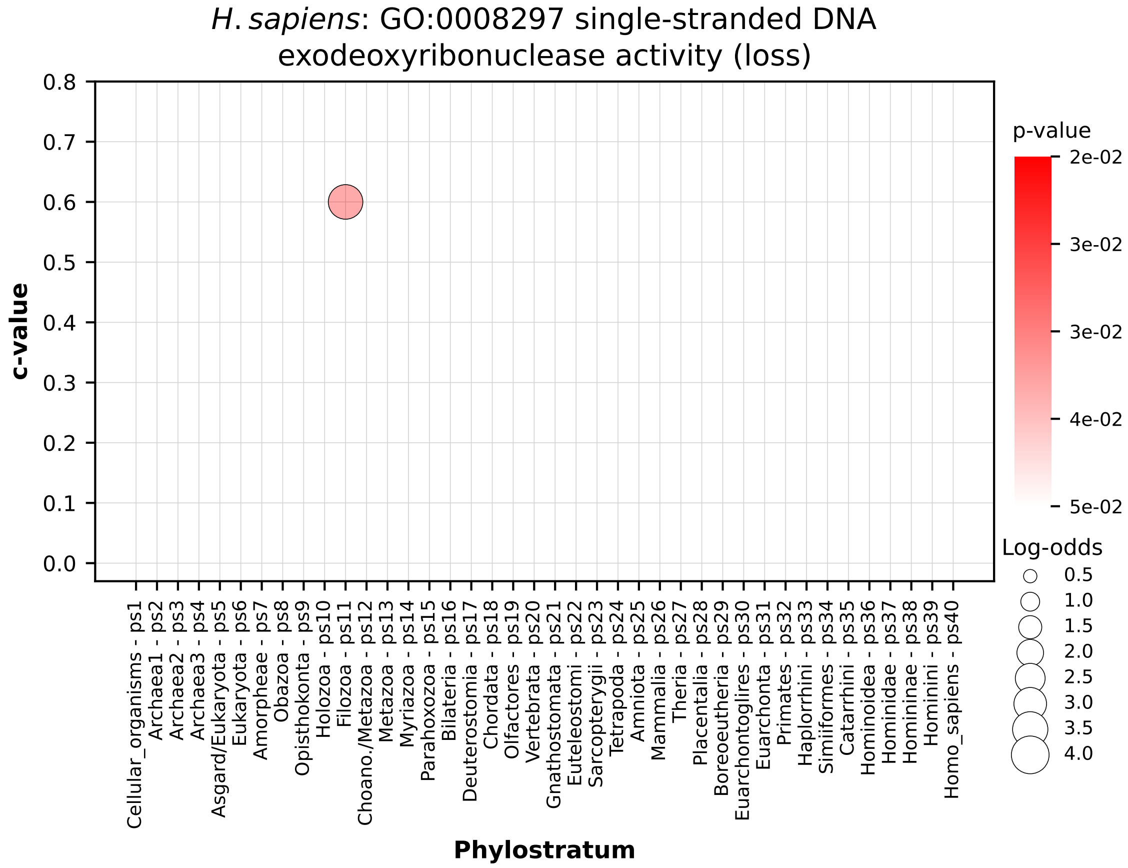

H. sapiens: GO:0008297 single-stranded DNA exodeoxyribonuclease activity (loss)
c-value
0.8
0.7
0.6
0.5
0.4
0.3
0.2
0.1
0.0
p-value
2e-02
3e-02
3e-02
4e-02
5e-02
Log-odds
0.5
1.0
1.5
2.0
2.5
3.0
3.5
4.0
Cellular_organisms - ps1
Archaea1 - ps2
Archaea2 - ps3
Archaea3 - ps4
Asgard/Eukaryota - ps5
Eukaryota - ps6
Amorpheae - ps7
Obazoa - ps8
Opisthokonta - ps9
Holozoa - ps10
Filozoa - ps11
Choano./Metazoa - ps12
Metazoa - ps13
Myriazoa - ps14
Parahoxozoa - ps15
Bilateria - ps16
Deuterostomia - ps17
Chordata - ps18
Olfactores - ps19
Vertebrata - ps20
Gnathostomata - ps21
Euteleostomi - ps22
Sarcopterygii - ps23
Tetrapoda - ps24
Amniota - ps25
Mammalia - ps26
Theria - ps27
Placentalia - ps28
Boreoeutheria - ps29
Euarchontoglires - ps30
Euarchonta - ps31
Primates - ps32
Haplorrhini - ps33
Simiiformes - ps34
Catarrhini - ps35
Hominoidea - ps36
Hominidae - ps37
Homininae - ps38
Hominini - ps39
Homo_sapiens - ps40
Phylostratum

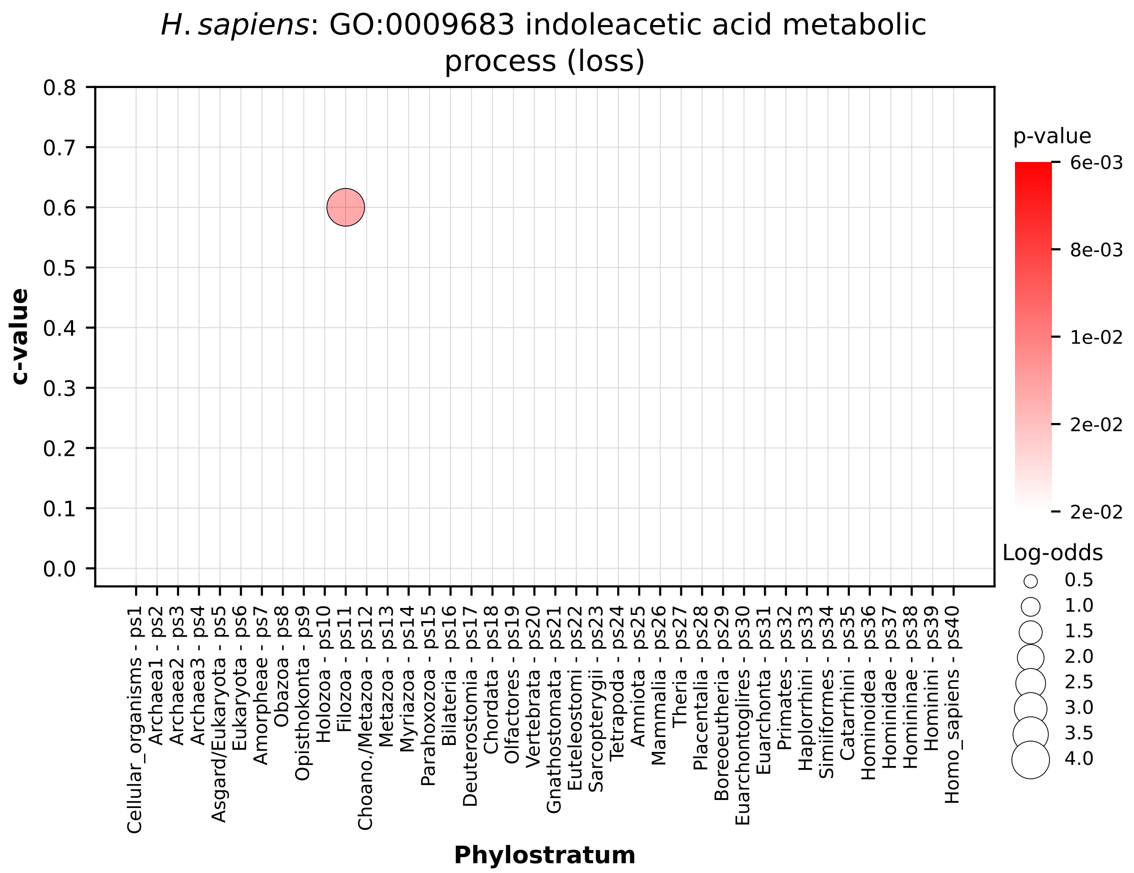

H. sapiens: GO:0009683 indoleacetic acid metabolic process (loss)
c-value
0.0
0.1
0.2
0.3
0.4
0.5
0.6
0.7
0.8
p-value
6e-03
8e-03
1e-02
2e-02
2e-02
Log-odds
0.5
1.0
1.5
2.0
2.5
3.0
3.5
4.0
Cellular_organisms - ps1
Archaea1 - ps2
Archaea2 - ps3
Archaea3 - ps4
Asgard/Eukaryota - ps5
Eukaryota - ps6
Amorpheae - ps7
Obazoa - ps8
Opisthokonta - ps9
Holozoa - ps10
Filozoa - ps11
Choano./Metazoa - ps12
Metazoa - ps13
Myriazoa - ps14
Parahoxozoa - ps15
Bilateria - ps16
Deuterostomia - ps17
Chordata - ps18
Olfactores - ps19
Vertebrata - ps20
Gnathostomata - ps21
Euteleostomi - ps22
Sarcopterygii - ps23
Tetrapoda - ps24
Amniota - ps25
Mammalia - ps26
Theria - ps27
Placentalia - ps28
Boreoeutheria - ps29
Euarchontoglires - ps30
Euarchonta - ps31
Primates - ps32
Haplorrhini - ps33
Simiiformes - ps34
Catarrhini - ps35
Hominoidea - ps36
Hominidae - ps37
Homininae - ps38
Hominini - ps39
Homo_sapiens - ps40
Phylostratum

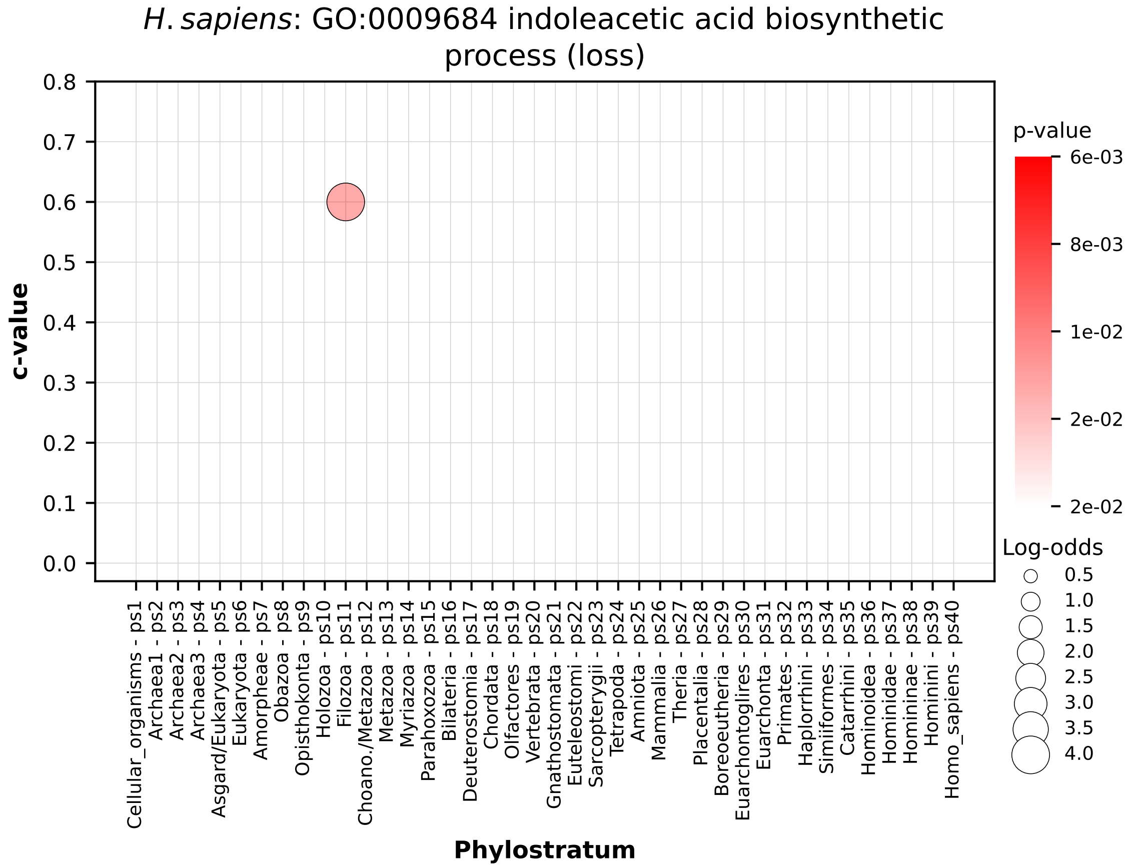

H. sapiens: GO:0009684 indoleacetic acid biosynthetic process (loss)
c-value
0.8
0.7
0.6
0.5
0.4
0.3
0.2
0.1
0.0
p-value
6e-03
8e-03
1e-02
2e-02
2e-02
Log-odds
0.5
1.0
1.5
2.0
2.5
3.0
3.5
4.0
Cellular_organisms - ps1
Archaea1 - ps2
Archaea2 - ps3
Archaea3 - ps4
Asgard/Eukaryota - ps5
Eukaryota - ps6
Amorpheae - ps7
Obazoa - ps8
Opisthokonta - ps9
Holozoa - ps10
Filozoa - ps11
Choano./Metazoa - ps12
Metazoa - ps13
Myriazoa - ps14
Parahoxozoa - ps15
Bilateria - ps16
Deuterostomia - ps17
Chordata - ps18
Olfactores - ps19
Vertebrata - ps20
Gnathostomata - ps21
Euteleostomi - ps22
Sarcopterygii - ps23
Tetrapoda - ps24
Amniota - ps25
Mammalia - ps26
Theria - ps27
Placentalia - ps28
Boreoeutheria - ps29
Euarchontoglires - ps30
Euarchonta - ps31
Primates - ps32
Haplorrhini - ps33
Simiiformes - ps34
Catarrhini - ps35
Hominoidea - ps36
Hominidae - ps37
Homininae - ps38
Hominini - ps39
Homo_sapiens - ps40
Phylostratum

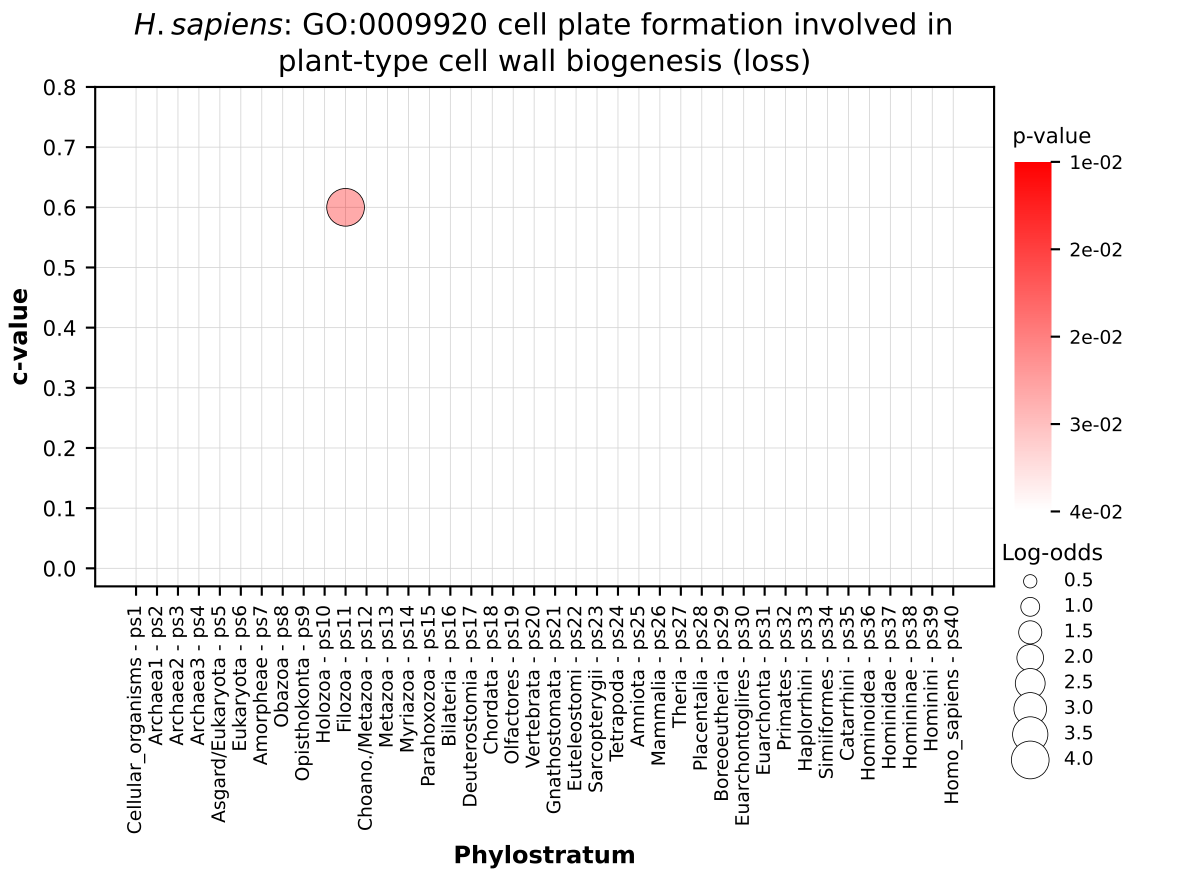

H. sapiens: GO:0009920 cell plate formation involved in plant-type cell wall biogenesis (loss)
c-value
0.8
0.7
0.6
0.5
0.4
0.3
0.2
0.1
0.0
Phylostratum
Cellular_organisms - ps1
Archaea1 - ps2
Archaea2 - ps3
Archaea3 - ps4
Asgard/Eukaryota - ps5
Eukaryota - ps6
Amorpheae - ps7
Obazoa - ps8
Opisthokonta - ps9
Holozoa - ps10
Filozoa - ps11
Choano./Metazoa - ps12
Metazoa - ps13
Myriazoa - ps14
Parahoxozoa - ps15
Bilateria - ps16
Deuterostomia - ps17
Chordata - ps18
Olfactores - ps19
Vertebrata - ps20
Gnathostomata - ps21
Euteleostomi - ps22
Sarcopterygii - ps23
Tetrapoda - ps24
Amniota - ps25
Mammalia - ps26
Theria - ps27
Placentalia - ps28
Boreoeutheria - ps29
Euarchontoglires - ps30
Euarchonta - ps31
Primates - ps32
Haplorrhini - ps33
Simiiformes - ps34
Catarrhini - ps35
Hominoidea - ps36
Hominidae - ps37
Homininae - ps38
Hominini - ps39
Homo_sapiens - ps40
p-value
1e-02
2e-02
2e-02
3e-02
4e-02
Log-odds
0.5
1.0
1.5
2.0
2.5
3.0
3.5
4.0

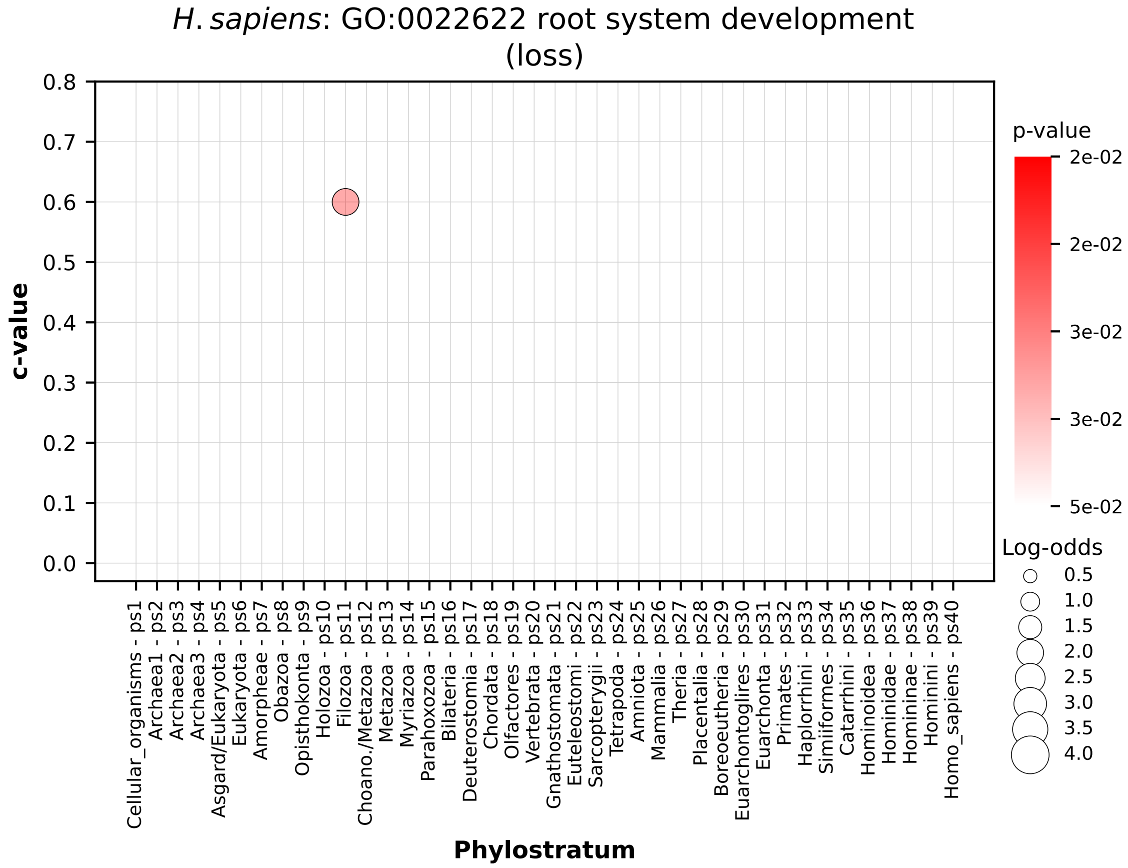

H. sapiens: GO:0022622 root system development
(loss)
c-value
0.8
0.7
0.6
0.5
0.4
0.3
0.2
0.1
0.0
p-value
2e-02
2e-02
3e-02
3e-02
5e-02
Log-odds
0.5
1.0
1.5
2.0
2.5
3.0
3.5
4.0
Cellular_organisms - ps1
Archaea1 - ps2
Archaea2 - ps3
Archaea3 - ps4
Asgard/Eukaryota - ps5
Eukaryota - ps6
Amorpheae - ps7
Obazoa - ps8
Opisthokonta - ps9
Holozoa - ps10
Filozoa - ps11
Choano./Metazoa - ps12
Metazoa - ps13
Myriazoa - ps14
Parahoxozoa - ps15
Bilateria - ps16
Deuterostomia - ps17
Chordata - ps18
Olfactores - ps19
Vertebrata - ps20
Gnathostomata - ps21
Euteleostomi - ps22
Sarcopterygii - ps23
Tetrapoda - ps24
Amniota - ps25
Mammalia - ps26
Theria - ps27
Placentalia - ps28
Boreoeutheria - ps29
Euarchontoglires - ps30
Euarchonta - ps31
Primates - ps32
Haplorrhini - ps33
Simiiformes - ps34
Catarrhini - ps35
Hominoidea - ps36
Hominidae - ps37
Homininae - ps38
Hominini - ps39
Homo_sapiens - ps40
Phylostratum

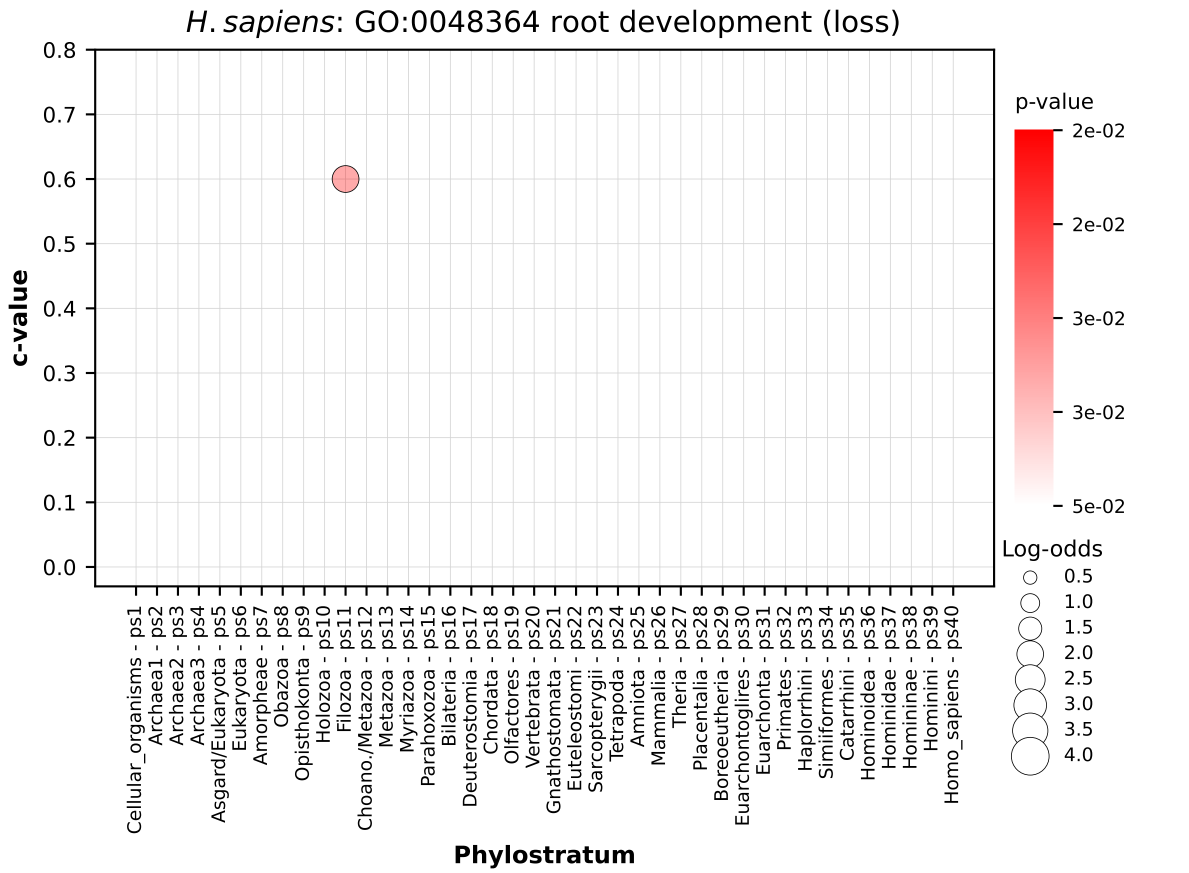

H. sapiens: GO:0048364 root development (loss)
c-value
0.8
0.7
0.6
0.5
0.4
0.3
0.2
0.1
0.0
Phylostratum
Cellular_organisms - ps1
Archaea1 - ps2
Archaea2 - ps3
Archaea3 - ps4
Asgard/Eukaryota - ps5
Eukaryota - ps6
Amorpheae - ps7
Obazoa - ps8
Opisthokonta - ps9
Holozoa - ps10
Filozoa - ps11
Choano./Metazoa - ps12
Metazoa - ps13
Myriazoa - ps14
Parahoxozoa - ps15
Bilateria - ps16
Deuterostomia - ps17
Chordata - ps18
Olfactores - ps19
Vertebrata - ps20
Gnathostomata - ps21
Euteleostomi - ps22
Sarcopterygii - ps23
Tetrapoda - ps24
Amniota - ps25
Mammalia - ps26
Theria - ps27
Placentalia - ps28
Boreoeutheria - ps29
Euarchontoglires - ps30
Euarchonta - ps31
Primates - ps32
Haplorrhini - ps33
Simiiformes - ps34
Catarrhini - ps35
Hominoidea - ps36
Hominidae - ps37
Homininae - ps38
Hominini - ps39
Homo_sapiens - ps40
p-value
2e-02
2e-02
3e-02
3e-02
5e-02
Log-odds
0.5
1.0
1.5
2.0
2.5
3.0
3.5
4.0

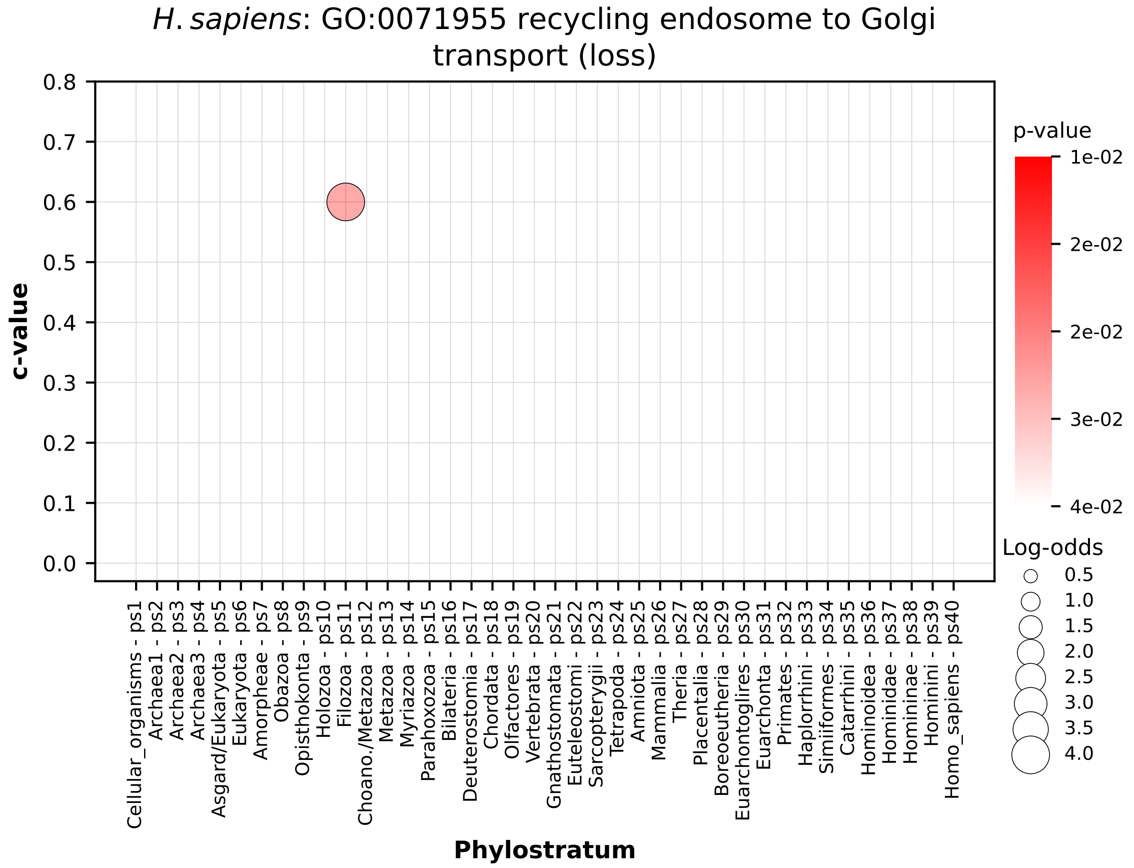

H. sapiens: GO:0071955 recycling endosome to Golgi transport (loss)
c-value
0.8
0.7
0.6
0.5
0.4
0.3
0.2
0.1
0.0
p-value
1e-02
2e-02
2e-02
3e-02
4e-02
Log-odds
0.5
1.0
1.5
2.0
2.5
3.0
3.5
4.0
Cellular_organisms - ps1
Archaea1 - ps2
Archaea2 - ps3
Archaea3 - ps4
Asgard/Eukaryota - ps5
Eukaryota - ps6
Amorpheae - ps7
Obazoa - ps8
Opisthokonta - ps9
Holozoa - ps10
Filozoa - ps11
Choano./Metazoa - ps12
Metazoa - ps13
Myriazoa - ps14
Parahoxozoa - ps15
Bilateria - ps16
Deuterostomia - ps17
Chordata - ps18
Olfactores - ps19
Vertebrata - ps20
Gnathostomata - ps21
Euteleostomi - ps22
Sarcopterygii - ps23
Tetrapoda - ps24
Amniota - ps25
Mammalia - ps26
Theria - ps27
Placentalia - ps28
Boreoeutheria - ps29
Euarchontoglires - ps30
Euarchonta - ps31
Primates - ps32
Haplorrhini - ps33
Simiiformes - ps34
Catarrhini - ps35
Hominoidea - ps36
Hominidae - ps37
Homininae - ps38
Hominini - ps39
Homo_sapiens - ps40
Phylostratum

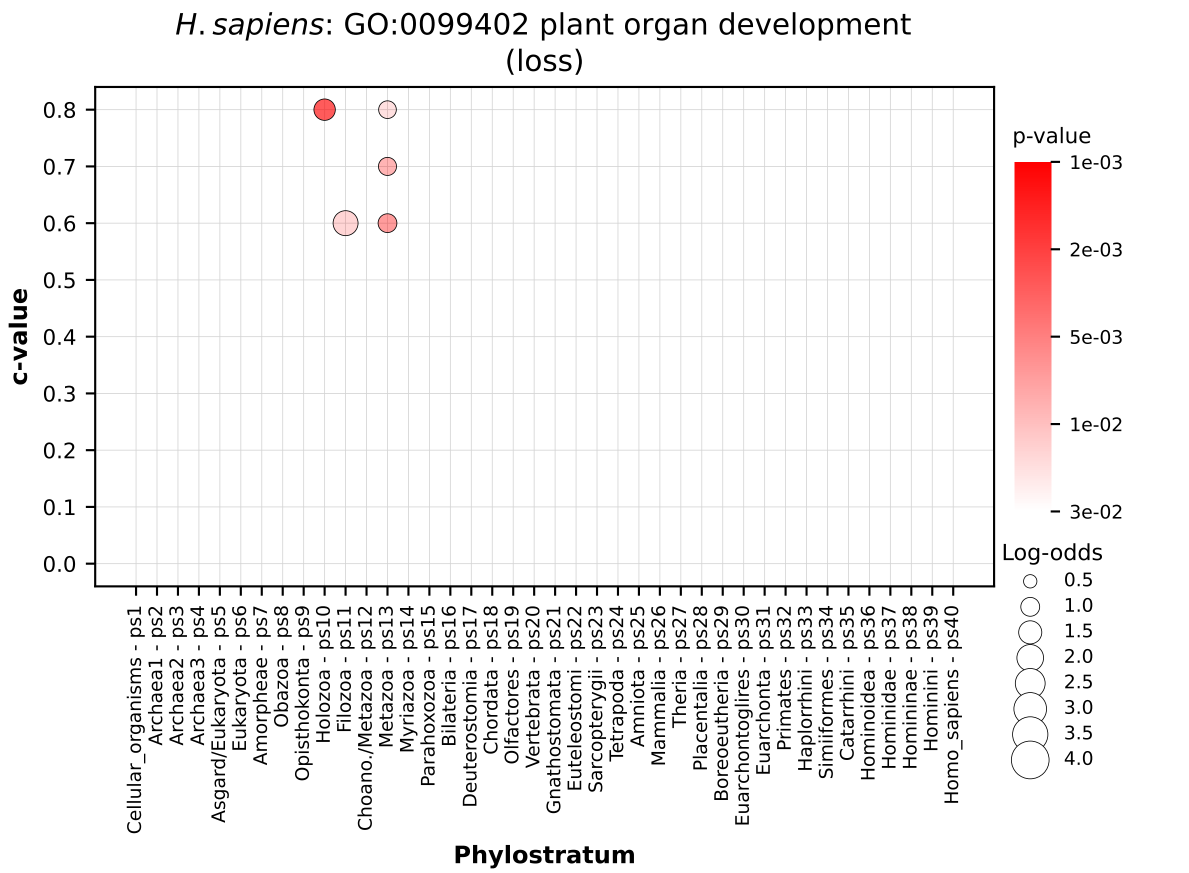

H. sapiens: GO:0099402 plant organ development
(loss)
c-value
0.0
0.1
0.2
0.3
0.4
0.5
0.6
0.7
0.8
Phylostratum
Cellular_organisms - ps1
Archaea1 - ps2
Archaea2 - ps3
Archaea3 - ps4
Asgard/Eukaryota - ps5
Eukaryota - ps6
Amorpheae - ps7
Obazoa - ps8
Opisthokonta - ps9
Holozoa - ps10
Filozoa - ps11
Choano./Metazoa - ps12
Metazoa - ps13
Myriazoa - ps14
Parahoxozoa - ps15
Bilateria - ps16
Deuterostomia - ps17
Chordata - ps18
Olfactores - ps19
Vertebrata - ps20
Gnathostomata - ps21
Euteleostomi - ps22
Sarcopterygii - ps23
Tetrapoda - ps24
Amniota - ps25
Mammalia - ps26
Theria - ps27
Placentalia - ps28
Boreoeutheria - ps29
Euarchontoglires - ps30
Euarchonta - ps31
Primates - ps32
Haplorrhini - ps33
Simiiformes - ps34
Catarrhini - ps35
Hominoidea - ps36
Hominidae - ps37
Homininae - ps38
Hominini - ps39
Homo_sapiens - ps40
p-value
1e-03
2e-03
5e-03
1e-02
3e-02
Log-odds
0.5
1.0
1.5
2.0
2.5
3.0
3.5
4.0

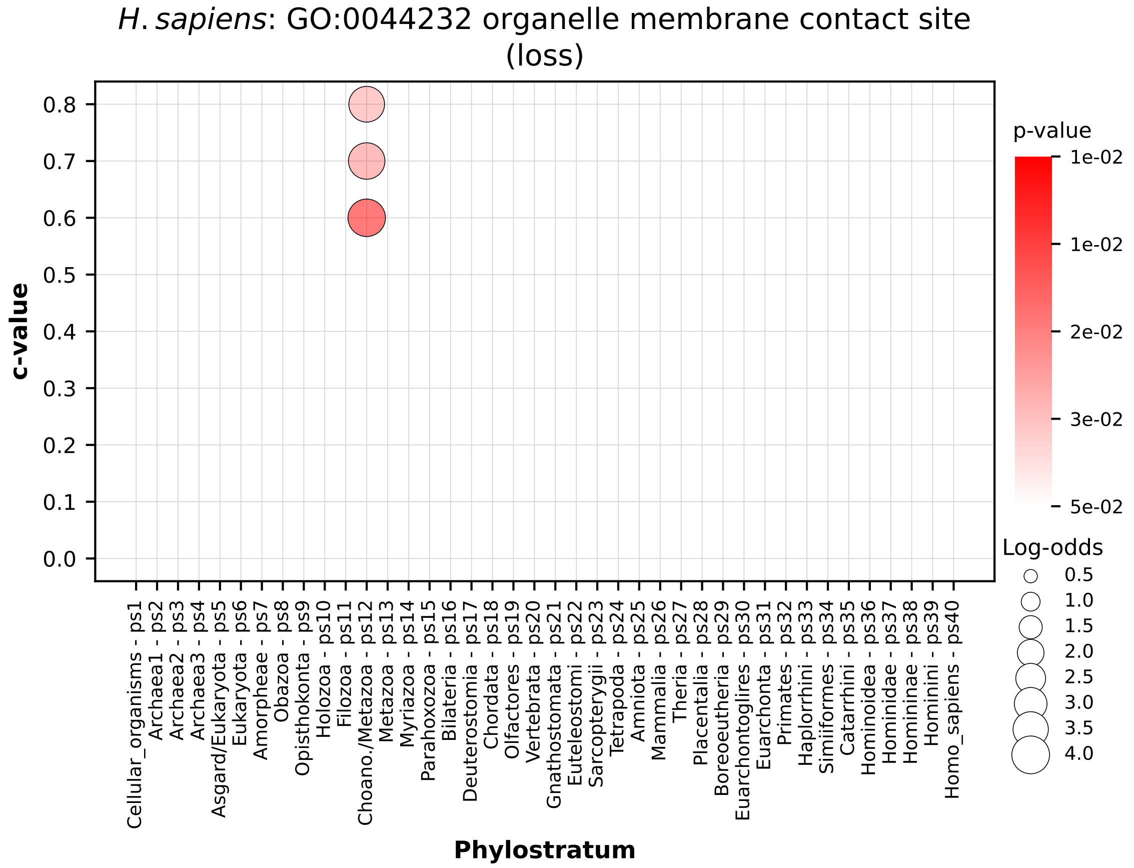
H. sapiens: GO:0044232 organelle membrane contact site
(loss)
c-value
0.0
0.1
0.2
0.3
0.4
0.5
0.6
0.7
0.8
p-value
1e-02
1e-02
2e-02
3e-02
5e-02
Log-odds
0.5
1.0
1.5
2.0
2.5
3.0
3.5
4.0
Cellular_organisms - ps1
Archaea1 - ps2
Archaea2 - ps3
Archaea3 - ps4
Asgard/Eukaryota - ps5
Eukaryota - ps6
Amorpheae - ps7
Obazoa - ps8
Opisthokonta - ps9
Holozoa - ps10
Filozoa - ps11
Choano./Metazoa - ps12
Metazoa - ps13
Myriazoa - ps14
Parahoxozoa - ps15
Bilateria - ps16
Deuterostomia - ps17
Chordata - ps18
Olfactores - ps19
Vertebrata - ps20
Gnathostomata - ps21
Euteleostomi - ps22
Sarcopterygii - ps23
Tetrapoda - ps24
Amniota - ps25
Mammalia - ps26
Theria - ps27
Placentalia - ps28
Boreoeutheria - ps29
Euarchontoglires - ps30
Euarchonta - ps31
Primates - ps32
Haplorrhini - ps33
Simiiformes - ps34
Catarrhini - ps35
Hominoidea - ps36
Hominidae - ps37
Homininae - ps38
Hominini - ps39
Homo_sapiens - ps40
Phylostratum

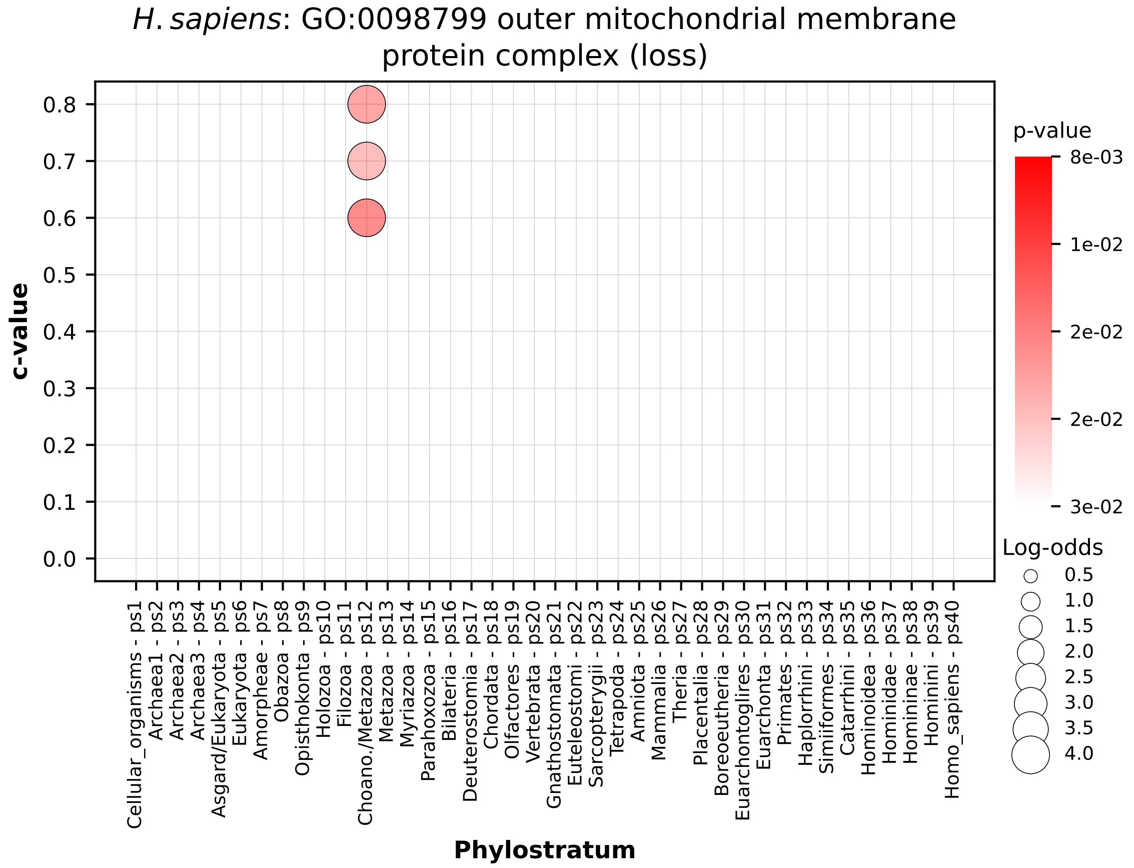

H. sapiens: GO:0098799 outer mitochondrial membrane protein complex (loss)
c-value
0.8
0.7
0.6
0.5
0.4
0.3
0.2
0.1
0.0
p-value
8e-03
1e-02
2e-02
2e-02
3e-02
Log-odds
0.5
1.0
1.5
2.0
2.5
3.0
3.5
4.0
Cellular_organisms - ps1
Archaea1 - ps2
Archaea2 - ps3
Archaea3 - ps4
Asgard/Eukaryota - ps5
Eukaryota - ps6
Amorpheae - ps7
Obazoa - ps8
Opisthokonta - ps9
Holozoa - ps10
Filozoa - ps11
Choano./Metazoa - ps12
Metazoa - ps13
Myriazoa - ps14
Parahoxozoa - ps15
Bilateria - ps16
Deuterostomia - ps17
Chordata - ps18
Olfactores - ps19
Vertebrata - ps20
Gnathostomata - ps21
Euteleostomi - ps22
Sarcopterygii - ps23
Tetrapoda - ps24
Amniota - ps25
Mammalia - ps26
Theria - ps27
Placentalia - ps28
Boreoeutheria - ps29
Euarchontoglires - ps30
Euarchonta - ps31
Primates - ps32
Haplorrhini - ps33
Simiiformes - ps34
Catarrhini - ps35
Hominoidea - ps36
Hominidae - ps37
Homininae - ps38
Hominini - ps39
Homo_sapiens - ps40
Phylostratum

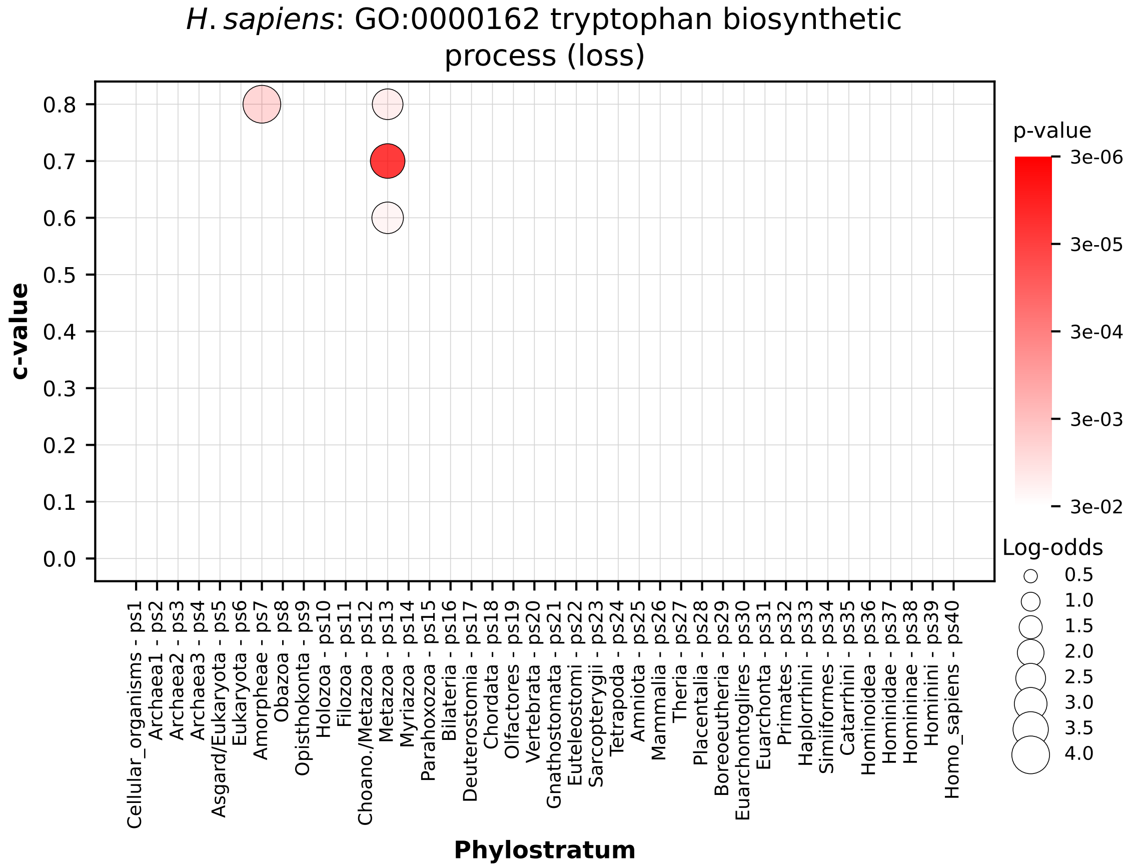

H. sapiens: GO:0000162 tryptophan biosynthetic process (loss)
c-value
0.0
0.1
0.2
0.3
0.4
0.5
0.6
0.7
0.8
Phylostratum
Cellular_organisms - ps1
Archaea1 - ps2
Archaea2 - ps3
Archaea3 - ps4
Asgard/Eukaryota - ps5
Eukaryota - ps6
Amorpheae - ps7
Obazoa - ps8
Opisthokonta - ps9
Holozoa - ps10
Filozoa - ps11
Choano./Metazoa - ps12
Metazoa - ps13
Myriazoa - ps14
Parahoxozoa - ps15
Bilateria - ps16
Deuterostomia - ps17
Chordata - ps18
Olfactores - ps19
Vertebrata - ps20
Gnathostomata - ps21
Euteleostomi - ps22
Sarcopterygii - ps23
Tetrapoda - ps24
Amniota - ps25
Mammalia - ps26
Theria - ps27
Placentalia - ps28
Boreoeutheria - ps29
Euarchontoglires - ps30
Euarchonta - ps31
Primates - ps32
Haplorrhini - ps33
Simiiformes - ps34
Catarrhini - ps35
Hominoidea - ps36
Hominidae - ps37
Homininae - ps38
Hominini - ps39
Homo_sapiens - ps40
p-value
3e-06
3e-05
3e-04
3e-03
3e-02
Log-odds
0.5
1.0
1.5
2.0
2.5
3.0
3.5
4.0

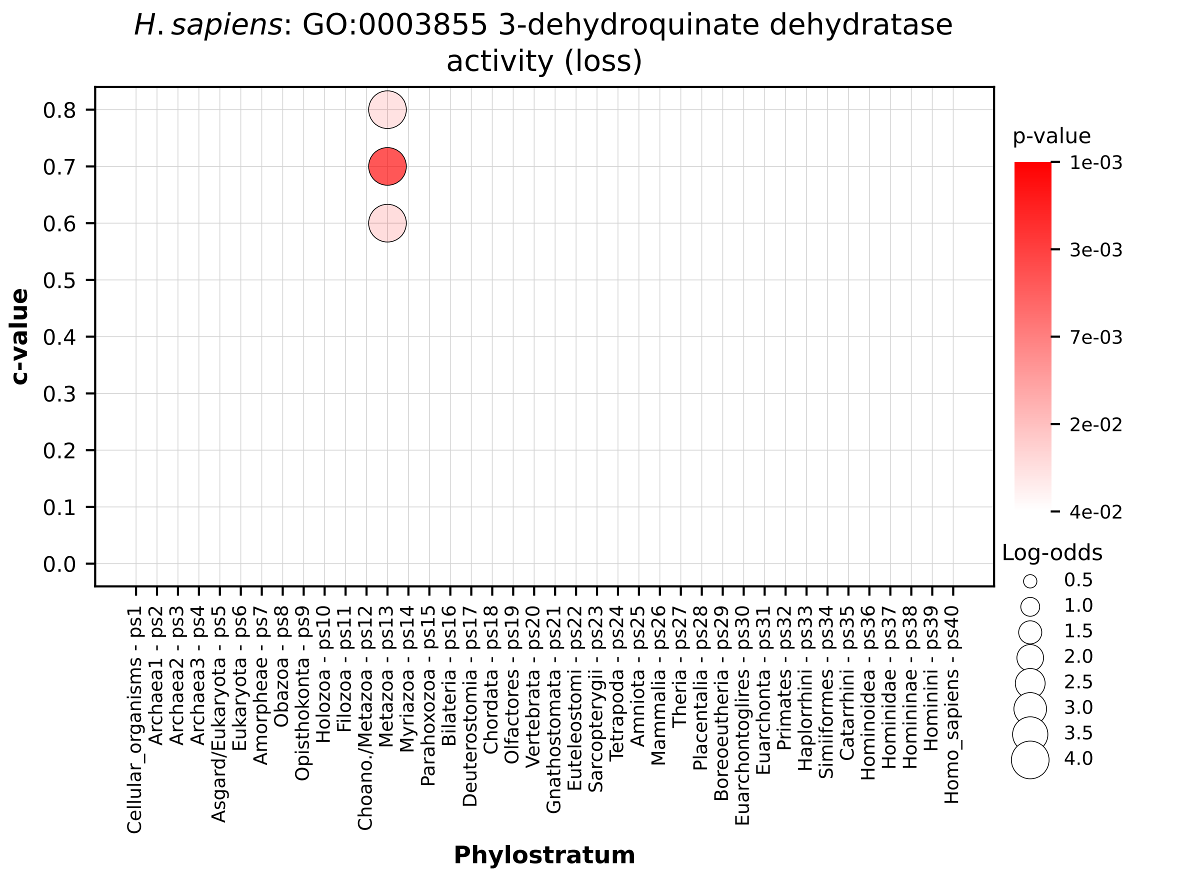
H. sapiens: GO:0003855 3-dehydroquinate dehydratase activity (loss)
c-value
0.0
0.1
0.2
0.3
0.4
0.5
0.6
0.7
0.8
Phylostratum
Cellular_organisms - ps1
Archaea1 - ps2
Archaea2 - ps3
Archaea3 - ps4
Asgard/Eukaryota - ps5
Eukaryota - ps6
Amorpheae - ps7
Obazoa - ps8
Opisthokonta - ps9
Holozoa - ps10
Filozoa - ps11
Choano./Metazoa - ps12
Metazoa - ps13
Myriazoa - ps14
Parahoxozoa - ps15
Bilateria - ps16
Deuterostomia - ps17
Chordata - ps18
Olfactores - ps19
Vertebrata - ps20
Gnathostomata - ps21
Euteleostomi - ps22
Sarcopterygii - ps23
Tetrapoda - ps24
Amniota - ps25
Mammalia - ps26
Theria - ps27
Placentalia - ps28
Boreoeutheria - ps29
Euarchontoglires - ps30
Euarchonta - ps31
Primates - ps32
Haplorrhini - ps33
Simiiformes - ps34
Catarrhini - ps35
Hominoidea - ps36
Hominidae - ps37
Homininae - ps38
Hominini - ps39
Homo_sapiens - ps40
p-value
1e-03
3e-03
7e-03
2e-02
4e-02
Log-odds
0.5
1.0
1.5
2.0
2.5
3.0
3.5
4.0

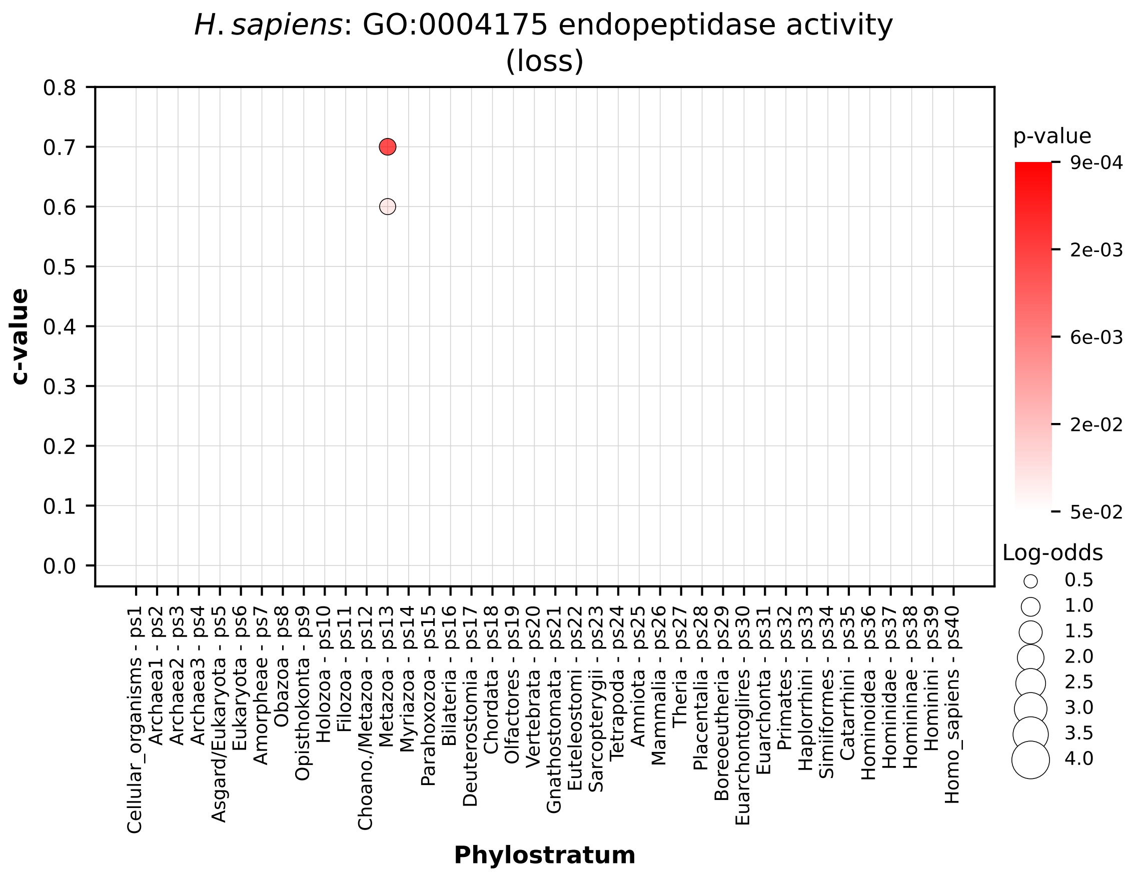
H. sapiens: GO:0004175 endopeptidase activity
(loss)
c-value
0.8
0.7
0.6
0.5
0.4
0.3
0.2
0.1
0.0
Phylostratum
Cellular_organisms - ps1
Archaea1 - ps2
Archaea2 - ps3
Archaea3 - ps4
Asgard/Eukaryota - ps5
Eukaryota - ps6
Amorpheae - ps7
Obazoa - ps8
Opisthokonta - ps9
Holozoa - ps10
Filozoa - ps11
Choano./Metazoa - ps12
Metazoa - ps13
Myriazoa - ps14
Parahoxozoa - ps15
Bilateria - ps16
Deuterostomia - ps17
Chordata - ps18
Olfactores - ps19
Vertebrata - ps20
Gnathostomata - ps21
Euteleostomi - ps22
Sarcopterygii - ps23
Tetrapoda - ps24
Amniota - ps25
Mammalia - ps26
Theria - ps27
Placentalia - ps28
Boreoeutheria - ps29
Euarchontoglires - ps30
Euarchonta - ps31
Primates - ps32
Haplorrhini - ps33
Simiiformes - ps34
Catarrhini - ps35
Hominoidea - ps36
Hominidae - ps37
Homininae - ps38
Hominini - ps39
Homo_sapiens - ps40
p-value
9e-04
2e-03
6e-03
2e-02
5e-02
Log-odds
0.5
1.0
1.5
2.0
2.5
3.0
3.5
4.0

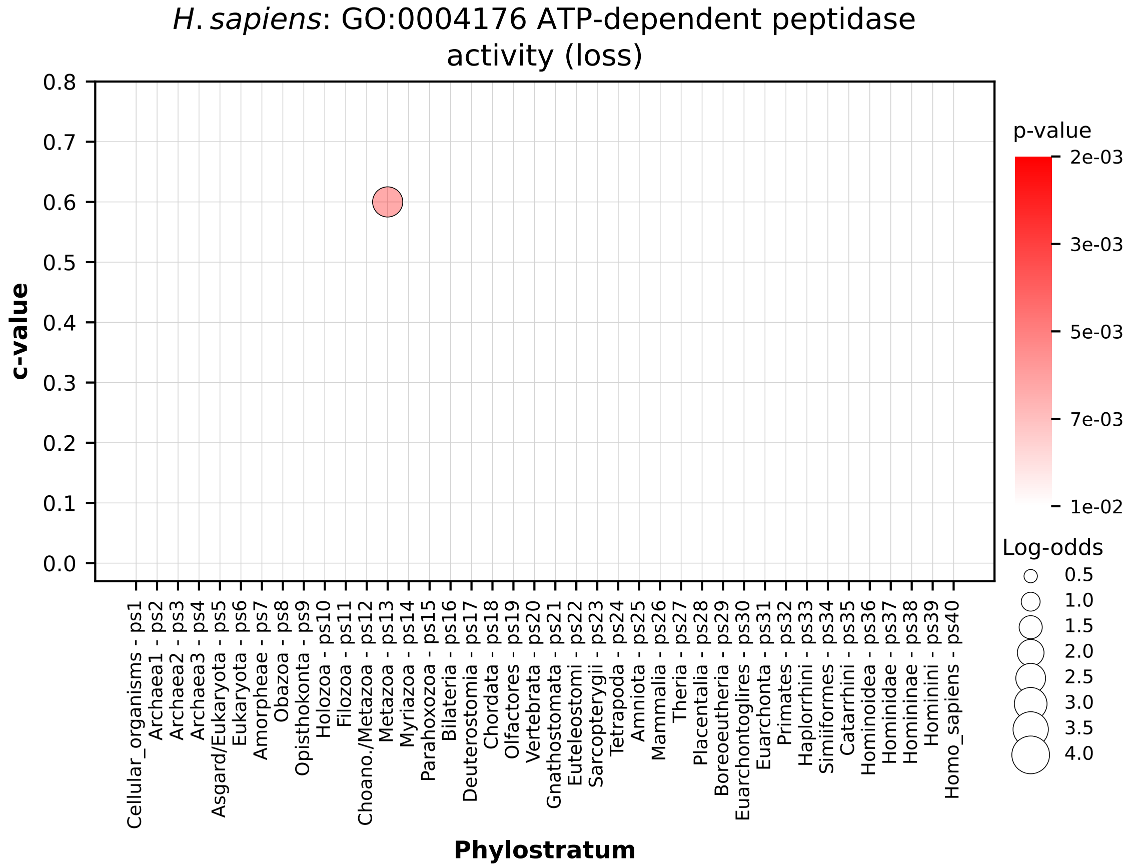
H. sapiens: GO:0004176 ATP-dependent peptidase activity (loss)
c-value
0.0
0.1
0.2
0.3
0.4
0.5
0.6
0.7
0.8
Phylostratum
Cellular_organisms - ps1
Archaea1 - ps2
Archaea2 - ps3
Archaea3 - ps4
Asgard/Eukaryota - ps5
Eukaryota - ps6
Amorpheae - ps7
Obazoa - ps8
Opisthokonta - ps9
Holozoa - ps10
Filozoa - ps11
Choano./Metazoa - ps12
Metazoa - ps13
Myriazoa - ps14
Parahoxozoa - ps15
Bilateria - ps16
Deuterostomia - ps17
Chordata - ps18
Olfactores - ps19
Vertebrata - ps20
Gnathostomata - ps21
Euteleostomi - ps22
Sarcopterygii - ps23
Tetrapoda - ps24
Amniota - ps25
Mammalia - ps26
Theria - ps27
Placentalia - ps28
Boreoeutheria - ps29
Euarchontoglires - ps30
Euarchonta - ps31
Primates - ps32
Haplorrhini - ps33
Simiiformes - ps34
Catarrhini - ps35
Hominoidea - ps36
Hominidae - ps37
Homininae - ps38
Hominini - ps39
Homo_sapiens - ps40
p-value
2e-03
3e-03
5e-03
7e-03
1e-02
Log-odds
0.5
1.0
1.5
2.0
2.5
3.0
3.5
4.0

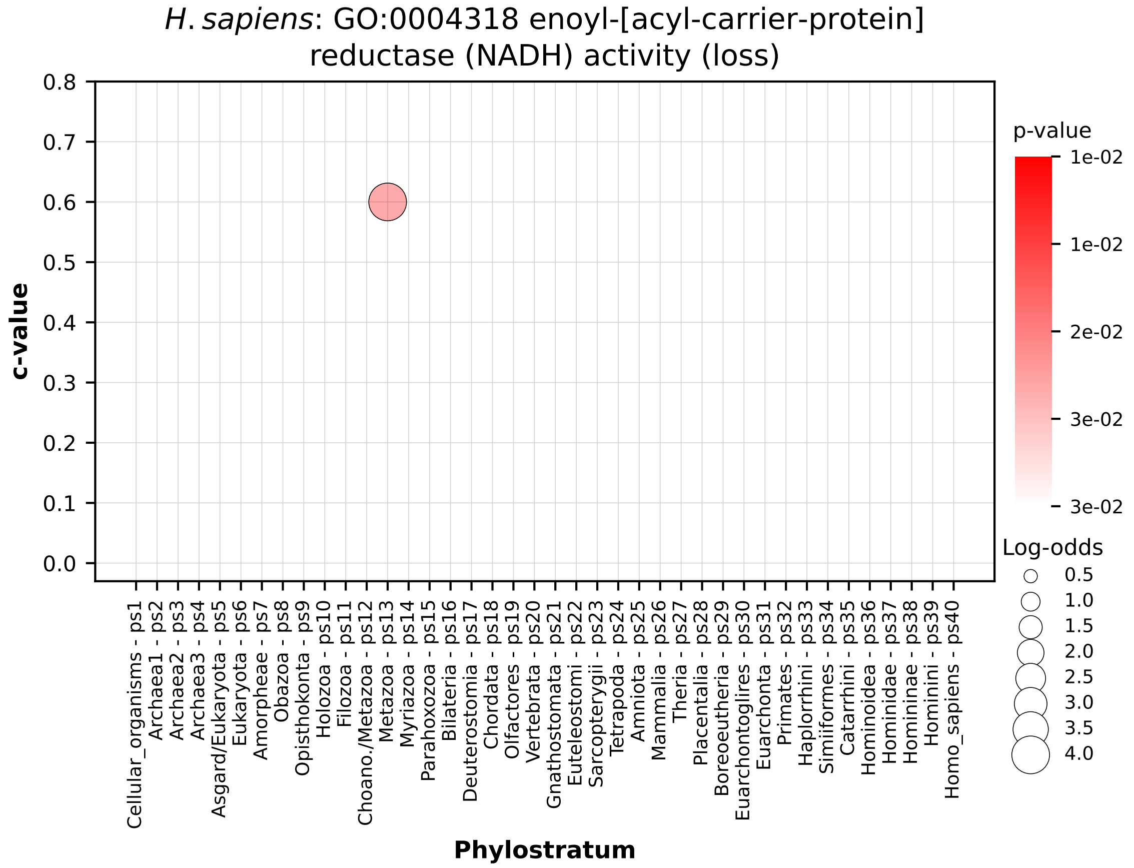

H. sapiens: GO:0004318 enoyl-[acyl-carrier-protein] reductase (NADH) activity (loss)
c-value
0.8
0.7
0.6
0.5
0.4
0.3
0.2
0.1
0.0
Phylostratum
Cellular_organisms - ps1
Archaea1 - ps2
Archaea2 - ps3
Archaea3 - ps4
Asgard/Eukaryota - ps5
Eukaryota - ps6
Amorpheae - ps7
Obazoa - ps8
Opisthokonta - ps9
Holozoa - ps10
Filozoa - ps11
Choano./Metazoa - ps12
Metazoa - ps13
Myriazoa - ps14
Parahoxozoa - ps15
Bilateria - ps16
Deuterostomia - ps17
Chordata - ps18
Olfactores - ps19
Vertebrata - ps20
Gnathostomata - ps21
Euteleostomi - ps22
Sarcopterygii - ps23
Tetrapoda - ps24
Amniota - ps25
Mammalia - ps26
Theria - ps27
Placentalia - ps28
Boreoeutheria - ps29
Euarchontoglires - ps30
Euarchonta - ps31
Primates - ps32
Haplorrhini - ps33
Simiiformes - ps34
Catarrhini - ps35
Hominoidea - ps36
Hominidae - ps37
Homininae - ps38
Hominini - ps39
Homo_sapiens - ps40
p-value
1e-02
1e-02
2e-02
3e-02
3e-02
Log-odds
0.5
1.0
1.5
2.0
2.5
3.0
3.5
4.0

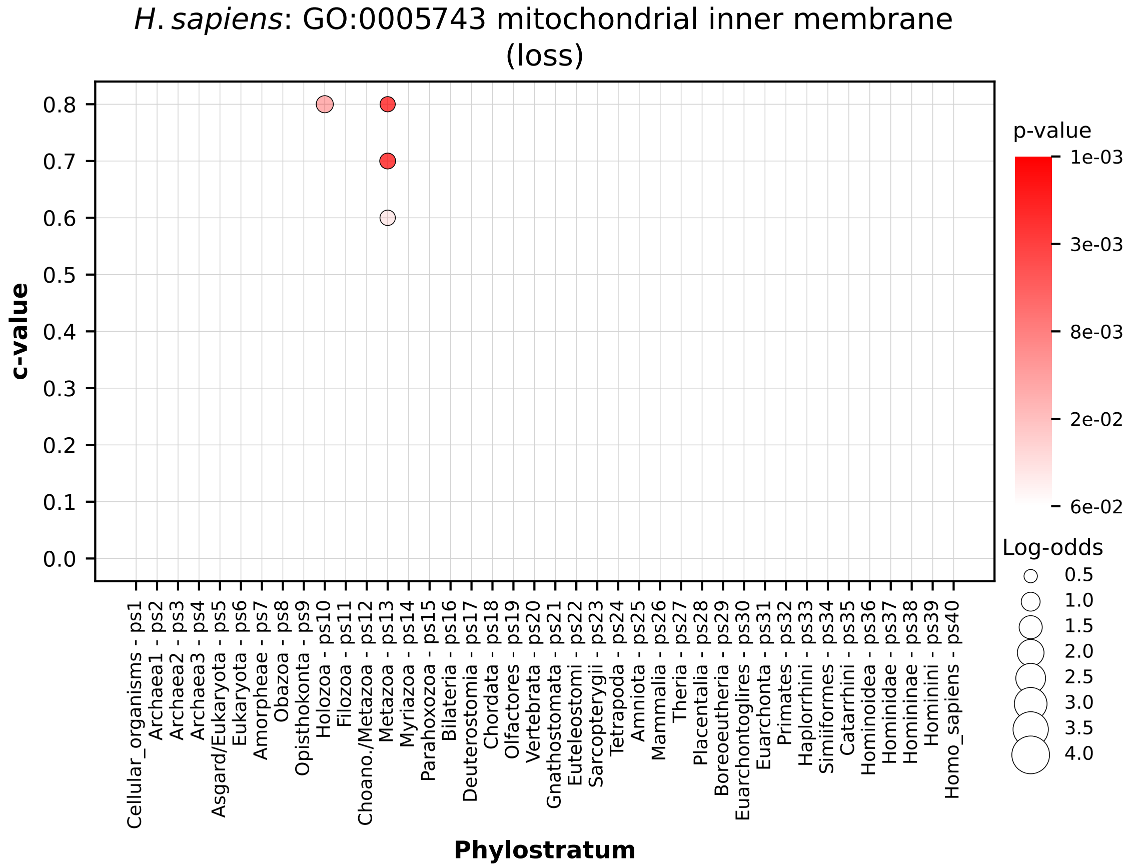

H. sapiens: GO:0005743 mitochondrial inner membrane
(loss)
c-value
0.8
0.7
0.6
0.5
0.4
0.3
0.2
0.1
0.0
p-value
1e-03
3e-03
8e-03
2e-02
6e-02
Log-odds
0.5
1.0
1.5
2.0
2.5
3.0
3.5
4.0
Cellular_organisms - ps1
Archaea1 - ps2
Archaea2 - ps3
Archaea3 - ps4
Asgard/Eukaryota - ps5
Eukaryota - ps6
Amorpheae - ps7
Obazoa - ps8
Opisthokonta - ps9
Holozoa - ps10
Filozoa - ps11
Choano./Metazoa - ps12
Metazoa - ps13
Myriazoa - ps14
Parahoxozoa - ps15
Bilateria - ps16
Deuterostomia - ps17
Chordata - ps18
Olfactores - ps19
Vertebrata - ps20
Gnathostomata - ps21
Euteleostomi - ps22
Sarcopterygii - ps23
Tetrapoda - ps24
Amniota - ps25
Mammalia - ps26
Theria - ps27
Placentalia - ps28
Boreoeutheria - ps29
Euarchontoglires - ps30
Euarchonta - ps31
Primates - ps32
Haplorrhini - ps33
Simiiformes - ps34
Catarrhini - ps35
Hominoidea - ps36
Hominidae - ps37
Homininae - ps38
Hominini - ps39
Homo_sapiens - ps40
Phylostratum

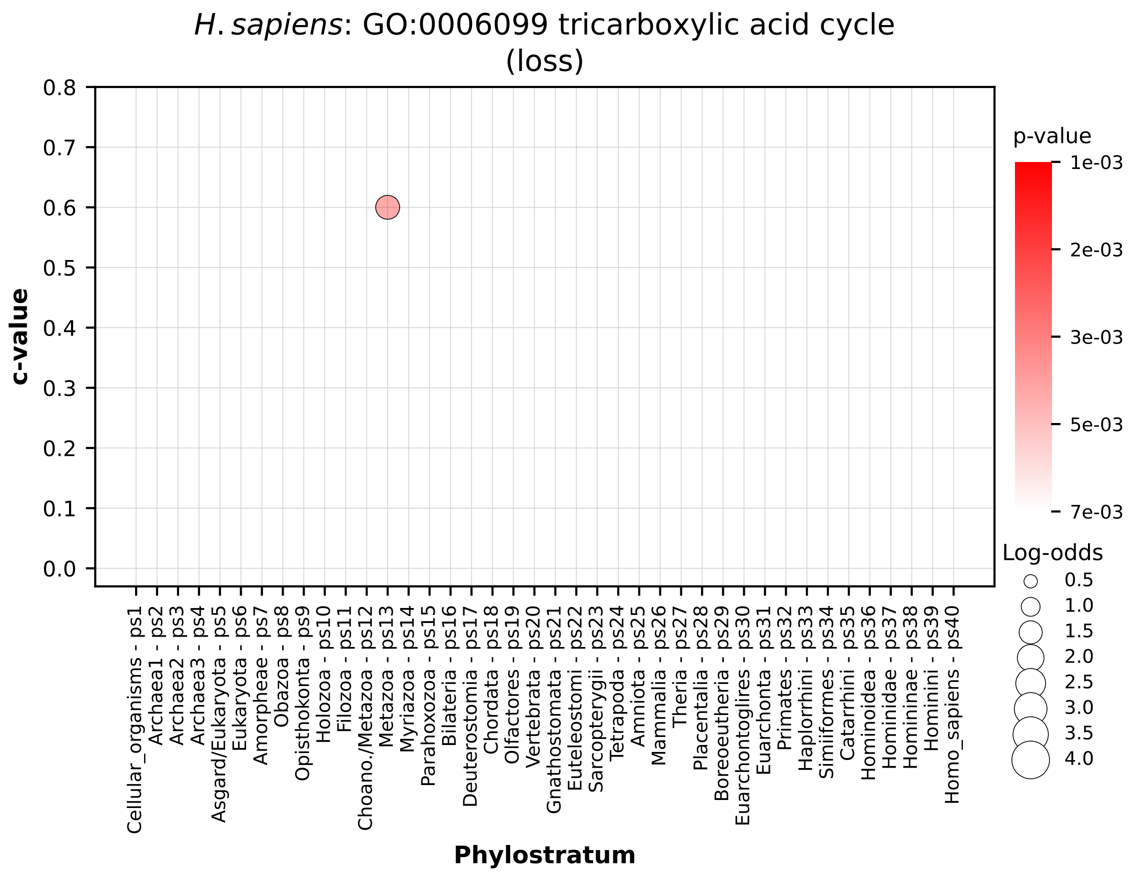
H. sapiens: GO:0006099 tricarboxylic acid cycle
(loss)
c-value
0.0
0.1
0.2
0.3
0.4
0.5
0.6
0.7
0.8
p-value
1e-03
2e-03
3e-03
5e-03
7e-03
Log-odds
0.5
1.0
1.5
2.0
2.5
3.0
3.5
4.0
Cellular_organisms - ps1
Archaea1 - ps2
Archaea2 - ps3
Archaea3 - ps4
Asgard/Eukaryota - ps5
Eukaryota - ps6
Amorpheae - ps7
Obazoa - ps8
Opisthokonta - ps9
Holozoa - ps10
Filozoa - ps11
Choano./Metazoa - ps12
Metazoa - ps13
Myriazoa - ps14
Parahoxozoa - ps15
Bilateria - ps16
Deuterostomia - ps17
Chordata - ps18
Olfactores - ps19
Vertebrata - ps20
Gnathostomata - ps21
Euteleostomi - ps22
Sarcopterygii - ps23
Tetrapoda - ps24
Amniota - ps25
Mammalia - ps26
Theria - ps27
Placentalia - ps28
Boreoeutheria - ps29
Euarchontoglires - ps30
Euarchonta - ps31
Primates - ps32
Haplorrhini - ps33
Simiiformes - ps34
Catarrhini - ps35
Hominoidea - ps36
Hominidae - ps37
Homininae - ps38
Hominini - ps39
Homo_sapiens - ps40
Phylostratum

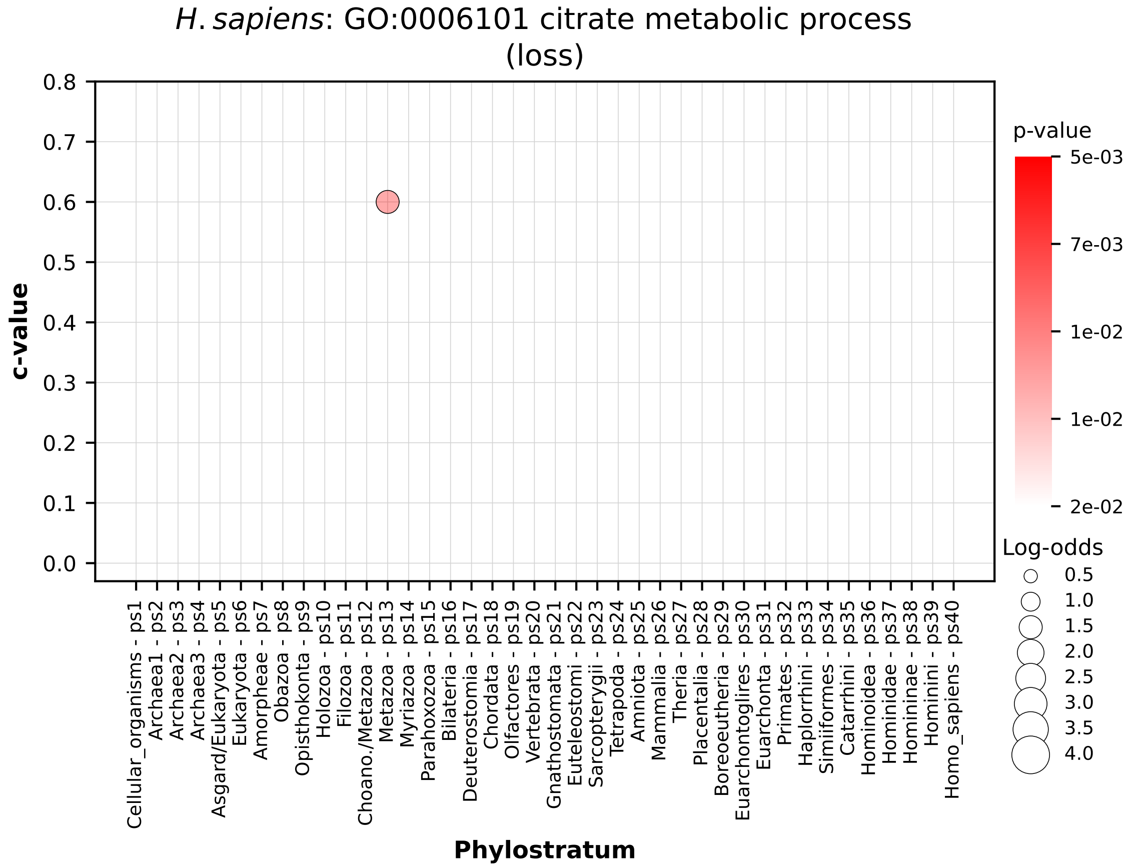

H. sapiens: GO:0006101 citrate metabolic process
(loss)
c-value
0.0
0.1
0.2
0.3
0.4
0.5
0.6
0.7
0.8
Phylostratum
Cellular_organisms - ps1
Archaea1 - ps2
Archaea2 - ps3
Archaea3 - ps4
Asgard/Eukaryota - ps5
Eukaryota - ps6
Amorpheae - ps7
Obazoa - ps8
Opisthokonta - ps9
Holozoa - ps10
Filozoa - ps11
Choano./Metazoa - ps12
Metazoa - ps13
Myriazoa - ps14
Parahoxozoa - ps15
Bilateria - ps16
Deuterostomia - ps17
Chordata - ps18
Olfactores - ps19
Vertebrata - ps20
Gnathostomata - ps21
Euteleostomi - ps22
Sarcopterygii - ps23
Tetrapoda - ps24
Amniota - ps25
Mammalia - ps26
Theria - ps27
Placentalia - ps28
Boreoeutheria - ps29
Euarchontoglires - ps30
Euarchonta - ps31
Primates - ps32
Haplorrhini - ps33
Simiiformes - ps34
Catarrhini - ps35
Hominoidea - ps36
Hominidae - ps37
Homininae - ps38
Hominini - ps39
Homo_sapiens - ps40
p-value
5e-03
7e-03
1e-02
1e-02
2e-02
Log-odds
0.5
1.0
1.5
2.0
2.5
3.0
3.5
4.0

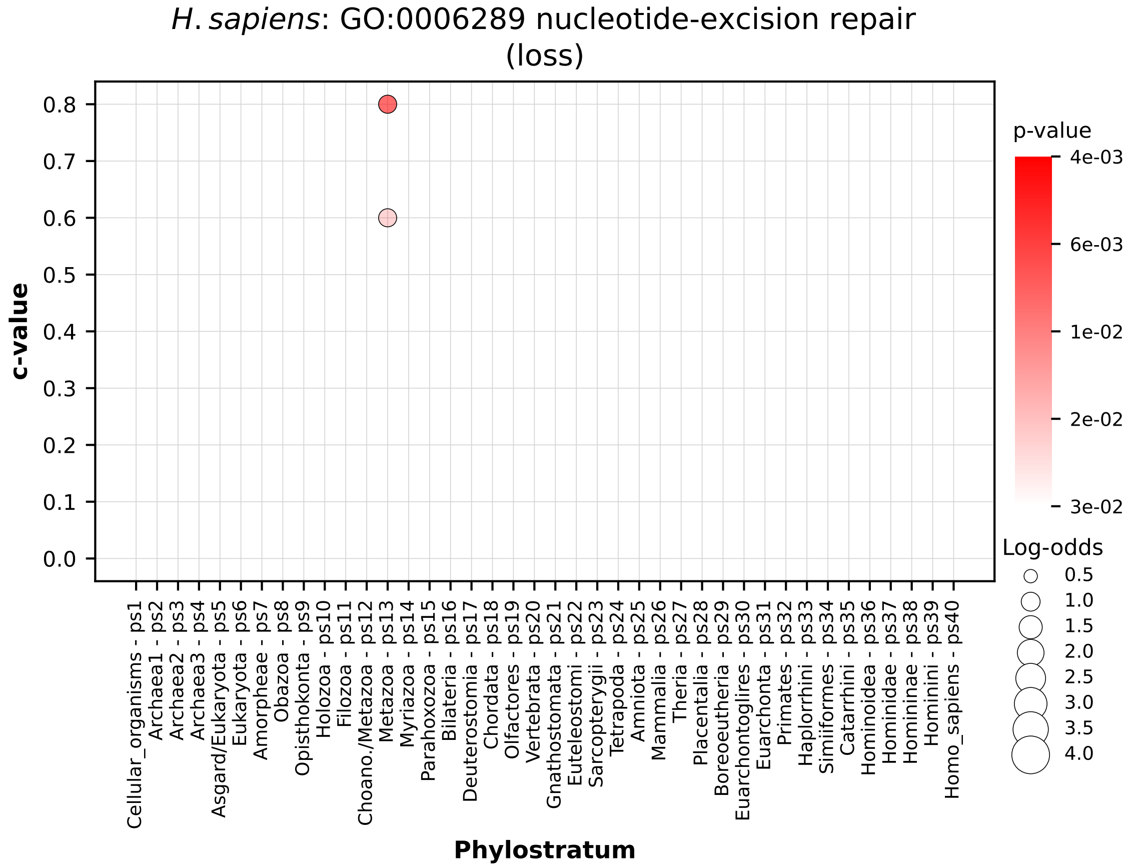

H. sapiens: GO:0006289 nucleotide-excision repair
(loss)
c-value
0.0
0.1
0.2
0.3
0.4
0.5
0.6
0.7
0.8
Phylostratum
Cellular_organisms - ps1
Archaea1 - ps2
Archaea2 - ps3
Archaea3 - ps4
Asgard/Eukaryota - ps5
Eukaryota - ps6
Amorpheae - ps7
Obazoa - ps8
Opisthokonta - ps9
Holozoa - ps10
Filozoa - ps11
Choano./Metazoa - ps12
Metazoa - ps13
Myriazoa - ps14
Parahoxozoa - ps15
Bilateria - ps16
Deuterostomia - ps17
Chordata - ps18
Olfactores - ps19
Vertebrata - ps20
Gnathostomata - ps21
Euteleostomi - ps22
Sarcopterygii - ps23
Tetrapoda - ps24
Amniota - ps25
Mammalia - ps26
Theria - ps27
Placentalia - ps28
Boreoeutheria - ps29
Euarchontoglires - ps30
Euarchonta - ps31
Primates - ps32
Haplorrhini - ps33
Simiiformes - ps34
Catarrhini - ps35
Hominoidea - ps36
Hominidae - ps37
Homininae - ps38
Hominini - ps39
Homo_sapiens - ps40
p-value
4e-03
6e-03
1e-02
2e-02
3e-02
Log-odds
0.5
1.0
1.5
2.0
2.5
3.0
3.5
4.0

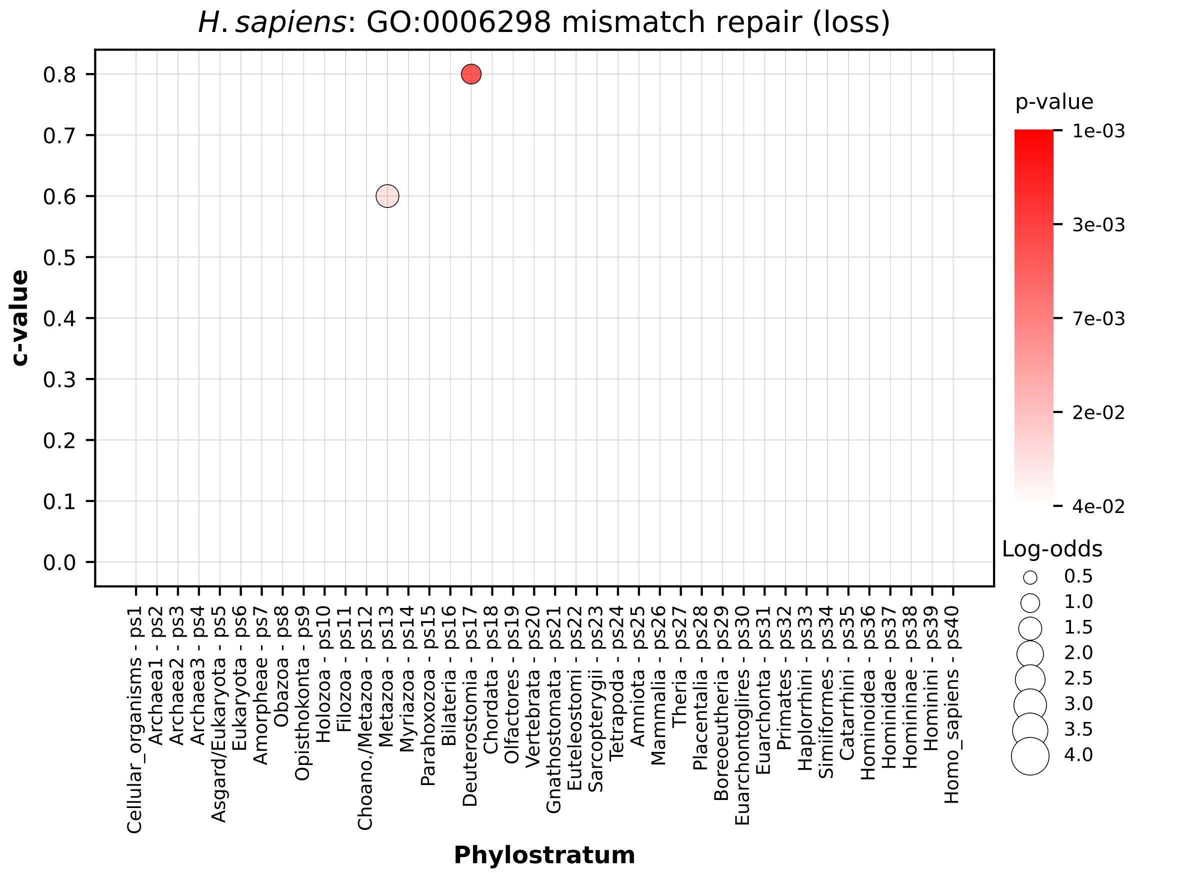

H. sapiens: GO:0006298 mismatch repair (loss)
c-value
0.0
0.1
0.2
0.3
0.4
0.5
0.6
0.7
0.8
Phylostratum
Cellular_organisms - ps1
Archaea1 - ps2
Archaea2 - ps3
Archaea3 - ps4
Asgard/Eukaryota - ps5
Eukaryota - ps6
Amorpheae - ps7
Obazoa - ps8
Opisthokonta - ps9
Holozoa - ps10
Filozoa - ps11
Choano./Metazoa - ps12
Metazoa - ps13
Myriazoa - ps14
Parahoxozoa - ps15
Bilateria - ps16
Deuterostomia - ps17
Chordata - ps18
Olfactores - ps19
Vertebrata - ps20
Gnathostomata - ps21
Euteleostomi - ps22
Sarcopterygii - ps23
Tetrapoda - ps24
Amniota - ps25
Mammalia - ps26
Theria - ps27
Placentalia - ps28
Boreoeutheria - ps29
Euarchontoglires - ps30
Euarchonta - ps31
Primates - ps32
Haplorrhini - ps33
Simiiformes - ps34
Catarrhini - ps35
Hominoidea - ps36
Hominidae - ps37
Homininae - ps38
Hominini - ps39
Homo_sapiens - ps40
p-value
1e-03
3e-03
7e-03
2e-02
4e-02
Log-odds
0.5
1.0
1.5
2.0
2.5
3.0
3.5
4.0

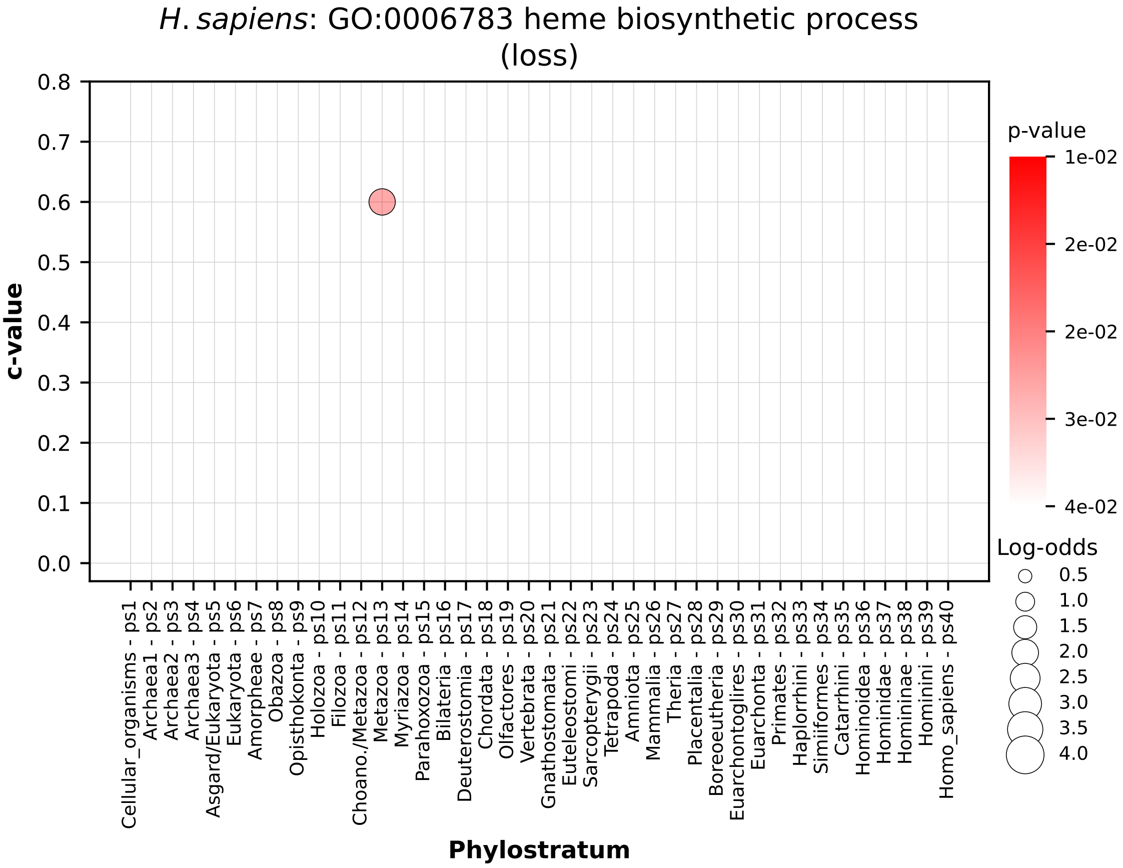

H. sapiens: GO:0006783 heme biosynthetic process (loss)
c-value
0.0
0.1
0.2
0.3
0.4
0.5
0.6
0.7
0.8
p-value
1e-02
2e-02
2e-02
3e-02
4e-02
Log-odds
0.5
1.0
1.5
2.0
2.5
3.0
3.5
4.0
Cellular_organisms - ps1
Archaea1 - ps2
Archaea2 - ps3
Archaea3 - ps4
Asgard/Eukaryota - ps5
Eukaryota - ps6
Amorpheae - ps7
Obazoa - ps8
Opisthokonta - ps9
Holozoa - ps10
Filozoa - ps11
Choano./Metazoa - ps12
Metazoa - ps13
Myriazoa - ps14
Parahoxozoa - ps15
Bilateria - ps16
Deuterostomia - ps17
Chordata - ps18
Olfactores - ps19
Vertebrata - ps20
Gnathostomata - ps21
Euteleostomi - ps22
Sarcopterygii - ps23
Tetrapoda - ps24
Amniota - ps25
Mammalia - ps26
Theria - ps27
Placentalia - ps28
Boreoeutheria - ps29
Euarchontoglires - ps30
Euarchonta - ps31
Primates - ps32
Haplorrhini - ps33
Simiiformes - ps34
Catarrhini - ps35
Hominoidea - ps36
Hominidae - ps37
Homininae - ps38
Hominini - ps39
Homo_sapiens - ps40
Phylostratum

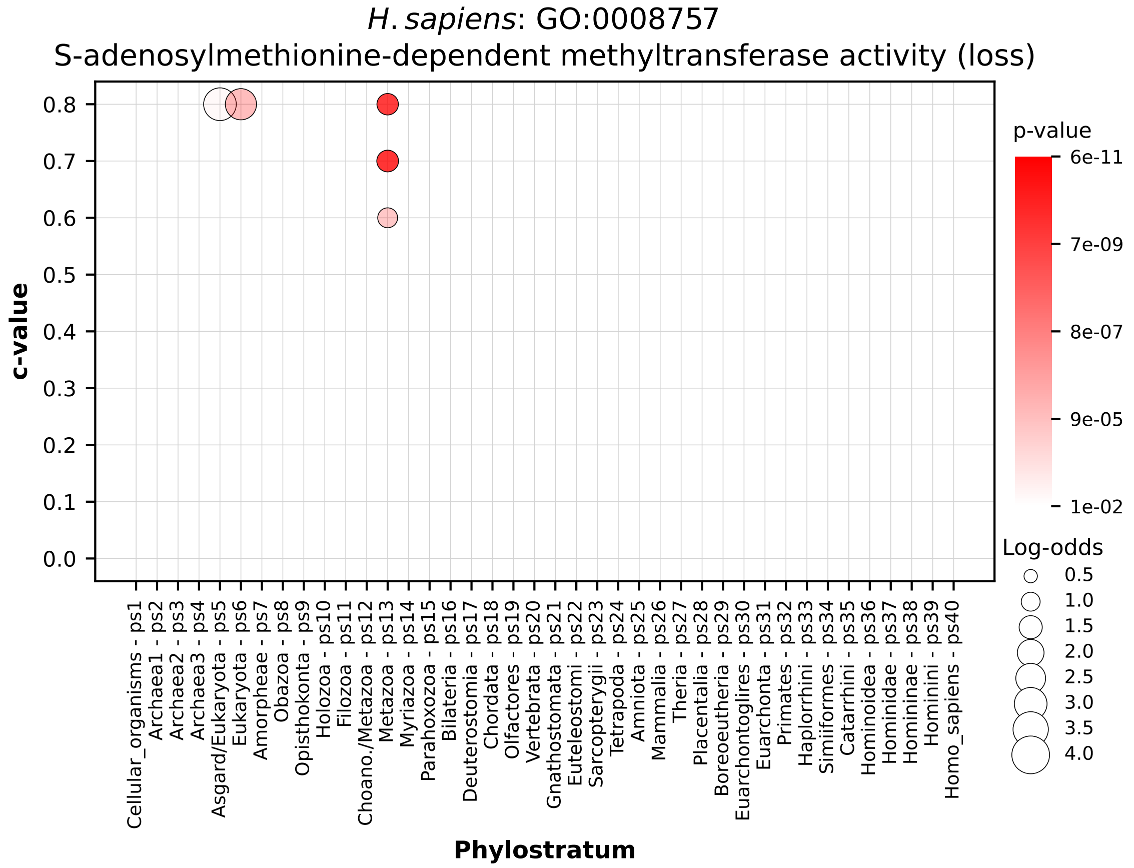

H. sapiens: GO:0008757
S-adenosylmethionine-dependent methyltransferase activity (loss)
c-value
0.0
0.1
0.2
0.3
0.4
0.5
0.6
0.7
0.8
Phylostratum
Cellular_organisms - ps1
Archaea1 - ps2
Archaea2 - ps3
Archaea3 - ps4
Asgard/Eukaryota - ps5
Eukaryota - ps6
Amorpheae - ps7
Obazoa - ps8
Opisthokonta - ps9
Holozoa - ps10
Filozoa - ps11
Choano./Metazoa - ps12
Metazoa - ps13
Myriazoa - ps14
Parahoxozoa - ps15
Bilateria - ps16
Deuterostomia - ps17
Chordata - ps18
Olfactores - ps19
Vertebrata - ps20
Gnathostomata - ps21
Euteleostomi - ps22
Sarcopterygii - ps23
Tetrapoda - ps24
Amniota - ps25
Mammalia - ps26
Theria - ps27
Placentalia - ps28
Boreoeutheria - ps29
Euarchontoglires - ps30
Euarchonta - ps31
Primates - ps32
Haplorrhini - ps33
Simiiformes - ps34
Catarrhini - ps35
Hominoidea - ps36
Hominidae - ps37
Homininae - ps38
Hominini - ps39
Homo_sapiens - ps40
p-value
6e-11
7e-09
8e-07
9e-05
1e-02
Log-odds
0.5
1.0
1.5
2.0
2.5
3.0
3.5
4.0

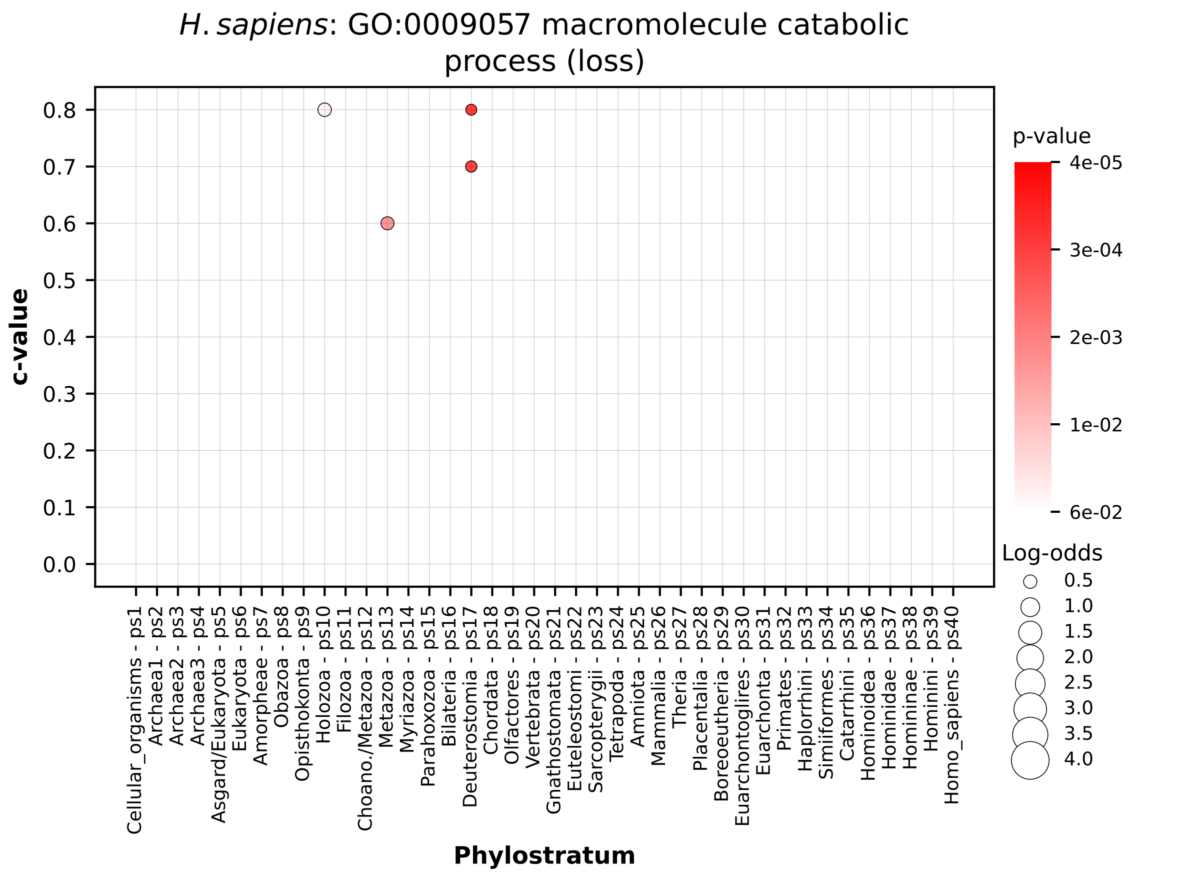
H. sapiens: GO:0009057 macromolecule catabolic process (loss)
c-value
0.8
0.7
0.6
0.5
0.4
0.3
0.2
0.1
0.0
Phylostratum
Cellular_organisms - ps1
Archaea1 - ps2
Archaea2 - ps3
Archaea3 - ps4
Asgard/Eukaryota - ps5
Eukaryota - ps6
Amorpheae - ps7
Obazoa - ps8
Opisthokonta - ps9
Holozoa - ps10
Filozoa - ps11
Choano./Metazoa - ps12
Metazoa - ps13
Myriazoa - ps14
Parahoxozoa - ps15
Bilateria - ps16
Deuterostomia - ps17
Chordata - ps18
Olfactores - ps19
Vertebrata - ps20
Gnathostomata - ps21
Euteleostomi - ps22
Sarcopterygii - ps23
Tetrapoda - ps24
Amniota - ps25
Mammalia - ps26
Theria - ps27
Placentalia - ps28
Boreoeutheria - ps29
Euarchontoglires - ps30
Euarchonta - ps31
Primates - ps32
Haplorrhini - ps33
Simiiformes - ps34
Catarrhini - ps35
Hominoidea - ps36
Hominidae - ps37
Homininae - ps38
Hominini - ps39
Homo_sapiens - ps40
p-value
4e-05
3e-04
2e-03
1e-02
6e-02
Log-odds
0.5
1.0
1.5
2.0
2.5
3.0
3.5
4.0

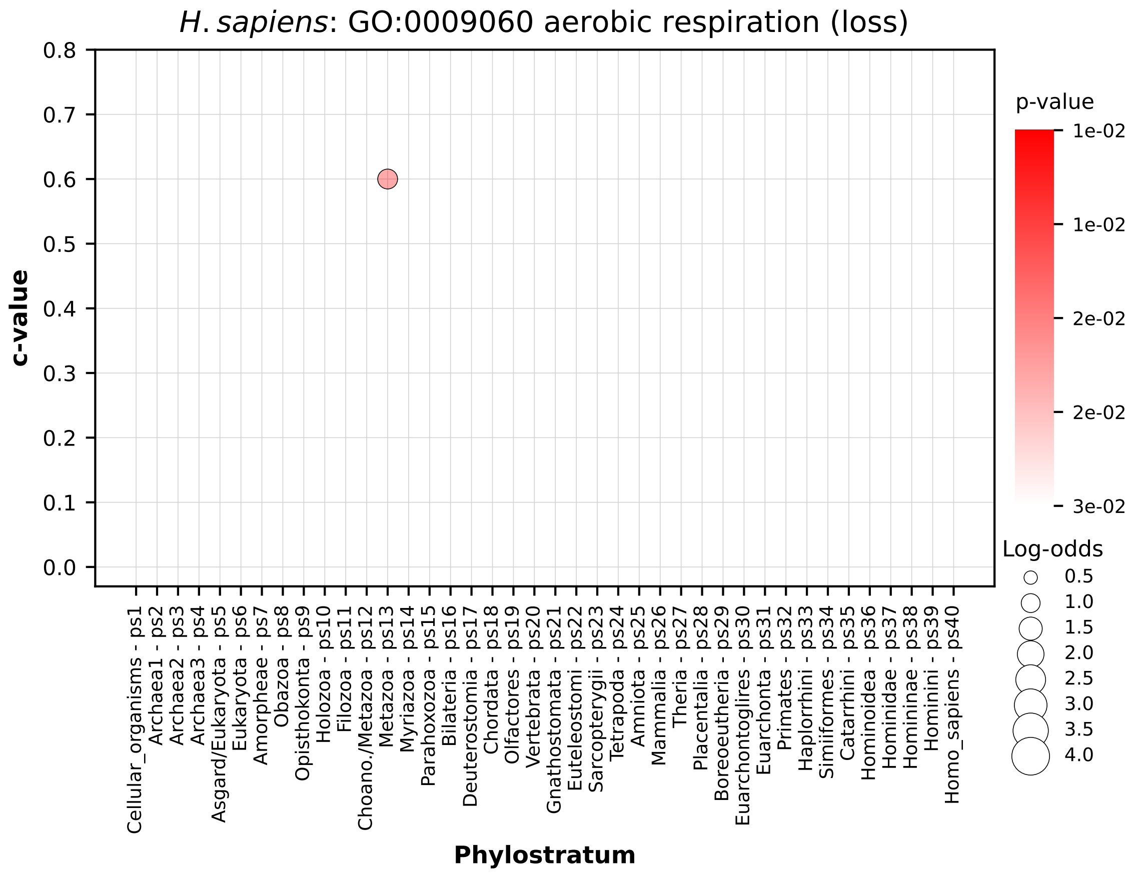

H. sapiens: GO:0009060 aerobic respiration (loss)
c-value
0.8
0.7
0.6
0.5
0.4
0.3
0.2
0.1
0.0
Phylostratum
Cellular_organisms - ps1
Archaea1 - ps2
Archaea2 - ps3
Archaea3 - ps4
Asgard/Eukaryota - ps5
Eukaryota - ps6
Amorpheae - ps7
Obazoa - ps8
Opisthokonta - ps9
Holozoa - ps10
Filozoa - ps11
Choano./Metazoa - ps12
Metazoa - ps13
Myriazoa - ps14
Parahoxozoa - ps15
Bilateria - ps16
Deuterostomia - ps17
Chordata - ps18
Olfactores - ps19
Vertebrata - ps20
Gnathostomata - ps21
Euteleostomi - ps22
Sarcopterygii - ps23
Tetrapoda - ps24
Amniota - ps25
Mammalia - ps26
Theria - ps27
Placentalia - ps28
Boreoeutheria - ps29
Euarchontoglires - ps30
Euarchonta - ps31
Primates - ps32
Haplorrhini - ps33
Simiiformes - ps34
Catarrhini - ps35
Hominoidea - ps36
Hominidae - ps37
Homininae - ps38
Hominini - ps39
Homo_sapiens - ps40
p-value
1e-02
1e-02
2e-02
2e-02
3e-02
Log-odds
0.5
1.0
1.5
2.0
2.5
3.0
3.5
4.0

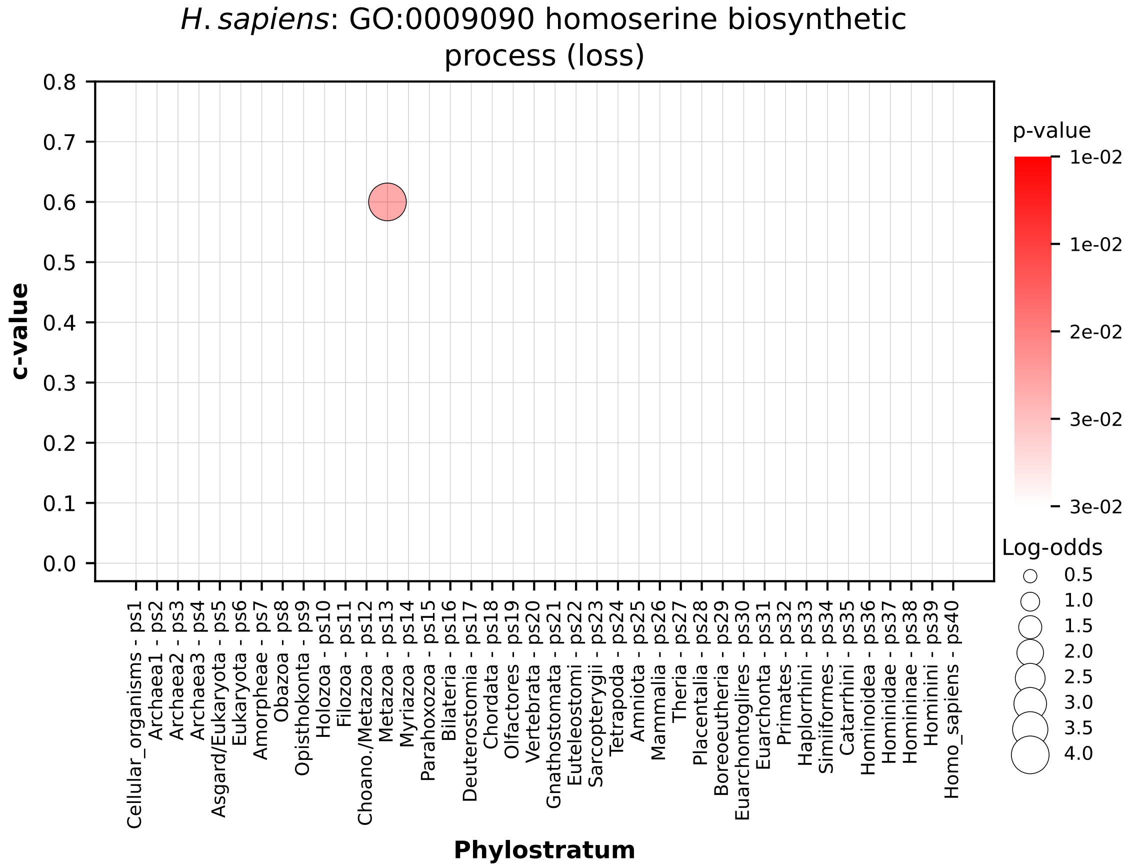

H. sapiens: GO:0009090 homoserine biosynthetic process (loss)
c-value
0.8
0.7
0.6
0.5
0.4
0.3
0.2
0.1
0.0
Phylostratum
Cellular_organisms - ps1
Archaea1 - ps2
Archaea2 - ps3
Archaea3 - ps4
Asgard/Eukaryota - ps5
Eukaryota - ps6
Amorpheae - ps7
Obazoa - ps8
Opisthokonta - ps9
Holozoa - ps10
Filozoa - ps11
Choano./Metazoa - ps12
Metazoa - ps13
Myriazoa - ps14
Parahoxozoa - ps15
Bilateria - ps16
Deuterostomia - ps17
Chordata - ps18
Olfactores - ps19
Vertebrata - ps20
Gnathostomata - ps21
Euteleostomi - ps22
Sarcopterygii - ps23
Tetrapoda - ps24
Amniota - ps25
Mammalia - ps26
Theria - ps27
Placentalia - ps28
Boreoeutheria - ps29
Euarchontoglires - ps30
Euarchonta - ps31
Primates - ps32
Haplorrhini - ps33
Simiiformes - ps34
Catarrhini - ps35
Hominoidea - ps36
Hominidae - ps37
Homininae - ps38
Hominini - ps39
Homo_sapiens - ps40
p-value
1e-02
1e-02
2e-02
3e-02
3e-02
Log-odds
0.5
1.0
1.5
2.0
2.5
3.0
3.5
4.0

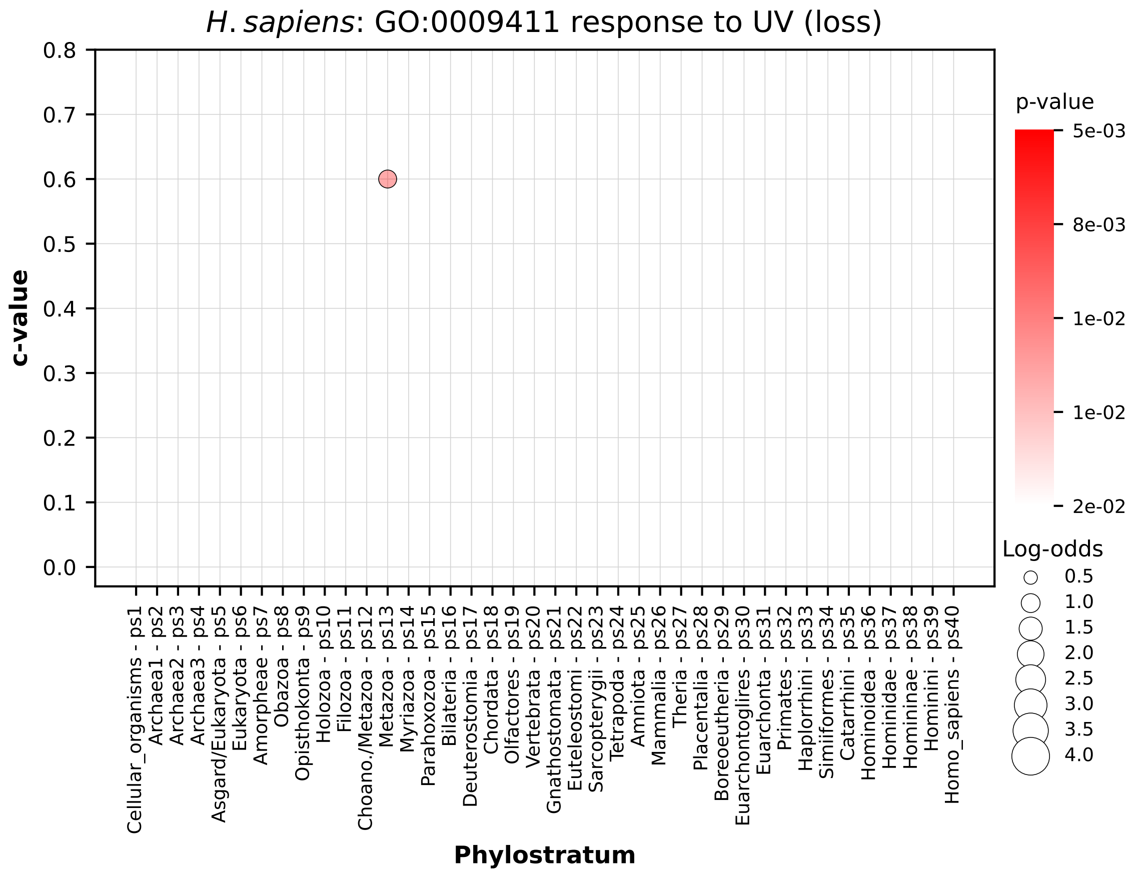

H. sapiens: GO:0009411 response to UV (loss)
c-value
0.8
0.7
0.6
0.5
0.4
0.3
0.2
0.1
0.0
p-value
5e-03
8e-03
1e-02
1e-02
2e-02
Log-odds
0.5
1.0
1.5
2.0
2.5
3.0
3.5
4.0
Cellular_organisms - ps1
Archaea1 - ps2
Archaea2 - ps3
Archaea3 - ps4
Asgard/Eukaryota - ps5
Eukaryota - ps6
Amorpheae - ps7
Obazoa - ps8
Opisthokonta - ps9
Holozoa - ps10
Filozoa - ps11
Choano./Metazoa - ps12
Metazoa - ps13
Myriazoa - ps14
Parahoxozoa - ps15
Bilateria - ps16
Deuterostomia - ps17
Chordata - ps18
Olfactores - ps19
Vertebrata - ps20
Gnathostomata - ps21
Euteleostomi - ps22
Sarcopterygii - ps23
Tetrapoda - ps24
Amniota - ps25
Mammalia - ps26
Theria - ps27
Placentalia - ps28
Boreoeutheria - ps29
Euarchontoglires - ps30
Euarchonta - ps31
Primates - ps32
Haplorrhini - ps33
Simiiformes - ps34
Catarrhini - ps35
Hominoidea - ps36
Hominidae - ps37
Homininae - ps38
Hominini - ps39
Homo_sapiens - ps40
Phylostratum

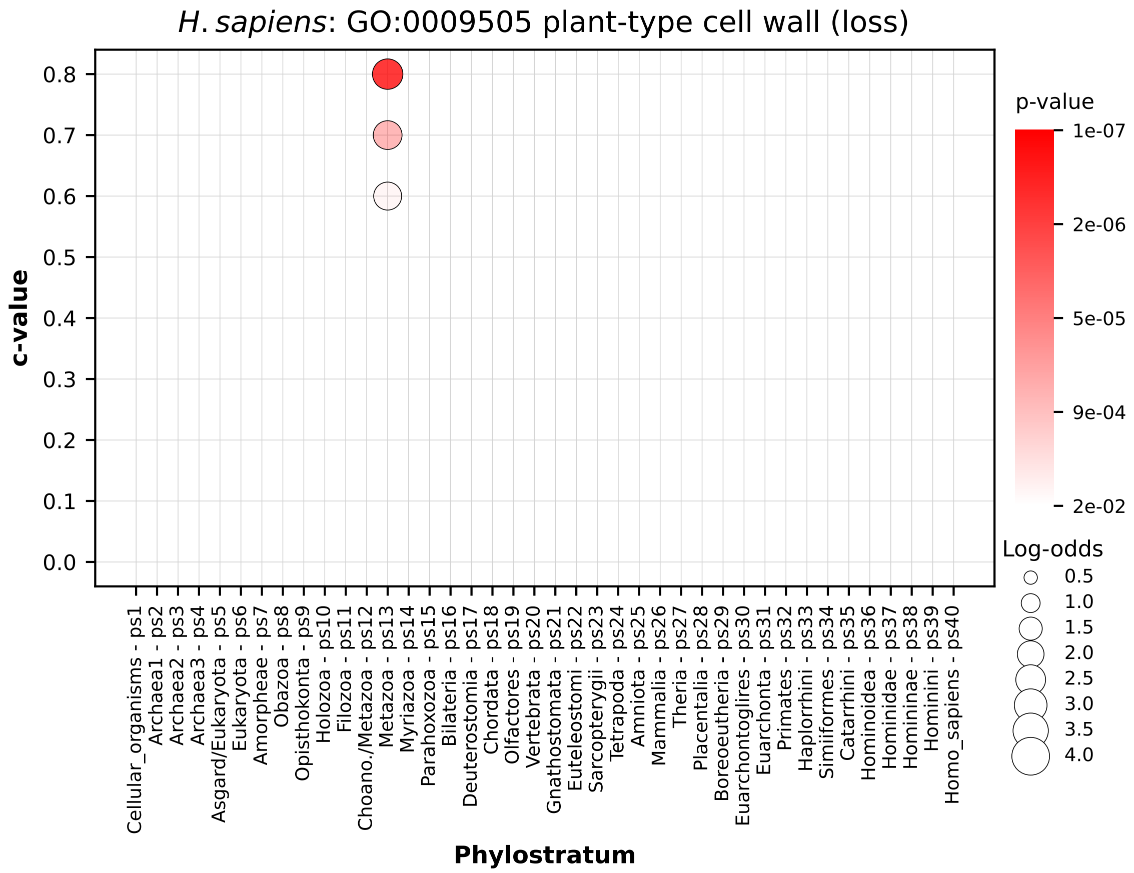
H. sapiens: GO:0009505 plant-type cell wall (loss)
c-value
0.0
0.1
0.2
0.3
0.4
0.5
0.6
0.7
0.8
p-value
1e-07
2e-06
5e-05
9e-04
2e-02
Log-odds
0.5
1.0
1.5
2.0
2.5
3.0
3.5
4.0
Cellular_organisms - ps1
Archaea1 - ps2
Archaea2 - ps3
Archaea3 - ps4
Asgard/Eukaryota - ps5
Eukaryota - ps6
Amorpheae - ps7
Obazoa - ps8
Opisthokonta - ps9
Holozoa - ps10
Filozoa - ps11
Choano./Metazoa - ps12
Metazoa - ps13
Myriazoa - ps14
Parahoxozoa - ps15
Bilateria - ps16
Deuterostomia - ps17
Chordata - ps18
Olfactores - ps19
Vertebrata - ps20
Gnathostomata - ps21
Euteleostomi - ps22
Sarcopterygii - ps23
Tetrapoda - ps24
Amniota - ps25
Mammalia - ps26
Theria - ps27
Placentalia - ps28
Boreoeutheria - ps29
Euarchontoglires - ps30
Euarchonta - ps31
Primates - ps32
Haplorrhini - ps33
Simiiformes - ps34
Catarrhini - ps35
Hominoidea - ps36
Hominidae - ps37
Homininae - ps38
Hominini - ps39
Homo_sapiens - ps40
Phylostratum

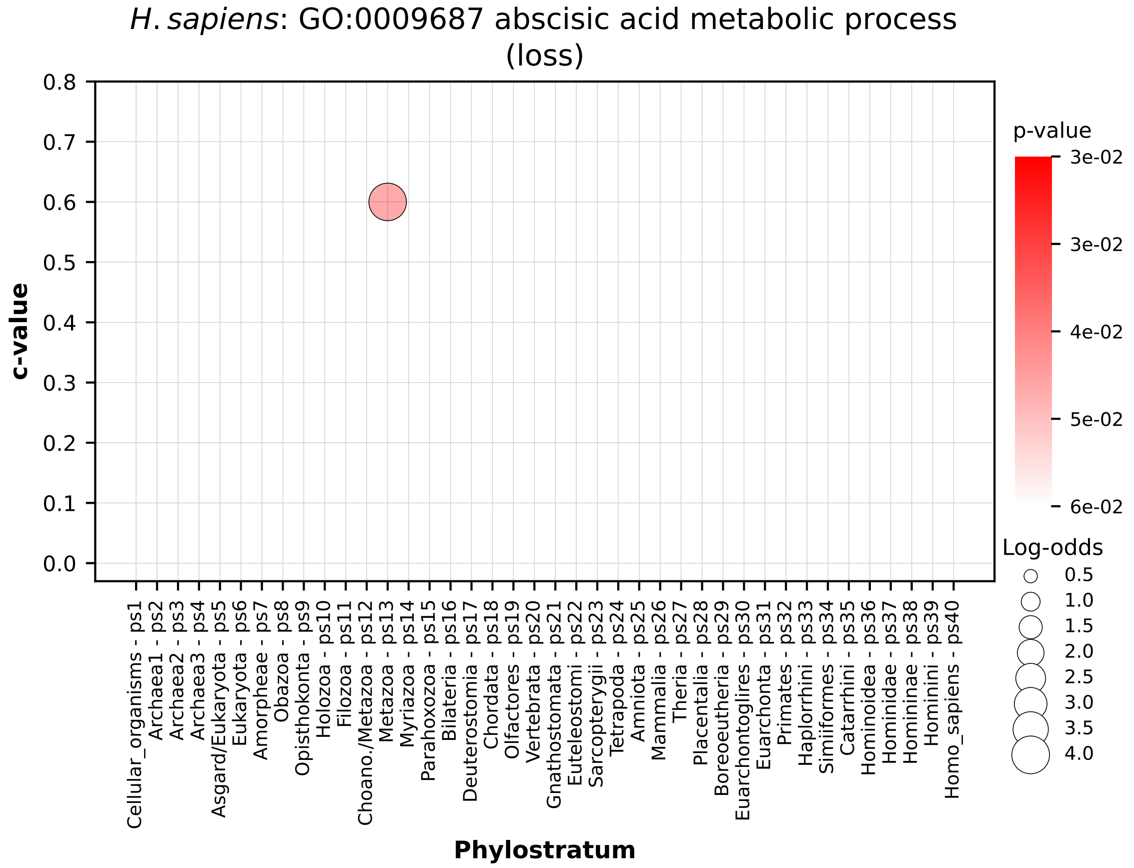

H. sapiens: GO:0009687 abscisic acid metabolic process
(loss)
c-value
0.8
0.7
0.6
0.5
0.4
0.3
0.2
0.1
0.0
p-value
3e-02
3e-02
4e-02
5e-02
6e-02
Log-odds
0.5
1.0
1.5
2.0
2.5
3.0
3.5
4.0
Cellular_organisms - ps1
Archaea1 - ps2
Archaea2 - ps3
Archaea3 - ps4
Asgard/Eukaryota - ps5
Eukaryota - ps6
Amorpheae - ps7
Obazoa - ps8
Opisthokonta - ps9
Holozoa - ps10
Filozoa - ps11
Choano./Metazoa - ps12
Metazoa - ps13
Myriazoa - ps14
Parahoxozoa - ps15
Bilateria - ps16
Deuterostomia - ps17
Chordata - ps18
Olfactores - ps19
Vertebrata - ps20
Gnathostomata - ps21
Euteleostomi - ps22
Sarcopterygii - ps23
Tetrapoda - ps24
Amniota - ps25
Mammalia - ps26
Theria - ps27
Placentalia - ps28
Boreoeutheria - ps29
Euarchontoglires - ps30
Euarchonta - ps31
Primates - ps32
Haplorrhini - ps33
Simiiformes - ps34
Catarrhini - ps35
Hominoidea - ps36
Hominidae - ps37
Homininae - ps38
Hominini - ps39
Homo_sapiens - ps40
Phylostratum

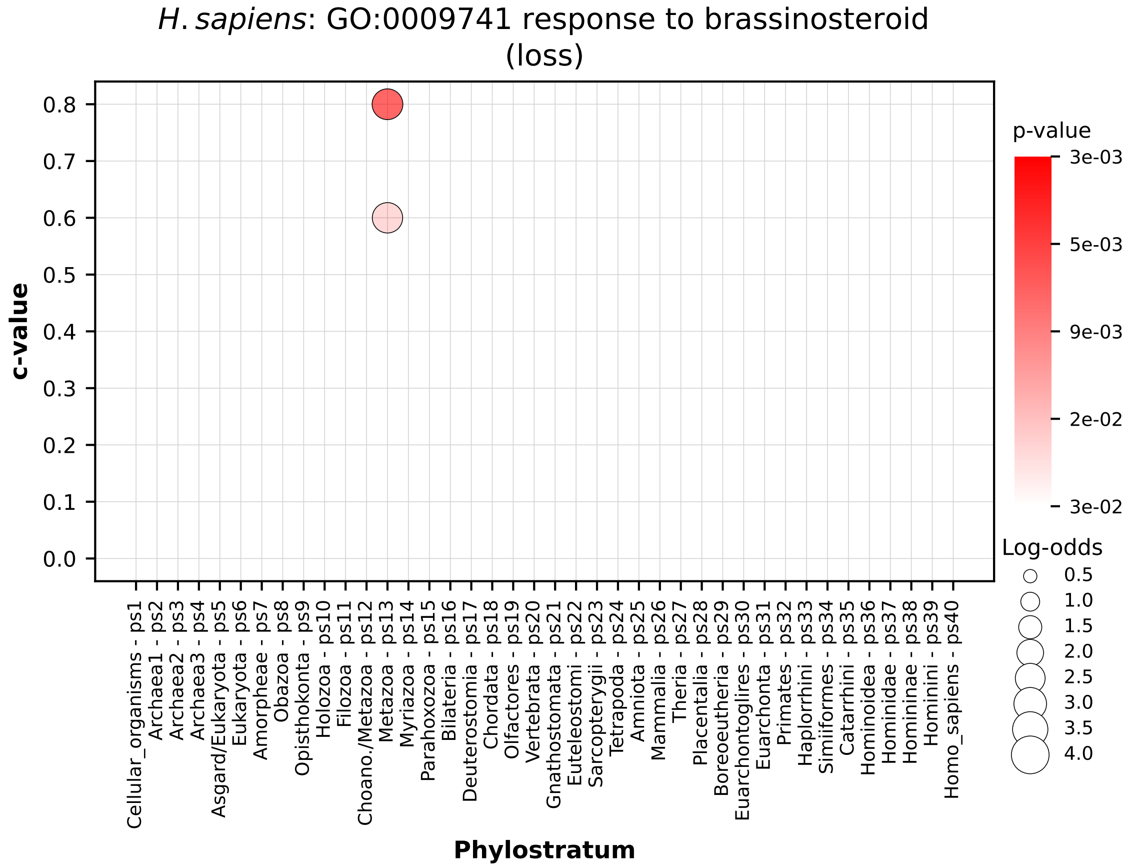

H. sapiens: GO:0009741 response to brassinosteroid
(loss)
c-value
0.0
0.1
0.2
0.3
0.4
0.5
0.6
0.7
0.8
p-value
3e-03
5e-03
9e-03
2e-02
3e-02
Log-odds
0.5
1.0
1.5
2.0
2.5
3.0
3.5
4.0
Cellular_organisms - ps1
Archaea1 - ps2
Archaea2 - ps3
Archaea3 - ps4
Asgard/Eukaryota - ps5
Eukaryota - ps6
Amorpheae - ps7
Obazoa - ps8
Opisthokonta - ps9
Holozoa - ps10
Filozoa - ps11
Choano./Metazoa - ps12
Metazoa - ps13
Myriazoa - ps14
Parahoxozoa - ps15
Bilateria - ps16
Deuterostomia - ps17
Chordata - ps18
Olfactores - ps19
Vertebrata - ps20
Gnathostomata - ps21
Euteleostomi - ps22
Sarcopterygii - ps23
Tetrapoda - ps24
Amniota - ps25
Mammalia - ps26
Theria - ps27
Placentalia - ps28
Boreoeutheria - ps29
Euarchontoglires - ps30
Euarchonta - ps31
Primates - ps32
Haplorrhini - ps33
Simiiformes - ps34
Catarrhini - ps35
Hominoidea - ps36
Hominidae - ps37
Homininae - ps38
Hominini - ps39
Homo_sapiens - ps40
Phylostratum

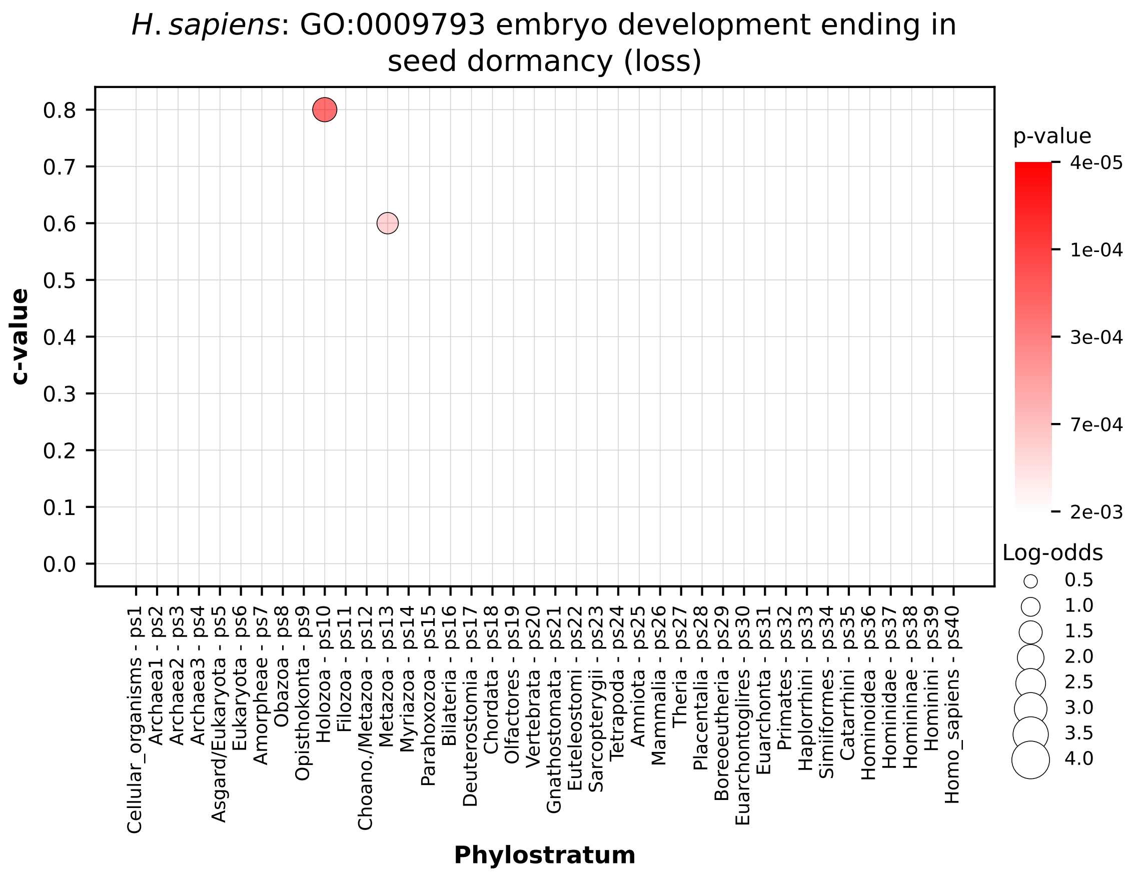

H. sapiens: GO:0009793 embryo development ending in seed dormancy (loss)
c-value
0.0
0.1
0.2
0.3
0.4
0.5
0.6
0.7
0.8
p-value
4e-05
1e-04
3e-04
7e-04
2e-03
Log-odds
0.5
1.0
1.5
2.0
2.5
3.0
3.5
4.0
Cellular_organisms - ps1
Archaea1 - ps2
Archaea2 - ps3
Archaea3 - ps4
Asgard/Eukaryota - ps5
Eukaryota - ps6
Amorpheae - ps7
Obazoa - ps8
Opisthokonta - ps9
Holozoa - ps10
Filozoa - ps11
Choano./Metazoa - ps12
Metazoa - ps13
Myriazoa - ps14
Parahoxozoa - ps15
Bilateria - ps16
Deuterostomia - ps17
Chordata - ps18
Olfactores - ps19
Vertebrata - ps20
Gnathostomata - ps21
Euteleostomi - ps22
Sarcopterygii - ps23
Tetrapoda - ps24
Amniota - ps25
Mammalia - ps26
Theria - ps27
Placentalia - ps28
Boreoeutheria - ps29
Euarchontoglires - ps30
Euarchonta - ps31
Primates - ps32
Haplorrhini - ps33
Simiiformes - ps34
Catarrhini - ps35
Hominoidea - ps36
Hominidae - ps37
Homininae - ps38
Hominini - ps39
Homo_sapiens - ps40
Phylostratum

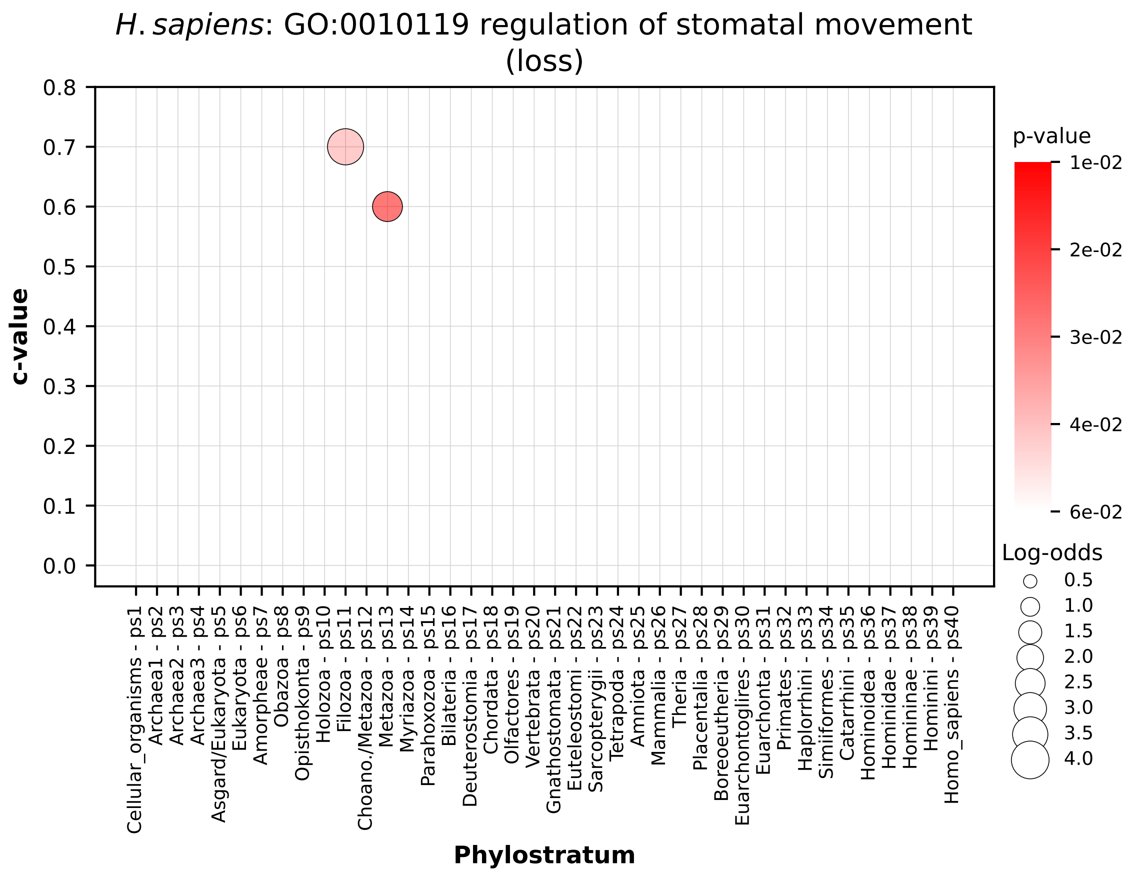

H. sapiens: GO:0010119 regulation of stomatal movement (loss)
c-value
0.8
0.7
0.6
0.5
0.4
0.3
0.2
0.1
0.0
p-value
1e-02
2e-02
3e-02
4e-02
6e-02
Log-odds
0.5
1.0
1.5
2.0
2.5
3.0
3.5
4.0
Cellular_organisms - ps1
Archaea1 - ps2
Archaea2 - ps3
Archaea3 - ps4
Asgard/Eukaryota - ps5
Eukaryota - ps6
Amorpheae - ps7
Obazoa - ps8
Opisthokonta - ps9
Holozoa - ps10
Filozoa - ps11
Choano./Metazoa - ps12
Metazoa - ps13
Myriazoa - ps14
Parahoxozoa - ps15
Bilateria - ps16
Deuterostomia - ps17
Chordata - ps18
Olfactores - ps19
Vertebrata - ps20
Gnathostomata - ps21
Euteleostomi - ps22
Sarcopterygii - ps23
Tetrapoda - ps24
Amniota - ps25
Mammalia - ps26
Theria - ps27
Placentalia - ps28
Boreoeutheria - ps29
Euarchontoglires - ps30
Euarchonta - ps31
Primates - ps32
Haplorrhini - ps33
Simiiformes - ps34
Catarrhini - ps35
Hominoidea - ps36
Hominidae - ps37
Homininae - ps38
Hominini - ps39
Homo_sapiens - ps40
Phylostratum

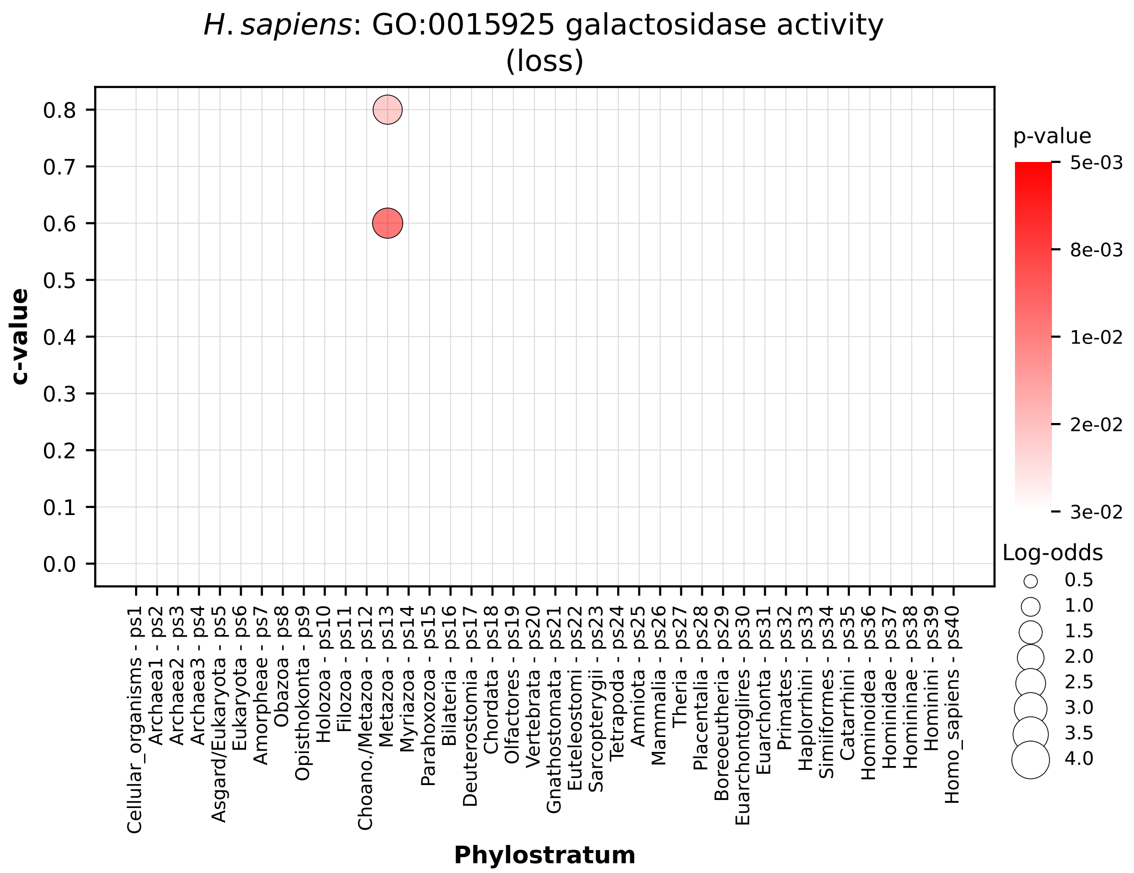
H. sapiens: GO:0015925 galactosidase activity
(loss)
c-value
0.0
0.1
0.2
0.3
0.4
0.5
0.6
0.7
0.8
p-value
5e-03
8e-03
1e-02
2e-02
3e-02
Log-odds
0.5
1.0
1.5
2.0
2.5
3.0
3.5
4.0
Cellular_organisms - ps1
Archaea1 - ps2
Archaea2 - ps3
Archaea3 - ps4
Asgard/Eukaryota - ps5
Eukaryota - ps6
Amorpheae - ps7
Obazoa - ps8
Opisthokonta - ps9
Holozoa - ps10
Filozoa - ps11
Choano./Metazoa - ps12
Metazoa - ps13
Myriazoa - ps14
Parahoxozoa - ps15
Bilateria - ps16
Deuterostomia - ps17
Chordata - ps18
Olfactores - ps19
Vertebrata - ps20
Gnathostomata - ps21
Euteleostomi - ps22
Sarcopterygii - ps23
Tetrapoda - ps24
Amniota - ps25
Mammalia - ps26
Theria - ps27
Placentalia - ps28
Boreoeutheria - ps29
Euarchontoglires - ps30
Euarchonta - ps31
Primates - ps32
Haplorrhini - ps33
Simiiformes - ps34
Catarrhini - ps35
Hominoidea - ps36
Hominidae - ps37
Homininae - ps38
Hominini - ps39
Homo_sapiens - ps40
Phylostratum

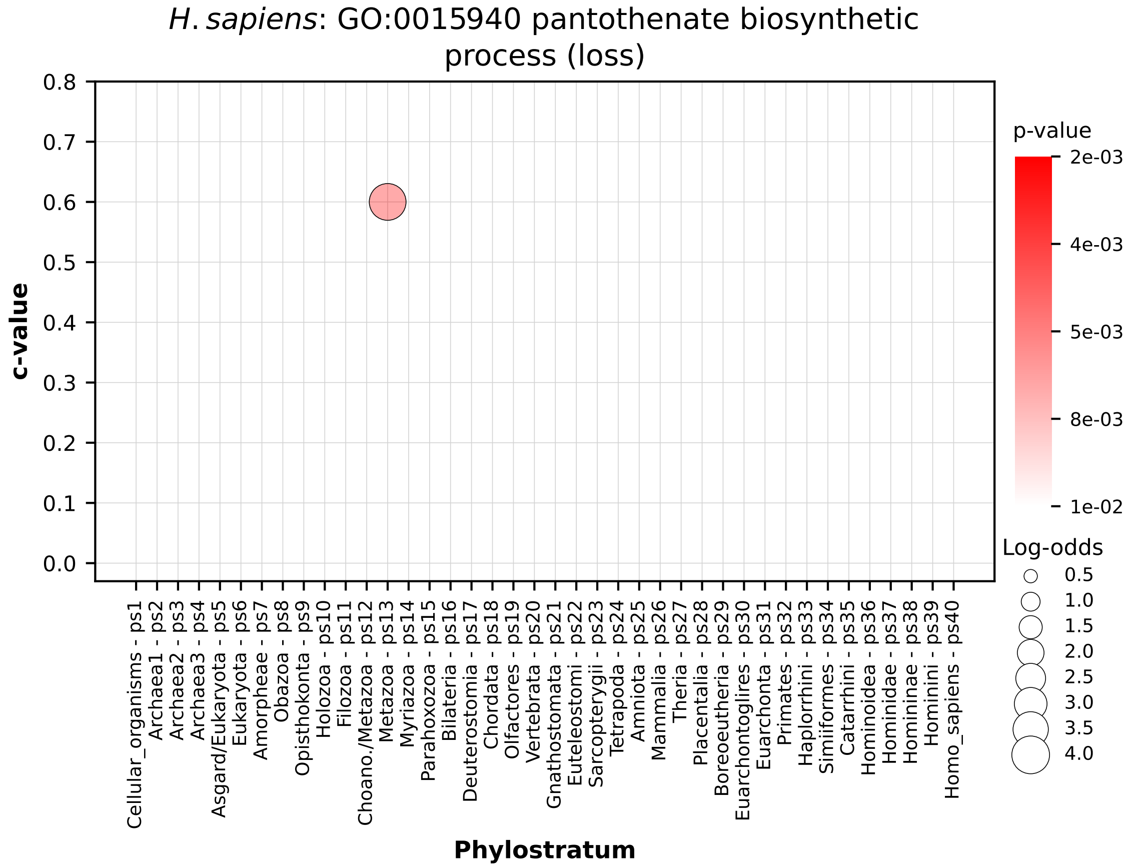

H. sapiens: GO:0015940 pantothenate biosynthetic process (loss)
c-value
0.8
0.7
0.6
0.5
0.4
0.3
0.2
0.1
0.0
Phylostratum
Cellular_organisms - ps1
Archaea1 - ps2
Archaea2 - ps3
Archaea3 - ps4
Asgard/Eukaryota - ps5
Eukaryota - ps6
Amorpheae - ps7
Obazoa - ps8
Opisthokonta - ps9
Holozoa - ps10
Filozoa - ps11
Choano./Metazoa - ps12
Metazoa - ps13
Myriazoa - ps14
Parahoxozoa - ps15
Bilateria - ps16
Deuterostomia - ps17
Chordata - ps18
Olfactores - ps19
Vertebrata - ps20
Gnathostomata - ps21
Euteleostomi - ps22
Sarcopterygii - ps23
Tetrapoda - ps24
Amniota - ps25
Mammalia - ps26
Theria - ps27
Placentalia - ps28
Boreoeutheria - ps29
Euarchontoglires - ps30
Euarchonta - ps31
Primates - ps32
Haplorrhini - ps33
Simiiformes - ps34
Catarrhini - ps35
Hominoidea - ps36
Hominidae - ps37
Homininae - ps38
Hominini - ps39
Homo_sapiens - ps40
p-value
2e-03
4e-03
5e-03
8e-03
1e-02
Log-odds
0.5
1.0
1.5
2.0
2.5
3.0
3.5
4.0

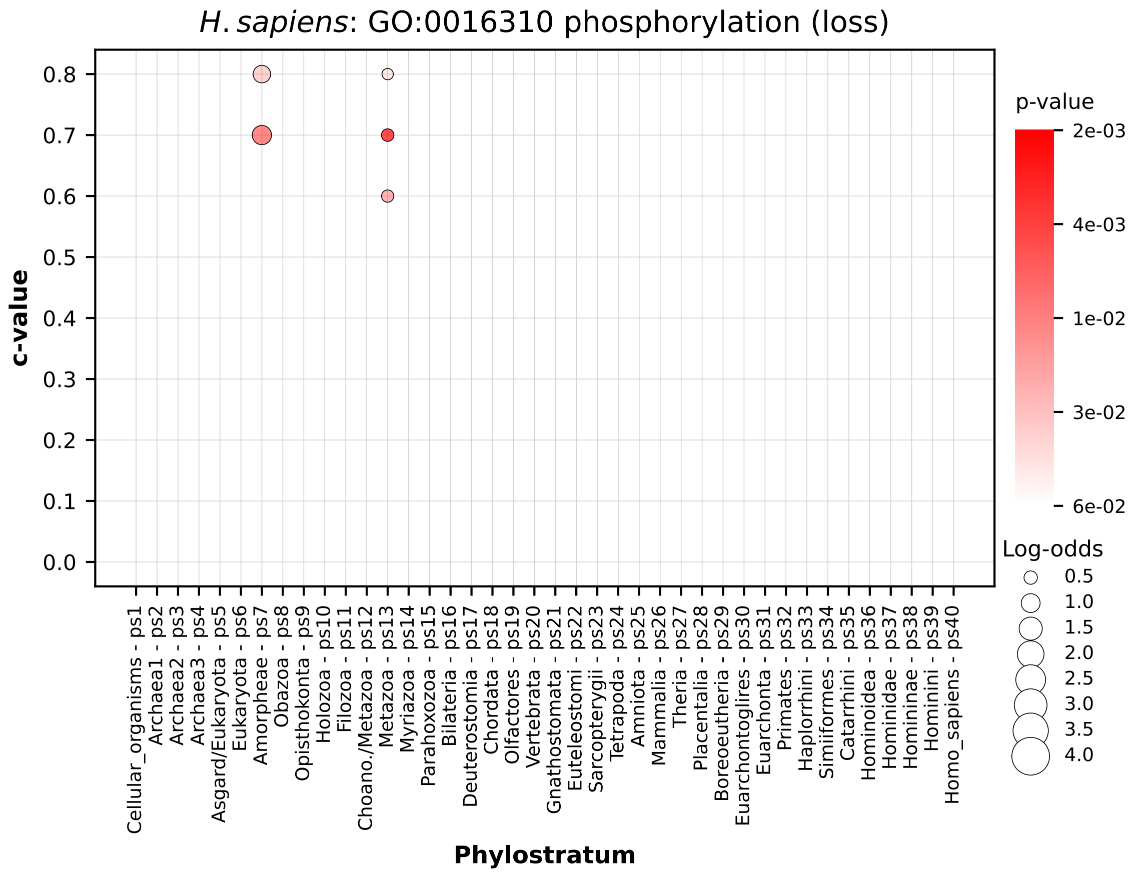

H. sapiens: GO:0016310 phosphorylation (loss)
c-value
0.8
0.7
0.6
0.5
0.4
0.3
0.2
0.1
0.0
p-value
2e-03
4e-03
1e-02
3e-02
6e-02
Log-odds
0.5
1.0
1.5
2.0
2.5
3.0
3.5
4.0
Cellular_organisms - ps1
Archaea1 - ps2
Archaea2 - ps3
Archaea3 - ps4
Asgard/Eukaryota - ps5
Eukaryota - ps6
Amorpheae - ps7
Obazoa - ps8
Opisthokonta - ps9
Holozoa - ps10
Filozoa - ps11
Choano./Metazoa - ps12
Metazoa - ps13
Myriazoa - ps14
Parahoxozoa - ps15
Bilateria - ps16
Deuterostomia - ps17
Chordata - ps18
Olfactores - ps19
Vertebrata - ps20
Gnathostomata - ps21
Euteleostomi - ps22
Sarcopterygii - ps23
Tetrapoda - ps24
Amniota - ps25
Mammalia - ps26
Theria - ps27
Placentalia - ps28
Boreoeutheria - ps29
Euarchontoglires - ps30
Euarchonta - ps31
Primates - ps32
Haplorrhini - ps33
Simiiformes - ps34
Catarrhini - ps35
Hominoidea - ps36
Hominidae - ps37
Homininae - ps38
Hominini - ps39
Homo_sapiens - ps40
Phylostratum

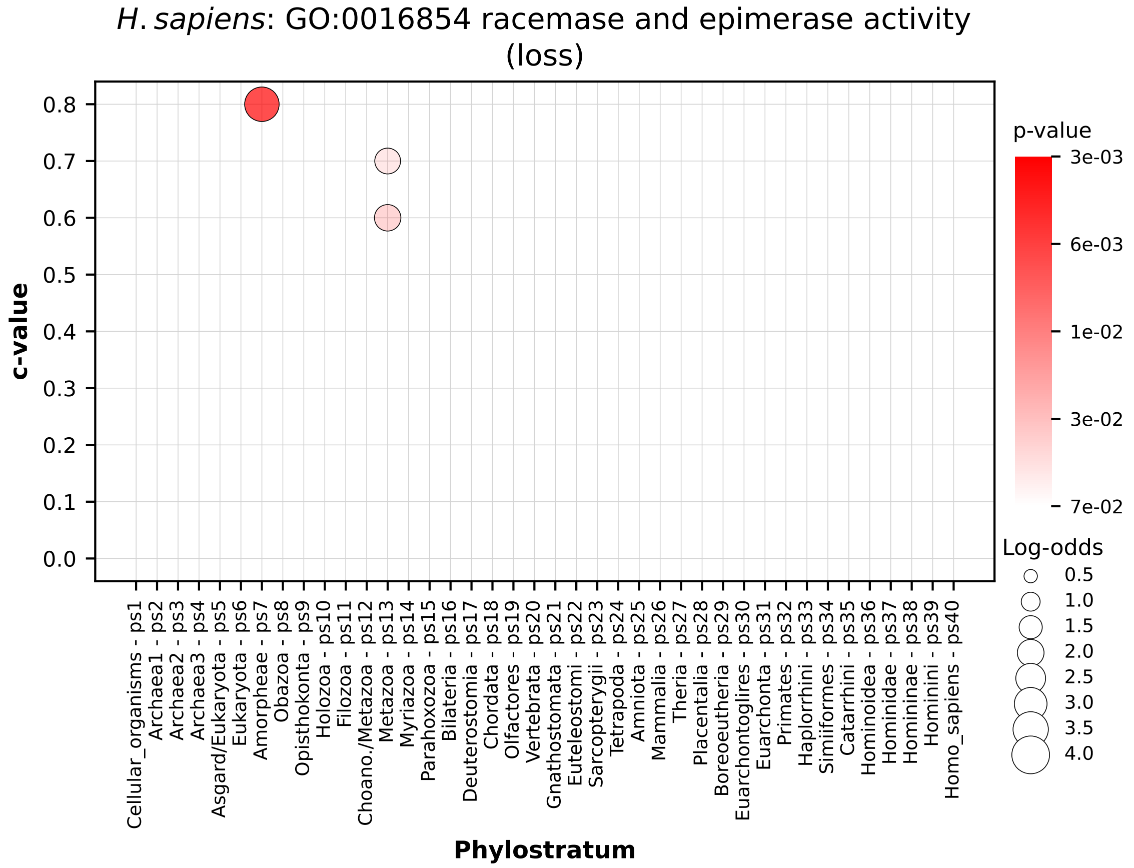
H. sapiens: GO:0016854 racemase and epimerase activity (loss)
c-value
0.8
0.7
0.6
0.5
0.4
0.3
0.2
0.1
0.0
Phylostratum
Cellular_organisms - ps1
Archaea1 - ps2
Archaea2 - ps3
Archaea3 - ps4
Asgard/Eukaryota - ps5
Eukaryota - ps6
Amorpheae - ps7
Obazoa - ps8
Opisthokonta - ps9
Holozoa - ps10
Filozoa - ps11
Choano./Metazoa - ps12
Metazoa - ps13
Myriazoa - ps14
Parahoxozoa - ps15
Bilateria - ps16
Deuterostomia - ps17
Chordata - ps18
Olfactores - ps19
Vertebrata - ps20
Gnathostomata - ps21
Euteleostomi - ps22
Sarcopterygii - ps23
Tetrapoda - ps24
Amniota - ps25
Mammalia - ps26
Theria - ps27
Placentalia - ps28
Boreoeutheria - ps29
Euarchontoglires - ps30
Euarchonta - ps31
Primates - ps32
Haplorrhini - ps33
Simiiformes - ps34
Catarrhini - ps35
Hominoidea - ps36
Hominidae - ps37
Homininae - ps38
Hominini - ps39
Homo_sapiens - ps40
p-value
3e-03
6e-03
1e-02
3e-02
7e-02
Log-odds
0.5
1.0
1.5
2.0
2.5
3.0
3.5
4.0

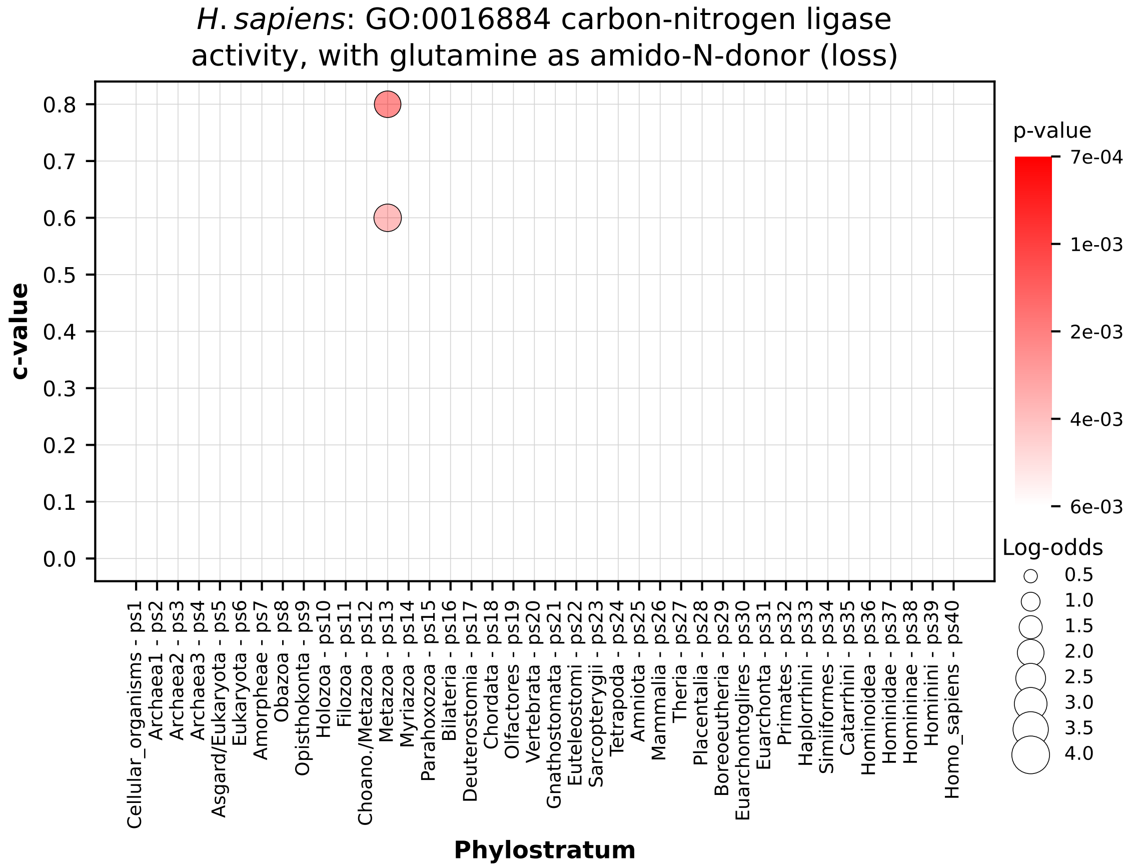

H. sapiens: GO:0016884 carbon-nitrogen ligase activity, with glutamine as amido-N-donor (loss)
c-value
0.0
0.1
0.2
0.3
0.4
0.5
0.6
0.7
0.8
Phylostratum
Cellular_organisms - ps1
Archaea1 - ps2
Archaea2 - ps3
Archaea3 - ps4
Asgard/Eukaryota - ps5
Eukaryota - ps6
Amorpheae - ps7
Obazoa - ps8
Opisthokonta - ps9
Holozoa - ps10
Filozoa - ps11
Choano./Metazoa - ps12
Metazoa - ps13
Myriazoa - ps14
Parahoxozoa - ps15
Bilateria - ps16
Deuterostomia - ps17
Chordata - ps18
Olfactores - ps19
Vertebrata - ps20
Gnathostomata - ps21
Euteleostomi - ps22
Sarcopterygii - ps23
Tetrapoda - ps24
Amniota - ps25
Mammalia - ps26
Theria - ps27
Placentalia - ps28
Boreoeutheria - ps29
Euarchontoglires - ps30
Euarchonta - ps31
Primates - ps32
Haplorrhini - ps33
Simiiformes - ps34
Catarrhini - ps35
Hominoidea - ps36
Hominidae - ps37
Homininae - ps38
Hominini - ps39
Homo_sapiens - ps40
p-value
7e-04
1e-03
2e-03
4e-03
6e-03
Log-odds
0.5
1.0
1.5
2.0
2.5
3.0
3.5
4.0

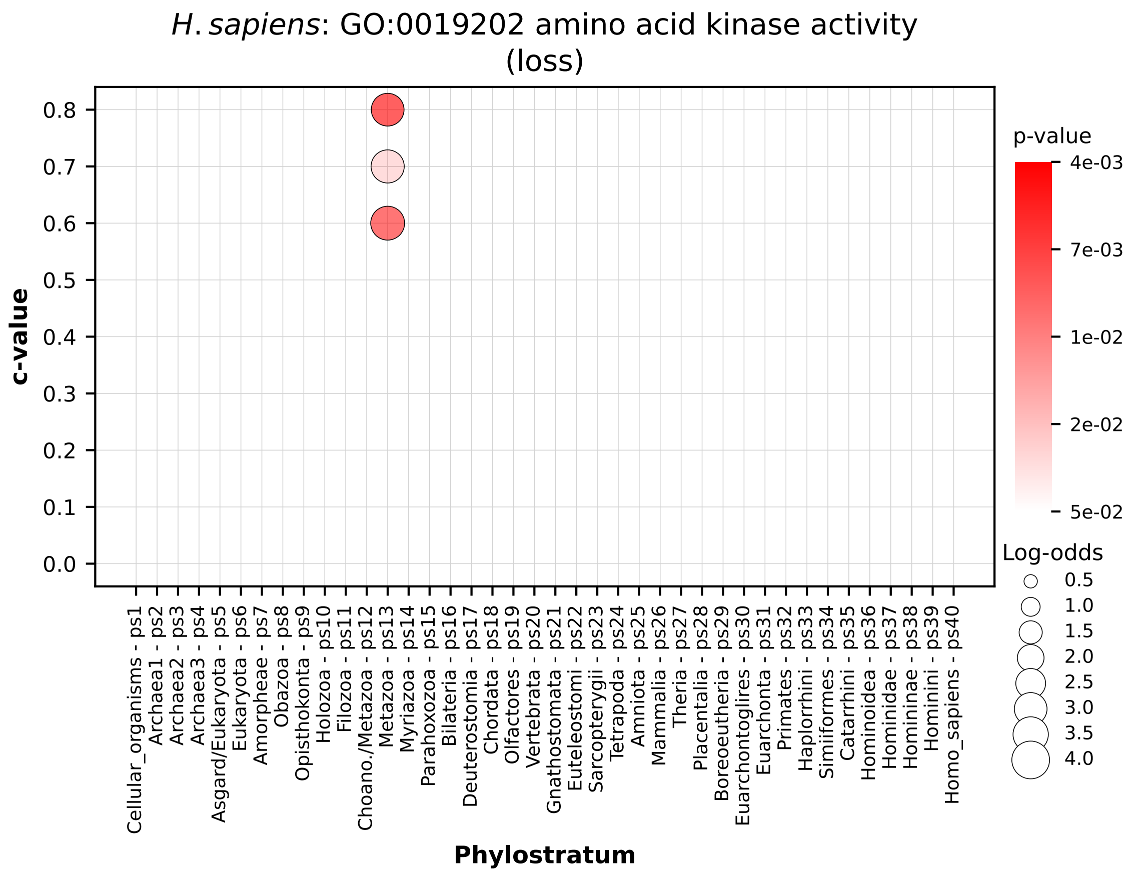

H. sapiens: GO:0019202 amino acid kinase activity
(loss)
c-value
0.8
0.7
0.6
0.5
0.4
0.3
0.2
0.1
0.0
p-value
4e-03
7e-03
1e-02
2e-02
5e-02
Log-odds
0.5
1.0
1.5
2.0
2.5
3.0
3.5
4.0
Cellular_organisms - ps1
Archaea1 - ps2
Archaea2 - ps3
Archaea3 - ps4
Asgard/Eukaryota - ps5
Eukaryota - ps6
Amorpheae - ps7
Obazoa - ps8
Opisthokonta - ps9
Holozoa - ps10
Filozoa - ps11
Choano./Metazoa - ps12
Metazoa - ps13
Myriazoa - ps14
Parahoxozoa - ps15
Bilateria - ps16
Deuterostomia - ps17
Chordata - ps18
Olfactores - ps19
Vertebrata - ps20
Gnathostomata - ps21
Euteleostomi - ps22
Sarcopterygii - ps23
Tetrapoda - ps24
Amniota - ps25
Mammalia - ps26
Theria - ps27
Placentalia - ps28
Boreoeutheria - ps29
Euarchontoglires - ps30
Euarchonta - ps31
Primates - ps32
Haplorrhini - ps33
Simiiformes - ps34
Catarrhini - ps35
Hominoidea - ps36
Hominidae - ps37
Homininae - ps38
Hominini - ps39
Homo_sapiens - ps40
Phylostratum

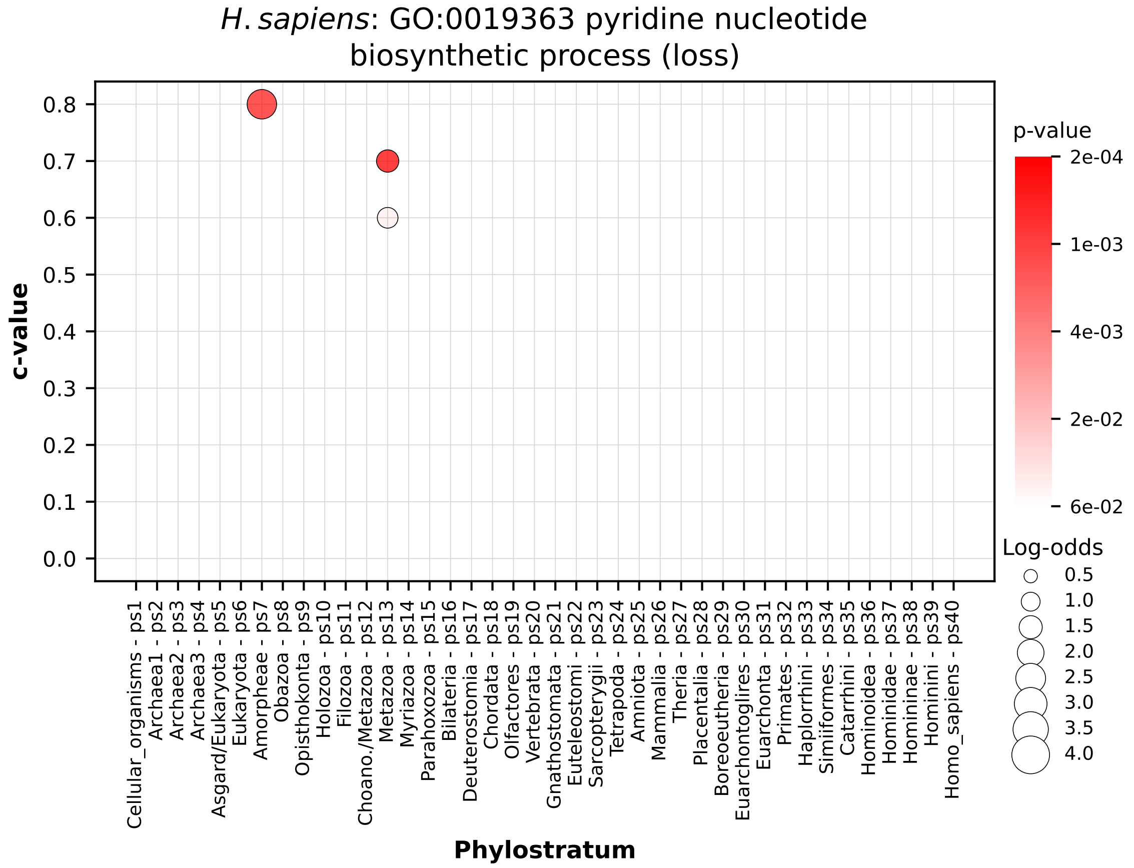

H. sapiens: GO:0019363 pyridine nucleotide biosynthetic process (loss)
c-value
0.8
0.7
0.6
0.5
0.4
0.3
0.2
0.1
0.0
Phylostratum
Cellular_organisms - ps1
Archaea1 - ps2
Archaea2 - ps3
Archaea3 - ps4
Asgard/Eukaryota - ps5
Eukaryota - ps6
Amorpheae - ps7
Obazoa - ps8
Opisthokonta - ps9
Holozoa - ps10
Filozoa - ps11
Choano./Metazoa - ps12
Metazoa - ps13
Myriazoa - ps14
Parahoxozoa - ps15
Bilateria - ps16
Deuterostomia - ps17
Chordata - ps18
Olfactores - ps19
Vertebrata - ps20
Gnathostomata - ps21
Euteleostomi - ps22
Sarcopterygii - ps23
Tetrapoda - ps24
Amniota - ps25
Mammalia - ps26
Theria - ps27
Placentalia - ps28
Boreoeutheria - ps29
Euarchontoglires - ps30
Euarchonta - ps31
Primates - ps32
Haplorrhini - ps33
Simiiformes - ps34
Catarrhini - ps35
Hominoidea - ps36
Hominidae - ps37
Homininae - ps38
Hominini - ps39
Homo_sapiens - ps40
p-value
2e-04
1e-03
4e-03
2e-02
6e-02
Log-odds
0.5
1.0
1.5
2.0
2.5
3.0
3.5
4.0

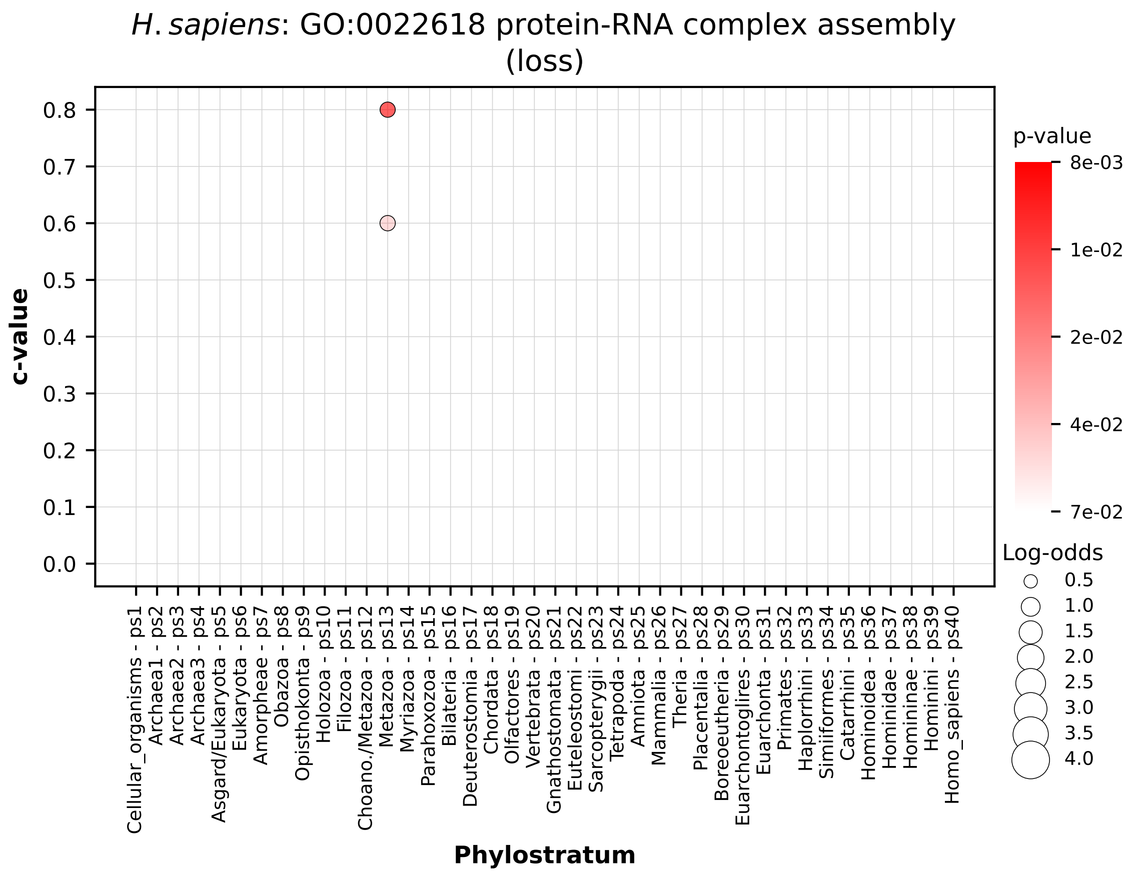

H. sapiens: GO:0022618 protein-RNA complex assembly (loss)
c-value
0.0
0.1
0.2
0.3
0.4
0.5
0.6
0.7
0.8
Phylostratum
Cellular_organisms - ps1
Archaea1 - ps2
Archaea2 - ps3
Archaea3 - ps4
Asgard/Eukaryota - ps5
Eukaryota - ps6
Amorpheae - ps7
Obazoa - ps8
Opisthokonta - ps9
Holozoa - ps10
Filozoa - ps11
Choano./Metazoa - ps12
Metazoa - ps13
Myriazoa - ps14
Parahoxozoa - ps15
Bilateria - ps16
Deuterostomia - ps17
Chordata - ps18
Olfactores - ps19
Vertebrata - ps20
Gnathostomata - ps21
Euteleostomi - ps22
Sarcopterygii - ps23
Tetrapoda - ps24
Amniota - ps25
Mammalia - ps26
Theria - ps27
Placentalia - ps28
Boreoeutheria - ps29
Euarchontoglires - ps30
Euarchonta - ps31
Primates - ps32
Haplorrhini - ps33
Simiiformes - ps34
Catarrhini - ps35
Hominoidea - ps36
Hominidae - ps37
Homininae - ps38
Hominini - ps39
Homo_sapiens - ps40
p-value
8e-03
1e-02
2e-02
4e-02
7e-02
Log-odds
0.5
1.0
1.5
2.0
2.5
3.0
3.5
4.0

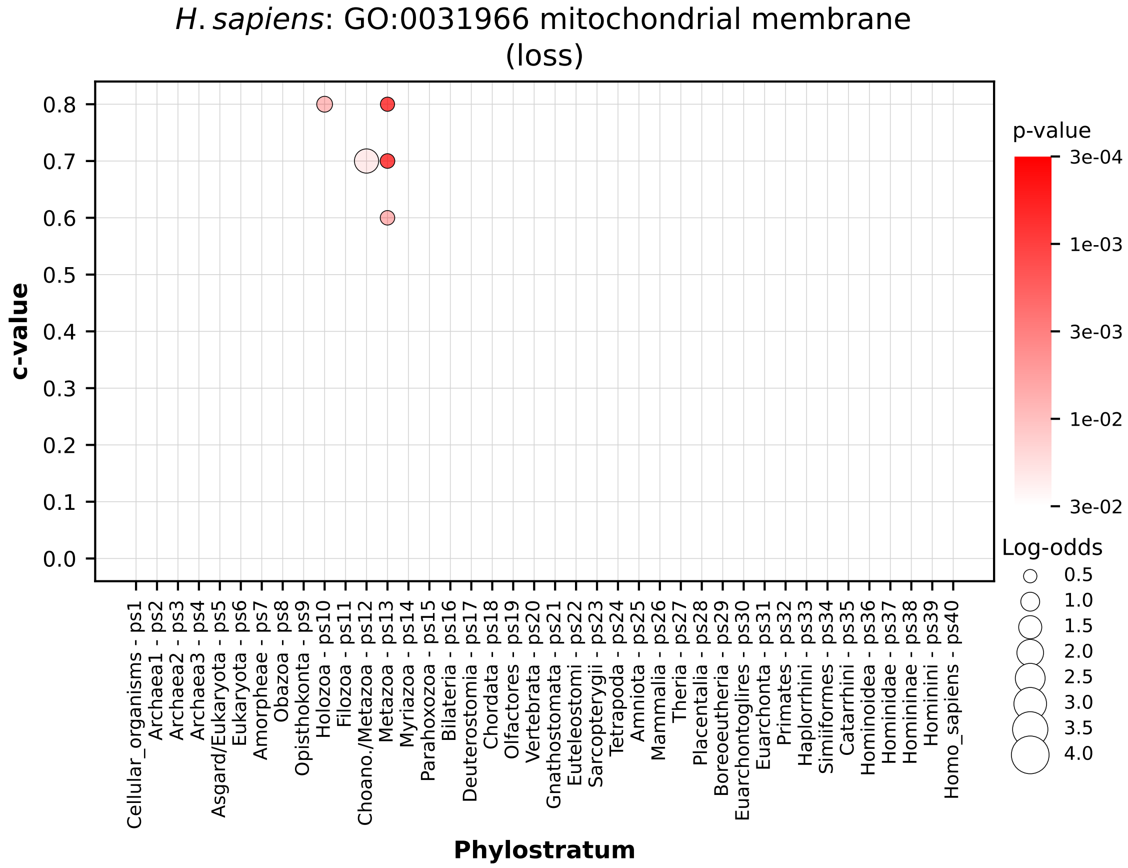

H. sapiens: GO:0031966 mitochondrial membrane
(loss)
c-value
0.0
0.1
0.2
0.3
0.4
0.5
0.6
0.7
0.8
p-value
3e-04
1e-03
3e-03
1e-02
3e-02
Log-odds
0.5
1.0
1.5
2.0
2.5
3.0
3.5
4.0
Cellular_organisms - ps1
Archaea1 - ps2
Archaea2 - ps3
Archaea3 - ps4
Asgard/Eukaryota - ps5
Eukaryota - ps6
Amorpheae - ps7
Obazoa - ps8
Opisthokonta - ps9
Holozoa - ps10
Filozoa - ps11
Choano./Metazoa - ps12
Metazoa - ps13
Myriazoa - ps14
Parahoxozoa - ps15
Bilateria - ps16
Deuterostomia - ps17
Chordata - ps18
Olfactores - ps19
Vertebrata - ps20
Gnathostomata - ps21
Euteleostomi - ps22
Sarcopterygii - ps23
Tetrapoda - ps24
Amniota - ps25
Mammalia - ps26
Theria - ps27
Placentalia - ps28
Boreoeutheria - ps29
Euarchontoglires - ps30
Euarchonta - ps31
Primates - ps32
Haplorrhini - ps33
Simiiformes - ps34
Catarrhini - ps35
Hominoidea - ps36
Hominidae - ps37
Homininae - ps38
Hominini - ps39
Homo_sapiens - ps40
Phylostratum

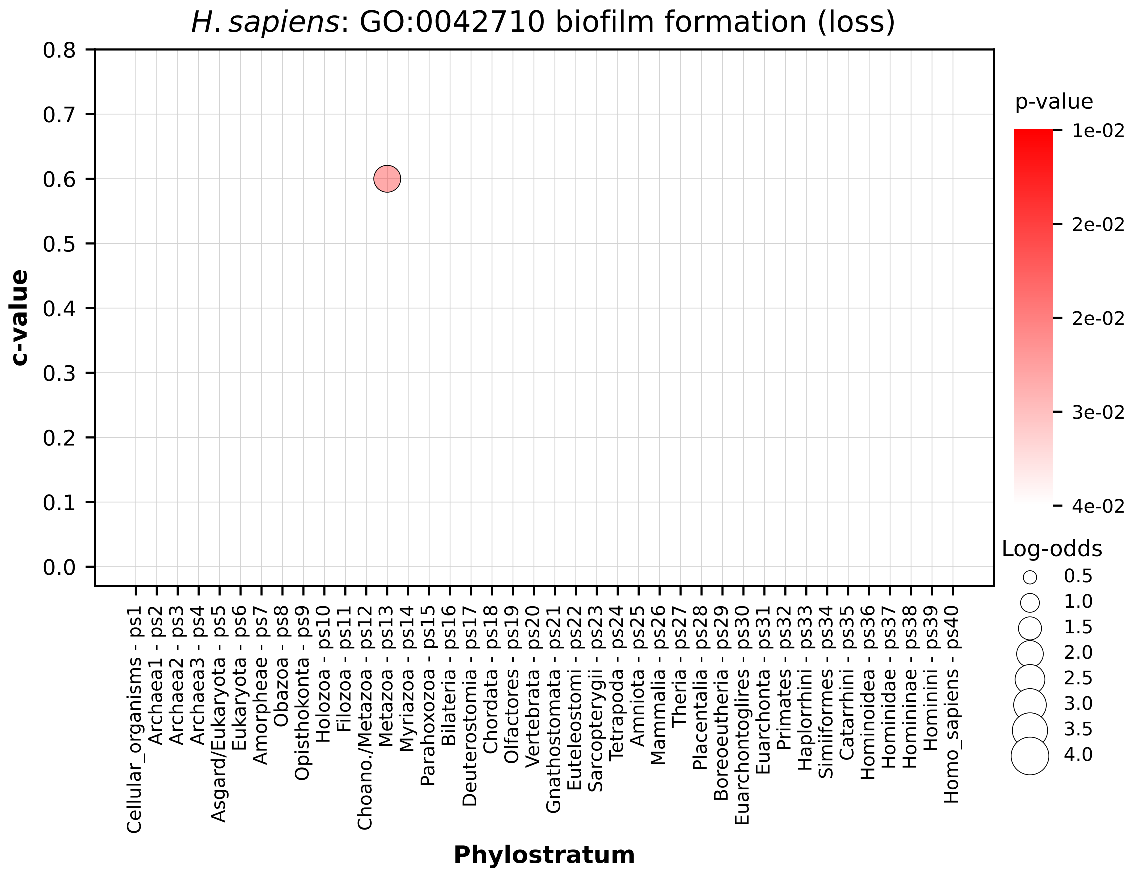
H. sapiens: GO:0042710 biofilm formation (loss)
c-value
0.0
0.1
0.2
0.3
0.4
0.5
0.6
0.7
0.8
Phylostratum
Cellular_organisms - ps1
Archaea1 - ps2
Archaea2 - ps3
Archaea3 - ps4
Asgard/Eukaryota - ps5
Eukaryota - ps6
Amorpheae - ps7
Obazoa - ps8
Opisthokonta - ps9
Holozoa - ps10
Filozoa - ps11
Choano./Metazoa - ps12
Metazoa - ps13
Myriazoa - ps14
Parahoxozoa - ps15
Bilateria - ps16
Deuterostomia - ps17
Chordata - ps18
Olfactores - ps19
Vertebrata - ps20
Gnathostomata - ps21
Euteleostomi - ps22
Sarcopterygii - ps23
Tetrapoda - ps24
Amniota - ps25
Mammalia - ps26
Theria - ps27
Placentalia - ps28
Boreoeutheria - ps29
Euarchontoglires - ps30
Euarchonta - ps31
Primates - ps32
Haplorrhini - ps33
Simiiformes - ps34
Catarrhini - ps35
Hominoidea - ps36
Hominidae - ps37
Homininae - ps38
Hominini - ps39
Homo_sapiens - ps40
p-value
1e-02
2e-02
2e-02
3e-02
4e-02
Log-odds
0.5
1.0
1.5
2.0
2.5
3.0
3.5
4.0

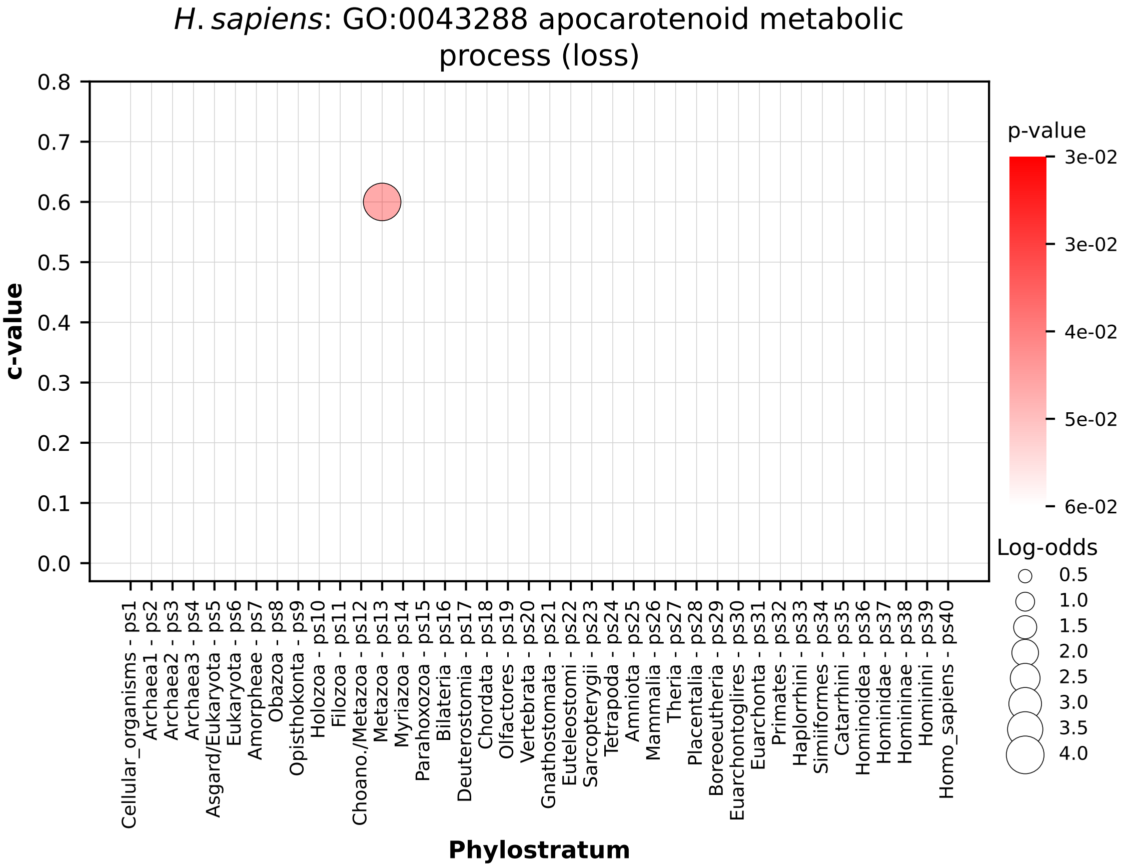

H. sapiens: GO:0043288 apocarotenoid metabolic process (loss)
c-value
0.8
0.7
0.6
0.5
0.4
0.3
0.2
0.1
0.0
p-value
3e-02
3e-02
4e-02
5e-02
6e-02
Log-odds
0.5
1.0
1.5
2.0
2.5
3.0
3.5
4.0
Cellular_organisms - ps1
Archaea1 - ps2
Archaea2 - ps3
Archaea3 - ps4
Asgard/Eukaryota - ps5
Eukaryota - ps6
Amorpheae - ps7
Obazoa - ps8
Opisthokonta - ps9
Holozoa - ps10
Filozoa - ps11
Choano./Metazoa - ps12
Metazoa - ps13
Myriazoa - ps14
Parahoxozoa - ps15
Bilateria - ps16
Deuterostomia - ps17
Chordata - ps18
Olfactores - ps19
Vertebrata - ps20
Gnathostomata - ps21
Euteleostomi - ps22
Sarcopterygii - ps23
Tetrapoda - ps24
Amniota - ps25
Mammalia - ps26
Theria - ps27
Placentalia - ps28
Boreoeutheria - ps29
Euarchontoglires - ps30
Euarchonta - ps31
Primates - ps32
Haplorrhini - ps33
Simiiformes - ps34
Catarrhini - ps35
Hominoidea - ps36
Hominidae - ps37
Homininae - ps38
Hominini - ps39
Homo_sapiens - ps40
Phylostratum

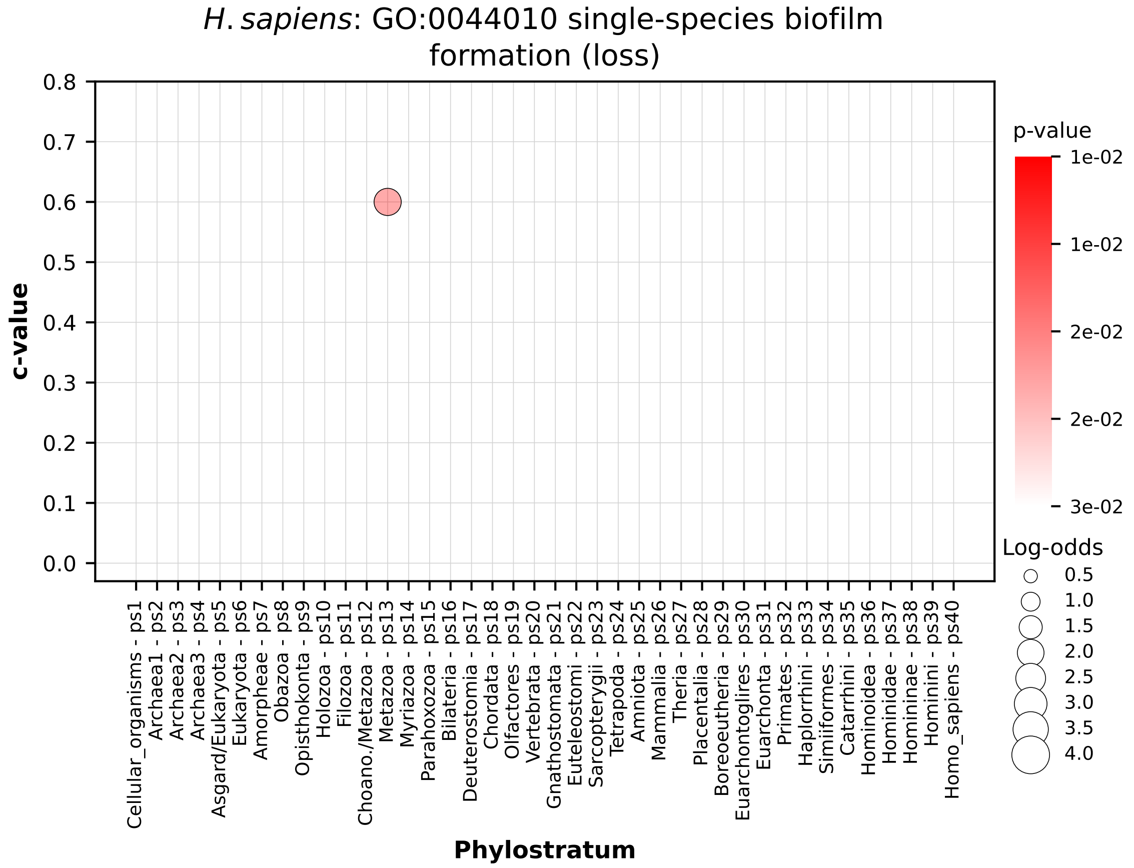

H. sapiens: GO:0044010 single-species biofilm formation (loss)
c-value
0.8
0.7
0.6
0.5
0.4
0.3
0.2
0.1
0.0
Phylostratum
Cellular_organisms - ps1
Archaea1 - ps2
Archaea2 - ps3
Archaea3 - ps4
Asgard/Eukaryota - ps5
Eukaryota - ps6
Amorpheae - ps7
Obazoa - ps8
Opisthokonta - ps9
Holozoa - ps10
Filozoa - ps11
Choano./Metazoa - ps12
Metazoa - ps13
Myriazoa - ps14
Parahoxozoa - ps15
Bilateria - ps16
Deuterostomia - ps17
Chordata - ps18
Olfactores - ps19
Vertebrata - ps20
Gnathostomata - ps21
Euteleostomi - ps22
Sarcopterygii - ps23
Tetrapoda - ps24
Amniota - ps25
Mammalia - ps26
Theria - ps27
Placentalia - ps28
Boreoeutheria - ps29
Euarchontoglires - ps30
Euarchonta - ps31
Primates - ps32
Haplorrhini - ps33
Simiiformes - ps34
Catarrhini - ps35
Hominoidea - ps36
Hominidae - ps37
Homininae - ps38
Hominini - ps39
Homo_sapiens - ps40
p-value
1e-02
1e-02
2e-02
2e-02
3e-02
Log-odds
0.5
1.0
1.5
2.0
2.5
3.0
3.5
4.0

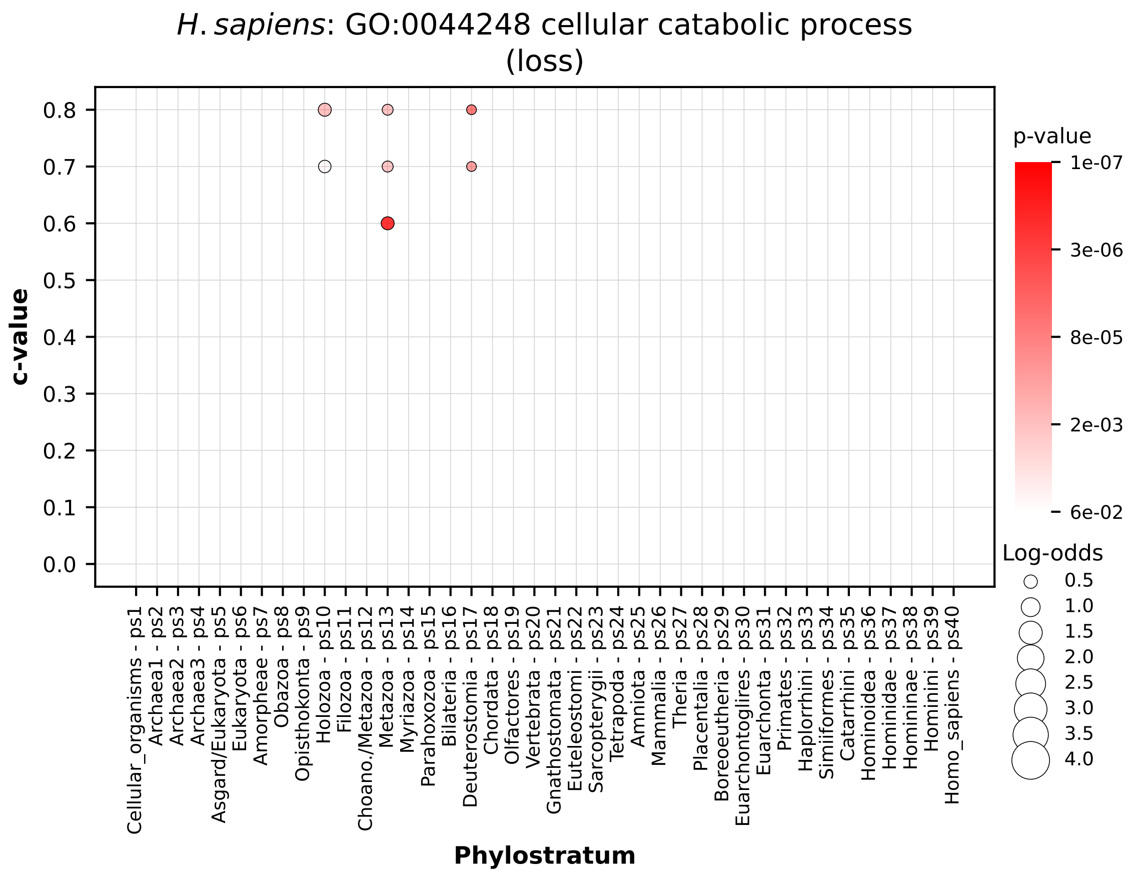

H. sapiens: GO:0044248 cellular catabolic process
(loss)
c-value
0.0
0.1
0.2
0.3
0.4
0.5
0.6
0.7
0.8
Phylostratum
Cellular_organisms - ps1
Archaea1 - ps2
Archaea2 - ps3
Archaea3 - ps4
Asgard/Eukaryota - ps5
Eukaryota - ps6
Amorpheae - ps7
Obazoa - ps8
Opisthokonta - ps9
Holozoa - ps10
Filozoa - ps11
Choano./Metazoa - ps12
Metazoa - ps13
Myriazoa - ps14
Parahoxozoa - ps15
Bilateria - ps16
Deuterostomia - ps17
Chordata - ps18
Olfactores - ps19
Vertebrata - ps20
Gnathostomata - ps21
Euteleostomi - ps22
Sarcopterygii - ps23
Tetrapoda - ps24
Amniota - ps25
Mammalia - ps26
Theria - ps27
Placentalia - ps28
Boreoeutheria - ps29
Euarchontoglires - ps30
Euarchonta - ps31
Primates - ps32
Haplorrhini - ps33
Simiiformes - ps34
Catarrhini - ps35
Hominoidea - ps36
Hominidae - ps37
Homininae - ps38
Hominini - ps39
Homo_sapiens - ps40
p-value
1e-07
3e-06
8e-05
2e-03
6e-02
Log-odds
0.5
1.0
1.5
2.0
2.5
3.0
3.5
4.0

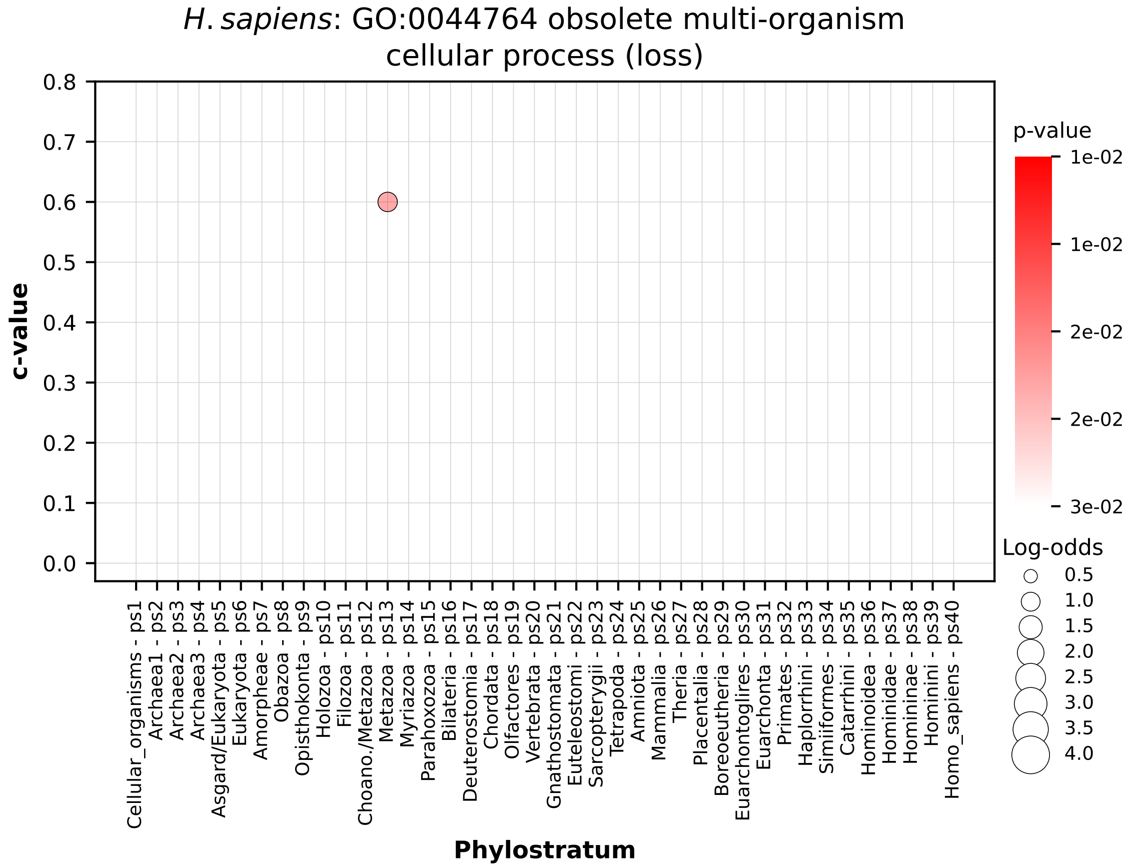

H. sapiens: GO:0044764 obsolete multi-organism cellular process (loss)
c-value
0.8
0.7
0.6
0.5
0.4
0.3
0.2
0.1
0.0
p-value
1e-02
1e-02
2e-02
2e-02
3e-02
Log-odds
0.5
1.0
1.5
2.0
2.5
3.0
3.5
4.0
Cellular_organisms - ps1
Archaea1 - ps2
Archaea2 - ps3
Archaea3 - ps4
Asgard/Eukaryota - ps5
Eukaryota - ps6
Amorpheae - ps7
Obazoa - ps8
Opisthokonta - ps9
Holozoa - ps10
Filozoa - ps11
Choano./Metazoa - ps12
Metazoa - ps13
Myriazoa - ps14
Parahoxozoa - ps15
Bilateria - ps16
Deuterostomia - ps17
Chordata - ps18
Olfactores - ps19
Vertebrata - ps20
Gnathostomata - ps21
Euteleostomi - ps22
Sarcopterygii - ps23
Tetrapoda - ps24
Amniota - ps25
Mammalia - ps26
Theria - ps27
Placentalia - ps28
Boreoeutheria - ps29
Euarchontoglires - ps30
Euarchonta - ps31
Primates - ps32
Haplorrhini - ps33
Simiiformes - ps34
Catarrhini - ps35
Hominoidea - ps36
Hominidae - ps37
Homininae - ps38
Hominini - ps39
Homo_sapiens - ps40
Phylostratum

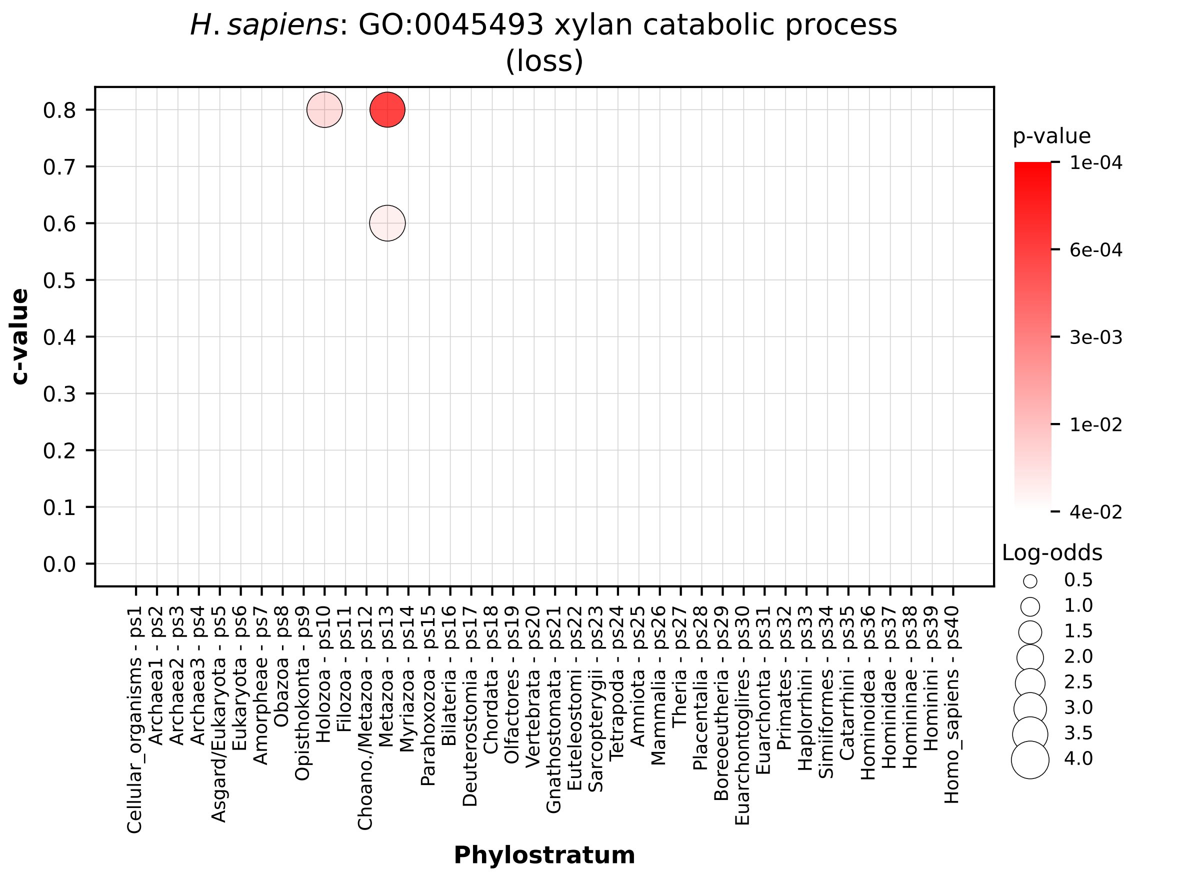
H. sapiens: GO:0045493 xylan catabolic process
(loss)
c-value
0.0
0.1
0.2
0.3
0.4
0.5
0.6
0.7
0.8
Phylostratum
Cellular_organisms - ps1
Archaea1 - ps2
Archaea2 - ps3
Archaea3 - ps4
Asgard/Eukaryota - ps5
Eukaryota - ps6
Amorpheae - ps7
Obazoa - ps8
Opisthokonta - ps9
Holozoa - ps10
Filozoa - ps11
Choano./Metazoa - ps12
Metazoa - ps13
Myriazoa - ps14
Parahoxozoa - ps15
Bilateria - ps16
Deuterostomia - ps17
Chordata - ps18
Olfactores - ps19
Vertebrata - ps20
Gnathostomata - ps21
Euteleostomi - ps22
Sarcopterygii - ps23
Tetrapoda - ps24
Amniota - ps25
Mammalia - ps26
Theria - ps27
Placentalia - ps28
Boreoeutheria - ps29
Euarchontoglires - ps30
Euarchonta - ps31
Primates - ps32
Haplorrhini - ps33
Simiiformes - ps34
Catarrhini - ps35
Hominoidea - ps36
Hominidae - ps37
Homininae - ps38
Hominini - ps39
Homo_sapiens - ps40
p-value
1e-04
6e-04
3e-03
1e-02
4e-02
Log-odds
0.5
1.0
1.5
2.0
2.5
3.0
3.5
4.0

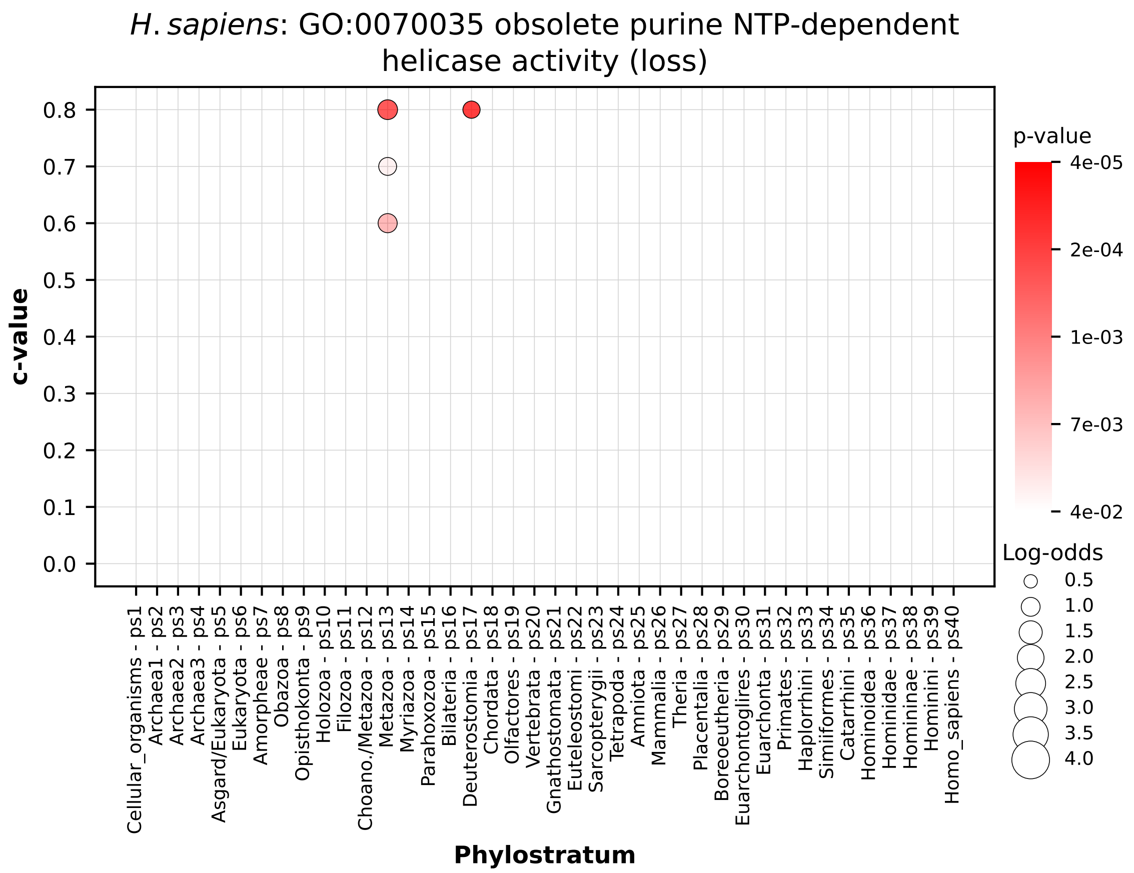
H. sapiens: GO:0070035 obsolete purine NTP-dependent helicase activity (loss)
c-value
0.0
0.1
0.2
0.3
0.4
0.5
0.6
0.7
0.8
p-value
4e-05
2e-04
1e-03
7e-03
4e-02
Log-odds
0.5
1.0
1.5
2.0
2.5
3.0
3.5
4.0
Phylostratum
Cellular_organisms - ps1
Archaea1 - ps2
Archaea2 - ps3
Archaea3 - ps4
Asgard/Eukaryota - ps5
Eukaryota - ps6
Amorpheae - ps7
Obazoa - ps8
Opisthokonta - ps9
Holozoa - ps10
Filozoa - ps11
Choano./Metazoa - ps12
Metazoa - ps13
Myriazoa - ps14
Parahoxozoa - ps15
Bilateria - ps16
Deuterostomia - ps17
Chordata - ps18
Olfactores - ps19
Vertebrata - ps20
Gnathostomata - ps21
Euteleostomi - ps22
Sarcopterygii - ps23
Tetrapoda - ps24
Amniota - ps25
Mammalia - ps26
Theria - ps27
Placentalia - ps28
Boreoeutheria - ps29
Euarchontoglires - ps30
Euarchonta - ps31
Primates - ps32
Haplorrhini - ps33
Simiiformes - ps34
Catarrhini - ps35
Hominoidea - ps36
Hominidae - ps37
Homininae - ps38
Hominini - ps39
Homo_sapiens - ps40

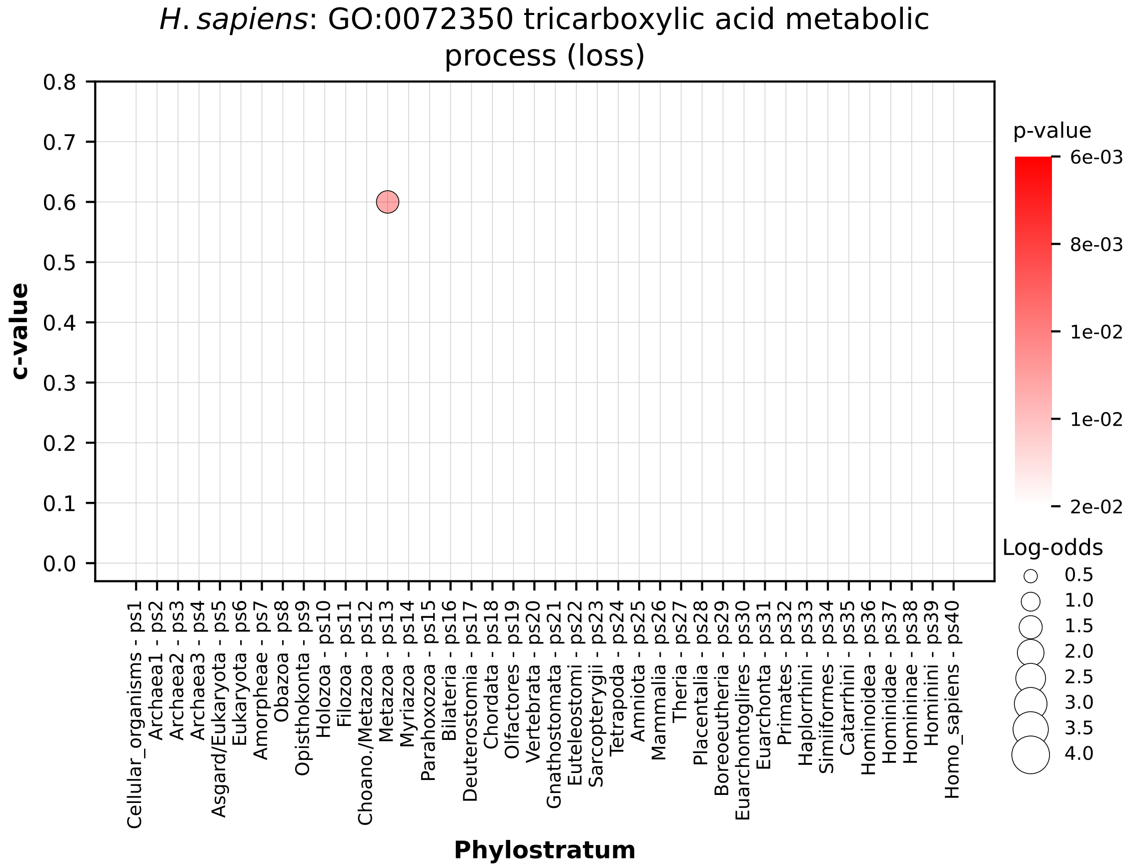

H. sapiens: GO:0072350 tricarboxylic acid metabolic process (loss)
c-value
0.8
0.7
0.6
0.5
0.4
0.3
0.2
0.1
0.0
Phylostratum
Cellular_organisms - ps1
Archaea1 - ps2
Archaea2 - ps3
Archaea3 - ps4
Asgard/Eukaryota - ps5
Eukaryota - ps6
Amorpheae - ps7
Obazoa - ps8
Opisthokonta - ps9
Holozoa - ps10
Filozoa - ps11
Choano./Metazoa - ps12
Metazoa - ps13
Myriazoa - ps14
Parahoxozoa - ps15
Bilateria - ps16
Deuterostomia - ps17
Chordata - ps18
Olfactores - ps19
Vertebrata - ps20
Gnathostomata - ps21
Euteleostomi - ps22
Sarcopterygii - ps23
Tetrapoda - ps24
Amniota - ps25
Mammalia - ps26
Theria - ps27
Placentalia - ps28
Boreoeutheria - ps29
Euarchontoglires - ps30
Euarchonta - ps31
Primates - ps32
Haplorrhini - ps33
Simiiformes - ps34
Catarrhini - ps35
Hominoidea - ps36
Hominidae - ps37
Homininae - ps38
Hominini - ps39
Homo_sapiens - ps40
p-value
6e-03
8e-03
1e-02
1e-02
2e-02
Log-odds
0.5
1.0
1.5
2.0
2.5
3.0
3.5
4.0

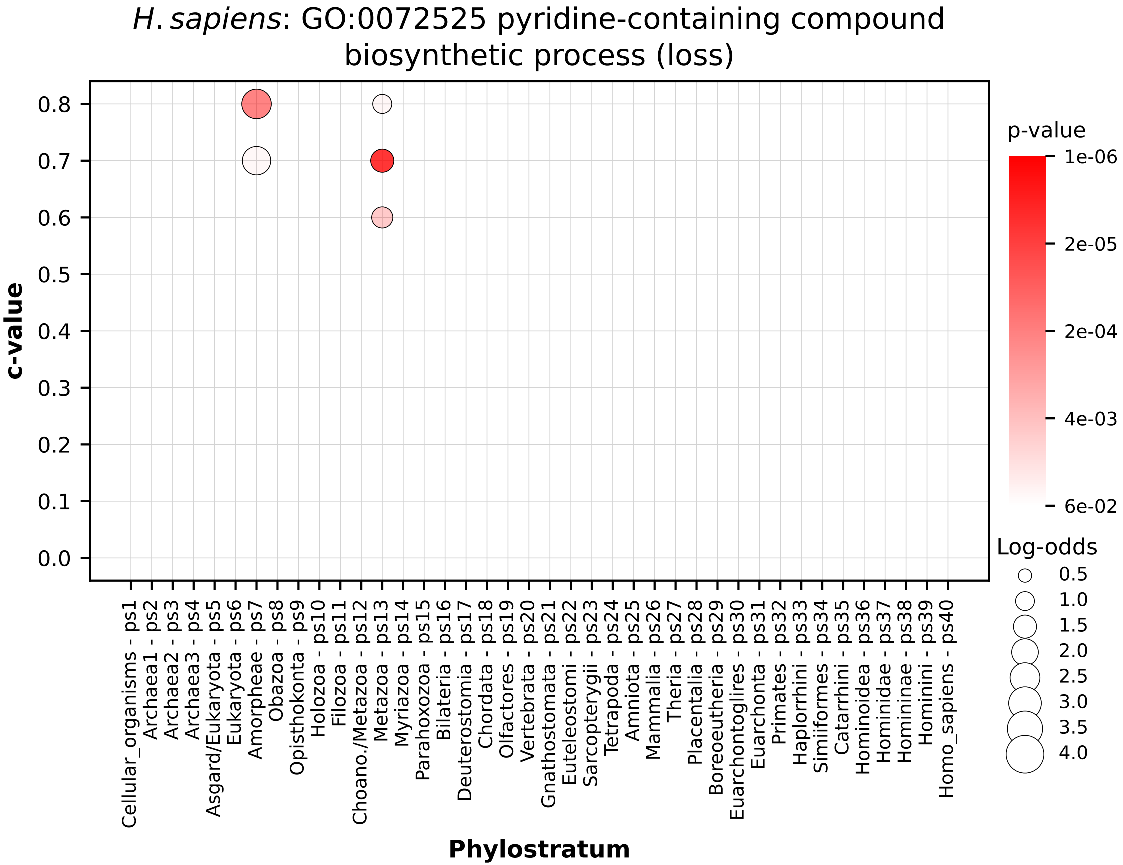

H. sapiens: GO:0072525 pyridine-containing compound biosynthetic process (loss)
c-value
0.8
0.7
0.6
0.5
0.4
0.3
0.2
0.1
0.0
p-value
1e-06
2e-05
2e-04
4e-03
6e-02
Log-odds
0.5
1.0
1.5
2.0
2.5
3.0
3.5
4.0
Cellular_organisms - ps1
Archaea1 - ps2
Archaea2 - ps3
Archaea3 - ps4
Asgard/Eukaryota - ps5
Eukaryota - ps6
Amorpheae - ps7
Obazoa - ps8
Opisthokonta - ps9
Holozoa - ps10
Filozoa - ps11
Choano./Metazoa - ps12
Metazoa - ps13
Myriazoa - ps14
Parahoxozoa - ps15
Bilateria - ps16
Deuterostomia - ps17
Chordata - ps18
Olfactores - ps19
Vertebrata - ps20
Gnathostomata - ps21
Euteleostomi - ps22
Sarcopterygii - ps23
Tetrapoda - ps24
Amniota - ps25
Mammalia - ps26
Theria - ps27
Placentalia - ps28
Boreoeutheria - ps29
Euarchontoglires - ps30
Euarchonta - ps31
Primates - ps32
Haplorrhini - ps33
Simiiformes - ps34
Catarrhini - ps35
Hominoidea - ps36
Hominidae - ps37
Homininae - ps38
Hominini - ps39
Homo_sapiens - ps40
Phylostratum

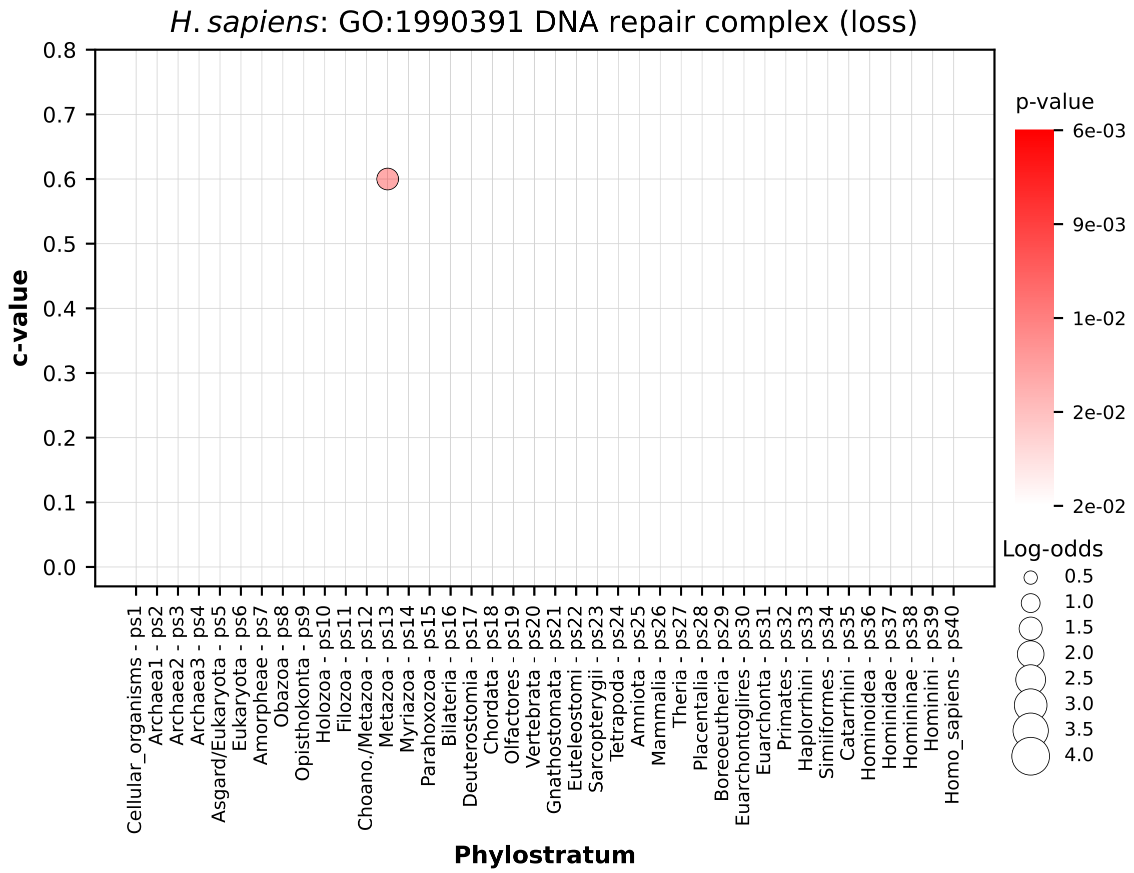
H. sapiens: GO:1990391 DNA repair complex (loss)
c-value
0.0
0.1
0.2
0.3
0.4
0.5
0.6
0.7
0.8
Phylostratum
Cellular_organisms - ps1
Archaea1 - ps2
Archaea2 - ps3
Archaea3 - ps4
Asgard/Eukaryota - ps5
Eukaryota - ps6
Amorpheae - ps7
Obazoa - ps8
Opisthokonta - ps9
Holozoa - ps10
Filozoa - ps11
Choano./Metazoa - ps12
Metazoa - ps13
Myriazoa - ps14
Parahoxozoa - ps15
Bilateria - ps16
Deuterostomia - ps17
Chordata - ps18
Olfactores - ps19
Vertebrata - ps20
Gnathostomata - ps21
Euteleostomi - ps22
Sarcopterygii - ps23
Tetrapoda - ps24
Amniota - ps25
Mammalia - ps26
Theria - ps27
Placentalia - ps28
Boreoeutheria - ps29
Euarchontoglires - ps30
Euarchonta - ps31
Primates - ps32
Haplorrhini - ps33
Simiiformes - ps34
Catarrhini - ps35
Hominoidea - ps36
Hominidae - ps37
Homininae - ps38
Hominini - ps39
Homo_sapiens - ps40
p-value
6e-03
9e-03
1e-02
2e-02
2e-02
Log-odds
0.5
1.0
1.5
2.0
2.5
3.0
3.5
4.0

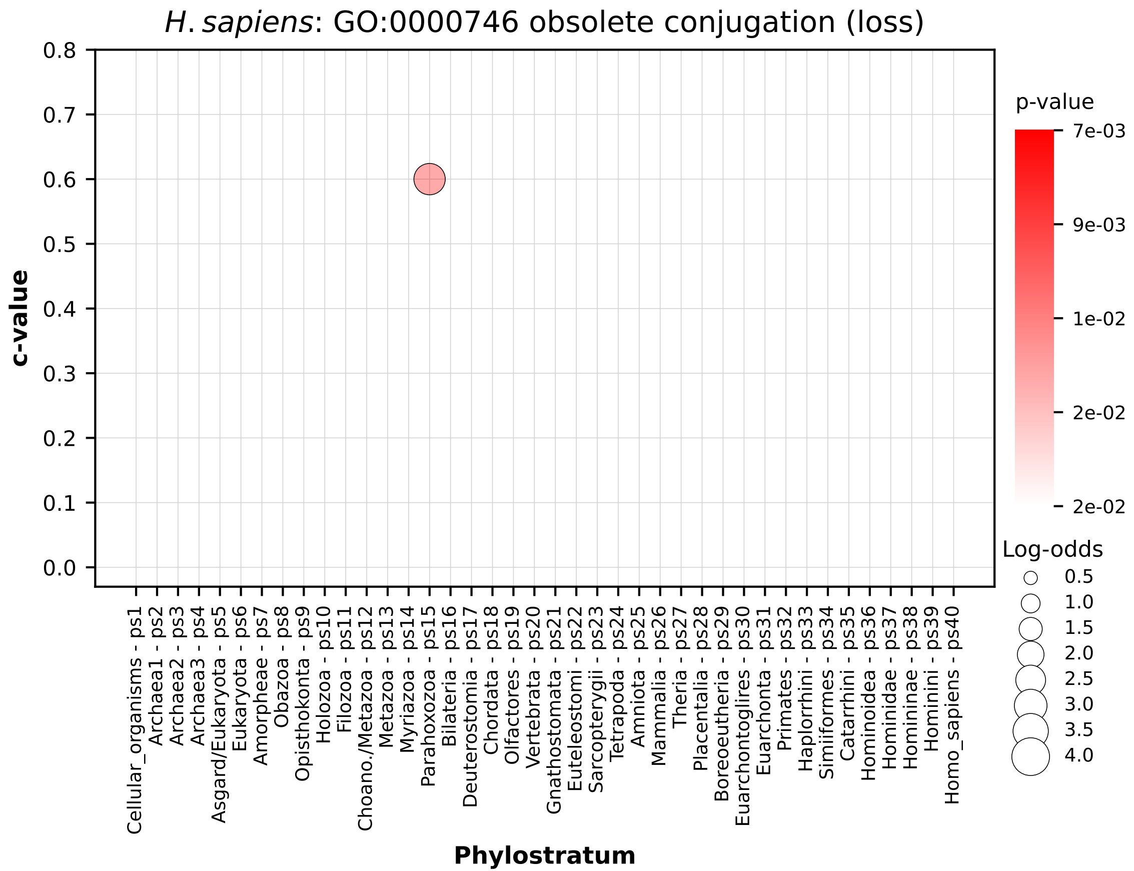

H. sapiens: GO:0000746 obsolete conjugation (loss)
c-value
0.8
0.7
0.6
0.5
0.4
0.3
0.2
0.1
0.0
Phylostratum
Cellular_organisms - ps1
Archaea1 - ps2
Archaea2 - ps3
Archaea3 - ps4
Asgard/Eukaryota - ps5
Eukaryota - ps6
Amorpheae - ps7
Obazoa - ps8
Opisthokonta - ps9
Holozoa - ps10
Filozoa - ps11
Choano./Metazoa - ps12
Metazoa - ps13
Myriazoa - ps14
Parahoxozoa - ps15
Bilateria - ps16
Deuterostomia - ps17
Chordata - ps18
Olfactores - ps19
Vertebrata - ps20
Gnathostomata - ps21
Euteleostomi - ps22
Sarcopterygii - ps23
Tetrapoda - ps24
Amniota - ps25
Mammalia - ps26
Theria - ps27
Placentalia - ps28
Boreoeutheria - ps29
Euarchontoglires - ps30
Euarchonta - ps31
Primates - ps32
Haplorrhini - ps33
Simiiformes - ps34
Catarrhini - ps35
Hominoidea - ps36
Hominidae - ps37
Homininae - ps38
Hominini - ps39
Homo_sapiens - ps40
p-value
7e-03
9e-03
1e-02
2e-02
2e-02
Log-odds
0.5
1.0
1.5
2.0
2.5
3.0
3.5
4.0

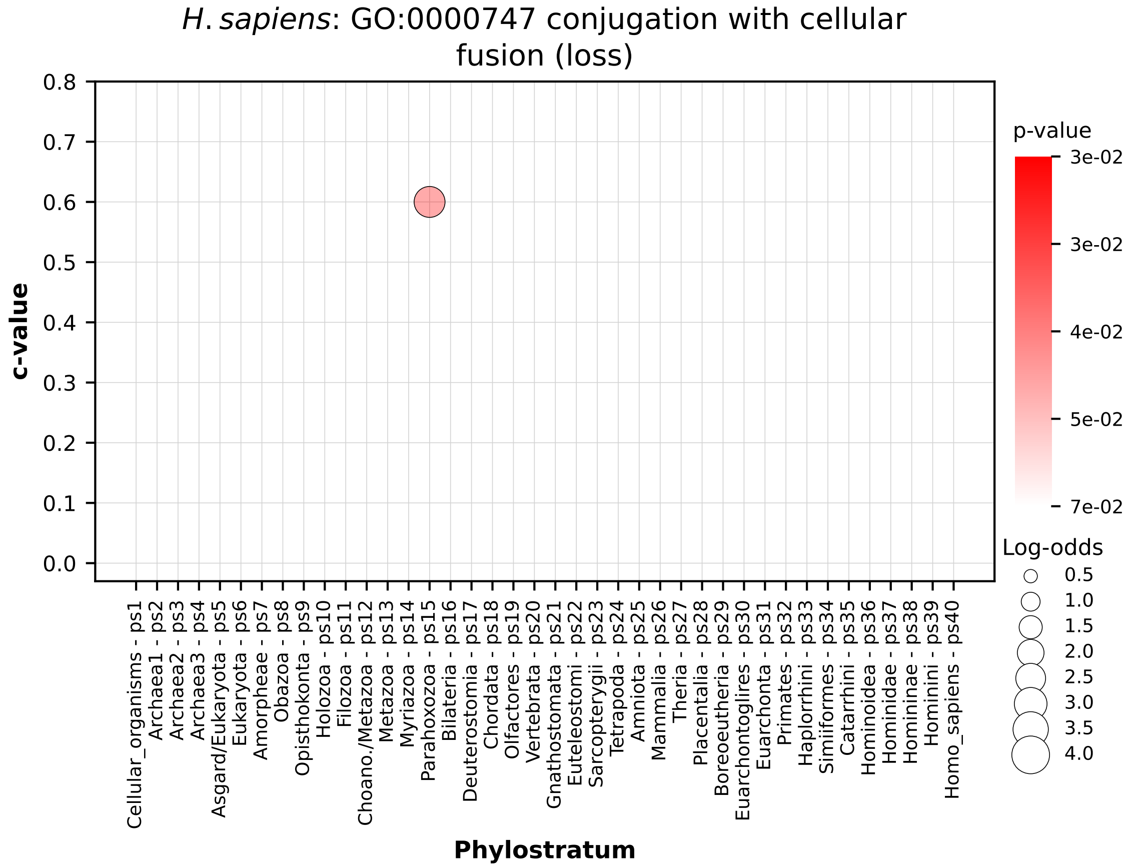

H. sapiens: GO:0000747 conjugation with cellular fusion (loss)
c-value
0.8
0.7
0.6
0.5
0.4
0.3
0.2
0.1
0.0
p-value
3e-02
3e-02
4e-02
5e-02
7e-02
Log-odds
0.5
1.0
1.5
2.0
2.5
3.0
3.5
4.0
Cellular_organisms - ps1
Archaea1 - ps2
Archaea2 - ps3
Archaea3 - ps4
Asgard/Eukaryota - ps5
Eukaryota - ps6
Amorpheae - ps7
Obazoa - ps8
Opisthokonta - ps9
Holozoa - ps10
Filozoa - ps11
Choano./Metazoa - ps12
Metazoa - ps13
Myriazoa - ps14
Parahoxozoa - ps15
Bilateria - ps16
Deuterostomia - ps17
Chordata - ps18
Olfactores - ps19
Vertebrata - ps20
Gnathostomata - ps21
Euteleostomi - ps22
Sarcopterygii - ps23
Tetrapoda - ps24
Amniota - ps25
Mammalia - ps26
Theria - ps27
Placentalia - ps28
Boreoeutheria - ps29
Euarchontoglires - ps30
Euarchonta - ps31
Primates - ps32
Haplorrhini - ps33
Simiiformes - ps34
Catarrhini - ps35
Hominoidea - ps36
Hominidae - ps37
Homininae - ps38
Hominini - ps39
Homo_sapiens - ps40
Phylostratum

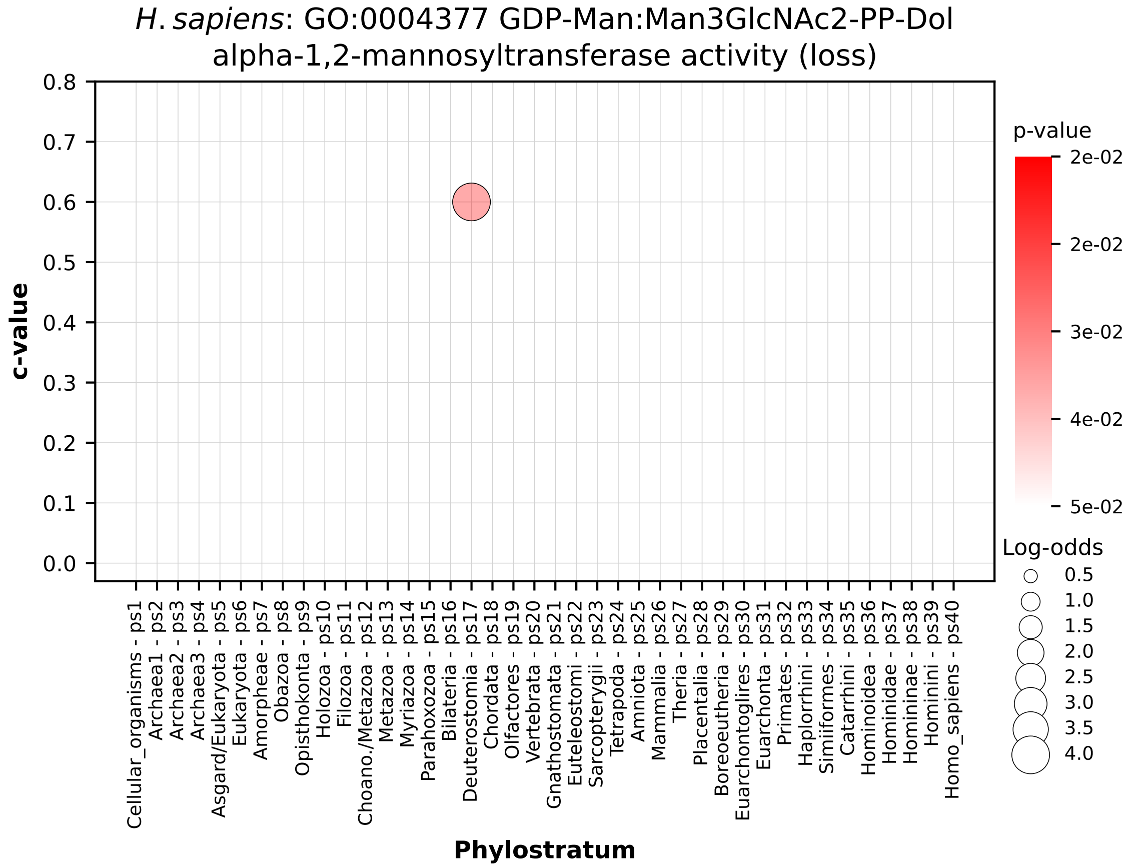
H. sapiens: GO:0004377 GDP-Man:Man3GlcNAc2-PP-Dol alpha-1,2-mannosyltransferase activity (loss)
c-value
0.8
0.7
0.6
0.5
0.4
0.3
0.2
0.1
0.0
Phylostratum
Cellular_organisms - ps1
Archaea1 - ps2
Archaea2 - ps3
Archaea3 - ps4
Asgard/Eukaryota - ps5
Eukaryota - ps6
Amorpheae - ps7
Obazoa - ps8
Opisthokonta - ps9
Holozoa - ps10
Filozoa - ps11
Choano./Metazoa - ps12
Metazoa - ps13
Myriazoa - ps14
Parahoxozoa - ps15
Bilateria - ps16
Deuterostomia - ps17
Chordata - ps18
Olfactores - ps19
Vertebrata - ps20
Gnathostomata - ps21
Euteleostomi - ps22
Sarcopterygii - ps23
Tetrapoda - ps24
Amniota - ps25
Mammalia - ps26
Theria - ps27
Placentalia - ps28
Boreoeutheria - ps29
Euarchontoglires - ps30
Euarchonta - ps31
Primates - ps32
Haplorrhini - ps33
Simiiformes - ps34
Catarrhini - ps35
Hominoidea - ps36
Hominidae - ps37
Homininae - ps38
Hominini - ps39
Homo_sapiens - ps40
p-value
2e-02
2e-02
3e-02
4e-02
5e-02
Log-odds
0.5
1.0
1.5
2.0
2.5
3.0
3.5
4.0

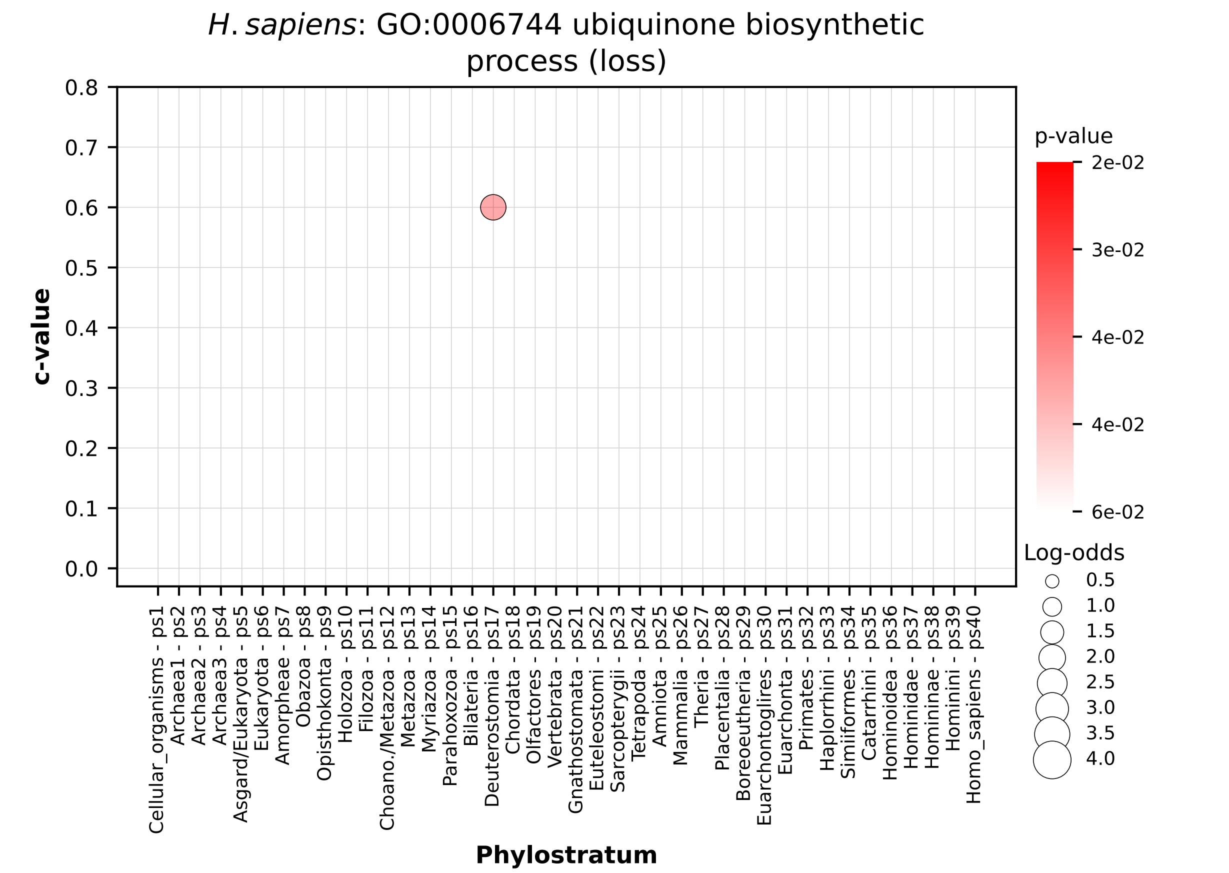

H. sapiens: GO:0006744 ubiquinone biosynthetic process (loss)
c-value
0.8
0.7
0.6
0.5
0.4
0.3
0.2
0.1
0.0
Phylostratum
Cellular_organisms - ps1
Archaea1 - ps2
Archaea2 - ps3
Archaea3 - ps4
Asgard/Eukaryota - ps5
Eukaryota - ps6
Amorpheae - ps7
Obazoa - ps8
Opisthokonta - ps9
Holozoa - ps10
Filozoa - ps11
Choano./Metazoa - ps12
Metazoa - ps13
Myriazoa - ps14
Parahoxozoa - ps15
Bilateria - ps16
Deuterostomia - ps17
Chordata - ps18
Olfactores - ps19
Vertebrata - ps20
Gnathostomata - ps21
Euteleostomi - ps22
Sarcopterygii - ps23
Tetrapoda - ps24
Amniota - ps25
Mammalia - ps26
Theria - ps27
Placentalia - ps28
Boreoeutheria - ps29
Euarchontoglires - ps30
Euarchonta - ps31
Primates - ps32
Haplorrhini - ps33
Simiiformes - ps34
Catarrhini - ps35
Hominoidea - ps36
Hominidae - ps37
Homininae - ps38
Hominini - ps39
Homo_sapiens - ps40
p-value
2e-02
3e-02
4e-02
4e-02
6e-02
Log-odds
0.5
1.0
1.5
2.0
2.5
3.0
3.5
4.0

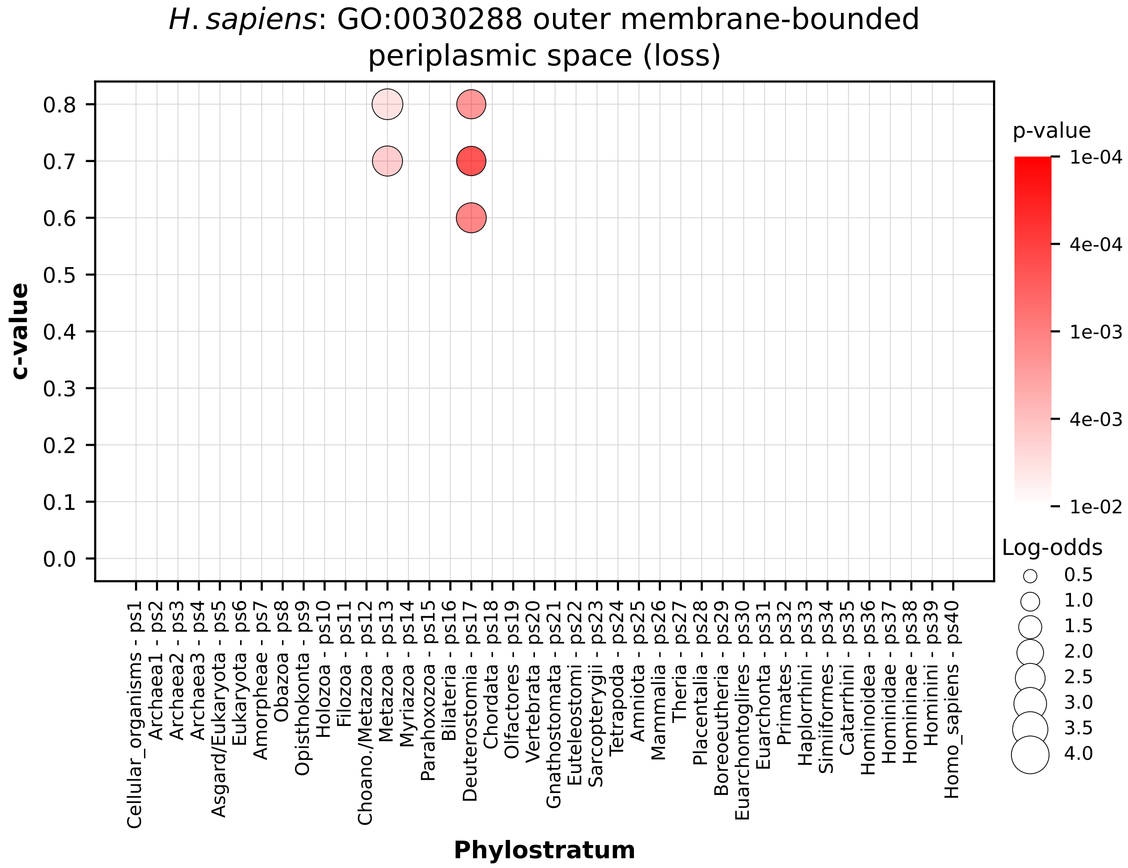

H. sapiens: GO:0030288 outer membrane-bounded periplasmic space (loss)
c-value
0.8
0.7
0.6
0.5
0.4
0.3
0.2
0.1
0.0
Phylostratum
Cellular_organisms - ps1
Archaea1 - ps2
Archaea2 - ps3
Archaea3 - ps4
Asgard/Eukaryota - ps5
Eukaryota - ps6
Amorpheae - ps7
Obazoa - ps8
Opisthokonta - ps9
Holozoa - ps10
Filozoa - ps11
Choano./Metazoa - ps12
Metazoa - ps13
Myriazoa - ps14
Parahoxozoa - ps15
Bilateria - ps16
Deuterostomia - ps17
Chordata - ps18
Olfactores - ps19
Vertebrata - ps20
Gnathostomata - ps21
Euteleostomi - ps22
Sarcopterygii - ps23
Tetrapoda - ps24
Amniota - ps25
Mammalia - ps26
Theria - ps27
Placentalia - ps28
Boreoeutheria - ps29
Euarchontoglires - ps30
Euarchonta - ps31
Primates - ps32
Haplorrhini - ps33
Simiiformes - ps34
Catarrhini - ps35
Hominoidea - ps36
Hominidae - ps37
Homininae - ps38
Hominini - ps39
Homo_sapiens - ps40
p-value
1e-04
4e-04
1e-03
4e-03
1e-02
Log-odds
0.5
1.0
1.5
2.0
2.5
3.0
3.5
4.0

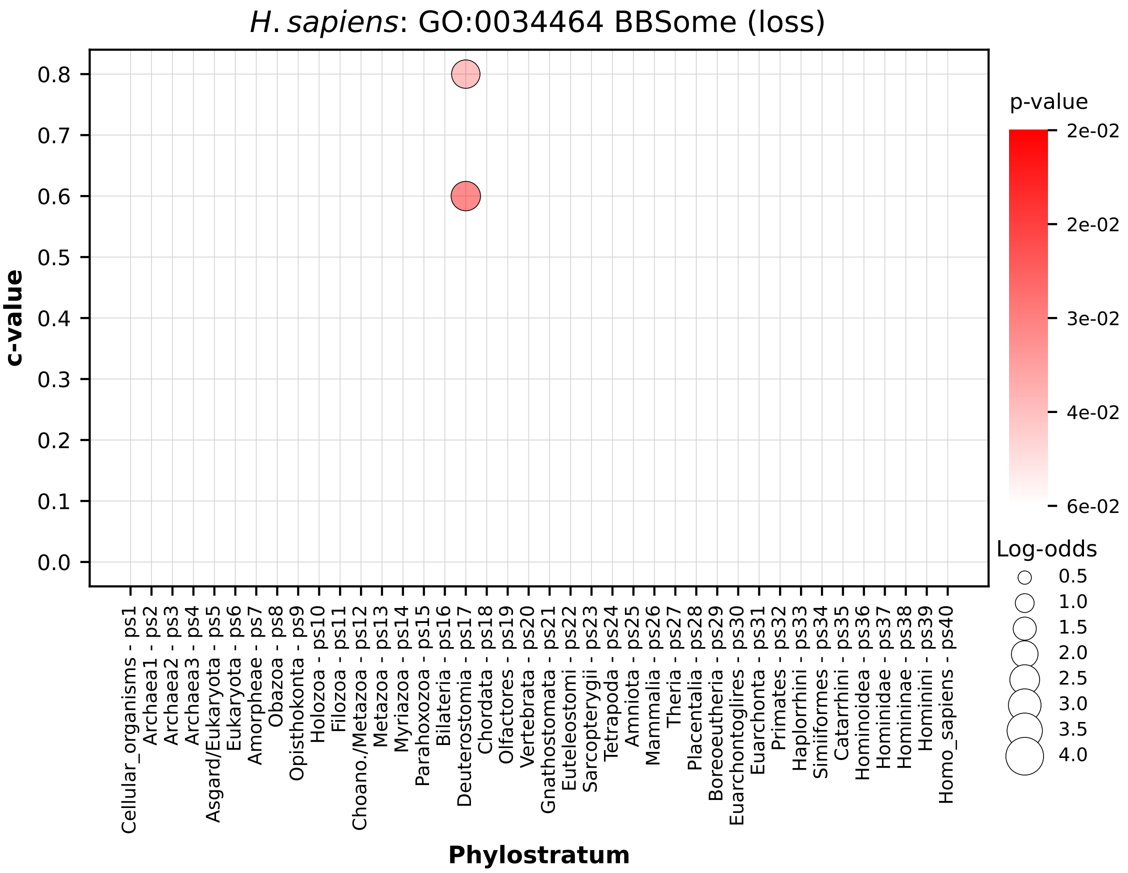

H. sapiens: GO:0034464 BBSome (loss)
c-value
0.0
0.1
0.2
0.3
0.4
0.5
0.6
0.7
0.8
Phylostratum
Cellular_organisms - ps1
Archaea1 - ps2
Archaea2 - ps3
Archaea3 - ps4
Asgard/Eukaryota - ps5
Eukaryota - ps6
Amorpheae - ps7
Obazoa - ps8
Opisthokonta - ps9
Holozoa - ps10
Filozoa - ps11
Choano./Metazoa - ps12
Metazoa - ps13
Myriazoa - ps14
Parahoxozoa - ps15
Bilateria - ps16
Deuterostomia - ps17
Chordata - ps18
Olfactores - ps19
Vertebrata - ps20
Gnathostomata - ps21
Euteleostomi - ps22
Sarcopterygii - ps23
Tetrapoda - ps24
Amniota - ps25
Mammalia - ps26
Theria - ps27
Placentalia - ps28
Boreoeutheria - ps29
Euarchontoglires - ps30
Euarchonta - ps31
Primates - ps32
Haplorrhini - ps33
Simiiformes - ps34
Catarrhini - ps35
Hominoidea - ps36
Hominidae - ps37
Homininae - ps38
Hominini - ps39
Homo_sapiens - ps40
p-value
2e-02
2e-02
3e-02
4e-02
6e-02
Log-odds
0.5
1.0
1.5
2.0
2.5
3.0
3.5
4.0

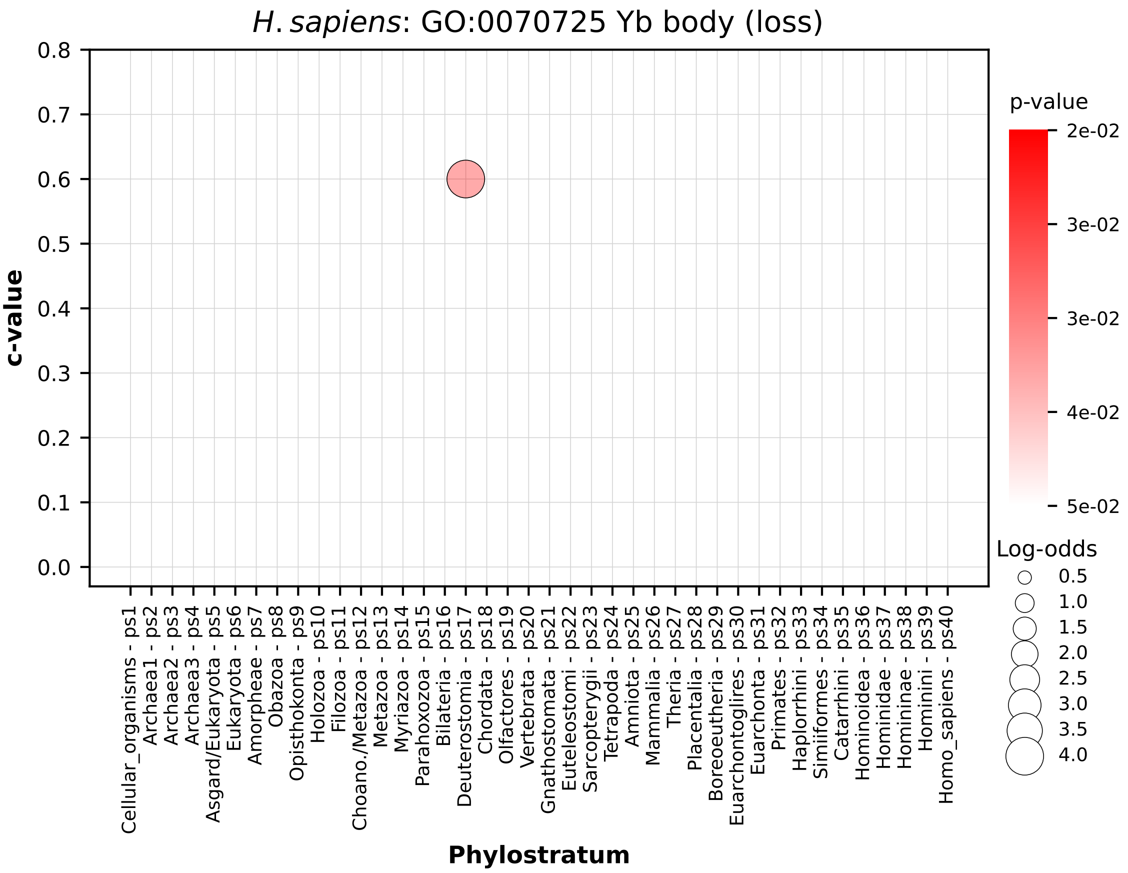
$H. sapiens$: GO:0070725 Yb body (loss)
c-value
0.0
0.1
0.2
0.3
0.4
0.5
0.6
0.7
0.8
Phylostratum
Cellular_organisms - ps1
Archaea1 - ps2
Archaea2 - ps3
Archaea3 - ps4
Asgard/Eukaryota - ps5
Eukaryota - ps6
Amorpheae - ps7
Obazoa - ps8
Opisthokonta - ps9
Holozoa - ps10
Filozoa - ps11
Choano./Metazoa - ps12
Metazoa - ps13
Myriazoa - ps14
Parahoxozoa - ps15
Bilateria - ps16
Deuterostomia - ps17
Chordata - ps18
Olfactores - ps19
Vertebrata - ps20
Gnathostomata - ps21
Euteleostomi - ps22
Sarcopterygii - ps23
Tetrapoda - ps24
Amniota - ps25
Mammalia - ps26
Theria - ps27
Placentalia - ps28
Boreoeutheria - ps29
Euarchontoglires - ps30
Euarchonta - ps31
Primates - ps32
Haplorrhini - ps33
Simiiformes - ps34
Catarrhini - ps35
Hominoidea - ps36
Hominidae - ps37
Homininae - ps38
Hominini - ps39
Homo_sapiens - ps40
p-value
2e-02
3e-02
3e-02
4e-02
5e-02
Log-odds
0.5
1.0
1.5
2.0
2.5
3.0
3.5
4.0

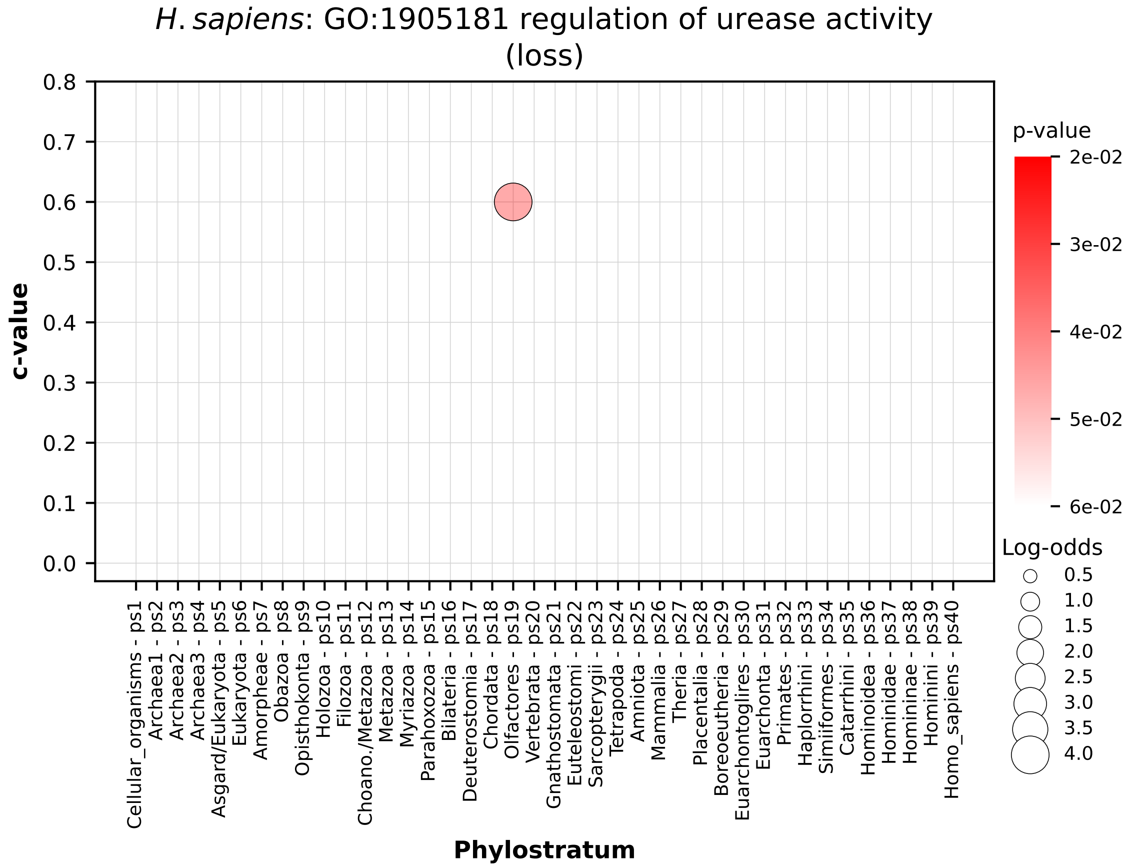

H. sapiens: GO:1905181 regulation of urease activity (loss)
c-value
0.8
0.7
0.6
0.5
0.4
0.3
0.2
0.1
0.0
Phylostratum
Cellular_organisms - ps1
Archaea1 - ps2
Archaea2 - ps3
Archaea3 - ps4
Asgard/Eukaryota - ps5
Eukaryota - ps6
Amorpheae - ps7
Obazoa - ps8
Opisthokonta - ps9
Holozoa - ps10
Filozoa - ps11
Choano./Metazoa - ps12
Metazoa - ps13
Myriazoa - ps14
Parahoxozoa - ps15
Bilateria - ps16
Deuterostomia - ps17
Chordata - ps18
Olfactores - ps19
Vertebrata - ps20
Gnathostomata - ps21
Euteleostomi - ps22
Sarcopterygii - ps23
Tetrapoda - ps24
Amniota - ps25
Mammalia - ps26
Theria - ps27
Placentalia - ps28
Boreoeutheria - ps29
Euarchontoglires - ps30
Euarchonta - ps31
Primates - ps32
Haplorrhini - ps33
Simiiformes - ps34
Catarrhini - ps35
Hominoidea - ps36
Hominidae - ps37
Homininae - ps38
Hominini - ps39
Homo_sapiens - ps40
p-value
2e-02
3e-02
4e-02
5e-02
6e-02
Log-odds
0.5
1.0
1.5
2.0
2.5
3.0
3.5
4.0

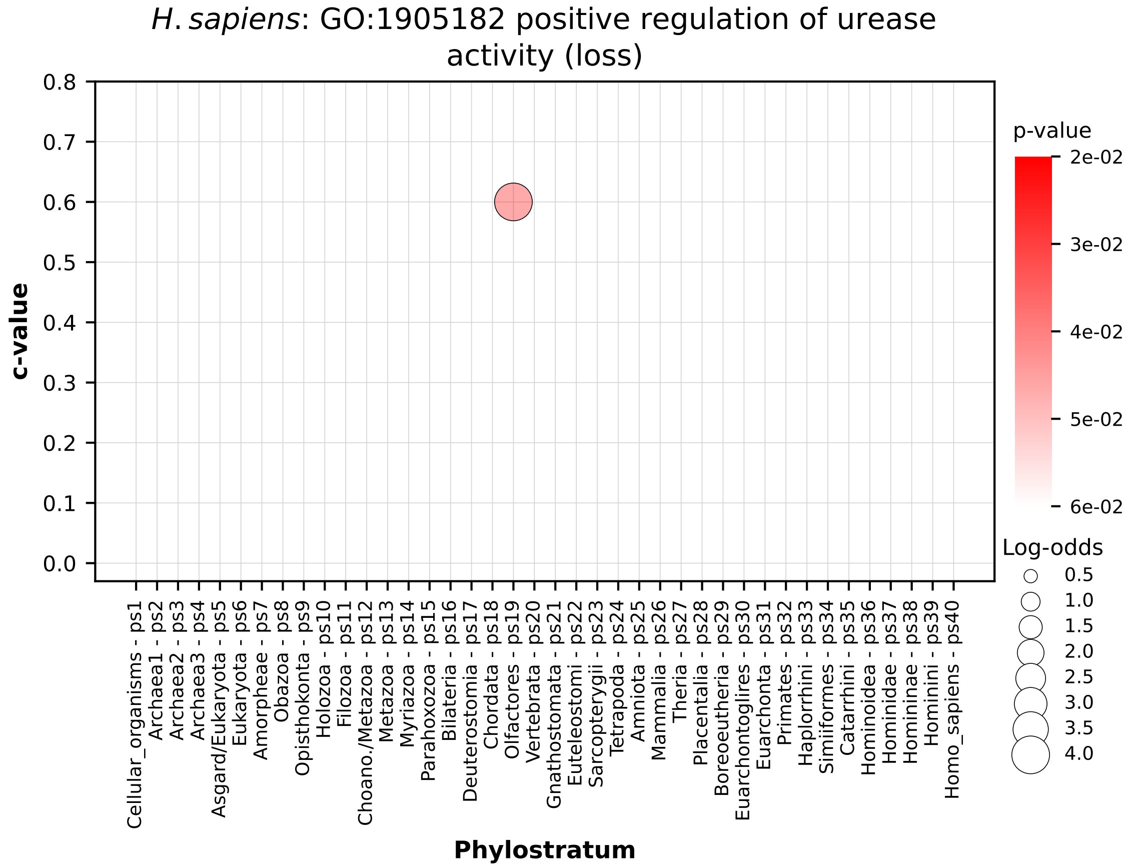

H. sapiens: GO:1905182 positive regulation of urease activity (loss)
c-value
0.8
0.7
0.6
0.5
0.4
0.3
0.2
0.1
0.0
p-value
2e-02
3e-02
4e-02
5e-02
6e-02
Log-odds
0.5
1.0
1.5
2.0
2.5
3.0
3.5
4.0
Cellular_organisms - ps1
Archaea1 - ps2
Archaea2 - ps3
Archaea3 - ps4
Asgard/Eukaryota - ps5
Eukaryota - ps6
Amorpheae - ps7
Obazoa - ps8
Opisthokonta - ps9
Holozoa - ps10
Filozoa - ps11
Choano./Metazoa - ps12
Metazoa - ps13
Myriazoa - ps14
Parahoxozoa - ps15
Bilateria - ps16
Deuterostomia - ps17
Chordata - ps18
Olfactores - ps19
Vertebrata - ps20
Gnathostomata - ps21
Euteleostomi - ps22
Sarcopterygii - ps23
Tetrapoda - ps24
Amniota - ps25
Mammalia - ps26
Theria - ps27
Placentalia - ps28
Boreoeutheria - ps29
Euarchontoglires - ps30
Euarchonta - ps31
Primates - ps32
Haplorrhini - ps33
Simiiformes - ps34
Catarrhini - ps35
Hominoidea - ps36
Hominidae - ps37
Homininae - ps38
Hominini - ps39
Homo_sapiens - ps40
Phylostratum

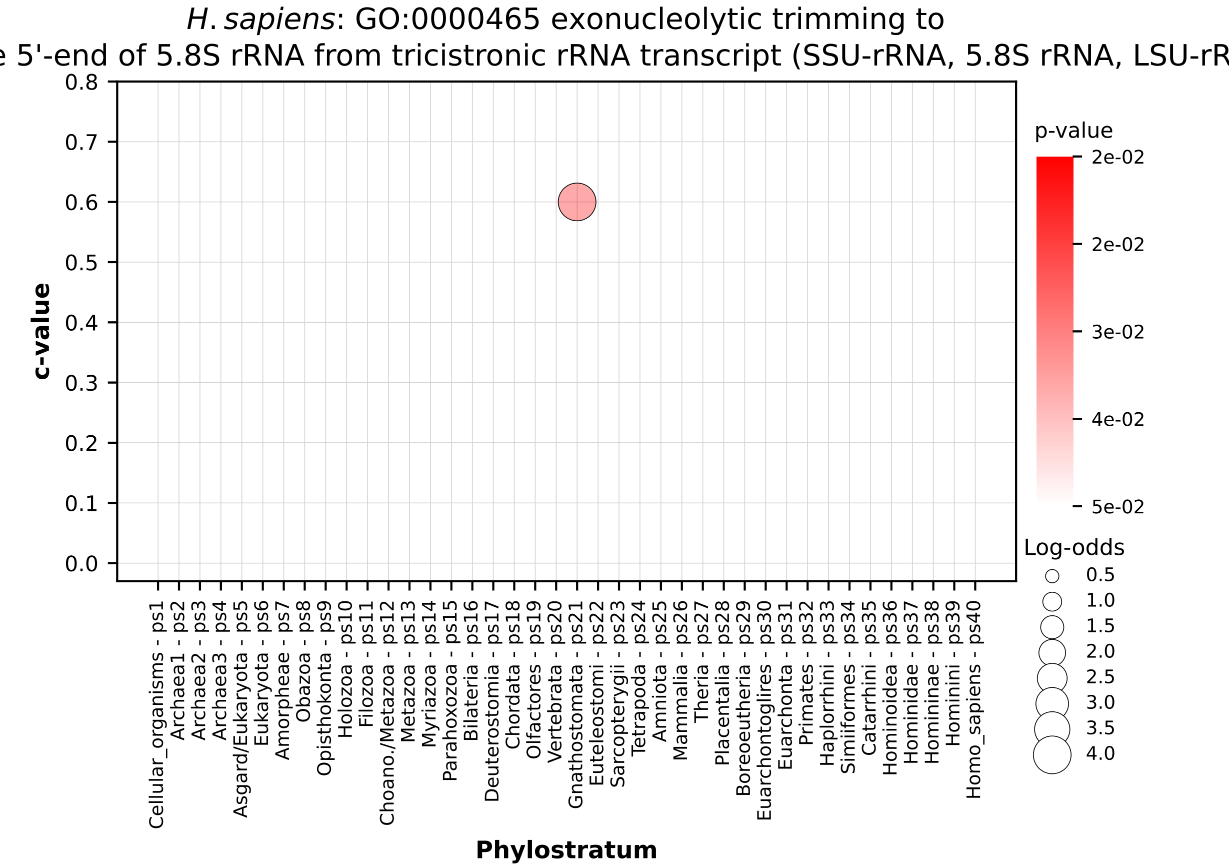

H. sapiens: GO:0000465 exonucleolytic trimming to
e 5'-end of 5.8S rRNA from tricistronic rRNA transcript (SSU-rRNA, 5.8S rRNA, LSU-rR
c-value
0.8
0.7
0.6
0.5
0.4
0.3
0.2
0.1
0.0
p-value
2e-02
2e-02
3e-02
4e-02
5e-02
Log-odds
0.5
1.0
1.5
2.0
2.5
3.0
3.5
4.0
Cellular_organisms - ps1
Archaea1 - ps2
Archaea2 - ps3
Archaea3 - ps4
Asgard/Eukaryota - ps5
Eukaryota - ps6
Amorpheae - ps7
Obazoa - ps8
Opisthokonta - ps9
Holozoa - ps10
Filozoa - ps11
Choano./Metazoa - ps12
Metazoa - ps13
Myriazoa - ps14
Parahoxozoa - ps15
Bilateria - ps16
Deuterostomia - ps17
Chordata - ps18
Olfactores - ps19
Vertebrata - ps20
Gnathostomata - ps21
Euteleostomi - ps22
Sarcopterygii - ps23
Tetrapoda - ps24
Amniota - ps25
Mammalia - ps26
Theria - ps27
Placentalia - ps28
Boreoeutheria - ps29
Euarchontoglires - ps30
Euarchonta - ps31
Primates - ps32
Haplorrhini - ps33
Simiiformes - ps34
Catarrhini - ps35
Hominoidea - ps36
Hominidae - ps37
Homininae - ps38
Hominini - ps39
Homo_sapiens - ps40
Phylostratum

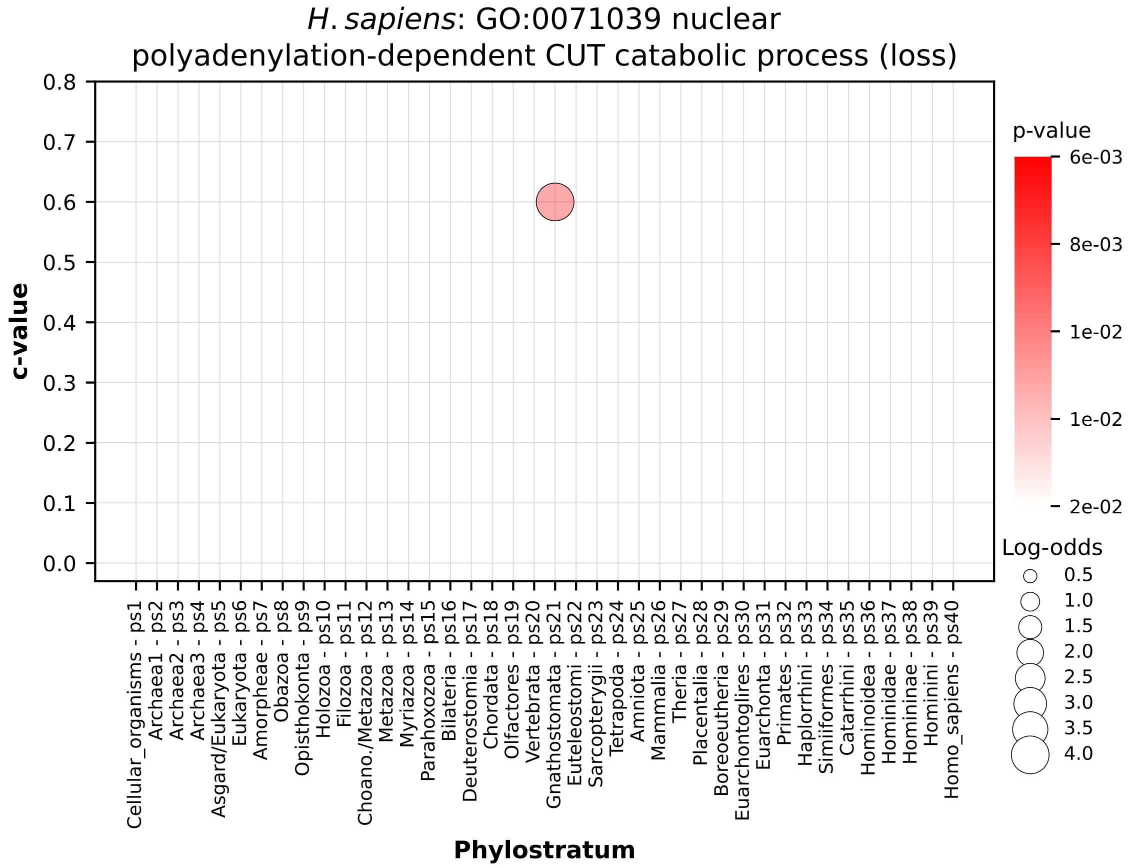

H. sapiens: GO:0071039 nuclear polyadenylation-dependent CUT catabolic process (loss)
c-value
0.8
0.7
0.6
0.5
0.4
0.3
0.2
0.1
0.0
Phylostratum
Cellular_organisms - ps1
Archaea1 - ps2
Archaea2 - ps3
Archaea3 - ps4
Asgard/Eukaryota - ps5
Eukaryota - ps6
Amorpheae - ps7
Obazoa - ps8
Opisthokonta - ps9
Holozoa - ps10
Filozoa - ps11
Choano./Metazoa - ps12
Metazoa - ps13
Myriazoa - ps14
Parahoxozoa - ps15
Bilateria - ps16
Deuterostomia - ps17
Chordata - ps18
Olfactores - ps19
Vertebrata - ps20
Gnathostomata - ps21
Euteleostomi - ps22
Sarcopterygii - ps23
Tetrapoda - ps24
Amniota - ps25
Mammalia - ps26
Theria - ps27
Placentalia - ps28
Boreoeutheria - ps29
Euarchontoglires - ps30
Euarchonta - ps31
Primates - ps32
Haplorrhini - ps33
Simiiformes - ps34
Catarrhini - ps35
Hominoidea - ps36
Hominidae - ps37
Homininae - ps38
Hominini - ps39
Homo_sapiens - ps40
p-value
6e-03
8e-03
1e-02
1e-02
2e-02
Log-odds
0.5
1.0
1.5
2.0
2.5
3.0
3.5
4.0

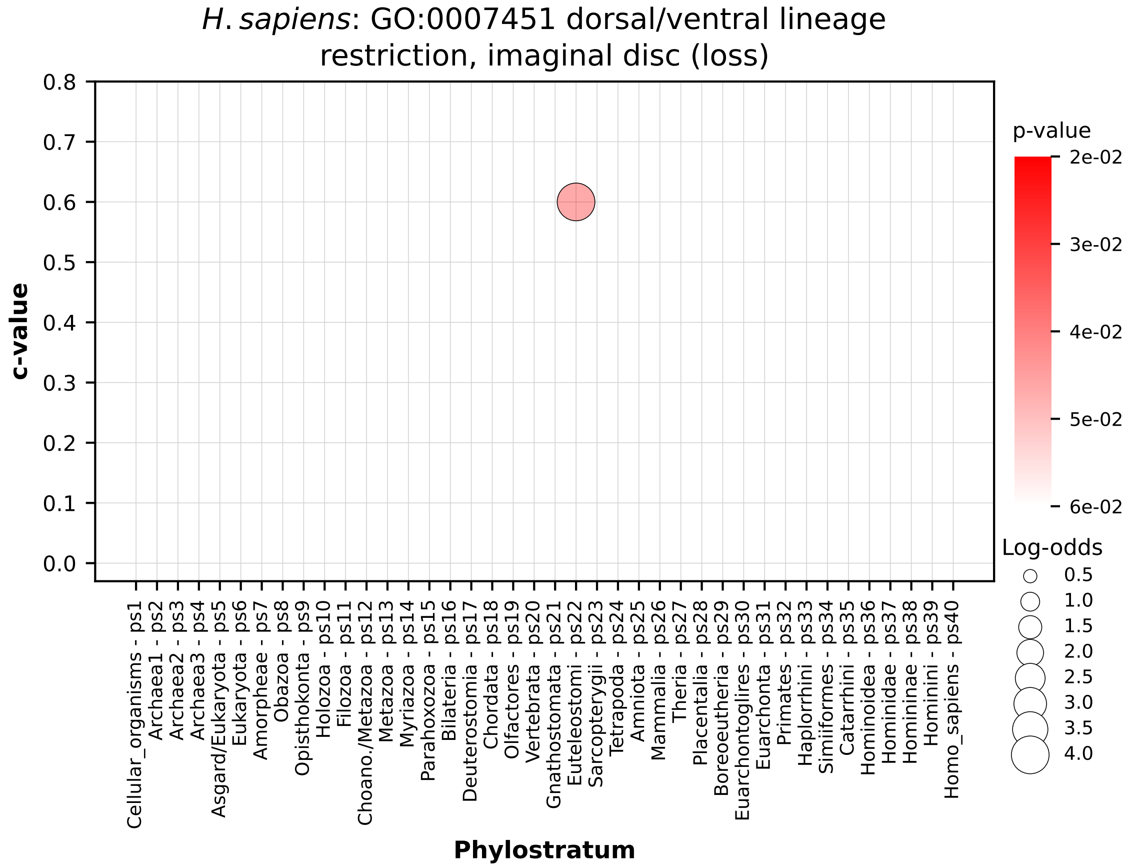
H. sapiens: GO:0007451 dorsal/ventral lineage restriction, imaginal disc (loss)
c-value
0.8
0.7
0.6
0.5
0.4
0.3
0.2
0.1
0.0
p-value
2e-02
3e-02
4e-02
5e-02
6e-02
Log-odds
0.5
1.0
1.5
2.0
2.5
3.0
3.5
4.0
Cellular_organisms - ps1
Archaea1 - ps2
Archaea2 - ps3
Archaea3 - ps4
Asgard/Eukaryota - ps5
Eukaryota - ps6
Amorpheae - ps7
Obazoa - ps8
Opisthokonta - ps9
Holozoa - ps10
Filozoa - ps11
Choano./Metazoa - ps12
Metazoa - ps13
Myriazoa - ps14
Parahoxozoa - ps15
Bilateria - ps16
Deuterostomia - ps17
Chordata - ps18
Olfactores - ps19
Vertebrata - ps20
Gnathostomata - ps21
Euteleostomi - ps22
Sarcopterygii - ps23
Tetrapoda - ps24
Amniota - ps25
Mammalia - ps26
Theria - ps27
Placentalia - ps28
Boreoeutheria - ps29
Euarchontoglires - ps30
Euarchonta - ps31
Primates - ps32
Haplorrhini - ps33
Simiiformes - ps34
Catarrhini - ps35
Hominoidea - ps36
Hominidae - ps37
Homininae - ps38
Hominini - ps39
Homo_sapiens - ps40
Phylostratum

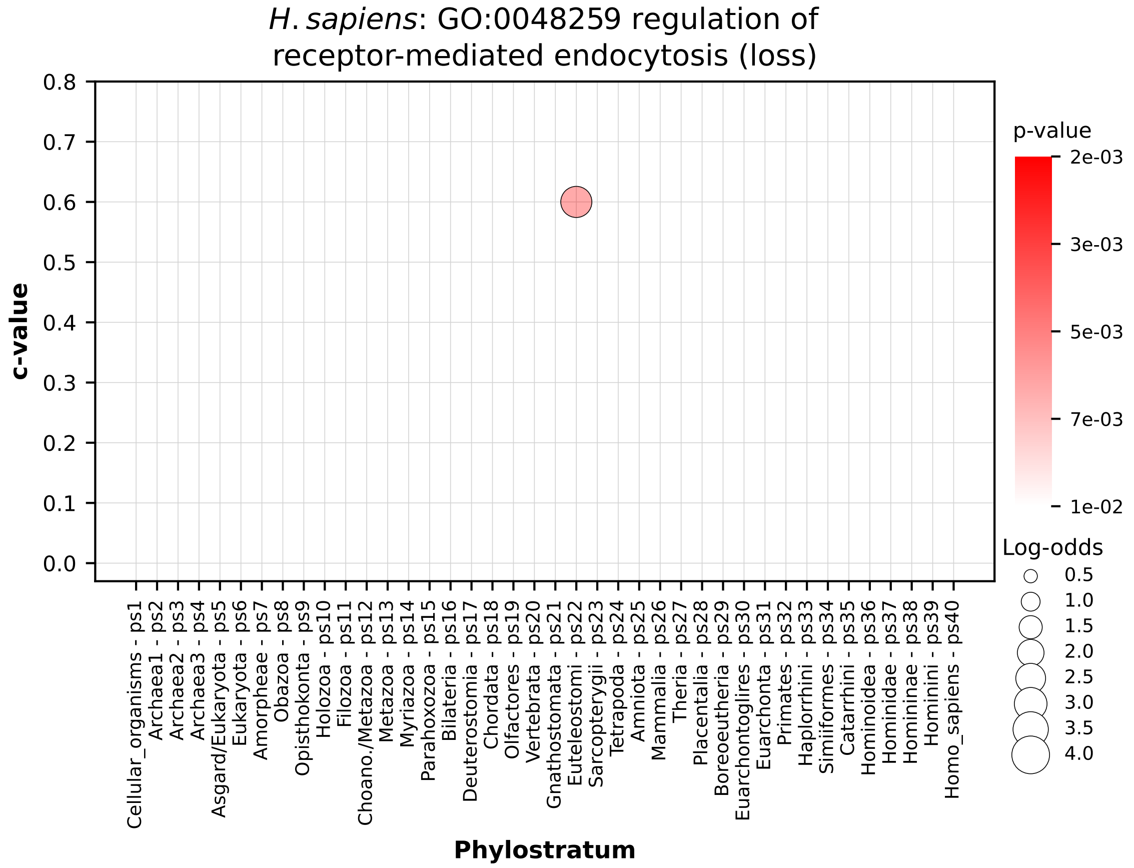

H. sapiens: GO:0048259 regulation of receptor-mediated endocytosis (loss)
c-value
0.8
0.7
0.6
0.5
0.4
0.3
0.2
0.1
0.0
Phylostratum
Cellular_organisms - ps1
Archaea1 - ps2
Archaea2 - ps3
Archaea3 - ps4
Asgard/Eukaryota - ps5
Eukaryota - ps6
Amorpheae - ps7
Obazoa - ps8
Opisthokonta - ps9
Holozoa - ps10
Filozoa - ps11
Choano./Metazoa - ps12
Metazoa - ps13
Myriazoa - ps14
Parahoxozoa - ps15
Bilateria - ps16
Deuterostomia - ps17
Chordata - ps18
Olfactores - ps19
Vertebrata - ps20
Gnathostomata - ps21
Euteleostomi - ps22
Sarcopterygii - ps23
Tetrapoda - ps24
Amniota - ps25
Mammalia - ps26
Theria - ps27
Placentalia - ps28
Boreoeutheria - ps29
Euarchontoglires - ps30
Euarchonta - ps31
Primates - ps32
Haplorrhini - ps33
Simiiformes - ps34
Catarrhini - ps35
Hominoidea - ps36
Hominidae - ps37
Homininae - ps38
Hominini - ps39
Homo_sapiens - ps40
p-value
2e-03
3e-03
5e-03
7e-03
1e-02
Log-odds
0.5
1.0
1.5
2.0
2.5
3.0
3.5
4.0

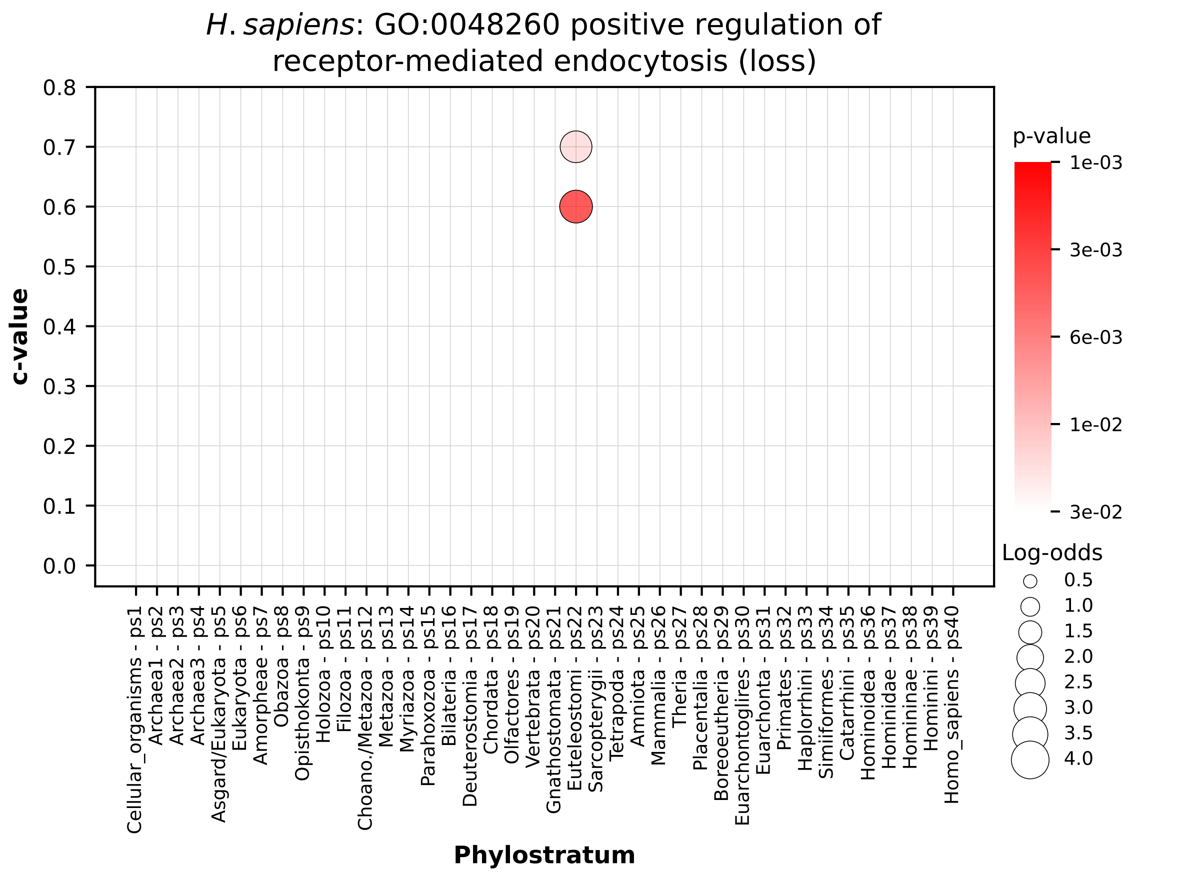

H. sapiens: GO:0048260 positive regulation of receptor-mediated endocytosis (loss)
c-value
0.8
0.7
0.6
0.5
0.4
0.3
0.2
0.1
0.0
Phylostratum
Cellular_organisms - ps1
Archaea1 - ps2
Archaea2 - ps3
Archaea3 - ps4
Asgard/Eukaryota - ps5
Eukaryota - ps6
Amorpheae - ps7
Obazoa - ps8
Opisthokonta - ps9
Holozoa - ps10
Filozoa - ps11
Choano./Metazoa - ps12
Metazoa - ps13
Myriazoa - ps14
Parahoxozoa - ps15
Bilateria - ps16
Deuterostomia - ps17
Chordata - ps18
Olfactores - ps19
Vertebrata - ps20
Gnathostomata - ps21
Euteleostomi - ps22
Sarcopterygii - ps23
Tetrapoda - ps24
Amniota - ps25
Mammalia - ps26
Theria - ps27
Placentalia - ps28
Boreoeutheria - ps29
Euarchontoglires - ps30
Euarchonta - ps31
Primates - ps32
Haplorrhini - ps33
Simiiformes - ps34
Catarrhini - ps35
Hominoidea - ps36
Hominidae - ps37
Homininae - ps38
Hominini - ps39
Homo_sapiens - ps40
p-value
1e-03
3e-03
6e-03
1e-02
3e-02
Log-odds
0.5
1.0
1.5
2.0
2.5
3.0
3.5
4.0

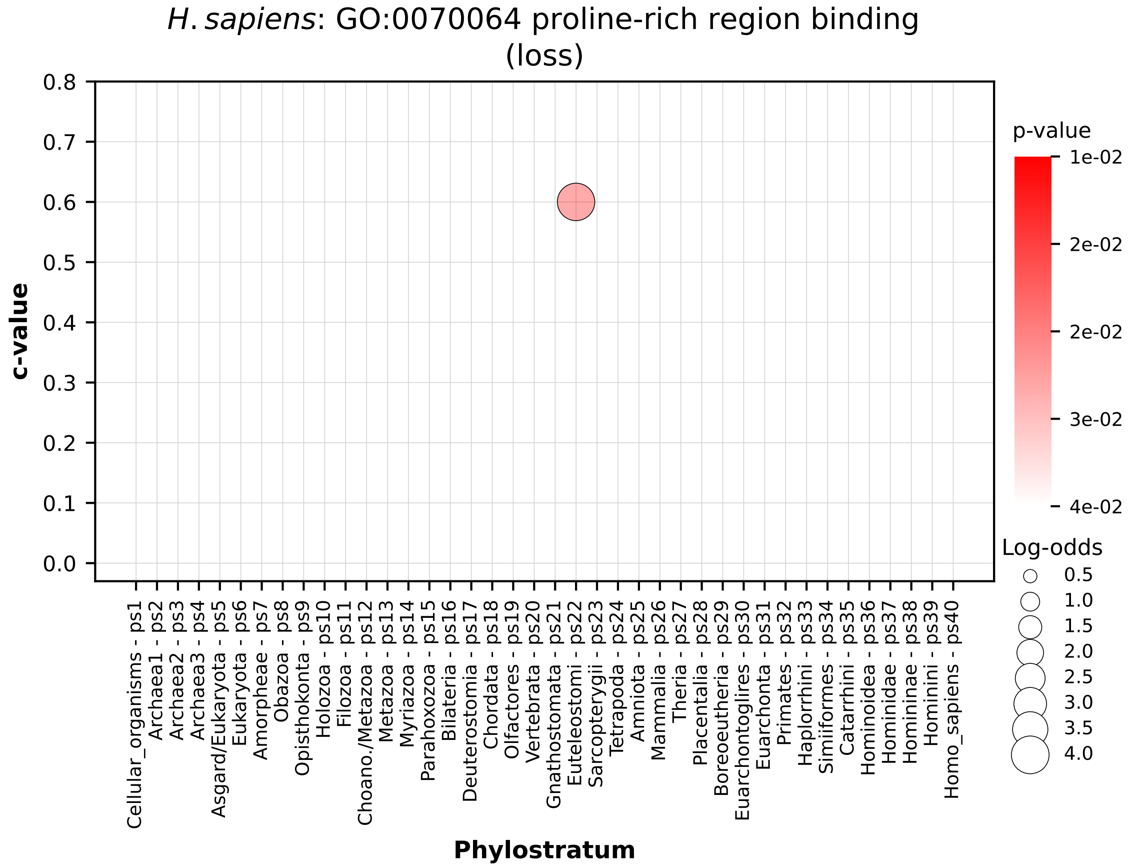

H. sapiens: GO:0070064 proline-rich region binding (loss)
c-value
0.8
0.7
0.6
0.5
0.4
0.3
0.2
0.1
0.0
p-value
1e-02
2e-02
2e-02
3e-02
4e-02
Log-odds
0.5
1.0
1.5
2.0
2.5
3.0
3.5
4.0
Cellular_organisms - ps1
Archaea1 - ps2
Archaea2 - ps3
Archaea3 - ps4
Asgard/Eukaryota - ps5
Eukaryota - ps6
Amorpheae - ps7
Obazoa - ps8
Opisthokonta - ps9
Holozoa - ps10
Filozoa - ps11
Choano./Metazoa - ps12
Metazoa - ps13
Myriazoa - ps14
Parahoxozoa - ps15
Bilateria - ps16
Deuterostomia - ps17
Chordata - ps18
Olfactores - ps19
Vertebrata - ps20
Gnathostomata - ps21
Euteleostomi - ps22
Sarcopterygii - ps23
Tetrapoda - ps24
Amniota - ps25
Mammalia - ps26
Theria - ps27
Placentalia - ps28
Boreoeutheria - ps29
Euarchontoglires - ps30
Euarchonta - ps31
Primates - ps32
Haplorrhini - ps33
Simiiformes - ps34
Catarrhini - ps35
Hominoidea - ps36
Hominidae - ps37
Homininae - ps38
Hominini - ps39
Homo_sapiens - ps40
Phylostratum

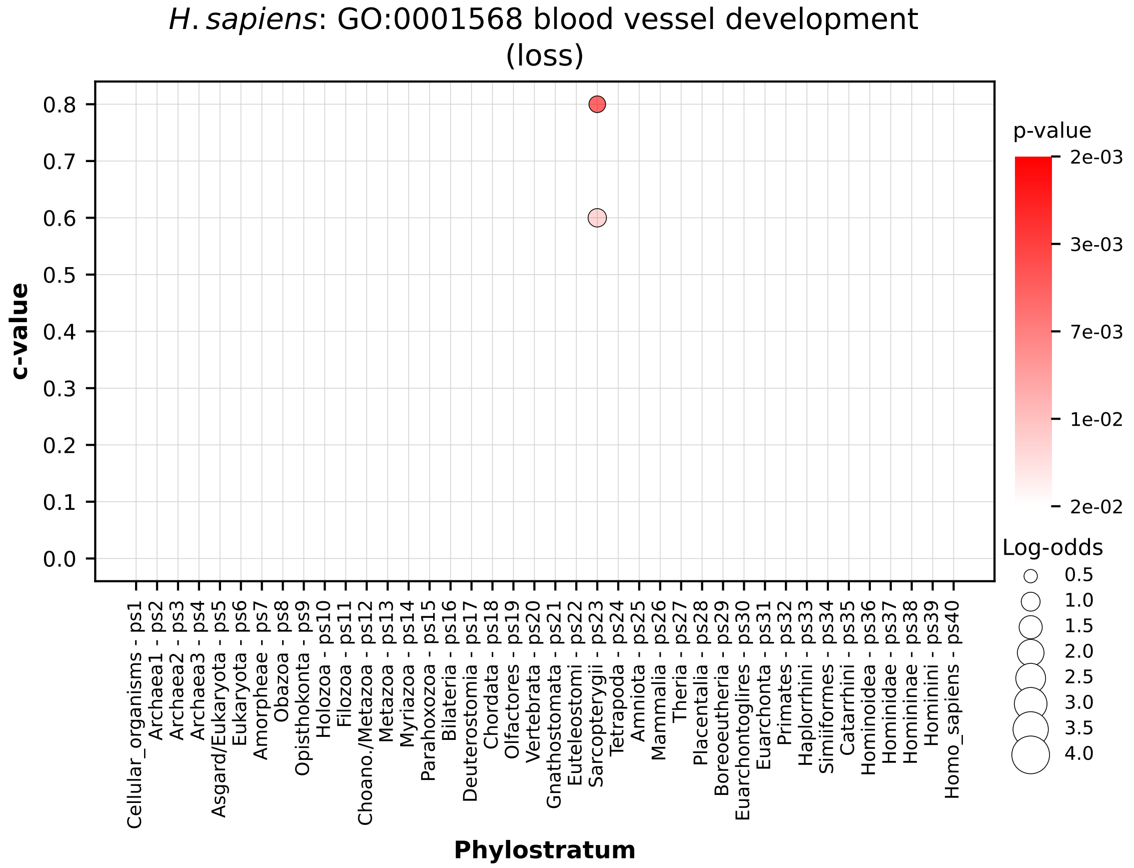

H. sapiens: GO:0001568 blood vessel development
(loss)
c-value
0.0
0.1
0.2
0.3
0.4
0.5
0.6
0.7
0.8
p-value
2e-03
3e-03
7e-03
1e-02
2e-02
Log-odds
0.5
1.0
1.5
2.0
2.5
3.0
3.5
4.0
Cellular_organisms - ps1
Archaea1 - ps2
Archaea2 - ps3
Archaea3 - ps4
Asgard/Eukaryota - ps5
Eukaryota - ps6
Amorpheae - ps7
Obazoa - ps8
Opisthokonta - ps9
Holozoa - ps10
Filozoa - ps11
Choano./Metazoa - ps12
Metazoa - ps13
Myriazoa - ps14
Parahoxozoa - ps15
Bilateria - ps16
Deuterostomia - ps17
Chordata - ps18
Olfactores - ps19
Vertebrata - ps20
Gnathostomata - ps21
Euteleostomi - ps22
Sarcopterygii - ps23
Tetrapoda - ps24
Amniota - ps25
Mammalia - ps26
Theria - ps27
Placentalia - ps28
Boreoeutheria - ps29
Euarchontoglires - ps30
Euarchonta - ps31
Primates - ps32
Haplorrhini - ps33
Simiiformes - ps34
Catarrhini - ps35
Hominoidea - ps36
Hominidae - ps37
Homininae - ps38
Hominini - ps39
Homo_sapiens - ps40
Phylostratum

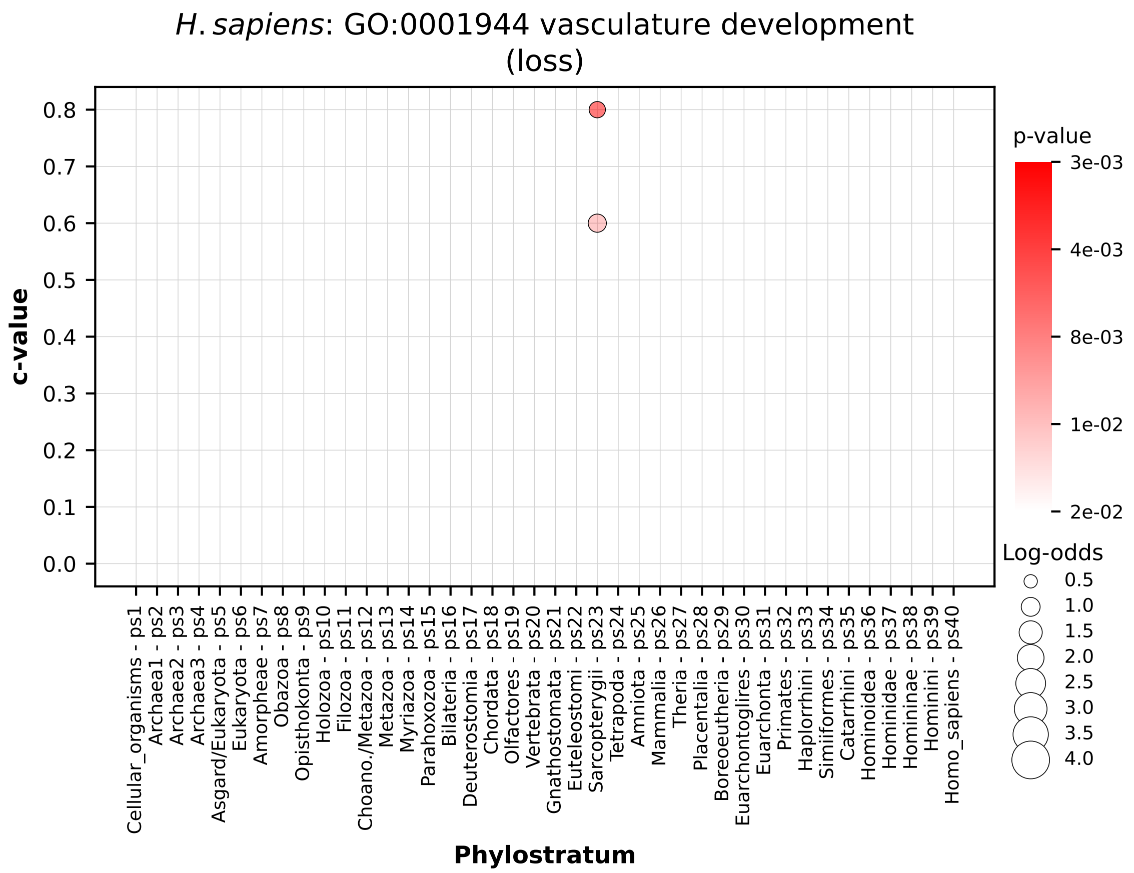

H. sapiens: GO:0001944 vasculature development
(loss)
c-value
0.0
0.1
0.2
0.3
0.4
0.5
0.6
0.7
0.8
Phylostratum
Cellular_organisms - ps1
Archaea1 - ps2
Archaea2 - ps3
Archaea3 - ps4
Asgard/Eukaryota - ps5
Eukaryota - ps6
Amorpheae - ps7
Obazoa - ps8
Opisthokonta - ps9
Holozoa - ps10
Filozoa - ps11
Choano./Metazoa - ps12
Metazoa - ps13
Myriazoa - ps14
Parahoxozoa - ps15
Bilateria - ps16
Deuterostomia - ps17
Chordata - ps18
Olfactores - ps19
Vertebrata - ps20
Gnathostomata - ps21
Euteleostomi - ps22
Sarcopterygii - ps23
Tetrapoda - ps24
Amniota - ps25
Mammalia - ps26
Theria - ps27
Placentalia - ps28
Boreoeutheria - ps29
Euarchontoglires - ps30
Euarchonta - ps31
Primates - ps32
Haplorrhini - ps33
Simiiformes - ps34
Catarrhini - ps35
Hominoidea - ps36
Hominidae - ps37
Homininae - ps38
Hominini - ps39
Homo_sapiens - ps40
p-value
3e-03
4e-03
8e-03
1e-02
2e-02
Log-odds
0.5
1.0
1.5
2.0
2.5
3.0
3.5
4.0

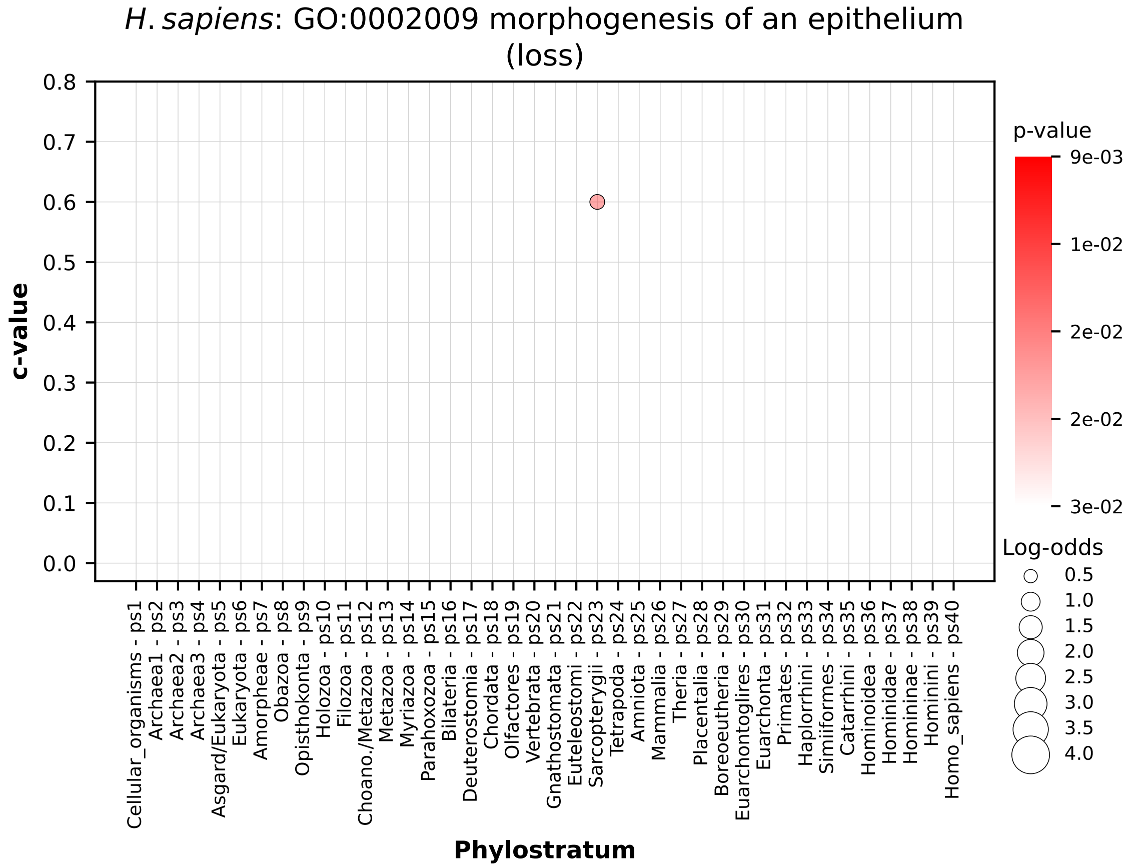

H. sapiens: GO:0002009 morphogenesis of an epithelium
(loss)
c-value
0.8
0.7
0.6
0.5
0.4
0.3
0.2
0.1
0.0
p-value
9e-03
1e-02
2e-02
2e-02
3e-02
Log-odds
0.5
1.0
1.5
2.0
2.5
3.0
3.5
4.0
Cellular_organisms - ps1
Archaea1 - ps2
Archaea2 - ps3
Archaea3 - ps4
Asgard/Eukaryota - ps5
Eukaryota - ps6
Amorpheae - ps7
Obazoa - ps8
Opisthokonta - ps9
Holozoa - ps10
Filozoa - ps11
Choano./Metazoa - ps12
Metazoa - ps13
Myriazoa - ps14
Parahoxozoa - ps15
Bilateria - ps16
Deuterostomia - ps17
Chordata - ps18
Olfactores - ps19
Vertebrata - ps20
Gnathostomata - ps21
Euteleostomi - ps22
Sarcopterygii - ps23
Tetrapoda - ps24
Amniota - ps25
Mammalia - ps26
Theria - ps27
Placentalia - ps28
Boreoeutheria - ps29
Euarchontoglires - ps30
Euarchonta - ps31
Primates - ps32
Haplorrhini - ps33
Simiiformes - ps34
Catarrhini - ps35
Hominoidea - ps36
Hominidae - ps37
Homininae - ps38
Hominini - ps39
Homo_sapiens - ps40
Phylostratum

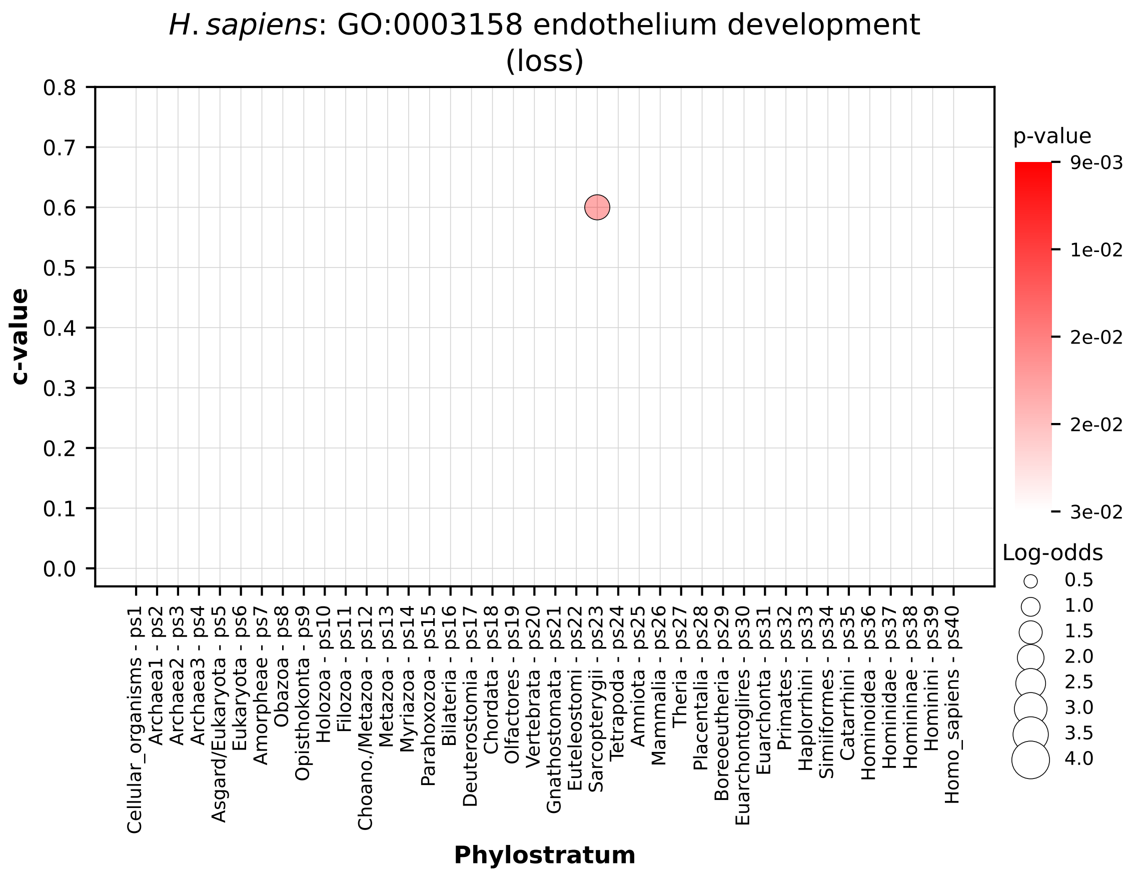

H. sapiens: GO:0003158 endothelium development
(loss)
c-value
0.8
0.7
0.6
0.5
0.4
0.3
0.2
0.1
0.0
Phylostratum
Cellular_organisms - ps1
Archaea1 - ps2
Archaea2 - ps3
Archaea3 - ps4
Asgard/Eukaryota - ps5
Eukaryota - ps6
Amorpheae - ps7
Obazoa - ps8
Opisthokonta - ps9
Holozoa - ps10
Filozoa - ps11
Choano./Metazoa - ps12
Metazoa - ps13
Myriazoa - ps14
Parahoxozoa - ps15
Bilateria - ps16
Deuterostomia - ps17
Chordata - ps18
Olfactores - ps19
Vertebrata - ps20
Gnathostomata - ps21
Euteleostomi - ps22
Sarcopterygii - ps23
Tetrapoda - ps24
Amniota - ps25
Mammalia - ps26
Theria - ps27
Placentalia - ps28
Boreoeutheria - ps29
Euarchontoglires - ps30
Euarchonta - ps31
Primates - ps32
Haplorrhini - ps33
Simiiformes - ps34
Catarrhini - ps35
Hominoidea - ps36
Hominidae - ps37
Homininae - ps38
Hominini - ps39
Homo_sapiens - ps40
p-value
9e-03
1e-02
2e-02
2e-02
3e-02
Log-odds
0.5
1.0
1.5
2.0
2.5
3.0
3.5
4.0

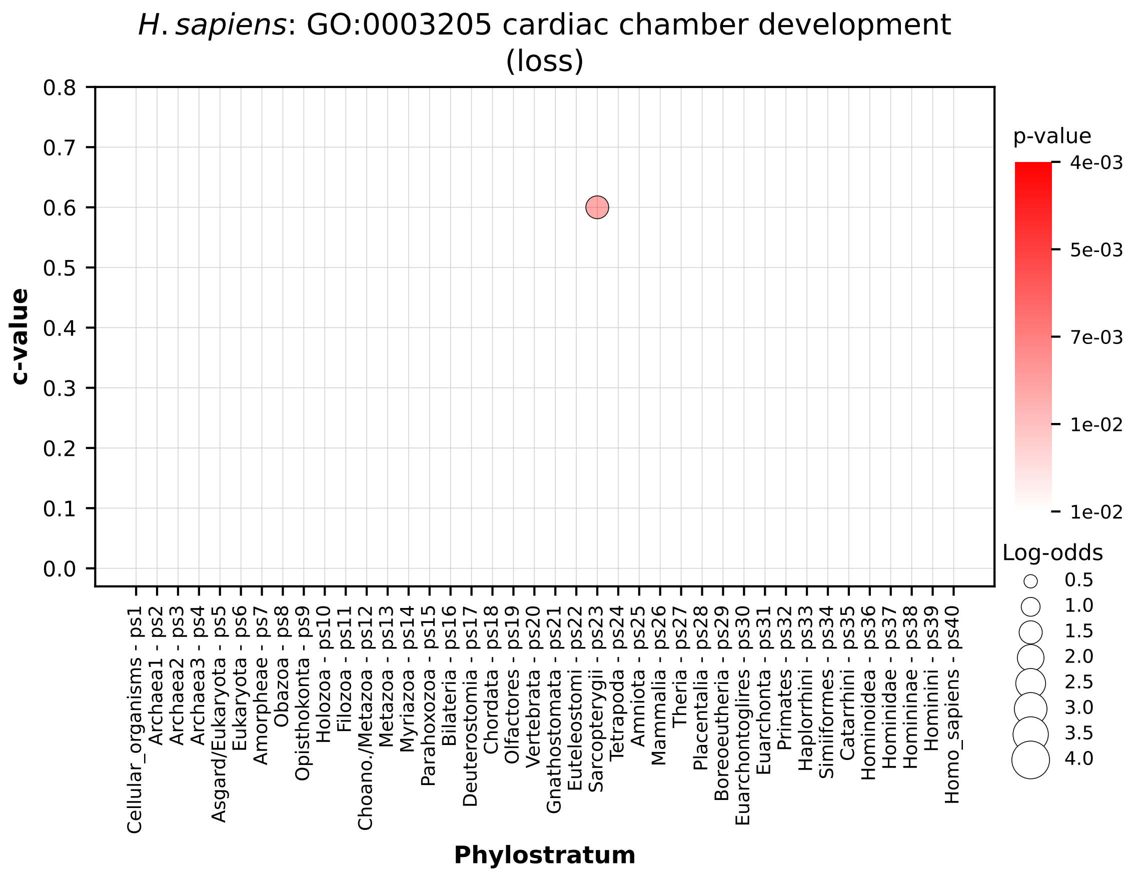

H. sapiens: GO:0003205 cardiac chamber development
(loss)
c-value
0.8
0.7
0.6
0.5
0.4
0.3
0.2
0.1
0.0
p-value
4e-03
5e-03
7e-03
1e-02
1e-02
Log-odds
0.5
1.0
1.5
2.0
2.5
3.0
3.5
4.0
Cellular_organisms - ps1
Archaea1 - ps2
Archaea2 - ps3
Archaea3 - ps4
Asgard/Eukaryota - ps5
Eukaryota - ps6
Amorpheae - ps7
Obazoa - ps8
Opisthokonta - ps9
Holozoa - ps10
Filozoa - ps11
Choano./Metazoa - ps12
Metazoa - ps13
Myriazoa - ps14
Parahoxozoa - ps15
Bilateria - ps16
Deuterostomia - ps17
Chordata - ps18
Olfactores - ps19
Vertebrata - ps20
Gnathostomata - ps21
Euteleostomi - ps22
Sarcopterygii - ps23
Tetrapoda - ps24
Amniota - ps25
Mammalia - ps26
Theria - ps27
Placentalia - ps28
Boreoeutheria - ps29
Euarchontoglires - ps30
Euarchonta - ps31
Primates - ps32
Haplorrhini - ps33
Simiiformes - ps34
Catarrhini - ps35
Hominoidea - ps36
Hominidae - ps37
Homininae - ps38
Hominini - ps39
Homo_sapiens - ps40
Phylostratum

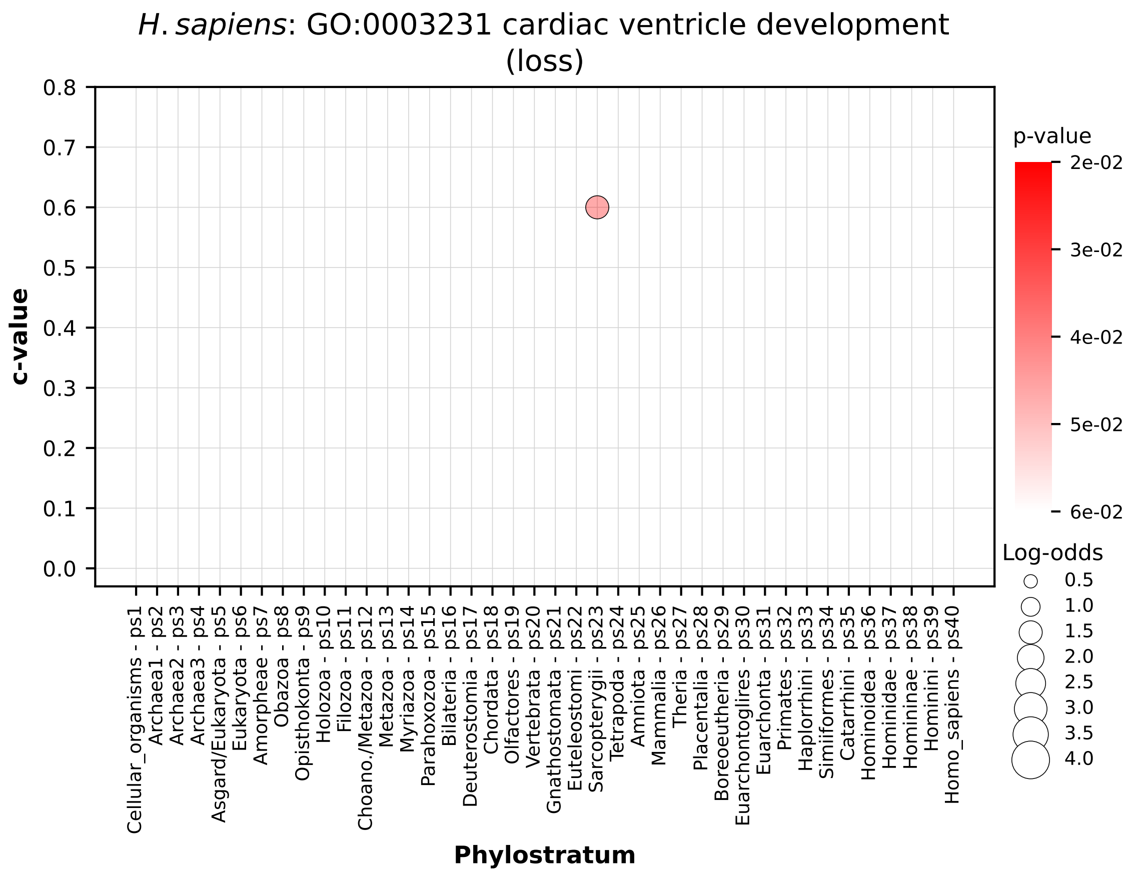

H. sapiens: GO:0003231 cardiac ventricle development
(loss)
c-value
0.8
0.7
0.6
0.5
0.4
0.3
0.2
0.1
0.0
p-value
2e-02
3e-02
4e-02
5e-02
6e-02
Log-odds
0.5
1.0
1.5
2.0
2.5
3.0
3.5
4.0
Cellular_organisms - ps1
Archaea1 - ps2
Archaea2 - ps3
Archaea3 - ps4
Asgard/Eukaryota - ps5
Eukaryota - ps6
Amorpheae - ps7
Obazoa - ps8
Opisthokonta - ps9
Holozoa - ps10
Filozoa - ps11
Choano./Metazoa - ps12
Metazoa - ps13
Myriazoa - ps14
Parahoxozoa - ps15
Bilateria - ps16
Deuterostomia - ps17
Chordata - ps18
Olfactores - ps19
Vertebrata - ps20
Gnathostomata - ps21
Euteleostomi - ps22
Sarcopterygii - ps23
Tetrapoda - ps24
Amniota - ps25
Mammalia - ps26
Theria - ps27
Placentalia - ps28
Boreoeutheria - ps29
Euarchontoglires - ps30
Euarchonta - ps31
Primates - ps32
Haplorrhini - ps33
Simiiformes - ps34
Catarrhini - ps35
Hominoidea - ps36
Hominidae - ps37
Homininae - ps38
Hominini - ps39
Homo_sapiens - ps40
Phylostratum

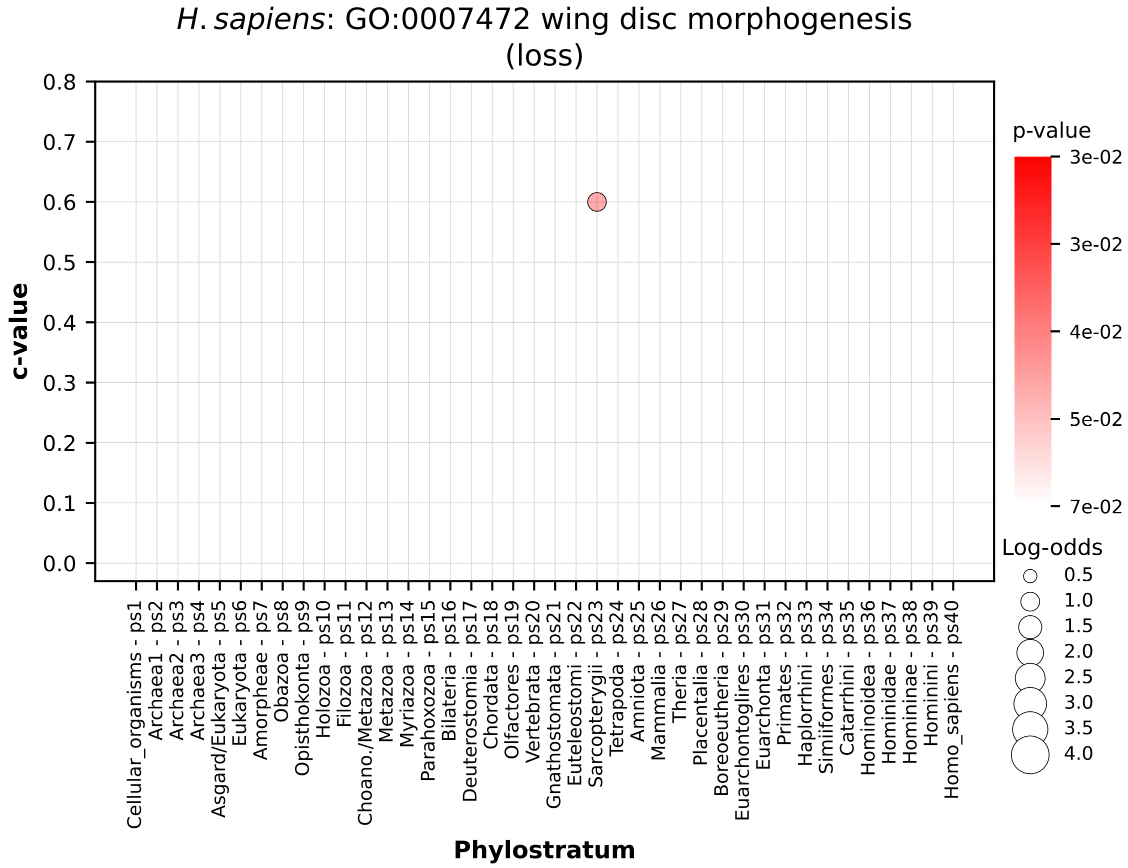

H. sapiens: GO:0007472 wing disc morphogenesis
(loss)
c-value
0.8
0.7
0.6
0.5
0.4
0.3
0.2
0.1
0.0
p-value
3e-02
3e-02
4e-02
5e-02
7e-02
Log-odds
0.5
1.0
1.5
2.0
2.5
3.0
3.5
4.0
Cellular_organisms - ps1
Archaea1 - ps2
Archaea2 - ps3
Archaea3 - ps4
Asgard/Eukaryota - ps5
Eukaryota - ps6
Amorpheae - ps7
Obazoa - ps8
Opisthokonta - ps9
Holozoa - ps10
Filozoa - ps11
Choano./Metazoa - ps12
Metazoa - ps13
Myriazoa - ps14
Parahoxozoa - ps15
Bilateria - ps16
Deuterostomia - ps17
Chordata - ps18
Olfactores - ps19
Vertebrata - ps20
Gnathostomata - ps21
Euteleostomi - ps22
Sarcopterygii - ps23
Tetrapoda - ps24
Amniota - ps25
Mammalia - ps26
Theria - ps27
Placentalia - ps28
Boreoeutheria - ps29
Euarchontoglires - ps30
Euarchonta - ps31
Primates - ps32
Haplorrhini - ps33
Simiiformes - ps34
Catarrhini - ps35
Hominoidea - ps36
Hominidae - ps37
Homininae - ps38
Hominini - ps39
Homo_sapiens - ps40
Phylostratum

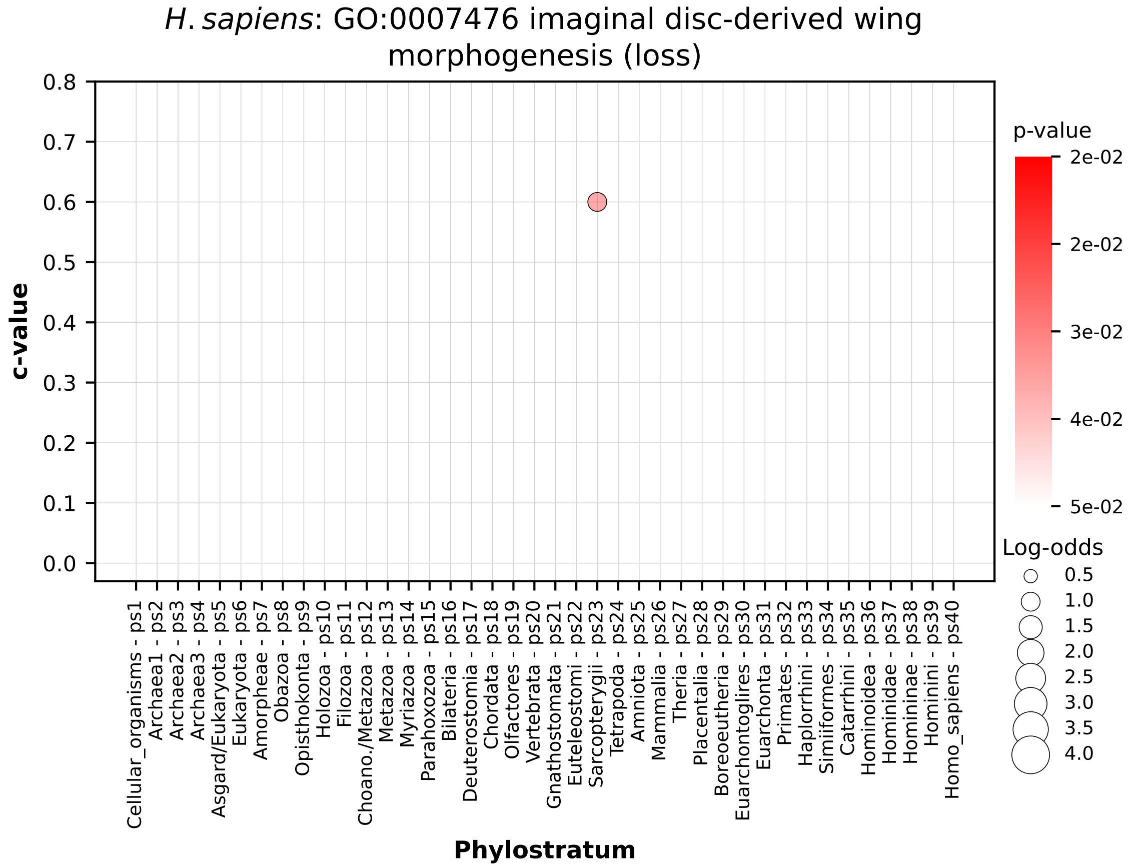

H. sapiens: GO:0007476 imaginal disc-derived wing morphogenesis (loss)
c-value
0.8
0.7
0.6
0.5
0.4
0.3
0.2
0.1
0.0
Phylostratum
Cellular_organisms - ps1
Archaea1 - ps2
Archaea2 - ps3
Archaea3 - ps4
Asgard/Eukaryota - ps5
Eukaryota - ps6
Amorpheae - ps7
Obazoa - ps8
Opisthokonta - ps9
Holozoa - ps10
Filozoa - ps11
Choano./Metazoa - ps12
Metazoa - ps13
Myriazoa - ps14
Parahoxozoa - ps15
Bilateria - ps16
Deuterostomia - ps17
Chordata - ps18
Olfactores - ps19
Vertebrata - ps20
Gnathostomata - ps21
Euteleostomi - ps22
Sarcopterygii - ps23
Tetrapoda - ps24
Amniota - ps25
Mammalia - ps26
Theria - ps27
Placentalia - ps28
Boreoeutheria - ps29
Euarchontoglires - ps30
Euarchonta - ps31
Primates - ps32
Haplorrhini - ps33
Simiiformes - ps34
Catarrhini - ps35
Hominoidea - ps36
Hominidae - ps37
Homininae - ps38
Hominini - ps39
Homo_sapiens - ps40
p-value
2e-02
2e-02
3e-02
4e-02
5e-02
Log-odds
0.5
1.0
1.5
2.0
2.5
3.0
3.5
4.0

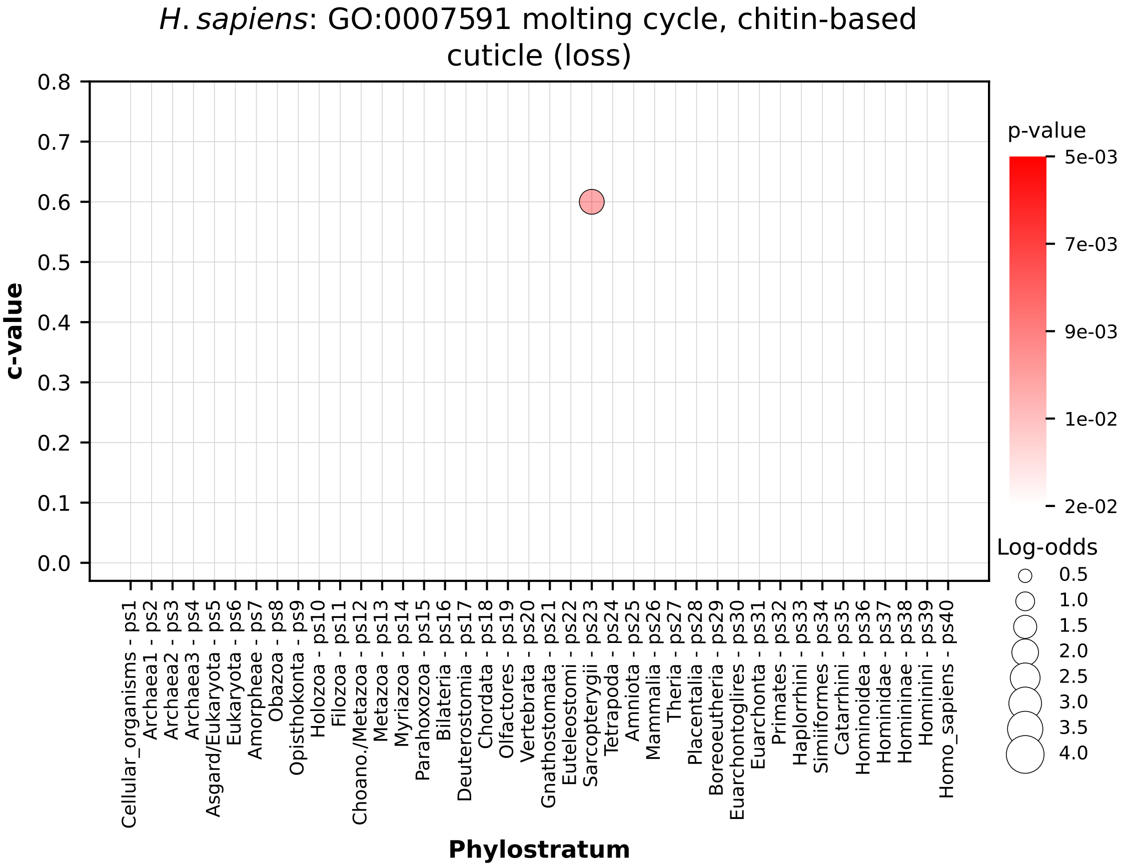

H. sapiens: GO:0007591 molting cycle, chitin-based cuticle (loss)
c-value
0.0
0.1
0.2
0.3
0.4
0.5
0.6
0.7
0.8
Phylostratum
Cellular_organisms - ps1
Archaea1 - ps2
Archaea2 - ps3
Archaea3 - ps4
Asgard/Eukaryota - ps5
Eukaryota - ps6
Amorpheae - ps7
Obazoa - ps8
Opisthokonta - ps9
Holozoa - ps10
Filozoa - ps11
Choano./Metazoa - ps12
Metazoa - ps13
Myriazoa - ps14
Parahoxozoa - ps15
Bilateria - ps16
Deuterostomia - ps17
Chordata - ps18
Olfactores - ps19
Vertebrata - ps20
Gnathostomata - ps21
Euteleostomi - ps22
Sarcopterygii - ps23
Tetrapoda - ps24
Amniota - ps25
Mammalia - ps26
Theria - ps27
Placentalia - ps28
Boreoeutheria - ps29
Euarchontoglires - ps30
Euarchonta - ps31
Primates - ps32
Haplorrhini - ps33
Simiiformes - ps34
Catarrhini - ps35
Hominoidea - ps36
Hominidae - ps37
Homininae - ps38
Hominini - ps39
Homo_sapiens - ps40
p-value
5e-03
7e-03
9e-03
1e-02
2e-02
Log-odds
0.5
1.0
1.5
2.0
2.5
3.0
3.5
4.0

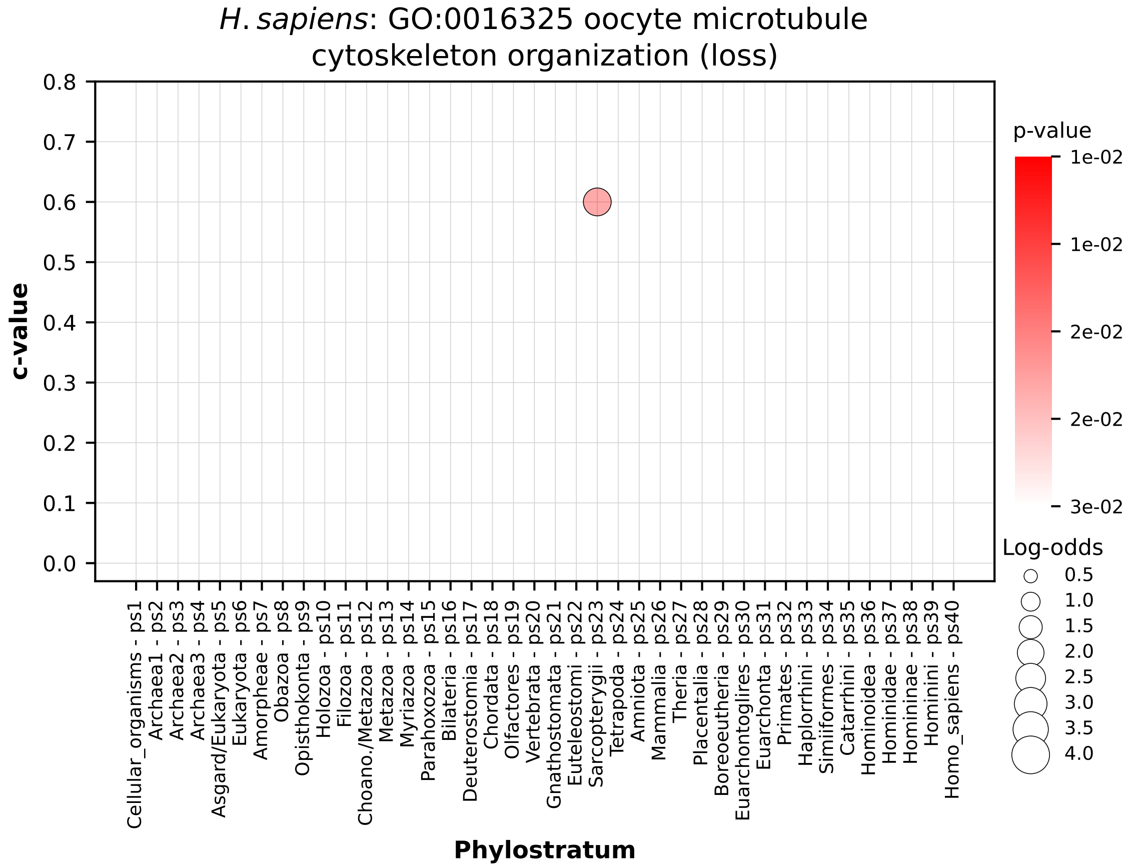

H. sapiens: GO:0016325 oocyte microtubule cytoskeleton organization (loss)
c-value
0.8
0.7
0.6
0.5
0.4
0.3
0.2
0.1
0.0
p-value
1e-02
1e-02
2e-02
2e-02
3e-02
Log-odds
0.5
1.0
1.5
2.0
2.5
3.0
3.5
4.0
Cellular_organisms - ps1
Archaea1 - ps2
Archaea2 - ps3
Archaea3 - ps4
Asgard/Eukaryota - ps5
Eukaryota - ps6
Amorpheae - ps7
Obazoa - ps8
Opisthokonta - ps9
Holozoa - ps10
Filozoa - ps11
Choano./Metazoa - ps12
Metazoa - ps13
Myriazoa - ps14
Parahoxozoa - ps15
Bilateria - ps16
Deuterostomia - ps17
Chordata - ps18
Olfactores - ps19
Vertebrata - ps20
Gnathostomata - ps21
Euteleostomi - ps22
Sarcopterygii - ps23
Tetrapoda - ps24
Amniota - ps25
Mammalia - ps26
Theria - ps27
Placentalia - ps28
Boreoeutheria - ps29
Euarchontoglires - ps30
Euarchonta - ps31
Primates - ps32
Haplorrhini - ps33
Simiiformes - ps34
Catarrhini - ps35
Hominoidea - ps36
Hominidae - ps37
Homininae - ps38
Hominini - ps39
Homo_sapiens - ps40
Phylostratum

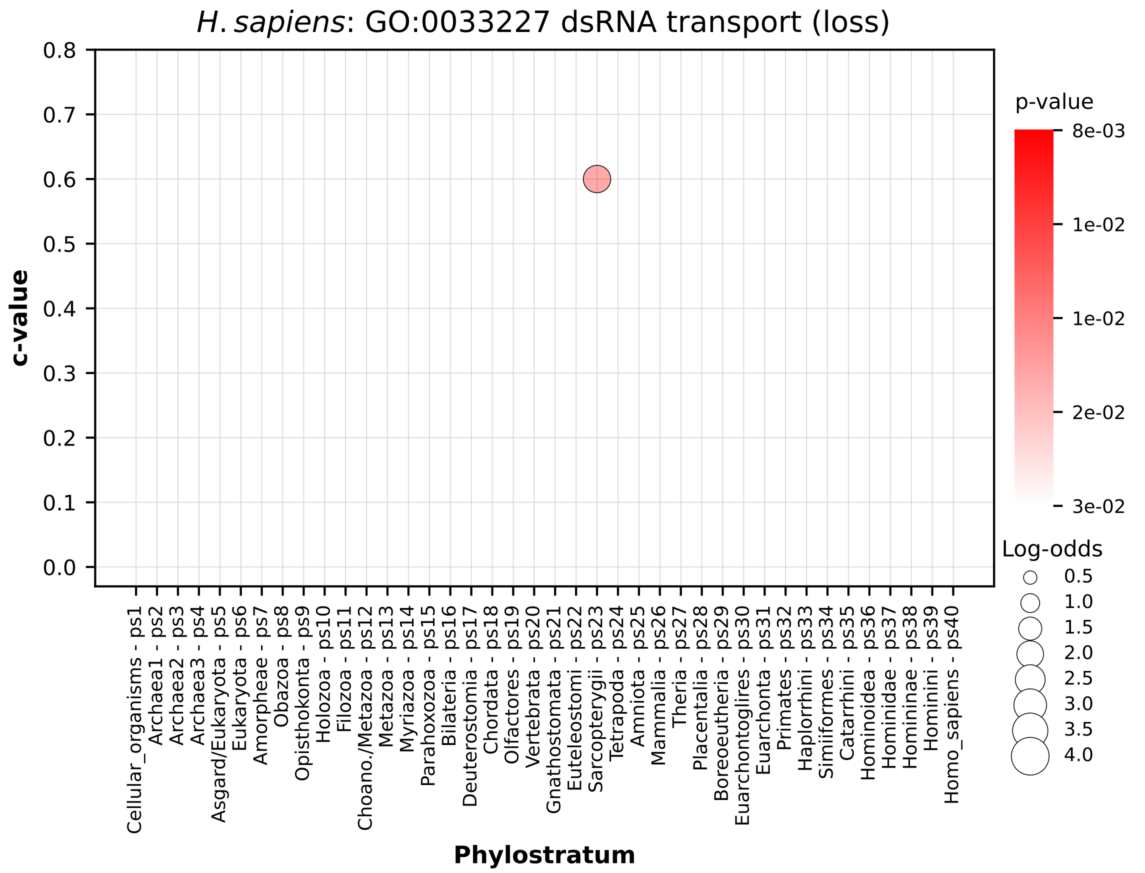

H. sapiens: GO:0033227 dsRNA transport (loss)
c-value
0.8
0.7
0.6
0.5
0.4
0.3
0.2
0.1
0.0
p-value
8e-03
1e-02
1e-02
2e-02
3e-02
Log-odds
0.5
1.0
1.5
2.0
2.5
3.0
3.5
4.0
Cellular_organisms - ps1
Archaea1 - ps2
Archaea2 - ps3
Archaea3 - ps4
Asgard/Eukaryota - ps5
Eukaryota - ps6
Amorpheae - ps7
Obazoa - ps8
Opisthokonta - ps9
Holozoa - ps10
Filozoa - ps11
Choano./Metazoa - ps12
Metazoa - ps13
Myriazoa - ps14
Parahoxozoa - ps15
Bilateria - ps16
Deuterostomia - ps17
Chordata - ps18
Olfactores - ps19
Vertebrata - ps20
Gnathostomata - ps21
Euteleostomi - ps22
Sarcopterygii - ps23
Tetrapoda - ps24
Amniota - ps25
Mammalia - ps26
Theria - ps27
Placentalia - ps28
Boreoeutheria - ps29
Euarchontoglires - ps30
Euarchonta - ps31
Primates - ps32
Haplorrhini - ps33
Simiiformes - ps34
Catarrhini - ps35
Hominoidea - ps36
Hominidae - ps37
Homininae - ps38
Hominini - ps39
Homo_sapiens - ps40
Phylostratum

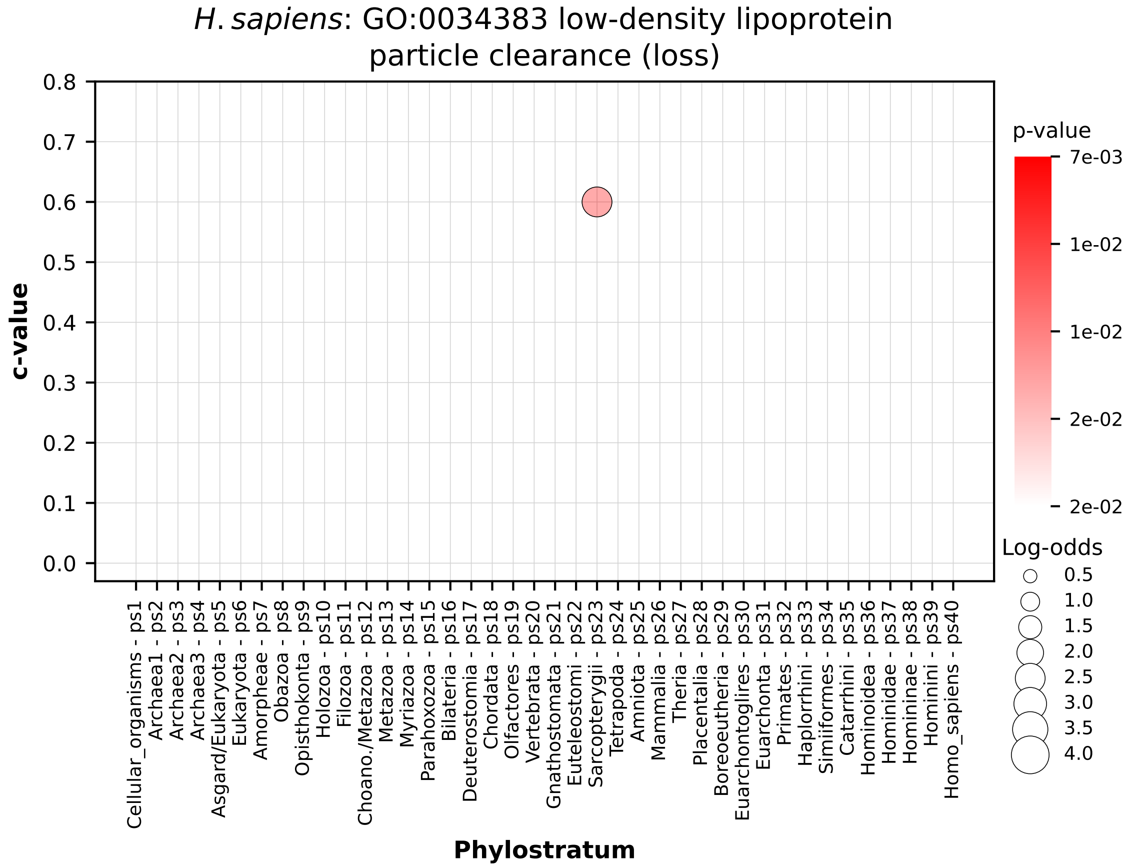

H. sapiens: GO:0034383 low-density lipoprotein particle clearance (loss)
c-value
0.8
0.7
0.6
0.5
0.4
0.3
0.2
0.1
0.0
p-value
7e-03
1e-02
1e-02
2e-02
2e-02
Log-odds
0.5
1.0
1.5
2.0
2.5
3.0
3.5
4.0
Cellular_organisms - ps1
Archaea1 - ps2
Archaea2 - ps3
Archaea3 - ps4
Asgard/Eukaryota - ps5
Eukaryota - ps6
Amorpheae - ps7
Obazoa - ps8
Opisthokonta - ps9
Holozoa - ps10
Filozoa - ps11
Choano./Metazoa - ps12
Metazoa - ps13
Myriazoa - ps14
Parahoxozoa - ps15
Bilateria - ps16
Deuterostomia - ps17
Chordata - ps18
Olfactores - ps19
Vertebrata - ps20
Gnathostomata - ps21
Euteleostomi - ps22
Sarcopterygii - ps23
Tetrapoda - ps24
Amniota - ps25
Mammalia - ps26
Theria - ps27
Placentalia - ps28
Boreoeutheria - ps29
Euarchontoglires - ps30
Euarchonta - ps31
Primates - ps32
Haplorrhini - ps33
Simiiformes - ps34
Catarrhini - ps35
Hominoidea - ps36
Hominidae - ps37
Homininae - ps38
Hominini - ps39
Homo_sapiens - ps40
Phylostratum

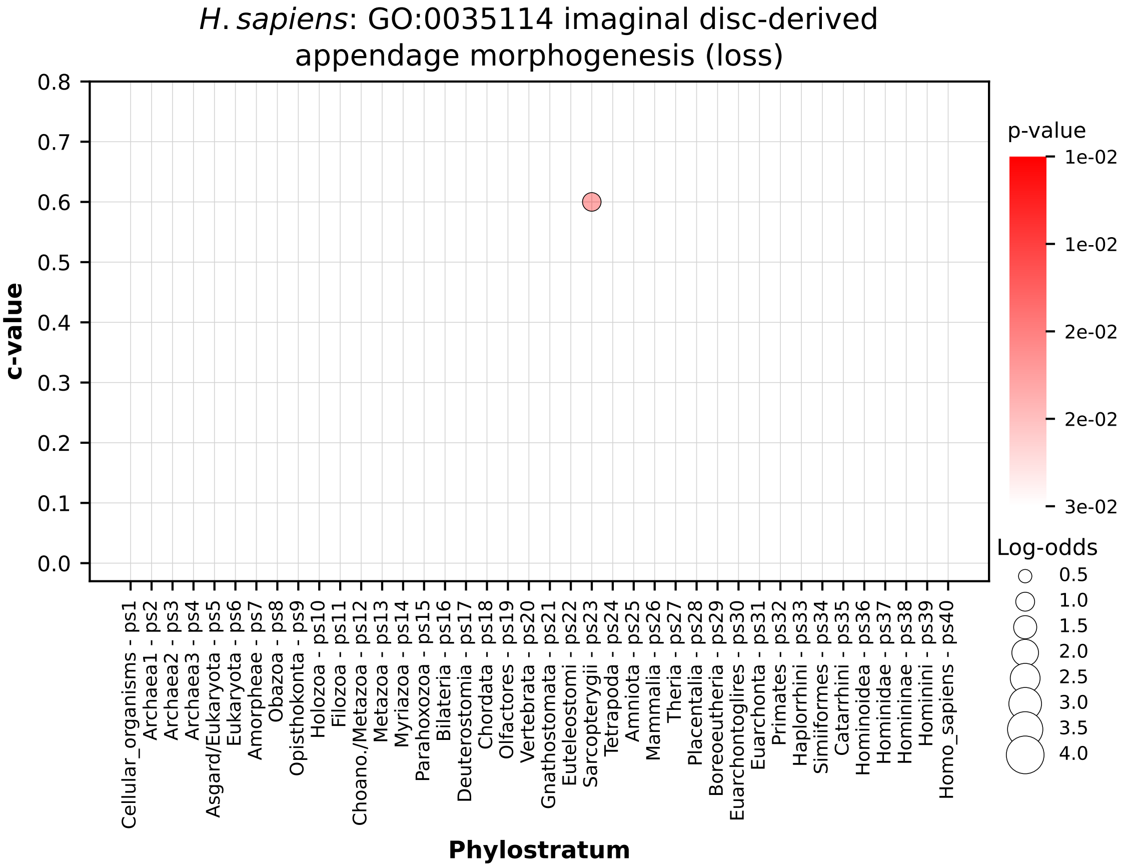

H. sapiens: GO:0035114 imaginal disc-derived appendage morphogenesis (loss)
c-value
0.8
0.7
0.6
0.5
0.4
0.3
0.2
0.1
0.0
Phylostratum
Cellular_organisms - ps1
Archaea1 - ps2
Archaea2 - ps3
Archaea3 - ps4
Asgard/Eukaryota - ps5
Eukaryota - ps6
Amorpheae - ps7
Obazoa - ps8
Opisthokonta - ps9
Holozoa - ps10
Filozoa - ps11
Choano./Metazoa - ps12
Metazoa - ps13
Myriazoa - ps14
Parahoxozoa - ps15
Bilateria - ps16
Deuterostomia - ps17
Chordata - ps18
Olfactores - ps19
Vertebrata - ps20
Gnathostomata - ps21
Euteleostomi - ps22
Sarcopterygii - ps23
Tetrapoda - ps24
Amniota - ps25
Mammalia - ps26
Theria - ps27
Placentalia - ps28
Boreoeutheria - ps29
Euarchontoglires - ps30
Euarchonta - ps31
Primates - ps32
Haplorrhini - ps33
Simiiformes - ps34
Catarrhini - ps35
Hominoidea - ps36
Hominidae - ps37
Homininae - ps38
Hominini - ps39
Homo_sapiens - ps40
p-value
1e-02
1e-02
2e-02
2e-02
3e-02
Log-odds
0.5
1.0
1.5
2.0
2.5
3.0
3.5
4.0

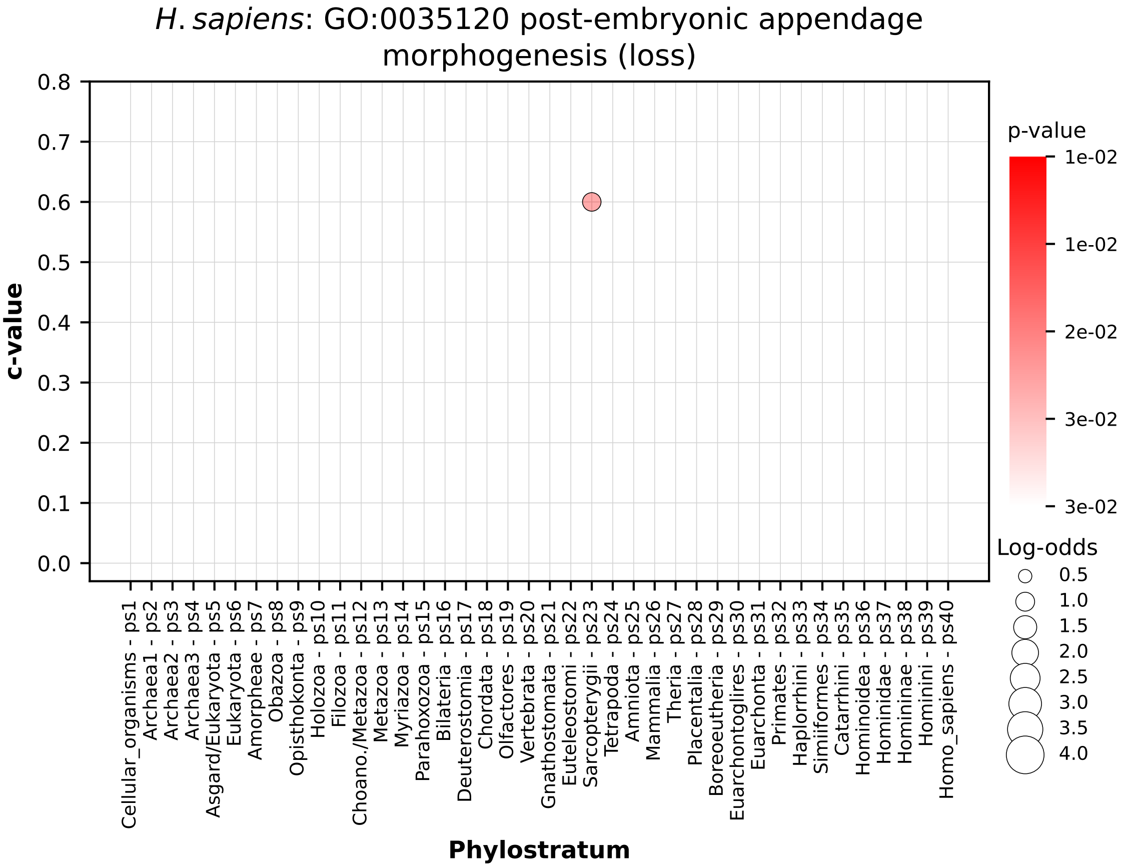
H. sapiens: GO:0035120 post-embryonic appendage morphogenesis (loss)
c-value
0.8
0.7
0.6
0.5
0.4
0.3
0.2
0.1
0.0
p-value
1e-02
1e-02
2e-02
3e-02
3e-02
Log-odds
0.5
1.0
1.5
2.0
2.5
3.0
3.5
4.0
Cellular_organisms - ps1
Archaea1 - ps2
Archaea2 - ps3
Archaea3 - ps4
Asgard/Eukaryota - ps5
Eukaryota - ps6
Amorpheae - ps7
Obazoa - ps8
Opisthokonta - ps9
Holozoa - ps10
Filozoa - ps11
Choano./Metazoa - ps12
Metazoa - ps13
Myriazoa - ps14
Parahoxozoa - ps15
Bilateria - ps16
Deuterostomia - ps17
Chordata - ps18
Olfactores - ps19
Vertebrata - ps20
Gnathostomata - ps21
Euteleostomi - ps22
Sarcopterygii - ps23
Tetrapoda - ps24
Amniota - ps25
Mammalia - ps26
Theria - ps27
Placentalia - ps28
Boreoeutheria - ps29
Euarchontoglires - ps30
Euarchonta - ps31
Primates - ps32
Haplorrhini - ps33
Simiiformes - ps34
Catarrhini - ps35
Hominoidea - ps36
Hominidae - ps37
Homininae - ps38
Hominini - ps39
Homo_sapiens - ps40
Phylostratum

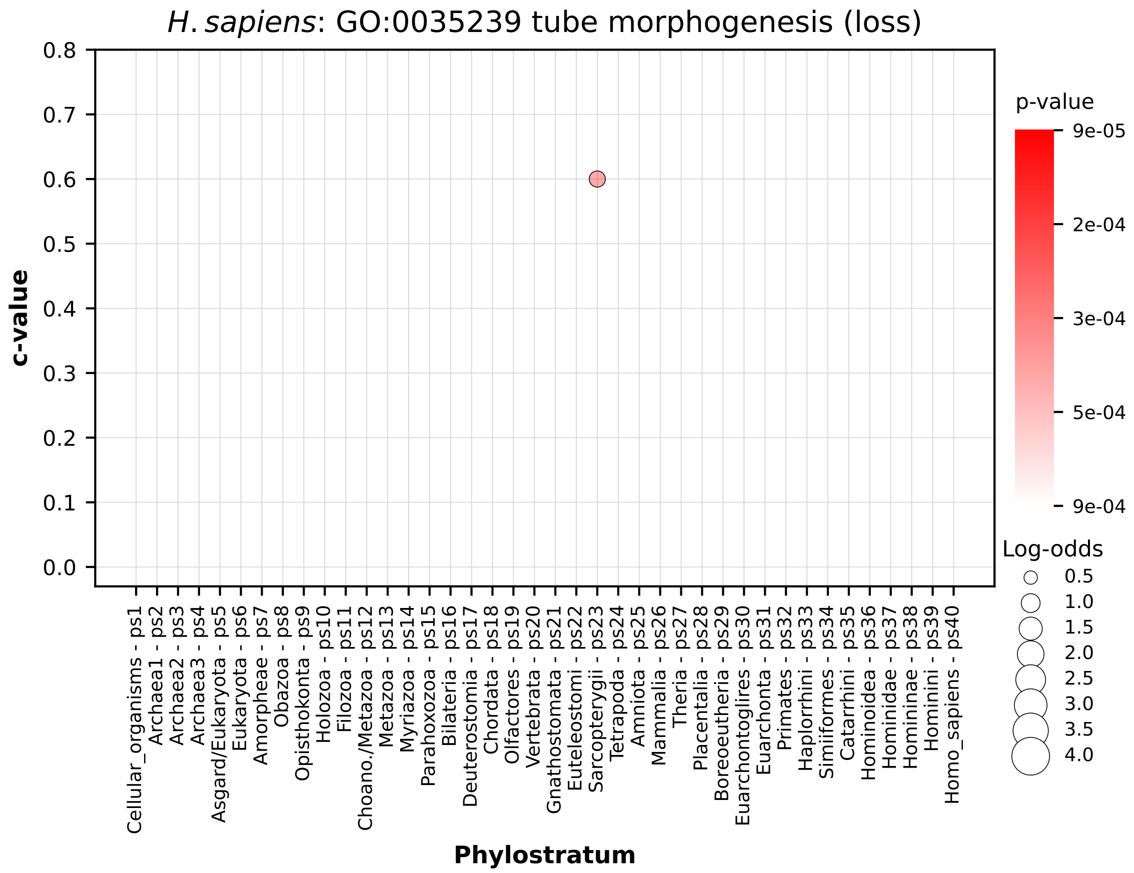

H. sapiens: GO:0035239 tube morphogenesis (loss)
c-value
0.8
0.7
0.6
0.5
0.4
0.3
0.2
0.1
0.0
Phylostratum
Cellular_organisms - ps1
Archaea1 - ps2
Archaea2 - ps3
Archaea3 - ps4
Asgard/Eukaryota - ps5
Eukaryota - ps6
Amorpheae - ps7
Obazoa - ps8
Opisthokonta - ps9
Holozoa - ps10
Filozoa - ps11
Choano./Metazoa - ps12
Metazoa - ps13
Myriazoa - ps14
Parahoxozoa - ps15
Bilateria - ps16
Deuterostomia - ps17
Chordata - ps18
Olfactores - ps19
Vertebrata - ps20
Gnathostomata - ps21
Euteleostomi - ps22
Sarcopterygii - ps23
Tetrapoda - ps24
Amniota - ps25
Mammalia - ps26
Theria - ps27
Placentalia - ps28
Boreoeutheria - ps29
Euarchontoglires - ps30
Euarchonta - ps31
Primates - ps32
Haplorrhini - ps33
Simiiformes - ps34
Catarrhini - ps35
Hominoidea - ps36
Hominidae - ps37
Homininae - ps38
Hominini - ps39
Homo_sapiens - ps40
p-value
9e-05
2e-04
3e-04
5e-04
9e-04
Log-odds
0.5
1.0
1.5
2.0
2.5
3.0
3.5
4.0

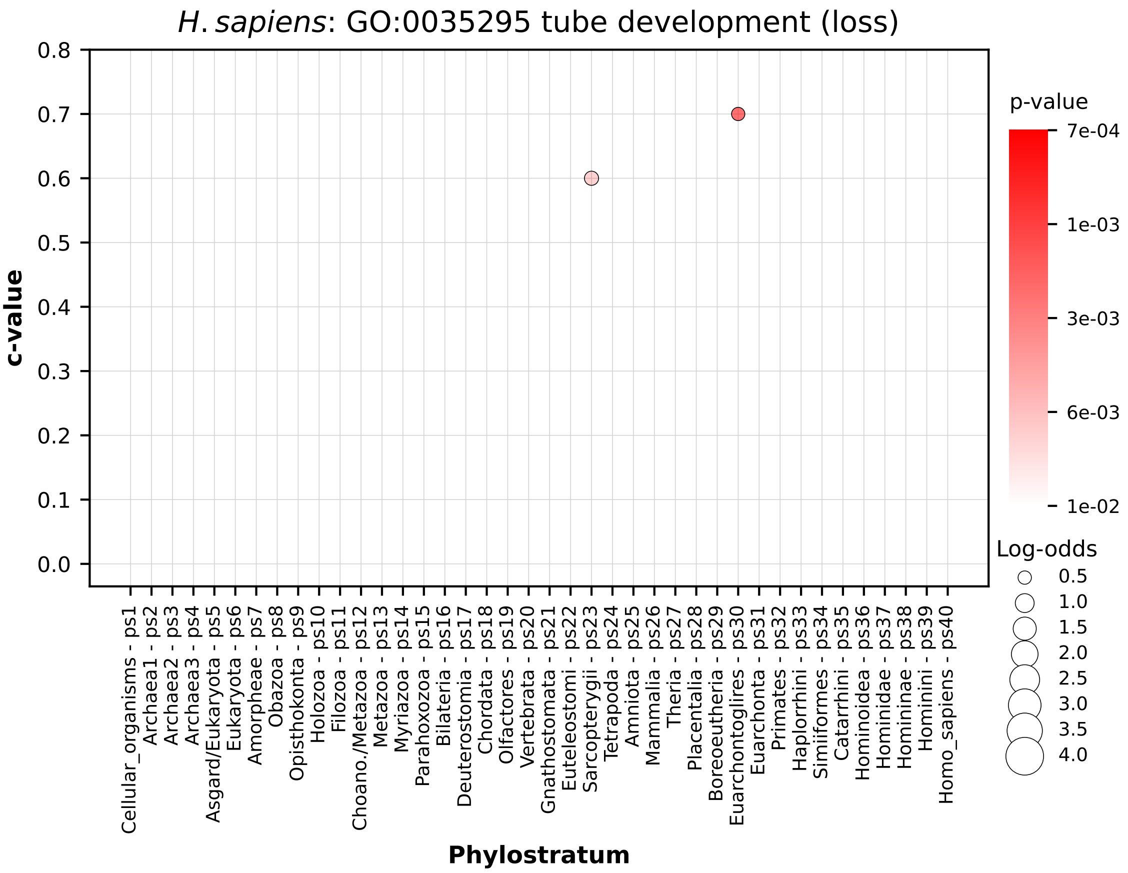

H. sapiens: GO:0035295 tube development (loss)
c-value
0.8
0.7
0.6
0.5
0.4
0.3
0.2
0.1
0.0
p-value
7e-04
1e-03
3e-03
6e-03
1e-02
Log-odds
0.5
1.0
1.5
2.0
2.5
3.0
3.5
4.0
Cellular_organisms - ps1
Archaea1 - ps2
Archaea2 - ps3
Archaea3 - ps4
Asgard/Eukaryota - ps5
Eukaryota - ps6
Amorpheae - ps7
Obazoa - ps8
Opisthokonta - ps9
Holozoa - ps10
Filozoa - ps11
Choano./Metazoa - ps12
Metazoa - ps13
Myriazoa - ps14
Parahoxozoa - ps15
Bilateria - ps16
Deuterostomia - ps17
Chordata - ps18
Olfactores - ps19
Vertebrata - ps20
Gnathostomata - ps21
Euteleostomi - ps22
Sarcopterygii - ps23
Tetrapoda - ps24
Amniota - ps25
Mammalia - ps26
Theria - ps27
Placentalia - ps28
Boreoeutheria - ps29
Euarchontoglires - ps30
Euarchonta - ps31
Primates - ps32
Haplorrhini - ps33
Simiiformes - ps34
Catarrhini - ps35
Hominoidea - ps36
Hominidae - ps37
Homininae - ps38
Hominini - ps39
Homo_sapiens - ps40
Phylostratum

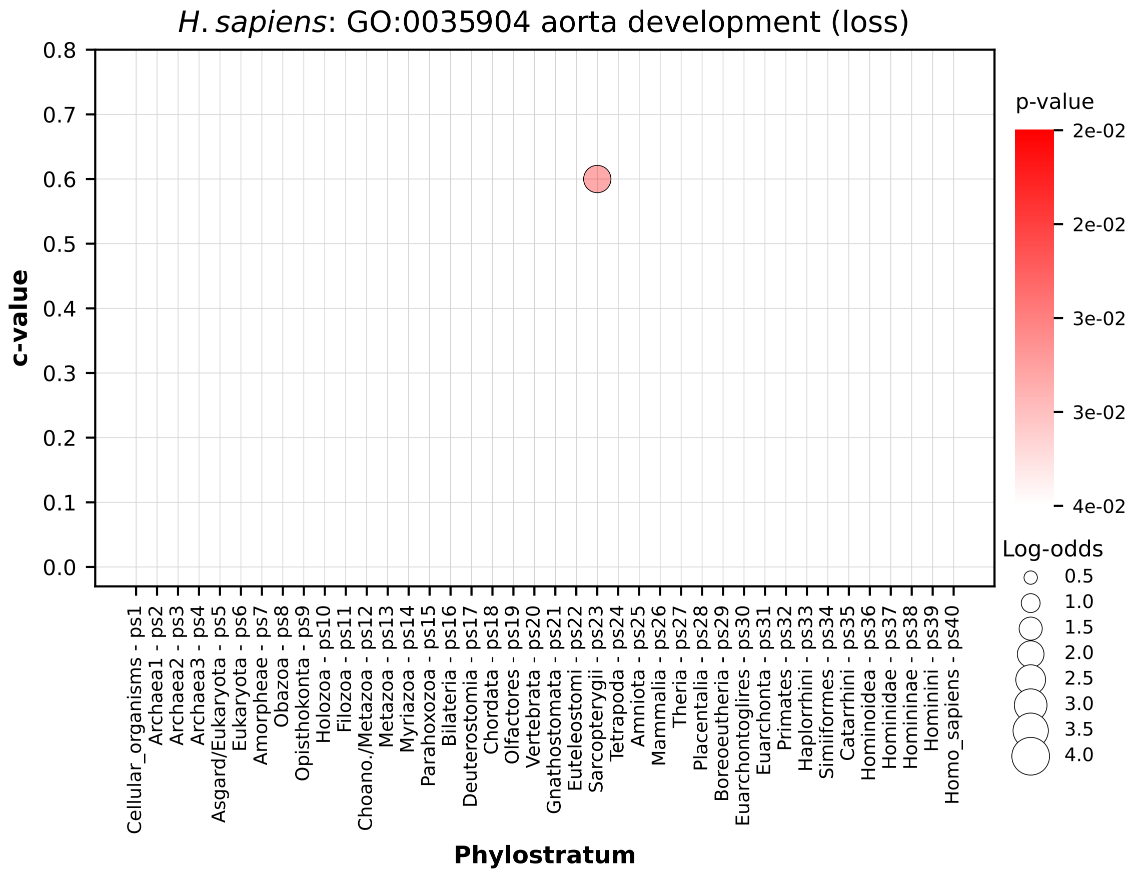

H. sapiens: GO:0035904 aorta development (loss)
c-value
0.8
0.7
0.6
0.5
0.4
0.3
0.2
0.1
0.0
p-value
2e-02
2e-02
3e-02
3e-02
4e-02
Log-odds
0.5
1.0
1.5
2.0
2.5
3.0
3.5
4.0
Cellular_organisms - ps1
Archaea1 - ps2
Archaea2 - ps3
Archaea3 - ps4
Asgard/Eukaryota - ps5
Eukaryota - ps6
Amorpheae - ps7
Obazoa - ps8
Opisthokonta - ps9
Holozoa - ps10
Filozoa - ps11
Choano./Metazoa - ps12
Metazoa - ps13
Myriazoa - ps14
Parahoxozoa - ps15
Bilateria - ps16
Deuterostomia - ps17
Chordata - ps18
Olfactores - ps19
Vertebrata - ps20
Gnathostomata - ps21
Euteleostomi - ps22
Sarcopterygii - ps23
Tetrapoda - ps24
Amniota - ps25
Mammalia - ps26
Theria - ps27
Placentalia - ps28
Boreoeutheria - ps29
Euarchontoglires - ps30
Euarchonta - ps31
Primates - ps32
Haplorrhini - ps33
Simiiformes - ps34
Catarrhini - ps35
Hominoidea - ps36
Hominidae - ps37
Homininae - ps38
Hominini - ps39
Homo_sapiens - ps40
Phylostratum

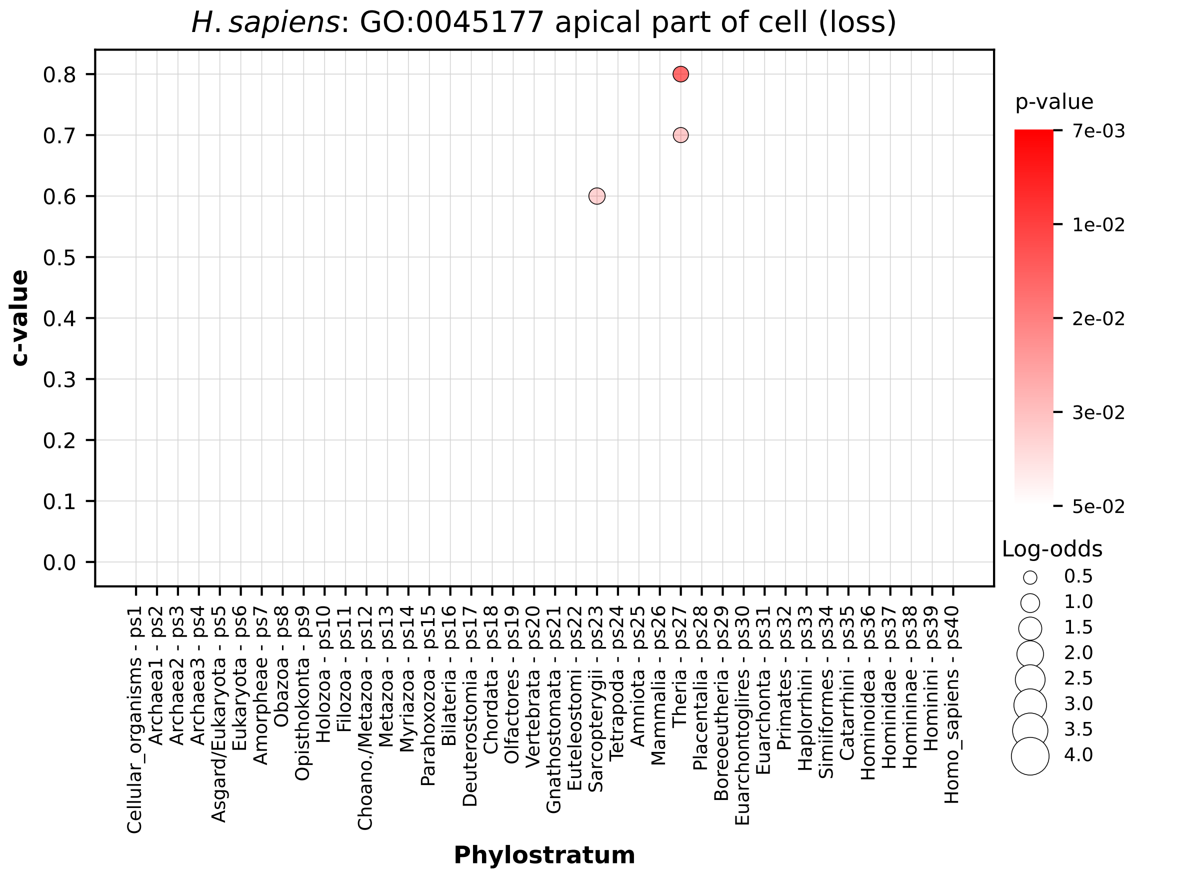

H. sapiens: GO:0045177 apical part of cell (loss)
c-value
0.8
0.7
0.6
0.5
0.4
0.3
0.2
0.1
0.0
p-value
7e-03
1e-02
2e-02
3e-02
5e-02
Log-odds
0.5
1.0
1.5
2.0
2.5
3.0
3.5
4.0
Cellular_organisms - ps1
Archaea1 - ps2
Archaea2 - ps3
Archaea3 - ps4
Asgard/Eukaryota - ps5
Eukaryota - ps6
Amorpheae - ps7
Obazoa - ps8
Opisthokonta - ps9
Holozoa - ps10
Filozoa - ps11
Choano./Metazoa - ps12
Metazoa - ps13
Myriazoa - ps14
Parahoxozoa - ps15
Bilateria - ps16
Deuterostomia - ps17
Chordata - ps18
Olfactores - ps19
Vertebrata - ps20
Gnathostomata - ps21
Euteleostomi - ps22
Sarcopterygii - ps23
Tetrapoda - ps24
Amniota - ps25
Mammalia - ps26
Theria - ps27
Placentalia - ps28
Boreoeutheria - ps29
Euarchontoglires - ps30
Euarchonta - ps31
Primates - ps32
Haplorrhini - ps33
Simiiformes - ps34
Catarrhini - ps35
Hominoidea - ps36
Hominidae - ps37
Homininae - ps38
Hominini - ps39
Homo_sapiens - ps40
Phylostratum

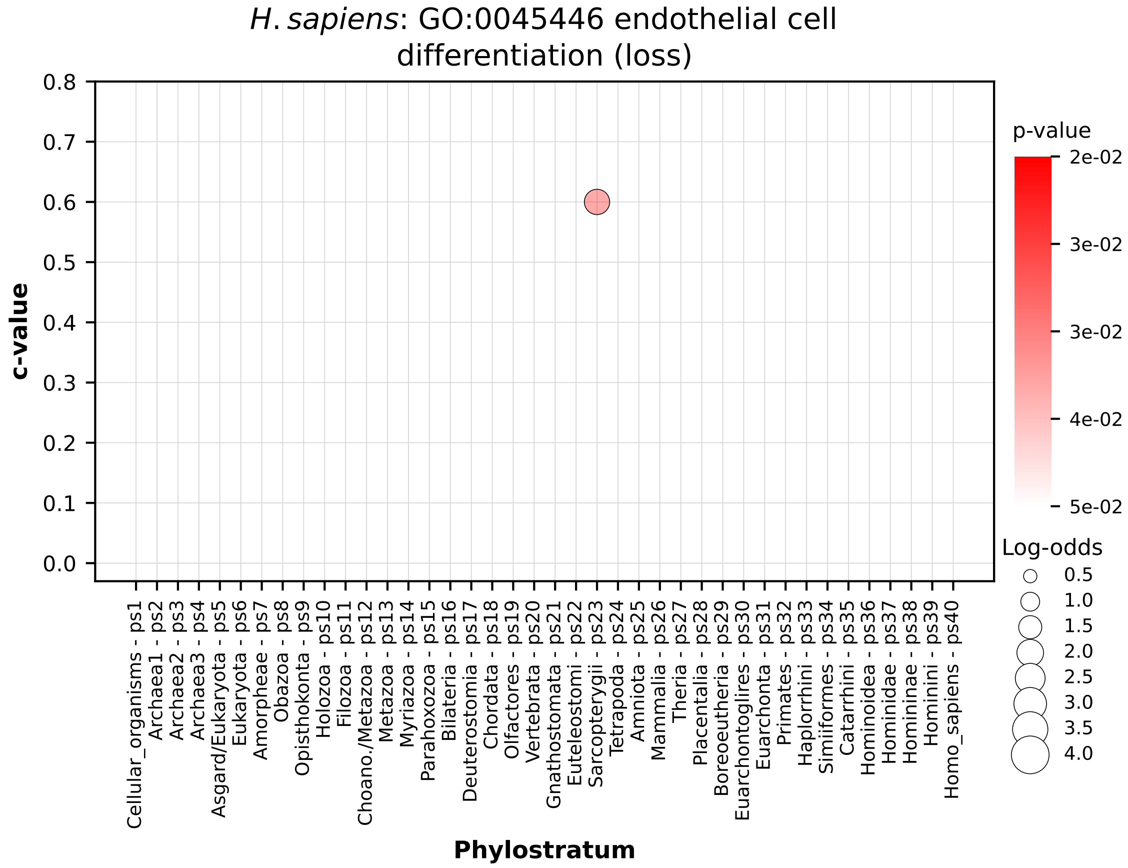
H. sapiens: GO:0045446 endothelial cell differentiation (loss)
c-value
0.8
0.7
0.6
0.5
0.4
0.3
0.2
0.1
0.0
Phylostratum
Cellular_organisms - ps1
Archaea1 - ps2
Archaea2 - ps3
Archaea3 - ps4
Asgard/Eukaryota - ps5
Eukaryota - ps6
Amorpheae - ps7
Obazoa - ps8
Opisthokonta - ps9
Holozoa - ps10
Filozoa - ps11
Choano./Metazoa - ps12
Metazoa - ps13
Myriazoa - ps14
Parahoxozoa - ps15
Bilateria - ps16
Deuterostomia - ps17
Chordata - ps18
Olfactores - ps19
Vertebrata - ps20
Gnathostomata - ps21
Euteleostomi - ps22
Sarcopterygii - ps23
Tetrapoda - ps24
Amniota - ps25
Mammalia - ps26
Theria - ps27
Placentalia - ps28
Boreoeutheria - ps29
Euarchontoglires - ps30
Euarchonta - ps31
Primates - ps32
Haplorrhini - ps33
Simiiformes - ps34
Catarrhini - ps35
Hominoidea - ps36
Hominidae - ps37
Homininae - ps38
Hominini - ps39
Homo_sapiens - ps40
p-value
2e-02
3e-02
3e-02
4e-02
5e-02
Log-odds
0.5
1.0
1.5
2.0
2.5
3.0
3.5
4.0

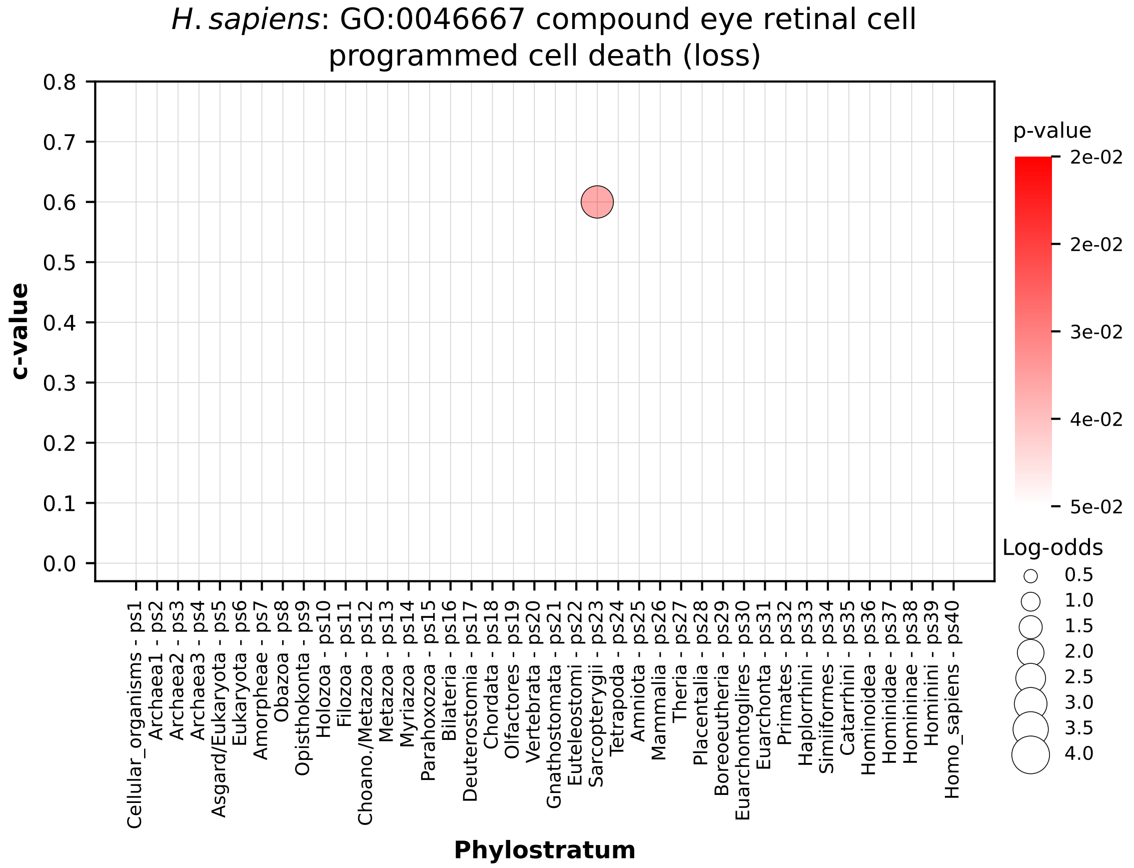

H. sapiens: GO:0046667 compound eye retinal cell programmed cell death (loss)
c-value
0.8
0.7
0.6
0.5
0.4
0.3
0.2
0.1
0.0
p-value
2e-02
2e-02
3e-02
4e-02
5e-02
Log-odds
0.5
1.0
1.5
2.0
2.5
3.0
3.5
4.0
Cellular_organisms - ps1
Archaea1 - ps2
Archaea2 - ps3
Archaea3 - ps4
Asgard/Eukaryota - ps5
Eukaryota - ps6
Amorpheae - ps7
Obazoa - ps8
Opisthokonta - ps9
Holozoa - ps10
Filozoa - ps11
Choano./Metazoa - ps12
Metazoa - ps13
Myriazoa - ps14
Parahoxozoa - ps15
Bilateria - ps16
Deuterostomia - ps17
Chordata - ps18
Olfactores - ps19
Vertebrata - ps20
Gnathostomata - ps21
Euteleostomi - ps22
Sarcopterygii - ps23
Tetrapoda - ps24
Amniota - ps25
Mammalia - ps26
Theria - ps27
Placentalia - ps28
Boreoeutheria - ps29
Euarchontoglires - ps30
Euarchonta - ps31
Primates - ps32
Haplorrhini - ps33
Simiiformes - ps34
Catarrhini - ps35
Hominoidea - ps36
Hominidae - ps37
Homininae - ps38
Hominini - ps39
Homo_sapiens - ps40
Phylostratum

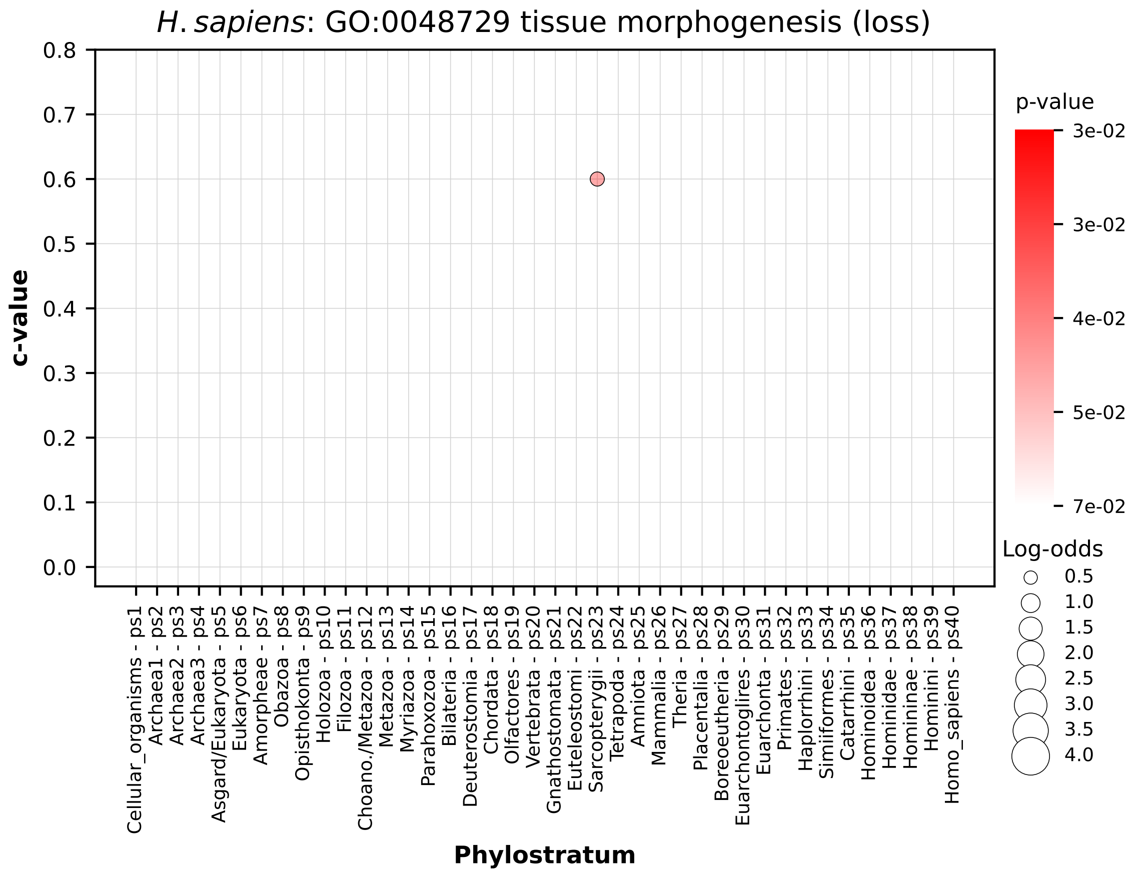

H. sapiens: GO:0048729 tissue morphogenesis (loss)
c-value
0.0
0.1
0.2
0.3
0.4
0.5
0.6
0.7
0.8
Phylostratum
Cellular_organisms - ps1
Archaea1 - ps2
Archaea2 - ps3
Archaea3 - ps4
Asgard/Eukaryota - ps5
Eukaryota - ps6
Amorpheae - ps7
Obazoa - ps8
Opisthokonta - ps9
Holozoa - ps10
Filozoa - ps11
Choano./Metazoa - ps12
Metazoa - ps13
Myriazoa - ps14
Parahoxozoa - ps15
Bilateria - ps16
Deuterostomia - ps17
Chordata - ps18
Olfactores - ps19
Vertebrata - ps20
Gnathostomata - ps21
Euteleostomi - ps22
Sarcopterygii - ps23
Tetrapoda - ps24
Amniota - ps25
Mammalia - ps26
Theria - ps27
Placentalia - ps28
Boreoeutheria - ps29
Euarchontoglires - ps30
Euarchonta - ps31
Primates - ps32
Haplorrhini - ps33
Simiiformes - ps34
Catarrhini - ps35
Hominoidea - ps36
Hominidae - ps37
Homininae - ps38
Hominini - ps39
Homo_sapiens - ps40
p-value
3e-02
3e-02
4e-02
5e-02
7e-02
Log-odds
0.5
1.0
1.5
2.0
2.5
3.0
3.5
4.0

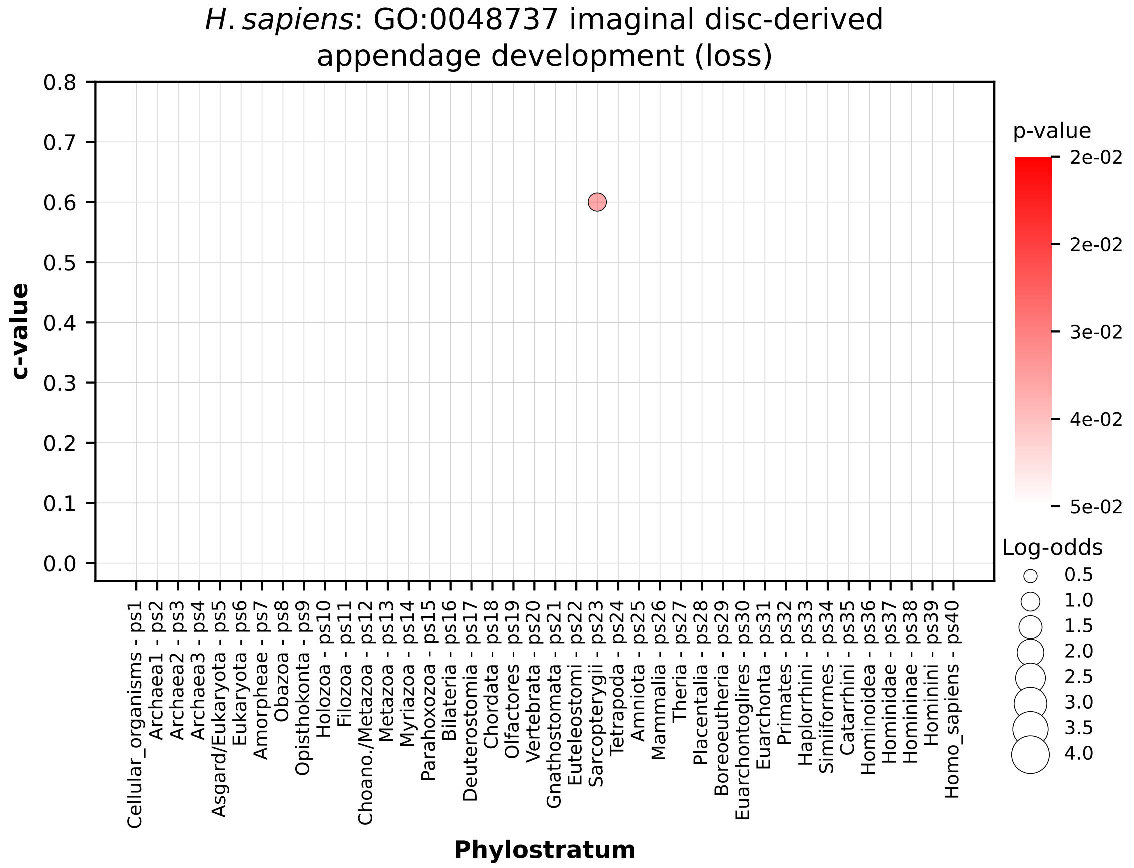

H. sapiens: GO:0048737 imaginal disc-derived appendage development (loss)
c-value
0.8
0.7
0.6
0.5
0.4
0.3
0.2
0.1
0.0
Phylostratum
Cellular_organisms - ps1
Archaea1 - ps2
Archaea2 - ps3
Archaea3 - ps4
Asgard/Eukaryota - ps5
Eukaryota - ps6
Amorpheae - ps7
Obazoa - ps8
Opisthokonta - ps9
Holozoa - ps10
Filozoa - ps11
Choano./Metazoa - ps12
Metazoa - ps13
Myriazoa - ps14
Parahoxozoa - ps15
Bilateria - ps16
Deuterostomia - ps17
Chordata - ps18
Olfactores - ps19
Vertebrata - ps20
Gnathostomata - ps21
Euteleostomi - ps22
Sarcopterygii - ps23
Tetrapoda - ps24
Amniota - ps25
Mammalia - ps26
Theria - ps27
Placentalia - ps28
Boreoeutheria - ps29
Euarchontoglires - ps30
Euarchonta - ps31
Primates - ps32
Haplorrhini - ps33
Simiiformes - ps34
Catarrhini - ps35
Hominoidea - ps36
Hominidae - ps37
Homininae - ps38
Hominini - ps39
Homo_sapiens - ps40
p-value
2e-02
2e-02
3e-02
4e-02
5e-02
Log-odds
0.5
1.0
1.5
2.0
2.5
3.0
3.5
4.0

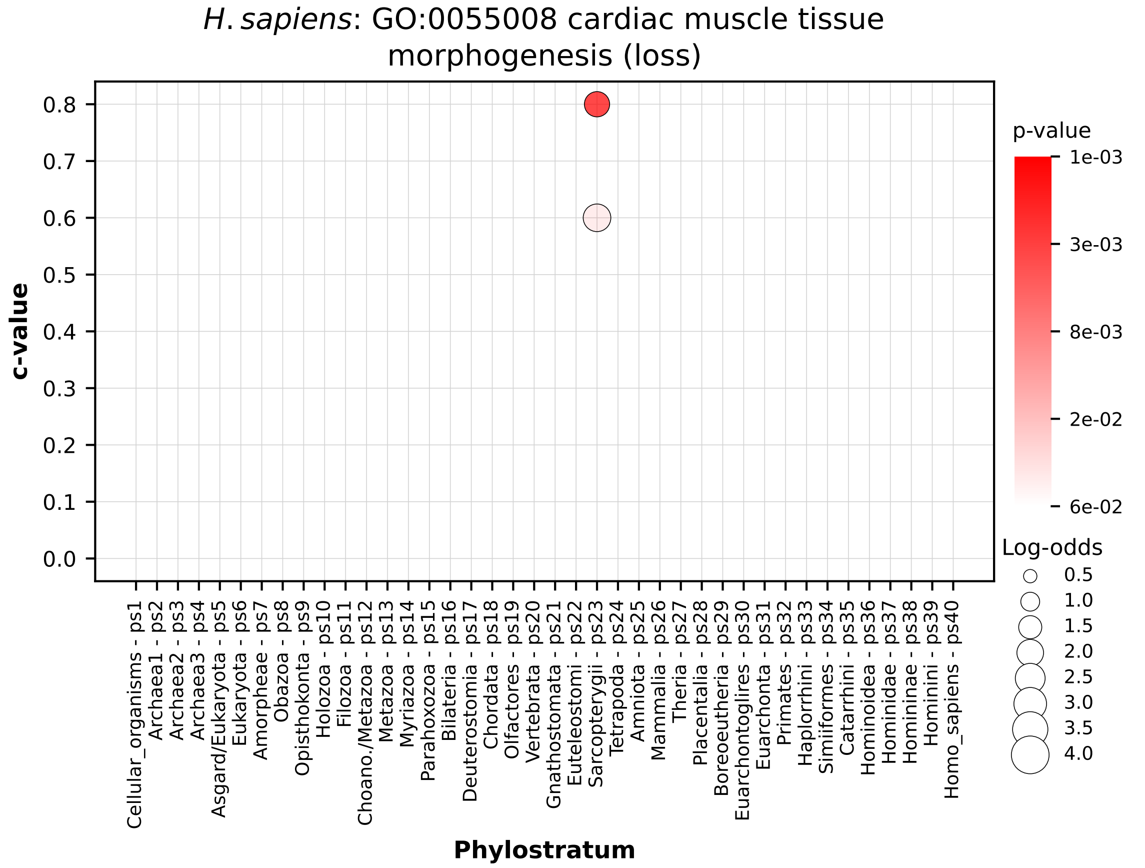

H. sapiens: GO:0055008 cardiac muscle tissue morphogenesis (loss)
c-value
0.8
0.7
0.6
0.5
0.4
0.3
0.2
0.1
0.0
p-value
1e-03
3e-03
8e-03
2e-02
6e-02
Log-odds
0.5
1.0
1.5
2.0
2.5
3.0
3.5
4.0
Cellular_organisms - ps1
Archaea1 - ps2
Archaea2 - ps3
Archaea3 - ps4
Asgard/Eukaryota - ps5
Eukaryota - ps6
Amorpheae - ps7
Obazoa - ps8
Opisthokonta - ps9
Holozoa - ps10
Filozoa - ps11
Choano./Metazoa - ps12
Metazoa - ps13
Myriazoa - ps14
Parahoxozoa - ps15
Bilateria - ps16
Deuterostomia - ps17
Chordata - ps18
Olfactores - ps19
Vertebrata - ps20
Gnathostomata - ps21
Euteleostomi - ps22
Sarcopterygii - ps23
Tetrapoda - ps24
Amniota - ps25
Mammalia - ps26
Theria - ps27
Placentalia - ps28
Boreoeutheria - ps29
Euarchontoglires - ps30
Euarchonta - ps31
Primates - ps32
Haplorrhini - ps33
Simiiformes - ps34
Catarrhini - ps35
Hominoidea - ps36
Hominidae - ps37
Homininae - ps38
Hominini - ps39
Homo_sapiens - ps40
Phylostratum

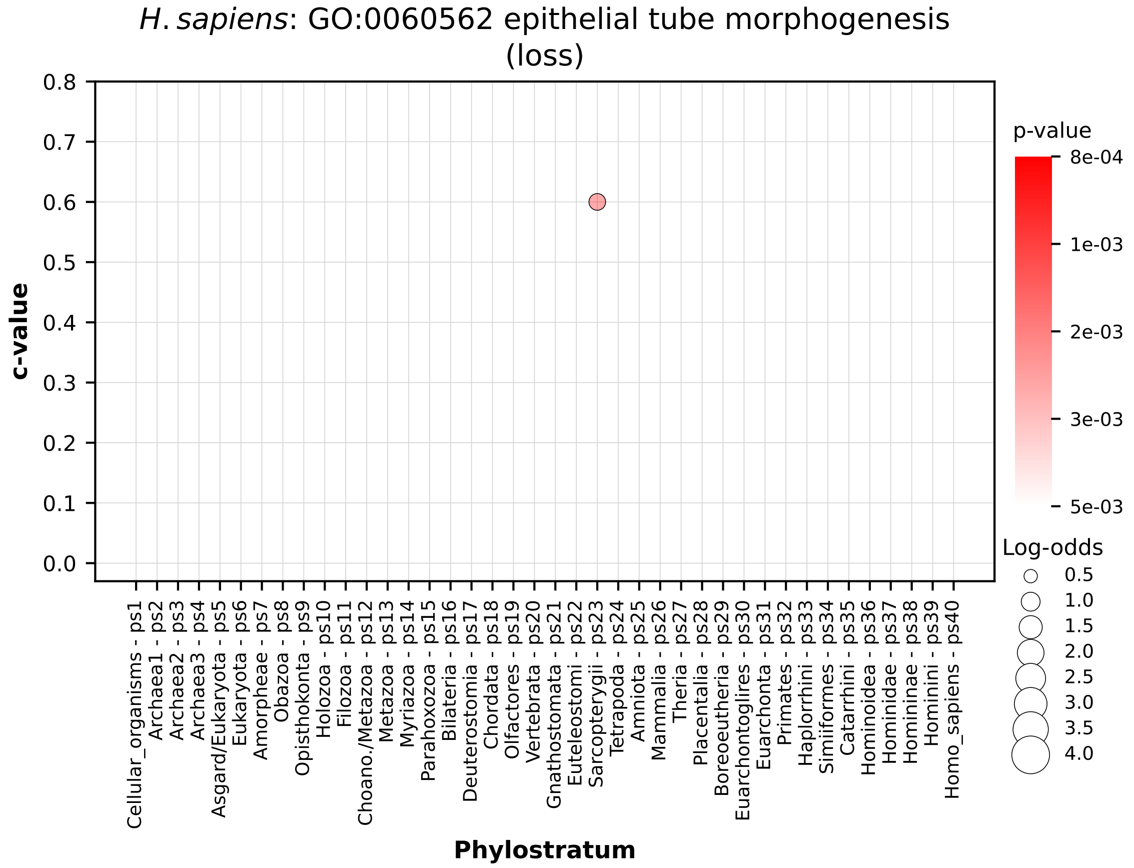
H. sapiens: GO:0060562 epithelial tube morphogenesis
(loss)
c-value
0.8
0.7
0.6
0.5
0.4
0.3
0.2
0.1
0.0
p-value
8e-04
1e-03
2e-03
3e-03
5e-03
Log-odds
0.5
1.0
1.5
2.0
2.5
3.0
3.5
4.0
Cellular_organisms - ps1
Archaea1 - ps2
Archaea2 - ps3
Archaea3 - ps4
Asgard/Eukaryota - ps5
Eukaryota - ps6
Amorpheae - ps7
Obazoa - ps8
Opisthokonta - ps9
Holozoa - ps10
Filozoa - ps11
Choano./Metazoa - ps12
Metazoa - ps13
Myriazoa - ps14
Parahoxozoa - ps15
Bilateria - ps16
Deuterostomia - ps17
Chordata - ps18
Olfactores - ps19
Vertebrata - ps20
Gnathostomata - ps21
Euteleostomi - ps22
Sarcopterygii - ps23
Tetrapoda - ps24
Amniota - ps25
Mammalia - ps26
Theria - ps27
Placentalia - ps28
Boreoeutheria - ps29
Euarchontoglires - ps30
Euarchonta - ps31
Primates - ps32
Haplorrhini - ps33
Simiiformes - ps34
Catarrhini - ps35
Hominoidea - ps36
Hominidae - ps37
Homininae - ps38
Hominini - ps39
Homo_sapiens - ps40
Phylostratum

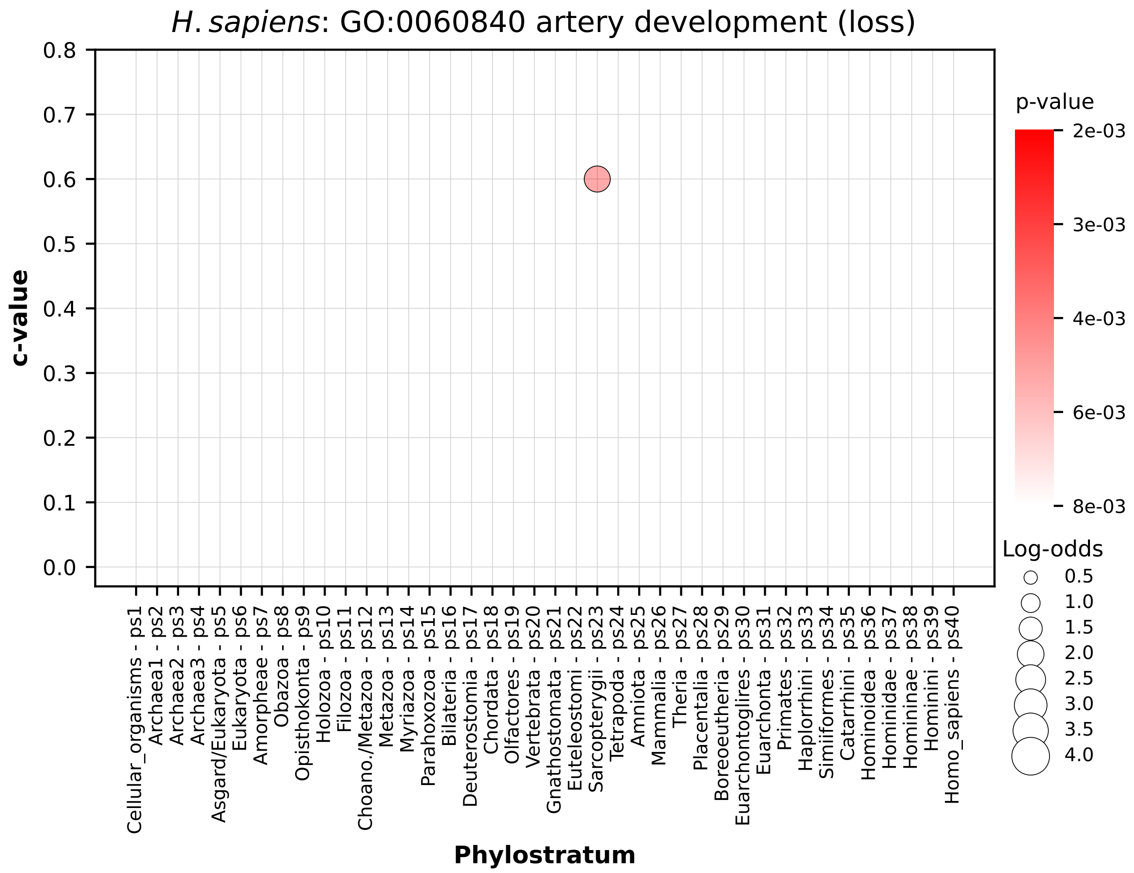

H. sapiens: GO:0060840 artery development (loss)
c-value
0.0
0.1
0.2
0.3
0.4
0.5
0.6
0.7
0.8
Phylostratum
Cellular_organisms - ps1
Archaea1 - ps2
Archaea2 - ps3
Archaea3 - ps4
Asgard/Eukaryota - ps5
Eukaryota - ps6
Amorpheae - ps7
Obazoa - ps8
Opisthokonta - ps9
Holozoa - ps10
Filozoa - ps11
Choano./Metazoa - ps12
Metazoa - ps13
Myriazoa - ps14
Parahoxozoa - ps15
Bilateria - ps16
Deuterostomia - ps17
Chordata - ps18
Olfactores - ps19
Vertebrata - ps20
Gnathostomata - ps21
Euteleostomi - ps22
Sarcopterygii - ps23
Tetrapoda - ps24
Amniota - ps25
Mammalia - ps26
Theria - ps27
Placentalia - ps28
Boreoeutheria - ps29
Euarchontoglires - ps30
Euarchonta - ps31
Primates - ps32
Haplorrhini - ps33
Simiiformes - ps34
Catarrhini - ps35
Hominoidea - ps36
Hominidae - ps37
Homininae - ps38
Hominini - ps39
Homo_sapiens - ps40
p-value
2e-03
3e-03
4e-03
6e-03
8e-03
Log-odds
0.5
1.0
1.5
2.0
2.5
3.0
3.5
4.0

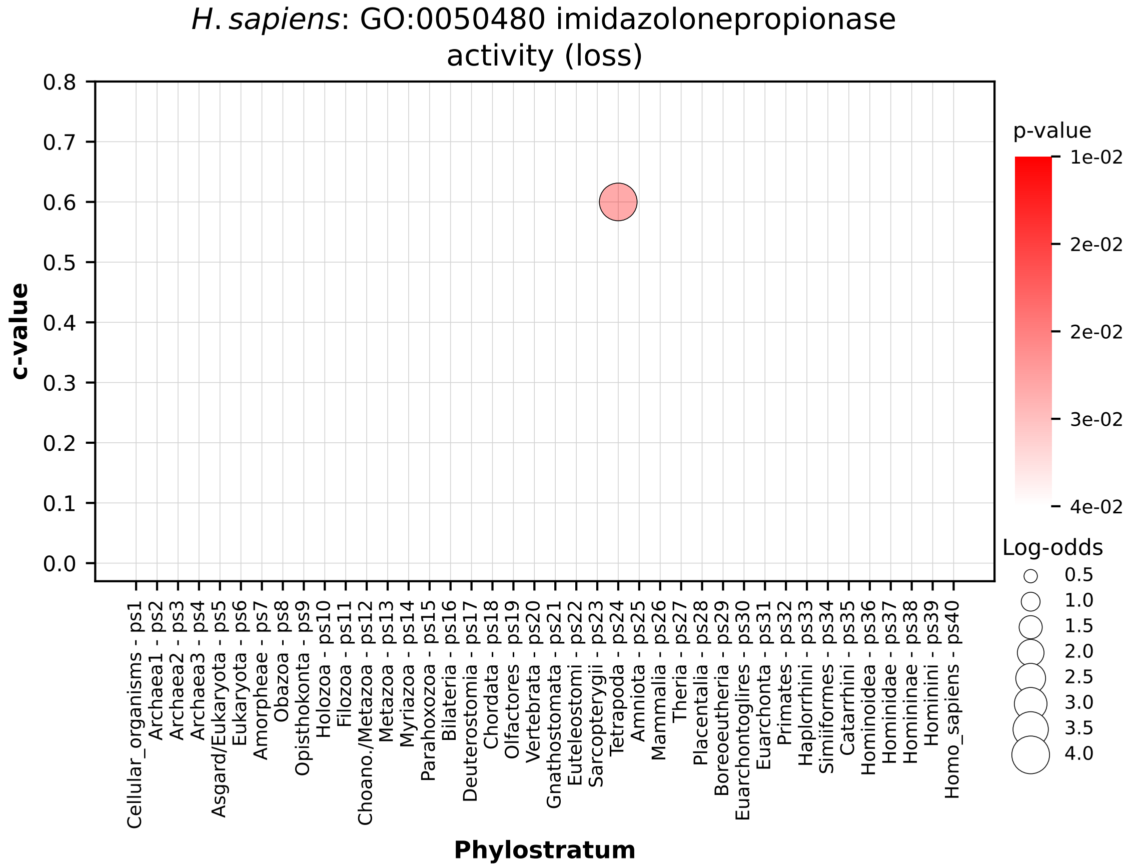

H. sapiens: GO:0050480 imidazolonepropionase activity (loss)
c-value
0.8
0.7
0.6
0.5
0.4
0.3
0.2
0.1
0.0
Phylostratum
Cellular_organisms - ps1
Archaea1 - ps2
Archaea2 - ps3
Archaea3 - ps4
Asgard/Eukaryota - ps5
Eukaryota - ps6
Amorpheae - ps7
Obazoa - ps8
Opisthokonta - ps9
Holozoa - ps10
Filozoa - ps11
Choano./Metazoa - ps12
Metazoa - ps13
Myriazoa - ps14
Parahoxozoa - ps15
Bilateria - ps16
Deuterostomia - ps17
Chordata - ps18
Olfactores - ps19
Vertebrata - ps20
Gnathostomata - ps21
Euteleostomi - ps22
Sarcopterygii - ps23
Tetrapoda - ps24
Amniota - ps25
Mammalia - ps26
Theria - ps27
Placentalia - ps28
Boreoeutheria - ps29
Euarchontoglires - ps30
Euarchonta - ps31
Primates - ps32
Haplorrhini - ps33
Simiiformes - ps34
Catarrhini - ps35
Hominoidea - ps36
Hominidae - ps37
Homininae - ps38
Hominini - ps39
Homo_sapiens - ps40
p-value
1e-02
2e-02
2e-02
3e-02
4e-02
Log-odds
0.5
1.0
1.5
2.0
2.5
3.0
3.5
4.0

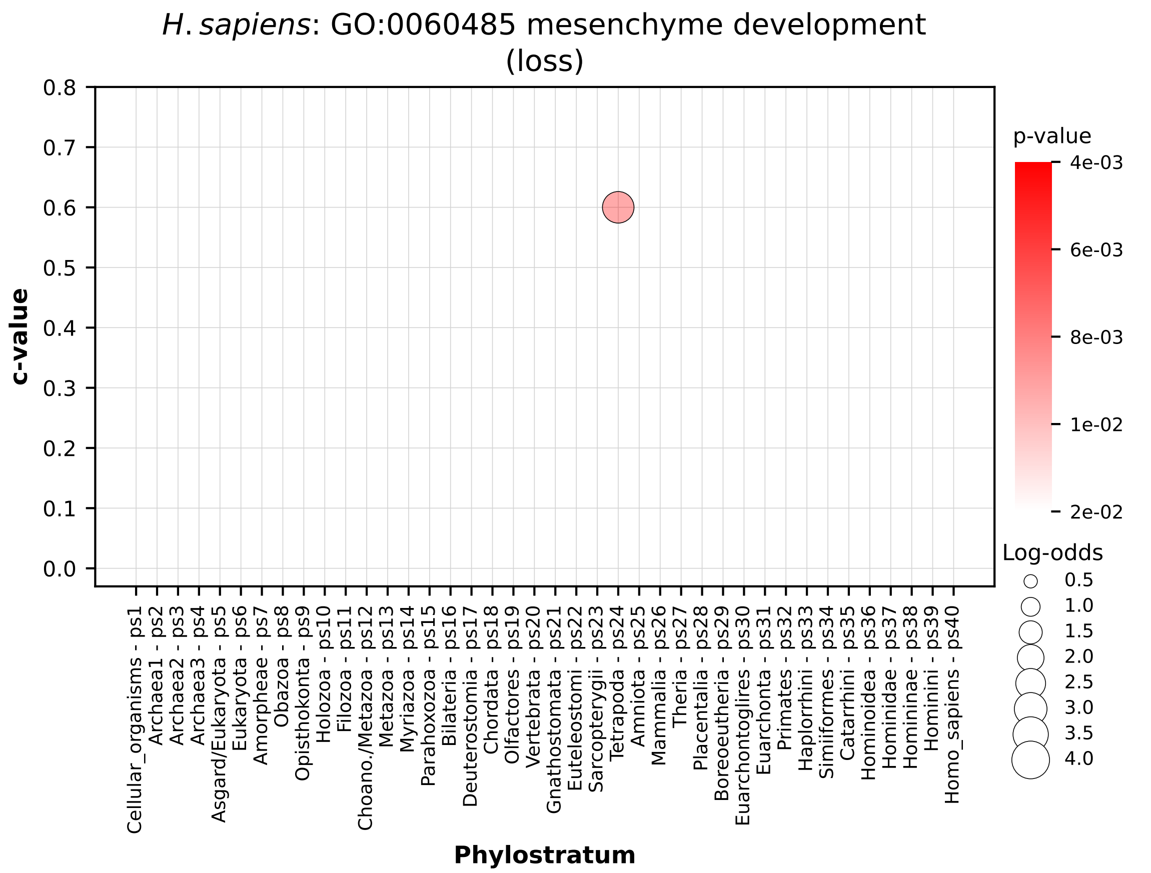
H. sapiens: GO:0060485 mesenchyme development
(loss)
c-value
0.8
0.7
0.6
0.5
0.4
0.3
0.2
0.1
0.0
p-value
4e-03
6e-03
8e-03
1e-02
2e-02
Log-odds
0.5
1.0
1.5
2.0
2.5
3.0
3.5
4.0
Cellular_organisms - ps1
Archaea1 - ps2
Archaea2 - ps3
Archaea3 - ps4
Asgard/Eukaryota - ps5
Eukaryota - ps6
Amorpheae - ps7
Obazoa - ps8
Opisthokonta - ps9
Holozoa - ps10
Filozoa - ps11
Choano./Metazoa - ps12
Metazoa - ps13
Myriazoa - ps14
Parahoxozoa - ps15
Bilateria - ps16
Deuterostomia - ps17
Chordata - ps18
Olfactores - ps19
Vertebrata - ps20
Gnathostomata - ps21
Euteleostomi - ps22
Sarcopterygii - ps23
Tetrapoda - ps24
Amniota - ps25
Mammalia - ps26
Theria - ps27
Placentalia - ps28
Boreoeutheria - ps29
Euarchontoglires - ps30
Euarchonta - ps31
Primates - ps32
Haplorrhini - ps33
Simiiformes - ps34
Catarrhini - ps35
Hominoidea - ps36
Hominidae - ps37
Homininae - ps38
Hominini - ps39
Homo_sapiens - ps40
Phylostratum

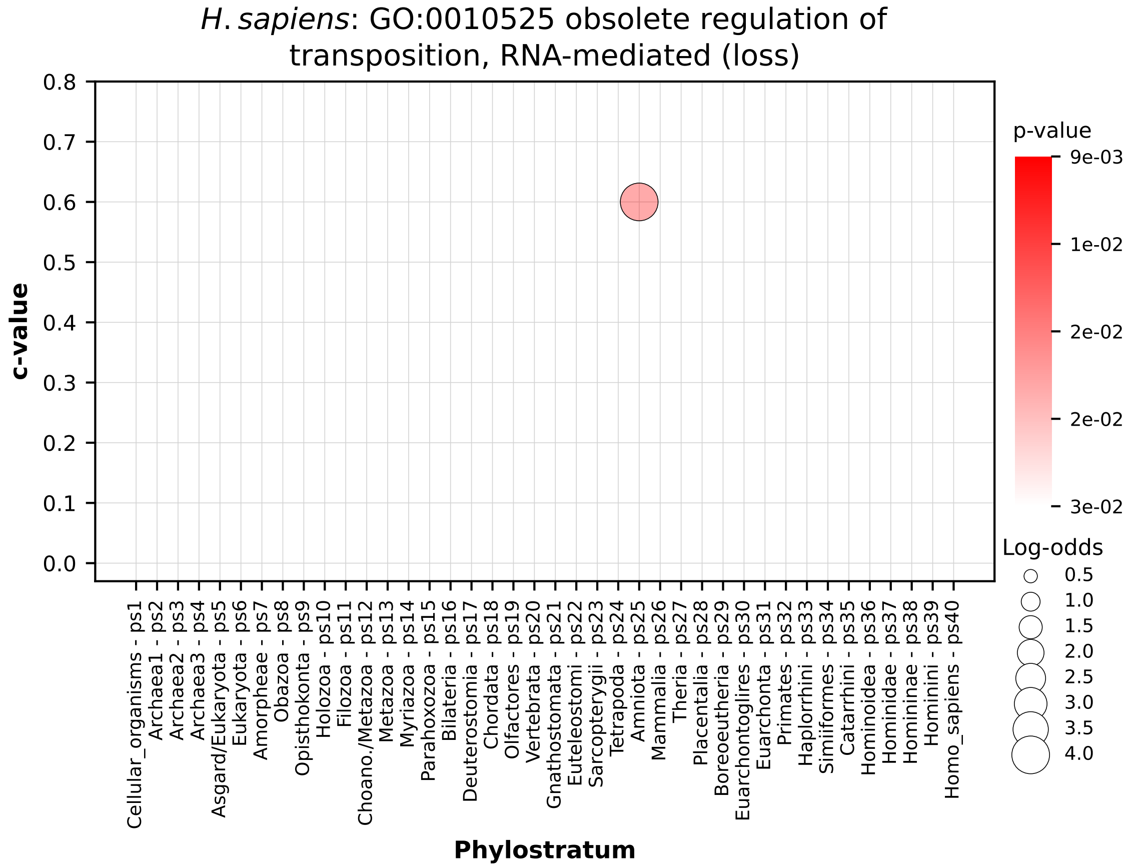
H. sapiens: GO:0010525 obsolete regulation of transposition, RNA-mediated (loss)
c-value
0.8
0.7
0.6
0.5
0.4
0.3
0.2
0.1
0.0
Phylostratum
Cellular_organisms - ps1
Archaea1 - ps2
Archaea2 - ps3
Archaea3 - ps4
Asgard/Eukaryota - ps5
Eukaryota - ps6
Amorpheae - ps7
Obazoa - ps8
Opisthokonta - ps9
Holozoa - ps10
Filozoa - ps11
Choano./Metazoa - ps12
Metazoa - ps13
Myriazoa - ps14
Parahoxozoa - ps15
Bilateria - ps16
Deuterostomia - ps17
Chordata - ps18
Olfactores - ps19
Vertebrata - ps20
Gnathostomata - ps21
Euteleostomi - ps22
Sarcopterygii - ps23
Tetrapoda - ps24
Amniota - ps25
Mammalia - ps26
Theria - ps27
Placentalia - ps28
Boreoeutheria - ps29
Euarchontoglires - ps30
Euarchonta - ps31
Primates - ps32
Haplorrhini - ps33
Simiiformes - ps34
Catarrhini - ps35
Hominoidea - ps36
Hominidae - ps37
Homininae - ps38
Hominini - ps39
Homo_sapiens - ps40
p-value
9e-03
1e-02
2e-02
2e-02
3e-02
Log-odds
0.5
1.0
1.5
2.0
2.5
3.0
3.5
4.0

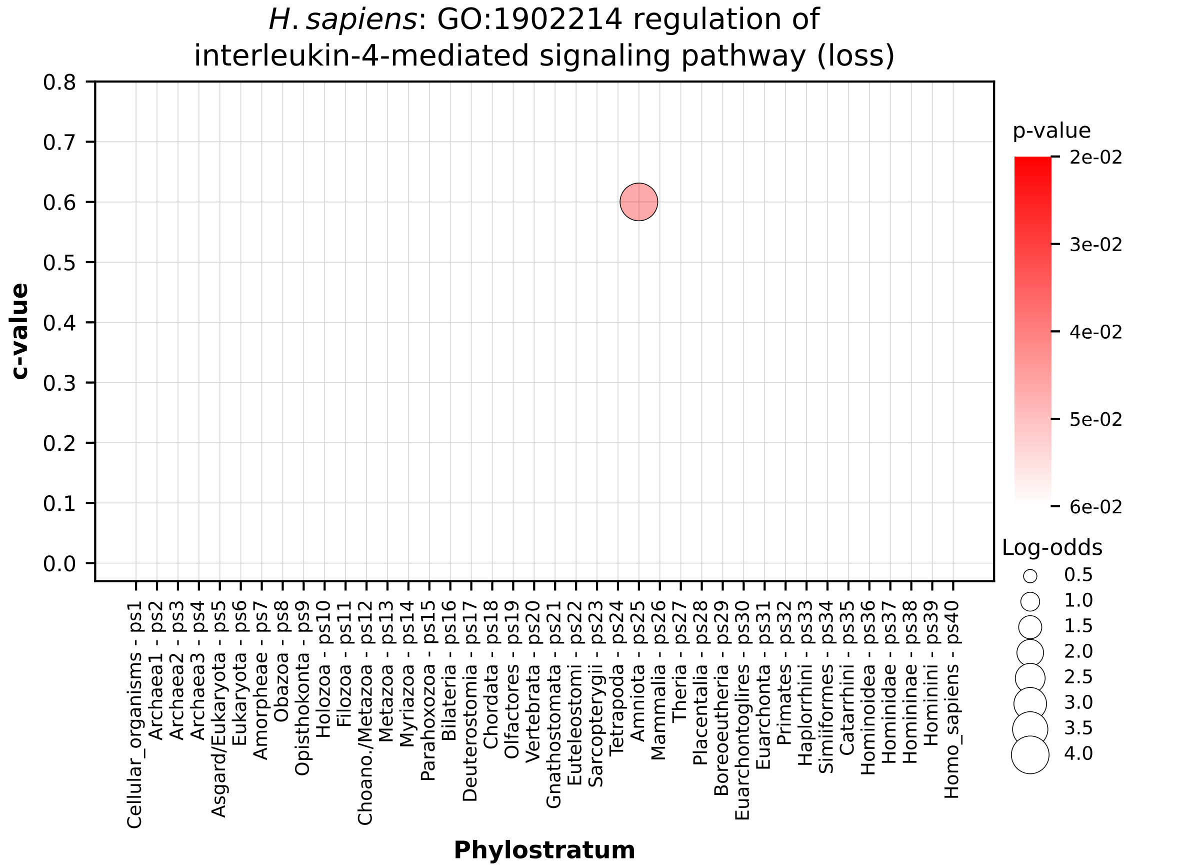
H. sapiens: GO:1902214 regulation of interleukin-4-mediated signaling pathway (loss)
c-value
0.8
0.7
0.6
0.5
0.4
0.3
0.2
0.1
0.0
p-value
2e-02
3e-02
4e-02
5e-02
6e-02
Log-odds
0.5
1.0
1.5
2.0
2.5
3.0
3.5
4.0
Cellular_organisms - ps1
Archaea1 - ps2
Archaea2 - ps3
Archaea3 - ps4
Asgard/Eukaryota - ps5
Eukaryota - ps6
Amorpheae - ps7
Obazoa - ps8
Opisthokonta - ps9
Holozoa - ps10
Filozoa - ps11
Choano./Metazoa - ps12
Metazoa - ps13
Myriazoa - ps14
Parahoxozoa - ps15
Bilateria - ps16
Deuterostomia - ps17
Chordata - ps18
Olfactores - ps19
Vertebrata - ps20
Gnathostomata - ps21
Euteleostomi - ps22
Sarcopterygii - ps23
Tetrapoda - ps24
Amniota - ps25
Mammalia - ps26
Theria - ps27
Placentalia - ps28
Boreoeutheria - ps29
Euarchontoglires - ps30
Euarchonta - ps31
Primates - ps32
Haplorrhini - ps33
Simiiformes - ps34
Catarrhini - ps35
Hominoidea - ps36
Hominidae - ps37
Homininae - ps38
Hominini - ps39
Homo_sapiens - ps40
Phylostratum

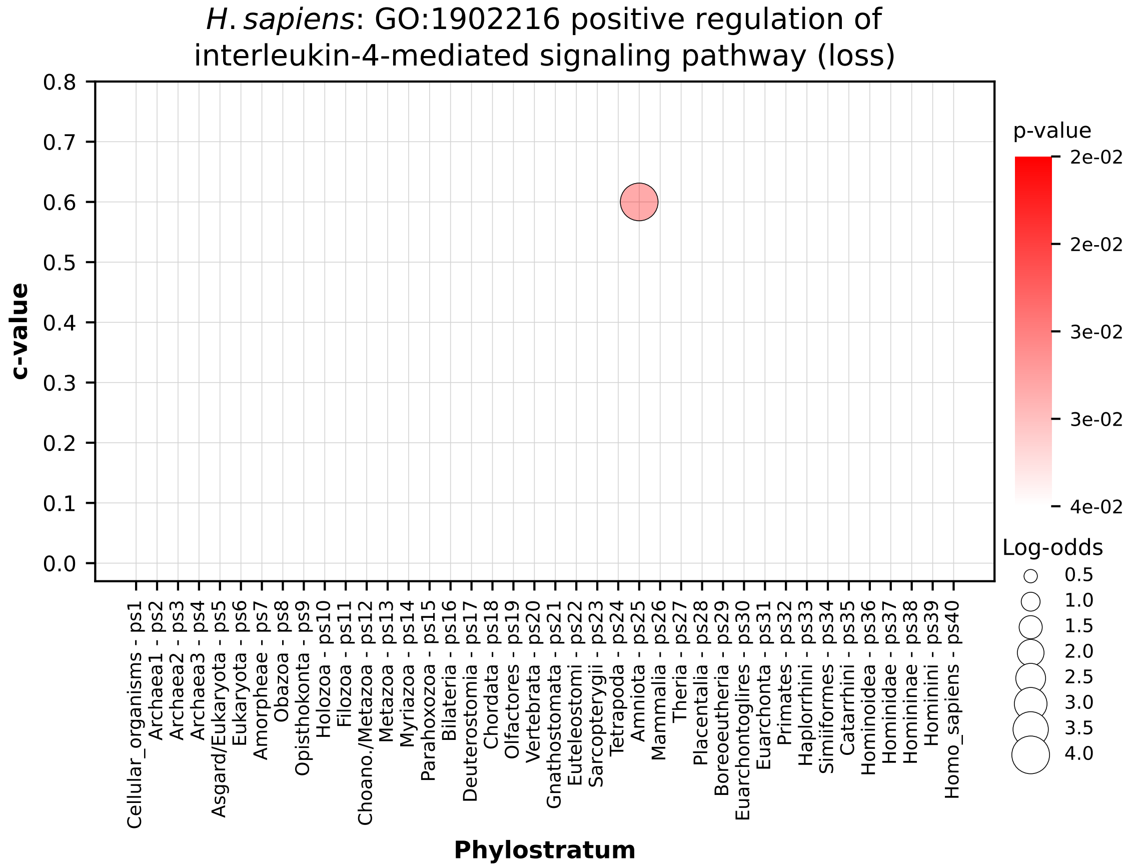
H. sapiens: GO:1902216 positive regulation of interleukin-4-mediated signaling pathway (loss)
c-value
0.8
0.7
0.6
0.5
0.4
0.3
0.2
0.1
0.0
p-value
2e-02
2e-02
3e-02
3e-02
4e-02
Log-odds
0.5
1.0
1.5
2.0
2.5
3.0
3.5
4.0
Cellular_organisms - ps1
Archaea1 - ps2
Archaea2 - ps3
Archaea3 - ps4
Asgard/Eukaryota - ps5
Eukaryota - ps6
Amorpheae - ps7
Obazoa - ps8
Opisthokonta - ps9
Holozoa - ps10
Filozoa - ps11
Choano./Metazoa - ps12
Metazoa - ps13
Myriazoa - ps14
Parahoxozoa - ps15
Bilateria - ps16
Deuterostomia - ps17
Chordata - ps18
Olfactores - ps19
Vertebrata - ps20
Gnathostomata - ps21
Euteleostomi - ps22
Sarcopterygii - ps23
Tetrapoda - ps24
Amniota - ps25
Mammalia - ps26
Theria - ps27
Placentalia - ps28
Boreoeutheria - ps29
Euarchontoglires - ps30
Euarchonta - ps31
Primates - ps32
Haplorrhini - ps33
Simiiformes - ps34
Catarrhini - ps35
Hominoidea - ps36
Hominidae - ps37
Homininae - ps38
Hominini - ps39
Homo_sapiens - ps40
Phylostratum

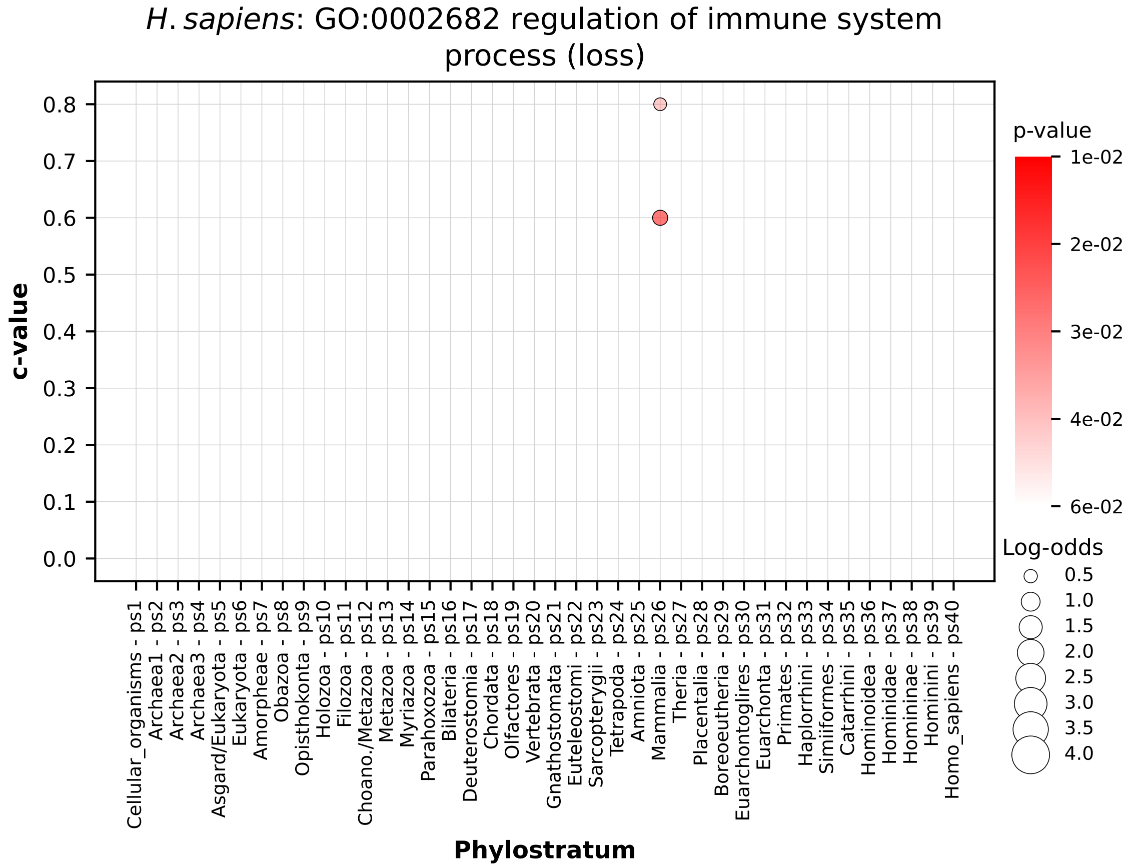

H. sapiens: GO:0002682 regulation of immune system process (loss)
c-value
0.8
0.7
0.6
0.5
0.4
0.3
0.2
0.1
0.0
Phylostratum
Cellular_organisms - ps1
Archaea1 - ps2
Archaea2 - ps3
Archaea3 - ps4
Asgard/Eukaryota - ps5
Eukaryota - ps6
Amorpheae - ps7
Obazoa - ps8
Opisthokonta - ps9
Holozoa - ps10
Filozoa - ps11
Choano./Metazoa - ps12
Metazoa - ps13
Myriazoa - ps14
Parahoxozoa - ps15
Bilateria - ps16
Deuterostomia - ps17
Chordata - ps18
Olfactores - ps19
Vertebrata - ps20
Gnathostomata - ps21
Euteleostomi - ps22
Sarcopterygii - ps23
Tetrapoda - ps24
Amniota - ps25
Mammalia - ps26
Theria - ps27
Placentalia - ps28
Boreoeutheria - ps29
Euarchontoglires - ps30
Euarchonta - ps31
Primates - ps32
Haplorrhini - ps33
Simiiformes - ps34
Catarrhini - ps35
Hominoidea - ps36
Hominidae - ps37
Homininae - ps38
Hominini - ps39
Homo_sapiens - ps40
p-value
1e-02
2e-02
3e-02
4e-02
6e-02
Log-odds
0.5
1.0
1.5
2.0
2.5
3.0
3.5
4.0

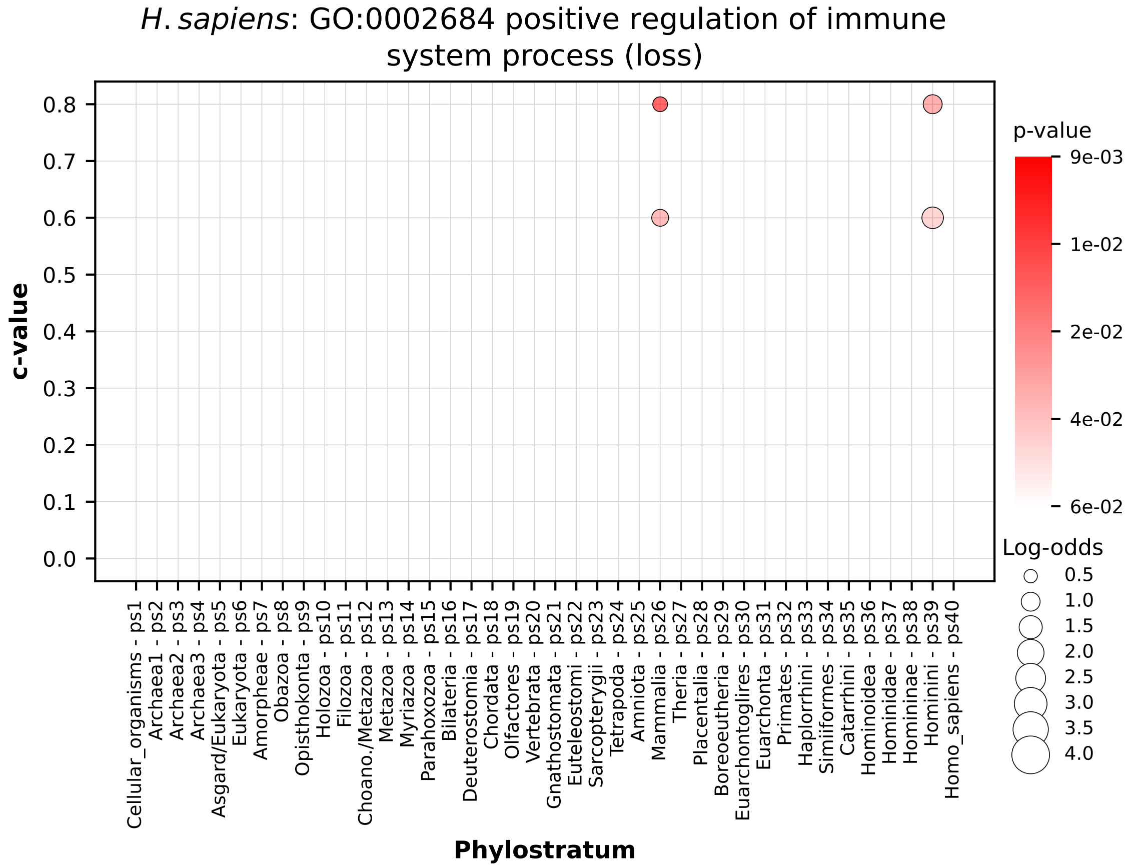
H. sapiens: GO:0002684 positive regulation of immune system process (loss)
c-value
0.8
0.7
0.6
0.5
0.4
0.3
0.2
0.1
0.0
Phylostratum
Cellular_organisms - ps1
Archaea1 - ps2
Archaea2 - ps3
Archaea3 - ps4
Asgard/Eukaryota - ps5
Eukaryota - ps6
Amorpheae - ps7
Obazoa - ps8
Opisthokonta - ps9
Holozoa - ps10
Filozoa - ps11
Choano./Metazoa - ps12
Metazoa - ps13
Myriazoa - ps14
Parahoxozoa - ps15
Bilateria - ps16
Deuterostomia - ps17
Chordata - ps18
Olfactores - ps19
Vertebrata - ps20
Gnathostomata - ps21
Euteleostomi - ps22
Sarcopterygii - ps23
Tetrapoda - ps24
Amniota - ps25
Mammalia - ps26
Theria - ps27
Placentalia - ps28
Boreoeutheria - ps29
Euarchontoglires - ps30
Euarchonta - ps31
Primates - ps32
Haplorrhini - ps33
Simiiformes - ps34
Catarrhini - ps35
Hominoidea - ps36
Hominidae - ps37
Homininae - ps38
Hominini - ps39
Homo_sapiens - ps40
p-value
9e-03
1e-02
2e-02
4e-02
6e-02
Log-odds
0.5
1.0
1.5
2.0
2.5
3.0
3.5
4.0

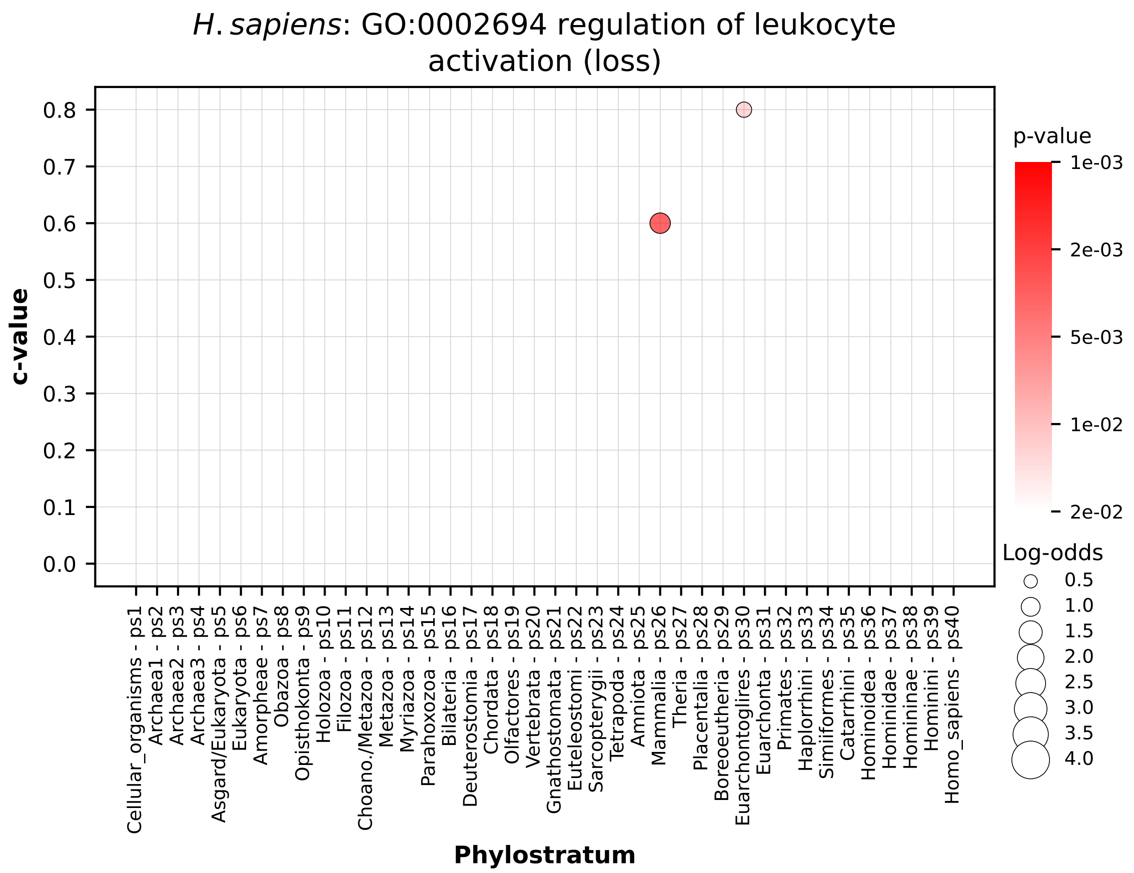

H. sapiens: GO:0002694 regulation of leukocyte activation (loss)
c-value
0.0
0.1
0.2
0.3
0.4
0.5
0.6
0.7
0.8
Phylostratum
Cellular_organisms - ps1
Archaea1 - ps2
Archaea2 - ps3
Archaea3 - ps4
Asgard/Eukaryota - ps5
Eukaryota - ps6
Amorpheae - ps7
Obazoa - ps8
Opisthokonta - ps9
Holozoa - ps10
Filozoa - ps11
Choano./Metazoa - ps12
Metazoa - ps13
Myriazoa - ps14
Parahoxozoa - ps15
Bilateria - ps16
Deuterostomia - ps17
Chordata - ps18
Olfactores - ps19
Vertebrata - ps20
Gnathostomata - ps21
Euteleostomi - ps22
Sarcopterygii - ps23
Tetrapoda - ps24
Amniota - ps25
Mammalia - ps26
Theria - ps27
Placentalia - ps28
Boreoeutheria - ps29
Euarchontoglires - ps30
Euarchonta - ps31
Primates - ps32
Haplorrhini - ps33
Simiiformes - ps34
Catarrhini - ps35
Hominoidea - ps36
Hominidae - ps37
Homininae - ps38
Hominini - ps39
Homo_sapiens - ps40
p-value
1e-03
2e-03
5e-03
1e-02
2e-02
Log-odds
0.5
1.0
1.5
2.0
2.5
3.0
3.5
4.0

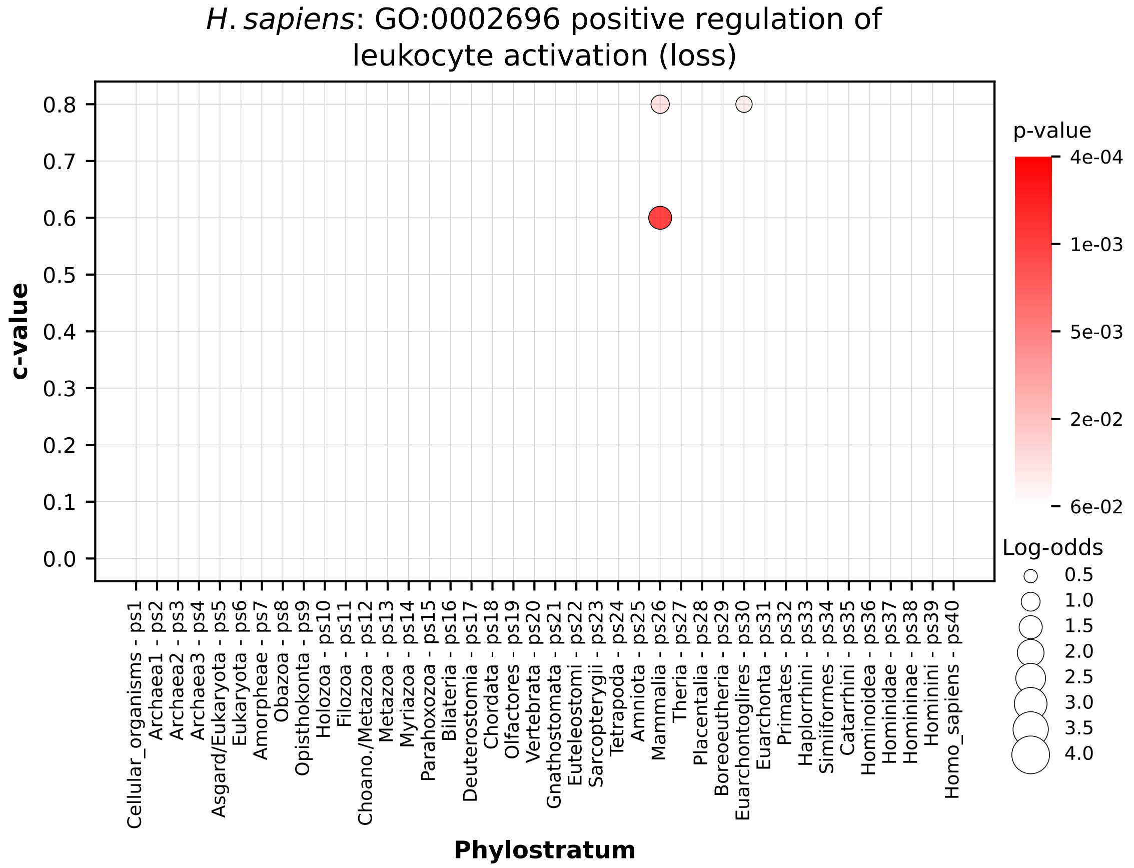
H. sapiens: GO:0002696 positive regulation of leukocyte activation (loss)
c-value
0.0
0.1
0.2
0.3
0.4
0.5
0.6
0.7
0.8
Phylostratum
Cellular_organisms - ps1
Archaea1 - ps2
Archaea2 - ps3
Archaea3 - ps4
Asgard/Eukaryota - ps5
Eukaryota - ps6
Amorpheae - ps7
Obazoa - ps8
Opisthokonta - ps9
Holozoa - ps10
Filozoa - ps11
Choano./Metazoa - ps12
Metazoa - ps13
Myriazoa - ps14
Parahoxozoa - ps15
Bilateria - ps16
Deuterostomia - ps17
Chordata - ps18
Olfactores - ps19
Vertebrata - ps20
Gnathostomata - ps21
Euteleostomi - ps22
Sarcopterygii - ps23
Tetrapoda - ps24
Amniota - ps25
Mammalia - ps26
Theria - ps27
Placentalia - ps28
Boreoeutheria - ps29
Euarchontoglires - ps30
Euarchonta - ps31
Primates - ps32
Haplorrhini - ps33
Simiiformes - ps34
Catarrhini - ps35
Hominoidea - ps36
Hominidae - ps37
Homininae - ps38
Hominini - ps39
Homo_sapiens - ps40
p-value
4e-04
1e-03
5e-03
2e-02
6e-02
Log-odds
0.5
1.0
1.5
2.0
2.5
3.0
3.5
4.0

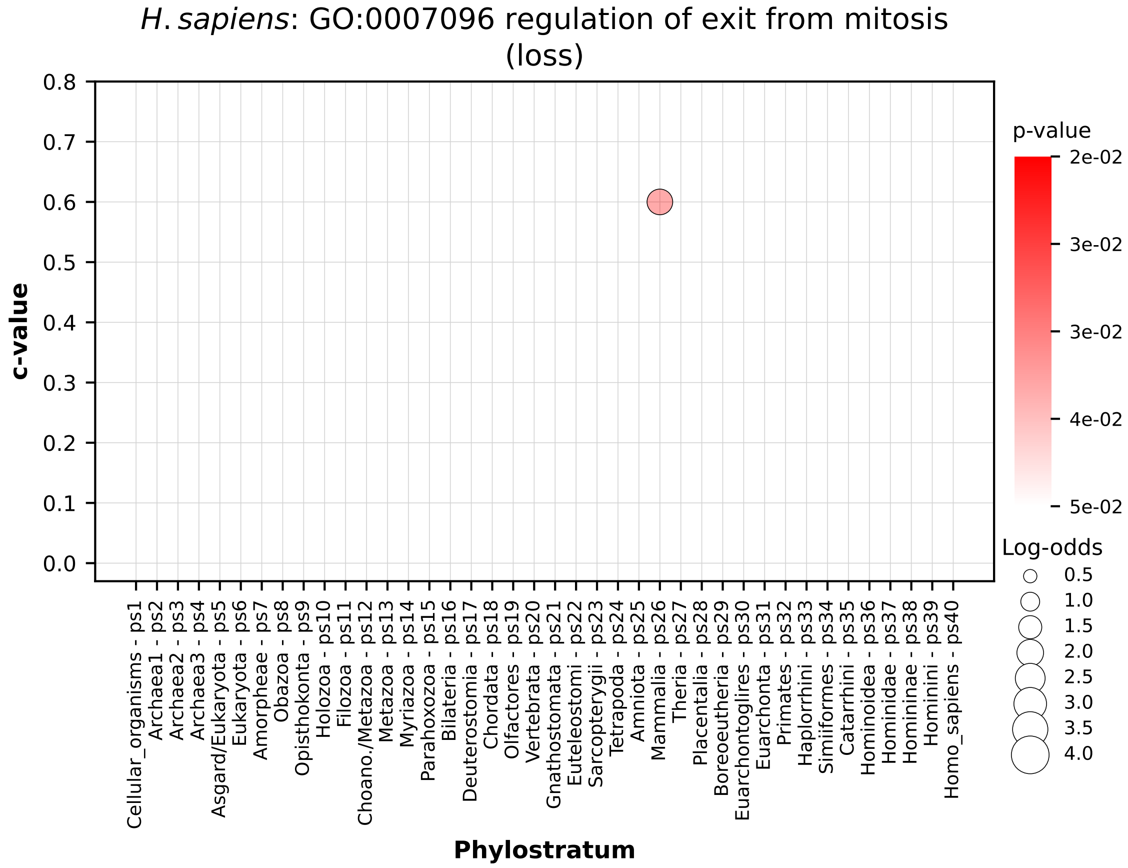

H. sapiens: GO:0007096 regulation of exit from mitosis
(loss)
c-value
0.8
0.7
0.6
0.5
0.4
0.3
0.2
0.1
0.0
p-value
2e-02
3e-02
3e-02
4e-02
5e-02
Log-odds
0.5
1.0
1.5
2.0
2.5
3.0
3.5
4.0
Cellular_organisms - ps1
Archaea1 - ps2
Archaea2 - ps3
Archaea3 - ps4
Asgard/Eukaryota - ps5
Eukaryota - ps6
Amorpheae - ps7
Obazoa - ps8
Opisthokonta - ps9
Holozoa - ps10
Filozoa - ps11
Choano./Metazoa - ps12
Metazoa - ps13
Myriazoa - ps14
Parahoxozoa - ps15
Bilateria - ps16
Deuterostomia - ps17
Chordata - ps18
Olfactores - ps19
Vertebrata - ps20
Gnathostomata - ps21
Euteleostomi - ps22
Sarcopterygii - ps23
Tetrapoda - ps24
Amniota - ps25
Mammalia - ps26
Theria - ps27
Placentalia - ps28
Boreoeutheria - ps29
Euarchontoglires - ps30
Euarchonta - ps31
Primates - ps32
Haplorrhini - ps33
Simiiformes - ps34
Catarrhini - ps35
Hominoidea - ps36
Hominidae - ps37
Homininae - ps38
Hominini - ps39
Homo_sapiens - ps40
Phylostratum

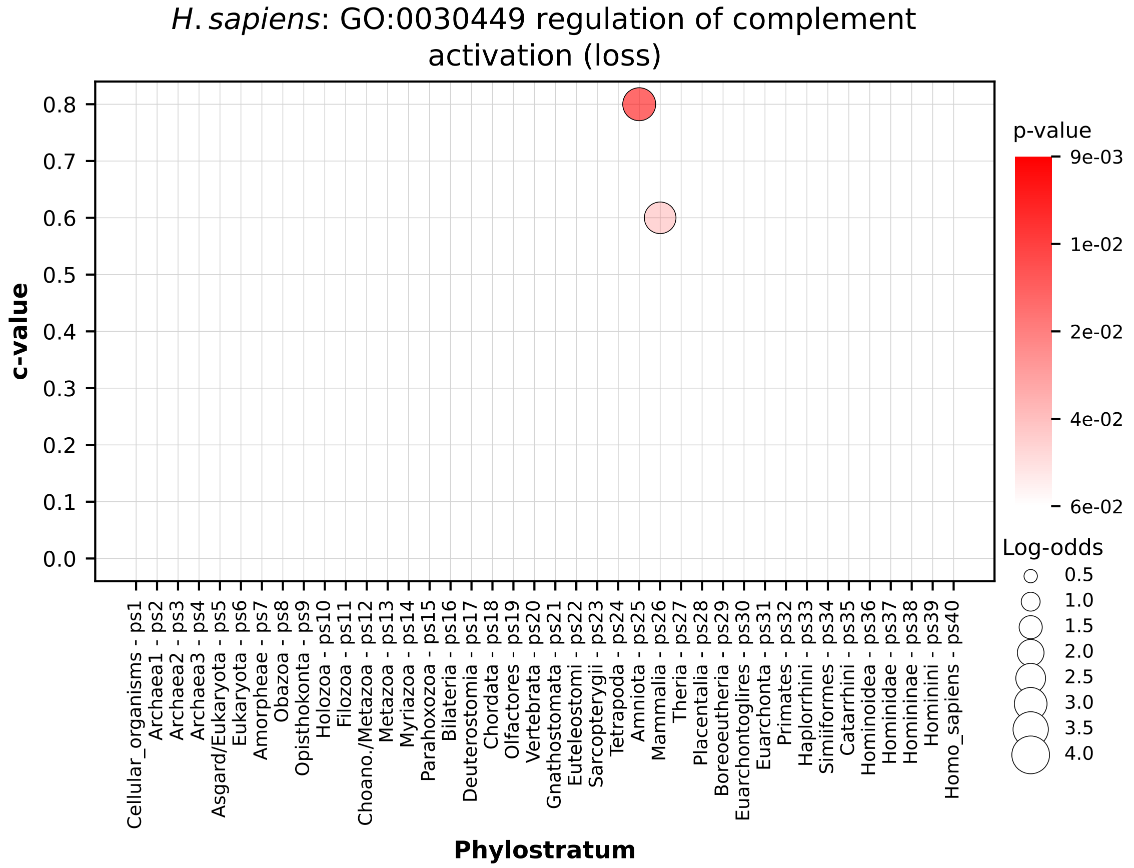

H. sapiens: GO:0030449 regulation of complement activation (loss)
c-value
0.0
0.1
0.2
0.3
0.4
0.5
0.6
0.7
0.8
p-value
9e-03
1e-02
2e-02
4e-02
6e-02
Log-odds
0.5
1.0
1.5
2.0
2.5
3.0
3.5
4.0
Cellular_organisms - ps1
Archaea1 - ps2
Archaea2 - ps3
Archaea3 - ps4
Asgard/Eukaryota - ps5
Eukaryota - ps6
Amorpheae - ps7
Obazoa - ps8
Opisthokonta - ps9
Holozoa - ps10
Filozoa - ps11
Choano./Metazoa - ps12
Metazoa - ps13
Myriazoa - ps14
Parahoxozoa - ps15
Bilateria - ps16
Deuterostomia - ps17
Chordata - ps18
Olfactores - ps19
Vertebrata - ps20
Gnathostomata - ps21
Euteleostomi - ps22
Sarcopterygii - ps23
Tetrapoda - ps24
Amniota - ps25
Mammalia - ps26
Theria - ps27
Placentalia - ps28
Boreoeutheria - ps29
Euarchontoglires - ps30
Euarchonta - ps31
Primates - ps32
Haplorrhini - ps33
Simiiformes - ps34
Catarrhini - ps35
Hominoidea - ps36
Hominidae - ps37
Homininae - ps38
Hominini - ps39
Homo_sapiens - ps40
Phylostratum

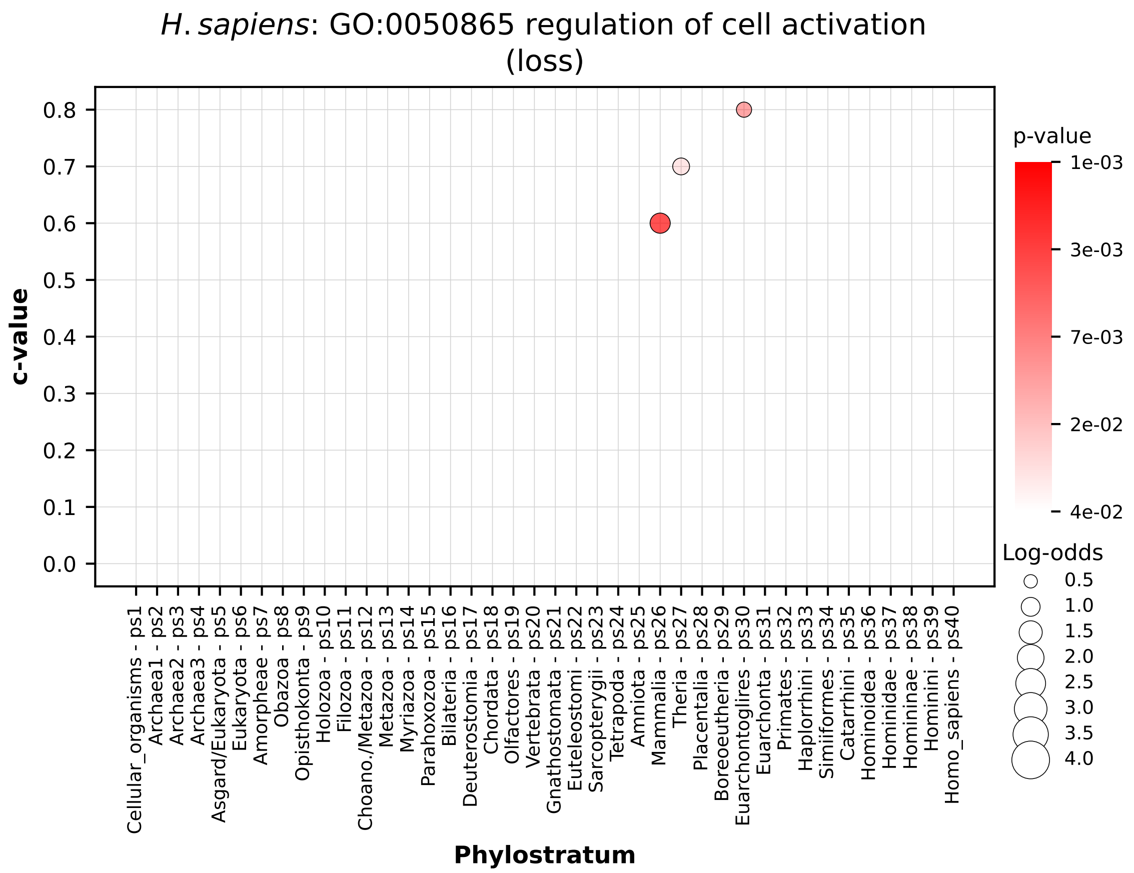
H. sapiens: GO:0050865 regulation of cell activation
(loss)
c-value
0.8
0.7
0.6
0.5
0.4
0.3
0.2
0.1
0.0
Phylostratum
Cellular_organisms - ps1
Archaea1 - ps2
Archaea2 - ps3
Archaea3 - ps4
Asgard/Eukaryota - ps5
Eukaryota - ps6
Amorpheae - ps7
Obazoa - ps8
Opisthokonta - ps9
Holozoa - ps10
Filozoa - ps11
Choano./Metazoa - ps12
Metazoa - ps13
Myriazoa - ps14
Parahoxozoa - ps15
Bilateria - ps16
Deuterostomia - ps17
Chordata - ps18
Olfactores - ps19
Vertebrata - ps20
Gnathostomata - ps21
Euteleostomi - ps22
Sarcopterygii - ps23
Tetrapoda - ps24
Amniota - ps25
Mammalia - ps26
Theria - ps27
Placentalia - ps28
Boreoeutheria - ps29
Euarchontoglires - ps30
Euarchonta - ps31
Primates - ps32
Haplorrhini - ps33
Simiiformes - ps34
Catarrhini - ps35
Hominoidea - ps36
Hominidae - ps37
Homininae - ps38
Hominini - ps39
Homo_sapiens - ps40
p-value
1e-03
3e-03
7e-03
2e-02
4e-02
Log-odds
0.5
1.0
1.5
2.0
2.5
3.0
3.5
4.0

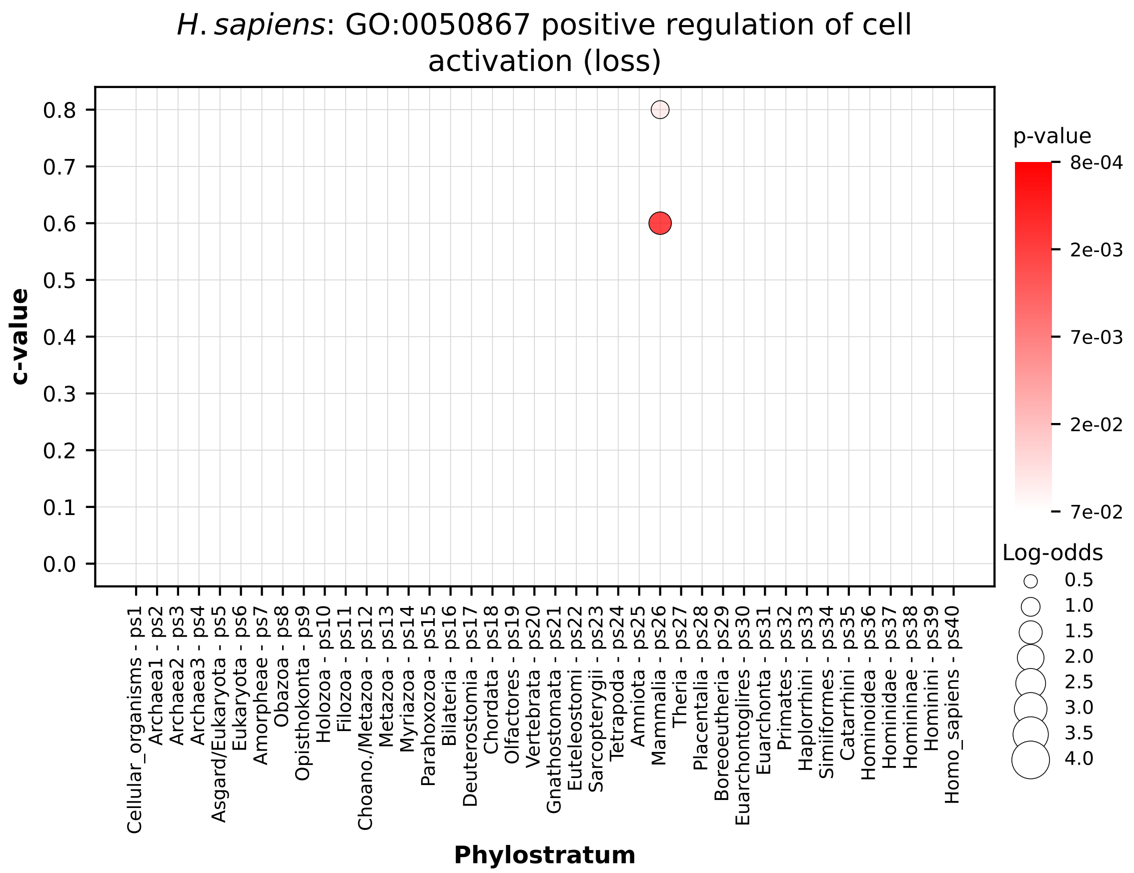

H. sapiens: GO:0050867 positive regulation of cell activation (loss)
c-value
0.8
0.7
0.6
0.5
0.4
0.3
0.2
0.1
0.0
Phylostratum
Cellular_organisms - ps1
Archaea1 - ps2
Archaea2 - ps3
Archaea3 - ps4
Asgard/Eukaryota - ps5
Eukaryota - ps6
Amorpheae - ps7
Obazoa - ps8
Opisthokonta - ps9
Holozoa - ps10
Filozoa - ps11
Choano./Metazoa - ps12
Metazoa - ps13
Myriazoa - ps14
Parahoxozoa - ps15
Bilateria - ps16
Deuterostomia - ps17
Chordata - ps18
Olfactores - ps19
Vertebrata - ps20
Gnathostomata - ps21
Euteleostomi - ps22
Sarcopterygii - ps23
Tetrapoda - ps24
Amniota - ps25
Mammalia - ps26
Theria - ps27
Placentalia - ps28
Boreoeutheria - ps29
Euarchontoglires - ps30
Euarchonta - ps31
Primates - ps32
Haplorrhini - ps33
Simiiformes - ps34
Catarrhini - ps35
Hominoidea - ps36
Hominidae - ps37
Homininae - ps38
Hominini - ps39
Homo_sapiens - ps40
p-value
8e-04
2e-03
7e-03
2e-02
7e-02
Log-odds
0.5
1.0
1.5
2.0
2.5
3.0
3.5
4.0

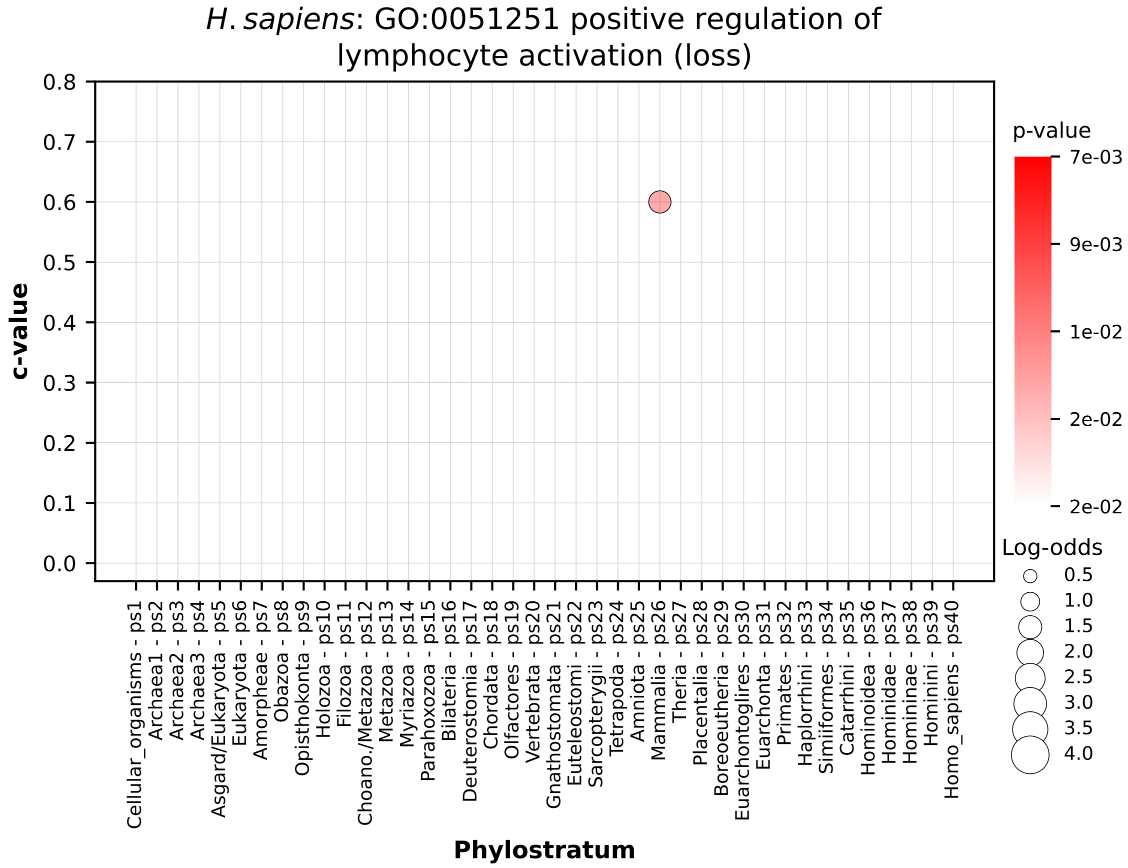

H. sapiens: GO:0051251 positive regulation of lymphocyte activation (loss)
c-value
0.8
0.7
0.6
0.5
0.4
0.3
0.2
0.1
0.0
p-value
7e-03
9e-03
1e-02
2e-02
2e-02
Log-odds
0.5
1.0
1.5
2.0
2.5
3.0
3.5
4.0
Cellular_organisms - ps1
Archaea1 - ps2
Archaea2 - ps3
Archaea3 - ps4
Asgard/Eukaryota - ps5
Eukaryota - ps6
Amorpheae - ps7
Obazoa - ps8
Opisthokonta - ps9
Holozoa - ps10
Filozoa - ps11
Choano./Metazoa - ps12
Metazoa - ps13
Myriazoa - ps14
Parahoxozoa - ps15
Bilateria - ps16
Deuterostomia - ps17
Chordata - ps18
Olfactores - ps19
Vertebrata - ps20
Gnathostomata - ps21
Euteleostomi - ps22
Sarcopterygii - ps23
Tetrapoda - ps24
Amniota - ps25
Mammalia - ps26
Theria - ps27
Placentalia - ps28
Boreoeutheria - ps29
Euarchontoglires - ps30
Euarchonta - ps31
Primates - ps32
Haplorrhini - ps33
Simiiformes - ps34
Catarrhini - ps35
Hominoidea - ps36
Hominidae - ps37
Homininae - ps38
Hominini - ps39
Homo_sapiens - ps40
Phylostratum

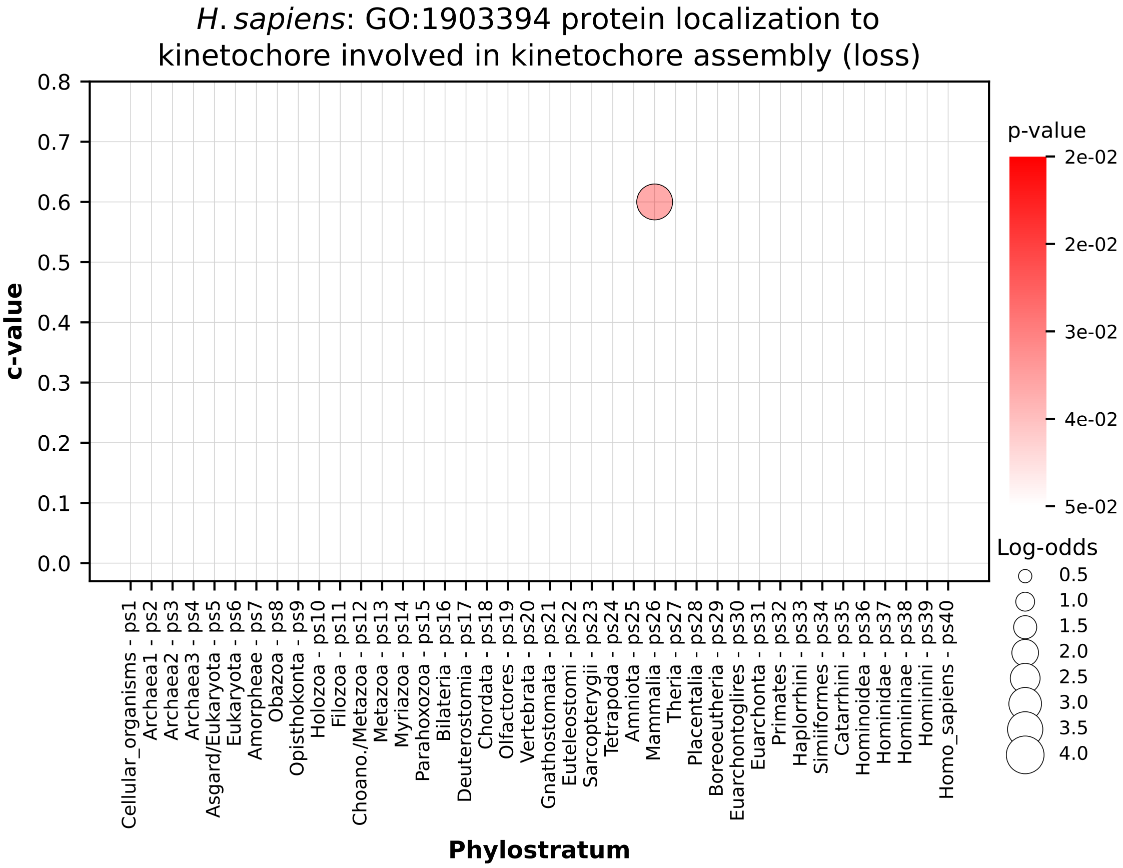
H. sapiens: GO:1903394 protein localization to kinetochore involved in kinetochore assembly (loss)
c-value
0.8
0.7
0.6
0.5
0.4
0.3
0.2
0.1
0.0
Phylostratum
Cellular_organisms - ps1
Archaea1 - ps2
Archaea2 - ps3
Archaea3 - ps4
Asgard/Eukaryota - ps5
Eukaryota - ps6
Amorpheae - ps7
Obazoa - ps8
Opisthokonta - ps9
Holozoa - ps10
Filozoa - ps11
Choano./Metazoa - ps12
Metazoa - ps13
Myriazoa - ps14
Parahoxozoa - ps15
Bilateria - ps16
Deuterostomia - ps17
Chordata - ps18
Olfactores - ps19
Vertebrata - ps20
Gnathostomata - ps21
Euteleostomi - ps22
Sarcopterygii - ps23
Tetrapoda - ps24
Amniota - ps25
Mammalia - ps26
Theria - ps27
Placentalia - ps28
Boreoeutheria - ps29
Euarchontoglires - ps30
Euarchonta - ps31
Primates - ps32
Haplorrhini - ps33
Simiiformes - ps34
Catarrhini - ps35
Hominoidea - ps36
Hominidae - ps37
Homininae - ps38
Hominini - ps39
Homo_sapiens - ps40
p-value
2e-02
2e-02
3e-02
4e-02
5e-02
Log-odds
0.5
1.0
1.5
2.0
2.5
3.0
3.5
4.0

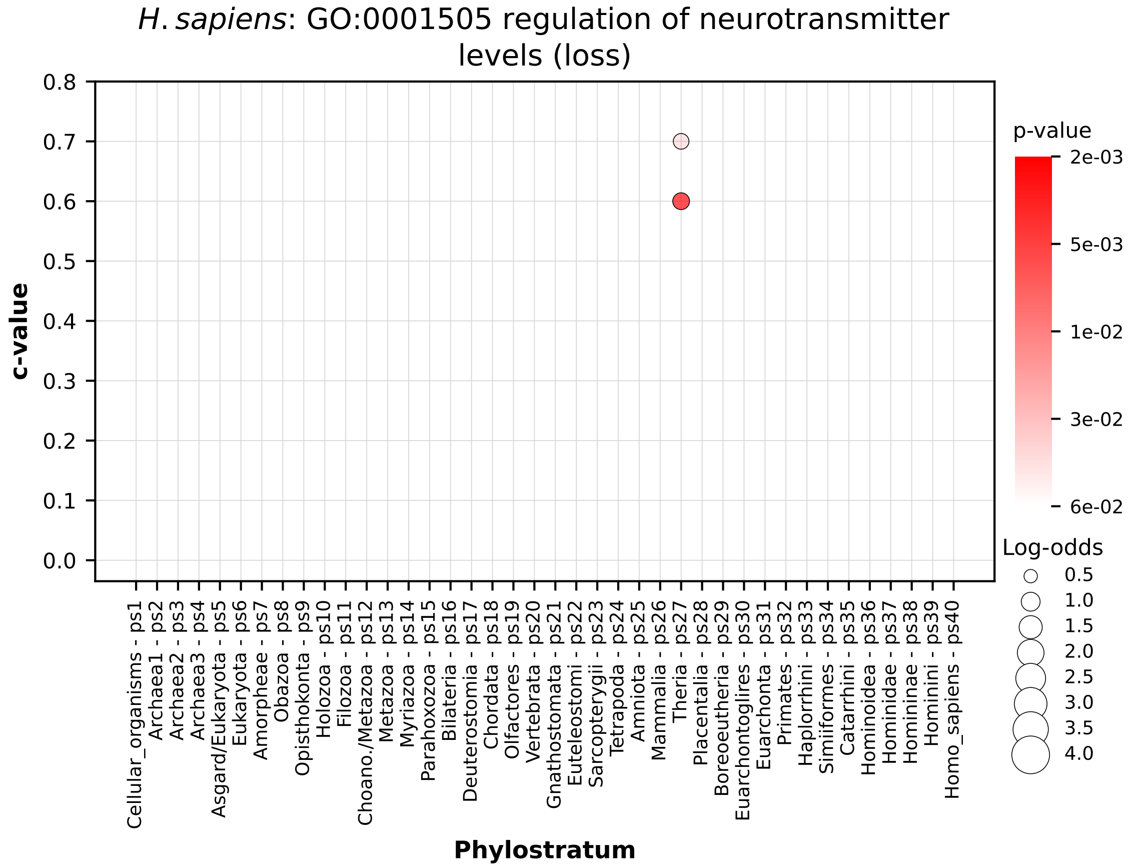

H. sapiens: GO:0001505 regulation of neurotransmitter levels (loss)
c-value
0.8
0.7
0.6
0.5
0.4
0.3
0.2
0.1
0.0
p-value
2e-03
5e-03
1e-02
3e-02
6e-02
Log-odds
0.5
1.0
1.5
2.0
2.5
3.0
3.5
4.0
Cellular_organisms - ps1
Archaea1 - ps2
Archaea2 - ps3
Archaea3 - ps4
Asgard/Eukaryota - ps5
Eukaryota - ps6
Amorpheae - ps7
Obazoa - ps8
Opisthokonta - ps9
Holozoa - ps10
Filozoa - ps11
Choano./Metazoa - ps12
Metazoa - ps13
Myriazoa - ps14
Parahoxozoa - ps15
Bilateria - ps16
Deuterostomia - ps17
Chordata - ps18
Olfactores - ps19
Vertebrata - ps20
Gnathostomata - ps21
Euteleostomi - ps22
Sarcopterygii - ps23
Tetrapoda - ps24
Amniota - ps25
Mammalia - ps26
Theria - ps27
Placentalia - ps28
Boreoeutheria - ps29
Euarchontoglires - ps30
Euarchonta - ps31
Primates - ps32
Haplorrhini - ps33
Simiiformes - ps34
Catarrhini - ps35
Hominoidea - ps36
Hominidae - ps37
Homininae - ps38
Hominini - ps39
Homo_sapiens - ps40
Phylostratum

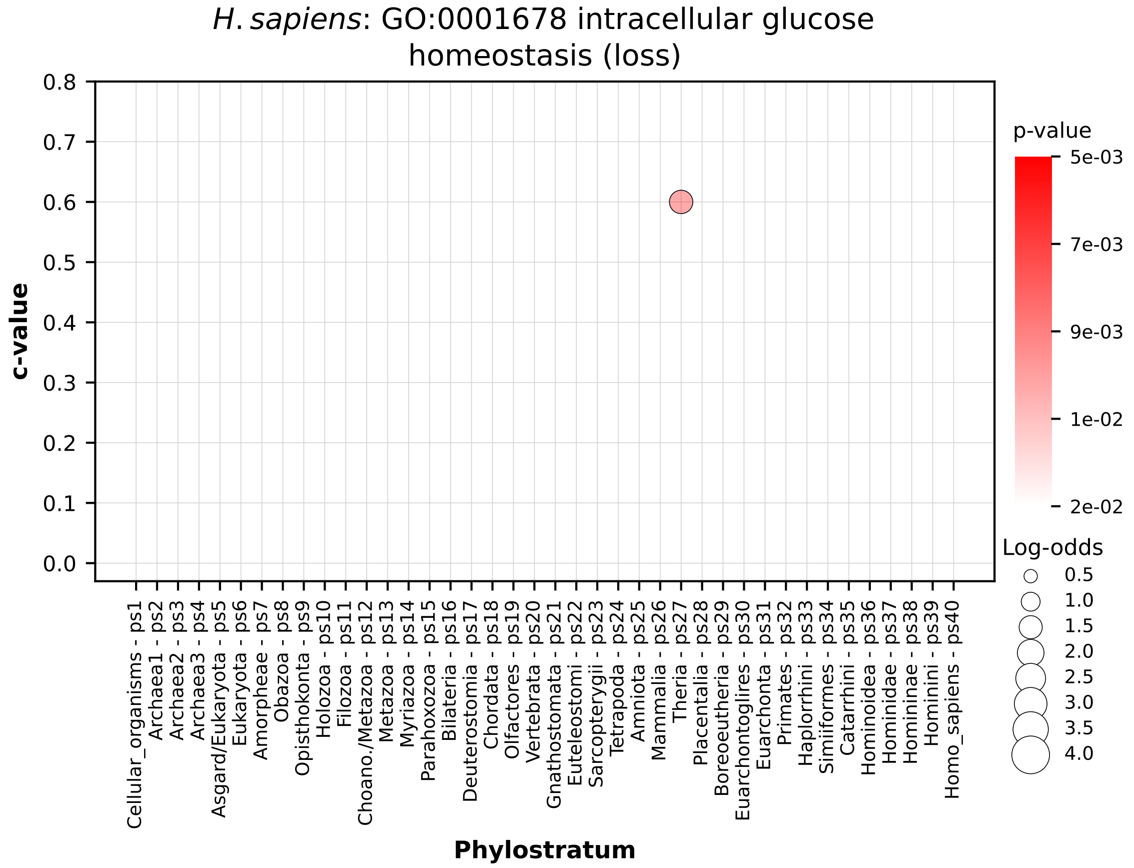

H. sapiens: GO:0001678 intracellular glucose homeostasis (loss)
c-value
0.0
0.1
0.2
0.3
0.4
0.5
0.6
0.7
0.8
Phylostratum
Cellular_organisms - ps1
Archaea1 - ps2
Archaea2 - ps3
Archaea3 - ps4
Asgard/Eukaryota - ps5
Eukaryota - ps6
Amorpheae - ps7
Obazoa - ps8
Opisthokonta - ps9
Holozoa - ps10
Filozoa - ps11
Choano./Metazoa - ps12
Metazoa - ps13
Myriazoa - ps14
Parahoxozoa - ps15
Bilateria - ps16
Deuterostomia - ps17
Chordata - ps18
Olfactores - ps19
Vertebrata - ps20
Gnathostomata - ps21
Euteleostomi - ps22
Sarcopterygii - ps23
Tetrapoda - ps24
Amniota - ps25
Mammalia - ps26
Theria - ps27
Placentalia - ps28
Boreoeutheria - ps29
Euarchontoglires - ps30
Euarchonta - ps31
Primates - ps32
Haplorrhini - ps33
Simiiformes - ps34
Catarrhini - ps35
Hominoidea - ps36
Hominidae - ps37
Homininae - ps38
Hominini - ps39
Homo_sapiens - ps40
p-value
5e-03
7e-03
9e-03
1e-02
2e-02
Log-odds
0.5
1.0
1.5
2.0
2.5
3.0
3.5
4.0

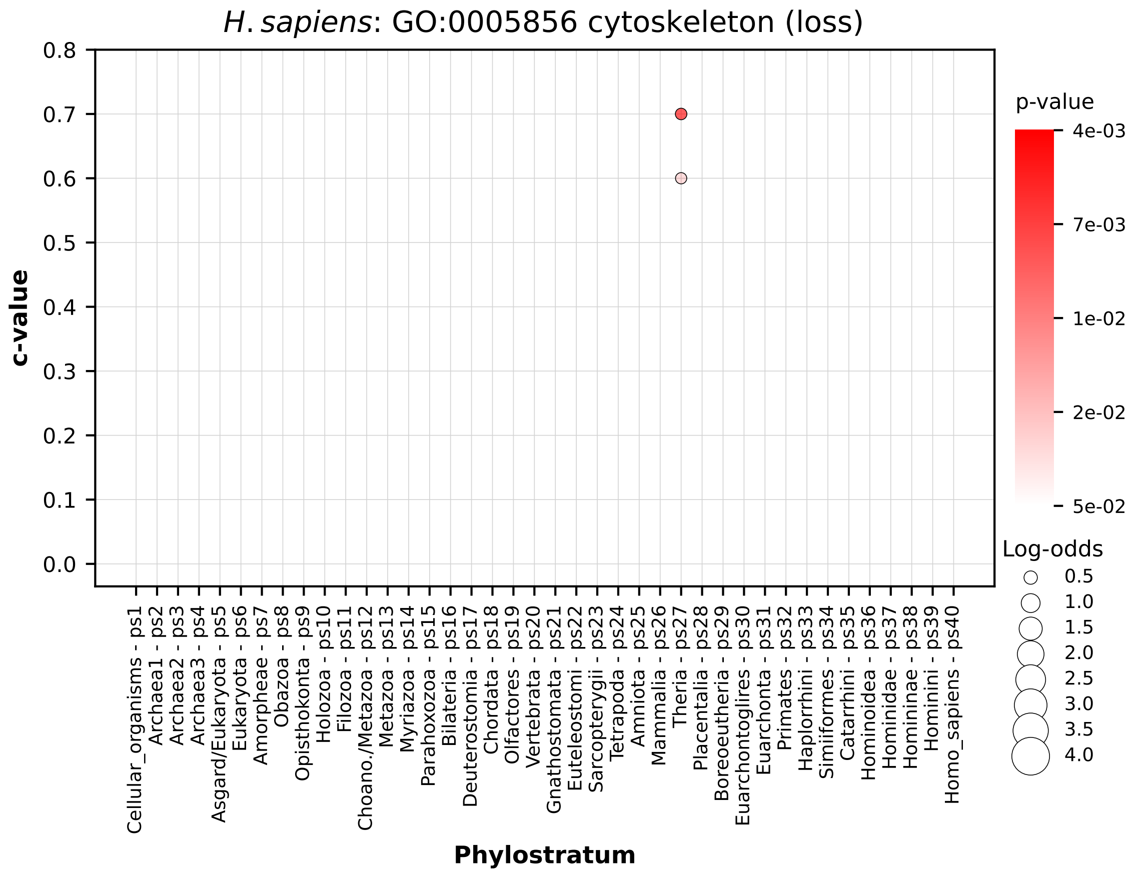

H. sapiens: GO:0005856 cytoskeleton (loss)
c-value
0.8
0.7
0.6
0.5
0.4
0.3
0.2
0.1
0.0
Phylostratum
Cellular_organisms - ps1
Archaea1 - ps2
Archaea2 - ps3
Archaea3 - ps4
Asgard/Eukaryota - ps5
Eukaryota - ps6
Amorpheae - ps7
Obazoa - ps8
Opisthokonta - ps9
Holozoa - ps10
Filozoa - ps11
Choano./Metazoa - ps12
Metazoa - ps13
Myriazoa - ps14
Parahoxozoa - ps15
Bilateria - ps16
Deuterostomia - ps17
Chordata - ps18
Olfactores - ps19
Vertebrata - ps20
Gnathostomata - ps21
Euteleostomi - ps22
Sarcopterygii - ps23
Tetrapoda - ps24
Amniota - ps25
Mammalia - ps26
Theria - ps27
Placentalia - ps28
Boreoeutheria - ps29
Euarchontoglires - ps30
Euarchonta - ps31
Primates - ps32
Haplorrhini - ps33
Simiiformes - ps34
Catarrhini - ps35
Hominoidea - ps36
Hominidae - ps37
Homininae - ps38
Hominini - ps39
Homo_sapiens - ps40
p-value
4e-03
7e-03
1e-02
2e-02
5e-02
Log-odds
0.5
1.0
1.5
2.0
2.5
3.0
3.5
4.0

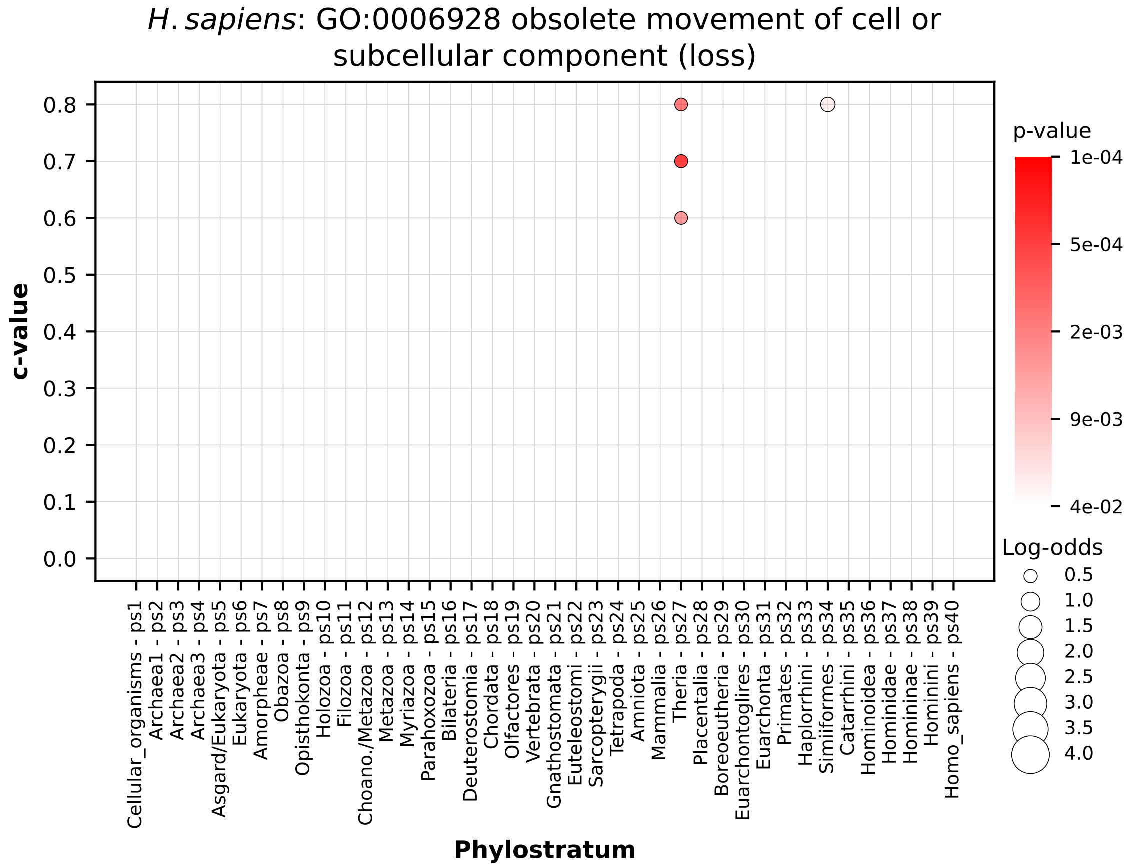

H. sapiens: GO:0006928 obsolete movement of cell or subcellular component (loss)
c-value
0.0
0.1
0.2
0.3
0.4
0.5
0.6
0.7
0.8
p-value
1e-04
5e-04
2e-03
9e-03
4e-02
Log-odds
0.5
1.0
1.5
2.0
2.5
3.0
3.5
4.0
Cellular_organisms - ps1
Archaea1 - ps2
Archaea2 - ps3
Archaea3 - ps4
Asgard/Eukaryota - ps5
Eukaryota - ps6
Amorpheae - ps7
Obazoa - ps8
Opisthokonta - ps9
Holozoa - ps10
Filozoa - ps11
Choano./Metazoa - ps12
Metazoa - ps13
Myriazoa - ps14
Parahoxozoa - ps15
Bilateria - ps16
Deuterostomia - ps17
Chordata - ps18
Olfactores - ps19
Vertebrata - ps20
Gnathostomata - ps21
Euteleostomi - ps22
Sarcopterygii - ps23
Tetrapoda - ps24
Amniota - ps25
Mammalia - ps26
Theria - ps27
Placentalia - ps28
Boreoeutheria - ps29
Euarchontoglires - ps30
Euarchonta - ps31
Primates - ps32
Haplorrhini - ps33
Simiiformes - ps34
Catarrhini - ps35
Hominoidea - ps36
Hominidae - ps37
Homininae - ps38
Hominini - ps39
Homo_sapiens - ps40
Phylostratum

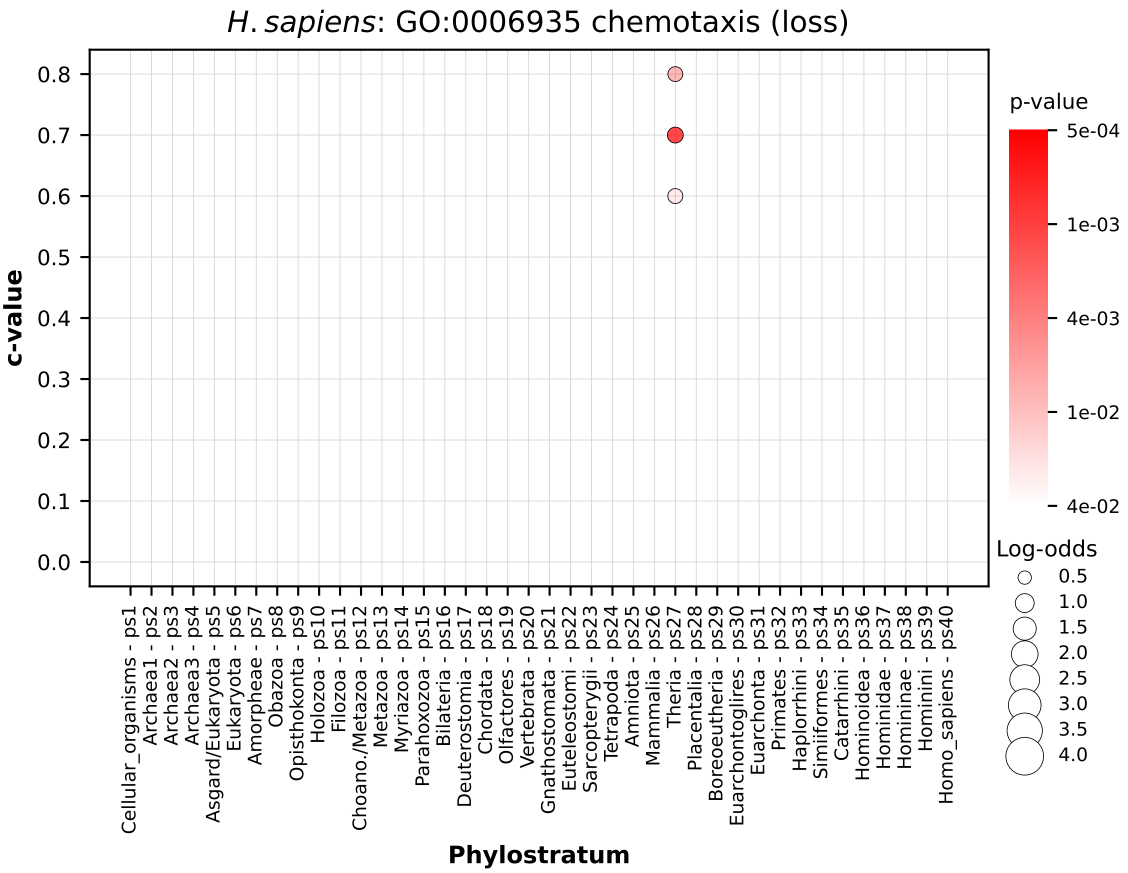

H. sapiens: GO:0006935 chemotaxis (loss)
c-value
0.8
0.7
0.6
0.5
0.4
0.3
0.2
0.1
0.0
p-value
5e-04
1e-03
4e-03
1e-02
4e-02
Log-odds
0.5
1.0
1.5
2.0
2.5
3.0
3.5
4.0
Cellular_organisms - ps1
Archaea1 - ps2
Archaea2 - ps3
Archaea3 - ps4
Asgard/Eukaryota - ps5
Eukaryota - ps6
Amorpheae - ps7
Obazoa - ps8
Opisthokonta - ps9
Holozoa - ps10
Filozoa - ps11
Choano./Metazoa - ps12
Metazoa - ps13
Myriazoa - ps14
Parahoxozoa - ps15
Bilateria - ps16
Deuterostomia - ps17
Chordata - ps18
Olfactores - ps19
Vertebrata - ps20
Gnathostomata - ps21
Euteleostomi - ps22
Sarcopterygii - ps23
Tetrapoda - ps24
Amniota - ps25
Mammalia - ps26
Theria - ps27
Placentalia - ps28
Boreoeutheria - ps29
Euarchontoglires - ps30
Euarchonta - ps31
Primates - ps32
Haplorrhini - ps33
Simiiformes - ps34
Catarrhini - ps35
Hominoidea - ps36
Hominidae - ps37
Homininae - ps38
Hominini - ps39
Homo_sapiens - ps40
Phylostratum

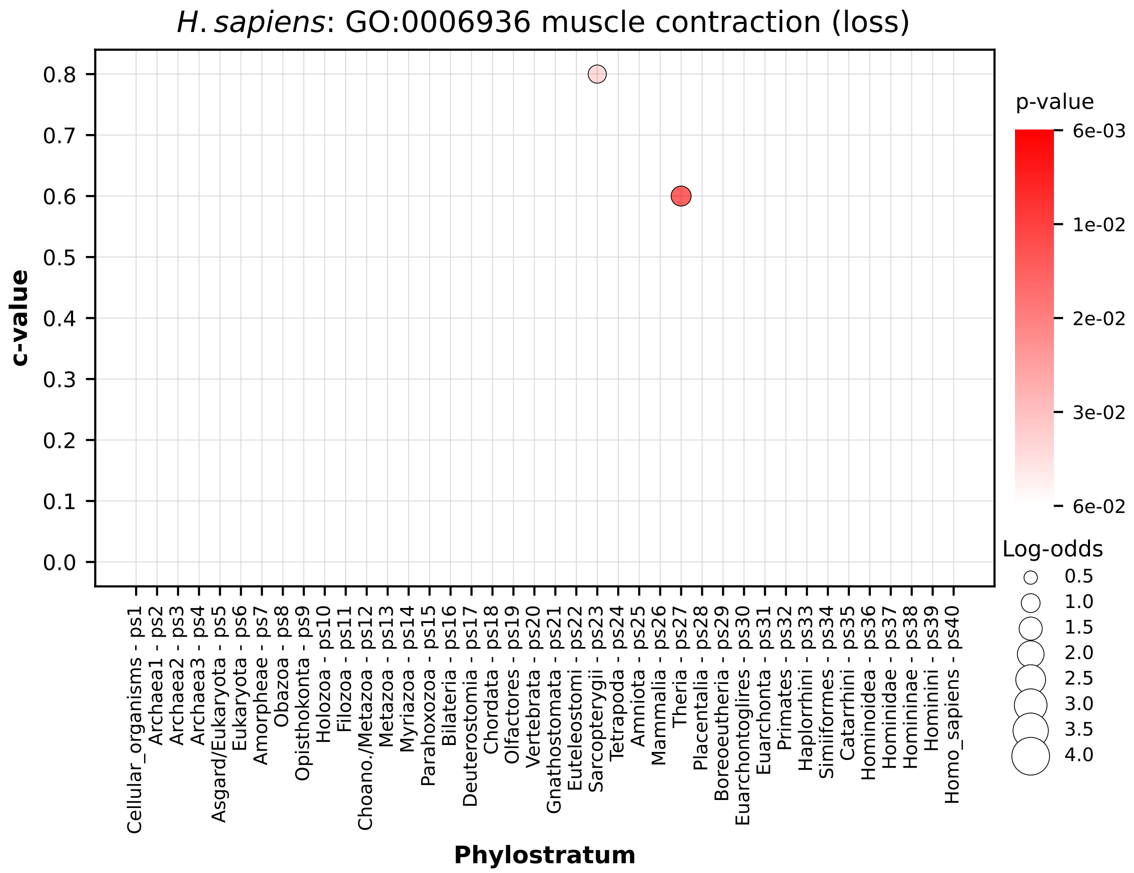

H. sapiens: GO:0006936 muscle contraction (loss)
c-value
0.8
0.7
0.6
0.5
0.4
0.3
0.2
0.1
0.0
p-value
6e-03
1e-02
2e-02
3e-02
6e-02
Log-odds
0.5
1.0
1.5
2.0
2.5
3.0
3.5
4.0
Cellular_organisms - ps1
Archaea1 - ps2
Archaea2 - ps3
Archaea3 - ps4
Asgard/Eukaryota - ps5
Eukaryota - ps6
Amorpheae - ps7
Obazoa - ps8
Opisthokonta - ps9
Holozoa - ps10
Filozoa - ps11
Choano./Metazoa - ps12
Metazoa - ps13
Myriazoa - ps14
Parahoxozoa - ps15
Bilateria - ps16
Deuterostomia - ps17
Chordata - ps18
Olfactores - ps19
Vertebrata - ps20
Gnathostomata - ps21
Euteleostomi - ps22
Sarcopterygii - ps23
Tetrapoda - ps24
Amniota - ps25
Mammalia - ps26
Theria - ps27
Placentalia - ps28
Boreoeutheria - ps29
Euarchontoglires - ps30
Euarchonta - ps31
Primates - ps32
Haplorrhini - ps33
Simiiformes - ps34
Catarrhini - ps35
Hominoidea - ps36
Hominidae - ps37
Homininae - ps38
Hominini - ps39
Homo_sapiens - ps40
Phylostratum

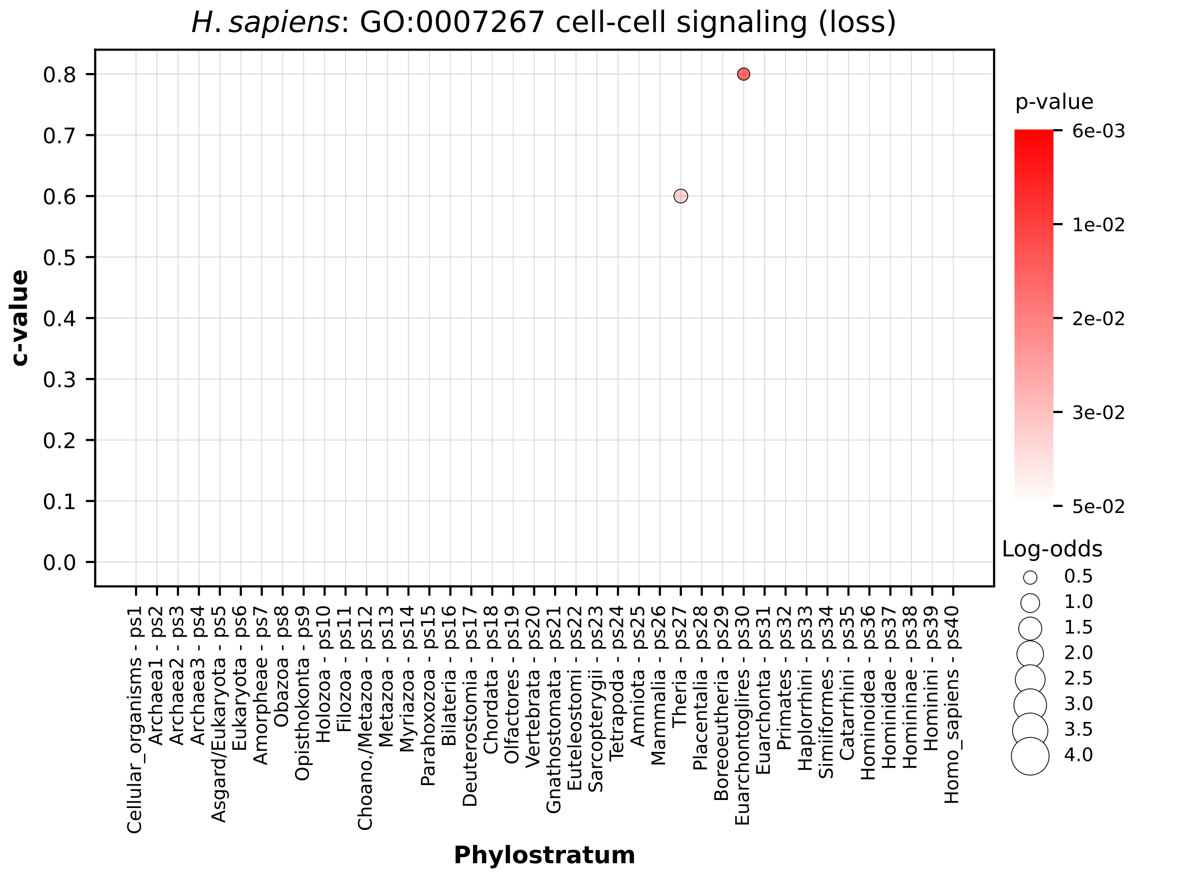

H. sapiens: GO:0007267 cell-cell signaling (loss)
c-value
0.0
0.1
0.2
0.3
0.4
0.5
0.6
0.7
0.8
Phylostratum
Cellular_organisms - ps1
Archaea1 - ps2
Archaea2 - ps3
Archaea3 - ps4
Asgard/Eukaryota - ps5
Eukaryota - ps6
Amorpheae - ps7
Obazoa - ps8
Opisthokonta - ps9
Holozoa - ps10
Filozoa - ps11
Choano./Metazoa - ps12
Metazoa - ps13
Myriazoa - ps14
Parahoxozoa - ps15
Bilateria - ps16
Deuterostomia - ps17
Chordata - ps18
Olfactores - ps19
Vertebrata - ps20
Gnathostomata - ps21
Euteleostomi - ps22
Sarcopterygii - ps23
Tetrapoda - ps24
Amniota - ps25
Mammalia - ps26
Theria - ps27
Placentalia - ps28
Boreoeutheria - ps29
Euarchontoglires - ps30
Euarchonta - ps31
Primates - ps32
Haplorrhini - ps33
Simiiformes - ps34
Catarrhini - ps35
Hominoidea - ps36
Hominidae - ps37
Homininae - ps38
Hominini - ps39
Homo_sapiens - ps40
p-value
6e-03
1e-02
2e-02
3e-02
5e-02
Log-odds
0.5
1.0
1.5
2.0
2.5
3.0
3.5
4.0

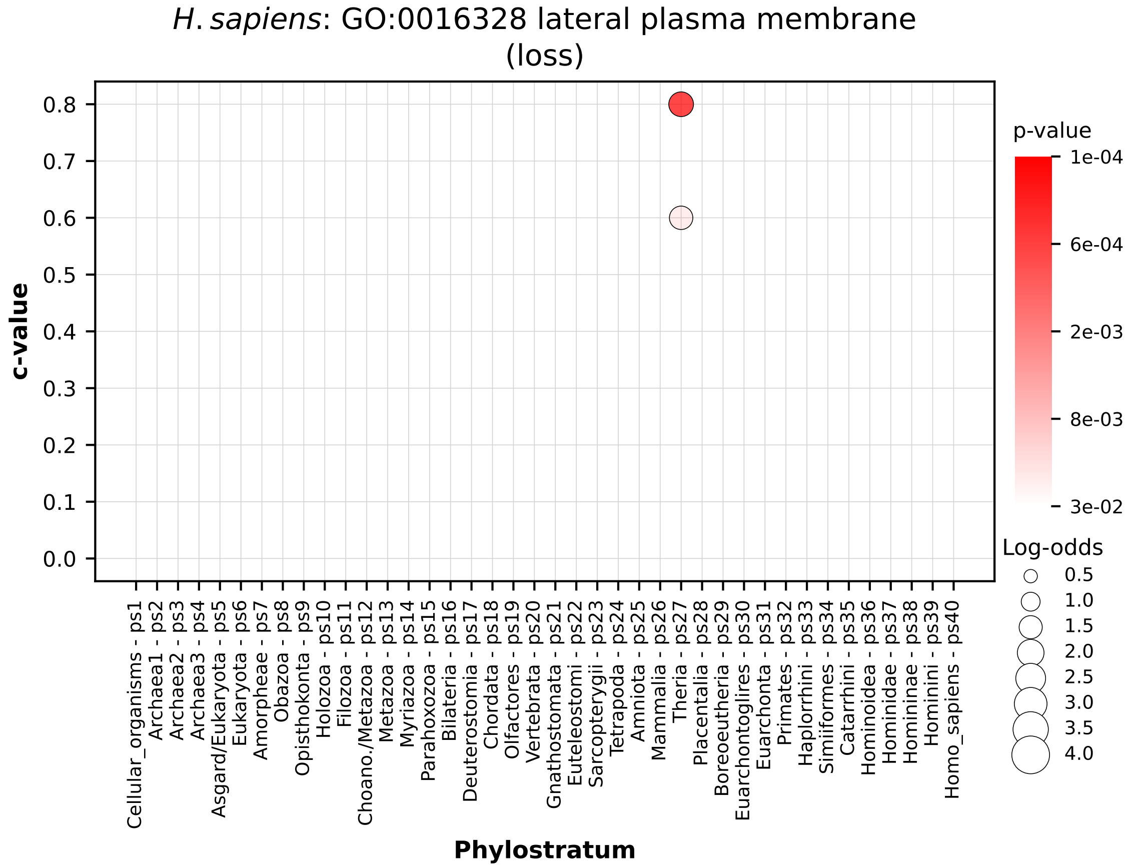
H. sapiens: GO:0016328 lateral plasma membrane
(loss)
c-value
0.8
0.7
0.6
0.5
0.4
0.3
0.2
0.1
0.0
p-value
1e-04
6e-04
2e-03
8e-03
3e-02
Log-odds
0.5
1.0
1.5
2.0
2.5
3.0
3.5
4.0
Cellular_organisms - ps1
Archaea1 - ps2
Archaea2 - ps3
Archaea3 - ps4
Asgard/Eukaryota - ps5
Eukaryota - ps6
Amorpheae - ps7
Obazoa - ps8
Opisthokonta - ps9
Holozoa - ps10
Filozoa - ps11
Choano./Metazoa - ps12
Metazoa - ps13
Myriazoa - ps14
Parahoxozoa - ps15
Bilateria - ps16
Deuterostomia - ps17
Chordata - ps18
Olfactores - ps19
Vertebrata - ps20
Gnathostomata - ps21
Euteleostomi - ps22
Sarcopterygii - ps23
Tetrapoda - ps24
Amniota - ps25
Mammalia - ps26
Theria - ps27
Placentalia - ps28
Boreoeutheria - ps29
Euarchontoglires - ps30
Euarchonta - ps31
Primates - ps32
Haplorrhini - ps33
Simiiformes - ps34
Catarrhini - ps35
Hominoidea - ps36
Hominidae - ps37
Homininae - ps38
Hominini - ps39
Homo_sapiens - ps40
Phylostratum

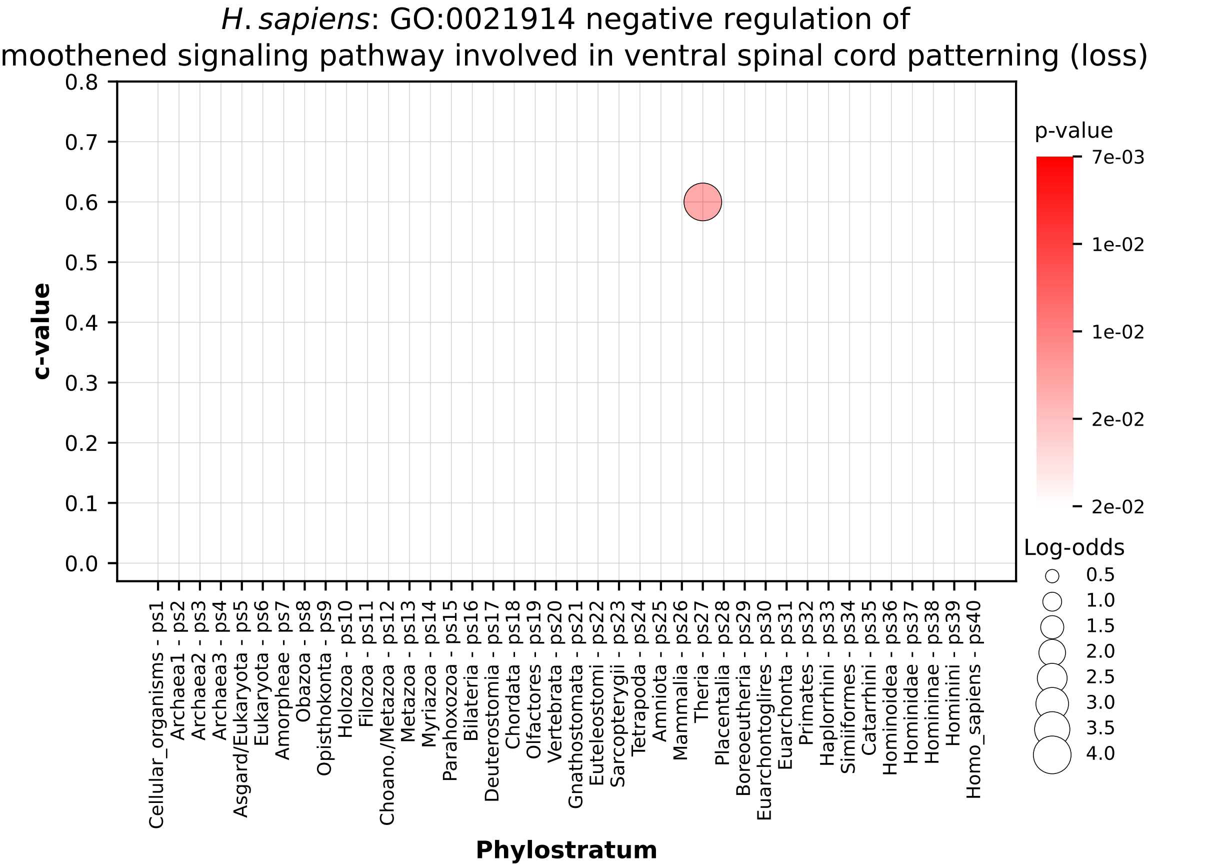
H. sapiens: GO:0021914 negative regulation of
smoothened signaling pathway involved in ventral spinal cord patterning (loss)
c-value
0.8
0.7
0.6
0.5
0.4
0.3
0.2
0.1
0.0
Phylostratum
Cellular_organisms - ps1
Archaea1 - ps2
Archaea2 - ps3
Archaea3 - ps4
Asgard/Eukaryota - ps5
Eukaryota - ps6
Amorpheae - ps7
Obazoa - ps8
Opisthokonta - ps9
Holozoa - ps10
Filozoa - ps11
Choano./Metazoa - ps12
Metazoa - ps13
Myriazoa - ps14
Parahoxozoa - ps15
Bilateria - ps16
Deuterostomia - ps17
Chordata - ps18
Olfactores - ps19
Vertebrata - ps20
Gnathostomata - ps21
Euteleostomi - ps22
Sarcopterygii - ps23
Tetrapoda - ps24
Amniota - ps25
Mammalia - ps26
Theria - ps27
Placentalia - ps28
Boreoeutheria - ps29
Euarchontoglires - ps30
Euarchonta - ps31
Primates - ps32
Haplorrhini - ps33
Simiiformes - ps34
Catarrhini - ps35
Hominoidea - ps36
Hominidae - ps37
Homininae - ps38
Hominini - ps39
Homo_sapiens - ps40
p-value
7e-03
1e-02
1e-02
2e-02
2e-02
Log-odds
0.5
1.0
1.5
2.0
2.5
3.0
3.5
4.0

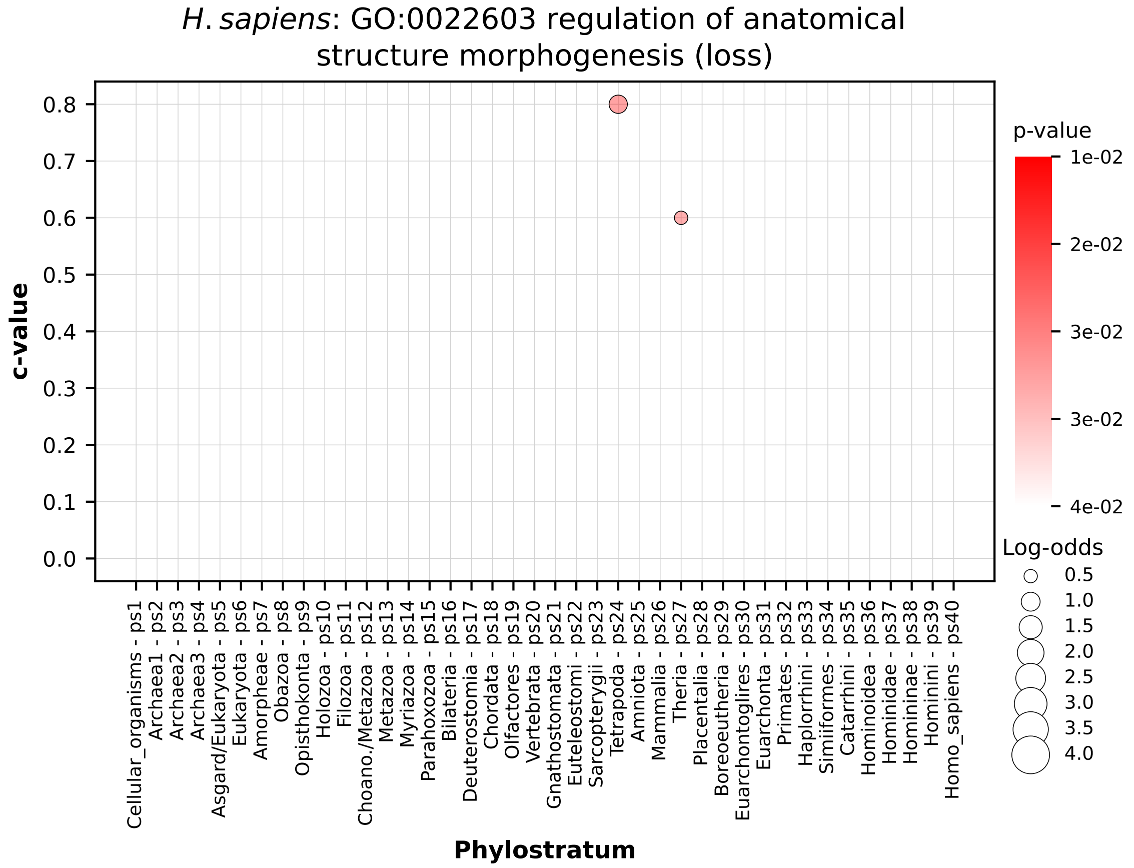

H. sapiens: GO:0022603 regulation of anatomical structure morphogenesis (loss)
c-value
0.0
0.1
0.2
0.3
0.4
0.5
0.6
0.7
0.8
Phylostratum
Cellular_organisms - ps1
Archaea1 - ps2
Archaea2 - ps3
Archaea3 - ps4
Asgard/Eukaryota - ps5
Eukaryota - ps6
Amorpheae - ps7
Obazoa - ps8
Opisthokonta - ps9
Holozoa - ps10
Filozoa - ps11
Choano./Metazoa - ps12
Metazoa - ps13
Myriazoa - ps14
Parahoxozoa - ps15
Bilateria - ps16
Deuterostomia - ps17
Chordata - ps18
Olfactores - ps19
Vertebrata - ps20
Gnathostomata - ps21
Euteleostomi - ps22
Sarcopterygii - ps23
Tetrapoda - ps24
Amniota - ps25
Mammalia - ps26
Theria - ps27
Placentalia - ps28
Boreoeutheria - ps29
Euarchontoglires - ps30
Euarchonta - ps31
Primates - ps32
Haplorrhini - ps33
Simiiformes - ps34
Catarrhini - ps35
Hominoidea - ps36
Hominidae - ps37
Homininae - ps38
Hominini - ps39
Homo_sapiens - ps40
p-value
1e-02
2e-02
3e-02
3e-02
4e-02
Log-odds
0.5
1.0
1.5
2.0
2.5
3.0
3.5
4.0

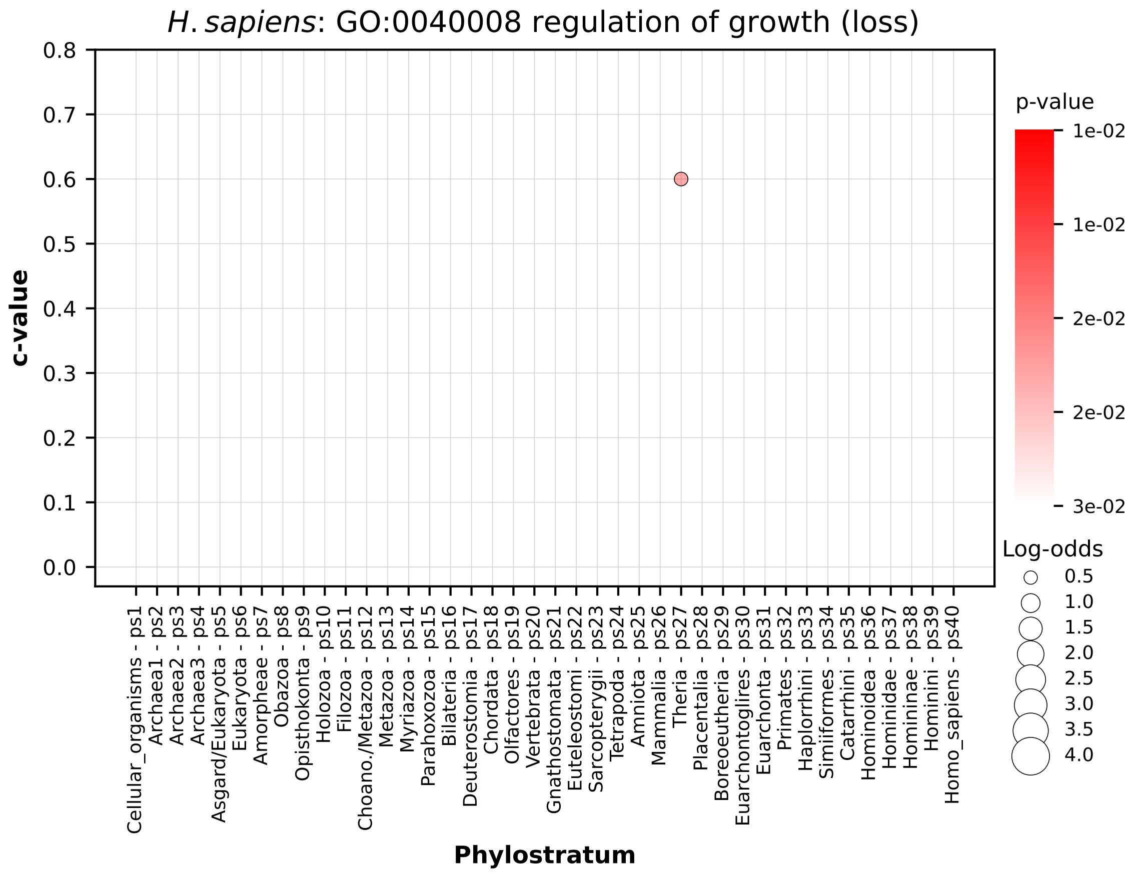
H. sapiens: GO:0040008 regulation of growth (loss)
c-value
0.8
0.7
0.6
0.5
0.4
0.3
0.2
0.1
0.0
Phylostratum
Cellular_organisms - ps1
Archaea1 - ps2
Archaea2 - ps3
Archaea3 - ps4
Asgard/Eukaryota - ps5
Eukaryota - ps6
Amorpheae - ps7
Obazoa - ps8
Opisthokonta - ps9
Holozoa - ps10
Filozoa - ps11
Choano./Metazoa - ps12
Metazoa - ps13
Myriazoa - ps14
Parahoxozoa - ps15
Bilateria - ps16
Deuterostomia - ps17
Chordata - ps18
Olfactores - ps19
Vertebrata - ps20
Gnathostomata - ps21
Euteleostomi - ps22
Sarcopterygii - ps23
Tetrapoda - ps24
Amniota - ps25
Mammalia - ps26
Theria - ps27
Placentalia - ps28
Boreoeutheria - ps29
Euarchontoglires - ps30
Euarchonta - ps31
Primates - ps32
Haplorrhini - ps33
Simiiformes - ps34
Catarrhini - ps35
Hominoidea - ps36
Hominidae - ps37
Homininae - ps38
Hominini - ps39
Homo_sapiens - ps40
p-value
1e-02
1e-02
2e-02
2e-02
3e-02
Log-odds
0.5
1.0
1.5
2.0
2.5
3.0
3.5
4.0

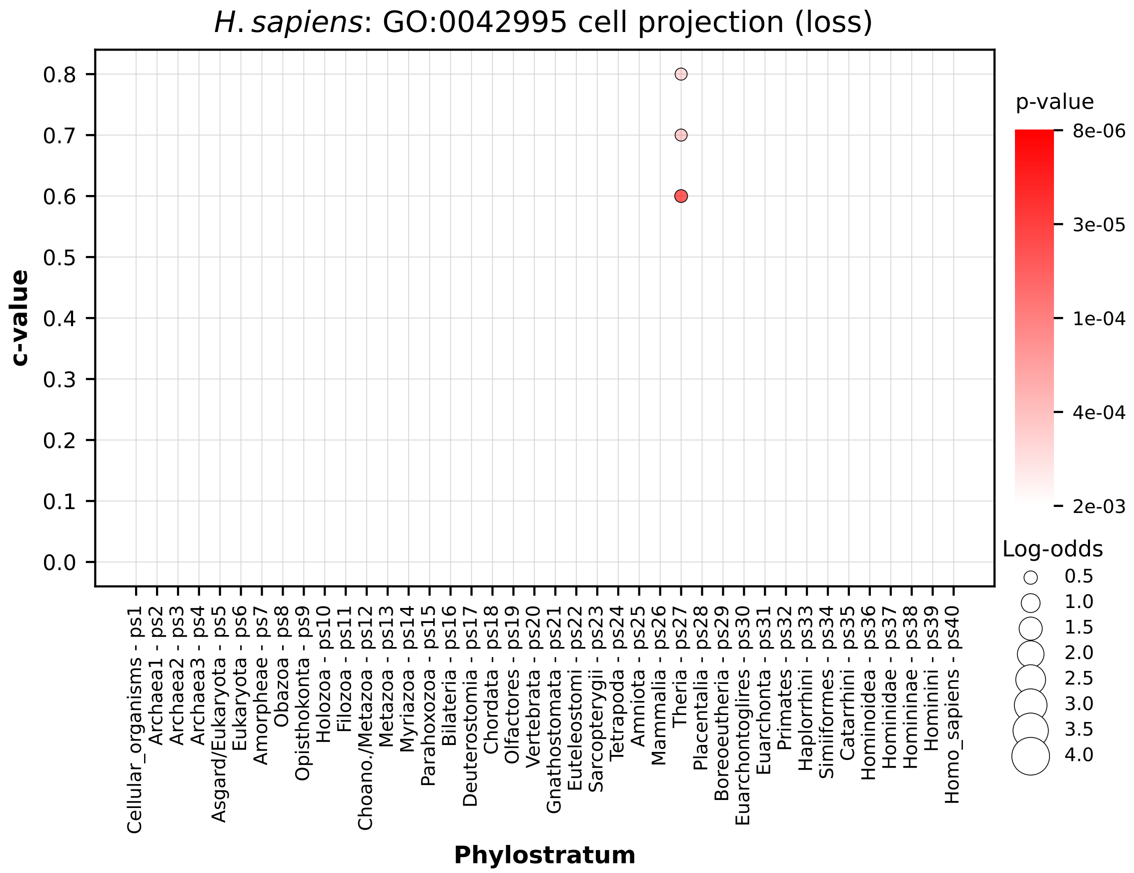

H. sapiens: GO:0042995 cell projection (loss)
c-value
0.8
0.7
0.6
0.5
0.4
0.3
0.2
0.1
0.0
p-value
8e-06
3e-05
1e-04
4e-04
2e-03
Log-odds
0.5
1.0
1.5
2.0
2.5
3.0
3.5
4.0
Cellular_organisms - ps1
Archaea1 - ps2
Archaea2 - ps3
Archaea3 - ps4
Asgard/Eukaryota - ps5
Eukaryota - ps6
Amorpheae - ps7
Obazoa - ps8
Opisthokonta - ps9
Holozoa - ps10
Filozoa - ps11
Choano./Metazoa - ps12
Metazoa - ps13
Myriazoa - ps14
Parahoxozoa - ps15
Bilateria - ps16
Deuterostomia - ps17
Chordata - ps18
Olfactores - ps19
Vertebrata - ps20
Gnathostomata - ps21
Euteleostomi - ps22
Sarcopterygii - ps23
Tetrapoda - ps24
Amniota - ps25
Mammalia - ps26
Theria - ps27
Placentalia - ps28
Boreoeutheria - ps29
Euarchontoglires - ps30
Euarchonta - ps31
Primates - ps32
Haplorrhini - ps33
Simiiformes - ps34
Catarrhini - ps35
Hominoidea - ps36
Hominidae - ps37
Homininae - ps38
Hominini - ps39
Homo_sapiens - ps40
Phylostratum

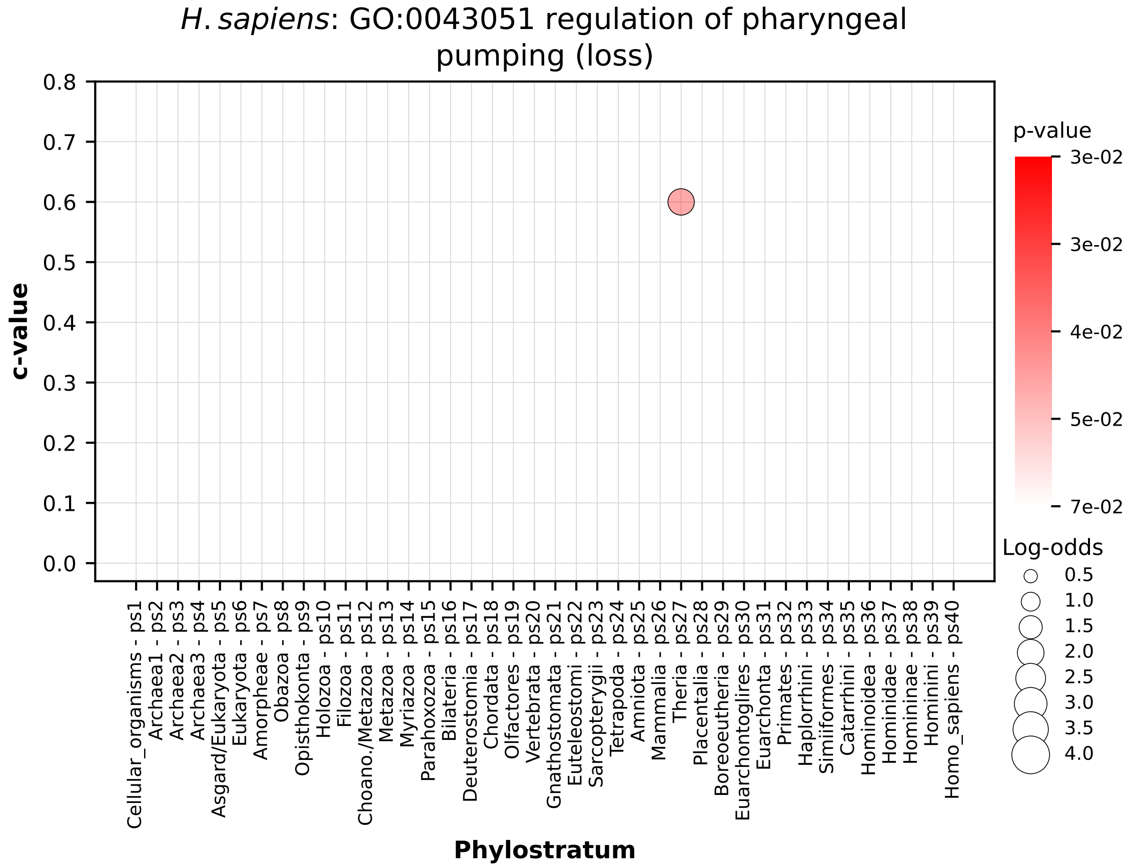
H. sapiens: GO:0043051 regulation of pharyngeal pumping (loss)
c-value
0.8
0.7
0.6
0.5
0.4
0.3
0.2
0.1
0.0
p-value
3e-02
3e-02
4e-02
5e-02
7e-02
Log-odds
0.5
1.0
1.5
2.0
2.5
3.0
3.5
4.0
Cellular_organisms - ps1
Archaea1 - ps2
Archaea2 - ps3
Archaea3 - ps4
Asgard/Eukaryota - ps5
Eukaryota - ps6
Amorpheae - ps7
Obazoa - ps8
Opisthokonta - ps9
Holozoa - ps10
Filozoa - ps11
Choano./Metazoa - ps12
Metazoa - ps13
Myriazoa - ps14
Parahoxozoa - ps15
Bilateria - ps16
Deuterostomia - ps17
Chordata - ps18
Olfactores - ps19
Vertebrata - ps20
Gnathostomata - ps21
Euteleostomi - ps22
Sarcopterygii - ps23
Tetrapoda - ps24
Amniota - ps25
Mammalia - ps26
Theria - ps27
Placentalia - ps28
Boreoeutheria - ps29
Euarchontoglires - ps30
Euarchonta - ps31
Primates - ps32
Haplorrhini - ps33
Simiiformes - ps34
Catarrhini - ps35
Hominoidea - ps36
Hominidae - ps37
Homininae - ps38
Hominini - ps39
Homo_sapiens - ps40
Phylostratum

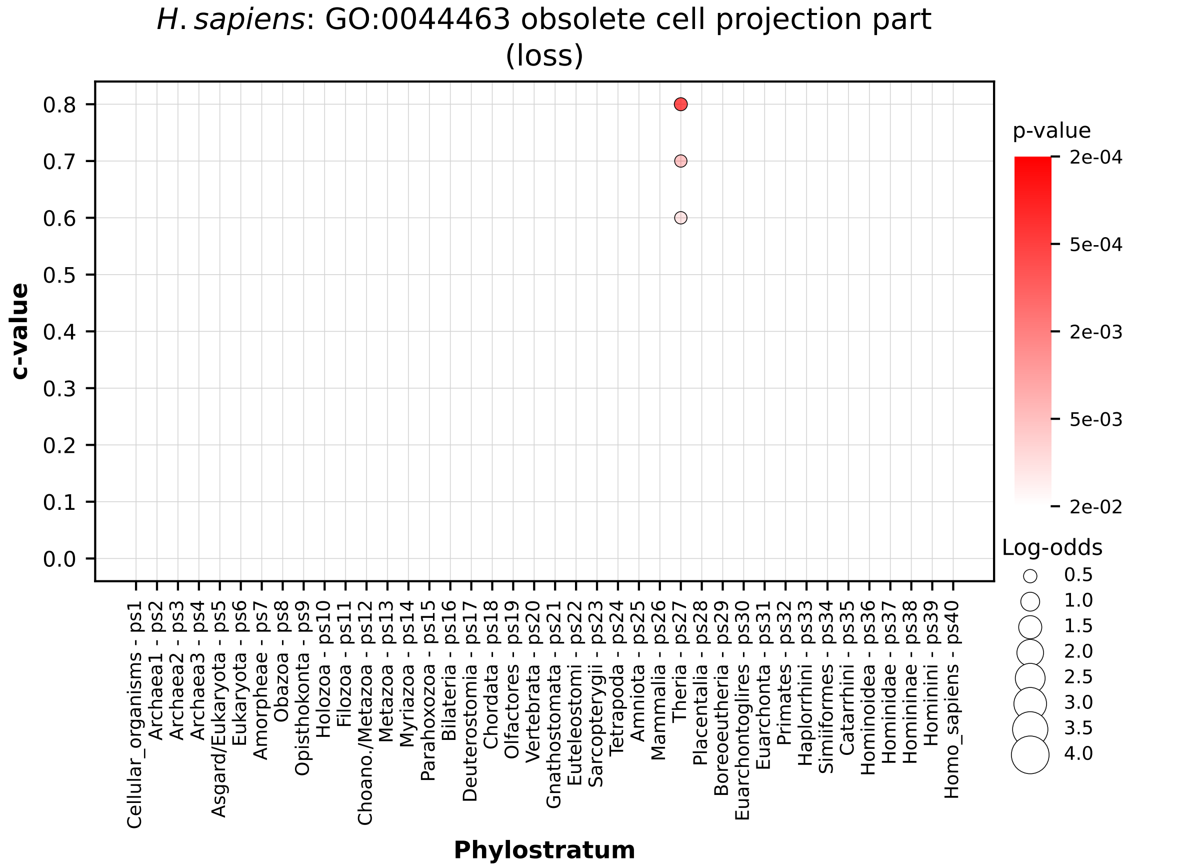

H. sapiens: GO:0044463 obsolete cell projection part
(loss)
c-value
0.0
0.1
0.2
0.3
0.4
0.5
0.6
0.7
0.8
p-value
2e-04
5e-04
2e-03
5e-03
2e-02
Log-odds
0.5
1.0
1.5
2.0
2.5
3.0
3.5
4.0
Cellular_organisms - ps1
Archaea1 - ps2
Archaea2 - ps3
Archaea3 - ps4
Asgard/Eukaryota - ps5
Eukaryota - ps6
Amorpheae - ps7
Obazoa - ps8
Opisthokonta - ps9
Holozoa - ps10
Filozoa - ps11
Choano./Metazoa - ps12
Metazoa - ps13
Myriazoa - ps14
Parahoxozoa - ps15
Bilateria - ps16
Deuterostomia - ps17
Chordata - ps18
Olfactores - ps19
Vertebrata - ps20
Gnathostomata - ps21
Euteleostomi - ps22
Sarcopterygii - ps23
Tetrapoda - ps24
Amniota - ps25
Mammalia - ps26
Theria - ps27
Placentalia - ps28
Boreoeutheria - ps29
Euarchontoglires - ps30
Euarchonta - ps31
Primates - ps32
Haplorrhini - ps33
Simiiformes - ps34
Catarrhini - ps35
Hominoidea - ps36
Hominidae - ps37
Homininae - ps38
Hominini - ps39
Homo_sapiens - ps40
Phylostratum

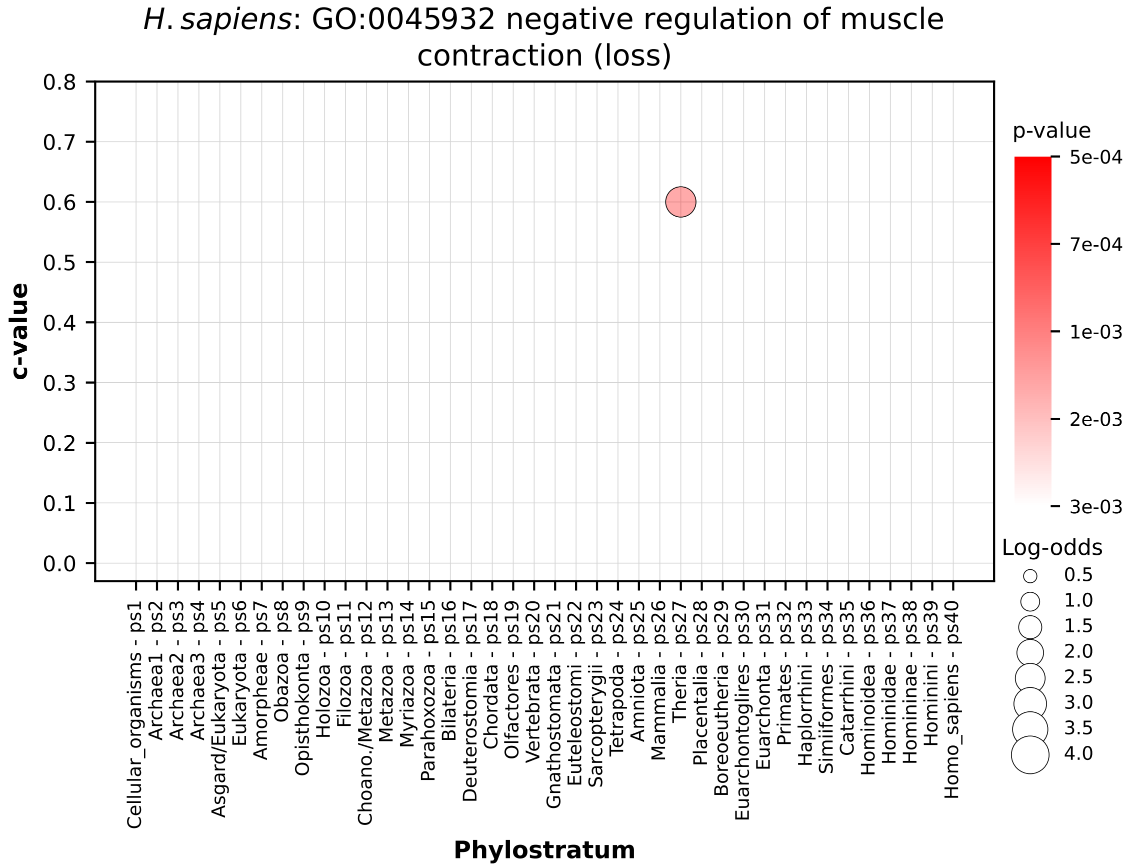
H. sapiens: GO:0045932 negative regulation of muscle contraction (loss)
c-value
0.8
0.7
0.6
0.5
0.4
0.3
0.2
0.1
0.0
p-value
5e-04
7e-04
1e-03
2e-03
3e-03
Log-odds
0.5
1.0
1.5
2.0
2.5
3.0
3.5
4.0
Cellular_organisms - ps1
Archaea1 - ps2
Archaea2 - ps3
Archaea3 - ps4
Asgard/Eukaryota - ps5
Eukaryota - ps6
Amorpheae - ps7
Obazoa - ps8
Opisthokonta - ps9
Holozoa - ps10
Filozoa - ps11
Choano./Metazoa - ps12
Metazoa - ps13
Myriazoa - ps14
Parahoxozoa - ps15
Bilateria - ps16
Deuterostomia - ps17
Chordata - ps18
Olfactores - ps19
Vertebrata - ps20
Gnathostomata - ps21
Euteleostomi - ps22
Sarcopterygii - ps23
Tetrapoda - ps24
Amniota - ps25
Mammalia - ps26
Theria - ps27
Placentalia - ps28
Boreoeutheria - ps29
Euarchontoglires - ps30
Euarchonta - ps31
Primates - ps32
Haplorrhini - ps33
Simiiformes - ps34
Catarrhini - ps35
Hominoidea - ps36
Hominidae - ps37
Homininae - ps38
Hominini - ps39
Homo_sapiens - ps40
Phylostratum

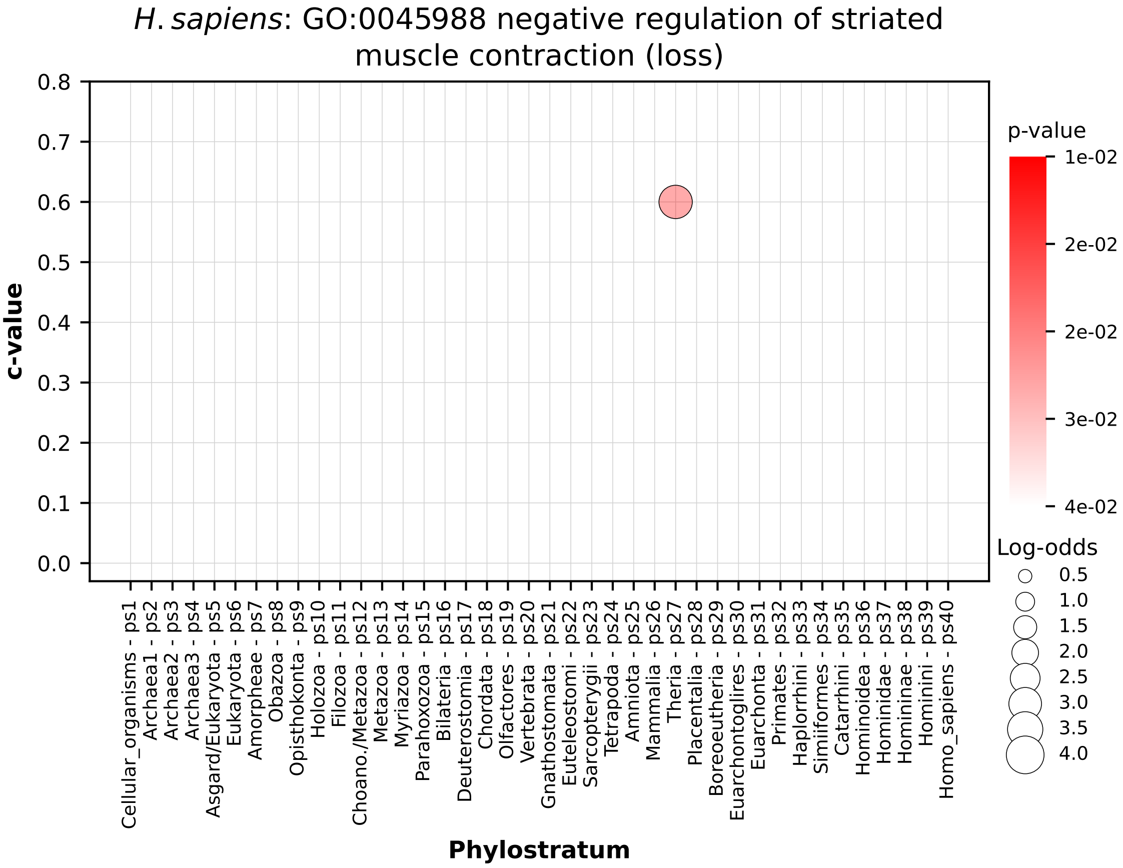

H. sapiens: GO:0045988 negative regulation of striated muscle contraction (loss)
c-value
0.8
0.7
0.6
0.5
0.4
0.3
0.2
0.1
0.0
p-value
1e-02
2e-02
2e-02
3e-02
4e-02
Log-odds
0.5
1.0
1.5
2.0
2.5
3.0
3.5
4.0
Cellular_organisms - ps1
Archaea1 - ps2
Archaea2 - ps3
Archaea3 - ps4
Asgard/Eukaryota - ps5
Eukaryota - ps6
Amorpheae - ps7
Obazoa - ps8
Opisthokonta - ps9
Holozoa - ps10
Filozoa - ps11
Choano./Metazoa - ps12
Metazoa - ps13
Myriazoa - ps14
Parahoxozoa - ps15
Bilateria - ps16
Deuterostomia - ps17
Chordata - ps18
Olfactores - ps19
Vertebrata - ps20
Gnathostomata - ps21
Euteleostomi - ps22
Sarcopterygii - ps23
Tetrapoda - ps24
Amniota - ps25
Mammalia - ps26
Theria - ps27
Placentalia - ps28
Boreoeutheria - ps29
Euarchontoglires - ps30
Euarchonta - ps31
Primates - ps32
Haplorrhini - ps33
Simiiformes - ps34
Catarrhini - ps35
Hominoidea - ps36
Hominidae - ps37
Homininae - ps38
Hominini - ps39
Homo_sapiens - ps40
Phylostratum

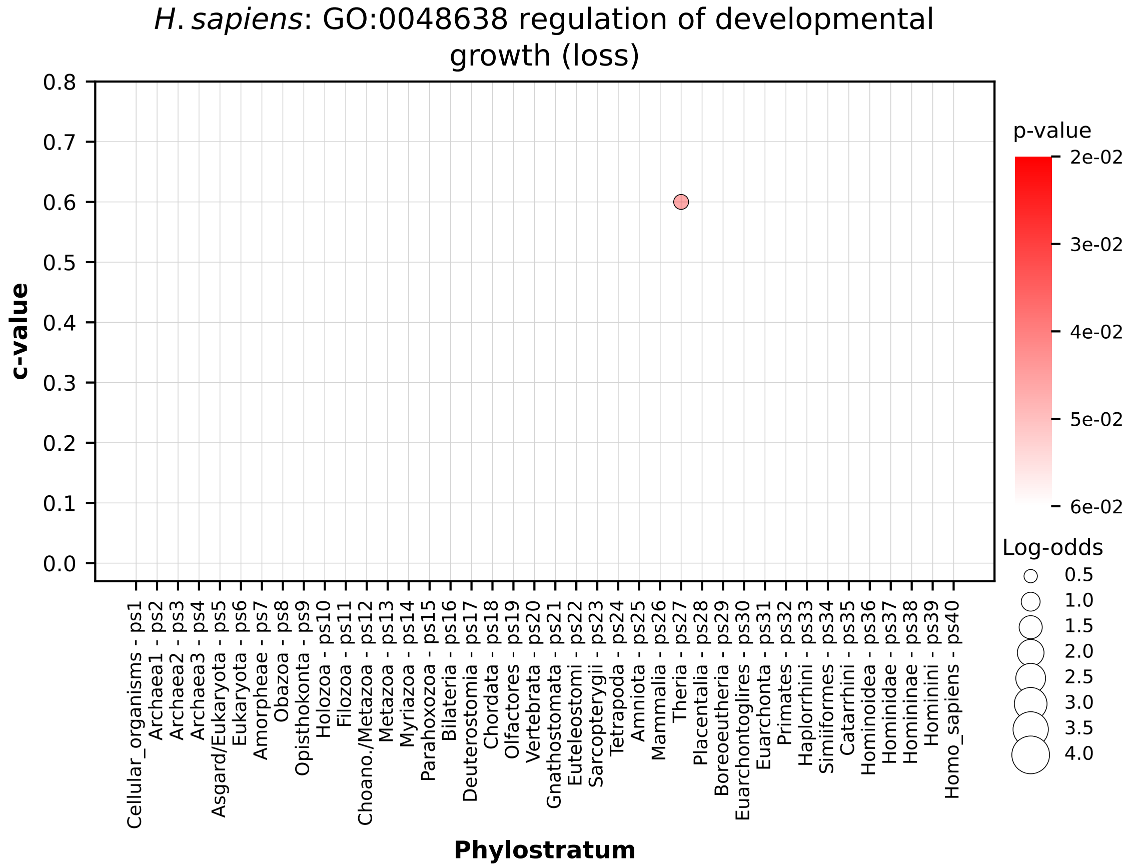
H. sapiens: GO:0048638 regulation of developmental growth (loss)
c-value
0.8
0.7
0.6
0.5
0.4
0.3
0.2
0.1
0.0
p-value
2e-02
3e-02
4e-02
5e-02
6e-02
Log-odds
0.5
1.0
1.5
2.0
2.5
3.0
3.5
4.0
Cellular_organisms - ps1
Archaea1 - ps2
Archaea2 - ps3
Archaea3 - ps4
Asgard/Eukaryota - ps5
Eukaryota - ps6
Amorpheae - ps7
Obazoa - ps8
Opisthokonta - ps9
Holozoa - ps10
Filozoa - ps11
Choano./Metazoa - ps12
Metazoa - ps13
Myriazoa - ps14
Parahoxozoa - ps15
Bilateria - ps16
Deuterostomia - ps17
Chordata - ps18
Olfactores - ps19
Vertebrata - ps20
Gnathostomata - ps21
Euteleostomi - ps22
Sarcopterygii - ps23
Tetrapoda - ps24
Amniota - ps25
Mammalia - ps26
Theria - ps27
Placentalia - ps28
Boreoeutheria - ps29
Euarchontoglires - ps30
Euarchonta - ps31
Primates - ps32
Haplorrhini - ps33
Simiiformes - ps34
Catarrhini - ps35
Hominoidea - ps36
Hominidae - ps37
Homininae - ps38
Hominini - ps39
Homo_sapiens - ps40
Phylostratum

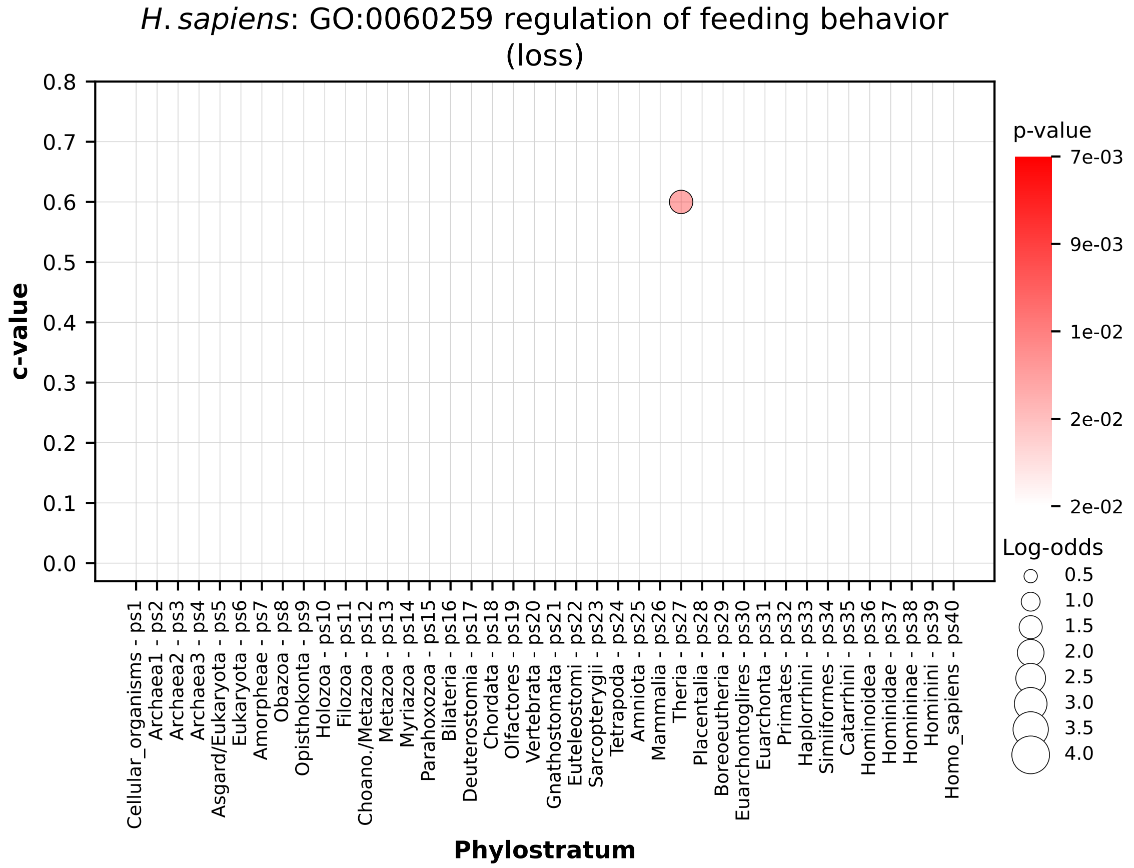

H. sapiens: GO:0060259 regulation of feeding behavior
(loss)
c-value
0.8
0.7
0.6
0.5
0.4
0.3
0.2
0.1
0.0
Phylostratum
Cellular_organisms - ps1
Archaea1 - ps2
Archaea2 - ps3
Archaea3 - ps4
Asgard/Eukaryota - ps5
Eukaryota - ps6
Amorpheae - ps7
Obazoa - ps8
Opisthokonta - ps9
Holozoa - ps10
Filozoa - ps11
Choano./Metazoa - ps12
Metazoa - ps13
Myriazoa - ps14
Parahoxozoa - ps15
Bilateria - ps16
Deuterostomia - ps17
Chordata - ps18
Olfactores - ps19
Vertebrata - ps20
Gnathostomata - ps21
Euteleostomi - ps22
Sarcopterygii - ps23
Tetrapoda - ps24
Amniota - ps25
Mammalia - ps26
Theria - ps27
Placentalia - ps28
Boreoeutheria - ps29
Euarchontoglires - ps30
Euarchonta - ps31
Primates - ps32
Haplorrhini - ps33
Simiiformes - ps34
Catarrhini - ps35
Hominoidea - ps36
Hominidae - ps37
Homininae - ps38
Hominini - ps39
Homo_sapiens - ps40
p-value
7e-03
9e-03
1e-02
2e-02
2e-02
Log-odds
0.5
1.0
1.5
2.0
2.5
3.0
3.5
4.0

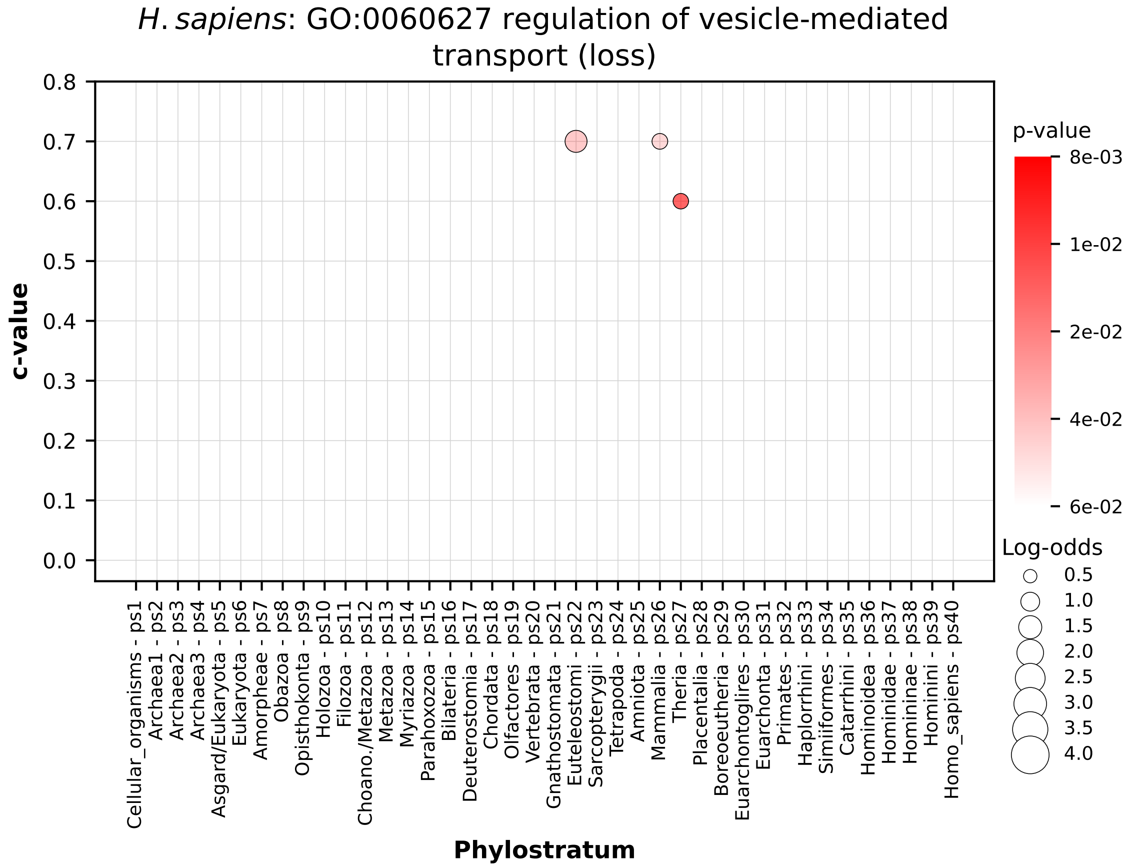

H. sapiens: GO:0060627 regulation of vesicle-mediated transport (loss)
c-value
0.8
0.7
0.6
0.5
0.4
0.3
0.2
0.1
0.0
Phylostratum
Cellular_organisms - ps1
Archaea1 - ps2
Archaea2 - ps3
Archaea3 - ps4
Asgard/Eukaryota - ps5
Eukaryota - ps6
Amorpheae - ps7
Obazoa - ps8
Opisthokonta - ps9
Holozoa - ps10
Filozoa - ps11
Choano./Metazoa - ps12
Metazoa - ps13
Myriazoa - ps14
Parahoxozoa - ps15
Bilateria - ps16
Deuterostomia - ps17
Chordata - ps18
Olfactores - ps19
Vertebrata - ps20
Gnathostomata - ps21
Euteleostomi - ps22
Sarcopterygii - ps23
Tetrapoda - ps24
Amniota - ps25
Mammalia - ps26
Theria - ps27
Placentalia - ps28
Boreoeutheria - ps29
Euarchontoglires - ps30
Euarchonta - ps31
Primates - ps32
Haplorrhini - ps33
Simiiformes - ps34
Catarrhini - ps35
Hominoidea - ps36
Hominidae - ps37
Homininae - ps38
Hominini - ps39
Homo_sapiens - ps40
p-value
8e-03
1e-02
2e-02
4e-02
6e-02
Log-odds
0.5
1.0
1.5
2.0
2.5
3.0
3.5
4.0

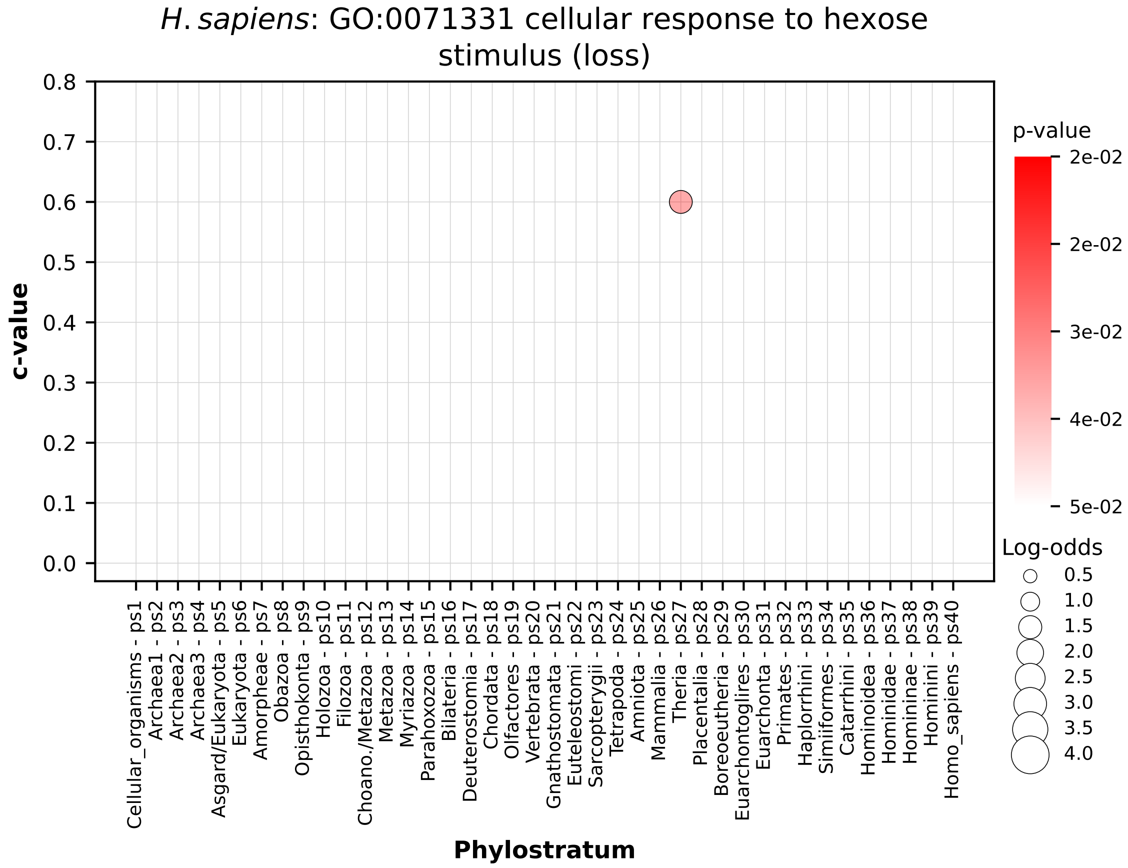

H. sapiens: GO:0071331 cellular response to hexose stimulus (loss)
c-value
0.8
0.7
0.6
0.5
0.4
0.3
0.2
0.1
0.0
Phylostratum
Cellular_organisms - ps1
Archaea1 - ps2
Archaea2 - ps3
Archaea3 - ps4
Asgard/Eukaryota - ps5
Eukaryota - ps6
Amorpheae - ps7
Obazoa - ps8
Opisthokonta - ps9
Holozoa - ps10
Filozoa - ps11
Choano./Metazoa - ps12
Metazoa - ps13
Myriazoa - ps14
Parahoxozoa - ps15
Bilateria - ps16
Deuterostomia - ps17
Chordata - ps18
Olfactores - ps19
Vertebrata - ps20
Gnathostomata - ps21
Euteleostomi - ps22
Sarcopterygii - ps23
Tetrapoda - ps24
Amniota - ps25
Mammalia - ps26
Theria - ps27
Placentalia - ps28
Boreoeutheria - ps29
Euarchontoglires - ps30
Euarchonta - ps31
Primates - ps32
Haplorrhini - ps33
Simiiformes - ps34
Catarrhini - ps35
Hominoidea - ps36
Hominidae - ps37
Homininae - ps38
Hominini - ps39
Homo_sapiens - ps40
p-value
2e-02
2e-02
3e-02
4e-02
5e-02
Log-odds
0.5
1.0
1.5
2.0
2.5
3.0
3.5
4.0

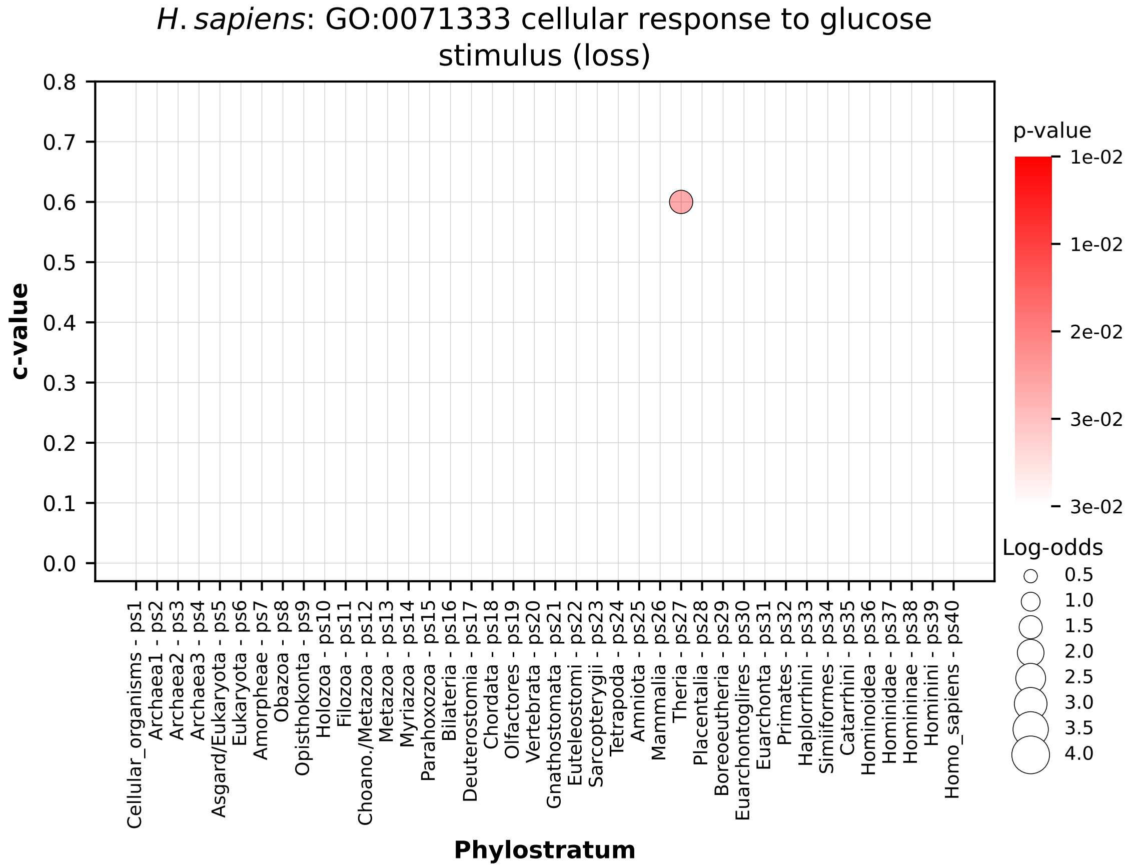
H. sapiens: GO:0071333 cellular response to glucose stimulus (loss)
c-value
0.8
0.7
0.6
0.5
0.4
0.3
0.2
0.1
0.0
p-value
1e-02
1e-02
2e-02
3e-02
3e-02
Log-odds
0.5
1.0
1.5
2.0
2.5
3.0
3.5
4.0
Cellular_organisms - ps1
Archaea1 - ps2
Archaea2 - ps3
Archaea3 - ps4
Asgard/Eukaryota - ps5
Eukaryota - ps6
Amorpheae - ps7
Obazoa - ps8
Opisthokonta - ps9
Holozoa - ps10
Filozoa - ps11
Choano./Metazoa - ps12
Metazoa - ps13
Myriazoa - ps14
Parahoxozoa - ps15
Bilateria - ps16
Deuterostomia - ps17
Chordata - ps18
Olfactores - ps19
Vertebrata - ps20
Gnathostomata - ps21
Euteleostomi - ps22
Sarcopterygii - ps23
Tetrapoda - ps24
Amniota - ps25
Mammalia - ps26
Theria - ps27
Placentalia - ps28
Boreoeutheria - ps29
Euarchontoglires - ps30
Euarchonta - ps31
Primates - ps32
Haplorrhini - ps33
Simiiformes - ps34
Catarrhini - ps35
Hominoidea - ps36
Hominidae - ps37
Homininae - ps38
Hominini - ps39
Homo_sapiens - ps40
Phylostratum

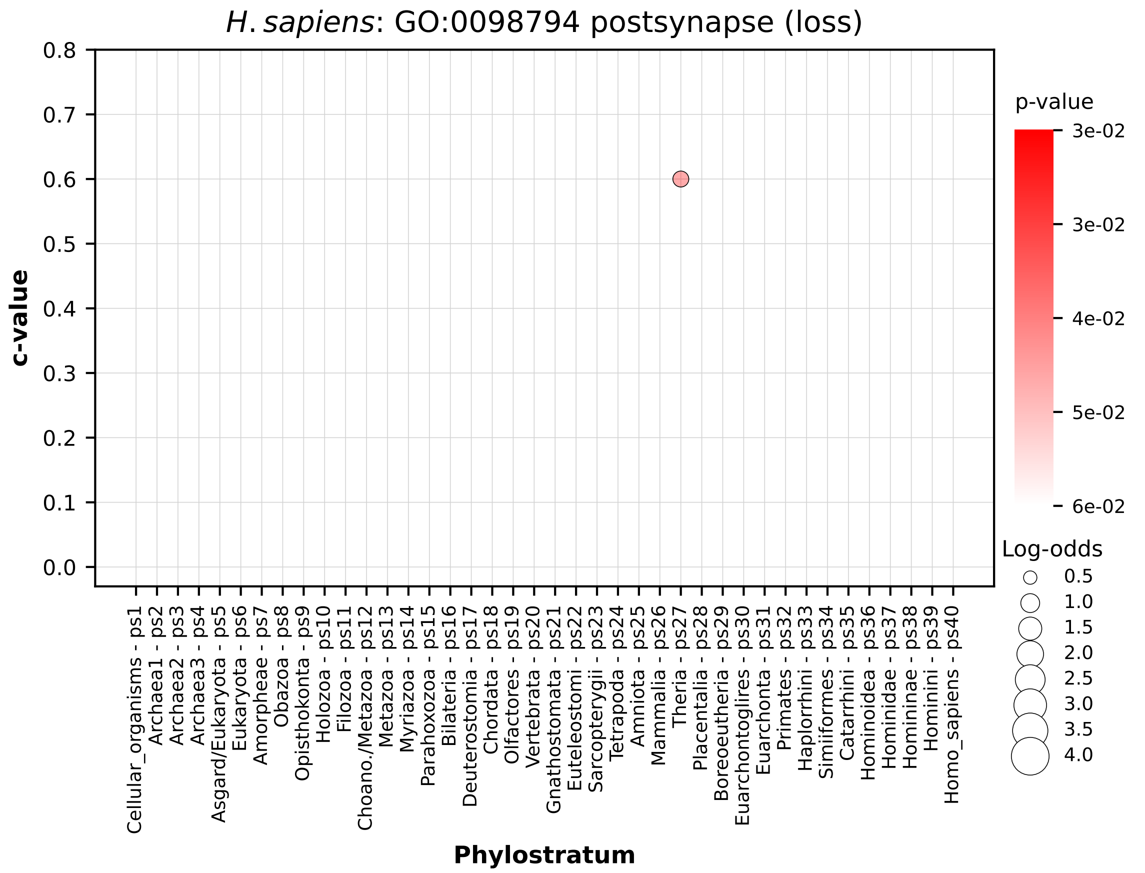

H. sapiens: GO:0098794 postsynapse (loss)
c-value
0.0
0.1
0.2
0.3
0.4
0.5
0.6
0.7
0.8
Phylostratum
Cellular_organisms - ps1
Archaea1 - ps2
Archaea2 - ps3
Archaea3 - ps4
Asgard/Eukaryota - ps5
Eukaryota - ps6
Amorpheae - ps7
Obazoa - ps8
Opisthokonta - ps9
Holozoa - ps10
Filozoa - ps11
Choano./Metazoa - ps12
Metazoa - ps13
Myriazoa - ps14
Parahoxozoa - ps15
Bilateria - ps16
Deuterostomia - ps17
Chordata - ps18
Olfactores - ps19
Vertebrata - ps20
Gnathostomata - ps21
Euteleostomi - ps22
Sarcopterygii - ps23
Tetrapoda - ps24
Amniota - ps25
Mammalia - ps26
Theria - ps27
Placentalia - ps28
Boreoeutheria - ps29
Euarchontoglires - ps30
Euarchonta - ps31
Primates - ps32
Haplorrhini - ps33
Simiiformes - ps34
Catarrhini - ps35
Hominoidea - ps36
Hominidae - ps37
Homininae - ps38
Hominini - ps39
Homo_sapiens - ps40
p-value
3e-02
3e-02
4e-02
5e-02
6e-02
Log-odds
0.5
1.0
1.5
2.0
2.5
3.0
3.5
4.0

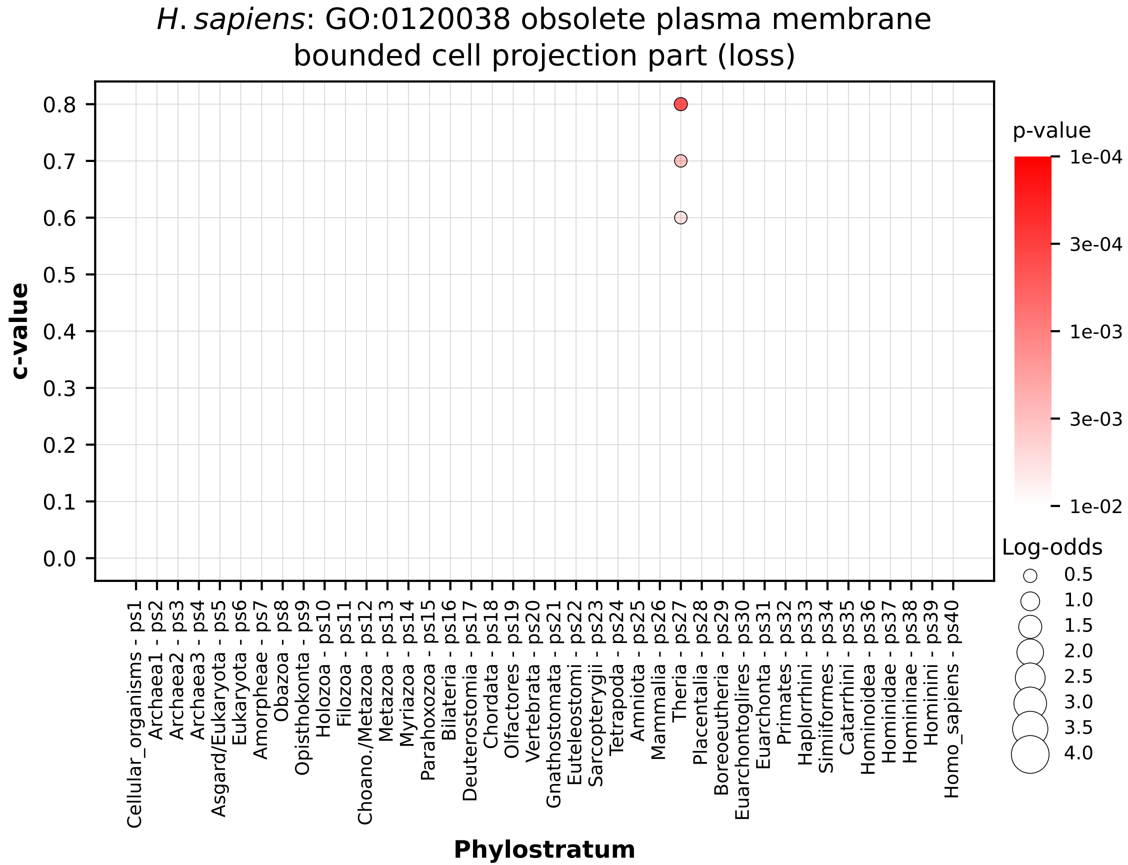

H. sapiens: GO:0120038 obsolete plasma membrane bounded cell projection part (loss)
c-value
0.0
0.1
0.2
0.3
0.4
0.5
0.6
0.7
0.8
Phylostratum
Cellular_organisms - ps1
Archaea1 - ps2
Archaea2 - ps3
Archaea3 - ps4
Asgard/Eukaryota - ps5
Eukaryota - ps6
Amorpheae - ps7
Obazoa - ps8
Opisthokonta - ps9
Holozoa - ps10
Filozoa - ps11
Choano./Metazoa - ps12
Metazoa - ps13
Myriazoa - ps14
Parahoxozoa - ps15
Bilateria - ps16
Deuterostomia - ps17
Chordata - ps18
Olfactores - ps19
Vertebrata - ps20
Gnathostomata - ps21
Euteleostomi - ps22
Sarcopterygii - ps23
Tetrapoda - ps24
Amniota - ps25
Mammalia - ps26
Theria - ps27
Placentalia - ps28
Boreoeutheria - ps29
Euarchontoglires - ps30
Euarchonta - ps31
Primates - ps32
Haplorrhini - ps33
Simiiformes - ps34
Catarrhini - ps35
Hominoidea - ps36
Hominidae - ps37
Homininae - ps38
Hominini - ps39
Homo_sapiens - ps40
p-value
1e-04
3e-04
1e-03
3e-03
1e-02
Log-odds
0.5
1.0
1.5
2.0
2.5
3.0
3.5
4.0

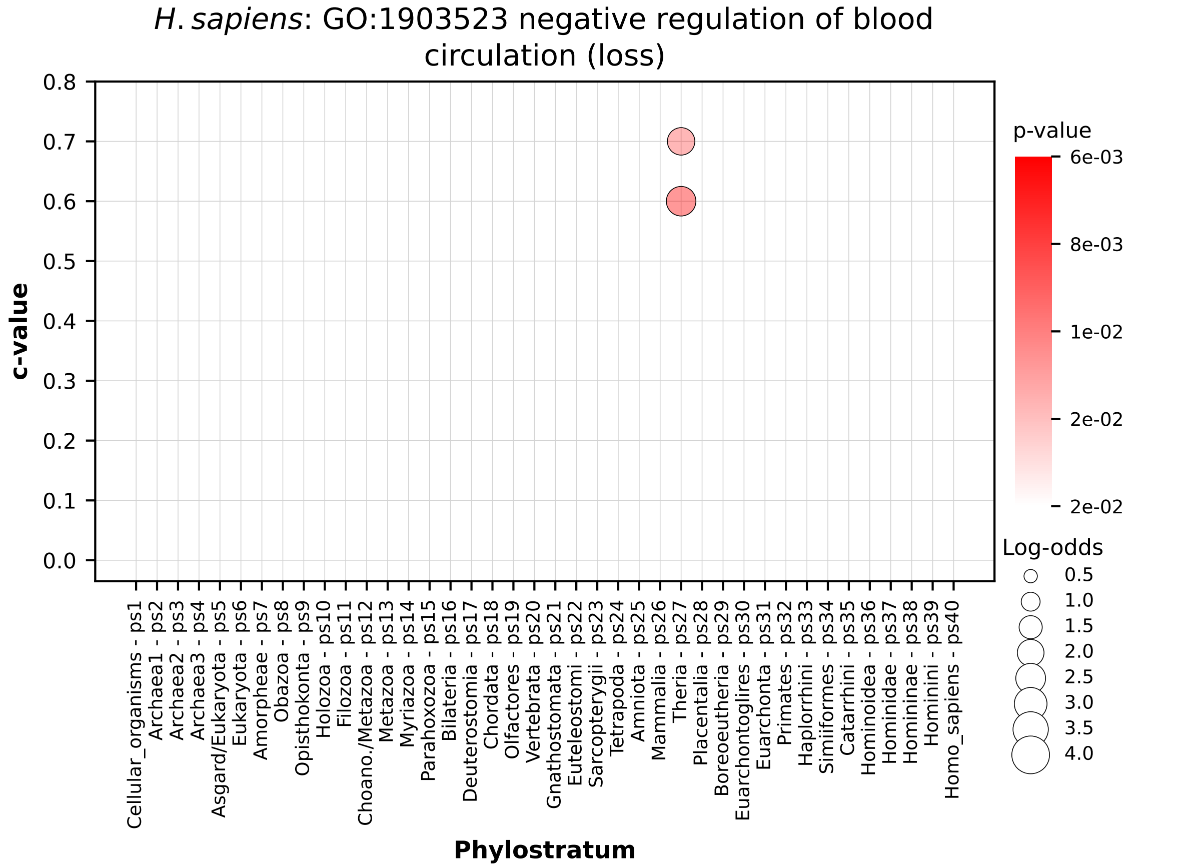

H. sapiens: GO:1903523 negative regulation of blood circulation (loss)
c-value
0.0
0.1
0.2
0.3
0.4
0.5
0.6
0.7
0.8
p-value
6e-03
8e-03
1e-02
2e-02
2e-02
Log-odds
0.5
1.0
1.5
2.0
2.5
3.0
3.5
4.0
Cellular_organisms - ps1
Archaea1 - ps2
Archaea2 - ps3
Archaea3 - ps4
Asgard/Eukaryota - ps5
Eukaryota - ps6
Amorpheae - ps7
Obazoa - ps8
Opisthokonta - ps9
Holozoa - ps10
Filozoa - ps11
Choano./Metazoa - ps12
Metazoa - ps13
Myriazoa - ps14
Parahoxozoa - ps15
Bilateria - ps16
Deuterostomia - ps17
Chordata - ps18
Olfactores - ps19
Vertebrata - ps20
Gnathostomata - ps21
Euteleostomi - ps22
Sarcopterygii - ps23
Tetrapoda - ps24
Amniota - ps25
Mammalia - ps26
Theria - ps27
Placentalia - ps28
Boreoeutheria - ps29
Euarchontoglires - ps30
Euarchonta - ps31
Primates - ps32
Haplorrhini - ps33
Simiiformes - ps34
Catarrhini - ps35
Hominoidea - ps36
Hominidae - ps37
Homininae - ps38
Hominini - ps39
Homo_sapiens - ps40
Phylostratum

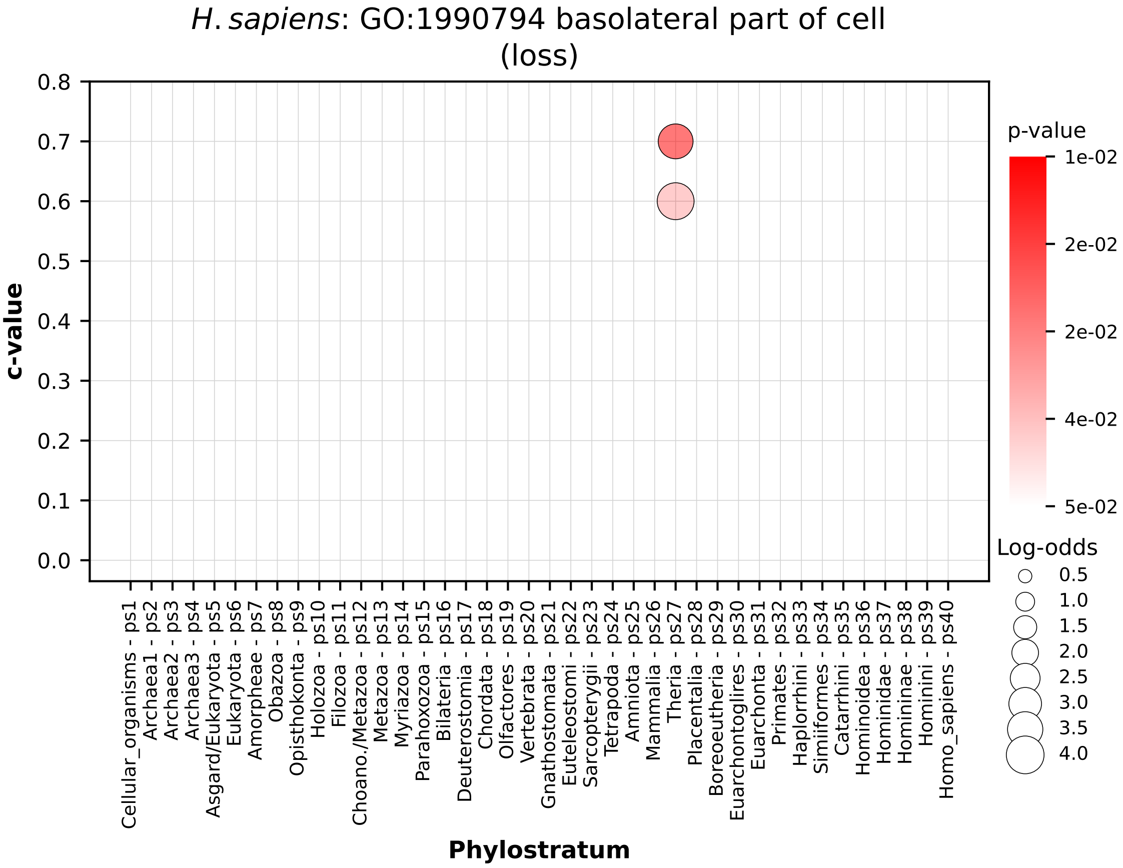

H. sapiens: GO:1990794 basolateral part of cell
(loss)
c-value
0.8
0.7
0.6
0.5
0.4
0.3
0.2
0.1
0.0
p-value
1e-02
2e-02
2e-02
4e-02
5e-02
Log-odds
0.5
1.0
1.5
2.0
2.5
3.0
3.5
4.0
Cellular_organisms - ps1
Archaea1 - ps2
Archaea2 - ps3
Archaea3 - ps4
Asgard/Eukaryota - ps5
Eukaryota - ps6
Amorpheae - ps7
Obazoa - ps8
Opisthokonta - ps9
Holozoa - ps10
Filozoa - ps11
Choano./Metazoa - ps12
Metazoa - ps13
Myriazoa - ps14
Parahoxozoa - ps15
Bilateria - ps16
Deuterostomia - ps17
Chordata - ps18
Olfactores - ps19
Vertebrata - ps20
Gnathostomata - ps21
Euteleostomi - ps22
Sarcopterygii - ps23
Tetrapoda - ps24
Amniota - ps25
Mammalia - ps26
Theria - ps27
Placentalia - ps28
Boreoeutheria - ps29
Euarchontoglires - ps30
Euarchonta - ps31
Primates - ps32
Haplorrhini - ps33
Simiiformes - ps34
Catarrhini - ps35
Hominoidea - ps36
Hominidae - ps37
Homininae - ps38
Hominini - ps39
Homo_sapiens - ps40
Phylostratum

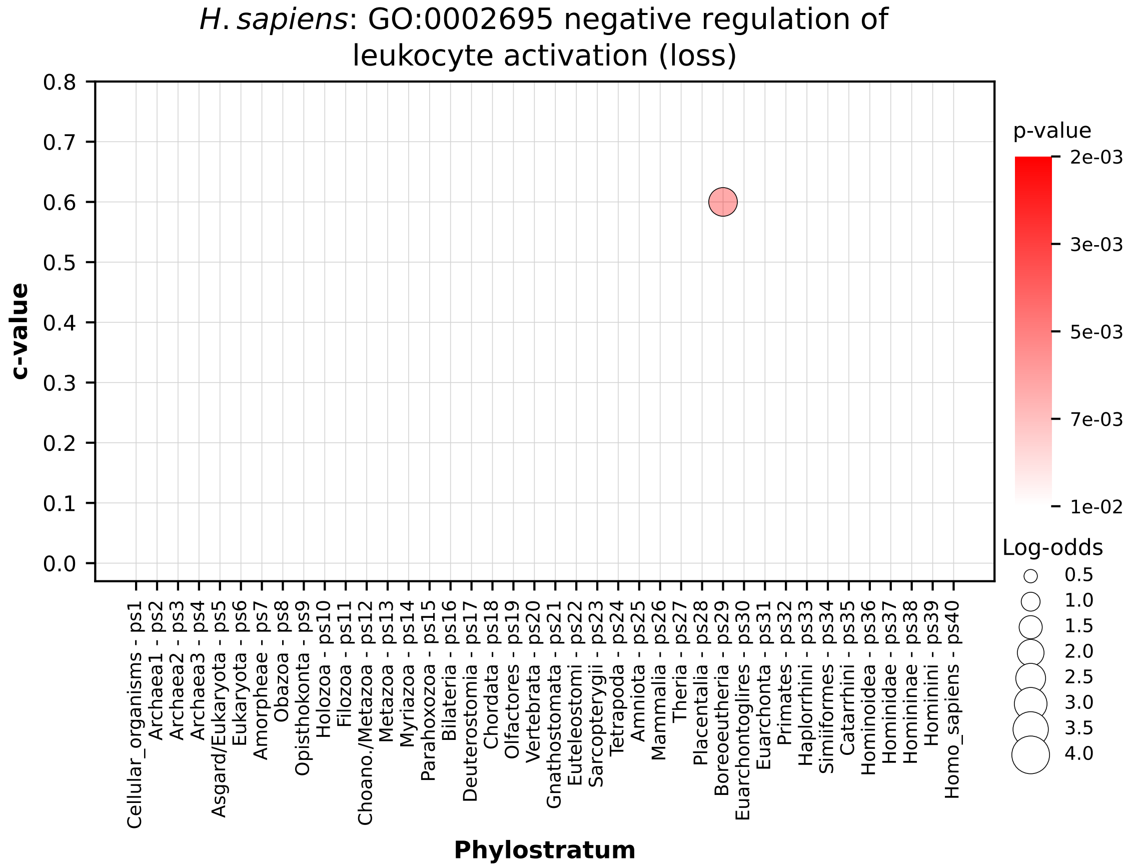

H. sapiens: GO:0002695 negative regulation of leukocyte activation (loss)
c-value
0.8
0.7
0.6
0.5
0.4
0.3
0.2
0.1
0.0
p-value
2e-03
3e-03
5e-03
7e-03
1e-02
Log-odds
0.5
1.0
1.5
2.0
2.5
3.0
3.5
4.0
Cellular_organisms - ps1
Archaea1 - ps2
Archaea2 - ps3
Archaea3 - ps4
Asgard/Eukaryota - ps5
Eukaryota - ps6
Amorpheae - ps7
Obazoa - ps8
Opisthokonta - ps9
Holozoa - ps10
Filozoa - ps11
Choano./Metazoa - ps12
Metazoa - ps13
Myriazoa - ps14
Parahoxozoa - ps15
Bilateria - ps16
Deuterostomia - ps17
Chordata - ps18
Olfactores - ps19
Vertebrata - ps20
Gnathostomata - ps21
Euteleostomi - ps22
Sarcopterygii - ps23
Tetrapoda - ps24
Amniota - ps25
Mammalia - ps26
Theria - ps27
Placentalia - ps28
Boreoeutheria - ps29
Euarchontoglires - ps30
Euarchonta - ps31
Primates - ps32
Haplorrhini - ps33
Simiiformes - ps34
Catarrhini - ps35
Hominoidea - ps36
Hominidae - ps37
Homininae - ps38
Hominini - ps39
Homo_sapiens - ps40
Phylostratum

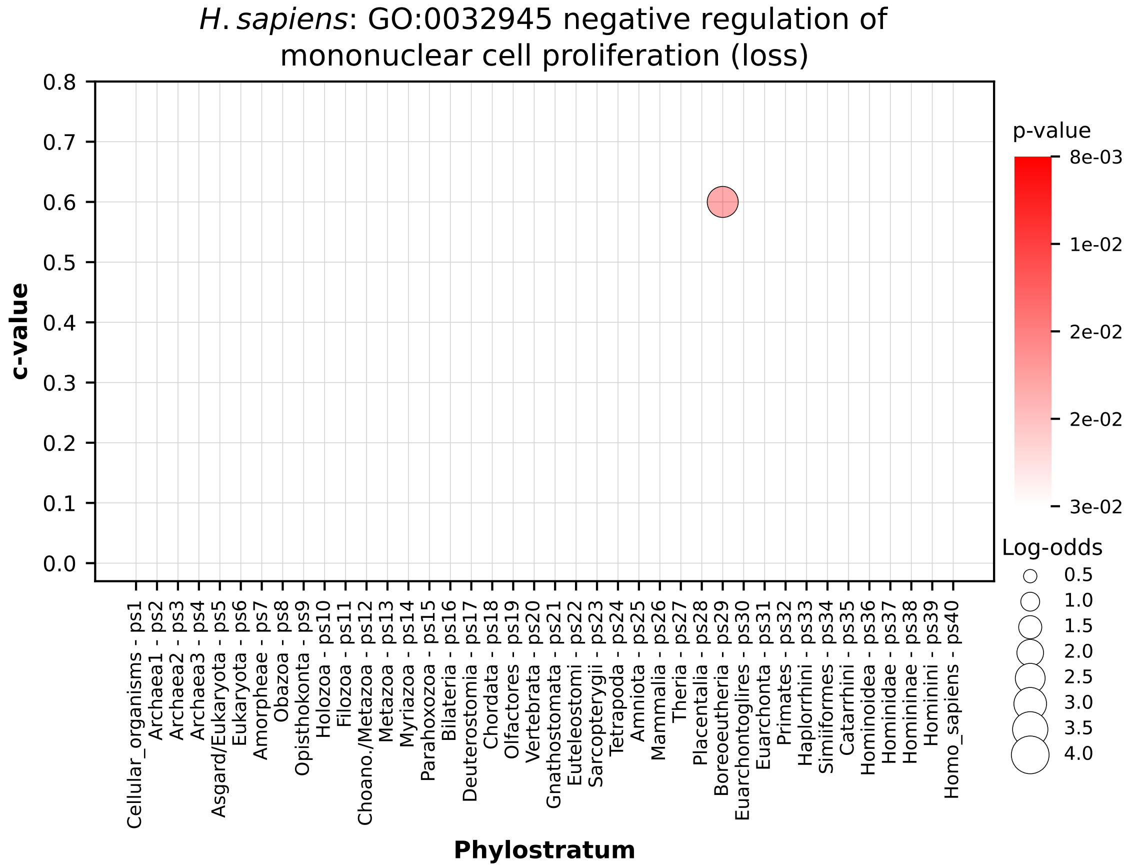

H. sapiens: GO:0032945 negative regulation of mononuclear cell proliferation (loss)
c-value
0.8
0.7
0.6
0.5
0.4
0.3
0.2
0.1
0.0
Phylostratum
Cellular_organisms - ps1
Archaea1 - ps2
Archaea2 - ps3
Archaea3 - ps4
Asgard/Eukaryota - ps5
Eukaryota - ps6
Amorpheae - ps7
Obazoa - ps8
Opisthokonta - ps9
Holozoa - ps10
Filozoa - ps11
Choano./Metazoa - ps12
Metazoa - ps13
Myriazoa - ps14
Parahoxozoa - ps15
Bilateria - ps16
Deuterostomia - ps17
Chordata - ps18
Olfactores - ps19
Vertebrata - ps20
Gnathostomata - ps21
Euteleostomi - ps22
Sarcopterygii - ps23
Tetrapoda - ps24
Amniota - ps25
Mammalia - ps26
Theria - ps27
Placentalia - ps28
Boreoeutheria - ps29
Euarchontoglires - ps30
Euarchonta - ps31
Primates - ps32
Haplorrhini - ps33
Simiiformes - ps34
Catarrhini - ps35
Hominoidea - ps36
Hominidae - ps37
Homininae - ps38
Hominini - ps39
Homo_sapiens - ps40
p-value
8e-03
1e-02
2e-02
2e-02
3e-02
Log-odds
0.5
1.0
1.5
2.0
2.5
3.0
3.5
4.0

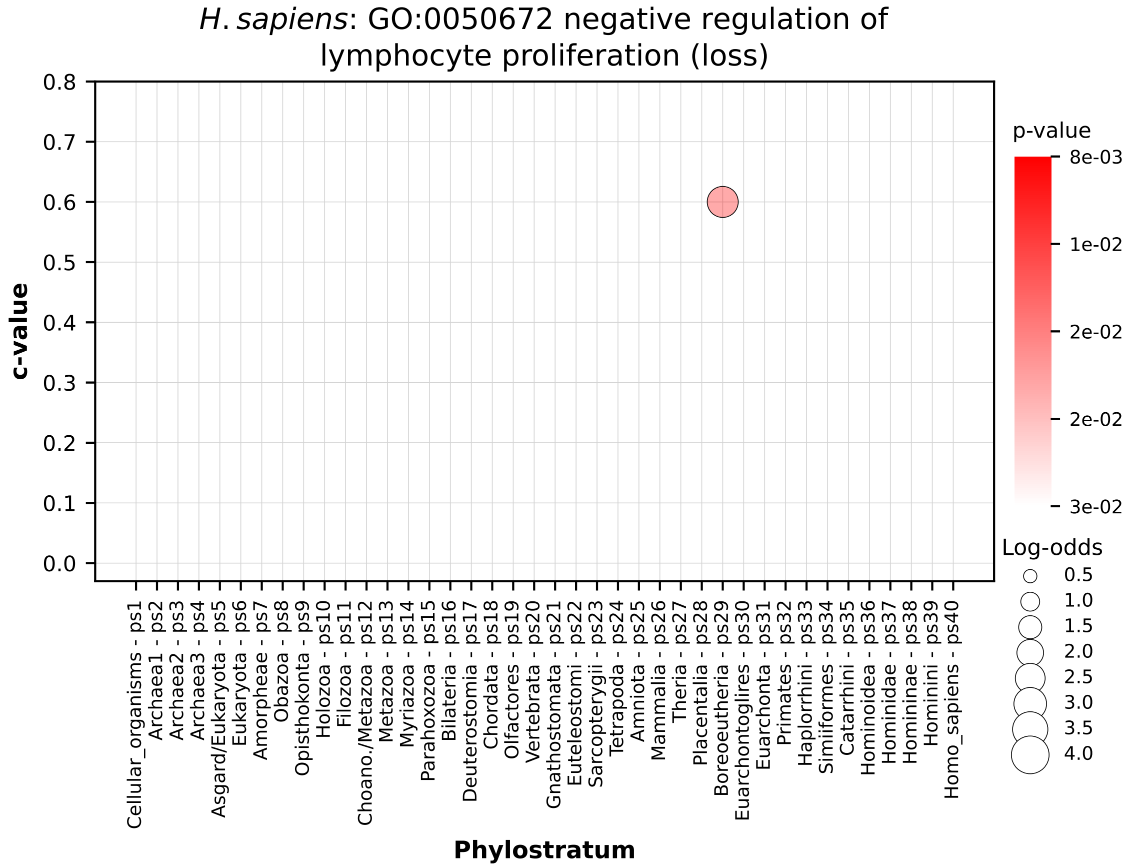

H. sapiens: GO:0050672 negative regulation of lymphocyte proliferation (loss)
c-value
0.8
0.7
0.6
0.5
0.4
0.3
0.2
0.1
0.0
p-value
8e-03
1e-02
2e-02
2e-02
3e-02
Log-odds
0.5
1.0
1.5
2.0
2.5
3.0
3.5
4.0
Cellular_organisms - ps1
Archaea1 - ps2
Archaea2 - ps3
Archaea3 - ps4
Asgard/Eukaryota - ps5
Eukaryota - ps6
Amorpheae - ps7
Obazoa - ps8
Opisthokonta - ps9
Holozoa - ps10
Filozoa - ps11
Choano./Metazoa - ps12
Metazoa - ps13
Myriazoa - ps14
Parahoxozoa - ps15
Bilateria - ps16
Deuterostomia - ps17
Chordata - ps18
Olfactores - ps19
Vertebrata - ps20
Gnathostomata - ps21
Euteleostomi - ps22
Sarcopterygii - ps23
Tetrapoda - ps24
Amniota - ps25
Mammalia - ps26
Theria - ps27
Placentalia - ps28
Boreoeutheria - ps29
Euarchontoglires - ps30
Euarchonta - ps31
Primates - ps32
Haplorrhini - ps33
Simiiformes - ps34
Catarrhini - ps35
Hominoidea - ps36
Hominidae - ps37
Homininae - ps38
Hominini - ps39
Homo_sapiens - ps40
Phylostratum

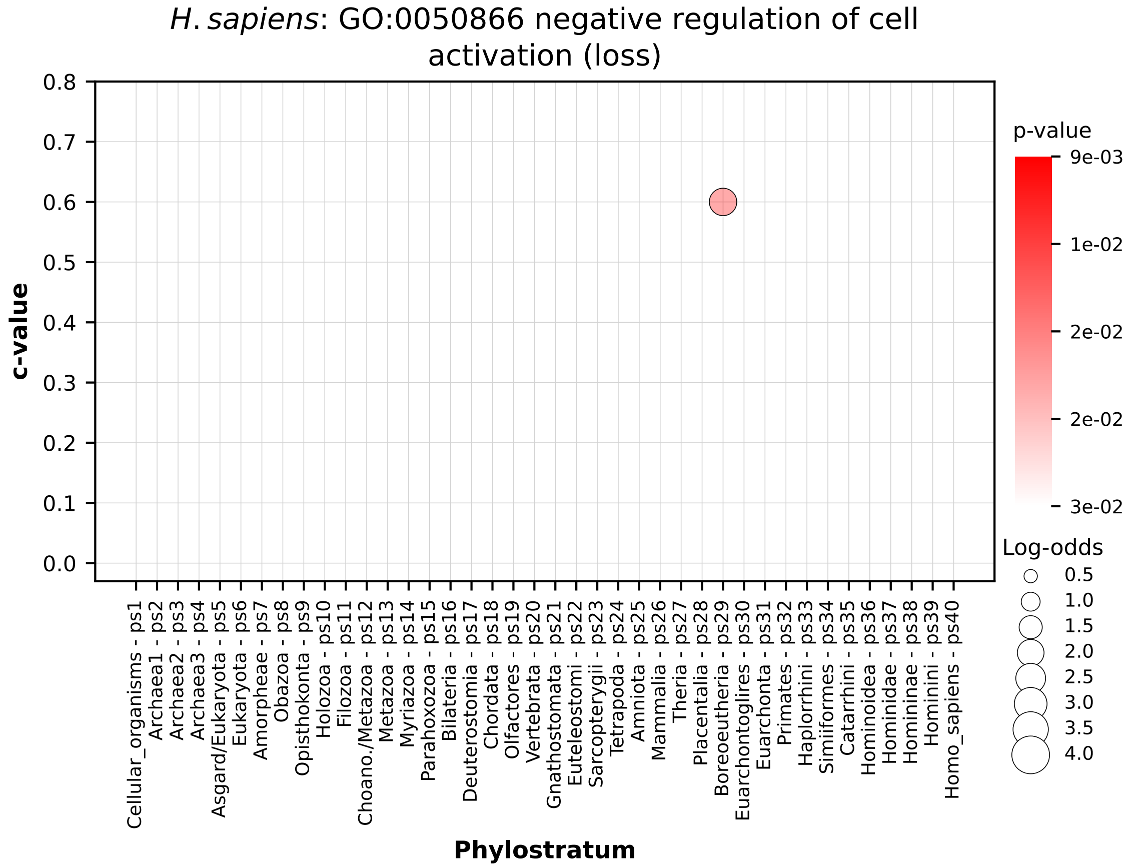

H. sapiens: GO:0050866 negative regulation of cell activation (loss)
c-value
0.0
0.1
0.2
0.3
0.4
0.5
0.6
0.7
0.8
Phylostratum
Cellular_organisms - ps1
Archaea1 - ps2
Archaea2 - ps3
Archaea3 - ps4
Asgard/Eukaryota - ps5
Eukaryota - ps6
Amorpheae - ps7
Obazoa - ps8
Opisthokonta - ps9
Holozoa - ps10
Filozoa - ps11
Choano./Metazoa - ps12
Metazoa - ps13
Myriazoa - ps14
Parahoxozoa - ps15
Bilateria - ps16
Deuterostomia - ps17
Chordata - ps18
Olfactores - ps19
Vertebrata - ps20
Gnathostomata - ps21
Euteleostomi - ps22
Sarcopterygii - ps23
Tetrapoda - ps24
Amniota - ps25
Mammalia - ps26
Theria - ps27
Placentalia - ps28
Boreoeutheria - ps29
Euarchontoglires - ps30
Euarchonta - ps31
Primates - ps32
Haplorrhini - ps33
Simiiformes - ps34
Catarrhini - ps35
Hominoidea - ps36
Hominidae - ps37
Homininae - ps38
Hominini - ps39
Homo_sapiens - ps40
p-value
9e-03
1e-02
2e-02
2e-02
3e-02
Log-odds
0.5
1.0
1.5
2.0
2.5
3.0
3.5
4.0

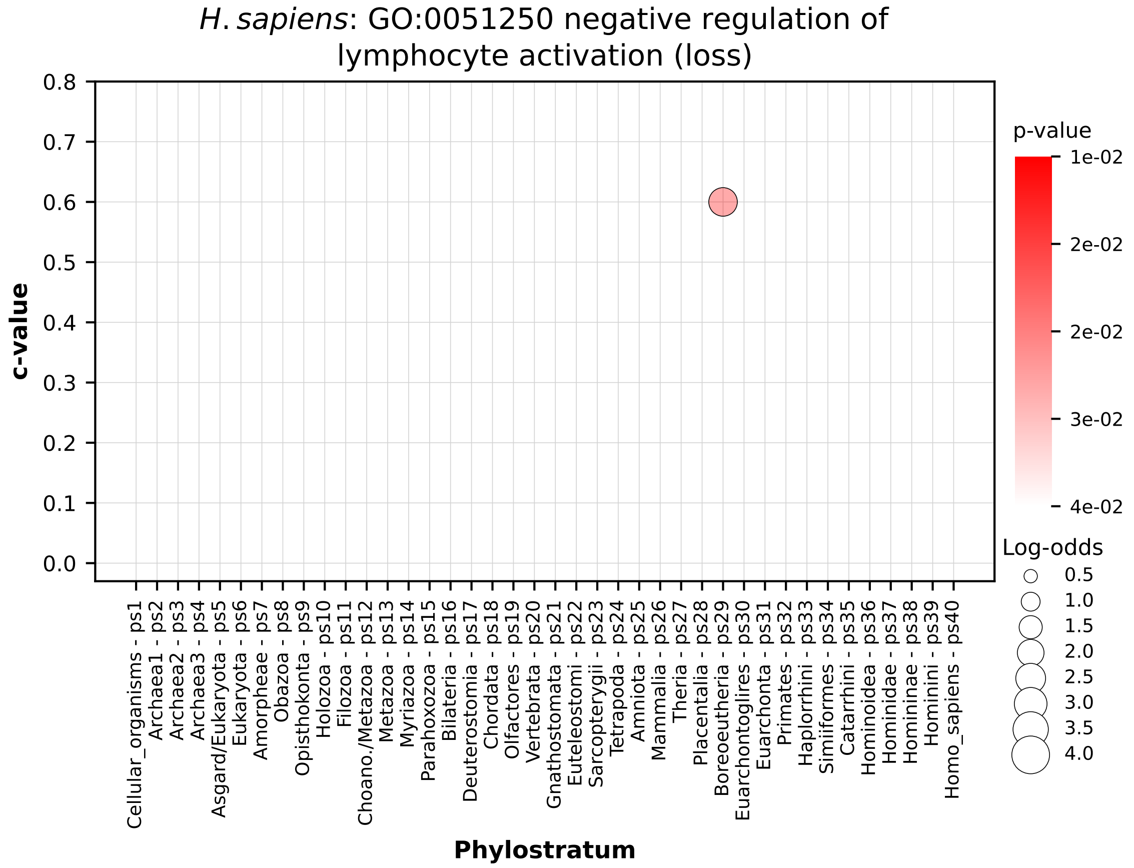
H. sapiens: GO:0051250 negative regulation of lymphocyte activation (loss)
c-value
0.8
0.7
0.6
0.5
0.4
0.3
0.2
0.1
0.0
p-value
1e-02
2e-02
2e-02
3e-02
4e-02
Log-odds
0.5
1.0
1.5
2.0
2.5
3.0
3.5
4.0
Cellular_organisms - ps1
Archaea1 - ps2
Archaea2 - ps3
Archaea3 - ps4
Asgard/Eukaryota - ps5
Eukaryota - ps6
Amorpheae - ps7
Obazoa - ps8
Opisthokonta - ps9
Holozoa - ps10
Filozoa - ps11
Choano./Metazoa - ps12
Metazoa - ps13
Myriazoa - ps14
Parahoxozoa - ps15
Bilateria - ps16
Deuterostomia - ps17
Chordata - ps18
Olfactores - ps19
Vertebrata - ps20
Gnathostomata - ps21
Euteleostomi - ps22
Sarcopterygii - ps23
Tetrapoda - ps24
Amniota - ps25
Mammalia - ps26
Theria - ps27
Placentalia - ps28
Boreoeutheria - ps29
Euarchontoglires - ps30
Euarchonta - ps31
Primates - ps32
Haplorrhini - ps33
Simiiformes - ps34
Catarrhini - ps35
Hominoidea - ps36
Hominidae - ps37
Homininae - ps38
Hominini - ps39
Homo_sapiens - ps40
Phylostratum

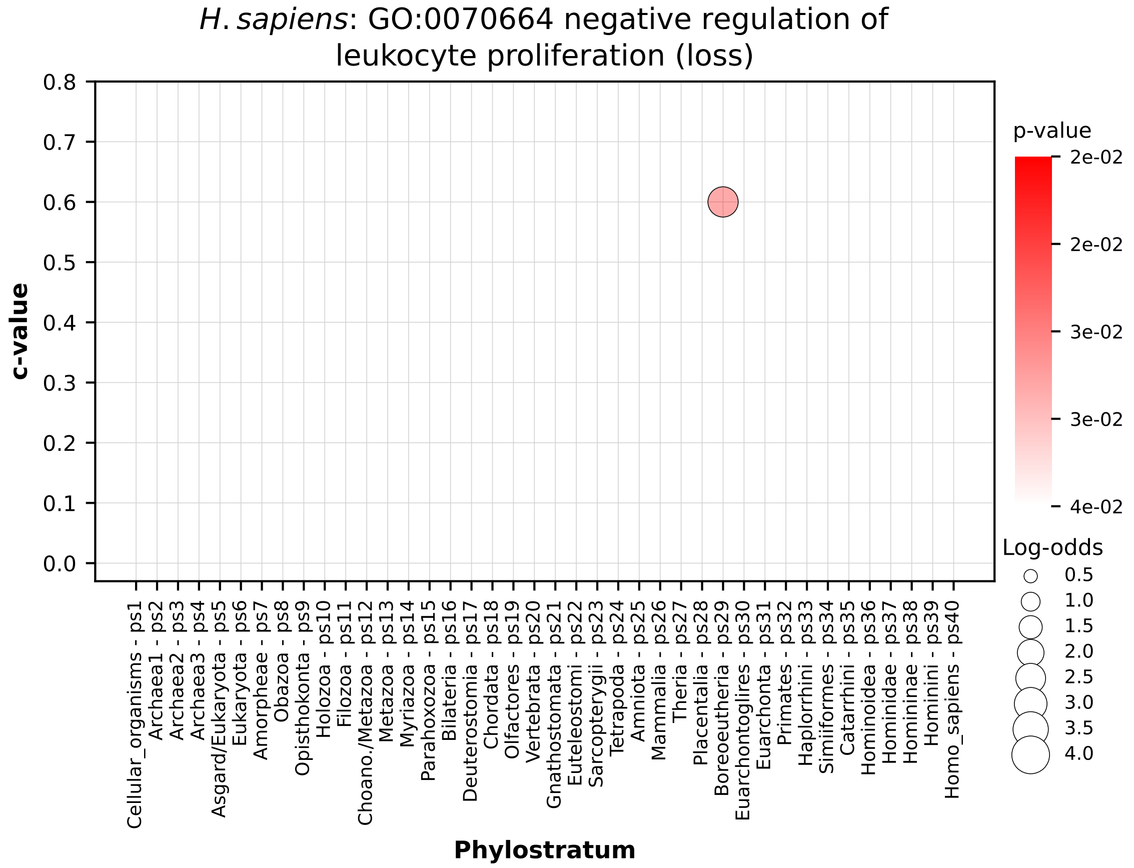

H. sapiens: GO:0070664 negative regulation of leukocyte proliferation (loss)
c-value
0.8
0.7
0.6
0.5
0.4
0.3
0.2
0.1
0.0
p-value
2e-02
2e-02
3e-02
3e-02
4e-02
Log-odds
0.5
1.0
1.5
2.0
2.5
3.0
3.5
4.0
Cellular_organisms - ps1
Archaea1 - ps2
Archaea2 - ps3
Archaea3 - ps4
Asgard/Eukaryota - ps5
Eukaryota - ps6
Amorpheae - ps7
Obazoa - ps8
Opisthokonta - ps9
Holozoa - ps10
Filozoa - ps11
Choano./Metazoa - ps12
Metazoa - ps13
Myriazoa - ps14
Parahoxozoa - ps15
Bilateria - ps16
Deuterostomia - ps17
Chordata - ps18
Olfactores - ps19
Vertebrata - ps20
Gnathostomata - ps21
Euteleostomi - ps22
Sarcopterygii - ps23
Tetrapoda - ps24
Amniota - ps25
Mammalia - ps26
Theria - ps27
Placentalia - ps28
Boreoeutheria - ps29
Euarchontoglires - ps30
Euarchonta - ps31
Primates - ps32
Haplorrhini - ps33
Simiiformes - ps34
Catarrhini - ps35
Hominoidea - ps36
Hominidae - ps37
Homininae - ps38
Hominini - ps39
Homo_sapiens - ps40
Phylostratum

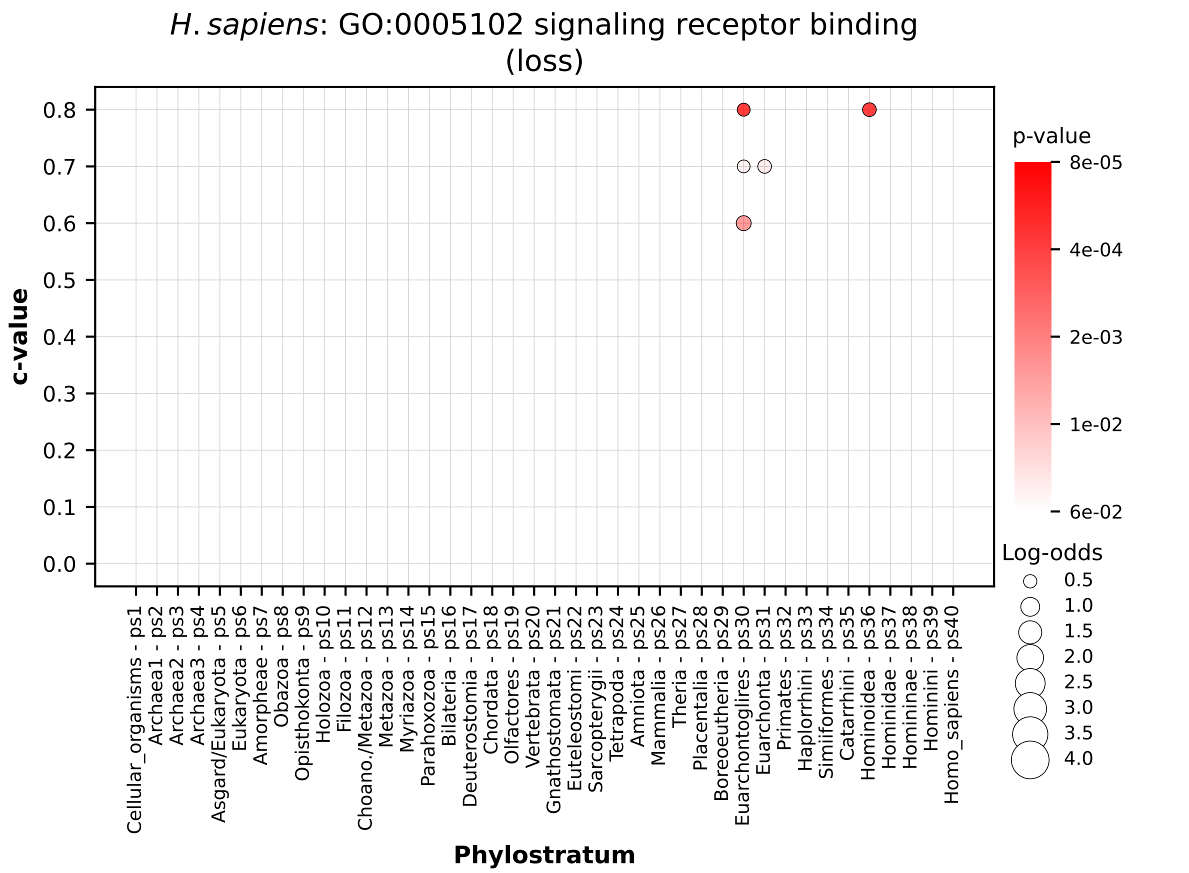

H. sapiens: GO:0005102 signaling receptor binding
(loss)
c-value
0.0
0.1
0.2
0.3
0.4
0.5
0.6
0.7
0.8
Phylostratum
Cellular_organisms - ps1
Archaea1 - ps2
Archaea2 - ps3
Archaea3 - ps4
Asgard/Eukaryota - ps5
Eukaryota - ps6
Amorpheae - ps7
Obazoa - ps8
Opisthokonta - ps9
Holozoa - ps10
Filozoa - ps11
Choano./Metazoa - ps12
Metazoa - ps13
Myriazoa - ps14
Parahoxozoa - ps15
Bilateria - ps16
Deuterostomia - ps17
Chordata - ps18
Olfactores - ps19
Vertebrata - ps20
Gnathostomata - ps21
Euteleostomi - ps22
Sarcopterygii - ps23
Tetrapoda - ps24
Amniota - ps25
Mammalia - ps26
Theria - ps27
Placentalia - ps28
Boreoeutheria - ps29
Euarchontoglires - ps30
Euarchonta - ps31
Primates - ps32
Haplorrhini - ps33
Simiiformes - ps34
Catarrhini - ps35
Hominoidea - ps36
Hominidae - ps37
Homininae - ps38
Hominini - ps39
Homo_sapiens - ps40
p-value
8e-05
4e-04
2e-03
1e-02
6e-02
Log-odds
0.5
1.0
1.5
2.0
2.5
3.0
3.5
4.0

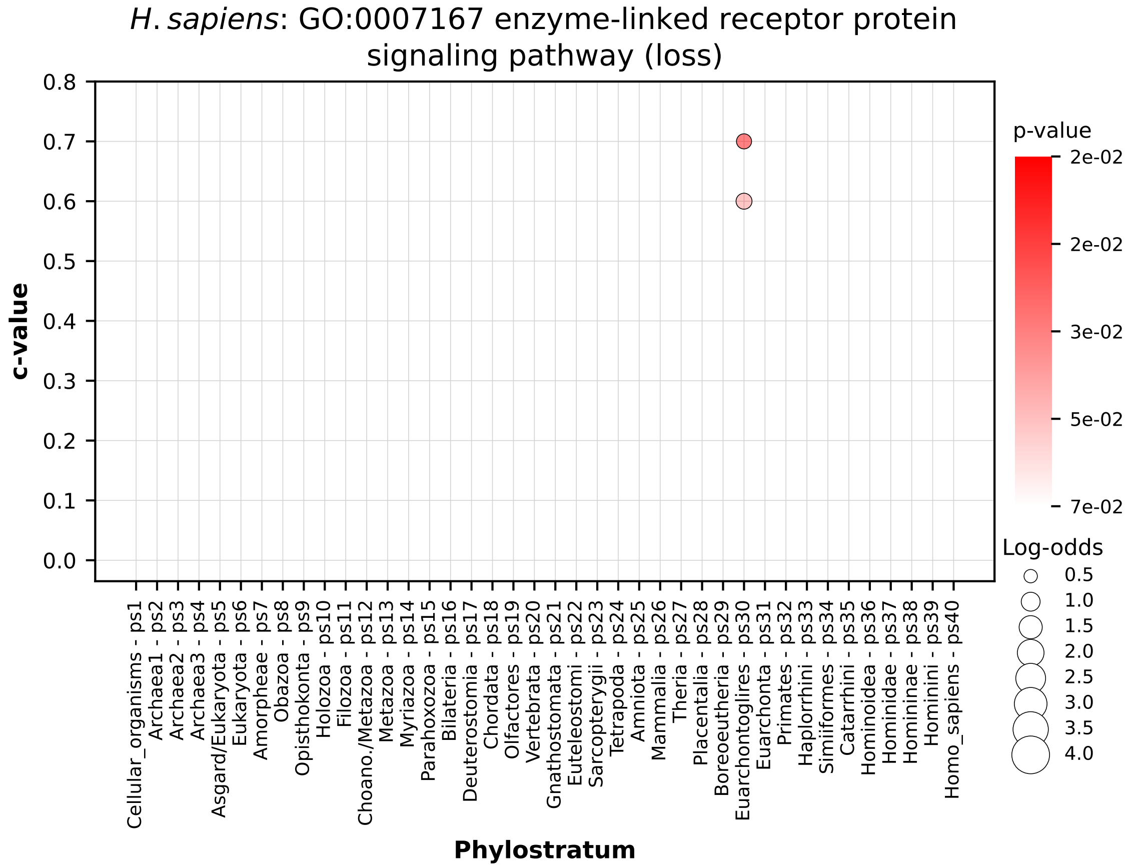
H. sapiens: GO:0007167 enzyme-linked receptor protein signaling pathway (loss)
c-value
0.8
0.7
0.6
0.5
0.4
0.3
0.2
0.1
0.0
p-value
2e-02
2e-02
3e-02
5e-02
7e-02
Log-odds
0.5
1.0
1.5
2.0
2.5
3.0
3.5
4.0
Cellular_organisms - ps1
Archaea1 - ps2
Archaea2 - ps3
Archaea3 - ps4
Asgard/Eukaryota - ps5
Eukaryota - ps6
Amorpheae - ps7
Obazoa - ps8
Opisthokonta - ps9
Holozoa - ps10
Filozoa - ps11
Choano./Metazoa - ps12
Metazoa - ps13
Myriazoa - ps14
Parahoxozoa - ps15
Bilateria - ps16
Deuterostomia - ps17
Chordata - ps18
Olfactores - ps19
Vertebrata - ps20
Gnathostomata - ps21
Euteleostomi - ps22
Sarcopterygii - ps23
Tetrapoda - ps24
Amniota - ps25
Mammalia - ps26
Theria - ps27
Placentalia - ps28
Boreoeutheria - ps29
Euarchontoglires - ps30
Euarchonta - ps31
Primates - ps32
Haplorrhini - ps33
Simiiformes - ps34
Catarrhini - ps35
Hominoidea - ps36
Hominidae - ps37
Homininae - ps38
Hominini - ps39
Homo_sapiens - ps40
Phylostratum

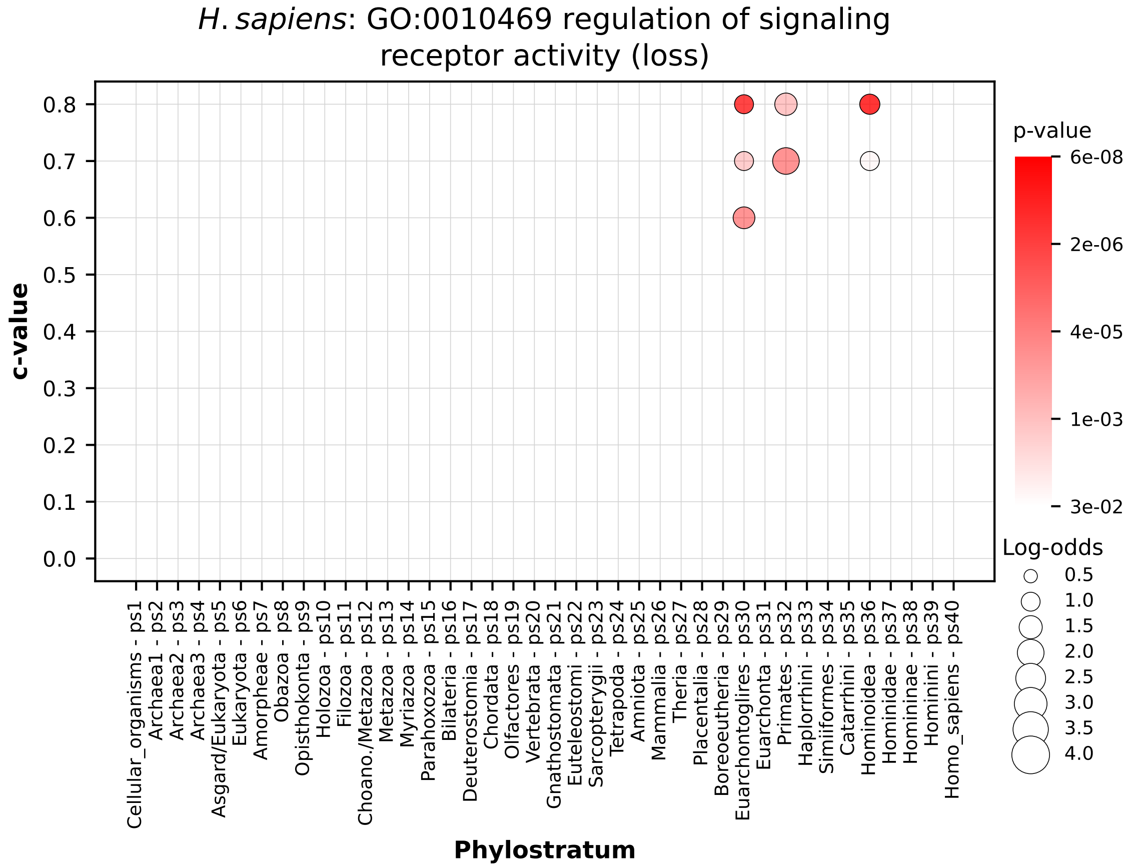
H. sapiens: GO:0010469 regulation of signaling receptor activity (loss)
c-value
0.0
0.1
0.2
0.3
0.4
0.5
0.6
0.7
0.8
Phylostratum
Cellular_organisms - ps1
Archaea1 - ps2
Archaea2 - ps3
Archaea3 - ps4
Asgard/Eukaryota - ps5
Eukaryota - ps6
Amorpheae - ps7
Obazoa - ps8
Opisthokonta - ps9
Holozoa - ps10
Filozoa - ps11
Choano./Metazoa - ps12
Metazoa - ps13
Myriazoa - ps14
Parahoxozoa - ps15
Bilateria - ps16
Deuterostomia - ps17
Chordata - ps18
Olfactores - ps19
Vertebrata - ps20
Gnathostomata - ps21
Euteleostomi - ps22
Sarcopterygii - ps23
Tetrapoda - ps24
Amniota - ps25
Mammalia - ps26
Theria - ps27
Placentalia - ps28
Boreoeutheria - ps29
Euarchontoglires - ps30
Euarchonta - ps31
Primates - ps32
Haplorrhini - ps33
Simiiformes - ps34
Catarrhini - ps35
Hominoidea - ps36
Hominidae - ps37
Homininae - ps38
Hominini - ps39
Homo_sapiens - ps40
p-value
6e-08
2e-06
4e-05
1e-03
3e-02
Log-odds
0.5
1.0
1.5
2.0
2.5
3.0
3.5
4.0

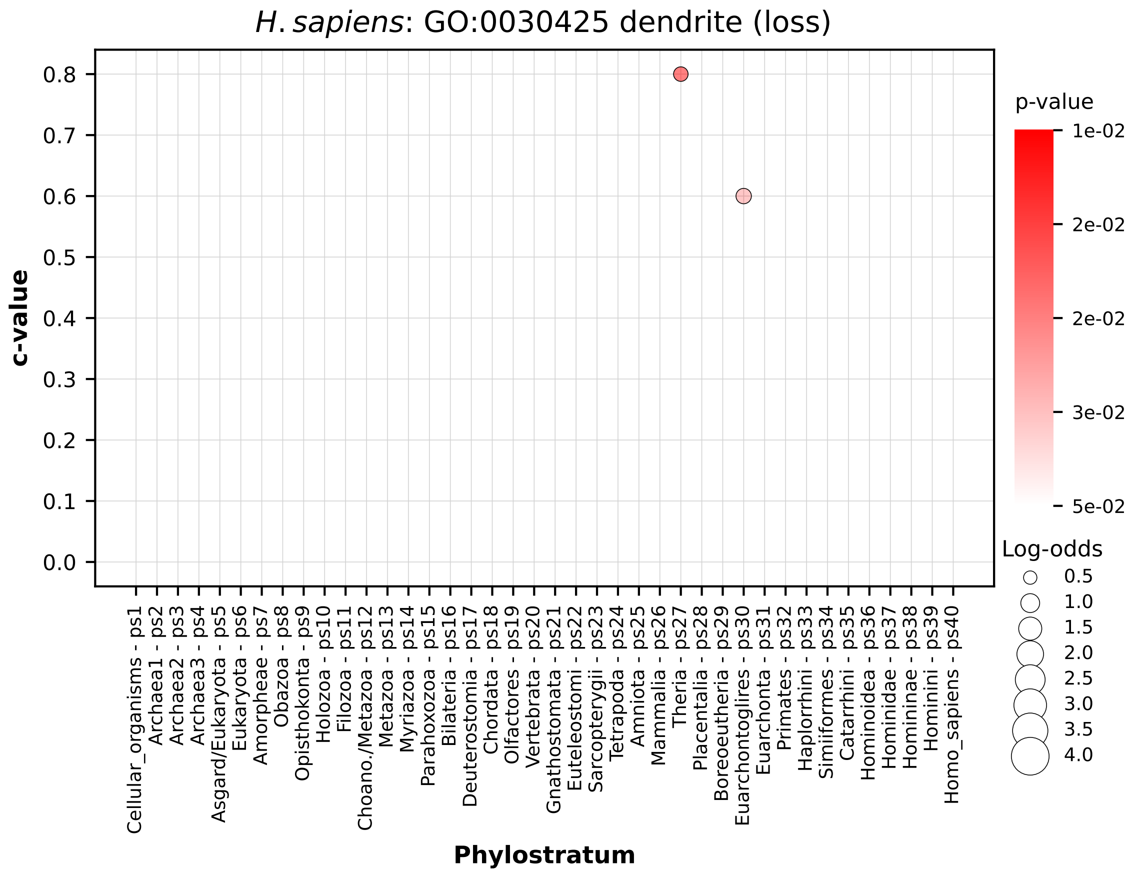

H. sapiens: GO:0030425 dendrite (loss)
c-value
0.0
0.1
0.2
0.3
0.4
0.5
0.6
0.7
0.8
p-value
1e-02
2e-02
2e-02
3e-02
5e-02
Log-odds
0.5
1.0
1.5
2.0
2.5
3.0
3.5
4.0
Cellular_organisms - ps1
Archaea1 - ps2
Archaea2 - ps3
Archaea3 - ps4
Asgard/Eukaryota - ps5
Eukaryota - ps6
Amorpheae - ps7
Obazoa - ps8
Opisthokonta - ps9
Holozoa - ps10
Filozoa - ps11
Choano./Metazoa - ps12
Metazoa - ps13
Myriazoa - ps14
Parahoxozoa - ps15
Bilateria - ps16
Deuterostomia - ps17
Chordata - ps18
Olfactores - ps19
Vertebrata - ps20
Gnathostomata - ps21
Euteleostomi - ps22
Sarcopterygii - ps23
Tetrapoda - ps24
Amniota - ps25
Mammalia - ps26
Theria - ps27
Placentalia - ps28
Boreoeutheria - ps29
Euarchontoglires - ps30
Euarchonta - ps31
Primates - ps32
Haplorrhini - ps33
Simiiformes - ps34
Catarrhini - ps35
Hominoidea - ps36
Hominidae - ps37
Homininae - ps38
Hominini - ps39
Homo_sapiens - ps40
Phylostratum

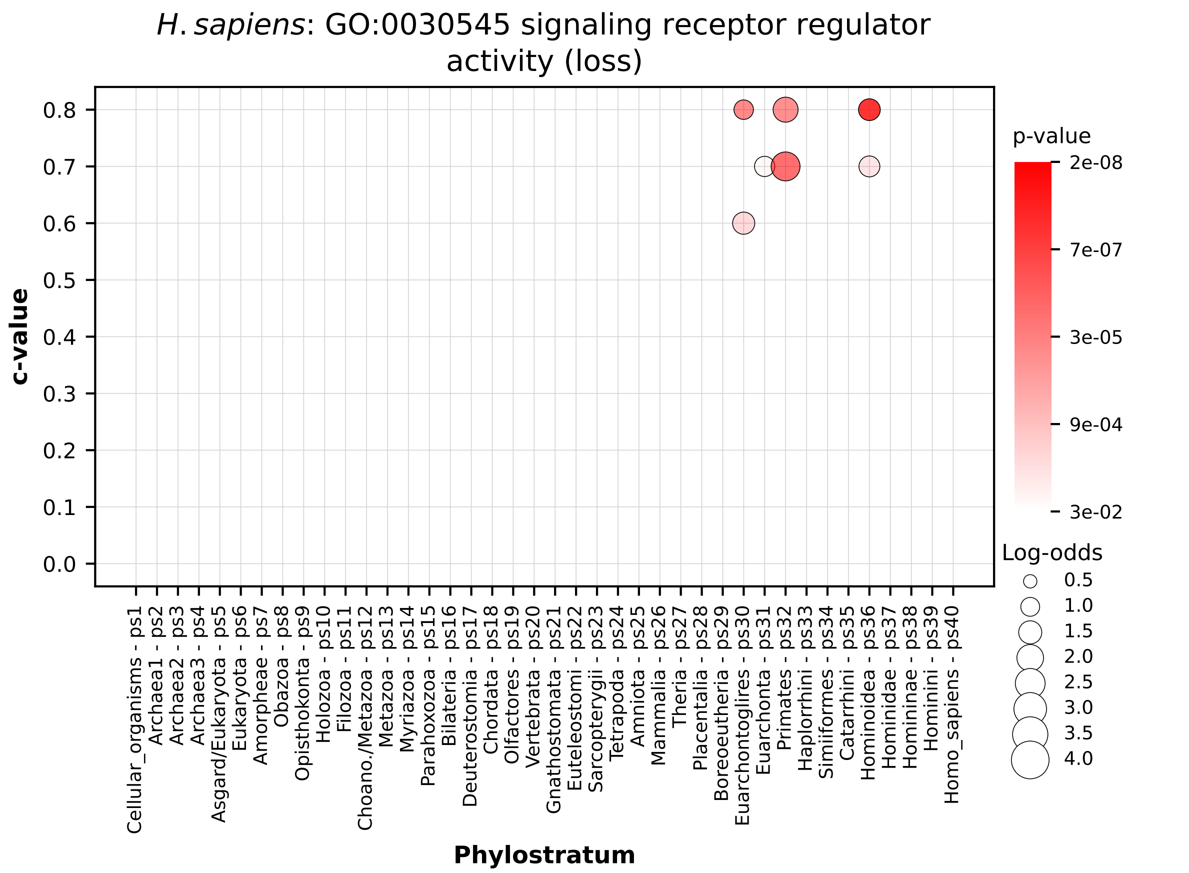

H. sapiens: GO:0030545 signaling receptor regulator activity (loss)
c-value
0.0
0.1
0.2
0.3
0.4
0.5
0.6
0.7
0.8
Phylostratum
Cellular_organisms - ps1
Archaea1 - ps2
Archaea2 - ps3
Archaea3 - ps4
Asgard/Eukaryota - ps5
Eukaryota - ps6
Amorpheae - ps7
Obazoa - ps8
Opisthokonta - ps9
Holozoa - ps10
Filozoa - ps11
Choano./Metazoa - ps12
Metazoa - ps13
Myriazoa - ps14
Parahoxozoa - ps15
Bilateria - ps16
Deuterostomia - ps17
Chordata - ps18
Olfactores - ps19
Vertebrata - ps20
Gnathostomata - ps21
Euteleostomi - ps22
Sarcopterygii - ps23
Tetrapoda - ps24
Amniota - ps25
Mammalia - ps26
Theria - ps27
Placentalia - ps28
Boreoeutheria - ps29
Euarchontoglires - ps30
Euarchonta - ps31
Primates - ps32
Haplorrhini - ps33
Simiiformes - ps34
Catarrhini - ps35
Hominoidea - ps36
Hominidae - ps37
Homininae - ps38
Hominini - ps39
Homo_sapiens - ps40
p-value
2e-08
7e-07
3e-05
9e-04
3e-02
Log-odds
0.5
1.0
1.5
2.0
2.5
3.0
3.5
4.0

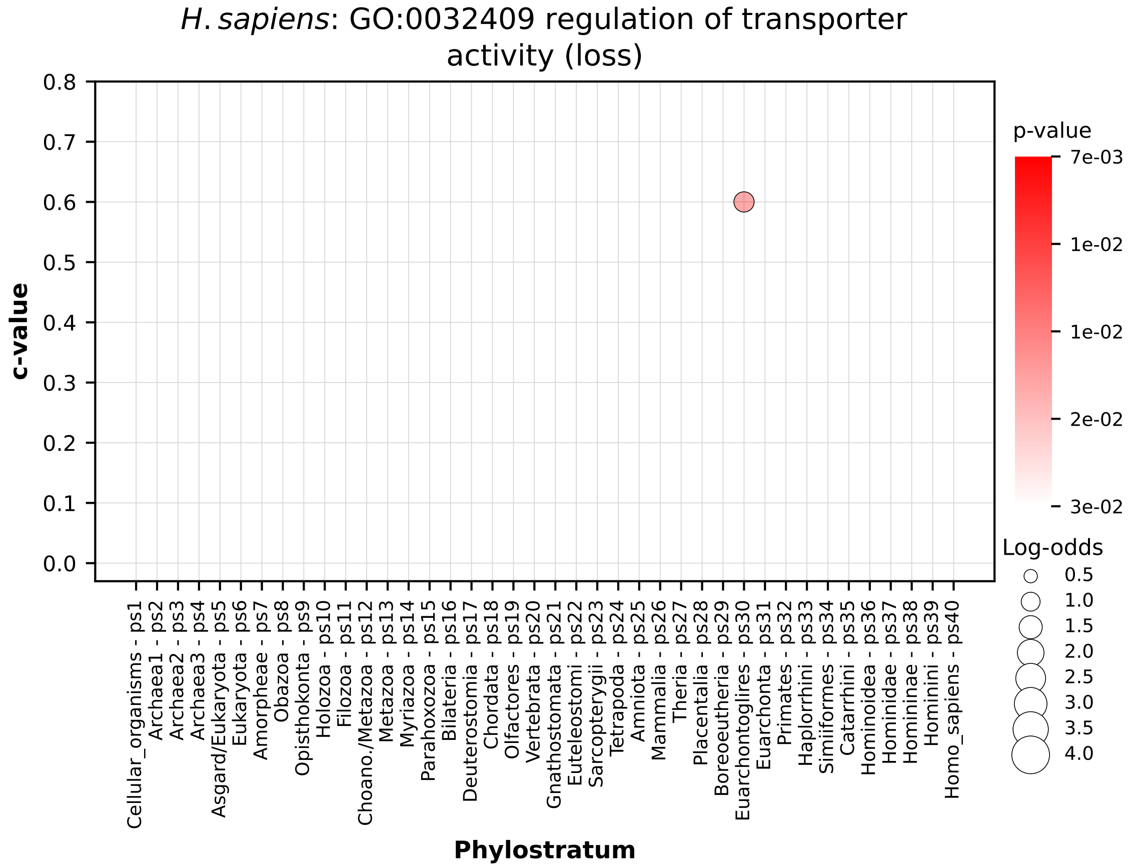
H. sapiens: GO:0032409 regulation of transporter activity (loss)
c-value
0.8
0.7
0.6
0.5
0.4
0.3
0.2
0.1
0.0
Phylostratum
Cellular_organisms - ps1
Archaea1 - ps2
Archaea2 - ps3
Archaea3 - ps4
Asgard/Eukaryota - ps5
Eukaryota - ps6
Amorpheae - ps7
Obazoa - ps8
Opisthokonta - ps9
Holozoa - ps10
Filozoa - ps11
Choano./Metazoa - ps12
Metazoa - ps13
Myriazoa - ps14
Parahoxozoa - ps15
Bilateria - ps16
Deuterostomia - ps17
Chordata - ps18
Olfactores - ps19
Vertebrata - ps20
Gnathostomata - ps21
Euteleostomi - ps22
Sarcopterygii - ps23
Tetrapoda - ps24
Amniota - ps25
Mammalia - ps26
Theria - ps27
Placentalia - ps28
Boreoeutheria - ps29
Euarchontoglires - ps30
Euarchonta - ps31
Primates - ps32
Haplorrhini - ps33
Simiiformes - ps34
Catarrhini - ps35
Hominoidea - ps36
Hominidae - ps37
Homininae - ps38
Hominini - ps39
Homo_sapiens - ps40
p-value
7e-03
1e-02
1e-02
2e-02
3e-02
Log-odds
0.5
1.0
1.5
2.0
2.5
3.0
3.5
4.0

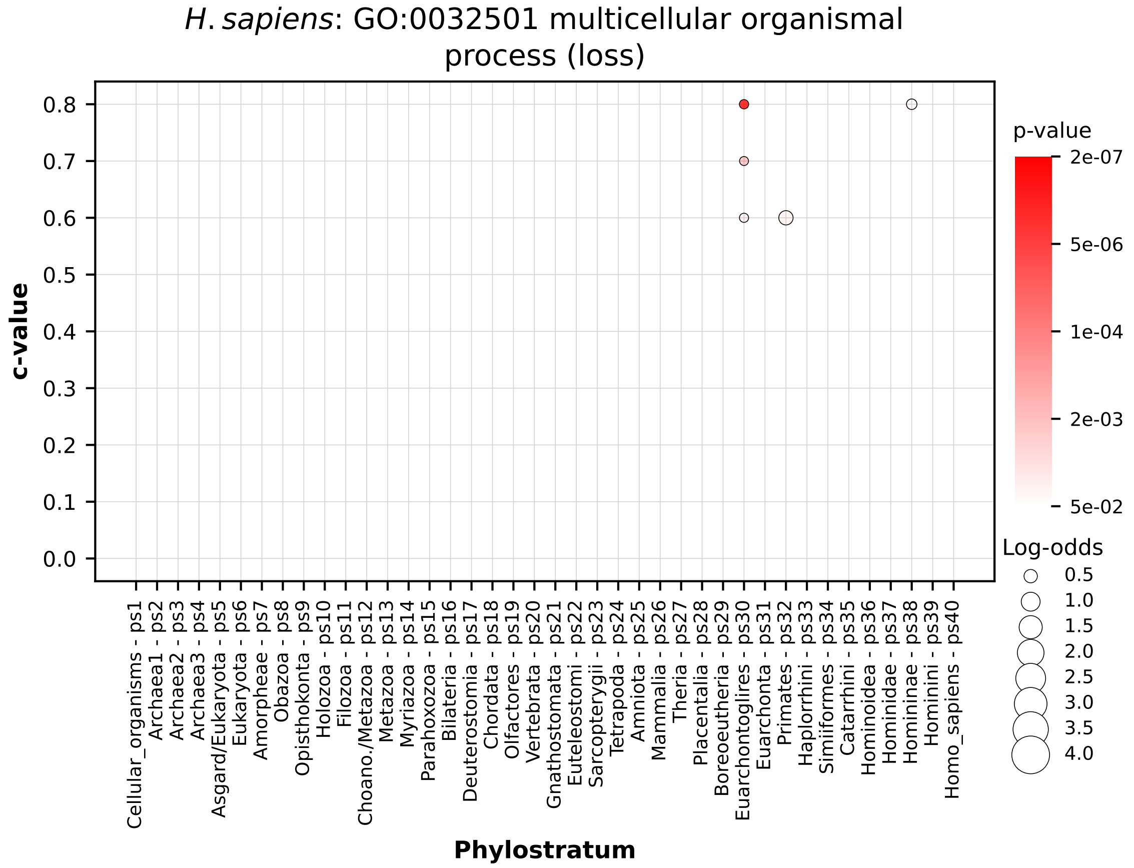
H. sapiens: GO:0032501 multicellular organismal process (loss)
c-value
0.8
0.7
0.6
0.5
0.4
0.3
0.2
0.1
0.0
p-value
2e-07
5e-06
1e-04
2e-03
5e-02
Log-odds
0.5
1.0
1.5
2.0
2.5
3.0
3.5
4.0
Cellular_organisms - ps1
Archaea1 - ps2
Archaea2 - ps3
Archaea3 - ps4
Asgard/Eukaryota - ps5
Eukaryota - ps6
Amorpheae - ps7
Obazoa - ps8
Opisthokonta - ps9
Holozoa - ps10
Filozoa - ps11
Choano./Metazoa - ps12
Metazoa - ps13
Myriazoa - ps14
Parahoxozoa - ps15
Bilateria - ps16
Deuterostomia - ps17
Chordata - ps18
Olfactores - ps19
Vertebrata - ps20
Gnathostomata - ps21
Euteleostomi - ps22
Sarcopterygii - ps23
Tetrapoda - ps24
Amniota - ps25
Mammalia - ps26
Theria - ps27
Placentalia - ps28
Boreoeutheria - ps29
Euarchontoglires - ps30
Euarchonta - ps31
Primates - ps32
Haplorrhini - ps33
Simiiformes - ps34
Catarrhini - ps35
Hominoidea - ps36
Hominidae - ps37
Homininae - ps38
Hominini - ps39
Homo_sapiens - ps40
Phylostratum

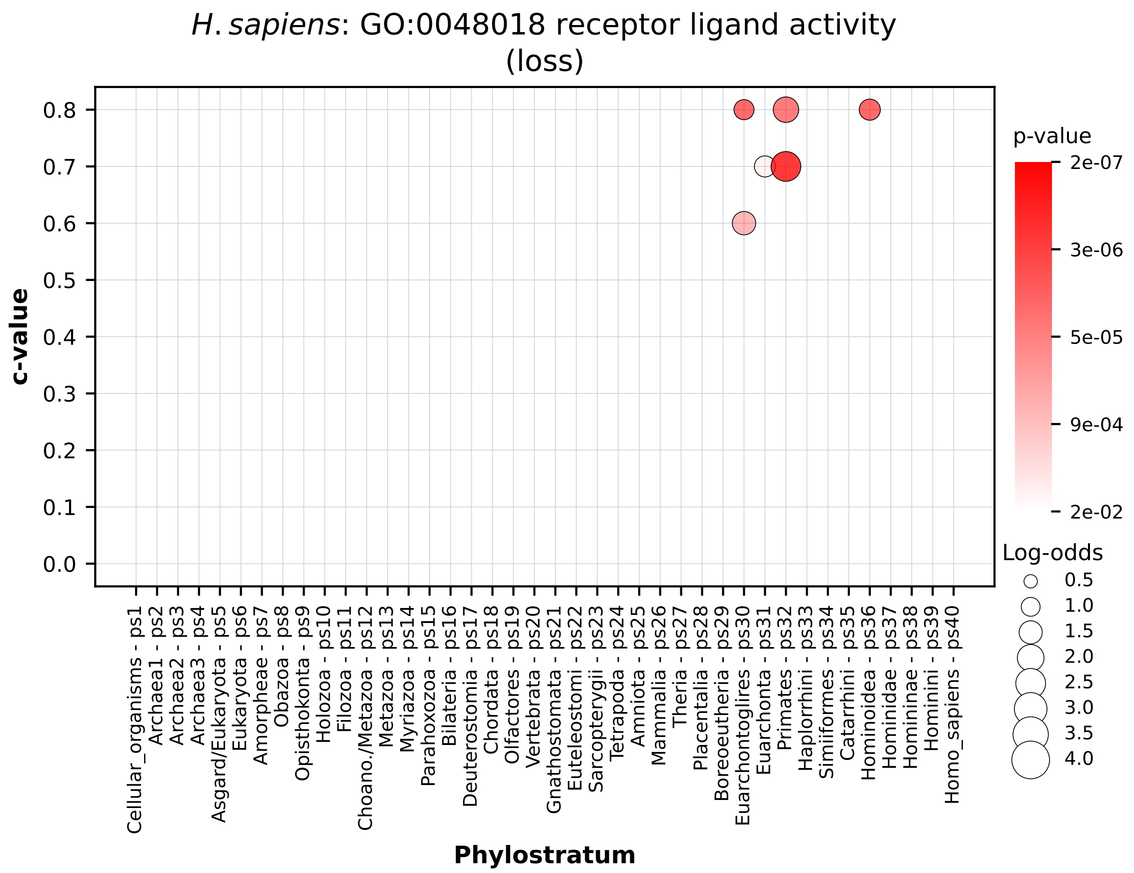

H. sapiens: GO:0048018 receptor ligand activity
(loss)
c-value
0.8
0.7
0.6
0.5
0.4
0.3
0.2
0.1
0.0
Phylostratum
Cellular_organisms - ps1
Archaea1 - ps2
Archaea2 - ps3
Archaea3 - ps4
Asgard/Eukaryota - ps5
Eukaryota - ps6
Amorpheae - ps7
Obazoa - ps8
Opisthokonta - ps9
Holozoa - ps10
Filozoa - ps11
Choano./Metazoa - ps12
Metazoa - ps13
Myriazoa - ps14
Parahoxozoa - ps15
Bilateria - ps16
Deuterostomia - ps17
Chordata - ps18
Olfactores - ps19
Vertebrata - ps20
Gnathostomata - ps21
Euteleostomi - ps22
Sarcopterygii - ps23
Tetrapoda - ps24
Amniota - ps25
Mammalia - ps26
Theria - ps27
Placentalia - ps28
Boreoeutheria - ps29
Euarchontoglires - ps30
Euarchonta - ps31
Primates - ps32
Haplorrhini - ps33
Simiiformes - ps34
Catarrhini - ps35
Hominoidea - ps36
Hominidae - ps37
Homininae - ps38
Hominini - ps39
Homo_sapiens - ps40
p-value
2e-07
3e-06
5e-05
9e-04
2e-02
Log-odds
0.5
1.0
1.5
2.0
2.5
3.0
3.5
4.0

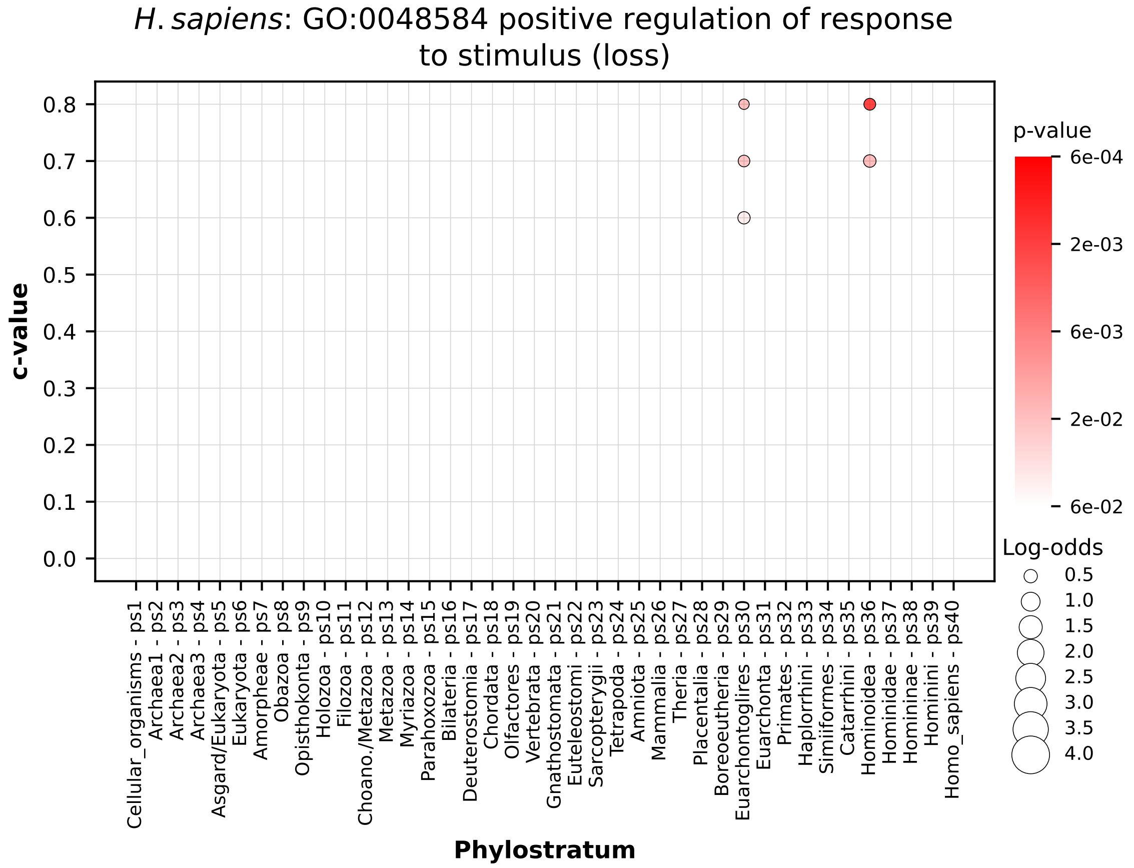

H. sapiens: GO:0048584 positive regulation of response to stimulus (loss)
c-value
0.0
0.1
0.2
0.3
0.4
0.5
0.6
0.7
0.8
Phylostratum
Cellular_organisms - ps1
Archaea1 - ps2
Archaea2 - ps3
Archaea3 - ps4
Asgard/Eukaryota - ps5
Eukaryota - ps6
Amorpheae - ps7
Obazoa - ps8
Opisthokonta - ps9
Holozoa - ps10
Filozoa - ps11
Choano./Metazoa - ps12
Metazoa - ps13
Myriazoa - ps14
Parahoxozoa - ps15
Bilateria - ps16
Deuterostomia - ps17
Chordata - ps18
Olfactores - ps19
Vertebrata - ps20
Gnathostomata - ps21
Euteleostomi - ps22
Sarcopterygii - ps23
Tetrapoda - ps24
Amniota - ps25
Mammalia - ps26
Theria - ps27
Placentalia - ps28
Boreoeutheria - ps29
Euarchontoglires - ps30
Euarchonta - ps31
Primates - ps32
Haplorrhini - ps33
Simiiformes - ps34
Catarrhini - ps35
Hominoidea - ps36
Hominidae - ps37
Homininae - ps38
Hominini - ps39
Homo_sapiens - ps40
p-value
6e-04
2e-03
6e-03
2e-02
6e-02
Log-odds
0.5
1.0
1.5
2.0
2.5
3.0
3.5
4.0

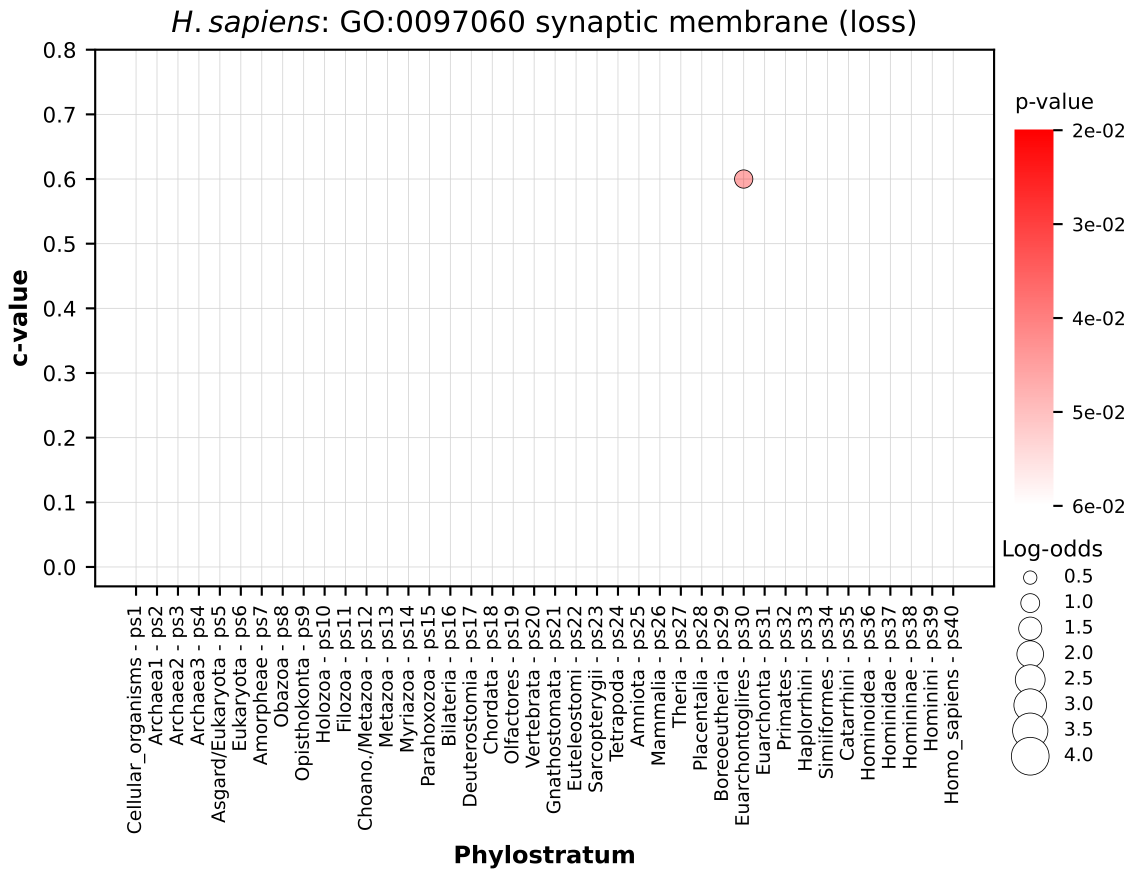

H. sapiens: GO:0097060 synaptic membrane (loss)
c-value
0.0
0.1
0.2
0.3
0.4
0.5
0.6
0.7
0.8
Phylostratum
Cellular_organisms - ps1
Archaea1 - ps2
Archaea2 - ps3
Archaea3 - ps4
Asgard/Eukaryota - ps5
Eukaryota - ps6
Amorpheae - ps7
Obazoa - ps8
Opisthokonta - ps9
Holozoa - ps10
Filozoa - ps11
Choano./Metazoa - ps12
Metazoa - ps13
Myriazoa - ps14
Parahoxozoa - ps15
Bilateria - ps16
Deuterostomia - ps17
Chordata - ps18
Olfactores - ps19
Vertebrata - ps20
Gnathostomata - ps21
Euteleostomi - ps22
Sarcopterygii - ps23
Tetrapoda - ps24
Amniota - ps25
Mammalia - ps26
Theria - ps27
Placentalia - ps28
Boreoeutheria - ps29
Euarchontoglires - ps30
Euarchonta - ps31
Primates - ps32
Haplorrhini - ps33
Simiiformes - ps34
Catarrhini - ps35
Hominoidea - ps36
Hominidae - ps37
Homininae - ps38
Hominini - ps39
Homo_sapiens - ps40
p-value
2e-02
3e-02
4e-02
5e-02
6e-02
Log-odds
0.5
1.0
1.5
2.0
2.5
3.0
3.5
4.0

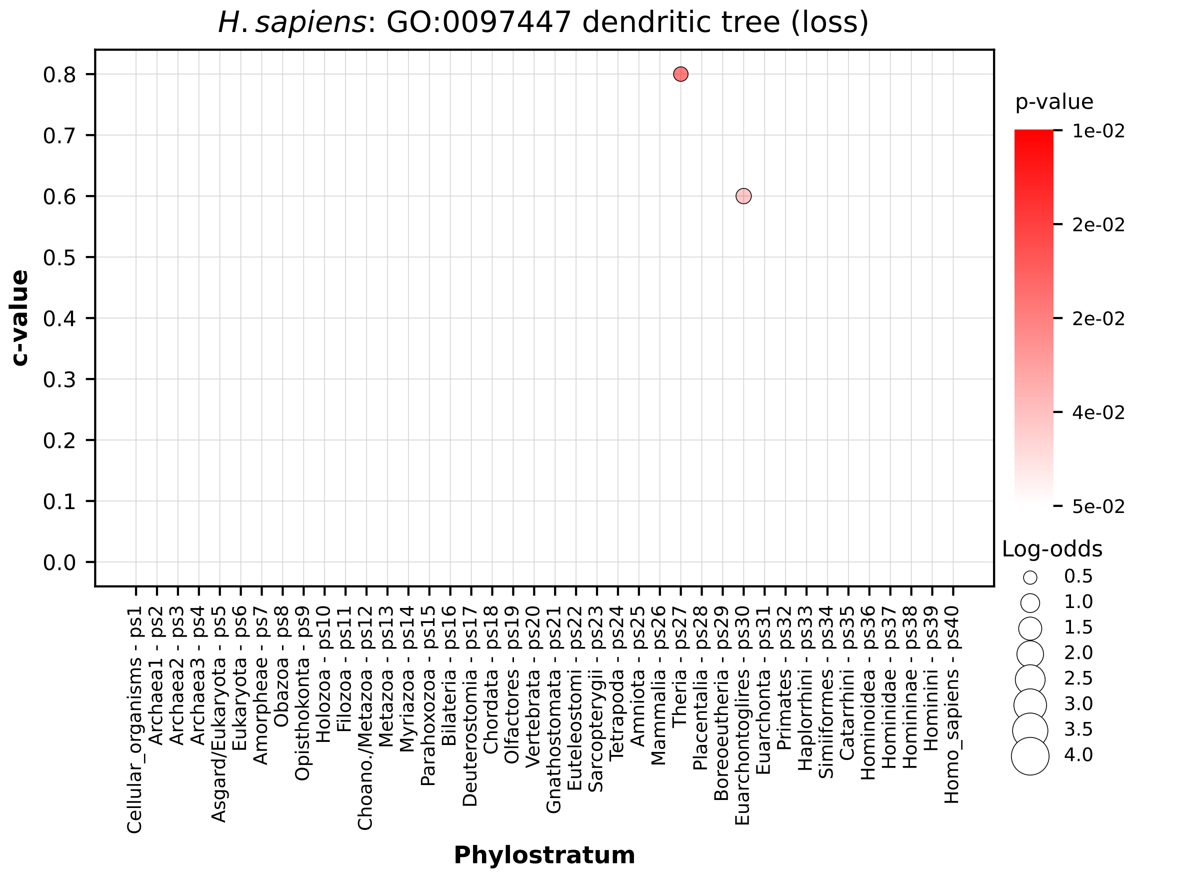
H. sapiens: GO:0097447 dendritic tree (loss)
c-value
0.0
0.1
0.2
0.3
0.4
0.5
0.6
0.7
0.8
Phylostratum
Cellular_organisms - ps1
Archaea1 - ps2
Archaea2 - ps3
Archaea3 - ps4
Asgard/Eukaryota - ps5
Eukaryota - ps6
Amorpheae - ps7
Obazoa - ps8
Opisthokonta - ps9
Holozoa - ps10
Filozoa - ps11
Choano./Metazoa - ps12
Metazoa - ps13
Myriazoa - ps14
Parahoxozoa - ps15
Bilateria - ps16
Deuterostomia - ps17
Chordata - ps18
Olfactores - ps19
Vertebrata - ps20
Gnathostomata - ps21
Euteleostomi - ps22
Sarcopterygii - ps23
Tetrapoda - ps24
Amniota - ps25
Mammalia - ps26
Theria - ps27
Placentalia - ps28
Boreoeutheria - ps29
Euarchontoglires - ps30
Euarchonta - ps31
Primates - ps32
Haplorrhini - ps33
Simiiformes - ps34
Catarrhini - ps35
Hominoidea - ps36
Hominidae - ps37
Homininae - ps38
Hominini - ps39
Homo_sapiens - ps40
p-value
1e-02
2e-02
2e-02
4e-02
5e-02
Log-odds
0.5
1.0
1.5
2.0
2.5
3.0
3.5
4.0

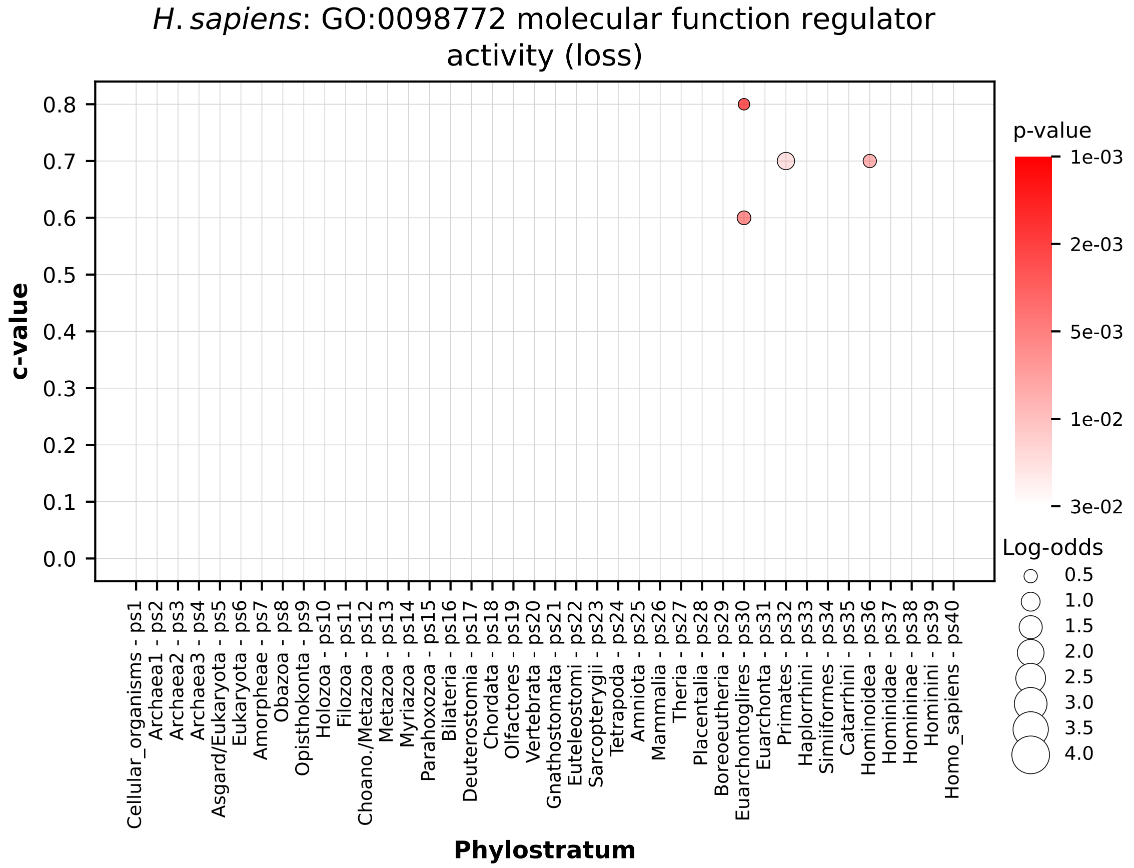

H. sapiens: GO:0098772 molecular function regulator activity (loss)
c-value
0.0
0.1
0.2
0.3
0.4
0.5
0.6
0.7
0.8
Phylostratum
Cellular_organisms - ps1
Archaea1 - ps2
Archaea2 - ps3
Archaea3 - ps4
Asgard/Eukaryota - ps5
Eukaryota - ps6
Amorpheae - ps7
Obazoa - ps8
Opisthokonta - ps9
Holozoa - ps10
Filozoa - ps11
Choano./Metazoa - ps12
Metazoa - ps13
Myriazoa - ps14
Parahoxozoa - ps15
Bilateria - ps16
Deuterostomia - ps17
Chordata - ps18
Olfactores - ps19
Vertebrata - ps20
Gnathostomata - ps21
Euteleostomi - ps22
Sarcopterygii - ps23
Tetrapoda - ps24
Amniota - ps25
Mammalia - ps26
Theria - ps27
Placentalia - ps28
Boreoeutheria - ps29
Euarchontoglires - ps30
Euarchonta - ps31
Primates - ps32
Haplorrhini - ps33
Simiiformes - ps34
Catarrhini - ps35
Hominoidea - ps36
Hominidae - ps37
Homininae - ps38
Hominini - ps39
Homo_sapiens - ps40
p-value
1e-03
2e-03
5e-03
1e-02
3e-02
Log-odds
0.5
1.0
1.5
2.0
2.5
3.0
3.5
4.0

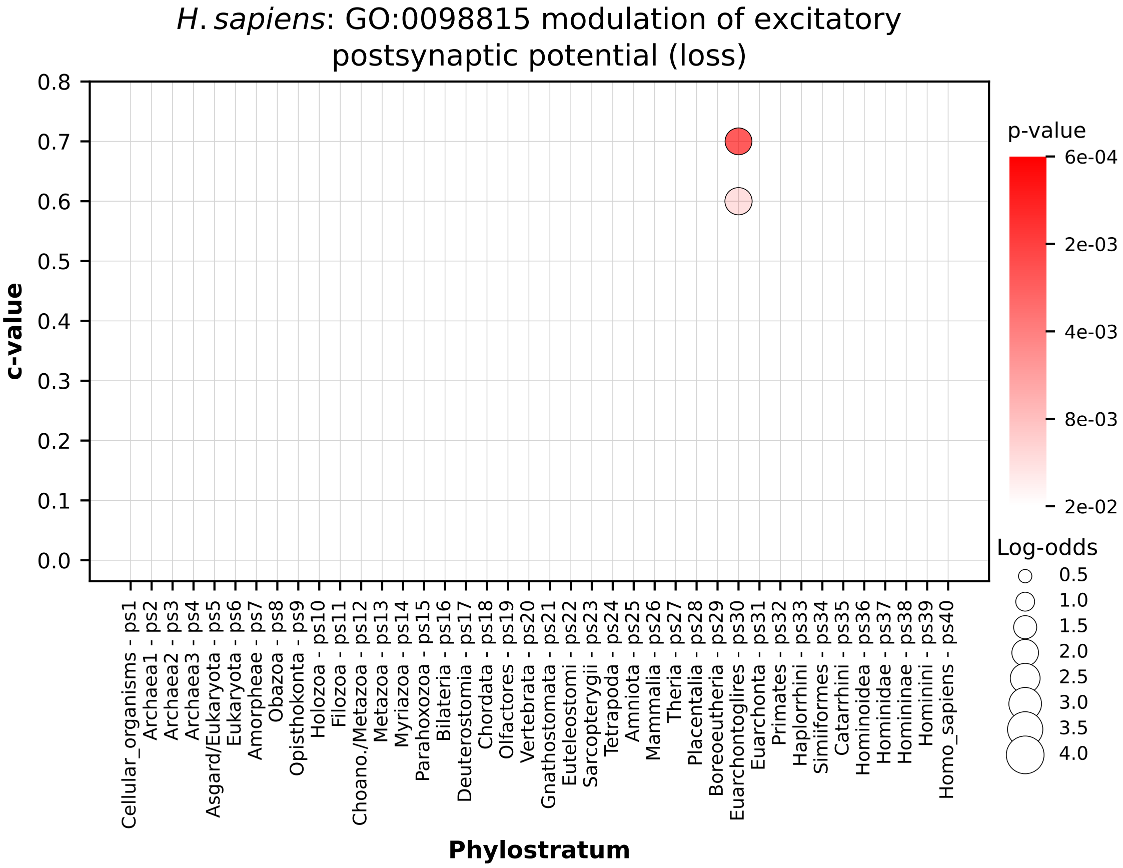

H. sapiens: GO:0098815 modulation of excitatory postsynaptic potential (loss)
c-value
0.8
0.7
0.6
0.5
0.4
0.3
0.2
0.1
0.0
p-value
6e-04
2e-03
4e-03
8e-03
2e-02
Log-odds
0.5
1.0
1.5
2.0
2.5
3.0
3.5
4.0
Cellular_organisms - ps1
Archaea1 - ps2
Archaea2 - ps3
Archaea3 - ps4
Asgard/Eukaryota - ps5
Eukaryota - ps6
Amorpheae - ps7
Obazoa - ps8
Opisthokonta - ps9
Holozoa - ps10
Filozoa - ps11
Choano./Metazoa - ps12
Metazoa - ps13
Myriazoa - ps14
Parahoxozoa - ps15
Bilateria - ps16
Deuterostomia - ps17
Chordata - ps18
Olfactores - ps19
Vertebrata - ps20
Gnathostomata - ps21
Euteleostomi - ps22
Sarcopterygii - ps23
Tetrapoda - ps24
Amniota - ps25
Mammalia - ps26
Theria - ps27
Placentalia - ps28
Boreoeutheria - ps29
Euarchontoglires - ps30
Euarchonta - ps31
Primates - ps32
Haplorrhini - ps33
Simiiformes - ps34
Catarrhini - ps35
Hominoidea - ps36
Hominidae - ps37
Homininae - ps38
Hominini - ps39
Homo_sapiens - ps40
Phylostratum

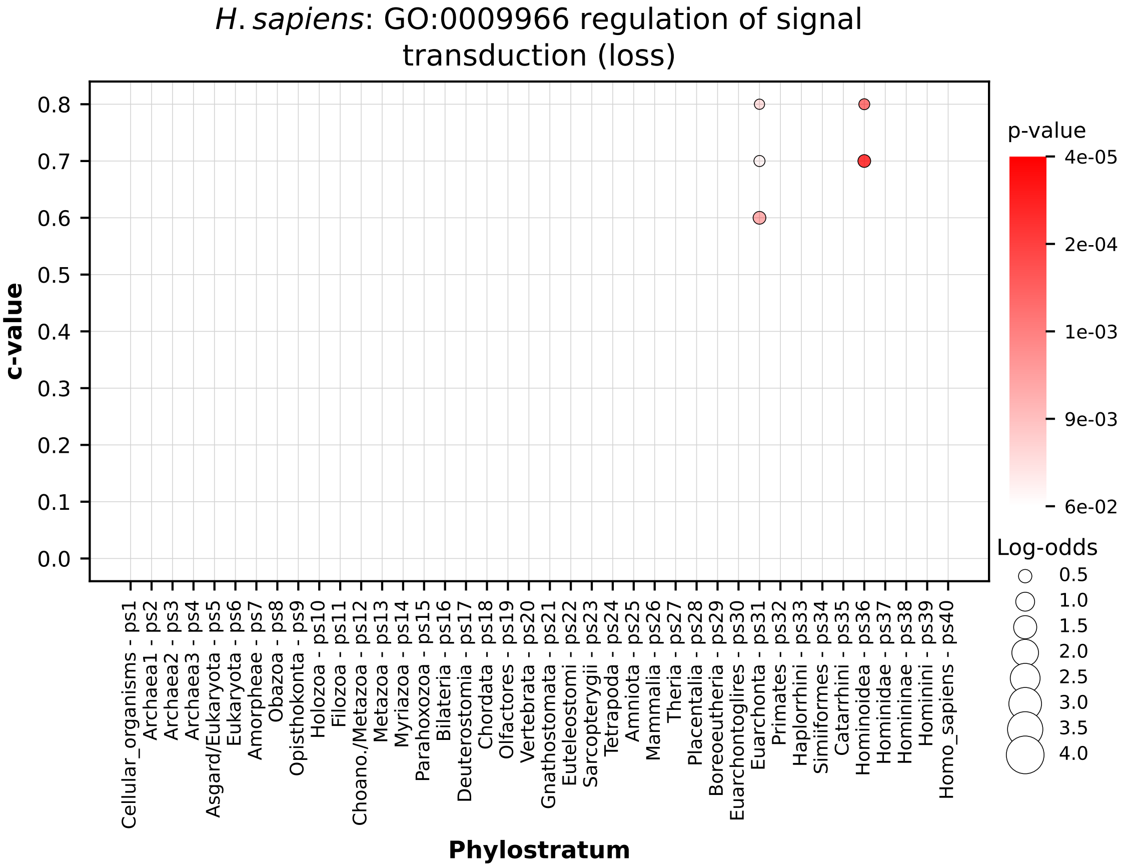

H. sapiens: GO:0009966 regulation of signal transduction (loss)
c-value
0.0
0.1
0.2
0.3
0.4
0.5
0.6
0.7
0.8
Phylostratum
Cellular_organisms - ps1
Archaea1 - ps2
Archaea2 - ps3
Archaea3 - ps4
Asgard/Eukaryota - ps5
Eukaryota - ps6
Amorpheae - ps7
Obazoa - ps8
Opisthokonta - ps9
Holozoa - ps10
Filozoa - ps11
Choano./Metazoa - ps12
Metazoa - ps13
Myriazoa - ps14
Parahoxozoa - ps15
Bilateria - ps16
Deuterostomia - ps17
Chordata - ps18
Olfactores - ps19
Vertebrata - ps20
Gnathostomata - ps21
Euteleostomi - ps22
Sarcopterygii - ps23
Tetrapoda - ps24
Amniota - ps25
Mammalia - ps26
Theria - ps27
Placentalia - ps28
Boreoeutheria - ps29
Euarchontoglires - ps30
Euarchonta - ps31
Primates - ps32
Haplorrhini - ps33
Simiiformes - ps34
Catarrhini - ps35
Hominoidea - ps36
Hominidae - ps37
Homininae - ps38
Hominini - ps39
Homo_sapiens - ps40
p-value
4e-05
2e-04
1e-03
9e-03
6e-02
Log-odds
0.5
1.0
1.5
2.0
2.5
3.0
3.5
4.0

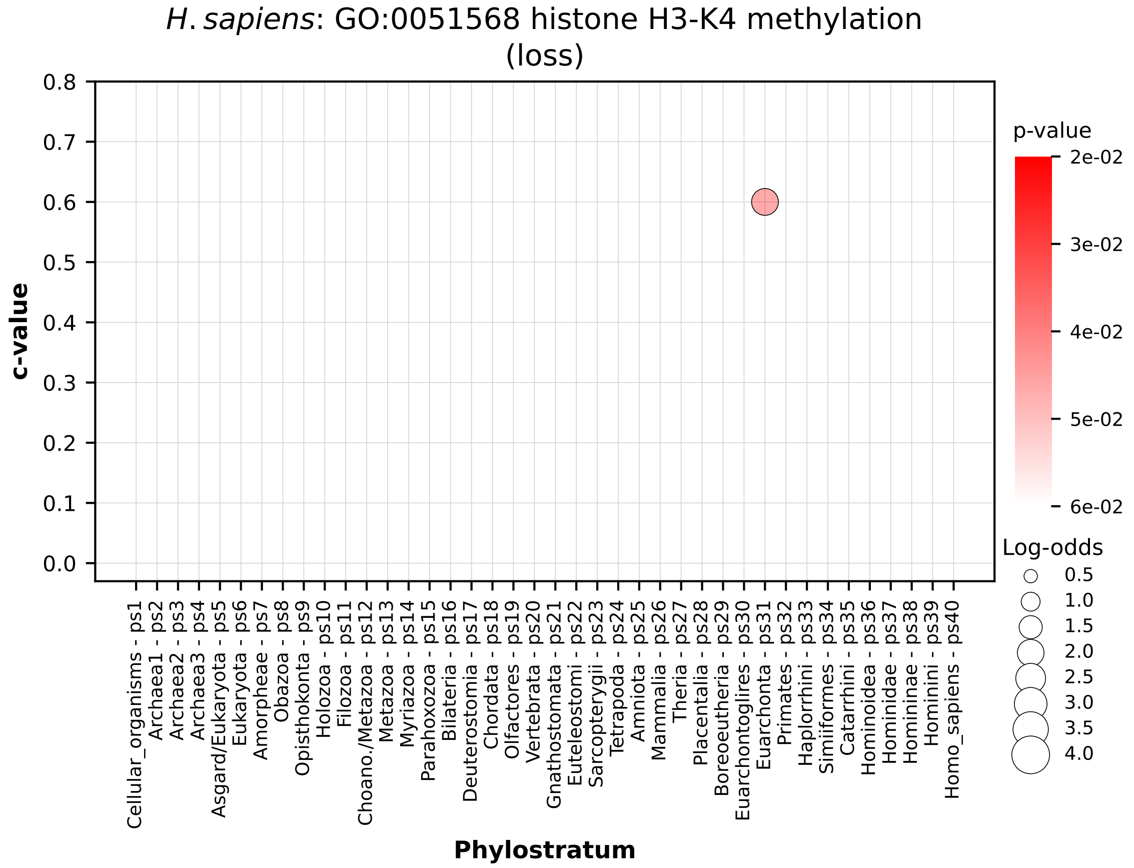

H. sapiens: GO:0051568 histone H3-K4 methylation (loss)
c-value
0.8
0.7
0.6
0.5
0.4
0.3
0.2
0.1
0.0
Cellular_organisms - ps1
Archaea1 - ps2
Archaea2 - ps3
Archaea3 - ps4
Asgard/Eukaryota - ps5
Eukaryota - ps6
Amorpheae - ps7
Obazoa - ps8
Opisthokonta - ps9
Holozoa - ps10
Filozoa - ps11
Choano./Metazoa - ps12
Metazoa - ps13
Myriazoa - ps14
Parahoxozoa - ps15
Bilateria - ps16
Deuterostomia - ps17
Chordata - ps18
Olfactores - ps19
Vertebrata - ps20
Gnathostomata - ps21
Euteleostomi - ps22
Sarcopterygii - ps23
Tetrapoda - ps24
Amniota - ps25
Mammalia - ps26
Theria - ps27
Placentalia - ps28
Boreoeutheria - ps29
Euarchontoglires - ps30
Euarchonta - ps31
Primates - ps32
Haplorrhini - ps33
Simiiformes - ps34
Catarrhini - ps35
Hominoidea - ps36
Hominidae - ps37
Homininae - ps38
Hominini - ps39
Homo_sapiens - ps40
Phylostratum
p-value
2e-02
3e-02
4e-02
5e-02
6e-02
Log-odds
0.5
1.0
1.5
2.0
2.5
3.0
3.5
4.0

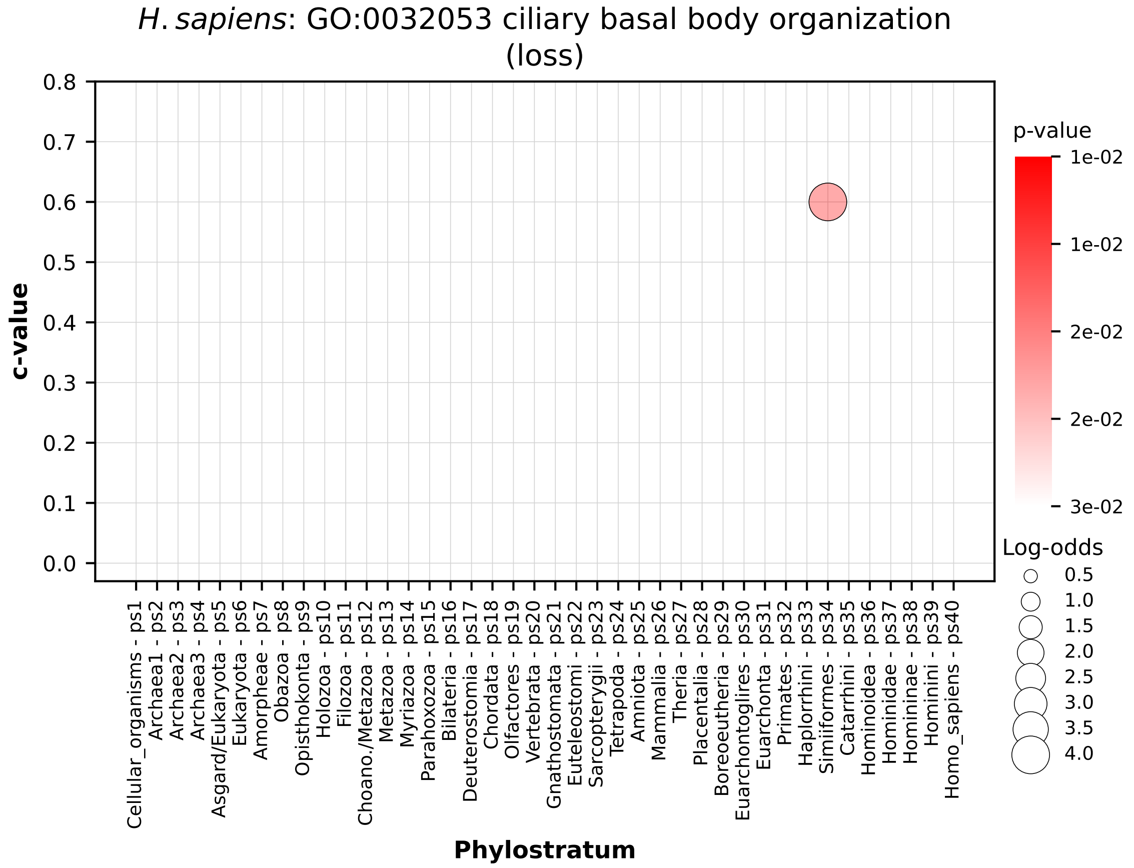

H. sapiens: GO:0032053 ciliary basal body organization
(loss)
c-value
0.8
0.7
0.6
0.5
0.4
0.3
0.2
0.1
0.0
p-value
1e-02
1e-02
2e-02
2e-02
3e-02
Log-odds
0.5
1.0
1.5
2.0
2.5
3.0
3.5
4.0
Cellular_organisms - ps1
Archaea1 - ps2
Archaea2 - ps3
Archaea3 - ps4
Asgard/Eukaryota - ps5
Eukaryota - ps6
Amorpheae - ps7
Obazoa - ps8
Opisthokonta - ps9
Holozoa - ps10
Filozoa - ps11
Choano./Metazoa - ps12
Metazoa - ps13
Myriazoa - ps14
Parahoxozoa - ps15
Bilateria - ps16
Deuterostomia - ps17
Chordata - ps18
Olfactores - ps19
Vertebrata - ps20
Gnathostomata - ps21
Euteleostomi - ps22
Sarcopterygii - ps23
Tetrapoda - ps24
Amniota - ps25
Mammalia - ps26
Theria - ps27
Placentalia - ps28
Boreoeutheria - ps29
Euarchontoglires - ps30
Euarchonta - ps31
Primates - ps32
Haplorrhini - ps33
Simiiformes - ps34
Catarrhini - ps35
Hominoidea - ps36
Hominidae - ps37
Homininae - ps38
Hominini - ps39
Homo_sapiens - ps40
Phylostratum

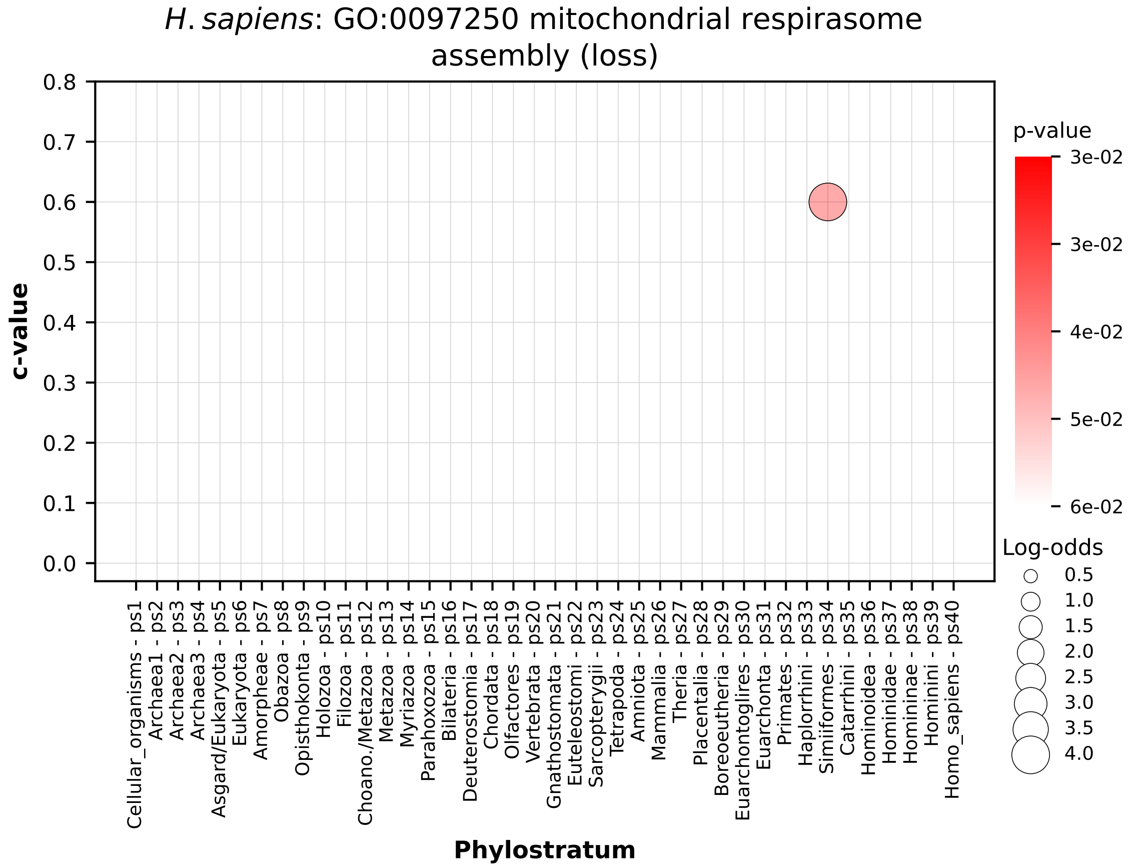

H. sapiens: GO:0097250 mitochondrial respirasome assembly (loss)
c-value
0.8
0.7
0.6
0.5
0.4
0.3
0.2
0.1
0.0
p-value
3e-02
3e-02
4e-02
5e-02
6e-02
Log-odds
0.5
1.0
1.5
2.0
2.5
3.0
3.5
4.0
Cellular_organisms - ps1
Archaea1 - ps2
Archaea2 - ps3
Archaea3 - ps4
Asgard/Eukaryota - ps5
Eukaryota - ps6
Amorpheae - ps7
Obazoa - ps8
Opisthokonta - ps9
Holozoa - ps10
Filozoa - ps11
Choano./Metazoa - ps12
Metazoa - ps13
Myriazoa - ps14
Parahoxozoa - ps15
Bilateria - ps16
Deuterostomia - ps17
Chordata - ps18
Olfactores - ps19
Vertebrata - ps20
Gnathostomata - ps21
Euteleostomi - ps22
Sarcopterygii - ps23
Tetrapoda - ps24
Amniota - ps25
Mammalia - ps26
Theria - ps27
Placentalia - ps28
Boreoeutheria - ps29
Euarchontoglires - ps30
Euarchonta - ps31
Primates - ps32
Haplorrhini - ps33
Simiiformes - ps34
Catarrhini - ps35
Hominoidea - ps36
Hominidae - ps37
Homininae - ps38
Hominini - ps39
Homo_sapiens - ps40
Phylostratum

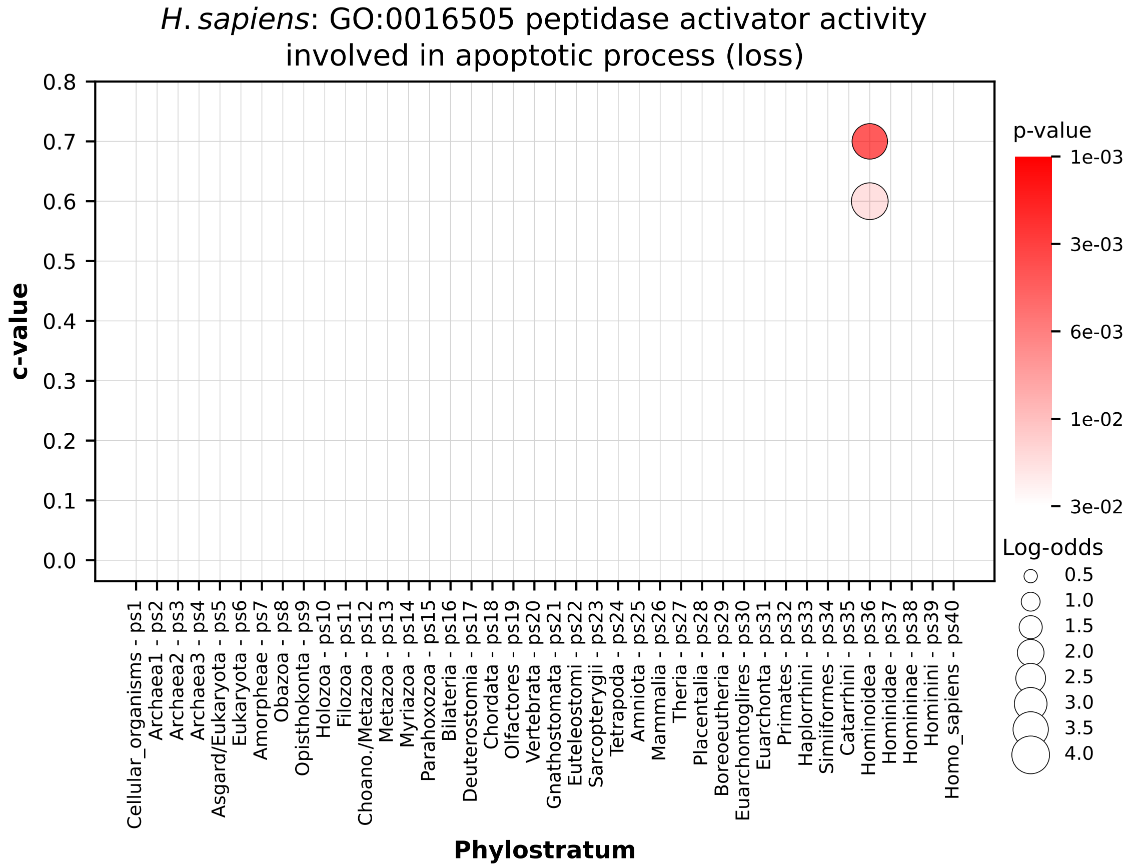

H. sapiens: GO:0016505 peptidase activator activity involved in apoptotic process (loss)
c-value
0.8
0.7
0.6
0.5
0.4
0.3
0.2
0.1
0.0
p-value
1e-03
3e-03
6e-03
1e-02
3e-02
Log-odds
0.5
1.0
1.5
2.0
2.5
3.0
3.5
4.0
Cellular_organisms - ps1
Archaea1 - ps2
Archaea2 - ps3
Archaea3 - ps4
Asgard/Eukaryota - ps5
Eukaryota - ps6
Amorpheae - ps7
Obazoa - ps8
Opisthokonta - ps9
Holozoa - ps10
Filozoa - ps11
Choano./Metazoa - ps12
Metazoa - ps13
Myriazoa - ps14
Parahoxozoa - ps15
Bilateria - ps16
Deuterostomia - ps17
Chordata - ps18
Olfactores - ps19
Vertebrata - ps20
Gnathostomata - ps21
Euteleostomi - ps22
Sarcopterygii - ps23
Tetrapoda - ps24
Amniota - ps25
Mammalia - ps26
Theria - ps27
Placentalia - ps28
Boreoeutheria - ps29
Euarchontoglires - ps30
Euarchonta - ps31
Primates - ps32
Haplorrhini - ps33
Simiiformes - ps34
Catarrhini - ps35
Hominoidea - ps36
Hominidae - ps37
Homininae - ps38
Hominini - ps39
Homo_sapiens - ps40
Phylostratum

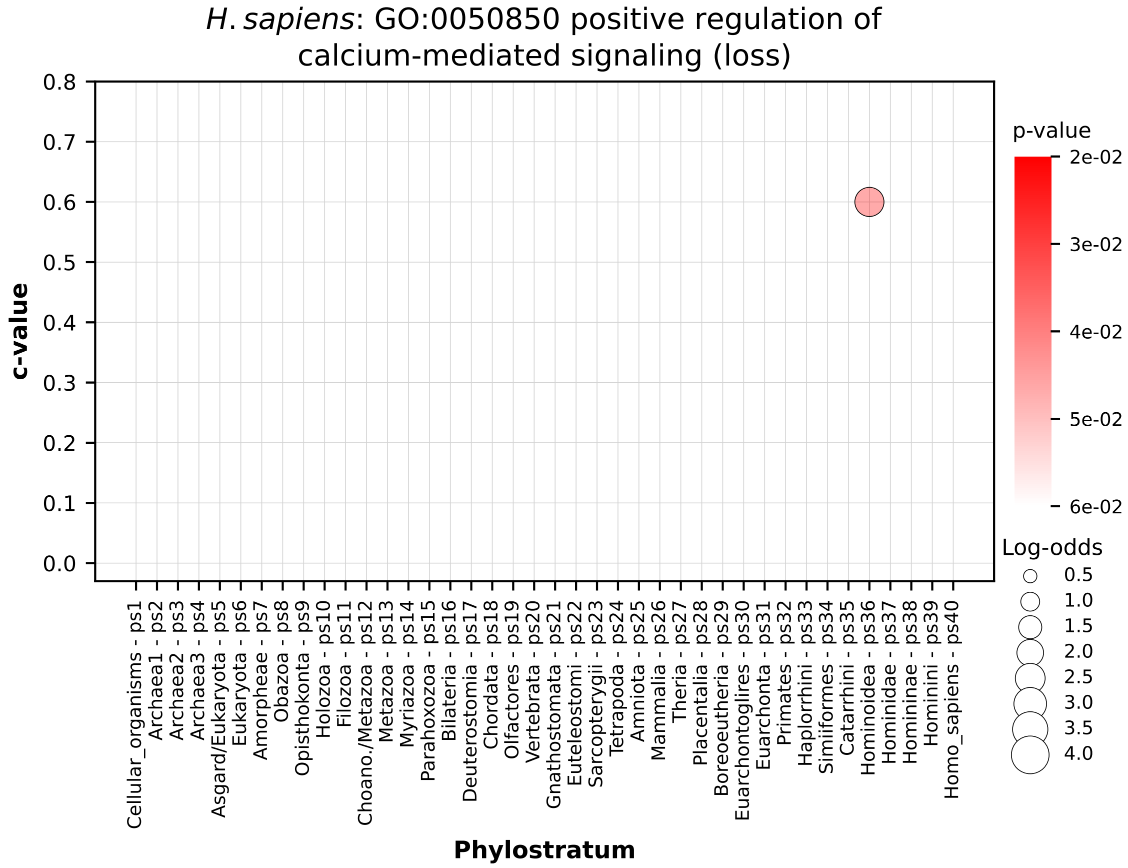

H. sapiens: GO:0050850 positive regulation of calcium-mediated signaling (loss)
c-value
0.8
0.7
0.6
0.5
0.4
0.3
0.2
0.1
0.0
p-value
2e-02
3e-02
4e-02
5e-02
6e-02
Log-odds
0.5
1.0
1.5
2.0
2.5
3.0
3.5
4.0
Cellular_organisms - ps1
Archaea1 - ps2
Archaea2 - ps3
Archaea3 - ps4
Asgard/Eukaryota - ps5
Eukaryota - ps6
Amorpheae - ps7
Obazoa - ps8
Opisthokonta - ps9
Holozoa - ps10
Filozoa - ps11
Choano./Metazoa - ps12
Metazoa - ps13
Myriazoa - ps14
Parahoxozoa - ps15
Bilateria - ps16
Deuterostomia - ps17
Chordata - ps18
Olfactores - ps19
Vertebrata - ps20
Gnathostomata - ps21
Euteleostomi - ps22
Sarcopterygii - ps23
Tetrapoda - ps24
Amniota - ps25
Mammalia - ps26
Theria - ps27
Placentalia - ps28
Boreoeutheria - ps29
Euarchontoglires - ps30
Euarchonta - ps31
Primates - ps32
Haplorrhini - ps33
Simiiformes - ps34
Catarrhini - ps35
Hominoidea - ps36
Hominidae - ps37
Homininae - ps38
Hominini - ps39
Homo_sapiens - ps40
Phylostratum

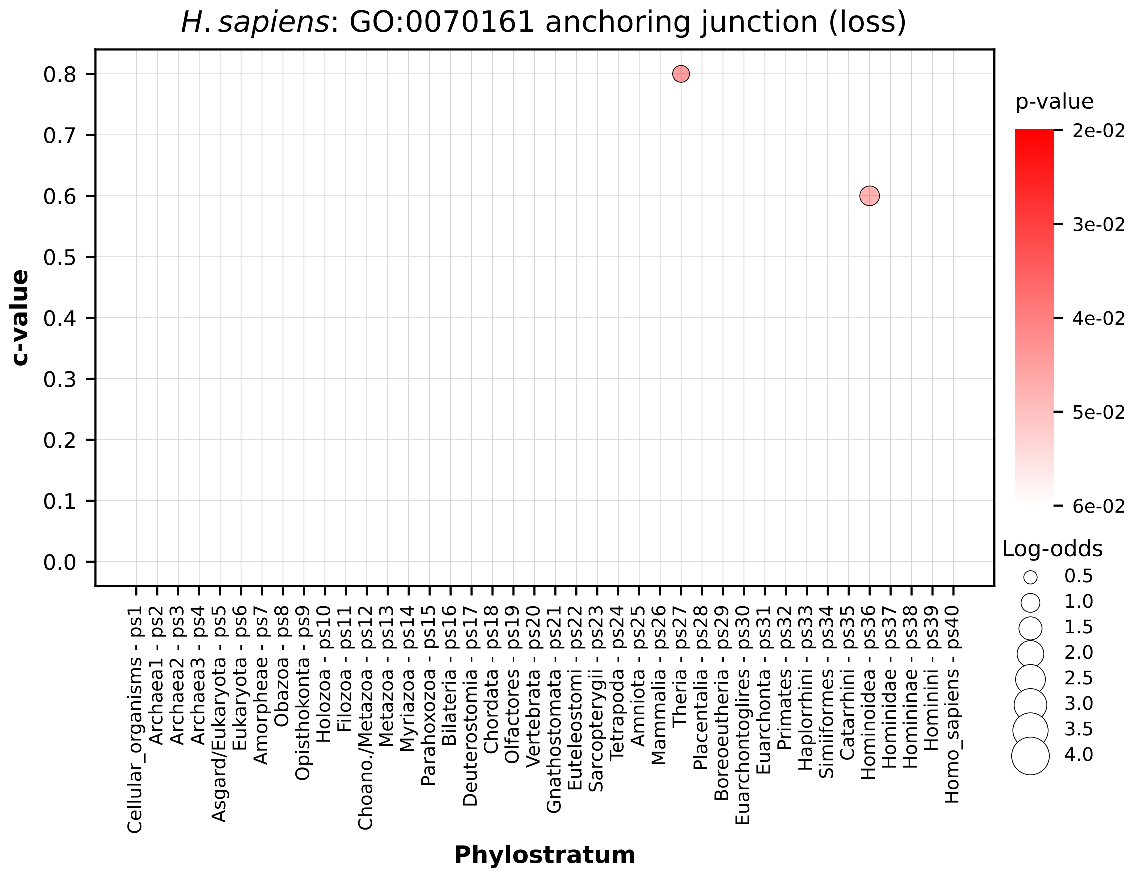

H. sapiens: GO:0070161 anchoring junction (loss)
c-value
0.0
0.1
0.2
0.3
0.4
0.5
0.6
0.7
0.8
Phylostratum
Cellular_organisms - ps1
Archaea1 - ps2
Archaea2 - ps3
Archaea3 - ps4
Asgard/Eukaryota - ps5
Eukaryota - ps6
Amorpheae - ps7
Obazoa - ps8
Opisthokonta - ps9
Holozoa - ps10
Filozoa - ps11
Choano./Metazoa - ps12
Metazoa - ps13
Myriazoa - ps14
Parahoxozoa - ps15
Bilateria - ps16
Deuterostomia - ps17
Chordata - ps18
Olfactores - ps19
Vertebrata - ps20
Gnathostomata - ps21
Euteleostomi - ps22
Sarcopterygii - ps23
Tetrapoda - ps24
Amniota - ps25
Mammalia - ps26
Theria - ps27
Placentalia - ps28
Boreoeutheria - ps29
Euarchontoglires - ps30
Euarchonta - ps31
Primates - ps32
Haplorrhini - ps33
Simiiformes - ps34
Catarrhini - ps35
Hominoidea - ps36
Hominidae - ps37
Homininae - ps38
Hominini - ps39
Homo_sapiens - ps40
p-value
2e-02
3e-02
4e-02
5e-02
6e-02
Log-odds
0.5
1.0
1.5
2.0
2.5
3.0
3.5
4.0

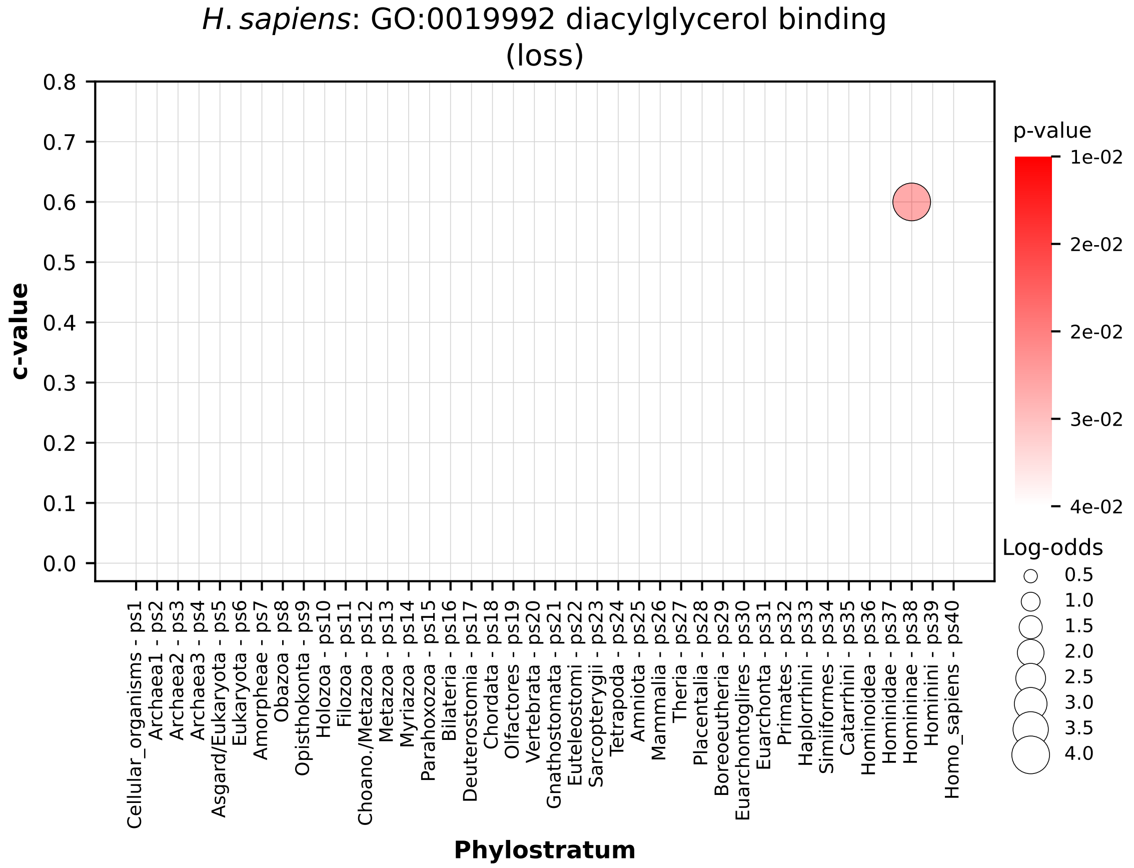
H. sapiens: GO:0019992 diacylglycerol binding
(loss)
c-value
0.8
0.7
0.6
0.5
0.4
0.3
0.2
0.1
0.0
p-value
1e-02
2e-02
2e-02
3e-02
4e-02
Log-odds
0.5
1.0
1.5
2.0
2.5
3.0
3.5
4.0
Cellular_organisms - ps1
Archaea1 - ps2
Archaea2 - ps3
Archaea3 - ps4
Asgard/Eukaryota - ps5
Eukaryota - ps6
Amorpheae - ps7
Obazoa - ps8
Opisthokonta - ps9
Holozoa - ps10
Filozoa - ps11
Choano./Metazoa - ps12
Metazoa - ps13
Myriazoa - ps14
Parahoxozoa - ps15
Bilateria - ps16
Deuterostomia - ps17
Chordata - ps18
Olfactores - ps19
Vertebrata - ps20
Gnathostomata - ps21
Euteleostomi - ps22
Sarcopterygii - ps23
Tetrapoda - ps24
Amniota - ps25
Mammalia - ps26
Theria - ps27
Placentalia - ps28
Boreoeutheria - ps29
Euarchontoglires - ps30
Euarchonta - ps31
Primates - ps32
Haplorrhini - ps33
Simiiformes - ps34
Catarrhini - ps35
Hominoidea - ps36
Hominidae - ps37
Homininae - ps38
Hominini - ps39
Homo_sapiens - ps40
Phylostratum

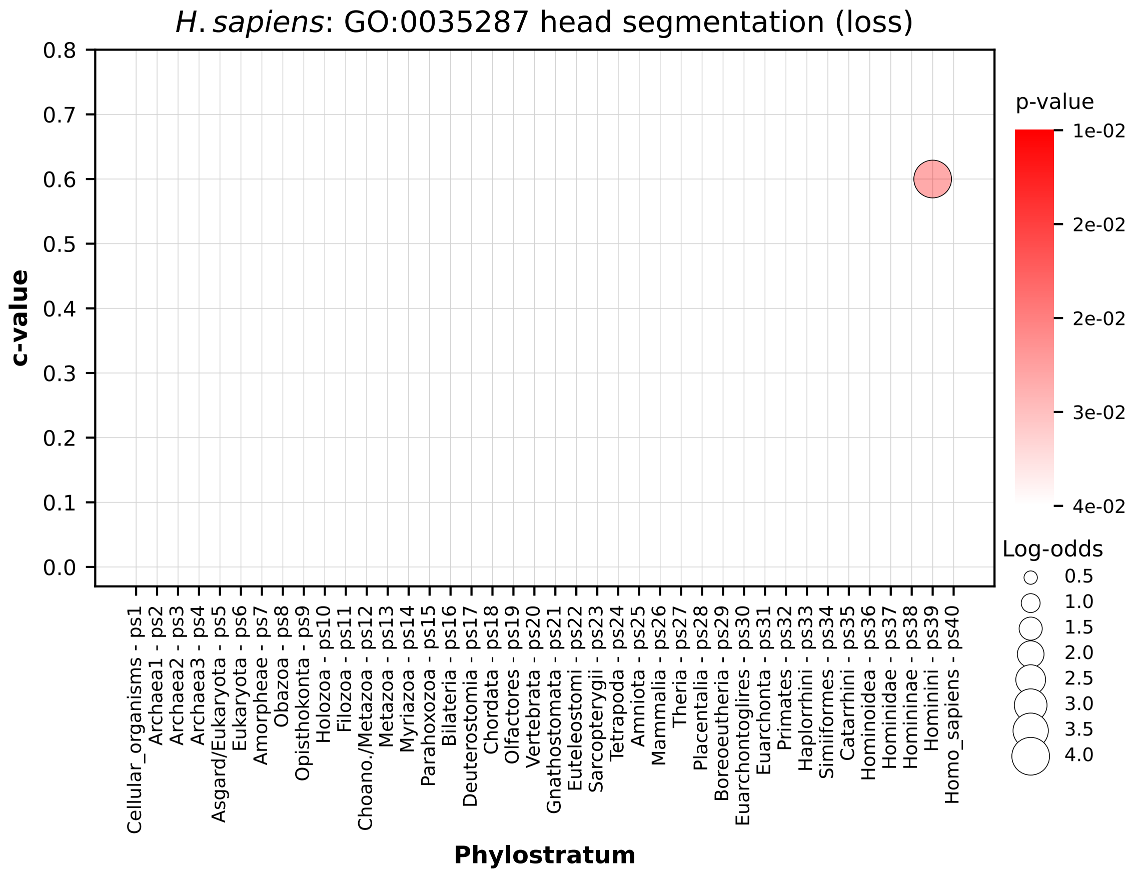

H. sapiens: GO:0035287 head segmentation (loss)
c-value
0.0
0.1
0.2
0.3
0.4
0.5
0.6
0.7
0.8
Phylostratum
Cellular_organisms - ps1
Archaea1 - ps2
Archaea2 - ps3
Archaea3 - ps4
Asgard/Eukaryota - ps5
Eukaryota - ps6
Amorpheae - ps7
Obazoa - ps8
Opisthokonta - ps9
Holozoa - ps10
Filozoa - ps11
Choano./Metazoa - ps12
Metazoa - ps13
Myriazoa - ps14
Parahoxozoa - ps15
Bilateria - ps16
Deuterostomia - ps17
Chordata - ps18
Olfactores - ps19
Vertebrata - ps20
Gnathostomata - ps21
Euteleostomi - ps22
Sarcopterygii - ps23
Tetrapoda - ps24
Amniota - ps25
Mammalia - ps26
Theria - ps27
Placentalia - ps28
Boreoeutheria - ps29
Euarchontoglires - ps30
Euarchonta - ps31
Primates - ps32
Haplorrhini - ps33
Simiiformes - ps34
Catarrhini - ps35
Hominoidea - ps36
Hominidae - ps37
Homininae - ps38
Hominini - ps39
Homo_sapiens - ps40
p-value
1e-02
2e-02
2e-02
3e-02
4e-02
Log-odds
0.5
1.0
1.5
2.0
2.5
3.0
3.5
4.0

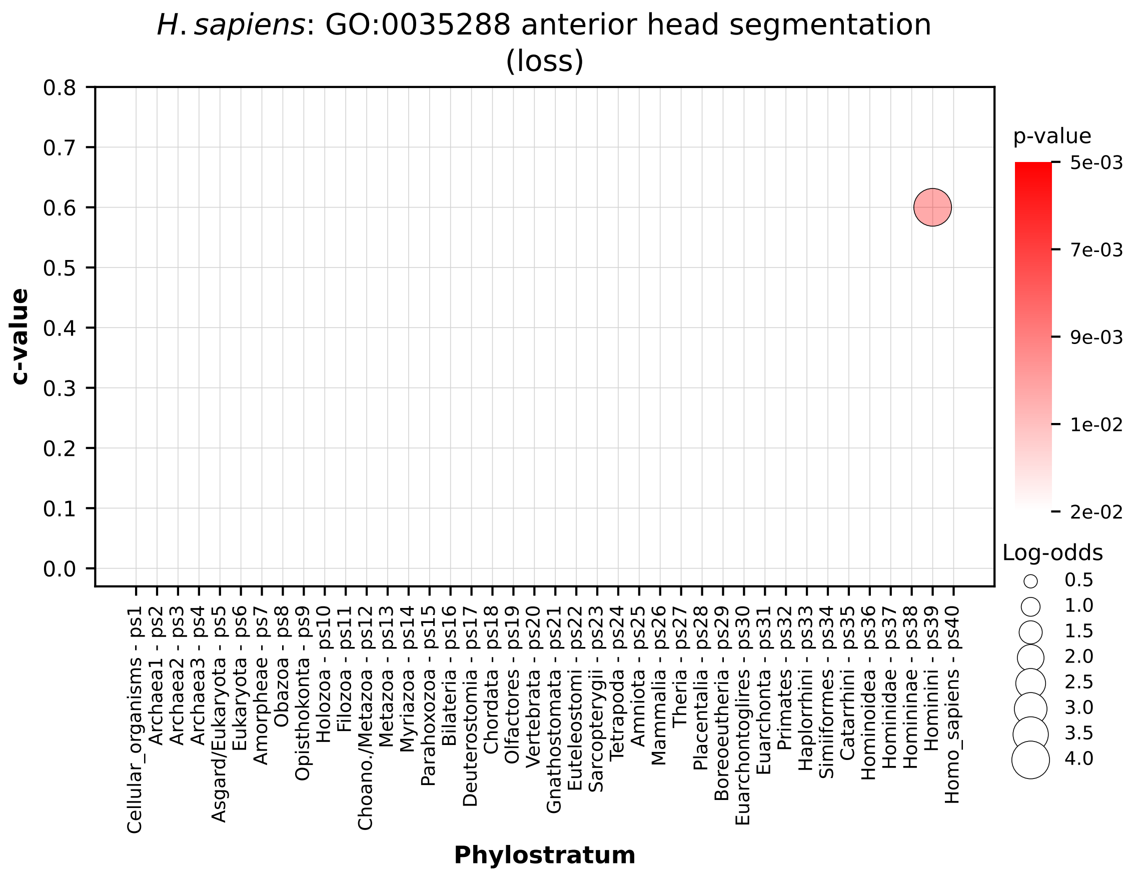

H. sapiens: GO:0035288 anterior head segmentation (loss)
c-value
0.8
0.7
0.6
0.5
0.4
0.3
0.2
0.1
0.0
p-value
5e-03
7e-03
9e-03
1e-02
2e-02
Log-odds
0.5
1.0
1.5
2.0
2.5
3.0
3.5
4.0
Cellular_organisms - ps1
Archaea1 - ps2
Archaea2 - ps3
Archaea3 - ps4
Asgard/Eukaryota - ps5
Eukaryota - ps6
Amorpheae - ps7
Obazoa - ps8
Opisthokonta - ps9
Holozoa - ps10
Filozoa - ps11
Choano./Metazoa - ps12
Metazoa - ps13
Myriazoa - ps14
Parahoxozoa - ps15
Bilateria - ps16
Deuterostomia - ps17
Chordata - ps18
Olfactores - ps19
Vertebrata - ps20
Gnathostomata - ps21
Euteleostomi - ps22
Sarcopterygii - ps23
Tetrapoda - ps24
Amniota - ps25
Mammalia - ps26
Theria - ps27
Placentalia - ps28
Boreoeutheria - ps29
Euarchontoglires - ps30
Euarchonta - ps31
Primates - ps32
Haplorrhini - ps33
Simiiformes - ps34
Catarrhini - ps35
Hominoidea - ps36
Hominidae - ps37
Homininae - ps38
Hominini - ps39
Homo_sapiens - ps40
Phylostratum

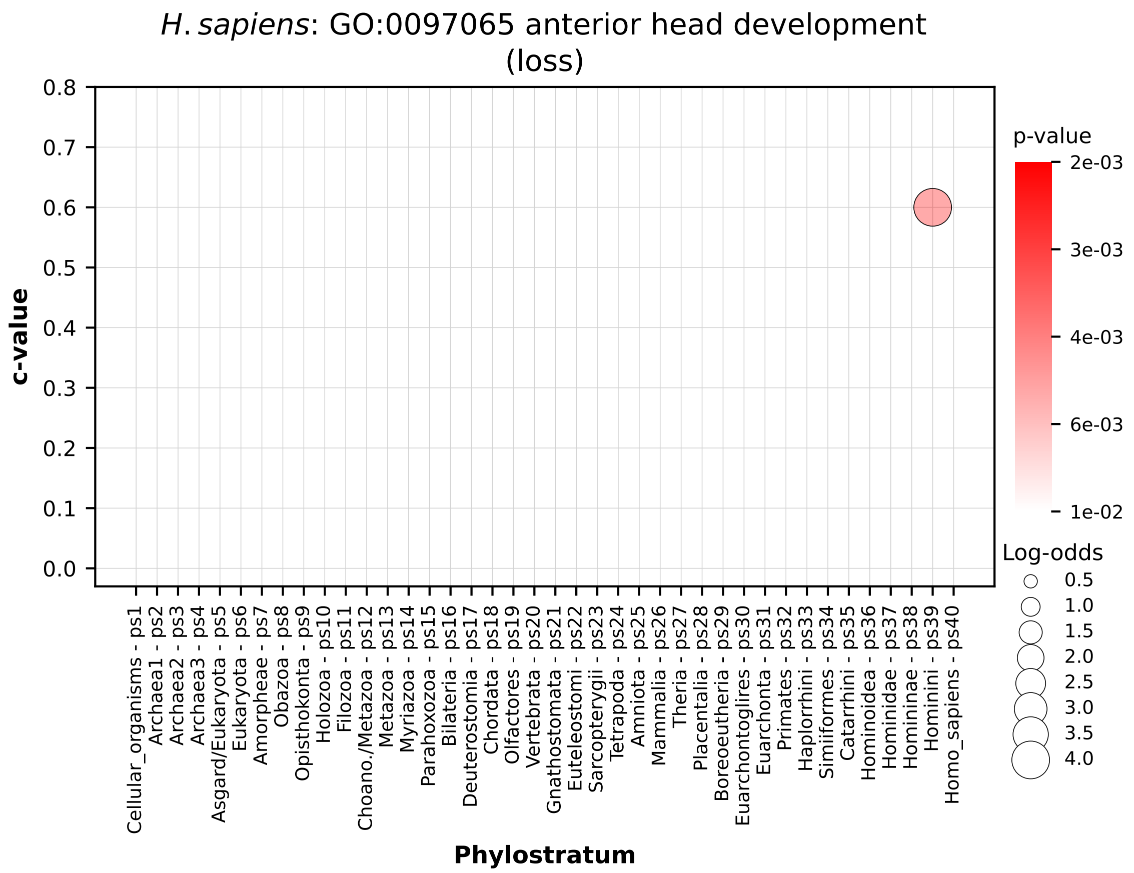

H. sapiens: GO:0097065 anterior head development (loss)
c-value
0.8
0.7
0.6
0.5
0.4
0.3
0.2
0.1
0.0
p-value
2e-03
3e-03
4e-03
6e-03
1e-02
Log-odds
0.5
1.0
1.5
2.0
2.5
3.0
3.5
4.0
Cellular_organisms - ps1
Archaea1 - ps2
Archaea2 - ps3
Archaea3 - ps4
Asgard/Eukaryota - ps5
Eukaryota - ps6
Amorpheae - ps7
Obazoa - ps8
Opisthokonta - ps9
Holozoa - ps10
Filozoa - ps11
Choano./Metazoa - ps12
Metazoa - ps13
Myriazoa - ps14
Parahoxozoa - ps15
Bilateria - ps16
Deuterostomia - ps17
Chordata - ps18
Olfactores - ps19
Vertebrata - ps20
Gnathostomata - ps21
Euteleostomi - ps22
Sarcopterygii - ps23
Tetrapoda - ps24
Amniota - ps25
Mammalia - ps26
Theria - ps27
Placentalia - ps28
Boreoeutheria - ps29
Euarchontoglires - ps30
Euarchonta - ps31
Primates - ps32
Haplorrhini - ps33
Simiiformes - ps34
Catarrhini - ps35
Hominoidea - ps36
Hominidae - ps37
Homininae - ps38
Hominini - ps39
Homo_sapiens - ps40
Phylostratum

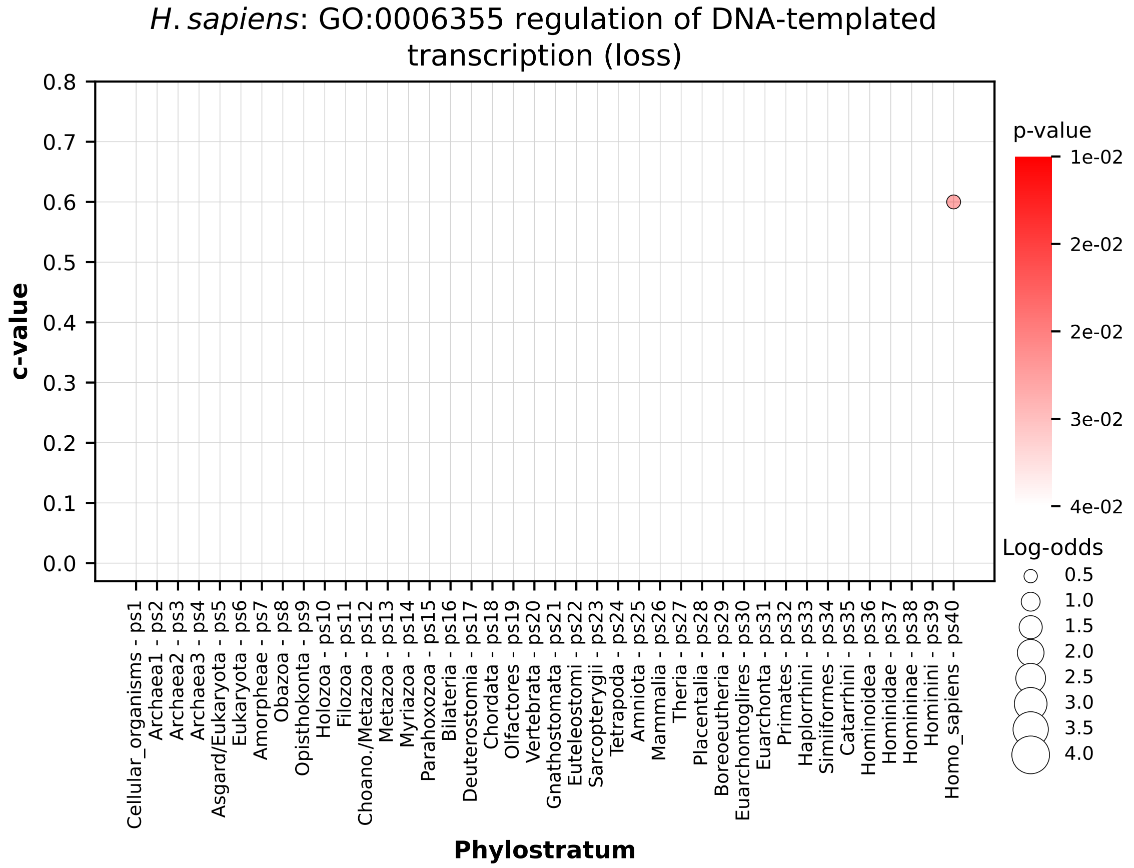

H. sapiens: GO:0006355 regulation of DNA-templated transcription (loss)
c-value
0.8
0.7
0.6
0.5
0.4
0.3
0.2
0.1
0.0
Phylostratum
Cellular_organisms - ps1
Archaea1 - ps2
Archaea2 - ps3
Archaea3 - ps4
Asgard/Eukaryota - ps5
Eukaryota - ps6
Amorpheae - ps7
Obazoa - ps8
Opisthokonta - ps9
Holozoa - ps10
Filozoa - ps11
Choano./Metazoa - ps12
Metazoa - ps13
Myriazoa - ps14
Parahoxozoa - ps15
Bilateria - ps16
Deuterostomia - ps17
Chordata - ps18
Olfactores - ps19
Vertebrata - ps20
Gnathostomata - ps21
Euteleostomi - ps22
Sarcopterygii - ps23
Tetrapoda - ps24
Amniota - ps25
Mammalia - ps26
Theria - ps27
Placentalia - ps28
Boreoeutheria - ps29
Euarchontoglires - ps30
Euarchonta - ps31
Primates - ps32
Haplorrhini - ps33
Simiiformes - ps34
Catarrhini - ps35
Hominoidea - ps36
Hominidae - ps37
Homininae - ps38
Hominini - ps39
Homo_sapiens - ps40
p-value
1e-02
2e-02
2e-02
3e-02
4e-02
Log-odds
0.5
1.0
1.5
2.0
2.5
3.0
3.5
4.0

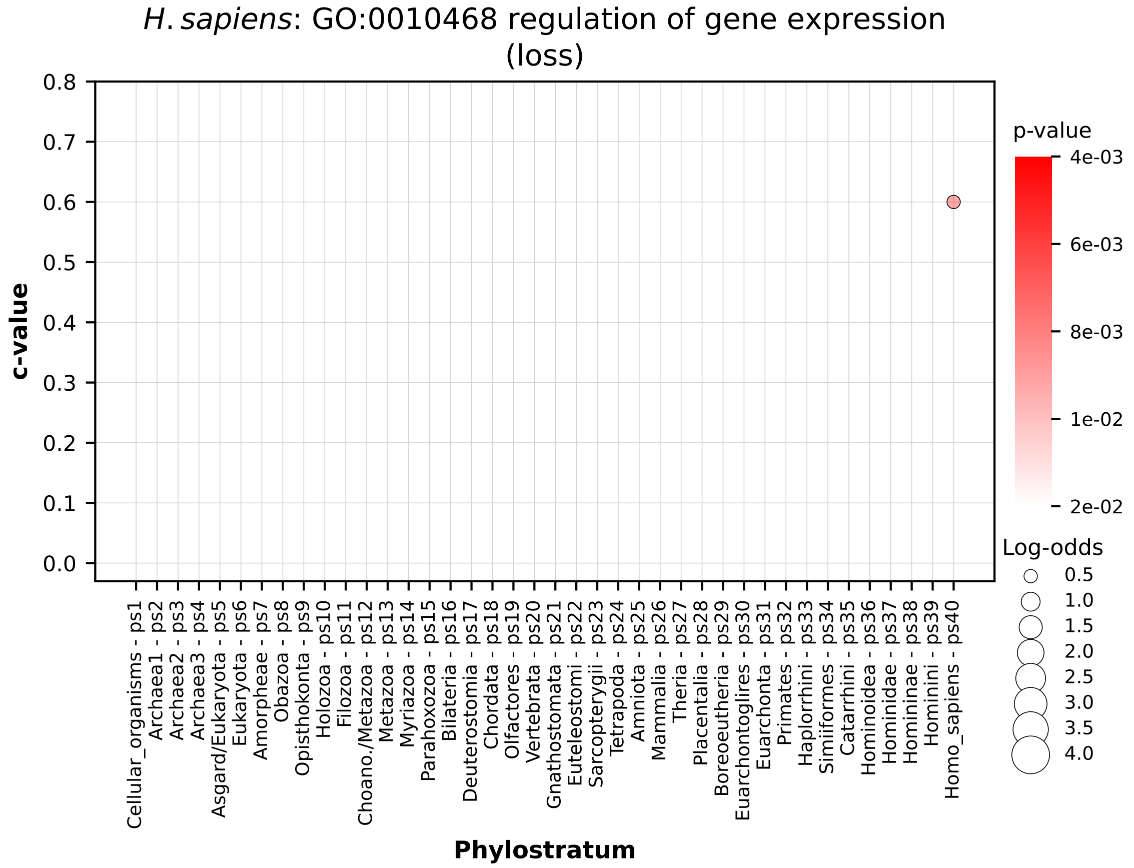

H. sapiens: GO:0010468 regulation of gene expression
(loss)
c-value
0.0
0.1
0.2
0.3
0.4
0.5
0.6
0.7
0.8
p-value
4e-03
6e-03
8e-03
1e-02
2e-02
Log-odds
0.5
1.0
1.5
2.0
2.5
3.0
3.5
4.0
Cellular_organisms - ps1
Archaea1 - ps2
Archaea2 - ps3
Archaea3 - ps4
Asgard/Eukaryota - ps5
Eukaryota - ps6
Amorpheae - ps7
Obazoa - ps8
Opisthokonta - ps9
Holozoa - ps10
Filozoa - ps11
Choano./Metazoa - ps12
Metazoa - ps13
Myriazoa - ps14
Parahoxozoa - ps15
Bilateria - ps16
Deuterostomia - ps17
Chordata - ps18
Olfactores - ps19
Vertebrata - ps20
Gnathostomata - ps21
Euteleostomi - ps22
Sarcopterygii - ps23
Tetrapoda - ps24
Amniota - ps25
Mammalia - ps26
Theria - ps27
Placentalia - ps28
Boreoeutheria - ps29
Euarchontoglires - ps30
Euarchonta - ps31
Primates - ps32
Haplorrhini - ps33
Simiiformes - ps34
Catarrhini - ps35
Hominoidea - ps36
Hominidae - ps37
Homininae - ps38
Hominini - ps39
Homo_sapiens - ps40
Phylostratum

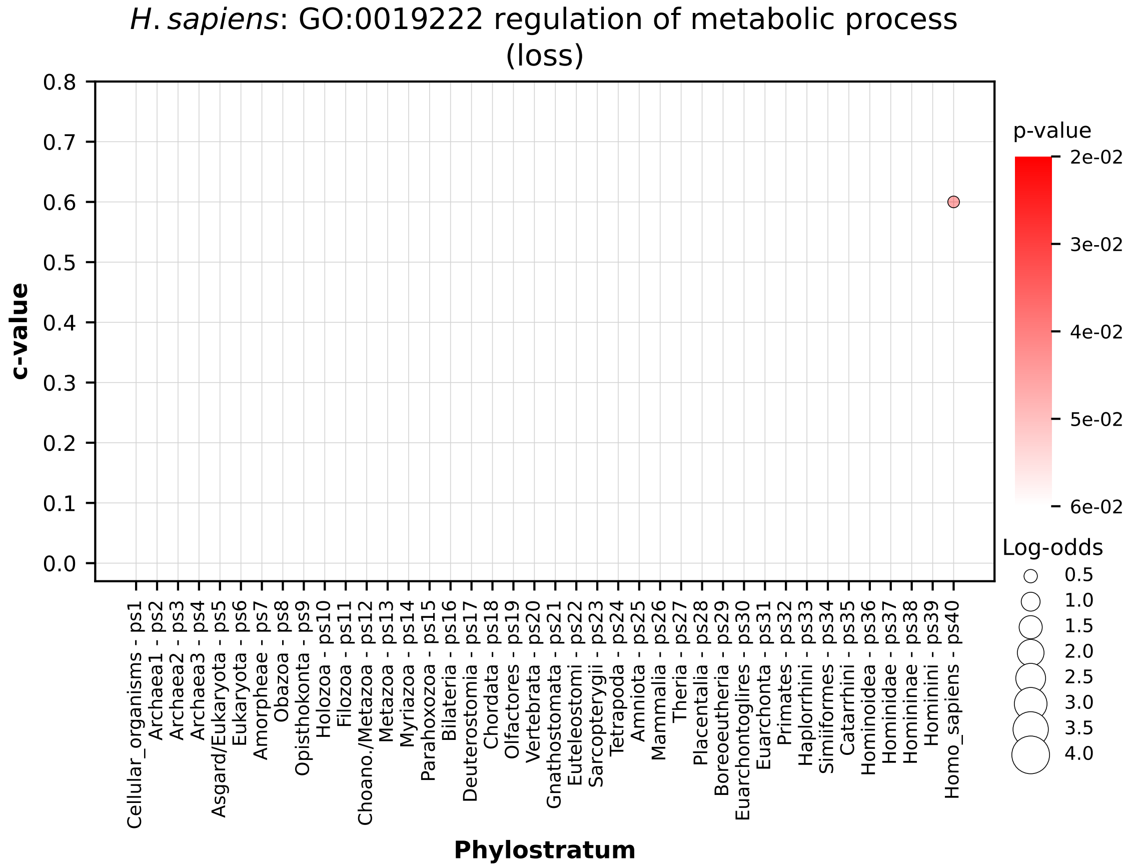

H. sapiens: GO:0019222 regulation of metabolic process (loss)
c-value
0.8
0.7
0.6
0.5
0.4
0.3
0.2
0.1
0.0
Phylostratum
Cellular_organisms - ps1
Archaea1 - ps2
Archaea2 - ps3
Archaea3 - ps4
Asgard/Eukaryota - ps5
Eukaryota - ps6
Amorpheae - ps7
Obazoa - ps8
Opisthokonta - ps9
Holozoa - ps10
Filozoa - ps11
Choano./Metazoa - ps12
Metazoa - ps13
Myriazoa - ps14
Parahoxozoa - ps15
Bilateria - ps16
Deuterostomia - ps17
Chordata - ps18
Olfactores - ps19
Vertebrata - ps20
Gnathostomata - ps21
Euteleostomi - ps22
Sarcopterygii - ps23
Tetrapoda - ps24
Amniota - ps25
Mammalia - ps26
Theria - ps27
Placentalia - ps28
Boreoeutheria - ps29
Euarchontoglires - ps30
Euarchonta - ps31
Primates - ps32
Haplorrhini - ps33
Simiiformes - ps34
Catarrhini - ps35
Hominoidea - ps36
Hominidae - ps37
Homininae - ps38
Hominini - ps39
Homo_sapiens - ps40
p-value
2e-02
3e-02
4e-02
5e-02
6e-02
Log-odds
0.5
1.0
1.5
2.0
2.5
3.0
3.5
4.0

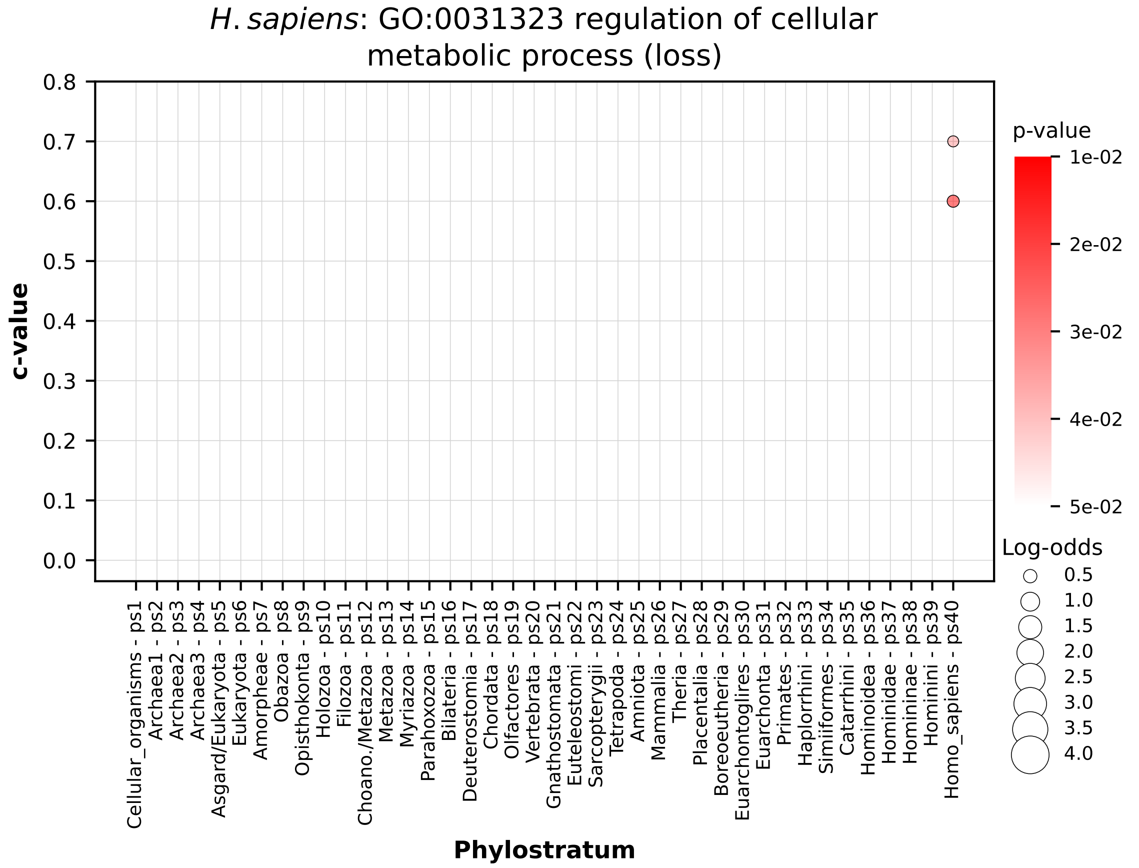

H. sapiens: GO:0031323 regulation of cellular metabolic process (loss)
c-value
0.8
0.7
0.6
0.5
0.4
0.3
0.2
0.1
0.0
Phylostratum
Cellular_organisms - ps1
Archaea1 - ps2
Archaea2 - ps3
Archaea3 - ps4
Asgard/Eukaryota - ps5
Eukaryota - ps6
Amorpheae - ps7
Obazoa - ps8
Opisthokonta - ps9
Holozoa - ps10
Filozoa - ps11
Choano./Metazoa - ps12
Metazoa - ps13
Myriazoa - ps14
Parahoxozoa - ps15
Bilateria - ps16
Deuterostomia - ps17
Chordata - ps18
Olfactores - ps19
Vertebrata - ps20
Gnathostomata - ps21
Euteleostomi - ps22
Sarcopterygii - ps23
Tetrapoda - ps24
Amniota - ps25
Mammalia - ps26
Theria - ps27
Placentalia - ps28
Boreoeutheria - ps29
Euarchontoglires - ps30
Euarchonta - ps31
Primates - ps32
Haplorrhini - ps33
Simiiformes - ps34
Catarrhini - ps35
Hominoidea - ps36
Hominidae - ps37
Homininae - ps38
Hominini - ps39
Homo_sapiens - ps40
p-value
1e-02
2e-02
3e-02
4e-02
5e-02
Log-odds
0.5
1.0
1.5
2.0
2.5
3.0
3.5
4.0

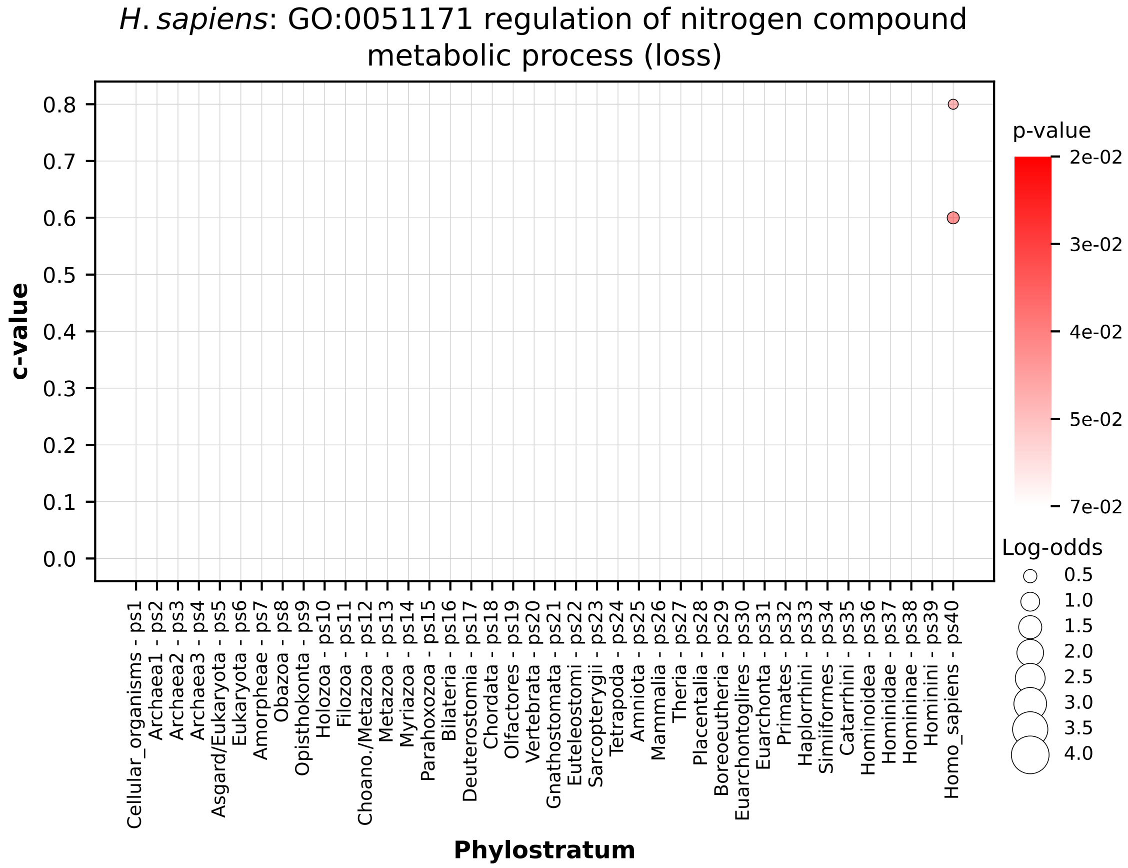

H. sapiens: GO:0051171 regulation of nitrogen compound metabolic process (loss)
c-value
0.8
0.7
0.6
0.5
0.4
0.3
0.2
0.1
0.0
p-value
2e-02
3e-02
4e-02
5e-02
7e-02
Log-odds
0.5
1.0
1.5
2.0
2.5
3.0
3.5
4.0
Cellular_organisms - ps1
Archaea1 - ps2
Archaea2 - ps3
Archaea3 - ps4
Asgard/Eukaryota - ps5
Eukaryota - ps6
Amorpheae - ps7
Obazoa - ps8
Opisthokonta - ps9
Holozoa - ps10
Filozoa - ps11
Choano./Metazoa - ps12
Metazoa - ps13
Myriazoa - ps14
Parahoxozoa - ps15
Bilateria - ps16
Deuterostomia - ps17
Chordata - ps18
Olfactores - ps19
Vertebrata - ps20
Gnathostomata - ps21
Euteleostomi - ps22
Sarcopterygii - ps23
Tetrapoda - ps24
Amniota - ps25
Mammalia - ps26
Theria - ps27
Placentalia - ps28
Boreoeutheria - ps29
Euarchontoglires - ps30
Euarchonta - ps31
Primates - ps32
Haplorrhini - ps33
Simiiformes - ps34
Catarrhini - ps35
Hominoidea - ps36
Hominidae - ps37
Homininae - ps38
Hominini - ps39
Homo_sapiens - ps40
Phylostratum

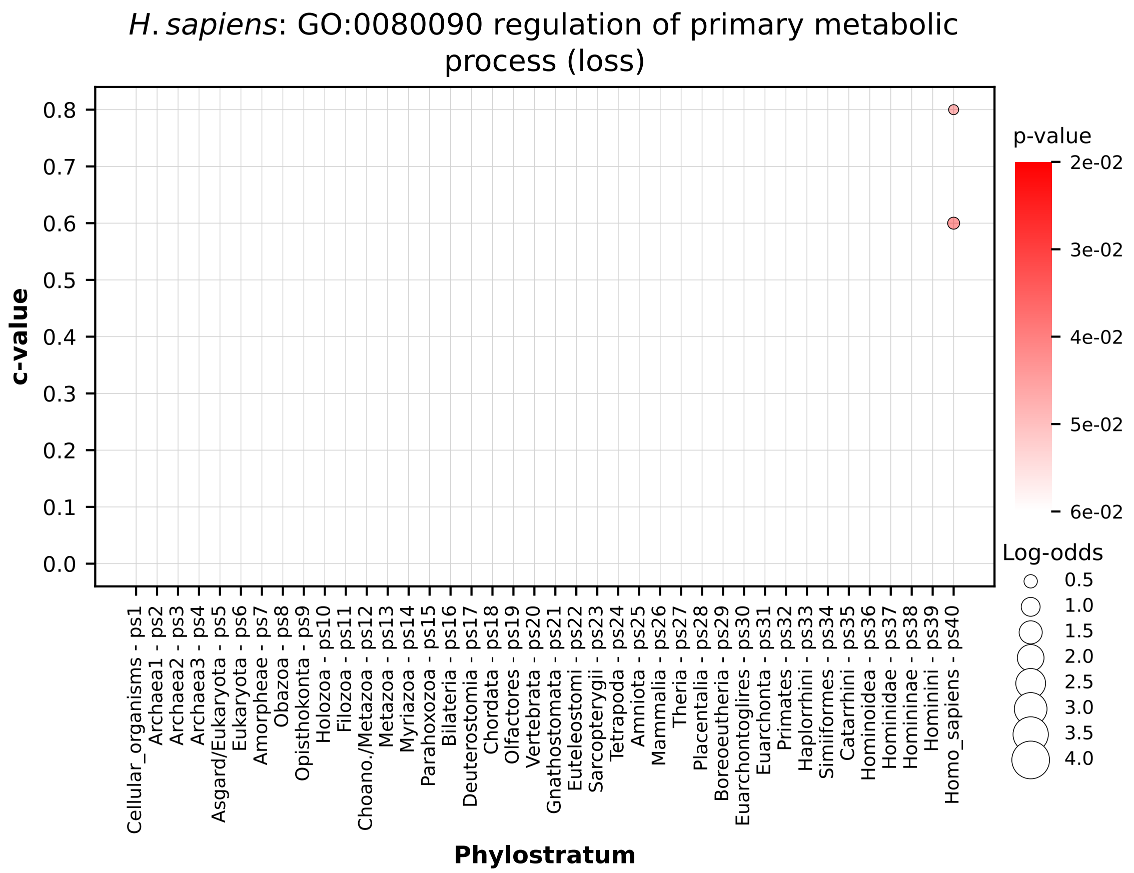

H. sapiens: GO:0080090 regulation of primary metabolic process (loss)
c-value
0.8
0.7
0.6
0.5
0.4
0.3
0.2
0.1
0.0
Phylostratum
Cellular_organisms - ps1
Archaea1 - ps2
Archaea2 - ps3
Archaea3 - ps4
Asgard/Eukaryota - ps5
Eukaryota - ps6
Amorpheae - ps7
Obazoa - ps8
Opisthokonta - ps9
Holozoa - ps10
Filozoa - ps11
Choano./Metazoa - ps12
Metazoa - ps13
Myriazoa - ps14
Parahoxozoa - ps15
Bilateria - ps16
Deuterostomia - ps17
Chordata - ps18
Olfactores - ps19
Vertebrata - ps20
Gnathostomata - ps21
Euteleostomi - ps22
Sarcopterygii - ps23
Tetrapoda - ps24
Amniota - ps25
Mammalia - ps26
Theria - ps27
Placentalia - ps28
Boreoeutheria - ps29
Euarchontoglires - ps30
Euarchonta - ps31
Primates - ps32
Haplorrhini - ps33
Simiiformes - ps34
Catarrhini - ps35
Hominoidea - ps36
Hominidae - ps37
Homininae - ps38
Hominini - ps39
Homo_sapiens - ps40
p-value
2e-02
3e-02
4e-02
5e-02
6e-02
Log-odds
0.5
1.0
1.5
2.0
2.5
3.0
3.5
4.0

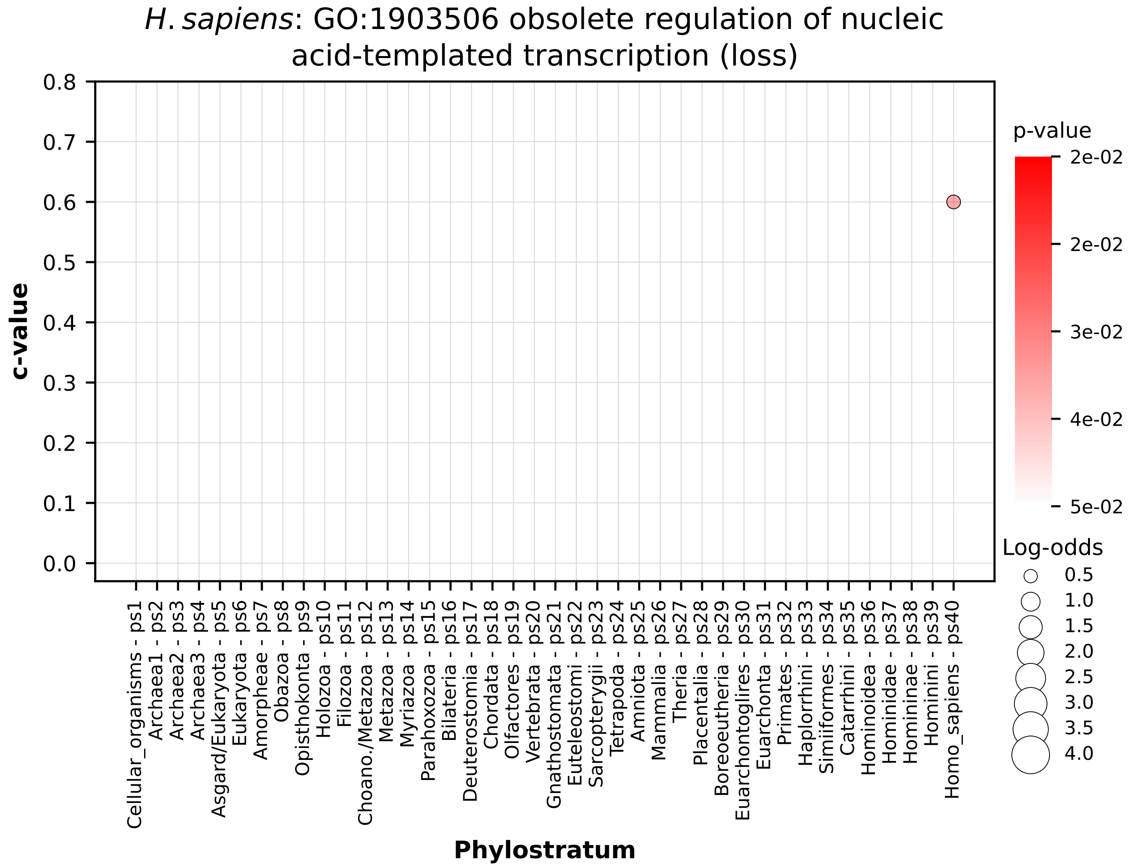

H. sapiens: GO:1903506 obsolete regulation of nucleic acid-templated transcription (loss)
c-value
0.0
0.1
0.2
0.3
0.4
0.5
0.6
0.7
0.8
Phylostratum
Cellular_organisms - ps1
Archaea1 - ps2
Archaea2 - ps3
Archaea3 - ps4
Asgard/Eukaryota - ps5
Eukaryota - ps6
Amorpheae - ps7
Obazoa - ps8
Opisthokonta - ps9
Holozoa - ps10
Filozoa - ps11
Choano./Metazoa - ps12
Metazoa - ps13
Myriazoa - ps14
Parahoxozoa - ps15
Bilateria - ps16
Deuterostomia - ps17
Chordata - ps18
Olfactores - ps19
Vertebrata - ps20
Gnathostomata - ps21
Euteleostomi - ps22
Sarcopterygii - ps23
Tetrapoda - ps24
Amniota - ps25
Mammalia - ps26
Theria - ps27
Placentalia - ps28
Boreoeutheria - ps29
Euarchontoglires - ps30
Euarchonta - ps31
Primates - ps32
Haplorrhini - ps33
Simiiformes - ps34
Catarrhini - ps35
Hominoidea - ps36
Hominidae - ps37
Homininae - ps38
Hominini - ps39
Homo_sapiens - ps40
p-value
2e-02
2e-02
3e-02
4e-02
5e-02
Log-odds
0.5
1.0
1.5
2.0
2.5
3.0
3.5
4.0

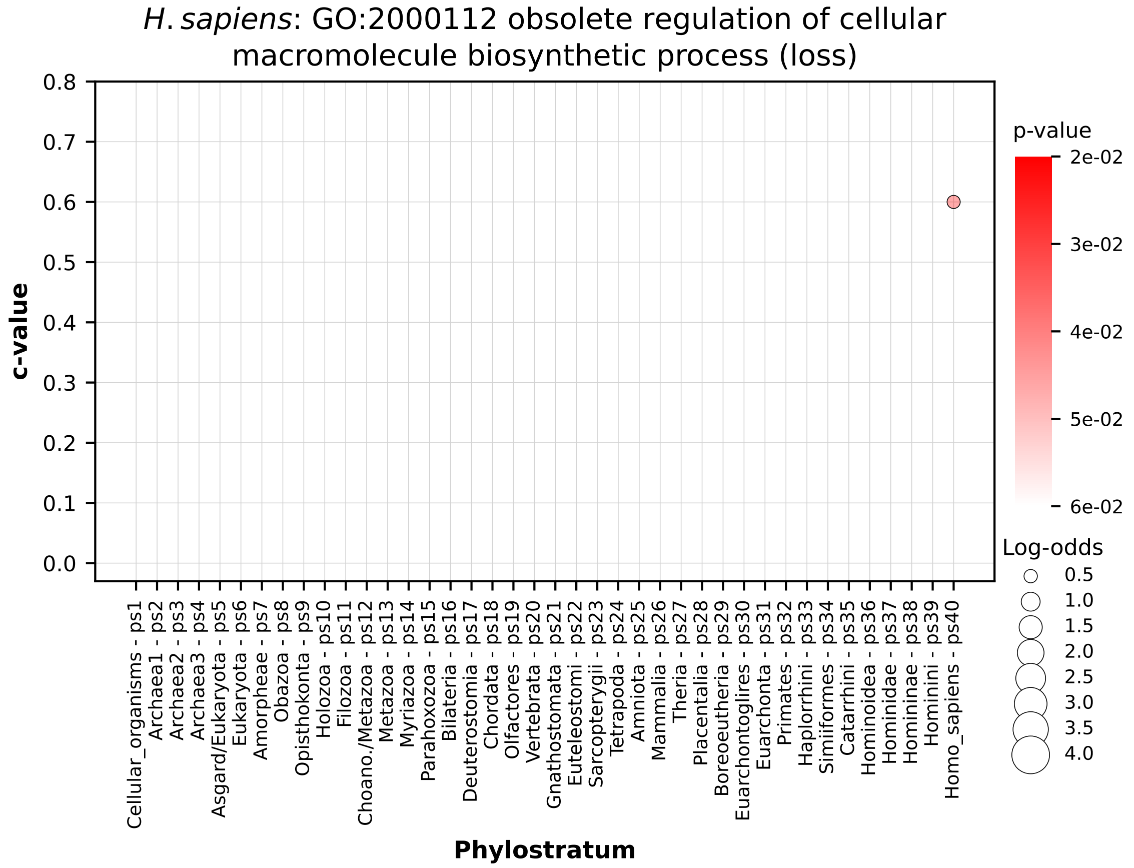
H. sapiens: GO:2000112 obsolete regulation of cellular macromolecule biosynthetic process (loss)
c-value
0.8
0.7
0.6
0.5
0.4
0.3
0.2
0.1
0.0
p-value
2e-02
3e-02
4e-02
5e-02
6e-02
Log-odds
0.5
1.0
1.5
2.0
2.5
3.0
3.5
4.0
Cellular_organisms - ps1
Archaea1 - ps2
Archaea2 - ps3
Archaea3 - ps4
Asgard/Eukaryota - ps5
Eukaryota - ps6
Amorpheae - ps7
Obazoa - ps8
Opisthokonta - ps9
Holozoa - ps10
Filozoa - ps11
Choano./Metazoa - ps12
Metazoa - ps13
Myriazoa - ps14
Parahoxozoa - ps15
Bilateria - ps16
Deuterostomia - ps17
Chordata - ps18
Olfactores - ps19
Vertebrata - ps20
Gnathostomata - ps21
Euteleostomi - ps22
Sarcopterygii - ps23
Tetrapoda - ps24
Amniota - ps25
Mammalia - ps26
Theria - ps27
Placentalia - ps28
Boreoeutheria - ps29
Euarchontoglires - ps30
Euarchonta - ps31
Primates - ps32
Haplorrhini - ps33
Simiiformes - ps34
Catarrhini - ps35
Hominoidea - ps36
Hominidae - ps37
Homininae - ps38
Hominini - ps39
Homo_sapiens - ps40
Phylostratum

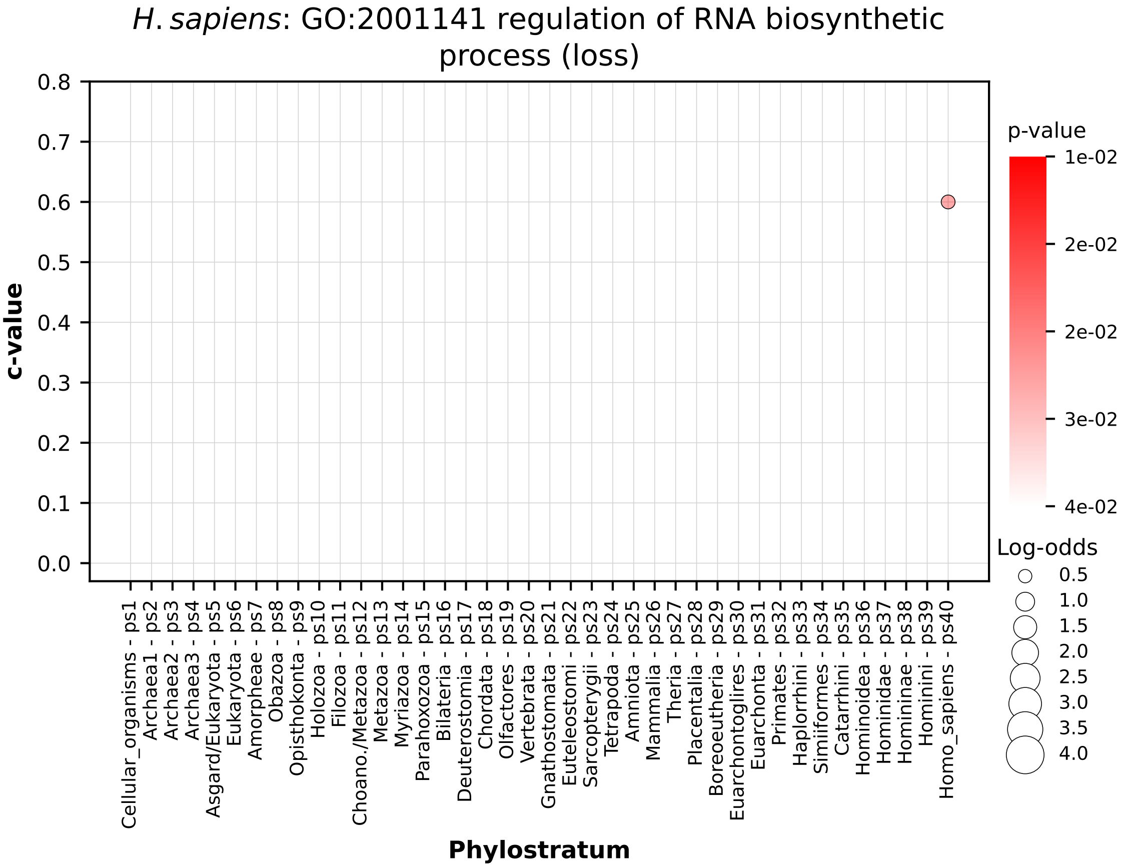

H. sapiens: GO:2001141 regulation of RNA biosynthetic process (loss)
c-value
0.8
0.7
0.6
0.5
0.4
0.3
0.2
0.1
0.0
Phylostratum
Cellular_organisms - ps1
Archaea1 - ps2
Archaea2 - ps3
Archaea3 - ps4
Asgard/Eukaryota - ps5
Eukaryota - ps6
Amorpheae - ps7
Obazoa - ps8
Opisthokonta - ps9
Holozoa - ps10
Filozoa - ps11
Choano./Metazoa - ps12
Metazoa - ps13
Myriazoa - ps14
Parahoxozoa - ps15
Bilateria - ps16
Deuterostomia - ps17
Chordata - ps18
Olfactores - ps19
Vertebrata - ps20
Gnathostomata - ps21
Euteleostomi - ps22
Sarcopterygii - ps23
Tetrapoda - ps24
Amniota - ps25
Mammalia - ps26
Theria - ps27
Placentalia - ps28
Boreoeutheria - ps29
Euarchontoglires - ps30
Euarchonta - ps31
Primates - ps32
Haplorrhini - ps33
Simiiformes - ps34
Catarrhini - ps35
Hominoidea - ps36
Hominidae - ps37
Homininae - ps38
Hominini - ps39
Homo_sapiens - ps40
p-value
1e-02
2e-02
2e-02
3e-02
4e-02
Log-odds
0.5
1.0
1.5
2.0
2.5
3.0
3.5
4.0

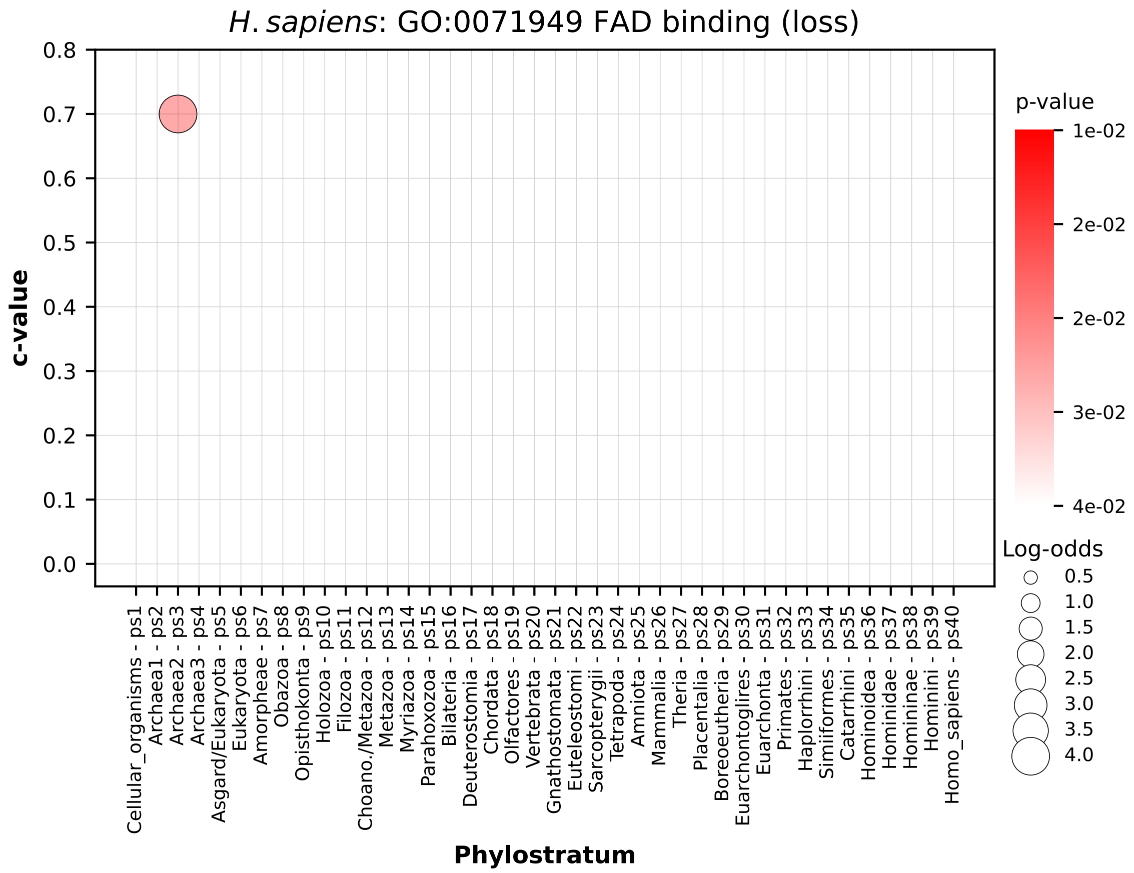

H. sapiens: GO:0071949 FAD binding (loss)
c-value
0.8
0.7
0.6
0.5
0.4
0.3
0.2
0.1
0.0
Cellular_organisms - ps1
Archaea1 - ps2
Archaea2 - ps3
Archaea3 - ps4
Asgard/Eukaryota - ps5
Eukaryota - ps6
Amorpheae - ps7
Obazoa - ps8
Opisthokonta - ps9
Holozoa - ps10
Filozoa - ps11
Choano./Metazoa - ps12
Metazoa - ps13
Myriazoa - ps14
Parahoxozoa - ps15
Bilateria - ps16
Deuterostomia - ps17
Chordata - ps18
Olfactores - ps19
Vertebrata - ps20
Gnathostomata - ps21
Euteleostomi - ps22
Sarcopterygii - ps23
Tetrapoda - ps24
Amniota - ps25
Mammalia - ps26
Theria - ps27
Placentalia - ps28
Boreoeutheria - ps29
Euarchontoglires - ps30
Euarchonta - ps31
Primates - ps32
Haplorrhini - ps33
Simiiformes - ps34
Catarrhini - ps35
Hominoidea - ps36
Hominidae - ps37
Homininae - ps38
Hominini - ps39
Homo_sapiens - ps40
Phylostratum
p-value
1e-02
2e-02
2e-02
3e-02
4e-02
Log-odds
0.5
1.0
1.5
2.0
2.5
3.0
3.5
4.0

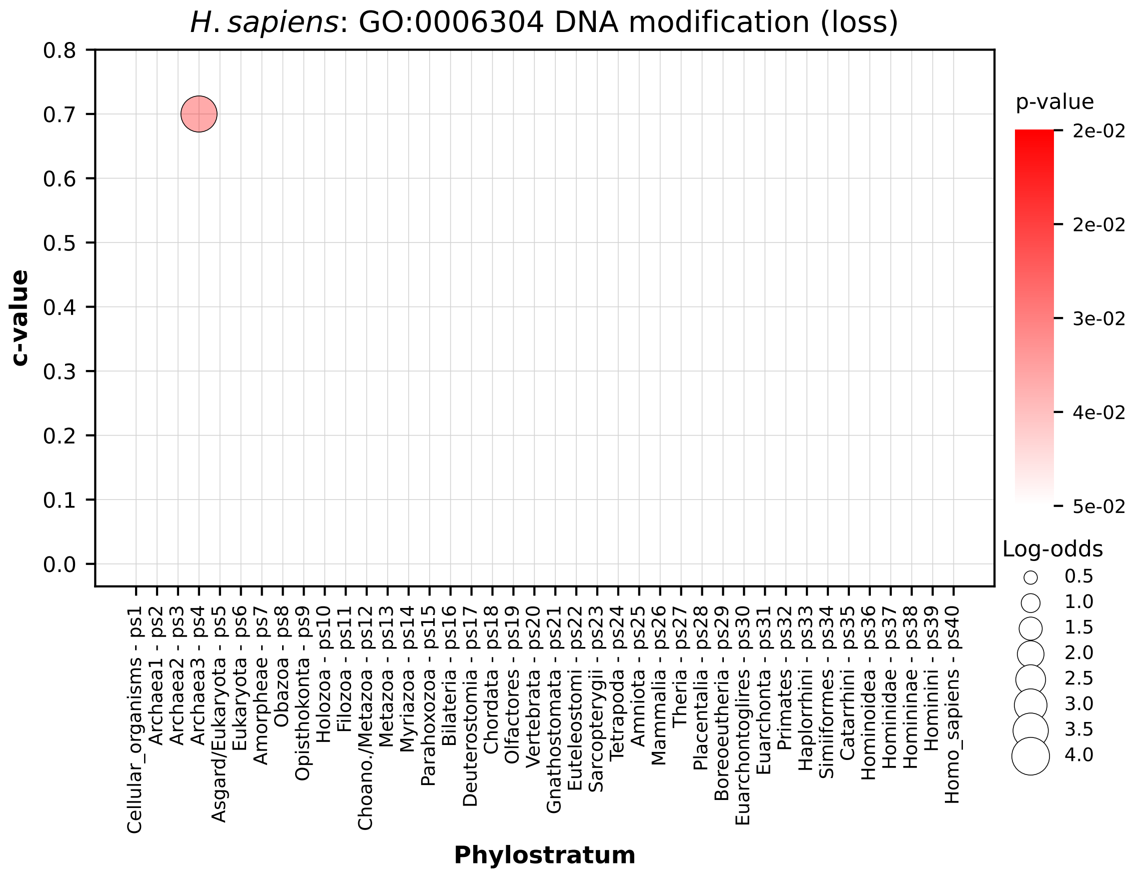

H. sapiens: GO:0006304 DNA modification (loss)
c-value
0.8
0.7
0.6
0.5
0.4
0.3
0.2
0.1
0.0
Phylostratum
Cellular_organisms - ps1
Archaea1 - ps2
Archaea2 - ps3
Archaea3 - ps4
Asgard/Eukaryota - ps5
Eukaryota - ps6
Amorpheae - ps7
Obazoa - ps8
Opisthokonta - ps9
Holozoa - ps10
Filozoa - ps11
Choano./Metazoa - ps12
Metazoa - ps13
Myriazoa - ps14
Parahoxozoa - ps15
Bilateria - ps16
Deuterostomia - ps17
Chordata - ps18
Olfactores - ps19
Vertebrata - ps20
Gnathostomata - ps21
Euteleostomi - ps22
Sarcopterygii - ps23
Tetrapoda - ps24
Amniota - ps25
Mammalia - ps26
Theria - ps27
Placentalia - ps28
Boreoeutheria - ps29
Euarchontoglires - ps30
Euarchonta - ps31
Primates - ps32
Haplorrhini - ps33
Simiiformes - ps34
Catarrhini - ps35
Hominoidea - ps36
Hominidae - ps37
Homininae - ps38
Hominini - ps39
Homo_sapiens - ps40
p-value
2e-02
2e-02
3e-02
4e-02
5e-02
Log-odds
0.5
1.0
1.5
2.0
2.5
3.0
3.5
4.0

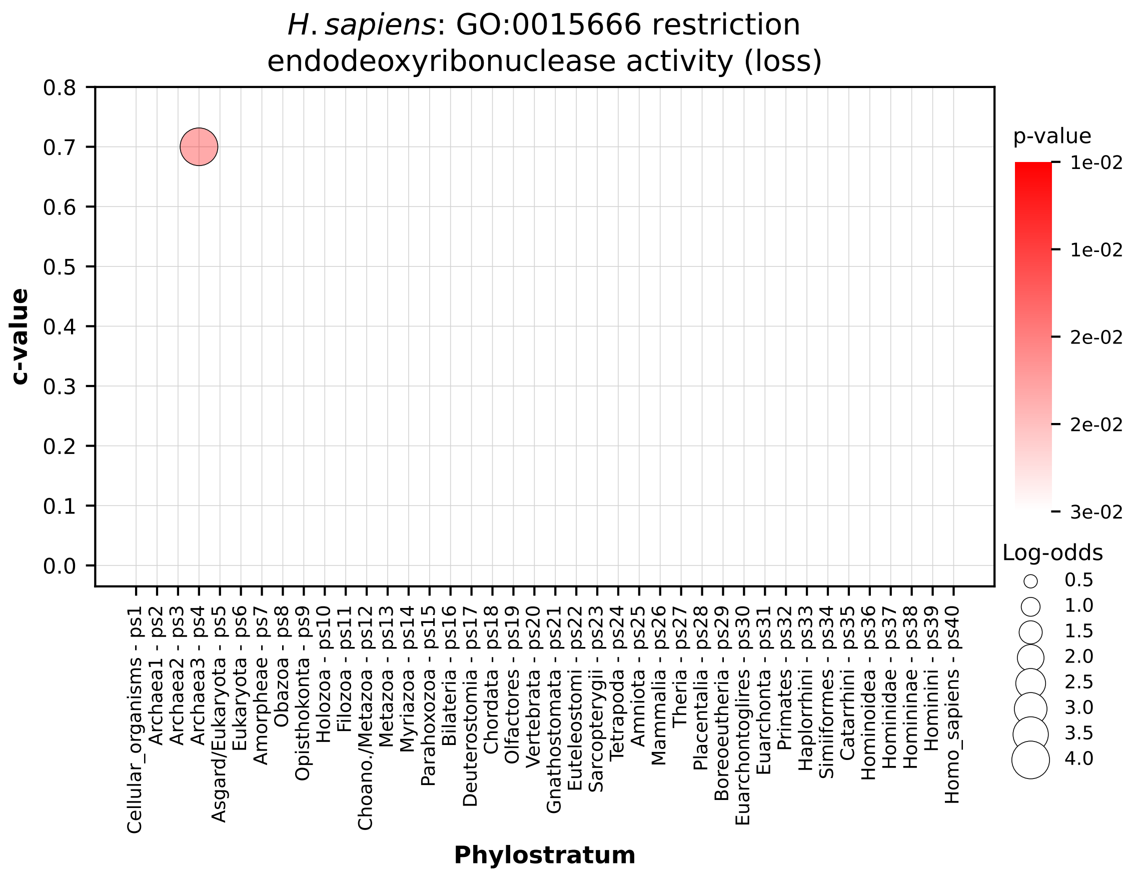
H. sapiens: GO:0015666 restriction endodeoxyribonuclease activity (loss)
c-value
0.8
0.7
0.6
0.5
0.4
0.3
0.2
0.1
0.0
Phylostratum
Cellular_organisms - ps1
Archaea1 - ps2
Archaea2 - ps3
Archaea3 - ps4
Asgard/Eukaryota - ps5
Eukaryota - ps6
Amorpheae - ps7
Obazoa - ps8
Opisthokonta - ps9
Holozoa - ps10
Filozoa - ps11
Choano./Metazoa - ps12
Metazoa - ps13
Myriazoa - ps14
Parahoxozoa - ps15
Bilateria - ps16
Deuterostomia - ps17
Chordata - ps18
Olfactores - ps19
Vertebrata - ps20
Gnathostomata - ps21
Euteleostomi - ps22
Sarcopterygii - ps23
Tetrapoda - ps24
Amniota - ps25
Mammalia - ps26
Theria - ps27
Placentalia - ps28
Boreoeutheria - ps29
Euarchontoglires - ps30
Euarchonta - ps31
Primates - ps32
Haplorrhini - ps33
Simiiformes - ps34
Catarrhini - ps35
Hominoidea - ps36
Hominidae - ps37
Homininae - ps38
Hominini - ps39
Homo_sapiens - ps40
p-value
1e-02
1e-02
2e-02
2e-02
3e-02
Log-odds
0.5
1.0
1.5
2.0
2.5
3.0
3.5
4.0

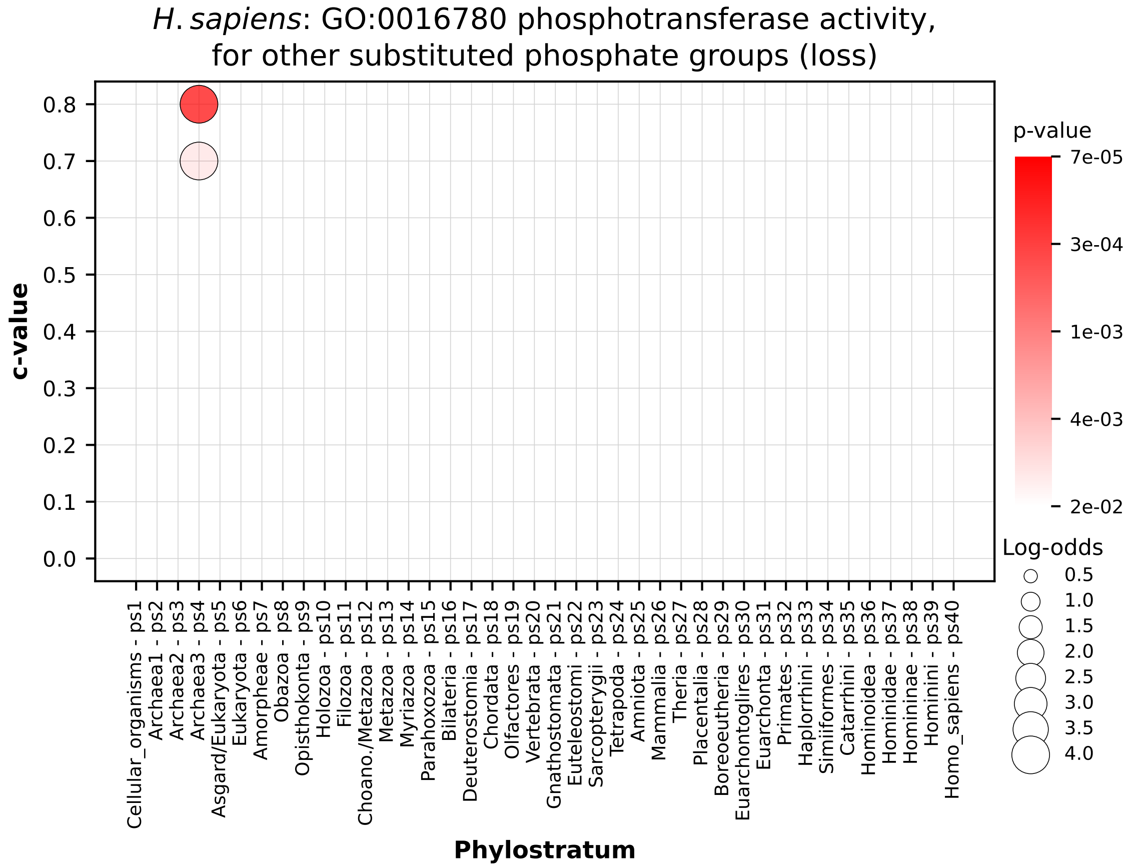

H. sapiens: GO:0016780 phosphotransferase activity, for other substituted phosphate groups (loss)
c-value
0.8
0.7
0.6
0.5
0.4
0.3
0.2
0.1
0.0
Phylostratum
Cellular_organisms - ps1
Archaea1 - ps2
Archaea2 - ps3
Archaea3 - ps4
Asgard/Eukaryota - ps5
Eukaryota - ps6
Amorpheae - ps7
Obazoa - ps8
Opisthokonta - ps9
Holozoa - ps10
Filozoa - ps11
Choano./Metazoa - ps12
Metazoa - ps13
Myriazoa - ps14
Parahoxozoa - ps15
Bilateria - ps16
Deuterostomia - ps17
Chordata - ps18
Olfactores - ps19
Vertebrata - ps20
Gnathostomata - ps21
Euteleostomi - ps22
Sarcopterygii - ps23
Tetrapoda - ps24
Amniota - ps25
Mammalia - ps26
Theria - ps27
Placentalia - ps28
Boreoeutheria - ps29
Euarchontoglires - ps30
Euarchonta - ps31
Primates - ps32
Haplorrhini - ps33
Simiiformes - ps34
Catarrhini - ps35
Hominoidea - ps36
Hominidae - ps37
Homininae - ps38
Hominini - ps39
Homo_sapiens - ps40
p-value
7e-05
3e-04
1e-03
4e-03
2e-02
Log-odds
0.5
1.0
1.5
2.0
2.5
3.0
3.5
4.0

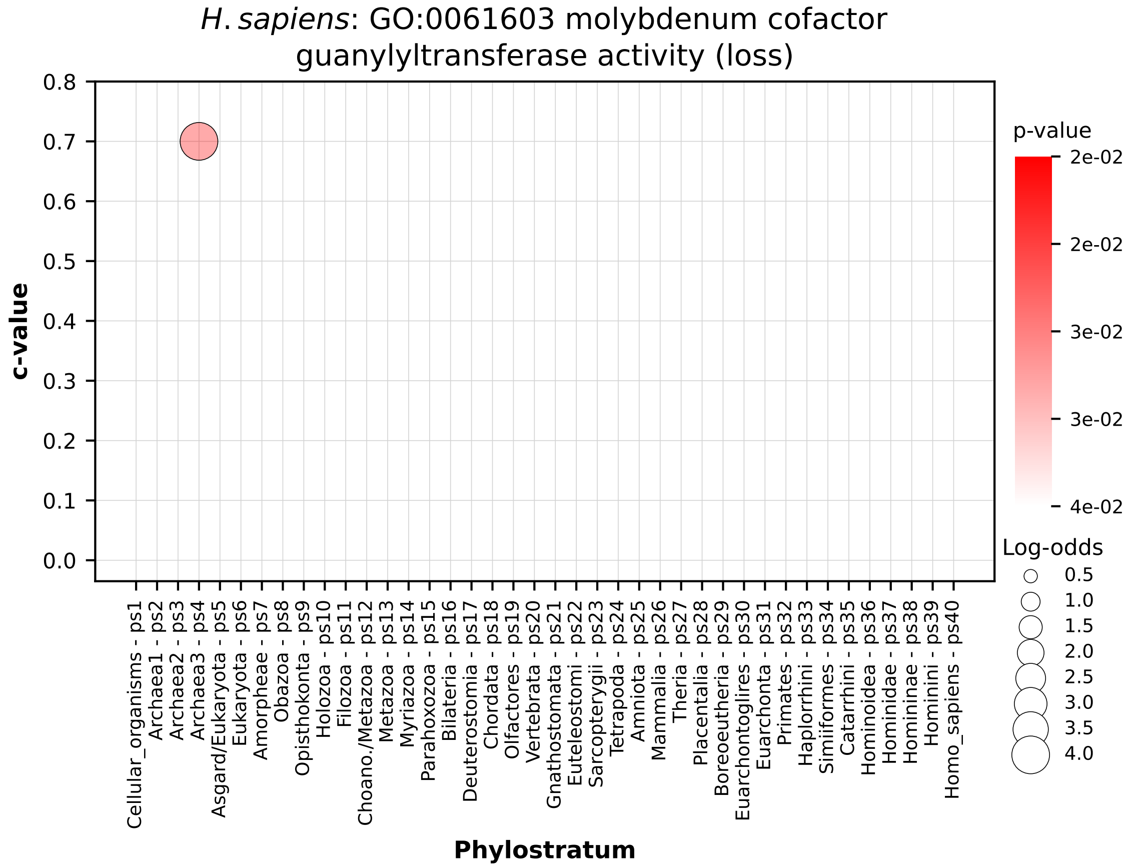

$H. sapiens$: GO:0061603 molybdenum cofactor guanylyltransferase activity (loss)
c-value
0.8
0.7
0.6
0.5
0.4
0.3
0.2
0.1
0.0
p-value
2e-02
2e-02
3e-02
3e-02
4e-02
Log-odds
0.5
1.0
1.5
2.0
2.5
3.0
3.5
4.0
Cellular_organisms - ps1
Archaea1 - ps2
Archaea2 - ps3
Archaea3 - ps4
Asgard/Eukaryota - ps5
Eukaryota - ps6
Amorpheae - ps7
Obazoa - ps8
Opisthokonta - ps9
Holozoa - ps10
Filozoa - ps11
Choano./Metazoa - ps12
Metazoa - ps13
Myriazoa - ps14
Parahoxozoa - ps15
Bilateria - ps16
Deuterostomia - ps17
Chordata - ps18
Olfactores - ps19
Vertebrata - ps20
Gnathostomata - ps21
Euteleostomi - ps22
Sarcopterygii - ps23
Tetrapoda - ps24
Amniota - ps25
Mammalia - ps26
Theria - ps27
Placentalia - ps28
Boreoeutheria - ps29
Euarchontoglires - ps30
Euarchonta - ps31
Primates - ps32
Haplorrhini - ps33
Simiiformes - ps34
Catarrhini - ps35
Hominoidea - ps36
Hominidae - ps37
Homininae - ps38
Hominini - ps39
Homo_sapiens - ps40
Phylostratum

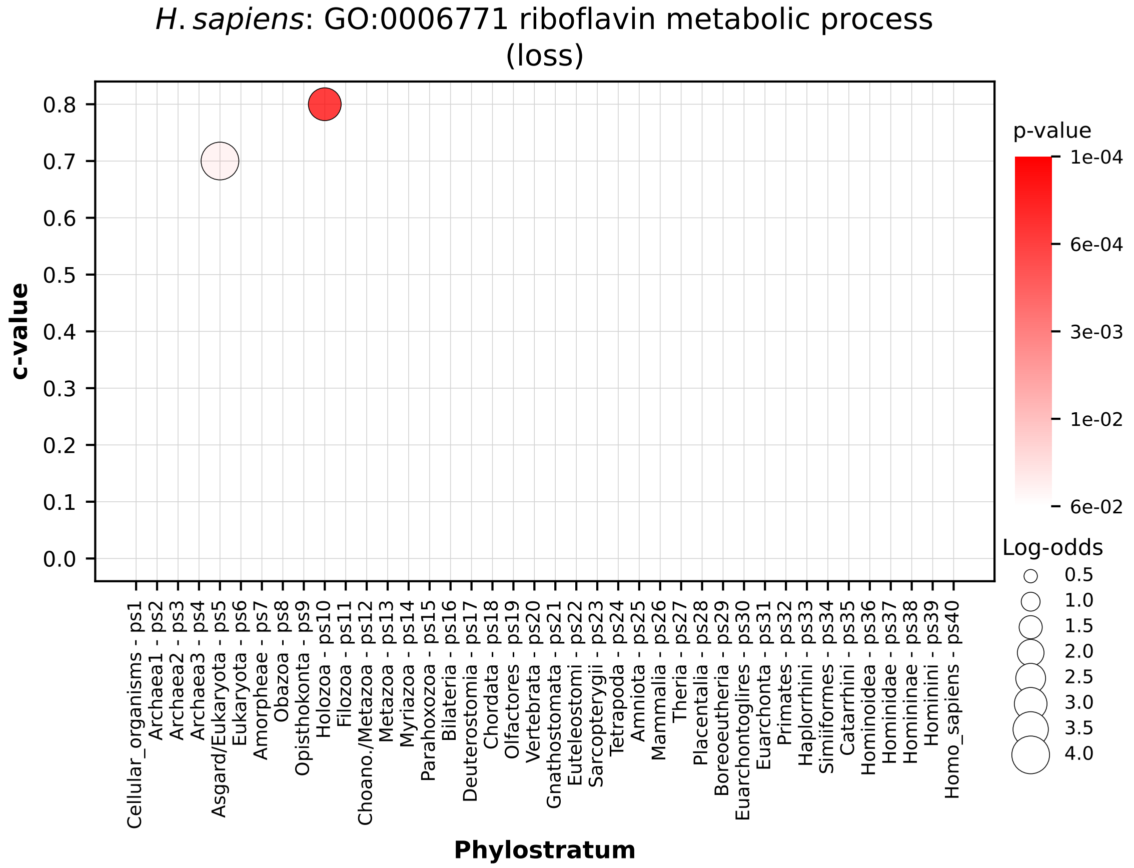

H. sapiens: GO:0006771 riboflavin metabolic process
(loss)
c-value
0.0
0.1
0.2
0.3
0.4
0.5
0.6
0.7
0.8
p-value
1e-04
6e-04
3e-03
1e-02
6e-02
Log-odds
0.5
1.0
1.5
2.0
2.5
3.0
3.5
4.0
Cellular_organisms - ps1
Archaea1 - ps2
Archaea2 - ps3
Archaea3 - ps4
Asgard/Eukaryota - ps5
Eukaryota - ps6
Amorpheae - ps7
Obazoa - ps8
Opisthokonta - ps9
Holozoa - ps10
Filozoa - ps11
Choano./Metazoa - ps12
Metazoa - ps13
Myriazoa - ps14
Parahoxozoa - ps15
Bilateria - ps16
Deuterostomia - ps17
Chordata - ps18
Olfactores - ps19
Vertebrata - ps20
Gnathostomata - ps21
Euteleostomi - ps22
Sarcopterygii - ps23
Tetrapoda - ps24
Amniota - ps25
Mammalia - ps26
Theria - ps27
Placentalia - ps28
Boreoeutheria - ps29
Euarchontoglires - ps30
Euarchonta - ps31
Primates - ps32
Haplorrhini - ps33
Simiiformes - ps34
Catarrhini - ps35
Hominoidea - ps36
Hominidae - ps37
Homininae - ps38
Hominini - ps39
Homo_sapiens - ps40
Phylostratum

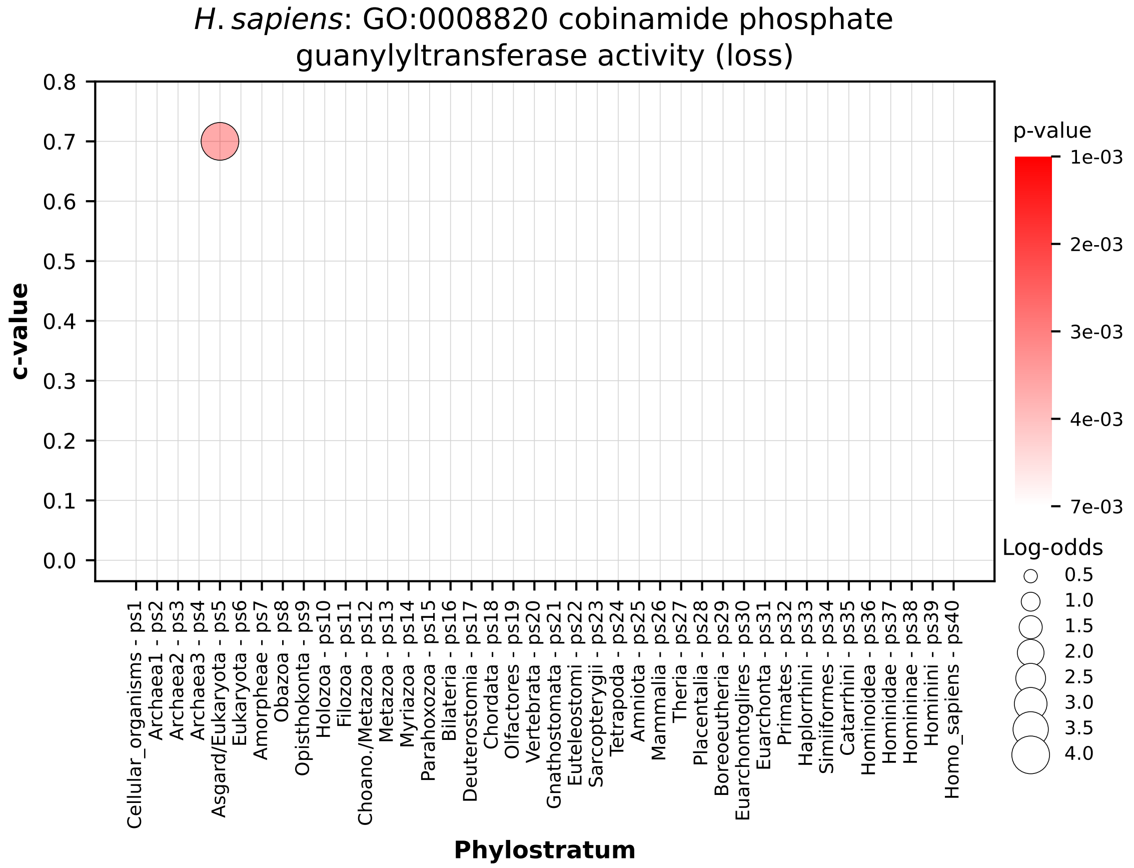

H. sapiens: GO:0008820 cobinamide phosphate guanylyltransferase activity (loss)
c-value
0.8
0.7
0.6
0.5
0.4
0.3
0.2
0.1
0.0
Cellular_organisms - ps1
Archaea1 - ps2
Archaea2 - ps3
Archaea3 - ps4
Asgard/Eukaryota - ps5
Eukaryota - ps6
Amorpheae - ps7
Obazoa - ps8
Opisthokonta - ps9
Holozoa - ps10
Filozoa - ps11
Choano./Metazoa - ps12
Metazoa - ps13
Myriazoa - ps14
Parahoxozoa - ps15
Bilateria - ps16
Deuterostomia - ps17
Chordata - ps18
Olfactores - ps19
Vertebrata - ps20
Gnathostomata - ps21
Euteleostomi - ps22
Sarcopterygii - ps23
Tetrapoda - ps24
Amniota - ps25
Mammalia - ps26
Theria - ps27
Placentalia - ps28
Boreoeutheria - ps29
Euarchontoglires - ps30
Euarchonta - ps31
Primates - ps32
Haplorrhini - ps33
Simiiformes - ps34
Catarrhini - ps35
Hominoidea - ps36
Hominidae - ps37
Homininae - ps38
Hominini - ps39
Homo_sapiens - ps40
Phylostratum
p-value
1e-03
2e-03
3e-03
4e-03
7e-03
Log-odds
0.5
1.0
1.5
2.0
2.5
3.0
3.5
4.0

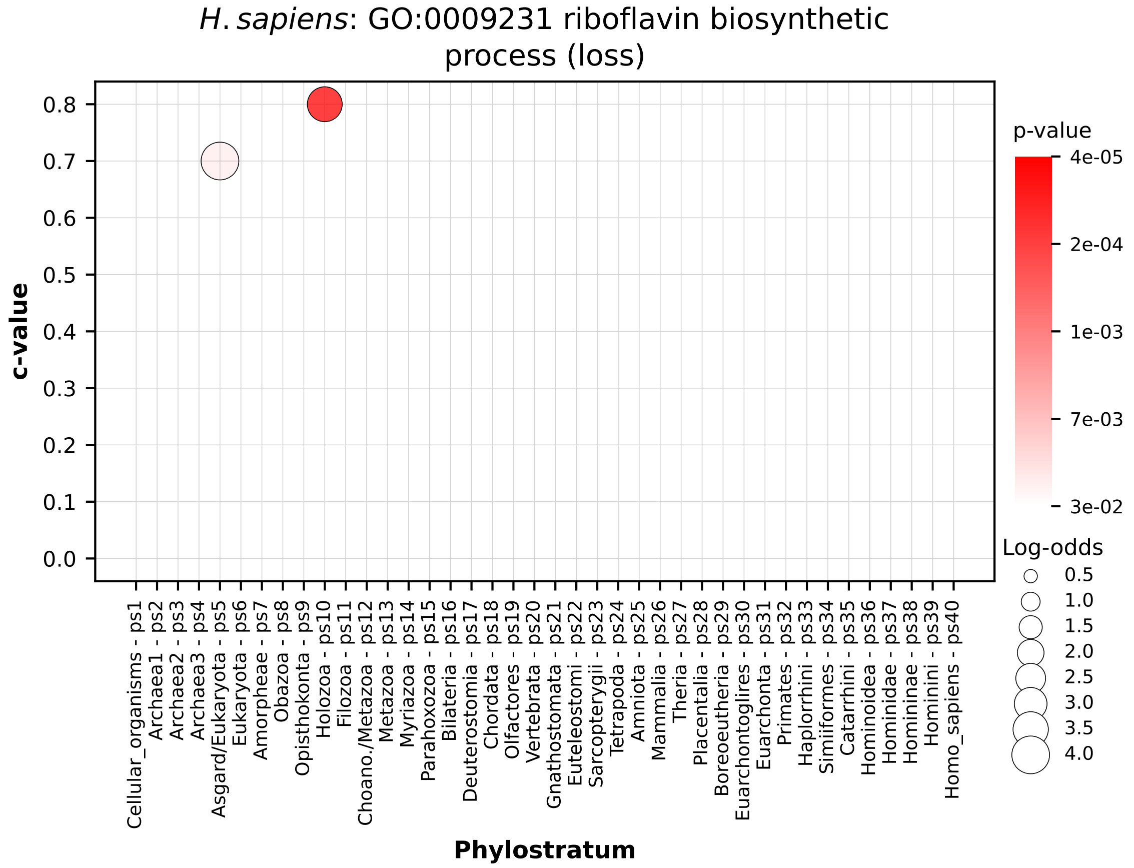

H. sapiens: GO:0009231 riboflavin biosynthetic process (loss)
c-value
0.8
0.7
0.6
0.5
0.4
0.3
0.2
0.1
0.0
p-value
4e-05
2e-04
1e-03
7e-03
3e-02
Log-odds
0.5
1.0
1.5
2.0
2.5
3.0
3.5
4.0
Cellular_organisms - ps1
Archaea1 - ps2
Archaea2 - ps3
Archaea3 - ps4
Asgard/Eukaryota - ps5
Eukaryota - ps6
Amorpheae - ps7
Obazoa - ps8
Opisthokonta - ps9
Holozoa - ps10
Filozoa - ps11
Choano./Metazoa - ps12
Metazoa - ps13
Myriazoa - ps14
Parahoxozoa - ps15
Bilateria - ps16
Deuterostomia - ps17
Chordata - ps18
Olfactores - ps19
Vertebrata - ps20
Gnathostomata - ps21
Euteleostomi - ps22
Sarcopterygii - ps23
Tetrapoda - ps24
Amniota - ps25
Mammalia - ps26
Theria - ps27
Placentalia - ps28
Boreoeutheria - ps29
Euarchontoglires - ps30
Euarchonta - ps31
Primates - ps32
Haplorrhini - ps33
Simiiformes - ps34
Catarrhini - ps35
Hominoidea - ps36
Hominidae - ps37
Homininae - ps38
Hominini - ps39
Homo_sapiens - ps40
Phylostratum

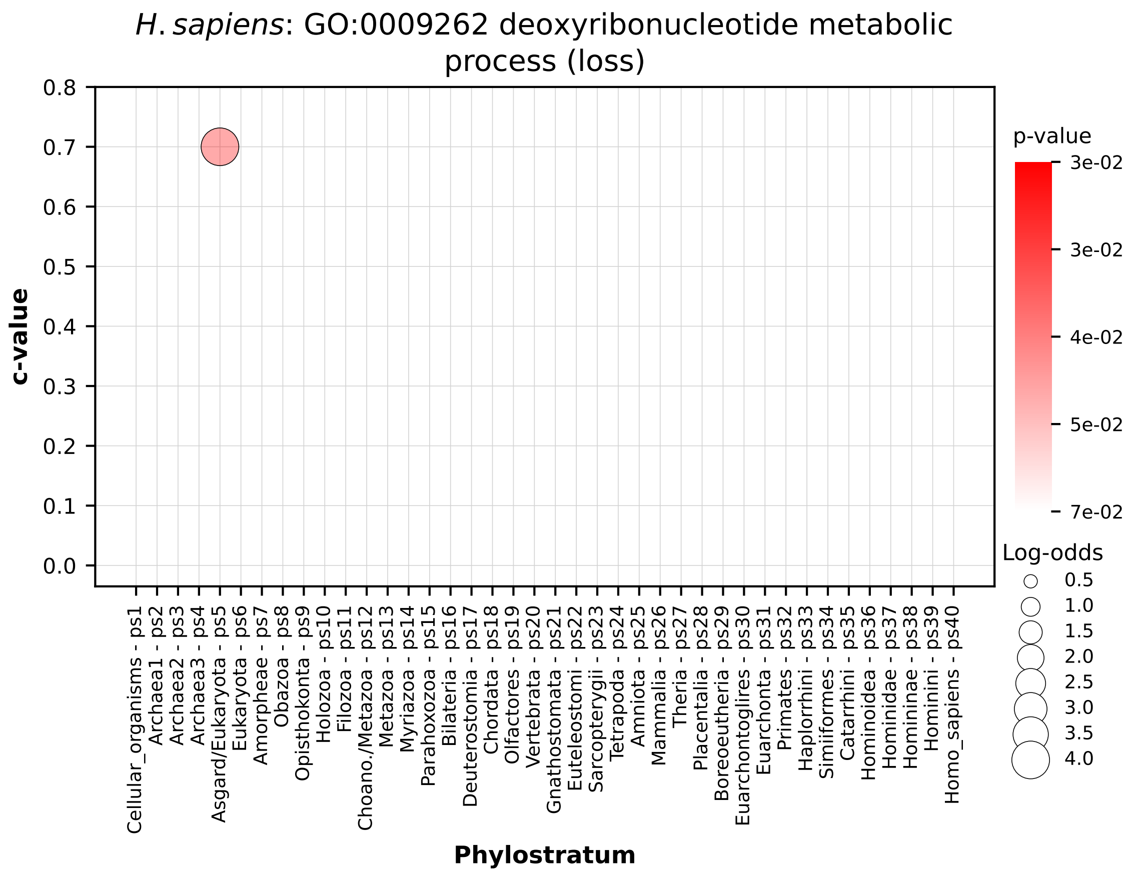

H. sapiens: GO:0009262 deoxyribonucleotide metabolic process (loss)
c-value
0.8
0.7
0.6
0.5
0.4
0.3
0.2
0.1
0.0
p-value
3e-02
3e-02
4e-02
5e-02
7e-02
Log-odds
0.5
1.0
1.5
2.0
2.5
3.0
3.5
4.0
Cellular_organisms - ps1
Archaea1 - ps2
Archaea2 - ps3
Archaea3 - ps4
Asgard/Eukaryota - ps5
Eukaryota - ps6
Amorpheae - ps7
Obazoa - ps8
Opisthokonta - ps9
Holozoa - ps10
Filozoa - ps11
Choano./Metazoa - ps12
Metazoa - ps13
Myriazoa - ps14
Parahoxozoa - ps15
Bilateria - ps16
Deuterostomia - ps17
Chordata - ps18
Olfactores - ps19
Vertebrata - ps20
Gnathostomata - ps21
Euteleostomi - ps22
Sarcopterygii - ps23
Tetrapoda - ps24
Amniota - ps25
Mammalia - ps26
Theria - ps27
Placentalia - ps28
Boreoeutheria - ps29
Euarchontoglires - ps30
Euarchonta - ps31
Primates - ps32
Haplorrhini - ps33
Simiiformes - ps34
Catarrhini - ps35
Hominoidea - ps36
Hominidae - ps37
Homininae - ps38
Hominini - ps39
Homo_sapiens - ps40
Phylostratum

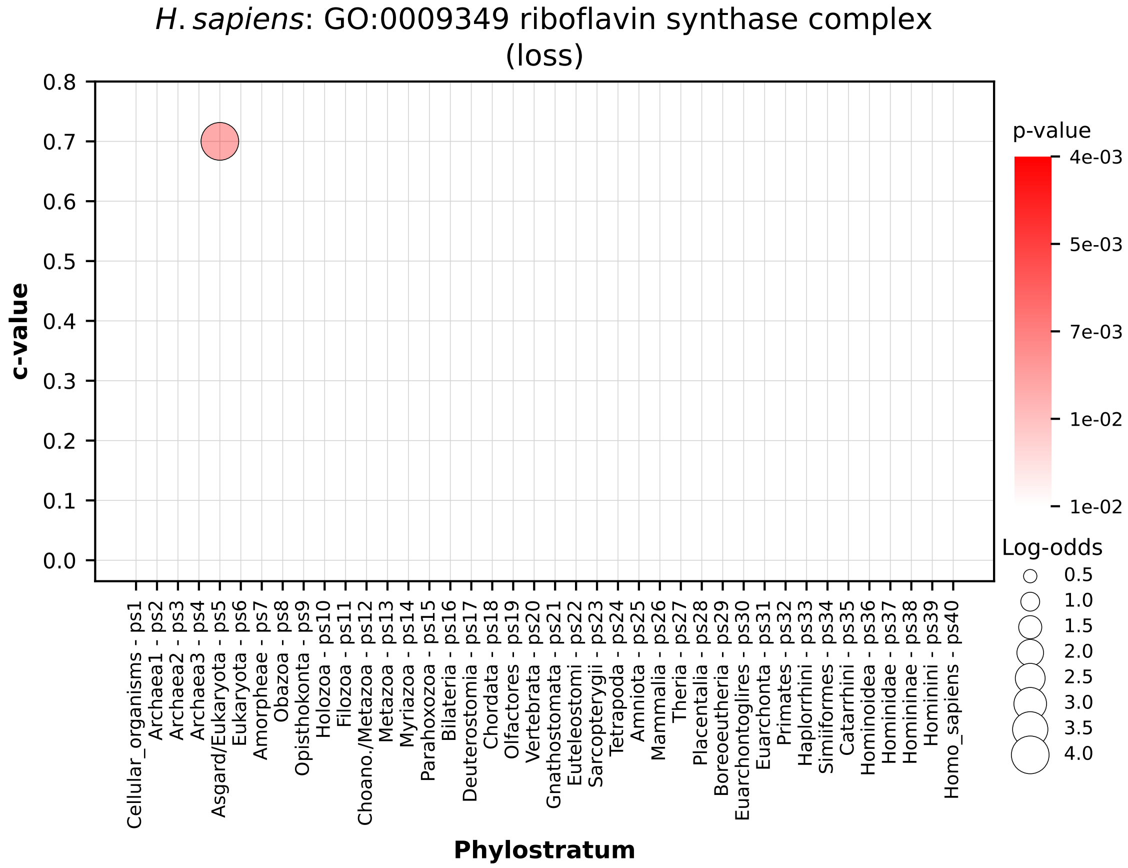

H. sapiens: GO:0009349 riboflavin synthase complex
(loss)
c-value
0.8
0.7
0.6
0.5
0.4
0.3
0.2
0.1
0.0
p-value
4e-03
5e-03
7e-03
1e-02
1e-02
Log-odds
0.5
1.0
1.5
2.0
2.5
3.0
3.5
4.0
Cellular_organisms - ps1
Archaea1 - ps2
Archaea2 - ps3
Archaea3 - ps4
Asgard/Eukaryota - ps5
Eukaryota - ps6
Amorpheae - ps7
Obazoa - ps8
Opisthokonta - ps9
Holozoa - ps10
Filozoa - ps11
Choano./Metazoa - ps12
Metazoa - ps13
Myriazoa - ps14
Parahoxozoa - ps15
Bilateria - ps16
Deuterostomia - ps17
Chordata - ps18
Olfactores - ps19
Vertebrata - ps20
Gnathostomata - ps21
Euteleostomi - ps22
Sarcopterygii - ps23
Tetrapoda - ps24
Amniota - ps25
Mammalia - ps26
Theria - ps27
Placentalia - ps28
Boreoeutheria - ps29
Euarchontoglires - ps30
Euarchonta - ps31
Primates - ps32
Haplorrhini - ps33
Simiiformes - ps34
Catarrhini - ps35
Hominoidea - ps36
Hominidae - ps37
Homininae - ps38
Hominini - ps39
Homo_sapiens - ps40
Phylostratum

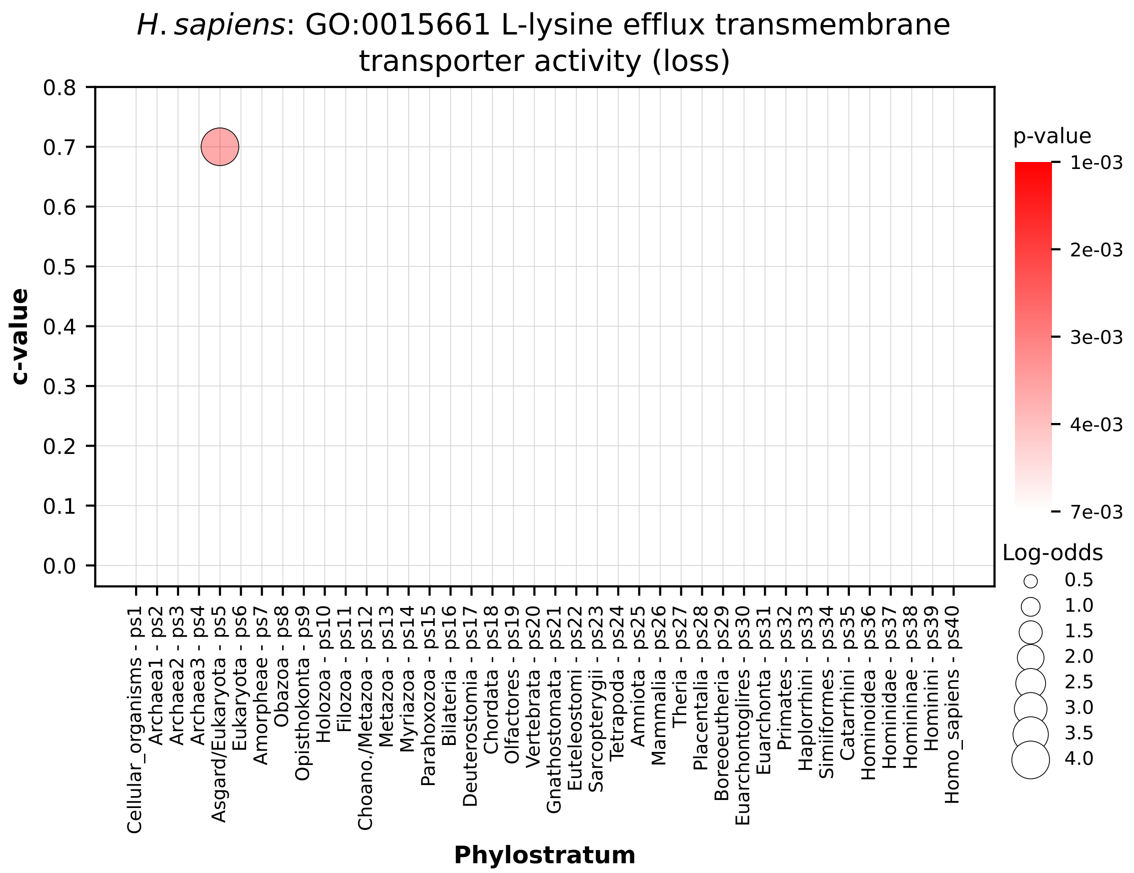

H. sapiens: GO:0015661 L-lysine efflux transmembrane transporter activity (loss)
c-value
0.8
0.7
0.6
0.5
0.4
0.3
0.2
0.1
0.0
Phylostratum
Cellular_organisms - ps1
Archaea1 - ps2
Archaea2 - ps3
Archaea3 - ps4
Asgard/Eukaryota - ps5
Eukaryota - ps6
Amorpheae - ps7
Obazoa - ps8
Opisthokonta - ps9
Holozoa - ps10
Filozoa - ps11
Choano./Metazoa - ps12
Metazoa - ps13
Myriazoa - ps14
Parahoxozoa - ps15
Bilateria - ps16
Deuterostomia - ps17
Chordata - ps18
Olfactores - ps19
Vertebrata - ps20
Gnathostomata - ps21
Euteleostomi - ps22
Sarcopterygii - ps23
Tetrapoda - ps24
Amniota - ps25
Mammalia - ps26
Theria - ps27
Placentalia - ps28
Boreoeutheria - ps29
Euarchontoglires - ps30
Euarchonta - ps31
Primates - ps32
Haplorrhini - ps33
Simiiformes - ps34
Catarrhini - ps35
Hominoidea - ps36
Hominidae - ps37
Homininae - ps38
Hominini - ps39
Homo_sapiens - ps40
p-value
1e-03
2e-03
3e-03
4e-03
7e-03
Log-odds
0.5
1.0
1.5
2.0
2.5
3.0
3.5
4.0

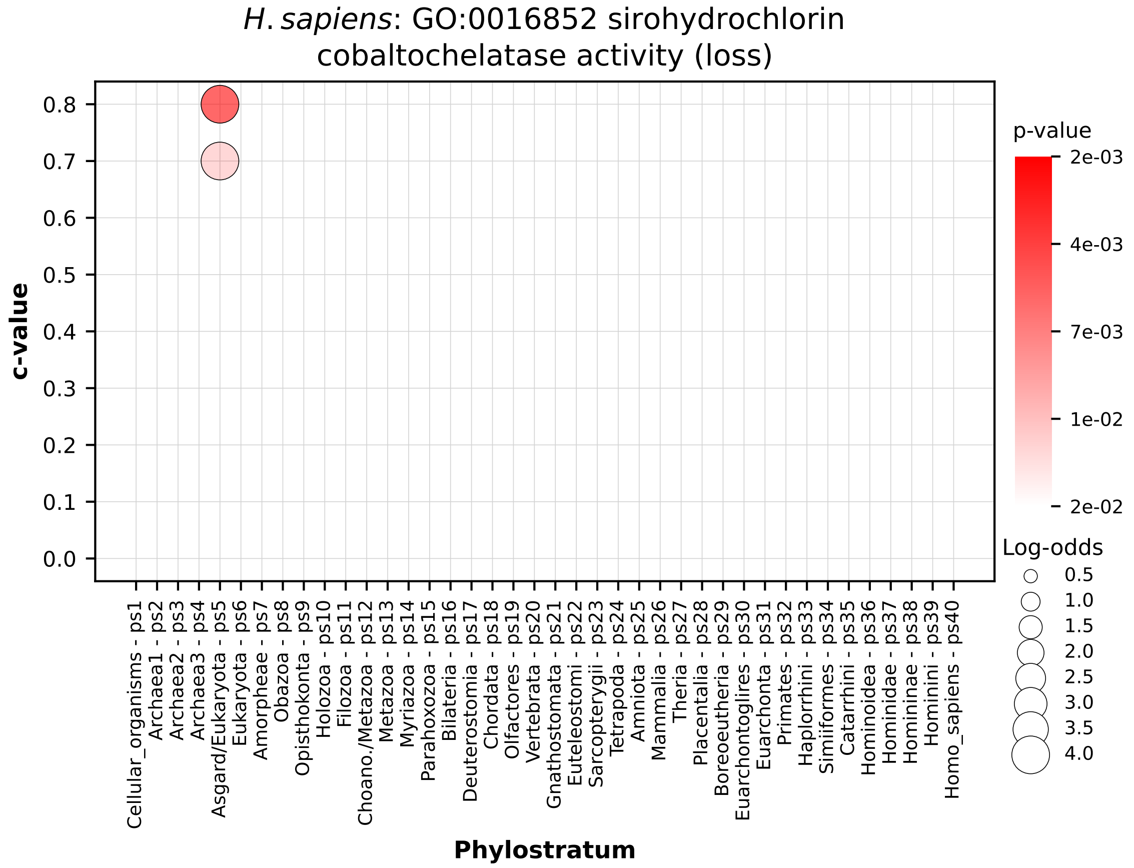

H. sapiens: GO:0016852 sirohydrochlorin cobaltochelatase activity (loss)
c-value
0.8
0.7
0.6
0.5
0.4
0.3
0.2
0.1
0.0
Phylostratum
Cellular_organisms - ps1
Archaea1 - ps2
Archaea2 - ps3
Archaea3 - ps4
Asgard/Eukaryota - ps5
Eukaryota - ps6
Amorpheae - ps7
Obazoa - ps8
Opisthokonta - ps9
Holozoa - ps10
Filozoa - ps11
Choano./Metazoa - ps12
Metazoa - ps13
Myriazoa - ps14
Parahoxozoa - ps15
Bilateria - ps16
Deuterostomia - ps17
Chordata - ps18
Olfactores - ps19
Vertebrata - ps20
Gnathostomata - ps21
Euteleostomi - ps22
Sarcopterygii - ps23
Tetrapoda - ps24
Amniota - ps25
Mammalia - ps26
Theria - ps27
Placentalia - ps28
Boreoeutheria - ps29
Euarchontoglires - ps30
Euarchonta - ps31
Primates - ps32
Haplorrhini - ps33
Simiiformes - ps34
Catarrhini - ps35
Hominoidea - ps36
Hominidae - ps37
Homininae - ps38
Hominini - ps39
Homo_sapiens - ps40
p-value
2e-03
4e-03
7e-03
1e-02
2e-02
Log-odds
0.5
1.0
1.5
2.0
2.5
3.0
3.5
4.0

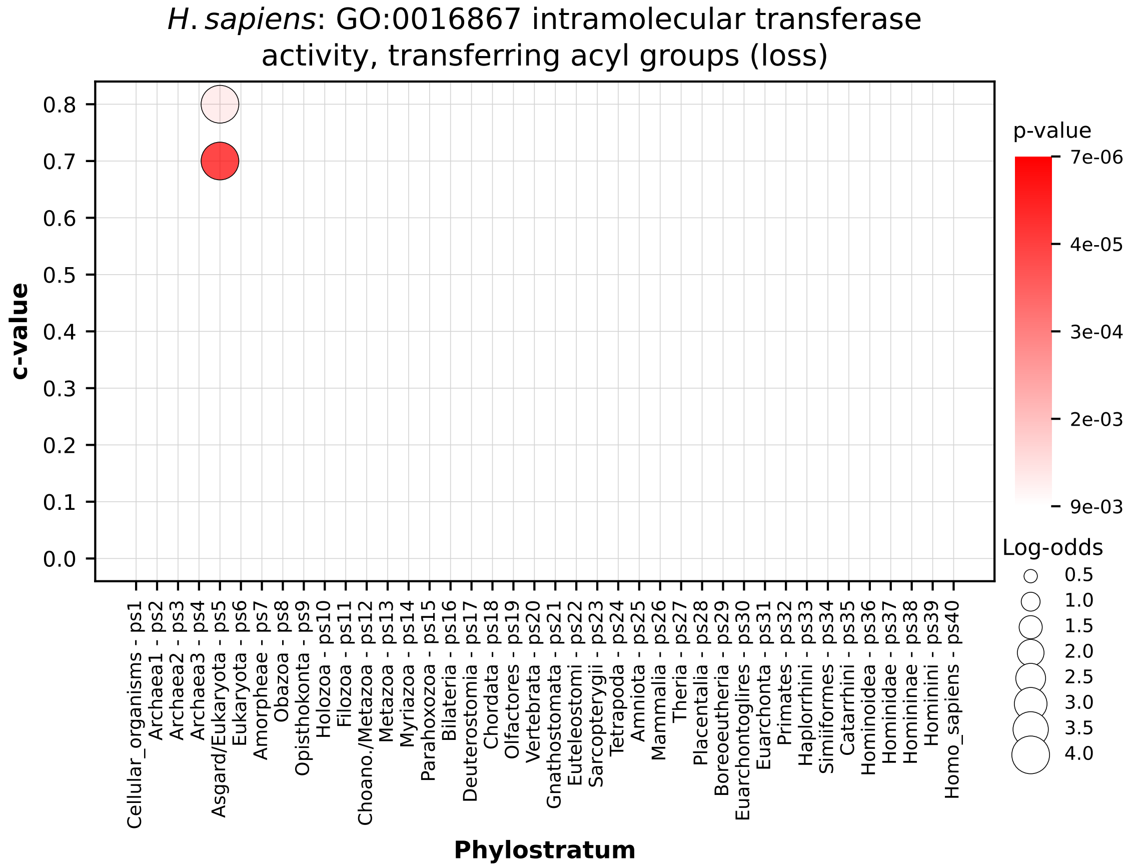

H. sapiens: GO:0016867 intramolecular transferase activity, transferring acyl groups (loss)
c-value
0.8
0.7
0.6
0.5
0.4
0.3
0.2
0.1
0.0
p-value
7e-06
4e-05
3e-04
2e-03
9e-03
Log-odds
0.5
1.0
1.5
2.0
2.5
3.0
3.5
4.0
Cellular_organisms - ps1
Archaea1 - ps2
Archaea2 - ps3
Archaea3 - ps4
Asgard/Eukaryota - ps5
Eukaryota - ps6
Amorpheae - ps7
Obazoa - ps8
Opisthokonta - ps9
Holozoa - ps10
Filozoa - ps11
Choano./Metazoa - ps12
Metazoa - ps13
Myriazoa - ps14
Parahoxozoa - ps15
Bilateria - ps16
Deuterostomia - ps17
Chordata - ps18
Olfactores - ps19
Vertebrata - ps20
Gnathostomata - ps21
Euteleostomi - ps22
Sarcopterygii - ps23
Tetrapoda - ps24
Amniota - ps25
Mammalia - ps26
Theria - ps27
Placentalia - ps28
Boreoeutheria - ps29
Euarchontoglires - ps30
Euarchonta - ps31
Primates - ps32
Haplorrhini - ps33
Simiiformes - ps34
Catarrhini - ps35
Hominoidea - ps36
Hominidae - ps37
Homininae - ps38
Hominini - ps39
Homo_sapiens - ps40
Phylostratum

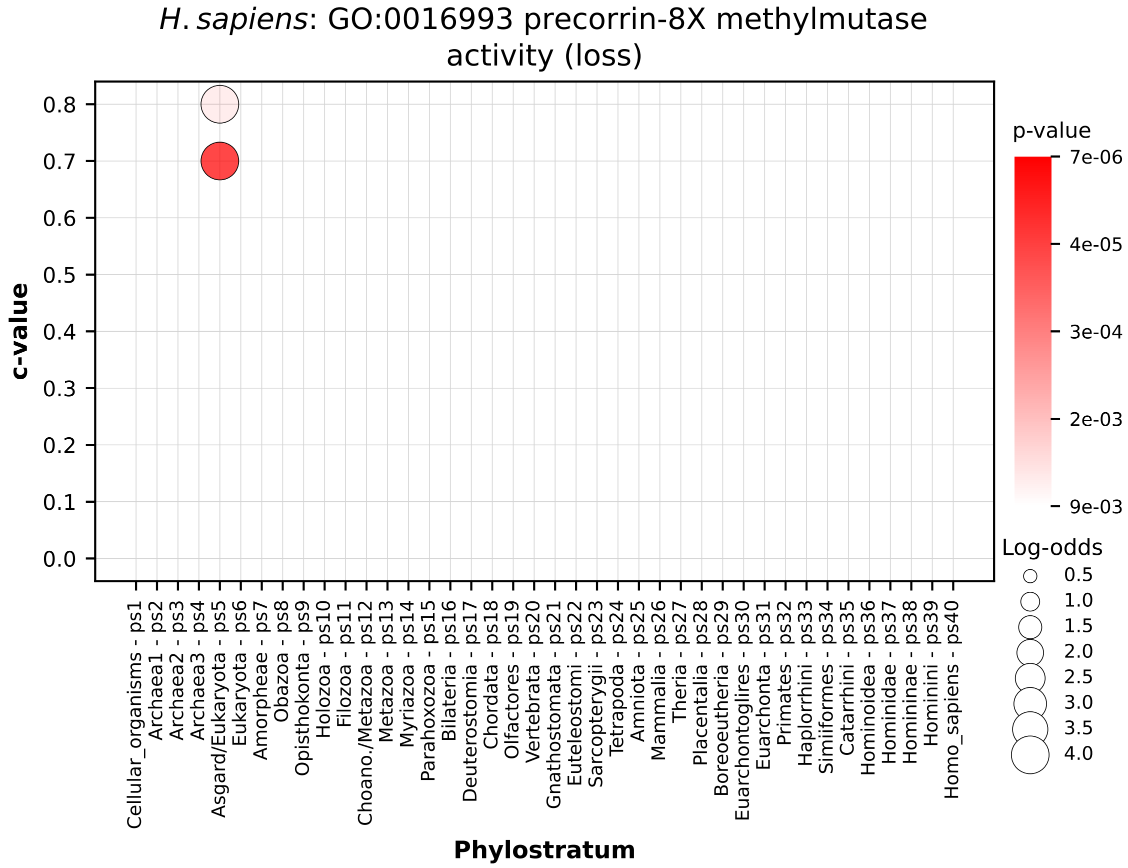

H. sapiens: GO:0016993 precorrin-8X methylmutase activity (loss)
c-value
0.8
0.7
0.6
0.5
0.4
0.3
0.2
0.1
0.0
p-value
7e-06
4e-05
3e-04
2e-03
9e-03
Log-odds
0.5
1.0
1.5
2.0
2.5
3.0
3.5
4.0
Cellular_organisms - ps1
Archaea1 - ps2
Archaea2 - ps3
Archaea3 - ps4
Asgard/Eukaryota - ps5
Eukaryota - ps6
Amorpheae - ps7
Obazoa - ps8
Opisthokonta - ps9
Holozoa - ps10
Filozoa - ps11
Choano./Metazoa - ps12
Metazoa - ps13
Myriazoa - ps14
Parahoxozoa - ps15
Bilateria - ps16
Deuterostomia - ps17
Chordata - ps18
Olfactores - ps19
Vertebrata - ps20
Gnathostomata - ps21
Euteleostomi - ps22
Sarcopterygii - ps23
Tetrapoda - ps24
Amniota - ps25
Mammalia - ps26
Theria - ps27
Placentalia - ps28
Boreoeutheria - ps29
Euarchontoglires - ps30
Euarchonta - ps31
Primates - ps32
Haplorrhini - ps33
Simiiformes - ps34
Catarrhini - ps35
Hominoidea - ps36
Hominidae - ps37
Homininae - ps38
Hominini - ps39
Homo_sapiens - ps40
Phylostratum

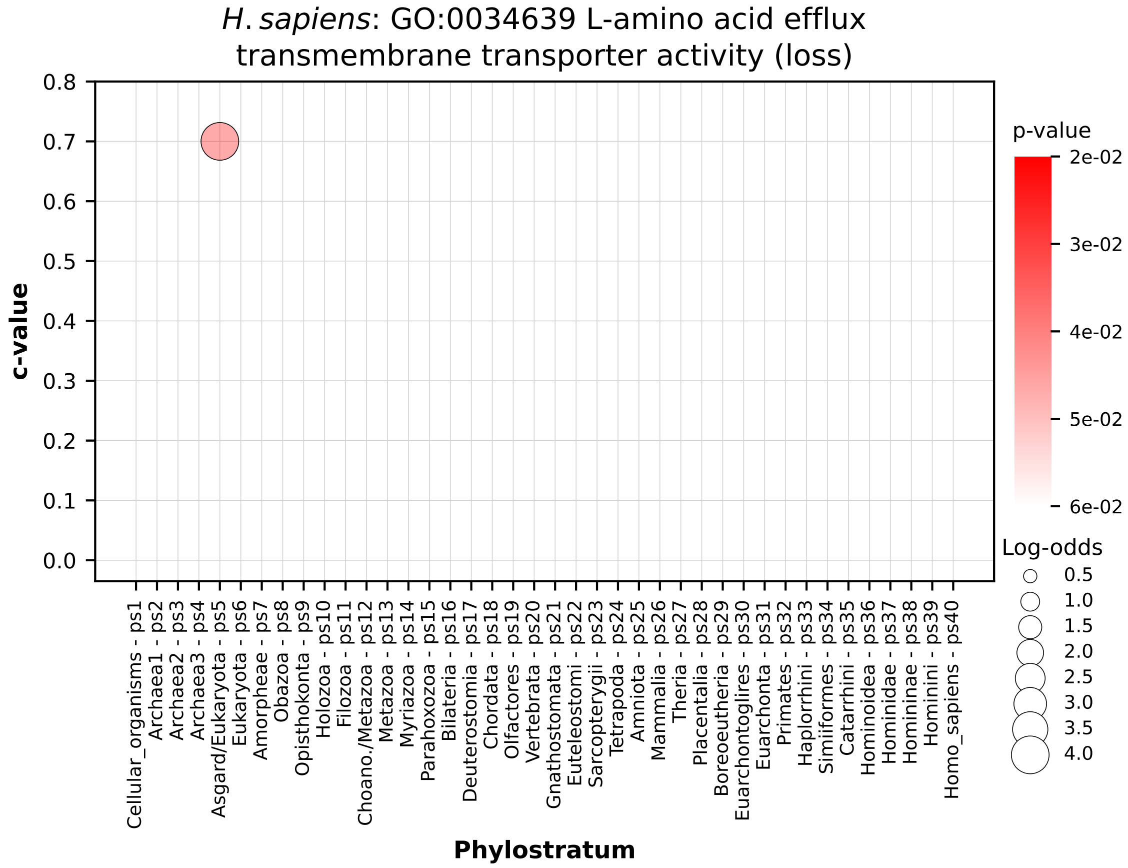
H. sapiens: GO:0034639 L-amino acid efflux transmembrane transporter activity (loss)
c-value
0.8
0.7
0.6
0.5
0.4
0.3
0.2
0.1
0.0
Cellular_organisms - ps1
Archaea1 - ps2
Archaea2 - ps3
Archaea3 - ps4
Asgard/Eukaryota - ps5
Eukaryota - ps6
Amorpheae - ps7
Obazoa - ps8
Opisthokonta - ps9
Holozoa - ps10
Filozoa - ps11
Choano./Metazoa - ps12
Metazoa - ps13
Myriazoa - ps14
Parahoxozoa - ps15
Bilateria - ps16
Deuterostomia - ps17
Chordata - ps18
Olfactores - ps19
Vertebrata - ps20
Gnathostomata - ps21
Euteleostomi - ps22
Sarcopterygii - ps23
Tetrapoda - ps24
Amniota - ps25
Mammalia - ps26
Theria - ps27
Placentalia - ps28
Boreoeutheria - ps29
Euarchontoglires - ps30
Euarchonta - ps31
Primates - ps32
Haplorrhini - ps33
Simiiformes - ps34
Catarrhini - ps35
Hominoidea - ps36
Hominidae - ps37
Homininae - ps38
Hominini - ps39
Homo_sapiens - ps40
Phylostratum
p-value
2e-02
3e-02
4e-02
5e-02
6e-02
Log-odds
0.5
1.0
1.5
2.0
2.5
3.0
3.5
4.0

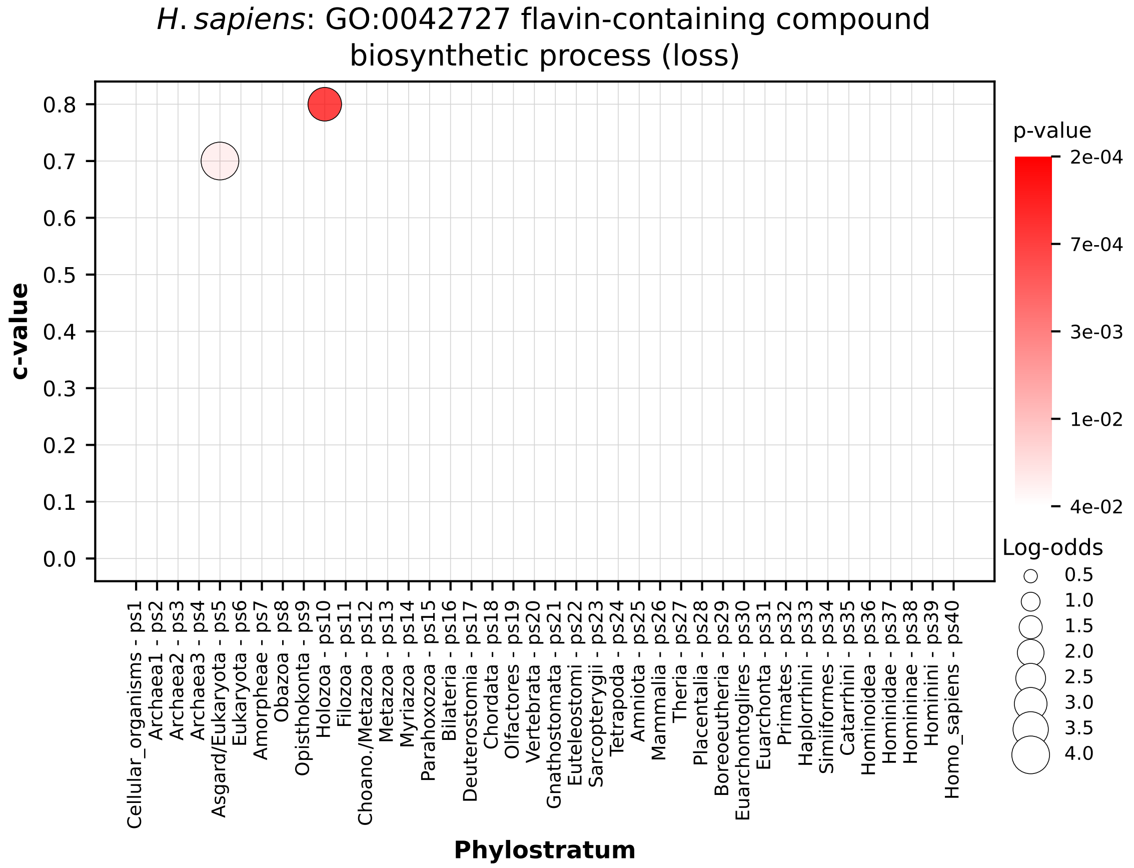

H. sapiens: GO:0042727 flavin-containing compound biosynthetic process (loss)
c-value
0.0
0.1
0.2
0.3
0.4
0.5
0.6
0.7
0.8
p-value
2e-04
7e-04
3e-03
1e-02
4e-02
Log-odds
0.5
1.0
1.5
2.0
2.5
3.0
3.5
4.0
Cellular_organisms - ps1
Archaea1 - ps2
Archaea2 - ps3
Archaea3 - ps4
Asgard/Eukaryota - ps5
Eukaryota - ps6
Amorpheae - ps7
Obazoa - ps8
Opisthokonta - ps9
Holozoa - ps10
Filozoa - ps11
Choano./Metazoa - ps12
Metazoa - ps13
Myriazoa - ps14
Parahoxozoa - ps15
Bilateria - ps16
Deuterostomia - ps17
Chordata - ps18
Olfactores - ps19
Vertebrata - ps20
Gnathostomata - ps21
Euteleostomi - ps22
Sarcopterygii - ps23
Tetrapoda - ps24
Amniota - ps25
Mammalia - ps26
Theria - ps27
Placentalia - ps28
Boreoeutheria - ps29
Euarchontoglires - ps30
Euarchonta - ps31
Primates - ps32
Haplorrhini - ps33
Simiiformes - ps34
Catarrhini - ps35
Hominoidea - ps36
Hominidae - ps37
Homininae - ps38
Hominini - ps39
Homo_sapiens - ps40
Phylostratum

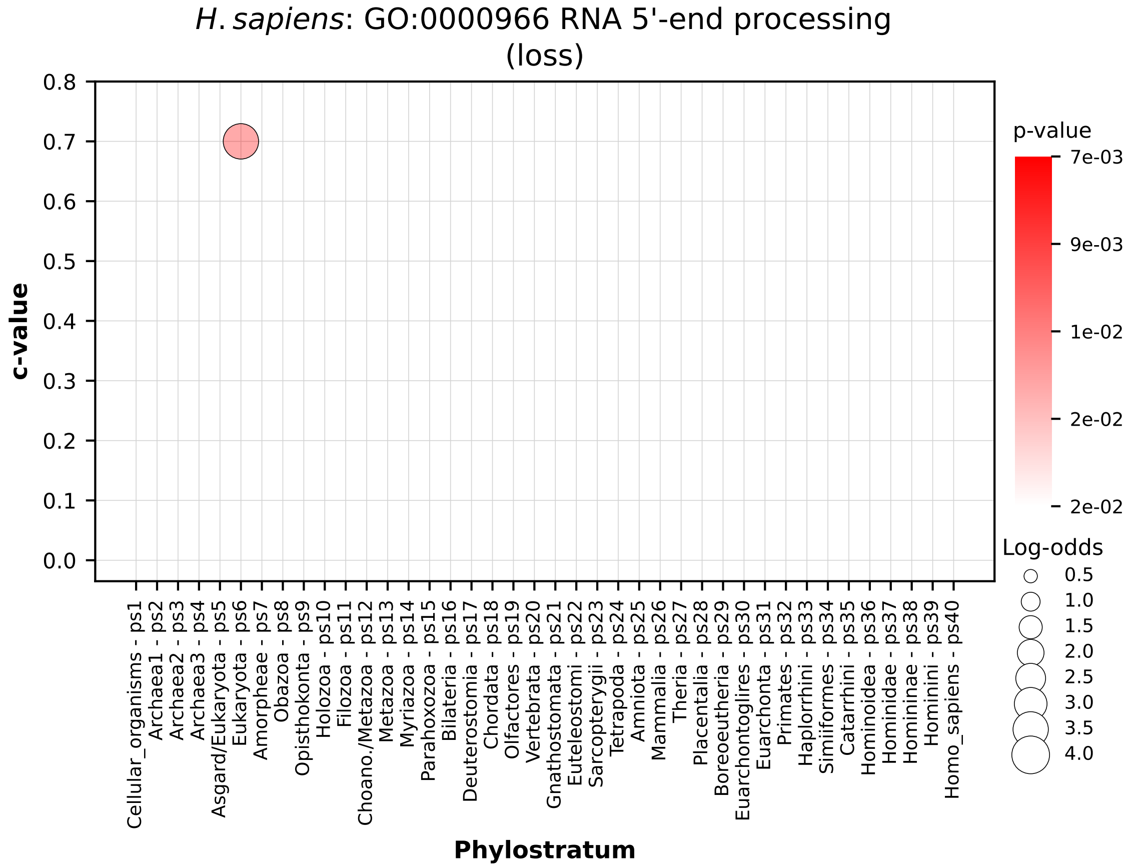

H. sapiens: GO:0000966 RNA 5'-end processing (loss)
c-value
0.8
0.7
0.6
0.5
0.4
0.3
0.2
0.1
0.0
Phylostratum
Cellular_organisms - ps1
Archaea1 - ps2
Archaea2 - ps3
Archaea3 - ps4
Asgard/Eukaryota - ps5
Eukaryota - ps6
Amorpheae - ps7
Obazoa - ps8
Opisthokonta - ps9
Holozoa - ps10
Filozoa - ps11
Choano./Metazoa - ps12
Metazoa - ps13
Myriazoa - ps14
Parahoxozoa - ps15
Bilateria - ps16
Deuterostomia - ps17
Chordata - ps18
Olfactores - ps19
Vertebrata - ps20
Gnathostomata - ps21
Euteleostomi - ps22
Sarcopterygii - ps23
Tetrapoda - ps24
Amniota - ps25
Mammalia - ps26
Theria - ps27
Placentalia - ps28
Boreoeutheria - ps29
Euarchontoglires - ps30
Euarchonta - ps31
Primates - ps32
Haplorrhini - ps33
Simiiformes - ps34
Catarrhini - ps35
Hominoidea - ps36
Hominidae - ps37
Homininae - ps38
Hominini - ps39
Homo_sapiens - ps40
p-value
7e-03
9e-03
1e-02
2e-02
2e-02
Log-odds
0.5
1.0
1.5
2.0
2.5
3.0
3.5
4.0

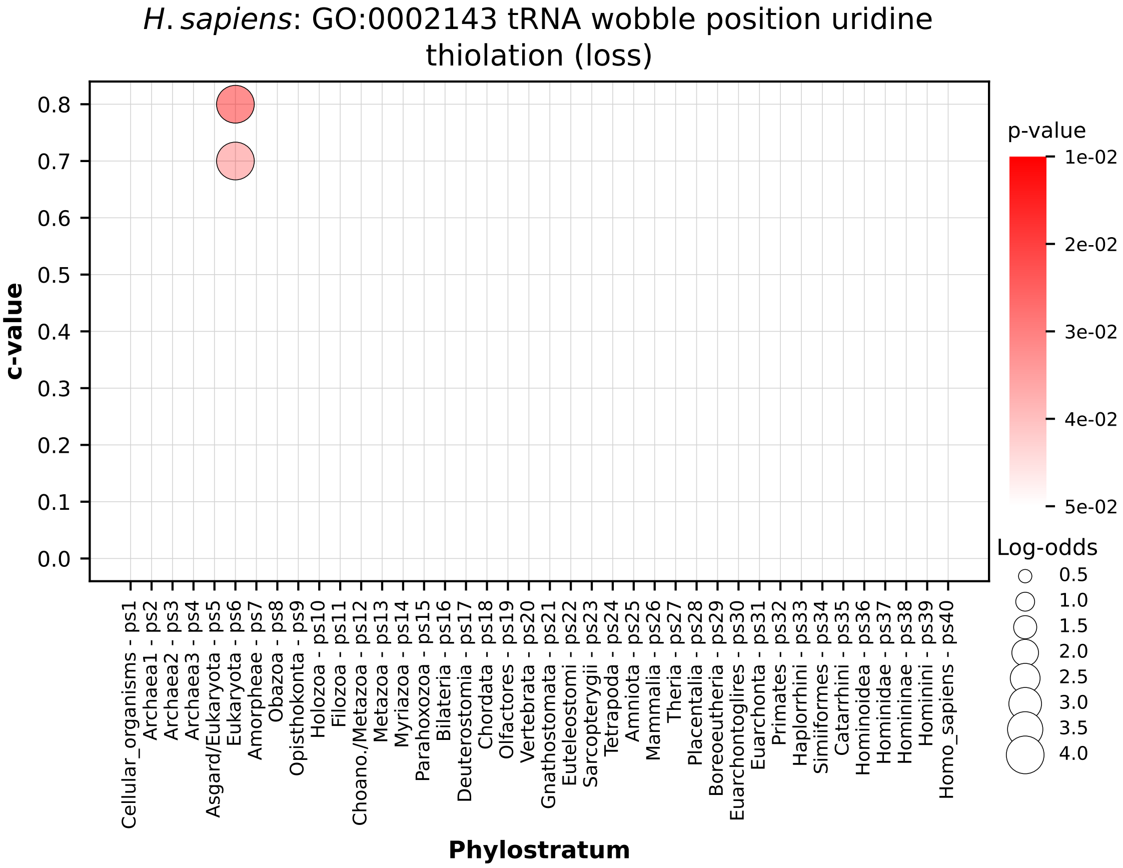

H. sapiens: GO:0002143 tRNA wobble position uridine thiolation (loss)
c-value
0.0
0.1
0.2
0.3
0.4
0.5
0.6
0.7
0.8
p-value
1e-02
2e-02
3e-02
4e-02
5e-02
Log-odds
0.5
1.0
1.5
2.0
2.5
3.0
3.5
4.0
Cellular_organisms - ps1
Archaea1 - ps2
Archaea2 - ps3
Archaea3 - ps4
Asgard/Eukaryota - ps5
Eukaryota - ps6
Amorpheae - ps7
Obazoa - ps8
Opisthokonta - ps9
Holozoa - ps10
Filozoa - ps11
Choano./Metazoa - ps12
Metazoa - ps13
Myriazoa - ps14
Parahoxozoa - ps15
Bilateria - ps16
Deuterostomia - ps17
Chordata - ps18
Olfactores - ps19
Vertebrata - ps20
Gnathostomata - ps21
Euteleostomi - ps22
Sarcopterygii - ps23
Tetrapoda - ps24
Amniota - ps25
Mammalia - ps26
Theria - ps27
Placentalia - ps28
Boreoeutheria - ps29
Euarchontoglires - ps30
Euarchonta - ps31
Primates - ps32
Haplorrhini - ps33
Simiiformes - ps34
Catarrhini - ps35
Hominoidea - ps36
Hominidae - ps37
Homininae - ps38
Hominini - ps39
Homo_sapiens - ps40
Phylostratum

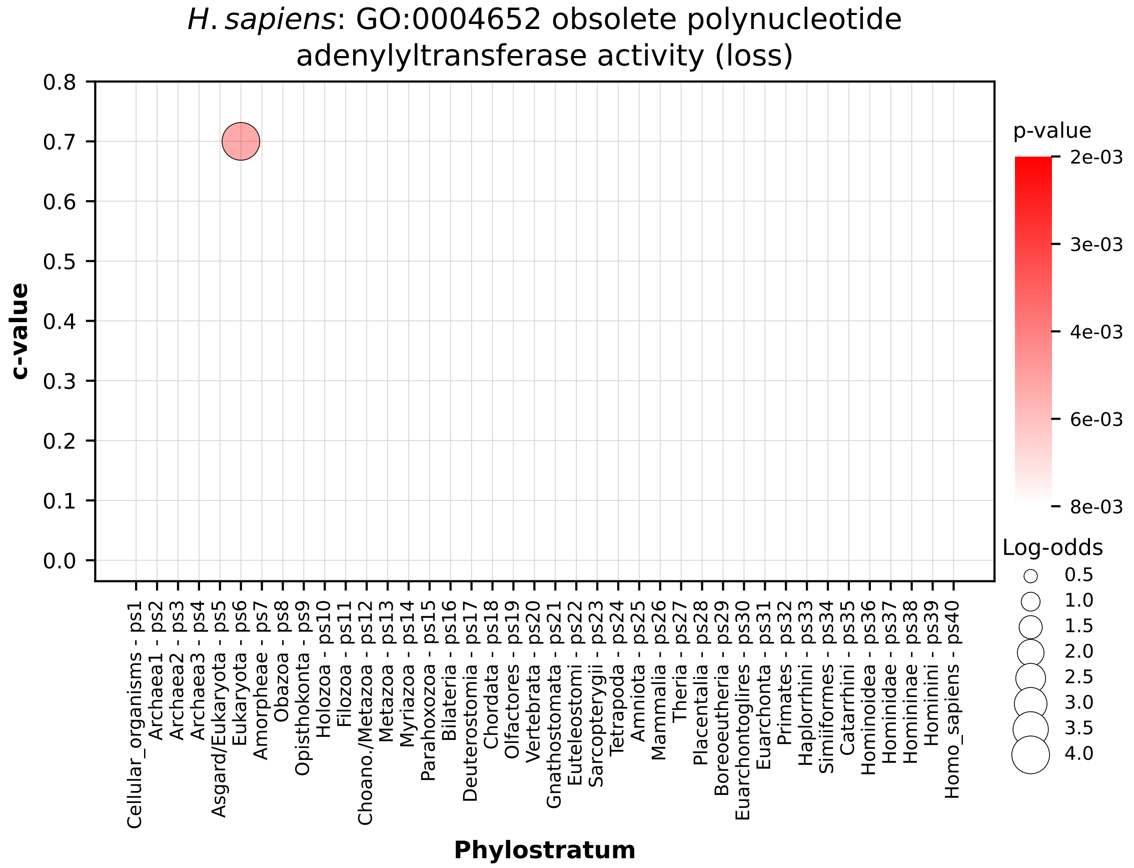
H. sapiens: GO:0004652 obsolete polynucleotide adenylyltransferase activity (loss)
c-value
0.8
0.7
0.6
0.5
0.4
0.3
0.2
0.1
0.0
Phylostratum
Cellular_organisms - ps1
Archaea1 - ps2
Archaea2 - ps3
Archaea3 - ps4
Asgard/Eukaryota - ps5
Eukaryota - ps6
Amorpheae - ps7
Obazoa - ps8
Opisthokonta - ps9
Holozoa - ps10
Filozoa - ps11
Choano./Metazoa - ps12
Metazoa - ps13
Myriazoa - ps14
Parahoxozoa - ps15
Bilateria - ps16
Deuterostomia - ps17
Chordata - ps18
Olfactores - ps19
Vertebrata - ps20
Gnathostomata - ps21
Euteleostomi - ps22
Sarcopterygii - ps23
Tetrapoda - ps24
Amniota - ps25
Mammalia - ps26
Theria - ps27
Placentalia - ps28
Boreoeutheria - ps29
Euarchontoglires - ps30
Euarchonta - ps31
Primates - ps32
Haplorrhini - ps33
Simiiformes - ps34
Catarrhini - ps35
Hominoidea - ps36
Hominidae - ps37
Homininae - ps38
Hominini - ps39
Homo_sapiens - ps40
p-value
2e-03
3e-03
4e-03
6e-03
8e-03
Log-odds
0.5
1.0
1.5
2.0
2.5
3.0
3.5
4.0

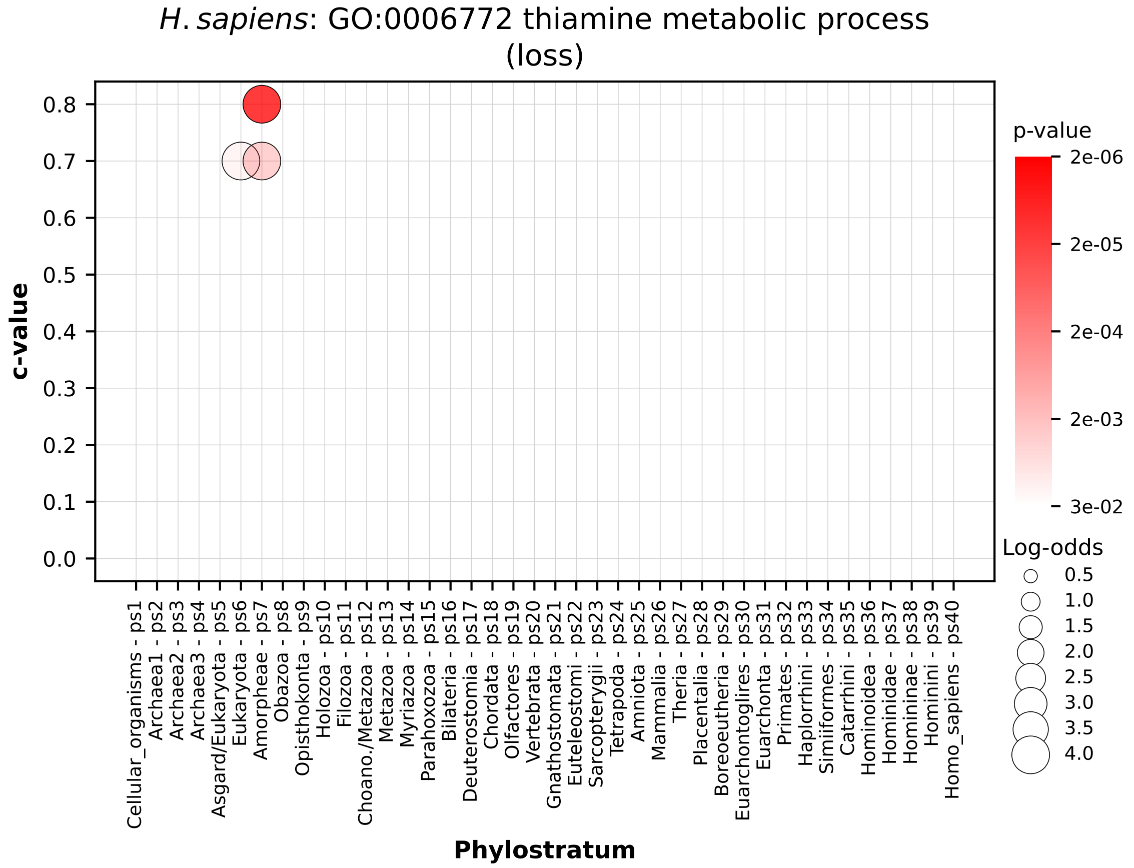

H. sapiens: GO:0006772 thiamine metabolic process
(loss)
c-value
0.8
0.7
0.6
0.5
0.4
0.3
0.2
0.1
0.0
p-value
2e-06
2e-05
2e-04
2e-03
3e-02
Log-odds
0.5
1.0
1.5
2.0
2.5
3.0
3.5
4.0
Cellular_organisms - ps1
Archaea1 - ps2
Archaea2 - ps3
Archaea3 - ps4
Asgard/Eukaryota - ps5
Eukaryota - ps6
Amorpheae - ps7
Obazoa - ps8
Opisthokonta - ps9
Holozoa - ps10
Filozoa - ps11
Choano./Metazoa - ps12
Metazoa - ps13
Myriazoa - ps14
Parahoxozoa - ps15
Bilateria - ps16
Deuterostomia - ps17
Chordata - ps18
Olfactores - ps19
Vertebrata - ps20
Gnathostomata - ps21
Euteleostomi - ps22
Sarcopterygii - ps23
Tetrapoda - ps24
Amniota - ps25
Mammalia - ps26
Theria - ps27
Placentalia - ps28
Boreoeutheria - ps29
Euarchontoglires - ps30
Euarchonta - ps31
Primates - ps32
Haplorrhini - ps33
Simiiformes - ps34
Catarrhini - ps35
Hominoidea - ps36
Hominidae - ps37
Homininae - ps38
Hominini - ps39
Homo_sapiens - ps40
Phylostratum

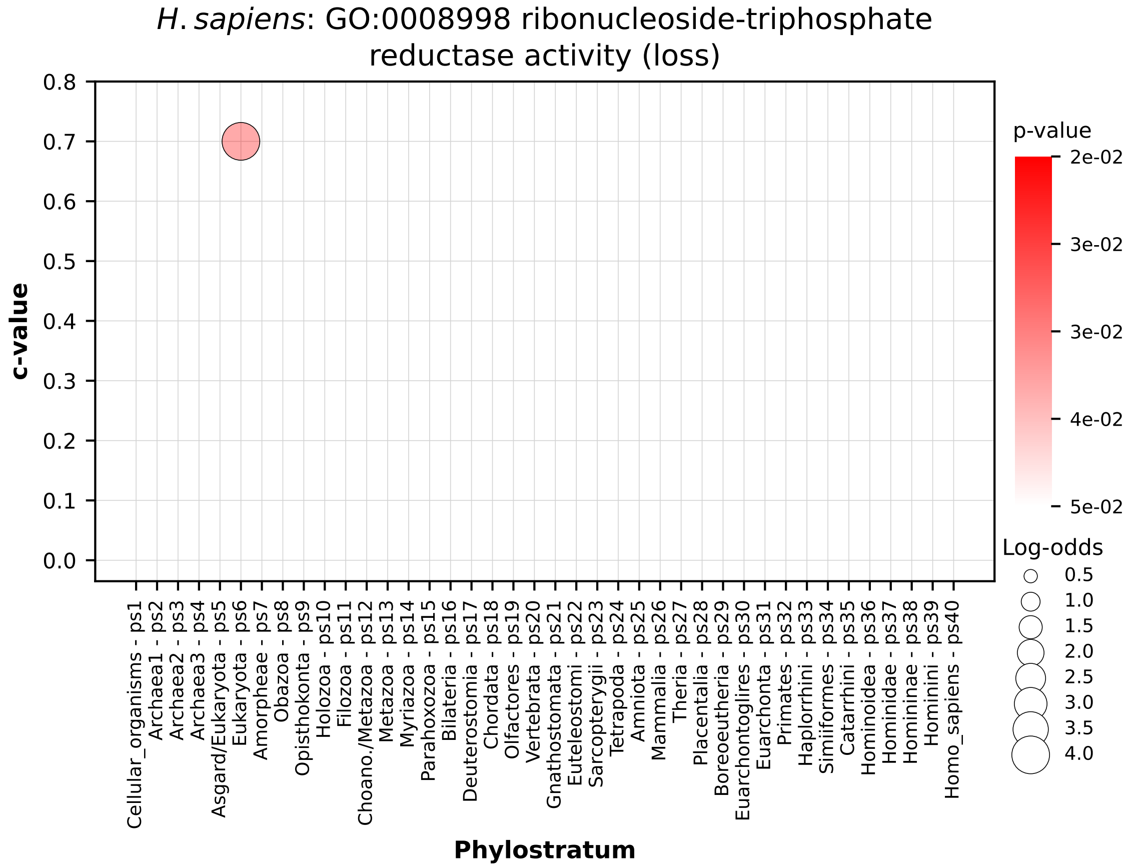

H. sapiens: GO:0008998 ribonucleoside-triphosphate reductase activity (loss)
c-value
0.8
0.7
0.6
0.5
0.4
0.3
0.2
0.1
0.0
p-value
2e-02
3e-02
3e-02
4e-02
5e-02
Log-odds
0.5
1.0
1.5
2.0
2.5
3.0
3.5
4.0
Cellular_organisms - ps1
Archaea1 - ps2
Archaea2 - ps3
Archaea3 - ps4
Asgard/Eukaryota - ps5
Eukaryota - ps6
Amorpheae - ps7
Obazoa - ps8
Opisthokonta - ps9
Holozoa - ps10
Filozoa - ps11
Choano./Metazoa - ps12
Metazoa - ps13
Myriazoa - ps14
Parahoxozoa - ps15
Bilateria - ps16
Deuterostomia - ps17
Chordata - ps18
Olfactores - ps19
Vertebrata - ps20
Gnathostomata - ps21
Euteleostomi - ps22
Sarcopterygii - ps23
Tetrapoda - ps24
Amniota - ps25
Mammalia - ps26
Theria - ps27
Placentalia - ps28
Boreoeutheria - ps29
Euarchontoglires - ps30
Euarchonta - ps31
Primates - ps32
Haplorrhini - ps33
Simiiformes - ps34
Catarrhini - ps35
Hominoidea - ps36
Hominidae - ps37
Homininae - ps38
Hominini - ps39
Homo_sapiens - ps40
Phylostratum

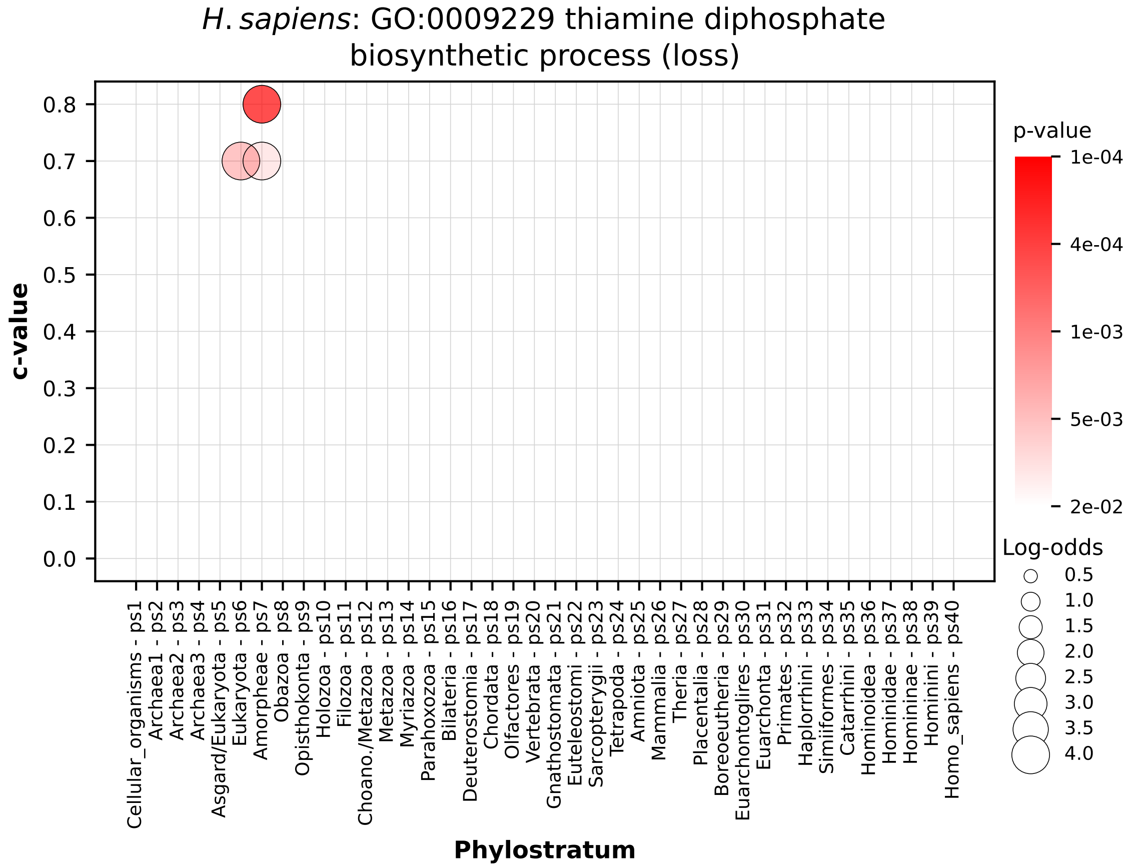

H. sapiens: GO:0009229 thiamine diphosphate biosynthetic process (loss)
c-value
0.0
0.1
0.2
0.3
0.4
0.5
0.6
0.7
0.8
Phylostratum
Cellular_organisms - ps1
Archaea1 - ps2
Archaea2 - ps3
Archaea3 - ps4
Asgard/Eukaryota - ps5
Eukaryota - ps6
Amorpheae - ps7
Obazoa - ps8
Opisthokonta - ps9
Holozoa - ps10
Filozoa - ps11
Choano./Metazoa - ps12
Metazoa - ps13
Myriazoa - ps14
Parahoxozoa - ps15
Bilateria - ps16
Deuterostomia - ps17
Chordata - ps18
Olfactores - ps19
Vertebrata - ps20
Gnathostomata - ps21
Euteleostomi - ps22
Sarcopterygii - ps23
Tetrapoda - ps24
Amniota - ps25
Mammalia - ps26
Theria - ps27
Placentalia - ps28
Boreoeutheria - ps29
Euarchontoglires - ps30
Euarchonta - ps31
Primates - ps32
Haplorrhini - ps33
Simiiformes - ps34
Catarrhini - ps35
Hominoidea - ps36
Hominidae - ps37
Homininae - ps38
Hominini - ps39
Homo_sapiens - ps40
p-value
1e-04
4e-04
1e-03
5e-03
2e-02
Log-odds
0.5
1.0
1.5
2.0
2.5
3.0
3.5
4.0

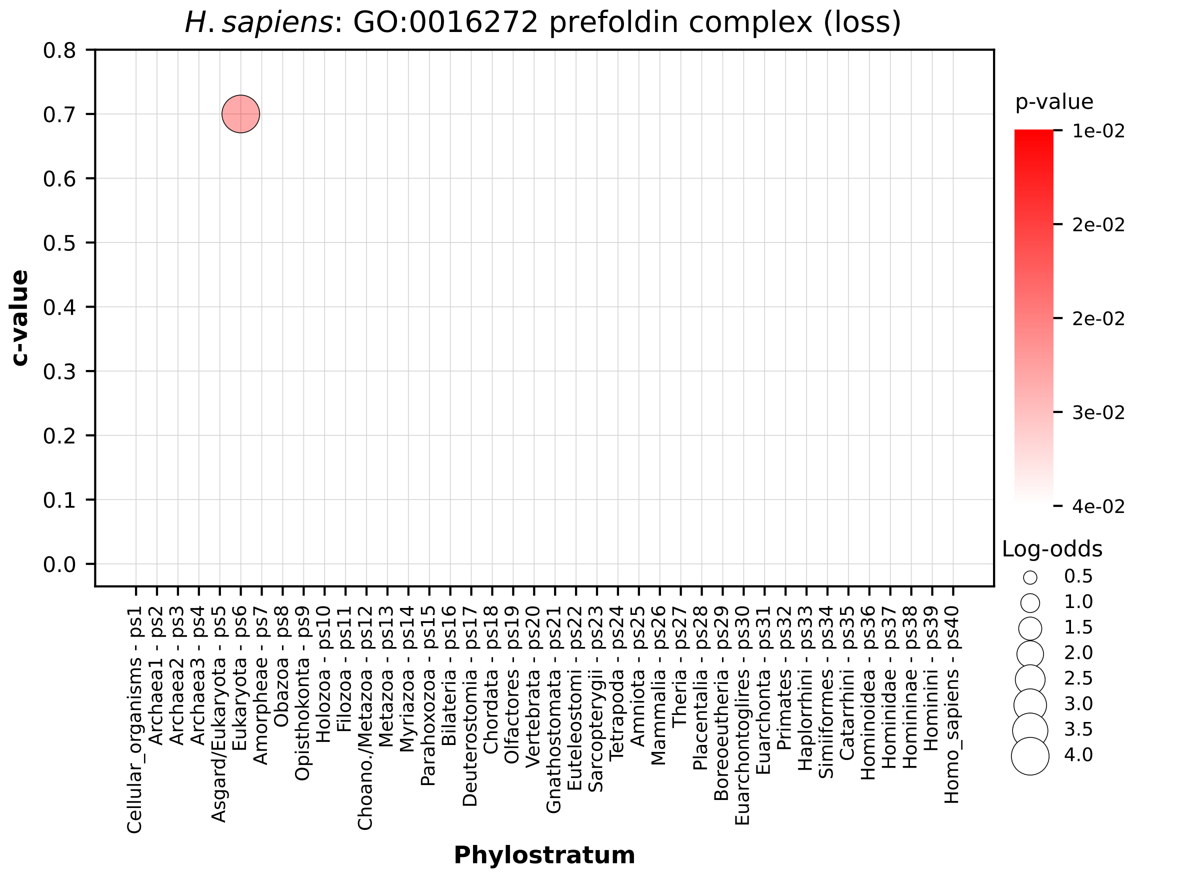

H. sapiens: GO:0016272 prefoldin complex (loss)
c-value
0.8
0.7
0.6
0.5
0.4
0.3
0.2
0.1
0.0
Phylostratum
Cellular_organisms - ps1
Archaea1 - ps2
Archaea2 - ps3
Archaea3 - ps4
Asgard/Eukaryota - ps5
Eukaryota - ps6
Amorpheae - ps7
Obazoa - ps8
Opisthokonta - ps9
Holozoa - ps10
Filozoa - ps11
Choano./Metazoa - ps12
Metazoa - ps13
Myriazoa - ps14
Parahoxozoa - ps15
Bilateria - ps16
Deuterostomia - ps17
Chordata - ps18
Olfactores - ps19
Vertebrata - ps20
Gnathostomata - ps21
Euteleostomi - ps22
Sarcopterygii - ps23
Tetrapoda - ps24
Amniota - ps25
Mammalia - ps26
Theria - ps27
Placentalia - ps28
Boreoeutheria - ps29
Euarchontoglires - ps30
Euarchonta - ps31
Primates - ps32
Haplorrhini - ps33
Simiiformes - ps34
Catarrhini - ps35
Hominoidea - ps36
Hominidae - ps37
Homininae - ps38
Hominini - ps39
Homo_sapiens - ps40
p-value
1e-02
2e-02
2e-02
3e-02
4e-02
Log-odds
0.5
1.0
1.5
2.0
2.5
3.0
3.5
4.0

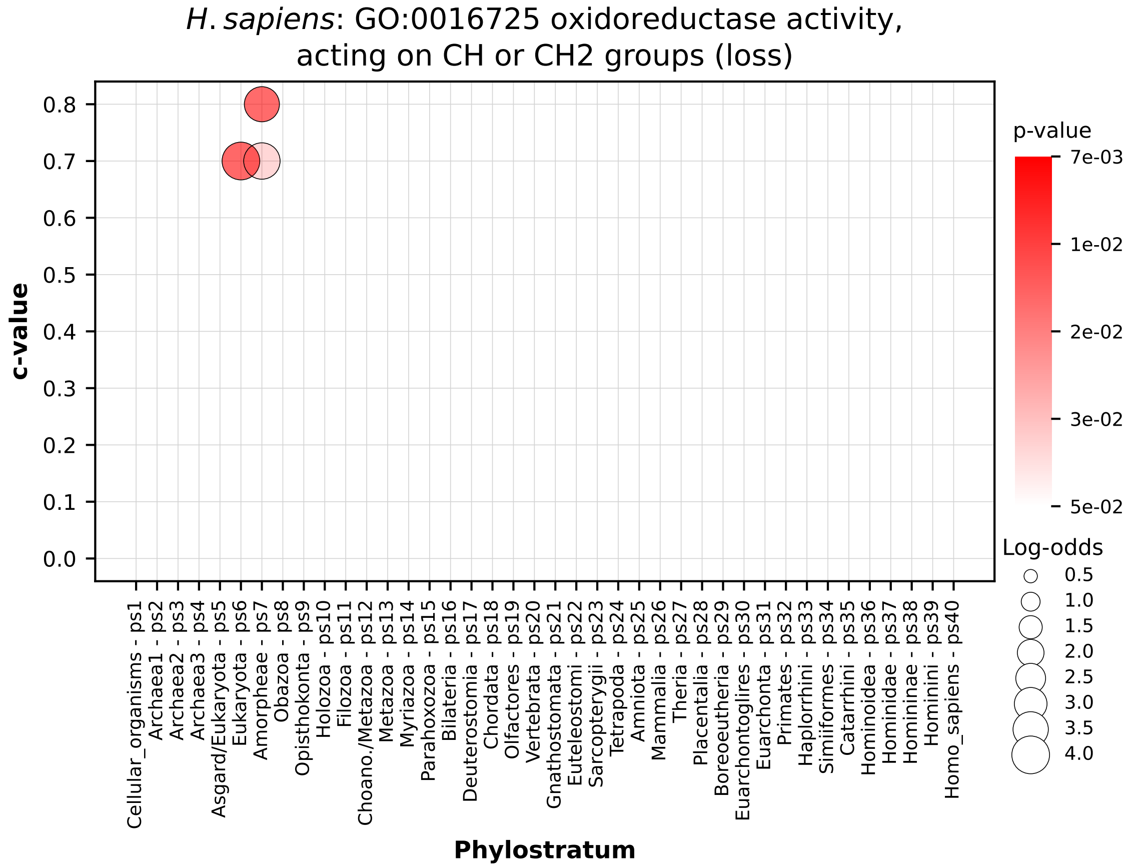

H. sapiens: GO:0016725 oxidoreductase activity, acting on CH or CH2 groups (loss)
c-value
0.0
0.1
0.2
0.3
0.4
0.5
0.6
0.7
0.8
Phylostratum
Cellular_organisms - ps1
Archaea1 - ps2
Archaea2 - ps3
Archaea3 - ps4
Asgard/Eukaryota - ps5
Eukaryota - ps6
Amorpheae - ps7
Obazoa - ps8
Opisthokonta - ps9
Holozoa - ps10
Filozoa - ps11
Choano./Metazoa - ps12
Metazoa - ps13
Myriazoa - ps14
Parahoxozoa - ps15
Bilateria - ps16
Deuterostomia - ps17
Chordata - ps18
Olfactores - ps19
Vertebrata - ps20
Gnathostomata - ps21
Euteleostomi - ps22
Sarcopterygii - ps23
Tetrapoda - ps24
Amniota - ps25
Mammalia - ps26
Theria - ps27
Placentalia - ps28
Boreoeutheria - ps29
Euarchontoglires - ps30
Euarchonta - ps31
Primates - ps32
Haplorrhini - ps33
Simiiformes - ps34
Catarrhini - ps35
Hominoidea - ps36
Hominidae - ps37
Homininae - ps38
Hominini - ps39
Homo_sapiens - ps40
p-value
7e-03
1e-02
2e-02
3e-02
5e-02
Log-odds
0.5
1.0
1.5
2.0
2.5
3.0
3.5
4.0

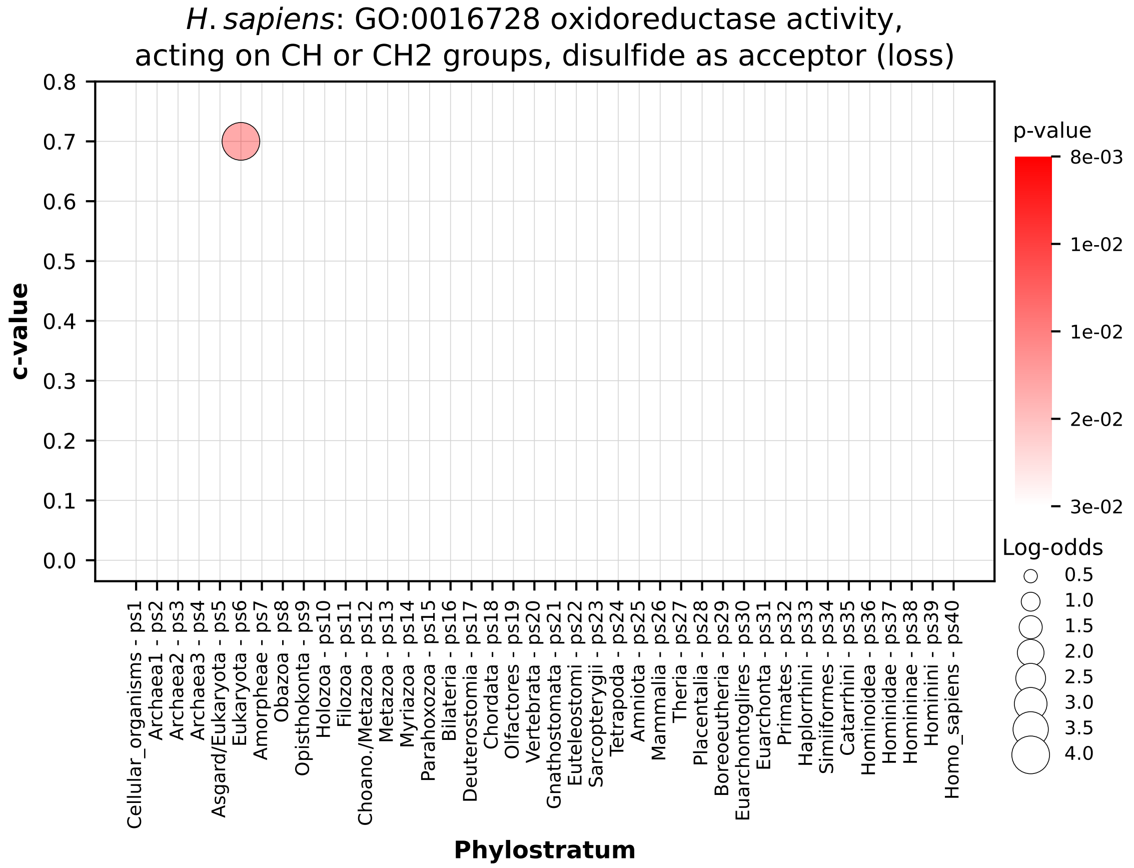

H. sapiens: GO:0016728 oxidoreductase activity, acting on CH or CH2 groups, disulfide as acceptor (loss)
c-value
0.8
0.7
0.6
0.5
0.4
0.3
0.2
0.1
0.0
p-value
8e-03
1e-02
1e-02
2e-02
3e-02
Log-odds
0.5
1.0
1.5
2.0
2.5
3.0
3.5
4.0
Cellular_organisms - ps1
Archaea1 - ps2
Archaea2 - ps3
Archaea3 - ps4
Asgard/Eukaryota - ps5
Eukaryota - ps6
Amorpheae - ps7
Obazoa - ps8
Opisthokonta - ps9
Holozoa - ps10
Filozoa - ps11
Choano./Metazoa - ps12
Metazoa - ps13
Myriazoa - ps14
Parahoxozoa - ps15
Bilateria - ps16
Deuterostomia - ps17
Chordata - ps18
Olfactores - ps19
Vertebrata - ps20
Gnathostomata - ps21
Euteleostomi - ps22
Sarcopterygii - ps23
Tetrapoda - ps24
Amniota - ps25
Mammalia - ps26
Theria - ps27
Placentalia - ps28
Boreoeutheria - ps29
Euarchontoglires - ps30
Euarchonta - ps31
Primates - ps32
Haplorrhini - ps33
Simiiformes - ps34
Catarrhini - ps35
Hominoidea - ps36
Hominidae - ps37
Homininae - ps38
Hominini - ps39
Homo_sapiens - ps40
Phylostratum

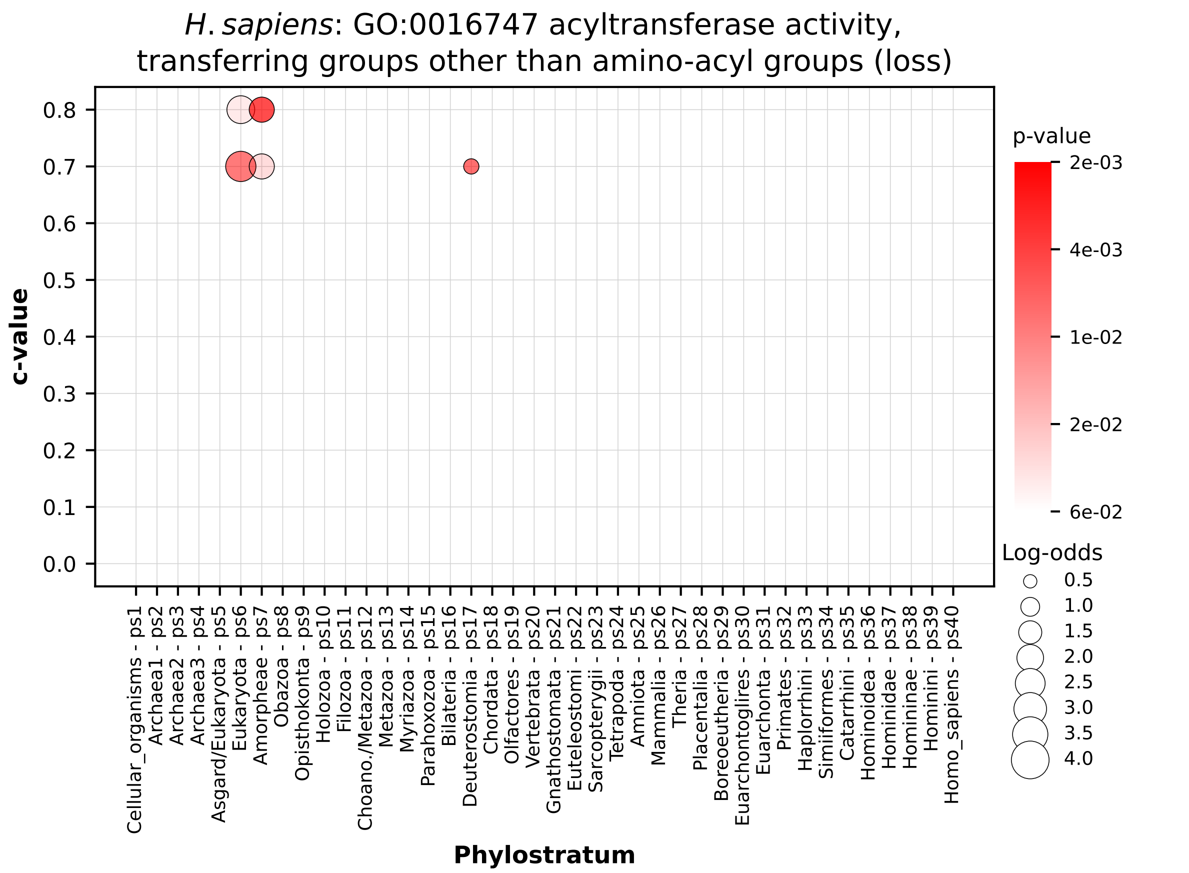

H. sapiens: GO:0016747 acyltransferase activity, transferring groups other than amino-acyl groups (loss)
c-value
0.0
0.1
0.2
0.3
0.4
0.5
0.6
0.7
0.8
Phylostratum
Cellular_organisms - ps1
Archaea1 - ps2
Archaea2 - ps3
Archaea3 - ps4
Asgard/Eukaryota - ps5
Eukaryota - ps6
Amorpheae - ps7
Obazoa - ps8
Opisthokonta - ps9
Holozoa - ps10
Filozoa - ps11
Choano./Metazoa - ps12
Metazoa - ps13
Myriazoa - ps14
Parahoxozoa - ps15
Bilateria - ps16
Deuterostomia - ps17
Chordata - ps18
Olfactores - ps19
Vertebrata - ps20
Gnathostomata - ps21
Euteleostomi - ps22
Sarcopterygii - ps23
Tetrapoda - ps24
Amniota - ps25
Mammalia - ps26
Theria - ps27
Placentalia - ps28
Boreoeutheria - ps29
Euarchontoglires - ps30
Euarchonta - ps31
Primates - ps32
Haplorrhini - ps33
Simiiformes - ps34
Catarrhini - ps35
Hominoidea - ps36
Hominidae - ps37
Homininae - ps38
Hominini - ps39
Homo_sapiens - ps40
p-value
2e-03
4e-03
1e-02
2e-02
6e-02
Log-odds
0.5
1.0
1.5
2.0
2.5
3.0
3.5
4.0

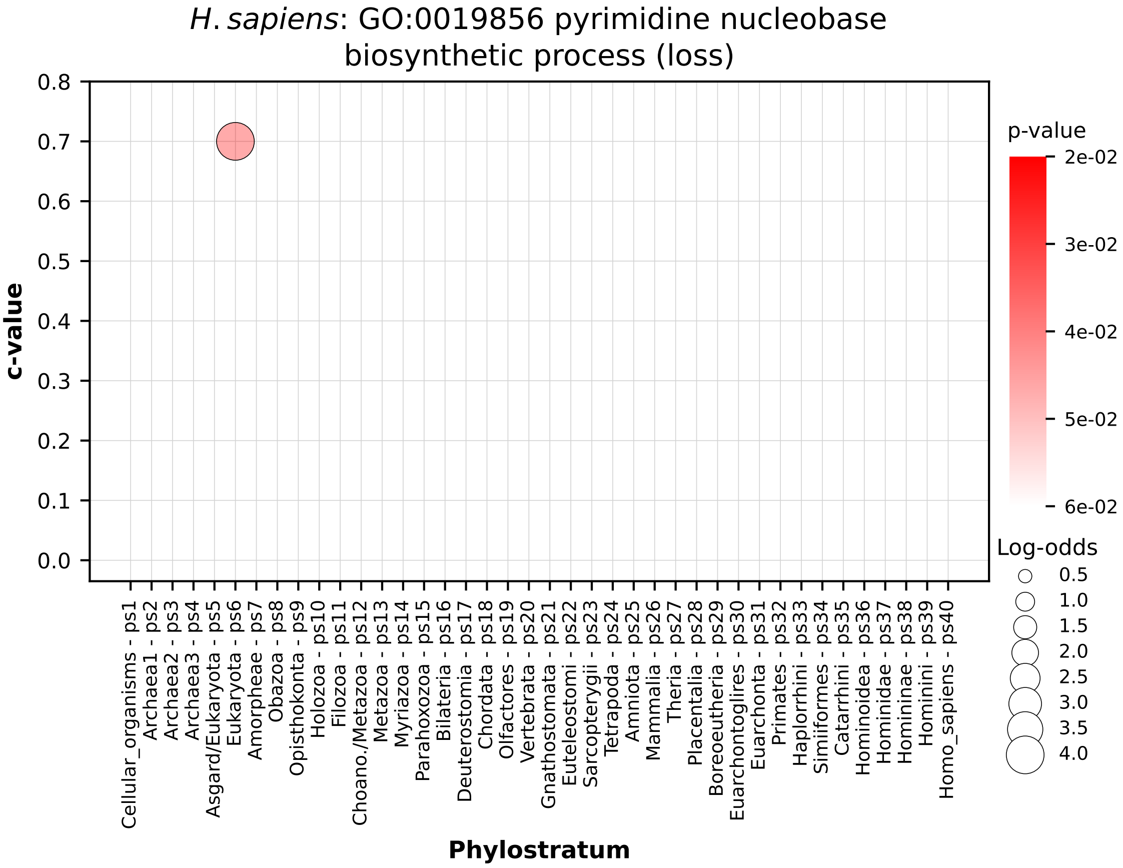

H. sapiens: GO:0019856 pyrimidine nucleobase biosynthetic process (loss)
c-value
0.8
0.7
0.6
0.5
0.4
0.3
0.2
0.1
0.0
p-value
2e-02
3e-02
4e-02
5e-02
6e-02
Log-odds
0.5
1.0
1.5
2.0
2.5
3.0
3.5
4.0
Cellular_organisms - ps1
Archaea1 - ps2
Archaea2 - ps3
Archaea3 - ps4
Asgard/Eukaryota - ps5
Eukaryota - ps6
Amorpheae - ps7
Obazoa - ps8
Opisthokonta - ps9
Holozoa - ps10
Filozoa - ps11
Choano./Metazoa - ps12
Metazoa - ps13
Myriazoa - ps14
Parahoxozoa - ps15
Bilateria - ps16
Deuterostomia - ps17
Chordata - ps18
Olfactores - ps19
Vertebrata - ps20
Gnathostomata - ps21
Euteleostomi - ps22
Sarcopterygii - ps23
Tetrapoda - ps24
Amniota - ps25
Mammalia - ps26
Theria - ps27
Placentalia - ps28
Boreoeutheria - ps29
Euarchontoglires - ps30
Euarchonta - ps31
Primates - ps32
Haplorrhini - ps33
Simiiformes - ps34
Catarrhini - ps35
Hominoidea - ps36
Hominidae - ps37
Homininae - ps38
Hominini - ps39
Homo_sapiens - ps40
Phylostratum

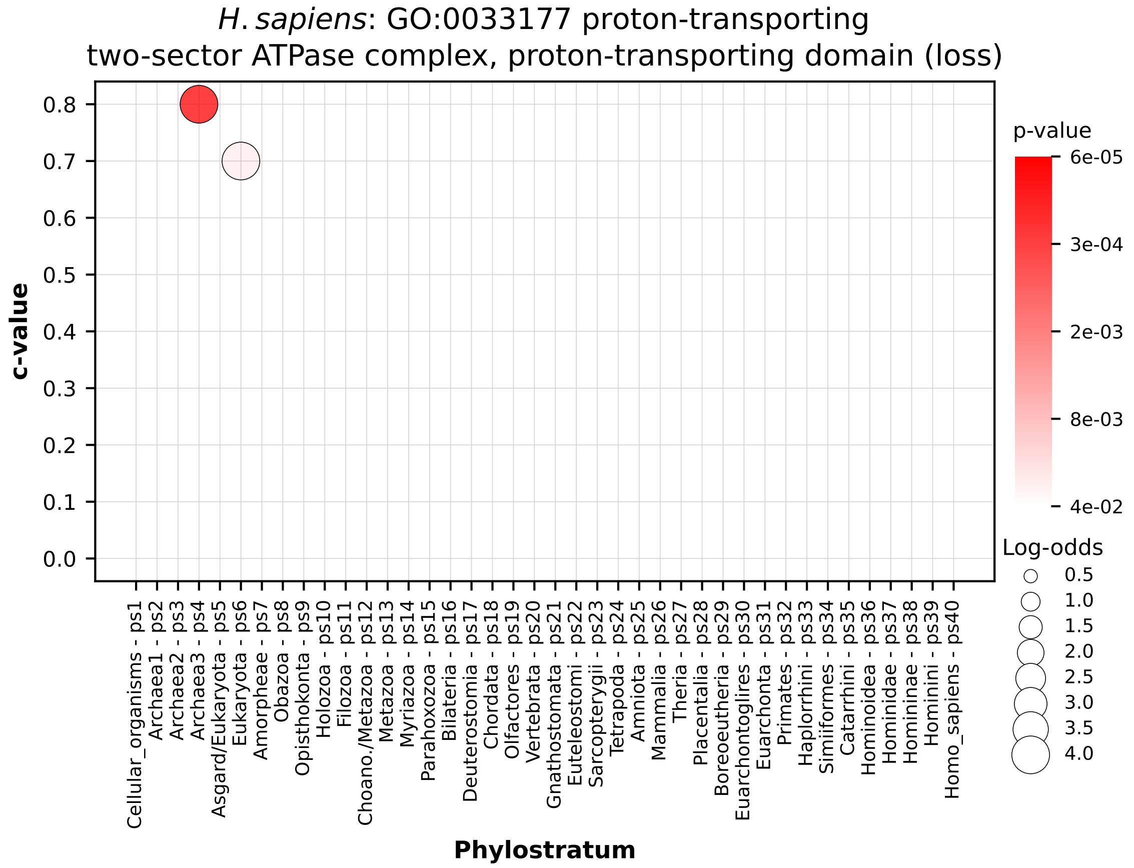

H. sapiens: GO:0033177 proton-transporting two-sector ATPase complex, proton-transporting domain (loss)
c-value
0.8
0.7
0.6
0.5
0.4
0.3
0.2
0.1
0.0
p-value
6e-05
3e-04
2e-03
8e-03
4e-02
Log-odds
0.5
1.0
1.5
2.0
2.5
3.0
3.5
4.0
Cellular_organisms - ps1
Archaea1 - ps2
Archaea2 - ps3
Archaea3 - ps4
Asgard/Eukaryota - ps5
Eukaryota - ps6
Amorpheae - ps7
Obazoa - ps8
Opisthokonta - ps9
Holozoa - ps10
Filozoa - ps11
Choano./Metazoa - ps12
Metazoa - ps13
Myriazoa - ps14
Parahoxozoa - ps15
Bilateria - ps16
Deuterostomia - ps17
Chordata - ps18
Olfactores - ps19
Vertebrata - ps20
Gnathostomata - ps21
Euteleostomi - ps22
Sarcopterygii - ps23
Tetrapoda - ps24
Amniota - ps25
Mammalia - ps26
Theria - ps27
Placentalia - ps28
Boreoeutheria - ps29
Euarchontoglires - ps30
Euarchonta - ps31
Primates - ps32
Haplorrhini - ps33
Simiiformes - ps34
Catarrhini - ps35
Hominoidea - ps36
Hominidae - ps37
Homininae - ps38
Hominini - ps39
Homo_sapiens - ps40
Phylostratum

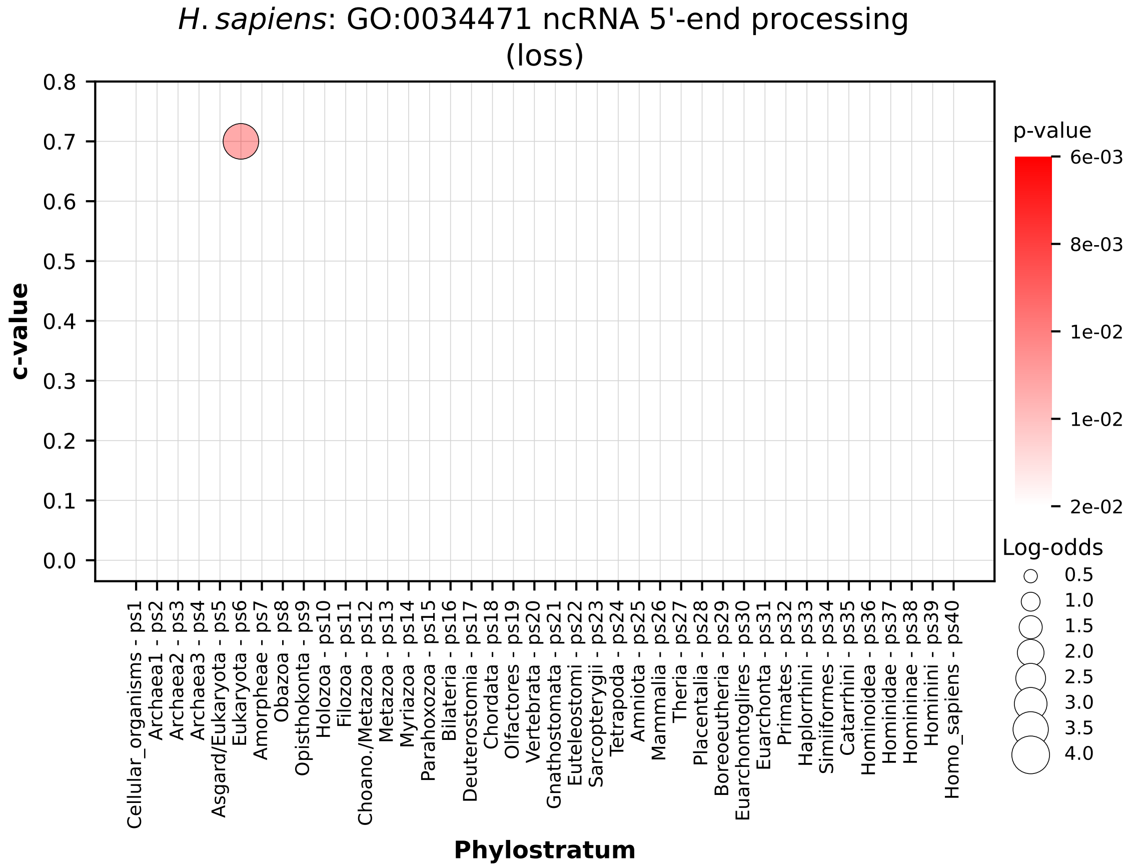

H. sapiens: GO:0034471 ncRNA 5'-end processing
(loss)
c-value
0.8
0.7
0.6
0.5
0.4
0.3
0.2
0.1
0.0
Phylostratum
Cellular_organisms - ps1
Archaea1 - ps2
Archaea2 - ps3
Archaea3 - ps4
Asgard/Eukaryota - ps5
Eukaryota - ps6
Amorpheae - ps7
Obazoa - ps8
Opisthokonta - ps9
Holozoa - ps10
Filozoa - ps11
Choano./Metazoa - ps12
Metazoa - ps13
Myriazoa - ps14
Parahoxozoa - ps15
Bilateria - ps16
Deuterostomia - ps17
Chordata - ps18
Olfactores - ps19
Vertebrata - ps20
Gnathostomata - ps21
Euteleostomi - ps22
Sarcopterygii - ps23
Tetrapoda - ps24
Amniota - ps25
Mammalia - ps26
Theria - ps27
Placentalia - ps28
Boreoeutheria - ps29
Euarchontoglires - ps30
Euarchonta - ps31
Primates - ps32
Haplorrhini - ps33
Simiiformes - ps34
Catarrhini - ps35
Hominoidea - ps36
Hominidae - ps37
Homininae - ps38
Hominini - ps39
Homo_sapiens - ps40
p-value
6e-03
8e-03
1e-02
1e-02
2e-02
Log-odds
0.5
1.0
1.5
2.0
2.5
3.0
3.5
4.0

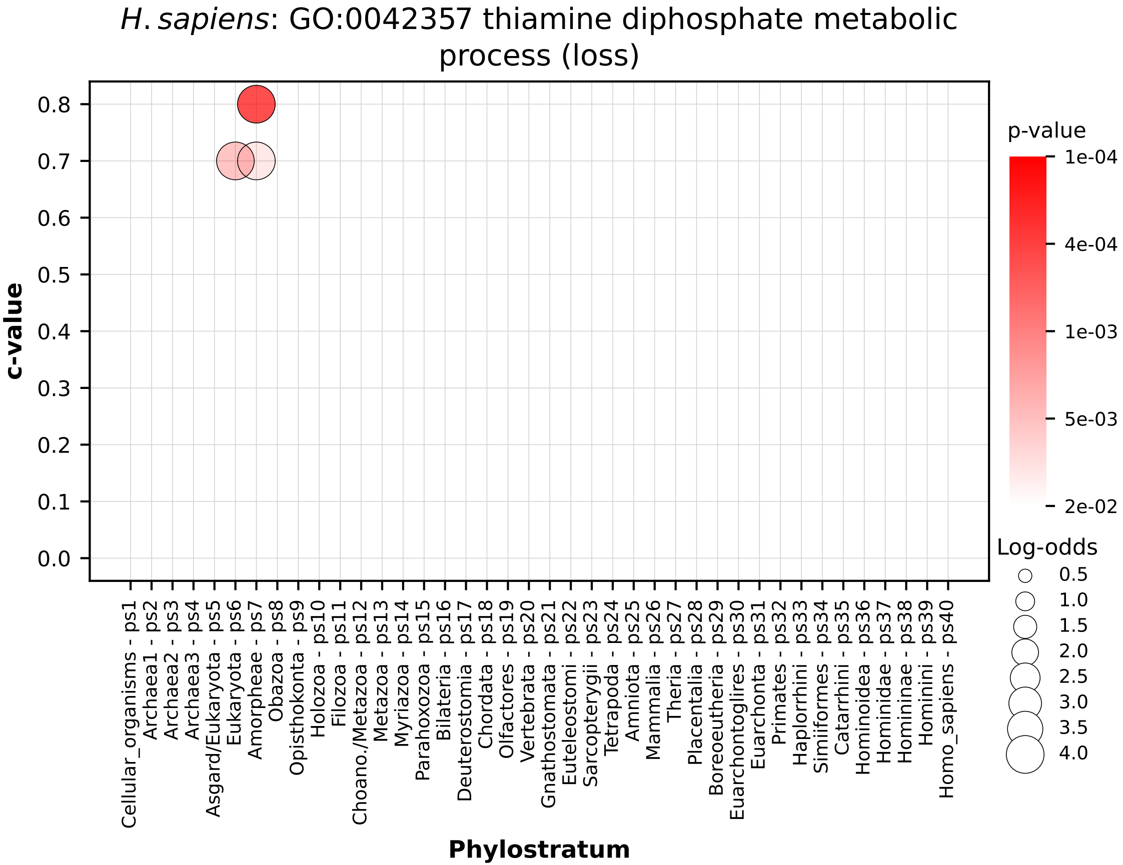

H. sapiens: GO:0042357 thiamine diphosphate metabolic process (loss)
c-value
0.0
0.1
0.2
0.3
0.4
0.5
0.6
0.7
0.8
Phylostratum
Cellular_organisms - ps1
Archaea1 - ps2
Archaea2 - ps3
Archaea3 - ps4
Asgard/Eukaryota - ps5
Eukaryota - ps6
Amorpheae - ps7
Obazoa - ps8
Opisthokonta - ps9
Holozoa - ps10
Filozoa - ps11
Choano./Metazoa - ps12
Metazoa - ps13
Myriazoa - ps14
Parahoxozoa - ps15
Bilateria - ps16
Deuterostomia - ps17
Chordata - ps18
Olfactores - ps19
Vertebrata - ps20
Gnathostomata - ps21
Euteleostomi - ps22
Sarcopterygii - ps23
Tetrapoda - ps24
Amniota - ps25
Mammalia - ps26
Theria - ps27
Placentalia - ps28
Boreoeutheria - ps29
Euarchontoglires - ps30
Euarchonta - ps31
Primates - ps32
Haplorrhini - ps33
Simiiformes - ps34
Catarrhini - ps35
Hominoidea - ps36
Hominidae - ps37
Homininae - ps38
Hominini - ps39
Homo_sapiens - ps40
p-value
1e-04
4e-04
1e-03
5e-03
2e-02
Log-odds
0.5
1.0
1.5
2.0
2.5
3.0
3.5
4.0

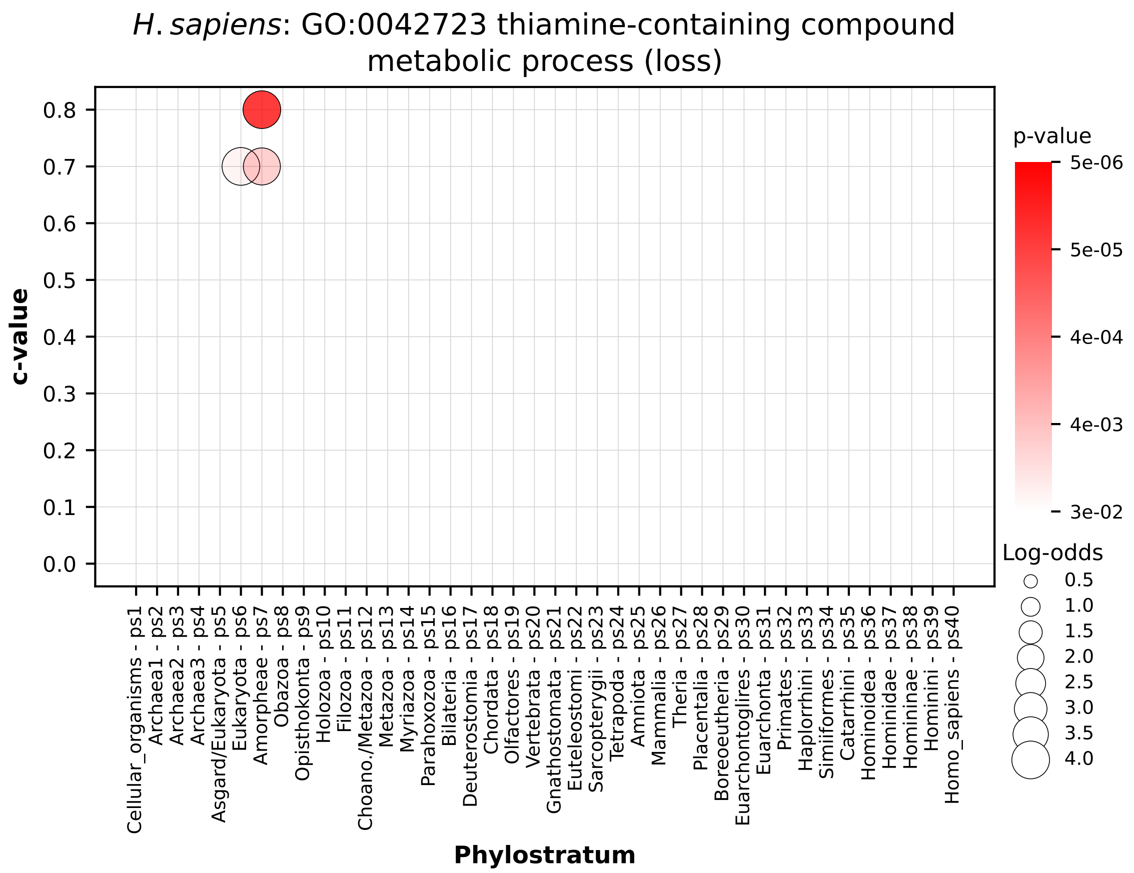

H. sapiens: GO:0042723 thiamine-containing compound metabolic process (loss)
c-value
0.8
0.7
0.6
0.5
0.4
0.3
0.2
0.1
0.0
p-value
5e-06
5e-05
4e-04
4e-03
3e-02
Log-odds
0.5
1.0
1.5
2.0
2.5
3.0
3.5
4.0
Cellular_organisms - ps1
Archaea1 - ps2
Archaea2 - ps3
Archaea3 - ps4
Asgard/Eukaryota - ps5
Eukaryota - ps6
Amorpheae - ps7
Obazoa - ps8
Opisthokonta - ps9
Holozoa - ps10
Filozoa - ps11
Choano./Metazoa - ps12
Metazoa - ps13
Myriazoa - ps14
Parahoxozoa - ps15
Bilateria - ps16
Deuterostomia - ps17
Chordata - ps18
Olfactores - ps19
Vertebrata - ps20
Gnathostomata - ps21
Euteleostomi - ps22
Sarcopterygii - ps23
Tetrapoda - ps24
Amniota - ps25
Mammalia - ps26
Theria - ps27
Placentalia - ps28
Boreoeutheria - ps29
Euarchontoglires - ps30
Euarchonta - ps31
Primates - ps32
Haplorrhini - ps33
Simiiformes - ps34
Catarrhini - ps35
Hominoidea - ps36
Hominidae - ps37
Homininae - ps38
Hominini - ps39
Homo_sapiens - ps40
Phylostratum

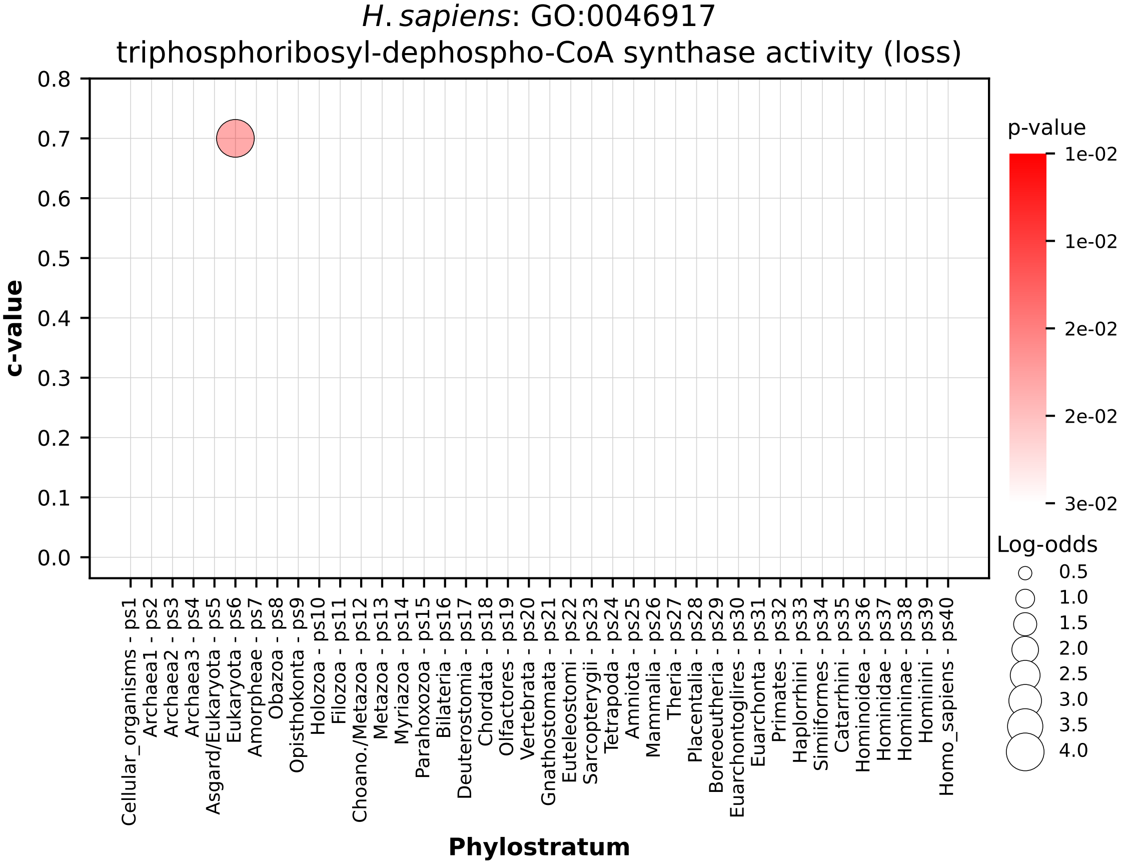

H. sapiens: GO:0046917
triphosphoribosyl-dephospho-CoA synthase activity (loss)
c-value
0.8
0.7
0.6
0.5
0.4
0.3
0.2
0.1
0.0
Phylostratum
Cellular_organisms - ps1
Archaea1 - ps2
Archaea2 - ps3
Archaea3 - ps4
Asgard/Eukaryota - ps5
Eukaryota - ps6
Amorpheae - ps7
Obazoa - ps8
Opisthokonta - ps9
Holozoa - ps10
Filozoa - ps11
Choano./Metazoa - ps12
Metazoa - ps13
Myriazoa - ps14
Parahoxozoa - ps15
Bilateria - ps16
Deuterostomia - ps17
Chordata - ps18
Olfactores - ps19
Vertebrata - ps20
Gnathostomata - ps21
Euteleostomi - ps22
Sarcopterygii - ps23
Tetrapoda - ps24
Amniota - ps25
Mammalia - ps26
Theria - ps27
Placentalia - ps28
Boreoeutheria - ps29
Euarchontoglires - ps30
Euarchonta - ps31
Primates - ps32
Haplorrhini - ps33
Simiiformes - ps34
Catarrhini - ps35
Hominoidea - ps36
Hominidae - ps37
Homininae - ps38
Hominini - ps39
Homo_sapiens - ps40
p-value
1e-02
1e-02
2e-02
2e-02
3e-02
Log-odds
0.5
1.0
1.5
2.0
2.5
3.0
3.5
4.0

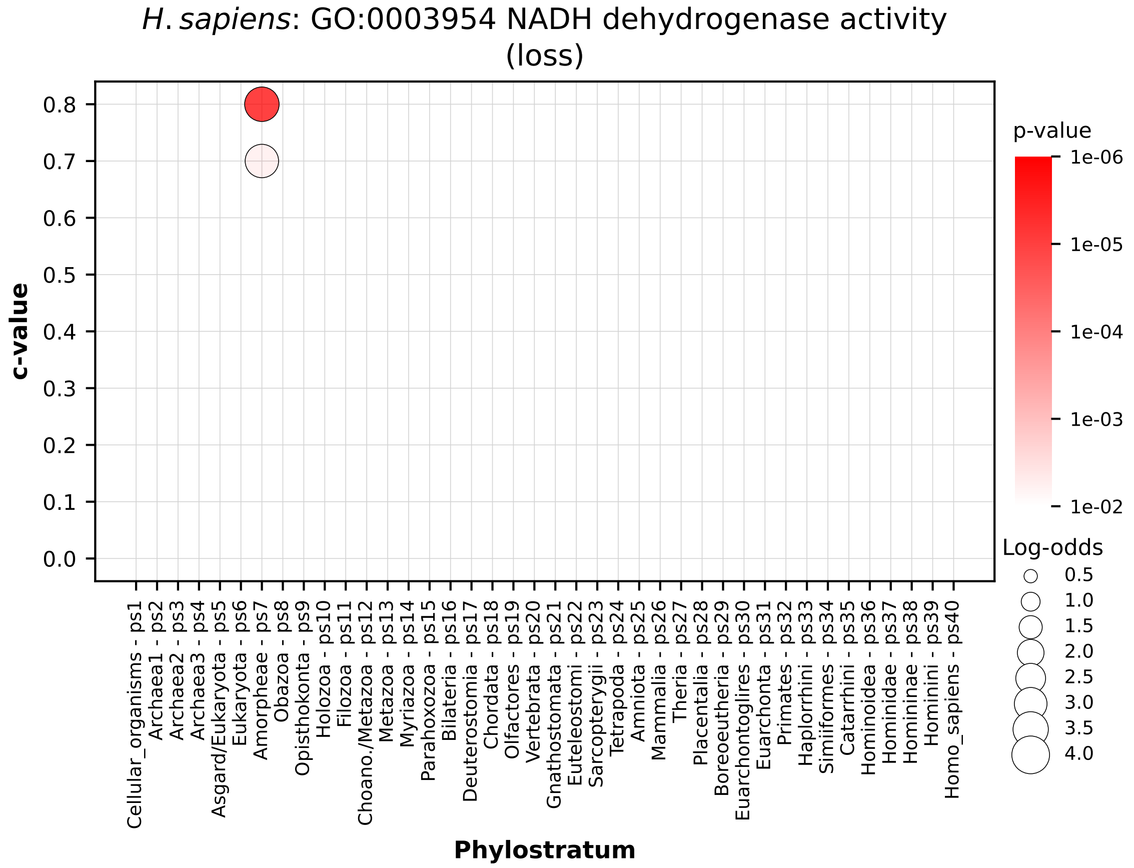
H. sapiens: GO:0003954 NADH dehydrogenase activity (loss)
c-value
0.0
0.1
0.2
0.3
0.4
0.5
0.6
0.7
0.8
Phylostratum
Cellular_organisms - ps1
Archaea1 - ps2
Archaea2 - ps3
Archaea3 - ps4
Asgard/Eukaryota - ps5
Eukaryota - ps6
Amorpheae - ps7
Obazoa - ps8
Opisthokonta - ps9
Holozoa - ps10
Filozoa - ps11
Choano./Metazoa - ps12
Metazoa - ps13
Myriazoa - ps14
Parahoxozoa - ps15
Bilateria - ps16
Deuterostomia - ps17
Chordata - ps18
Olfactores - ps19
Vertebrata - ps20
Gnathostomata - ps21
Euteleostomi - ps22
Sarcopterygii - ps23
Tetrapoda - ps24
Amniota - ps25
Mammalia - ps26
Theria - ps27
Placentalia - ps28
Boreoeutheria - ps29
Euarchontoglires - ps30
Euarchonta - ps31
Primates - ps32
Haplorrhini - ps33
Simiiformes - ps34
Catarrhini - ps35
Hominoidea - ps36
Hominidae - ps37
Homininae - ps38
Hominini - ps39
Homo_sapiens - ps40
p-value
1e-06
1e-05
1e-04
1e-03
1e-02
Log-odds
0.5
1.0
1.5
2.0
2.5
3.0
3.5
4.0

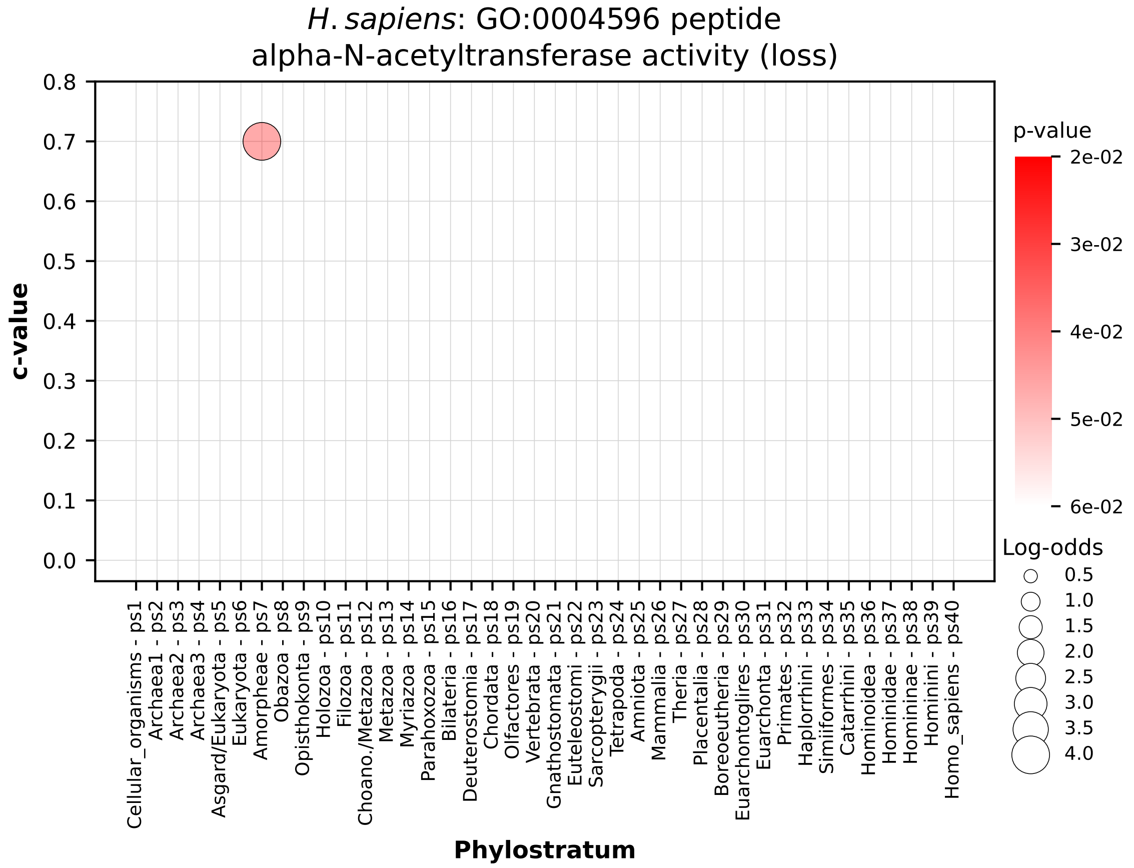
H. sapiens: GO:0004596 peptide alpha-N-acetyltransferase activity (loss)
c-value
0.8
0.7
0.6
0.5
0.4
0.3
0.2
0.1
0.0
p-value
2e-02
3e-02
4e-02
5e-02
6e-02
Log-odds
0.5
1.0
1.5
2.0
2.5
3.0
3.5
4.0
Cellular_organisms - ps1
Archaea1 - ps2
Archaea2 - ps3
Archaea3 - ps4
Asgard/Eukaryota - ps5
Eukaryota - ps6
Amorpheae - ps7
Obazoa - ps8
Opisthokonta - ps9
Holozoa - ps10
Filozoa - ps11
Choano./Metazoa - ps12
Metazoa - ps13
Myriazoa - ps14
Parahoxozoa - ps15
Bilateria - ps16
Deuterostomia - ps17
Chordata - ps18
Olfactores - ps19
Vertebrata - ps20
Gnathostomata - ps21
Euteleostomi - ps22
Sarcopterygii - ps23
Tetrapoda - ps24
Amniota - ps25
Mammalia - ps26
Theria - ps27
Placentalia - ps28
Boreoeutheria - ps29
Euarchontoglires - ps30
Euarchonta - ps31
Primates - ps32
Haplorrhini - ps33
Simiiformes - ps34
Catarrhini - ps35
Hominoidea - ps36
Hominidae - ps37
Homininae - ps38
Hominini - ps39
Homo_sapiens - ps40
Phylostratum

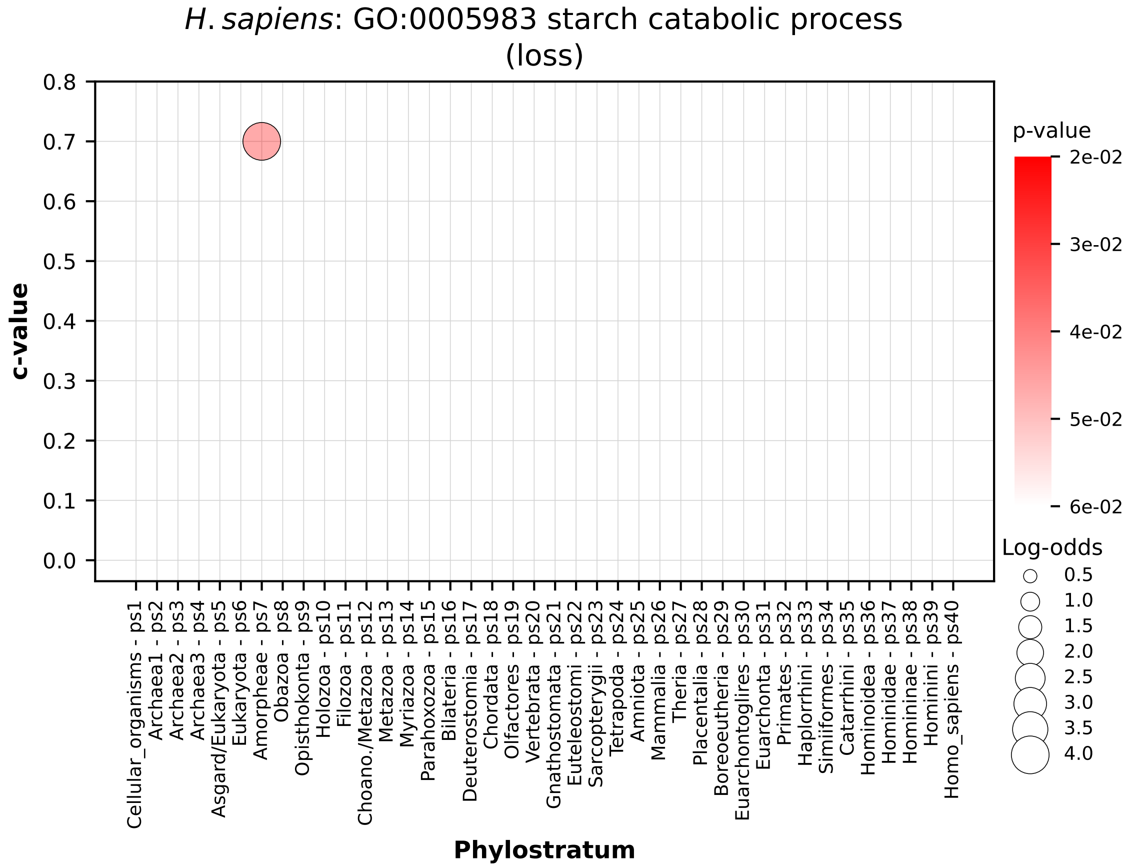

H. sapiens: GO:0005983 starch catabolic process
(loss)
c-value
0.8
0.7
0.6
0.5
0.4
0.3
0.2
0.1
0.0
p-value
2e-02
3e-02
4e-02
5e-02
6e-02
Log-odds
0.5
1.0
1.5
2.0
2.5
3.0
3.5
4.0
Cellular_organisms - ps1
Archaea1 - ps2
Archaea2 - ps3
Archaea3 - ps4
Asgard/Eukaryota - ps5
Eukaryota - ps6
Amorpheae - ps7
Obazoa - ps8
Opisthokonta - ps9
Holozoa - ps10
Filozoa - ps11
Choano./Metazoa - ps12
Metazoa - ps13
Myriazoa - ps14
Parahoxozoa - ps15
Bilateria - ps16
Deuterostomia - ps17
Chordata - ps18
Olfactores - ps19
Vertebrata - ps20
Gnathostomata - ps21
Euteleostomi - ps22
Sarcopterygii - ps23
Tetrapoda - ps24
Amniota - ps25
Mammalia - ps26
Theria - ps27
Placentalia - ps28
Boreoeutheria - ps29
Euarchontoglires - ps30
Euarchonta - ps31
Primates - ps32
Haplorrhini - ps33
Simiiformes - ps34
Catarrhini - ps35
Hominoidea - ps36
Hominidae - ps37
Homininae - ps38
Hominini - ps39
Homo_sapiens - ps40
Phylostratum

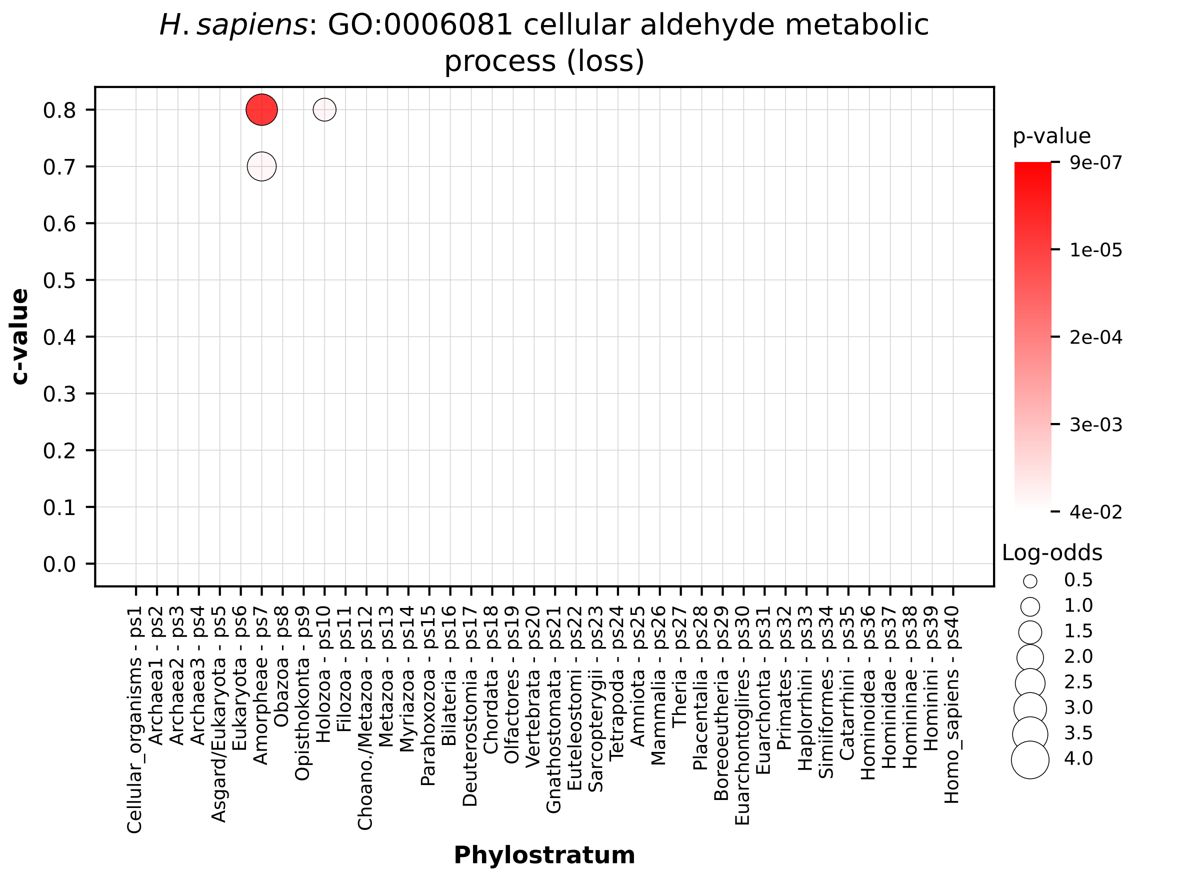

H. sapiens: GO:0006081 cellular aldehyde metabolic process (loss)
c-value
0.0
0.1
0.2
0.3
0.4
0.5
0.6
0.7
0.8
Phylostratum
Cellular_organisms - ps1
Archaea1 - ps2
Archaea2 - ps3
Archaea3 - ps4
Asgard/Eukaryota - ps5
Eukaryota - ps6
Amorpheae - ps7
Obazoa - ps8
Opisthokonta - ps9
Holozoa - ps10
Filozoa - ps11
Choano./Metazoa - ps12
Metazoa - ps13
Myriazoa - ps14
Parahoxozoa - ps15
Bilateria - ps16
Deuterostomia - ps17
Chordata - ps18
Olfactores - ps19
Vertebrata - ps20
Gnathostomata - ps21
Euteleostomi - ps22
Sarcopterygii - ps23
Tetrapoda - ps24
Amniota - ps25
Mammalia - ps26
Theria - ps27
Placentalia - ps28
Boreoeutheria - ps29
Euarchontoglires - ps30
Euarchonta - ps31
Primates - ps32
Haplorrhini - ps33
Simiiformes - ps34
Catarrhini - ps35
Hominoidea - ps36
Hominidae - ps37
Homininae - ps38
Hominini - ps39
Homo_sapiens - ps40
p-value
9e-07
1e-05
2e-04
3e-03
4e-02
Log-odds
0.5
1.0
1.5
2.0
2.5
3.0
3.5
4.0

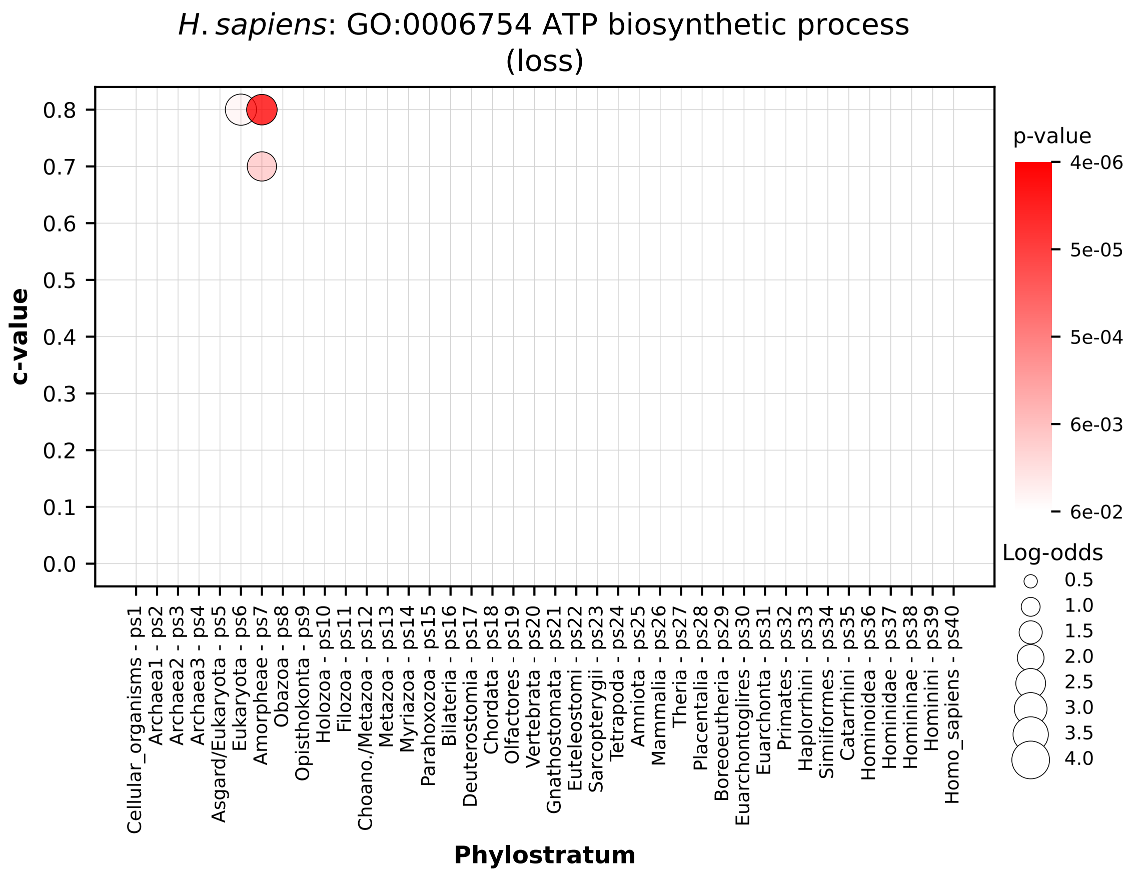

H. sapiens: GO:0006754 ATP biosynthetic process
(loss)
c-value
0.0
0.1
0.2
0.3
0.4
0.5
0.6
0.7
0.8
Phylostratum
Cellular_organisms - ps1
Archaea1 - ps2
Archaea2 - ps3
Archaea3 - ps4
Asgard/Eukaryota - ps5
Eukaryota - ps6
Amorpheae - ps7
Obazoa - ps8
Opisthokonta - ps9
Holozoa - ps10
Filozoa - ps11
Choano./Metazoa - ps12
Metazoa - ps13
Myriazoa - ps14
Parahoxozoa - ps15
Bilateria - ps16
Deuterostomia - ps17
Chordata - ps18
Olfactores - ps19
Vertebrata - ps20
Gnathostomata - ps21
Euteleostomi - ps22
Sarcopterygii - ps23
Tetrapoda - ps24
Amniota - ps25
Mammalia - ps26
Theria - ps27
Placentalia - ps28
Boreoeutheria - ps29
Euarchontoglires - ps30
Euarchonta - ps31
Primates - ps32
Haplorrhini - ps33
Simiiformes - ps34
Catarrhini - ps35
Hominoidea - ps36
Hominidae - ps37
Homininae - ps38
Hominini - ps39
Homo_sapiens - ps40
p-value
4e-06
5e-05
5e-04
6e-03
6e-02
Log-odds
0.5
1.0
1.5
2.0
2.5
3.0
3.5
4.0

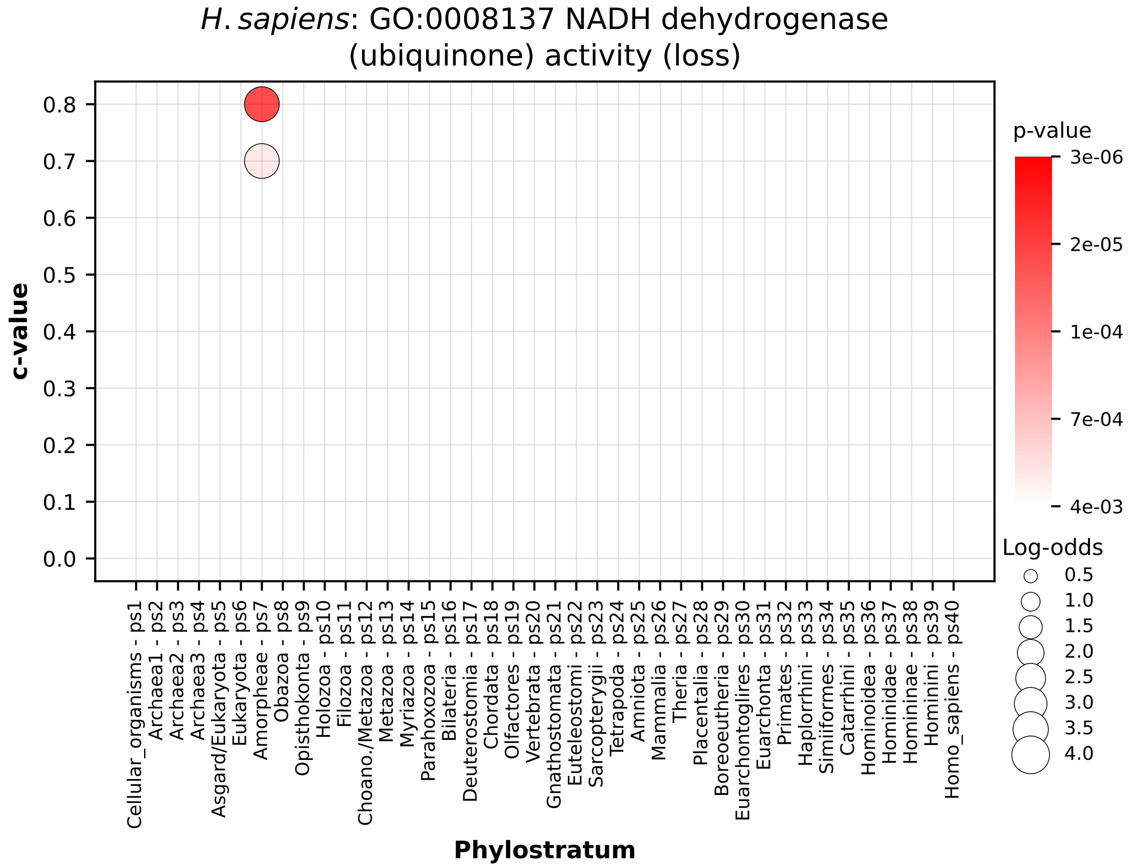

H. sapiens: GO:0008137 NADH dehydrogenase (ubiquinone) activity (loss)
c-value
0.0
0.1
0.2
0.3
0.4
0.5
0.6
0.7
0.8
p-value
3e-06
2e-05
1e-04
7e-04
4e-03
Log-odds
0.5
1.0
1.5
2.0
2.5
3.0
3.5
4.0
Cellular_organisms - ps1
Archaea1 - ps2
Archaea2 - ps3
Archaea3 - ps4
Asgard/Eukaryota - ps5
Eukaryota - ps6
Amorpheae - ps7
Obazoa - ps8
Opisthokonta - ps9
Holozoa - ps10
Filozoa - ps11
Choano./Metazoa - ps12
Metazoa - ps13
Myriazoa - ps14
Parahoxozoa - ps15
Bilateria - ps16
Deuterostomia - ps17
Chordata - ps18
Olfactores - ps19
Vertebrata - ps20
Gnathostomata - ps21
Euteleostomi - ps22
Sarcopterygii - ps23
Tetrapoda - ps24
Amniota - ps25
Mammalia - ps26
Theria - ps27
Placentalia - ps28
Boreoeutheria - ps29
Euarchontoglires - ps30
Euarchonta - ps31
Primates - ps32
Haplorrhini - ps33
Simiiformes - ps34
Catarrhini - ps35
Hominoidea - ps36
Hominidae - ps37
Homininae - ps38
Hominini - ps39
Homo_sapiens - ps40
Phylostratum

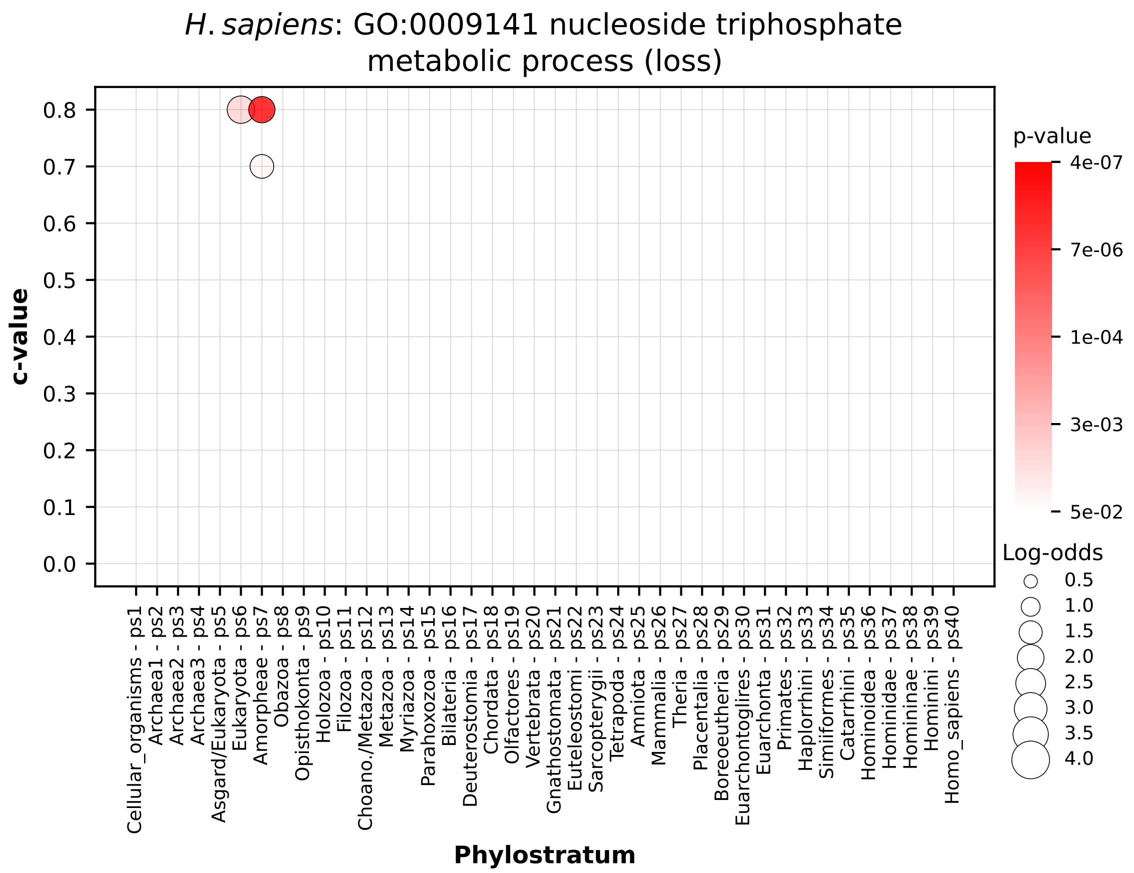

H. sapiens: GO:0009141 nucleoside triphosphate metabolic process (loss)
c-value
0.8
0.7
0.6
0.5
0.4
0.3
0.2
0.1
0.0
Phylostratum
Cellular_organisms - ps1
Archaea1 - ps2
Archaea2 - ps3
Archaea3 - ps4
Asgard/Eukaryota - ps5
Eukaryota - ps6
Amorpheae - ps7
Obazoa - ps8
Opisthokonta - ps9
Holozoa - ps10
Filozoa - ps11
Choano./Metazoa - ps12
Metazoa - ps13
Myriazoa - ps14
Parahoxozoa - ps15
Bilateria - ps16
Deuterostomia - ps17
Chordata - ps18
Olfactores - ps19
Vertebrata - ps20
Gnathostomata - ps21
Euteleostomi - ps22
Sarcopterygii - ps23
Tetrapoda - ps24
Amniota - ps25
Mammalia - ps26
Theria - ps27
Placentalia - ps28
Boreoeutheria - ps29
Euarchontoglires - ps30
Euarchonta - ps31
Primates - ps32
Haplorrhini - ps33
Simiiformes - ps34
Catarrhini - ps35
Hominoidea - ps36
Hominidae - ps37
Homininae - ps38
Hominini - ps39
Homo_sapiens - ps40
p-value
4e-07
7e-06
1e-04
3e-03
5e-02
Log-odds
0.5
1.0
1.5
2.0
2.5
3.0
3.5
4.0

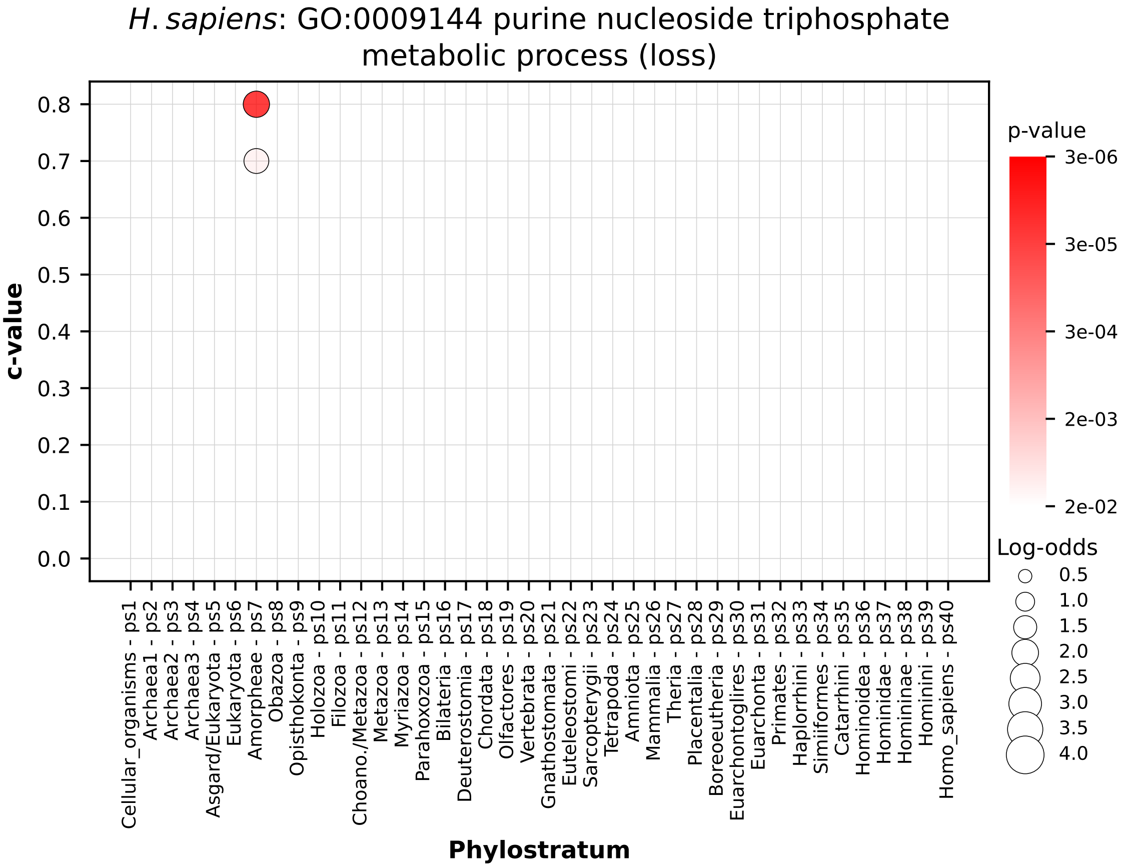

H. sapiens: GO:0009144 purine nucleoside triphosphate metabolic process (loss)
c-value
0.0
0.1
0.2
0.3
0.4
0.5
0.6
0.7
0.8
Phylostratum
Cellular_organisms - ps1
Archaea1 - ps2
Archaea2 - ps3
Archaea3 - ps4
Asgard/Eukaryota - ps5
Eukaryota - ps6
Amorpheae - ps7
Obazoa - ps8
Opisthokonta - ps9
Holozoa - ps10
Filozoa - ps11
Choano./Metazoa - ps12
Metazoa - ps13
Myriazoa - ps14
Parahoxozoa - ps15
Bilateria - ps16
Deuterostomia - ps17
Chordata - ps18
Olfactores - ps19
Vertebrata - ps20
Gnathostomata - ps21
Euteleostomi - ps22
Sarcopterygii - ps23
Tetrapoda - ps24
Amniota - ps25
Mammalia - ps26
Theria - ps27
Placentalia - ps28
Boreoeutheria - ps29
Euarchontoglires - ps30
Euarchonta - ps31
Primates - ps32
Haplorrhini - ps33
Simiiformes - ps34
Catarrhini - ps35
Hominoidea - ps36
Hominidae - ps37
Homininae - ps38
Hominini - ps39
Homo_sapiens - ps40
p-value
3e-06
3e-05
3e-04
2e-03
2e-02
Log-odds
0.5
1.0
1.5
2.0
2.5
3.0
3.5
4.0

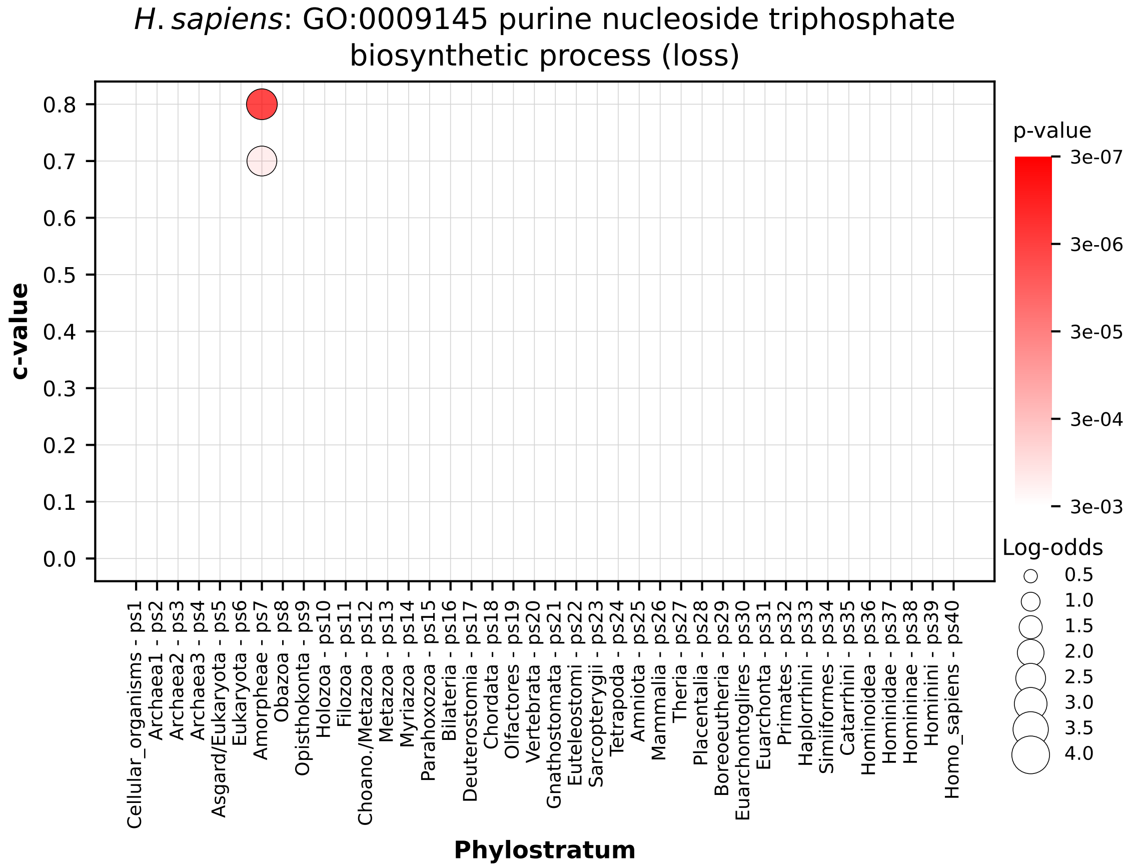

H. sapiens: GO:0009145 purine nucleoside triphosphate biosynthetic process (loss)
c-value
0.8
0.7
0.6
0.5
0.4
0.3
0.2
0.1
0.0
p-value
3e-07
3e-06
3e-05
3e-04
3e-03
Log-odds
0.5
1.0
1.5
2.0
2.5
3.0
3.5
4.0
Cellular_organisms - ps1
Archaea1 - ps2
Archaea2 - ps3
Archaea3 - ps4
Asgard/Eukaryota - ps5
Eukaryota - ps6
Amorpheae - ps7
Obazoa - ps8
Opisthokonta - ps9
Holozoa - ps10
Filozoa - ps11
Choano./Metazoa - ps12
Metazoa - ps13
Myriazoa - ps14
Parahoxozoa - ps15
Bilateria - ps16
Deuterostomia - ps17
Chordata - ps18
Olfactores - ps19
Vertebrata - ps20
Gnathostomata - ps21
Euteleostomi - ps22
Sarcopterygii - ps23
Tetrapoda - ps24
Amniota - ps25
Mammalia - ps26
Theria - ps27
Placentalia - ps28
Boreoeutheria - ps29
Euarchontoglires - ps30
Euarchonta - ps31
Primates - ps32
Haplorrhini - ps33
Simiiformes - ps34
Catarrhini - ps35
Hominoidea - ps36
Hominidae - ps37
Homininae - ps38
Hominini - ps39
Homo_sapiens - ps40
Phylostratum

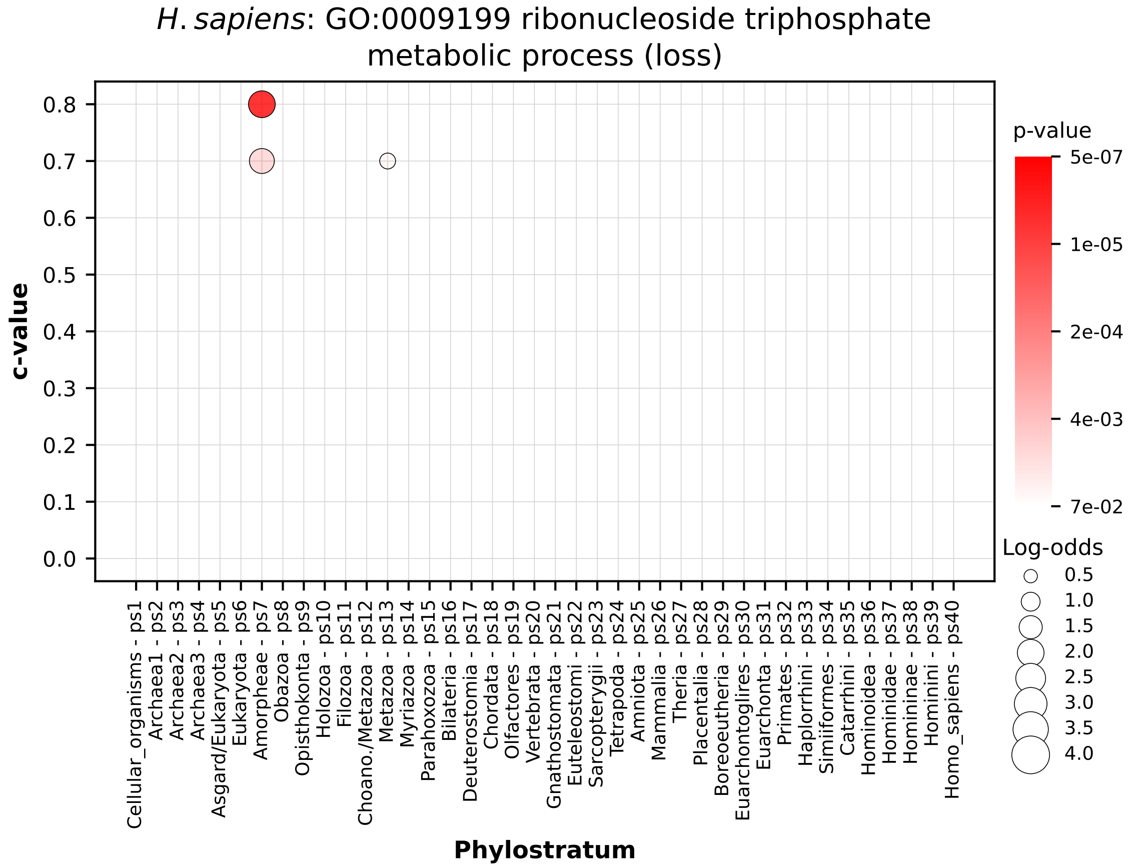

H. sapiens: GO:0009199 ribonucleoside triphosphate metabolic process (loss)
c-value
0.0
0.1
0.2
0.3
0.4
0.5
0.6
0.7
0.8
Phylostratum
Cellular_organisms - ps1
Archaea1 - ps2
Archaea2 - ps3
Archaea3 - ps4
Asgard/Eukaryota - ps5
Eukaryota - ps6
Amorpheae - ps7
Obazoa - ps8
Opisthokonta - ps9
Holozoa - ps10
Filozoa - ps11
Choano./Metazoa - ps12
Metazoa - ps13
Myriazoa - ps14
Parahoxozoa - ps15
Bilateria - ps16
Deuterostomia - ps17
Chordata - ps18
Olfactores - ps19
Vertebrata - ps20
Gnathostomata - ps21
Euteleostomi - ps22
Sarcopterygii - ps23
Tetrapoda - ps24
Amniota - ps25
Mammalia - ps26
Theria - ps27
Placentalia - ps28
Boreoeutheria - ps29
Euarchontoglires - ps30
Euarchonta - ps31
Primates - ps32
Haplorrhini - ps33
Simiiformes - ps34
Catarrhini - ps35
Hominoidea - ps36
Hominidae - ps37
Homininae - ps38
Hominini - ps39
Homo_sapiens - ps40
p-value
5e-07
1e-05
2e-04
4e-03
7e-02
Log-odds
0.5
1.0
1.5
2.0
2.5
3.0
3.5
4.0

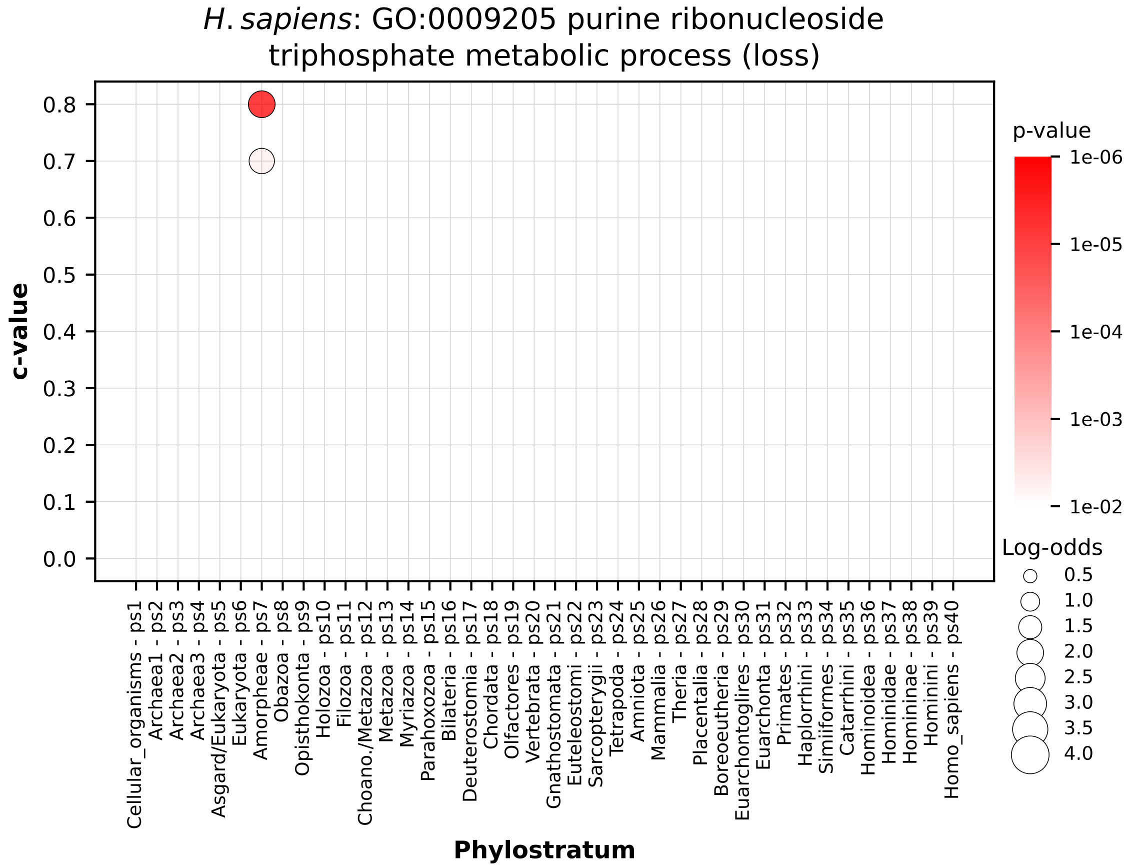

H. sapiens: GO:0009205 purine ribonucleoside triphosphate metabolic process (loss)
c-value
0.0
0.1
0.2
0.3
0.4
0.5
0.6
0.7
0.8
p-value
1e-06
1e-05
1e-04
1e-03
1e-02
Log-odds
0.5
1.0
1.5
2.0
2.5
3.0
3.5
4.0
Cellular_organisms - ps1
Archaea1 - ps2
Archaea2 - ps3
Archaea3 - ps4
Asgard/Eukaryota - ps5
Eukaryota - ps6
Amorpheae - ps7
Obazoa - ps8
Opisthokonta - ps9
Holozoa - ps10
Filozoa - ps11
Choano./Metazoa - ps12
Metazoa - ps13
Myriazoa - ps14
Parahoxozoa - ps15
Bilateria - ps16
Deuterostomia - ps17
Chordata - ps18
Olfactores - ps19
Vertebrata - ps20
Gnathostomata - ps21
Euteleostomi - ps22
Sarcopterygii - ps23
Tetrapoda - ps24
Amniota - ps25
Mammalia - ps26
Theria - ps27
Placentalia - ps28
Boreoeutheria - ps29
Euarchontoglires - ps30
Euarchonta - ps31
Primates - ps32
Haplorrhini - ps33
Simiiformes - ps34
Catarrhini - ps35
Hominoidea - ps36
Hominidae - ps37
Homininae - ps38
Hominini - ps39
Homo_sapiens - ps40
Phylostratum

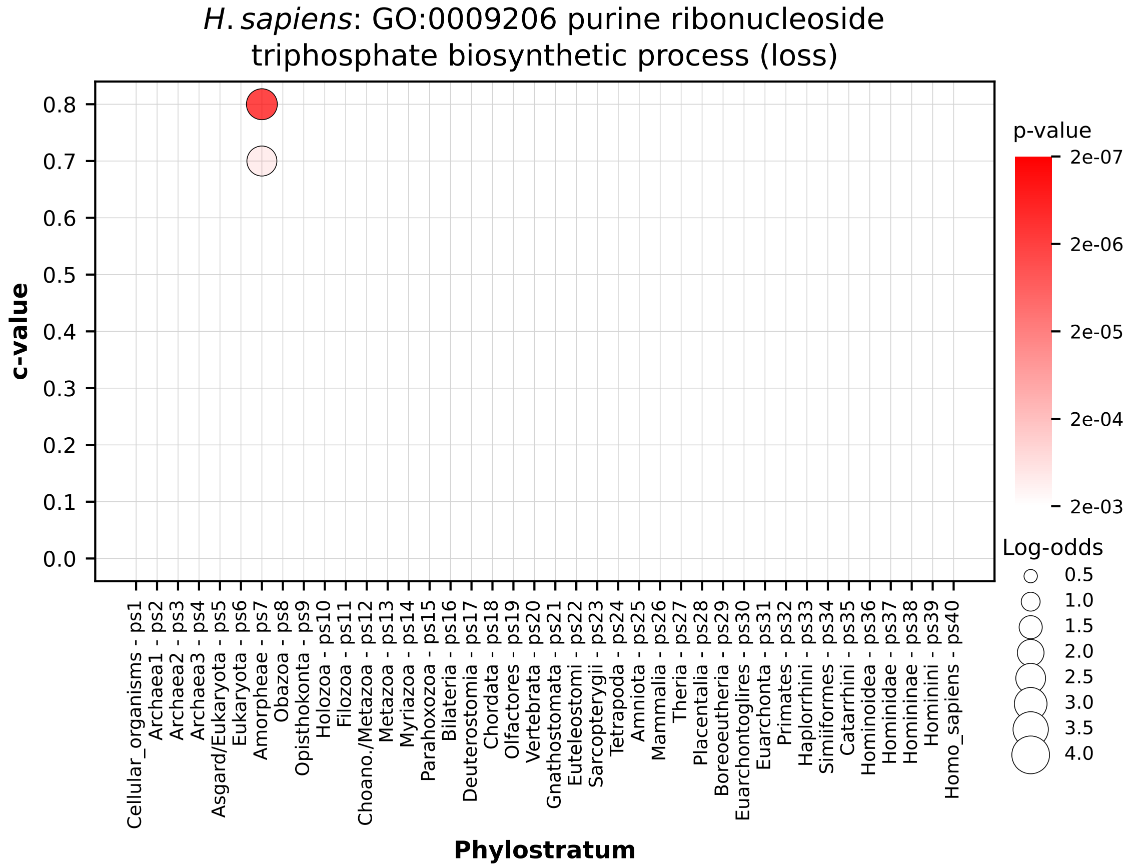

H. sapiens: GO:0009206 purine ribonucleoside triphosphate biosynthetic process (loss)
c-value
0.0
0.1
0.2
0.3
0.4
0.5
0.6
0.7
0.8
p-value
2e-07
2e-06
2e-05
2e-04
2e-03
Log-odds
0.5
1.0
1.5
2.0
2.5
3.0
3.5
4.0
Cellular_organisms - ps1
Archaea1 - ps2
Archaea2 - ps3
Archaea3 - ps4
Asgard/Eukaryota - ps5
Eukaryota - ps6
Amorpheae - ps7
Obazoa - ps8
Opisthokonta - ps9
Holozoa - ps10
Filozoa - ps11
Choano./Metazoa - ps12
Metazoa - ps13
Myriazoa - ps14
Parahoxozoa - ps15
Bilateria - ps16
Deuterostomia - ps17
Chordata - ps18
Olfactores - ps19
Vertebrata - ps20
Gnathostomata - ps21
Euteleostomi - ps22
Sarcopterygii - ps23
Tetrapoda - ps24
Amniota - ps25
Mammalia - ps26
Theria - ps27
Placentalia - ps28
Boreoeutheria - ps29
Euarchontoglires - ps30
Euarchonta - ps31
Primates - ps32
Haplorrhini - ps33
Simiiformes - ps34
Catarrhini - ps35
Hominoidea - ps36
Hominidae - ps37
Homininae - ps38
Hominini - ps39
Homo_sapiens - ps40
Phylostratum

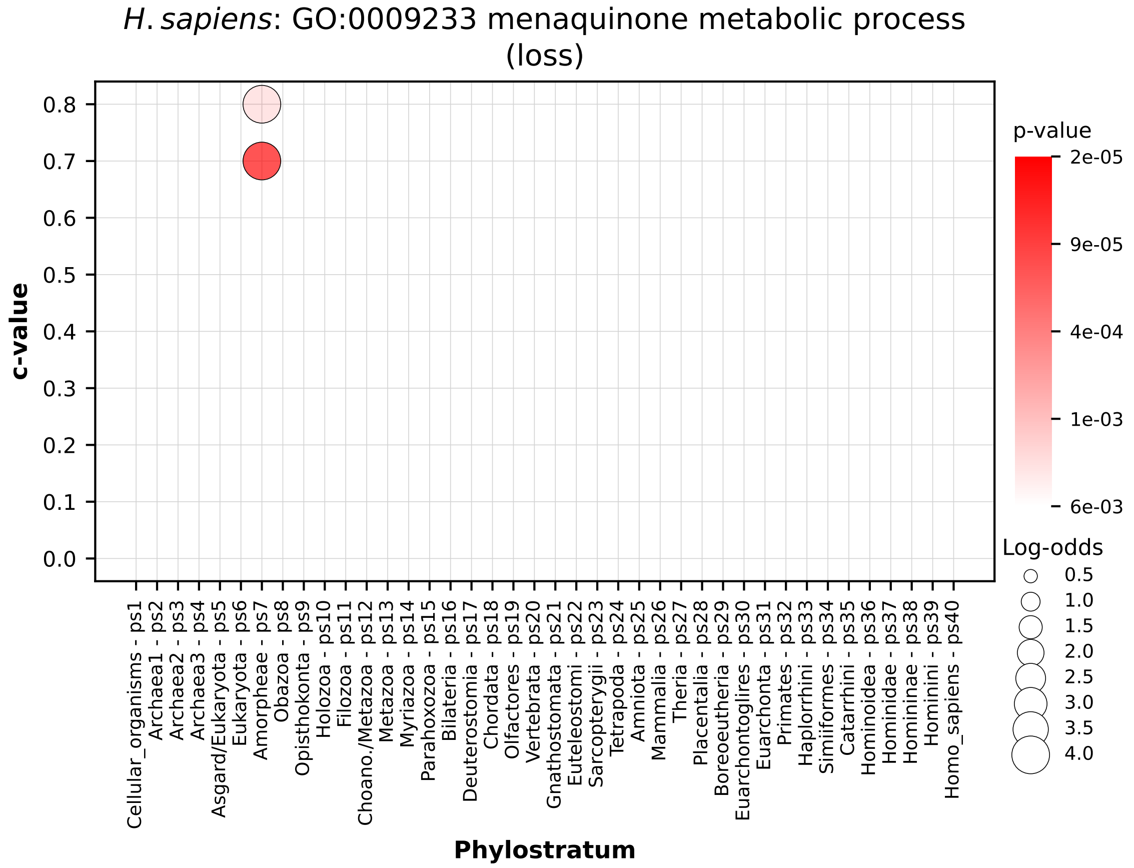

H. sapiens: GO:0009233 menaquinone metabolic process (loss)
c-value
0.8
0.7
0.6
0.5
0.4
0.3
0.2
0.1
0.0
p-value
2e-05
9e-05
4e-04
1e-03
6e-03
Log-odds
0.5
1.0
1.5
2.0
2.5
3.0
3.5
4.0
Cellular_organisms - ps1
Archaea1 - ps2
Archaea2 - ps3
Archaea3 - ps4
Asgard/Eukaryota - ps5
Eukaryota - ps6
Amorpheae - ps7
Obazoa - ps8
Opisthokonta - ps9
Holozoa - ps10
Filozoa - ps11
Choano./Metazoa - ps12
Metazoa - ps13
Myriazoa - ps14
Parahoxozoa - ps15
Bilateria - ps16
Deuterostomia - ps17
Chordata - ps18
Olfactores - ps19
Vertebrata - ps20
Gnathostomata - ps21
Euteleostomi - ps22
Sarcopterygii - ps23
Tetrapoda - ps24
Amniota - ps25
Mammalia - ps26
Theria - ps27
Placentalia - ps28
Boreoeutheria - ps29
Euarchontoglires - ps30
Euarchonta - ps31
Primates - ps32
Haplorrhini - ps33
Simiiformes - ps34
Catarrhini - ps35
Hominoidea - ps36
Hominidae - ps37
Homininae - ps38
Hominini - ps39
Homo_sapiens - ps40
Phylostratum

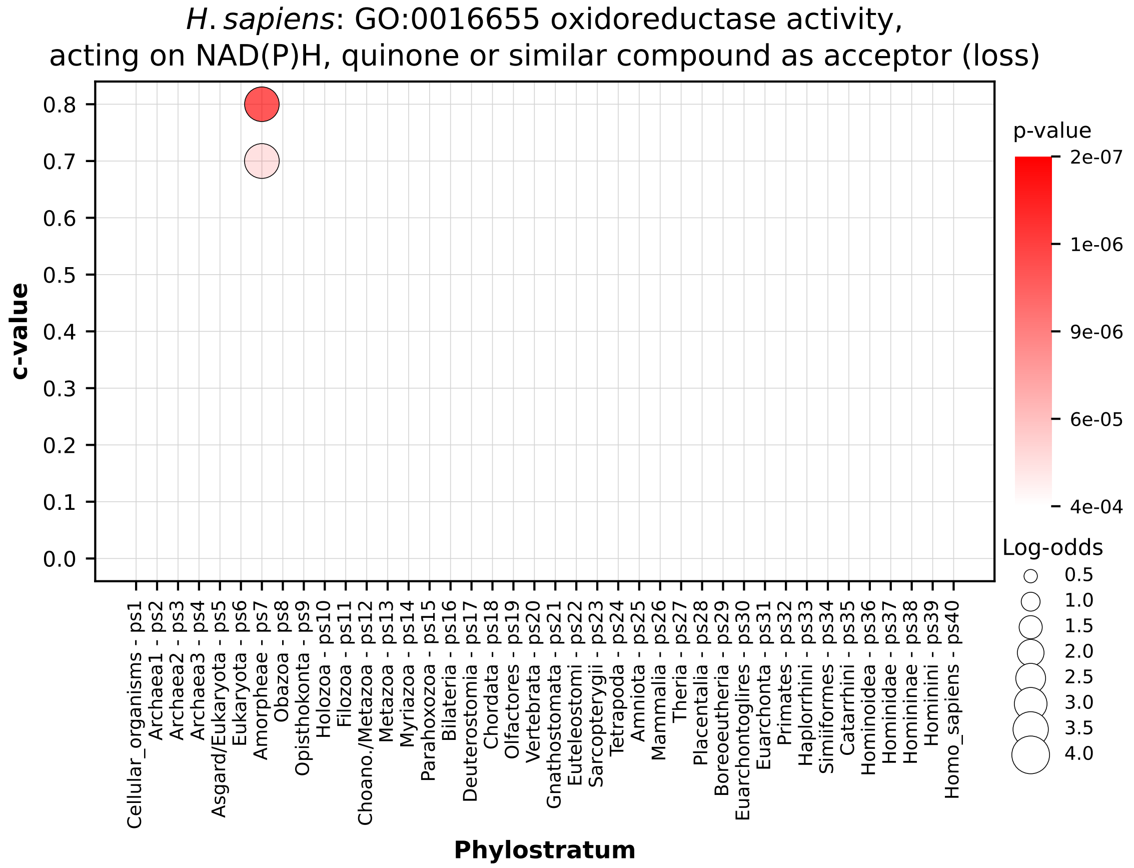
H. sapiens: GO:0016655 oxidoreductase activity,
acting on NAD(P)H, quinone or similar compound as acceptor (loss)
c-value
Phylostratum
p-value
2e-07
1e-06
9e-06
6e-05
4e-04
Log-odds
0.5
1.0
1.5
2.0
2.5
3.0
3.5
4.0
Cellular_organisms - ps1
Archaea1 - ps2
Archaea2 - ps3
Archaea3 - ps4
Asgard/Eukaryota - ps5
Eukaryota - ps6
Amorpheae - ps7
Obazoa - ps8
Opisthokonta - ps9
Holozoa - ps10
Filozoa - ps11
Choano./Metazoa - ps12
Metazoa - ps13
Myriazoa - ps14
Parahoxozoa - ps15
Bilateria - ps16
Deuterostomia - ps17
Chordata - ps18
Olfactores - ps19
Vertebrata - ps20
Gnathostomata - ps21
Euteleostomi - ps22
Sarcopterygii - ps23
Tetrapoda - ps24
Amniota - ps25
Mammalia - ps26
Theria - ps27
Placentalia - ps28
Boreoeutheria - ps29
Euarchontoglires - ps30
Euarchonta - ps31
Primates - ps32
Haplorrhini - ps33
Simiiformes - ps34
Catarrhini - ps35
Hominoidea - ps36
Hominidae - ps37
Homininae - ps38
Hominini - ps39
Homo_sapiens - ps40

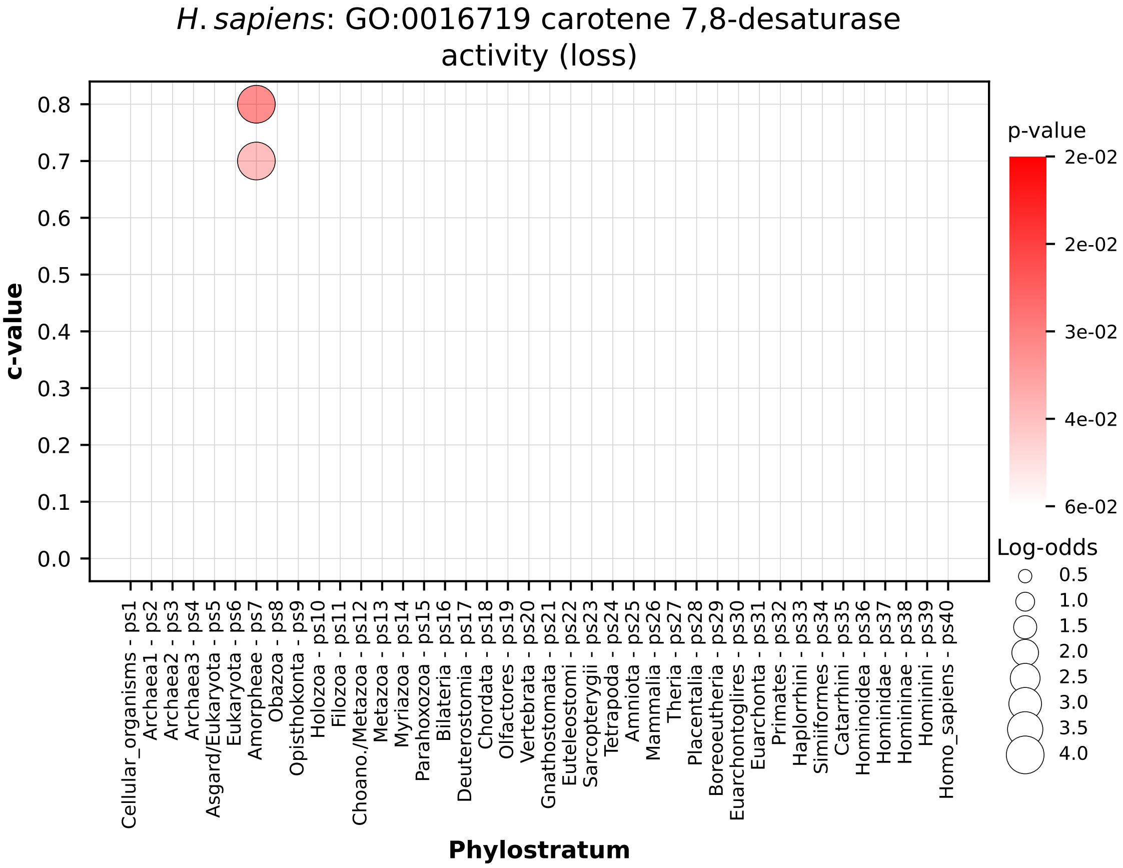

H. sapiens: GO:0016719 carotene 7,8-desaturase activity (loss)
c-value
0.8
0.7
0.6
0.5
0.4
0.3
0.2
0.1
0.0
p-value
2e-02
2e-02
3e-02
4e-02
6e-02
Log-odds
0.5
1.0
1.5
2.0
2.5
3.0
3.5
4.0
Cellular_organisms - ps1
Archaea1 - ps2
Archaea2 - ps3
Archaea3 - ps4
Asgard/Eukaryota - ps5
Eukaryota - ps6
Amorpheae - ps7
Obazoa - ps8
Opisthokonta - ps9
Holozoa - ps10
Filozoa - ps11
Choano./Metazoa - ps12
Metazoa - ps13
Myriazoa - ps14
Parahoxozoa - ps15
Bilateria - ps16
Deuterostomia - ps17
Chordata - ps18
Olfactores - ps19
Vertebrata - ps20
Gnathostomata - ps21
Euteleostomi - ps22
Sarcopterygii - ps23
Tetrapoda - ps24
Amniota - ps25
Mammalia - ps26
Theria - ps27
Placentalia - ps28
Boreoeutheria - ps29
Euarchontoglires - ps30
Euarchonta - ps31
Primates - ps32
Haplorrhini - ps33
Simiiformes - ps34
Catarrhini - ps35
Hominoidea - ps36
Hominidae - ps37
Homininae - ps38
Hominini - ps39
Homo_sapiens - ps40
Phylostratum

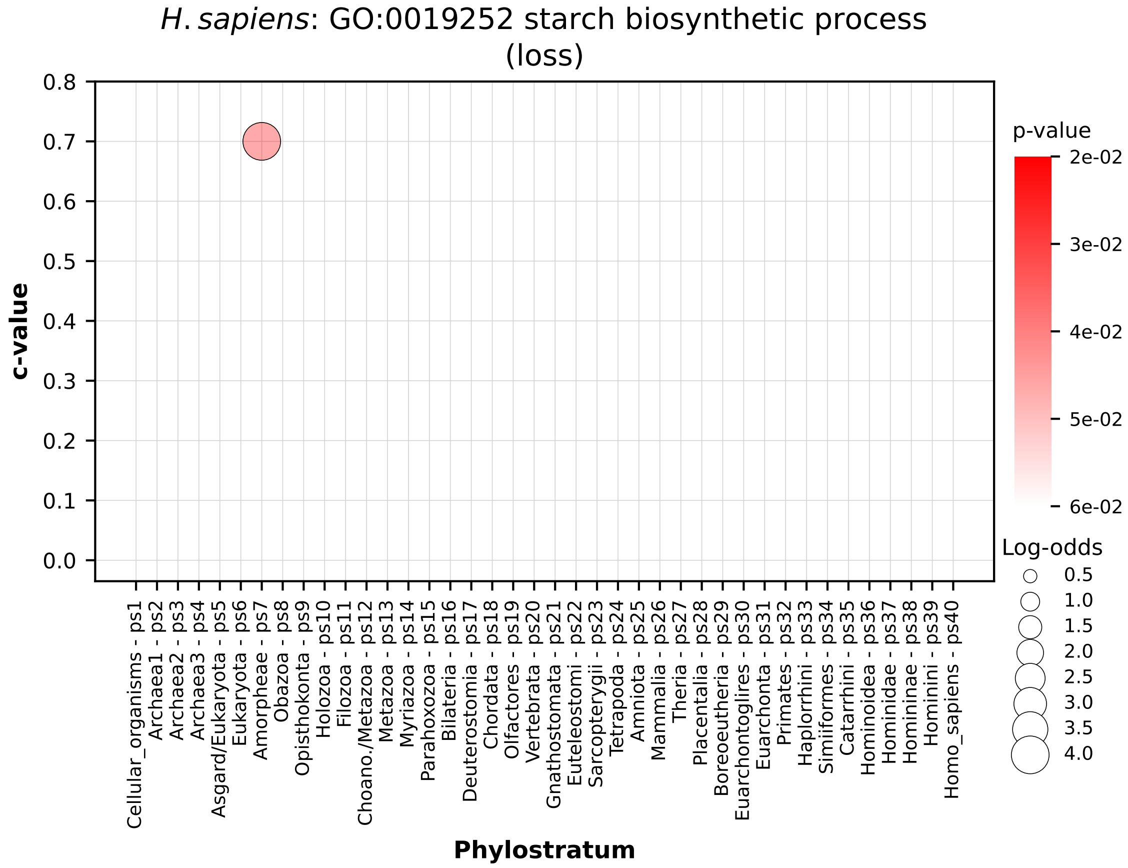

H. sapiens: GO:0019252 starch biosynthetic process
(loss)
c-value
0.8
0.7
0.6
0.5
0.4
0.3
0.2
0.1
0.0
p-value
2e-02
3e-02
4e-02
5e-02
6e-02
Log-odds
0.5
1.0
1.5
2.0
2.5
3.0
3.5
4.0
Cellular_organisms - ps1
Archaea1 - ps2
Archaea2 - ps3
Archaea3 - ps4
Asgard/Eukaryota - ps5
Eukaryota - ps6
Amorpheae - ps7
Obazoa - ps8
Opisthokonta - ps9
Holozoa - ps10
Filozoa - ps11
Choano./Metazoa - ps12
Metazoa - ps13
Myriazoa - ps14
Parahoxozoa - ps15
Bilateria - ps16
Deuterostomia - ps17
Chordata - ps18
Olfactores - ps19
Vertebrata - ps20
Gnathostomata - ps21
Euteleostomi - ps22
Sarcopterygii - ps23
Tetrapoda - ps24
Amniota - ps25
Mammalia - ps26
Theria - ps27
Placentalia - ps28
Boreoeutheria - ps29
Euarchontoglires - ps30
Euarchonta - ps31
Primates - ps32
Haplorrhini - ps33
Simiiformes - ps34
Catarrhini - ps35
Hominoidea - ps36
Hominidae - ps37
Homininae - ps38
Hominini - ps39
Homo_sapiens - ps40
Phylostratum

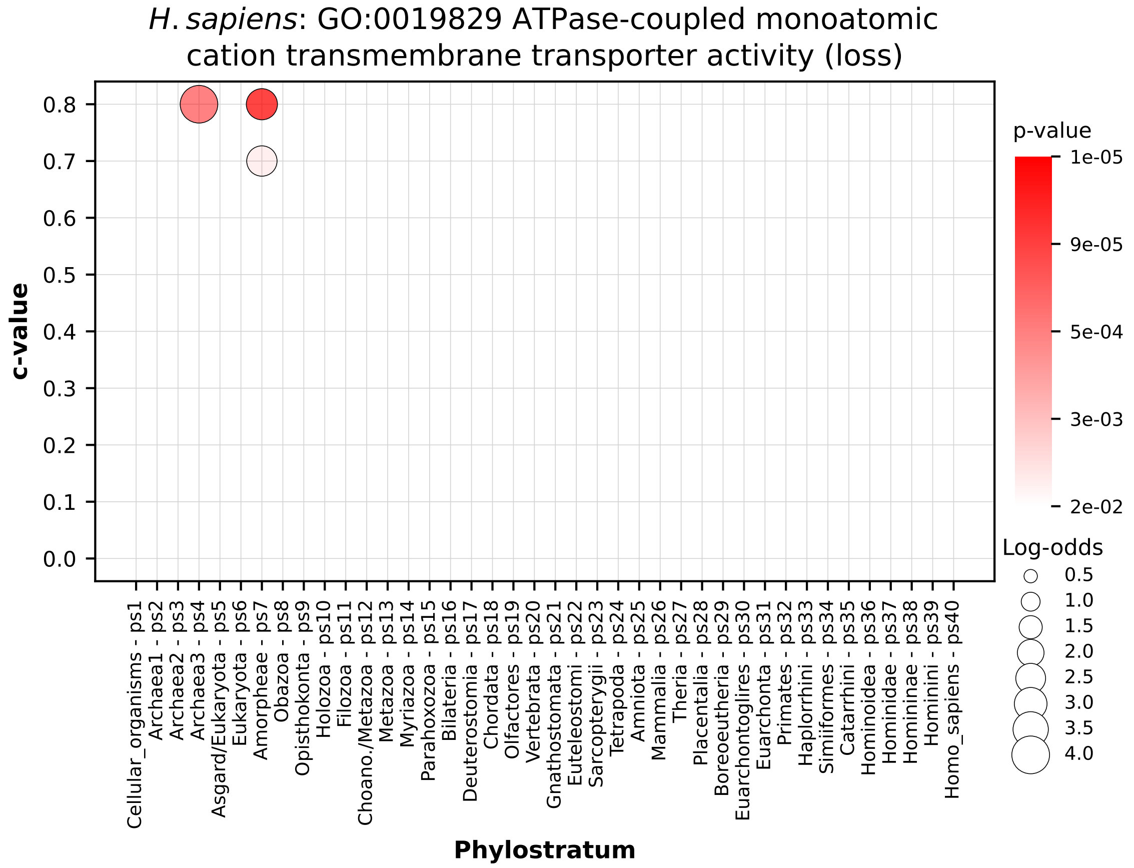

H. sapiens: GO:0019829 ATPase-coupled monoatomic cation transmembrane transporter activity (loss)
c-value
0.0
0.1
0.2
0.3
0.4
0.5
0.6
0.7
0.8
Phylostratum
Cellular_organisms - ps1
Archaea1 - ps2
Archaea2 - ps3
Archaea3 - ps4
Asgard/Eukaryota - ps5
Eukaryota - ps6
Amorpheae - ps7
Obazoa - ps8
Opisthokonta - ps9
Holozoa - ps10
Filozoa - ps11
Choano./Metazoa - ps12
Metazoa - ps13
Myriazoa - ps14
Parahoxozoa - ps15
Bilateria - ps16
Deuterostomia - ps17
Chordata - ps18
Olfactores - ps19
Vertebrata - ps20
Gnathostomata - ps21
Euteleostomi - ps22
Sarcopterygii - ps23
Tetrapoda - ps24
Amniota - ps25
Mammalia - ps26
Theria - ps27
Placentalia - ps28
Boreoeutheria - ps29
Euarchontoglires - ps30
Euarchonta - ps31
Primates - ps32
Haplorrhini - ps33
Simiiformes - ps34
Catarrhini - ps35
Hominoidea - ps36
Hominidae - ps37
Homininae - ps38
Hominini - ps39
Homo_sapiens - ps40
p-value
1e-05
9e-05
5e-04
3e-03
2e-02
Log-odds
0.5
1.0
1.5
2.0
2.5
3.0
3.5
4.0

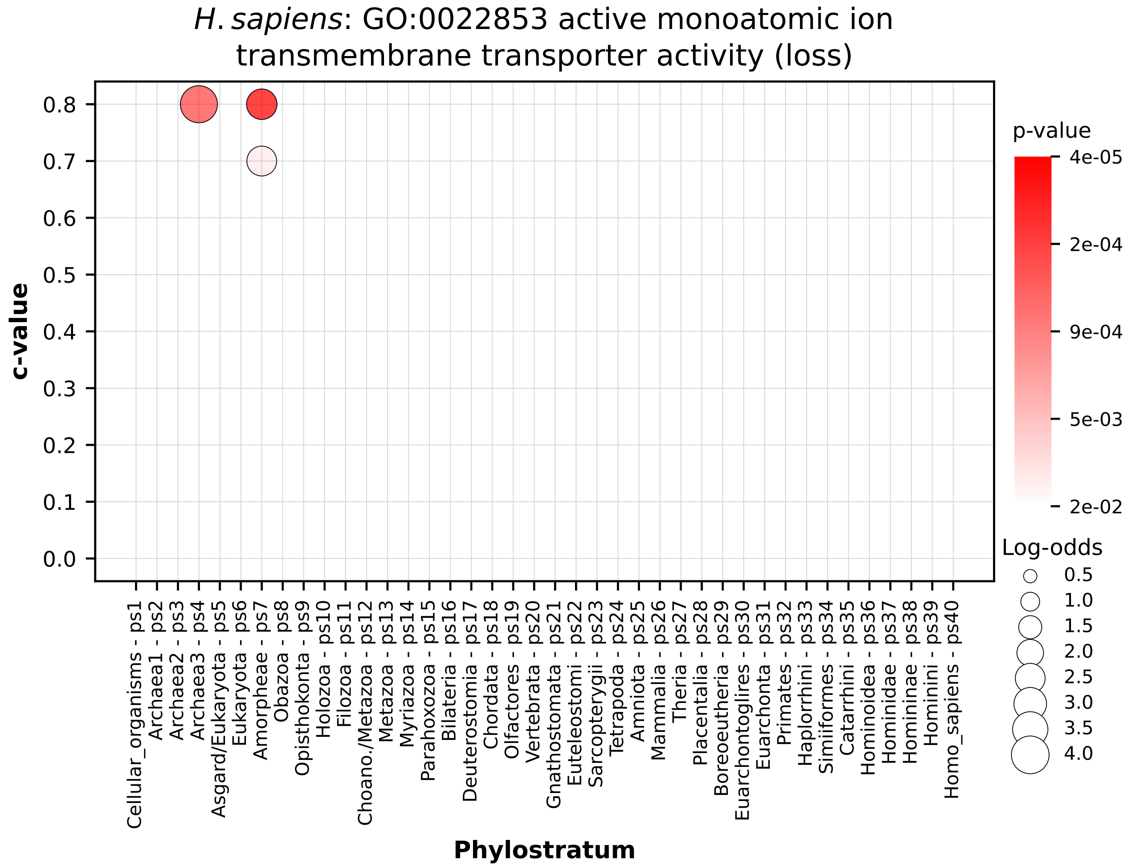

H. sapiens: GO:0022853 active monoatomic ion transmembrane transporter activity (loss)
c-value
0.8
0.7
0.6
0.5
0.4
0.3
0.2
0.1
0.0
p-value
4e-05
2e-04
9e-04
5e-03
2e-02
Log-odds
0.5
1.0
1.5
2.0
2.5
3.0
3.5
4.0
Cellular_organisms - ps1
Archaea1 - ps2
Archaea2 - ps3
Archaea3 - ps4
Asgard/Eukaryota - ps5
Eukaryota - ps6
Amorpheae - ps7
Obazoa - ps8
Opisthokonta - ps9
Holozoa - ps10
Filozoa - ps11
Choano./Metazoa - ps12
Metazoa - ps13
Myriazoa - ps14
Parahoxozoa - ps15
Bilateria - ps16
Deuterostomia - ps17
Chordata - ps18
Olfactores - ps19
Vertebrata - ps20
Gnathostomata - ps21
Euteleostomi - ps22
Sarcopterygii - ps23
Tetrapoda - ps24
Amniota - ps25
Mammalia - ps26
Theria - ps27
Placentalia - ps28
Boreoeutheria - ps29
Euarchontoglires - ps30
Euarchonta - ps31
Primates - ps32
Haplorrhini - ps33
Simiiformes - ps34
Catarrhini - ps35
Hominoidea - ps36
Hominidae - ps37
Homininae - ps38
Hominini - ps39
Homo_sapiens - ps40
Phylostratum

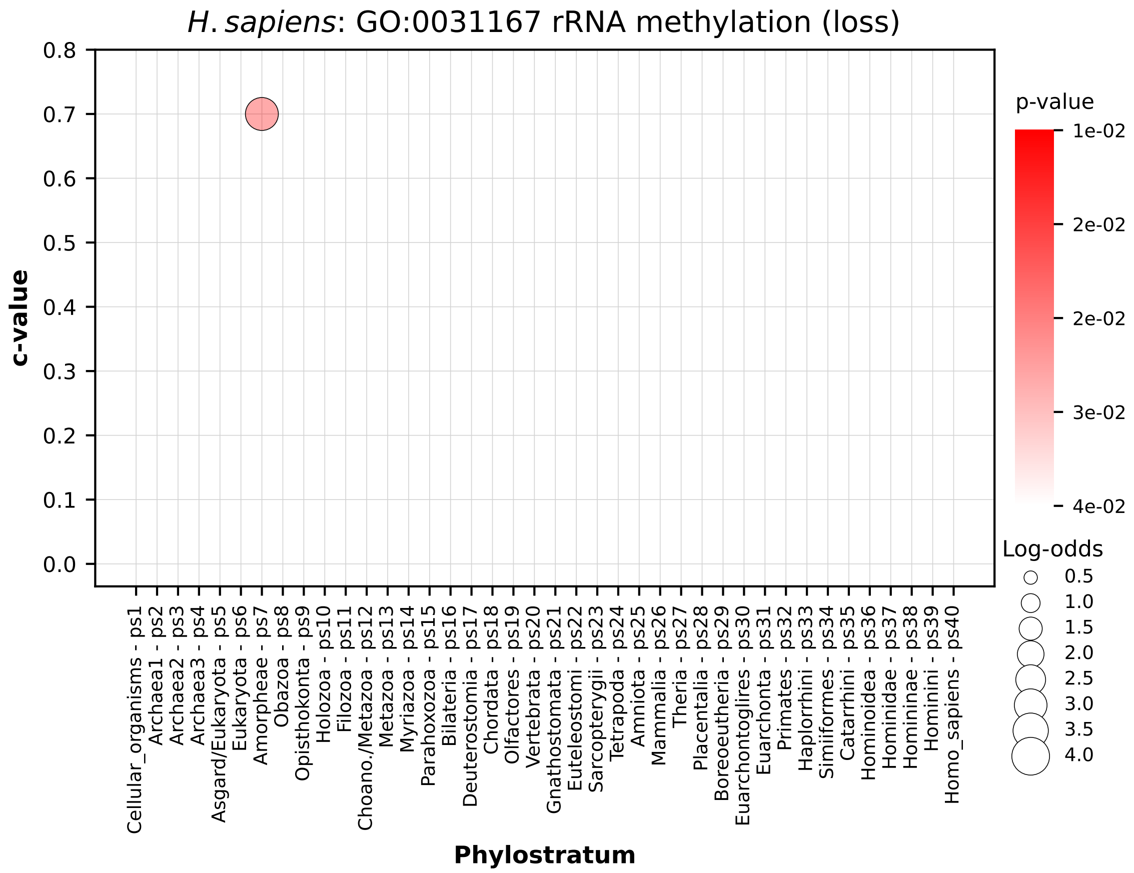

H. sapiens: GO:0031167 rRNA methylation (loss)
c-value
0.8
0.7
0.6
0.5
0.4
0.3
0.2
0.1
0.0
p-value
1e-02
2e-02
2e-02
3e-02
4e-02
Log-odds
0.5
1.0
1.5
2.0
2.5
3.0
3.5
4.0
Cellular_organisms - ps1
Archaea1 - ps2
Archaea2 - ps3
Archaea3 - ps4
Asgard/Eukaryota - ps5
Eukaryota - ps6
Amorpheae - ps7
Obazoa - ps8
Opisthokonta - ps9
Holozoa - ps10
Filozoa - ps11
Choano./Metazoa - ps12
Metazoa - ps13
Myriazoa - ps14
Parahoxozoa - ps15
Bilateria - ps16
Deuterostomia - ps17
Chordata - ps18
Olfactores - ps19
Vertebrata - ps20
Gnathostomata - ps21
Euteleostomi - ps22
Sarcopterygii - ps23
Tetrapoda - ps24
Amniota - ps25
Mammalia - ps26
Theria - ps27
Placentalia - ps28
Boreoeutheria - ps29
Euarchontoglires - ps30
Euarchonta - ps31
Primates - ps32
Haplorrhini - ps33
Simiiformes - ps34
Catarrhini - ps35
Hominoidea - ps36
Hominidae - ps37
Homininae - ps38
Hominini - ps39
Homo_sapiens - ps40
Phylostratum

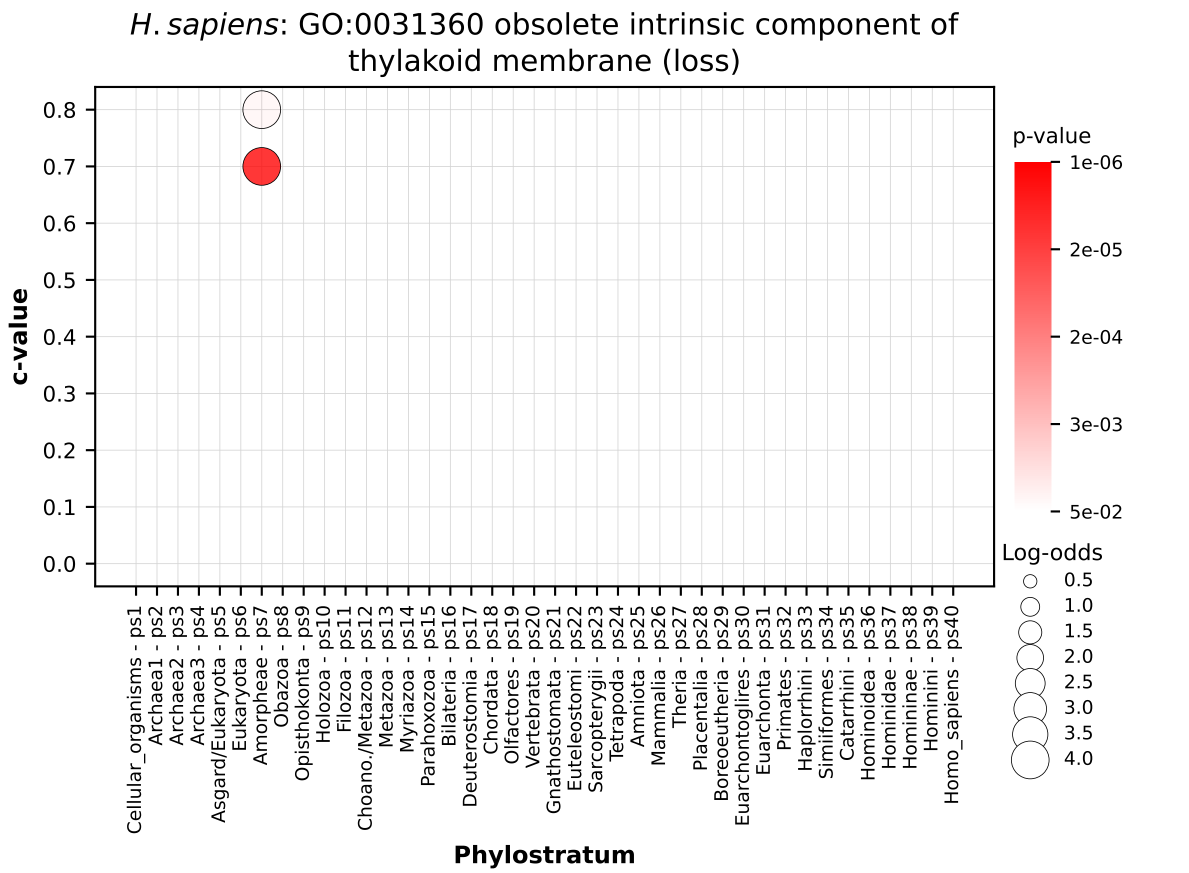

H. sapiens: GO:0031360 obsolete intrinsic component of thylakoid membrane (loss)
c-value
0.8
0.7
0.6
0.5
0.4
0.3
0.2
0.1
0.0
Phylostratum
Cellular_organisms - ps1
Archaea1 - ps2
Archaea2 - ps3
Archaea3 - ps4
Asgard/Eukaryota - ps5
Eukaryota - ps6
Amorpheae - ps7
Obazoa - ps8
Opisthokonta - ps9
Holozoa - ps10
Filozoa - ps11
Choano./Metazoa - ps12
Metazoa - ps13
Myriazoa - ps14
Parahoxozoa - ps15
Bilateria - ps16
Deuterostomia - ps17
Chordata - ps18
Olfactores - ps19
Vertebrata - ps20
Gnathostomata - ps21
Euteleostomi - ps22
Sarcopterygii - ps23
Tetrapoda - ps24
Amniota - ps25
Mammalia - ps26
Theria - ps27
Placentalia - ps28
Boreoeutheria - ps29
Euarchontoglires - ps30
Euarchonta - ps31
Primates - ps32
Haplorrhini - ps33
Simiiformes - ps34
Catarrhini - ps35
Hominoidea - ps36
Hominidae - ps37
Homininae - ps38
Hominini - ps39
Homo_sapiens - ps40
p-value
1e-06
2e-05
2e-04
3e-03
5e-02
Log-odds
0.5
1.0
1.5
2.0
2.5
3.0
3.5
4.0

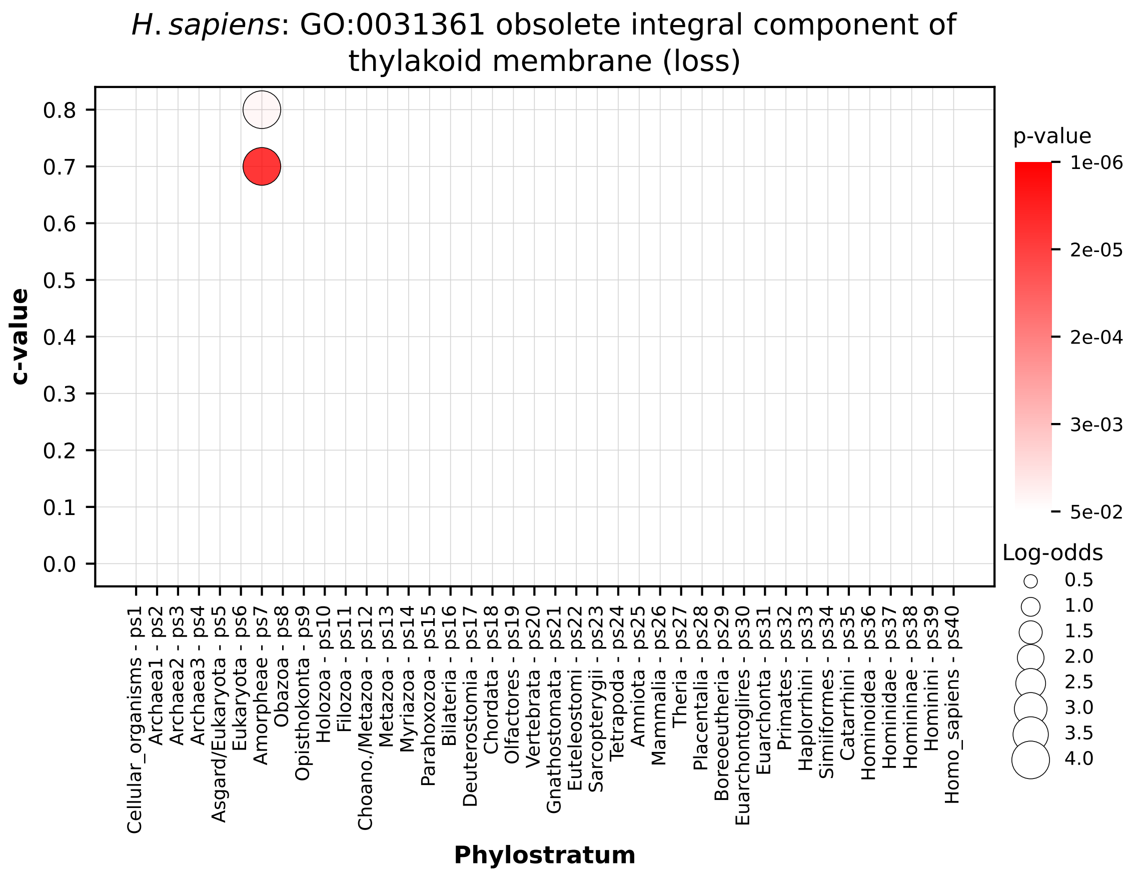

H. sapiens: GO:0031361 obsolete integral component of thylakoid membrane (loss)
c-value
0.0
0.1
0.2
0.3
0.4
0.5
0.6
0.7
0.8
Phylostratum
Cellular_organisms - ps1
Archaea1 - ps2
Archaea2 - ps3
Archaea3 - ps4
Asgard/Eukaryota - ps5
Eukaryota - ps6
Amorpheae - ps7
Obazoa - ps8
Opisthokonta - ps9
Holozoa - ps10
Filozoa - ps11
Choano./Metazoa - ps12
Metazoa - ps13
Myriazoa - ps14
Parahoxozoa - ps15
Bilateria - ps16
Deuterostomia - ps17
Chordata - ps18
Olfactores - ps19
Vertebrata - ps20
Gnathostomata - ps21
Euteleostomi - ps22
Sarcopterygii - ps23
Tetrapoda - ps24
Amniota - ps25
Mammalia - ps26
Theria - ps27
Placentalia - ps28
Boreoeutheria - ps29
Euarchontoglires - ps30
Euarchonta - ps31
Primates - ps32
Haplorrhini - ps33
Simiiformes - ps34
Catarrhini - ps35
Hominoidea - ps36
Hominidae - ps37
Homininae - ps38
Hominini - ps39
Homo_sapiens - ps40
p-value
1e-06
2e-05
2e-04
3e-03
5e-02
Log-odds
0.5
1.0
1.5
2.0
2.5
3.0
3.5
4.0

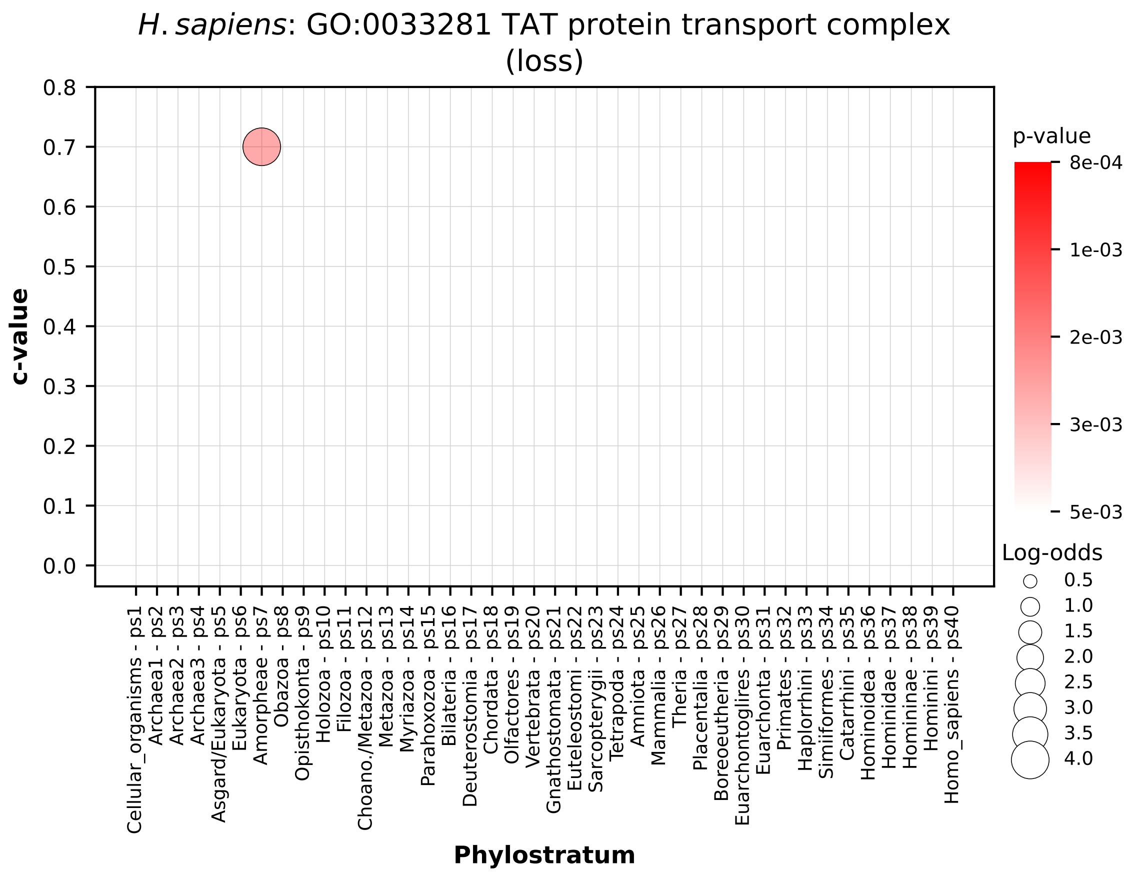

H. sapiens: GO:0033281 TAT protein transport complex
(loss)
c-value
0.8
0.7
0.6
0.5
0.4
0.3
0.2
0.1
0.0
p-value
8e-04
1e-03
2e-03
3e-03
5e-03
Log-odds
0.5
1.0
1.5
2.0
2.5
3.0
3.5
4.0
Cellular_organisms - ps1
Archaea1 - ps2
Archaea2 - ps3
Archaea3 - ps4
Asgard/Eukaryota - ps5
Eukaryota - ps6
Amorpheae - ps7
Obazoa - ps8
Opisthokonta - ps9
Holozoa - ps10
Filozoa - ps11
Choano./Metazoa - ps12
Metazoa - ps13
Myriazoa - ps14
Parahoxozoa - ps15
Bilateria - ps16
Deuterostomia - ps17
Chordata - ps18
Olfactores - ps19
Vertebrata - ps20
Gnathostomata - ps21
Euteleostomi - ps22
Sarcopterygii - ps23
Tetrapoda - ps24
Amniota - ps25
Mammalia - ps26
Theria - ps27
Placentalia - ps28
Boreoeutheria - ps29
Euarchontoglires - ps30
Euarchonta - ps31
Primates - ps32
Haplorrhini - ps33
Simiiformes - ps34
Catarrhini - ps35
Hominoidea - ps36
Hominidae - ps37
Homininae - ps38
Hominini - ps39
Homo_sapiens - ps40
Phylostratum

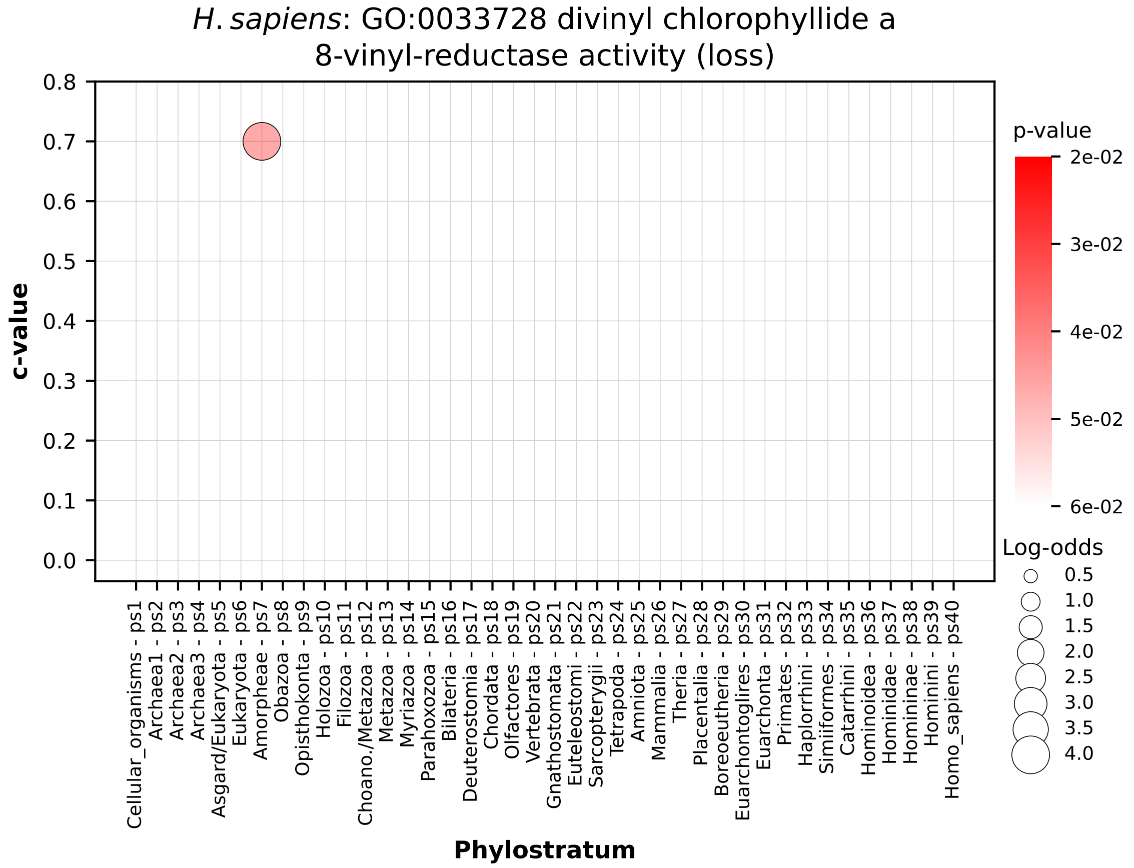

H. sapiens: GO:0033728 divinyl chlorophyllide a 8-vinyl-reductase activity (loss)
c-value
0.8
0.7
0.6
0.5
0.4
0.3
0.2
0.1
0.0
Phylostratum
Cellular_organisms - ps1
Archaea1 - ps2
Archaea2 - ps3
Archaea3 - ps4
Asgard/Eukaryota - ps5
Eukaryota - ps6
Amorpheae - ps7
Obazoa - ps8
Opisthokonta - ps9
Holozoa - ps10
Filozoa - ps11
Choano./Metazoa - ps12
Metazoa - ps13
Myriazoa - ps14
Parahoxozoa - ps15
Bilateria - ps16
Deuterostomia - ps17
Chordata - ps18
Olfactores - ps19
Vertebrata - ps20
Gnathostomata - ps21
Euteleostomi - ps22
Sarcopterygii - ps23
Tetrapoda - ps24
Amniota - ps25
Mammalia - ps26
Theria - ps27
Placentalia - ps28
Boreoeutheria - ps29
Euarchontoglires - ps30
Euarchonta - ps31
Primates - ps32
Haplorrhini - ps33
Simiiformes - ps34
Catarrhini - ps35
Hominoidea - ps36
Hominidae - ps37
Homininae - ps38
Hominini - ps39
Homo_sapiens - ps40
p-value
2e-02
3e-02
4e-02
5e-02
6e-02
Log-odds
0.5
1.0
1.5
2.0
2.5
3.0
3.5
4.0

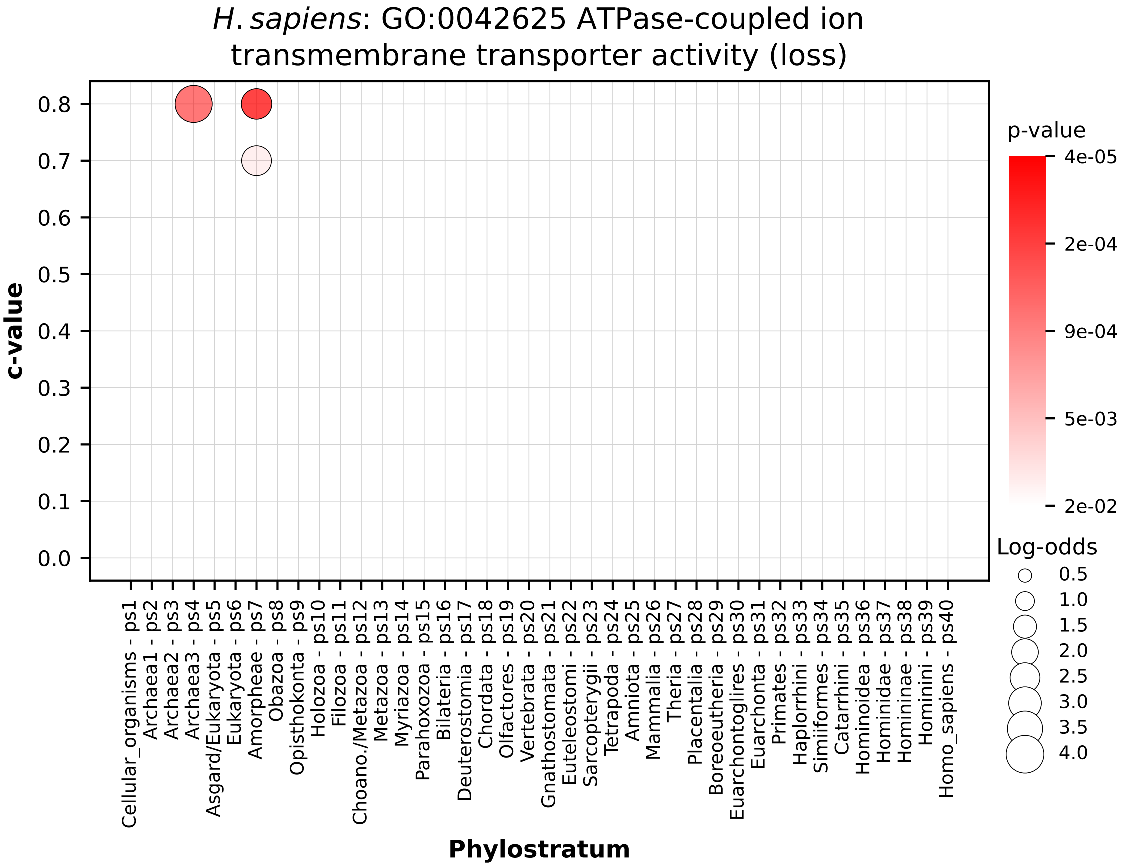

H. sapiens: GO:0042625 ATPase-coupled ion transmembrane transporter activity (loss)
c-value
0.8
0.7
0.6
0.5
0.4
0.3
0.2
0.1
0.0
p-value
4e-05
2e-04
9e-04
5e-03
2e-02
Log-odds
0.5
1.0
1.5
2.0
2.5
3.0
3.5
4.0
Cellular_organisms - ps1
Archaea1 - ps2
Archaea2 - ps3
Archaea3 - ps4
Asgard/Eukaryota - ps5
Eukaryota - ps6
Amorpheae - ps7
Obazoa - ps8
Opisthokonta - ps9
Holozoa - ps10
Filozoa - ps11
Choano./Metazoa - ps12
Metazoa - ps13
Myriazoa - ps14
Parahoxozoa - ps15
Bilateria - ps16
Deuterostomia - ps17
Chordata - ps18
Olfactores - ps19
Vertebrata - ps20
Gnathostomata - ps21
Euteleostomi - ps22
Sarcopterygii - ps23
Tetrapoda - ps24
Amniota - ps25
Mammalia - ps26
Theria - ps27
Placentalia - ps28
Boreoeutheria - ps29
Euarchontoglires - ps30
Euarchonta - ps31
Primates - ps32
Haplorrhini - ps33
Simiiformes - ps34
Catarrhini - ps35
Hominoidea - ps36
Hominidae - ps37
Homininae - ps38
Hominini - ps39
Homo_sapiens - ps40
Phylostratum

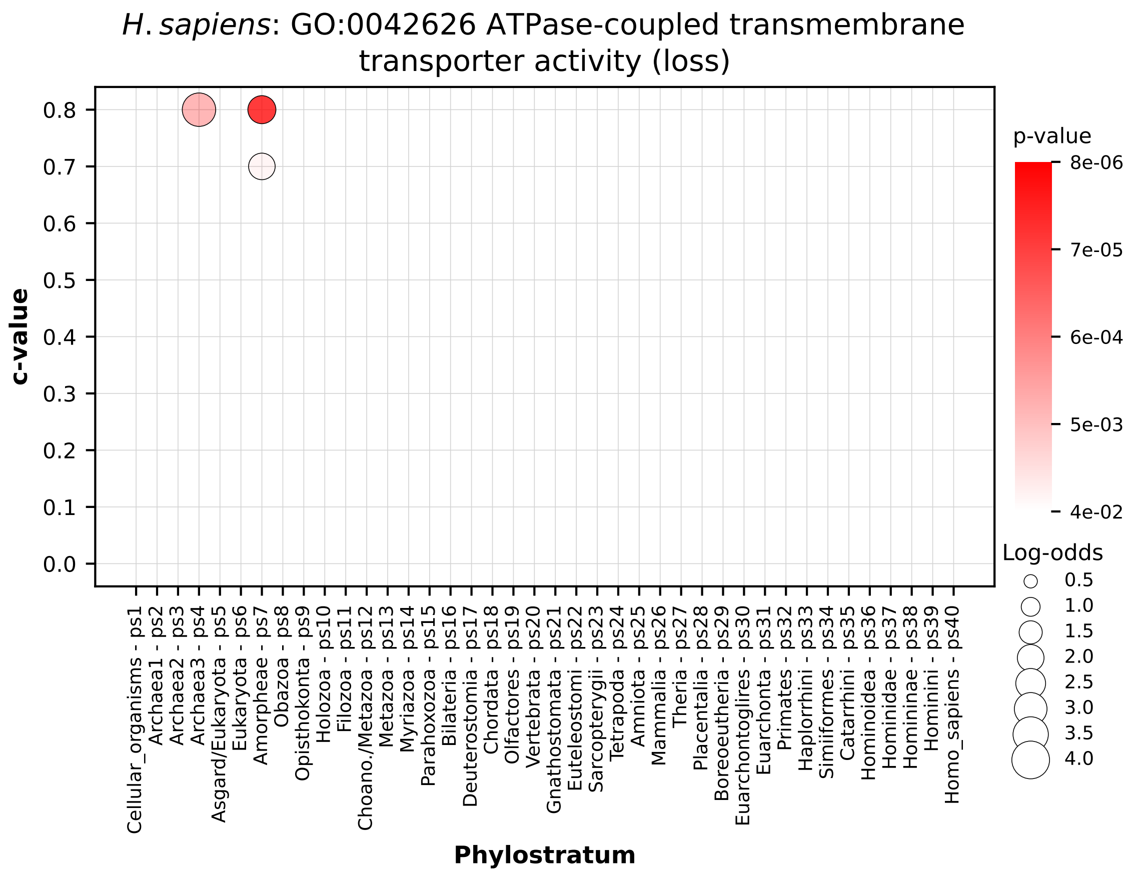

H. sapiens: GO:0042626 ATPase-coupled transmembrane transporter activity (loss)
c-value
0.0
0.1
0.2
0.3
0.4
0.5
0.6
0.7
0.8
Phylostratum
Cellular_organisms - ps1
Archaea1 - ps2
Archaea2 - ps3
Archaea3 - ps4
Asgard/Eukaryota - ps5
Eukaryota - ps6
Amorpheae - ps7
Obazoa - ps8
Opisthokonta - ps9
Holozoa - ps10
Filozoa - ps11
Choano./Metazoa - ps12
Metazoa - ps13
Myriazoa - ps14
Parahoxozoa - ps15
Bilateria - ps16
Deuterostomia - ps17
Chordata - ps18
Olfactores - ps19
Vertebrata - ps20
Gnathostomata - ps21
Euteleostomi - ps22
Sarcopterygii - ps23
Tetrapoda - ps24
Amniota - ps25
Mammalia - ps26
Theria - ps27
Placentalia - ps28
Boreoeutheria - ps29
Euarchontoglires - ps30
Euarchonta - ps31
Primates - ps32
Haplorrhini - ps33
Simiiformes - ps34
Catarrhini - ps35
Hominoidea - ps36
Hominidae - ps37
Homininae - ps38
Hominini - ps39
Homo_sapiens - ps40
p-value
8e-06
7e-05
6e-04
5e-03
4e-02
Log-odds
0.5
1.0
1.5
2.0
2.5
3.0
3.5
4.0

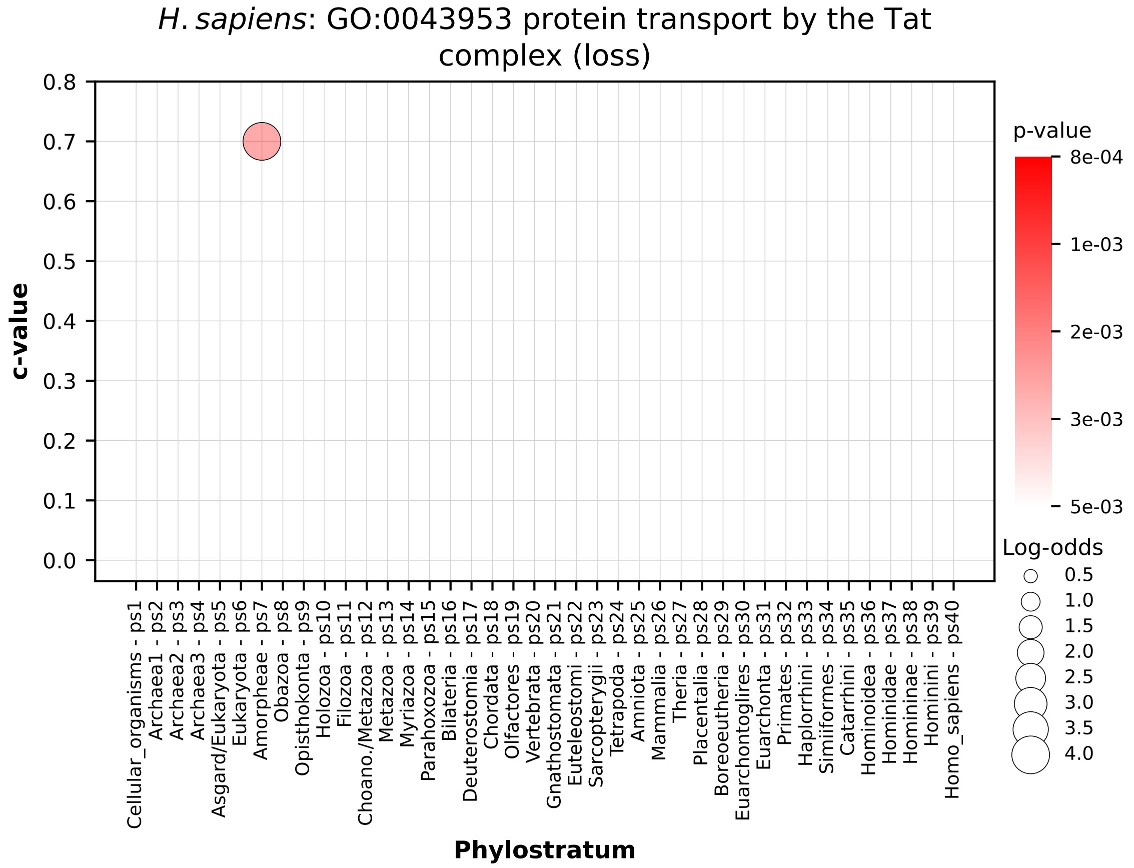

H. sapiens: GO:0043953 protein transport by the Tat complex (loss)
c-value
0.8
0.7
0.6
0.5
0.4
0.3
0.2
0.1
0.0
p-value
8e-04
1e-03
2e-03
3e-03
5e-03
Log-odds
0.5
1.0
1.5
2.0
2.5
3.0
3.5
4.0
Cellular_organisms - ps1
Archaea1 - ps2
Archaea2 - ps3
Archaea3 - ps4
Asgard/Eukaryota - ps5
Eukaryota - ps6
Amorpheae - ps7
Obazoa - ps8
Opisthokonta - ps9
Holozoa - ps10
Filozoa - ps11
Choano./Metazoa - ps12
Metazoa - ps13
Myriazoa - ps14
Parahoxozoa - ps15
Bilateria - ps16
Deuterostomia - ps17
Chordata - ps18
Olfactores - ps19
Vertebrata - ps20
Gnathostomata - ps21
Euteleostomi - ps22
Sarcopterygii - ps23
Tetrapoda - ps24
Amniota - ps25
Mammalia - ps26
Theria - ps27
Placentalia - ps28
Boreoeutheria - ps29
Euarchontoglires - ps30
Euarchonta - ps31
Primates - ps32
Haplorrhini - ps33
Simiiformes - ps34
Catarrhini - ps35
Hominoidea - ps36
Hominidae - ps37
Homininae - ps38
Hominini - ps39
Homo_sapiens - ps40
Phylostratum

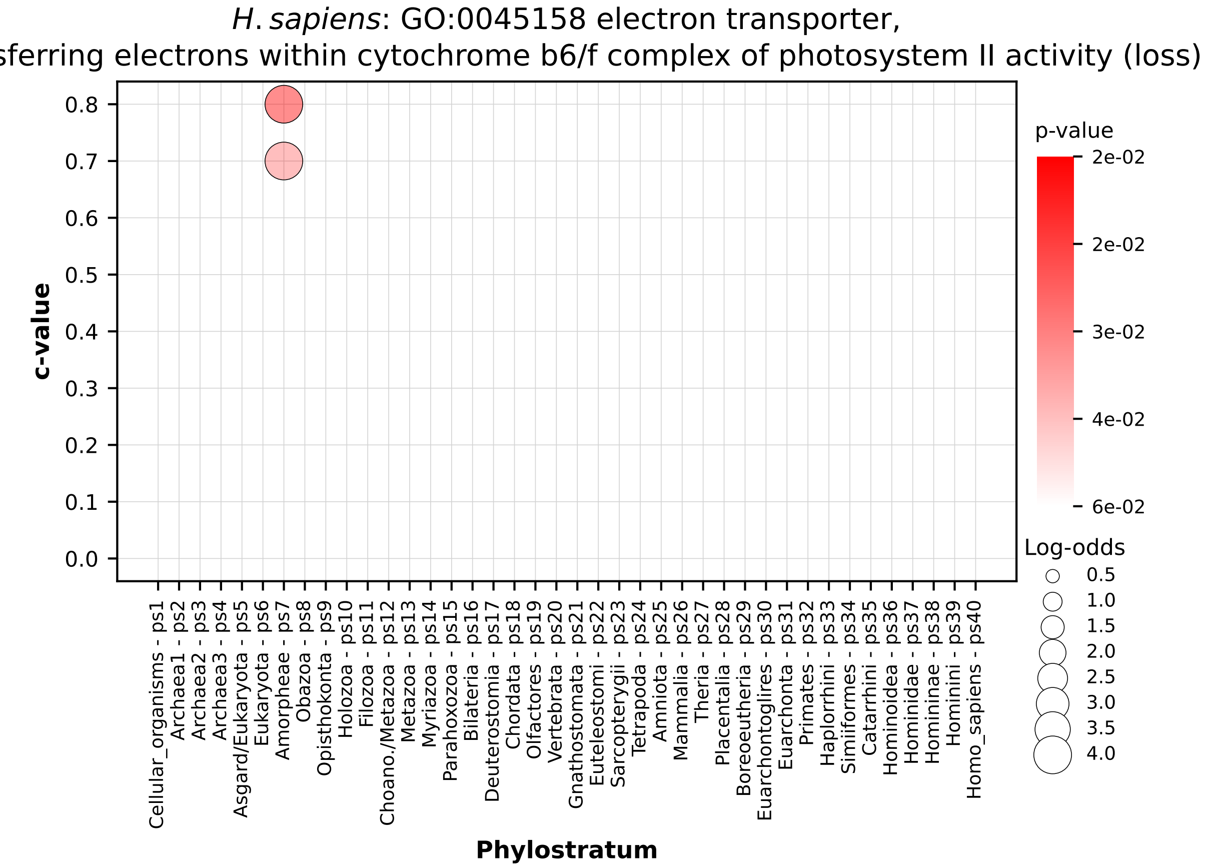

H. sapiens: GO:0045158 electron transporter,
sferring electrons within cytochrome b6/f complex of photosystem II activity (loss)
c-value
0.8
0.7
0.6
0.5
0.4
0.3
0.2
0.1
0.0
p-value
2e-02
2e-02
3e-02
4e-02
6e-02
Log-odds
0.5
1.0
1.5
2.0
2.5
3.0
3.5
4.0
Cellular_organisms - ps1
Archaea1 - ps2
Archaea2 - ps3
Archaea3 - ps4
Asgard/Eukaryota - ps5
Eukaryota - ps6
Amorpheae - ps7
Obazoa - ps8
Opisthokonta - ps9
Holozoa - ps10
Filozoa - ps11
Choano./Metazoa - ps12
Metazoa - ps13
Myriazoa - ps14
Parahoxozoa - ps15
Bilateria - ps16
Deuterostomia - ps17
Chordata - ps18
Olfactores - ps19
Vertebrata - ps20
Gnathostomata - ps21
Euteleostomi - ps22
Sarcopterygii - ps23
Tetrapoda - ps24
Amniota - ps25
Mammalia - ps26
Theria - ps27
Placentalia - ps28
Boreoeutheria - ps29
Euarchontoglires - ps30
Euarchonta - ps31
Primates - ps32
Haplorrhini - ps33
Simiiformes - ps34
Catarrhini - ps35
Hominoidea - ps36
Hominidae - ps37
Homininae - ps38
Hominini - ps39
Homo_sapiens - ps40
Phylostratum

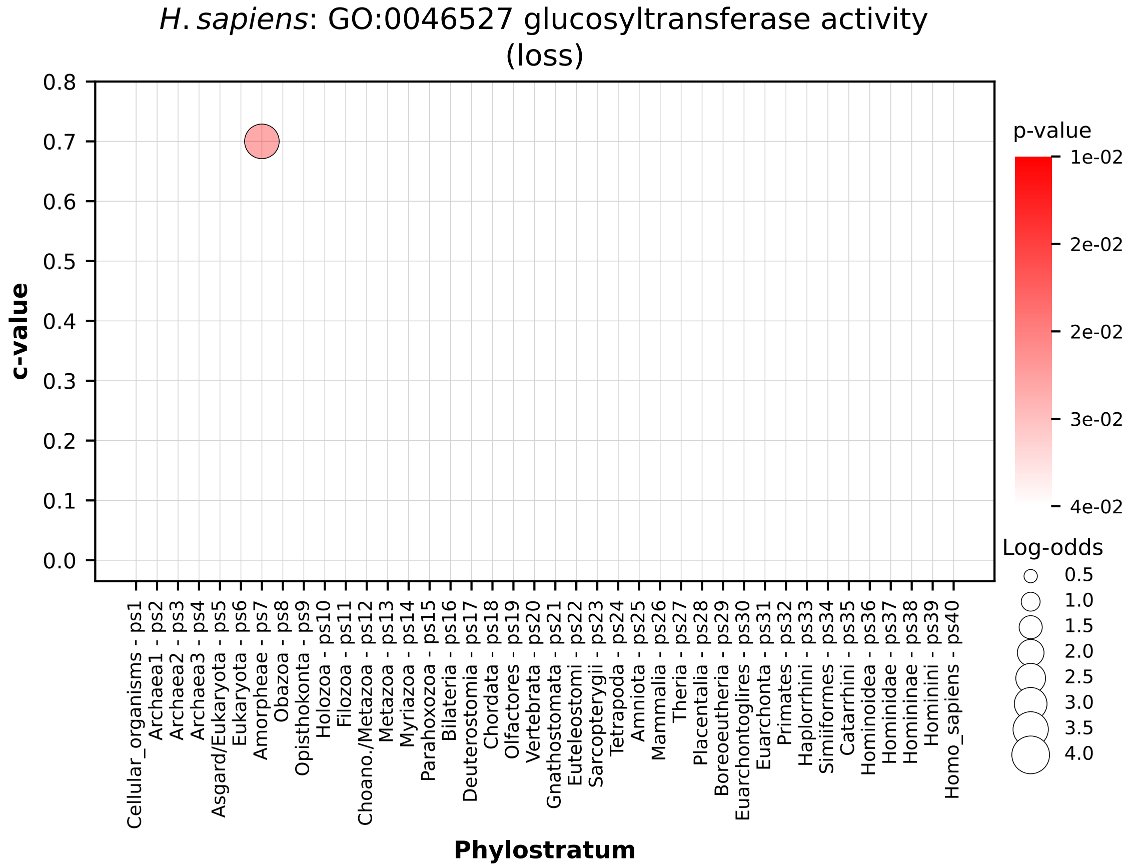
H. sapiens: GO:0046527 glucosyltransferase activity
(loss)
c-value
0.8
0.7
0.6
0.5
0.4
0.3
0.2
0.1
0.0
p-value
1e-02
2e-02
2e-02
3e-02
4e-02
Log-odds
0.5
1.0
1.5
2.0
2.5
3.0
3.5
4.0
Cellular_organisms - ps1
Archaea1 - ps2
Archaea2 - ps3
Archaea3 - ps4
Asgard/Eukaryota - ps5
Eukaryota - ps6
Amorpheae - ps7
Obazoa - ps8
Opisthokonta - ps9
Holozoa - ps10
Filozoa - ps11
Choano./Metazoa - ps12
Metazoa - ps13
Myriazoa - ps14
Parahoxozoa - ps15
Bilateria - ps16
Deuterostomia - ps17
Chordata - ps18
Olfactores - ps19
Vertebrata - ps20
Gnathostomata - ps21
Euteleostomi - ps22
Sarcopterygii - ps23
Tetrapoda - ps24
Amniota - ps25
Mammalia - ps26
Theria - ps27
Placentalia - ps28
Boreoeutheria - ps29
Euarchontoglires - ps30
Euarchonta - ps31
Primates - ps32
Haplorrhini - ps33
Simiiformes - ps34
Catarrhini - ps35
Hominoidea - ps36
Hominidae - ps37
Homininae - ps38
Hominini - ps39
Homo_sapiens - ps40
Phylostratum

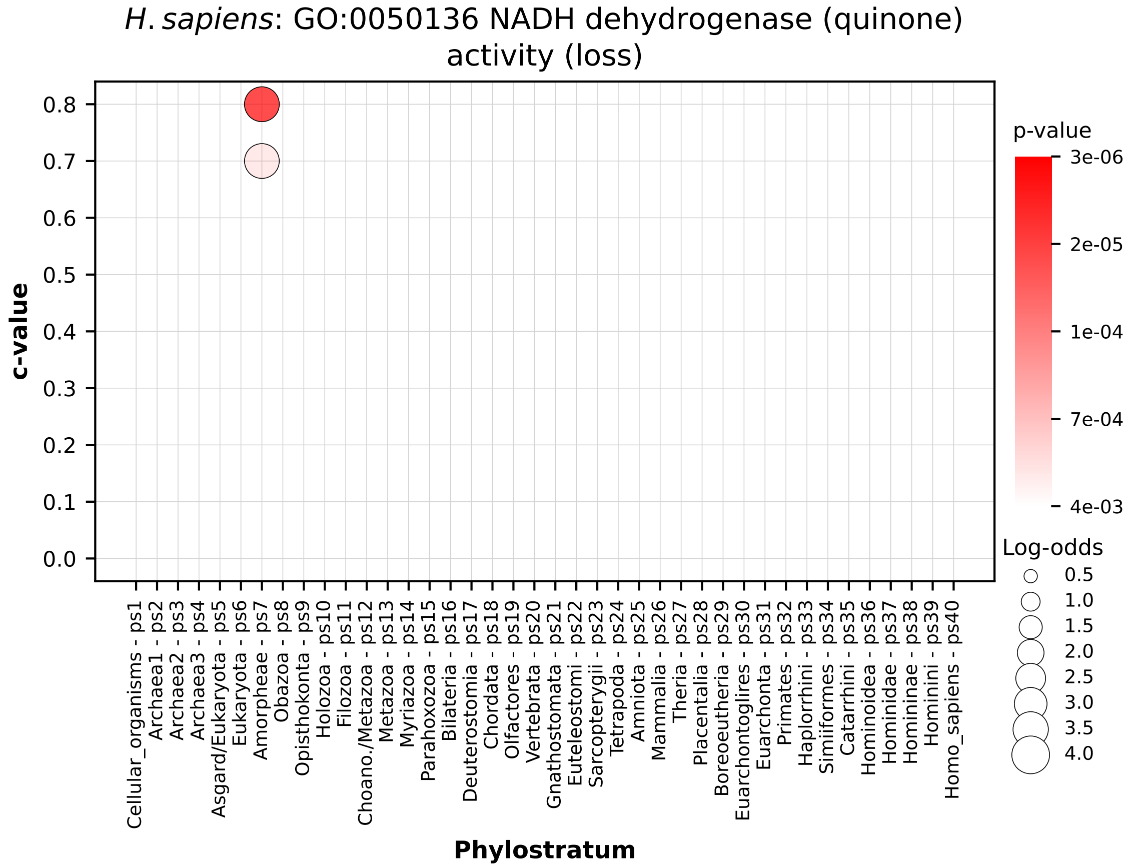

H. sapiens: GO:0050136 NADH dehydrogenase (quinone) activity (loss)
c-value
0.8
0.7
0.6
0.5
0.4
0.3
0.2
0.1
0.0
p-value
3e-06
2e-05
1e-04
7e-04
4e-03
Log-odds
0.5
1.0
1.5
2.0
2.5
3.0
3.5
4.0
Cellular_organisms - ps1
Archaea1 - ps2
Archaea2 - ps3
Archaea3 - ps4
Asgard/Eukaryota - ps5
Eukaryota - ps6
Amorpheae - ps7
Obazoa - ps8
Opisthokonta - ps9
Holozoa - ps10
Filozoa - ps11
Choano./Metazoa - ps12
Metazoa - ps13
Myriazoa - ps14
Parahoxozoa - ps15
Bilateria - ps16
Deuterostomia - ps17
Chordata - ps18
Olfactores - ps19
Vertebrata - ps20
Gnathostomata - ps21
Euteleostomi - ps22
Sarcopterygii - ps23
Tetrapoda - ps24
Amniota - ps25
Mammalia - ps26
Theria - ps27
Placentalia - ps28
Boreoeutheria - ps29
Euarchontoglires - ps30
Euarchonta - ps31
Primates - ps32
Haplorrhini - ps33
Simiiformes - ps34
Catarrhini - ps35
Hominoidea - ps36
Hominidae - ps37
Homininae - ps38
Hominini - ps39
Homo_sapiens - ps40
Phylostratum

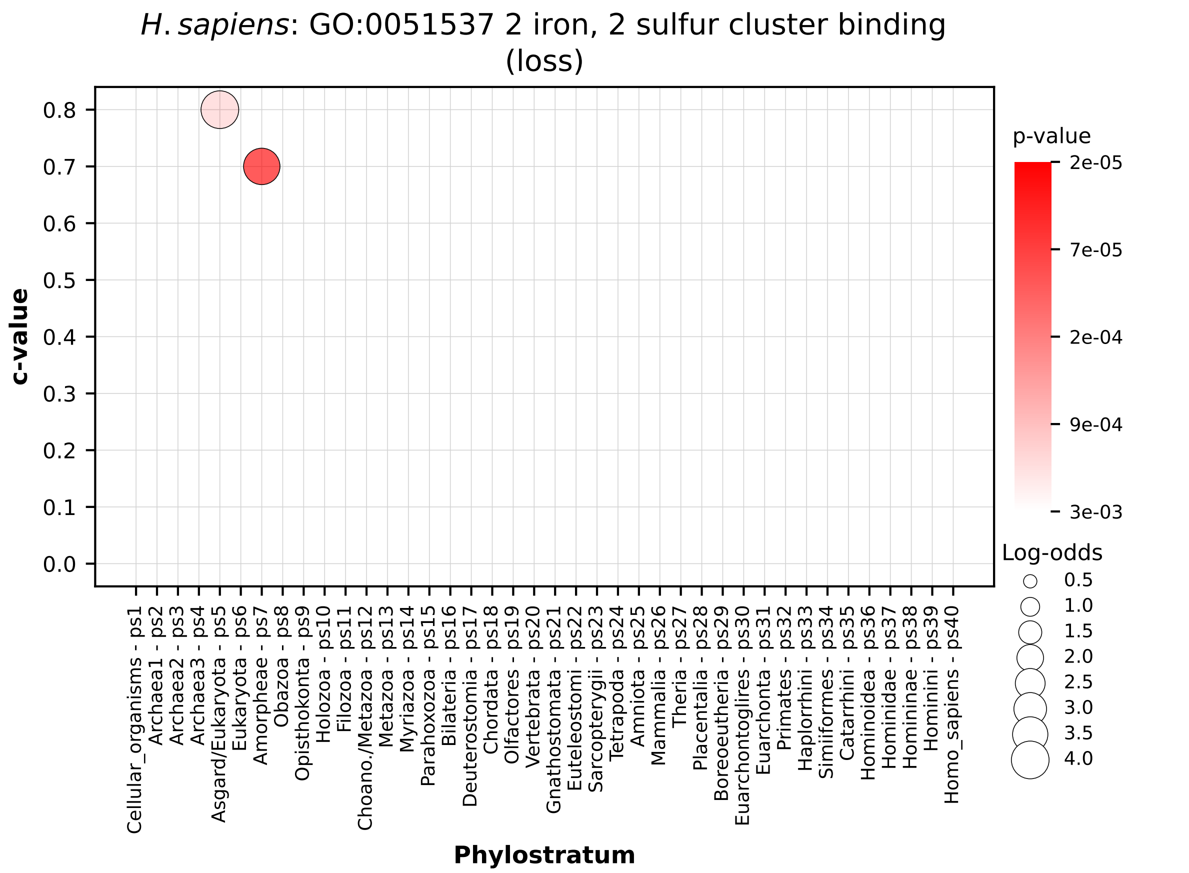
H. sapiens: GO:0051537 2 iron, 2 sulfur cluster binding
(loss)
c-value
0.8
0.7
0.6
0.5
0.4
0.3
0.2
0.1
0.0
p-value
2e-05
7e-05
2e-04
9e-04
3e-03
Log-odds
0.5
1.0
1.5
2.0
2.5
3.0
3.5
4.0
Cellular_organisms - ps1
Archaea1 - ps2
Archaea2 - ps3
Archaea3 - ps4
Asgard/Eukaryota - ps5
Eukaryota - ps6
Amorpheae - ps7
Obazoa - ps8
Opisthokonta - ps9
Holozoa - ps10
Filozoa - ps11
Choano./Metazoa - ps12
Metazoa - ps13
Myriazoa - ps14
Parahoxozoa - ps15
Bilateria - ps16
Deuterostomia - ps17
Chordata - ps18
Olfactores - ps19
Vertebrata - ps20
Gnathostomata - ps21
Euteleostomi - ps22
Sarcopterygii - ps23
Tetrapoda - ps24
Amniota - ps25
Mammalia - ps26
Theria - ps27
Placentalia - ps28
Boreoeutheria - ps29
Euarchontoglires - ps30
Euarchonta - ps31
Primates - ps32
Haplorrhini - ps33
Simiiformes - ps34
Catarrhini - ps35
Hominoidea - ps36
Hominidae - ps37
Homininae - ps38
Hominini - ps39
Homo_sapiens - ps40
Phylostratum

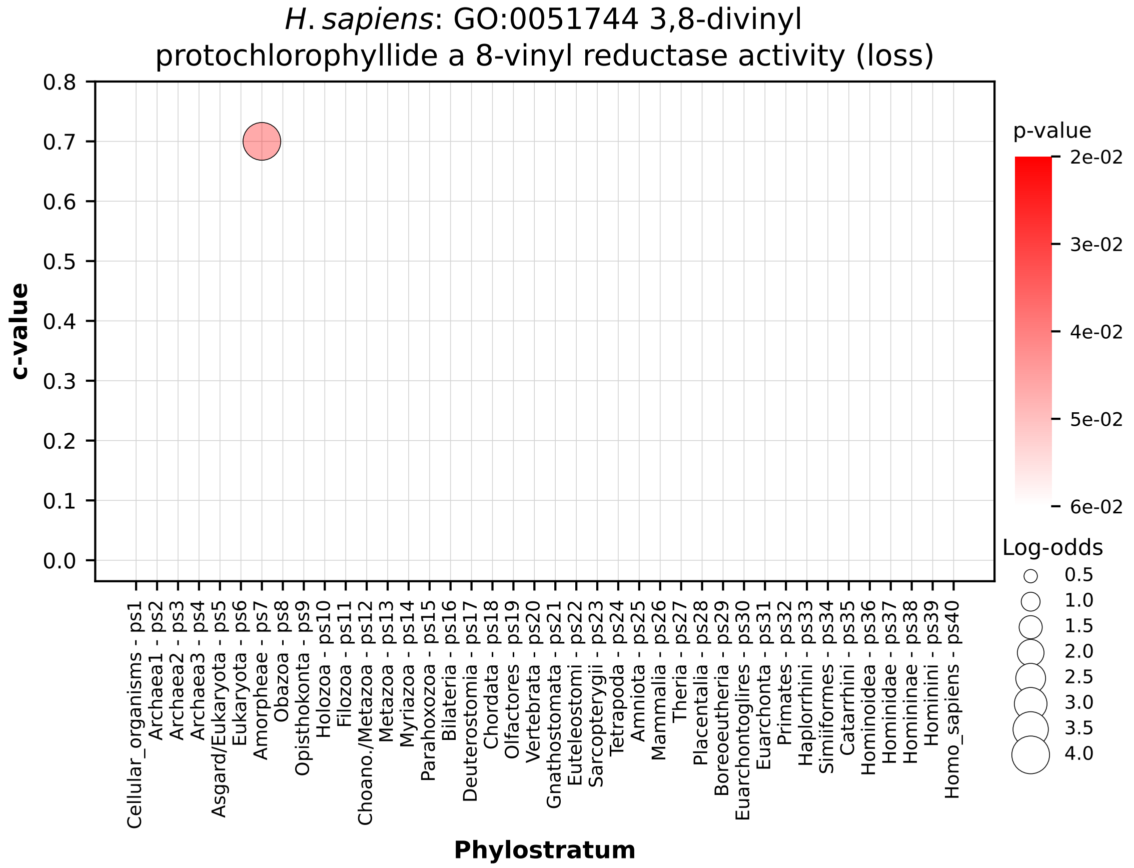
H. sapiens: GO:0051744 3,8-divinyl protochlorophyllide a 8-vinyl reductase activity (loss)
c-value
0.8
0.7
0.6
0.5
0.4
0.3
0.2
0.1
0.0
Phylostratum
Cellular_organisms - ps1
Archaea1 - ps2
Archaea2 - ps3
Archaea3 - ps4
Asgard/Eukaryota - ps5
Eukaryota - ps6
Amorpheae - ps7
Obazoa - ps8
Opisthokonta - ps9
Holozoa - ps10
Filozoa - ps11
Choano./Metazoa - ps12
Metazoa - ps13
Myriazoa - ps14
Parahoxozoa - ps15
Bilateria - ps16
Deuterostomia - ps17
Chordata - ps18
Olfactores - ps19
Vertebrata - ps20
Gnathostomata - ps21
Euteleostomi - ps22
Sarcopterygii - ps23
Tetrapoda - ps24
Amniota - ps25
Mammalia - ps26
Theria - ps27
Placentalia - ps28
Boreoeutheria - ps29
Euarchontoglires - ps30
Euarchonta - ps31
Primates - ps32
Haplorrhini - ps33
Simiiformes - ps34
Catarrhini - ps35
Hominoidea - ps36
Hominidae - ps37
Homininae - ps38
Hominini - ps39
Homo_sapiens - ps40
p-value
2e-02
3e-02
4e-02
5e-02
6e-02
Log-odds
0.5
1.0
1.5
2.0
2.5
3.0
3.5
4.0

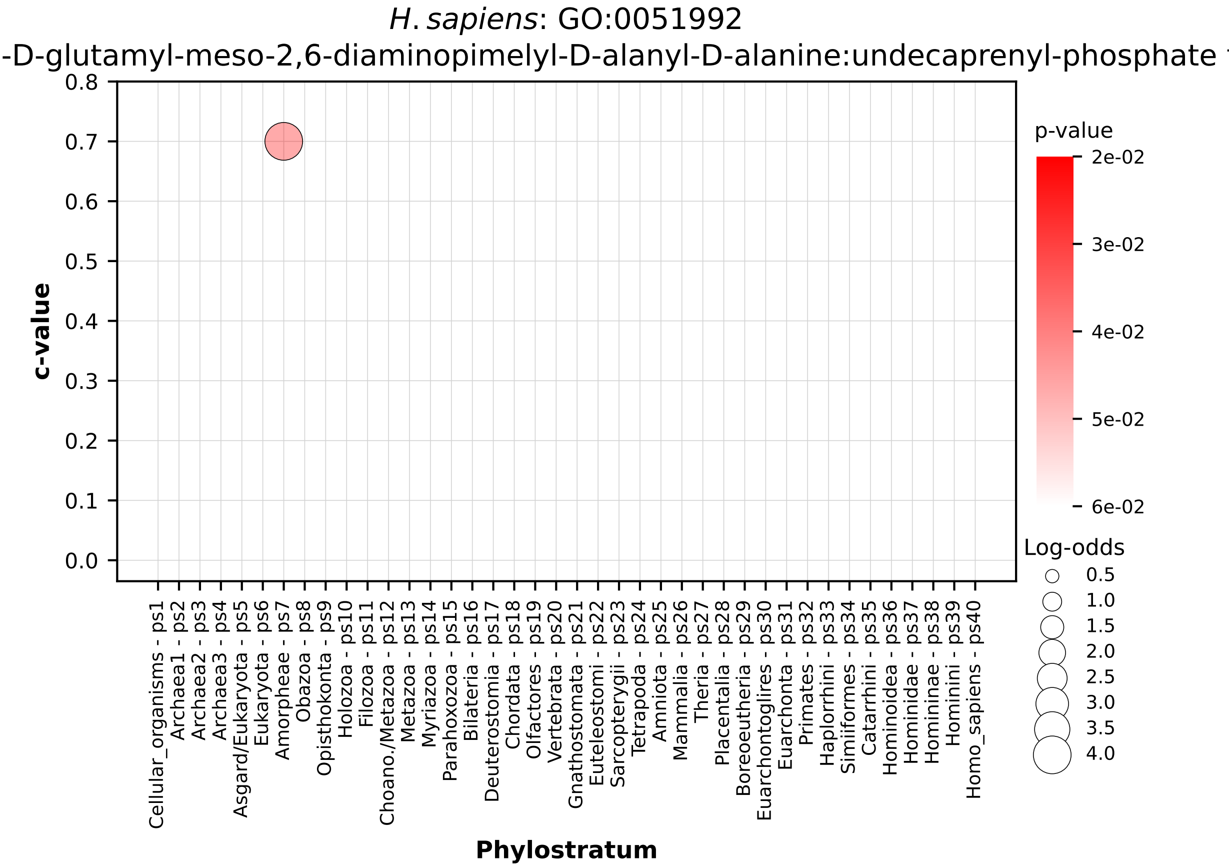

$H. sapiens$: GO:0051992
-D-glutamyl-meso-2,6-diaminopimelyl-D-alanyl-D-alanine:undecaprenyl-phosphate
c-value
0.8
0.7
0.6
0.5
0.4
0.3
0.2
0.1
0.0
Phylostratum
Cellular_organisms - ps1
Archaea1 - ps2
Archaea2 - ps3
Archaea3 - ps4
Asgard/Eukaryota - ps5
Eukaryota - ps6
Amorpheae - ps7
Obazoa - ps8
Opisthokonta - ps9
Holozoa - ps10
Filozoa - ps11
Choano./Metazoa - ps12
Metazoa - ps13
Myriazoa - ps14
Parahoxozoa - ps15
Bilateria - ps16
Deuterostomia - ps17
Chordata - ps18
Olfactores - ps19
Vertebrata - ps20
Gnathostomata - ps21
Euteleostomi - ps22
Sarcopterygii - ps23
Tetrapoda - ps24
Amniota - ps25
Mammalia - ps26
Theria - ps27
Placentalia - ps28
Boreoeutheria - ps29
Euarchontoglires - ps30
Euarchonta - ps31
Primates - ps32
Haplorrhini - ps33
Simiiformes - ps34
Catarrhini - ps35
Hominoidea - ps36
Hominidae - ps37
Homininae - ps38
Hominini - ps39
Homo_sapiens - ps40
p-value
2e-02
3e-02
4e-02
5e-02
6e-02
Log-odds
0.5
1.0
1.5
2.0
2.5
3.0
3.5
4.0

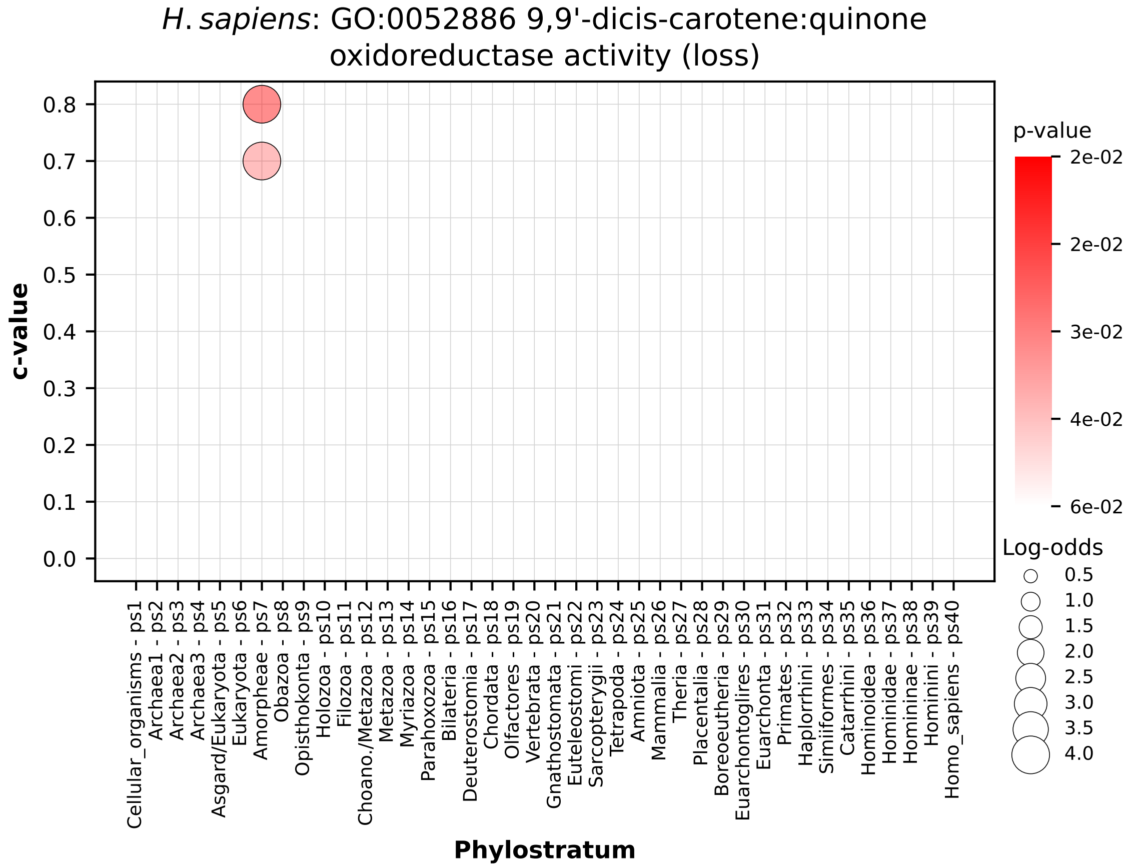

H. sapiens: GO:0052886 9,9'-dicis-carotene:quinone oxidoreductase activity (loss)
c-value
0.8
0.7
0.6
0.5
0.4
0.3
0.2
0.1
0.0
p-value
2e-02
2e-02
3e-02
4e-02
6e-02
Log-odds
0.5
1.0
1.5
2.0
2.5
3.0
3.5
4.0
Cellular_organisms - ps1
Archaea1 - ps2
Archaea2 - ps3
Archaea3 - ps4
Asgard/Eukaryota - ps5
Eukaryota - ps6
Amorpheae - ps7
Obazoa - ps8
Opisthokonta - ps9
Holozoa - ps10
Filozoa - ps11
Choano./Metazoa - ps12
Metazoa - ps13
Myriazoa - ps14
Parahoxozoa - ps15
Bilateria - ps16
Deuterostomia - ps17
Chordata - ps18
Olfactores - ps19
Vertebrata - ps20
Gnathostomata - ps21
Euteleostomi - ps22
Sarcopterygii - ps23
Tetrapoda - ps24
Amniota - ps25
Mammalia - ps26
Theria - ps27
Placentalia - ps28
Boreoeutheria - ps29
Euarchontoglires - ps30
Euarchonta - ps31
Primates - ps32
Haplorrhini - ps33
Simiiformes - ps34
Catarrhini - ps35
Hominoidea - ps36
Hominidae - ps37
Homininae - ps38
Hominini - ps39
Homo_sapiens - ps40
Phylostratum

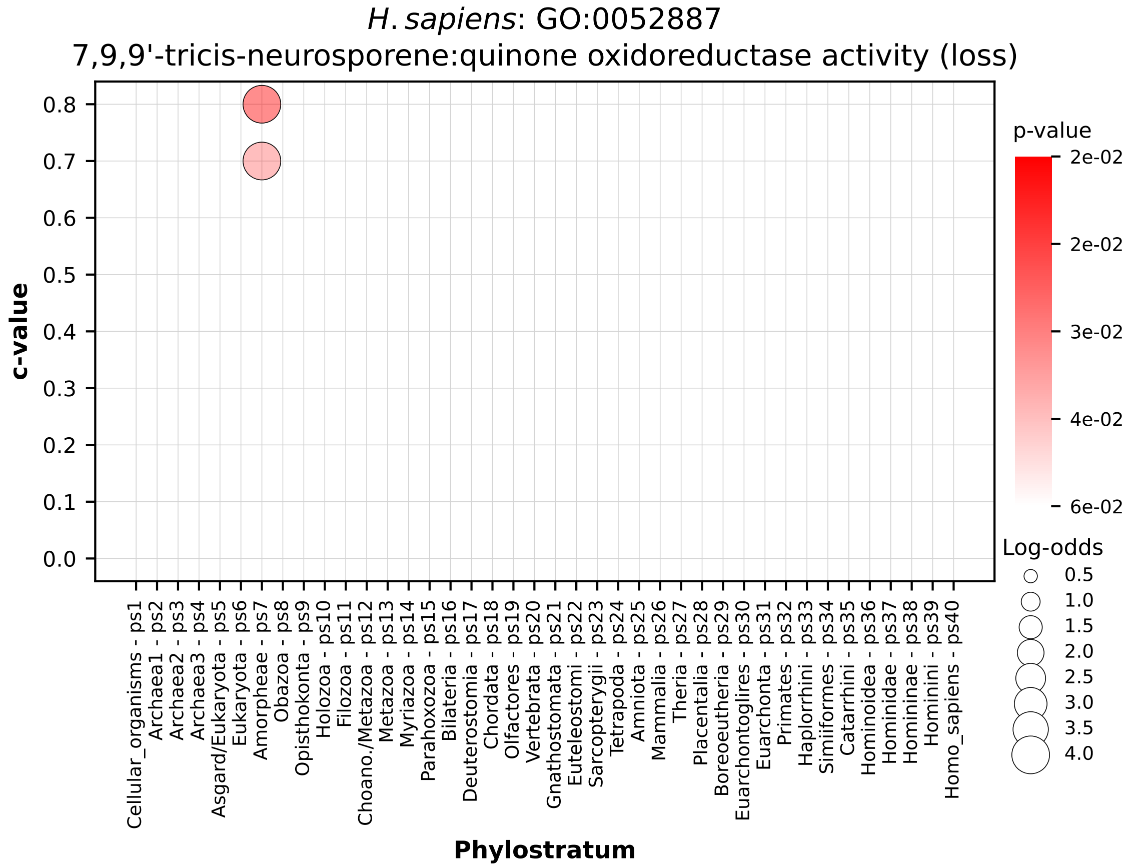

H. sapiens: GO:0052887
7,9,9'-tricis-neurosporene:quinone oxidoreductase activity (loss)
c-value
0.8
0.7
0.6
0.5
0.4
0.3
0.2
0.1
0.0
p-value
2e-02
2e-02
3e-02
4e-02
6e-02
Log-odds
0.5
1.0
1.5
2.0
2.5
3.0
3.5
4.0
Cellular_organisms - ps1
Archaea1 - ps2
Archaea2 - ps3
Archaea3 - ps4
Asgard/Eukaryota - ps5
Eukaryota - ps6
Amorpheae - ps7
Obazoa - ps8
Opisthokonta - ps9
Holozoa - ps10
Filozoa - ps11
Choano./Metazoa - ps12
Metazoa - ps13
Myriazoa - ps14
Parahoxozoa - ps15
Bilateria - ps16
Deuterostomia - ps17
Chordata - ps18
Olfactores - ps19
Vertebrata - ps20
Gnathostomata - ps21
Euteleostomi - ps22
Sarcopterygii - ps23
Tetrapoda - ps24
Amniota - ps25
Mammalia - ps26
Theria - ps27
Placentalia - ps28
Boreoeutheria - ps29
Euarchontoglires - ps30
Euarchonta - ps31
Primates - ps32
Haplorrhini - ps33
Simiiformes - ps34
Catarrhini - ps35
Hominoidea - ps36
Hominidae - ps37
Homininae - ps38
Hominini - ps39
Homo_sapiens - ps40
Phylostratum

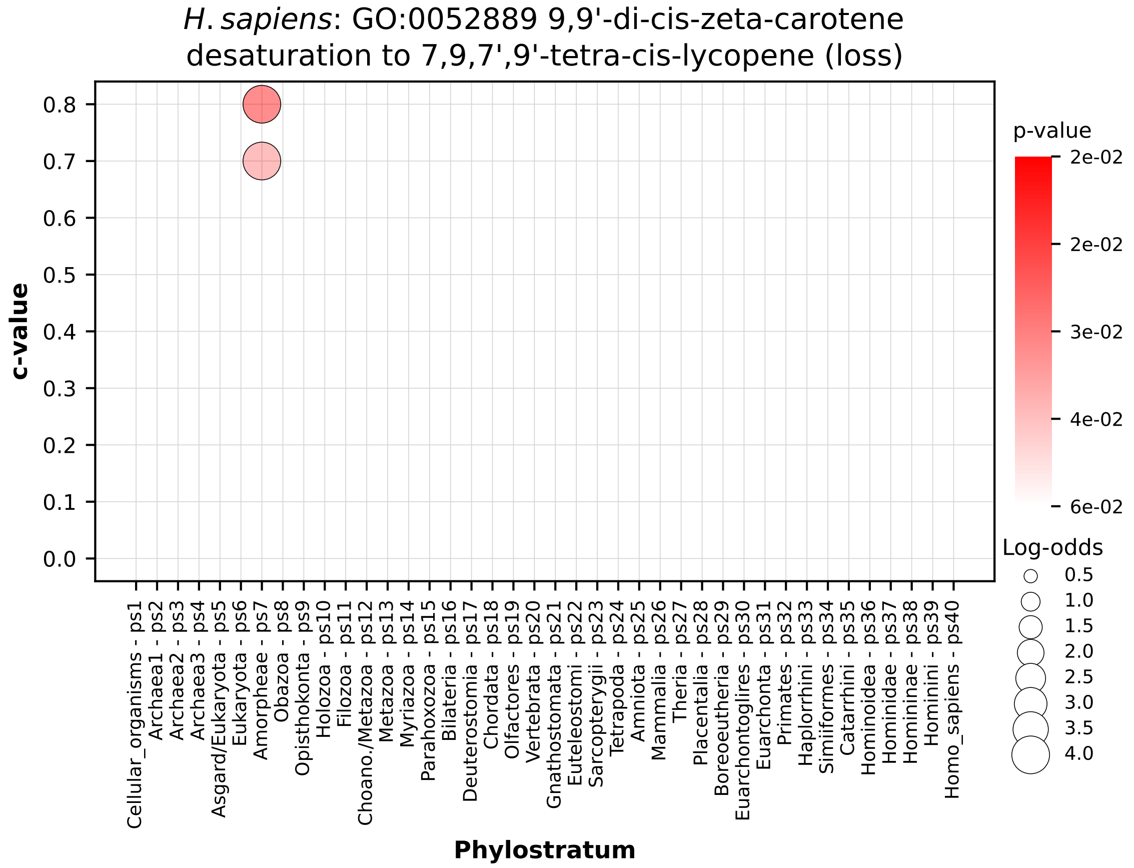

H. sapiens: GO:0052889 9,9'-di-cis-zeta-carotene desaturation to 7,9,7',9'-tetra-cis-lycopene (loss)
c-value
0.8
0.7
0.6
0.5
0.4
0.3
0.2
0.1
0.0
p-value
2e-02
2e-02
3e-02
4e-02
6e-02
Log-odds
0.5
1.0
1.5
2.0
2.5
3.0
3.5
4.0
Cellular_organisms - ps1
Archaea1 - ps2
Archaea2 - ps3
Archaea3 - ps4
Asgard/Eukaryota - ps5
Eukaryota - ps6
Amorpheae - ps7
Obazoa - ps8
Opisthokonta - ps9
Holozoa - ps10
Filozoa - ps11
Choano./Metazoa - ps12
Metazoa - ps13
Myriazoa - ps14
Parahoxozoa - ps15
Bilateria - ps16
Deuterostomia - ps17
Chordata - ps18
Olfactores - ps19
Vertebrata - ps20
Gnathostomata - ps21
Euteleostomi - ps22
Sarcopterygii - ps23
Tetrapoda - ps24
Amniota - ps25
Mammalia - ps26
Theria - ps27
Placentalia - ps28
Boreoeutheria - ps29
Euarchontoglires - ps30
Euarchonta - ps31
Primates - ps32
Haplorrhini - ps33
Simiiformes - ps34
Catarrhini - ps35
Hominoidea - ps36
Hominidae - ps37
Homininae - ps38
Hominini - ps39
Homo_sapiens - ps40
Phylostratum

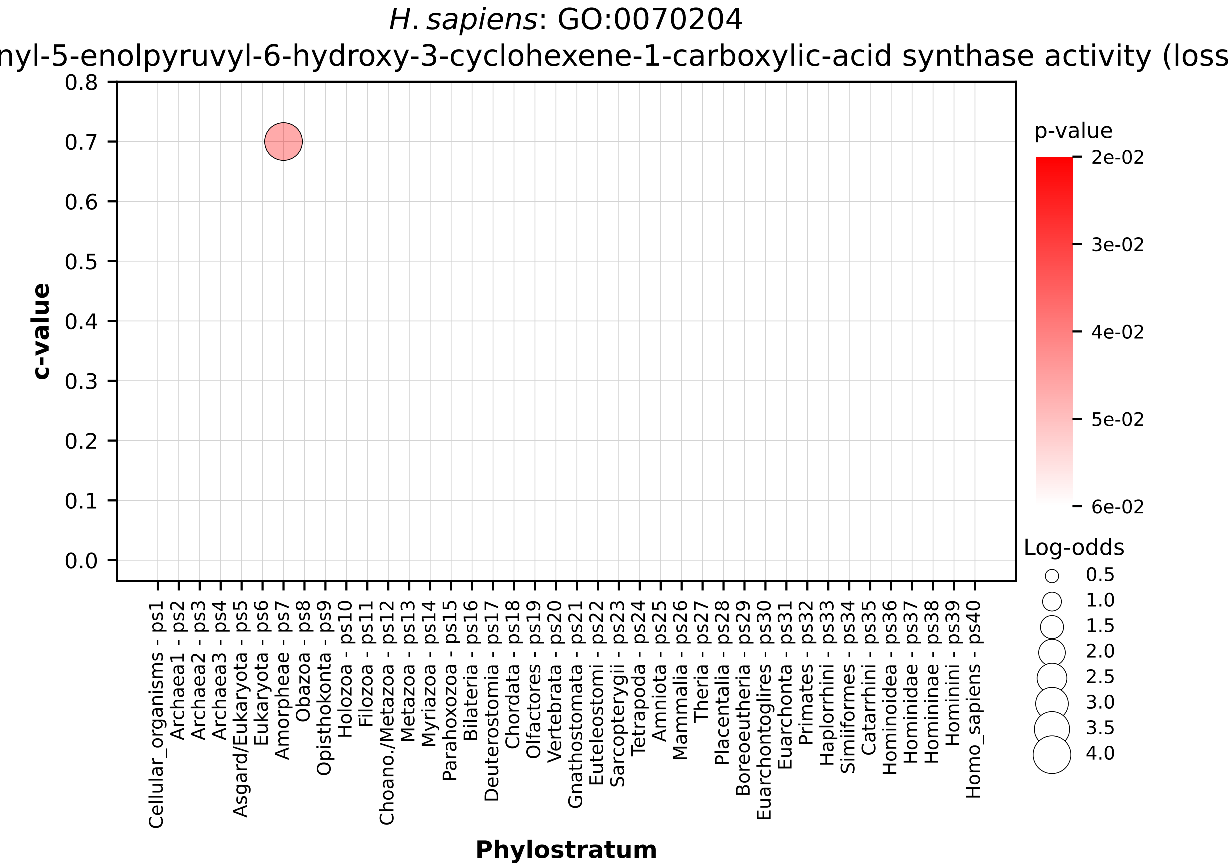

H. sapiens: GO:0070204
nyl-5-enolpyruvyl-6-hydroxy-3-cyclohexene-1-carboxylic-acid synthase activity (loss
c-value
0.8
0.7
0.6
0.5
0.4
0.3
0.2
0.1
0.0
p-value
2e-02
3e-02
4e-02
5e-02
6e-02
Log-odds
0.5
1.0
1.5
2.0
2.5
3.0
3.5
4.0
Cellular_organisms - ps1
Archaea1 - ps2
Archaea2 - ps3
Archaea3 - ps4
Asgard/Eukaryota - ps5
Eukaryota - ps6
Amorpheae - ps7
Obazoa - ps8
Opisthokonta - ps9
Holozoa - ps10
Filozoa - ps11
Choano./Metazoa - ps12
Metazoa - ps13
Myriazoa - ps14
Parahoxozoa - ps15
Bilateria - ps16
Deuterostomia - ps17
Chordata - ps18
Olfactores - ps19
Vertebrata - ps20
Gnathostomata - ps21
Euteleostomi - ps22
Sarcopterygii - ps23
Tetrapoda - ps24
Amniota - ps25
Mammalia - ps26
Theria - ps27
Placentalia - ps28
Boreoeutheria - ps29
Euarchontoglires - ps30
Euarchonta - ps31
Primates - ps32
Haplorrhini - ps33
Simiiformes - ps34
Catarrhini - ps35
Hominoidea - ps36
Hominidae - ps37
Homininae - ps38
Hominini - ps39
Homo_sapiens - ps40
Phylostratum

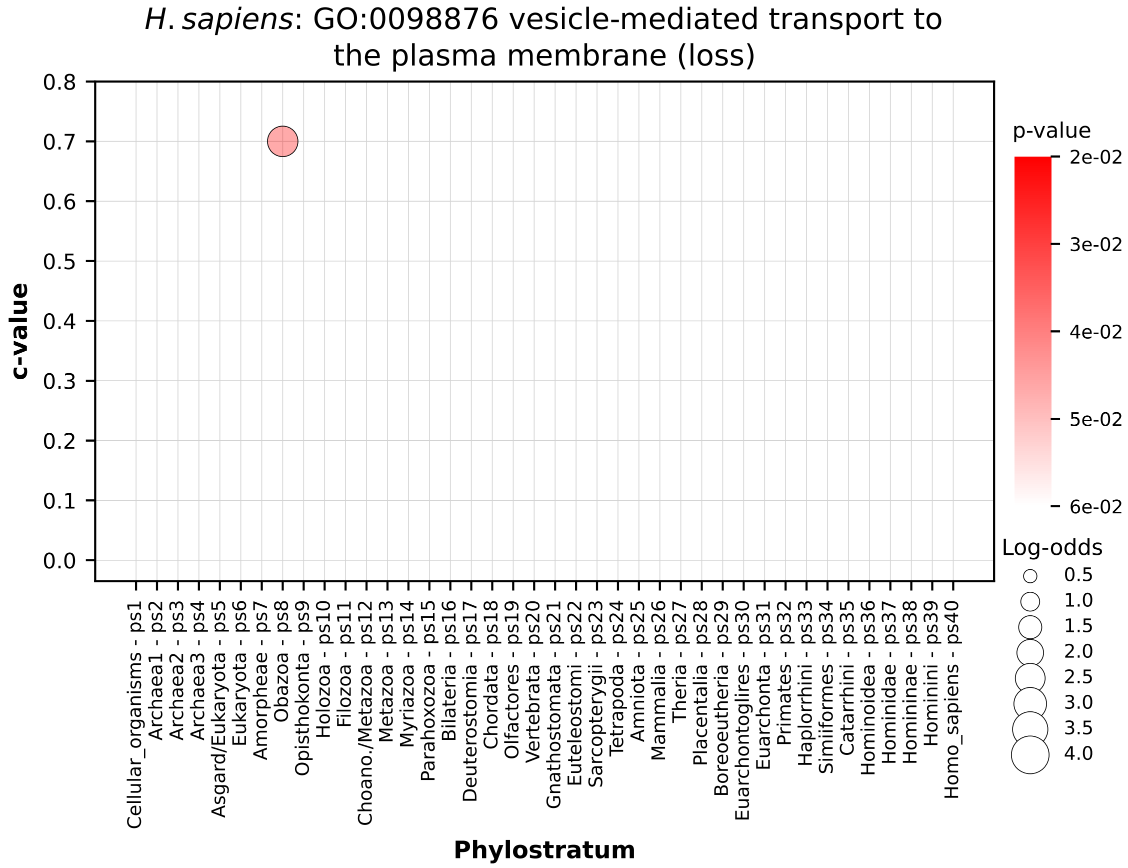

H. sapiens: GO:0098876 vesicle-mediated transport to the plasma membrane (loss)
c-value
0.8
0.7
0.6
0.5
0.4
0.3
0.2
0.1
0.0
p-value
2e-02
3e-02
4e-02
5e-02
6e-02
Log-odds
0.5
1.0
1.5
2.0
2.5
3.0
3.5
4.0
Cellular_organisms - ps1
Archaea1 - ps2
Archaea2 - ps3
Archaea3 - ps4
Asgard/Eukaryota - ps5
Eukaryota - ps6
Amorpheae - ps7
Obazoa - ps8
Opisthokonta - ps9
Holozoa - ps10
Filozoa - ps11
Choano./Metazoa - ps12
Metazoa - ps13
Myriazoa - ps14
Parahoxozoa - ps15
Bilateria - ps16
Deuterostomia - ps17
Chordata - ps18
Olfactores - ps19
Vertebrata - ps20
Gnathostomata - ps21
Euteleostomi - ps22
Sarcopterygii - ps23
Tetrapoda - ps24
Amniota - ps25
Mammalia - ps26
Theria - ps27
Placentalia - ps28
Boreoeutheria - ps29
Euarchontoglires - ps30
Euarchonta - ps31
Primates - ps32
Haplorrhini - ps33
Simiiformes - ps34
Catarrhini - ps35
Hominoidea - ps36
Hominidae - ps37
Homininae - ps38
Hominini - ps39
Homo_sapiens - ps40
Phylostratum

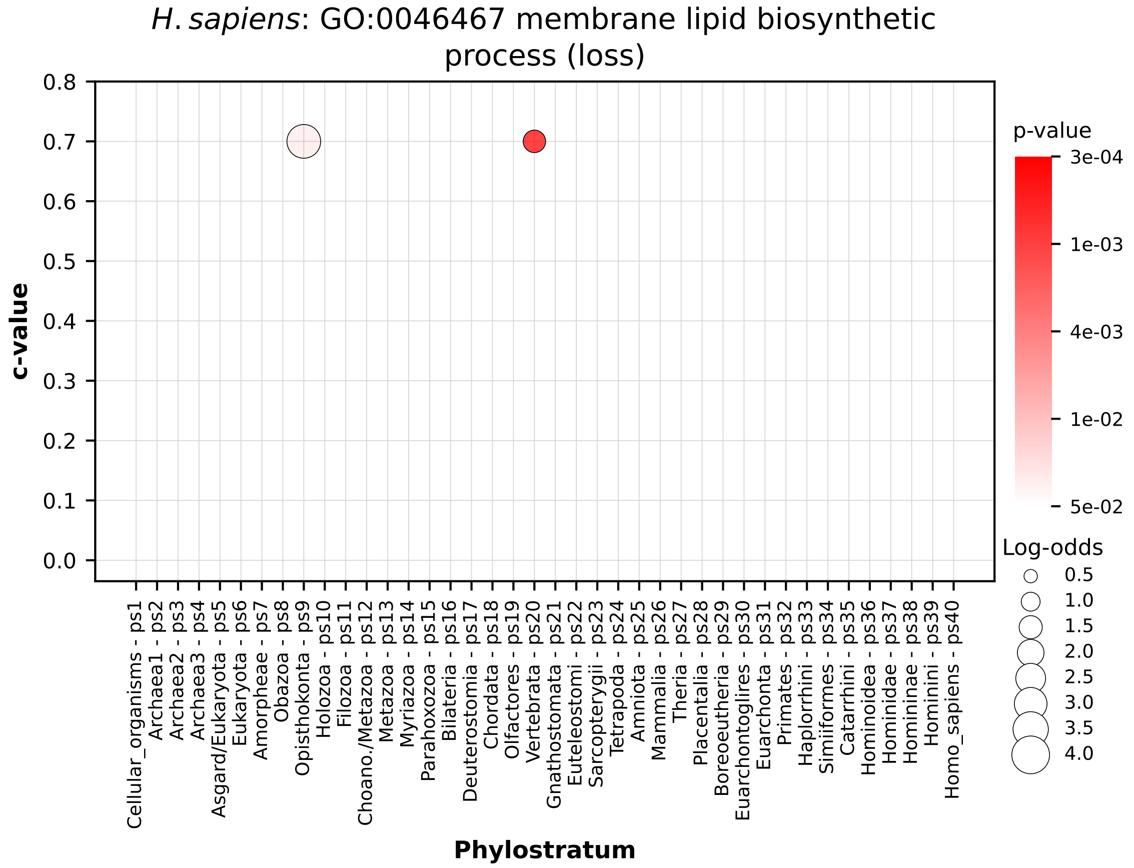

H. sapiens: GO:0046467 membrane lipid biosynthetic process (loss)
c-value
0.8
0.7
0.6
0.5
0.4
0.3
0.2
0.1
0.0
Phylostratum
Cellular_organisms - ps1
Archaea1 - ps2
Archaea2 - ps3
Archaea3 - ps4
Asgard/Eukaryota - ps5
Eukaryota - ps6
Amorpheae - ps7
Obazoa - ps8
Opisthokonta - ps9
Holozoa - ps10
Filozoa - ps11
Choano./Metazoa - ps12
Metazoa - ps13
Myriazoa - ps14
Parahoxozoa - ps15
Bilateria - ps16
Deuterostomia - ps17
Chordata - ps18
Olfactores - ps19
Vertebrata - ps20
Gnathostomata - ps21
Euteleostomi - ps22
Sarcopterygii - ps23
Tetrapoda - ps24
Amniota - ps25
Mammalia - ps26
Theria - ps27
Placentalia - ps28
Boreoeutheria - ps29
Euarchontoglires - ps30
Euarchonta - ps31
Primates - ps32
Haplorrhini - ps33
Simiiformes - ps34
Catarrhini - ps35
Hominoidea - ps36
Hominidae - ps37
Homininae - ps38
Hominini - ps39
Homo_sapiens - ps40
p-value
3e-04
1e-03
4e-03
1e-02
5e-02
Log-odds
0.5
1.0
1.5
2.0
2.5
3.0
3.5
4.0

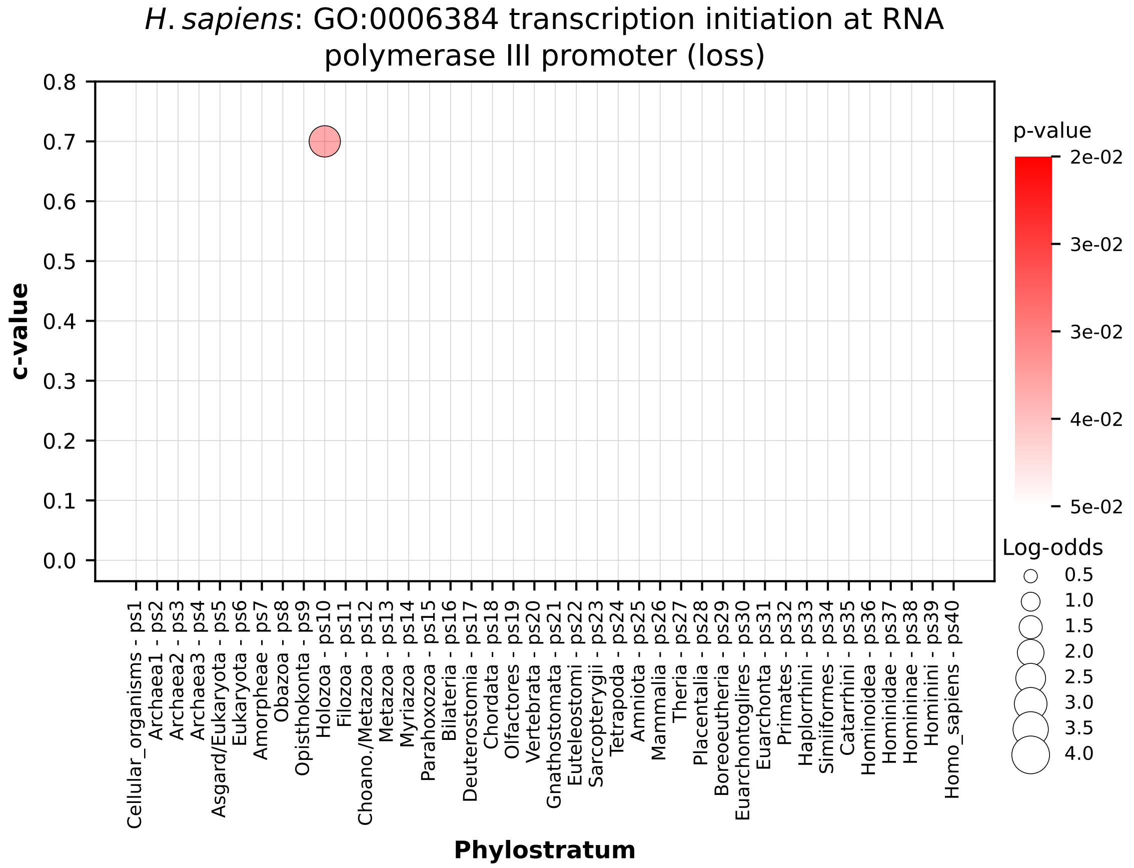

H. sapiens: GO:0006384 transcription initiation at RNA polymerase III promoter (loss)
c-value
0.8
0.7
0.6
0.5
0.4
0.3
0.2
0.1
0.0
Phylostratum
Cellular_organisms - ps1
Archaea1 - ps2
Archaea2 - ps3
Archaea3 - ps4
Asgard/Eukaryota - ps5
Eukaryota - ps6
Amorpheae - ps7
Obazoa - ps8
Opisthokonta - ps9
Holozoa - ps10
Filozoa - ps11
Choano./Metazoa - ps12
Metazoa - ps13
Myriazoa - ps14
Parahoxozoa - ps15
Bilateria - ps16
Deuterostomia - ps17
Chordata - ps18
Olfactores - ps19
Vertebrata - ps20
Gnathostomata - ps21
Euteleostomi - ps22
Sarcopterygii - ps23
Tetrapoda - ps24
Amniota - ps25
Mammalia - ps26
Theria - ps27
Placentalia - ps28
Boreoeutheria - ps29
Euarchontoglires - ps30
Euarchonta - ps31
Primates - ps32
Haplorrhini - ps33
Simiiformes - ps34
Catarrhini - ps35
Hominoidea - ps36
Hominidae - ps37
Homininae - ps38
Hominini - ps39
Homo_sapiens - ps40
p-value
2e-02
3e-02
3e-02
4e-02
5e-02
Log-odds
0.5
1.0
1.5
2.0
2.5
3.0
3.5
4.0

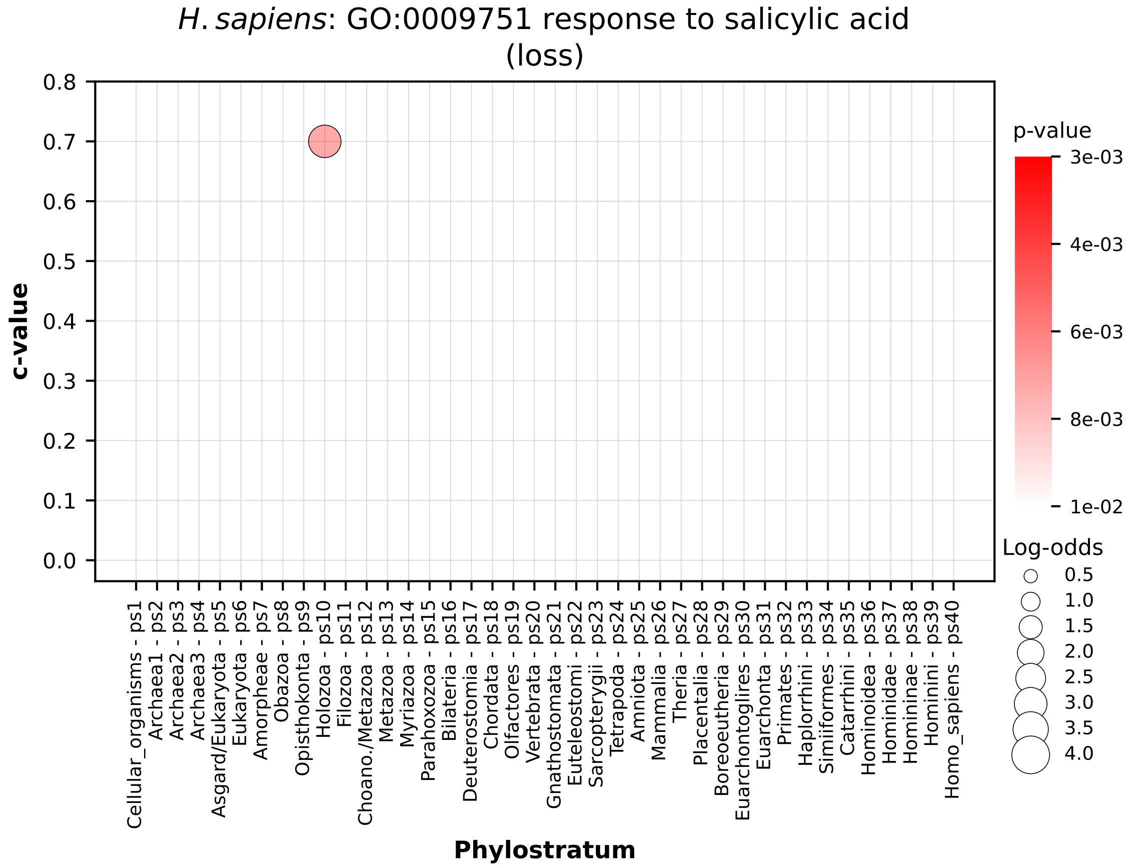

H. sapiens: GO:0009751 response to salicylic acid
(loss)
c-value
0.8
0.7
0.6
0.5
0.4
0.3
0.2
0.1
0.0
p-value
3e-03
4e-03
6e-03
8e-03
1e-02
Log-odds
0.5
1.0
1.5
2.0
2.5
3.0
3.5
4.0
Cellular_organisms - ps1
Archaea1 - ps2
Archaea2 - ps3
Archaea3 - ps4
Asgard/Eukaryota - ps5
Eukaryota - ps6
Amorpheae - ps7
Obazoa - ps8
Opisthokonta - ps9
Holozoa - ps10
Filozoa - ps11
Choano./Metazoa - ps12
Metazoa - ps13
Myriazoa - ps14
Parahoxozoa - ps15
Bilateria - ps16
Deuterostomia - ps17
Chordata - ps18
Olfactores - ps19
Vertebrata - ps20
Gnathostomata - ps21
Euteleostomi - ps22
Sarcopterygii - ps23
Tetrapoda - ps24
Amniota - ps25
Mammalia - ps26
Theria - ps27
Placentalia - ps28
Boreoeutheria - ps29
Euarchontoglires - ps30
Euarchonta - ps31
Primates - ps32
Haplorrhini - ps33
Simiiformes - ps34
Catarrhini - ps35
Hominoidea - ps36
Hominidae - ps37
Homininae - ps38
Hominini - ps39
Homo_sapiens - ps40
Phylostratum

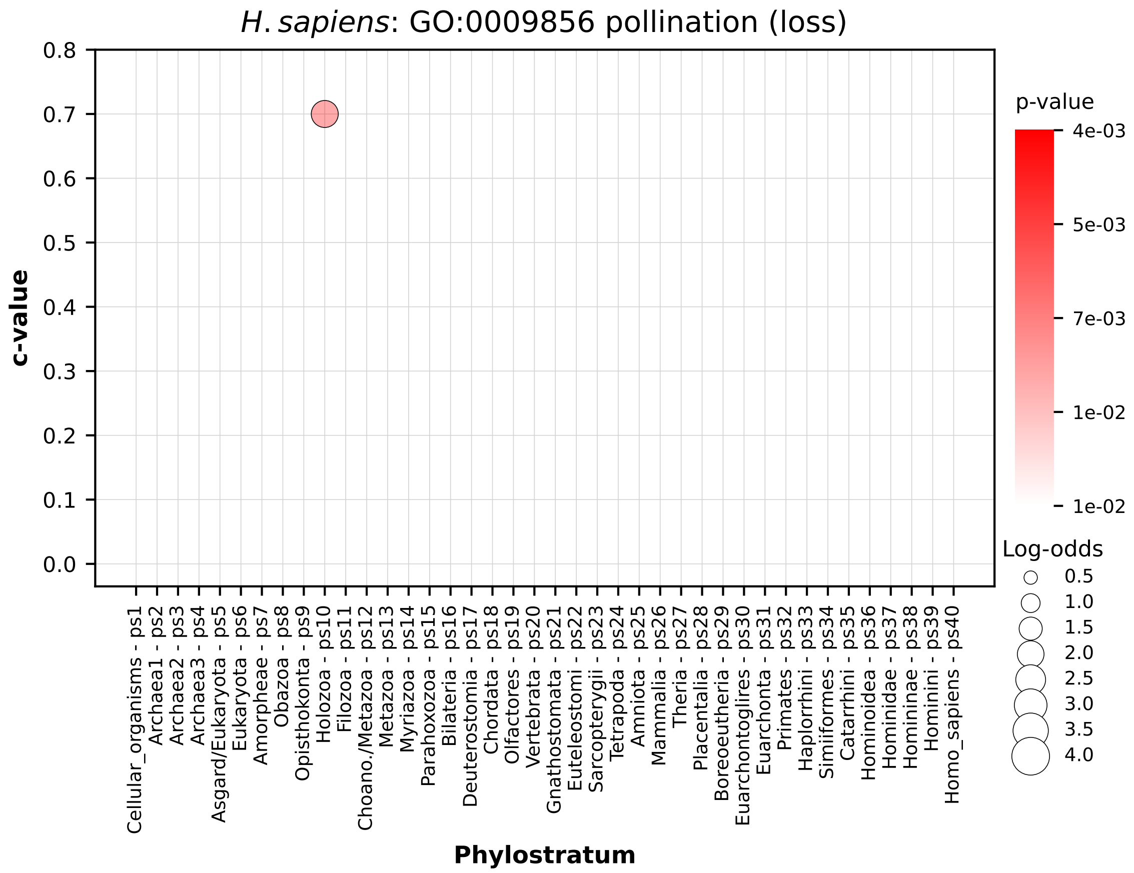
H. sapiens: GO:0009856 pollination (loss)
c-value
0.8
0.7
0.6
0.5
0.4
0.3
0.2
0.1
0.0
Phylostratum
Cellular_organisms - ps1
Archaea1 - ps2
Archaea2 - ps3
Archaea3 - ps4
Asgard/Eukaryota - ps5
Eukaryota - ps6
Amorpheae - ps7
Obazoa - ps8
Opisthokonta - ps9
Holozoa - ps10
Filozoa - ps11
Choano./Metazoa - ps12
Metazoa - ps13
Myriazoa - ps14
Parahoxozoa - ps15
Bilateria - ps16
Deuterostomia - ps17
Chordata - ps18
Olfactores - ps19
Vertebrata - ps20
Gnathostomata - ps21
Euteleostomi - ps22
Sarcopterygii - ps23
Tetrapoda - ps24
Amniota - ps25
Mammalia - ps26
Theria - ps27
Placentalia - ps28
Boreoeutheria - ps29
Euarchontoglires - ps30
Euarchonta - ps31
Primates - ps32
Haplorrhini - ps33
Simiiformes - ps34
Catarrhini - ps35
Hominoidea - ps36
Hominidae - ps37
Homininae - ps38
Hominini - ps39
Homo_sapiens - ps40
p-value
4e-03
5e-03
7e-03
1e-02
1e-02
Log-odds
0.5
1.0
1.5
2.0
2.5
3.0
3.5
4.0

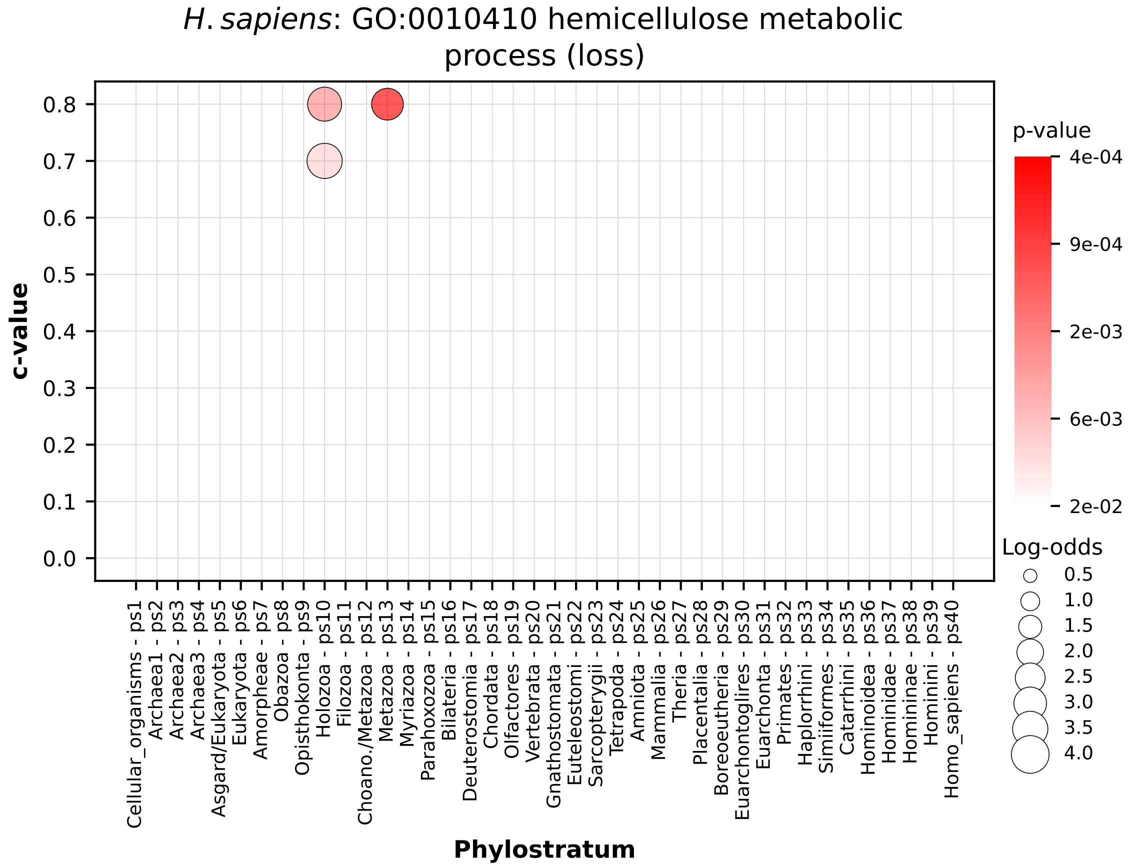

H. sapiens: GO:0010410 hemicellulose metabolic process (loss)
c-value
0.0
0.1
0.2
0.3
0.4
0.5
0.6
0.7
0.8
Phylostratum
Cellular_organisms - ps1
Archaea1 - ps2
Archaea2 - ps3
Archaea3 - ps4
Asgard/Eukaryota - ps5
Eukaryota - ps6
Amorpheae - ps7
Obazoa - ps8
Opisthokonta - ps9
Holozoa - ps10
Filozoa - ps11
Choano./Metazoa - ps12
Metazoa - ps13
Myriazoa - ps14
Parahoxozoa - ps15
Bilateria - ps16
Deuterostomia - ps17
Chordata - ps18
Olfactores - ps19
Vertebrata - ps20
Gnathostomata - ps21
Euteleostomi - ps22
Sarcopterygii - ps23
Tetrapoda - ps24
Amniota - ps25
Mammalia - ps26
Theria - ps27
Placentalia - ps28
Boreoeutheria - ps29
Euarchontoglires - ps30
Euarchonta - ps31
Primates - ps32
Haplorrhini - ps33
Simiiformes - ps34
Catarrhini - ps35
Hominoidea - ps36
Hominidae - ps37
Homininae - ps38
Hominini - ps39
Homo_sapiens - ps40
p-value
4e-04
9e-04
2e-03
6e-03
2e-02
Log-odds
0.5
1.0
1.5
2.0
2.5
3.0
3.5
4.0

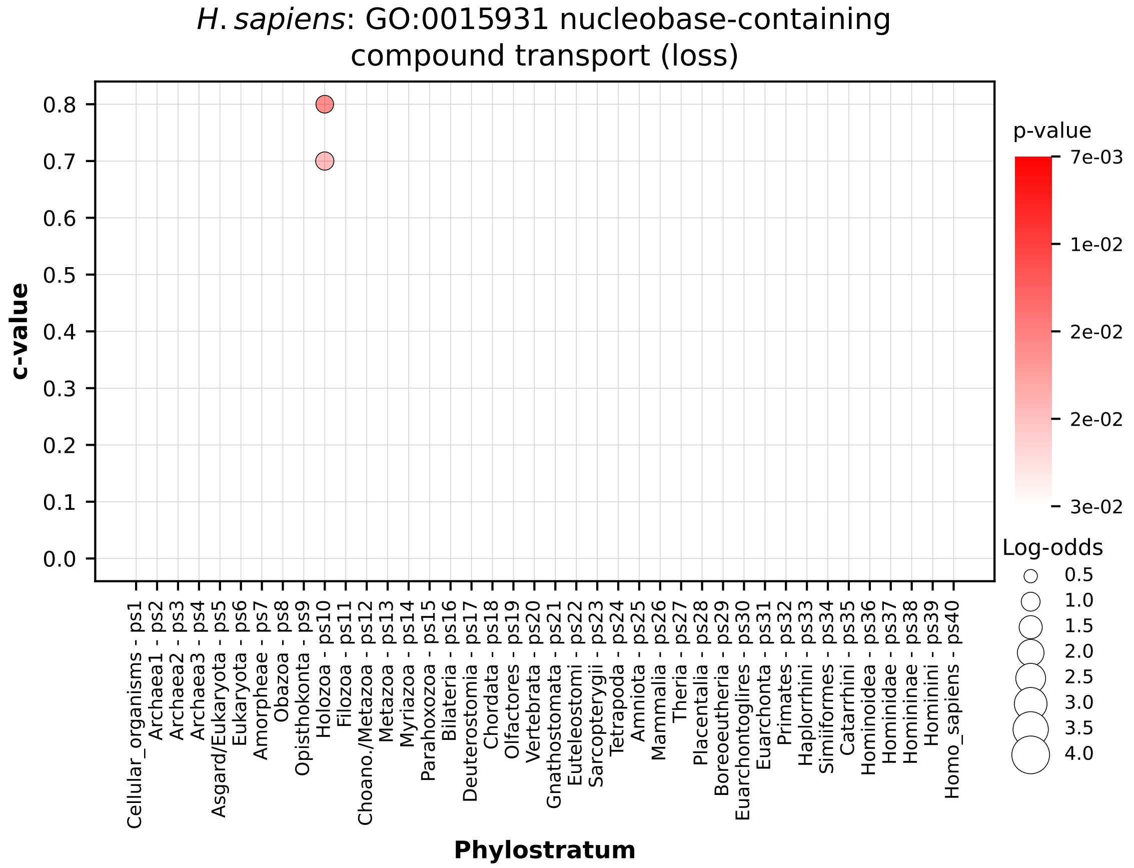
H. sapiens: GO:0015931 nucleobase-containing compound transport (loss)
c-value
0.8
0.7
0.6
0.5
0.4
0.3
0.2
0.1
0.0
Phylostratum
Cellular_organisms - ps1
Archaea1 - ps2
Archaea2 - ps3
Archaea3 - ps4
Asgard/Eukaryota - ps5
Eukaryota - ps6
Amorpheae - ps7
Obazoa - ps8
Opisthokonta - ps9
Holozoa - ps10
Filozoa - ps11
Choano./Metazoa - ps12
Metazoa - ps13
Myriazoa - ps14
Parahoxozoa - ps15
Bilateria - ps16
Deuterostomia - ps17
Chordata - ps18
Olfactores - ps19
Vertebrata - ps20
Gnathostomata - ps21
Euteleostomi - ps22
Sarcopterygii - ps23
Tetrapoda - ps24
Amniota - ps25
Mammalia - ps26
Theria - ps27
Placentalia - ps28
Boreoeutheria - ps29
Euarchontoglires - ps30
Euarchonta - ps31
Primates - ps32
Haplorrhini - ps33
Simiiformes - ps34
Catarrhini - ps35
Hominoidea - ps36
Hominidae - ps37
Homininae - ps38
Hominini - ps39
Homo_sapiens - ps40
p-value
7e-03
1e-02
2e-02
2e-02
3e-02
Log-odds
0.5
1.0
1.5
2.0
2.5
3.0
3.5
4.0

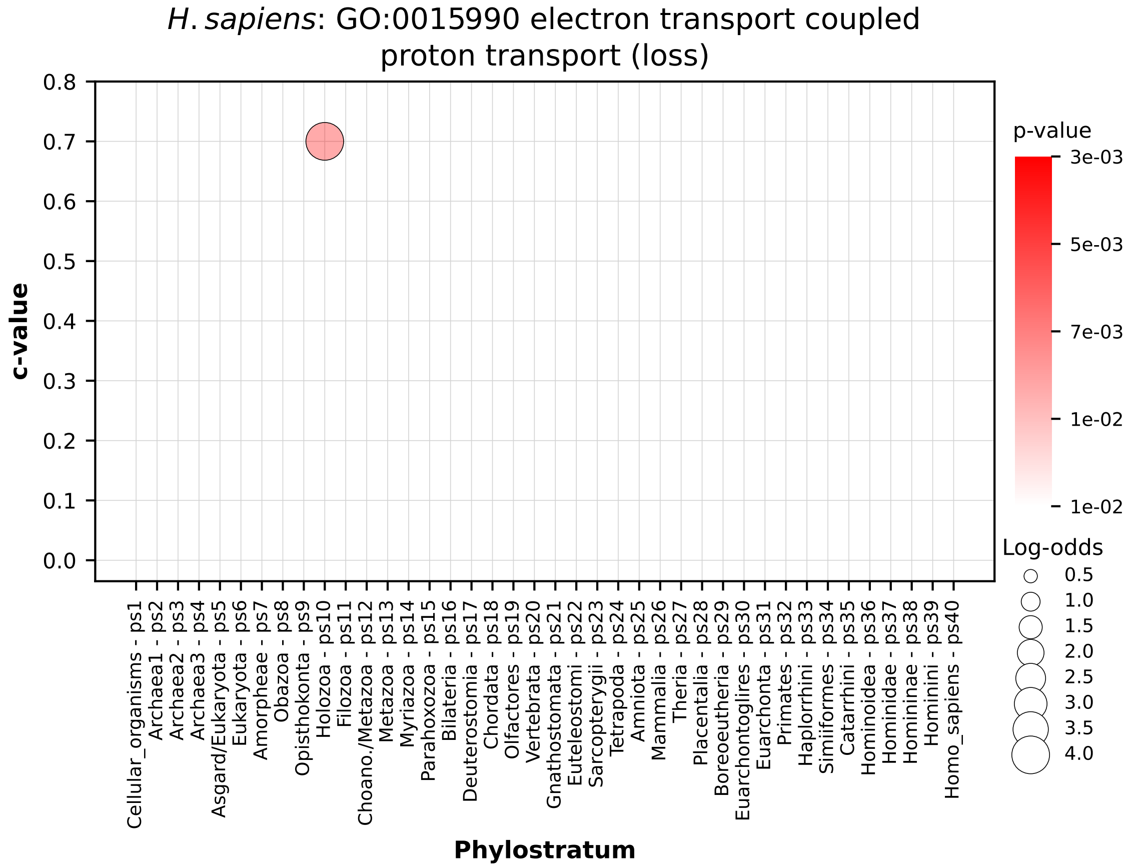

H. sapiens: GO:0015990 electron transport coupled proton transport (loss)
c-value
0.8
0.7
0.6
0.5
0.4
0.3
0.2
0.1
0.0
p-value
3e-03
5e-03
7e-03
1e-02
1e-02
Log-odds
0.5
1.0
1.5
2.0
2.5
3.0
3.5
4.0
Cellular_organisms - ps1
Archaea1 - ps2
Archaea2 - ps3
Archaea3 - ps4
Asgard/Eukaryota - ps5
Eukaryota - ps6
Amorpheae - ps7
Obazoa - ps8
Opisthokonta - ps9
Holozoa - ps10
Filozoa - ps11
Choano./Metazoa - ps12
Metazoa - ps13
Myriazoa - ps14
Parahoxozoa - ps15
Bilateria - ps16
Deuterostomia - ps17
Chordata - ps18
Olfactores - ps19
Vertebrata - ps20
Gnathostomata - ps21
Euteleostomi - ps22
Sarcopterygii - ps23
Tetrapoda - ps24
Amniota - ps25
Mammalia - ps26
Theria - ps27
Placentalia - ps28
Boreoeutheria - ps29
Euarchontoglires - ps30
Euarchonta - ps31
Primates - ps32
Haplorrhini - ps33
Simiiformes - ps34
Catarrhini - ps35
Hominoidea - ps36
Hominidae - ps37
Homininae - ps38
Hominini - ps39
Homo_sapiens - ps40
Phylostratum

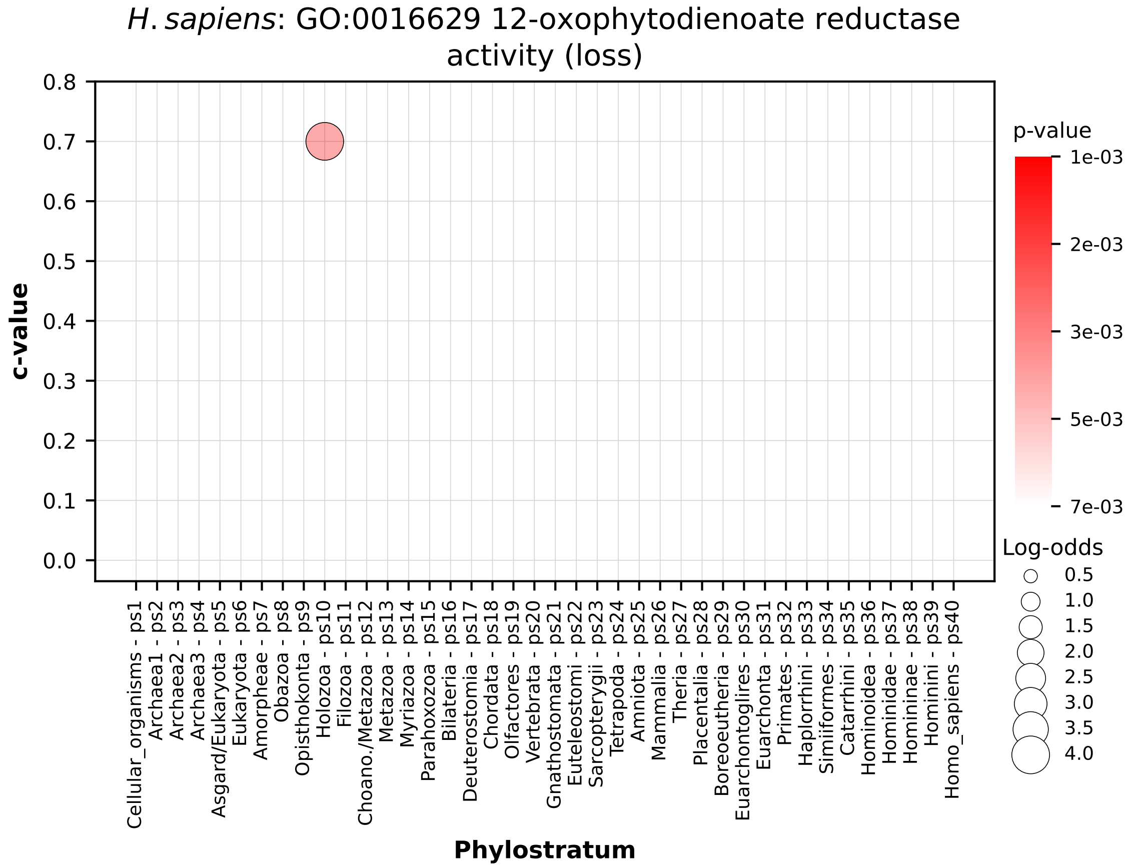

H. sapiens: GO:0016629 12-oxophytodienoate reductase activity (loss)
c-value
0.8
0.7
0.6
0.5
0.4
0.3
0.2
0.1
0.0
Phylostratum
Cellular_organisms - ps1
Archaea1 - ps2
Archaea2 - ps3
Archaea3 - ps4
Asgard/Eukaryota - ps5
Eukaryota - ps6
Amorpheae - ps7
Obazoa - ps8
Opisthokonta - ps9
Holozoa - ps10
Filozoa - ps11
Choano./Metazoa - ps12
Metazoa - ps13
Myriazoa - ps14
Parahoxozoa - ps15
Bilateria - ps16
Deuterostomia - ps17
Chordata - ps18
Olfactores - ps19
Vertebrata - ps20
Gnathostomata - ps21
Euteleostomi - ps22
Sarcopterygii - ps23
Tetrapoda - ps24
Amniota - ps25
Mammalia - ps26
Theria - ps27
Placentalia - ps28
Boreoeutheria - ps29
Euarchontoglires - ps30
Euarchonta - ps31
Primates - ps32
Haplorrhini - ps33
Simiiformes - ps34
Catarrhini - ps35
Hominoidea - ps36
Hominidae - ps37
Homininae - ps38
Hominini - ps39
Homo_sapiens - ps40
p-value
1e-03
2e-03
3e-03
5e-03
7e-03
Log-odds
0.5
1.0
1.5
2.0
2.5
3.0
3.5
4.0

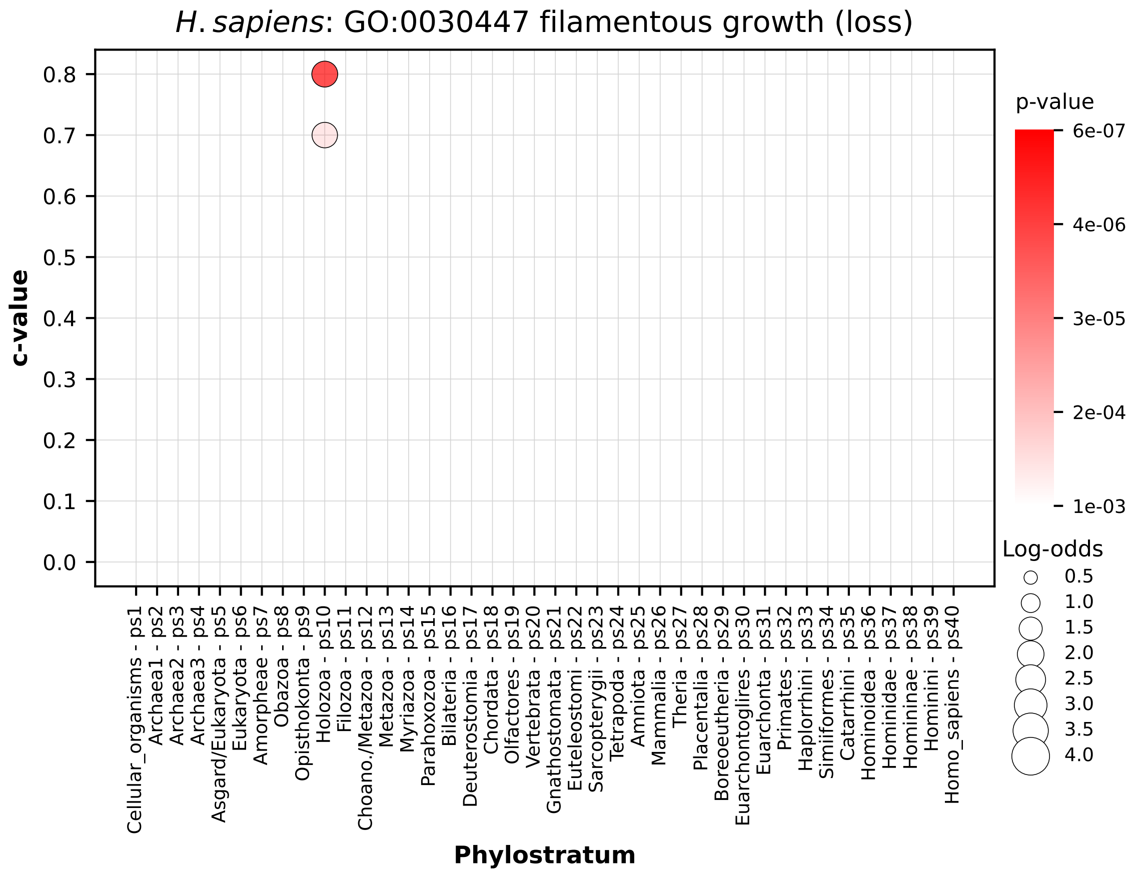

H. sapiens: GO:0030447 filamentous growth (loss)
c-value
0.0
0.1
0.2
0.3
0.4
0.5
0.6
0.7
0.8
Phylostratum
Cellular_organisms - ps1
Archaea1 - ps2
Archaea2 - ps3
Archaea3 - ps4
Asgard/Eukaryota - ps5
Eukaryota - ps6
Amorpheae - ps7
Obazoa - ps8
Opisthokonta - ps9
Holozoa - ps10
Filozoa - ps11
Choano./Metazoa - ps12
Metazoa - ps13
Myriazoa - ps14
Parahoxozoa - ps15
Bilateria - ps16
Deuterostomia - ps17
Chordata - ps18
Olfactores - ps19
Vertebrata - ps20
Gnathostomata - ps21
Euteleostomi - ps22
Sarcopterygii - ps23
Tetrapoda - ps24
Amniota - ps25
Mammalia - ps26
Theria - ps27
Placentalia - ps28
Boreoeutheria - ps29
Euarchontoglires - ps30
Euarchonta - ps31
Primates - ps32
Haplorrhini - ps33
Simiiformes - ps34
Catarrhini - ps35
Hominoidea - ps36
Hominidae - ps37
Homininae - ps38
Hominini - ps39
Homo_sapiens - ps40
p-value
6e-07
4e-06
3e-05
2e-04
1e-03
Log-odds
0.5
1.0
1.5
2.0
2.5
3.0
3.5
4.0

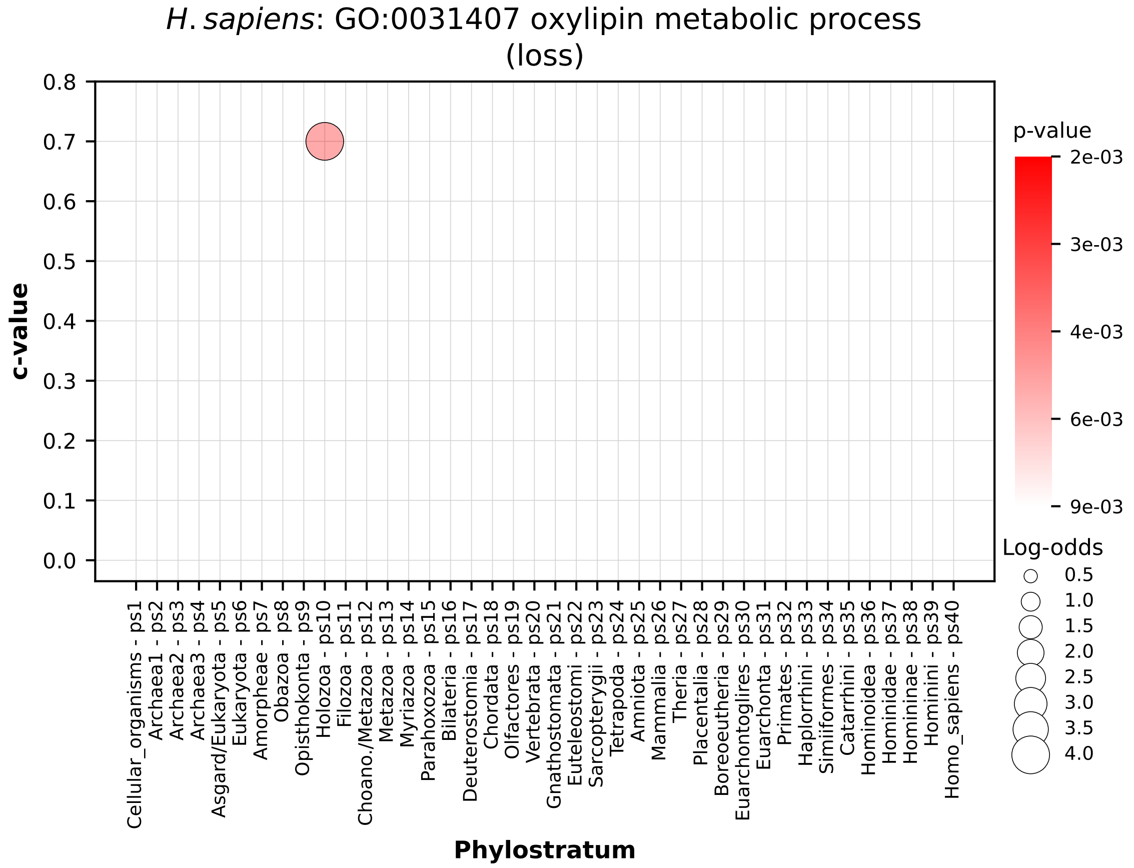

H. sapiens: GO:0031407 oxylipin metabolic process
(loss)
c-value
0.8
0.7
0.6
0.5
0.4
0.3
0.2
0.1
0.0
p-value
2e-03
3e-03
4e-03
6e-03
9e-03
Log-odds
0.5
1.0
1.5
2.0
2.5
3.0
3.5
4.0
Cellular_organisms - ps1
Archaea1 - ps2
Archaea2 - ps3
Archaea3 - ps4
Asgard/Eukaryota - ps5
Eukaryota - ps6
Amorpheae - ps7
Obazoa - ps8
Opisthokonta - ps9
Holozoa - ps10
Filozoa - ps11
Choano./Metazoa - ps12
Metazoa - ps13
Myriazoa - ps14
Parahoxozoa - ps15
Bilateria - ps16
Deuterostomia - ps17
Chordata - ps18
Olfactores - ps19
Vertebrata - ps20
Gnathostomata - ps21
Euteleostomi - ps22
Sarcopterygii - ps23
Tetrapoda - ps24
Amniota - ps25
Mammalia - ps26
Theria - ps27
Placentalia - ps28
Boreoeutheria - ps29
Euarchontoglires - ps30
Euarchonta - ps31
Primates - ps32
Haplorrhini - ps33
Simiiformes - ps34
Catarrhini - ps35
Hominoidea - ps36
Hominidae - ps37
Homininae - ps38
Hominini - ps39
Homo_sapiens - ps40
Phylostratum

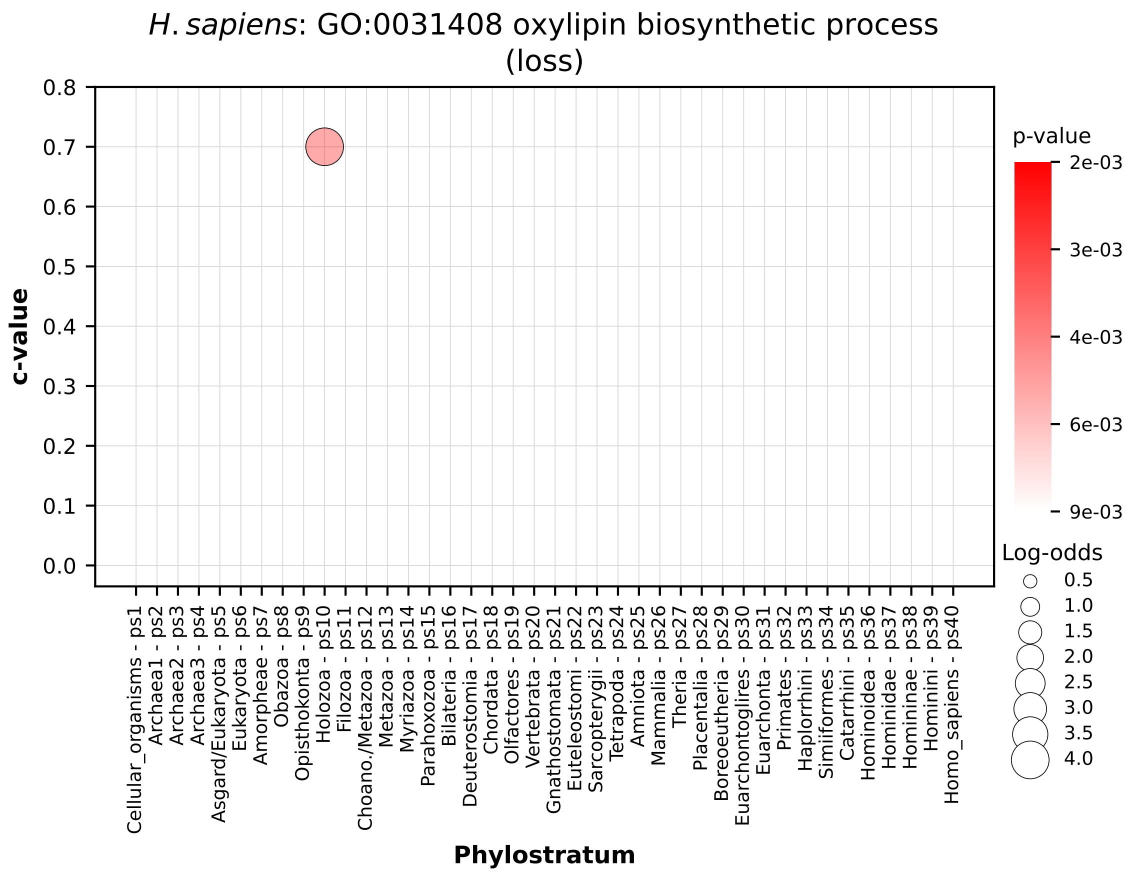

H. sapiens: GO:0031408 oxylipin biosynthetic process (loss)
c-value
0.0
0.1
0.2
0.3
0.4
0.5
0.6
0.7
0.8
Phylostratum
Cellular_organisms - ps1
Archaea1 - ps2
Archaea2 - ps3
Archaea3 - ps4
Asgard/Eukaryota - ps5
Eukaryota - ps6
Amorpheae - ps7
Obazoa - ps8
Opisthokonta - ps9
Holozoa - ps10
Filozoa - ps11
Choano./Metazoa - ps12
Metazoa - ps13
Myriazoa - ps14
Parahoxozoa - ps15
Bilateria - ps16
Deuterostomia - ps17
Chordata - ps18
Olfactores - ps19
Vertebrata - ps20
Gnathostomata - ps21
Euteleostomi - ps22
Sarcopterygii - ps23
Tetrapoda - ps24
Amniota - ps25
Mammalia - ps26
Theria - ps27
Placentalia - ps28
Boreoeutheria - ps29
Euarchontoglires - ps30
Euarchonta - ps31
Primates - ps32
Haplorrhini - ps33
Simiiformes - ps34
Catarrhini - ps35
Hominoidea - ps36
Hominidae - ps37
Homininae - ps38
Hominini - ps39
Homo_sapiens - ps40
p-value
2e-03
3e-03
4e-03
6e-03
9e-03
Log-odds
0.5
1.0
1.5
2.0
2.5
3.0
3.5
4.0

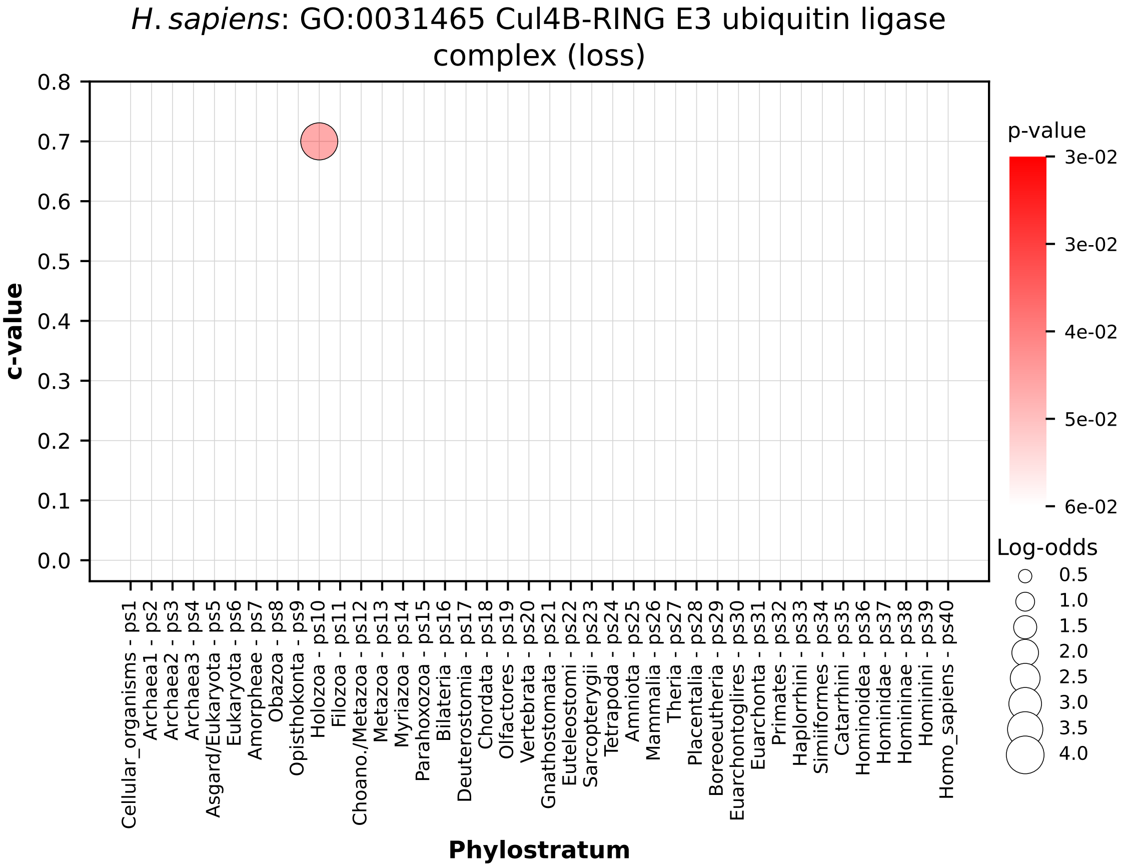

H. sapiens: GO:0031465 Cul4B-RING E3 ubiquitin ligase complex (loss)
c-value
0.8
0.7
0.6
0.5
0.4
0.3
0.2
0.1
0.0
Phylostratum
Cellular_organisms - ps1
Archaea1 - ps2
Archaea2 - ps3
Archaea3 - ps4
Asgard/Eukaryota - ps5
Eukaryota - ps6
Amorpheae - ps7
Obazoa - ps8
Opisthokonta - ps9
Holozoa - ps10
Filozoa - ps11
Choano./Metazoa - ps12
Metazoa - ps13
Myriazoa - ps14
Parahoxozoa - ps15
Bilateria - ps16
Deuterostomia - ps17
Chordata - ps18
Olfactores - ps19
Vertebrata - ps20
Gnathostomata - ps21
Euteleostomi - ps22
Sarcopterygii - ps23
Tetrapoda - ps24
Amniota - ps25
Mammalia - ps26
Theria - ps27
Placentalia - ps28
Boreoeutheria - ps29
Euarchontoglires - ps30
Euarchonta - ps31
Primates - ps32
Haplorrhini - ps33
Simiiformes - ps34
Catarrhini - ps35
Hominoidea - ps36
Hominidae - ps37
Homininae - ps38
Hominini - ps39
Homo_sapiens - ps40
p-value
3e-02
3e-02
4e-02
5e-02
6e-02
Log-odds
0.5
1.0
1.5
2.0
2.5
3.0
3.5
4.0

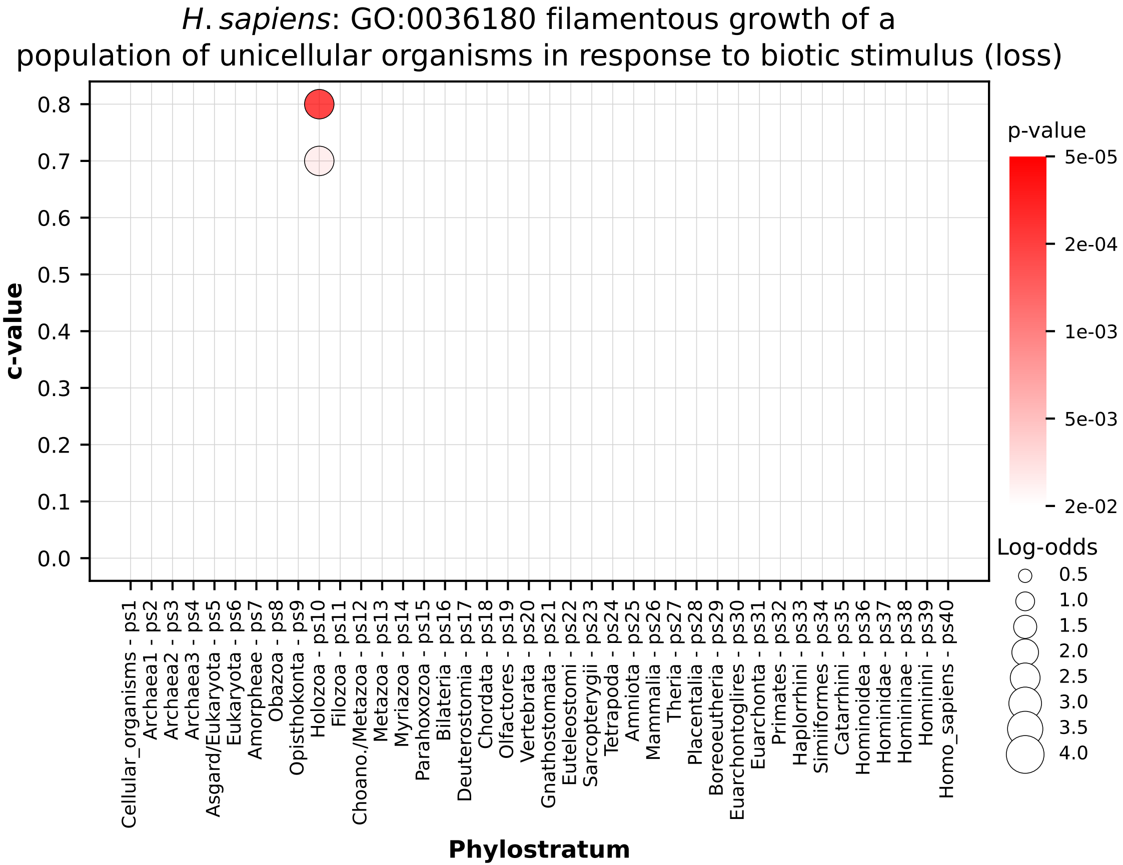

H. sapiens: GO:0036180 filamentous growth of a population of unicellular organisms in response to biotic stimulus (loss)
c-value
0.0
0.1
0.2
0.3
0.4
0.5
0.6
0.7
0.8
Phylostratum
Cellular_organisms - ps1
Archaea1 - ps2
Archaea2 - ps3
Archaea3 - ps4
Asgard/Eukaryota - ps5
Eukaryota - ps6
Amorpheae - ps7
Obazoa - ps8
Opisthokonta - ps9
Holozoa - ps10
Filozoa - ps11
Choano./Metazoa - ps12
Metazoa - ps13
Myriazoa - ps14
Parahoxozoa - ps15
Bilateria - ps16
Deuterostomia - ps17
Chordata - ps18
Olfactores - ps19
Vertebrata - ps20
Gnathostomata - ps21
Euteleostomi - ps22
Sarcopterygii - ps23
Tetrapoda - ps24
Amniota - ps25
Mammalia - ps26
Theria - ps27
Placentalia - ps28
Boreoeutheria - ps29
Euarchontoglires - ps30
Euarchonta - ps31
Primates - ps32
Haplorrhini - ps33
Simiiformes - ps34
Catarrhini - ps35
Hominoidea - ps36
Hominidae - ps37
Homininae - ps38
Hominini - ps39
Homo_sapiens - ps40
p-value
5e-05
2e-04
1e-03
5e-03
2e-02
Log-odds
0.5
1.0
1.5
2.0
2.5
3.0
3.5
4.0

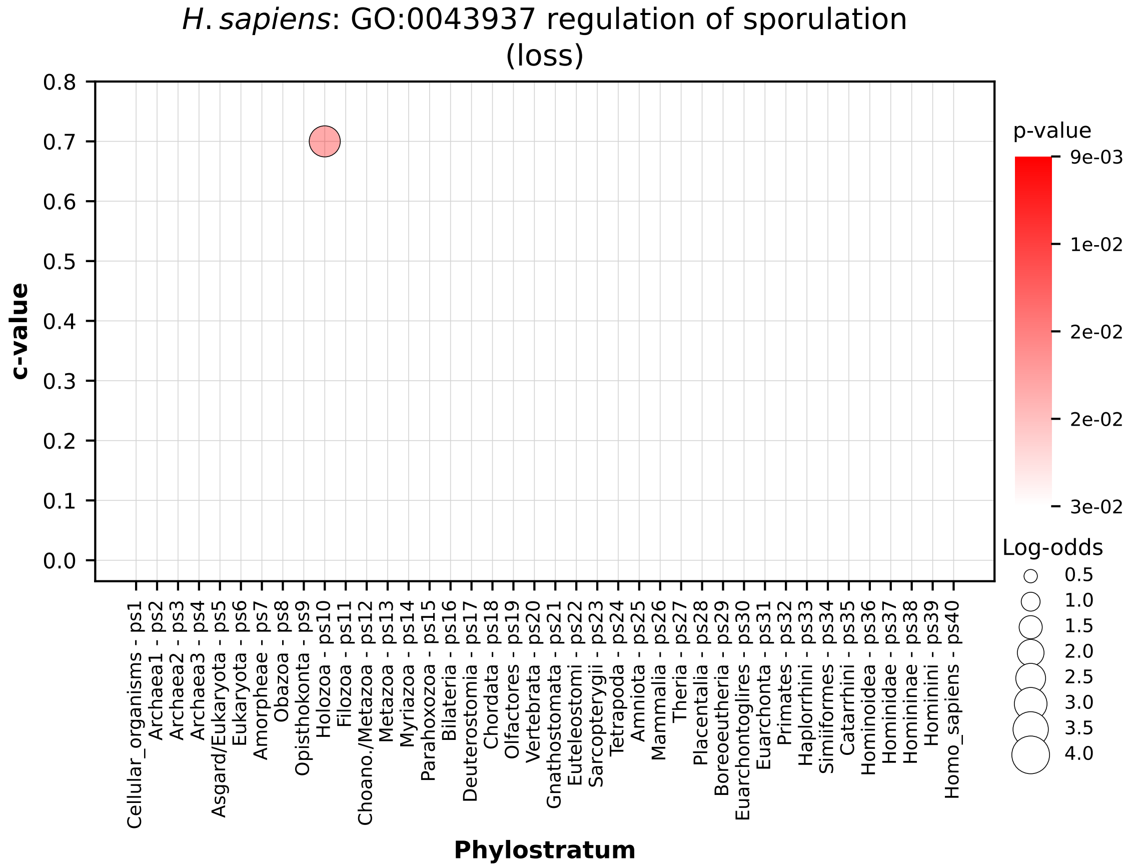
H. sapiens: GO:0043937 regulation of sporulation
(loss)
c-value
0.8
0.7
0.6
0.5
0.4
0.3
0.2
0.1
0.0
Phylostratum
Cellular_organisms - ps1
Archaea1 - ps2
Archaea2 - ps3
Archaea3 - ps4
Asgard/Eukaryota - ps5
Eukaryota - ps6
Amorpheae - ps7
Obazoa - ps8
Opisthokonta - ps9
Holozoa - ps10
Filozoa - ps11
Choano./Metazoa - ps12
Metazoa - ps13
Myriazoa - ps14
Parahoxozoa - ps15
Bilateria - ps16
Deuterostomia - ps17
Chordata - ps18
Olfactores - ps19
Vertebrata - ps20
Gnathostomata - ps21
Euteleostomi - ps22
Sarcopterygii - ps23
Tetrapoda - ps24
Amniota - ps25
Mammalia - ps26
Theria - ps27
Placentalia - ps28
Boreoeutheria - ps29
Euarchontoglires - ps30
Euarchonta - ps31
Primates - ps32
Haplorrhini - ps33
Simiiformes - ps34
Catarrhini - ps35
Hominoidea - ps36
Hominidae - ps37
Homininae - ps38
Hominini - ps39
Homo_sapiens - ps40
p-value
9e-03
1e-02
2e-02
2e-02
3e-02
Log-odds
0.5
1.0
1.5
2.0
2.5
3.0
3.5
4.0

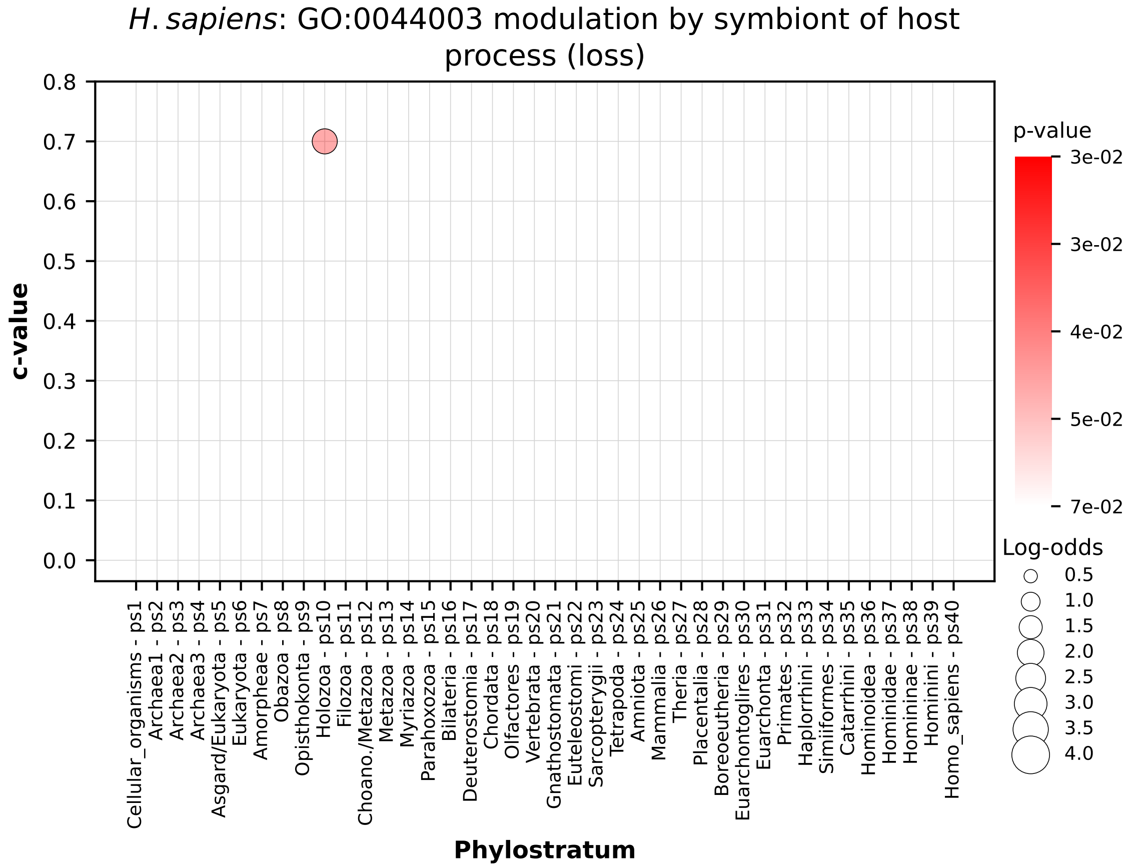

H. sapiens: GO:0044003 modulation by symbiont of host process (loss)
c-value
0.8
0.7
0.6
0.5
0.4
0.3
0.2
0.1
0.0
p-value
3e-02
3e-02
4e-02
5e-02
7e-02
Log-odds
0.5
1.0
1.5
2.0
2.5
3.0
3.5
4.0
Cellular_organisms - ps1
Archaea1 - ps2
Archaea2 - ps3
Archaea3 - ps4
Asgard/Eukaryota - ps5
Eukaryota - ps6
Amorpheae - ps7
Obazoa - ps8
Opisthokonta - ps9
Holozoa - ps10
Filozoa - ps11
Choano./Metazoa - ps12
Metazoa - ps13
Myriazoa - ps14
Parahoxozoa - ps15
Bilateria - ps16
Deuterostomia - ps17
Chordata - ps18
Olfactores - ps19
Vertebrata - ps20
Gnathostomata - ps21
Euteleostomi - ps22
Sarcopterygii - ps23
Tetrapoda - ps24
Amniota - ps25
Mammalia - ps26
Theria - ps27
Placentalia - ps28
Boreoeutheria - ps29
Euarchontoglires - ps30
Euarchonta - ps31
Primates - ps32
Haplorrhini - ps33
Simiiformes - ps34
Catarrhini - ps35
Hominoidea - ps36
Hominidae - ps37
Homininae - ps38
Hominini - ps39
Homo_sapiens - ps40
Phylostratum

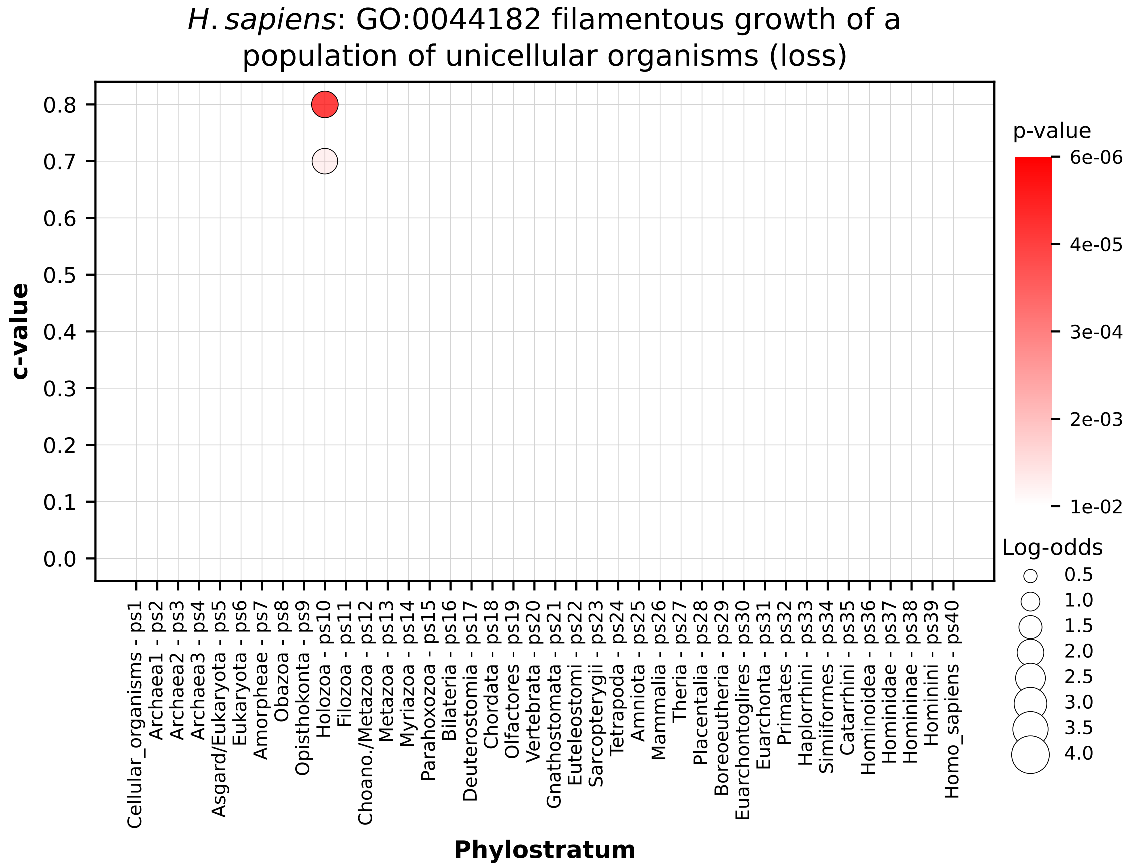

H. sapiens: GO:0044182 filamentous growth of a population of unicellular organisms (loss)
c-value
0.0
0.1
0.2
0.3
0.4
0.5
0.6
0.7
0.8
Phylostratum
Cellular_organisms - ps1
Archaea1 - ps2
Archaea2 - ps3
Archaea3 - ps4
Asgard/Eukaryota - ps5
Eukaryota - ps6
Amorpheae - ps7
Obazoa - ps8
Opisthokonta - ps9
Holozoa - ps10
Filozoa - ps11
Choano./Metazoa - ps12
Metazoa - ps13
Myriazoa - ps14
Parahoxozoa - ps15
Bilateria - ps16
Deuterostomia - ps17
Chordata - ps18
Olfactores - ps19
Vertebrata - ps20
Gnathostomata - ps21
Euteleostomi - ps22
Sarcopterygii - ps23
Tetrapoda - ps24
Amniota - ps25
Mammalia - ps26
Theria - ps27
Placentalia - ps28
Boreoeutheria - ps29
Euarchontoglires - ps30
Euarchonta - ps31
Primates - ps32
Haplorrhini - ps33
Simiiformes - ps34
Catarrhini - ps35
Hominoidea - ps36
Hominidae - ps37
Homininae - ps38
Hominini - ps39
Homo_sapiens - ps40
p-value
6e-06
4e-05
3e-04
2e-03
1e-02
Log-odds
0.5
1.0
1.5
2.0
2.5
3.0
3.5
4.0

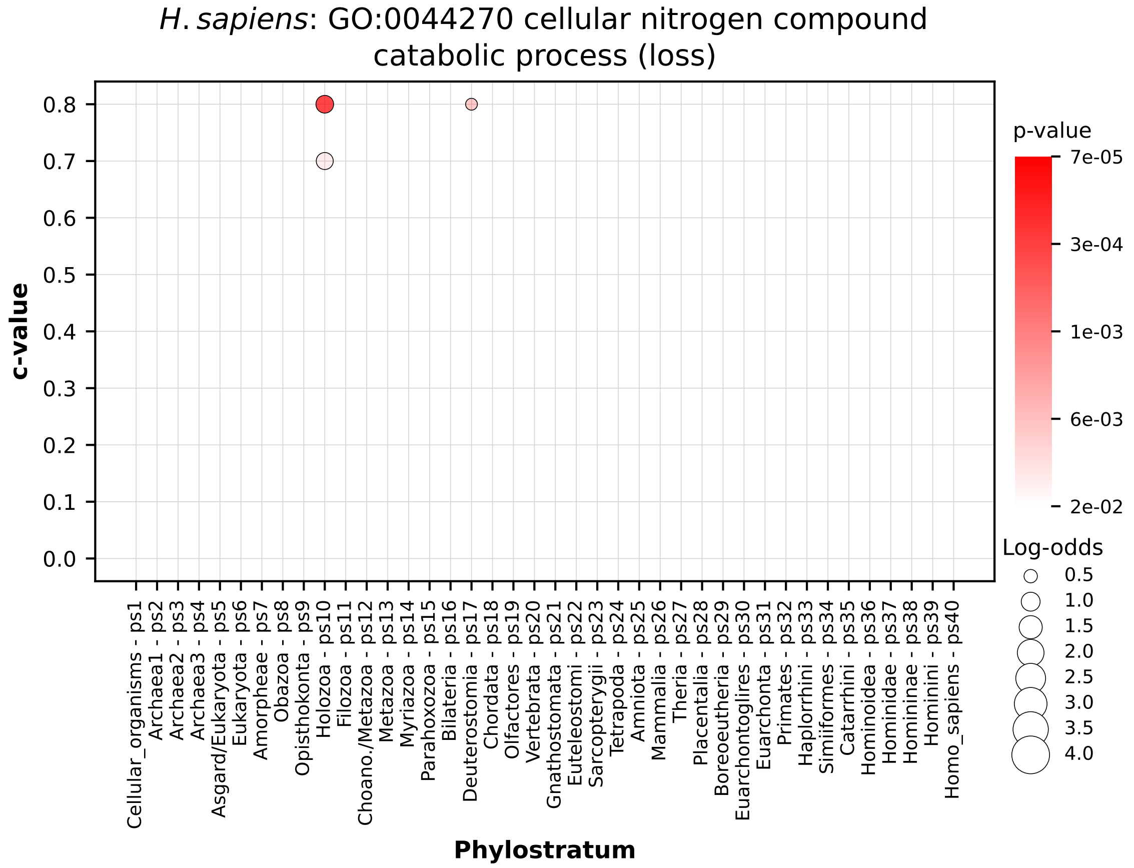
H. sapiens: GO:0044270 cellular nitrogen compound catabolic process (loss)
c-value
0.0
0.1
0.2
0.3
0.4
0.5
0.6
0.7
0.8
Phylostratum
Cellular_organisms - ps1
Archaea1 - ps2
Archaea2 - ps3
Archaea3 - ps4
Asgard/Eukaryota - ps5
Eukaryota - ps6
Amorpheae - ps7
Obazoa - ps8
Opisthokonta - ps9
Holozoa - ps10
Filozoa - ps11
Choano./Metazoa - ps12
Metazoa - ps13
Myriazoa - ps14
Parahoxozoa - ps15
Bilateria - ps16
Deuterostomia - ps17
Chordata - ps18
Olfactores - ps19
Vertebrata - ps20
Gnathostomata - ps21
Euteleostomi - ps22
Sarcopterygii - ps23
Tetrapoda - ps24
Amniota - ps25
Mammalia - ps26
Theria - ps27
Placentalia - ps28
Boreoeutheria - ps29
Euarchontoglires - ps30
Euarchonta - ps31
Primates - ps32
Haplorrhini - ps33
Simiiformes - ps34
Catarrhini - ps35
Hominoidea - ps36
Hominidae - ps37
Homininae - ps38
Hominini - ps39
Homo_sapiens - ps40
p-value
7e-05
3e-04
1e-03
6e-03
2e-02
Log-odds
0.5
1.0
1.5
2.0
2.5
3.0
3.5
4.0

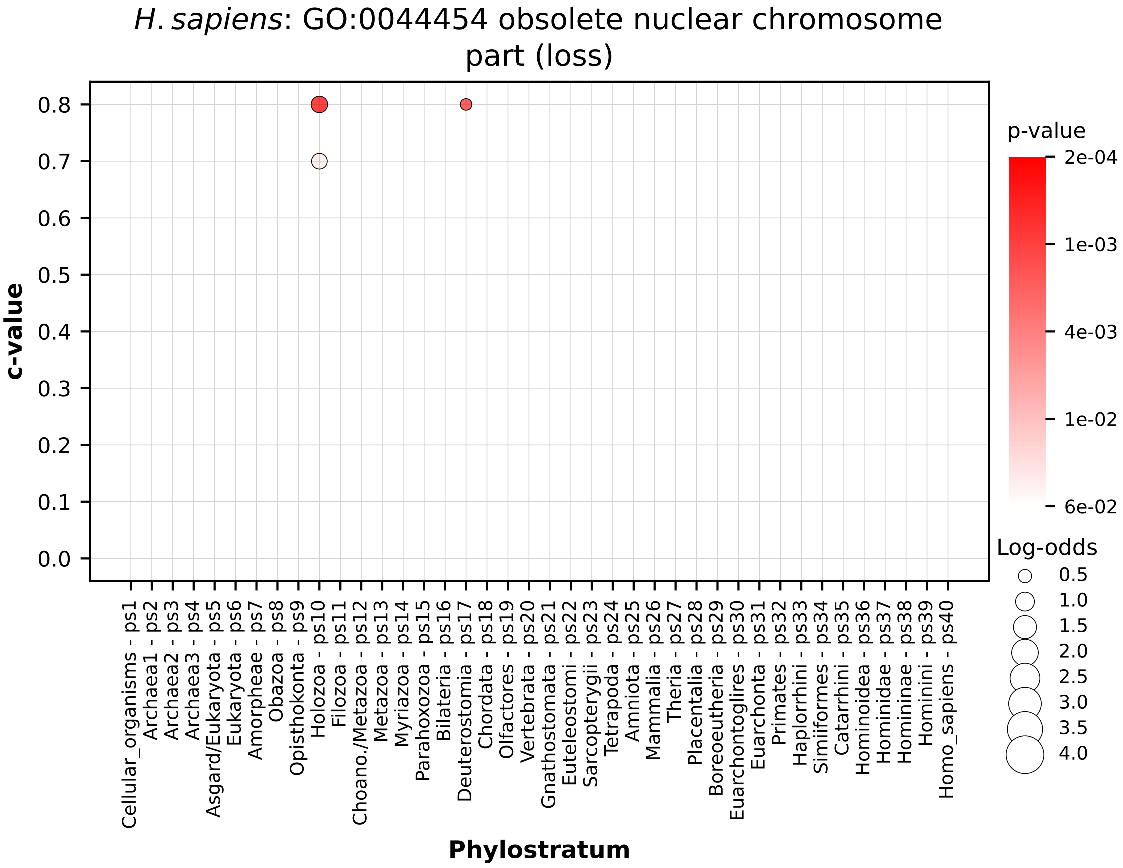
H. sapiens: GO:0044454 obsolete nuclear chromosome part (loss)
c-value
0.0
0.1
0.2
0.3
0.4
0.5
0.6
0.7
0.8
p-value
2e-04
1e-03
4e-03
1e-02
6e-02
Log-odds
0.5
1.0
1.5
2.0
2.5
3.0
3.5
4.0
Cellular_organisms - ps1
Archaea1 - ps2
Archaea2 - ps3
Archaea3 - ps4
Asgard/Eukaryota - ps5
Eukaryota - ps6
Amorpheae - ps7
Obazoa - ps8
Opisthokonta - ps9
Holozoa - ps10
Filozoa - ps11
Choano./Metazoa - ps12
Metazoa - ps13
Myriazoa - ps14
Parahoxozoa - ps15
Bilateria - ps16
Deuterostomia - ps17
Chordata - ps18
Olfactores - ps19
Vertebrata - ps20
Gnathostomata - ps21
Euteleostomi - ps22
Sarcopterygii - ps23
Tetrapoda - ps24
Amniota - ps25
Mammalia - ps26
Theria - ps27
Placentalia - ps28
Boreoeutheria - ps29
Euarchontoglires - ps30
Euarchonta - ps31
Primates - ps32
Haplorrhini - ps33
Simiiformes - ps34
Catarrhini - ps35
Hominoidea - ps36
Hominidae - ps37
Homininae - ps38
Hominini - ps39
Homo_sapiens - ps40
Phylostratum

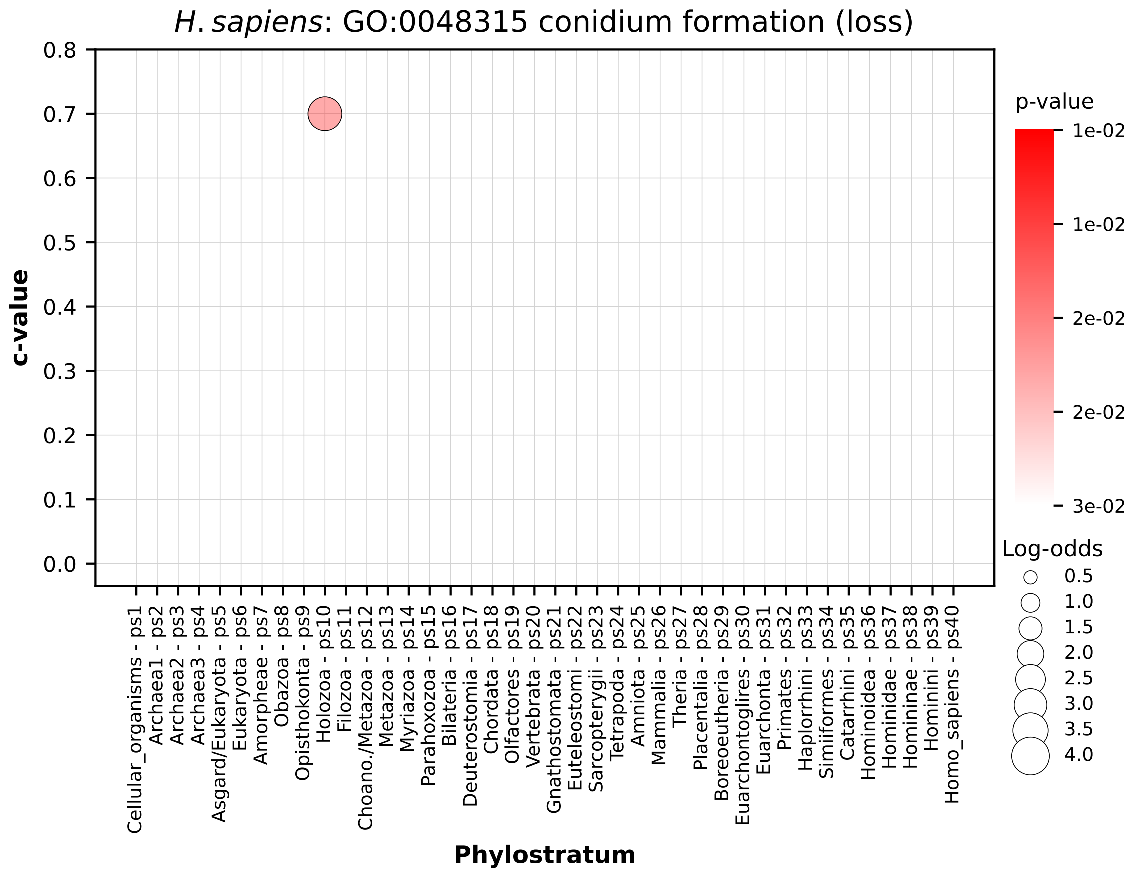

H. sapiens: GO:0048315 conidium formation (loss)
c-value
0.8
0.7
0.6
0.5
0.4
0.3
0.2
0.1
0.0
p-value
1e-02
1e-02
2e-02
2e-02
3e-02
Log-odds
0.5
1.0
1.5
2.0
2.5
3.0
3.5
4.0
Cellular_organisms - ps1
Archaea1 - ps2
Archaea2 - ps3
Archaea3 - ps4
Asgard/Eukaryota - ps5
Eukaryota - ps6
Amorpheae - ps7
Obazoa - ps8
Opisthokonta - ps9
Holozoa - ps10
Filozoa - ps11
Choano./Metazoa - ps12
Metazoa - ps13
Myriazoa - ps14
Parahoxozoa - ps15
Bilateria - ps16
Deuterostomia - ps17
Chordata - ps18
Olfactores - ps19
Vertebrata - ps20
Gnathostomata - ps21
Euteleostomi - ps22
Sarcopterygii - ps23
Tetrapoda - ps24
Amniota - ps25
Mammalia - ps26
Theria - ps27
Placentalia - ps28
Boreoeutheria - ps29
Euarchontoglires - ps30
Euarchonta - ps31
Primates - ps32
Haplorrhini - ps33
Simiiformes - ps34
Catarrhini - ps35
Hominoidea - ps36
Hominidae - ps37
Homininae - ps38
Hominini - ps39
Homo_sapiens - ps40
Phylostratum

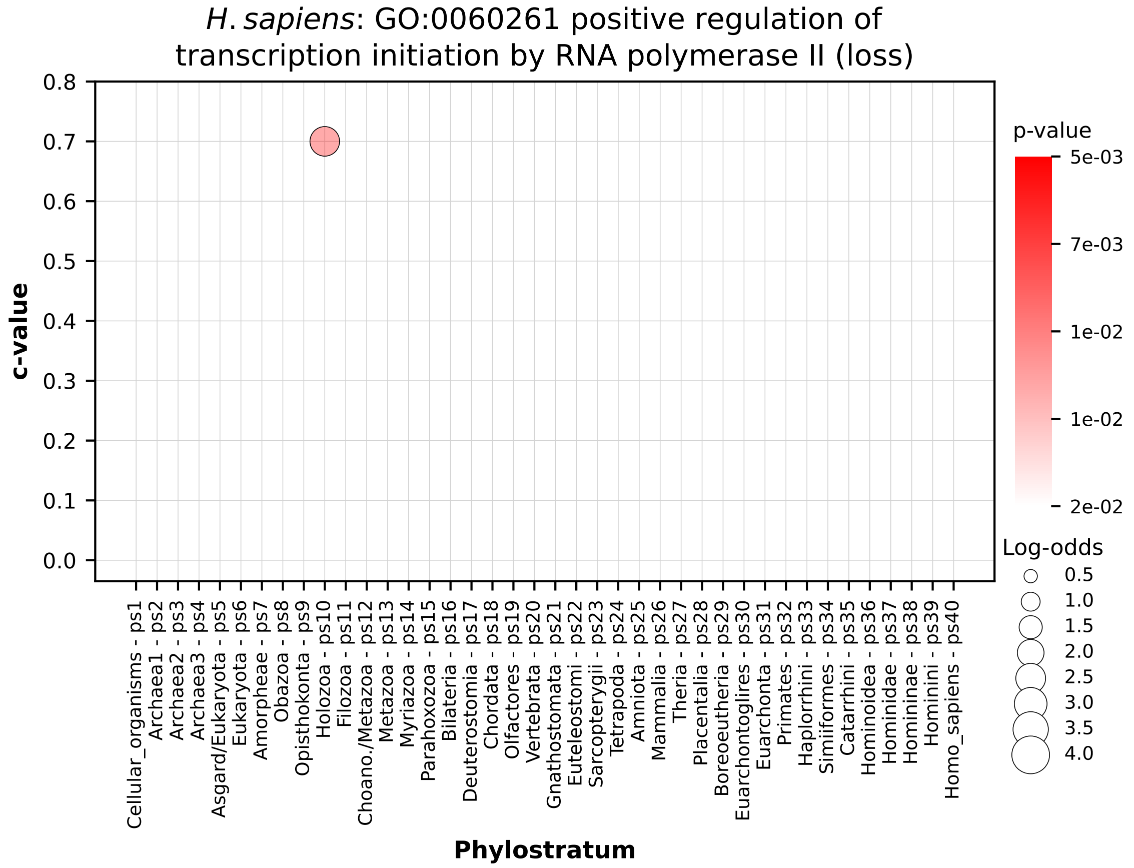
H. sapiens: GO:0060261 positive regulation of transcription initiation by RNA polymerase II (loss)
c-value
0.8
0.7
0.6
0.5
0.4
0.3
0.2
0.1
0.0
Phylostratum
Cellular_organisms - ps1
Archaea1 - ps2
Archaea2 - ps3
Archaea3 - ps4
Asgard/Eukaryota - ps5
Eukaryota - ps6
Amorpheae - ps7
Obazoa - ps8
Opisthokonta - ps9
Holozoa - ps10
Filozoa - ps11
Choano./Metazoa - ps12
Metazoa - ps13
Myriazoa - ps14
Parahoxozoa - ps15
Bilateria - ps16
Deuterostomia - ps17
Chordata - ps18
Olfactores - ps19
Vertebrata - ps20
Gnathostomata - ps21
Euteleostomi - ps22
Sarcopterygii - ps23
Tetrapoda - ps24
Amniota - ps25
Mammalia - ps26
Theria - ps27
Placentalia - ps28
Boreoeutheria - ps29
Euarchontoglires - ps30
Euarchonta - ps31
Primates - ps32
Haplorrhini - ps33
Simiiformes - ps34
Catarrhini - ps35
Hominoidea - ps36
Hominidae - ps37
Homininae - ps38
Hominini - ps39
Homo_sapiens - ps40
p-value
5e-03
7e-03
1e-02
1e-02
2e-02
Log-odds
0.5
1.0
1.5
2.0
2.5
3.0
3.5
4.0

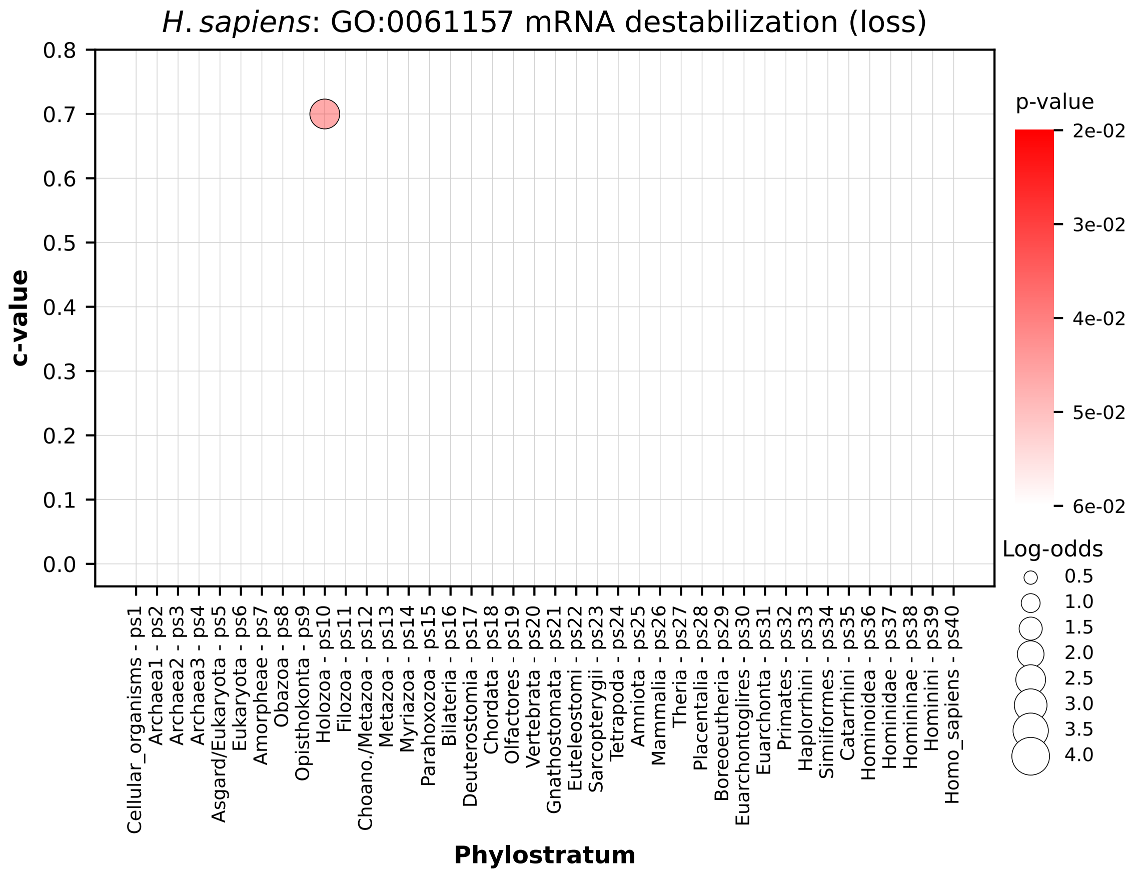

H. sapiens: GO:0061157 mRNA destabilization (loss)
c-value
0.8
0.7
0.6
0.5
0.4
0.3
0.2
0.1
0.0
p-value
2e-02
3e-02
4e-02
5e-02
6e-02
Log-odds
0.5
1.0
1.5
2.0
2.5
3.0
3.5
4.0
Cellular_organisms - ps1
Archaea1 - ps2
Archaea2 - ps3
Archaea3 - ps4
Asgard/Eukaryota - ps5
Eukaryota - ps6
Amorpheae - ps7
Obazoa - ps8
Opisthokonta - ps9
Holozoa - ps10
Filozoa - ps11
Choano./Metazoa - ps12
Metazoa - ps13
Myriazoa - ps14
Parahoxozoa - ps15
Bilateria - ps16
Deuterostomia - ps17
Chordata - ps18
Olfactores - ps19
Vertebrata - ps20
Gnathostomata - ps21
Euteleostomi - ps22
Sarcopterygii - ps23
Tetrapoda - ps24
Amniota - ps25
Mammalia - ps26
Theria - ps27
Placentalia - ps28
Boreoeutheria - ps29
Euarchontoglires - ps30
Euarchonta - ps31
Primates - ps32
Haplorrhini - ps33
Simiiformes - ps34
Catarrhini - ps35
Hominoidea - ps36
Hominidae - ps37
Homininae - ps38
Hominini - ps39
Homo_sapiens - ps40
Phylostratum

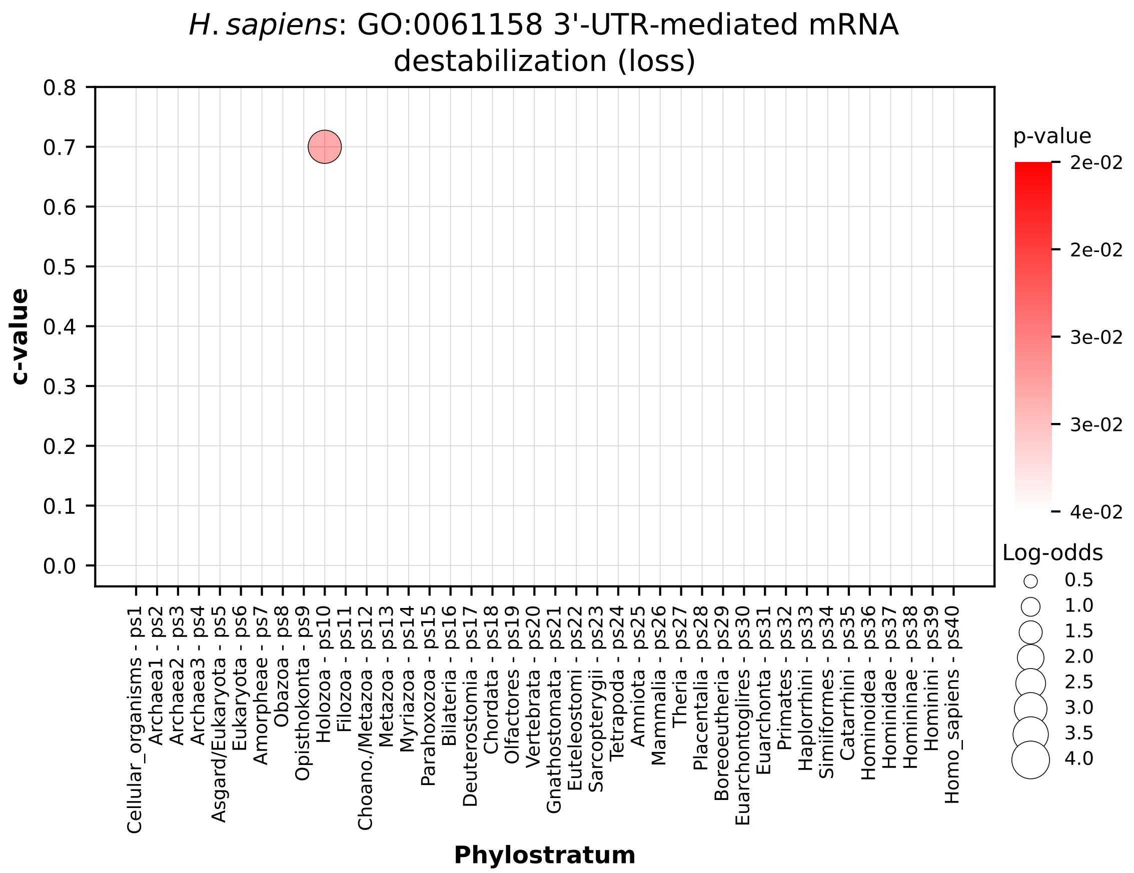

H. sapiens: GO:0061158 3'-UTR-mediated mRNA destabilization (loss)
c-value
0.0
0.1
0.2
0.3
0.4
0.5
0.6
0.7
0.8
Phylostratum
Cellular_organisms - ps1
Archaea1 - ps2
Archaea2 - ps3
Archaea3 - ps4
Asgard/Eukaryota - ps5
Eukaryota - ps6
Amorpheae - ps7
Obazoa - ps8
Opisthokonta - ps9
Holozoa - ps10
Filozoa - ps11
Choano./Metazoa - ps12
Metazoa - ps13
Myriazoa - ps14
Parahoxozoa - ps15
Bilateria - ps16
Deuterostomia - ps17
Chordata - ps18
Olfactores - ps19
Vertebrata - ps20
Gnathostomata - ps21
Euteleostomi - ps22
Sarcopterygii - ps23
Tetrapoda - ps24
Amniota - ps25
Mammalia - ps26
Theria - ps27
Placentalia - ps28
Boreoeutheria - ps29
Euarchontoglires - ps30
Euarchonta - ps31
Primates - ps32
Haplorrhini - ps33
Simiiformes - ps34
Catarrhini - ps35
Hominoidea - ps36
Hominidae - ps37
Homininae - ps38
Hominini - ps39
Homo_sapiens - ps40
p-value
2e-02
2e-02
3e-02
3e-02
4e-02
Log-odds
0.5
1.0
1.5
2.0
2.5
3.0
3.5
4.0

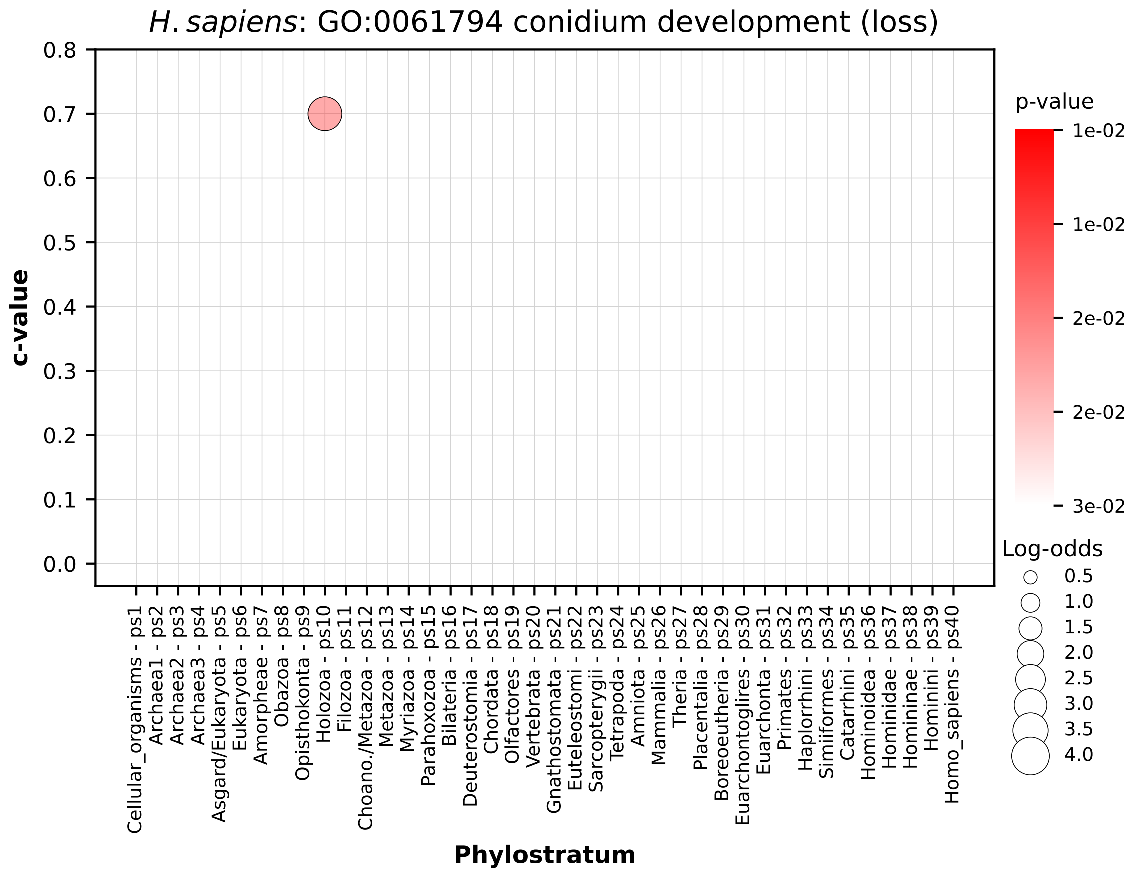

H. sapiens: GO:0061794 conidium development (loss)
c-value
0.8
0.7
0.6
0.5
0.4
0.3
0.2
0.1
0.0
p-value
1e-02
1e-02
2e-02
2e-02
3e-02
Log-odds
0.5
1.0
1.5
2.0
2.5
3.0
3.5
4.0
Cellular_organisms - ps1
Archaea1 - ps2
Archaea2 - ps3
Archaea3 - ps4
Asgard/Eukaryota - ps5
Eukaryota - ps6
Amorpheae - ps7
Obazoa - ps8
Opisthokonta - ps9
Holozoa - ps10
Filozoa - ps11
Choano./Metazoa - ps12
Metazoa - ps13
Myriazoa - ps14
Parahoxozoa - ps15
Bilateria - ps16
Deuterostomia - ps17
Chordata - ps18
Olfactores - ps19
Vertebrata - ps20
Gnathostomata - ps21
Euteleostomi - ps22
Sarcopterygii - ps23
Tetrapoda - ps24
Amniota - ps25
Mammalia - ps26
Theria - ps27
Placentalia - ps28
Boreoeutheria - ps29
Euarchontoglires - ps30
Euarchonta - ps31
Primates - ps32
Haplorrhini - ps33
Simiiformes - ps34
Catarrhini - ps35
Hominoidea - ps36
Hominidae - ps37
Homininae - ps38
Hominini - ps39
Homo_sapiens - ps40
Phylostratum

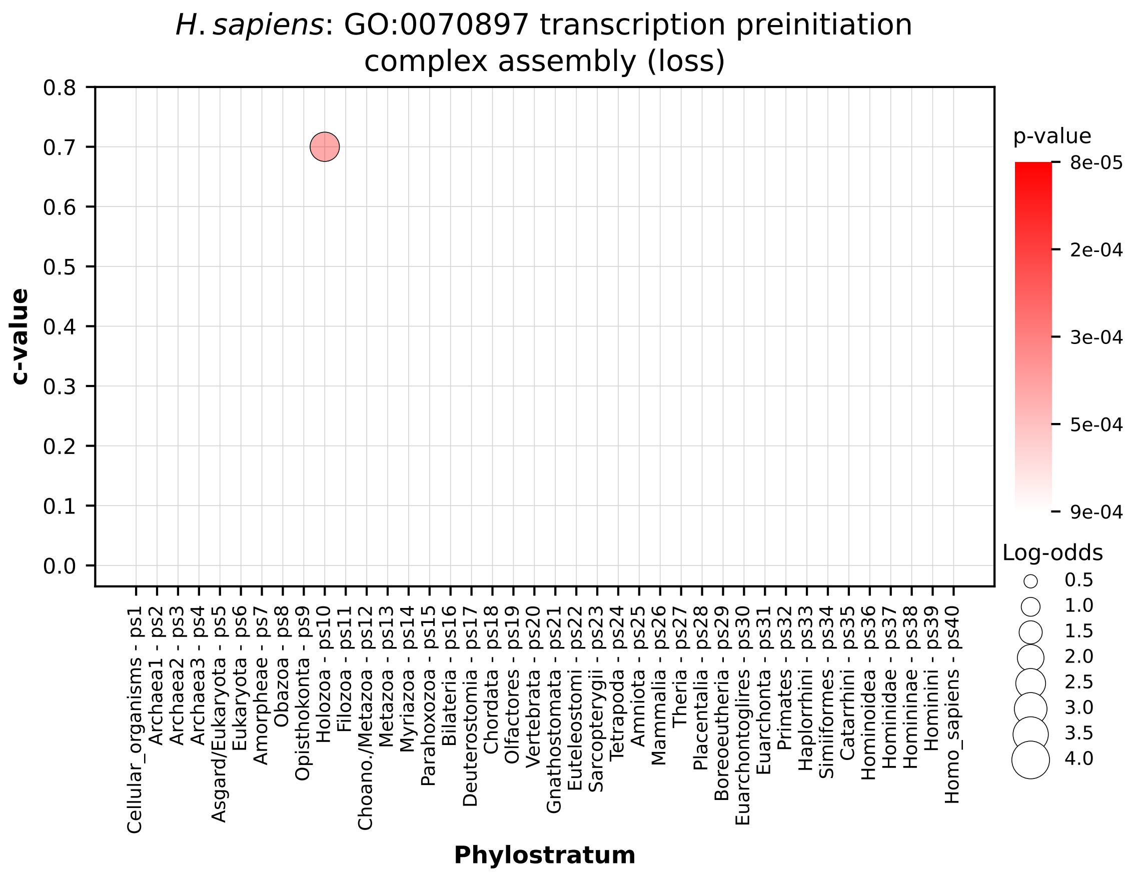

H. sapiens: GO:0070897 transcription preinitiation complex assembly (loss)
c-value
0.8
0.7
0.6
0.5
0.4
0.3
0.2
0.1
0.0
p-value
8e-05
2e-04
3e-04
5e-04
9e-04
Log-odds
0.5
1.0
1.5
2.0
2.5
3.0
3.5
4.0
Cellular_organisms - ps1
Archaea1 - ps2
Archaea2 - ps3
Archaea3 - ps4
Asgard/Eukaryota - ps5
Eukaryota - ps6
Amorpheae - ps7
Obazoa - ps8
Opisthokonta - ps9
Holozoa - ps10
Filozoa - ps11
Choano./Metazoa - ps12
Metazoa - ps13
Myriazoa - ps14
Parahoxozoa - ps15
Bilateria - ps16
Deuterostomia - ps17
Chordata - ps18
Olfactores - ps19
Vertebrata - ps20
Gnathostomata - ps21
Euteleostomi - ps22
Sarcopterygii - ps23
Tetrapoda - ps24
Amniota - ps25
Mammalia - ps26
Theria - ps27
Placentalia - ps28
Boreoeutheria - ps29
Euarchontoglires - ps30
Euarchonta - ps31
Primates - ps32
Haplorrhini - ps33
Simiiformes - ps34
Catarrhini - ps35
Hominoidea - ps36
Hominidae - ps37
Homininae - ps38
Hominini - ps39
Homo_sapiens - ps40
Phylostratum

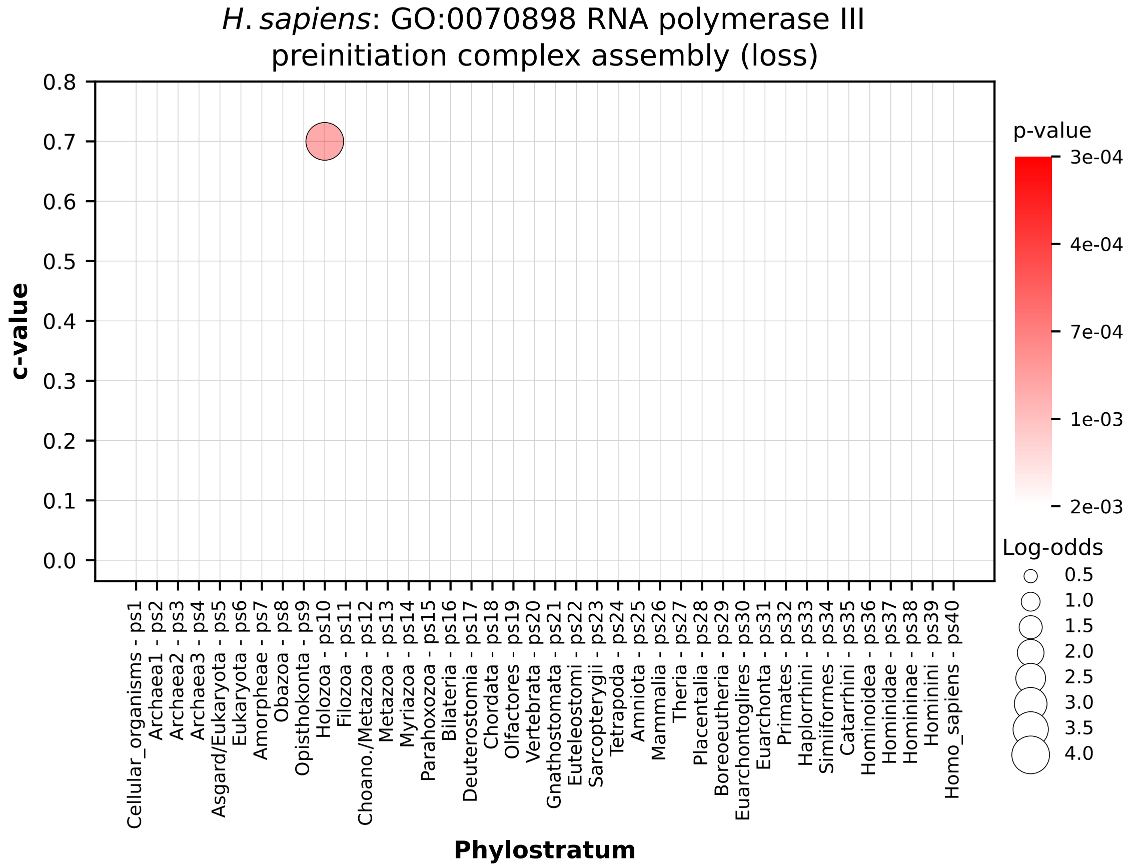

H. sapiens: GO:0070898 RNA polymerase III preinitiation complex assembly (loss)
c-value
0.8
0.7
0.6
0.5
0.4
0.3
0.2
0.1
0.0
p-value
3e-04
4e-04
7e-04
1e-03
2e-03
Log-odds
0.5
1.0
1.5
2.0
2.5
3.0
3.5
4.0
Cellular_organisms - ps1
Archaea1 - ps2
Archaea2 - ps3
Archaea3 - ps4
Asgard/Eukaryota - ps5
Eukaryota - ps6
Amorpheae - ps7
Obazoa - ps8
Opisthokonta - ps9
Holozoa - ps10
Filozoa - ps11
Choano./Metazoa - ps12
Metazoa - ps13
Myriazoa - ps14
Parahoxozoa - ps15
Bilateria - ps16
Deuterostomia - ps17
Chordata - ps18
Olfactores - ps19
Vertebrata - ps20
Gnathostomata - ps21
Euteleostomi - ps22
Sarcopterygii - ps23
Tetrapoda - ps24
Amniota - ps25
Mammalia - ps26
Theria - ps27
Placentalia - ps28
Boreoeutheria - ps29
Euarchontoglires - ps30
Euarchonta - ps31
Primates - ps32
Haplorrhini - ps33
Simiiformes - ps34
Catarrhini - ps35
Hominoidea - ps36
Hominidae - ps37
Homininae - ps38
Hominini - ps39
Homo_sapiens - ps40
Phylostratum

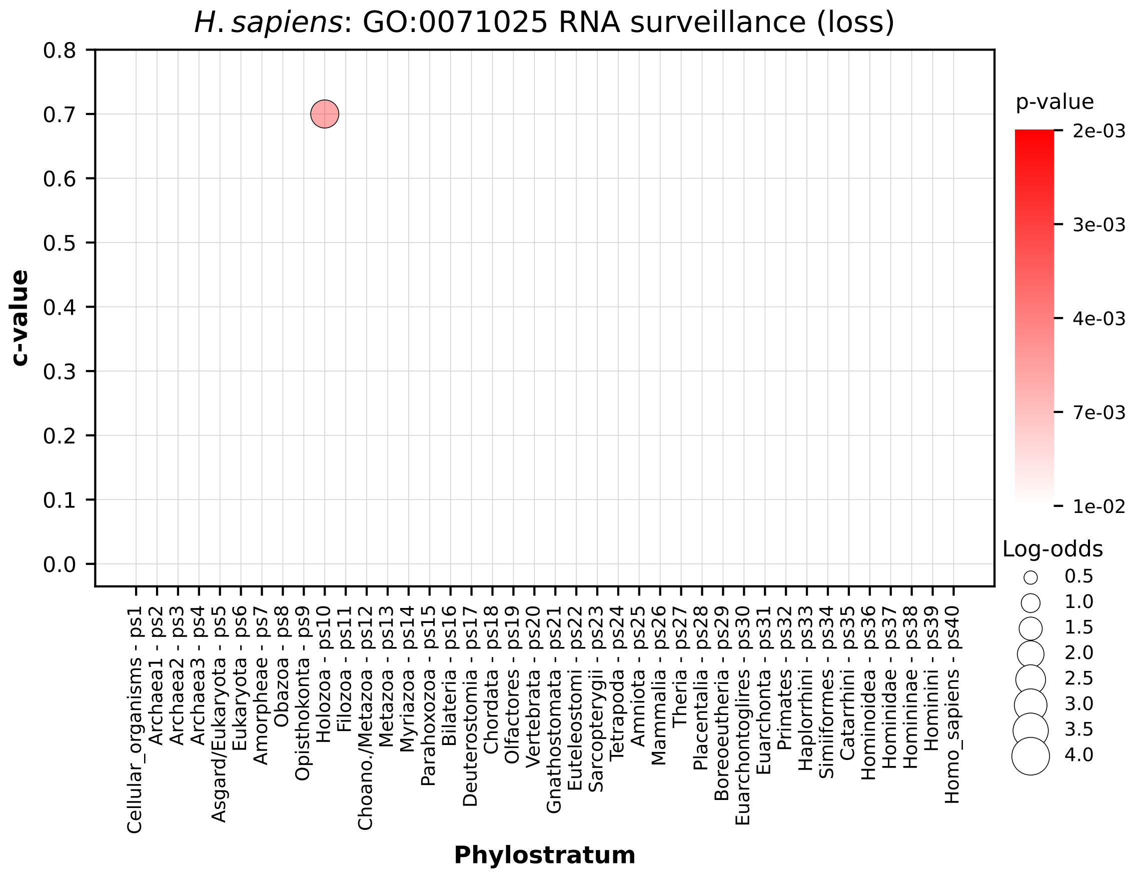

H. sapiens: GO:0071025 RNA surveillance (loss)
c-value
0.8
0.7
0.6
0.5
0.4
0.3
0.2
0.1
0.0
Phylostratum
Cellular_organisms - ps1
Archaea1 - ps2
Archaea2 - ps3
Archaea3 - ps4
Asgard/Eukaryota - ps5
Eukaryota - ps6
Amorpheae - ps7
Obazoa - ps8
Opisthokonta - ps9
Holozoa - ps10
Filozoa - ps11
Choano./Metazoa - ps12
Metazoa - ps13
Myriazoa - ps14
Parahoxozoa - ps15
Bilateria - ps16
Deuterostomia - ps17
Chordata - ps18
Olfactores - ps19
Vertebrata - ps20
Gnathostomata - ps21
Euteleostomi - ps22
Sarcopterygii - ps23
Tetrapoda - ps24
Amniota - ps25
Mammalia - ps26
Theria - ps27
Placentalia - ps28
Boreoeutheria - ps29
Euarchontoglires - ps30
Euarchonta - ps31
Primates - ps32
Haplorrhini - ps33
Simiiformes - ps34
Catarrhini - ps35
Hominoidea - ps36
Hominidae - ps37
Homininae - ps38
Hominini - ps39
Homo_sapiens - ps40
p-value
2e-03
3e-03
4e-03
7e-03
1e-02
Log-odds
0.5
1.0
1.5
2.0
2.5
3.0
3.5
4.0

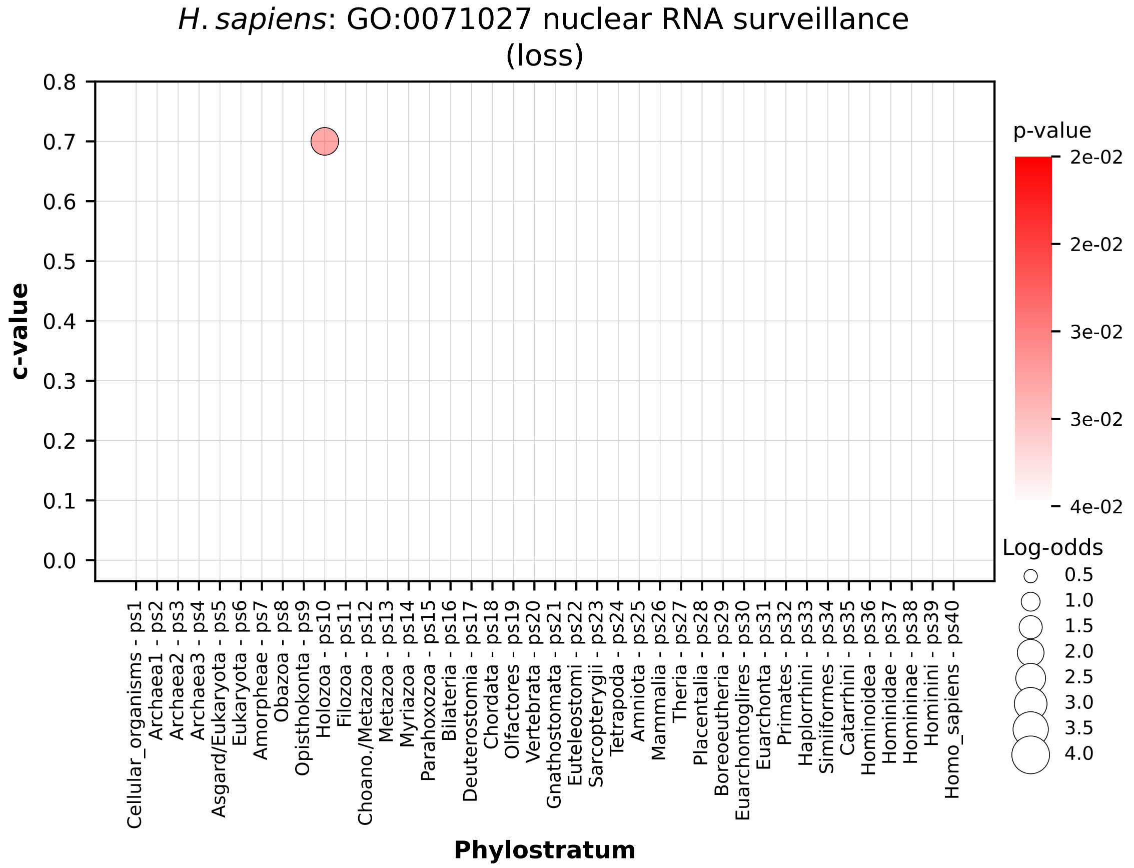

H. sapiens: GO:0071027 nuclear RNA surveillance
(loss)
c-value
0.8
0.7
0.6
0.5
0.4
0.3
0.2
0.1
0.0
Phylostratum
Cellular_organisms - ps1
Archaea1 - ps2
Archaea2 - ps3
Archaea3 - ps4
Asgard/Eukaryota - ps5
Eukaryota - ps6
Amorpheae - ps7
Obazoa - ps8
Opisthokonta - ps9
Holozoa - ps10
Filozoa - ps11
Choano./Metazoa - ps12
Metazoa - ps13
Myriazoa - ps14
Parahoxozoa - ps15
Bilateria - ps16
Deuterostomia - ps17
Chordata - ps18
Olfactores - ps19
Vertebrata - ps20
Gnathostomata - ps21
Euteleostomi - ps22
Sarcopterygii - ps23
Tetrapoda - ps24
Amniota - ps25
Mammalia - ps26
Theria - ps27
Placentalia - ps28
Boreoeutheria - ps29
Euarchontoglires - ps30
Euarchonta - ps31
Primates - ps32
Haplorrhini - ps33
Simiiformes - ps34
Catarrhini - ps35
Hominoidea - ps36
Hominidae - ps37
Homininae - ps38
Hominini - ps39
Homo_sapiens - ps40
p-value
2e-02
2e-02
3e-02
3e-02
4e-02
Log-odds
0.5
1.0
1.5
2.0
2.5
3.0
3.5
4.0

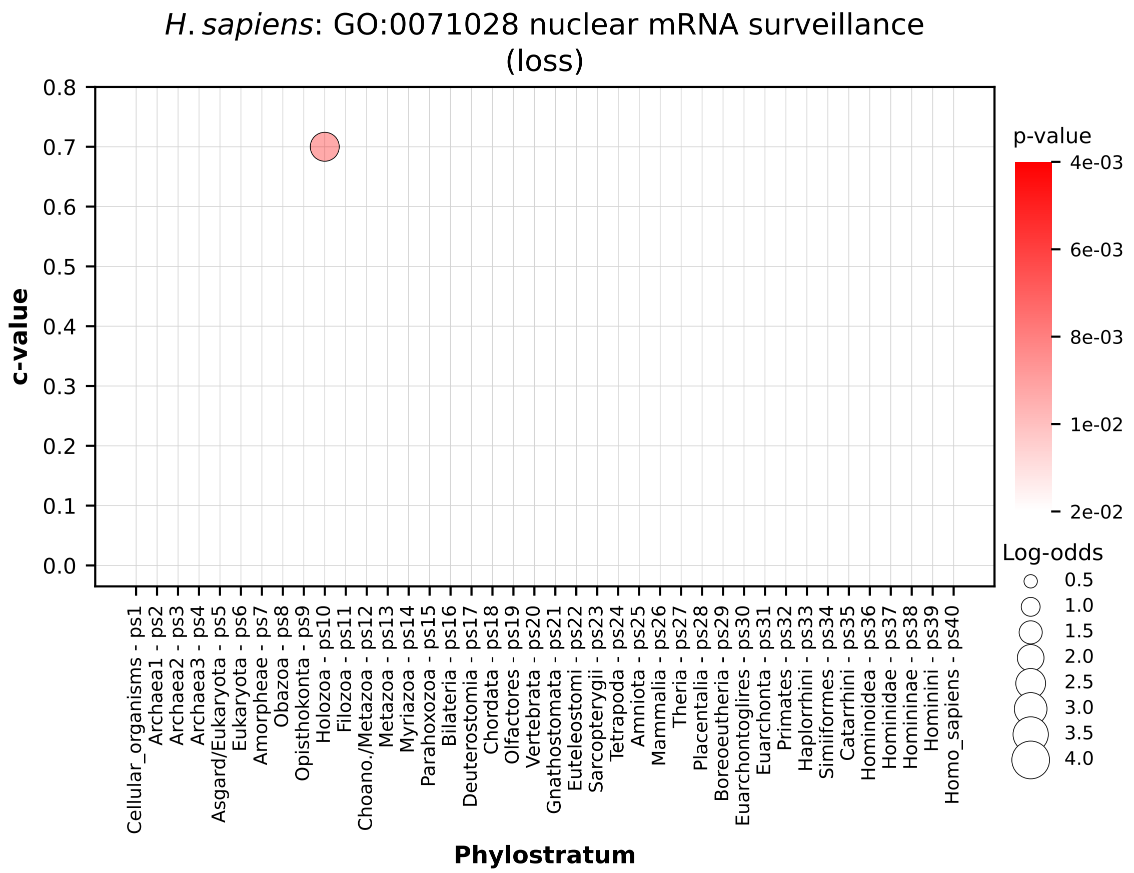

H. sapiens: GO:0071028 nuclear mRNA surveillance (loss)
c-value
0.8
0.7
0.6
0.5
0.4
0.3
0.2
0.1
0.0
p-value
4e-03
6e-03
8e-03
1e-02
2e-02
Log-odds
0.5
1.0
1.5
2.0
2.5
3.0
3.5
4.0
Cellular_organisms - ps1
Archaea1 - ps2
Archaea2 - ps3
Archaea3 - ps4
Asgard/Eukaryota - ps5
Eukaryota - ps6
Amorpheae - ps7
Obazoa - ps8
Opisthokonta - ps9
Holozoa - ps10
Filozoa - ps11
Choano./Metazoa - ps12
Metazoa - ps13
Myriazoa - ps14
Parahoxozoa - ps15
Bilateria - ps16
Deuterostomia - ps17
Chordata - ps18
Olfactores - ps19
Vertebrata - ps20
Gnathostomata - ps21
Euteleostomi - ps22
Sarcopterygii - ps23
Tetrapoda - ps24
Amniota - ps25
Mammalia - ps26
Theria - ps27
Placentalia - ps28
Boreoeutheria - ps29
Euarchontoglires - ps30
Euarchonta - ps31
Primates - ps32
Haplorrhini - ps33
Simiiformes - ps34
Catarrhini - ps35
Hominoidea - ps36
Hominidae - ps37
Homininae - ps38
Hominini - ps39
Homo_sapiens - ps40
Phylostratum

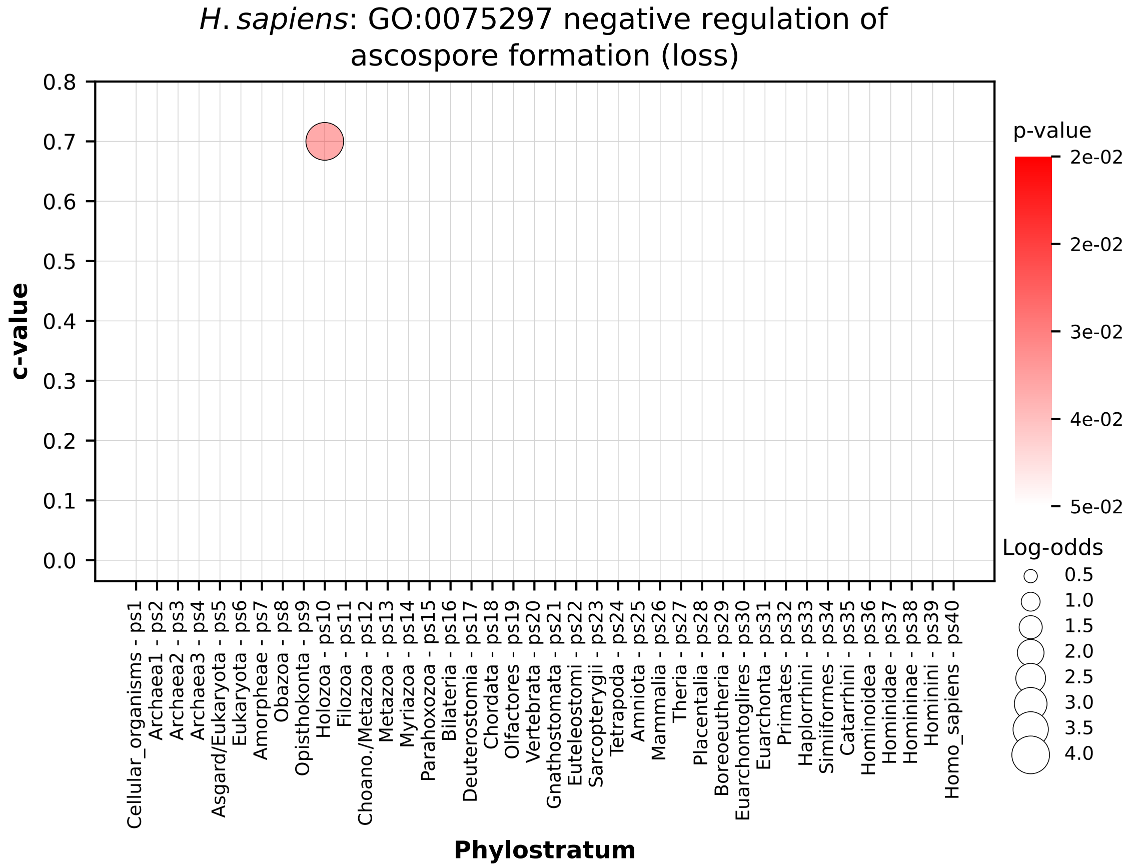

H. sapiens: GO:0075297 negative regulation of ascospore formation (loss)
c-value
0.8
0.7
0.6
0.5
0.4
0.3
0.2
0.1
0.0
Phylostratum
Cellular_organisms - ps1
Archaea1 - ps2
Archaea2 - ps3
Archaea3 - ps4
Asgard/Eukaryota - ps5
Eukaryota - ps6
Amorpheae - ps7
Obazoa - ps8
Opisthokonta - ps9
Holozoa - ps10
Filozoa - ps11
Choano./Metazoa - ps12
Metazoa - ps13
Myriazoa - ps14
Parahoxozoa - ps15
Bilateria - ps16
Deuterostomia - ps17
Chordata - ps18
Olfactores - ps19
Vertebrata - ps20
Gnathostomata - ps21
Euteleostomi - ps22
Sarcopterygii - ps23
Tetrapoda - ps24
Amniota - ps25
Mammalia - ps26
Theria - ps27
Placentalia - ps28
Boreoeutheria - ps29
Euarchontoglires - ps30
Euarchonta - ps31
Primates - ps32
Haplorrhini - ps33
Simiiformes - ps34
Catarrhini - ps35
Hominoidea - ps36
Hominidae - ps37
Homininae - ps38
Hominini - ps39
Homo_sapiens - ps40
p-value
2e-02
2e-02
3e-02
4e-02
5e-02
Log-odds
0.5
1.0
1.5
2.0
2.5
3.0
3.5
4.0

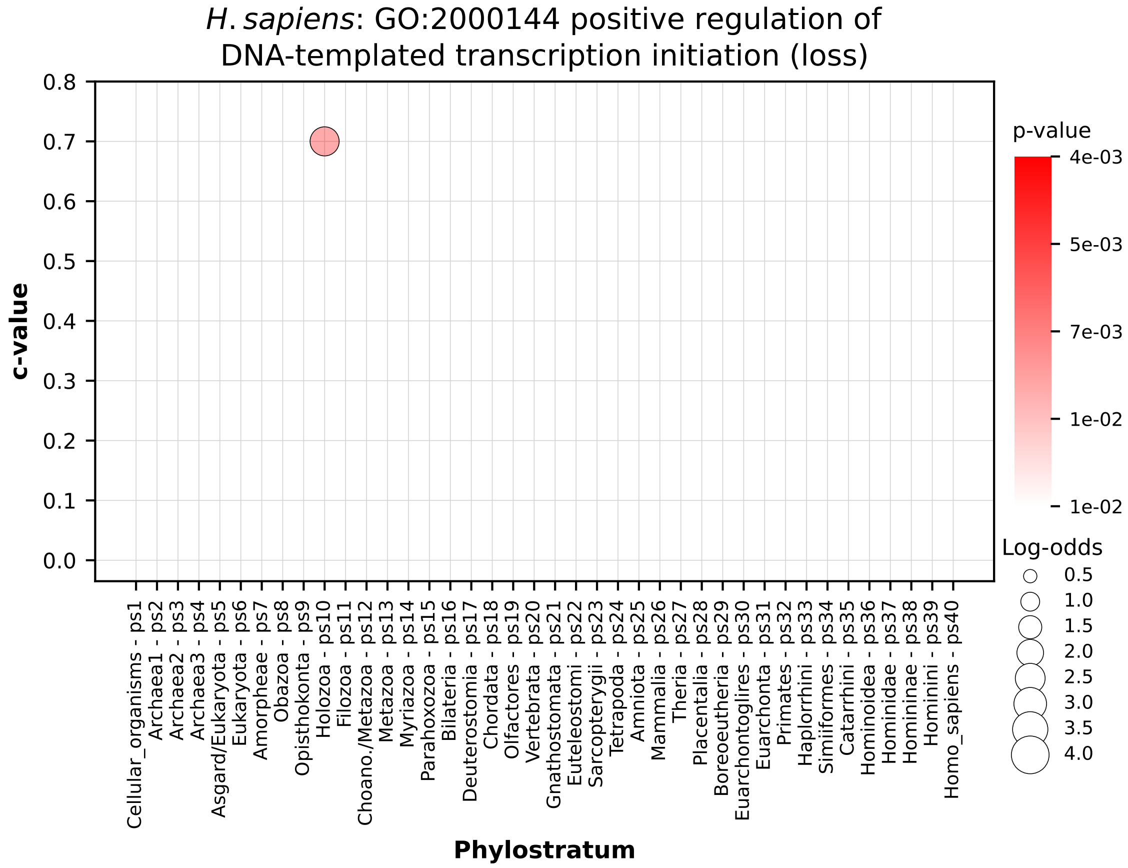

H. sapiens: GO:2000144 positive regulation of DNA-templated transcription initiation (loss)
c-value
0.0
0.1
0.2
0.3
0.4
0.5
0.6
0.7
0.8
Phylostratum
Cellular_organisms - ps1
Archaea1 - ps2
Archaea2 - ps3
Archaea3 - ps4
Asgard/Eukaryota - ps5
Eukaryota - ps6
Amorpheae - ps7
Obazoa - ps8
Opisthokonta - ps9
Holozoa - ps10
Filozoa - ps11
Choano./Metazoa - ps12
Metazoa - ps13
Myriazoa - ps14
Parahoxozoa - ps15
Bilateria - ps16
Deuterostomia - ps17
Chordata - ps18
Olfactores - ps19
Vertebrata - ps20
Gnathostomata - ps21
Euteleostomi - ps22
Sarcopterygii - ps23
Tetrapoda - ps24
Amniota - ps25
Mammalia - ps26
Theria - ps27
Placentalia - ps28
Boreoeutheria - ps29
Euarchontoglires - ps30
Euarchonta - ps31
Primates - ps32
Haplorrhini - ps33
Simiiformes - ps34
Catarrhini - ps35
Hominoidea - ps36
Hominidae - ps37
Homininae - ps38
Hominini - ps39
Homo_sapiens - ps40
p-value
4e-03
5e-03
7e-03
1e-02
1e-02
Log-odds
0.5
1.0
1.5
2.0
2.5
3.0
3.5
4.0

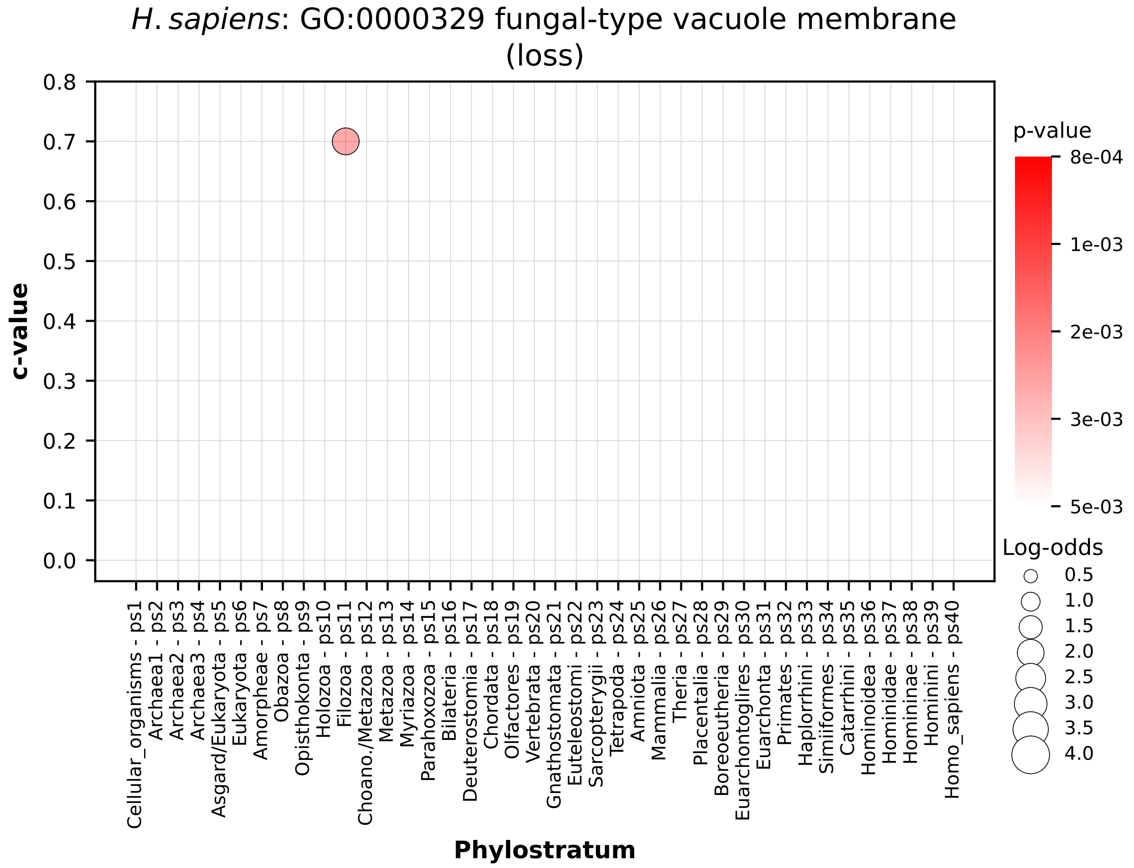
H. sapiens: GO:0000329 fungal-type vacuole membrane
(loss)
c-value
0.8
0.7
0.6
0.5
0.4
0.3
0.2
0.1
0.0
p-value
8e-04
1e-03
2e-03
3e-03
5e-03
Log-odds
0.5
1.0
1.5
2.0
2.5
3.0
3.5
4.0
Cellular_organisms - ps1
Archaea1 - ps2
Archaea2 - ps3
Archaea3 - ps4
Asgard/Eukaryota - ps5
Eukaryota - ps6
Amorpheae - ps7
Obazoa - ps8
Opisthokonta - ps9
Holozoa - ps10
Filozoa - ps11
Choano./Metazoa - ps12
Metazoa - ps13
Myriazoa - ps14
Parahoxozoa - ps15
Bilateria - ps16
Deuterostomia - ps17
Chordata - ps18
Olfactores - ps19
Vertebrata - ps20
Gnathostomata - ps21
Euteleostomi - ps22
Sarcopterygii - ps23
Tetrapoda - ps24
Amniota - ps25
Mammalia - ps26
Theria - ps27
Placentalia - ps28
Boreoeutheria - ps29
Euarchontoglires - ps30
Euarchonta - ps31
Primates - ps32
Haplorrhini - ps33
Simiiformes - ps34
Catarrhini - ps35
Hominoidea - ps36
Hominidae - ps37
Homininae - ps38
Hominini - ps39
Homo_sapiens - ps40
Phylostratum

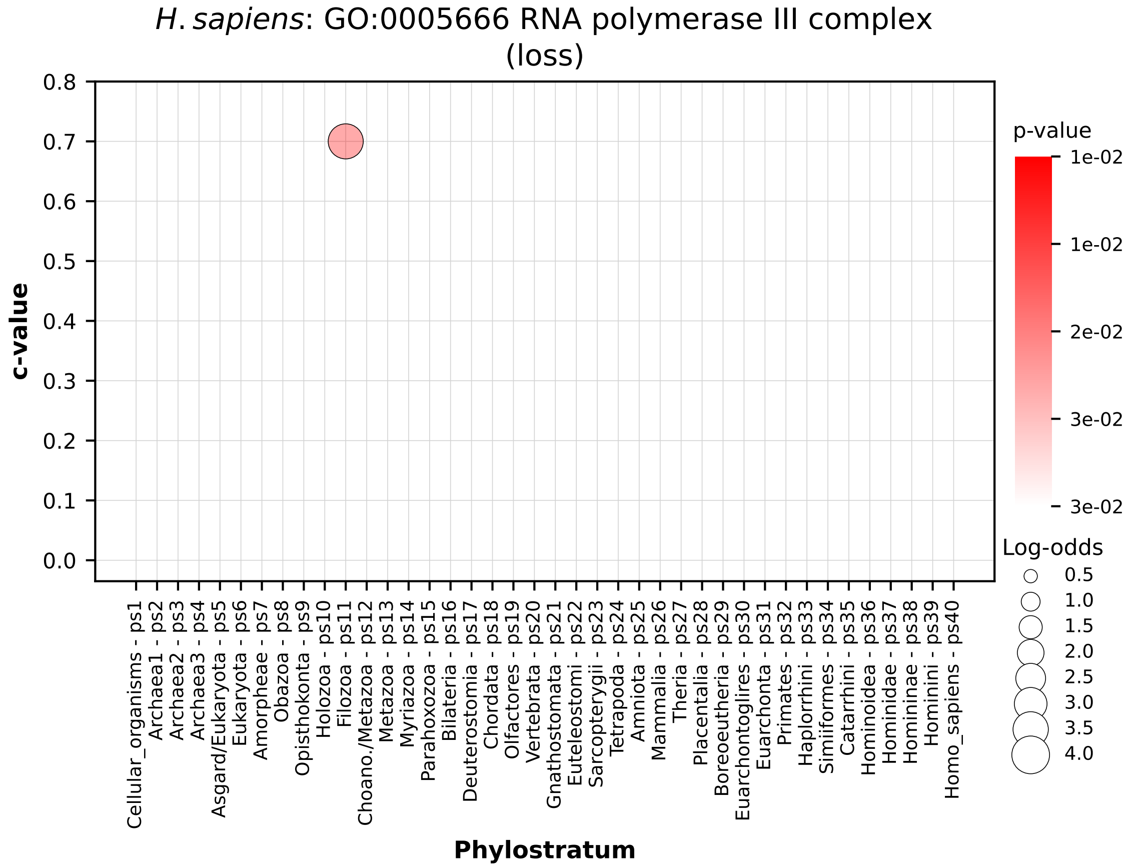
H. sapiens: GO:0005666 RNA polymerase III complex
(loss)
c-value
0.0
0.1
0.2
0.3
0.4
0.5
0.6
0.7
0.8
Phylostratum
Cellular_organisms - ps1
Archaea1 - ps2
Archaea2 - ps3
Archaea3 - ps4
Asgard/Eukaryota - ps5
Eukaryota - ps6
Amorpheae - ps7
Obazoa - ps8
Opisthokonta - ps9
Holozoa - ps10
Filozoa - ps11
Choano./Metazoa - ps12
Metazoa - ps13
Myriazoa - ps14
Parahoxozoa - ps15
Bilateria - ps16
Deuterostomia - ps17
Chordata - ps18
Olfactores - ps19
Vertebrata - ps20
Gnathostomata - ps21
Euteleostomi - ps22
Sarcopterygii - ps23
Tetrapoda - ps24
Amniota - ps25
Mammalia - ps26
Theria - ps27
Placentalia - ps28
Boreoeutheria - ps29
Euarchontoglires - ps30
Euarchonta - ps31
Primates - ps32
Haplorrhini - ps33
Simiiformes - ps34
Catarrhini - ps35
Hominoidea - ps36
Hominidae - ps37
Homininae - ps38
Hominini - ps39
Homo_sapiens - ps40
p-value
1e-02
1e-02
2e-02
3e-02
3e-02
Log-odds
0.5
1.0
1.5
2.0
2.5
3.0
3.5
4.0

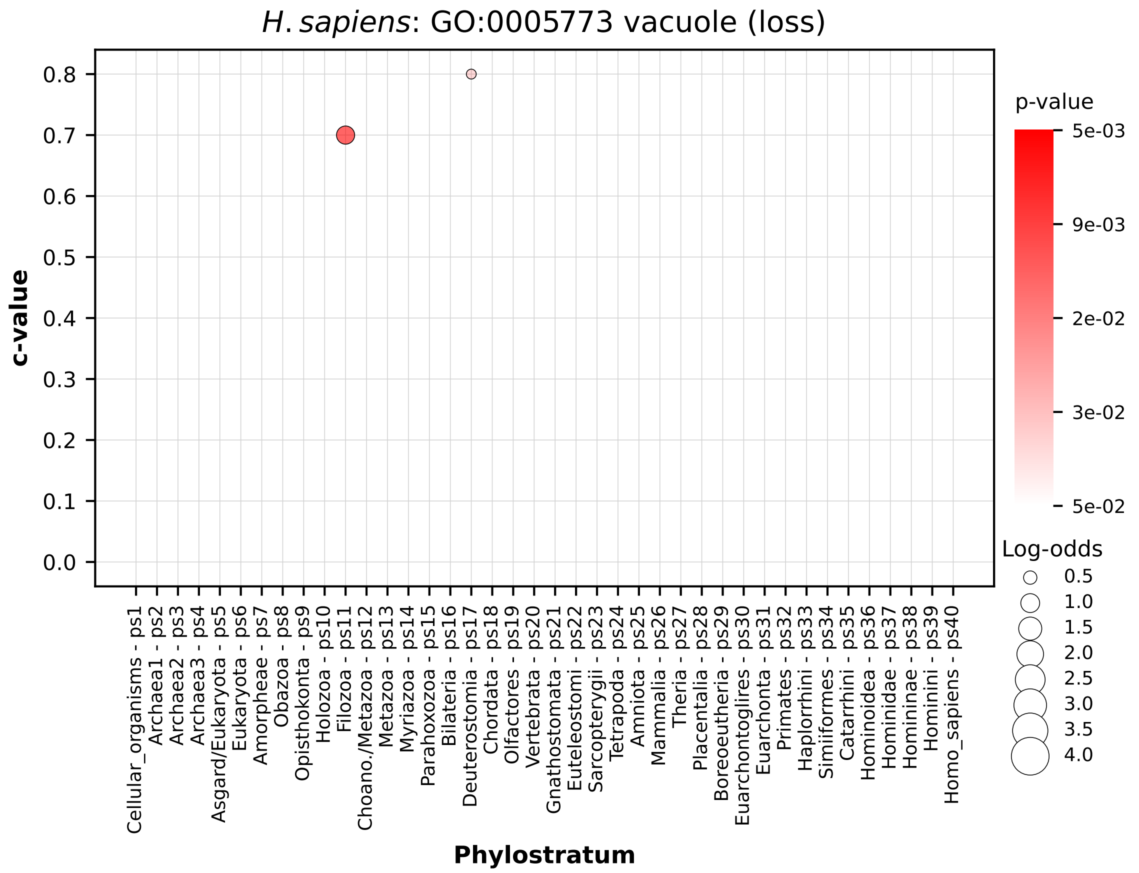

H. sapiens: GO:0005773 vacuole (loss)
c-value
0.8
0.7
0.6
0.5
0.4
0.3
0.2
0.1
0.0
p-value
5e-03
9e-03
2e-02
3e-02
5e-02
Log-odds
0.5
1.0
1.5
2.0
2.5
3.0
3.5
4.0
Cellular_organisms - ps1
Archaea1 - ps2
Archaea2 - ps3
Archaea3 - ps4
Asgard/Eukaryota - ps5
Eukaryota - ps6
Amorpheae - ps7
Obazoa - ps8
Opisthokonta - ps9
Holozoa - ps10
Filozoa - ps11
Choano./Metazoa - ps12
Metazoa - ps13
Myriazoa - ps14
Parahoxozoa - ps15
Bilateria - ps16
Deuterostomia - ps17
Chordata - ps18
Olfactores - ps19
Vertebrata - ps20
Gnathostomata - ps21
Euteleostomi - ps22
Sarcopterygii - ps23
Tetrapoda - ps24
Amniota - ps25
Mammalia - ps26
Theria - ps27
Placentalia - ps28
Boreoeutheria - ps29
Euarchontoglires - ps30
Euarchonta - ps31
Primates - ps32
Haplorrhini - ps33
Simiiformes - ps34
Catarrhini - ps35
Hominoidea - ps36
Hominidae - ps37
Homininae - ps38
Hominini - ps39
Homo_sapiens - ps40
Phylostratum

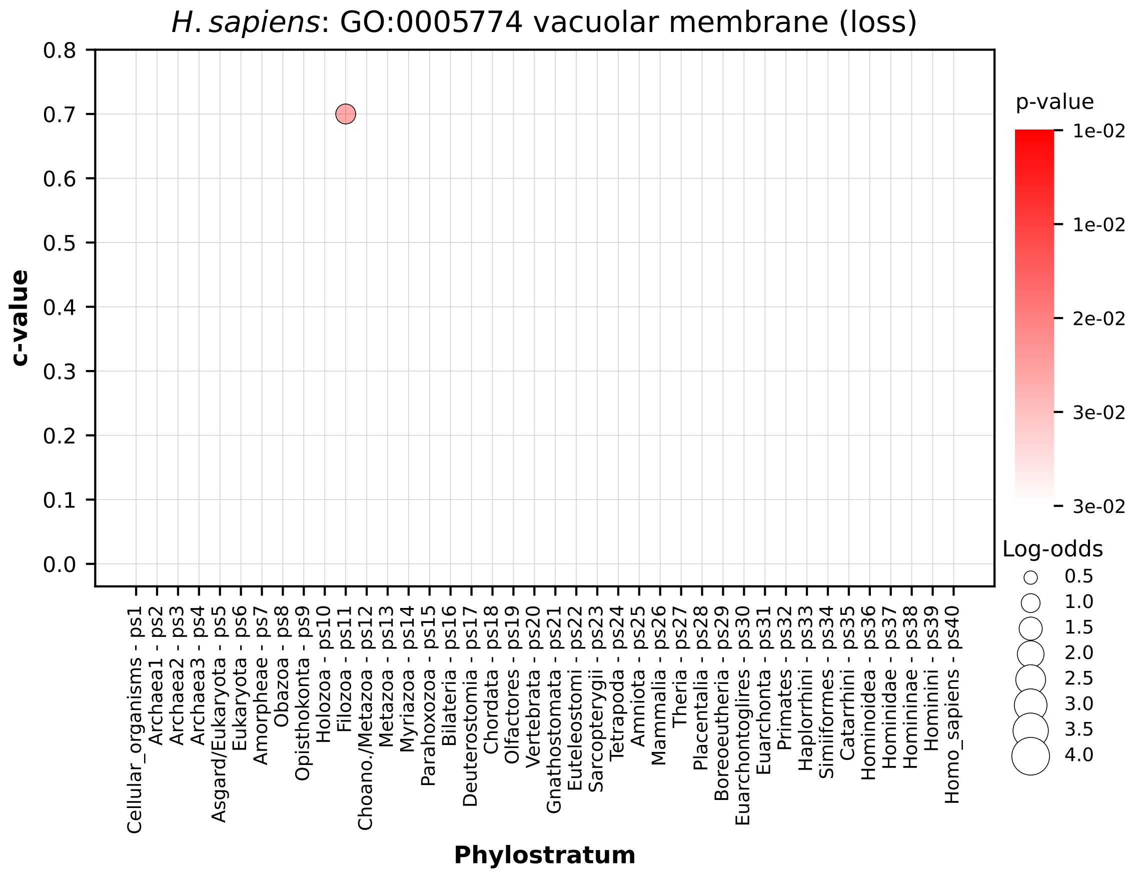

H. sapiens: GO:0005774 vacuolar membrane (loss)
c-value
0.0
0.1
0.2
0.3
0.4
0.5
0.6
0.7
0.8
Phylostratum
Cellular_organisms - ps1
Archaea1 - ps2
Archaea2 - ps3
Archaea3 - ps4
Asgard/Eukaryota - ps5
Eukaryota - ps6
Amorpheae - ps7
Obazoa - ps8
Opisthokonta - ps9
Holozoa - ps10
Filozoa - ps11
Choano./Metazoa - ps12
Metazoa - ps13
Myriazoa - ps14
Parahoxozoa - ps15
Bilateria - ps16
Deuterostomia - ps17
Chordata - ps18
Olfactores - ps19
Vertebrata - ps20
Gnathostomata - ps21
Euteleostomi - ps22
Sarcopterygii - ps23
Tetrapoda - ps24
Amniota - ps25
Mammalia - ps26
Theria - ps27
Placentalia - ps28
Boreoeutheria - ps29
Euarchontoglires - ps30
Euarchonta - ps31
Primates - ps32
Haplorrhini - ps33
Simiiformes - ps34
Catarrhini - ps35
Hominoidea - ps36
Hominidae - ps37
Homininae - ps38
Hominini - ps39
Homo_sapiens - ps40
p-value
1e-02
1e-02
2e-02
3e-02
3e-02
Log-odds
0.5
1.0
1.5
2.0
2.5
3.0
3.5
4.0

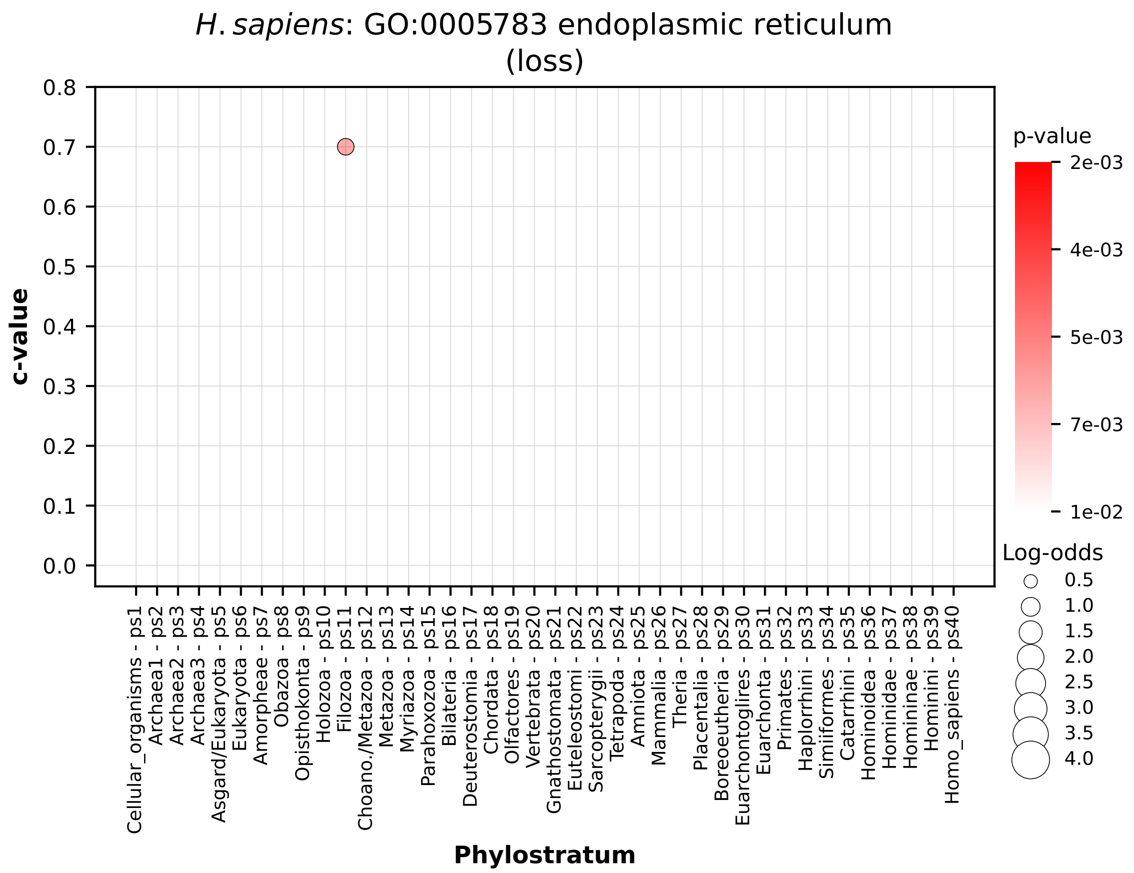
H. sapiens: GO:0005783 endoplasmic reticulum
(loss)
c-value
0.8
0.7
0.6
0.5
0.4
0.3
0.2
0.1
0.0
p-value
2e-03
4e-03
5e-03
7e-03
1e-02
Log-odds
0.5
1.0
1.5
2.0
2.5
3.0
3.5
4.0
Cellular_organisms - ps1
Archaea1 - ps2
Archaea2 - ps3
Archaea3 - ps4
Asgard/Eukaryota - ps5
Eukaryota - ps6
Amorpheae - ps7
Obazoa - ps8
Opisthokonta - ps9
Holozoa - ps10
Filozoa - ps11
Choano./Metazoa - ps12
Metazoa - ps13
Myriazoa - ps14
Parahoxozoa - ps15
Bilateria - ps16
Deuterostomia - ps17
Chordata - ps18
Olfactores - ps19
Vertebrata - ps20
Gnathostomata - ps21
Euteleostomi - ps22
Sarcopterygii - ps23
Tetrapoda - ps24
Amniota - ps25
Mammalia - ps26
Theria - ps27
Placentalia - ps28
Boreoeutheria - ps29
Euarchontoglires - ps30
Euarchonta - ps31
Primates - ps32
Haplorrhini - ps33
Simiiformes - ps34
Catarrhini - ps35
Hominoidea - ps36
Hominidae - ps37
Homininae - ps38
Hominini - ps39
Homo_sapiens - ps40
Phylostratum

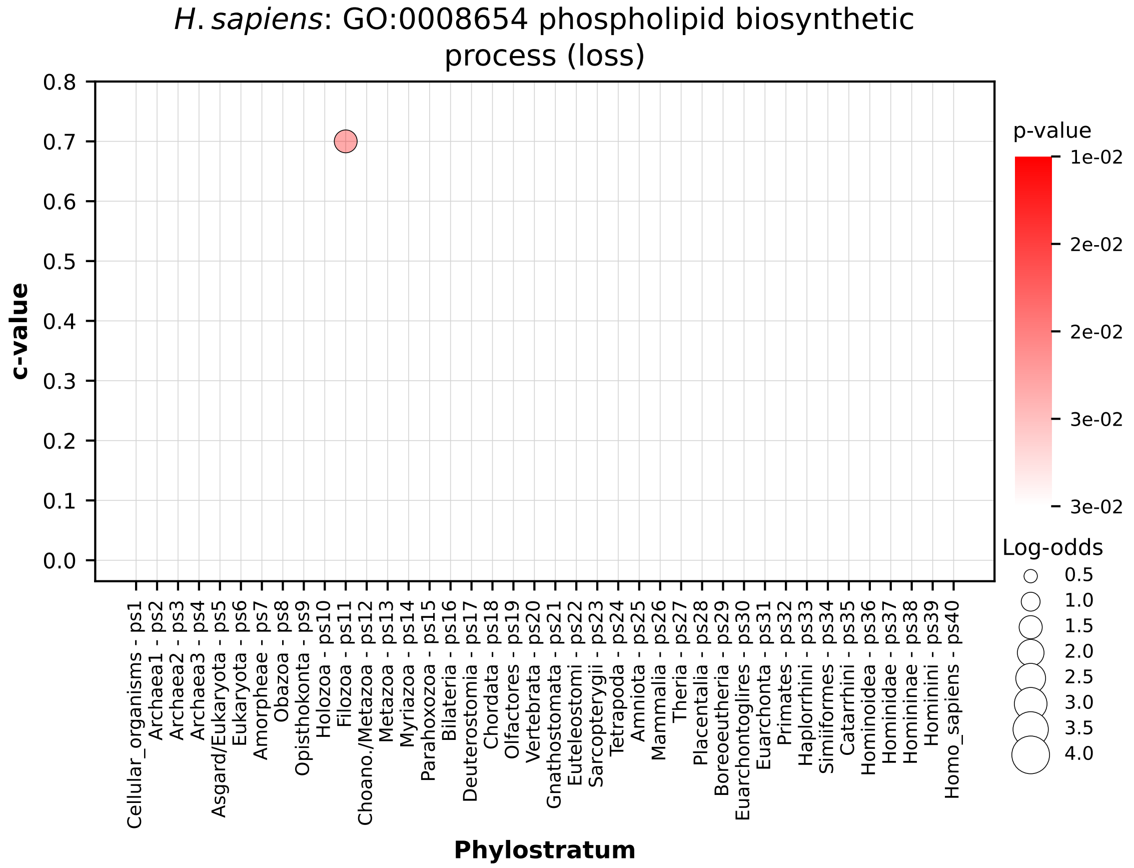

H. sapiens: GO:0008654 phospholipid biosynthetic process (loss)
c-value
0.0
0.1
0.2
0.3
0.4
0.5
0.6
0.7
0.8
Phylostratum
Cellular_organisms - ps1
Archaea1 - ps2
Archaea2 - ps3
Archaea3 - ps4
Asgard/Eukaryota - ps5
Eukaryota - ps6
Amorpheae - ps7
Obazoa - ps8
Opisthokonta - ps9
Holozoa - ps10
Filozoa - ps11
Choano./Metazoa - ps12
Metazoa - ps13
Myriazoa - ps14
Parahoxozoa - ps15
Bilateria - ps16
Deuterostomia - ps17
Chordata - ps18
Olfactores - ps19
Vertebrata - ps20
Gnathostomata - ps21
Euteleostomi - ps22
Sarcopterygii - ps23
Tetrapoda - ps24
Amniota - ps25
Mammalia - ps26
Theria - ps27
Placentalia - ps28
Boreoeutheria - ps29
Euarchontoglires - ps30
Euarchonta - ps31
Primates - ps32
Haplorrhini - ps33
Simiiformes - ps34
Catarrhini - ps35
Hominoidea - ps36
Hominidae - ps37
Homininae - ps38
Hominini - ps39
Homo_sapiens - ps40
p-value
1e-02
2e-02
2e-02
3e-02
3e-02
Log-odds
0.5
1.0
1.5
2.0
2.5
3.0
3.5
4.0

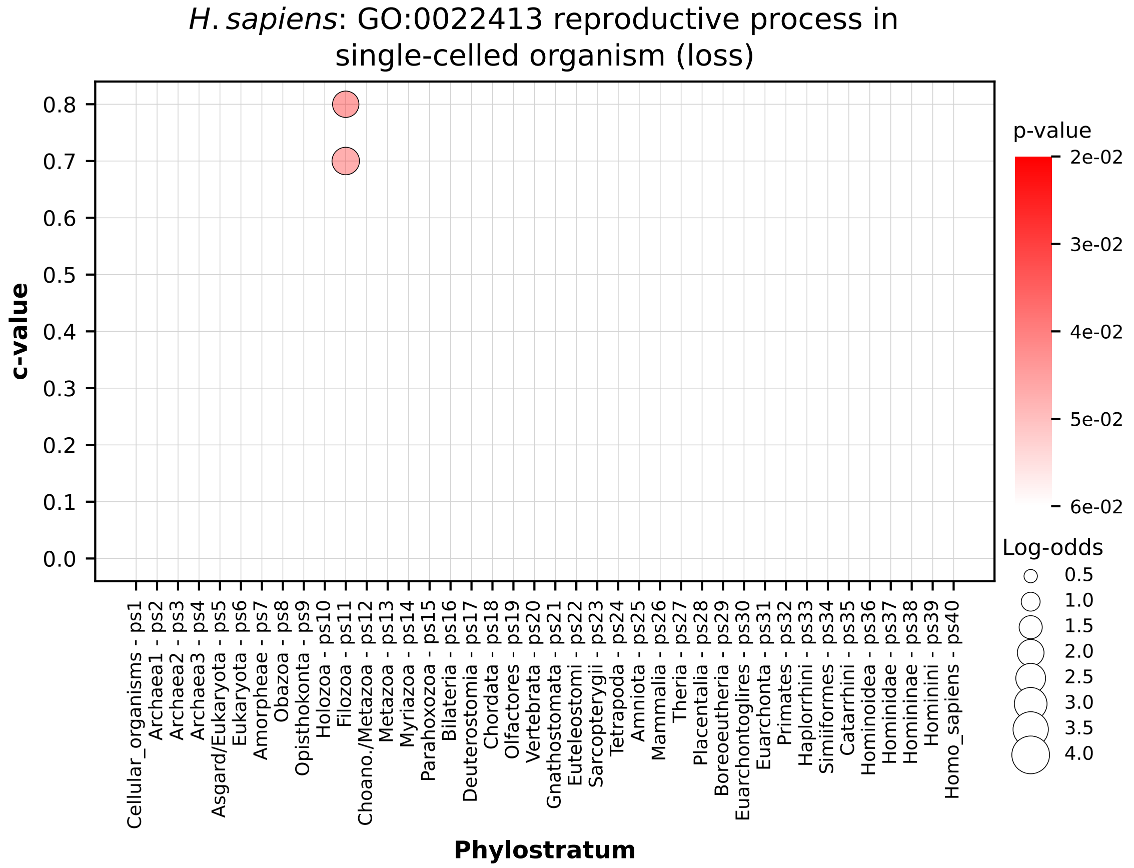
H. sapiens: GO:0022413 reproductive process in single-celled organism (loss)
c-value
0.0
0.1
0.2
0.3
0.4
0.5
0.6
0.7
0.8
Phylostratum
Cellular_organisms - ps1
Archaea1 - ps2
Archaea2 - ps3
Archaea3 - ps4
Asgard/Eukaryota - ps5
Eukaryota - ps6
Amorpheae - ps7
Obazoa - ps8
Opisthokonta - ps9
Holozoa - ps10
Filozoa - ps11
Choano./Metazoa - ps12
Metazoa - ps13
Myriazoa - ps14
Parahoxozoa - ps15
Bilateria - ps16
Deuterostomia - ps17
Chordata - ps18
Olfactores - ps19
Vertebrata - ps20
Gnathostomata - ps21
Euteleostomi - ps22
Sarcopterygii - ps23
Tetrapoda - ps24
Amniota - ps25
Mammalia - ps26
Theria - ps27
Placentalia - ps28
Boreoeutheria - ps29
Euarchontoglires - ps30
Euarchonta - ps31
Primates - ps32
Haplorrhini - ps33
Simiiformes - ps34
Catarrhini - ps35
Hominoidea - ps36
Hominidae - ps37
Homininae - ps38
Hominini - ps39
Homo_sapiens - ps40
p-value
2e-02
3e-02
4e-02
5e-02
6e-02
Log-odds
0.5
1.0
1.5
2.0
2.5
3.0
3.5
4.0

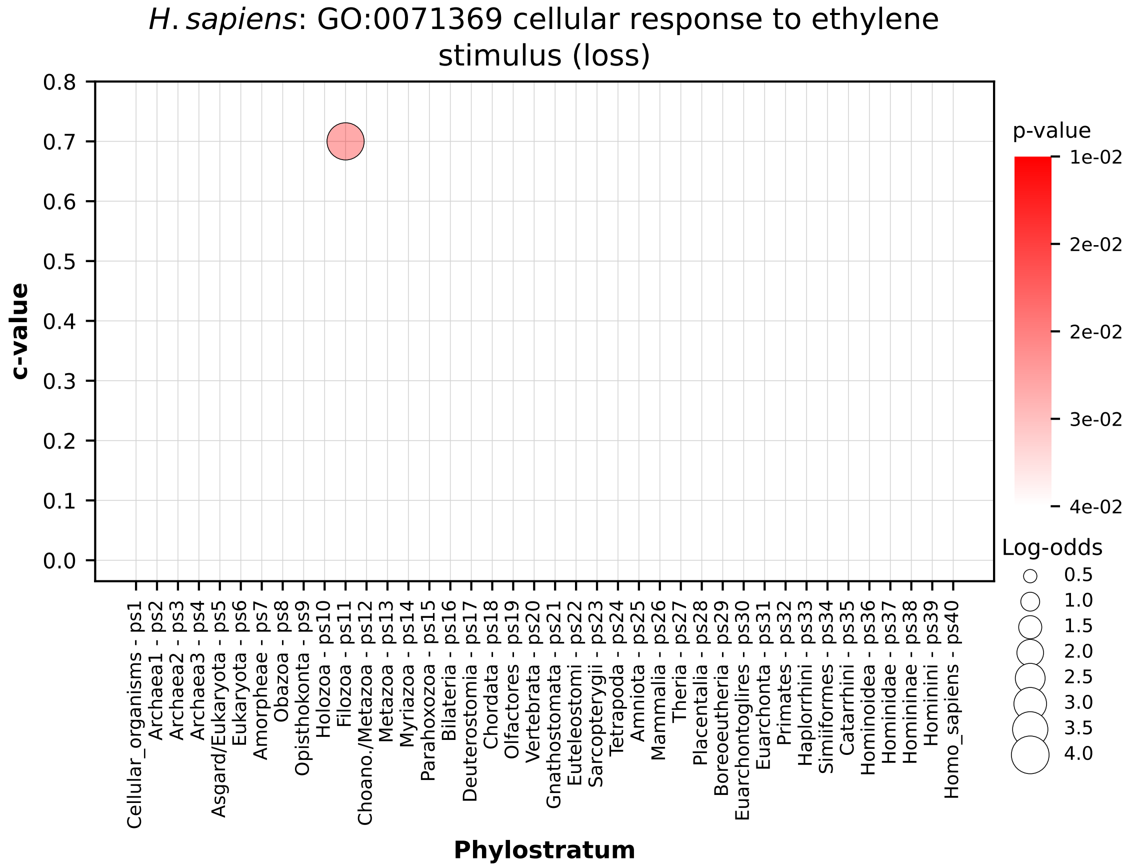

H. sapiens: GO:0071369 cellular response to ethylene stimulus (loss)
c-value
0.8
0.7
0.6
0.5
0.4
0.3
0.2
0.1
0.0
Phylostratum
Cellular_organisms - ps1
Archaea1 - ps2
Archaea2 - ps3
Archaea3 - ps4
Asgard/Eukaryota - ps5
Eukaryota - ps6
Amorpheae - ps7
Obazoa - ps8
Opisthokonta - ps9
Holozoa - ps10
Filozoa - ps11
Choano./Metazoa - ps12
Metazoa - ps13
Myriazoa - ps14
Parahoxozoa - ps15
Bilateria - ps16
Deuterostomia - ps17
Chordata - ps18
Olfactores - ps19
Vertebrata - ps20
Gnathostomata - ps21
Euteleostomi - ps22
Sarcopterygii - ps23
Tetrapoda - ps24
Amniota - ps25
Mammalia - ps26
Theria - ps27
Placentalia - ps28
Boreoeutheria - ps29
Euarchontoglires - ps30
Euarchonta - ps31
Primates - ps32
Haplorrhini - ps33
Simiiformes - ps34
Catarrhini - ps35
Hominoidea - ps36
Hominidae - ps37
Homininae - ps38
Hominini - ps39
Homo_sapiens - ps40
p-value
1e-02
2e-02
2e-02
3e-02
4e-02
Log-odds
0.5
1.0
1.5
2.0
2.5
3.0
3.5
4.0

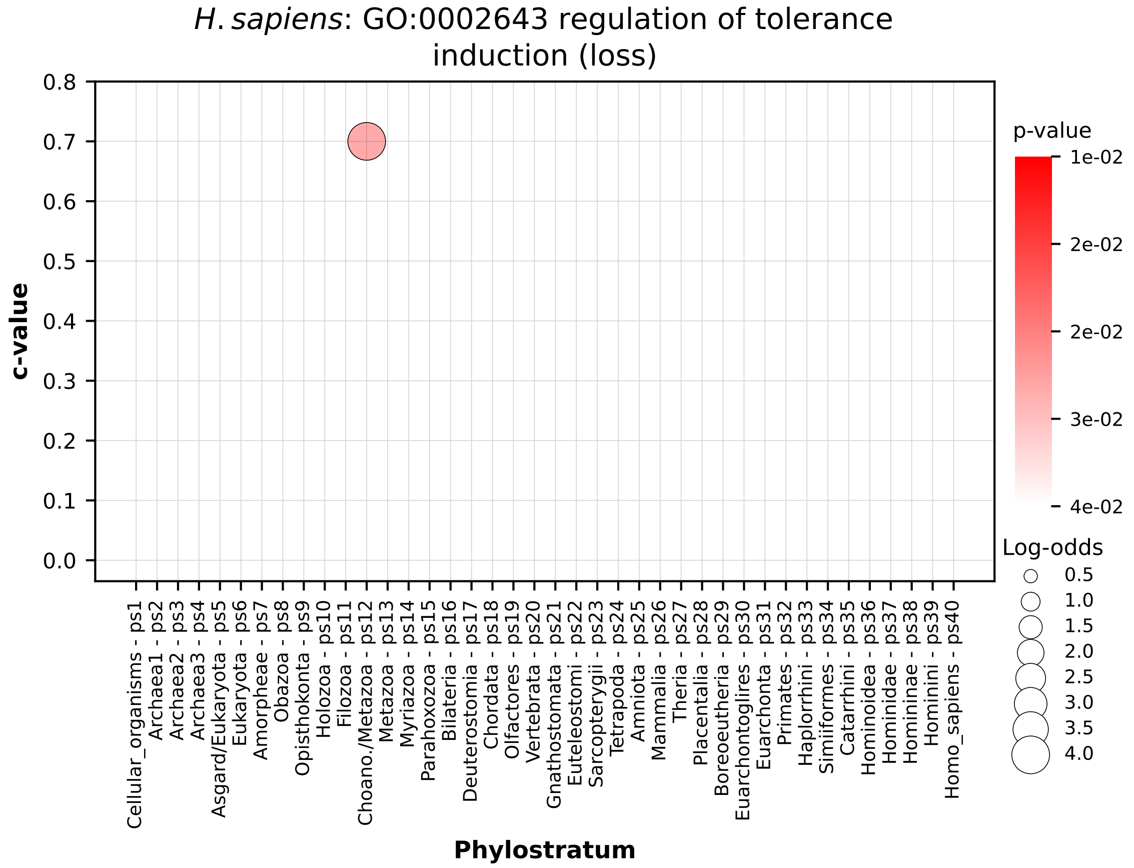

H. sapiens: GO:0002643 regulation of tolerance induction (loss)
c-value
0.8
0.7
0.6
0.5
0.4
0.3
0.2
0.1
0.0
p-value
1e-02
2e-02
2e-02
3e-02
4e-02
Log-odds
0.5
1.0
1.5
2.0
2.5
3.0
3.5
4.0
Cellular_organisms - ps1
Archaea1 - ps2
Archaea2 - ps3
Archaea3 - ps4
Asgard/Eukaryota - ps5
Eukaryota - ps6
Amorpheae - ps7
Obazoa - ps8
Opisthokonta - ps9
Holozoa - ps10
Filozoa - ps11
Choano./Metazoa - ps12
Metazoa - ps13
Myriazoa - ps14
Parahoxozoa - ps15
Bilateria - ps16
Deuterostomia - ps17
Chordata - ps18
Olfactores - ps19
Vertebrata - ps20
Gnathostomata - ps21
Euteleostomi - ps22
Sarcopterygii - ps23
Tetrapoda - ps24
Amniota - ps25
Mammalia - ps26
Theria - ps27
Placentalia - ps28
Boreoeutheria - ps29
Euarchontoglires - ps30
Euarchonta - ps31
Primates - ps32
Haplorrhini - ps33
Simiiformes - ps34
Catarrhini - ps35
Hominoidea - ps36
Hominidae - ps37
Homininae - ps38
Hominini - ps39
Homo_sapiens - ps40
Phylostratum

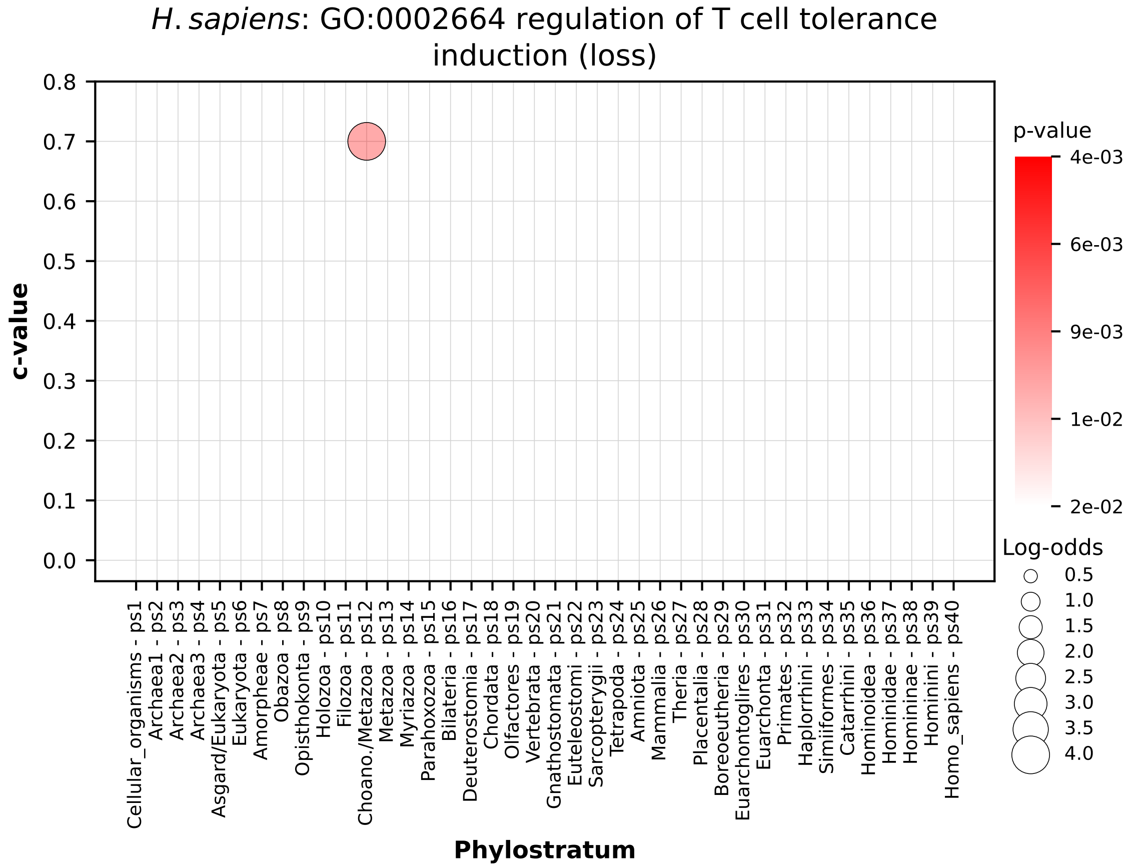

H. sapiens: GO:0002664 regulation of T cell tolerance induction (loss)
c-value
0.8
0.7
0.6
0.5
0.4
0.3
0.2
0.1
0.0
Phylostratum
Cellular_organisms - ps1
Archaea1 - ps2
Archaea2 - ps3
Archaea3 - ps4
Asgard/Eukaryota - ps5
Eukaryota - ps6
Amorpheae - ps7
Obazoa - ps8
Opisthokonta - ps9
Holozoa - ps10
Filozoa - ps11
Choano./Metazoa - ps12
Metazoa - ps13
Myriazoa - ps14
Parahoxozoa - ps15
Bilateria - ps16
Deuterostomia - ps17
Chordata - ps18
Olfactores - ps19
Vertebrata - ps20
Gnathostomata - ps21
Euteleostomi - ps22
Sarcopterygii - ps23
Tetrapoda - ps24
Amniota - ps25
Mammalia - ps26
Theria - ps27
Placentalia - ps28
Boreoeutheria - ps29
Euarchontoglires - ps30
Euarchonta - ps31
Primates - ps32
Haplorrhini - ps33
Simiiformes - ps34
Catarrhini - ps35
Hominoidea - ps36
Hominidae - ps37
Homininae - ps38
Hominini - ps39
Homo_sapiens - ps40
p-value
4e-03
6e-03
9e-03
1e-02
2e-02
Log-odds
0.5
1.0
1.5
2.0
2.5
3.0
3.5
4.0

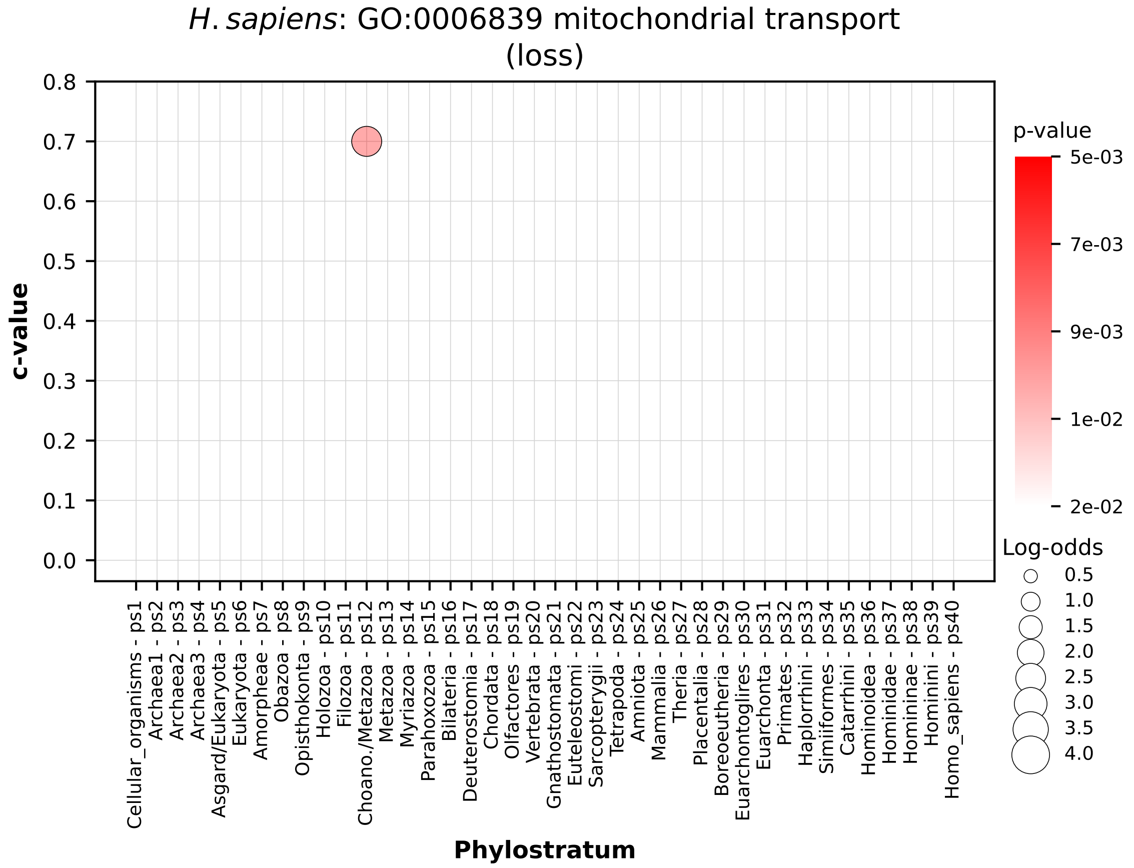
H. sapiens: GO:0006839 mitochondrial transport
(loss)
c-value
0.8
0.7
0.6
0.5
0.4
0.3
0.2
0.1
0.0
Phylostratum
Cellular_organisms - ps1
Archaea1 - ps2
Archaea2 - ps3
Archaea3 - ps4
Asgard/Eukaryota - ps5
Eukaryota - ps6
Amorpheae - ps7
Obazoa - ps8
Opisthokonta - ps9
Holozoa - ps10
Filozoa - ps11
Choano./Metazoa - ps12
Metazoa - ps13
Myriazoa - ps14
Parahoxozoa - ps15
Bilateria - ps16
Deuterostomia - ps17
Chordata - ps18
Olfactores - ps19
Vertebrata - ps20
Gnathostomata - ps21
Euteleostomi - ps22
Sarcopterygii - ps23
Tetrapoda - ps24
Amniota - ps25
Mammalia - ps26
Theria - ps27
Placentalia - ps28
Boreoeutheria - ps29
Euarchontoglires - ps30
Euarchonta - ps31
Primates - ps32
Haplorrhini - ps33
Simiiformes - ps34
Catarrhini - ps35
Hominoidea - ps36
Hominidae - ps37
Homininae - ps38
Hominini - ps39
Homo_sapiens - ps40
p-value
5e-03
7e-03
9e-03
1e-02
2e-02
Log-odds
0.5
1.0
1.5
2.0
2.5
3.0
3.5
4.0

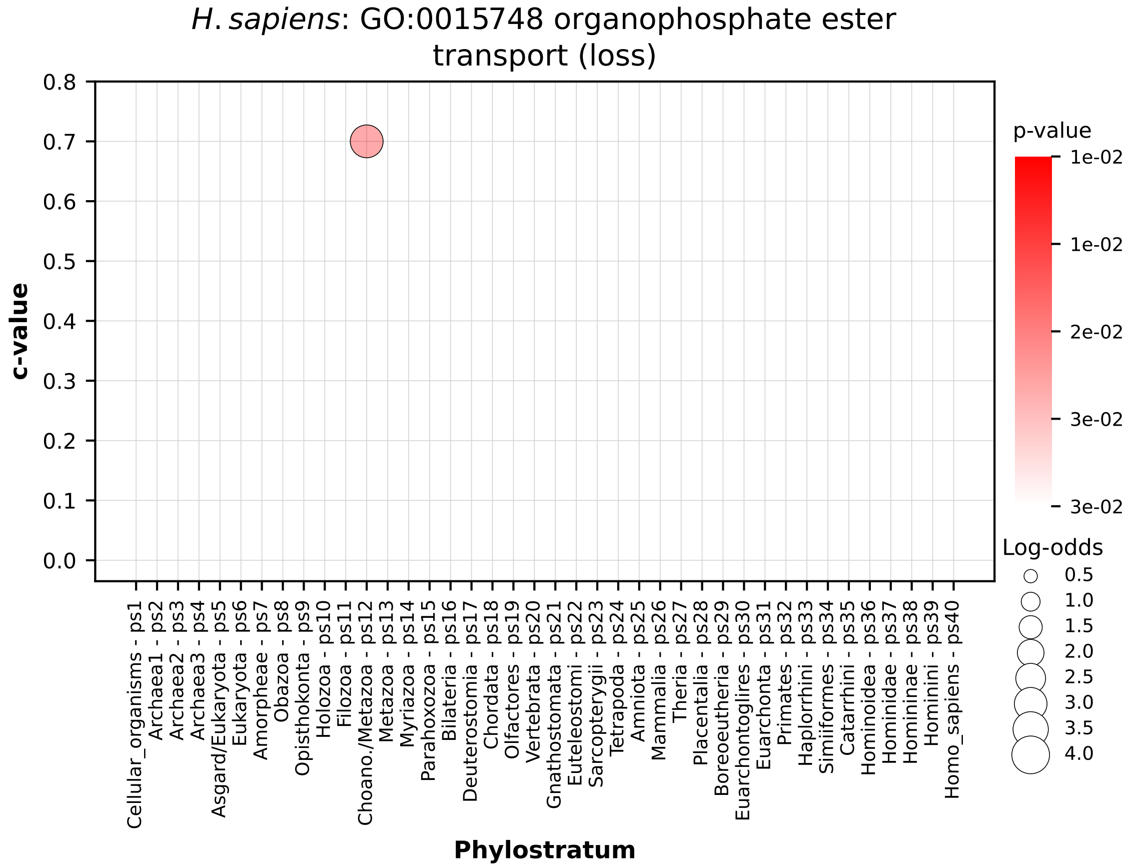

H. sapiens: GO:0015748 organophosphate ester transport (loss)
c-value
0.8
0.7
0.6
0.5
0.4
0.3
0.2
0.1
0.0
p-value
1e-02
1e-02
2e-02
3e-02
3e-02
Log-odds
0.5
1.0
1.5
2.0
2.5
3.0
3.5
4.0
Cellular_organisms - ps1
Archaea1 - ps2
Archaea2 - ps3
Archaea3 - ps4
Asgard/Eukaryota - ps5
Eukaryota - ps6
Amorpheae - ps7
Obazoa - ps8
Opisthokonta - ps9
Holozoa - ps10
Filozoa - ps11
Choano./Metazoa - ps12
Metazoa - ps13
Myriazoa - ps14
Parahoxozoa - ps15
Bilateria - ps16
Deuterostomia - ps17
Chordata - ps18
Olfactores - ps19
Vertebrata - ps20
Gnathostomata - ps21
Euteleostomi - ps22
Sarcopterygii - ps23
Tetrapoda - ps24
Amniota - ps25
Mammalia - ps26
Theria - ps27
Placentalia - ps28
Boreoeutheria - ps29
Euarchontoglires - ps30
Euarchonta - ps31
Primates - ps32
Haplorrhini - ps33
Simiiformes - ps34
Catarrhini - ps35
Hominoidea - ps36
Hominidae - ps37
Homininae - ps38
Hominini - ps39
Homo_sapiens - ps40
Phylostratum

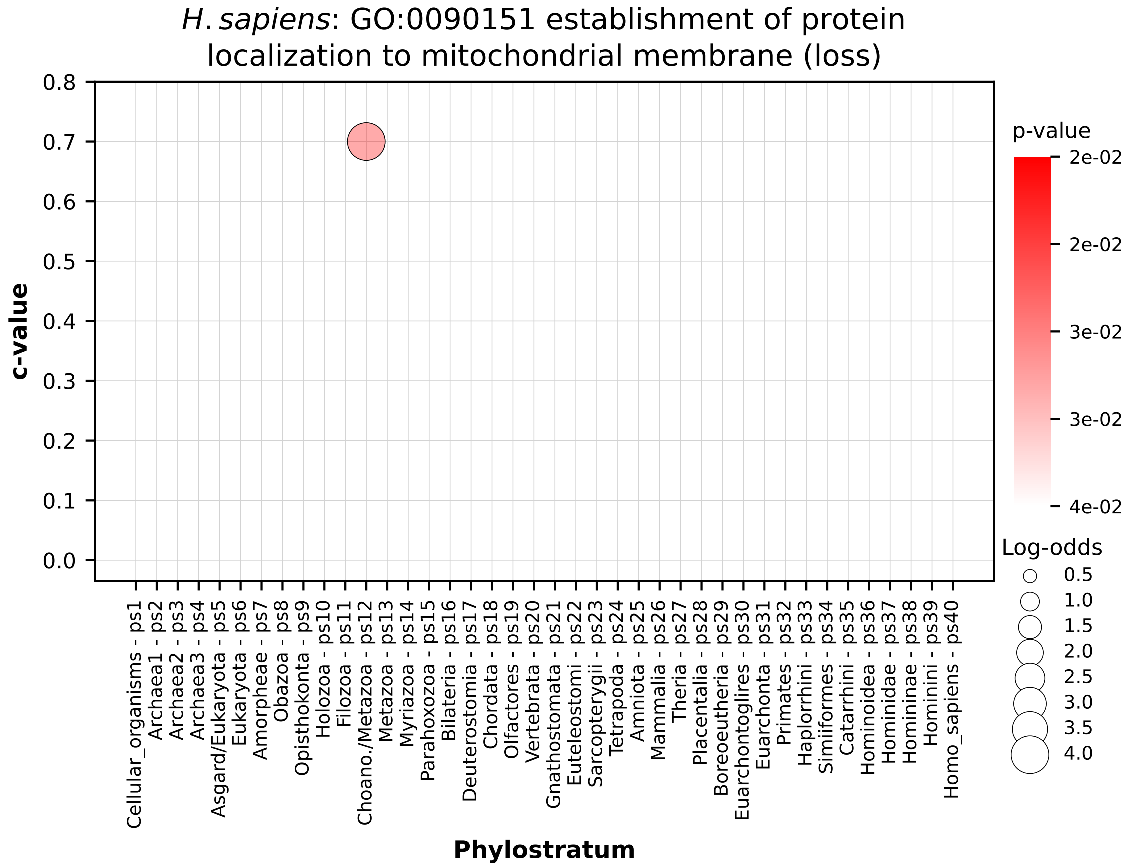

H. sapiens: GO:0090151 establishment of protein localization to mitochondrial membrane (loss)
c-value
0.8
0.7
0.6
0.5
0.4
0.3
0.2
0.1
0.0
Phylostratum
Cellular_organisms - ps1
Archaea1 - ps2
Archaea2 - ps3
Archaea3 - ps4
Asgard/Eukaryota - ps5
Eukaryota - ps6
Amorpheae - ps7
Obazoa - ps8
Opisthokonta - ps9
Holozoa - ps10
Filozoa - ps11
Choano./Metazoa - ps12
Metazoa - ps13
Myriazoa - ps14
Parahoxozoa - ps15
Bilateria - ps16
Deuterostomia - ps17
Chordata - ps18
Olfactores - ps19
Vertebrata - ps20
Gnathostomata - ps21
Euteleostomi - ps22
Sarcopterygii - ps23
Tetrapoda - ps24
Amniota - ps25
Mammalia - ps26
Theria - ps27
Placentalia - ps28
Boreoeutheria - ps29
Euarchontoglires - ps30
Euarchonta - ps31
Primates - ps32
Haplorrhini - ps33
Simiiformes - ps34
Catarrhini - ps35
Hominoidea - ps36
Hominidae - ps37
Homininae - ps38
Hominini - ps39
Homo_sapiens - ps40
p-value
2e-02
2e-02
3e-02
3e-02
4e-02
Log-odds
0.5
1.0
1.5
2.0
2.5
3.0
3.5
4.0

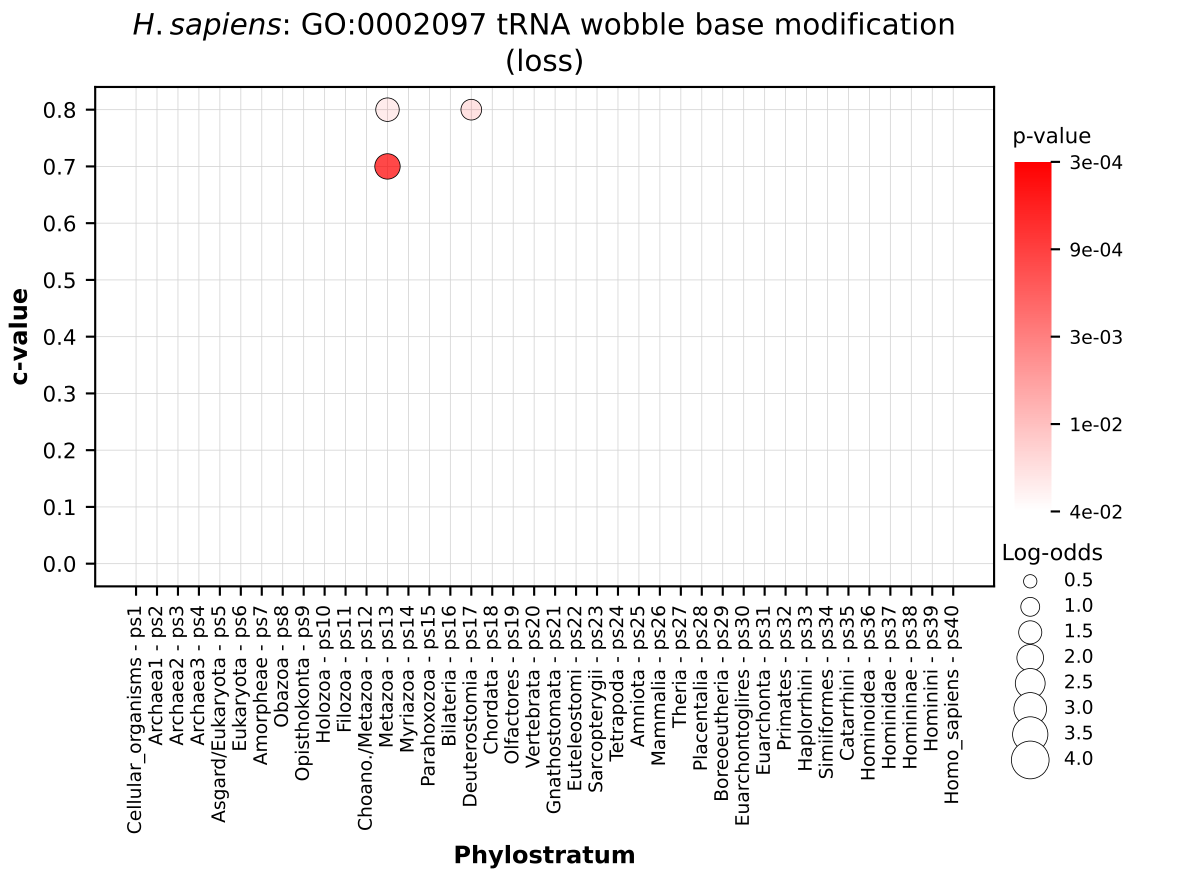

H. sapiens: GO:0002097 tRNA wobble base modification (loss)
c-value
0.0
0.1
0.2
0.3
0.4
0.5
0.6
0.7
0.8
Phylostratum
Cellular_organisms - ps1
Archaea1 - ps2
Archaea2 - ps3
Archaea3 - ps4
Asgard/Eukaryota - ps5
Eukaryota - ps6
Amorpheae - ps7
Obazoa - ps8
Opisthokonta - ps9
Holozoa - ps10
Filozoa - ps11
Choano./Metazoa - ps12
Metazoa - ps13
Myriazoa - ps14
Parahoxozoa - ps15
Bilateria - ps16
Deuterostomia - ps17
Chordata - ps18
Olfactores - ps19
Vertebrata - ps20
Gnathostomata - ps21
Euteleostomi - ps22
Sarcopterygii - ps23
Tetrapoda - ps24
Amniota - ps25
Mammalia - ps26
Theria - ps27
Placentalia - ps28
Boreoeutheria - ps29
Euarchontoglires - ps30
Euarchonta - ps31
Primates - ps32
Haplorrhini - ps33
Simiiformes - ps34
Catarrhini - ps35
Hominoidea - ps36
Hominidae - ps37
Homininae - ps38
Hominini - ps39
Homo_sapiens - ps40
p-value
3e-04
9e-04
3e-03
1e-02
4e-02
Log-odds
0.5
1.0
1.5
2.0
2.5
3.0
3.5
4.0

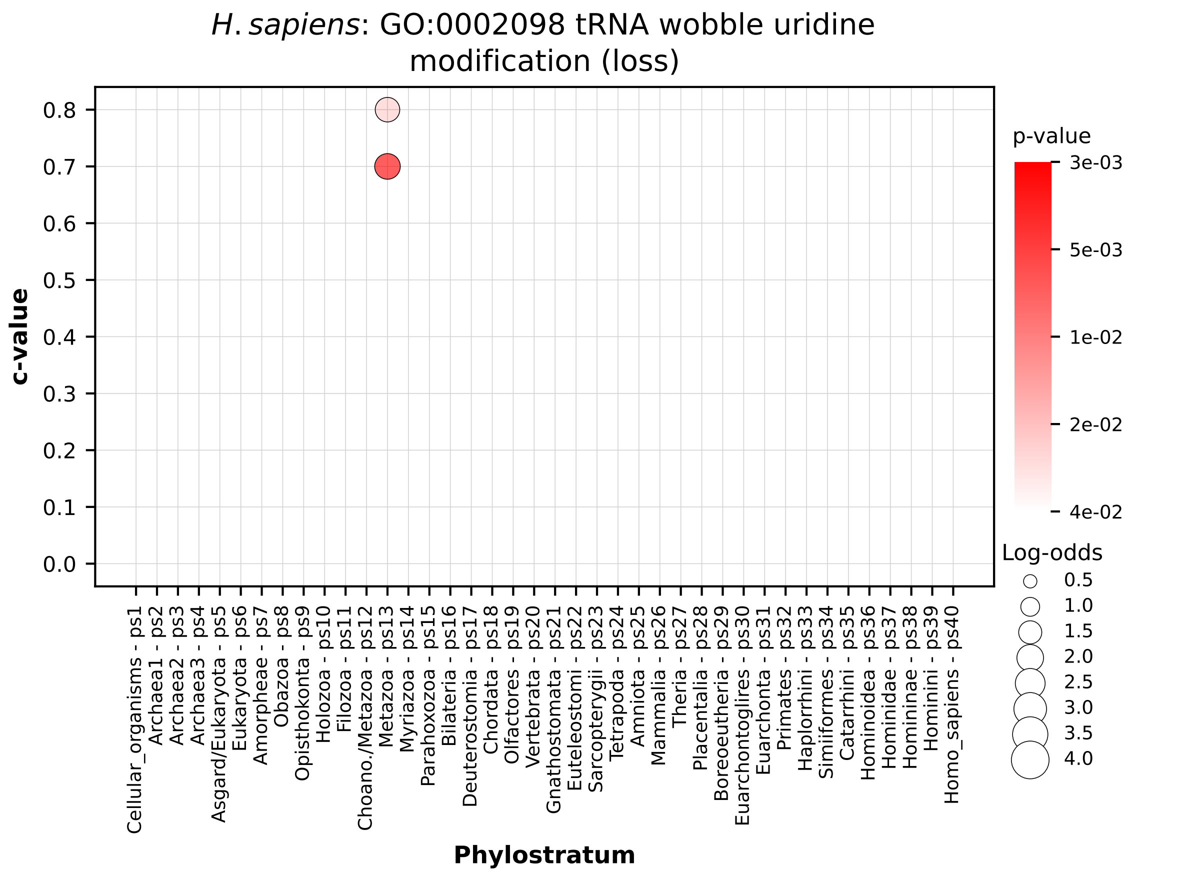
H. sapiens: GO:0002098 tRNA wobble uridine modification (loss)
c-value
0.0
0.1
0.2
0.3
0.4
0.5
0.6
0.7
0.8
Phylostratum
Cellular_organisms - ps1
Archaea1 - ps2
Archaea2 - ps3
Archaea3 - ps4
Asgard/Eukaryota - ps5
Eukaryota - ps6
Amorpheae - ps7
Obazoa - ps8
Opisthokonta - ps9
Holozoa - ps10
Filozoa - ps11
Choano./Metazoa - ps12
Metazoa - ps13
Myriazoa - ps14
Parahoxozoa - ps15
Bilateria - ps16
Deuterostomia - ps17
Chordata - ps18
Olfactores - ps19
Vertebrata - ps20
Gnathostomata - ps21
Euteleostomi - ps22
Sarcopterygii - ps23
Tetrapoda - ps24
Amniota - ps25
Mammalia - ps26
Theria - ps27
Placentalia - ps28
Boreoeutheria - ps29
Euarchontoglires - ps30
Euarchonta - ps31
Primates - ps32
Haplorrhini - ps33
Simiiformes - ps34
Catarrhini - ps35
Hominoidea - ps36
Hominidae - ps37
Homininae - ps38
Hominini - ps39
Homo_sapiens - ps40
p-value
3e-03
5e-03
1e-02
2e-02
4e-02
Log-odds
0.5
1.0
1.5
2.0
2.5
3.0
3.5
4.0

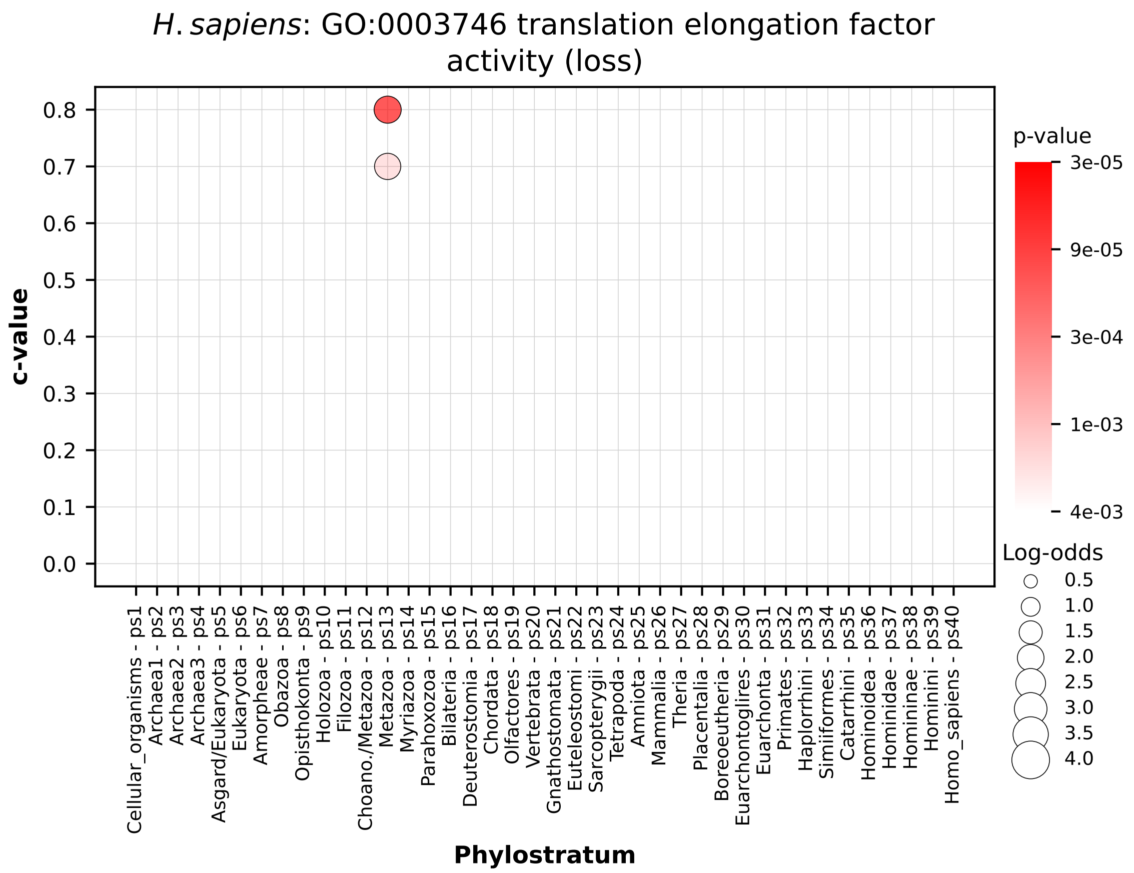

H. sapiens: GO:0003746 translation elongation factor activity (loss)
c-value
0.0
0.1
0.2
0.3
0.4
0.5
0.6
0.7
0.8
p-value
3e-05
9e-05
3e-04
1e-03
4e-03
Log-odds
0.5
1.0
1.5
2.0
2.5
3.0
3.5
4.0
Cellular_organisms - ps1
Archaea1 - ps2
Archaea2 - ps3
Archaea3 - ps4
Asgard/Eukaryota - ps5
Eukaryota - ps6
Amorpheae - ps7
Obazoa - ps8
Opisthokonta - ps9
Holozoa - ps10
Filozoa - ps11
Choano./Metazoa - ps12
Metazoa - ps13
Myriazoa - ps14
Parahoxozoa - ps15
Bilateria - ps16
Deuterostomia - ps17
Chordata - ps18
Olfactores - ps19
Vertebrata - ps20
Gnathostomata - ps21
Euteleostomi - ps22
Sarcopterygii - ps23
Tetrapoda - ps24
Amniota - ps25
Mammalia - ps26
Theria - ps27
Placentalia - ps28
Boreoeutheria - ps29
Euarchontoglires - ps30
Euarchonta - ps31
Primates - ps32
Haplorrhini - ps33
Simiiformes - ps34
Catarrhini - ps35
Hominoidea - ps36
Hominidae - ps37
Homininae - ps38
Hominini - ps39
Homo_sapiens - ps40
Phylostratum

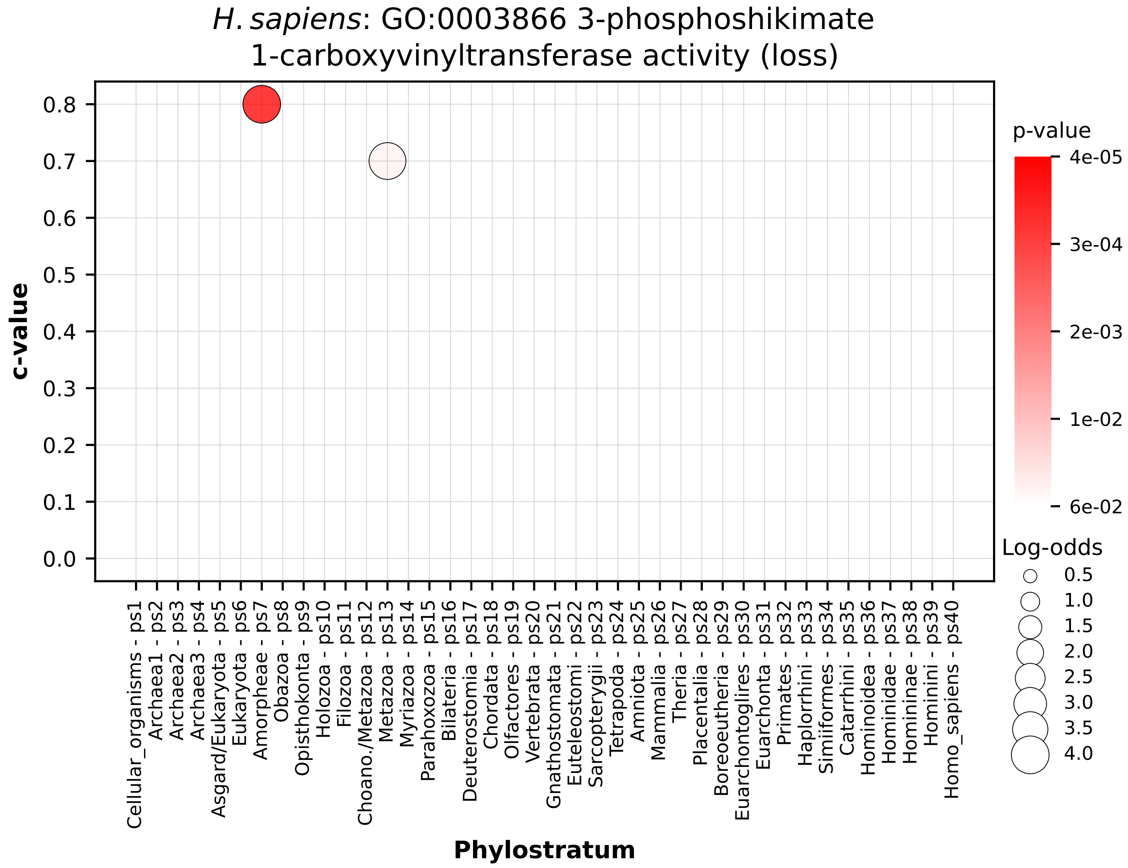
H. sapiens: GO:0003866 3-phosphoshikimate 1-carboxyvinyltransferase activity (loss)
c-value
0.0
0.1
0.2
0.3
0.4
0.5
0.6
0.7
0.8
p-value
4e-05
3e-04
2e-03
1e-02
6e-02
Log-odds
0.5
1.0
1.5
2.0
2.5
3.0
3.5
4.0
Cellular_organisms - ps1
Archaea1 - ps2
Archaea2 - ps3
Archaea3 - ps4
Asgard/Eukaryota - ps5
Eukaryota - ps6
Amorpheae - ps7
Obazoa - ps8
Opisthokonta - ps9
Holozoa - ps10
Filozoa - ps11
Choano./Metazoa - ps12
Metazoa - ps13
Myriazoa - ps14
Parahoxozoa - ps15
Bilateria - ps16
Deuterostomia - ps17
Chordata - ps18
Olfactores - ps19
Vertebrata - ps20
Gnathostomata - ps21
Euteleostomi - ps22
Sarcopterygii - ps23
Tetrapoda - ps24
Amniota - ps25
Mammalia - ps26
Theria - ps27
Placentalia - ps28
Boreoeutheria - ps29
Euarchontoglires - ps30
Euarchonta - ps31
Primates - ps32
Haplorrhini - ps33
Simiiformes - ps34
Catarrhini - ps35
Hominoidea - ps36
Hominidae - ps37
Homininae - ps38
Hominini - ps39
Homo_sapiens - ps40
Phylostratum

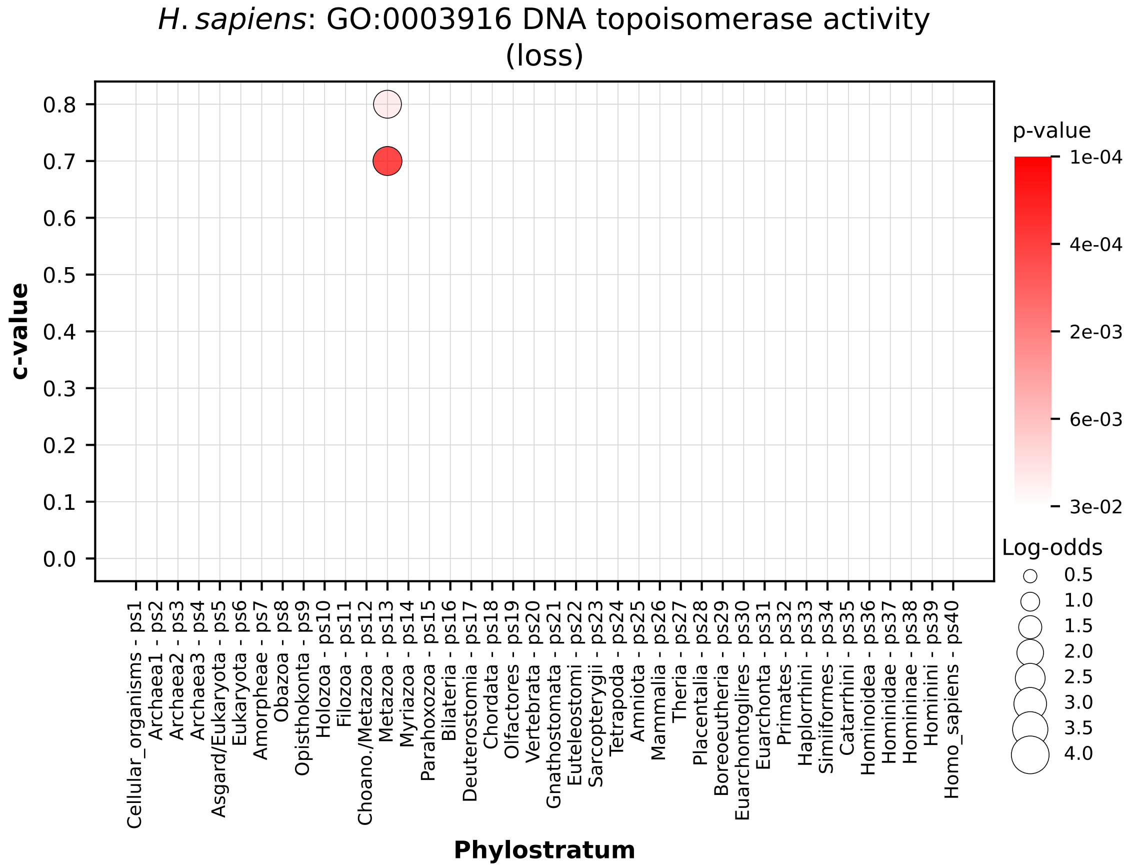

H. sapiens: GO:0003916 DNA topoisomerase activity (loss)
c-value
0.8
0.7
0.6
0.5
0.4
0.3
0.2
0.1
0.0
p-value
1e-04
4e-04
2e-03
6e-03
3e-02
Log-odds
0.5
1.0
1.5
2.0
2.5
3.0
3.5
4.0
Cellular_organisms - ps1
Archaea1 - ps2
Archaea2 - ps3
Archaea3 - ps4
Asgard/Eukaryota - ps5
Eukaryota - ps6
Amorpheae - ps7
Obazoa - ps8
Opisthokonta - ps9
Holozoa - ps10
Filozoa - ps11
Choano./Metazoa - ps12
Metazoa - ps13
Myriazoa - ps14
Parahoxozoa - ps15
Bilateria - ps16
Deuterostomia - ps17
Chordata - ps18
Olfactores - ps19
Vertebrata - ps20
Gnathostomata - ps21
Euteleostomi - ps22
Sarcopterygii - ps23
Tetrapoda - ps24
Amniota - ps25
Mammalia - ps26
Theria - ps27
Placentalia - ps28
Boreoeutheria - ps29
Euarchontoglires - ps30
Euarchonta - ps31
Primates - ps32
Haplorrhini - ps33
Simiiformes - ps34
Catarrhini - ps35
Hominoidea - ps36
Hominidae - ps37
Homininae - ps38
Hominini - ps39
Homo_sapiens - ps40
Phylostratum

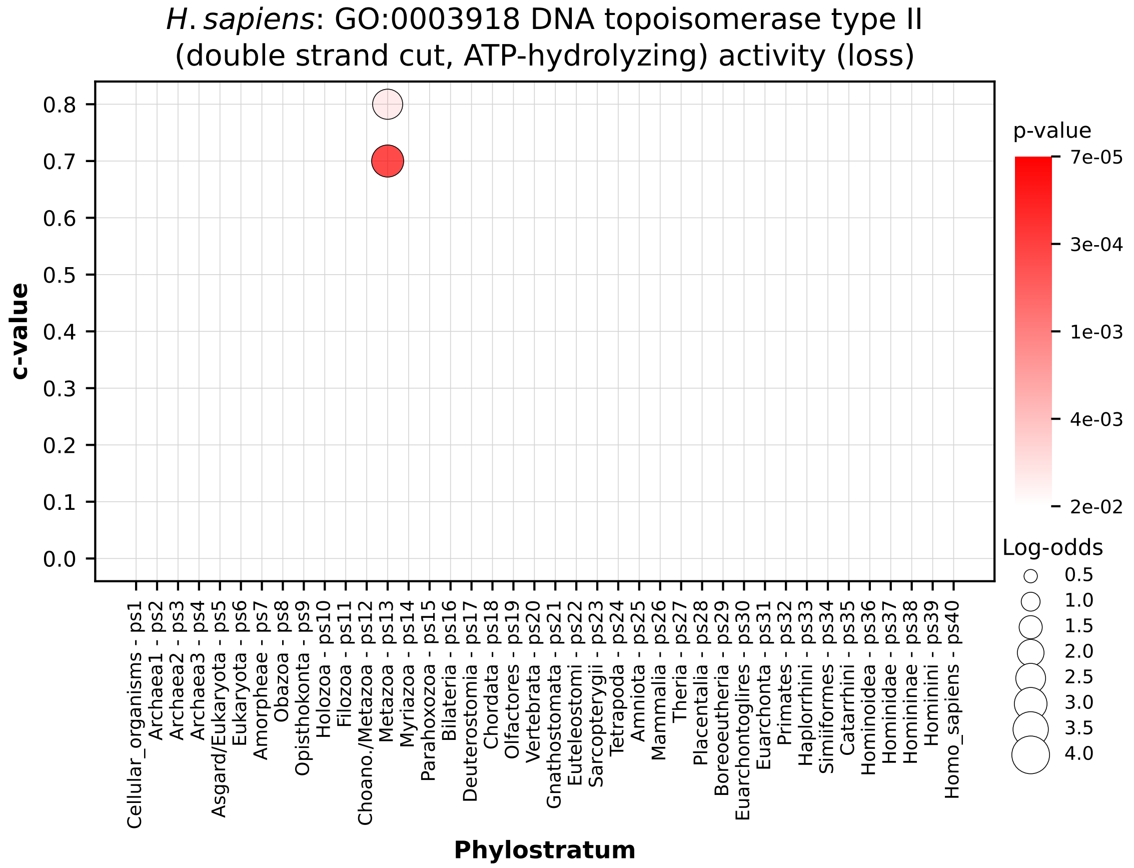

H. sapiens: GO:0003918 DNA topoisomerase type II
(double strand cut, ATP-hydrolyzing) activity (loss)
c-value
0.0
0.1
0.2
0.3
0.4
0.5
0.6
0.7
0.8
p-value
7e-05
3e-04
1e-03
4e-03
2e-02
Log-odds
0.5
1.0
1.5
2.0
2.5
3.0
3.5
4.0
Cellular_organisms - ps1
Archaea1 - ps2
Archaea2 - ps3
Archaea3 - ps4
Asgard/Eukaryota - ps5
Eukaryota - ps6
Amorpheae - ps7
Obazoa - ps8
Opisthokonta - ps9
Holozoa - ps10
Filozoa - ps11
Choano./Metazoa - ps12
Metazoa - ps13
Myriazoa - ps14
Parahoxozoa - ps15
Bilateria - ps16
Deuterostomia - ps17
Chordata - ps18
Olfactores - ps19
Vertebrata - ps20
Gnathostomata - ps21
Euteleostomi - ps22
Sarcopterygii - ps23
Tetrapoda - ps24
Amniota - ps25
Mammalia - ps26
Theria - ps27
Placentalia - ps28
Boreoeutheria - ps29
Euarchontoglires - ps30
Euarchonta - ps31
Primates - ps32
Haplorrhini - ps33
Simiiformes - ps34
Catarrhini - ps35
Hominoidea - ps36
Hominidae - ps37
Homininae - ps38
Hominini - ps39
Homo_sapiens - ps40
Phylostratum

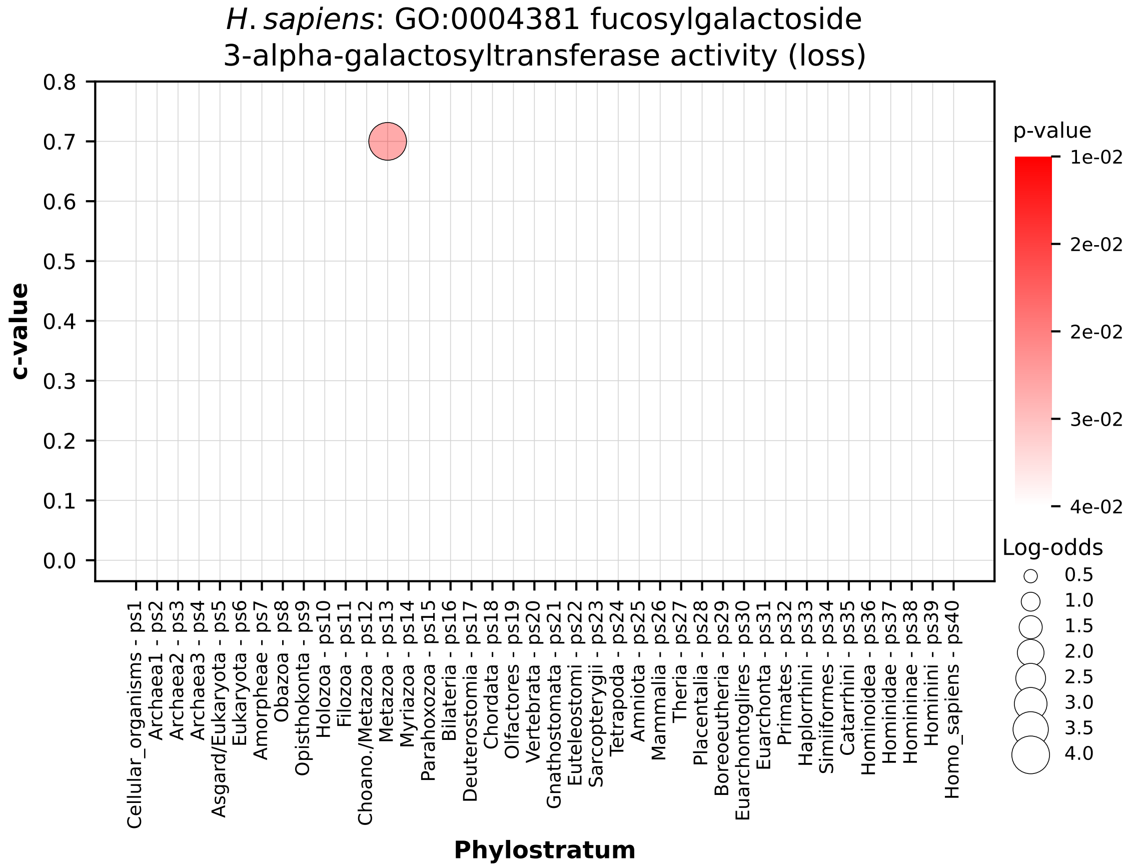
H. sapiens: GO:0004381 fucosylgalactoside 3-alpha-galactosyltransferase activity (loss)
c-value
0.8
0.7
0.6
0.5
0.4
0.3
0.2
0.1
0.0
p-value
1e-02
2e-02
2e-02
3e-02
4e-02
Log-odds
0.5
1.0
1.5
2.0
2.5
3.0
3.5
4.0
Cellular_organisms - ps1
Archaea1 - ps2
Archaea2 - ps3
Archaea3 - ps4
Asgard/Eukaryota - ps5
Eukaryota - ps6
Amorpheae - ps7
Obazoa - ps8
Opisthokonta - ps9
Holozoa - ps10
Filozoa - ps11
Choano./Metazoa - ps12
Metazoa - ps13
Myriazoa - ps14
Parahoxozoa - ps15
Bilateria - ps16
Deuterostomia - ps17
Chordata - ps18
Olfactores - ps19
Vertebrata - ps20
Gnathostomata - ps21
Euteleostomi - ps22
Sarcopterygii - ps23
Tetrapoda - ps24
Amniota - ps25
Mammalia - ps26
Theria - ps27
Placentalia - ps28
Boreoeutheria - ps29
Euarchontoglires - ps30
Euarchonta - ps31
Primates - ps32
Haplorrhini - ps33
Simiiformes - ps34
Catarrhini - ps35
Hominoidea - ps36
Hominidae - ps37
Homininae - ps38
Hominini - ps39
Homo_sapiens - ps40
Phylostratum

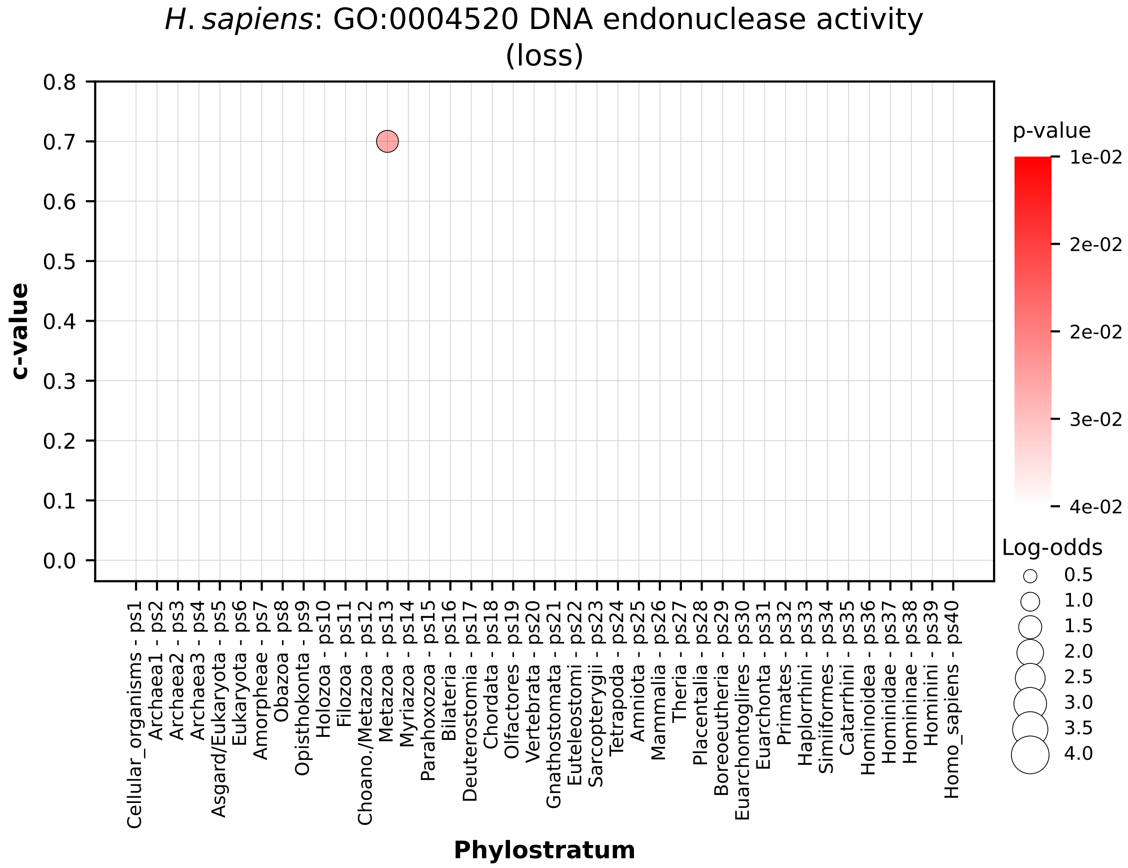

H. sapiens: GO:0004520 DNA endonuclease activity (loss)
c-value
0.8
0.7
0.6
0.5
0.4
0.3
0.2
0.1
0.0
p-value
1e-02
2e-02
2e-02
3e-02
4e-02
Log-odds
0.5
1.0
1.5
2.0
2.5
3.0
3.5
4.0
Cellular_organisms - ps1
Archaea1 - ps2
Archaea2 - ps3
Archaea3 - ps4
Asgard/Eukaryota - ps5
Eukaryota - ps6
Amorpheae - ps7
Obazoa - ps8
Opisthokonta - ps9
Holozoa - ps10
Filozoa - ps11
Choano./Metazoa - ps12
Metazoa - ps13
Myriazoa - ps14
Parahoxozoa - ps15
Bilateria - ps16
Deuterostomia - ps17
Chordata - ps18
Olfactores - ps19
Vertebrata - ps20
Gnathostomata - ps21
Euteleostomi - ps22
Sarcopterygii - ps23
Tetrapoda - ps24
Amniota - ps25
Mammalia - ps26
Theria - ps27
Placentalia - ps28
Boreoeutheria - ps29
Euarchontoglires - ps30
Euarchonta - ps31
Primates - ps32
Haplorrhini - ps33
Simiiformes - ps34
Catarrhini - ps35
Hominoidea - ps36
Hominidae - ps37
Homininae - ps38
Hominini - ps39
Homo_sapiens - ps40
Phylostratum

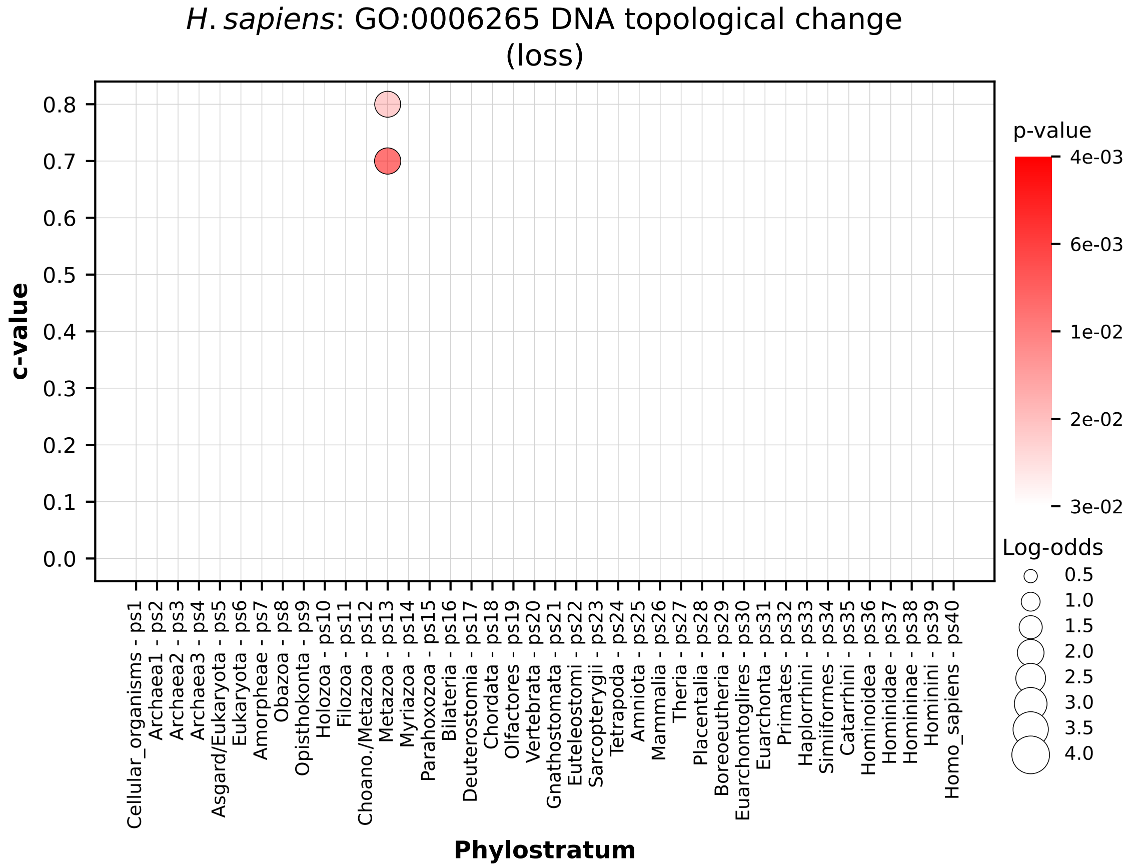

H. sapiens: GO:0006265 DNA topological change (loss)
c-value
0.0
0.1
0.2
0.3
0.4
0.5
0.6
0.7
0.8
Phylostratum
Cellular_organisms - ps1
Archaea1 - ps2
Archaea2 - ps3
Archaea3 - ps4
Asgard/Eukaryota - ps5
Eukaryota - ps6
Amorpheae - ps7
Obazoa - ps8
Opisthokonta - ps9
Holozoa - ps10
Filozoa - ps11
Choano./Metazoa - ps12
Metazoa - ps13
Myriazoa - ps14
Parahoxozoa - ps15
Bilateria - ps16
Deuterostomia - ps17
Chordata - ps18
Olfactores - ps19
Vertebrata - ps20
Gnathostomata - ps21
Euteleostomi - ps22
Sarcopterygii - ps23
Tetrapoda - ps24
Amniota - ps25
Mammalia - ps26
Theria - ps27
Placentalia - ps28
Boreoeutheria - ps29
Euarchontoglires - ps30
Euarchonta - ps31
Primates - ps32
Haplorrhini - ps33
Simiiformes - ps34
Catarrhini - ps35
Hominoidea - ps36
Hominidae - ps37
Homininae - ps38
Hominini - ps39
Homo_sapiens - ps40
p-value
4e-03
6e-03
1e-02
2e-02
3e-02
Log-odds
0.5
1.0
1.5
2.0
2.5
3.0
3.5
4.0

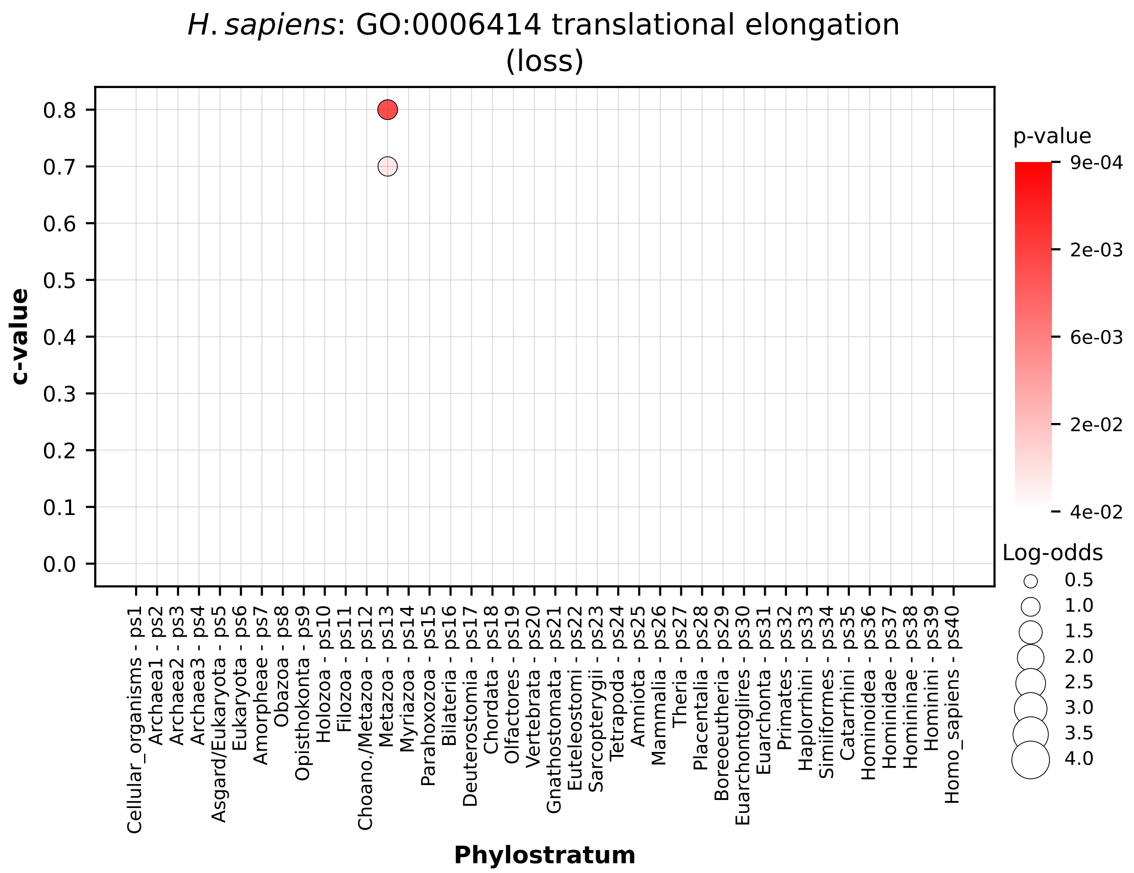

H. sapiens: GO:0006414 translational elongation
(loss)
c-value
0.8
0.7
0.6
0.5
0.4
0.3
0.2
0.1
0.0
p-value
9e-04
2e-03
6e-03
2e-02
4e-02
Log-odds
0.5
1.0
1.5
2.0
2.5
3.0
3.5
4.0
Cellular_organisms - ps1
Archaea1 - ps2
Archaea2 - ps3
Archaea3 - ps4
Asgard/Eukaryota - ps5
Eukaryota - ps6
Amorpheae - ps7
Obazoa - ps8
Opisthokonta - ps9
Holozoa - ps10
Filozoa - ps11
Choano./Metazoa - ps12
Metazoa - ps13
Myriazoa - ps14
Parahoxozoa - ps15
Bilateria - ps16
Deuterostomia - ps17
Chordata - ps18
Olfactores - ps19
Vertebrata - ps20
Gnathostomata - ps21
Euteleostomi - ps22
Sarcopterygii - ps23
Tetrapoda - ps24
Amniota - ps25
Mammalia - ps26
Theria - ps27
Placentalia - ps28
Boreoeutheria - ps29
Euarchontoglires - ps30
Euarchonta - ps31
Primates - ps32
Haplorrhini - ps33
Simiiformes - ps34
Catarrhini - ps35
Hominoidea - ps36
Hominidae - ps37
Homininae - ps38
Hominini - ps39
Homo_sapiens - ps40
Phylostratum

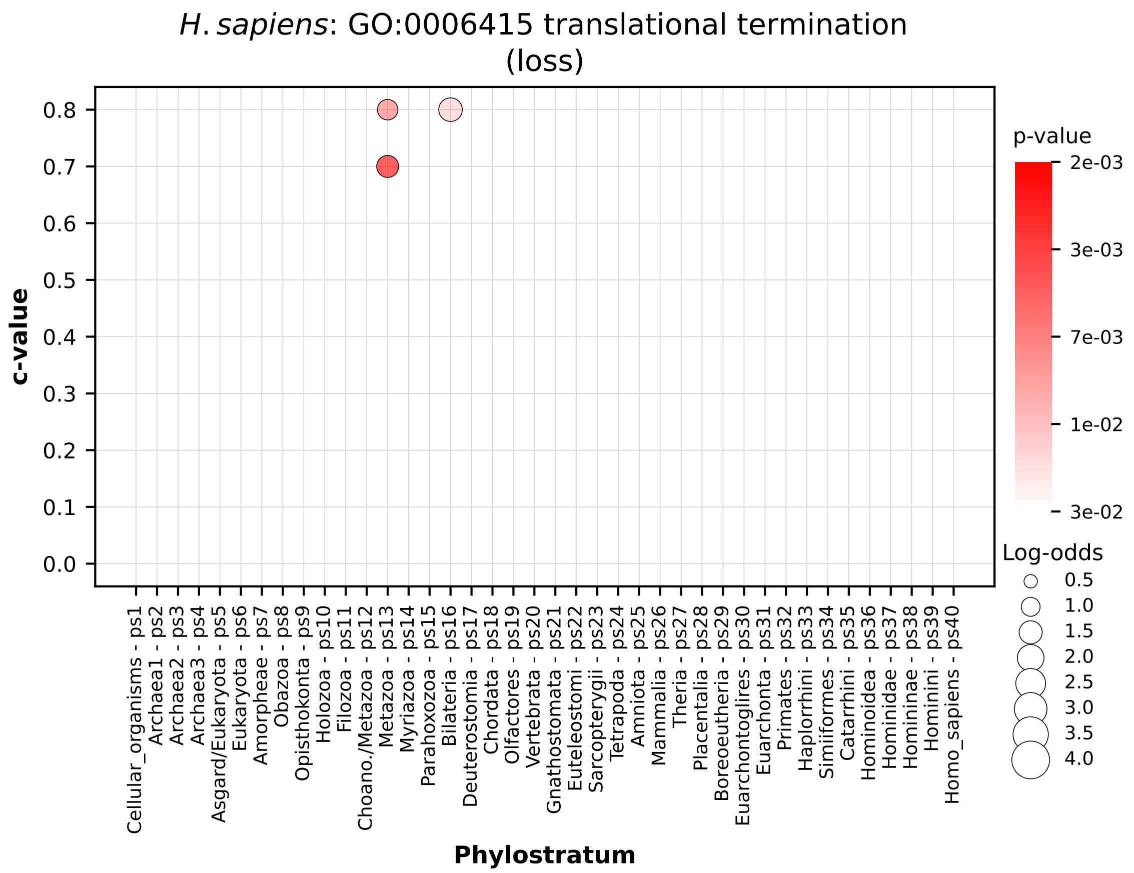
H. sapiens: GO:0006415 translational termination
(loss)
c-value
0.0
0.1
0.2
0.3
0.4
0.5
0.6
0.7
0.8
p-value
2e-03
3e-03
7e-03
1e-02
3e-02
Log-odds
0.5
1.0
1.5
2.0
2.5
3.0
3.5
4.0
Cellular_organisms - ps1
Archaea1 - ps2
Archaea2 - ps3
Archaea3 - ps4
Asgard/Eukaryota - ps5
Eukaryota - ps6
Amorpheae - ps7
Obazoa - ps8
Opisthokonta - ps9
Holozoa - ps10
Filozoa - ps11
Choano./Metazoa - ps12
Metazoa - ps13
Myriazoa - ps14
Parahoxozoa - ps15
Bilateria - ps16
Deuterostomia - ps17
Chordata - ps18
Olfactores - ps19
Vertebrata - ps20
Gnathostomata - ps21
Euteleostomi - ps22
Sarcopterygii - ps23
Tetrapoda - ps24
Amniota - ps25
Mammalia - ps26
Theria - ps27
Placentalia - ps28
Boreoeutheria - ps29
Euarchontoglires - ps30
Euarchonta - ps31
Primates - ps32
Haplorrhini - ps33
Simiiformes - ps34
Catarrhini - ps35
Hominoidea - ps36
Hominidae - ps37
Homininae - ps38
Hominini - ps39
Homo_sapiens - ps40
Phylostratum

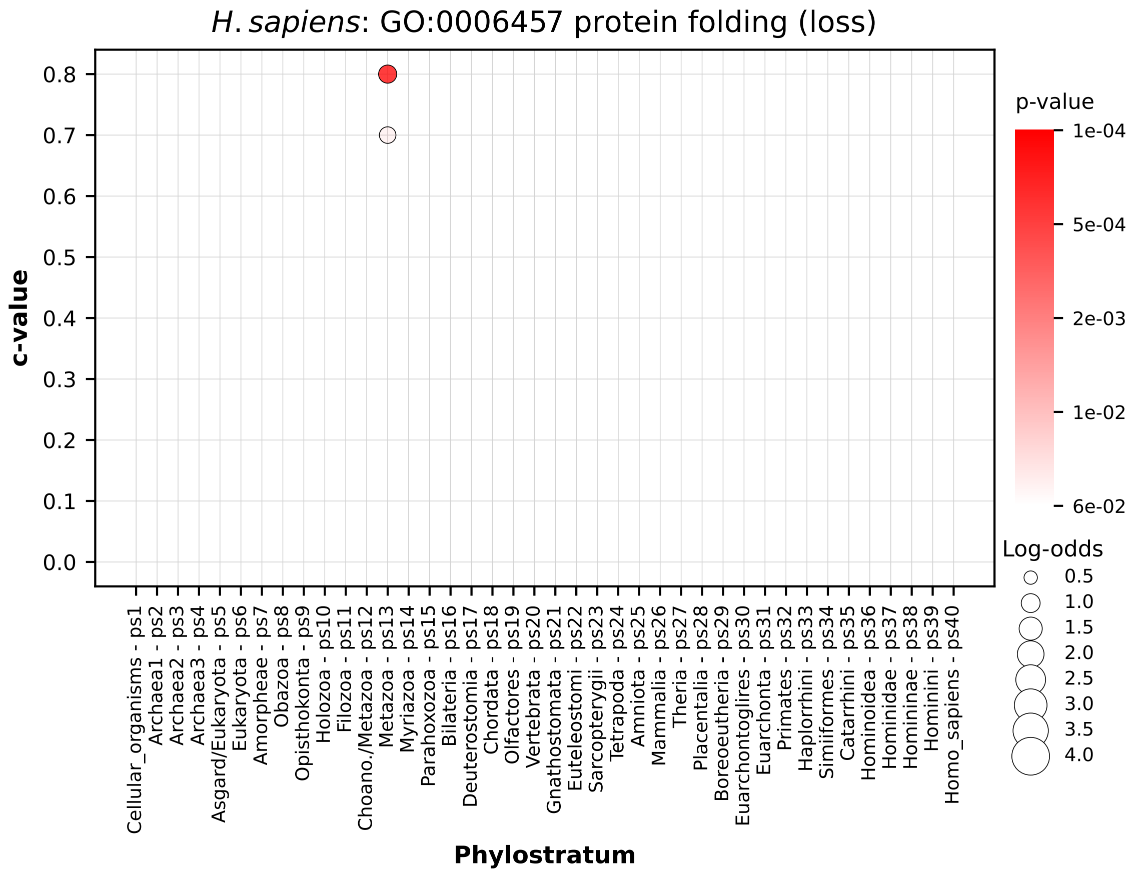
H. sapiens: GO:0006457 protein folding (loss)
c-value
0.0
0.1
0.2
0.3
0.4
0.5
0.6
0.7
0.8
p-value
1e-04
5e-04
2e-03
1e-02
6e-02
Log-odds
0.5
1.0
1.5
2.0
2.5
3.0
3.5
4.0
Phylostratum
Cellular_organisms - ps1
Archaea1 - ps2
Archaea2 - ps3
Archaea3 - ps4
Asgard/Eukaryota - ps5
Eukaryota - ps6
Amorpheae - ps7
Obazoa - ps8
Opisthokonta - ps9
Holozoa - ps10
Filozoa - ps11
Choano./Metazoa - ps12
Metazoa - ps13
Myriazoa - ps14
Parahoxozoa - ps15
Bilateria - ps16
Deuterostomia - ps17
Chordata - ps18
Olfactores - ps19
Vertebrata - ps20
Gnathostomata - ps21
Euteleostomi - ps22
Sarcopterygii - ps23
Tetrapoda - ps24
Amniota - ps25
Mammalia - ps26
Theria - ps27
Placentalia - ps28
Boreoeutheria - ps29
Euarchontoglires - ps30
Euarchonta - ps31
Primates - ps32
Haplorrhini - ps33
Simiiformes - ps34
Catarrhini - ps35
Hominoidea - ps36
Hominidae - ps37
Homininae - ps38
Hominini - ps39
Homo_sapiens - ps40

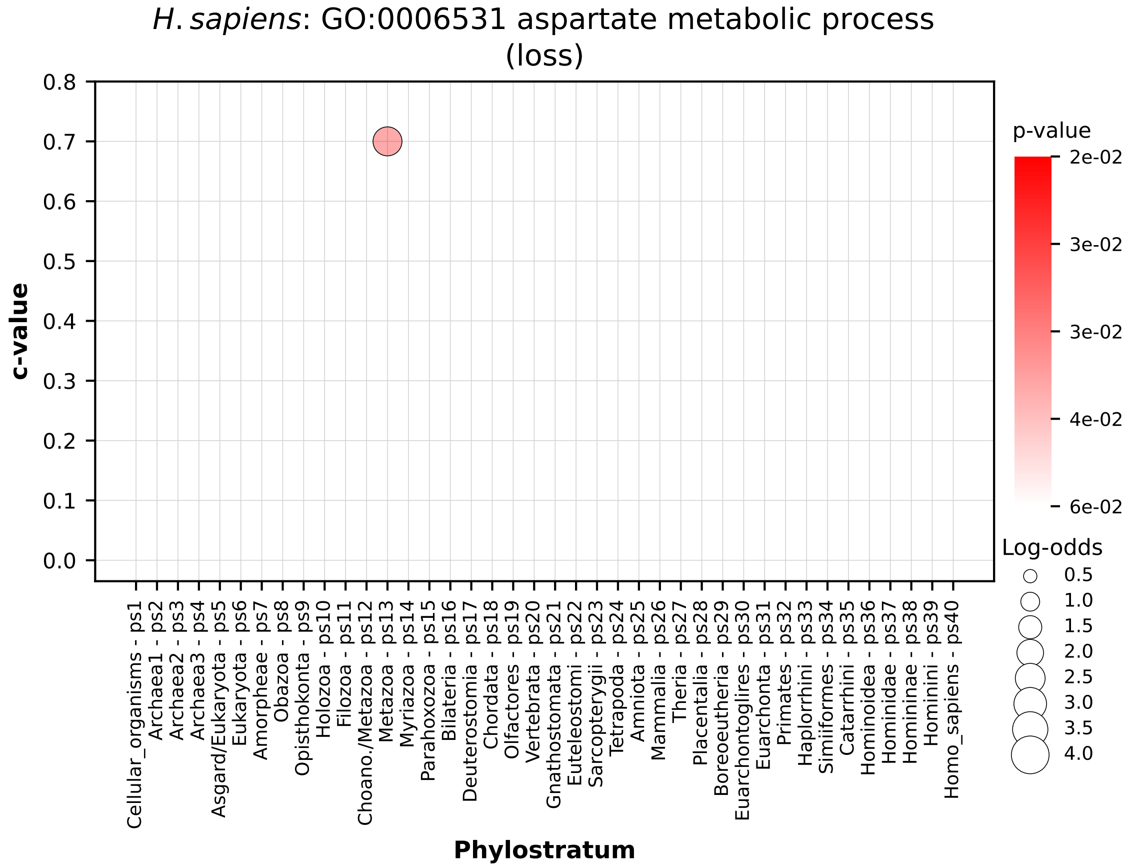

H. sapiens: GO:0006531 aspartate metabolic process (loss)
c-value
0.8
0.7
0.6
0.5
0.4
0.3
0.2
0.1
0.0
p-value
2e-02
3e-02
3e-02
4e-02
6e-02
Log-odds
0.5
1.0
1.5
2.0
2.5
3.0
3.5
4.0
Cellular_organisms - ps1
Archaea1 - ps2
Archaea2 - ps3
Archaea3 - ps4
Asgard/Eukaryota - ps5
Eukaryota - ps6
Amorpheae - ps7
Obazoa - ps8
Opisthokonta - ps9
Holozoa - ps10
Filozoa - ps11
Choano./Metazoa - ps12
Metazoa - ps13
Myriazoa - ps14
Parahoxozoa - ps15
Bilateria - ps16
Deuterostomia - ps17
Chordata - ps18
Olfactores - ps19
Vertebrata - ps20
Gnathostomata - ps21
Euteleostomi - ps22
Sarcopterygii - ps23
Tetrapoda - ps24
Amniota - ps25
Mammalia - ps26
Theria - ps27
Placentalia - ps28
Boreoeutheria - ps29
Euarchontoglires - ps30
Euarchonta - ps31
Primates - ps32
Haplorrhini - ps33
Simiiformes - ps34
Catarrhini - ps35
Hominoidea - ps36
Hominidae - ps37
Homininae - ps38
Hominini - ps39
Homo_sapiens - ps40
Phylostratum

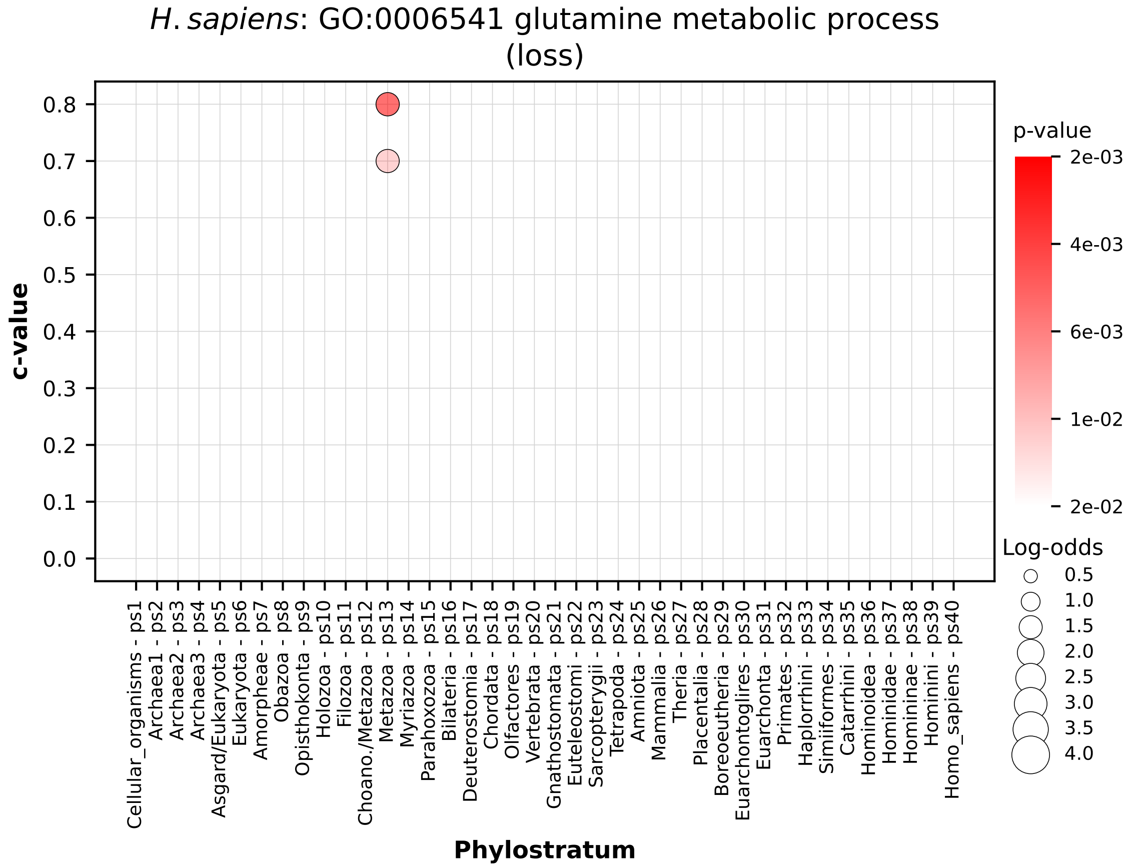

H. sapiens: GO:0006541 glutamine metabolic process
(loss)
c-value
0.0
0.1
0.2
0.3
0.4
0.5
0.6
0.7
0.8
Phylostratum
Cellular_organisms - ps1
Archaea1 - ps2
Archaea2 - ps3
Archaea3 - ps4
Asgard/Eukaryota - ps5
Eukaryota - ps6
Amorpheae - ps7
Obazoa - ps8
Opisthokonta - ps9
Holozoa - ps10
Filozoa - ps11
Choano./Metazoa - ps12
Metazoa - ps13
Myriazoa - ps14
Parahoxozoa - ps15
Bilateria - ps16
Deuterostomia - ps17
Chordata - ps18
Olfactores - ps19
Vertebrata - ps20
Gnathostomata - ps21
Euteleostomi - ps22
Sarcopterygii - ps23
Tetrapoda - ps24
Amniota - ps25
Mammalia - ps26
Theria - ps27
Placentalia - ps28
Boreoeutheria - ps29
Euarchontoglires - ps30
Euarchonta - ps31
Primates - ps32
Haplorrhini - ps33
Simiiformes - ps34
Catarrhini - ps35
Hominoidea - ps36
Hominidae - ps37
Homininae - ps38
Hominini - ps39
Homo_sapiens - ps40
p-value
2e-03
4e-03
6e-03
1e-02
2e-02
Log-odds
0.5
1.0
1.5
2.0
2.5
3.0
3.5
4.0

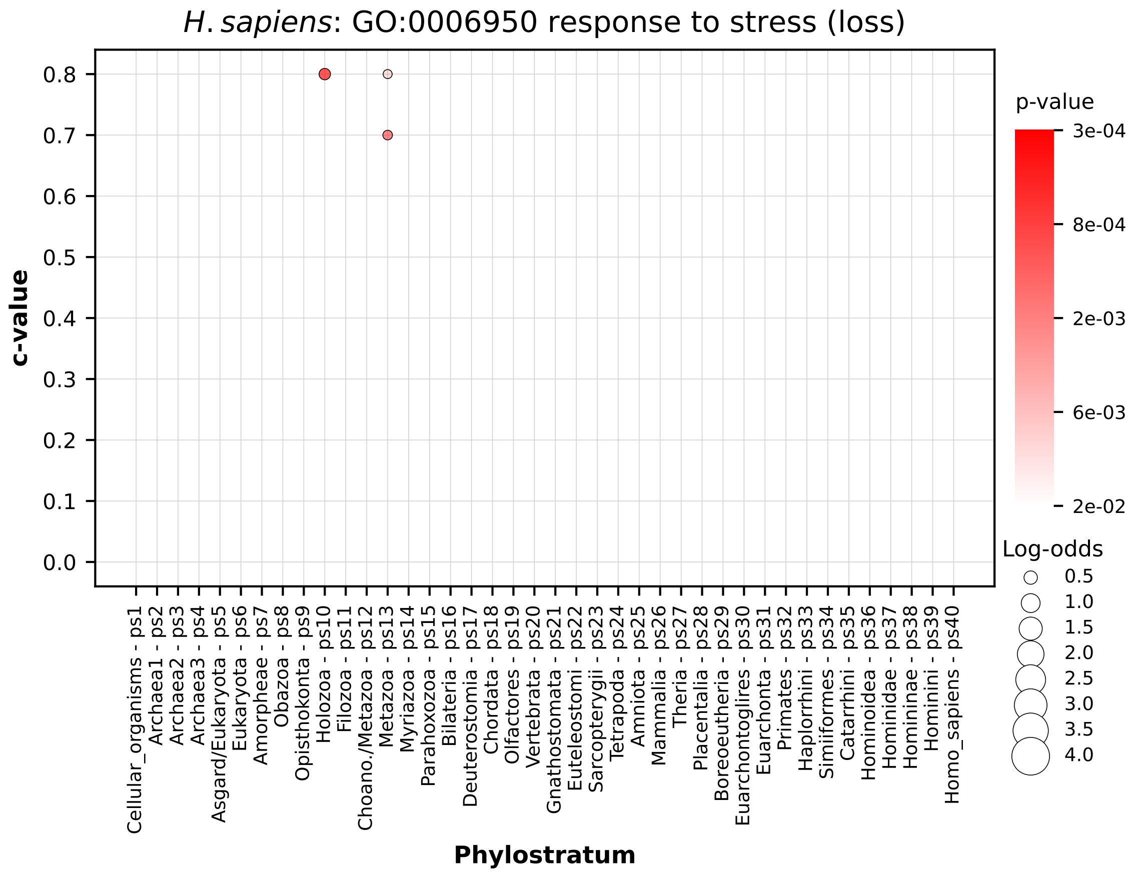

H. sapiens: GO:0006950 response to stress (loss)
c-value
0.0
0.1
0.2
0.3
0.4
0.5
0.6
0.7
0.8
p-value
3e-04
8e-04
2e-03
6e-03
2e-02
Log-odds
0.5
1.0
1.5
2.0
2.5
3.0
3.5
4.0
Cellular_organisms - ps1
Archaea1 - ps2
Archaea2 - ps3
Archaea3 - ps4
Asgard/Eukaryota - ps5
Eukaryota - ps6
Amorpheae - ps7
Obazoa - ps8
Opisthokonta - ps9
Holozoa - ps10
Filozoa - ps11
Choano./Metazoa - ps12
Metazoa - ps13
Myriazoa - ps14
Parahoxozoa - ps15
Bilateria - ps16
Deuterostomia - ps17
Chordata - ps18
Olfactores - ps19
Vertebrata - ps20
Gnathostomata - ps21
Euteleostomi - ps22
Sarcopterygii - ps23
Tetrapoda - ps24
Amniota - ps25
Mammalia - ps26
Theria - ps27
Placentalia - ps28
Boreoeutheria - ps29
Euarchontoglires - ps30
Euarchonta - ps31
Primates - ps32
Haplorrhini - ps33
Simiiformes - ps34
Catarrhini - ps35
Hominoidea - ps36
Hominidae - ps37
Homininae - ps38
Hominini - ps39
Homo_sapiens - ps40
Phylostratum

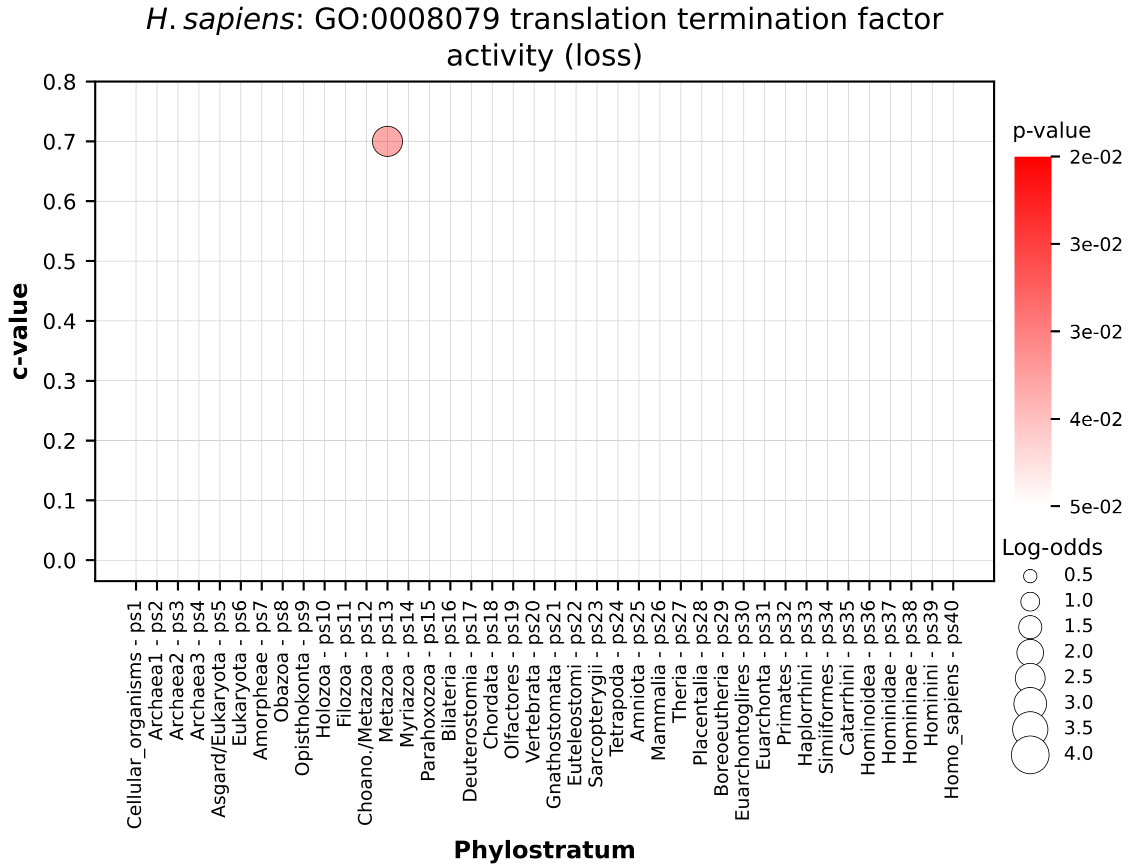

H. sapiens: GO:0008079 translation termination factor activity (loss)
c-value
0.8
0.7
0.6
0.5
0.4
0.3
0.2
0.1
0.0
p-value
2e-02
3e-02
3e-02
4e-02
5e-02
Log-odds
0.5
1.0
1.5
2.0
2.5
3.0
3.5
4.0
Cellular_organisms - ps1
Archaea1 - ps2
Archaea2 - ps3
Archaea3 - ps4
Asgard/Eukaryota - ps5
Eukaryota - ps6
Amorpheae - ps7
Obazoa - ps8
Opisthokonta - ps9
Holozoa - ps10
Filozoa - ps11
Choano./Metazoa - ps12
Metazoa - ps13
Myriazoa - ps14
Parahoxozoa - ps15
Bilateria - ps16
Deuterostomia - ps17
Chordata - ps18
Olfactores - ps19
Vertebrata - ps20
Gnathostomata - ps21
Euteleostomi - ps22
Sarcopterygii - ps23
Tetrapoda - ps24
Amniota - ps25
Mammalia - ps26
Theria - ps27
Placentalia - ps28
Boreoeutheria - ps29
Euarchontoglires - ps30
Euarchonta - ps31
Primates - ps32
Haplorrhini - ps33
Simiiformes - ps34
Catarrhini - ps35
Hominoidea - ps36
Hominidae - ps37
Homininae - ps38
Hominini - ps39
Homo_sapiens - ps40
Phylostratum

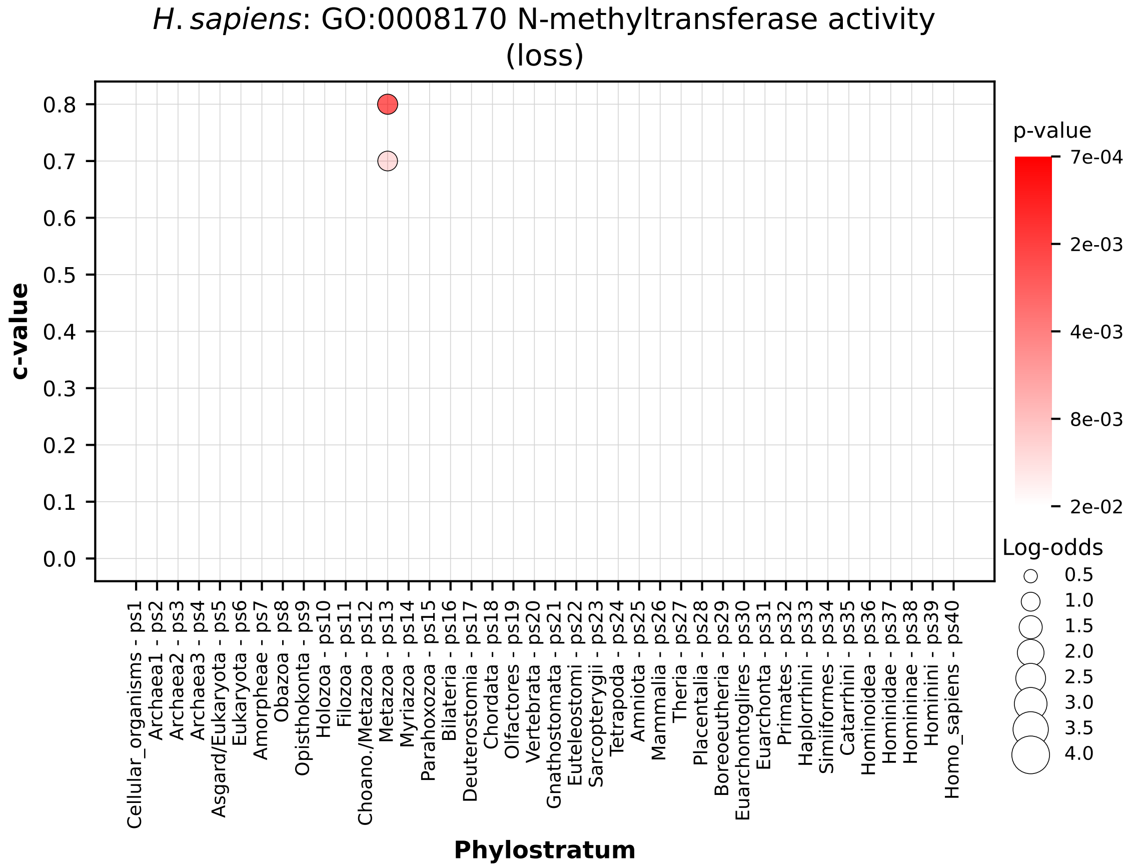
H. sapiens: GO:0008170 N-methyltransferase activity
(loss)
c-value
0.8
0.7
0.6
0.5
0.4
0.3
0.2
0.1
0.0
p-value
7e-04
2e-03
4e-03
8e-03
2e-02
Log-odds
0.5
1.0
1.5
2.0
2.5
3.0
3.5
4.0
Cellular_organisms - ps1
Archaea1 - ps2
Archaea2 - ps3
Archaea3 - ps4
Asgard/Eukaryota - ps5
Eukaryota - ps6
Amorpheae - ps7
Obazoa - ps8
Opisthokonta - ps9
Holozoa - ps10
Filozoa - ps11
Choano./Metazoa - ps12
Metazoa - ps13
Myriazoa - ps14
Parahoxozoa - ps15
Bilateria - ps16
Deuterostomia - ps17
Chordata - ps18
Olfactores - ps19
Vertebrata - ps20
Gnathostomata - ps21
Euteleostomi - ps22
Sarcopterygii - ps23
Tetrapoda - ps24
Amniota - ps25
Mammalia - ps26
Theria - ps27
Placentalia - ps28
Boreoeutheria - ps29
Euarchontoglires - ps30
Euarchonta - ps31
Primates - ps32
Haplorrhini - ps33
Simiiformes - ps34
Catarrhini - ps35
Hominoidea - ps36
Hominidae - ps37
Homininae - ps38
Hominini - ps39
Homo_sapiens - ps40
Phylostratum

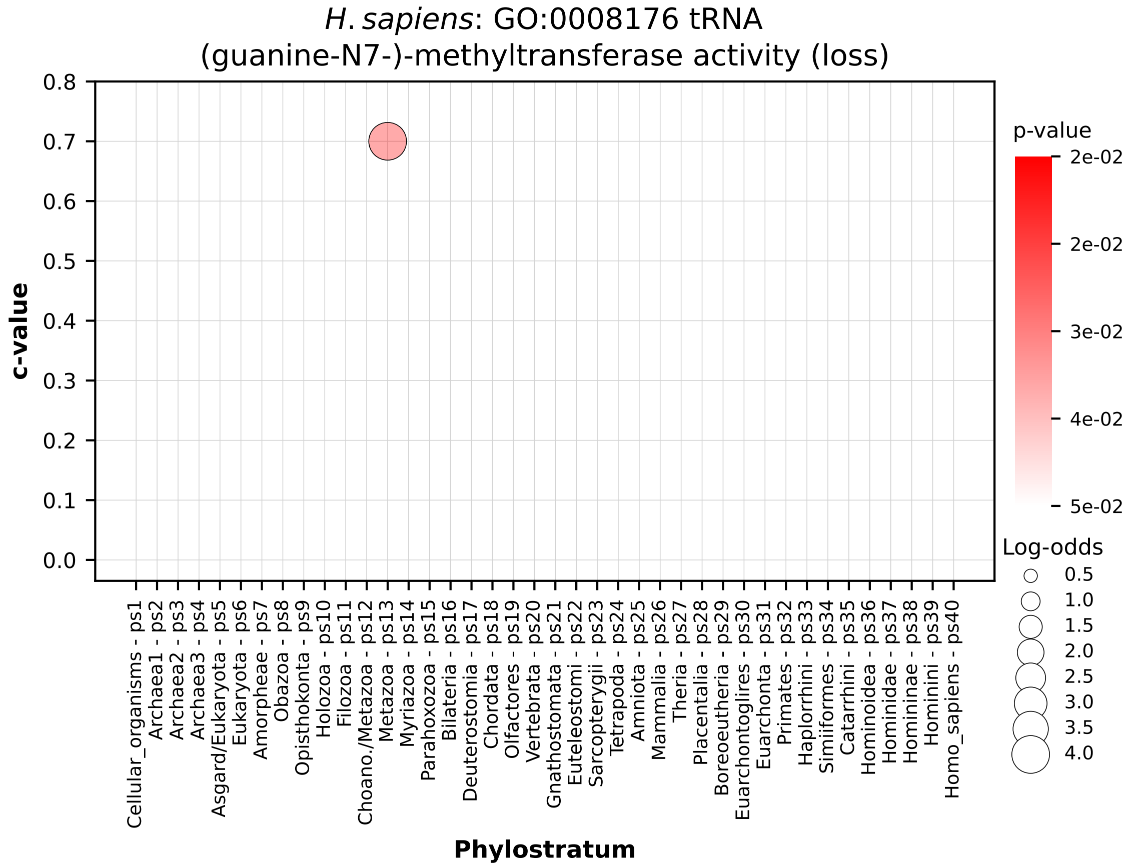

H. sapiens: GO:0008176 tRNA (guanine-N7-)-methyltransferase activity (loss)
c-value
0.8
0.7
0.6
0.5
0.4
0.3
0.2
0.1
0.0
Phylostratum
Cellular_organisms - ps1
Archaea1 - ps2
Archaea2 - ps3
Archaea3 - ps4
Asgard/Eukaryota - ps5
Eukaryota - ps6
Amorpheae - ps7
Obazoa - ps8
Opisthokonta - ps9
Holozoa - ps10
Filozoa - ps11
Choano./Metazoa - ps12
Metazoa - ps13
Myriazoa - ps14
Parahoxozoa - ps15
Bilateria - ps16
Deuterostomia - ps17
Chordata - ps18
Olfactores - ps19
Vertebrata - ps20
Gnathostomata - ps21
Euteleostomi - ps22
Sarcopterygii - ps23
Tetrapoda - ps24
Amniota - ps25
Mammalia - ps26
Theria - ps27
Placentalia - ps28
Boreoeutheria - ps29
Euarchontoglires - ps30
Euarchonta - ps31
Primates - ps32
Haplorrhini - ps33
Simiiformes - ps34
Catarrhini - ps35
Hominoidea - ps36
Hominidae - ps37
Homininae - ps38
Hominini - ps39
Homo_sapiens - ps40
p-value
2e-02
2e-02
3e-02
4e-02
5e-02
Log-odds
0.5
1.0
1.5
2.0
2.5
3.0
3.5
4.0

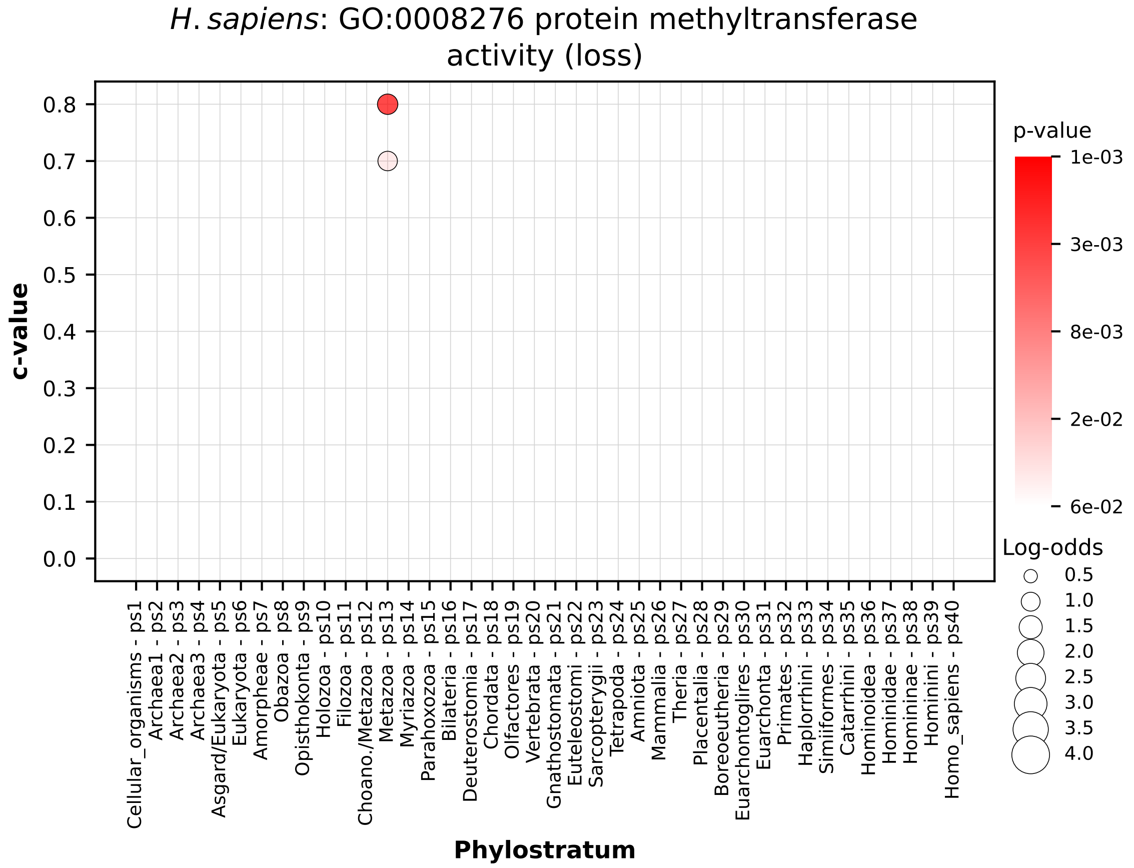
H. sapiens: GO:0008276 protein methyltransferase activity (loss)
c-value
0.0
0.1
0.2
0.3
0.4
0.5
0.6
0.7
0.8
Phylostratum
Cellular_organisms - ps1
Archaea1 - ps2
Archaea2 - ps3
Archaea3 - ps4
Asgard/Eukaryota - ps5
Eukaryota - ps6
Amorpheae - ps7
Obazoa - ps8
Opisthokonta - ps9
Holozoa - ps10
Filozoa - ps11
Choano./Metazoa - ps12
Metazoa - ps13
Myriazoa - ps14
Parahoxozoa - ps15
Bilateria - ps16
Deuterostomia - ps17
Chordata - ps18
Olfactores - ps19
Vertebrata - ps20
Gnathostomata - ps21
Euteleostomi - ps22
Sarcopterygii - ps23
Tetrapoda - ps24
Amniota - ps25
Mammalia - ps26
Theria - ps27
Placentalia - ps28
Boreoeutheria - ps29
Euarchontoglires - ps30
Euarchonta - ps31
Primates - ps32
Haplorrhini - ps33
Simiiformes - ps34
Catarrhini - ps35
Hominoidea - ps36
Hominidae - ps37
Homininae - ps38
Hominini - ps39
Homo_sapiens - ps40
p-value
1e-03
3e-03
8e-03
2e-02
6e-02
Log-odds
0.5
1.0
1.5
2.0
2.5
3.0
3.5
4.0

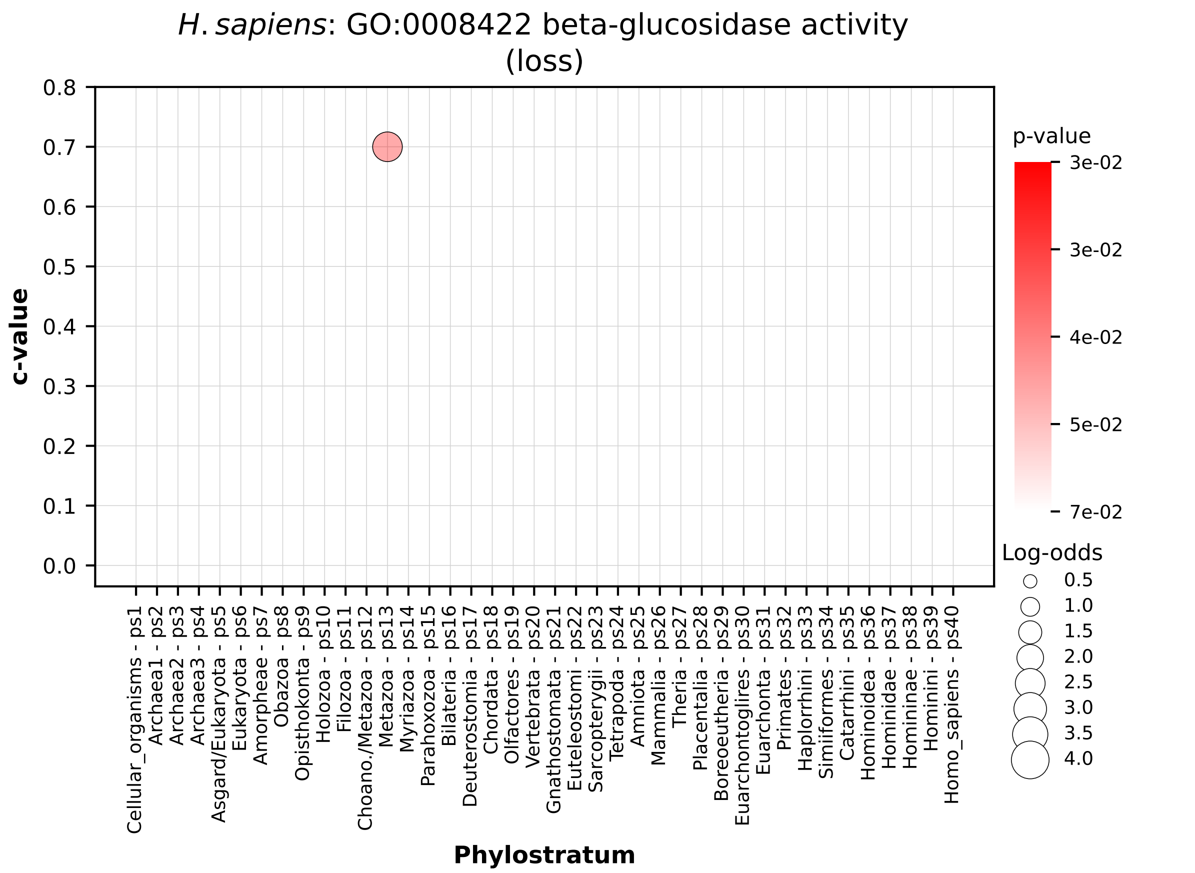

H. sapiens: GO:0008422 beta-glucosidase activity
(loss)
c-value
0.0
0.1
0.2
0.3
0.4
0.5
0.6
0.7
0.8
Phylostratum
Cellular_organisms - ps1
Archaea1 - ps2
Archaea2 - ps3
Archaea3 - ps4
Asgard/Eukaryota - ps5
Eukaryota - ps6
Amorpheae - ps7
Obazoa - ps8
Opisthokonta - ps9
Holozoa - ps10
Filozoa - ps11
Choano./Metazoa - ps12
Metazoa - ps13
Myriazoa - ps14
Parahoxozoa - ps15
Bilateria - ps16
Deuterostomia - ps17
Chordata - ps18
Olfactores - ps19
Vertebrata - ps20
Gnathostomata - ps21
Euteleostomi - ps22
Sarcopterygii - ps23
Tetrapoda - ps24
Amniota - ps25
Mammalia - ps26
Theria - ps27
Placentalia - ps28
Boreoeutheria - ps29
Euarchontoglires - ps30
Euarchonta - ps31
Primates - ps32
Haplorrhini - ps33
Simiiformes - ps34
Catarrhini - ps35
Hominoidea - ps36
Hominidae - ps37
Homininae - ps38
Hominini - ps39
Homo_sapiens - ps40
p-value
3e-02
3e-02
4e-02
5e-02
7e-02
Log-odds
0.5
1.0
1.5
2.0
2.5
3.0
3.5
4.0

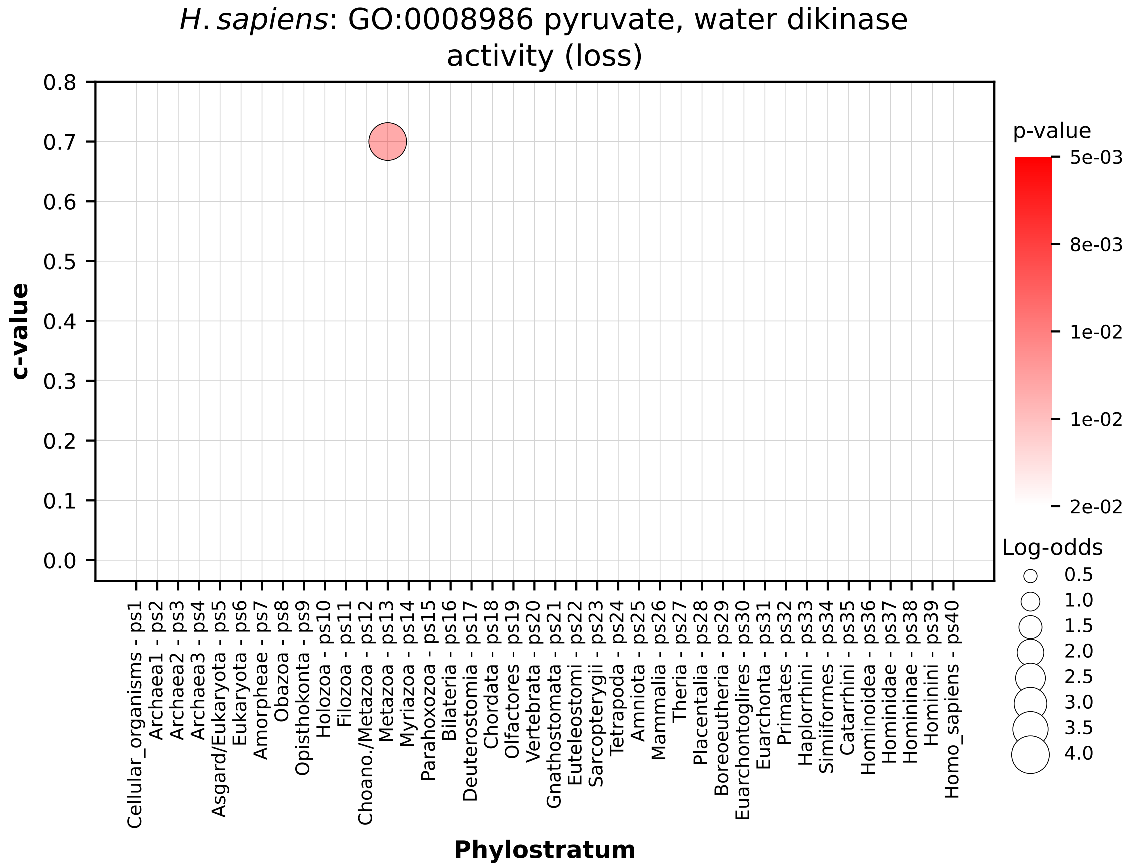

H. sapiens: GO:0008986 pyruvate, water dikinase activity (loss)
c-value
0.8
0.7
0.6
0.5
0.4
0.3
0.2
0.1
0.0
p-value
5e-03
8e-03
1e-02
1e-02
2e-02
Log-odds
0.5
1.0
1.5
2.0
2.5
3.0
3.5
4.0
Cellular_organisms - ps1
Archaea1 - ps2
Archaea2 - ps3
Archaea3 - ps4
Asgard/Eukaryota - ps5
Eukaryota - ps6
Amorpheae - ps7
Obazoa - ps8
Opisthokonta - ps9
Holozoa - ps10
Filozoa - ps11
Choano./Metazoa - ps12
Metazoa - ps13
Myriazoa - ps14
Parahoxozoa - ps15
Bilateria - ps16
Deuterostomia - ps17
Chordata - ps18
Olfactores - ps19
Vertebrata - ps20
Gnathostomata - ps21
Euteleostomi - ps22
Sarcopterygii - ps23
Tetrapoda - ps24
Amniota - ps25
Mammalia - ps26
Theria - ps27
Placentalia - ps28
Boreoeutheria - ps29
Euarchontoglires - ps30
Euarchonta - ps31
Primates - ps32
Haplorrhini - ps33
Simiiformes - ps34
Catarrhini - ps35
Hominoidea - ps36
Hominidae - ps37
Homininae - ps38
Hominini - ps39
Homo_sapiens - ps40
Phylostratum

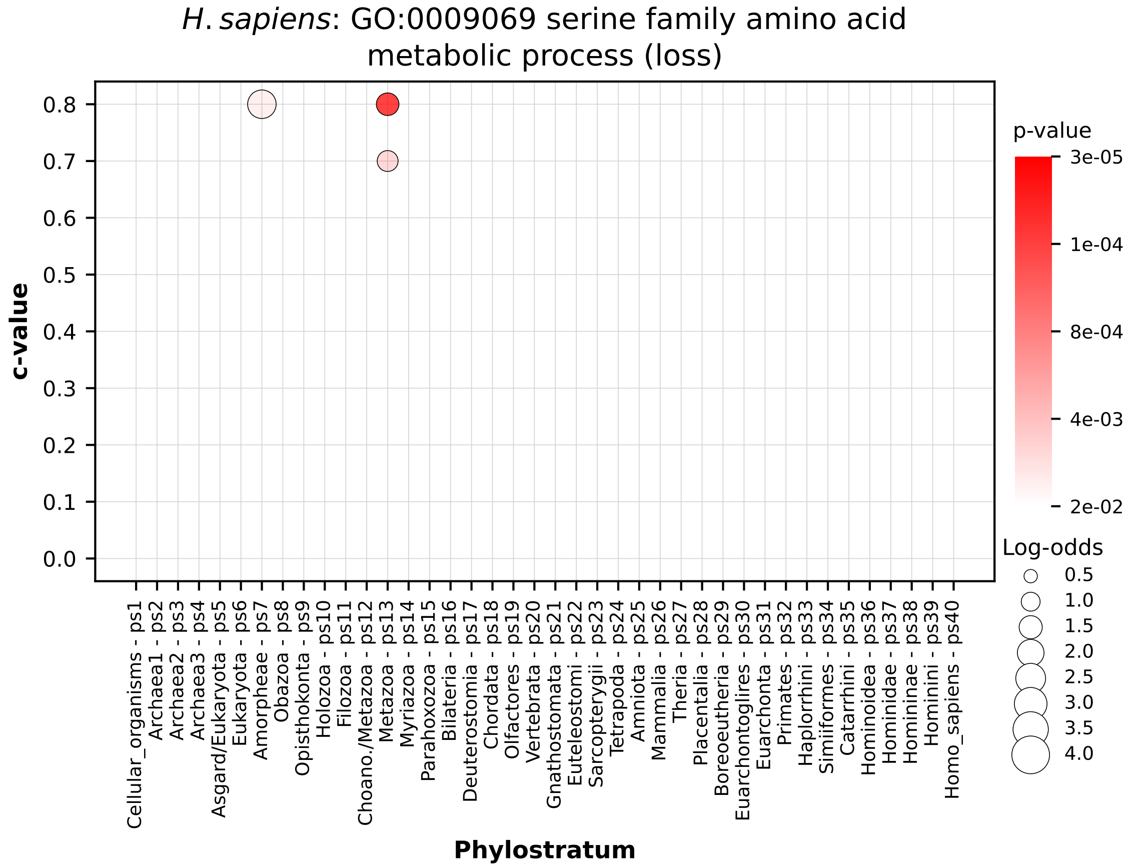

H. sapiens: GO:0009069 serine family amino acid metabolic process (loss)
c-value
0.0
0.1
0.2
0.3
0.4
0.5
0.6
0.7
0.8
p-value
3e-05
1e-04
8e-04
4e-03
2e-02
Log-odds
0.5
1.0
1.5
2.0
2.5
3.0
3.5
4.0
Cellular_organisms - ps1
Archaea1 - ps2
Archaea2 - ps3
Archaea3 - ps4
Asgard/Eukaryota - ps5
Eukaryota - ps6
Amorpheae - ps7
Obazoa - ps8
Opisthokonta - ps9
Holozoa - ps10
Filozoa - ps11
Choano./Metazoa - ps12
Metazoa - ps13
Myriazoa - ps14
Parahoxozoa - ps15
Bilateria - ps16
Deuterostomia - ps17
Chordata - ps18
Olfactores - ps19
Vertebrata - ps20
Gnathostomata - ps21
Euteleostomi - ps22
Sarcopterygii - ps23
Tetrapoda - ps24
Amniota - ps25
Mammalia - ps26
Theria - ps27
Placentalia - ps28
Boreoeutheria - ps29
Euarchontoglires - ps30
Euarchonta - ps31
Primates - ps32
Haplorrhini - ps33
Simiiformes - ps34
Catarrhini - ps35
Hominoidea - ps36
Hominidae - ps37
Homininae - ps38
Hominini - ps39
Homo_sapiens - ps40
Phylostratum

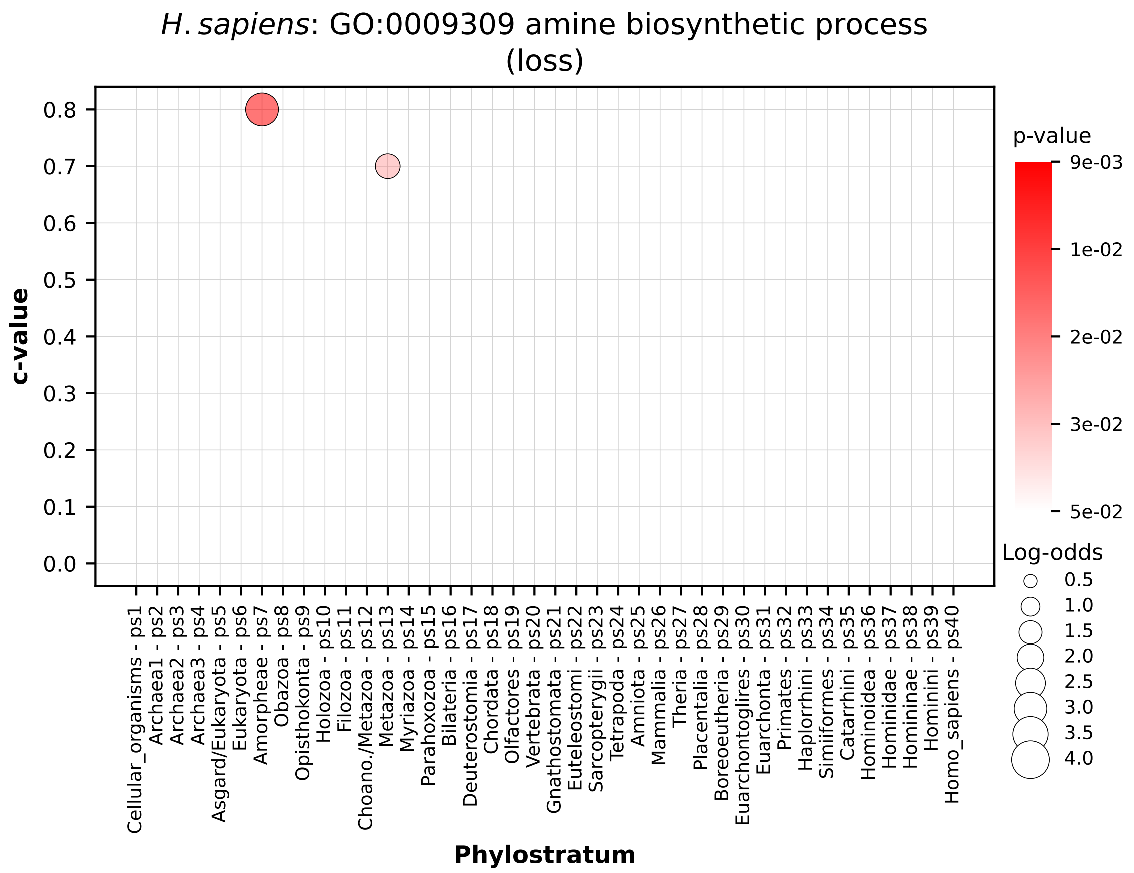

H. sapiens: GO:0009309 amine biosynthetic process
(loss)
c-value
0.0
0.1
0.2
0.3
0.4
0.5
0.6
0.7
0.8
p-value
9e-03
1e-02
2e-02
3e-02
5e-02
Log-odds
0.5
1.0
1.5
2.0
2.5
3.0
3.5
4.0
Cellular_organisms - ps1
Archaea1 - ps2
Archaea2 - ps3
Archaea3 - ps4
Asgard/Eukaryota - ps5
Eukaryota - ps6
Amorpheae - ps7
Obazoa - ps8
Opisthokonta - ps9
Holozoa - ps10
Filozoa - ps11
Choano./Metazoa - ps12
Metazoa - ps13
Myriazoa - ps14
Parahoxozoa - ps15
Bilateria - ps16
Deuterostomia - ps17
Chordata - ps18
Olfactores - ps19
Vertebrata - ps20
Gnathostomata - ps21
Euteleostomi - ps22
Sarcopterygii - ps23
Tetrapoda - ps24
Amniota - ps25
Mammalia - ps26
Theria - ps27
Placentalia - ps28
Boreoeutheria - ps29
Euarchontoglires - ps30
Euarchonta - ps31
Primates - ps32
Haplorrhini - ps33
Simiiformes - ps34
Catarrhini - ps35
Hominoidea - ps36
Hominidae - ps37
Homininae - ps38
Hominini - ps39
Homo_sapiens - ps40
Phylostratum

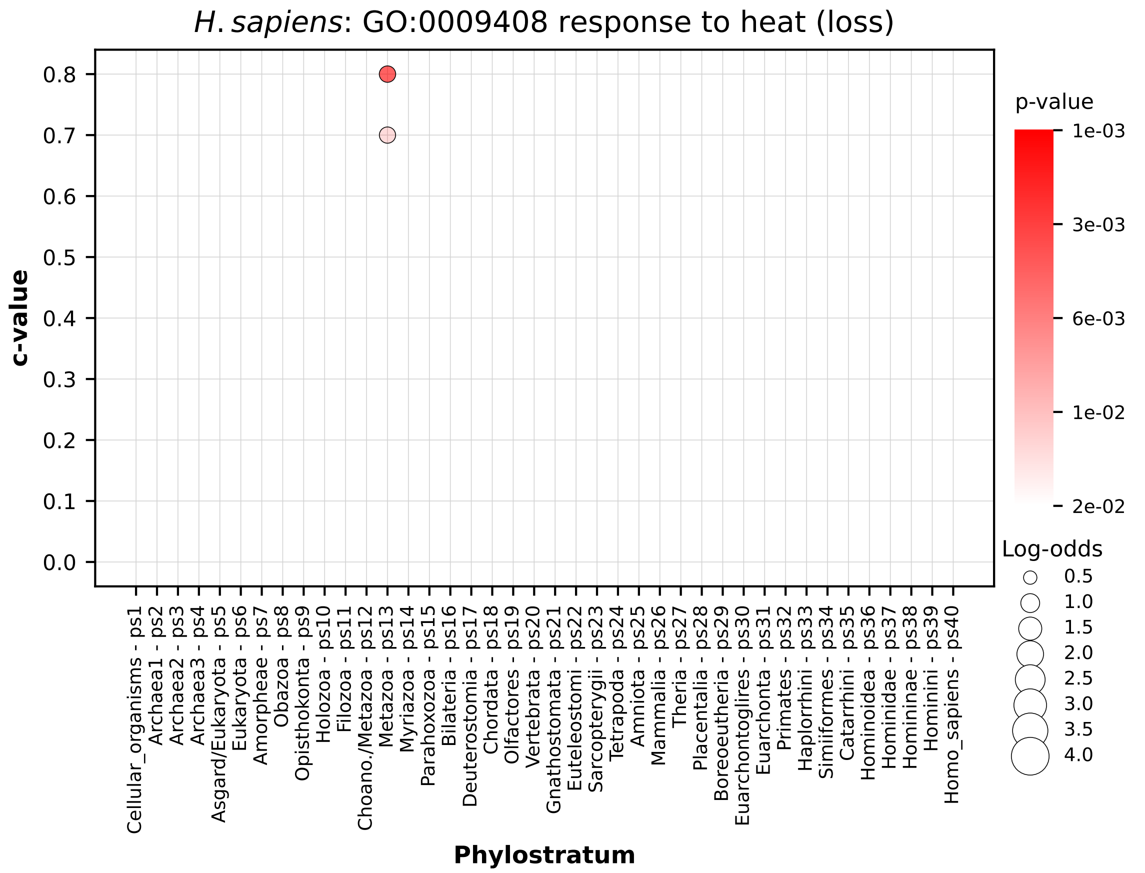
H. sapiens: GO:0009408 response to heat (loss)
c-value
0.0
0.1
0.2
0.3
0.4
0.5
0.6
0.7
0.8
Phylostratum
Cellular_organisms - ps1
Archaea1 - ps2
Archaea2 - ps3
Archaea3 - ps4
Asgard/Eukaryota - ps5
Eukaryota - ps6
Amorpheae - ps7
Obazoa - ps8
Opisthokonta - ps9
Holozoa - ps10
Filozoa - ps11
Choano./Metazoa - ps12
Metazoa - ps13
Myriazoa - ps14
Parahoxozoa - ps15
Bilateria - ps16
Deuterostomia - ps17
Chordata - ps18
Olfactores - ps19
Vertebrata - ps20
Gnathostomata - ps21
Euteleostomi - ps22
Sarcopterygii - ps23
Tetrapoda - ps24
Amniota - ps25
Mammalia - ps26
Theria - ps27
Placentalia - ps28
Boreoeutheria - ps29
Euarchontoglires - ps30
Euarchonta - ps31
Primates - ps32
Haplorrhini - ps33
Simiiformes - ps34
Catarrhini - ps35
Hominoidea - ps36
Hominidae - ps37
Homininae - ps38
Hominini - ps39
Homo_sapiens - ps40
p-value
1e-03
3e-03
6e-03
1e-02
2e-02
Log-odds
0.5
1.0
1.5
2.0
2.5
3.0
3.5
4.0

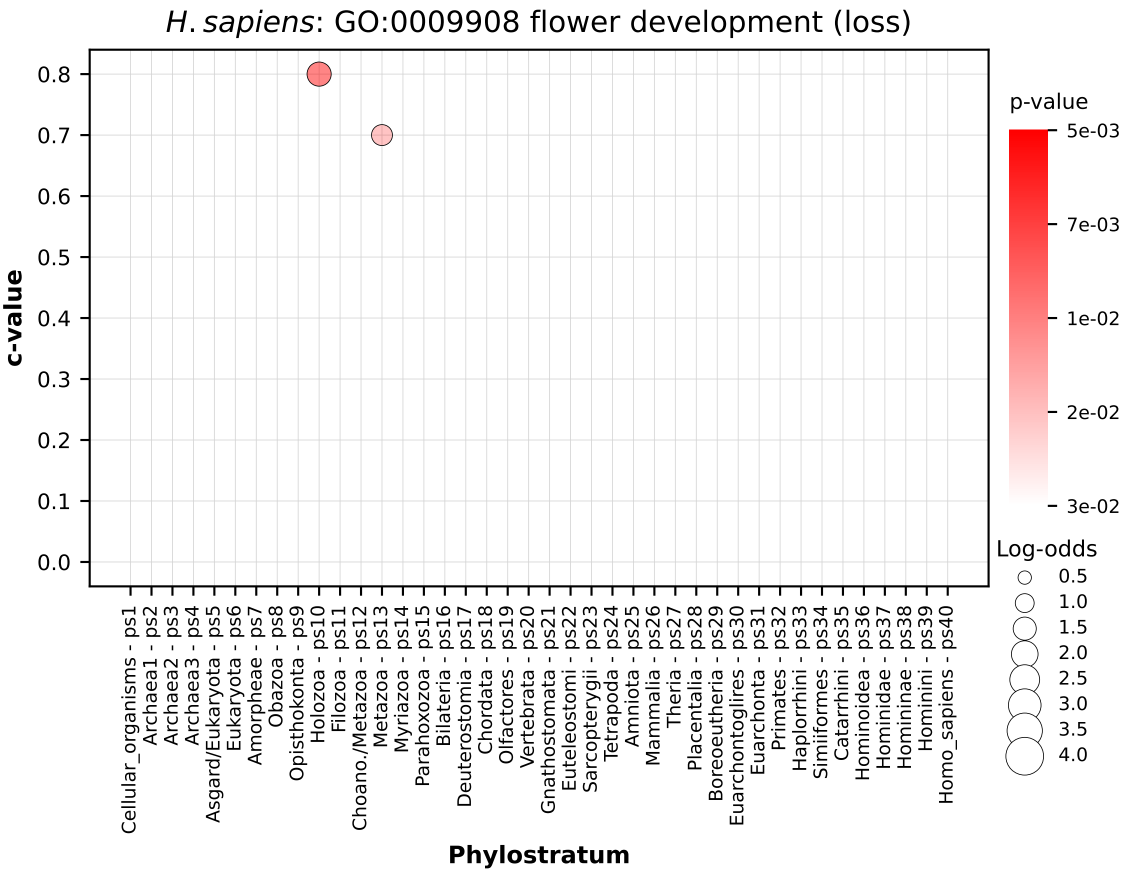

H. sapiens: GO:0009908 flower development (loss)
c-value
0.0
0.1
0.2
0.3
0.4
0.5
0.6
0.7
0.8
Phylostratum
Cellular_organisms - ps1
Archaea1 - ps2
Archaea2 - ps3
Archaea3 - ps4
Asgard/Eukaryota - ps5
Eukaryota - ps6
Amorpheae - ps7
Obazoa - ps8
Opisthokonta - ps9
Holozoa - ps10
Filozoa - ps11
Choano./Metazoa - ps12
Metazoa - ps13
Myriazoa - ps14
Parahoxozoa - ps15
Bilateria - ps16
Deuterostomia - ps17
Chordata - ps18
Olfactores - ps19
Vertebrata - ps20
Gnathostomata - ps21
Euteleostomi - ps22
Sarcopterygii - ps23
Tetrapoda - ps24
Amniota - ps25
Mammalia - ps26
Theria - ps27
Placentalia - ps28
Boreoeutheria - ps29
Euarchontoglires - ps30
Euarchonta - ps31
Primates - ps32
Haplorrhini - ps33
Simiiformes - ps34
Catarrhini - ps35
Hominoidea - ps36
Hominidae - ps37
Homininae - ps38
Hominini - ps39
Homo_sapiens - ps40
p-value
5e-03
7e-03
1e-02
2e-02
3e-02
Log-odds
0.5
1.0
1.5
2.0
2.5
3.0
3.5
4.0

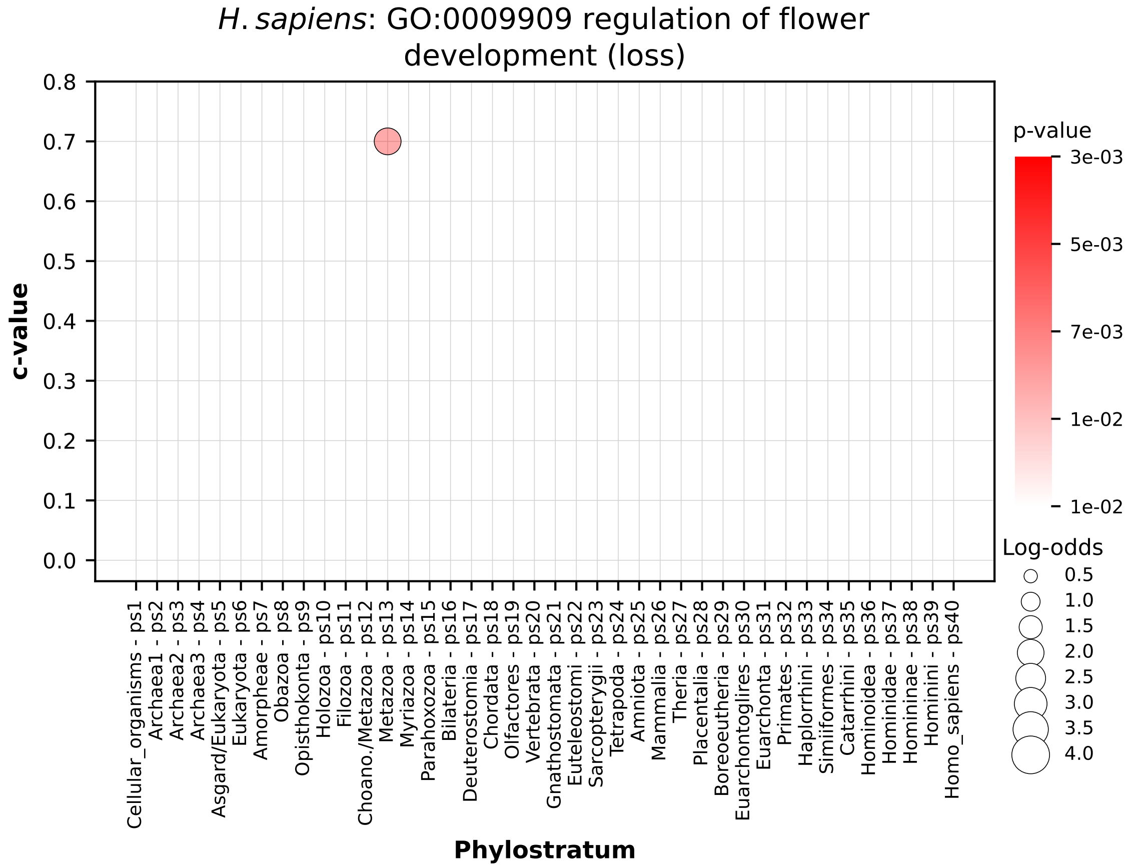

H. sapiens: GO:0009909 regulation of flower development (loss)
c-value
0.8
0.7
0.6
0.5
0.4
0.3
0.2
0.1
0.0
p-value
3e-03
5e-03
7e-03
1e-02
1e-02
Log-odds
0.5
1.0
1.5
2.0
2.5
3.0
3.5
4.0
Cellular_organisms - ps1
Archaea1 - ps2
Archaea2 - ps3
Archaea3 - ps4
Asgard/Eukaryota - ps5
Eukaryota - ps6
Amorpheae - ps7
Obazoa - ps8
Opisthokonta - ps9
Holozoa - ps10
Filozoa - ps11
Choano./Metazoa - ps12
Metazoa - ps13
Myriazoa - ps14
Parahoxozoa - ps15
Bilateria - ps16
Deuterostomia - ps17
Chordata - ps18
Olfactores - ps19
Vertebrata - ps20
Gnathostomata - ps21
Euteleostomi - ps22
Sarcopterygii - ps23
Tetrapoda - ps24
Amniota - ps25
Mammalia - ps26
Theria - ps27
Placentalia - ps28
Boreoeutheria - ps29
Euarchontoglires - ps30
Euarchonta - ps31
Primates - ps32
Haplorrhini - ps33
Simiiformes - ps34
Catarrhini - ps35
Hominoidea - ps36
Hominidae - ps37
Homininae - ps38
Hominini - ps39
Homo_sapiens - ps40
Phylostratum

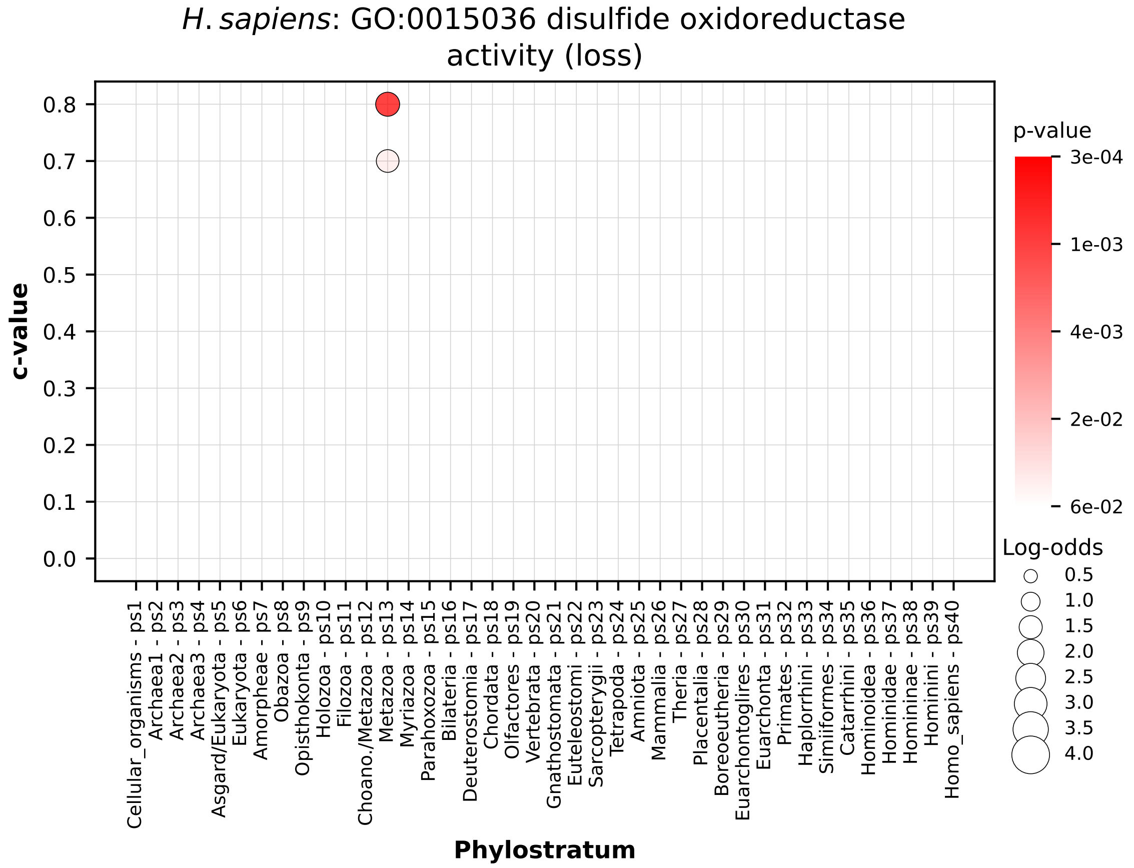
H. sapiens: GO:0015036 disulfide oxidoreductase activity (loss)
c-value
0.8
0.7
0.6
0.5
0.4
0.3
0.2
0.1
0.0
p-value
3e-04
1e-03
4e-03
2e-02
6e-02
Log-odds
0.5
1.0
1.5
2.0
2.5
3.0
3.5
4.0
Cellular_organisms - ps1
Archaea1 - ps2
Archaea2 - ps3
Archaea3 - ps4
Asgard/Eukaryota - ps5
Eukaryota - ps6
Amorpheae - ps7
Obazoa - ps8
Opisthokonta - ps9
Holozoa - ps10
Filozoa - ps11
Choano./Metazoa - ps12
Metazoa - ps13
Myriazoa - ps14
Parahoxozoa - ps15
Bilateria - ps16
Deuterostomia - ps17
Chordata - ps18
Olfactores - ps19
Vertebrata - ps20
Gnathostomata - ps21
Euteleostomi - ps22
Sarcopterygii - ps23
Tetrapoda - ps24
Amniota - ps25
Mammalia - ps26
Theria - ps27
Placentalia - ps28
Boreoeutheria - ps29
Euarchontoglires - ps30
Euarchonta - ps31
Primates - ps32
Haplorrhini - ps33
Simiiformes - ps34
Catarrhini - ps35
Hominoidea - ps36
Hominidae - ps37
Homininae - ps38
Hominini - ps39
Homo_sapiens - ps40
Phylostratum

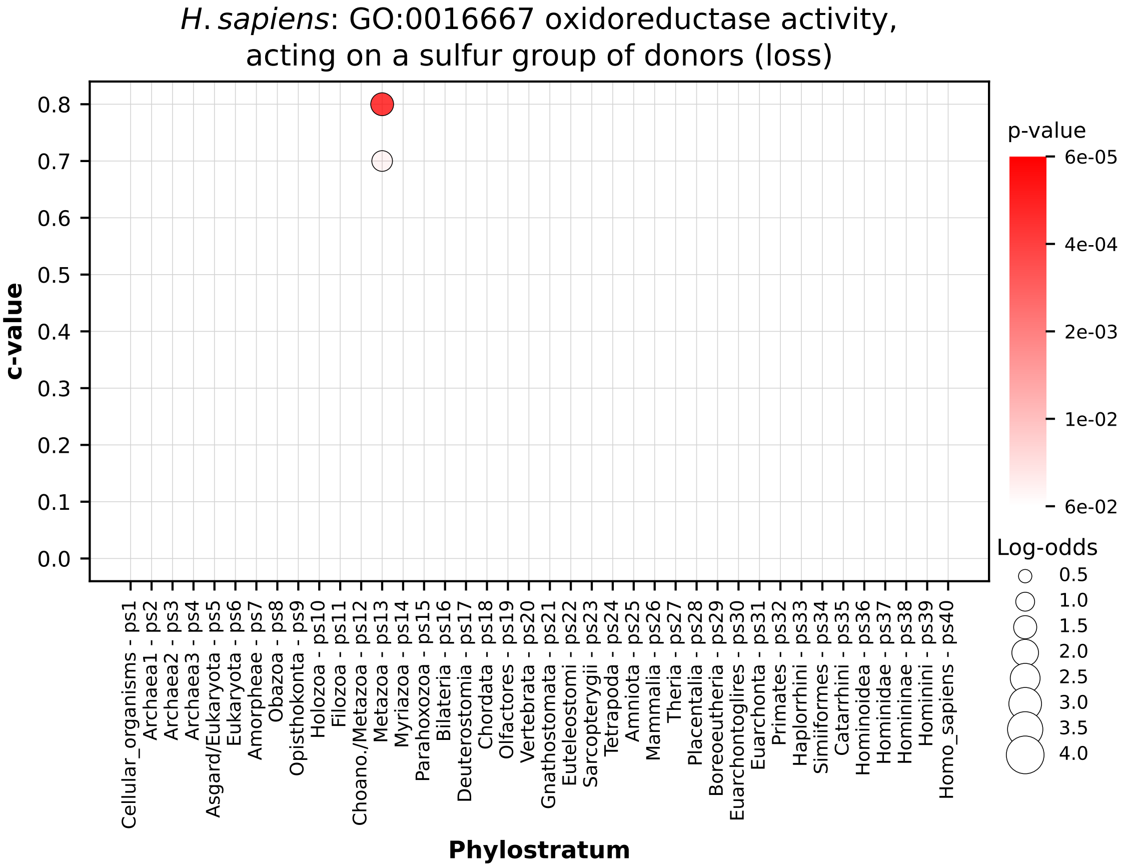
H. sapiens: GO:0016667 oxidoreductase activity, acting on a sulfur group of donors (loss)
c-value
0.8
0.7
0.6
0.5
0.4
0.3
0.2
0.1
0.0
Phylostratum
Cellular_organisms - ps1
Archaea1 - ps2
Archaea2 - ps3
Archaea3 - ps4
Asgard/Eukaryota - ps5
Eukaryota - ps6
Amorpheae - ps7
Obazoa - ps8
Opisthokonta - ps9
Holozoa - ps10
Filozoa - ps11
Choano./Metazoa - ps12
Metazoa - ps13
Myriazoa - ps14
Parahoxozoa - ps15
Bilateria - ps16
Deuterostomia - ps17
Chordata - ps18
Olfactores - ps19
Vertebrata - ps20
Gnathostomata - ps21
Euteleostomi - ps22
Sarcopterygii - ps23
Tetrapoda - ps24
Amniota - ps25
Mammalia - ps26
Theria - ps27
Placentalia - ps28
Boreoeutheria - ps29
Euarchontoglires - ps30
Euarchonta - ps31
Primates - ps32
Haplorrhini - ps33
Simiiformes - ps34
Catarrhini - ps35
Hominoidea - ps36
Hominidae - ps37
Homininae - ps38
Hominini - ps39
Homo_sapiens - ps40
p-value
6e-05
4e-04
2e-03
1e-02
6e-02
Log-odds
0.5
1.0
1.5
2.0
2.5
3.0
3.5
4.0

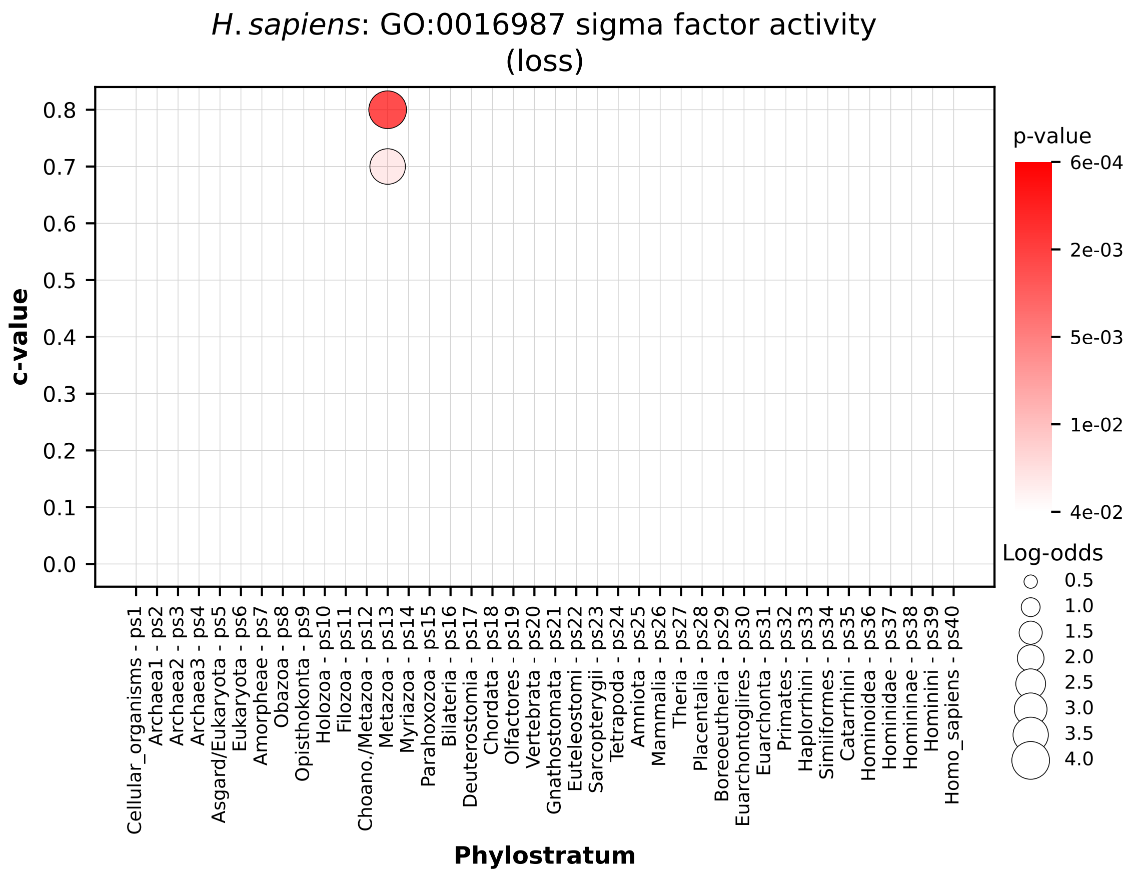

H. sapiens: GO:0016987 sigma factor activity
(loss)
c-value
0.0
0.1
0.2
0.3
0.4
0.5
0.6
0.7
0.8
p-value
6e-04
2e-03
5e-03
1e-02
4e-02
Log-odds
0.5
1.0
1.5
2.0
2.5
3.0
3.5
4.0
Cellular_organisms - ps1
Archaea1 - ps2
Archaea2 - ps3
Archaea3 - ps4
Asgard/Eukaryota - ps5
Eukaryota - ps6
Amorpheae - ps7
Obazoa - ps8
Opisthokonta - ps9
Holozoa - ps10
Filozoa - ps11
Choano./Metazoa - ps12
Metazoa - ps13
Myriazoa - ps14
Parahoxozoa - ps15
Bilateria - ps16
Deuterostomia - ps17
Chordata - ps18
Olfactores - ps19
Vertebrata - ps20
Gnathostomata - ps21
Euteleostomi - ps22
Sarcopterygii - ps23
Tetrapoda - ps24
Amniota - ps25
Mammalia - ps26
Theria - ps27
Placentalia - ps28
Boreoeutheria - ps29
Euarchontoglires - ps30
Euarchonta - ps31
Primates - ps32
Haplorrhini - ps33
Simiiformes - ps34
Catarrhini - ps35
Hominoidea - ps36
Hominidae - ps37
Homininae - ps38
Hominini - ps39
Homo_sapiens - ps40
Phylostratum

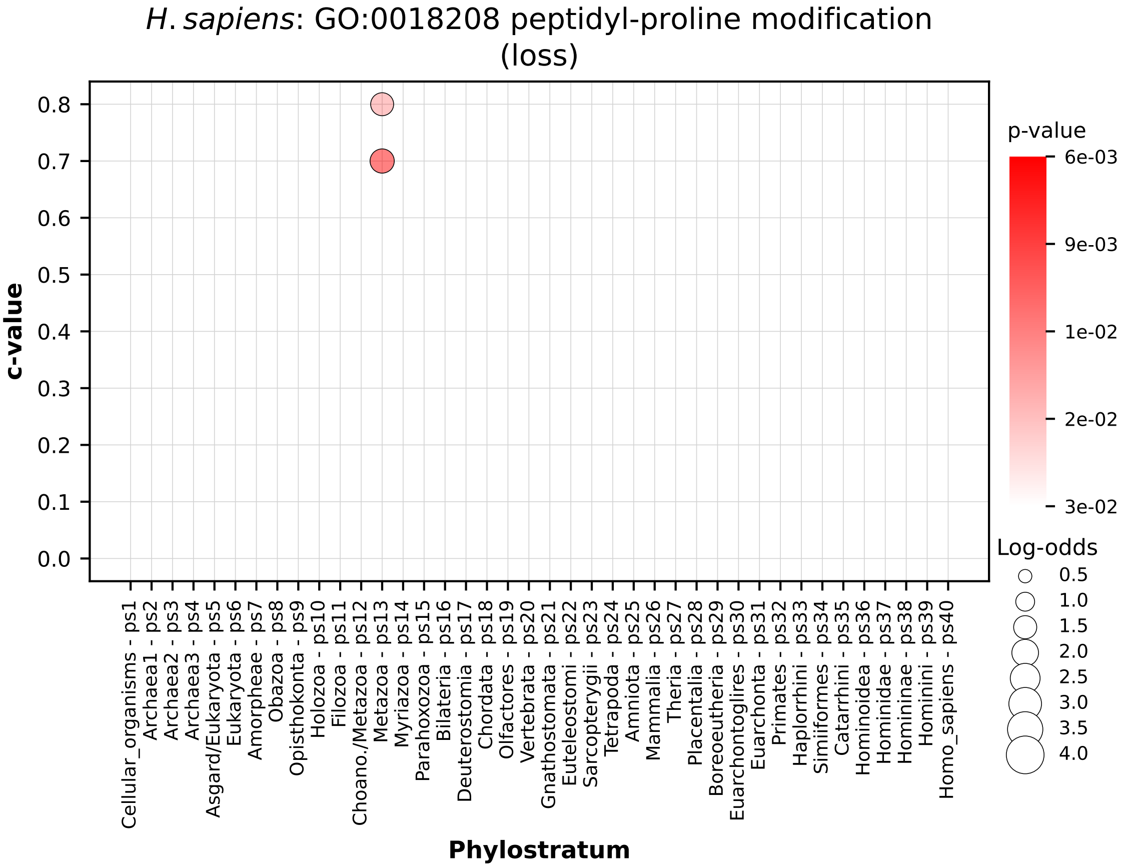

H. sapiens: GO:0018208 peptidyl-proline modification (loss)
c-value
0.0
0.1
0.2
0.3
0.4
0.5
0.6
0.7
0.8
Phylostratum
Cellular_organisms - ps1
Archaea1 - ps2
Archaea2 - ps3
Archaea3 - ps4
Asgard/Eukaryota - ps5
Eukaryota - ps6
Amorpheae - ps7
Obazoa - ps8
Opisthokonta - ps9
Holozoa - ps10
Filozoa - ps11
Choano./Metazoa - ps12
Metazoa - ps13
Myriazoa - ps14
Parahoxozoa - ps15
Bilateria - ps16
Deuterostomia - ps17
Chordata - ps18
Olfactores - ps19
Vertebrata - ps20
Gnathostomata - ps21
Euteleostomi - ps22
Sarcopterygii - ps23
Tetrapoda - ps24
Amniota - ps25
Mammalia - ps26
Theria - ps27
Placentalia - ps28
Boreoeutheria - ps29
Euarchontoglires - ps30
Euarchonta - ps31
Primates - ps32
Haplorrhini - ps33
Simiiformes - ps34
Catarrhini - ps35
Hominoidea - ps36
Hominidae - ps37
Homininae - ps38
Hominini - ps39
Homo_sapiens - ps40
p-value
6e-03
9e-03
1e-02
2e-02
3e-02
Log-odds
0.5
1.0
1.5
2.0
2.5
3.0
3.5
4.0

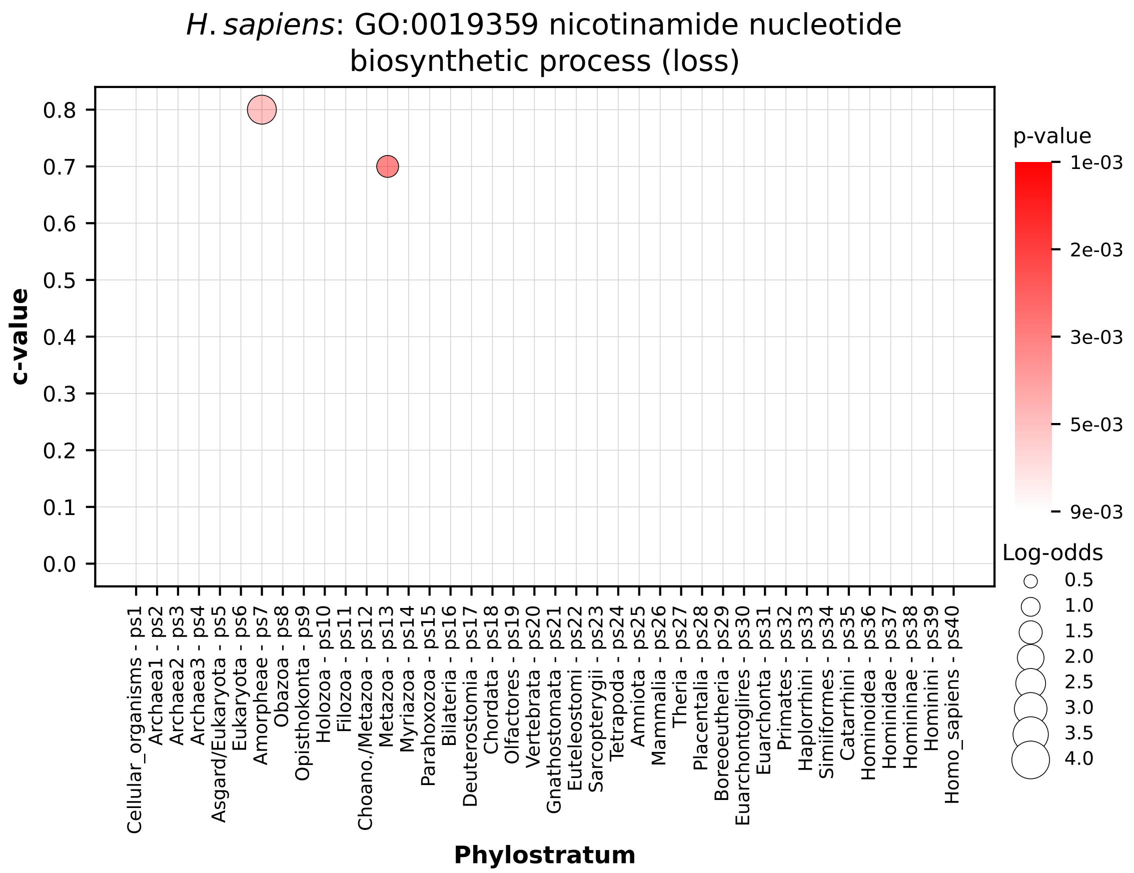

H. sapiens: GO:0019359 nicotinamide nucleotide biosynthetic process (loss)
c-value
0.8
0.7
0.6
0.5
0.4
0.3
0.2
0.1
0.0
p-value
1e-03
2e-03
3e-03
5e-03
9e-03
Log-odds
0.5
1.0
1.5
2.0
2.5
3.0
3.5
4.0
Cellular_organisms - ps1
Archaea1 - ps2
Archaea2 - ps3
Archaea3 - ps4
Asgard/Eukaryota - ps5
Eukaryota - ps6
Amorpheae - ps7
Obazoa - ps8
Opisthokonta - ps9
Holozoa - ps10
Filozoa - ps11
Choano./Metazoa - ps12
Metazoa - ps13
Myriazoa - ps14
Parahoxozoa - ps15
Bilateria - ps16
Deuterostomia - ps17
Chordata - ps18
Olfactores - ps19
Vertebrata - ps20
Gnathostomata - ps21
Euteleostomi - ps22
Sarcopterygii - ps23
Tetrapoda - ps24
Amniota - ps25
Mammalia - ps26
Theria - ps27
Placentalia - ps28
Boreoeutheria - ps29
Euarchontoglires - ps30
Euarchonta - ps31
Primates - ps32
Haplorrhini - ps33
Simiiformes - ps34
Catarrhini - ps35
Hominoidea - ps36
Hominidae - ps37
Homininae - ps38
Hominini - ps39
Homo_sapiens - ps40
Phylostratum

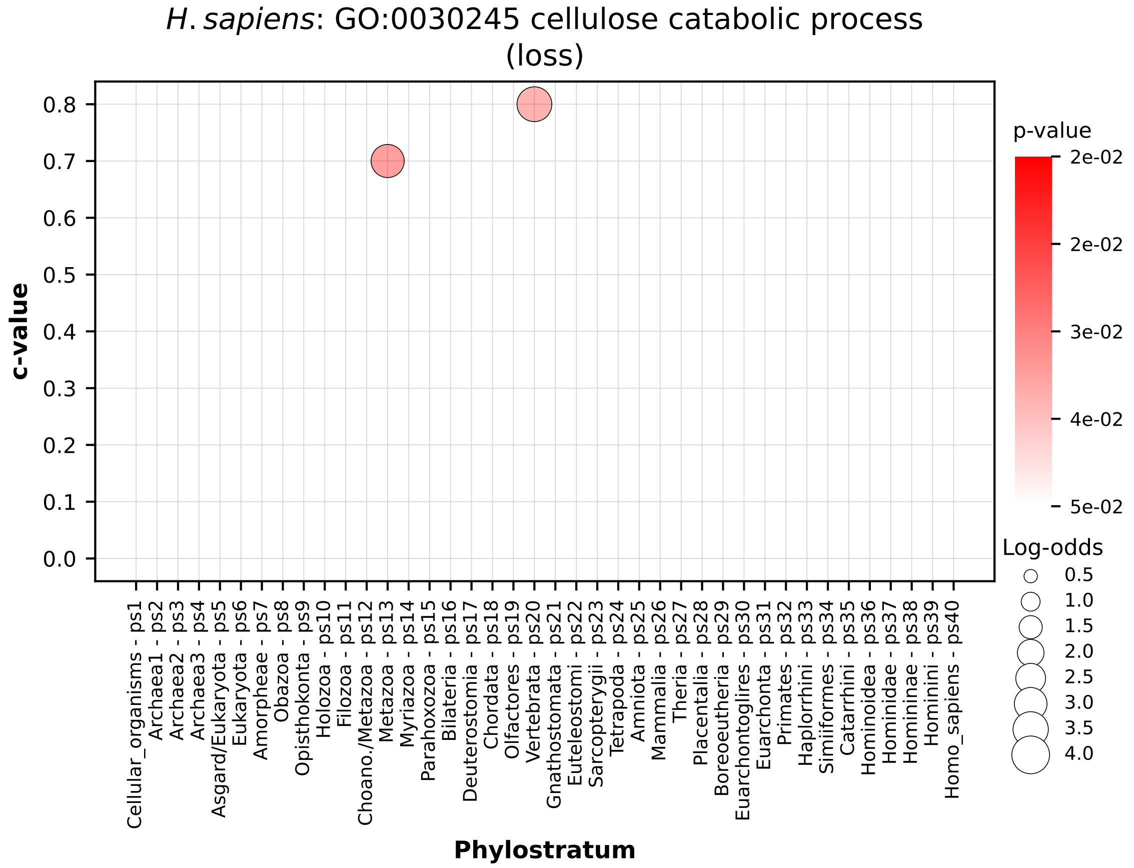

H. sapiens: GO:0030245 cellulose catabolic process
(loss)
c-value
0.0
0.1
0.2
0.3
0.4
0.5
0.6
0.7
0.8
p-value
2e-02
2e-02
3e-02
4e-02
5e-02
Log-odds
0.5
1.0
1.5
2.0
2.5
3.0
3.5
4.0
Cellular_organisms - ps1
Archaea1 - ps2
Archaea2 - ps3
Archaea3 - ps4
Asgard/Eukaryota - ps5
Eukaryota - ps6
Amorpheae - ps7
Obazoa - ps8
Opisthokonta - ps9
Holozoa - ps10
Filozoa - ps11
Choano./Metazoa - ps12
Metazoa - ps13
Myriazoa - ps14
Parahoxozoa - ps15
Bilateria - ps16
Deuterostomia - ps17
Chordata - ps18
Olfactores - ps19
Vertebrata - ps20
Gnathostomata - ps21
Euteleostomi - ps22
Sarcopterygii - ps23
Tetrapoda - ps24
Amniota - ps25
Mammalia - ps26
Theria - ps27
Placentalia - ps28
Boreoeutheria - ps29
Euarchontoglires - ps30
Euarchonta - ps31
Primates - ps32
Haplorrhini - ps33
Simiiformes - ps34
Catarrhini - ps35
Hominoidea - ps36
Hominidae - ps37
Homininae - ps38
Hominini - ps39
Homo_sapiens - ps40
Phylostratum

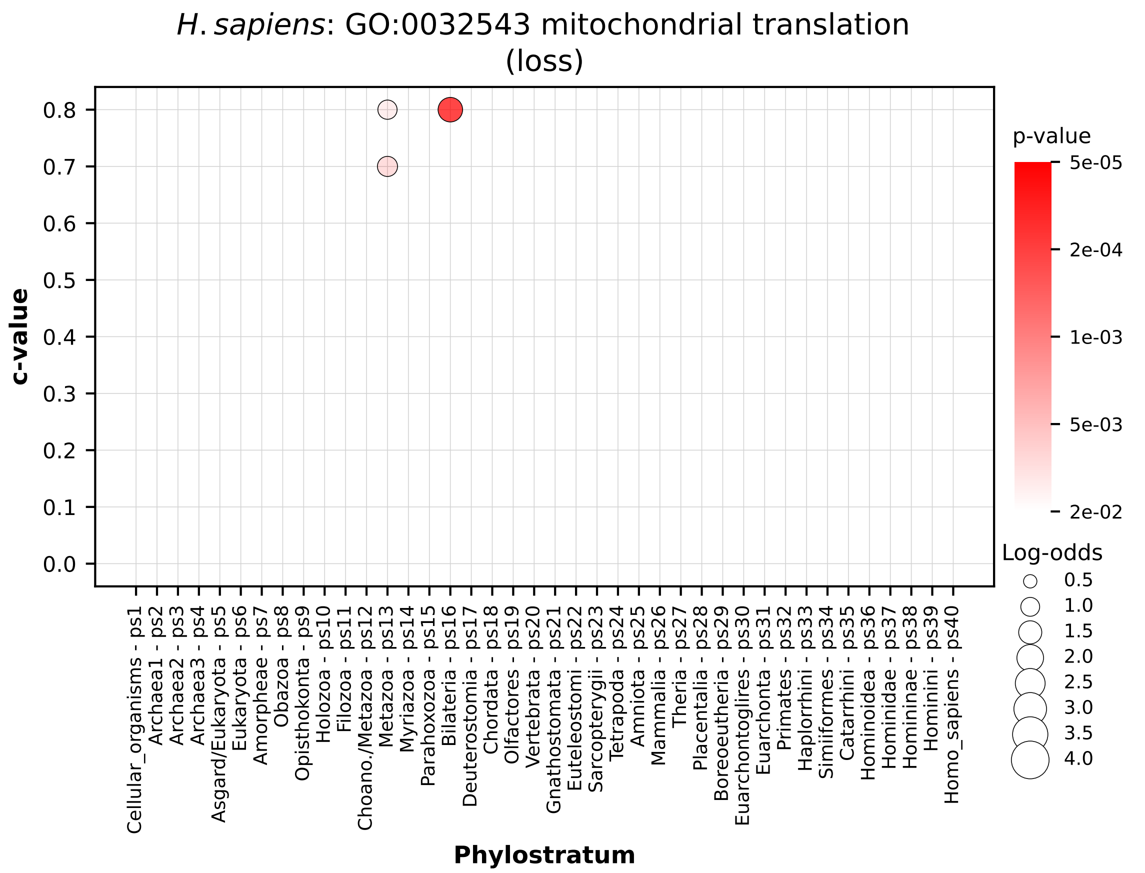

H. sapiens: GO:0032543 mitochondrial translation
(loss)
c-value
0.0
0.1
0.2
0.3
0.4
0.5
0.6
0.7
0.8
p-value
5e-05
2e-04
1e-03
5e-03
2e-02
Log-odds
0.5
1.0
1.5
2.0
2.5
3.0
3.5
4.0
Cellular_organisms - ps1
Archaea1 - ps2
Archaea2 - ps3
Archaea3 - ps4
Asgard/Eukaryota - ps5
Eukaryota - ps6
Amorpheae - ps7
Obazoa - ps8
Opisthokonta - ps9
Holozoa - ps10
Filozoa - ps11
Choano./Metazoa - ps12
Metazoa - ps13
Myriazoa - ps14
Parahoxozoa - ps15
Bilateria - ps16
Deuterostomia - ps17
Chordata - ps18
Olfactores - ps19
Vertebrata - ps20
Gnathostomata - ps21
Euteleostomi - ps22
Sarcopterygii - ps23
Tetrapoda - ps24
Amniota - ps25
Mammalia - ps26
Theria - ps27
Placentalia - ps28
Boreoeutheria - ps29
Euarchontoglires - ps30
Euarchonta - ps31
Primates - ps32
Haplorrhini - ps33
Simiiformes - ps34
Catarrhini - ps35
Hominoidea - ps36
Hominidae - ps37
Homininae - ps38
Hominini - ps39
Homo_sapiens - ps40
Phylostratum

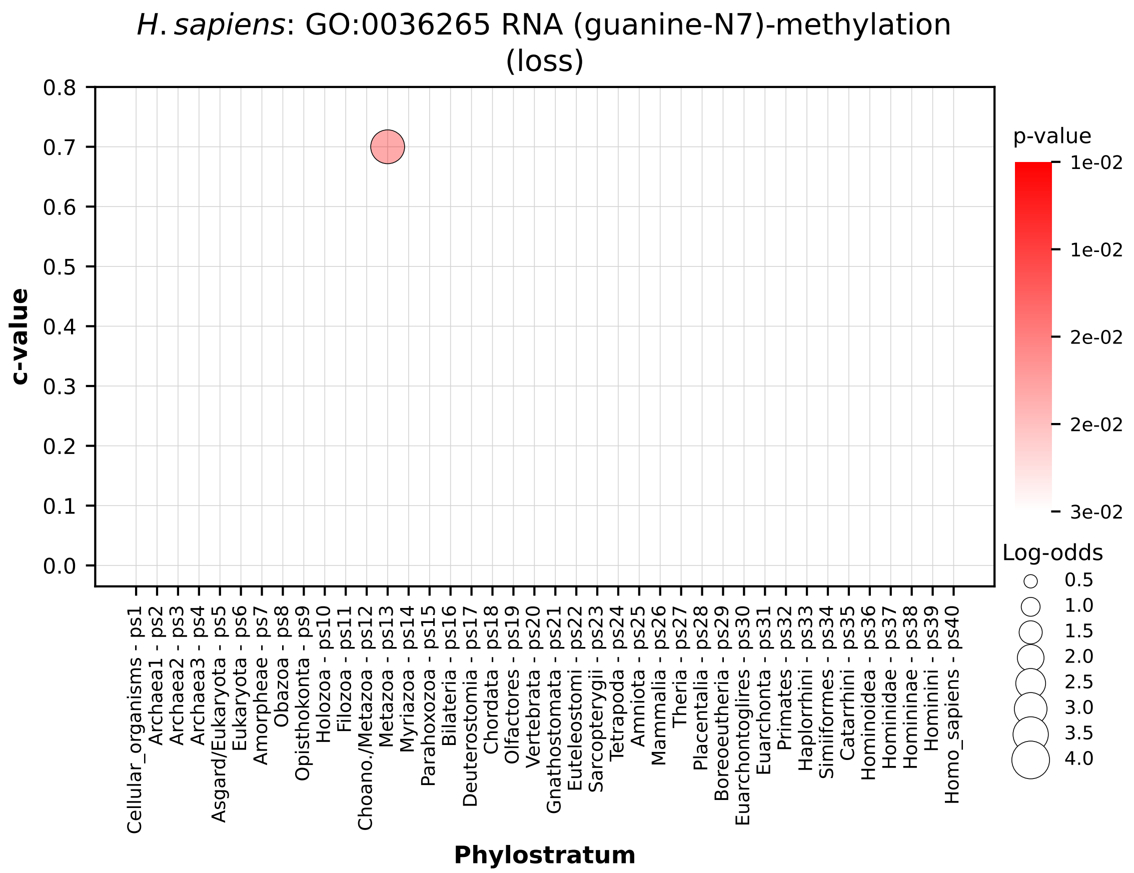

H. sapiens: GO:0036265 RNA (guanine-N7)-methylation (loss)
c-value
0.8
0.7
0.6
0.5
0.4
0.3
0.2
0.1
0.0
p-value
1e-02
1e-02
2e-02
2e-02
3e-02
Log-odds
0.5
1.0
1.5
2.0
2.5
3.0
3.5
4.0
Cellular_organisms - ps1
Archaea1 - ps2
Archaea2 - ps3
Archaea3 - ps4
Asgard/Eukaryota - ps5
Eukaryota - ps6
Amorpheae - ps7
Obazoa - ps8
Opisthokonta - ps9
Holozoa - ps10
Filozoa - ps11
Choano./Metazoa - ps12
Metazoa - ps13
Myriazoa - ps14
Parahoxozoa - ps15
Bilateria - ps16
Deuterostomia - ps17
Chordata - ps18
Olfactores - ps19
Vertebrata - ps20
Gnathostomata - ps21
Euteleostomi - ps22
Sarcopterygii - ps23
Tetrapoda - ps24
Amniota - ps25
Mammalia - ps26
Theria - ps27
Placentalia - ps28
Boreoeutheria - ps29
Euarchontoglires - ps30
Euarchonta - ps31
Primates - ps32
Haplorrhini - ps33
Simiiformes - ps34
Catarrhini - ps35
Hominoidea - ps36
Hominidae - ps37
Homininae - ps38
Hominini - ps39
Homo_sapiens - ps40
Phylostratum

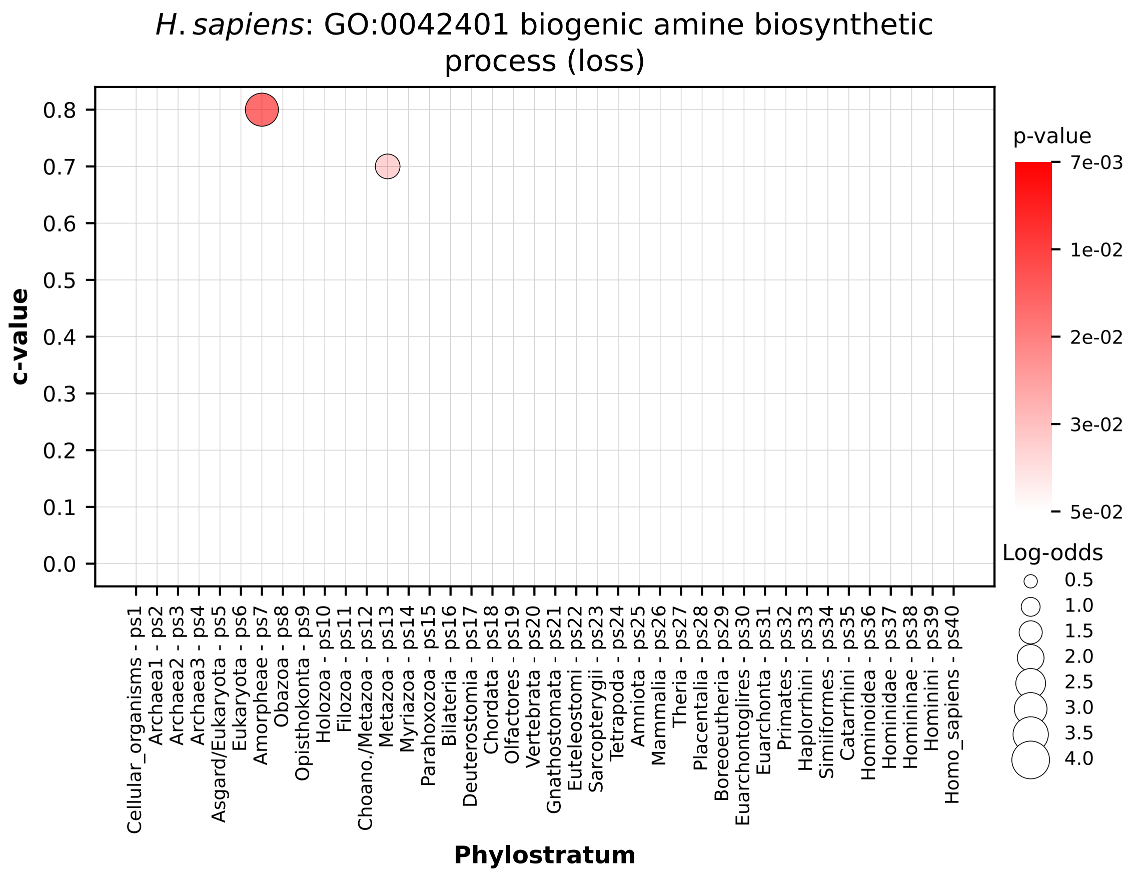
H. sapiens: GO:0042401 biogenic amine biosynthetic process (loss)
c-value
0.8
0.7
0.6
0.5
0.4
0.3
0.2
0.1
0.0
p-value
7e-03
1e-02
2e-02
3e-02
5e-02
Log-odds
0.5
1.0
1.5
2.0
2.5
3.0
3.5
4.0
Cellular_organisms - ps1
Archaea1 - ps2
Archaea2 - ps3
Archaea3 - ps4
Asgard/Eukaryota - ps5
Eukaryota - ps6
Amorpheae - ps7
Obazoa - ps8
Opisthokonta - ps9
Holozoa - ps10
Filozoa - ps11
Choano./Metazoa - ps12
Metazoa - ps13
Myriazoa - ps14
Parahoxozoa - ps15
Bilateria - ps16
Deuterostomia - ps17
Chordata - ps18
Olfactores - ps19
Vertebrata - ps20
Gnathostomata - ps21
Euteleostomi - ps22
Sarcopterygii - ps23
Tetrapoda - ps24
Amniota - ps25
Mammalia - ps26
Theria - ps27
Placentalia - ps28
Boreoeutheria - ps29
Euarchontoglires - ps30
Euarchonta - ps31
Primates - ps32
Haplorrhini - ps33
Simiiformes - ps34
Catarrhini - ps35
Hominoidea - ps36
Hominidae - ps37
Homininae - ps38
Hominini - ps39
Homo_sapiens - ps40
Phylostratum

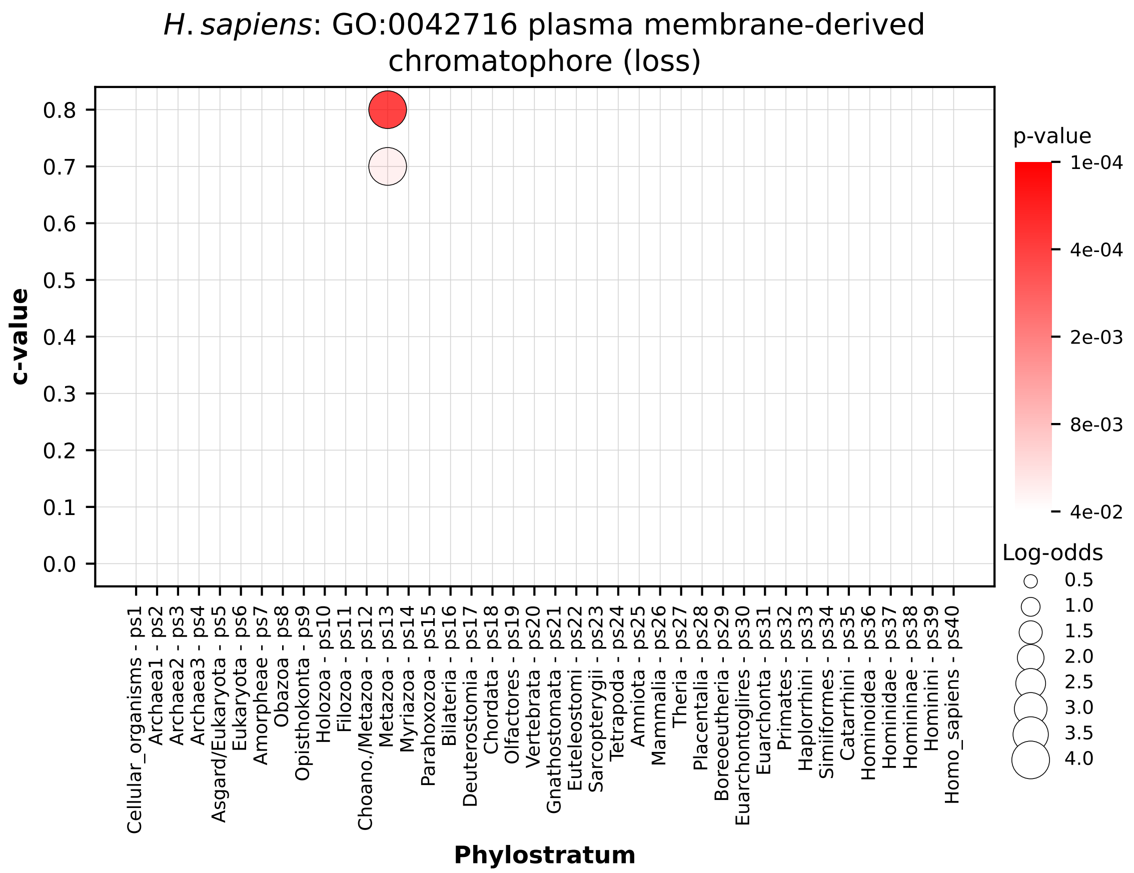

H. sapiens: GO:0042716 plasma membrane-derived chromatophore (loss)
c-value
0.8
0.7
0.6
0.5
0.4
0.3
0.2
0.1
0.0
p-value
1e-04
4e-04
2e-03
8e-03
4e-02
Log-odds
0.5
1.0
1.5
2.0
2.5
3.0
3.5
4.0
Cellular_organisms - ps1
Archaea1 - ps2
Archaea2 - ps3
Archaea3 - ps4
Asgard/Eukaryota - ps5
Eukaryota - ps6
Amorpheae - ps7
Obazoa - ps8
Opisthokonta - ps9
Holozoa - ps10
Filozoa - ps11
Choano./Metazoa - ps12
Metazoa - ps13
Myriazoa - ps14
Parahoxozoa - ps15
Bilateria - ps16
Deuterostomia - ps17
Chordata - ps18
Olfactores - ps19
Vertebrata - ps20
Gnathostomata - ps21
Euteleostomi - ps22
Sarcopterygii - ps23
Tetrapoda - ps24
Amniota - ps25
Mammalia - ps26
Theria - ps27
Placentalia - ps28
Boreoeutheria - ps29
Euarchontoglires - ps30
Euarchonta - ps31
Primates - ps32
Haplorrhini - ps33
Simiiformes - ps34
Catarrhini - ps35
Hominoidea - ps36
Hominidae - ps37
Homininae - ps38
Hominini - ps39
Homo_sapiens - ps40
Phylostratum

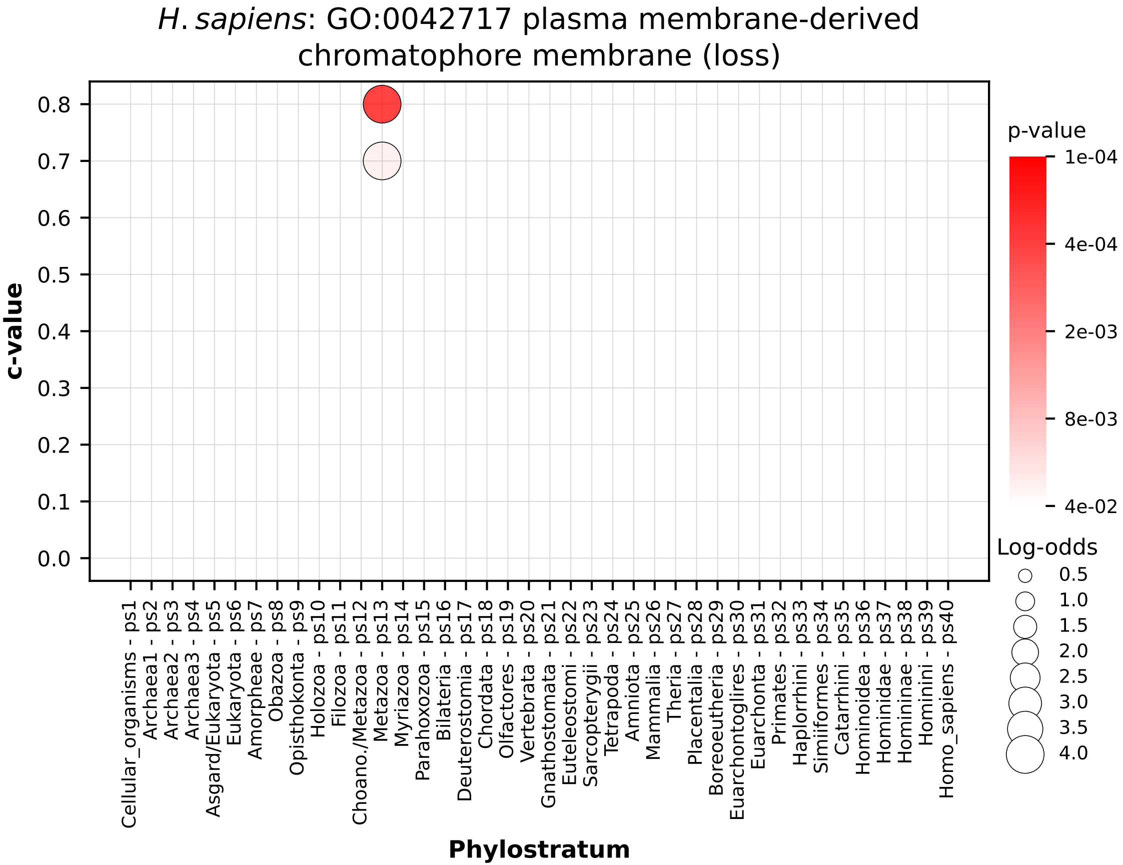

H. sapiens: GO:0042717 plasma membrane-derived chromatophore membrane (loss)
c-value
0.8
0.7
0.6
0.5
0.4
0.3
0.2
0.1
0.0
p-value
1e-04
4e-04
2e-03
8e-03
4e-02
Log-odds
0.5
1.0
1.5
2.0
2.5
3.0
3.5
4.0
Cellular_organisms - ps1
Archaea1 - ps2
Archaea2 - ps3
Archaea3 - ps4
Asgard/Eukaryota - ps5
Eukaryota - ps6
Amorpheae - ps7
Obazoa - ps8
Opisthokonta - ps9
Holozoa - ps10
Filozoa - ps11
Choano./Metazoa - ps12
Metazoa - ps13
Myriazoa - ps14
Parahoxozoa - ps15
Bilateria - ps16
Deuterostomia - ps17
Chordata - ps18
Olfactores - ps19
Vertebrata - ps20
Gnathostomata - ps21
Euteleostomi - ps22
Sarcopterygii - ps23
Tetrapoda - ps24
Amniota - ps25
Mammalia - ps26
Theria - ps27
Placentalia - ps28
Boreoeutheria - ps29
Euarchontoglires - ps30
Euarchonta - ps31
Primates - ps32
Haplorrhini - ps33
Simiiformes - ps34
Catarrhini - ps35
Hominoidea - ps36
Hominidae - ps37
Homininae - ps38
Hominini - ps39
Homo_sapiens - ps40
Phylostratum

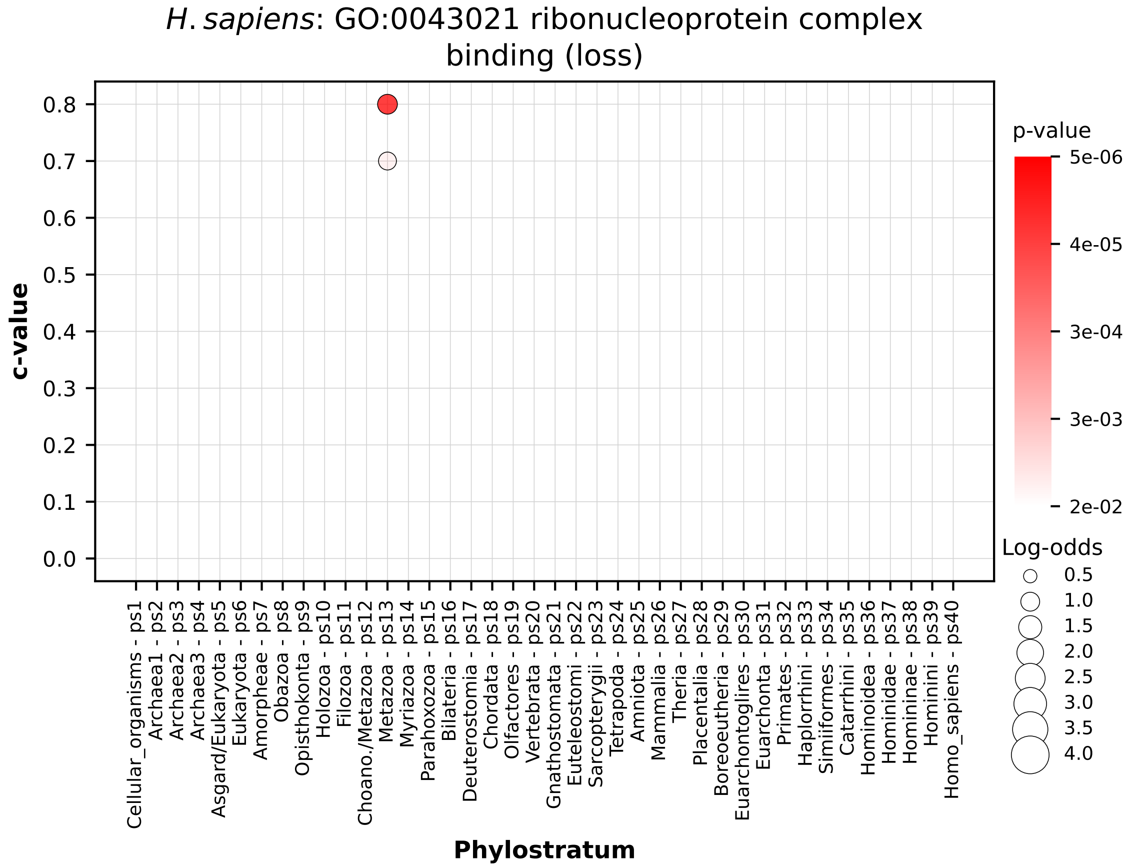
H. sapiens: GO:0043021 ribonucleoprotein complex binding (loss)
c-value
0.0
0.1
0.2
0.3
0.4
0.5
0.6
0.7
0.8
Phylostratum
Cellular_organisms - ps1
Archaea1 - ps2
Archaea2 - ps3
Archaea3 - ps4
Asgard/Eukaryota - ps5
Eukaryota - ps6
Amorpheae - ps7
Obazoa - ps8
Opisthokonta - ps9
Holozoa - ps10
Filozoa - ps11
Choano./Metazoa - ps12
Metazoa - ps13
Myriazoa - ps14
Parahoxozoa - ps15
Bilateria - ps16
Deuterostomia - ps17
Chordata - ps18
Olfactores - ps19
Vertebrata - ps20
Gnathostomata - ps21
Euteleostomi - ps22
Sarcopterygii - ps23
Tetrapoda - ps24
Amniota - ps25
Mammalia - ps26
Theria - ps27
Placentalia - ps28
Boreoeutheria - ps29
Euarchontoglires - ps30
Euarchonta - ps31
Primates - ps32
Haplorrhini - ps33
Simiiformes - ps34
Catarrhini - ps35
Hominoidea - ps36
Hominidae - ps37
Homininae - ps38
Hominini - ps39
Homo_sapiens - ps40
p-value
5e-06
4e-05
3e-04
3e-03
2e-02
Log-odds
0.5
1.0
1.5
2.0
2.5
3.0
3.5
4.0

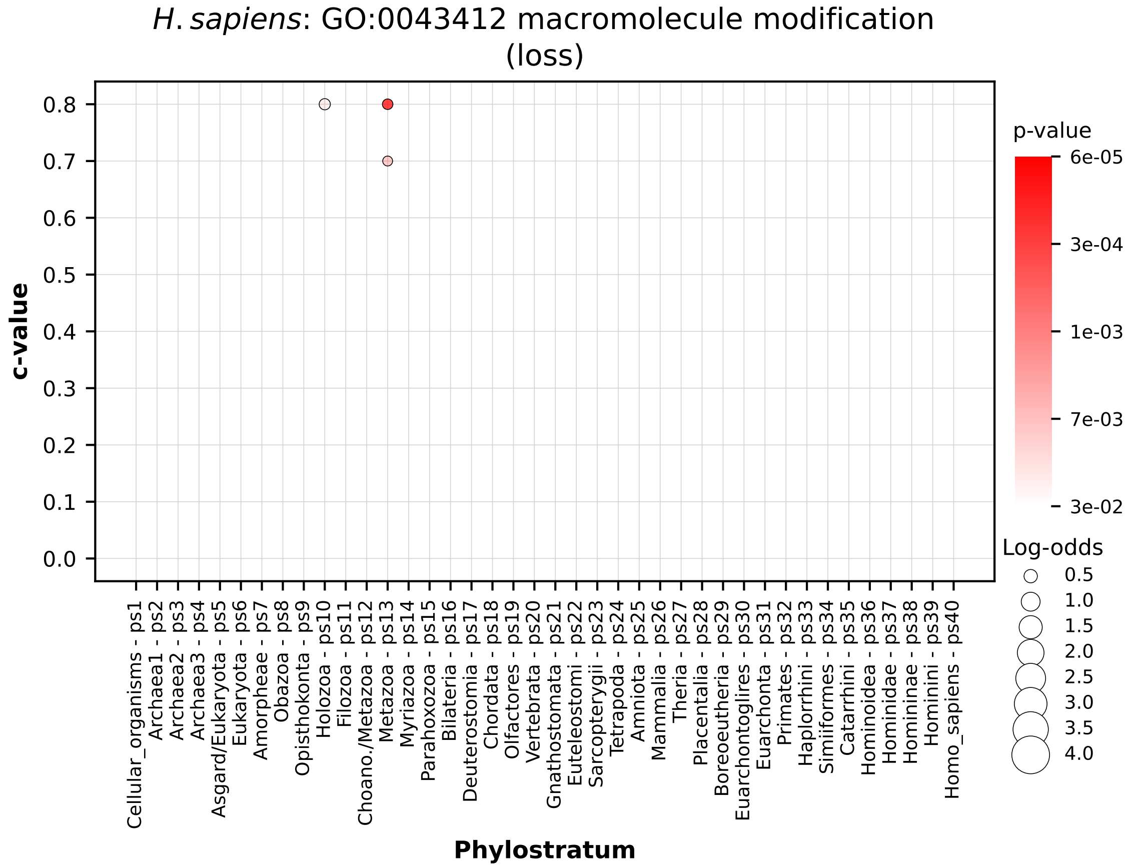

H. sapiens: GO:0043412 macromolecule modification (loss)
c-value
0.8
0.7
0.6
0.5
0.4
0.3
0.2
0.1
0.0
p-value
6e-05
3e-04
1e-03
7e-03
3e-02
Log-odds
0.5
1.0
1.5
2.0
2.5
3.0
3.5
4.0
Cellular_organisms - ps1
Archaea1 - ps2
Archaea2 - ps3
Archaea3 - ps4
Asgard/Eukaryota - ps5
Eukaryota - ps6
Amorpheae - ps7
Obazoa - ps8
Opisthokonta - ps9
Holozoa - ps10
Filozoa - ps11
Choano./Metazoa - ps12
Metazoa - ps13
Myriazoa - ps14
Parahoxozoa - ps15
Bilateria - ps16
Deuterostomia - ps17
Chordata - ps18
Olfactores - ps19
Vertebrata - ps20
Gnathostomata - ps21
Euteleostomi - ps22
Sarcopterygii - ps23
Tetrapoda - ps24
Amniota - ps25
Mammalia - ps26
Theria - ps27
Placentalia - ps28
Boreoeutheria - ps29
Euarchontoglires - ps30
Euarchonta - ps31
Primates - ps32
Haplorrhini - ps33
Simiiformes - ps34
Catarrhini - ps35
Hominoidea - ps36
Hominidae - ps37
Homininae - ps38
Hominini - ps39
Homo_sapiens - ps40
Phylostratum

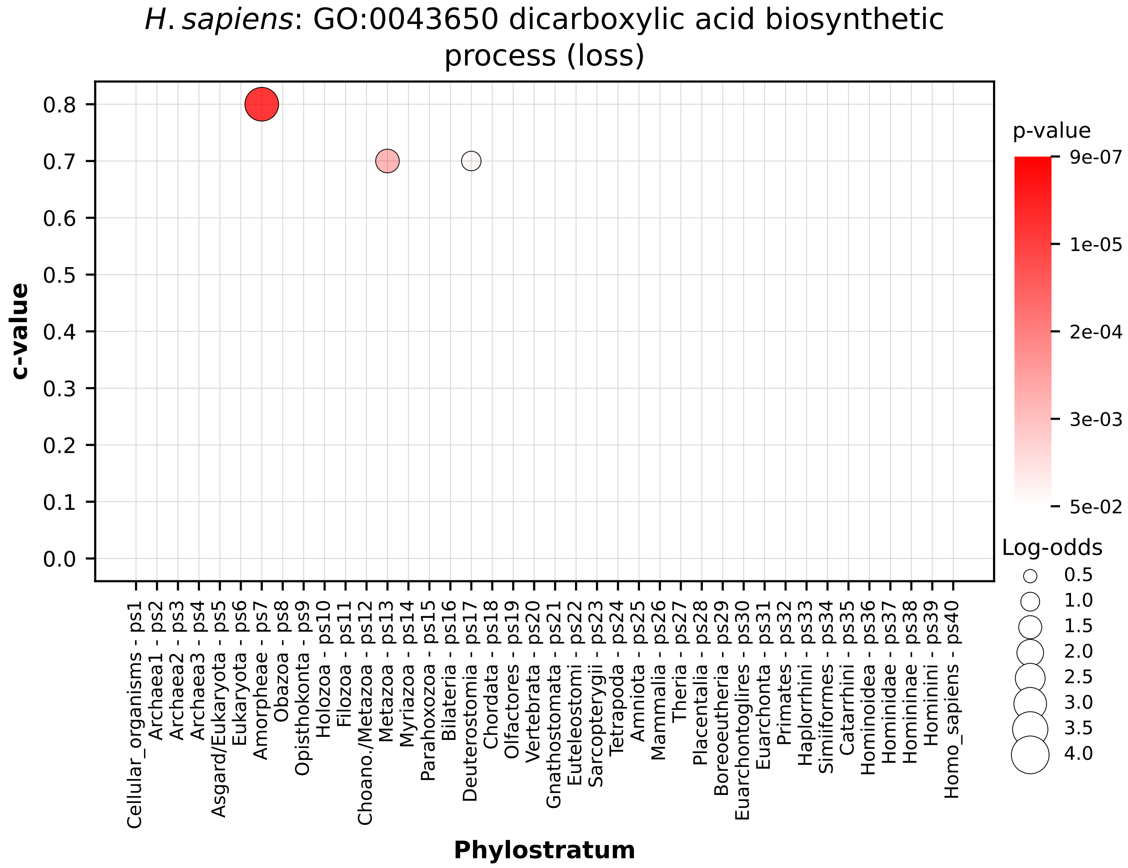

H. sapiens: GO:0043650 dicarboxylic acid biosynthetic process (loss)
c-value
0.8
0.7
0.6
0.5
0.4
0.3
0.2
0.1
0.0
p-value
9e-07
1e-05
2e-04
3e-03
5e-02
Log-odds
0.5
1.0
1.5
2.0
2.5
3.0
3.5
4.0
Cellular_organisms - ps1
Archaea1 - ps2
Archaea2 - ps3
Archaea3 - ps4
Asgard/Eukaryota - ps5
Eukaryota - ps6
Amorpheae - ps7
Obazoa - ps8
Opisthokonta - ps9
Holozoa - ps10
Filozoa - ps11
Choano./Metazoa - ps12
Metazoa - ps13
Myriazoa - ps14
Parahoxozoa - ps15
Bilateria - ps16
Deuterostomia - ps17
Chordata - ps18
Olfactores - ps19
Vertebrata - ps20
Gnathostomata - ps21
Euteleostomi - ps22
Sarcopterygii - ps23
Tetrapoda - ps24
Amniota - ps25
Mammalia - ps26
Theria - ps27
Placentalia - ps28
Boreoeutheria - ps29
Euarchontoglires - ps30
Euarchonta - ps31
Primates - ps32
Haplorrhini - ps33
Simiiformes - ps34
Catarrhini - ps35
Hominoidea - ps36
Hominidae - ps37
Homininae - ps38
Hominini - ps39
Homo_sapiens - ps40
Phylostratum

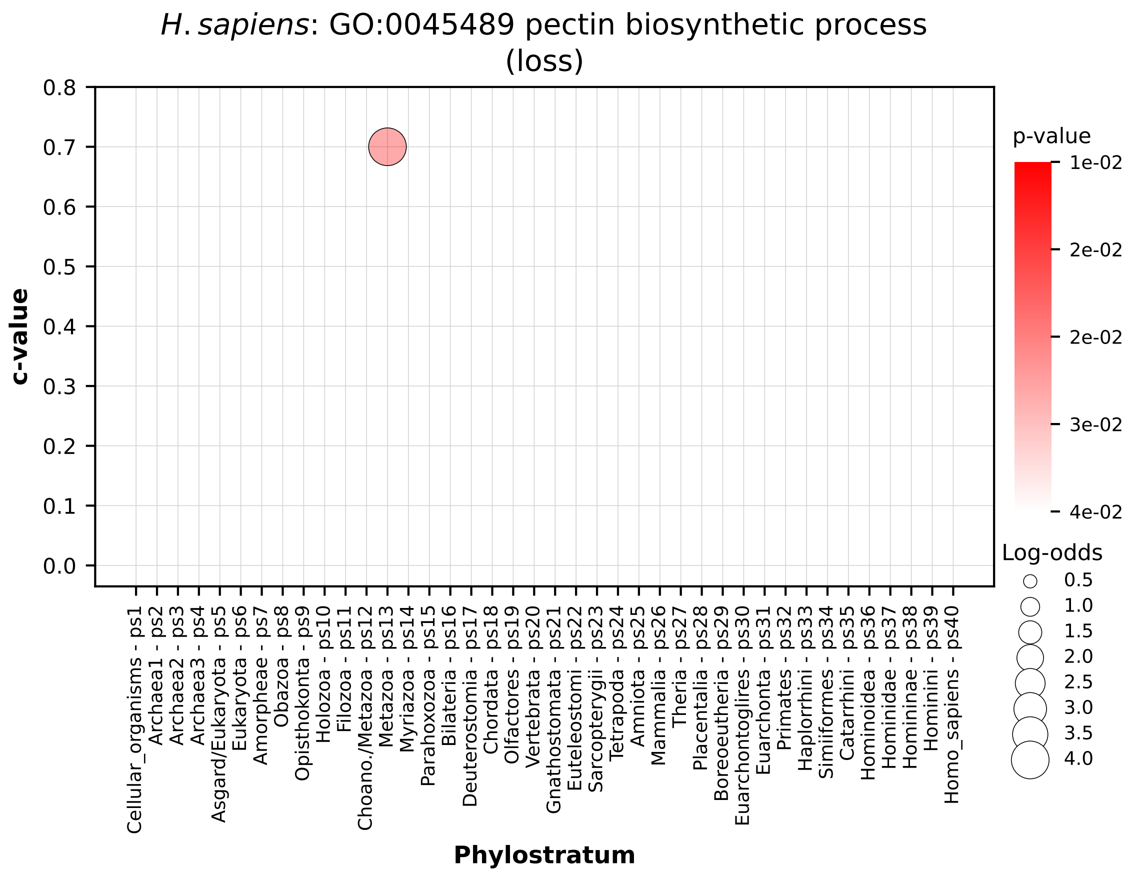

H. sapiens: GO:0045489 pectin biosynthetic process
(loss)
c-value
0.8
0.7
0.6
0.5
0.4
0.3
0.2
0.1
0.0
p-value
1e-02
2e-02
2e-02
3e-02
4e-02
Log-odds
0.5
1.0
1.5
2.0
2.5
3.0
3.5
4.0
Cellular_organisms - ps1
Archaea1 - ps2
Archaea2 - ps3
Archaea3 - ps4
Asgard/Eukaryota - ps5
Eukaryota - ps6
Amorpheae - ps7
Obazoa - ps8
Opisthokonta - ps9
Holozoa - ps10
Filozoa - ps11
Choano./Metazoa - ps12
Metazoa - ps13
Myriazoa - ps14
Parahoxozoa - ps15
Bilateria - ps16
Deuterostomia - ps17
Chordata - ps18
Olfactores - ps19
Vertebrata - ps20
Gnathostomata - ps21
Euteleostomi - ps22
Sarcopterygii - ps23
Tetrapoda - ps24
Amniota - ps25
Mammalia - ps26
Theria - ps27
Placentalia - ps28
Boreoeutheria - ps29
Euarchontoglires - ps30
Euarchonta - ps31
Primates - ps32
Haplorrhini - ps33
Simiiformes - ps34
Catarrhini - ps35
Hominoidea - ps36
Hominidae - ps37
Homininae - ps38
Hominini - ps39
Homo_sapiens - ps40
Phylostratum

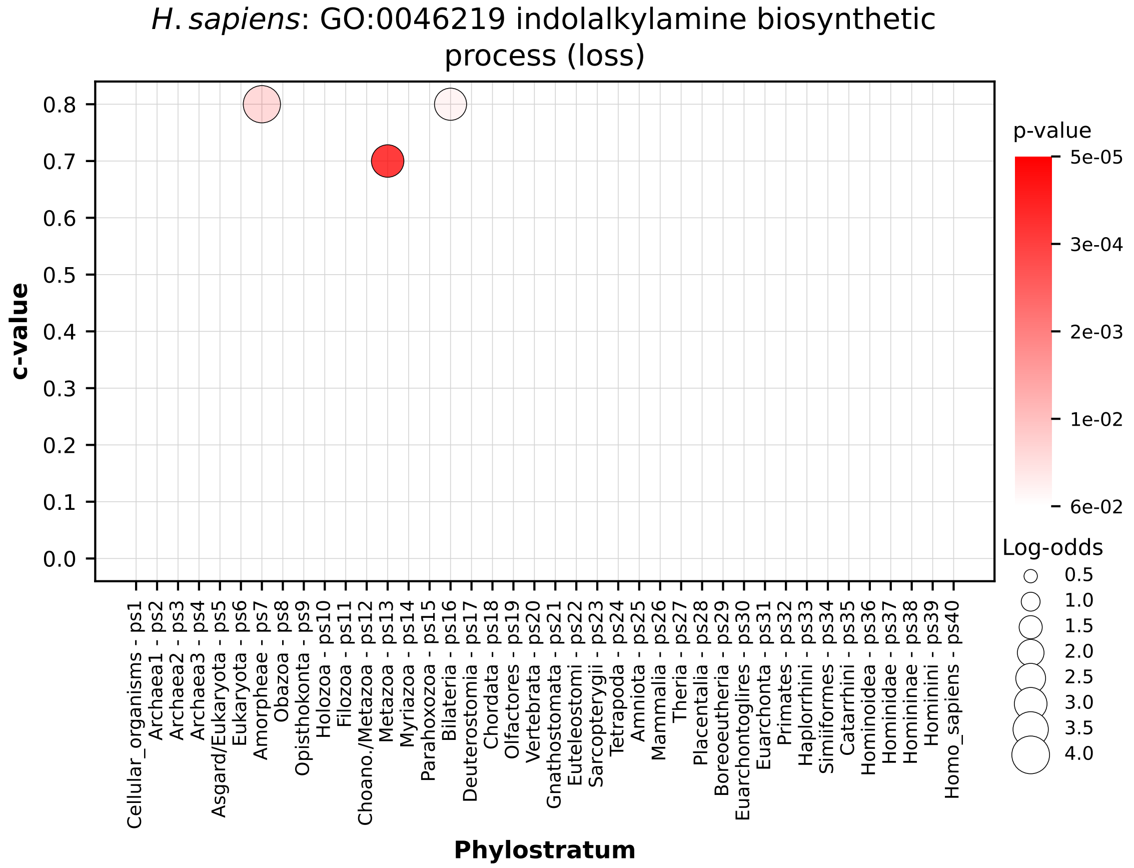

H. sapiens: GO:0046219 indolalkylamine biosynthetic process (loss)
c-value
0.0
0.1
0.2
0.3
0.4
0.5
0.6
0.7
0.8
p-value
5e-05
3e-04
2e-03
1e-02
6e-02
Log-odds
0.5
1.0
1.5
2.0
2.5
3.0
3.5
4.0
Cellular_organisms - ps1
Archaea1 - ps2
Archaea2 - ps3
Archaea3 - ps4
Asgard/Eukaryota - ps5
Eukaryota - ps6
Amorpheae - ps7
Obazoa - ps8
Opisthokonta - ps9
Holozoa - ps10
Filozoa - ps11
Choano./Metazoa - ps12
Metazoa - ps13
Myriazoa - ps14
Parahoxozoa - ps15
Bilateria - ps16
Deuterostomia - ps17
Chordata - ps18
Olfactores - ps19
Vertebrata - ps20
Gnathostomata - ps21
Euteleostomi - ps22
Sarcopterygii - ps23
Tetrapoda - ps24
Amniota - ps25
Mammalia - ps26
Theria - ps27
Placentalia - ps28
Boreoeutheria - ps29
Euarchontoglires - ps30
Euarchonta - ps31
Primates - ps32
Haplorrhini - ps33
Simiiformes - ps34
Catarrhini - ps35
Hominoidea - ps36
Hominidae - ps37
Homininae - ps38
Hominini - ps39
Homo_sapiens - ps40
Phylostratum

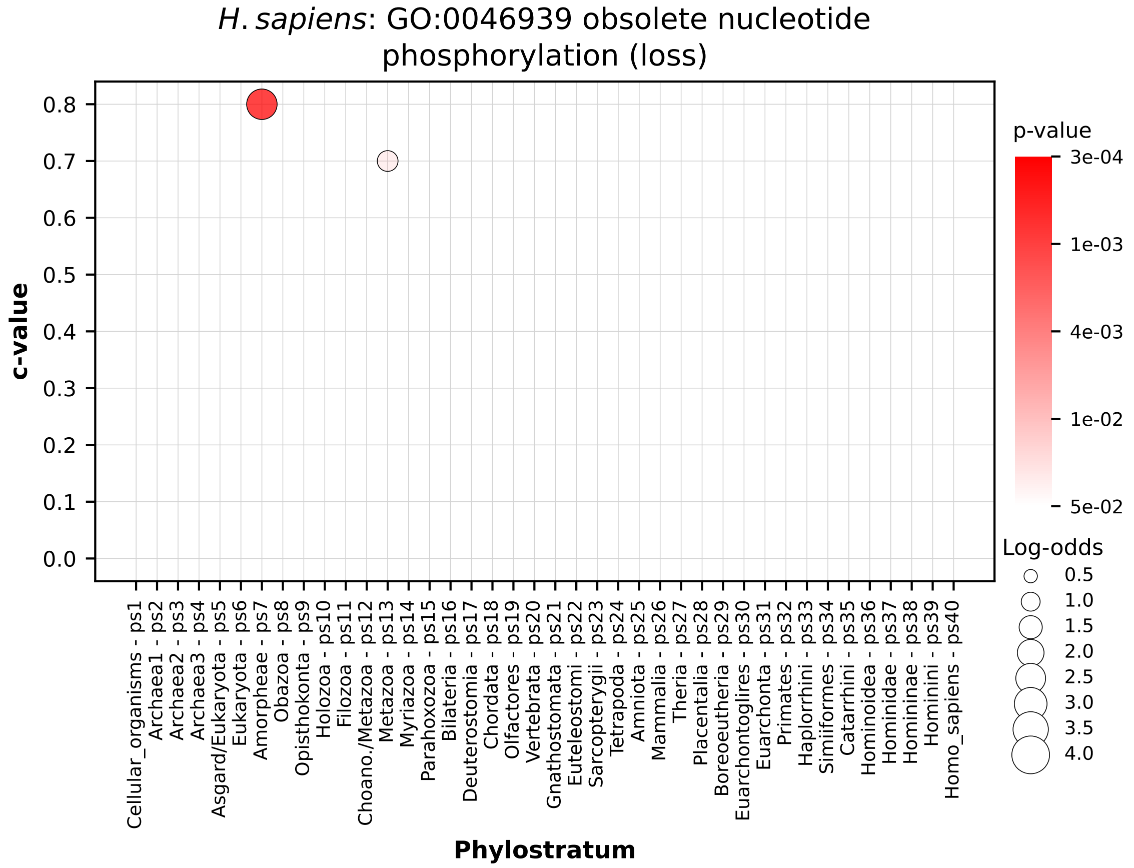

H. sapiens: GO:0046939 obsolete nucleotide phosphorylation (loss)
c-value
0.8
0.7
0.6
0.5
0.4
0.3
0.2
0.1
0.0
p-value
3e-04
1e-03
4e-03
1e-02
5e-02
Log-odds
0.5
1.0
1.5
2.0
2.5
3.0
3.5
4.0
Cellular_organisms - ps1
Archaea1 - ps2
Archaea2 - ps3
Archaea3 - ps4
Asgard/Eukaryota - ps5
Eukaryota - ps6
Amorpheae - ps7
Obazoa - ps8
Opisthokonta - ps9
Holozoa - ps10
Filozoa - ps11
Choano./Metazoa - ps12
Metazoa - ps13
Myriazoa - ps14
Parahoxozoa - ps15
Bilateria - ps16
Deuterostomia - ps17
Chordata - ps18
Olfactores - ps19
Vertebrata - ps20
Gnathostomata - ps21
Euteleostomi - ps22
Sarcopterygii - ps23
Tetrapoda - ps24
Amniota - ps25
Mammalia - ps26
Theria - ps27
Placentalia - ps28
Boreoeutheria - ps29
Euarchontoglires - ps30
Euarchonta - ps31
Primates - ps32
Haplorrhini - ps33
Simiiformes - ps34
Catarrhini - ps35
Hominoidea - ps36
Hominidae - ps37
Homininae - ps38
Hominini - ps39
Homo_sapiens - ps40
Phylostratum

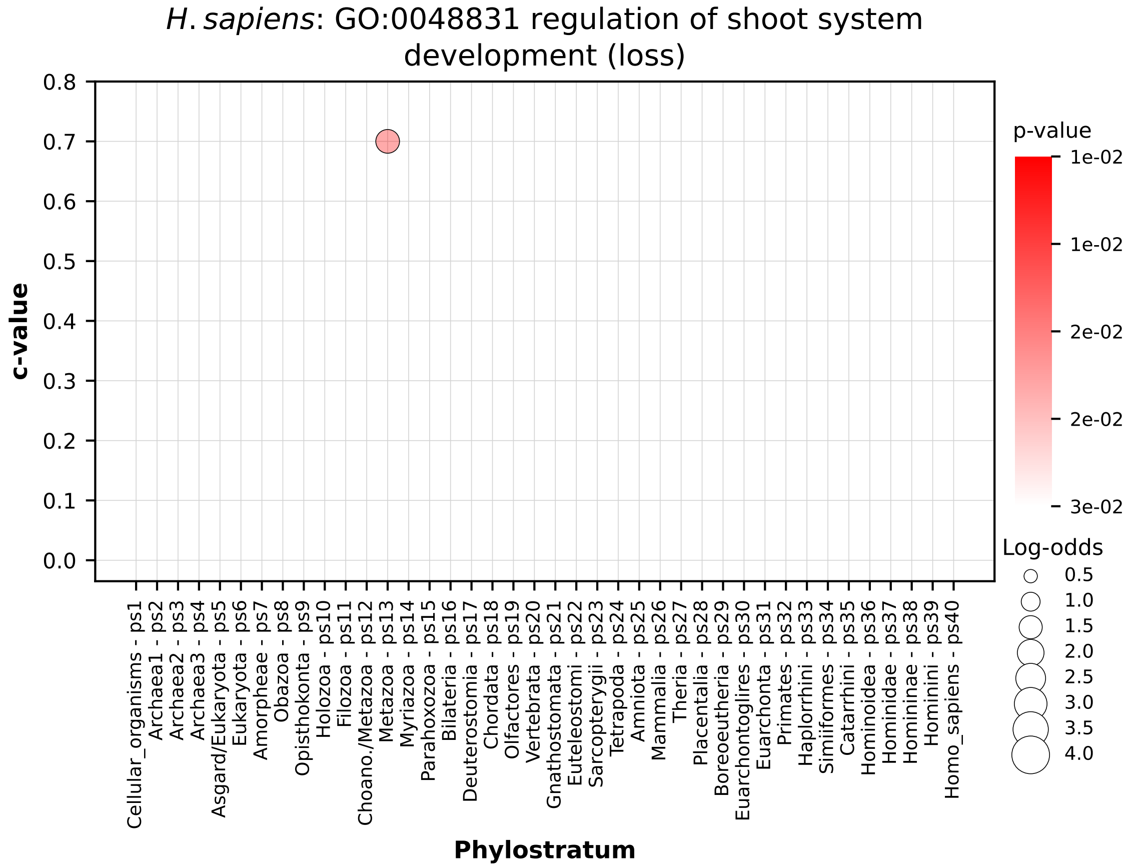

H. sapiens: GO:0048831 regulation of shoot system development (loss)
c-value
0.8
0.7
0.6
0.5
0.4
0.3
0.2
0.1
0.0
p-value
1e-02
1e-02
2e-02
2e-02
3e-02
Log-odds
0.5
1.0
1.5
2.0
2.5
3.0
3.5
4.0
Cellular_organisms - ps1
Archaea1 - ps2
Archaea2 - ps3
Archaea3 - ps4
Asgard/Eukaryota - ps5
Eukaryota - ps6
Amorpheae - ps7
Obazoa - ps8
Opisthokonta - ps9
Holozoa - ps10
Filozoa - ps11
Choano./Metazoa - ps12
Metazoa - ps13
Myriazoa - ps14
Parahoxozoa - ps15
Bilateria - ps16
Deuterostomia - ps17
Chordata - ps18
Olfactores - ps19
Vertebrata - ps20
Gnathostomata - ps21
Euteleostomi - ps22
Sarcopterygii - ps23
Tetrapoda - ps24
Amniota - ps25
Mammalia - ps26
Theria - ps27
Placentalia - ps28
Boreoeutheria - ps29
Euarchontoglires - ps30
Euarchonta - ps31
Primates - ps32
Haplorrhini - ps33
Simiiformes - ps34
Catarrhini - ps35
Hominoidea - ps36
Hominidae - ps37
Homininae - ps38
Hominini - ps39
Homo_sapiens - ps40
Phylostratum

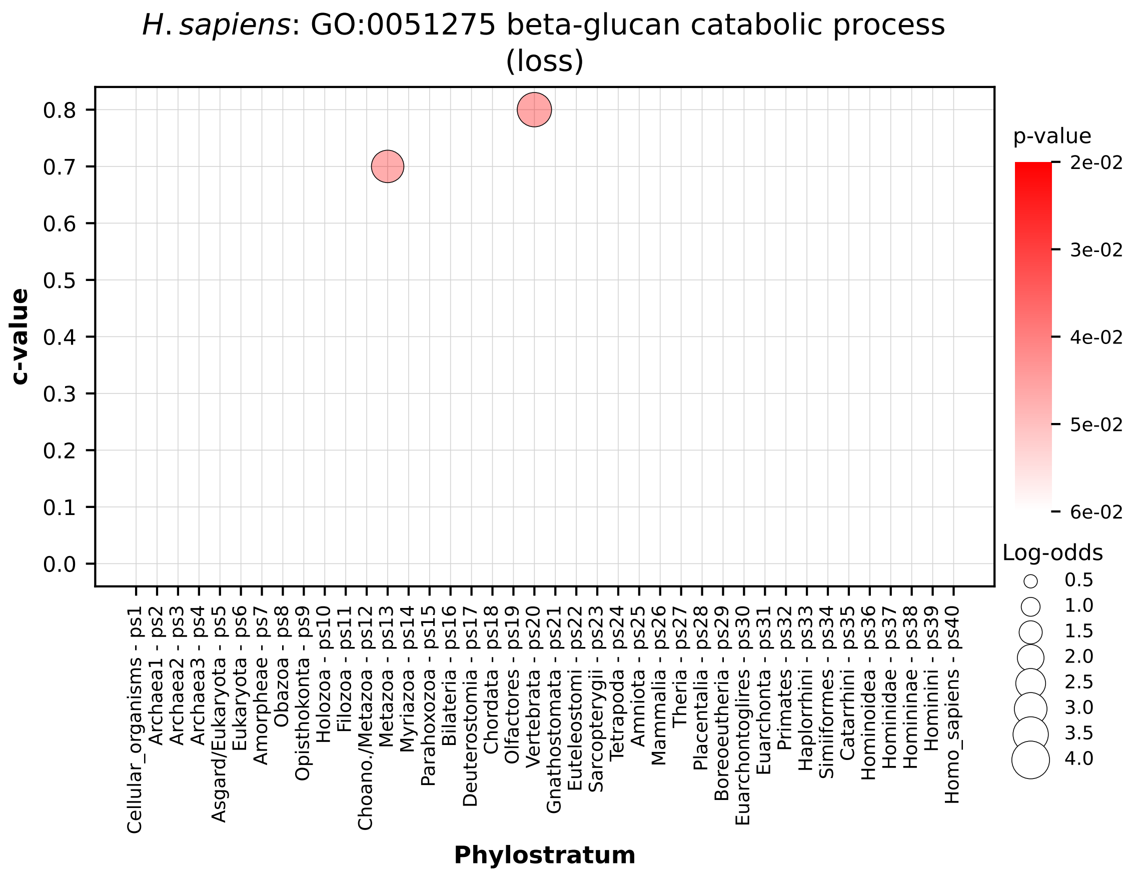

H. sapiens: GO:0051275 beta-glucan catabolic process (loss)
c-value
0.8
0.7
0.6
0.5
0.4
0.3
0.2
0.1
0.0
p-value
2e-02
3e-02
4e-02
5e-02
6e-02
Log-odds
0.5
1.0
1.5
2.0
2.5
3.0
3.5
4.0
Cellular_organisms - ps1
Archaea1 - ps2
Archaea2 - ps3
Archaea3 - ps4
Asgard/Eukaryota - ps5
Eukaryota - ps6
Amorpheae - ps7
Obazoa - ps8
Opisthokonta - ps9
Holozoa - ps10
Filozoa - ps11
Choano./Metazoa - ps12
Metazoa - ps13
Myriazoa - ps14
Parahoxozoa - ps15
Bilateria - ps16
Deuterostomia - ps17
Chordata - ps18
Olfactores - ps19
Vertebrata - ps20
Gnathostomata - ps21
Euteleostomi - ps22
Sarcopterygii - ps23
Tetrapoda - ps24
Amniota - ps25
Mammalia - ps26
Theria - ps27
Placentalia - ps28
Boreoeutheria - ps29
Euarchontoglires - ps30
Euarchonta - ps31
Primates - ps32
Haplorrhini - ps33
Simiiformes - ps34
Catarrhini - ps35
Hominoidea - ps36
Hominidae - ps37
Homininae - ps38
Hominini - ps39
Homo_sapiens - ps40
Phylostratum

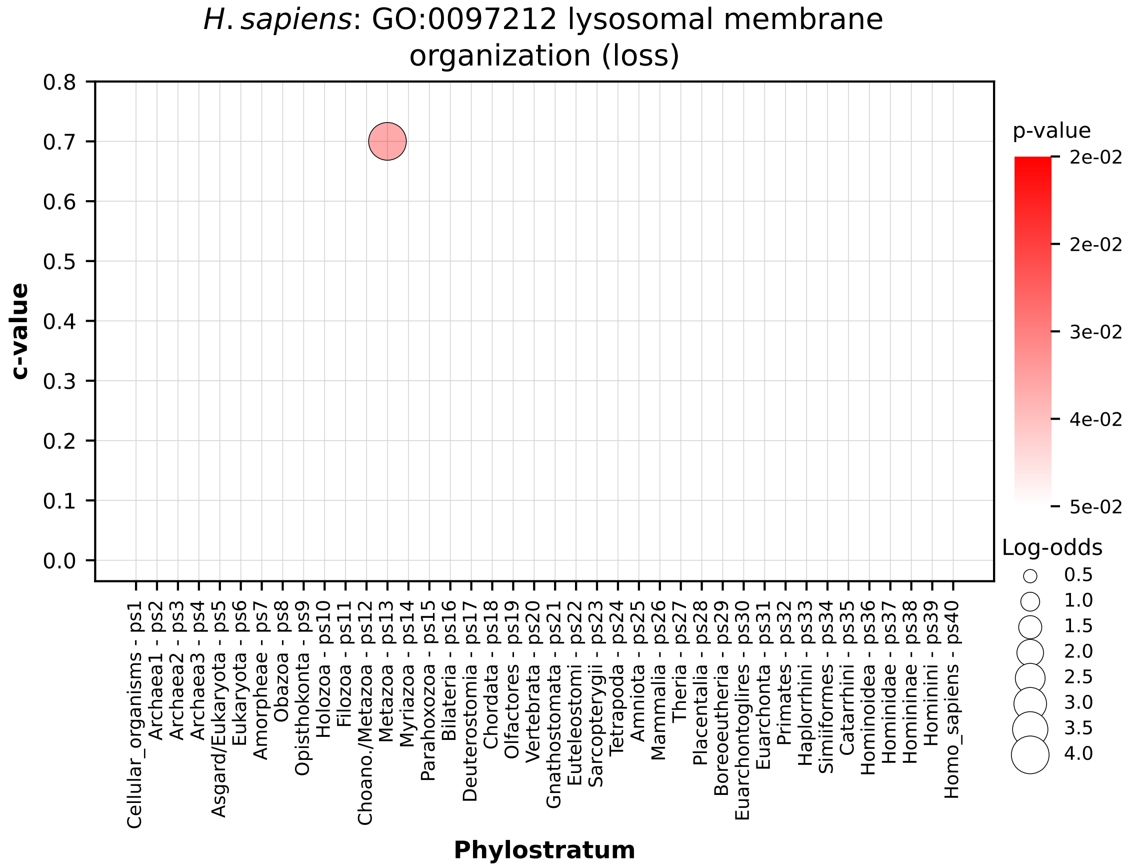
H. sapiens: GO:0097212 lysosomal membrane organization (loss)
c-value
0.0
0.1
0.2
0.3
0.4
0.5
0.6
0.7
0.8
Phylostratum
Cellular_organisms - ps1
Archaea1 - ps2
Archaea2 - ps3
Archaea3 - ps4
Asgard/Eukaryota - ps5
Eukaryota - ps6
Amorpheae - ps7
Obazoa - ps8
Opisthokonta - ps9
Holozoa - ps10
Filozoa - ps11
Choano./Metazoa - ps12
Metazoa - ps13
Myriazoa - ps14
Parahoxozoa - ps15
Bilateria - ps16
Deuterostomia - ps17
Chordata - ps18
Olfactores - ps19
Vertebrata - ps20
Gnathostomata - ps21
Euteleostomi - ps22
Sarcopterygii - ps23
Tetrapoda - ps24
Amniota - ps25
Mammalia - ps26
Theria - ps27
Placentalia - ps28
Boreoeutheria - ps29
Euarchontoglires - ps30
Euarchonta - ps31
Primates - ps32
Haplorrhini - ps33
Simiiformes - ps34
Catarrhini - ps35
Hominoidea - ps36
Hominidae - ps37
Homininae - ps38
Hominini - ps39
Homo_sapiens - ps40
p-value
2e-02
2e-02
3e-02
4e-02
5e-02
Log-odds
0.5
1.0
1.5
2.0
2.5
3.0
3.5
4.0

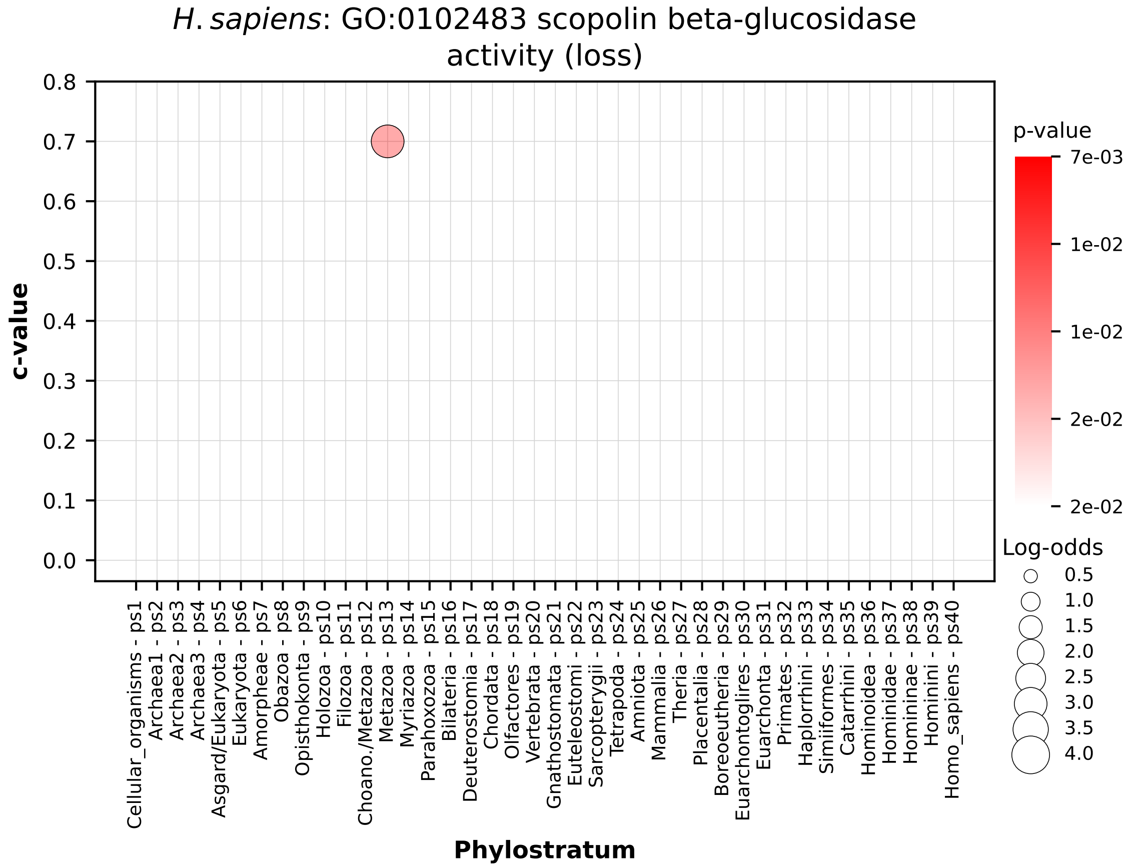

H. sapiens: GO:0102483 scopolin beta-glucosidase activity (loss)
c-value
0.8
0.7
0.6
0.5
0.4
0.3
0.2
0.1
0.0
p-value
7e-03
1e-02
1e-02
2e-02
2e-02
Log-odds
0.5
1.0
1.5
2.0
2.5
3.0
3.5
4.0
Cellular_organisms - ps1
Archaea1 - ps2
Archaea2 - ps3
Archaea3 - ps4
Asgard/Eukaryota - ps5
Eukaryota - ps6
Amorpheae - ps7
Obazoa - ps8
Opisthokonta - ps9
Holozoa - ps10
Filozoa - ps11
Choano./Metazoa - ps12
Metazoa - ps13
Myriazoa - ps14
Parahoxozoa - ps15
Bilateria - ps16
Deuterostomia - ps17
Chordata - ps18
Olfactores - ps19
Vertebrata - ps20
Gnathostomata - ps21
Euteleostomi - ps22
Sarcopterygii - ps23
Tetrapoda - ps24
Amniota - ps25
Mammalia - ps26
Theria - ps27
Placentalia - ps28
Boreoeutheria - ps29
Euarchontoglires - ps30
Euarchonta - ps31
Primates - ps32
Haplorrhini - ps33
Simiiformes - ps34
Catarrhini - ps35
Hominoidea - ps36
Hominidae - ps37
Homininae - ps38
Hominini - ps39
Homo_sapiens - ps40
Phylostratum

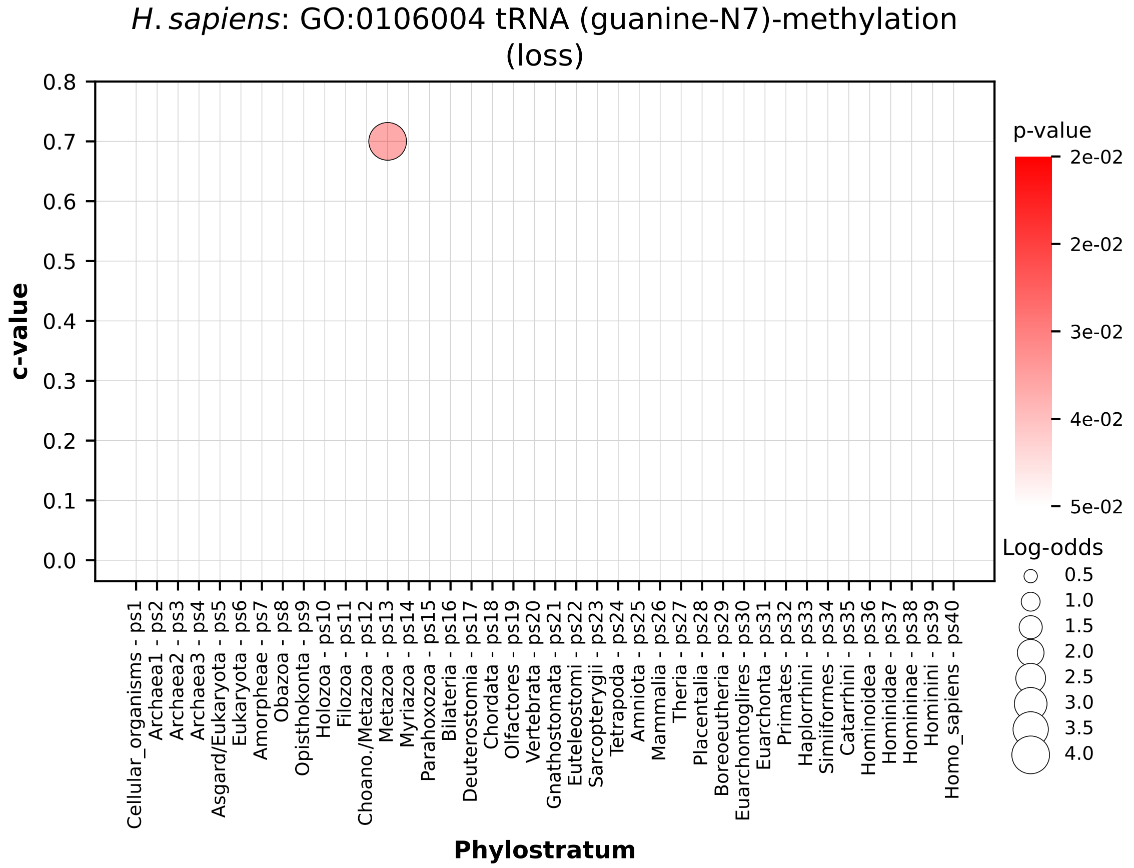

H. sapiens: GO:0106004 tRNA (guanine-N7)-methylation
(loss)
c-value
0.8
0.7
0.6
0.5
0.4
0.3
0.2
0.1
0.0
Phylostratum
Cellular_organisms - ps1
Archaea1 - ps2
Archaea2 - ps3
Archaea3 - ps4
Asgard/Eukaryota - ps5
Eukaryota - ps6
Amorpheae - ps7
Obazoa - ps8
Opisthokonta - ps9
Holozoa - ps10
Filozoa - ps11
Choano./Metazoa - ps12
Metazoa - ps13
Myriazoa - ps14
Parahoxozoa - ps15
Bilateria - ps16
Deuterostomia - ps17
Chordata - ps18
Olfactores - ps19
Vertebrata - ps20
Gnathostomata - ps21
Euteleostomi - ps22
Sarcopterygii - ps23
Tetrapoda - ps24
Amniota - ps25
Mammalia - ps26
Theria - ps27
Placentalia - ps28
Boreoeutheria - ps29
Euarchontoglires - ps30
Euarchonta - ps31
Primates - ps32
Haplorrhini - ps33
Simiiformes - ps34
Catarrhini - ps35
Hominoidea - ps36
Hominidae - ps37
Homininae - ps38
Hominini - ps39
Homo_sapiens - ps40
p-value
2e-02
2e-02
3e-02
4e-02
5e-02
Log-odds
0.5
1.0
1.5
2.0
2.5
3.0
3.5
4.0

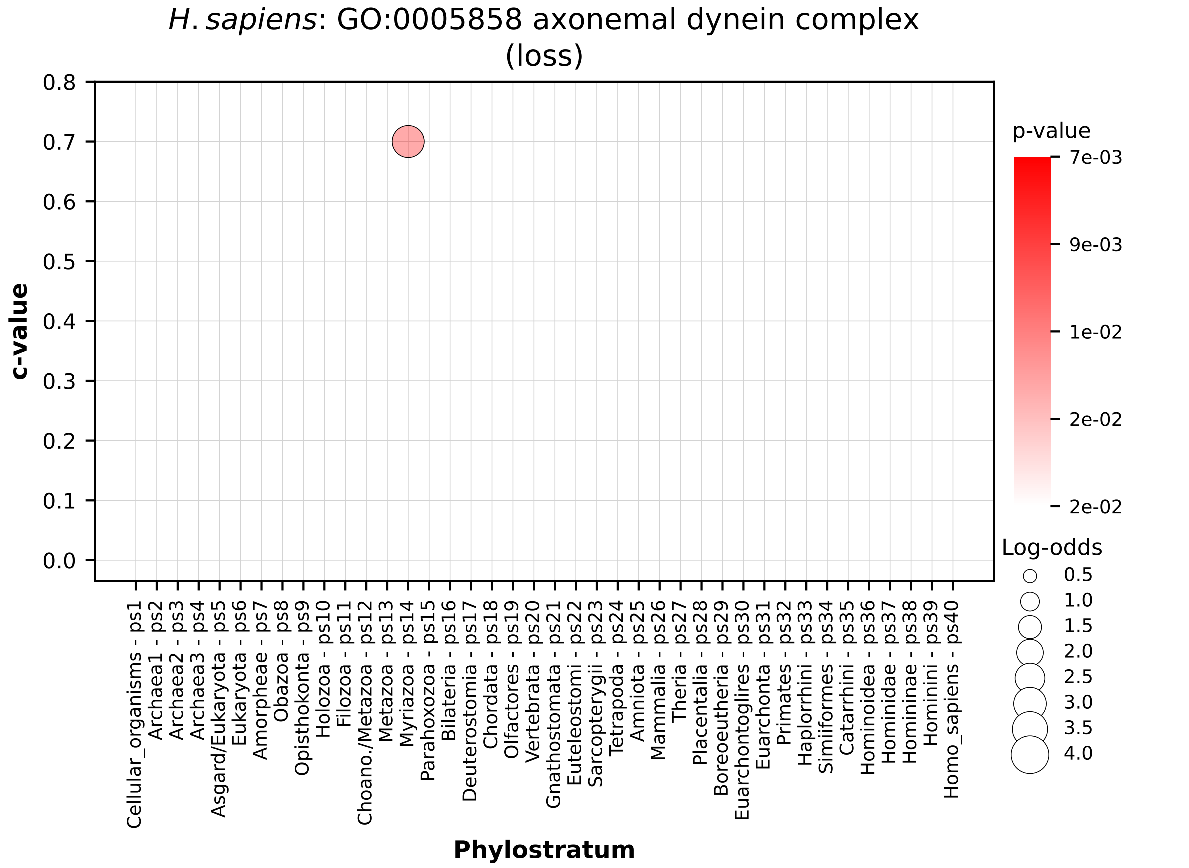

H. sapiens: GO:0005858 axonemal dynein complex
(loss)
c-value
0.8
0.7
0.6
0.5
0.4
0.3
0.2
0.1
0.0
p-value
7e-03
9e-03
1e-02
2e-02
2e-02
Log-odds
0.5
1.0
1.5
2.0
2.5
3.0
3.5
4.0
Cellular_organisms - ps1
Archaea1 - ps2
Archaea2 - ps3
Archaea3 - ps4
Asgard/Eukaryota - ps5
Eukaryota - ps6
Amorpheae - ps7
Obazoa - ps8
Opisthokonta - ps9
Holozoa - ps10
Filozoa - ps11
Choano./Metazoa - ps12
Metazoa - ps13
Myriazoa - ps14
Parahoxozoa - ps15
Bilateria - ps16
Deuterostomia - ps17
Chordata - ps18
Olfactores - ps19
Vertebrata - ps20
Gnathostomata - ps21
Euteleostomi - ps22
Sarcopterygii - ps23
Tetrapoda - ps24
Amniota - ps25
Mammalia - ps26
Theria - ps27
Placentalia - ps28
Boreoeutheria - ps29
Euarchontoglires - ps30
Euarchonta - ps31
Primates - ps32
Haplorrhini - ps33
Simiiformes - ps34
Catarrhini - ps35
Hominoidea - ps36
Hominidae - ps37
Homininae - ps38
Hominini - ps39
Homo_sapiens - ps40
Phylostratum

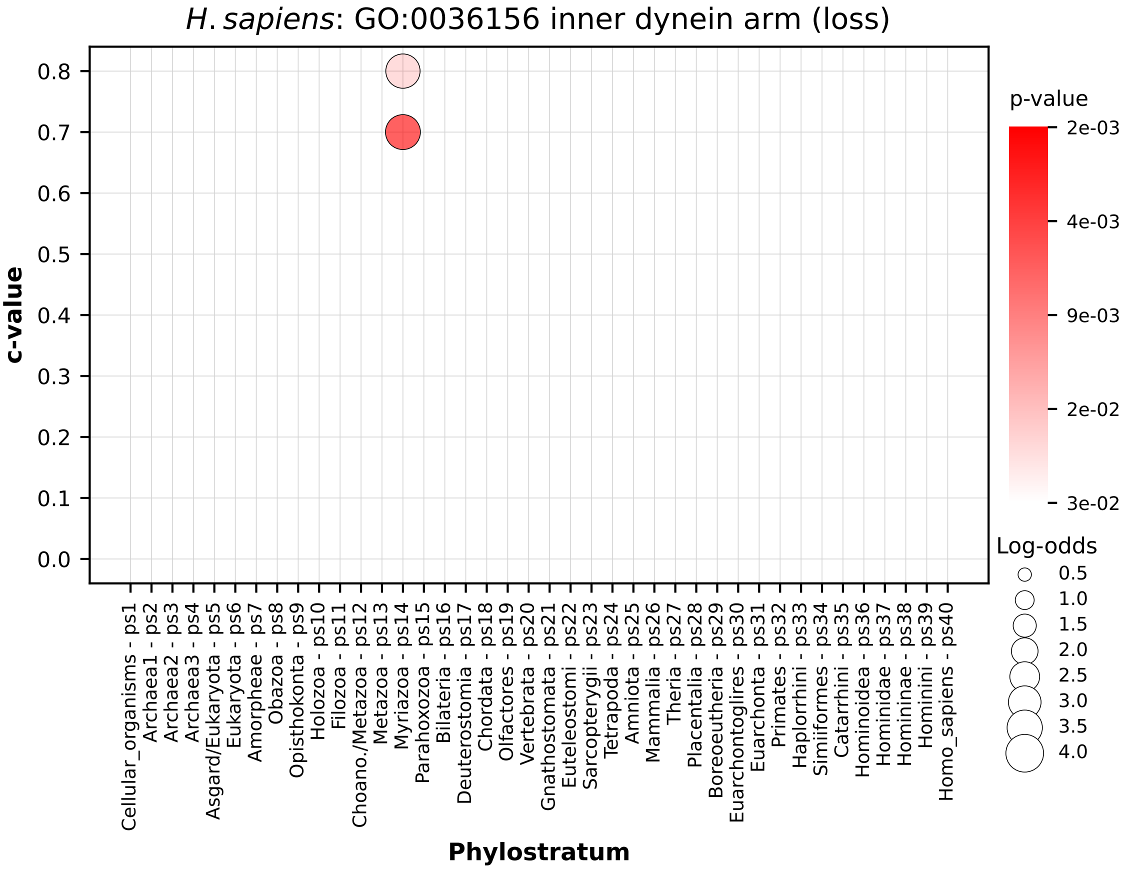

H. sapiens: GO:0036156 inner dynein arm (loss)
c-value
0.0
0.1
0.2
0.3
0.4
0.5
0.6
0.7
0.8
Phylostratum
Cellular_organisms - ps1
Archaea1 - ps2
Archaea2 - ps3
Archaea3 - ps4
Asgard/Eukaryota - ps5
Eukaryota - ps6
Amorpheae - ps7
Obazoa - ps8
Opisthokonta - ps9
Holozoa - ps10
Filozoa - ps11
Choano./Metazoa - ps12
Metazoa - ps13
Myriazoa - ps14
Parahoxozoa - ps15
Bilateria - ps16
Deuterostomia - ps17
Chordata - ps18
Olfactores - ps19
Vertebrata - ps20
Gnathostomata - ps21
Euteleostomi - ps22
Sarcopterygii - ps23
Tetrapoda - ps24
Amniota - ps25
Mammalia - ps26
Theria - ps27
Placentalia - ps28
Boreoeutheria - ps29
Euarchontoglires - ps30
Euarchonta - ps31
Primates - ps32
Haplorrhini - ps33
Simiiformes - ps34
Catarrhini - ps35
Hominoidea - ps36
Hominidae - ps37
Homininae - ps38
Hominini - ps39
Homo_sapiens - ps40
p-value
2e-03
4e-03
9e-03
2e-02
3e-02
Log-odds
0.5
1.0
1.5
2.0
2.5
3.0
3.5
4.0

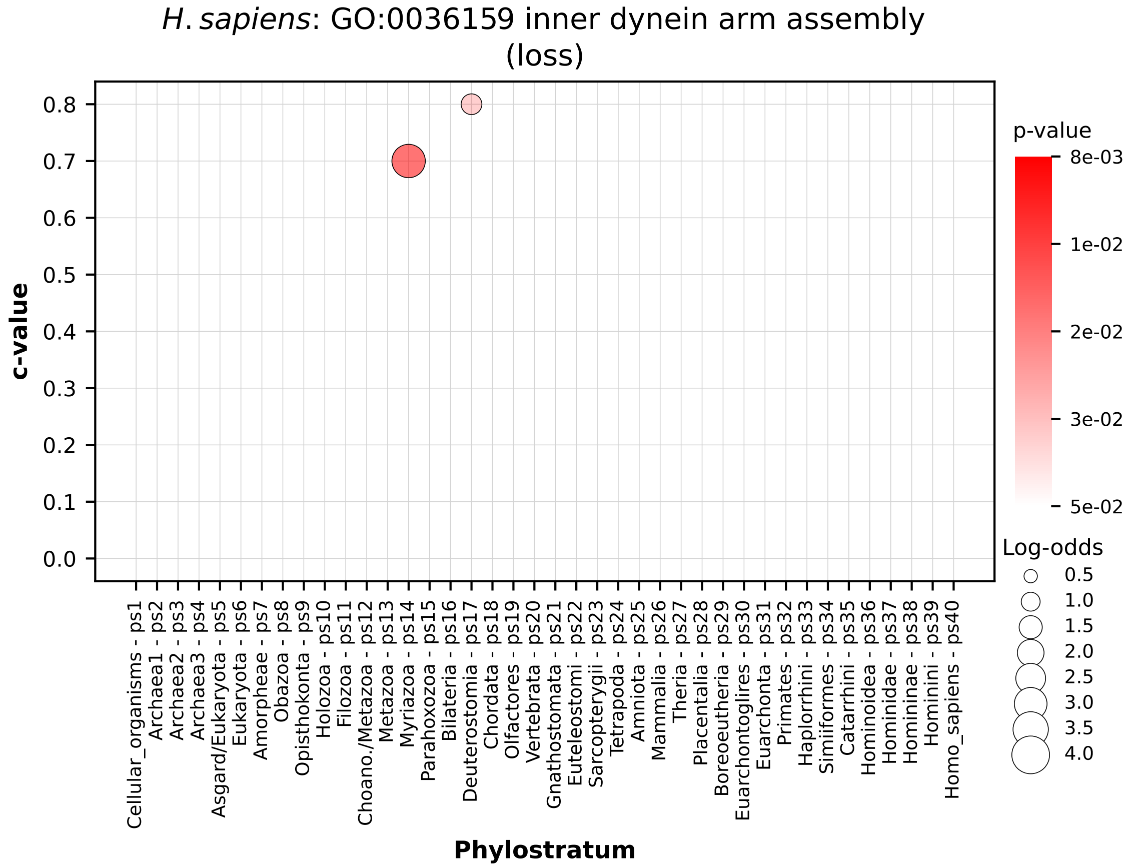

H. sapiens: GO:0036159 inner dynein arm assembly
(loss)
c-value
0.0
0.1
0.2
0.3
0.4
0.5
0.6
0.7
0.8
Phylostratum
Cellular_organisms - ps1
Archaea1 - ps2
Archaea2 - ps3
Archaea3 - ps4
Asgard/Eukaryota - ps5
Eukaryota - ps6
Amorpheae - ps7
Obazoa - ps8
Opisthokonta - ps9
Holozoa - ps10
Filozoa - ps11
Choano./Metazoa - ps12
Metazoa - ps13
Myriazoa - ps14
Parahoxozoa - ps15
Bilateria - ps16
Deuterostomia - ps17
Chordata - ps18
Olfactores - ps19
Vertebrata - ps20
Gnathostomata - ps21
Euteleostomi - ps22
Sarcopterygii - ps23
Tetrapoda - ps24
Amniota - ps25
Mammalia - ps26
Theria - ps27
Placentalia - ps28
Boreoeutheria - ps29
Euarchontoglires - ps30
Euarchonta - ps31
Primates - ps32
Haplorrhini - ps33
Simiiformes - ps34
Catarrhini - ps35
Hominoidea - ps36
Hominidae - ps37
Homininae - ps38
Hominini - ps39
Homo_sapiens - ps40
p-value
8e-03
1e-02
2e-02
3e-02
5e-02
Log-odds
0.5
1.0
1.5
2.0
2.5
3.0
3.5
4.0

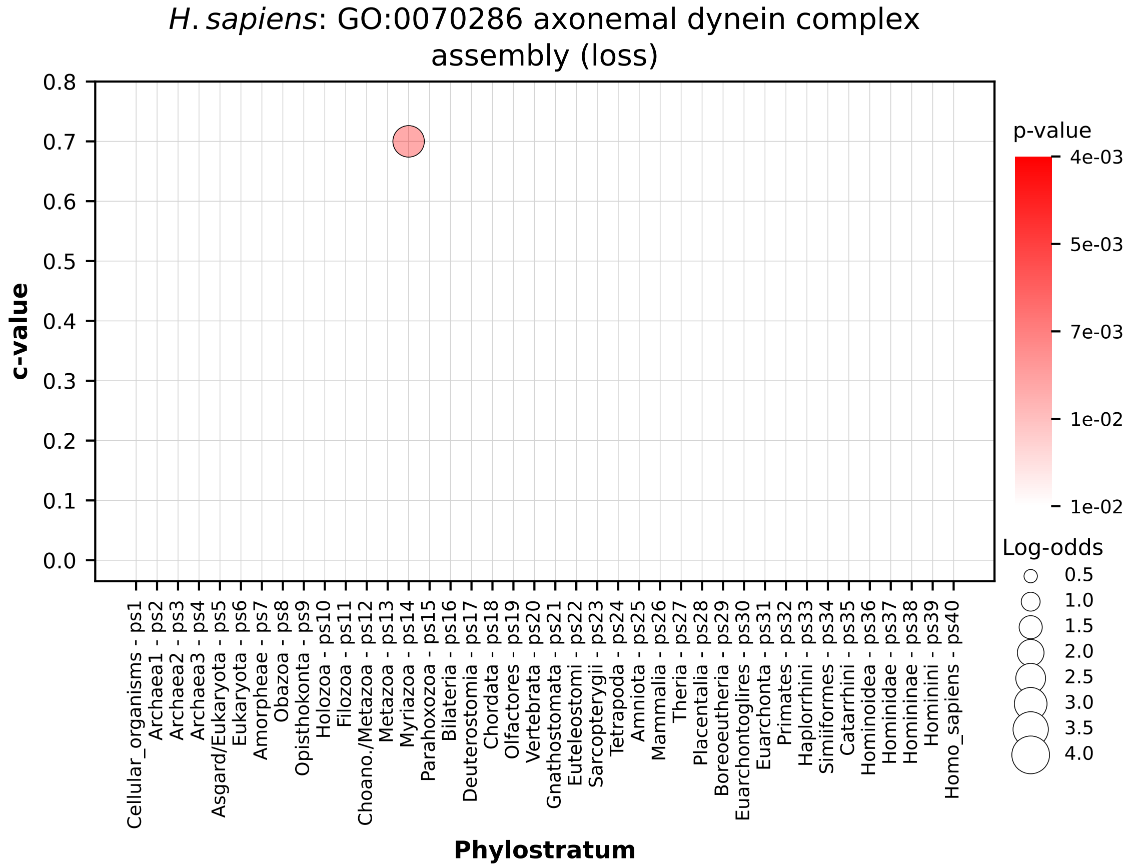

H. sapiens: GO:0070286 axonemal dynein complex assembly (loss)
c-value
0.8
0.7
0.6
0.5
0.4
0.3
0.2
0.1
0.0
p-value
4e-03
5e-03
7e-03
1e-02
1e-02
Log-odds
0.5
1.0
1.5
2.0
2.5
3.0
3.5
4.0
Cellular_organisms - ps1
Archaea1 - ps2
Archaea2 - ps3
Archaea3 - ps4
Asgard/Eukaryota - ps5
Eukaryota - ps6
Amorpheae - ps7
Obazoa - ps8
Opisthokonta - ps9
Holozoa - ps10
Filozoa - ps11
Choano./Metazoa - ps12
Metazoa - ps13
Myriazoa - ps14
Parahoxozoa - ps15
Bilateria - ps16
Deuterostomia - ps17
Chordata - ps18
Olfactores - ps19
Vertebrata - ps20
Gnathostomata - ps21
Euteleostomi - ps22
Sarcopterygii - ps23
Tetrapoda - ps24
Amniota - ps25
Mammalia - ps26
Theria - ps27
Placentalia - ps28
Boreoeutheria - ps29
Euarchontoglires - ps30
Euarchonta - ps31
Primates - ps32
Haplorrhini - ps33
Simiiformes - ps34
Catarrhini - ps35
Hominoidea - ps36
Hominidae - ps37
Homininae - ps38
Hominini - ps39
Homo_sapiens - ps40
Phylostratum

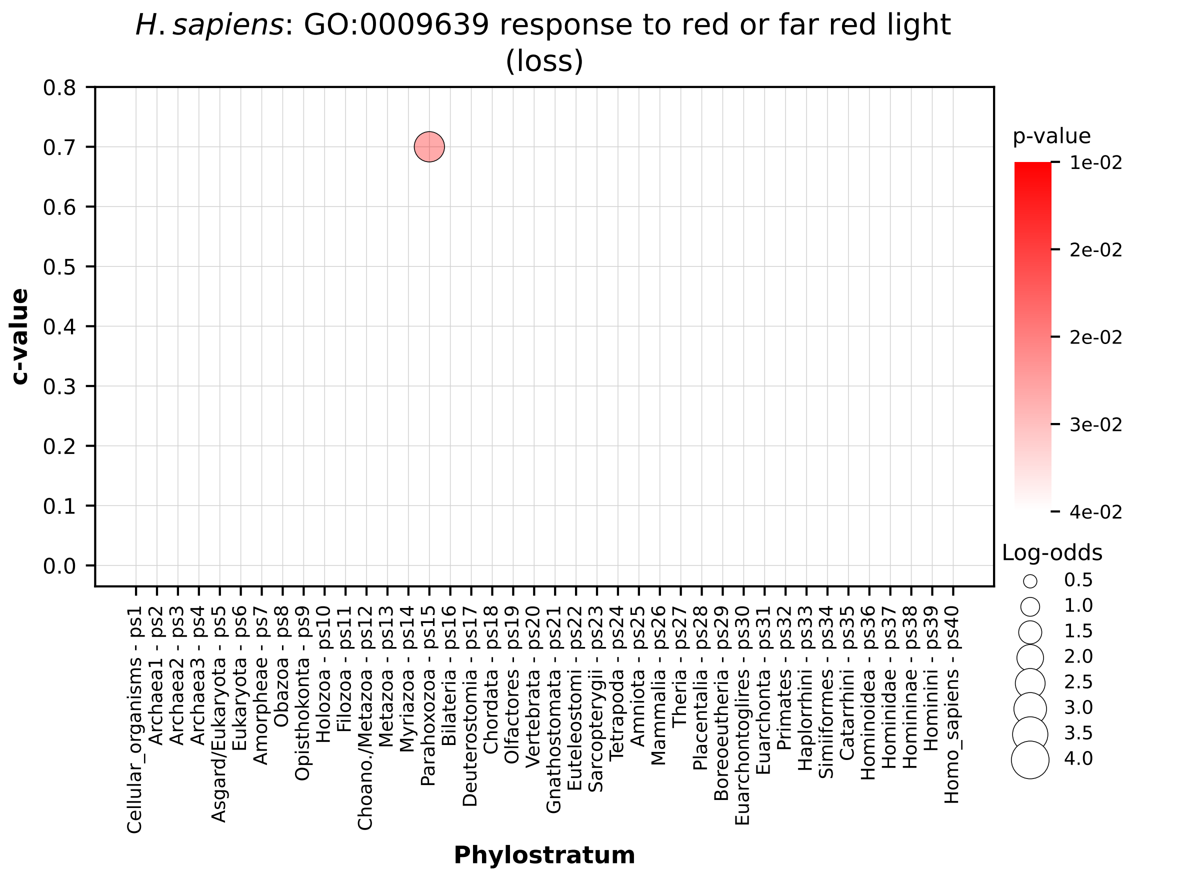

H. sapiens: GO:0009639 response to red or far red light
(loss)
c-value
0.8
0.7
0.6
0.5
0.4
0.3
0.2
0.1
0.0
p-value
1e-02
2e-02
2e-02
3e-02
4e-02
Log-odds
0.5
1.0
1.5
2.0
2.5
3.0
3.5
4.0
Cellular_organisms - ps1
Archaea1 - ps2
Archaea2 - ps3
Archaea3 - ps4
Asgard/Eukaryota - ps5
Eukaryota - ps6
Amorpheae - ps7
Obazoa - ps8
Opisthokonta - ps9
Holozoa - ps10
Filozoa - ps11
Choano./Metazoa - ps12
Metazoa - ps13
Myriazoa - ps14
Parahoxozoa - ps15
Bilateria - ps16
Deuterostomia - ps17
Chordata - ps18
Olfactores - ps19
Vertebrata - ps20
Gnathostomata - ps21
Euteleostomi - ps22
Sarcopterygii - ps23
Tetrapoda - ps24
Amniota - ps25
Mammalia - ps26
Theria - ps27
Placentalia - ps28
Boreoeutheria - ps29
Euarchontoglires - ps30
Euarchonta - ps31
Primates - ps32
Haplorrhini - ps33
Simiiformes - ps34
Catarrhini - ps35
Hominoidea - ps36
Hominidae - ps37
Homininae - ps38
Hominini - ps39
Homo_sapiens - ps40
Phylostratum

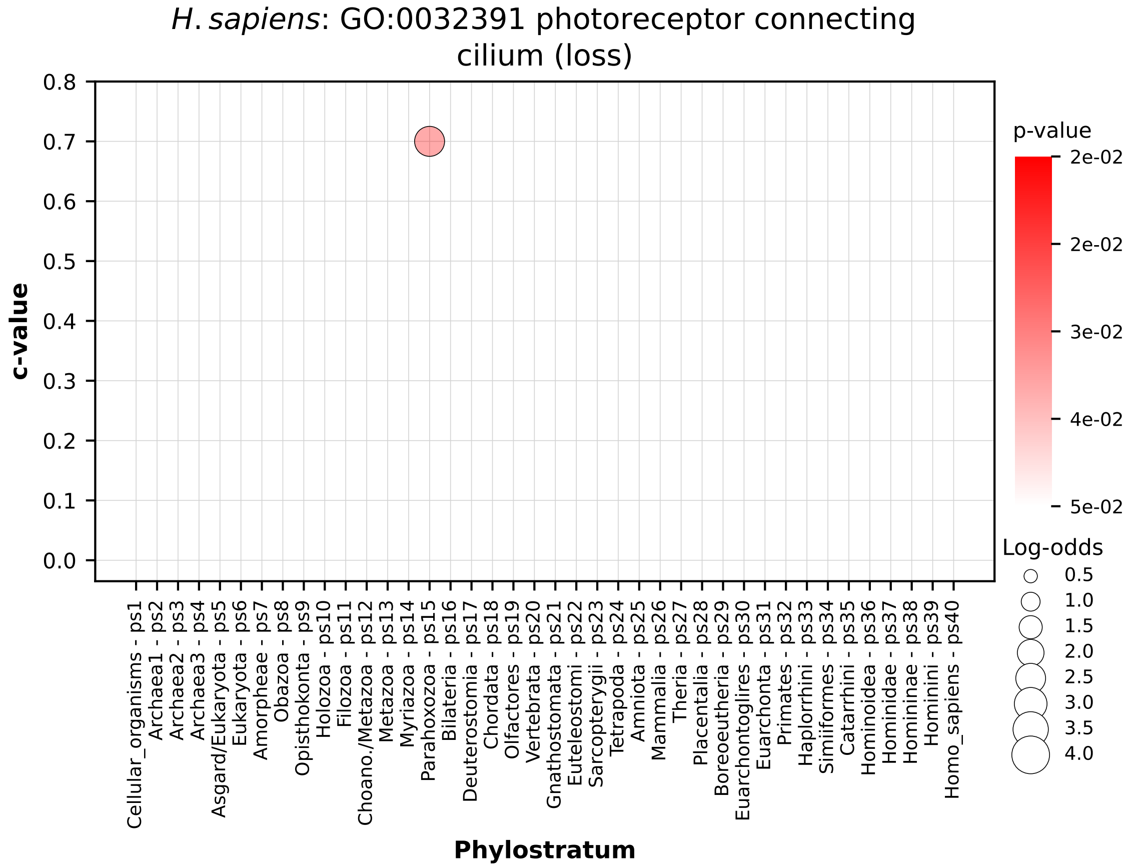

H. sapiens: GO:0032391 photoreceptor connecting cilium (loss)
c-value
0.8
0.7
0.6
0.5
0.4
0.3
0.2
0.1
0.0
p-value
2e-02
2e-02
3e-02
4e-02
5e-02
Log-odds
0.5
1.0
1.5
2.0
2.5
3.0
3.5
4.0
Cellular_organisms - ps1
Archaea1 - ps2
Archaea2 - ps3
Archaea3 - ps4
Asgard/Eukaryota - ps5
Eukaryota - ps6
Amorpheae - ps7
Obazoa - ps8
Opisthokonta - ps9
Holozoa - ps10
Filozoa - ps11
Choano./Metazoa - ps12
Metazoa - ps13
Myriazoa - ps14
Parahoxozoa - ps15
Bilateria - ps16
Deuterostomia - ps17
Chordata - ps18
Olfactores - ps19
Vertebrata - ps20
Gnathostomata - ps21
Euteleostomi - ps22
Sarcopterygii - ps23
Tetrapoda - ps24
Amniota - ps25
Mammalia - ps26
Theria - ps27
Placentalia - ps28
Boreoeutheria - ps29
Euarchontoglires - ps30
Euarchonta - ps31
Primates - ps32
Haplorrhini - ps33
Simiiformes - ps34
Catarrhini - ps35
Hominoidea - ps36
Hominidae - ps37
Homininae - ps38
Hominini - ps39
Homo_sapiens - ps40
Phylostratum

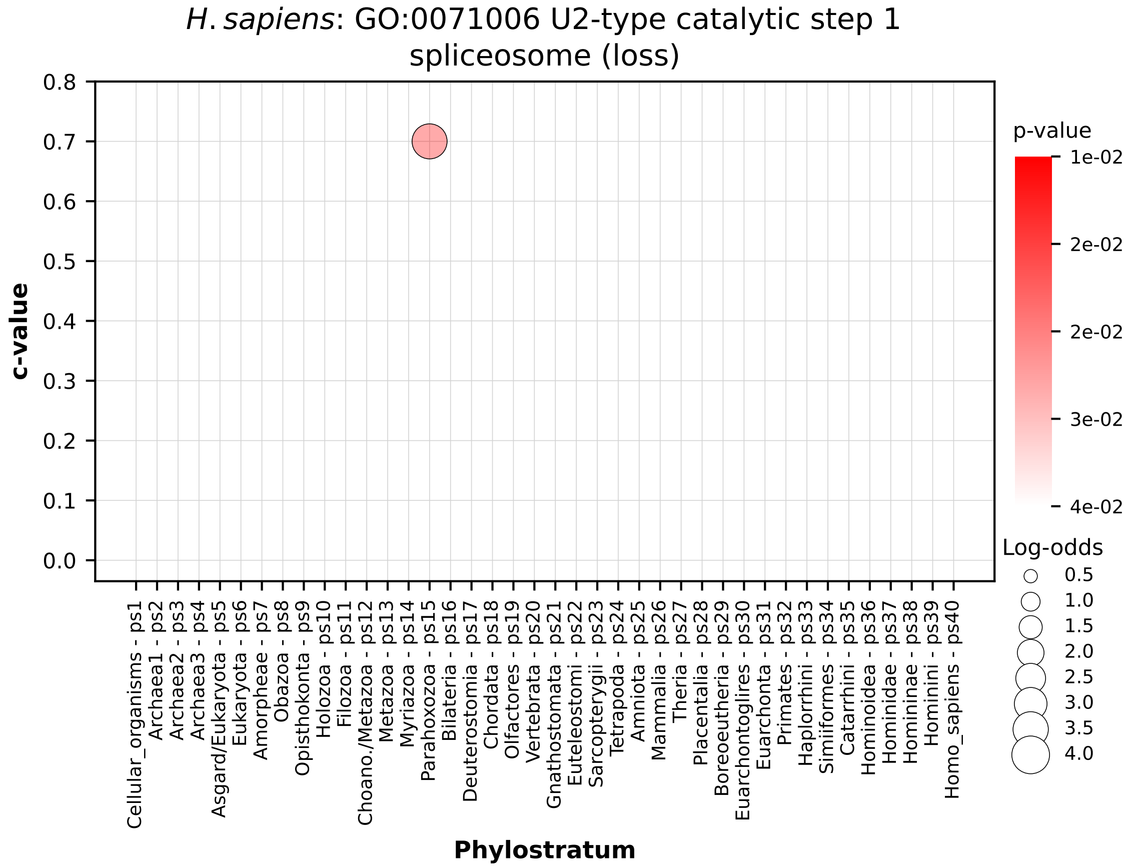
H. sapiens: GO:0071006 U2-type catalytic step 1
spliceosome (loss)
c-value
0.8
0.7
0.6
0.5
0.4
0.3
0.2
0.1
0.0
p-value
1e-02
2e-02
2e-02
3e-02
4e-02
Log-odds
0.5
1.0
1.5
2.0
2.5
3.0
3.5
4.0
Cellular_organisms - ps1
Archaea1 - ps2
Archaea2 - ps3
Archaea3 - ps4
Asgard/Eukaryota - ps5
Eukaryota - ps6
Amorpheae - ps7
Obazoa - ps8
Opisthokonta - ps9
Holozoa - ps10
Filozoa - ps11
Choano./Metazoa - ps12
Metazoa - ps13
Myriazoa - ps14
Parahoxozoa - ps15
Bilateria - ps16
Deuterostomia - ps17
Chordata - ps18
Olfactores - ps19
Vertebrata - ps20
Gnathostomata - ps21
Euteleostomi - ps22
Sarcopterygii - ps23
Tetrapoda - ps24
Amniota - ps25
Mammalia - ps26
Theria - ps27
Placentalia - ps28
Boreoeutheria - ps29
Euarchontoglires - ps30
Euarchonta - ps31
Primates - ps32
Haplorrhini - ps33
Simiiformes - ps34
Catarrhini - ps35
Hominoidea - ps36
Hominidae - ps37
Homininae - ps38
Hominini - ps39
Homo_sapiens - ps40
Phylostratum

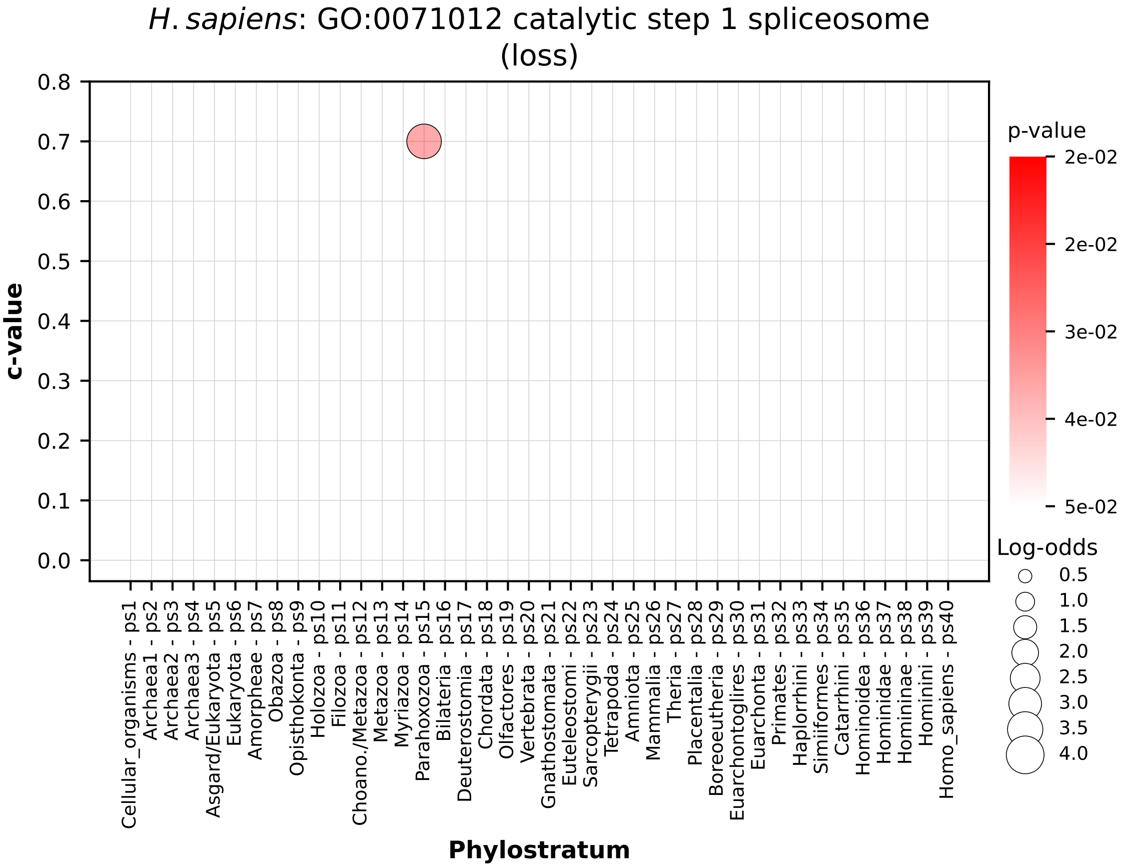
H. sapiens: GO:0071012 catalytic step 1 spliceosome
(loss)
c-value
0.8
0.7
0.6
0.5
0.4
0.3
0.2
0.1
0.0
p-value
2e-02
2e-02
3e-02
4e-02
5e-02
Log-odds
0.5
1.0
1.5
2.0
2.5
3.0
3.5
4.0
Cellular_organisms - ps1
Archaea1 - ps2
Archaea2 - ps3
Archaea3 - ps4
Asgard/Eukaryota - ps5
Eukaryota - ps6
Amorpheae - ps7
Obazoa - ps8
Opisthokonta - ps9
Holozoa - ps10
Filozoa - ps11
Choano./Metazoa - ps12
Metazoa - ps13
Myriazoa - ps14
Parahoxozoa - ps15
Bilateria - ps16
Deuterostomia - ps17
Chordata - ps18
Olfactores - ps19
Vertebrata - ps20
Gnathostomata - ps21
Euteleostomi - ps22
Sarcopterygii - ps23
Tetrapoda - ps24
Amniota - ps25
Mammalia - ps26
Theria - ps27
Placentalia - ps28
Boreoeutheria - ps29
Euarchontoglires - ps30
Euarchonta - ps31
Primates - ps32
Haplorrhini - ps33
Simiiformes - ps34
Catarrhini - ps35
Hominoidea - ps36
Hominidae - ps37
Homininae - ps38
Hominini - ps39
Homo_sapiens - ps40
Phylostratum

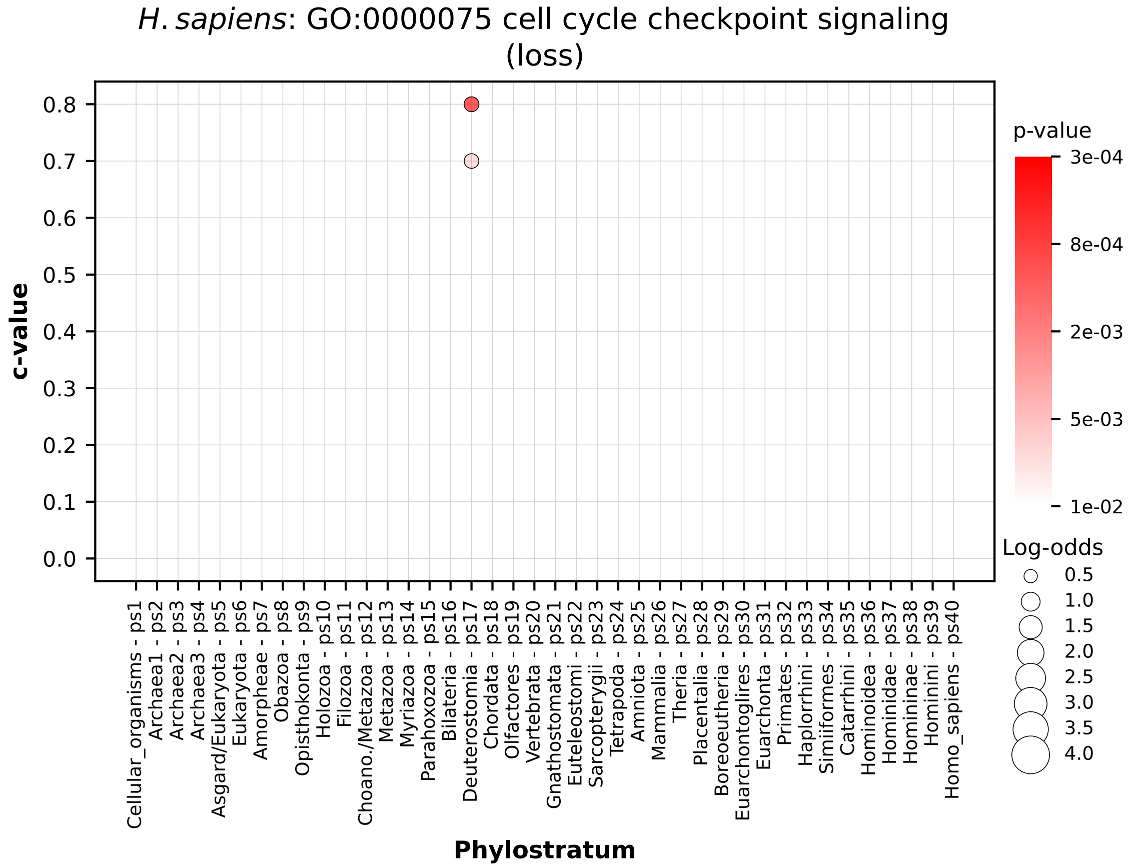
H. sapiens: GO:0000075 cell cycle checkpoint signaling
(loss)
c-value
0.8
0.7
0.6
0.5
0.4
0.3
0.2
0.1
0.0
Phylostratum
Cellular_organisms - ps1
Archaea1 - ps2
Archaea2 - ps3
Archaea3 - ps4
Asgard/Eukaryota - ps5
Eukaryota - ps6
Amorpheae - ps7
Obazoa - ps8
Opisthokonta - ps9
Holozoa - ps10
Filozoa - ps11
Choano./Metazoa - ps12
Metazoa - ps13
Myriazoa - ps14
Parahoxozoa - ps15
Bilateria - ps16
Deuterostomia - ps17
Chordata - ps18
Olfactores - ps19
Vertebrata - ps20
Gnathostomata - ps21
Euteleostomi - ps22
Sarcopterygii - ps23
Tetrapoda - ps24
Amniota - ps25
Mammalia - ps26
Theria - ps27
Placentalia - ps28
Boreoeutheria - ps29
Euarchontoglires - ps30
Euarchonta - ps31
Primates - ps32
Haplorrhini - ps33
Simiiformes - ps34
Catarrhini - ps35
Hominoidea - ps36
Hominidae - ps37
Homininae - ps38
Hominini - ps39
Homo_sapiens - ps40
p-value
3e-04
8e-04
2e-03
5e-03
1e-02
Log-odds
0.5
1.0
1.5
2.0
2.5
3.0
3.5
4.0

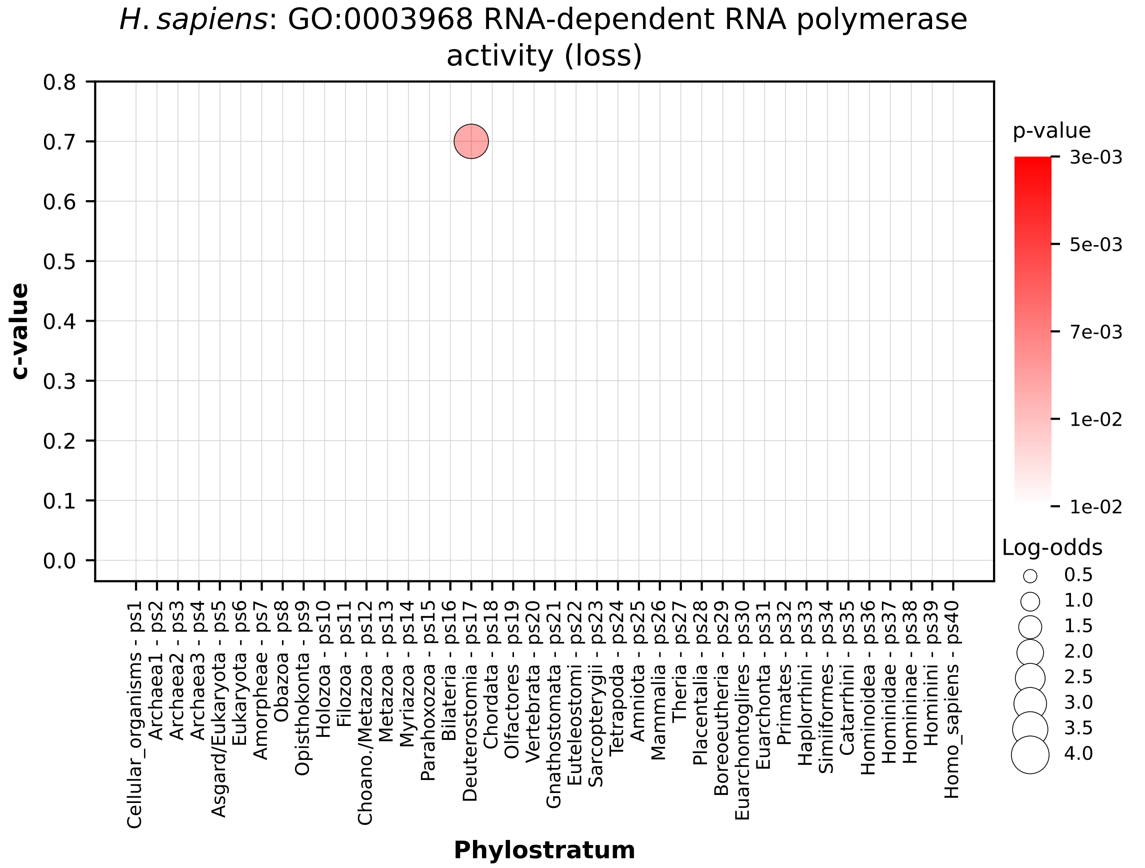

H. sapiens: GO:0003968 RNA-dependent RNA polymerase activity (loss)
c-value
0.8
0.7
0.6
0.5
0.4
0.3
0.2
0.1
0.0
Phylostratum
Cellular_organisms - ps1
Archaea1 - ps2
Archaea2 - ps3
Archaea3 - ps4
Asgard/Eukaryota - ps5
Eukaryota - ps6
Amorpheae - ps7
Obazoa - ps8
Opisthokonta - ps9
Holozoa - ps10
Filozoa - ps11
Choano./Metazoa - ps12
Metazoa - ps13
Myriazoa - ps14
Parahoxozoa - ps15
Bilateria - ps16
Deuterostomia - ps17
Chordata - ps18
Olfactores - ps19
Vertebrata - ps20
Gnathostomata - ps21
Euteleostomi - ps22
Sarcopterygii - ps23
Tetrapoda - ps24
Amniota - ps25
Mammalia - ps26
Theria - ps27
Placentalia - ps28
Boreoeutheria - ps29
Euarchontoglires - ps30
Euarchonta - ps31
Primates - ps32
Haplorrhini - ps33
Simiiformes - ps34
Catarrhini - ps35
Hominoidea - ps36
Hominidae - ps37
Homininae - ps38
Hominini - ps39
Homo_sapiens - ps40
p-value
3e-03
5e-03
7e-03
1e-02
1e-02
Log-odds
0.5
1.0
1.5
2.0
2.5
3.0
3.5
4.0

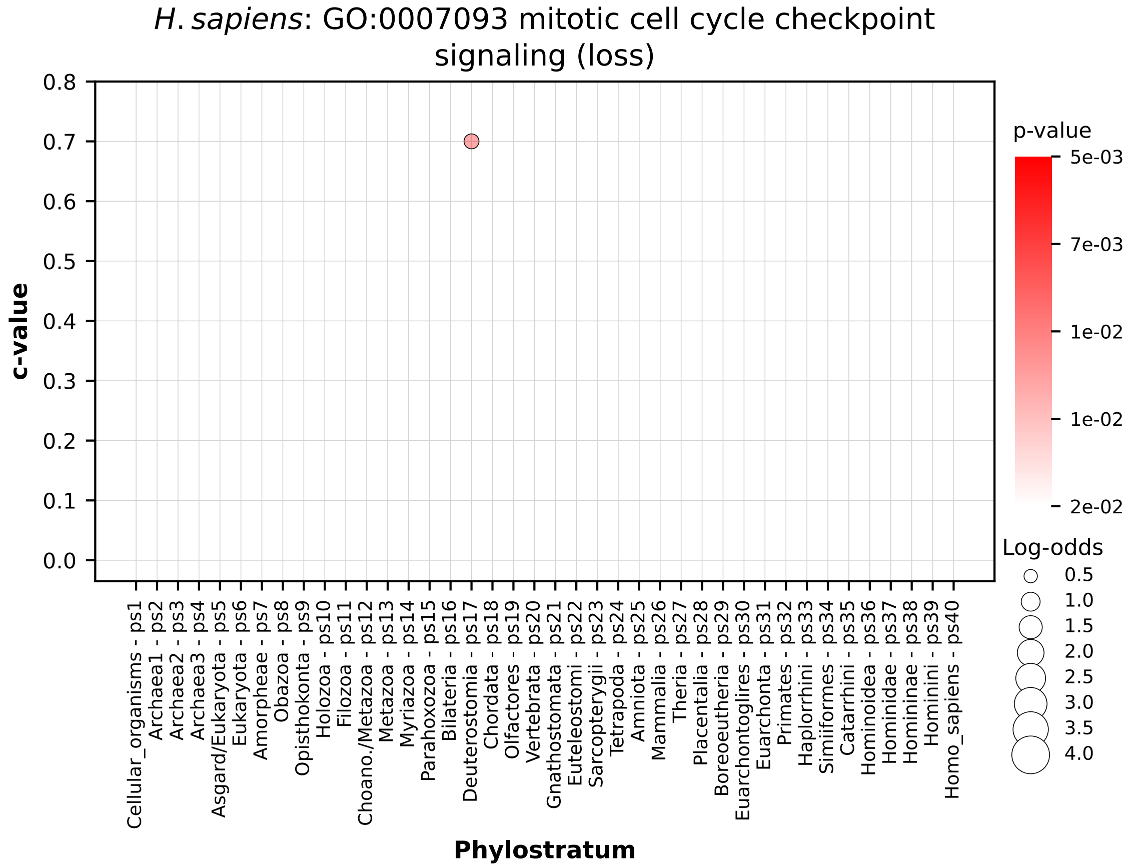

H. sapiens: GO:0007093 mitotic cell cycle checkpoint signaling (loss)
c-value
0.8
0.7
0.6
0.5
0.4
0.3
0.2
0.1
0.0
p-value
5e-03
7e-03
1e-02
1e-02
2e-02
Log-odds
0.5
1.0
1.5
2.0
2.5
3.0
3.5
4.0
Cellular_organisms - ps1
Archaea1 - ps2
Archaea2 - ps3
Archaea3 - ps4
Asgard/Eukaryota - ps5
Eukaryota - ps6
Amorpheae - ps7
Obazoa - ps8
Opisthokonta - ps9
Holozoa - ps10
Filozoa - ps11
Choano./Metazoa - ps12
Metazoa - ps13
Myriazoa - ps14
Parahoxozoa - ps15
Bilateria - ps16
Deuterostomia - ps17
Chordata - ps18
Olfactores - ps19
Vertebrata - ps20
Gnathostomata - ps21
Euteleostomi - ps22
Sarcopterygii - ps23
Tetrapoda - ps24
Amniota - ps25
Mammalia - ps26
Theria - ps27
Placentalia - ps28
Boreoeutheria - ps29
Euarchontoglires - ps30
Euarchonta - ps31
Primates - ps32
Haplorrhini - ps33
Simiiformes - ps34
Catarrhini - ps35
Hominoidea - ps36
Hominidae - ps37
Homininae - ps38
Hominini - ps39
Homo_sapiens - ps40
Phylostratum

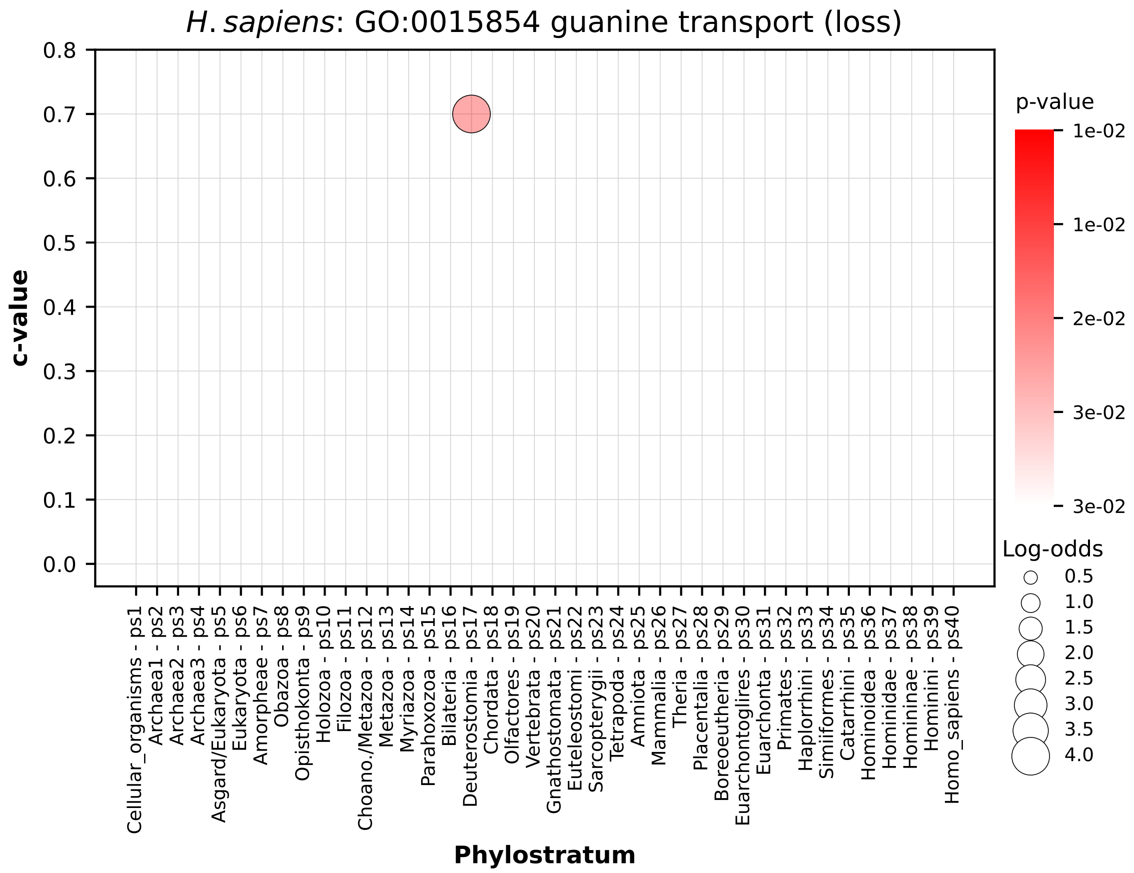
H. sapiens: GO:0015854 guanine transport (loss)
c-value
0.8
0.7
0.6
0.5
0.4
0.3
0.2
0.1
0.0
Phylostratum
Cellular_organisms - ps1
Archaea1 - ps2
Archaea2 - ps3
Archaea3 - ps4
Asgard/Eukaryota - ps5
Eukaryota - ps6
Amorpheae - ps7
Obazoa - ps8
Opisthokonta - ps9
Holozoa - ps10
Filozoa - ps11
Choano./Metazoa - ps12
Metazoa - ps13
Myriazoa - ps14
Parahoxozoa - ps15
Bilateria - ps16
Deuterostomia - ps17
Chordata - ps18
Olfactores - ps19
Vertebrata - ps20
Gnathostomata - ps21
Euteleostomi - ps22
Sarcopterygii - ps23
Tetrapoda - ps24
Amniota - ps25
Mammalia - ps26
Theria - ps27
Placentalia - ps28
Boreoeutheria - ps29
Euarchontoglires - ps30
Euarchonta - ps31
Primates - ps32
Haplorrhini - ps33
Simiiformes - ps34
Catarrhini - ps35
Hominoidea - ps36
Hominidae - ps37
Homininae - ps38
Hominini - ps39
Homo_sapiens - ps40
p-value
1e-02
1e-02
2e-02
3e-02
3e-02
Log-odds
0.5
1.0
1.5
2.0
2.5
3.0
3.5
4.0

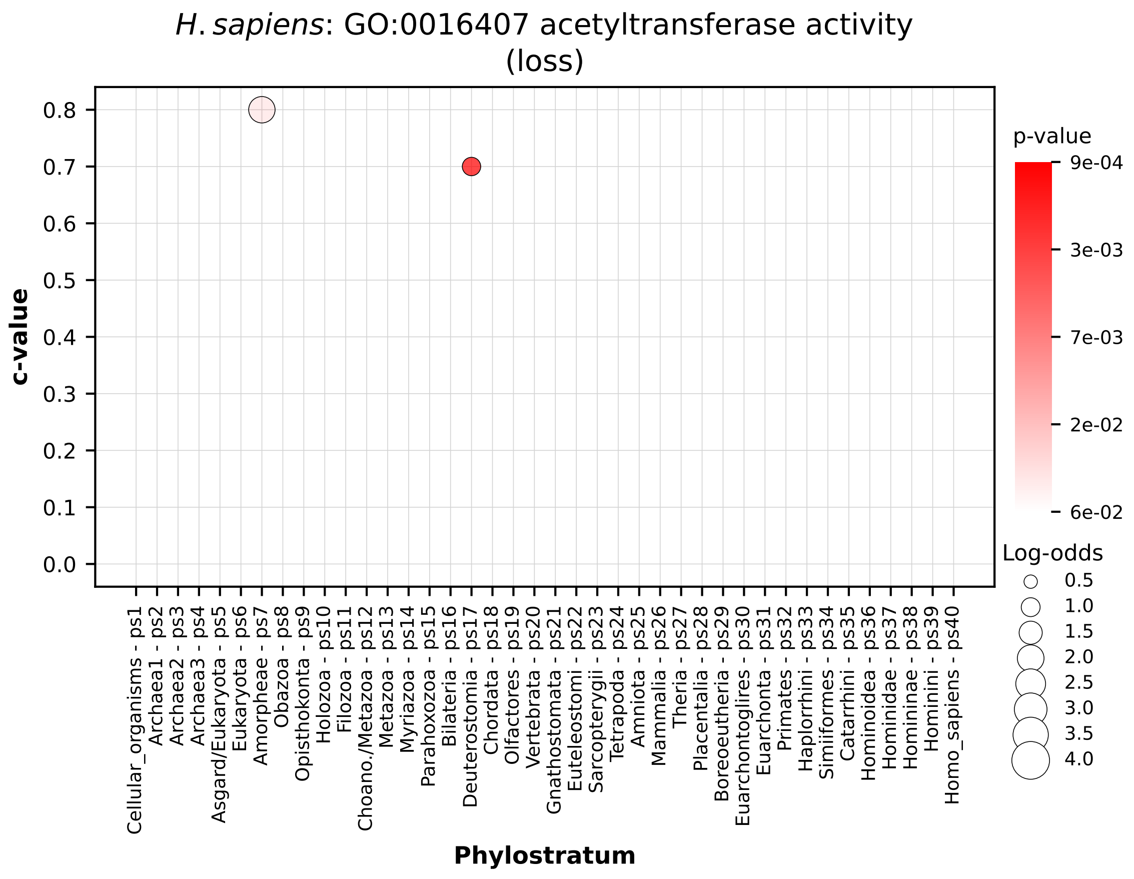

H. sapiens: GO:0016407 acetyltransferase activity
(loss)
c-value
0.8
0.7
0.6
0.5
0.4
0.3
0.2
0.1
0.0
p-value
9e-04
3e-03
7e-03
2e-02
6e-02
Log-odds
0.5
1.0
1.5
2.0
2.5
3.0
3.5
4.0
Cellular_organisms - ps1
Archaea1 - ps2
Archaea2 - ps3
Archaea3 - ps4
Asgard/Eukaryota - ps5
Eukaryota - ps6
Amorpheae - ps7
Obazoa - ps8
Opisthokonta - ps9
Holozoa - ps10
Filozoa - ps11
Choano./Metazoa - ps12
Metazoa - ps13
Myriazoa - ps14
Parahoxozoa - ps15
Bilateria - ps16
Deuterostomia - ps17
Chordata - ps18
Olfactores - ps19
Vertebrata - ps20
Gnathostomata - ps21
Euteleostomi - ps22
Sarcopterygii - ps23
Tetrapoda - ps24
Amniota - ps25
Mammalia - ps26
Theria - ps27
Placentalia - ps28
Boreoeutheria - ps29
Euarchontoglires - ps30
Euarchonta - ps31
Primates - ps32
Haplorrhini - ps33
Simiiformes - ps34
Catarrhini - ps35
Hominoidea - ps36
Hominidae - ps37
Homininae - ps38
Hominini - ps39
Homo_sapiens - ps40
Phylostratum

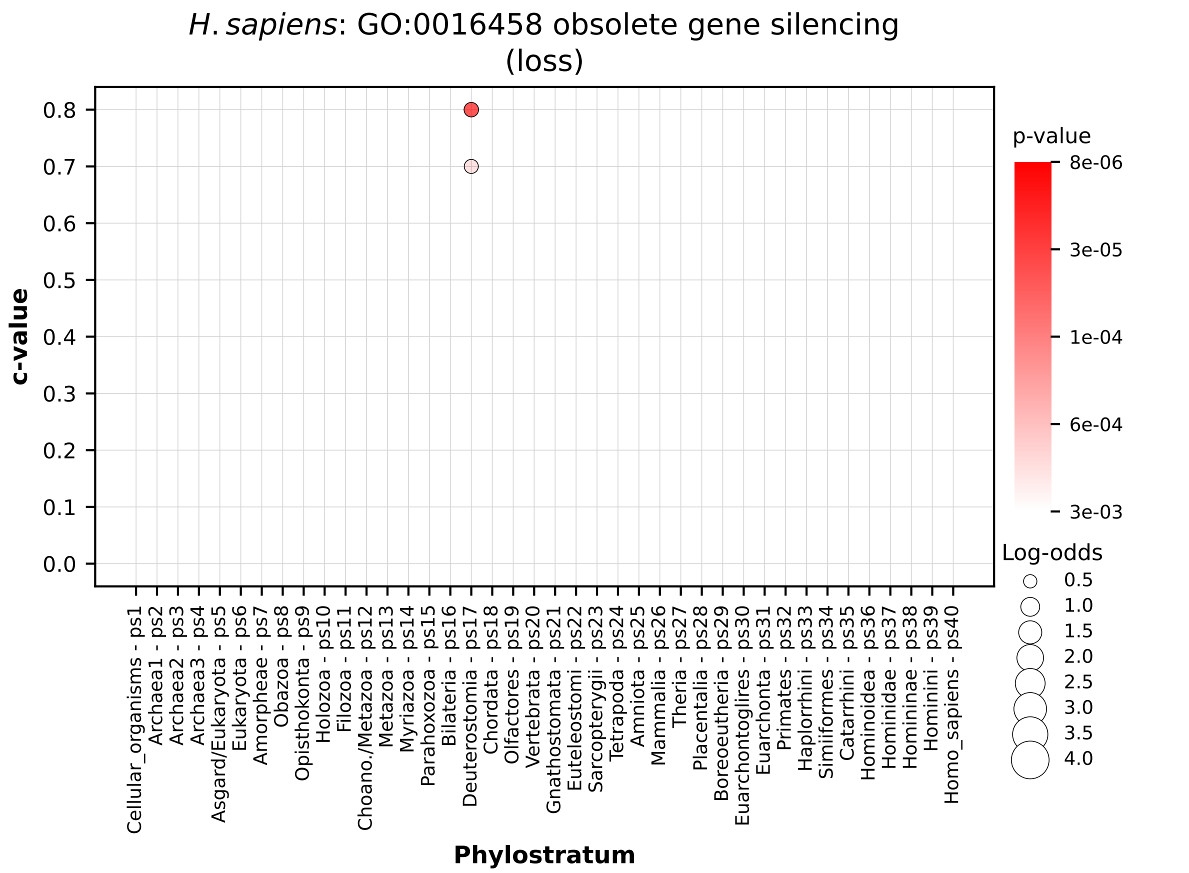

H. sapiens: GO:0016458 obsolete gene silencing
(loss)
c-value
0.0
0.1
0.2
0.3
0.4
0.5
0.6
0.7
0.8
p-value
8e-06
3e-05
1e-04
6e-04
3e-03
Log-odds
0.5
1.0
1.5
2.0
2.5
3.0
3.5
4.0
Cellular_organisms - ps1
Archaea1 - ps2
Archaea2 - ps3
Archaea3 - ps4
Asgard/Eukaryota - ps5
Eukaryota - ps6
Amorpheae - ps7
Obazoa - ps8
Opisthokonta - ps9
Holozoa - ps10
Filozoa - ps11
Choano./Metazoa - ps12
Metazoa - ps13
Myriazoa - ps14
Parahoxozoa - ps15
Bilateria - ps16
Deuterostomia - ps17
Chordata - ps18
Olfactores - ps19
Vertebrata - ps20
Gnathostomata - ps21
Euteleostomi - ps22
Sarcopterygii - ps23
Tetrapoda - ps24
Amniota - ps25
Mammalia - ps26
Theria - ps27
Placentalia - ps28
Boreoeutheria - ps29
Euarchontoglires - ps30
Euarchonta - ps31
Primates - ps32
Haplorrhini - ps33
Simiiformes - ps34
Catarrhini - ps35
Hominoidea - ps36
Hominidae - ps37
Homininae - ps38
Hominini - ps39
Homo_sapiens - ps40
Phylostratum

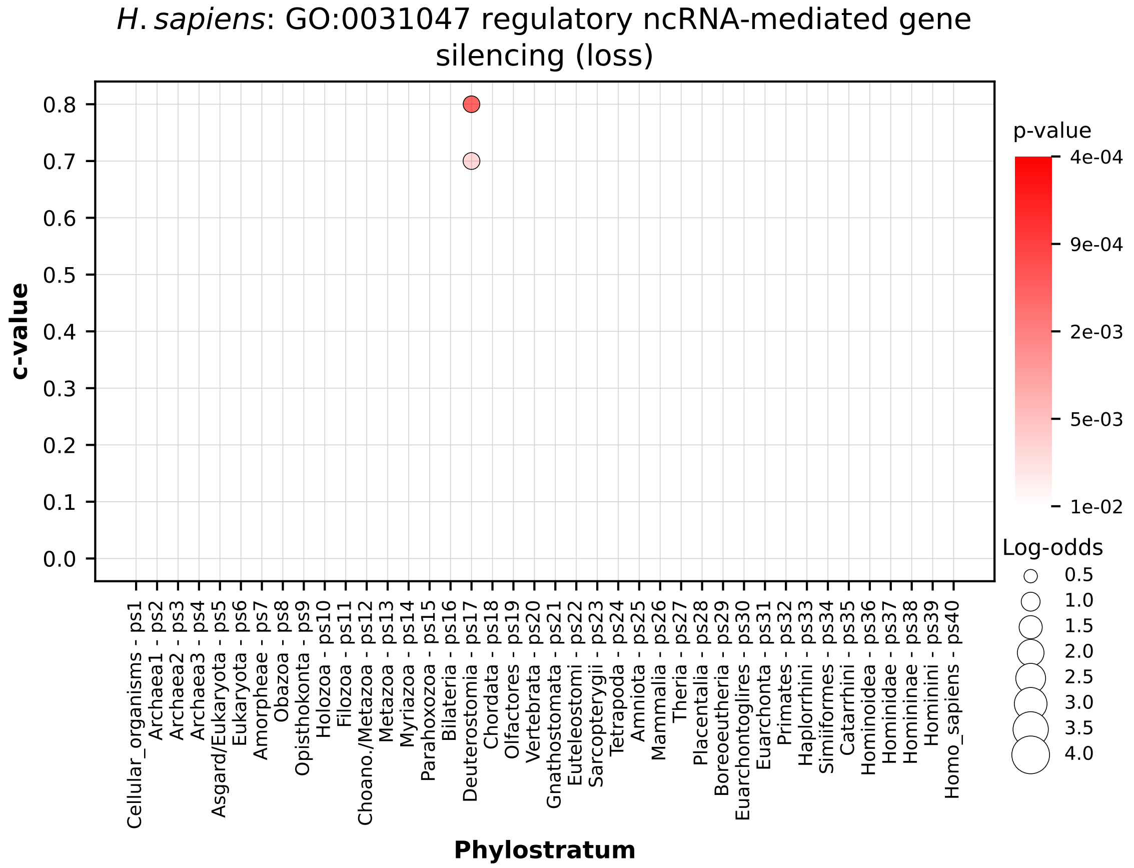

H. sapiens: GO:0031047 regulatory ncRNA-mediated gene silencing (loss)
c-value
0.8
0.7
0.6
0.5
0.4
0.3
0.2
0.1
0.0
p-value
4e-04
9e-04
2e-03
5e-03
1e-02
Log-odds
0.5
1.0
1.5
2.0
2.5
3.0
3.5
4.0
Cellular_organisms - ps1
Archaea1 - ps2
Archaea2 - ps3
Archaea3 - ps4
Asgard/Eukaryota - ps5
Eukaryota - ps6
Amorpheae - ps7
Obazoa - ps8
Opisthokonta - ps9
Holozoa - ps10
Filozoa - ps11
Choano./Metazoa - ps12
Metazoa - ps13
Myriazoa - ps14
Parahoxozoa - ps15
Bilateria - ps16
Deuterostomia - ps17
Chordata - ps18
Olfactores - ps19
Vertebrata - ps20
Gnathostomata - ps21
Euteleostomi - ps22
Sarcopterygii - ps23
Tetrapoda - ps24
Amniota - ps25
Mammalia - ps26
Theria - ps27
Placentalia - ps28
Boreoeutheria - ps29
Euarchontoglires - ps30
Euarchonta - ps31
Primates - ps32
Haplorrhini - ps33
Simiiformes - ps34
Catarrhini - ps35
Hominoidea - ps36
Hominidae - ps37
Homininae - ps38
Hominini - ps39
Homo_sapiens - ps40
Phylostratum

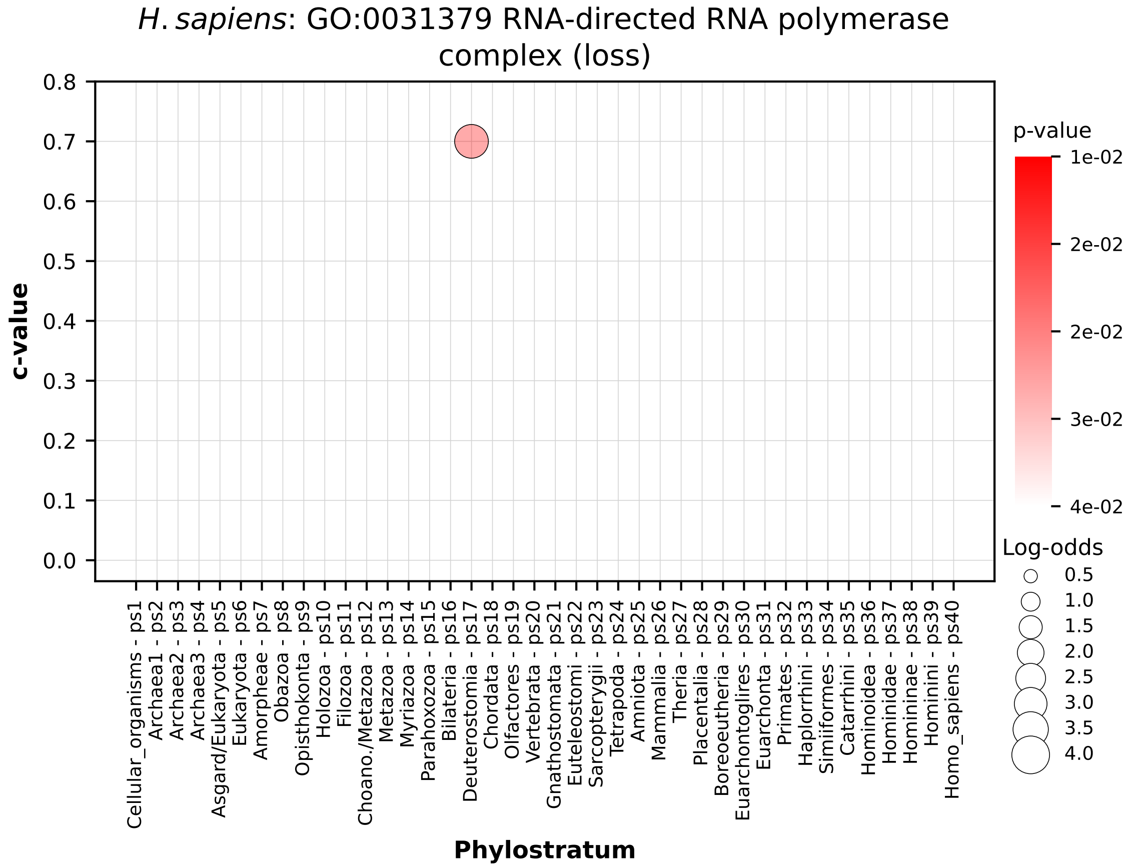
H. sapiens: GO:0031379 RNA-directed RNA polymerase complex (loss)
c-value
0.0
0.1
0.2
0.3
0.4
0.5
0.6
0.7
0.8
Phylostratum
Cellular_organisms - ps1
Archaea1 - ps2
Archaea2 - ps3
Archaea3 - ps4
Asgard/Eukaryota - ps5
Eukaryota - ps6
Amorpheae - ps7
Obazoa - ps8
Opisthokonta - ps9
Holozoa - ps10
Filozoa - ps11
Choano./Metazoa - ps12
Metazoa - ps13
Myriazoa - ps14
Parahoxozoa - ps15
Bilateria - ps16
Deuterostomia - ps17
Chordata - ps18
Olfactores - ps19
Vertebrata - ps20
Gnathostomata - ps21
Euteleostomi - ps22
Sarcopterygii - ps23
Tetrapoda - ps24
Amniota - ps25
Mammalia - ps26
Theria - ps27
Placentalia - ps28
Boreoeutheria - ps29
Euarchontoglires - ps30
Euarchonta - ps31
Primates - ps32
Haplorrhini - ps33
Simiiformes - ps34
Catarrhini - ps35
Hominoidea - ps36
Hominidae - ps37
Homininae - ps38
Hominini - ps39
Homo_sapiens - ps40
p-value
1e-02
2e-02
2e-02
3e-02
4e-02
Log-odds
0.5
1.0
1.5
2.0
2.5
3.0
3.5
4.0

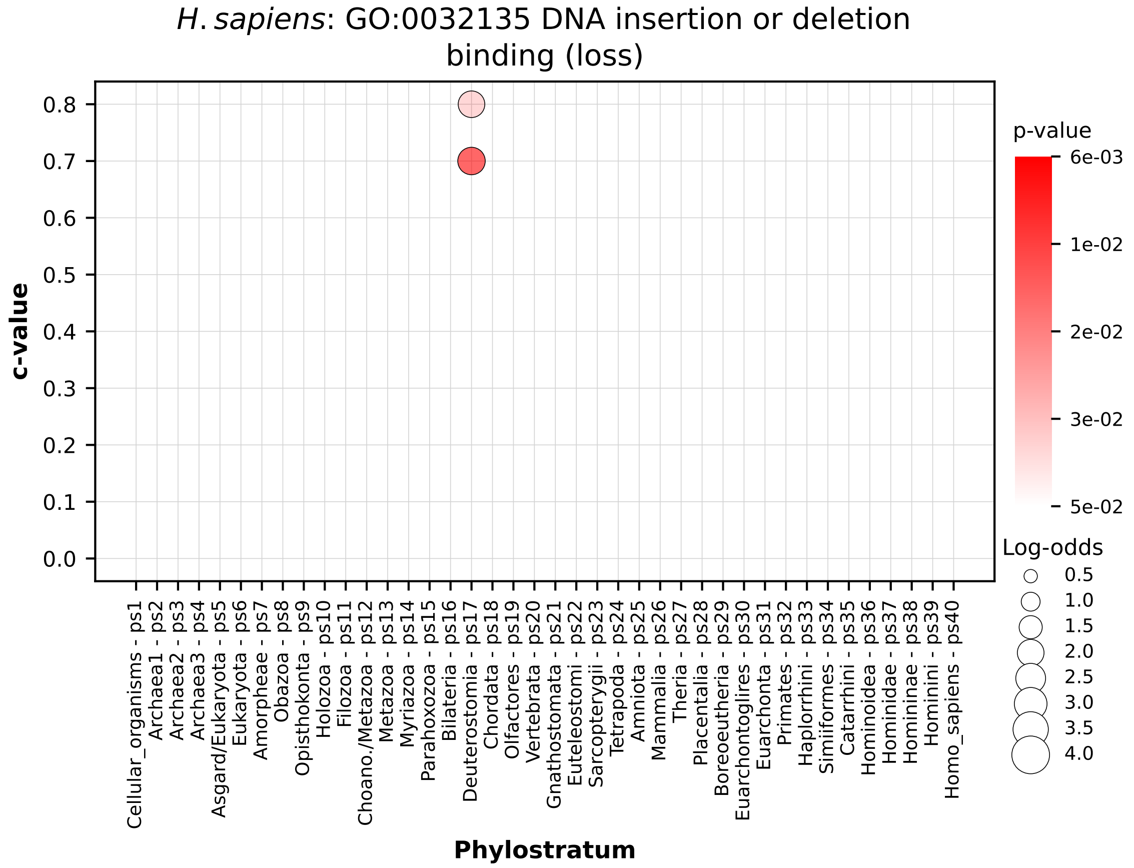
H. sapiens: GO:0032135 DNA insertion or deletion binding (loss)
c-value
0.0
0.1
0.2
0.3
0.4
0.5
0.6
0.7
0.8
p-value
6e-03
1e-02
2e-02
3e-02
5e-02
Log-odds
0.5
1.0
1.5
2.0
2.5
3.0
3.5
4.0
Cellular_organisms - ps1
Archaea1 - ps2
Archaea2 - ps3
Archaea3 - ps4
Asgard/Eukaryota - ps5
Eukaryota - ps6
Amorpheae - ps7
Obazoa - ps8
Opisthokonta - ps9
Holozoa - ps10
Filozoa - ps11
Choano./Metazoa - ps12
Metazoa - ps13
Myriazoa - ps14
Parahoxozoa - ps15
Bilateria - ps16
Deuterostomia - ps17
Chordata - ps18
Olfactores - ps19
Vertebrata - ps20
Gnathostomata - ps21
Euteleostomi - ps22
Sarcopterygii - ps23
Tetrapoda - ps24
Amniota - ps25
Mammalia - ps26
Theria - ps27
Placentalia - ps28
Boreoeutheria - ps29
Euarchontoglires - ps30
Euarchonta - ps31
Primates - ps32
Haplorrhini - ps33
Simiiformes - ps34
Catarrhini - ps35
Hominoidea - ps36
Hominidae - ps37
Homininae - ps38
Hominini - ps39
Homo_sapiens - ps40
Phylostratum

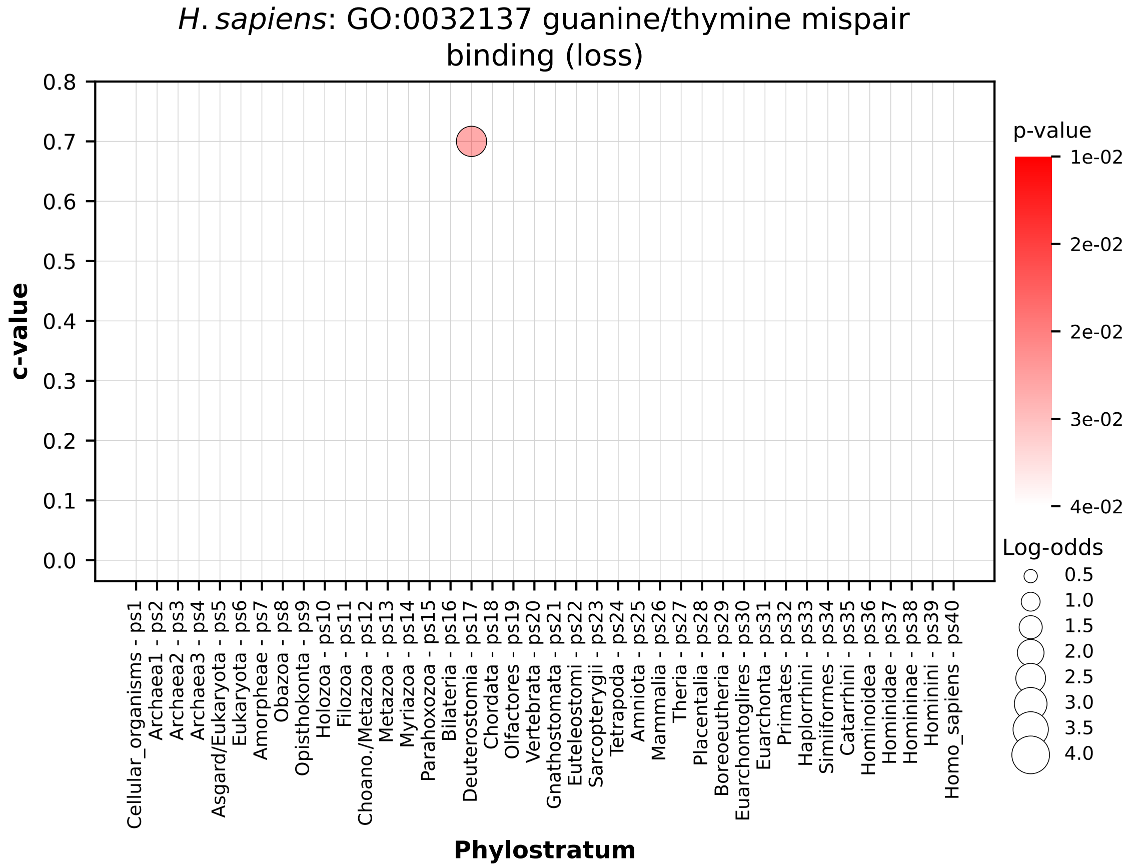

H. sapiens: GO:0032137 guanine/thymine mispair binding (loss)
c-value
0.8
0.7
0.6
0.5
0.4
0.3
0.2
0.1
0.0
Phylostratum
Cellular_organisms - ps1
Archaea1 - ps2
Archaea2 - ps3
Archaea3 - ps4
Asgard/Eukaryota - ps5
Eukaryota - ps6
Amorpheae - ps7
Obazoa - ps8
Opisthokonta - ps9
Holozoa - ps10
Filozoa - ps11
Choano./Metazoa - ps12
Metazoa - ps13
Myriazoa - ps14
Parahoxozoa - ps15
Bilateria - ps16
Deuterostomia - ps17
Chordata - ps18
Olfactores - ps19
Vertebrata - ps20
Gnathostomata - ps21
Euteleostomi - ps22
Sarcopterygii - ps23
Tetrapoda - ps24
Amniota - ps25
Mammalia - ps26
Theria - ps27
Placentalia - ps28
Boreoeutheria - ps29
Euarchontoglires - ps30
Euarchonta - ps31
Primates - ps32
Haplorrhini - ps33
Simiiformes - ps34
Catarrhini - ps35
Hominoidea - ps36
Hominidae - ps37
Homininae - ps38
Hominini - ps39
Homo_sapiens - ps40
p-value
1e-02
2e-02
2e-02
3e-02
4e-02
Log-odds
0.5
1.0
1.5
2.0
2.5
3.0
3.5
4.0

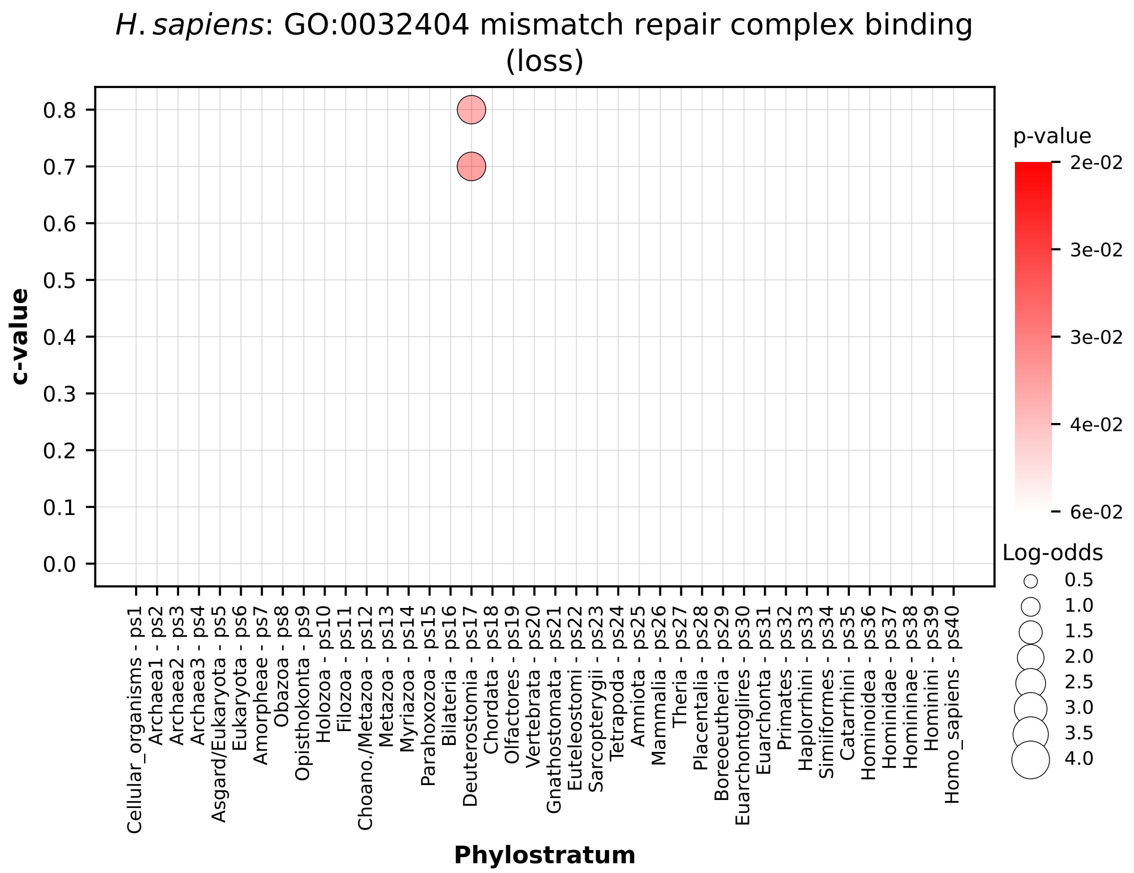

H. sapiens: GO:0032404 mismatch repair complex binding (loss)
c-value
0.8
0.7
0.6
0.5
0.4
0.3
0.2
0.1
0.0
p-value
2e-02
3e-02
3e-02
4e-02
6e-02
Log-odds
0.5
1.0
1.5
2.0
2.5
3.0
3.5
4.0
Cellular_organisms - ps1
Archaea1 - ps2
Archaea2 - ps3
Archaea3 - ps4
Asgard/Eukaryota - ps5
Eukaryota - ps6
Amorpheae - ps7
Obazoa - ps8
Opisthokonta - ps9
Holozoa - ps10
Filozoa - ps11
Choano./Metazoa - ps12
Metazoa - ps13
Myriazoa - ps14
Parahoxozoa - ps15
Bilateria - ps16
Deuterostomia - ps17
Chordata - ps18
Olfactores - ps19
Vertebrata - ps20
Gnathostomata - ps21
Euteleostomi - ps22
Sarcopterygii - ps23
Tetrapoda - ps24
Amniota - ps25
Mammalia - ps26
Theria - ps27
Placentalia - ps28
Boreoeutheria - ps29
Euarchontoglires - ps30
Euarchonta - ps31
Primates - ps32
Haplorrhini - ps33
Simiiformes - ps34
Catarrhini - ps35
Hominoidea - ps36
Hominidae - ps37
Homininae - ps38
Hominini - ps39
Homo_sapiens - ps40
Phylostratum

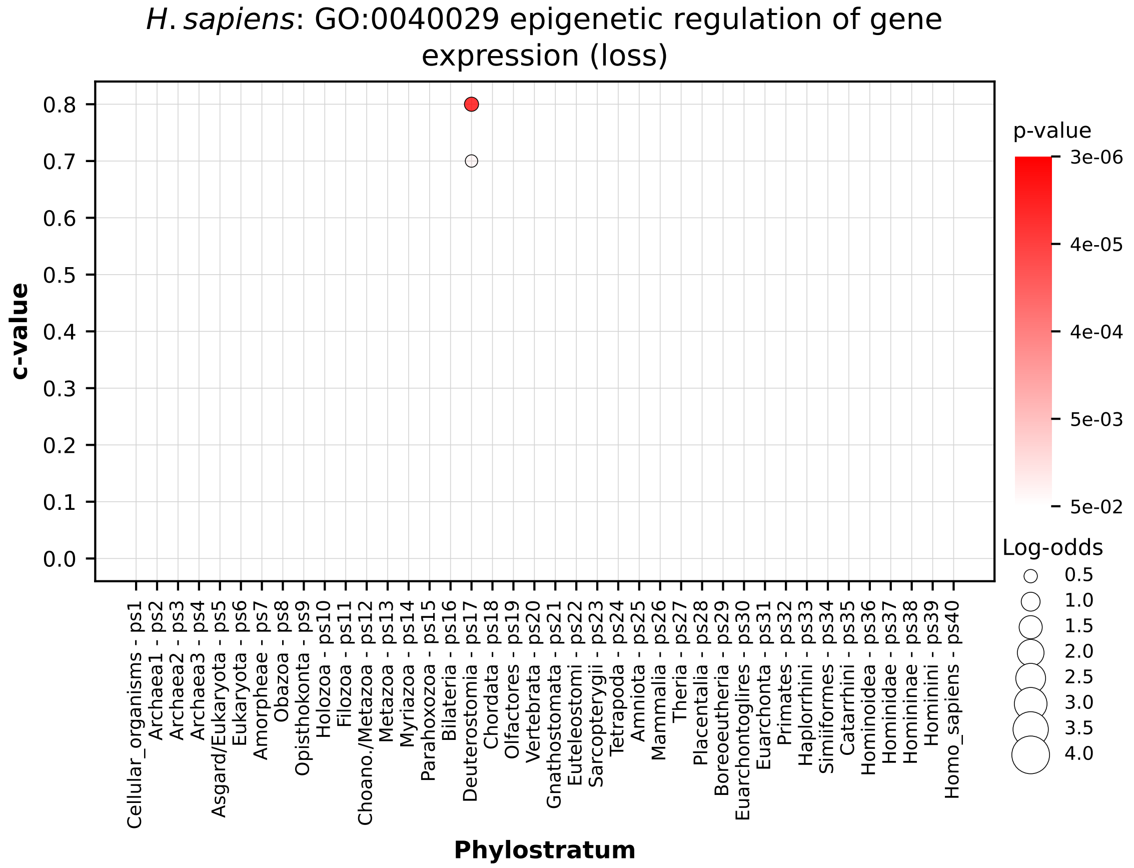

H. sapiens: GO:0040029 epigenetic regulation of gene expression (loss)
c-value
0.0
0.1
0.2
0.3
0.4
0.5
0.6
0.7
0.8
Phylostratum
Cellular_organisms - ps1
Archaea1 - ps2
Archaea2 - ps3
Archaea3 - ps4
Asgard/Eukaryota - ps5
Eukaryota - ps6
Amorpheae - ps7
Obazoa - ps8
Opisthokonta - ps9
Holozoa - ps10
Filozoa - ps11
Choano./Metazoa - ps12
Metazoa - ps13
Myriazoa - ps14
Parahoxozoa - ps15
Bilateria - ps16
Deuterostomia - ps17
Chordata - ps18
Olfactores - ps19
Vertebrata - ps20
Gnathostomata - ps21
Euteleostomi - ps22
Sarcopterygii - ps23
Tetrapoda - ps24
Amniota - ps25
Mammalia - ps26
Theria - ps27
Placentalia - ps28
Boreoeutheria - ps29
Euarchontoglires - ps30
Euarchonta - ps31
Primates - ps32
Haplorrhini - ps33
Simiiformes - ps34
Catarrhini - ps35
Hominoidea - ps36
Hominidae - ps37
Homininae - ps38
Hominini - ps39
Homo_sapiens - ps40
p-value
3e-06
4e-05
4e-04
5e-03
5e-02
Log-odds
0.5
1.0
1.5
2.0
2.5
3.0
3.5
4.0

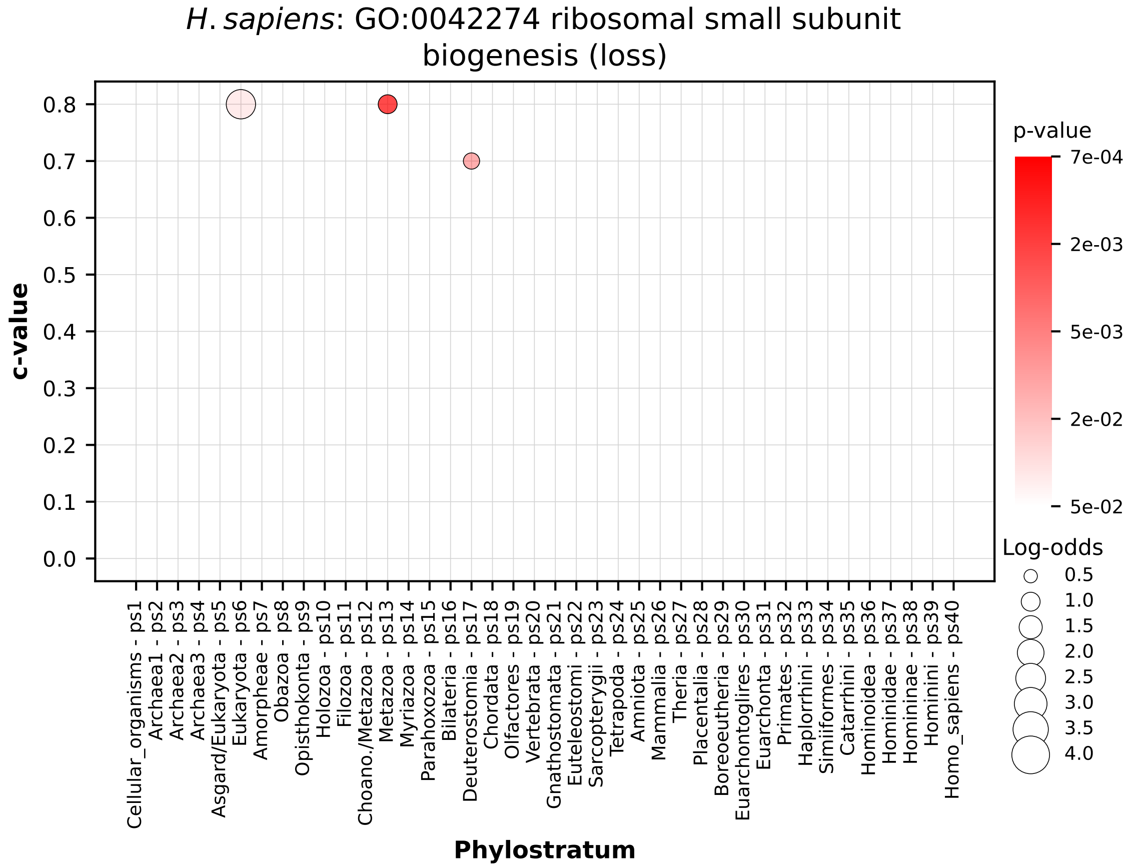

H. sapiens: GO:0042274 ribosomal small subunit biogenesis (loss)
c-value
0.0
0.1
0.2
0.3
0.4
0.5
0.6
0.7
0.8
p-value
7e-04
2e-03
5e-03
2e-02
5e-02
Log-odds
0.5
1.0
1.5
2.0
2.5
3.0
3.5
4.0
Cellular_organisms - ps1
Archaea1 - ps2
Archaea2 - ps3
Archaea3 - ps4
Asgard/Eukaryota - ps5
Eukaryota - ps6
Amorpheae - ps7
Obazoa - ps8
Opisthokonta - ps9
Holozoa - ps10
Filozoa - ps11
Choano./Metazoa - ps12
Metazoa - ps13
Myriazoa - ps14
Parahoxozoa - ps15
Bilateria - ps16
Deuterostomia - ps17
Chordata - ps18
Olfactores - ps19
Vertebrata - ps20
Gnathostomata - ps21
Euteleostomi - ps22
Sarcopterygii - ps23
Tetrapoda - ps24
Amniota - ps25
Mammalia - ps26
Theria - ps27
Placentalia - ps28
Boreoeutheria - ps29
Euarchontoglires - ps30
Euarchonta - ps31
Primates - ps32
Haplorrhini - ps33
Simiiformes - ps34
Catarrhini - ps35
Hominoidea - ps36
Hominidae - ps37
Homininae - ps38
Hominini - ps39
Homo_sapiens - ps40
Phylostratum

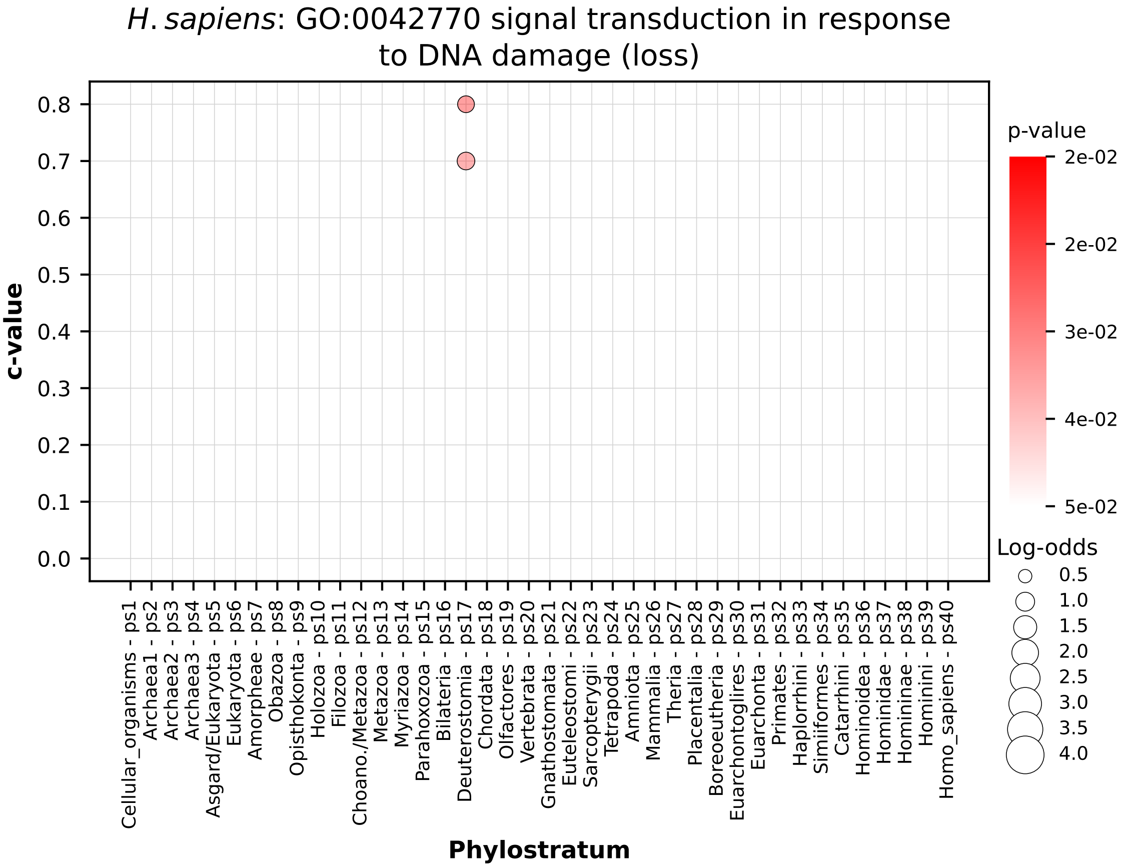
H. sapiens: GO:0042770 signal transduction in response to DNA damage (loss)
c-value
0.0
0.1
0.2
0.3
0.4
0.5
0.6
0.7
0.8
Phylostratum
Cellular_organisms - ps1
Archaea1 - ps2
Archaea2 - ps3
Archaea3 - ps4
Asgard/Eukaryota - ps5
Eukaryota - ps6
Amorpheae - ps7
Obazoa - ps8
Opisthokonta - ps9
Holozoa - ps10
Filozoa - ps11
Choano./Metazoa - ps12
Metazoa - ps13
Myriazoa - ps14
Parahoxozoa - ps15
Bilateria - ps16
Deuterostomia - ps17
Chordata - ps18
Olfactores - ps19
Vertebrata - ps20
Gnathostomata - ps21
Euteleostomi - ps22
Sarcopterygii - ps23
Tetrapoda - ps24
Amniota - ps25
Mammalia - ps26
Theria - ps27
Placentalia - ps28
Boreoeutheria - ps29
Euarchontoglires - ps30
Euarchonta - ps31
Primates - ps32
Haplorrhini - ps33
Simiiformes - ps34
Catarrhini - ps35
Hominoidea - ps36
Hominidae - ps37
Homininae - ps38
Hominini - ps39
Homo_sapiens - ps40
p-value
2e-02
2e-02
3e-02
4e-02
5e-02
Log-odds
0.5
1.0
1.5
2.0
2.5
3.0
3.5
4.0

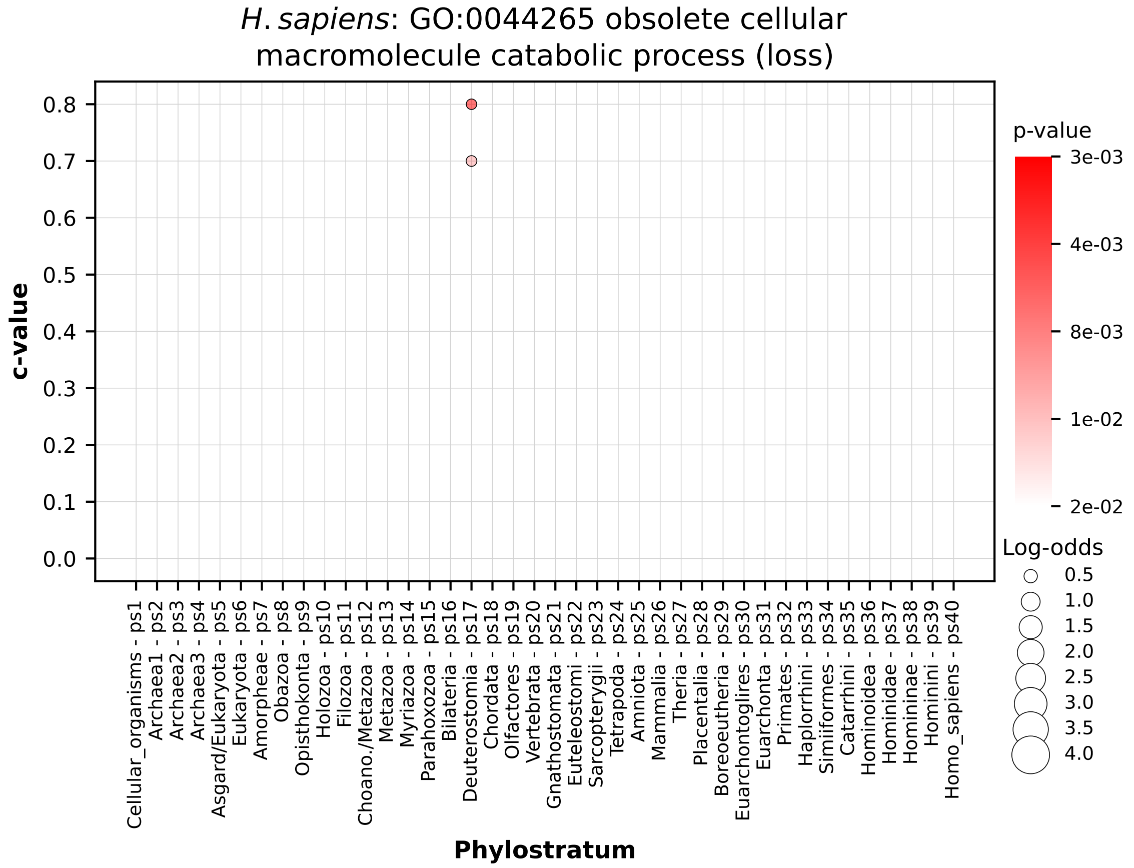

H. sapiens: GO:0044265 obsolete cellular macromolecule catabolic process (loss)
c-value
0.0
0.1
0.2
0.3
0.4
0.5
0.6
0.7
0.8
Phylostratum
Cellular_organisms - ps1
Archaea1 - ps2
Archaea2 - ps3
Archaea3 - ps4
Asgard/Eukaryota - ps5
Eukaryota - ps6
Amorpheae - ps7
Obazoa - ps8
Opisthokonta - ps9
Holozoa - ps10
Filozoa - ps11
Choano./Metazoa - ps12
Metazoa - ps13
Myriazoa - ps14
Parahoxozoa - ps15
Bilateria - ps16
Deuterostomia - ps17
Chordata - ps18
Olfactores - ps19
Vertebrata - ps20
Gnathostomata - ps21
Euteleostomi - ps22
Sarcopterygii - ps23
Tetrapoda - ps24
Amniota - ps25
Mammalia - ps26
Theria - ps27
Placentalia - ps28
Boreoeutheria - ps29
Euarchontoglires - ps30
Euarchonta - ps31
Primates - ps32
Haplorrhini - ps33
Simiiformes - ps34
Catarrhini - ps35
Hominoidea - ps36
Hominidae - ps37
Homininae - ps38
Hominini - ps39
Homo_sapiens - ps40
p-value
3e-03
4e-03
8e-03
1e-02
2e-02
Log-odds
0.5
1.0
1.5
2.0
2.5
3.0
3.5
4.0

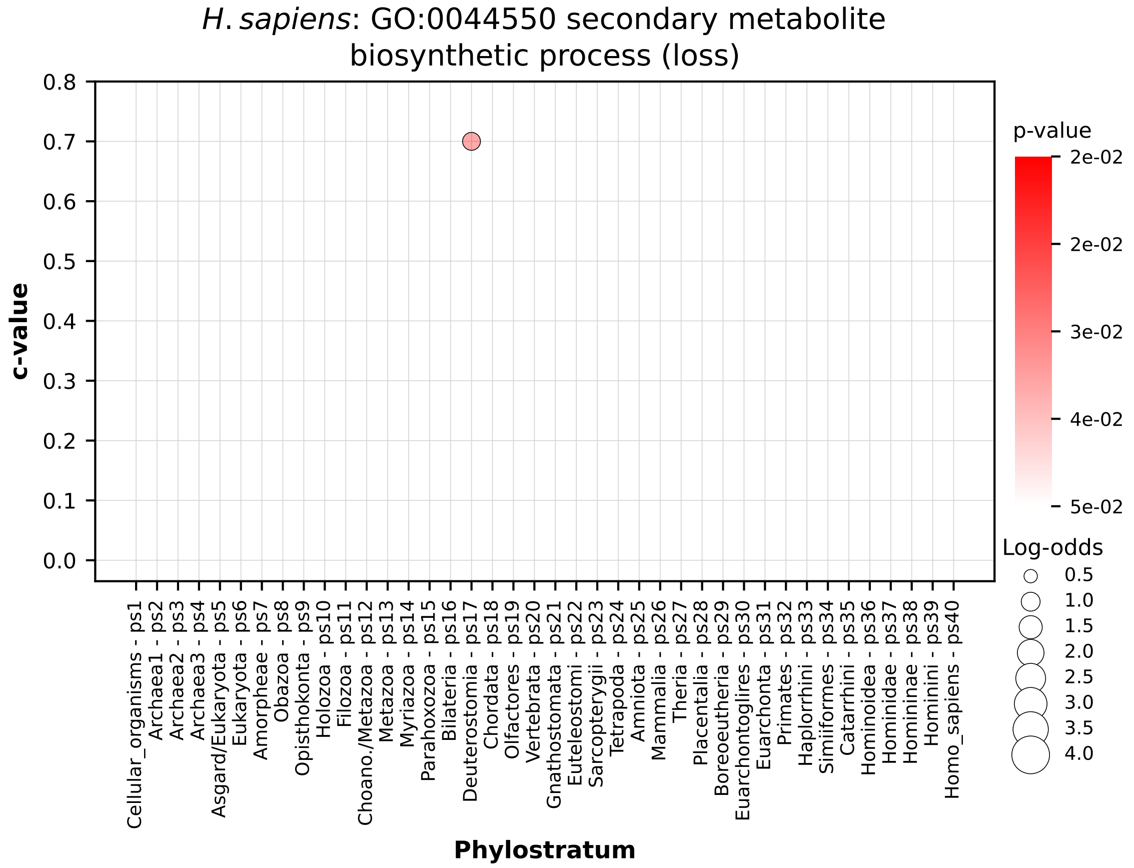

H. sapiens: GO:0044550 secondary metabolite biosynthetic process (loss)
c-value
0.8
0.7
0.6
0.5
0.4
0.3
0.2
0.1
0.0
p-value
2e-02
2e-02
3e-02
4e-02
5e-02
Log-odds
0.5
1.0
1.5
2.0
2.5
3.0
3.5
4.0
Cellular_organisms - ps1
Archaea1 - ps2
Archaea2 - ps3
Archaea3 - ps4
Asgard/Eukaryota - ps5
Eukaryota - ps6
Amorpheae - ps7
Obazoa - ps8
Opisthokonta - ps9
Holozoa - ps10
Filozoa - ps11
Choano./Metazoa - ps12
Metazoa - ps13
Myriazoa - ps14
Parahoxozoa - ps15
Bilateria - ps16
Deuterostomia - ps17
Chordata - ps18
Olfactores - ps19
Vertebrata - ps20
Gnathostomata - ps21
Euteleostomi - ps22
Sarcopterygii - ps23
Tetrapoda - ps24
Amniota - ps25
Mammalia - ps26
Theria - ps27
Placentalia - ps28
Boreoeutheria - ps29
Euarchontoglires - ps30
Euarchonta - ps31
Primates - ps32
Haplorrhini - ps33
Simiiformes - ps34
Catarrhini - ps35
Hominoidea - ps36
Hominidae - ps37
Homininae - ps38
Hominini - ps39
Homo_sapiens - ps40
Phylostratum

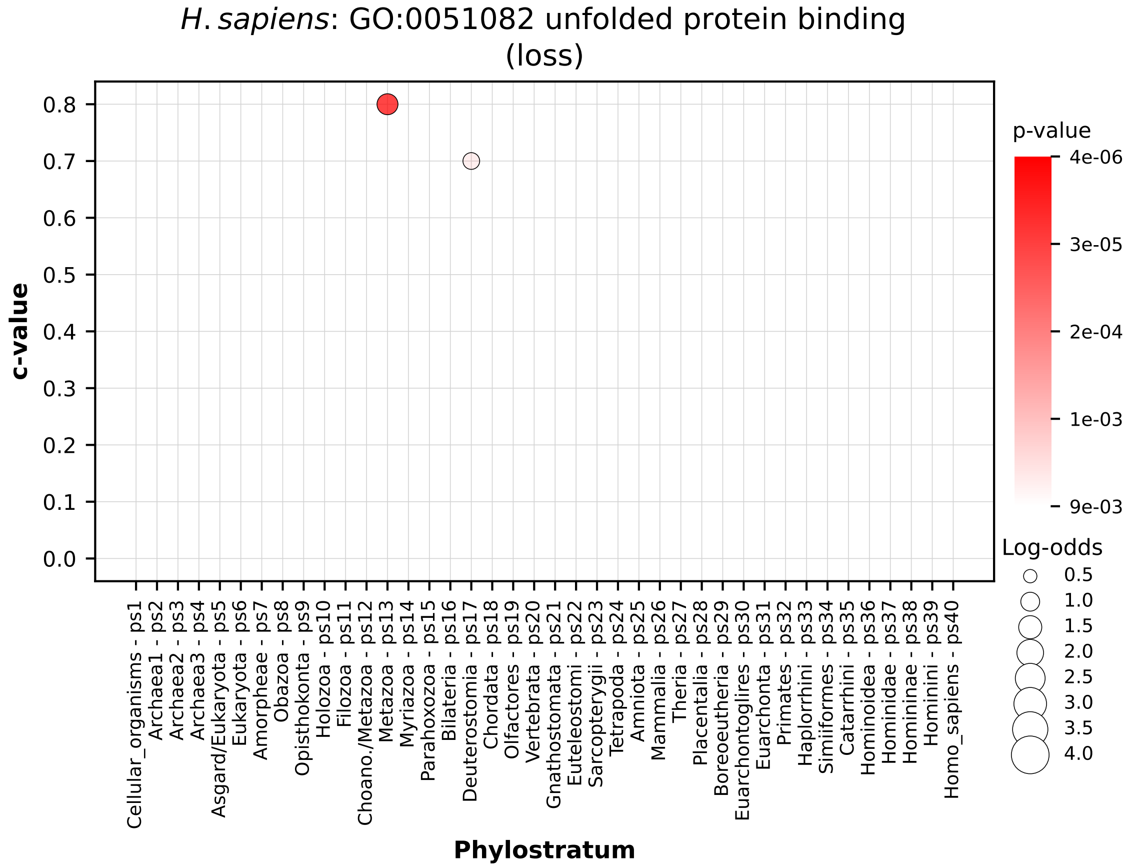

H. sapiens: GO:0051082 unfolded protein binding
(loss)
c-value
0.8
0.7
0.6
0.5
0.4
0.3
0.2
0.1
0.0
p-value
4e-06
3e-05
2e-04
1e-03
9e-03
Log-odds
0.5
1.0
1.5
2.0
2.5
3.0
3.5
4.0
Cellular_organisms - ps1
Archaea1 - ps2
Archaea2 - ps3
Archaea3 - ps4
Asgard/Eukaryota - ps5
Eukaryota - ps6
Amorpheae - ps7
Obazoa - ps8
Opisthokonta - ps9
Holozoa - ps10
Filozoa - ps11
Choano./Metazoa - ps12
Metazoa - ps13
Myriazoa - ps14
Parahoxozoa - ps15
Bilateria - ps16
Deuterostomia - ps17
Chordata - ps18
Olfactores - ps19
Vertebrata - ps20
Gnathostomata - ps21
Euteleostomi - ps22
Sarcopterygii - ps23
Tetrapoda - ps24
Amniota - ps25
Mammalia - ps26
Theria - ps27
Placentalia - ps28
Boreoeutheria - ps29
Euarchontoglires - ps30
Euarchonta - ps31
Primates - ps32
Haplorrhini - ps33
Simiiformes - ps34
Catarrhini - ps35
Hominoidea - ps36
Hominidae - ps37
Homininae - ps38
Hominini - ps39
Homo_sapiens - ps40
Phylostratum

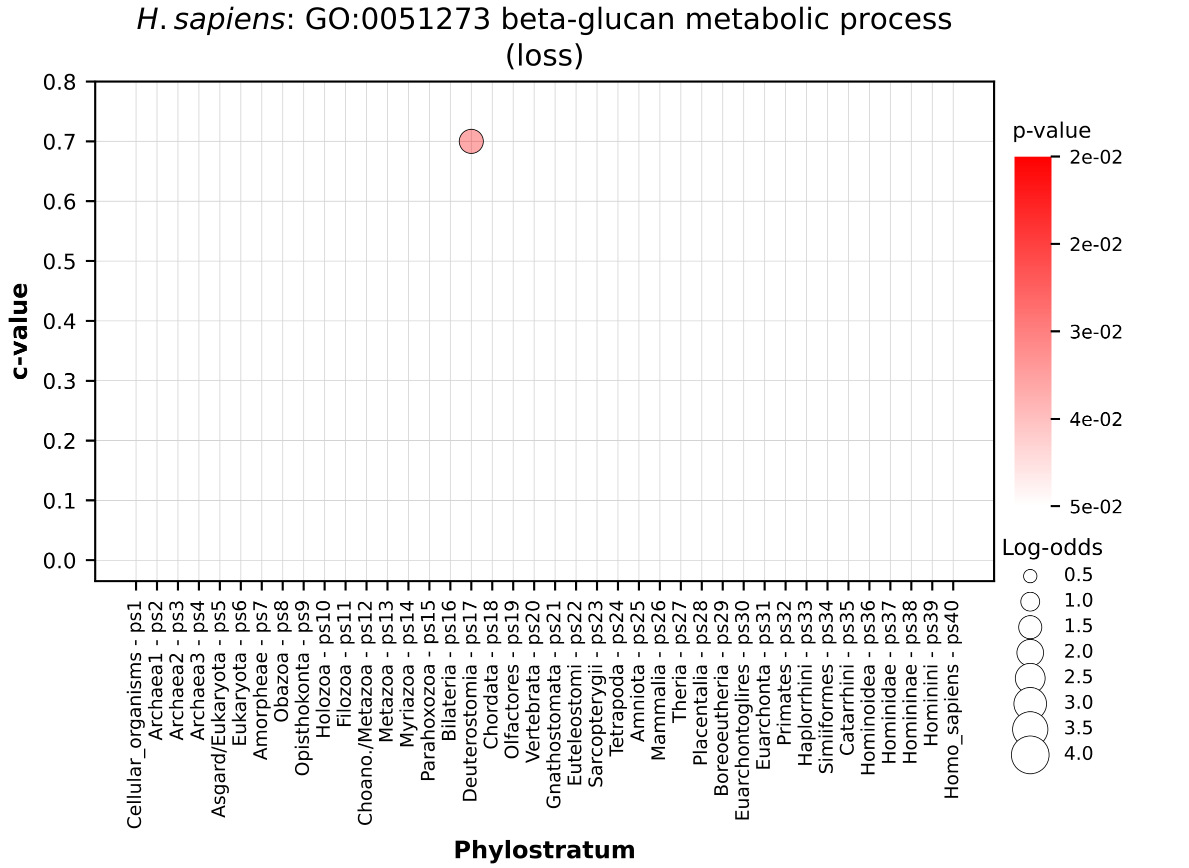

H. sapiens: GO:0051273 beta-glucan metabolic process
(loss)
c-value
0.8
0.7
0.6
0.5
0.4
0.3
0.2
0.1
0.0
p-value
2e-02
2e-02
3e-02
4e-02
5e-02
Log-odds
0.5
1.0
1.5
2.0
2.5
3.0
3.5
4.0
Cellular_organisms - ps1
Archaea1 - ps2
Archaea2 - ps3
Archaea3 - ps4
Asgard/Eukaryota - ps5
Eukaryota - ps6
Amorpheae - ps7
Obazoa - ps8
Opisthokonta - ps9
Holozoa - ps10
Filozoa - ps11
Choano./Metazoa - ps12
Metazoa - ps13
Myriazoa - ps14
Parahoxozoa - ps15
Bilateria - ps16
Deuterostomia - ps17
Chordata - ps18
Olfactores - ps19
Vertebrata - ps20
Gnathostomata - ps21
Euteleostomi - ps22
Sarcopterygii - ps23
Tetrapoda - ps24
Amniota - ps25
Mammalia - ps26
Theria - ps27
Placentalia - ps28
Boreoeutheria - ps29
Euarchontoglires - ps30
Euarchonta - ps31
Primates - ps32
Haplorrhini - ps33
Simiiformes - ps34
Catarrhini - ps35
Hominoidea - ps36
Hominidae - ps37
Homininae - ps38
Hominini - ps39
Homo_sapiens - ps40
Phylostratum

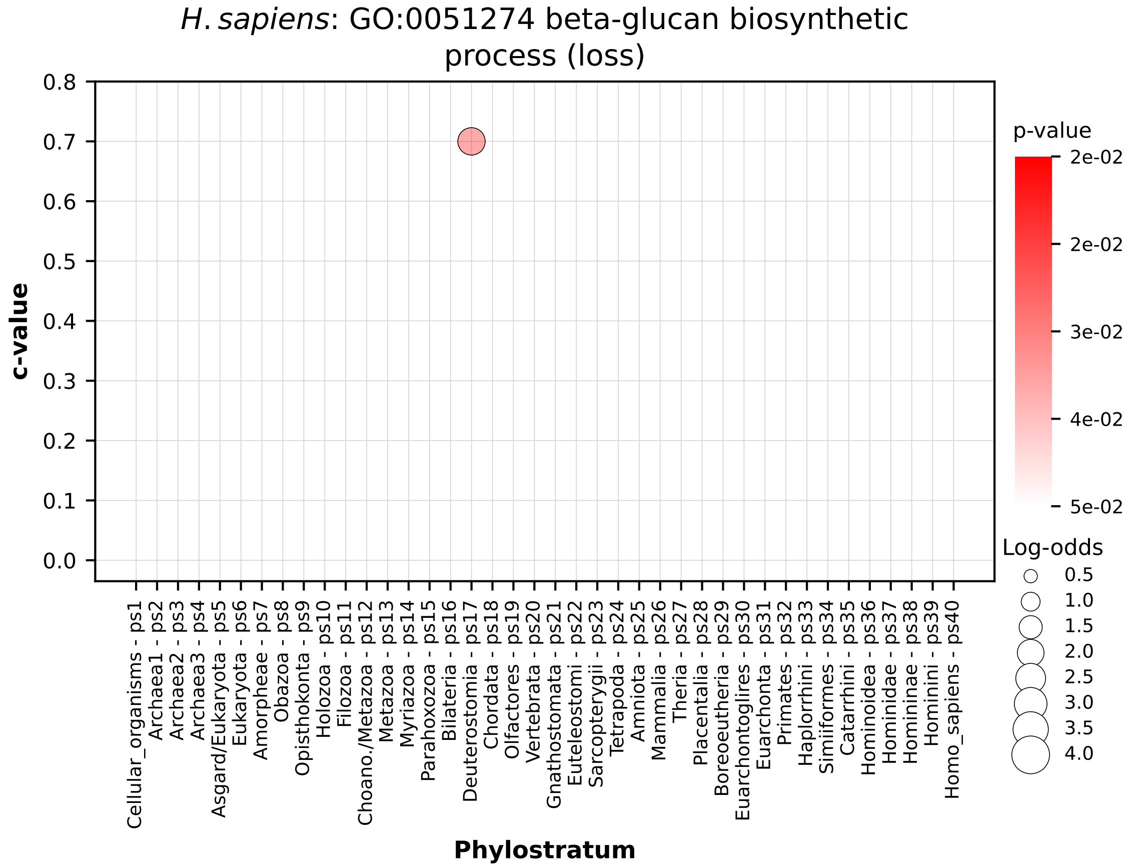

H. sapiens: GO:0051274 beta-glucan biosynthetic process (loss)
c-value
0.8
0.7
0.6
0.5
0.4
0.3
0.2
0.1
0.0
Phylostratum
Cellular_organisms - ps1
Archaea1 - ps2
Archaea2 - ps3
Archaea3 - ps4
Asgard/Eukaryota - ps5
Eukaryota - ps6
Amorpheae - ps7
Obazoa - ps8
Opisthokonta - ps9
Holozoa - ps10
Filozoa - ps11
Choano./Metazoa - ps12
Metazoa - ps13
Myriazoa - ps14
Parahoxozoa - ps15
Bilateria - ps16
Deuterostomia - ps17
Chordata - ps18
Olfactores - ps19
Vertebrata - ps20
Gnathostomata - ps21
Euteleostomi - ps22
Sarcopterygii - ps23
Tetrapoda - ps24
Amniota - ps25
Mammalia - ps26
Theria - ps27
Placentalia - ps28
Boreoeutheria - ps29
Euarchontoglires - ps30
Euarchonta - ps31
Primates - ps32
Haplorrhini - ps33
Simiiformes - ps34
Catarrhini - ps35
Hominoidea - ps36
Hominidae - ps37
Homininae - ps38
Hominini - ps39
Homo_sapiens - ps40
p-value
2e-02
2e-02
3e-02
4e-02
5e-02
Log-odds
0.5
1.0
1.5
2.0
2.5
3.0
3.5
4.0

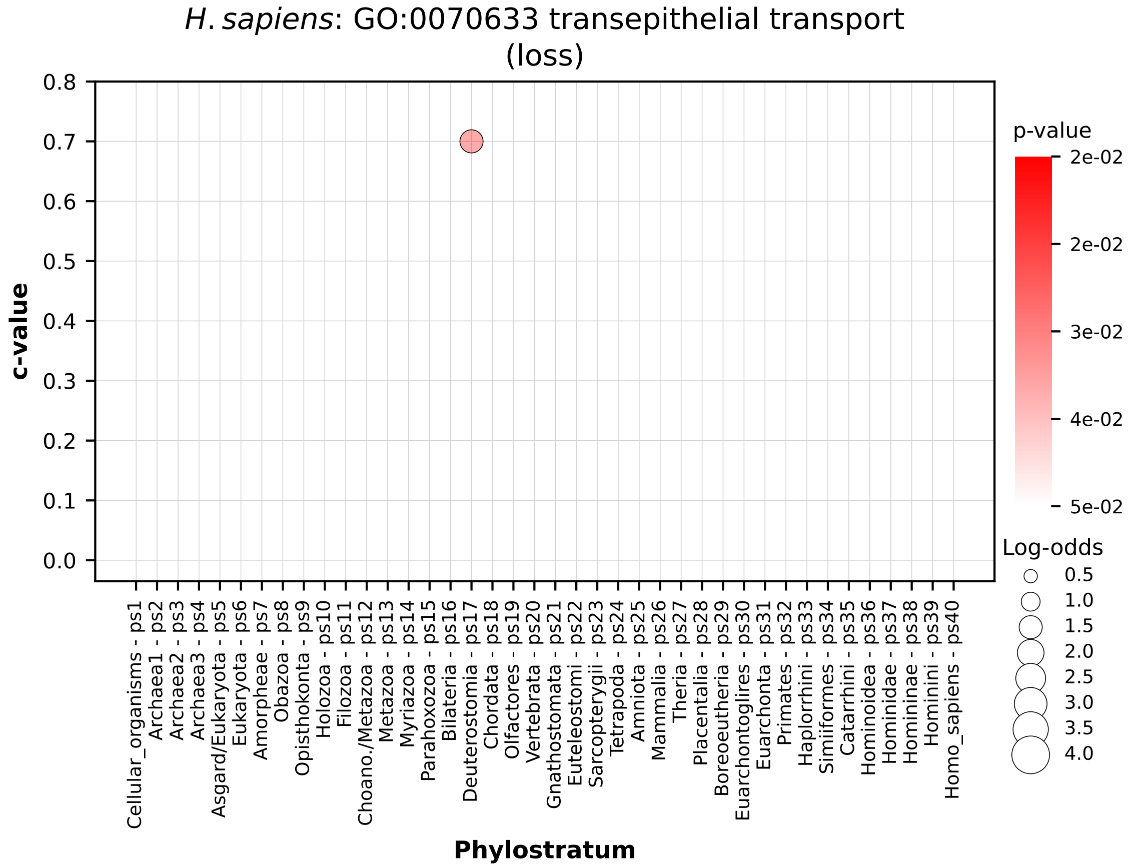

H. sapiens: GO:0070633 transepithelial transport
(loss)
c-value
0.8
0.7
0.6
0.5
0.4
0.3
0.2
0.1
0.0
p-value
2e-02
2e-02
3e-02
4e-02
5e-02
Log-odds
0.5
1.0
1.5
2.0
2.5
3.0
3.5
4.0
Cellular_organisms - ps1
Archaea1 - ps2
Archaea2 - ps3
Archaea3 - ps4
Asgard/Eukaryota - ps5
Eukaryota - ps6
Amorpheae - ps7
Obazoa - ps8
Opisthokonta - ps9
Holozoa - ps10
Filozoa - ps11
Choano./Metazoa - ps12
Metazoa - ps13
Myriazoa - ps14
Parahoxozoa - ps15
Bilateria - ps16
Deuterostomia - ps17
Chordata - ps18
Olfactores - ps19
Vertebrata - ps20
Gnathostomata - ps21
Euteleostomi - ps22
Sarcopterygii - ps23
Tetrapoda - ps24
Amniota - ps25
Mammalia - ps26
Theria - ps27
Placentalia - ps28
Boreoeutheria - ps29
Euarchontoglires - ps30
Euarchonta - ps31
Primates - ps32
Haplorrhini - ps33
Simiiformes - ps34
Catarrhini - ps35
Hominoidea - ps36
Hominidae - ps37
Homininae - ps38
Hominini - ps39
Homo_sapiens - ps40
Phylostratum

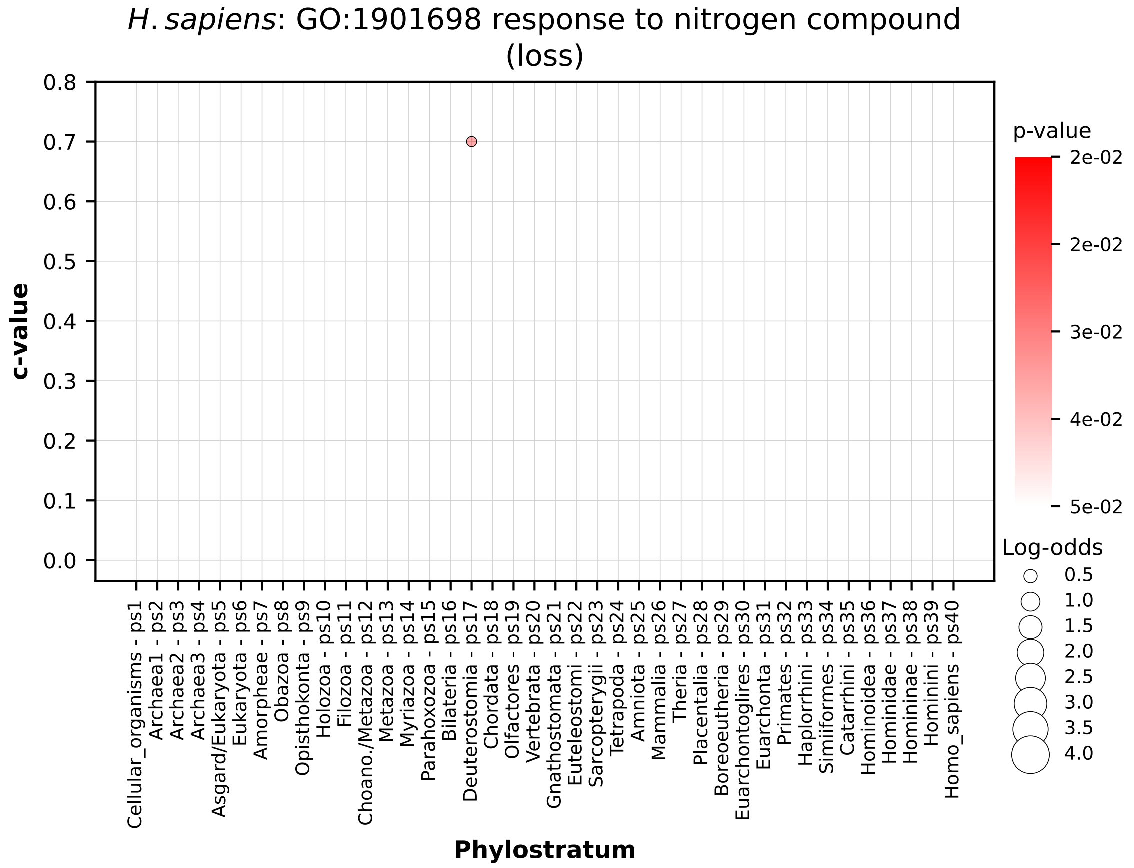

H. sapiens: GO:1901698 response to nitrogen compound (loss)
c-value
0.8
0.7
0.6
0.5
0.4
0.3
0.2
0.1
0.0
Phylostratum
Cellular_organisms - ps1
Archaea1 - ps2
Archaea2 - ps3
Archaea3 - ps4
Asgard/Eukaryota - ps5
Eukaryota - ps6
Amorpheae - ps7
Obazoa - ps8
Opisthokonta - ps9
Holozoa - ps10
Filozoa - ps11
Choano./Metazoa - ps12
Metazoa - ps13
Myriazoa - ps14
Parahoxozoa - ps15
Bilateria - ps16
Deuterostomia - ps17
Chordata - ps18
Olfactores - ps19
Vertebrata - ps20
Gnathostomata - ps21
Euteleostomi - ps22
Sarcopterygii - ps23
Tetrapoda - ps24
Amniota - ps25
Mammalia - ps26
Theria - ps27
Placentalia - ps28
Boreoeutheria - ps29
Euarchontoglires - ps30
Euarchonta - ps31
Primates - ps32
Haplorrhini - ps33
Simiiformes - ps34
Catarrhini - ps35
Hominoidea - ps36
Hominidae - ps37
Homininae - ps38
Hominini - ps39
Homo_sapiens - ps40
p-value
2e-02
2e-02
3e-02
4e-02
5e-02
Log-odds
0.5
1.0
1.5
2.0
2.5
3.0
3.5
4.0

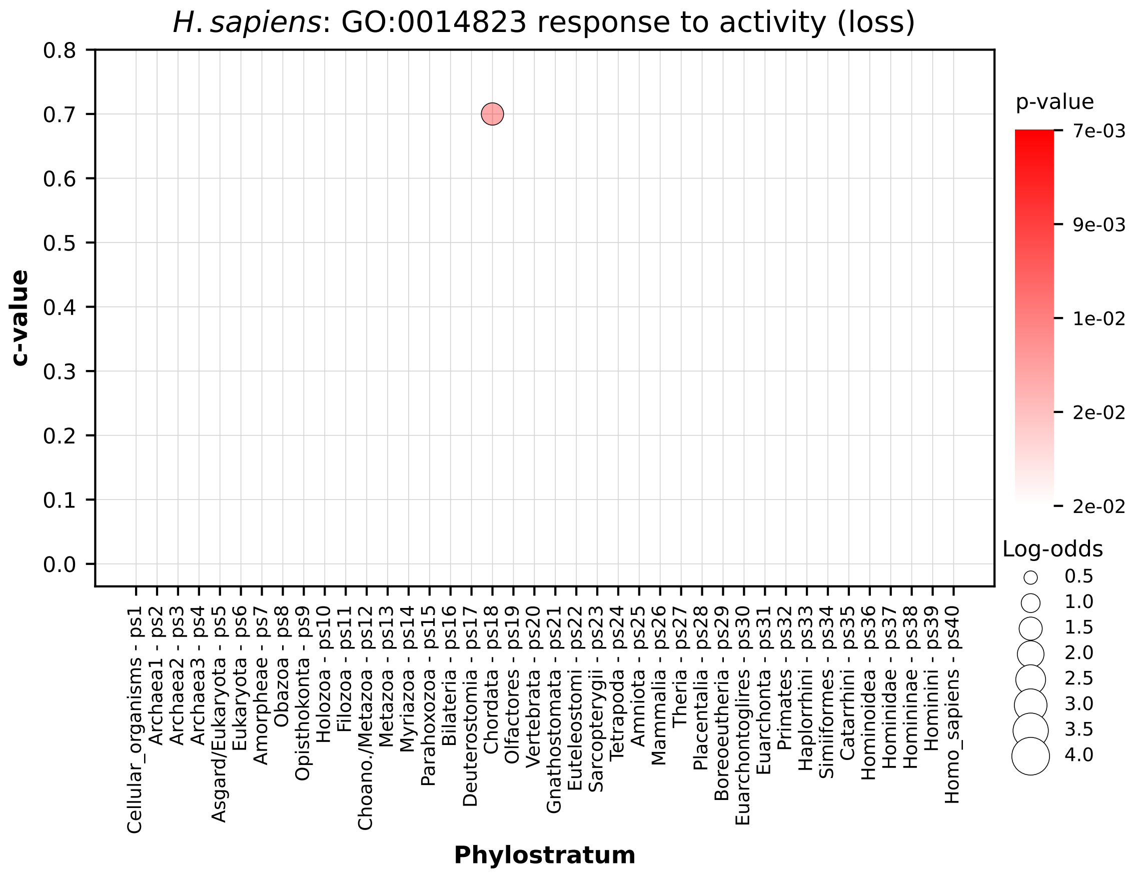

H. sapiens: GO:0014823 response to activity (loss)
c-value
0.8
0.7
0.6
0.5
0.4
0.3
0.2
0.1
0.0
Phylostratum
Cellular_organisms - ps1
Archaea1 - ps2
Archaea2 - ps3
Archaea3 - ps4
Asgard/Eukaryota - ps5
Eukaryota - ps6
Amorpheae - ps7
Obazoa - ps8
Opisthokonta - ps9
Holozoa - ps10
Filozoa - ps11
Choano./Metazoa - ps12
Metazoa - ps13
Myriazoa - ps14
Parahoxozoa - ps15
Bilateria - ps16
Deuterostomia - ps17
Chordata - ps18
Olfactores - ps19
Vertebrata - ps20
Gnathostomata - ps21
Euteleostomi - ps22
Sarcopterygii - ps23
Tetrapoda - ps24
Amniota - ps25
Mammalia - ps26
Theria - ps27
Placentalia - ps28
Boreoeutheria - ps29
Euarchontoglires - ps30
Euarchonta - ps31
Primates - ps32
Haplorrhini - ps33
Simiiformes - ps34
Catarrhini - ps35
Hominoidea - ps36
Hominidae - ps37
Homininae - ps38
Hominini - ps39
Homo_sapiens - ps40
p-value
7e-03
9e-03
1e-02
2e-02
2e-02
Log-odds
0.5
1.0
1.5
2.0
2.5
3.0
3.5
4.0

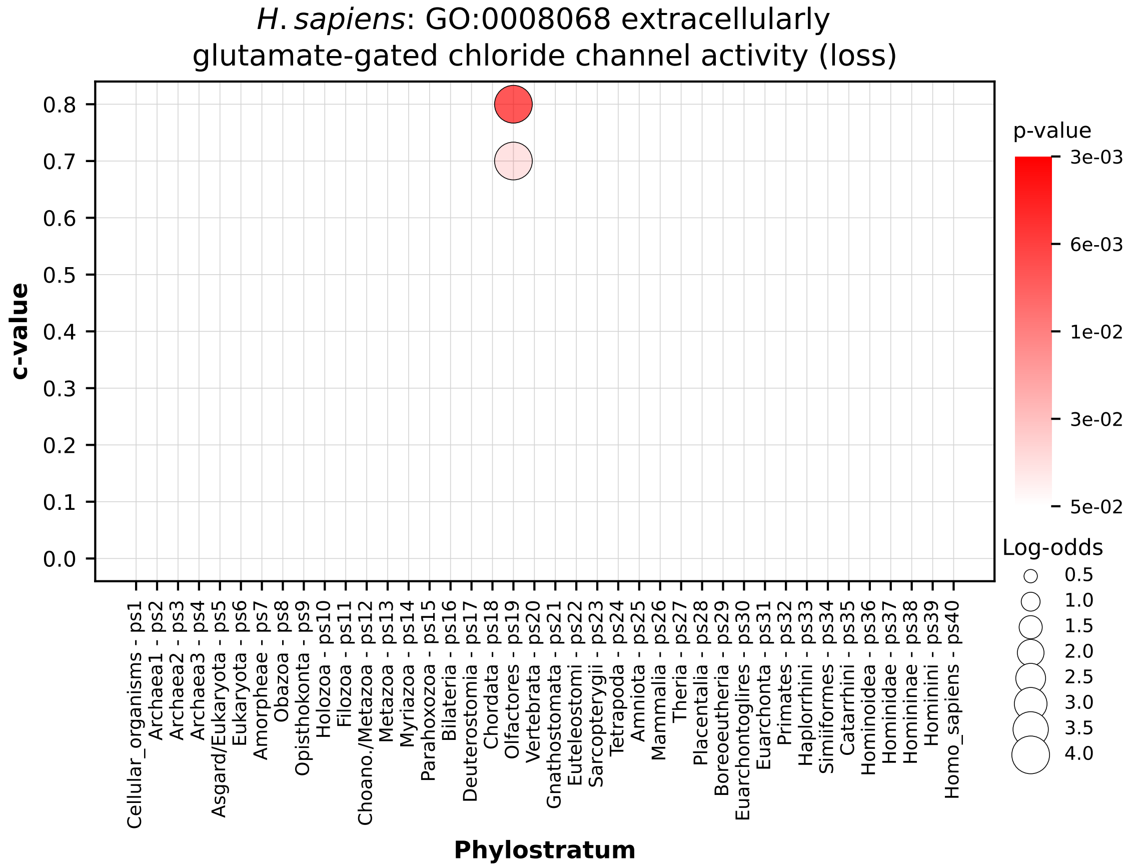

H. sapiens: GO:0008068 extracellularly glutamate-gated chloride channel activity (loss)
c-value
0.8
0.7
0.6
0.5
0.4
0.3
0.2
0.1
0.0
p-value
3e-03
6e-03
1e-02
3e-02
5e-02
Log-odds
0.5
1.0
1.5
2.0
2.5
3.0
3.5
4.0
Cellular_organisms - ps1
Archaea1 - ps2
Archaea2 - ps3
Archaea3 - ps4
Asgard/Eukaryota - ps5
Eukaryota - ps6
Amorpheae - ps7
Obazoa - ps8
Opisthokonta - ps9
Holozoa - ps10
Filozoa - ps11
Choano./Metazoa - ps12
Metazoa - ps13
Myriazoa - ps14
Parahoxozoa - ps15
Bilateria - ps16
Deuterostomia - ps17
Chordata - ps18
Olfactores - ps19
Vertebrata - ps20
Gnathostomata - ps21
Euteleostomi - ps22
Sarcopterygii - ps23
Tetrapoda - ps24
Amniota - ps25
Mammalia - ps26
Theria - ps27
Placentalia - ps28
Boreoeutheria - ps29
Euarchontoglires - ps30
Euarchonta - ps31
Primates - ps32
Haplorrhini - ps33
Simiiformes - ps34
Catarrhini - ps35
Hominoidea - ps36
Hominidae - ps37
Homininae - ps38
Hominini - ps39
Homo_sapiens - ps40
Phylostratum

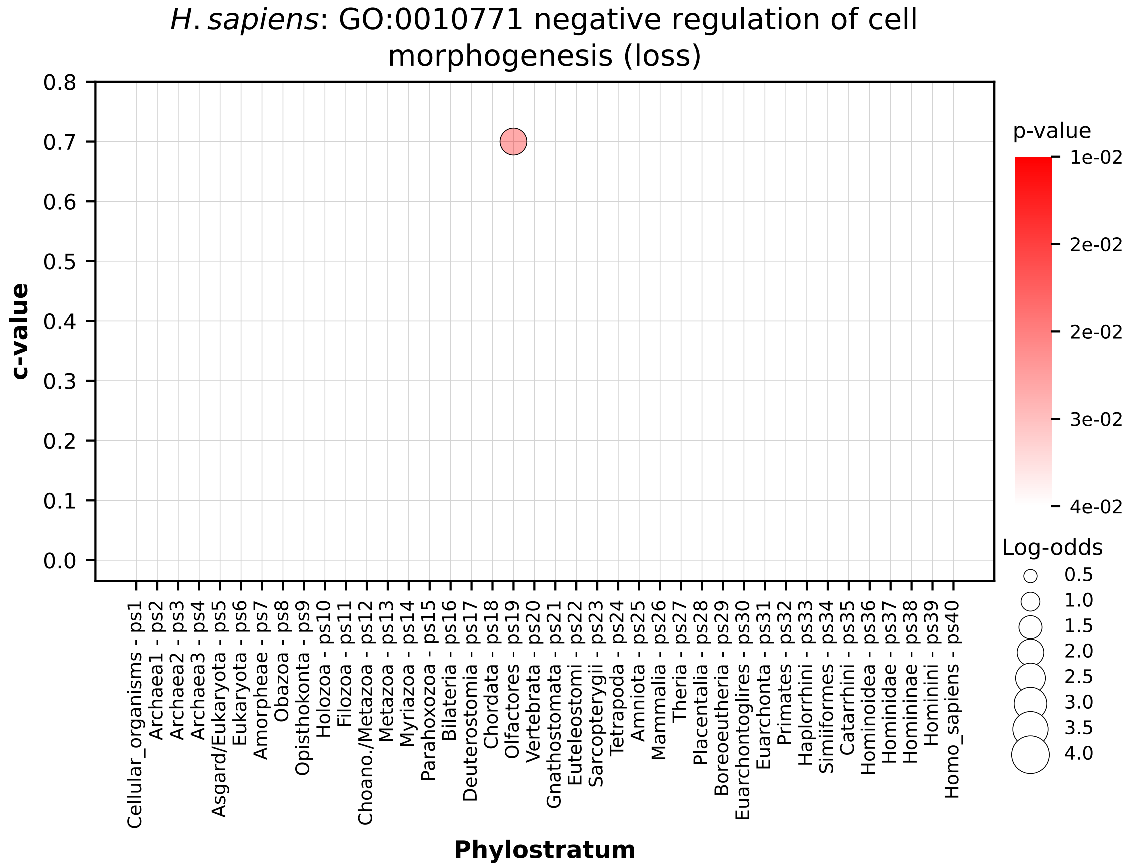

H. sapiens: GO:0010771 negative regulation of cell morphogenesis (loss)
c-value
0.8
0.7
0.6
0.5
0.4
0.3
0.2
0.1
0.0
p-value
1e-02
2e-02
2e-02
3e-02
4e-02
Log-odds
0.5
1.0
1.5
2.0
2.5
3.0
3.5
4.0
Cellular_organisms - ps1
Archaea1 - ps2
Archaea2 - ps3
Archaea3 - ps4
Asgard/Eukaryota - ps5
Eukaryota - ps6
Amorpheae - ps7
Obazoa - ps8
Opisthokonta - ps9
Holozoa - ps10
Filozoa - ps11
Choano./Metazoa - ps12
Metazoa - ps13
Myriazoa - ps14
Parahoxozoa - ps15
Bilateria - ps16
Deuterostomia - ps17
Chordata - ps18
Olfactores - ps19
Vertebrata - ps20
Gnathostomata - ps21
Euteleostomi - ps22
Sarcopterygii - ps23
Tetrapoda - ps24
Amniota - ps25
Mammalia - ps26
Theria - ps27
Placentalia - ps28
Boreoeutheria - ps29
Euarchontoglires - ps30
Euarchonta - ps31
Primates - ps32
Haplorrhini - ps33
Simiiformes - ps34
Catarrhini - ps35
Hominoidea - ps36
Hominidae - ps37
Homininae - ps38
Hominini - ps39
Homo_sapiens - ps40
Phylostratum

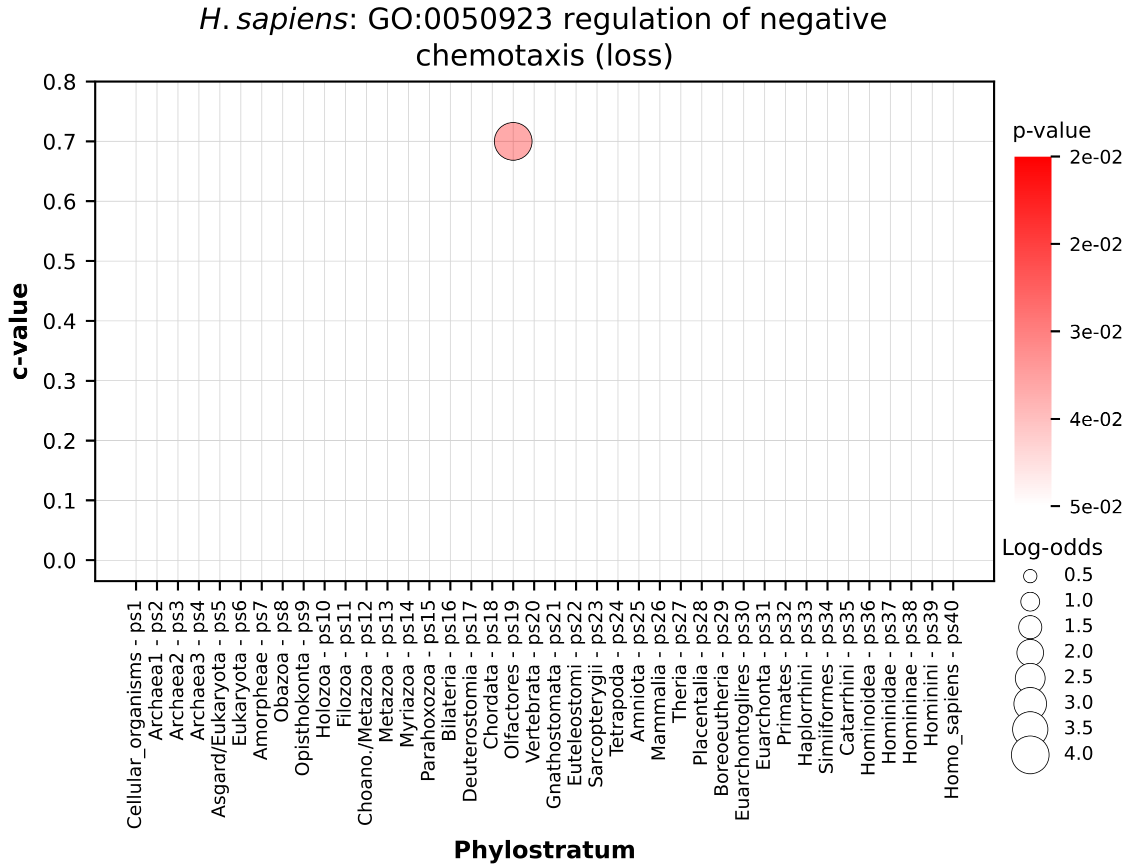

H. sapiens: GO:0050923 regulation of negative chemotaxis (loss)
c-value
0.8
0.7
0.6
0.5
0.4
0.3
0.2
0.1
0.0
p-value
2e-02
2e-02
3e-02
4e-02
5e-02
Log-odds
0.5
1.0
1.5
2.0
2.5
3.0
3.5
4.0
Cellular_organisms - ps1
Archaea1 - ps2
Archaea2 - ps3
Archaea3 - ps4
Asgard/Eukaryota - ps5
Eukaryota - ps6
Amorpheae - ps7
Obazoa - ps8
Opisthokonta - ps9
Holozoa - ps10
Filozoa - ps11
Choano./Metazoa - ps12
Metazoa - ps13
Myriazoa - ps14
Parahoxozoa - ps15
Bilateria - ps16
Deuterostomia - ps17
Chordata - ps18
Olfactores - ps19
Vertebrata - ps20
Gnathostomata - ps21
Euteleostomi - ps22
Sarcopterygii - ps23
Tetrapoda - ps24
Amniota - ps25
Mammalia - ps26
Theria - ps27
Placentalia - ps28
Boreoeutheria - ps29
Euarchontoglires - ps30
Euarchonta - ps31
Primates - ps32
Haplorrhini - ps33
Simiiformes - ps34
Catarrhini - ps35
Hominoidea - ps36
Hominidae - ps37
Homininae - ps38
Hominini - ps39
Homo_sapiens - ps40
Phylostratum

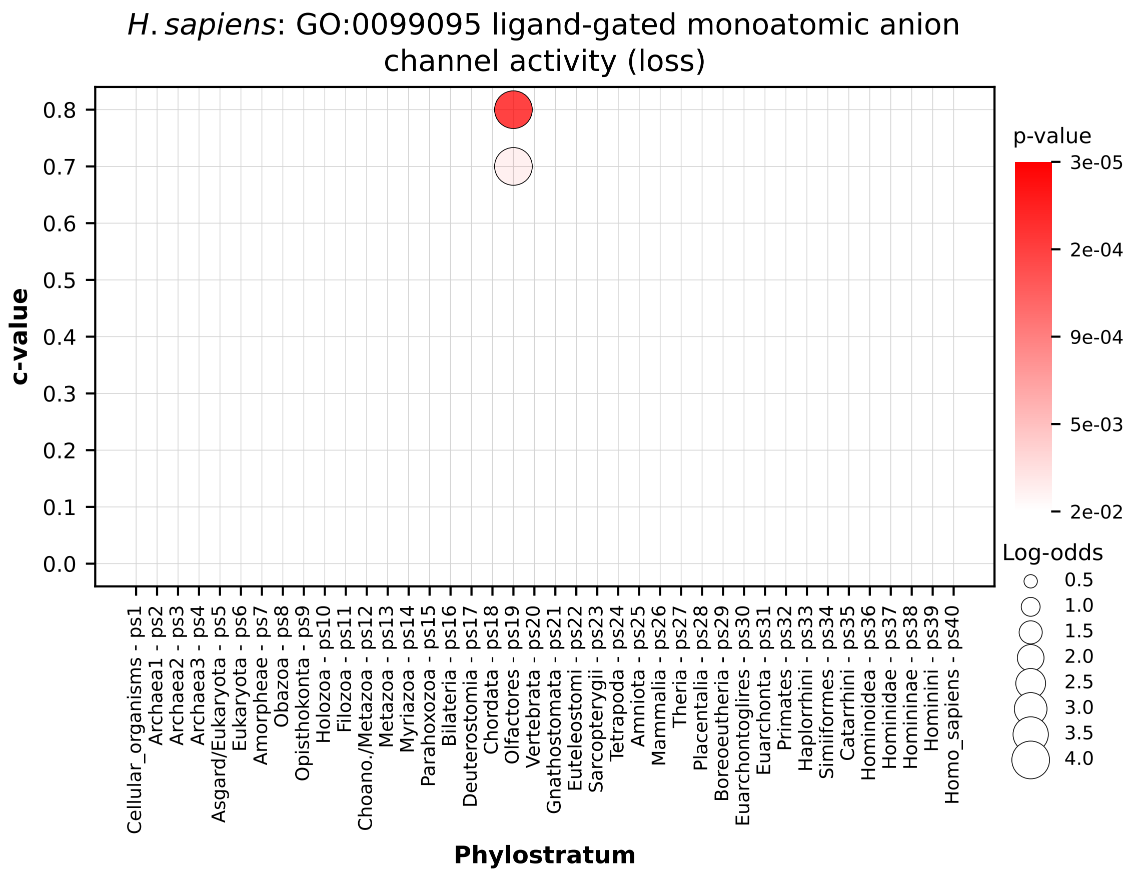

H. sapiens: GO:0099095 ligand-gated monoatomic anion channel activity (loss)
c-value
0.0
0.1
0.2
0.3
0.4
0.5
0.6
0.7
0.8
p-value
3e-05
2e-04
9e-04
5e-03
2e-02
Log-odds
0.5
1.0
1.5
2.0
2.5
3.0
3.5
4.0
Cellular_organisms - ps1
Archaea1 - ps2
Archaea2 - ps3
Archaea3 - ps4
Asgard/Eukaryota - ps5
Eukaryota - ps6
Amorpheae - ps7
Obazoa - ps8
Opisthokonta - ps9
Holozoa - ps10
Filozoa - ps11
Choano./Metazoa - ps12
Metazoa - ps13
Myriazoa - ps14
Parahoxozoa - ps15
Bilateria - ps16
Deuterostomia - ps17
Chordata - ps18
Olfactores - ps19
Vertebrata - ps20
Gnathostomata - ps21
Euteleostomi - ps22
Sarcopterygii - ps23
Tetrapoda - ps24
Amniota - ps25
Mammalia - ps26
Theria - ps27
Placentalia - ps28
Boreoeutheria - ps29
Euarchontoglires - ps30
Euarchonta - ps31
Primates - ps32
Haplorrhini - ps33
Simiiformes - ps34
Catarrhini - ps35
Hominoidea - ps36
Hominidae - ps37
Homininae - ps38
Hominini - ps39
Homo_sapiens - ps40
Phylostratum

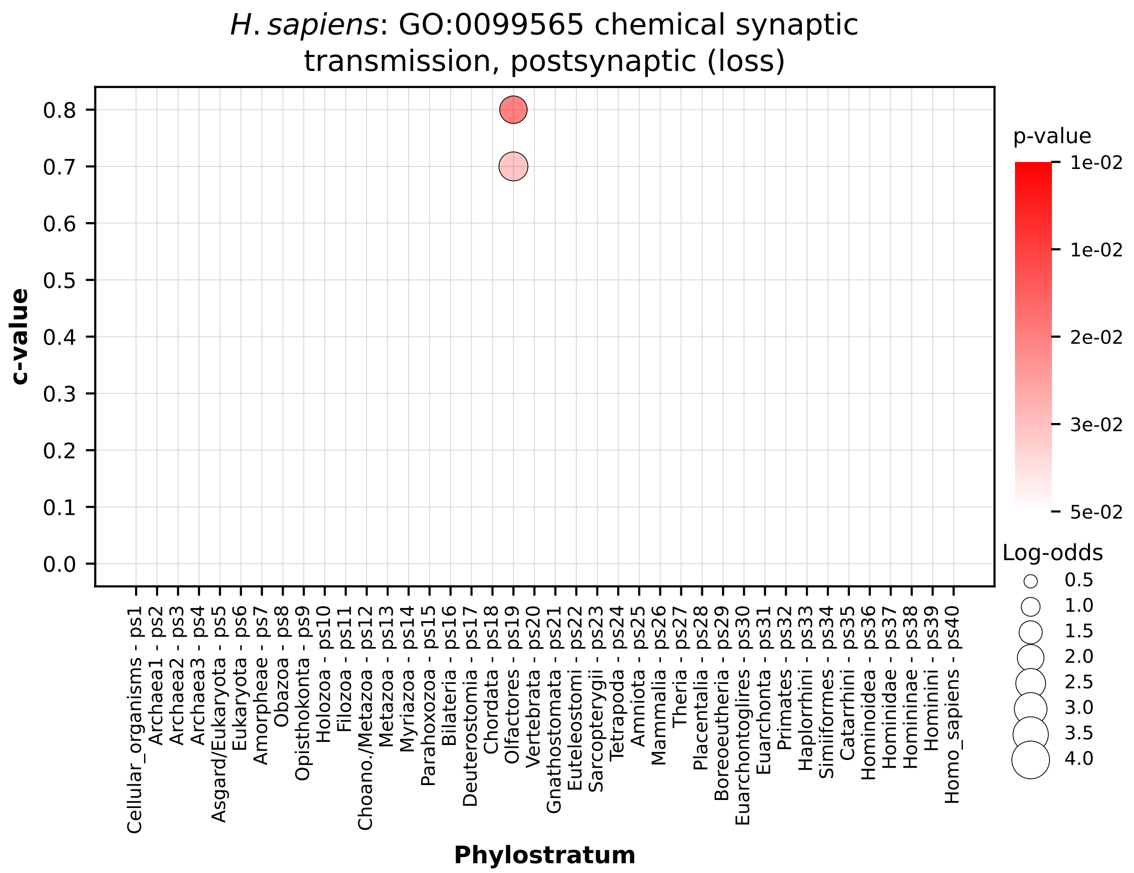

H. sapiens: GO:0099565 chemical synaptic transmission, postsynaptic (loss)
c-value
0.0
0.1
0.2
0.3
0.4
0.5
0.6
0.7
0.8
Phylostratum
Cellular_organisms - ps1
Archaea1 - ps2
Archaea2 - ps3
Archaea3 - ps4
Asgard/Eukaryota - ps5
Eukaryota - ps6
Amorpheae - ps7
Obazoa - ps8
Opisthokonta - ps9
Holozoa - ps10
Filozoa - ps11
Choano./Metazoa - ps12
Metazoa - ps13
Myriazoa - ps14
Parahoxozoa - ps15
Bilateria - ps16
Deuterostomia - ps17
Chordata - ps18
Olfactores - ps19
Vertebrata - ps20
Gnathostomata - ps21
Euteleostomi - ps22
Sarcopterygii - ps23
Tetrapoda - ps24
Amniota - ps25
Mammalia - ps26
Theria - ps27
Placentalia - ps28
Boreoeutheria - ps29
Euarchontoglires - ps30
Euarchonta - ps31
Primates - ps32
Haplorrhini - ps33
Simiiformes - ps34
Catarrhini - ps35
Hominoidea - ps36
Hominidae - ps37
Homininae - ps38
Hominini - ps39
Homo_sapiens - ps40
p-value
1e-02
1e-02
2e-02
3e-02
5e-02
Log-odds
0.5
1.0
1.5
2.0
2.5
3.0
3.5
4.0

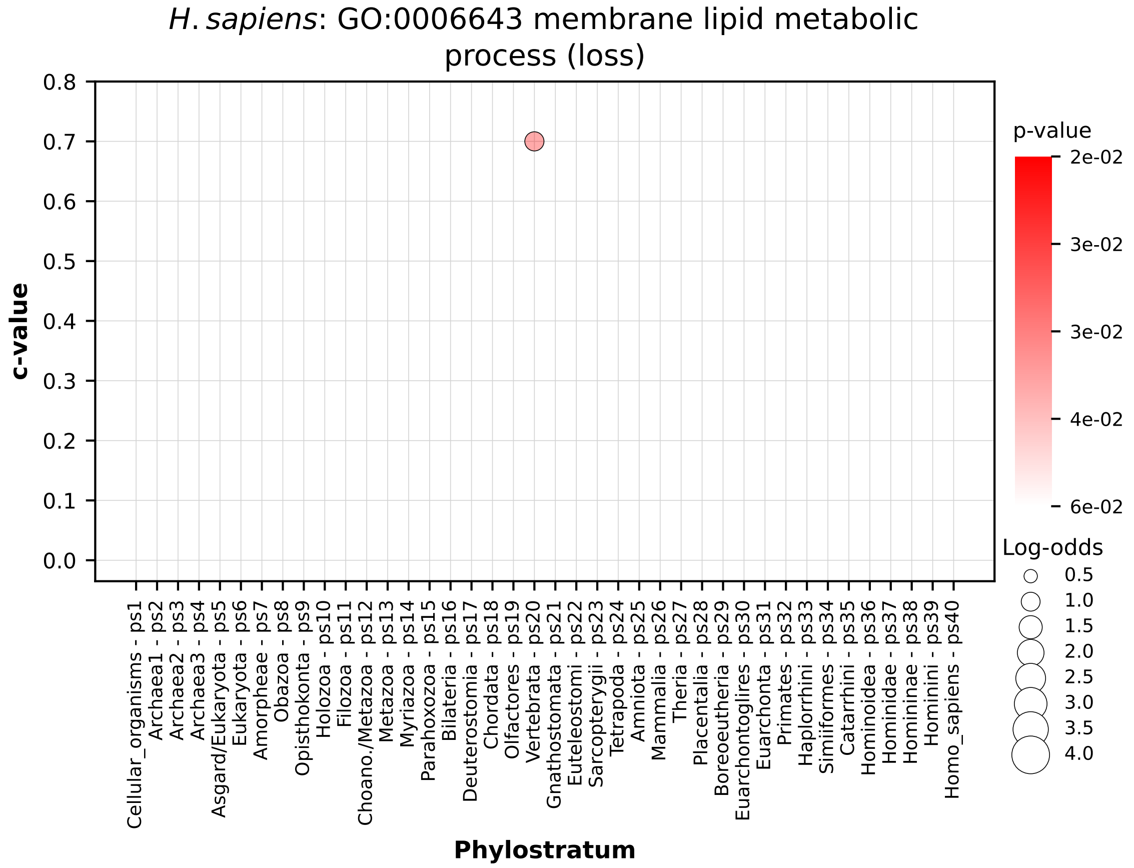

H. sapiens: GO:0006643 membrane lipid metabolic process (loss)
c-value
0.8
0.7
0.6
0.5
0.4
0.3
0.2
0.1
0.0
p-value
2e-02
3e-02
3e-02
4e-02
6e-02
Log-odds
0.5
1.0
1.5
2.0
2.5
3.0
3.5
4.0
Cellular_organisms - ps1
Archaea1 - ps2
Archaea2 - ps3
Archaea3 - ps4
Asgard/Eukaryota - ps5
Eukaryota - ps6
Amorpheae - ps7
Obazoa - ps8
Opisthokonta - ps9
Holozoa - ps10
Filozoa - ps11
Choano./Metazoa - ps12
Metazoa - ps13
Myriazoa - ps14
Parahoxozoa - ps15
Bilateria - ps16
Deuterostomia - ps17
Chordata - ps18
Olfactores - ps19
Vertebrata - ps20
Gnathostomata - ps21
Euteleostomi - ps22
Sarcopterygii - ps23
Tetrapoda - ps24
Amniota - ps25
Mammalia - ps26
Theria - ps27
Placentalia - ps28
Boreoeutheria - ps29
Euarchontoglires - ps30
Euarchonta - ps31
Primates - ps32
Haplorrhini - ps33
Simiiformes - ps34
Catarrhini - ps35
Hominoidea - ps36
Hominidae - ps37
Homininae - ps38
Hominini - ps39
Homo_sapiens - ps40
Phylostratum

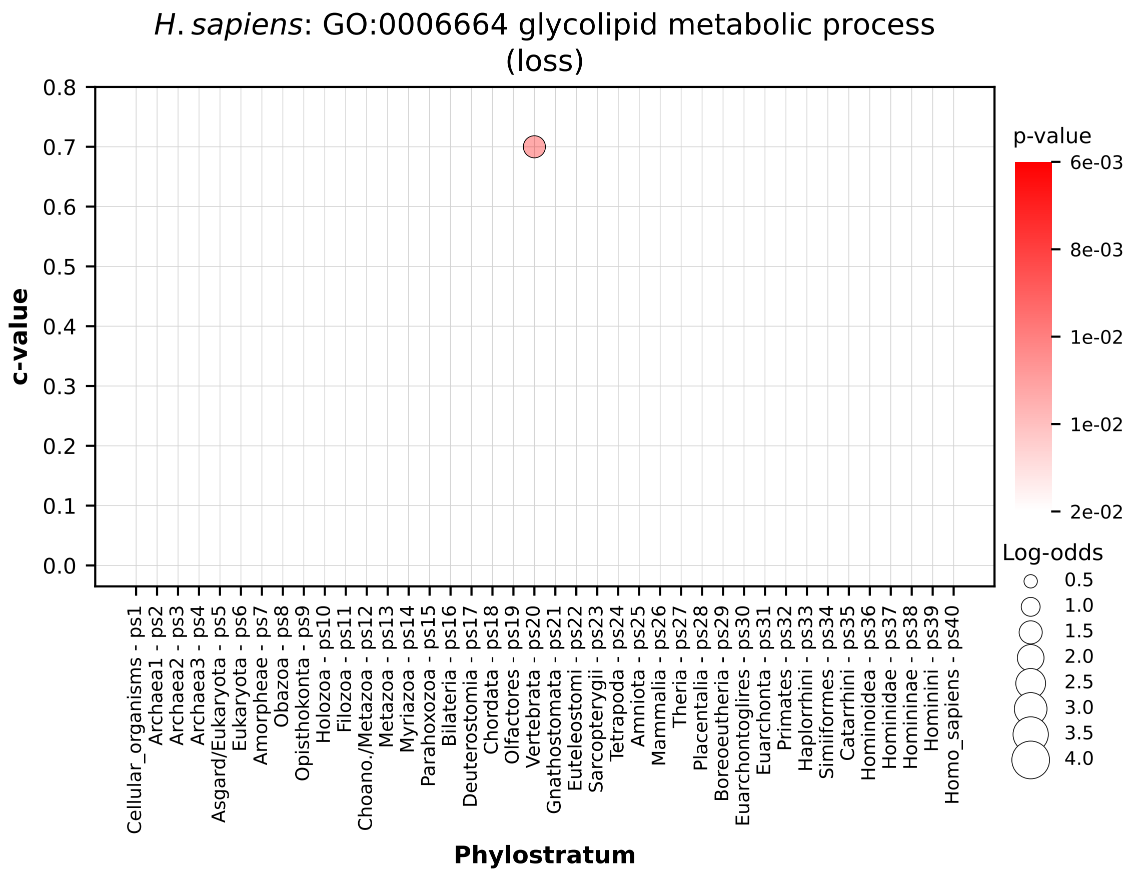

H. sapiens: GO:0006664 glycolipid metabolic process
(loss)
c-value
0.8
0.7
0.6
0.5
0.4
0.3
0.2
0.1
0.0
p-value
6e-03
8e-03
1e-02
1e-02
2e-02
Log-odds
0.5
1.0
1.5
2.0
2.5
3.0
3.5
4.0
Cellular_organisms - ps1
Archaea1 - ps2
Archaea2 - ps3
Archaea3 - ps4
Asgard/Eukaryota - ps5
Eukaryota - ps6
Amorpheae - ps7
Obazoa - ps8
Opisthokonta - ps9
Holozoa - ps10
Filozoa - ps11
Choano./Metazoa - ps12
Metazoa - ps13
Myriazoa - ps14
Parahoxozoa - ps15
Bilateria - ps16
Deuterostomia - ps17
Chordata - ps18
Olfactores - ps19
Vertebrata - ps20
Gnathostomata - ps21
Euteleostomi - ps22
Sarcopterygii - ps23
Tetrapoda - ps24
Amniota - ps25
Mammalia - ps26
Theria - ps27
Placentalia - ps28
Boreoeutheria - ps29
Euarchontoglires - ps30
Euarchonta - ps31
Primates - ps32
Haplorrhini - ps33
Simiiformes - ps34
Catarrhini - ps35
Hominoidea - ps36
Hominidae - ps37
Homininae - ps38
Hominini - ps39
Homo_sapiens - ps40
Phylostratum

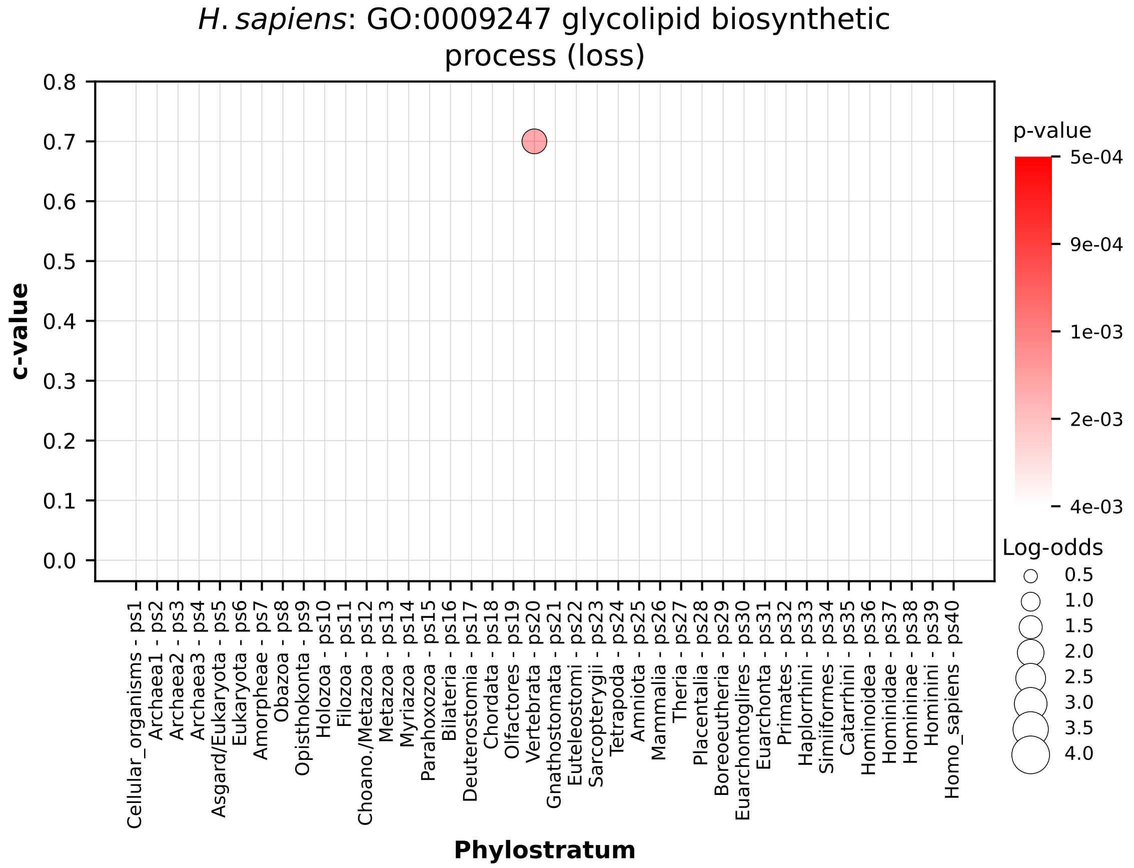

H. sapiens: GO:0009247 glycolipid biosynthetic process (loss)
c-value
0.0
0.1
0.2
0.3
0.4
0.5
0.6
0.7
0.8
Phylostratum
Cellular_organisms - ps1
Archaea1 - ps2
Archaea2 - ps3
Archaea3 - ps4
Asgard/Eukaryota - ps5
Eukaryota - ps6
Amorpheae - ps7
Obazoa - ps8
Opisthokonta - ps9
Holozoa - ps10
Filozoa - ps11
Choano./Metazoa - ps12
Metazoa - ps13
Myriazoa - ps14
Parahoxozoa - ps15
Bilateria - ps16
Deuterostomia - ps17
Chordata - ps18
Olfactores - ps19
Vertebrata - ps20
Gnathostomata - ps21
Euteleostomi - ps22
Sarcopterygii - ps23
Tetrapoda - ps24
Amniota - ps25
Mammalia - ps26
Theria - ps27
Placentalia - ps28
Boreoeutheria - ps29
Euarchontoglires - ps30
Euarchonta - ps31
Primates - ps32
Haplorrhini - ps33
Simiiformes - ps34
Catarrhini - ps35
Hominoidea - ps36
Hominidae - ps37
Homininae - ps38
Hominini - ps39
Homo_sapiens - ps40
p-value
5e-04
9e-04
1e-03
2e-03
4e-03
Log-odds
0.5
1.0
1.5
2.0
2.5
3.0
3.5
4.0

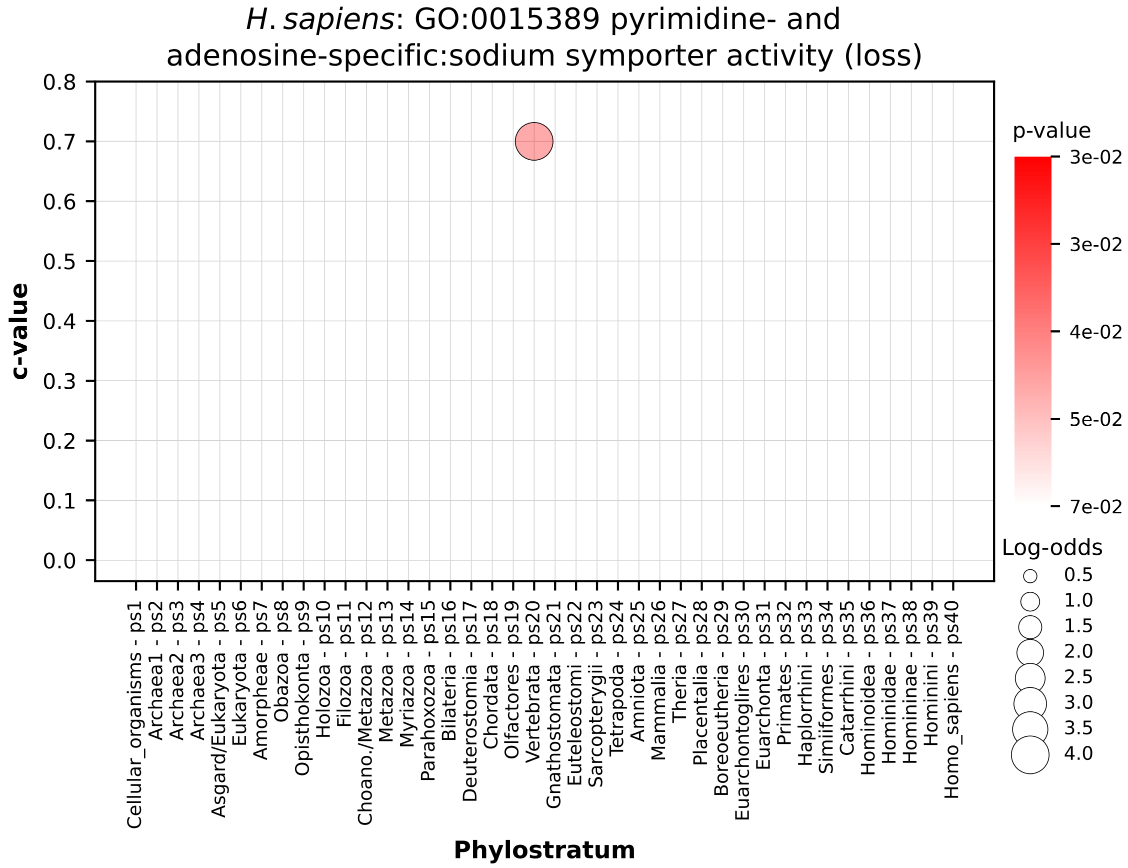

H. sapiens: GO:0015389 pyrimidine- and adenosine-specific:sodium symporter activity (loss)
c-value
0.8
0.7
0.6
0.5
0.4
0.3
0.2
0.1
0.0
Phylostratum
Cellular_organisms - ps1
Archaea1 - ps2
Archaea2 - ps3
Archaea3 - ps4
Asgard/Eukaryota - ps5
Eukaryota - ps6
Amorpheae - ps7
Obazoa - ps8
Opisthokonta - ps9
Holozoa - ps10
Filozoa - ps11
Choano./Metazoa - ps12
Metazoa - ps13
Myriazoa - ps14
Parahoxozoa - ps15
Bilateria - ps16
Deuterostomia - ps17
Chordata - ps18
Olfactores - ps19
Vertebrata - ps20
Gnathostomata - ps21
Euteleostomi - ps22
Sarcopterygii - ps23
Tetrapoda - ps24
Amniota - ps25
Mammalia - ps26
Theria - ps27
Placentalia - ps28
Boreoeutheria - ps29
Euarchontoglires - ps30
Euarchonta - ps31
Primates - ps32
Haplorrhini - ps33
Simiiformes - ps34
Catarrhini - ps35
Hominoidea - ps36
Hominidae - ps37
Homininae - ps38
Hominini - ps39
Homo_sapiens - ps40
p-value
3e-02
3e-02
4e-02
5e-02
7e-02
Log-odds
0.5
1.0
1.5
2.0
2.5
3.0
3.5
4.0

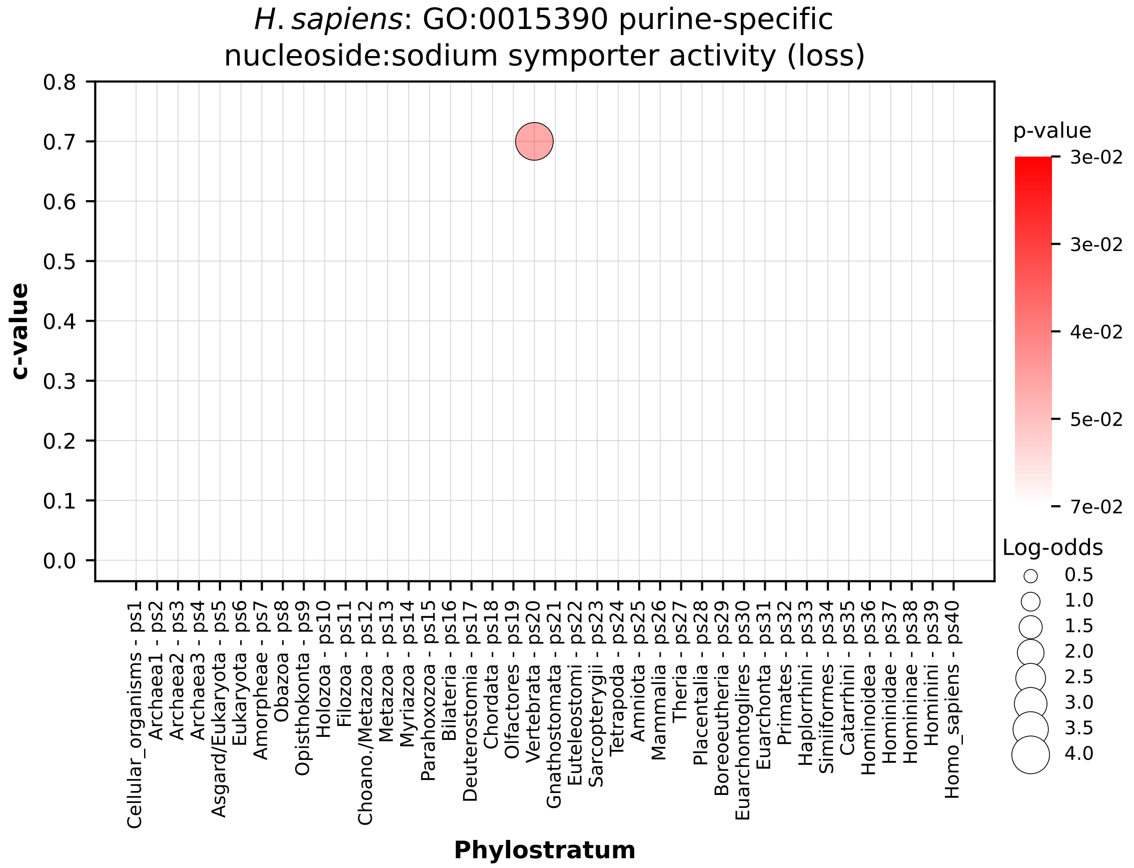
H. sapiens: GO:0015390 purine-specific nucleoside:sodium symporter activity (loss)
c-value
0.8
0.7
0.6
0.5
0.4
0.3
0.2
0.1
0.0
p-value
3e-02
3e-02
4e-02
5e-02
7e-02
Log-odds
0.5
1.0
1.5
2.0
2.5
3.0
3.5
4.0
Cellular_organisms - ps1
Archaea1 - ps2
Archaea2 - ps3
Archaea3 - ps4
Asgard/Eukaryota - ps5
Eukaryota - ps6
Amorpheae - ps7
Obazoa - ps8
Opisthokonta - ps9
Holozoa - ps10
Filozoa - ps11
Choano./Metazoa - ps12
Metazoa - ps13
Myriazoa - ps14
Parahoxozoa - ps15
Bilateria - ps16
Deuterostomia - ps17
Chordata - ps18
Olfactores - ps19
Vertebrata - ps20
Gnathostomata - ps21
Euteleostomi - ps22
Sarcopterygii - ps23
Tetrapoda - ps24
Amniota - ps25
Mammalia - ps26
Theria - ps27
Placentalia - ps28
Boreoeutheria - ps29
Euarchontoglires - ps30
Euarchonta - ps31
Primates - ps32
Haplorrhini - ps33
Simiiformes - ps34
Catarrhini - ps35
Hominoidea - ps36
Hominidae - ps37
Homininae - ps38
Hominini - ps39
Homo_sapiens - ps40
Phylostratum

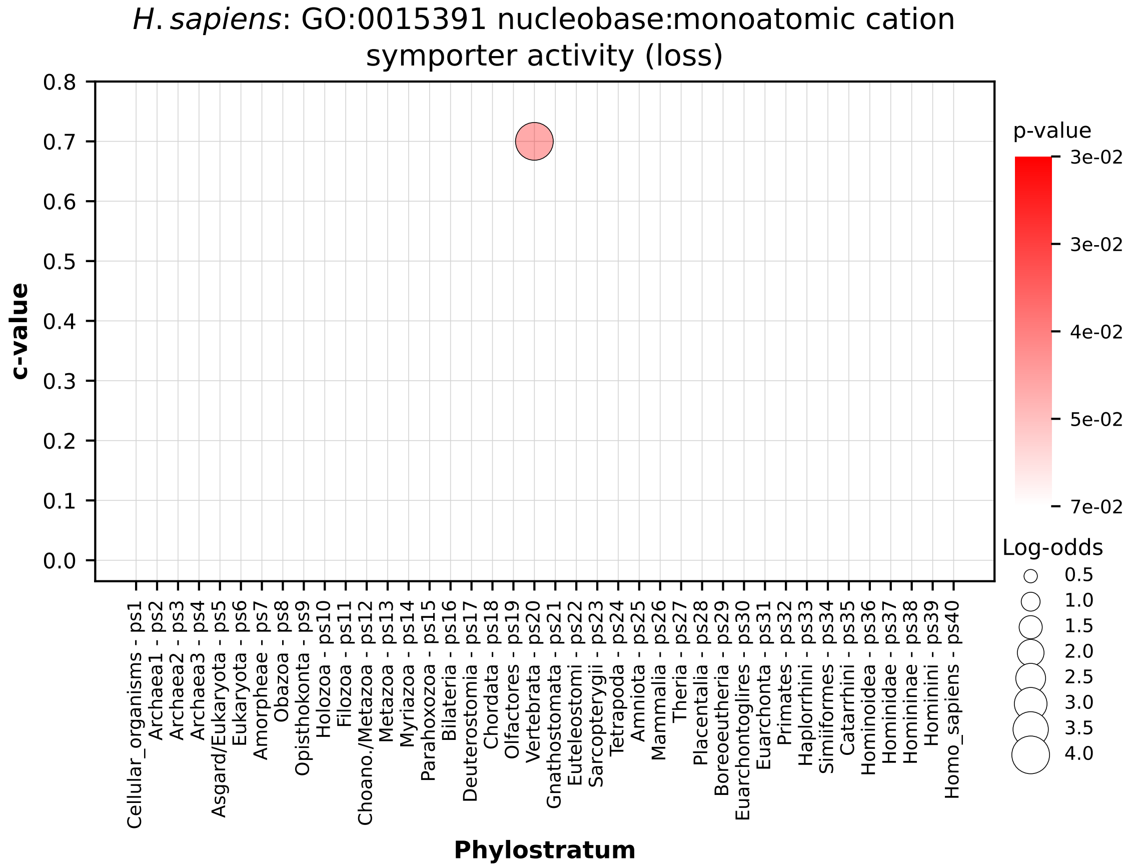

H. sapiens: GO:0015391 nucleobase:monoatomic cation symporter activity (loss)
c-value
0.8
0.7
0.6
0.5
0.4
0.3
0.2
0.1
0.0
p-value
3e-02
3e-02
4e-02
5e-02
7e-02
Log-odds
0.5
1.0
1.5
2.0
2.5
3.0
3.5
4.0
Cellular_organisms - ps1
Archaea1 - ps2
Archaea2 - ps3
Archaea3 - ps4
Asgard/Eukaryota - ps5
Eukaryota - ps6
Amorpheae - ps7
Obazoa - ps8
Opisthokonta - ps9
Holozoa - ps10
Filozoa - ps11
Choano./Metazoa - ps12
Metazoa - ps13
Myriazoa - ps14
Parahoxozoa - ps15
Bilateria - ps16
Deuterostomia - ps17
Chordata - ps18
Olfactores - ps19
Vertebrata - ps20
Gnathostomata - ps21
Euteleostomi - ps22
Sarcopterygii - ps23
Tetrapoda - ps24
Amniota - ps25
Mammalia - ps26
Theria - ps27
Placentalia - ps28
Boreoeutheria - ps29
Euarchontoglires - ps30
Euarchonta - ps31
Primates - ps32
Haplorrhini - ps33
Simiiformes - ps34
Catarrhini - ps35
Hominoidea - ps36
Hominidae - ps37
Homininae - ps38
Hominini - ps39
Homo_sapiens - ps40
Phylostratum

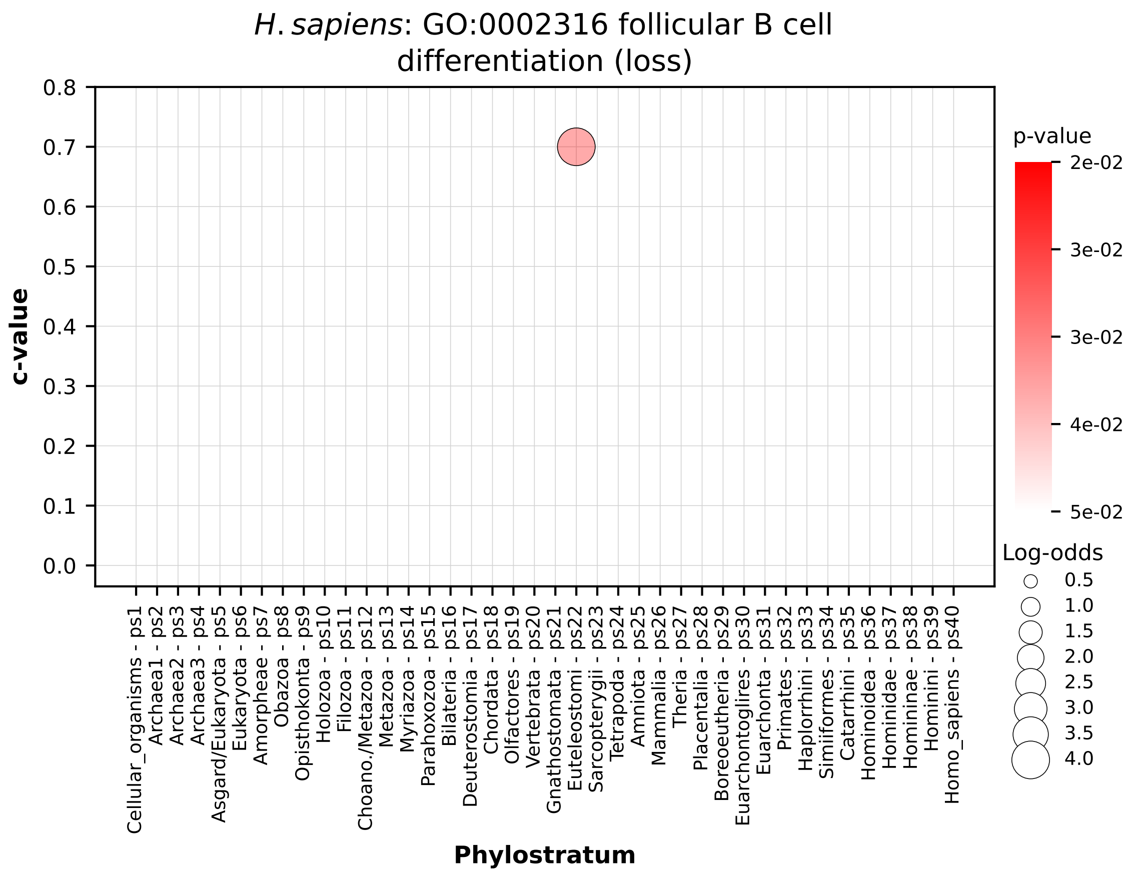

H. sapiens: GO:0002316 follicular B cell differentiation (loss)
c-value
0.8
0.7
0.6
0.5
0.4
0.3
0.2
0.1
0.0
p-value
2e-02
3e-02
3e-02
4e-02
5e-02
Log-odds
0.5
1.0
1.5
2.0
2.5
3.0
3.5
4.0
Cellular_organisms - ps1
Archaea1 - ps2
Archaea2 - ps3
Archaea3 - ps4
Asgard/Eukaryota - ps5
Eukaryota - ps6
Amorpheae - ps7
Obazoa - ps8
Opisthokonta - ps9
Holozoa - ps10
Filozoa - ps11
Choano./Metazoa - ps12
Metazoa - ps13
Myriazoa - ps14
Parahoxozoa - ps15
Bilateria - ps16
Deuterostomia - ps17
Chordata - ps18
Olfactores - ps19
Vertebrata - ps20
Gnathostomata - ps21
Euteleostomi - ps22
Sarcopterygii - ps23
Tetrapoda - ps24
Amniota - ps25
Mammalia - ps26
Theria - ps27
Placentalia - ps28
Boreoeutheria - ps29
Euarchontoglires - ps30
Euarchonta - ps31
Primates - ps32
Haplorrhini - ps33
Simiiformes - ps34
Catarrhini - ps35
Hominoidea - ps36
Hominidae - ps37
Homininae - ps38
Hominini - ps39
Homo_sapiens - ps40
Phylostratum

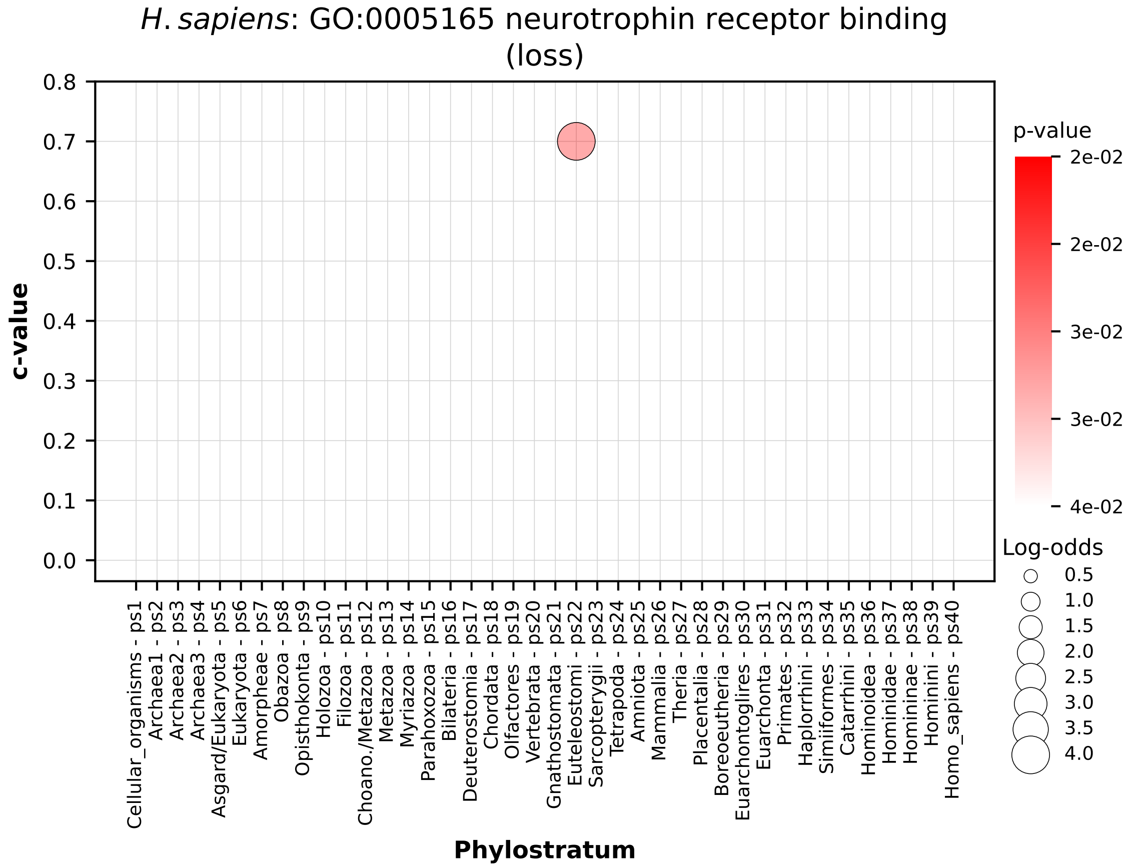
H. sapiens: GO:0005165 neurotrophin receptor binding (loss)
c-value
0.8
0.7
0.6
0.5
0.4
0.3
0.2
0.1
0.0
p-value
2e-02
2e-02
3e-02
3e-02
4e-02
Log-odds
0.5
1.0
1.5
2.0
2.5
3.0
3.5
4.0
Cellular_organisms - ps1
Archaea1 - ps2
Archaea2 - ps3
Archaea3 - ps4
Asgard/Eukaryota - ps5
Eukaryota - ps6
Amorpheae - ps7
Obazoa - ps8
Opisthokonta - ps9
Holozoa - ps10
Filozoa - ps11
Choano./Metazoa - ps12
Metazoa - ps13
Myriazoa - ps14
Parahoxozoa - ps15
Bilateria - ps16
Deuterostomia - ps17
Chordata - ps18
Olfactores - ps19
Vertebrata - ps20
Gnathostomata - ps21
Euteleostomi - ps22
Sarcopterygii - ps23
Tetrapoda - ps24
Amniota - ps25
Mammalia - ps26
Theria - ps27
Placentalia - ps28
Boreoeutheria - ps29
Euarchontoglires - ps30
Euarchonta - ps31
Primates - ps32
Haplorrhini - ps33
Simiiformes - ps34
Catarrhini - ps35
Hominoidea - ps36
Hominidae - ps37
Homininae - ps38
Hominini - ps39
Homo_sapiens - ps40
Phylostratum

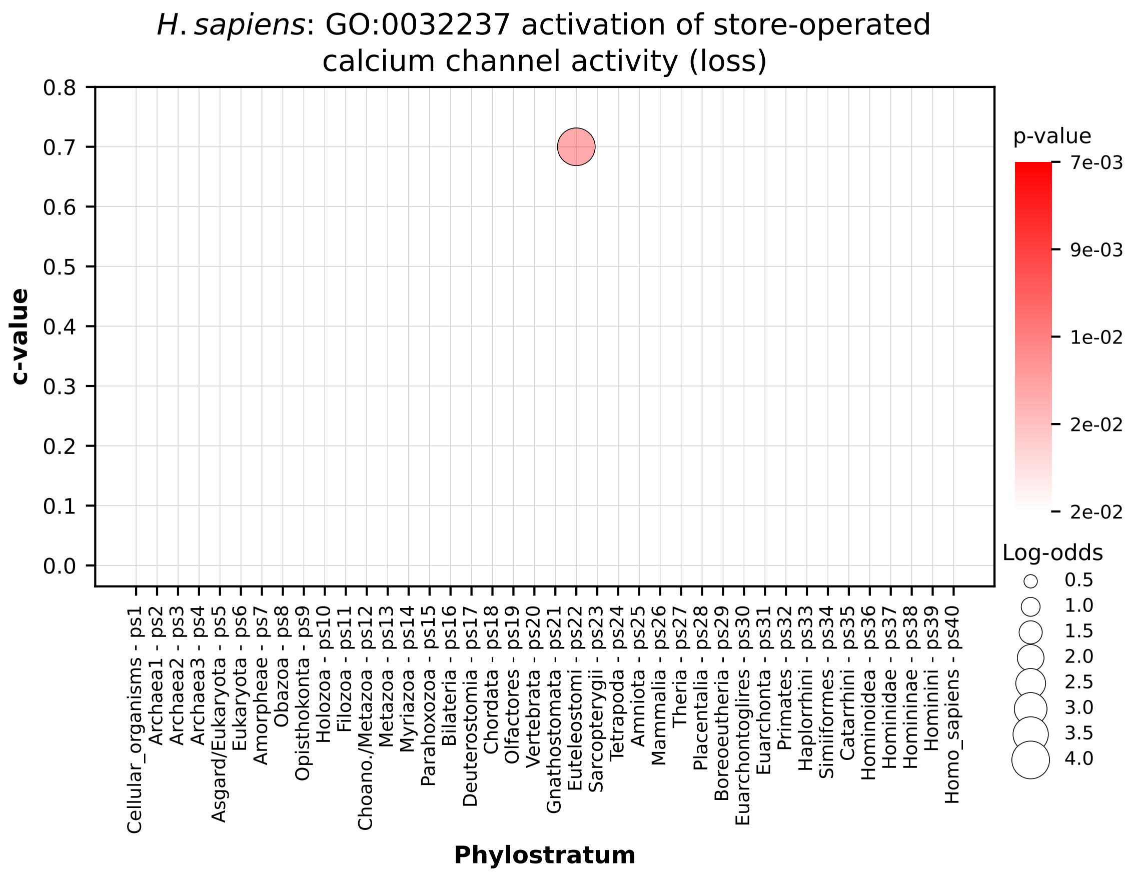
H. sapiens: GO:0032237 activation of store-operated calcium channel activity (loss)
c-value
Phylostratum
p-value
7e-03
9e-03
1e-02
2e-02
2e-02
Log-odds
0.5
1.0
1.5
2.0
2.5
3.0
3.5
4.0
Cellular_organisms - ps1
Archaea1 - ps2
Archaea2 - ps3
Archaea3 - ps4
Asgard/Eukaryota - ps5
Eukaryota - ps6
Amorpheae - ps7
Obazoa - ps8
Opisthokonta - ps9
Holozoa - ps10
Filozoa - ps11
Choano./Metazoa - ps12
Metazoa - ps13
Myriazoa - ps14
Parahoxozoa - ps15
Bilateria - ps16
Deuterostomia - ps17
Chordata - ps18
Olfactores - ps19
Vertebrata - ps20
Gnathostomata - ps21
Euteleostomi - ps22
Sarcopterygii - ps23
Tetrapoda - ps24
Amniota - ps25
Mammalia - ps26
Theria - ps27
Placentalia - ps28
Boreoeutheria - ps29
Euarchontoglires - ps30
Euarchonta - ps31
Primates - ps32
Haplorrhini - ps33
Simiiformes - ps34
Catarrhini - ps35
Hominoidea - ps36
Hominidae - ps37
Homininae - ps38
Hominini - ps39
Homo_sapiens - ps40

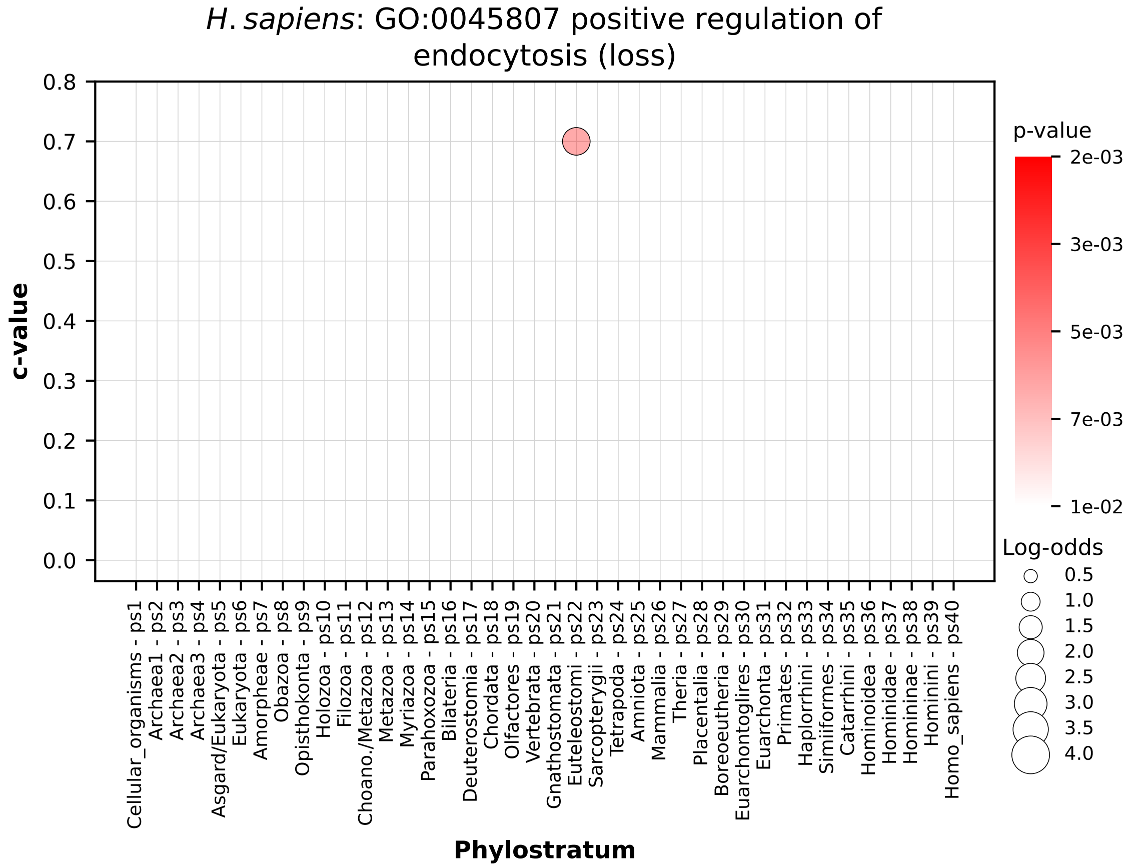

H. sapiens: GO:0045807 positive regulation of endocytosis (loss)
c-value
0.8
0.7
0.6
0.5
0.4
0.3
0.2
0.1
0.0
p-value
2e-03
3e-03
5e-03
7e-03
1e-02
Log-odds
0.5
1.0
1.5
2.0
2.5
3.0
3.5
4.0
Cellular_organisms - ps1
Archaea1 - ps2
Archaea2 - ps3
Archaea3 - ps4
Asgard/Eukaryota - ps5
Eukaryota - ps6
Amorpheae - ps7
Obazoa - ps8
Opisthokonta - ps9
Holozoa - ps10
Filozoa - ps11
Choano./Metazoa - ps12
Metazoa - ps13
Myriazoa - ps14
Parahoxozoa - ps15
Bilateria - ps16
Deuterostomia - ps17
Chordata - ps18
Olfactores - ps19
Vertebrata - ps20
Gnathostomata - ps21
Euteleostomi - ps22
Sarcopterygii - ps23
Tetrapoda - ps24
Amniota - ps25
Mammalia - ps26
Theria - ps27
Placentalia - ps28
Boreoeutheria - ps29
Euarchontoglires - ps30
Euarchonta - ps31
Primates - ps32
Haplorrhini - ps33
Simiiformes - ps34
Catarrhini - ps35
Hominoidea - ps36
Hominidae - ps37
Homininae - ps38
Hominini - ps39
Homo_sapiens - ps40
Phylostratum

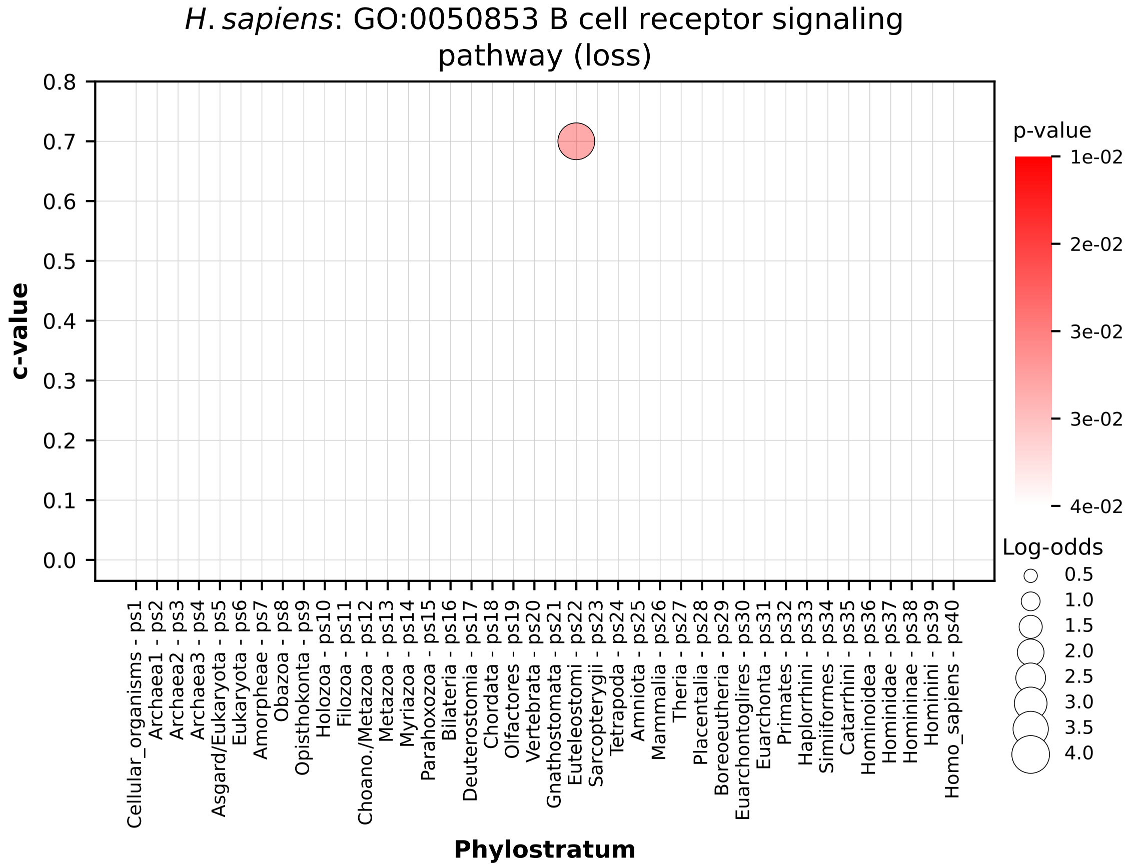

H. sapiens: GO:0050853 B cell receptor signaling pathway (loss)
c-value
0.0
0.1
0.2
0.3
0.4
0.5
0.6
0.7
0.8
Phylostratum
Cellular_organisms - ps1
Archaea1 - ps2
Archaea2 - ps3
Archaea3 - ps4
Asgard/Eukaryota - ps5
Eukaryota - ps6
Amorpheae - ps7
Obazoa - ps8
Opisthokonta - ps9
Holozoa - ps10
Filozoa - ps11
Choano./Metazoa - ps12
Metazoa - ps13
Myriazoa - ps14
Parahoxozoa - ps15
Bilateria - ps16
Deuterostomia - ps17
Chordata - ps18
Olfactores - ps19
Vertebrata - ps20
Gnathostomata - ps21
Euteleostomi - ps22
Sarcopterygii - ps23
Tetrapoda - ps24
Amniota - ps25
Mammalia - ps26
Theria - ps27
Placentalia - ps28
Boreoeutheria - ps29
Euarchontoglires - ps30
Euarchonta - ps31
Primates - ps32
Haplorrhini - ps33
Simiiformes - ps34
Catarrhini - ps35
Hominoidea - ps36
Hominidae - ps37
Homininae - ps38
Hominini - ps39
Homo_sapiens - ps40
p-value
1e-02
2e-02
3e-02
3e-02
4e-02
Log-odds
0.5
1.0
1.5
2.0
2.5
3.0
3.5
4.0

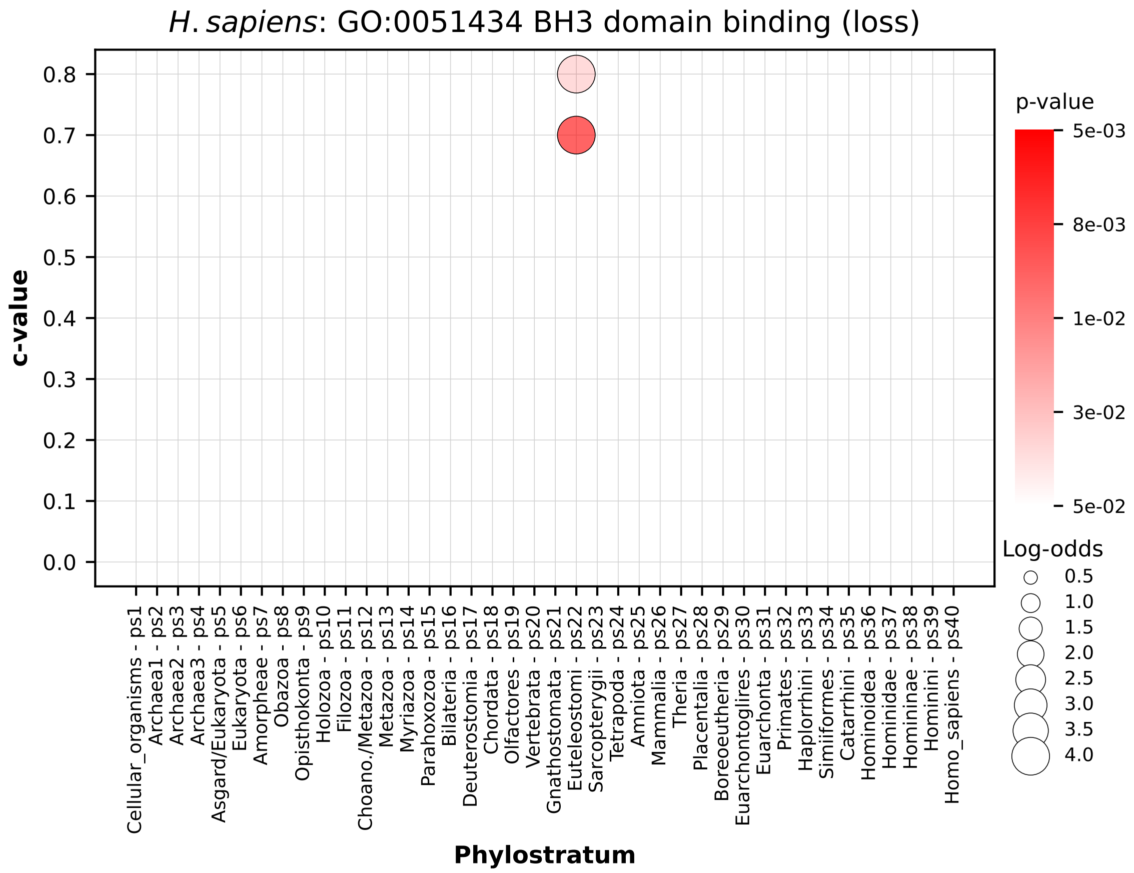

H. sapiens: GO:0051434 BH3 domain binding (loss)
c-value
Phylostratum
p-value
5e-03
8e-03
1e-02
3e-02
5e-02
Log-odds
0.5
1.0
1.5
2.0
2.5
3.0
3.5
4.0
0.0
0.1
0.2
0.3
0.4
0.5
0.6
0.7
0.8
Cellular_organisms - ps1
Archaea1 - ps2
Archaea2 - ps3
Archaea3 - ps4
Asgard/Eukaryota - ps5
Eukaryota - ps6
Amorpheae - ps7
Obazoa - ps8
Opisthokonta - ps9
Holozoa - ps10
Filozoa - ps11
Choano./Metazoa - ps12
Metazoa - ps13
Myriazoa - ps14
Parahoxozoa - ps15
Bilateria - ps16
Deuterostomia - ps17
Chordata - ps18
Olfactores - ps19
Vertebrata - ps20
Gnathostomata - ps21
Euteleostomi - ps22
Sarcopterygii - ps23
Tetrapoda - ps24
Amniota - ps25
Mammalia - ps26
Theria - ps27
Placentalia - ps28
Boreoeutheria - ps29
Euarchontoglires - ps30
Euarchonta - ps31
Primates - ps32
Haplorrhini - ps33
Simiiformes - ps34
Catarrhini - ps35
Hominoidea - ps36
Hominidae - ps37
Homininae - ps38
Hominini - ps39
Homo_sapiens - ps40

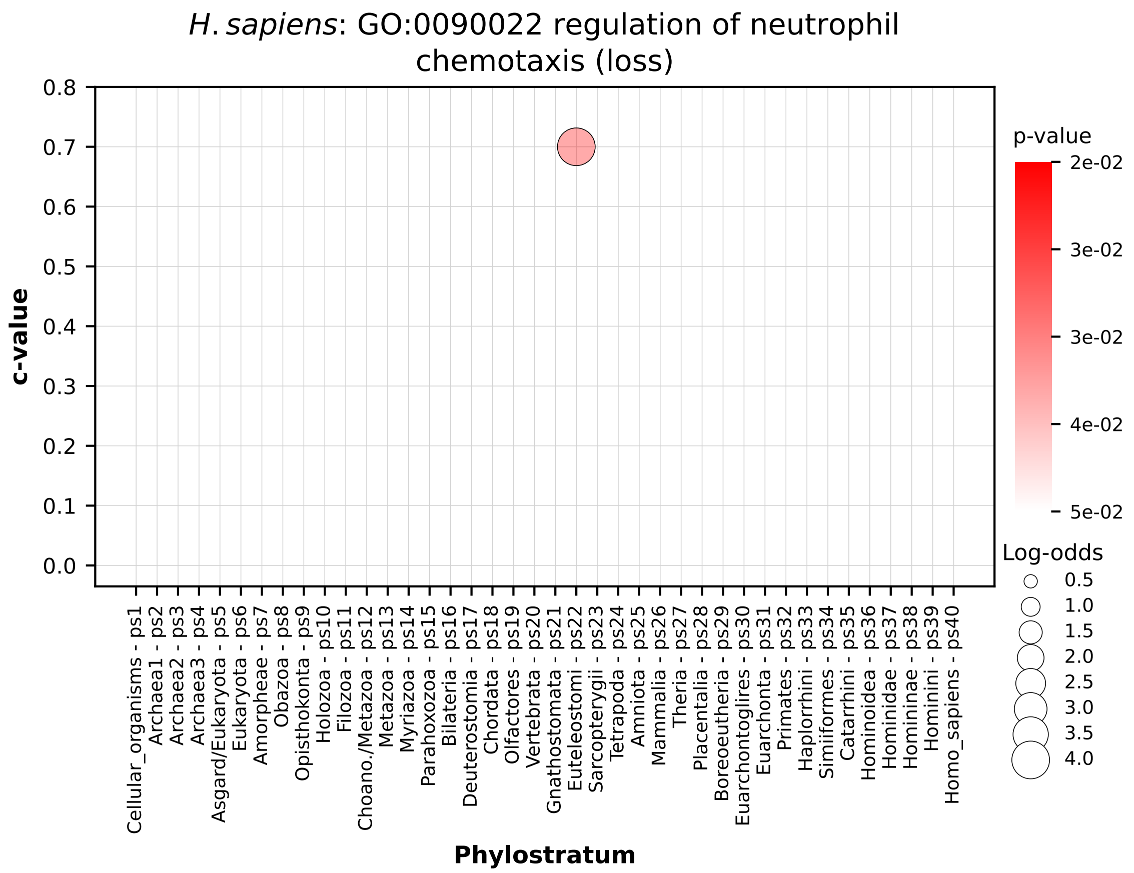
H. sapiens: GO:0090022 regulation of neutrophil chemotaxis (loss)
c-value
0.8
0.7
0.6
0.5
0.4
0.3
0.2
0.1
0.0
p-value
2e-02
3e-02
3e-02
4e-02
5e-02
Log-odds
0.5
1.0
1.5
2.0
2.5
3.0
3.5
4.0
Cellular_organisms - ps1
Archaea1 - ps2
Archaea2 - ps3
Archaea3 - ps4
Asgard/Eukaryota - ps5
Eukaryota - ps6
Amorpheae - ps7
Obazoa - ps8
Opisthokonta - ps9
Holozoa - ps10
Filozoa - ps11
Choano./Metazoa - ps12
Metazoa - ps13
Myriazoa - ps14
Parahoxozoa - ps15
Bilateria - ps16
Deuterostomia - ps17
Chordata - ps18
Olfactores - ps19
Vertebrata - ps20
Gnathostomata - ps21
Euteleostomi - ps22
Sarcopterygii - ps23
Tetrapoda - ps24
Amniota - ps25
Mammalia - ps26
Theria - ps27
Placentalia - ps28
Boreoeutheria - ps29
Euarchontoglires - ps30
Euarchonta - ps31
Primates - ps32
Haplorrhini - ps33
Simiiformes - ps34
Catarrhini - ps35
Hominoidea - ps36
Hominidae - ps37
Homininae - ps38
Hominini - ps39
Homo_sapiens - ps40
Phylostratum

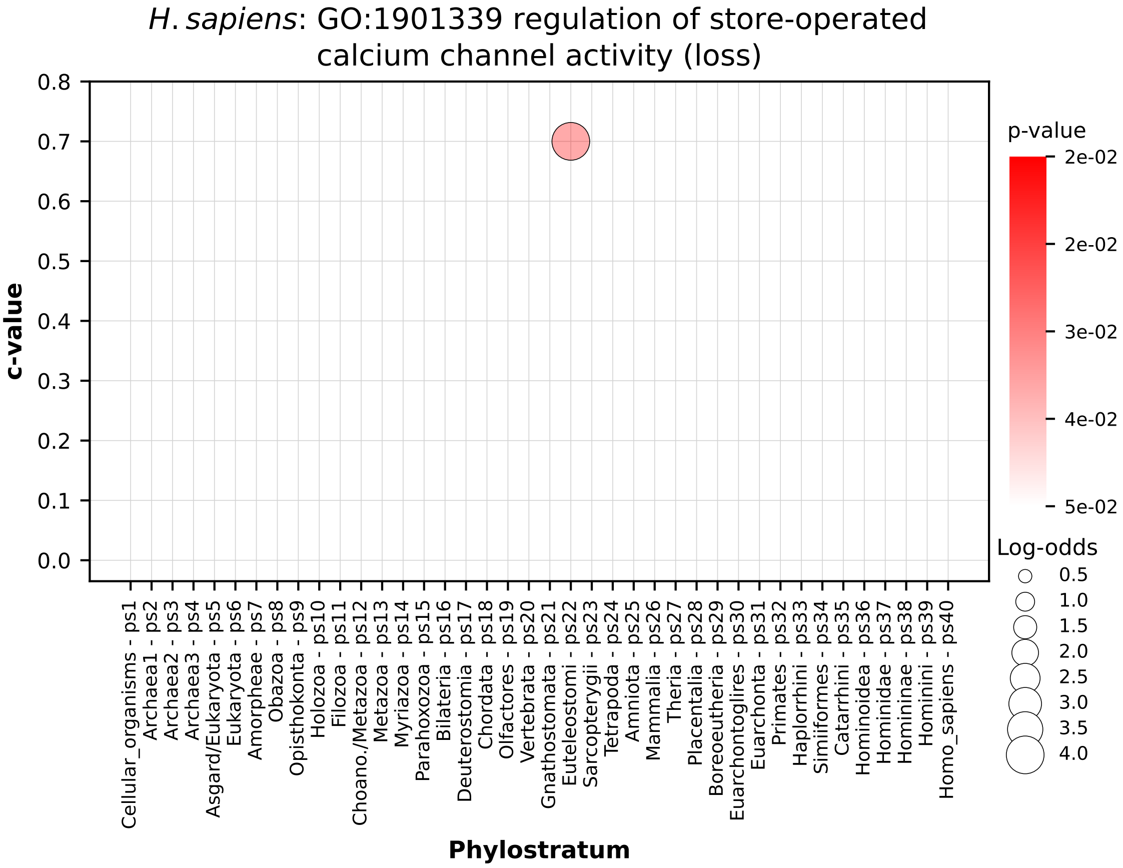

H. sapiens: GO:1901339 regulation of store-operated calcium channel activity (loss)
c-value
0.8
0.7
0.6
0.5
0.4
0.3
0.2
0.1
0.0
p-value
2e-02
2e-02
3e-02
4e-02
5e-02
Log-odds
0.5
1.0
1.5
2.0
2.5
3.0
3.5
4.0
Cellular_organisms - ps1
Archaea1 - ps2
Archaea2 - ps3
Archaea3 - ps4
Asgard/Eukaryota - ps5
Eukaryota - ps6
Amorpheae - ps7
Obazoa - ps8
Opisthokonta - ps9
Holozoa - ps10
Filozoa - ps11
Choano./Metazoa - ps12
Metazoa - ps13
Myriazoa - ps14
Parahoxozoa - ps15
Bilateria - ps16
Deuterostomia - ps17
Chordata - ps18
Olfactores - ps19
Vertebrata - ps20
Gnathostomata - ps21
Euteleostomi - ps22
Sarcopterygii - ps23
Tetrapoda - ps24
Amniota - ps25
Mammalia - ps26
Theria - ps27
Placentalia - ps28
Boreoeutheria - ps29
Euarchontoglires - ps30
Euarchonta - ps31
Primates - ps32
Haplorrhini - ps33
Simiiformes - ps34
Catarrhini - ps35
Hominoidea - ps36
Hominidae - ps37
Homininae - ps38
Hominini - ps39
Homo_sapiens - ps40
Phylostratum

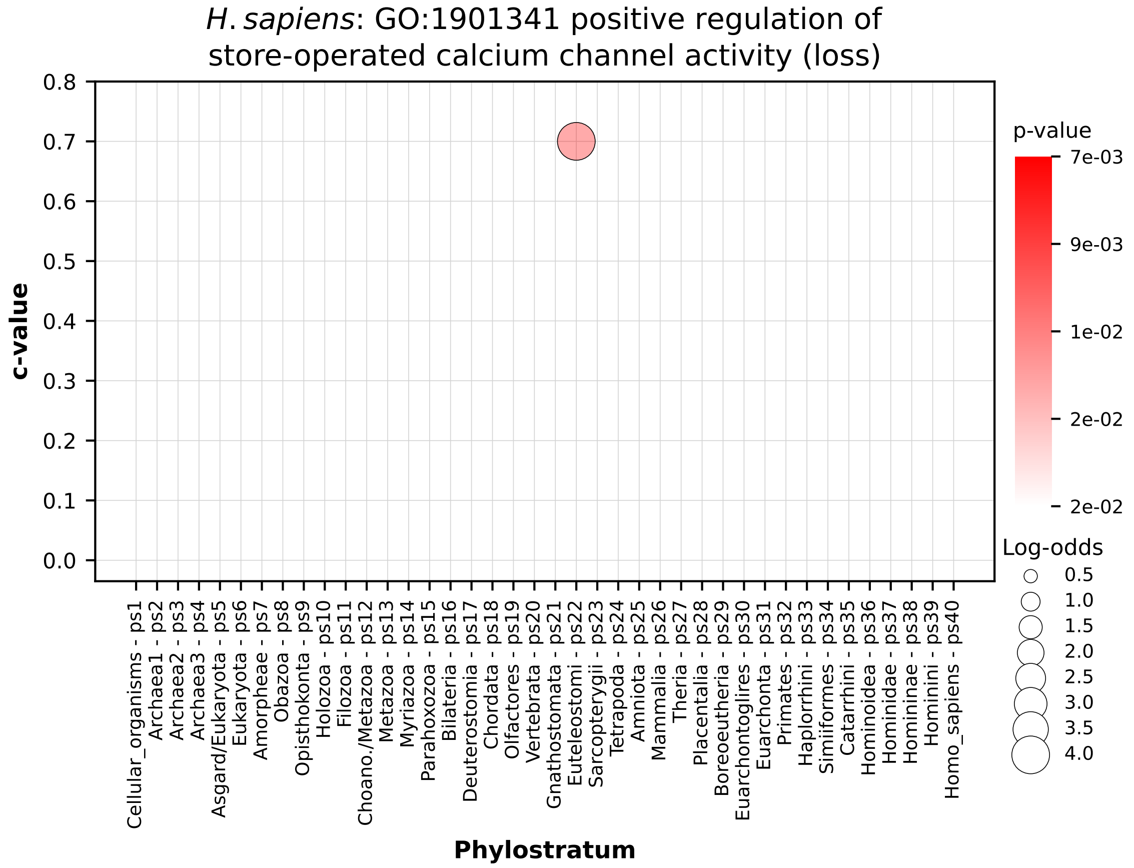

H. sapiens: GO:1901341 positive regulation of store-operated calcium channel activity (loss)
c-value
0.0
0.1
0.2
0.3
0.4
0.5
0.6
0.7
0.8
p-value
7e-03
9e-03
1e-02
2e-02
2e-02
Log-odds
0.5
1.0
1.5
2.0
2.5
3.0
3.5
4.0
Cellular_organisms - ps1
Archaea1 - ps2
Archaea2 - ps3
Archaea3 - ps4
Asgard/Eukaryota - ps5
Eukaryota - ps6
Amorpheae - ps7
Obazoa - ps8
Opisthokonta - ps9
Holozoa - ps10
Filozoa - ps11
Choano./Metazoa - ps12
Metazoa - ps13
Myriazoa - ps14
Parahoxozoa - ps15
Bilateria - ps16
Deuterostomia - ps17
Chordata - ps18
Olfactores - ps19
Vertebrata - ps20
Gnathostomata - ps21
Euteleostomi - ps22
Sarcopterygii - ps23
Tetrapoda - ps24
Amniota - ps25
Mammalia - ps26
Theria - ps27
Placentalia - ps28
Boreoeutheria - ps29
Euarchontoglires - ps30
Euarchonta - ps31
Primates - ps32
Haplorrhini - ps33
Simiiformes - ps34
Catarrhini - ps35
Hominoidea - ps36
Hominidae - ps37
Homininae - ps38
Hominini - ps39
Homo_sapiens - ps40
Phylostratum

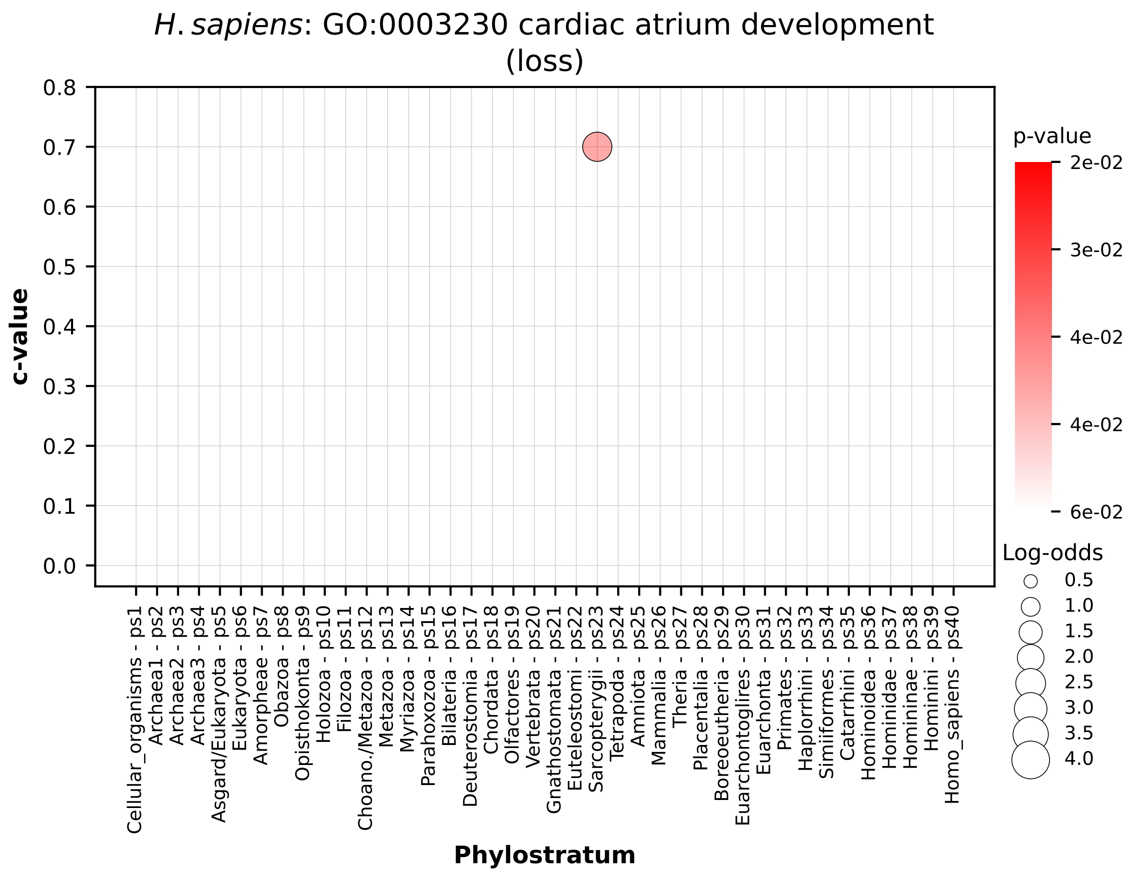

H. sapiens: GO:0003230 cardiac atrium development
(loss)
c-value
0.8
0.7
0.6
0.5
0.4
0.3
0.2
0.1
0.0
p-value
2e-02
3e-02
4e-02
4e-02
6e-02
Log-odds
0.5
1.0
1.5
2.0
2.5
3.0
3.5
4.0
Cellular_organisms - ps1
Archaea1 - ps2
Archaea2 - ps3
Archaea3 - ps4
Asgard/Eukaryota - ps5
Eukaryota - ps6
Amorpheae - ps7
Obazoa - ps8
Opisthokonta - ps9
Holozoa - ps10
Filozoa - ps11
Choano./Metazoa - ps12
Metazoa - ps13
Myriazoa - ps14
Parahoxozoa - ps15
Bilateria - ps16
Deuterostomia - ps17
Chordata - ps18
Olfactores - ps19
Vertebrata - ps20
Gnathostomata - ps21
Euteleostomi - ps22
Sarcopterygii - ps23
Tetrapoda - ps24
Amniota - ps25
Mammalia - ps26
Theria - ps27
Placentalia - ps28
Boreoeutheria - ps29
Euarchontoglires - ps30
Euarchonta - ps31
Primates - ps32
Haplorrhini - ps33
Simiiformes - ps34
Catarrhini - ps35
Hominoidea - ps36
Hominidae - ps37
Homininae - ps38
Hominini - ps39
Homo_sapiens - ps40
Phylostratum

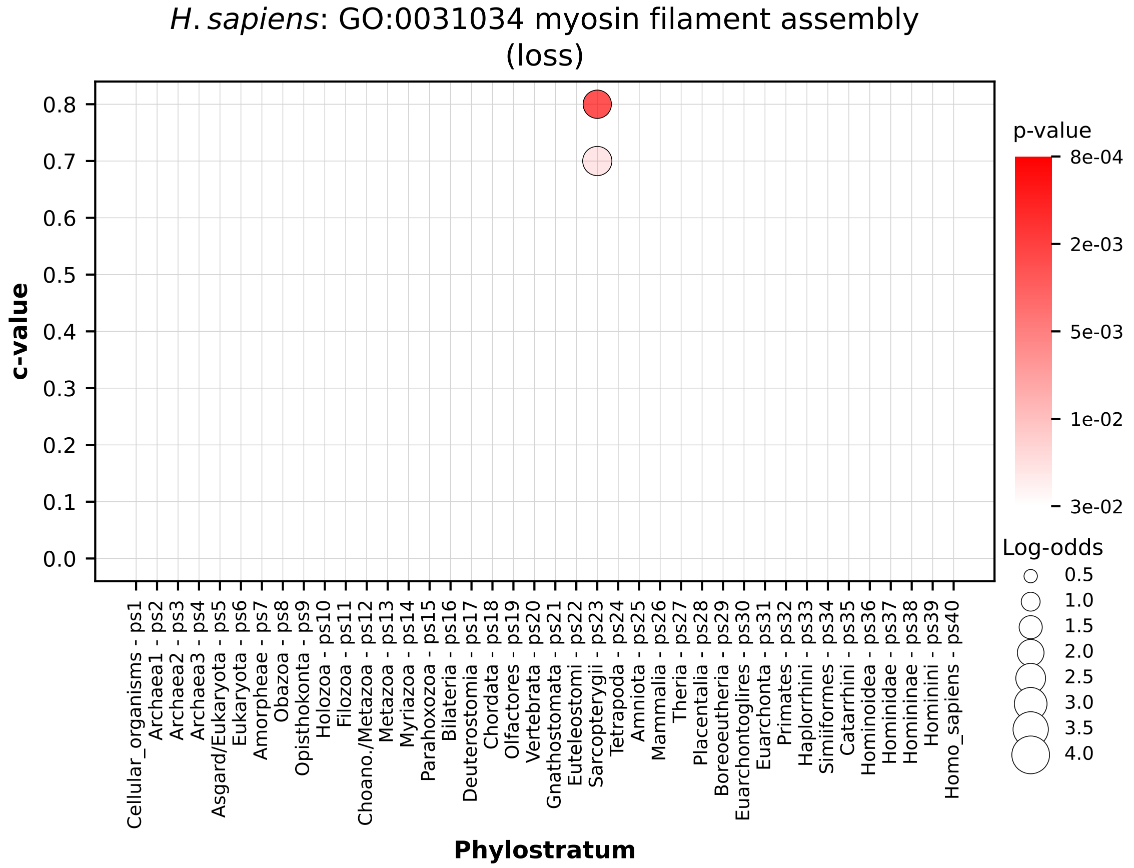

H. sapiens: GO:0031034 myosin filament assembly
(loss)
c-value
0.0
0.1
0.2
0.3
0.4
0.5
0.6
0.7
0.8
p-value
8e-04
2e-03
5e-03
1e-02
3e-02
Log-odds
0.5
1.0
1.5
2.0
2.5
3.0
3.5
4.0
Cellular_organisms - ps1
Archaea1 - ps2
Archaea2 - ps3
Archaea3 - ps4
Asgard/Eukaryota - ps5
Eukaryota - ps6
Amorpheae - ps7
Obazoa - ps8
Opisthokonta - ps9
Holozoa - ps10
Filozoa - ps11
Choano./Metazoa - ps12
Metazoa - ps13
Myriazoa - ps14
Parahoxozoa - ps15
Bilateria - ps16
Deuterostomia - ps17
Chordata - ps18
Olfactores - ps19
Vertebrata - ps20
Gnathostomata - ps21
Euteleostomi - ps22
Sarcopterygii - ps23
Tetrapoda - ps24
Amniota - ps25
Mammalia - ps26
Theria - ps27
Placentalia - ps28
Boreoeutheria - ps29
Euarchontoglires - ps30
Euarchonta - ps31
Primates - ps32
Haplorrhini - ps33
Simiiformes - ps34
Catarrhini - ps35
Hominoidea - ps36
Hominidae - ps37
Homininae - ps38
Hominini - ps39
Homo_sapiens - ps40
Phylostratum

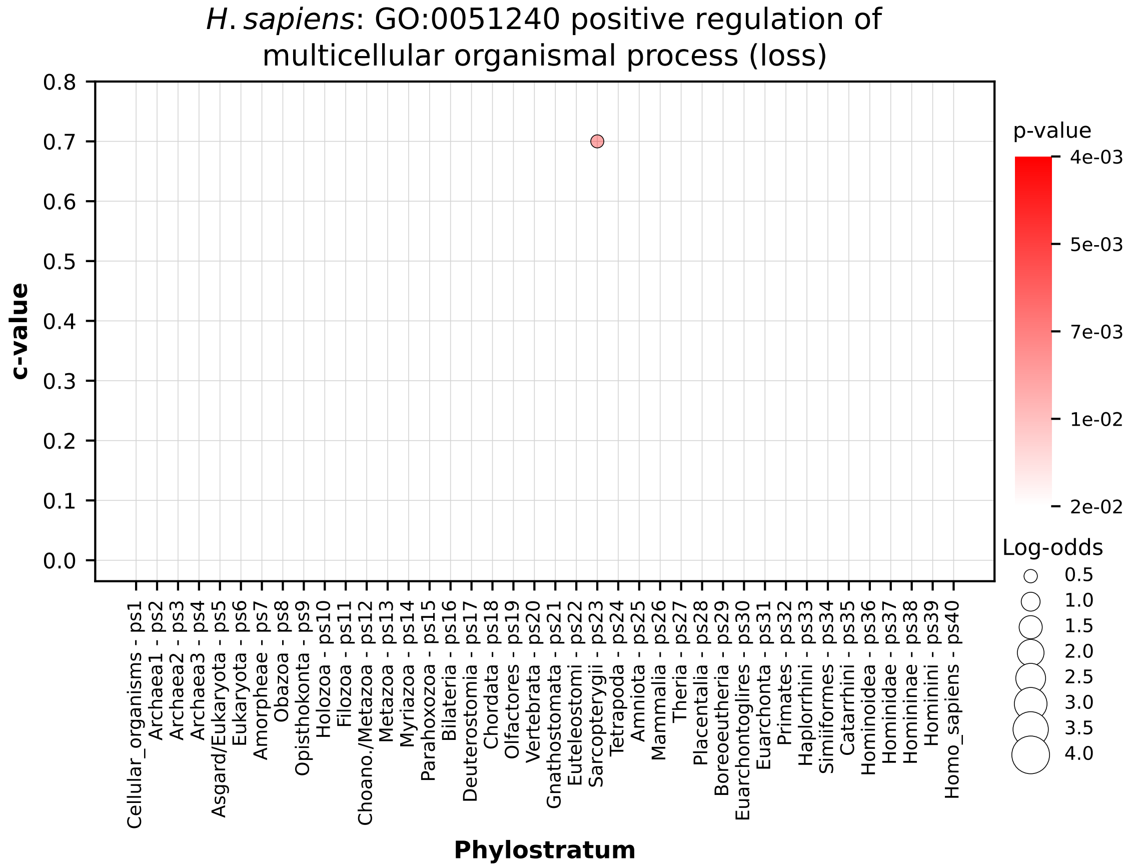

H. sapiens: GO:0051240 positive regulation of multicellular organismal process (loss)
c-value
0.8
0.7
0.6
0.5
0.4
0.3
0.2
0.1
0.0
p-value
4e-03
5e-03
7e-03
1e-02
2e-02
Log-odds
0.5
1.0
1.5
2.0
2.5
3.0
3.5
4.0
Cellular_organisms - ps1
Archaea1 - ps2
Archaea2 - ps3
Archaea3 - ps4
Asgard/Eukaryota - ps5
Eukaryota - ps6
Amorpheae - ps7
Obazoa - ps8
Opisthokonta - ps9
Holozoa - ps10
Filozoa - ps11
Choano./Metazoa - ps12
Metazoa - ps13
Myriazoa - ps14
Parahoxozoa - ps15
Bilateria - ps16
Deuterostomia - ps17
Chordata - ps18
Olfactores - ps19
Vertebrata - ps20
Gnathostomata - ps21
Euteleostomi - ps22
Sarcopterygii - ps23
Tetrapoda - ps24
Amniota - ps25
Mammalia - ps26
Theria - ps27
Placentalia - ps28
Boreoeutheria - ps29
Euarchontoglires - ps30
Euarchonta - ps31
Primates - ps32
Haplorrhini - ps33
Simiiformes - ps34
Catarrhini - ps35
Hominoidea - ps36
Hominidae - ps37
Homininae - ps38
Hominini - ps39
Homo_sapiens - ps40
Phylostratum

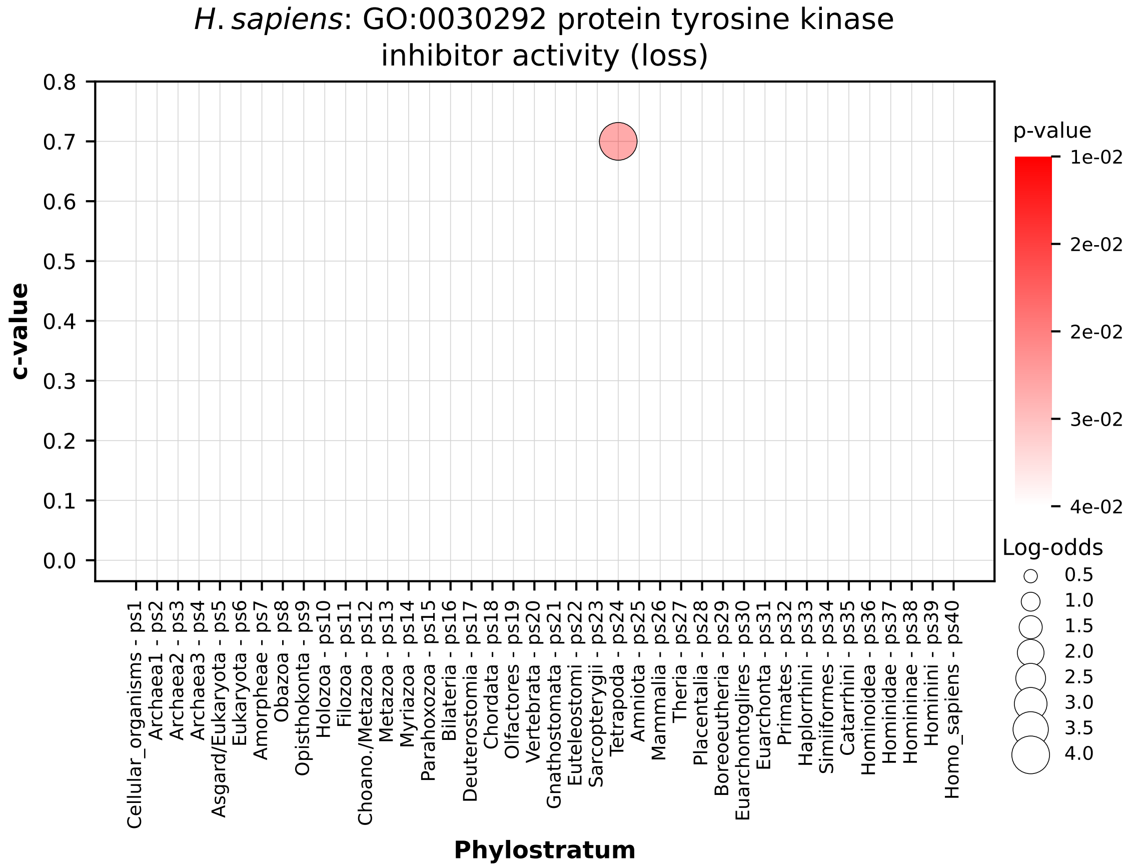
H. sapiens: GO:0030292 protein tyrosine kinase inhibitor activity (loss)
c-value
0.8
0.7
0.6
0.5
0.4
0.3
0.2
0.1
0.0
Phylostratum
Cellular_organisms - ps1
Archaea1 - ps2
Archaea2 - ps3
Archaea3 - ps4
Asgard/Eukaryota - ps5
Eukaryota - ps6
Amorpheae - ps7
Obazoa - ps8
Opisthokonta - ps9
Holozoa - ps10
Filozoa - ps11
Choano./Metazoa - ps12
Metazoa - ps13
Myriazoa - ps14
Parahoxozoa - ps15
Bilateria - ps16
Deuterostomia - ps17
Chordata - ps18
Olfactores - ps19
Vertebrata - ps20
Gnathostomata - ps21
Euteleostomi - ps22
Sarcopterygii - ps23
Tetrapoda - ps24
Amniota - ps25
Mammalia - ps26
Theria - ps27
Placentalia - ps28
Boreoeutheria - ps29
Euarchontoglires - ps30
Euarchonta - ps31
Primates - ps32
Haplorrhini - ps33
Simiiformes - ps34
Catarrhini - ps35
Hominoidea - ps36
Hominidae - ps37
Homininae - ps38
Hominini - ps39
Homo_sapiens - ps40
p-value
1e-02
2e-02
2e-02
3e-02
4e-02
Log-odds
0.5
1.0
1.5
2.0
2.5
3.0
3.5
4.0

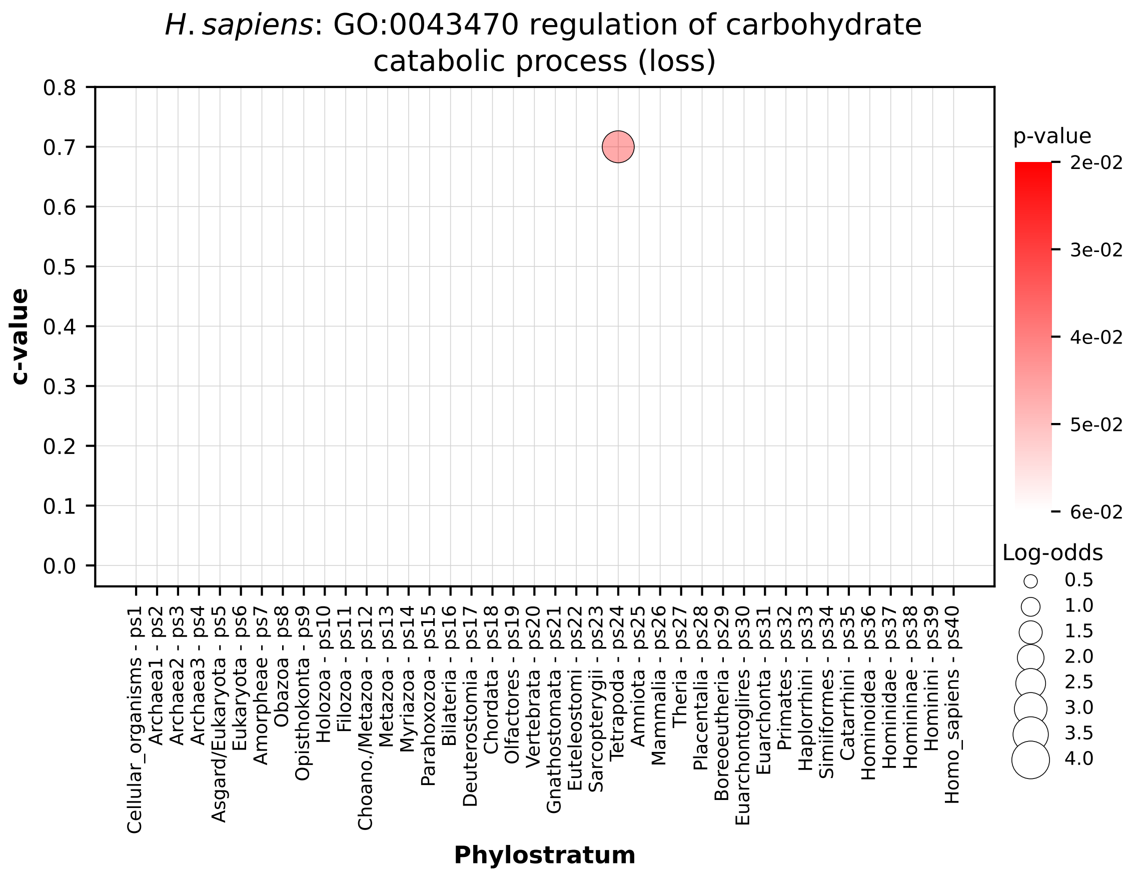

H. sapiens: GO:0043470 regulation of carbohydrate catabolic process (loss)
c-value
0.8
0.7
0.6
0.5
0.4
0.3
0.2
0.1
0.0
p-value
2e-02
3e-02
4e-02
5e-02
6e-02
Log-odds
0.5
1.0
1.5
2.0
2.5
3.0
3.5
4.0
Cellular_organisms - ps1
Archaea1 - ps2
Archaea2 - ps3
Archaea3 - ps4
Asgard/Eukaryota - ps5
Eukaryota - ps6
Amorpheae - ps7
Obazoa - ps8
Opisthokonta - ps9
Holozoa - ps10
Filozoa - ps11
Choano./Metazoa - ps12
Metazoa - ps13
Myriazoa - ps14
Parahoxozoa - ps15
Bilateria - ps16
Deuterostomia - ps17
Chordata - ps18
Olfactores - ps19
Vertebrata - ps20
Gnathostomata - ps21
Euteleostomi - ps22
Sarcopterygii - ps23
Tetrapoda - ps24
Amniota - ps25
Mammalia - ps26
Theria - ps27
Placentalia - ps28
Boreoeutheria - ps29
Euarchontoglires - ps30
Euarchonta - ps31
Primates - ps32
Haplorrhini - ps33
Simiiformes - ps34
Catarrhini - ps35
Hominoidea - ps36
Hominidae - ps37
Homininae - ps38
Hominini - ps39
Homo_sapiens - ps40
Phylostratum

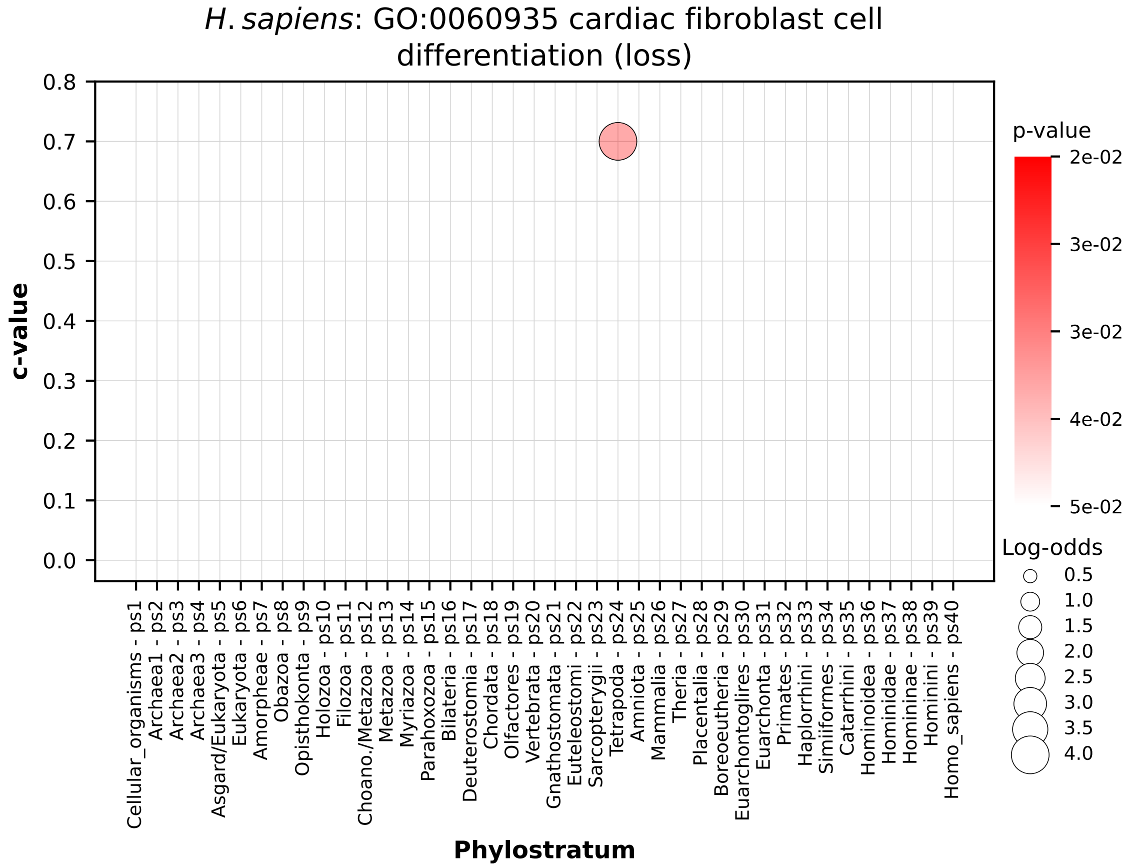
H. sapiens: GO:0060935 cardiac fibroblast cell differentiation (loss)
c-value
0.8
0.7
0.6
0.5
0.4
0.3
0.2
0.1
0.0
p-value
2e-02
3e-02
3e-02
4e-02
5e-02
Log-odds
0.5
1.0
1.5
2.0
2.5
3.0
3.5
4.0
Cellular_organisms - ps1
Archaea1 - ps2
Archaea2 - ps3
Archaea3 - ps4
Asgard/Eukaryota - ps5
Eukaryota - ps6
Amorpheae - ps7
Obazoa - ps8
Opisthokonta - ps9
Holozoa - ps10
Filozoa - ps11
Choano./Metazoa - ps12
Metazoa - ps13
Myriazoa - ps14
Parahoxozoa - ps15
Bilateria - ps16
Deuterostomia - ps17
Chordata - ps18
Olfactores - ps19
Vertebrata - ps20
Gnathostomata - ps21
Euteleostomi - ps22
Sarcopterygii - ps23
Tetrapoda - ps24
Amniota - ps25
Mammalia - ps26
Theria - ps27
Placentalia - ps28
Boreoeutheria - ps29
Euarchontoglires - ps30
Euarchonta - ps31
Primates - ps32
Haplorrhini - ps33
Simiiformes - ps34
Catarrhini - ps35
Hominoidea - ps36
Hominidae - ps37
Homininae - ps38
Hominini - ps39
Homo_sapiens - ps40
Phylostratum

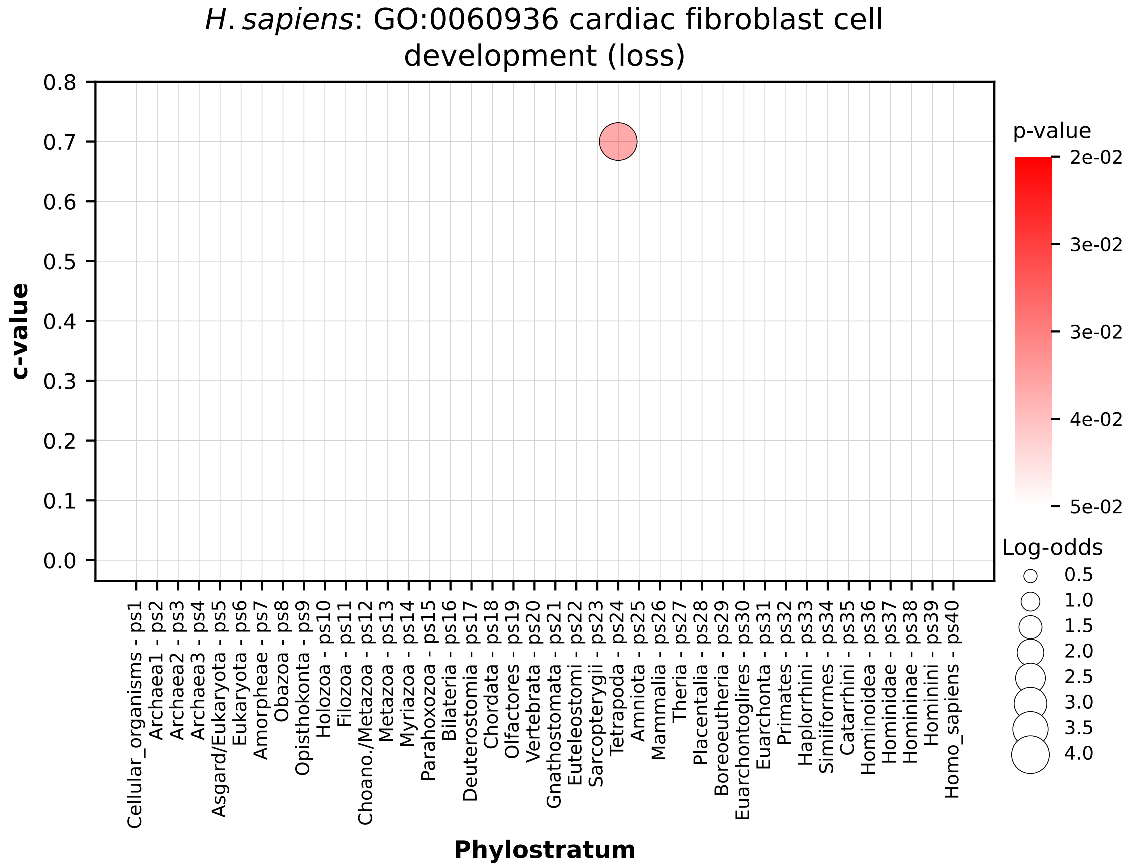

H. sapiens: GO:0060936 cardiac fibroblast cell development (loss)
c-value
0.8
0.7
0.6
0.5
0.4
0.3
0.2
0.1
0.0
p-value
2e-02
3e-02
3e-02
4e-02
5e-02
Log-odds
0.5
1.0
1.5
2.0
2.5
3.0
3.5
4.0
Cellular_organisms - ps1
Archaea1 - ps2
Archaea2 - ps3
Archaea3 - ps4
Asgard/Eukaryota - ps5
Eukaryota - ps6
Amorpheae - ps7
Obazoa - ps8
Opisthokonta - ps9
Holozoa - ps10
Filozoa - ps11
Choano./Metazoa - ps12
Metazoa - ps13
Myriazoa - ps14
Parahoxozoa - ps15
Bilateria - ps16
Deuterostomia - ps17
Chordata - ps18
Olfactores - ps19
Vertebrata - ps20
Gnathostomata - ps21
Euteleostomi - ps22
Sarcopterygii - ps23
Tetrapoda - ps24
Amniota - ps25
Mammalia - ps26
Theria - ps27
Placentalia - ps28
Boreoeutheria - ps29
Euarchontoglires - ps30
Euarchonta - ps31
Primates - ps32
Haplorrhini - ps33
Simiiformes - ps34
Catarrhini - ps35
Hominoidea - ps36
Hominidae - ps37
Homininae - ps38
Hominini - ps39
Homo_sapiens - ps40
Phylostratum

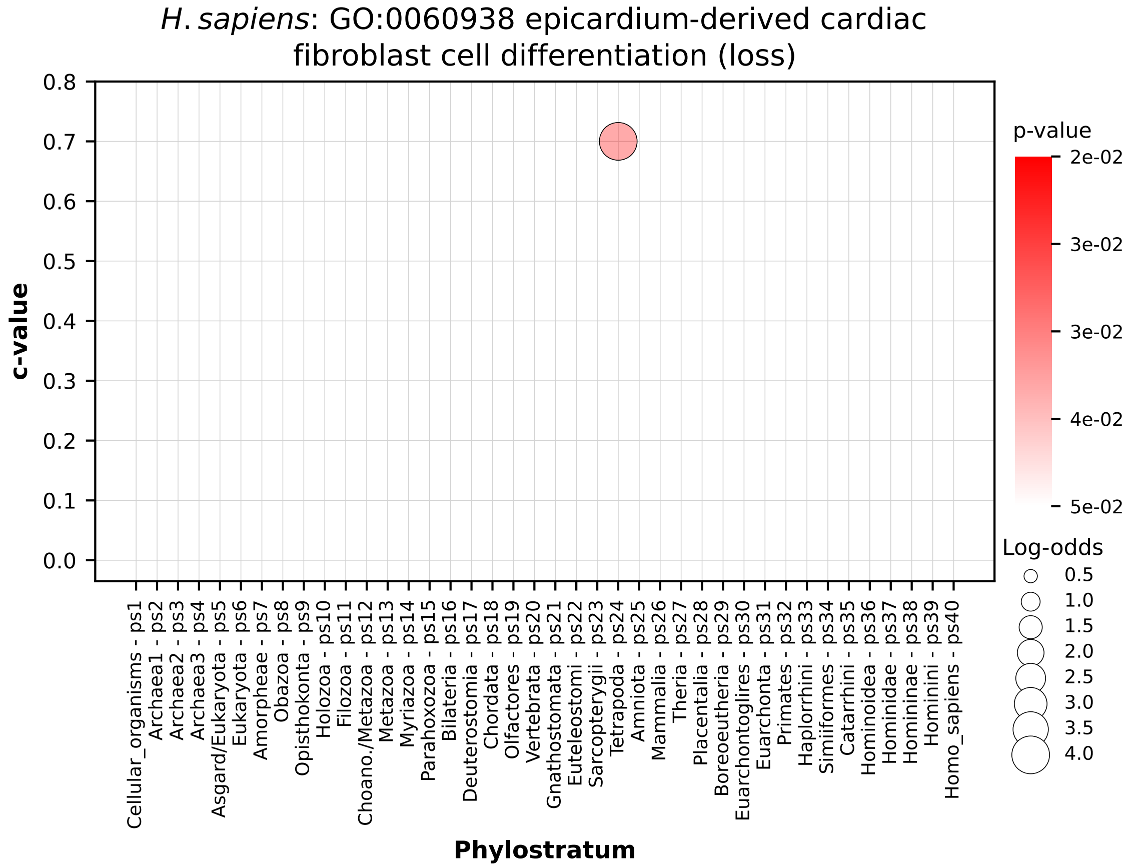

H. sapiens: GO:0060938 epicardium-derived cardiac fibroblast cell differentiation (loss)
c-value
0.8
0.7
0.6
0.5
0.4
0.3
0.2
0.1
0.0
Phylostratum
Cellular_organisms - ps1
Archaea1 - ps2
Archaea2 - ps3
Archaea3 - ps4
Asgard/Eukaryota - ps5
Eukaryota - ps6
Amorpheae - ps7
Obazoa - ps8
Opisthokonta - ps9
Holozoa - ps10
Filozoa - ps11
Choano./Metazoa - ps12
Metazoa - ps13
Myriazoa - ps14
Parahoxozoa - ps15
Bilateria - ps16
Deuterostomia - ps17
Chordata - ps18
Olfactores - ps19
Vertebrata - ps20
Gnathostomata - ps21
Euteleostomi - ps22
Sarcopterygii - ps23
Tetrapoda - ps24
Amniota - ps25
Mammalia - ps26
Theria - ps27
Placentalia - ps28
Boreoeutheria - ps29
Euarchontoglires - ps30
Euarchonta - ps31
Primates - ps32
Haplorrhini - ps33
Simiiformes - ps34
Catarrhini - ps35
Hominoidea - ps36
Hominidae - ps37
Homininae - ps38
Hominini - ps39
Homo_sapiens - ps40
p-value
2e-02
3e-02
3e-02
4e-02
5e-02
Log-odds
0.5
1.0
1.5
2.0
2.5
3.0
3.5
4.0

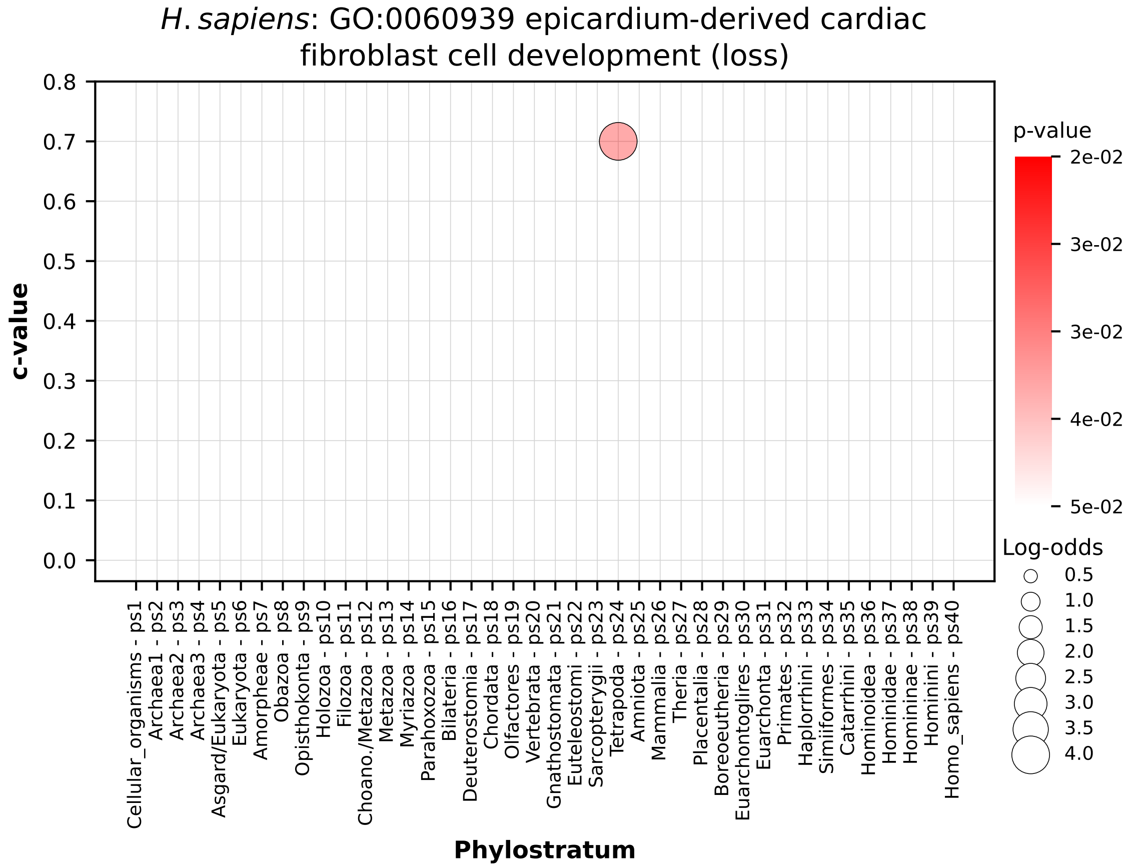

H. sapiens: GO:0060939 epicardium-derived cardiac fibroblast cell development (loss)
c-value
0.8
0.7
0.6
0.5
0.4
0.3
0.2
0.1
0.0
p-value
2e-02
3e-02
3e-02
4e-02
5e-02
Log-odds
0.5
1.0
1.5
2.0
2.5
3.0
3.5
4.0
Cellular_organisms - ps1
Archaea1 - ps2
Archaea2 - ps3
Archaea3 - ps4
Asgard/Eukaryota - ps5
Eukaryota - ps6
Amorpheae - ps7
Obazoa - ps8
Opisthokonta - ps9
Holozoa - ps10
Filozoa - ps11
Choano./Metazoa - ps12
Metazoa - ps13
Myriazoa - ps14
Parahoxozoa - ps15
Bilateria - ps16
Deuterostomia - ps17
Chordata - ps18
Olfactores - ps19
Vertebrata - ps20
Gnathostomata - ps21
Euteleostomi - ps22
Sarcopterygii - ps23
Tetrapoda - ps24
Amniota - ps25
Mammalia - ps26
Theria - ps27
Placentalia - ps28
Boreoeutheria - ps29
Euarchontoglires - ps30
Euarchonta - ps31
Primates - ps32
Haplorrhini - ps33
Simiiformes - ps34
Catarrhini - ps35
Hominoidea - ps36
Hominidae - ps37
Homininae - ps38
Hominini - ps39
Homo_sapiens - ps40
Phylostratum

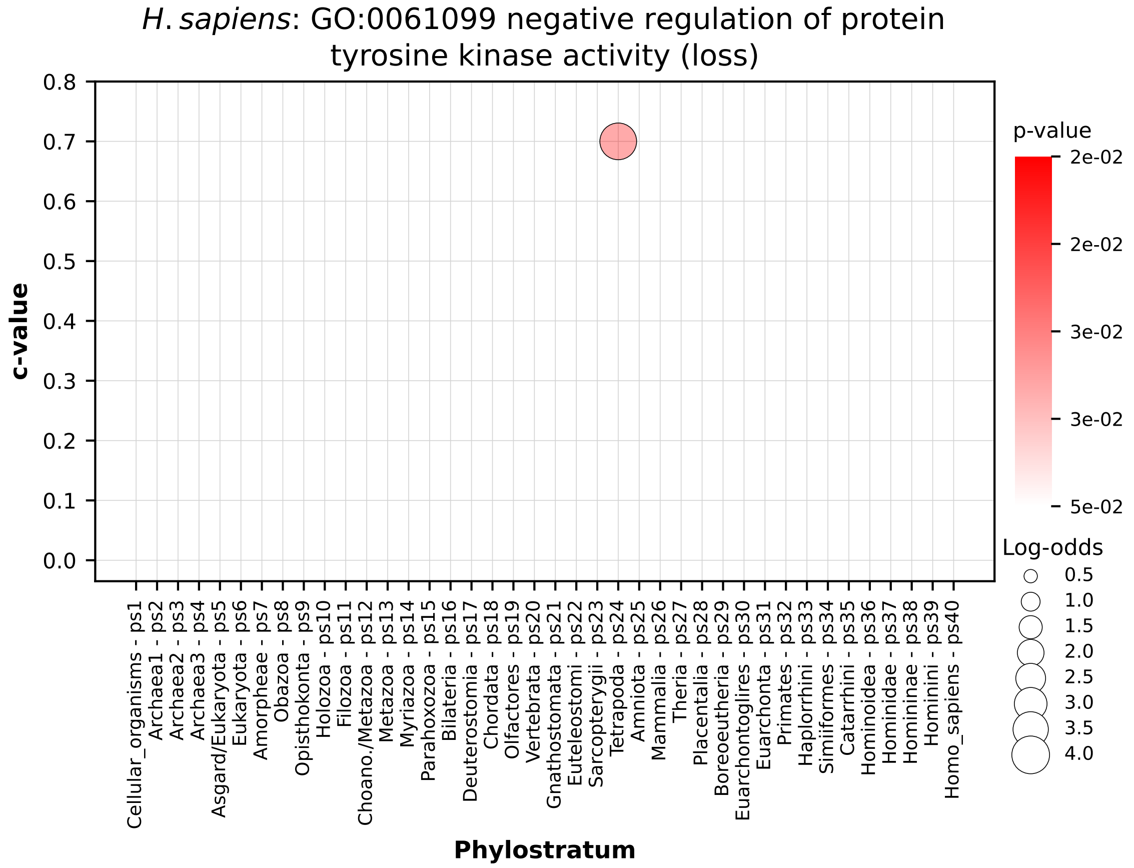

H. sapiens: GO:0061099 negative regulation of protein tyrosine kinase activity (loss)
c-value
0.8
0.7
0.6
0.5
0.4
0.3
0.2
0.1
0.0
p-value
2e-02
2e-02
3e-02
3e-02
5e-02
Log-odds
0.5
1.0
1.5
2.0
2.5
3.0
3.5
4.0
Cellular_organisms - ps1
Archaea1 - ps2
Archaea2 - ps3
Archaea3 - ps4
Asgard/Eukaryota - ps5
Eukaryota - ps6
Amorpheae - ps7
Obazoa - ps8
Opisthokonta - ps9
Holozoa - ps10
Filozoa - ps11
Choano./Metazoa - ps12
Metazoa - ps13
Myriazoa - ps14
Parahoxozoa - ps15
Bilateria - ps16
Deuterostomia - ps17
Chordata - ps18
Olfactores - ps19
Vertebrata - ps20
Gnathostomata - ps21
Euteleostomi - ps22
Sarcopterygii - ps23
Tetrapoda - ps24
Amniota - ps25
Mammalia - ps26
Theria - ps27
Placentalia - ps28
Boreoeutheria - ps29
Euarchontoglires - ps30
Euarchonta - ps31
Primates - ps32
Haplorrhini - ps33
Simiiformes - ps34
Catarrhini - ps35
Hominoidea - ps36
Hominidae - ps37
Homininae - ps38
Hominini - ps39
Homo_sapiens - ps40
Phylostratum

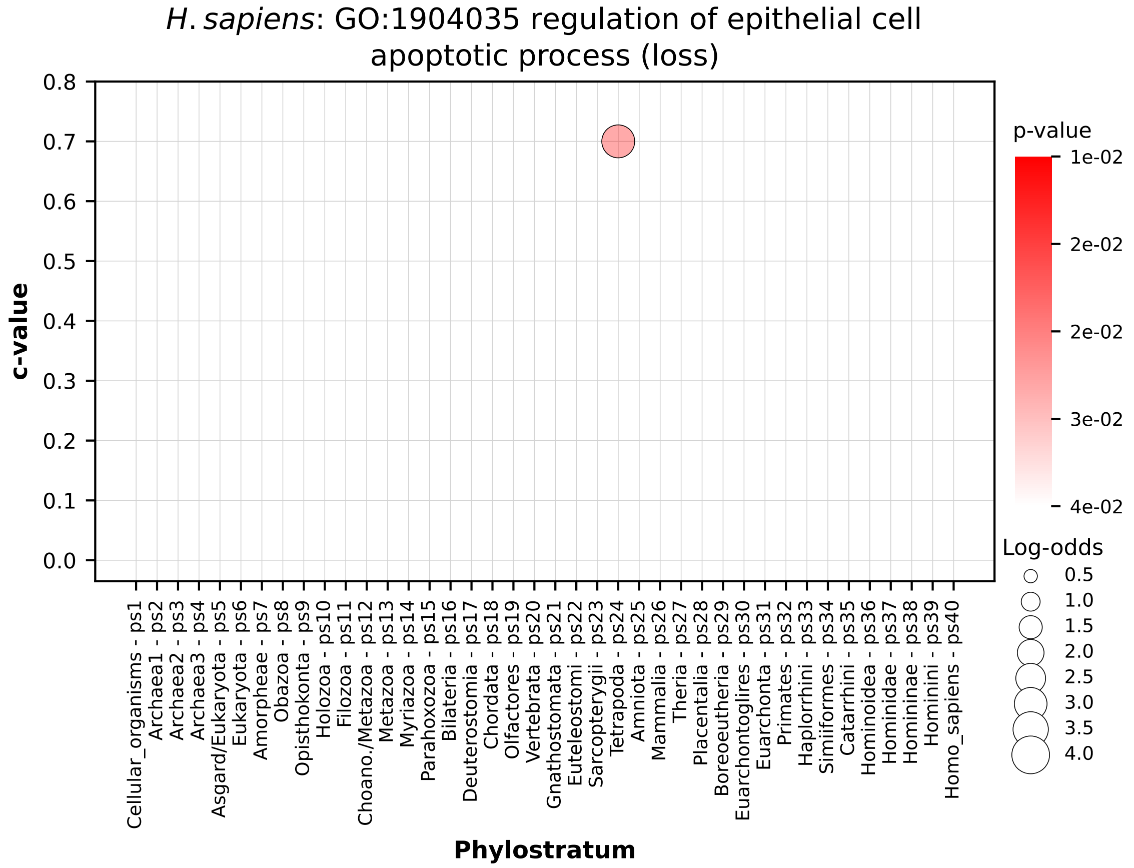

H. sapiens: GO:1904035 regulation of epithelial cell apoptotic process (loss)
c-value
0.8
0.7
0.6
0.5
0.4
0.3
0.2
0.1
0.0
Phylostratum
Cellular_organisms - ps1
Archaea1 - ps2
Archaea2 - ps3
Archaea3 - ps4
Asgard/Eukaryota - ps5
Eukaryota - ps6
Amorpheae - ps7
Obazoa - ps8
Opisthokonta - ps9
Holozoa - ps10
Filozoa - ps11
Choano./Metazoa - ps12
Metazoa - ps13
Myriazoa - ps14
Parahoxozoa - ps15
Bilateria - ps16
Deuterostomia - ps17
Chordata - ps18
Olfactores - ps19
Vertebrata - ps20
Gnathostomata - ps21
Euteleostomi - ps22
Sarcopterygii - ps23
Tetrapoda - ps24
Amniota - ps25
Mammalia - ps26
Theria - ps27
Placentalia - ps28
Boreoeutheria - ps29
Euarchontoglires - ps30
Euarchonta - ps31
Primates - ps32
Haplorrhini - ps33
Simiiformes - ps34
Catarrhini - ps35
Hominoidea - ps36
Hominidae - ps37
Homininae - ps38
Hominini - ps39
Homo_sapiens - ps40
p-value
1e-02
2e-02
2e-02
3e-02
4e-02
Log-odds
0.5
1.0
1.5
2.0
2.5
3.0
3.5
4.0

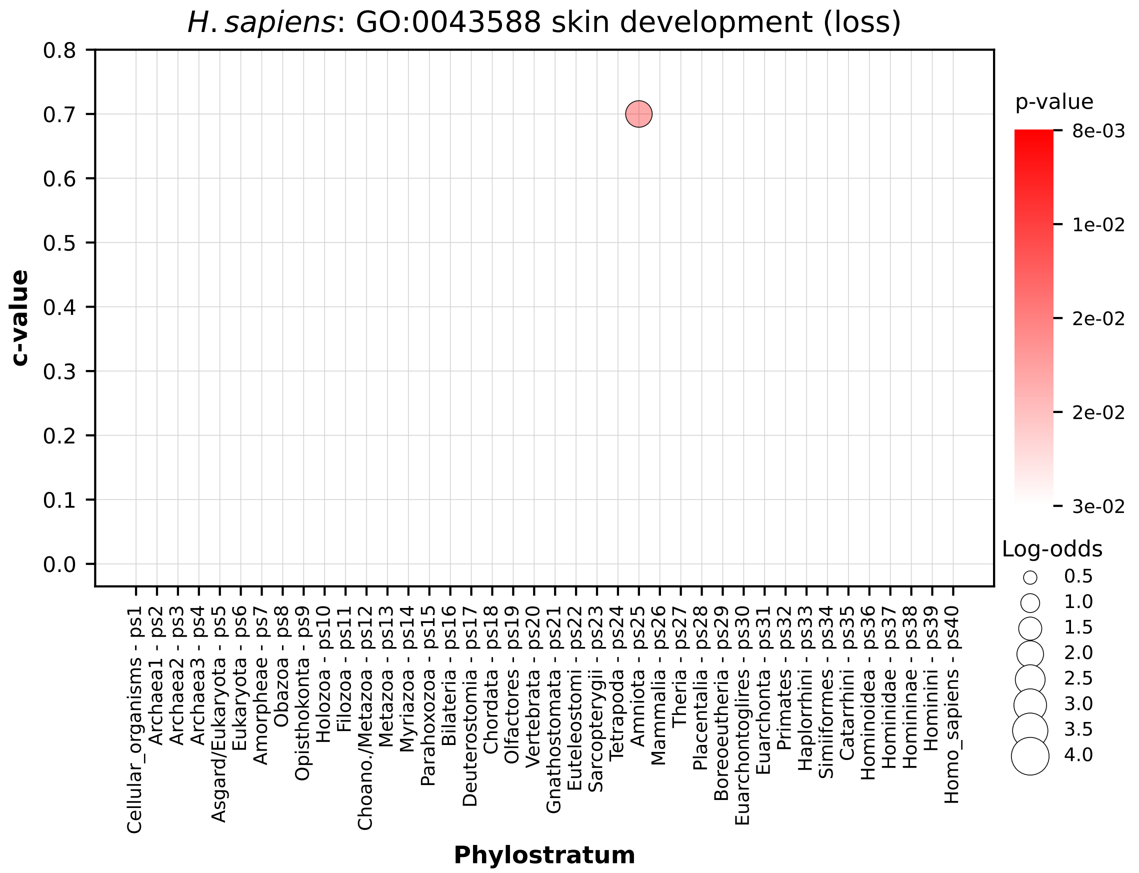

H. sapiens: GO:0043588 skin development (loss)
c-value
0.0
0.1
0.2
0.3
0.4
0.5
0.6
0.7
0.8
p-value
8e-03
1e-02
2e-02
2e-02
3e-02
Log-odds
0.5
1.0
1.5
2.0
2.5
3.0
3.5
4.0
Cellular_organisms - ps1
Archaea1 - ps2
Archaea2 - ps3
Archaea3 - ps4
Asgard/Eukaryota - ps5
Eukaryota - ps6
Amorpheae - ps7
Obazoa - ps8
Opisthokonta - ps9
Holozoa - ps10
Filozoa - ps11
Choano./Metazoa - ps12
Metazoa - ps13
Myriazoa - ps14
Parahoxozoa - ps15
Bilateria - ps16
Deuterostomia - ps17
Chordata - ps18
Olfactores - ps19
Vertebrata - ps20
Gnathostomata - ps21
Euteleostomi - ps22
Sarcopterygii - ps23
Tetrapoda - ps24
Amniota - ps25
Mammalia - ps26
Theria - ps27
Placentalia - ps28
Boreoeutheria - ps29
Euarchontoglires - ps30
Euarchonta - ps31
Primates - ps32
Haplorrhini - ps33
Simiiformes - ps34
Catarrhini - ps35
Hominoidea - ps36
Hominidae - ps37
Homininae - ps38
Hominini - ps39
Homo_sapiens - ps40
Phylostratum

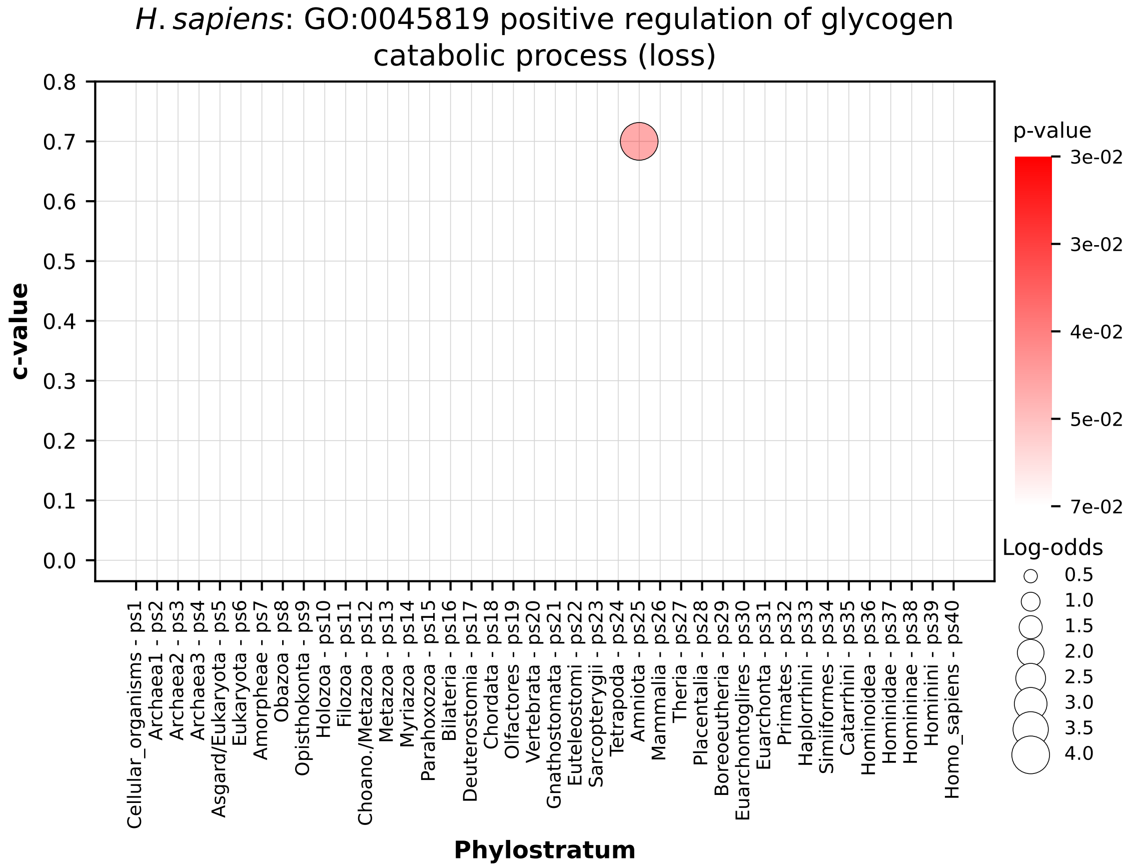

H. sapiens: GO:0045819 positive regulation of glycogen catabolic process (loss)
c-value
0.0
0.1
0.2
0.3
0.4
0.5
0.6
0.7
0.8
Phylostratum
Cellular_organisms - ps1
Archaea1 - ps2
Archaea2 - ps3
Archaea3 - ps4
Asgard/Eukaryota - ps5
Eukaryota - ps6
Amorpheae - ps7
Obazoa - ps8
Opisthokonta - ps9
Holozoa - ps10
Filozoa - ps11
Choano./Metazoa - ps12
Metazoa - ps13
Myriazoa - ps14
Parahoxozoa - ps15
Bilateria - ps16
Deuterostomia - ps17
Chordata - ps18
Olfactores - ps19
Vertebrata - ps20
Gnathostomata - ps21
Euteleostomi - ps22
Sarcopterygii - ps23
Tetrapoda - ps24
Amniota - ps25
Mammalia - ps26
Theria - ps27
Placentalia - ps28
Boreoeutheria - ps29
Euarchontoglires - ps30
Euarchonta - ps31
Primates - ps32
Haplorrhini - ps33
Simiiformes - ps34
Catarrhini - ps35
Hominoidea - ps36
Hominidae - ps37
Homininae - ps38
Hominini - ps39
Homo_sapiens - ps40
p-value
3e-02
3e-02
4e-02
5e-02
7e-02
Log-odds
0.5
1.0
1.5
2.0
2.5
3.0
3.5
4.0

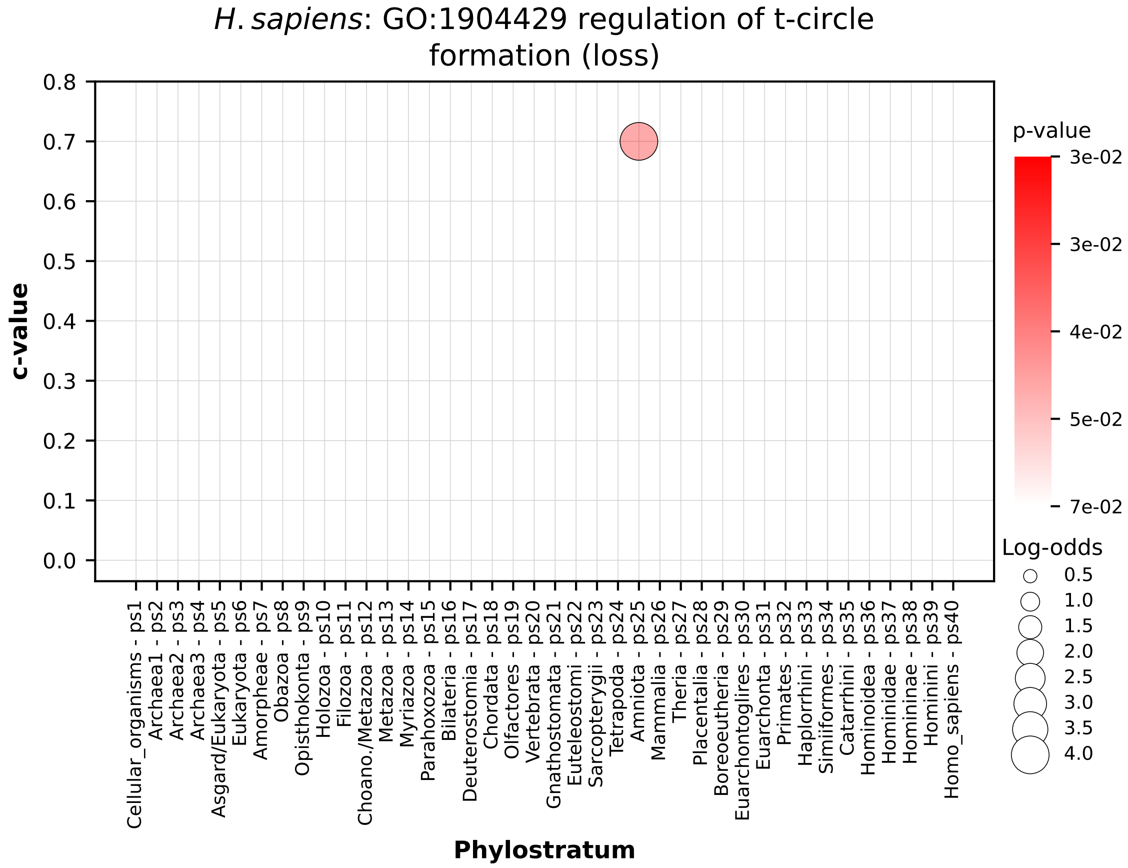

H. sapiens: GO:1904429 regulation of t-circle formation (loss)
c-value
0.8
0.7
0.6
0.5
0.4
0.3
0.2
0.1
0.0
Phylostratum
Cellular_organisms - ps1
Archaea1 - ps2
Archaea2 - ps3
Archaea3 - ps4
Asgard/Eukaryota - ps5
Eukaryota - ps6
Amorpheae - ps7
Obazoa - ps8
Opisthokonta - ps9
Holozoa - ps10
Filozoa - ps11
Choano./Metazoa - ps12
Metazoa - ps13
Myriazoa - ps14
Parahoxozoa - ps15
Bilateria - ps16
Deuterostomia - ps17
Chordata - ps18
Olfactores - ps19
Vertebrata - ps20
Gnathostomata - ps21
Euteleostomi - ps22
Sarcopterygii - ps23
Tetrapoda - ps24
Amniota - ps25
Mammalia - ps26
Theria - ps27
Placentalia - ps28
Boreoeutheria - ps29
Euarchontoglires - ps30
Euarchonta - ps31
Primates - ps32
Haplorrhini - ps33
Simiiformes - ps34
Catarrhini - ps35
Hominoidea - ps36
Hominidae - ps37
Homininae - ps38
Hominini - ps39
Homo_sapiens - ps40
p-value
3e-02
3e-02
4e-02
5e-02
7e-02
Log-odds
0.5
1.0
1.5
2.0
2.5
3.0
3.5
4.0

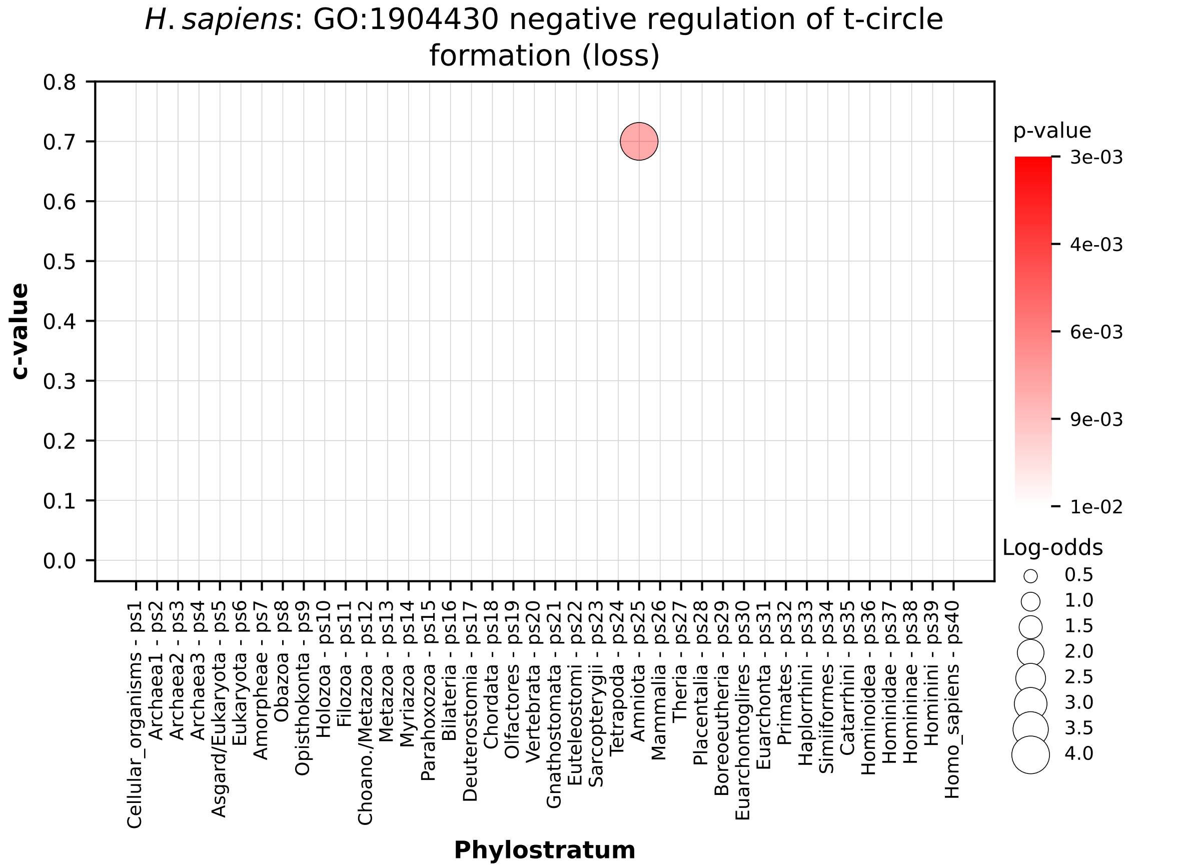

H. sapiens: GO:1904430 negative regulation of t-circle formation (loss)
c-value
0.8
0.7
0.6
0.5
0.4
0.3
0.2
0.1
0.0
Phylostratum
Cellular_organisms - ps1
Archaea1 - ps2
Archaea2 - ps3
Archaea3 - ps4
Asgard/Eukaryota - ps5
Eukaryota - ps6
Amorpheae - ps7
Obazoa - ps8
Opisthokonta - ps9
Holozoa - ps10
Filozoa - ps11
Choano./Metazoa - ps12
Metazoa - ps13
Myriazoa - ps14
Parahoxozoa - ps15
Bilateria - ps16
Deuterostomia - ps17
Chordata - ps18
Olfactores - ps19
Vertebrata - ps20
Gnathostomata - ps21
Euteleostomi - ps22
Sarcopterygii - ps23
Tetrapoda - ps24
Amniota - ps25
Mammalia - ps26
Theria - ps27
Placentalia - ps28
Boreoeutheria - ps29
Euarchontoglires - ps30
Euarchonta - ps31
Primates - ps32
Haplorrhini - ps33
Simiiformes - ps34
Catarrhini - ps35
Hominoidea - ps36
Hominidae - ps37
Homininae - ps38
Hominini - ps39
Homo_sapiens - ps40
p-value
3e-03
4e-03
6e-03
9e-03
1e-02
Log-odds
0.5
1.0
1.5
2.0
2.5
3.0
3.5
4.0

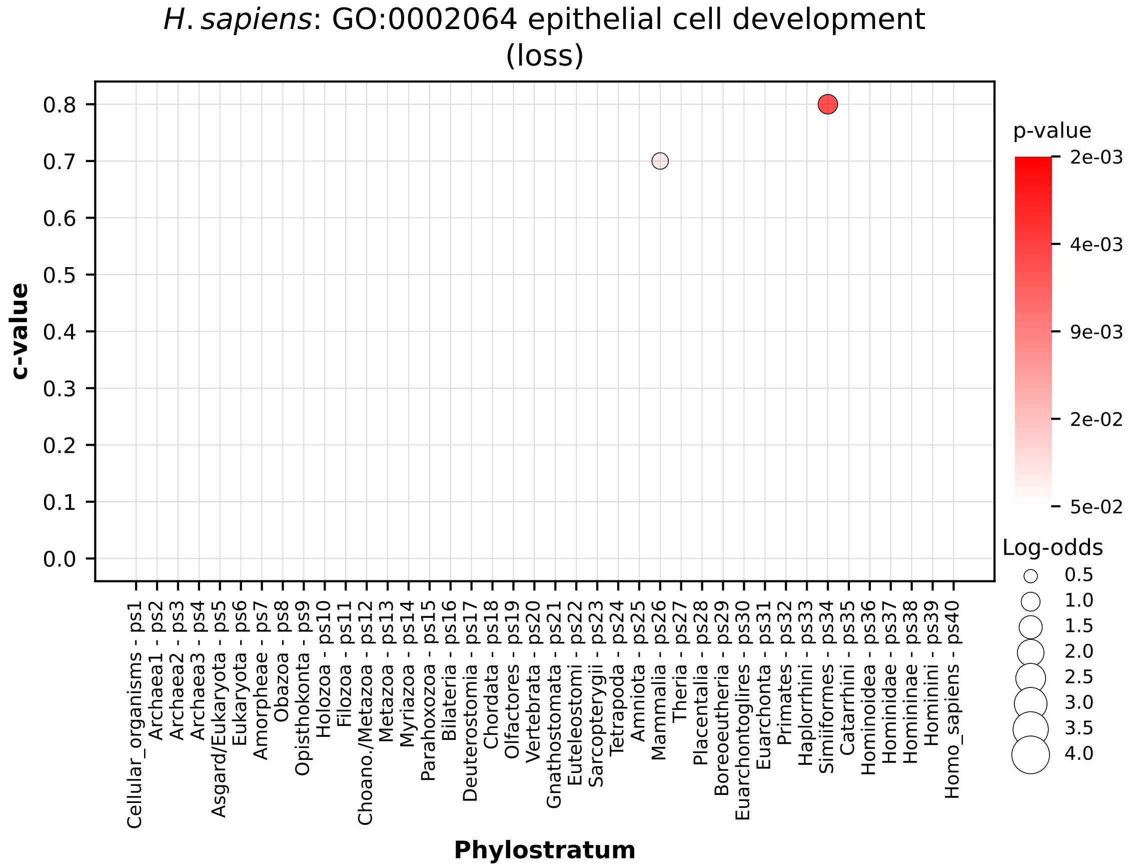

H. sapiens: GO:0002064 epithelial cell development
(loss)
c-value
0.0
0.1
0.2
0.3
0.4
0.5
0.6
0.7
0.8
p-value
2e-03
4e-03
9e-03
2e-02
5e-02
Log-odds
0.5
1.0
1.5
2.0
2.5
3.0
3.5
4.0
Cellular_organisms - ps1
Archaea1 - ps2
Archaea2 - ps3
Archaea3 - ps4
Asgard/Eukaryota - ps5
Eukaryota - ps6
Amorpheae - ps7
Obazoa - ps8
Opisthokonta - ps9
Holozoa - ps10
Filozoa - ps11
Choano./Metazoa - ps12
Metazoa - ps13
Myriazoa - ps14
Parahoxozoa - ps15
Bilateria - ps16
Deuterostomia - ps17
Chordata - ps18
Olfactores - ps19
Vertebrata - ps20
Gnathostomata - ps21
Euteleostomi - ps22
Sarcopterygii - ps23
Tetrapoda - ps24
Amniota - ps25
Mammalia - ps26
Theria - ps27
Placentalia - ps28
Boreoeutheria - ps29
Euarchontoglires - ps30
Euarchonta - ps31
Primates - ps32
Haplorrhini - ps33
Simiiformes - ps34
Catarrhini - ps35
Hominoidea - ps36
Hominidae - ps37
Homininae - ps38
Hominini - ps39
Homo_sapiens - ps40
Phylostratum

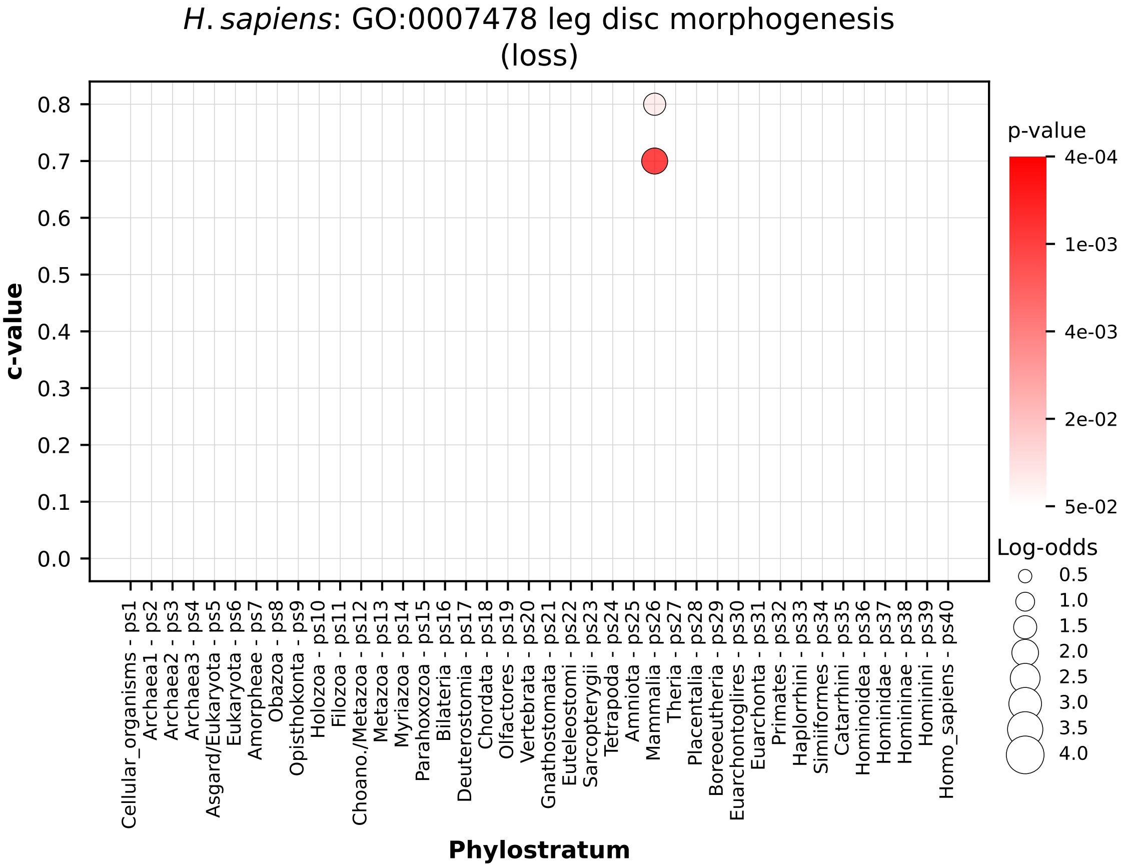

H. sapiens: GO:0007478 leg disc morphogenesis
(loss)
c-value
0.8
0.7
0.6
0.5
0.4
0.3
0.2
0.1
0.0
p-value
4e-04
1e-03
4e-03
2e-02
5e-02
Log-odds
0.5
1.0
1.5
2.0
2.5
3.0
3.5
4.0
Cellular_organisms - ps1
Archaea1 - ps2
Archaea2 - ps3
Archaea3 - ps4
Asgard/Eukaryota - ps5
Eukaryota - ps6
Amorpheae - ps7
Obazoa - ps8
Opisthokonta - ps9
Holozoa - ps10
Filozoa - ps11
Choano./Metazoa - ps12
Metazoa - ps13
Myriazoa - ps14
Parahoxozoa - ps15
Bilateria - ps16
Deuterostomia - ps17
Chordata - ps18
Olfactores - ps19
Vertebrata - ps20
Gnathostomata - ps21
Euteleostomi - ps22
Sarcopterygii - ps23
Tetrapoda - ps24
Amniota - ps25
Mammalia - ps26
Theria - ps27
Placentalia - ps28
Boreoeutheria - ps29
Euarchontoglires - ps30
Euarchonta - ps31
Primates - ps32
Haplorrhini - ps33
Simiiformes - ps34
Catarrhini - ps35
Hominoidea - ps36
Hominidae - ps37
Homininae - ps38
Hominini - ps39
Homo_sapiens - ps40
Phylostratum

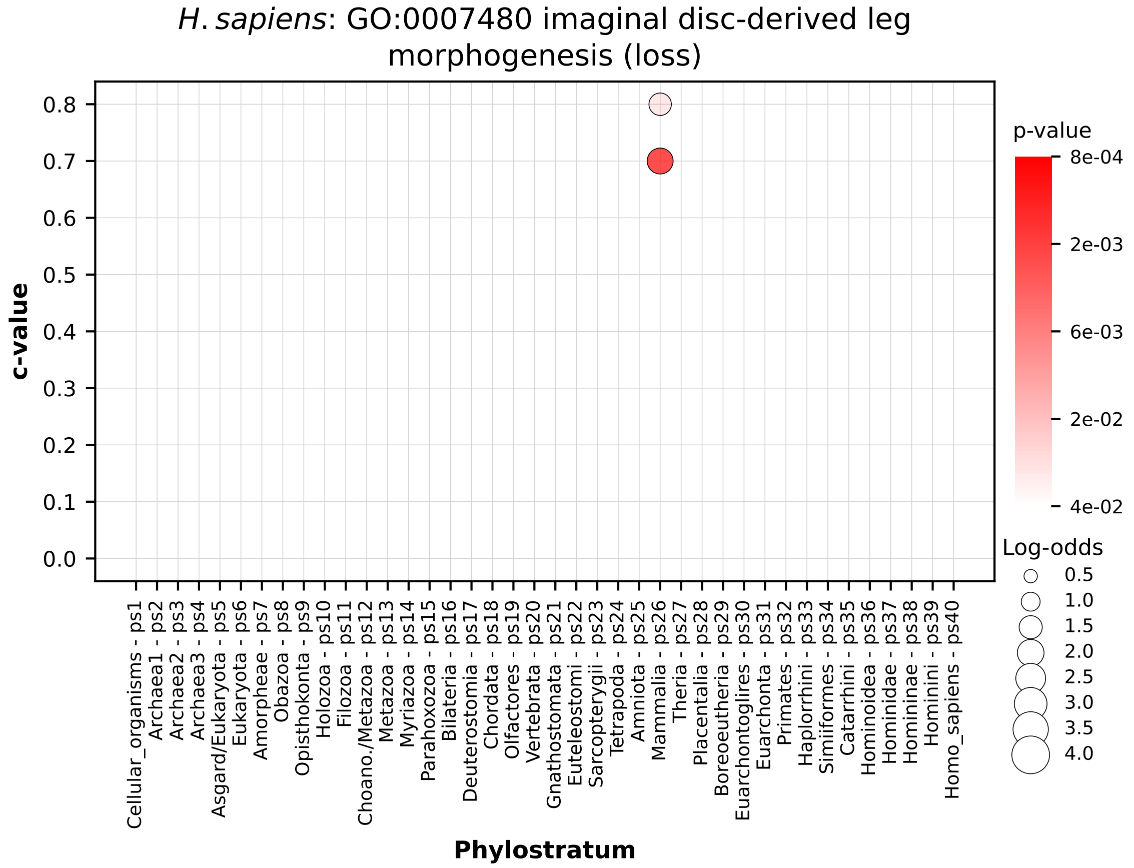

H. sapiens: GO:0007480 imaginal disc-derived leg morphogenesis (loss)
c-value
0.8
0.7
0.6
0.5
0.4
0.3
0.2
0.1
0.0
p-value
8e-04
2e-03
6e-03
2e-02
4e-02
Log-odds
0.5
1.0
1.5
2.0
2.5
3.0
3.5
4.0
Cellular_organisms - ps1
Archaea1 - ps2
Archaea2 - ps3
Archaea3 - ps4
Asgard/Eukaryota - ps5
Eukaryota - ps6
Amorpheae - ps7
Obazoa - ps8
Opisthokonta - ps9
Holozoa - ps10
Filozoa - ps11
Choano./Metazoa - ps12
Metazoa - ps13
Myriazoa - ps14
Parahoxozoa - ps15
Bilateria - ps16
Deuterostomia - ps17
Chordata - ps18
Olfactores - ps19
Vertebrata - ps20
Gnathostomata - ps21
Euteleostomi - ps22
Sarcopterygii - ps23
Tetrapoda - ps24
Amniota - ps25
Mammalia - ps26
Theria - ps27
Placentalia - ps28
Boreoeutheria - ps29
Euarchontoglires - ps30
Euarchonta - ps31
Primates - ps32
Haplorrhini - ps33
Simiiformes - ps34
Catarrhini - ps35
Hominoidea - ps36
Hominidae - ps37
Homininae - ps38
Hominini - ps39
Homo_sapiens - ps40
Phylostratum

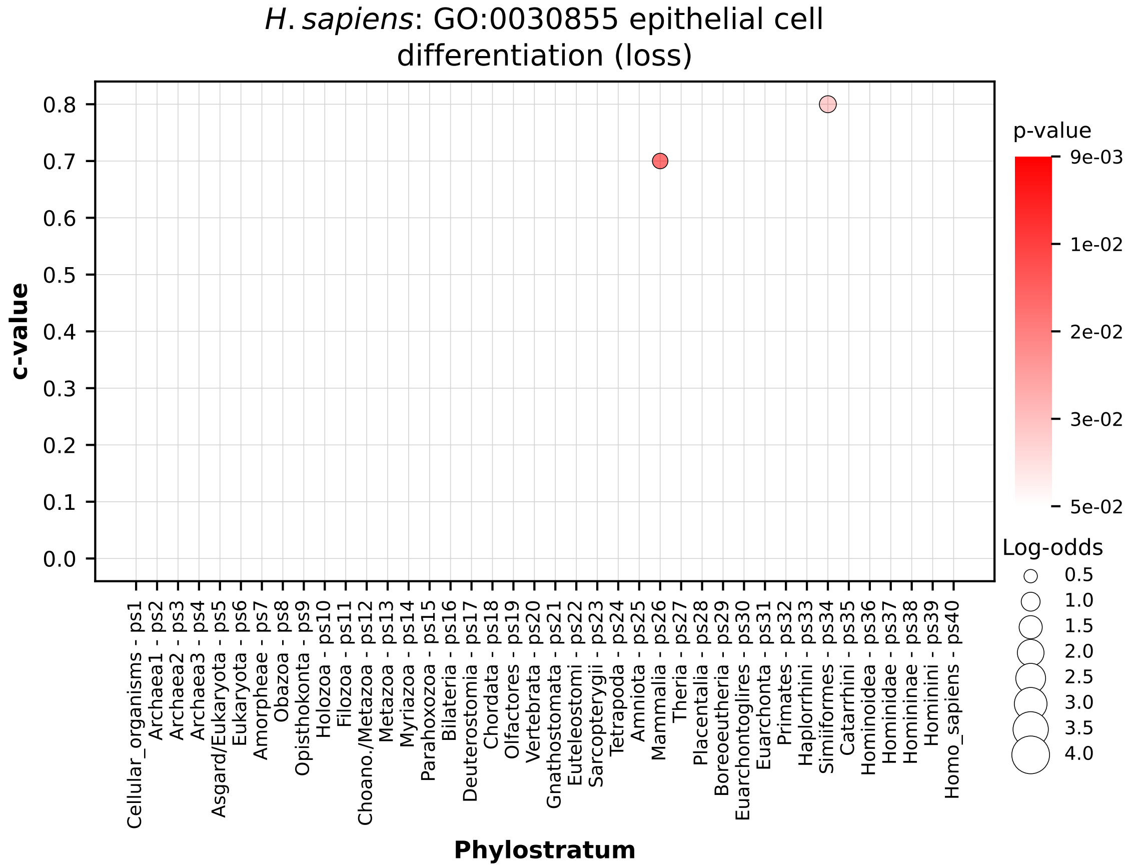
H. sapiens: GO:0030855 epithelial cell differentiation (loss)
c-value
0.0
0.1
0.2
0.3
0.4
0.5
0.6
0.7
0.8
Phylostratum
Cellular_organisms - ps1
Archaea1 - ps2
Archaea2 - ps3
Archaea3 - ps4
Asgard/Eukaryota - ps5
Eukaryota - ps6
Amorpheae - ps7
Obazoa - ps8
Opisthokonta - ps9
Holozoa - ps10
Filozoa - ps11
Choano./Metazoa - ps12
Metazoa - ps13
Myriazoa - ps14
Parahoxozoa - ps15
Bilateria - ps16
Deuterostomia - ps17
Chordata - ps18
Olfactores - ps19
Vertebrata - ps20
Gnathostomata - ps21
Euteleostomi - ps22
Sarcopterygii - ps23
Tetrapoda - ps24
Amniota - ps25
Mammalia - ps26
Theria - ps27
Placentalia - ps28
Boreoeutheria - ps29
Euarchontoglires - ps30
Euarchonta - ps31
Primates - ps32
Haplorrhini - ps33
Simiiformes - ps34
Catarrhini - ps35
Hominoidea - ps36
Hominidae - ps37
Homininae - ps38
Hominini - ps39
Homo_sapiens - ps40
p-value
9e-03
1e-02
2e-02
3e-02
5e-02
Log-odds
0.5
1.0
1.5
2.0
2.5
3.0
3.5
4.0

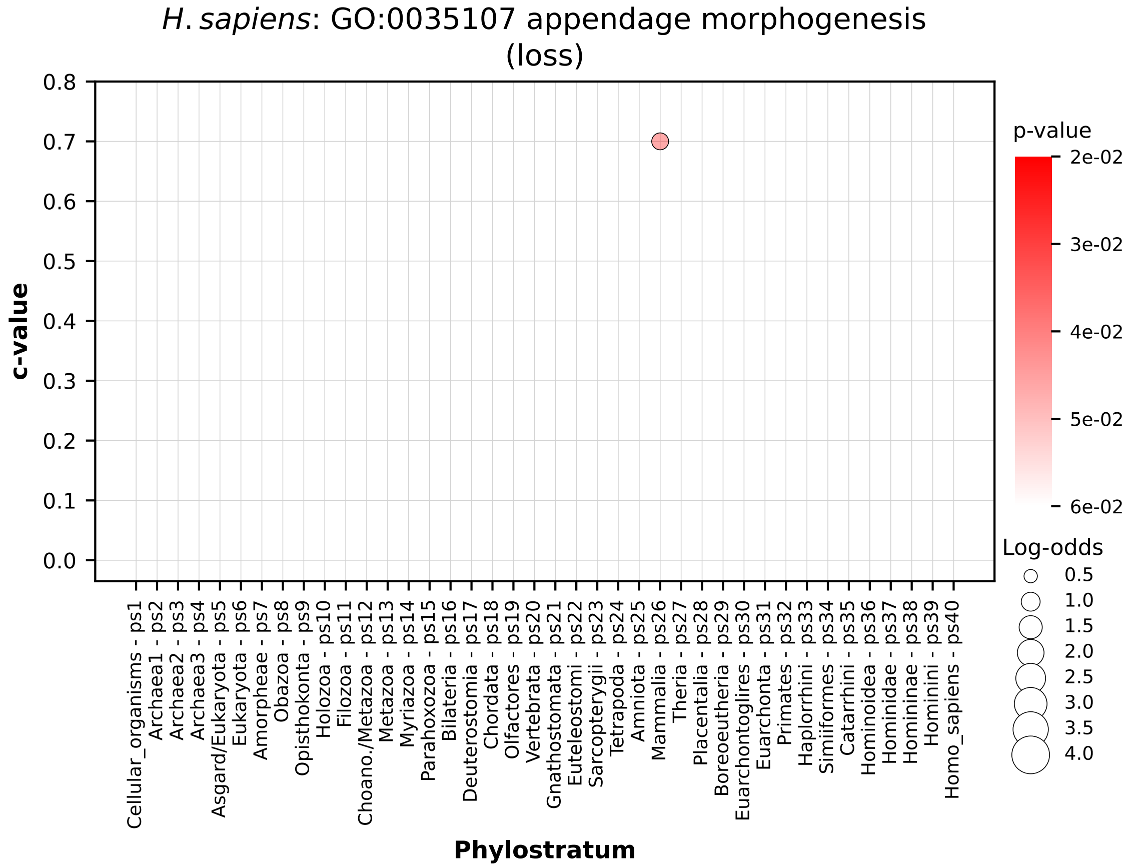

H. sapiens: GO:0035107 appendage morphogenesis
(loss)
c-value
0.8
0.7
0.6
0.5
0.4
0.3
0.2
0.1
0.0
p-value
2e-02
3e-02
4e-02
5e-02
6e-02
Log-odds
0.5
1.0
1.5
2.0
2.5
3.0
3.5
4.0
Cellular_organisms - ps1
Archaea1 - ps2
Archaea2 - ps3
Archaea3 - ps4
Asgard/Eukaryota - ps5
Eukaryota - ps6
Amorpheae - ps7
Obazoa - ps8
Opisthokonta - ps9
Holozoa - ps10
Filozoa - ps11
Choano./Metazoa - ps12
Metazoa - ps13
Myriazoa - ps14
Parahoxozoa - ps15
Bilateria - ps16
Deuterostomia - ps17
Chordata - ps18
Olfactores - ps19
Vertebrata - ps20
Gnathostomata - ps21
Euteleostomi - ps22
Sarcopterygii - ps23
Tetrapoda - ps24
Amniota - ps25
Mammalia - ps26
Theria - ps27
Placentalia - ps28
Boreoeutheria - ps29
Euarchontoglires - ps30
Euarchonta - ps31
Primates - ps32
Haplorrhini - ps33
Simiiformes - ps34
Catarrhini - ps35
Hominoidea - ps36
Hominidae - ps37
Homininae - ps38
Hominini - ps39
Homo_sapiens - ps40
Phylostratum

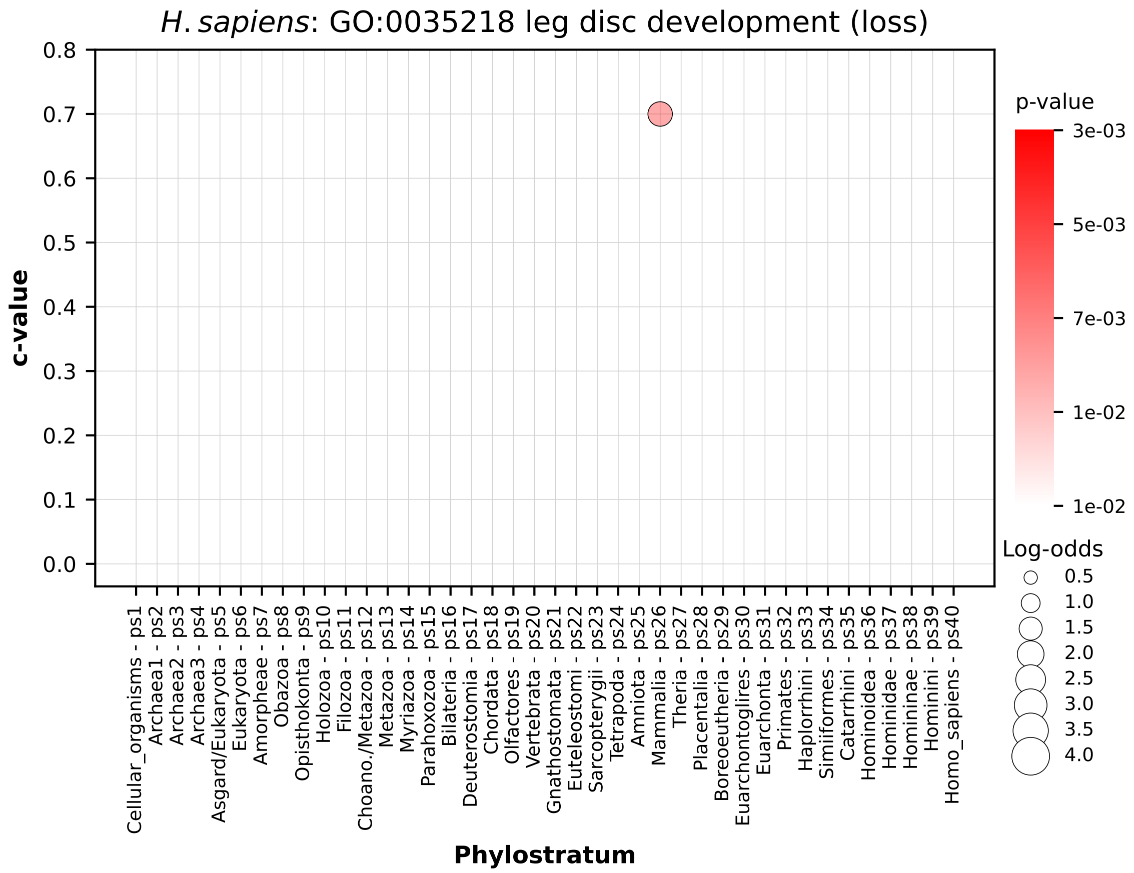

H. sapiens: GO:0035218 leg disc development (loss)
c-value
0.8
0.7
0.6
0.5
0.4
0.3
0.2
0.1
0.0
Phylostratum
Cellular_organisms - ps1
Archaea1 - ps2
Archaea2 - ps3
Archaea3 - ps4
Asgard/Eukaryota - ps5
Eukaryota - ps6
Amorpheae - ps7
Obazoa - ps8
Opisthokonta - ps9
Holozoa - ps10
Filozoa - ps11
Choano./Metazoa - ps12
Metazoa - ps13
Myriazoa - ps14
Parahoxozoa - ps15
Bilateria - ps16
Deuterostomia - ps17
Chordata - ps18
Olfactores - ps19
Vertebrata - ps20
Gnathostomata - ps21
Euteleostomi - ps22
Sarcopterygii - ps23
Tetrapoda - ps24
Amniota - ps25
Mammalia - ps26
Theria - ps27
Placentalia - ps28
Boreoeutheria - ps29
Euarchontoglires - ps30
Euarchonta - ps31
Primates - ps32
Haplorrhini - ps33
Simiiformes - ps34
Catarrhini - ps35
Hominoidea - ps36
Hominidae - ps37
Homininae - ps38
Hominini - ps39
Homo_sapiens - ps40
p-value
3e-03
5e-03
7e-03
1e-02
1e-02
Log-odds
0.5
1.0
1.5
2.0
2.5
3.0
3.5
4.0

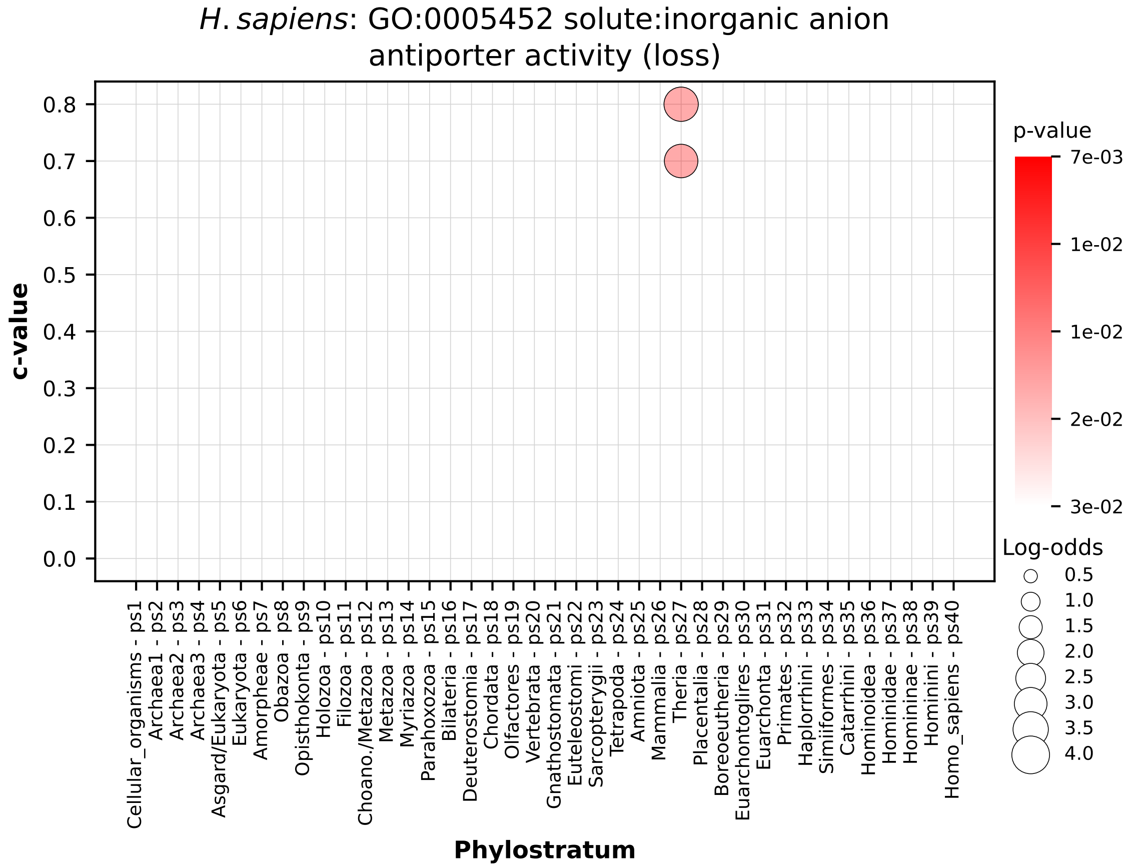

H. sapiens: GO:0005452 solute:inorganic anion antiporter activity (loss)
c-value
0.0
0.1
0.2
0.3
0.4
0.5
0.6
0.7
0.8
Phylostratum
Cellular_organisms - ps1
Archaea1 - ps2
Archaea2 - ps3
Archaea3 - ps4
Asgard/Eukaryota - ps5
Eukaryota - ps6
Amorpheae - ps7
Obazoa - ps8
Opisthokonta - ps9
Holozoa - ps10
Filozoa - ps11
Choano./Metazoa - ps12
Metazoa - ps13
Myriazoa - ps14
Parahoxozoa - ps15
Bilateria - ps16
Deuterostomia - ps17
Chordata - ps18
Olfactores - ps19
Vertebrata - ps20
Gnathostomata - ps21
Euteleostomi - ps22
Sarcopterygii - ps23
Tetrapoda - ps24
Amniota - ps25
Mammalia - ps26
Theria - ps27
Placentalia - ps28
Boreoeutheria - ps29
Euarchontoglires - ps30
Euarchonta - ps31
Primates - ps32
Haplorrhini - ps33
Simiiformes - ps34
Catarrhini - ps35
Hominoidea - ps36
Hominidae - ps37
Homininae - ps38
Hominini - ps39
Homo_sapiens - ps40
p-value
7e-03
1e-02
1e-02
2e-02
3e-02
Log-odds
0.5
1.0
1.5
2.0
2.5
3.0
3.5
4.0

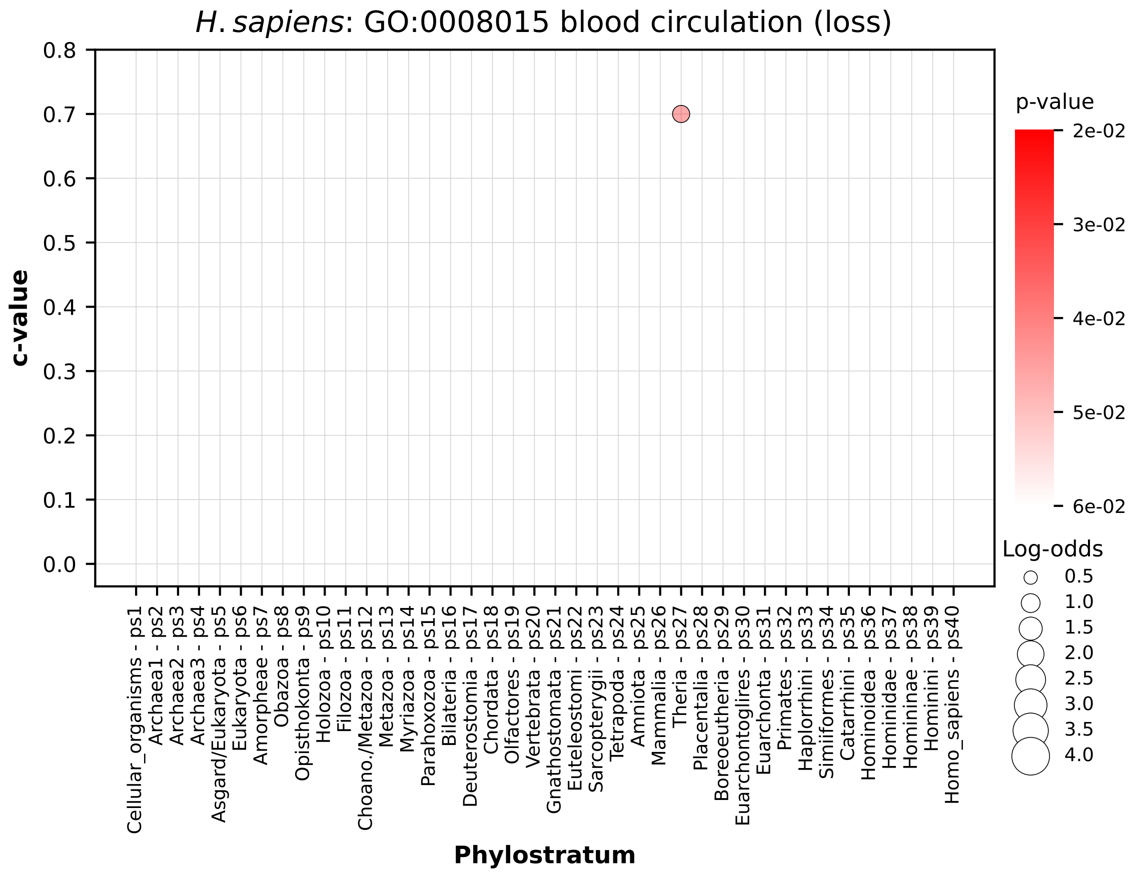

H. sapiens: GO:0008015 blood circulation (loss)
c-value
0.8
0.7
0.6
0.5
0.4
0.3
0.2
0.1
0.0
p-value
2e-02
3e-02
4e-02
5e-02
6e-02
Log-odds
0.5
1.0
1.5
2.0
2.5
3.0
3.5
4.0
Cellular_organisms - ps1
Archaea1 - ps2
Archaea2 - ps3
Archaea3 - ps4
Asgard/Eukaryota - ps5
Eukaryota - ps6
Amorpheae - ps7
Obazoa - ps8
Opisthokonta - ps9
Holozoa - ps10
Filozoa - ps11
Choano./Metazoa - ps12
Metazoa - ps13
Myriazoa - ps14
Parahoxozoa - ps15
Bilateria - ps16
Deuterostomia - ps17
Chordata - ps18
Olfactores - ps19
Vertebrata - ps20
Gnathostomata - ps21
Euteleostomi - ps22
Sarcopterygii - ps23
Tetrapoda - ps24
Amniota - ps25
Mammalia - ps26
Theria - ps27
Placentalia - ps28
Boreoeutheria - ps29
Euarchontoglires - ps30
Euarchonta - ps31
Primates - ps32
Haplorrhini - ps33
Simiiformes - ps34
Catarrhini - ps35
Hominoidea - ps36
Hominidae - ps37
Homininae - ps38
Hominini - ps39
Homo_sapiens - ps40
Phylostratum

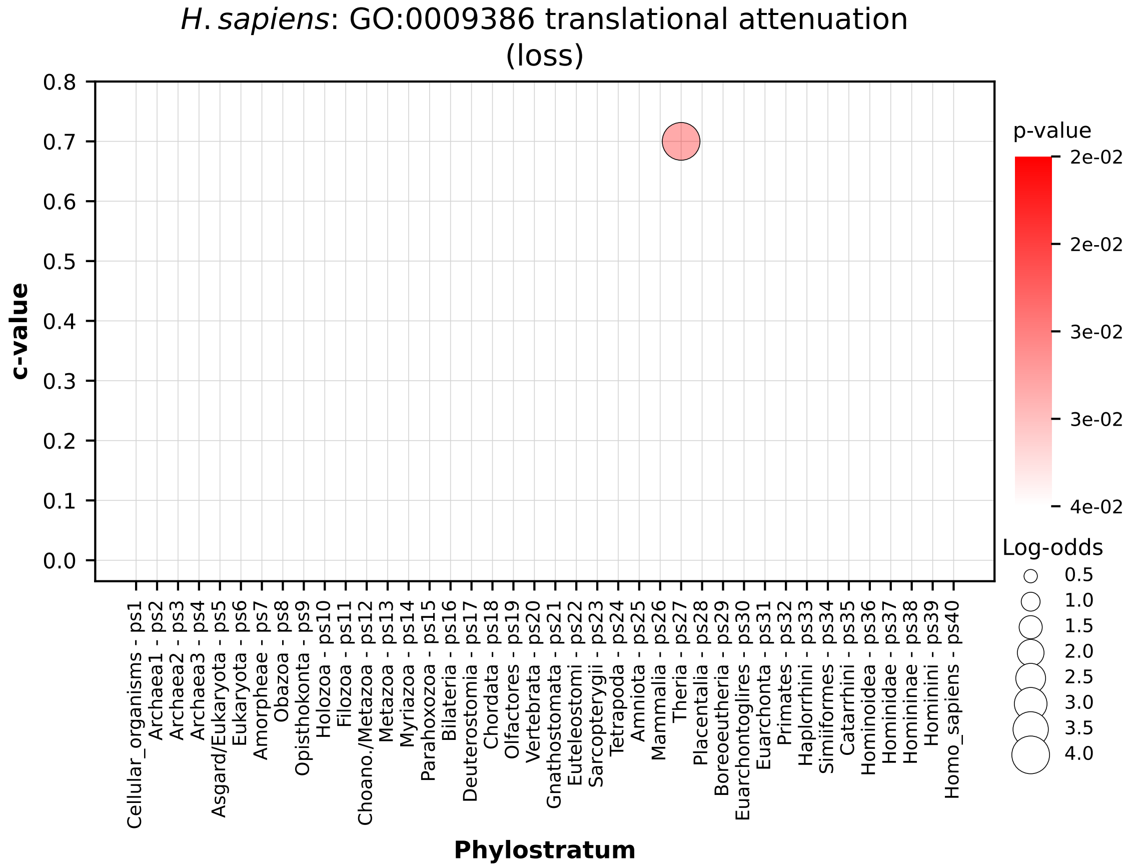

H. sapiens: GO:0009386 translational attenuation
(loss)
c-value
0.8
0.7
0.6
0.5
0.4
0.3
0.2
0.1
0.0
p-value
2e-02
2e-02
3e-02
3e-02
4e-02
Log-odds
0.5
1.0
1.5
2.0
2.5
3.0
3.5
4.0
Cellular_organisms - ps1
Archaea1 - ps2
Archaea2 - ps3
Archaea3 - ps4
Asgard/Eukaryota - ps5
Eukaryota - ps6
Amorpheae - ps7
Obazoa - ps8
Opisthokonta - ps9
Holozoa - ps10
Filozoa - ps11
Choano./Metazoa - ps12
Metazoa - ps13
Myriazoa - ps14
Parahoxozoa - ps15
Bilateria - ps16
Deuterostomia - ps17
Chordata - ps18
Olfactores - ps19
Vertebrata - ps20
Gnathostomata - ps21
Euteleostomi - ps22
Sarcopterygii - ps23
Tetrapoda - ps24
Amniota - ps25
Mammalia - ps26
Theria - ps27
Placentalia - ps28
Boreoeutheria - ps29
Euarchontoglires - ps30
Euarchonta - ps31
Primates - ps32
Haplorrhini - ps33
Simiiformes - ps34
Catarrhini - ps35
Hominoidea - ps36
Hominidae - ps37
Homininae - ps38
Hominini - ps39
Homo_sapiens - ps40
Phylostratum

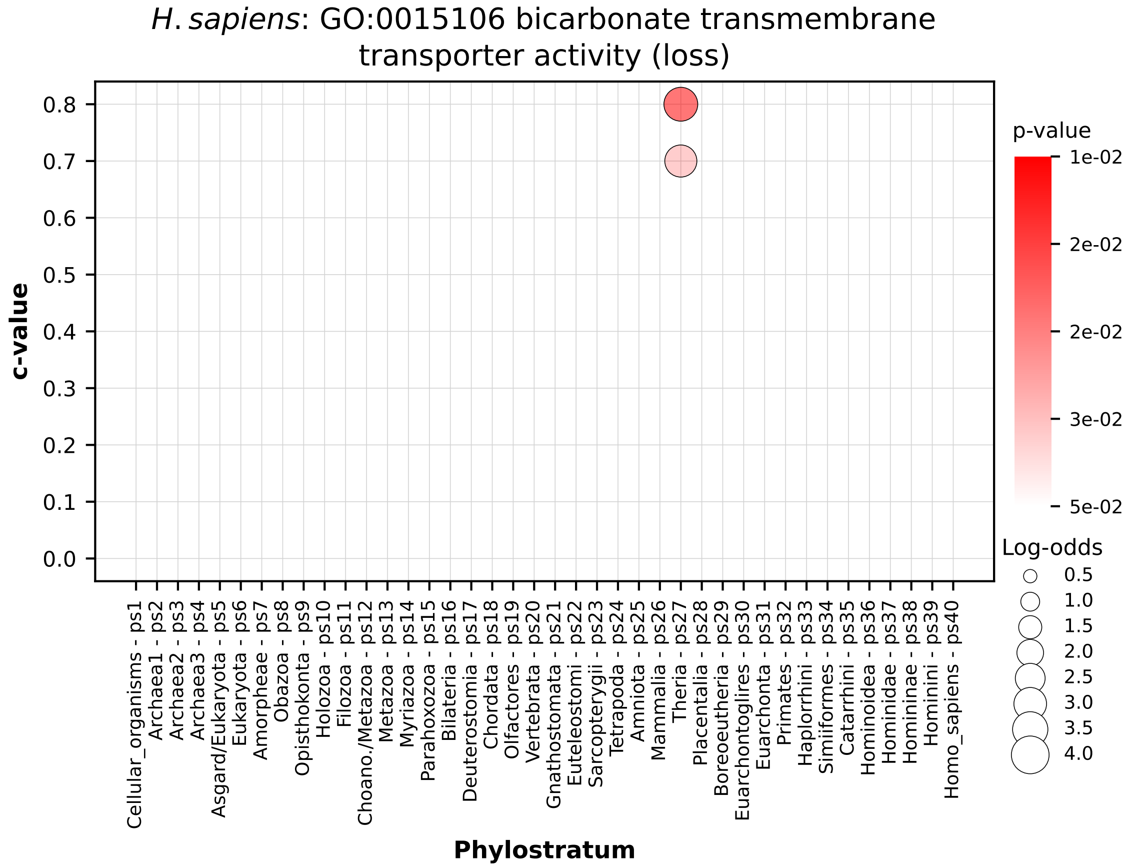
H. sapiens: GO:0015106 bicarbonate transmembrane transporter activity (loss)
c-value
0.8
0.7
0.6
0.5
0.4
0.3
0.2
0.1
0.0
Phylostratum
Cellular_organisms - ps1
Archaea1 - ps2
Archaea2 - ps3
Archaea3 - ps4
Asgard/Eukaryota - ps5
Eukaryota - ps6
Amorpheae - ps7
Obazoa - ps8
Opisthokonta - ps9
Holozoa - ps10
Filozoa - ps11
Choano./Metazoa - ps12
Metazoa - ps13
Myriazoa - ps14
Parahoxozoa - ps15
Bilateria - ps16
Deuterostomia - ps17
Chordata - ps18
Olfactores - ps19
Vertebrata - ps20
Gnathostomata - ps21
Euteleostomi - ps22
Sarcopterygii - ps23
Tetrapoda - ps24
Amniota - ps25
Mammalia - ps26
Theria - ps27
Placentalia - ps28
Boreoeutheria - ps29
Euarchontoglires - ps30
Euarchonta - ps31
Primates - ps32
Haplorrhini - ps33
Simiiformes - ps34
Catarrhini - ps35
Hominoidea - ps36
Hominidae - ps37
Homininae - ps38
Hominini - ps39
Homo_sapiens - ps40
p-value
1e-02
2e-02
2e-02
3e-02
5e-02
Log-odds
0.5
1.0
1.5
2.0
2.5
3.0
3.5
4.0

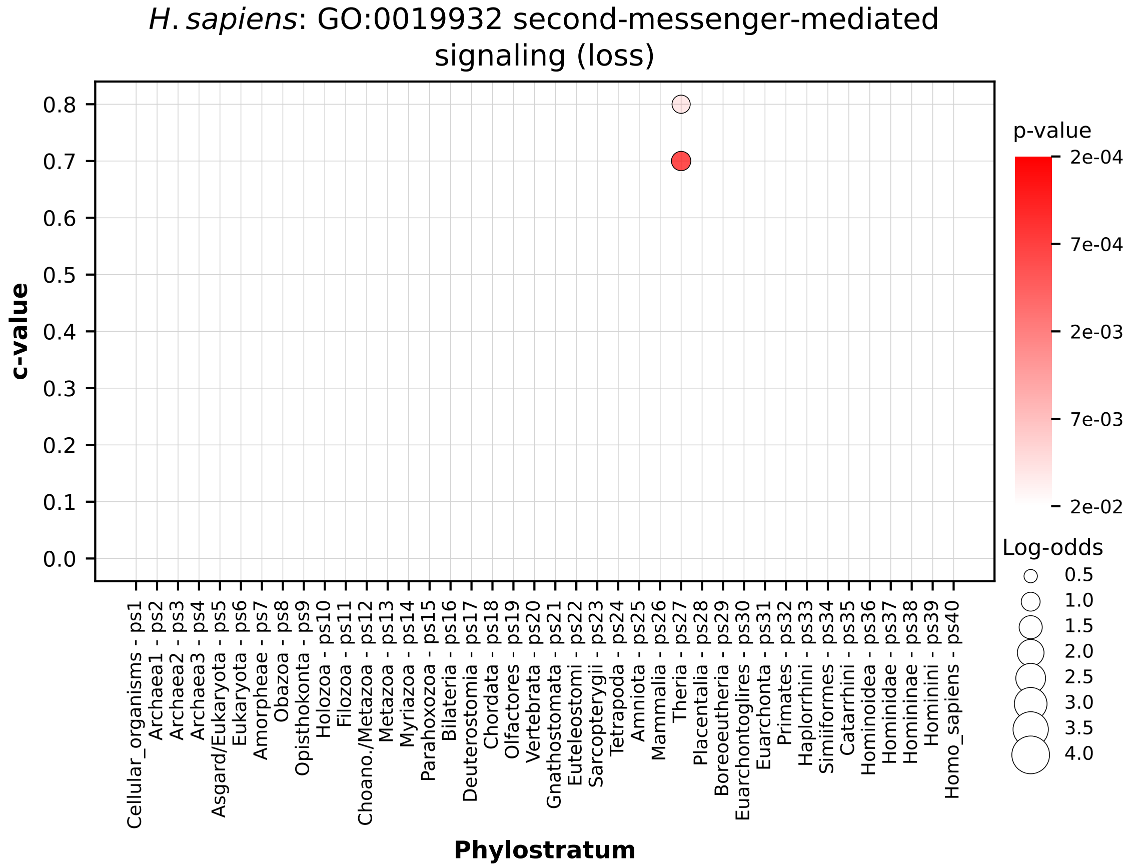
H. sapiens: GO:0019932 second-messenger-mediated signaling (loss)
c-value
Phylostratum
p-value
2e-04
7e-04
2e-03
7e-03
2e-02
Log-odds
0.5
1.0
1.5
2.0
2.5
3.0
3.5
4.0
Cellular_organisms - ps1
Archaea1 - ps2
Archaea2 - ps3
Archaea3 - ps4
Asgard/Eukaryota - ps5
Eukaryota - ps6
Amorpheae - ps7
Obazoa - ps8
Opisthokonta - ps9
Holozoa - ps10
Filozoa - ps11
Choano./Metazoa - ps12
Metazoa - ps13
Myriazoa - ps14
Parahoxozoa - ps15
Bilateria - ps16
Deuterostomia - ps17
Chordata - ps18
Olfactores - ps19
Vertebrata - ps20
Gnathostomata - ps21
Euteleostomi - ps22
Sarcopterygii - ps23
Tetrapoda - ps24
Amniota - ps25
Mammalia - ps26
Theria - ps27
Placentalia - ps28
Boreoeutheria - ps29
Euarchontoglires - ps30
Euarchonta - ps31
Primates - ps32
Haplorrhini - ps33
Simiiformes - ps34
Catarrhini - ps35
Hominoidea - ps36
Hominidae - ps37
Homininae - ps38
Hominini - ps39
Homo_sapiens - ps40

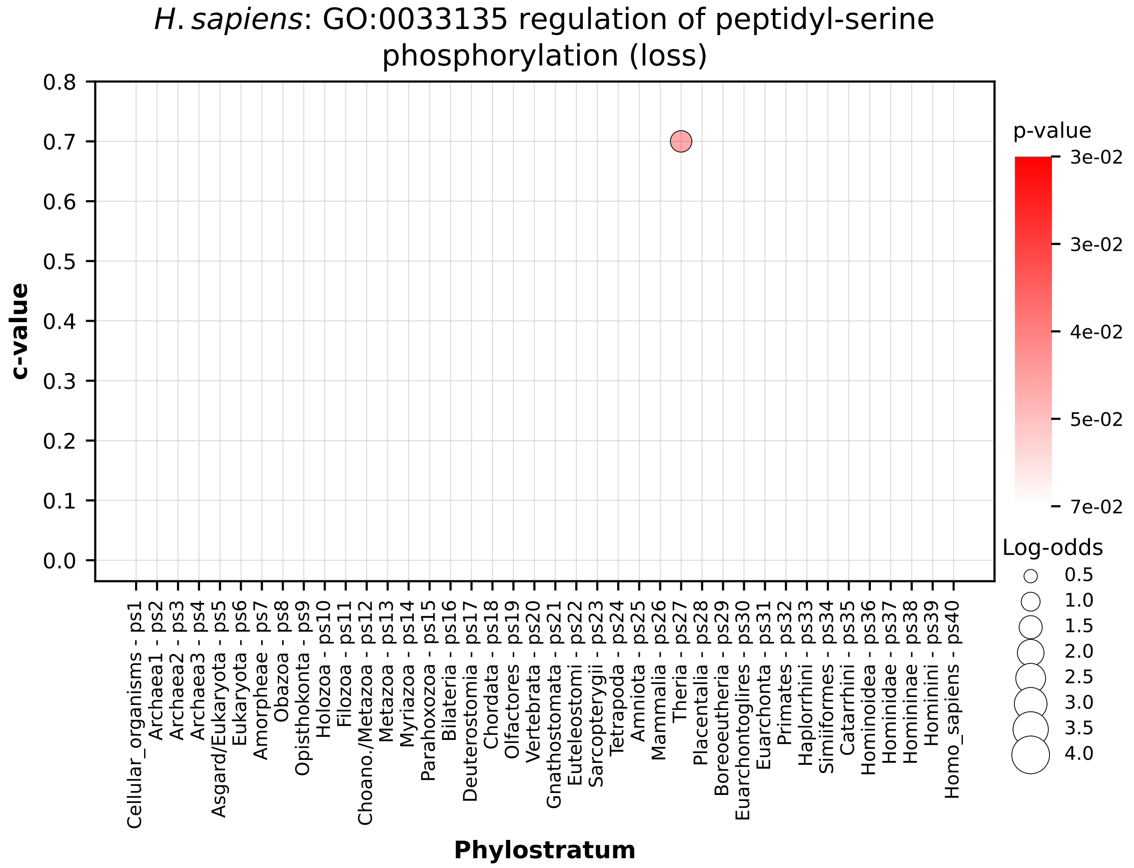

H. sapiens: GO:0033135 regulation of peptidyl-serine phosphorylation (loss)
c-value
0.8
0.7
0.6
0.5
0.4
0.3
0.2
0.1
0.0
p-value
3e-02
3e-02
4e-02
5e-02
7e-02
Log-odds
0.5
1.0
1.5
2.0
2.5
3.0
3.5
4.0
Cellular_organisms - ps1
Archaea1 - ps2
Archaea2 - ps3
Archaea3 - ps4
Asgard/Eukaryota - ps5
Eukaryota - ps6
Amorpheae - ps7
Obazoa - ps8
Opisthokonta - ps9
Holozoa - ps10
Filozoa - ps11
Choano./Metazoa - ps12
Metazoa - ps13
Myriazoa - ps14
Parahoxozoa - ps15
Bilateria - ps16
Deuterostomia - ps17
Chordata - ps18
Olfactores - ps19
Vertebrata - ps20
Gnathostomata - ps21
Euteleostomi - ps22
Sarcopterygii - ps23
Tetrapoda - ps24
Amniota - ps25
Mammalia - ps26
Theria - ps27
Placentalia - ps28
Boreoeutheria - ps29
Euarchontoglires - ps30
Euarchonta - ps31
Primates - ps32
Haplorrhini - ps33
Simiiformes - ps34
Catarrhini - ps35
Hominoidea - ps36
Hominidae - ps37
Homininae - ps38
Hominini - ps39
Homo_sapiens - ps40
Phylostratum

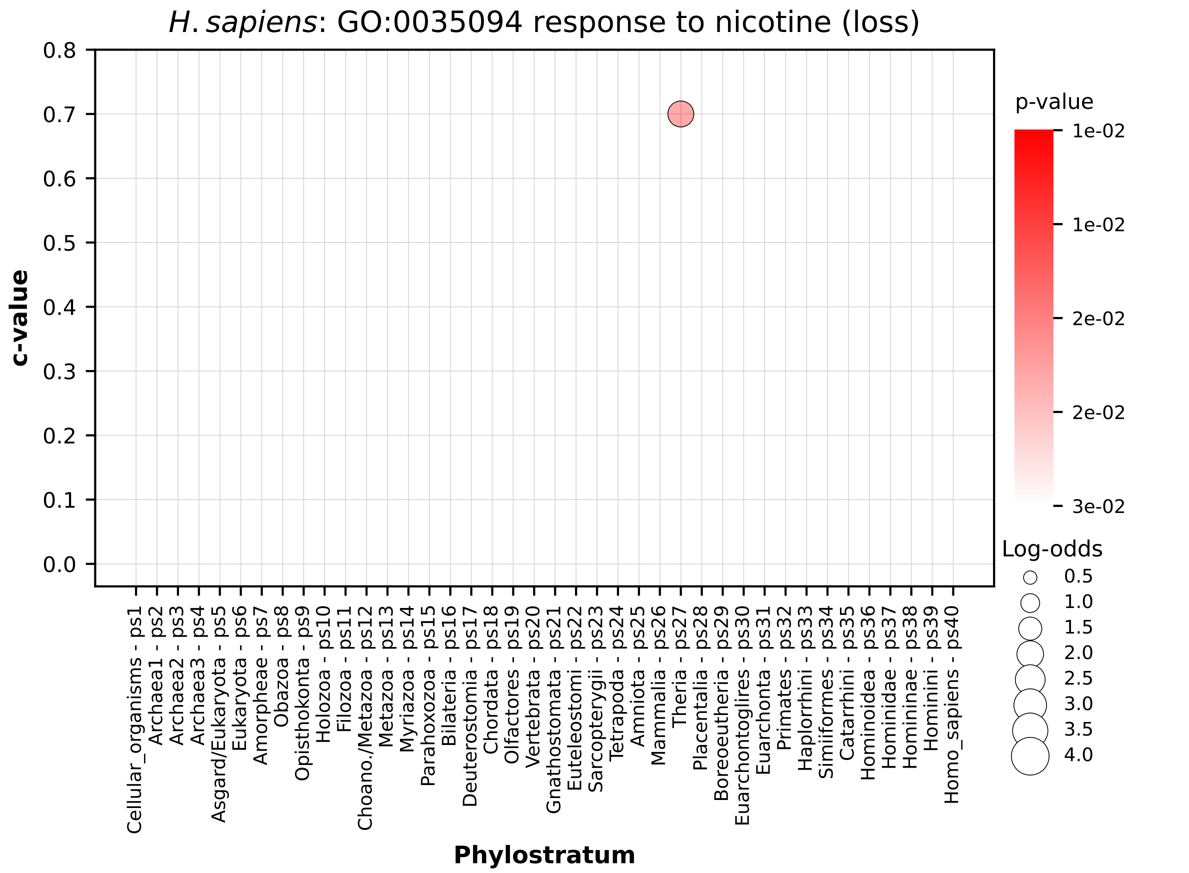

H. sapiens: GO:0035094 response to nicotine (loss)
c-value
0.8
0.7
0.6
0.5
0.4
0.3
0.2
0.1
0.0
p-value
1e-02
1e-02
2e-02
2e-02
3e-02
Log-odds
0.5
1.0
1.5
2.0
2.5
3.0
3.5
4.0
Cellular_organisms - ps1
Archaea1 - ps2
Archaea2 - ps3
Archaea3 - ps4
Asgard/Eukaryota - ps5
Eukaryota - ps6
Amorpheae - ps7
Obazoa - ps8
Opisthokonta - ps9
Holozoa - ps10
Filozoa - ps11
Choano./Metazoa - ps12
Metazoa - ps13
Myriazoa - ps14
Parahoxozoa - ps15
Bilateria - ps16
Deuterostomia - ps17
Chordata - ps18
Olfactores - ps19
Vertebrata - ps20
Gnathostomata - ps21
Euteleostomi - ps22
Sarcopterygii - ps23
Tetrapoda - ps24
Amniota - ps25
Mammalia - ps26
Theria - ps27
Placentalia - ps28
Boreoeutheria - ps29
Euarchontoglires - ps30
Euarchonta - ps31
Primates - ps32
Haplorrhini - ps33
Simiiformes - ps34
Catarrhini - ps35
Hominoidea - ps36
Hominidae - ps37
Homininae - ps38
Hominini - ps39
Homo_sapiens - ps40
Phylostratum

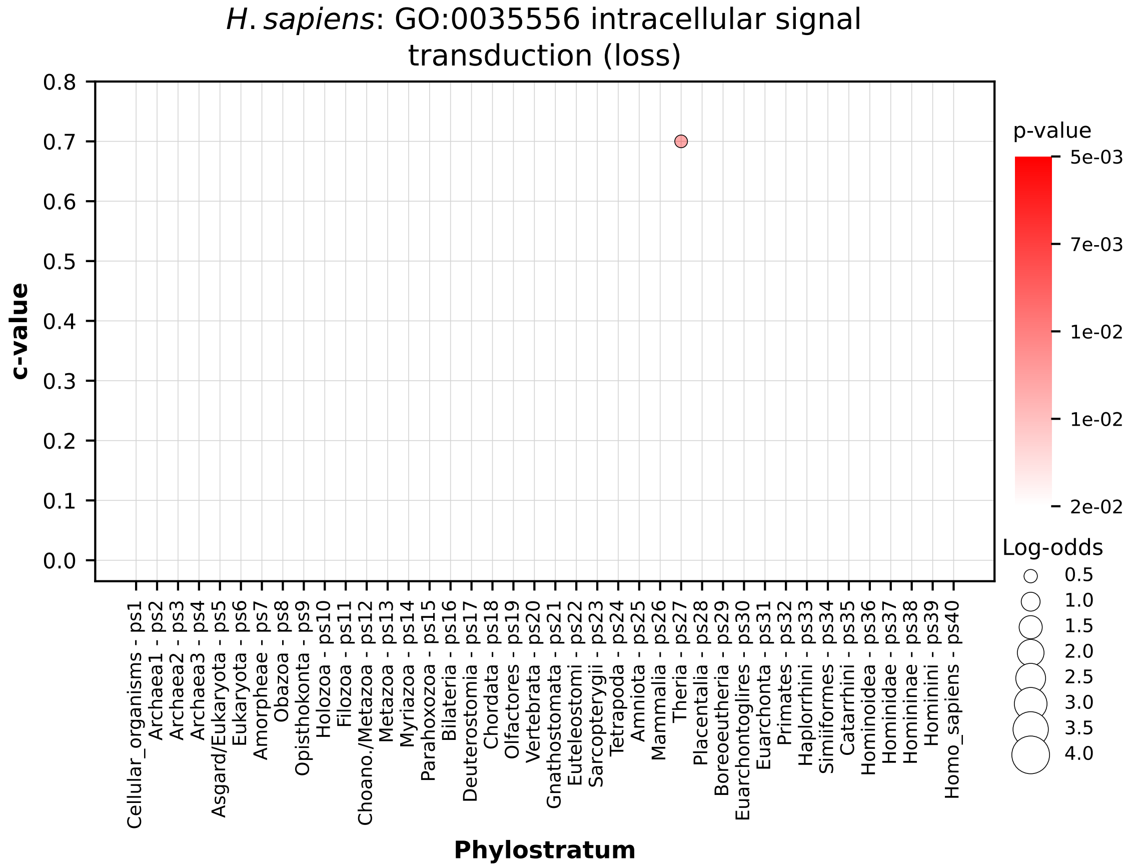
H. sapiens: GO:0035556 intracellular signal transduction (loss)
c-value
0.8
0.7
0.6
0.5
0.4
0.3
0.2
0.1
0.0
p-value
5e-03
7e-03
1e-02
1e-02
2e-02
Log-odds
0.5
1.0
1.5
2.0
2.5
3.0
3.5
4.0
Cellular_organisms - ps1
Archaea1 - ps2
Archaea2 - ps3
Archaea3 - ps4
Asgard/Eukaryota - ps5
Eukaryota - ps6
Amorpheae - ps7
Obazoa - ps8
Opisthokonta - ps9
Holozoa - ps10
Filozoa - ps11
Choano./Metazoa - ps12
Metazoa - ps13
Myriazoa - ps14
Parahoxozoa - ps15
Bilateria - ps16
Deuterostomia - ps17
Chordata - ps18
Olfactores - ps19
Vertebrata - ps20
Gnathostomata - ps21
Euteleostomi - ps22
Sarcopterygii - ps23
Tetrapoda - ps24
Amniota - ps25
Mammalia - ps26
Theria - ps27
Placentalia - ps28
Boreoeutheria - ps29
Euarchontoglires - ps30
Euarchonta - ps31
Primates - ps32
Haplorrhini - ps33
Simiiformes - ps34
Catarrhini - ps35
Hominoidea - ps36
Hominidae - ps37
Homininae - ps38
Hominini - ps39
Homo_sapiens - ps40
Phylostratum

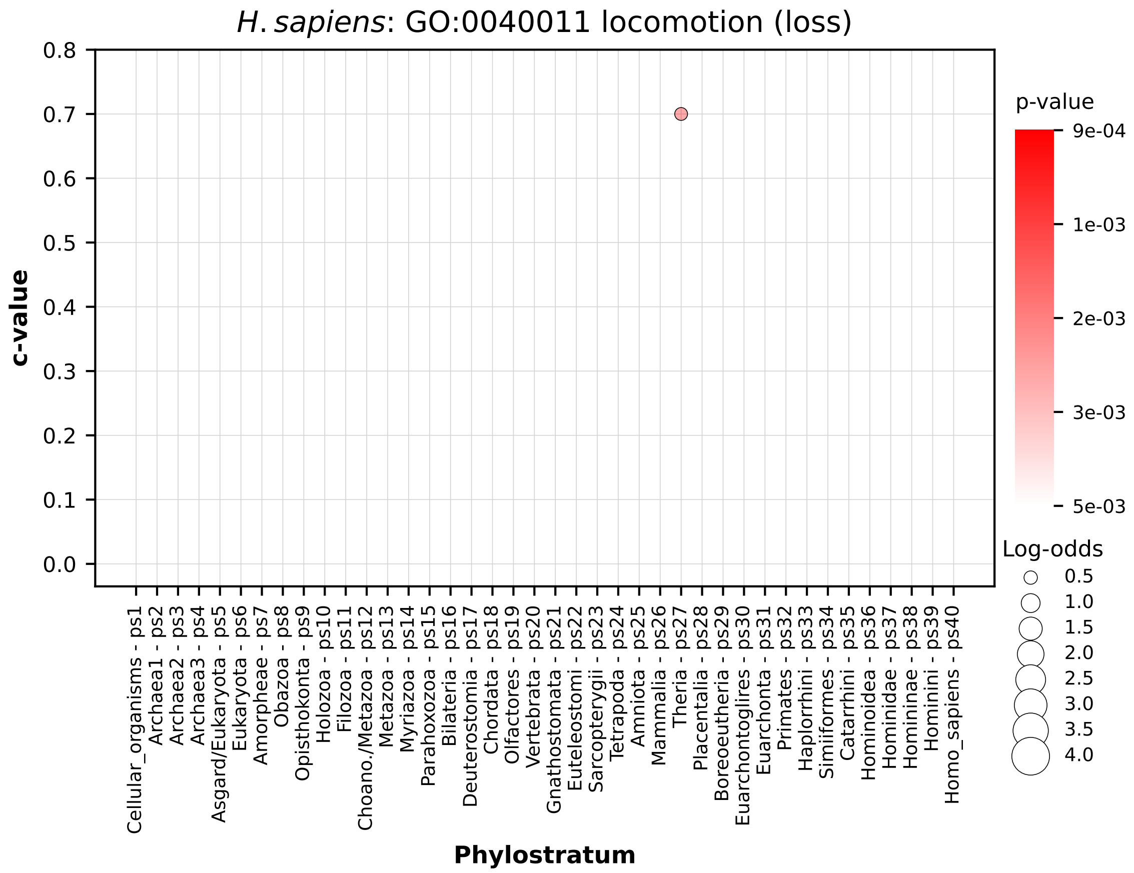

H. sapiens: GO:0040011 locomotion (loss)
c-value
0.0
0.1
0.2
0.3
0.4
0.5
0.6
0.7
0.8
Phylostratum
Cellular_organisms - ps1
Archaea1 - ps2
Archaea2 - ps3
Archaea3 - ps4
Asgard/Eukaryota - ps5
Eukaryota - ps6
Amorpheae - ps7
Obazoa - ps8
Opisthokonta - ps9
Holozoa - ps10
Filozoa - ps11
Choano./Metazoa - ps12
Metazoa - ps13
Myriazoa - ps14
Parahoxozoa - ps15
Bilateria - ps16
Deuterostomia - ps17
Chordata - ps18
Olfactores - ps19
Vertebrata - ps20
Gnathostomata - ps21
Euteleostomi - ps22
Sarcopterygii - ps23
Tetrapoda - ps24
Amniota - ps25
Mammalia - ps26
Theria - ps27
Placentalia - ps28
Boreoeutheria - ps29
Euarchontoglires - ps30
Euarchonta - ps31
Primates - ps32
Haplorrhini - ps33
Simiiformes - ps34
Catarrhini - ps35
Hominoidea - ps36
Hominidae - ps37
Homininae - ps38
Hominini - ps39
Homo_sapiens - ps40
p-value
9e-04
1e-03
2e-03
3e-03
5e-03
Log-odds
0.5
1.0
1.5
2.0
2.5
3.0
3.5
4.0

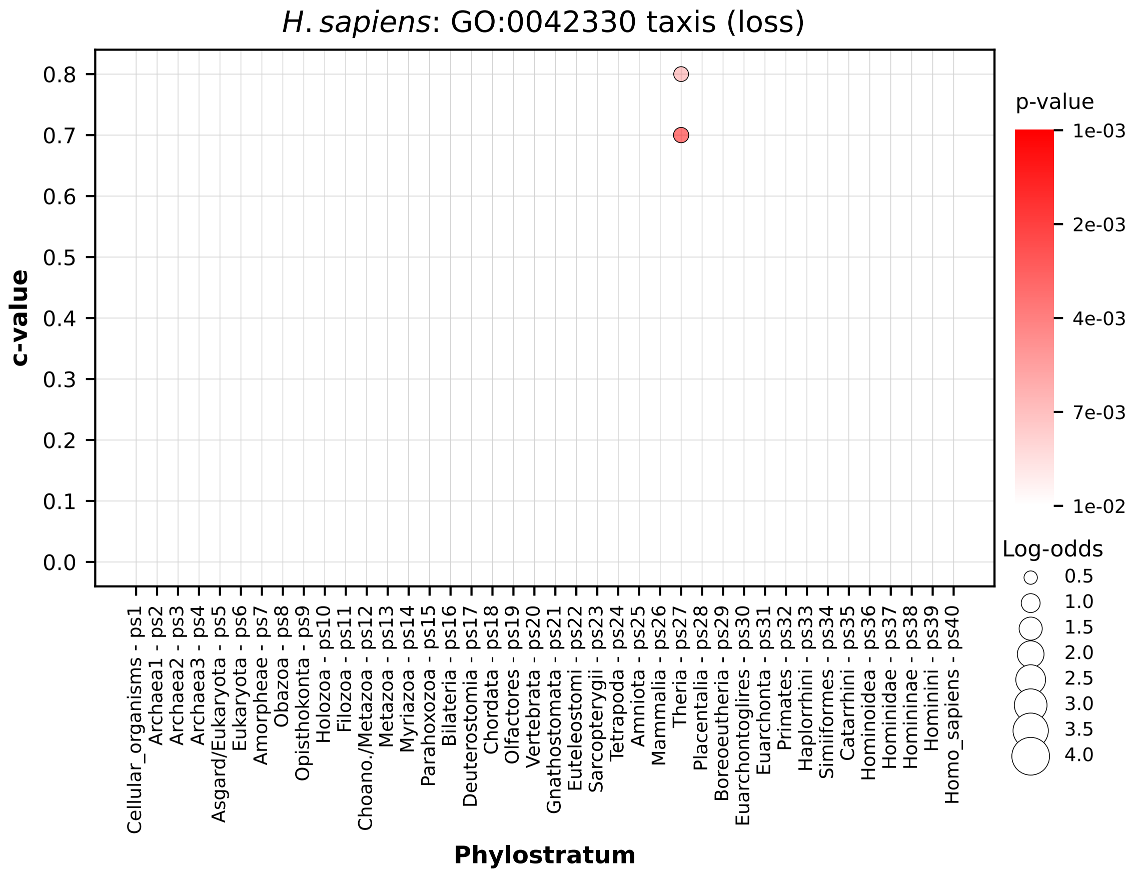

H. sapiens: GO:0042330 taxis (loss)
c-value
0.0
0.1
0.2
0.3
0.4
0.5
0.6
0.7
0.8
Phylostratum
Cellular_organisms - ps1
Archaea1 - ps2
Archaea2 - ps3
Archaea3 - ps4
Asgard/Eukaryota - ps5
Eukaryota - ps6
Amorpheae - ps7
Obazoa - ps8
Opisthokonta - ps9
Holozoa - ps10
Filozoa - ps11
Choano./Metazoa - ps12
Metazoa - ps13
Myriazoa - ps14
Parahoxozoa - ps15
Bilateria - ps16
Deuterostomia - ps17
Chordata - ps18
Olfactores - ps19
Vertebrata - ps20
Gnathostomata - ps21
Euteleostomi - ps22
Sarcopterygii - ps23
Tetrapoda - ps24
Amniota - ps25
Mammalia - ps26
Theria - ps27
Placentalia - ps28
Boreoeutheria - ps29
Euarchontoglires - ps30
Euarchonta - ps31
Primates - ps32
Haplorrhini - ps33
Simiiformes - ps34
Catarrhini - ps35
Hominoidea - ps36
Hominidae - ps37
Homininae - ps38
Hominini - ps39
Homo_sapiens - ps40
p-value
1e-03
2e-03
4e-03
7e-03
1e-02
Log-odds
0.5
1.0
1.5
2.0
2.5
3.0
3.5
4.0

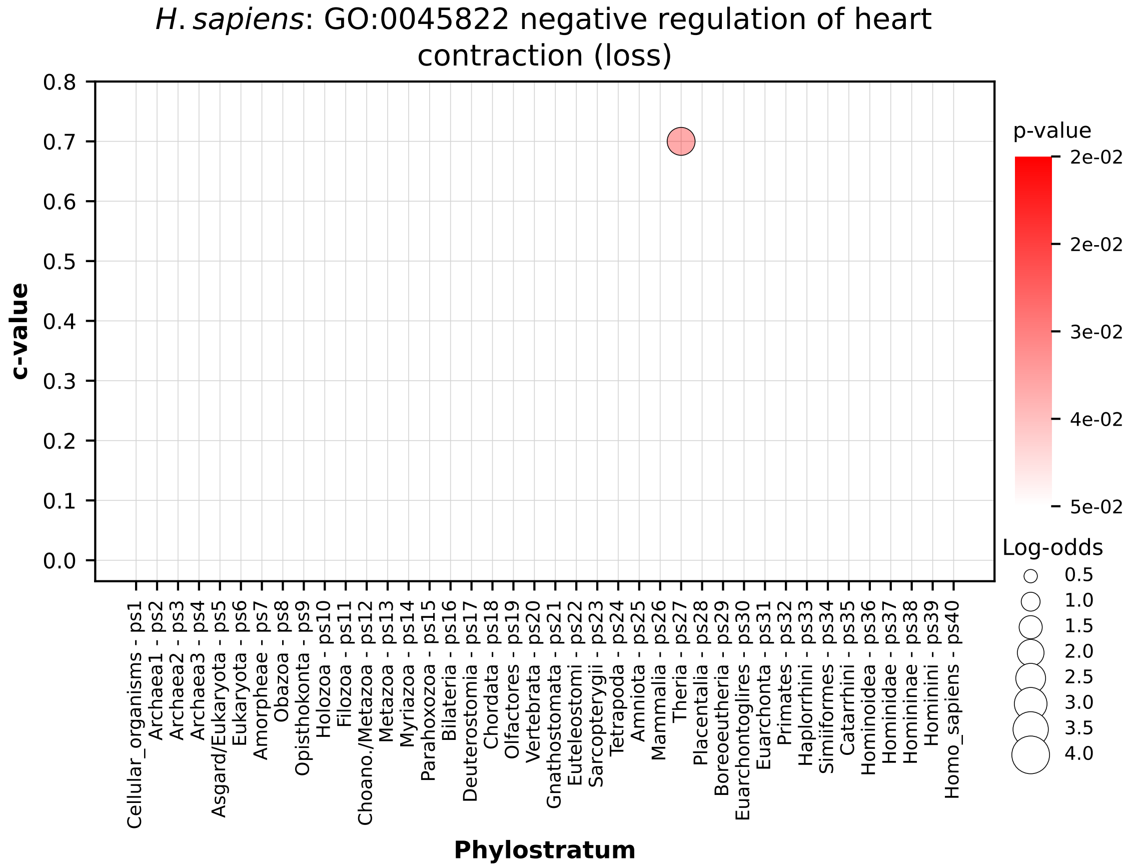
H. sapiens: GO:0045822 negative regulation of heart contraction (loss)
c-value
0.8
0.7
0.6
0.5
0.4
0.3
0.2
0.1
0.0
p-value
2e-02
2e-02
3e-02
4e-02
5e-02
Log-odds
0.5
1.0
1.5
2.0
2.5
3.0
3.5
4.0
Cellular_organisms - ps1
Archaea1 - ps2
Archaea2 - ps3
Archaea3 - ps4
Asgard/Eukaryota - ps5
Eukaryota - ps6
Amorpheae - ps7
Obazoa - ps8
Opisthokonta - ps9
Holozoa - ps10
Filozoa - ps11
Choano./Metazoa - ps12
Metazoa - ps13
Myriazoa - ps14
Parahoxozoa - ps15
Bilateria - ps16
Deuterostomia - ps17
Chordata - ps18
Olfactores - ps19
Vertebrata - ps20
Gnathostomata - ps21
Euteleostomi - ps22
Sarcopterygii - ps23
Tetrapoda - ps24
Amniota - ps25
Mammalia - ps26
Theria - ps27
Placentalia - ps28
Boreoeutheria - ps29
Euarchontoglires - ps30
Euarchonta - ps31
Primates - ps32
Haplorrhini - ps33
Simiiformes - ps34
Catarrhini - ps35
Hominoidea - ps36
Hominidae - ps37
Homininae - ps38
Hominini - ps39
Homo_sapiens - ps40
Phylostratum

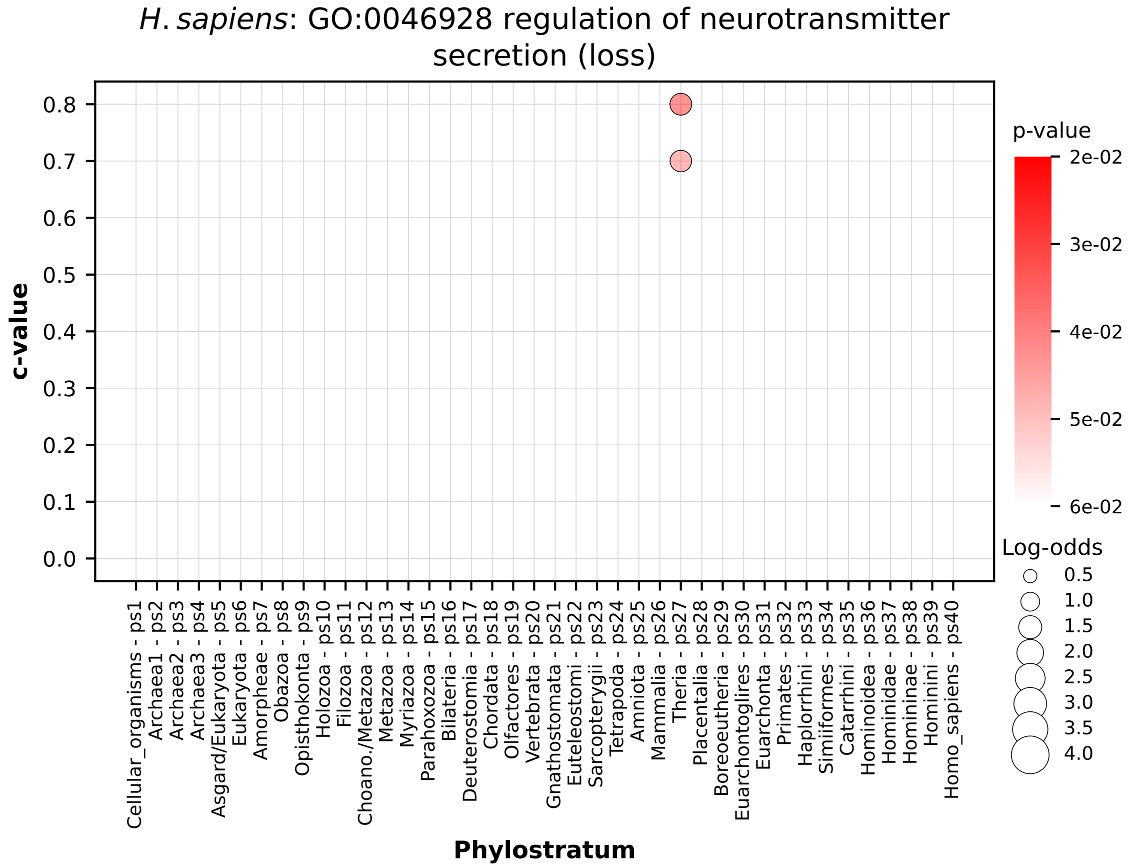

H. sapiens: GO:0046928 regulation of neurotransmitter secretion (loss)
c-value
0.8
0.7
0.6
0.5
0.4
0.3
0.2
0.1
0.0
p-value
2e-02
3e-02
4e-02
5e-02
6e-02
Log-odds
0.5
1.0
1.5
2.0
2.5
3.0
3.5
4.0
Cellular_organisms - ps1
Archaea1 - ps2
Archaea2 - ps3
Archaea3 - ps4
Asgard/Eukaryota - ps5
Eukaryota - ps6
Amorpheae - ps7
Obazoa - ps8
Opisthokonta - ps9
Holozoa - ps10
Filozoa - ps11
Choano./Metazoa - ps12
Metazoa - ps13
Myriazoa - ps14
Parahoxozoa - ps15
Bilateria - ps16
Deuterostomia - ps17
Chordata - ps18
Olfactores - ps19
Vertebrata - ps20
Gnathostomata - ps21
Euteleostomi - ps22
Sarcopterygii - ps23
Tetrapoda - ps24
Amniota - ps25
Mammalia - ps26
Theria - ps27
Placentalia - ps28
Boreoeutheria - ps29
Euarchontoglires - ps30
Euarchonta - ps31
Primates - ps32
Haplorrhini - ps33
Simiiformes - ps34
Catarrhini - ps35
Hominoidea - ps36
Hominidae - ps37
Homininae - ps38
Hominini - ps39
Homo_sapiens - ps40
Phylostratum

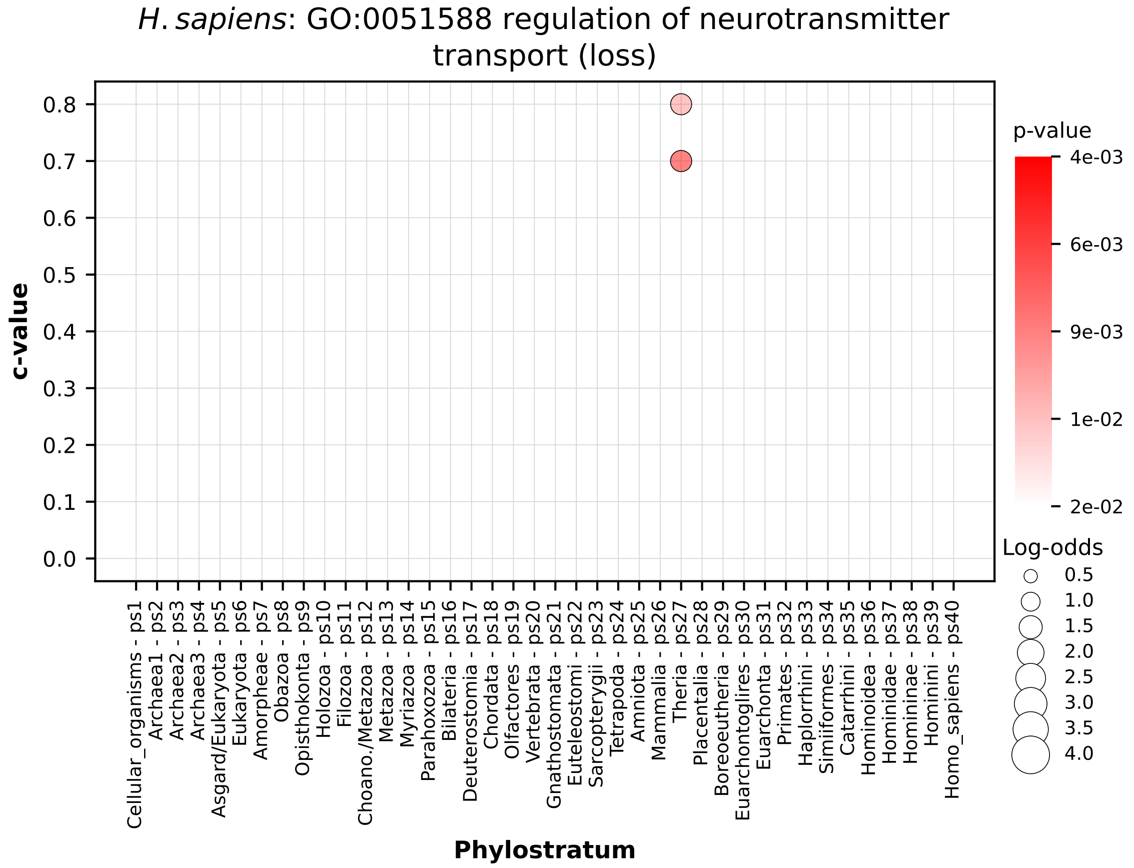

H. sapiens: GO:0051588 regulation of neurotransmitter transport (loss)
c-value
0.8
0.7
0.6
0.5
0.4
0.3
0.2
0.1
0.0
p-value
4e-03
6e-03
9e-03
1e-02
2e-02
Log-odds
0.5
1.0
1.5
2.0
2.5
3.0
3.5
4.0
Cellular_organisms - ps1
Archaea1 - ps2
Archaea2 - ps3
Archaea3 - ps4
Asgard/Eukaryota - ps5
Eukaryota - ps6
Amorpheae - ps7
Obazoa - ps8
Opisthokonta - ps9
Holozoa - ps10
Filozoa - ps11
Choano./Metazoa - ps12
Metazoa - ps13
Myriazoa - ps14
Parahoxozoa - ps15
Bilateria - ps16
Deuterostomia - ps17
Chordata - ps18
Olfactores - ps19
Vertebrata - ps20
Gnathostomata - ps21
Euteleostomi - ps22
Sarcopterygii - ps23
Tetrapoda - ps24
Amniota - ps25
Mammalia - ps26
Theria - ps27
Placentalia - ps28
Boreoeutheria - ps29
Euarchontoglires - ps30
Euarchonta - ps31
Primates - ps32
Haplorrhini - ps33
Simiiformes - ps34
Catarrhini - ps35
Hominoidea - ps36
Hominidae - ps37
Homininae - ps38
Hominini - ps39
Homo_sapiens - ps40
Phylostratum

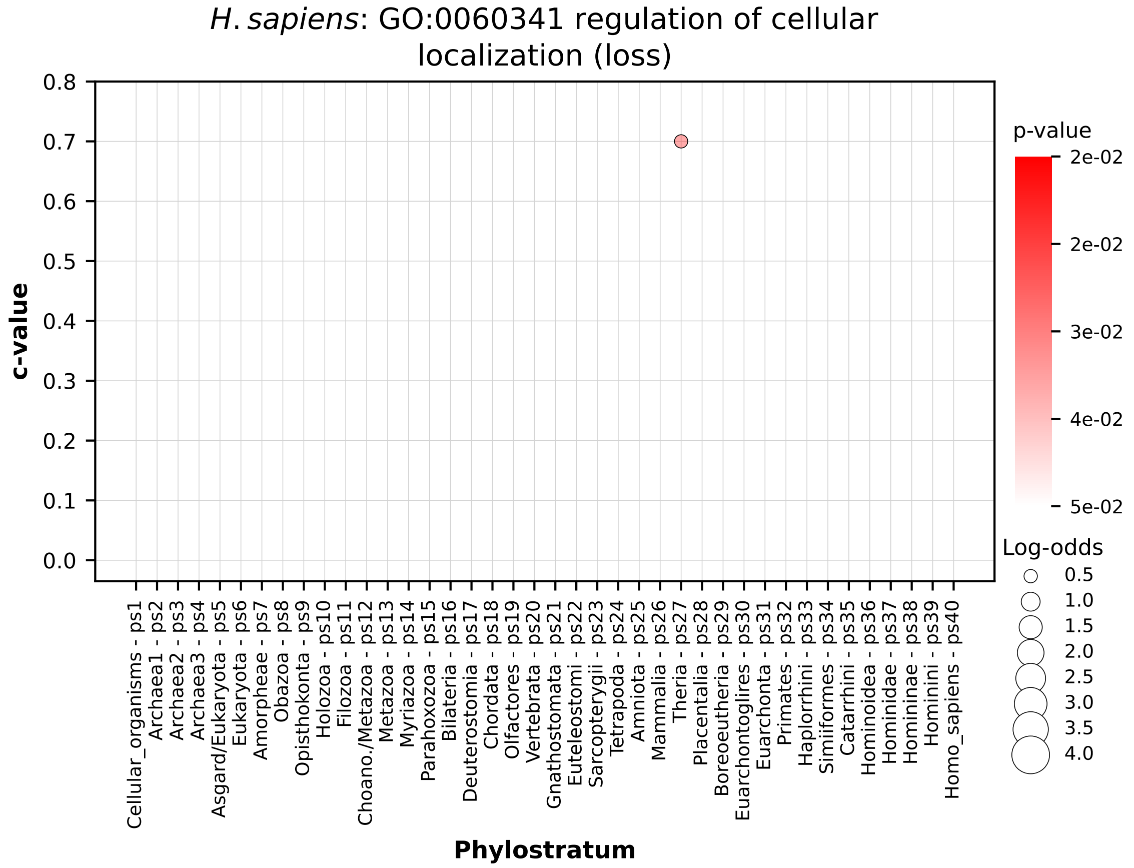

H. sapiens: GO:0060341 regulation of cellular localization (loss)
c-value
0.8
0.7
0.6
0.5
0.4
0.3
0.2
0.1
0.0
p-value
2e-02
2e-02
3e-02
4e-02
5e-02
Log-odds
0.5
1.0
1.5
2.0
2.5
3.0
3.5
4.0
Cellular_organisms - ps1
Archaea1 - ps2
Archaea2 - ps3
Archaea3 - ps4
Asgard/Eukaryota - ps5
Eukaryota - ps6
Amorpheae - ps7
Obazoa - ps8
Opisthokonta - ps9
Holozoa - ps10
Filozoa - ps11
Choano./Metazoa - ps12
Metazoa - ps13
Myriazoa - ps14
Parahoxozoa - ps15
Bilateria - ps16
Deuterostomia - ps17
Chordata - ps18
Olfactores - ps19
Vertebrata - ps20
Gnathostomata - ps21
Euteleostomi - ps22
Sarcopterygii - ps23
Tetrapoda - ps24
Amniota - ps25
Mammalia - ps26
Theria - ps27
Placentalia - ps28
Boreoeutheria - ps29
Euarchontoglires - ps30
Euarchonta - ps31
Primates - ps32
Haplorrhini - ps33
Simiiformes - ps34
Catarrhini - ps35
Hominoidea - ps36
Hominidae - ps37
Homininae - ps38
Hominini - ps39
Homo_sapiens - ps40
Phylostratum

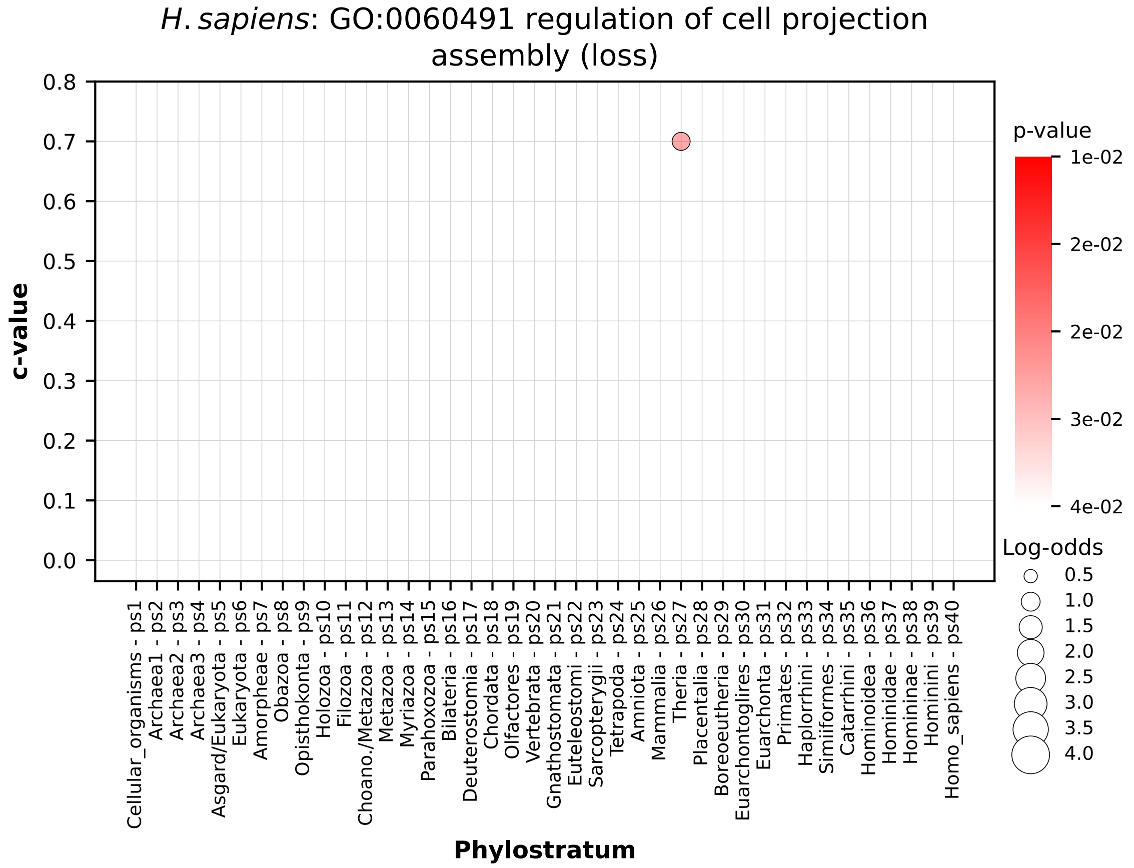

H. sapiens: GO:0060491 regulation of cell projection assembly (loss)
c-value
0.8
0.7
0.6
0.5
0.4
0.3
0.2
0.1
0.0
p-value
1e-02
2e-02
2e-02
3e-02
4e-02
Log-odds
0.5
1.0
1.5
2.0
2.5
3.0
3.5
4.0
Cellular_organisms - ps1
Archaea1 - ps2
Archaea2 - ps3
Archaea3 - ps4
Asgard/Eukaryota - ps5
Eukaryota - ps6
Amorpheae - ps7
Obazoa - ps8
Opisthokonta - ps9
Holozoa - ps10
Filozoa - ps11
Choano./Metazoa - ps12
Metazoa - ps13
Myriazoa - ps14
Parahoxozoa - ps15
Bilateria - ps16
Deuterostomia - ps17
Chordata - ps18
Olfactores - ps19
Vertebrata - ps20
Gnathostomata - ps21
Euteleostomi - ps22
Sarcopterygii - ps23
Tetrapoda - ps24
Amniota - ps25
Mammalia - ps26
Theria - ps27
Placentalia - ps28
Boreoeutheria - ps29
Euarchontoglires - ps30
Euarchonta - ps31
Primates - ps32
Haplorrhini - ps33
Simiiformes - ps34
Catarrhini - ps35
Hominoidea - ps36
Hominidae - ps37
Homininae - ps38
Hominini - ps39
Homo_sapiens - ps40
Phylostratum

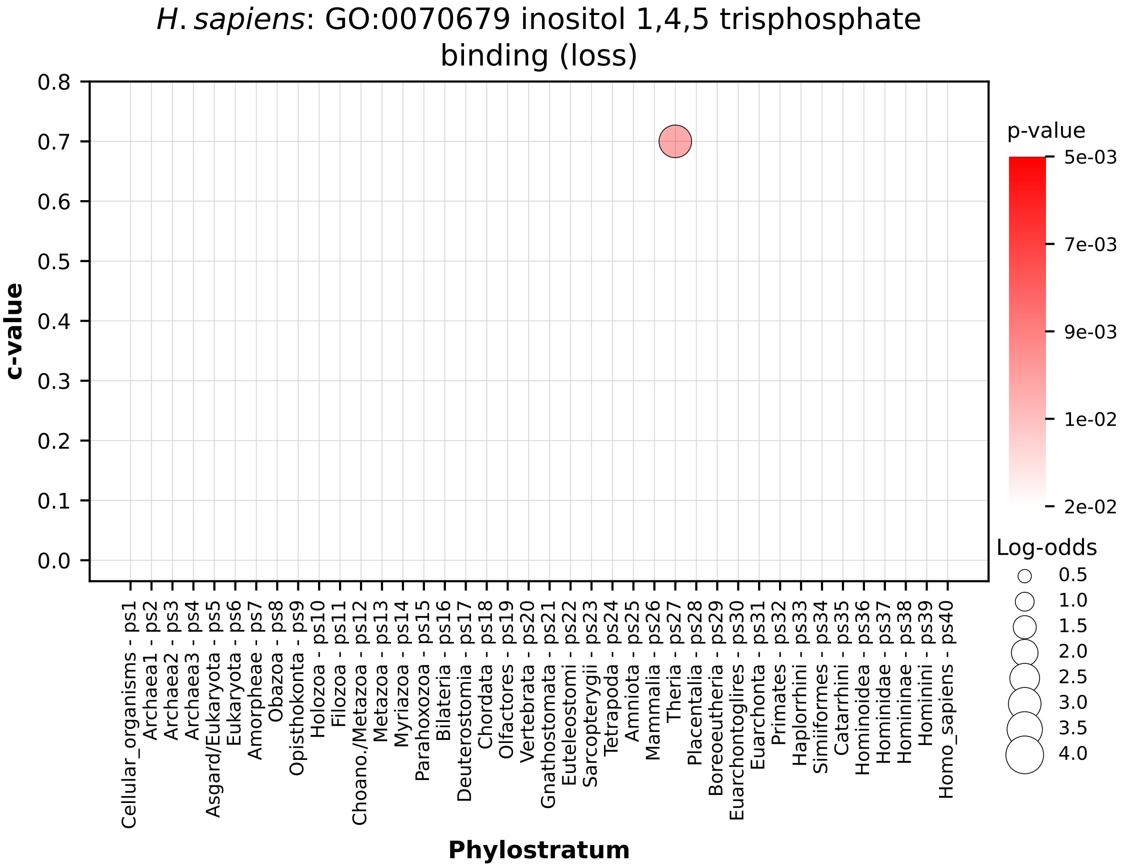
H. sapiens: GO:0070679 inositol 1,4,5 trisphosphate binding (loss)
c-value
0.8
0.7
0.6
0.5
0.4
0.3
0.2
0.1
0.0
Phylostratum
Cellular_organisms - ps1
Archaea1 - ps2
Archaea2 - ps3
Archaea3 - ps4
Asgard/Eukaryota - ps5
Eukaryota - ps6
Amorpheae - ps7
Obazoa - ps8
Opisthokonta - ps9
Holozoa - ps10
Filozoa - ps11
Choano./Metazoa - ps12
Metazoa - ps13
Myriazoa - ps14
Parahoxozoa - ps15
Bilateria - ps16
Deuterostomia - ps17
Chordata - ps18
Olfactores - ps19
Vertebrata - ps20
Gnathostomata - ps21
Euteleostomi - ps22
Sarcopterygii - ps23
Tetrapoda - ps24
Amniota - ps25
Mammalia - ps26
Theria - ps27
Placentalia - ps28
Boreoeutheria - ps29
Euarchontoglires - ps30
Euarchonta - ps31
Primates - ps32
Haplorrhini - ps33
Simiiformes - ps34
Catarrhini - ps35
Hominoidea - ps36
Hominidae - ps37
Homininae - ps38
Hominini - ps39
Homo_sapiens - ps40
p-value
5e-03
7e-03
9e-03
1e-02
2e-02
Log-odds
0.5
1.0
1.5
2.0
2.5
3.0
3.5
4.0

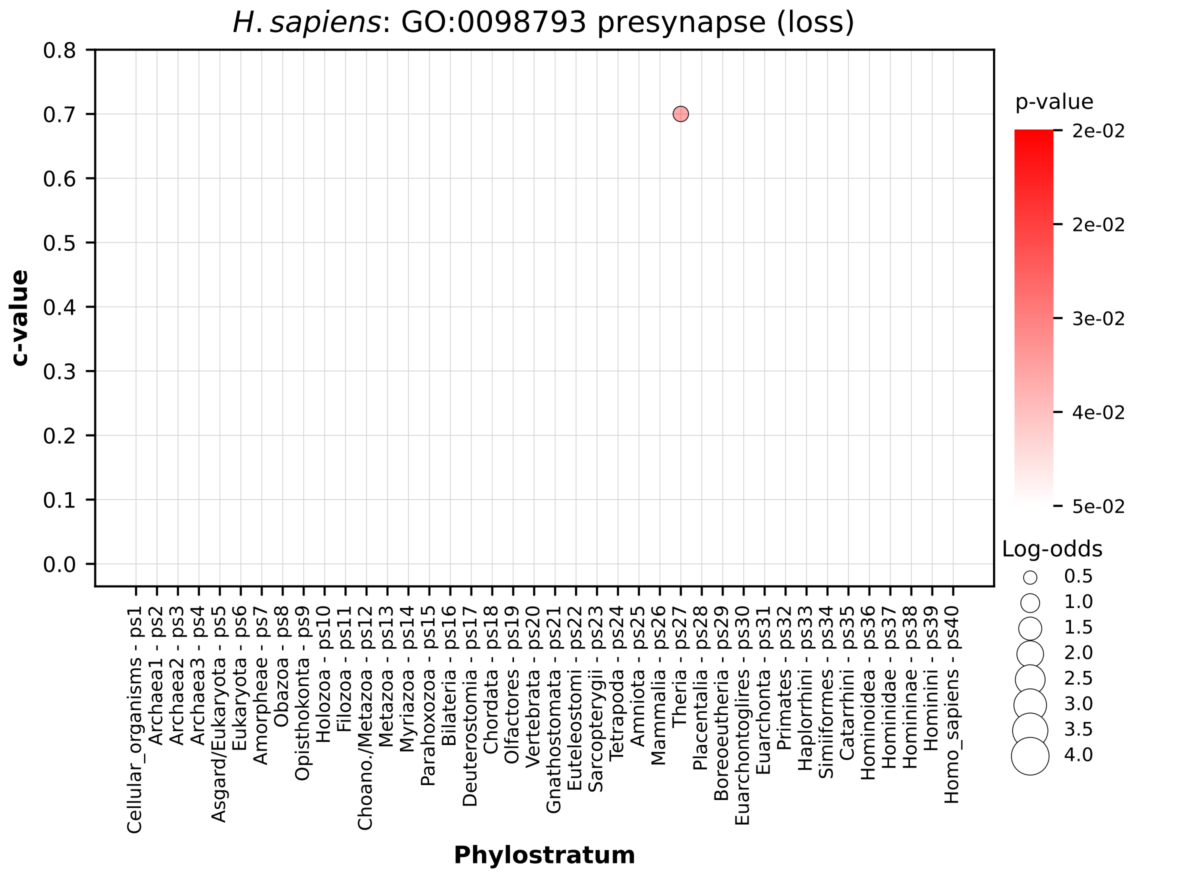

H. sapiens: GO:0098793 presynapse (loss)
c-value
0.8
0.7
0.6
0.5
0.4
0.3
0.2
0.1
0.0
Phylostratum
Cellular_organisms - ps1
Archaea1 - ps2
Archaea2 - ps3
Archaea3 - ps4
Asgard/Eukaryota - ps5
Eukaryota - ps6
Amorpheae - ps7
Obazoa - ps8
Opisthokonta - ps9
Holozoa - ps10
Filozoa - ps11
Choano./Metazoa - ps12
Metazoa - ps13
Myriazoa - ps14
Parahoxozoa - ps15
Bilateria - ps16
Deuterostomia - ps17
Chordata - ps18
Olfactores - ps19
Vertebrata - ps20
Gnathostomata - ps21
Euteleostomi - ps22
Sarcopterygii - ps23
Tetrapoda - ps24
Amniota - ps25
Mammalia - ps26
Theria - ps27
Placentalia - ps28
Boreoeutheria - ps29
Euarchontoglires - ps30
Euarchonta - ps31
Primates - ps32
Haplorrhini - ps33
Simiiformes - ps34
Catarrhini - ps35
Hominoidea - ps36
Hominidae - ps37
Homininae - ps38
Hominini - ps39
Homo_sapiens - ps40
p-value
2e-02
2e-02
3e-02
4e-02
5e-02
Log-odds
0.5
1.0
1.5
2.0
2.5
3.0
3.5
4.0

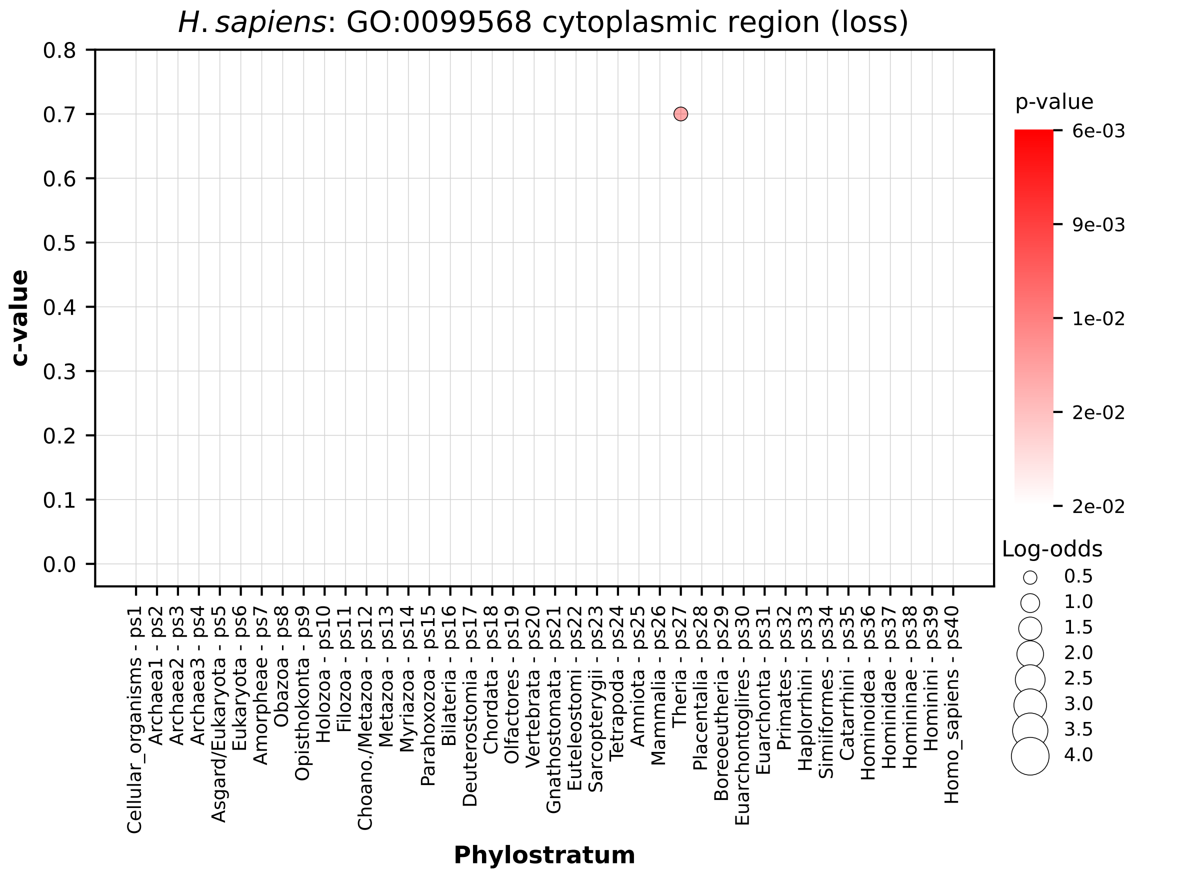

H. sapiens: GO:0099568 cytoplasmic region (loss)
c-value
0.8
0.7
0.6
0.5
0.4
0.3
0.2
0.1
0.0
Phylostratum
Cellular_organisms - ps1
Archaea1 - ps2
Archaea2 - ps3
Archaea3 - ps4
Asgard/Eukaryota - ps5
Eukaryota - ps6
Amorpheae - ps7
Obazoa - ps8
Opisthokonta - ps9
Holozoa - ps10
Filozoa - ps11
Choano./Metazoa - ps12
Metazoa - ps13
Myriazoa - ps14
Parahoxozoa - ps15
Bilateria - ps16
Deuterostomia - ps17
Chordata - ps18
Olfactores - ps19
Vertebrata - ps20
Gnathostomata - ps21
Euteleostomi - ps22
Sarcopterygii - ps23
Tetrapoda - ps24
Amniota - ps25
Mammalia - ps26
Theria - ps27
Placentalia - ps28
Boreoeutheria - ps29
Euarchontoglires - ps30
Euarchonta - ps31
Primates - ps32
Haplorrhini - ps33
Simiiformes - ps34
Catarrhini - ps35
Hominoidea - ps36
Hominidae - ps37
Homininae - ps38
Hominini - ps39
Homo_sapiens - ps40
p-value
6e-03
9e-03
1e-02
2e-02
2e-02
Log-odds
0.5
1.0
1.5
2.0
2.5
3.0
3.5
4.0

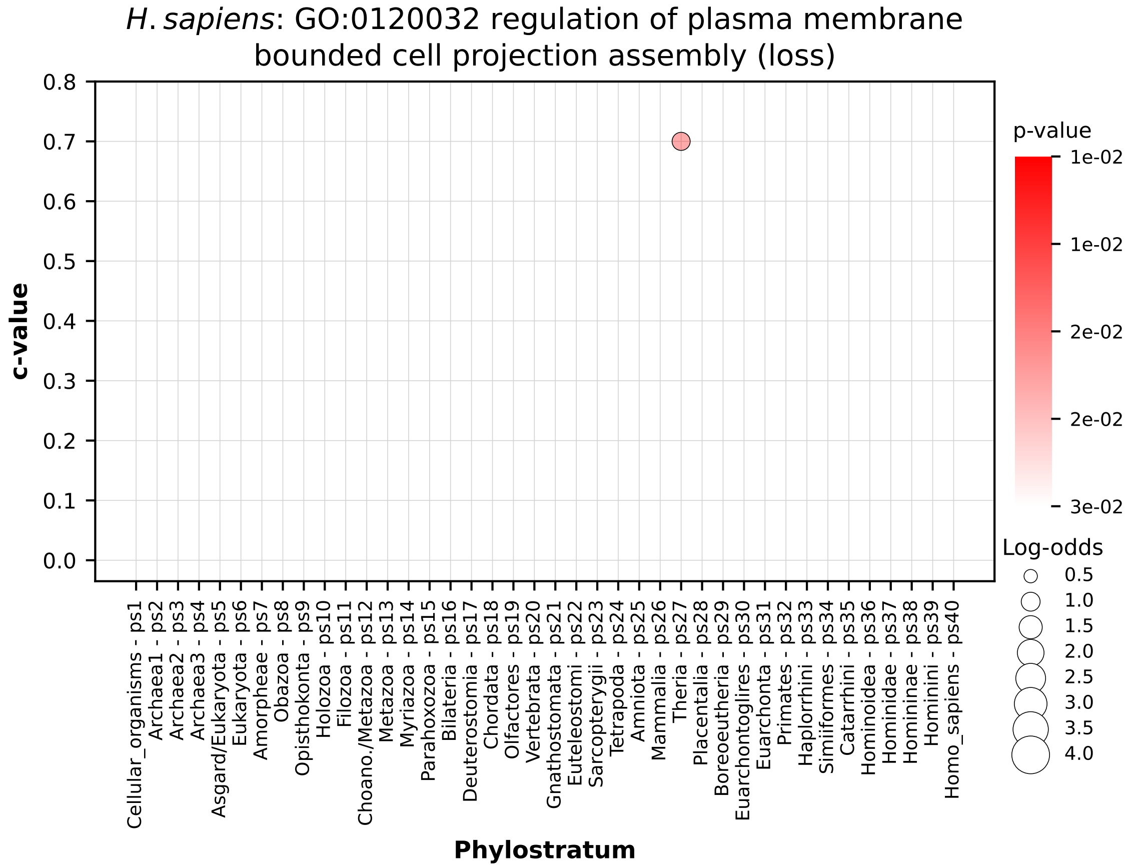

H. sapiens: GO:0120032 regulation of plasma membrane bounded cell projection assembly (loss)
c-value
0.8
0.7
0.6
0.5
0.4
0.3
0.2
0.1
0.0
p-value
1e-02
1e-02
2e-02
2e-02
3e-02
Log-odds
0.5
1.0
1.5
2.0
2.5
3.0
3.5
4.0
Cellular_organisms - ps1
Archaea1 - ps2
Archaea2 - ps3
Archaea3 - ps4
Asgard/Eukaryota - ps5
Eukaryota - ps6
Amorpheae - ps7
Obazoa - ps8
Opisthokonta - ps9
Holozoa - ps10
Filozoa - ps11
Choano./Metazoa - ps12
Metazoa - ps13
Myriazoa - ps14
Parahoxozoa - ps15
Bilateria - ps16
Deuterostomia - ps17
Chordata - ps18
Olfactores - ps19
Vertebrata - ps20
Gnathostomata - ps21
Euteleostomi - ps22
Sarcopterygii - ps23
Tetrapoda - ps24
Amniota - ps25
Mammalia - ps26
Theria - ps27
Placentalia - ps28
Boreoeutheria - ps29
Euarchontoglires - ps30
Euarchonta - ps31
Primates - ps32
Haplorrhini - ps33
Simiiformes - ps34
Catarrhini - ps35
Hominoidea - ps36
Hominidae - ps37
Homininae - ps38
Hominini - ps39
Homo_sapiens - ps40
Phylostratum

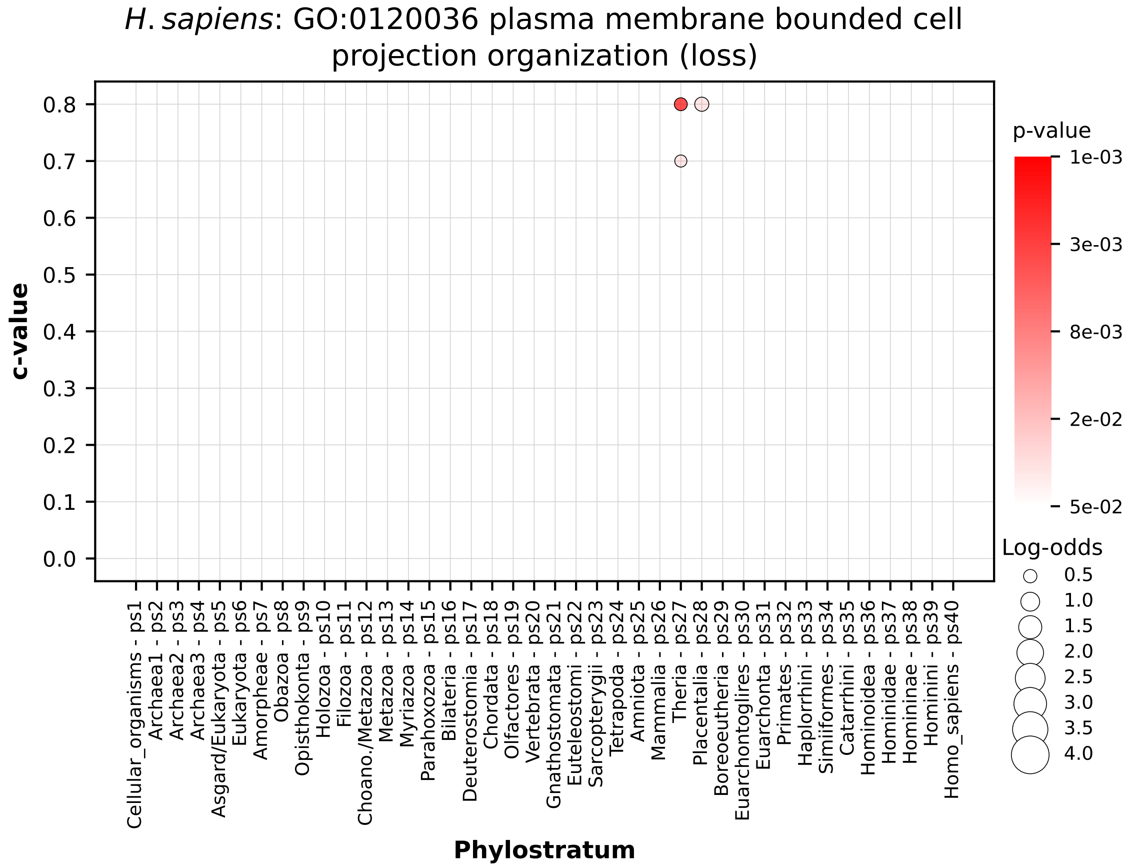

H. sapiens: GO:0120036 plasma membrane bounded cell projection organization (loss)
c-value
0.0
0.1
0.2
0.3
0.4
0.5
0.6
0.7
0.8
p-value
1e-03
3e-03
8e-03
2e-02
5e-02
Log-odds
0.5
1.0
1.5
2.0
2.5
3.0
3.5
4.0
Cellular_organisms - ps1
Archaea1 - ps2
Archaea2 - ps3
Archaea3 - ps4
Asgard/Eukaryota - ps5
Eukaryota - ps6
Amorpheae - ps7
Obazoa - ps8
Opisthokonta - ps9
Holozoa - ps10
Filozoa - ps11
Choano./Metazoa - ps12
Metazoa - ps13
Myriazoa - ps14
Parahoxozoa - ps15
Bilateria - ps16
Deuterostomia - ps17
Chordata - ps18
Olfactores - ps19
Vertebrata - ps20
Gnathostomata - ps21
Euteleostomi - ps22
Sarcopterygii - ps23
Tetrapoda - ps24
Amniota - ps25
Mammalia - ps26
Theria - ps27
Placentalia - ps28
Boreoeutheria - ps29
Euarchontoglires - ps30
Euarchonta - ps31
Primates - ps32
Haplorrhini - ps33
Simiiformes - ps34
Catarrhini - ps35
Hominoidea - ps36
Hominidae - ps37
Homininae - ps38
Hominini - ps39
Homo_sapiens - ps40
Phylostratum

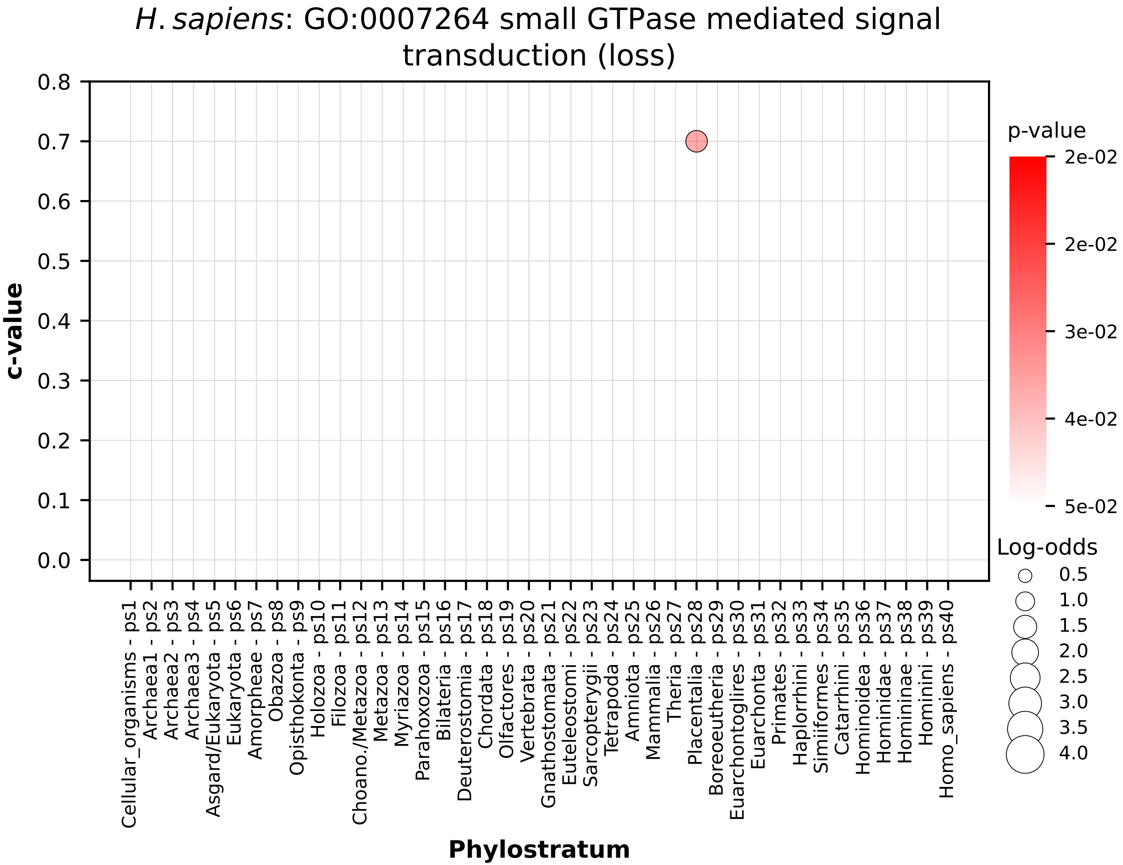
H. sapiens: GO:0007264 small GTPase mediated signal transduction (loss)
c-value
0.8
0.7
0.6
0.5
0.4
0.3
0.2
0.1
0.0
Phylostratum
Cellular_organisms - ps1
Archaea1 - ps2
Archaea2 - ps3
Archaea3 - ps4
Asgard/Eukaryota - ps5
Eukaryota - ps6
Amorpheae - ps7
Obazoa - ps8
Opisthokonta - ps9
Holozoa - ps10
Filozoa - ps11
Choano./Metazoa - ps12
Metazoa - ps13
Myriazoa - ps14
Parahoxozoa - ps15
Bilateria - ps16
Deuterostomia - ps17
Chordata - ps18
Olfactores - ps19
Vertebrata - ps20
Gnathostomata - ps21
Euteleostomi - ps22
Sarcopterygii - ps23
Tetrapoda - ps24
Amniota - ps25
Mammalia - ps26
Theria - ps27
Placentalia - ps28
Boreoeutheria - ps29
Euarchontoglires - ps30
Euarchonta - ps31
Primates - ps32
Haplorrhini - ps33
Simiiformes - ps34
Catarrhini - ps35
Hominoidea - ps36
Hominidae - ps37
Homininae - ps38
Hominini - ps39
Homo_sapiens - ps40
p-value
2e-02
2e-02
3e-02
4e-02
5e-02
Log-odds
0.5
1.0
1.5
2.0
2.5
3.0
3.5
4.0

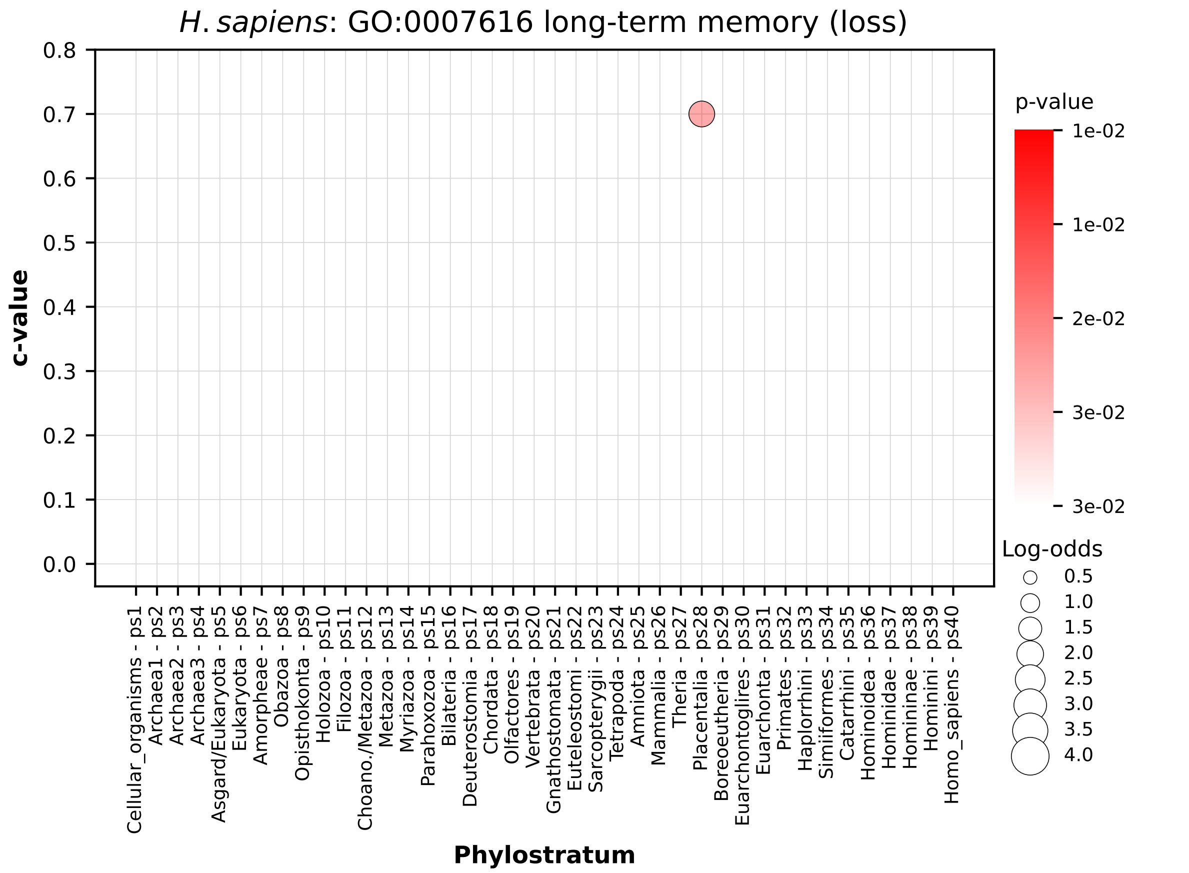
H. sapiens: GO:0007616 long-term memory (loss)
c-value
0.8
0.7
0.6
0.5
0.4
0.3
0.2
0.1
0.0
Phylostratum
Cellular_organisms - ps1
Archaea1 - ps2
Archaea2 - ps3
Archaea3 - ps4
Asgard/Eukaryota - ps5
Eukaryota - ps6
Amorpheae - ps7
Obazoa - ps8
Opisthokonta - ps9
Holozoa - ps10
Filozoa - ps11
Choano./Metazoa - ps12
Metazoa - ps13
Myriazoa - ps14
Parahoxozoa - ps15
Bilateria - ps16
Deuterostomia - ps17
Chordata - ps18
Olfactores - ps19
Vertebrata - ps20
Gnathostomata - ps21
Euteleostomi - ps22
Sarcopterygii - ps23
Tetrapoda - ps24
Amniota - ps25
Mammalia - ps26
Theria - ps27
Placentalia - ps28
Boreoeutheria - ps29
Euarchontoglires - ps30
Euarchonta - ps31
Primates - ps32
Haplorrhini - ps33
Simiiformes - ps34
Catarrhini - ps35
Hominoidea - ps36
Hominidae - ps37
Homininae - ps38
Hominini - ps39
Homo_sapiens - ps40
p-value
1e-02
1e-02
2e-02
3e-02
3e-02
Log-odds
0.5
1.0
1.5
2.0
2.5
3.0
3.5
4.0

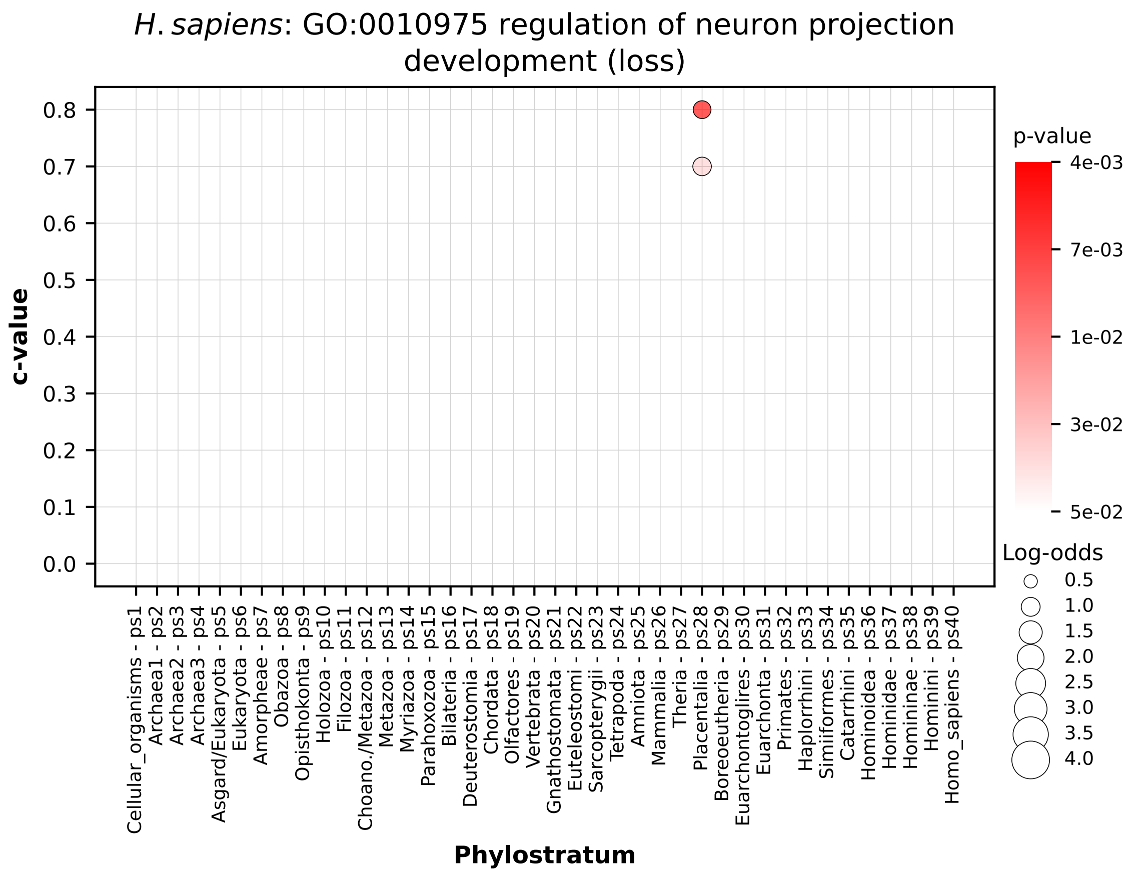

H. sapiens: GO:0010975 regulation of neuron projection development (loss)
c-value
0.0
0.1
0.2
0.3
0.4
0.5
0.6
0.7
0.8
Phylostratum
Cellular_organisms - ps1
Archaea1 - ps2
Archaea2 - ps3
Archaea3 - ps4
Asgard/Eukaryota - ps5
Eukaryota - ps6
Amorpheae - ps7
Obazoa - ps8
Opisthokonta - ps9
Holozoa - ps10
Filozoa - ps11
Choano./Metazoa - ps12
Metazoa - ps13
Myriazoa - ps14
Parahoxozoa - ps15
Bilateria - ps16
Deuterostomia - ps17
Chordata - ps18
Olfactores - ps19
Vertebrata - ps20
Gnathostomata - ps21
Euteleostomi - ps22
Sarcopterygii - ps23
Tetrapoda - ps24
Amniota - ps25
Mammalia - ps26
Theria - ps27
Placentalia - ps28
Boreoeutheria - ps29
Euarchontoglires - ps30
Euarchonta - ps31
Primates - ps32
Haplorrhini - ps33
Simiiformes - ps34
Catarrhini - ps35
Hominoidea - ps36
Hominidae - ps37
Homininae - ps38
Hominini - ps39
Homo_sapiens - ps40
p-value
4e-03
7e-03
1e-02
3e-02
5e-02
Log-odds
0.5
1.0
1.5
2.0
2.5
3.0
3.5
4.0

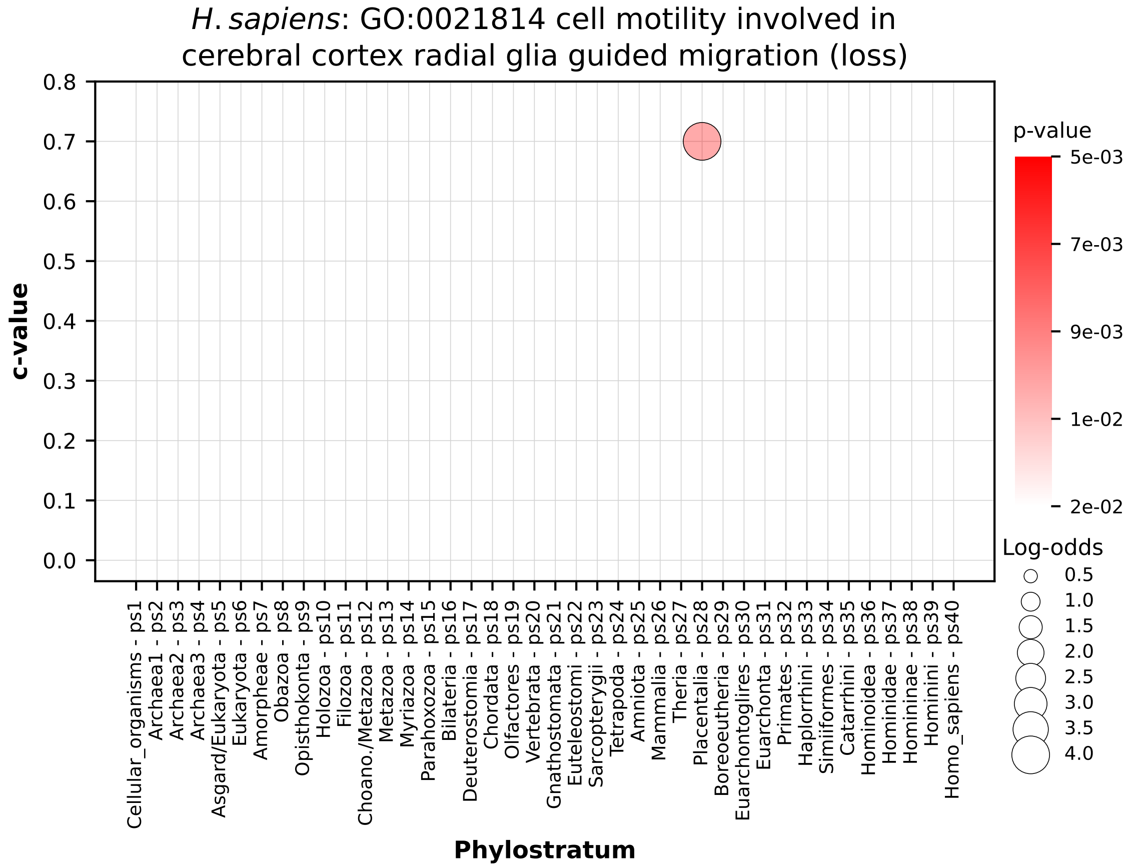

H. sapiens: GO:0021814 cell motility involved in cerebral cortex radial glia guided migration (loss)
c-value
0.8
0.7
0.6
0.5
0.4
0.3
0.2
0.1
0.0
p-value
5e-03
7e-03
9e-03
1e-02
2e-02
Log-odds
0.5
1.0
1.5
2.0
2.5
3.0
3.5
4.0
Cellular_organisms - ps1
Archaea1 - ps2
Archaea2 - ps3
Archaea3 - ps4
Asgard/Eukaryota - ps5
Eukaryota - ps6
Amorpheae - ps7
Obazoa - ps8
Opisthokonta - ps9
Holozoa - ps10
Filozoa - ps11
Choano./Metazoa - ps12
Metazoa - ps13
Myriazoa - ps14
Parahoxozoa - ps15
Bilateria - ps16
Deuterostomia - ps17
Chordata - ps18
Olfactores - ps19
Vertebrata - ps20
Gnathostomata - ps21
Euteleostomi - ps22
Sarcopterygii - ps23
Tetrapoda - ps24
Amniota - ps25
Mammalia - ps26
Theria - ps27
Placentalia - ps28
Boreoeutheria - ps29
Euarchontoglires - ps30
Euarchonta - ps31
Primates - ps32
Haplorrhini - ps33
Simiiformes - ps34
Catarrhini - ps35
Hominoidea - ps36
Hominidae - ps37
Homininae - ps38
Hominini - ps39
Homo_sapiens - ps40
Phylostratum

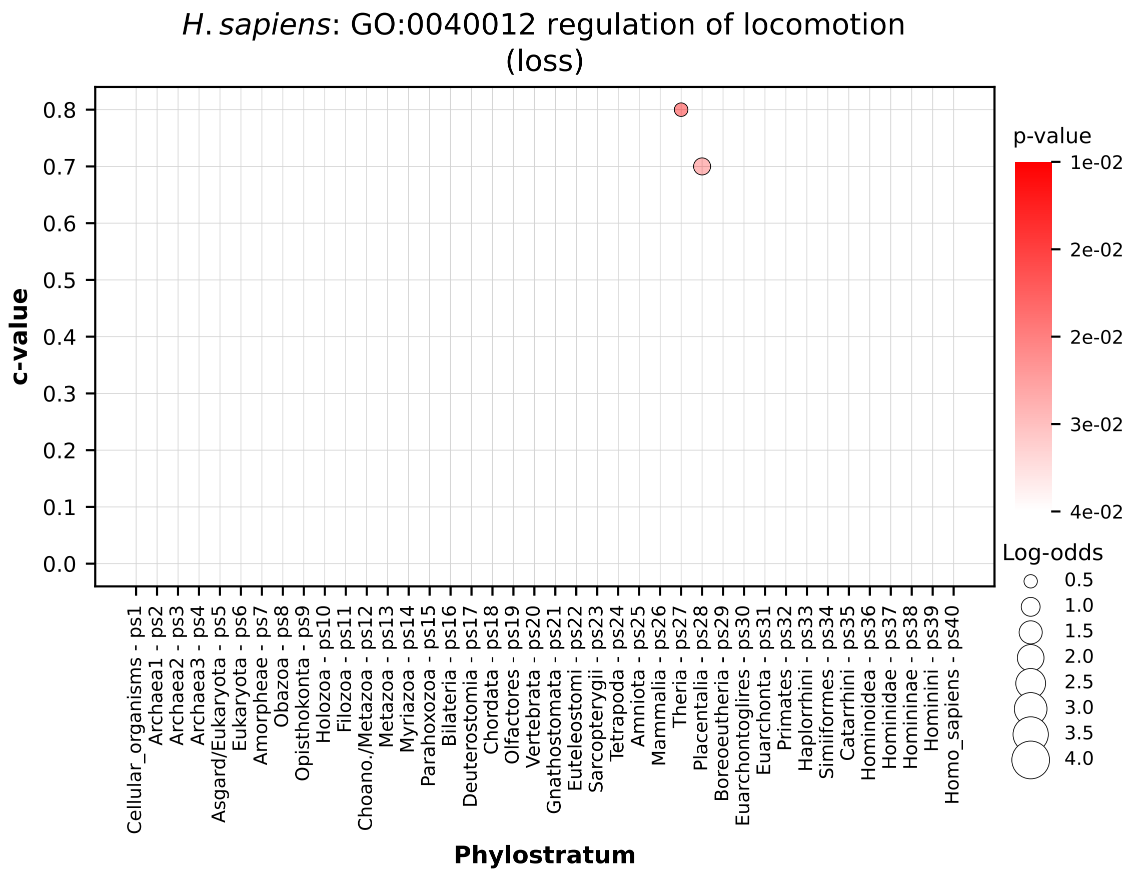

H. sapiens: GO:0040012 regulation of locomotion
(loss)
c-value
0.0
0.1
0.2
0.3
0.4
0.5
0.6
0.7
0.8
Phylostratum
Cellular_organisms - ps1
Archaea1 - ps2
Archaea2 - ps3
Archaea3 - ps4
Asgard/Eukaryota - ps5
Eukaryota - ps6
Amorpheae - ps7
Obazoa - ps8
Opisthokonta - ps9
Holozoa - ps10
Filozoa - ps11
Choano./Metazoa - ps12
Metazoa - ps13
Myriazoa - ps14
Parahoxozoa - ps15
Bilateria - ps16
Deuterostomia - ps17
Chordata - ps18
Olfactores - ps19
Vertebrata - ps20
Gnathostomata - ps21
Euteleostomi - ps22
Sarcopterygii - ps23
Tetrapoda - ps24
Amniota - ps25
Mammalia - ps26
Theria - ps27
Placentalia - ps28
Boreoeutheria - ps29
Euarchontoglires - ps30
Euarchonta - ps31
Primates - ps32
Haplorrhini - ps33
Simiiformes - ps34
Catarrhini - ps35
Hominoidea - ps36
Hominidae - ps37
Homininae - ps38
Hominini - ps39
Homo_sapiens - ps40
p-value
1e-02
2e-02
2e-02
3e-02
4e-02
Log-odds
0.5
1.0
1.5
2.0
2.5
3.0
3.5
4.0

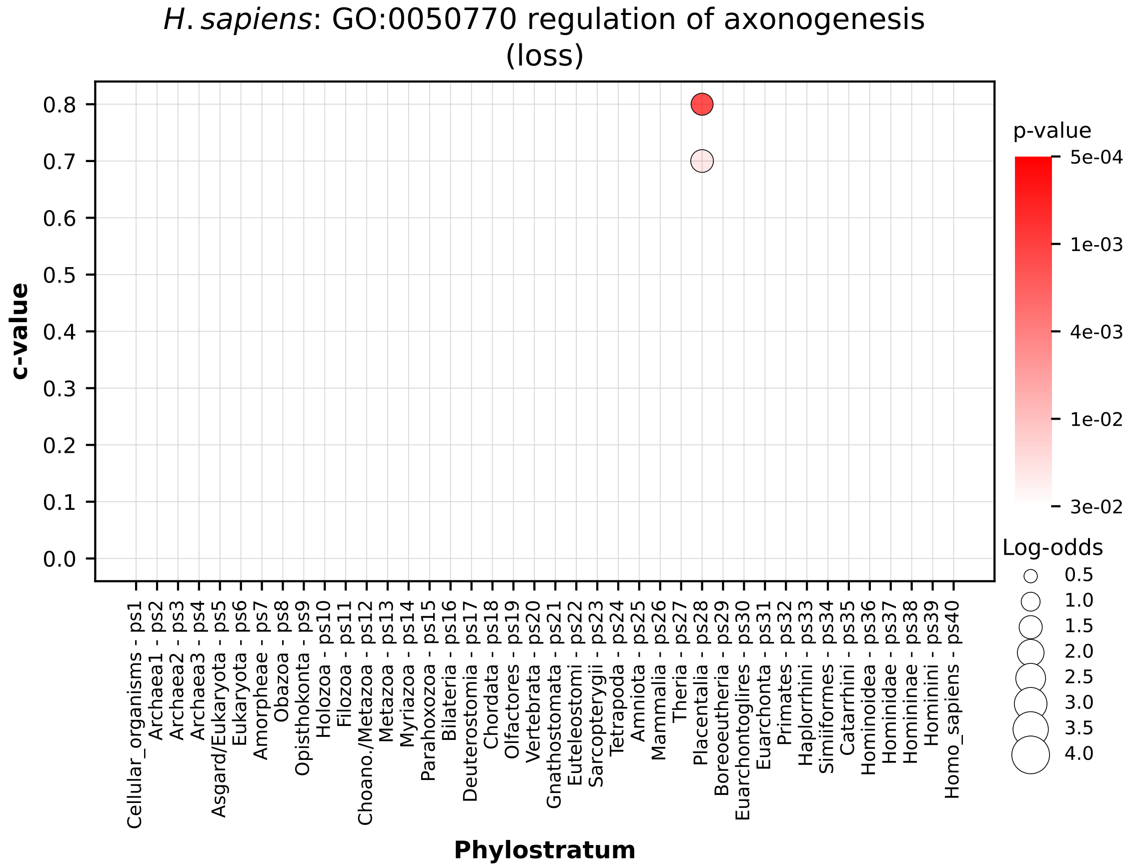

H. sapiens: GO:0050770 regulation of axonogenesis
(loss)
c-value
0.0
0.1
0.2
0.3
0.4
0.5
0.6
0.7
0.8
p-value
5e-04
1e-03
4e-03
1e-02
3e-02
Log-odds
0.5
1.0
1.5
2.0
2.5
3.0
3.5
4.0
Cellular_organisms - ps1
Archaea1 - ps2
Archaea2 - ps3
Archaea3 - ps4
Asgard/Eukaryota - ps5
Eukaryota - ps6
Amorpheae - ps7
Obazoa - ps8
Opisthokonta - ps9
Holozoa - ps10
Filozoa - ps11
Choano./Metazoa - ps12
Metazoa - ps13
Myriazoa - ps14
Parahoxozoa - ps15
Bilateria - ps16
Deuterostomia - ps17
Chordata - ps18
Olfactores - ps19
Vertebrata - ps20
Gnathostomata - ps21
Euteleostomi - ps22
Sarcopterygii - ps23
Tetrapoda - ps24
Amniota - ps25
Mammalia - ps26
Theria - ps27
Placentalia - ps28
Boreoeutheria - ps29
Euarchontoglires - ps30
Euarchonta - ps31
Primates - ps32
Haplorrhini - ps33
Simiiformes - ps34
Catarrhini - ps35
Hominoidea - ps36
Hominidae - ps37
Homininae - ps38
Hominini - ps39
Homo_sapiens - ps40
Phylostratum

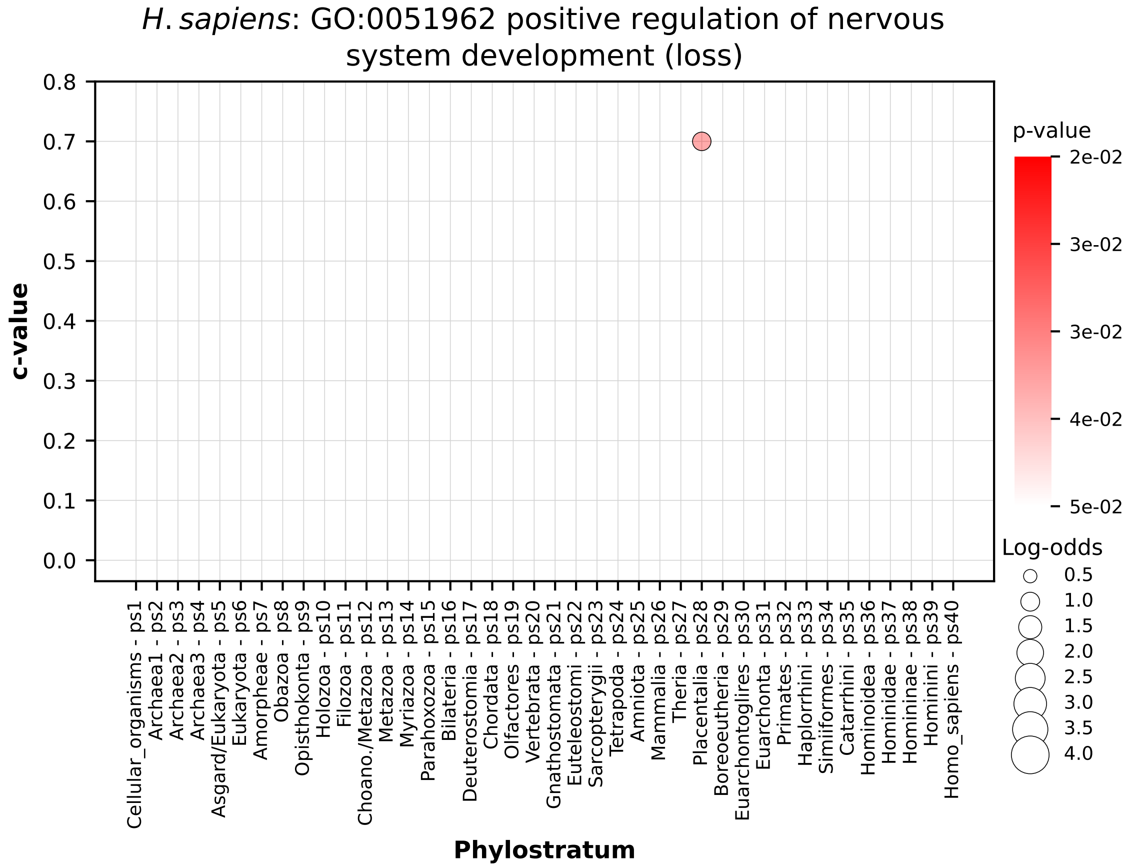

H. sapiens: GO:0051962 positive regulation of nervous system development (loss)
c-value
0.8
0.7
0.6
0.5
0.4
0.3
0.2
0.1
0.0
Phylostratum
Cellular_organisms - ps1
Archaea1 - ps2
Archaea2 - ps3
Archaea3 - ps4
Asgard/Eukaryota - ps5
Eukaryota - ps6
Amorpheae - ps7
Obazoa - ps8
Opisthokonta - ps9
Holozoa - ps10
Filozoa - ps11
Choano./Metazoa - ps12
Metazoa - ps13
Myriazoa - ps14
Parahoxozoa - ps15
Bilateria - ps16
Deuterostomia - ps17
Chordata - ps18
Olfactores - ps19
Vertebrata - ps20
Gnathostomata - ps21
Euteleostomi - ps22
Sarcopterygii - ps23
Tetrapoda - ps24
Amniota - ps25
Mammalia - ps26
Theria - ps27
Placentalia - ps28
Boreoeutheria - ps29
Euarchontoglires - ps30
Euarchonta - ps31
Primates - ps32
Haplorrhini - ps33
Simiiformes - ps34
Catarrhini - ps35
Hominoidea - ps36
Hominidae - ps37
Homininae - ps38
Hominini - ps39
Homo_sapiens - ps40
p-value
2e-02
3e-02
3e-02
4e-02
5e-02
Log-odds
0.5
1.0
1.5
2.0
2.5
3.0
3.5
4.0

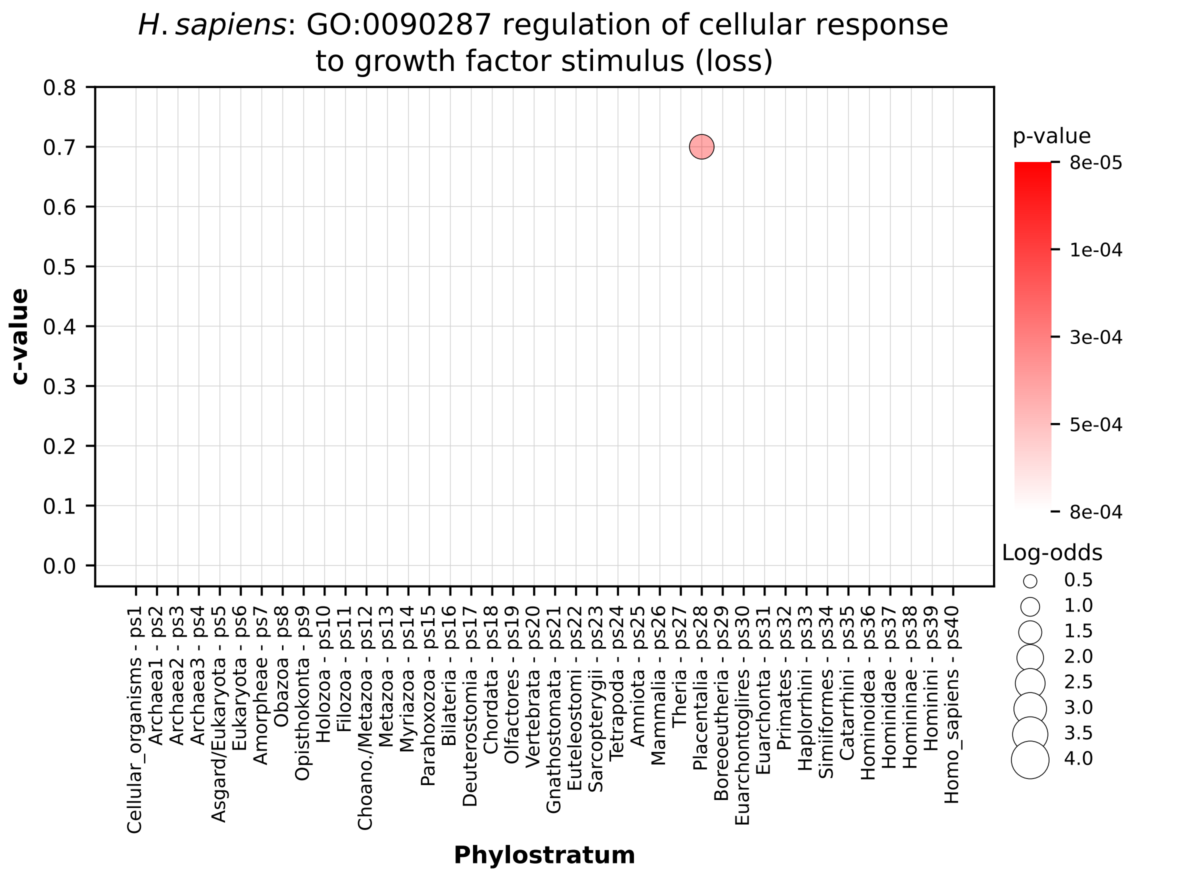

H. sapiens: GO:0090287 regulation of cellular response to growth factor stimulus (loss)
c-value
0.8
0.7
0.6
0.5
0.4
0.3
0.2
0.1
0.0
p-value
8e-05
1e-04
3e-04
5e-04
8e-04
Log-odds
0.5
1.0
1.5
2.0
2.5
3.0
3.5
4.0
Cellular_organisms - ps1
Archaea1 - ps2
Archaea2 - ps3
Archaea3 - ps4
Asgard/Eukaryota - ps5
Eukaryota - ps6
Amorpheae - ps7
Obazoa - ps8
Opisthokonta - ps9
Holozoa - ps10
Filozoa - ps11
Choano./Metazoa - ps12
Metazoa - ps13
Myriazoa - ps14
Parahoxozoa - ps15
Bilateria - ps16
Deuterostomia - ps17
Chordata - ps18
Olfactores - ps19
Vertebrata - ps20
Gnathostomata - ps21
Euteleostomi - ps22
Sarcopterygii - ps23
Tetrapoda - ps24
Amniota - ps25
Mammalia - ps26
Theria - ps27
Placentalia - ps28
Boreoeutheria - ps29
Euarchontoglires - ps30
Euarchonta - ps31
Primates - ps32
Haplorrhini - ps33
Simiiformes - ps34
Catarrhini - ps35
Hominoidea - ps36
Hominidae - ps37
Homininae - ps38
Hominini - ps39
Homo_sapiens - ps40
Phylostratum

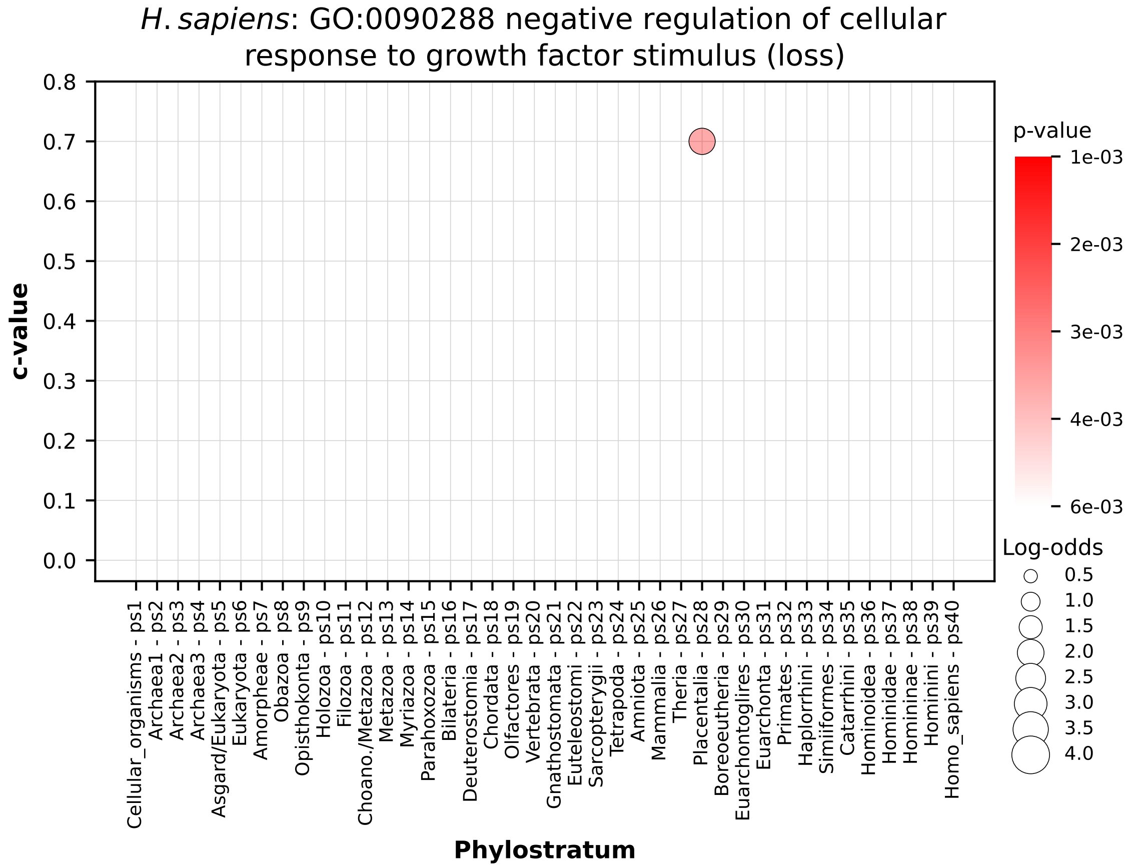
H. sapiens: GO:0090288 negative regulation of cellular response to growth factor stimulus (loss)
c-value
Phylostratum
p-value
1e-03
2e-03
3e-03
4e-03
6e-03
Log-odds
0.5
1.0
1.5
2.0
2.5
3.0
3.5
4.0
Cellular_organisms - ps1
Archaea1 - ps2
Archaea2 - ps3
Archaea3 - ps4
Asgard/Eukaryota - ps5
Eukaryota - ps6
Amorpheae - ps7
Obazoa - ps8
Opisthokonta - ps9
Holozoa - ps10
Filozoa - ps11
Choano./Metazoa - ps12
Metazoa - ps13
Myriazoa - ps14
Parahoxozoa - ps15
Bilateria - ps16
Deuterostomia - ps17
Chordata - ps18
Olfactores - ps19
Vertebrata - ps20
Gnathostomata - ps21
Euteleostomi - ps22
Sarcopterygii - ps23
Tetrapoda - ps24
Amniota - ps25
Mammalia - ps26
Theria - ps27
Placentalia - ps28
Boreoeutheria - ps29
Euarchontoglires - ps30
Euarchonta - ps31
Primates - ps32
Haplorrhini - ps33
Simiiformes - ps34
Catarrhini - ps35
Hominoidea - ps36
Hominidae - ps37
Homininae - ps38
Hominini - ps39
Homo_sapiens - ps40

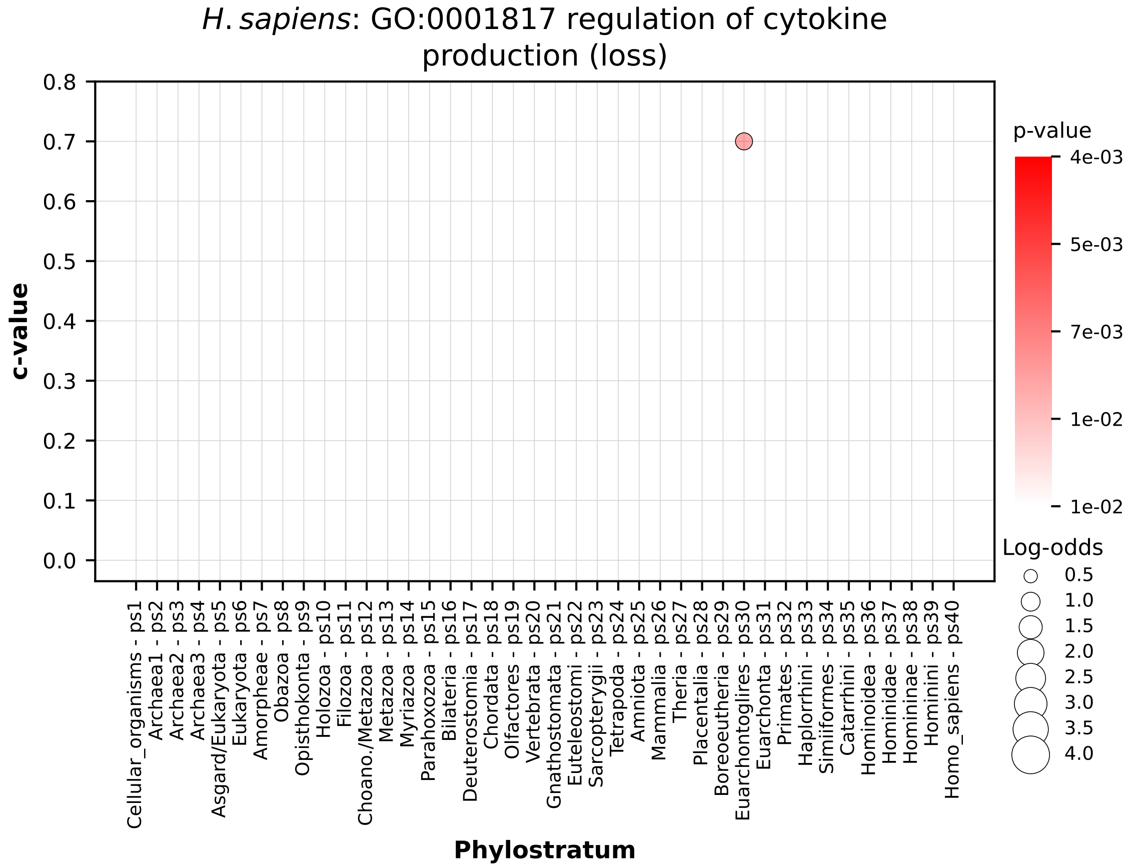

H. sapiens: GO:0001817 regulation of cytokine production (loss)
c-value
0.8
0.7
0.6
0.5
0.4
0.3
0.2
0.1
0.0
p-value
4e-03
5e-03
7e-03
1e-02
1e-02
Log-odds
0.5
1.0
1.5
2.0
2.5
3.0
3.5
4.0
Cellular_organisms - ps1
Archaea1 - ps2
Archaea2 - ps3
Archaea3 - ps4
Asgard/Eukaryota - ps5
Eukaryota - ps6
Amorpheae - ps7
Obazoa - ps8
Opisthokonta - ps9
Holozoa - ps10
Filozoa - ps11
Choano./Metazoa - ps12
Metazoa - ps13
Myriazoa - ps14
Parahoxozoa - ps15
Bilateria - ps16
Deuterostomia - ps17
Chordata - ps18
Olfactores - ps19
Vertebrata - ps20
Gnathostomata - ps21
Euteleostomi - ps22
Sarcopterygii - ps23
Tetrapoda - ps24
Amniota - ps25
Mammalia - ps26
Theria - ps27
Placentalia - ps28
Boreoeutheria - ps29
Euarchontoglires - ps30
Euarchonta - ps31
Primates - ps32
Haplorrhini - ps33
Simiiformes - ps34
Catarrhini - ps35
Hominoidea - ps36
Hominidae - ps37
Homininae - ps38
Hominini - ps39
Homo_sapiens - ps40
Phylostratum

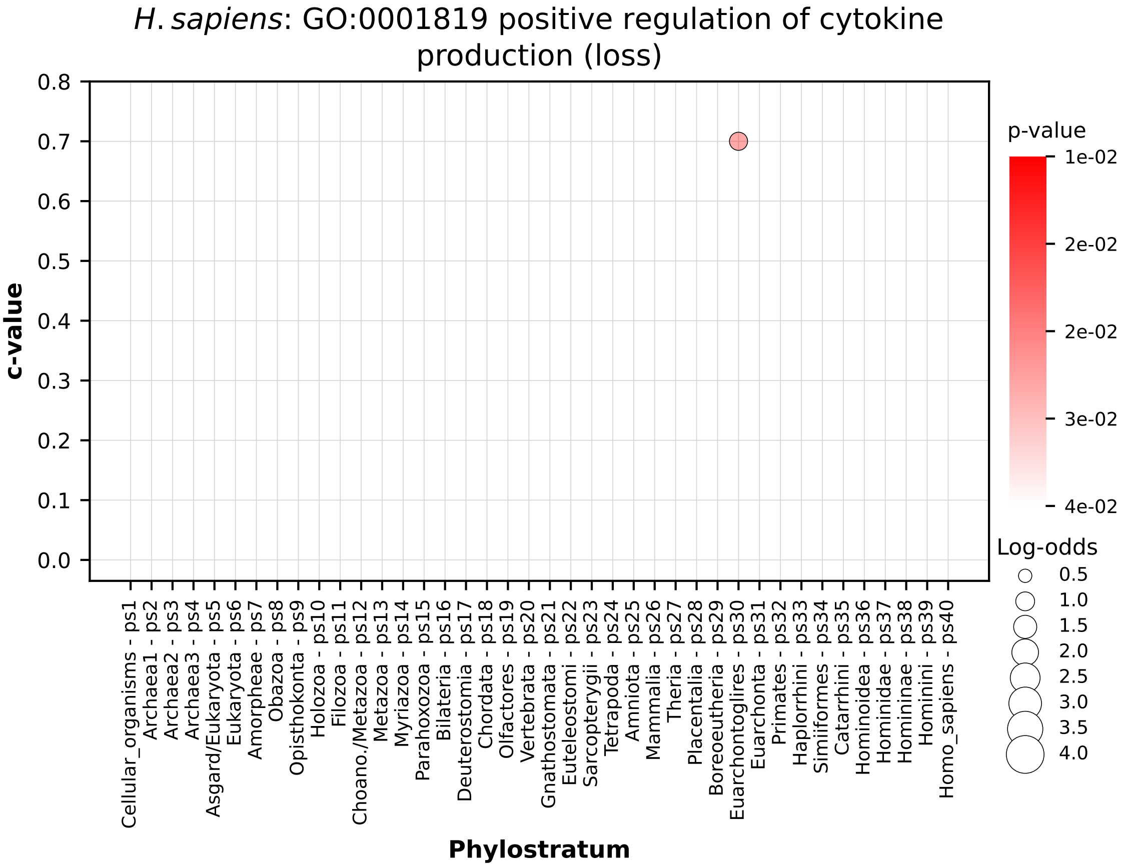
H. sapiens: GO:0001819 positive regulation of cytokine production (loss)
c-value
0.8
0.7
0.6
0.5
0.4
0.3
0.2
0.1
0.0
p-value
1e-02
2e-02
2e-02
3e-02
4e-02
Log-odds
0.5
1.0
1.5
2.0
2.5
3.0
3.5
4.0
Cellular_organisms - ps1
Archaea1 - ps2
Archaea2 - ps3
Archaea3 - ps4
Asgard/Eukaryota - ps5
Eukaryota - ps6
Amorpheae - ps7
Obazoa - ps8
Opisthokonta - ps9
Holozoa - ps10
Filozoa - ps11
Choano./Metazoa - ps12
Metazoa - ps13
Myriazoa - ps14
Parahoxozoa - ps15
Bilateria - ps16
Deuterostomia - ps17
Chordata - ps18
Olfactores - ps19
Vertebrata - ps20
Gnathostomata - ps21
Euteleostomi - ps22
Sarcopterygii - ps23
Tetrapoda - ps24
Amniota - ps25
Mammalia - ps26
Theria - ps27
Placentalia - ps28
Boreoeutheria - ps29
Euarchontoglires - ps30
Euarchonta - ps31
Primates - ps32
Haplorrhini - ps33
Simiiformes - ps34
Catarrhini - ps35
Hominoidea - ps36
Hominidae - ps37
Homininae - ps38
Hominini - ps39
Homo_sapiens - ps40
Phylostratum

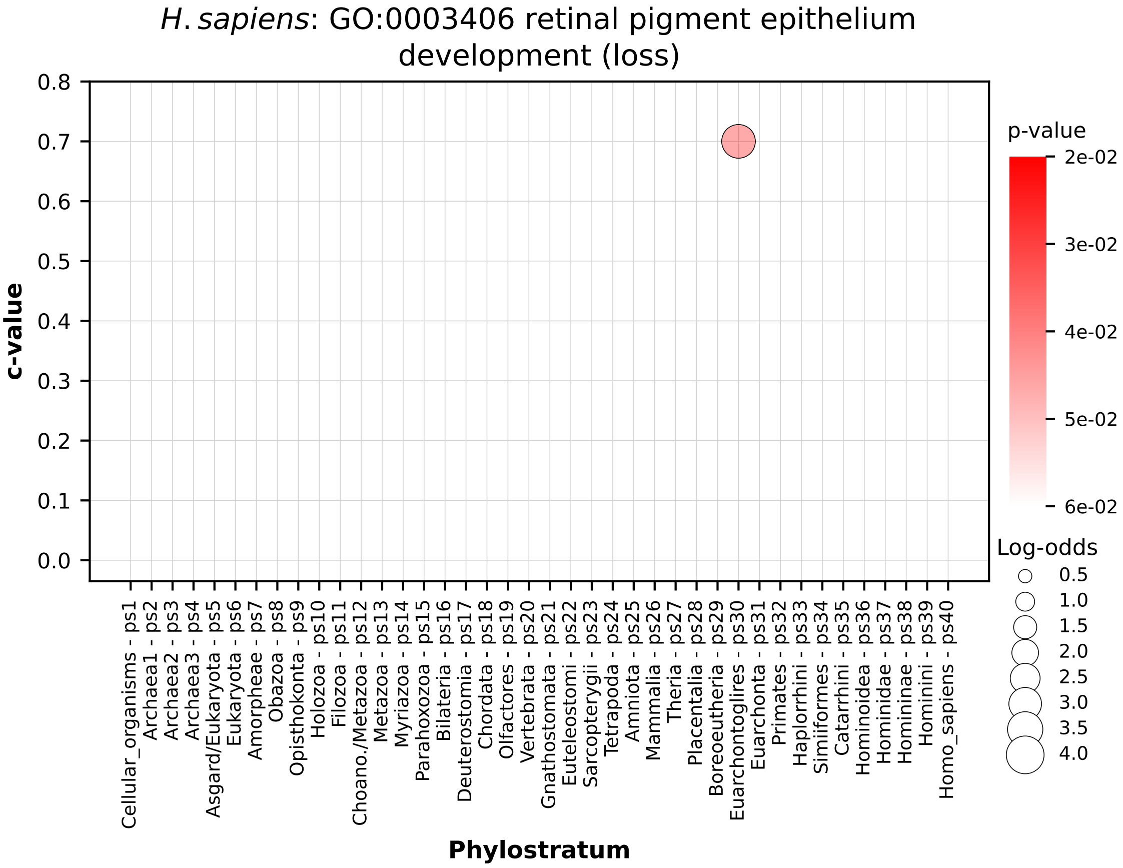

H. sapiens: GO:0003406 retinal pigment epithelium development (loss)
c-value
0.8
0.7
0.6
0.5
0.4
0.3
0.2
0.1
0.0
p-value
2e-02
3e-02
4e-02
5e-02
6e-02
Log-odds
0.5
1.0
1.5
2.0
2.5
3.0
3.5
4.0
Cellular_organisms - ps1
Archaea1 - ps2
Archaea2 - ps3
Archaea3 - ps4
Asgard/Eukaryota - ps5
Eukaryota - ps6
Amorpheae - ps7
Obazoa - ps8
Opisthokonta - ps9
Holozoa - ps10
Filozoa - ps11
Choano./Metazoa - ps12
Metazoa - ps13
Myriazoa - ps14
Parahoxozoa - ps15
Bilateria - ps16
Deuterostomia - ps17
Chordata - ps18
Olfactores - ps19
Vertebrata - ps20
Gnathostomata - ps21
Euteleostomi - ps22
Sarcopterygii - ps23
Tetrapoda - ps24
Amniota - ps25
Mammalia - ps26
Theria - ps27
Placentalia - ps28
Boreoeutheria - ps29
Euarchontoglires - ps30
Euarchonta - ps31
Primates - ps32
Haplorrhini - ps33
Simiiformes - ps34
Catarrhini - ps35
Hominoidea - ps36
Hominidae - ps37
Homininae - ps38
Hominini - ps39
Homo_sapiens - ps40
Phylostratum

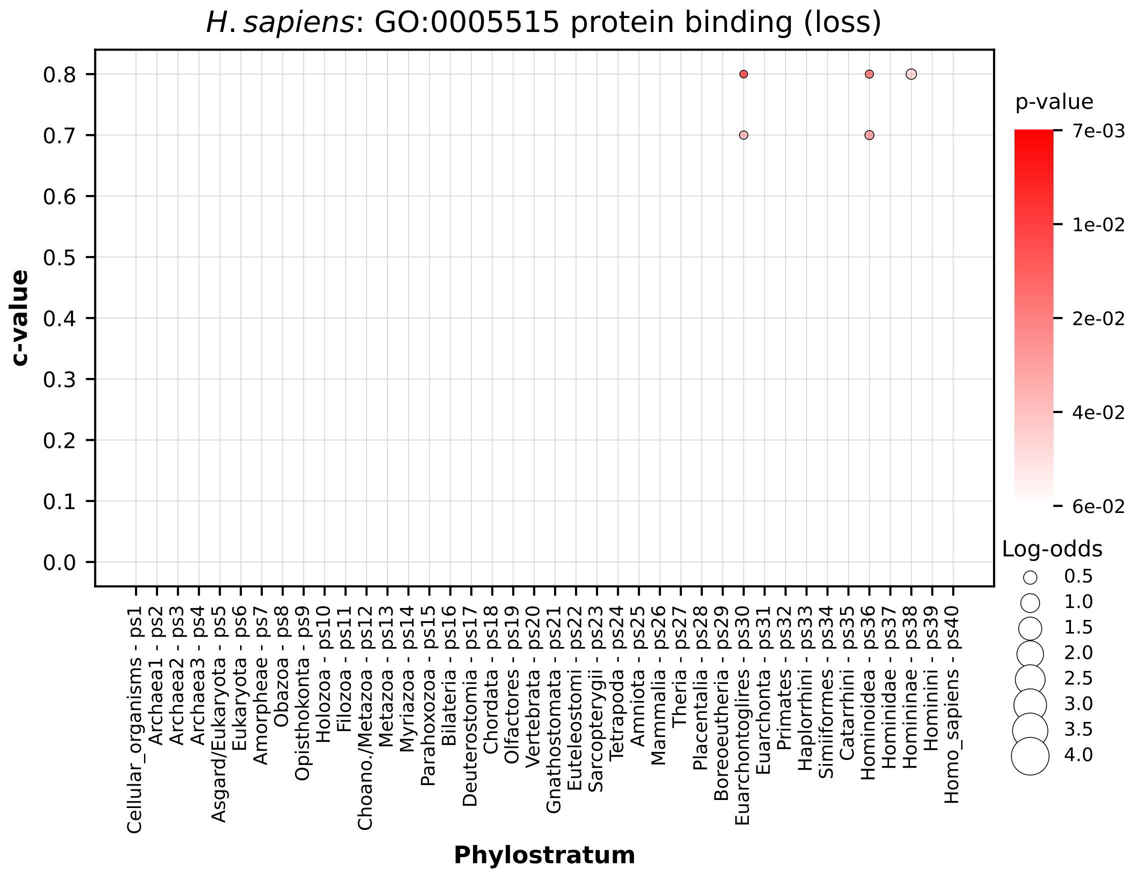

H. sapiens: GO:0005515 protein binding (loss)
c-value
0.0
0.1
0.2
0.3
0.4
0.5
0.6
0.7
0.8
Phylostratum
Cellular_organisms - ps1
Archaea1 - ps2
Archaea2 - ps3
Archaea3 - ps4
Asgard/Eukaryota - ps5
Eukaryota - ps6
Amorpheae - ps7
Obazoa - ps8
Opisthokonta - ps9
Holozoa - ps10
Filozoa - ps11
Choano./Metazoa - ps12
Metazoa - ps13
Myriazoa - ps14
Parahoxozoa - ps15
Bilateria - ps16
Deuterostomia - ps17
Chordata - ps18
Olfactores - ps19
Vertebrata - ps20
Gnathostomata - ps21
Euteleostomi - ps22
Sarcopterygii - ps23
Tetrapoda - ps24
Amniota - ps25
Mammalia - ps26
Theria - ps27
Placentalia - ps28
Boreoeutheria - ps29
Euarchontoglires - ps30
Euarchonta - ps31
Primates - ps32
Haplorrhini - ps33
Simiiformes - ps34
Catarrhini - ps35
Hominoidea - ps36
Hominidae - ps37
Homininae - ps38
Hominini - ps39
Homo_sapiens - ps40
p-value
7e-03
1e-02
2e-02
4e-02
6e-02
Log-odds
0.5
1.0
1.5
2.0
2.5
3.0
3.5
4.0

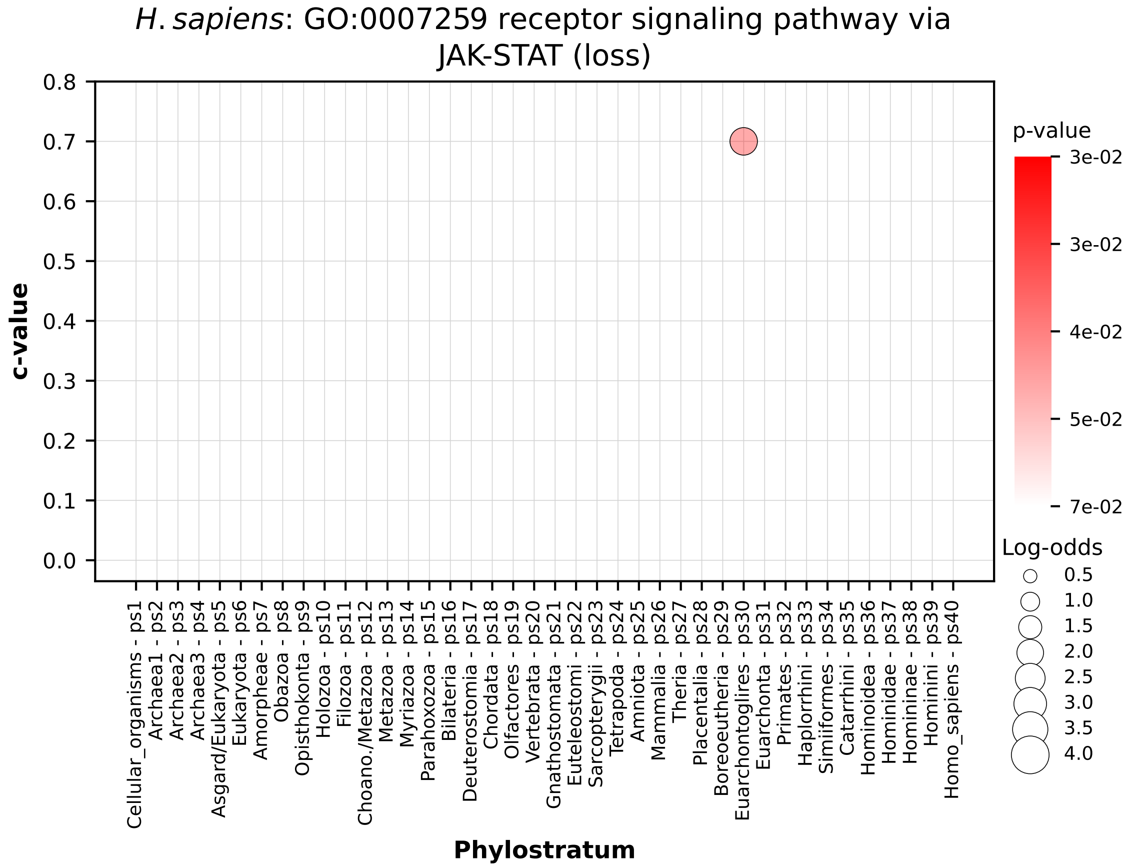

H. sapiens: GO:0007259 receptor signaling pathway via JAK-STAT (loss)
c-value
0.8
0.7
0.6
0.5
0.4
0.3
0.2
0.1
0.0
p-value
3e-02
3e-02
4e-02
5e-02
7e-02
Log-odds
0.5
1.0
1.5
2.0
2.5
3.0
3.5
4.0
Cellular_organisms - ps1
Archaea1 - ps2
Archaea2 - ps3
Archaea3 - ps4
Asgard/Eukaryota - ps5
Eukaryota - ps6
Amorpheae - ps7
Obazoa - ps8
Opisthokonta - ps9
Holozoa - ps10
Filozoa - ps11
Choano./Metazoa - ps12
Metazoa - ps13
Myriazoa - ps14
Parahoxozoa - ps15
Bilateria - ps16
Deuterostomia - ps17
Chordata - ps18
Olfactores - ps19
Vertebrata - ps20
Gnathostomata - ps21
Euteleostomi - ps22
Sarcopterygii - ps23
Tetrapoda - ps24
Amniota - ps25
Mammalia - ps26
Theria - ps27
Placentalia - ps28
Boreoeutheria - ps29
Euarchontoglires - ps30
Euarchonta - ps31
Primates - ps32
Haplorrhini - ps33
Simiiformes - ps34
Catarrhini - ps35
Hominoidea - ps36
Hominidae - ps37
Homininae - ps38
Hominini - ps39
Homo_sapiens - ps40
Phylostratum

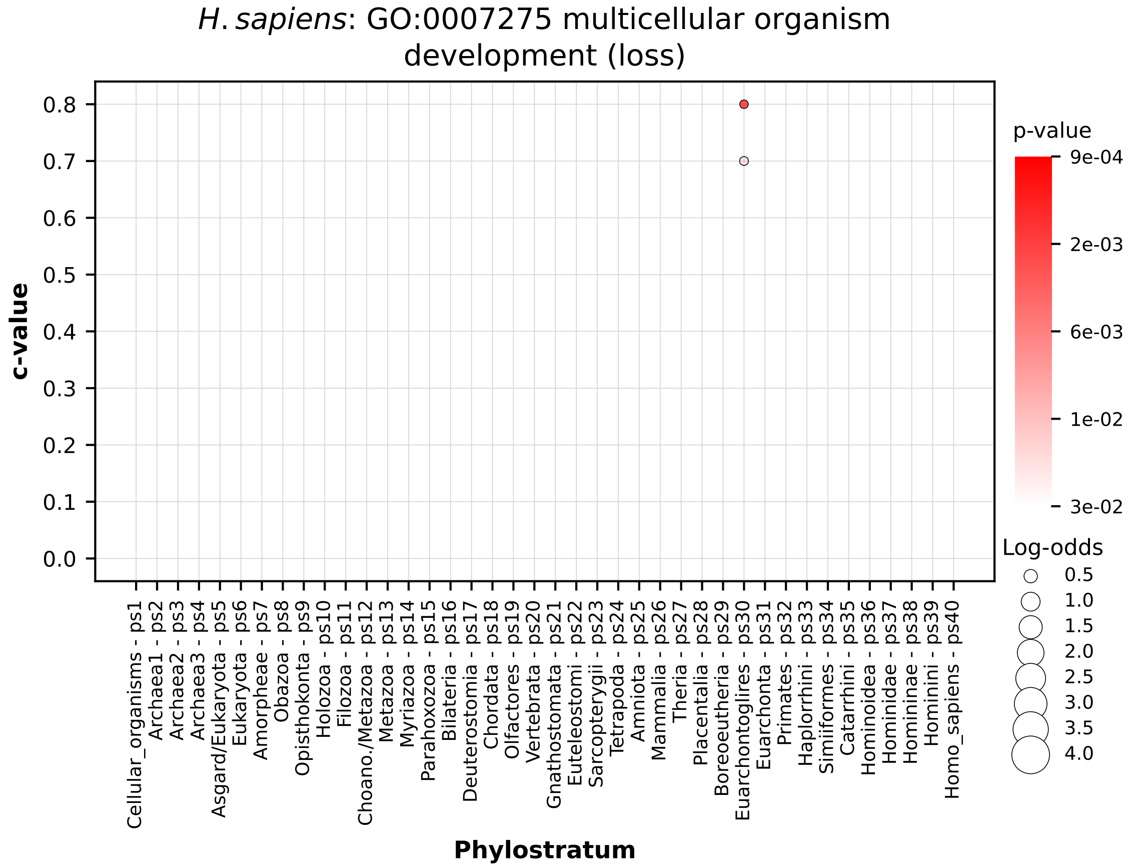

H. sapiens: GO:0007275 multicellular organism development (loss)
c-value
0.8
0.7
0.6
0.5
0.4
0.3
0.2
0.1
0.0
p-value
9e-04
2e-03
6e-03
1e-02
3e-02
Log-odds
0.5
1.0
1.5
2.0
2.5
3.0
3.5
4.0
Cellular_organisms - ps1
Archaea1 - ps2
Archaea2 - ps3
Archaea3 - ps4
Asgard/Eukaryota - ps5
Eukaryota - ps6
Amorpheae - ps7
Obazoa - ps8
Opisthokonta - ps9
Holozoa - ps10
Filozoa - ps11
Choano./Metazoa - ps12
Metazoa - ps13
Myriazoa - ps14
Parahoxozoa - ps15
Bilateria - ps16
Deuterostomia - ps17
Chordata - ps18
Olfactores - ps19
Vertebrata - ps20
Gnathostomata - ps21
Euteleostomi - ps22
Sarcopterygii - ps23
Tetrapoda - ps24
Amniota - ps25
Mammalia - ps26
Theria - ps27
Placentalia - ps28
Boreoeutheria - ps29
Euarchontoglires - ps30
Euarchonta - ps31
Primates - ps32
Haplorrhini - ps33
Simiiformes - ps34
Catarrhini - ps35
Hominoidea - ps36
Hominidae - ps37
Homininae - ps38
Hominini - ps39
Homo_sapiens - ps40
Phylostratum

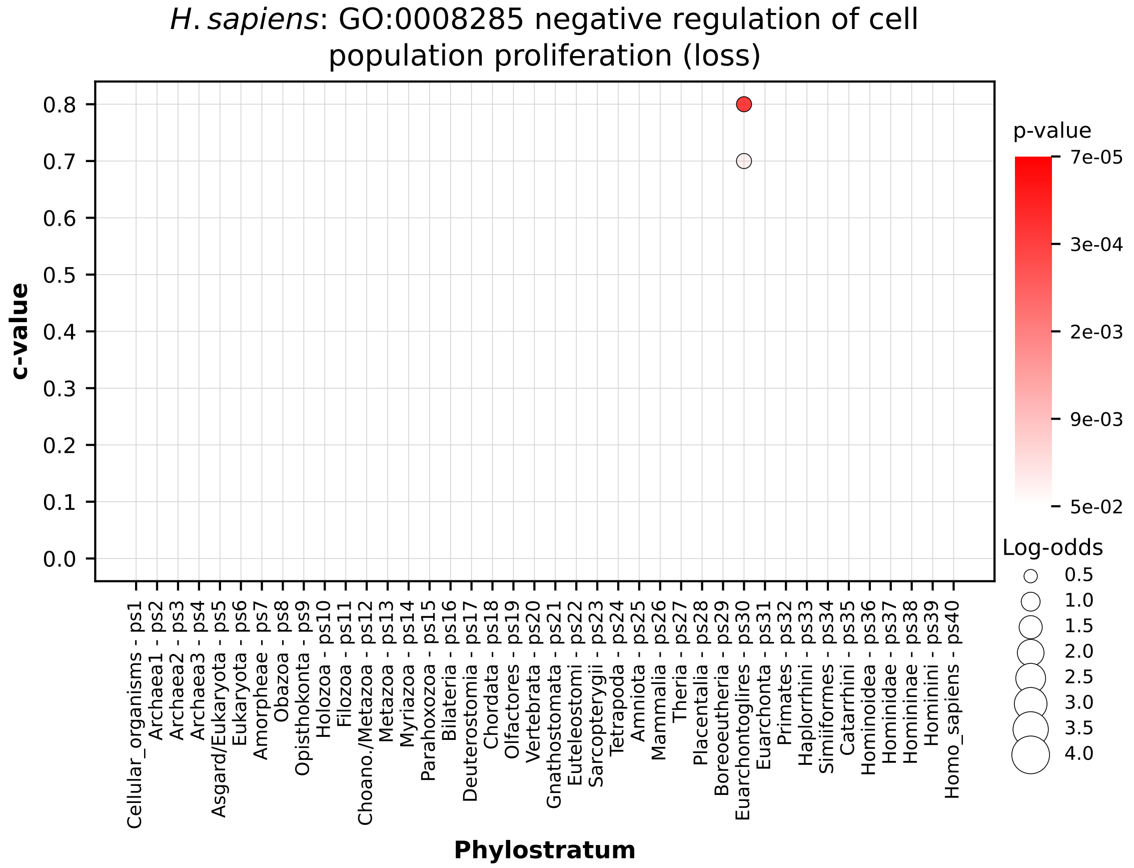
H. sapiens: GO:0008285 negative regulation of cell population proliferation (loss)
c-value
0.0
0.1
0.2
0.3
0.4
0.5
0.6
0.7
0.8
Phylostratum
Cellular_organisms - ps1
Archaea1 - ps2
Archaea2 - ps3
Archaea3 - ps4
Asgard/Eukaryota - ps5
Eukaryota - ps6
Amorpheae - ps7
Obazoa - ps8
Opisthokonta - ps9
Holozoa - ps10
Filozoa - ps11
Choano./Metazoa - ps12
Metazoa - ps13
Myriazoa - ps14
Parahoxozoa - ps15
Bilateria - ps16
Deuterostomia - ps17
Chordata - ps18
Olfactores - ps19
Vertebrata - ps20
Gnathostomata - ps21
Euteleostomi - ps22
Sarcopterygii - ps23
Tetrapoda - ps24
Amniota - ps25
Mammalia - ps26
Theria - ps27
Placentalia - ps28
Boreoeutheria - ps29
Euarchontoglires - ps30
Euarchonta - ps31
Primates - ps32
Haplorrhini - ps33
Simiiformes - ps34
Catarrhini - ps35
Hominoidea - ps36
Hominidae - ps37
Homininae - ps38
Hominini - ps39
Homo_sapiens - ps40
p-value
7e-05
3e-04
2e-03
9e-03
5e-02
Log-odds
0.5
1.0
1.5
2.0
2.5
3.0
3.5
4.0

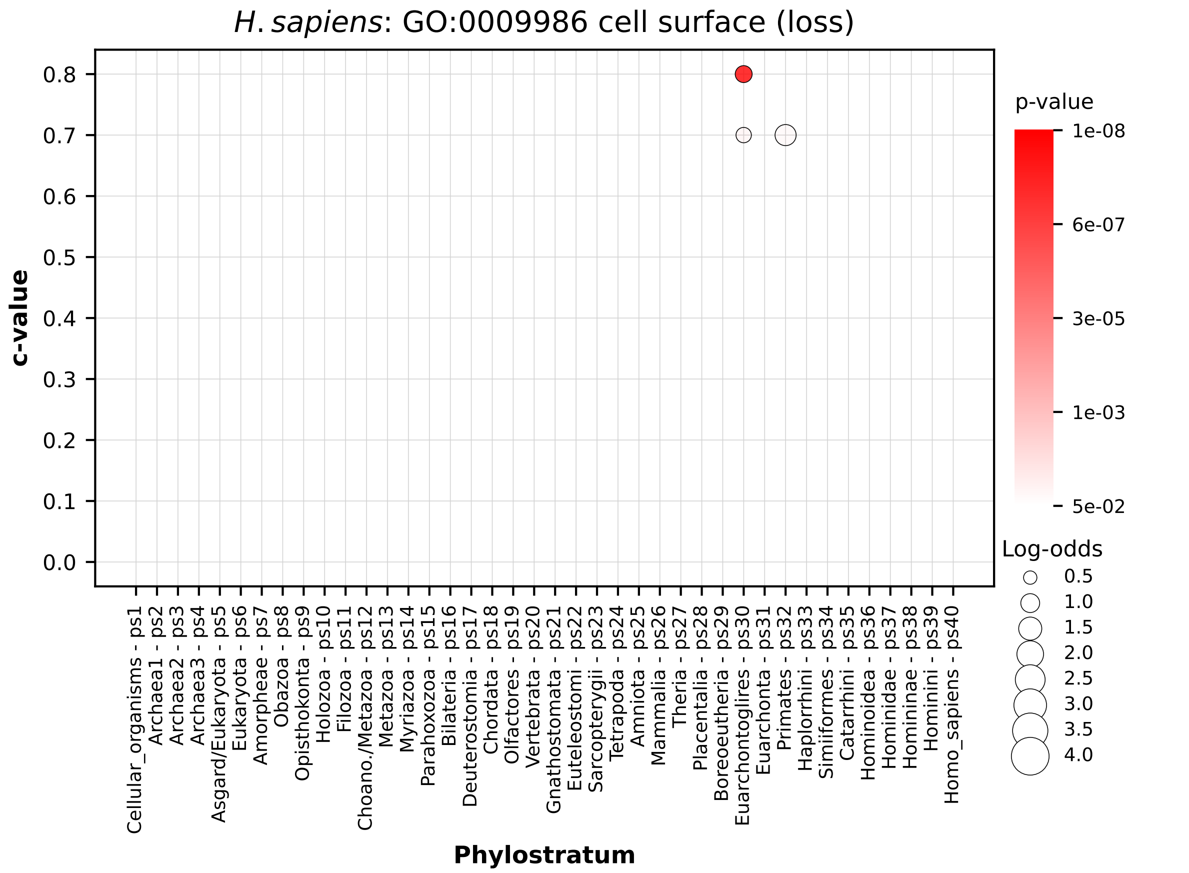

H. sapiens: GO:0009986 cell surface (loss)
c-value
0.8
0.7
0.6
0.5
0.4
0.3
0.2
0.1
0.0
p-value
1e-08
6e-07
3e-05
1e-03
5e-02
Log-odds
0.5
1.0
1.5
2.0
2.5
3.0
3.5
4.0
Cellular_organisms - ps1
Archaea1 - ps2
Archaea2 - ps3
Archaea3 - ps4
Asgard/Eukaryota - ps5
Eukaryota - ps6
Amorpheae - ps7
Obazoa - ps8
Opisthokonta - ps9
Holozoa - ps10
Filozoa - ps11
Choano./Metazoa - ps12
Metazoa - ps13
Myriazoa - ps14
Parahoxozoa - ps15
Bilateria - ps16
Deuterostomia - ps17
Chordata - ps18
Olfactores - ps19
Vertebrata - ps20
Gnathostomata - ps21
Euteleostomi - ps22
Sarcopterygii - ps23
Tetrapoda - ps24
Amniota - ps25
Mammalia - ps26
Theria - ps27
Placentalia - ps28
Boreoeutheria - ps29
Euarchontoglires - ps30
Euarchonta - ps31
Primates - ps32
Haplorrhini - ps33
Simiiformes - ps34
Catarrhini - ps35
Hominoidea - ps36
Hominidae - ps37
Homininae - ps38
Hominini - ps39
Homo_sapiens - ps40
Phylostratum

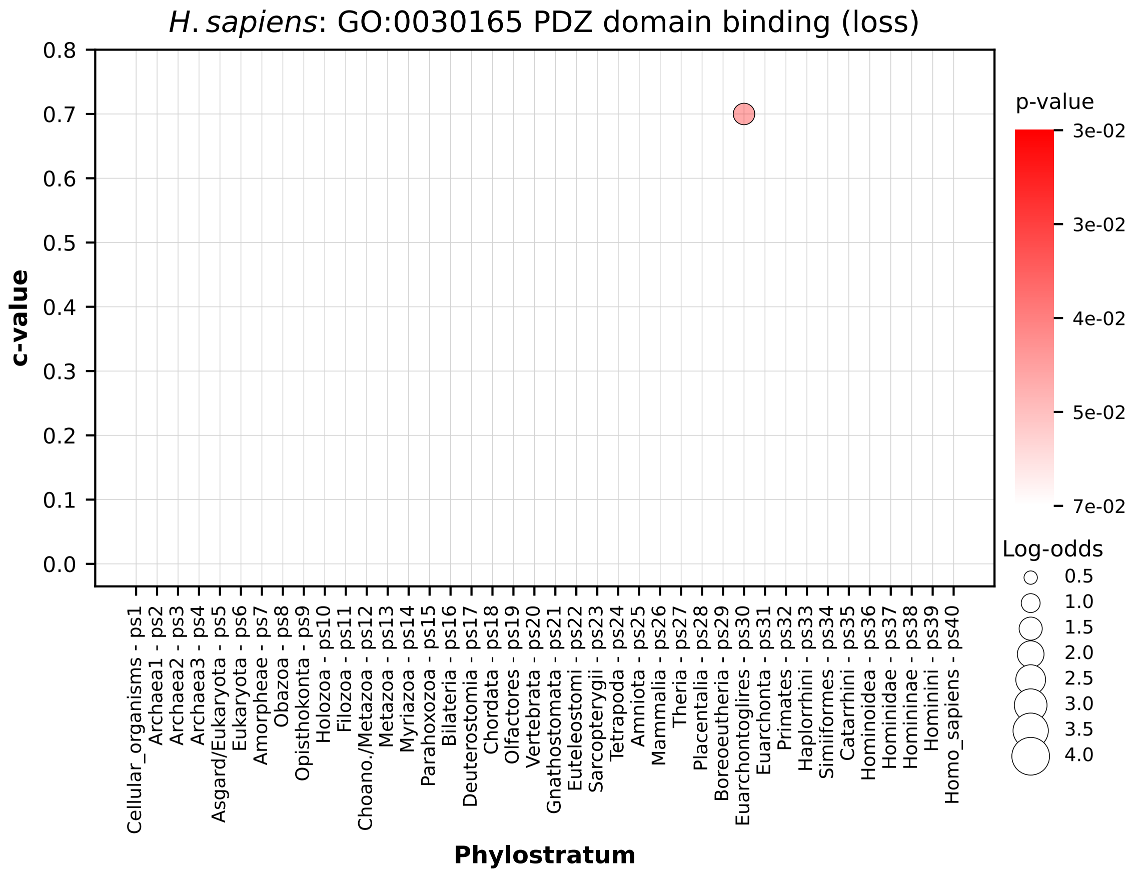

H. sapiens: GO:0030165 PDZ domain binding (loss)
c-value
0.8
0.7
0.6
0.5
0.4
0.3
0.2
0.1
0.0
p-value
3e-02
3e-02
4e-02
5e-02
7e-02
Log-odds
0.5
1.0
1.5
2.0
2.5
3.0
3.5
4.0
Cellular_organisms - ps1
Archaea1 - ps2
Archaea2 - ps3
Archaea3 - ps4
Asgard/Eukaryota - ps5
Eukaryota - ps6
Amorpheae - ps7
Obazoa - ps8
Opisthokonta - ps9
Holozoa - ps10
Filozoa - ps11
Choano./Metazoa - ps12
Metazoa - ps13
Myriazoa - ps14
Parahoxozoa - ps15
Bilateria - ps16
Deuterostomia - ps17
Chordata - ps18
Olfactores - ps19
Vertebrata - ps20
Gnathostomata - ps21
Euteleostomi - ps22
Sarcopterygii - ps23
Tetrapoda - ps24
Amniota - ps25
Mammalia - ps26
Theria - ps27
Placentalia - ps28
Boreoeutheria - ps29
Euarchontoglires - ps30
Euarchonta - ps31
Primates - ps32
Haplorrhini - ps33
Simiiformes - ps34
Catarrhini - ps35
Hominoidea - ps36
Hominidae - ps37
Homininae - ps38
Hominini - ps39
Homo_sapiens - ps40
Phylostratum

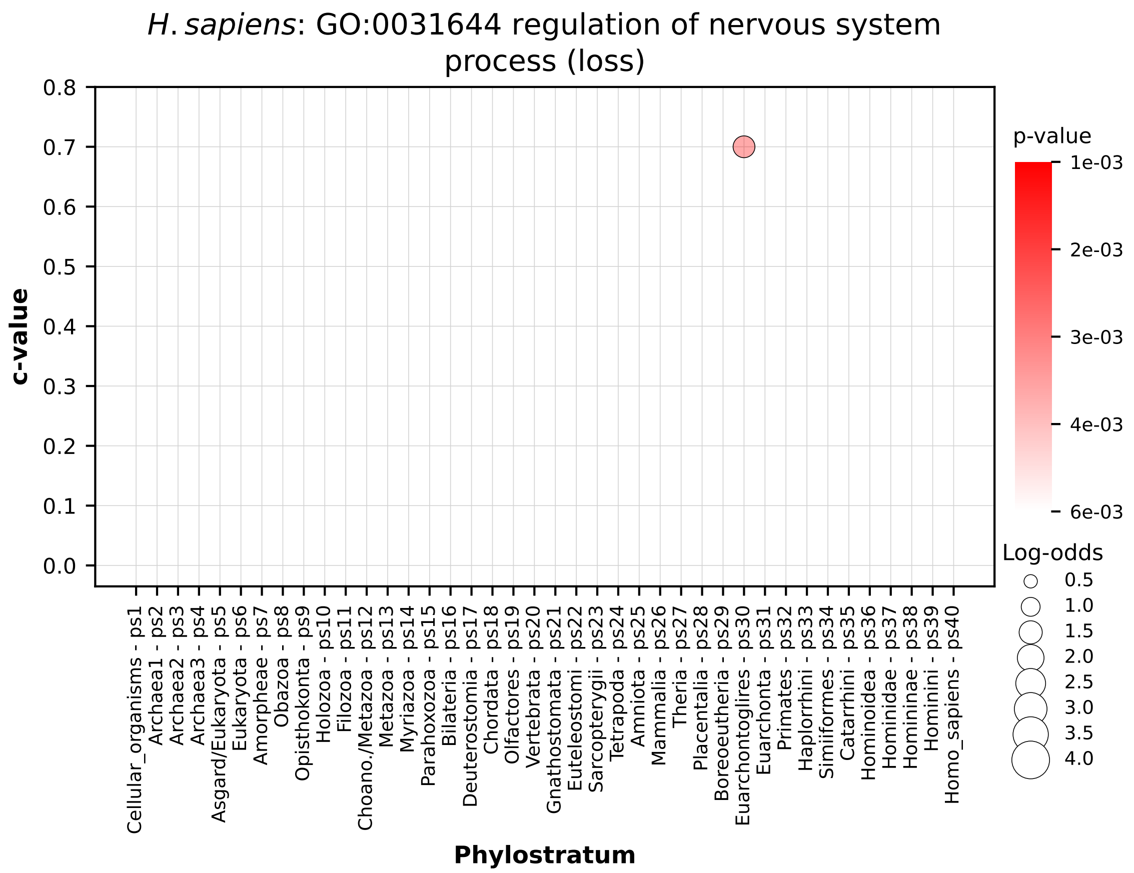

H. sapiens: GO:0031644 regulation of nervous system process (loss)
c-value
0.8
0.7
0.6
0.5
0.4
0.3
0.2
0.1
0.0
p-value
1e-03
2e-03
3e-03
4e-03
6e-03
Log-odds
0.5
1.0
1.5
2.0
2.5
3.0
3.5
4.0
Cellular_organisms - ps1
Archaea1 - ps2
Archaea2 - ps3
Archaea3 - ps4
Asgard/Eukaryota - ps5
Eukaryota - ps6
Amorpheae - ps7
Obazoa - ps8
Opisthokonta - ps9
Holozoa - ps10
Filozoa - ps11
Choano./Metazoa - ps12
Metazoa - ps13
Myriazoa - ps14
Parahoxozoa - ps15
Bilateria - ps16
Deuterostomia - ps17
Chordata - ps18
Olfactores - ps19
Vertebrata - ps20
Gnathostomata - ps21
Euteleostomi - ps22
Sarcopterygii - ps23
Tetrapoda - ps24
Amniota - ps25
Mammalia - ps26
Theria - ps27
Placentalia - ps28
Boreoeutheria - ps29
Euarchontoglires - ps30
Euarchonta - ps31
Primates - ps32
Haplorrhini - ps33
Simiiformes - ps34
Catarrhini - ps35
Hominoidea - ps36
Hominidae - ps37
Homininae - ps38
Hominini - ps39
Homo_sapiens - ps40
Phylostratum

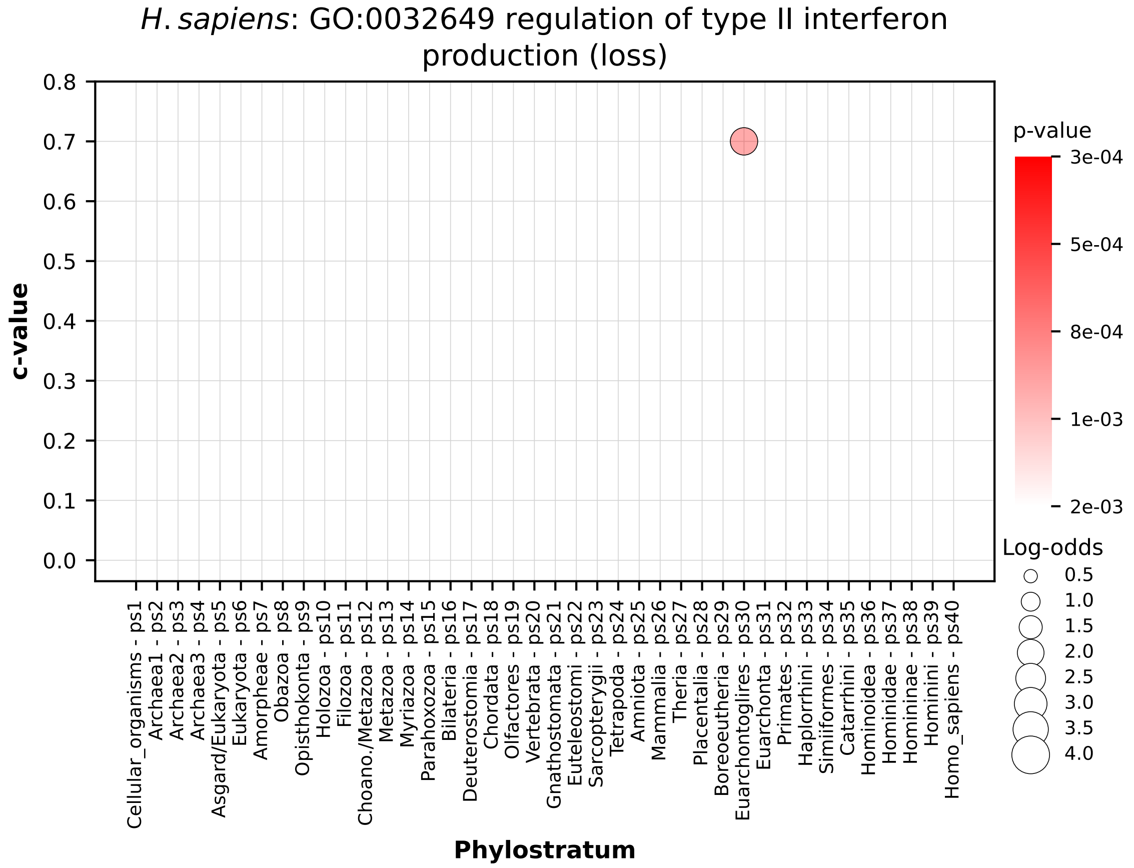

H. sapiens: GO:0032649 regulation of type II interferon production (loss)
c-value
0.8
0.7
0.6
0.5
0.4
0.3
0.2
0.1
0.0
p-value
3e-04
5e-04
8e-04
1e-03
2e-03
Log-odds
0.5
1.0
1.5
2.0
2.5
3.0
3.5
4.0
Cellular_organisms - ps1
Archaea1 - ps2
Archaea2 - ps3
Archaea3 - ps4
Asgard/Eukaryota - ps5
Eukaryota - ps6
Amorpheae - ps7
Obazoa - ps8
Opisthokonta - ps9
Holozoa - ps10
Filozoa - ps11
Choano./Metazoa - ps12
Metazoa - ps13
Myriazoa - ps14
Parahoxozoa - ps15
Bilateria - ps16
Deuterostomia - ps17
Chordata - ps18
Olfactores - ps19
Vertebrata - ps20
Gnathostomata - ps21
Euteleostomi - ps22
Sarcopterygii - ps23
Tetrapoda - ps24
Amniota - ps25
Mammalia - ps26
Theria - ps27
Placentalia - ps28
Boreoeutheria - ps29
Euarchontoglires - ps30
Euarchonta - ps31
Primates - ps32
Haplorrhini - ps33
Simiiformes - ps34
Catarrhini - ps35
Hominoidea - ps36
Hominidae - ps37
Homininae - ps38
Hominini - ps39
Homo_sapiens - ps40
Phylostratum

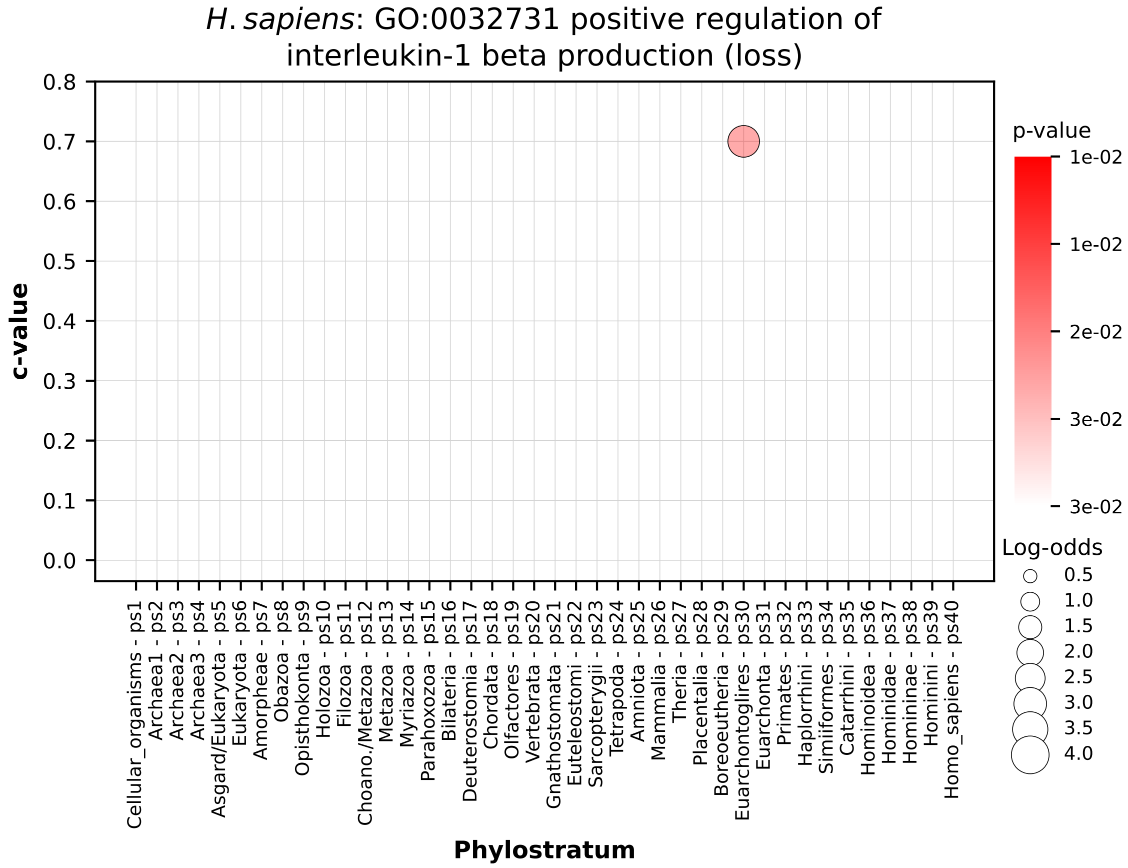
H. sapiens: GO:0032731 positive regulation of interleukin-1 beta production (loss)
c-value
0.8
0.7
0.6
0.5
0.4
0.3
0.2
0.1
0.0
p-value
1e-02
1e-02
2e-02
3e-02
3e-02
Log-odds
0.5
1.0
1.5
2.0
2.5
3.0
3.5
4.0
Cellular_organisms - ps1
Archaea1 - ps2
Archaea2 - ps3
Archaea3 - ps4
Asgard/Eukaryota - ps5
Eukaryota - ps6
Amorpheae - ps7
Obazoa - ps8
Opisthokonta - ps9
Holozoa - ps10
Filozoa - ps11
Choano./Metazoa - ps12
Metazoa - ps13
Myriazoa - ps14
Parahoxozoa - ps15
Bilateria - ps16
Deuterostomia - ps17
Chordata - ps18
Olfactores - ps19
Vertebrata - ps20
Gnathostomata - ps21
Euteleostomi - ps22
Sarcopterygii - ps23
Tetrapoda - ps24
Amniota - ps25
Mammalia - ps26
Theria - ps27
Placentalia - ps28
Boreoeutheria - ps29
Euarchontoglires - ps30
Euarchonta - ps31
Primates - ps32
Haplorrhini - ps33
Simiiformes - ps34
Catarrhini - ps35
Hominoidea - ps36
Hominidae - ps37
Homininae - ps38
Hominini - ps39
Homo_sapiens - ps40
Phylostratum

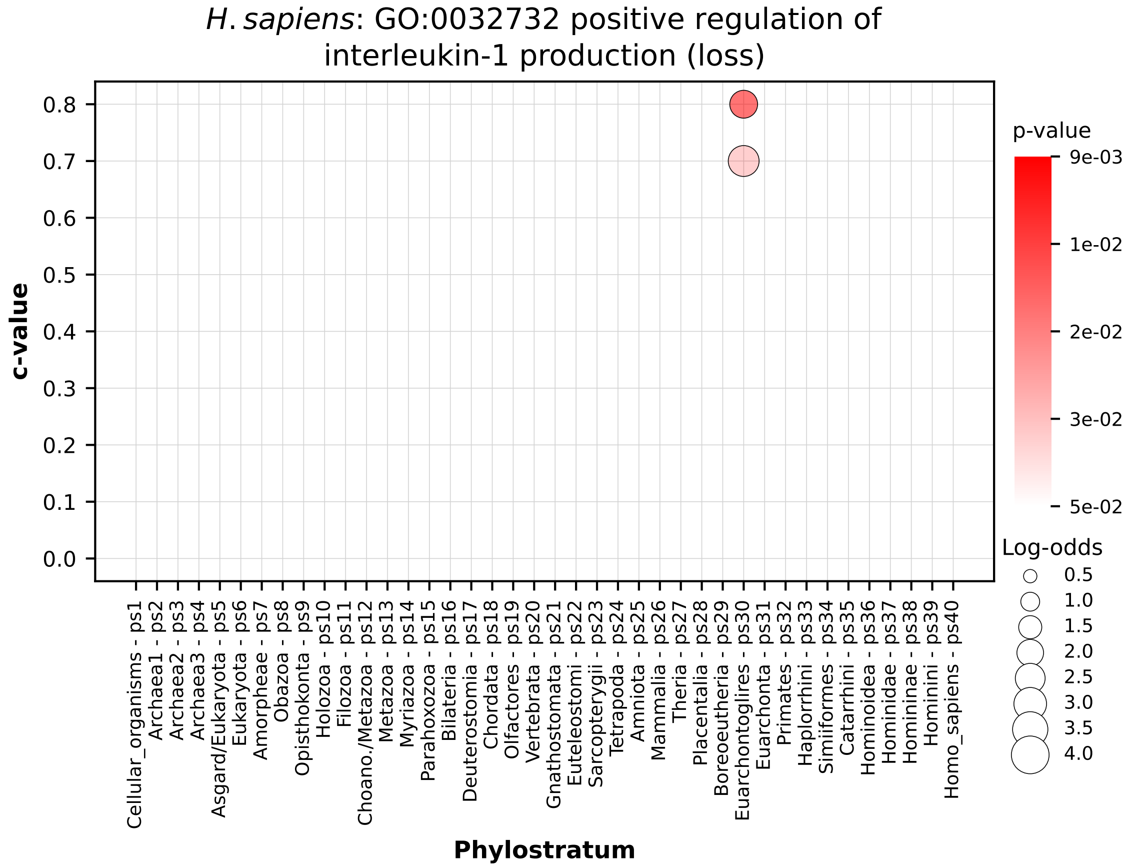

H. sapiens: GO:0032732 positive regulation of interleukin-1 production (loss)
c-value
0.8
0.7
0.6
0.5
0.4
0.3
0.2
0.1
0.0
p-value
9e-03
1e-02
2e-02
3e-02
5e-02
Log-odds
0.5
1.0
1.5
2.0
2.5
3.0
3.5
4.0
Cellular_organisms - ps1
Archaea1 - ps2
Archaea2 - ps3
Archaea3 - ps4
Asgard/Eukaryota - ps5
Eukaryota - ps6
Amorpheae - ps7
Obazoa - ps8
Opisthokonta - ps9
Holozoa - ps10
Filozoa - ps11
Choano./Metazoa - ps12
Metazoa - ps13
Myriazoa - ps14
Parahoxozoa - ps15
Bilateria - ps16
Deuterostomia - ps17
Chordata - ps18
Olfactores - ps19
Vertebrata - ps20
Gnathostomata - ps21
Euteleostomi - ps22
Sarcopterygii - ps23
Tetrapoda - ps24
Amniota - ps25
Mammalia - ps26
Theria - ps27
Placentalia - ps28
Boreoeutheria - ps29
Euarchontoglires - ps30
Euarchonta - ps31
Primates - ps32
Haplorrhini - ps33
Simiiformes - ps34
Catarrhini - ps35
Hominoidea - ps36
Hominidae - ps37
Homininae - ps38
Hominini - ps39
Homo_sapiens - ps40
Phylostratum

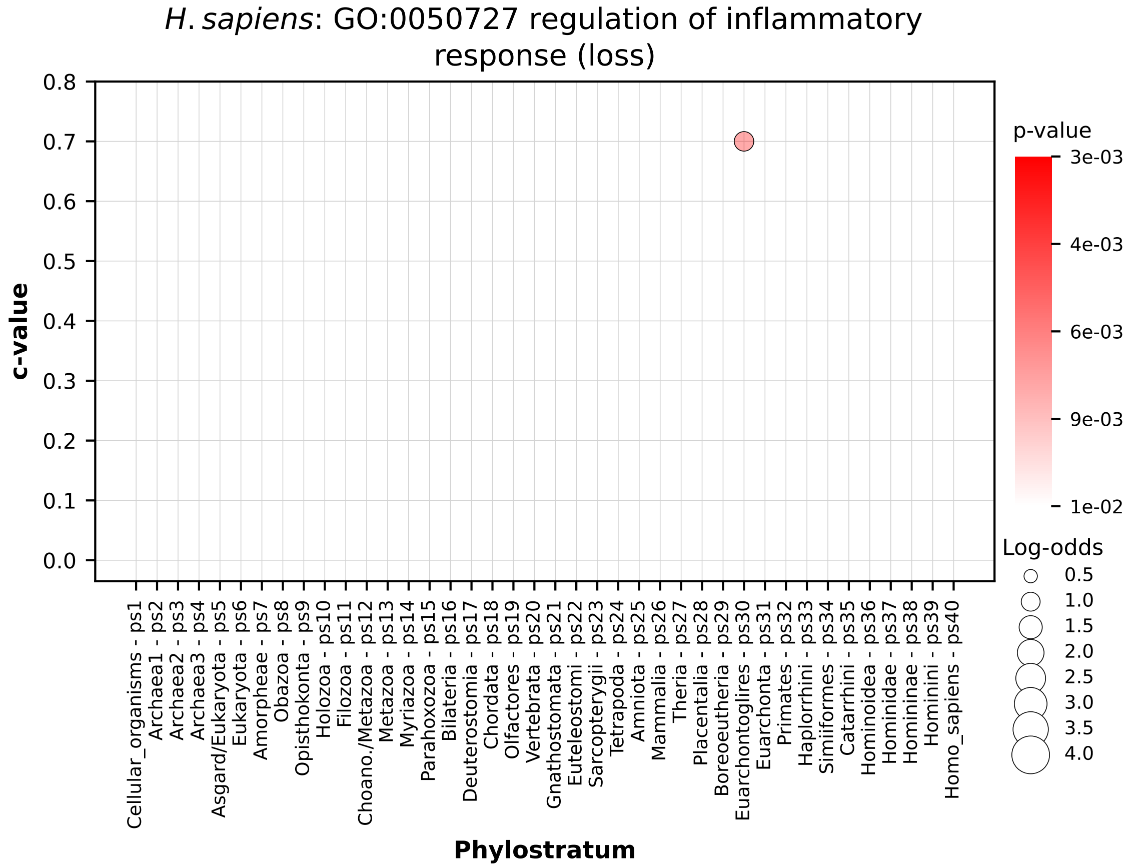

H. sapiens: GO:0050727 regulation of inflammatory response (loss)
c-value
0.0
0.1
0.2
0.3
0.4
0.5
0.6
0.7
0.8
p-value
3e-03
4e-03
6e-03
9e-03
1e-02
Log-odds
0.5
1.0
1.5
2.0
2.5
3.0
3.5
4.0
Cellular_organisms - ps1
Archaea1 - ps2
Archaea2 - ps3
Archaea3 - ps4
Asgard/Eukaryota - ps5
Eukaryota - ps6
Amorpheae - ps7
Obazoa - ps8
Opisthokonta - ps9
Holozoa - ps10
Filozoa - ps11
Choano./Metazoa - ps12
Metazoa - ps13
Myriazoa - ps14
Parahoxozoa - ps15
Bilateria - ps16
Deuterostomia - ps17
Chordata - ps18
Olfactores - ps19
Vertebrata - ps20
Gnathostomata - ps21
Euteleostomi - ps22
Sarcopterygii - ps23
Tetrapoda - ps24
Amniota - ps25
Mammalia - ps26
Theria - ps27
Placentalia - ps28
Boreoeutheria - ps29
Euarchontoglires - ps30
Euarchonta - ps31
Primates - ps32
Haplorrhini - ps33
Simiiformes - ps34
Catarrhini - ps35
Hominoidea - ps36
Hominidae - ps37
Homininae - ps38
Hominini - ps39
Homo_sapiens - ps40
Phylostratum

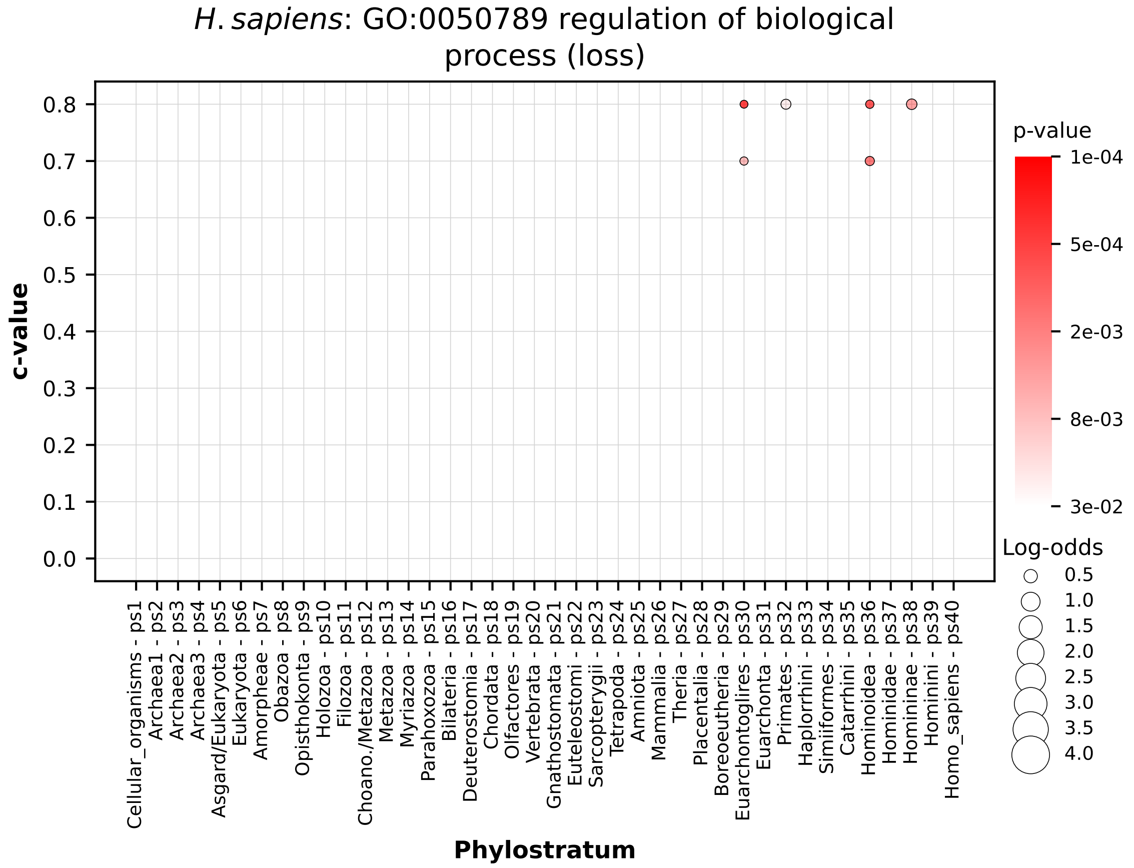

H. sapiens: GO:0050789 regulation of biological process (loss)
c-value
0.0
0.1
0.2
0.3
0.4
0.5
0.6
0.7
0.8
Phylostratum
Cellular_organisms - ps1
Archaea1 - ps2
Archaea2 - ps3
Archaea3 - ps4
Asgard/Eukaryota - ps5
Eukaryota - ps6
Amorpheae - ps7
Obazoa - ps8
Opisthokonta - ps9
Holozoa - ps10
Filozoa - ps11
Choano./Metazoa - ps12
Metazoa - ps13
Myriazoa - ps14
Parahoxozoa - ps15
Bilateria - ps16
Deuterostomia - ps17
Chordata - ps18
Olfactores - ps19
Vertebrata - ps20
Gnathostomata - ps21
Euteleostomi - ps22
Sarcopterygii - ps23
Tetrapoda - ps24
Amniota - ps25
Mammalia - ps26
Theria - ps27
Placentalia - ps28
Boreoeutheria - ps29
Euarchontoglires - ps30
Euarchonta - ps31
Primates - ps32
Haplorrhini - ps33
Simiiformes - ps34
Catarrhini - ps35
Hominoidea - ps36
Hominidae - ps37
Homininae - ps38
Hominini - ps39
Homo_sapiens - ps40
p-value
1e-04
5e-04
2e-03
8e-03
3e-02
Log-odds
0.5
1.0
1.5
2.0
2.5
3.0
3.5
4.0

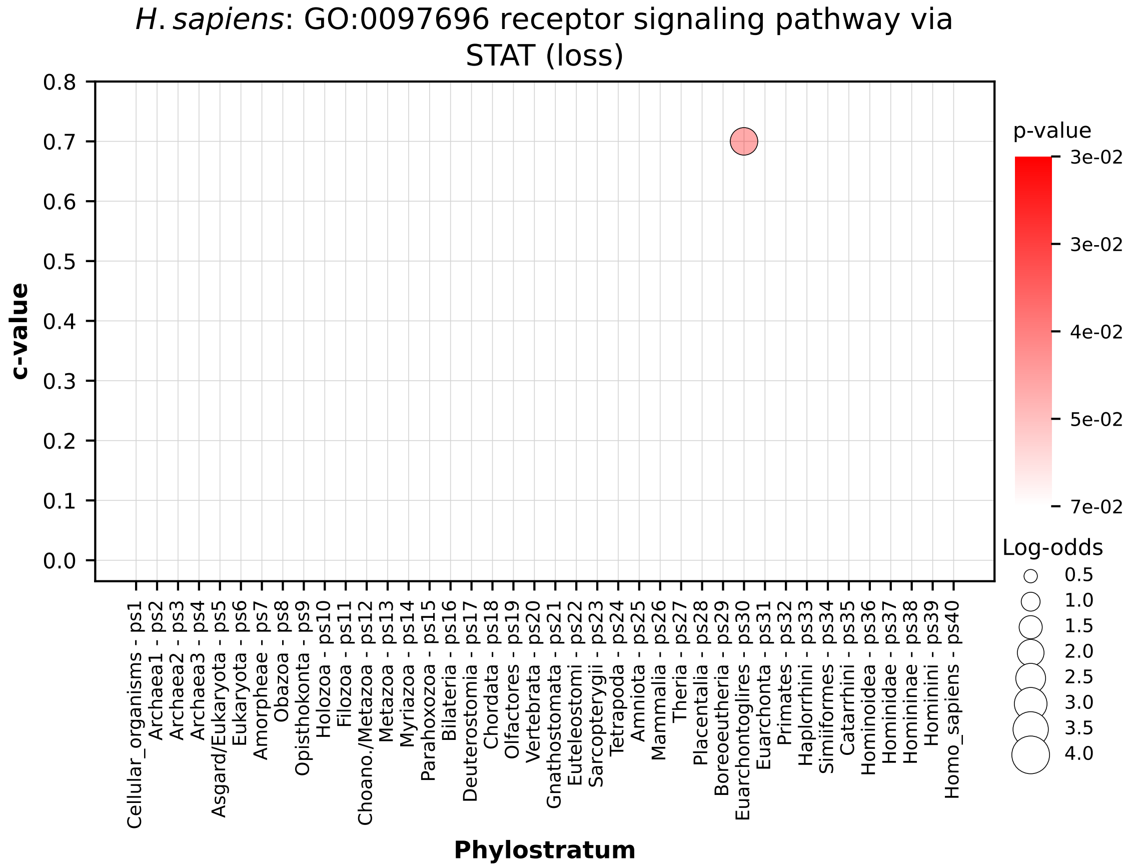

H. sapiens: GO:0097696 receptor signaling pathway via STAT (loss)
c-value
0.8
0.7
0.6
0.5
0.4
0.3
0.2
0.1
0.0
p-value
3e-02
3e-02
4e-02
5e-02
7e-02
Log-odds
0.5
1.0
1.5
2.0
2.5
3.0
3.5
4.0
Cellular_organisms - ps1
Archaea1 - ps2
Archaea2 - ps3
Archaea3 - ps4
Asgard/Eukaryota - ps5
Eukaryota - ps6
Amorpheae - ps7
Obazoa - ps8
Opisthokonta - ps9
Holozoa - ps10
Filozoa - ps11
Choano./Metazoa - ps12
Metazoa - ps13
Myriazoa - ps14
Parahoxozoa - ps15
Bilateria - ps16
Deuterostomia - ps17
Chordata - ps18
Olfactores - ps19
Vertebrata - ps20
Gnathostomata - ps21
Euteleostomi - ps22
Sarcopterygii - ps23
Tetrapoda - ps24
Amniota - ps25
Mammalia - ps26
Theria - ps27
Placentalia - ps28
Boreoeutheria - ps29
Euarchontoglires - ps30
Euarchonta - ps31
Primates - ps32
Haplorrhini - ps33
Simiiformes - ps34
Catarrhini - ps35
Hominoidea - ps36
Hominidae - ps37
Homininae - ps38
Hominini - ps39
Homo_sapiens - ps40
Phylostratum

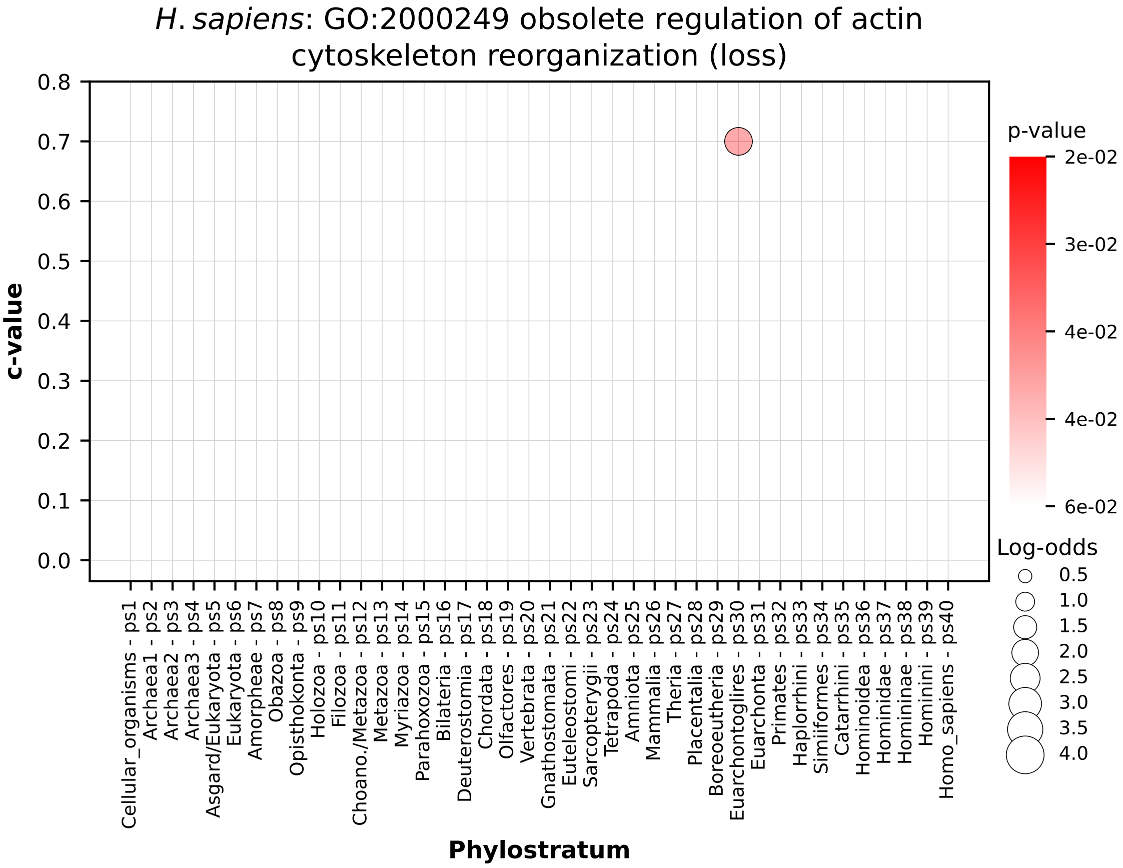
H. sapiens: GO:2000249 obsolete regulation of actin cytoskeleton reorganization (loss)
c-value
0.8
0.7
0.6
0.5
0.4
0.3
0.2
0.1
0.0
p-value
2e-02
3e-02
4e-02
4e-02
6e-02
Log-odds
0.5
1.0
1.5
2.0
2.5
3.0
3.5
4.0
Cellular_organisms - ps1
Archaea1 - ps2
Archaea2 - ps3
Archaea3 - ps4
Asgard/Eukaryota - ps5
Eukaryota - ps6
Amorpheae - ps7
Obazoa - ps8
Opisthokonta - ps9
Holozoa - ps10
Filozoa - ps11
Choano./Metazoa - ps12
Metazoa - ps13
Myriazoa - ps14
Parahoxozoa - ps15
Bilateria - ps16
Deuterostomia - ps17
Chordata - ps18
Olfactores - ps19
Vertebrata - ps20
Gnathostomata - ps21
Euteleostomi - ps22
Sarcopterygii - ps23
Tetrapoda - ps24
Amniota - ps25
Mammalia - ps26
Theria - ps27
Placentalia - ps28
Boreoeutheria - ps29
Euarchontoglires - ps30
Euarchonta - ps31
Primates - ps32
Haplorrhini - ps33
Simiiformes - ps34
Catarrhini - ps35
Hominoidea - ps36
Hominidae - ps37
Homininae - ps38
Hominini - ps39
Homo_sapiens - ps40
Phylostratum

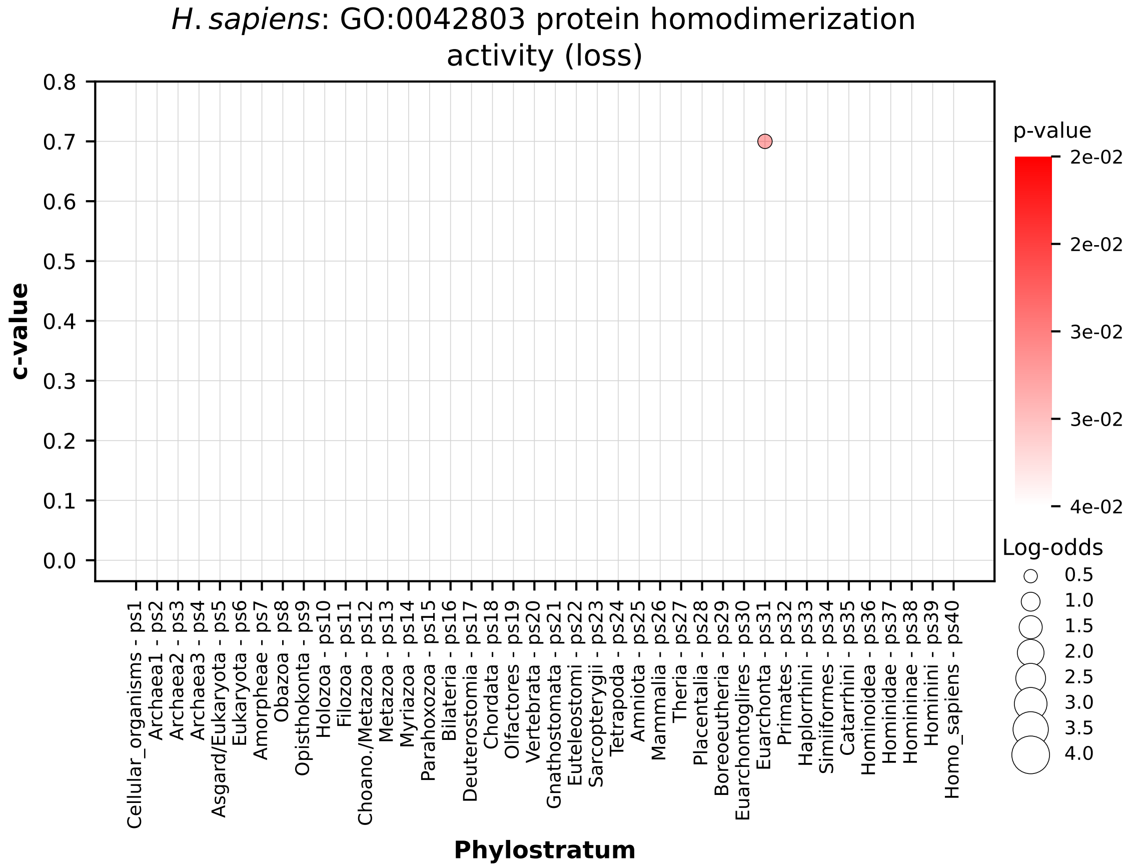
H. sapiens: GO:0042803 protein homodimerization activity (loss)
c-value
0.8
0.7
0.6
0.5
0.4
0.3
0.2
0.1
0.0
p-value
2e-02
2e-02
3e-02
3e-02
4e-02
Log-odds
0.5
1.0
1.5
2.0
2.5
3.0
3.5
4.0
Cellular_organisms - ps1
Archaea1 - ps2
Archaea2 - ps3
Archaea3 - ps4
Asgard/Eukaryota - ps5
Eukaryota - ps6
Amorpheae - ps7
Obazoa - ps8
Opisthokonta - ps9
Holozoa - ps10
Filozoa - ps11
Choano./Metazoa - ps12
Metazoa - ps13
Myriazoa - ps14
Parahoxozoa - ps15
Bilateria - ps16
Deuterostomia - ps17
Chordata - ps18
Olfactores - ps19
Vertebrata - ps20
Gnathostomata - ps21
Euteleostomi - ps22
Sarcopterygii - ps23
Tetrapoda - ps24
Amniota - ps25
Mammalia - ps26
Theria - ps27
Placentalia - ps28
Boreoeutheria - ps29
Euarchontoglires - ps30
Euarchonta - ps31
Primates - ps32
Haplorrhini - ps33
Simiiformes - ps34
Catarrhini - ps35
Hominoidea - ps36
Hominidae - ps37
Homininae - ps38
Hominini - ps39
Homo_sapiens - ps40
Phylostratum

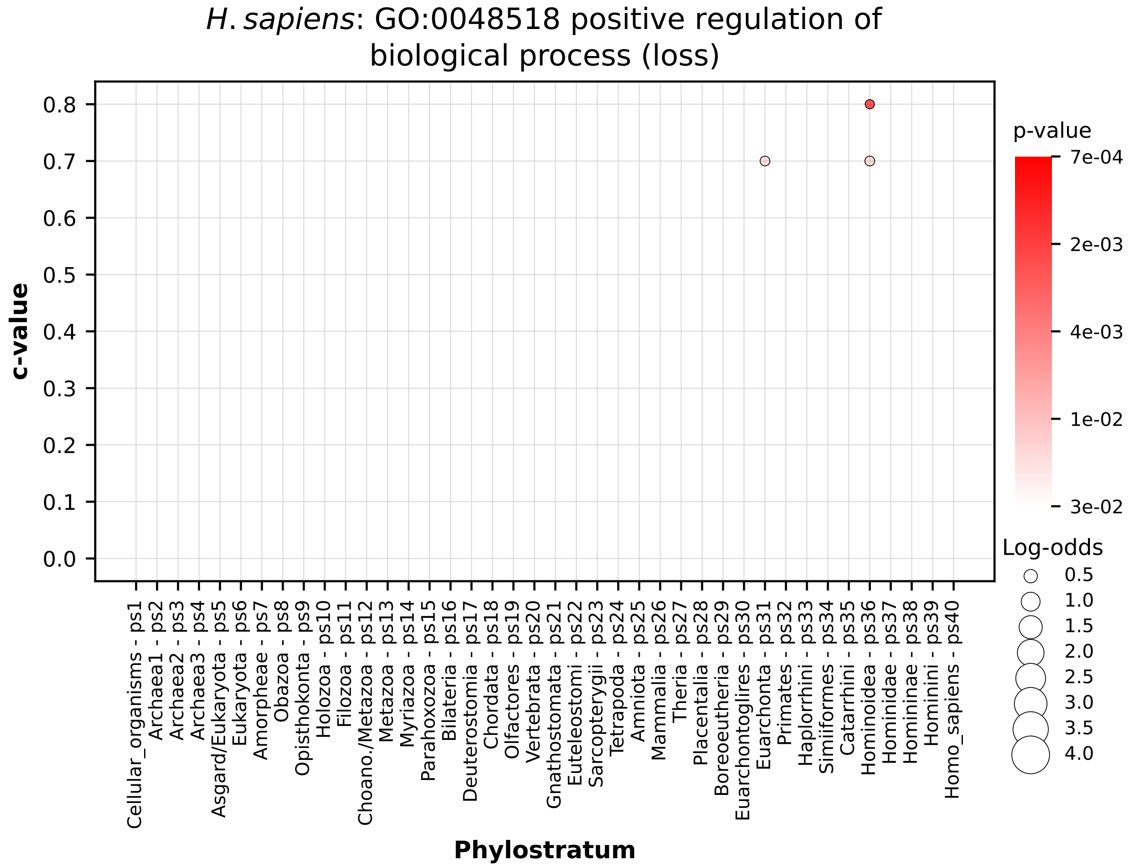

H. sapiens: GO:0048518 positive regulation of biological process (loss)
c-value
0.0
0.1
0.2
0.3
0.4
0.5
0.6
0.7
0.8
Phylostratum
Cellular_organisms - ps1
Archaea1 - ps2
Archaea2 - ps3
Archaea3 - ps4
Asgard/Eukaryota - ps5
Eukaryota - ps6
Amorpheae - ps7
Obazoa - ps8
Opisthokonta - ps9
Holozoa - ps10
Filozoa - ps11
Choano./Metazoa - ps12
Metazoa - ps13
Myriazoa - ps14
Parahoxozoa - ps15
Bilateria - ps16
Deuterostomia - ps17
Chordata - ps18
Olfactores - ps19
Vertebrata - ps20
Gnathostomata - ps21
Euteleostomi - ps22
Sarcopterygii - ps23
Tetrapoda - ps24
Amniota - ps25
Mammalia - ps26
Theria - ps27
Placentalia - ps28
Boreoeutheria - ps29
Euarchontoglires - ps30
Euarchonta - ps31
Primates - ps32
Haplorrhini - ps33
Simiiformes - ps34
Catarrhini - ps35
Hominoidea - ps36
Hominidae - ps37
Homininae - ps38
Hominini - ps39
Homo_sapiens - ps40
p-value
7e-04
2e-03
4e-03
1e-02
3e-02
Log-odds
0.5
1.0
1.5
2.0
2.5
3.0
3.5
4.0

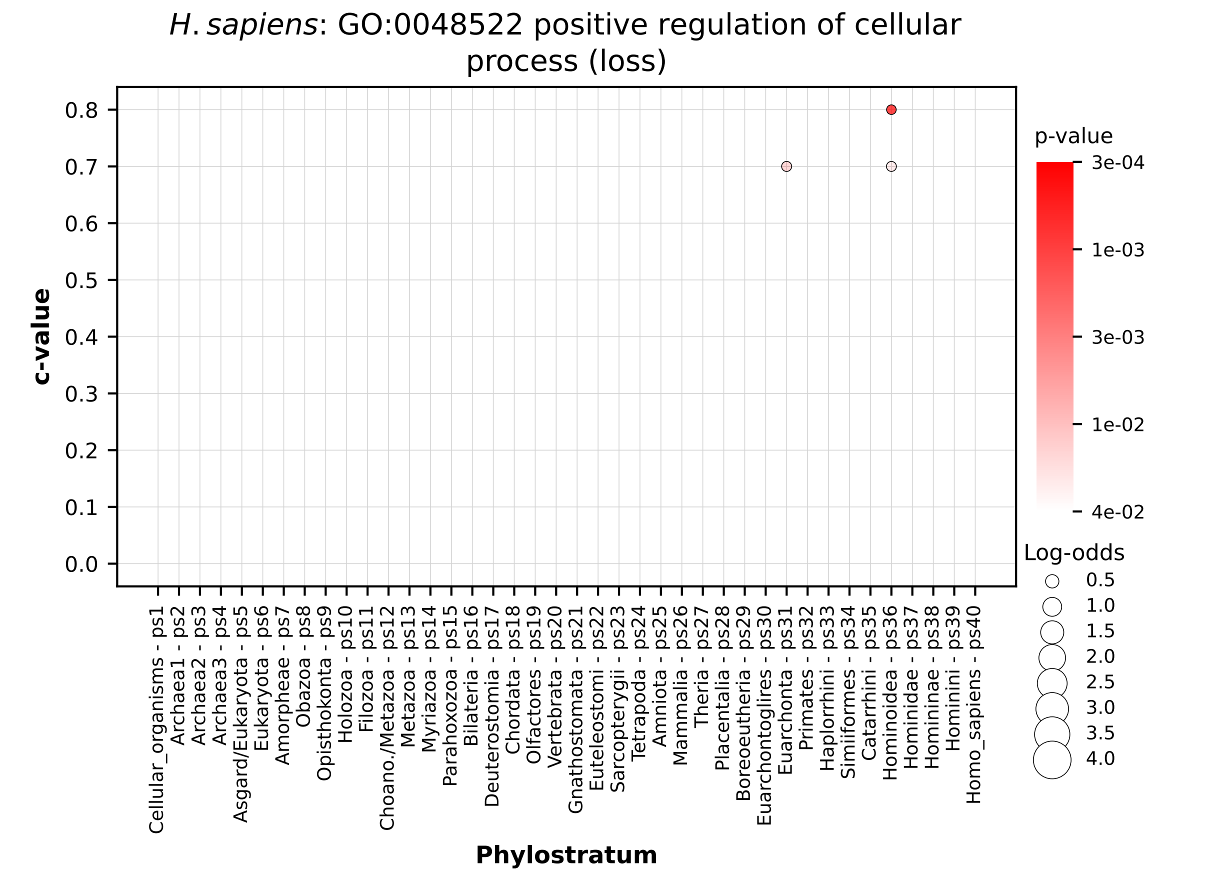

H. sapiens: GO:0048522 positive regulation of cellular process (loss)
c-value
0.0
0.1
0.2
0.3
0.4
0.5
0.6
0.7
0.8
Phylostratum
Cellular_organisms - ps1
Archaea1 - ps2
Archaea2 - ps3
Archaea3 - ps4
Asgard/Eukaryota - ps5
Eukaryota - ps6
Amorpheae - ps7
Obazoa - ps8
Opisthokonta - ps9
Holozoa - ps10
Filozoa - ps11
Choano./Metazoa - ps12
Metazoa - ps13
Myriazoa - ps14
Parahoxozoa - ps15
Bilateria - ps16
Deuterostomia - ps17
Chordata - ps18
Olfactores - ps19
Vertebrata - ps20
Gnathostomata - ps21
Euteleostomi - ps22
Sarcopterygii - ps23
Tetrapoda - ps24
Amniota - ps25
Mammalia - ps26
Theria - ps27
Placentalia - ps28
Boreoeutheria - ps29
Euarchontoglires - ps30
Euarchonta - ps31
Primates - ps32
Haplorrhini - ps33
Simiiformes - ps34
Catarrhini - ps35
Hominoidea - ps36
Hominidae - ps37
Homininae - ps38
Hominini - ps39
Homo_sapiens - ps40
p-value
3e-04
1e-03
3e-03
1e-02
4e-02
Log-odds
0.5
1.0
1.5
2.0
2.5
3.0
3.5
4.0

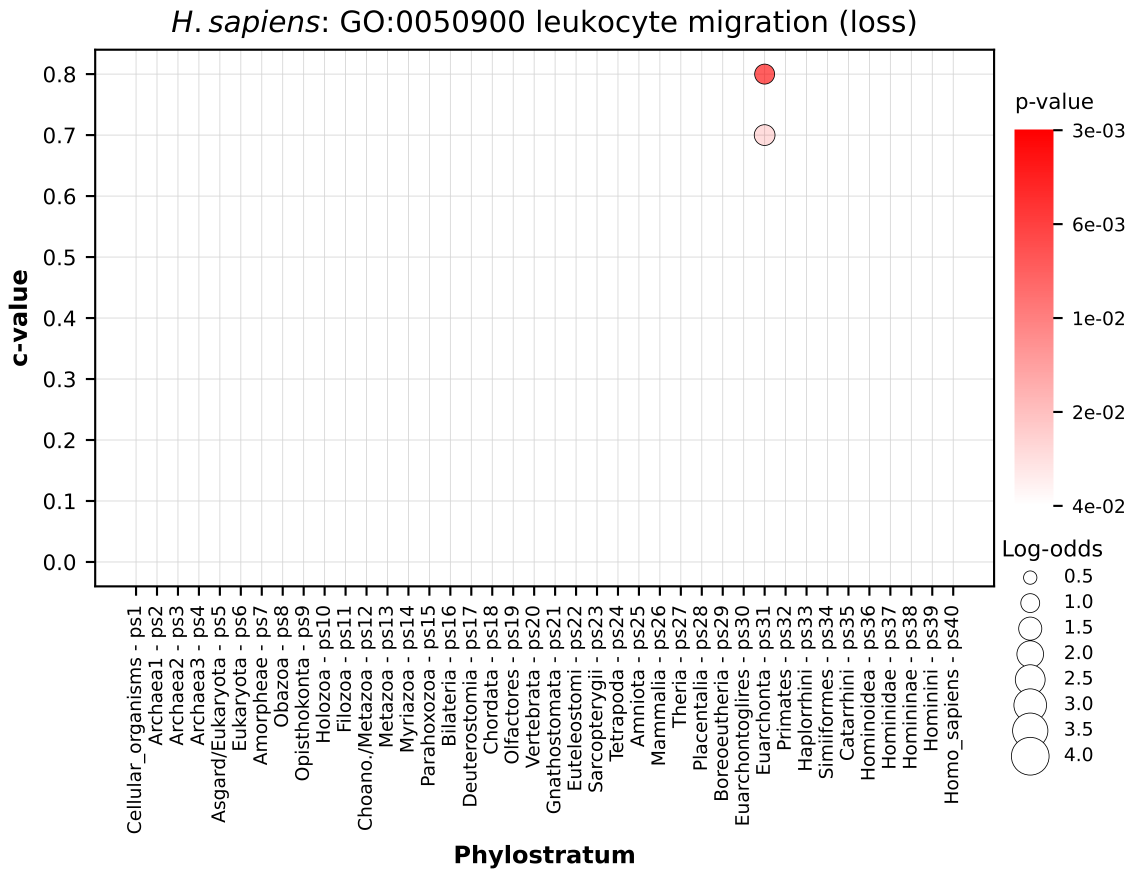
H. sapiens: GO:0050900 leukocyte migration (loss)
c-value
0.0
0.1
0.2
0.3
0.4
0.5
0.6
0.7
0.8
Phylostratum
Cellular_organisms - ps1
Archaea1 - ps2
Archaea2 - ps3
Archaea3 - ps4
Asgard/Eukaryota - ps5
Eukaryota - ps6
Amorpheae - ps7
Obazoa - ps8
Opisthokonta - ps9
Holozoa - ps10
Filozoa - ps11
Choano./Metazoa - ps12
Metazoa - ps13
Myriazoa - ps14
Parahoxozoa - ps15
Bilateria - ps16
Deuterostomia - ps17
Chordata - ps18
Olfactores - ps19
Vertebrata - ps20
Gnathostomata - ps21
Euteleostomi - ps22
Sarcopterygii - ps23
Tetrapoda - ps24
Amniota - ps25
Mammalia - ps26
Theria - ps27
Placentalia - ps28
Boreoeutheria - ps29
Euarchontoglires - ps30
Euarchonta - ps31
Primates - ps32
Haplorrhini - ps33
Simiiformes - ps34
Catarrhini - ps35
Hominoidea - ps36
Hominidae - ps37
Homininae - ps38
Hominini - ps39
Homo_sapiens - ps40
p-value
3e-03
6e-03
1e-02
2e-02
4e-02
Log-odds
0.5
1.0
1.5
2.0
2.5
3.0
3.5
4.0

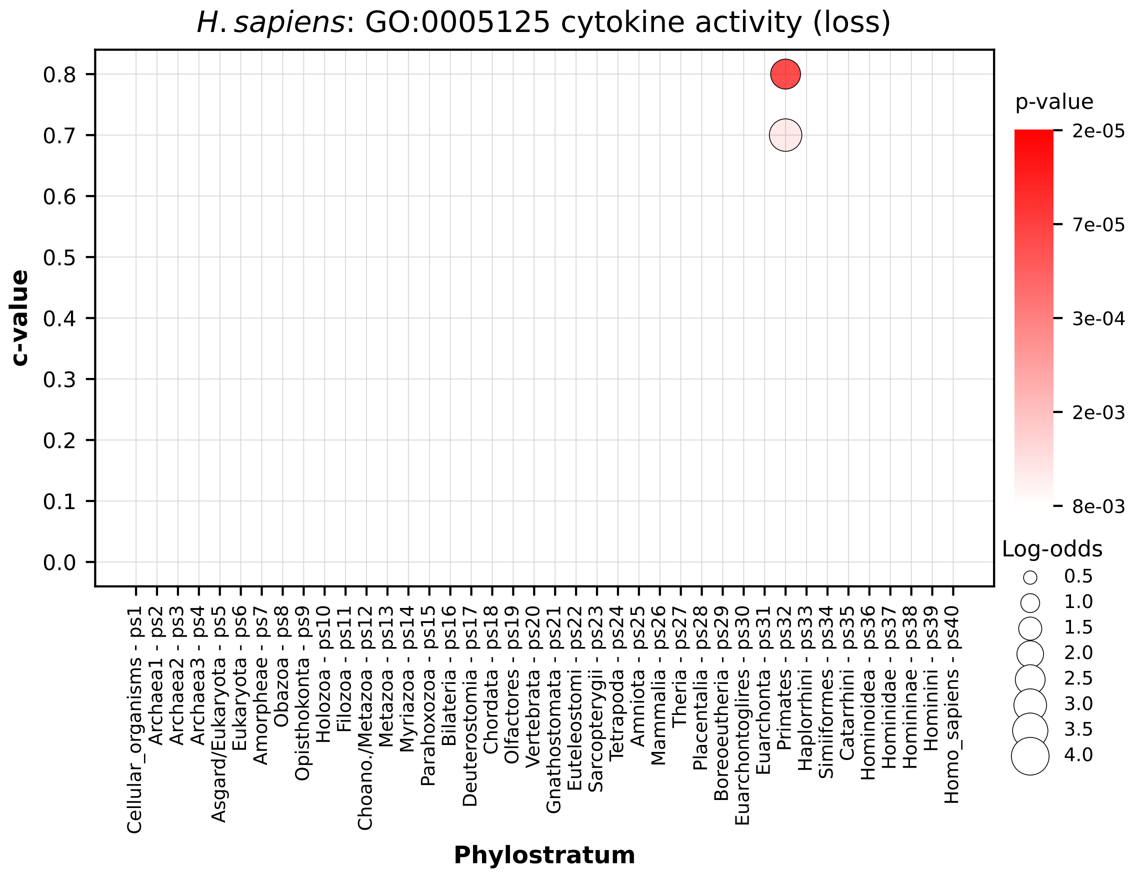
H. sapiens: GO:0005125 cytokine activity (loss)
c-value
0.0
0.1
0.2
0.3
0.4
0.5
0.6
0.7
0.8
Phylostratum
Cellular_organisms - ps1
Archaea1 - ps2
Archaea2 - ps3
Archaea3 - ps4
Asgard/Eukaryota - ps5
Eukaryota - ps6
Amorpheae - ps7
Obazoa - ps8
Opisthokonta - ps9
Holozoa - ps10
Filozoa - ps11
Choano./Metazoa - ps12
Metazoa - ps13
Myriazoa - ps14
Parahoxozoa - ps15
Bilateria - ps16
Deuterostomia - ps17
Chordata - ps18
Olfactores - ps19
Vertebrata - ps20
Gnathostomata - ps21
Euteleostomi - ps22
Sarcopterygii - ps23
Tetrapoda - ps24
Amniota - ps25
Mammalia - ps26
Theria - ps27
Placentalia - ps28
Boreoeutheria - ps29
Euarchontoglires - ps30
Euarchonta - ps31
Primates - ps32
Haplorrhini - ps33
Simiiformes - ps34
Catarrhini - ps35
Hominoidea - ps36
Hominidae - ps37
Homininae - ps38
Hominini - ps39
Homo_sapiens - ps40
p-value
2e-05
7e-05
3e-04
2e-03
8e-03
Log-odds
0.5
1.0
1.5
2.0
2.5
3.0
3.5
4.0

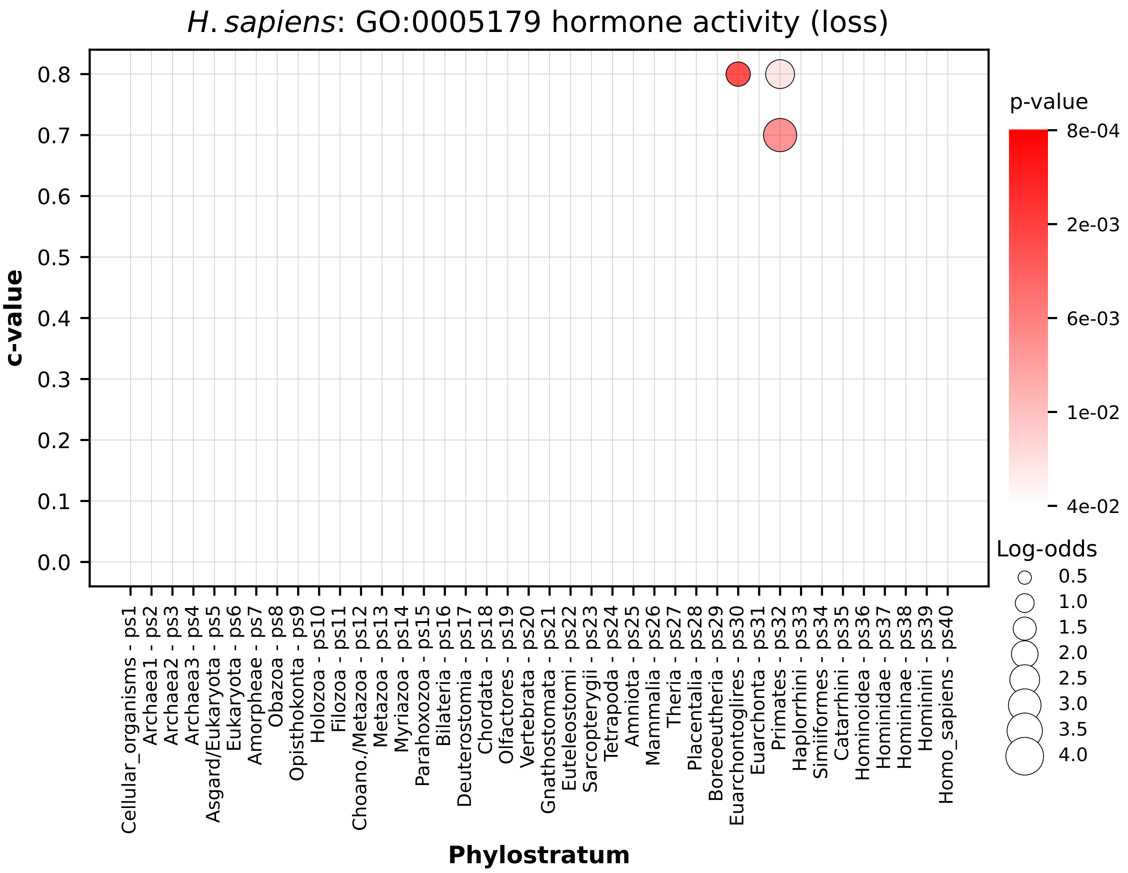
H. sapiens: GO:0005179 hormone activity (loss)
c-value
0.0
0.1
0.2
0.3
0.4
0.5
0.6
0.7
0.8
p-value
8e-04
2e-03
6e-03
1e-02
4e-02
Log-odds
0.5
1.0
1.5
2.0
2.5
3.0
3.5
4.0
Cellular_organisms - ps1
Archaea1 - ps2
Archaea2 - ps3
Archaea3 - ps4
Asgard/Eukaryota - ps5
Eukaryota - ps6
Amorpheae - ps7
Obazoa - ps8
Opisthokonta - ps9
Holozoa - ps10
Filozoa - ps11
Choano./Metazoa - ps12
Metazoa - ps13
Myriazoa - ps14
Parahoxozoa - ps15
Bilateria - ps16
Deuterostomia - ps17
Chordata - ps18
Olfactores - ps19
Vertebrata - ps20
Gnathostomata - ps21
Euteleostomi - ps22
Sarcopterygii - ps23
Tetrapoda - ps24
Amniota - ps25
Mammalia - ps26
Theria - ps27
Placentalia - ps28
Boreoeutheria - ps29
Euarchontoglires - ps30
Euarchonta - ps31
Primates - ps32
Haplorrhini - ps33
Simiiformes - ps34
Catarrhini - ps35
Hominoidea - ps36
Hominidae - ps37
Homininae - ps38
Hominini - ps39
Homo_sapiens - ps40
Phylostratum

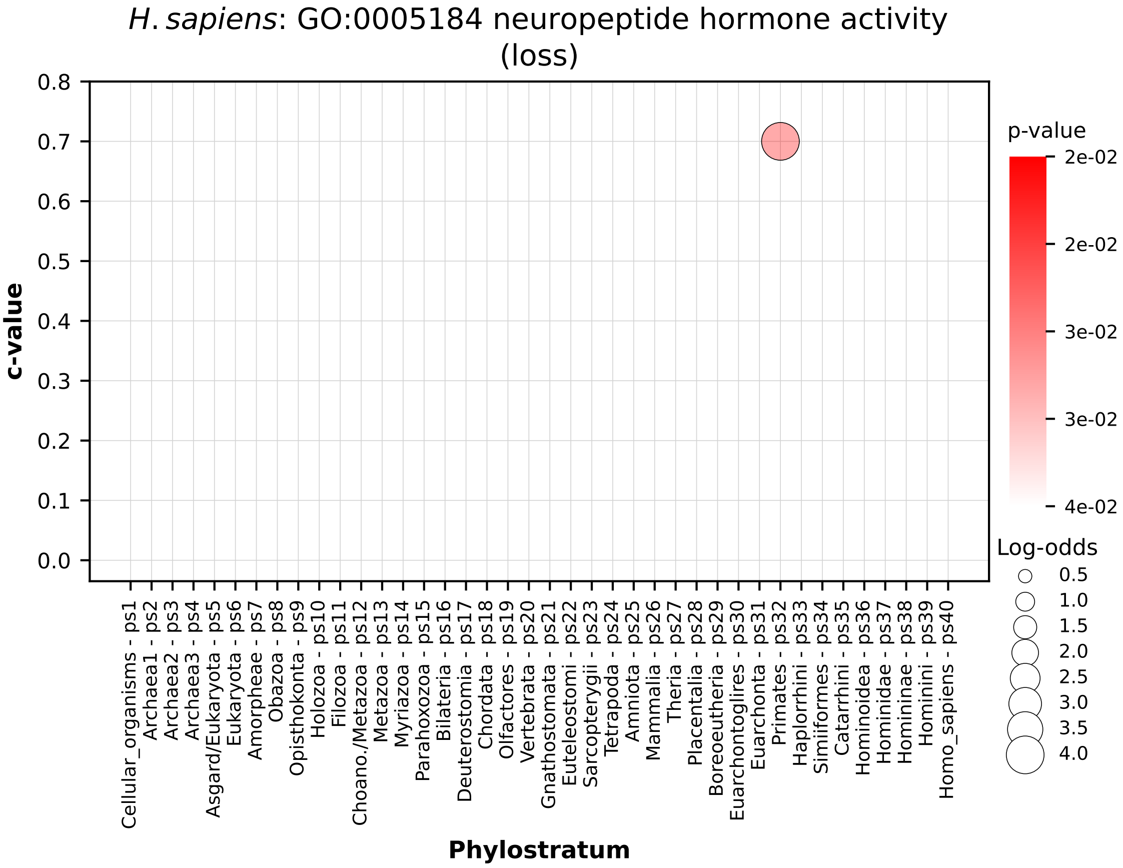

H. sapiens: GO:0005184 neuropeptide hormone activity
(loss)
c-value
0.8
0.7
0.6
0.5
0.4
0.3
0.2
0.1
0.0
p-value
2e-02
2e-02
3e-02
3e-02
4e-02
Log-odds
0.5
1.0
1.5
2.0
2.5
3.0
3.5
4.0
Cellular_organisms - ps1
Archaea1 - ps2
Archaea2 - ps3
Archaea3 - ps4
Asgard/Eukaryota - ps5
Eukaryota - ps6
Amorpheae - ps7
Obazoa - ps8
Opisthokonta - ps9
Holozoa - ps10
Filozoa - ps11
Choano./Metazoa - ps12
Metazoa - ps13
Myriazoa - ps14
Parahoxozoa - ps15
Bilateria - ps16
Deuterostomia - ps17
Chordata - ps18
Olfactores - ps19
Vertebrata - ps20
Gnathostomata - ps21
Euteleostomi - ps22
Sarcopterygii - ps23
Tetrapoda - ps24
Amniota - ps25
Mammalia - ps26
Theria - ps27
Placentalia - ps28
Boreoeutheria - ps29
Euarchontoglires - ps30
Euarchonta - ps31
Primates - ps32
Haplorrhini - ps33
Simiiformes - ps34
Catarrhini - ps35
Hominoidea - ps36
Hominidae - ps37
Homininae - ps38
Hominini - ps39
Homo_sapiens - ps40
Phylostratum

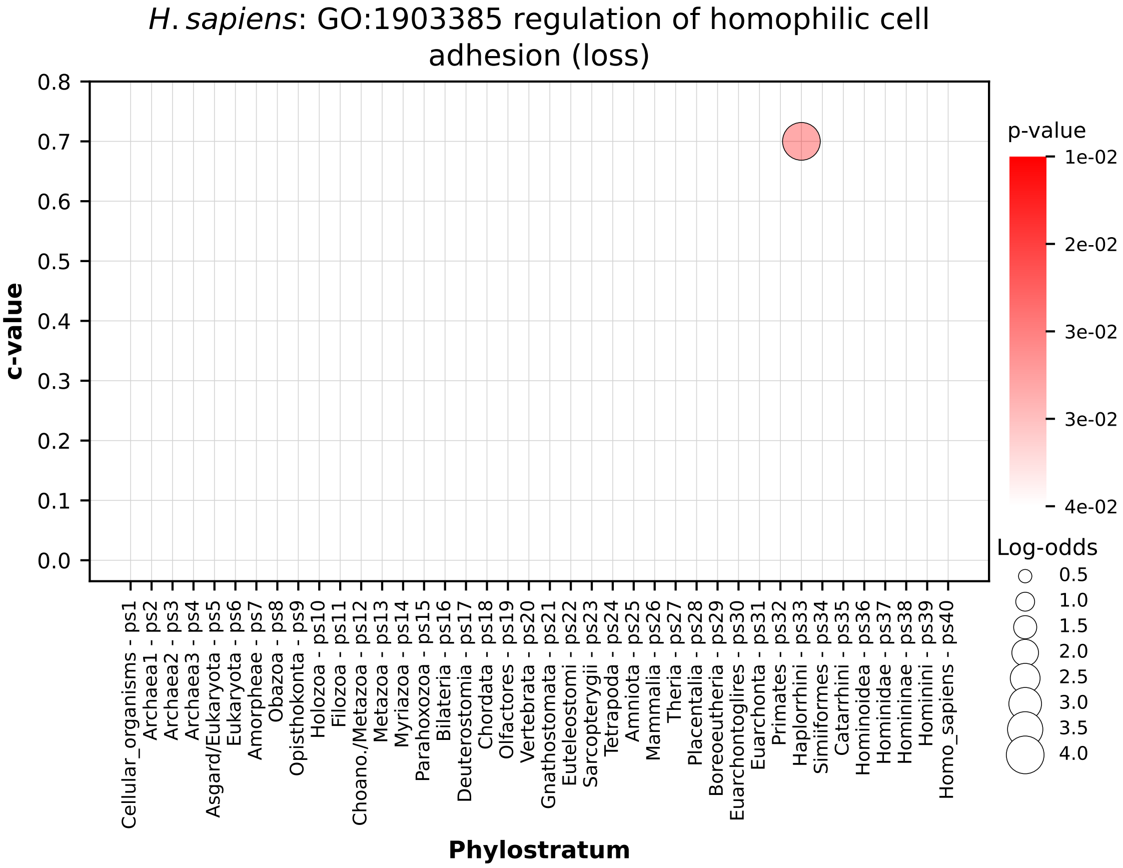

H. sapiens: GO:1903385 regulation of homophilic cell adhesion (loss)
c-value
0.8
0.7
0.6
0.5
0.4
0.3
0.2
0.1
0.0
Phylostratum
Cellular_organisms - ps1
Archaea1 - ps2
Archaea2 - ps3
Archaea3 - ps4
Asgard/Eukaryota - ps5
Eukaryota - ps6
Amorpheae - ps7
Obazoa - ps8
Opisthokonta - ps9
Holozoa - ps10
Filozoa - ps11
Choano./Metazoa - ps12
Metazoa - ps13
Myriazoa - ps14
Parahoxozoa - ps15
Bilateria - ps16
Deuterostomia - ps17
Chordata - ps18
Olfactores - ps19
Vertebrata - ps20
Gnathostomata - ps21
Euteleostomi - ps22
Sarcopterygii - ps23
Tetrapoda - ps24
Amniota - ps25
Mammalia - ps26
Theria - ps27
Placentalia - ps28
Boreoeutheria - ps29
Euarchontoglires - ps30
Euarchonta - ps31
Primates - ps32
Haplorrhini - ps33
Simiiformes - ps34
Catarrhini - ps35
Hominoidea - ps36
Hominidae - ps37
Homininae - ps38
Hominini - ps39
Homo_sapiens - ps40
p-value
1e-02
2e-02
3e-02
3e-02
4e-02
Log-odds
0.5
1.0
1.5
2.0
2.5
3.0
3.5
4.0

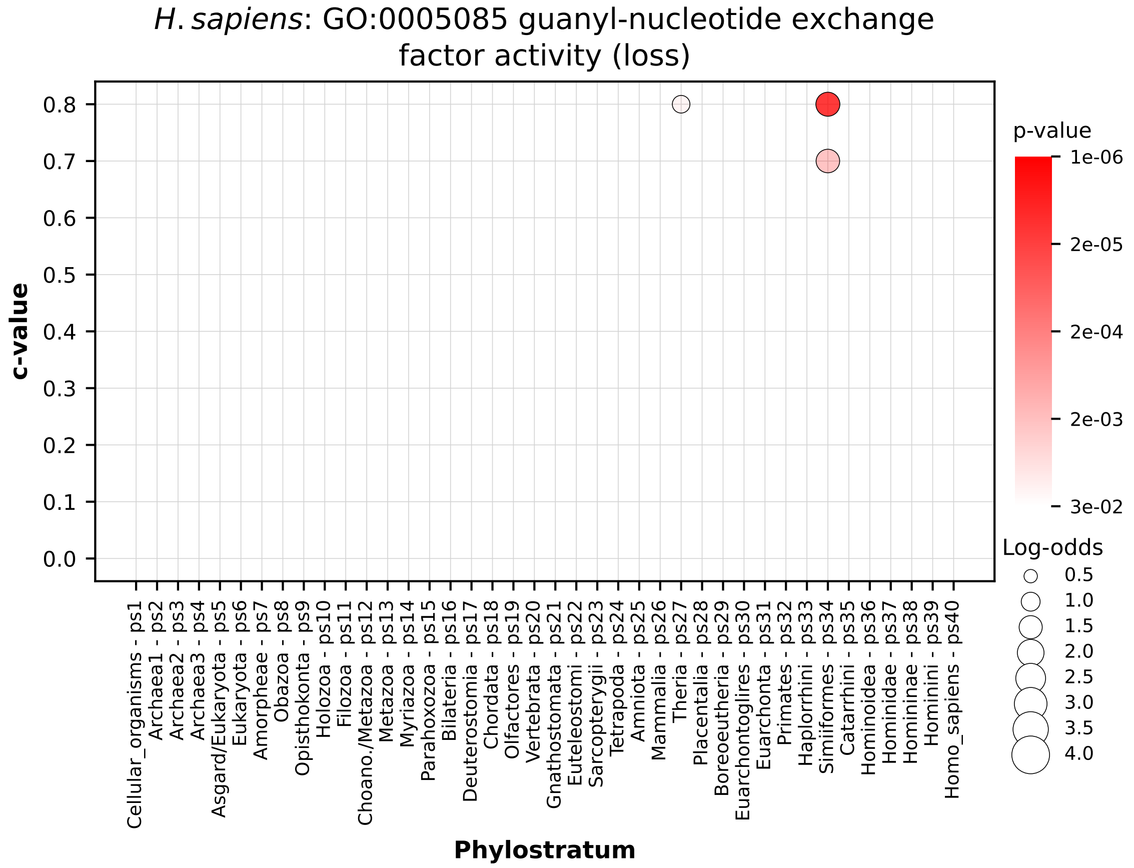
H. sapiens: GO:0005085 guanyl-nucleotide exchange factor activity (loss)
c-value
0.0
0.1
0.2
0.3
0.4
0.5
0.6
0.7
0.8
Phylostratum
Cellular_organisms - ps1
Archaea1 - ps2
Archaea2 - ps3
Archaea3 - ps4
Asgard/Eukaryota - ps5
Eukaryota - ps6
Amorpheae - ps7
Obazoa - ps8
Opisthokonta - ps9
Holozoa - ps10
Filozoa - ps11
Choano./Metazoa - ps12
Metazoa - ps13
Myriazoa - ps14
Parahoxozoa - ps15
Bilateria - ps16
Deuterostomia - ps17
Chordata - ps18
Olfactores - ps19
Vertebrata - ps20
Gnathostomata - ps21
Euteleostomi - ps22
Sarcopterygii - ps23
Tetrapoda - ps24
Amniota - ps25
Mammalia - ps26
Theria - ps27
Placentalia - ps28
Boreoeutheria - ps29
Euarchontoglires - ps30
Euarchonta - ps31
Primates - ps32
Haplorrhini - ps33
Simiiformes - ps34
Catarrhini - ps35
Hominoidea - ps36
Hominidae - ps37
Homininae - ps38
Hominini - ps39
Homo_sapiens - ps40
p-value
1e-06
2e-05
2e-04
2e-03
3e-02
Log-odds
0.5
1.0
1.5
2.0
2.5
3.0
3.5
4.0

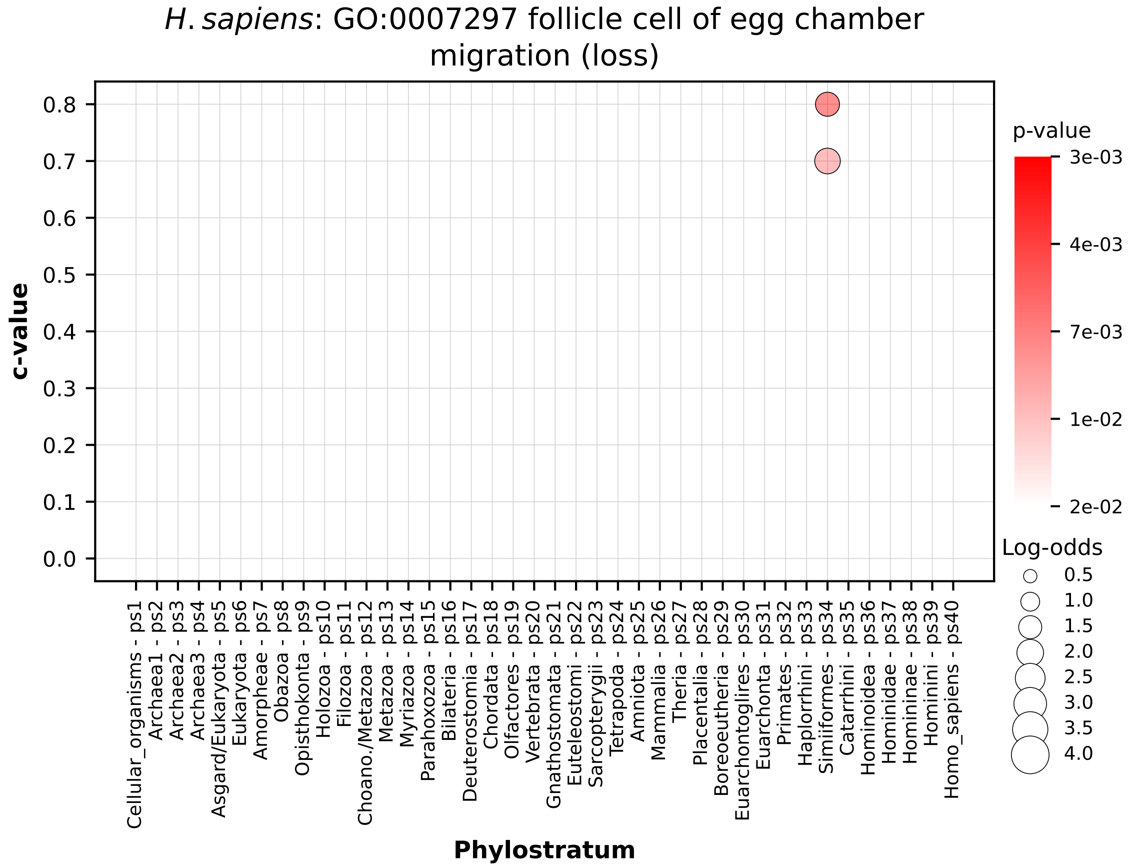

H. sapiens: GO:0007297 follicle cell of egg chamber migration (loss)
c-value
0.8
0.7
0.6
0.5
0.4
0.3
0.2
0.1
0.0
p-value
3e-03
4e-03
7e-03
1e-02
2e-02
Log-odds
0.5
1.0
1.5
2.0
2.5
3.0
3.5
4.0
Cellular_organisms - ps1
Archaea1 - ps2
Archaea2 - ps3
Archaea3 - ps4
Asgard/Eukaryota - ps5
Eukaryota - ps6
Amorpheae - ps7
Obazoa - ps8
Opisthokonta - ps9
Holozoa - ps10
Filozoa - ps11
Choano./Metazoa - ps12
Metazoa - ps13
Myriazoa - ps14
Parahoxozoa - ps15
Bilateria - ps16
Deuterostomia - ps17
Chordata - ps18
Olfactores - ps19
Vertebrata - ps20
Gnathostomata - ps21
Euteleostomi - ps22
Sarcopterygii - ps23
Tetrapoda - ps24
Amniota - ps25
Mammalia - ps26
Theria - ps27
Placentalia - ps28
Boreoeutheria - ps29
Euarchontoglires - ps30
Euarchonta - ps31
Primates - ps32
Haplorrhini - ps33
Simiiformes - ps34
Catarrhini - ps35
Hominoidea - ps36
Hominidae - ps37
Homininae - ps38
Hominini - ps39
Homo_sapiens - ps40
Phylostratum

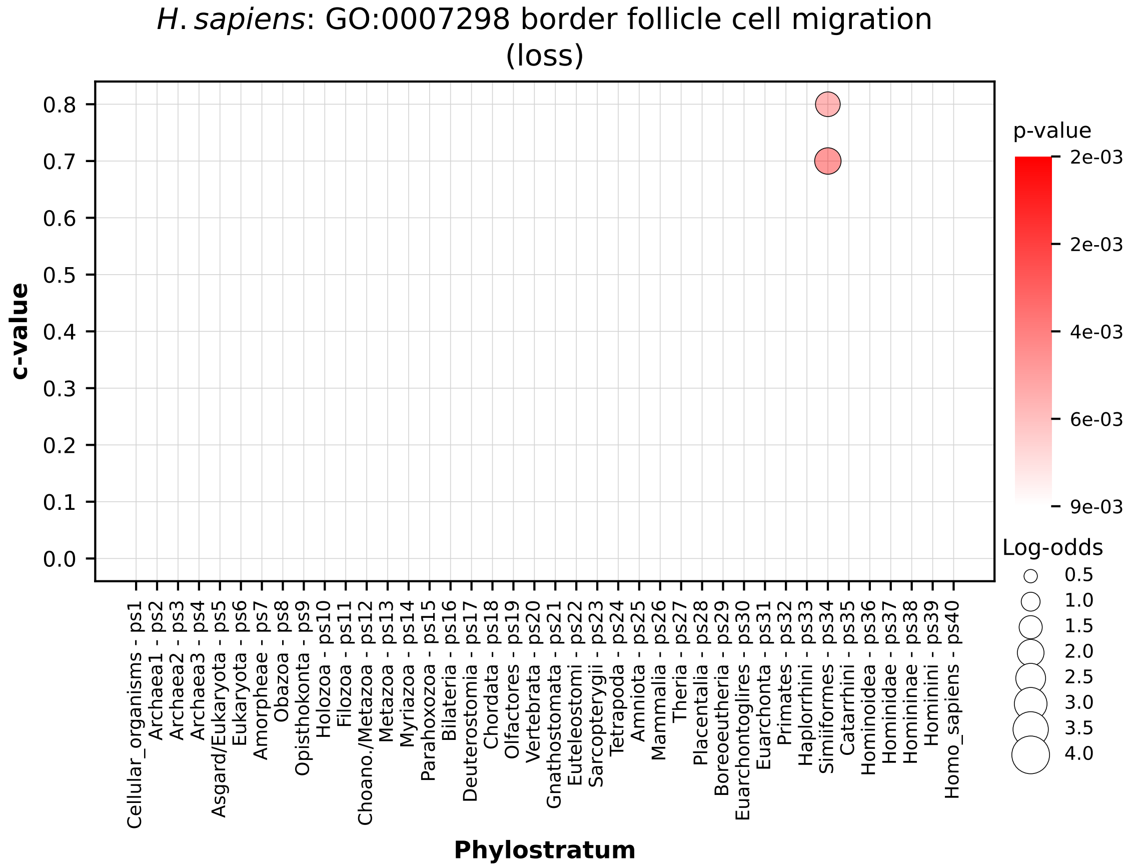

H. sapiens: GO:0007298 border follicle cell migration
(loss)
c-value
0.0
0.1
0.2
0.3
0.4
0.5
0.6
0.7
0.8
p-value
2e-03
2e-03
4e-03
6e-03
9e-03
Log-odds
0.5
1.0
1.5
2.0
2.5
3.0
3.5
4.0
Cellular_organisms - ps1
Archaea1 - ps2
Archaea2 - ps3
Archaea3 - ps4
Asgard/Eukaryota - ps5
Eukaryota - ps6
Amorpheae - ps7
Obazoa - ps8
Opisthokonta - ps9
Holozoa - ps10
Filozoa - ps11
Choano./Metazoa - ps12
Metazoa - ps13
Myriazoa - ps14
Parahoxozoa - ps15
Bilateria - ps16
Deuterostomia - ps17
Chordata - ps18
Olfactores - ps19
Vertebrata - ps20
Gnathostomata - ps21
Euteleostomi - ps22
Sarcopterygii - ps23
Tetrapoda - ps24
Amniota - ps25
Mammalia - ps26
Theria - ps27
Placentalia - ps28
Boreoeutheria - ps29
Euarchontoglires - ps30
Euarchonta - ps31
Primates - ps32
Haplorrhini - ps33
Simiiformes - ps34
Catarrhini - ps35
Hominoidea - ps36
Hominidae - ps37
Homininae - ps38
Hominini - ps39
Homo_sapiens - ps40
Phylostratum

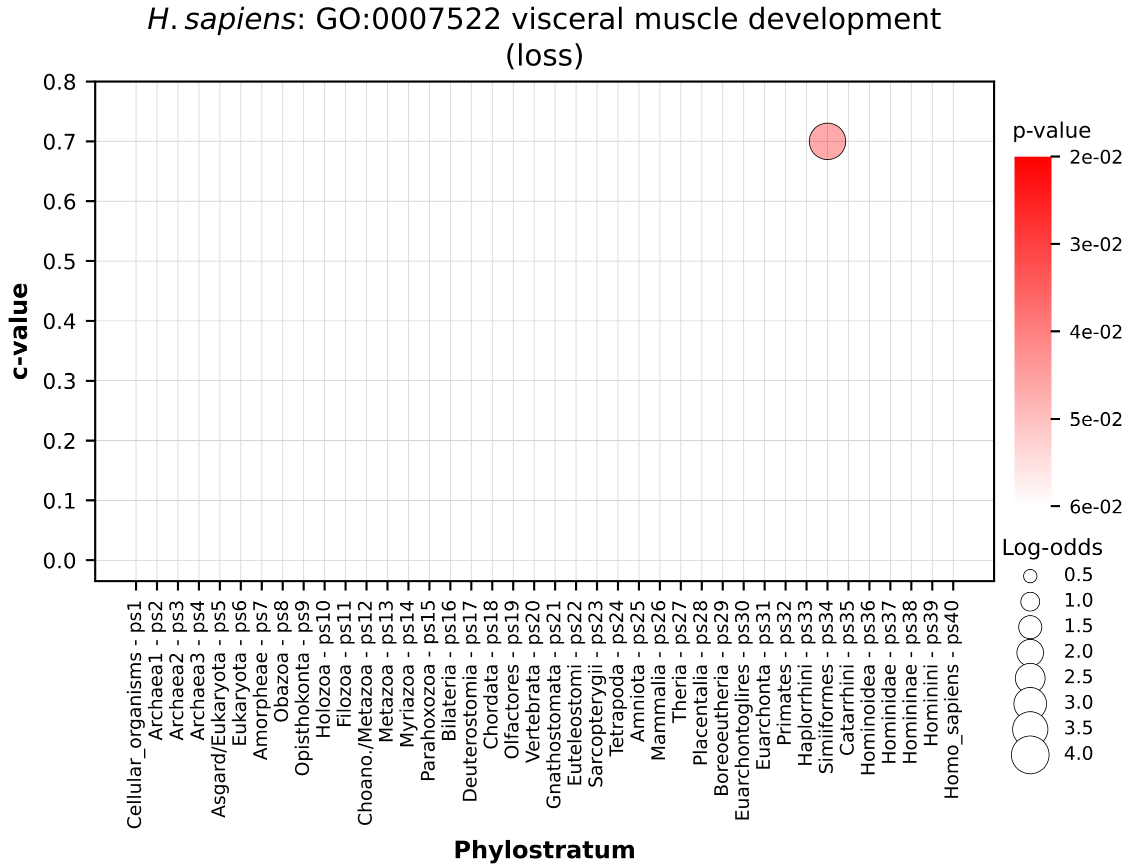

H. sapiens: GO:0007522 visceral muscle development
(loss)
c-value
0.0
0.1
0.2
0.3
0.4
0.5
0.6
0.7
0.8
Phylostratum
Cellular_organisms - ps1
Archaea1 - ps2
Archaea2 - ps3
Archaea3 - ps4
Asgard/Eukaryota - ps5
Eukaryota - ps6
Amorpheae - ps7
Obazoa - ps8
Opisthokonta - ps9
Holozoa - ps10
Filozoa - ps11
Choano./Metazoa - ps12
Metazoa - ps13
Myriazoa - ps14
Parahoxozoa - ps15
Bilateria - ps16
Deuterostomia - ps17
Chordata - ps18
Olfactores - ps19
Vertebrata - ps20
Gnathostomata - ps21
Euteleostomi - ps22
Sarcopterygii - ps23
Tetrapoda - ps24
Amniota - ps25
Mammalia - ps26
Theria - ps27
Placentalia - ps28
Boreoeutheria - ps29
Euarchontoglires - ps30
Euarchonta - ps31
Primates - ps32
Haplorrhini - ps33
Simiiformes - ps34
Catarrhini - ps35
Hominoidea - ps36
Hominidae - ps37
Homininae - ps38
Hominini - ps39
Homo_sapiens - ps40
p-value
2e-02
3e-02
4e-02
5e-02
6e-02
Log-odds
0.5
1.0
1.5
2.0
2.5
3.0
3.5
4.0

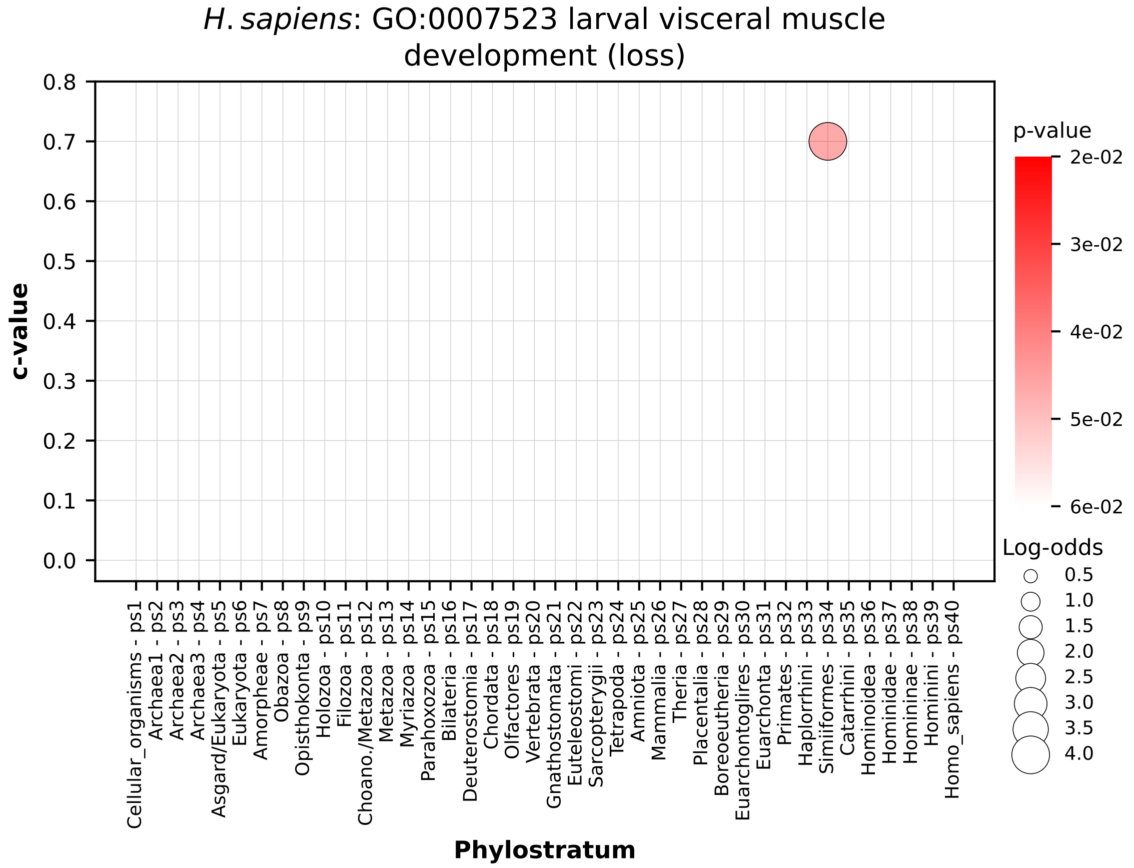
H. sapiens: GO:0007523 larval visceral muscle development (loss)
c-value
0.8
0.7
0.6
0.5
0.4
0.3
0.2
0.1
0.0
p-value
2e-02
3e-02
4e-02
5e-02
6e-02
Log-odds
0.5
1.0
1.5
2.0
2.5
3.0
3.5
4.0
Cellular_organisms - ps1
Archaea1 - ps2
Archaea2 - ps3
Archaea3 - ps4
Asgard/Eukaryota - ps5
Eukaryota - ps6
Amorpheae - ps7
Obazoa - ps8
Opisthokonta - ps9
Holozoa - ps10
Filozoa - ps11
Choano./Metazoa - ps12
Metazoa - ps13
Myriazoa - ps14
Parahoxozoa - ps15
Bilateria - ps16
Deuterostomia - ps17
Chordata - ps18
Olfactores - ps19
Vertebrata - ps20
Gnathostomata - ps21
Euteleostomi - ps22
Sarcopterygii - ps23
Tetrapoda - ps24
Amniota - ps25
Mammalia - ps26
Theria - ps27
Placentalia - ps28
Boreoeutheria - ps29
Euarchontoglires - ps30
Euarchonta - ps31
Primates - ps32
Haplorrhini - ps33
Simiiformes - ps34
Catarrhini - ps35
Hominoidea - ps36
Hominidae - ps37
Homininae - ps38
Hominini - ps39
Homo_sapiens - ps40
Phylostratum

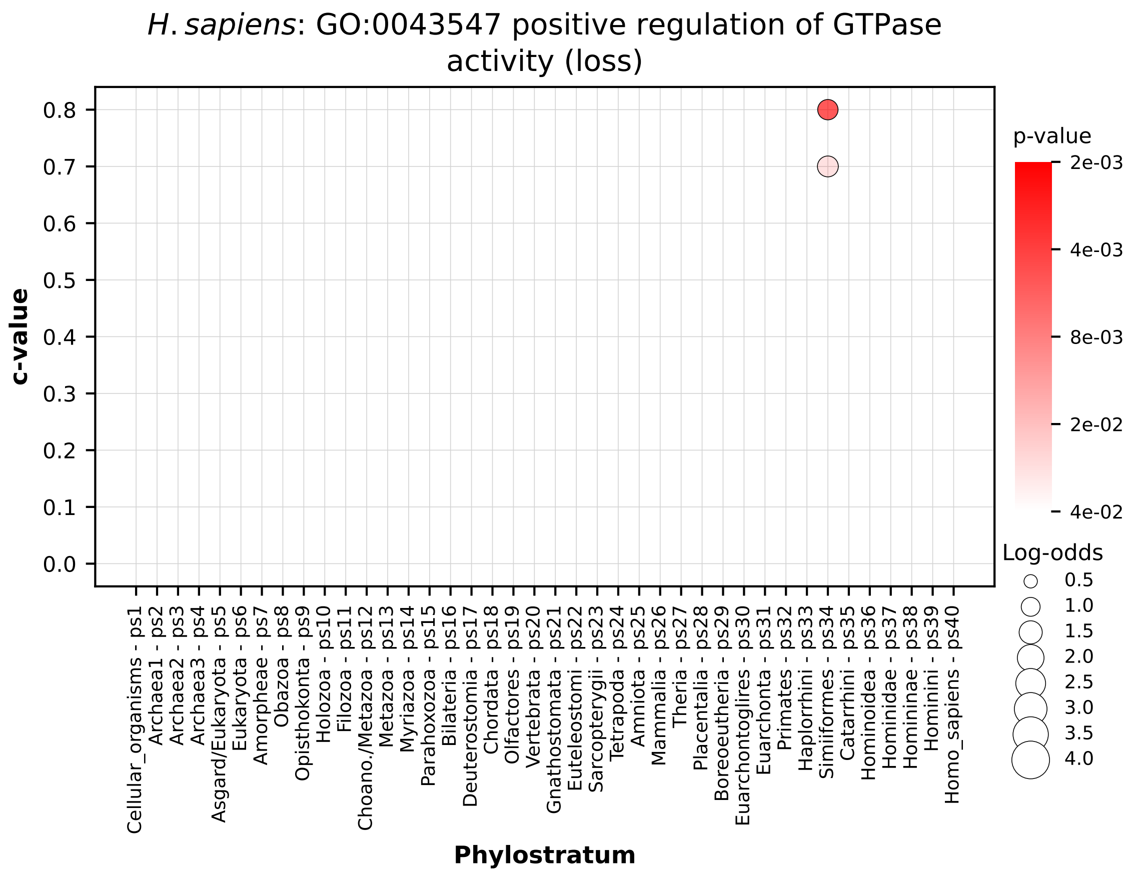

H. sapiens: GO:0043547 positive regulation of GTPase activity (loss)
c-value
0.0
0.1
0.2
0.3
0.4
0.5
0.6
0.7
0.8
p-value
2e-03
4e-03
8e-03
2e-02
4e-02
Log-odds
0.5
1.0
1.5
2.0
2.5
3.0
3.5
4.0
Cellular_organisms - ps1
Archaea1 - ps2
Archaea2 - ps3
Archaea3 - ps4
Asgard/Eukaryota - ps5
Eukaryota - ps6
Amorpheae - ps7
Obazoa - ps8
Opisthokonta - ps9
Holozoa - ps10
Filozoa - ps11
Choano./Metazoa - ps12
Metazoa - ps13
Myriazoa - ps14
Parahoxozoa - ps15
Bilateria - ps16
Deuterostomia - ps17
Chordata - ps18
Olfactores - ps19
Vertebrata - ps20
Gnathostomata - ps21
Euteleostomi - ps22
Sarcopterygii - ps23
Tetrapoda - ps24
Amniota - ps25
Mammalia - ps26
Theria - ps27
Placentalia - ps28
Boreoeutheria - ps29
Euarchontoglires - ps30
Euarchonta - ps31
Primates - ps32
Haplorrhini - ps33
Simiiformes - ps34
Catarrhini - ps35
Hominoidea - ps36
Hominidae - ps37
Homininae - ps38
Hominini - ps39
Homo_sapiens - ps40
Phylostratum

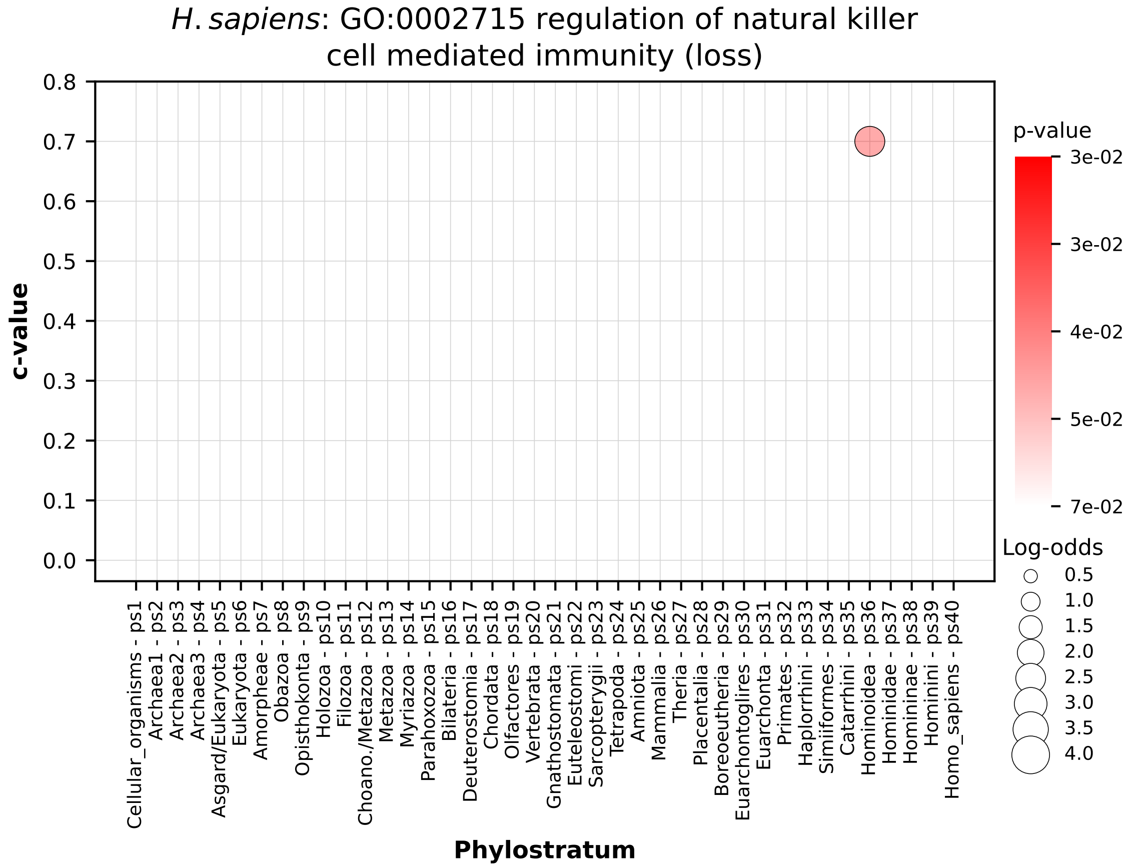

H. sapiens: GO:0002715 regulation of natural killer cell mediated immunity (loss)
c-value
0.8
0.7
0.6
0.5
0.4
0.3
0.2
0.1
0.0
Phylostratum
Cellular_organisms - ps1
Archaea1 - ps2
Archaea2 - ps3
Archaea3 - ps4
Asgard/Eukaryota - ps5
Eukaryota - ps6
Amorpheae - ps7
Obazoa - ps8
Opisthokonta - ps9
Holozoa - ps10
Filozoa - ps11
Choano./Metazoa - ps12
Metazoa - ps13
Myriazoa - ps14
Parahoxozoa - ps15
Bilateria - ps16
Deuterostomia - ps17
Chordata - ps18
Olfactores - ps19
Vertebrata - ps20
Gnathostomata - ps21
Euteleostomi - ps22
Sarcopterygii - ps23
Tetrapoda - ps24
Amniota - ps25
Mammalia - ps26
Theria - ps27
Placentalia - ps28
Boreoeutheria - ps29
Euarchontoglires - ps30
Euarchonta - ps31
Primates - ps32
Haplorrhini - ps33
Simiiformes - ps34
Catarrhini - ps35
Hominoidea - ps36
Hominidae - ps37
Homininae - ps38
Hominini - ps39
Homo_sapiens - ps40
p-value
3e-02
3e-02
4e-02
5e-02
7e-02
Log-odds
0.5
1.0
1.5
2.0
2.5
3.0
3.5
4.0

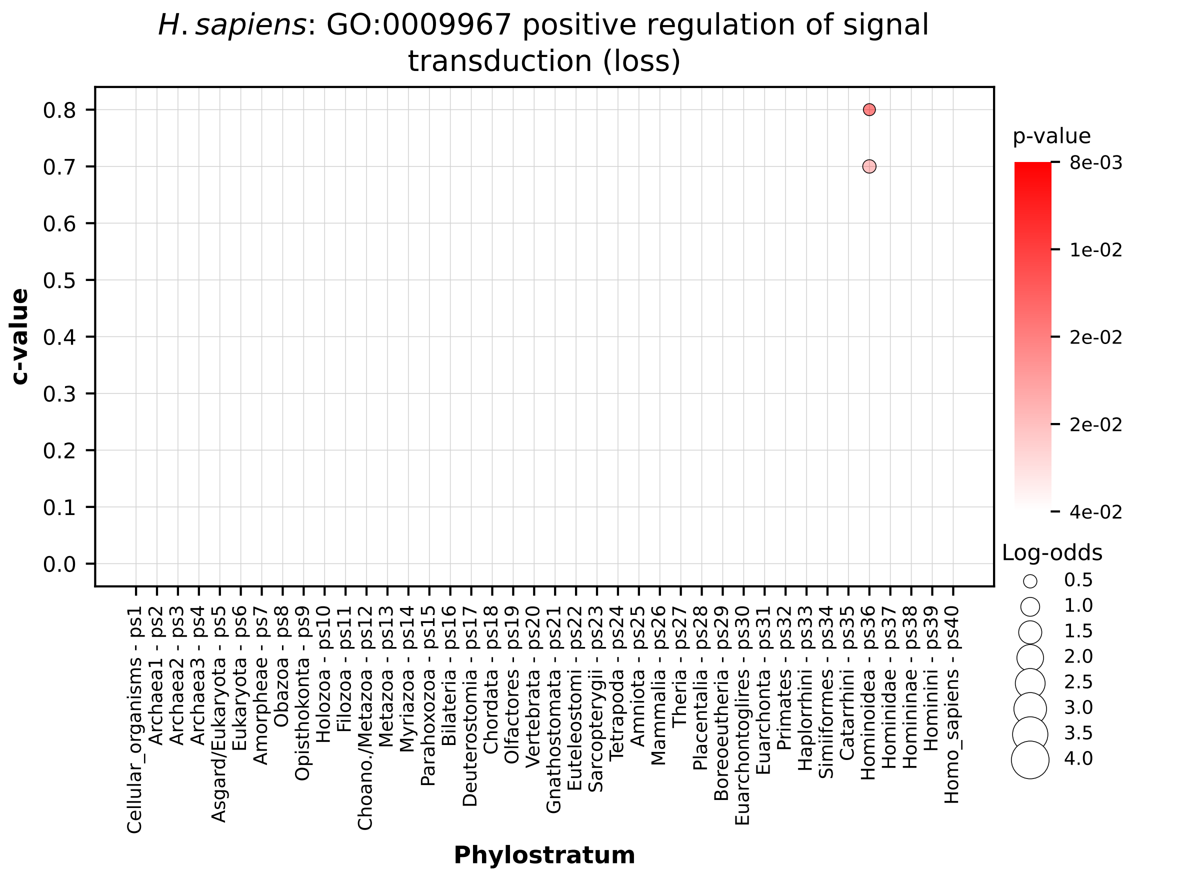
H. sapiens: GO:0009967 positive regulation of signal transduction (loss)
c-value
0.0
0.1
0.2
0.3
0.4
0.5
0.6
0.7
0.8
p-value
8e-03
1e-02
2e-02
2e-02
4e-02
Log-odds
0.5
1.0
1.5
2.0
2.5
3.0
3.5
4.0
Cellular_organisms - ps1
Archaea1 - ps2
Archaea2 - ps3
Archaea3 - ps4
Asgard/Eukaryota - ps5
Eukaryota - ps6
Amorpheae - ps7
Obazoa - ps8
Opisthokonta - ps9
Holozoa - ps10
Filozoa - ps11
Choano./Metazoa - ps12
Metazoa - ps13
Myriazoa - ps14
Parahoxozoa - ps15
Bilateria - ps16
Deuterostomia - ps17
Chordata - ps18
Olfactores - ps19
Vertebrata - ps20
Gnathostomata - ps21
Euteleostomi - ps22
Sarcopterygii - ps23
Tetrapoda - ps24
Amniota - ps25
Mammalia - ps26
Theria - ps27
Placentalia - ps28
Boreoeutheria - ps29
Euarchontoglires - ps30
Euarchonta - ps31
Primates - ps32
Haplorrhini - ps33
Simiiformes - ps34
Catarrhini - ps35
Hominoidea - ps36
Hominidae - ps37
Homininae - ps38
Hominini - ps39
Homo_sapiens - ps40
Phylostratum

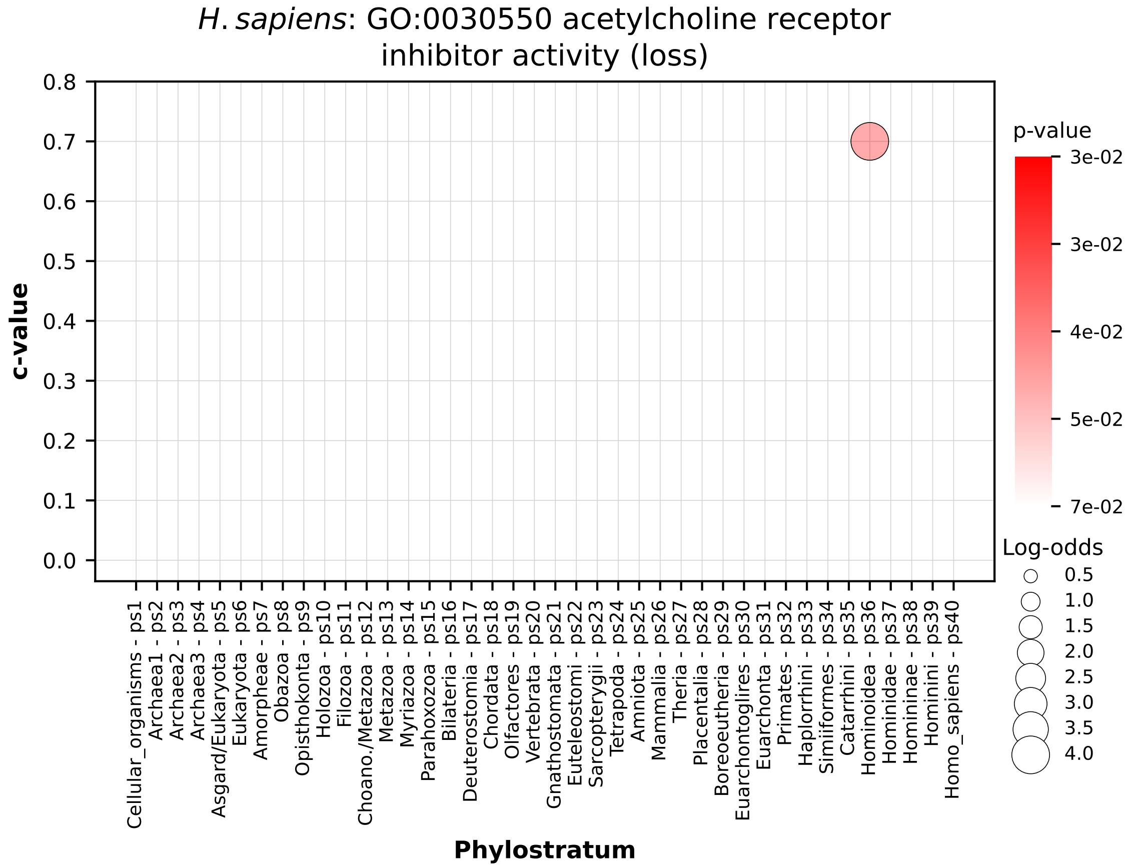

H. sapiens: GO:0030550 acetylcholine receptor inhibitor activity (loss)
c-value
0.8
0.7
0.6
0.5
0.4
0.3
0.2
0.1
0.0
p-value
3e-02
3e-02
4e-02
5e-02
7e-02
Log-odds
0.5
1.0
1.5
2.0
2.5
3.0
3.5
4.0
Cellular_organisms - ps1
Archaea1 - ps2
Archaea2 - ps3
Archaea3 - ps4
Asgard/Eukaryota - ps5
Eukaryota - ps6
Amorpheae - ps7
Obazoa - ps8
Opisthokonta - ps9
Holozoa - ps10
Filozoa - ps11
Choano./Metazoa - ps12
Metazoa - ps13
Myriazoa - ps14
Parahoxozoa - ps15
Bilateria - ps16
Deuterostomia - ps17
Chordata - ps18
Olfactores - ps19
Vertebrata - ps20
Gnathostomata - ps21
Euteleostomi - ps22
Sarcopterygii - ps23
Tetrapoda - ps24
Amniota - ps25
Mammalia - ps26
Theria - ps27
Placentalia - ps28
Boreoeutheria - ps29
Euarchontoglires - ps30
Euarchonta - ps31
Primates - ps32
Haplorrhini - ps33
Simiiformes - ps34
Catarrhini - ps35
Hominoidea - ps36
Hominidae - ps37
Homininae - ps38
Hominini - ps39
Homo_sapiens - ps40
Phylostratum

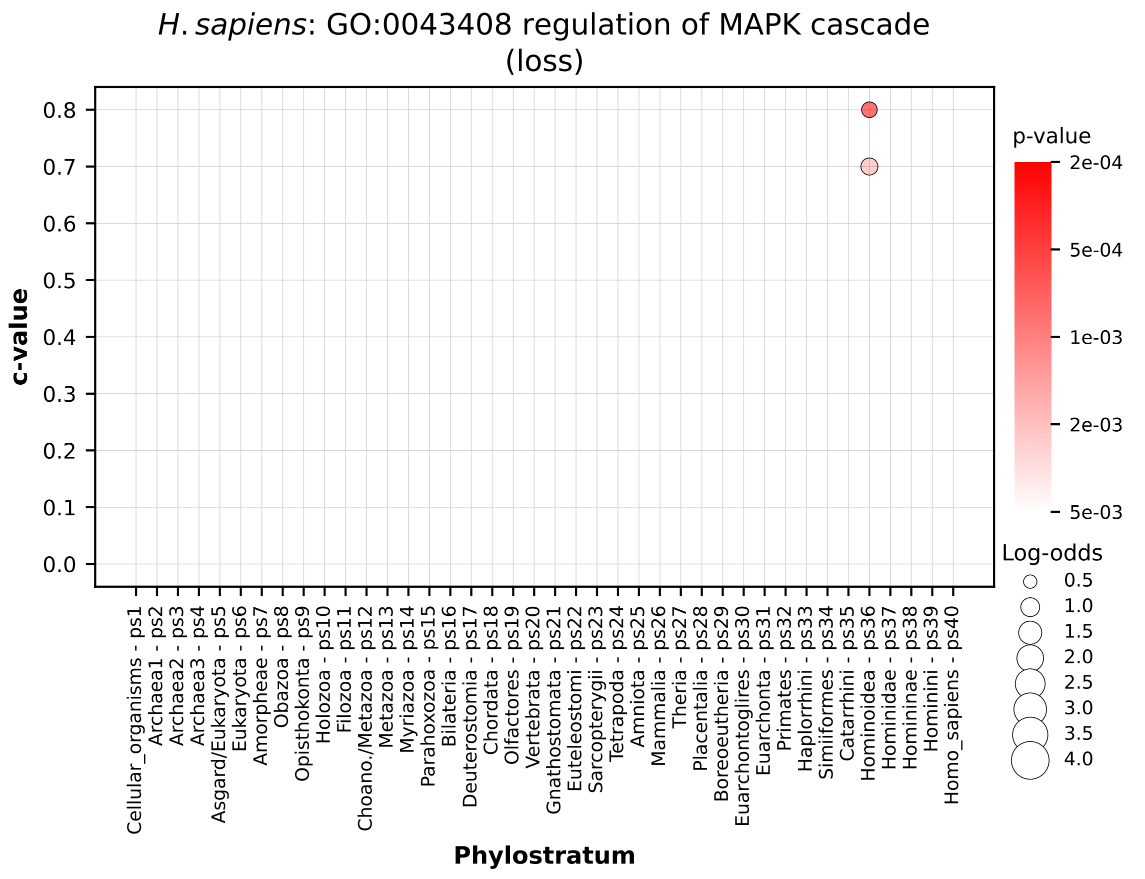
H. sapiens: GO:0043408 regulation of MAPK cascade (loss)
c-value
0.0
0.1
0.2
0.3
0.4
0.5
0.6
0.7
0.8
p-value
2e-04
5e-04
1e-03
2e-03
5e-03
Log-odds
0.5
1.0
1.5
2.0
2.5
3.0
3.5
4.0
Cellular_organisms - ps1
Archaea1 - ps2
Archaea2 - ps3
Archaea3 - ps4
Asgard/Eukaryota - ps5
Eukaryota - ps6
Amorpheae - ps7
Obazoa - ps8
Opisthokonta - ps9
Holozoa - ps10
Filozoa - ps11
Choano./Metazoa - ps12
Metazoa - ps13
Myriazoa - ps14
Parahoxozoa - ps15
Bilateria - ps16
Deuterostomia - ps17
Chordata - ps18
Olfactores - ps19
Vertebrata - ps20
Gnathostomata - ps21
Euteleostomi - ps22
Sarcopterygii - ps23
Tetrapoda - ps24
Amniota - ps25
Mammalia - ps26
Theria - ps27
Placentalia - ps28
Boreoeutheria - ps29
Euarchontoglires - ps30
Euarchonta - ps31
Primates - ps32
Haplorrhini - ps33
Simiiformes - ps34
Catarrhini - ps35
Hominoidea - ps36
Hominidae - ps37
Homininae - ps38
Hominini - ps39
Homo_sapiens - ps40
Phylostratum

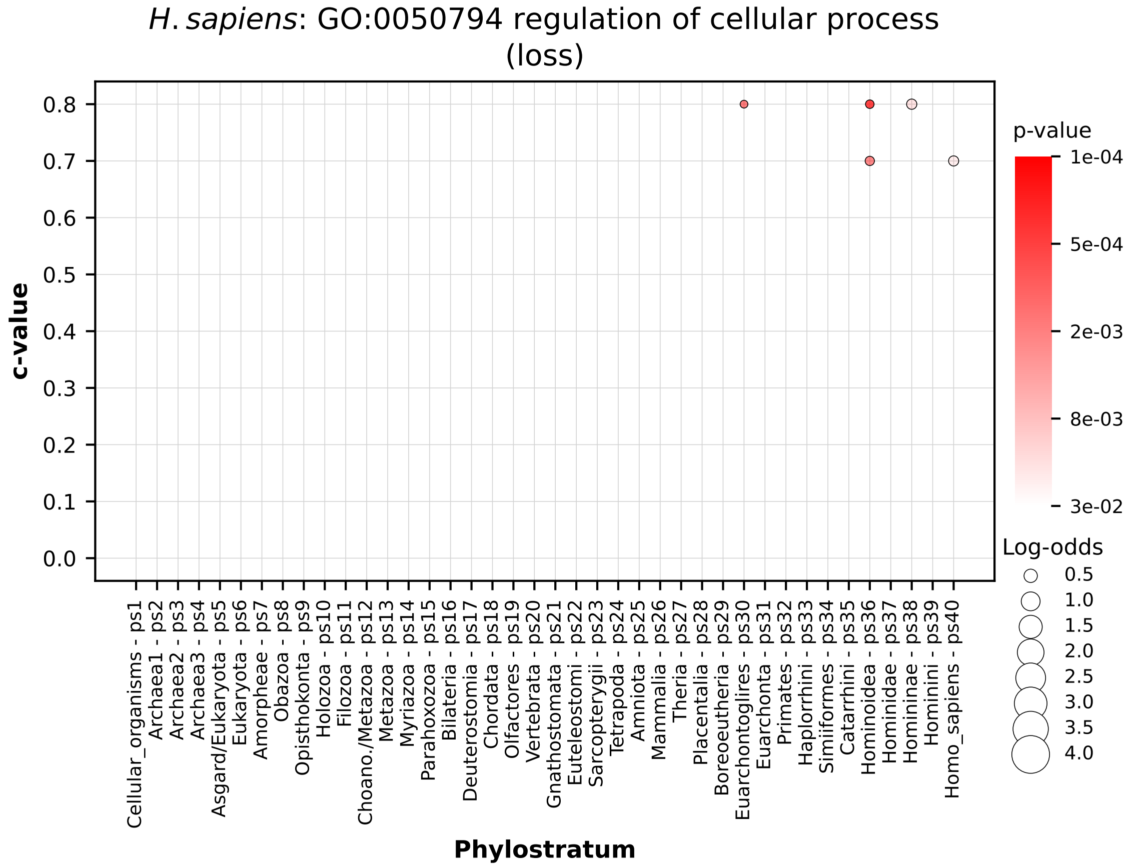

H. sapiens: GO:0050794 regulation of cellular process
(loss)
c-value
0.0
0.1
0.2
0.3
0.4
0.5
0.6
0.7
0.8
Phylostratum
Cellular_organisms - ps1
Archaea1 - ps2
Archaea2 - ps3
Archaea3 - ps4
Asgard/Eukaryota - ps5
Eukaryota - ps6
Amorpheae - ps7
Obazoa - ps8
Opisthokonta - ps9
Holozoa - ps10
Filozoa - ps11
Choano./Metazoa - ps12
Metazoa - ps13
Myriazoa - ps14
Parahoxozoa - ps15
Bilateria - ps16
Deuterostomia - ps17
Chordata - ps18
Olfactores - ps19
Vertebrata - ps20
Gnathostomata - ps21
Euteleostomi - ps22
Sarcopterygii - ps23
Tetrapoda - ps24
Amniota - ps25
Mammalia - ps26
Theria - ps27
Placentalia - ps28
Boreoeutheria - ps29
Euarchontoglires - ps30
Euarchonta - ps31
Primates - ps32
Haplorrhini - ps33
Simiiformes - ps34
Catarrhini - ps35
Hominoidea - ps36
Hominidae - ps37
Homininae - ps38
Hominini - ps39
Homo_sapiens - ps40
p-value
1e-04
5e-04
2e-03
8e-03
3e-02
Log-odds
0.5
1.0
1.5
2.0
2.5
3.0
3.5
4.0

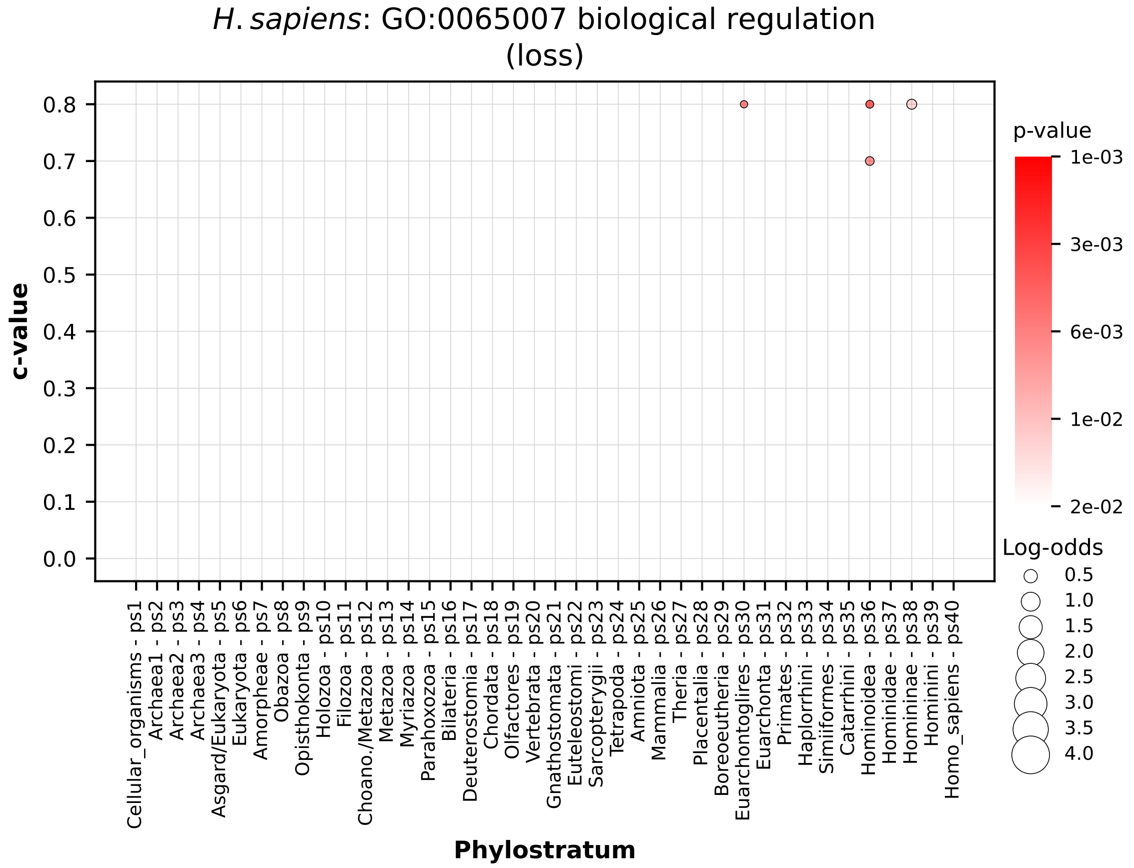

H. sapiens: GO:0065007 biological regulation
(loss)
c-value
0.0
0.1
0.2
0.3
0.4
0.5
0.6
0.7
0.8
Phylostratum
Cellular_organisms - ps1
Archaea1 - ps2
Archaea2 - ps3
Archaea3 - ps4
Asgard/Eukaryota - ps5
Eukaryota - ps6
Amorpheae - ps7
Obazoa - ps8
Opisthokonta - ps9
Holozoa - ps10
Filozoa - ps11
Choano./Metazoa - ps12
Metazoa - ps13
Myriazoa - ps14
Parahoxozoa - ps15
Bilateria - ps16
Deuterostomia - ps17
Chordata - ps18
Olfactores - ps19
Vertebrata - ps20
Gnathostomata - ps21
Euteleostomi - ps22
Sarcopterygii - ps23
Tetrapoda - ps24
Amniota - ps25
Mammalia - ps26
Theria - ps27
Placentalia - ps28
Boreoeutheria - ps29
Euarchontoglires - ps30
Euarchonta - ps31
Primates - ps32
Haplorrhini - ps33
Simiiformes - ps34
Catarrhini - ps35
Hominoidea - ps36
Hominidae - ps37
Homininae - ps38
Hominini - ps39
Homo_sapiens - ps40
p-value
1e-03
3e-03
6e-03
1e-02
2e-02
Log-odds
0.5
1.0
1.5
2.0
2.5
3.0
3.5
4.0

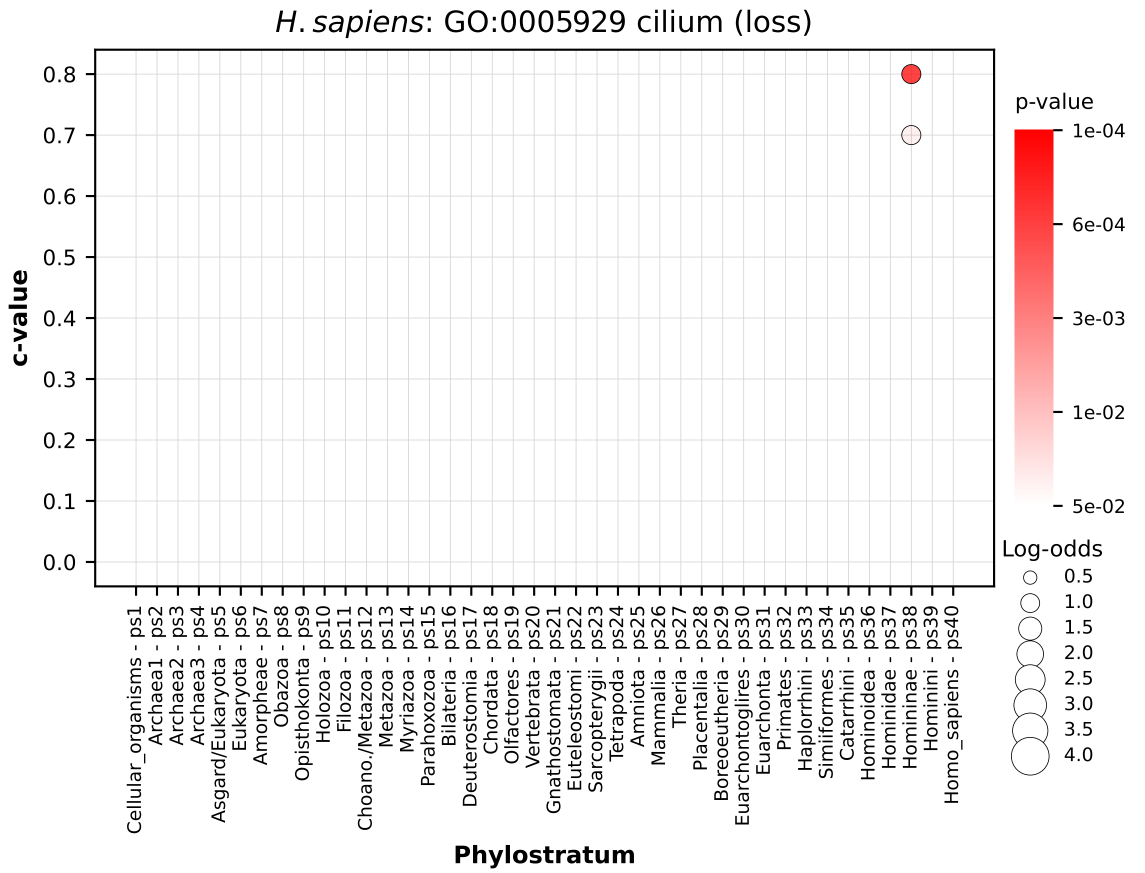

H. sapiens: GO:0005929 cilium (loss)
c-value
0.0
0.1
0.2
0.3
0.4
0.5
0.6
0.7
0.8
Phylostratum
Cellular_organisms - ps1
Archaea1 - ps2
Archaea2 - ps3
Archaea3 - ps4
Asgard/Eukaryota - ps5
Eukaryota - ps6
Amorpheae - ps7
Obazoa - ps8
Opisthokonta - ps9
Holozoa - ps10
Filozoa - ps11
Choano./Metazoa - ps12
Metazoa - ps13
Myriazoa - ps14
Parahoxozoa - ps15
Bilateria - ps16
Deuterostomia - ps17
Chordata - ps18
Olfactores - ps19
Vertebrata - ps20
Gnathostomata - ps21
Euteleostomi - ps22
Sarcopterygii - ps23
Tetrapoda - ps24
Amniota - ps25
Mammalia - ps26
Theria - ps27
Placentalia - ps28
Boreoeutheria - ps29
Euarchontoglires - ps30
Euarchonta - ps31
Primates - ps32
Haplorrhini - ps33
Simiiformes - ps34
Catarrhini - ps35
Hominoidea - ps36
Hominidae - ps37
Homininae - ps38
Hominini - ps39
Homo_sapiens - ps40
p-value
1e-04
6e-04
3e-03
1e-02
5e-02
Log-odds
0.5
1.0
1.5
2.0
2.5
3.0
3.5
4.0

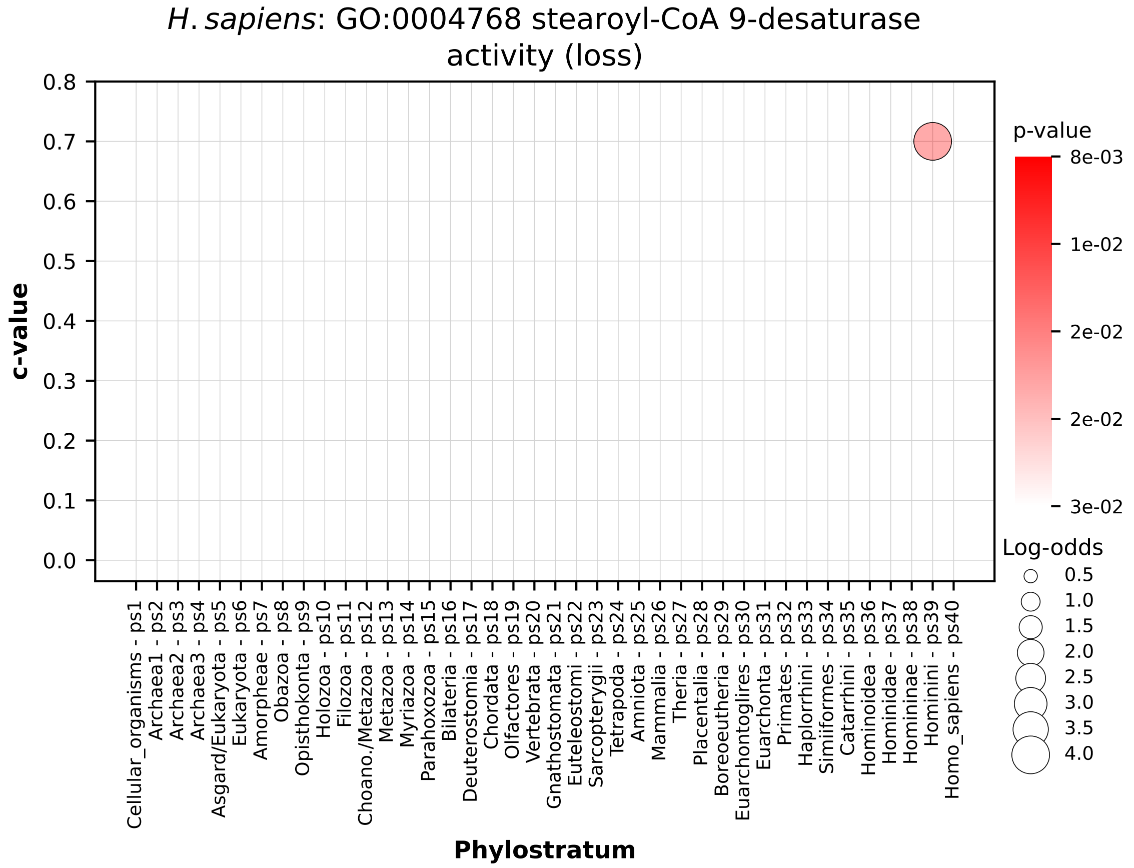

H. sapiens: GO:0004768 stearoyl-CoA 9-desaturase activity (loss)
c-value
0.8
0.7
0.6
0.5
0.4
0.3
0.2
0.1
0.0
p-value
8e-03
1e-02
2e-02
2e-02
3e-02
Log-odds
0.5
1.0
1.5
2.0
2.5
3.0
3.5
4.0
Cellular_organisms - ps1
Archaea1 - ps2
Archaea2 - ps3
Archaea3 - ps4
Asgard/Eukaryota - ps5
Eukaryota - ps6
Amorpheae - ps7
Obazoa - ps8
Opisthokonta - ps9
Holozoa - ps10
Filozoa - ps11
Choano./Metazoa - ps12
Metazoa - ps13
Myriazoa - ps14
Parahoxozoa - ps15
Bilateria - ps16
Deuterostomia - ps17
Chordata - ps18
Olfactores - ps19
Vertebrata - ps20
Gnathostomata - ps21
Euteleostomi - ps22
Sarcopterygii - ps23
Tetrapoda - ps24
Amniota - ps25
Mammalia - ps26
Theria - ps27
Placentalia - ps28
Boreoeutheria - ps29
Euarchontoglires - ps30
Euarchonta - ps31
Primates - ps32
Haplorrhini - ps33
Simiiformes - ps34
Catarrhini - ps35
Hominoidea - ps36
Hominidae - ps37
Homininae - ps38
Hominini - ps39
Homo_sapiens - ps40
Phylostratum

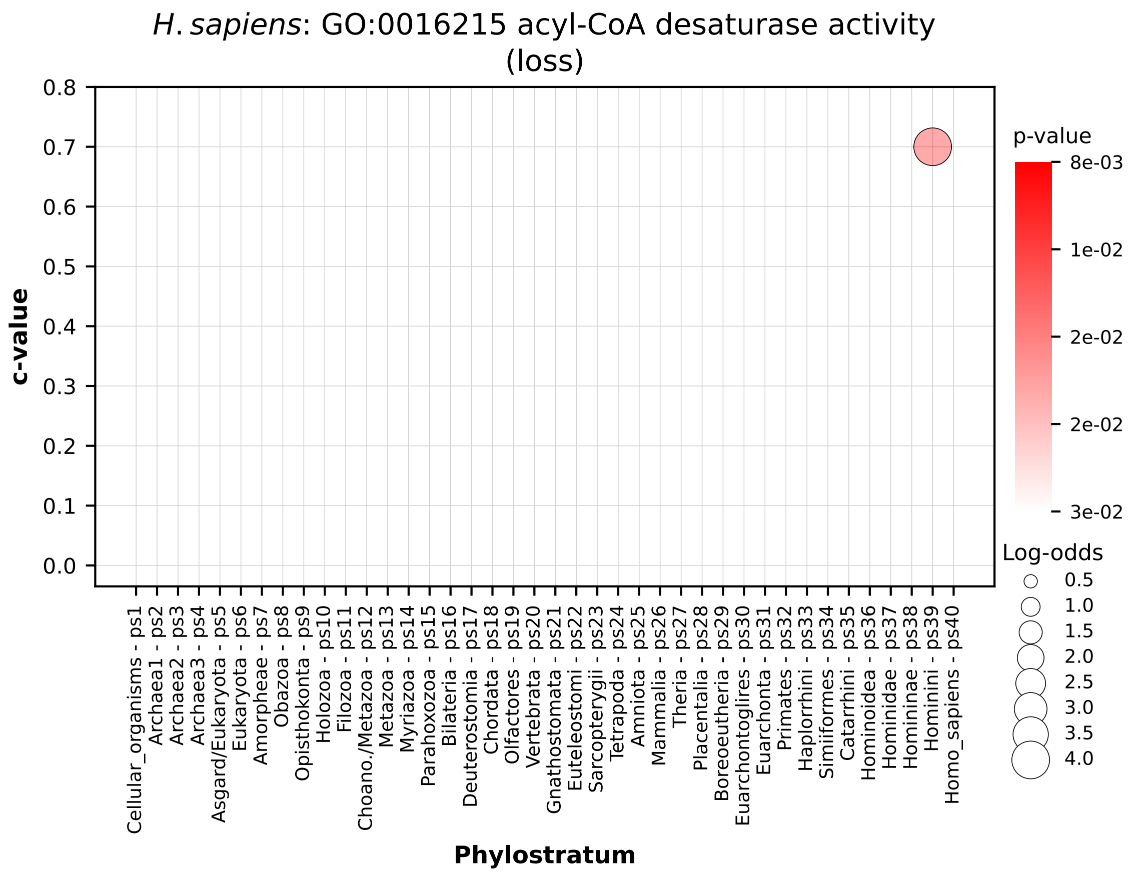

H. sapiens: GO:0016215 acyl-CoA desaturase activity
(loss)
c-value
0.8
0.7
0.6
0.5
0.4
0.3
0.2
0.1
0.0
p-value
8e-03
1e-02
2e-02
2e-02
3e-02
Log-odds
0.5
1.0
1.5
2.0
2.5
3.0
3.5
4.0
Cellular_organisms - ps1
Archaea1 - ps2
Archaea2 - ps3
Archaea3 - ps4
Asgard/Eukaryota - ps5
Eukaryota - ps6
Amorpheae - ps7
Obazoa - ps8
Opisthokonta - ps9
Holozoa - ps10
Filozoa - ps11
Choano./Metazoa - ps12
Metazoa - ps13
Myriazoa - ps14
Parahoxozoa - ps15
Bilateria - ps16
Deuterostomia - ps17
Chordata - ps18
Olfactores - ps19
Vertebrata - ps20
Gnathostomata - ps21
Euteleostomi - ps22
Sarcopterygii - ps23
Tetrapoda - ps24
Amniota - ps25
Mammalia - ps26
Theria - ps27
Placentalia - ps28
Boreoeutheria - ps29
Euarchontoglires - ps30
Euarchonta - ps31
Primates - ps32
Haplorrhini - ps33
Simiiformes - ps34
Catarrhini - ps35
Hominoidea - ps36
Hominidae - ps37
Homininae - ps38
Hominini - ps39
Homo_sapiens - ps40
Phylostratum

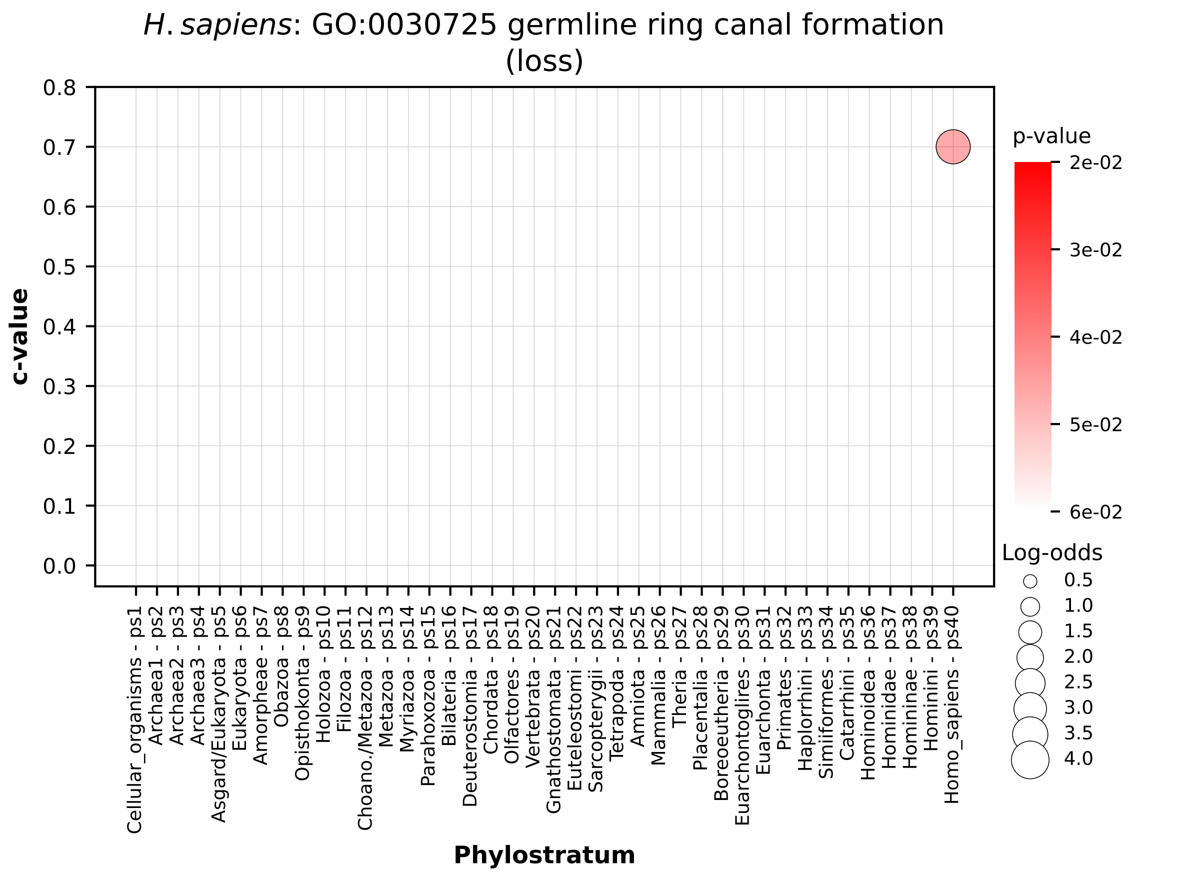

H. sapiens: GO:0030725 germline ring canal formation
(loss)
c-value
0.8
0.7
0.6
0.5
0.4
0.3
0.2
0.1
0.0
p-value
2e-02
3e-02
4e-02
5e-02
6e-02
Log-odds
0.5
1.0
1.5
2.0
2.5
3.0
3.5
4.0
Cellular_organisms - ps1
Archaea1 - ps2
Archaea2 - ps3
Archaea3 - ps4
Asgard/Eukaryota - ps5
Eukaryota - ps6
Amorpheae - ps7
Obazoa - ps8
Opisthokonta - ps9
Holozoa - ps10
Filozoa - ps11
Choano./Metazoa - ps12
Metazoa - ps13
Myriazoa - ps14
Parahoxozoa - ps15
Bilateria - ps16
Deuterostomia - ps17
Chordata - ps18
Olfactores - ps19
Vertebrata - ps20
Gnathostomata - ps21
Euteleostomi - ps22
Sarcopterygii - ps23
Tetrapoda - ps24
Amniota - ps25
Mammalia - ps26
Theria - ps27
Placentalia - ps28
Boreoeutheria - ps29
Euarchontoglires - ps30
Euarchonta - ps31
Primates - ps32
Haplorrhini - ps33
Simiiformes - ps34
Catarrhini - ps35
Hominoidea - ps36
Hominidae - ps37
Homininae - ps38
Hominini - ps39
Homo_sapiens - ps40
Phylostratum

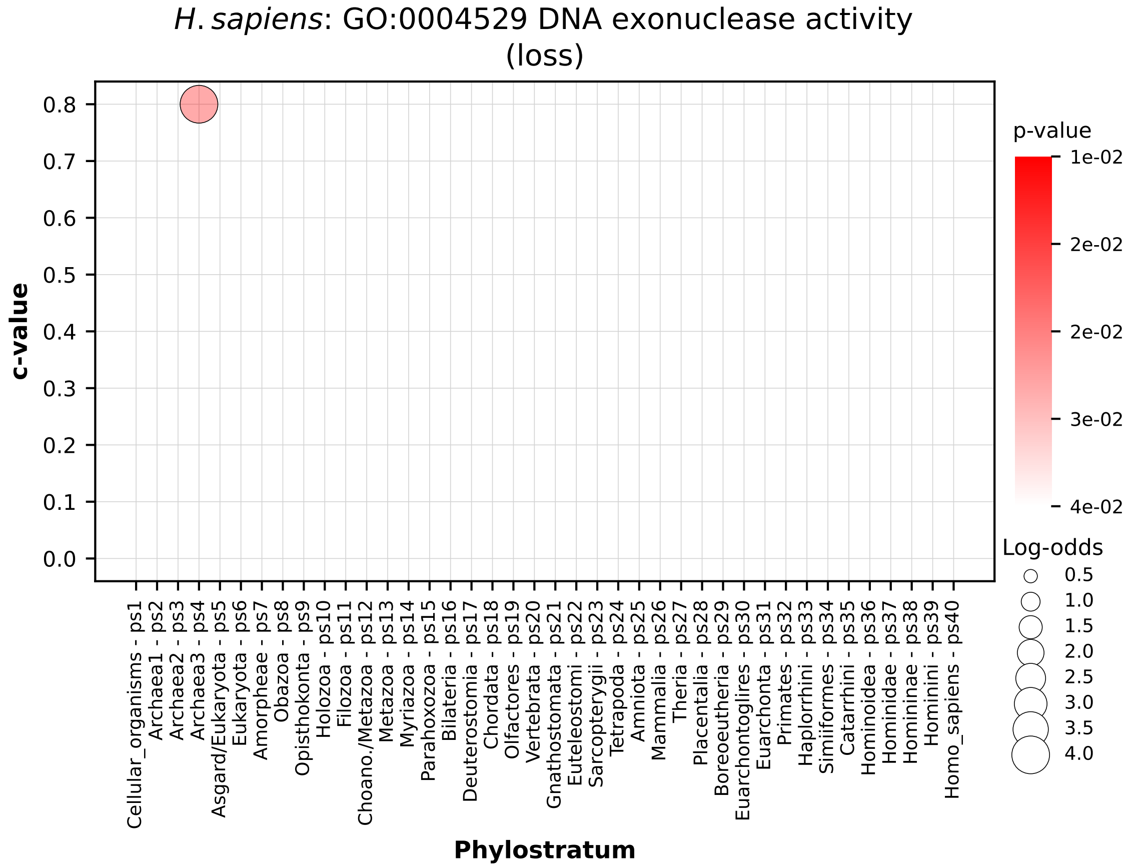

H. sapiens: GO:0004529 DNA exonuclease activity
(loss)
c-value
0.8
0.7
0.6
0.5
0.4
0.3
0.2
0.1
0.0
p-value
1e-02
2e-02
2e-02
3e-02
4e-02
Log-odds
0.5
1.0
1.5
2.0
2.5
3.0
3.5
4.0
Cellular_organisms - ps1
Archaea1 - ps2
Archaea2 - ps3
Archaea3 - ps4
Asgard/Eukaryota - ps5
Eukaryota - ps6
Amorpheae - ps7
Obazoa - ps8
Opisthokonta - ps9
Holozoa - ps10
Filozoa - ps11
Choano./Metazoa - ps12
Metazoa - ps13
Myriazoa - ps14
Parahoxozoa - ps15
Bilateria - ps16
Deuterostomia - ps17
Chordata - ps18
Olfactores - ps19
Vertebrata - ps20
Gnathostomata - ps21
Euteleostomi - ps22
Sarcopterygii - ps23
Tetrapoda - ps24
Amniota - ps25
Mammalia - ps26
Theria - ps27
Placentalia - ps28
Boreoeutheria - ps29
Euarchontoglires - ps30
Euarchonta - ps31
Primates - ps32
Haplorrhini - ps33
Simiiformes - ps34
Catarrhini - ps35
Hominoidea - ps36
Hominidae - ps37
Homininae - ps38
Hominini - ps39
Homo_sapiens - ps40
Phylostratum

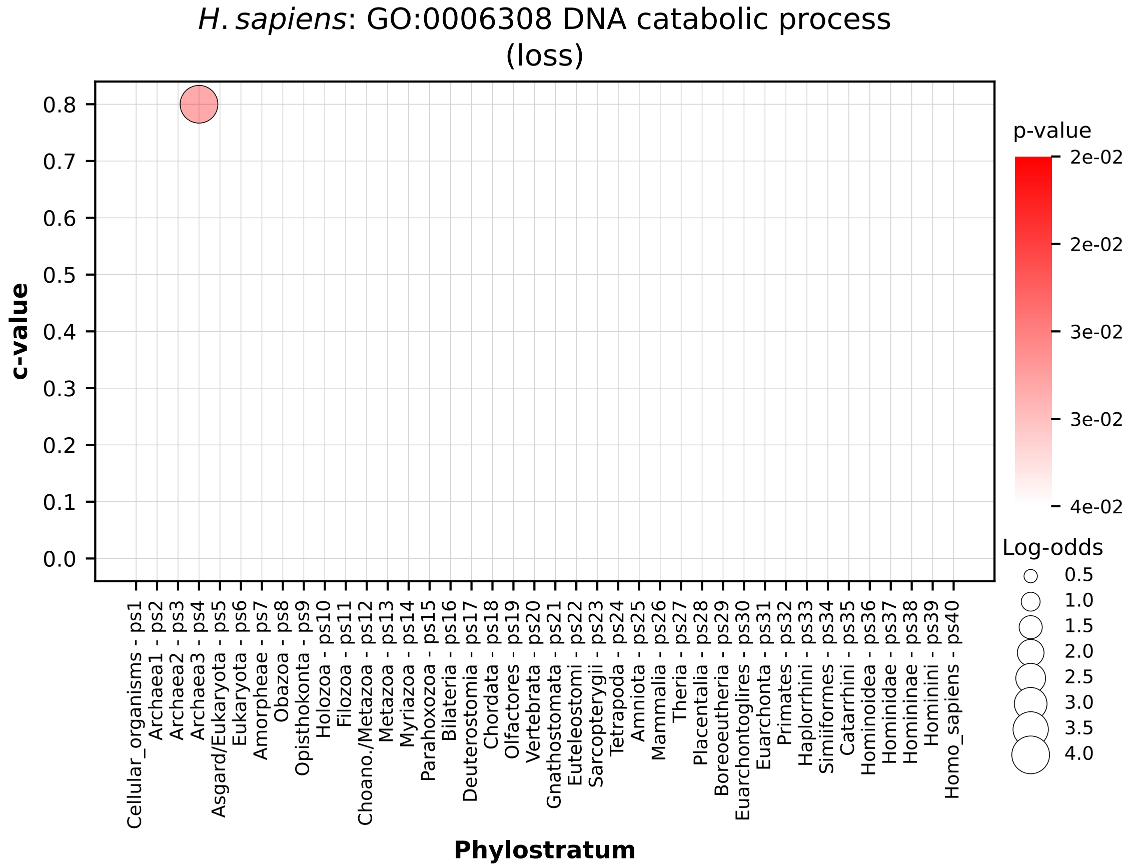

H. sapiens: GO:0006308 DNA catabolic process
(loss)
c-value
0.8
0.7
0.6
0.5
0.4
0.3
0.2
0.1
0.0
p-value
2e-02
2e-02
3e-02
3e-02
4e-02
Log-odds
0.5
1.0
1.5
2.0
2.5
3.0
3.5
4.0
Cellular_organisms - ps1
Archaea1 - ps2
Archaea2 - ps3
Archaea3 - ps4
Asgard/Eukaryota - ps5
Eukaryota - ps6
Amorpheae - ps7
Obazoa - ps8
Opisthokonta - ps9
Holozoa - ps10
Filozoa - ps11
Choano./Metazoa - ps12
Metazoa - ps13
Myriazoa - ps14
Parahoxozoa - ps15
Bilateria - ps16
Deuterostomia - ps17
Chordata - ps18
Olfactores - ps19
Vertebrata - ps20
Gnathostomata - ps21
Euteleostomi - ps22
Sarcopterygii - ps23
Tetrapoda - ps24
Amniota - ps25
Mammalia - ps26
Theria - ps27
Placentalia - ps28
Boreoeutheria - ps29
Euarchontoglires - ps30
Euarchonta - ps31
Primates - ps32
Haplorrhini - ps33
Simiiformes - ps34
Catarrhini - ps35
Hominoidea - ps36
Hominidae - ps37
Homininae - ps38
Hominini - ps39
Homo_sapiens - ps40
Phylostratum

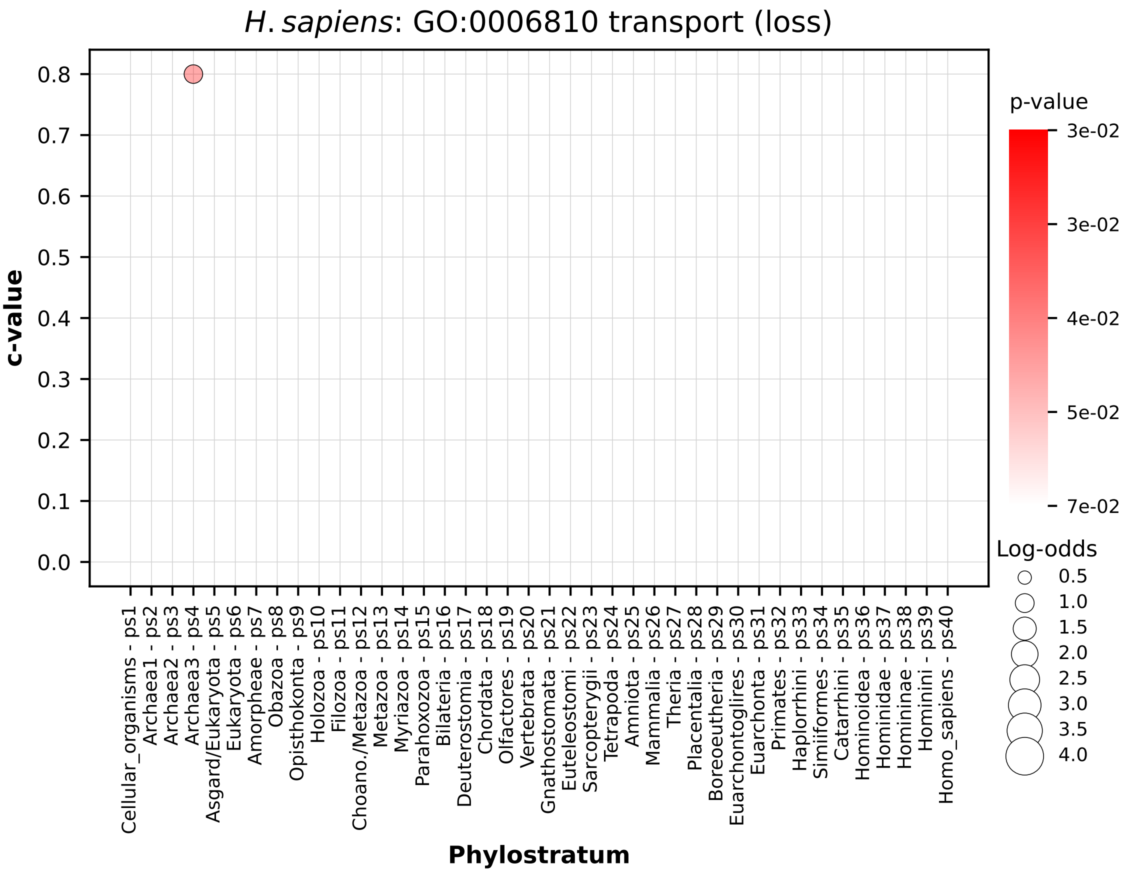

H. sapiens: GO:0006810 transport (loss)
c-value
0.8
0.7
0.6
0.5
0.4
0.3
0.2
0.1
0.0
p-value
3e-02
3e-02
4e-02
5e-02
7e-02
Log-odds
0.5
1.0
1.5
2.0
2.5
3.0
3.5
4.0
Cellular_organisms - ps1
Archaea1 - ps2
Archaea2 - ps3
Archaea3 - ps4
Asgard/Eukaryota - ps5
Eukaryota - ps6
Amorpheae - ps7
Obazoa - ps8
Opisthokonta - ps9
Holozoa - ps10
Filozoa - ps11
Choano./Metazoa - ps12
Metazoa - ps13
Myriazoa - ps14
Parahoxozoa - ps15
Bilateria - ps16
Deuterostomia - ps17
Chordata - ps18
Olfactores - ps19
Vertebrata - ps20
Gnathostomata - ps21
Euteleostomi - ps22
Sarcopterygii - ps23
Tetrapoda - ps24
Amniota - ps25
Mammalia - ps26
Theria - ps27
Placentalia - ps28
Boreoeutheria - ps29
Euarchontoglires - ps30
Euarchonta - ps31
Primates - ps32
Haplorrhini - ps33
Simiiformes - ps34
Catarrhini - ps35
Hominoidea - ps36
Hominidae - ps37
Homininae - ps38
Hominini - ps39
Homo_sapiens - ps40
Phylostratum

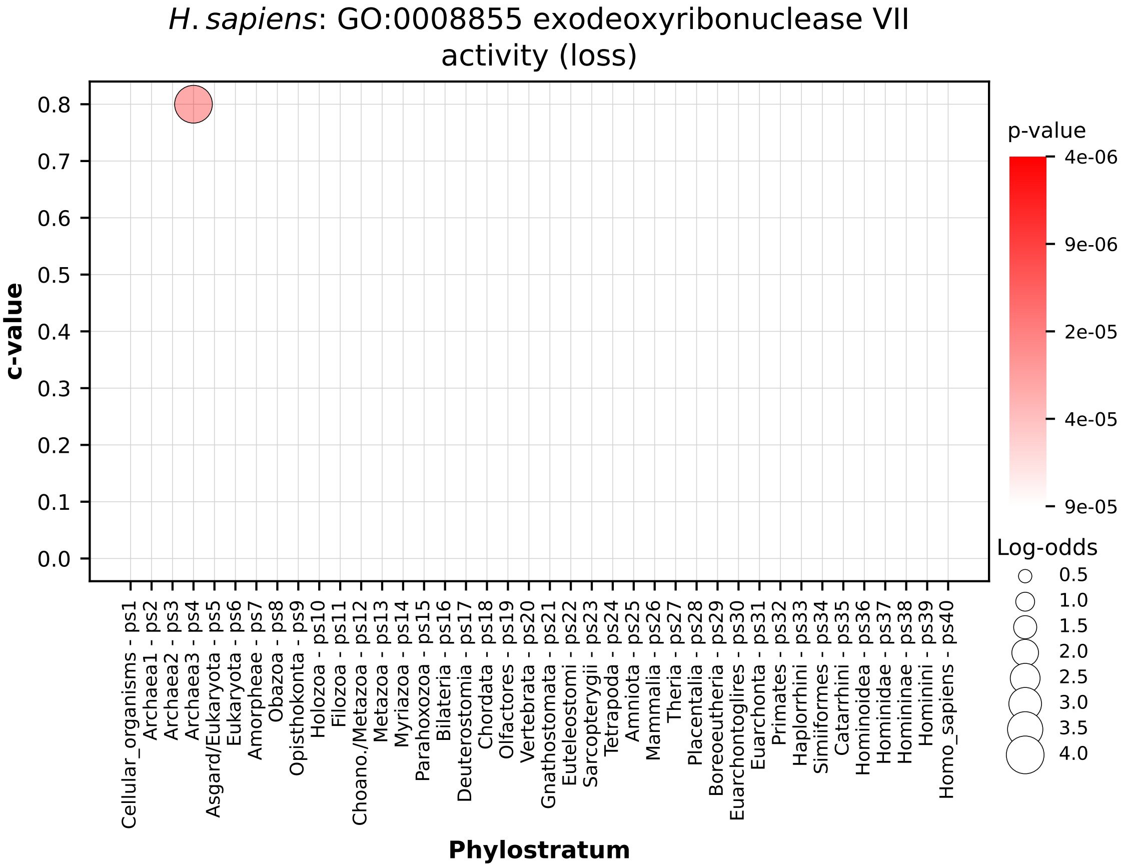

H. sapiens: GO:0008855 exodeoxyribonuclease VII activity (loss)
c-value
0.8
0.7
0.6
0.5
0.4
0.3
0.2
0.1
0.0
p-value
4e-06
9e-06
2e-05
4e-05
9e-05
Log-odds
0.5
1.0
1.5
2.0
2.5
3.0
3.5
4.0
Cellular_organisms - ps1
Archaea1 - ps2
Archaea2 - ps3
Archaea3 - ps4
Asgard/Eukaryota - ps5
Eukaryota - ps6
Amorpheae - ps7
Obazoa - ps8
Opisthokonta - ps9
Holozoa - ps10
Filozoa - ps11
Choano./Metazoa - ps12
Metazoa - ps13
Myriazoa - ps14
Parahoxozoa - ps15
Bilateria - ps16
Deuterostomia - ps17
Chordata - ps18
Olfactores - ps19
Vertebrata - ps20
Gnathostomata - ps21
Euteleostomi - ps22
Sarcopterygii - ps23
Tetrapoda - ps24
Amniota - ps25
Mammalia - ps26
Theria - ps27
Placentalia - ps28
Boreoeutheria - ps29
Euarchontoglires - ps30
Euarchonta - ps31
Primates - ps32
Haplorrhini - ps33
Simiiformes - ps34
Catarrhini - ps35
Hominoidea - ps36
Hominidae - ps37
Homininae - ps38
Hominini - ps39
Homo_sapiens - ps40
Phylostratum

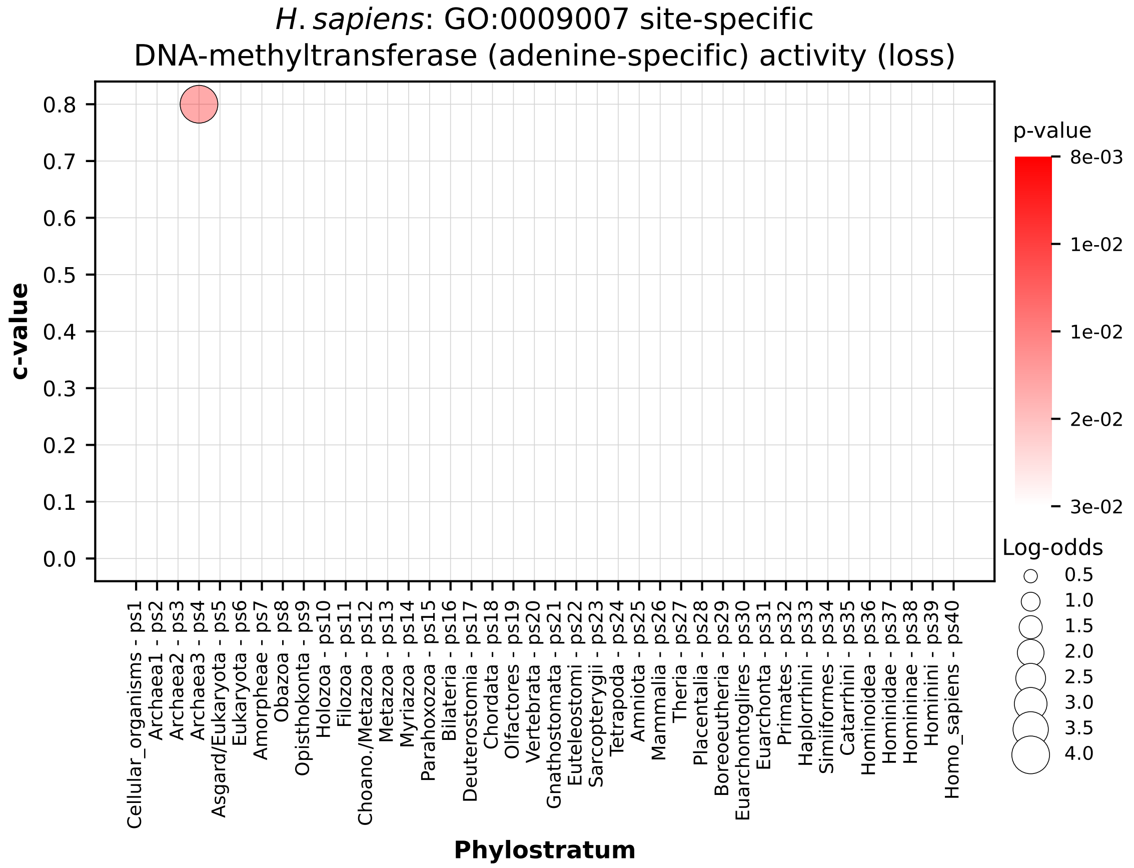
H. sapiens: GO:0009007 site-specific
DNA-methyltransferase (adenine-specific) activity (loss)
c-value
0.0
0.1
0.2
0.3
0.4
0.5
0.6
0.7
0.8
Phylostratum
Cellular_organisms - ps1
Archaea1 - ps2
Archaea2 - ps3
Archaea3 - ps4
Asgard/Eukaryota - ps5
Eukaryota - ps6
Amorpheae - ps7
Obazoa - ps8
Opisthokonta - ps9
Holozoa - ps10
Filozoa - ps11
Choano./Metazoa - ps12
Metazoa - ps13
Myriazoa - ps14
Parahoxozoa - ps15
Bilateria - ps16
Deuterostomia - ps17
Chordata - ps18
Olfactores - ps19
Vertebrata - ps20
Gnathostomata - ps21
Euteleostomi - ps22
Sarcopterygii - ps23
Tetrapoda - ps24
Amniota - ps25
Mammalia - ps26
Theria - ps27
Placentalia - ps28
Boreoeutheria - ps29
Euarchontoglires - ps30
Euarchonta - ps31
Primates - ps32
Haplorrhini - ps33
Simiiformes - ps34
Catarrhini - ps35
Hominoidea - ps36
Hominidae - ps37
Homininae - ps38
Hominini - ps39
Homo_sapiens - ps40
p-value
8e-03
1e-02
1e-02
2e-02
3e-02
Log-odds
0.5
1.0
1.5
2.0
2.5
3.0
3.5
4.0

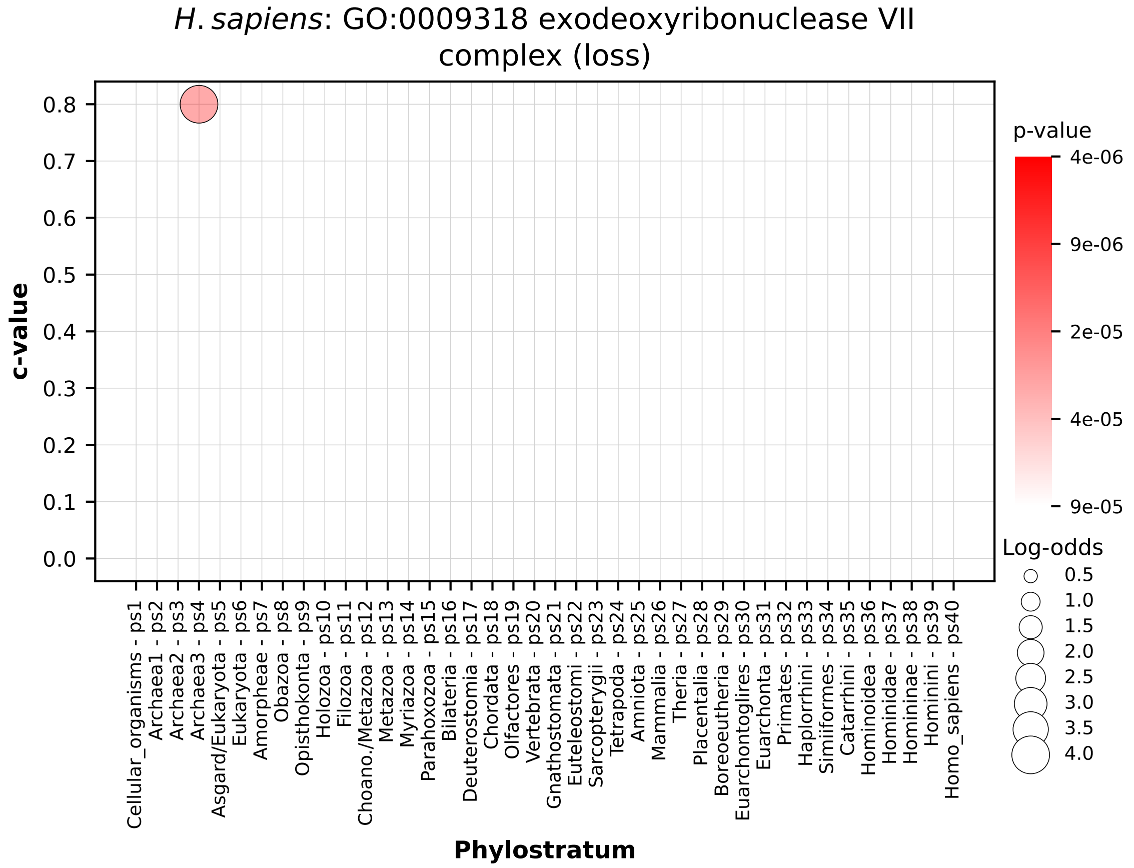

H. sapiens: GO:0009318 exodeoxyribonuclease VII complex (loss)
c-value
0.8
0.7
0.6
0.5
0.4
0.3
0.2
0.1
0.0
p-value
4e-06
9e-06
2e-05
4e-05
9e-05
Log-odds
0.5
1.0
1.5
2.0
2.5
3.0
3.5
4.0
Cellular_organisms - ps1
Archaea1 - ps2
Archaea2 - ps3
Archaea3 - ps4
Asgard/Eukaryota - ps5
Eukaryota - ps6
Amorpheae - ps7
Obazoa - ps8
Opisthokonta - ps9
Holozoa - ps10
Filozoa - ps11
Choano./Metazoa - ps12
Metazoa - ps13
Myriazoa - ps14
Parahoxozoa - ps15
Bilateria - ps16
Deuterostomia - ps17
Chordata - ps18
Olfactores - ps19
Vertebrata - ps20
Gnathostomata - ps21
Euteleostomi - ps22
Sarcopterygii - ps23
Tetrapoda - ps24
Amniota - ps25
Mammalia - ps26
Theria - ps27
Placentalia - ps28
Boreoeutheria - ps29
Euarchontoglires - ps30
Euarchonta - ps31
Primates - ps32
Haplorrhini - ps33
Simiiformes - ps34
Catarrhini - ps35
Hominoidea - ps36
Hominidae - ps37
Homininae - ps38
Hominini - ps39
Homo_sapiens - ps40
Phylostratum

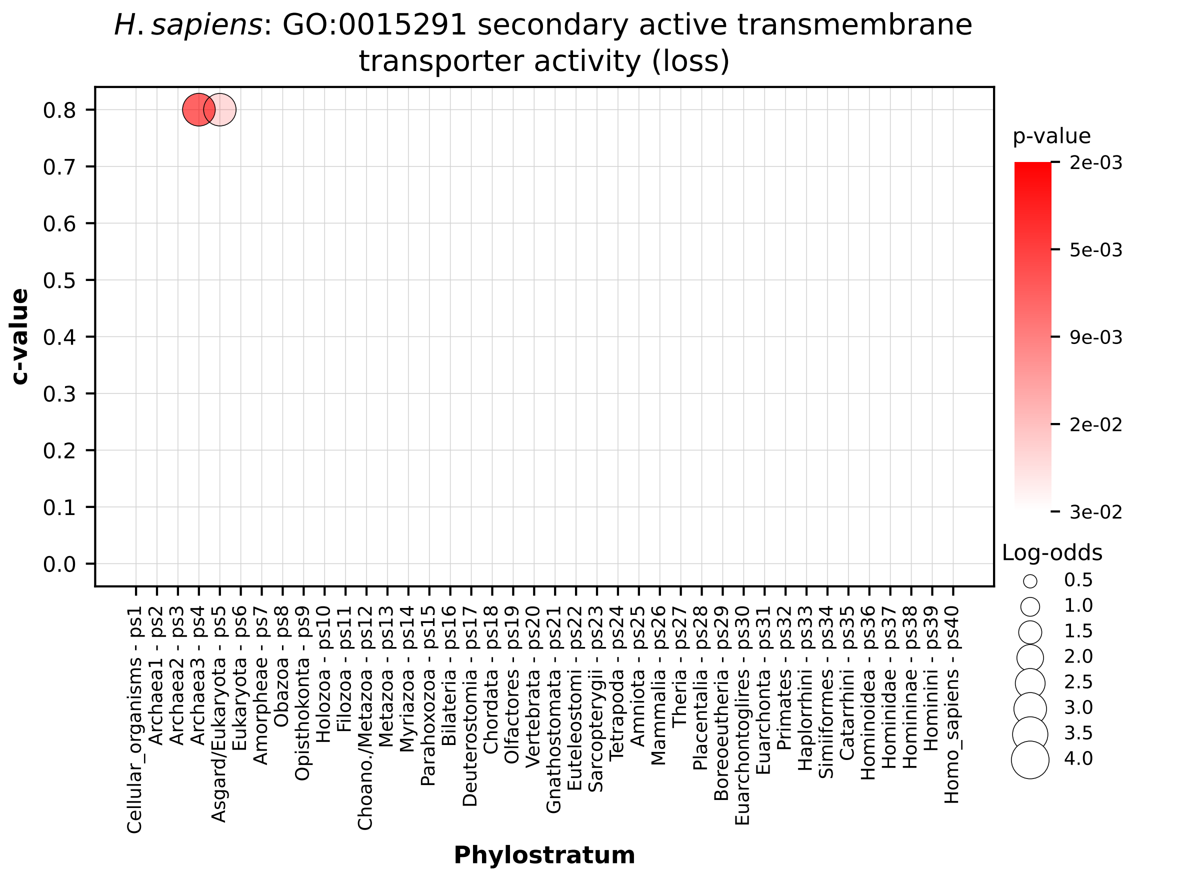
H. sapiens: GO:0015291 secondary active transmembrane transporter activity (loss)
c-value
0.8
0.7
0.6
0.5
0.4
0.3
0.2
0.1
0.0
p-value
2e-03
5e-03
9e-03
2e-02
3e-02
Log-odds
0.5
1.0
1.5
2.0
2.5
3.0
3.5
4.0
Cellular_organisms - ps1
Archaea1 - ps2
Archaea2 - ps3
Archaea3 - ps4
Asgard/Eukaryota - ps5
Eukaryota - ps6
Amorpheae - ps7
Obazoa - ps8
Opisthokonta - ps9
Holozoa - ps10
Filozoa - ps11
Choano./Metazoa - ps12
Metazoa - ps13
Myriazoa - ps14
Parahoxozoa - ps15
Bilateria - ps16
Deuterostomia - ps17
Chordata - ps18
Olfactores - ps19
Vertebrata - ps20
Gnathostomata - ps21
Euteleostomi - ps22
Sarcopterygii - ps23
Tetrapoda - ps24
Amniota - ps25
Mammalia - ps26
Theria - ps27
Placentalia - ps28
Boreoeutheria - ps29
Euarchontoglires - ps30
Euarchonta - ps31
Primates - ps32
Haplorrhini - ps33
Simiiformes - ps34
Catarrhini - ps35
Hominoidea - ps36
Hominidae - ps37
Homininae - ps38
Hominini - ps39
Homo_sapiens - ps40
Phylostratum

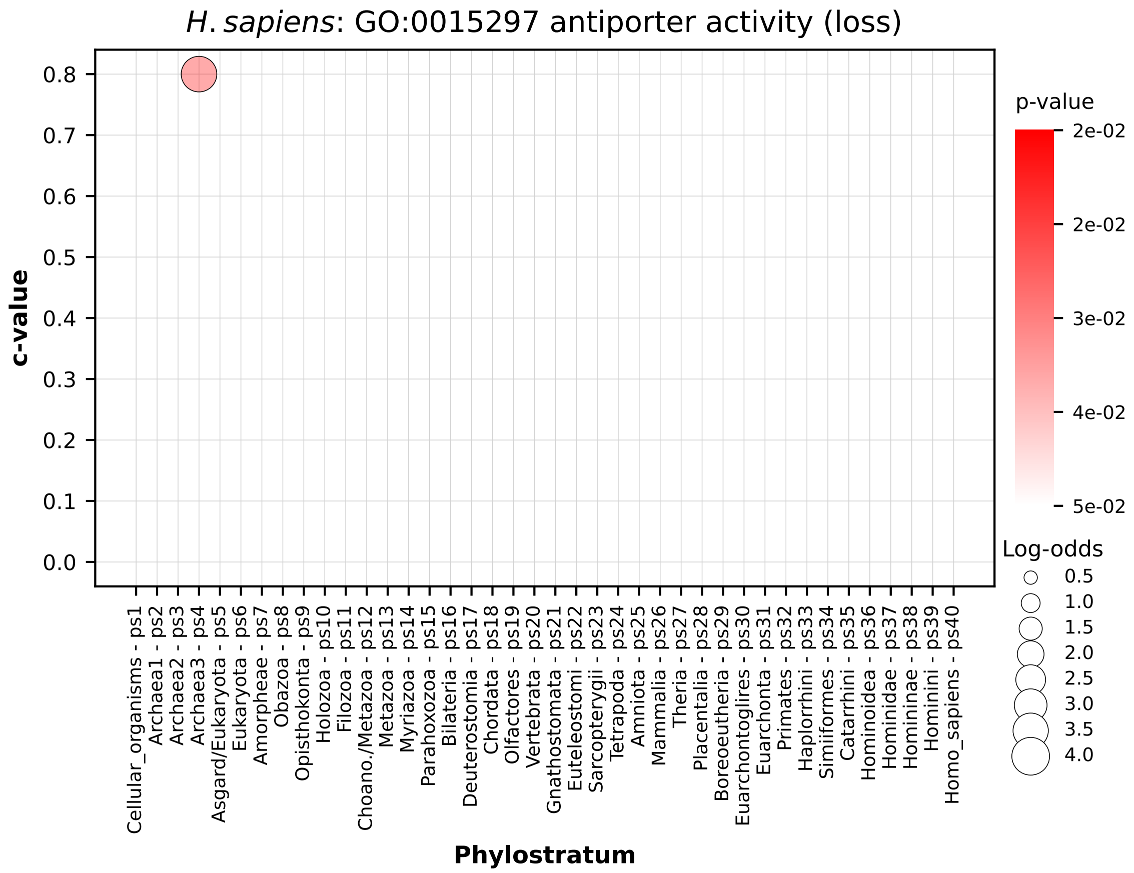
H. sapiens: GO:0015297 antiporter activity (loss)
c-value
0.0
0.1
0.2
0.3
0.4
0.5
0.6
0.7
0.8
Phylostratum
Cellular_organisms - ps1
Archaea1 - ps2
Archaea2 - ps3
Archaea3 - ps4
Asgard/Eukaryota - ps5
Eukaryota - ps6
Amorpheae - ps7
Obazoa - ps8
Opisthokonta - ps9
Holozoa - ps10
Filozoa - ps11
Choano./Metazoa - ps12
Metazoa - ps13
Myriazoa - ps14
Parahoxozoa - ps15
Bilateria - ps16
Deuterostomia - ps17
Chordata - ps18
Olfactores - ps19
Vertebrata - ps20
Gnathostomata - ps21
Euteleostomi - ps22
Sarcopterygii - ps23
Tetrapoda - ps24
Amniota - ps25
Mammalia - ps26
Theria - ps27
Placentalia - ps28
Boreoeutheria - ps29
Euarchontoglires - ps30
Euarchonta - ps31
Primates - ps32
Haplorrhini - ps33
Simiiformes - ps34
Catarrhini - ps35
Hominoidea - ps36
Hominidae - ps37
Homininae - ps38
Hominini - ps39
Homo_sapiens - ps40
p-value
2e-02
2e-02
3e-02
4e-02
5e-02
Log-odds
0.5
1.0
1.5
2.0
2.5
3.0
3.5
4.0

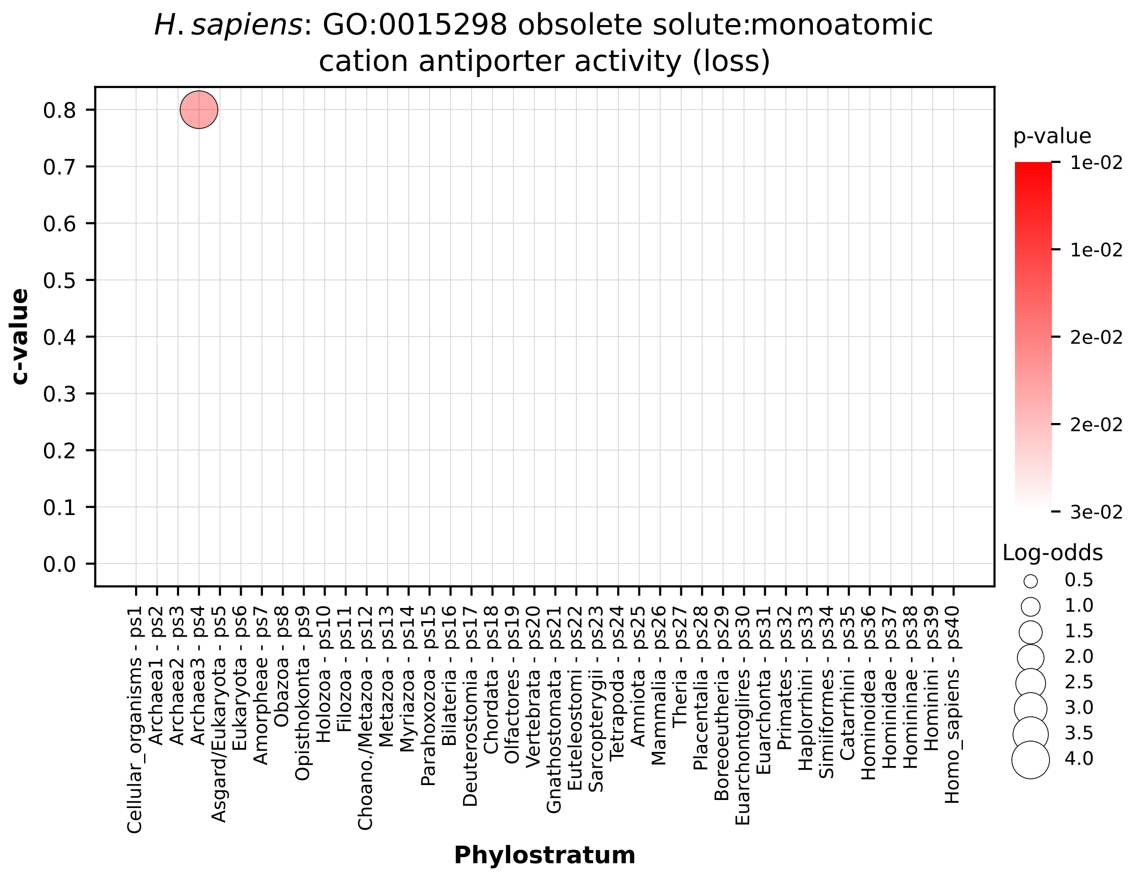

H. sapiens: GO:0015298 obsolete solute:monoatomic cation antiporter activity (loss)
c-value
0.0
0.1
0.2
0.3
0.4
0.5
0.6
0.7
0.8
Phylostratum
Cellular_organisms - ps1
Archaea1 - ps2
Archaea2 - ps3
Archaea3 - ps4
Asgard/Eukaryota - ps5
Eukaryota - ps6
Amorpheae - ps7
Obazoa - ps8
Opisthokonta - ps9
Holozoa - ps10
Filozoa - ps11
Choano./Metazoa - ps12
Metazoa - ps13
Myriazoa - ps14
Parahoxozoa - ps15
Bilateria - ps16
Deuterostomia - ps17
Chordata - ps18
Olfactores - ps19
Vertebrata - ps20
Gnathostomata - ps21
Euteleostomi - ps22
Sarcopterygii - ps23
Tetrapoda - ps24
Amniota - ps25
Mammalia - ps26
Theria - ps27
Placentalia - ps28
Boreoeutheria - ps29
Euarchontoglires - ps30
Euarchonta - ps31
Primates - ps32
Haplorrhini - ps33
Simiiformes - ps34
Catarrhini - ps35
Hominoidea - ps36
Hominidae - ps37
Homininae - ps38
Hominini - ps39
Homo_sapiens - ps40
p-value
1e-02
1e-02
2e-02
2e-02
3e-02
Log-odds
0.5
1.0
1.5
2.0
2.5
3.0
3.5
4.0

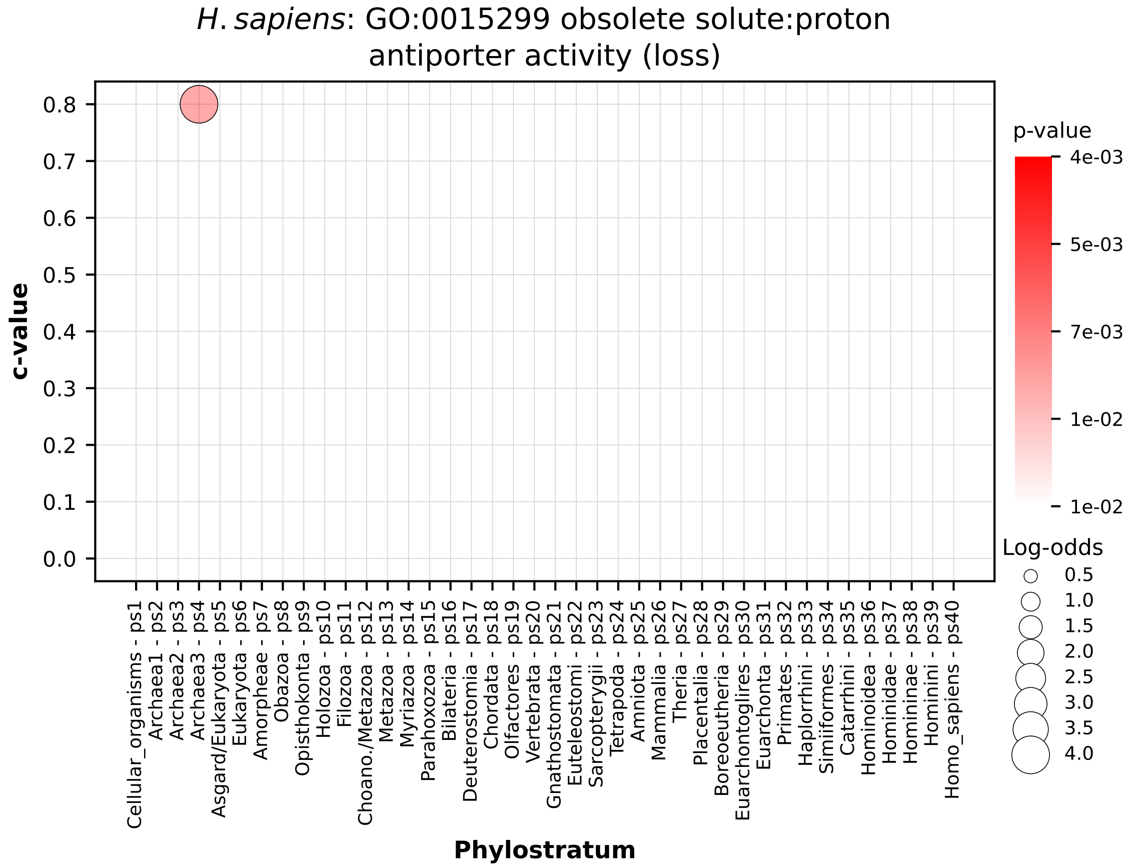

H. sapiens: GO:0015299 obsolete solute:proton antiporter activity (loss)
c-value
0.0
0.1
0.2
0.3
0.4
0.5
0.6
0.7
0.8
Phylostratum
Cellular_organisms - ps1
Archaea1 - ps2
Archaea2 - ps3
Archaea3 - ps4
Asgard/Eukaryota - ps5
Eukaryota - ps6
Amorpheae - ps7
Obazoa - ps8
Opisthokonta - ps9
Holozoa - ps10
Filozoa - ps11
Choano./Metazoa - ps12
Metazoa - ps13
Myriazoa - ps14
Parahoxozoa - ps15
Bilateria - ps16
Deuterostomia - ps17
Chordata - ps18
Olfactores - ps19
Vertebrata - ps20
Gnathostomata - ps21
Euteleostomi - ps22
Sarcopterygii - ps23
Tetrapoda - ps24
Amniota - ps25
Mammalia - ps26
Theria - ps27
Placentalia - ps28
Boreoeutheria - ps29
Euarchontoglires - ps30
Euarchonta - ps31
Primates - ps32
Haplorrhini - ps33
Simiiformes - ps34
Catarrhini - ps35
Hominoidea - ps36
Hominidae - ps37
Homininae - ps38
Hominini - ps39
Homo_sapiens - ps40
p-value
4e-03
5e-03
7e-03
1e-02
1e-02
Log-odds
0.5
1.0
1.5
2.0
2.5
3.0
3.5
4.0

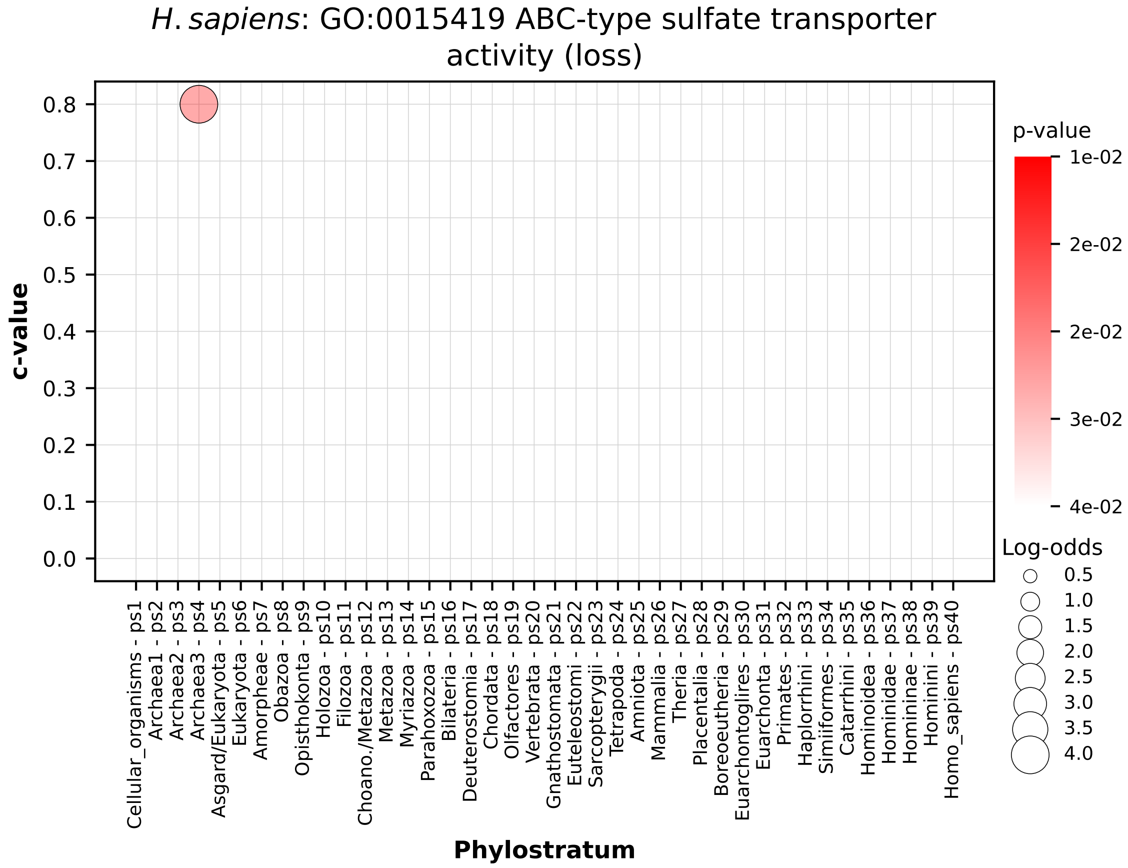

H. sapiens: GO:0015419 ABC-type sulfate transporter activity (loss)
c-value
0.8
0.7
0.6
0.5
0.4
0.3
0.2
0.1
0.0
p-value
1e-02
2e-02
2e-02
3e-02
4e-02
Log-odds
0.5
1.0
1.5
2.0
2.5
3.0
3.5
4.0
Cellular_organisms - ps1
Archaea1 - ps2
Archaea2 - ps3
Archaea3 - ps4
Asgard/Eukaryota - ps5
Eukaryota - ps6
Amorpheae - ps7
Obazoa - ps8
Opisthokonta - ps9
Holozoa - ps10
Filozoa - ps11
Choano./Metazoa - ps12
Metazoa - ps13
Myriazoa - ps14
Parahoxozoa - ps15
Bilateria - ps16
Deuterostomia - ps17
Chordata - ps18
Olfactores - ps19
Vertebrata - ps20
Gnathostomata - ps21
Euteleostomi - ps22
Sarcopterygii - ps23
Tetrapoda - ps24
Amniota - ps25
Mammalia - ps26
Theria - ps27
Placentalia - ps28
Boreoeutheria - ps29
Euarchontoglires - ps30
Euarchonta - ps31
Primates - ps32
Haplorrhini - ps33
Simiiformes - ps34
Catarrhini - ps35
Hominoidea - ps36
Hominidae - ps37
Homininae - ps38
Hominini - ps39
Homo_sapiens - ps40
Phylostratum

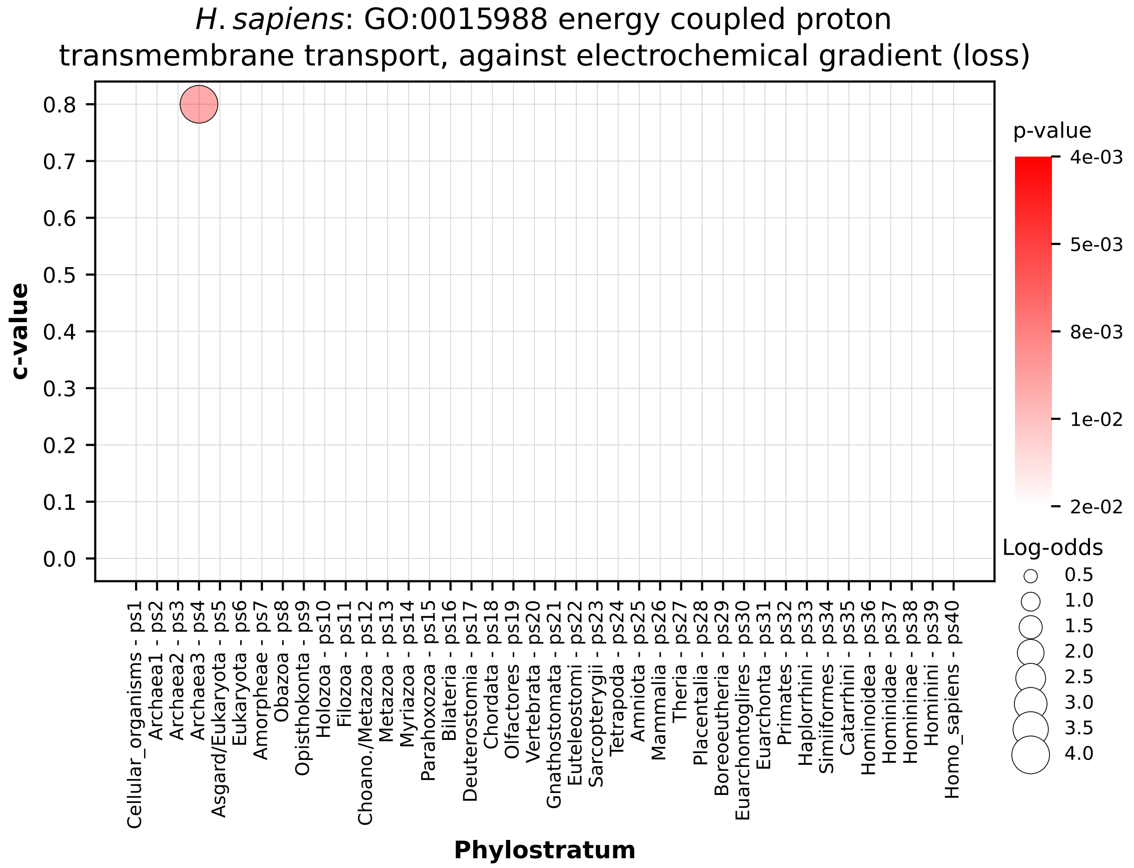

H. sapiens: GO:0015988 energy coupled proton transmembrane transport, against electrochemical gradient (loss)
c-value
0.0
0.1
0.2
0.3
0.4
0.5
0.6
0.7
0.8
p-value
4e-03
5e-03
8e-03
1e-02
2e-02
Log-odds
0.5
1.0
1.5
2.0
2.5
3.0
3.5
4.0
Cellular_organisms - ps1
Archaea1 - ps2
Archaea2 - ps3
Archaea3 - ps4
Asgard/Eukaryota - ps5
Eukaryota - ps6
Amorpheae - ps7
Obazoa - ps8
Opisthokonta - ps9
Holozoa - ps10
Filozoa - ps11
Choano./Metazoa - ps12
Metazoa - ps13
Myriazoa - ps14
Parahoxozoa - ps15
Bilateria - ps16
Deuterostomia - ps17
Chordata - ps18
Olfactores - ps19
Vertebrata - ps20
Gnathostomata - ps21
Euteleostomi - ps22
Sarcopterygii - ps23
Tetrapoda - ps24
Amniota - ps25
Mammalia - ps26
Theria - ps27
Placentalia - ps28
Boreoeutheria - ps29
Euarchontoglires - ps30
Euarchonta - ps31
Primates - ps32
Haplorrhini - ps33
Simiiformes - ps34
Catarrhini - ps35
Hominoidea - ps36
Hominidae - ps37
Homininae - ps38
Hominini - ps39
Homo_sapiens - ps40
Phylostratum

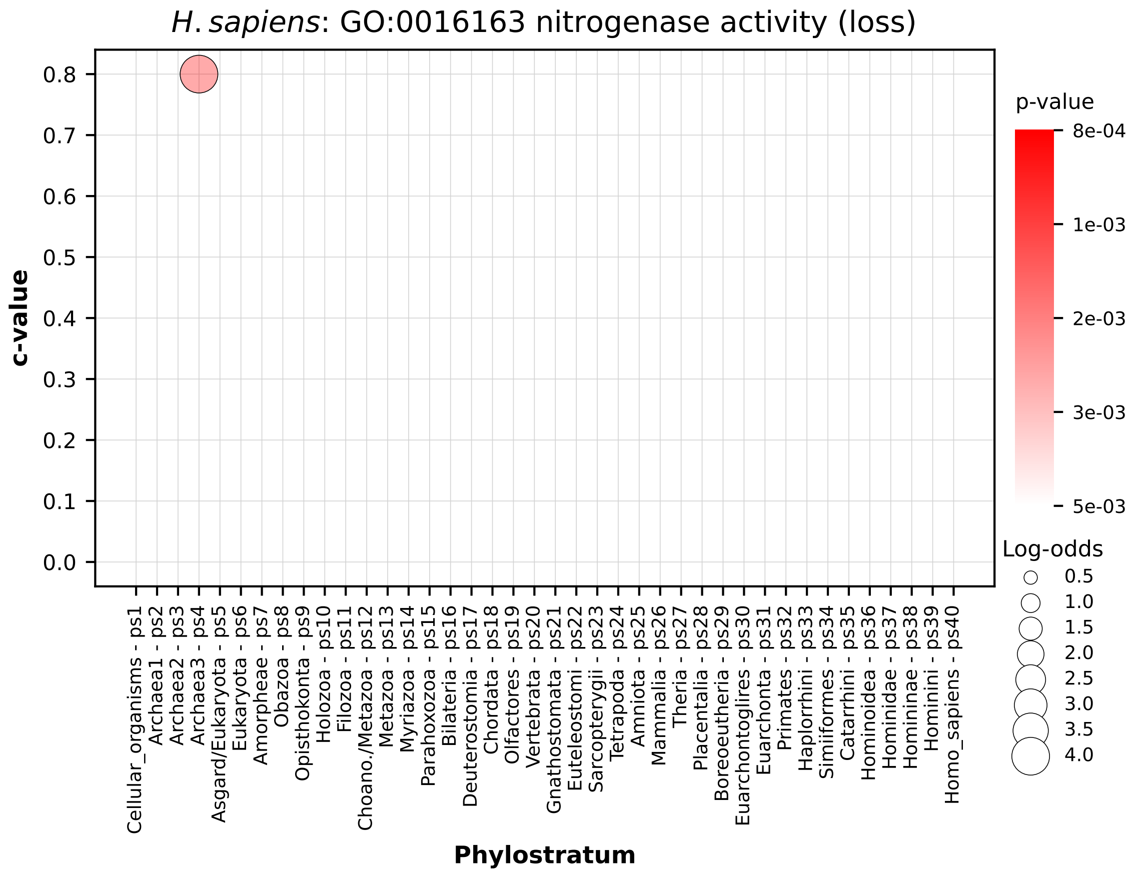

H. sapiens: GO:0016163 nitrogenase activity (loss)
c-value
0.8
0.7
0.6
0.5
0.4
0.3
0.2
0.1
0.0
Phylostratum
Cellular_organisms - ps1
Archaea1 - ps2
Archaea2 - ps3
Archaea3 - ps4
Asgard/Eukaryota - ps5
Eukaryota - ps6
Amorpheae - ps7
Obazoa - ps8
Opisthokonta - ps9
Holozoa - ps10
Filozoa - ps11
Choano./Metazoa - ps12
Metazoa - ps13
Myriazoa - ps14
Parahoxozoa - ps15
Bilateria - ps16
Deuterostomia - ps17
Chordata - ps18
Olfactores - ps19
Vertebrata - ps20
Gnathostomata - ps21
Euteleostomi - ps22
Sarcopterygii - ps23
Tetrapoda - ps24
Amniota - ps25
Mammalia - ps26
Theria - ps27
Placentalia - ps28
Boreoeutheria - ps29
Euarchontoglires - ps30
Euarchonta - ps31
Primates - ps32
Haplorrhini - ps33
Simiiformes - ps34
Catarrhini - ps35
Hominoidea - ps36
Hominidae - ps37
Homininae - ps38
Hominini - ps39
Homo_sapiens - ps40
p-value
8e-04
1e-03
2e-03
3e-03
5e-03
Log-odds
0.5
1.0
1.5
2.0
2.5
3.0
3.5
4.0

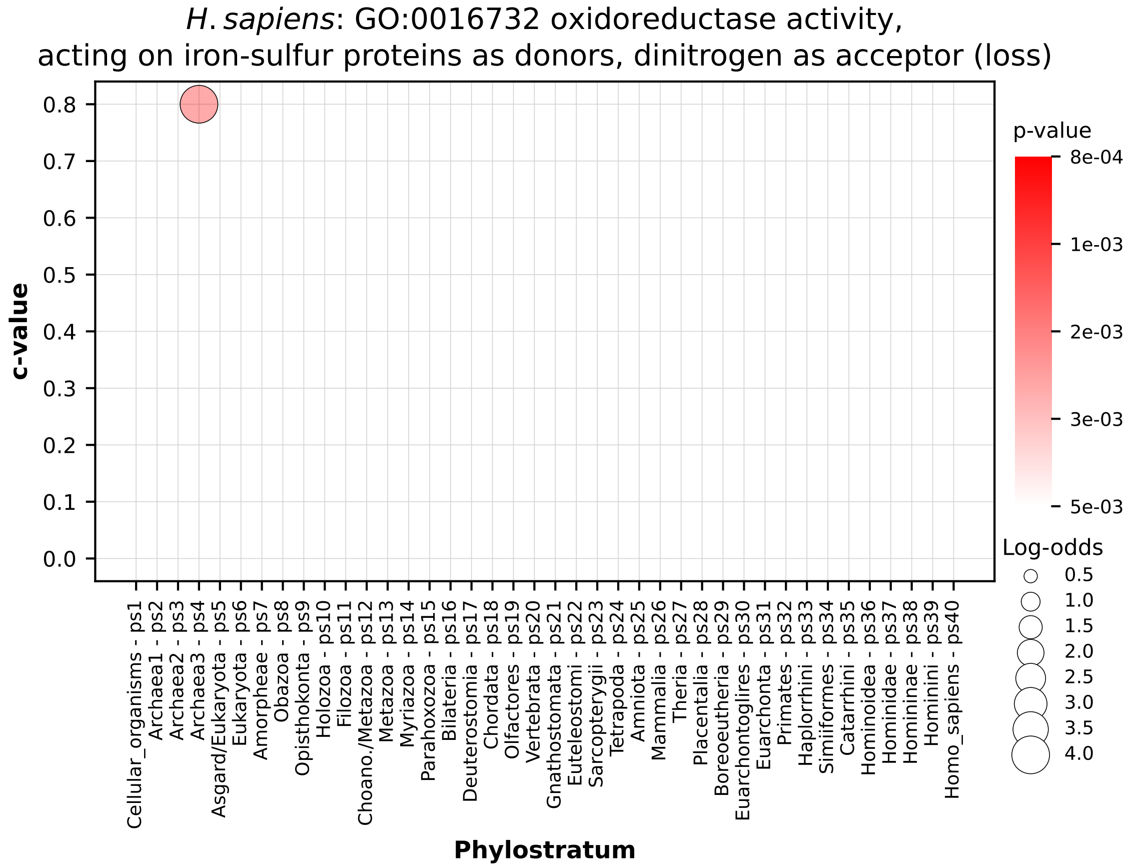

H. sapiens: GO:0016732 oxidoreductase activity,
acting on iron-sulfur proteins as donors, dinitrogen as acceptor (loss)
c-value
0.8
0.7
0.6
0.5
0.4
0.3
0.2
0.1
0.0
Phylostratum
Cellular_organisms - ps1
Archaea1 - ps2
Archaea2 - ps3
Archaea3 - ps4
Asgard/Eukaryota - ps5
Eukaryota - ps6
Amorpheae - ps7
Obazoa - ps8
Opisthokonta - ps9
Holozoa - ps10
Filozoa - ps11
Choano./Metazoa - ps12
Metazoa - ps13
Myriazoa - ps14
Parahoxozoa - ps15
Bilateria - ps16
Deuterostomia - ps17
Chordata - ps18
Olfactores - ps19
Vertebrata - ps20
Gnathostomata - ps21
Euteleostomi - ps22
Sarcopterygii - ps23
Tetrapoda - ps24
Amniota - ps25
Mammalia - ps26
Theria - ps27
Placentalia - ps28
Boreoeutheria - ps29
Euarchontoglires - ps30
Euarchonta - ps31
Primates - ps32
Haplorrhini - ps33
Simiiformes - ps34
Catarrhini - ps35
Hominoidea - ps36
Hominidae - ps37
Homininae - ps38
Hominini - ps39
Homo_sapiens - ps40
p-value
8e-04
1e-03
2e-03
3e-03
5e-03
Log-odds
0.5
1.0
1.5
2.0
2.5
3.0
3.5
4.0

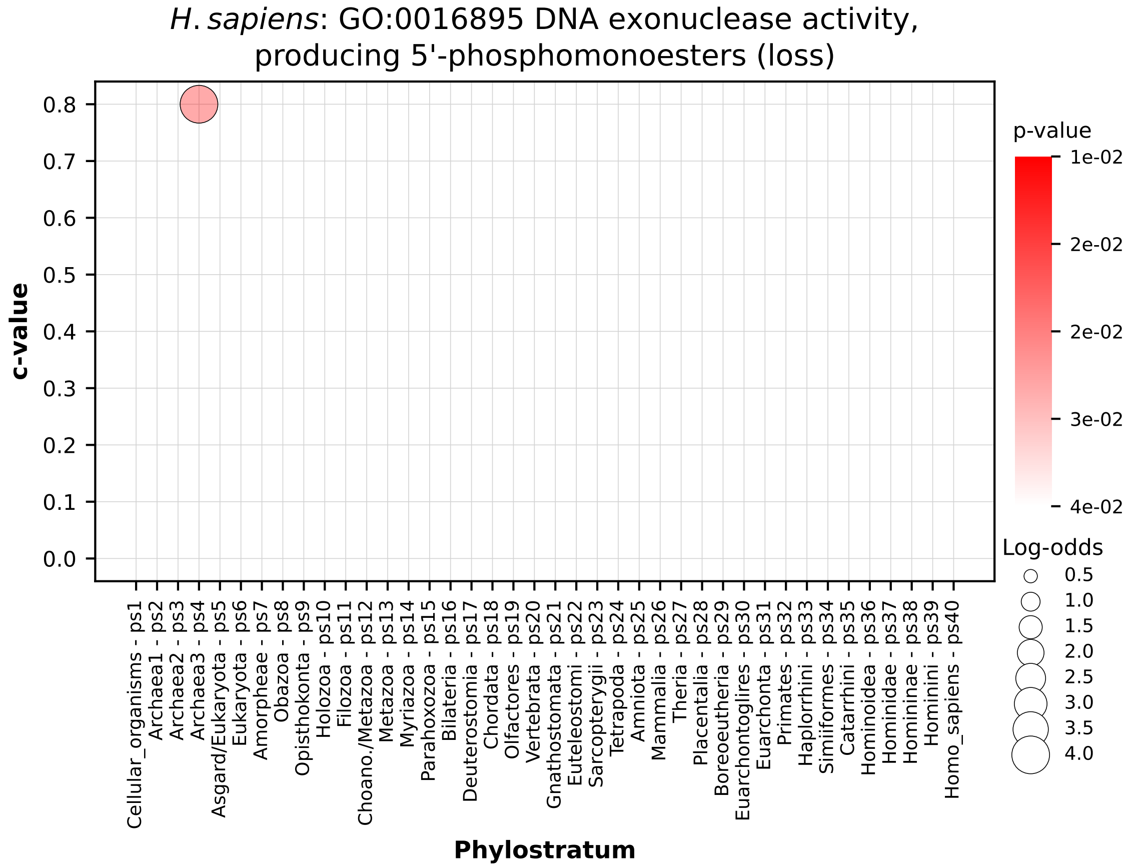

H. sapiens: GO:0016895 DNA exonuclease activity, producing 5'-phosphomonoesters (loss)
c-value
0.8
0.7
0.6
0.5
0.4
0.3
0.2
0.1
0.0
Phylostratum
Cellular_organisms - ps1
Archaea1 - ps2
Archaea2 - ps3
Archaea3 - ps4
Asgard/Eukaryota - ps5
Eukaryota - ps6
Amorpheae - ps7
Obazoa - ps8
Opisthokonta - ps9
Holozoa - ps10
Filozoa - ps11
Choano./Metazoa - ps12
Metazoa - ps13
Myriazoa - ps14
Parahoxozoa - ps15
Bilateria - ps16
Deuterostomia - ps17
Chordata - ps18
Olfactores - ps19
Vertebrata - ps20
Gnathostomata - ps21
Euteleostomi - ps22
Sarcopterygii - ps23
Tetrapoda - ps24
Amniota - ps25
Mammalia - ps26
Theria - ps27
Placentalia - ps28
Boreoeutheria - ps29
Euarchontoglires - ps30
Euarchonta - ps31
Primates - ps32
Haplorrhini - ps33
Simiiformes - ps34
Catarrhini - ps35
Hominoidea - ps36
Hominidae - ps37
Homininae - ps38
Hominini - ps39
Homo_sapiens - ps40
p-value
1e-02
2e-02
2e-02
3e-02
4e-02
Log-odds
0.5
1.0
1.5
2.0
2.5
3.0
3.5
4.0

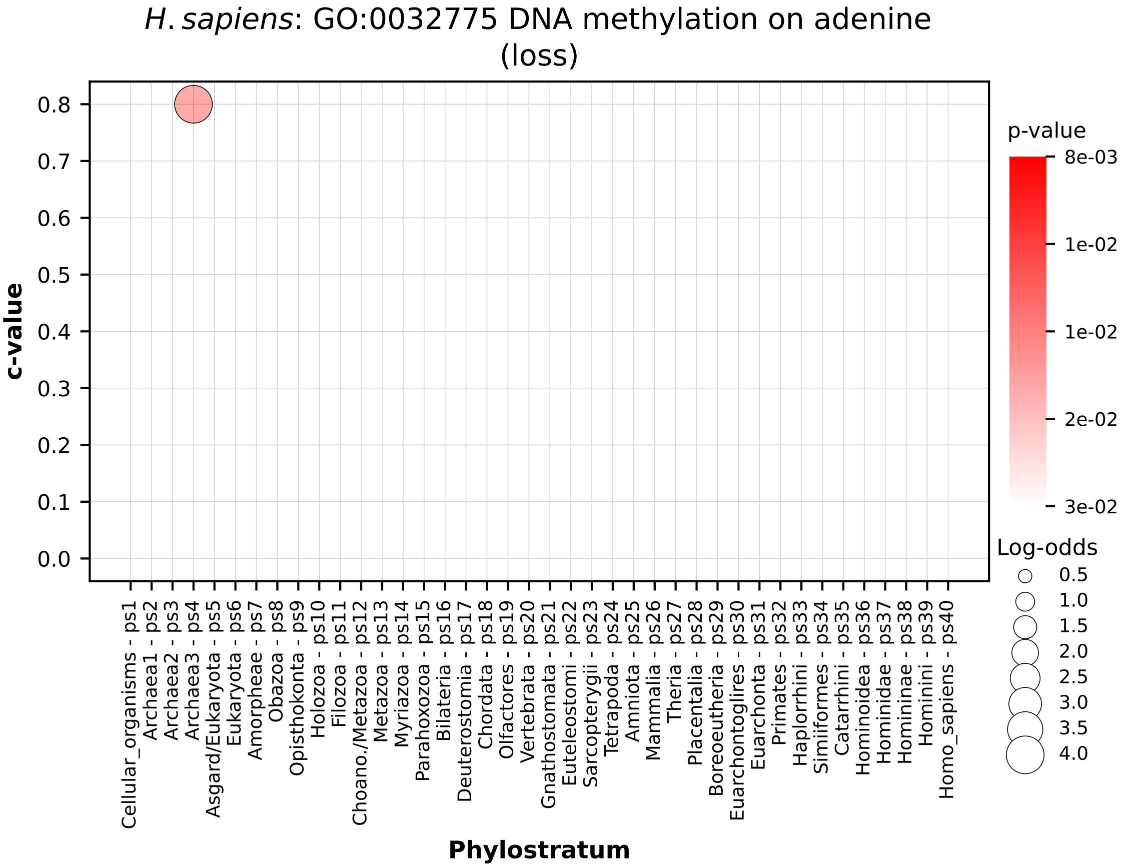

H. sapiens: GO:0032775 DNA methylation on adenine
(loss)
c-value
0.8
0.7
0.6
0.5
0.4
0.3
0.2
0.1
0.0
p-value
8e-03
1e-02
1e-02
2e-02
3e-02
Log-odds
0.5
1.0
1.5
2.0
2.5
3.0
3.5
4.0
Cellular_organisms - ps1
Archaea1 - ps2
Archaea2 - ps3
Archaea3 - ps4
Asgard/Eukaryota - ps5
Eukaryota - ps6
Amorpheae - ps7
Obazoa - ps8
Opisthokonta - ps9
Holozoa - ps10
Filozoa - ps11
Choano./Metazoa - ps12
Metazoa - ps13
Myriazoa - ps14
Parahoxozoa - ps15
Bilateria - ps16
Deuterostomia - ps17
Chordata - ps18
Olfactores - ps19
Vertebrata - ps20
Gnathostomata - ps21
Euteleostomi - ps22
Sarcopterygii - ps23
Tetrapoda - ps24
Amniota - ps25
Mammalia - ps26
Theria - ps27
Placentalia - ps28
Boreoeutheria - ps29
Euarchontoglires - ps30
Euarchonta - ps31
Primates - ps32
Haplorrhini - ps33
Simiiformes - ps34
Catarrhini - ps35
Hominoidea - ps36
Hominidae - ps37
Homininae - ps38
Hominini - ps39
Homo_sapiens - ps40
Phylostratum

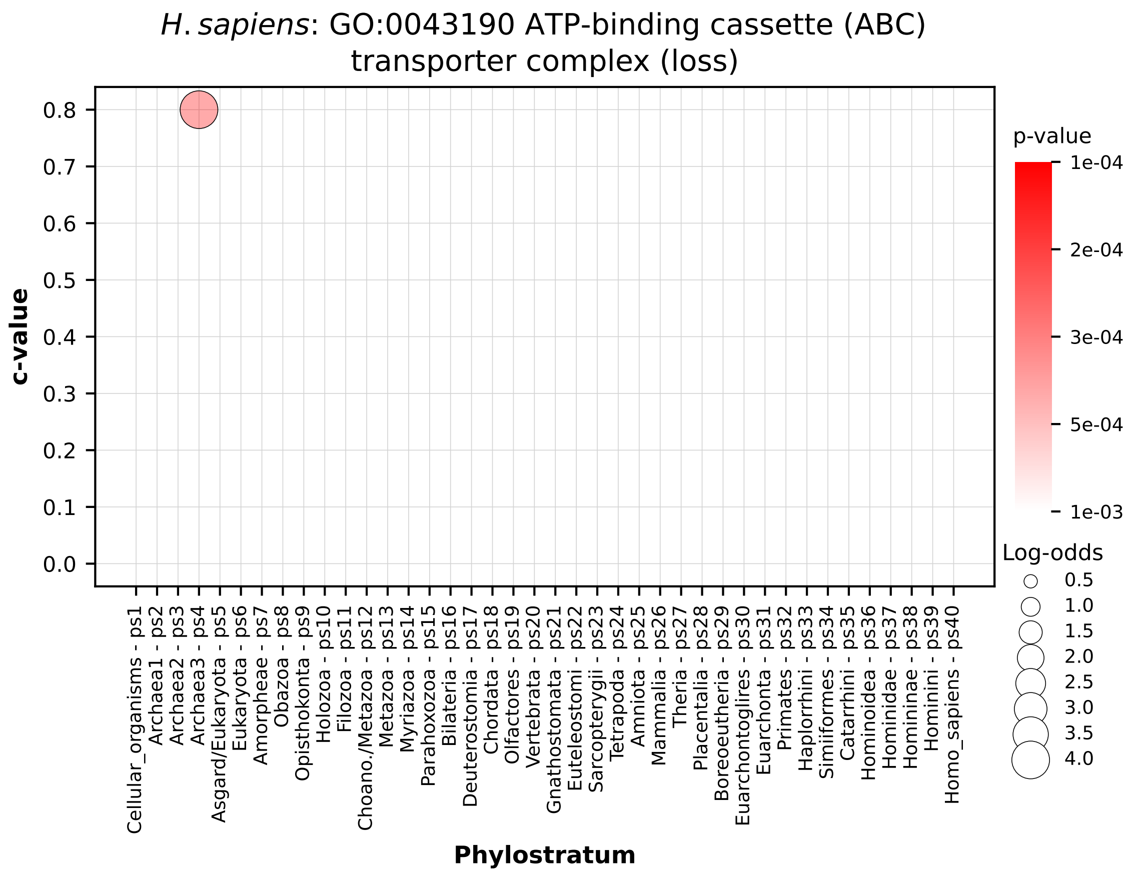
H. sapiens: GO:0043190 ATP-binding cassette (ABC) transporter complex (loss)
c-value
0.0
0.1
0.2
0.3
0.4
0.5
0.6
0.7
0.8
Phylostratum
Cellular_organisms - ps1
Archaea1 - ps2
Archaea2 - ps3
Archaea3 - ps4
Asgard/Eukaryota - ps5
Eukaryota - ps6
Amorpheae - ps7
Obazoa - ps8
Opisthokonta - ps9
Holozoa - ps10
Filozoa - ps11
Choano./Metazoa - ps12
Metazoa - ps13
Myriazoa - ps14
Parahoxozoa - ps15
Bilateria - ps16
Deuterostomia - ps17
Chordata - ps18
Olfactores - ps19
Vertebrata - ps20
Gnathostomata - ps21
Euteleostomi - ps22
Sarcopterygii - ps23
Tetrapoda - ps24
Amniota - ps25
Mammalia - ps26
Theria - ps27
Placentalia - ps28
Boreoeutheria - ps29
Euarchontoglires - ps30
Euarchonta - ps31
Primates - ps32
Haplorrhini - ps33
Simiiformes - ps34
Catarrhini - ps35
Hominoidea - ps36
Hominidae - ps37
Homininae - ps38
Hominini - ps39
Homo_sapiens - ps40
p-value
1e-04
2e-04
3e-04
5e-04
1e-03
Log-odds
0.5
1.0
1.5
2.0
2.5
3.0
3.5
4.0

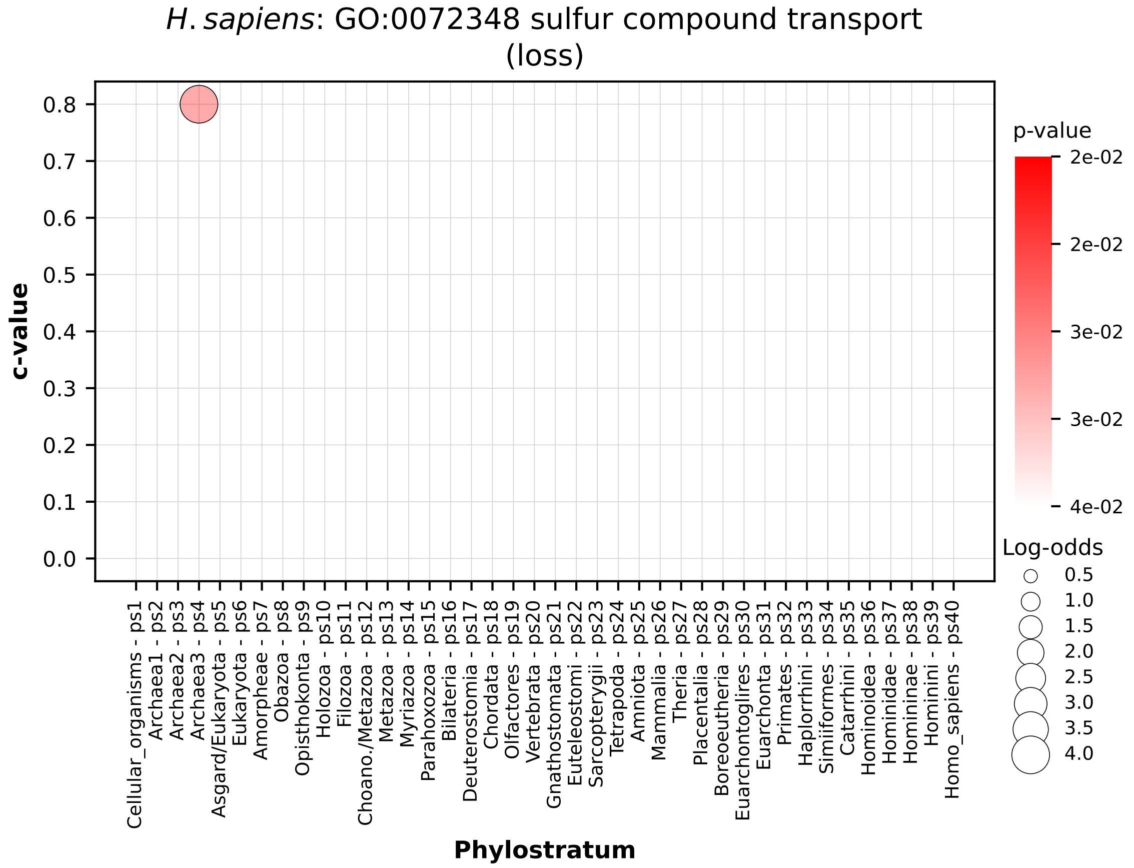

H. sapiens: GO:0072348 sulfur compound transport
(loss)
c-value
0.0
0.1
0.2
0.3
0.4
0.5
0.6
0.7
0.8
p-value
2e-02
2e-02
3e-02
3e-02
4e-02
Log-odds
0.5
1.0
1.5
2.0
2.5
3.0
3.5
4.0
Cellular_organisms - ps1
Archaea1 - ps2
Archaea2 - ps3
Archaea3 - ps4
Asgard/Eukaryota - ps5
Eukaryota - ps6
Amorpheae - ps7
Obazoa - ps8
Opisthokonta - ps9
Holozoa - ps10
Filozoa - ps11
Choano./Metazoa - ps12
Metazoa - ps13
Myriazoa - ps14
Parahoxozoa - ps15
Bilateria - ps16
Deuterostomia - ps17
Chordata - ps18
Olfactores - ps19
Vertebrata - ps20
Gnathostomata - ps21
Euteleostomi - ps22
Sarcopterygii - ps23
Tetrapoda - ps24
Amniota - ps25
Mammalia - ps26
Theria - ps27
Placentalia - ps28
Boreoeutheria - ps29
Euarchontoglires - ps30
Euarchonta - ps31
Primates - ps32
Haplorrhini - ps33
Simiiformes - ps34
Catarrhini - ps35
Hominoidea - ps36
Hominidae - ps37
Homininae - ps38
Hominini - ps39
Homo_sapiens - ps40
Phylostratum

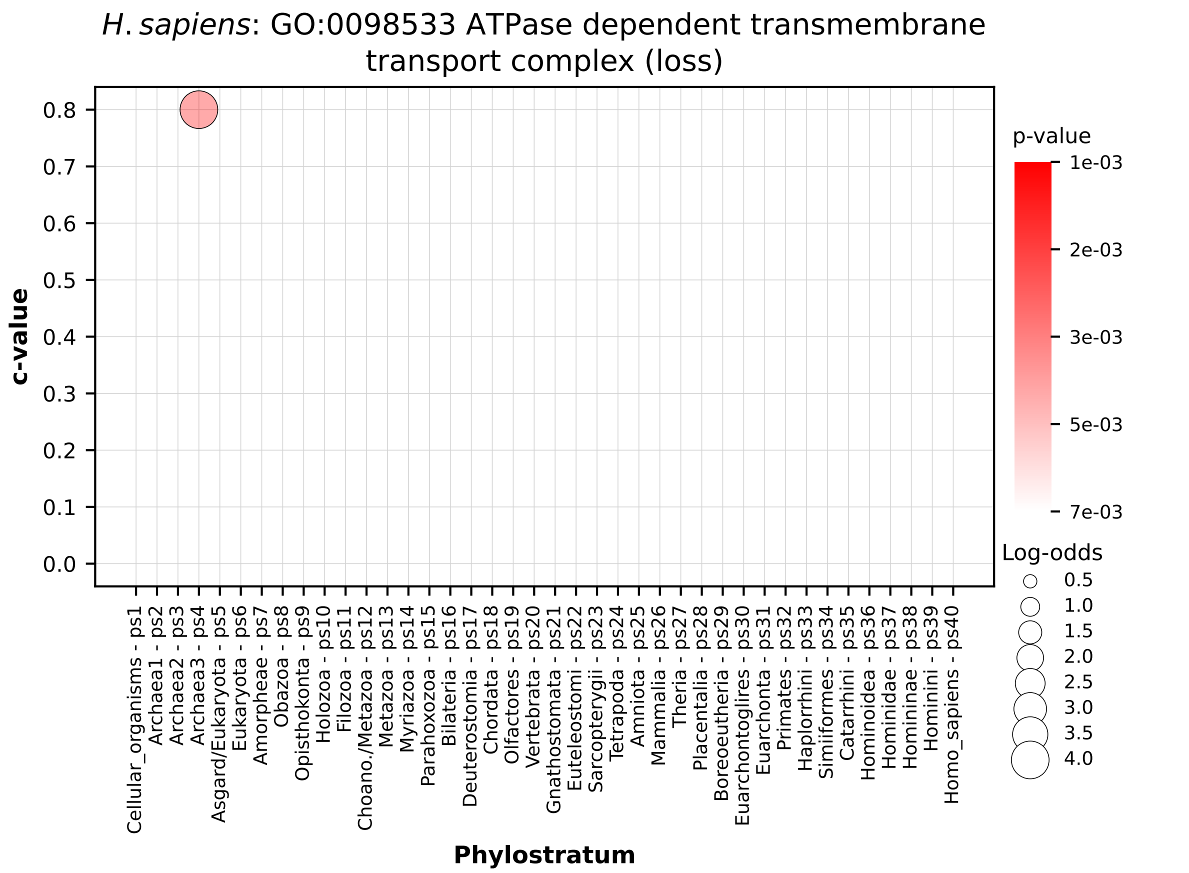

H. sapiens: GO:0098533 ATPase dependent transmembrane transport complex (loss)
c-value
0.0
0.1
0.2
0.3
0.4
0.5
0.6
0.7
0.8
Phylostratum
Cellular_organisms - ps1
Archaea1 - ps2
Archaea2 - ps3
Archaea3 - ps4
Asgard/Eukaryota - ps5
Eukaryota - ps6
Amorpheae - ps7
Obazoa - ps8
Opisthokonta - ps9
Holozoa - ps10
Filozoa - ps11
Choano./Metazoa - ps12
Metazoa - ps13
Myriazoa - ps14
Parahoxozoa - ps15
Bilateria - ps16
Deuterostomia - ps17
Chordata - ps18
Olfactores - ps19
Vertebrata - ps20
Gnathostomata - ps21
Euteleostomi - ps22
Sarcopterygii - ps23
Tetrapoda - ps24
Amniota - ps25
Mammalia - ps26
Theria - ps27
Placentalia - ps28
Boreoeutheria - ps29
Euarchontoglires - ps30
Euarchonta - ps31
Primates - ps32
Haplorrhini - ps33
Simiiformes - ps34
Catarrhini - ps35
Hominoidea - ps36
Hominidae - ps37
Homininae - ps38
Hominini - ps39
Homo_sapiens - ps40
p-value
1e-03
2e-03
3e-03
5e-03
7e-03
Log-odds
0.5
1.0
1.5
2.0
2.5
3.0
3.5
4.0

H. sapiens: GO:0098656 monoatomic anion transmembrane transport (loss)

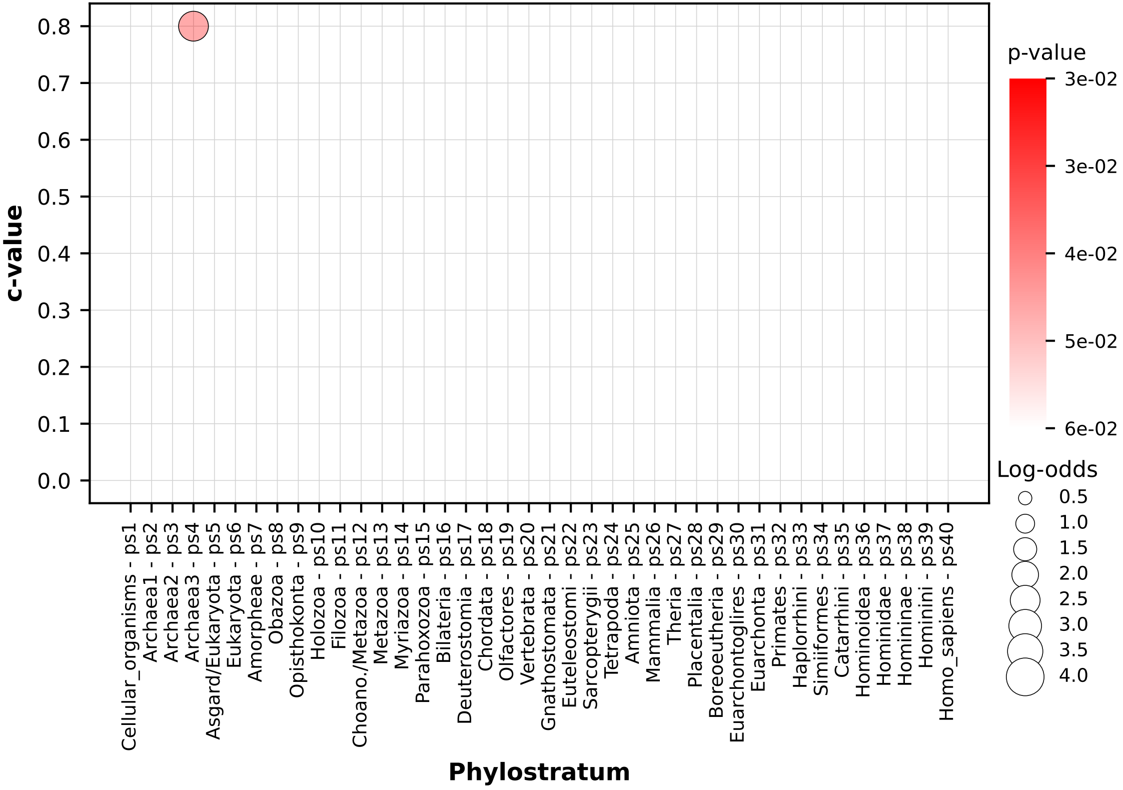

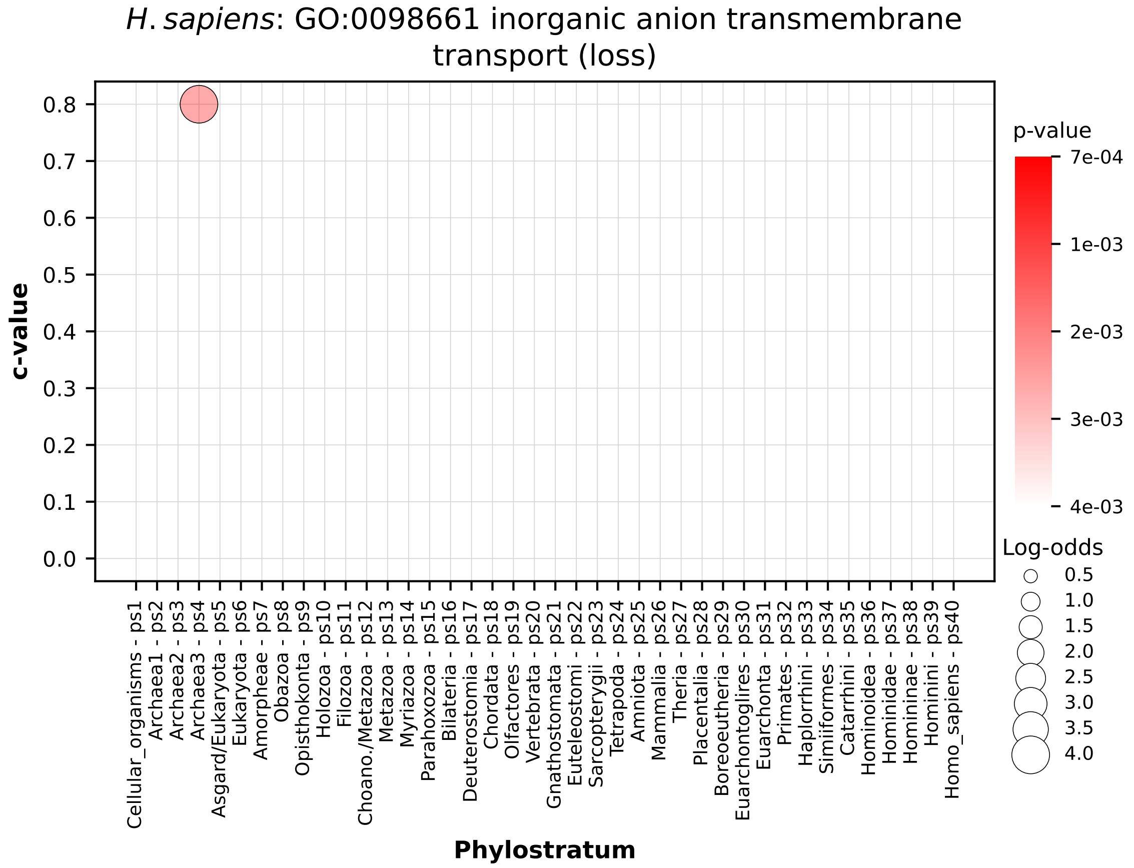

H. sapiens: GO:0098661 inorganic anion transmembrane transport (loss)
c-value
0.0
0.1
0.2
0.3
0.4
0.5
0.6
0.7
0.8
Phylostratum
Cellular_organisms - ps1
Archaea1 - ps2
Archaea2 - ps3
Archaea3 - ps4
Asgard/Eukaryota - ps5
Eukaryota - ps6
Amorpheae - ps7
Obazoa - ps8
Opisthokonta - ps9
Holozoa - ps10
Filozoa - ps11
Choano./Metazoa - ps12
Metazoa - ps13
Myriazoa - ps14
Parahoxozoa - ps15
Bilateria - ps16
Deuterostomia - ps17
Chordata - ps18
Olfactores - ps19
Vertebrata - ps20
Gnathostomata - ps21
Euteleostomi - ps22
Sarcopterygii - ps23
Tetrapoda - ps24
Amniota - ps25
Mammalia - ps26
Theria - ps27
Placentalia - ps28
Boreoeutheria - ps29
Euarchontoglires - ps30
Euarchonta - ps31
Primates - ps32
Haplorrhini - ps33
Simiiformes - ps34
Catarrhini - ps35
Hominoidea - ps36
Hominidae - ps37
Homininae - ps38
Hominini - ps39
Homo_sapiens - ps40
p-value
7e-04
1e-03
2e-03
3e-03
4e-03
Log-odds
0.5
1.0
1.5
2.0
2.5
3.0
3.5
4.0

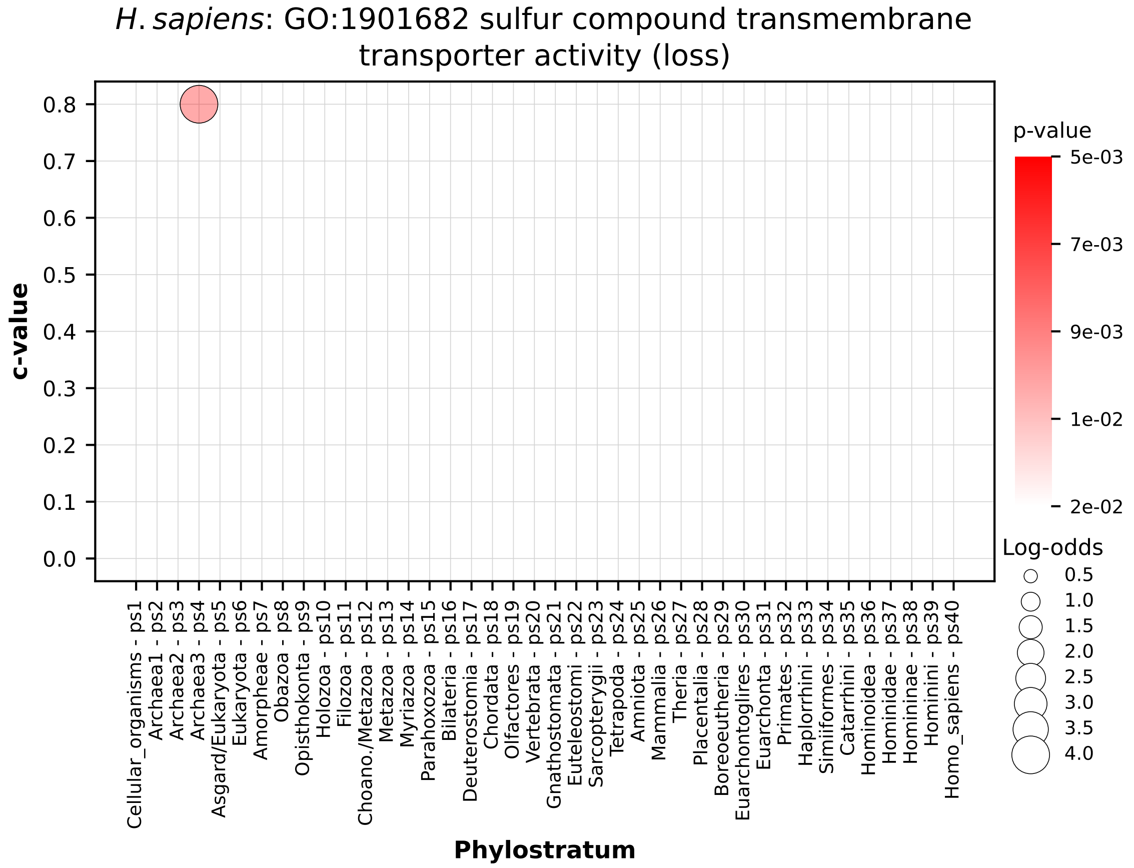
H. sapiens: GO:1901682 sulfur compound transmembrane transporter activity (loss)
c-value
0.0
0.1
0.2
0.3
0.4
0.5
0.6
0.7
0.8
Phylostratum
Cellular_organisms - ps1
Archaea1 - ps2
Archaea2 - ps3
Archaea3 - ps4
Asgard/Eukaryota - ps5
Eukaryota - ps6
Amorpheae - ps7
Obazoa - ps8
Opisthokonta - ps9
Holozoa - ps10
Filozoa - ps11
Choano./Metazoa - ps12
Metazoa - ps13
Myriazoa - ps14
Parahoxozoa - ps15
Bilateria - ps16
Deuterostomia - ps17
Chordata - ps18
Olfactores - ps19
Vertebrata - ps20
Gnathostomata - ps21
Euteleostomi - ps22
Sarcopterygii - ps23
Tetrapoda - ps24
Amniota - ps25
Mammalia - ps26
Theria - ps27
Placentalia - ps28
Boreoeutheria - ps29
Euarchontoglires - ps30
Euarchonta - ps31
Primates - ps32
Haplorrhini - ps33
Simiiformes - ps34
Catarrhini - ps35
Hominoidea - ps36
Hominidae - ps37
Homininae - ps38
Hominini - ps39
Homo_sapiens - ps40
p-value
5e-03
7e-03
9e-03
1e-02
2e-02
Log-odds
0.5
1.0
1.5
2.0
2.5
3.0
3.5
4.0

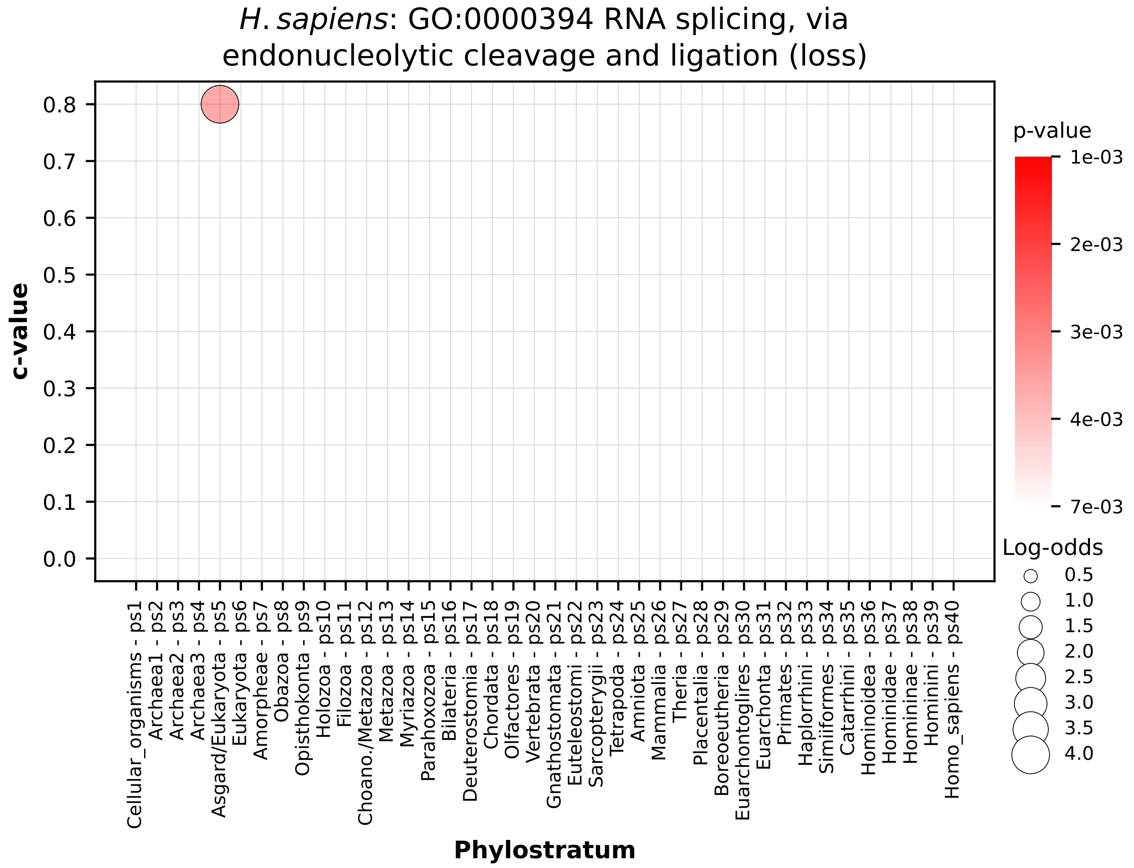

H. sapiens: GO:0000394 RNA splicing, via endonucleolytic cleavage and ligation (loss)
c-value
0.8
0.7
0.6
0.5
0.4
0.3
0.2
0.1
0.0
p-value
1e-03
2e-03
3e-03
4e-03
7e-03
Log-odds
0.5
1.0
1.5
2.0
2.5
3.0
3.5
4.0
Cellular_organisms - ps1
Archaea1 - ps2
Archaea2 - ps3
Archaea3 - ps4
Asgard/Eukaryota - ps5
Eukaryota - ps6
Amorpheae - ps7
Obazoa - ps8
Opisthokonta - ps9
Holozoa - ps10
Filozoa - ps11
Choano./Metazoa - ps12
Metazoa - ps13
Myriazoa - ps14
Parahoxozoa - ps15
Bilateria - ps16
Deuterostomia - ps17
Chordata - ps18
Olfactores - ps19
Vertebrata - ps20
Gnathostomata - ps21
Euteleostomi - ps22
Sarcopterygii - ps23
Tetrapoda - ps24
Amniota - ps25
Mammalia - ps26
Theria - ps27
Placentalia - ps28
Boreoeutheria - ps29
Euarchontoglires - ps30
Euarchonta - ps31
Primates - ps32
Haplorrhini - ps33
Simiiformes - ps34
Catarrhini - ps35
Hominoidea - ps36
Hominidae - ps37
Homininae - ps38
Hominini - ps39
Homo_sapiens - ps40
Phylostratum

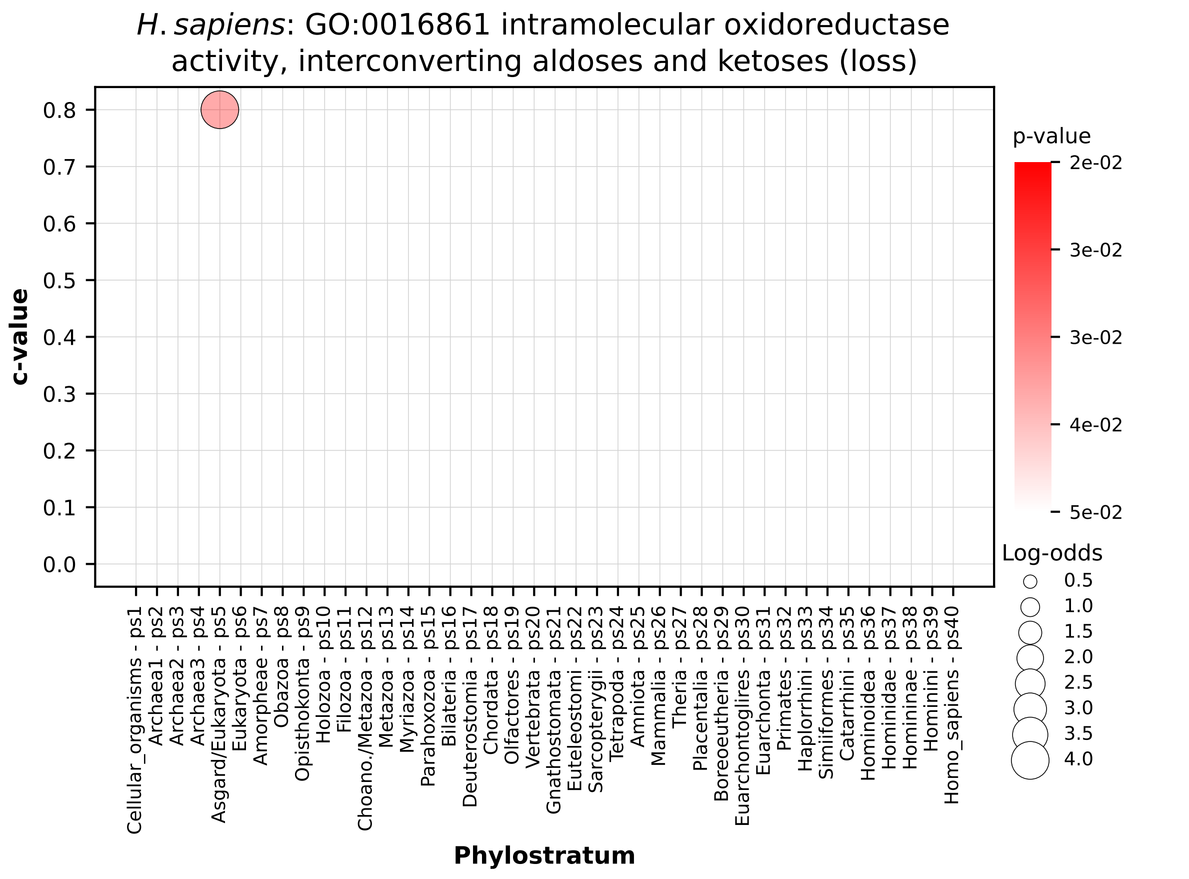

H. sapiens: GO:0016861 intramolecular oxidoreductase activity, interconverting aldoses and ketoses (loss)
c-value
0.0
0.1
0.2
0.3
0.4
0.5
0.6
0.7
0.8
p-value
2e-02
3e-02
3e-02
4e-02
5e-02
Log-odds
0.5
1.0
1.5
2.0
2.5
3.0
3.5
4.0
Cellular_organisms - ps1
Archaea1 - ps2
Archaea2 - ps3
Archaea3 - ps4
Asgard/Eukaryota - ps5
Eukaryota - ps6
Amorpheae - ps7
Obazoa - ps8
Opisthokonta - ps9
Holozoa - ps10
Filozoa - ps11
Choano./Metazoa - ps12
Metazoa - ps13
Myriazoa - ps14
Parahoxozoa - ps15
Bilateria - ps16
Deuterostomia - ps17
Chordata - ps18
Olfactores - ps19
Vertebrata - ps20
Gnathostomata - ps21
Euteleostomi - ps22
Sarcopterygii - ps23
Tetrapoda - ps24
Amniota - ps25
Mammalia - ps26
Theria - ps27
Placentalia - ps28
Boreoeutheria - ps29
Euarchontoglires - ps30
Euarchonta - ps31
Primates - ps32
Haplorrhini - ps33
Simiiformes - ps34
Catarrhini - ps35
Hominoidea - ps36
Hominidae - ps37
Homininae - ps38
Hominini - ps39
Homo_sapiens - ps40
Phylostratum

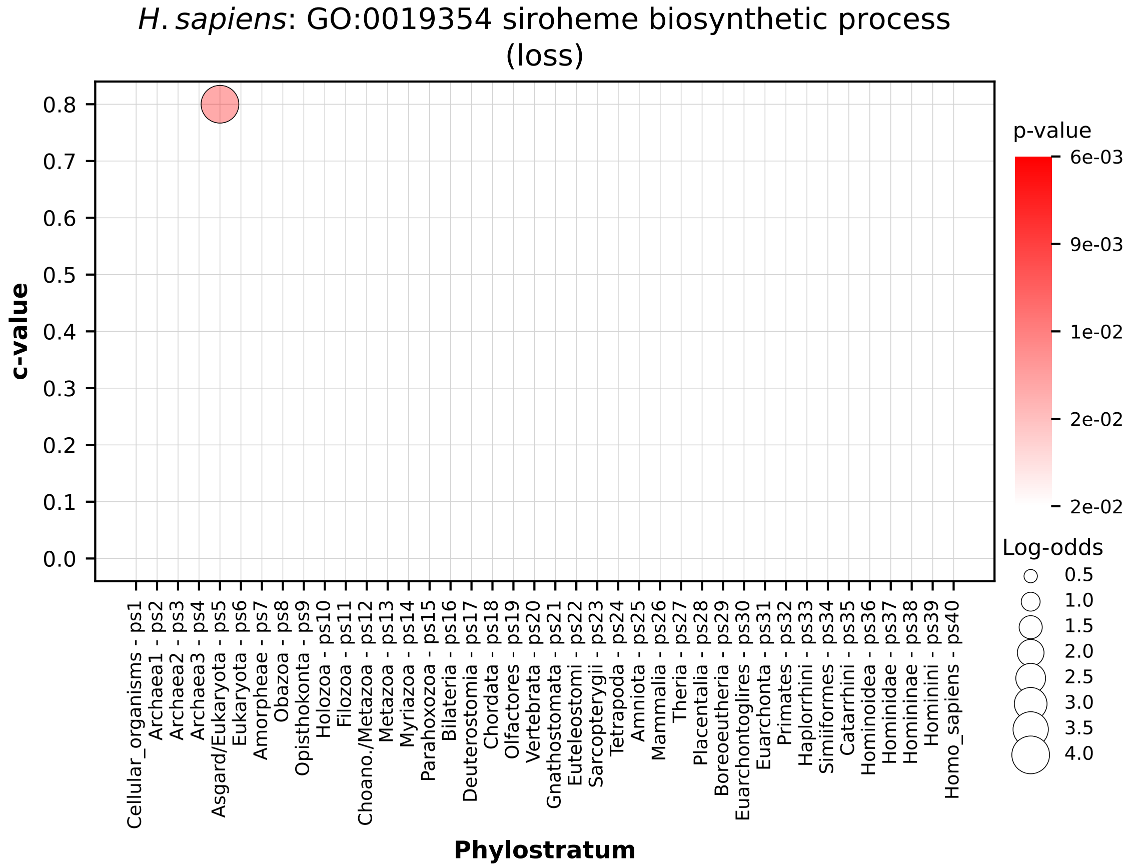
H. sapiens: GO:0019354 siroheme biosynthetic process
(loss)
c-value
0.0
0.1
0.2
0.3
0.4
0.5
0.6
0.7
0.8
Phylostratum
Cellular_organisms - ps1
Archaea1 - ps2
Archaea2 - ps3
Archaea3 - ps4
Asgard/Eukaryota - ps5
Eukaryota - ps6
Amorpheae - ps7
Obazoa - ps8
Opisthokonta - ps9
Holozoa - ps10
Filozoa - ps11
Choano./Metazoa - ps12
Metazoa - ps13
Myriazoa - ps14
Parahoxozoa - ps15
Bilateria - ps16
Deuterostomia - ps17
Chordata - ps18
Olfactores - ps19
Vertebrata - ps20
Gnathostomata - ps21
Euteleostomi - ps22
Sarcopterygii - ps23
Tetrapoda - ps24
Amniota - ps25
Mammalia - ps26
Theria - ps27
Placentalia - ps28
Boreoeutheria - ps29
Euarchontoglires - ps30
Euarchonta - ps31
Primates - ps32
Haplorrhini - ps33
Simiiformes - ps34
Catarrhini - ps35
Hominoidea - ps36
Hominidae - ps37
Homininae - ps38
Hominini - ps39
Homo_sapiens - ps40
p-value
6e-03
9e-03
1e-02
2e-02
2e-02
Log-odds
0.5
1.0
1.5
2.0
2.5
3.0
3.5
4.0

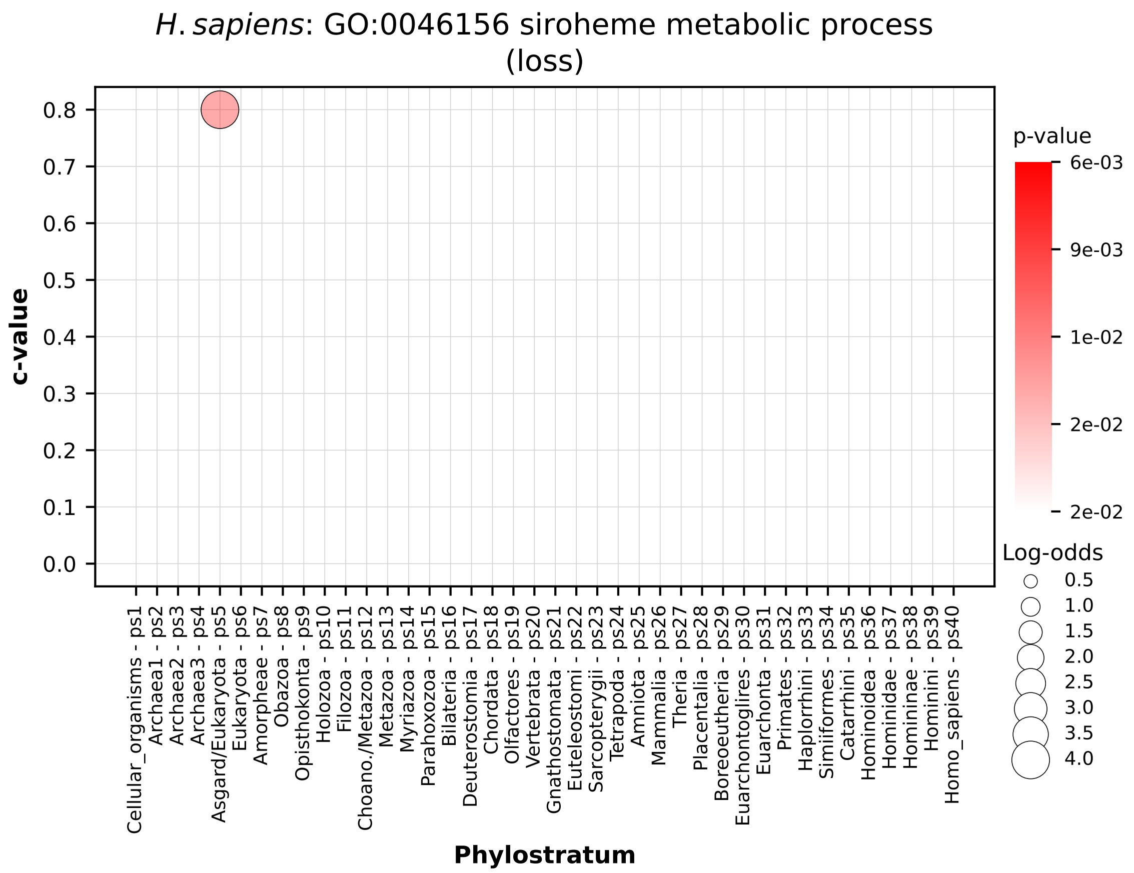

$H. sapiens$: GO:0046156 siroheme metabolic process
(loss)
c-value
0.0
0.1
0.2
0.3
0.4
0.5
0.6
0.7
0.8
p-value
6e-03
9e-03
1e-02
2e-02
2e-02
Log-odds
0.5
1.0
1.5
2.0
2.5
3.0
3.5
4.0
Cellular_organisms - ps1
Archaea1 - ps2
Archaea2 - ps3
Archaea3 - ps4
Asgard/Eukaryota - ps5
Eukaryota - ps6
Amorpheae - ps7
Obazoa - ps8
Opisthokonta - ps9
Holozoa - ps10
Filozoa - ps11
Choano./Metazoa - ps12
Metazoa - ps13
Myriazoa - ps14
Parahoxozoa - ps15
Bilateria - ps16
Deuterostomia - ps17
Chordata - ps18
Olfactores - ps19
Vertebrata - ps20
Gnathostomata - ps21
Euteleostomi - ps22
Sarcopterygii - ps23
Tetrapoda - ps24
Amniota - ps25
Mammalia - ps26
Theria - ps27
Placentalia - ps28
Boreoeutheria - ps29
Euarchontoglires - ps30
Euarchonta - ps31
Primates - ps32
Haplorrhini - ps33
Simiiformes - ps34
Catarrhini - ps35
Hominoidea - ps36
Hominidae - ps37
Homininae - ps38
Hominini - ps39
Homo_sapiens - ps40
Phylostratum

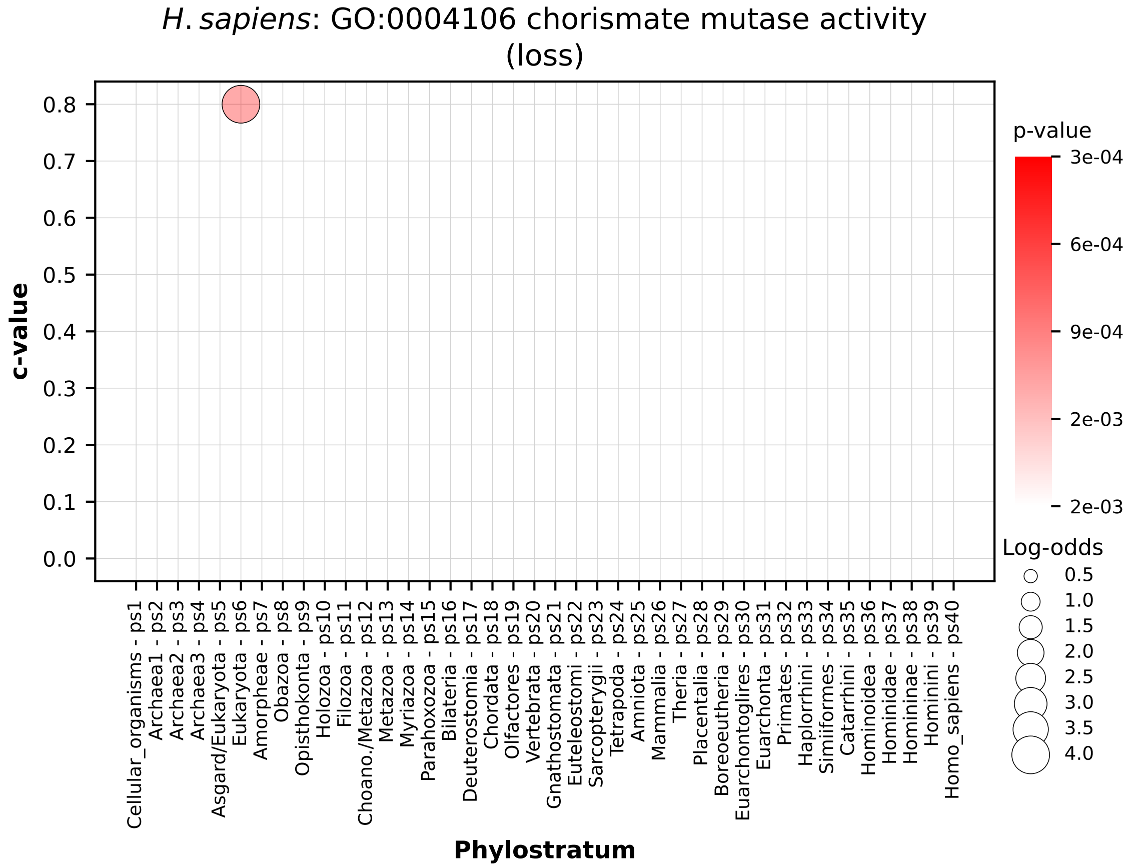

H. sapiens: GO:0004106 chorismate mutase activity
(loss)
c-value
0.0
0.1
0.2
0.3
0.4
0.5
0.6
0.7
0.8
p-value
3e-04
6e-04
9e-04
2e-03
2e-03
Log-odds
0.5
1.0
1.5
2.0
2.5
3.0
3.5
4.0
Cellular_organisms - ps1
Archaea1 - ps2
Archaea2 - ps3
Archaea3 - ps4
Asgard/Eukaryota - ps5
Eukaryota - ps6
Amorpheae - ps7
Obazoa - ps8
Opisthokonta - ps9
Holozoa - ps10
Filozoa - ps11
Choano./Metazoa - ps12
Metazoa - ps13
Myriazoa - ps14
Parahoxozoa - ps15
Bilateria - ps16
Deuterostomia - ps17
Chordata - ps18
Olfactores - ps19
Vertebrata - ps20
Gnathostomata - ps21
Euteleostomi - ps22
Sarcopterygii - ps23
Tetrapoda - ps24
Amniota - ps25
Mammalia - ps26
Theria - ps27
Placentalia - ps28
Boreoeutheria - ps29
Euarchontoglires - ps30
Euarchonta - ps31
Primates - ps32
Haplorrhini - ps33
Simiiformes - ps34
Catarrhini - ps35
Hominoidea - ps36
Hominidae - ps37
Homininae - ps38
Hominini - ps39
Homo_sapiens - ps40
Phylostratum

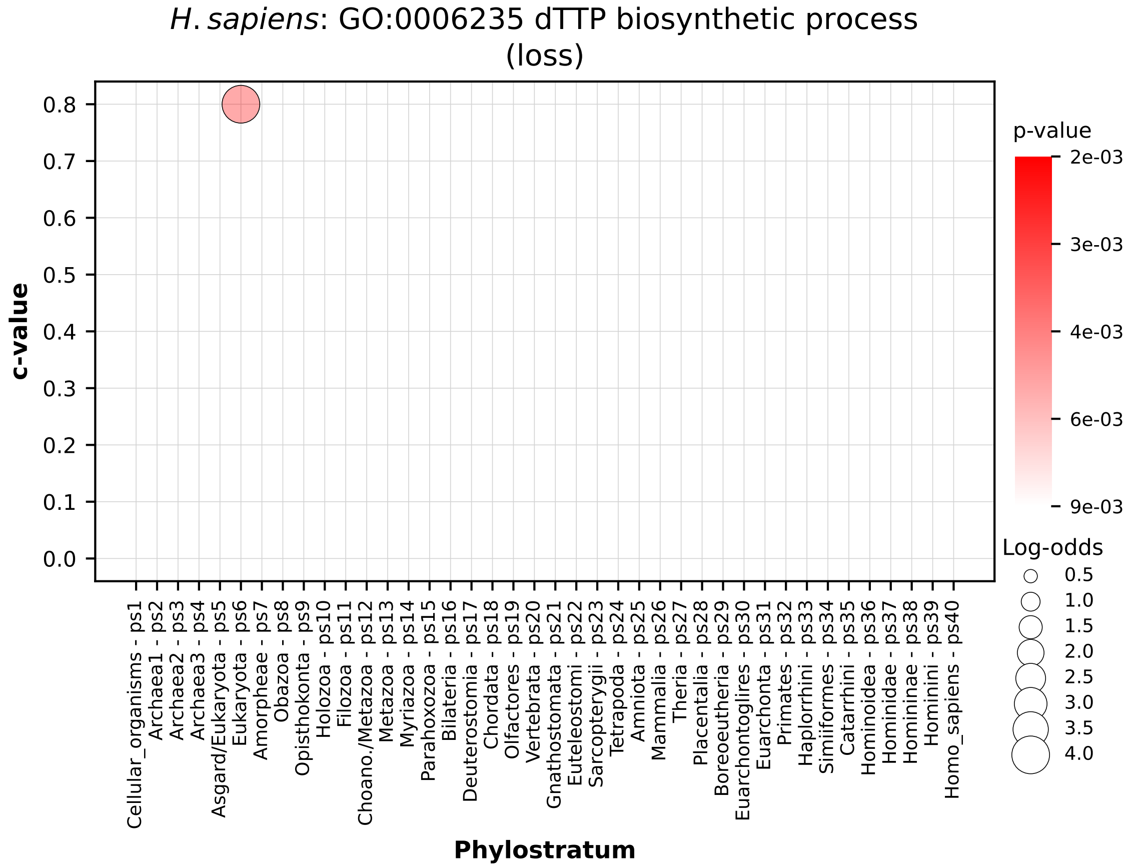

H. sapiens: GO:0006235 dTTP biosynthetic process
(loss)
c-value
0.0
0.1
0.2
0.3
0.4
0.5
0.6
0.7
0.8
Phylostratum
Cellular_organisms - ps1
Archaea1 - ps2
Archaea2 - ps3
Archaea3 - ps4
Asgard/Eukaryota - ps5
Eukaryota - ps6
Amorpheae - ps7
Obazoa - ps8
Opisthokonta - ps9
Holozoa - ps10
Filozoa - ps11
Choano./Metazoa - ps12
Metazoa - ps13
Myriazoa - ps14
Parahoxozoa - ps15
Bilateria - ps16
Deuterostomia - ps17
Chordata - ps18
Olfactores - ps19
Vertebrata - ps20
Gnathostomata - ps21
Euteleostomi - ps22
Sarcopterygii - ps23
Tetrapoda - ps24
Amniota - ps25
Mammalia - ps26
Theria - ps27
Placentalia - ps28
Boreoeutheria - ps29
Euarchontoglires - ps30
Euarchonta - ps31
Primates - ps32
Haplorrhini - ps33
Simiiformes - ps34
Catarrhini - ps35
Hominoidea - ps36
Hominidae - ps37
Homininae - ps38
Hominini - ps39
Homo_sapiens - ps40
p-value
2e-03
3e-03
4e-03
6e-03
9e-03
Log-odds
0.5
1.0
1.5
2.0
2.5
3.0
3.5
4.0

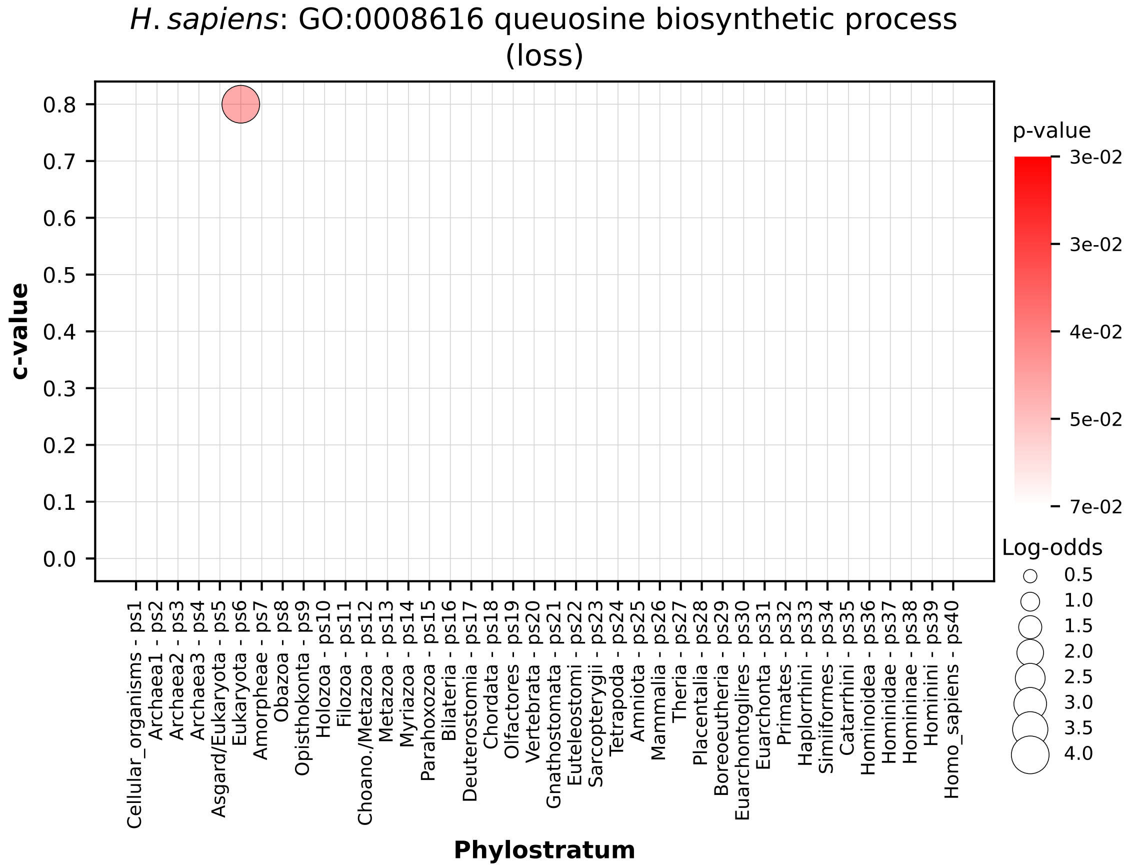
H. sapiens: GO:0008616 queuosine biosynthetic process (loss)
c-value
0.0
0.1
0.2
0.3
0.4
0.5
0.6
0.7
0.8
Phylostratum
Cellular_organisms - ps1
Archaea1 - ps2
Archaea2 - ps3
Archaea3 - ps4
Asgard/Eukaryota - ps5
Eukaryota - ps6
Amorpheae - ps7
Obazoa - ps8
Opisthokonta - ps9
Holozoa - ps10
Filozoa - ps11
Choano./Metazoa - ps12
Metazoa - ps13
Myriazoa - ps14
Parahoxozoa - ps15
Bilateria - ps16
Deuterostomia - ps17
Chordata - ps18
Olfactores - ps19
Vertebrata - ps20
Gnathostomata - ps21
Euteleostomi - ps22
Sarcopterygii - ps23
Tetrapoda - ps24
Amniota - ps25
Mammalia - ps26
Theria - ps27
Placentalia - ps28
Boreoeutheria - ps29
Euarchontoglires - ps30
Euarchonta - ps31
Primates - ps32
Haplorrhini - ps33
Simiiformes - ps34
Catarrhini - ps35
Hominoidea - ps36
Hominidae - ps37
Homininae - ps38
Hominini - ps39
Homo_sapiens - ps40
p-value
3e-02
3e-02
4e-02
5e-02
7e-02
Log-odds
0.5
1.0
1.5
2.0
2.5
3.0
3.5
4.0

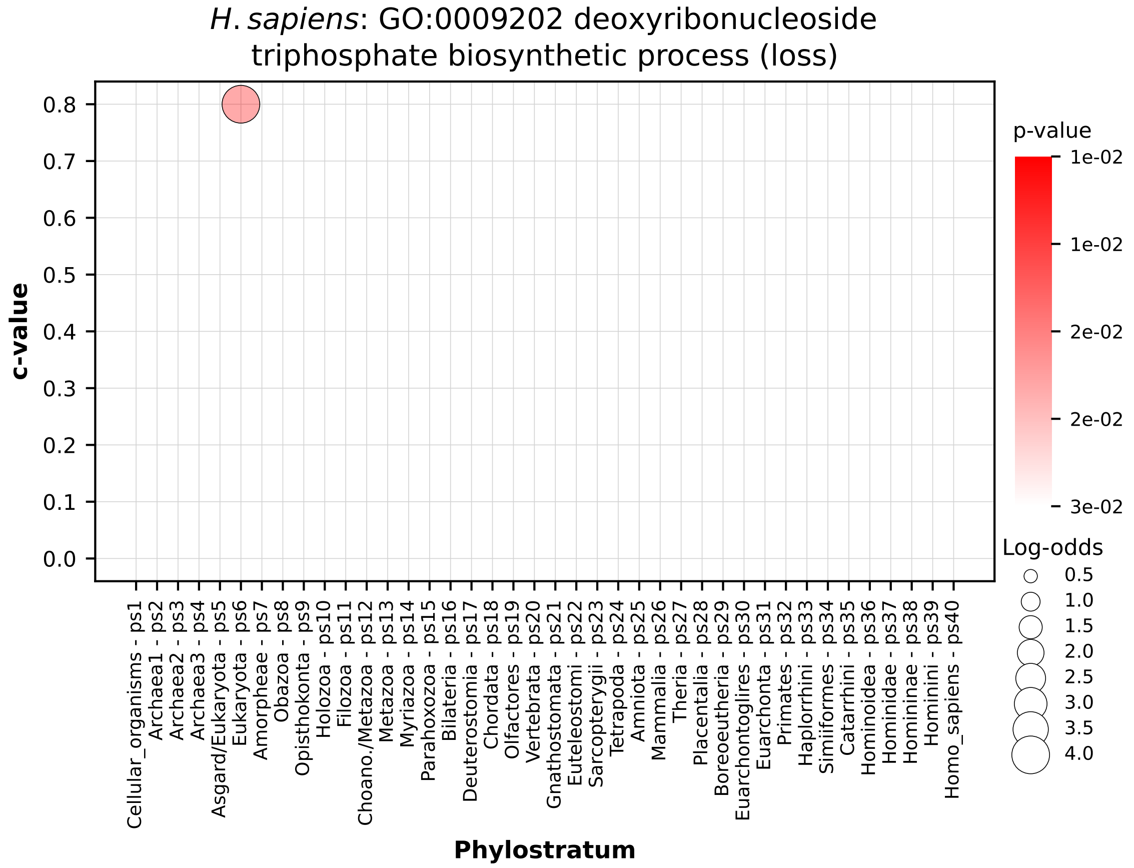

H. sapiens: GO:0009202 deoxyribonucleoside triphosphate biosynthetic process (loss)
c-value
0.8
0.7
0.6
0.5
0.4
0.3
0.2
0.1
0.0
p-value
1e-02
1e-02
2e-02
2e-02
3e-02
Log-odds
0.5
1.0
1.5
2.0
2.5
3.0
3.5
4.0
Cellular_organisms - ps1
Archaea1 - ps2
Archaea2 - ps3
Archaea3 - ps4
Asgard/Eukaryota - ps5
Eukaryota - ps6
Amorpheae - ps7
Obazoa - ps8
Opisthokonta - ps9
Holozoa - ps10
Filozoa - ps11
Choano./Metazoa - ps12
Metazoa - ps13
Myriazoa - ps14
Parahoxozoa - ps15
Bilateria - ps16
Deuterostomia - ps17
Chordata - ps18
Olfactores - ps19
Vertebrata - ps20
Gnathostomata - ps21
Euteleostomi - ps22
Sarcopterygii - ps23
Tetrapoda - ps24
Amniota - ps25
Mammalia - ps26
Theria - ps27
Placentalia - ps28
Boreoeutheria - ps29
Euarchontoglires - ps30
Euarchonta - ps31
Primates - ps32
Haplorrhini - ps33
Simiiformes - ps34
Catarrhini - ps35
Hominoidea - ps36
Hominidae - ps37
Homininae - ps38
Hominini - ps39
Homo_sapiens - ps40
Phylostratum

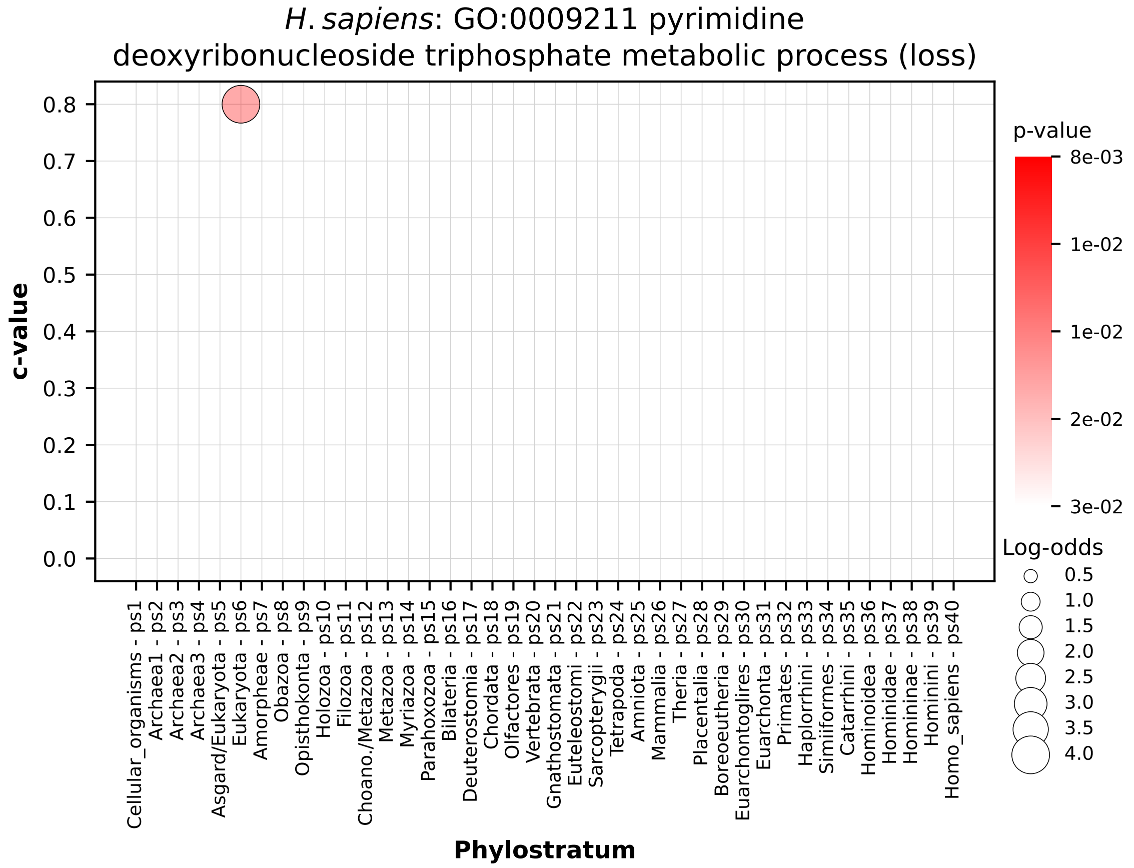
H. sapiens: GO:0009211 pyrimidine deoxyribonucleoside triphosphate metabolic process (loss)
c-value
0.0
0.1
0.2
0.3
0.4
0.5
0.6
0.7
0.8
Phylostratum
Cellular_organisms - ps1
Archaea1 - ps2
Archaea2 - ps3
Archaea3 - ps4
Asgard/Eukaryota - ps5
Eukaryota - ps6
Amorpheae - ps7
Obazoa - ps8
Opisthokonta - ps9
Holozoa - ps10
Filozoa - ps11
Choano./Metazoa - ps12
Metazoa - ps13
Myriazoa - ps14
Parahoxozoa - ps15
Bilateria - ps16
Deuterostomia - ps17
Chordata - ps18
Olfactores - ps19
Vertebrata - ps20
Gnathostomata - ps21
Euteleostomi - ps22
Sarcopterygii - ps23
Tetrapoda - ps24
Amniota - ps25
Mammalia - ps26
Theria - ps27
Placentalia - ps28
Boreoeutheria - ps29
Euarchontoglires - ps30
Euarchonta - ps31
Primates - ps32
Haplorrhini - ps33
Simiiformes - ps34
Catarrhini - ps35
Hominoidea - ps36
Hominidae - ps37
Homininae - ps38
Hominini - ps39
Homo_sapiens - ps40
p-value
8e-03
1e-02
1e-02
2e-02
3e-02
Log-odds
0.5
1.0
1.5
2.0
2.5
3.0
3.5
4.0

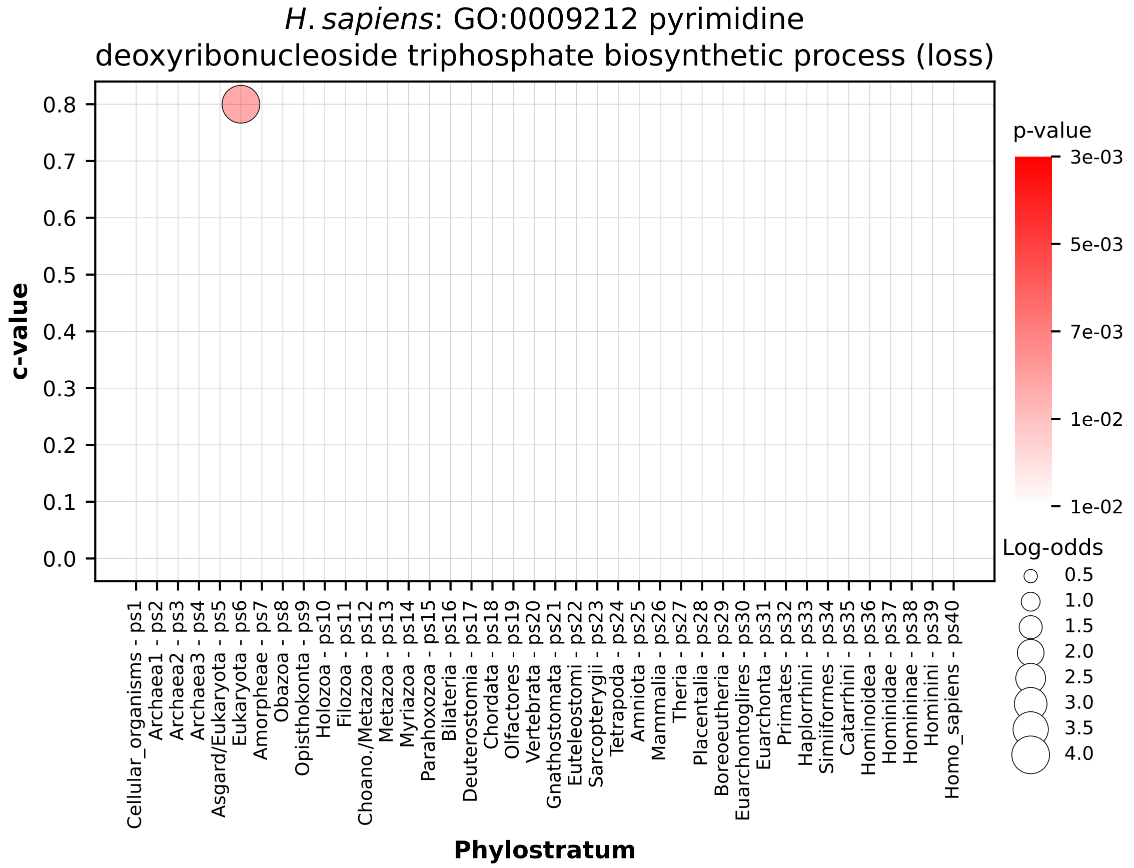

H. sapiens: GO:0009212 pyrimidine deoxyribonucleoside triphosphate biosynthetic process (loss)
c-value
0.0
0.1
0.2
0.3
0.4
0.5
0.6
0.7
0.8
Phylostratum
Cellular_organisms - ps1
Archaea1 - ps2
Archaea2 - ps3
Archaea3 - ps4
Asgard/Eukaryota - ps5
Eukaryota - ps6
Amorpheae - ps7
Obazoa - ps8
Opisthokonta - ps9
Holozoa - ps10
Filozoa - ps11
Choano./Metazoa - ps12
Metazoa - ps13
Myriazoa - ps14
Parahoxozoa - ps15
Bilateria - ps16
Deuterostomia - ps17
Chordata - ps18
Olfactores - ps19
Vertebrata - ps20
Gnathostomata - ps21
Euteleostomi - ps22
Sarcopterygii - ps23
Tetrapoda - ps24
Amniota - ps25
Mammalia - ps26
Theria - ps27
Placentalia - ps28
Boreoeutheria - ps29
Euarchontoglires - ps30
Euarchonta - ps31
Primates - ps32
Haplorrhini - ps33
Simiiformes - ps34
Catarrhini - ps35
Hominoidea - ps36
Hominidae - ps37
Homininae - ps38
Hominini - ps39
Homo_sapiens - ps40
p-value
3e-03
5e-03
7e-03
1e-02
1e-02
Log-odds
0.5
1.0
1.5
2.0
2.5
3.0
3.5
4.0

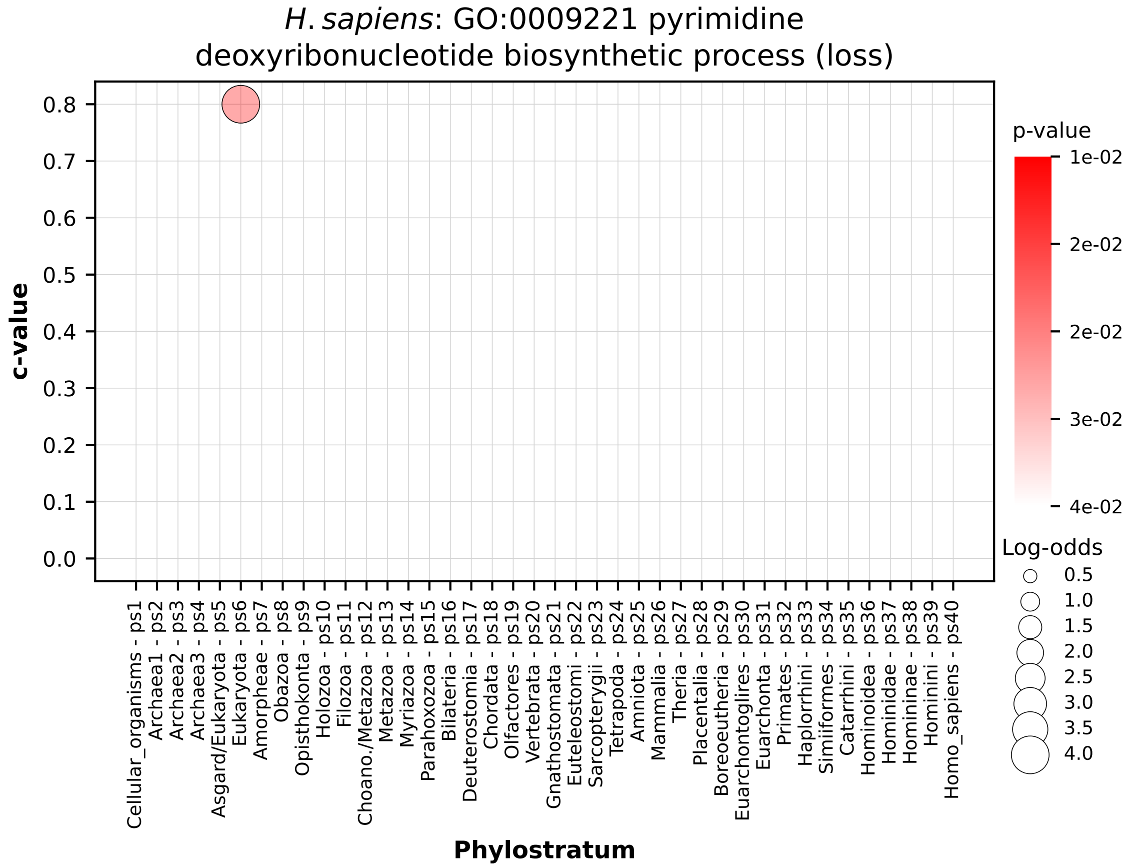

H. sapiens: GO:0009221 pyrimidine deoxyribonucleotide biosynthetic process (loss)
c-value
0.8
0.7
0.6
0.5
0.4
0.3
0.2
0.1
0.0
p-value
1e-02
2e-02
2e-02
3e-02
4e-02
Log-odds
0.5
1.0
1.5
2.0
2.5
3.0
3.5
4.0
Cellular_organisms - ps1
Archaea1 - ps2
Archaea2 - ps3
Archaea3 - ps4
Asgard/Eukaryota - ps5
Eukaryota - ps6
Amorpheae - ps7
Obazoa - ps8
Opisthokonta - ps9
Holozoa - ps10
Filozoa - ps11
Choano./Metazoa - ps12
Metazoa - ps13
Myriazoa - ps14
Parahoxozoa - ps15
Bilateria - ps16
Deuterostomia - ps17
Chordata - ps18
Olfactores - ps19
Vertebrata - ps20
Gnathostomata - ps21
Euteleostomi - ps22
Sarcopterygii - ps23
Tetrapoda - ps24
Amniota - ps25
Mammalia - ps26
Theria - ps27
Placentalia - ps28
Boreoeutheria - ps29
Euarchontoglires - ps30
Euarchonta - ps31
Primates - ps32
Haplorrhini - ps33
Simiiformes - ps34
Catarrhini - ps35
Hominoidea - ps36
Hominidae - ps37
Homininae - ps38
Hominini - ps39
Homo_sapiens - ps40
Phylostratum

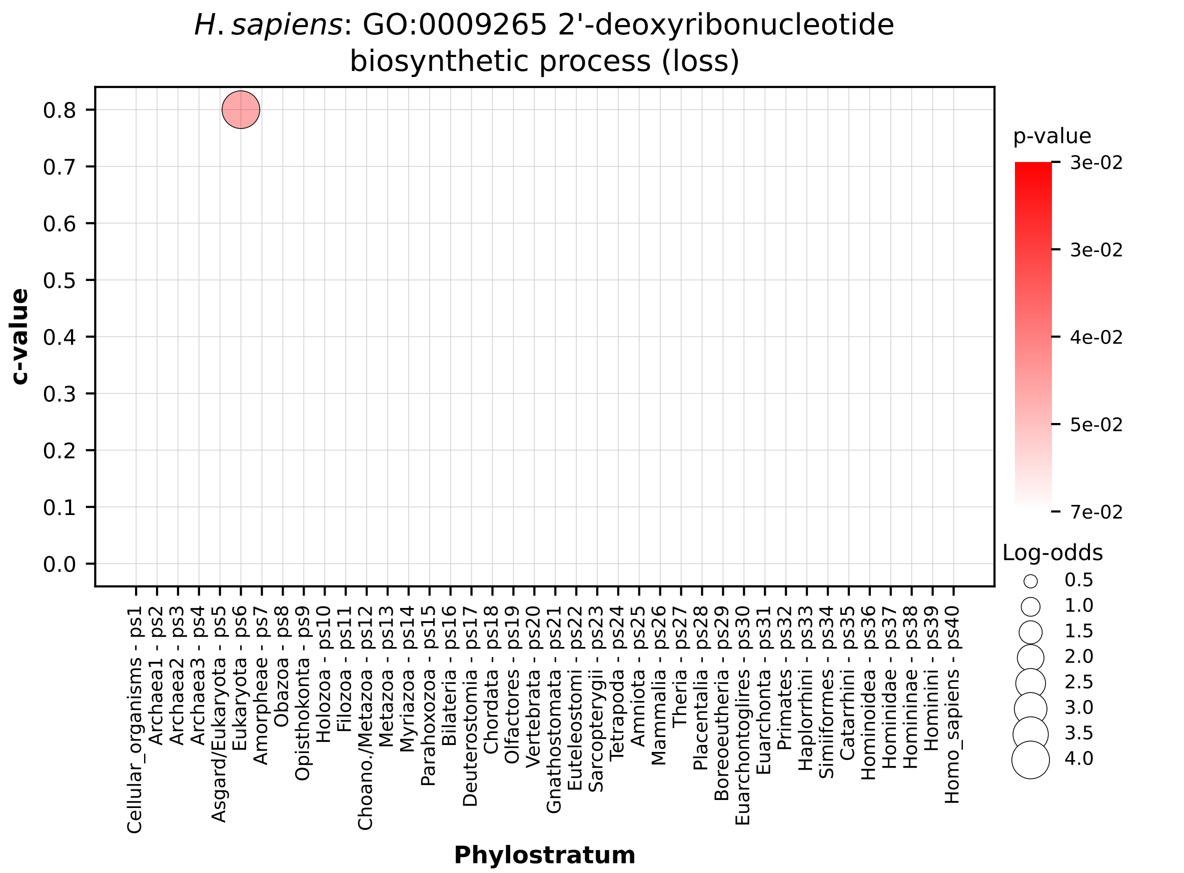

H. sapiens: GO:0009265 2'-deoxyribonucleotide biosynthetic process (loss)
c-value
0.8
0.7
0.6
0.5
0.4
0.3
0.2
0.1
0.0
p-value
3e-02
3e-02
4e-02
5e-02
7e-02
Log-odds
0.5
1.0
1.5
2.0
2.5
3.0
3.5
4.0
Cellular_organisms - ps1
Archaea1 - ps2
Archaea2 - ps3
Archaea3 - ps4
Asgard/Eukaryota - ps5
Eukaryota - ps6
Amorpheae - ps7
Obazoa - ps8
Opisthokonta - ps9
Holozoa - ps10
Filozoa - ps11
Choano./Metazoa - ps12
Metazoa - ps13
Myriazoa - ps14
Parahoxozoa - ps15
Bilateria - ps16
Deuterostomia - ps17
Chordata - ps18
Olfactores - ps19
Vertebrata - ps20
Gnathostomata - ps21
Euteleostomi - ps22
Sarcopterygii - ps23
Tetrapoda - ps24
Amniota - ps25
Mammalia - ps26
Theria - ps27
Placentalia - ps28
Boreoeutheria - ps29
Euarchontoglires - ps30
Euarchonta - ps31
Primates - ps32
Haplorrhini - ps33
Simiiformes - ps34
Catarrhini - ps35
Hominoidea - ps36
Hominidae - ps37
Homininae - ps38
Hominini - ps39
Homo_sapiens - ps40
Phylostratum

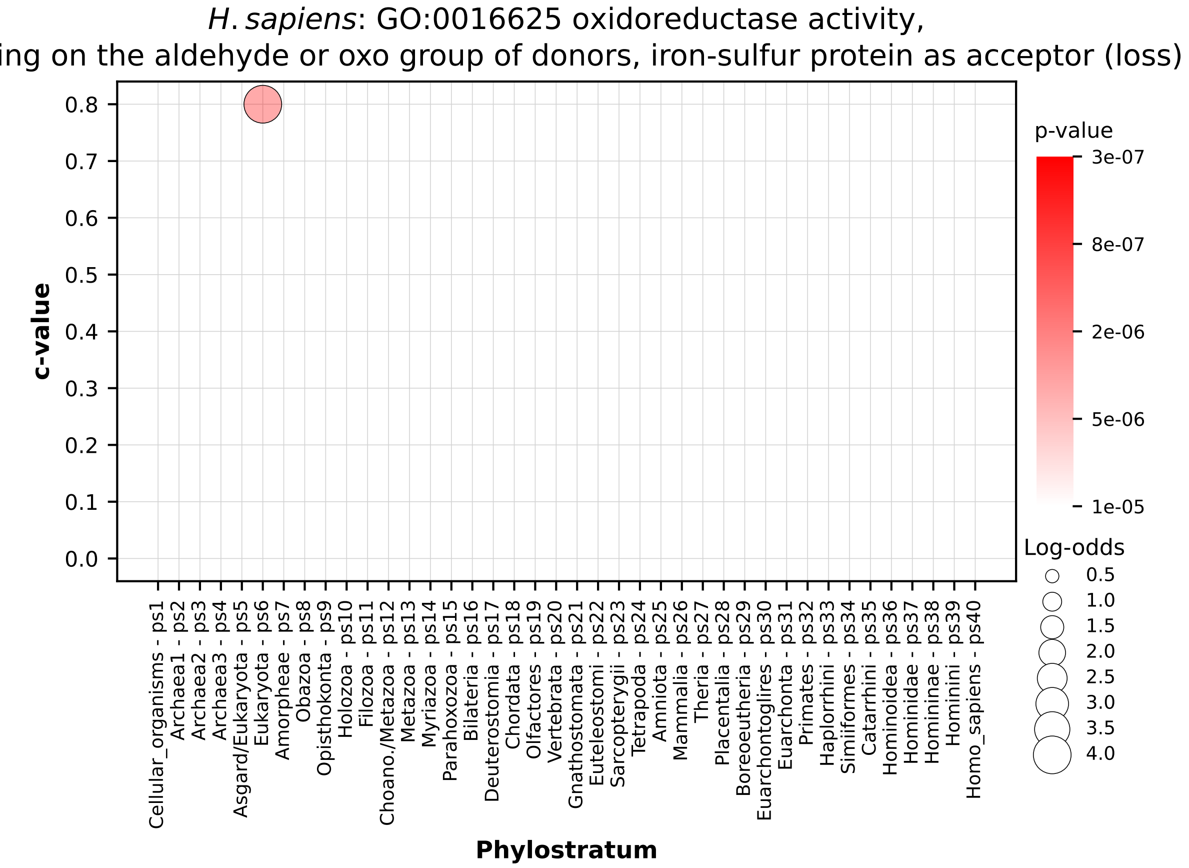

H. sapiens: GO:0016625 oxidoreductase activity,
ing on the aldehyde or oxo group of donors, iron-sulfur protein as acceptor (loss)
c-value
0.0
0.1
0.2
0.3
0.4
0.5
0.6
0.7
0.8
Phylostratum
Cellular_organisms - ps1
Archaea1 - ps2
Archaea2 - ps3
Archaea3 - ps4
Asgard/Eukaryota - ps5
Eukaryota - ps6
Amorpheae - ps7
Obazoa - ps8
Opisthokonta - ps9
Holozoa - ps10
Filozoa - ps11
Choano./Metazoa - ps12
Metazoa - ps13
Myriazoa - ps14
Parahoxozoa - ps15
Bilateria - ps16
Deuterostomia - ps17
Chordata - ps18
Olfactores - ps19
Vertebrata - ps20
Gnathostomata - ps21
Euteleostomi - ps22
Sarcopterygii - ps23
Tetrapoda - ps24
Amniota - ps25
Mammalia - ps26
Theria - ps27
Placentalia - ps28
Boreoeutheria - ps29
Euarchontoglires - ps30
Euarchonta - ps31
Primates - ps32
Haplorrhini - ps33
Simiiformes - ps34
Catarrhini - ps35
Hominoidea - ps36
Hominidae - ps37
Homininae - ps38
Hominini - ps39
Homo_sapiens - ps40
p-value
3e-07
8e-07
2e-06
5e-06
1e-05
Log-odds
0.5
1.0
1.5
2.0
2.5
3.0
3.5
4.0

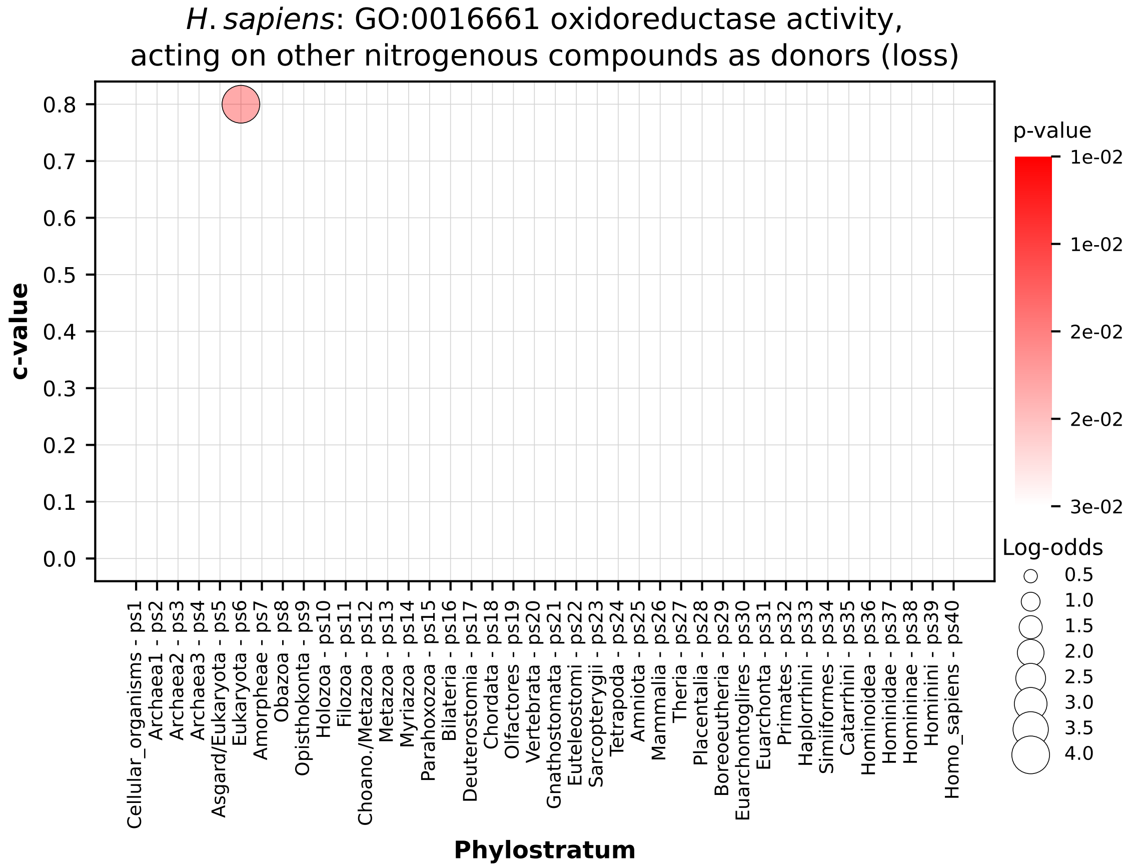
H. sapiens: GO:0016661 oxidoreductase activity, acting on other nitrogenous compounds as donors (loss)
c-value
0.8
0.7
0.6
0.5
0.4
0.3
0.2
0.1
0.0
p-value
1e-02
1e-02
2e-02
2e-02
3e-02
Log-odds
0.5
1.0
1.5
2.0
2.5
3.0
3.5
4.0
Cellular_organisms - ps1
Archaea1 - ps2
Archaea2 - ps3
Archaea3 - ps4
Asgard/Eukaryota - ps5
Eukaryota - ps6
Amorpheae - ps7
Obazoa - ps8
Opisthokonta - ps9
Holozoa - ps10
Filozoa - ps11
Choano./Metazoa - ps12
Metazoa - ps13
Myriazoa - ps14
Parahoxozoa - ps15
Bilateria - ps16
Deuterostomia - ps17
Chordata - ps18
Olfactores - ps19
Vertebrata - ps20
Gnathostomata - ps21
Euteleostomi - ps22
Sarcopterygii - ps23
Tetrapoda - ps24
Amniota - ps25
Mammalia - ps26
Theria - ps27
Placentalia - ps28
Boreoeutheria - ps29
Euarchontoglires - ps30
Euarchonta - ps31
Primates - ps32
Haplorrhini - ps33
Simiiformes - ps34
Catarrhini - ps35
Hominoidea - ps36
Hominidae - ps37
Homininae - ps38
Hominini - ps39
Homo_sapiens - ps40
Phylostratum

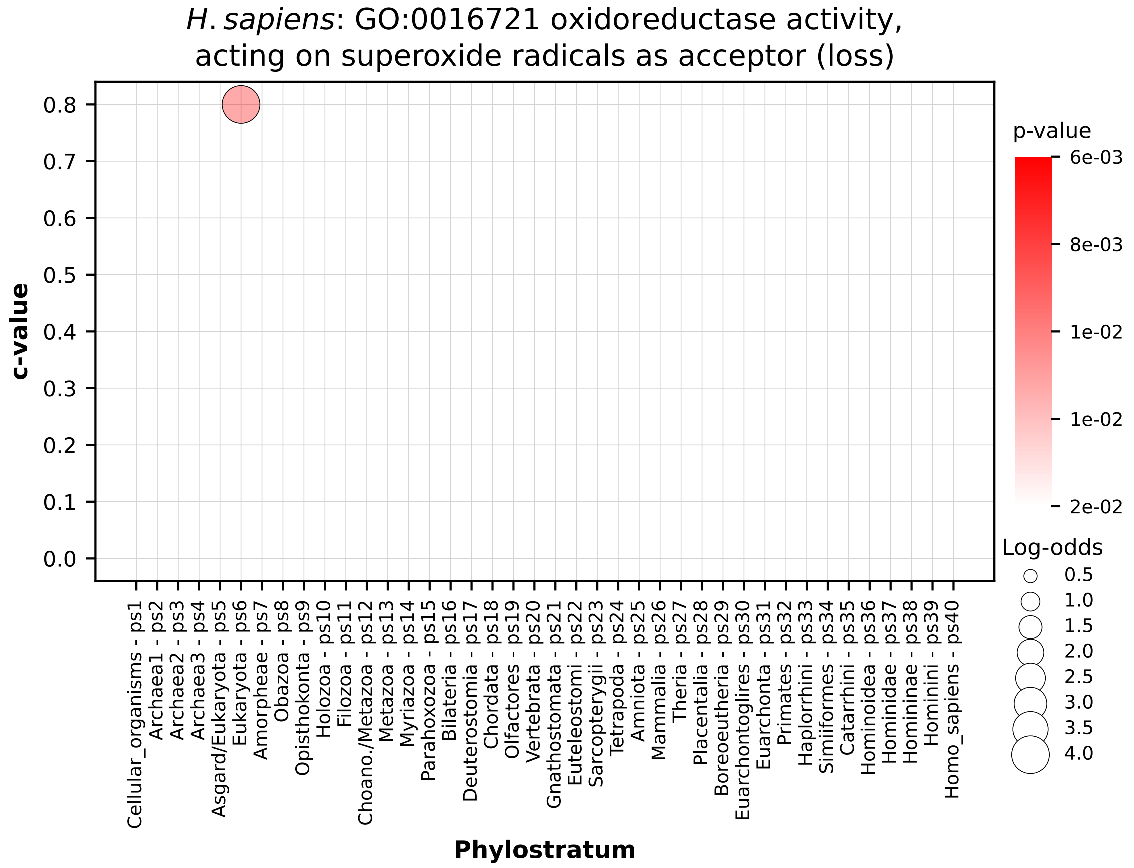

H. sapiens: GO:0016721 oxidoreductase activity, acting on superoxide radicals as acceptor (loss)
c-value
0.0
0.1
0.2
0.3
0.4
0.5
0.6
0.7
0.8
Phylostratum
Cellular_organisms - ps1
Archaea1 - ps2
Archaea2 - ps3
Archaea3 - ps4
Asgard/Eukaryota - ps5
Eukaryota - ps6
Amorpheae - ps7
Obazoa - ps8
Opisthokonta - ps9
Holozoa - ps10
Filozoa - ps11
Choano./Metazoa - ps12
Metazoa - ps13
Myriazoa - ps14
Parahoxozoa - ps15
Bilateria - ps16
Deuterostomia - ps17
Chordata - ps18
Olfactores - ps19
Vertebrata - ps20
Gnathostomata - ps21
Euteleostomi - ps22
Sarcopterygii - ps23
Tetrapoda - ps24
Amniota - ps25
Mammalia - ps26
Theria - ps27
Placentalia - ps28
Boreoeutheria - ps29
Euarchontoglires - ps30
Euarchonta - ps31
Primates - ps32
Haplorrhini - ps33
Simiiformes - ps34
Catarrhini - ps35
Hominoidea - ps36
Hominidae - ps37
Homininae - ps38
Hominini - ps39
Homo_sapiens - ps40
p-value
6e-03
8e-03
1e-02
1e-02
2e-02
Log-odds
0.5
1.0
1.5
2.0
2.5
3.0
3.5
4.0

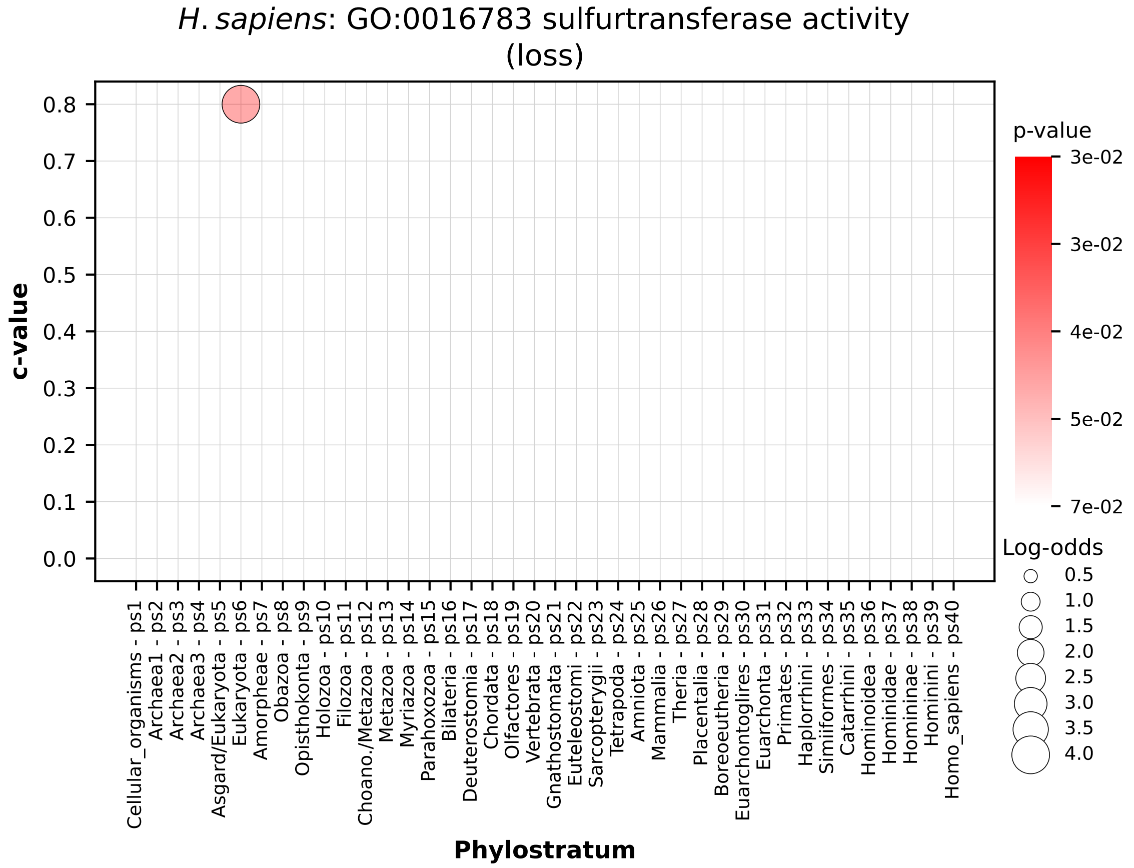
H. sapiens: GO:0016783 sulfurtransferase activity
(loss)
c-value
0.8
0.7
0.6
0.5
0.4
0.3
0.2
0.1
0.0
p-value
3e-02
3e-02
4e-02
5e-02
7e-02
Log-odds
0.5
1.0
1.5
2.0
2.5
3.0
3.5
4.0
Cellular_organisms - ps1
Archaea1 - ps2
Archaea2 - ps3
Archaea3 - ps4
Asgard/Eukaryota - ps5
Eukaryota - ps6
Amorpheae - ps7
Obazoa - ps8
Opisthokonta - ps9
Holozoa - ps10
Filozoa - ps11
Choano./Metazoa - ps12
Metazoa - ps13
Myriazoa - ps14
Parahoxozoa - ps15
Bilateria - ps16
Deuterostomia - ps17
Chordata - ps18
Olfactores - ps19
Vertebrata - ps20
Gnathostomata - ps21
Euteleostomi - ps22
Sarcopterygii - ps23
Tetrapoda - ps24
Amniota - ps25
Mammalia - ps26
Theria - ps27
Placentalia - ps28
Boreoeutheria - ps29
Euarchontoglires - ps30
Euarchonta - ps31
Primates - ps32
Haplorrhini - ps33
Simiiformes - ps34
Catarrhini - ps35
Hominoidea - ps36
Hominidae - ps37
Homininae - ps38
Hominini - ps39
Homo_sapiens - ps40
Phylostratum

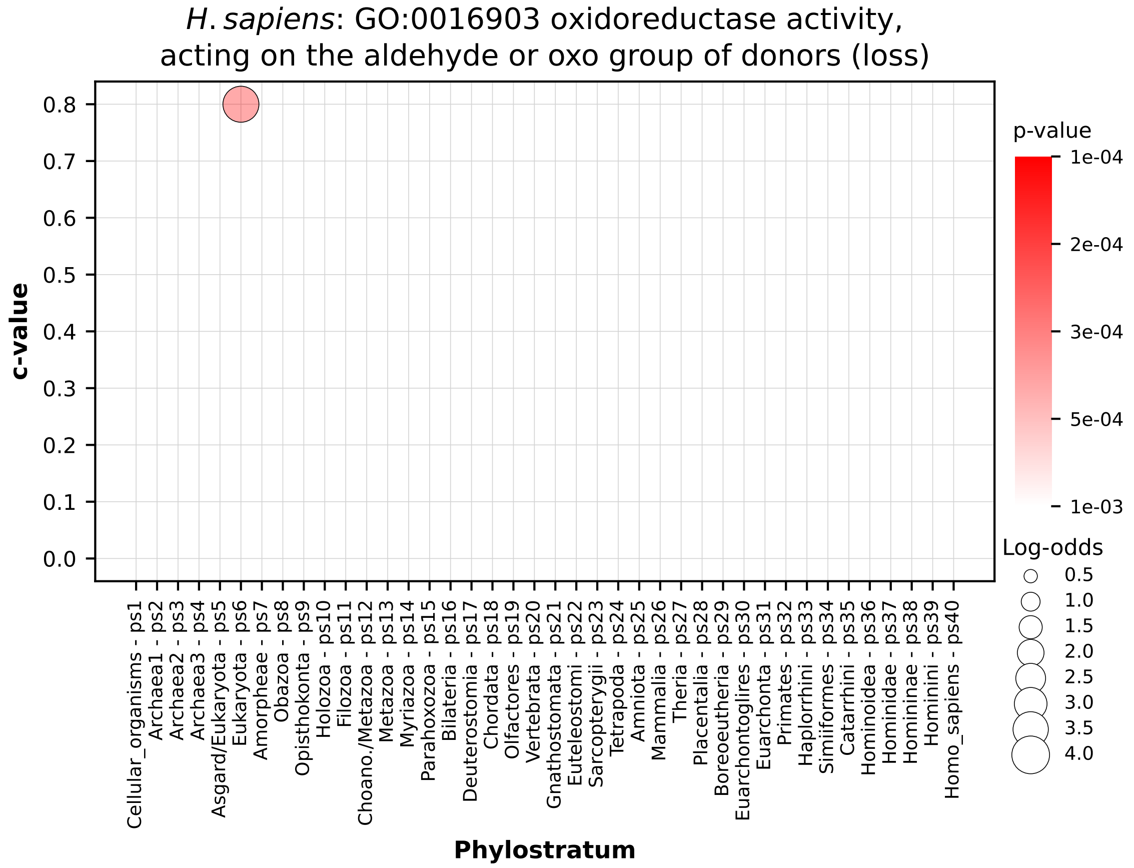
H. sapiens: GO:0016903 oxidoreductase activity,
acting on the aldehyde or oxo group of donors (loss)
c-value
0.0
0.1
0.2
0.3
0.4
0.5
0.6
0.7
0.8
p-value
1e-04
2e-04
3e-04
5e-04
1e-03
Log-odds
0.5
1.0
1.5
2.0
2.5
3.0
3.5
4.0
Cellular_organisms - ps1
Archaea1 - ps2
Archaea2 - ps3
Archaea3 - ps4
Asgard/Eukaryota - ps5
Eukaryota - ps6
Amorpheae - ps7
Obazoa - ps8
Opisthokonta - ps9
Holozoa - ps10
Filozoa - ps11
Choano./Metazoa - ps12
Metazoa - ps13
Myriazoa - ps14
Parahoxozoa - ps15
Bilateria - ps16
Deuterostomia - ps17
Chordata - ps18
Olfactores - ps19
Vertebrata - ps20
Gnathostomata - ps21
Euteleostomi - ps22
Sarcopterygii - ps23
Tetrapoda - ps24
Amniota - ps25
Mammalia - ps26
Theria - ps27
Placentalia - ps28
Boreoeutheria - ps29
Euarchontoglires - ps30
Euarchonta - ps31
Primates - ps32
Haplorrhini - ps33
Simiiformes - ps34
Catarrhini - ps35
Hominoidea - ps36
Hominidae - ps37
Homininae - ps38
Hominini - ps39
Homo_sapiens - ps40
Phylostratum

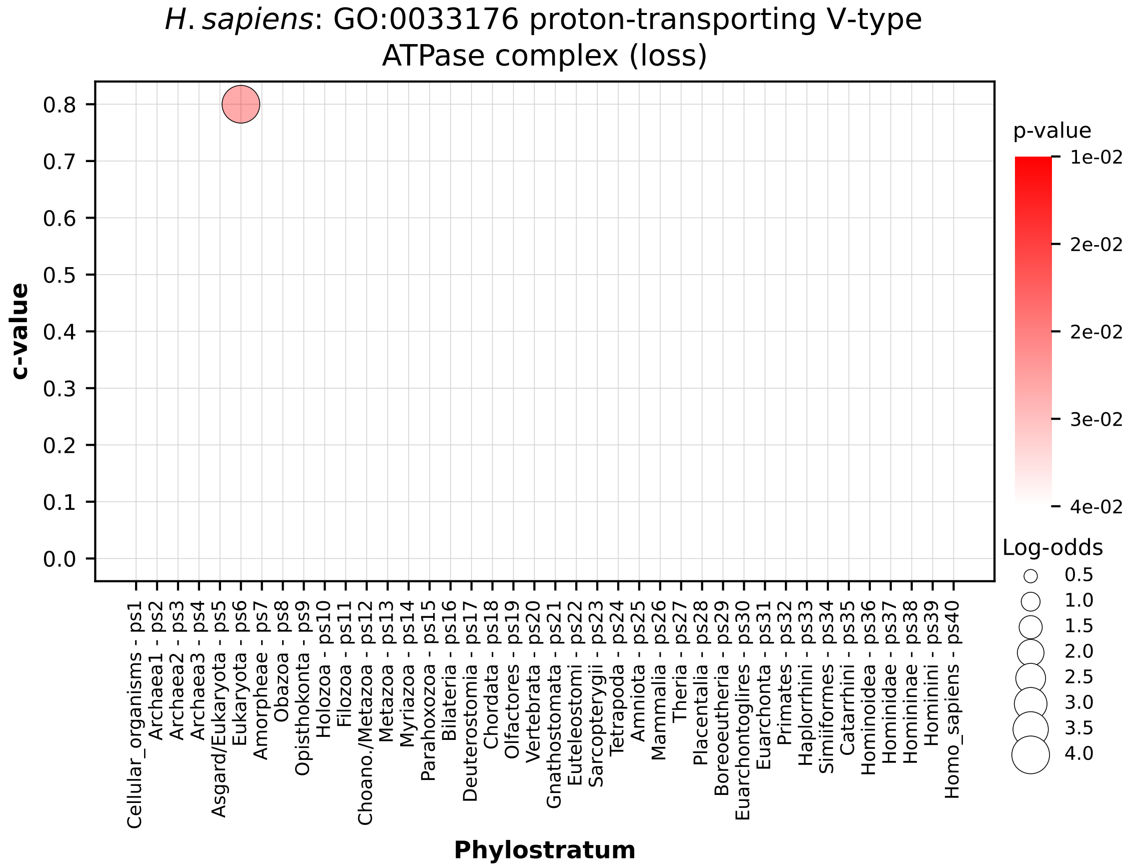

H. sapiens: GO:0033176 proton-transporting V-type ATPase complex (loss)
c-value
0.0
0.1
0.2
0.3
0.4
0.5
0.6
0.7
0.8
Phylostratum
Cellular_organisms - ps1
Archaea1 - ps2
Archaea2 - ps3
Archaea3 - ps4
Asgard/Eukaryota - ps5
Eukaryota - ps6
Amorpheae - ps7
Obazoa - ps8
Opisthokonta - ps9
Holozoa - ps10
Filozoa - ps11
Choano./Metazoa - ps12
Metazoa - ps13
Myriazoa - ps14
Parahoxozoa - ps15
Bilateria - ps16
Deuterostomia - ps17
Chordata - ps18
Olfactores - ps19
Vertebrata - ps20
Gnathostomata - ps21
Euteleostomi - ps22
Sarcopterygii - ps23
Tetrapoda - ps24
Amniota - ps25
Mammalia - ps26
Theria - ps27
Placentalia - ps28
Boreoeutheria - ps29
Euarchontoglires - ps30
Euarchonta - ps31
Primates - ps32
Haplorrhini - ps33
Simiiformes - ps34
Catarrhini - ps35
Hominoidea - ps36
Hominidae - ps37
Homininae - ps38
Hominini - ps39
Homo_sapiens - ps40
p-value
1e-02
2e-02
2e-02
3e-02
4e-02
Log-odds
0.5
1.0
1.5
2.0
2.5
3.0
3.5
4.0

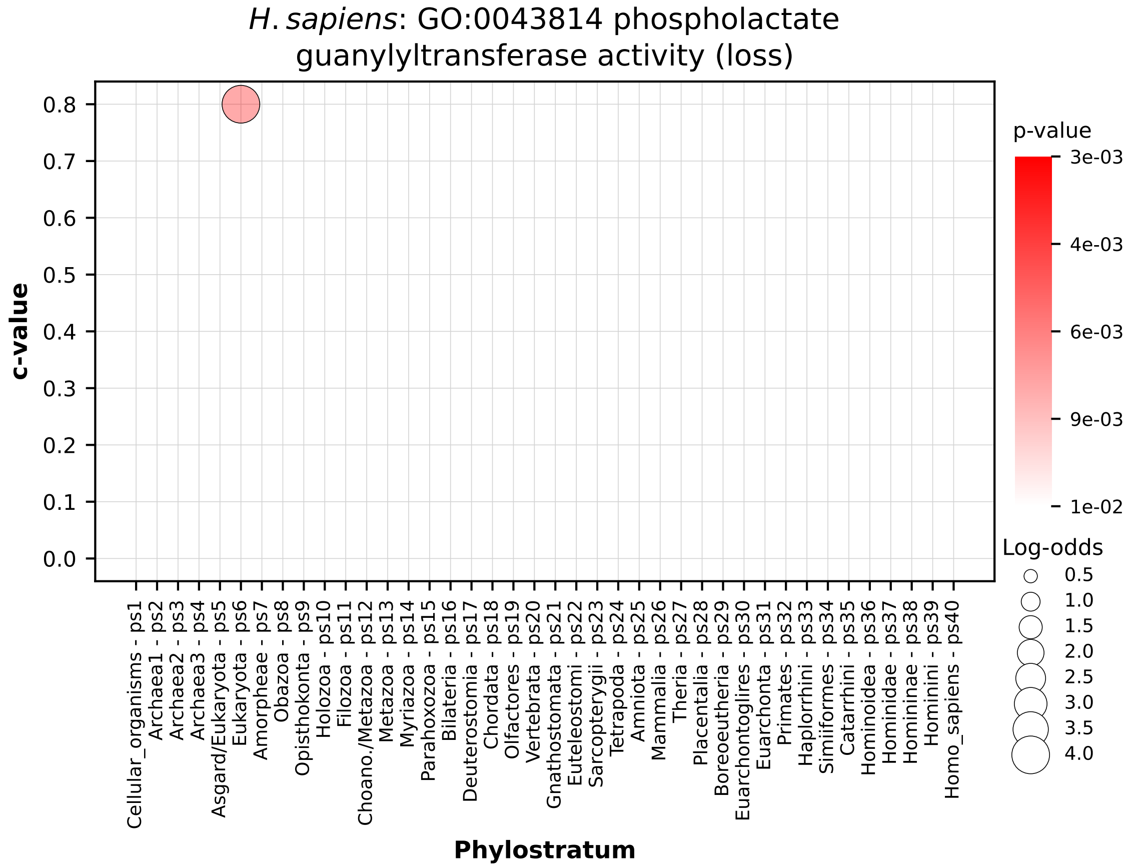

H. sapiens: GO:0043814 phospholactate guanylyltransferase activity (loss)
c-value
0.0
0.1
0.2
0.3
0.4
0.5
0.6
0.7
0.8
p-value
3e-03
4e-03
6e-03
9e-03
1e-02
Log-odds
0.5
1.0
1.5
2.0
2.5
3.0
3.5
4.0
Cellular_organisms - ps1
Archaea1 - ps2
Archaea2 - ps3
Archaea3 - ps4
Asgard/Eukaryota - ps5
Eukaryota - ps6
Amorpheae - ps7
Obazoa - ps8
Opisthokonta - ps9
Holozoa - ps10
Filozoa - ps11
Choano./Metazoa - ps12
Metazoa - ps13
Myriazoa - ps14
Parahoxozoa - ps15
Bilateria - ps16
Deuterostomia - ps17
Chordata - ps18
Olfactores - ps19
Vertebrata - ps20
Gnathostomata - ps21
Euteleostomi - ps22
Sarcopterygii - ps23
Tetrapoda - ps24
Amniota - ps25
Mammalia - ps26
Theria - ps27
Placentalia - ps28
Boreoeutheria - ps29
Euarchontoglires - ps30
Euarchonta - ps31
Primates - ps32
Haplorrhini - ps33
Simiiformes - ps34
Catarrhini - ps35
Hominoidea - ps36
Hominidae - ps37
Homininae - ps38
Hominini - ps39
Homo_sapiens - ps40
Phylostratum

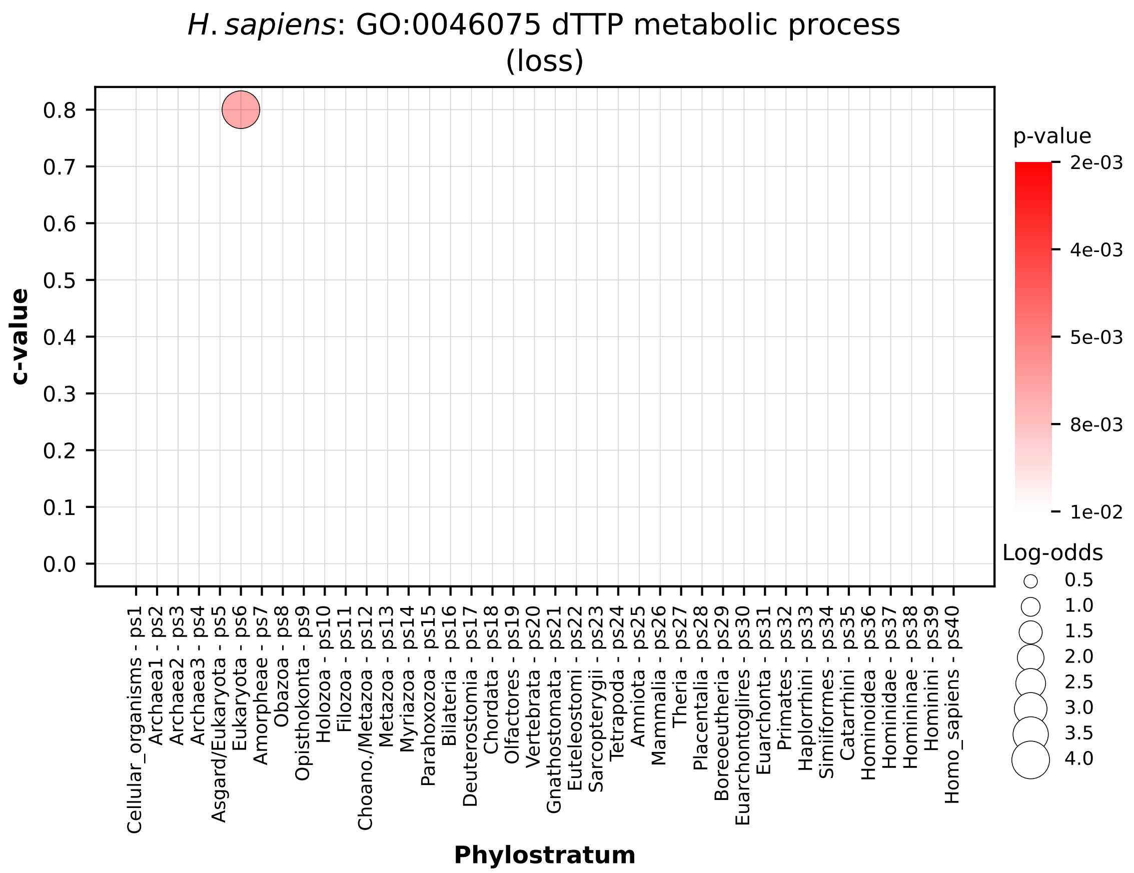

H. sapiens: GO:0046075 dTTP metabolic process
(loss)
c-value
0.0
0.1
0.2
0.3
0.4
0.5
0.6
0.7
0.8
p-value
2e-03
4e-03
5e-03
8e-03
1e-02
Log-odds
0.5
1.0
1.5
2.0
2.5
3.0
3.5
4.0
Cellular_organisms - ps1
Archaea1 - ps2
Archaea2 - ps3
Archaea3 - ps4
Asgard/Eukaryota - ps5
Eukaryota - ps6
Amorpheae - ps7
Obazoa - ps8
Opisthokonta - ps9
Holozoa - ps10
Filozoa - ps11
Choano./Metazoa - ps12
Metazoa - ps13
Myriazoa - ps14
Parahoxozoa - ps15
Bilateria - ps16
Deuterostomia - ps17
Chordata - ps18
Olfactores - ps19
Vertebrata - ps20
Gnathostomata - ps21
Euteleostomi - ps22
Sarcopterygii - ps23
Tetrapoda - ps24
Amniota - ps25
Mammalia - ps26
Theria - ps27
Placentalia - ps28
Boreoeutheria - ps29
Euarchontoglires - ps30
Euarchonta - ps31
Primates - ps32
Haplorrhini - ps33
Simiiformes - ps34
Catarrhini - ps35
Hominoidea - ps36
Hominidae - ps37
Homininae - ps38
Hominini - ps39
Homo_sapiens - ps40
Phylostratum

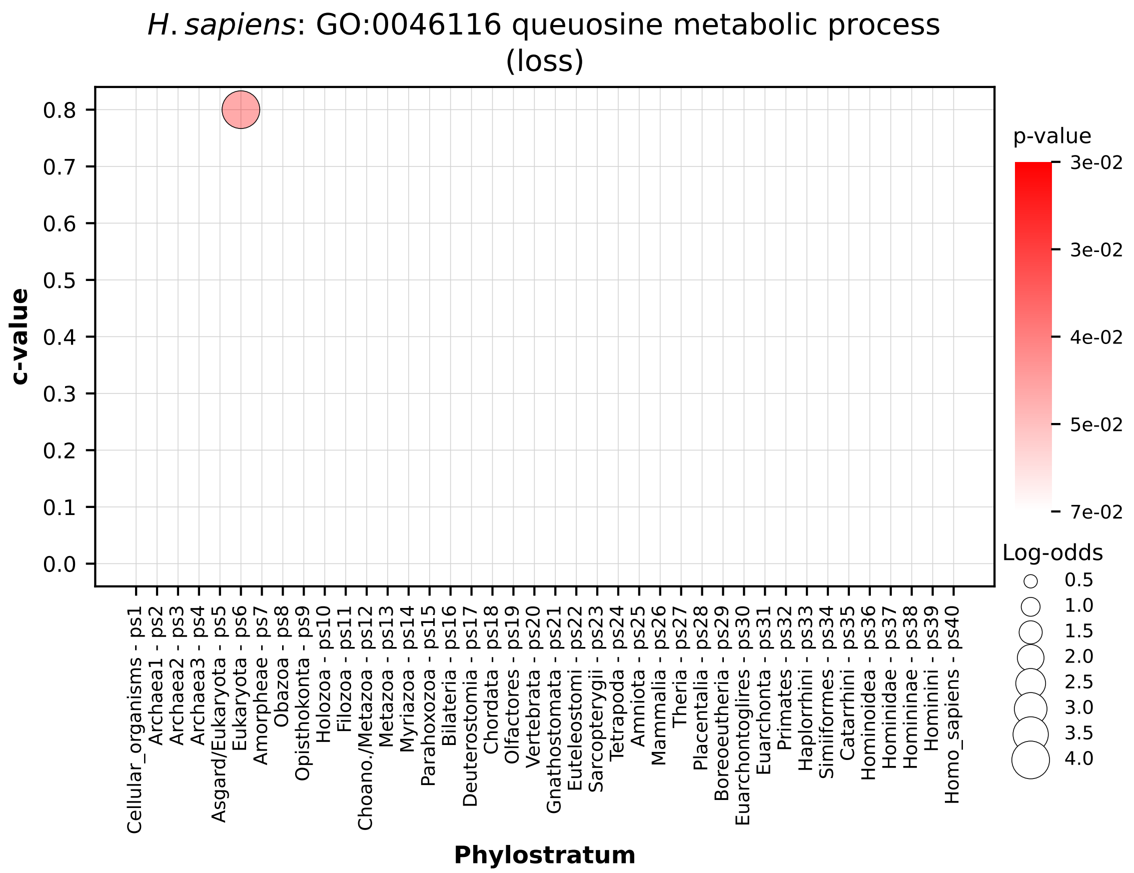
H. sapiens: GO:0046116 queuosine metabolic process
(loss)
c-value
0.0
0.1
0.2
0.3
0.4
0.5
0.6
0.7
0.8
Phylostratum
Cellular_organisms - ps1
Archaea1 - ps2
Archaea2 - ps3
Archaea3 - ps4
Asgard/Eukaryota - ps5
Eukaryota - ps6
Amorpheae - ps7
Obazoa - ps8
Opisthokonta - ps9
Holozoa - ps10
Filozoa - ps11
Choano./Metazoa - ps12
Metazoa - ps13
Myriazoa - ps14
Parahoxozoa - ps15
Bilateria - ps16
Deuterostomia - ps17
Chordata - ps18
Olfactores - ps19
Vertebrata - ps20
Gnathostomata - ps21
Euteleostomi - ps22
Sarcopterygii - ps23
Tetrapoda - ps24
Amniota - ps25
Mammalia - ps26
Theria - ps27
Placentalia - ps28
Boreoeutheria - ps29
Euarchontoglires - ps30
Euarchonta - ps31
Primates - ps32
Haplorrhini - ps33
Simiiformes - ps34
Catarrhini - ps35
Hominoidea - ps36
Hominidae - ps37
Homininae - ps38
Hominini - ps39
Homo_sapiens - ps40
p-value
3e-02
3e-02
4e-02
5e-02
7e-02
Log-odds
0.5
1.0
1.5
2.0
2.5
3.0
3.5
4.0

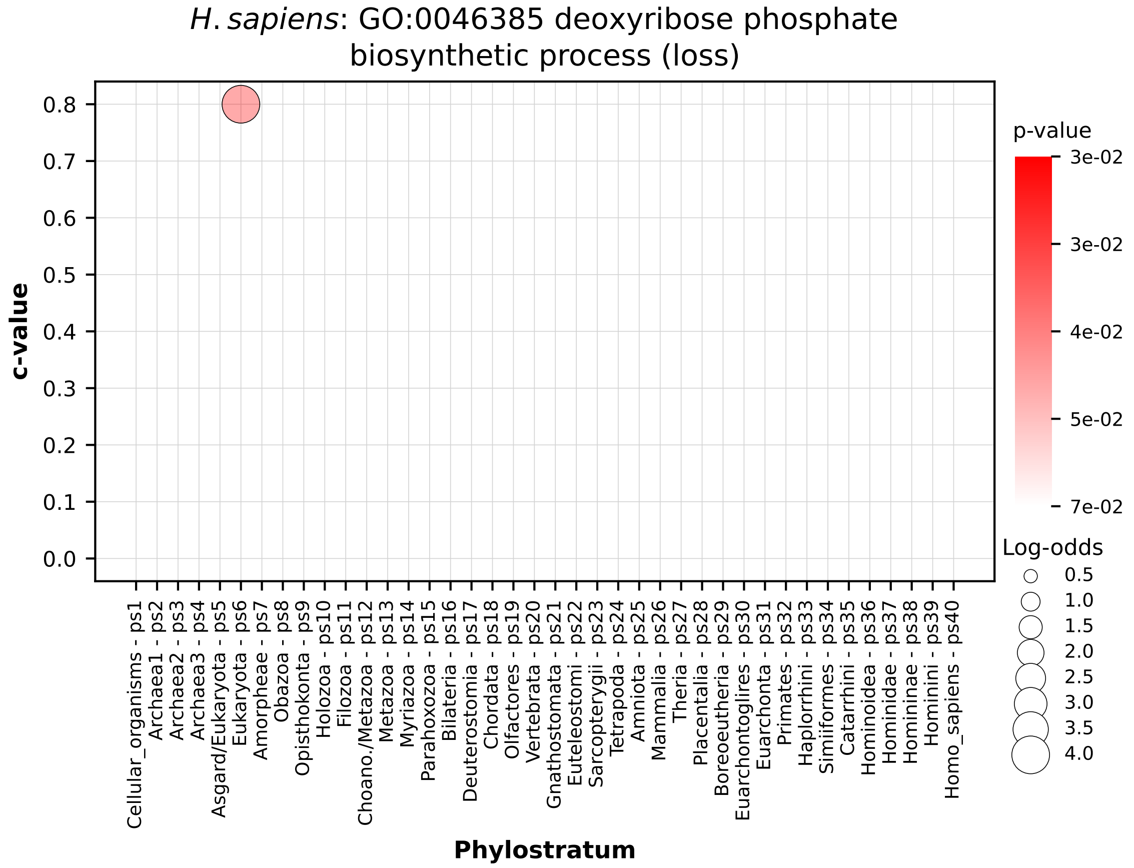
H. sapiens: GO:0046385 deoxyribose phosphate biosynthetic process (loss)
c-value
0.0
0.1
0.2
0.3
0.4
0.5
0.6
0.7
0.8
p-value
3e-02
3e-02
4e-02
5e-02
7e-02
Log-odds
0.5
1.0
1.5
2.0
2.5
3.0
3.5
4.0
Cellular_organisms - ps1
Archaea1 - ps2
Archaea2 - ps3
Archaea3 - ps4
Asgard/Eukaryota - ps5
Eukaryota - ps6
Amorpheae - ps7
Obazoa - ps8
Opisthokonta - ps9
Holozoa - ps10
Filozoa - ps11
Choano./Metazoa - ps12
Metazoa - ps13
Myriazoa - ps14
Parahoxozoa - ps15
Bilateria - ps16
Deuterostomia - ps17
Chordata - ps18
Olfactores - ps19
Vertebrata - ps20
Gnathostomata - ps21
Euteleostomi - ps22
Sarcopterygii - ps23
Tetrapoda - ps24
Amniota - ps25
Mammalia - ps26
Theria - ps27
Placentalia - ps28
Boreoeutheria - ps29
Euarchontoglires - ps30
Euarchonta - ps31
Primates - ps32
Haplorrhini - ps33
Simiiformes - ps34
Catarrhini - ps35
Hominoidea - ps36
Hominidae - ps37
Homininae - ps38
Hominini - ps39
Homo_sapiens - ps40
Phylostratum

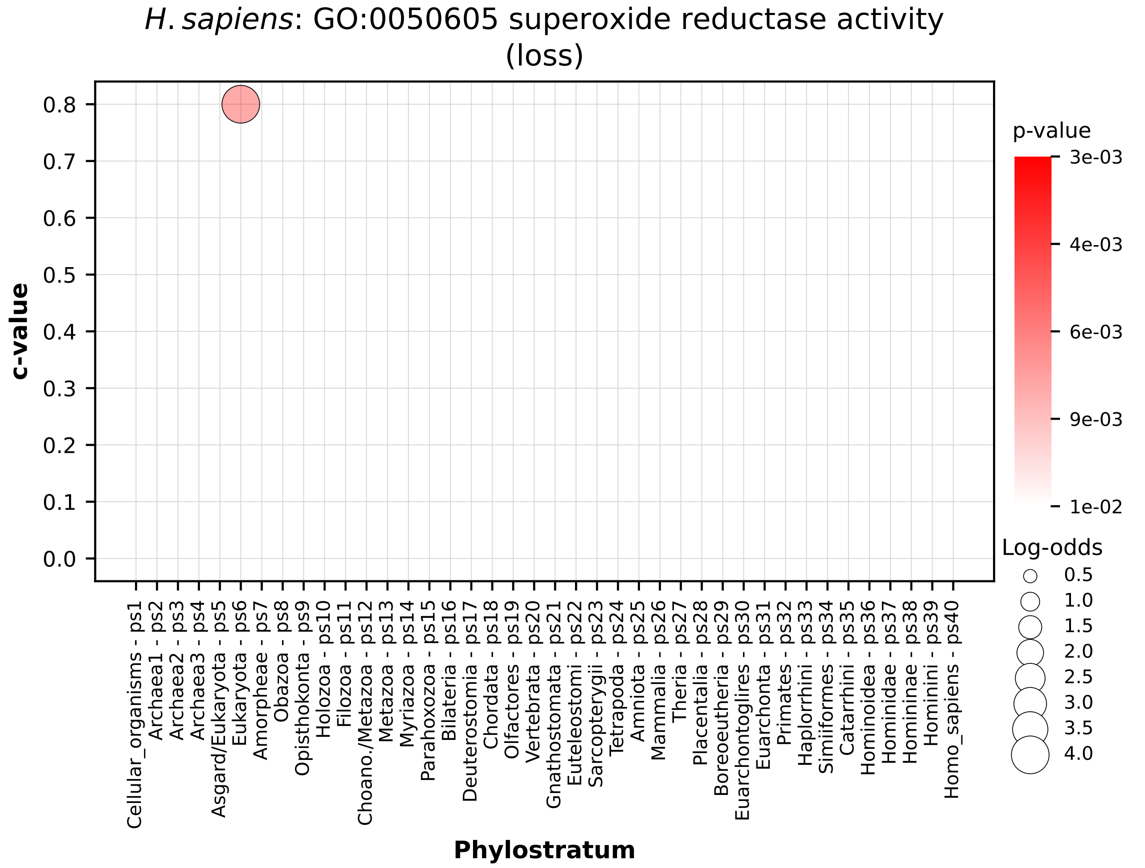

H. sapiens: GO:0050605 superoxide reductase activity
(loss)
c-value
0.0
0.1
0.2
0.3
0.4
0.5
0.6
0.7
0.8
Phylostratum
Cellular_organisms - ps1
Archaea1 - ps2
Archaea2 - ps3
Archaea3 - ps4
Asgard/Eukaryota - ps5
Eukaryota - ps6
Amorpheae - ps7
Obazoa - ps8
Opisthokonta - ps9
Holozoa - ps10
Filozoa - ps11
Choano./Metazoa - ps12
Metazoa - ps13
Myriazoa - ps14
Parahoxozoa - ps15
Bilateria - ps16
Deuterostomia - ps17
Chordata - ps18
Olfactores - ps19
Vertebrata - ps20
Gnathostomata - ps21
Euteleostomi - ps22
Sarcopterygii - ps23
Tetrapoda - ps24
Amniota - ps25
Mammalia - ps26
Theria - ps27
Placentalia - ps28
Boreoeutheria - ps29
Euarchontoglires - ps30
Euarchonta - ps31
Primates - ps32
Haplorrhini - ps33
Simiiformes - ps34
Catarrhini - ps35
Hominoidea - ps36
Hominidae - ps37
Homininae - ps38
Hominini - ps39
Homo_sapiens - ps40
p-value
3e-03
4e-03
6e-03
9e-03
1e-02
Log-odds
0.5
1.0
1.5
2.0
2.5
3.0
3.5
4.0

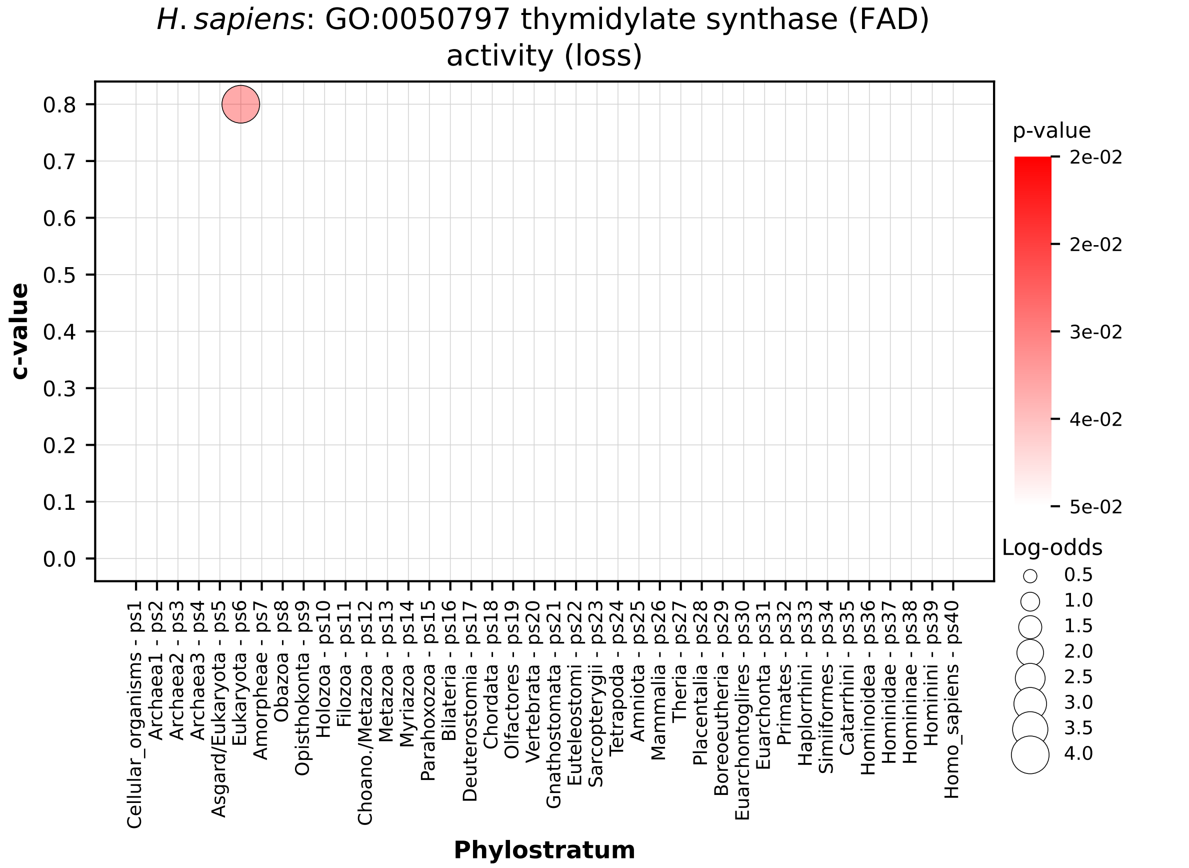

H. sapiens: GO:0050797 thymidylate synthase (FAD) activity (loss)
c-value
0.8
0.7
0.6
0.5
0.4
0.3
0.2
0.1
0.0
p-value
2e-02
2e-02
3e-02
4e-02
5e-02
Log-odds
0.5
1.0
1.5
2.0
2.5
3.0
3.5
4.0
Cellular_organisms - ps1
Archaea1 - ps2
Archaea2 - ps3
Archaea3 - ps4
Asgard/Eukaryota - ps5
Eukaryota - ps6
Amorpheae - ps7
Obazoa - ps8
Opisthokonta - ps9
Holozoa - ps10
Filozoa - ps11
Choano./Metazoa - ps12
Metazoa - ps13
Myriazoa - ps14
Parahoxozoa - ps15
Bilateria - ps16
Deuterostomia - ps17
Chordata - ps18
Olfactores - ps19
Vertebrata - ps20
Gnathostomata - ps21
Euteleostomi - ps22
Sarcopterygii - ps23
Tetrapoda - ps24
Amniota - ps25
Mammalia - ps26
Theria - ps27
Placentalia - ps28
Boreoeutheria - ps29
Euarchontoglires - ps30
Euarchonta - ps31
Primates - ps32
Haplorrhini - ps33
Simiiformes - ps34
Catarrhini - ps35
Hominoidea - ps36
Hominidae - ps37
Homininae - ps38
Hominini - ps39
Homo_sapiens - ps40
Phylostratum

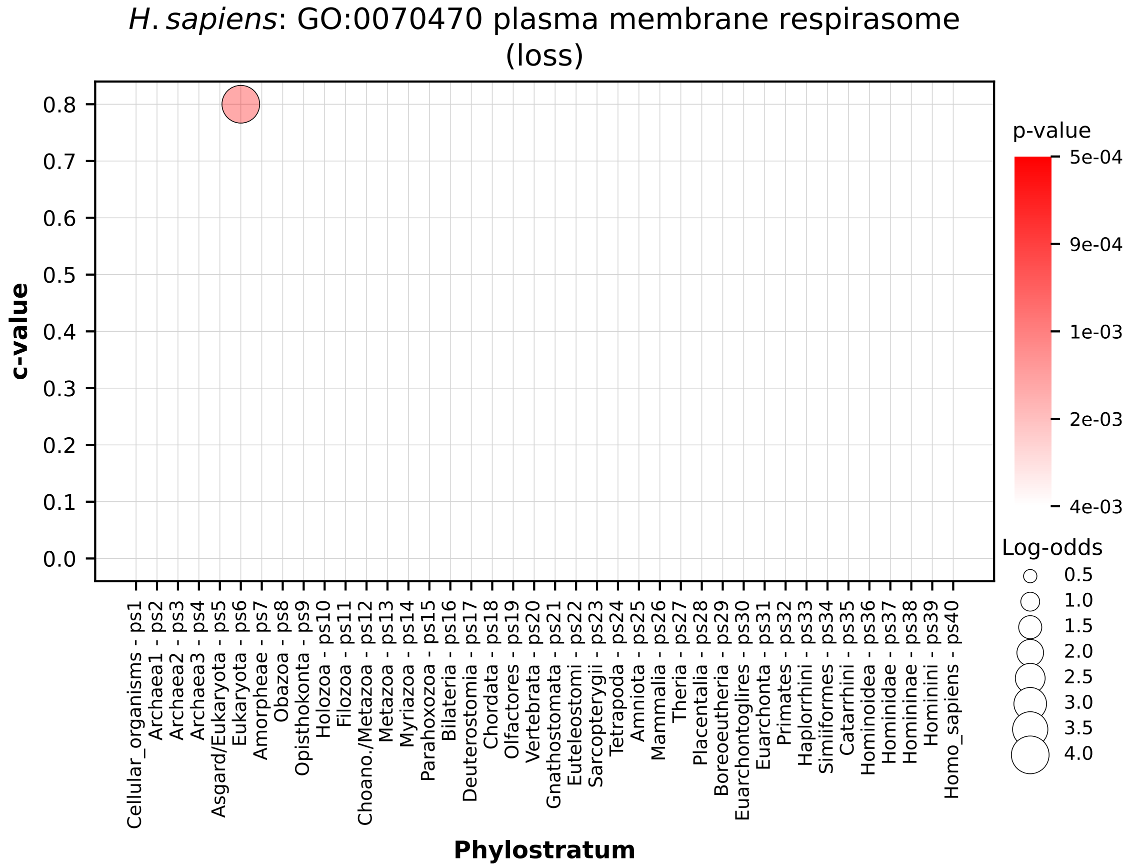
H. sapiens: GO:0070470 plasma membrane respirasome
(loss)
c-value
0.8
0.7
0.6
0.5
0.4
0.3
0.2
0.1
0.0
Phylostratum
Cellular_organisms - ps1
Archaea1 - ps2
Archaea2 - ps3
Archaea3 - ps4
Asgard/Eukaryota - ps5
Eukaryota - ps6
Amorpheae - ps7
Obazoa - ps8
Opisthokonta - ps9
Holozoa - ps10
Filozoa - ps11
Choano./Metazoa - ps12
Metazoa - ps13
Myriazoa - ps14
Parahoxozoa - ps15
Bilateria - ps16
Deuterostomia - ps17
Chordata - ps18
Olfactores - ps19
Vertebrata - ps20
Gnathostomata - ps21
Euteleostomi - ps22
Sarcopterygii - ps23
Tetrapoda - ps24
Amniota - ps25
Mammalia - ps26
Theria - ps27
Placentalia - ps28
Boreoeutheria - ps29
Euarchontoglires - ps30
Euarchonta - ps31
Primates - ps32
Haplorrhini - ps33
Simiiformes - ps34
Catarrhini - ps35
Hominoidea - ps36
Hominidae - ps37
Homininae - ps38
Hominini - ps39
Homo_sapiens - ps40
p-value
5e-04
9e-04
1e-03
2e-03
4e-03
Log-odds
0.5
1.0
1.5
2.0
2.5
3.0
3.5
4.0

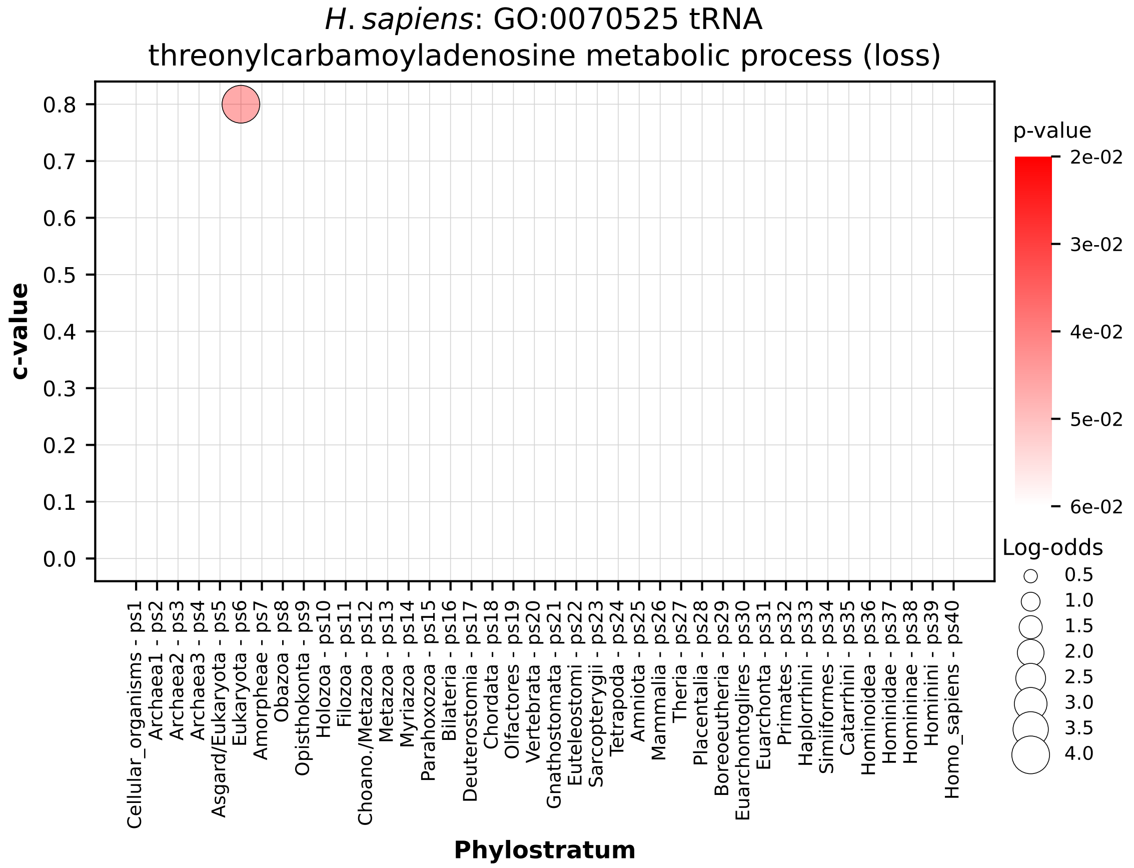
H. sapiens: GO:0070525 tRNA threonylcarbamoyladenosine metabolic process (loss)
c-value
0.0
0.1
0.2
0.3
0.4
0.5
0.6
0.7
0.8
Phylostratum
Cellular_organisms - ps1
Archaea1 - ps2
Archaea2 - ps3
Archaea3 - ps4
Asgard/Eukaryota - ps5
Eukaryota - ps6
Amorpheae - ps7
Obazoa - ps8
Opisthokonta - ps9
Holozoa - ps10
Filozoa - ps11
Choano./Metazoa - ps12
Metazoa - ps13
Myriazoa - ps14
Parahoxozoa - ps15
Bilateria - ps16
Deuterostomia - ps17
Chordata - ps18
Olfactores - ps19
Vertebrata - ps20
Gnathostomata - ps21
Euteleostomi - ps22
Sarcopterygii - ps23
Tetrapoda - ps24
Amniota - ps25
Mammalia - ps26
Theria - ps27
Placentalia - ps28
Boreoeutheria - ps29
Euarchontoglires - ps30
Euarchonta - ps31
Primates - ps32
Haplorrhini - ps33
Simiiformes - ps34
Catarrhini - ps35
Hominoidea - ps36
Hominidae - ps37
Homininae - ps38
Hominini - ps39
Homo_sapiens - ps40
p-value
2e-02
3e-02
4e-02
5e-02
6e-02
Log-odds
0.5
1.0
1.5
2.0
2.5
3.0
3.5
4.0

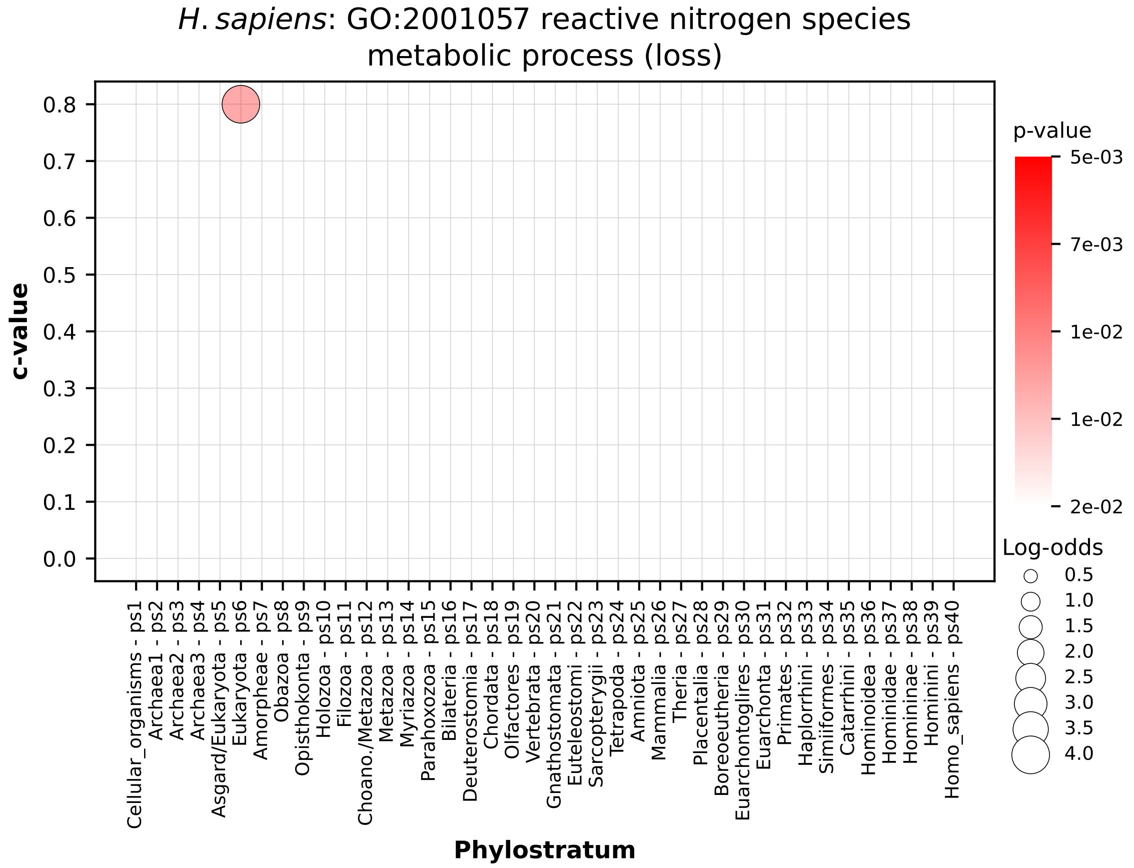

H. sapiens: GO:2001057 reactive nitrogen species metabolic process (loss)
c-value
0.8
0.7
0.6
0.5
0.4
0.3
0.2
0.1
0.0
p-value
5e-03
7e-03
1e-02
1e-02
2e-02
Log-odds
0.5
1.0
1.5
2.0
2.5
3.0
3.5
4.0
Cellular_organisms - ps1
Archaea1 - ps2
Archaea2 - ps3
Archaea3 - ps4
Asgard/Eukaryota - ps5
Eukaryota - ps6
Amorpheae - ps7
Obazoa - ps8
Opisthokonta - ps9
Holozoa - ps10
Filozoa - ps11
Choano./Metazoa - ps12
Metazoa - ps13
Myriazoa - ps14
Parahoxozoa - ps15
Bilateria - ps16
Deuterostomia - ps17
Chordata - ps18
Olfactores - ps19
Vertebrata - ps20
Gnathostomata - ps21
Euteleostomi - ps22
Sarcopterygii - ps23
Tetrapoda - ps24
Amniota - ps25
Mammalia - ps26
Theria - ps27
Placentalia - ps28
Boreoeutheria - ps29
Euarchontoglires - ps30
Euarchonta - ps31
Primates - ps32
Haplorrhini - ps33
Simiiformes - ps34
Catarrhini - ps35
Hominoidea - ps36
Hominidae - ps37
Homininae - ps38
Hominini - ps39
Homo_sapiens - ps40
Phylostratum

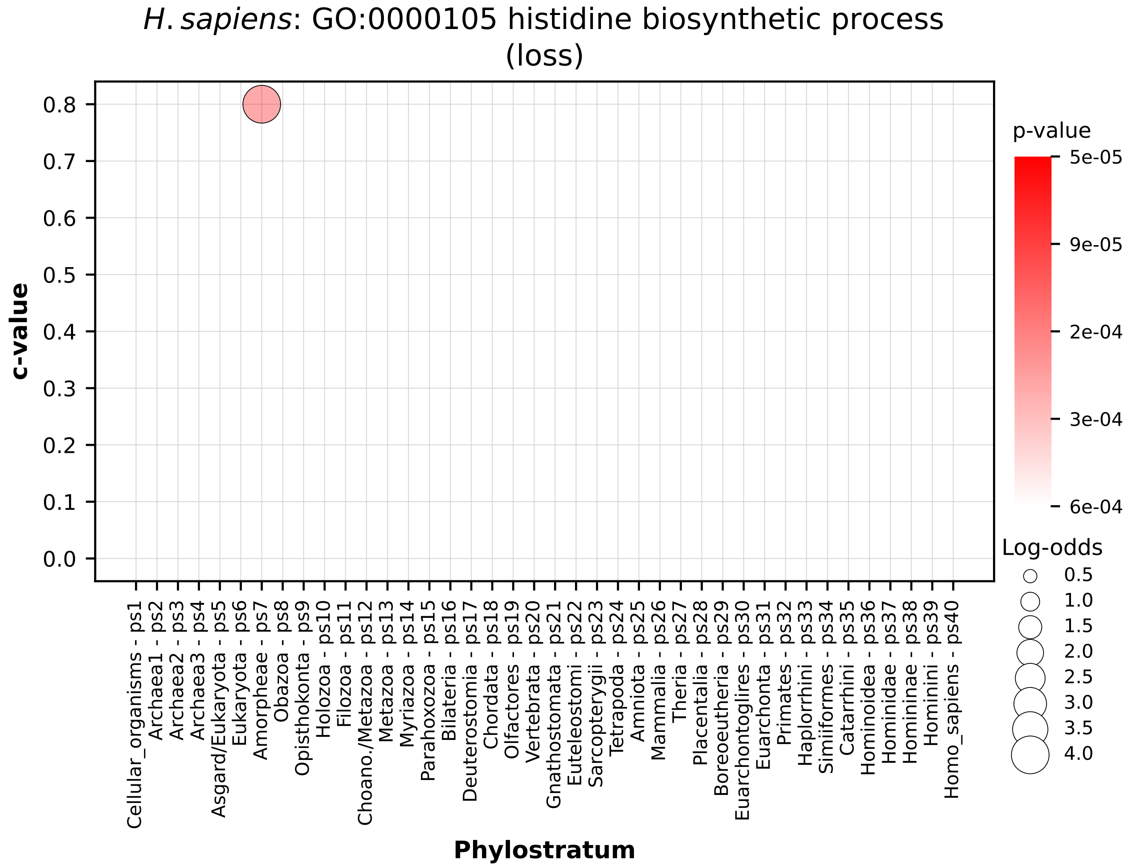

H. sapiens: GO:0000105 histidine biosynthetic process
(loss)
c-value
0.8
0.7
0.6
0.5
0.4
0.3
0.2
0.1
0.0
p-value
5e-05
9e-05
2e-04
3e-04
6e-04
Log-odds
0.5
1.0
1.5
2.0
2.5
3.0
3.5
4.0
Cellular_organisms - ps1
Archaea1 - ps2
Archaea2 - ps3
Archaea3 - ps4
Asgard/Eukaryota - ps5
Eukaryota - ps6
Amorpheae - ps7
Obazoa - ps8
Opisthokonta - ps9
Holozoa - ps10
Filozoa - ps11
Choano./Metazoa - ps12
Metazoa - ps13
Myriazoa - ps14
Parahoxozoa - ps15
Bilateria - ps16
Deuterostomia - ps17
Chordata - ps18
Olfactores - ps19
Vertebrata - ps20
Gnathostomata - ps21
Euteleostomi - ps22
Sarcopterygii - ps23
Tetrapoda - ps24
Amniota - ps25
Mammalia - ps26
Theria - ps27
Placentalia - ps28
Boreoeutheria - ps29
Euarchontoglires - ps30
Euarchonta - ps31
Primates - ps32
Haplorrhini - ps33
Simiiformes - ps34
Catarrhini - ps35
Hominoidea - ps36
Hominidae - ps37
Homininae - ps38
Hominini - ps39
Homo_sapiens - ps40
Phylostratum

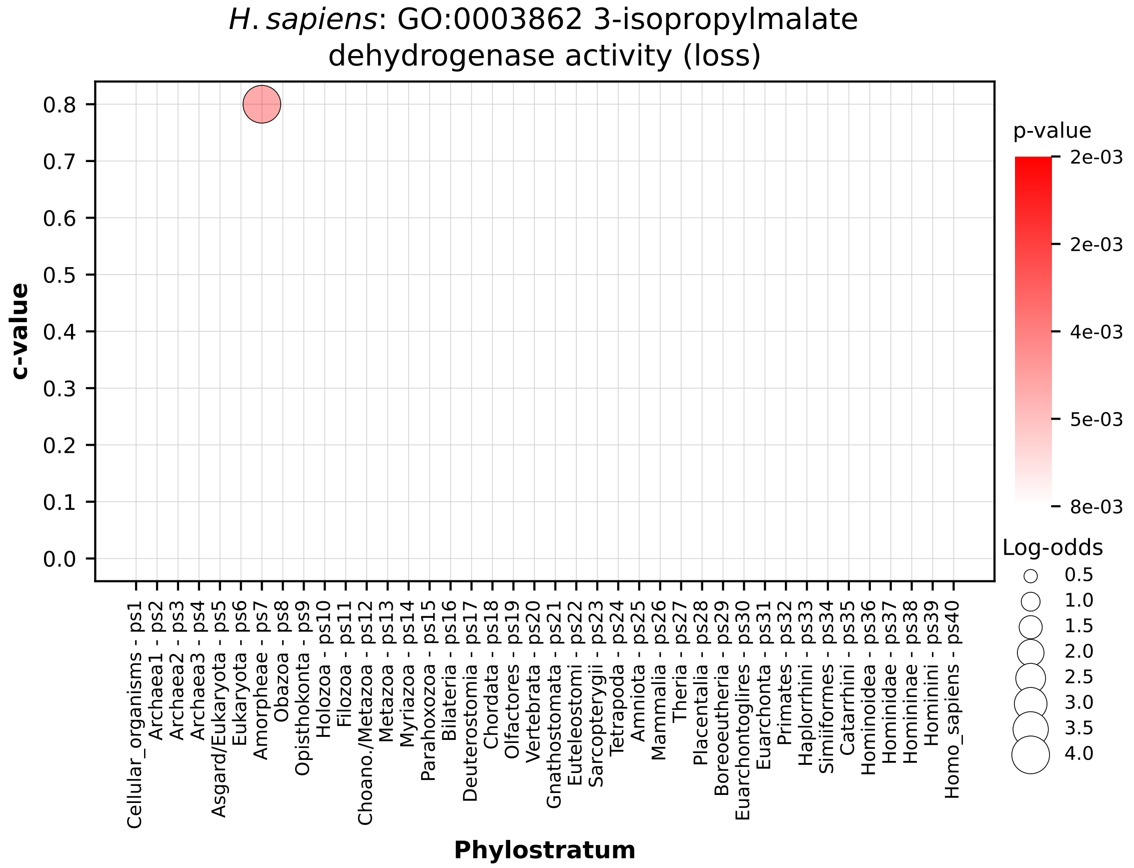
H. sapiens: GO:0003862 3-isopropylmalate dehydrogenase activity (loss)
c-value
0.8
0.7
0.6
0.5
0.4
0.3
0.2
0.1
0.0
p-value
2e-03
2e-03
4e-03
5e-03
8e-03
Log-odds
0.5
1.0
1.5
2.0
2.5
3.0
3.5
4.0
Cellular_organisms - ps1
Archaea1 - ps2
Archaea2 - ps3
Archaea3 - ps4
Asgard/Eukaryota - ps5
Eukaryota - ps6
Amorpheae - ps7
Obazoa - ps8
Opisthokonta - ps9
Holozoa - ps10
Filozoa - ps11
Choano./Metazoa - ps12
Metazoa - ps13
Myriazoa - ps14
Parahoxozoa - ps15
Bilateria - ps16
Deuterostomia - ps17
Chordata - ps18
Olfactores - ps19
Vertebrata - ps20
Gnathostomata - ps21
Euteleostomi - ps22
Sarcopterygii - ps23
Tetrapoda - ps24
Amniota - ps25
Mammalia - ps26
Theria - ps27
Placentalia - ps28
Boreoeutheria - ps29
Euarchontoglires - ps30
Euarchonta - ps31
Primates - ps32
Haplorrhini - ps33
Simiiformes - ps34
Catarrhini - ps35
Hominoidea - ps36
Hominidae - ps37
Homininae - ps38
Hominini - ps39
Homo_sapiens - ps40
Phylostratum

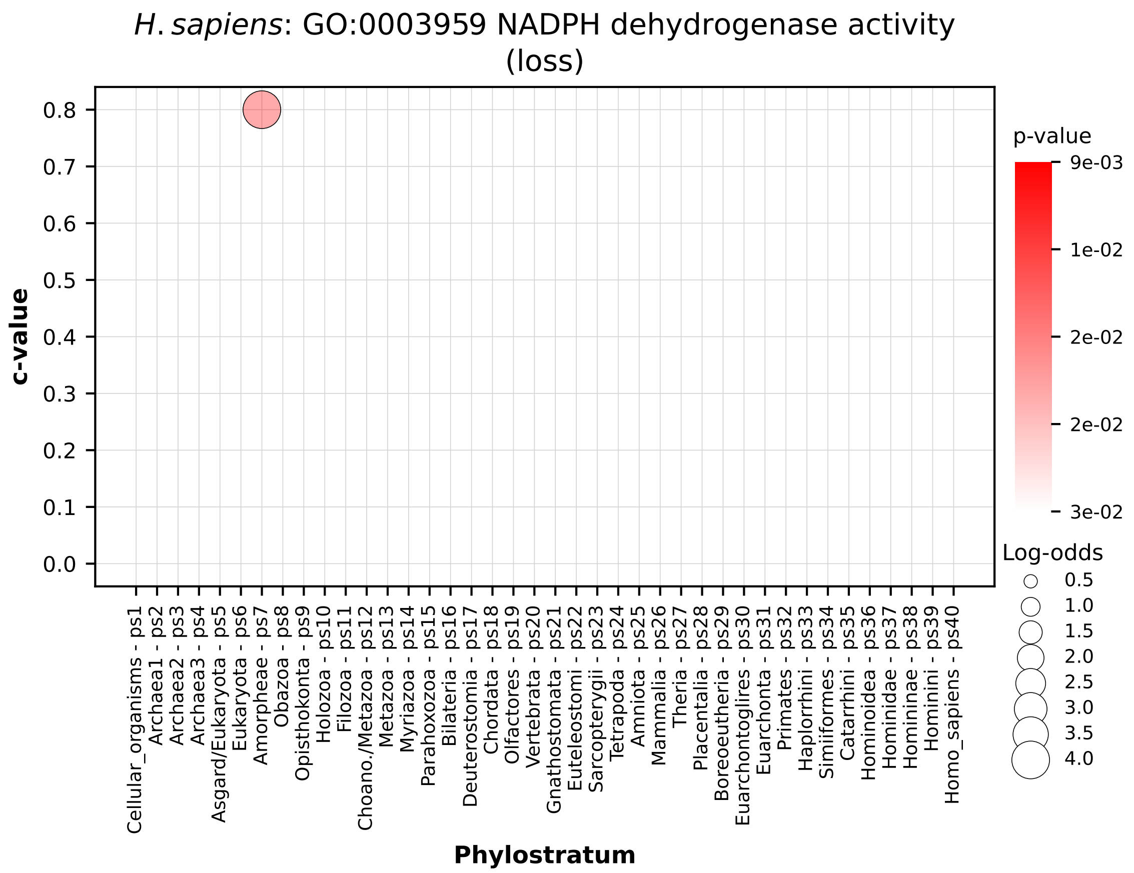

H. sapiens: GO:0003959 NADPH dehydrogenase activity
(loss)
c-value
0.8
0.7
0.6
0.5
0.4
0.3
0.2
0.1
0.0
p-value
9e-03
1e-02
2e-02
2e-02
3e-02
Log-odds
0.5
1.0
1.5
2.0
2.5
3.0
3.5
4.0
Cellular_organisms - ps1
Archaea1 - ps2
Archaea2 - ps3
Archaea3 - ps4
Asgard/Eukaryota - ps5
Eukaryota - ps6
Amorpheae - ps7
Obazoa - ps8
Opisthokonta - ps9
Holozoa - ps10
Filozoa - ps11
Choano./Metazoa - ps12
Metazoa - ps13
Myriazoa - ps14
Parahoxozoa - ps15
Bilateria - ps16
Deuterostomia - ps17
Chordata - ps18
Olfactores - ps19
Vertebrata - ps20
Gnathostomata - ps21
Euteleostomi - ps22
Sarcopterygii - ps23
Tetrapoda - ps24
Amniota - ps25
Mammalia - ps26
Theria - ps27
Placentalia - ps28
Boreoeutheria - ps29
Euarchontoglires - ps30
Euarchonta - ps31
Primates - ps32
Haplorrhini - ps33
Simiiformes - ps34
Catarrhini - ps35
Hominoidea - ps36
Hominidae - ps37
Homininae - ps38
Hominini - ps39
Homo_sapiens - ps40
Phylostratum

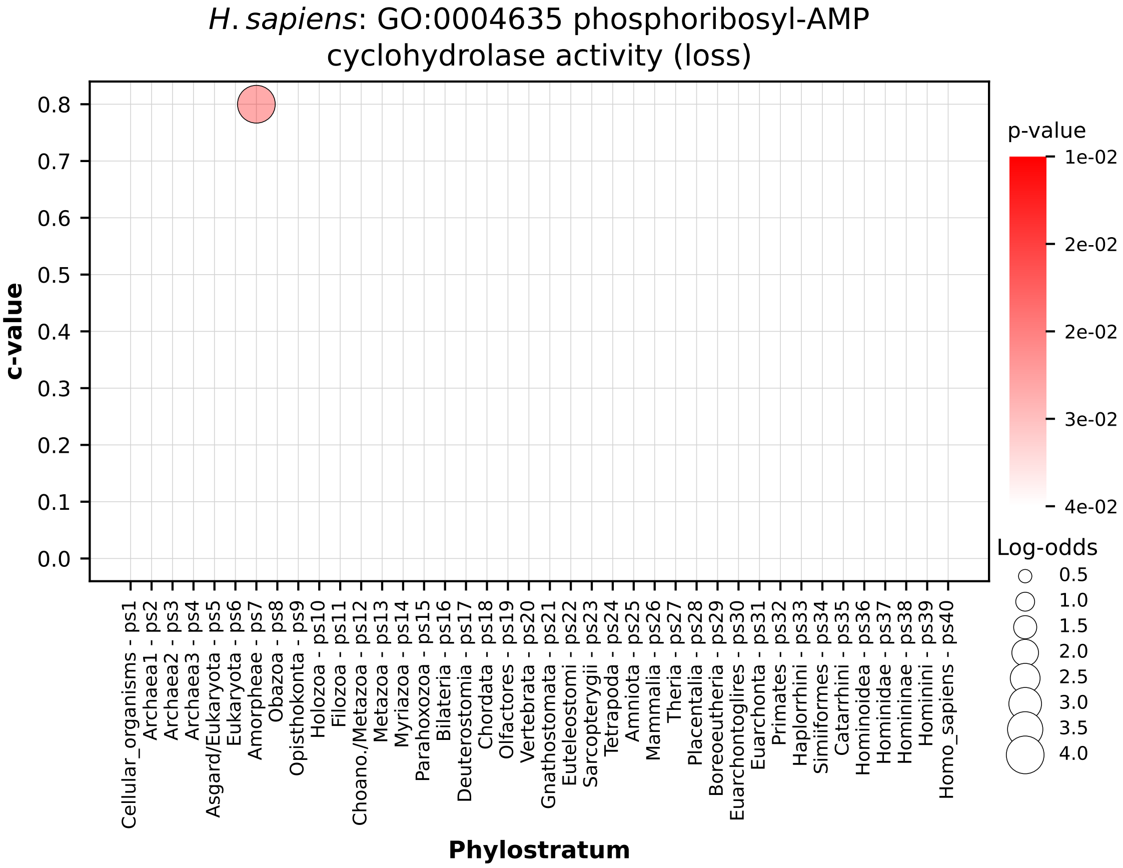

H. sapiens: GO:0004635 phosphoribosyl-AMP cyclohydrolase activity (loss)
c-value
0.0
0.1
0.2
0.3
0.4
0.5
0.6
0.7
0.8
p-value
1e-02
2e-02
2e-02
3e-02
4e-02
Log-odds
0.5
1.0
1.5
2.0
2.5
3.0
3.5
4.0
Cellular_organisms - ps1
Archaea1 - ps2
Archaea2 - ps3
Archaea3 - ps4
Asgard/Eukaryota - ps5
Eukaryota - ps6
Amorpheae - ps7
Obazoa - ps8
Opisthokonta - ps9
Holozoa - ps10
Filozoa - ps11
Choano./Metazoa - ps12
Metazoa - ps13
Myriazoa - ps14
Parahoxozoa - ps15
Bilateria - ps16
Deuterostomia - ps17
Chordata - ps18
Olfactores - ps19
Vertebrata - ps20
Gnathostomata - ps21
Euteleostomi - ps22
Sarcopterygii - ps23
Tetrapoda - ps24
Amniota - ps25
Mammalia - ps26
Theria - ps27
Placentalia - ps28
Boreoeutheria - ps29
Euarchontoglires - ps30
Euarchonta - ps31
Primates - ps32
Haplorrhini - ps33
Simiiformes - ps34
Catarrhini - ps35
Hominoidea - ps36
Hominidae - ps37
Homininae - ps38
Hominini - ps39
Homo_sapiens - ps40
Phylostratum

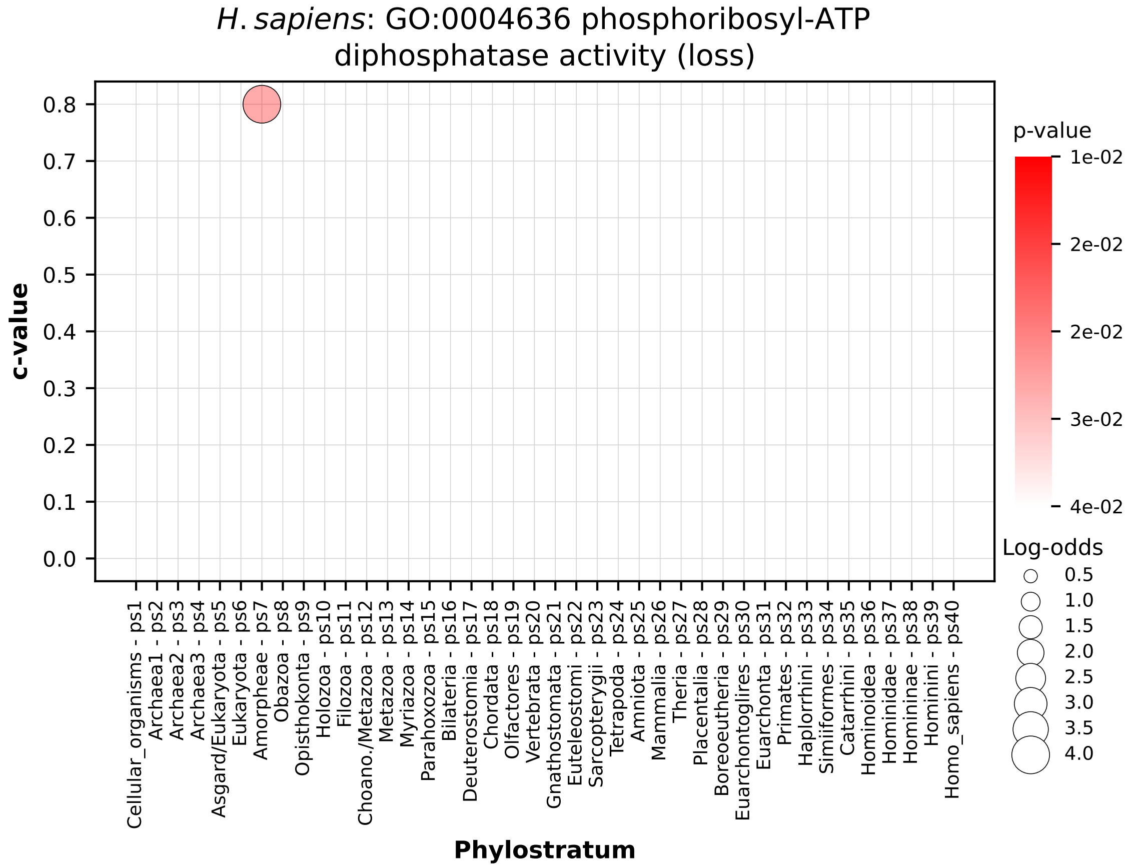

H. sapiens: GO:0004636 phosphoribosyl-ATP diphosphatase activity (loss)
c-value
0.0
0.1
0.2
0.3
0.4
0.5
0.6
0.7
0.8
p-value
1e-02
2e-02
2e-02
3e-02
4e-02
Log-odds
0.5
1.0
1.5
2.0
2.5
3.0
3.5
4.0
Cellular_organisms - ps1
Archaea1 - ps2
Archaea2 - ps3
Archaea3 - ps4
Asgard/Eukaryota - ps5
Eukaryota - ps6
Amorpheae - ps7
Obazoa - ps8
Opisthokonta - ps9
Holozoa - ps10
Filozoa - ps11
Choano./Metazoa - ps12
Metazoa - ps13
Myriazoa - ps14
Parahoxozoa - ps15
Bilateria - ps16
Deuterostomia - ps17
Chordata - ps18
Olfactores - ps19
Vertebrata - ps20
Gnathostomata - ps21
Euteleostomi - ps22
Sarcopterygii - ps23
Tetrapoda - ps24
Amniota - ps25
Mammalia - ps26
Theria - ps27
Placentalia - ps28
Boreoeutheria - ps29
Euarchontoglires - ps30
Euarchonta - ps31
Primates - ps32
Haplorrhini - ps33
Simiiformes - ps34
Catarrhini - ps35
Hominoidea - ps36
Hominidae - ps37
Homininae - ps38
Hominini - ps39
Homo_sapiens - ps40
Phylostratum

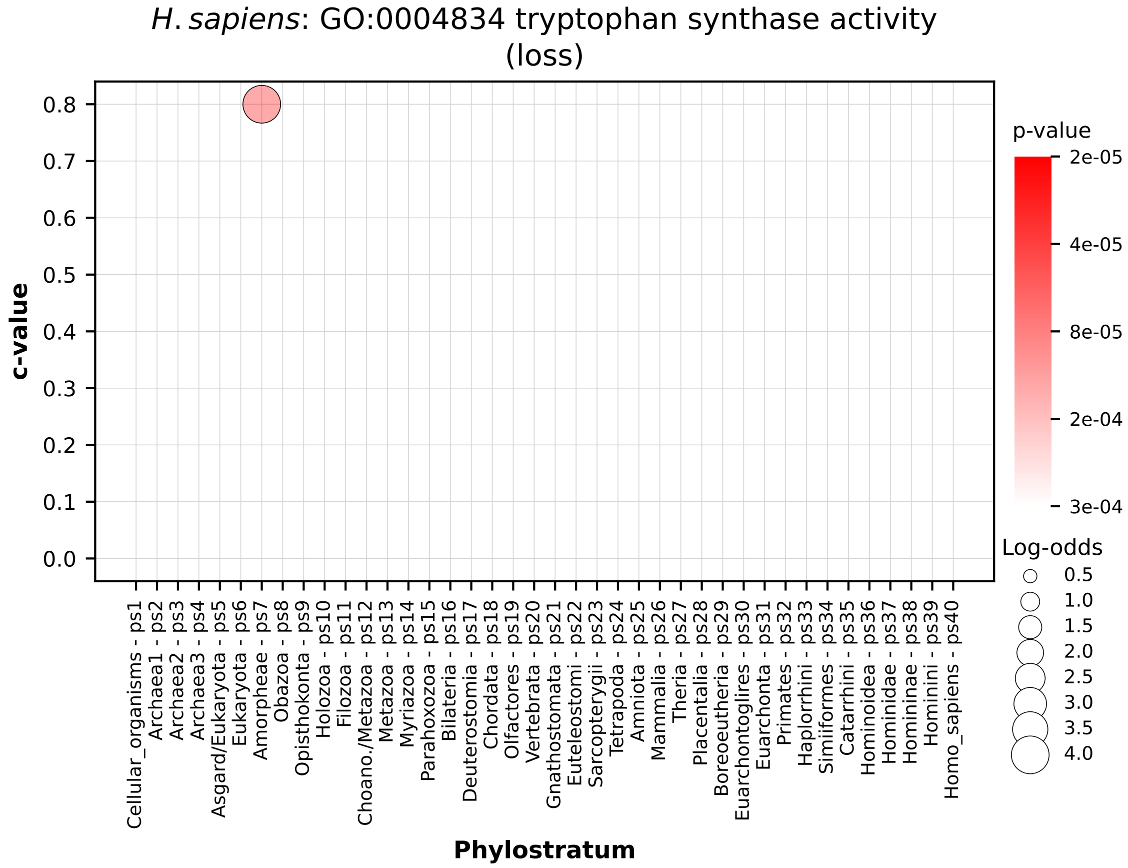

H. sapiens: GO:0004834 tryptophan synthase activity
(loss)
c-value
0.8
0.7
0.6
0.5
0.4
0.3
0.2
0.1
0.0
Phylostratum
Cellular_organisms - ps1
Archaea1 - ps2
Archaea2 - ps3
Archaea3 - ps4
Asgard/Eukaryota - ps5
Eukaryota - ps6
Amorpheae - ps7
Obazoa - ps8
Opisthokonta - ps9
Holozoa - ps10
Filozoa - ps11
Choano./Metazoa - ps12
Metazoa - ps13
Myriazoa - ps14
Parahoxozoa - ps15
Bilateria - ps16
Deuterostomia - ps17
Chordata - ps18
Olfactores - ps19
Vertebrata - ps20
Gnathostomata - ps21
Euteleostomi - ps22
Sarcopterygii - ps23
Tetrapoda - ps24
Amniota - ps25
Mammalia - ps26
Theria - ps27
Placentalia - ps28
Boreoeutheria - ps29
Euarchontoglires - ps30
Euarchonta - ps31
Primates - ps32
Haplorrhini - ps33
Simiiformes - ps34
Catarrhini - ps35
Hominoidea - ps36
Hominidae - ps37
Homininae - ps38
Hominini - ps39
Homo_sapiens - ps40
p-value
2e-05
4e-05
8e-05
2e-04
3e-04
Log-odds
0.5
1.0
1.5
2.0
2.5
3.0
3.5
4.0

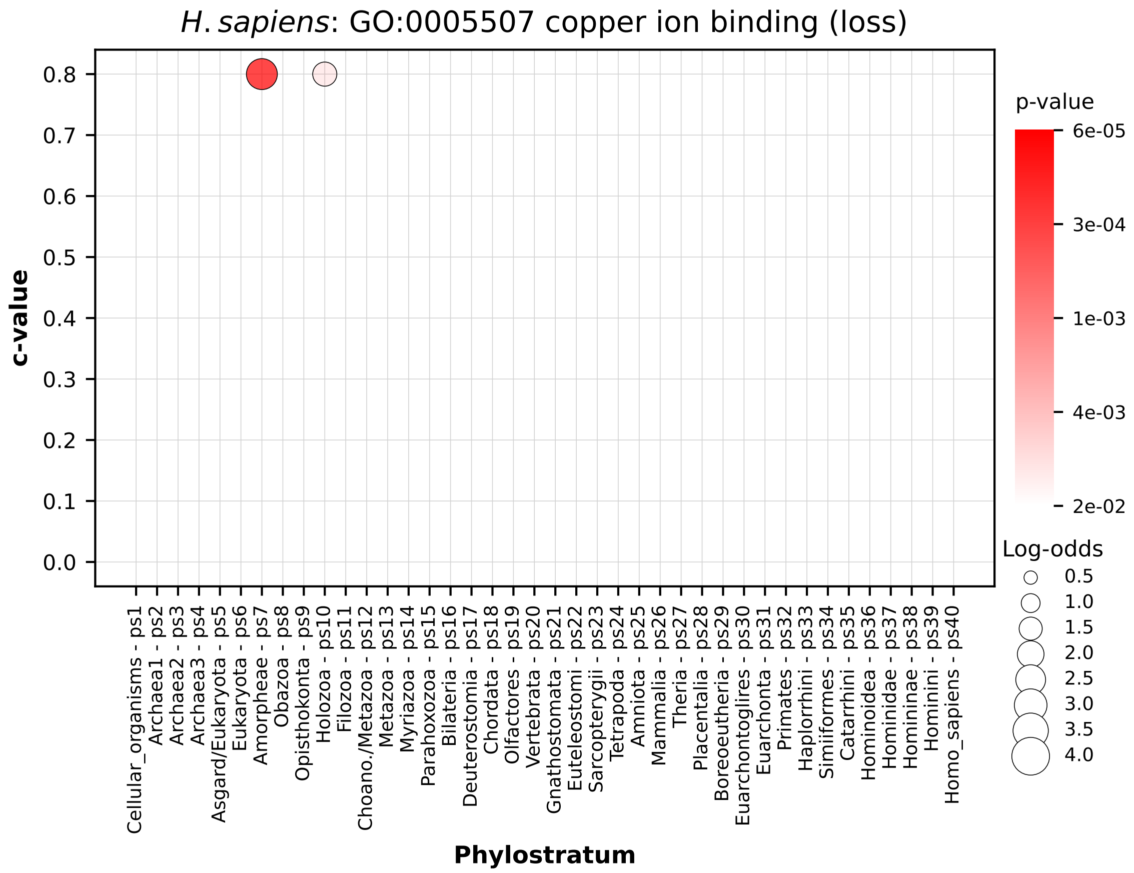

H. sapiens: GO:0005507 copper ion binding (loss)
c-value
0.0
0.1
0.2
0.3
0.4
0.5
0.6
0.7
0.8
Phylostratum
Cellular_organisms - ps1
Archaea1 - ps2
Archaea2 - ps3
Archaea3 - ps4
Asgard/Eukaryota - ps5
Eukaryota - ps6
Amorpheae - ps7
Obazoa - ps8
Opisthokonta - ps9
Holozoa - ps10
Filozoa - ps11
Choano./Metazoa - ps12
Metazoa - ps13
Myriazoa - ps14
Parahoxozoa - ps15
Bilateria - ps16
Deuterostomia - ps17
Chordata - ps18
Olfactores - ps19
Vertebrata - ps20
Gnathostomata - ps21
Euteleostomi - ps22
Sarcopterygii - ps23
Tetrapoda - ps24
Amniota - ps25
Mammalia - ps26
Theria - ps27
Placentalia - ps28
Boreoeutheria - ps29
Euarchontoglires - ps30
Euarchonta - ps31
Primates - ps32
Haplorrhini - ps33
Simiiformes - ps34
Catarrhini - ps35
Hominoidea - ps36
Hominidae - ps37
Homininae - ps38
Hominini - ps39
Homo_sapiens - ps40
p-value
6e-05
3e-04
1e-03
4e-03
2e-02
Log-odds
0.5
1.0
1.5
2.0
2.5
3.0
3.5
4.0

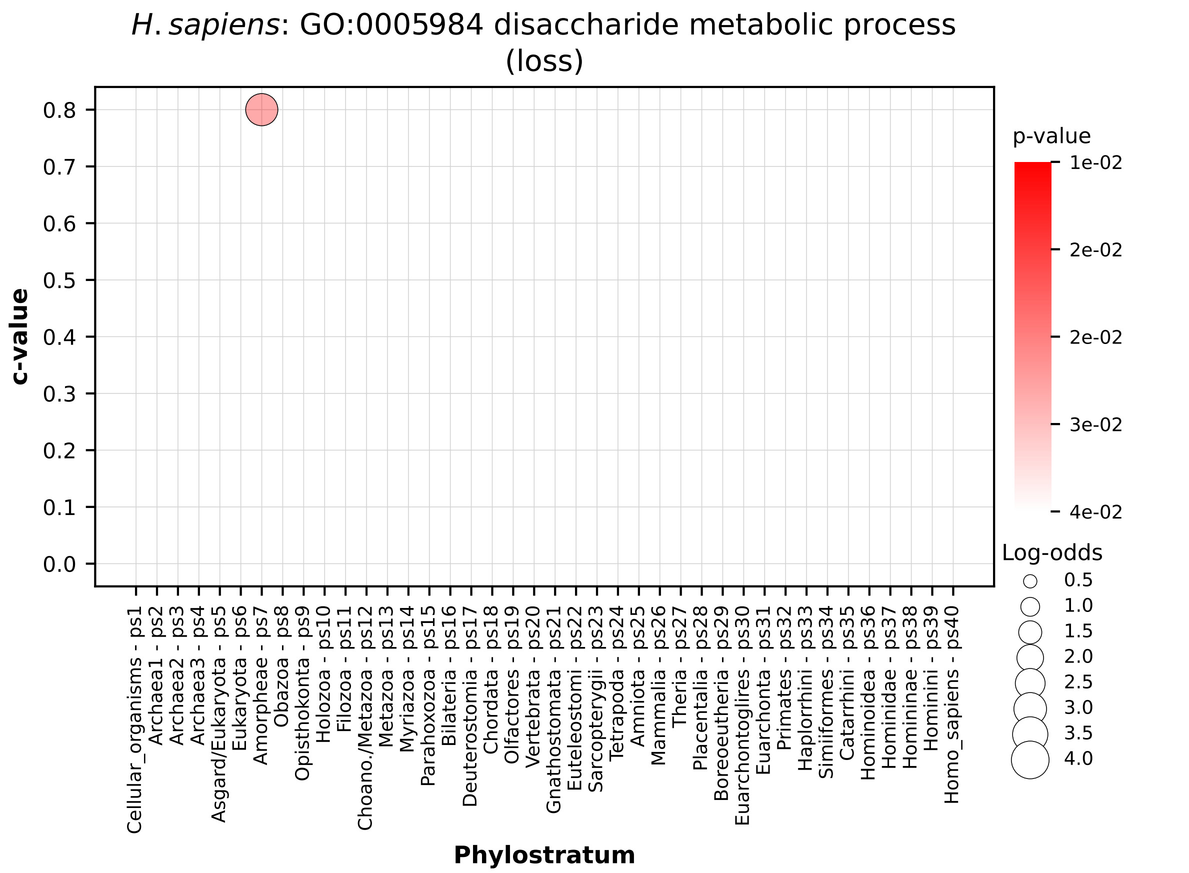

H. sapiens: GO:0005984 disaccharide metabolic process
(loss)
c-value
0.8
0.7
0.6
0.5
0.4
0.3
0.2
0.1
0.0
p-value
1e-02
2e-02
2e-02
3e-02
4e-02
Log-odds
0.5
1.0
1.5
2.0
2.5
3.0
3.5
4.0
Cellular_organisms - ps1
Archaea1 - ps2
Archaea2 - ps3
Archaea3 - ps4
Asgard/Eukaryota - ps5
Eukaryota - ps6
Amorpheae - ps7
Obazoa - ps8
Opisthokonta - ps9
Holozoa - ps10
Filozoa - ps11
Choano./Metazoa - ps12
Metazoa - ps13
Myriazoa - ps14
Parahoxozoa - ps15
Bilateria - ps16
Deuterostomia - ps17
Chordata - ps18
Olfactores - ps19
Vertebrata - ps20
Gnathostomata - ps21
Euteleostomi - ps22
Sarcopterygii - ps23
Tetrapoda - ps24
Amniota - ps25
Mammalia - ps26
Theria - ps27
Placentalia - ps28
Boreoeutheria - ps29
Euarchontoglires - ps30
Euarchonta - ps31
Primates - ps32
Haplorrhini - ps33
Simiiformes - ps34
Catarrhini - ps35
Hominoidea - ps36
Hominidae - ps37
Homininae - ps38
Hominini - ps39
Homo_sapiens - ps40
Phylostratum

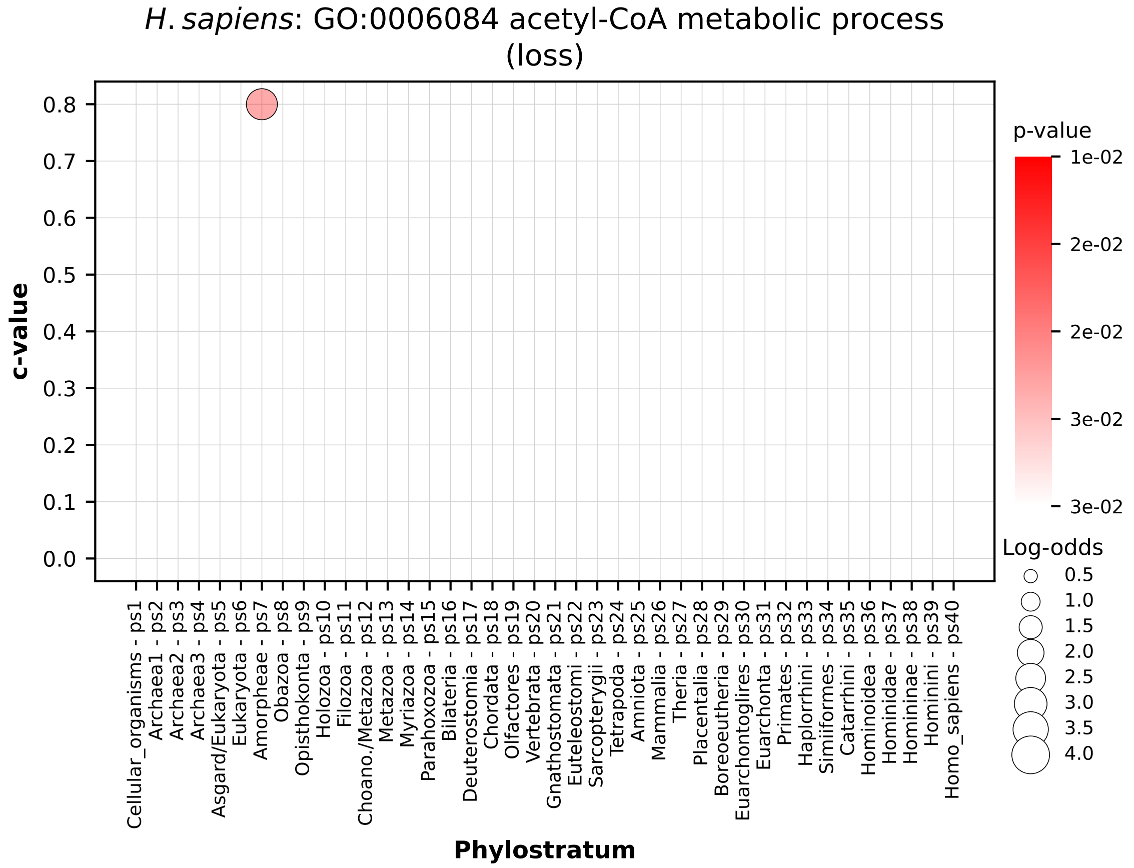

H. sapiens: GO:0006084 acetyl-CoA metabolic process (loss)
c-value
0.8
0.7
0.6
0.5
0.4
0.3
0.2
0.1
0.0
p-value
1e-02
2e-02
2e-02
3e-02
3e-02
Log-odds
0.5
1.0
1.5
2.0
2.5
3.0
3.5
4.0
Cellular_organisms - ps1
Archaea1 - ps2
Archaea2 - ps3
Archaea3 - ps4
Asgard/Eukaryota - ps5
Eukaryota - ps6
Amorpheae - ps7
Obazoa - ps8
Opisthokonta - ps9
Holozoa - ps10
Filozoa - ps11
Choano./Metazoa - ps12
Metazoa - ps13
Myriazoa - ps14
Parahoxozoa - ps15
Bilateria - ps16
Deuterostomia - ps17
Chordata - ps18
Olfactores - ps19
Vertebrata - ps20
Gnathostomata - ps21
Euteleostomi - ps22
Sarcopterygii - ps23
Tetrapoda - ps24
Amniota - ps25
Mammalia - ps26
Theria - ps27
Placentalia - ps28
Boreoeutheria - ps29
Euarchontoglires - ps30
Euarchonta - ps31
Primates - ps32
Haplorrhini - ps33
Simiiformes - ps34
Catarrhini - ps35
Hominoidea - ps36
Hominidae - ps37
Homininae - ps38
Hominini - ps39
Homo_sapiens - ps40
Phylostratum

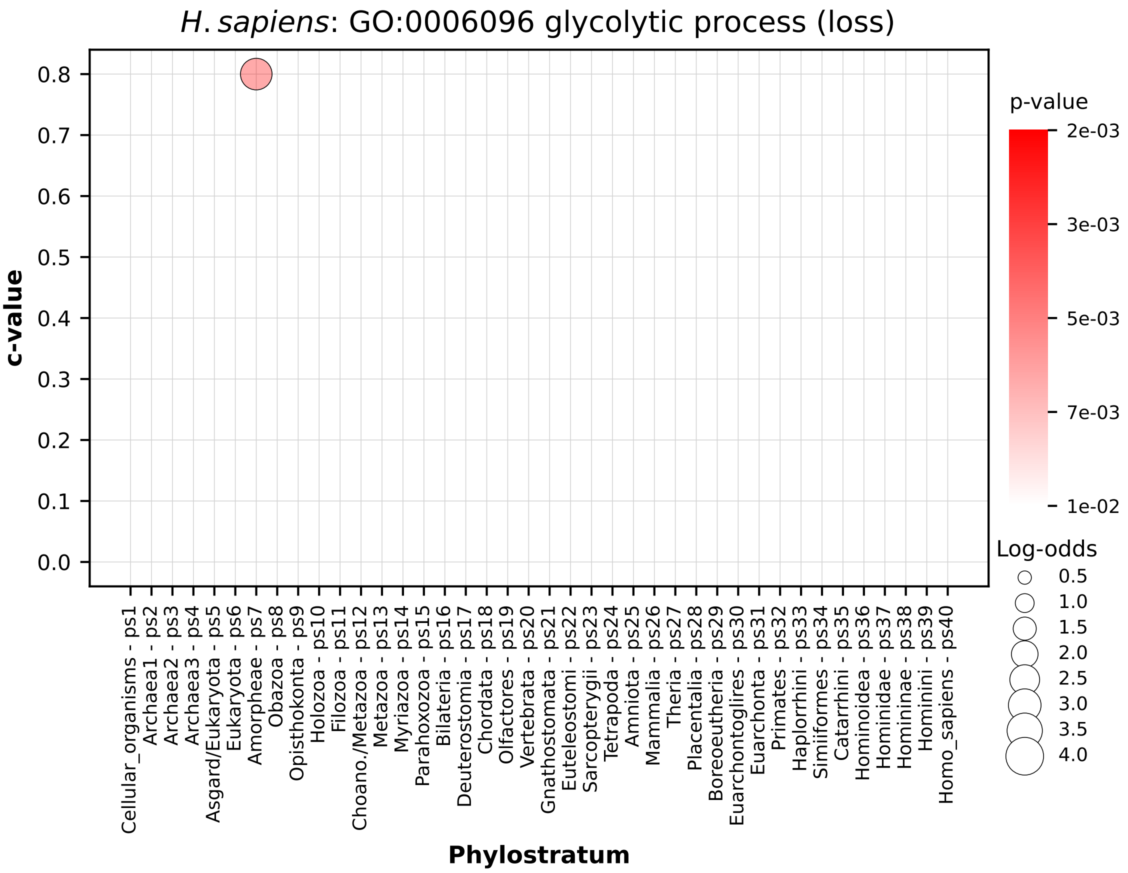

H. sapiens: GO:0006096 glycolytic process (loss)
c-value
0.8
0.7
0.6
0.5
0.4
0.3
0.2
0.1
0.0
p-value
2e-03
3e-03
5e-03
7e-03
1e-02
Log-odds
0.5
1.0
1.5
2.0
2.5
3.0
3.5
4.0
Cellular_organisms - ps1
Archaea1 - ps2
Archaea2 - ps3
Archaea3 - ps4
Asgard/Eukaryota - ps5
Eukaryota - ps6
Amorpheae - ps7
Obazoa - ps8
Opisthokonta - ps9
Holozoa - ps10
Filozoa - ps11
Choano./Metazoa - ps12
Metazoa - ps13
Myriazoa - ps14
Parahoxozoa - ps15
Bilateria - ps16
Deuterostomia - ps17
Chordata - ps18
Olfactores - ps19
Vertebrata - ps20
Gnathostomata - ps21
Euteleostomi - ps22
Sarcopterygii - ps23
Tetrapoda - ps24
Amniota - ps25
Mammalia - ps26
Theria - ps27
Placentalia - ps28
Boreoeutheria - ps29
Euarchontoglires - ps30
Euarchonta - ps31
Primates - ps32
Haplorrhini - ps33
Simiiformes - ps34
Catarrhini - ps35
Hominoidea - ps36
Hominidae - ps37
Homininae - ps38
Hominini - ps39
Homo_sapiens - ps40
Phylostratum

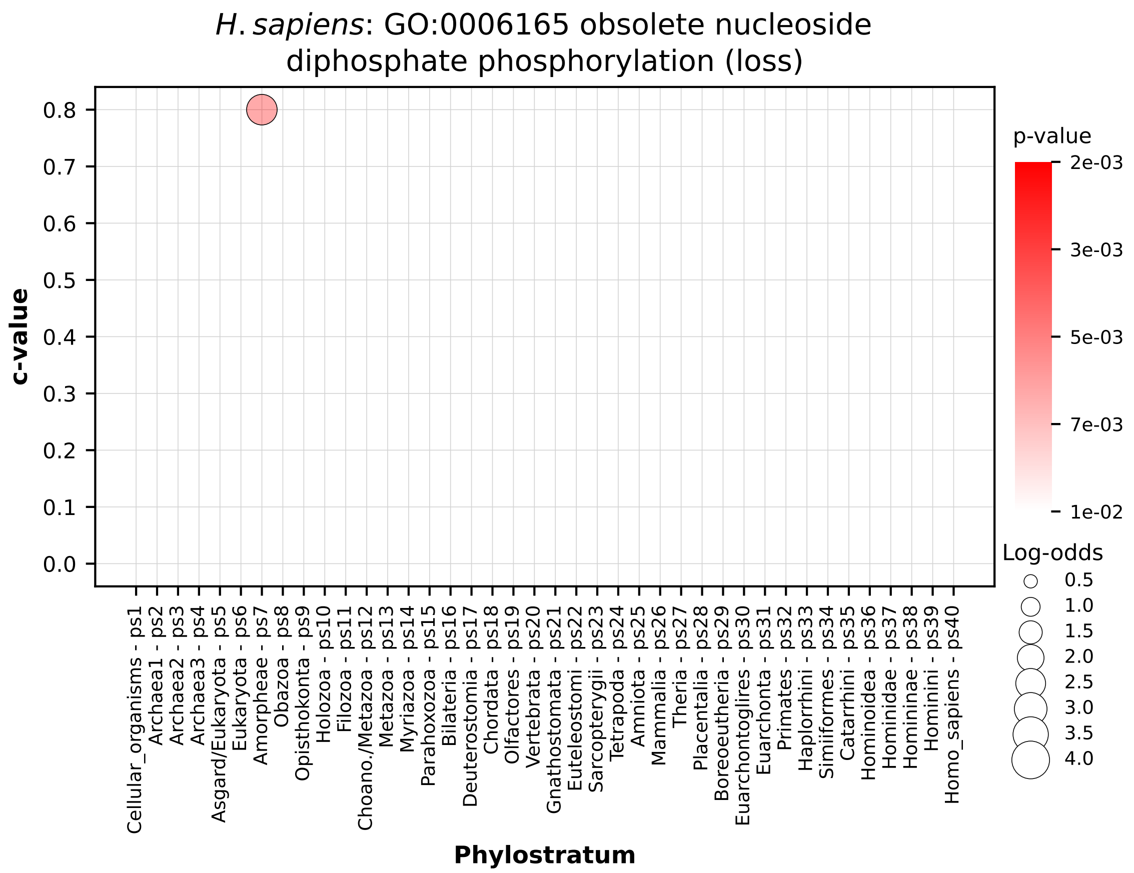
H. sapiens: GO:0006165 obsolete nucleoside diphosphate phosphorylation (loss)
c-value
0.0
0.1
0.2
0.3
0.4
0.5
0.6
0.7
0.8
Phylostratum
Cellular_organisms - ps1
Archaea1 - ps2
Archaea2 - ps3
Archaea3 - ps4
Asgard/Eukaryota - ps5
Eukaryota - ps6
Amorpheae - ps7
Obazoa - ps8
Opisthokonta - ps9
Holozoa - ps10
Filozoa - ps11
Choano./Metazoa - ps12
Metazoa - ps13
Myriazoa - ps14
Parahoxozoa - ps15
Bilateria - ps16
Deuterostomia - ps17
Chordata - ps18
Olfactores - ps19
Vertebrata - ps20
Gnathostomata - ps21
Euteleostomi - ps22
Sarcopterygii - ps23
Tetrapoda - ps24
Amniota - ps25
Mammalia - ps26
Theria - ps27
Placentalia - ps28
Boreoeutheria - ps29
Euarchontoglires - ps30
Euarchonta - ps31
Primates - ps32
Haplorrhini - ps33
Simiiformes - ps34
Catarrhini - ps35
Hominoidea - ps36
Hominidae - ps37
Homininae - ps38
Hominini - ps39
Homo_sapiens - ps40
p-value
2e-03
3e-03
5e-03
7e-03
1e-02
Log-odds
0.5
1.0
1.5
2.0
2.5
3.0
3.5
4.0

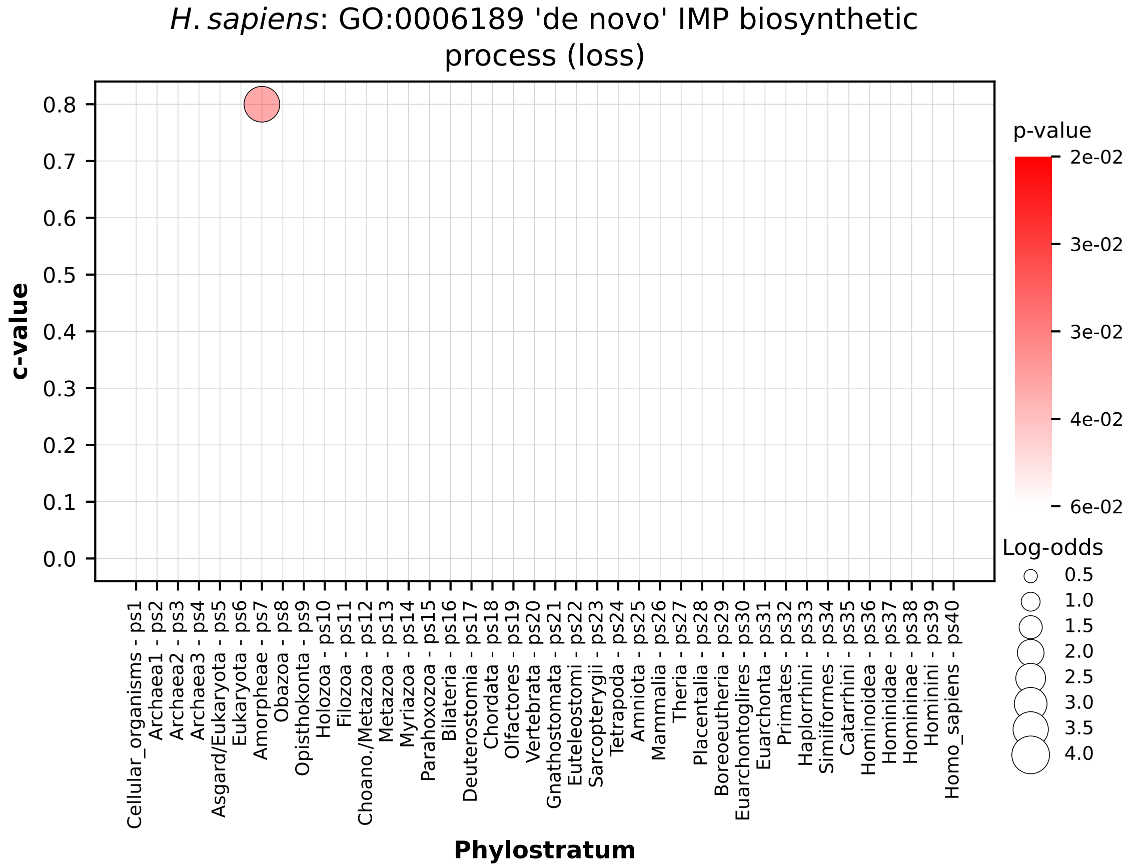
H. sapiens: GO:0006189 'de novo' IMP biosynthetic process (loss)
c-value
0.0
0.1
0.2
0.3
0.4
0.5
0.6
0.7
0.8
p-value
2e-02
3e-02
3e-02
4e-02
6e-02
Log-odds
0.5
1.0
1.5
2.0
2.5
3.0
3.5
4.0
Cellular_organisms - ps1
Archaea1 - ps2
Archaea2 - ps3
Archaea3 - ps4
Asgard/Eukaryota - ps5
Eukaryota - ps6
Amorpheae - ps7
Obazoa - ps8
Opisthokonta - ps9
Holozoa - ps10
Filozoa - ps11
Choano./Metazoa - ps12
Metazoa - ps13
Myriazoa - ps14
Parahoxozoa - ps15
Bilateria - ps16
Deuterostomia - ps17
Chordata - ps18
Olfactores - ps19
Vertebrata - ps20
Gnathostomata - ps21
Euteleostomi - ps22
Sarcopterygii - ps23
Tetrapoda - ps24
Amniota - ps25
Mammalia - ps26
Theria - ps27
Placentalia - ps28
Boreoeutheria - ps29
Euarchontoglires - ps30
Euarchonta - ps31
Primates - ps32
Haplorrhini - ps33
Simiiformes - ps34
Catarrhini - ps35
Hominoidea - ps36
Hominidae - ps37
Homininae - ps38
Hominini - ps39
Homo_sapiens - ps40
Phylostratum

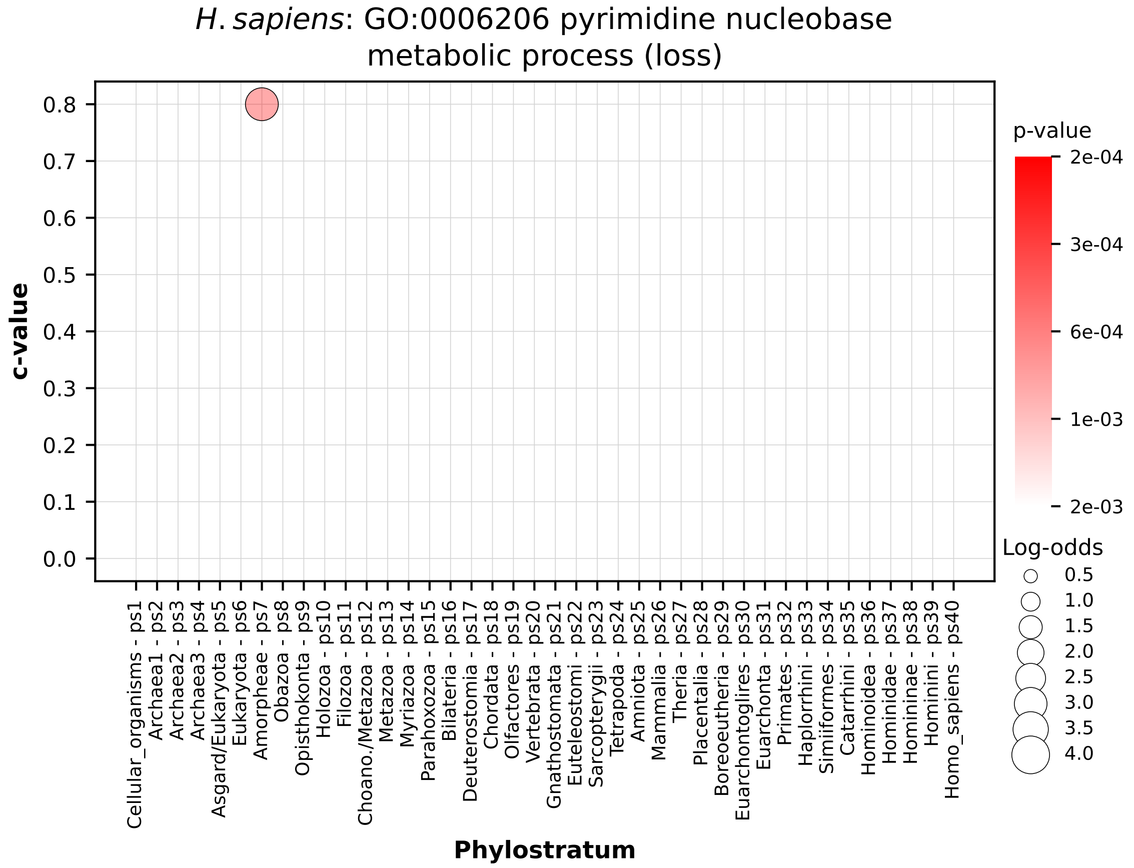
H. sapiens: GO:0006206 pyrimidine nucleobase metabolic process (loss)
c-value
0.0
0.1
0.2
0.3
0.4
0.5
0.6
0.7
0.8
Phylostratum
Cellular_organisms - ps1
Archaea1 - ps2
Archaea2 - ps3
Archaea3 - ps4
Asgard/Eukaryota - ps5
Eukaryota - ps6
Amorpheae - ps7
Obazoa - ps8
Opisthokonta - ps9
Holozoa - ps10
Filozoa - ps11
Choano./Metazoa - ps12
Metazoa - ps13
Myriazoa - ps14
Parahoxozoa - ps15
Bilateria - ps16
Deuterostomia - ps17
Chordata - ps18
Olfactores - ps19
Vertebrata - ps20
Gnathostomata - ps21
Euteleostomi - ps22
Sarcopterygii - ps23
Tetrapoda - ps24
Amniota - ps25
Mammalia - ps26
Theria - ps27
Placentalia - ps28
Boreoeutheria - ps29
Euarchontoglires - ps30
Euarchonta - ps31
Primates - ps32
Haplorrhini - ps33
Simiiformes - ps34
Catarrhini - ps35
Hominoidea - ps36
Hominidae - ps37
Homininae - ps38
Hominini - ps39
Homo_sapiens - ps40
p-value
2e-04
3e-04
6e-04
1e-03
2e-03
Log-odds
0.5
1.0
1.5
2.0
2.5
3.0
3.5
4.0

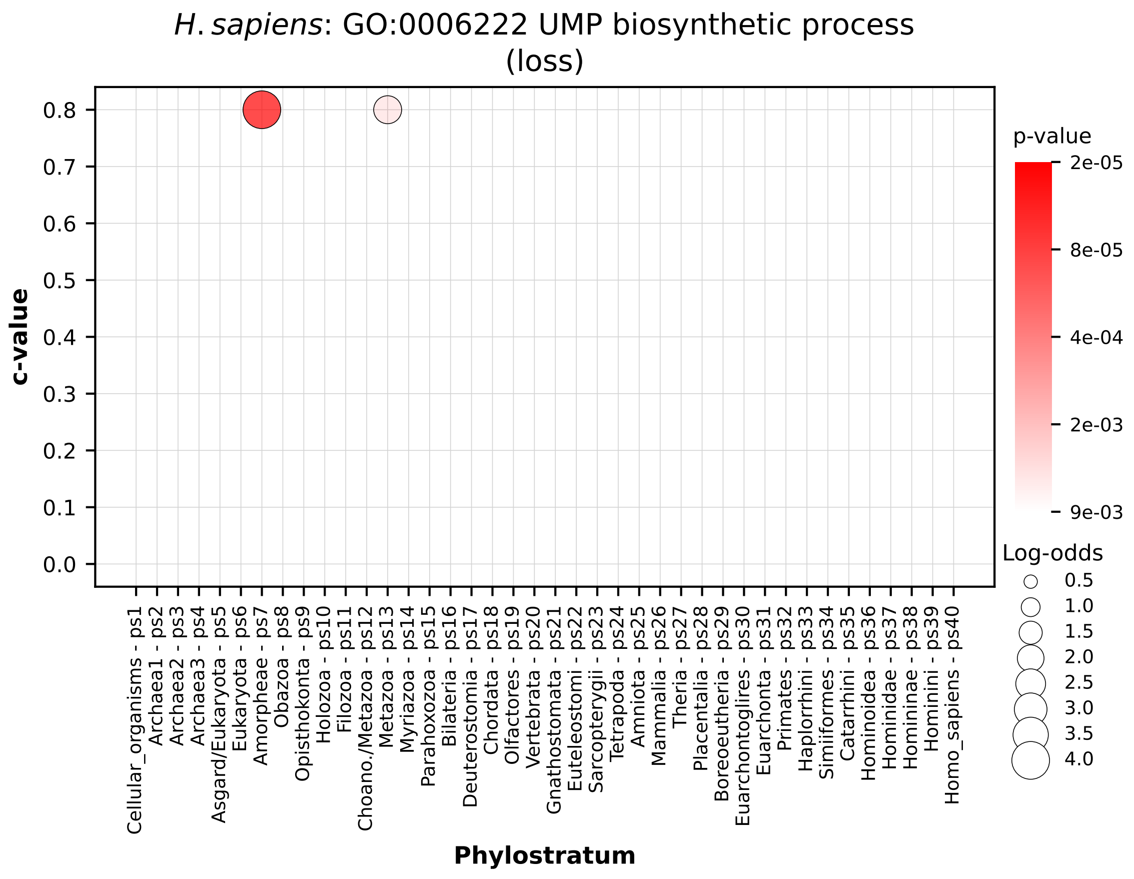
H. sapiens: GO:0006222 UMP biosynthetic process
(loss)
c-value
0.0
0.1
0.2
0.3
0.4
0.5
0.6
0.7
0.8
p-value
2e-05
8e-05
4e-04
2e-03
9e-03
Log-odds
0.5
1.0
1.5
2.0
2.5
3.0
3.5
4.0
Cellular_organisms - ps1
Archaea1 - ps2
Archaea2 - ps3
Archaea3 - ps4
Asgard/Eukaryota - ps5
Eukaryota - ps6
Amorpheae - ps7
Obazoa - ps8
Opisthokonta - ps9
Holozoa - ps10
Filozoa - ps11
Choano./Metazoa - ps12
Metazoa - ps13
Myriazoa - ps14
Parahoxozoa - ps15
Bilateria - ps16
Deuterostomia - ps17
Chordata - ps18
Olfactores - ps19
Vertebrata - ps20
Gnathostomata - ps21
Euteleostomi - ps22
Sarcopterygii - ps23
Tetrapoda - ps24
Amniota - ps25
Mammalia - ps26
Theria - ps27
Placentalia - ps28
Boreoeutheria - ps29
Euarchontoglires - ps30
Euarchonta - ps31
Primates - ps32
Haplorrhini - ps33
Simiiformes - ps34
Catarrhini - ps35
Hominoidea - ps36
Hominidae - ps37
Homininae - ps38
Hominini - ps39
Homo_sapiens - ps40
Phylostratum

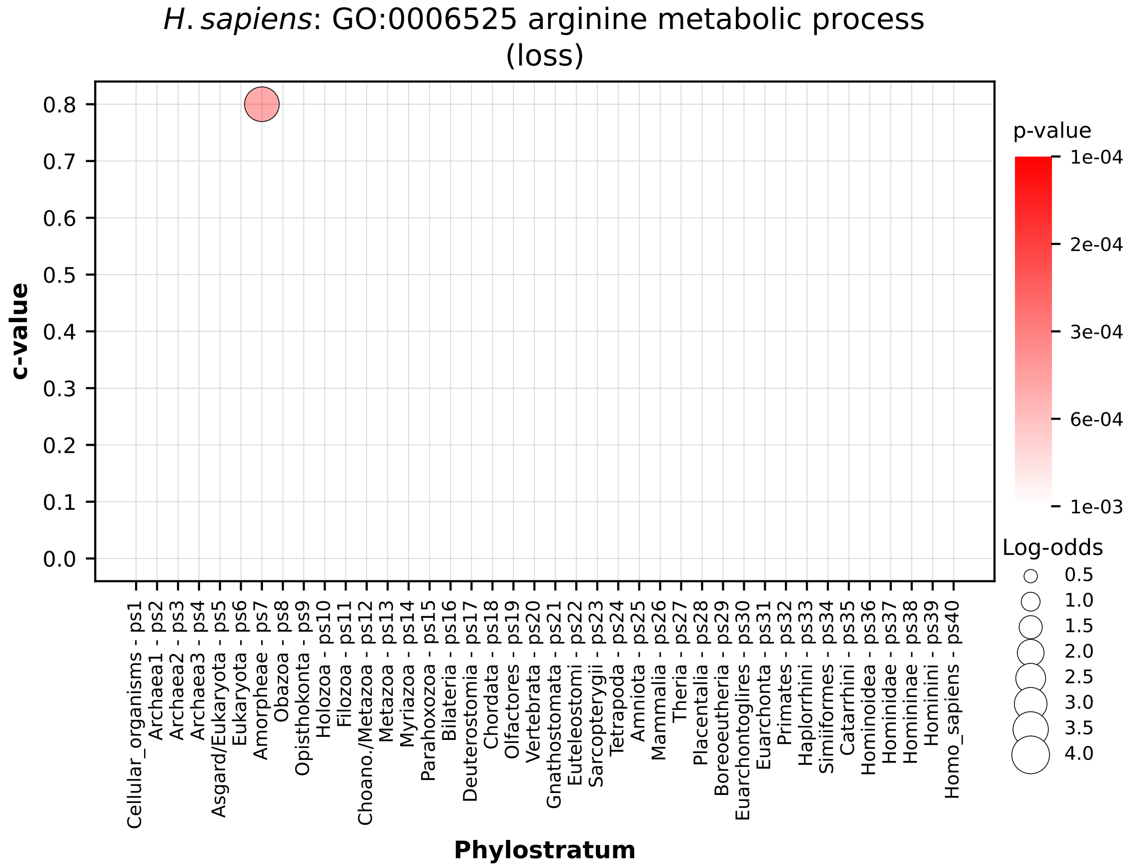

H. sapiens: GO:0006525 arginine metabolic process
(loss)
c-value
0.8
0.7
0.6
0.5
0.4
0.3
0.2
0.1
0.0
p-value
1e-04
2e-04
3e-04
6e-04
1e-03
Log-odds
0.5
1.0
1.5
2.0
2.5
3.0
3.5
4.0
Cellular_organisms - ps1
Archaea1 - ps2
Archaea2 - ps3
Archaea3 - ps4
Asgard/Eukaryota - ps5
Eukaryota - ps6
Amorpheae - ps7
Obazoa - ps8
Opisthokonta - ps9
Holozoa - ps10
Filozoa - ps11
Choano./Metazoa - ps12
Metazoa - ps13
Myriazoa - ps14
Parahoxozoa - ps15
Bilateria - ps16
Deuterostomia - ps17
Chordata - ps18
Olfactores - ps19
Vertebrata - ps20
Gnathostomata - ps21
Euteleostomi - ps22
Sarcopterygii - ps23
Tetrapoda - ps24
Amniota - ps25
Mammalia - ps26
Theria - ps27
Placentalia - ps28
Boreoeutheria - ps29
Euarchontoglires - ps30
Euarchonta - ps31
Primates - ps32
Haplorrhini - ps33
Simiiformes - ps34
Catarrhini - ps35
Hominoidea - ps36
Hominidae - ps37
Homininae - ps38
Hominini - ps39
Homo_sapiens - ps40
Phylostratum

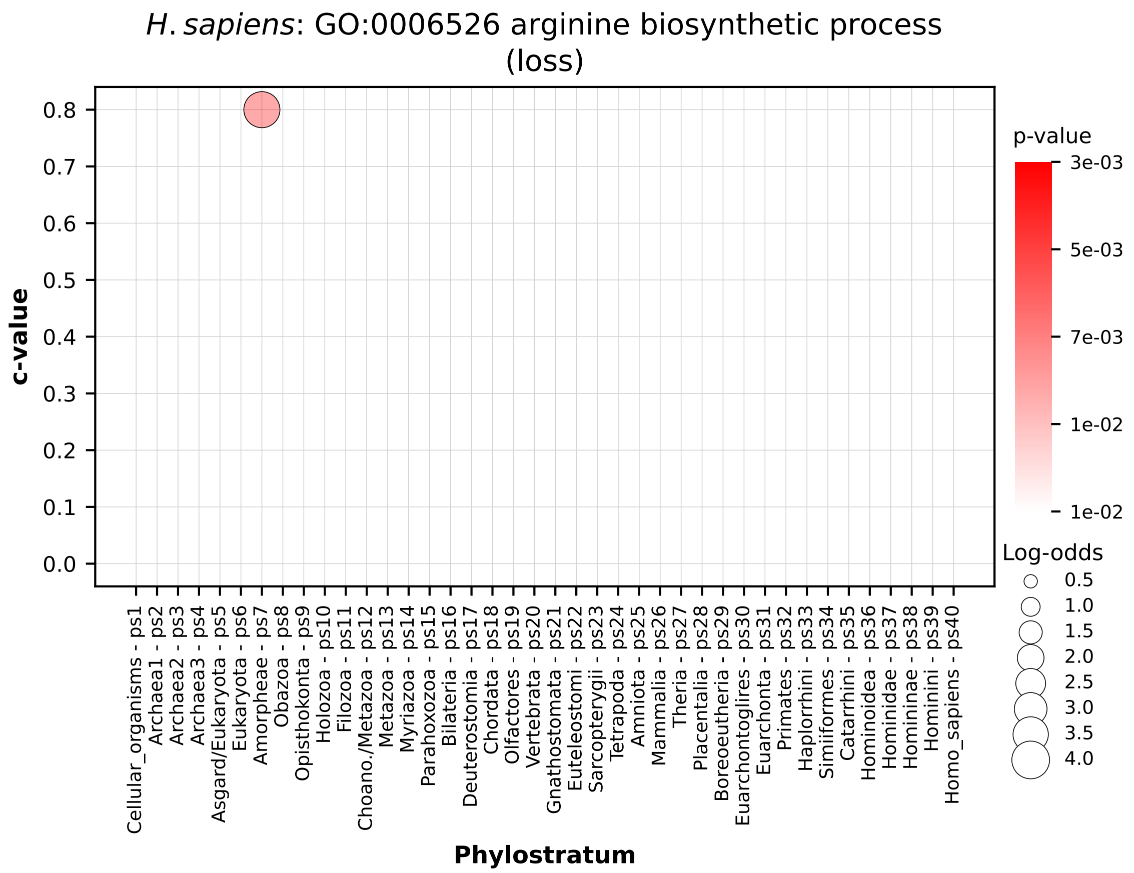

H. sapiens: GO:0006526 arginine biosynthetic process
(loss)
c-value
0.0
0.1
0.2
0.3
0.4
0.5
0.6
0.7
0.8
Phylostratum
Cellular_organisms - ps1
Archaea1 - ps2
Archaea2 - ps3
Archaea3 - ps4
Asgard/Eukaryota - ps5
Eukaryota - ps6
Amorpheae - ps7
Obazoa - ps8
Opisthokonta - ps9
Holozoa - ps10
Filozoa - ps11
Choano./Metazoa - ps12
Metazoa - ps13
Myriazoa - ps14
Parahoxozoa - ps15
Bilateria - ps16
Deuterostomia - ps17
Chordata - ps18
Olfactores - ps19
Vertebrata - ps20
Gnathostomata - ps21
Euteleostomi - ps22
Sarcopterygii - ps23
Tetrapoda - ps24
Amniota - ps25
Mammalia - ps26
Theria - ps27
Placentalia - ps28
Boreoeutheria - ps29
Euarchontoglires - ps30
Euarchonta - ps31
Primates - ps32
Haplorrhini - ps33
Simiiformes - ps34
Catarrhini - ps35
Hominoidea - ps36
Hominidae - ps37
Homininae - ps38
Hominini - ps39
Homo_sapiens - ps40
p-value
3e-03
5e-03
7e-03
1e-02
1e-02
Log-odds
0.5
1.0
1.5
2.0
2.5
3.0
3.5
4.0

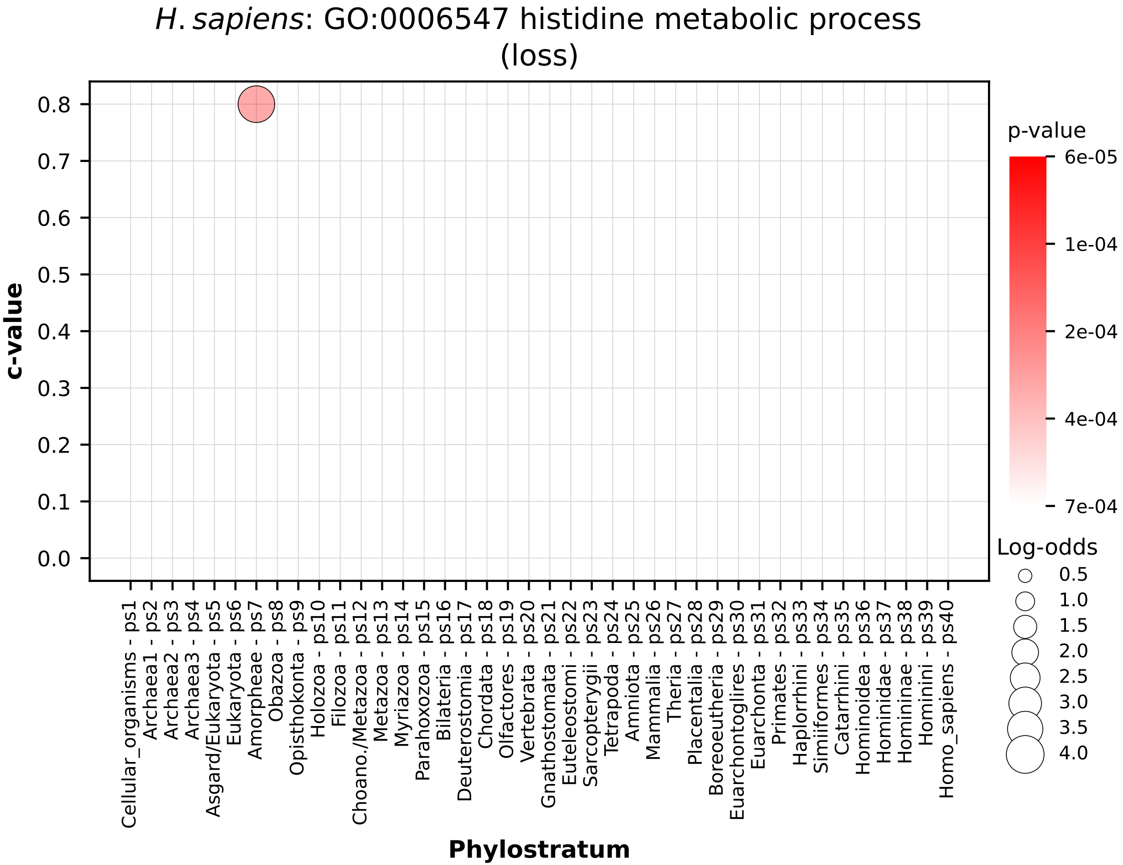

H. sapiens: GO:0006547 histidine metabolic process
(loss)
c-value
0.0
0.1
0.2
0.3
0.4
0.5
0.6
0.7
0.8
p-value
6e-05
1e-04
2e-04
4e-04
7e-04
Log-odds
0.5
1.0
1.5
2.0
2.5
3.0
3.5
4.0
Cellular_organisms - ps1
Archaea1 - ps2
Archaea2 - ps3
Archaea3 - ps4
Asgard/Eukaryota - ps5
Eukaryota - ps6
Amorpheae - ps7
Obazoa - ps8
Opisthokonta - ps9
Holozoa - ps10
Filozoa - ps11
Choano./Metazoa - ps12
Metazoa - ps13
Myriazoa - ps14
Parahoxozoa - ps15
Bilateria - ps16
Deuterostomia - ps17
Chordata - ps18
Olfactores - ps19
Vertebrata - ps20
Gnathostomata - ps21
Euteleostomi - ps22
Sarcopterygii - ps23
Tetrapoda - ps24
Amniota - ps25
Mammalia - ps26
Theria - ps27
Placentalia - ps28
Boreoeutheria - ps29
Euarchontoglires - ps30
Euarchonta - ps31
Primates - ps32
Haplorrhini - ps33
Simiiformes - ps34
Catarrhini - ps35
Hominoidea - ps36
Hominidae - ps37
Homininae - ps38
Hominini - ps39
Homo_sapiens - ps40
Phylostratum

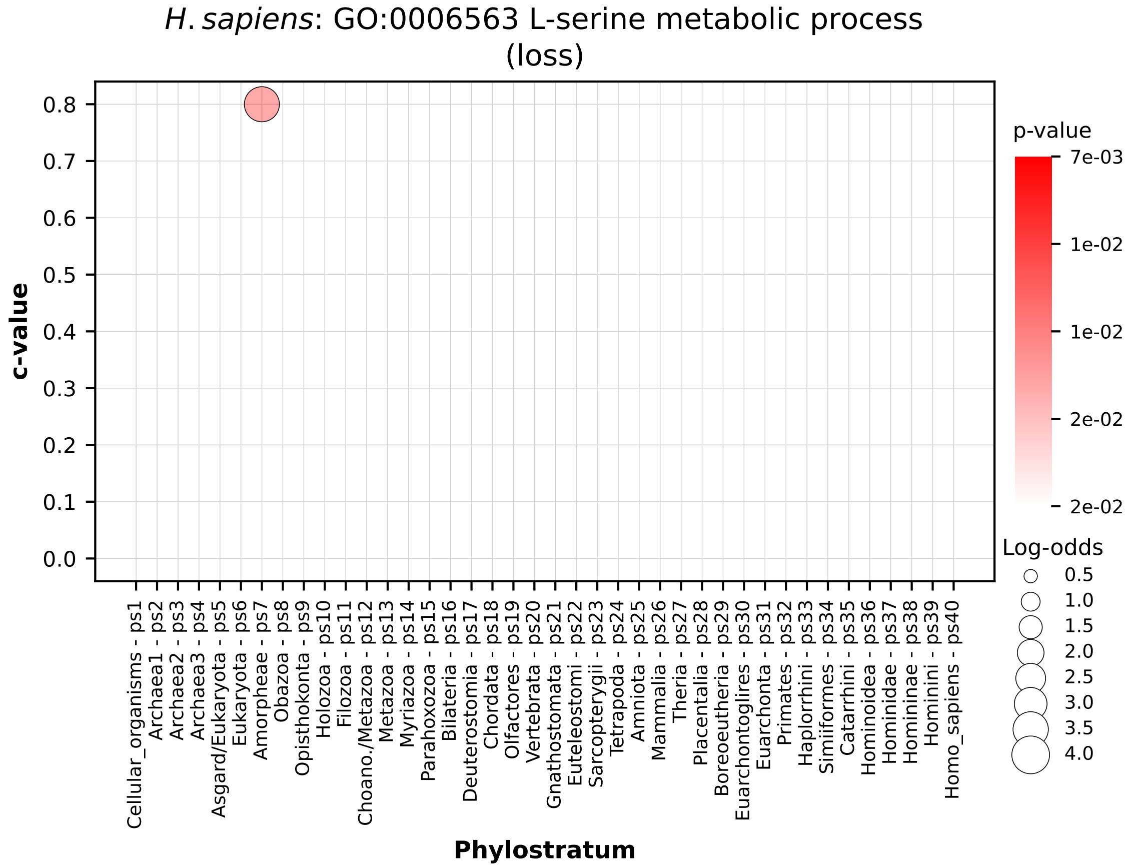
H. sapiens: GO:0006563 L-serine metabolic process
(loss)
c-value
0.0
0.1
0.2
0.3
0.4
0.5
0.6
0.7
0.8
p-value
7e-03
1e-02
1e-02
2e-02
2e-02
Log-odds
0.5
1.0
1.5
2.0
2.5
3.0
3.5
4.0
Cellular_organisms - ps1
Archaea1 - ps2
Archaea2 - ps3
Archaea3 - ps4
Asgard/Eukaryota - ps5
Eukaryota - ps6
Amorpheae - ps7
Obazoa - ps8
Opisthokonta - ps9
Holozoa - ps10
Filozoa - ps11
Choano./Metazoa - ps12
Metazoa - ps13
Myriazoa - ps14
Parahoxozoa - ps15
Bilateria - ps16
Deuterostomia - ps17
Chordata - ps18
Olfactores - ps19
Vertebrata - ps20
Gnathostomata - ps21
Euteleostomi - ps22
Sarcopterygii - ps23
Tetrapoda - ps24
Amniota - ps25
Mammalia - ps26
Theria - ps27
Placentalia - ps28
Boreoeutheria - ps29
Euarchontoglires - ps30
Euarchonta - ps31
Primates - ps32
Haplorrhini - ps33
Simiiformes - ps34
Catarrhini - ps35
Hominoidea - ps36
Hominidae - ps37
Homininae - ps38
Hominini - ps39
Homo_sapiens - ps40
Phylostratum

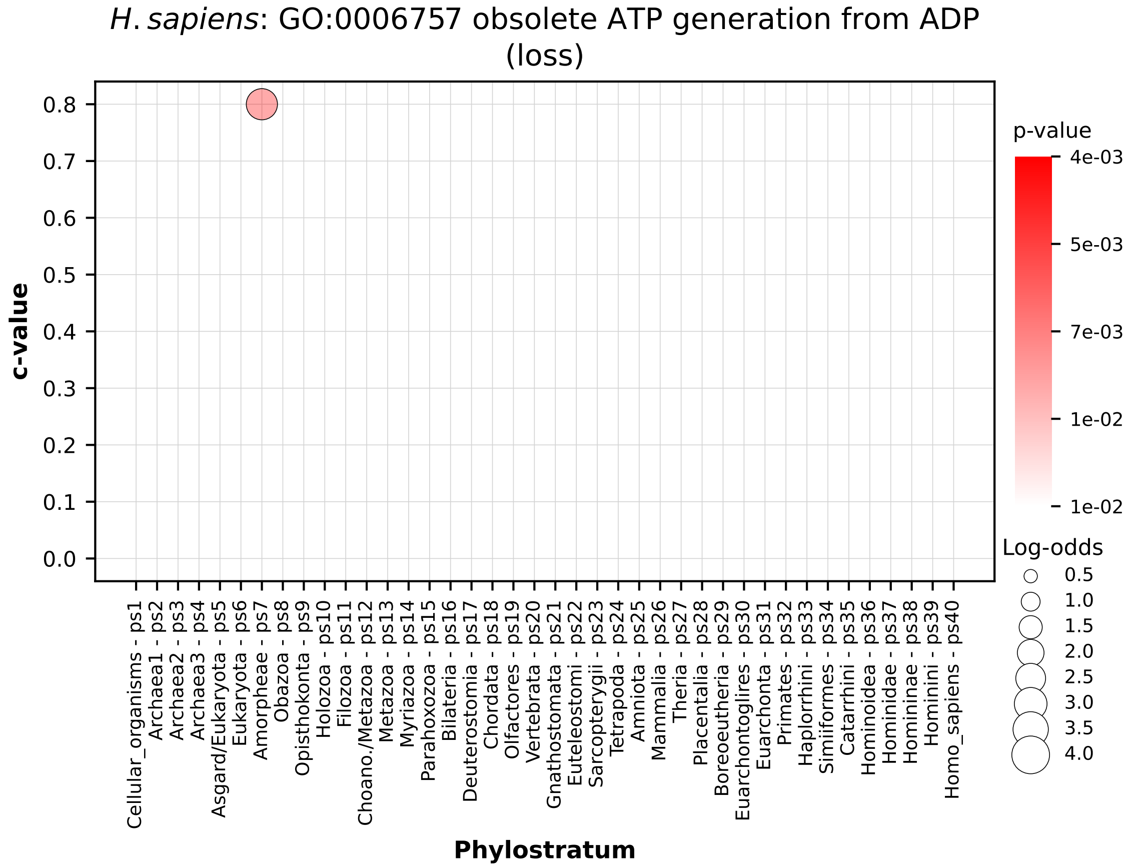

H. sapiens: GO:0006757 obsolete ATP generation from ADP (loss)
c-value
0.0
0.1
0.2
0.3
0.4
0.5
0.6
0.7
0.8
Phylostratum
Cellular_organisms - ps1
Archaea1 - ps2
Archaea2 - ps3
Archaea3 - ps4
Asgard/Eukaryota - ps5
Eukaryota - ps6
Amorpheae - ps7
Obazoa - ps8
Opisthokonta - ps9
Holozoa - ps10
Filozoa - ps11
Choano./Metazoa - ps12
Metazoa - ps13
Myriazoa - ps14
Parahoxozoa - ps15
Bilateria - ps16
Deuterostomia - ps17
Chordata - ps18
Olfactores - ps19
Vertebrata - ps20
Gnathostomata - ps21
Euteleostomi - ps22
Sarcopterygii - ps23
Tetrapoda - ps24
Amniota - ps25
Mammalia - ps26
Theria - ps27
Placentalia - ps28
Boreoeutheria - ps29
Euarchontoglires - ps30
Euarchonta - ps31
Primates - ps32
Haplorrhini - ps33
Simiiformes - ps34
Catarrhini - ps35
Hominoidea - ps36
Hominidae - ps37
Homininae - ps38
Hominini - ps39
Homo_sapiens - ps40
p-value
4e-03
5e-03
7e-03
1e-02
1e-02
Log-odds
0.5
1.0
1.5
2.0
2.5
3.0
3.5
4.0

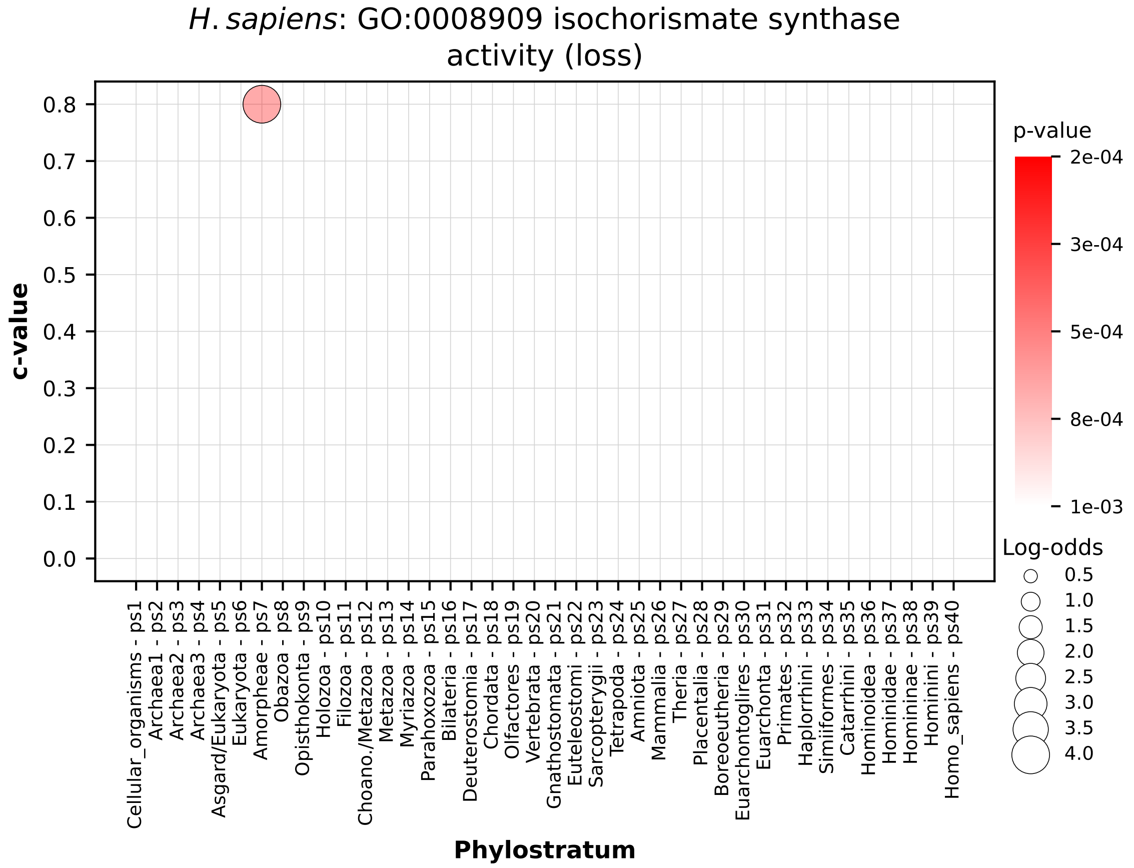

H. sapiens: GO:0008909 isochorismate synthase activity (loss)
c-value
0.8
0.7
0.6
0.5
0.4
0.3
0.2
0.1
0.0
p-value
2e-04
3e-04
5e-04
8e-04
1e-03
Log-odds
0.5
1.0
1.5
2.0
2.5
3.0
3.5
4.0
Cellular_organisms - ps1
Archaea1 - ps2
Archaea2 - ps3
Archaea3 - ps4
Asgard/Eukaryota - ps5
Eukaryota - ps6
Amorpheae - ps7
Obazoa - ps8
Opisthokonta - ps9
Holozoa - ps10
Filozoa - ps11
Choano./Metazoa - ps12
Metazoa - ps13
Myriazoa - ps14
Parahoxozoa - ps15
Bilateria - ps16
Deuterostomia - ps17
Chordata - ps18
Olfactores - ps19
Vertebrata - ps20
Gnathostomata - ps21
Euteleostomi - ps22
Sarcopterygii - ps23
Tetrapoda - ps24
Amniota - ps25
Mammalia - ps26
Theria - ps27
Placentalia - ps28
Boreoeutheria - ps29
Euarchontoglires - ps30
Euarchonta - ps31
Primates - ps32
Haplorrhini - ps33
Simiiformes - ps34
Catarrhini - ps35
Hominoidea - ps36
Hominidae - ps37
Homininae - ps38
Hominini - ps39
Homo_sapiens - ps40
Phylostratum

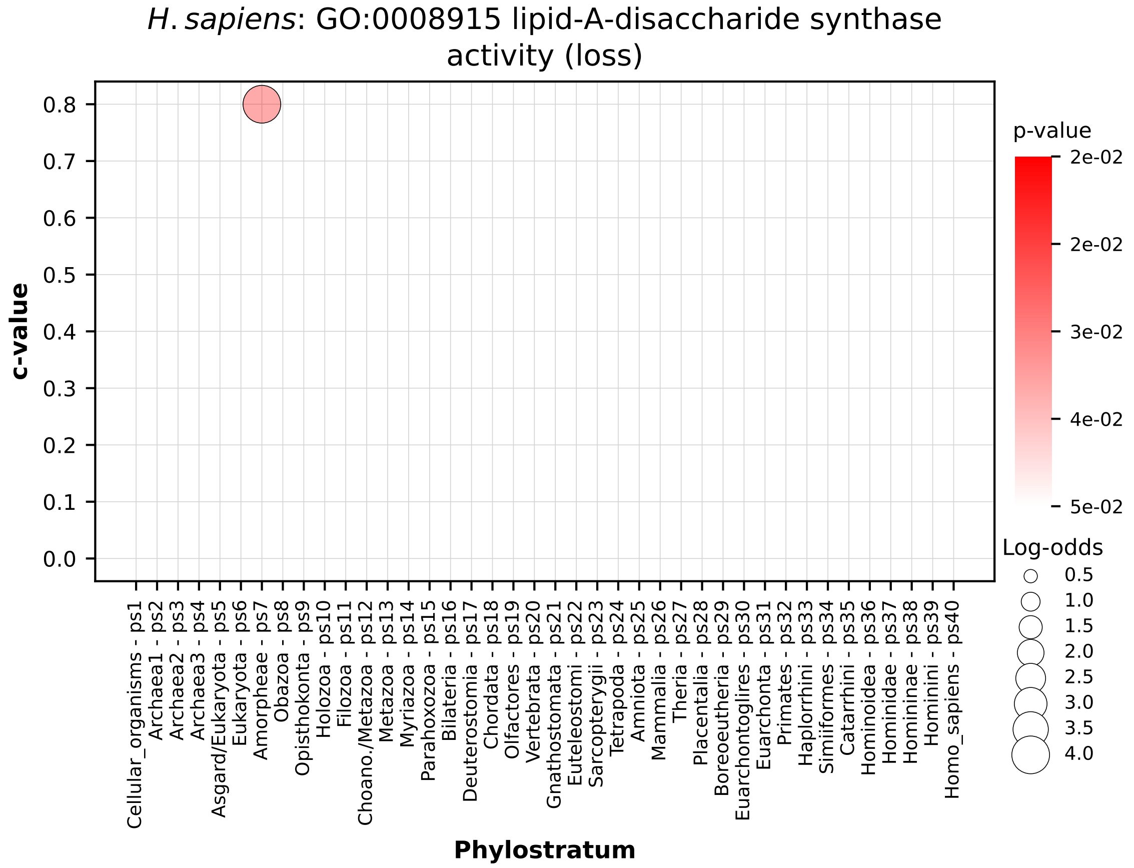
H. sapiens: GO:0008915 lipid-A-disaccharide synthase activity (loss)
c-value
0.8
0.7
0.6
0.5
0.4
0.3
0.2
0.1
0.0
Phylostratum
Cellular_organisms - ps1
Archaea1 - ps2
Archaea2 - ps3
Archaea3 - ps4
Asgard/Eukaryota - ps5
Eukaryota - ps6
Amorpheae - ps7
Obazoa - ps8
Opisthokonta - ps9
Holozoa - ps10
Filozoa - ps11
Choano./Metazoa - ps12
Metazoa - ps13
Myriazoa - ps14
Parahoxozoa - ps15
Bilateria - ps16
Deuterostomia - ps17
Chordata - ps18
Olfactores - ps19
Vertebrata - ps20
Gnathostomata - ps21
Euteleostomi - ps22
Sarcopterygii - ps23
Tetrapoda - ps24
Amniota - ps25
Mammalia - ps26
Theria - ps27
Placentalia - ps28
Boreoeutheria - ps29
Euarchontoglires - ps30
Euarchonta - ps31
Primates - ps32
Haplorrhini - ps33
Simiiformes - ps34
Catarrhini - ps35
Hominoidea - ps36
Hominidae - ps37
Homininae - ps38
Hominini - ps39
Homo_sapiens - ps40
p-value
2e-02
2e-02
3e-02
4e-02
5e-02
Log-odds
0.5
1.0
1.5
2.0
2.5
3.0
3.5
4.0

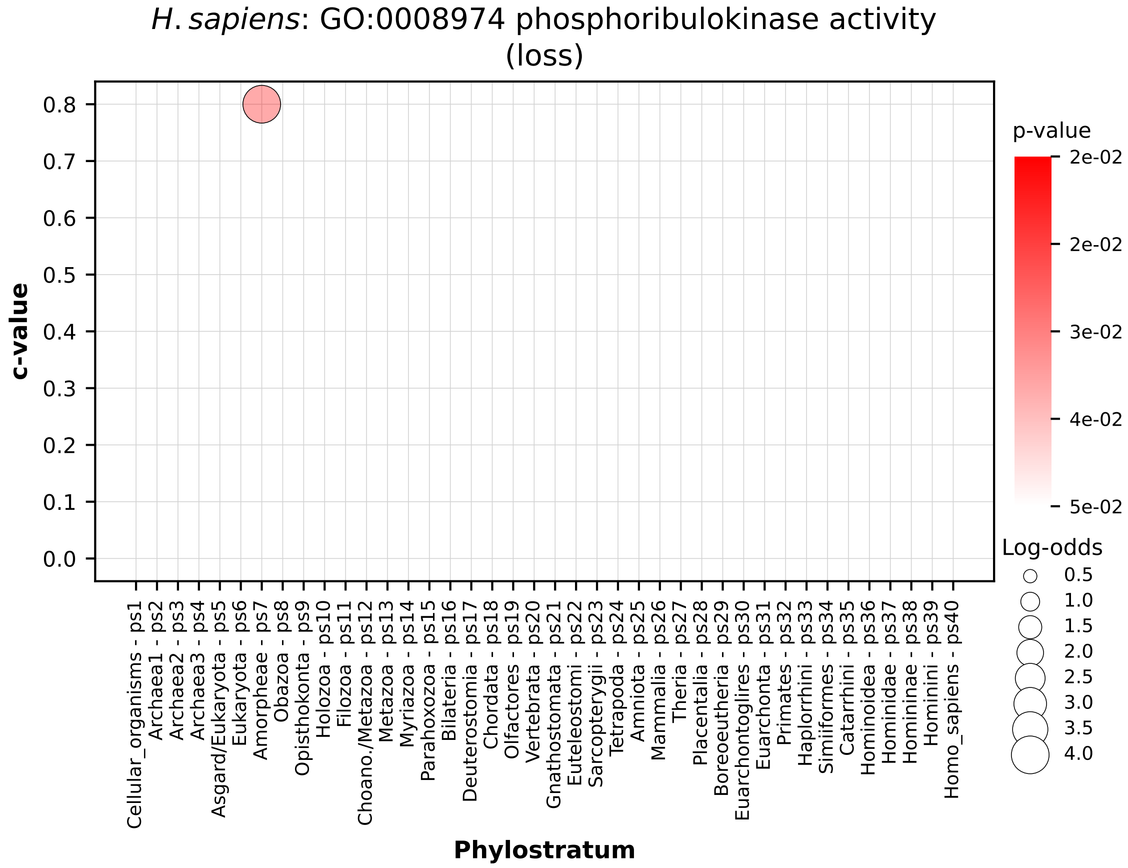

H. sapiens: GO:0008974 phosphoribulokinase activity
(loss)
c-value
0.0
0.1
0.2
0.3
0.4
0.5
0.6
0.7
0.8
p-value
2e-02
2e-02
3e-02
4e-02
5e-02
Log-odds
0.5
1.0
1.5
2.0
2.5
3.0
3.5
4.0
Cellular_organisms - ps1
Archaea1 - ps2
Archaea2 - ps3
Archaea3 - ps4
Asgard/Eukaryota - ps5
Eukaryota - ps6
Amorpheae - ps7
Obazoa - ps8
Opisthokonta - ps9
Holozoa - ps10
Filozoa - ps11
Choano./Metazoa - ps12
Metazoa - ps13
Myriazoa - ps14
Parahoxozoa - ps15
Bilateria - ps16
Deuterostomia - ps17
Chordata - ps18
Olfactores - ps19
Vertebrata - ps20
Gnathostomata - ps21
Euteleostomi - ps22
Sarcopterygii - ps23
Tetrapoda - ps24
Amniota - ps25
Mammalia - ps26
Theria - ps27
Placentalia - ps28
Boreoeutheria - ps29
Euarchontoglires - ps30
Euarchonta - ps31
Primates - ps32
Haplorrhini - ps33
Simiiformes - ps34
Catarrhini - ps35
Hominoidea - ps36
Hominidae - ps37
Homininae - ps38
Hominini - ps39
Homo_sapiens - ps40
Phylostratum

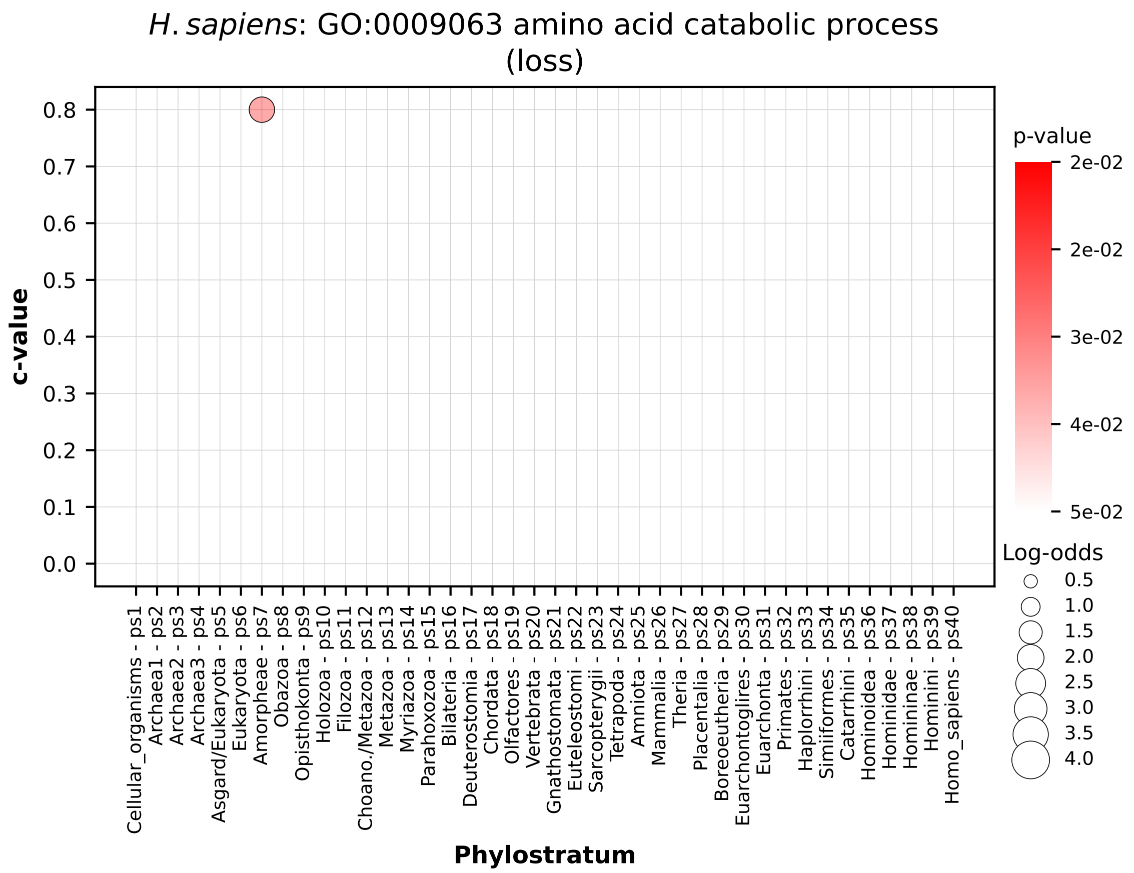
H. sapiens: GO:0009063 amino acid catabolic process (loss)
c-value
0.0
0.1
0.2
0.3
0.4
0.5
0.6
0.7
0.8
p-value
2e-02
2e-02
3e-02
4e-02
5e-02
Log-odds
0.5
1.0
1.5
2.0
2.5
3.0
3.5
4.0
Cellular_organisms - ps1
Archaea1 - ps2
Archaea2 - ps3
Archaea3 - ps4
Asgard/Eukaryota - ps5
Eukaryota - ps6
Amorpheae - ps7
Obazoa - ps8
Opisthokonta - ps9
Holozoa - ps10
Filozoa - ps11
Choano./Metazoa - ps12
Metazoa - ps13
Myriazoa - ps14
Parahoxozoa - ps15
Bilateria - ps16
Deuterostomia - ps17
Chordata - ps18
Olfactores - ps19
Vertebrata - ps20
Gnathostomata - ps21
Euteleostomi - ps22
Sarcopterygii - ps23
Tetrapoda - ps24
Amniota - ps25
Mammalia - ps26
Theria - ps27
Placentalia - ps28
Boreoeutheria - ps29
Euarchontoglires - ps30
Euarchonta - ps31
Primates - ps32
Haplorrhini - ps33
Simiiformes - ps34
Catarrhini - ps35
Hominoidea - ps36
Hominidae - ps37
Homininae - ps38
Hominini - ps39
Homo_sapiens - ps40
Phylostratum

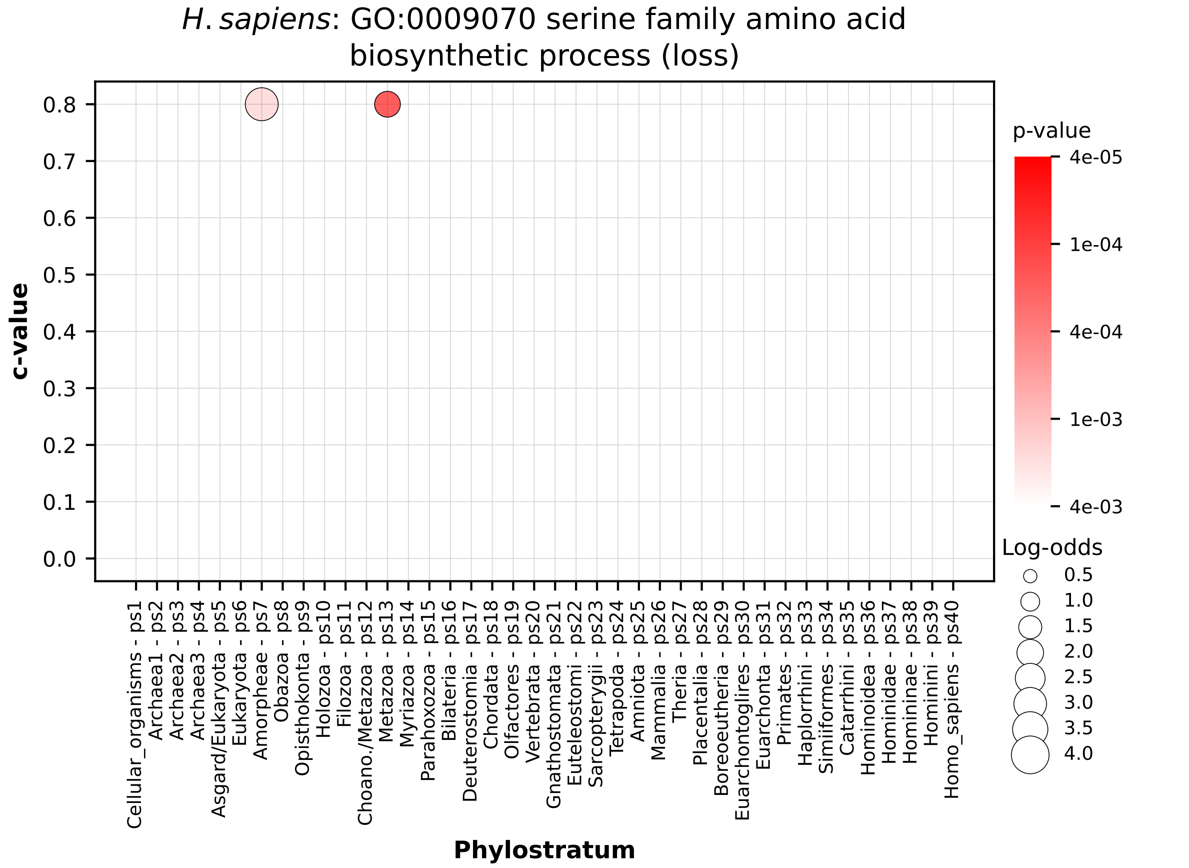

H. sapiens: GO:0009070 serine family amino acid biosynthetic process (loss)
c-value
0.8
0.7
0.6
0.5
0.4
0.3
0.2
0.1
0.0
p-value
4e-05
1e-04
4e-04
1e-03
4e-03
Log-odds
0.5
1.0
1.5
2.0
2.5
3.0
3.5
4.0
Cellular_organisms - ps1
Archaea1 - ps2
Archaea2 - ps3
Archaea3 - ps4
Asgard/Eukaryota - ps5
Eukaryota - ps6
Amorpheae - ps7
Obazoa - ps8
Opisthokonta - ps9
Holozoa - ps10
Filozoa - ps11
Choano./Metazoa - ps12
Metazoa - ps13
Myriazoa - ps14
Parahoxozoa - ps15
Bilateria - ps16
Deuterostomia - ps17
Chordata - ps18
Olfactores - ps19
Vertebrata - ps20
Gnathostomata - ps21
Euteleostomi - ps22
Sarcopterygii - ps23
Tetrapoda - ps24
Amniota - ps25
Mammalia - ps26
Theria - ps27
Placentalia - ps28
Boreoeutheria - ps29
Euarchontoglires - ps30
Euarchonta - ps31
Primates - ps32
Haplorrhini - ps33
Simiiformes - ps34
Catarrhini - ps35
Hominoidea - ps36
Hominidae - ps37
Homininae - ps38
Hominini - ps39
Homo_sapiens - ps40
Phylostratum

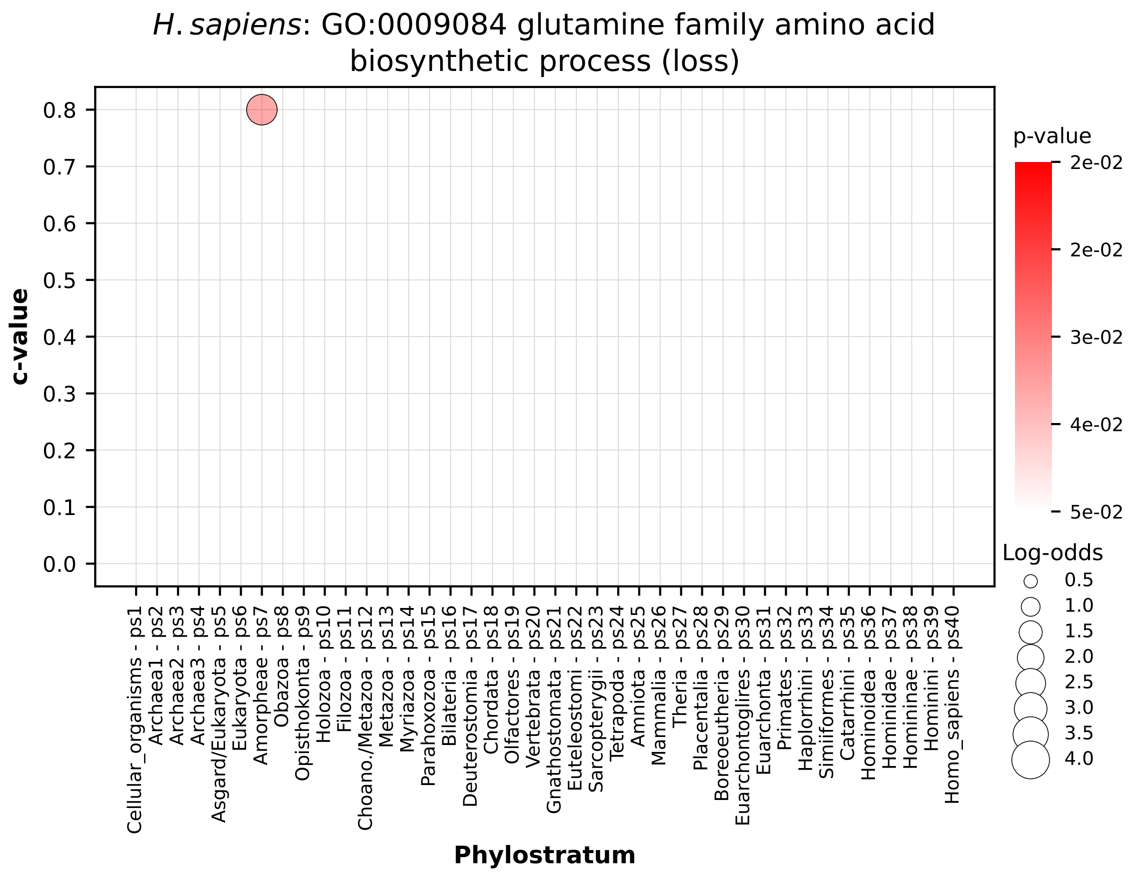

H. sapiens: GO:0009084 glutamine family amino acid biosynthetic process (loss)
c-value
0.0
0.1
0.2
0.3
0.4
0.5
0.6
0.7
0.8
Phylostratum
Cellular_organisms - ps1
Archaea1 - ps2
Archaea2 - ps3
Archaea3 - ps4
Asgard/Eukaryota - ps5
Eukaryota - ps6
Amorpheae - ps7
Obazoa - ps8
Opisthokonta - ps9
Holozoa - ps10
Filozoa - ps11
Choano./Metazoa - ps12
Metazoa - ps13
Myriazoa - ps14
Parahoxozoa - ps15
Bilateria - ps16
Deuterostomia - ps17
Chordata - ps18
Olfactores - ps19
Vertebrata - ps20
Gnathostomata - ps21
Euteleostomi - ps22
Sarcopterygii - ps23
Tetrapoda - ps24
Amniota - ps25
Mammalia - ps26
Theria - ps27
Placentalia - ps28
Boreoeutheria - ps29
Euarchontoglires - ps30
Euarchonta - ps31
Primates - ps32
Haplorrhini - ps33
Simiiformes - ps34
Catarrhini - ps35
Hominoidea - ps36
Hominidae - ps37
Homininae - ps38
Hominini - ps39
Homo_sapiens - ps40
p-value
2e-02
2e-02
3e-02
4e-02
5e-02
Log-odds
0.5
1.0
1.5
2.0
2.5
3.0
3.5
4.0

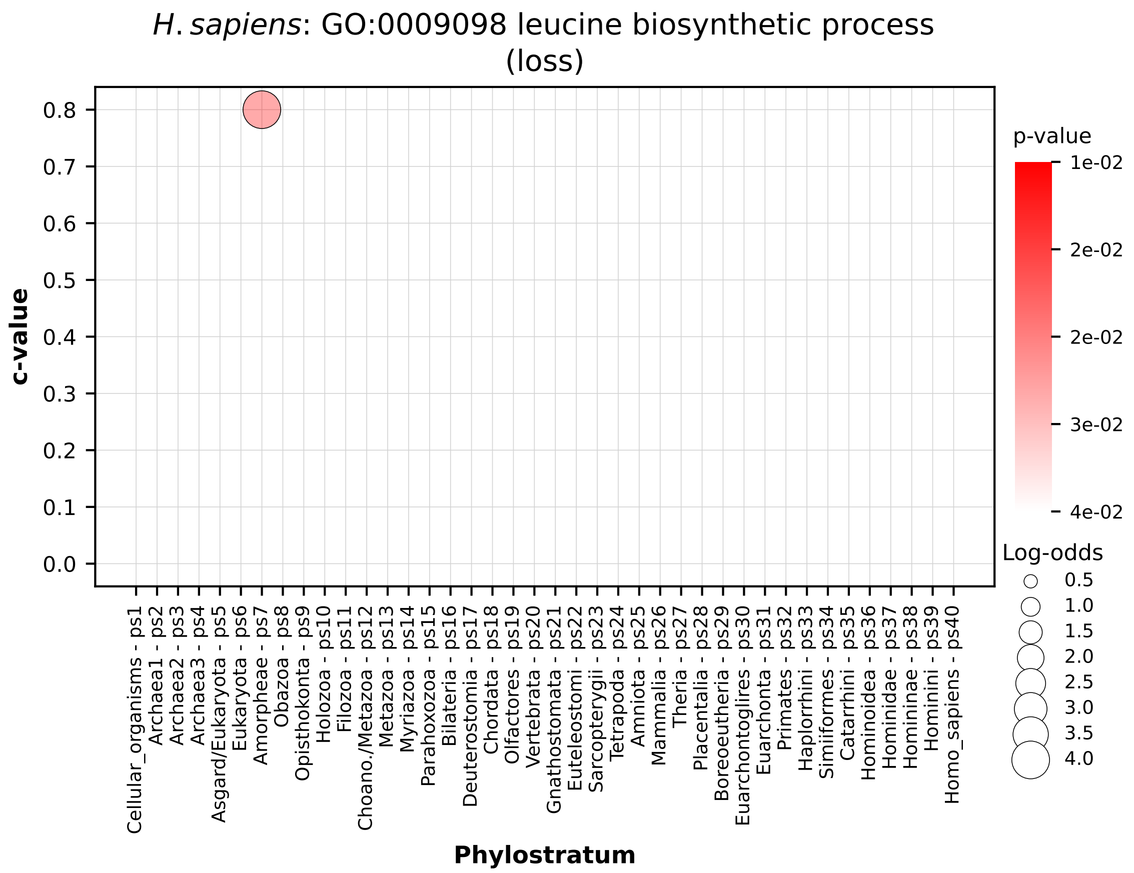

H. sapiens: GO:0009098 leucine biosynthetic process
(loss)
c-value
0.0
0.1
0.2
0.3
0.4
0.5
0.6
0.7
0.8
Phylostratum
Cellular_organisms - ps1
Archaea1 - ps2
Archaea2 - ps3
Archaea3 - ps4
Asgard/Eukaryota - ps5
Eukaryota - ps6
Amorpheae - ps7
Obazoa - ps8
Opisthokonta - ps9
Holozoa - ps10
Filozoa - ps11
Choano./Metazoa - ps12
Metazoa - ps13
Myriazoa - ps14
Parahoxozoa - ps15
Bilateria - ps16
Deuterostomia - ps17
Chordata - ps18
Olfactores - ps19
Vertebrata - ps20
Gnathostomata - ps21
Euteleostomi - ps22
Sarcopterygii - ps23
Tetrapoda - ps24
Amniota - ps25
Mammalia - ps26
Theria - ps27
Placentalia - ps28
Boreoeutheria - ps29
Euarchontoglires - ps30
Euarchonta - ps31
Primates - ps32
Haplorrhini - ps33
Simiiformes - ps34
Catarrhini - ps35
Hominoidea - ps36
Hominidae - ps37
Homininae - ps38
Hominini - ps39
Homo_sapiens - ps40
p-value
1e-02
2e-02
2e-02
3e-02
4e-02
Log-odds
0.5
1.0
1.5
2.0
2.5
3.0
3.5
4.0

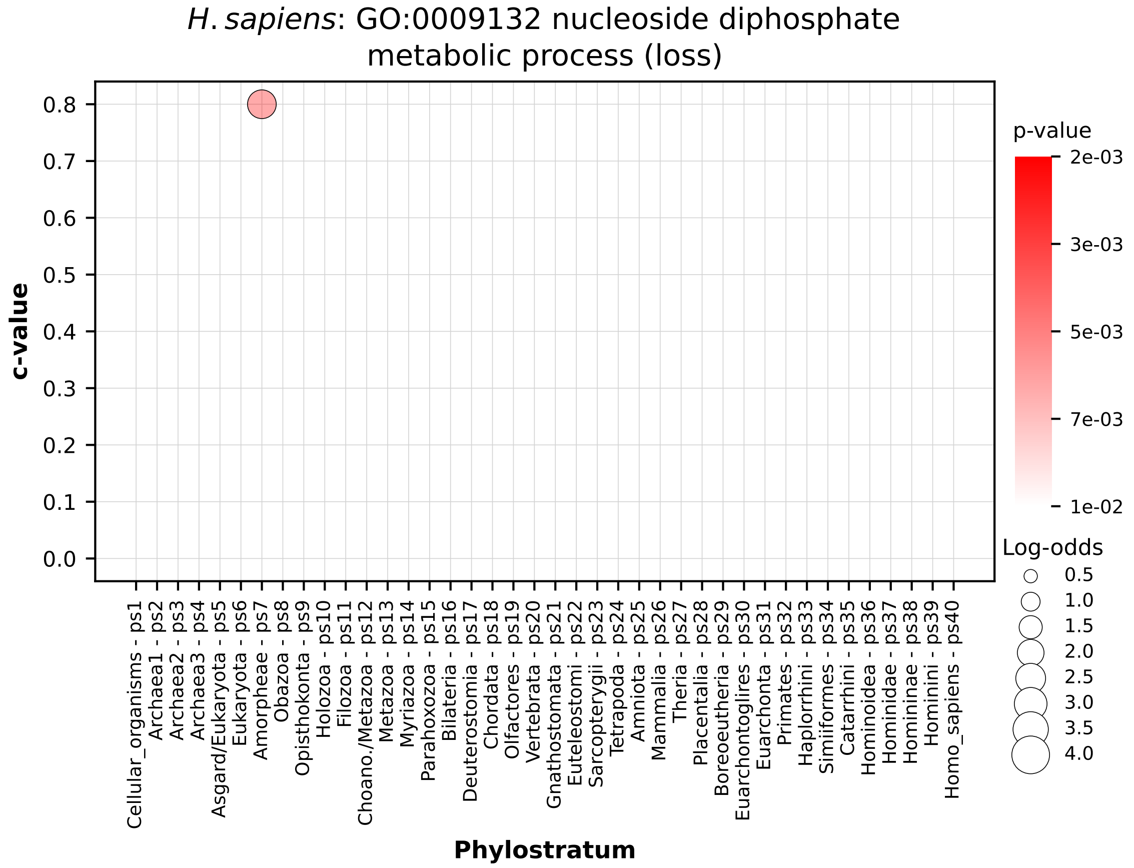

H. sapiens: GO:0009132 nucleoside diphosphate metabolic process (loss)
c-value
0.8
0.7
0.6
0.5
0.4
0.3
0.2
0.1
0.0
p-value
2e-03
3e-03
5e-03
7e-03
1e-02
Log-odds
0.5
1.0
1.5
2.0
2.5
3.0
3.5
4.0
Cellular_organisms - ps1
Archaea1 - ps2
Archaea2 - ps3
Archaea3 - ps4
Asgard/Eukaryota - ps5
Eukaryota - ps6
Amorpheae - ps7
Obazoa - ps8
Opisthokonta - ps9
Holozoa - ps10
Filozoa - ps11
Choano./Metazoa - ps12
Metazoa - ps13
Myriazoa - ps14
Parahoxozoa - ps15
Bilateria - ps16
Deuterostomia - ps17
Chordata - ps18
Olfactores - ps19
Vertebrata - ps20
Gnathostomata - ps21
Euteleostomi - ps22
Sarcopterygii - ps23
Tetrapoda - ps24
Amniota - ps25
Mammalia - ps26
Theria - ps27
Placentalia - ps28
Boreoeutheria - ps29
Euarchontoglires - ps30
Euarchonta - ps31
Primates - ps32
Haplorrhini - ps33
Simiiformes - ps34
Catarrhini - ps35
Hominoidea - ps36
Hominidae - ps37
Homininae - ps38
Hominini - ps39
Homo_sapiens - ps40
Phylostratum

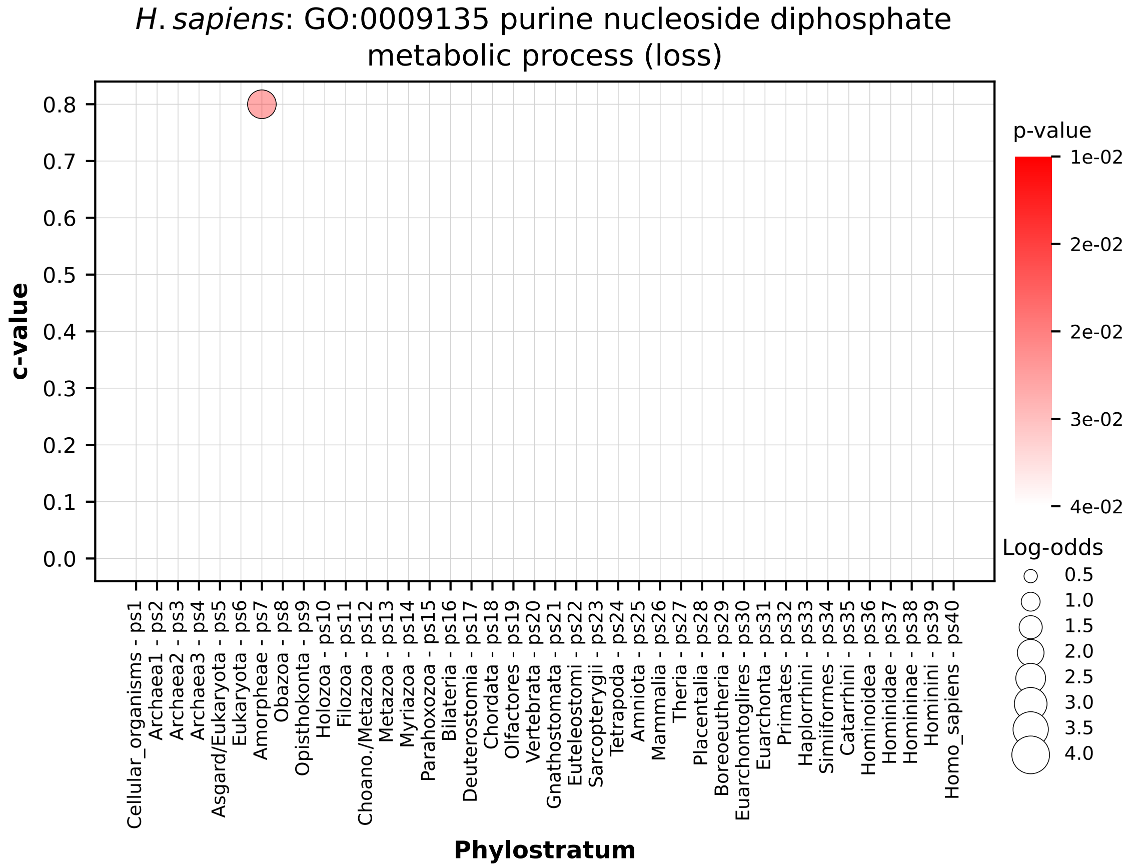

H. sapiens: GO:0009135 purine nucleoside diphosphate metabolic process (loss)
c-value
0.0
0.1
0.2
0.3
0.4
0.5
0.6
0.7
0.8
p-value
1e-02
2e-02
2e-02
3e-02
4e-02
Log-odds
0.5
1.0
1.5
2.0
2.5
3.0
3.5
4.0
Cellular_organisms - ps1
Archaea1 - ps2
Archaea2 - ps3
Archaea3 - ps4
Asgard/Eukaryota - ps5
Eukaryota - ps6
Amorpheae - ps7
Obazoa - ps8
Opisthokonta - ps9
Holozoa - ps10
Filozoa - ps11
Choano./Metazoa - ps12
Metazoa - ps13
Myriazoa - ps14
Parahoxozoa - ps15
Bilateria - ps16
Deuterostomia - ps17
Chordata - ps18
Olfactores - ps19
Vertebrata - ps20
Gnathostomata - ps21
Euteleostomi - ps22
Sarcopterygii - ps23
Tetrapoda - ps24
Amniota - ps25
Mammalia - ps26
Theria - ps27
Placentalia - ps28
Boreoeutheria - ps29
Euarchontoglires - ps30
Euarchonta - ps31
Primates - ps32
Haplorrhini - ps33
Simiiformes - ps34
Catarrhini - ps35
Hominoidea - ps36
Hominidae - ps37
Homininae - ps38
Hominini - ps39
Homo_sapiens - ps40
Phylostratum

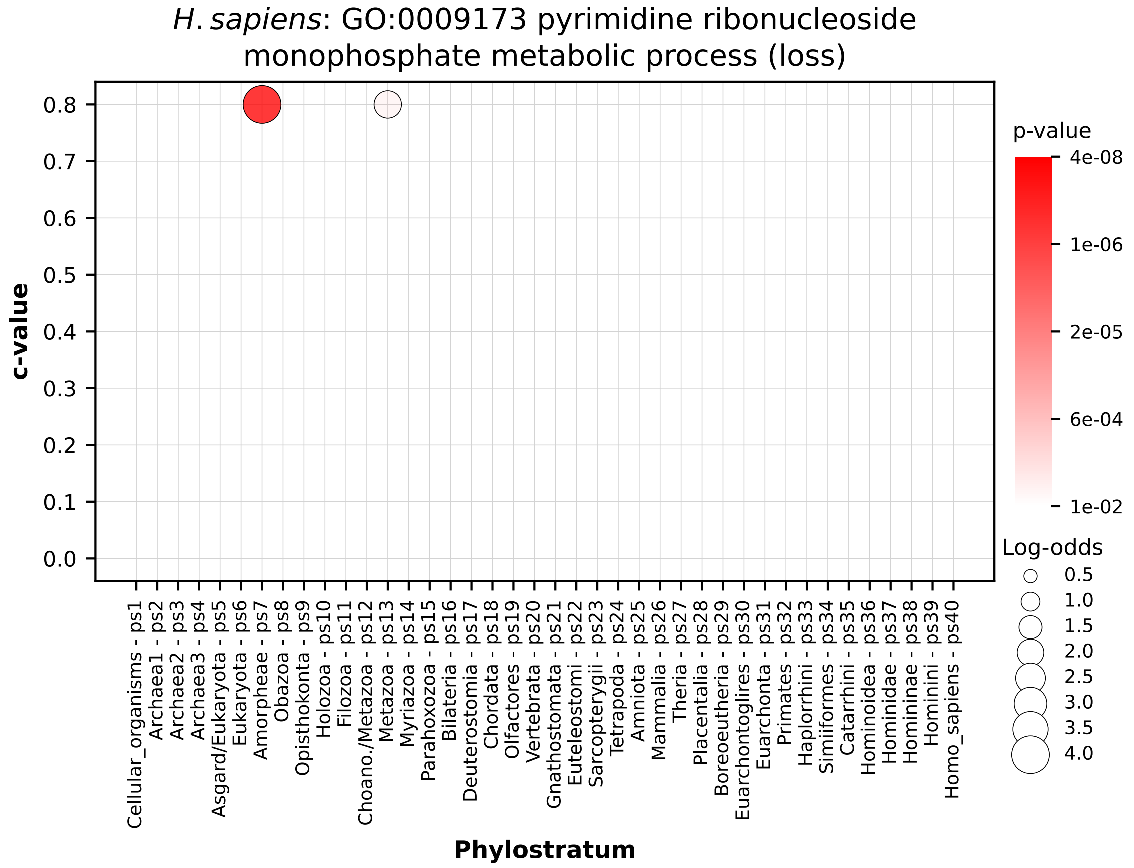

H. sapiens: GO:0009173 pyrimidine ribonucleoside monophosphate metabolic process (loss)
c-value
0.0
0.1
0.2
0.3
0.4
0.5
0.6
0.7
0.8
Phylostratum
Cellular_organisms - ps1
Archaea1 - ps2
Archaea2 - ps3
Archaea3 - ps4
Asgard/Eukaryota - ps5
Eukaryota - ps6
Amorpheae - ps7
Obazoa - ps8
Opisthokonta - ps9
Holozoa - ps10
Filozoa - ps11
Choano./Metazoa - ps12
Metazoa - ps13
Myriazoa - ps14
Parahoxozoa - ps15
Bilateria - ps16
Deuterostomia - ps17
Chordata - ps18
Olfactores - ps19
Vertebrata - ps20
Gnathostomata - ps21
Euteleostomi - ps22
Sarcopterygii - ps23
Tetrapoda - ps24
Amniota - ps25
Mammalia - ps26
Theria - ps27
Placentalia - ps28
Boreoeutheria - ps29
Euarchontoglires - ps30
Euarchonta - ps31
Primates - ps32
Haplorrhini - ps33
Simiiformes - ps34
Catarrhini - ps35
Hominoidea - ps36
Hominidae - ps37
Homininae - ps38
Hominini - ps39
Homo_sapiens - ps40
p-value
4e-08
1e-06
2e-05
6e-04
1e-02
Log-odds
0.5
1.0
1.5
2.0
2.5
3.0
3.5
4.0

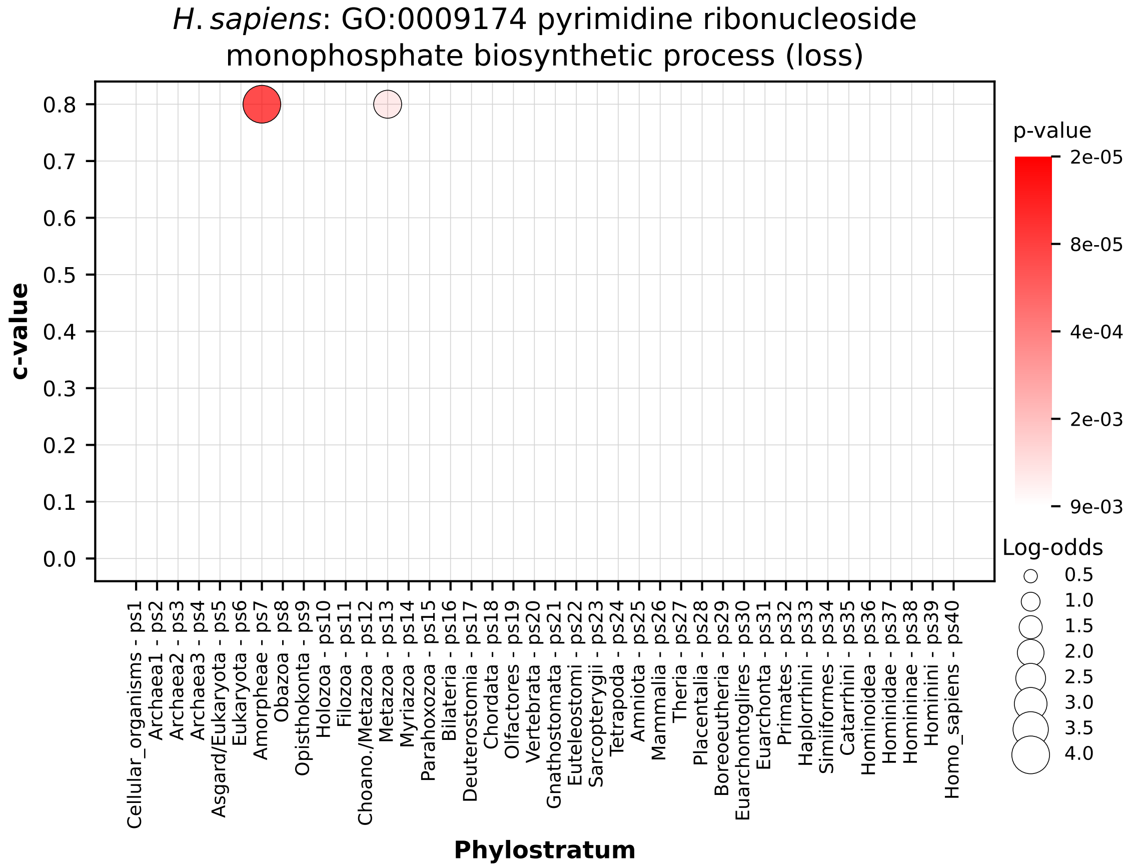

H. sapiens: GO:0009174 pyrimidine ribonucleoside monophosphate biosynthetic process (loss)
c-value
0.0
0.1
0.2
0.3
0.4
0.5
0.6
0.7
0.8
Phylostratum
Cellular_organisms - ps1
Archaea1 - ps2
Archaea2 - ps3
Archaea3 - ps4
Asgard/Eukaryota - ps5
Eukaryota - ps6
Amorpheae - ps7
Obazoa - ps8
Opisthokonta - ps9
Holozoa - ps10
Filozoa - ps11
Choano./Metazoa - ps12
Metazoa - ps13
Myriazoa - ps14
Parahoxozoa - ps15
Bilateria - ps16
Deuterostomia - ps17
Chordata - ps18
Olfactores - ps19
Vertebrata - ps20
Gnathostomata - ps21
Euteleostomi - ps22
Sarcopterygii - ps23
Tetrapoda - ps24
Amniota - ps25
Mammalia - ps26
Theria - ps27
Placentalia - ps28
Boreoeutheria - ps29
Euarchontoglires - ps30
Euarchonta - ps31
Primates - ps32
Haplorrhini - ps33
Simiiformes - ps34
Catarrhini - ps35
Hominoidea - ps36
Hominidae - ps37
Homininae - ps38
Hominini - ps39
Homo_sapiens - ps40
p-value
2e-05
8e-05
4e-04
2e-03
9e-03
Log-odds
0.5
1.0
1.5
2.0
2.5
3.0
3.5
4.0

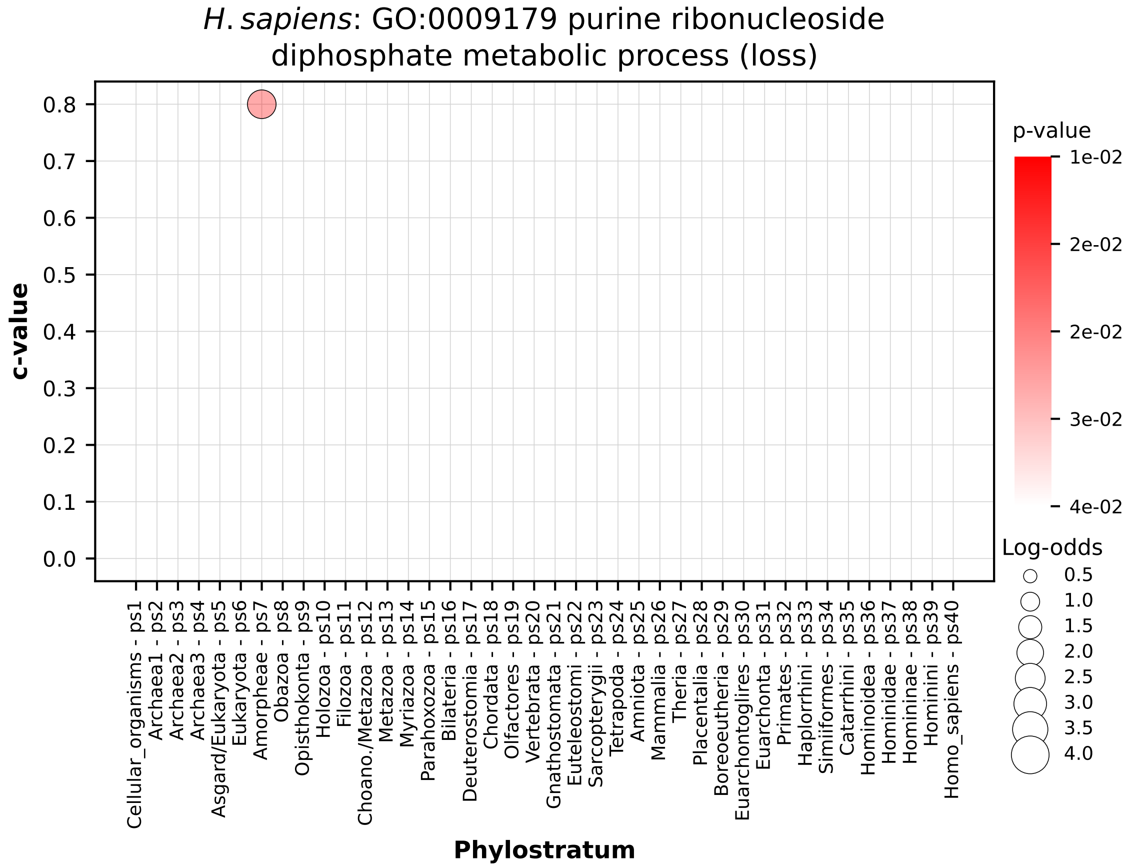

H. sapiens: GO:0009179 purine ribonucleoside diphosphate metabolic process (loss)
c-value
0.0
0.1
0.2
0.3
0.4
0.5
0.6
0.7
0.8
p-value
1e-02
2e-02
2e-02
3e-02
4e-02
Log-odds
0.5
1.0
1.5
2.0
2.5
3.0
3.5
4.0
Cellular_organisms - ps1
Archaea1 - ps2
Archaea2 - ps3
Archaea3 - ps4
Asgard/Eukaryota - ps5
Eukaryota - ps6
Amorpheae - ps7
Obazoa - ps8
Opisthokonta - ps9
Holozoa - ps10
Filozoa - ps11
Choano./Metazoa - ps12
Metazoa - ps13
Myriazoa - ps14
Parahoxozoa - ps15
Bilateria - ps16
Deuterostomia - ps17
Chordata - ps18
Olfactores - ps19
Vertebrata - ps20
Gnathostomata - ps21
Euteleostomi - ps22
Sarcopterygii - ps23
Tetrapoda - ps24
Amniota - ps25
Mammalia - ps26
Theria - ps27
Placentalia - ps28
Boreoeutheria - ps29
Euarchontoglires - ps30
Euarchonta - ps31
Primates - ps32
Haplorrhini - ps33
Simiiformes - ps34
Catarrhini - ps35
Hominoidea - ps36
Hominidae - ps37
Homininae - ps38
Hominini - ps39
Homo_sapiens - ps40
Phylostratum

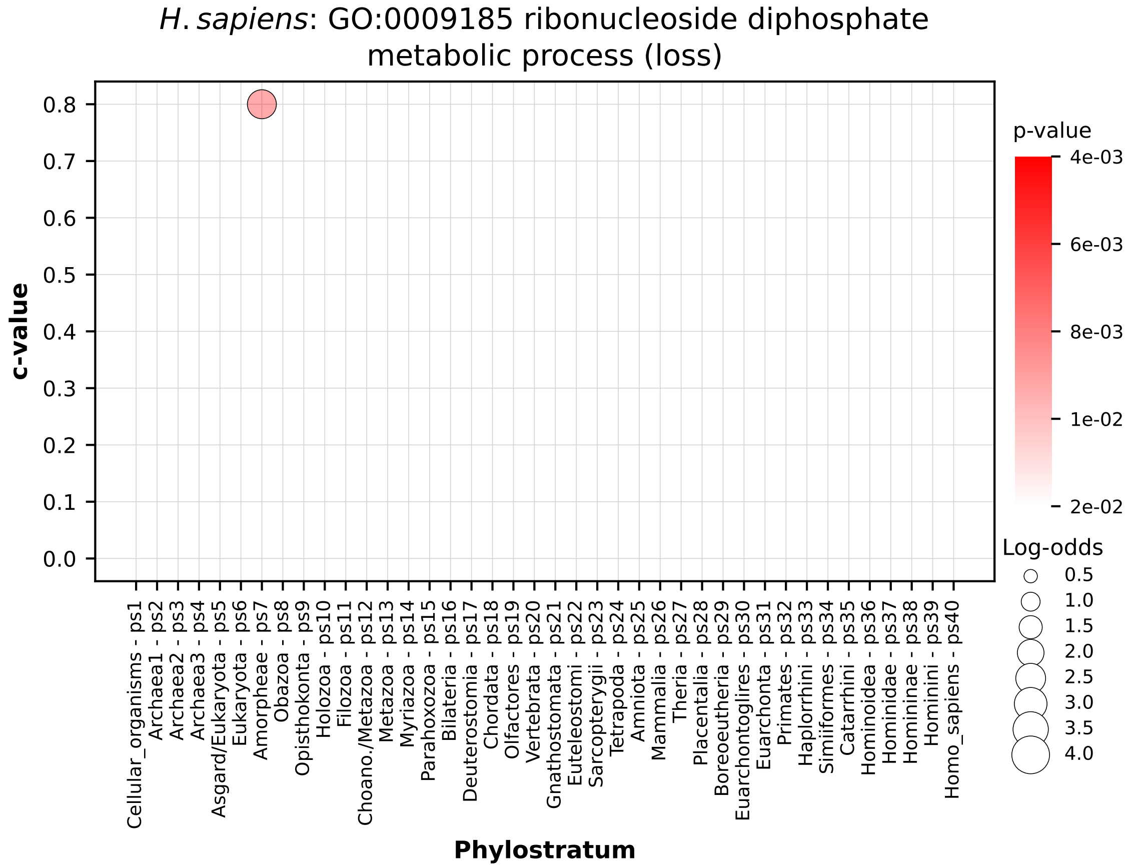

H. sapiens: GO:0009185 ribonucleoside diphosphate metabolic process (loss)
c-value
0.8
0.7
0.6
0.5
0.4
0.3
0.2
0.1
0.0
Phylostratum
Cellular_organisms - ps1
Archaea1 - ps2
Archaea2 - ps3
Archaea3 - ps4
Asgard/Eukaryota - ps5
Eukaryota - ps6
Amorpheae - ps7
Obazoa - ps8
Opisthokonta - ps9
Holozoa - ps10
Filozoa - ps11
Choano./Metazoa - ps12
Metazoa - ps13
Myriazoa - ps14
Parahoxozoa - ps15
Bilateria - ps16
Deuterostomia - ps17
Chordata - ps18
Olfactores - ps19
Vertebrata - ps20
Gnathostomata - ps21
Euteleostomi - ps22
Sarcopterygii - ps23
Tetrapoda - ps24
Amniota - ps25
Mammalia - ps26
Theria - ps27
Placentalia - ps28
Boreoeutheria - ps29
Euarchontoglires - ps30
Euarchonta - ps31
Primates - ps32
Haplorrhini - ps33
Simiiformes - ps34
Catarrhini - ps35
Hominoidea - ps36
Hominidae - ps37
Homininae - ps38
Hominini - ps39
Homo_sapiens - ps40
p-value
4e-03
6e-03
8e-03
1e-02
2e-02
Log-odds
0.5
1.0
1.5
2.0
2.5
3.0
3.5
4.0

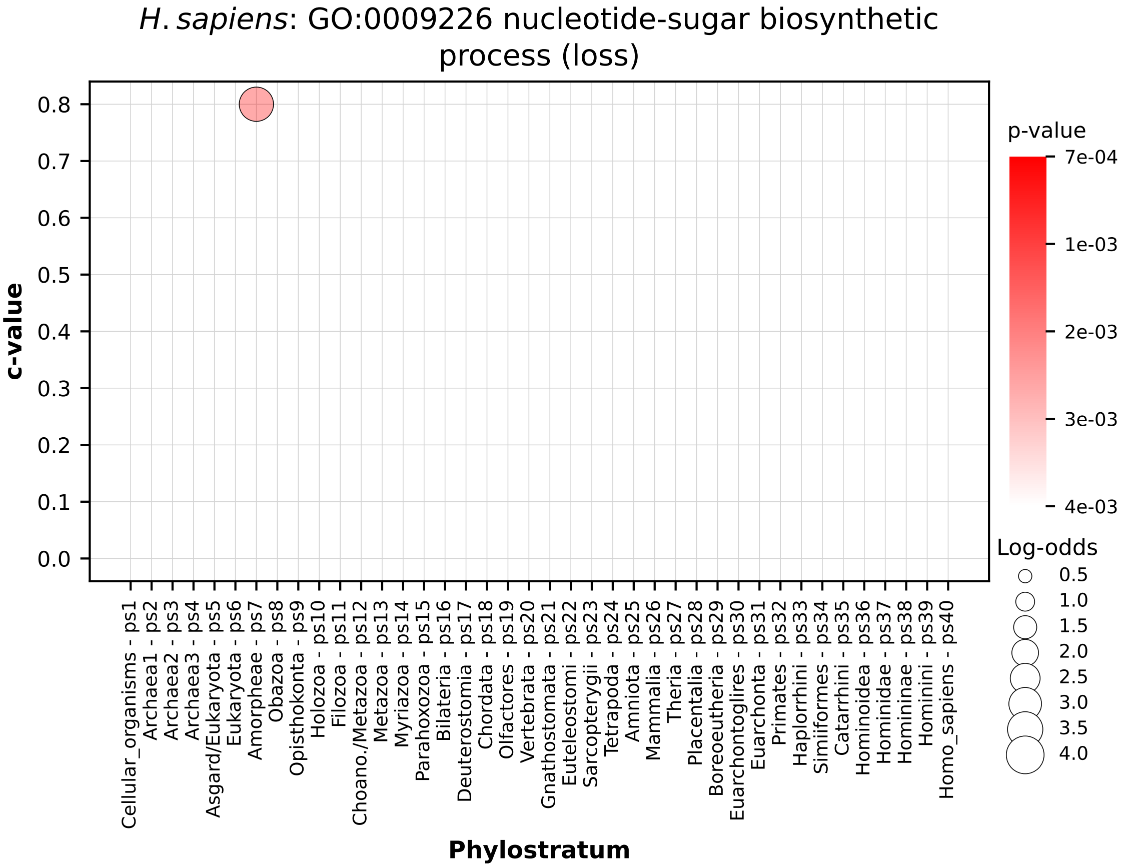
H. sapiens: GO:0009226 nucleotide-sugar biosynthetic process (loss)
c-value
0.0
0.1
0.2
0.3
0.4
0.5
0.6
0.7
0.8
Phylostratum
Cellular_organisms - ps1
Archaea1 - ps2
Archaea2 - ps3
Archaea3 - ps4
Asgard/Eukaryota - ps5
Eukaryota - ps6
Amorpheae - ps7
Obazoa - ps8
Opisthokonta - ps9
Holozoa - ps10
Filozoa - ps11
Choano./Metazoa - ps12
Metazoa - ps13
Myriazoa - ps14
Parahoxozoa - ps15
Bilateria - ps16
Deuterostomia - ps17
Chordata - ps18
Olfactores - ps19
Vertebrata - ps20
Gnathostomata - ps21
Euteleostomi - ps22
Sarcopterygii - ps23
Tetrapoda - ps24
Amniota - ps25
Mammalia - ps26
Theria - ps27
Placentalia - ps28
Boreoeutheria - ps29
Euarchontoglires - ps30
Euarchonta - ps31
Primates - ps32
Haplorrhini - ps33
Simiiformes - ps34
Catarrhini - ps35
Hominoidea - ps36
Hominidae - ps37
Homininae - ps38
Hominini - ps39
Homo_sapiens - ps40
p-value
7e-04
1e-03
2e-03
3e-03
4e-03
Log-odds
0.5
1.0
1.5
2.0
2.5
3.0
3.5
4.0

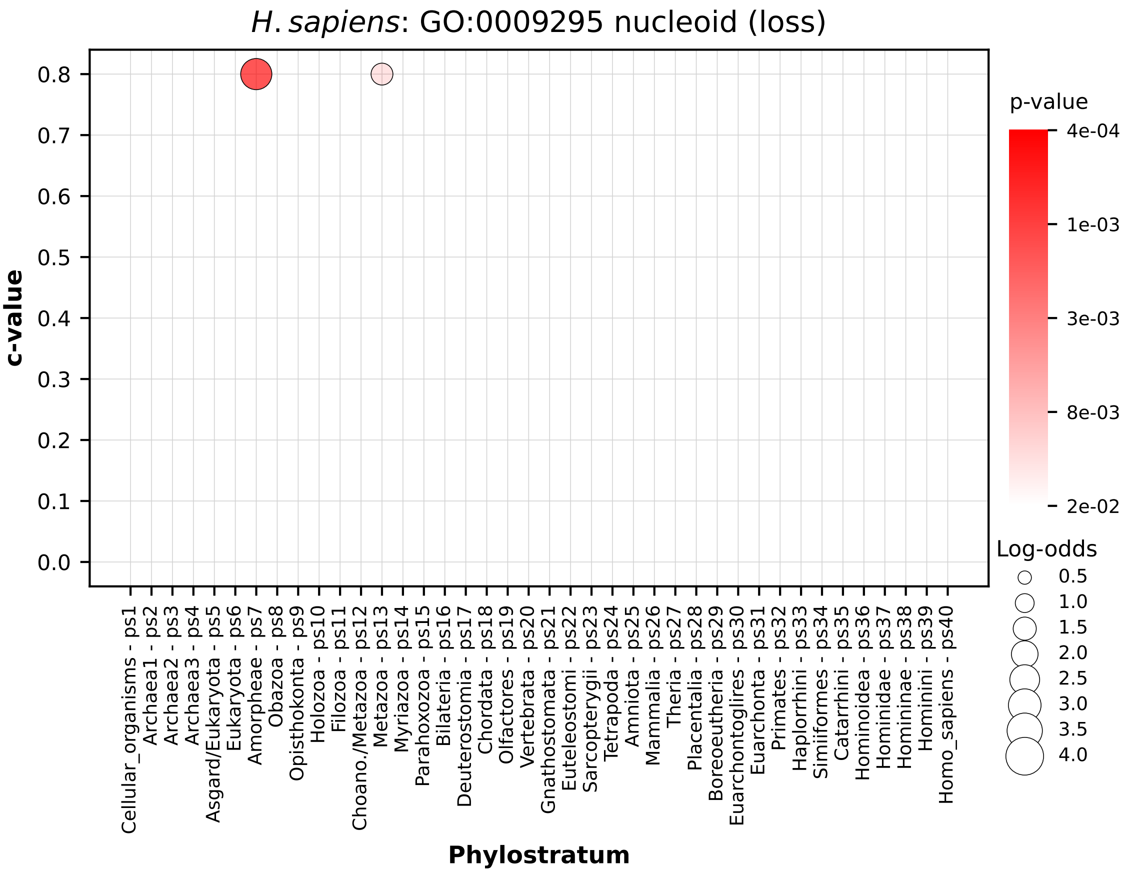

H. sapiens: GO:0009295 nucleoid (loss)
c-value
0.0
0.1
0.2
0.3
0.4
0.5
0.6
0.7
0.8
Phylostratum
Cellular_organisms - ps1
Archaea1 - ps2
Archaea2 - ps3
Archaea3 - ps4
Asgard/Eukaryota - ps5
Eukaryota - ps6
Amorpheae - ps7
Obazoa - ps8
Opisthokonta - ps9
Holozoa - ps10
Filozoa - ps11
Choano./Metazoa - ps12
Metazoa - ps13
Myriazoa - ps14
Parahoxozoa - ps15
Bilateria - ps16
Deuterostomia - ps17
Chordata - ps18
Olfactores - ps19
Vertebrata - ps20
Gnathostomata - ps21
Euteleostomi - ps22
Sarcopterygii - ps23
Tetrapoda - ps24
Amniota - ps25
Mammalia - ps26
Theria - ps27
Placentalia - ps28
Boreoeutheria - ps29
Euarchontoglires - ps30
Euarchonta - ps31
Primates - ps32
Haplorrhini - ps33
Simiiformes - ps34
Catarrhini - ps35
Hominoidea - ps36
Hominidae - ps37
Homininae - ps38
Hominini - ps39
Homo_sapiens - ps40
p-value
4e-04
1e-03
3e-03
8e-03
2e-02
Log-odds
0.5
1.0
1.5
2.0
2.5
3.0
3.5
4.0

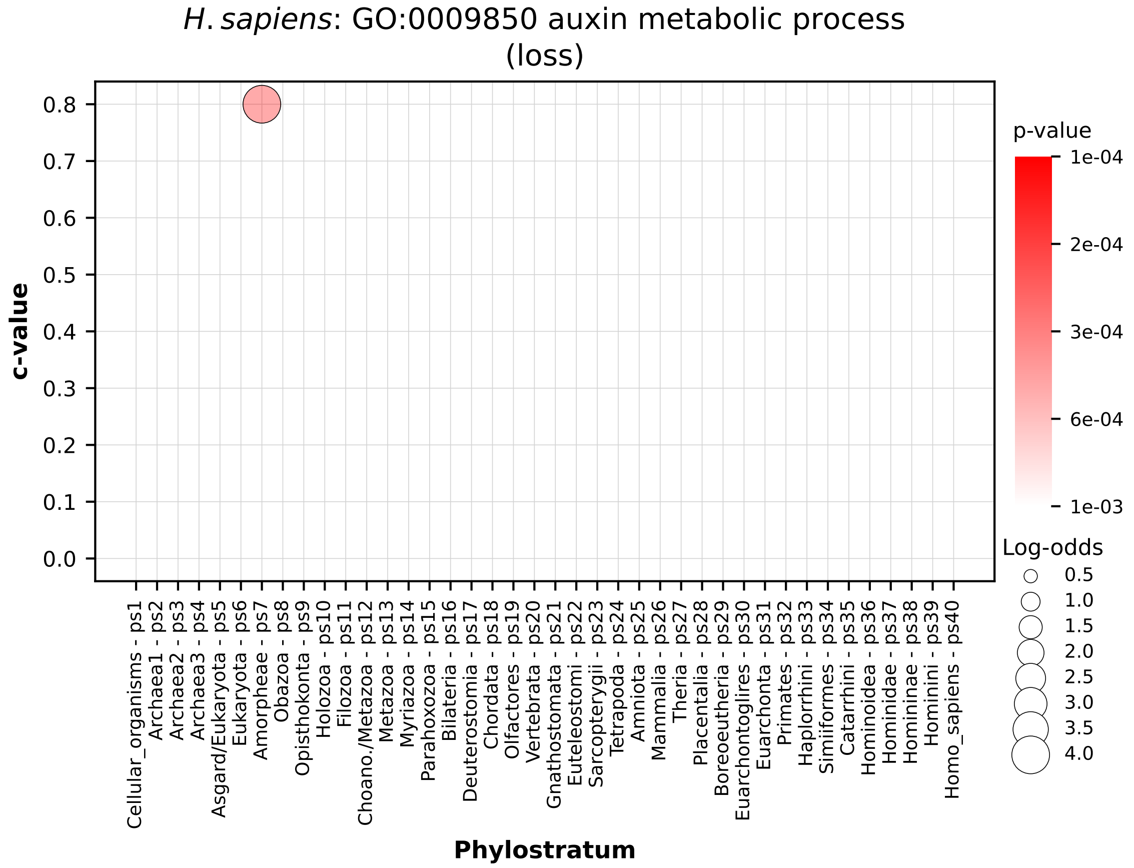

H. sapiens: GO:0009850 auxin metabolic process
(loss)
c-value
0.0
0.1
0.2
0.3
0.4
0.5
0.6
0.7
0.8
Phylostratum
Cellular_organisms - ps1
Archaea1 - ps2
Archaea2 - ps3
Archaea3 - ps4
Asgard/Eukaryota - ps5
Eukaryota - ps6
Amorpheae - ps7
Obazoa - ps8
Opisthokonta - ps9
Holozoa - ps10
Filozoa - ps11
Choano./Metazoa - ps12
Metazoa - ps13
Myriazoa - ps14
Parahoxozoa - ps15
Bilateria - ps16
Deuterostomia - ps17
Chordata - ps18
Olfactores - ps19
Vertebrata - ps20
Gnathostomata - ps21
Euteleostomi - ps22
Sarcopterygii - ps23
Tetrapoda - ps24
Amniota - ps25
Mammalia - ps26
Theria - ps27
Placentalia - ps28
Boreoeutheria - ps29
Euarchontoglires - ps30
Euarchonta - ps31
Primates - ps32
Haplorrhini - ps33
Simiiformes - ps34
Catarrhini - ps35
Hominoidea - ps36
Hominidae - ps37
Homininae - ps38
Hominini - ps39
Homo_sapiens - ps40
p-value
1e-04
2e-04
3e-04
6e-04
1e-03
Log-odds
0.5
1.0
1.5
2.0
2.5
3.0
3.5
4.0

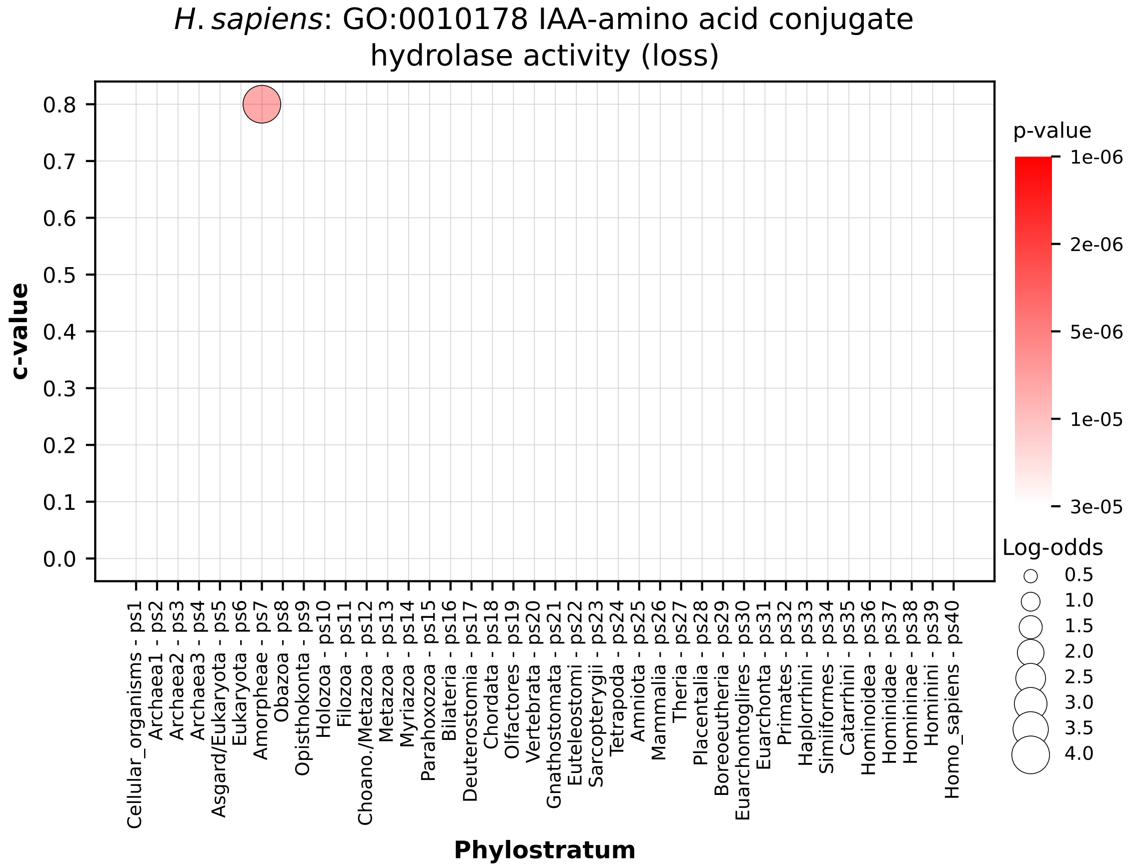

H. sapiens: GO:0010178 IAA-amino acid conjugate hydrolase activity (loss)
c-value
0.8
0.7
0.6
0.5
0.4
0.3
0.2
0.1
0.0
p-value
1e-06
2e-06
5e-06
1e-05
3e-05
Log-odds
0.5
1.0
1.5
2.0
2.5
3.0
3.5
4.0
Cellular_organisms - ps1
Archaea1 - ps2
Archaea2 - ps3
Archaea3 - ps4
Asgard/Eukaryota - ps5
Eukaryota - ps6
Amorpheae - ps7
Obazoa - ps8
Opisthokonta - ps9
Holozoa - ps10
Filozoa - ps11
Choano./Metazoa - ps12
Metazoa - ps13
Myriazoa - ps14
Parahoxozoa - ps15
Bilateria - ps16
Deuterostomia - ps17
Chordata - ps18
Olfactores - ps19
Vertebrata - ps20
Gnathostomata - ps21
Euteleostomi - ps22
Sarcopterygii - ps23
Tetrapoda - ps24
Amniota - ps25
Mammalia - ps26
Theria - ps27
Placentalia - ps28
Boreoeutheria - ps29
Euarchontoglires - ps30
Euarchonta - ps31
Primates - ps32
Haplorrhini - ps33
Simiiformes - ps34
Catarrhini - ps35
Hominoidea - ps36
Hominidae - ps37
Homininae - ps38
Hominini - ps39
Homo_sapiens - ps40
Phylostratum

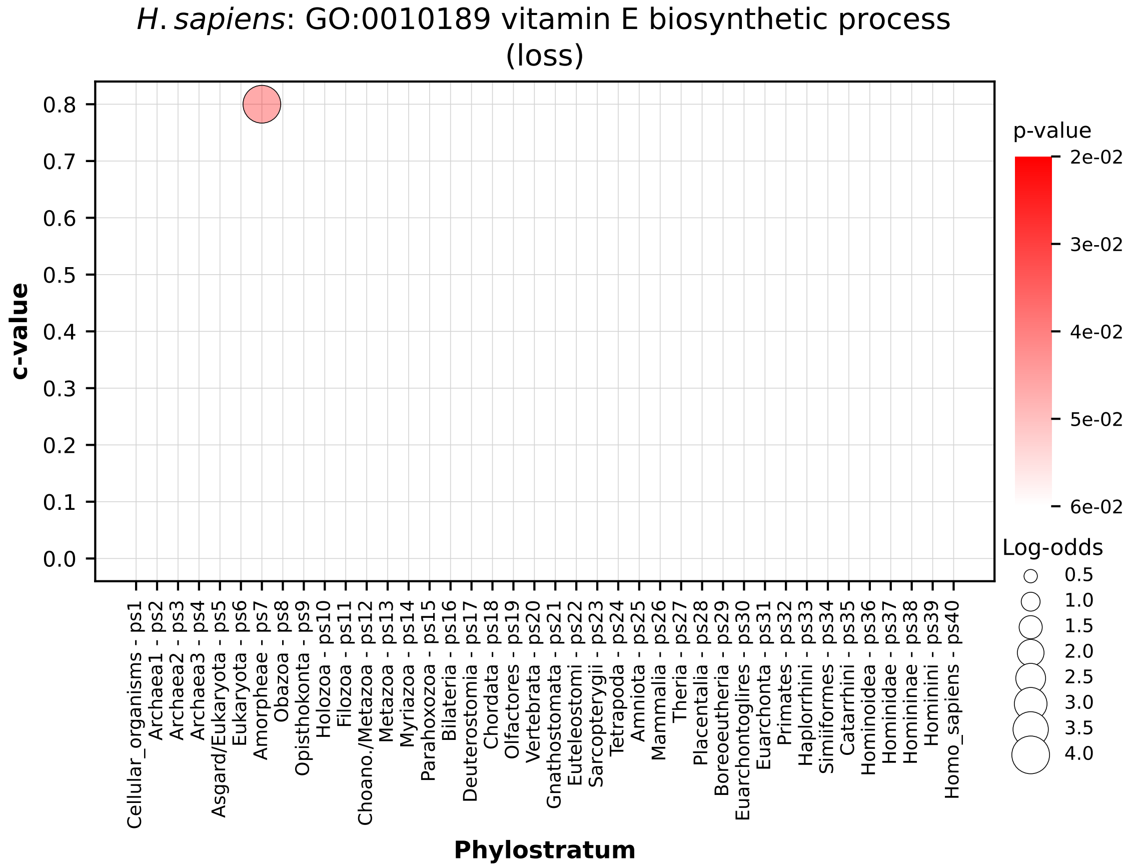

H. sapiens: GO:0010189 vitamin E biosynthetic process
(loss)
c-value
0.8
0.7
0.6
0.5
0.4
0.3
0.2
0.1
0.0
p-value
2e-02
3e-02
4e-02
5e-02
6e-02
Log-odds
0.5
1.0
1.5
2.0
2.5
3.0
3.5
4.0
Cellular_organisms - ps1
Archaea1 - ps2
Archaea2 - ps3
Archaea3 - ps4
Asgard/Eukaryota - ps5
Eukaryota - ps6
Amorpheae - ps7
Obazoa - ps8
Opisthokonta - ps9
Holozoa - ps10
Filozoa - ps11
Choano./Metazoa - ps12
Metazoa - ps13
Myriazoa - ps14
Parahoxozoa - ps15
Bilateria - ps16
Deuterostomia - ps17
Chordata - ps18
Olfactores - ps19
Vertebrata - ps20
Gnathostomata - ps21
Euteleostomi - ps22
Sarcopterygii - ps23
Tetrapoda - ps24
Amniota - ps25
Mammalia - ps26
Theria - ps27
Placentalia - ps28
Boreoeutheria - ps29
Euarchontoglires - ps30
Euarchonta - ps31
Primates - ps32
Haplorrhini - ps33
Simiiformes - ps34
Catarrhini - ps35
Hominoidea - ps36
Hominidae - ps37
Homininae - ps38
Hominini - ps39
Homo_sapiens - ps40
Phylostratum

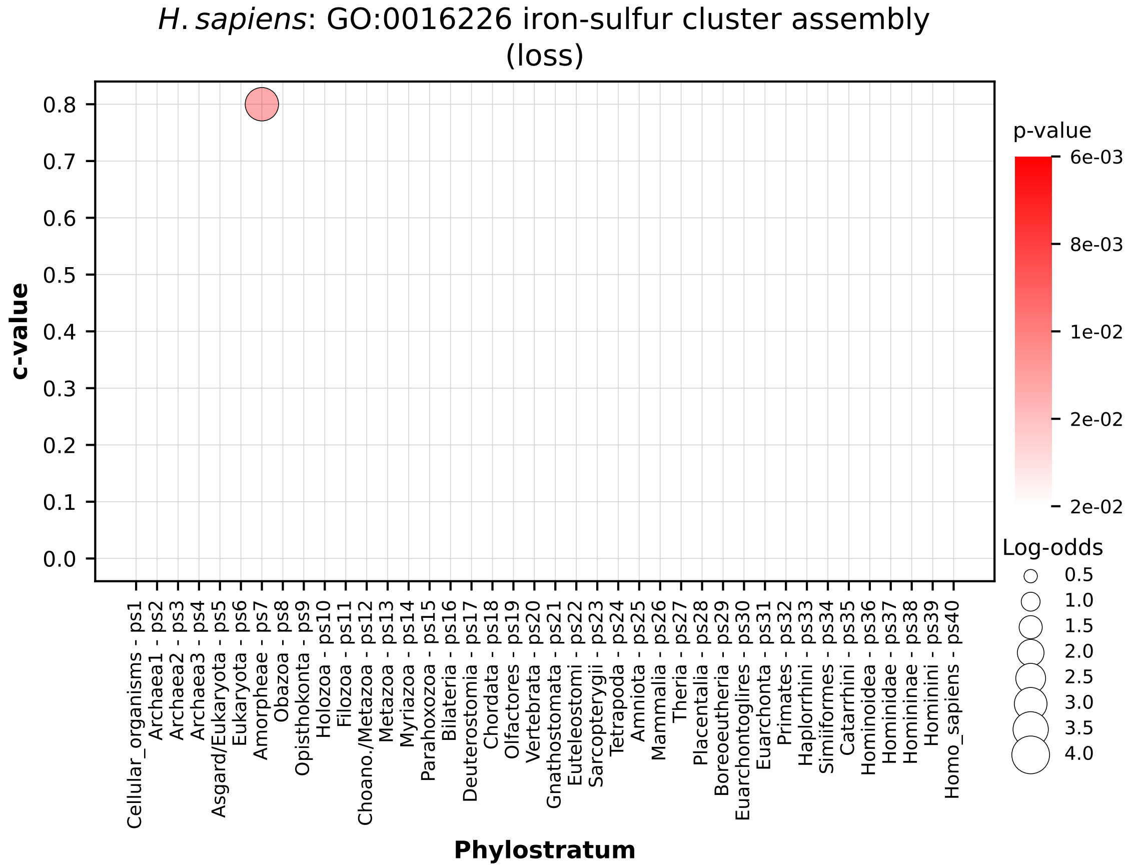

H. sapiens: GO:0016226 iron-sulfur cluster assembly
(loss)
c-value
0.8
0.7
0.6
0.5
0.4
0.3
0.2
0.1
0.0
p-value
6e-03
8e-03
1e-02
2e-02
2e-02
Log-odds
0.5
1.0
1.5
2.0
2.5
3.0
3.5
4.0
Cellular_organisms - ps1
Archaea1 - ps2
Archaea2 - ps3
Archaea3 - ps4
Asgard/Eukaryota - ps5
Eukaryota - ps6
Amorpheae - ps7
Obazoa - ps8
Opisthokonta - ps9
Holozoa - ps10
Filozoa - ps11
Choano./Metazoa - ps12
Metazoa - ps13
Myriazoa - ps14
Parahoxozoa - ps15
Bilateria - ps16
Deuterostomia - ps17
Chordata - ps18
Olfactores - ps19
Vertebrata - ps20
Gnathostomata - ps21
Euteleostomi - ps22
Sarcopterygii - ps23
Tetrapoda - ps24
Amniota - ps25
Mammalia - ps26
Theria - ps27
Placentalia - ps28
Boreoeutheria - ps29
Euarchontoglires - ps30
Euarchonta - ps31
Primates - ps32
Haplorrhini - ps33
Simiiformes - ps34
Catarrhini - ps35
Hominoidea - ps36
Hominidae - ps37
Homininae - ps38
Hominini - ps39
Homo_sapiens - ps40
Phylostratum

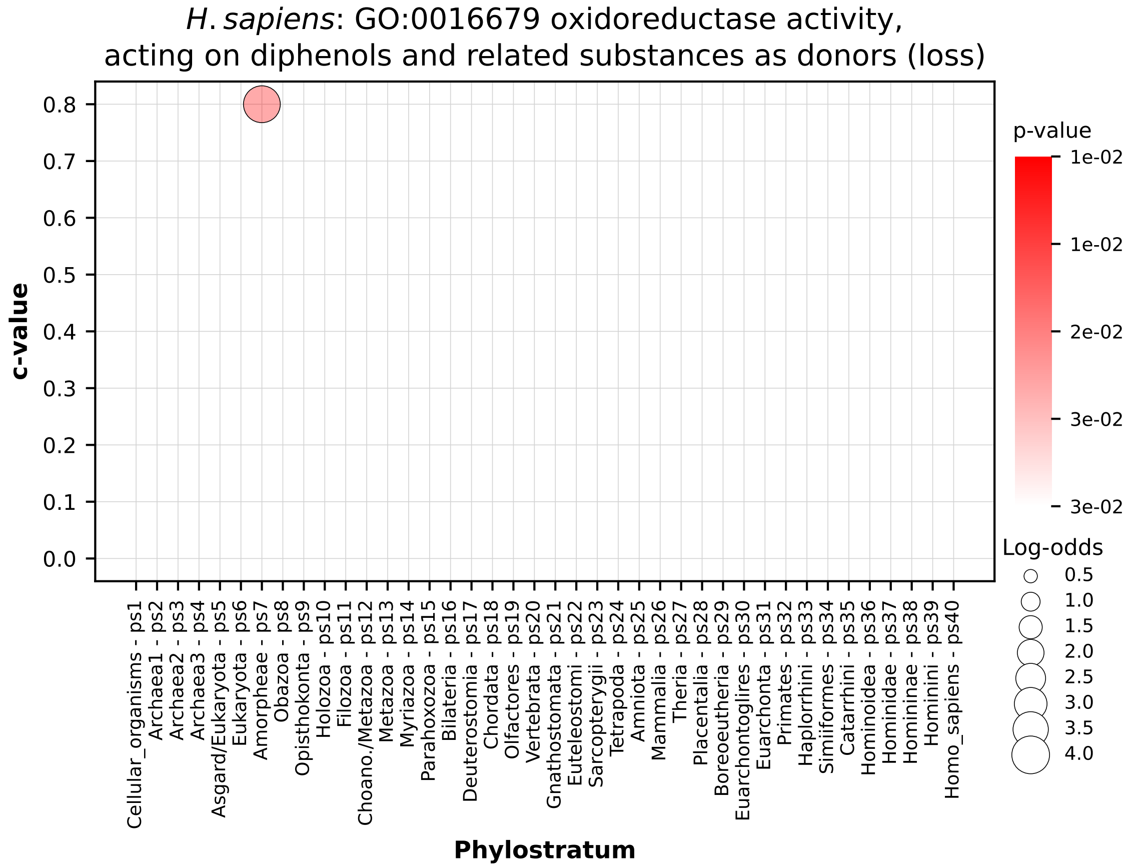

H. sapiens: GO:0016679 oxidoreductase activity,
acting on diphenols and related substances as donors (loss)
c-value
0.0
0.1
0.2
0.3
0.4
0.5
0.6
0.7
0.8
Phylostratum
Cellular_organisms - ps1
Archaea1 - ps2
Archaea2 - ps3
Archaea3 - ps4
Asgard/Eukaryota - ps5
Eukaryota - ps6
Amorpheae - ps7
Obazoa - ps8
Opisthokonta - ps9
Holozoa - ps10
Filozoa - ps11
Choano./Metazoa - ps12
Metazoa - ps13
Myriazoa - ps14
Parahoxozoa - ps15
Bilateria - ps16
Deuterostomia - ps17
Chordata - ps18
Olfactores - ps19
Vertebrata - ps20
Gnathostomata - ps21
Euteleostomi - ps22
Sarcopterygii - ps23
Tetrapoda - ps24
Amniota - ps25
Mammalia - ps26
Theria - ps27
Placentalia - ps28
Boreoeutheria - ps29
Euarchontoglires - ps30
Euarchonta - ps31
Primates - ps32
Haplorrhini - ps33
Simiiformes - ps34
Catarrhini - ps35
Hominoidea - ps36
Hominidae - ps37
Homininae - ps38
Hominini - ps39
Homo_sapiens - ps40
p-value
1e-02
1e-02
2e-02
3e-02
3e-02
Log-odds
0.5
1.0
1.5
2.0
2.5
3.0
3.5
4.0

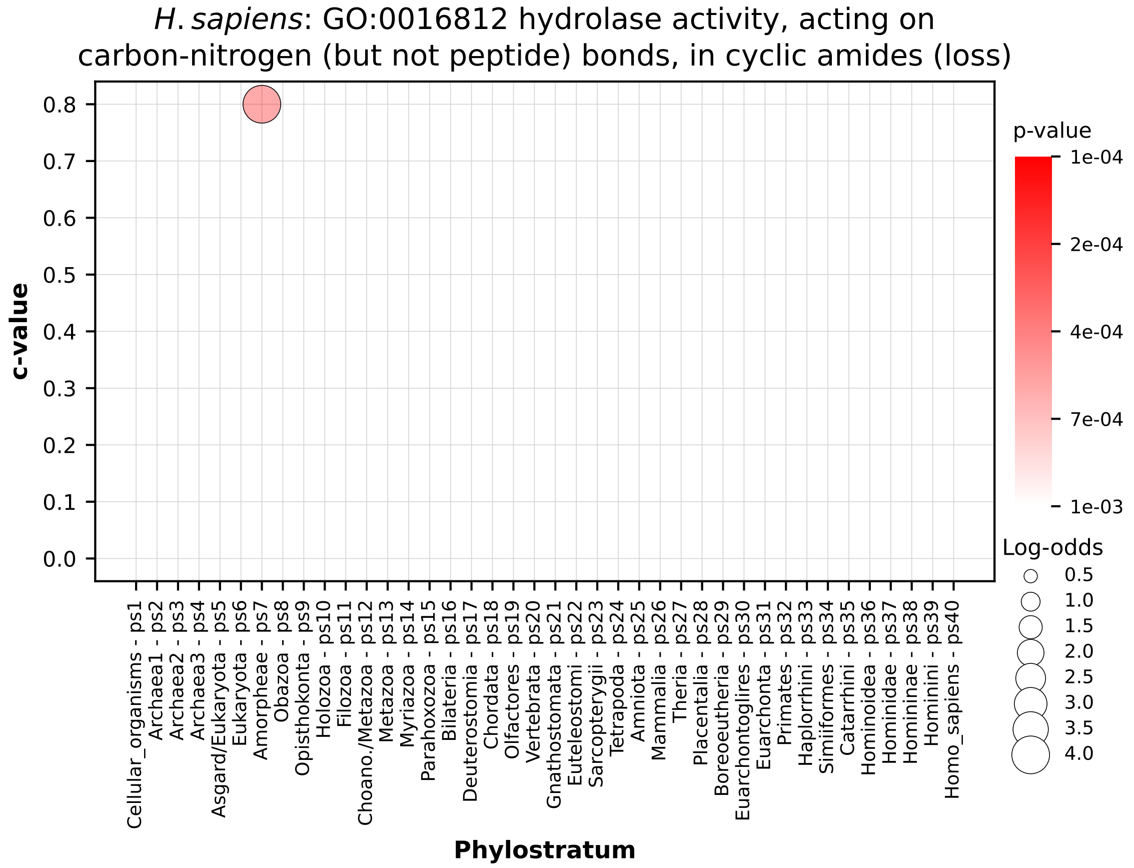

H. sapiens: GO:0016812 hydrolase activity, acting on carbon-nitrogen (but not peptide) bonds, in cyclic amides (loss)
c-value
0.0
0.1
0.2
0.3
0.4
0.5
0.6
0.7
0.8
Phylostratum
Cellular_organisms - ps1
Archaea1 - ps2
Archaea2 - ps3
Archaea3 - ps4
Asgard/Eukaryota - ps5
Eukaryota - ps6
Amorpheae - ps7
Obazoa - ps8
Opisthokonta - ps9
Holozoa - ps10
Filozoa - ps11
Choano./Metazoa - ps12
Metazoa - ps13
Myriazoa - ps14
Parahoxozoa - ps15
Bilateria - ps16
Deuterostomia - ps17
Chordata - ps18
Olfactores - ps19
Vertebrata - ps20
Gnathostomata - ps21
Euteleostomi - ps22
Sarcopterygii - ps23
Tetrapoda - ps24
Amniota - ps25
Mammalia - ps26
Theria - ps27
Placentalia - ps28
Boreoeutheria - ps29
Euarchontoglires - ps30
Euarchonta - ps31
Primates - ps32
Haplorrhini - ps33
Simiiformes - ps34
Catarrhini - ps35
Hominoidea - ps36
Hominidae - ps37
Homininae - ps38
Hominini - ps39
Homo_sapiens - ps40
p-value
1e-04
2e-04
4e-04
7e-04
1e-03
Log-odds
0.5
1.0
1.5
2.0
2.5
3.0
3.5
4.0

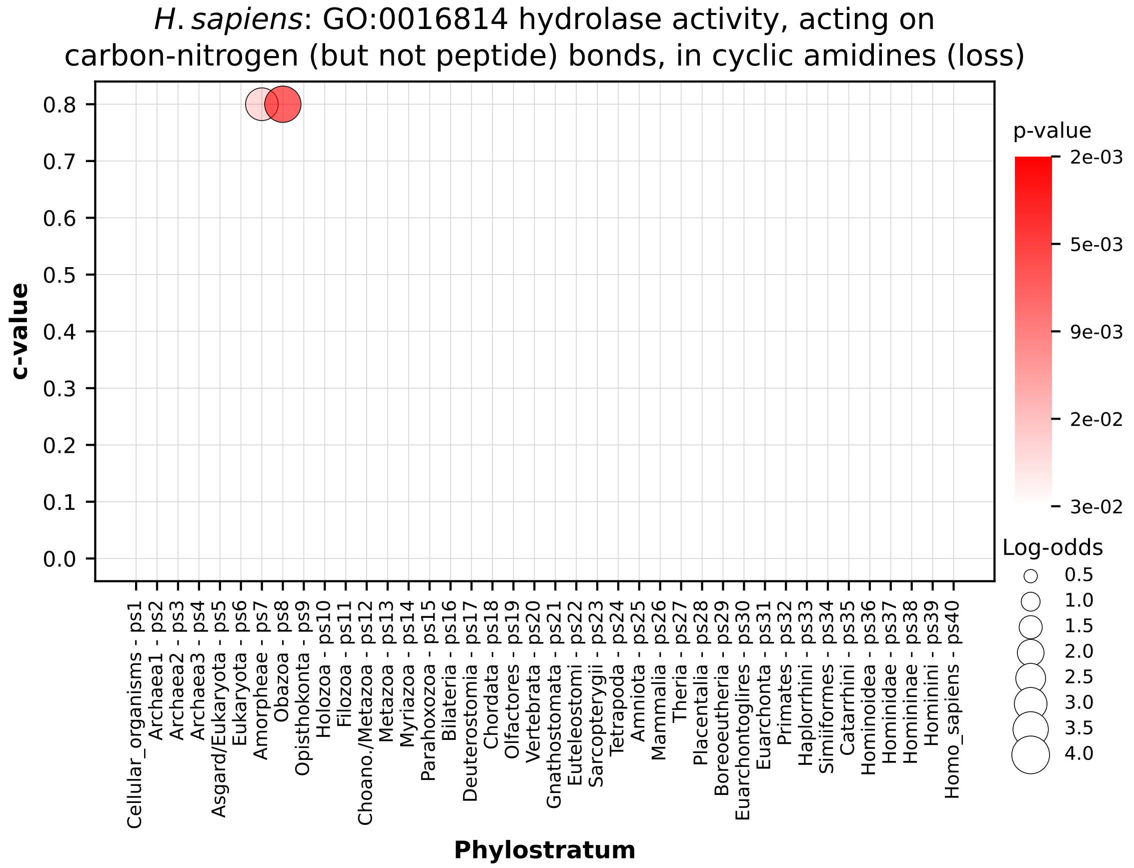

H. sapiens: GO:0016814 hydrolase activity, acting on carbon-nitrogen (but not peptide) bonds, in cyclic amidines (loss)
c-value
0.0
0.1
0.2
0.3
0.4
0.5
0.6
0.7
0.8
Phylostratum
Cellular_organisms - ps1
Archaea1 - ps2
Archaea2 - ps3
Archaea3 - ps4
Asgard/Eukaryota - ps5
Eukaryota - ps6
Amorpheae - ps7
Obazoa - ps8
Opisthokonta - ps9
Holozoa - ps10
Filozoa - ps11
Choano./Metazoa - ps12
Metazoa - ps13
Myriazoa - ps14
Parahoxozoa - ps15
Bilateria - ps16
Deuterostomia - ps17
Chordata - ps18
Olfactores - ps19
Vertebrata - ps20
Gnathostomata - ps21
Euteleostomi - ps22
Sarcopterygii - ps23
Tetrapoda - ps24
Amniota - ps25
Mammalia - ps26
Theria - ps27
Placentalia - ps28
Boreoeutheria - ps29
Euarchontoglires - ps30
Euarchonta - ps31
Primates - ps32
Haplorrhini - ps33
Simiiformes - ps34
Catarrhini - ps35
Hominoidea - ps36
Hominidae - ps37
Homininae - ps38
Hominini - ps39
Homo_sapiens - ps40
p-value
2e-03
5e-03
9e-03
2e-02
3e-02
Log-odds
0.5
1.0
1.5
2.0
2.5
3.0
3.5
4.0

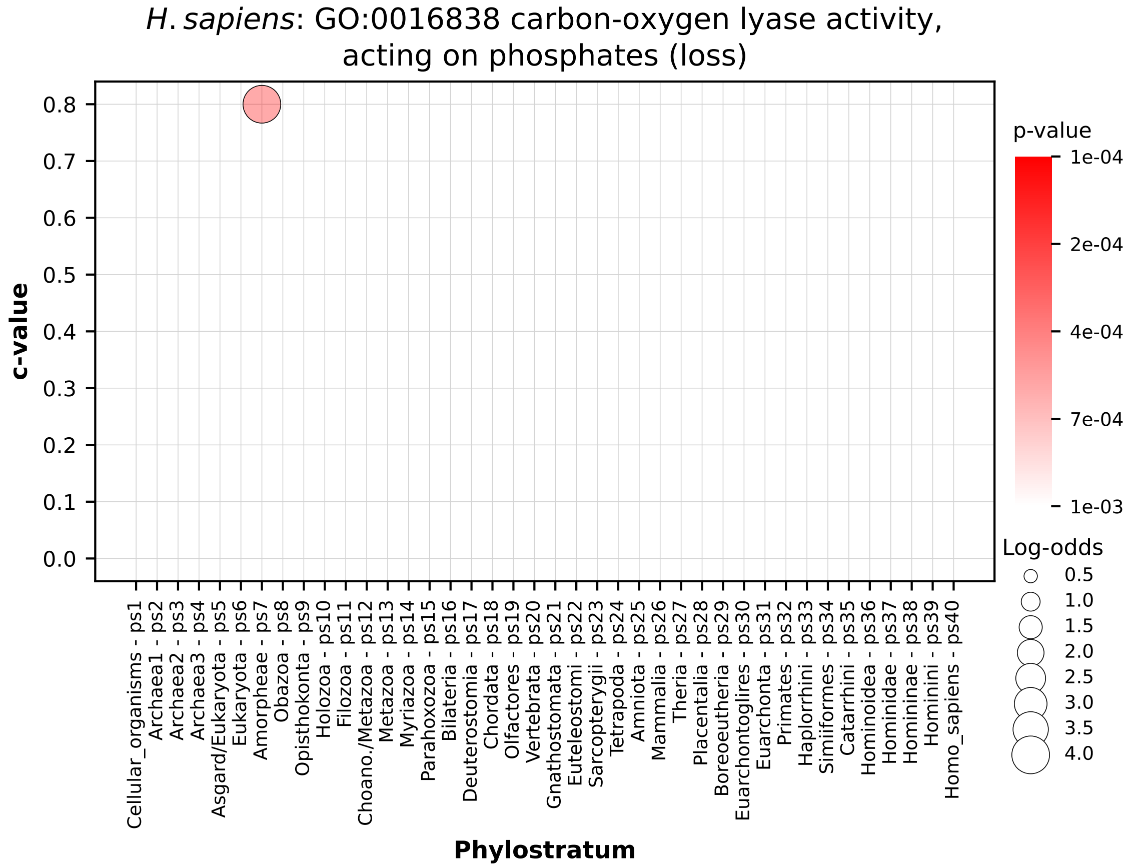
H. sapiens: GO:0016838 carbon-oxygen lyase activity, acting on phosphates (loss)
c-value
0.0
0.1
0.2
0.3
0.4
0.5
0.6
0.7
0.8
p-value
1e-04
2e-04
4e-04
7e-04
1e-03
Log-odds
0.5
1.0
1.5
2.0
2.5
3.0
3.5
4.0
Cellular_organisms - ps1
Archaea1 - ps2
Archaea2 - ps3
Archaea3 - ps4
Asgard/Eukaryota - ps5
Eukaryota - ps6
Amorpheae - ps7
Obazoa - ps8
Opisthokonta - ps9
Holozoa - ps10
Filozoa - ps11
Choano./Metazoa - ps12
Metazoa - ps13
Myriazoa - ps14
Parahoxozoa - ps15
Bilateria - ps16
Deuterostomia - ps17
Chordata - ps18
Olfactores - ps19
Vertebrata - ps20
Gnathostomata - ps21
Euteleostomi - ps22
Sarcopterygii - ps23
Tetrapoda - ps24
Amniota - ps25
Mammalia - ps26
Theria - ps27
Placentalia - ps28
Boreoeutheria - ps29
Euarchontoglires - ps30
Euarchonta - ps31
Primates - ps32
Haplorrhini - ps33
Simiiformes - ps34
Catarrhini - ps35
Hominoidea - ps36
Hominidae - ps37
Homininae - ps38
Hominini - ps39
Homo_sapiens - ps40
Phylostratum

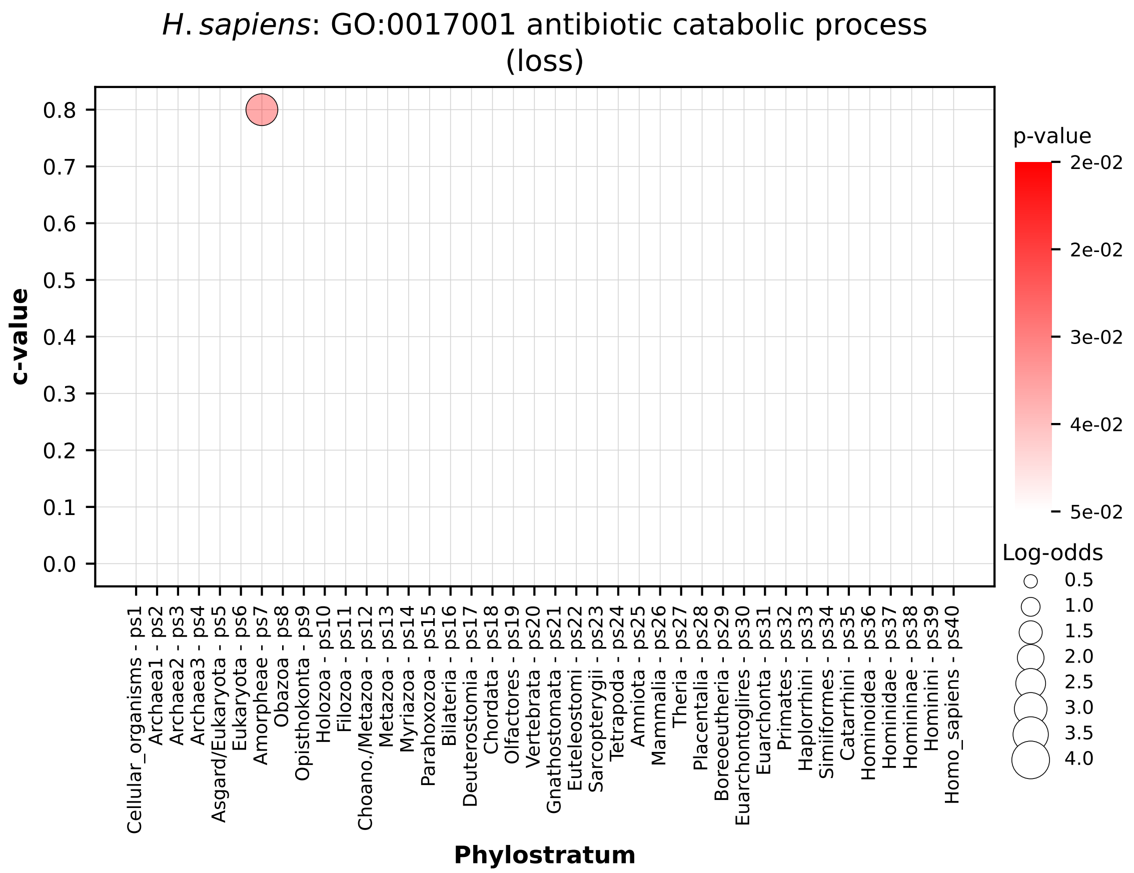

H. sapiens: GO:0017001 antibiotic catabolic process
(loss)
c-value
0.0
0.1
0.2
0.3
0.4
0.5
0.6
0.7
0.8
p-value
2e-02
2e-02
3e-02
4e-02
5e-02
Log-odds
0.5
1.0
1.5
2.0
2.5
3.0
3.5
4.0
Cellular_organisms - ps1
Archaea1 - ps2
Archaea2 - ps3
Archaea3 - ps4
Asgard/Eukaryota - ps5
Eukaryota - ps6
Amorpheae - ps7
Obazoa - ps8
Opisthokonta - ps9
Holozoa - ps10
Filozoa - ps11
Choano./Metazoa - ps12
Metazoa - ps13
Myriazoa - ps14
Parahoxozoa - ps15
Bilateria - ps16
Deuterostomia - ps17
Chordata - ps18
Olfactores - ps19
Vertebrata - ps20
Gnathostomata - ps21
Euteleostomi - ps22
Sarcopterygii - ps23
Tetrapoda - ps24
Amniota - ps25
Mammalia - ps26
Theria - ps27
Placentalia - ps28
Boreoeutheria - ps29
Euarchontoglires - ps30
Euarchonta - ps31
Primates - ps32
Haplorrhini - ps33
Simiiformes - ps34
Catarrhini - ps35
Hominoidea - ps36
Hominidae - ps37
Homininae - ps38
Hominini - ps39
Homo_sapiens - ps40
Phylostratum

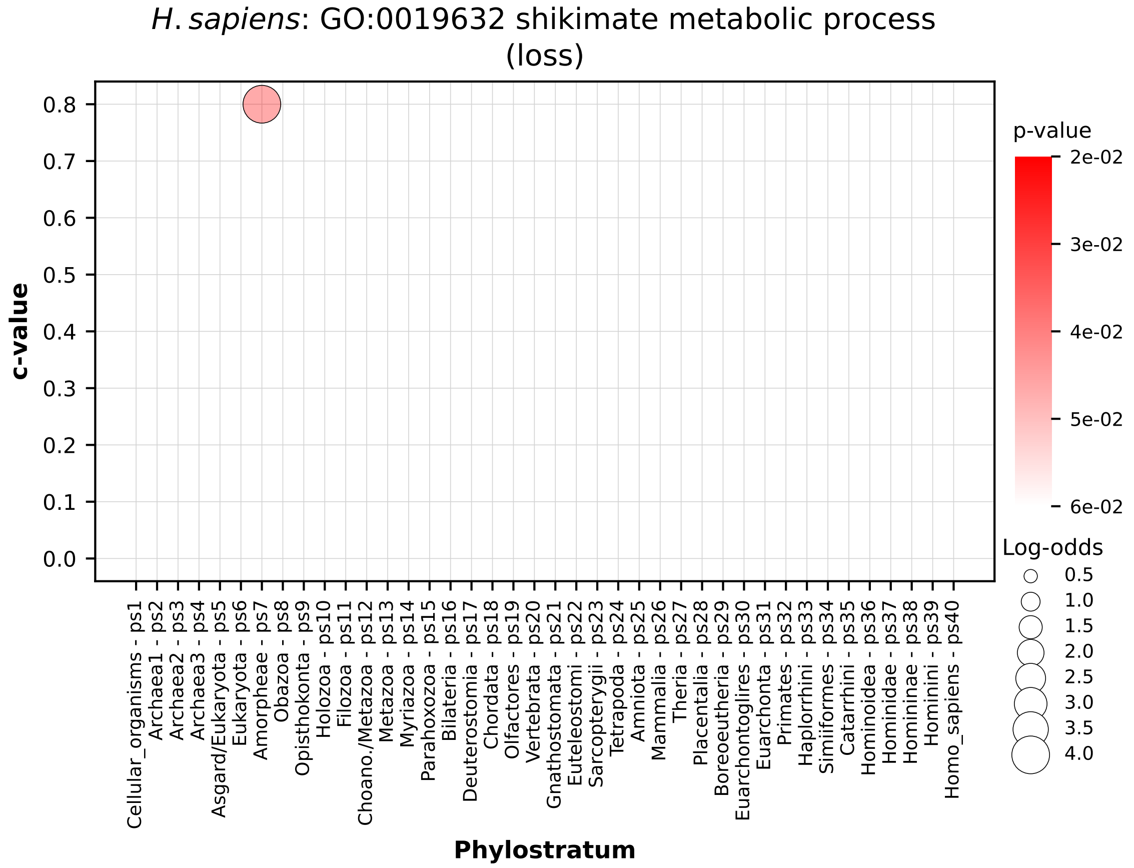

H. sapiens: GO:0019632 shikimate metabolic process
(loss)
c-value
0.8
0.7
0.6
0.5
0.4
0.3
0.2
0.1
0.0
p-value
2e-02
3e-02
4e-02
5e-02
6e-02
Log-odds
0.5
1.0
1.5
2.0
2.5
3.0
3.5
4.0
Cellular_organisms - ps1
Archaea1 - ps2
Archaea2 - ps3
Archaea3 - ps4
Asgard/Eukaryota - ps5
Eukaryota - ps6
Amorpheae - ps7
Obazoa - ps8
Opisthokonta - ps9
Holozoa - ps10
Filozoa - ps11
Choano./Metazoa - ps12
Metazoa - ps13
Myriazoa - ps14
Parahoxozoa - ps15
Bilateria - ps16
Deuterostomia - ps17
Chordata - ps18
Olfactores - ps19
Vertebrata - ps20
Gnathostomata - ps21
Euteleostomi - ps22
Sarcopterygii - ps23
Tetrapoda - ps24
Amniota - ps25
Mammalia - ps26
Theria - ps27
Placentalia - ps28
Boreoeutheria - ps29
Euarchontoglires - ps30
Euarchonta - ps31
Primates - ps32
Haplorrhini - ps33
Simiiformes - ps34
Catarrhini - ps35
Hominoidea - ps36
Hominidae - ps37
Homininae - ps38
Hominini - ps39
Homo_sapiens - ps40
Phylostratum

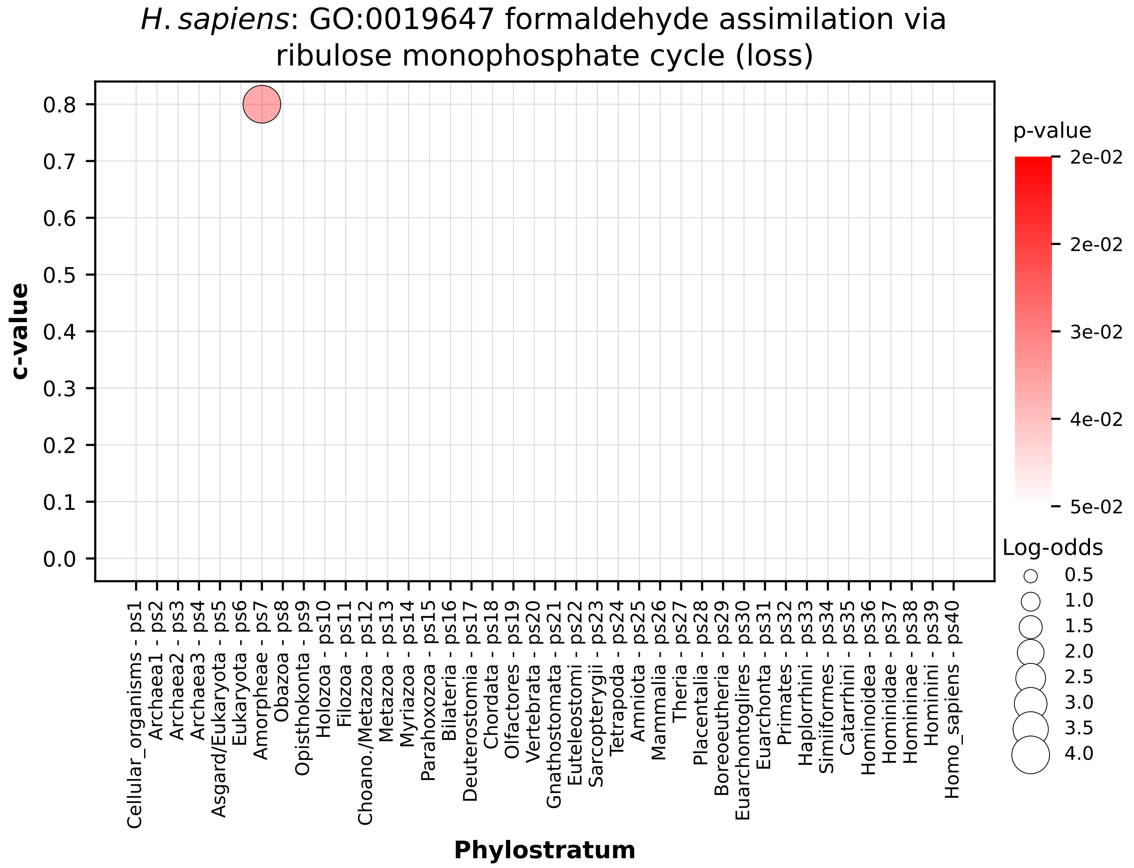

H. sapiens: GO:0019647 formaldehyde assimilation via ribulose monophosphate cycle (loss)
c-value
0.8
0.7
0.6
0.5
0.4
0.3
0.2
0.1
0.0
p-value
2e-02
2e-02
3e-02
4e-02
5e-02
Log-odds
0.5
1.0
1.5
2.0
2.5
3.0
3.5
4.0
Cellular_organisms - ps1
Archaea1 - ps2
Archaea2 - ps3
Archaea3 - ps4
Asgard/Eukaryota - ps5
Eukaryota - ps6
Amorpheae - ps7
Obazoa - ps8
Opisthokonta - ps9
Holozoa - ps10
Filozoa - ps11
Choano./Metazoa - ps12
Metazoa - ps13
Myriazoa - ps14
Parahoxozoa - ps15
Bilateria - ps16
Deuterostomia - ps17
Chordata - ps18
Olfactores - ps19
Vertebrata - ps20
Gnathostomata - ps21
Euteleostomi - ps22
Sarcopterygii - ps23
Tetrapoda - ps24
Amniota - ps25
Mammalia - ps26
Theria - ps27
Placentalia - ps28
Boreoeutheria - ps29
Euarchontoglires - ps30
Euarchonta - ps31
Primates - ps32
Haplorrhini - ps33
Simiiformes - ps34
Catarrhini - ps35
Hominoidea - ps36
Hominidae - ps37
Homininae - ps38
Hominini - ps39
Homo_sapiens - ps40
Phylostratum

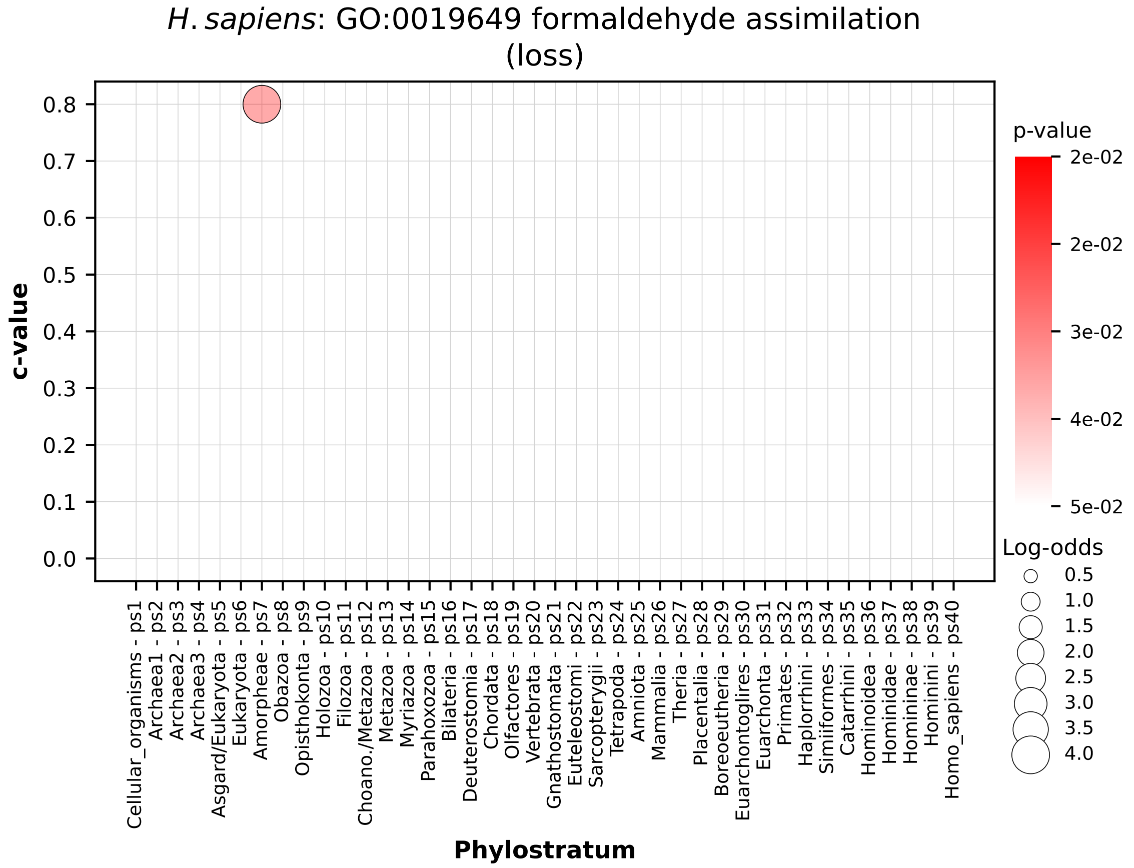

H. sapiens: GO:0019649 formaldehyde assimilation
(loss)
c-value
0.0
0.1
0.2
0.3
0.4
0.5
0.6
0.7
0.8
Phylostratum
Cellular_organisms - ps1
Archaea1 - ps2
Archaea2 - ps3
Archaea3 - ps4
Asgard/Eukaryota - ps5
Eukaryota - ps6
Amorpheae - ps7
Obazoa - ps8
Opisthokonta - ps9
Holozoa - ps10
Filozoa - ps11
Choano./Metazoa - ps12
Metazoa - ps13
Myriazoa - ps14
Parahoxozoa - ps15
Bilateria - ps16
Deuterostomia - ps17
Chordata - ps18
Olfactores - ps19
Vertebrata - ps20
Gnathostomata - ps21
Euteleostomi - ps22
Sarcopterygii - ps23
Tetrapoda - ps24
Amniota - ps25
Mammalia - ps26
Theria - ps27
Placentalia - ps28
Boreoeutheria - ps29
Euarchontoglires - ps30
Euarchonta - ps31
Primates - ps32
Haplorrhini - ps33
Simiiformes - ps34
Catarrhini - ps35
Hominoidea - ps36
Hominidae - ps37
Homininae - ps38
Hominini - ps39
Homo_sapiens - ps40
p-value
2e-02
2e-02
3e-02
4e-02
5e-02
Log-odds
0.5
1.0
1.5
2.0
2.5
3.0
3.5
4.0

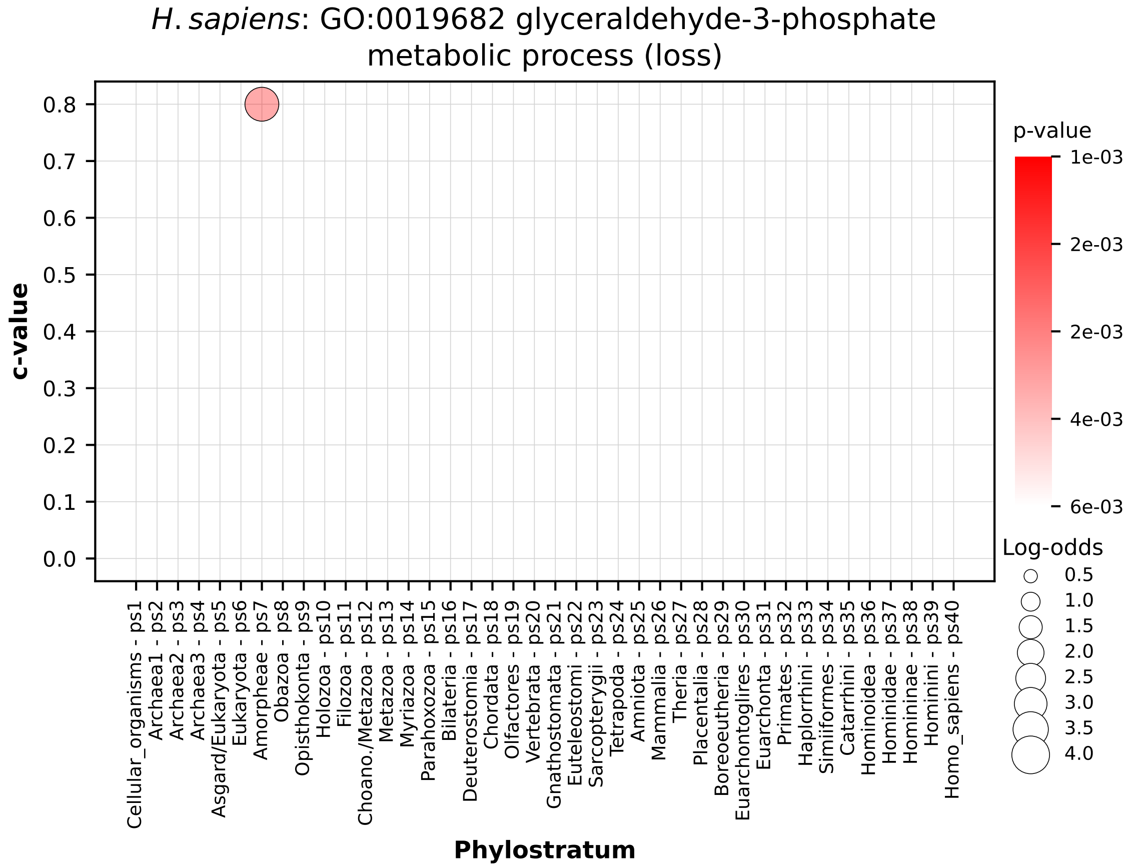

H. sapiens: GO:0019682 glyceraldehyde-3-phosphate metabolic process (loss)
c-value
0.0
0.1
0.2
0.3
0.4
0.5
0.6
0.7
0.8
p-value
1e-03
2e-03
2e-03
4e-03
6e-03
Log-odds
0.5
1.0
1.5
2.0
2.5
3.0
3.5
4.0
Cellular_organisms - ps1
Archaea1 - ps2
Archaea2 - ps3
Archaea3 - ps4
Asgard/Eukaryota - ps5
Eukaryota - ps6
Amorpheae - ps7
Obazoa - ps8
Opisthokonta - ps9
Holozoa - ps10
Filozoa - ps11
Choano./Metazoa - ps12
Metazoa - ps13
Myriazoa - ps14
Parahoxozoa - ps15
Bilateria - ps16
Deuterostomia - ps17
Chordata - ps18
Olfactores - ps19
Vertebrata - ps20
Gnathostomata - ps21
Euteleostomi - ps22
Sarcopterygii - ps23
Tetrapoda - ps24
Amniota - ps25
Mammalia - ps26
Theria - ps27
Placentalia - ps28
Boreoeutheria - ps29
Euarchontoglires - ps30
Euarchonta - ps31
Primates - ps32
Haplorrhini - ps33
Simiiformes - ps34
Catarrhini - ps35
Hominoidea - ps36
Hominidae - ps37
Homininae - ps38
Hominini - ps39
Homo_sapiens - ps40
Phylostratum

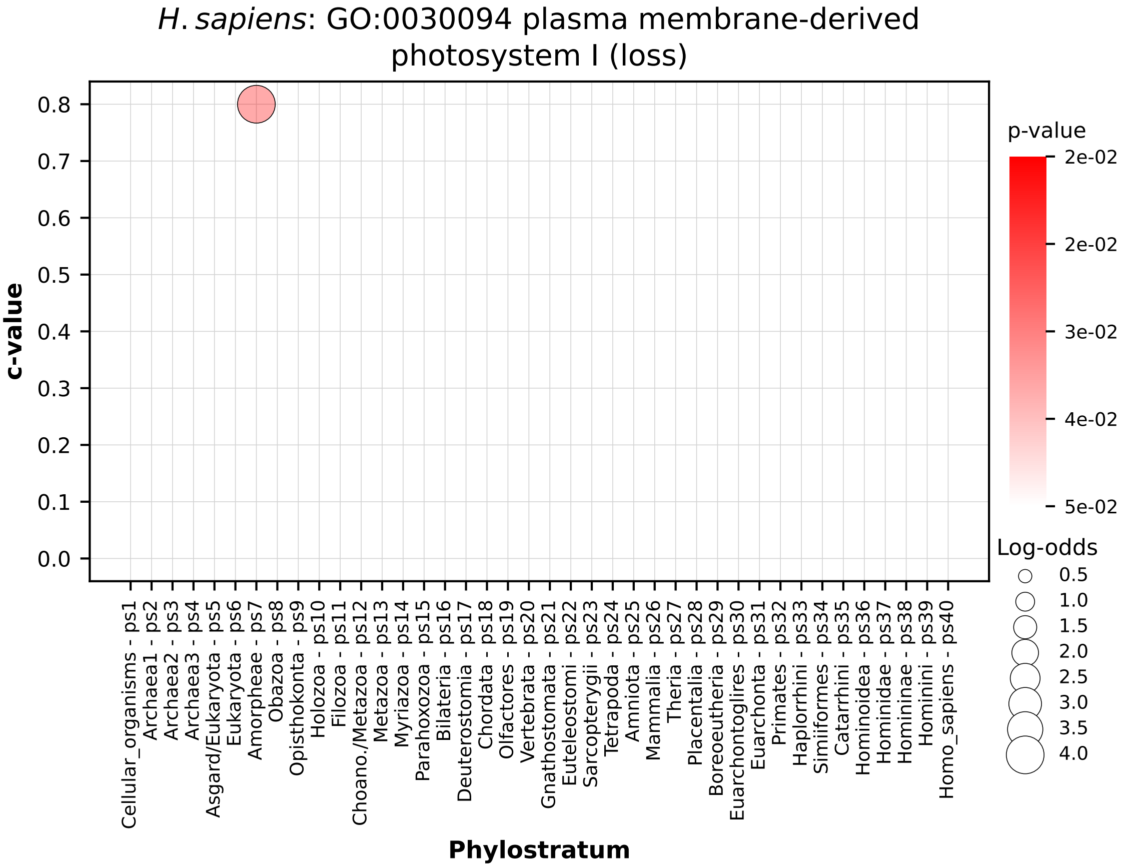

H. sapiens: GO:0030094 plasma membrane-derived photosystem I (loss)
c-value
0.8
0.7
0.6
0.5
0.4
0.3
0.2
0.1
0.0
p-value
2e-02
2e-02
3e-02
4e-02
5e-02
Log-odds
0.5
1.0
1.5
2.0
2.5
3.0
3.5
4.0
Cellular_organisms - ps1
Archaea1 - ps2
Archaea2 - ps3
Archaea3 - ps4
Asgard/Eukaryota - ps5
Eukaryota - ps6
Amorpheae - ps7
Obazoa - ps8
Opisthokonta - ps9
Holozoa - ps10
Filozoa - ps11
Choano./Metazoa - ps12
Metazoa - ps13
Myriazoa - ps14
Parahoxozoa - ps15
Bilateria - ps16
Deuterostomia - ps17
Chordata - ps18
Olfactores - ps19
Vertebrata - ps20
Gnathostomata - ps21
Euteleostomi - ps22
Sarcopterygii - ps23
Tetrapoda - ps24
Amniota - ps25
Mammalia - ps26
Theria - ps27
Placentalia - ps28
Boreoeutheria - ps29
Euarchontoglires - ps30
Euarchonta - ps31
Primates - ps32
Haplorrhini - ps33
Simiiformes - ps34
Catarrhini - ps35
Hominoidea - ps36
Hominidae - ps37
Homininae - ps38
Hominini - ps39
Homo_sapiens - ps40
Phylostratum

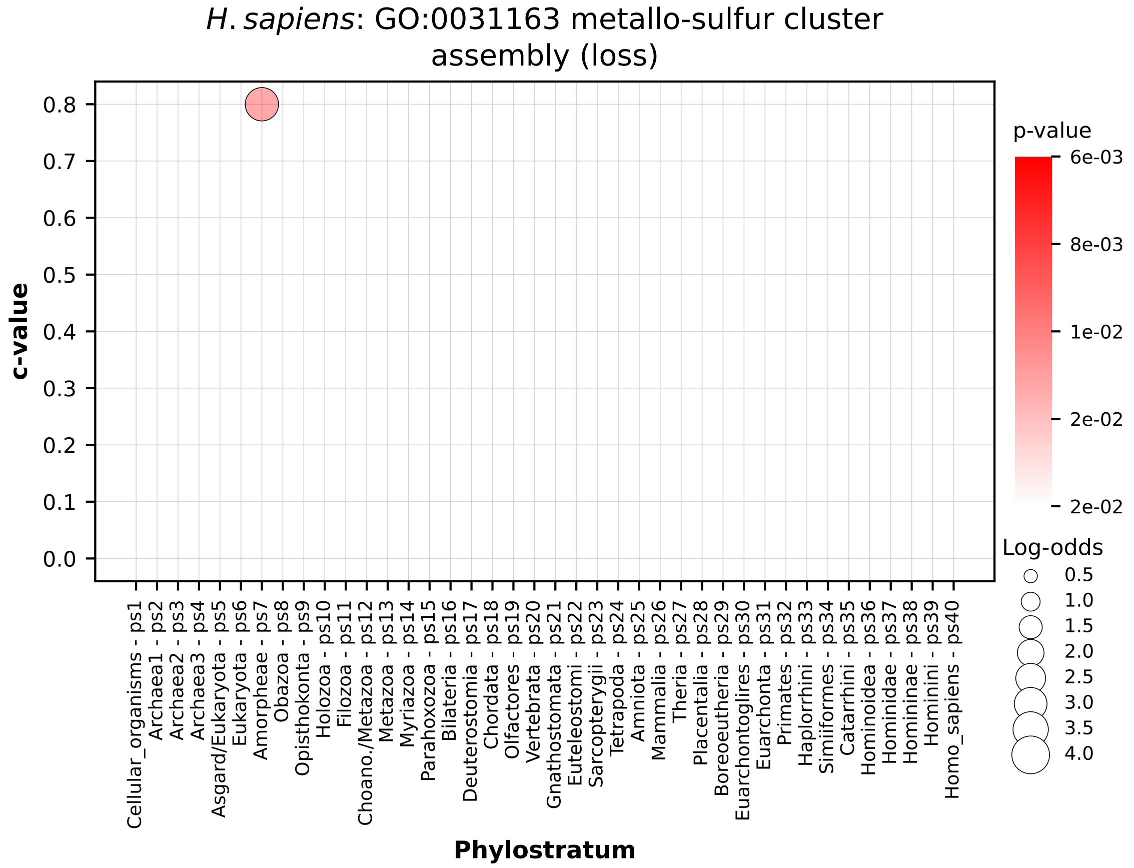

H. sapiens: GO:0031163 metallo-sulfur cluster assembly (loss)
c-value
0.8
0.7
0.6
0.5
0.4
0.3
0.2
0.1
0.0
p-value
6e-03
8e-03
1e-02
2e-02
2e-02
Log-odds
0.5
1.0
1.5
2.0
2.5
3.0
3.5
4.0
Cellular_organisms - ps1
Archaea1 - ps2
Archaea2 - ps3
Archaea3 - ps4
Asgard/Eukaryota - ps5
Eukaryota - ps6
Amorpheae - ps7
Obazoa - ps8
Opisthokonta - ps9
Holozoa - ps10
Filozoa - ps11
Choano./Metazoa - ps12
Metazoa - ps13
Myriazoa - ps14
Parahoxozoa - ps15
Bilateria - ps16
Deuterostomia - ps17
Chordata - ps18
Olfactores - ps19
Vertebrata - ps20
Gnathostomata - ps21
Euteleostomi - ps22
Sarcopterygii - ps23
Tetrapoda - ps24
Amniota - ps25
Mammalia - ps26
Theria - ps27
Placentalia - ps28
Boreoeutheria - ps29
Euarchontoglires - ps30
Euarchonta - ps31
Primates - ps32
Haplorrhini - ps33
Simiiformes - ps34
Catarrhini - ps35
Hominoidea - ps36
Hominidae - ps37
Homininae - ps38
Hominini - ps39
Homo_sapiens - ps40
Phylostratum

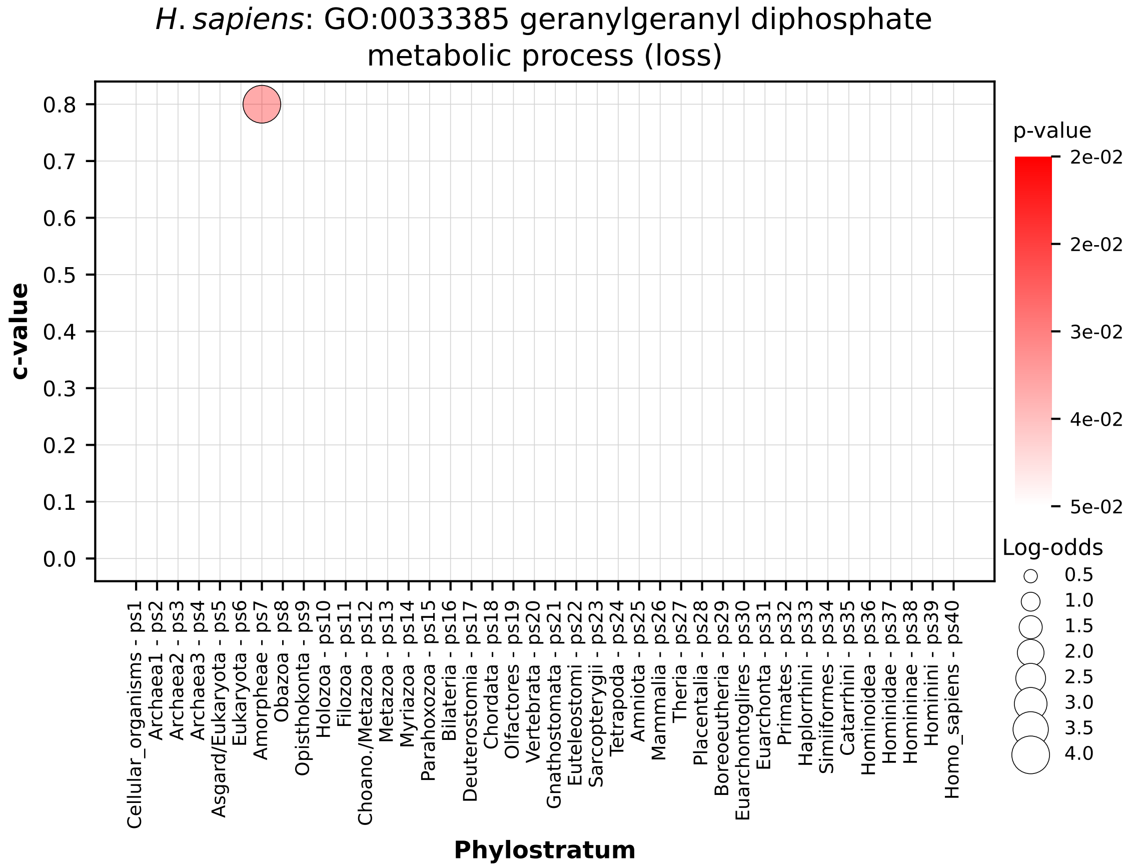

H. sapiens: GO:0033385 geranylgeranyl diphosphate metabolic process (loss)
c-value
0.8
0.7
0.6
0.5
0.4
0.3
0.2
0.1
0.0
p-value
2e-02
2e-02
3e-02
4e-02
5e-02
Log-odds
0.5
1.0
1.5
2.0
2.5
3.0
3.5
4.0
Cellular_organisms - ps1
Archaea1 - ps2
Archaea2 - ps3
Archaea3 - ps4
Asgard/Eukaryota - ps5
Eukaryota - ps6
Amorpheae - ps7
Obazoa - ps8
Opisthokonta - ps9
Holozoa - ps10
Filozoa - ps11
Choano./Metazoa - ps12
Metazoa - ps13
Myriazoa - ps14
Parahoxozoa - ps15
Bilateria - ps16
Deuterostomia - ps17
Chordata - ps18
Olfactores - ps19
Vertebrata - ps20
Gnathostomata - ps21
Euteleostomi - ps22
Sarcopterygii - ps23
Tetrapoda - ps24
Amniota - ps25
Mammalia - ps26
Theria - ps27
Placentalia - ps28
Boreoeutheria - ps29
Euarchontoglires - ps30
Euarchonta - ps31
Primates - ps32
Haplorrhini - ps33
Simiiformes - ps34
Catarrhini - ps35
Hominoidea - ps36
Hominidae - ps37
Homininae - ps38
Hominini - ps39
Homo_sapiens - ps40
Phylostratum

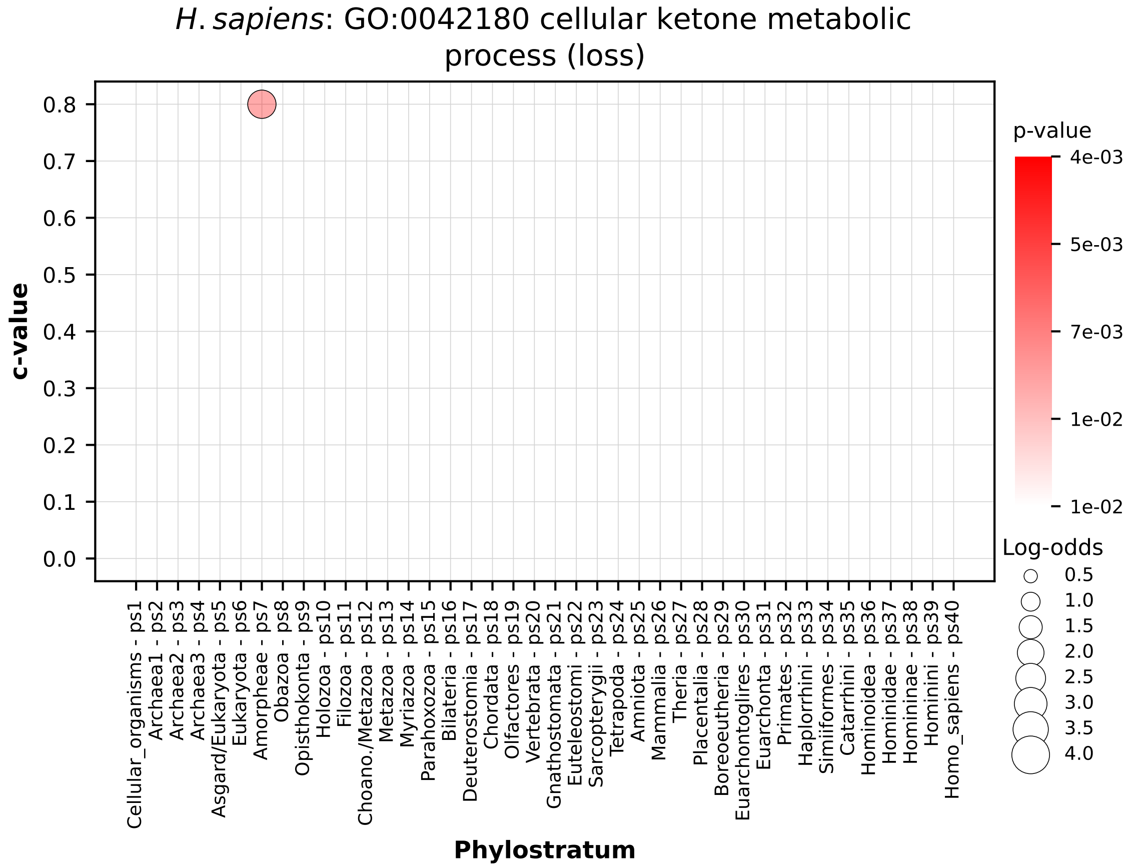

H. sapiens: GO:0042180 cellular ketone metabolic process (loss)
c-value
0.0
0.1
0.2
0.3
0.4
0.5
0.6
0.7
0.8
Phylostratum
Cellular_organisms - ps1
Archaea1 - ps2
Archaea2 - ps3
Archaea3 - ps4
Asgard/Eukaryota - ps5
Eukaryota - ps6
Amorpheae - ps7
Obazoa - ps8
Opisthokonta - ps9
Holozoa - ps10
Filozoa - ps11
Choano./Metazoa - ps12
Metazoa - ps13
Myriazoa - ps14
Parahoxozoa - ps15
Bilateria - ps16
Deuterostomia - ps17
Chordata - ps18
Olfactores - ps19
Vertebrata - ps20
Gnathostomata - ps21
Euteleostomi - ps22
Sarcopterygii - ps23
Tetrapoda - ps24
Amniota - ps25
Mammalia - ps26
Theria - ps27
Placentalia - ps28
Boreoeutheria - ps29
Euarchontoglires - ps30
Euarchonta - ps31
Primates - ps32
Haplorrhini - ps33
Simiiformes - ps34
Catarrhini - ps35
Hominoidea - ps36
Hominidae - ps37
Homininae - ps38
Hominini - ps39
Homo_sapiens - ps40
p-value
4e-03
5e-03
7e-03
1e-02
1e-02
Log-odds
0.5
1.0
1.5
2.0
2.5
3.0
3.5
4.0

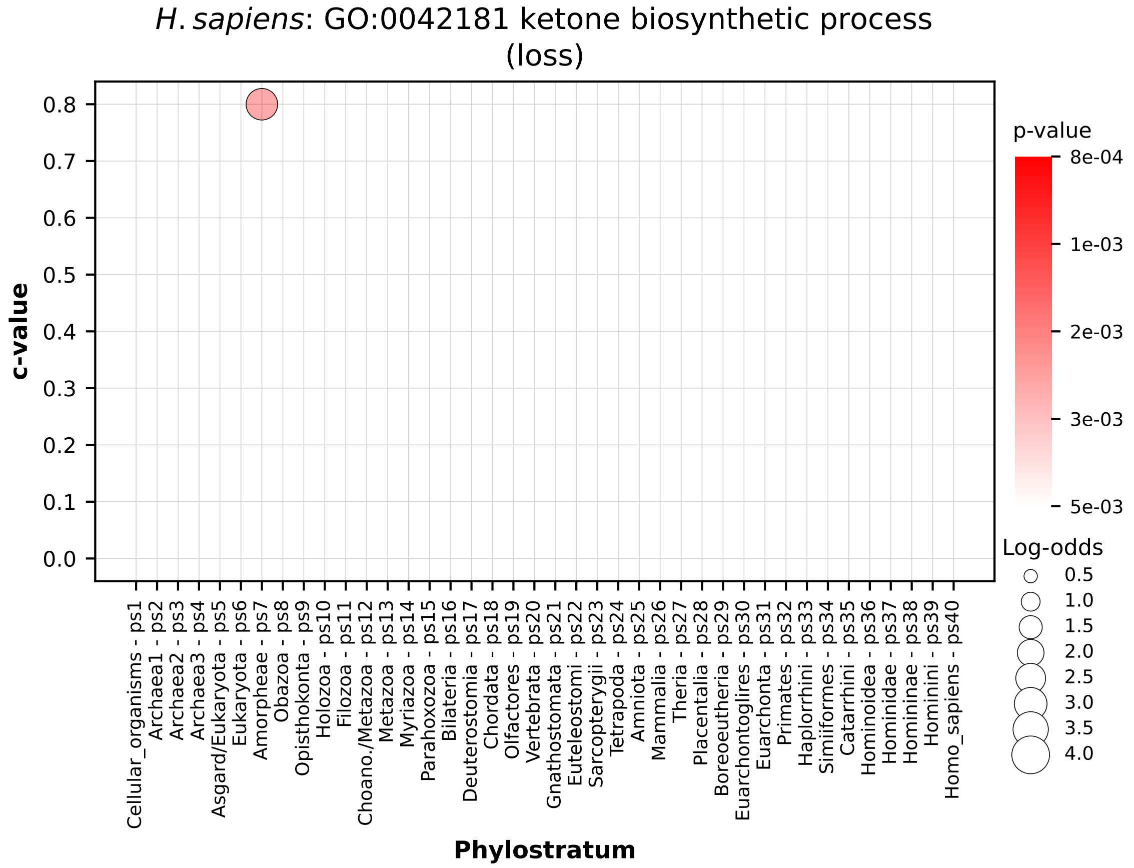
H. sapiens: GO:0042181 ketone biosynthetic process
(loss)
c-value
0.8
0.7
0.6
0.5
0.4
0.3
0.2
0.1
0.0
p-value
8e-04
1e-03
2e-03
3e-03
5e-03
Log-odds
0.5
1.0
1.5
2.0
2.5
3.0
3.5
4.0
Cellular_organisms - ps1
Archaea1 - ps2
Archaea2 - ps3
Archaea3 - ps4
Asgard/Eukaryota - ps5
Eukaryota - ps6
Amorpheae - ps7
Obazoa - ps8
Opisthokonta - ps9
Holozoa - ps10
Filozoa - ps11
Choano./Metazoa - ps12
Metazoa - ps13
Myriazoa - ps14
Parahoxozoa - ps15
Bilateria - ps16
Deuterostomia - ps17
Chordata - ps18
Olfactores - ps19
Vertebrata - ps20
Gnathostomata - ps21
Euteleostomi - ps22
Sarcopterygii - ps23
Tetrapoda - ps24
Amniota - ps25
Mammalia - ps26
Theria - ps27
Placentalia - ps28
Boreoeutheria - ps29
Euarchontoglires - ps30
Euarchonta - ps31
Primates - ps32
Haplorrhini - ps33
Simiiformes - ps34
Catarrhini - ps35
Hominoidea - ps36
Hominidae - ps37
Homininae - ps38
Hominini - ps39
Homo_sapiens - ps40
Phylostratum

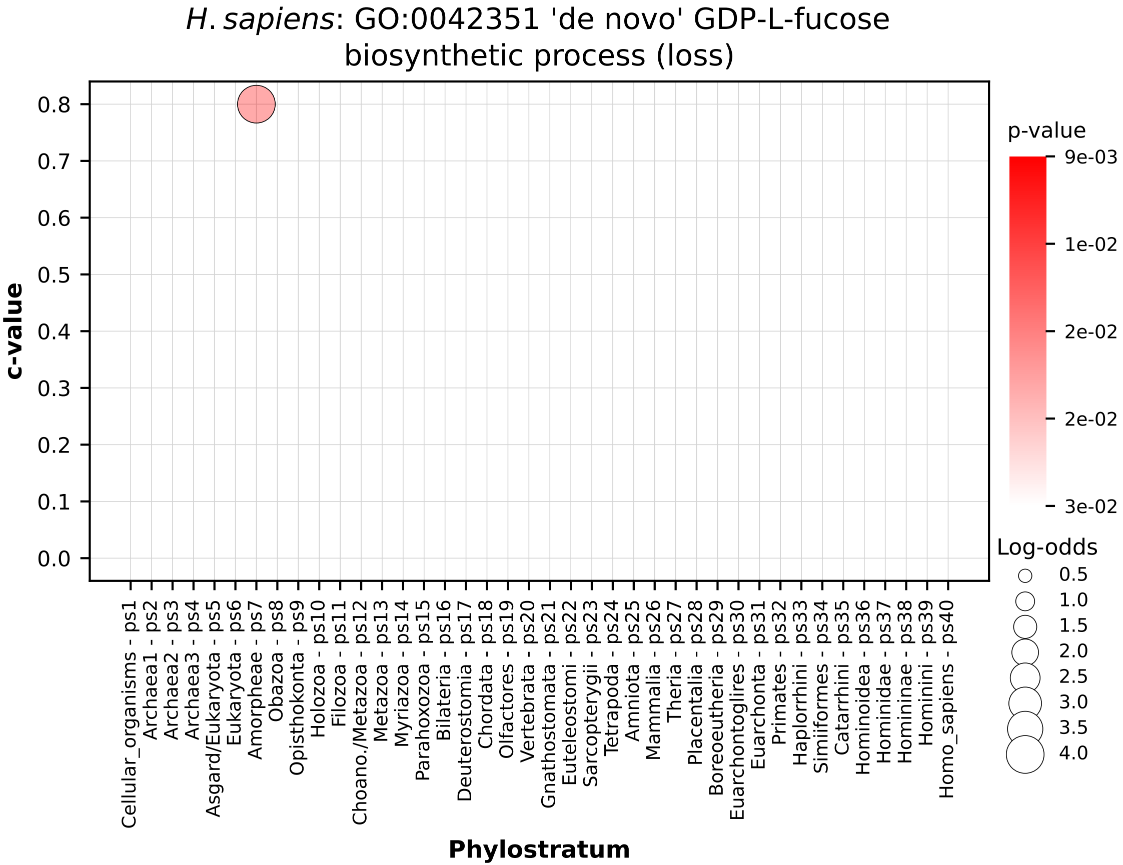

H. sapiens: GO:0042351 'de novo' GDP-L-fucose biosynthetic process (loss)
c-value
0.0
0.1
0.2
0.3
0.4
0.5
0.6
0.7
0.8
Phylostratum
Cellular_organisms - ps1
Archaea1 - ps2
Archaea2 - ps3
Archaea3 - ps4
Asgard/Eukaryota - ps5
Eukaryota - ps6
Amorpheae - ps7
Obazoa - ps8
Opisthokonta - ps9
Holozoa - ps10
Filozoa - ps11
Choano./Metazoa - ps12
Metazoa - ps13
Myriazoa - ps14
Parahoxozoa - ps15
Bilateria - ps16
Deuterostomia - ps17
Chordata - ps18
Olfactores - ps19
Vertebrata - ps20
Gnathostomata - ps21
Euteleostomi - ps22
Sarcopterygii - ps23
Tetrapoda - ps24
Amniota - ps25
Mammalia - ps26
Theria - ps27
Placentalia - ps28
Boreoeutheria - ps29
Euarchontoglires - ps30
Euarchonta - ps31
Primates - ps32
Haplorrhini - ps33
Simiiformes - ps34
Catarrhini - ps35
Hominoidea - ps36
Hominidae - ps37
Homininae - ps38
Hominini - ps39
Homo_sapiens - ps40
p-value
9e-03
1e-02
2e-02
2e-02
3e-02
Log-odds
0.5
1.0
1.5
2.0
2.5
3.0
3.5
4.0

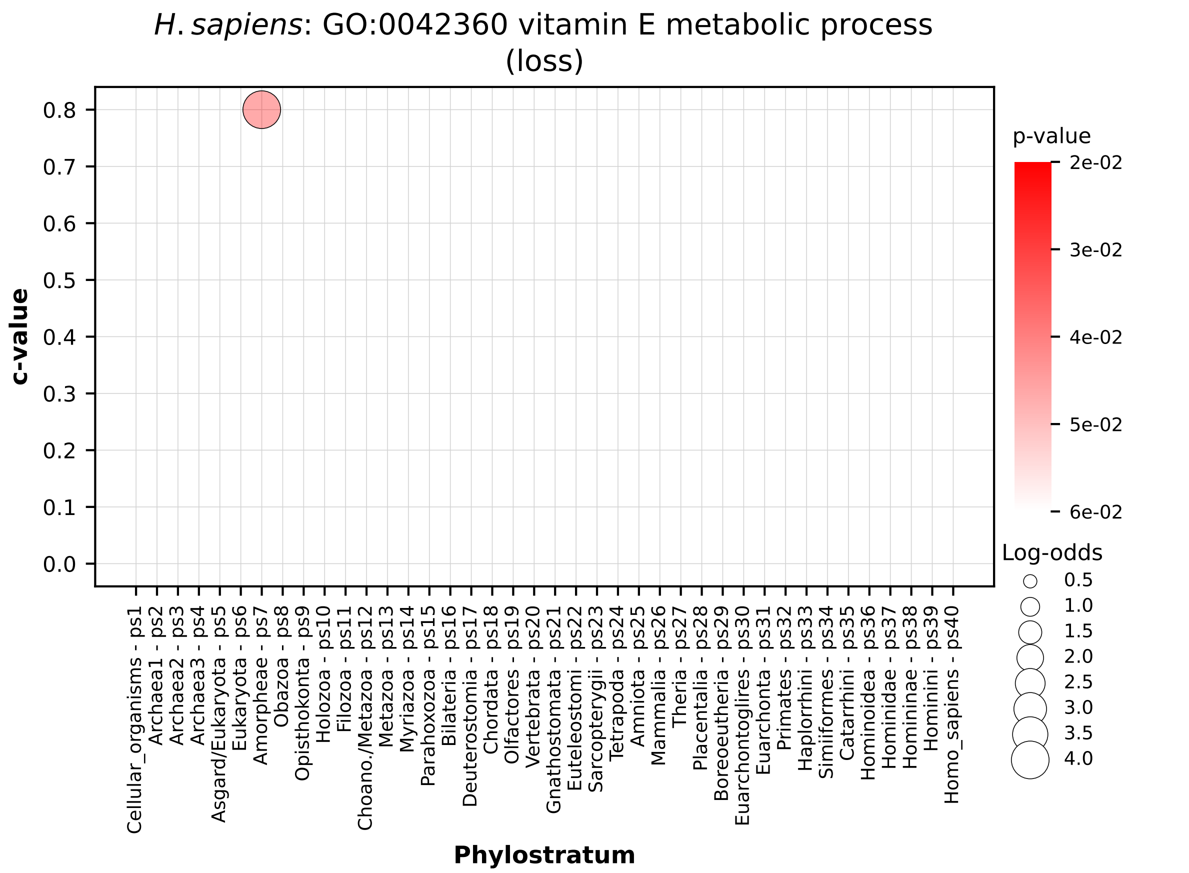

H. sapiens: GO:0042360 vitamin E metabolic process
(loss)
c-value
0.8
0.7
0.6
0.5
0.4
0.3
0.2
0.1
0.0
Phylostratum
Cellular_organisms - ps1
Archaea1 - ps2
Archaea2 - ps3
Archaea3 - ps4
Asgard/Eukaryota - ps5
Eukaryota - ps6
Amorpheae - ps7
Obazoa - ps8
Opisthokonta - ps9
Holozoa - ps10
Filozoa - ps11
Choano./Metazoa - ps12
Metazoa - ps13
Myriazoa - ps14
Parahoxozoa - ps15
Bilateria - ps16
Deuterostomia - ps17
Chordata - ps18
Olfactores - ps19
Vertebrata - ps20
Gnathostomata - ps21
Euteleostomi - ps22
Sarcopterygii - ps23
Tetrapoda - ps24
Amniota - ps25
Mammalia - ps26
Theria - ps27
Placentalia - ps28
Boreoeutheria - ps29
Euarchontoglires - ps30
Euarchonta - ps31
Primates - ps32
Haplorrhini - ps33
Simiiformes - ps34
Catarrhini - ps35
Hominoidea - ps36
Hominidae - ps37
Homininae - ps38
Hominini - ps39
Homo_sapiens - ps40
p-value
2e-02
3e-02
4e-02
5e-02
6e-02
Log-odds
0.5
1.0
1.5
2.0
2.5
3.0
3.5
4.0

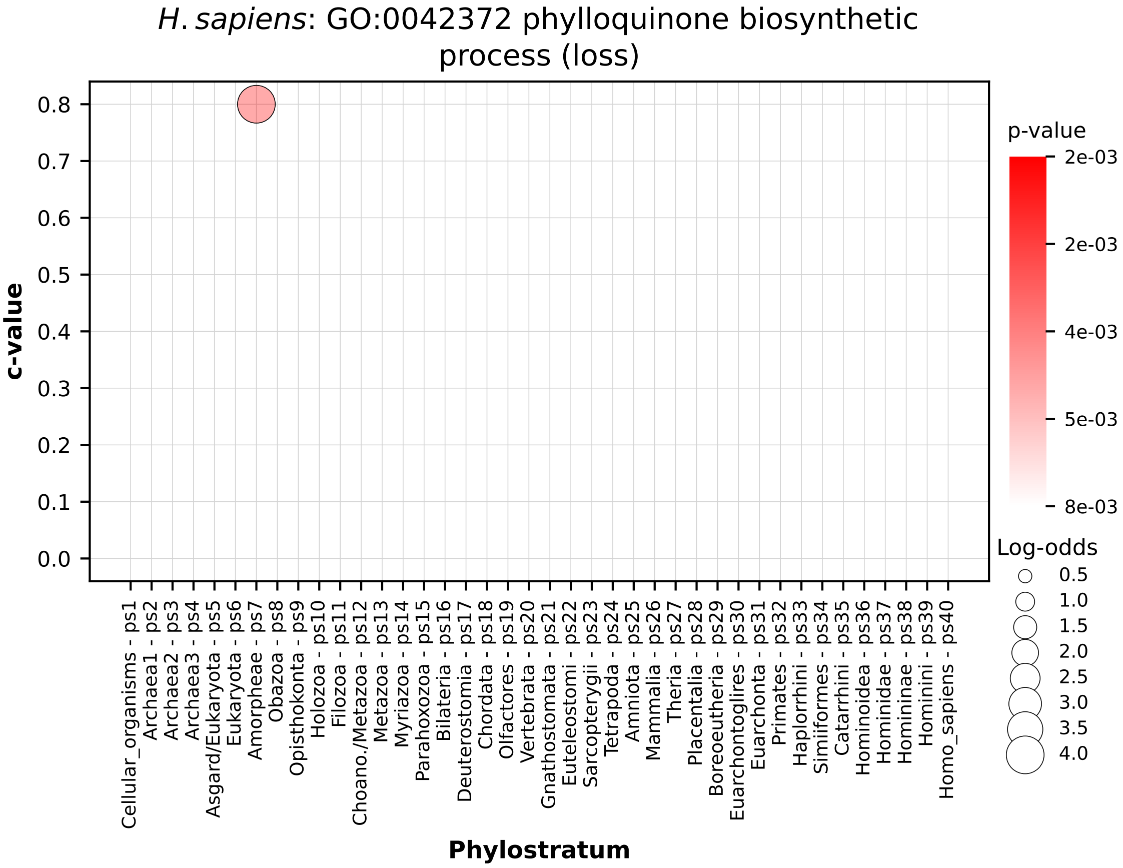
H. sapiens: GO:0042372 phylloquinone biosynthetic process (loss)
c-value
0.8
0.7
0.6
0.5
0.4
0.3
0.2
0.1
0.0
p-value
2e-03
2e-03
4e-03
5e-03
8e-03
Log-odds
0.5
1.0
1.5
2.0
2.5
3.0
3.5
4.0
Cellular_organisms - ps1
Archaea1 - ps2
Archaea2 - ps3
Archaea3 - ps4
Asgard/Eukaryota - ps5
Eukaryota - ps6
Amorpheae - ps7
Obazoa - ps8
Opisthokonta - ps9
Holozoa - ps10
Filozoa - ps11
Choano./Metazoa - ps12
Metazoa - ps13
Myriazoa - ps14
Parahoxozoa - ps15
Bilateria - ps16
Deuterostomia - ps17
Chordata - ps18
Olfactores - ps19
Vertebrata - ps20
Gnathostomata - ps21
Euteleostomi - ps22
Sarcopterygii - ps23
Tetrapoda - ps24
Amniota - ps25
Mammalia - ps26
Theria - ps27
Placentalia - ps28
Boreoeutheria - ps29
Euarchontoglires - ps30
Euarchonta - ps31
Primates - ps32
Haplorrhini - ps33
Simiiformes - ps34
Catarrhini - ps35
Hominoidea - ps36
Hominidae - ps37
Homininae - ps38
Hominini - ps39
Homo_sapiens - ps40
Phylostratum

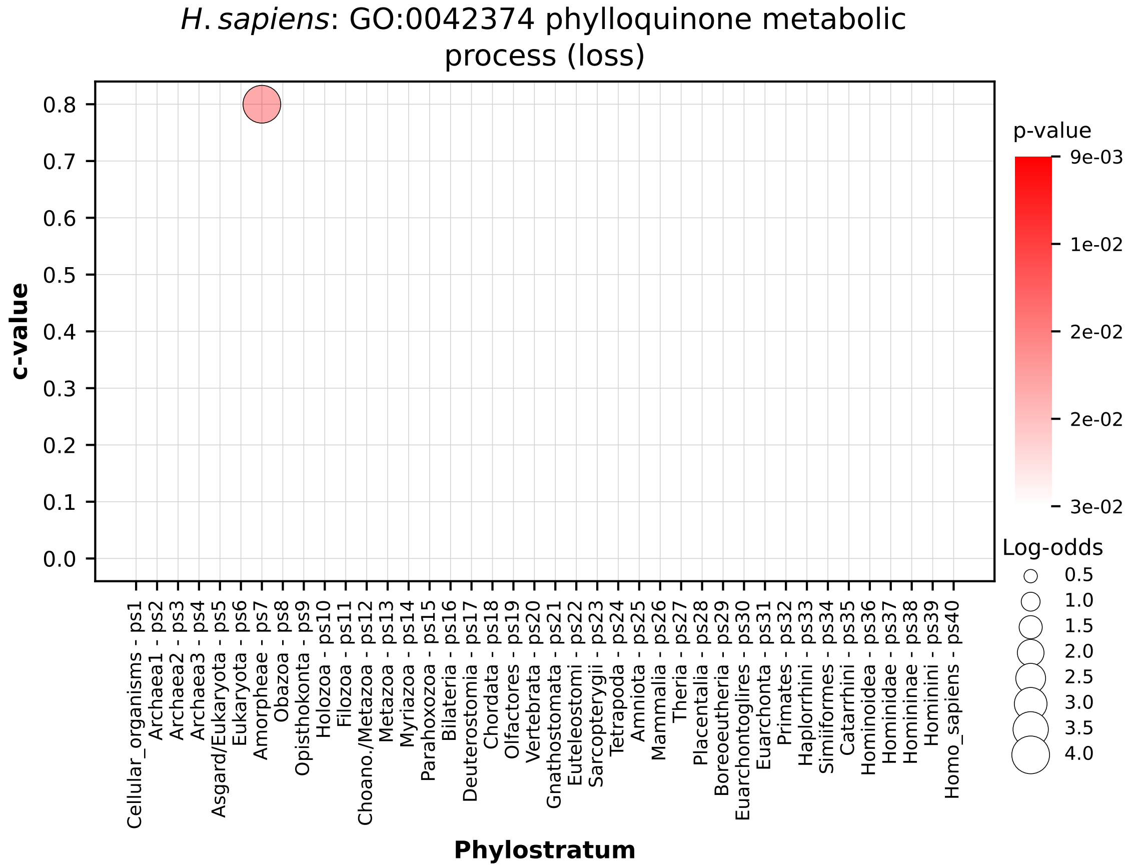

H. sapiens: GO:0042374 phylloquinone metabolic process (loss)
c-value
0.0
0.1
0.2
0.3
0.4
0.5
0.6
0.7
0.8
p-value
9e-03
1e-02
2e-02
2e-02
3e-02
Log-odds
0.5
1.0
1.5
2.0
2.5
3.0
3.5
4.0
Cellular_organisms - ps1
Archaea1 - ps2
Archaea2 - ps3
Archaea3 - ps4
Asgard/Eukaryota - ps5
Eukaryota - ps6
Amorpheae - ps7
Obazoa - ps8
Opisthokonta - ps9
Holozoa - ps10
Filozoa - ps11
Choano./Metazoa - ps12
Metazoa - ps13
Myriazoa - ps14
Parahoxozoa - ps15
Bilateria - ps16
Deuterostomia - ps17
Chordata - ps18
Olfactores - ps19
Vertebrata - ps20
Gnathostomata - ps21
Euteleostomi - ps22
Sarcopterygii - ps23
Tetrapoda - ps24
Amniota - ps25
Mammalia - ps26
Theria - ps27
Placentalia - ps28
Boreoeutheria - ps29
Euarchontoglires - ps30
Euarchonta - ps31
Primates - ps32
Haplorrhini - ps33
Simiiformes - ps34
Catarrhini - ps35
Hominoidea - ps36
Hominidae - ps37
Homininae - ps38
Hominini - ps39
Homo_sapiens - ps40
Phylostratum

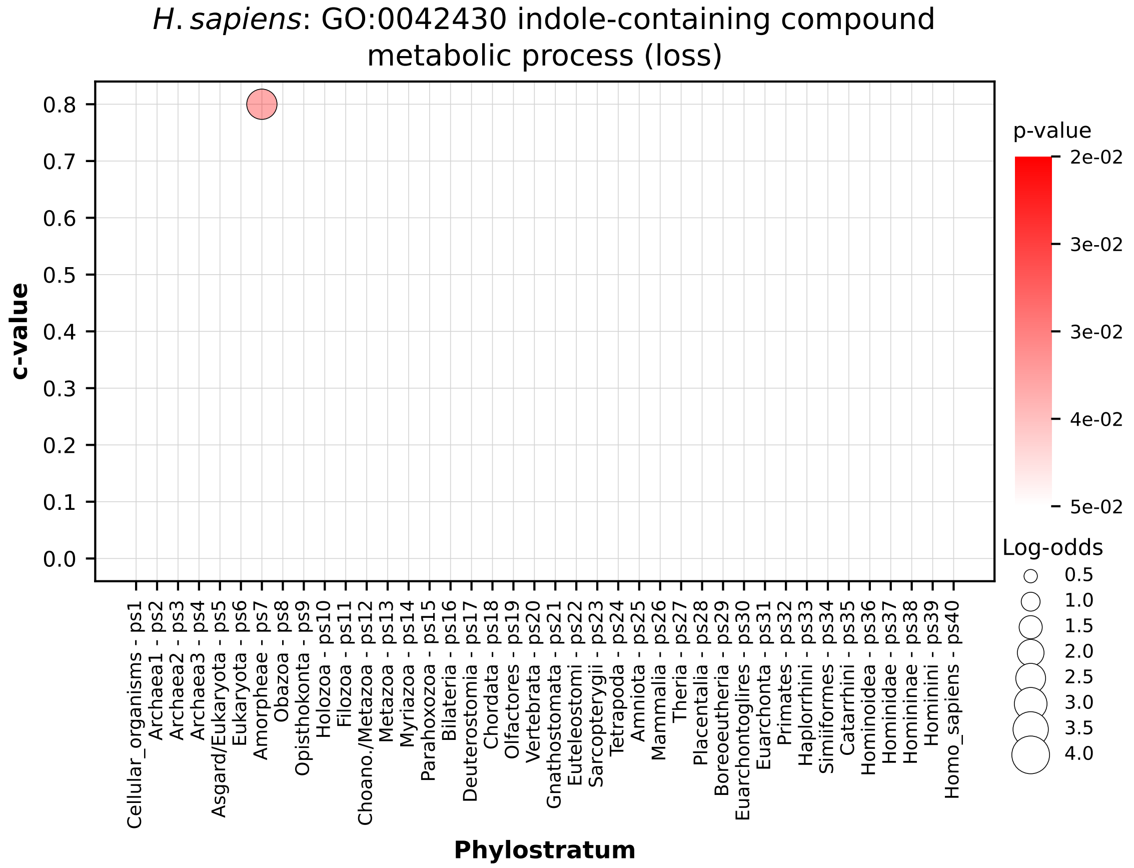

H. sapiens: GO:0042430 indole-containing compound metabolic process (loss)
c-value
0.8
0.7
0.6
0.5
0.4
0.3
0.2
0.1
0.0
p-value
2e-02
3e-02
3e-02
4e-02
5e-02
Log-odds
0.5
1.0
1.5
2.0
2.5
3.0
3.5
4.0
Cellular_organisms - ps1
Archaea1 - ps2
Archaea2 - ps3
Archaea3 - ps4
Asgard/Eukaryota - ps5
Eukaryota - ps6
Amorpheae - ps7
Obazoa - ps8
Opisthokonta - ps9
Holozoa - ps10
Filozoa - ps11
Choano./Metazoa - ps12
Metazoa - ps13
Myriazoa - ps14
Parahoxozoa - ps15
Bilateria - ps16
Deuterostomia - ps17
Chordata - ps18
Olfactores - ps19
Vertebrata - ps20
Gnathostomata - ps21
Euteleostomi - ps22
Sarcopterygii - ps23
Tetrapoda - ps24
Amniota - ps25
Mammalia - ps26
Theria - ps27
Placentalia - ps28
Boreoeutheria - ps29
Euarchontoglires - ps30
Euarchonta - ps31
Primates - ps32
Haplorrhini - ps33
Simiiformes - ps34
Catarrhini - ps35
Hominoidea - ps36
Hominidae - ps37
Homininae - ps38
Hominini - ps39
Homo_sapiens - ps40
Phylostratum

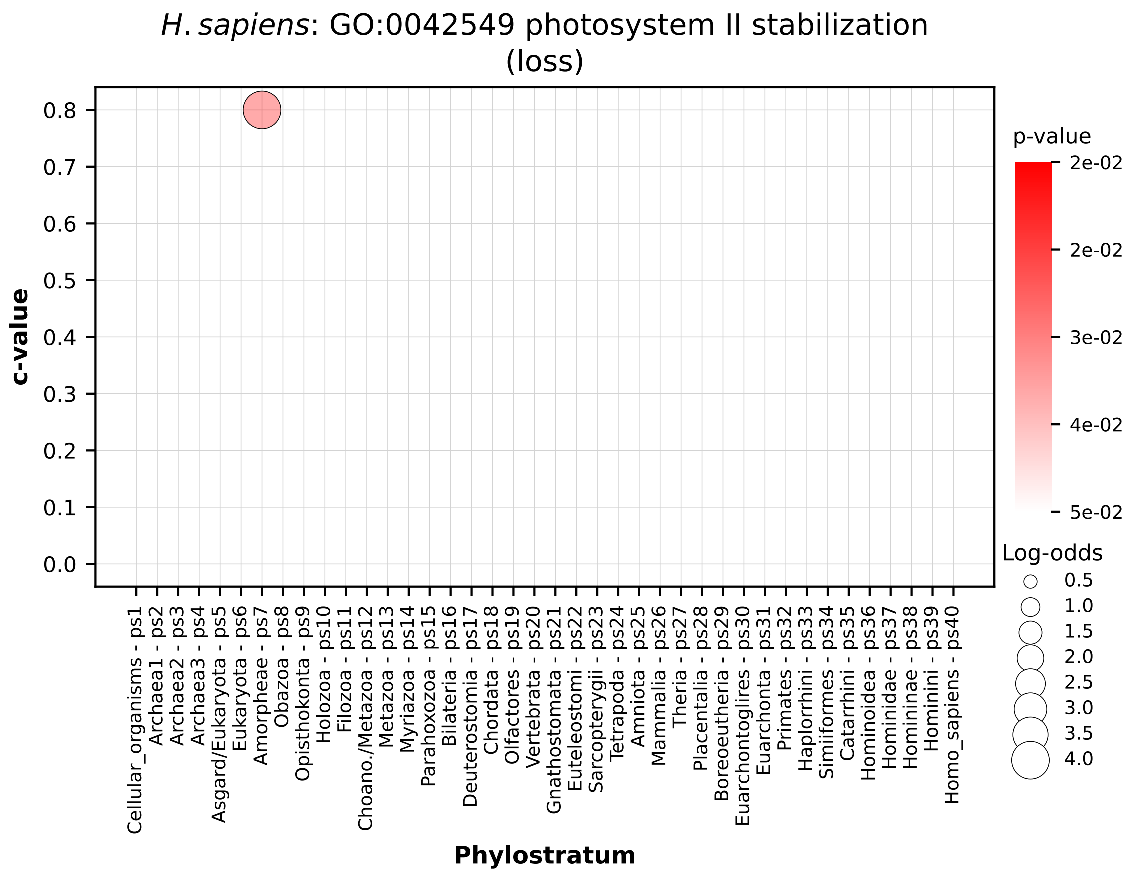
H. sapiens: GO:0042549 photosystem II stabilization
(loss)
c-value
0.0
0.1
0.2
0.3
0.4
0.5
0.6
0.7
0.8
Phylostratum
Cellular_organisms - ps1
Archaea1 - ps2
Archaea2 - ps3
Archaea3 - ps4
Asgard/Eukaryota - ps5
Eukaryota - ps6
Amorpheae - ps7
Obazoa - ps8
Opisthokonta - ps9
Holozoa - ps10
Filozoa - ps11
Choano./Metazoa - ps12
Metazoa - ps13
Myriazoa - ps14
Parahoxozoa - ps15
Bilateria - ps16
Deuterostomia - ps17
Chordata - ps18
Olfactores - ps19
Vertebrata - ps20
Gnathostomata - ps21
Euteleostomi - ps22
Sarcopterygii - ps23
Tetrapoda - ps24
Amniota - ps25
Mammalia - ps26
Theria - ps27
Placentalia - ps28
Boreoeutheria - ps29
Euarchontoglires - ps30
Euarchonta - ps31
Primates - ps32
Haplorrhini - ps33
Simiiformes - ps34
Catarrhini - ps35
Hominoidea - ps36
Hominidae - ps37
Homininae - ps38
Hominini - ps39
Homo_sapiens - ps40
p-value
2e-02
2e-02
3e-02
4e-02
5e-02
Log-odds
0.5
1.0
1.5
2.0
2.5
3.0
3.5
4.0

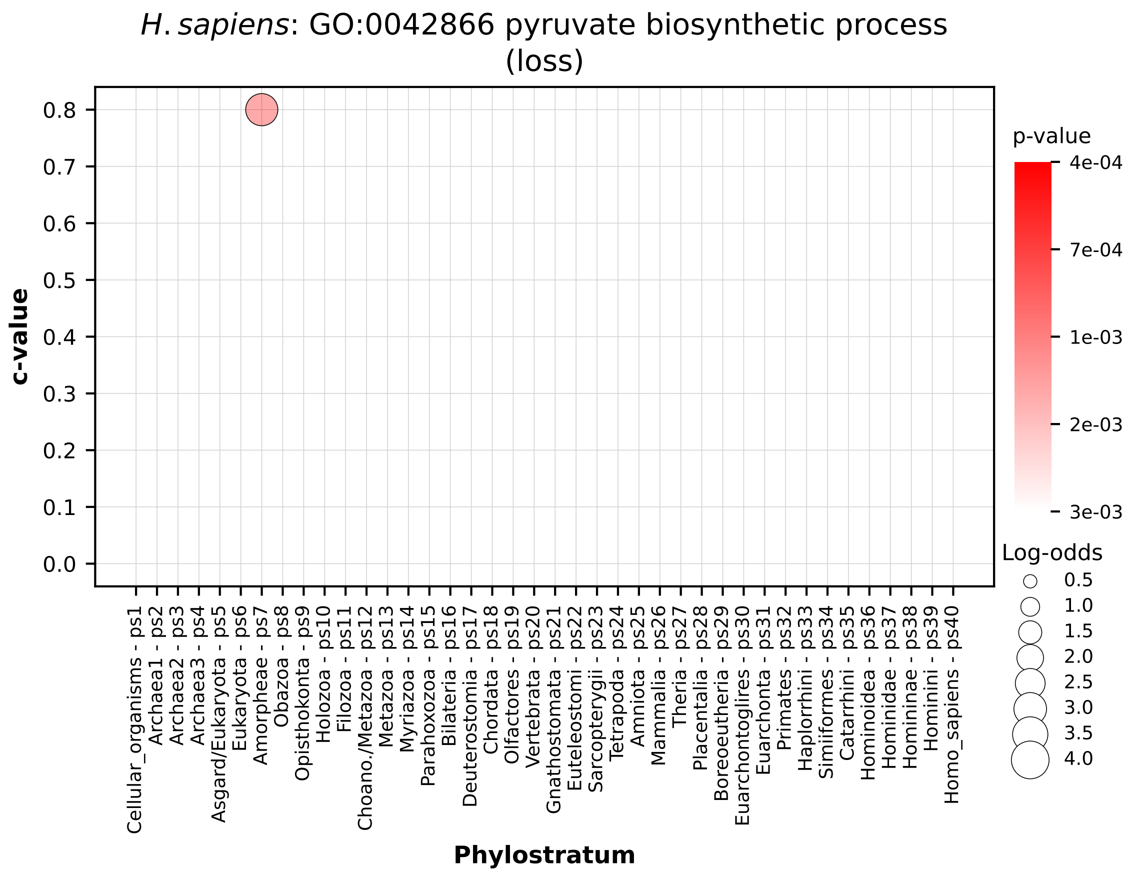

H. sapiens: GO:0042866 pyruvate biosynthetic process
(loss)
c-value
0.8
0.7
0.6
0.5
0.4
0.3
0.2
0.1
0.0
p-value
4e-04
7e-04
1e-03
2e-03
3e-03
Log-odds
0.5
1.0
1.5
2.0
2.5
3.0
3.5
4.0
Cellular_organisms - ps1
Archaea1 - ps2
Archaea2 - ps3
Archaea3 - ps4
Asgard/Eukaryota - ps5
Eukaryota - ps6
Amorpheae - ps7
Obazoa - ps8
Opisthokonta - ps9
Holozoa - ps10
Filozoa - ps11
Choano./Metazoa - ps12
Metazoa - ps13
Myriazoa - ps14
Parahoxozoa - ps15
Bilateria - ps16
Deuterostomia - ps17
Chordata - ps18
Olfactores - ps19
Vertebrata - ps20
Gnathostomata - ps21
Euteleostomi - ps22
Sarcopterygii - ps23
Tetrapoda - ps24
Amniota - ps25
Mammalia - ps26
Theria - ps27
Placentalia - ps28
Boreoeutheria - ps29
Euarchontoglires - ps30
Euarchonta - ps31
Primates - ps32
Haplorrhini - ps33
Simiiformes - ps34
Catarrhini - ps35
Hominoidea - ps36
Hominidae - ps37
Homininae - ps38
Hominini - ps39
Homo_sapiens - ps40
Phylostratum

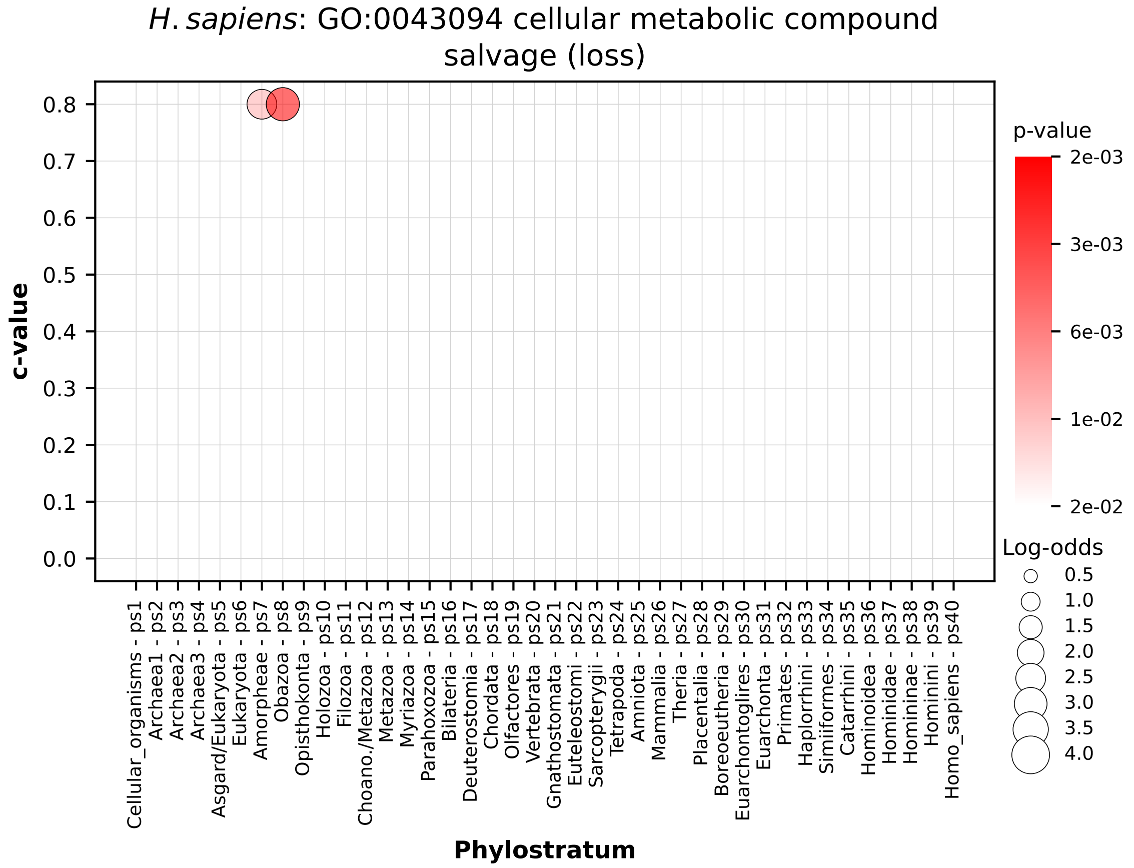
H. sapiens: GO:0043094 cellular metabolic compound salvage (loss)
c-value
0.0
0.1
0.2
0.3
0.4
0.5
0.6
0.7
0.8
Phylostratum
Cellular_organisms - ps1
Archaea1 - ps2
Archaea2 - ps3
Archaea3 - ps4
Asgard/Eukaryota - ps5
Eukaryota - ps6
Amorpheae - ps7
Obazoa - ps8
Opisthokonta - ps9
Holozoa - ps10
Filozoa - ps11
Choano./Metazoa - ps12
Metazoa - ps13
Myriazoa - ps14
Parahoxozoa - ps15
Bilateria - ps16
Deuterostomia - ps17
Chordata - ps18
Olfactores - ps19
Vertebrata - ps20
Gnathostomata - ps21
Euteleostomi - ps22
Sarcopterygii - ps23
Tetrapoda - ps24
Amniota - ps25
Mammalia - ps26
Theria - ps27
Placentalia - ps28
Boreoeutheria - ps29
Euarchontoglires - ps30
Euarchonta - ps31
Primates - ps32
Haplorrhini - ps33
Simiiformes - ps34
Catarrhini - ps35
Hominoidea - ps36
Hominidae - ps37
Homininae - ps38
Hominini - ps39
Homo_sapiens - ps40
p-value
2e-03
3e-03
6e-03
1e-02
2e-02
Log-odds
0.5
1.0
1.5
2.0
2.5
3.0
3.5
4.0

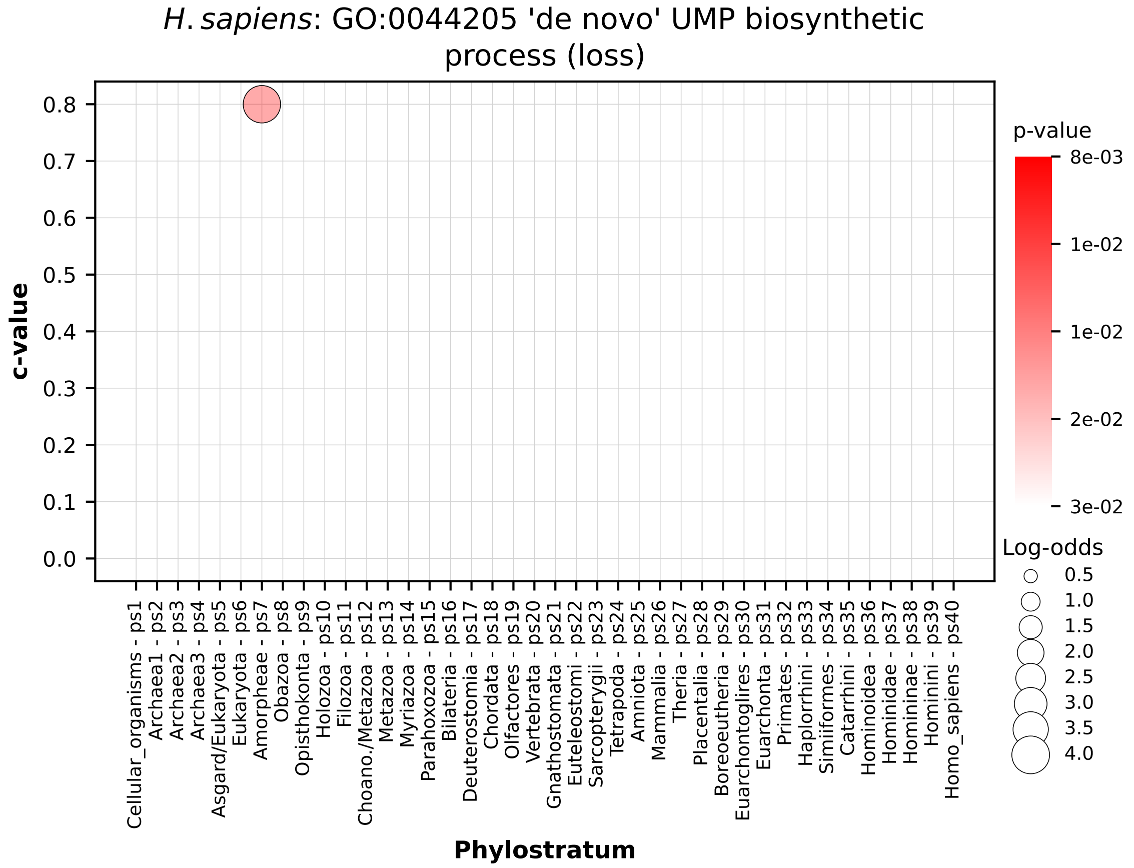

H. sapiens: GO:0044205 'de novo' UMP biosynthetic process (loss)
c-value
0.0
0.1
0.2
0.3
0.4
0.5
0.6
0.7
0.8
Phylostratum
Cellular_organisms - ps1
Archaea1 - ps2
Archaea2 - ps3
Archaea3 - ps4
Asgard/Eukaryota - ps5
Eukaryota - ps6
Amorpheae - ps7
Obazoa - ps8
Opisthokonta - ps9
Holozoa - ps10
Filozoa - ps11
Choano./Metazoa - ps12
Metazoa - ps13
Myriazoa - ps14
Parahoxozoa - ps15
Bilateria - ps16
Deuterostomia - ps17
Chordata - ps18
Olfactores - ps19
Vertebrata - ps20
Gnathostomata - ps21
Euteleostomi - ps22
Sarcopterygii - ps23
Tetrapoda - ps24
Amniota - ps25
Mammalia - ps26
Theria - ps27
Placentalia - ps28
Boreoeutheria - ps29
Euarchontoglires - ps30
Euarchonta - ps31
Primates - ps32
Haplorrhini - ps33
Simiiformes - ps34
Catarrhini - ps35
Hominoidea - ps36
Hominidae - ps37
Homininae - ps38
Hominini - ps39
Homo_sapiens - ps40
p-value
8e-03
1e-02
1e-02
2e-02
3e-02
Log-odds
0.5
1.0
1.5
2.0
2.5
3.0
3.5
4.0

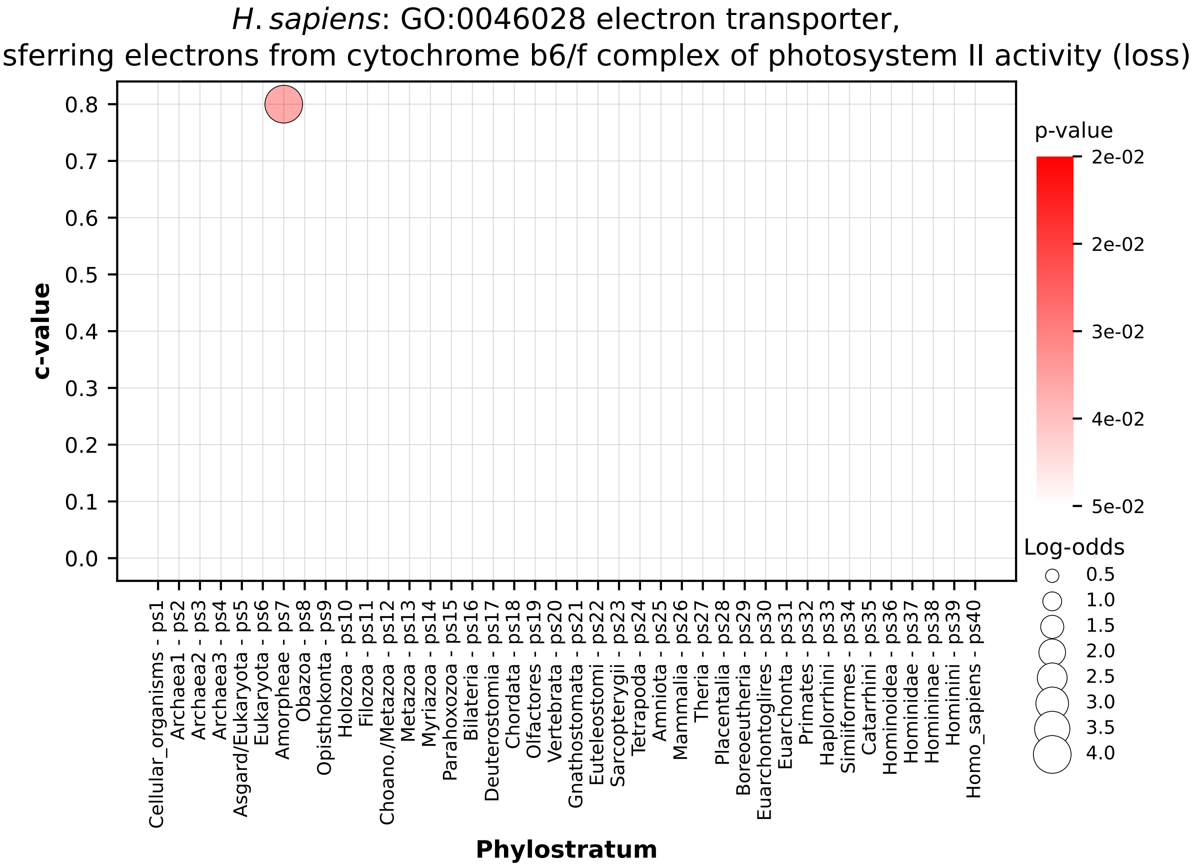

H. sapiens: GO:0046028 electron transporter,
sferring electrons from cytochrome b6/f complex of photosystem II activity (loss)
c-value
0.0
0.1
0.2
0.3
0.4
0.5
0.6
0.7
0.8
Phylostratum
Cellular_organisms - ps1
Archaea1 - ps2
Archaea2 - ps3
Archaea3 - ps4
Asgard/Eukaryota - ps5
Eukaryota - ps6
Amorpheae - ps7
Obazoa - ps8
Opisthokonta - ps9
Holozoa - ps10
Filozoa - ps11
Choano./Metazoa - ps12
Metazoa - ps13
Myriazoa - ps14
Parahoxozoa - ps15
Bilateria - ps16
Deuterostomia - ps17
Chordata - ps18
Olfactores - ps19
Vertebrata - ps20
Gnathostomata - ps21
Euteleostomi - ps22
Sarcopterygii - ps23
Tetrapoda - ps24
Amniota - ps25
Mammalia - ps26
Theria - ps27
Placentalia - ps28
Boreoeutheria - ps29
Euarchontoglires - ps30
Euarchonta - ps31
Primates - ps32
Haplorrhini - ps33
Simiiformes - ps34
Catarrhini - ps35
Hominoidea - ps36
Hominidae - ps37
Homininae - ps38
Hominini - ps39
Homo_sapiens - ps40
p-value
2e-02
2e-02
3e-02
4e-02
5e-02
Log-odds
0.5
1.0
1.5
2.0
2.5
3.0
3.5
4.0

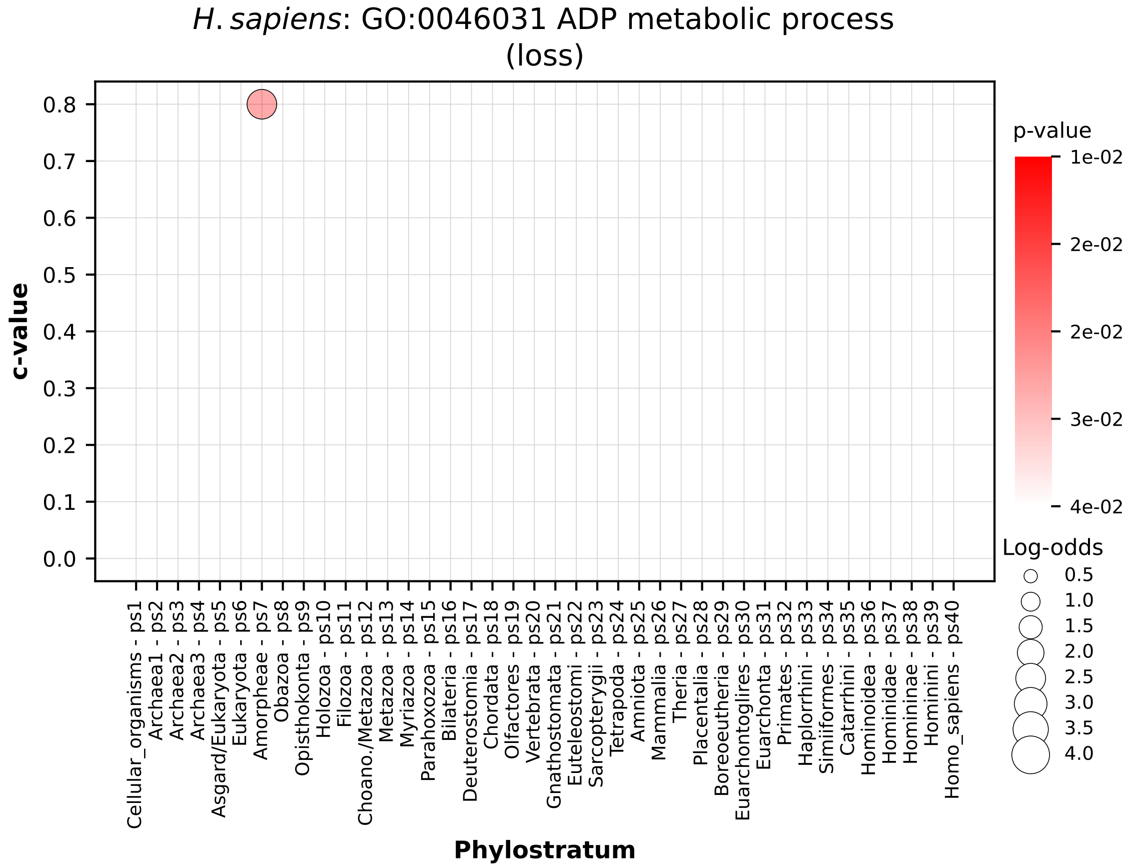

H. sapiens: GO:0046031 ADP metabolic process
(loss)
c-value
0.8
0.7
0.6
0.5
0.4
0.3
0.2
0.1
0.0
p-value
1e-02
2e-02
2e-02
3e-02
4e-02
Log-odds
0.5
1.0
1.5
2.0
2.5
3.0
3.5
4.0
Cellular_organisms - ps1
Archaea1 - ps2
Archaea2 - ps3
Archaea3 - ps4
Asgard/Eukaryota - ps5
Eukaryota - ps6
Amorpheae - ps7
Obazoa - ps8
Opisthokonta - ps9
Holozoa - ps10
Filozoa - ps11
Choano./Metazoa - ps12
Metazoa - ps13
Myriazoa - ps14
Parahoxozoa - ps15
Bilateria - ps16
Deuterostomia - ps17
Chordata - ps18
Olfactores - ps19
Vertebrata - ps20
Gnathostomata - ps21
Euteleostomi - ps22
Sarcopterygii - ps23
Tetrapoda - ps24
Amniota - ps25
Mammalia - ps26
Theria - ps27
Placentalia - ps28
Boreoeutheria - ps29
Euarchontoglires - ps30
Euarchonta - ps31
Primates - ps32
Haplorrhini - ps33
Simiiformes - ps34
Catarrhini - ps35
Hominoidea - ps36
Hominidae - ps37
Homininae - ps38
Hominini - ps39
Homo_sapiens - ps40
Phylostratum

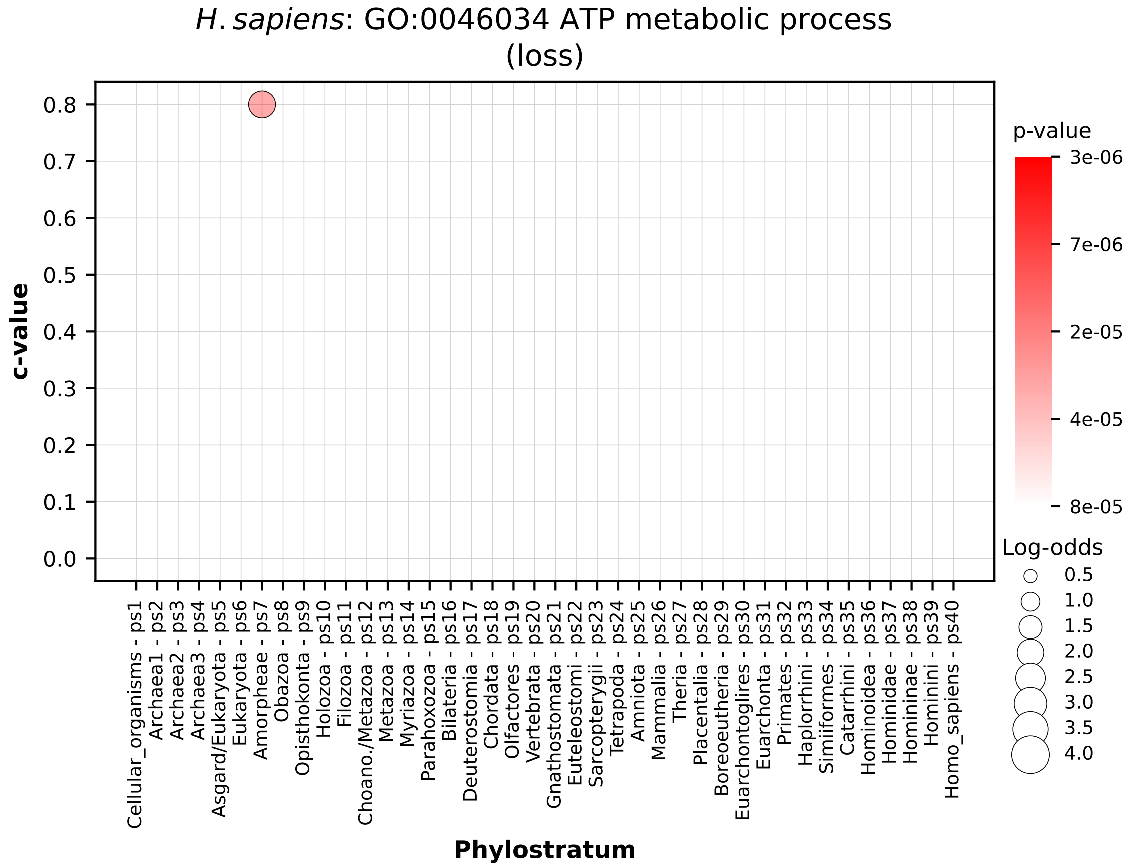

H. sapiens: GO:0046034 ATP metabolic process
(loss)
c-value
0.8
0.7
0.6
0.5
0.4
0.3
0.2
0.1
0.0
p-value
3e-06
7e-06
2e-05
4e-05
8e-05
Log-odds
0.5
1.0
1.5
2.0
2.5
3.0
3.5
4.0
Cellular_organisms - ps1
Archaea1 - ps2
Archaea2 - ps3
Archaea3 - ps4
Asgard/Eukaryota - ps5
Eukaryota - ps6
Amorpheae - ps7
Obazoa - ps8
Opisthokonta - ps9
Holozoa - ps10
Filozoa - ps11
Choano./Metazoa - ps12
Metazoa - ps13
Myriazoa - ps14
Parahoxozoa - ps15
Bilateria - ps16
Deuterostomia - ps17
Chordata - ps18
Olfactores - ps19
Vertebrata - ps20
Gnathostomata - ps21
Euteleostomi - ps22
Sarcopterygii - ps23
Tetrapoda - ps24
Amniota - ps25
Mammalia - ps26
Theria - ps27
Placentalia - ps28
Boreoeutheria - ps29
Euarchontoglires - ps30
Euarchonta - ps31
Primates - ps32
Haplorrhini - ps33
Simiiformes - ps34
Catarrhini - ps35
Hominoidea - ps36
Hominidae - ps37
Homininae - ps38
Hominini - ps39
Homo_sapiens - ps40
Phylostratum

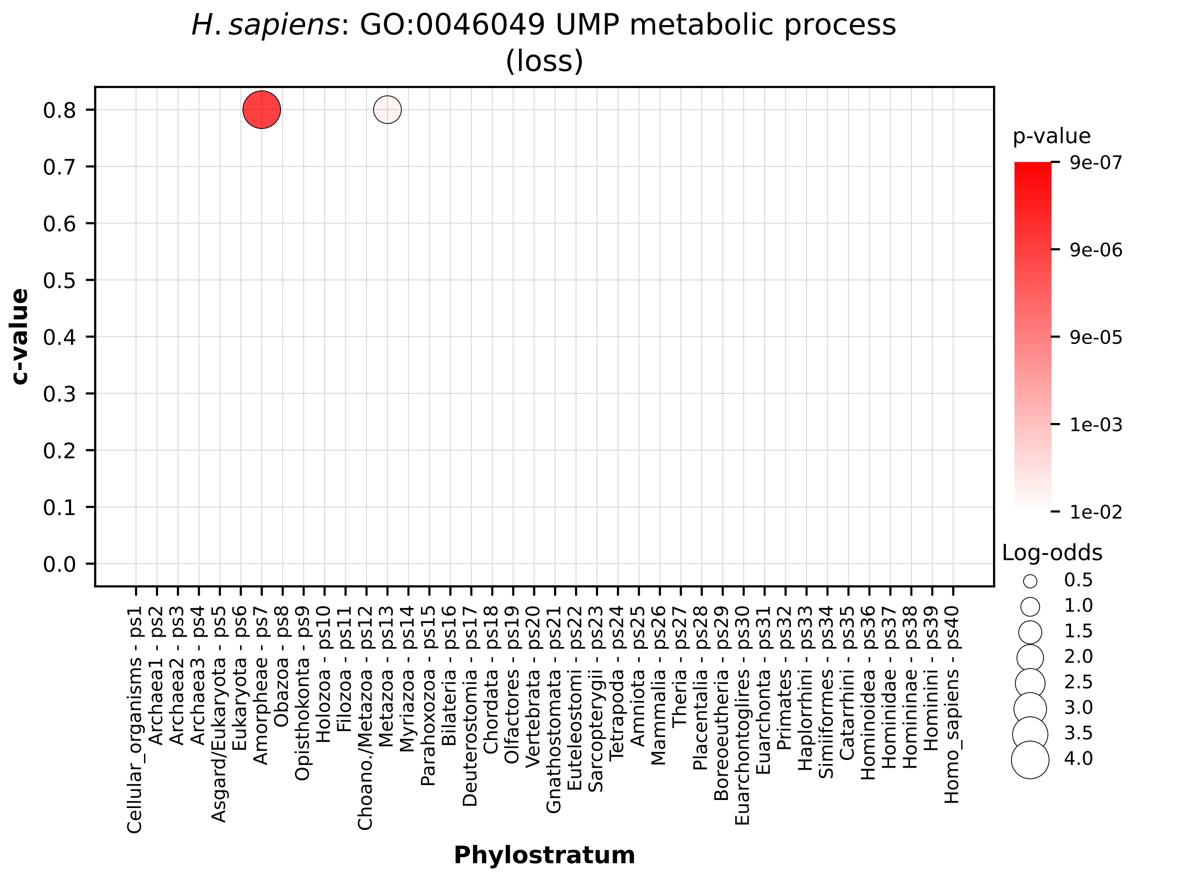
H. sapiens: GO:0046049 UMP metabolic process
(loss)
c-value
0.0
0.1
0.2
0.3
0.4
0.5
0.6
0.7
0.8
Phylostratum
Cellular_organisms - ps1
Archaea1 - ps2
Archaea2 - ps3
Archaea3 - ps4
Asgard/Eukaryota - ps5
Eukaryota - ps6
Amorpheae - ps7
Obazoa - ps8
Opisthokonta - ps9
Holozoa - ps10
Filozoa - ps11
Choano./Metazoa - ps12
Metazoa - ps13
Myriazoa - ps14
Parahoxozoa - ps15
Bilateria - ps16
Deuterostomia - ps17
Chordata - ps18
Olfactores - ps19
Vertebrata - ps20
Gnathostomata - ps21
Euteleostomi - ps22
Sarcopterygii - ps23
Tetrapoda - ps24
Amniota - ps25
Mammalia - ps26
Theria - ps27
Placentalia - ps28
Boreoeutheria - ps29
Euarchontoglires - ps30
Euarchonta - ps31
Primates - ps32
Haplorrhini - ps33
Simiiformes - ps34
Catarrhini - ps35
Hominoidea - ps36
Hominidae - ps37
Homininae - ps38
Hominini - ps39
Homo_sapiens - ps40
p-value
9e-07
9e-06
9e-05
1e-03
1e-02
Log-odds
0.5
1.0
1.5
2.0
2.5
3.0
3.5
4.0

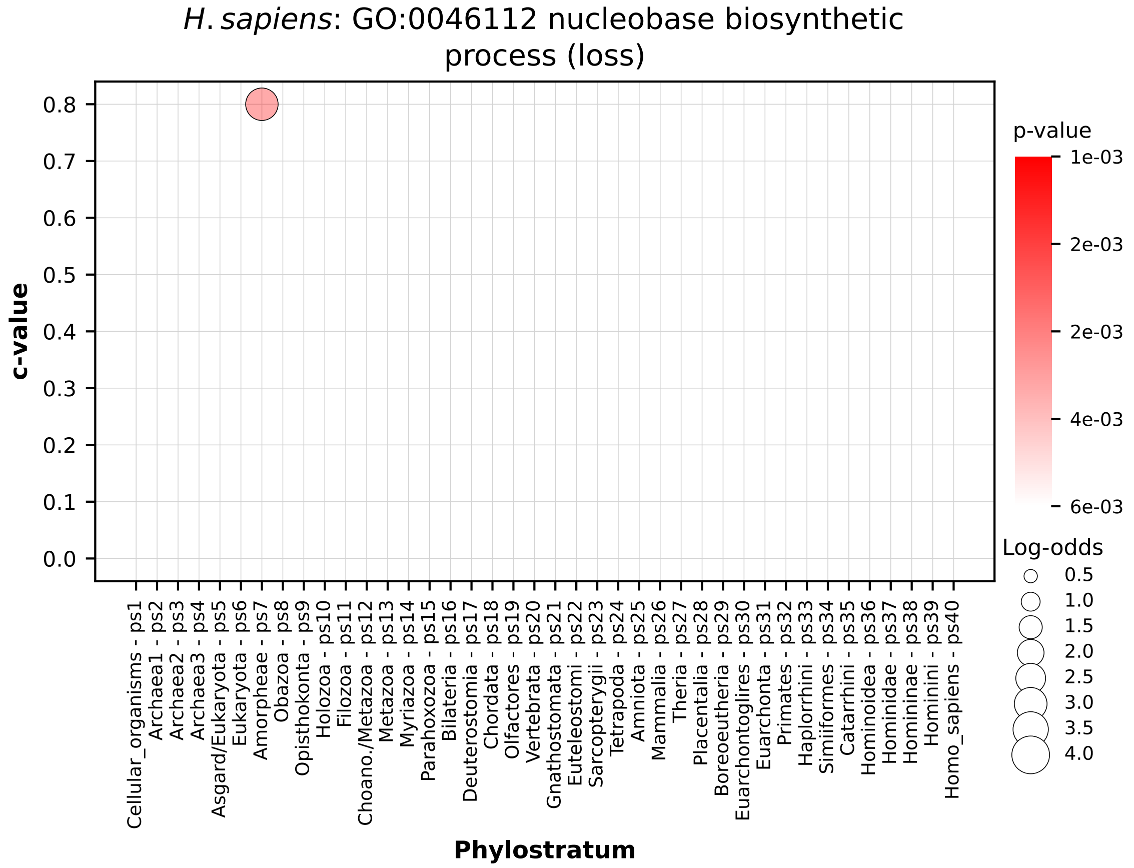

H. sapiens: GO:0046112 nucleobase biosynthetic process (loss)
c-value
0.8
0.7
0.6
0.5
0.4
0.3
0.2
0.1
0.0
Phylostratum
Cellular_organisms - ps1
Archaea1 - ps2
Archaea2 - ps3
Archaea3 - ps4
Asgard/Eukaryota - ps5
Eukaryota - ps6
Amorpheae - ps7
Obazoa - ps8
Opisthokonta - ps9
Holozoa - ps10
Filozoa - ps11
Choano./Metazoa - ps12
Metazoa - ps13
Myriazoa - ps14
Parahoxozoa - ps15
Bilateria - ps16
Deuterostomia - ps17
Chordata - ps18
Olfactores - ps19
Vertebrata - ps20
Gnathostomata - ps21
Euteleostomi - ps22
Sarcopterygii - ps23
Tetrapoda - ps24
Amniota - ps25
Mammalia - ps26
Theria - ps27
Placentalia - ps28
Boreoeutheria - ps29
Euarchontoglires - ps30
Euarchonta - ps31
Primates - ps32
Haplorrhini - ps33
Simiiformes - ps34
Catarrhini - ps35
Hominoidea - ps36
Hominidae - ps37
Homininae - ps38
Hominini - ps39
Homo_sapiens - ps40
p-value
1e-03
2e-03
2e-03
4e-03
6e-03
Log-odds
0.5
1.0
1.5
2.0
2.5
3.0
3.5
4.0

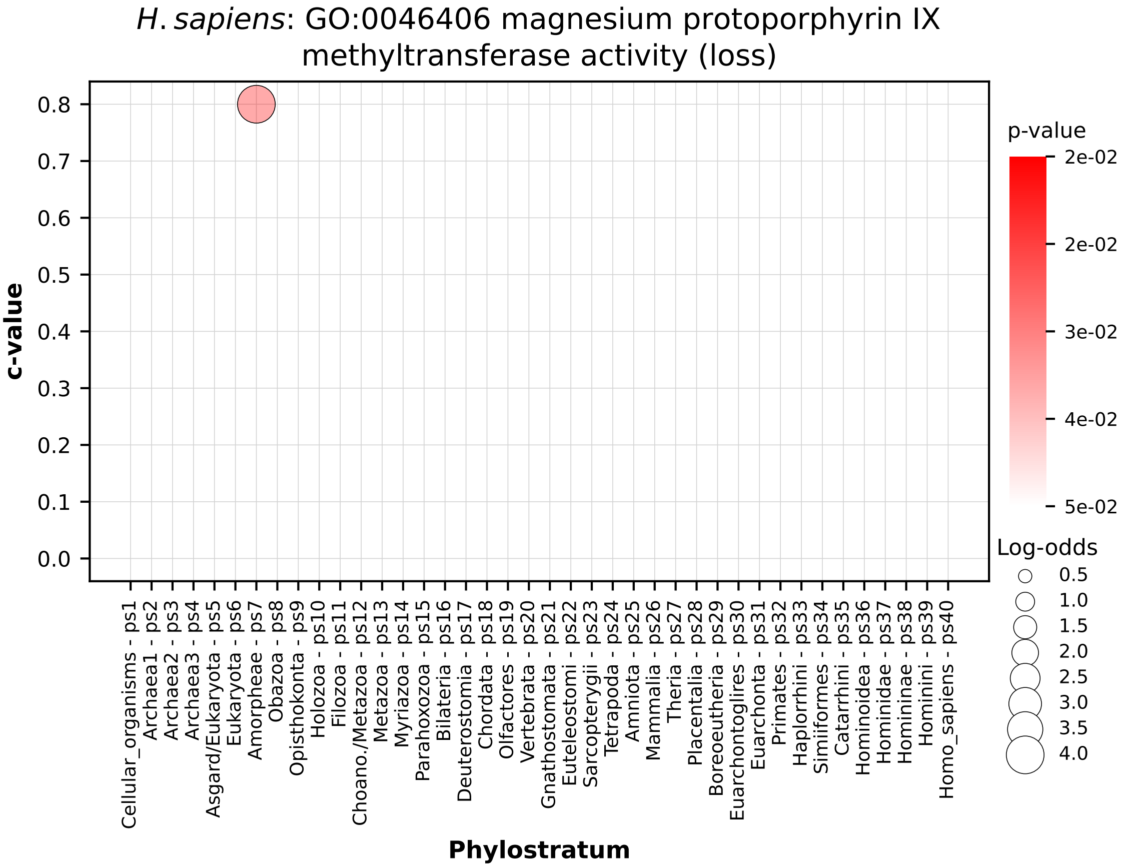

H. sapiens: GO:0046406 magnesium protoporphyrin IX methyltransferase activity (loss)
c-value
0.0
0.1
0.2
0.3
0.4
0.5
0.6
0.7
0.8
Phylostratum
Cellular_organisms - ps1
Archaea1 - ps2
Archaea2 - ps3
Archaea3 - ps4
Asgard/Eukaryota - ps5
Eukaryota - ps6
Amorpheae - ps7
Obazoa - ps8
Opisthokonta - ps9
Holozoa - ps10
Filozoa - ps11
Choano./Metazoa - ps12
Metazoa - ps13
Myriazoa - ps14
Parahoxozoa - ps15
Bilateria - ps16
Deuterostomia - ps17
Chordata - ps18
Olfactores - ps19
Vertebrata - ps20
Gnathostomata - ps21
Euteleostomi - ps22
Sarcopterygii - ps23
Tetrapoda - ps24
Amniota - ps25
Mammalia - ps26
Theria - ps27
Placentalia - ps28
Boreoeutheria - ps29
Euarchontoglires - ps30
Euarchonta - ps31
Primates - ps32
Haplorrhini - ps33
Simiiformes - ps34
Catarrhini - ps35
Hominoidea - ps36
Hominidae - ps37
Homininae - ps38
Hominini - ps39
Homo_sapiens - ps40
p-value
2e-02
2e-02
3e-02
4e-02
5e-02
Log-odds
0.5
1.0
1.5
2.0
2.5
3.0
3.5
4.0

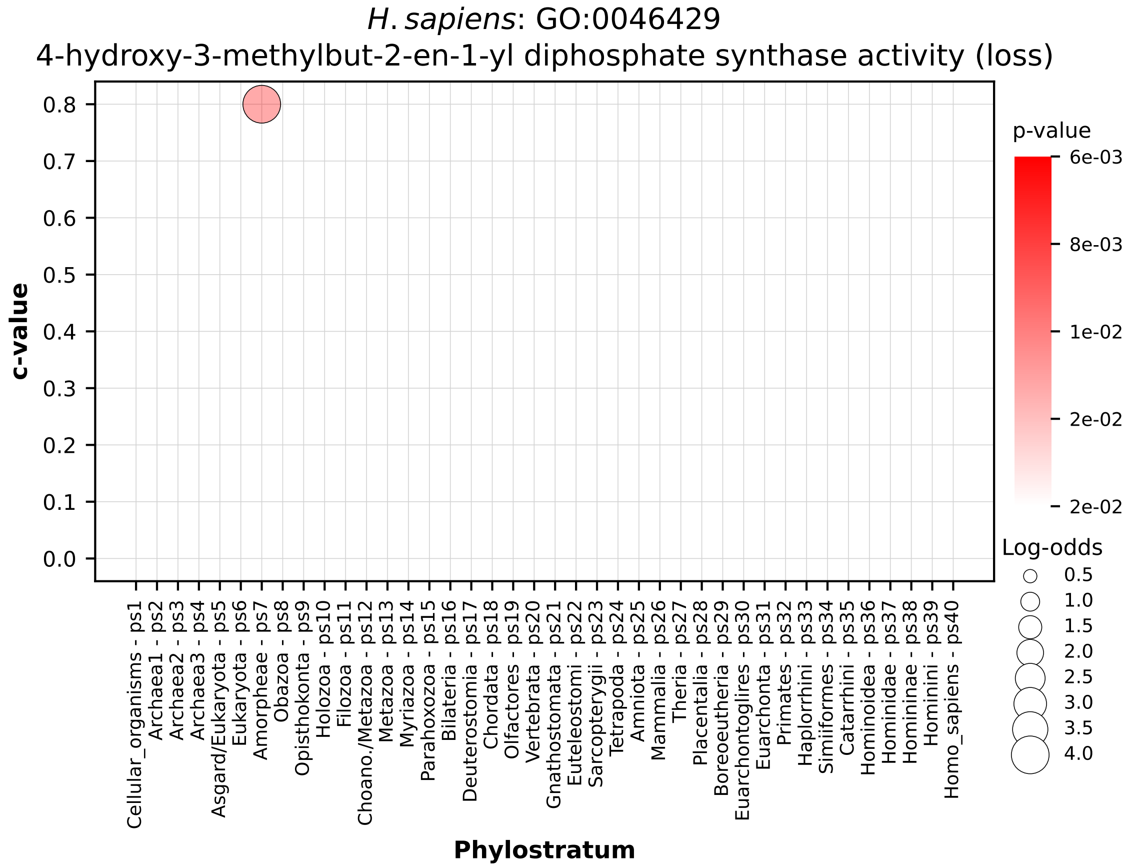

H. sapiens: GO:0046429
4-hydroxy-3-methylbut-2-en-1-yl diphosphate synthase activity (loss)
c-value
0.0
0.1
0.2
0.3
0.4
0.5
0.6
0.7
0.8
Phylostratum
Cellular_organisms - ps1
Archaea1 - ps2
Archaea2 - ps3
Archaea3 - ps4
Asgard/Eukaryota - ps5
Eukaryota - ps6
Amorpheae - ps7
Obazoa - ps8
Opisthokonta - ps9
Holozoa - ps10
Filozoa - ps11
Choano./Metazoa - ps12
Metazoa - ps13
Myriazoa - ps14
Parahoxozoa - ps15
Bilateria - ps16
Deuterostomia - ps17
Chordata - ps18
Olfactores - ps19
Vertebrata - ps20
Gnathostomata - ps21
Euteleostomi - ps22
Sarcopterygii - ps23
Tetrapoda - ps24
Amniota - ps25
Mammalia - ps26
Theria - ps27
Placentalia - ps28
Boreoeutheria - ps29
Euarchontoglires - ps30
Euarchonta - ps31
Primates - ps32
Haplorrhini - ps33
Simiiformes - ps34
Catarrhini - ps35
Hominoidea - ps36
Hominidae - ps37
Homininae - ps38
Hominini - ps39
Homo_sapiens - ps40
p-value
6e-03
8e-03
1e-02
2e-02
2e-02
Log-odds
0.5
1.0
1.5
2.0
2.5
3.0
3.5
4.0

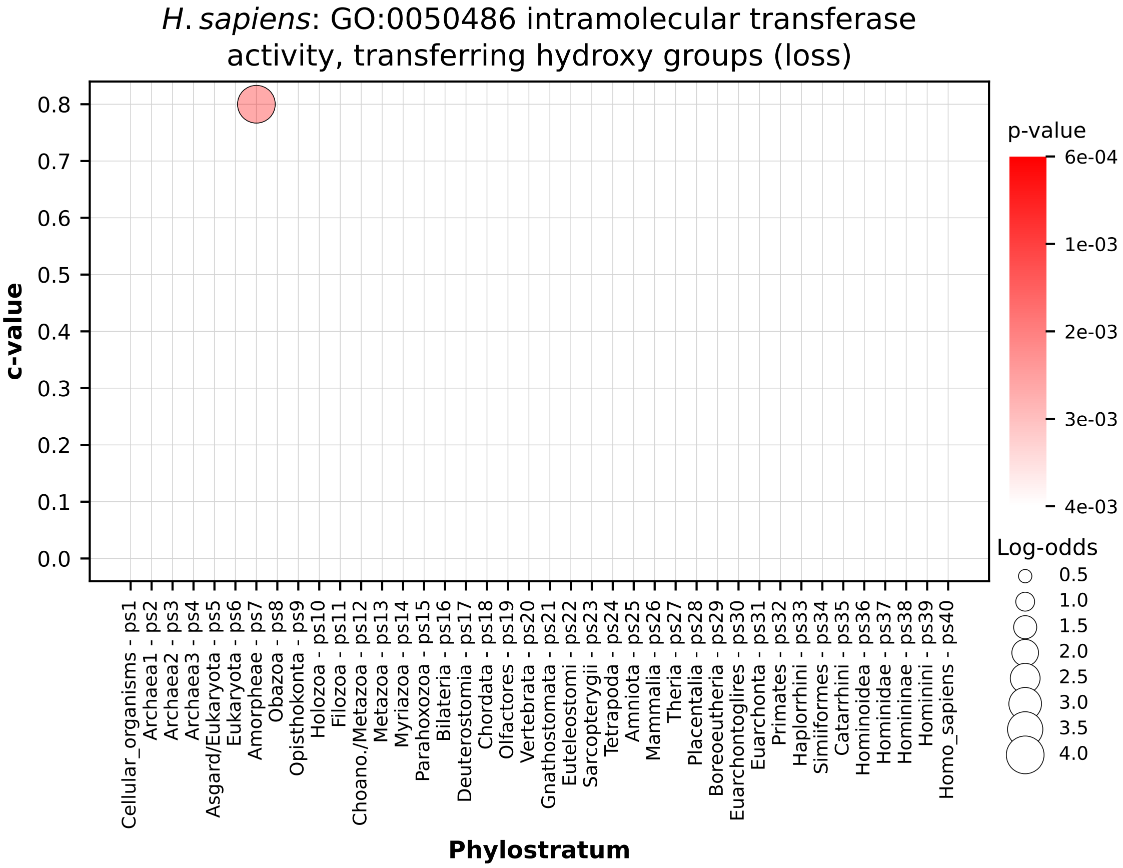
H. sapiens: GO:0050486 intramolecular transferase activity, transferring hydroxy groups (loss)
c-value
0.8
0.7
0.6
0.5
0.4
0.3
0.2
0.1
0.0
p-value
6e-04
1e-03
2e-03
3e-03
4e-03
Log-odds
0.5
1.0
1.5
2.0
2.5
3.0
3.5
4.0
Cellular_organisms - ps1
Archaea1 - ps2
Archaea2 - ps3
Archaea3 - ps4
Asgard/Eukaryota - ps5
Eukaryota - ps6
Amorpheae - ps7
Obazoa - ps8
Opisthokonta - ps9
Holozoa - ps10
Filozoa - ps11
Choano./Metazoa - ps12
Metazoa - ps13
Myriazoa - ps14
Parahoxozoa - ps15
Bilateria - ps16
Deuterostomia - ps17
Chordata - ps18
Olfactores - ps19
Vertebrata - ps20
Gnathostomata - ps21
Euteleostomi - ps22
Sarcopterygii - ps23
Tetrapoda - ps24
Amniota - ps25
Mammalia - ps26
Theria - ps27
Placentalia - ps28
Boreoeutheria - ps29
Euarchontoglires - ps30
Euarchonta - ps31
Primates - ps32
Haplorrhini - ps33
Simiiformes - ps34
Catarrhini - ps35
Hominoidea - ps36
Hominidae - ps37
Homininae - ps38
Hominini - ps39
Homo_sapiens - ps40
Phylostratum

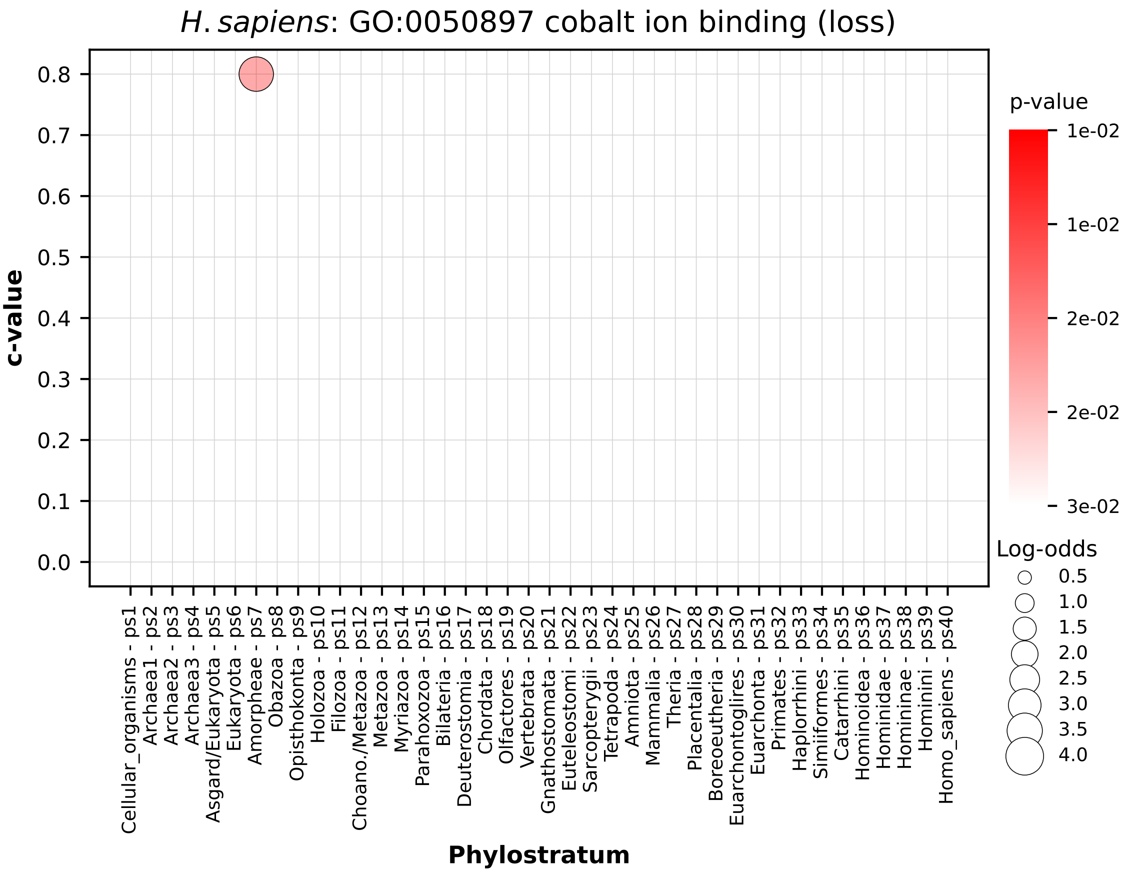

H. sapiens: GO:0050897 cobalt ion binding (loss)
c-value
0.0
0.1
0.2
0.3
0.4
0.5
0.6
0.7
0.8
Phylostratum
Cellular_organisms - ps1
Archaea1 - ps2
Archaea2 - ps3
Archaea3 - ps4
Asgard/Eukaryota - ps5
Eukaryota - ps6
Amorpheae - ps7
Obazoa - ps8
Opisthokonta - ps9
Holozoa - ps10
Filozoa - ps11
Choano./Metazoa - ps12
Metazoa - ps13
Myriazoa - ps14
Parahoxozoa - ps15
Bilateria - ps16
Deuterostomia - ps17
Chordata - ps18
Olfactores - ps19
Vertebrata - ps20
Gnathostomata - ps21
Euteleostomi - ps22
Sarcopterygii - ps23
Tetrapoda - ps24
Amniota - ps25
Mammalia - ps26
Theria - ps27
Placentalia - ps28
Boreoeutheria - ps29
Euarchontoglires - ps30
Euarchonta - ps31
Primates - ps32
Haplorrhini - ps33
Simiiformes - ps34
Catarrhini - ps35
Hominoidea - ps36
Hominidae - ps37
Homininae - ps38
Hominini - ps39
Homo_sapiens - ps40
p-value
1e-02
1e-02
2e-02
2e-02
3e-02
Log-odds
0.5
1.0
1.5
2.0
2.5
3.0
3.5
4.0

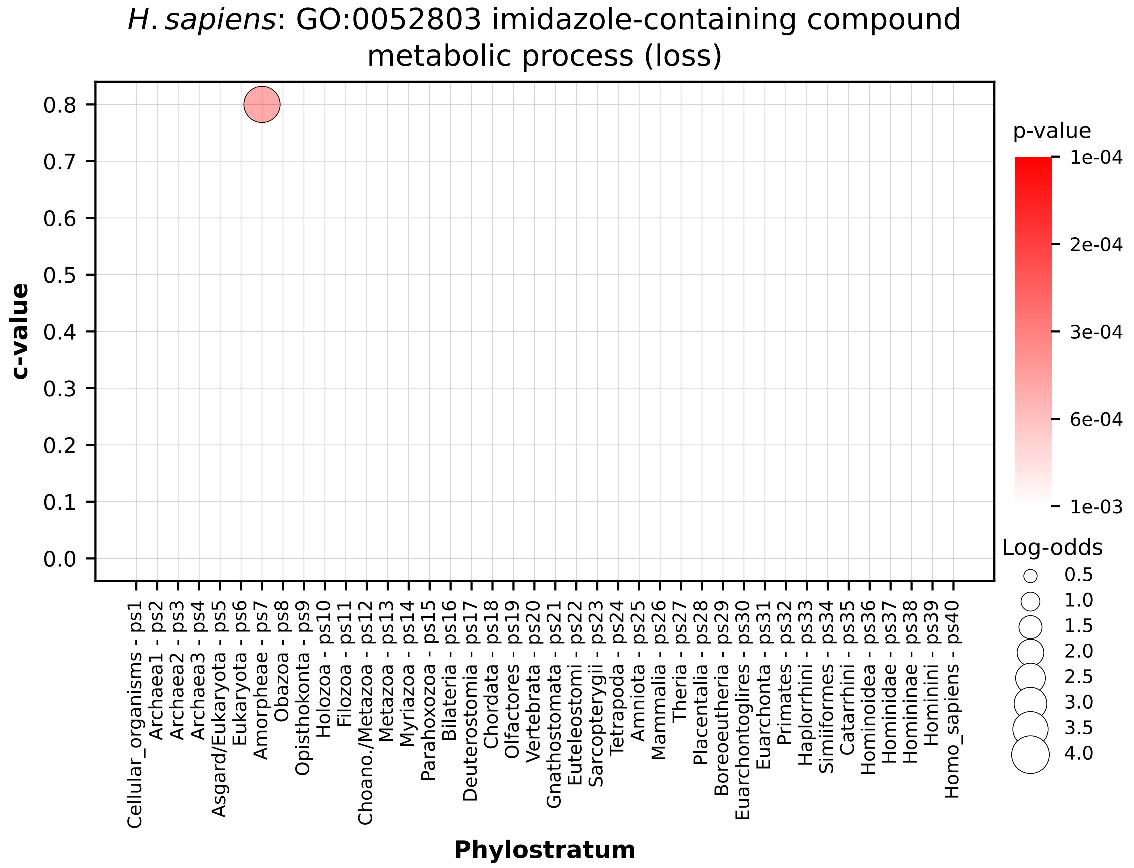
H. sapiens: GO:0052803 imidazole-containing compound metabolic process (loss)
c-value
0.8
0.7
0.6
0.5
0.4
0.3
0.2
0.1
0.0
Phylostratum
Cellular_organisms - ps1
Archaea1 - ps2
Archaea2 - ps3
Archaea3 - ps4
Asgard/Eukaryota - ps5
Eukaryota - ps6
Amorpheae - ps7
Obazoa - ps8
Opisthokonta - ps9
Holozoa - ps10
Filozoa - ps11
Choano./Metazoa - ps12
Metazoa - ps13
Myriazoa - ps14
Parahoxozoa - ps15
Bilateria - ps16
Deuterostomia - ps17
Chordata - ps18
Olfactores - ps19
Vertebrata - ps20
Gnathostomata - ps21
Euteleostomi - ps22
Sarcopterygii - ps23
Tetrapoda - ps24
Amniota - ps25
Mammalia - ps26
Theria - ps27
Placentalia - ps28
Boreoeutheria - ps29
Euarchontoglires - ps30
Euarchonta - ps31
Primates - ps32
Haplorrhini - ps33
Simiiformes - ps34
Catarrhini - ps35
Hominoidea - ps36
Hominidae - ps37
Homininae - ps38
Hominini - ps39
Homo_sapiens - ps40
p-value
1e-04
2e-04
3e-04
6e-04
1e-03
Log-odds
0.5
1.0
1.5
2.0
2.5
3.0
3.5
4.0

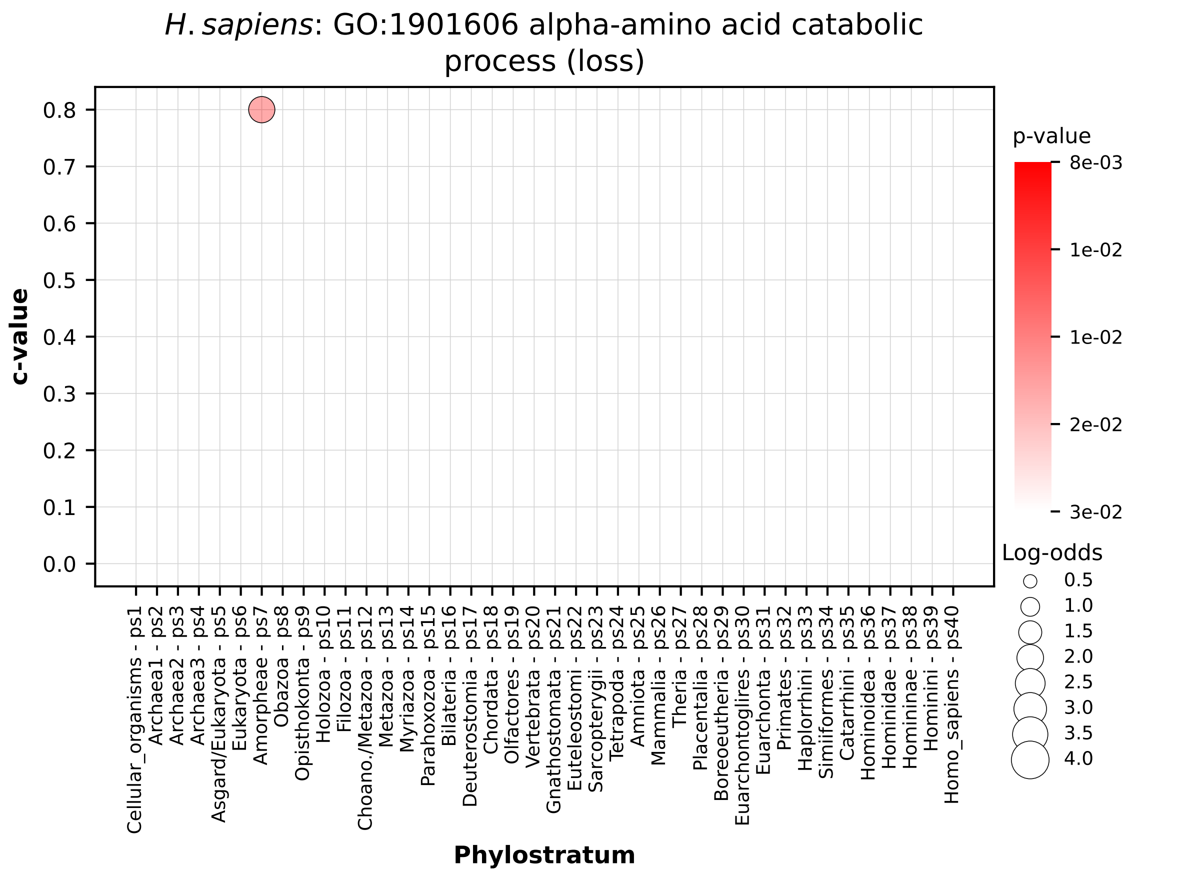

H. sapiens: GO:1901606 alpha-amino acid catabolic process (loss)
c-value
0.0
0.1
0.2
0.3
0.4
0.5
0.6
0.7
0.8
Phylostratum
Cellular_organisms - ps1
Archaea1 - ps2
Archaea2 - ps3
Archaea3 - ps4
Asgard/Eukaryota - ps5
Eukaryota - ps6
Amorpheae - ps7
Obazoa - ps8
Opisthokonta - ps9
Holozoa - ps10
Filozoa - ps11
Choano./Metazoa - ps12
Metazoa - ps13
Myriazoa - ps14
Parahoxozoa - ps15
Bilateria - ps16
Deuterostomia - ps17
Chordata - ps18
Olfactores - ps19
Vertebrata - ps20
Gnathostomata - ps21
Euteleostomi - ps22
Sarcopterygii - ps23
Tetrapoda - ps24
Amniota - ps25
Mammalia - ps26
Theria - ps27
Placentalia - ps28
Boreoeutheria - ps29
Euarchontoglires - ps30
Euarchonta - ps31
Primates - ps32
Haplorrhini - ps33
Simiiformes - ps34
Catarrhini - ps35
Hominoidea - ps36
Hominidae - ps37
Homininae - ps38
Hominini - ps39
Homo_sapiens - ps40
p-value
8e-03
1e-02
1e-02
2e-02
3e-02
Log-odds
0.5
1.0
1.5
2.0
2.5
3.0
3.5
4.0

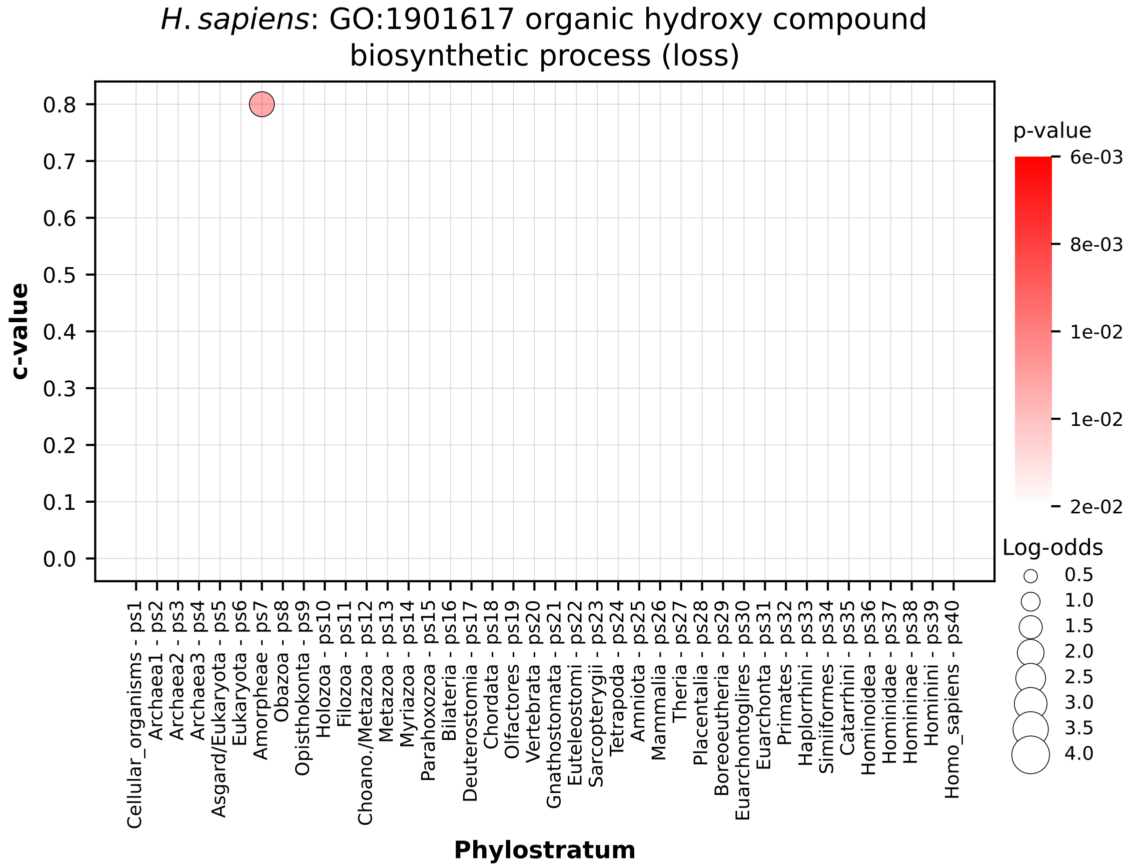
H. sapiens: GO:1901617 organic hydroxy compound biosynthetic process (loss)
c-value
0.0
0.1
0.2
0.3
0.4
0.5
0.6
0.7
0.8
Phylostratum
Cellular_organisms - ps1
Archaea1 - ps2
Archaea2 - ps3
Archaea3 - ps4
Asgard/Eukaryota - ps5
Eukaryota - ps6
Amorpheae - ps7
Obazoa - ps8
Opisthokonta - ps9
Holozoa - ps10
Filozoa - ps11
Choano./Metazoa - ps12
Metazoa - ps13
Myriazoa - ps14
Parahoxozoa - ps15
Bilateria - ps16
Deuterostomia - ps17
Chordata - ps18
Olfactores - ps19
Vertebrata - ps20
Gnathostomata - ps21
Euteleostomi - ps22
Sarcopterygii - ps23
Tetrapoda - ps24
Amniota - ps25
Mammalia - ps26
Theria - ps27
Placentalia - ps28
Boreoeutheria - ps29
Euarchontoglires - ps30
Euarchonta - ps31
Primates - ps32
Haplorrhini - ps33
Simiiformes - ps34
Catarrhini - ps35
Hominoidea - ps36
Hominidae - ps37
Homininae - ps38
Hominini - ps39
Homo_sapiens - ps40
p-value
6e-03
8e-03
1e-02
1e-02
2e-02
Log-odds
0.5
1.0
1.5
2.0
2.5
3.0
3.5
4.0

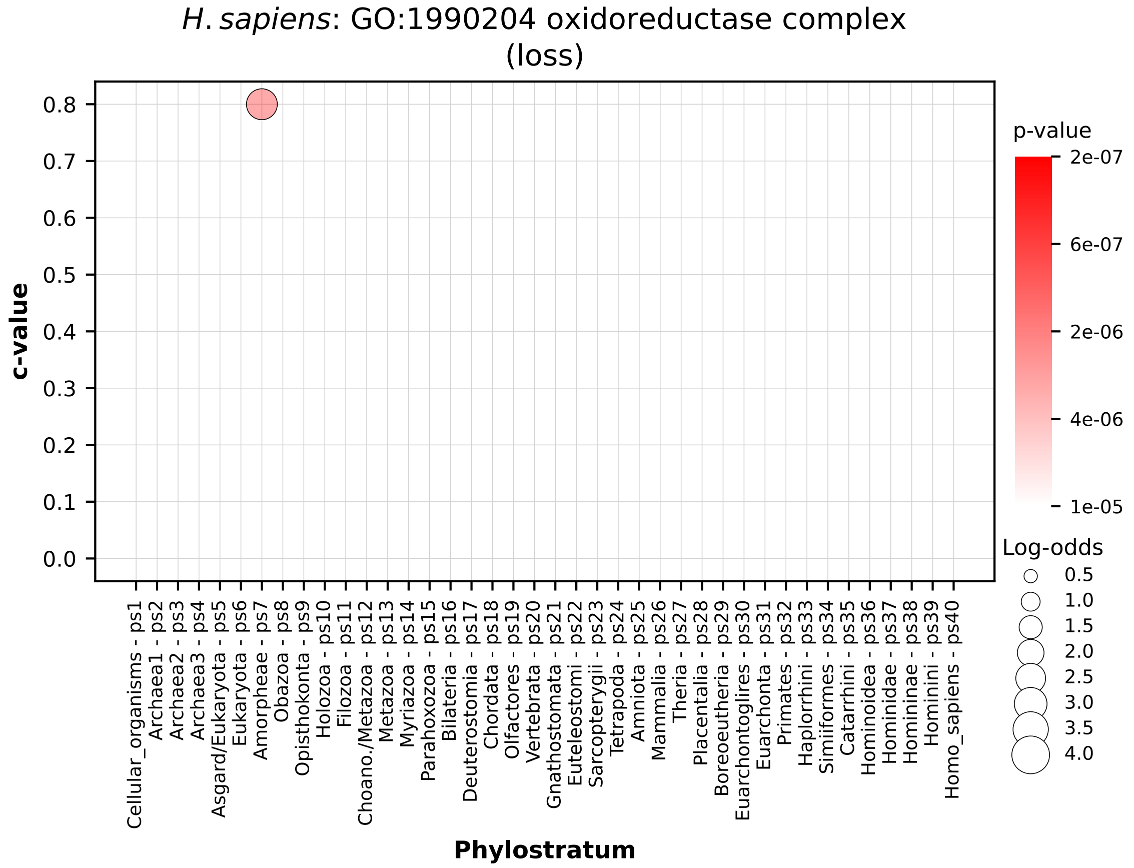

H. sapiens: GO:1990204 oxidoreductase complex
(loss)
c-value
0.8
0.7
0.6
0.5
0.4
0.3
0.2
0.1
0.0
p-value
2e-07
6e-07
2e-06
4e-06
1e-05
Log-odds
0.5
1.0
1.5
2.0
2.5
3.0
3.5
4.0
Cellular_organisms - ps1
Archaea1 - ps2
Archaea2 - ps3
Archaea3 - ps4
Asgard/Eukaryota - ps5
Eukaryota - ps6
Amorpheae - ps7
Obazoa - ps8
Opisthokonta - ps9
Holozoa - ps10
Filozoa - ps11
Choano./Metazoa - ps12
Metazoa - ps13
Myriazoa - ps14
Parahoxozoa - ps15
Bilateria - ps16
Deuterostomia - ps17
Chordata - ps18
Olfactores - ps19
Vertebrata - ps20
Gnathostomata - ps21
Euteleostomi - ps22
Sarcopterygii - ps23
Tetrapoda - ps24
Amniota - ps25
Mammalia - ps26
Theria - ps27
Placentalia - ps28
Boreoeutheria - ps29
Euarchontoglires - ps30
Euarchonta - ps31
Primates - ps32
Haplorrhini - ps33
Simiiformes - ps34
Catarrhini - ps35
Hominoidea - ps36
Hominidae - ps37
Homininae - ps38
Hominini - ps39
Homo_sapiens - ps40
Phylostratum

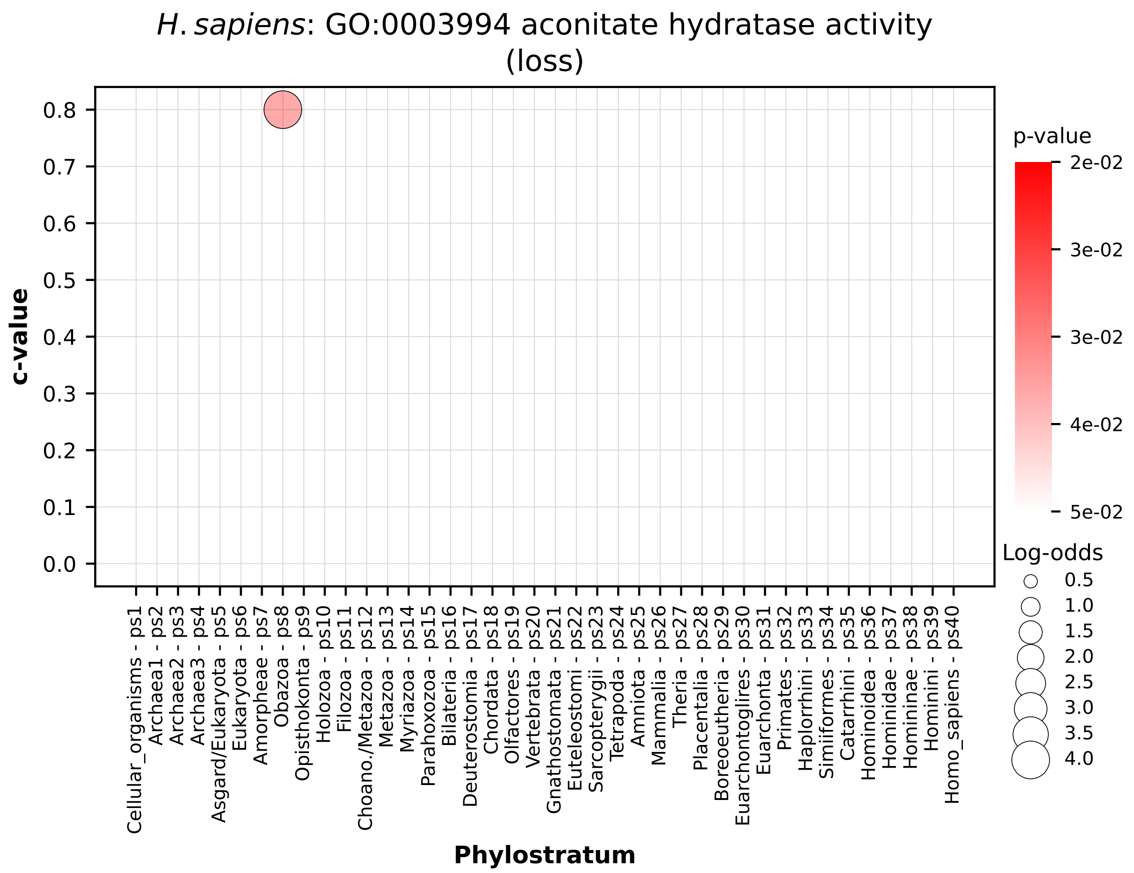

H. sapiens: GO:0003994 aconitate hydratase activity
(loss)
c-value
0.0
0.1
0.2
0.3
0.4
0.5
0.6
0.7
0.8
Phylostratum
Cellular_organisms - ps1
Archaea1 - ps2
Archaea2 - ps3
Archaea3 - ps4
Asgard/Eukaryota - ps5
Eukaryota - ps6
Amorpheae - ps7
Obazoa - ps8
Opisthokonta - ps9
Holozoa - ps10
Filozoa - ps11
Choano./Metazoa - ps12
Metazoa - ps13
Myriazoa - ps14
Parahoxozoa - ps15
Bilateria - ps16
Deuterostomia - ps17
Chordata - ps18
Olfactores - ps19
Vertebrata - ps20
Gnathostomata - ps21
Euteleostomi - ps22
Sarcopterygii - ps23
Tetrapoda - ps24
Amniota - ps25
Mammalia - ps26
Theria - ps27
Placentalia - ps28
Boreoeutheria - ps29
Euarchontoglires - ps30
Euarchonta - ps31
Primates - ps32
Haplorrhini - ps33
Simiiformes - ps34
Catarrhini - ps35
Hominoidea - ps36
Hominidae - ps37
Homininae - ps38
Hominini - ps39
Homo_sapiens - ps40
p-value
2e-02
3e-02
3e-02
4e-02
5e-02
Log-odds
0.5
1.0
1.5
2.0
2.5
3.0
3.5
4.0

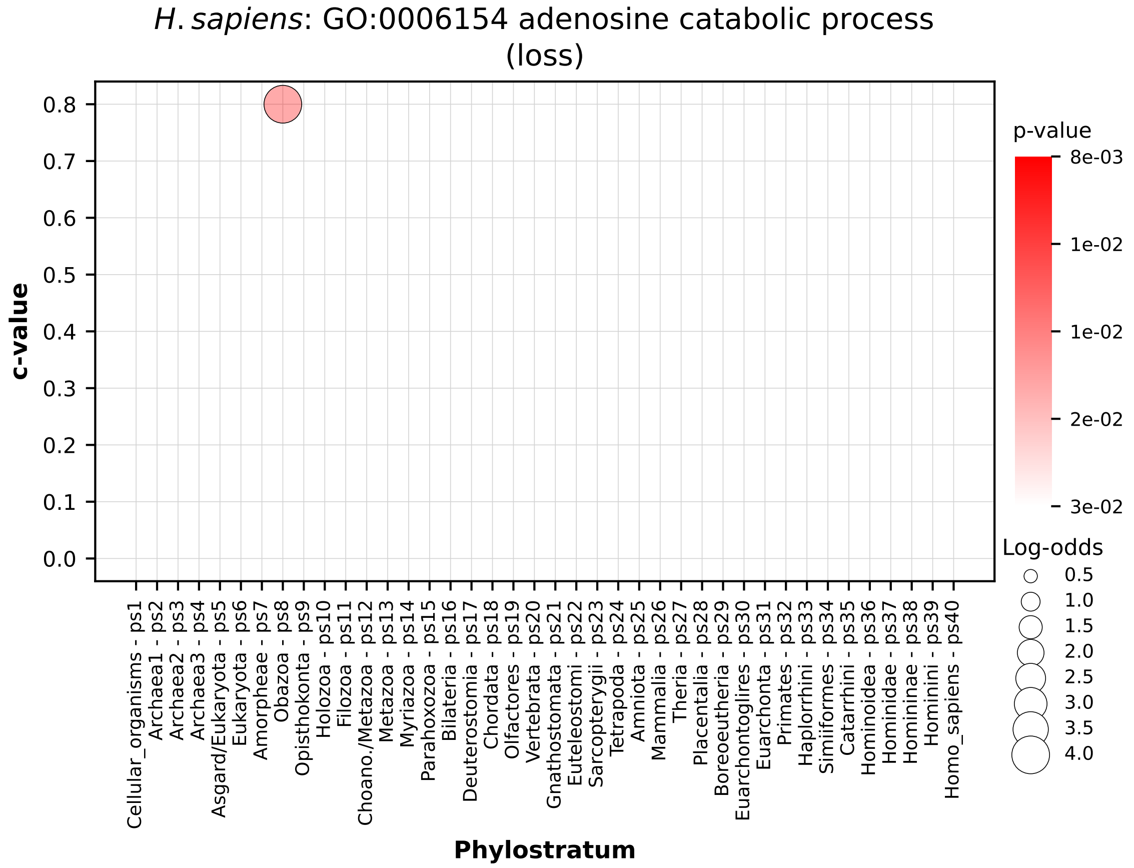

H. sapiens: GO:0006154 adenosine catabolic process
(loss)
c-value
0.8
0.7
0.6
0.5
0.4
0.3
0.2
0.1
0.0
p-value
8e-03
1e-02
1e-02
2e-02
3e-02
Log-odds
0.5
1.0
1.5
2.0
2.5
3.0
3.5
4.0
Cellular_organisms - ps1
Archaea1 - ps2
Archaea2 - ps3
Archaea3 - ps4
Asgard/Eukaryota - ps5
Eukaryota - ps6
Amorpheae - ps7
Obazoa - ps8
Opisthokonta - ps9
Holozoa - ps10
Filozoa - ps11
Choano./Metazoa - ps12
Metazoa - ps13
Myriazoa - ps14
Parahoxozoa - ps15
Bilateria - ps16
Deuterostomia - ps17
Chordata - ps18
Olfactores - ps19
Vertebrata - ps20
Gnathostomata - ps21
Euteleostomi - ps22
Sarcopterygii - ps23
Tetrapoda - ps24
Amniota - ps25
Mammalia - ps26
Theria - ps27
Placentalia - ps28
Boreoeutheria - ps29
Euarchontoglires - ps30
Euarchonta - ps31
Primates - ps32
Haplorrhini - ps33
Simiiformes - ps34
Catarrhini - ps35
Hominoidea - ps36
Hominidae - ps37
Homininae - ps38
Hominini - ps39
Homo_sapiens - ps40
Phylostratum

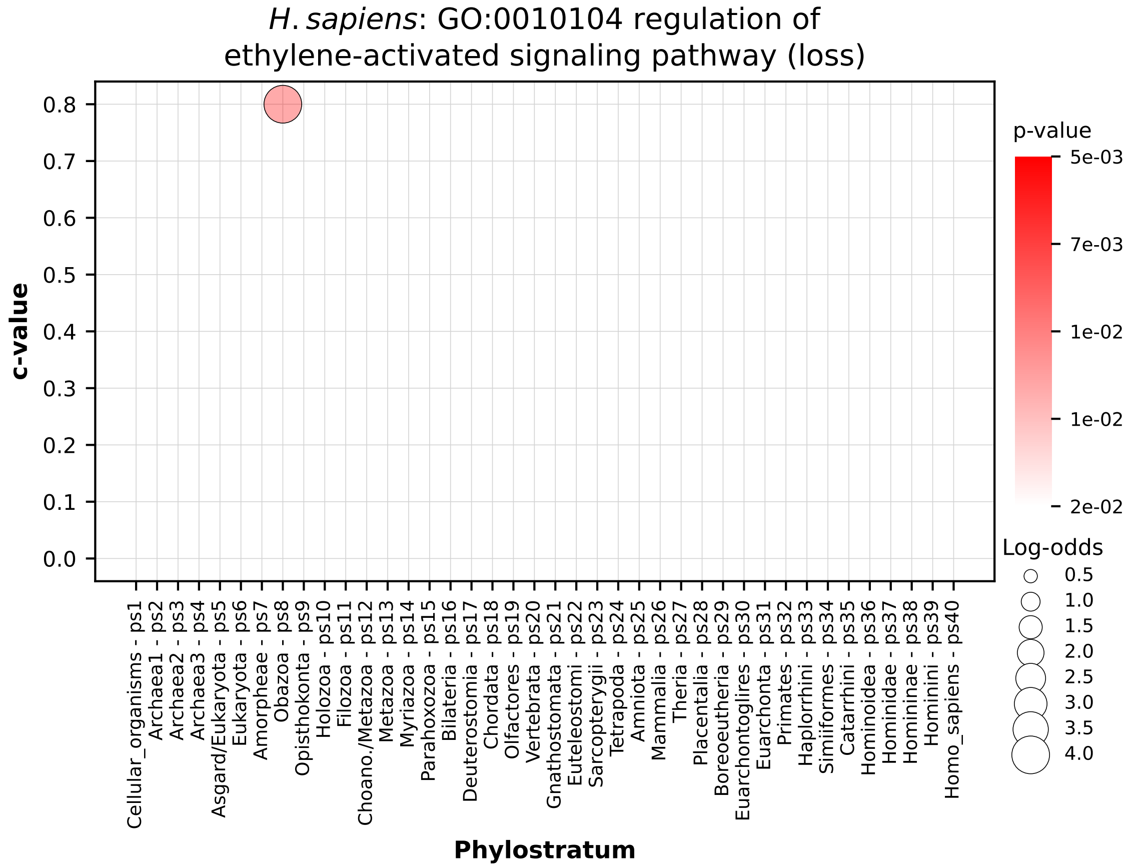

H. sapiens: GO:0010104 regulation of
ethylene-activated signaling pathway (loss)
c-value
0.0
0.1
0.2
0.3
0.4
0.5
0.6
0.7
0.8
Phylostratum
Cellular_organisms - ps1
Archaea1 - ps2
Archaea2 - ps3
Archaea3 - ps4
Asgard/Eukaryota - ps5
Eukaryota - ps6
Amorpheae - ps7
Obazoa - ps8
Opisthokonta - ps9
Holozoa - ps10
Filozoa - ps11
Choano./Metazoa - ps12
Metazoa - ps13
Myriazoa - ps14
Parahoxozoa - ps15
Bilateria - ps16
Deuterostomia - ps17
Chordata - ps18
Olfactores - ps19
Vertebrata - ps20
Gnathostomata - ps21
Euteleostomi - ps22
Sarcopterygii - ps23
Tetrapoda - ps24
Amniota - ps25
Mammalia - ps26
Theria - ps27
Placentalia - ps28
Boreoeutheria - ps29
Euarchontoglires - ps30
Euarchonta - ps31
Primates - ps32
Haplorrhini - ps33
Simiiformes - ps34
Catarrhini - ps35
Hominoidea - ps36
Hominidae - ps37
Homininae - ps38
Hominini - ps39
Homo_sapiens - ps40
p-value
5e-03
7e-03
1e-02
1e-02
2e-02
Log-odds
0.5
1.0
1.5
2.0
2.5
3.0
3.5
4.0

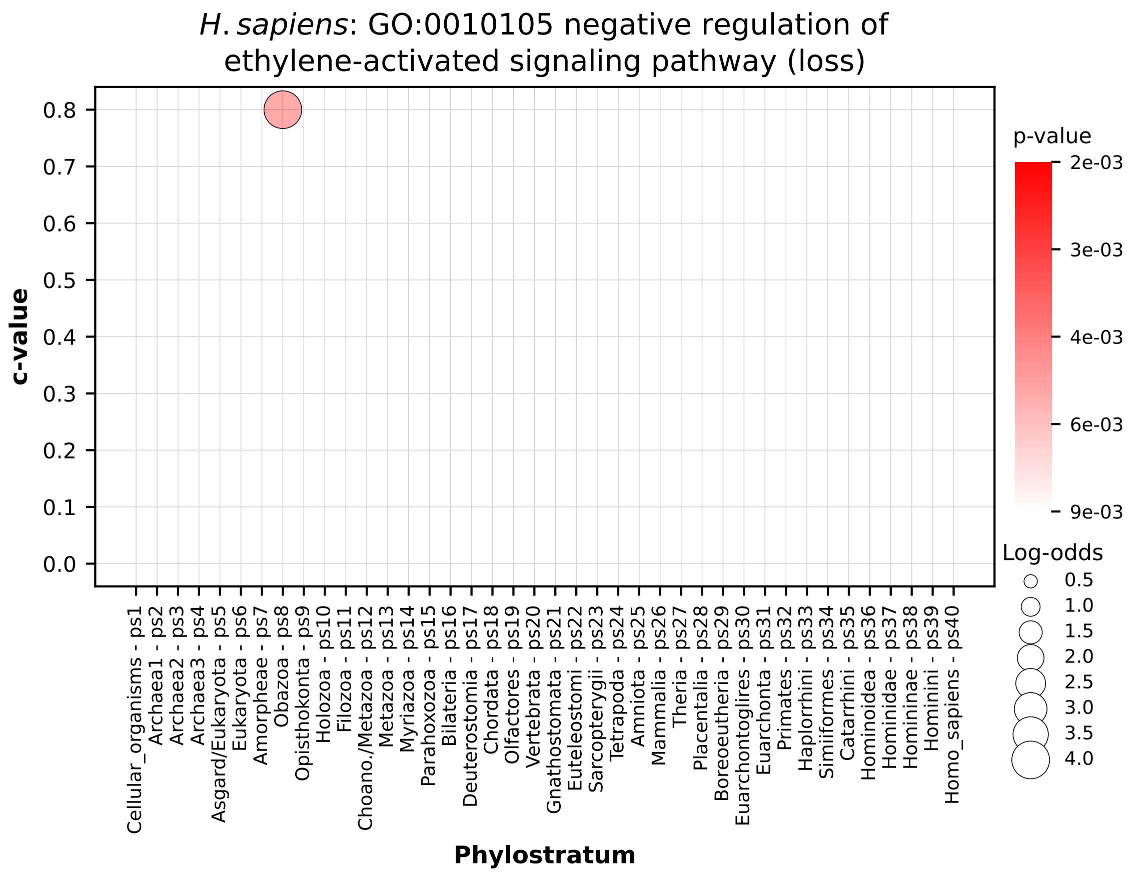
H. sapiens: GO:0010105 negative regulation of ethylene-activated signaling pathway (loss)
c-value
0.8
0.7
0.6
0.5
0.4
0.3
0.2
0.1
0.0
Phylostratum
Cellular_organisms - ps1
Archaea1 - ps2
Archaea2 - ps3
Archaea3 - ps4
Asgard/Eukaryota - ps5
Eukaryota - ps6
Amorpheae - ps7
Obazoa - ps8
Opisthokonta - ps9
Holozoa - ps10
Filozoa - ps11
Choano./Metazoa - ps12
Metazoa - ps13
Myriazoa - ps14
Parahoxozoa - ps15
Bilateria - ps16
Deuterostomia - ps17
Chordata - ps18
Olfactores - ps19
Vertebrata - ps20
Gnathostomata - ps21
Euteleostomi - ps22
Sarcopterygii - ps23
Tetrapoda - ps24
Amniota - ps25
Mammalia - ps26
Theria - ps27
Placentalia - ps28
Boreoeutheria - ps29
Euarchontoglires - ps30
Euarchonta - ps31
Primates - ps32
Haplorrhini - ps33
Simiiformes - ps34
Catarrhini - ps35
Hominoidea - ps36
Hominidae - ps37
Homininae - ps38
Hominini - ps39
Homo_sapiens - ps40
p-value
2e-03
3e-03
4e-03
6e-03
9e-03
Log-odds
0.5
1.0
1.5
2.0
2.5
3.0
3.5
4.0

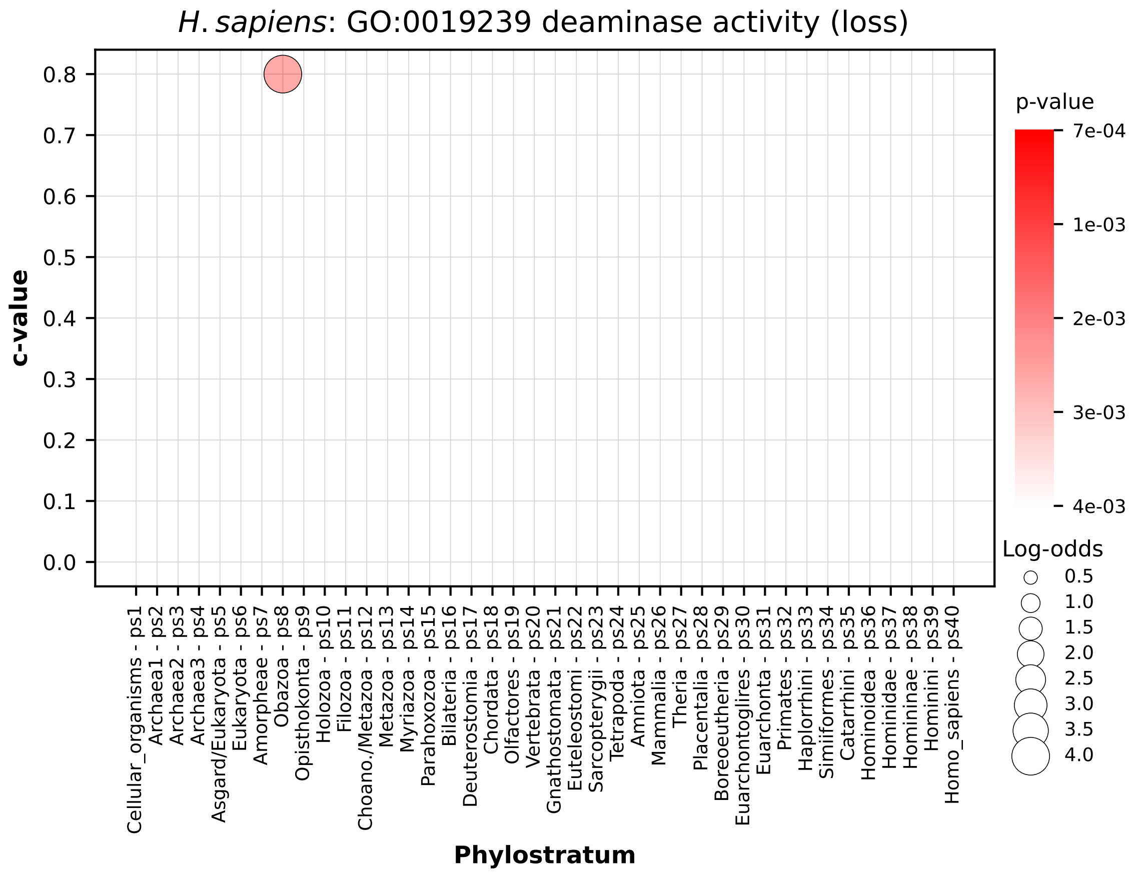

H. sapiens: GO:0019239 deaminase activity (loss)
c-value
0.0
0.1
0.2
0.3
0.4
0.5
0.6
0.7
0.8
Phylostratum
Cellular_organisms - ps1
Archaea1 - ps2
Archaea2 - ps3
Archaea3 - ps4
Asgard/Eukaryota - ps5
Eukaryota - ps6
Amorpheae - ps7
Obazoa - ps8
Opisthokonta - ps9
Holozoa - ps10
Filozoa - ps11
Choano./Metazoa - ps12
Metazoa - ps13
Myriazoa - ps14
Parahoxozoa - ps15
Bilateria - ps16
Deuterostomia - ps17
Chordata - ps18
Olfactores - ps19
Vertebrata - ps20
Gnathostomata - ps21
Euteleostomi - ps22
Sarcopterygii - ps23
Tetrapoda - ps24
Amniota - ps25
Mammalia - ps26
Theria - ps27
Placentalia - ps28
Boreoeutheria - ps29
Euarchontoglires - ps30
Euarchonta - ps31
Primates - ps32
Haplorrhini - ps33
Simiiformes - ps34
Catarrhini - ps35
Hominoidea - ps36
Hominidae - ps37
Homininae - ps38
Hominini - ps39
Homo_sapiens - ps40
p-value
7e-04
1e-03
2e-03
3e-03
4e-03
Log-odds
0.5
1.0
1.5
2.0
2.5
3.0
3.5
4.0

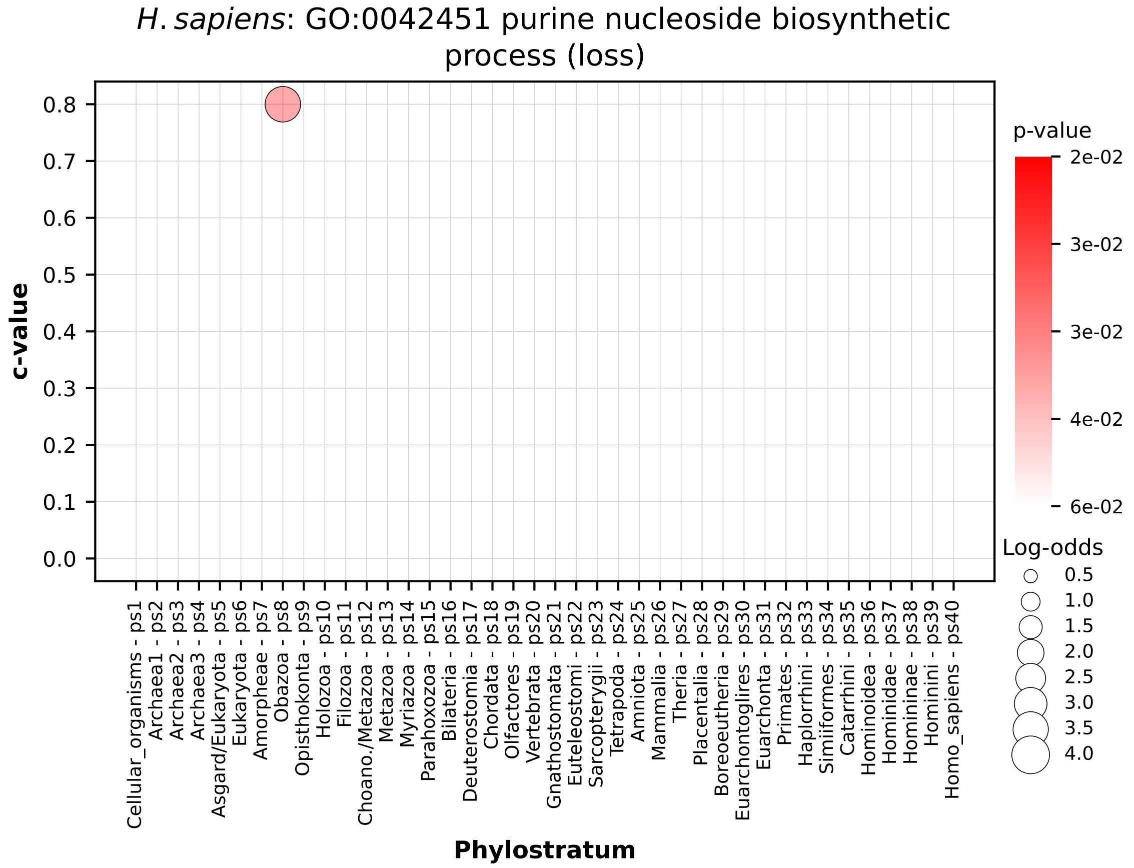

H. sapiens: GO:0042451 purine nucleoside biosynthetic process (loss)
c-value
0.8
0.7
0.6
0.5
0.4
0.3
0.2
0.1
0.0
p-value
2e-02
3e-02
3e-02
4e-02
6e-02
Log-odds
0.5
1.0
1.5
2.0
2.5
3.0
3.5
4.0
Cellular_organisms - ps1
Archaea1 - ps2
Archaea2 - ps3
Archaea3 - ps4
Asgard/Eukaryota - ps5
Eukaryota - ps6
Amorpheae - ps7
Obazoa - ps8
Opisthokonta - ps9
Holozoa - ps10
Filozoa - ps11
Choano./Metazoa - ps12
Metazoa - ps13
Myriazoa - ps14
Parahoxozoa - ps15
Bilateria - ps16
Deuterostomia - ps17
Chordata - ps18
Olfactores - ps19
Vertebrata - ps20
Gnathostomata - ps21
Euteleostomi - ps22
Sarcopterygii - ps23
Tetrapoda - ps24
Amniota - ps25
Mammalia - ps26
Theria - ps27
Placentalia - ps28
Boreoeutheria - ps29
Euarchontoglires - ps30
Euarchonta - ps31
Primates - ps32
Haplorrhini - ps33
Simiiformes - ps34
Catarrhini - ps35
Hominoidea - ps36
Hominidae - ps37
Homininae - ps38
Hominini - ps39
Homo_sapiens - ps40
Phylostratum

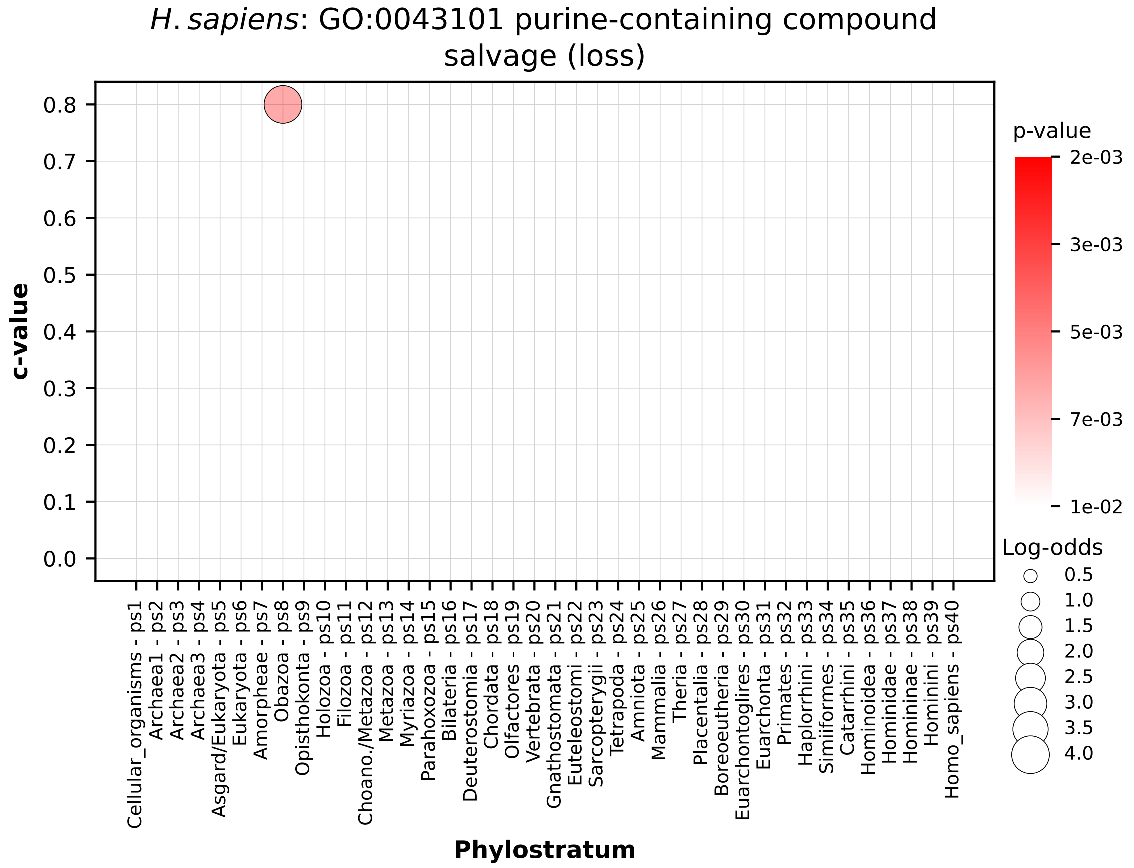

H. sapiens: GO:0043101 purine-containing compound salvage (loss)
c-value
0.0
0.1
0.2
0.3
0.4
0.5
0.6
0.7
0.8
p-value
2e-03
3e-03
5e-03
7e-03
1e-02
Log-odds
0.5
1.0
1.5
2.0
2.5
3.0
3.5
4.0
Cellular_organisms - ps1
Archaea1 - ps2
Archaea2 - ps3
Archaea3 - ps4
Asgard/Eukaryota - ps5
Eukaryota - ps6
Amorpheae - ps7
Obazoa - ps8
Opisthokonta - ps9
Holozoa - ps10
Filozoa - ps11
Choano./Metazoa - ps12
Metazoa - ps13
Myriazoa - ps14
Parahoxozoa - ps15
Bilateria - ps16
Deuterostomia - ps17
Chordata - ps18
Olfactores - ps19
Vertebrata - ps20
Gnathostomata - ps21
Euteleostomi - ps22
Sarcopterygii - ps23
Tetrapoda - ps24
Amniota - ps25
Mammalia - ps26
Theria - ps27
Placentalia - ps28
Boreoeutheria - ps29
Euarchontoglires - ps30
Euarchonta - ps31
Primates - ps32
Haplorrhini - ps33
Simiiformes - ps34
Catarrhini - ps35
Hominoidea - ps36
Hominidae - ps37
Homininae - ps38
Hominini - ps39
Homo_sapiens - ps40
Phylostratum

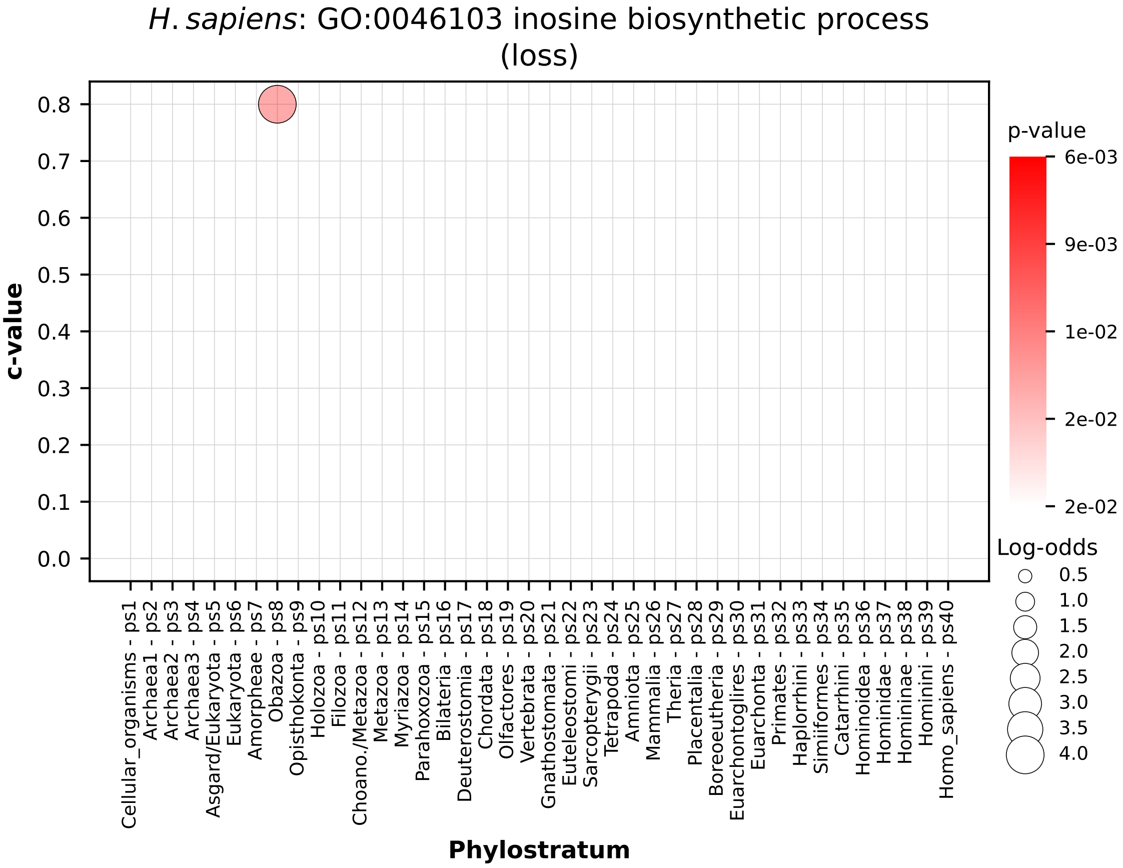

H. sapiens: GO:0046103 inosine biosynthetic process (loss)
c-value
0.8
0.7
0.6
0.5
0.4
0.3
0.2
0.1
0.0
Phylostratum
Cellular_organisms - ps1
Archaea1 - ps2
Archaea2 - ps3
Archaea3 - ps4
Asgard/Eukaryota - ps5
Eukaryota - ps6
Amorpheae - ps7
Obazoa - ps8
Opisthokonta - ps9
Holozoa - ps10
Filozoa - ps11
Choano./Metazoa - ps12
Metazoa - ps13
Myriazoa - ps14
Parahoxozoa - ps15
Bilateria - ps16
Deuterostomia - ps17
Chordata - ps18
Olfactores - ps19
Vertebrata - ps20
Gnathostomata - ps21
Euteleostomi - ps22
Sarcopterygii - ps23
Tetrapoda - ps24
Amniota - ps25
Mammalia - ps26
Theria - ps27
Placentalia - ps28
Boreoeutheria - ps29
Euarchontoglires - ps30
Euarchonta - ps31
Primates - ps32
Haplorrhini - ps33
Simiiformes - ps34
Catarrhini - ps35
Hominoidea - ps36
Hominidae - ps37
Homininae - ps38
Hominini - ps39
Homo_sapiens - ps40
p-value
6e-03
9e-03
1e-02
2e-02
2e-02
Log-odds
0.5
1.0
1.5
2.0
2.5
3.0
3.5
4.0

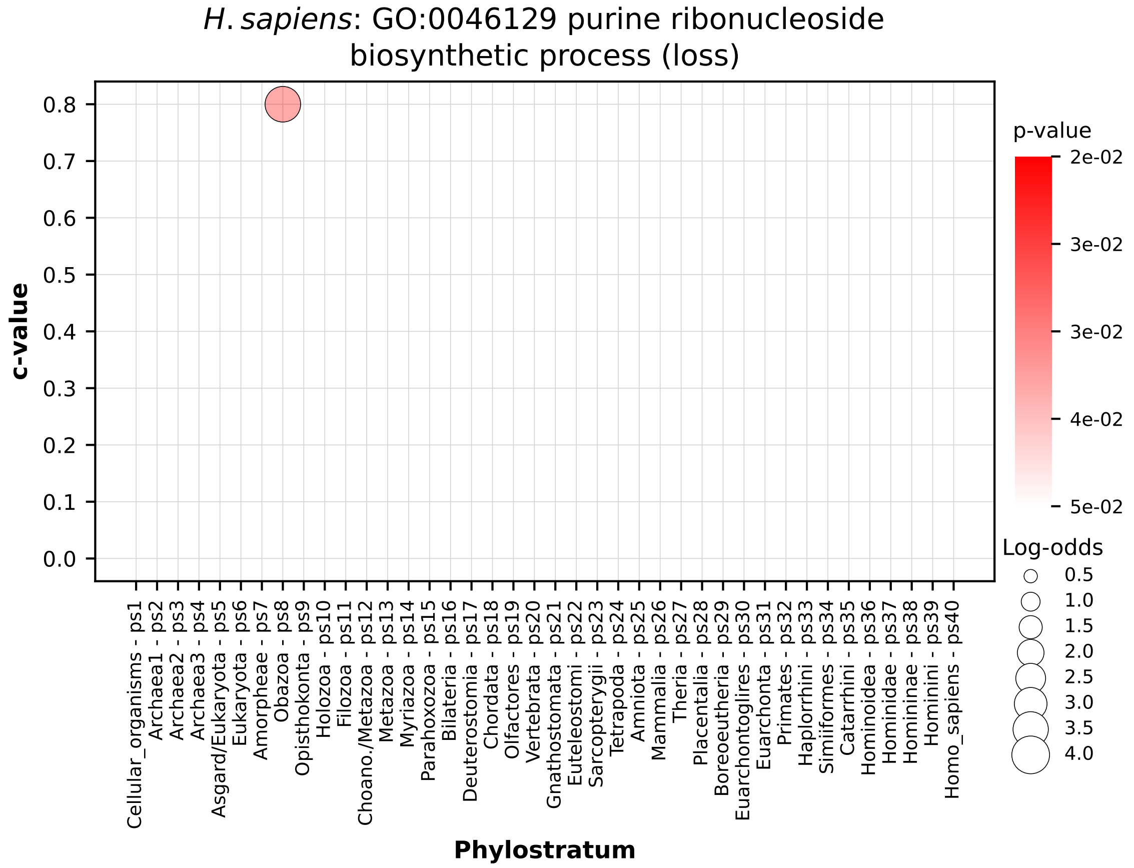
H. sapiens: GO:0046129 purine ribonucleoside biosynthetic process (loss)
c-value
0.0
0.1
0.2
0.3
0.4
0.5
0.6
0.7
0.8
Phylostratum
Cellular_organisms - ps1
Archaea1 - ps2
Archaea2 - ps3
Archaea3 - ps4
Asgard/Eukaryota - ps5
Eukaryota - ps6
Amorpheae - ps7
Obazoa - ps8
Opisthokonta - ps9
Holozoa - ps10
Filozoa - ps11
Choano./Metazoa - ps12
Metazoa - ps13
Myriazoa - ps14
Parahoxozoa - ps15
Bilateria - ps16
Deuterostomia - ps17
Chordata - ps18
Olfactores - ps19
Vertebrata - ps20
Gnathostomata - ps21
Euteleostomi - ps22
Sarcopterygii - ps23
Tetrapoda - ps24
Amniota - ps25
Mammalia - ps26
Theria - ps27
Placentalia - ps28
Boreoeutheria - ps29
Euarchontoglires - ps30
Euarchonta - ps31
Primates - ps32
Haplorrhini - ps33
Simiiformes - ps34
Catarrhini - ps35
Hominoidea - ps36
Hominidae - ps37
Homininae - ps38
Hominini - ps39
Homo_sapiens - ps40
p-value
2e-02
3e-02
3e-02
4e-02
5e-02
Log-odds
0.5
1.0
1.5
2.0
2.5
3.0
3.5
4.0

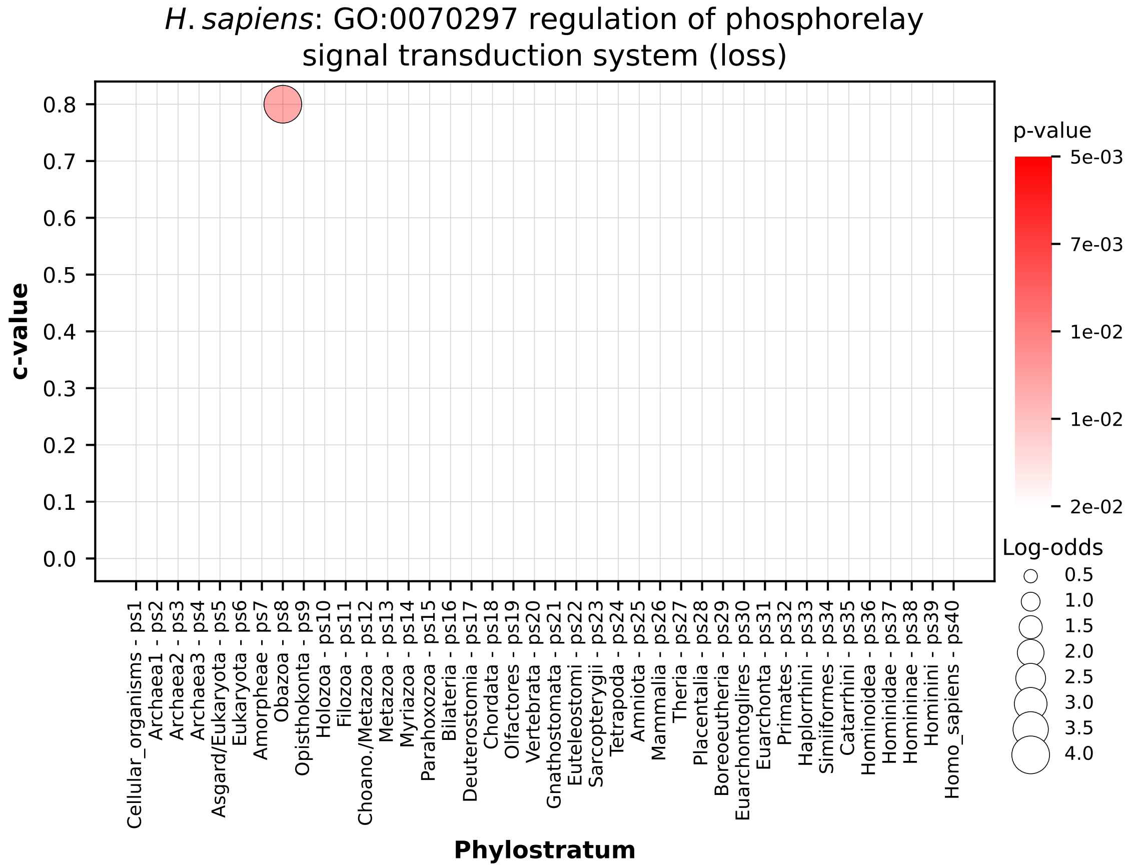
H. sapiens: GO:0070297 regulation of phosphorelay signal transduction system (loss)
c-value
0.0
0.1
0.2
0.3
0.4
0.5
0.6
0.7
0.8
Phylostratum
Cellular_organisms - ps1
Archaea1 - ps2
Archaea2 - ps3
Archaea3 - ps4
Asgard/Eukaryota - ps5
Eukaryota - ps6
Amorpheae - ps7
Obazoa - ps8
Opisthokonta - ps9
Holozoa - ps10
Filozoa - ps11
Choano./Metazoa - ps12
Metazoa - ps13
Myriazoa - ps14
Parahoxozoa - ps15
Bilateria - ps16
Deuterostomia - ps17
Chordata - ps18
Olfactores - ps19
Vertebrata - ps20
Gnathostomata - ps21
Euteleostomi - ps22
Sarcopterygii - ps23
Tetrapoda - ps24
Amniota - ps25
Mammalia - ps26
Theria - ps27
Placentalia - ps28
Boreoeutheria - ps29
Euarchontoglires - ps30
Euarchonta - ps31
Primates - ps32
Haplorrhini - ps33
Simiiformes - ps34
Catarrhini - ps35
Hominoidea - ps36
Hominidae - ps37
Homininae - ps38
Hominini - ps39
Homo_sapiens - ps40
p-value
5e-03
7e-03
1e-02
1e-02
2e-02
Log-odds
0.5
1.0
1.5
2.0
2.5
3.0
3.5
4.0

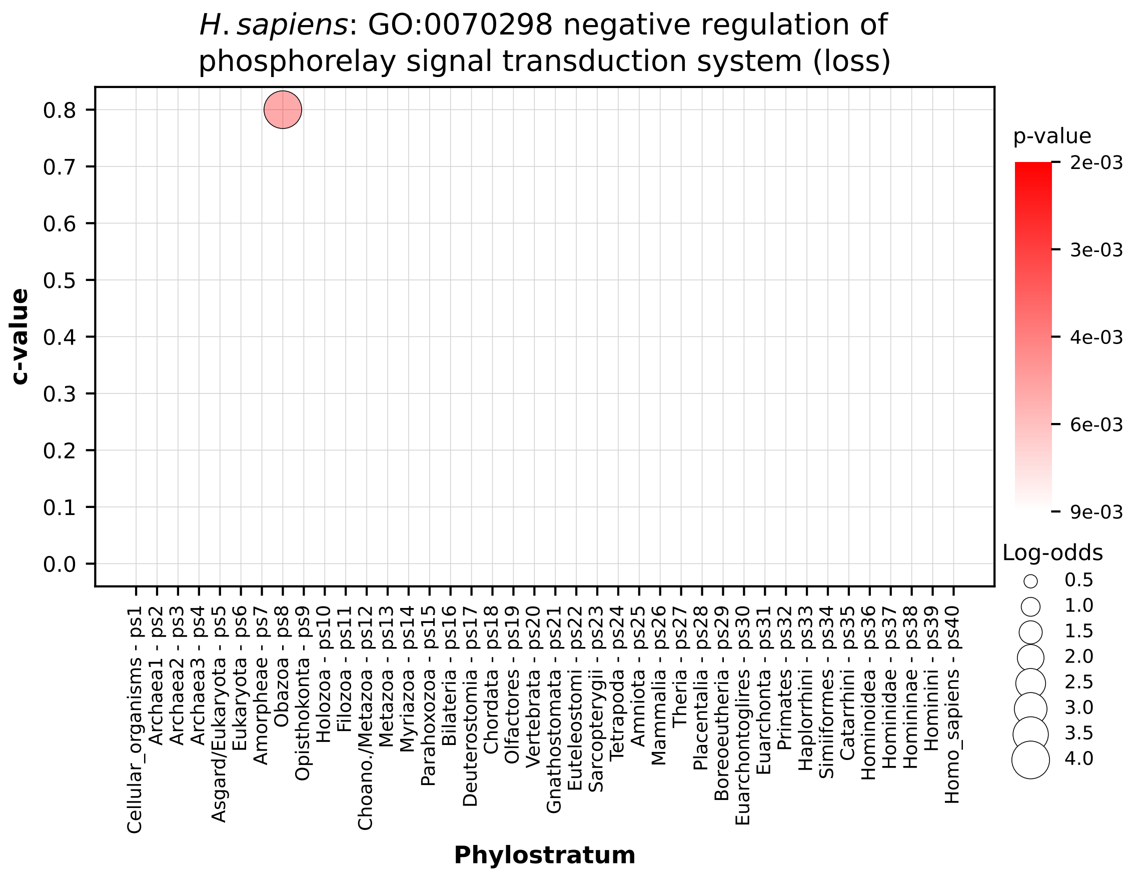

H. sapiens: GO:0070298 negative regulation of phosphorelay signal transduction system (loss)
c-value
0.0
0.1
0.2
0.3
0.4
0.5
0.6
0.7
0.8
Phylostratum
Cellular_organisms - ps1
Archaea1 - ps2
Archaea2 - ps3
Archaea3 - ps4
Asgard/Eukaryota - ps5
Eukaryota - ps6
Amorpheae - ps7
Obazoa - ps8
Opisthokonta - ps9
Holozoa - ps10
Filozoa - ps11
Choano./Metazoa - ps12
Metazoa - ps13
Myriazoa - ps14
Parahoxozoa - ps15
Bilateria - ps16
Deuterostomia - ps17
Chordata - ps18
Olfactores - ps19
Vertebrata - ps20
Gnathostomata - ps21
Euteleostomi - ps22
Sarcopterygii - ps23
Tetrapoda - ps24
Amniota - ps25
Mammalia - ps26
Theria - ps27
Placentalia - ps28
Boreoeutheria - ps29
Euarchontoglires - ps30
Euarchonta - ps31
Primates - ps32
Haplorrhini - ps33
Simiiformes - ps34
Catarrhini - ps35
Hominoidea - ps36
Hominidae - ps37
Homininae - ps38
Hominini - ps39
Homo_sapiens - ps40
p-value
2e-03
3e-03
4e-03
6e-03
9e-03
Log-odds
0.5
1.0
1.5
2.0
2.5
3.0
3.5
4.0

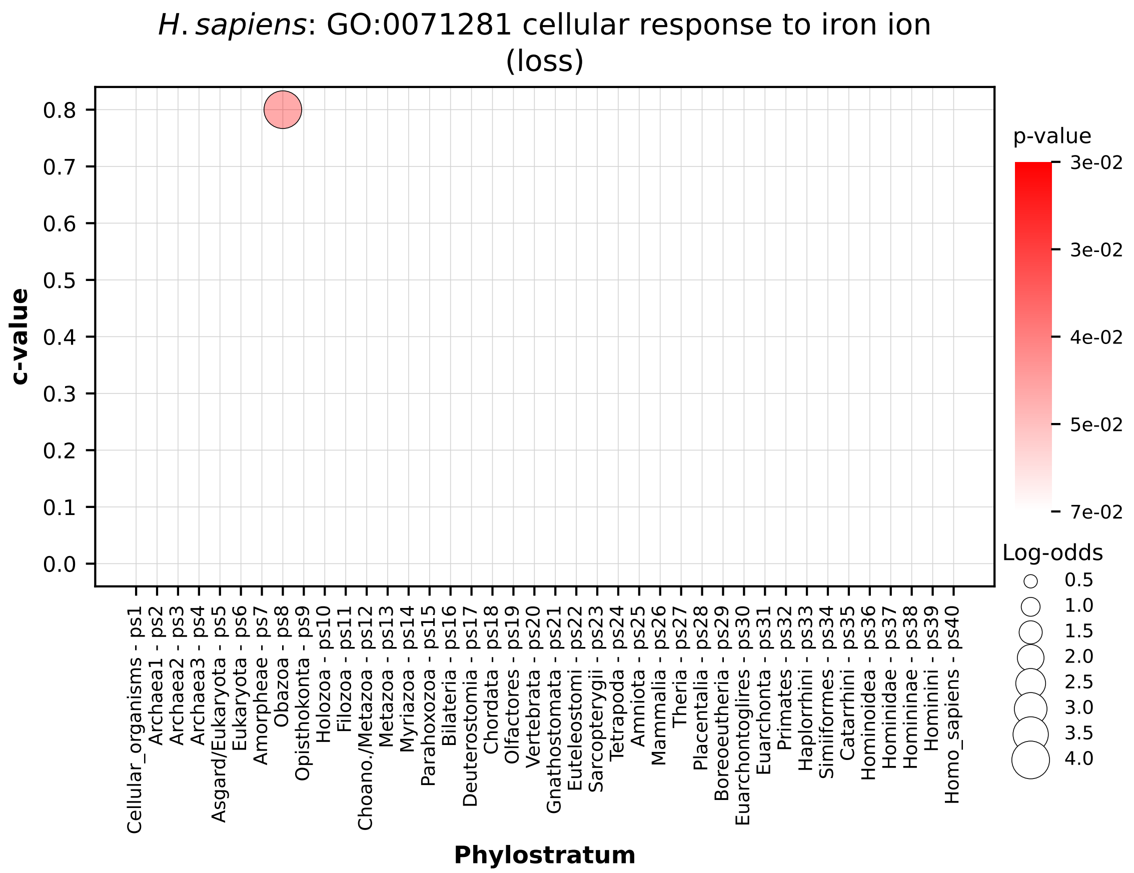

H. sapiens: GO:0071281 cellular response to iron ion
(loss)
c-value
0.0
0.1
0.2
0.3
0.4
0.5
0.6
0.7
0.8
Phylostratum
Cellular_organisms - ps1
Archaea1 - ps2
Archaea2 - ps3
Archaea3 - ps4
Asgard/Eukaryota - ps5
Eukaryota - ps6
Amorpheae - ps7
Obazoa - ps8
Opisthokonta - ps9
Holozoa - ps10
Filozoa - ps11
Choano./Metazoa - ps12
Metazoa - ps13
Myriazoa - ps14
Parahoxozoa - ps15
Bilateria - ps16
Deuterostomia - ps17
Chordata - ps18
Olfactores - ps19
Vertebrata - ps20
Gnathostomata - ps21
Euteleostomi - ps22
Sarcopterygii - ps23
Tetrapoda - ps24
Amniota - ps25
Mammalia - ps26
Theria - ps27
Placentalia - ps28
Boreoeutheria - ps29
Euarchontoglires - ps30
Euarchonta - ps31
Primates - ps32
Haplorrhini - ps33
Simiiformes - ps34
Catarrhini - ps35
Hominoidea - ps36
Hominidae - ps37
Homininae - ps38
Hominini - ps39
Homo_sapiens - ps40
p-value
3e-02
3e-02
4e-02
5e-02
7e-02
Log-odds
0.5
1.0
1.5
2.0
2.5
3.0
3.5
4.0

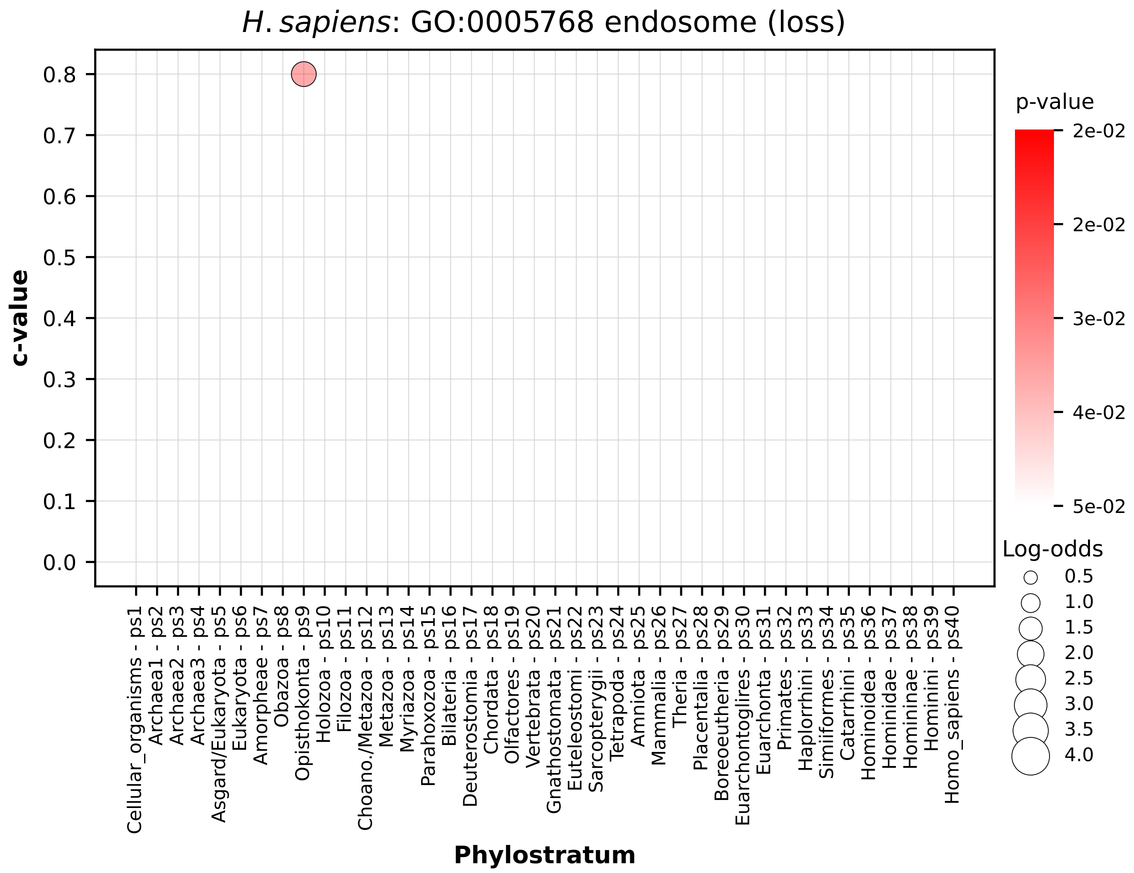

H. sapiens: GO:0005768 endosome (loss)
c-value
0.0
0.1
0.2
0.3
0.4
0.5
0.6
0.7
0.8
Phylostratum
Cellular_organisms - ps1
Archaea1 - ps2
Archaea2 - ps3
Archaea3 - ps4
Asgard/Eukaryota - ps5
Eukaryota - ps6
Amorpheae - ps7
Obazoa - ps8
Opisthokonta - ps9
Holozoa - ps10
Filozoa - ps11
Choano./Metazoa - ps12
Metazoa - ps13
Myriazoa - ps14
Parahoxozoa - ps15
Bilateria - ps16
Deuterostomia - ps17
Chordata - ps18
Olfactores - ps19
Vertebrata - ps20
Gnathostomata - ps21
Euteleostomi - ps22
Sarcopterygii - ps23
Tetrapoda - ps24
Amniota - ps25
Mammalia - ps26
Theria - ps27
Placentalia - ps28
Boreoeutheria - ps29
Euarchontoglires - ps30
Euarchonta - ps31
Primates - ps32
Haplorrhini - ps33
Simiiformes - ps34
Catarrhini - ps35
Hominoidea - ps36
Hominidae - ps37
Homininae - ps38
Hominini - ps39
Homo_sapiens - ps40
p-value
2e-02
2e-02
3e-02
4e-02
5e-02
Log-odds
0.5
1.0
1.5
2.0
2.5
3.0
3.5
4.0

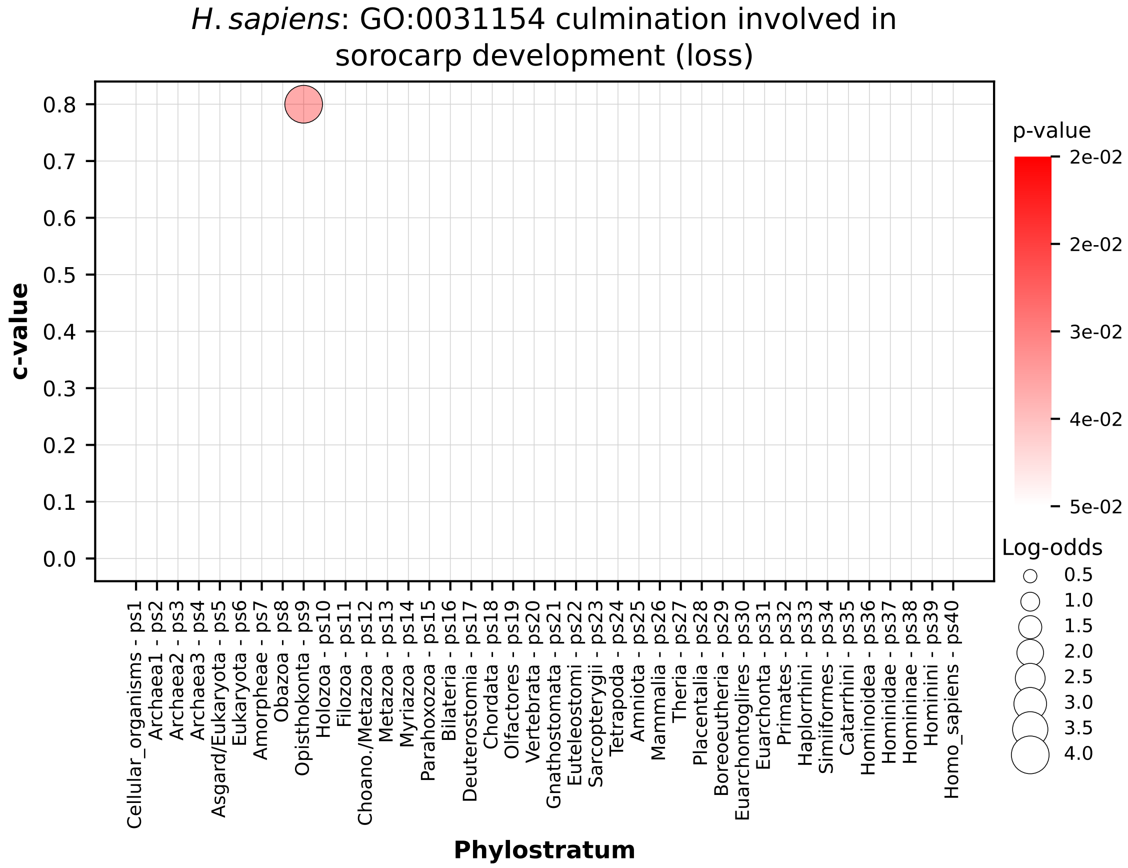
H. sapiens: GO:0031154 culmination involved in sorocarp development (loss)
c-value
0.8
0.7
0.6
0.5
0.4
0.3
0.2
0.1
0.0
p-value
2e-02
2e-02
3e-02
4e-02
5e-02
Log-odds
0.5
1.0
1.5
2.0
2.5
3.0
3.5
4.0
Cellular_organisms - ps1
Archaea1 - ps2
Archaea2 - ps3
Archaea3 - ps4
Asgard/Eukaryota - ps5
Eukaryota - ps6
Amorpheae - ps7
Obazoa - ps8
Opisthokonta - ps9
Holozoa - ps10
Filozoa - ps11
Choano./Metazoa - ps12
Metazoa - ps13
Myriazoa - ps14
Parahoxozoa - ps15
Bilateria - ps16
Deuterostomia - ps17
Chordata - ps18
Olfactores - ps19
Vertebrata - ps20
Gnathostomata - ps21
Euteleostomi - ps22
Sarcopterygii - ps23
Tetrapoda - ps24
Amniota - ps25
Mammalia - ps26
Theria - ps27
Placentalia - ps28
Boreoeutheria - ps29
Euarchontoglires - ps30
Euarchonta - ps31
Primates - ps32
Haplorrhini - ps33
Simiiformes - ps34
Catarrhini - ps35
Hominoidea - ps36
Hominidae - ps37
Homininae - ps38
Hominini - ps39
Homo_sapiens - ps40
Phylostratum

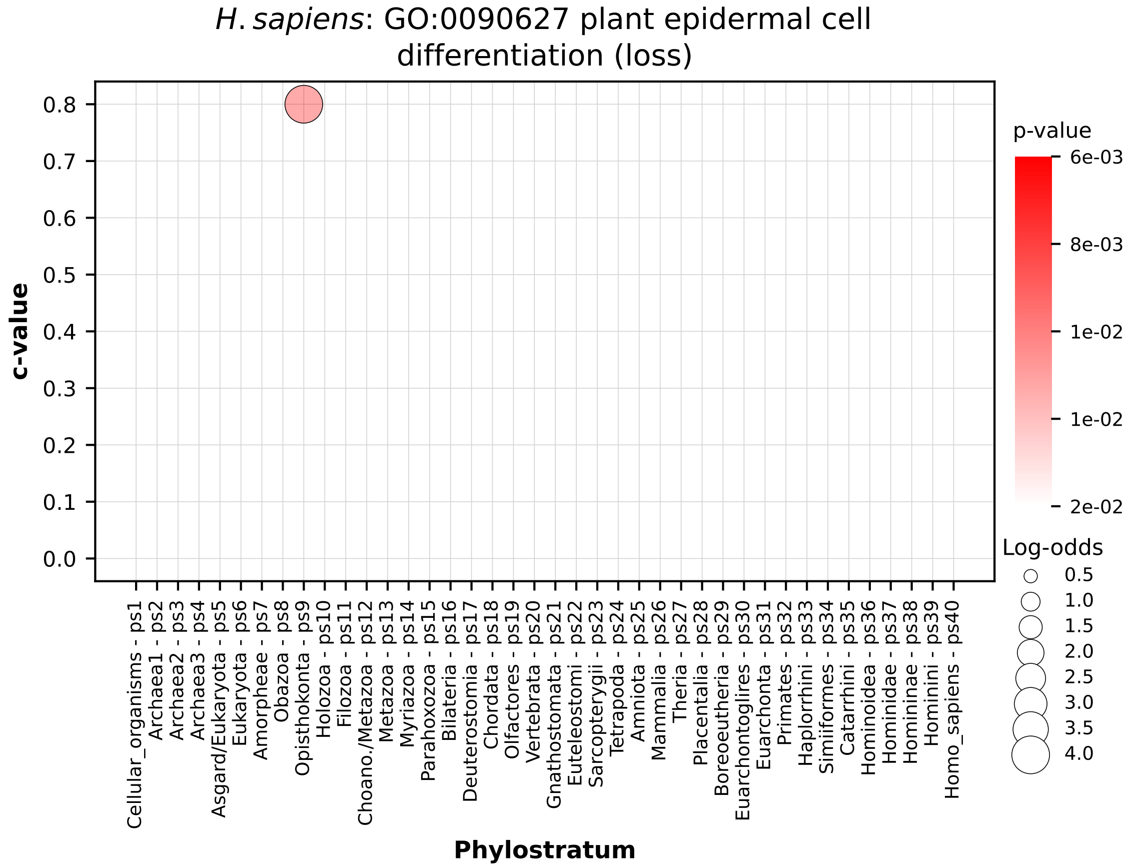

H. sapiens: GO:0090627 plant epidermal cell differentiation (loss)
c-value
0.0
0.1
0.2
0.3
0.4
0.5
0.6
0.7
0.8
p-value
6e-03
8e-03
1e-02
1e-02
2e-02
Log-odds
0.5
1.0
1.5
2.0
2.5
3.0
3.5
4.0
Cellular_organisms - ps1
Archaea1 - ps2
Archaea2 - ps3
Archaea3 - ps4
Asgard/Eukaryota - ps5
Eukaryota - ps6
Amorpheae - ps7
Obazoa - ps8
Opisthokonta - ps9
Holozoa - ps10
Filozoa - ps11
Choano./Metazoa - ps12
Metazoa - ps13
Myriazoa - ps14
Parahoxozoa - ps15
Bilateria - ps16
Deuterostomia - ps17
Chordata - ps18
Olfactores - ps19
Vertebrata - ps20
Gnathostomata - ps21
Euteleostomi - ps22
Sarcopterygii - ps23
Tetrapoda - ps24
Amniota - ps25
Mammalia - ps26
Theria - ps27
Placentalia - ps28
Boreoeutheria - ps29
Euarchontoglires - ps30
Euarchonta - ps31
Primates - ps32
Haplorrhini - ps33
Simiiformes - ps34
Catarrhini - ps35
Hominoidea - ps36
Hominidae - ps37
Homininae - ps38
Hominini - ps39
Homo_sapiens - ps40
Phylostratum

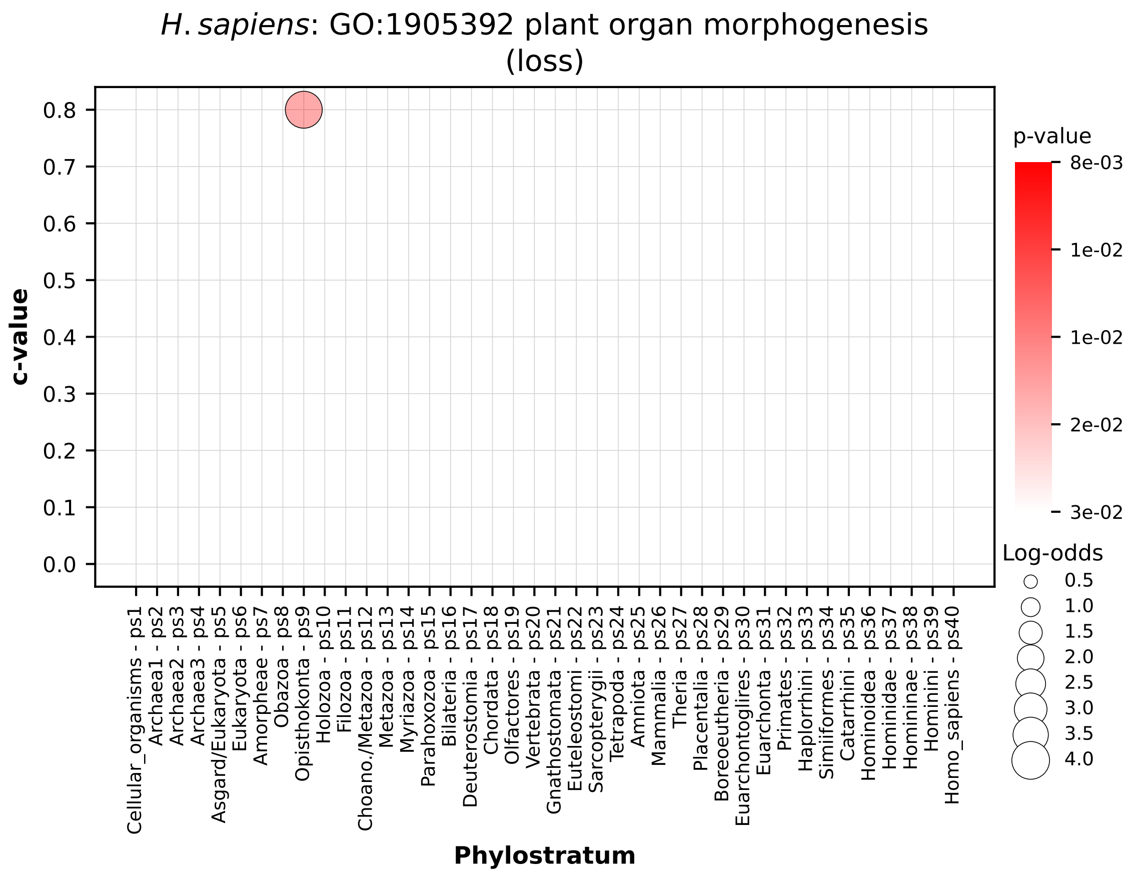

H. sapiens: GO:1905392 plant organ morphogenesis
(loss)
c-value
0.8
0.7
0.6
0.5
0.4
0.3
0.2
0.1
0.0
p-value
8e-03
1e-02
1e-02
2e-02
3e-02
Log-odds
0.5
1.0
1.5
2.0
2.5
3.0
3.5
4.0
Cellular_organisms - ps1
Archaea1 - ps2
Archaea2 - ps3
Archaea3 - ps4
Asgard/Eukaryota - ps5
Eukaryota - ps6
Amorpheae - ps7
Obazoa - ps8
Opisthokonta - ps9
Holozoa - ps10
Filozoa - ps11
Choano./Metazoa - ps12
Metazoa - ps13
Myriazoa - ps14
Parahoxozoa - ps15
Bilateria - ps16
Deuterostomia - ps17
Chordata - ps18
Olfactores - ps19
Vertebrata - ps20
Gnathostomata - ps21
Euteleostomi - ps22
Sarcopterygii - ps23
Tetrapoda - ps24
Amniota - ps25
Mammalia - ps26
Theria - ps27
Placentalia - ps28
Boreoeutheria - ps29
Euarchontoglires - ps30
Euarchonta - ps31
Primates - ps32
Haplorrhini - ps33
Simiiformes - ps34
Catarrhini - ps35
Hominoidea - ps36
Hominidae - ps37
Homininae - ps38
Hominini - ps39
Homo_sapiens - ps40
Phylostratum

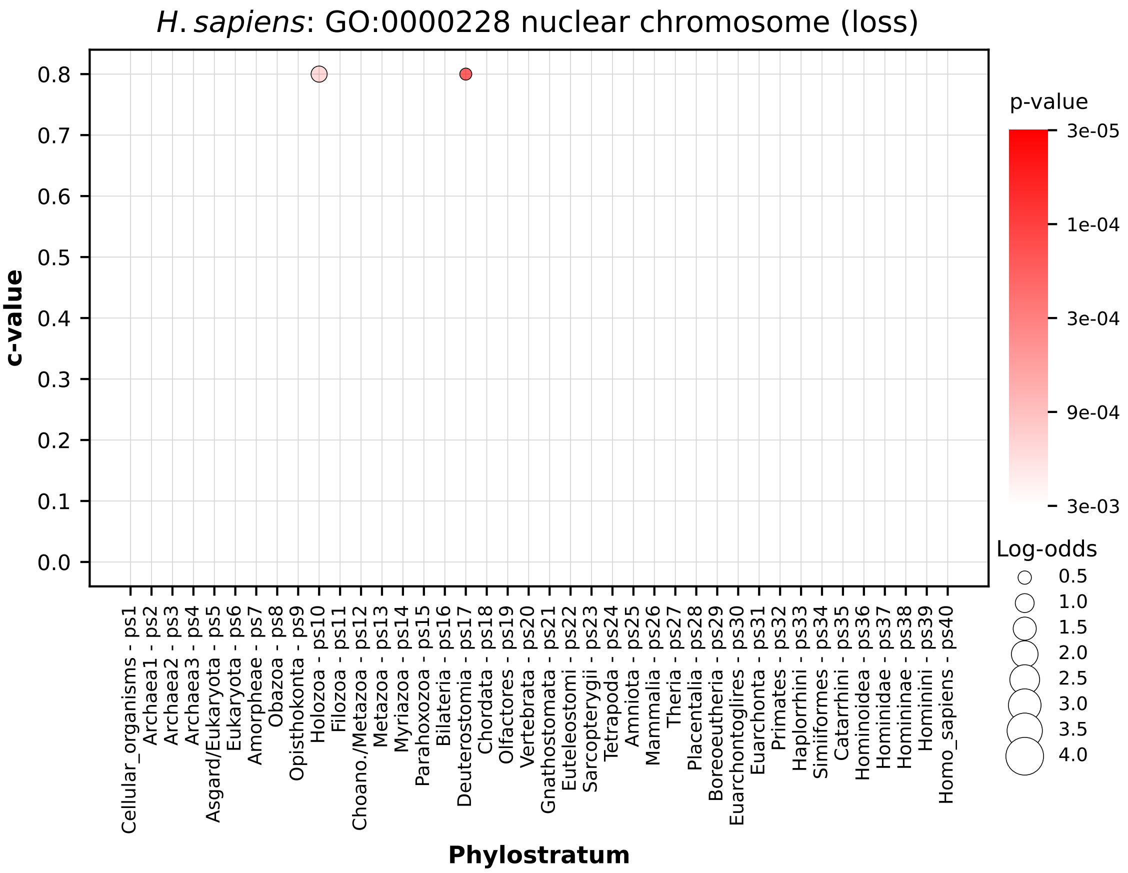

H. sapiens: GO:0000228 nuclear chromosome (loss)
c-value
0.8
0.7
0.6
0.5
0.4
0.3
0.2
0.1
0.0
p-value
3e-05
1e-04
3e-04
9e-04
3e-03
Log-odds
0.5
1.0
1.5
2.0
2.5
3.0
3.5
4.0
Cellular_organisms - ps1
Archaea1 - ps2
Archaea2 - ps3
Archaea3 - ps4
Asgard/Eukaryota - ps5
Eukaryota - ps6
Amorpheae - ps7
Obazoa - ps8
Opisthokonta - ps9
Holozoa - ps10
Filozoa - ps11
Choano./Metazoa - ps12
Metazoa - ps13
Myriazoa - ps14
Parahoxozoa - ps15
Bilateria - ps16
Deuterostomia - ps17
Chordata - ps18
Olfactores - ps19
Vertebrata - ps20
Gnathostomata - ps21
Euteleostomi - ps22
Sarcopterygii - ps23
Tetrapoda - ps24
Amniota - ps25
Mammalia - ps26
Theria - ps27
Placentalia - ps28
Boreoeutheria - ps29
Euarchontoglires - ps30
Euarchonta - ps31
Primates - ps32
Haplorrhini - ps33
Simiiformes - ps34
Catarrhini - ps35
Hominoidea - ps36
Hominidae - ps37
Homininae - ps38
Hominini - ps39
Homo_sapiens - ps40
Phylostratum

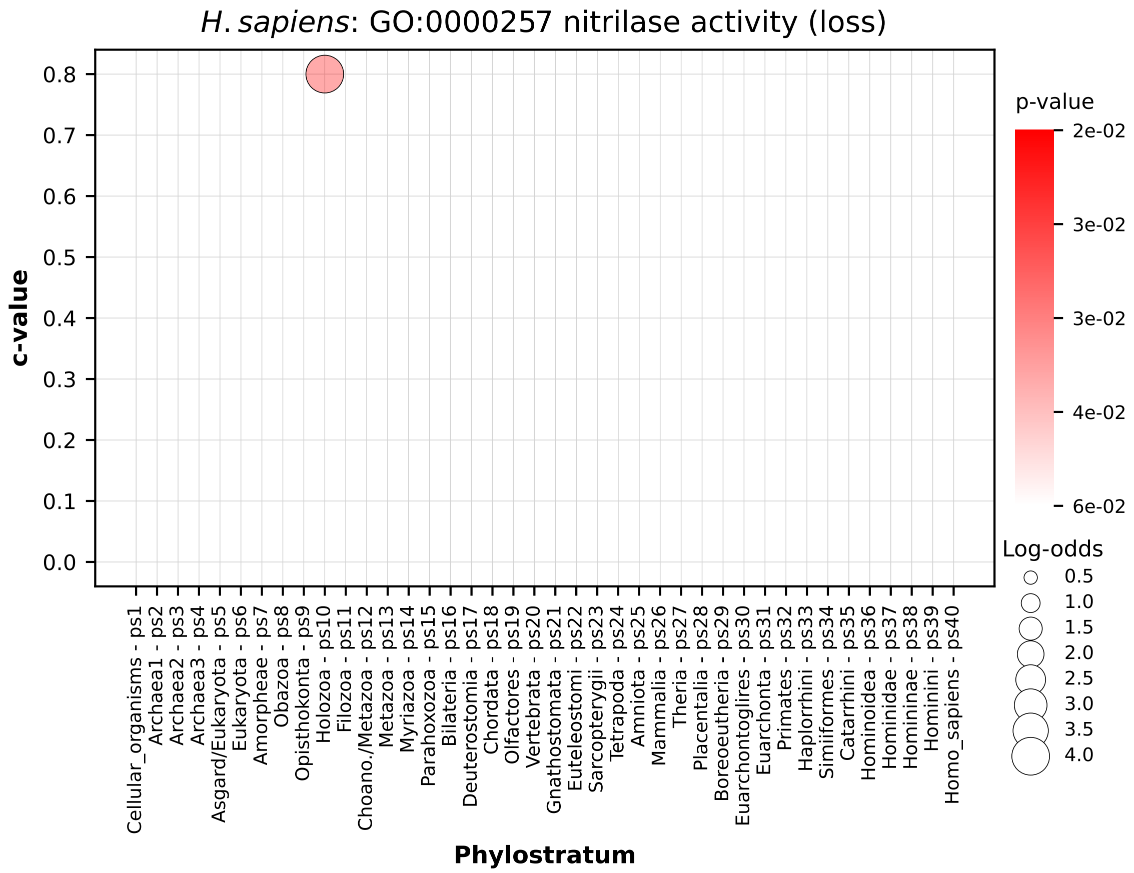
H. sapiens: GO:0000257 nitrilase activity (loss)
c-value
0.8
0.7
0.6
0.5
0.4
0.3
0.2
0.1
0.0
p-value
2e-02
3e-02
3e-02
4e-02
6e-02
Log-odds
0.5
1.0
1.5
2.0
2.5
3.0
3.5
4.0
Cellular_organisms - ps1
Archaea1 - ps2
Archaea2 - ps3
Archaea3 - ps4
Asgard/Eukaryota - ps5
Eukaryota - ps6
Amorpheae - ps7
Obazoa - ps8
Opisthokonta - ps9
Holozoa - ps10
Filozoa - ps11
Choano./Metazoa - ps12
Metazoa - ps13
Myriazoa - ps14
Parahoxozoa - ps15
Bilateria - ps16
Deuterostomia - ps17
Chordata - ps18
Olfactores - ps19
Vertebrata - ps20
Gnathostomata - ps21
Euteleostomi - ps22
Sarcopterygii - ps23
Tetrapoda - ps24
Amniota - ps25
Mammalia - ps26
Theria - ps27
Placentalia - ps28
Boreoeutheria - ps29
Euarchontoglires - ps30
Euarchonta - ps31
Primates - ps32
Haplorrhini - ps33
Simiiformes - ps34
Catarrhini - ps35
Hominoidea - ps36
Hominidae - ps37
Homininae - ps38
Hominini - ps39
Homo_sapiens - ps40
Phylostratum

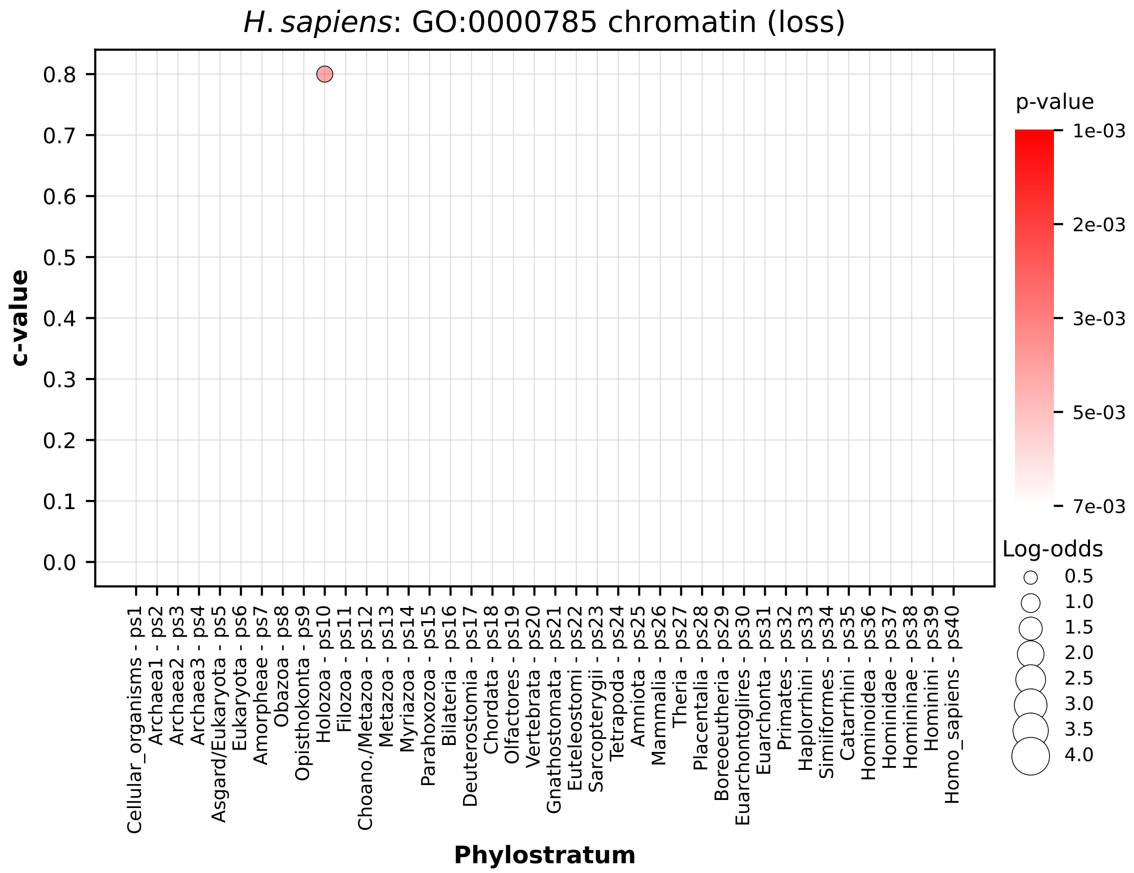

H. sapiens: GO:0000785 chromatin (loss)
c-value
0.8
0.7
0.6
0.5
0.4
0.3
0.2
0.1
0.0
p-value
1e-03
2e-03
3e-03
5e-03
7e-03
Log-odds
0.5
1.0
1.5
2.0
2.5
3.0
3.5
4.0
Cellular_organisms - ps1
Archaea1 - ps2
Archaea2 - ps3
Archaea3 - ps4
Asgard/Eukaryota - ps5
Eukaryota - ps6
Amorpheae - ps7
Obazoa - ps8
Opisthokonta - ps9
Holozoa - ps10
Filozoa - ps11
Choano./Metazoa - ps12
Metazoa - ps13
Myriazoa - ps14
Parahoxozoa - ps15
Bilateria - ps16
Deuterostomia - ps17
Chordata - ps18
Olfactores - ps19
Vertebrata - ps20
Gnathostomata - ps21
Euteleostomi - ps22
Sarcopterygii - ps23
Tetrapoda - ps24
Amniota - ps25
Mammalia - ps26
Theria - ps27
Placentalia - ps28
Boreoeutheria - ps29
Euarchontoglires - ps30
Euarchonta - ps31
Primates - ps32
Haplorrhini - ps33
Simiiformes - ps34
Catarrhini - ps35
Hominoidea - ps36
Hominidae - ps37
Homininae - ps38
Hominini - ps39
Homo_sapiens - ps40
Phylostratum

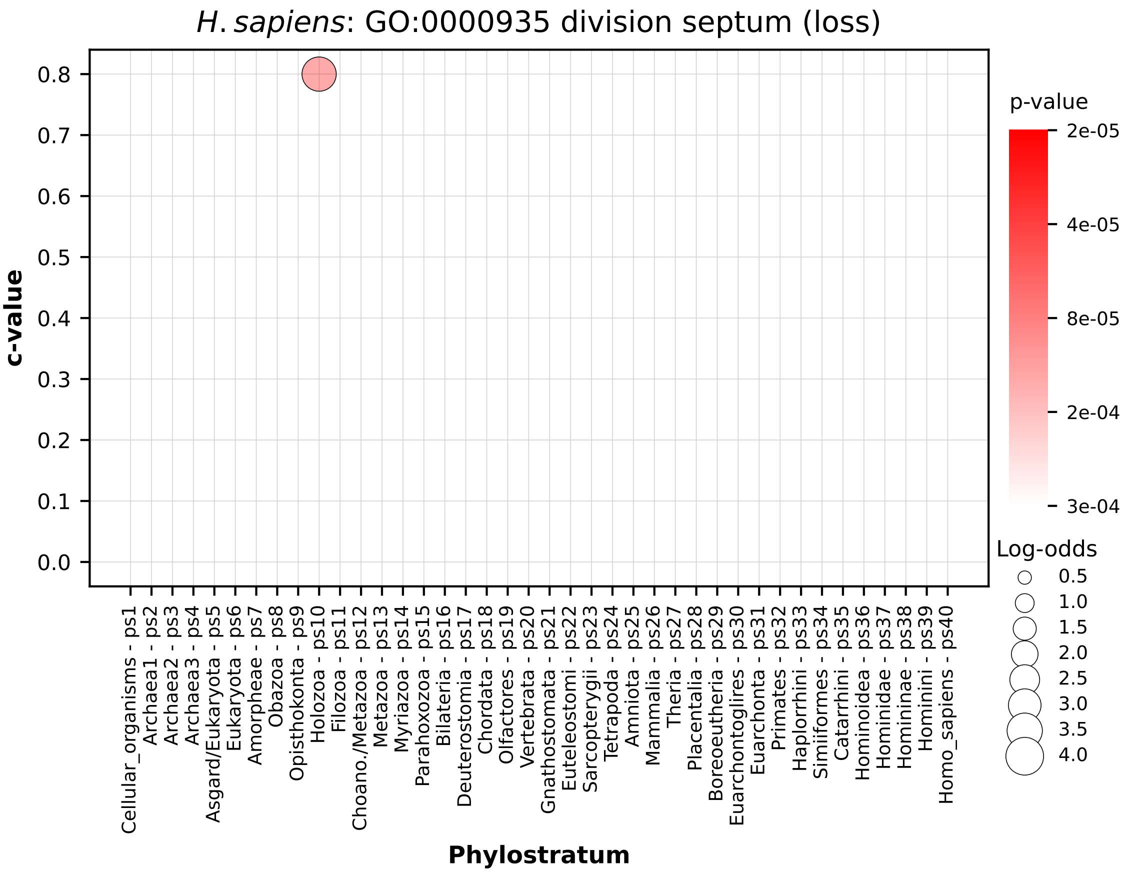

H. sapiens: GO:0000935 division septum (loss)
c-value
0.8
0.7
0.6
0.5
0.4
0.3
0.2
0.1
0.0
Phylostratum
Cellular_organisms - ps1
Archaea1 - ps2
Archaea2 - ps3
Archaea3 - ps4
Asgard/Eukaryota - ps5
Eukaryota - ps6
Amorpheae - ps7
Obazoa - ps8
Opisthokonta - ps9
Holozoa - ps10
Filozoa - ps11
Choano./Metazoa - ps12
Metazoa - ps13
Myriazoa - ps14
Parahoxozoa - ps15
Bilateria - ps16
Deuterostomia - ps17
Chordata - ps18
Olfactores - ps19
Vertebrata - ps20
Gnathostomata - ps21
Euteleostomi - ps22
Sarcopterygii - ps23
Tetrapoda - ps24
Amniota - ps25
Mammalia - ps26
Theria - ps27
Placentalia - ps28
Boreoeutheria - ps29
Euarchontoglires - ps30
Euarchonta - ps31
Primates - ps32
Haplorrhini - ps33
Simiiformes - ps34
Catarrhini - ps35
Hominoidea - ps36
Hominidae - ps37
Homininae - ps38
Hominini - ps39
Homo_sapiens - ps40
p-value
2e-05
4e-05
8e-05
2e-04
3e-04
Log-odds
0.5
1.0
1.5
2.0
2.5
3.0
3.5
4.0

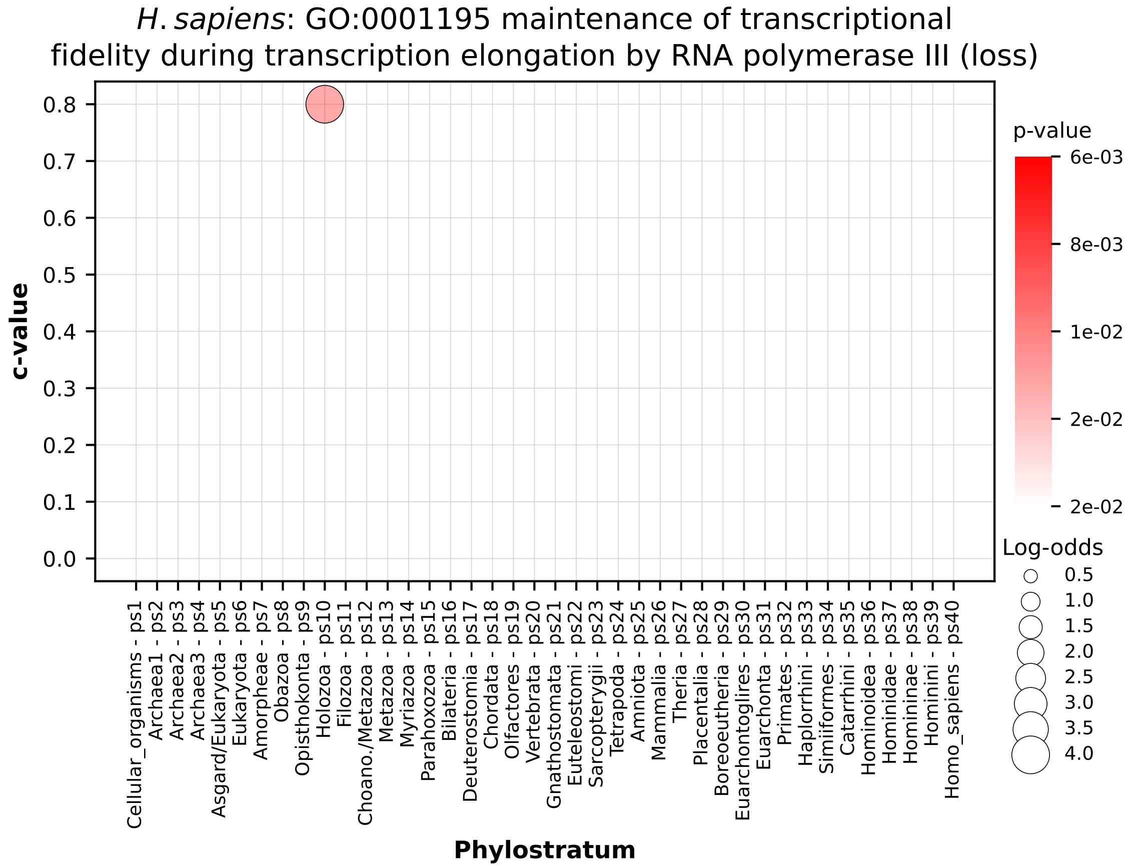

H. sapiens: GO:0001195 maintenance of transcriptional fidelity during transcription elongation by RNA polymerase III (loss)
c-value
0.8
0.7
0.6
0.5
0.4
0.3
0.2
0.1
0.0
p-value
6e-03
8e-03
1e-02
2e-02
2e-02
Log-odds
0.5
1.0
1.5
2.0
2.5
3.0
3.5
4.0
Cellular_organisms - ps1
Archaea1 - ps2
Archaea2 - ps3
Archaea3 - ps4
Asgard/Eukaryota - ps5
Eukaryota - ps6
Amorpheae - ps7
Obazoa - ps8
Opisthokonta - ps9
Holozoa - ps10
Filozoa - ps11
Choano./Metazoa - ps12
Metazoa - ps13
Myriazoa - ps14
Parahoxozoa - ps15
Bilateria - ps16
Deuterostomia - ps17
Chordata - ps18
Olfactores - ps19
Vertebrata - ps20
Gnathostomata - ps21
Euteleostomi - ps22
Sarcopterygii - ps23
Tetrapoda - ps24
Amniota - ps25
Mammalia - ps26
Theria - ps27
Placentalia - ps28
Boreoeutheria - ps29
Euarchontoglires - ps30
Euarchonta - ps31
Primates - ps32
Haplorrhini - ps33
Simiiformes - ps34
Catarrhini - ps35
Hominoidea - ps36
Hominidae - ps37
Homininae - ps38
Hominini - ps39
Homo_sapiens - ps40
Phylostratum

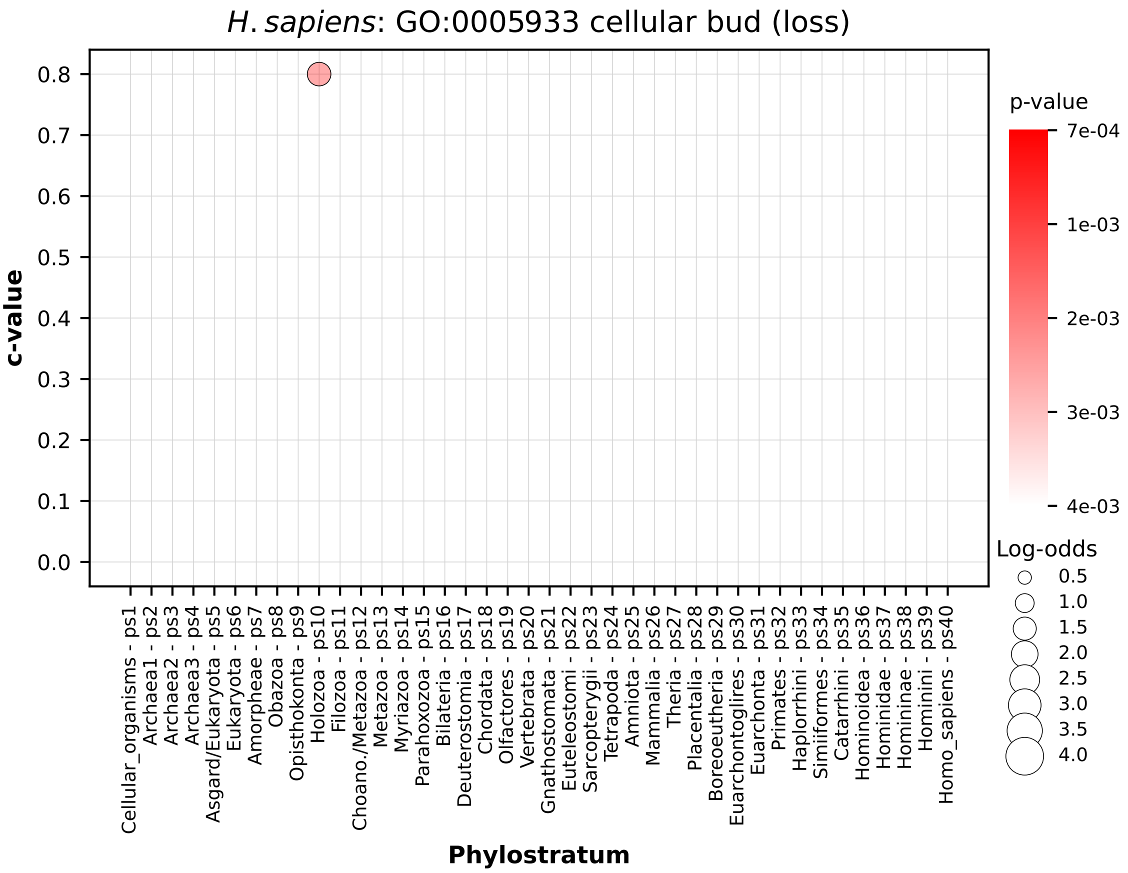

H. sapiens: GO:0005933 cellular bud (loss)
c-value
0.0
0.1
0.2
0.3
0.4
0.5
0.6
0.7
0.8
Phylostratum
Cellular_organisms - ps1
Archaea1 - ps2
Archaea2 - ps3
Archaea3 - ps4
Asgard/Eukaryota - ps5
Eukaryota - ps6
Amorpheae - ps7
Obazoa - ps8
Opisthokonta - ps9
Holozoa - ps10
Filozoa - ps11
Choano./Metazoa - ps12
Metazoa - ps13
Myriazoa - ps14
Parahoxozoa - ps15
Bilateria - ps16
Deuterostomia - ps17
Chordata - ps18
Olfactores - ps19
Vertebrata - ps20
Gnathostomata - ps21
Euteleostomi - ps22
Sarcopterygii - ps23
Tetrapoda - ps24
Amniota - ps25
Mammalia - ps26
Theria - ps27
Placentalia - ps28
Boreoeutheria - ps29
Euarchontoglires - ps30
Euarchonta - ps31
Primates - ps32
Haplorrhini - ps33
Simiiformes - ps34
Catarrhini - ps35
Hominoidea - ps36
Hominidae - ps37
Homininae - ps38
Hominini - ps39
Homo_sapiens - ps40
p-value
7e-04
1e-03
2e-03
3e-03
4e-03
Log-odds
0.5
1.0
1.5
2.0
2.5
3.0
3.5
4.0

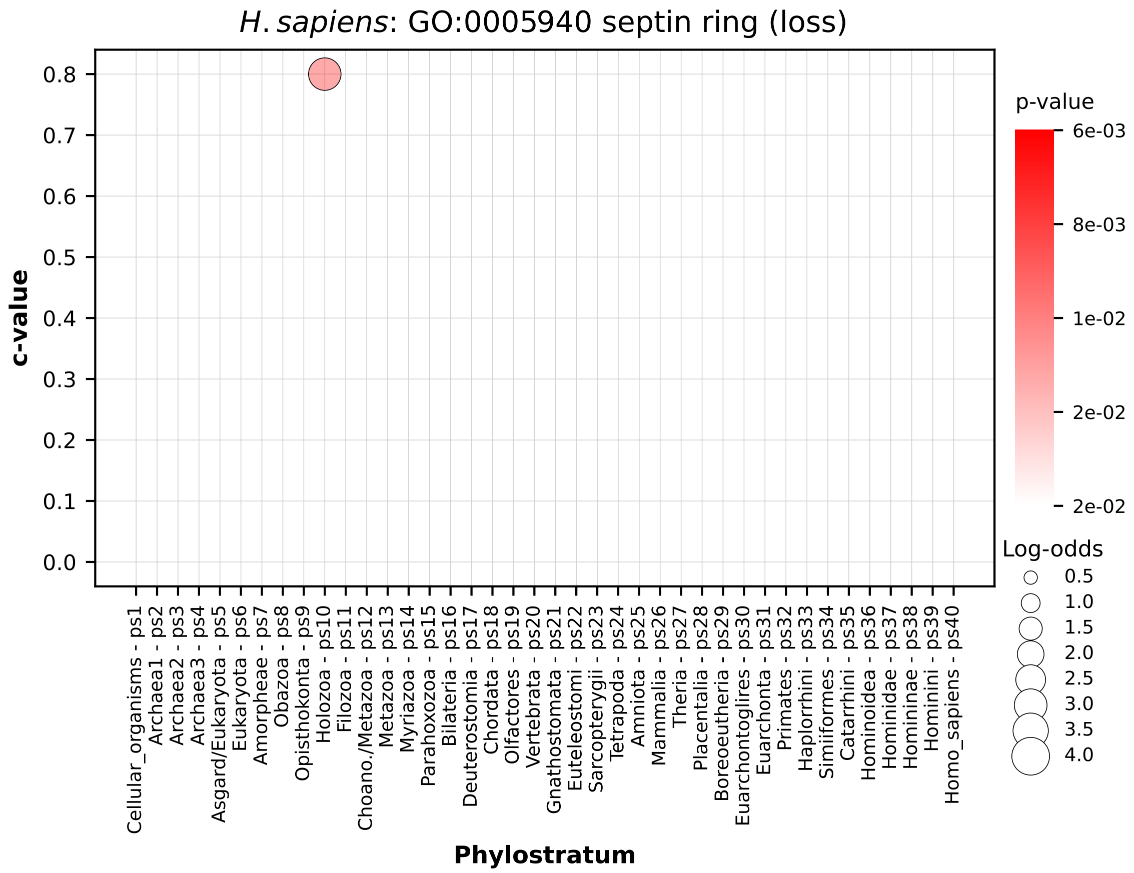

H. sapiens: GO:0005940 septin ring (loss)
c-value
0.0
0.1
0.2
0.3
0.4
0.5
0.6
0.7
0.8
p-value
6e-03
8e-03
1e-02
2e-02
2e-02
Log-odds
0.5
1.0
1.5
2.0
2.5
3.0
3.5
4.0
Cellular_organisms - ps1
Archaea1 - ps2
Archaea2 - ps3
Archaea3 - ps4
Asgard/Eukaryota - ps5
Eukaryota - ps6
Amorpheae - ps7
Obazoa - ps8
Opisthokonta - ps9
Holozoa - ps10
Filozoa - ps11
Choano./Metazoa - ps12
Metazoa - ps13
Myriazoa - ps14
Parahoxozoa - ps15
Bilateria - ps16
Deuterostomia - ps17
Chordata - ps18
Olfactores - ps19
Vertebrata - ps20
Gnathostomata - ps21
Euteleostomi - ps22
Sarcopterygii - ps23
Tetrapoda - ps24
Amniota - ps25
Mammalia - ps26
Theria - ps27
Placentalia - ps28
Boreoeutheria - ps29
Euarchontoglires - ps30
Euarchonta - ps31
Primates - ps32
Haplorrhini - ps33
Simiiformes - ps34
Catarrhini - ps35
Hominoidea - ps36
Hominidae - ps37
Homininae - ps38
Hominini - ps39
Homo_sapiens - ps40
Phylostratum

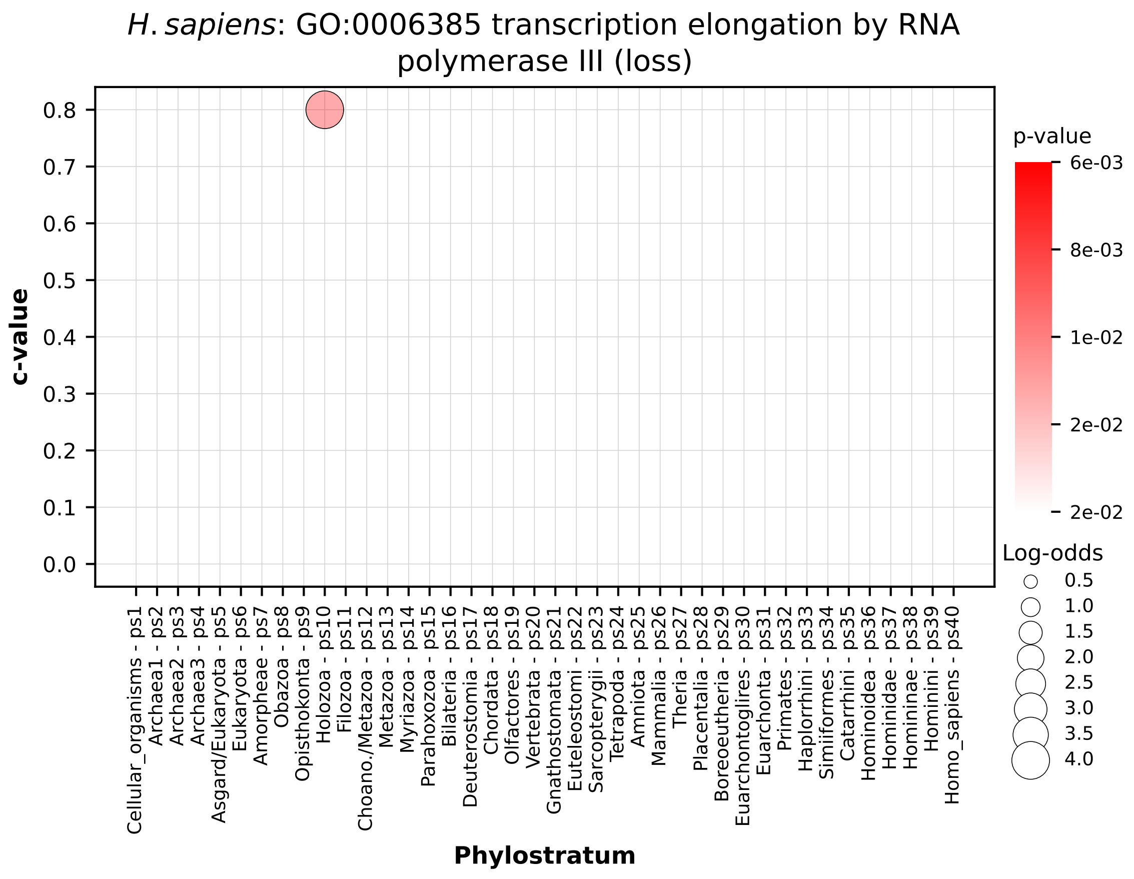

H. sapiens: GO:0006385 transcription elongation by RNA polymerase III (loss)
c-value
0.8
0.7
0.6
0.5
0.4
0.3
0.2
0.1
0.0
Phylostratum
Cellular_organisms - ps1
Archaea1 - ps2
Archaea2 - ps3
Archaea3 - ps4
Asgard/Eukaryota - ps5
Eukaryota - ps6
Amorpheae - ps7
Obazoa - ps8
Opisthokonta - ps9
Holozoa - ps10
Filozoa - ps11
Choano./Metazoa - ps12
Metazoa - ps13
Myriazoa - ps14
Parahoxozoa - ps15
Bilateria - ps16
Deuterostomia - ps17
Chordata - ps18
Olfactores - ps19
Vertebrata - ps20
Gnathostomata - ps21
Euteleostomi - ps22
Sarcopterygii - ps23
Tetrapoda - ps24
Amniota - ps25
Mammalia - ps26
Theria - ps27
Placentalia - ps28
Boreoeutheria - ps29
Euarchontoglires - ps30
Euarchonta - ps31
Primates - ps32
Haplorrhini - ps33
Simiiformes - ps34
Catarrhini - ps35
Hominoidea - ps36
Hominidae - ps37
Homininae - ps38
Hominini - ps39
Homo_sapiens - ps40
p-value
6e-03
8e-03
1e-02
2e-02
2e-02
Log-odds
0.5
1.0
1.5
2.0
2.5
3.0
3.5
4.0

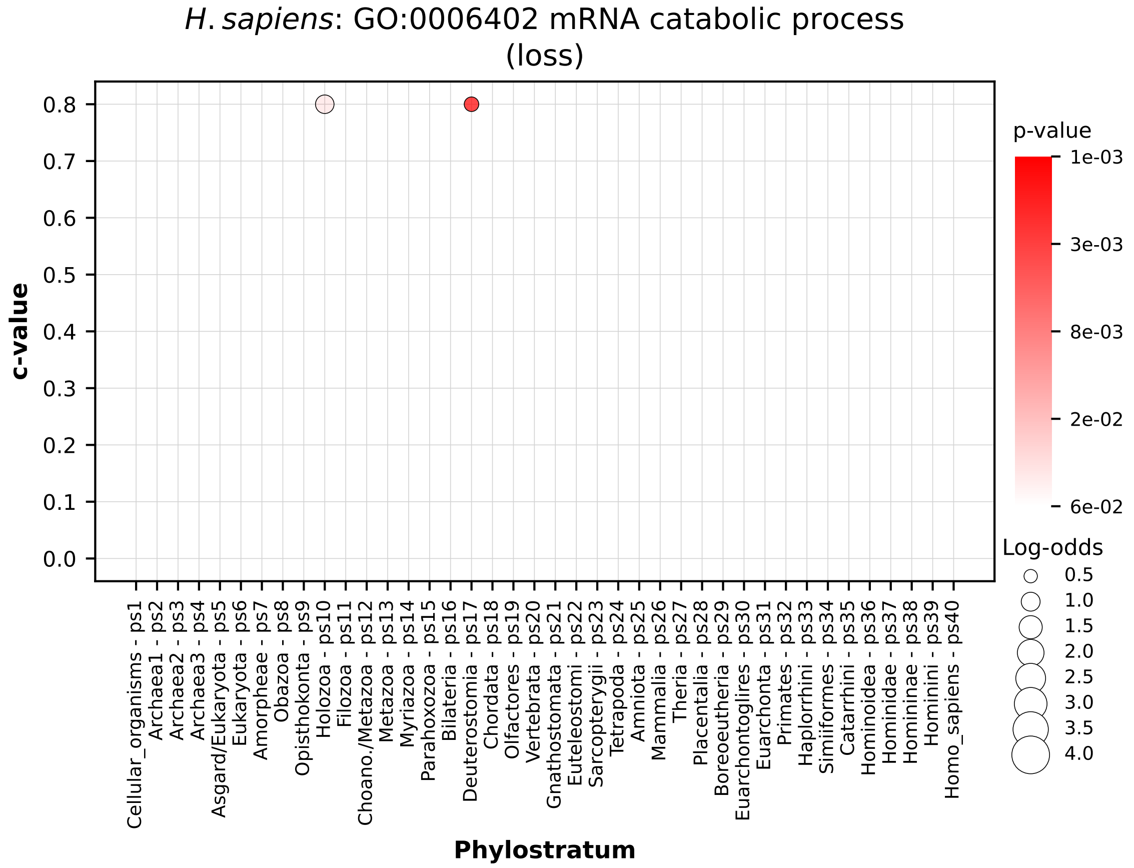

H. sapiens: GO:0006402 mRNA catabolic process
(loss)
c-value
0.0
0.1
0.2
0.3
0.4
0.5
0.6
0.7
0.8
Phylostratum
Cellular_organisms - ps1
Archaea1 - ps2
Archaea2 - ps3
Archaea3 - ps4
Asgard/Eukaryota - ps5
Eukaryota - ps6
Amorpheae - ps7
Obazoa - ps8
Opisthokonta - ps9
Holozoa - ps10
Filozoa - ps11
Choano./Metazoa - ps12
Metazoa - ps13
Myriazoa - ps14
Parahoxozoa - ps15
Bilateria - ps16
Deuterostomia - ps17
Chordata - ps18
Olfactores - ps19
Vertebrata - ps20
Gnathostomata - ps21
Euteleostomi - ps22
Sarcopterygii - ps23
Tetrapoda - ps24
Amniota - ps25
Mammalia - ps26
Theria - ps27
Placentalia - ps28
Boreoeutheria - ps29
Euarchontoglires - ps30
Euarchonta - ps31
Primates - ps32
Haplorrhini - ps33
Simiiformes - ps34
Catarrhini - ps35
Hominoidea - ps36
Hominidae - ps37
Homininae - ps38
Hominini - ps39
Homo_sapiens - ps40
p-value
1e-03
3e-03
8e-03
2e-02
6e-02
Log-odds
0.5
1.0
1.5
2.0
2.5
3.0
3.5
4.0

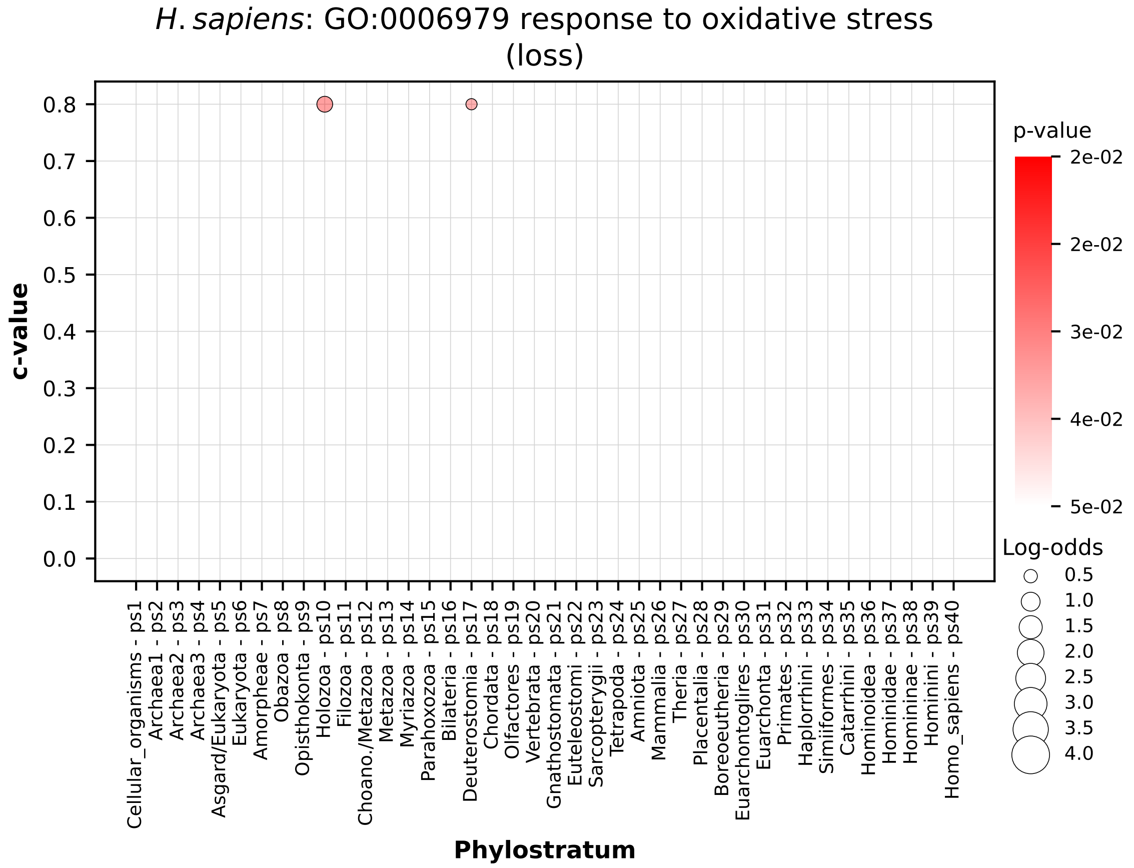

H. sapiens: GO:0006979 response to oxidative stress
(loss)
c-value
0.0
0.1
0.2
0.3
0.4
0.5
0.6
0.7
0.8
Phylostratum
Cellular_organisms - ps1
Archaea1 - ps2
Archaea2 - ps3
Archaea3 - ps4
Asgard/Eukaryota - ps5
Eukaryota - ps6
Amorpheae - ps7
Obazoa - ps8
Opisthokonta - ps9
Holozoa - ps10
Filozoa - ps11
Choano./Metazoa - ps12
Metazoa - ps13
Myriazoa - ps14
Parahoxozoa - ps15
Bilateria - ps16
Deuterostomia - ps17
Chordata - ps18
Olfactores - ps19
Vertebrata - ps20
Gnathostomata - ps21
Euteleostomi - ps22
Sarcopterygii - ps23
Tetrapoda - ps24
Amniota - ps25
Mammalia - ps26
Theria - ps27
Placentalia - ps28
Boreoeutheria - ps29
Euarchontoglires - ps30
Euarchonta - ps31
Primates - ps32
Haplorrhini - ps33
Simiiformes - ps34
Catarrhini - ps35
Hominoidea - ps36
Hominidae - ps37
Homininae - ps38
Hominini - ps39
Homo_sapiens - ps40
p-value
2e-02
2e-02
3e-02
4e-02
5e-02
Log-odds
0.5
1.0
1.5
2.0
2.5
3.0
3.5
4.0

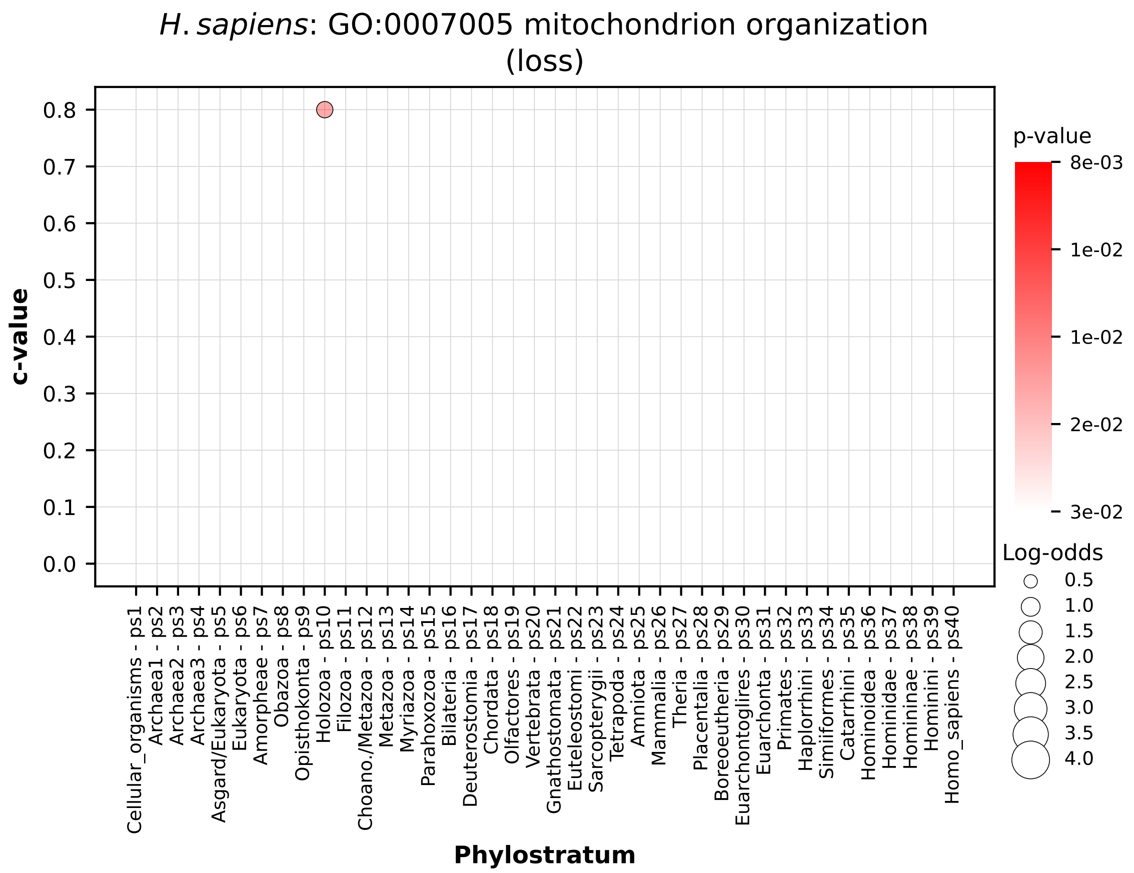

H. sapiens: GO:0007005 mitochondrion organization
(loss)
c-value
0.8
0.7
0.6
0.5
0.4
0.3
0.2
0.1
0.0
p-value
8e-03
1e-02
1e-02
2e-02
3e-02
Log-odds
0.5
1.0
1.5
2.0
2.5
3.0
3.5
4.0
Cellular_organisms - ps1
Archaea1 - ps2
Archaea2 - ps3
Archaea3 - ps4
Asgard/Eukaryota - ps5
Eukaryota - ps6
Amorpheae - ps7
Obazoa - ps8
Opisthokonta - ps9
Holozoa - ps10
Filozoa - ps11
Choano./Metazoa - ps12
Metazoa - ps13
Myriazoa - ps14
Parahoxozoa - ps15
Bilateria - ps16
Deuterostomia - ps17
Chordata - ps18
Olfactores - ps19
Vertebrata - ps20
Gnathostomata - ps21
Euteleostomi - ps22
Sarcopterygii - ps23
Tetrapoda - ps24
Amniota - ps25
Mammalia - ps26
Theria - ps27
Placentalia - ps28
Boreoeutheria - ps29
Euarchontoglires - ps30
Euarchonta - ps31
Primates - ps32
Haplorrhini - ps33
Simiiformes - ps34
Catarrhini - ps35
Hominoidea - ps36
Hominidae - ps37
Homininae - ps38
Hominini - ps39
Homo_sapiens - ps40
Phylostratum

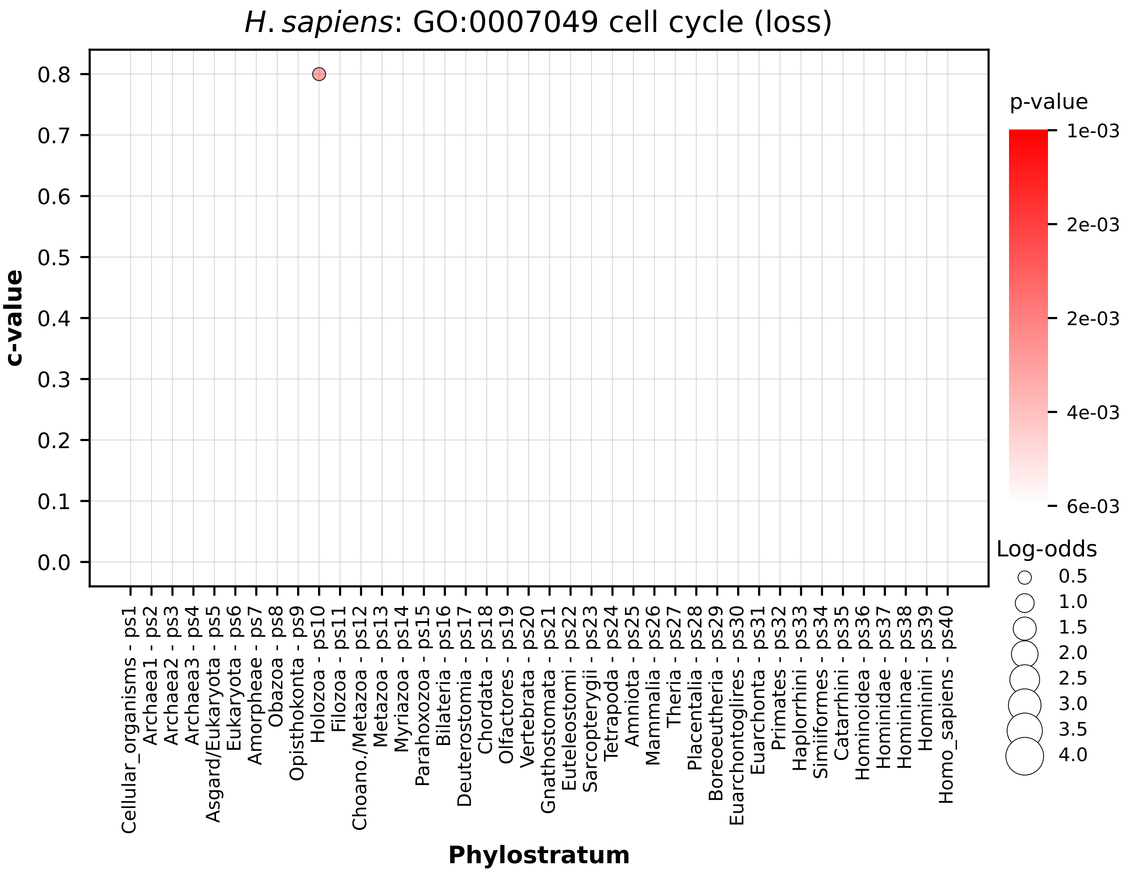

H. sapiens: GO:0007049 cell cycle (loss)
c-value
0.0
0.1
0.2
0.3
0.4
0.5
0.6
0.7
0.8
Phylostratum
Cellular_organisms - ps1
Archaea1 - ps2
Archaea2 - ps3
Archaea3 - ps4
Asgard/Eukaryota - ps5
Eukaryota - ps6
Amorpheae - ps7
Obazoa - ps8
Opisthokonta - ps9
Holozoa - ps10
Filozoa - ps11
Choano./Metazoa - ps12
Metazoa - ps13
Myriazoa - ps14
Parahoxozoa - ps15
Bilateria - ps16
Deuterostomia - ps17
Chordata - ps18
Olfactores - ps19
Vertebrata - ps20
Gnathostomata - ps21
Euteleostomi - ps22
Sarcopterygii - ps23
Tetrapoda - ps24
Amniota - ps25
Mammalia - ps26
Theria - ps27
Placentalia - ps28
Boreoeutheria - ps29
Euarchontoglires - ps30
Euarchonta - ps31
Primates - ps32
Haplorrhini - ps33
Simiiformes - ps34
Catarrhini - ps35
Hominoidea - ps36
Hominidae - ps37
Homininae - ps38
Hominini - ps39
Homo_sapiens - ps40
p-value
1e-03
2e-03
2e-03
4e-03
6e-03
Log-odds
0.5
1.0
1.5
2.0
2.5
3.0
3.5
4.0

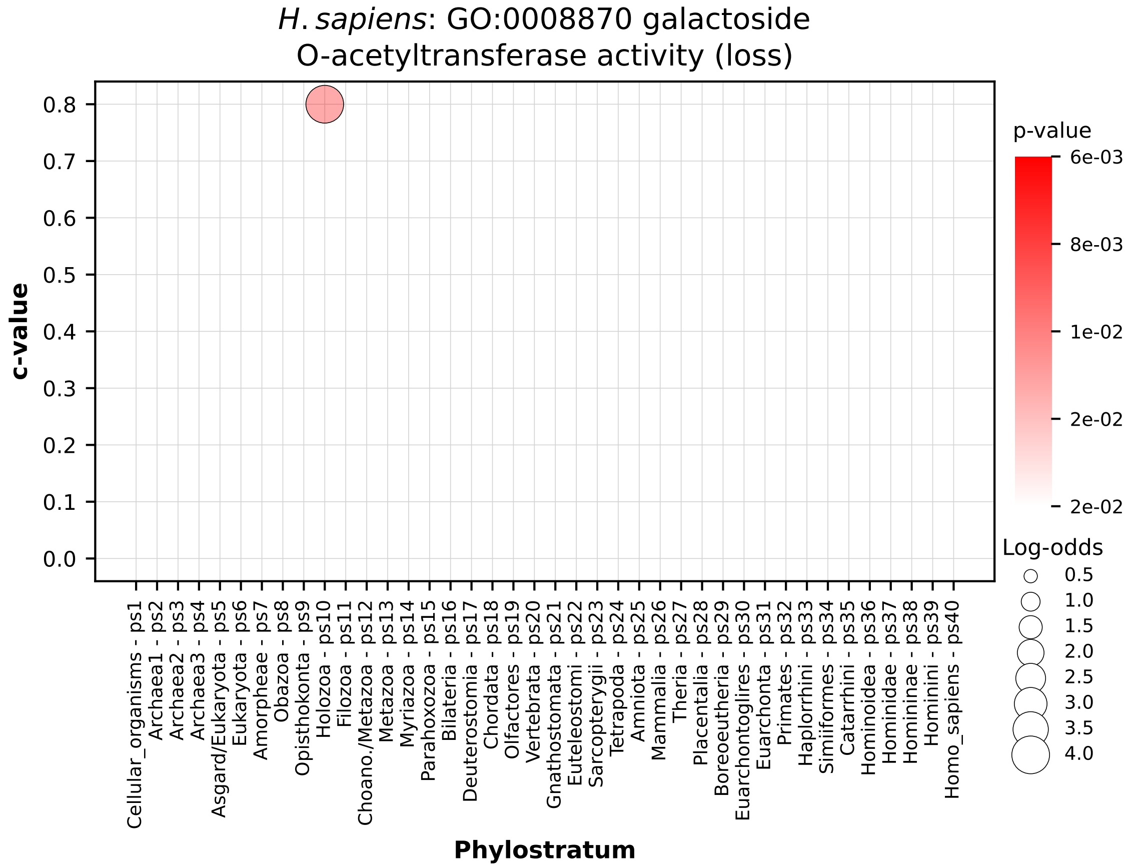
H. sapiens: GO:0008870 galactoside O-acetyltransferase activity (loss)
c-value
0.0
0.1
0.2
0.3
0.4
0.5
0.6
0.7
0.8
Phylostratum
Cellular_organisms - ps1
Archaea1 - ps2
Archaea2 - ps3
Archaea3 - ps4
Asgard/Eukaryota - ps5
Eukaryota - ps6
Amorpheae - ps7
Obazoa - ps8
Opisthokonta - ps9
Holozoa - ps10
Filozoa - ps11
Choano./Metazoa - ps12
Metazoa - ps13
Myriazoa - ps14
Parahoxozoa - ps15
Bilateria - ps16
Deuterostomia - ps17
Chordata - ps18
Olfactores - ps19
Vertebrata - ps20
Gnathostomata - ps21
Euteleostomi - ps22
Sarcopterygii - ps23
Tetrapoda - ps24
Amniota - ps25
Mammalia - ps26
Theria - ps27
Placentalia - ps28
Boreoeutheria - ps29
Euarchontoglires - ps30
Euarchonta - ps31
Primates - ps32
Haplorrhini - ps33
Simiiformes - ps34
Catarrhini - ps35
Hominoidea - ps36
Hominidae - ps37
Homininae - ps38
Hominini - ps39
Homo_sapiens - ps40
p-value
6e-03
8e-03
1e-02
2e-02
2e-02
Log-odds
0.5
1.0
1.5
2.0
2.5
3.0
3.5
4.0

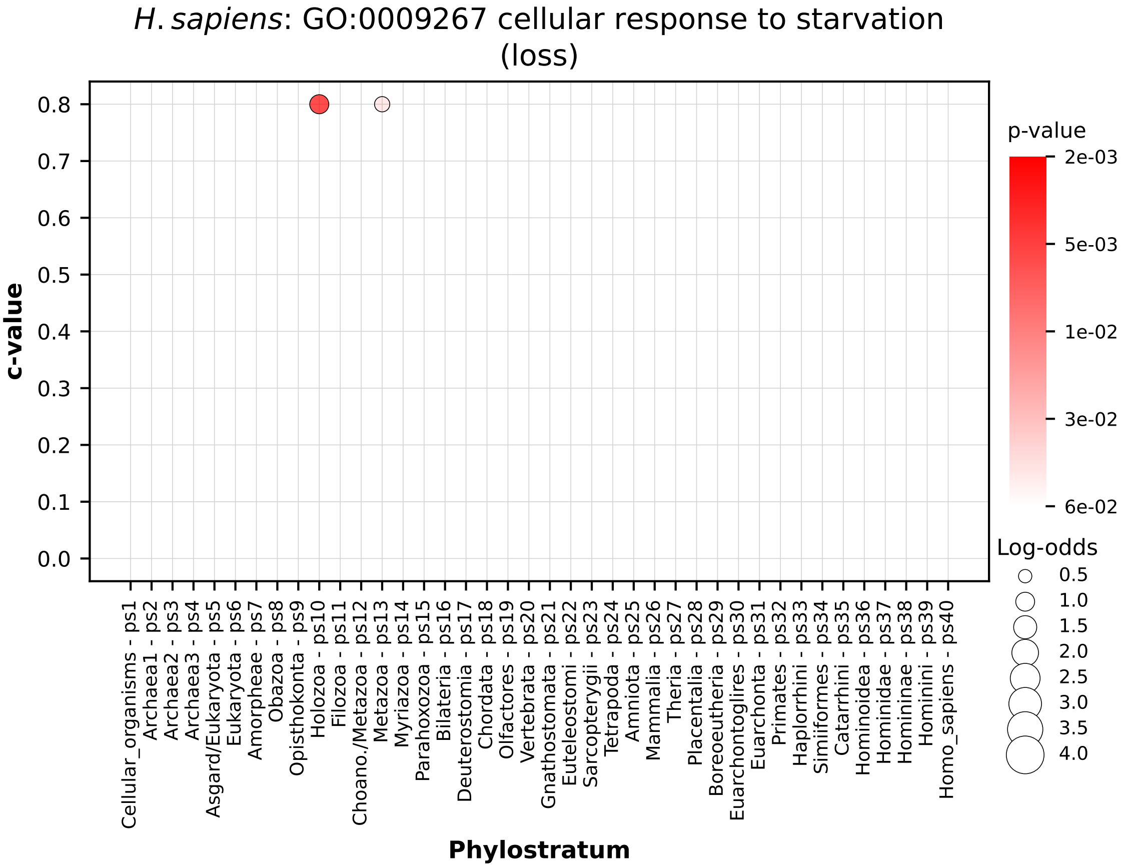

H. sapiens: GO:0009267 cellular response to starvation (loss)
c-value
0.8
0.7
0.6
0.5
0.4
0.3
0.2
0.1
0.0
Phylostratum
Cellular_organisms - ps1
Archaea1 - ps2
Archaea2 - ps3
Archaea3 - ps4
Asgard/Eukaryota - ps5
Eukaryota - ps6
Amorpheae - ps7
Obazoa - ps8
Opisthokonta - ps9
Holozoa - ps10
Filozoa - ps11
Choano./Metazoa - ps12
Metazoa - ps13
Myriazoa - ps14
Parahoxozoa - ps15
Bilateria - ps16
Deuterostomia - ps17
Chordata - ps18
Olfactores - ps19
Vertebrata - ps20
Gnathostomata - ps21
Euteleostomi - ps22
Sarcopterygii - ps23
Tetrapoda - ps24
Amniota - ps25
Mammalia - ps26
Theria - ps27
Placentalia - ps28
Boreoeutheria - ps29
Euarchontoglires - ps30
Euarchonta - ps31
Primates - ps32
Haplorrhini - ps33
Simiiformes - ps34
Catarrhini - ps35
Hominoidea - ps36
Hominidae - ps37
Homininae - ps38
Hominini - ps39
Homo_sapiens - ps40
p-value
2e-03
5e-03
1e-02
3e-02
6e-02
Log-odds
0.5
1.0
1.5
2.0
2.5
3.0
3.5
4.0

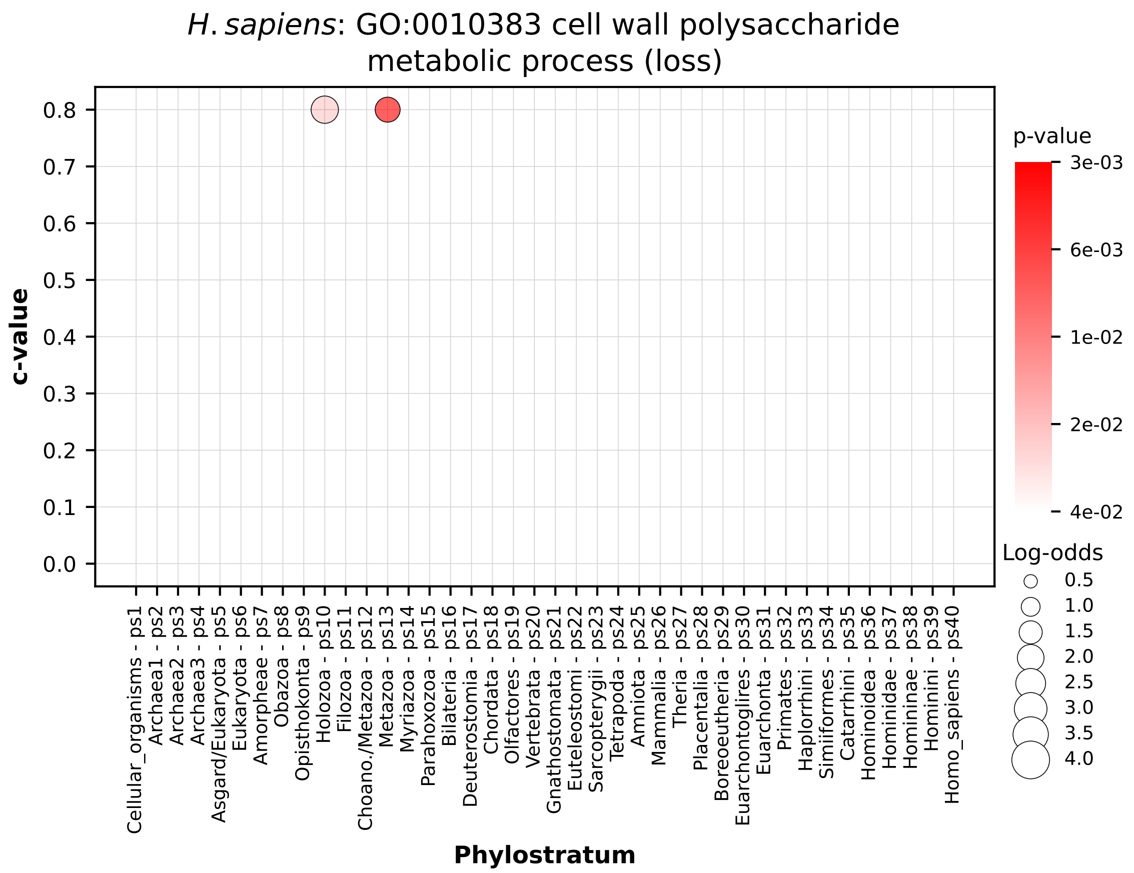
H. sapiens: GO:0010383 cell wall polysaccharide metabolic process (loss)
c-value
0.8
0.7
0.6
0.5
0.4
0.3
0.2
0.1
0.0
Phylostratum
Cellular_organisms - ps1
Archaea1 - ps2
Archaea2 - ps3
Archaea3 - ps4
Asgard/Eukaryota - ps5
Eukaryota - ps6
Amorpheae - ps7
Obazoa - ps8
Opisthokonta - ps9
Holozoa - ps10
Filozoa - ps11
Choano./Metazoa - ps12
Metazoa - ps13
Myriazoa - ps14
Parahoxozoa - ps15
Bilateria - ps16
Deuterostomia - ps17
Chordata - ps18
Olfactores - ps19
Vertebrata - ps20
Gnathostomata - ps21
Euteleostomi - ps22
Sarcopterygii - ps23
Tetrapoda - ps24
Amniota - ps25
Mammalia - ps26
Theria - ps27
Placentalia - ps28
Boreoeutheria - ps29
Euarchontoglires - ps30
Euarchonta - ps31
Primates - ps32
Haplorrhini - ps33
Simiiformes - ps34
Catarrhini - ps35
Hominoidea - ps36
Hominidae - ps37
Homininae - ps38
Hominini - ps39
Homo_sapiens - ps40
p-value
3e-03
6e-03
1e-02
2e-02
4e-02
Log-odds
0.5
1.0
1.5
2.0
2.5
3.0
3.5
4.0

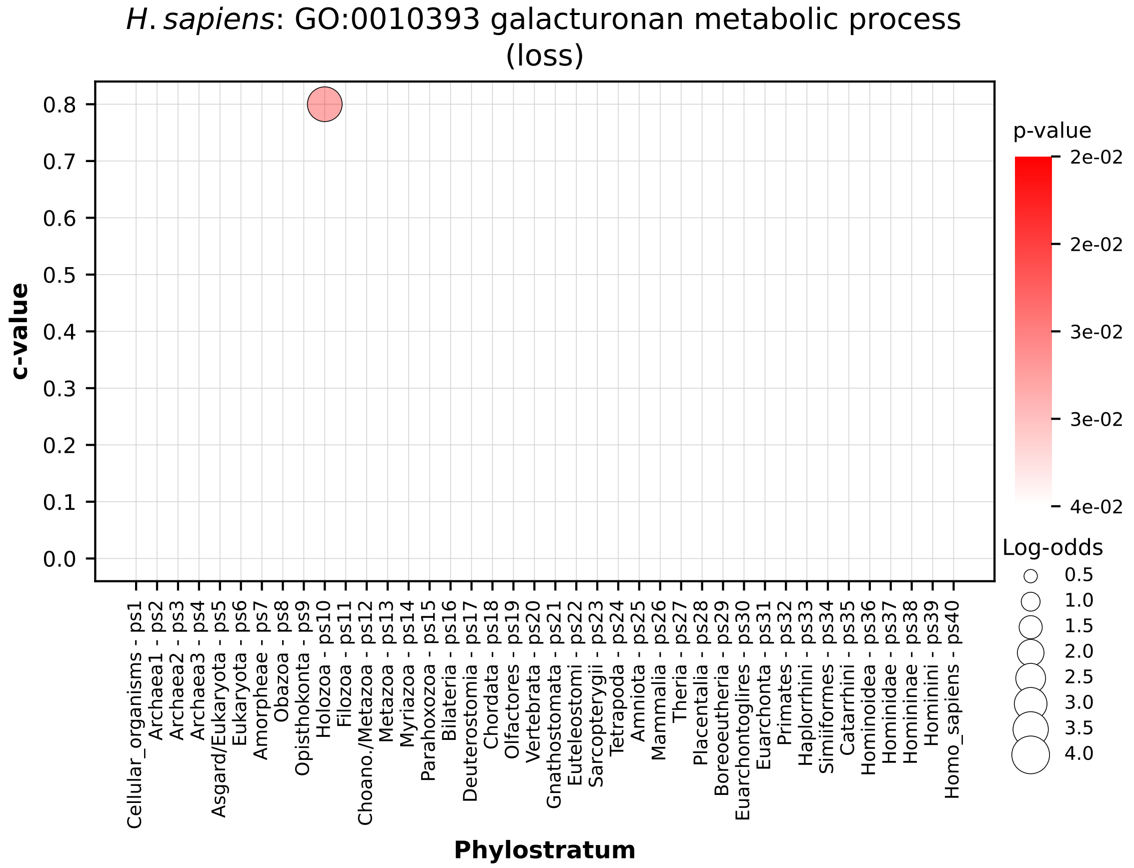

H. sapiens: GO:0010393 galacturonan metabolic process (loss)
c-value
0.0
0.1
0.2
0.3
0.4
0.5
0.6
0.7
0.8
p-value
2e-02
2e-02
3e-02
3e-02
4e-02
Log-odds
0.5
1.0
1.5
2.0
2.5
3.0
3.5
4.0
Cellular_organisms - ps1
Archaea1 - ps2
Archaea2 - ps3
Archaea3 - ps4
Asgard/Eukaryota - ps5
Eukaryota - ps6
Amorpheae - ps7
Obazoa - ps8
Opisthokonta - ps9
Holozoa - ps10
Filozoa - ps11
Choano./Metazoa - ps12
Metazoa - ps13
Myriazoa - ps14
Parahoxozoa - ps15
Bilateria - ps16
Deuterostomia - ps17
Chordata - ps18
Olfactores - ps19
Vertebrata - ps20
Gnathostomata - ps21
Euteleostomi - ps22
Sarcopterygii - ps23
Tetrapoda - ps24
Amniota - ps25
Mammalia - ps26
Theria - ps27
Placentalia - ps28
Boreoeutheria - ps29
Euarchontoglires - ps30
Euarchonta - ps31
Primates - ps32
Haplorrhini - ps33
Simiiformes - ps34
Catarrhini - ps35
Hominoidea - ps36
Hominidae - ps37
Homininae - ps38
Hominini - ps39
Homo_sapiens - ps40
Phylostratum

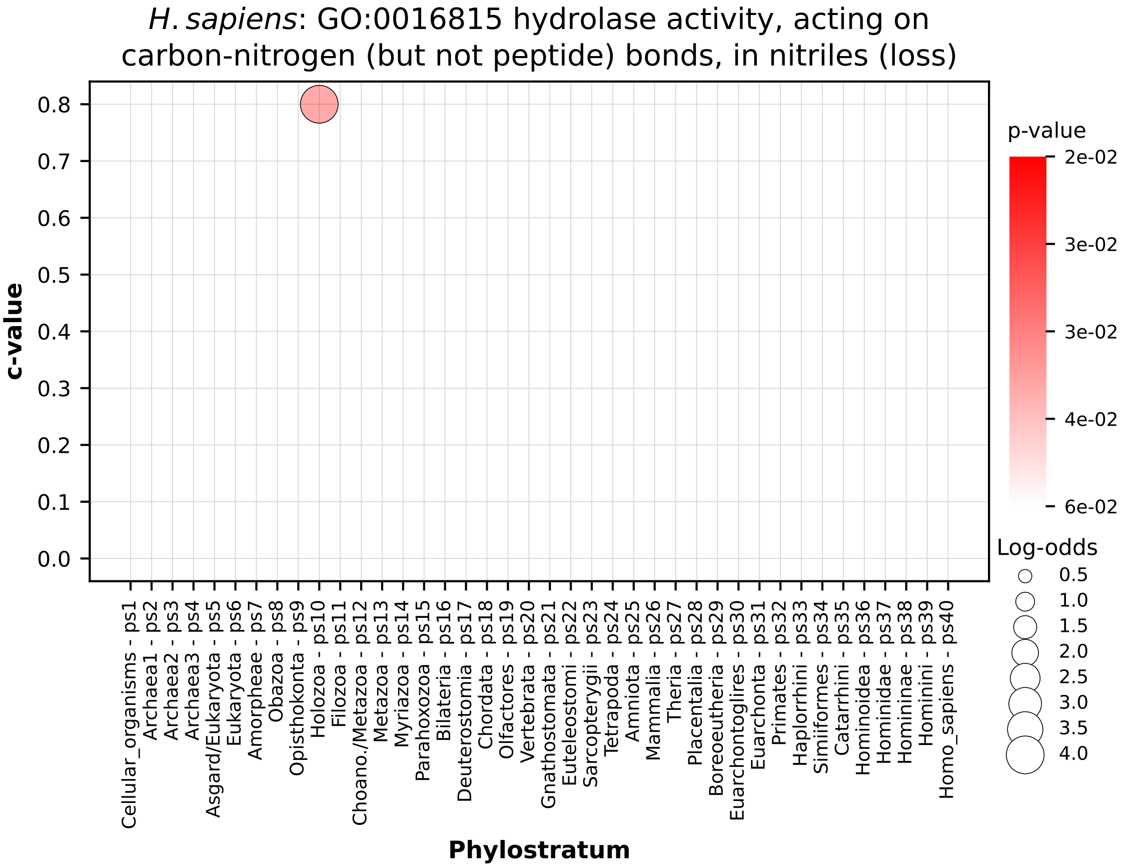
H. sapiens: GO:0016815 hydrolase activity, acting on carbon-nitrogen (but not peptide) bonds, in nitriles (loss)
c-value
0.8
0.7
0.6
0.5
0.4
0.3
0.2
0.1
0.0
p-value
2e-02
3e-02
3e-02
4e-02
6e-02
Log-odds
0.5
1.0
1.5
2.0
2.5
3.0
3.5
4.0
Cellular_organisms - ps1
Archaea1 - ps2
Archaea2 - ps3
Archaea3 - ps4
Asgard/Eukaryota - ps5
Eukaryota - ps6
Amorpheae - ps7
Obazoa - ps8
Opisthokonta - ps9
Holozoa - ps10
Filozoa - ps11
Choano./Metazoa - ps12
Metazoa - ps13
Myriazoa - ps14
Parahoxozoa - ps15
Bilateria - ps16
Deuterostomia - ps17
Chordata - ps18
Olfactores - ps19
Vertebrata - ps20
Gnathostomata - ps21
Euteleostomi - ps22
Sarcopterygii - ps23
Tetrapoda - ps24
Amniota - ps25
Mammalia - ps26
Theria - ps27
Placentalia - ps28
Boreoeutheria - ps29
Euarchontoglires - ps30
Euarchonta - ps31
Primates - ps32
Haplorrhini - ps33
Simiiformes - ps34
Catarrhini - ps35
Hominoidea - ps36
Hominidae - ps37
Homininae - ps38
Hominini - ps39
Homo_sapiens - ps40
Phylostratum

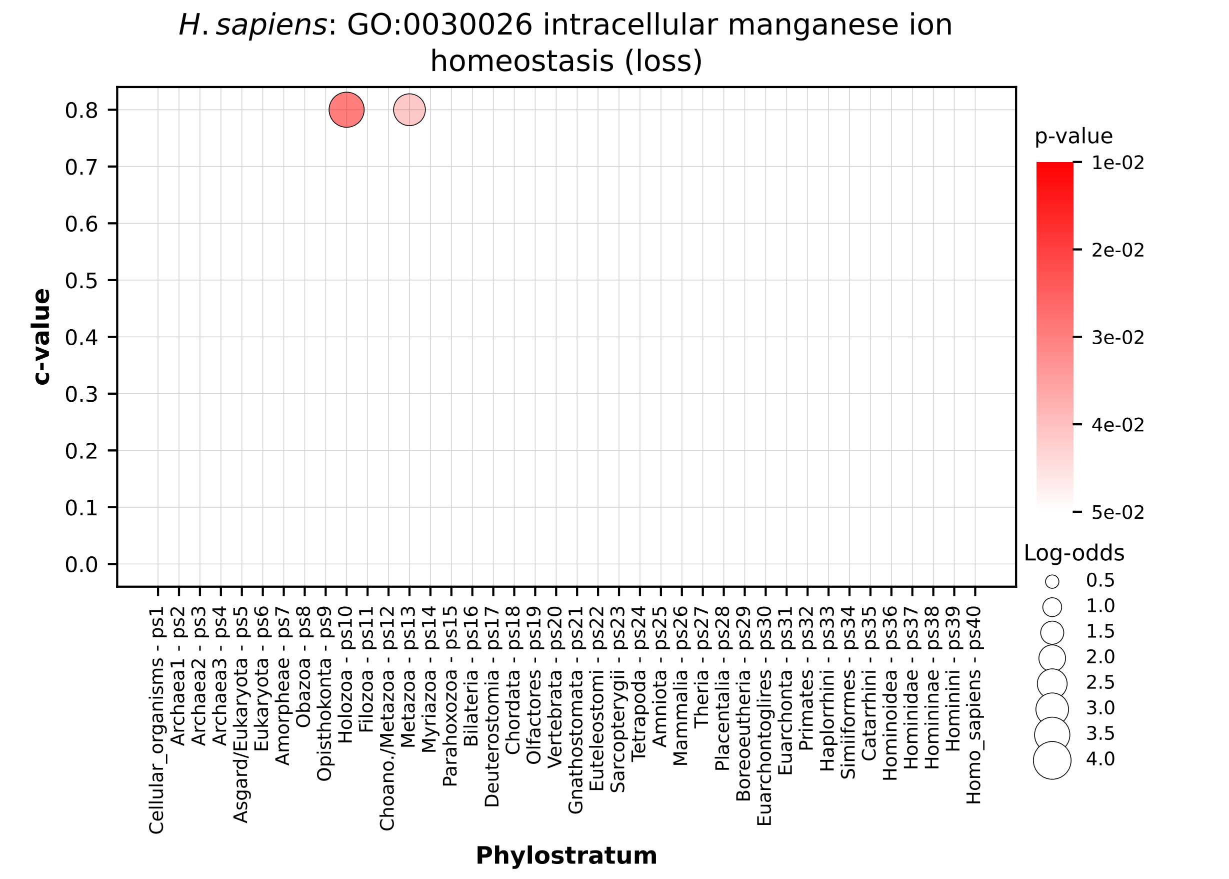

H. sapiens: GO:0030026 intracellular manganese ion homeostasis (loss)
c-value
0.8
0.7
0.6
0.5
0.4
0.3
0.2
0.1
0.0
p-value
1e-02
2e-02
3e-02
4e-02
5e-02
Log-odds
0.5
1.0
1.5
2.0
2.5
3.0
3.5
4.0
Cellular_organisms - ps1
Archaea1 - ps2
Archaea2 - ps3
Archaea3 - ps4
Asgard/Eukaryota - ps5
Eukaryota - ps6
Amorpheae - ps7
Obazoa - ps8
Opisthokonta - ps9
Holozoa - ps10
Filozoa - ps11
Choano./Metazoa - ps12
Metazoa - ps13
Myriazoa - ps14
Parahoxozoa - ps15
Bilateria - ps16
Deuterostomia - ps17
Chordata - ps18
Olfactores - ps19
Vertebrata - ps20
Gnathostomata - ps21
Euteleostomi - ps22
Sarcopterygii - ps23
Tetrapoda - ps24
Amniota - ps25
Mammalia - ps26
Theria - ps27
Placentalia - ps28
Boreoeutheria - ps29
Euarchontoglires - ps30
Euarchonta - ps31
Primates - ps32
Haplorrhini - ps33
Simiiformes - ps34
Catarrhini - ps35
Hominoidea - ps36
Hominidae - ps37
Homininae - ps38
Hominini - ps39
Homo_sapiens - ps40
Phylostratum

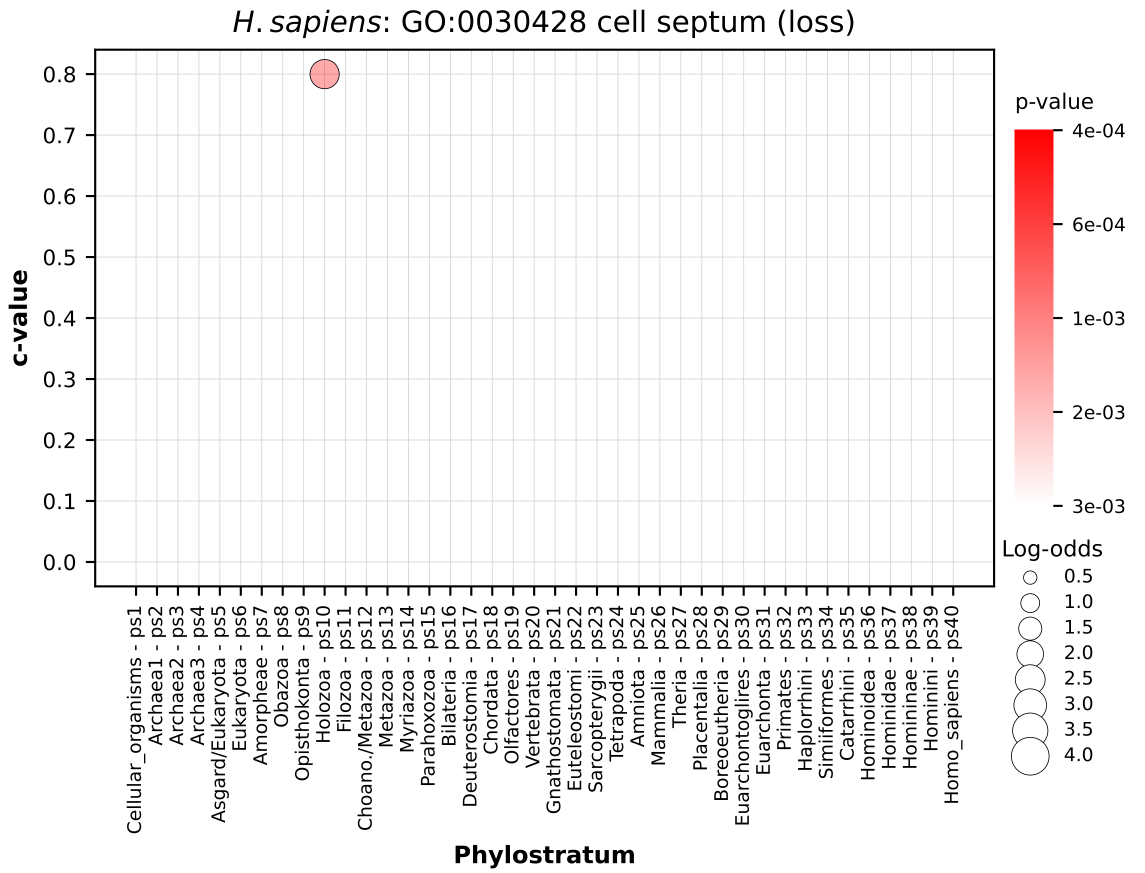
H. sapiens: GO:0030428 cell septum (loss)
c-value
0.0
0.1
0.2
0.3
0.4
0.5
0.6
0.7
0.8
Phylostratum
Cellular_organisms - ps1
Archaea1 - ps2
Archaea2 - ps3
Archaea3 - ps4
Asgard/Eukaryota - ps5
Eukaryota - ps6
Amorpheae - ps7
Obazoa - ps8
Opisthokonta - ps9
Holozoa - ps10
Filozoa - ps11
Choano./Metazoa - ps12
Metazoa - ps13
Myriazoa - ps14
Parahoxozoa - ps15
Bilateria - ps16
Deuterostomia - ps17
Chordata - ps18
Olfactores - ps19
Vertebrata - ps20
Gnathostomata - ps21
Euteleostomi - ps22
Sarcopterygii - ps23
Tetrapoda - ps24
Amniota - ps25
Mammalia - ps26
Theria - ps27
Placentalia - ps28
Boreoeutheria - ps29
Euarchontoglires - ps30
Euarchonta - ps31
Primates - ps32
Haplorrhini - ps33
Simiiformes - ps34
Catarrhini - ps35
Hominoidea - ps36
Hominidae - ps37
Homininae - ps38
Hominini - ps39
Homo_sapiens - ps40
p-value
4e-04
6e-04
1e-03
2e-03
3e-03
Log-odds
0.5
1.0
1.5
2.0
2.5
3.0
3.5
4.0

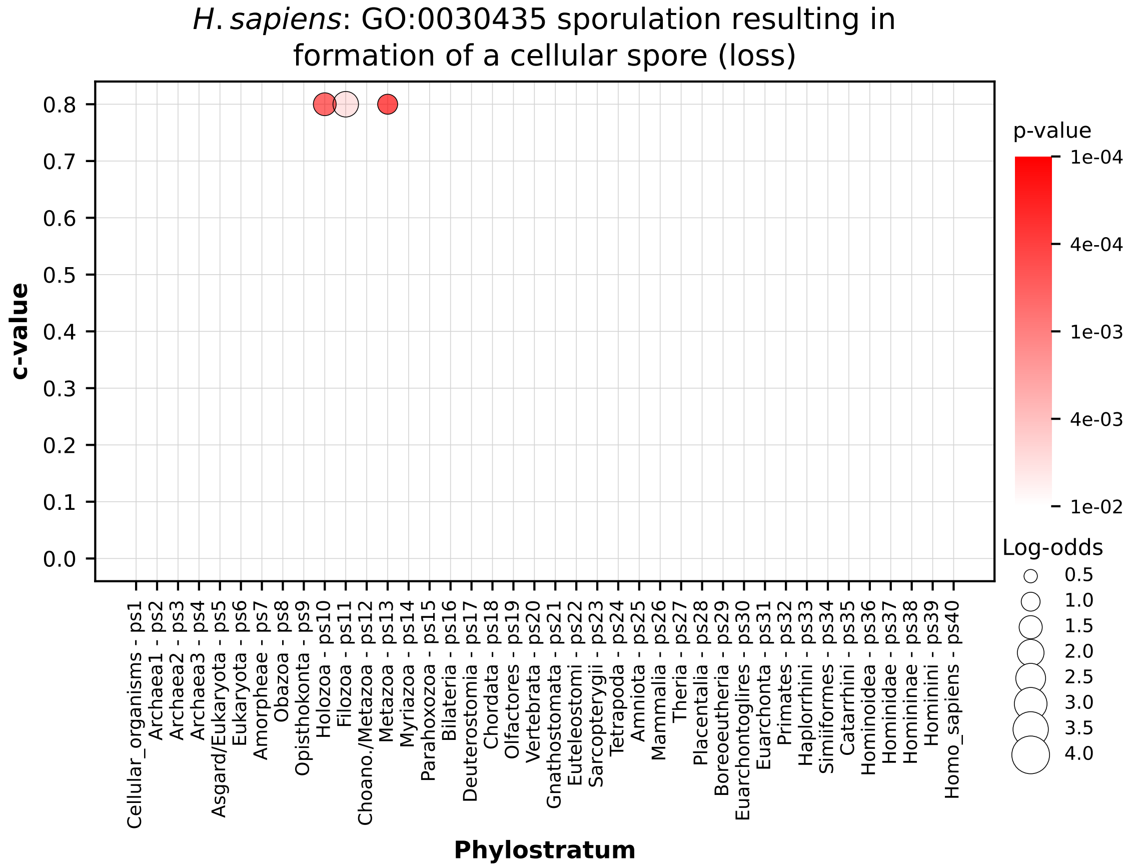

H. sapiens: GO:0030435 sporulation resulting in formation of a cellular spore (loss)
c-value
0.8
0.7
0.6
0.5
0.4
0.3
0.2
0.1
0.0
p-value
1e-04
4e-04
1e-03
4e-03
1e-02
Log-odds
0.5
1.0
1.5
2.0
2.5
3.0
3.5
4.0
Cellular_organisms - ps1
Archaea1 - ps2
Archaea2 - ps3
Archaea3 - ps4
Asgard/Eukaryota - ps5
Eukaryota - ps6
Amorpheae - ps7
Obazoa - ps8
Opisthokonta - ps9
Holozoa - ps10
Filozoa - ps11
Choano./Metazoa - ps12
Metazoa - ps13
Myriazoa - ps14
Parahoxozoa - ps15
Bilateria - ps16
Deuterostomia - ps17
Chordata - ps18
Olfactores - ps19
Vertebrata - ps20
Gnathostomata - ps21
Euteleostomi - ps22
Sarcopterygii - ps23
Tetrapoda - ps24
Amniota - ps25
Mammalia - ps26
Theria - ps27
Placentalia - ps28
Boreoeutheria - ps29
Euarchontoglires - ps30
Euarchonta - ps31
Primates - ps32
Haplorrhini - ps33
Simiiformes - ps34
Catarrhini - ps35
Hominoidea - ps36
Hominidae - ps37
Homininae - ps38
Hominini - ps39
Homo_sapiens - ps40
Phylostratum

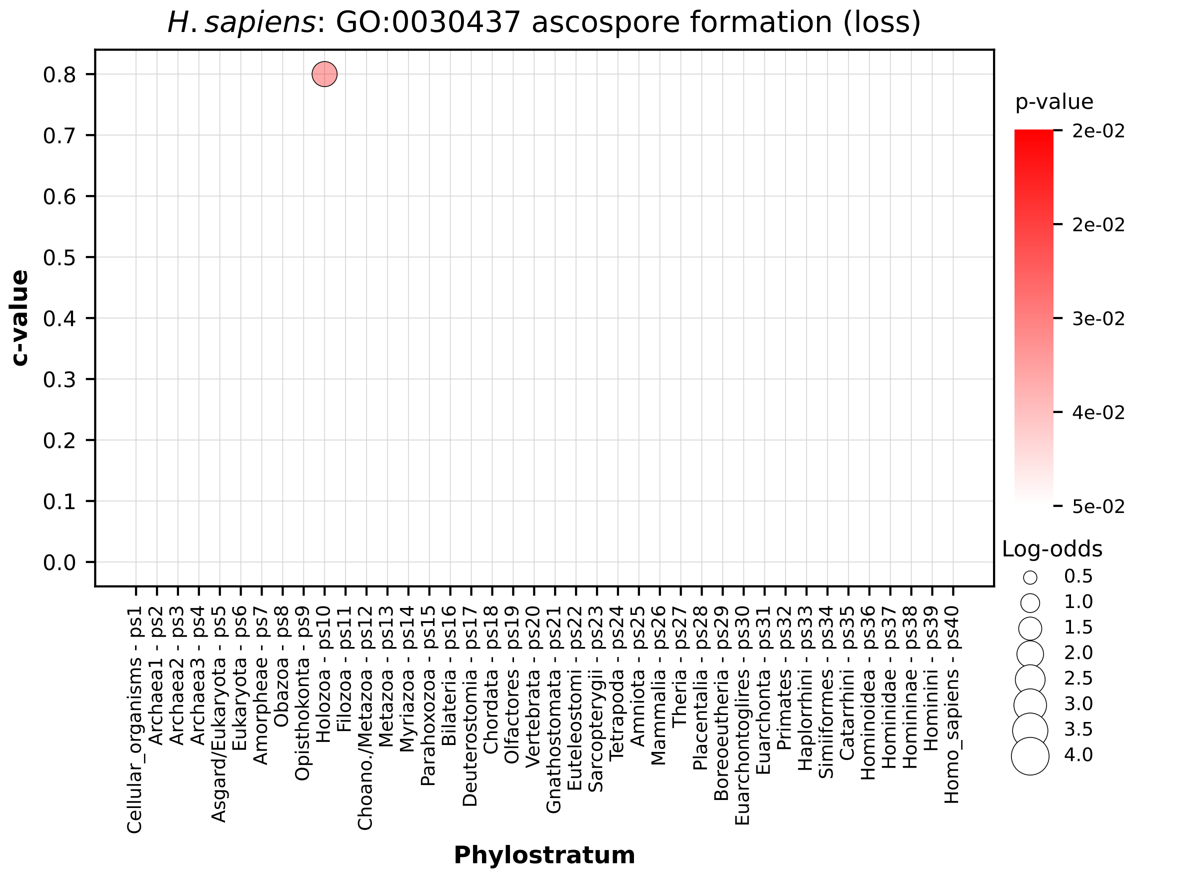

H. sapiens: GO:0030437 ascospore formation (loss)
c-value
0.8
0.7
0.6
0.5
0.4
0.3
0.2
0.1
0.0
p-value
2e-02
2e-02
3e-02
4e-02
5e-02
Log-odds
0.5
1.0
1.5
2.0
2.5
3.0
3.5
4.0
Cellular_organisms - ps1
Archaea1 - ps2
Archaea2 - ps3
Archaea3 - ps4
Asgard/Eukaryota - ps5
Eukaryota - ps6
Amorpheae - ps7
Obazoa - ps8
Opisthokonta - ps9
Holozoa - ps10
Filozoa - ps11
Choano./Metazoa - ps12
Metazoa - ps13
Myriazoa - ps14
Parahoxozoa - ps15
Bilateria - ps16
Deuterostomia - ps17
Chordata - ps18
Olfactores - ps19
Vertebrata - ps20
Gnathostomata - ps21
Euteleostomi - ps22
Sarcopterygii - ps23
Tetrapoda - ps24
Amniota - ps25
Mammalia - ps26
Theria - ps27
Placentalia - ps28
Boreoeutheria - ps29
Euarchontoglires - ps30
Euarchonta - ps31
Primates - ps32
Haplorrhini - ps33
Simiiformes - ps34
Catarrhini - ps35
Hominoidea - ps36
Hominidae - ps37
Homininae - ps38
Hominini - ps39
Homo_sapiens - ps40
Phylostratum

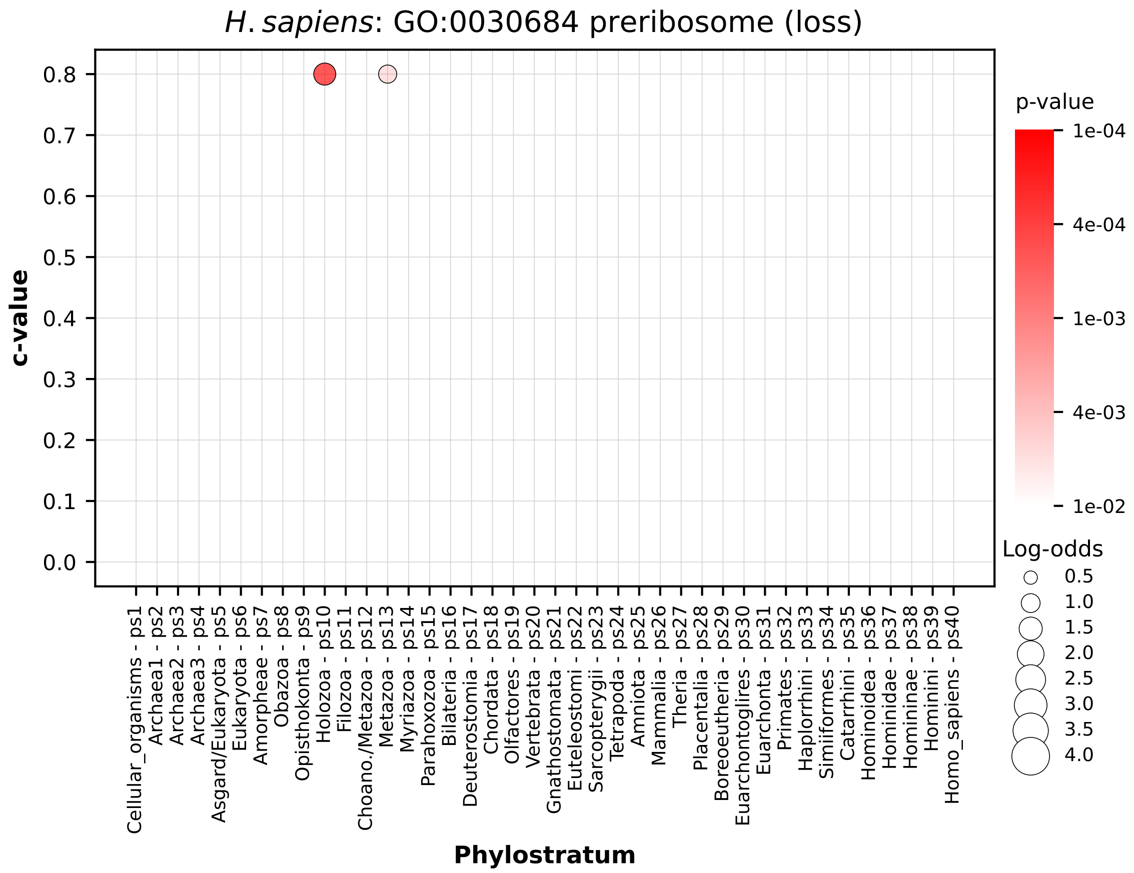

H. sapiens: GO:0030684 preribosome (loss)
c-value
0.8
0.7
0.6
0.5
0.4
0.3
0.2
0.1
0.0
Phylostratum
Cellular_organisms - ps1
Archaea1 - ps2
Archaea2 - ps3
Archaea3 - ps4
Asgard/Eukaryota - ps5
Eukaryota - ps6
Amorpheae - ps7
Obazoa - ps8
Opisthokonta - ps9
Holozoa - ps10
Filozoa - ps11
Choano./Metazoa - ps12
Metazoa - ps13
Myriazoa - ps14
Parahoxozoa - ps15
Bilateria - ps16
Deuterostomia - ps17
Chordata - ps18
Olfactores - ps19
Vertebrata - ps20
Gnathostomata - ps21
Euteleostomi - ps22
Sarcopterygii - ps23
Tetrapoda - ps24
Amniota - ps25
Mammalia - ps26
Theria - ps27
Placentalia - ps28
Boreoeutheria - ps29
Euarchontoglires - ps30
Euarchonta - ps31
Primates - ps32
Haplorrhini - ps33
Simiiformes - ps34
Catarrhini - ps35
Hominoidea - ps36
Hominidae - ps37
Homininae - ps38
Hominini - ps39
Homo_sapiens - ps40
p-value
1e-04
4e-04
1e-03
4e-03
1e-02
Log-odds
0.5
1.0
1.5
2.0
2.5
3.0
3.5
4.0

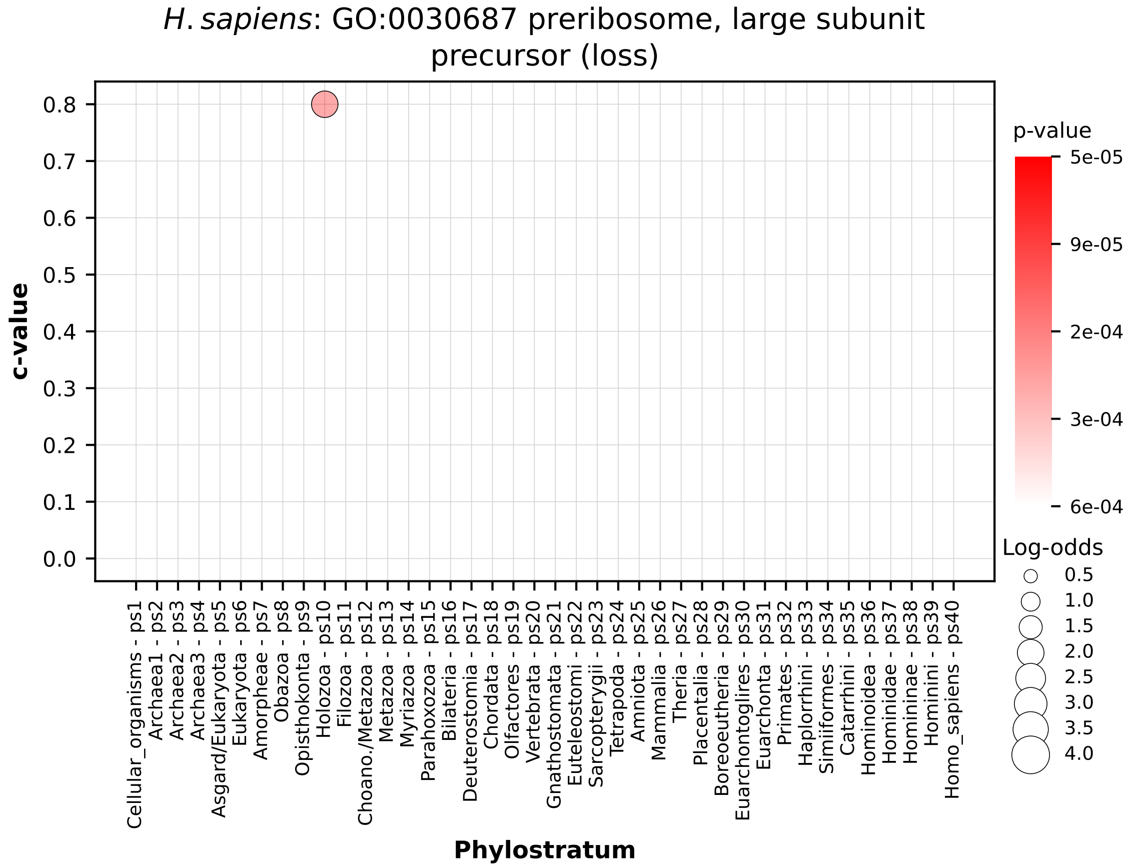

H. sapiens: GO:0030687 preribosome, large subunit precursor (loss)
c-value
0.8
0.7
0.6
0.5
0.4
0.3
0.2
0.1
0.0
p-value
5e-05
9e-05
2e-04
3e-04
6e-04
Log-odds
0.5
1.0
1.5
2.0
2.5
3.0
3.5
4.0
Cellular_organisms - ps1
Archaea1 - ps2
Archaea2 - ps3
Archaea3 - ps4
Asgard/Eukaryota - ps5
Eukaryota - ps6
Amorpheae - ps7
Obazoa - ps8
Opisthokonta - ps9
Holozoa - ps10
Filozoa - ps11
Choano./Metazoa - ps12
Metazoa - ps13
Myriazoa - ps14
Parahoxozoa - ps15
Bilateria - ps16
Deuterostomia - ps17
Chordata - ps18
Olfactores - ps19
Vertebrata - ps20
Gnathostomata - ps21
Euteleostomi - ps22
Sarcopterygii - ps23
Tetrapoda - ps24
Amniota - ps25
Mammalia - ps26
Theria - ps27
Placentalia - ps28
Boreoeutheria - ps29
Euarchontoglires - ps30
Euarchonta - ps31
Primates - ps32
Haplorrhini - ps33
Simiiformes - ps34
Catarrhini - ps35
Hominoidea - ps36
Hominidae - ps37
Homininae - ps38
Hominini - ps39
Homo_sapiens - ps40
Phylostratum

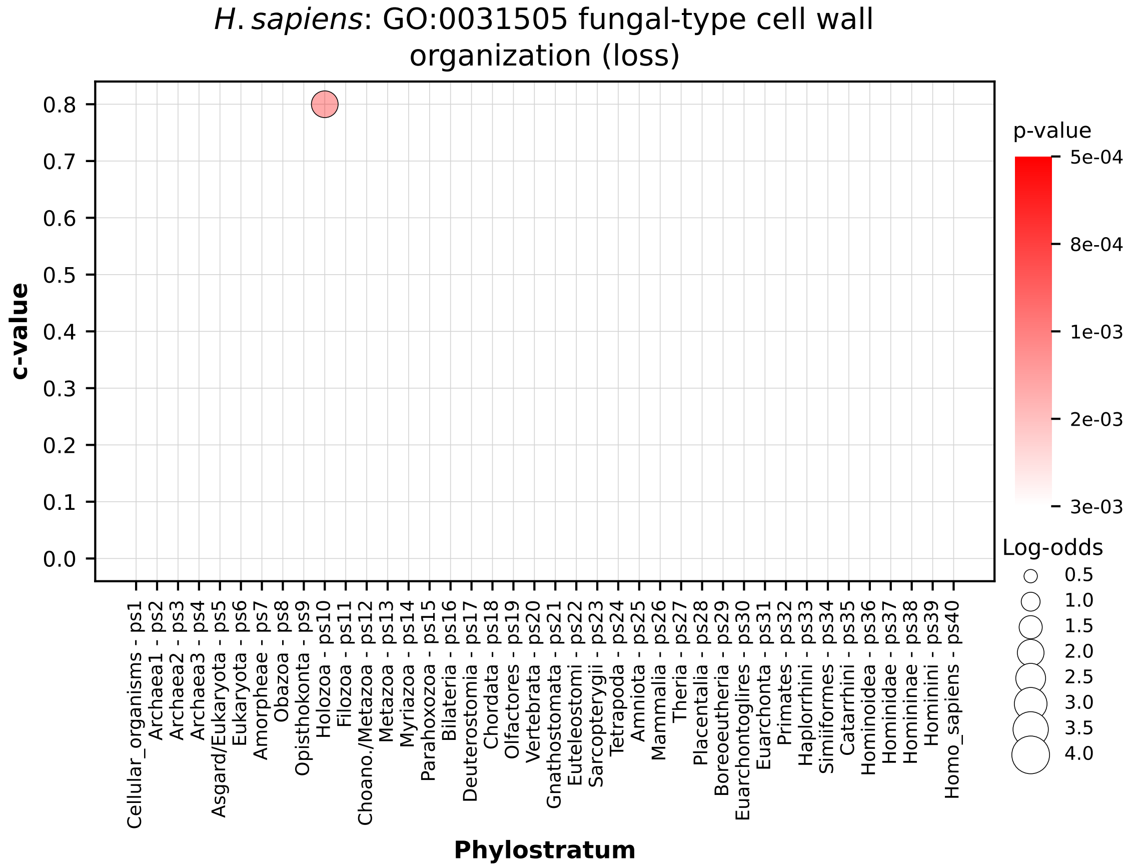

H. sapiens: GO:0031505 fungal-type cell wall organization (loss)
c-value
0.8
0.7
0.6
0.5
0.4
0.3
0.2
0.1
0.0
p-value
5e-04
8e-04
1e-03
2e-03
3e-03
Log-odds
0.5
1.0
1.5
2.0
2.5
3.0
3.5
4.0
Cellular_organisms - ps1
Archaea1 - ps2
Archaea2 - ps3
Archaea3 - ps4
Asgard/Eukaryota - ps5
Eukaryota - ps6
Amorpheae - ps7
Obazoa - ps8
Opisthokonta - ps9
Holozoa - ps10
Filozoa - ps11
Choano./Metazoa - ps12
Metazoa - ps13
Myriazoa - ps14
Parahoxozoa - ps15
Bilateria - ps16
Deuterostomia - ps17
Chordata - ps18
Olfactores - ps19
Vertebrata - ps20
Gnathostomata - ps21
Euteleostomi - ps22
Sarcopterygii - ps23
Tetrapoda - ps24
Amniota - ps25
Mammalia - ps26
Theria - ps27
Placentalia - ps28
Boreoeutheria - ps29
Euarchontoglires - ps30
Euarchonta - ps31
Primates - ps32
Haplorrhini - ps33
Simiiformes - ps34
Catarrhini - ps35
Hominoidea - ps36
Hominidae - ps37
Homininae - ps38
Hominini - ps39
Homo_sapiens - ps40
Phylostratum

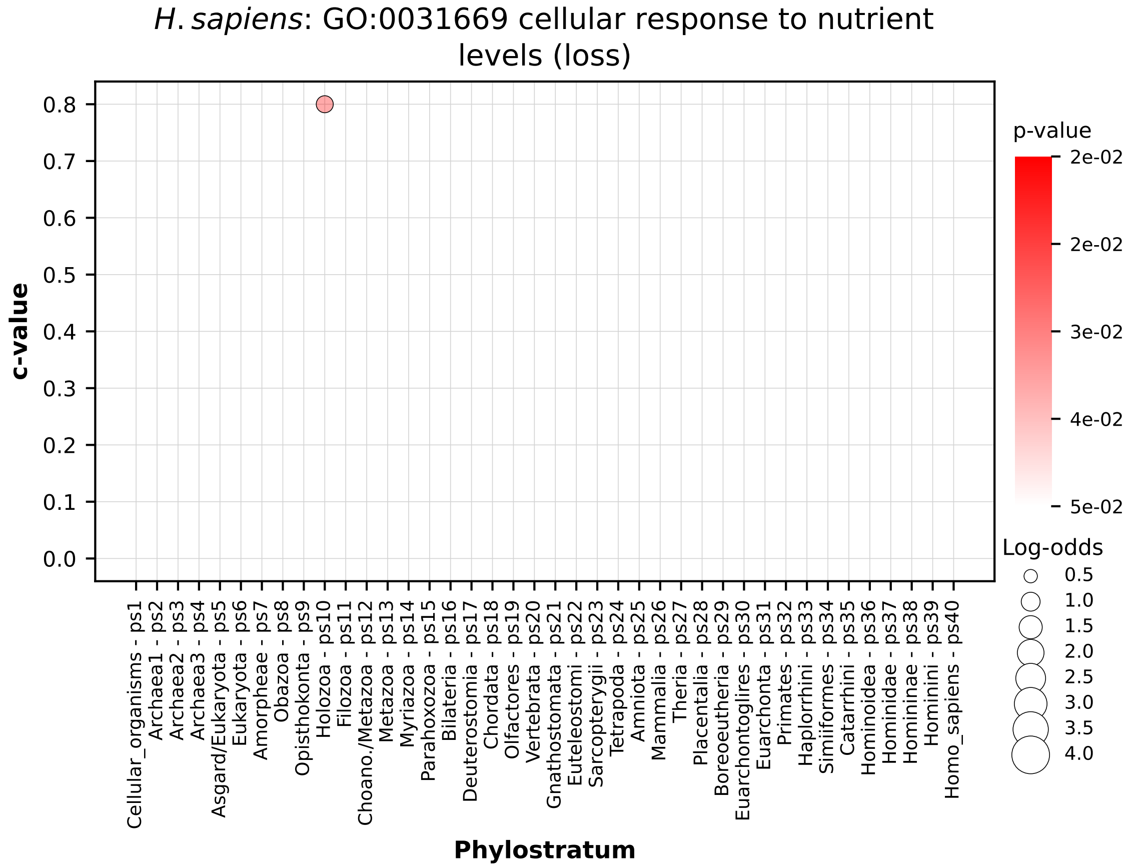
H. sapiens: GO:0031669 cellular response to nutrient levels (loss)
c-value
0.0
0.1
0.2
0.3
0.4
0.5
0.6
0.7
0.8
Phylostratum
Cellular_organisms - ps1
Archaea1 - ps2
Archaea2 - ps3
Archaea3 - ps4
Asgard/Eukaryota - ps5
Eukaryota - ps6
Amorpheae - ps7
Obazoa - ps8
Opisthokonta - ps9
Holozoa - ps10
Filozoa - ps11
Choano./Metazoa - ps12
Metazoa - ps13
Myriazoa - ps14
Parahoxozoa - ps15
Bilateria - ps16
Deuterostomia - ps17
Chordata - ps18
Olfactores - ps19
Vertebrata - ps20
Gnathostomata - ps21
Euteleostomi - ps22
Sarcopterygii - ps23
Tetrapoda - ps24
Amniota - ps25
Mammalia - ps26
Theria - ps27
Placentalia - ps28
Boreoeutheria - ps29
Euarchontoglires - ps30
Euarchonta - ps31
Primates - ps32
Haplorrhini - ps33
Simiiformes - ps34
Catarrhini - ps35
Hominoidea - ps36
Hominidae - ps37
Homininae - ps38
Hominini - ps39
Homo_sapiens - ps40
p-value
2e-02
2e-02
3e-02
4e-02
5e-02
Log-odds
0.5
1.0
1.5
2.0
2.5
3.0
3.5
4.0

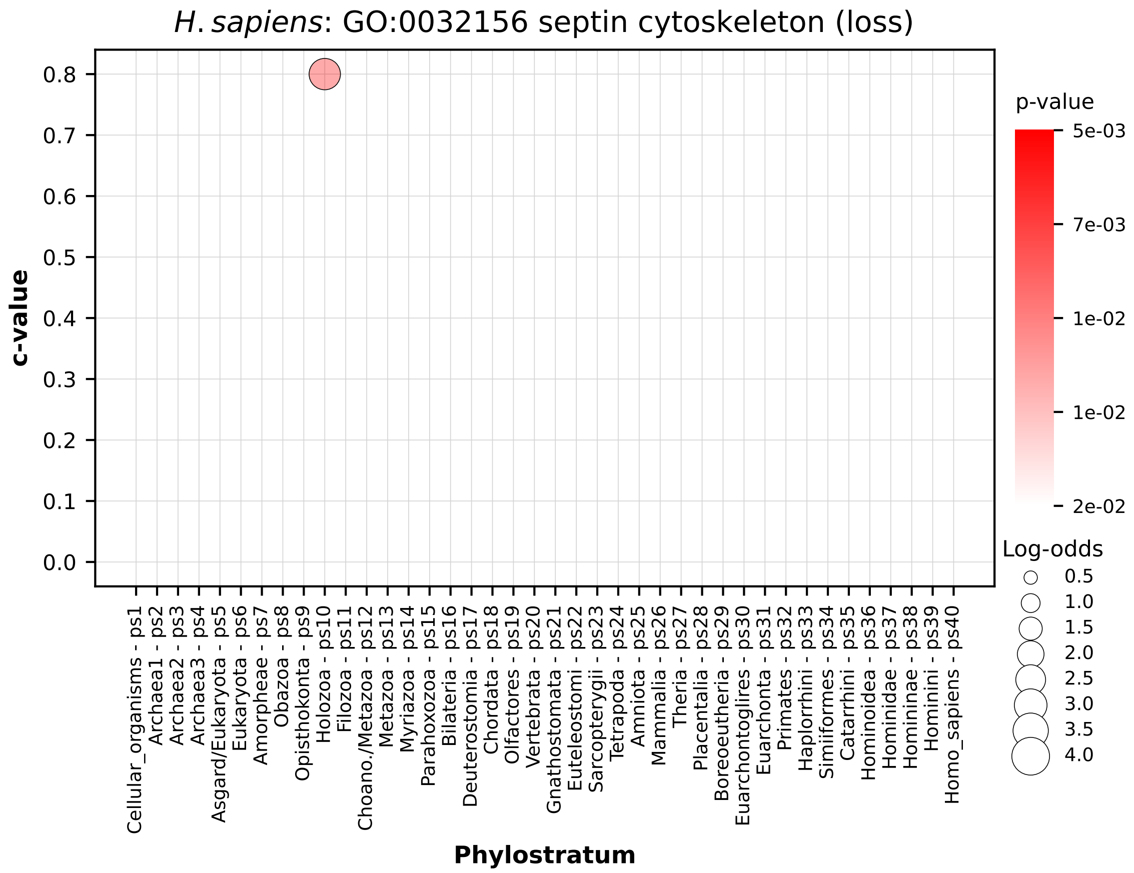

H. sapiens: GO:0032156 septin cytoskeleton (loss)
c-value
0.0
0.1
0.2
0.3
0.4
0.5
0.6
0.7
0.8
Phylostratum
Cellular_organisms - ps1
Archaea1 - ps2
Archaea2 - ps3
Archaea3 - ps4
Asgard/Eukaryota - ps5
Eukaryota - ps6
Amorpheae - ps7
Obazoa - ps8
Opisthokonta - ps9
Holozoa - ps10
Filozoa - ps11
Choano./Metazoa - ps12
Metazoa - ps13
Myriazoa - ps14
Parahoxozoa - ps15
Bilateria - ps16
Deuterostomia - ps17
Chordata - ps18
Olfactores - ps19
Vertebrata - ps20
Gnathostomata - ps21
Euteleostomi - ps22
Sarcopterygii - ps23
Tetrapoda - ps24
Amniota - ps25
Mammalia - ps26
Theria - ps27
Placentalia - ps28
Boreoeutheria - ps29
Euarchontoglires - ps30
Euarchonta - ps31
Primates - ps32
Haplorrhini - ps33
Simiiformes - ps34
Catarrhini - ps35
Hominoidea - ps36
Hominidae - ps37
Homininae - ps38
Hominini - ps39
Homo_sapiens - ps40
p-value
5e-03
7e-03
1e-02
1e-02
2e-02
Log-odds
0.5
1.0
1.5
2.0
2.5
3.0
3.5
4.0

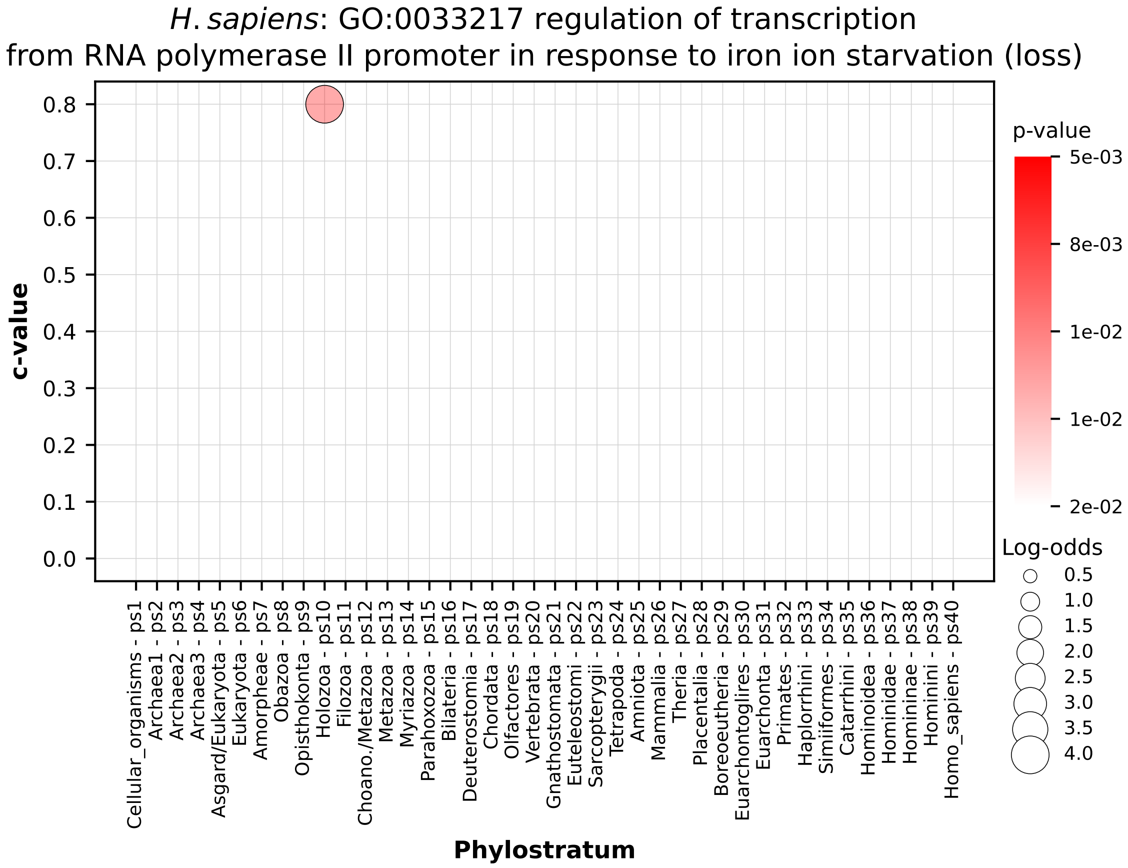

H. sapiens: GO:0033217 regulation of transcription
from RNA polymerase II promoter in response to iron ion starvation (loss)
c-value
0.0
0.1
0.2
0.3
0.4
0.5
0.6
0.7
0.8
Phylostratum
Cellular_organisms - ps1
Archaea1 - ps2
Archaea2 - ps3
Archaea3 - ps4
Asgard/Eukaryota - ps5
Eukaryota - ps6
Amorpheae - ps7
Obazoa - ps8
Opisthokonta - ps9
Holozoa - ps10
Filozoa - ps11
Choano./Metazoa - ps12
Metazoa - ps13
Myriazoa - ps14
Parahoxozoa - ps15
Bilateria - ps16
Deuterostomia - ps17
Chordata - ps18
Olfactores - ps19
Vertebrata - ps20
Gnathostomata - ps21
Euteleostomi - ps22
Sarcopterygii - ps23
Tetrapoda - ps24
Amniota - ps25
Mammalia - ps26
Theria - ps27
Placentalia - ps28
Boreoeutheria - ps29
Euarchontoglires - ps30
Euarchonta - ps31
Primates - ps32
Haplorrhini - ps33
Simiiformes - ps34
Catarrhini - ps35
Hominoidea - ps36
Hominidae - ps37
Homininae - ps38
Hominini - ps39
Homo_sapiens - ps40
p-value
5e-03
8e-03
1e-02
1e-02
2e-02
Log-odds
0.5
1.0
1.5
2.0
2.5
3.0
3.5
4.0

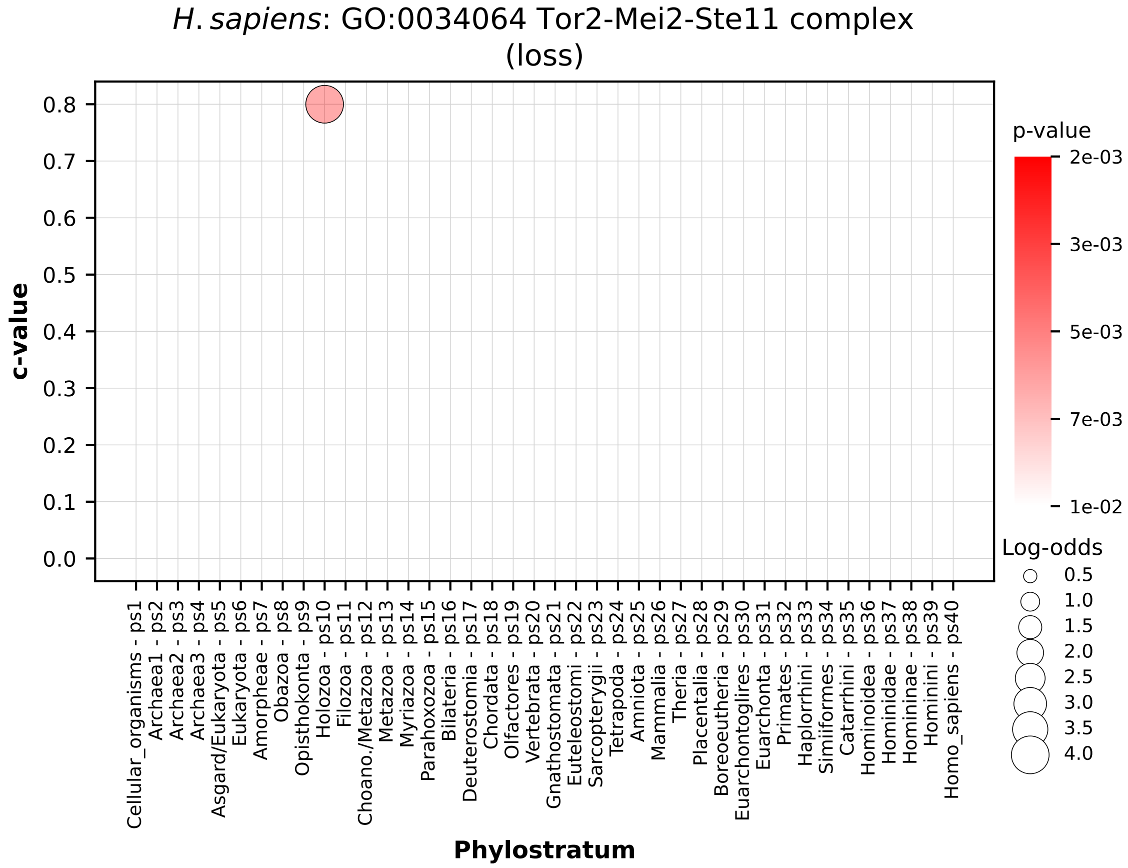

H. sapiens: GO:0034064 Tor2-Mei2-Ste11 complex
(loss)
c-value
0.8
0.7
0.6
0.5
0.4
0.3
0.2
0.1
0.0
p-value
2e-03
3e-03
5e-03
7e-03
1e-02
Log-odds
0.5
1.0
1.5
2.0
2.5
3.0
3.5
4.0
Cellular_organisms - ps1
Archaea1 - ps2
Archaea2 - ps3
Archaea3 - ps4
Asgard/Eukaryota - ps5
Eukaryota - ps6
Amorpheae - ps7
Obazoa - ps8
Opisthokonta - ps9
Holozoa - ps10
Filozoa - ps11
Choano./Metazoa - ps12
Metazoa - ps13
Myriazoa - ps14
Parahoxozoa - ps15
Bilateria - ps16
Deuterostomia - ps17
Chordata - ps18
Olfactores - ps19
Vertebrata - ps20
Gnathostomata - ps21
Euteleostomi - ps22
Sarcopterygii - ps23
Tetrapoda - ps24
Amniota - ps25
Mammalia - ps26
Theria - ps27
Placentalia - ps28
Boreoeutheria - ps29
Euarchontoglires - ps30
Euarchonta - ps31
Primates - ps32
Haplorrhini - ps33
Simiiformes - ps34
Catarrhini - ps35
Hominoidea - ps36
Hominidae - ps37
Homininae - ps38
Hominini - ps39
Homo_sapiens - ps40
Phylostratum

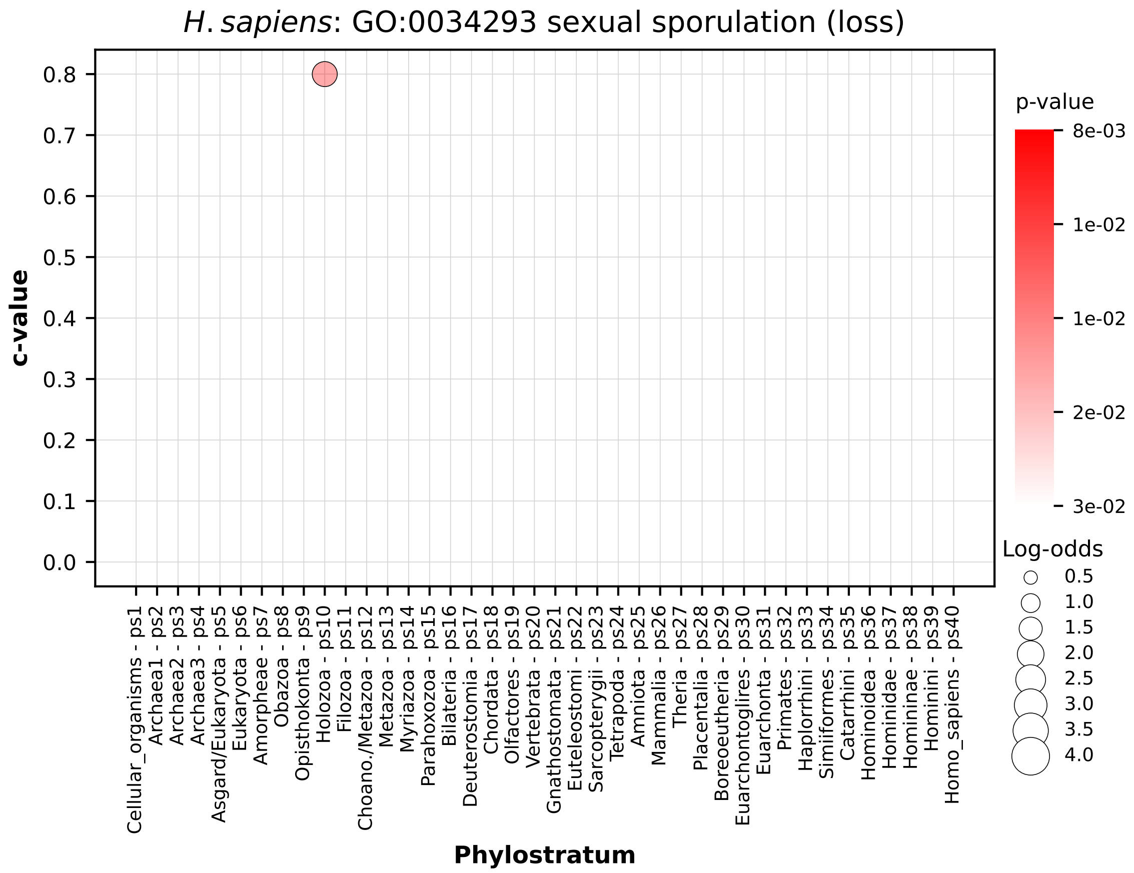

H. sapiens: GO:0034293 sexual sporulation (loss)
c-value
0.8
0.7
0.6
0.5
0.4
0.3
0.2
0.1
0.0
p-value
8e-03
1e-02
1e-02
2e-02
3e-02
Log-odds
0.5
1.0
1.5
2.0
2.5
3.0
3.5
4.0
Cellular_organisms - ps1
Archaea1 - ps2
Archaea2 - ps3
Archaea3 - ps4
Asgard/Eukaryota - ps5
Eukaryota - ps6
Amorpheae - ps7
Obazoa - ps8
Opisthokonta - ps9
Holozoa - ps10
Filozoa - ps11
Choano./Metazoa - ps12
Metazoa - ps13
Myriazoa - ps14
Parahoxozoa - ps15
Bilateria - ps16
Deuterostomia - ps17
Chordata - ps18
Olfactores - ps19
Vertebrata - ps20
Gnathostomata - ps21
Euteleostomi - ps22
Sarcopterygii - ps23
Tetrapoda - ps24
Amniota - ps25
Mammalia - ps26
Theria - ps27
Placentalia - ps28
Boreoeutheria - ps29
Euarchontoglires - ps30
Euarchonta - ps31
Primates - ps32
Haplorrhini - ps33
Simiiformes - ps34
Catarrhini - ps35
Hominoidea - ps36
Hominidae - ps37
Homininae - ps38
Hominini - ps39
Homo_sapiens - ps40
Phylostratum

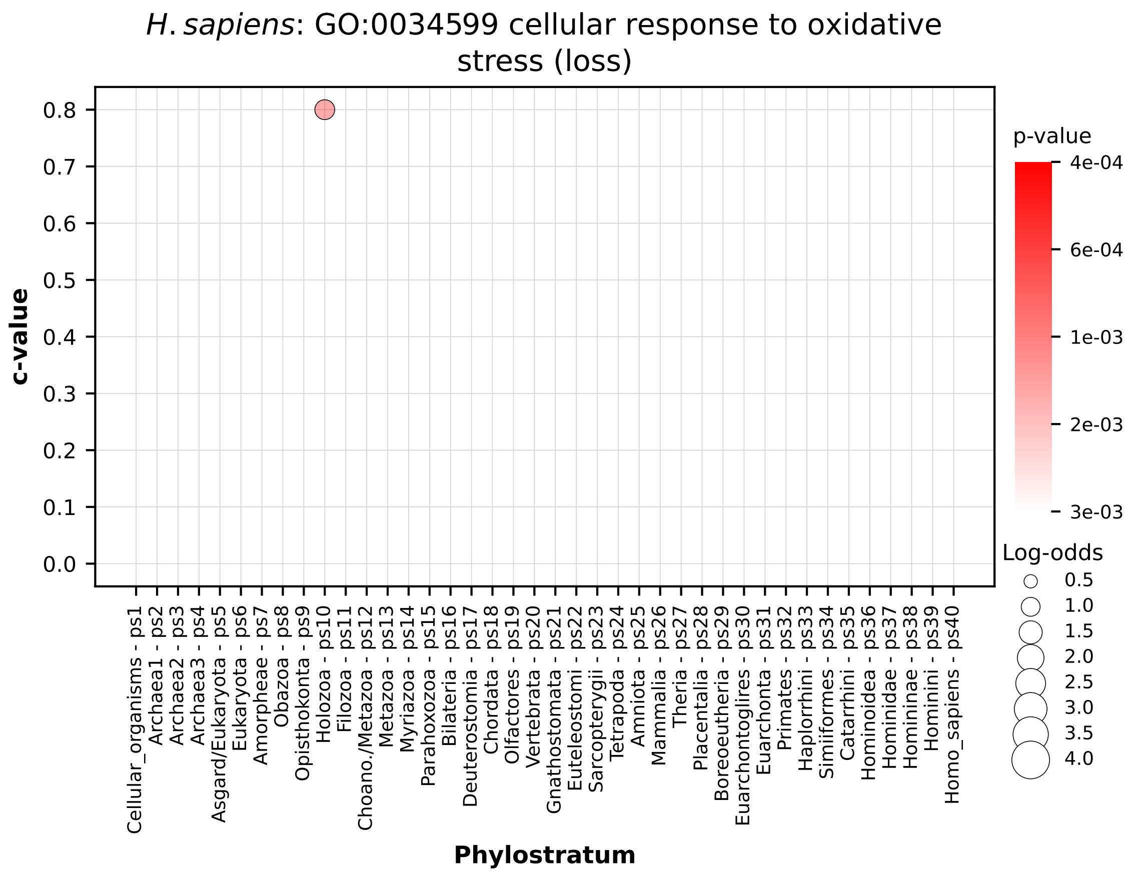

H. sapiens: GO:0034599 cellular response to oxidative stress (loss)
c-value
0.8
0.7
0.6
0.5
0.4
0.3
0.2
0.1
0.0
p-value
4e-04
6e-04
1e-03
2e-03
3e-03
Log-odds
0.5
1.0
1.5
2.0
2.5
3.0
3.5
4.0
Cellular_organisms - ps1
Archaea1 - ps2
Archaea2 - ps3
Archaea3 - ps4
Asgard/Eukaryota - ps5
Eukaryota - ps6
Amorpheae - ps7
Obazoa - ps8
Opisthokonta - ps9
Holozoa - ps10
Filozoa - ps11
Choano./Metazoa - ps12
Metazoa - ps13
Myriazoa - ps14
Parahoxozoa - ps15
Bilateria - ps16
Deuterostomia - ps17
Chordata - ps18
Olfactores - ps19
Vertebrata - ps20
Gnathostomata - ps21
Euteleostomi - ps22
Sarcopterygii - ps23
Tetrapoda - ps24
Amniota - ps25
Mammalia - ps26
Theria - ps27
Placentalia - ps28
Boreoeutheria - ps29
Euarchontoglires - ps30
Euarchonta - ps31
Primates - ps32
Haplorrhini - ps33
Simiiformes - ps34
Catarrhini - ps35
Hominoidea - ps36
Hominidae - ps37
Homininae - ps38
Hominini - ps39
Homo_sapiens - ps40
Phylostratum

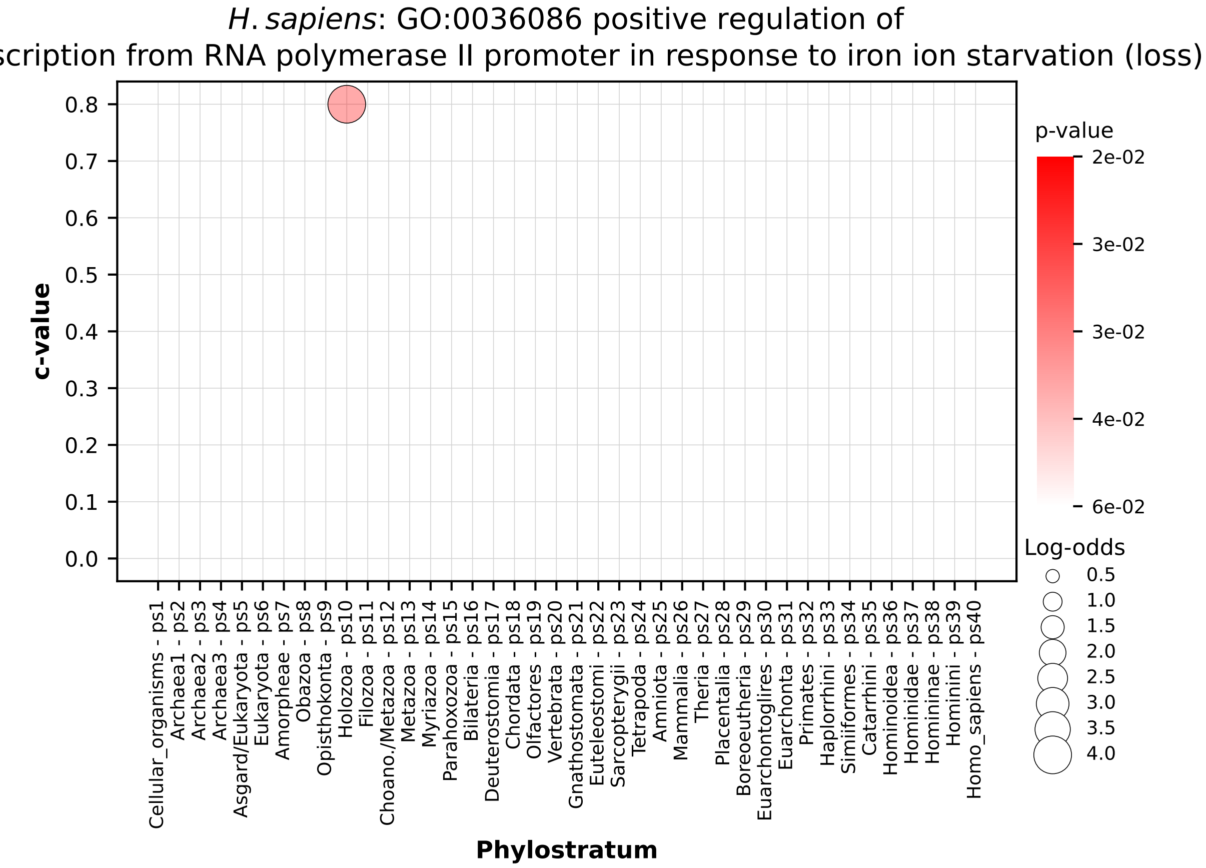

H. sapiens: GO:0036086 positive regulation of
scription from RNA polymerase II promoter in response to iron ion starvation (loss)
c-value
0.8
0.7
0.6
0.5
0.4
0.3
0.2
0.1
0.0
p-value
2e-02
3e-02
3e-02
4e-02
6e-02
Log-odds
0.5
1.0
1.5
2.0
2.5
3.0
3.5
4.0
Cellular_organisms - ps1
Archaea1 - ps2
Archaea2 - ps3
Archaea3 - ps4
Asgard/Eukaryota - ps5
Eukaryota - ps6
Amorpheae - ps7
Obazoa - ps8
Opisthokonta - ps9
Holozoa - ps10
Filozoa - ps11
Choano./Metazoa - ps12
Metazoa - ps13
Myriazoa - ps14
Parahoxozoa - ps15
Bilateria - ps16
Deuterostomia - ps17
Chordata - ps18
Olfactores - ps19
Vertebrata - ps20
Gnathostomata - ps21
Euteleostomi - ps22
Sarcopterygii - ps23
Tetrapoda - ps24
Amniota - ps25
Mammalia - ps26
Theria - ps27
Placentalia - ps28
Boreoeutheria - ps29
Euarchontoglires - ps30
Euarchonta - ps31
Primates - ps32
Haplorrhini - ps33
Simiiformes - ps34
Catarrhini - ps35
Hominoidea - ps36
Hominidae - ps37
Homininae - ps38
Hominini - ps39
Homo_sapiens - ps40
Phylostratum

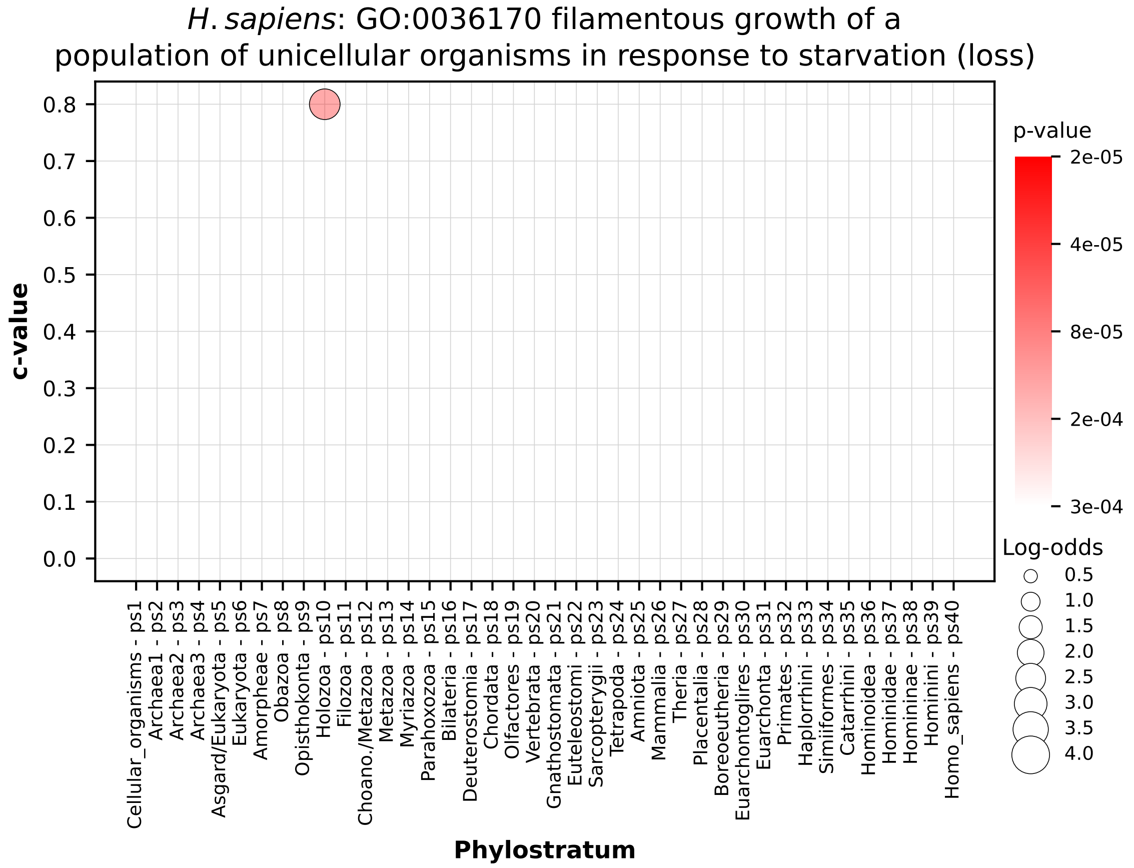

H. sapiens: GO:0036170 filamentous growth of a population of unicellular organisms in response to starvation (loss)
c-value
0.0
0.1
0.2
0.3
0.4
0.5
0.6
0.7
0.8
Phylostratum
Cellular_organisms - ps1
Archaea1 - ps2
Archaea2 - ps3
Archaea3 - ps4
Asgard/Eukaryota - ps5
Eukaryota - ps6
Amorpheae - ps7
Obazoa - ps8
Opisthokonta - ps9
Holozoa - ps10
Filozoa - ps11
Choano./Metazoa - ps12
Metazoa - ps13
Myriazoa - ps14
Parahoxozoa - ps15
Bilateria - ps16
Deuterostomia - ps17
Chordata - ps18
Olfactores - ps19
Vertebrata - ps20
Gnathostomata - ps21
Euteleostomi - ps22
Sarcopterygii - ps23
Tetrapoda - ps24
Amniota - ps25
Mammalia - ps26
Theria - ps27
Placentalia - ps28
Boreoeutheria - ps29
Euarchontoglires - ps30
Euarchonta - ps31
Primates - ps32
Haplorrhini - ps33
Simiiformes - ps34
Catarrhini - ps35
Hominoidea - ps36
Hominidae - ps37
Homininae - ps38
Hominini - ps39
Homo_sapiens - ps40
p-value
2e-05
4e-05
8e-05
2e-04
3e-04
Log-odds
0.5
1.0
1.5
2.0
2.5
3.0
3.5
4.0

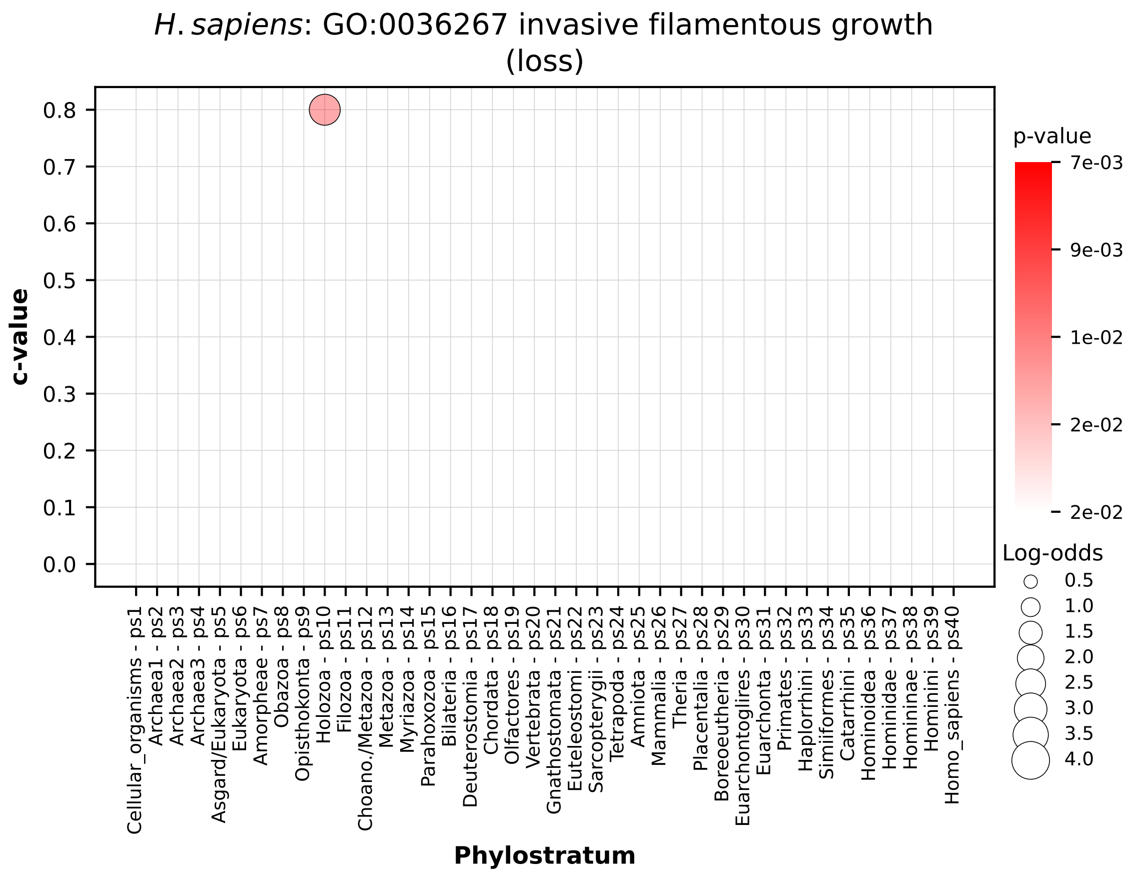

H. sapiens: GO:0036267 invasive filamentous growth (loss)
c-value
0.8
0.7
0.6
0.5
0.4
0.3
0.2
0.1
0.0
p-value
7e-03
9e-03
1e-02
2e-02
2e-02
Log-odds
0.5
1.0
1.5
2.0
2.5
3.0
3.5
4.0
Cellular_organisms - ps1
Archaea1 - ps2
Archaea2 - ps3
Archaea3 - ps4
Asgard/Eukaryota - ps5
Eukaryota - ps6
Amorpheae - ps7
Obazoa - ps8
Opisthokonta - ps9
Holozoa - ps10
Filozoa - ps11
Choano./Metazoa - ps12
Metazoa - ps13
Myriazoa - ps14
Parahoxozoa - ps15
Bilateria - ps16
Deuterostomia - ps17
Chordata - ps18
Olfactores - ps19
Vertebrata - ps20
Gnathostomata - ps21
Euteleostomi - ps22
Sarcopterygii - ps23
Tetrapoda - ps24
Amniota - ps25
Mammalia - ps26
Theria - ps27
Placentalia - ps28
Boreoeutheria - ps29
Euarchontoglires - ps30
Euarchonta - ps31
Primates - ps32
Haplorrhini - ps33
Simiiformes - ps34
Catarrhini - ps35
Hominoidea - ps36
Hominidae - ps37
Homininae - ps38
Hominini - ps39
Homo_sapiens - ps40
Phylostratum

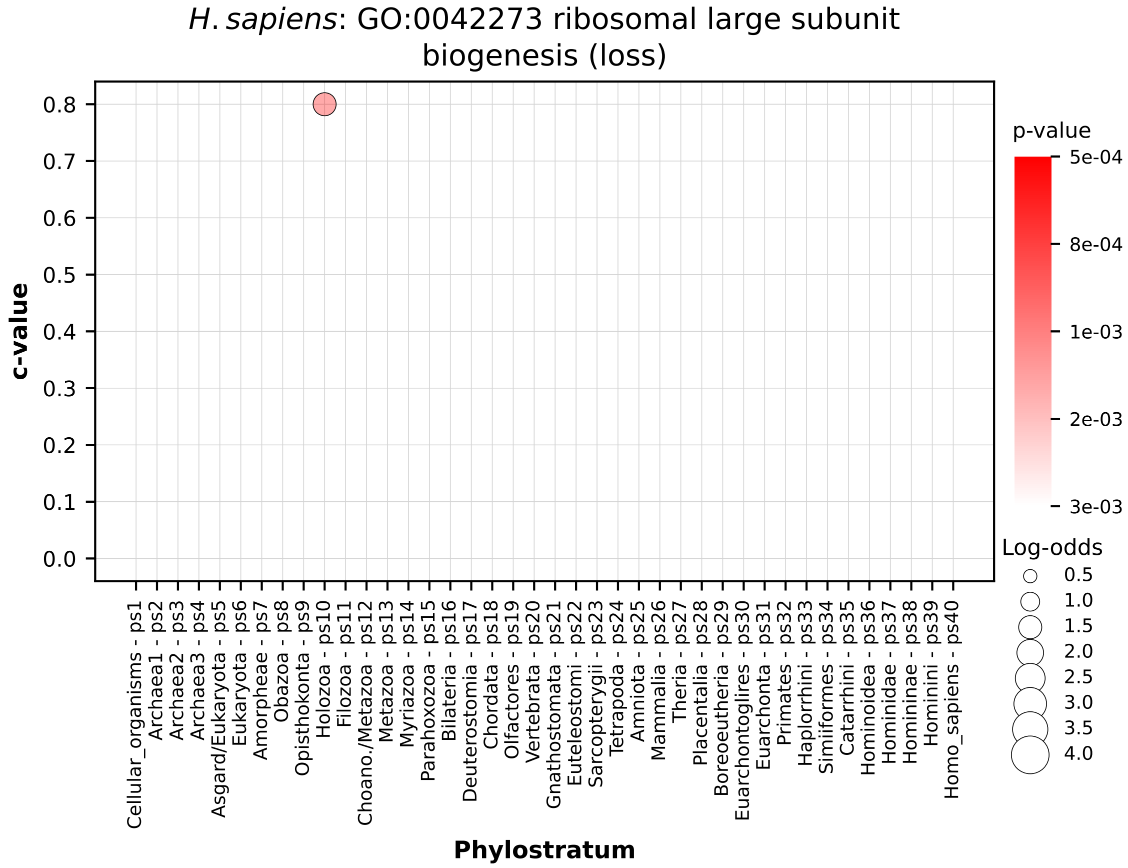
H. sapiens: GO:0042273 ribosomal large subunit biogenesis (loss)
c-value
0.8
0.7
0.6
0.5
0.4
0.3
0.2
0.1
0.0
p-value
5e-04
8e-04
1e-03
2e-03
3e-03
Log-odds
0.5
1.0
1.5
2.0
2.5
3.0
3.5
4.0
Cellular_organisms - ps1
Archaea1 - ps2
Archaea2 - ps3
Archaea3 - ps4
Asgard/Eukaryota - ps5
Eukaryota - ps6
Amorpheae - ps7
Obazoa - ps8
Opisthokonta - ps9
Holozoa - ps10
Filozoa - ps11
Choano./Metazoa - ps12
Metazoa - ps13
Myriazoa - ps14
Parahoxozoa - ps15
Bilateria - ps16
Deuterostomia - ps17
Chordata - ps18
Olfactores - ps19
Vertebrata - ps20
Gnathostomata - ps21
Euteleostomi - ps22
Sarcopterygii - ps23
Tetrapoda - ps24
Amniota - ps25
Mammalia - ps26
Theria - ps27
Placentalia - ps28
Boreoeutheria - ps29
Euarchontoglires - ps30
Euarchonta - ps31
Primates - ps32
Haplorrhini - ps33
Simiiformes - ps34
Catarrhini - ps35
Hominoidea - ps36
Hominidae - ps37
Homininae - ps38
Hominini - ps39
Homo_sapiens - ps40
Phylostratum

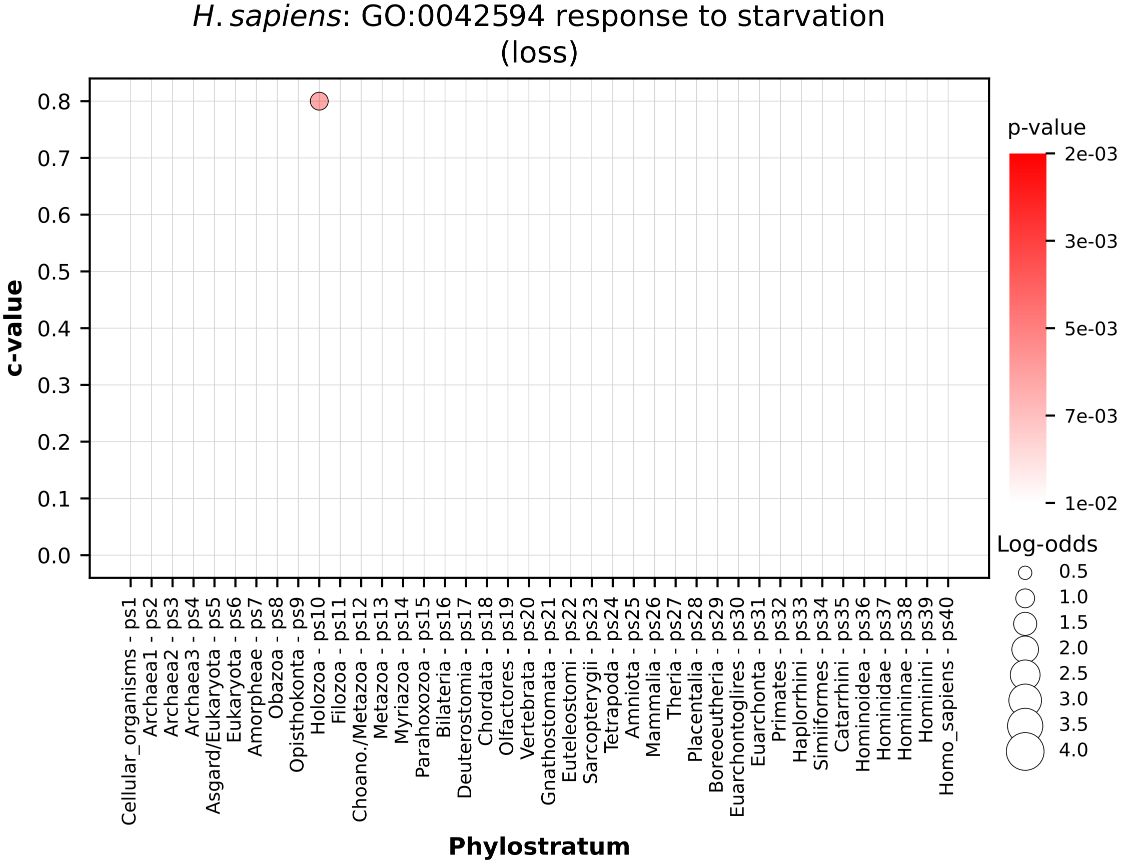

H. sapiens: GO:0042594 response to starvation (loss)
c-value
0.8
0.7
0.6
0.5
0.4
0.3
0.2
0.1
0.0
p-value
2e-03
3e-03
5e-03
7e-03
1e-02
Log-odds
0.5
1.0
1.5
2.0
2.5
3.0
3.5
4.0
Cellular_organisms - ps1
Archaea1 - ps2
Archaea2 - ps3
Archaea3 - ps4
Asgard/Eukaryota - ps5
Eukaryota - ps6
Amorpheae - ps7
Obazoa - ps8
Opisthokonta - ps9
Holozoa - ps10
Filozoa - ps11
Choano./Metazoa - ps12
Metazoa - ps13
Myriazoa - ps14
Parahoxozoa - ps15
Bilateria - ps16
Deuterostomia - ps17
Chordata - ps18
Olfactores - ps19
Vertebrata - ps20
Gnathostomata - ps21
Euteleostomi - ps22
Sarcopterygii - ps23
Tetrapoda - ps24
Amniota - ps25
Mammalia - ps26
Theria - ps27
Placentalia - ps28
Boreoeutheria - ps29
Euarchontoglires - ps30
Euarchonta - ps31
Primates - ps32
Haplorrhini - ps33
Simiiformes - ps34
Catarrhini - ps35
Hominoidea - ps36
Hominidae - ps37
Homininae - ps38
Hominini - ps39
Homo_sapiens - ps40
Phylostratum

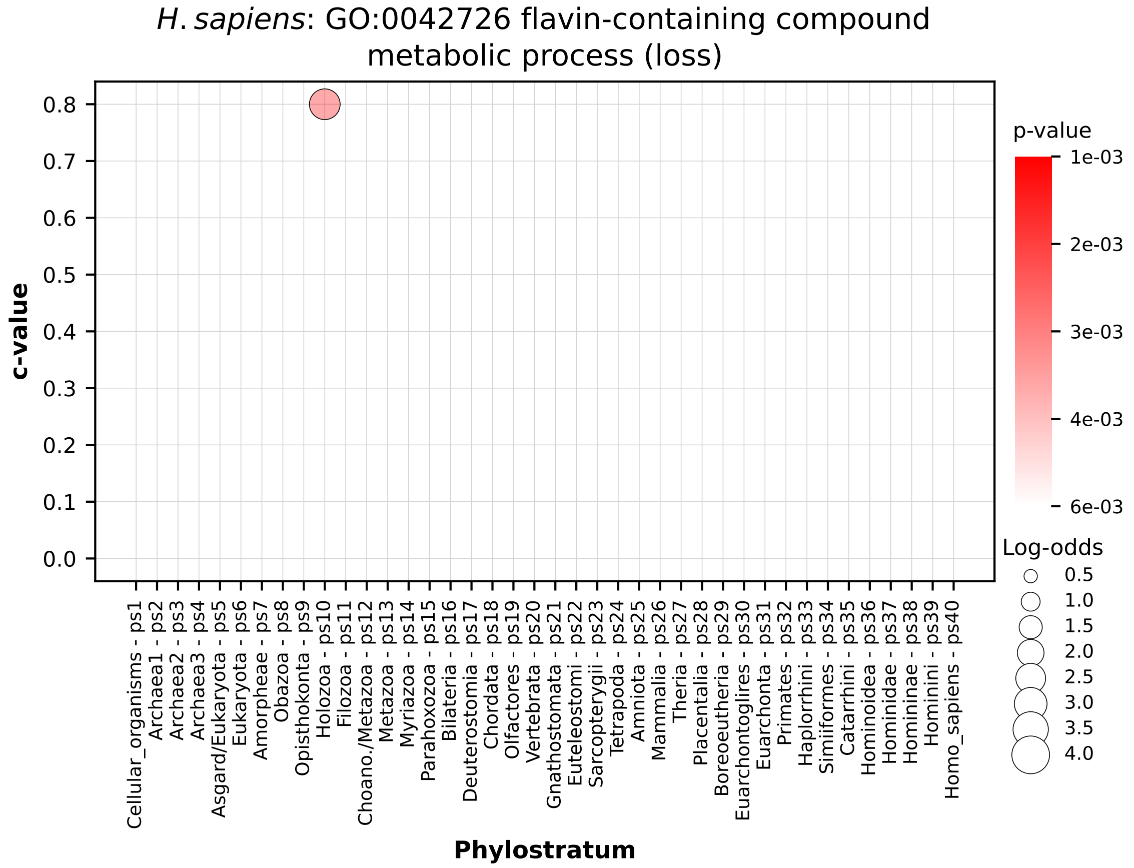
H. sapiens: GO:0042726 flavin-containing compound metabolic process (loss)
c-value
0.0
0.1
0.2
0.3
0.4
0.5
0.6
0.7
0.8
Phylostratum
Cellular_organisms - ps1
Archaea1 - ps2
Archaea2 - ps3
Archaea3 - ps4
Asgard/Eukaryota - ps5
Eukaryota - ps6
Amorpheae - ps7
Obazoa - ps8
Opisthokonta - ps9
Holozoa - ps10
Filozoa - ps11
Choano./Metazoa - ps12
Metazoa - ps13
Myriazoa - ps14
Parahoxozoa - ps15
Bilateria - ps16
Deuterostomia - ps17
Chordata - ps18
Olfactores - ps19
Vertebrata - ps20
Gnathostomata - ps21
Euteleostomi - ps22
Sarcopterygii - ps23
Tetrapoda - ps24
Amniota - ps25
Mammalia - ps26
Theria - ps27
Placentalia - ps28
Boreoeutheria - ps29
Euarchontoglires - ps30
Euarchonta - ps31
Primates - ps32
Haplorrhini - ps33
Simiiformes - ps34
Catarrhini - ps35
Hominoidea - ps36
Hominidae - ps37
Homininae - ps38
Hominini - ps39
Homo_sapiens - ps40
p-value
1e-03
2e-03
3e-03
4e-03
6e-03
Log-odds
0.5
1.0
1.5
2.0
2.5
3.0
3.5
4.0

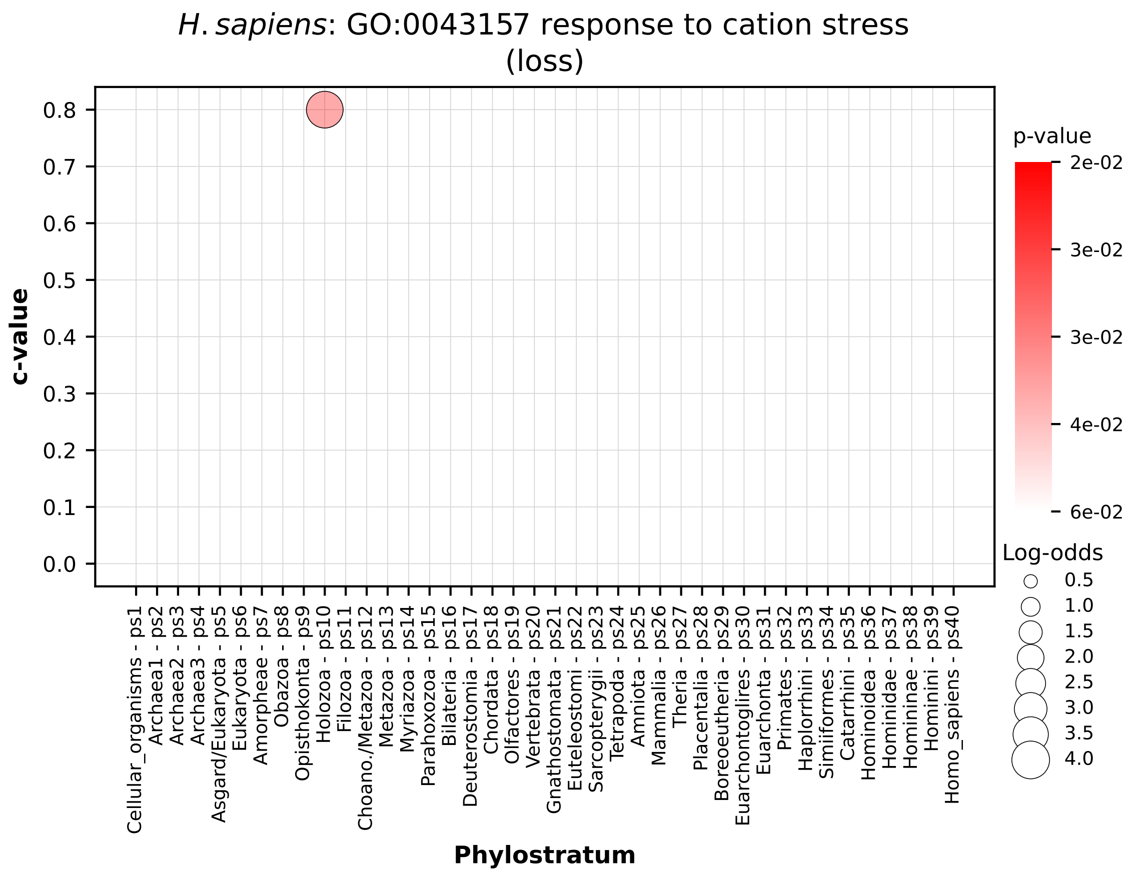

H. sapiens: GO:0043157 response to cation stress
(loss)
c-value
0.8
0.7
0.6
0.5
0.4
0.3
0.2
0.1
0.0
p-value
2e-02
3e-02
3e-02
4e-02
6e-02
Log-odds
0.5
1.0
1.5
2.0
2.5
3.0
3.5
4.0
Cellular_organisms - ps1
Archaea1 - ps2
Archaea2 - ps3
Archaea3 - ps4
Asgard/Eukaryota - ps5
Eukaryota - ps6
Amorpheae - ps7
Obazoa - ps8
Opisthokonta - ps9
Holozoa - ps10
Filozoa - ps11
Choano./Metazoa - ps12
Metazoa - ps13
Myriazoa - ps14
Parahoxozoa - ps15
Bilateria - ps16
Deuterostomia - ps17
Chordata - ps18
Olfactores - ps19
Vertebrata - ps20
Gnathostomata - ps21
Euteleostomi - ps22
Sarcopterygii - ps23
Tetrapoda - ps24
Amniota - ps25
Mammalia - ps26
Theria - ps27
Placentalia - ps28
Boreoeutheria - ps29
Euarchontoglires - ps30
Euarchonta - ps31
Primates - ps32
Haplorrhini - ps33
Simiiformes - ps34
Catarrhini - ps35
Hominoidea - ps36
Hominidae - ps37
Homininae - ps38
Hominini - ps39
Homo_sapiens - ps40
Phylostratum

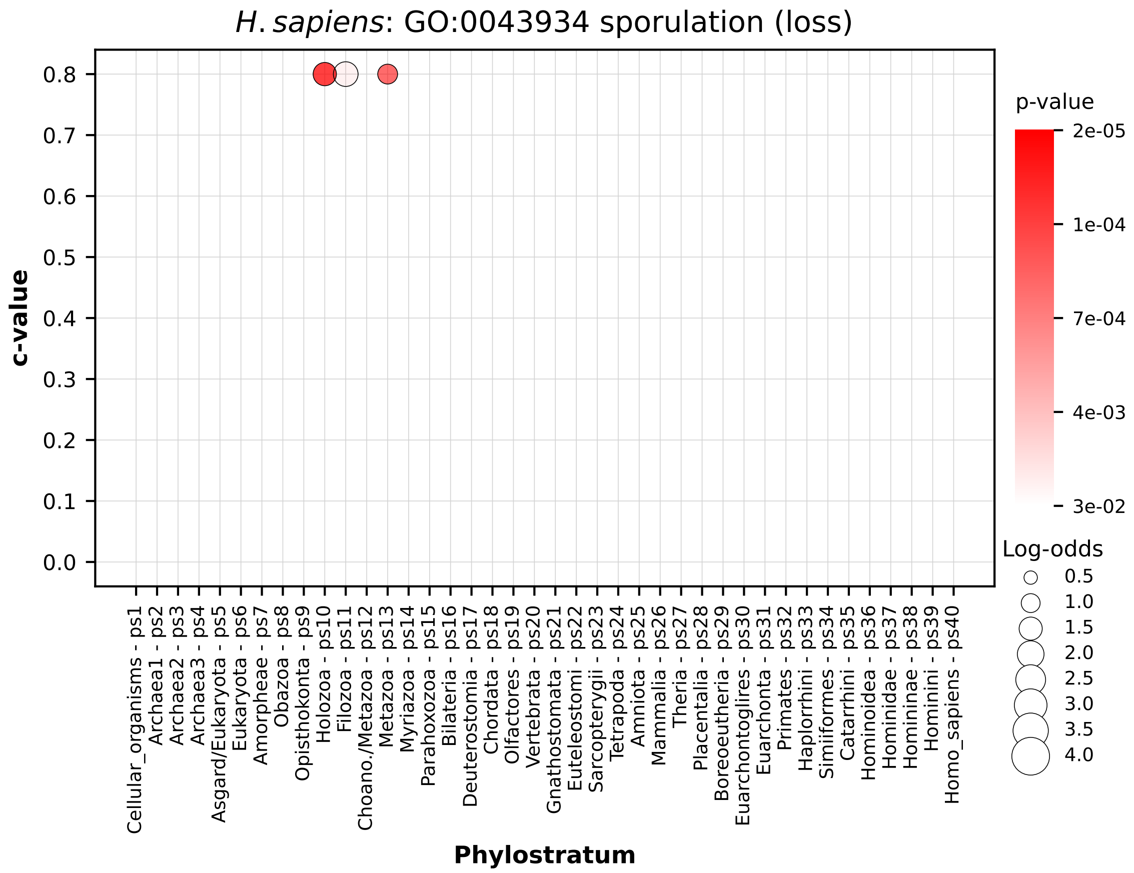
H. sapiens: GO:0043934 sporulation (loss)
c-value
0.0
0.1
0.2
0.3
0.4
0.5
0.6
0.7
0.8
Phylostratum
Cellular_organisms - ps1
Archaea1 - ps2
Archaea2 - ps3
Archaea3 - ps4
Asgard/Eukaryota - ps5
Eukaryota - ps6
Amorpheae - ps7
Obazoa - ps8
Opisthokonta - ps9
Holozoa - ps10
Filozoa - ps11
Choano./Metazoa - ps12
Metazoa - ps13
Myriazoa - ps14
Parahoxozoa - ps15
Bilateria - ps16
Deuterostomia - ps17
Chordata - ps18
Olfactores - ps19
Vertebrata - ps20
Gnathostomata - ps21
Euteleostomi - ps22
Sarcopterygii - ps23
Tetrapoda - ps24
Amniota - ps25
Mammalia - ps26
Theria - ps27
Placentalia - ps28
Boreoeutheria - ps29
Euarchontoglires - ps30
Euarchonta - ps31
Primates - ps32
Haplorrhini - ps33
Simiiformes - ps34
Catarrhini - ps35
Hominoidea - ps36
Hominidae - ps37
Homininae - ps38
Hominini - ps39
Homo_sapiens - ps40
p-value
2e-05
1e-04
7e-04
4e-03
3e-02
Log-odds
0.5
1.0
1.5
2.0
2.5
3.0
3.5
4.0

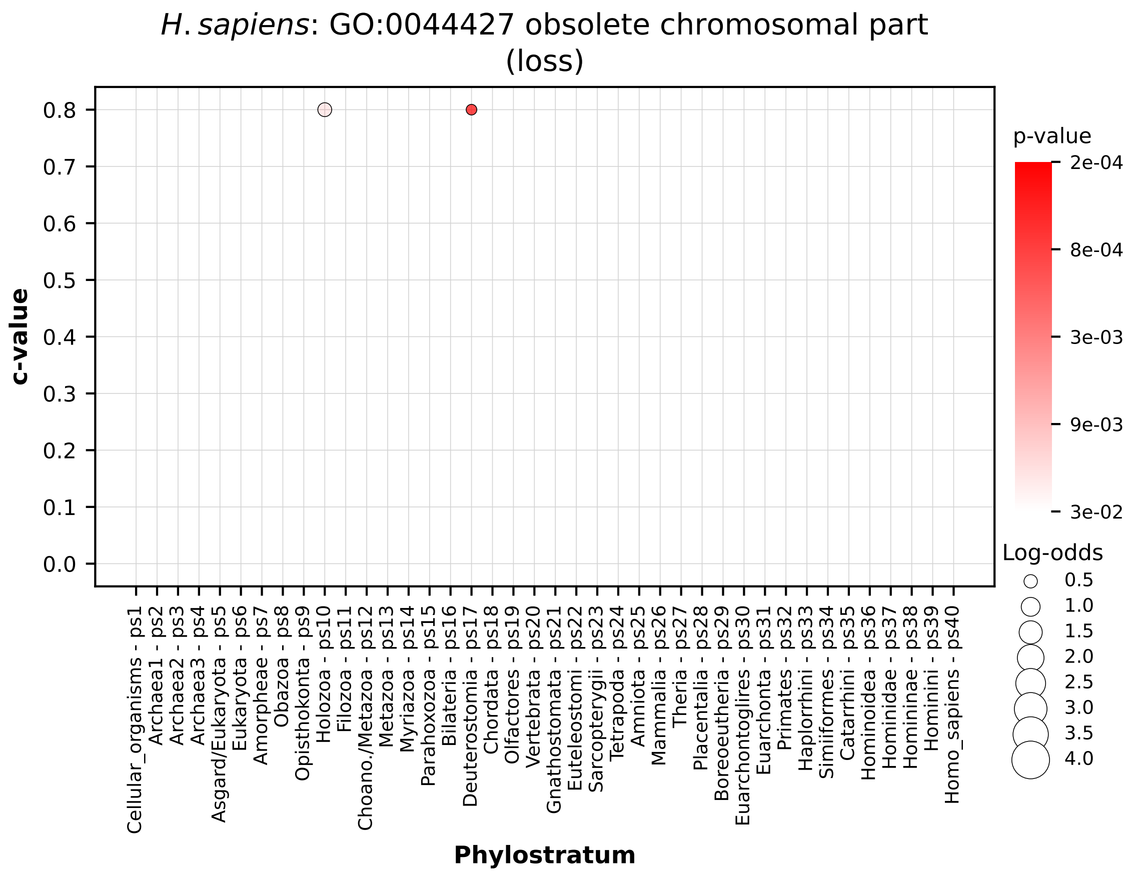

H. sapiens: GO:0044427 obsolete chromosomal part
(loss)
c-value
0.0
0.1
0.2
0.3
0.4
0.5
0.6
0.7
0.8
p-value
2e-04
8e-04
3e-03
9e-03
3e-02
Log-odds
0.5
1.0
1.5
2.0
2.5
3.0
3.5
4.0
Cellular_organisms - ps1
Archaea1 - ps2
Archaea2 - ps3
Archaea3 - ps4
Asgard/Eukaryota - ps5
Eukaryota - ps6
Amorpheae - ps7
Obazoa - ps8
Opisthokonta - ps9
Holozoa - ps10
Filozoa - ps11
Choano./Metazoa - ps12
Metazoa - ps13
Myriazoa - ps14
Parahoxozoa - ps15
Bilateria - ps16
Deuterostomia - ps17
Chordata - ps18
Olfactores - ps19
Vertebrata - ps20
Gnathostomata - ps21
Euteleostomi - ps22
Sarcopterygii - ps23
Tetrapoda - ps24
Amniota - ps25
Mammalia - ps26
Theria - ps27
Placentalia - ps28
Boreoeutheria - ps29
Euarchontoglires - ps30
Euarchonta - ps31
Primates - ps32
Haplorrhini - ps33
Simiiformes - ps34
Catarrhini - ps35
Hominoidea - ps36
Hominidae - ps37
Homininae - ps38
Hominini - ps39
Homo_sapiens - ps40
Phylostratum

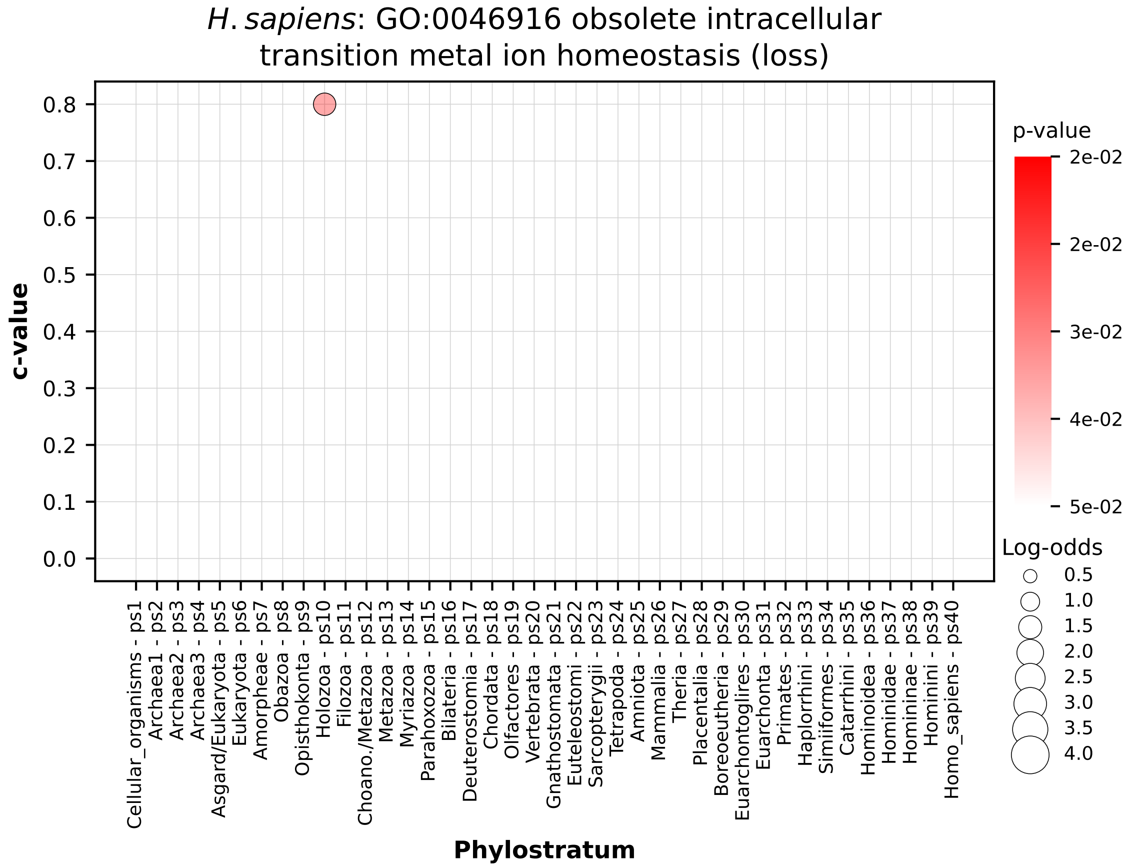

H. sapiens: GO:0046916 obsolete intracellular transition metal ion homeostasis (loss)
c-value
0.0
0.1
0.2
0.3
0.4
0.5
0.6
0.7
0.8
Phylostratum
Cellular_organisms - ps1
Archaea1 - ps2
Archaea2 - ps3
Archaea3 - ps4
Asgard/Eukaryota - ps5
Eukaryota - ps6
Amorpheae - ps7
Obazoa - ps8
Opisthokonta - ps9
Holozoa - ps10
Filozoa - ps11
Choano./Metazoa - ps12
Metazoa - ps13
Myriazoa - ps14
Parahoxozoa - ps15
Bilateria - ps16
Deuterostomia - ps17
Chordata - ps18
Olfactores - ps19
Vertebrata - ps20
Gnathostomata - ps21
Euteleostomi - ps22
Sarcopterygii - ps23
Tetrapoda - ps24
Amniota - ps25
Mammalia - ps26
Theria - ps27
Placentalia - ps28
Boreoeutheria - ps29
Euarchontoglires - ps30
Euarchonta - ps31
Primates - ps32
Haplorrhini - ps33
Simiiformes - ps34
Catarrhini - ps35
Hominoidea - ps36
Hominidae - ps37
Homininae - ps38
Hominini - ps39
Homo_sapiens - ps40
p-value
2e-02
2e-02
3e-02
4e-02
5e-02
Log-odds
0.5
1.0
1.5
2.0
2.5
3.0
3.5
4.0

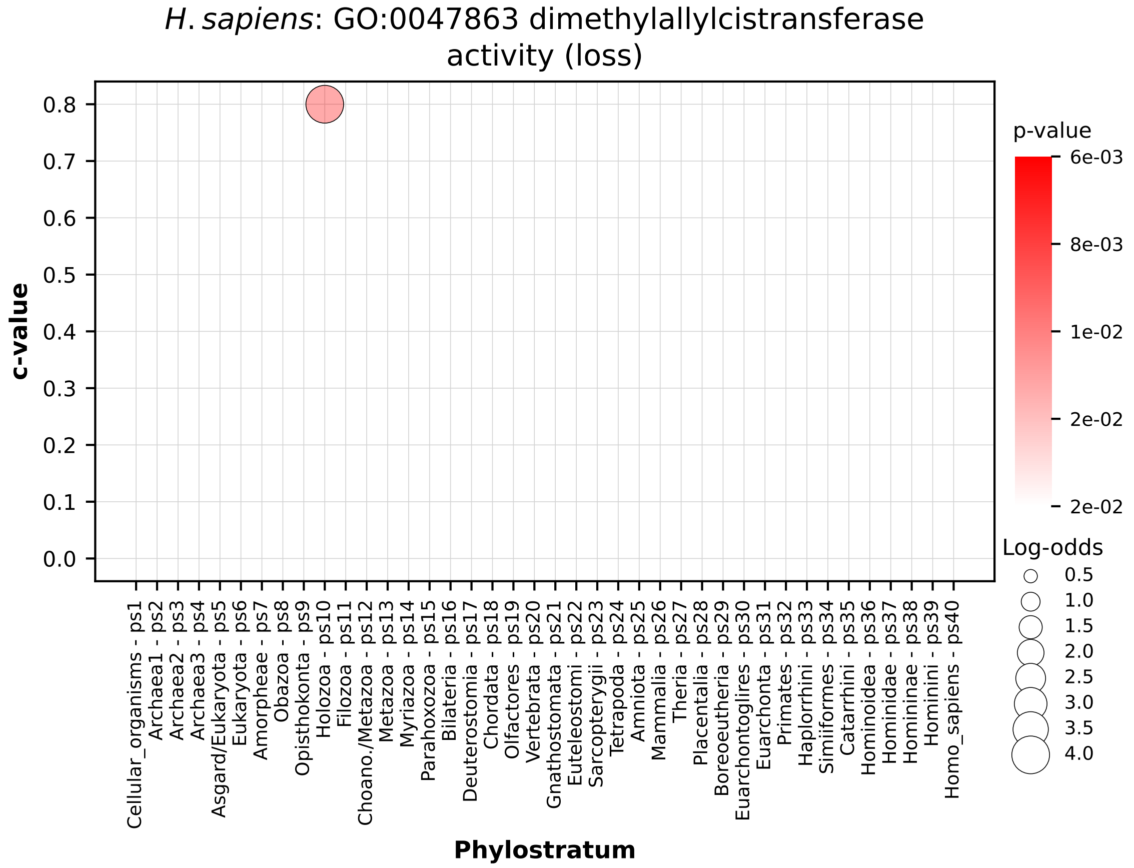

H. sapiens: GO:0047863 dimethylallylcistransferase activity (loss)
c-value
0.8
0.7
0.6
0.5
0.4
0.3
0.2
0.1
0.0
p-value
6e-03
8e-03
1e-02
2e-02
2e-02
Log-odds
0.5
1.0
1.5
2.0
2.5
3.0
3.5
4.0
Cellular_organisms - ps1
Archaea1 - ps2
Archaea2 - ps3
Archaea3 - ps4
Asgard/Eukaryota - ps5
Eukaryota - ps6
Amorpheae - ps7
Obazoa - ps8
Opisthokonta - ps9
Holozoa - ps10
Filozoa - ps11
Choano./Metazoa - ps12
Metazoa - ps13
Myriazoa - ps14
Parahoxozoa - ps15
Bilateria - ps16
Deuterostomia - ps17
Chordata - ps18
Olfactores - ps19
Vertebrata - ps20
Gnathostomata - ps21
Euteleostomi - ps22
Sarcopterygii - ps23
Tetrapoda - ps24
Amniota - ps25
Mammalia - ps26
Theria - ps27
Placentalia - ps28
Boreoeutheria - ps29
Euarchontoglires - ps30
Euarchonta - ps31
Primates - ps32
Haplorrhini - ps33
Simiiformes - ps34
Catarrhini - ps35
Hominoidea - ps36
Hominidae - ps37
Homininae - ps38
Hominini - ps39
Homo_sapiens - ps40
Phylostratum

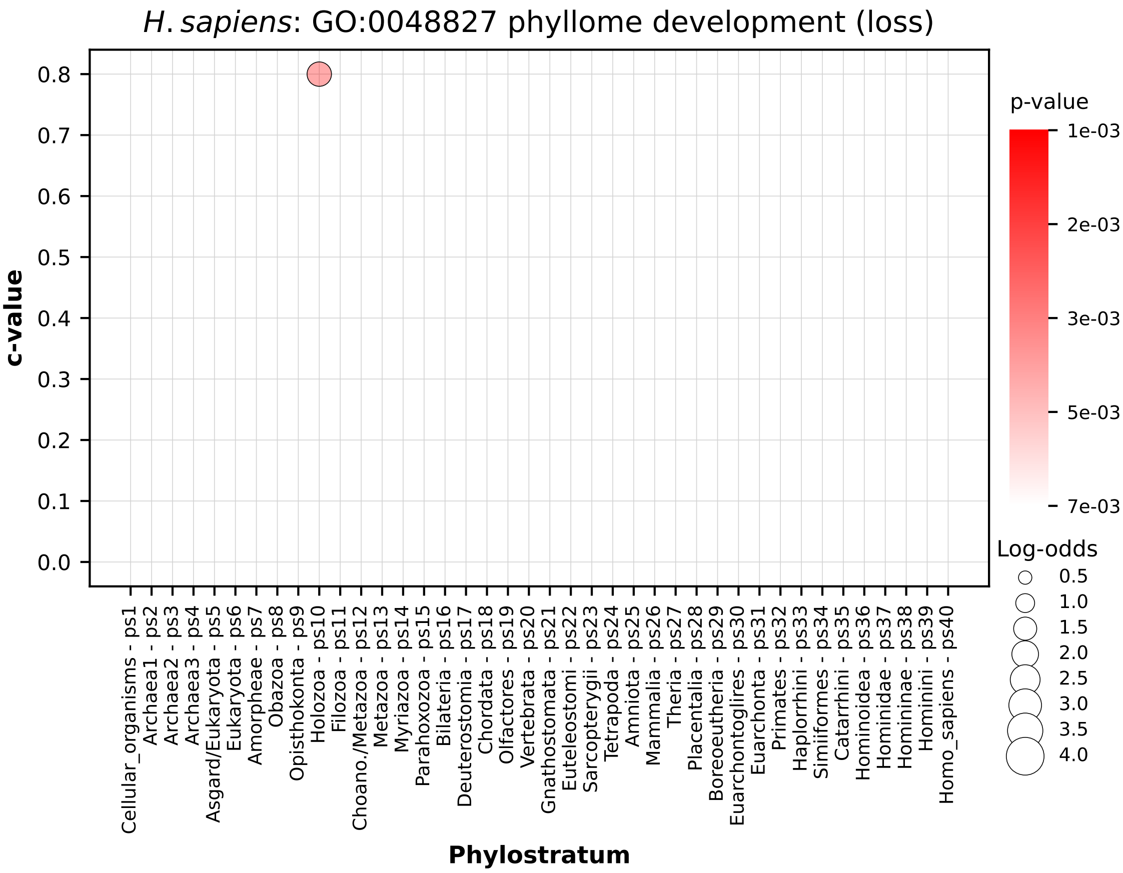
H. sapiens: GO:0048827 phyllome development (loss)
c-value
0.0
0.1
0.2
0.3
0.4
0.5
0.6
0.7
0.8
Phylostratum
Cellular_organisms - ps1
Archaea1 - ps2
Archaea2 - ps3
Archaea3 - ps4
Asgard/Eukaryota - ps5
Eukaryota - ps6
Amorpheae - ps7
Obazoa - ps8
Opisthokonta - ps9
Holozoa - ps10
Filozoa - ps11
Choano./Metazoa - ps12
Metazoa - ps13
Myriazoa - ps14
Parahoxozoa - ps15
Bilateria - ps16
Deuterostomia - ps17
Chordata - ps18
Olfactores - ps19
Vertebrata - ps20
Gnathostomata - ps21
Euteleostomi - ps22
Sarcopterygii - ps23
Tetrapoda - ps24
Amniota - ps25
Mammalia - ps26
Theria - ps27
Placentalia - ps28
Boreoeutheria - ps29
Euarchontoglires - ps30
Euarchonta - ps31
Primates - ps32
Haplorrhini - ps33
Simiiformes - ps34
Catarrhini - ps35
Hominoidea - ps36
Hominidae - ps37
Homininae - ps38
Hominini - ps39
Homo_sapiens - ps40
p-value
1e-03
2e-03
3e-03
5e-03
7e-03
Log-odds
0.5
1.0
1.5
2.0
2.5
3.0
3.5
4.0

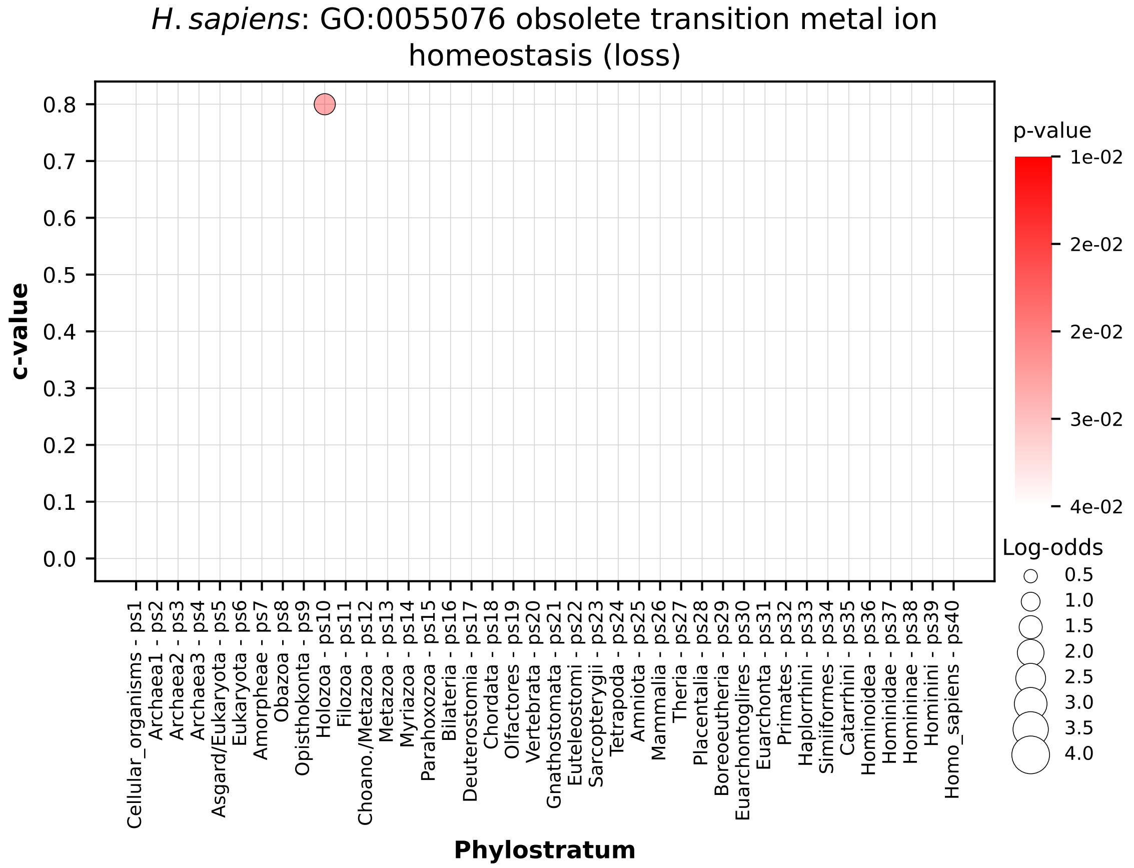
H. sapiens: GO:0055076 obsolete transition metal ion homeostasis (loss)
c-value
0.0
0.1
0.2
0.3
0.4
0.5
0.6
0.7
0.8
p-value
1e-02
2e-02
2e-02
3e-02
4e-02
Log-odds
0.5
1.0
1.5
2.0
2.5
3.0
3.5
4.0
Cellular_organisms - ps1
Archaea1 - ps2
Archaea2 - ps3
Archaea3 - ps4
Asgard/Eukaryota - ps5
Eukaryota - ps6
Amorpheae - ps7
Obazoa - ps8
Opisthokonta - ps9
Holozoa - ps10
Filozoa - ps11
Choano./Metazoa - ps12
Metazoa - ps13
Myriazoa - ps14
Parahoxozoa - ps15
Bilateria - ps16
Deuterostomia - ps17
Chordata - ps18
Olfactores - ps19
Vertebrata - ps20
Gnathostomata - ps21
Euteleostomi - ps22
Sarcopterygii - ps23
Tetrapoda - ps24
Amniota - ps25
Mammalia - ps26
Theria - ps27
Placentalia - ps28
Boreoeutheria - ps29
Euarchontoglires - ps30
Euarchonta - ps31
Primates - ps32
Haplorrhini - ps33
Simiiformes - ps34
Catarrhini - ps35
Hominoidea - ps36
Hominidae - ps37
Homininae - ps38
Hominini - ps39
Homo_sapiens - ps40
Phylostratum

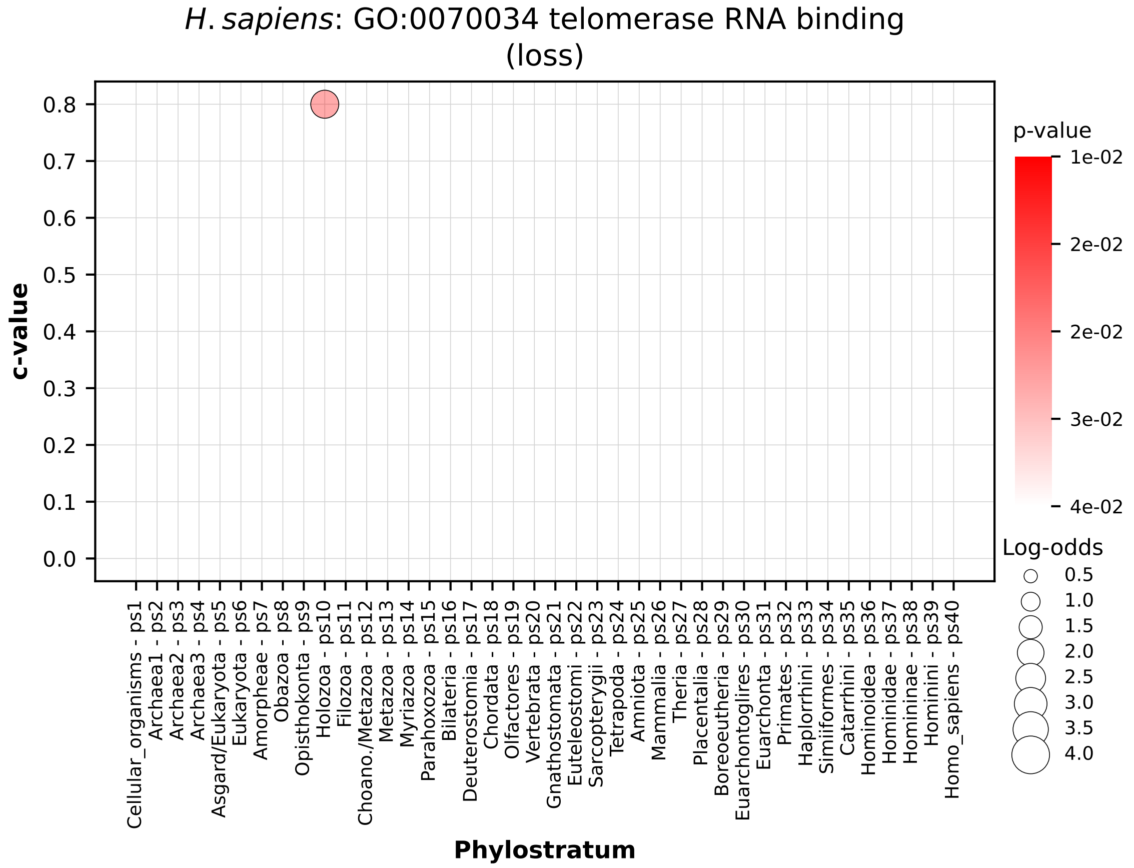

H. sapiens: GO:0070034 telomerase RNA binding
(loss)
c-value
0.8
0.7
0.6
0.5
0.4
0.3
0.2
0.1
0.0
p-value
1e-02
2e-02
2e-02
3e-02
4e-02
Log-odds
0.5
1.0
1.5
2.0
2.5
3.0
3.5
4.0
Cellular_organisms - ps1
Archaea1 - ps2
Archaea2 - ps3
Archaea3 - ps4
Asgard/Eukaryota - ps5
Eukaryota - ps6
Amorpheae - ps7
Obazoa - ps8
Opisthokonta - ps9
Holozoa - ps10
Filozoa - ps11
Choano./Metazoa - ps12
Metazoa - ps13
Myriazoa - ps14
Parahoxozoa - ps15
Bilateria - ps16
Deuterostomia - ps17
Chordata - ps18
Olfactores - ps19
Vertebrata - ps20
Gnathostomata - ps21
Euteleostomi - ps22
Sarcopterygii - ps23
Tetrapoda - ps24
Amniota - ps25
Mammalia - ps26
Theria - ps27
Placentalia - ps28
Boreoeutheria - ps29
Euarchontoglires - ps30
Euarchonta - ps31
Primates - ps32
Haplorrhini - ps33
Simiiformes - ps34
Catarrhini - ps35
Hominoidea - ps36
Hominidae - ps37
Homininae - ps38
Hominini - ps39
Homo_sapiens - ps40
Phylostratum

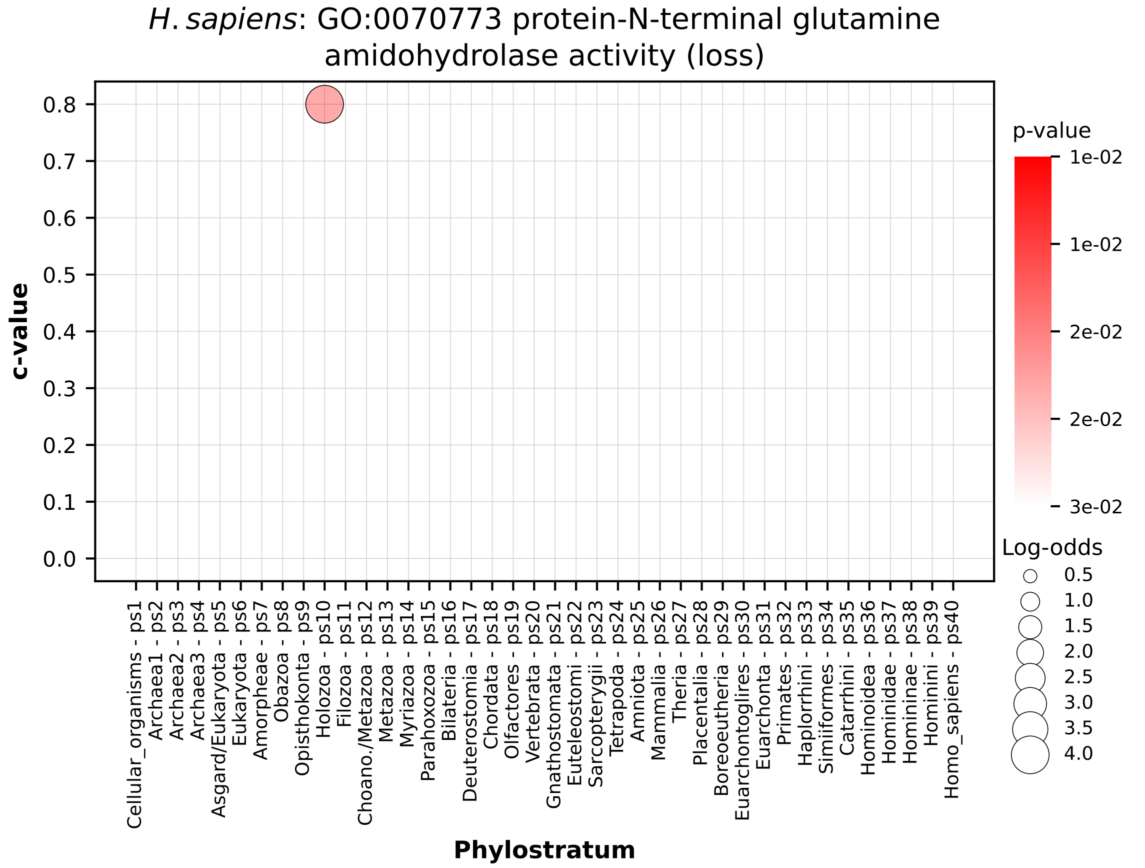
H. sapiens: GO:0070773 protein-N-terminal glutamine amidohydrolase activity (loss)
c-value
0.0
0.1
0.2
0.3
0.4
0.5
0.6
0.7
0.8
Phylostratum
Cellular_organisms - ps1
Archaea1 - ps2
Archaea2 - ps3
Archaea3 - ps4
Asgard/Eukaryota - ps5
Eukaryota - ps6
Amorpheae - ps7
Obazoa - ps8
Opisthokonta - ps9
Holozoa - ps10
Filozoa - ps11
Choano./Metazoa - ps12
Metazoa - ps13
Myriazoa - ps14
Parahoxozoa - ps15
Bilateria - ps16
Deuterostomia - ps17
Chordata - ps18
Olfactores - ps19
Vertebrata - ps20
Gnathostomata - ps21
Euteleostomi - ps22
Sarcopterygii - ps23
Tetrapoda - ps24
Amniota - ps25
Mammalia - ps26
Theria - ps27
Placentalia - ps28
Boreoeutheria - ps29
Euarchontoglires - ps30
Euarchonta - ps31
Primates - ps32
Haplorrhini - ps33
Simiiformes - ps34
Catarrhini - ps35
Hominoidea - ps36
Hominidae - ps37
Homininae - ps38
Hominini - ps39
Homo_sapiens - ps40
p-value
1e-02
1e-02
2e-02
2e-02
3e-02
Log-odds
0.5
1.0
1.5
2.0
2.5
3.0
3.5
4.0

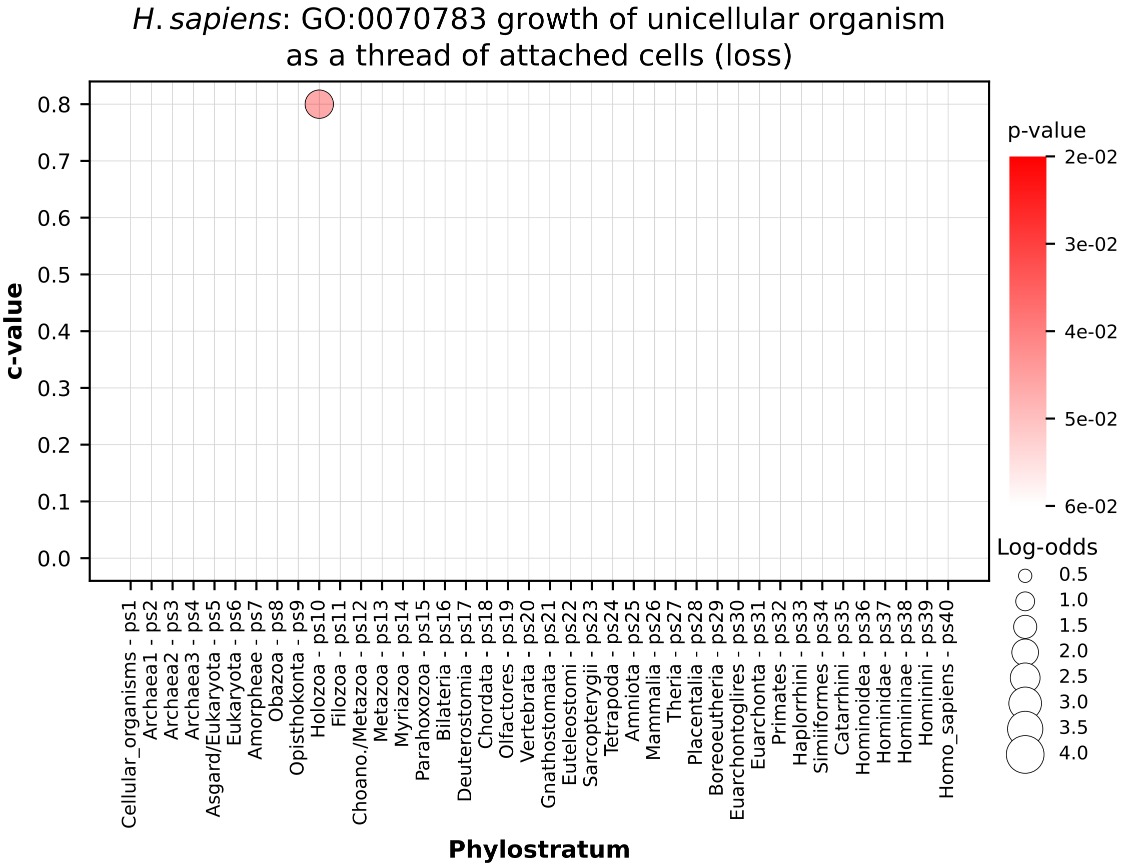

H. sapiens: GO:0070783 growth of unicellular organism as a thread of attached cells (loss)
c-value
0.0
0.1
0.2
0.3
0.4
0.5
0.6
0.7
0.8
Phylostratum
Cellular_organisms - ps1
Archaea1 - ps2
Archaea2 - ps3
Archaea3 - ps4
Asgard/Eukaryota - ps5
Eukaryota - ps6
Amorpheae - ps7
Obazoa - ps8
Opisthokonta - ps9
Holozoa - ps10
Filozoa - ps11
Choano./Metazoa - ps12
Metazoa - ps13
Myriazoa - ps14
Parahoxozoa - ps15
Bilateria - ps16
Deuterostomia - ps17
Chordata - ps18
Olfactores - ps19
Vertebrata - ps20
Gnathostomata - ps21
Euteleostomi - ps22
Sarcopterygii - ps23
Tetrapoda - ps24
Amniota - ps25
Mammalia - ps26
Theria - ps27
Placentalia - ps28
Boreoeutheria - ps29
Euarchontoglires - ps30
Euarchonta - ps31
Primates - ps32
Haplorrhini - ps33
Simiiformes - ps34
Catarrhini - ps35
Hominoidea - ps36
Hominidae - ps37
Homininae - ps38
Hominini - ps39
Homo_sapiens - ps40
p-value
2e-02
3e-02
4e-02
5e-02
6e-02
Log-odds
0.5
1.0
1.5
2.0
2.5
3.0
3.5
4.0

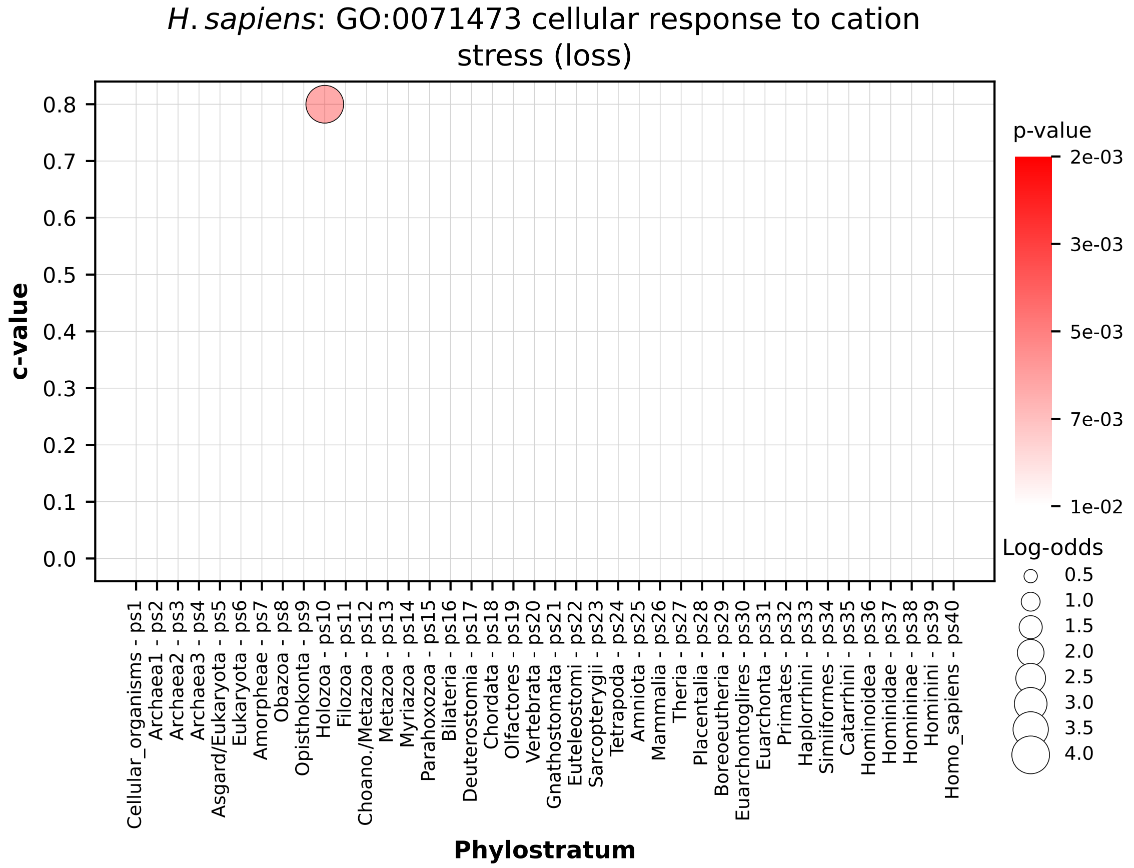

H. sapiens: GO:0071473 cellular response to cation stress (loss)
c-value
0.8
0.7
0.6
0.5
0.4
0.3
0.2
0.1
0.0
Phylostratum
Cellular_organisms - ps1
Archaea1 - ps2
Archaea2 - ps3
Archaea3 - ps4
Asgard/Eukaryota - ps5
Eukaryota - ps6
Amorpheae - ps7
Obazoa - ps8
Opisthokonta - ps9
Holozoa - ps10
Filozoa - ps11
Choano./Metazoa - ps12
Metazoa - ps13
Myriazoa - ps14
Parahoxozoa - ps15
Bilateria - ps16
Deuterostomia - ps17
Chordata - ps18
Olfactores - ps19
Vertebrata - ps20
Gnathostomata - ps21
Euteleostomi - ps22
Sarcopterygii - ps23
Tetrapoda - ps24
Amniota - ps25
Mammalia - ps26
Theria - ps27
Placentalia - ps28
Boreoeutheria - ps29
Euarchontoglires - ps30
Euarchonta - ps31
Primates - ps32
Haplorrhini - ps33
Simiiformes - ps34
Catarrhini - ps35
Hominoidea - ps36
Hominidae - ps37
Homininae - ps38
Hominini - ps39
Homo_sapiens - ps40
p-value
2e-03
3e-03
5e-03
7e-03
1e-02
Log-odds
0.5
1.0
1.5
2.0
2.5
3.0
3.5
4.0

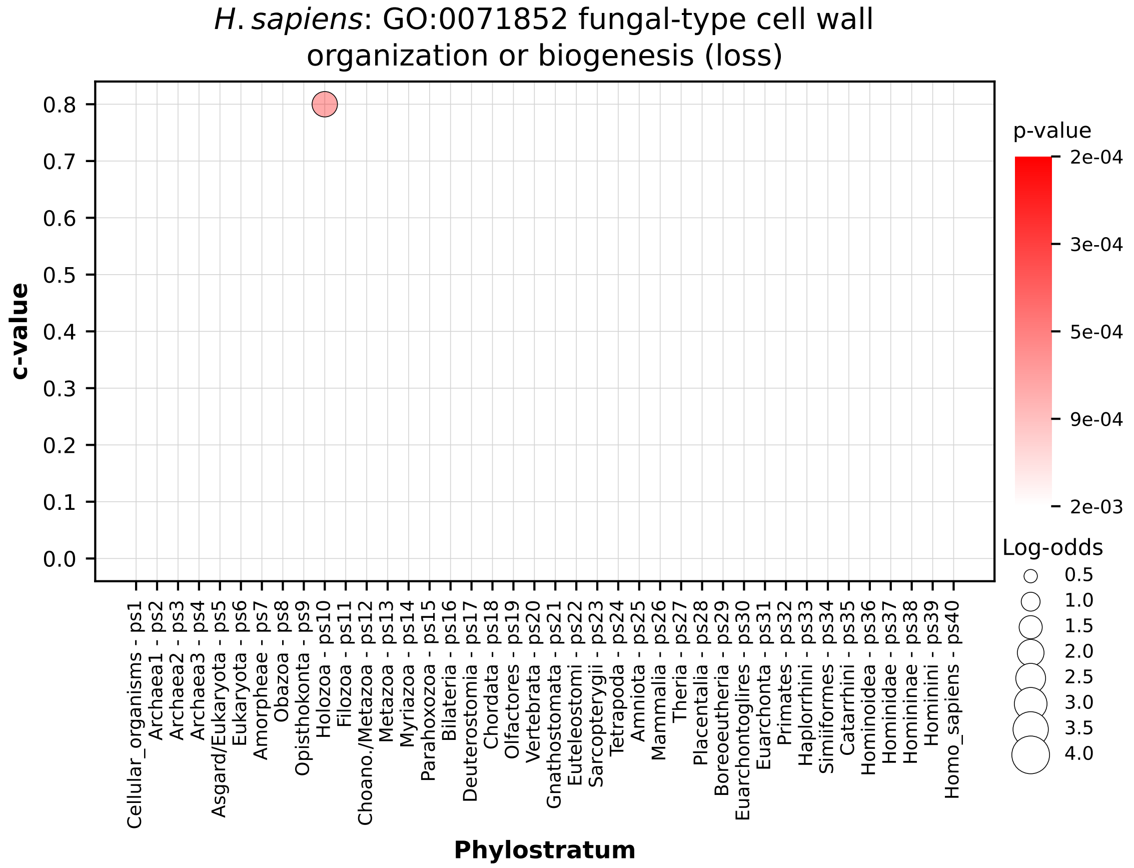

$H. sapiens$: GO:0071852 fungal-type cell wall organization or biogenesis (loss)
c-value
0.0
0.1
0.2
0.3
0.4
0.5
0.6
0.7
0.8
Phylostratum
Cellular_organisms - ps1
Archaea1 - ps2
Archaea2 - ps3
Archaea3 - ps4
Asgard/Eukaryota - ps5
Eukaryota - ps6
Amorpheae - ps7
Obazoa - ps8
Opisthokonta - ps9
Holozoa - ps10
Filozoa - ps11
Choano./Metazoa - ps12
Metazoa - ps13
Myriazoa - ps14
Parahoxozoa - ps15
Bilateria - ps16
Deuterostomia - ps17
Chordata - ps18
Olfactores - ps19
Vertebrata - ps20
Gnathostomata - ps21
Euteleostomi - ps22
Sarcopterygii - ps23
Tetrapoda - ps24
Amniota - ps25
Mammalia - ps26
Theria - ps27
Placentalia - ps28
Boreoeutheria - ps29
Euarchontoglires - ps30
Euarchonta - ps31
Primates - ps32
Haplorrhini - ps33
Simiiformes - ps34
Catarrhini - ps35
Hominoidea - ps36
Hominidae - ps37
Homininae - ps38
Hominini - ps39
Homo_sapiens - ps40
p-value
2e-04
3e-04
5e-04
9e-04
2e-03
Log-odds
0.5
1.0
1.5
2.0
2.5
3.0
3.5
4.0

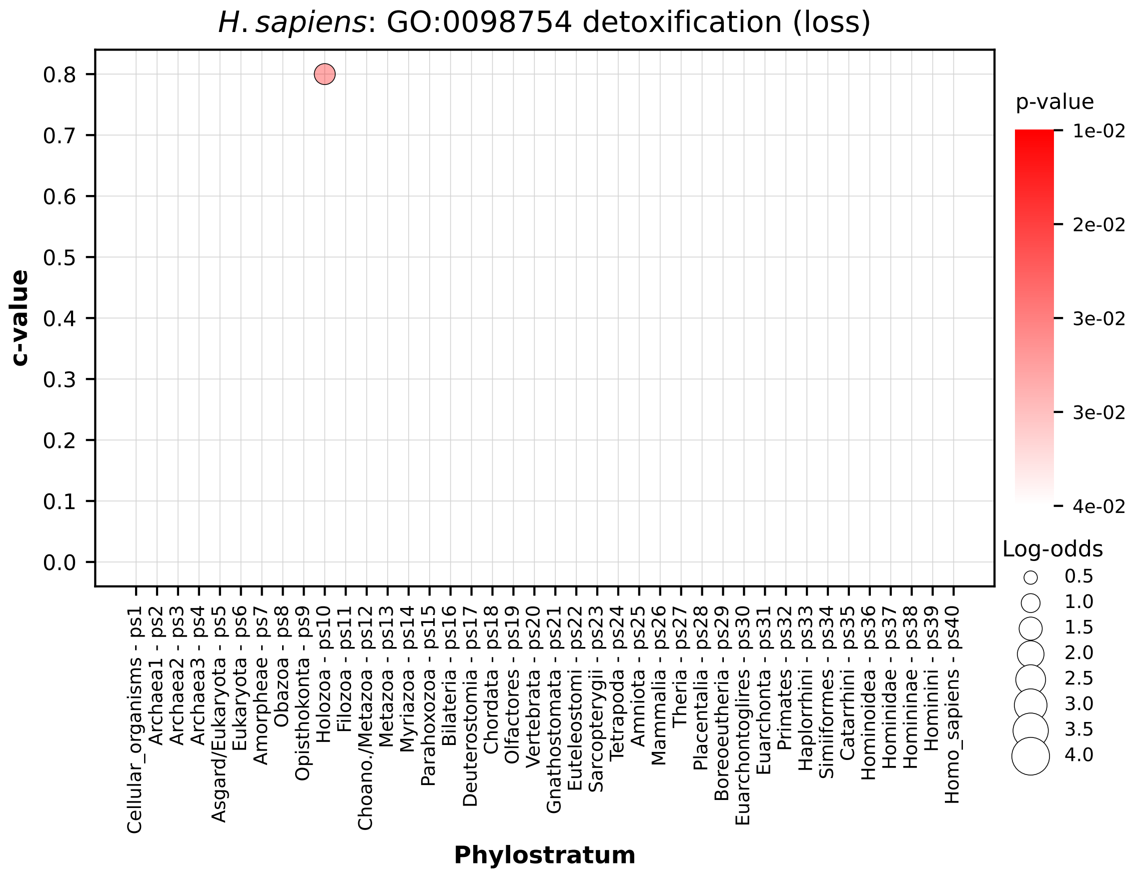

H. sapiens: GO:0098754 detoxification (loss)
c-value
0.0
0.1
0.2
0.3
0.4
0.5
0.6
0.7
0.8
Phylostratum
Cellular_organisms - ps1
Archaea1 - ps2
Archaea2 - ps3
Archaea3 - ps4
Asgard/Eukaryota - ps5
Eukaryota - ps6
Amorpheae - ps7
Obazoa - ps8
Opisthokonta - ps9
Holozoa - ps10
Filozoa - ps11
Choano./Metazoa - ps12
Metazoa - ps13
Myriazoa - ps14
Parahoxozoa - ps15
Bilateria - ps16
Deuterostomia - ps17
Chordata - ps18
Olfactores - ps19
Vertebrata - ps20
Gnathostomata - ps21
Euteleostomi - ps22
Sarcopterygii - ps23
Tetrapoda - ps24
Amniota - ps25
Mammalia - ps26
Theria - ps27
Placentalia - ps28
Boreoeutheria - ps29
Euarchontoglires - ps30
Euarchonta - ps31
Primates - ps32
Haplorrhini - ps33
Simiiformes - ps34
Catarrhini - ps35
Hominoidea - ps36
Hominidae - ps37
Homininae - ps38
Hominini - ps39
Homo_sapiens - ps40
p-value
1e-02
2e-02
3e-02
3e-02
4e-02
Log-odds
0.5
1.0
1.5
2.0
2.5
3.0
3.5
4.0

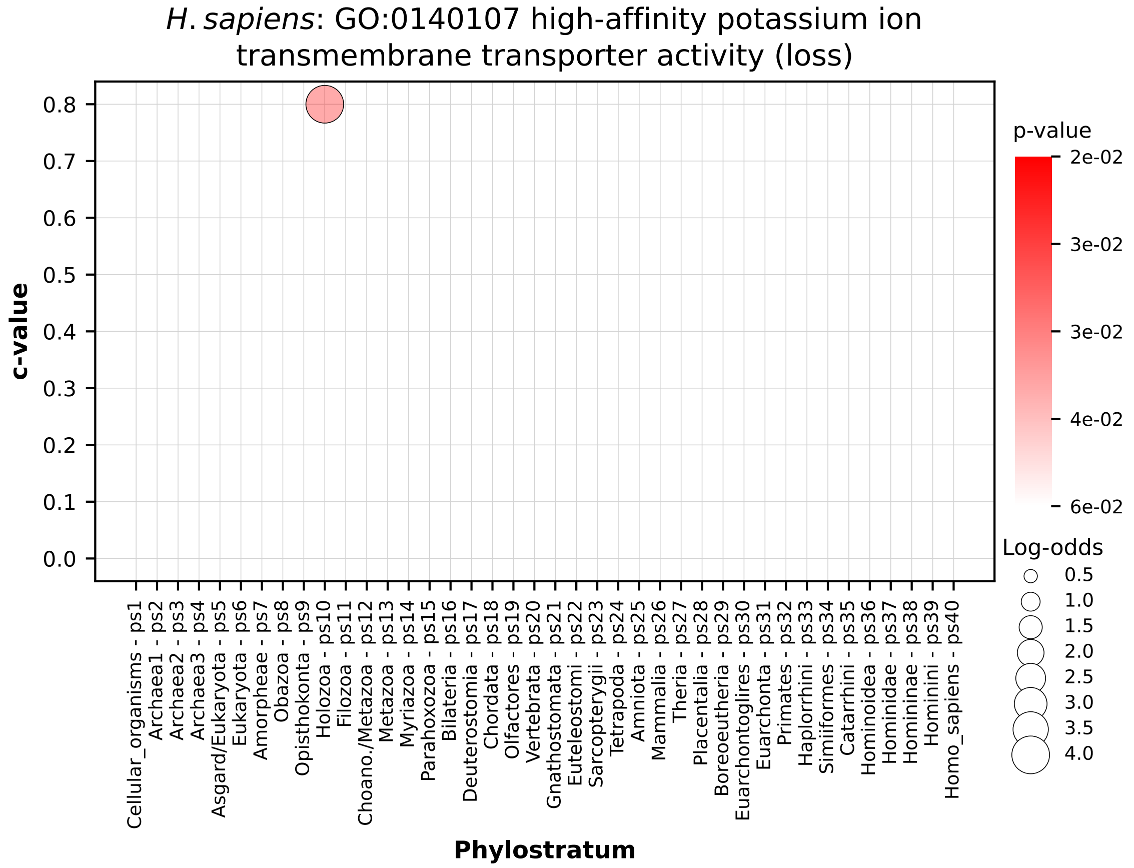

H. sapiens: GO:0140107 high-affinity potassium ion transmembrane transporter activity (loss)
c-value
0.0
0.1
0.2
0.3
0.4
0.5
0.6
0.7
0.8
p-value
2e-02
3e-02
3e-02
4e-02
6e-02
Log-odds
0.5
1.0
1.5
2.0
2.5
3.0
3.5
4.0
Cellular_organisms - ps1
Archaea1 - ps2
Archaea2 - ps3
Archaea3 - ps4
Asgard/Eukaryota - ps5
Eukaryota - ps6
Amorpheae - ps7
Obazoa - ps8
Opisthokonta - ps9
Holozoa - ps10
Filozoa - ps11
Choano./Metazoa - ps12
Metazoa - ps13
Myriazoa - ps14
Parahoxozoa - ps15
Bilateria - ps16
Deuterostomia - ps17
Chordata - ps18
Olfactores - ps19
Vertebrata - ps20
Gnathostomata - ps21
Euteleostomi - ps22
Sarcopterygii - ps23
Tetrapoda - ps24
Amniota - ps25
Mammalia - ps26
Theria - ps27
Placentalia - ps28
Boreoeutheria - ps29
Euarchontoglires - ps30
Euarchonta - ps31
Primates - ps32
Haplorrhini - ps33
Simiiformes - ps34
Catarrhini - ps35
Hominoidea - ps36
Hominidae - ps37
Homininae - ps38
Hominini - ps39
Homo_sapiens - ps40
Phylostratum

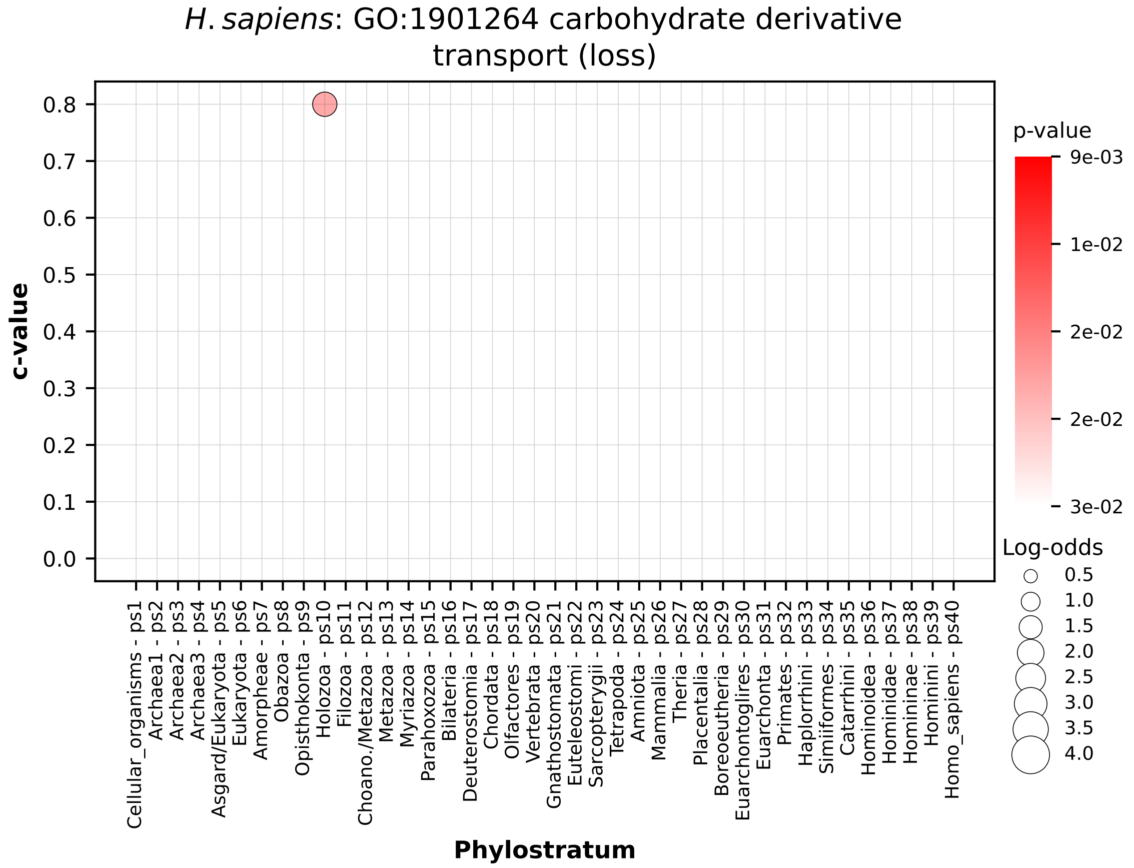

H. sapiens: GO:1901264 carbohydrate derivative transport (loss)
c-value
0.0
0.1
0.2
0.3
0.4
0.5
0.6
0.7
0.8
p-value
9e-03
1e-02
2e-02
2e-02
3e-02
Log-odds
0.5
1.0
1.5
2.0
2.5
3.0
3.5
4.0
Cellular_organisms - ps1
Archaea1 - ps2
Archaea2 - ps3
Archaea3 - ps4
Asgard/Eukaryota - ps5
Eukaryota - ps6
Amorpheae - ps7
Obazoa - ps8
Opisthokonta - ps9
Holozoa - ps10
Filozoa - ps11
Choano./Metazoa - ps12
Metazoa - ps13
Myriazoa - ps14
Parahoxozoa - ps15
Bilateria - ps16
Deuterostomia - ps17
Chordata - ps18
Olfactores - ps19
Vertebrata - ps20
Gnathostomata - ps21
Euteleostomi - ps22
Sarcopterygii - ps23
Tetrapoda - ps24
Amniota - ps25
Mammalia - ps26
Theria - ps27
Placentalia - ps28
Boreoeutheria - ps29
Euarchontoglires - ps30
Euarchonta - ps31
Primates - ps32
Haplorrhini - ps33
Simiiformes - ps34
Catarrhini - ps35
Hominoidea - ps36
Hominidae - ps37
Homininae - ps38
Hominini - ps39
Homo_sapiens - ps40
Phylostratum

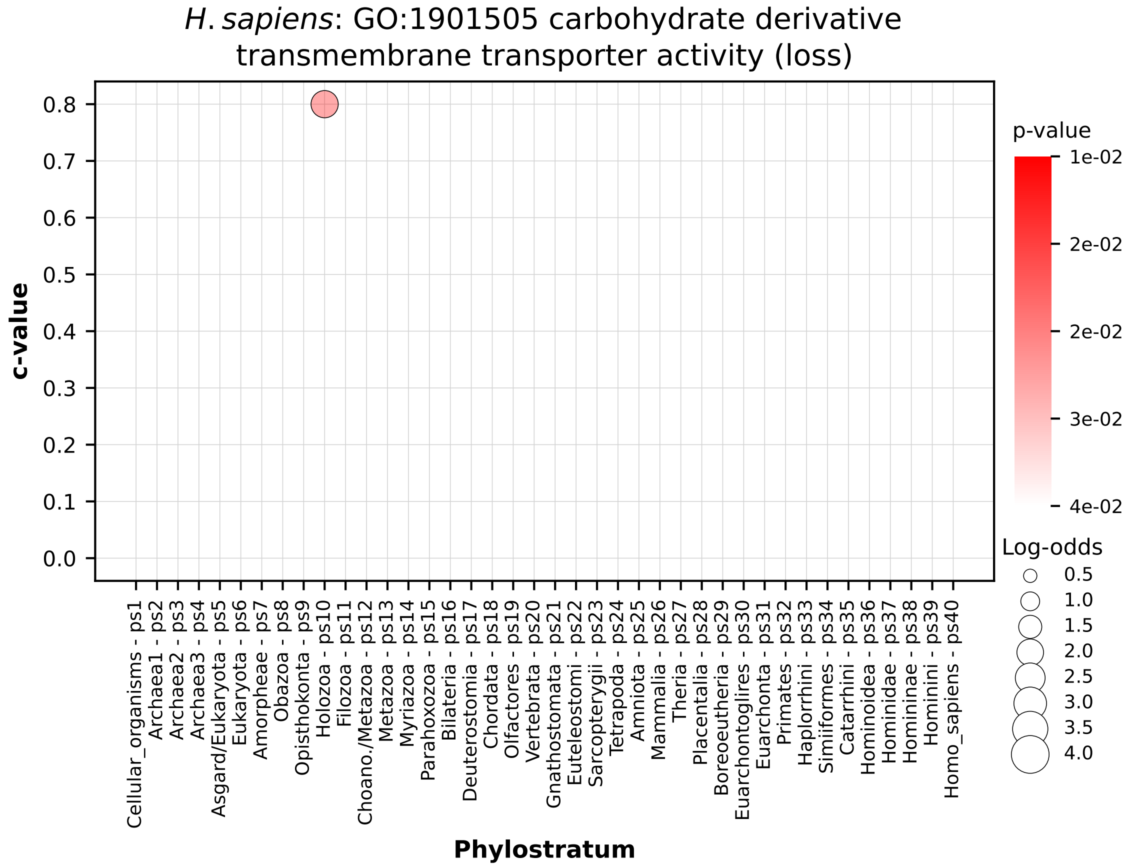
H. sapiens: GO:1901505 carbohydrate derivative transmembrane transporter activity (loss)
c-value
0.0
0.1
0.2
0.3
0.4
0.5
0.6
0.7
0.8
Phylostratum
Cellular_organisms - ps1
Archaea1 - ps2
Archaea2 - ps3
Archaea3 - ps4
Asgard/Eukaryota - ps5
Eukaryota - ps6
Amorpheae - ps7
Obazoa - ps8
Opisthokonta - ps9
Holozoa - ps10
Filozoa - ps11
Choano./Metazoa - ps12
Metazoa - ps13
Myriazoa - ps14
Parahoxozoa - ps15
Bilateria - ps16
Deuterostomia - ps17
Chordata - ps18
Olfactores - ps19
Vertebrata - ps20
Gnathostomata - ps21
Euteleostomi - ps22
Sarcopterygii - ps23
Tetrapoda - ps24
Amniota - ps25
Mammalia - ps26
Theria - ps27
Placentalia - ps28
Boreoeutheria - ps29
Euarchontoglires - ps30
Euarchonta - ps31
Primates - ps32
Haplorrhini - ps33
Simiiformes - ps34
Catarrhini - ps35
Hominoidea - ps36
Hominidae - ps37
Homininae - ps38
Hominini - ps39
Homo_sapiens - ps40
p-value
1e-02
2e-02
2e-02
3e-02
4e-02
Log-odds
0.5
1.0
1.5
2.0
2.5
3.0
3.5
4.0

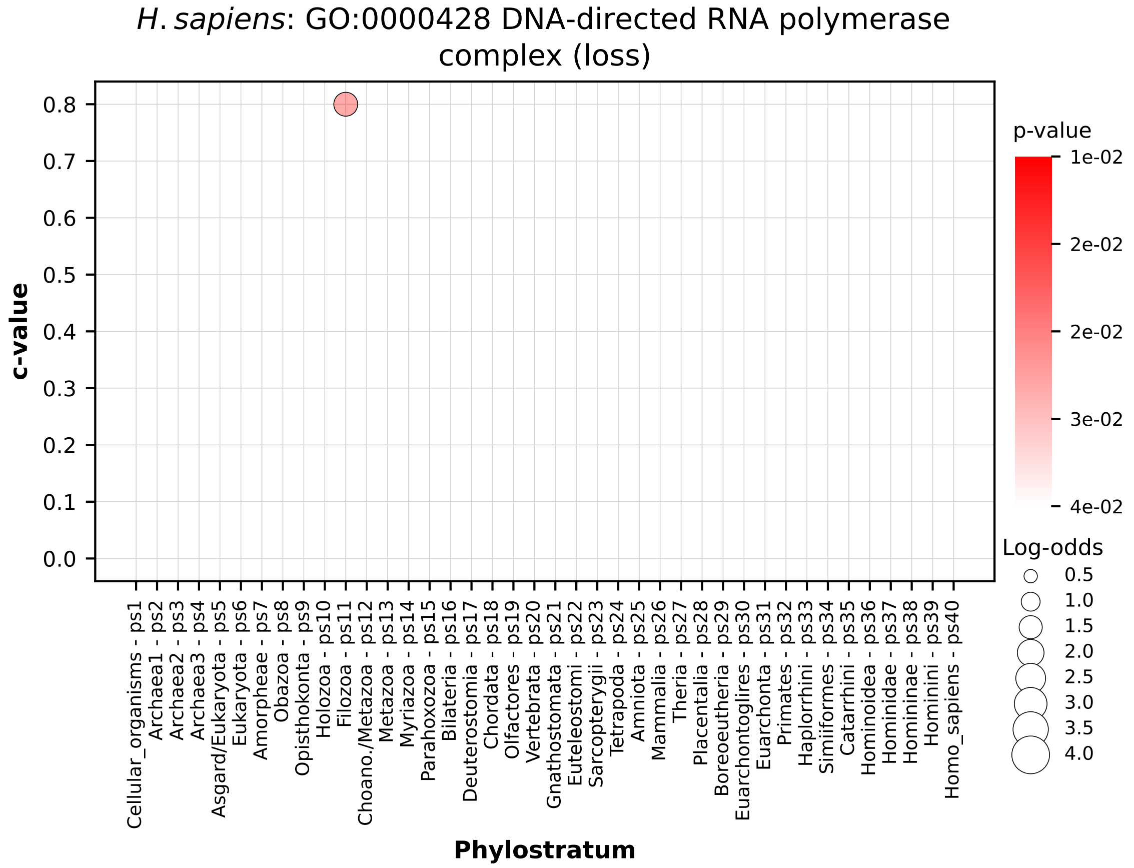

H. sapiens: GO:0000428 DNA-directed RNA polymerase complex (loss)
c-value
0.0
0.1
0.2
0.3
0.4
0.5
0.6
0.7
0.8
p-value
1e-02
2e-02
2e-02
3e-02
4e-02
Log-odds
0.5
1.0
1.5
2.0
2.5
3.0
3.5
4.0
Cellular_organisms - ps1
Archaea1 - ps2
Archaea2 - ps3
Archaea3 - ps4
Asgard/Eukaryota - ps5
Eukaryota - ps6
Amorpheae - ps7
Obazoa - ps8
Opisthokonta - ps9
Holozoa - ps10
Filozoa - ps11
Choano./Metazoa - ps12
Metazoa - ps13
Myriazoa - ps14
Parahoxozoa - ps15
Bilateria - ps16
Deuterostomia - ps17
Chordata - ps18
Olfactores - ps19
Vertebrata - ps20
Gnathostomata - ps21
Euteleostomi - ps22
Sarcopterygii - ps23
Tetrapoda - ps24
Amniota - ps25
Mammalia - ps26
Theria - ps27
Placentalia - ps28
Boreoeutheria - ps29
Euarchontoglires - ps30
Euarchonta - ps31
Primates - ps32
Haplorrhini - ps33
Simiiformes - ps34
Catarrhini - ps35
Hominoidea - ps36
Hominidae - ps37
Homininae - ps38
Hominini - ps39
Homo_sapiens - ps40
Phylostratum

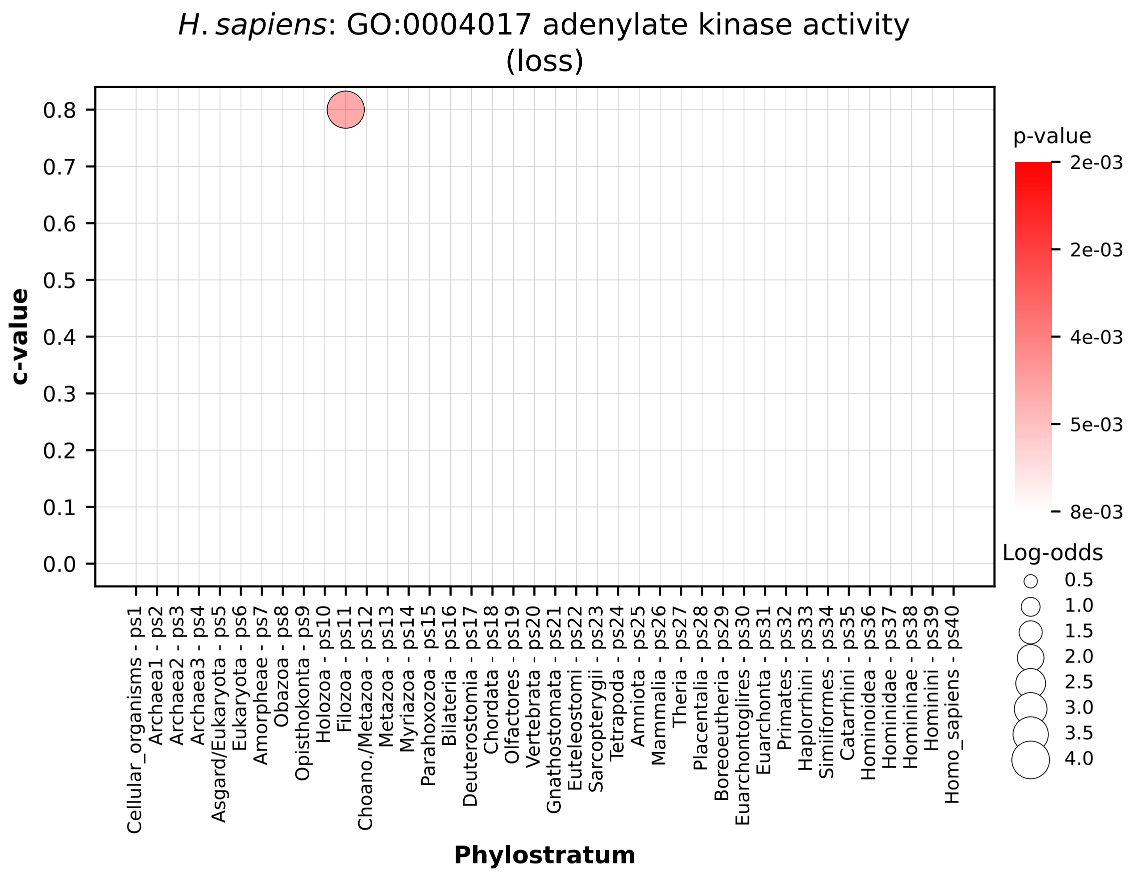
H. sapiens: GO:0004017 adenylate kinase activity
(loss)
c-value
0.8
0.7
0.6
0.5
0.4
0.3
0.2
0.1
0.0
p-value
2e-03
2e-03
4e-03
5e-03
8e-03
Log-odds
0.5
1.0
1.5
2.0
2.5
3.0
3.5
4.0
Cellular_organisms - ps1
Archaea1 - ps2
Archaea2 - ps3
Archaea3 - ps4
Asgard/Eukaryota - ps5
Eukaryota - ps6
Amorpheae - ps7
Obazoa - ps8
Opisthokonta - ps9
Holozoa - ps10
Filozoa - ps11
Choano./Metazoa - ps12
Metazoa - ps13
Myriazoa - ps14
Parahoxozoa - ps15
Bilateria - ps16
Deuterostomia - ps17
Chordata - ps18
Olfactores - ps19
Vertebrata - ps20
Gnathostomata - ps21
Euteleostomi - ps22
Sarcopterygii - ps23
Tetrapoda - ps24
Amniota - ps25
Mammalia - ps26
Theria - ps27
Placentalia - ps28
Boreoeutheria - ps29
Euarchontoglires - ps30
Euarchonta - ps31
Primates - ps32
Haplorrhini - ps33
Simiiformes - ps34
Catarrhini - ps35
Hominoidea - ps36
Hominidae - ps37
Homininae - ps38
Hominini - ps39
Homo_sapiens - ps40
Phylostratum

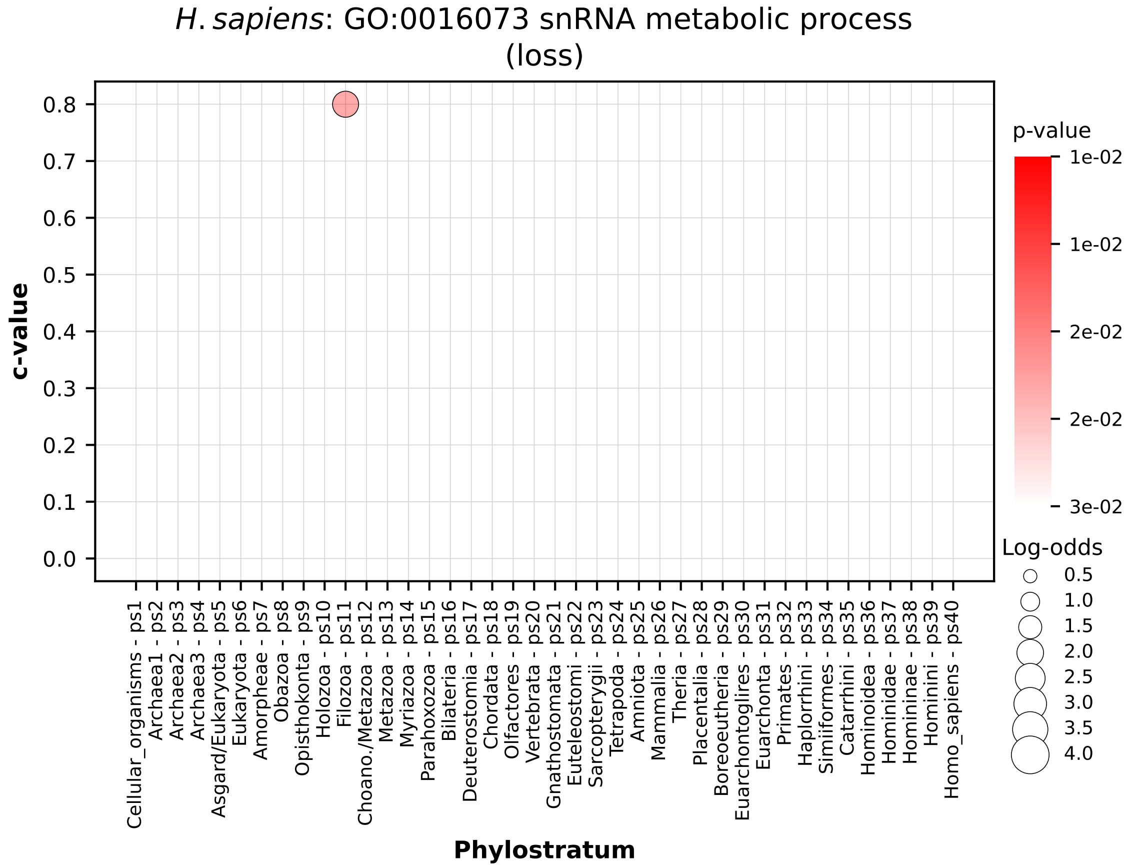

H. sapiens: GO:0016073 snRNA metabolic process
(loss)
c-value
0.8
0.7
0.6
0.5
0.4
0.3
0.2
0.1
0.0
p-value
1e-02
1e-02
2e-02
2e-02
3e-02
Log-odds
0.5
1.0
1.5
2.0
2.5
3.0
3.5
4.0
Cellular_organisms - ps1
Archaea1 - ps2
Archaea2 - ps3
Archaea3 - ps4
Asgard/Eukaryota - ps5
Eukaryota - ps6
Amorpheae - ps7
Obazoa - ps8
Opisthokonta - ps9
Holozoa - ps10
Filozoa - ps11
Choano./Metazoa - ps12
Metazoa - ps13
Myriazoa - ps14
Parahoxozoa - ps15
Bilateria - ps16
Deuterostomia - ps17
Chordata - ps18
Olfactores - ps19
Vertebrata - ps20
Gnathostomata - ps21
Euteleostomi - ps22
Sarcopterygii - ps23
Tetrapoda - ps24
Amniota - ps25
Mammalia - ps26
Theria - ps27
Placentalia - ps28
Boreoeutheria - ps29
Euarchontoglires - ps30
Euarchonta - ps31
Primates - ps32
Haplorrhini - ps33
Simiiformes - ps34
Catarrhini - ps35
Hominoidea - ps36
Hominidae - ps37
Homininae - ps38
Hominini - ps39
Homo_sapiens - ps40
Phylostratum

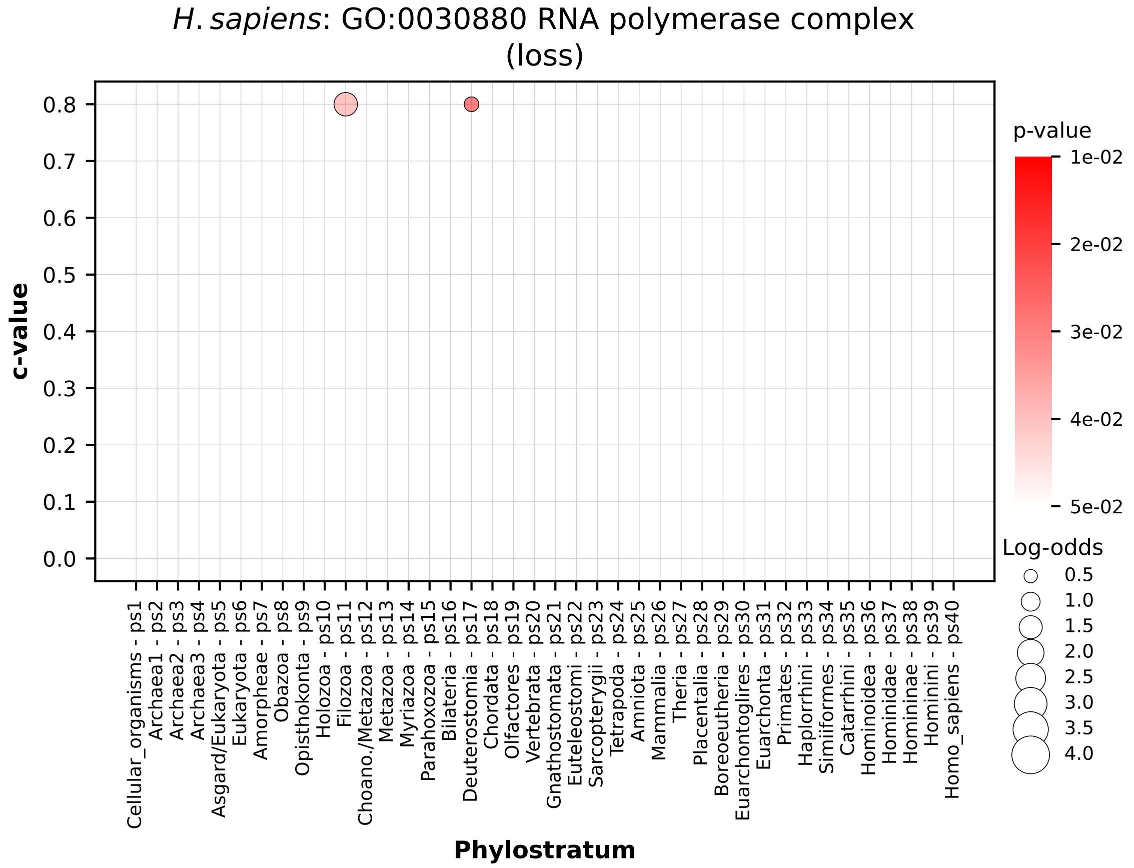

H. sapiens: GO:0030880 RNA polymerase complex
(loss)
c-value
0.0
0.1
0.2
0.3
0.4
0.5
0.6
0.7
0.8
p-value
1e-02
2e-02
3e-02
4e-02
5e-02
Log-odds
0.5
1.0
1.5
2.0
2.5
3.0
3.5
4.0
Cellular_organisms - ps1
Archaea1 - ps2
Archaea2 - ps3
Archaea3 - ps4
Asgard/Eukaryota - ps5
Eukaryota - ps6
Amorpheae - ps7
Obazoa - ps8
Opisthokonta - ps9
Holozoa - ps10
Filozoa - ps11
Choano./Metazoa - ps12
Metazoa - ps13
Myriazoa - ps14
Parahoxozoa - ps15
Bilateria - ps16
Deuterostomia - ps17
Chordata - ps18
Olfactores - ps19
Vertebrata - ps20
Gnathostomata - ps21
Euteleostomi - ps22
Sarcopterygii - ps23
Tetrapoda - ps24
Amniota - ps25
Mammalia - ps26
Theria - ps27
Placentalia - ps28
Boreoeutheria - ps29
Euarchontoglires - ps30
Euarchonta - ps31
Primates - ps32
Haplorrhini - ps33
Simiiformes - ps34
Catarrhini - ps35
Hominoidea - ps36
Hominidae - ps37
Homininae - ps38
Hominini - ps39
Homo_sapiens - ps40
Phylostratum

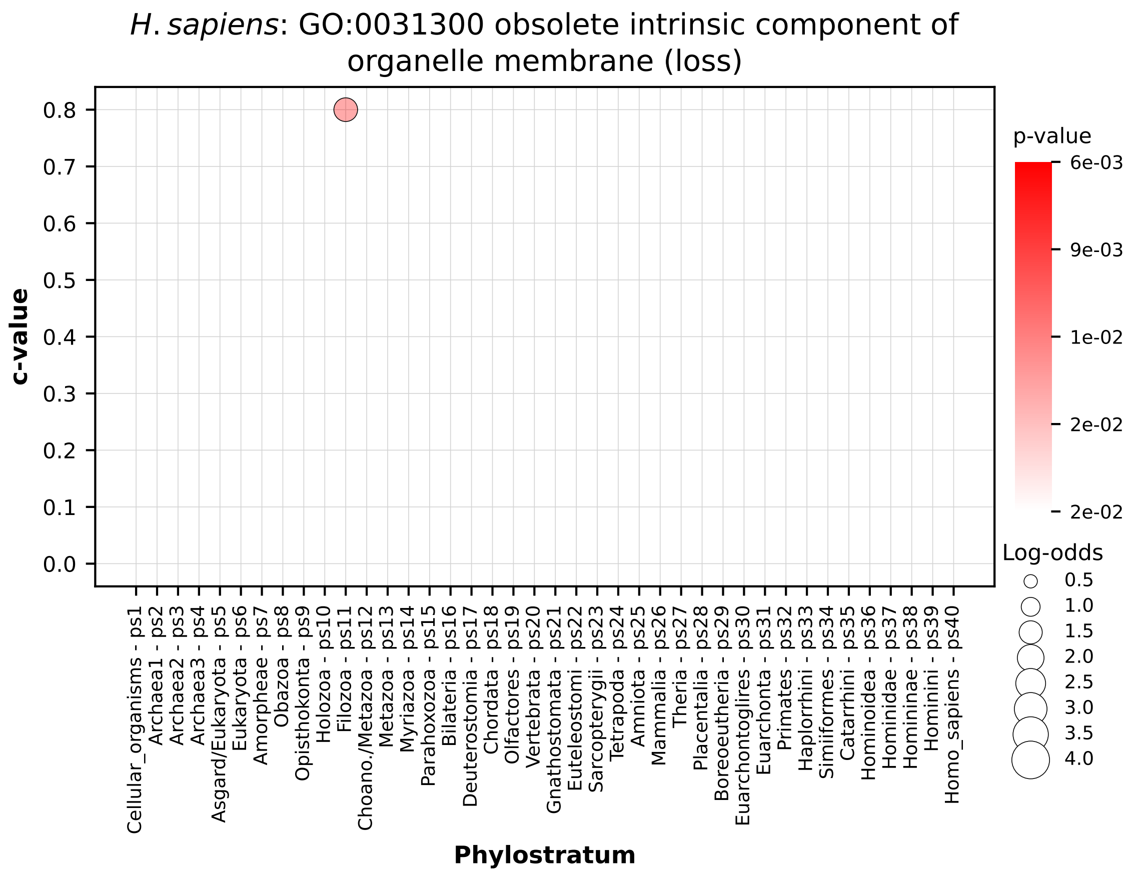

H. sapiens: GO:0031300 obsolete intrinsic component of organelle membrane (loss)
c-value
0.8
0.7
0.6
0.5
0.4
0.3
0.2
0.1
0.0
p-value
6e-03
9e-03
1e-02
2e-02
2e-02
Log-odds
0.5
1.0
1.5
2.0
2.5
3.0
3.5
4.0
Cellular_organisms - ps1
Archaea1 - ps2
Archaea2 - ps3
Archaea3 - ps4
Asgard/Eukaryota - ps5
Eukaryota - ps6
Amorpheae - ps7
Obazoa - ps8
Opisthokonta - ps9
Holozoa - ps10
Filozoa - ps11
Choano./Metazoa - ps12
Metazoa - ps13
Myriazoa - ps14
Parahoxozoa - ps15
Bilateria - ps16
Deuterostomia - ps17
Chordata - ps18
Olfactores - ps19
Vertebrata - ps20
Gnathostomata - ps21
Euteleostomi - ps22
Sarcopterygii - ps23
Tetrapoda - ps24
Amniota - ps25
Mammalia - ps26
Theria - ps27
Placentalia - ps28
Boreoeutheria - ps29
Euarchontoglires - ps30
Euarchonta - ps31
Primates - ps32
Haplorrhini - ps33
Simiiformes - ps34
Catarrhini - ps35
Hominoidea - ps36
Hominidae - ps37
Homininae - ps38
Hominini - ps39
Homo_sapiens - ps40
Phylostratum

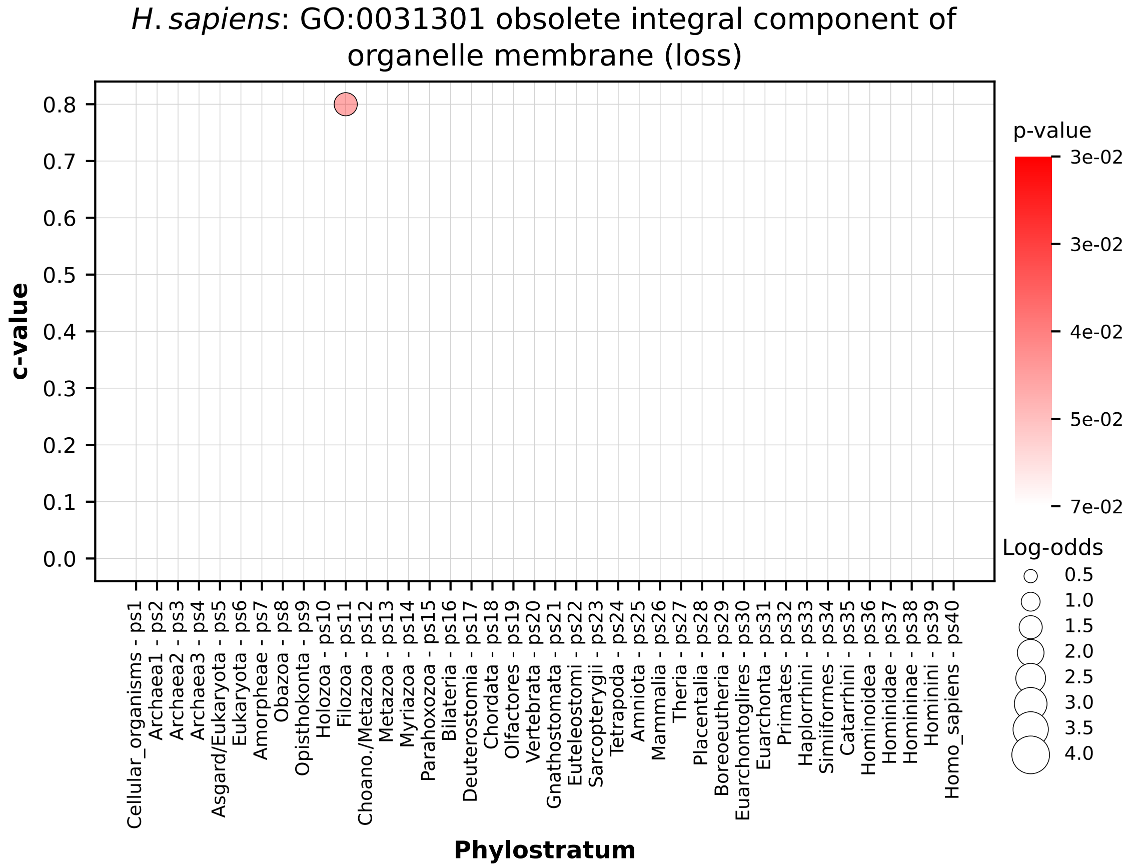

H. sapiens: GO:0031301 obsolete integral component of organelle membrane (loss)
c-value
0.0
0.1
0.2
0.3
0.4
0.5
0.6
0.7
0.8
Phylostratum
Cellular_organisms - ps1
Archaea1 - ps2
Archaea2 - ps3
Archaea3 - ps4
Asgard/Eukaryota - ps5
Eukaryota - ps6
Amorpheae - ps7
Obazoa - ps8
Opisthokonta - ps9
Holozoa - ps10
Filozoa - ps11
Choano./Metazoa - ps12
Metazoa - ps13
Myriazoa - ps14
Parahoxozoa - ps15
Bilateria - ps16
Deuterostomia - ps17
Chordata - ps18
Olfactores - ps19
Vertebrata - ps20
Gnathostomata - ps21
Euteleostomi - ps22
Sarcopterygii - ps23
Tetrapoda - ps24
Amniota - ps25
Mammalia - ps26
Theria - ps27
Placentalia - ps28
Boreoeutheria - ps29
Euarchontoglires - ps30
Euarchonta - ps31
Primates - ps32
Haplorrhini - ps33
Simiiformes - ps34
Catarrhini - ps35
Hominoidea - ps36
Hominidae - ps37
Homininae - ps38
Hominini - ps39
Homo_sapiens - ps40
p-value
3e-02
3e-02
4e-02
5e-02
7e-02
Log-odds
0.5
1.0
1.5
2.0
2.5
3.0
3.5
4.0

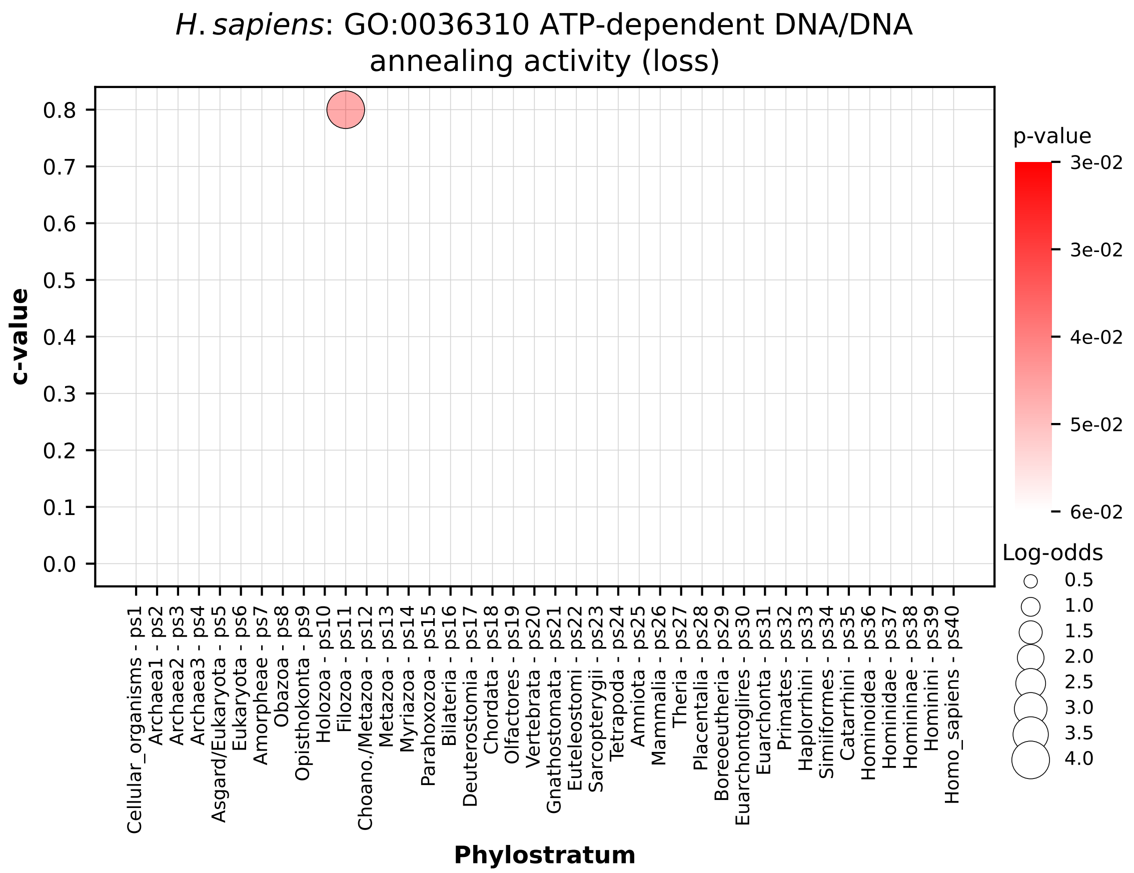

H. sapiens: GO:0036310 ATP-dependent DNA/DNA annealing activity (loss)
c-value
0.0
0.1
0.2
0.3
0.4
0.5
0.6
0.7
0.8
Phylostratum
Cellular_organisms - ps1
Archaea1 - ps2
Archaea2 - ps3
Archaea3 - ps4
Asgard/Eukaryota - ps5
Eukaryota - ps6
Amorpheae - ps7
Obazoa - ps8
Opisthokonta - ps9
Holozoa - ps10
Filozoa - ps11
Choano./Metazoa - ps12
Metazoa - ps13
Myriazoa - ps14
Parahoxozoa - ps15
Bilateria - ps16
Deuterostomia - ps17
Chordata - ps18
Olfactores - ps19
Vertebrata - ps20
Gnathostomata - ps21
Euteleostomi - ps22
Sarcopterygii - ps23
Tetrapoda - ps24
Amniota - ps25
Mammalia - ps26
Theria - ps27
Placentalia - ps28
Boreoeutheria - ps29
Euarchontoglires - ps30
Euarchonta - ps31
Primates - ps32
Haplorrhini - ps33
Simiiformes - ps34
Catarrhini - ps35
Hominoidea - ps36
Hominidae - ps37
Homininae - ps38
Hominini - ps39
Homo_sapiens - ps40
p-value
3e-02
3e-02
4e-02
5e-02
6e-02
Log-odds
0.5
1.0
1.5
2.0
2.5
3.0
3.5
4.0

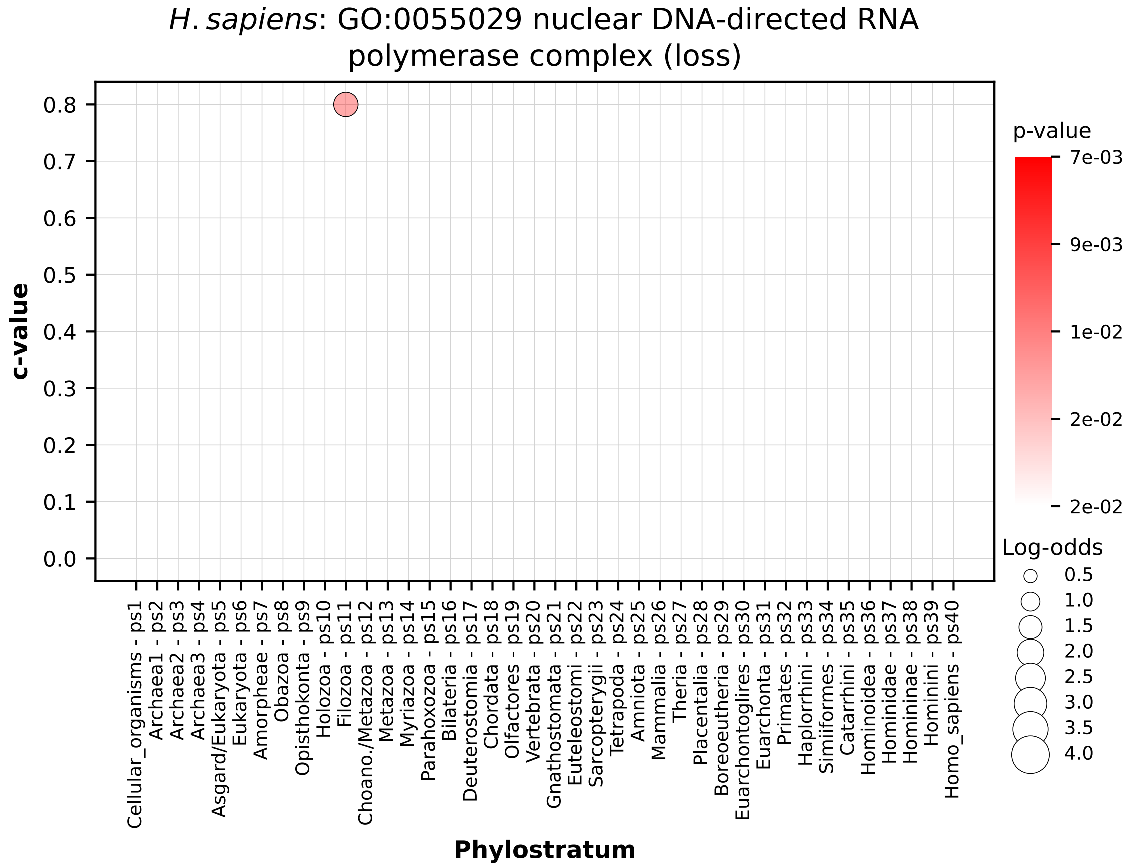

H. sapiens: GO:0055029 nuclear DNA-directed RNA polymerase complex (loss)
c-value
0.0
0.1
0.2
0.3
0.4
0.5
0.6
0.7
0.8
p-value
7e-03
9e-03
1e-02
2e-02
2e-02
Log-odds
0.5
1.0
1.5
2.0
2.5
3.0
3.5
4.0
Cellular_organisms - ps1
Archaea1 - ps2
Archaea2 - ps3
Archaea3 - ps4
Asgard/Eukaryota - ps5
Eukaryota - ps6
Amorpheae - ps7
Obazoa - ps8
Opisthokonta - ps9
Holozoa - ps10
Filozoa - ps11
Choano./Metazoa - ps12
Metazoa - ps13
Myriazoa - ps14
Parahoxozoa - ps15
Bilateria - ps16
Deuterostomia - ps17
Chordata - ps18
Olfactores - ps19
Vertebrata - ps20
Gnathostomata - ps21
Euteleostomi - ps22
Sarcopterygii - ps23
Tetrapoda - ps24
Amniota - ps25
Mammalia - ps26
Theria - ps27
Placentalia - ps28
Boreoeutheria - ps29
Euarchontoglires - ps30
Euarchonta - ps31
Primates - ps32
Haplorrhini - ps33
Simiiformes - ps34
Catarrhini - ps35
Hominoidea - ps36
Hominidae - ps37
Homininae - ps38
Hominini - ps39
Homo_sapiens - ps40
Phylostratum

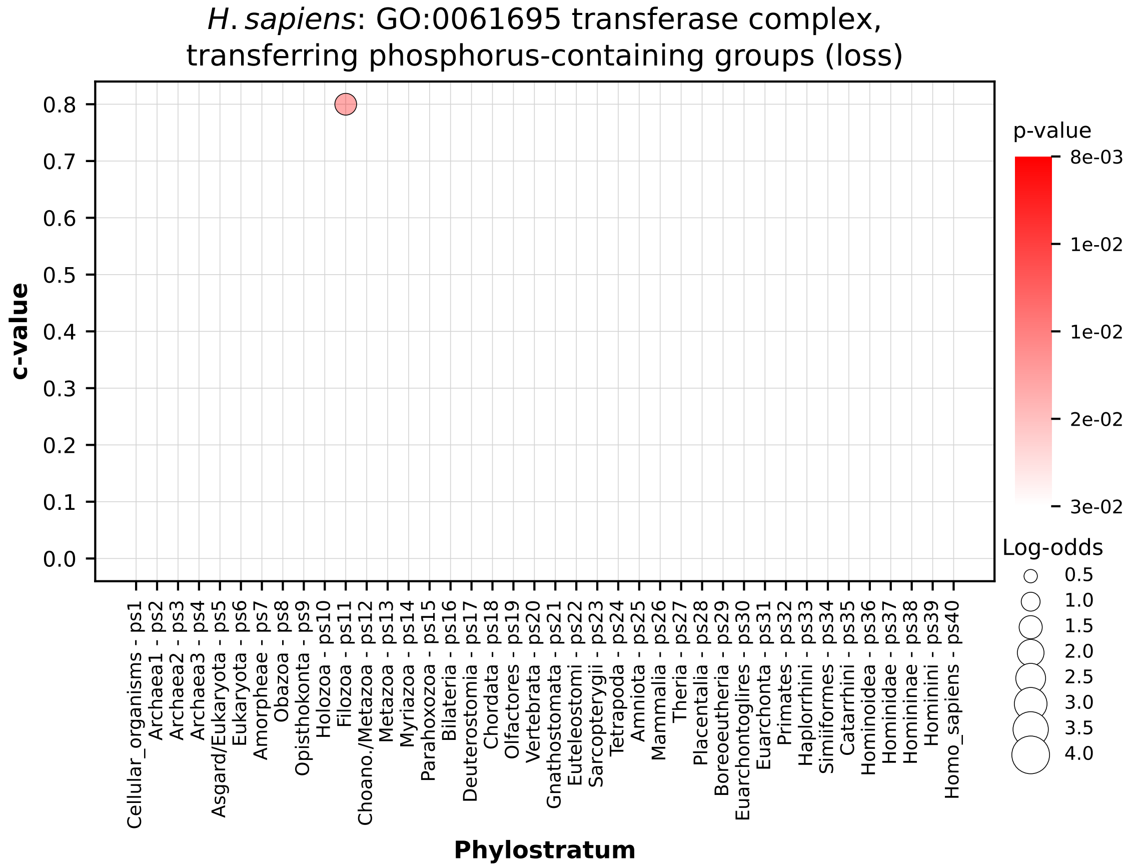

H. sapiens: GO:0061695 transferase complex, transferring phosphorus-containing groups (loss)
c-value
0.0
0.1
0.2
0.3
0.4
0.5
0.6
0.7
0.8
Phylostratum
Cellular_organisms - ps1
Archaea1 - ps2
Archaea2 - ps3
Archaea3 - ps4
Asgard/Eukaryota - ps5
Eukaryota - ps6
Amorpheae - ps7
Obazoa - ps8
Opisthokonta - ps9
Holozoa - ps10
Filozoa - ps11
Choano./Metazoa - ps12
Metazoa - ps13
Myriazoa - ps14
Parahoxozoa - ps15
Bilateria - ps16
Deuterostomia - ps17
Chordata - ps18
Olfactores - ps19
Vertebrata - ps20
Gnathostomata - ps21
Euteleostomi - ps22
Sarcopterygii - ps23
Tetrapoda - ps24
Amniota - ps25
Mammalia - ps26
Theria - ps27
Placentalia - ps28
Boreoeutheria - ps29
Euarchontoglires - ps30
Euarchonta - ps31
Primates - ps32
Haplorrhini - ps33
Simiiformes - ps34
Catarrhini - ps35
Hominoidea - ps36
Hominidae - ps37
Homininae - ps38
Hominini - ps39
Homo_sapiens - ps40
p-value
8e-03
1e-02
1e-02
2e-02
3e-02
Log-odds
0.5
1.0
1.5
2.0
2.5
3.0
3.5
4.0

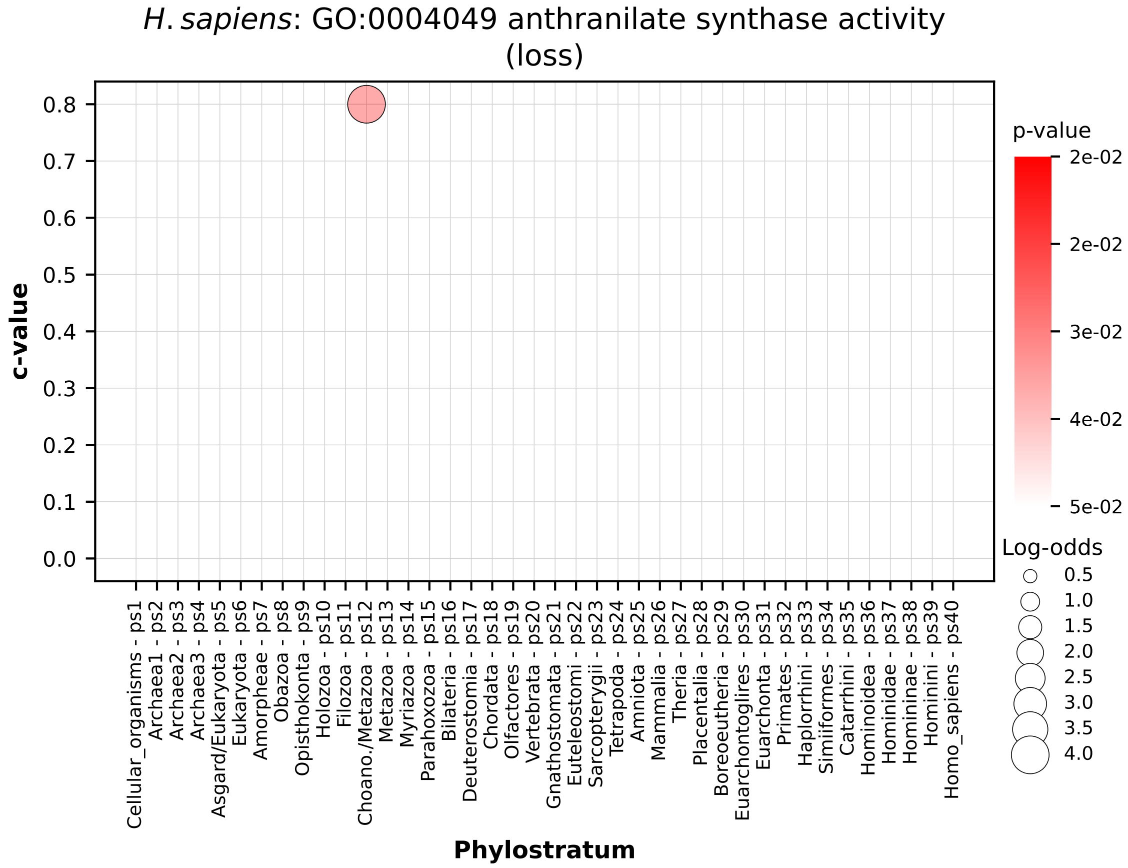

H. sapiens: GO:0004049 anthranilate synthase activity (loss)
c-value
0.0
0.1
0.2
0.3
0.4
0.5
0.6
0.7
0.8
Phylostratum
Cellular_organisms - ps1
Archaea1 - ps2
Archaea2 - ps3
Archaea3 - ps4
Asgard/Eukaryota - ps5
Eukaryota - ps6
Amorpheae - ps7
Obazoa - ps8
Opisthokonta - ps9
Holozoa - ps10
Filozoa - ps11
Choano./Metazoa - ps12
Metazoa - ps13
Myriazoa - ps14
Parahoxozoa - ps15
Bilateria - ps16
Deuterostomia - ps17
Chordata - ps18
Olfactores - ps19
Vertebrata - ps20
Gnathostomata - ps21
Euteleostomi - ps22
Sarcopterygii - ps23
Tetrapoda - ps24
Amniota - ps25
Mammalia - ps26
Theria - ps27
Placentalia - ps28
Boreoeutheria - ps29
Euarchontoglires - ps30
Euarchonta - ps31
Primates - ps32
Haplorrhini - ps33
Simiiformes - ps34
Catarrhini - ps35
Hominoidea - ps36
Hominidae - ps37
Homininae - ps38
Hominini - ps39
Homo_sapiens - ps40
p-value
2e-02
2e-02
3e-02
4e-02
5e-02
Log-odds
0.5
1.0
1.5
2.0
2.5
3.0
3.5
4.0

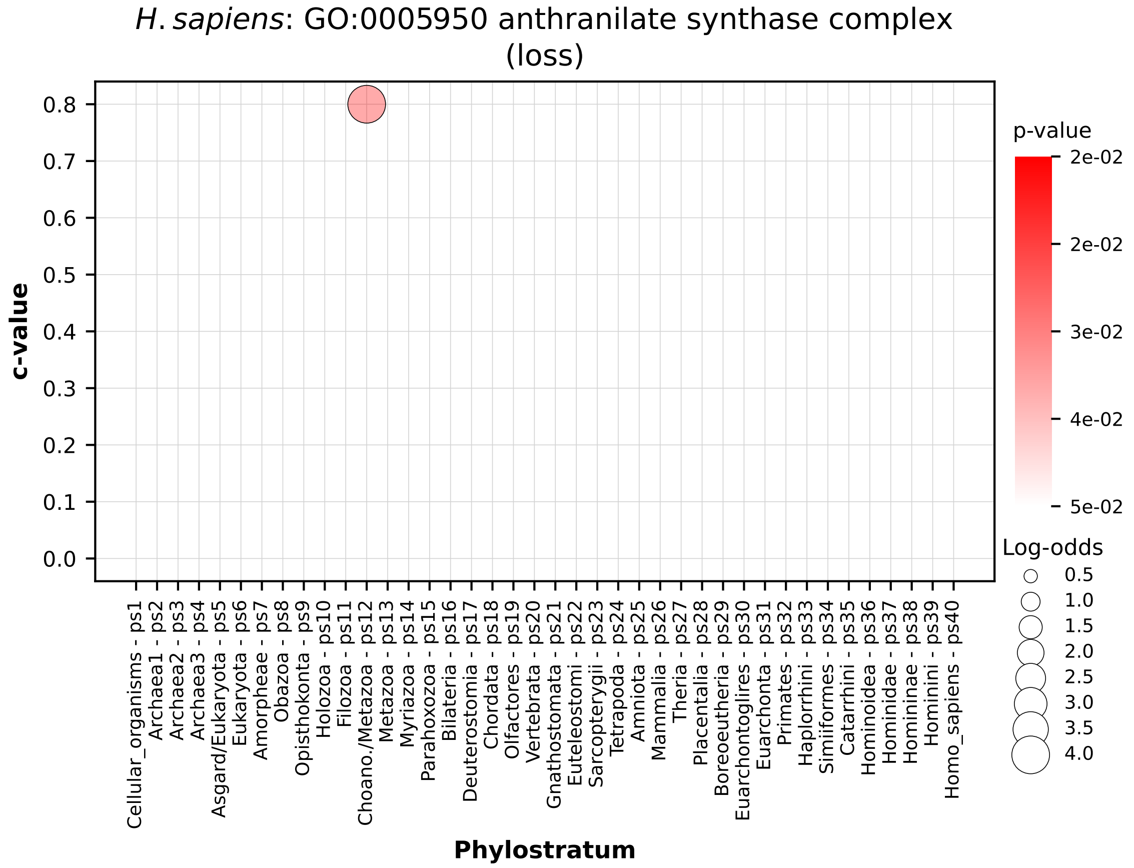
H. sapiens: GO:0005950 anthranilate synthase complex
(loss)
c-value
0.0
0.1
0.2
0.3
0.4
0.5
0.6
0.7
0.8
p-value
2e-02
2e-02
3e-02
4e-02
5e-02
Log-odds
0.5
1.0
1.5
2.0
2.5
3.0
3.5
4.0
Cellular_organisms - ps1
Archaea1 - ps2
Archaea2 - ps3
Archaea3 - ps4
Asgard/Eukaryota - ps5
Eukaryota - ps6
Amorpheae - ps7
Obazoa - ps8
Opisthokonta - ps9
Holozoa - ps10
Filozoa - ps11
Choano./Metazoa - ps12
Metazoa - ps13
Myriazoa - ps14
Parahoxozoa - ps15
Bilateria - ps16
Deuterostomia - ps17
Chordata - ps18
Olfactores - ps19
Vertebrata - ps20
Gnathostomata - ps21
Euteleostomi - ps22
Sarcopterygii - ps23
Tetrapoda - ps24
Amniota - ps25
Mammalia - ps26
Theria - ps27
Placentalia - ps28
Boreoeutheria - ps29
Euarchontoglires - ps30
Euarchonta - ps31
Primates - ps32
Haplorrhini - ps33
Simiiformes - ps34
Catarrhini - ps35
Hominoidea - ps36
Hominidae - ps37
Homininae - ps38
Hominini - ps39
Homo_sapiens - ps40
Phylostratum

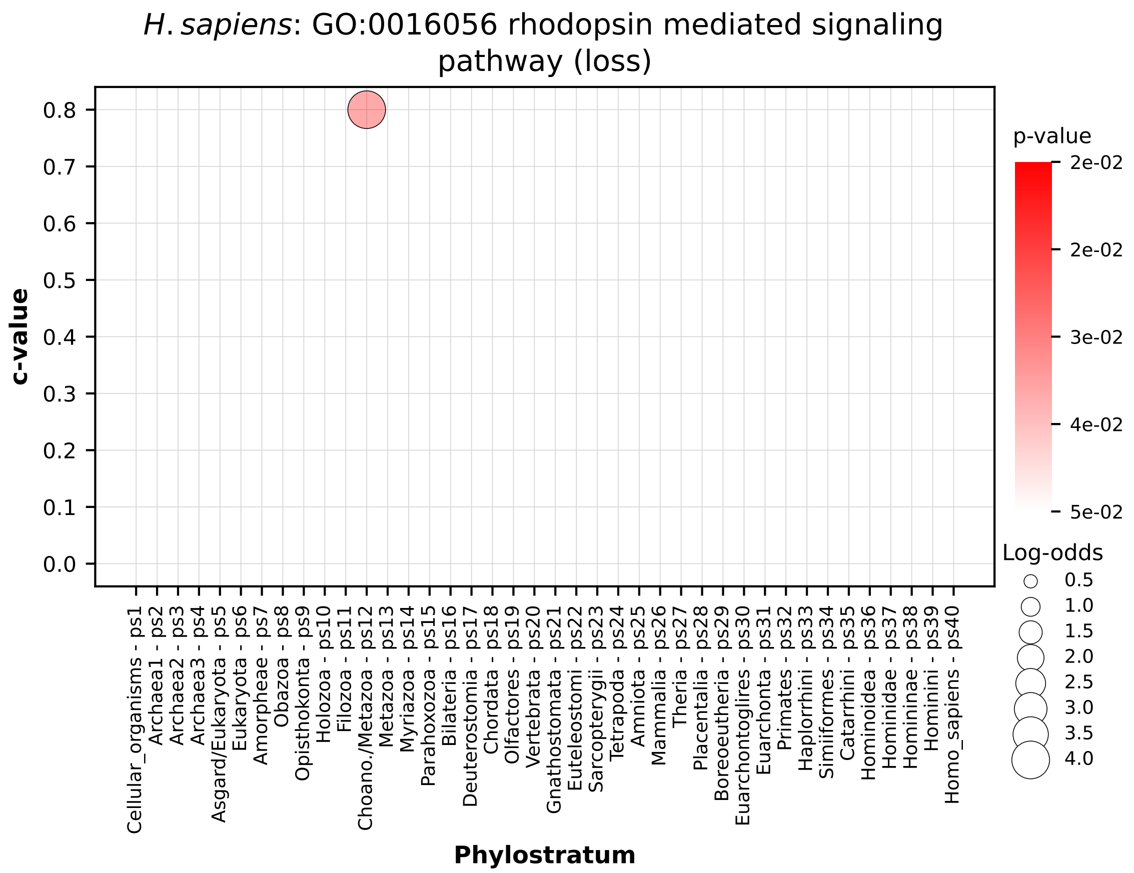

H. sapiens: GO:0016056 rhodopsin mediated signaling pathway (loss)
c-value
0.0
0.1
0.2
0.3
0.4
0.5
0.6
0.7
0.8
Phylostratum
Cellular_organisms - ps1
Archaea1 - ps2
Archaea2 - ps3
Archaea3 - ps4
Asgard/Eukaryota - ps5
Eukaryota - ps6
Amorpheae - ps7
Obazoa - ps8
Opisthokonta - ps9
Holozoa - ps10
Filozoa - ps11
Choano./Metazoa - ps12
Metazoa - ps13
Myriazoa - ps14
Parahoxozoa - ps15
Bilateria - ps16
Deuterostomia - ps17
Chordata - ps18
Olfactores - ps19
Vertebrata - ps20
Gnathostomata - ps21
Euteleostomi - ps22
Sarcopterygii - ps23
Tetrapoda - ps24
Amniota - ps25
Mammalia - ps26
Theria - ps27
Placentalia - ps28
Boreoeutheria - ps29
Euarchontoglires - ps30
Euarchonta - ps31
Primates - ps32
Haplorrhini - ps33
Simiiformes - ps34
Catarrhini - ps35
Hominoidea - ps36
Hominidae - ps37
Homininae - ps38
Hominini - ps39
Homo_sapiens - ps40
p-value
2e-02
2e-02
3e-02
4e-02
5e-02
Log-odds
0.5
1.0
1.5
2.0
2.5
3.0
3.5
4.0

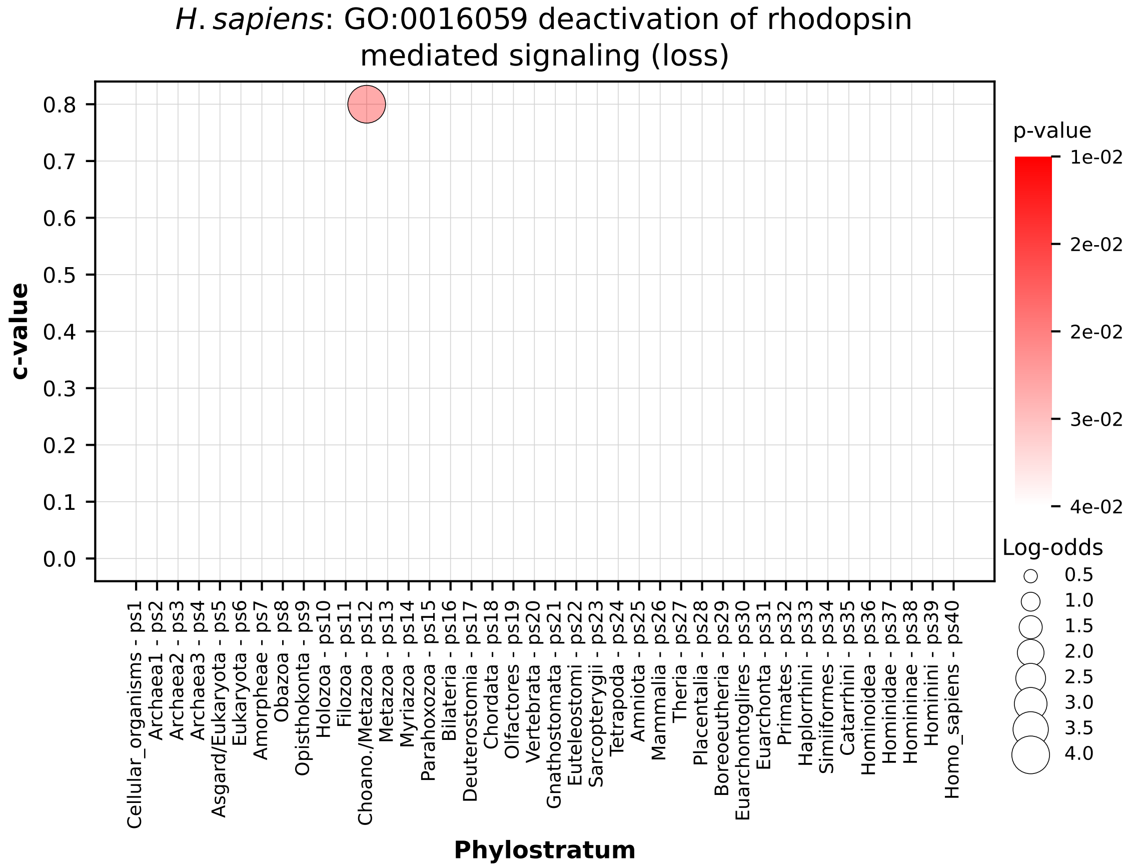

H. sapiens: GO:0016059 deactivation of rhodopsin mediated signaling (loss)
c-value
0.0
0.1
0.2
0.3
0.4
0.5
0.6
0.7
0.8
p-value
1e-02
2e-02
2e-02
3e-02
4e-02
Log-odds
0.5
1.0
1.5
2.0
2.5
3.0
3.5
4.0
Cellular_organisms - ps1
Archaea1 - ps2
Archaea2 - ps3
Archaea3 - ps4
Asgard/Eukaryota - ps5
Eukaryota - ps6
Amorpheae - ps7
Obazoa - ps8
Opisthokonta - ps9
Holozoa - ps10
Filozoa - ps11
Choano./Metazoa - ps12
Metazoa - ps13
Myriazoa - ps14
Parahoxozoa - ps15
Bilateria - ps16
Deuterostomia - ps17
Chordata - ps18
Olfactores - ps19
Vertebrata - ps20
Gnathostomata - ps21
Euteleostomi - ps22
Sarcopterygii - ps23
Tetrapoda - ps24
Amniota - ps25
Mammalia - ps26
Theria - ps27
Placentalia - ps28
Boreoeutheria - ps29
Euarchontoglires - ps30
Euarchonta - ps31
Primates - ps32
Haplorrhini - ps33
Simiiformes - ps34
Catarrhini - ps35
Hominoidea - ps36
Hominidae - ps37
Homininae - ps38
Hominini - ps39
Homo_sapiens - ps40
Phylostratum

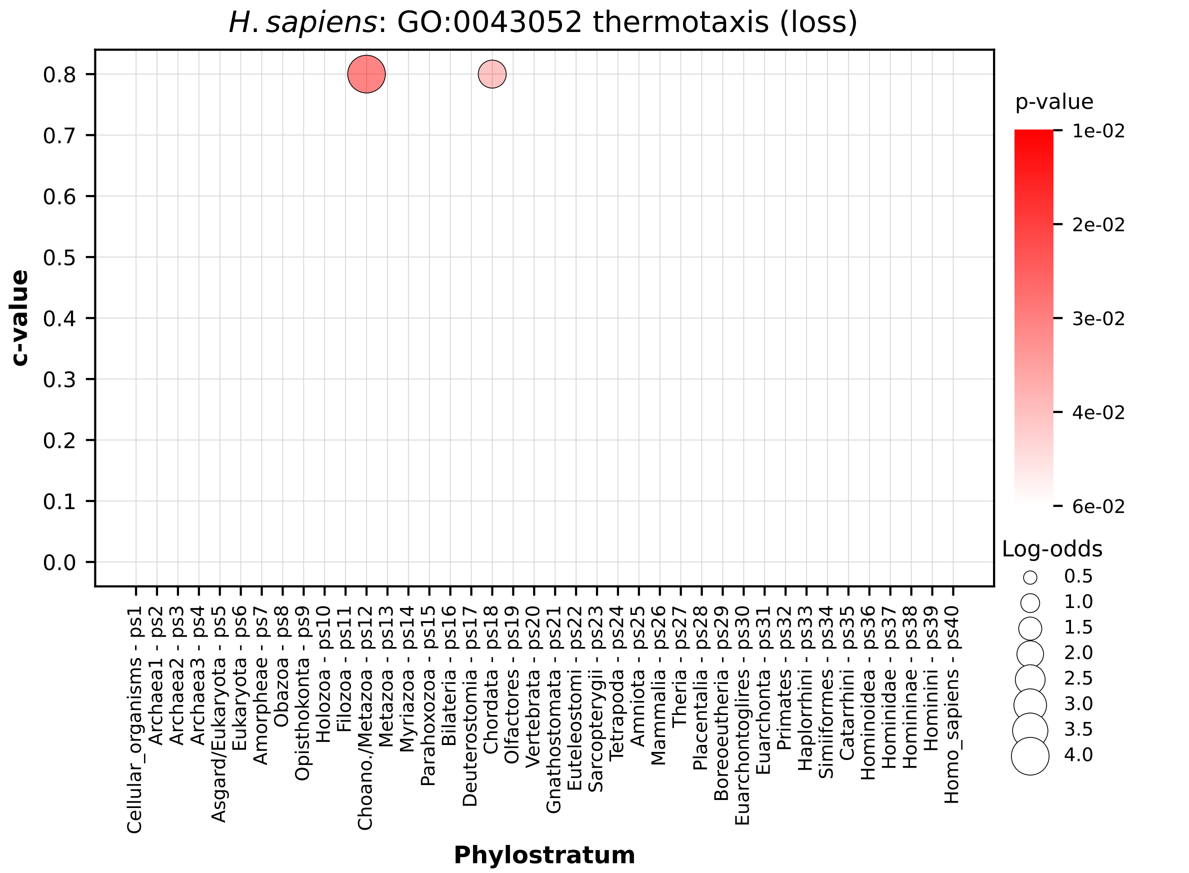

H. sapiens: GO:0043052 thermotaxis (loss)
c-value
0.0
0.1
0.2
0.3
0.4
0.5
0.6
0.7
0.8
Phylostratum
Cellular_organisms - ps1
Archaea1 - ps2
Archaea2 - ps3
Archaea3 - ps4
Asgard/Eukaryota - ps5
Eukaryota - ps6
Amorpheae - ps7
Obazoa - ps8
Opisthokonta - ps9
Holozoa - ps10
Filozoa - ps11
Choano./Metazoa - ps12
Metazoa - ps13
Myriazoa - ps14
Parahoxozoa - ps15
Bilateria - ps16
Deuterostomia - ps17
Chordata - ps18
Olfactores - ps19
Vertebrata - ps20
Gnathostomata - ps21
Euteleostomi - ps22
Sarcopterygii - ps23
Tetrapoda - ps24
Amniota - ps25
Mammalia - ps26
Theria - ps27
Placentalia - ps28
Boreoeutheria - ps29
Euarchontoglires - ps30
Euarchonta - ps31
Primates - ps32
Haplorrhini - ps33
Simiiformes - ps34
Catarrhini - ps35
Hominoidea - ps36
Hominidae - ps37
Homininae - ps38
Hominini - ps39
Homo_sapiens - ps40
p-value
1e-02
2e-02
3e-02
4e-02
6e-02
Log-odds
0.5
1.0
1.5
2.0
2.5
3.0
3.5
4.0

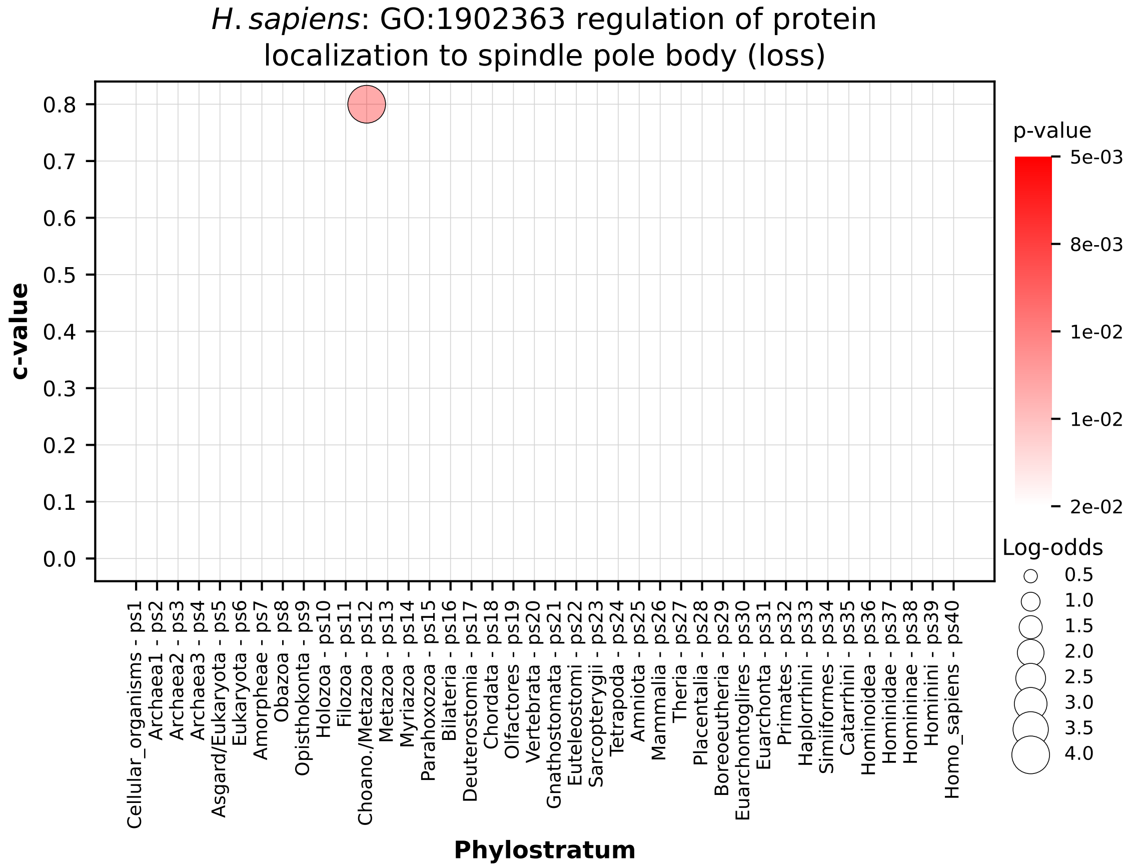

H. sapiens: GO:1902363 regulation of protein localization to spindle pole body (loss)
c-value
0.8
0.7
0.6
0.5
0.4
0.3
0.2
0.1
0.0
p-value
5e-03
8e-03
1e-02
1e-02
2e-02
Log-odds
0.5
1.0
1.5
2.0
2.5
3.0
3.5
4.0
Cellular_organisms - ps1
Archaea1 - ps2
Archaea2 - ps3
Archaea3 - ps4
Asgard/Eukaryota - ps5
Eukaryota - ps6
Amorpheae - ps7
Obazoa - ps8
Opisthokonta - ps9
Holozoa - ps10
Filozoa - ps11
Choano./Metazoa - ps12
Metazoa - ps13
Myriazoa - ps14
Parahoxozoa - ps15
Bilateria - ps16
Deuterostomia - ps17
Chordata - ps18
Olfactores - ps19
Vertebrata - ps20
Gnathostomata - ps21
Euteleostomi - ps22
Sarcopterygii - ps23
Tetrapoda - ps24
Amniota - ps25
Mammalia - ps26
Theria - ps27
Placentalia - ps28
Boreoeutheria - ps29
Euarchontoglires - ps30
Euarchonta - ps31
Primates - ps32
Haplorrhini - ps33
Simiiformes - ps34
Catarrhini - ps35
Hominoidea - ps36
Hominidae - ps37
Homininae - ps38
Hominini - ps39
Homo_sapiens - ps40
Phylostratum

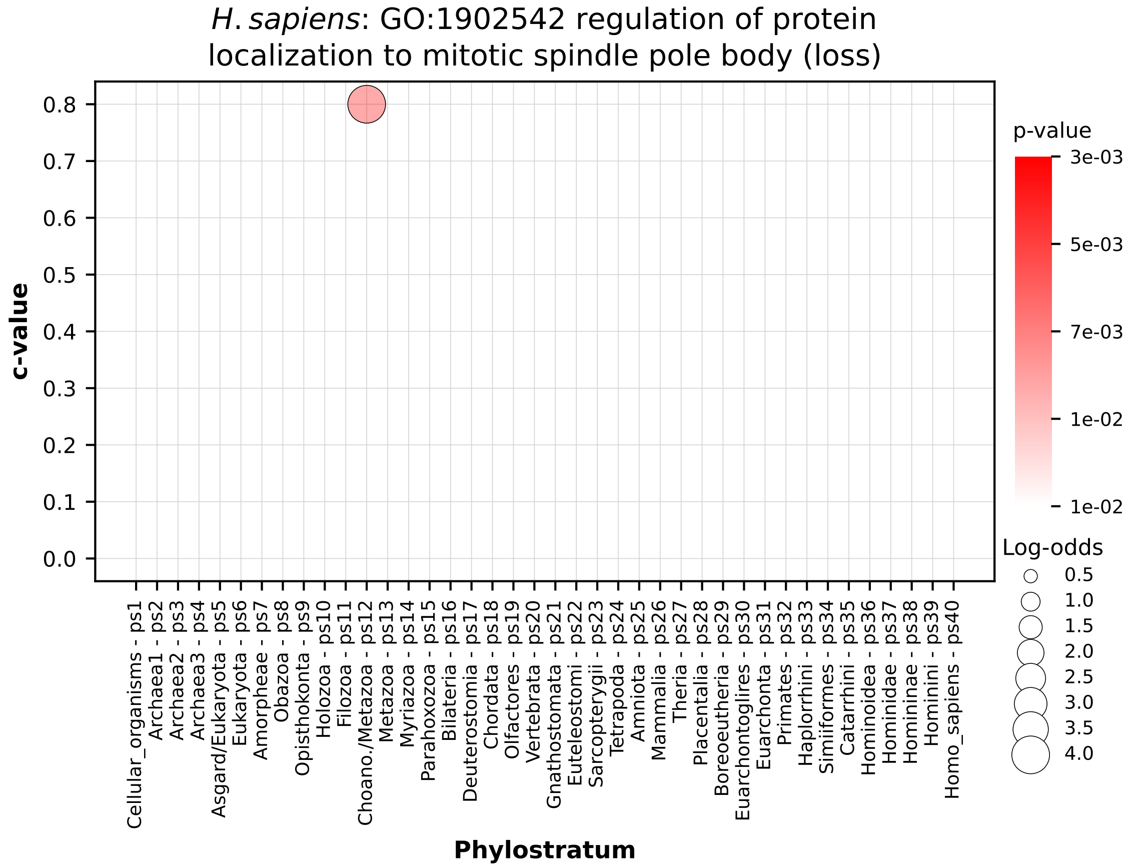

H. sapiens: GO:1902542 regulation of protein localization to mitotic spindle pole body (loss)
c-value
0.0
0.1
0.2
0.3
0.4
0.5
0.6
0.7
0.8
p-value
3e-03
5e-03
7e-03
1e-02
1e-02
Log-odds
0.5
1.0
1.5
2.0
2.5
3.0
3.5
4.0
Cellular_organisms - ps1
Archaea1 - ps2
Archaea2 - ps3
Archaea3 - ps4
Asgard/Eukaryota - ps5
Eukaryota - ps6
Amorpheae - ps7
Obazoa - ps8
Opisthokonta - ps9
Holozoa - ps10
Filozoa - ps11
Choano./Metazoa - ps12
Metazoa - ps13
Myriazoa - ps14
Parahoxozoa - ps15
Bilateria - ps16
Deuterostomia - ps17
Chordata - ps18
Olfactores - ps19
Vertebrata - ps20
Gnathostomata - ps21
Euteleostomi - ps22
Sarcopterygii - ps23
Tetrapoda - ps24
Amniota - ps25
Mammalia - ps26
Theria - ps27
Placentalia - ps28
Boreoeutheria - ps29
Euarchontoglires - ps30
Euarchonta - ps31
Primates - ps32
Haplorrhini - ps33
Simiiformes - ps34
Catarrhini - ps35
Hominoidea - ps36
Hominidae - ps37
Homininae - ps38
Hominini - ps39
Homo_sapiens - ps40
Phylostratum

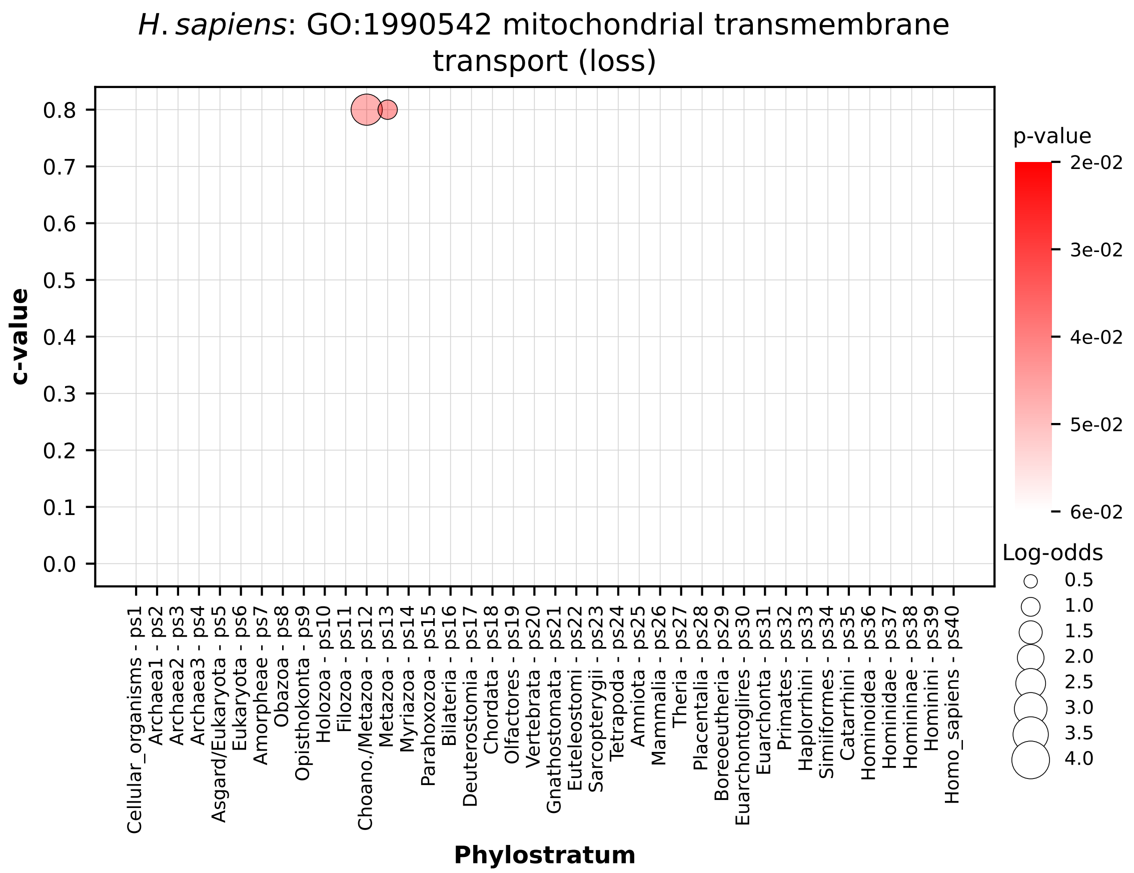
H. sapiens: GO:1990542 mitochondrial transmembrane transport (loss)
c-value
0.0
0.1
0.2
0.3
0.4
0.5
0.6
0.7
0.8
p-value
2e-02
3e-02
4e-02
5e-02
6e-02
Log-odds
0.5
1.0
1.5
2.0
2.5
3.0
3.5
4.0
Cellular_organisms - ps1
Archaea1 - ps2
Archaea2 - ps3
Archaea3 - ps4
Asgard/Eukaryota - ps5
Eukaryota - ps6
Amorpheae - ps7
Obazoa - ps8
Opisthokonta - ps9
Holozoa - ps10
Filozoa - ps11
Choano./Metazoa - ps12
Metazoa - ps13
Myriazoa - ps14
Parahoxozoa - ps15
Bilateria - ps16
Deuterostomia - ps17
Chordata - ps18
Olfactores - ps19
Vertebrata - ps20
Gnathostomata - ps21
Euteleostomi - ps22
Sarcopterygii - ps23
Tetrapoda - ps24
Amniota - ps25
Mammalia - ps26
Theria - ps27
Placentalia - ps28
Boreoeutheria - ps29
Euarchontoglires - ps30
Euarchonta - ps31
Primates - ps32
Haplorrhini - ps33
Simiiformes - ps34
Catarrhini - ps35
Hominoidea - ps36
Hominidae - ps37
Homininae - ps38
Hominini - ps39
Homo_sapiens - ps40
Phylostratum

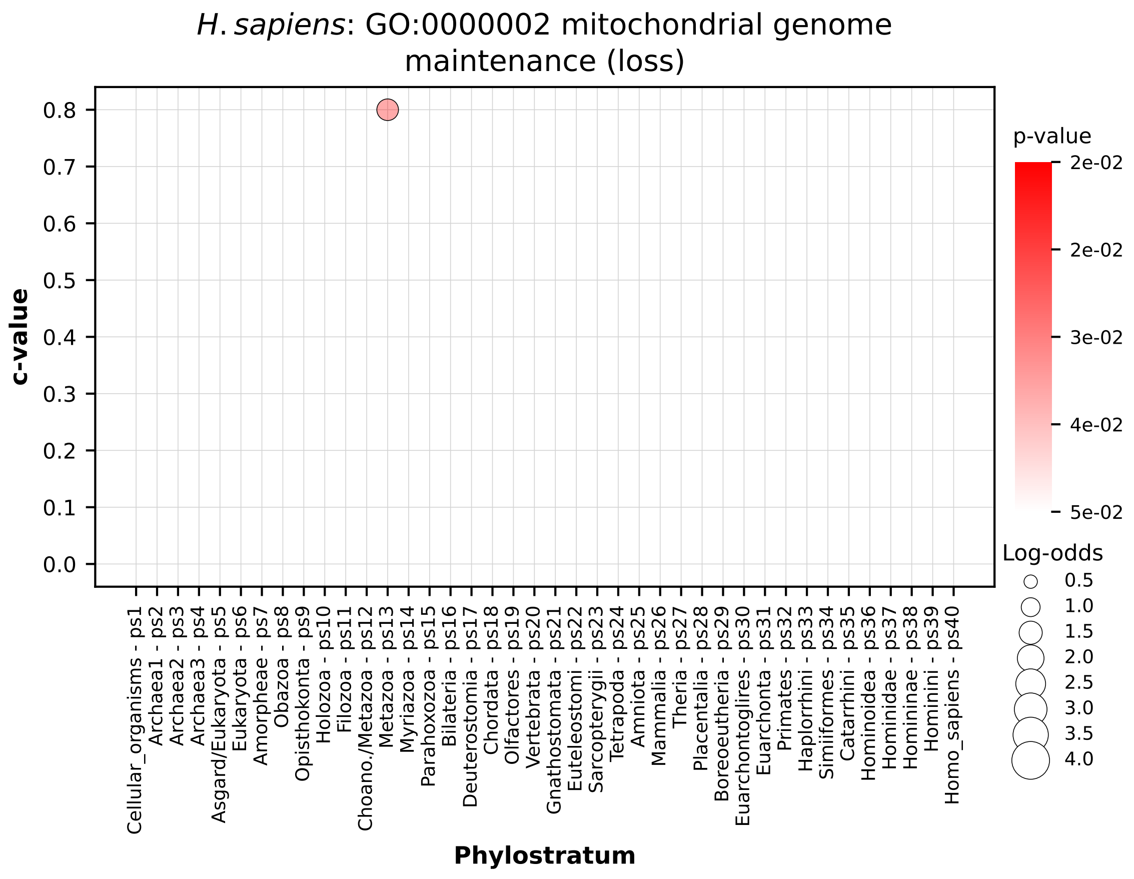
H. sapiens: GO:0000002 mitochondrial genome maintenance (loss)
c-value
0.8
0.7
0.6
0.5
0.4
0.3
0.2
0.1
0.0
p-value
2e-02
2e-02
3e-02
4e-02
5e-02
Log-odds
0.5
1.0
1.5
2.0
2.5
3.0
3.5
4.0
Cellular_organisms - ps1
Archaea1 - ps2
Archaea2 - ps3
Archaea3 - ps4
Asgard/Eukaryota - ps5
Eukaryota - ps6
Amorpheae - ps7
Obazoa - ps8
Opisthokonta - ps9
Holozoa - ps10
Filozoa - ps11
Choano./Metazoa - ps12
Metazoa - ps13
Myriazoa - ps14
Parahoxozoa - ps15
Bilateria - ps16
Deuterostomia - ps17
Chordata - ps18
Olfactores - ps19
Vertebrata - ps20
Gnathostomata - ps21
Euteleostomi - ps22
Sarcopterygii - ps23
Tetrapoda - ps24
Amniota - ps25
Mammalia - ps26
Theria - ps27
Placentalia - ps28
Boreoeutheria - ps29
Euarchontoglires - ps30
Euarchonta - ps31
Primates - ps32
Haplorrhini - ps33
Similformes - ps34
Catarrhini - ps35
Hominoidea - ps36
Hominidae - ps37
Homininae - ps38
Hominini - ps39
Homo_sapiens - ps40
Phylostratum

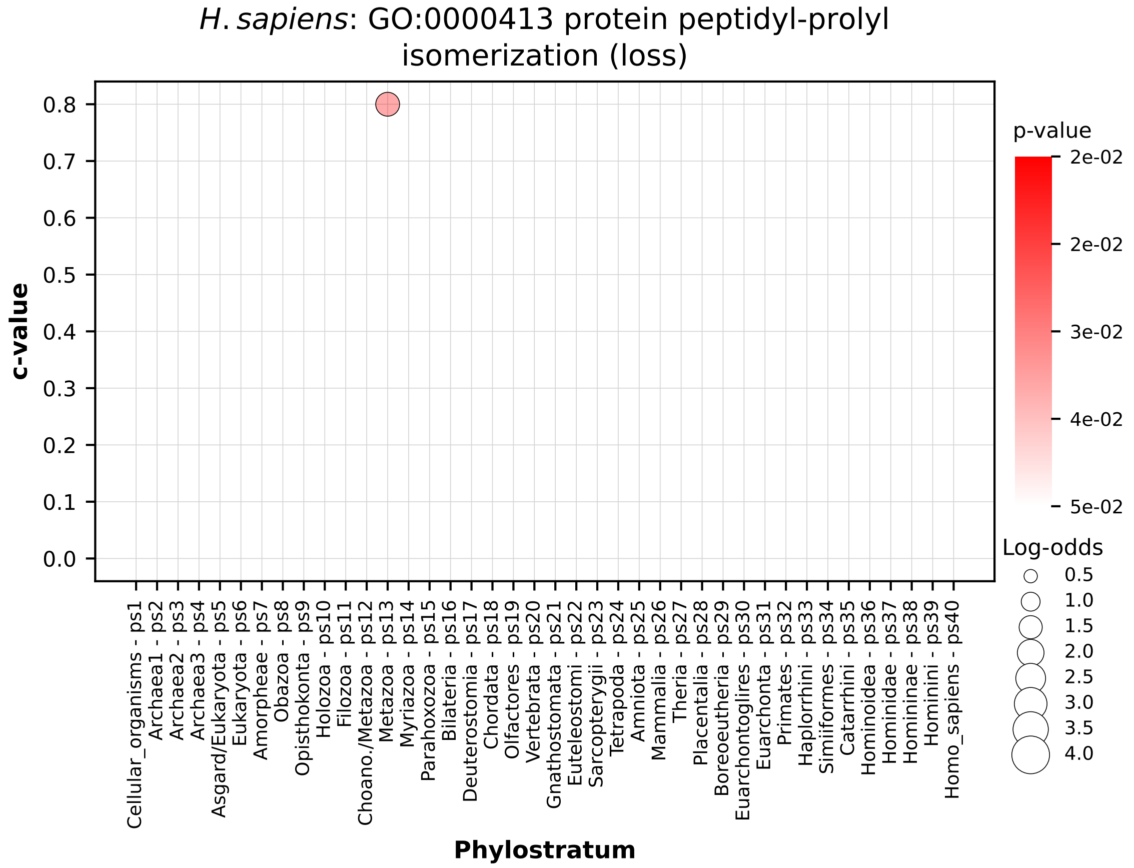

H. sapiens: GO:0000413 protein peptidyl-prolyl isomerization (loss)
c-value
0.0
0.1
0.2
0.3
0.4
0.5
0.6
0.7
0.8
p-value
2e-02
2e-02
3e-02
4e-02
5e-02
Log-odds
0.5
1.0
1.5
2.0
2.5
3.0
3.5
4.0
Cellular_organisms - ps1
Archaea1 - ps2
Archaea2 - ps3
Archaea3 - ps4
Asgard/Eukaryota - ps5
Eukaryota - ps6
Amorpheae - ps7
Obazoa - ps8
Opisthokonta - ps9
Holozoa - ps10
Filozoa - ps11
Choano./Metazoa - ps12
Metazoa - ps13
Myriazoa - ps14
Parahoxozoa - ps15
Bilateria - ps16
Deuterostomia - ps17
Chordata - ps18
Olfactores - ps19
Vertebrata - ps20
Gnathostomata - ps21
Euteleostomi - ps22
Sarcopterygii - ps23
Tetrapoda - ps24
Amniota - ps25
Mammalia - ps26
Theria - ps27
Placentalia - ps28
Boreoeutheria - ps29
Euarchontoglires - ps30
Euarchonta - ps31
Primates - ps32
Haplorrhini - ps33
Simiiformes - ps34
Catarrhini - ps35
Hominoidea - ps36
Hominidae - ps37
Homininae - ps38
Hominini - ps39
Homo_sapiens - ps40
Phylostratum

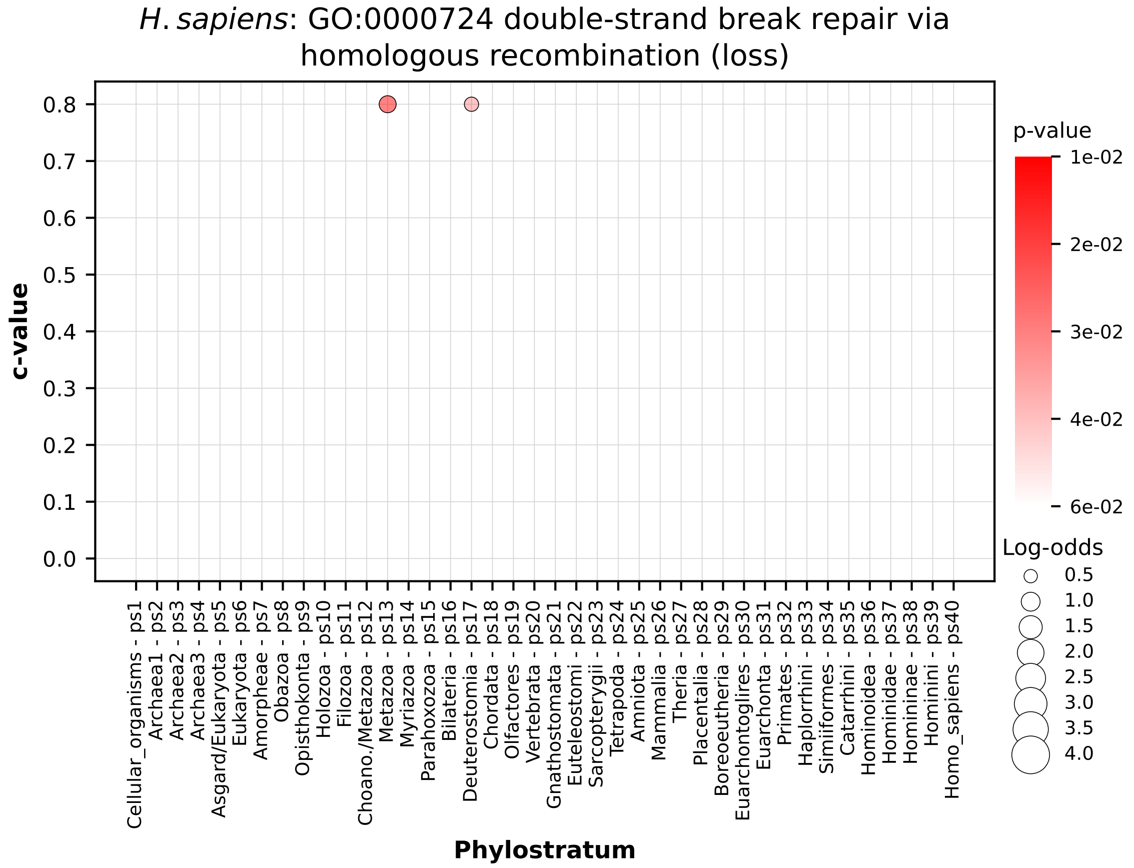

H. sapiens: GO:0000724 double-strand break repair via homologous recombination (loss)
c-value
0.8
0.7
0.6
0.5
0.4
0.3
0.2
0.1
0.0
p-value
1e-02
2e-02
3e-02
4e-02
6e-02
Log-odds
0.5
1.0
1.5
2.0
2.5
3.0
3.5
4.0
Cellular_organisms - ps1
Archaea1 - ps2
Archaea2 - ps3
Archaea3 - ps4
Asgard/Eukaryota - ps5
Eukaryota - ps6
Amorpheae - ps7
Obazoa - ps8
Opisthokonta - ps9
Holozoa - ps10
Filozoa - ps11
Choano./Metazoa - ps12
Metazoa - ps13
Myriazoa - ps14
Parahoxozoa - ps15
Bilateria - ps16
Deuterostomia - ps17
Chordata - ps18
Olfactores - ps19
Vertebrata - ps20
Gnathostomata - ps21
Euteleostomi - ps22
Sarcopterygii - ps23
Tetrapoda - ps24
Amniota - ps25
Mammalia - ps26
Theria - ps27
Placentalia - ps28
Boreoeutheria - ps29
Euarchontoglires - ps30
Euarchonta - ps31
Primates - ps32
Haplorrhini - ps33
Simiiformes - ps34
Catarrhini - ps35
Hominoidea - ps36
Hominidae - ps37
Homininae - ps38
Hominini - ps39
Homo_sapiens - ps40
Phylostratum

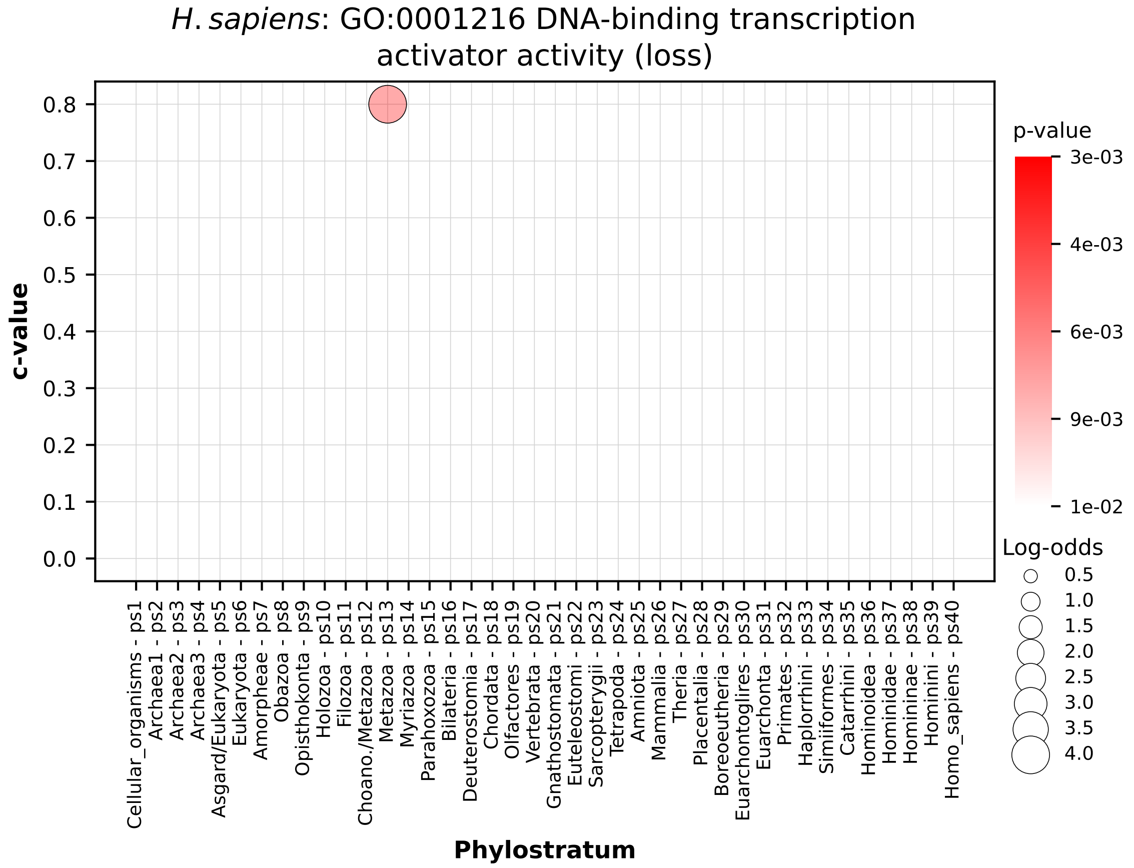

H. sapiens: GO:0001216 DNA-binding transcription activator activity (loss)
c-value
0.8
0.7
0.6
0.5
0.4
0.3
0.2
0.1
0.0
p-value
3e-03
4e-03
6e-03
9e-03
1e-02
Log-odds
0.5
1.0
1.5
2.0
2.5
3.0
3.5
4.0
Cellular_organisms - ps1
Archaea1 - ps2
Archaea2 - ps3
Archaea3 - ps4
Asgard/Eukaryota - ps5
Eukaryota - ps6
Amorpheae - ps7
Obazoa - ps8
Opisthokonta - ps9
Holozoa - ps10
Filozoa - ps11
Choano./Metazoa - ps12
Metazoa - ps13
Myriazoa - ps14
Parahoxozoa - ps15
Bilateria - ps16
Deuterostomia - ps17
Chordata - ps18
Olfactores - ps19
Vertebrata - ps20
Gnathostomata - ps21
Euteleostomi - ps22
Sarcopterygii - ps23
Tetrapoda - ps24
Amniota - ps25
Mammalia - ps26
Theria - ps27
Placentalia - ps28
Boreoeutheria - ps29
Euarchontoglires - ps30
Euarchonta - ps31
Primates - ps32
Haplorrhini - ps33
Simiiformes - ps34
Catarrhini - ps35
Hominoidea - ps36
Hominidae - ps37
Homininae - ps38
Hominini - ps39
Homo_sapiens - ps40
Phylostratum

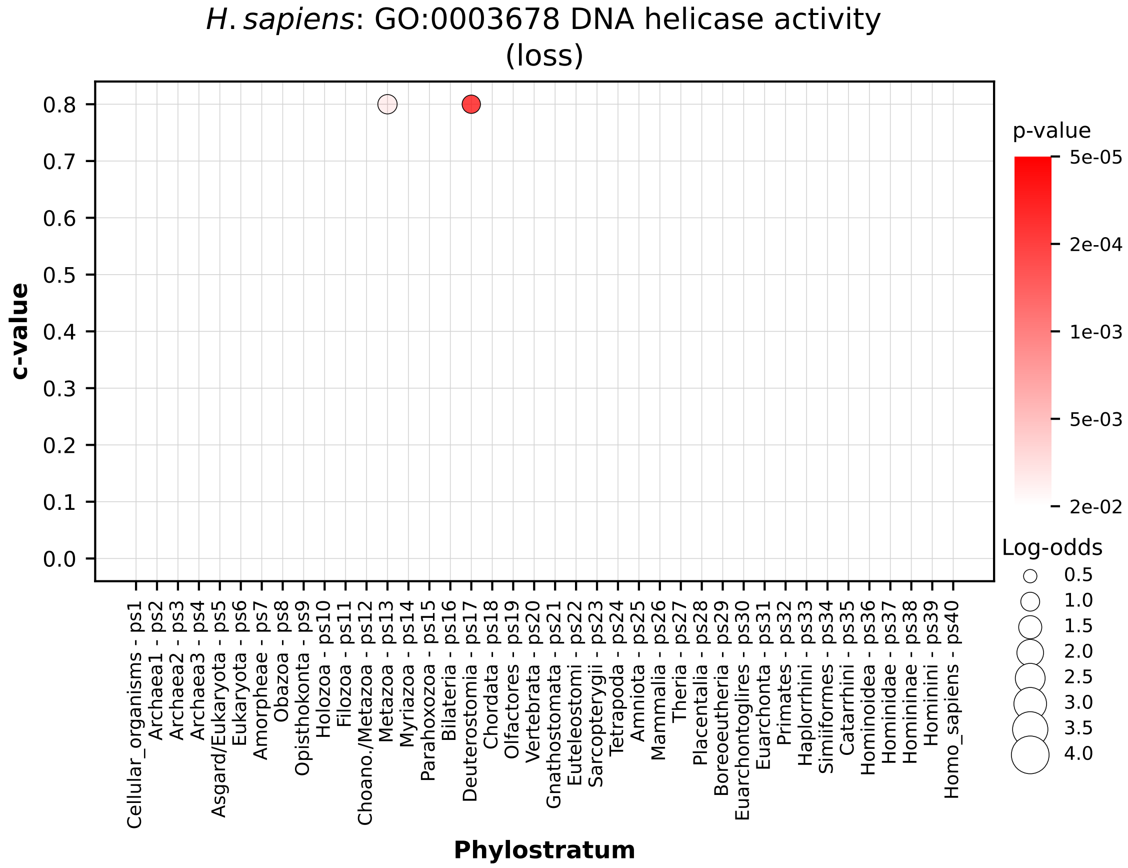

H. sapiens: GO:0003678 DNA helicase activity (loss)
c-value
0.8
0.7
0.6
0.5
0.4
0.3
0.2
0.1
0.0
Phylostratum
Cellular_organisms - ps1
Archaea1 - ps2
Archaea2 - ps3
Archaea3 - ps4
Asgard/Eukaryota - ps5
Eukaryota - ps6
Amorpheae - ps7
Obazoa - ps8
Opisthokonta - ps9
Holozoa - ps10
Filozoa - ps11
Choano./Metazoa - ps12
Metazoa - ps13
Myriazoa - ps14
Parahoxozoa - ps15
Bilateria - ps16
Deuterostomia - ps17
Chordata - ps18
Olfactores - ps19
Vertebrata - ps20
Gnathostomata - ps21
Euteleostomi - ps22
Sarcopterygii - ps23
Tetrapoda - ps24
Amniota - ps25
Mammalia - ps26
Theria - ps27
Placentalia - ps28
Boreoeutheria - ps29
Euarchontoglires - ps30
Euarchonta - ps31
Primates - ps32
Haplorrhini - ps33
Simiiformes - ps34
Catarrhini - ps35
Hominoidea - ps36
Hominidae - ps37
Homininae - ps38
Hominini - ps39
Homo_sapiens - ps40
p-value
5e-05
2e-04
1e-03
5e-03
2e-02
Log-odds
0.5
1.0
1.5
2.0
2.5
3.0
3.5
4.0

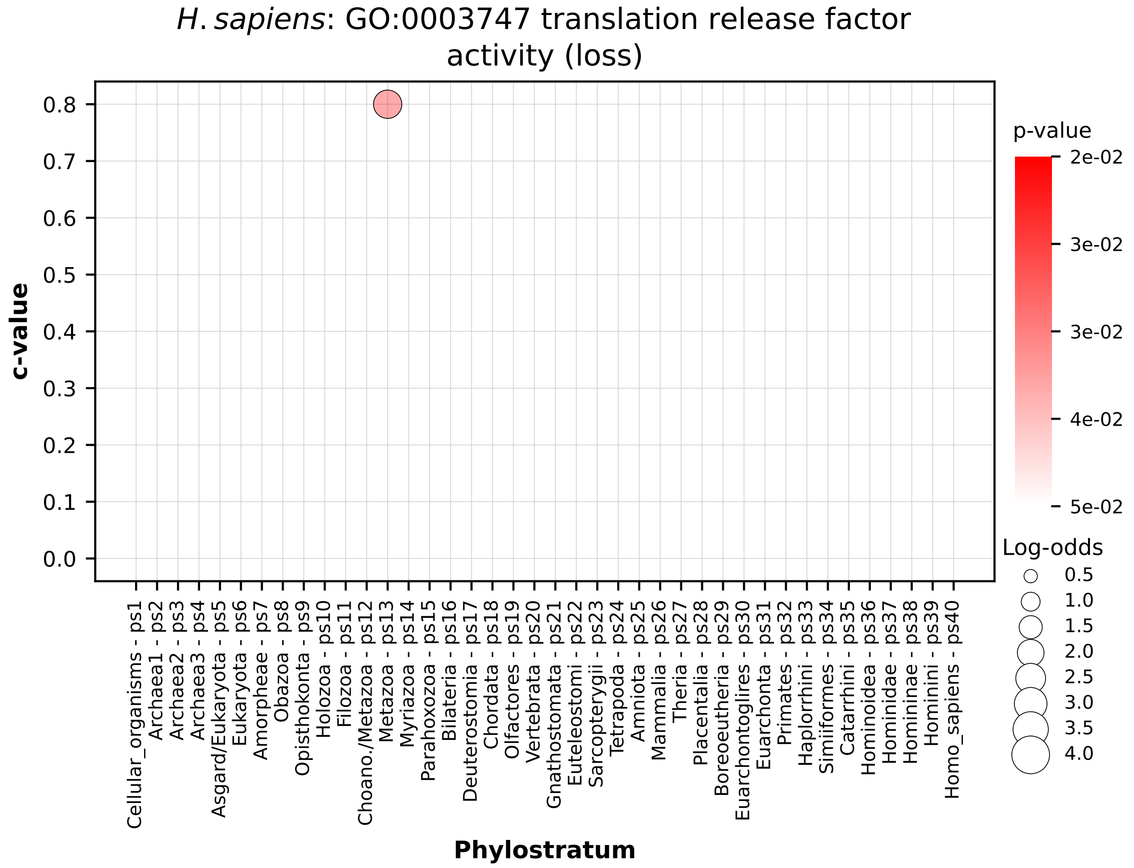
H. sapiens: GO:0003747 translation release factor activity (loss)
c-value
0.0
0.1
0.2
0.3
0.4
0.5
0.6
0.7
0.8
p-value
2e-02
3e-02
3e-02
4e-02
5e-02
Log-odds
0.5
1.0
1.5
2.0
2.5
3.0
3.5
4.0
Cellular_organisms - ps1
Archaea1 - ps2
Archaea2 - ps3
Archaea3 - ps4
Asgard/Eukaryota - ps5
Eukaryota - ps6
Amorpheae - ps7
Obazoa - ps8
Opisthokonta - ps9
Holozoa - ps10
Filozoa - ps11
Choano./Metazoa - ps12
Metazoa - ps13
Myriazoa - ps14
Parahoxozoa - ps15
Bilateria - ps16
Deuterostomia - ps17
Chordata - ps18
Olfactores - ps19
Vertebrata - ps20
Gnathostomata - ps21
Euteleostomi - ps22
Sarcopterygii - ps23
Tetrapoda - ps24
Amniota - ps25
Mammalia - ps26
Theria - ps27
Placentalia - ps28
Boreoeutheria - ps29
Euarchontoglires - ps30
Euarchonta - ps31
Primates - ps32
Haplorrhini - ps33
Simiiformes - ps34
Catarrhini - ps35
Hominoidea - ps36
Hominidae - ps37
Homininae - ps38
Hominini - ps39
Homo_sapiens - ps40
Phylostratum

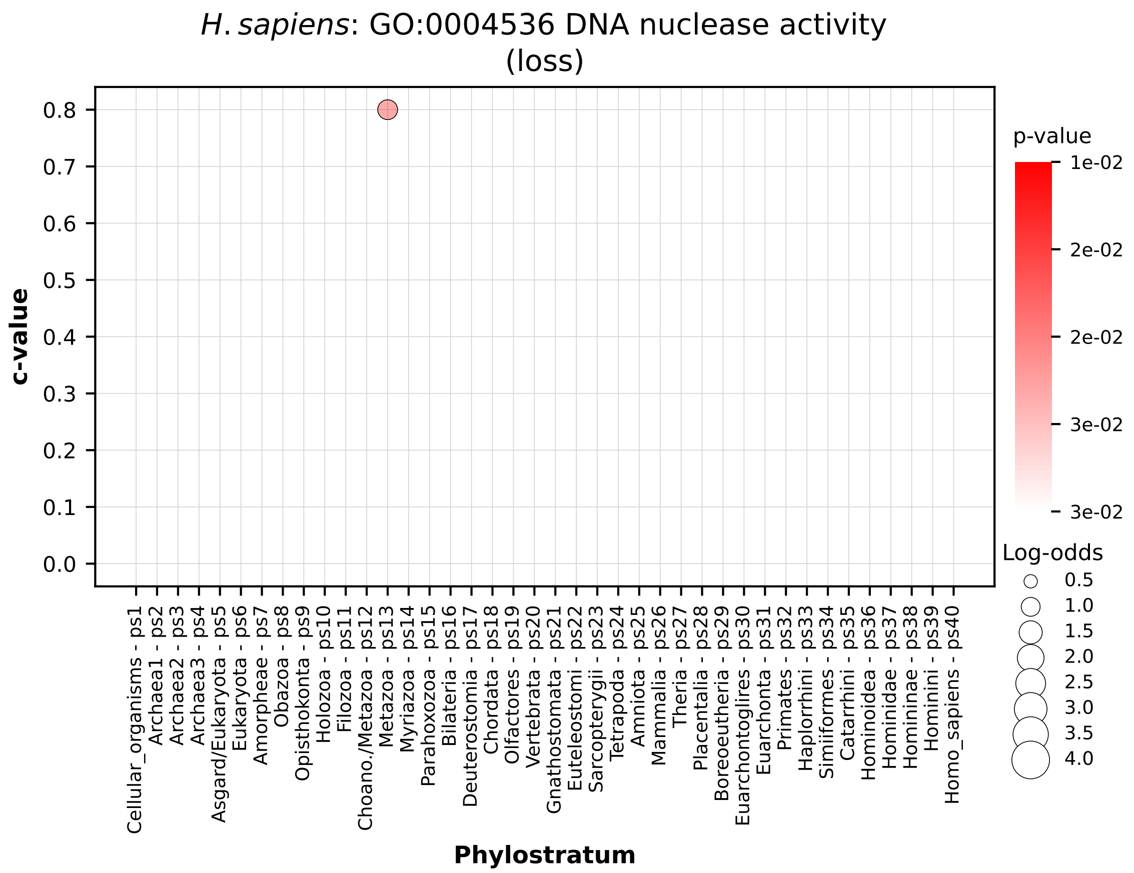

H. sapiens: GO:0004536 DNA nuclease activity
(loss)
c-value
0.0
0.1
0.2
0.3
0.4
0.5
0.6
0.7
0.8
Phylostratum
Cellular_organisms - ps1
Archaea1 - ps2
Archaea2 - ps3
Archaea3 - ps4
Asgard/Eukaryota - ps5
Eukaryota - ps6
Amorpheae - ps7
Obazoa - ps8
Opisthokonta - ps9
Holozoa - ps10
Filozoa - ps11
Choano./Metazoa - ps12
Metazoa - ps13
Myriazoa - ps14
Parahoxozoa - ps15
Bilateria - ps16
Deuterostomia - ps17
Chordata - ps18
Olfactores - ps19
Vertebrata - ps20
Gnathostomata - ps21
Euteleostomi - ps22
Sarcopterygii - ps23
Tetrapoda - ps24
Amniota - ps25
Mammalia - ps26
Theria - ps27
Placentalia - ps28
Boreoeutheria - ps29
Euarchontoglires - ps30
Euarchonta - ps31
Primates - ps32
Haplorrhini - ps33
Simiiformes - ps34
Catarrhini - ps35
Hominoidea - ps36
Hominidae - ps37
Homininae - ps38
Hominini - ps39
Homo_sapiens - ps40
p-value
1e-02
2e-02
2e-02
3e-02
3e-02
Log-odds
0.5
1.0
1.5
2.0
2.5
3.0
3.5
4.0

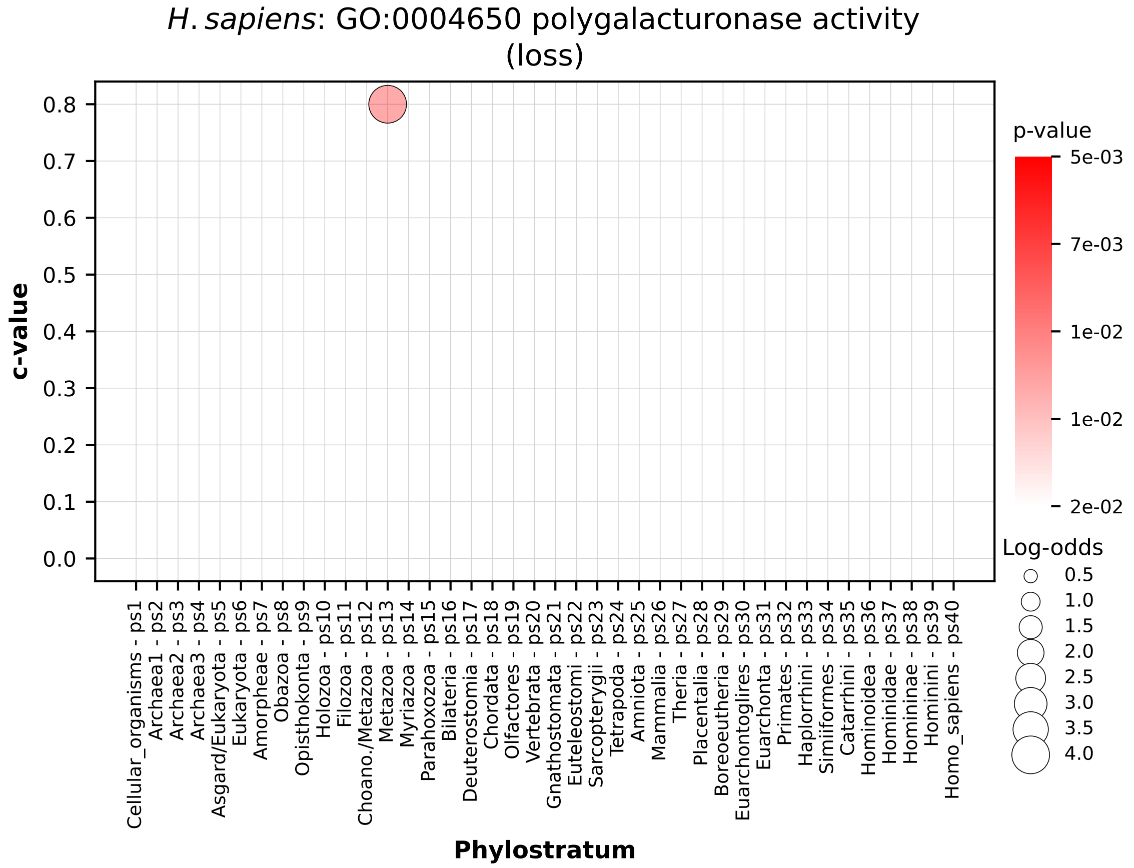

H. sapiens: GO:0004650 polygalacturonase activity (loss)
c-value
0.0
0.1
0.2
0.3
0.4
0.5
0.6
0.7
0.8
Phylostratum
Cellular_organisms - ps1
Archaea1 - ps2
Archaea2 - ps3
Archaea3 - ps4
Asgard/Eukaryota - ps5
Eukaryota - ps6
Amorpheae - ps7
Obazoa - ps8
Opisthokonta - ps9
Holozoa - ps10
Filozoa - ps11
Choano./Metazoa - ps12
Metazoa - ps13
Myriazoa - ps14
Parahoxozoa - ps15
Bilateria - ps16
Deuterostomia - ps17
Chordata - ps18
Olfactores - ps19
Vertebrata - ps20
Gnathostomata - ps21
Euteleostomi - ps22
Sarcopterygii - ps23
Tetrapoda - ps24
Amniota - ps25
Mammalia - ps26
Theria - ps27
Placentalia - ps28
Boreoeutheria - ps29
Euarchontoglires - ps30
Euarchonta - ps31
Primates - ps32
Haplorrhini - ps33
Simiiformes - ps34
Catarrhini - ps35
Hominoidea - ps36
Hominidae - ps37
Homininae - ps38
Hominini - ps39
Homo_sapiens - ps40
p-value
5e-03
7e-03
1e-02
1e-02
2e-02
Log-odds
0.5
1.0
1.5
2.0
2.5
3.0
3.5
4.0

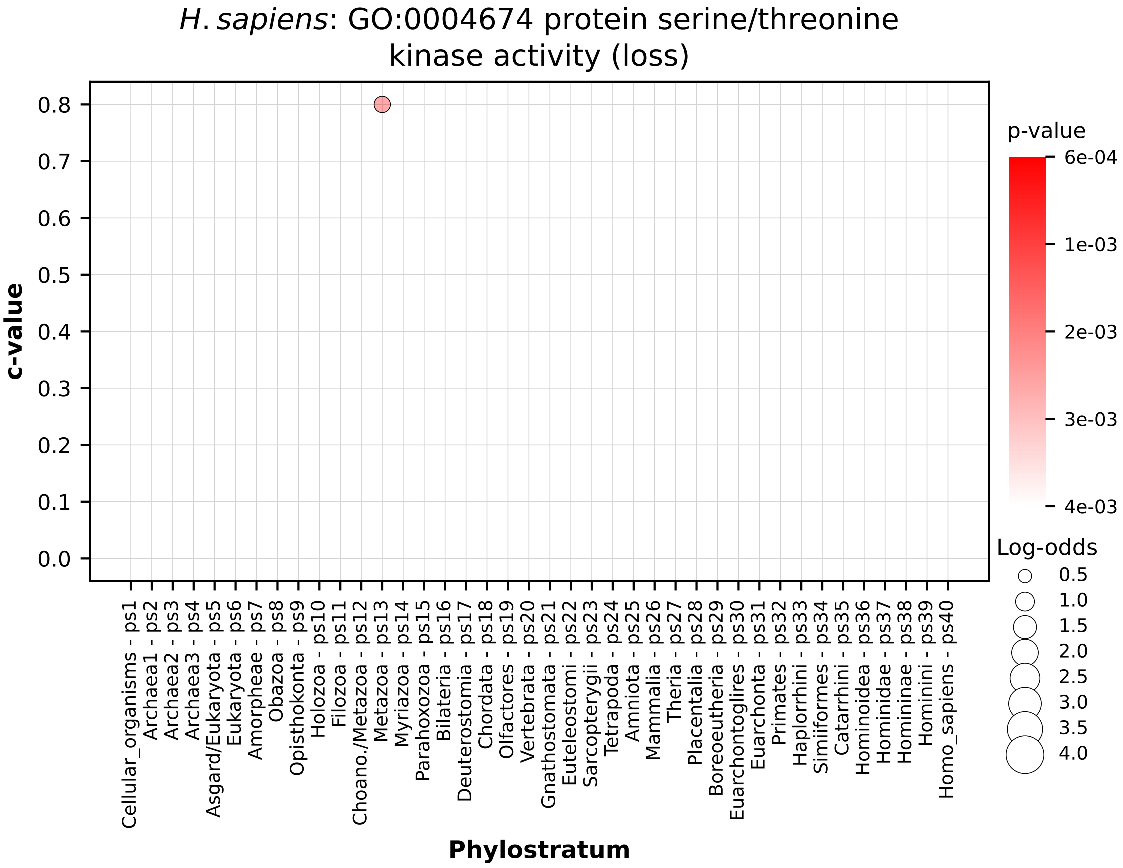
H. sapiens: GO:0004674 protein serine/threonine kinase activity (loss)
c-value
0.0
0.1
0.2
0.3
0.4
0.5
0.6
0.7
0.8
Phylostratum
Cellular_organisms - ps1
Archaea1 - ps2
Archaea2 - ps3
Archaea3 - ps4
Asgard/Eukaryota - ps5
Eukaryota - ps6
Amorpheae - ps7
Obazoa - ps8
Opisthokonta - ps9
Holozoa - ps10
Filozoa - ps11
Choano./Metazoa - ps12
Metazoa - ps13
Myriazoa - ps14
Parahoxozoa - ps15
Bilateria - ps16
Deuterostomia - ps17
Chordata - ps18
Olfactores - ps19
Vertebrata - ps20
Gnathostomata - ps21
Euteleostomi - ps22
Sarcopterygii - ps23
Tetrapoda - ps24
Amniota - ps25
Mammalia - ps26
Theria - ps27
Placentalia - ps28
Boreoeutheria - ps29
Euarchontoglires - ps30
Euarchonta - ps31
Primates - ps32
Haplorrhini - ps33
Simiiformes - ps34
Catarrhini - ps35
Hominoidea - ps36
Hominidae - ps37
Homininae - ps38
Hominini - ps39
Homo_sapiens - ps40
p-value
6e-04
1e-03
2e-03
3e-03
4e-03
Log-odds
0.5
1.0
1.5
2.0
2.5
3.0
3.5
4.0

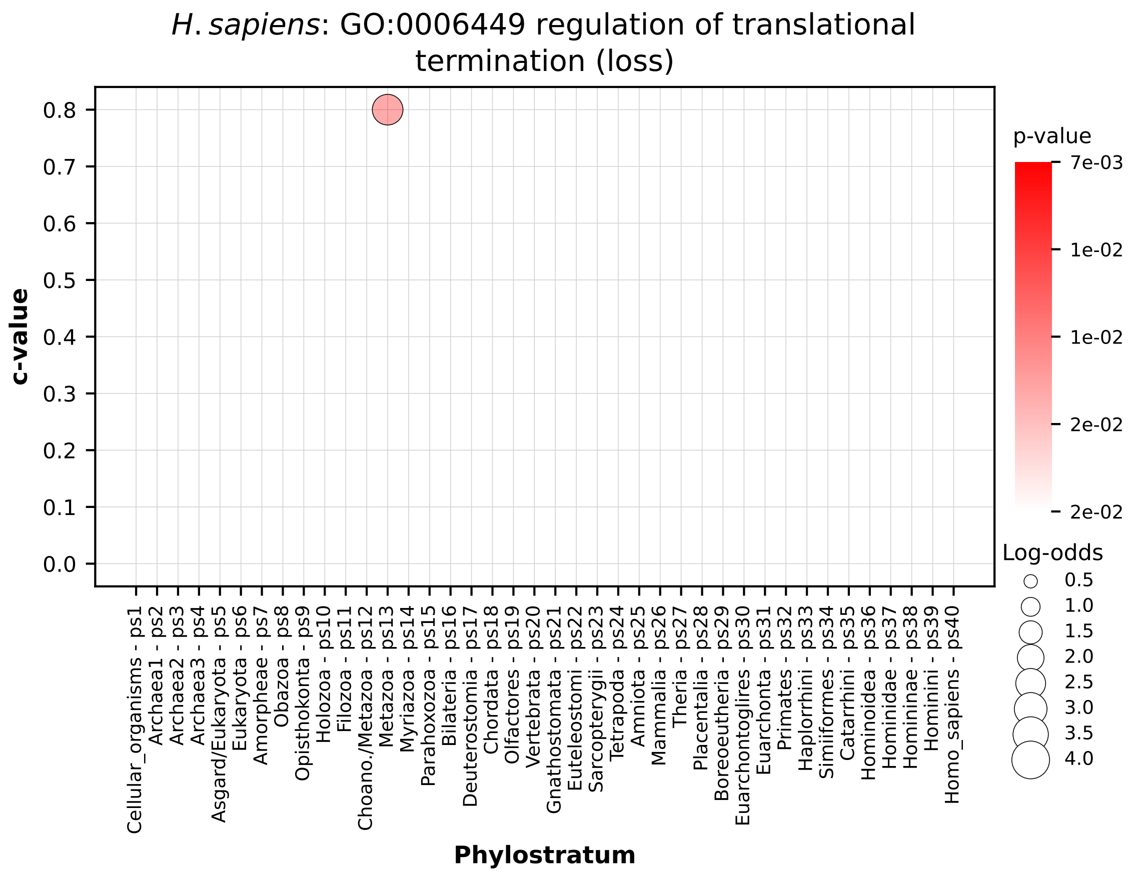

H. sapiens: GO:0006449 regulation of translational termination (loss)
c-value
0.8
0.7
0.6
0.5
0.4
0.3
0.2
0.1
0.0
p-value
7e-03
1e-02
1e-02
2e-02
2e-02
Log-odds
0.5
1.0
1.5
2.0
2.5
3.0
3.5
4.0
Cellular_organisms - ps1
Archaea1 - ps2
Archaea2 - ps3
Archaea3 - ps4
Asgard/Eukaryota - ps5
Eukaryota - ps6
Amorpheae - ps7
Obazoa - ps8
Opisthokonta - ps9
Holozoa - ps10
Filozoa - ps11
Choano./Metazoa - ps12
Metazoa - ps13
Myriazoa - ps14
Parahoxozoa - ps15
Bilateria - ps16
Deuterostomia - ps17
Chordata - ps18
Olfactores - ps19
Vertebrata - ps20
Gnathostomata - ps21
Euteleostomi - ps22
Sarcopterygii - ps23
Tetrapoda - ps24
Amniota - ps25
Mammalia - ps26
Theria - ps27
Placentalia - ps28
Boreoeutheria - ps29
Euarchontoglires - ps30
Euarchonta - ps31
Primates - ps32
Haplorrhini - ps33
Simiiformes - ps34
Catarrhini - ps35
Hominoidea - ps36
Hominidae - ps37
Homininae - ps38
Hominini - ps39
Homo_sapiens - ps40
Phylostratum

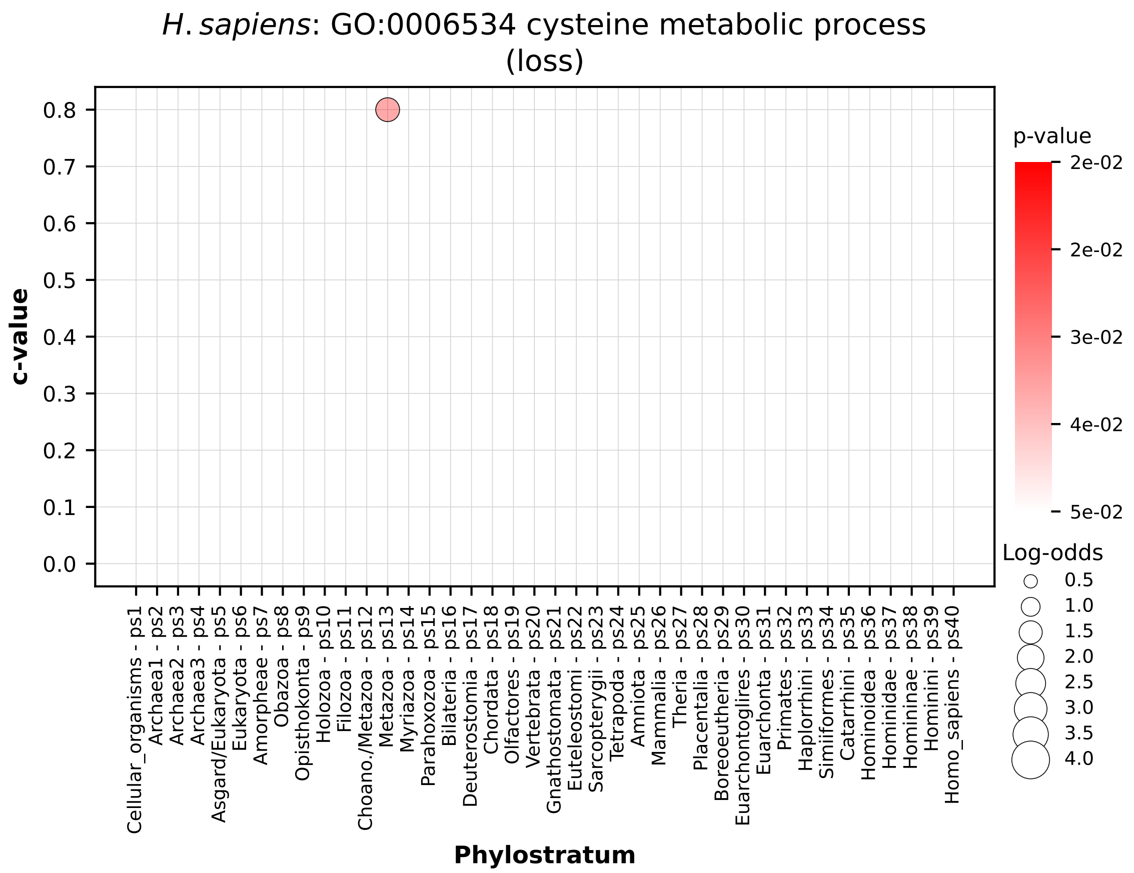
H. sapiens: GO:0006534 cysteine metabolic process
(loss)
c-value
0.0
0.1
0.2
0.3
0.4
0.5
0.6
0.7
0.8
p-value
2e-02
2e-02
3e-02
4e-02
5e-02
Log-odds
0.5
1.0
1.5
2.0
2.5
3.0
3.5
4.0
Cellular_organisms - ps1
Archaea1 - ps2
Archaea2 - ps3
Archaea3 - ps4
Asgard/Eukaryota - ps5
Eukaryota - ps6
Amorpheae - ps7
Obazoa - ps8
Opisthokonta - ps9
Holozoa - ps10
Filozoa - ps11
Choano./Metazoa - ps12
Metazoa - ps13
Myriazoa - ps14
Parahoxozoa - ps15
Bilateria - ps16
Deuterostomia - ps17
Chordata - ps18
Olfactores - ps19
Vertebrata - ps20
Gnathostomata - ps21
Euteleostomi - ps22
Sarcopterygii - ps23
Tetrapoda - ps24
Amniota - ps25
Mammalia - ps26
Theria - ps27
Placentalia - ps28
Boreoeutheria - ps29
Euarchontoglires - ps30
Euarchonta - ps31
Primates - ps32
Haplorrhini - ps33
Simiiformes - ps34
Catarrhini - ps35
Hominoidea - ps36
Hominidae - ps37
Homininae - ps38
Hominini - ps39
Homo_sapiens - ps40
Phylostratum

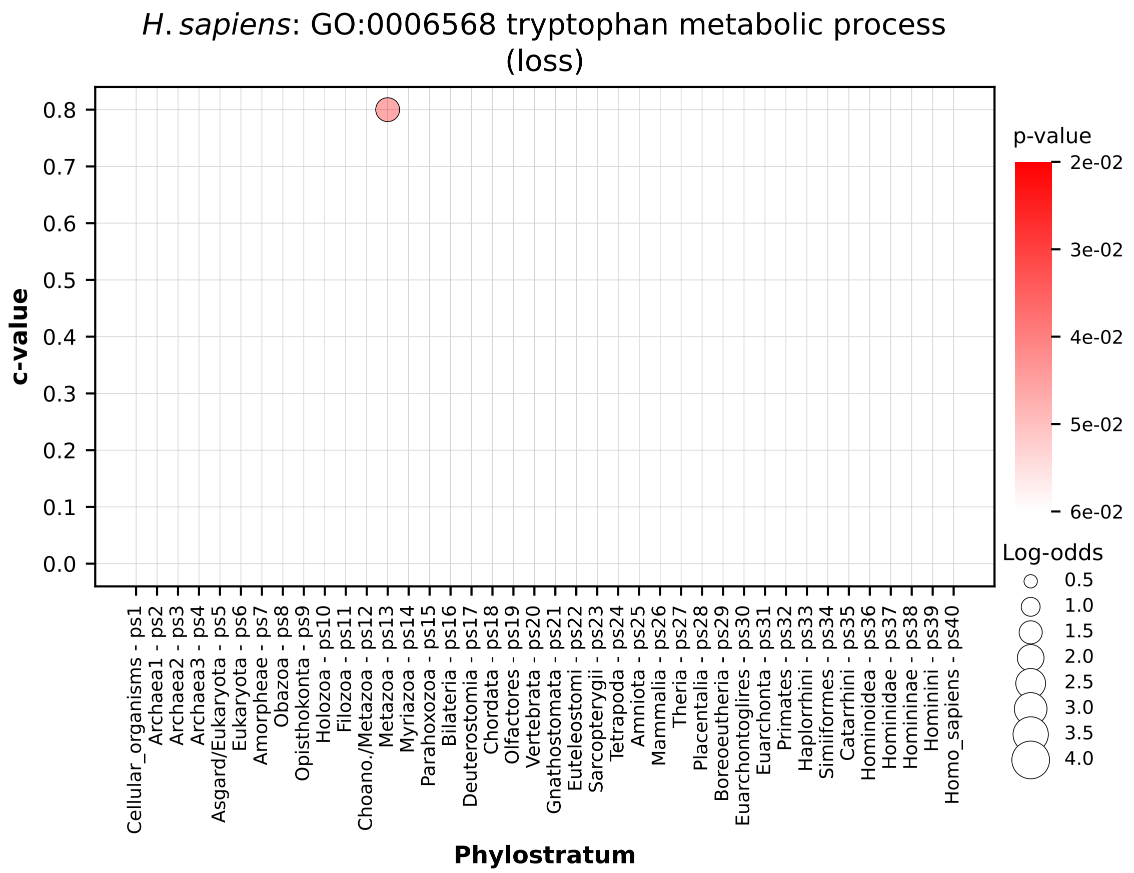

H. sapiens: GO:0006568 tryptophan metabolic process
(loss)
c-value
0.8
0.7
0.6
0.5
0.4
0.3
0.2
0.1
0.0
Phylostratum
Cellular_organisms - ps1
Archaea1 - ps2
Archaea2 - ps3
Archaea3 - ps4
Asgard/Eukaryota - ps5
Eukaryota - ps6
Amorpheae - ps7
Obazoa - ps8
Opisthokonta - ps9
Holozoa - ps10
Filozoa - ps11
Choano./Metazoa - ps12
Metazoa - ps13
Myriazoa - ps14
Parahoxozoa - ps15
Bilateria - ps16
Deuterostomia - ps17
Chordata - ps18
Olfactores - ps19
Vertebrata - ps20
Gnathostomata - ps21
Euteleostomi - ps22
Sarcopterygii - ps23
Tetrapoda - ps24
Amniota - ps25
Mammalia - ps26
Theria - ps27
Placentalia - ps28
Boreoeutheria - ps29
Euarchontoglires - ps30
Euarchonta - ps31
Primates - ps32
Haplorrhini - ps33
Simiiformes - ps34
Catarrhini - ps35
Hominoidea - ps36
Hominidae - ps37
Homininae - ps38
Hominini - ps39
Homo_sapiens - ps40
p-value
2e-02
3e-02
4e-02
5e-02
6e-02
Log-odds
0.5
1.0
1.5
2.0
2.5
3.0
3.5
4.0

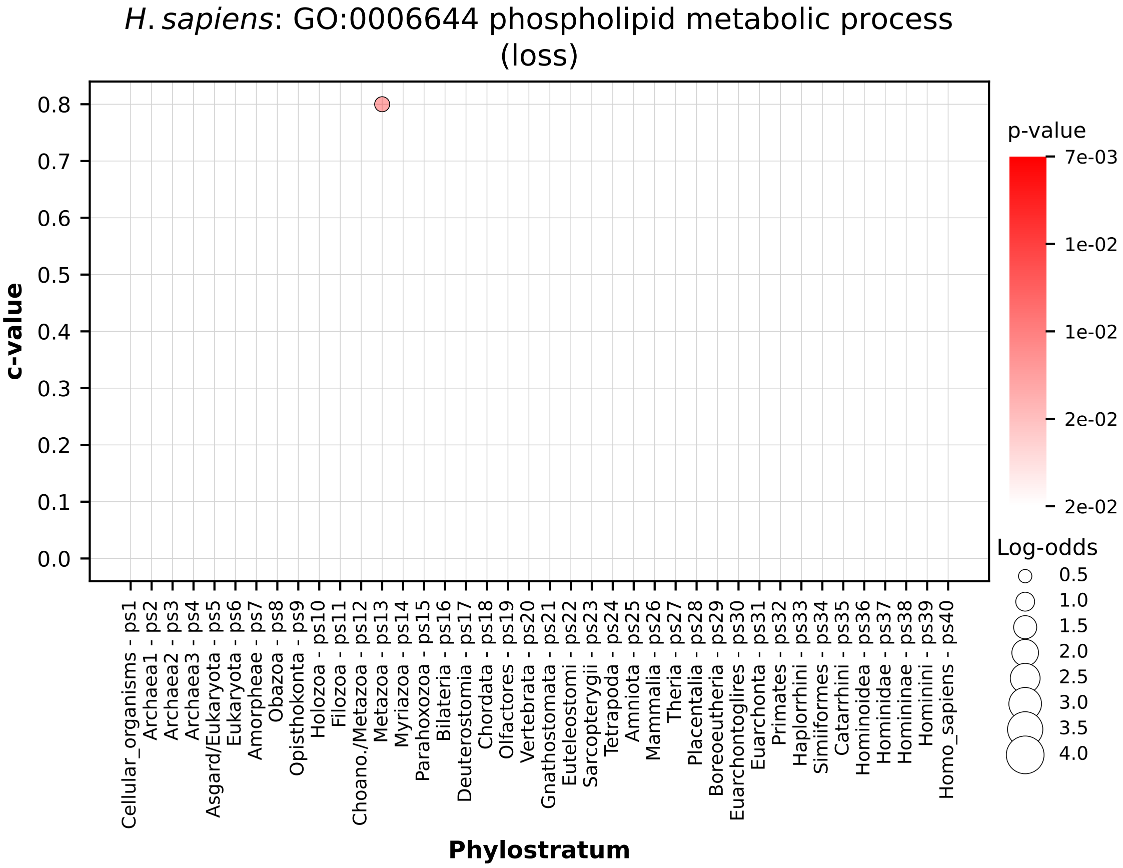

H. sapiens: GO:0006644 phospholipid metabolic process
(loss)
c-value
0.8
0.7
0.6
0.5
0.4
0.3
0.2
0.1
0.0
p-value
7e-03
1e-02
1e-02
2e-02
2e-02
Log-odds
0.5
1.0
1.5
2.0
2.5
3.0
3.5
4.0
Cellular_organisms - ps1
Archaea1 - ps2
Archaea2 - ps3
Archaea3 - ps4
Asgard/Eukaryota - ps5
Eukaryota - ps6
Amorpheae - ps7
Obazoa - ps8
Opisthokonta - ps9
Holozoa - ps10
Filozoa - ps11
Choano./Metazoa - ps12
Metazoa - ps13
Myriazoa - ps14
Parahoxozoa - ps15
Bilateria - ps16
Deuterostomia - ps17
Chordata - ps18
Olfactores - ps19
Vertebrata - ps20
Gnathostomata - ps21
Euteleostomi - ps22
Sarcopterygii - ps23
Tetrapoda - ps24
Amniota - ps25
Mammalia - ps26
Theria - ps27
Placentalia - ps28
Boreoeutheria - ps29
Euarchontoglires - ps30
Euarchonta - ps31
Primates - ps32
Haplorrhini - ps33
Simiiformes - ps34
Catarrhini - ps35
Hominoidea - ps36
Hominidae - ps37
Homininae - ps38
Hominini - ps39
Homo_sapiens - ps40
Phylostratum

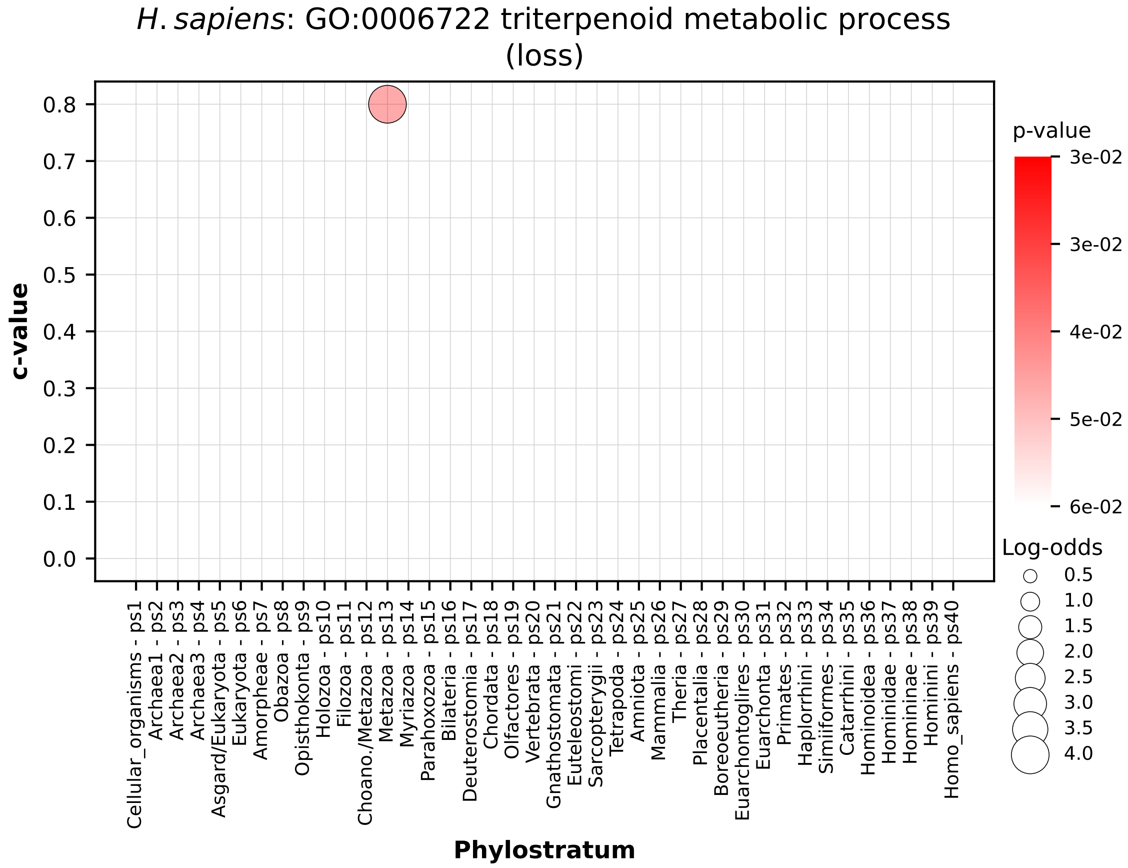

H. sapiens: GO:0006722 triterpenoid metabolic process
(loss)
c-value
0.0
0.1
0.2
0.3
0.4
0.5
0.6
0.7
0.8
p-value
3e-02
3e-02
4e-02
5e-02
6e-02
Log-odds
0.5
1.0
1.5
2.0
2.5
3.0
3.5
4.0
Cellular_organisms - ps1
Archaea1 - ps2
Archaea2 - ps3
Archaea3 - ps4
Asgard/Eukaryota - ps5
Eukaryota - ps6
Amorpheae - ps7
Obazoa - ps8
Opisthokonta - ps9
Holozoa - ps10
Filozoa - ps11
Choano./Metazoa - ps12
Metazoa - ps13
Myriazoa - ps14
Parahoxozoa - ps15
Bilateria - ps16
Deuterostomia - ps17
Chordata - ps18
Olfactores - ps19
Vertebrata - ps20
Gnathostomata - ps21
Euteleostomi - ps22
Sarcopterygii - ps23
Tetrapoda - ps24
Amniota - ps25
Mammalia - ps26
Theria - ps27
Placentalia - ps28
Boreoeutheria - ps29
Euarchontoglires - ps30
Euarchonta - ps31
Primates - ps32
Haplorrhini - ps33
Simiiformes - ps34
Catarrhini - ps35
Hominoidea - ps36
Hominidae - ps37
Homininae - ps38
Hominini - ps39
Homo_sapiens - ps40
Phylostratum

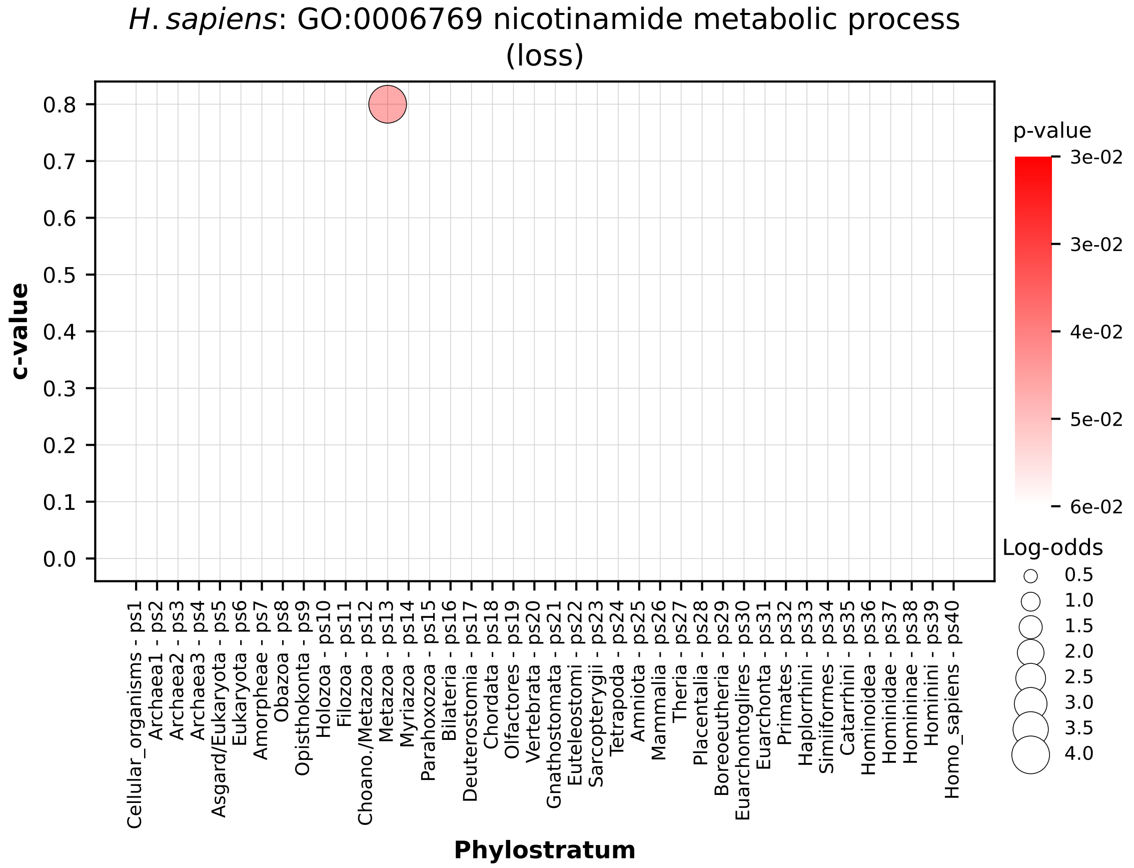

H. sapiens: GO:0006769 nicotinamide metabolic process
(loss)
c-value
0.0
0.1
0.2
0.3
0.4
0.5
0.6
0.7
0.8
p-value
3e-02
3e-02
4e-02
5e-02
6e-02
Log-odds
0.5
1.0
1.5
2.0
2.5
3.0
3.5
4.0
Cellular_organisms - ps1
Archaea1 - ps2
Archaea2 - ps3
Archaea3 - ps4
Asgard/Eukaryota - ps5
Eukaryota - ps6
Amorpheae - ps7
Obazoa - ps8
Opisthokonta - ps9
Holozoa - ps10
Filozoa - ps11
Choano./Metazoa - ps12
Metazoa - ps13
Myriazoa - ps14
Parahoxozoa - ps15
Bilateria - ps16
Deuterostomia - ps17
Chordata - ps18
Olfactores - ps19
Vertebrata - ps20
Gnathostomata - ps21
Euteleostomi - ps22
Sarcopterygii - ps23
Tetrapoda - ps24
Amniota - ps25
Mammalia - ps26
Theria - ps27
Placentalia - ps28
Boreoeutheria - ps29
Euarchontoglires - ps30
Euarchonta - ps31
Primates - ps32
Haplorrhini - ps33
Simiiformes - ps34
Catarrhini - ps35
Hominoidea - ps36
Hominidae - ps37
Homininae - ps38
Hominini - ps39
Homo_sapiens - ps40
Phylostratum

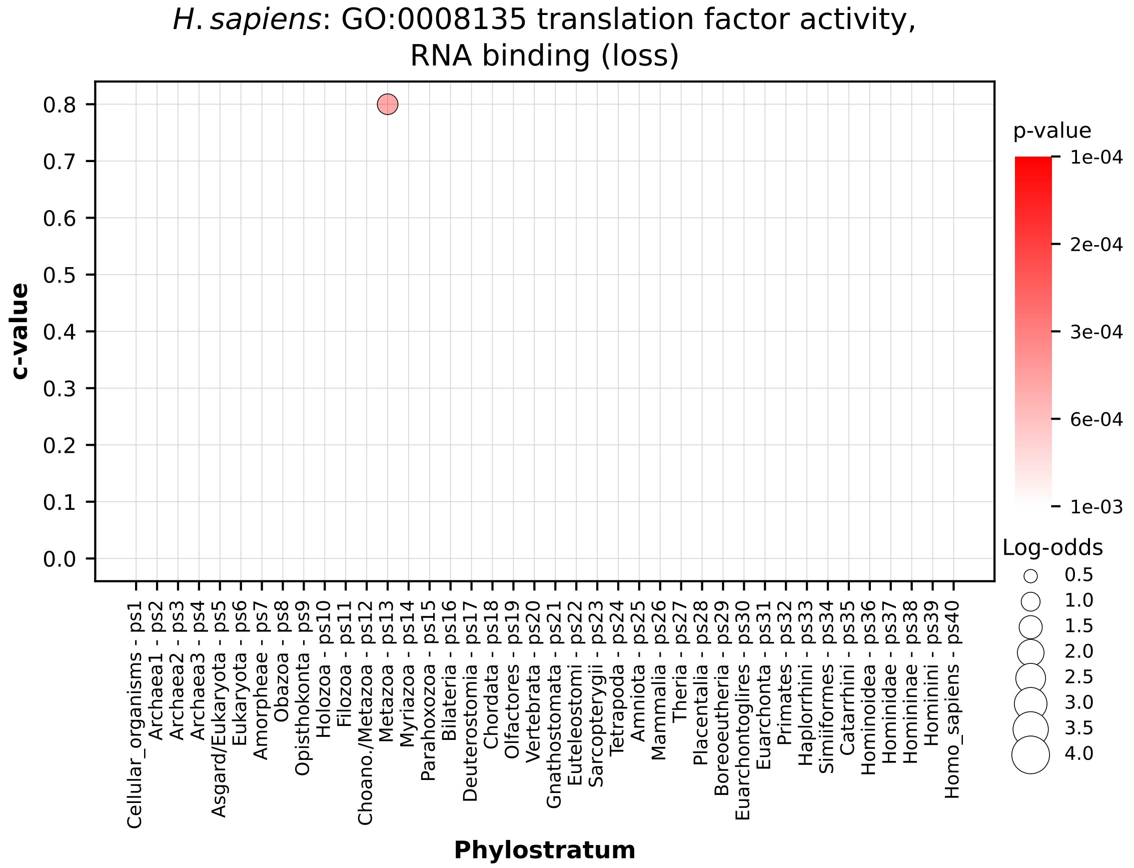

H. sapiens: GO:0008135 translation factor activity, RNA binding (loss)
c-value
0.8
0.7
0.6
0.5
0.4
0.3
0.2
0.1
0.0
p-value
1e-04
2e-04
3e-04
6e-04
1e-03
Log-odds
0.5
1.0
1.5
2.0
2.5
3.0
3.5
4.0
Cellular_organisms - ps1
Archaea1 - ps2
Archaea2 - ps3
Archaea3 - ps4
Asgard/Eukaryota - ps5
Eukaryota - ps6
Amorpheae - ps7
Obazoa - ps8
Opisthokonta - ps9
Holozoa - ps10
Filozoa - ps11
Choano./Metazoa - ps12
Metazoa - ps13
Myriazoa - ps14
Parahoxozoa - ps15
Bilateria - ps16
Deuterostomia - ps17
Chordata - ps18
Olfactores - ps19
Vertebrata - ps20
Gnathostomata - ps21
Euteleostomi - ps22
Sarcopterygii - ps23
Tetrapoda - ps24
Amniota - ps25
Mammalia - ps26
Theria - ps27
Placentalia - ps28
Boreoeutheria - ps29
Euarchontoglires - ps30
Euarchonta - ps31
Primates - ps32
Haplorrhini - ps33
Simiiformes - ps34
Catarrhini - ps35
Hominoidea - ps36
Hominidae - ps37
Homininae - ps38
Hominini - ps39
Homo_sapiens - ps40
Phylostratum

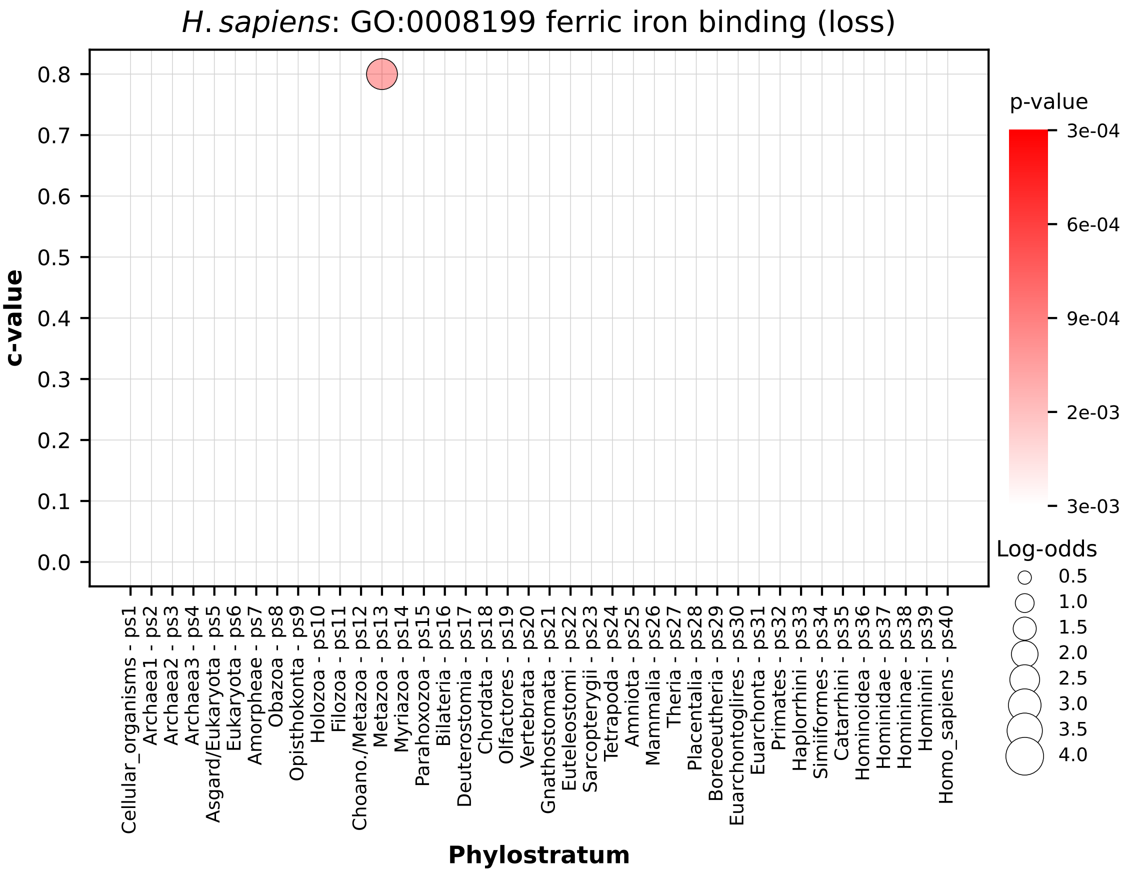

H. sapiens: GO:0008199 ferric iron binding (loss)
c-value
0.8
0.7
0.6
0.5
0.4
0.3
0.2
0.1
0.0
p-value
3e-04
6e-04
9e-04
2e-03
3e-03
Log-odds
0.5
1.0
1.5
2.0
2.5
3.0
3.5
4.0
Cellular_organisms - ps1
Archaea1 - ps2
Archaea2 - ps3
Archaea3 - ps4
Asgard/Eukaryota - ps5
Eukaryota - ps6
Amorpheae - ps7
Obazoa - ps8
Opisthokonta - ps9
Holozoa - ps10
Filozoa - ps11
Choano./Metazoa - ps12
Metazoa - ps13
Myriazoa - ps14
Parahoxozoa - ps15
Bilateria - ps16
Deuterostomia - ps17
Chordata - ps18
Olfactores - ps19
Vertebrata - ps20
Gnathostomata - ps21
Euteleostomi - ps22
Sarcopterygii - ps23
Tetrapoda - ps24
Amniota - ps25
Mammalia - ps26
Theria - ps27
Placentalia - ps28
Boreoeutheria - ps29
Euarchontoglires - ps30
Euarchonta - ps31
Primates - ps32
Haplorrhini - ps33
Simiiformes - ps34
Catarrhini - ps35
Hominoidea - ps36
Hominidae - ps37
Homininae - ps38
Hominini - ps39
Homo_sapiens - ps40
Phylostratum

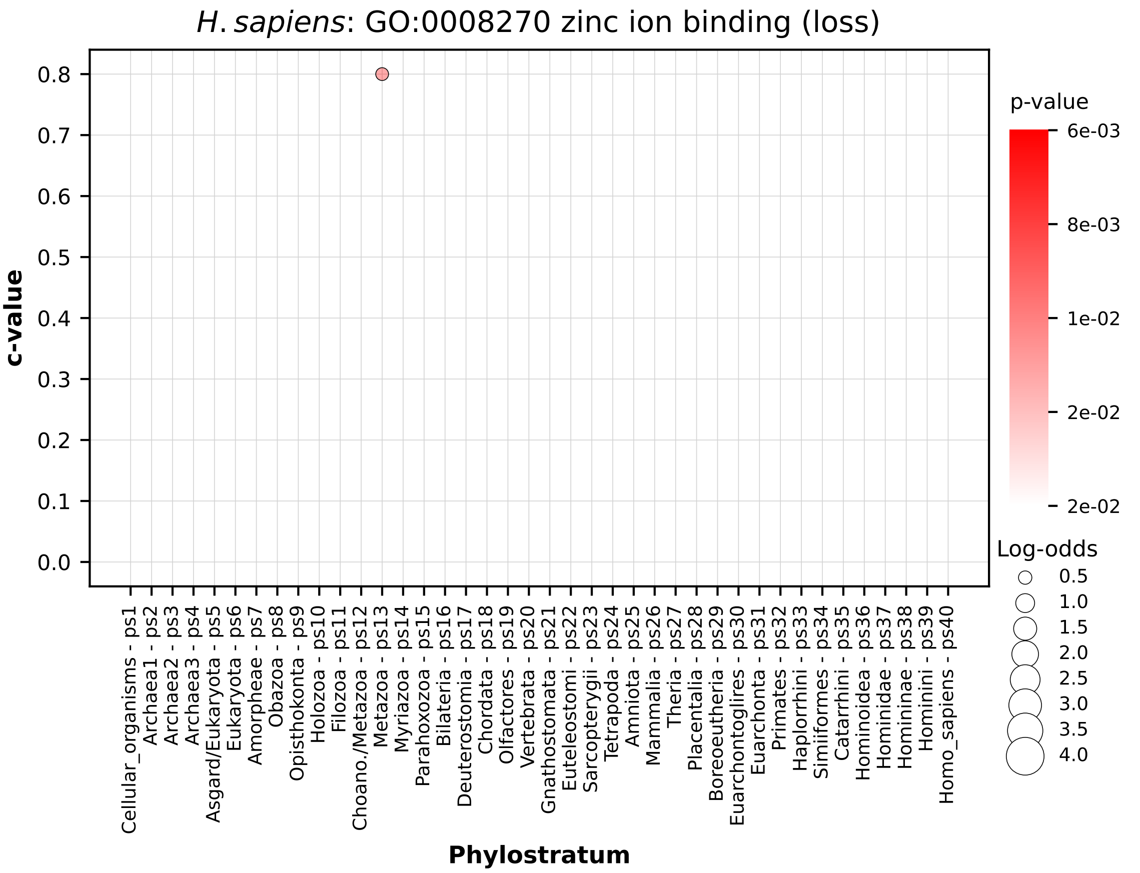
H. sapiens: GO:0008270 zinc ion binding (loss)
c-value
0.8
0.7
0.6
0.5
0.4
0.3
0.2
0.1
0.0
Phylostratum
Cellular_organisms - ps1
Archaea1 - ps2
Archaea2 - ps3
Archaea3 - ps4
Asgard/Eukaryota - ps5
Eukaryota - ps6
Amorpheae - ps7
Obazoa - ps8
Opisthokonta - ps9
Holozoa - ps10
Filozoa - ps11
Choano./Metazoa - ps12
Metazoa - ps13
Myriazoa - ps14
Parahoxozoa - ps15
Bilateria - ps16
Deuterostomia - ps17
Chordata - ps18
Olfactores - ps19
Vertebrata - ps20
Gnathostomata - ps21
Euteleostomi - ps22
Sarcopterygii - ps23
Tetrapoda - ps24
Amniota - ps25
Mammalia - ps26
Theria - ps27
Placentalia - ps28
Boreoeutheria - ps29
Euarchontoglires - ps30
Euarchonta - ps31
Primates - ps32
Haplorrhini - ps33
Simiiformes - ps34
Catarrhini - ps35
Hominoidea - ps36
Hominidae - ps37
Homininae - ps38
Hominini - ps39
Homo_sapiens - ps40
p-value
6e-03
8e-03
1e-02
2e-02
2e-02
Log-odds
0.5
1.0
1.5
2.0
2.5
3.0
3.5
4.0

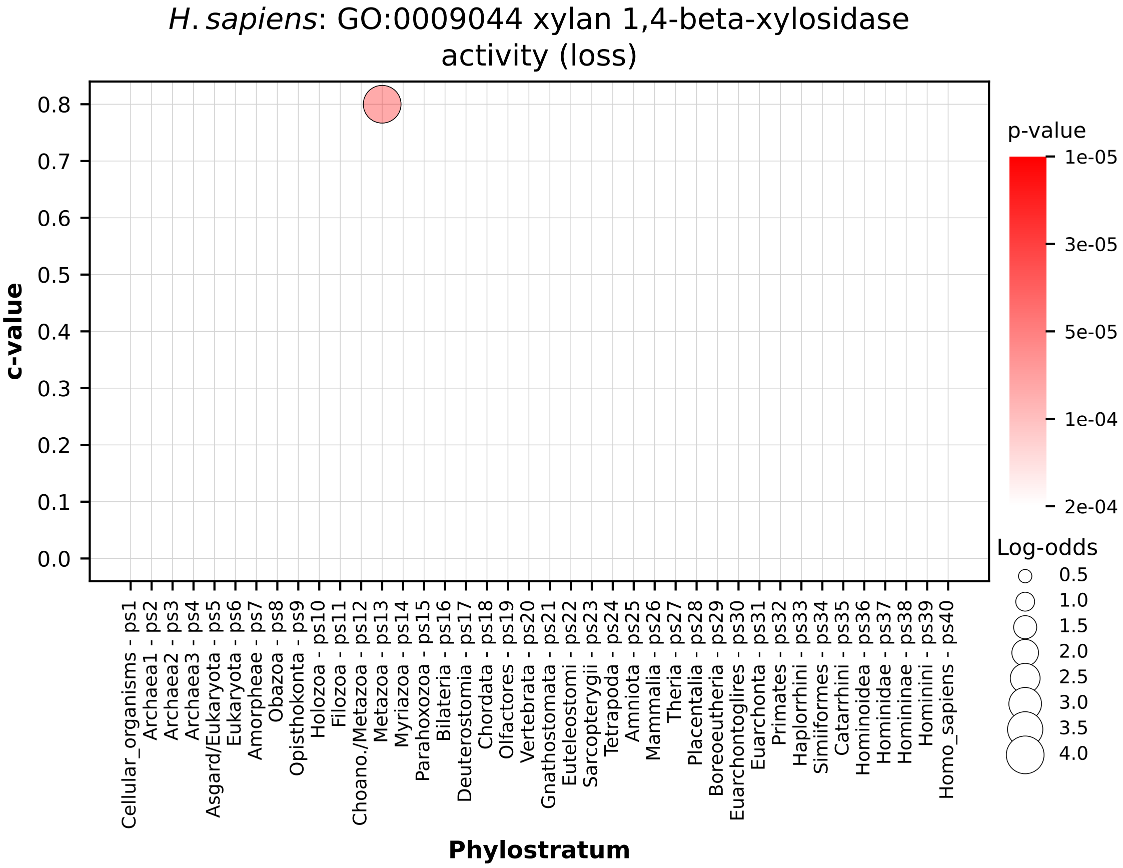

H. sapiens: GO:0009044 xylan 1,4-beta-xylosidase activity (loss)
c-value
0.0
0.1
0.2
0.3
0.4
0.5
0.6
0.7
0.8
Phylostratum
Cellular_organisms - ps1
Archaea1 - ps2
Archaea2 - ps3
Archaea3 - ps4
Asgard/Eukaryota - ps5
Eukaryota - ps6
Amorpheae - ps7
Obazoa - ps8
Opisthokonta - ps9
Holozoa - ps10
Filozoa - ps11
Choano./Metazoa - ps12
Metazoa - ps13
Myriazoa - ps14
Parahoxozoa - ps15
Bilateria - ps16
Deuterostomia - ps17
Chordata - ps18
Olfactores - ps19
Vertebrata - ps20
Gnathostomata - ps21
Euteleostomi - ps22
Sarcopterygii - ps23
Tetrapoda - ps24
Amniota - ps25
Mammalia - ps26
Theria - ps27
Placentalia - ps28
Boreoeutheria - ps29
Euarchontoglires - ps30
Euarchonta - ps31
Primates - ps32
Haplorrhini - ps33
Simiiformes - ps34
Catarrhini - ps35
Hominoidea - ps36
Hominidae - ps37
Homininae - ps38
Hominini - ps39
Homo_sapiens - ps40
p-value
1e-05
3e-05
5e-05
1e-04
2e-04
Log-odds
0.5
1.0
1.5
2.0
2.5
3.0
3.5
4.0

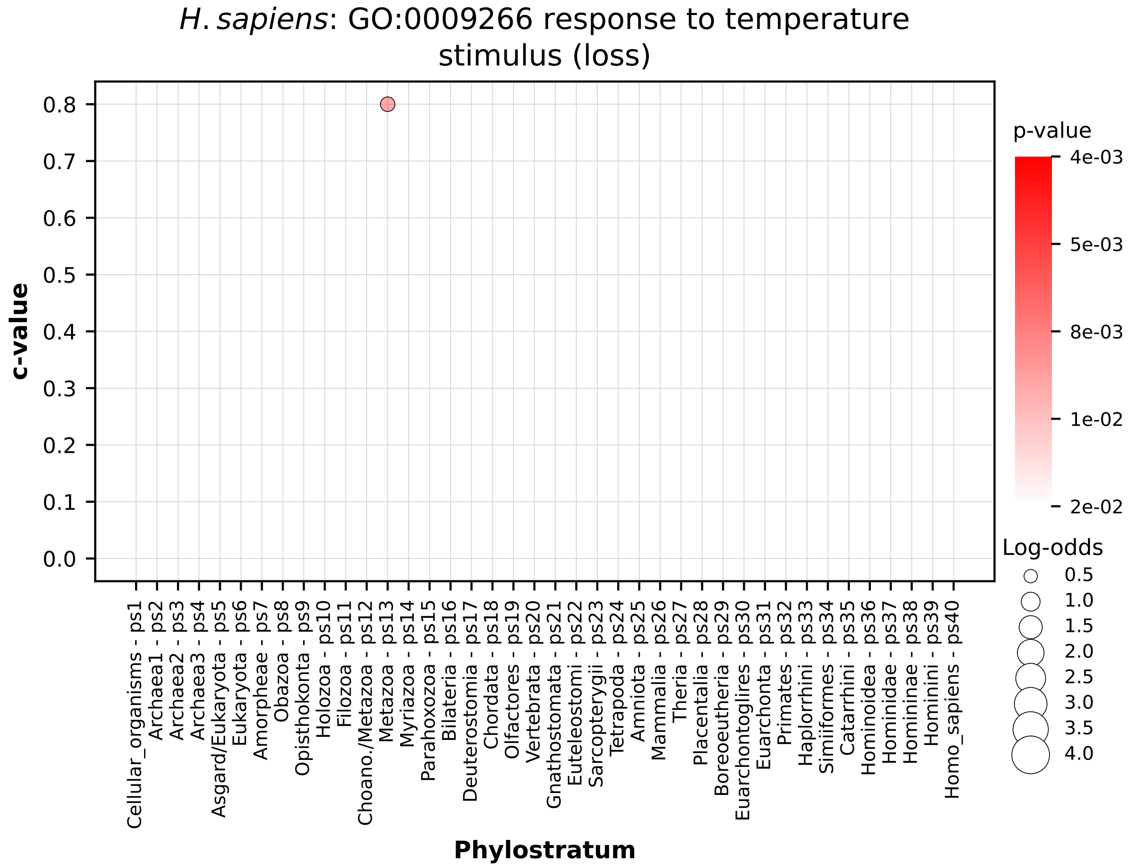
H. sapiens: GO:0009266 response to temperature stimulus (loss)
c-value
0.8
0.7
0.6
0.5
0.4
0.3
0.2
0.1
0.0
Phylostratum
Cellular_organisms - ps1
Archaea1 - ps2
Archaea2 - ps3
Archaea3 - ps4
Asgard/Eukaryota - ps5
Eukaryota - ps6
Amorpheae - ps7
Obazoa - ps8
Opisthokonta - ps9
Holozoa - ps10
Filozoa - ps11
Choano./Metazoa - ps12
Metazoa - ps13
Myriazoa - ps14
Parahoxozoa - ps15
Bilateria - ps16
Deuterostomia - ps17
Chordata - ps18
Olfactores - ps19
Vertebrata - ps20
Gnathostomata - ps21
Euteleostomi - ps22
Sarcopterygii - ps23
Tetrapoda - ps24
Amniota - ps25
Mammalia - ps26
Theria - ps27
Placentalia - ps28
Boreoeutheria - ps29
Euarchontoglires - ps30
Euarchonta - ps31
Primates - ps32
Haplorrhini - ps33
Simiiformes - ps34
Catarrhini - ps35
Hominoidea - ps36
Hominidae - ps37
Homininae - ps38
Hominini - ps39
Homo_sapiens - ps40
p-value
4e-03
5e-03
8e-03
1e-02
2e-02
Log-odds
0.5
1.0
1.5
2.0
2.5
3.0
3.5
4.0

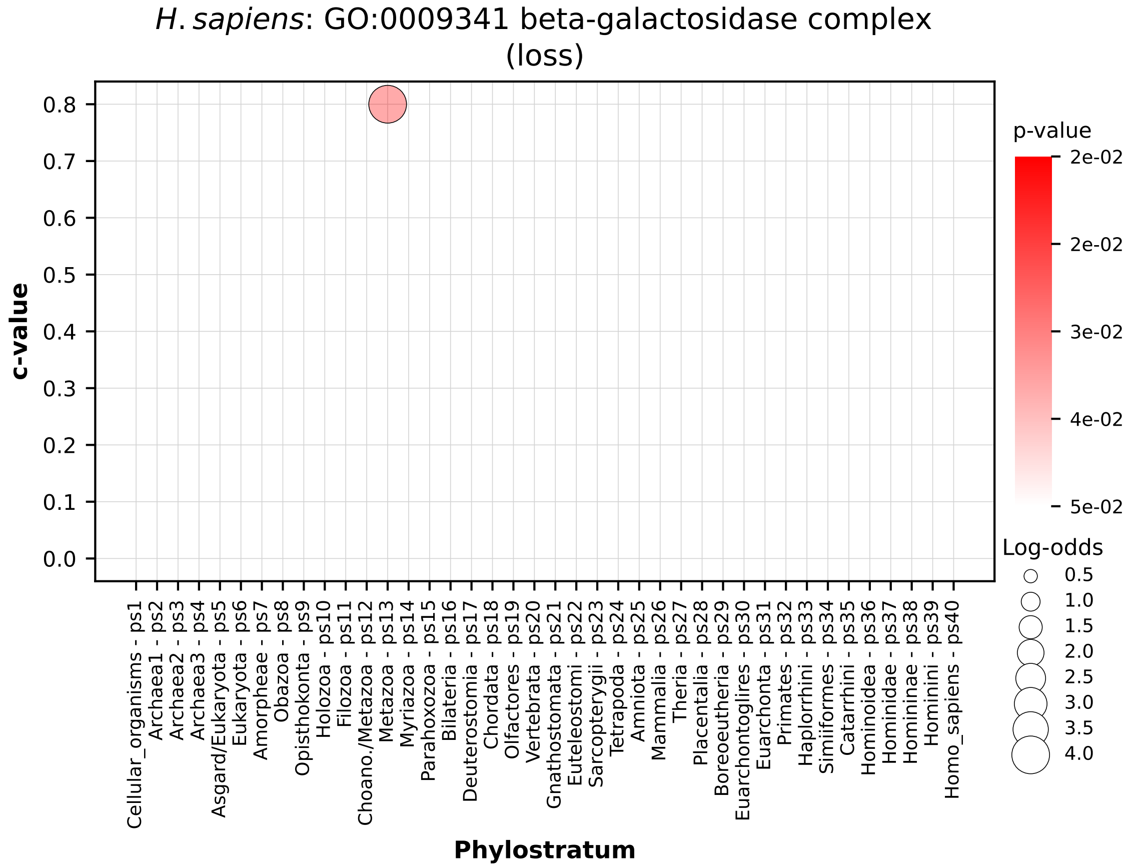

H. sapiens: GO:0009341 beta-galactosidase complex
(loss)
c-value
0.8
0.7
0.6
0.5
0.4
0.3
0.2
0.1
0.0
p-value
2e-02
2e-02
3e-02
4e-02
5e-02
Log-odds
0.5
1.0
1.5
2.0
2.5
3.0
3.5
4.0
Cellular_organisms - ps1
Archaea1 - ps2
Archaea2 - ps3
Archaea3 - ps4
Asgard/Eukaryota - ps5
Eukaryota - ps6
Amorpheae - ps7
Obazoa - ps8
Opisthokonta - ps9
Holozoa - ps10
Filozoa - ps11
Choano./Metazoa - ps12
Metazoa - ps13
Myriazoa - ps14
Parahoxozoa - ps15
Bilateria - ps16
Deuterostomia - ps17
Chordata - ps18
Olfactores - ps19
Vertebrata - ps20
Gnathostomata - ps21
Euteleostomi - ps22
Sarcopterygii - ps23
Tetrapoda - ps24
Amniota - ps25
Mammalia - ps26
Theria - ps27
Placentalia - ps28
Boreoeutheria - ps29
Euarchontoglires - ps30
Euarchonta - ps31
Primates - ps32
Haplorrhini - ps33
Simiiformes - ps34
Catarrhini - ps35
Hominoidea - ps36
Hominidae - ps37
Homininae - ps38
Hominini - ps39
Homo_sapiens - ps40
Phylostratum

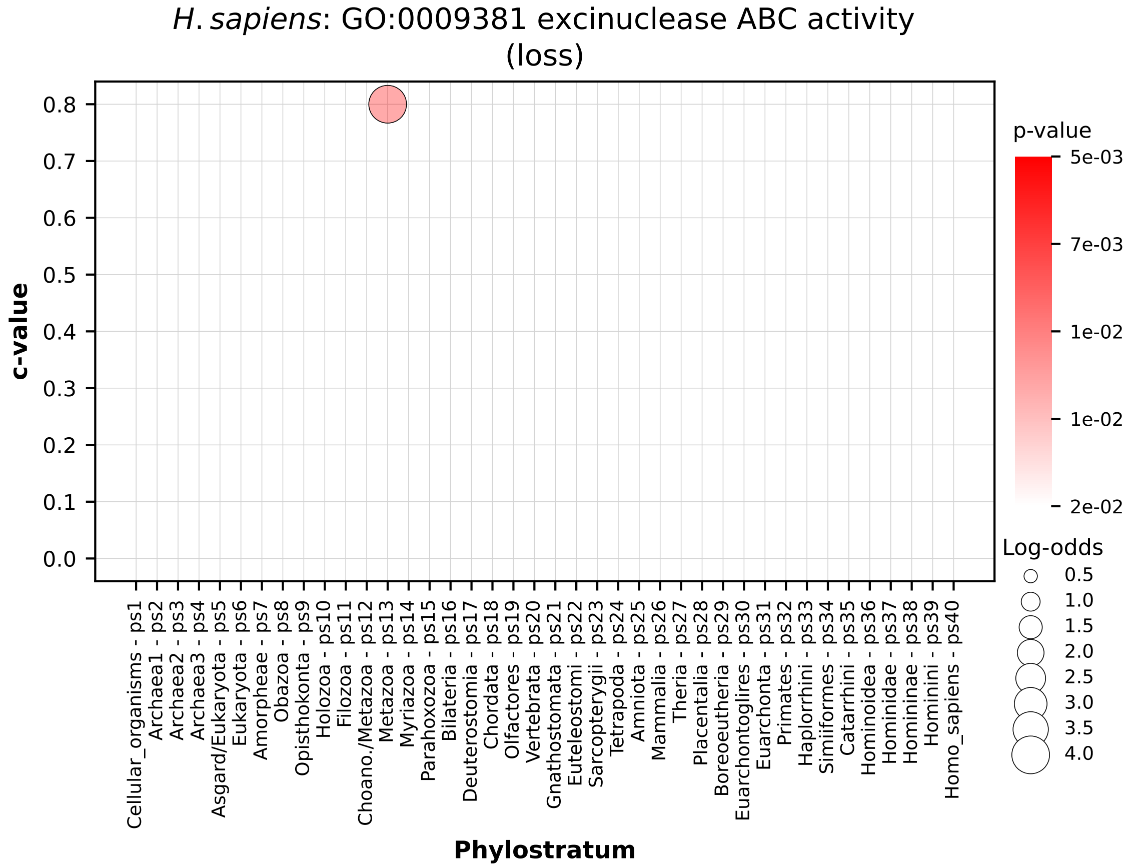

H. sapiens: GO:0009381 excinuclease ABC activity
(loss)
c-value
0.8
0.7
0.6
0.5
0.4
0.3
0.2
0.1
0.0
p-value
5e-03
7e-03
1e-02
1e-02
2e-02
Log-odds
0.5
1.0
1.5
2.0
2.5
3.0
3.5
4.0
Cellular_organisms - ps1
Archaea1 - ps2
Archaea2 - ps3
Archaea3 - ps4
Asgard/Eukaryota - ps5
Eukaryota - ps6
Amorpheae - ps7
Obazoa - ps8
Opisthokonta - ps9
Holozoa - ps10
Filozoa - ps11
Choano./Metazoa - ps12
Metazoa - ps13
Myriazoa - ps14
Parahoxozoa - ps15
Bilateria - ps16
Deuterostomia - ps17
Chordata - ps18
Olfactores - ps19
Vertebrata - ps20
Gnathostomata - ps21
Euteleostomi - ps22
Sarcopterygii - ps23
Tetrapoda - ps24
Amniota - ps25
Mammalia - ps26
Theria - ps27
Placentalia - ps28
Boreoeutheria - ps29
Euarchontoglires - ps30
Euarchonta - ps31
Primates - ps32
Haplorrhini - ps33
Simiiformes - ps34
Catarrhini - ps35
Hominoidea - ps36
Hominidae - ps37
Homininae - ps38
Hominini - ps39
Homo_sapiens - ps40
Phylostratum

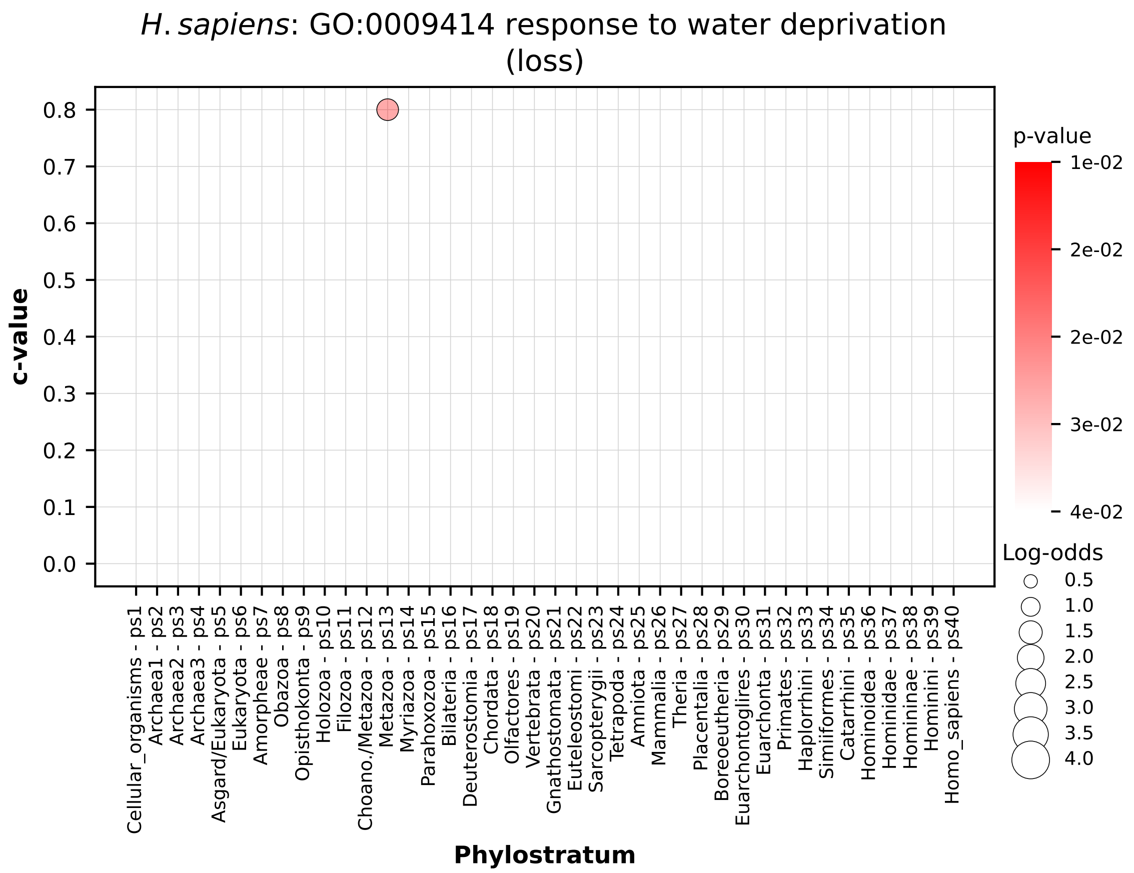

H. sapiens: GO:0009414 response to water deprivation (loss)
c-value
0.0
0.1
0.2
0.3
0.4
0.5
0.6
0.7
0.8
Phylostratum
Cellular_organisms - ps1
Archaea1 - ps2
Archaea2 - ps3
Archaea3 - ps4
Asgard/Eukaryota - ps5
Eukaryota - ps6
Amorpheae - ps7
Obazoa - ps8
Opisthokonta - ps9
Holozoa - ps10
Filozoa - ps11
Choano./Metazoa - ps12
Metazoa - ps13
Myriazoa - ps14
Parahoxozoa - ps15
Bilateria - ps16
Deuterostomia - ps17
Chordata - ps18
Olfactores - ps19
Vertebrata - ps20
Gnathostomata - ps21
Euteleostomi - ps22
Sarcopterygii - ps23
Tetrapoda - ps24
Amniota - ps25
Mammalia - ps26
Theria - ps27
Placentalia - ps28
Boreoeutheria - ps29
Euarchontoglires - ps30
Euarchonta - ps31
Primates - ps32
Haplorrhini - ps33
Simiiformes - ps34
Catarrhini - ps35
Hominoidea - ps36
Hominidae - ps37
Homininae - ps38
Hominini - ps39
Homo_sapiens - ps40
p-value
1e-02
2e-02
2e-02
3e-02
4e-02
Log-odds
0.5
1.0
1.5
2.0
2.5
3.0
3.5
4.0

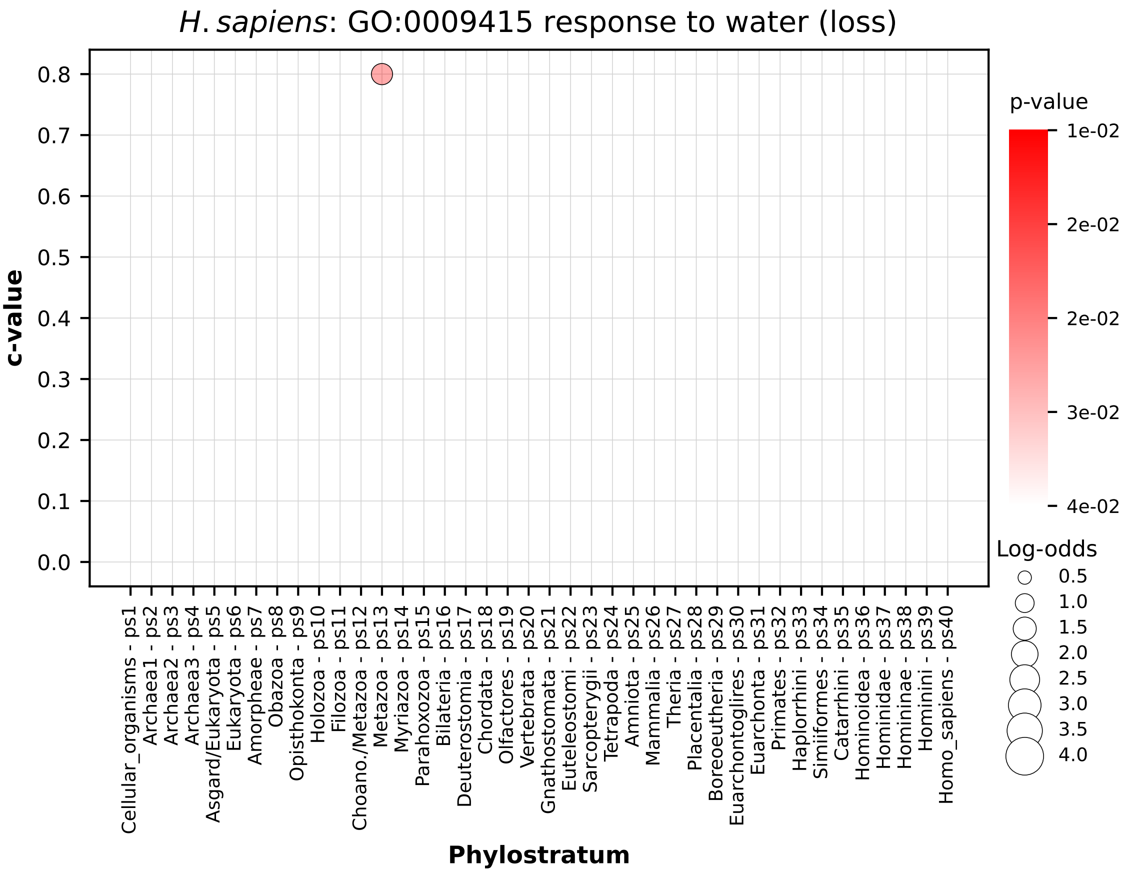

H. sapiens: GO:0009415 response to water (loss)
c-value
0.8
0.7
0.6
0.5
0.4
0.3
0.2
0.1
0.0
p-value
1e-02
2e-02
2e-02
3e-02
4e-02
Log-odds
0.5
1.0
1.5
2.0
2.5
3.0
3.5
4.0
Cellular_organisms - ps1
Archaea1 - ps2
Archaea2 - ps3
Archaea3 - ps4
Asgard/Eukaryota - ps5
Eukaryota - ps6
Amorpheae - ps7
Obazoa - ps8
Opisthokonta - ps9
Holozoa - ps10
Filozoa - ps11
Choano./Metazoa - ps12
Metazoa - ps13
Myriazoa - ps14
Parahoxozoa - ps15
Bilateria - ps16
Deuterostomia - ps17
Chordata - ps18
Olfactores - ps19
Vertebrata - ps20
Gnathostomata - ps21
Euteleostomi - ps22
Sarcopterygii - ps23
Tetrapoda - ps24
Amniota - ps25
Mammalia - ps26
Theria - ps27
Placentalia - ps28
Boreoeutheria - ps29
Euarchontoglires - ps30
Euarchonta - ps31
Primates - ps32
Haplorrhini - ps33
Simiiformes - ps34
Catarrhini - ps35
Hominoidea - ps36
Hominidae - ps37
Homininae - ps38
Hominini - ps39
Homo_sapiens - ps40
Phylostratum

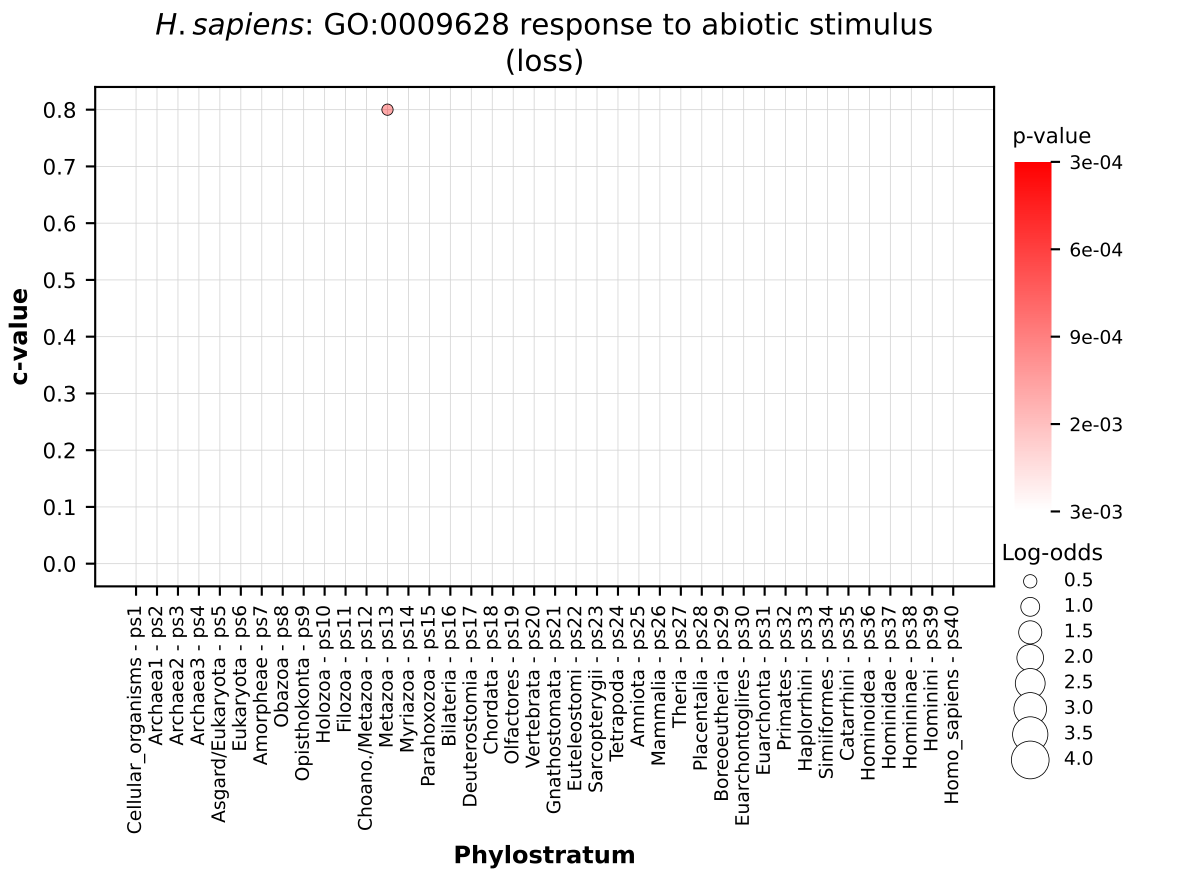

H. sapiens: GO:0009628 response to abiotic stimulus (loss)
c-value
0.0
0.1
0.2
0.3
0.4
0.5
0.6
0.7
0.8
Phylostratum
Cellular_organisms - ps1
Archaea1 - ps2
Archaea2 - ps3
Archaea3 - ps4
Asgard/Eukaryota - ps5
Eukaryota - ps6
Amorpheae - ps7
Obazoa - ps8
Opisthokonta - ps9
Holozoa - ps10
Filozoa - ps11
Choano./Metazoa - ps12
Metazoa - ps13
Myriazoa - ps14
Parahoxozoa - ps15
Bilateria - ps16
Deuterostomia - ps17
Chordata - ps18
Olfactores - ps19
Vertebrata - ps20
Gnathostomata - ps21
Euteleostomi - ps22
Sarcopterygii - ps23
Tetrapoda - ps24
Amniota - ps25
Mammalia - ps26
Theria - ps27
Placentalia - ps28
Boreoeutheria - ps29
Euarchontoglires - ps30
Euarchonta - ps31
Primates - ps32
Haplorrhini - ps33
Simiiformes - ps34
Catarrhini - ps35
Hominoidea - ps36
Hominidae - ps37
Homininae - ps38
Hominini - ps39
Homo_sapiens - ps40
p-value
3e-04
6e-04
9e-04
2e-03
3e-03
Log-odds
0.5
1.0
1.5
2.0
2.5
3.0
3.5
4.0

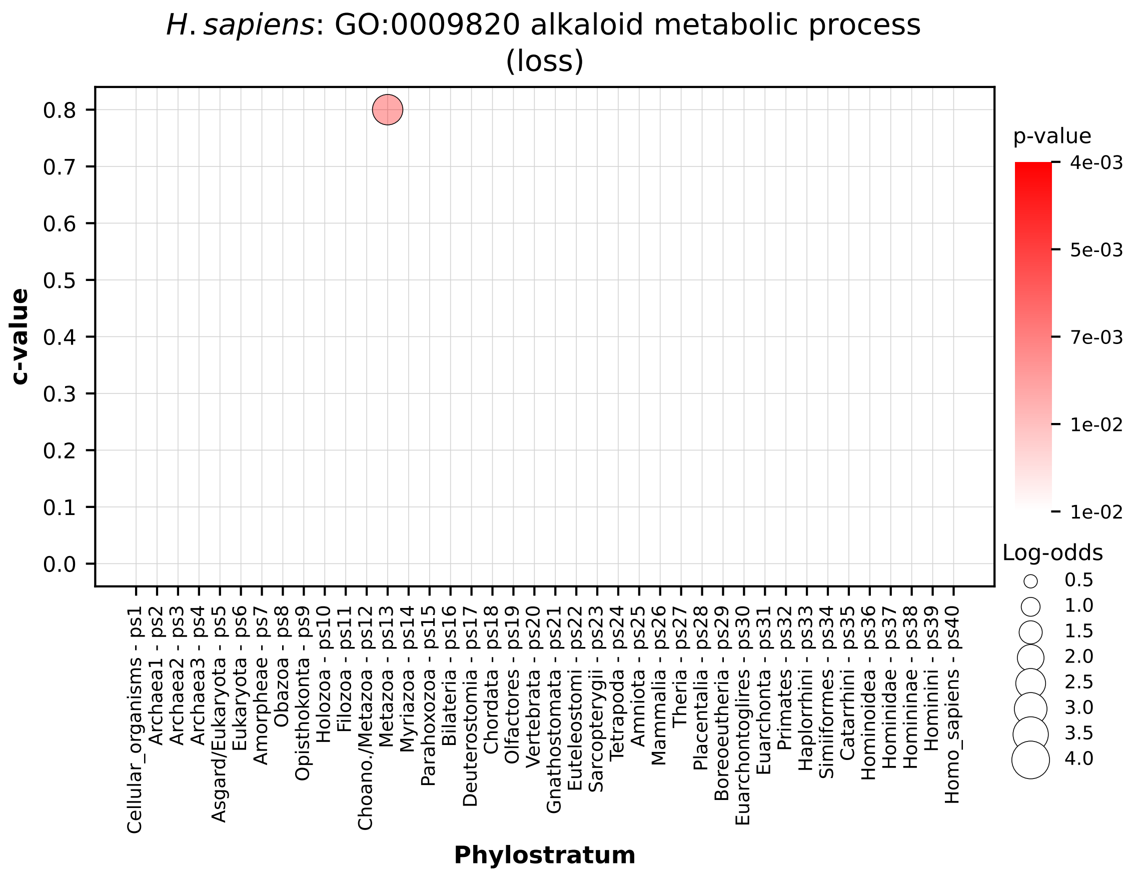

H. sapiens: GO:0009820 alkaloid metabolic process
(loss)
c-value
0.0
0.1
0.2
0.3
0.4
0.5
0.6
0.7
0.8
p-value
4e-03
5e-03
7e-03
1e-02
1e-02
Log-odds
0.5
1.0
1.5
2.0
2.5
3.0
3.5
4.0
Cellular_organisms - ps1
Archaea1 - ps2
Archaea2 - ps3
Archaea3 - ps4
Asgard/Eukaryota - ps5
Eukaryota - ps6
Amorpheae - ps7
Obazoa - ps8
Opisthokonta - ps9
Holozoa - ps10
Filozoa - ps11
Choano./Metazoa - ps12
Metazoa - ps13
Myriazoa - ps14
Parahoxozoa - ps15
Bilateria - ps16
Deuterostomia - ps17
Chordata - ps18
Olfactores - ps19
Vertebrata - ps20
Gnathostomata - ps21
Euteleostomi - ps22
Sarcopterygii - ps23
Tetrapoda - ps24
Amniota - ps25
Mammalia - ps26
Theria - ps27
Placentalia - ps28
Boreoeutheria - ps29
Euarchontoglires - ps30
Euarchonta - ps31
Primates - ps32
Haplorrhini - ps33
Simiiformes - ps34
Catarrhini - ps35
Hominoidea - ps36
Hominidae - ps37
Homininae - ps38
Hominini - ps39
Homo_sapiens - ps40
Phylostratum

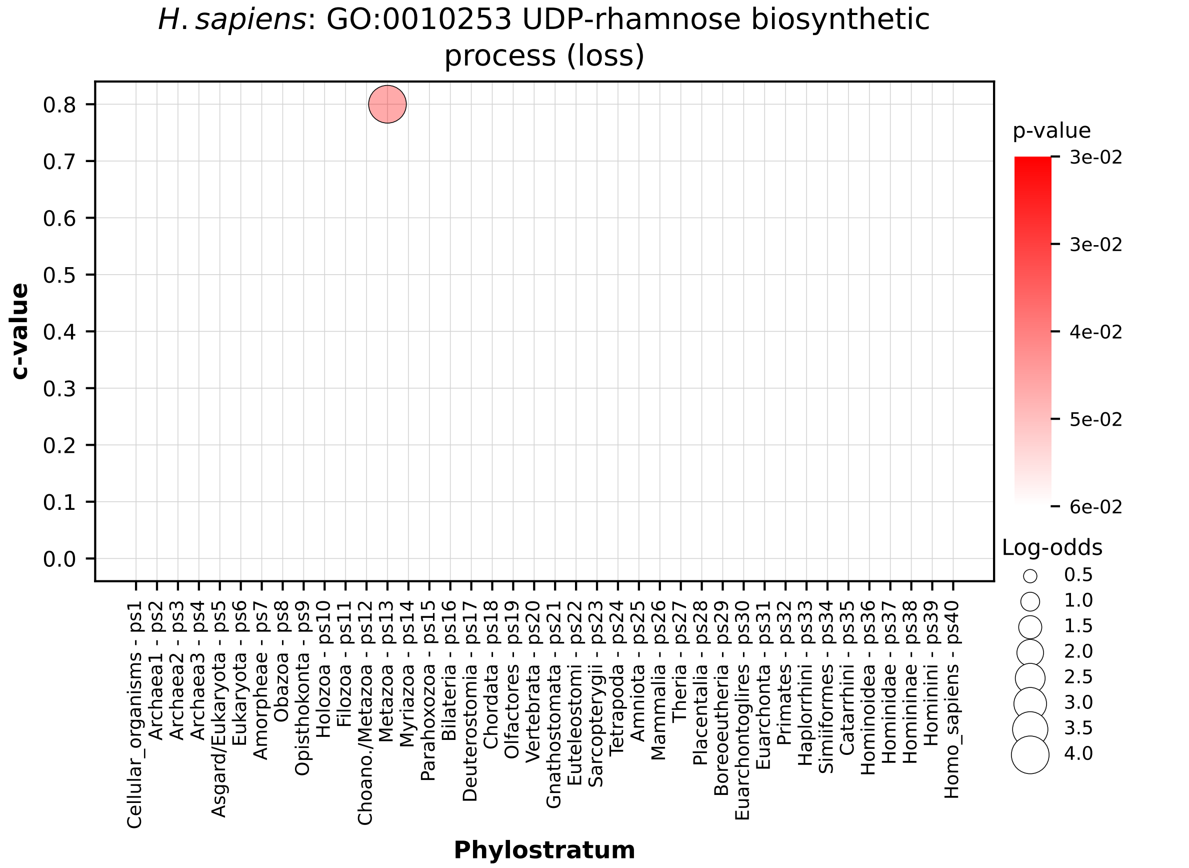
H. sapiens: GO:0010253 UDP-rhamnose biosynthetic process (loss)
c-value
0.8
0.7
0.6
0.5
0.4
0.3
0.2
0.1
0.0
Phylostratum
Cellular_organisms - ps1
Archaea1 - ps2
Archaea2 - ps3
Archaea3 - ps4
Asgard/Eukaryota - ps5
Eukaryota - ps6
Amorpheae - ps7
Obazoa - ps8
Opisthokonta - ps9
Holozoa - ps10
Filozoa - ps11
Choano./Metazoa - ps12
Metazoa - ps13
Myriazoa - ps14
Parahoxozoa - ps15
Bilateria - ps16
Deuterostomia - ps17
Chordata - ps18
Olfactores - ps19
Vertebrata - ps20
Gnathostomata - ps21
Euteleostomi - ps22
Sarcopterygii - ps23
Tetrapoda - ps24
Amniota - ps25
Mammalia - ps26
Theria - ps27
Placentalia - ps28
Boreoeutheria - ps29
Euarchontoglires - ps30
Euarchonta - ps31
Primates - ps32
Haplorrhini - ps33
Simiiformes - ps34
Catarrhini - ps35
Hominoidea - ps36
Hominidae - ps37
Homininae - ps38
Hominini - ps39
Homo_sapiens - ps40
p-value
3e-02
3e-02
4e-02
5e-02
6e-02
Log-odds
0.5
1.0
1.5
2.0
2.5
3.0
3.5
4.0

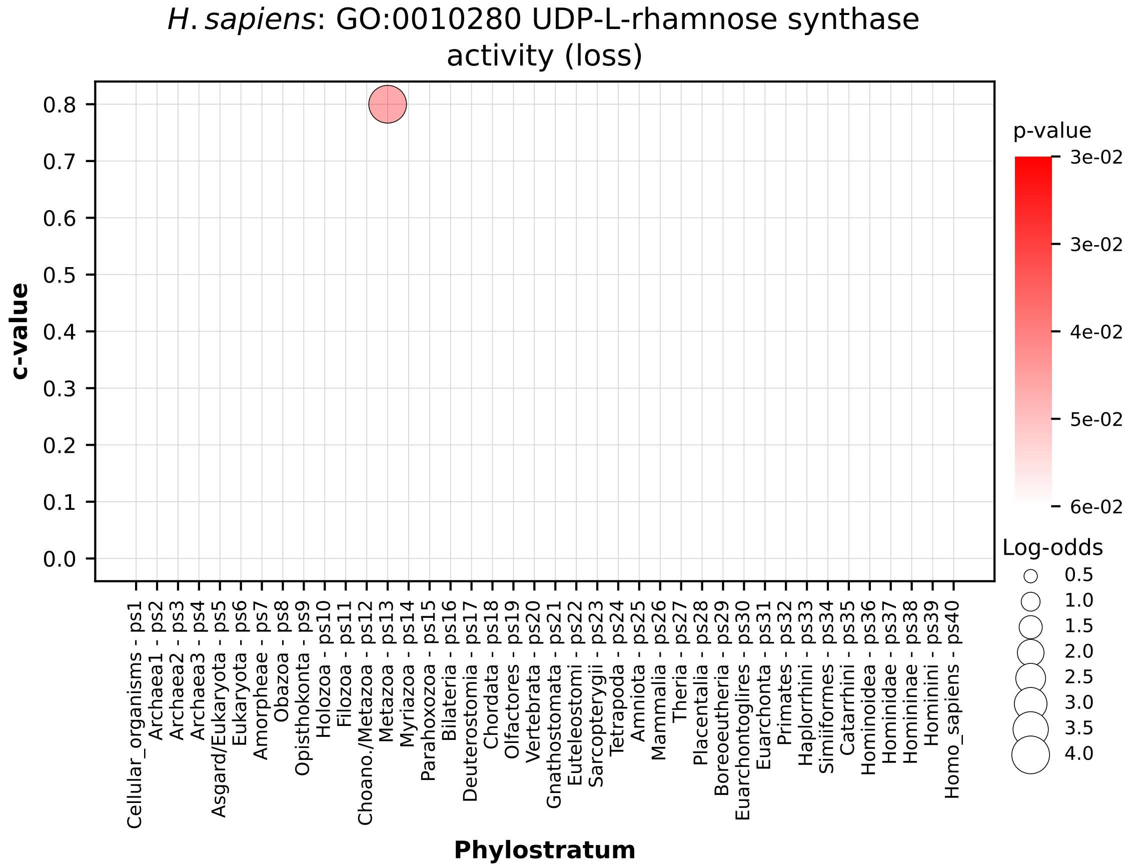
H. sapiens: GO:0010280 UDP-L-rhamnose synthase activity (loss)
c-value
0.0
0.1
0.2
0.3
0.4
0.5
0.6
0.7
0.8
p-value
3e-02
3e-02
4e-02
5e-02
6e-02
Log-odds
0.5
1.0
1.5
2.0
2.5
3.0
3.5
4.0
Cellular_organisms - ps1
Archaea1 - ps2
Archaea2 - ps3
Archaea3 - ps4
Asgard/Eukaryota - ps5
Eukaryota - ps6
Amorpheae - ps7
Obazoa - ps8
Opisthokonta - ps9
Holozoa - ps10
Filozoa - ps11
Choano./Metazoa - ps12
Metazoa - ps13
Myriazoa - ps14
Parahoxozoa - ps15
Bilateria - ps16
Deuterostomia - ps17
Chordata - ps18
Olfactores - ps19
Vertebrata - ps20
Gnathostomata - ps21
Euteleostomi - ps22
Sarcopterygii - ps23
Tetrapoda - ps24
Amniota - ps25
Mammalia - ps26
Theria - ps27
Placentalia - ps28
Boreoeutheria - ps29
Euarchontoglires - ps30
Euarchonta - ps31
Primates - ps32
Haplorrhini - ps33
Simiiformes - ps34
Catarrhini - ps35
Hominoidea - ps36
Hominidae - ps37
Homininae - ps38
Hominini - ps39
Homo_sapiens - ps40
Phylostratum

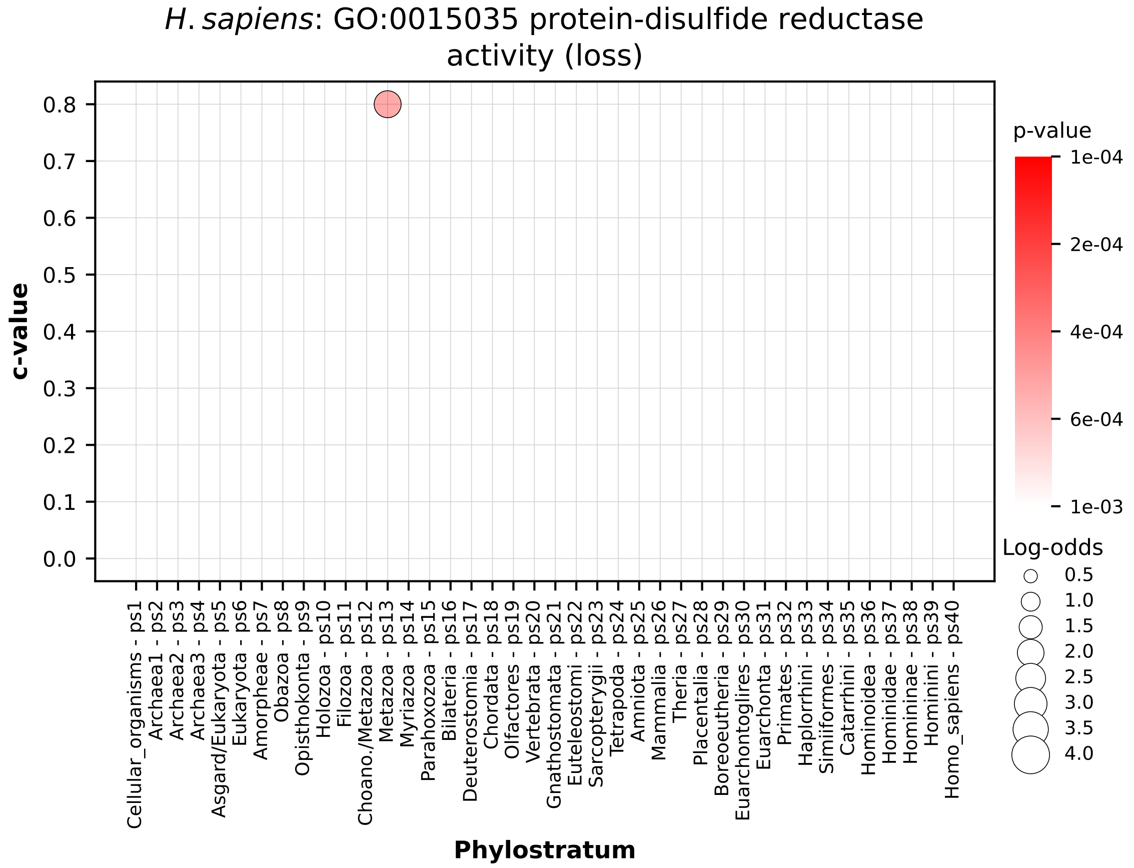
H. sapiens: GO:0015035 protein-disulfide reductase activity (loss)
c-value
0.8
0.7
0.6
0.5
0.4
0.3
0.2
0.1
0.0
p-value
1e-04
2e-04
4e-04
6e-04
1e-03
Log-odds
0.5
1.0
1.5
2.0
2.5
3.0
3.5
4.0
Cellular_organisms - ps1
Archaea1 - ps2
Archaea2 - ps3
Archaea3 - ps4
Asgard/Eukaryota - ps5
Eukaryota - ps6
Amorpheae - ps7
Obazoa - ps8
Opisthokonta - ps9
Holozoa - ps10
Filozoa - ps11
Choano./Metazoa - ps12
Metazoa - ps13
Myriazoa - ps14
Parahoxozoa - ps15
Bilateria - ps16
Deuterostomia - ps17
Chordata - ps18
Olfactores - ps19
Vertebrata - ps20
Gnathostomata - ps21
Euteleostomi - ps22
Sarcopterygii - ps23
Tetrapoda - ps24
Amniota - ps25
Mammalia - ps26
Theria - ps27
Placentalia - ps28
Boreoeutheria - ps29
Euarchontoglires - ps30
Euarchonta - ps31
Primates - ps32
Haplorrhini - ps33
Simiiformes - ps34
Catarrhini - ps35
Hominoidea - ps36
Hominidae - ps37
Homininae - ps38
Hominini - ps39
Homo_sapiens - ps40
Phylostratum

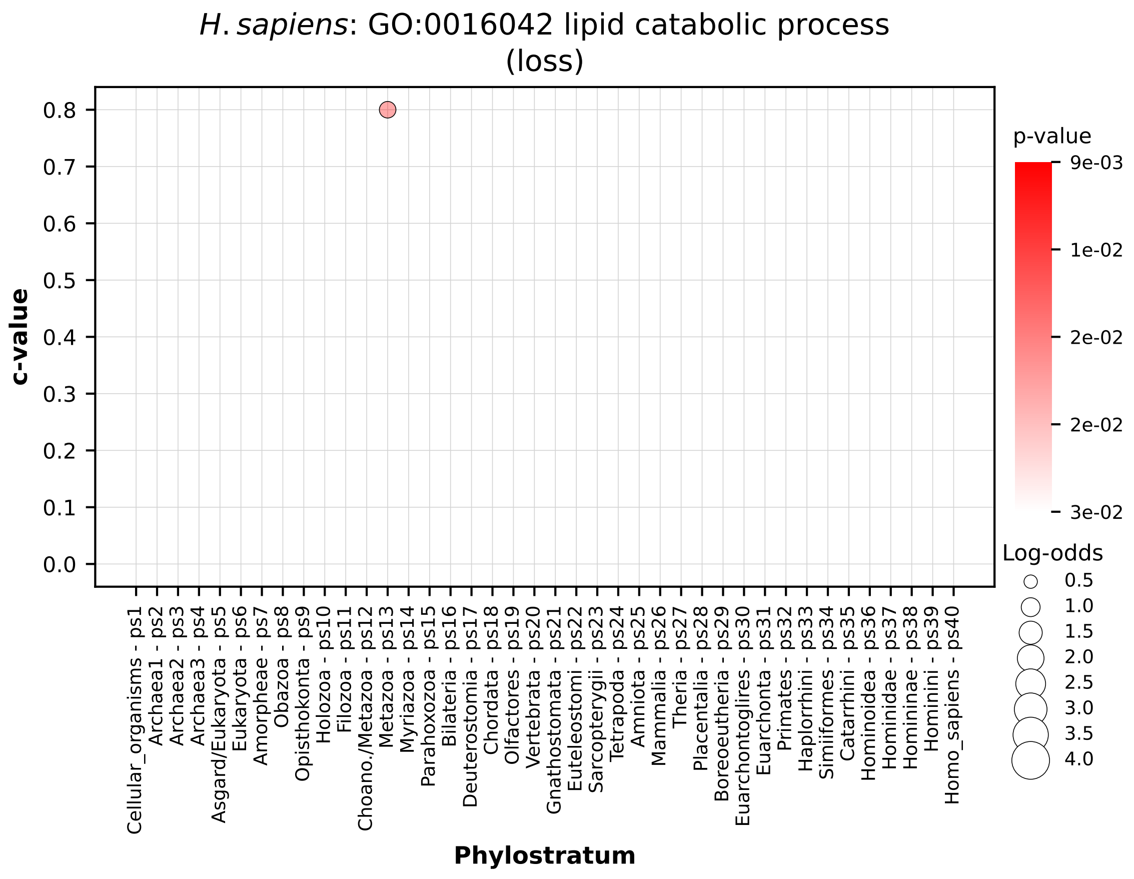
H. sapiens: GO:0016042 lipid catabolic process
(loss)
c-value
0.0
0.1
0.2
0.3
0.4
0.5
0.6
0.7
0.8
Phylostratum
Cellular_organisms - ps1
Archaea1 - ps2
Archaea2 - ps3
Archaea3 - ps4
Asgard/Eukaryota - ps5
Eukaryota - ps6
Amorpheae - ps7
Obazoa - ps8
Opisthokonta - ps9
Holozoa - ps10
Filozoa - ps11
Choano./Metazoa - ps12
Metazoa - ps13
Myriazoa - ps14
Parahoxozoa - ps15
Bilateria - ps16
Deuterostomia - ps17
Chordata - ps18
Olfactores - ps19
Vertebrata - ps20
Gnathostomata - ps21
Euteleostomi - ps22
Sarcopterygii - ps23
Tetrapoda - ps24
Amniota - ps25
Mammalia - ps26
Theria - ps27
Placentalia - ps28
Boreoeutheria - ps29
Euarchontoglires - ps30
Euarchonta - ps31
Primates - ps32
Haplorrhini - ps33
Simiiformes - ps34
Catarrhini - ps35
Hominoidea - ps36
Hominidae - ps37
Homininae - ps38
Hominini - ps39
Homo_sapiens - ps40
p-value
9e-03
1e-02
2e-02
2e-02
3e-02
Log-odds
0.5
1.0
1.5
2.0
2.5
3.0
3.5
4.0

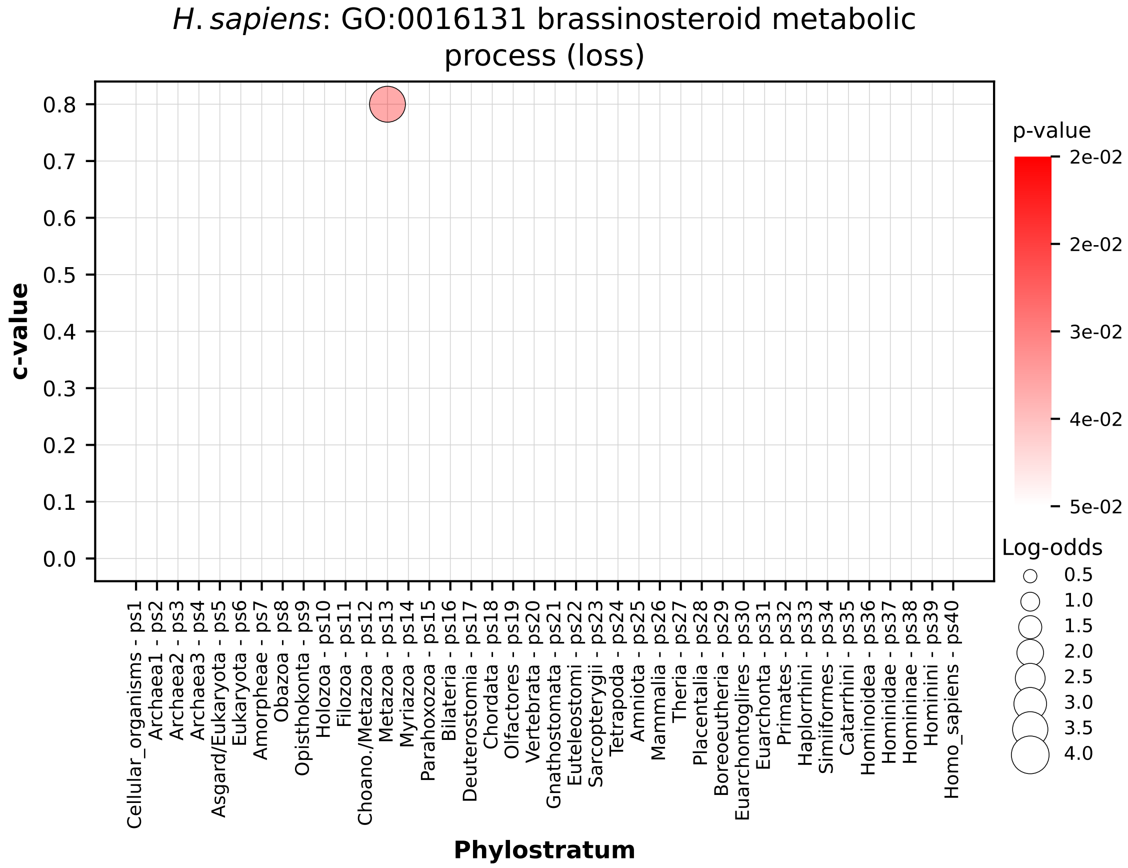

H. sapiens: GO:0016131 brassinosteroid metabolic process (loss)
c-value
0.8
0.7
0.6
0.5
0.4
0.3
0.2
0.1
0.0
p-value
2e-02
2e-02
3e-02
4e-02
5e-02
Log-odds
0.5
1.0
1.5
2.0
2.5
3.0
3.5
4.0
Cellular_organisms - ps1
Archaea1 - ps2
Archaea2 - ps3
Archaea3 - ps4
Asgard/Eukaryota - ps5
Eukaryota - ps6
Amorpheae - ps7
Obazoa - ps8
Opisthokonta - ps9
Holozoa - ps10
Filozoa - ps11
Choano./Metazoa - ps12
Metazoa - ps13
Myriazoa - ps14
Parahoxozoa - ps15
Bilateria - ps16
Deuterostomia - ps17
Chordata - ps18
Olfactores - ps19
Vertebrata - ps20
Gnathostomata - ps21
Euteleostomi - ps22
Sarcopterygii - ps23
Tetrapoda - ps24
Amniota - ps25
Mammalia - ps26
Theria - ps27
Placentalia - ps28
Boreoeutheria - ps29
Euarchontoglires - ps30
Euarchonta - ps31
Primates - ps32
Haplorrhini - ps33
Simiiformes - ps34
Catarrhini - ps35
Hominoidea - ps36
Hominidae - ps37
Homininae - ps38
Hominini - ps39
Homo_sapiens - ps40
Phylostratum

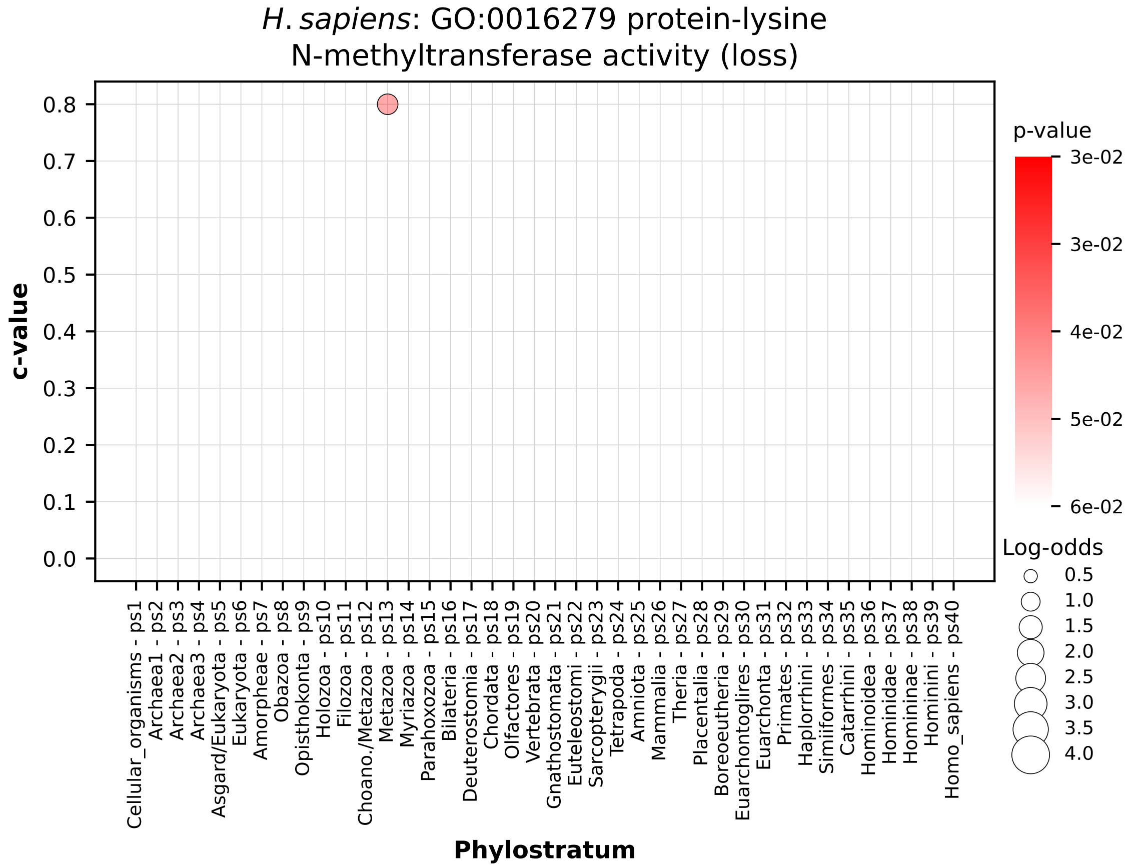

H. sapiens: GO:0016279 protein-lysine N-methyltransferase activity (loss)
c-value
0.0
0.1
0.2
0.3
0.4
0.5
0.6
0.7
0.8
Phylostratum
Cellular_organisms - ps1
Archaea1 - ps2
Archaea2 - ps3
Archaea3 - ps4
Asgard/Eukaryota - ps5
Eukaryota - ps6
Amorpheae - ps7
Obazoa - ps8
Opisthokonta - ps9
Holozoa - ps10
Filozoa - ps11
Choano./Metazoa - ps12
Metazoa - ps13
Myriazoa - ps14
Parahoxozoa - ps15
Bilateria - ps16
Deuterostomia - ps17
Chordata - ps18
Olfactores - ps19
Vertebrata - ps20
Gnathostomata - ps21
Euteleostomi - ps22
Sarcopterygii - ps23
Tetrapoda - ps24
Amniota - ps25
Mammalia - ps26
Theria - ps27
Placentalia - ps28
Boreoeutheria - ps29
Euarchontoglires - ps30
Euarchonta - ps31
Primates - ps32
Haplorrhini - ps33
Simiiformes - ps34
Catarrhini - ps35
Hominoidea - ps36
Hominidae - ps37
Homininae - ps38
Hominini - ps39
Homo_sapiens - ps40
p-value
3e-02
3e-02
4e-02
5e-02
6e-02
Log-odds
0.5
1.0
1.5
2.0
2.5
3.0
3.5
4.0

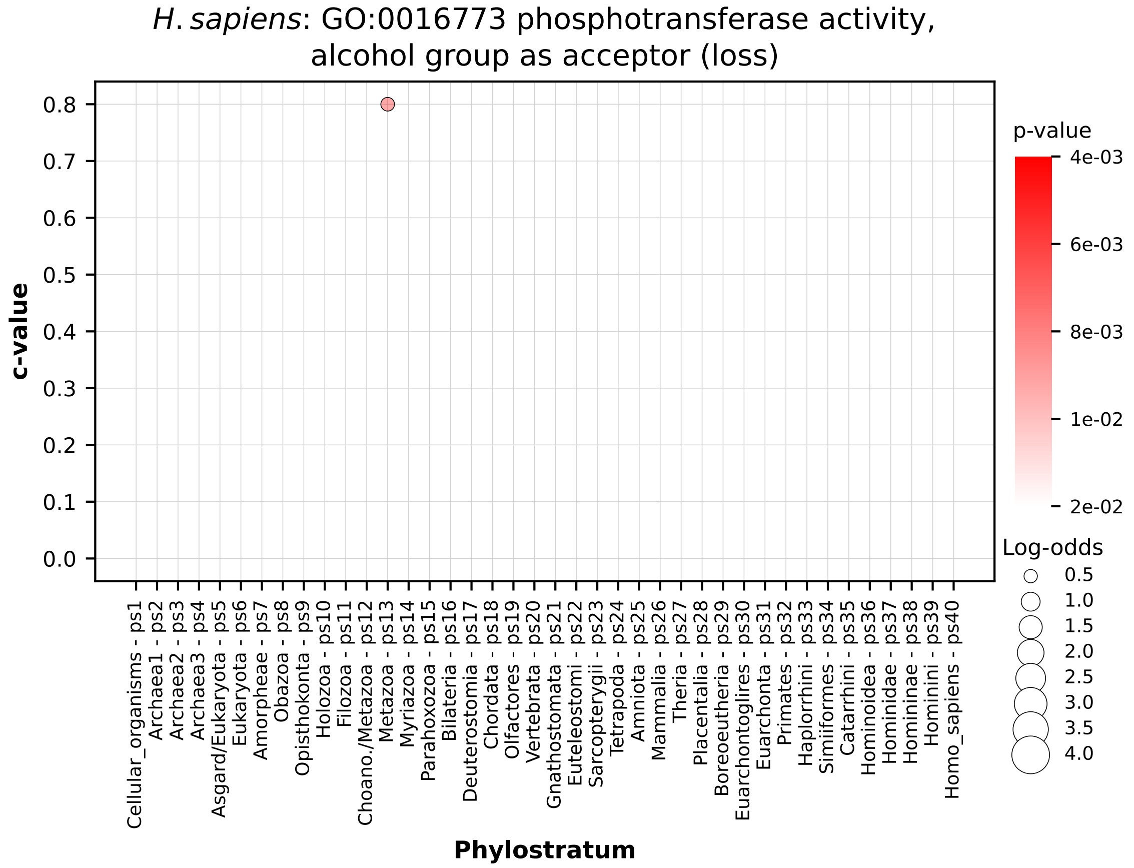

H. sapiens: GO:0016773 phosphotransferase activity, alcohol group as acceptor (loss)
c-value
0.8
0.7
0.6
0.5
0.4
0.3
0.2
0.1
0.0
p-value
4e-03
6e-03
8e-03
1e-02
2e-02
Log-odds
0.5
1.0
1.5
2.0
2.5
3.0
3.5
4.0
Cellular_organisms - ps1
Archaea1 - ps2
Archaea2 - ps3
Archaea3 - ps4
Asgard/Eukaryota - ps5
Eukaryota - ps6
Amorpheae - ps7
Obazoa - ps8
Opisthokonta - ps9
Holozoa - ps10
Filozoa - ps11
Choano./Metazoa - ps12
Metazoa - ps13
Myriazoa - ps14
Parahoxozoa - ps15
Bilateria - ps16
Deuterostomia - ps17
Chordata - ps18
Olfactores - ps19
Vertebrata - ps20
Gnathostomata - ps21
Euteleostomi - ps22
Sarcopterygii - ps23
Tetrapoda - ps24
Amniota - ps25
Mammalia - ps26
Theria - ps27
Placentalia - ps28
Boreoeutheria - ps29
Euarchontoglires - ps30
Euarchonta - ps31
Primates - ps32
Haplorrhini - ps33
Simiiformes - ps34
Catarrhini - ps35
Hominoidea - ps36
Hominidae - ps37
Homininae - ps38
Hominini - ps39
Homo_sapiens - ps40
Phylostratum

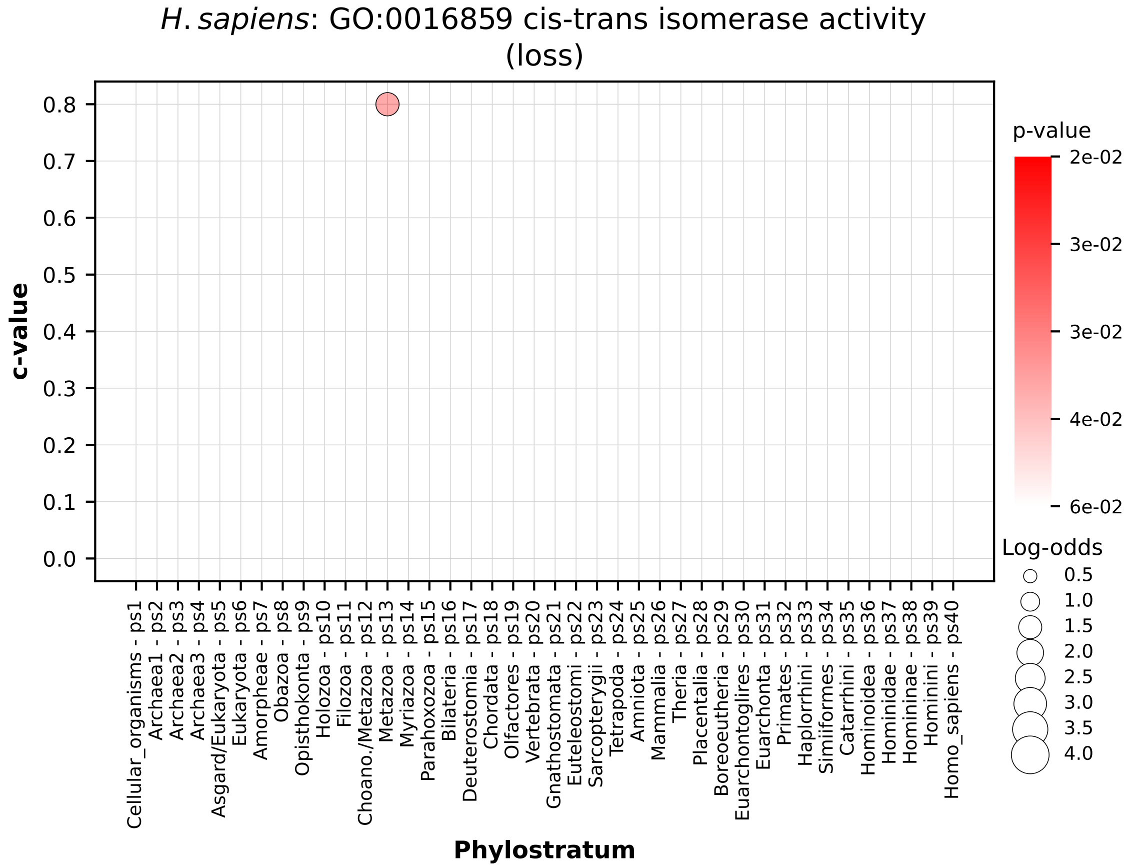

H. sapiens: GO:0016859 cis-trans isomerase activity (loss)
c-value
0.0
0.1
0.2
0.3
0.4
0.5
0.6
0.7
0.8
p-value
2e-02
3e-02
3e-02
4e-02
6e-02
Log-odds
0.5
1.0
1.5
2.0
2.5
3.0
3.5
4.0
Cellular_organisms - ps1
Archaea1 - ps2
Archaea2 - ps3
Archaea3 - ps4
Asgard/Eukaryota - ps5
Eukaryota - ps6
Amorpheae - ps7
Obazoa - ps8
Opisthokonta - ps9
Holozoa - ps10
Filozoa - ps11
Choano./Metazoa - ps12
Metazoa - ps13
Myriazoa - ps14
Parahoxozoa - ps15
Bilateria - ps16
Deuterostomia - ps17
Chordata - ps18
Olfactores - ps19
Vertebrata - ps20
Gnathostomata - ps21
Euteleostomi - ps22
Sarcopterygii - ps23
Tetrapoda - ps24
Amniota - ps25
Mammalia - ps26
Theria - ps27
Placentalia - ps28
Boreoeutheria - ps29
Euarchontoglires - ps30
Euarchonta - ps31
Primates - ps32
Haplorrhini - ps33
Simiiformes - ps34
Catarrhini - ps35
Hominoidea - ps36
Hominidae - ps37
Homininae - ps38
Hominini - ps39
Homo_sapiens - ps40
Phylostratum

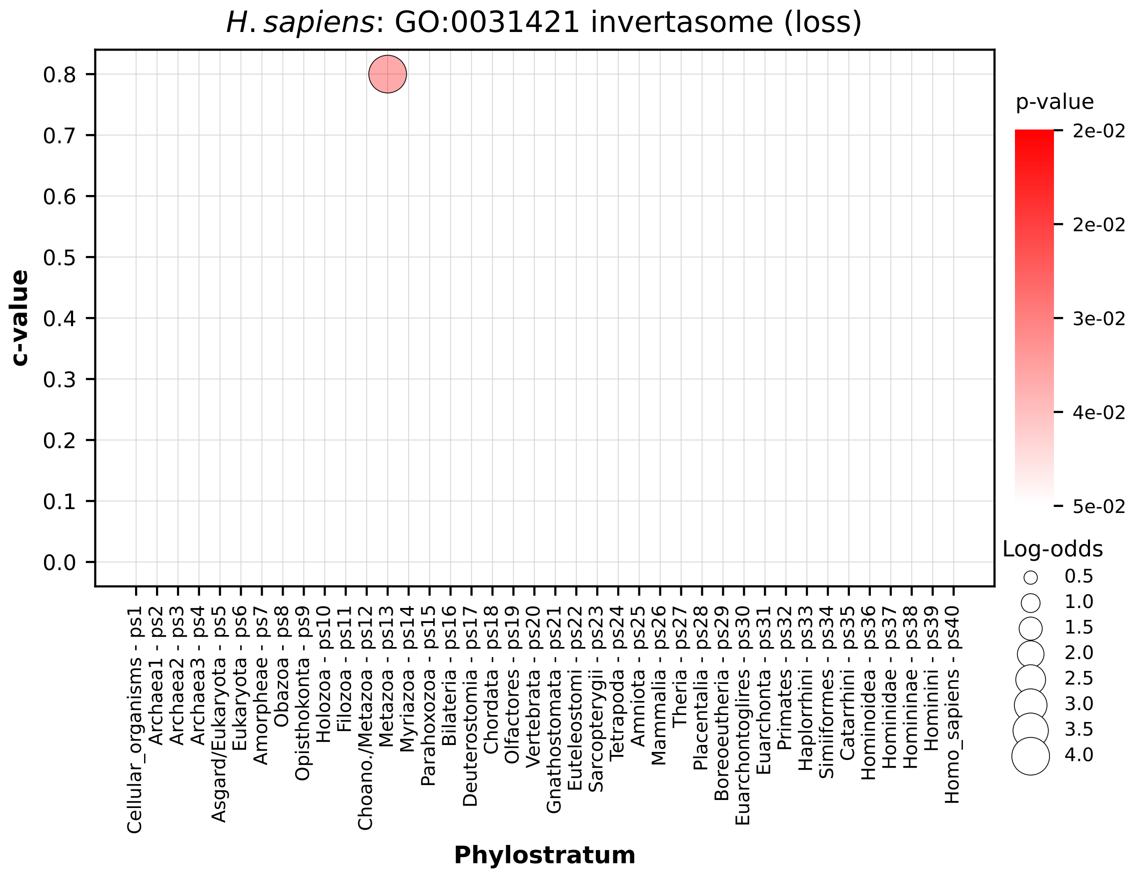
H. sapiens: GO:0031421 invertasome (loss)
c-value
0.0
0.1
0.2
0.3
0.4
0.5
0.6
0.7
0.8
Phylostratum
Cellular_organisms - ps1
Archaea1 - ps2
Archaea2 - ps3
Archaea3 - ps4
Asgard/Eukaryota - ps5
Eukaryota - ps6
Amorpheae - ps7
Obazoa - ps8
Opisthokonta - ps9
Holozoa - ps10
Filozoa - ps11
Choano./Metazoa - ps12
Metazoa - ps13
Myriazoa - ps14
Parahoxozoa - ps15
Bilateria - ps16
Deuterostomia - ps17
Chordata - ps18
Olfactores - ps19
Vertebrata - ps20
Gnathostomata - ps21
Euteleostomi - ps22
Sarcopterygii - ps23
Tetrapoda - ps24
Amniota - ps25
Mammalia - ps26
Theria - ps27
Placentalia - ps28
Boreoeutheria - ps29
Euarchontoglires - ps30
Euarchonta - ps31
Primates - ps32
Haplorrhini - ps33
Simiiformes - ps34
Catarrhini - ps35
Hominoidea - ps36
Hominidae - ps37
Homininae - ps38
Hominini - ps39
Homo_sapiens - ps40
p-value
2e-02
2e-02
3e-02
4e-02
5e-02
Log-odds
0.5
1.0
1.5
2.0
2.5
3.0
3.5
4.0

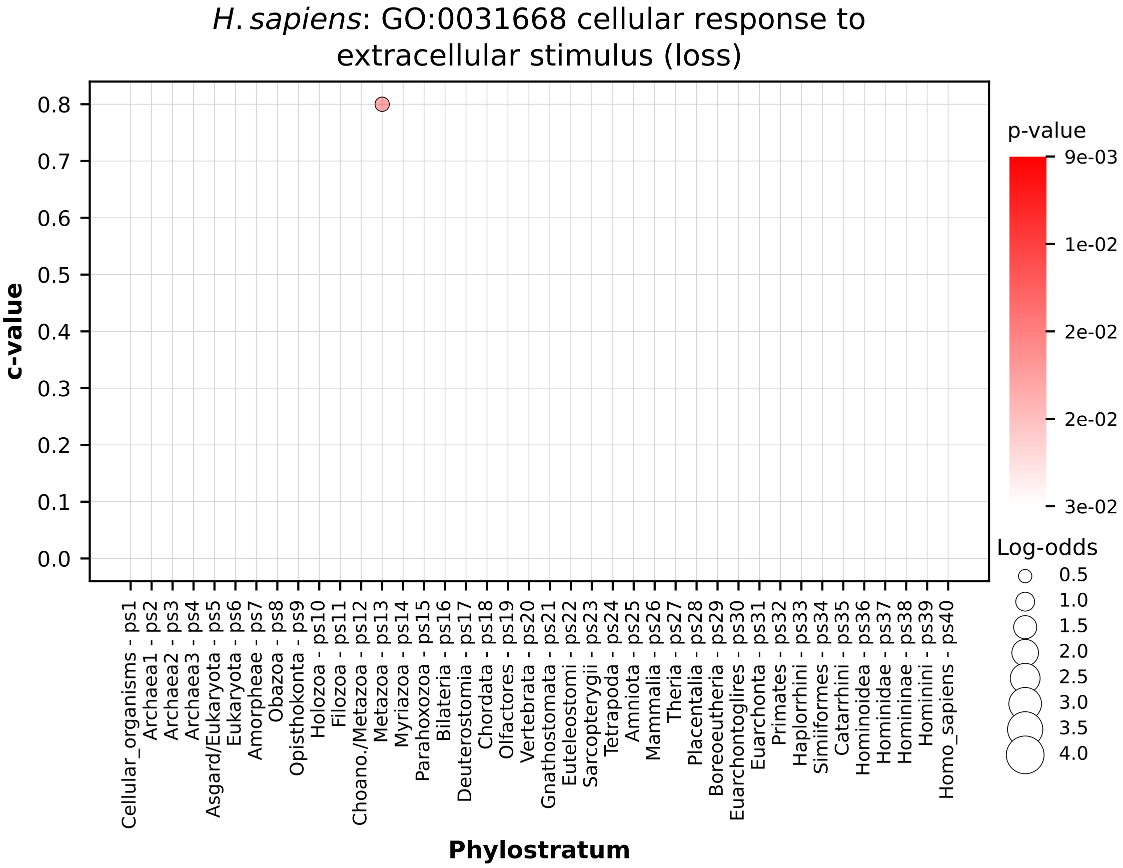

H. sapiens: GO:0031668 cellular response to extracellular stimulus (loss)
c-value
0.8
0.7
0.6
0.5
0.4
0.3
0.2
0.1
0.0
p-value
9e-03
1e-02
2e-02
2e-02
3e-02
Log-odds
0.5
1.0
1.5
2.0
2.5
3.0
3.5
4.0
Cellular_organisms - ps1
Archaea1 - ps2
Archaea2 - ps3
Archaea3 - ps4
Asgard/Eukaryota - ps5
Eukaryota - ps6
Amorpheae - ps7
Obazoa - ps8
Opisthokonta - ps9
Holozoa - ps10
Filozoa - ps11
Choano./Metazoa - ps12
Metazoa - ps13
Myriazoa - ps14
Parahoxozoa - ps15
Bilateria - ps16
Deuterostomia - ps17
Chordata - ps18
Olfactores - ps19
Vertebrata - ps20
Gnathostomata - ps21
Euteleostomi - ps22
Sarcopterygii - ps23
Tetrapoda - ps24
Amniota - ps25
Mammalia - ps26
Theria - ps27
Placentalia - ps28
Boreoeutheria - ps29
Euarchontoglires - ps30
Euarchonta - ps31
Primates - ps32
Haplorrhini - ps33
Simiiformes - ps34
Catarrhini - ps35
Hominoidea - ps36
Hominidae - ps37
Homininae - ps38
Hominini - ps39
Homo_sapiens - ps40
Phylostratum

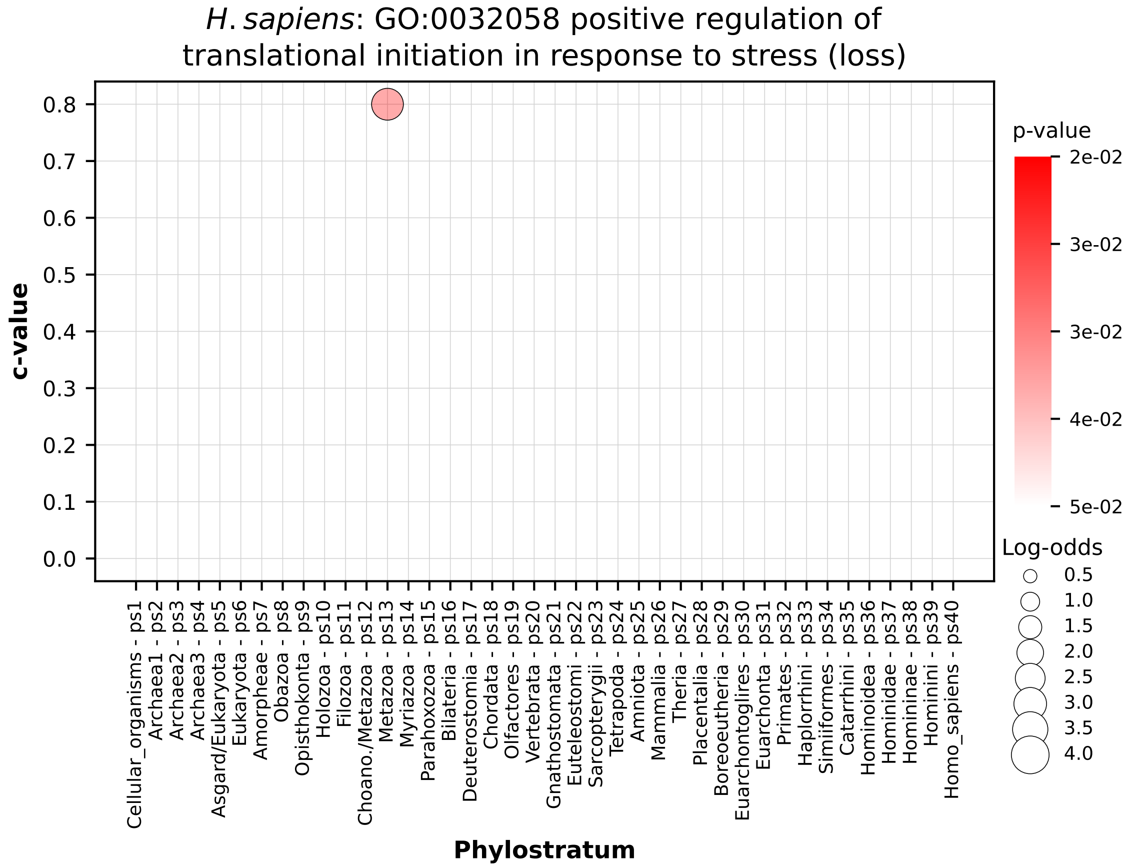

H. sapiens: GO:0032058 positive regulation of translational initiation in response to stress (loss)
c-value
0.8
0.7
0.6
0.5
0.4
0.3
0.2
0.1
0.0
Phylostratum
Cellular_organisms - ps1
Archaea1 - ps2
Archaea2 - ps3
Archaea3 - ps4
Asgard/Eukaryota - ps5
Eukaryota - ps6
Amorpheae - ps7
Obazoa - ps8
Opisthokonta - ps9
Holozoa - ps10
Filozoa - ps11
Choano./Metazoa - ps12
Metazoa - ps13
Myriazoa - ps14
Parahoxozoa - ps15
Bilateria - ps16
Deuterostomia - ps17
Chordata - ps18
Olfactores - ps19
Vertebrata - ps20
Gnathostomata - ps21
Euteleostomi - ps22
Sarcopterygii - ps23
Tetrapoda - ps24
Amniota - ps25
Mammalia - ps26
Theria - ps27
Placentalia - ps28
Boreoeutheria - ps29
Euarchontoglires - ps30
Euarchonta - ps31
Primates - ps32
Haplorrhini - ps33
Simiiformes - ps34
Catarrhini - ps35
Hominoidea - ps36
Hominidae - ps37
Homininae - ps38
Hominini - ps39
Homo_sapiens - ps40
p-value
2e-02
3e-02
3e-02
4e-02
5e-02
Log-odds
0.5
1.0
1.5
2.0
2.5
3.0
3.5
4.0

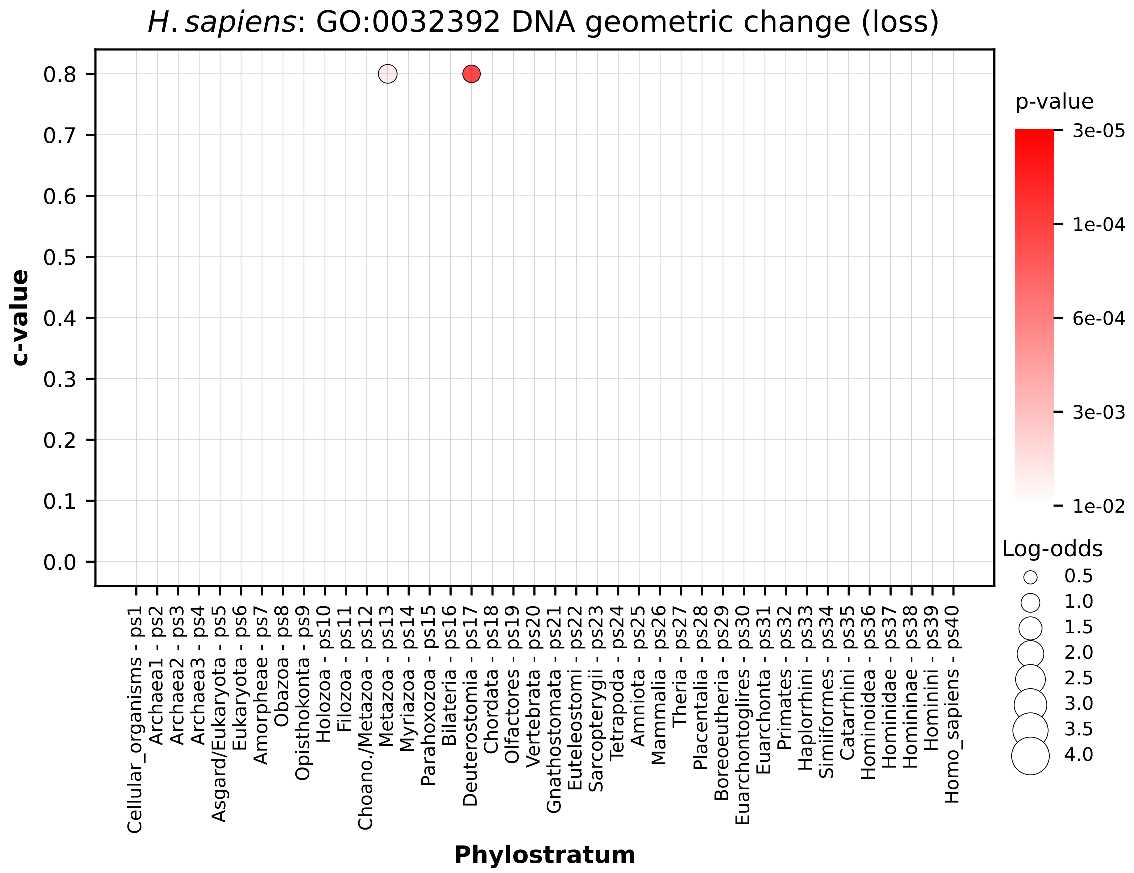

H. sapiens: GO:0032392 DNA geometric change (loss)
c-value
0.0
0.1
0.2
0.3
0.4
0.5
0.6
0.7
0.8
Phylostratum
Cellular_organisms - ps1
Archaea1 - ps2
Archaea2 - ps3
Archaea3 - ps4
Asgard/Eukaryota - ps5
Eukaryota - ps6
Amorpheae - ps7
Obazoa - ps8
Opisthokonta - ps9
Holozoa - ps10
Filozoa - ps11
Choano./Metazoa - ps12
Metazoa - ps13
Myriazoa - ps14
Parahoxozoa - ps15
Bilateria - ps16
Deuterostomia - ps17
Chordata - ps18
Olfactores - ps19
Vertebrata - ps20
Gnathostomata - ps21
Euteleostomi - ps22
Sarcopterygii - ps23
Tetrapoda - ps24
Amniota - ps25
Mammalia - ps26
Theria - ps27
Placentalia - ps28
Boreoeutheria - ps29
Euarchontoglires - ps30
Euarchonta - ps31
Primates - ps32
Haplorrhini - ps33
Simiiformes - ps34
Catarrhini - ps35
Hominoidea - ps36
Hominidae - ps37
Homininae - ps38
Hominini - ps39
Homo_sapiens - ps40
p-value
3e-05
1e-04
6e-04
3e-03
1e-02
Log-odds
0.5
1.0
1.5
2.0
2.5
3.0
3.5
4.0

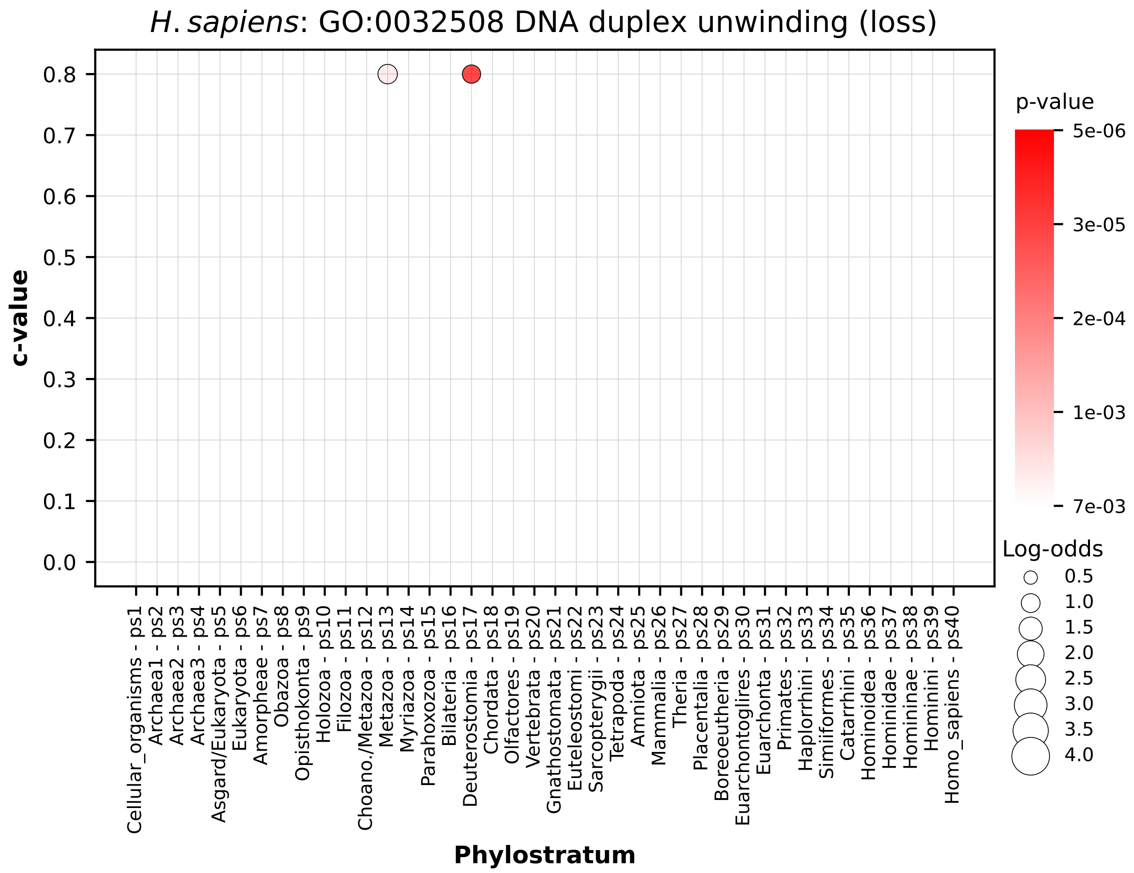
H. sapiens: GO:0032508 DNA duplex unwinding (loss)
c-value
0.0
0.1
0.2
0.3
0.4
0.5
0.6
0.7
0.8
Phylostratum
Cellular_organisms - ps1
Archaea1 - ps2
Archaea2 - ps3
Archaea3 - ps4
Asgard/Eukaryota - ps5
Eukaryota - ps6
Amorpheae - ps7
Obazoa - ps8
Opisthokonta - ps9
Holozoa - ps10
Filozoa - ps11
Choano./Metazoa - ps12
Metazoa - ps13
Myriazoa - ps14
Parahoxozoa - ps15
Bilateria - ps16
Deuterostomia - ps17
Chordata - ps18
Olfactores - ps19
Vertebrata - ps20
Gnathostomata - ps21
Euteleostomi - ps22
Sarcopterygii - ps23
Tetrapoda - ps24
Amniota - ps25
Mammalia - ps26
Theria - ps27
Placentalia - ps28
Boreoeutheria - ps29
Euarchontoglires - ps30
Euarchonta - ps31
Primates - ps32
Haplorrhini - ps33
Simiiformes - ps34
Catarrhini - ps35
Hominoidea - ps36
Hominidae - ps37
Homininae - ps38
Hominini - ps39
Homo_sapiens - ps40
p-value
5e-06
3e-05
2e-04
1e-03
7e-03
Log-odds
0.5
1.0
1.5
2.0
2.5
3.0
3.5
4.0

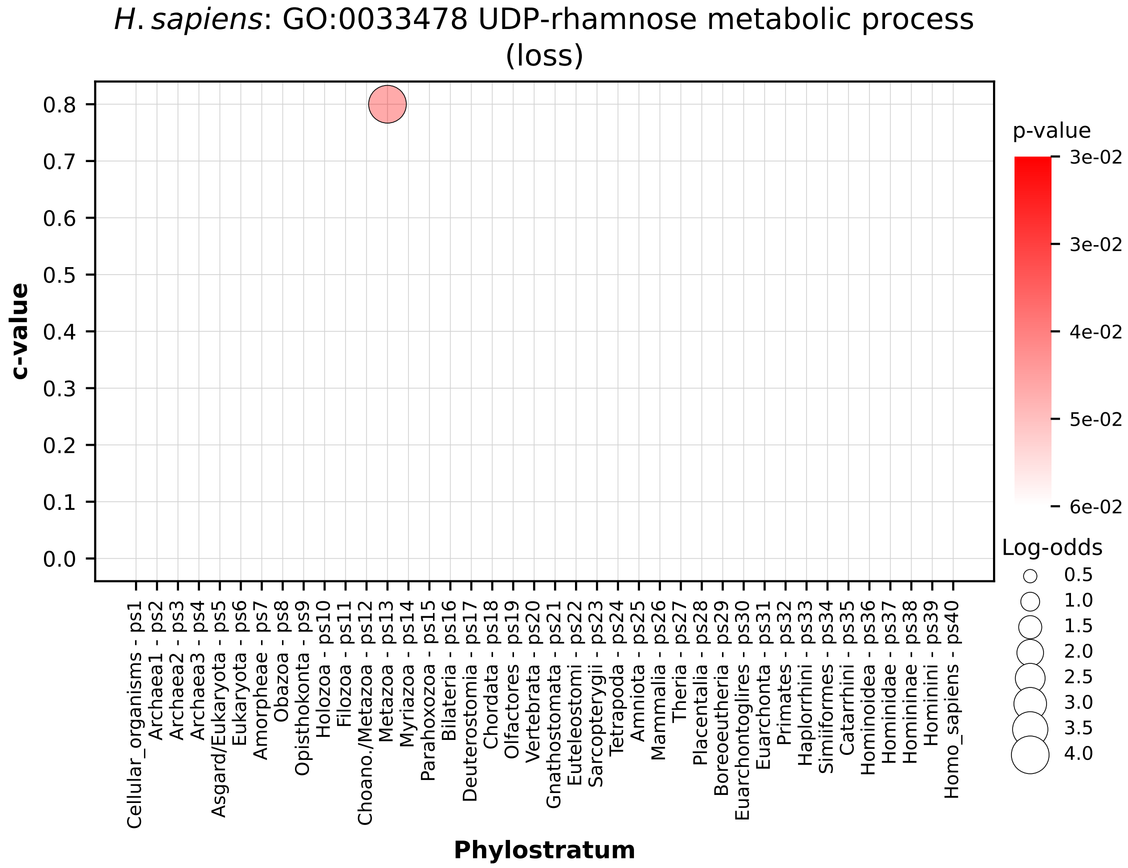

H. sapiens: GO:0033478 UDP-rhamnose metabolic process
(loss)
c-value
0.8
0.7
0.6
0.5
0.4
0.3
0.2
0.1
0.0
p-value
3e-02
3e-02
4e-02
5e-02
6e-02
Log-odds
0.5
1.0
1.5
2.0
2.5
3.0
3.5
4.0
Cellular_organisms - ps1
Archaea1 - ps2
Archaea2 - ps3
Archaea3 - ps4
Asgard/Eukaryota - ps5
Eukaryota - ps6
Amorpheae - ps7
Obazoa - ps8
Opisthokonta - ps9
Holozoa - ps10
Filozoa - ps11
Choano./Metazoa - ps12
Metazoa - ps13
Myriazoa - ps14
Parahoxozoa - ps15
Bilateria - ps16
Deuterostomia - ps17
Chordata - ps18
Olfactores - ps19
Vertebrata - ps20
Gnathostomata - ps21
Euteleostomi - ps22
Sarcopterygii - ps23
Tetrapoda - ps24
Amniota - ps25
Mammalia - ps26
Theria - ps27
Placentalia - ps28
Boreoeutheria - ps29
Euarchontoglires - ps30
Euarchonta - ps31
Primates - ps32
Haplorrhini - ps33
Simiiformes - ps34
Catarrhini - ps35
Hominoidea - ps36
Hominidae - ps37
Homininae - ps38
Hominini - ps39
Homo_sapiens - ps40
Phylostratum

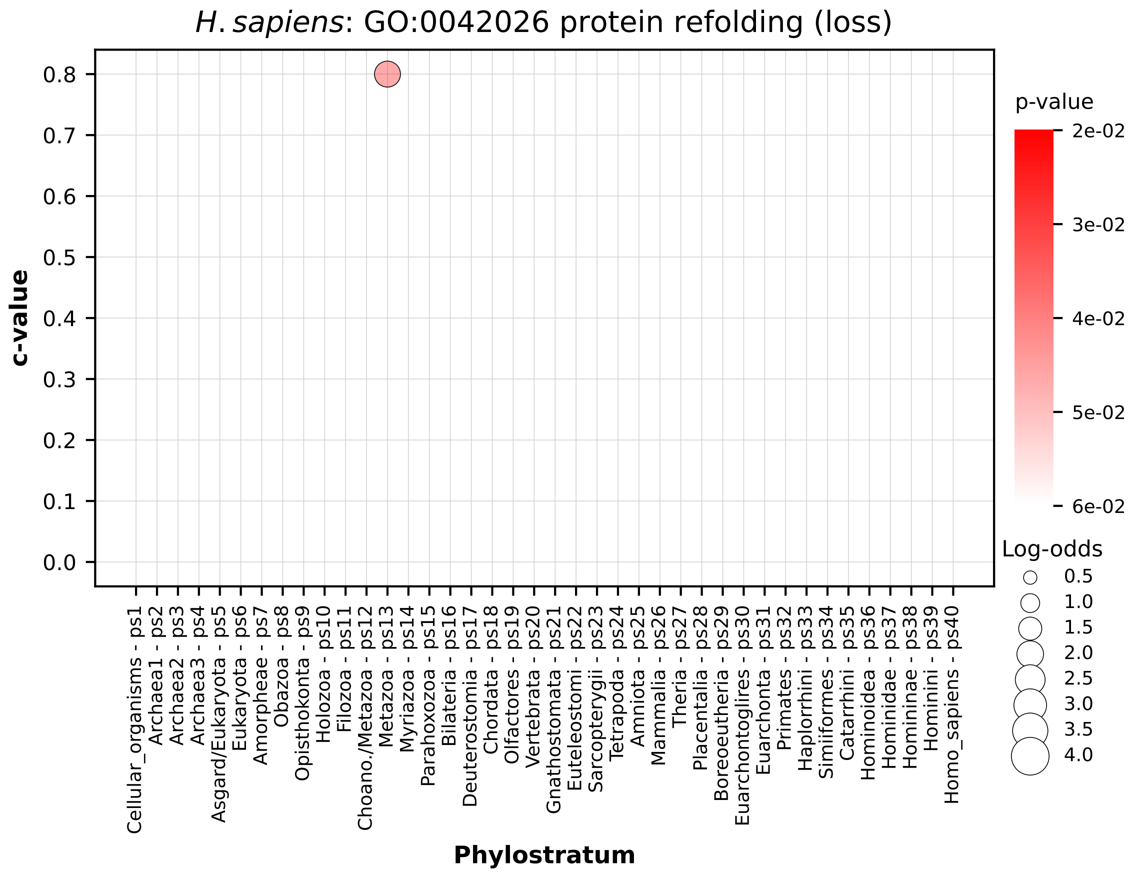

H. sapiens: GO:0042026 protein refolding (loss)
c-value
0.0
0.1
0.2
0.3
0.4
0.5
0.6
0.7
0.8
Phylostratum
Cellular_organisms - ps1
Archaea1 - ps2
Archaea2 - ps3
Archaea3 - ps4
Asgard/Eukaryota - ps5
Eukaryota - ps6
Amorpheae - ps7
Obazoa - ps8
Opisthokonta - ps9
Holozoa - ps10
Filozoa - ps11
Choano./Metazoa - ps12
Metazoa - ps13
Myriazoa - ps14
Parahoxozoa - ps15
Bilateria - ps16
Deuterostomia - ps17
Chordata - ps18
Olfactores - ps19
Vertebrata - ps20
Gnathostomata - ps21
Euteleostomi - ps22
Sarcopterygii - ps23
Tetrapoda - ps24
Amniota - ps25
Mammalia - ps26
Theria - ps27
Placentalia - ps28
Boreoeutheria - ps29
Euarchontoglires - ps30
Euarchonta - ps31
Primates - ps32
Haplorrhini - ps33
Simiiformes - ps34
Catarrhini - ps35
Hominoidea - ps36
Hominidae - ps37
Homininae - ps38
Hominini - ps39
Homo_sapiens - ps40
p-value
2e-02
3e-02
4e-02
5e-02
6e-02
Log-odds
0.5
1.0
1.5
2.0
2.5
3.0
3.5
4.0

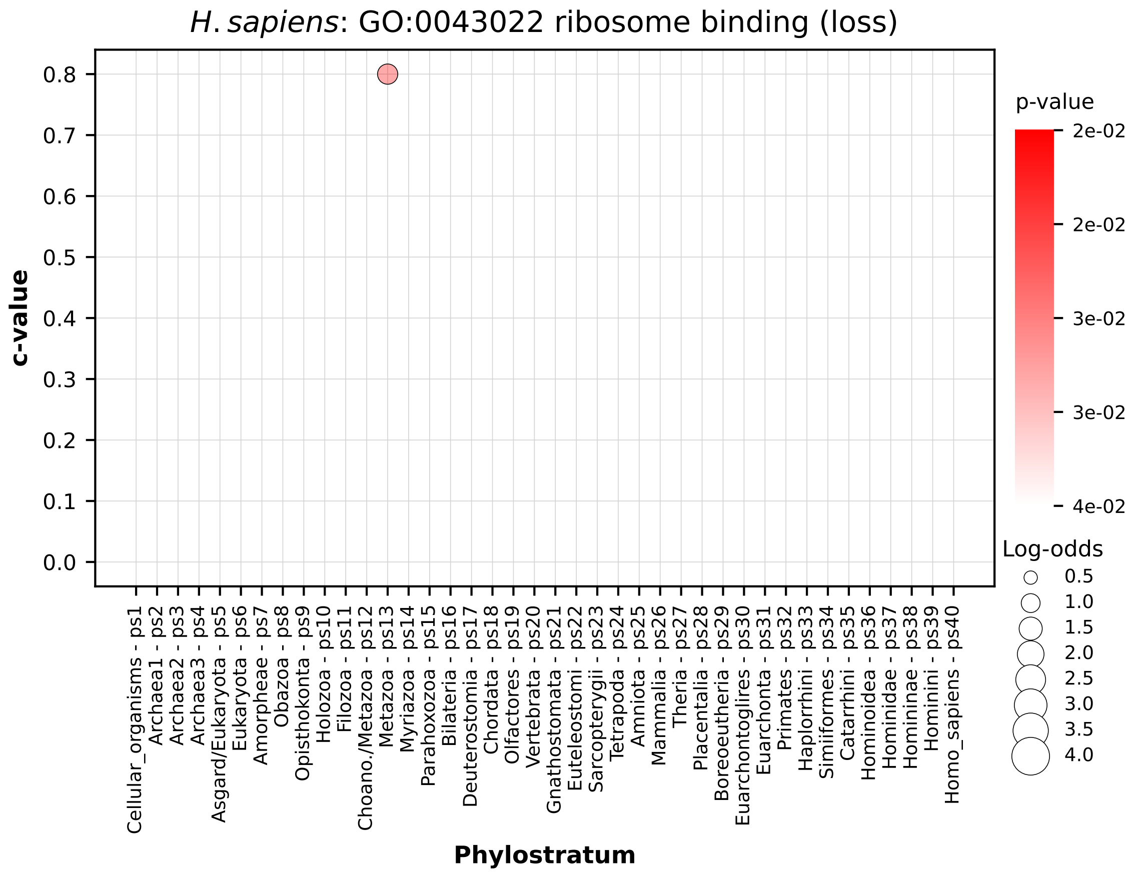

H. sapiens: GO:0043022 ribosome binding (loss)
c-value
0.0
0.1
0.2
0.3
0.4
0.5
0.6
0.7
0.8
Phylostratum
Cellular_organisms - ps1
Archaea1 - ps2
Archaea2 - ps3
Archaea3 - ps4
Asgard/Eukaryota - ps5
Eukaryota - ps6
Amorpheae - ps7
Obazoa - ps8
Opisthokonta - ps9
Holozoa - ps10
Filozoa - ps11
Choano./Metazoa - ps12
Metazoa - ps13
Myriazoa - ps14
Parahoxozoa - ps15
Bilateria - ps16
Deuterostomia - ps17
Chordata - ps18
Olfactores - ps19
Vertebrata - ps20
Gnathostomata - ps21
Euteleostomi - ps22
Sarcopterygii - ps23
Tetrapoda - ps24
Amniota - ps25
Mammalia - ps26
Theria - ps27
Placentalia - ps28
Boreoeutheria - ps29
Euarchontoglires - ps30
Euarchonta - ps31
Primates - ps32
Haplorrhini - ps33
Simiiformes - ps34
Catarrhini - ps35
Hominoidea - ps36
Hominidae - ps37
Homininae - ps38
Hominini - ps39
Homo_sapiens - ps40
p-value
2e-02
2e-02
3e-02
3e-02
4e-02
Log-odds
0.5
1.0
1.5
2.0
2.5
3.0
3.5
4.0

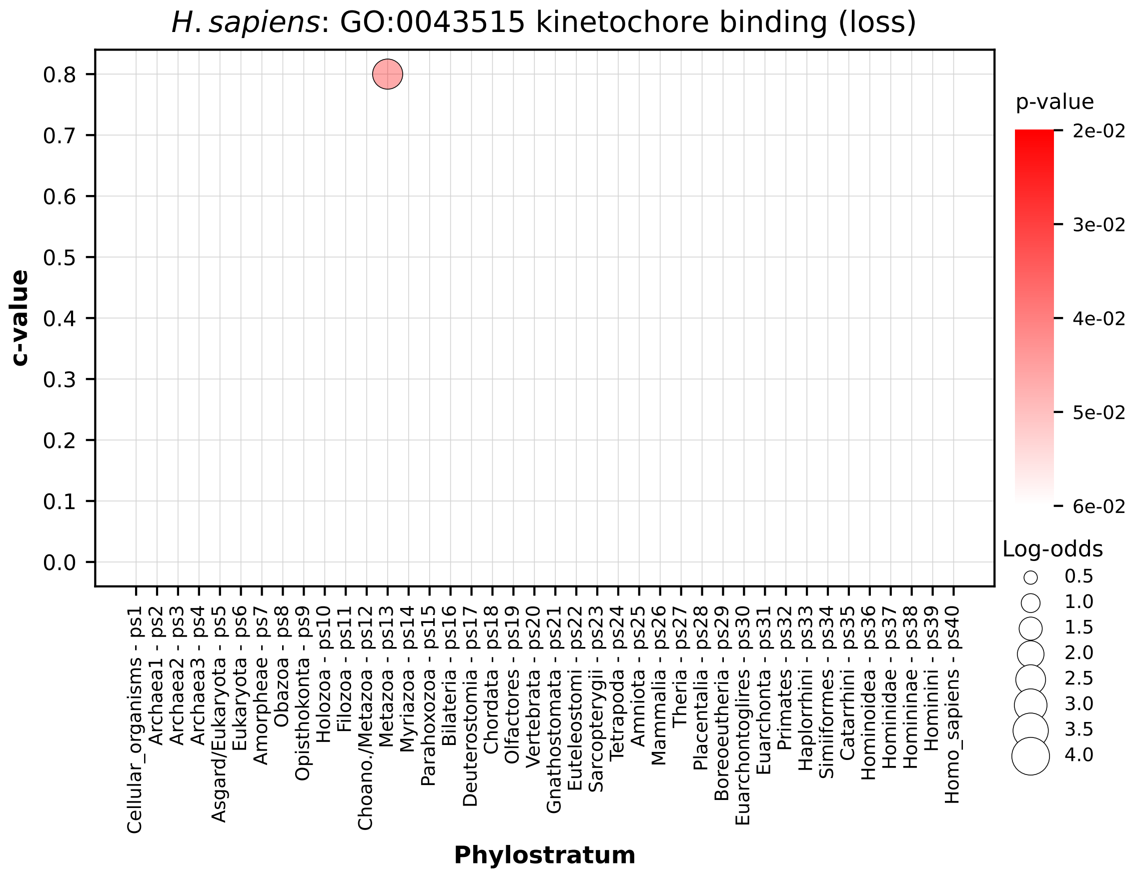

H. sapiens: GO:0043515 kinetochore binding (loss)
c-value
0.0
0.1
0.2
0.3
0.4
0.5
0.6
0.7
0.8
Phylostratum
Cellular_organisms - ps1
Archaea1 - ps2
Archaea2 - ps3
Archaea3 - ps4
Asgard/Eukaryota - ps5
Eukaryota - ps6
Amorpheae - ps7
Obazoa - ps8
Opisthokonta - ps9
Holozoa - ps10
Filozoa - ps11
Choano./Metazoa - ps12
Metazoa - ps13
Myriazoa - ps14
Parahoxozoa - ps15
Bilateria - ps16
Deuterostomia - ps17
Chordata - ps18
Olfactores - ps19
Vertebrata - ps20
Gnathostomata - ps21
Euteleostomi - ps22
Sarcopterygii - ps23
Tetrapoda - ps24
Amniota - ps25
Mammalia - ps26
Theria - ps27
Placentalia - ps28
Boreoeutheria - ps29
Euarchontoglires - ps30
Euarchonta - ps31
Primates - ps32
Haplorrhini - ps33
Simiiformes - ps34
Catarrhini - ps35
Hominoidea - ps36
Hominidae - ps37
Homininae - ps38
Hominini - ps39
Homo_sapiens - ps40
p-value
2e-02
3e-02
4e-02
5e-02
6e-02
Log-odds
0.5
1.0
1.5
2.0
2.5
3.0
3.5
4.0

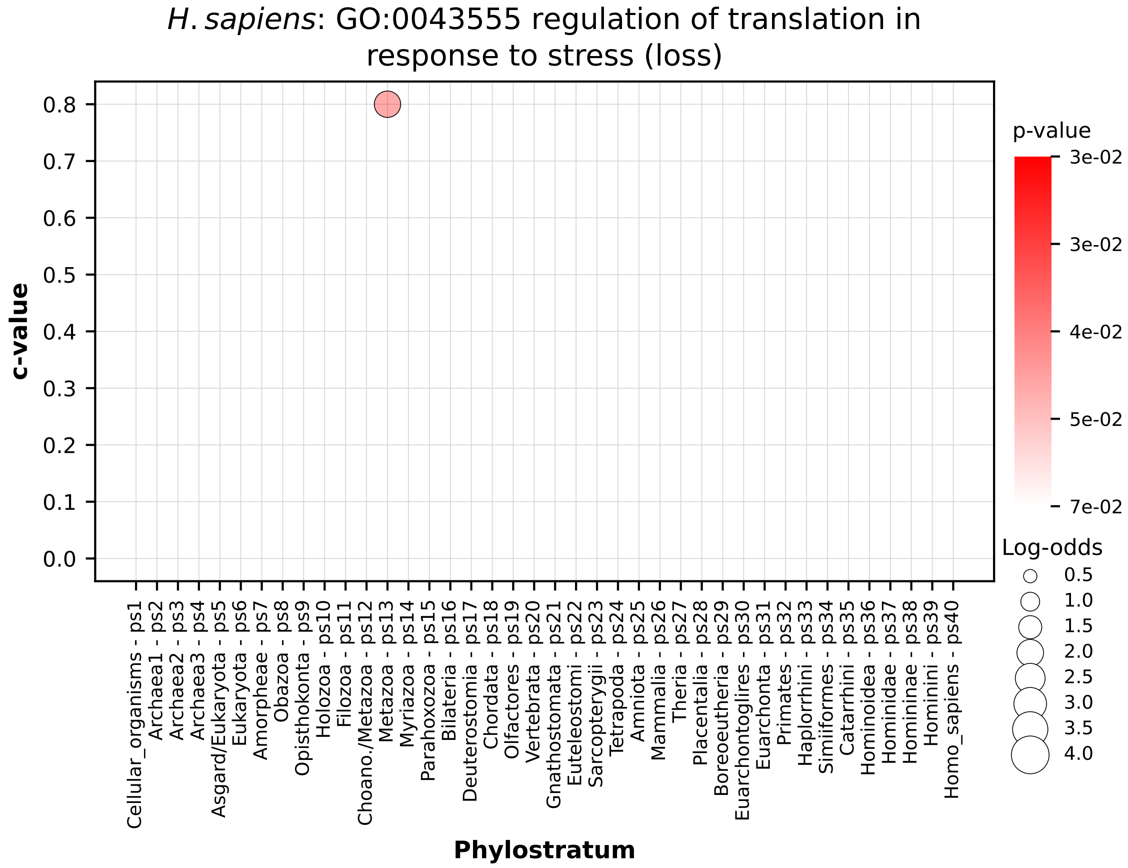

H. sapiens: GO:0043555 regulation of translation in response to stress (loss)
c-value
0.0
0.1
0.2
0.3
0.4
0.5
0.6
0.7
0.8
Phylostratum
Cellular_organisms - ps1
Archaea1 - ps2
Archaea2 - ps3
Archaea3 - ps4
Asgard/Eukaryota - ps5
Eukaryota - ps6
Amorpheae - ps7
Obazoa - ps8
Opisthokonta - ps9
Holozoa - ps10
Filozoa - ps11
Choano./Metazoa - ps12
Metazoa - ps13
Myriazoa - ps14
Parahoxozoa - ps15
Bilateria - ps16
Deuterostomia - ps17
Chordata - ps18
Olfactores - ps19
Vertebrata - ps20
Gnathostomata - ps21
Euteleostomi - ps22
Sarcopterygii - ps23
Tetrapoda - ps24
Amniota - ps25
Mammalia - ps26
Theria - ps27
Placentalia - ps28
Boreoeutheria - ps29
Euarchontoglires - ps30
Euarchonta - ps31
Primates - ps32
Haplorrhini - ps33
Simiiformes - ps34
Catarrhini - ps35
Hominoidea - ps36
Hominidae - ps37
Homininae - ps38
Hominini - ps39
Homo_sapiens - ps40
p-value
3e-02
3e-02
4e-02
5e-02
7e-02
Log-odds
0.5
1.0
1.5
2.0
2.5
3.0
3.5
4.0

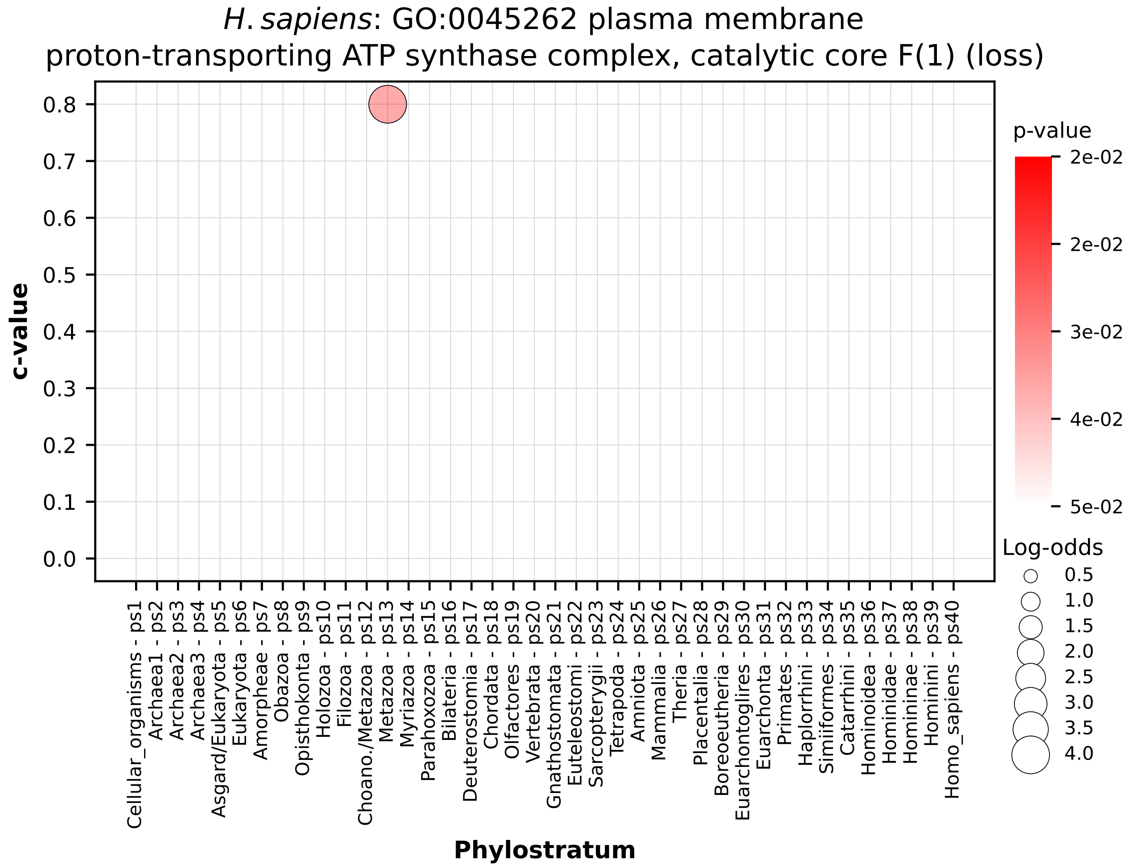

H. sapiens: GO:0045262 plasma membrane proton-transporting ATP synthase complex, catalytic core F(1) (loss)
c-value
0.0
0.1
0.2
0.3
0.4
0.5
0.6
0.7
0.8
Phylostratum
Cellular_organisms - ps1
Archaea1 - ps2
Archaea2 - ps3
Archaea3 - ps4
Asgard/Eukaryota - ps5
Eukaryota - ps6
Amorpheae - ps7
Obazoa - ps8
Opisthokonta - ps9
Holozoa - ps10
Filozoa - ps11
Choano./Metazoa - ps12
Metazoa - ps13
Myriazoa - ps14
Parahoxozoa - ps15
Bilateria - ps16
Deuterostomia - ps17
Chordata - ps18
Olfactores - ps19
Vertebrata - ps20
Gnathostomata - ps21
Euteleostomi - ps22
Sarcopterygii - ps23
Tetrapoda - ps24
Amniota - ps25
Mammalia - ps26
Theria - ps27
Placentalia - ps28
Boreoeutheria - ps29
Euarchontoglires - ps30
Euarchonta - ps31
Primates - ps32
Haplorrhini - ps33
Simiiformes - ps34
Catarrhini - ps35
Hominoidea - ps36
Hominidae - ps37
Homininae - ps38
Hominini - ps39
Homo_sapiens - ps40
p-value
2e-02
2e-02
3e-02
4e-02
5e-02
Log-odds
0.5
1.0
1.5
2.0
2.5
3.0
3.5
4.0

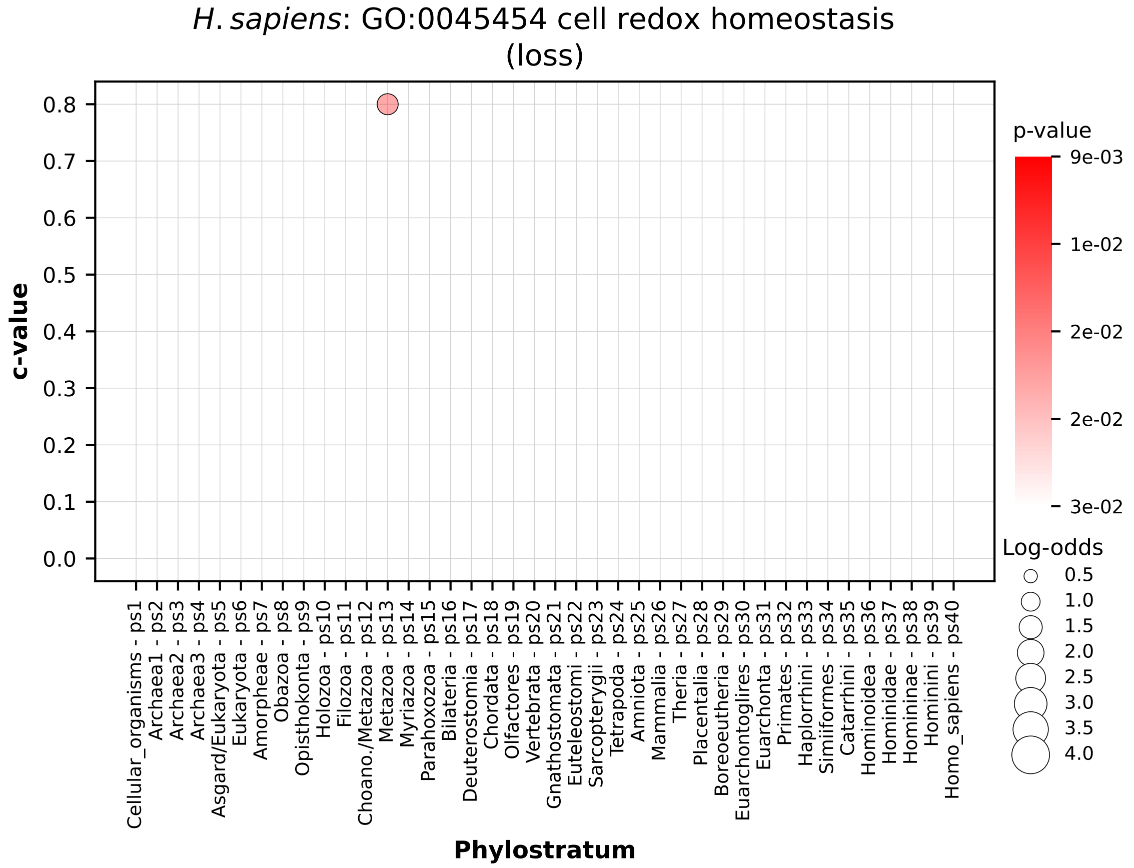

H. sapiens: GO:0045454 cell redox homeostasis
(loss)
c-value
0.8
0.7
0.6
0.5
0.4
0.3
0.2
0.1
0.0
p-value
9e-03
1e-02
2e-02
2e-02
3e-02
Log-odds
0.5
1.0
1.5
2.0
2.5
3.0
3.5
4.0
Cellular_organisms - ps1
Archaea1 - ps2
Archaea2 - ps3
Archaea3 - ps4
Asgard/Eukaryota - ps5
Eukaryota - ps6
Amorpheae - ps7
Obazoa - ps8
Opisthokonta - ps9
Holozoa - ps10
Filozoa - ps11
Choano./Metazoa - ps12
Metazoa - ps13
Myriazoa - ps14
Parahoxozoa - ps15
Bilateria - ps16
Deuterostomia - ps17
Chordata - ps18
Olfactores - ps19
Vertebrata - ps20
Gnathostomata - ps21
Euteleostomi - ps22
Sarcopterygii - ps23
Tetrapoda - ps24
Amniota - ps25
Mammalia - ps26
Theria - ps27
Placentalia - ps28
Boreoeutheria - ps29
Euarchontoglires - ps30
Euarchonta - ps31
Primates - ps32
Haplorrhini - ps33
Simiiformes - ps34
Catarrhini - ps35
Hominoidea - ps36
Hominidae - ps37
Homininae - ps38
Hominini - ps39
Homo_sapiens - ps40
Phylostratum

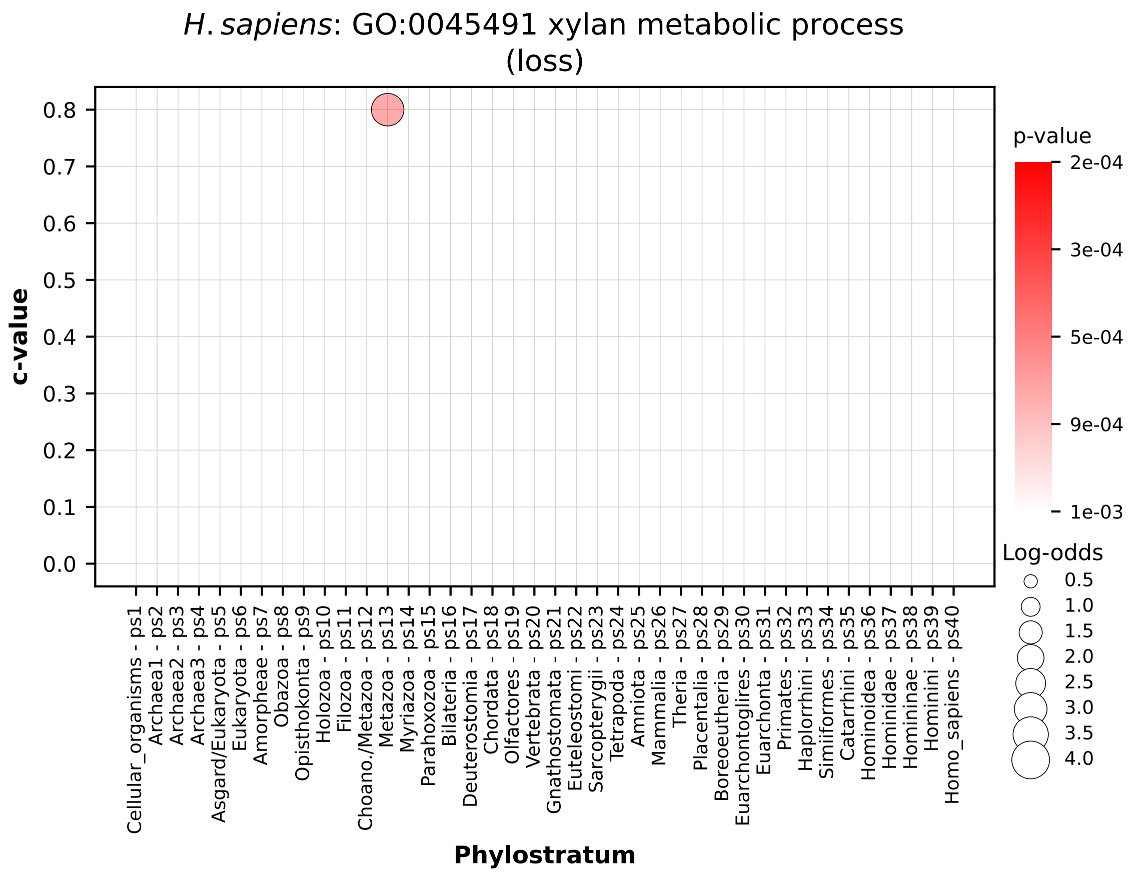

H. sapiens: GO:0045491 xylan metabolic process
(loss)
c-value
0.0
0.1
0.2
0.3
0.4
0.5
0.6
0.7
0.8
Phylostratum
Cellular_organisms - ps1
Archaea1 - ps2
Archaea2 - ps3
Archaea3 - ps4
Asgard/Eukaryota - ps5
Eukaryota - ps6
Amorpheae - ps7
Obazoa - ps8
Opisthokonta - ps9
Holozoa - ps10
Filozoa - ps11
Choano./Metazoa - ps12
Metazoa - ps13
Myriazoa - ps14
Parahoxozoa - ps15
Bilateria - ps16
Deuterostomia - ps17
Chordata - ps18
Olfactores - ps19
Vertebrata - ps20
Gnathostomata - ps21
Euteleostomi - ps22
Sarcopterygii - ps23
Tetrapoda - ps24
Amniota - ps25
Mammalia - ps26
Theria - ps27
Placentalia - ps28
Boreoeutheria - ps29
Euarchontoglires - ps30
Euarchonta - ps31
Primates - ps32
Haplorrhini - ps33
Simiiformes - ps34
Catarrhini - ps35
Hominoidea - ps36
Hominidae - ps37
Homininae - ps38
Hominini - ps39
Homo_sapiens - ps40
p-value
2e-04
3e-04
5e-04
9e-04
1e-03
Log-odds
0.5
1.0
1.5
2.0
2.5
3.0
3.5
4.0

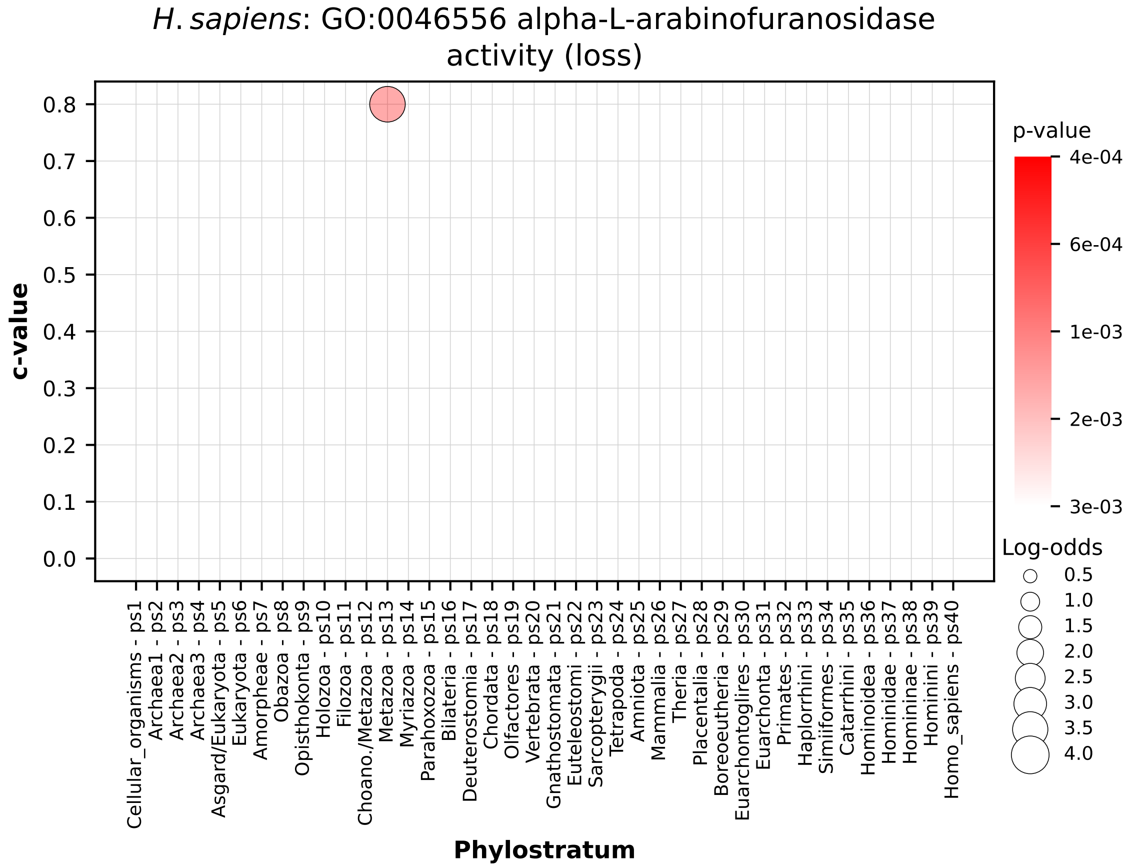

H. sapiens: GO:0046556 alpha-L-arabinofuranosidase activity (loss)
c-value
0.8
0.7
0.6
0.5
0.4
0.3
0.2
0.1
0.0
p-value
4e-04
6e-04
1e-03
2e-03
3e-03
Log-odds
0.5
1.0
1.5
2.0
2.5
3.0
3.5
4.0
Cellular_organisms - ps1
Archaea1 - ps2
Archaea2 - ps3
Archaea3 - ps4
Asgard/Eukaryota - ps5
Eukaryota - ps6
Amorpheae - ps7
Obazoa - ps8
Opisthokonta - ps9
Holozoa - ps10
Filozoa - ps11
Choano./Metazoa - ps12
Metazoa - ps13
Myriazoa - ps14
Parahoxozoa - ps15
Bilateria - ps16
Deuterostomia - ps17
Chordata - ps18
Olfactores - ps19
Vertebrata - ps20
Gnathostomata - ps21
Euteleostomi - ps22
Sarcopterygii - ps23
Tetrapoda - ps24
Amniota - ps25
Mammalia - ps26
Theria - ps27
Placentalia - ps28
Boreoeutheria - ps29
Euarchontoglires - ps30
Euarchonta - ps31
Primates - ps32
Haplorrhini - ps33
Simiiformes - ps34
Catarrhini - ps35
Hominoidea - ps36
Hominidae - ps37
Homininae - ps38
Hominini - ps39
Homo_sapiens - ps40
Phylostratum

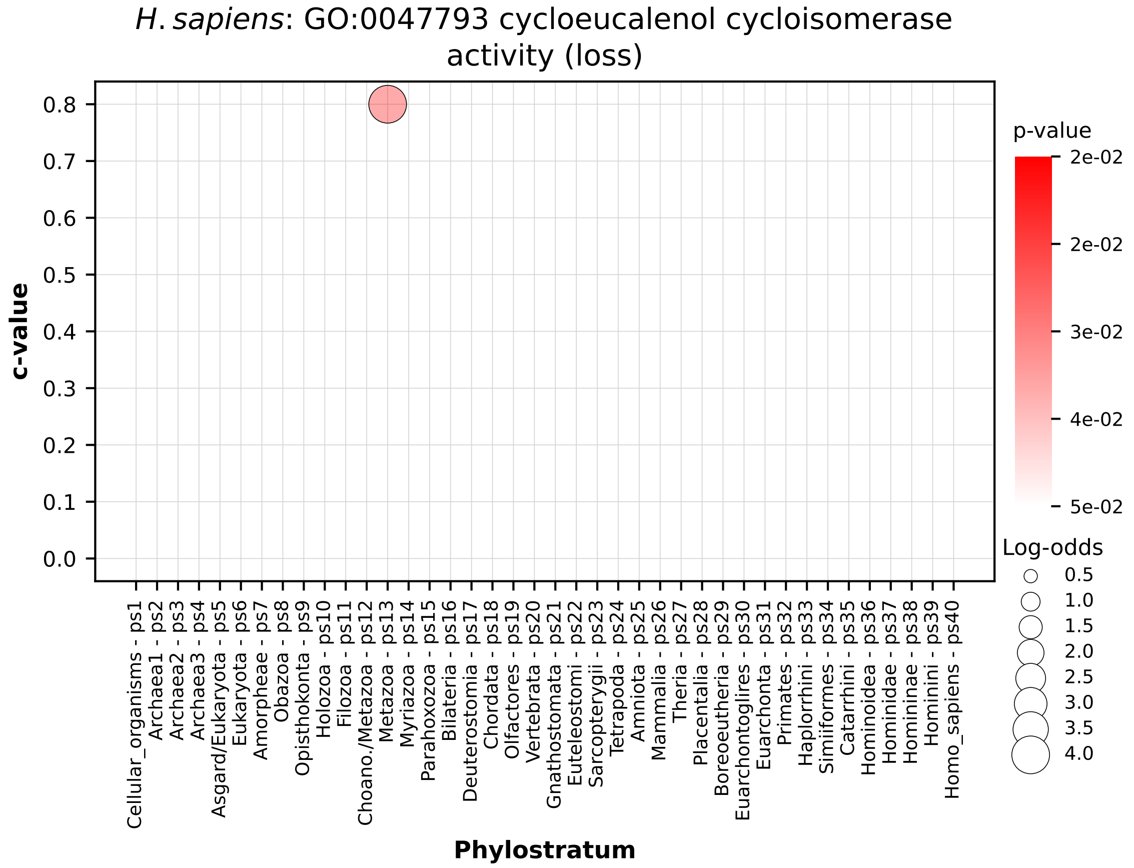
H. sapiens: GO:0047793 cycloeucalenol cycloisomerase activity (loss)
c-value
0.0
0.1
0.2
0.3
0.4
0.5
0.6
0.7
0.8
p-value
2e-02
2e-02
3e-02
4e-02
5e-02
Log-odds
0.5
1.0
1.5
2.0
2.5
3.0
3.5
4.0
Cellular_organisms - ps1
Archaea1 - ps2
Archaea2 - ps3
Archaea3 - ps4
Asgard/Eukaryota - ps5
Eukaryota - ps6
Amorpheae - ps7
Obazoa - ps8
Opisthokonta - ps9
Holozoa - ps10
Filozoa - ps11
Choano./Metazoa - ps12
Metazoa - ps13
Myriazoa - ps14
Parahoxozoa - ps15
Bilateria - ps16
Deuterostomia - ps17
Chordata - ps18
Olfactores - ps19
Vertebrata - ps20
Gnathostomata - ps21
Euteleostomi - ps22
Sarcopterygii - ps23
Tetrapoda - ps24
Amniota - ps25
Mammalia - ps26
Theria - ps27
Placentalia - ps28
Boreoeutheria - ps29
Euarchontoglires - ps30
Euarchonta - ps31
Primates - ps32
Haplorrhini - ps33
Simiiformes - ps34
Catarrhini - ps35
Hominoidea - ps36
Hominidae - ps37
Homininae - ps38
Hominini - ps39
Homo_sapiens - ps40
Phylostratum

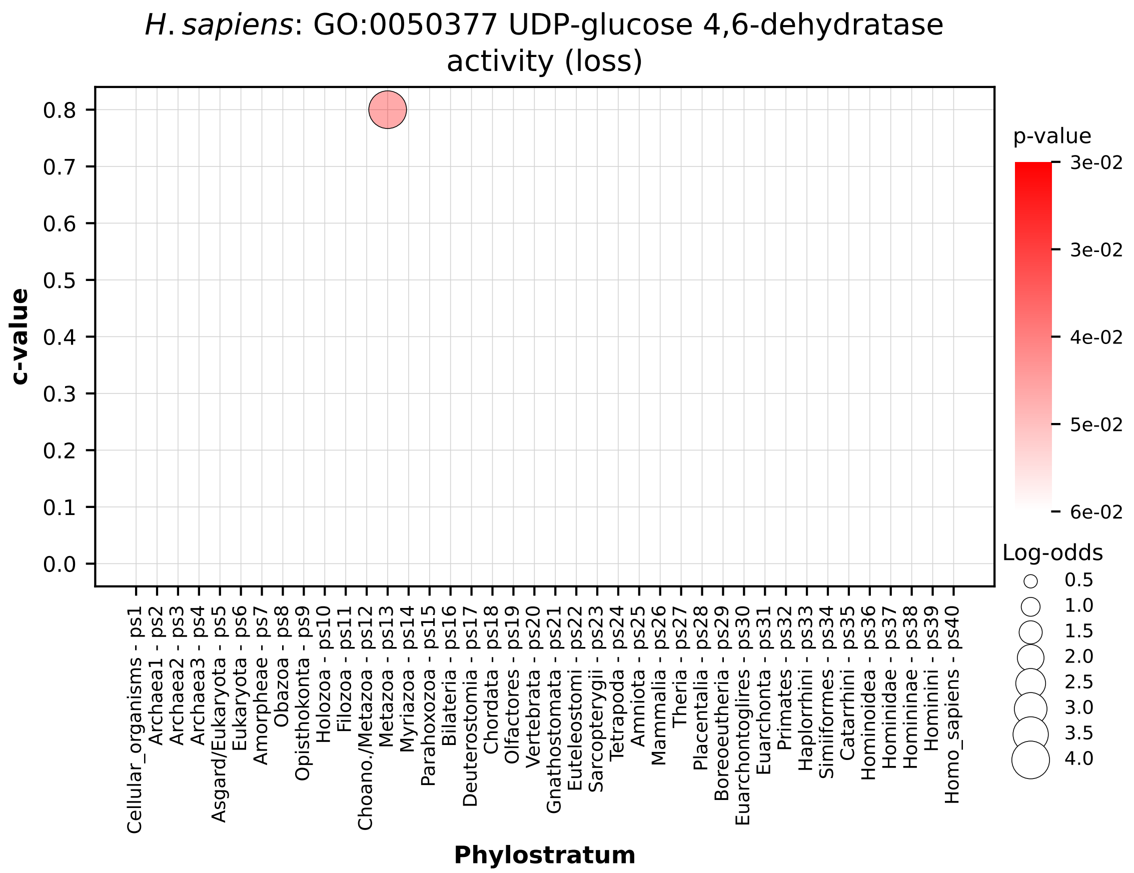
H. sapiens: GO:0050377 UDP-glucose 4,6-dehydratase activity (loss)
c-value
0.8
0.7
0.6
0.5
0.4
0.3
0.2
0.1
0.0
p-value
3e-02
3e-02
4e-02
5e-02
6e-02
Log-odds
0.5
1.0
1.5
2.0
2.5
3.0
3.5
4.0
Cellular_organisms - ps1
Archaea1 - ps2
Archaea2 - ps3
Archaea3 - ps4
Asgard/Eukaryota - ps5
Eukaryota - ps6
Amorpheae - ps7
Obazoa - ps8
Opisthokonta - ps9
Holozoa - ps10
Filozoa - ps11
Choano./Metazoa - ps12
Metazoa - ps13
Myriazoa - ps14
Parahoxozoa - ps15
Bilateria - ps16
Deuterostomia - ps17
Chordata - ps18
Olfactores - ps19
Vertebrata - ps20
Gnathostomata - ps21
Euteleostomi - ps22
Sarcopterygii - ps23
Tetrapoda - ps24
Amniota - ps25
Mammalia - ps26
Theria - ps27
Placentalia - ps28
Boreoeutheria - ps29
Euarchontoglires - ps30
Euarchonta - ps31
Primates - ps32
Haplorrhini - ps33
Simiiformes - ps34
Catarrhini - ps35
Hominoidea - ps36
Hominidae - ps37
Homininae - ps38
Hominini - ps39
Homo_sapiens - ps40
Phylostratum

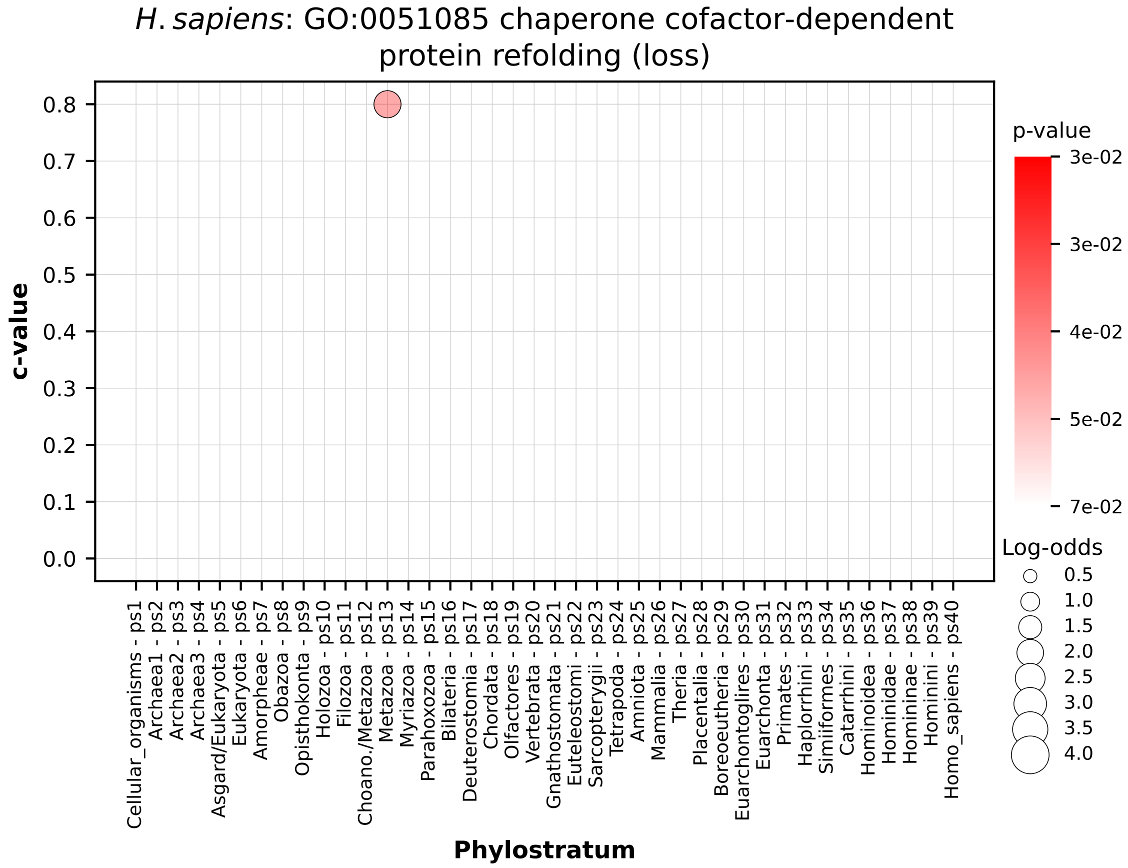

H. sapiens: GO:0051085 chaperone cofactor-dependent protein refolding (loss)
c-value
0.8
0.7
0.6
0.5
0.4
0.3
0.2
0.1
0.0
p-value
3e-02
3e-02
4e-02
5e-02
7e-02
Log-odds
0.5
1.0
1.5
2.0
2.5
3.0
3.5
4.0
Cellular_organisms - ps1
Archaea1 - ps2
Archaea2 - ps3
Archaea3 - ps4
Asgard/Eukaryota - ps5
Eukaryota - ps6
Amorpheae - ps7
Obazoa - ps8
Opisthokonta - ps9
Holozoa - ps10
Filozoa - ps11
Choano./Metazoa - ps12
Metazoa - ps13
Myriazoa - ps14
Parahoxozoa - ps15
Bilateria - ps16
Deuterostomia - ps17
Chordata - ps18
Olfactores - ps19
Vertebrata - ps20
Gnathostomata - ps21
Euteleostomi - ps22
Sarcopterygii - ps23
Tetrapoda - ps24
Amniota - ps25
Mammalia - ps26
Theria - ps27
Placentalia - ps28
Boreoeutheria - ps29
Euarchontoglires - ps30
Euarchonta - ps31
Primates - ps32
Haplorrhini - ps33
Simiiformes - ps34
Catarrhini - ps35
Hominoidea - ps36
Hominidae - ps37
Homininae - ps38
Hominini - ps39
Homo_sapiens - ps40
Phylostratum

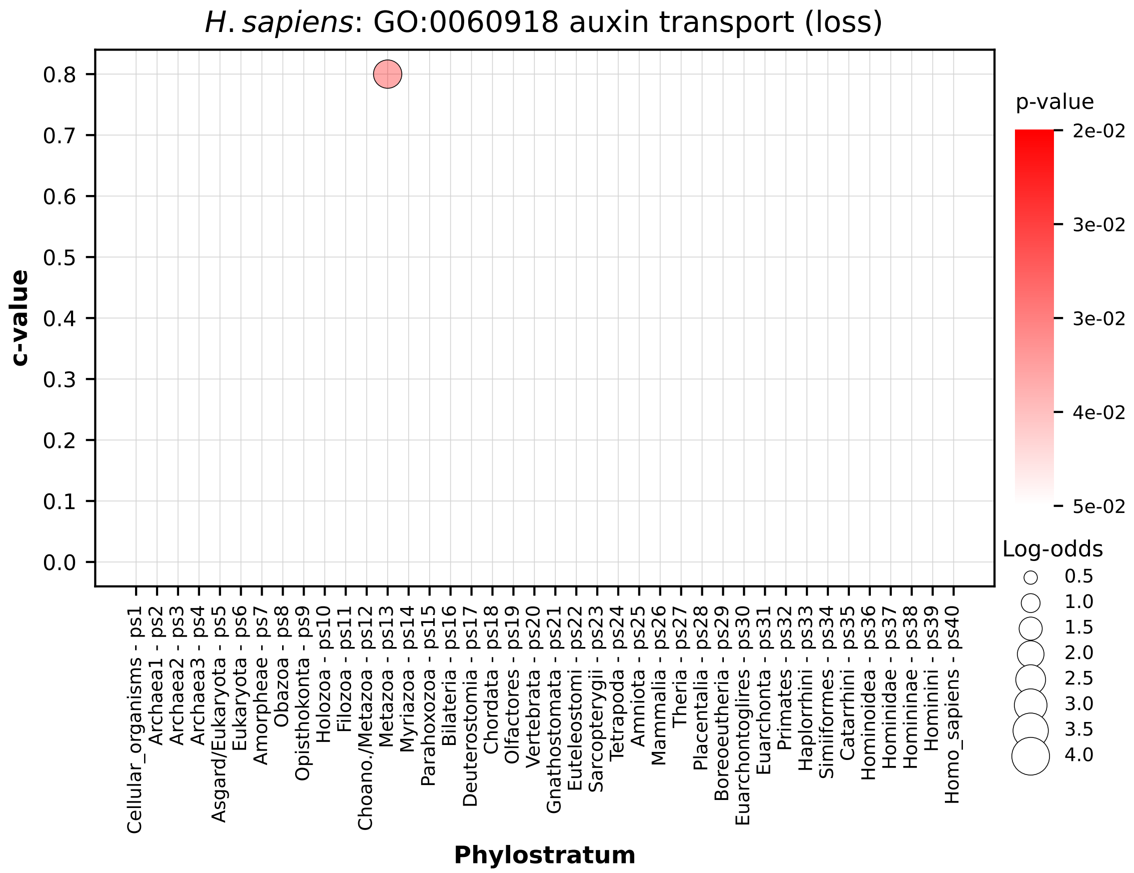

H. sapiens: GO:0060918 auxin transport (loss)
c-value
0.8
0.7
0.6
0.5
0.4
0.3
0.2
0.1
0.0
p-value
2e-02
3e-02
3e-02
4e-02
5e-02
Log-odds
0.5
1.0
1.5
2.0
2.5
3.0
3.5
4.0
Cellular_organisms - ps1
Archaea1 - ps2
Archaea2 - ps3
Archaea3 - ps4
Asgard/Eukaryota - ps5
Eukaryota - ps6
Amorpheae - ps7
Obazoa - ps8
Opisthokonta - ps9
Holozoa - ps10
Filozoa - ps11
Choano./Metazoa - ps12
Metazoa - ps13
Myriazoa - ps14
Parahoxozoa - ps15
Bilateria - ps16
Deuterostomia - ps17
Chordata - ps18
Olfactores - ps19
Vertebrata - ps20
Gnathostomata - ps21
Euteleostomi - ps22
Sarcopterygii - ps23
Tetrapoda - ps24
Amniota - ps25
Mammalia - ps26
Theria - ps27
Placentalia - ps28
Boreoeutheria - ps29
Euarchontoglires - ps30
Euarchonta - ps31
Primates - ps32
Haplorrhini - ps33
Simiiformes - ps34
Catarrhini - ps35
Hominoidea - ps36
Hominidae - ps37
Homininae - ps38
Hominini - ps39
Homo_sapiens - ps40
Phylostratum

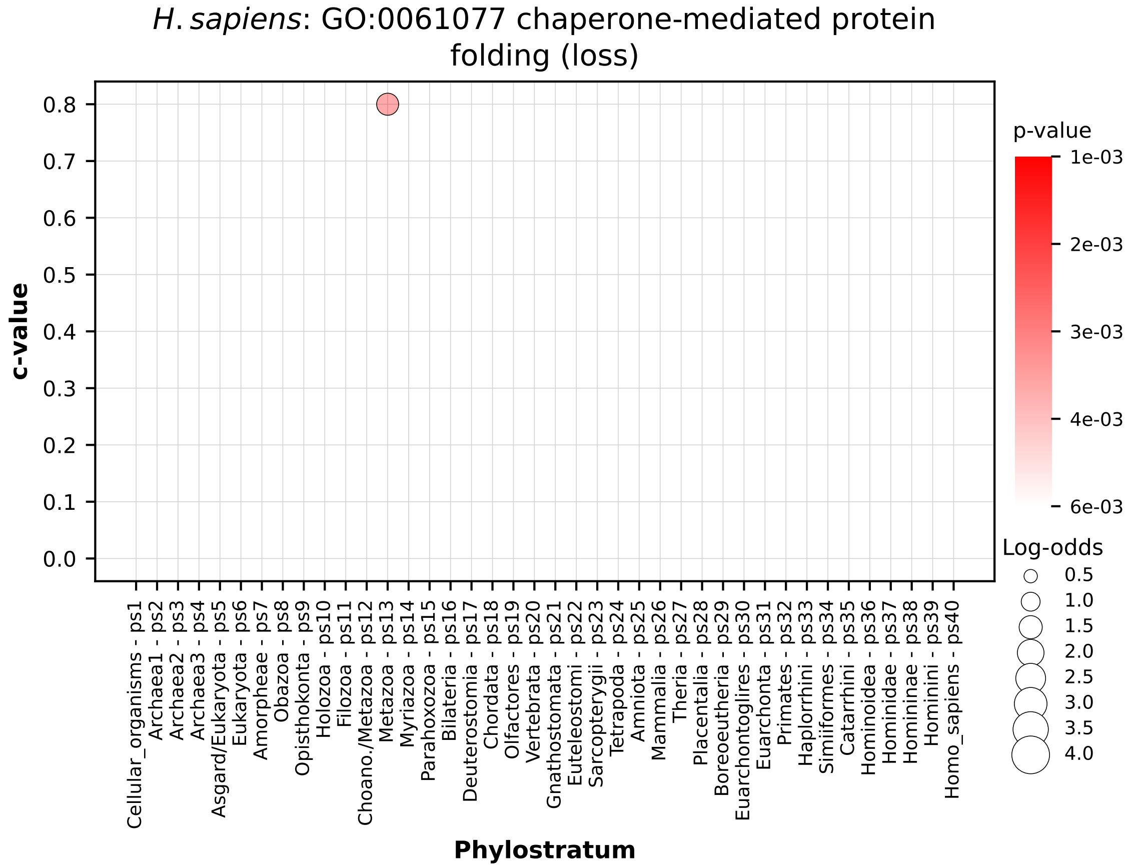

H. sapiens: GO:0061077 chaperone-mediated protein folding (loss)
c-value
0.0
0.1
0.2
0.3
0.4
0.5
0.6
0.7
0.8
p-value
1e-03
2e-03
3e-03
4e-03
6e-03
Log-odds
0.5
1.0
1.5
2.0
2.5
3.0
3.5
4.0
Cellular_organisms - ps1
Archaea1 - ps2
Archaea2 - ps3
Archaea3 - ps4
Asgard/Eukaryota - ps5
Eukaryota - ps6
Amorpheae - ps7
Obazoa - ps8
Opisthokonta - ps9
Holozoa - ps10
Filozoa - ps11
Choano./Metazoa - ps12
Metazoa - ps13
Myriazoa - ps14
Parahoxozoa - ps15
Bilateria - ps16
Deuterostomia - ps17
Chordata - ps18
Olfactores - ps19
Vertebrata - ps20
Gnathostomata - ps21
Euteleostomi - ps22
Sarcopterygii - ps23
Tetrapoda - ps24
Amniota - ps25
Mammalia - ps26
Theria - ps27
Placentalia - ps28
Boreoeutheria - ps29
Euarchontoglires - ps30
Euarchonta - ps31
Primates - ps32
Haplorrhini - ps33
Simiiformes - ps34
Catarrhini - ps35
Hominoidea - ps36
Hominidae - ps37
Homininae - ps38
Hominini - ps39
Homo_sapiens - ps40
Phylostratum

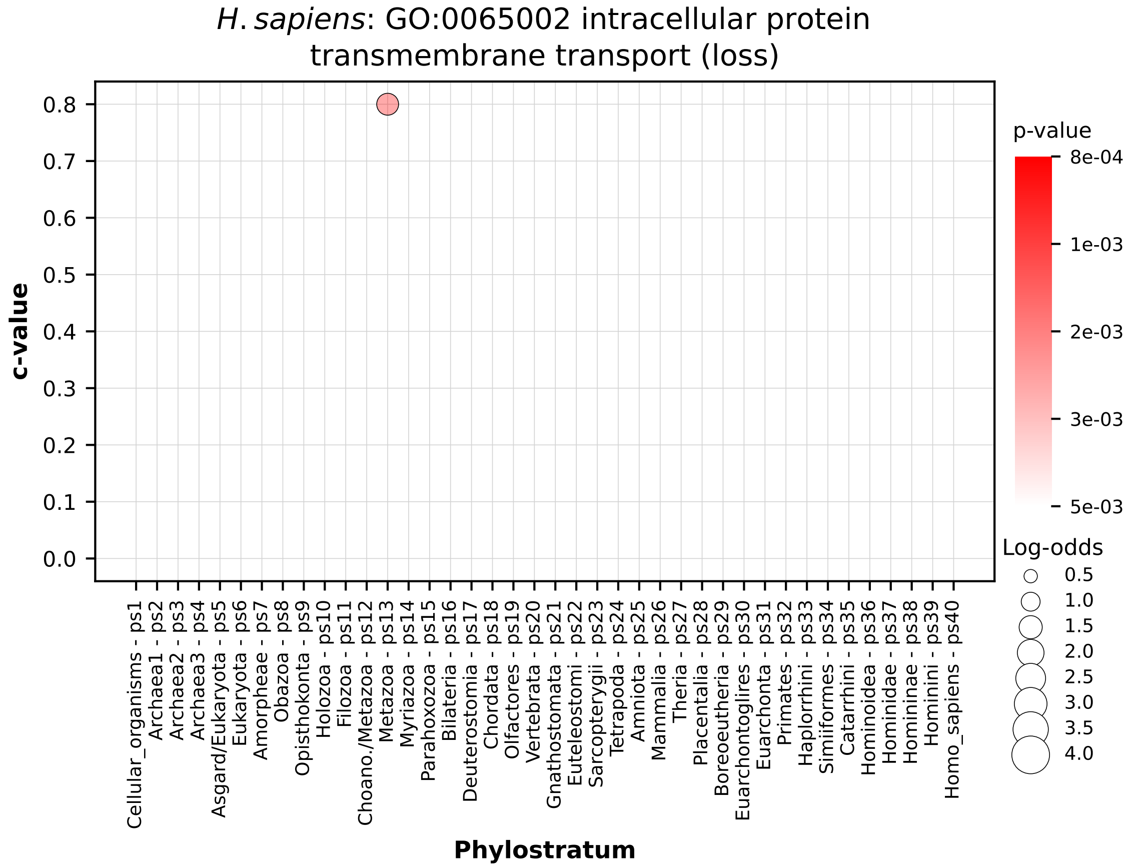

H. sapiens: GO:0065002 intracellular protein transmembrane transport (loss)
c-value
0.0
0.1
0.2
0.3
0.4
0.5
0.6
0.7
0.8
Phylostratum
Cellular_organisms - ps1
Archaea1 - ps2
Archaea2 - ps3
Archaea3 - ps4
Asgard/Eukaryota - ps5
Eukaryota - ps6
Amorpheae - ps7
Obazoa - ps8
Opisthokonta - ps9
Holozoa - ps10
Filozoa - ps11
Choano./Metazoa - ps12
Metazoa - ps13
Myriazoa - ps14
Parahoxozoa - ps15
Bilateria - ps16
Deuterostomia - ps17
Chordata - ps18
Olfactores - ps19
Vertebrata - ps20
Gnathostomata - ps21
Euteleostomi - ps22
Sarcopterygii - ps23
Tetrapoda - ps24
Amniota - ps25
Mammalia - ps26
Theria - ps27
Placentalia - ps28
Boreoeutheria - ps29
Euarchontoglires - ps30
Euarchonta - ps31
Primates - ps32
Haplorrhini - ps33
Simiiformes - ps34
Catarrhini - ps35
Hominoidea - ps36
Hominidae - ps37
Homininae - ps38
Hominini - ps39
Homo_sapiens - ps40
p-value
8e-04
1e-03
2e-03
3e-03
5e-03
Log-odds
0.5
1.0
1.5
2.0
2.5
3.0
3.5
4.0

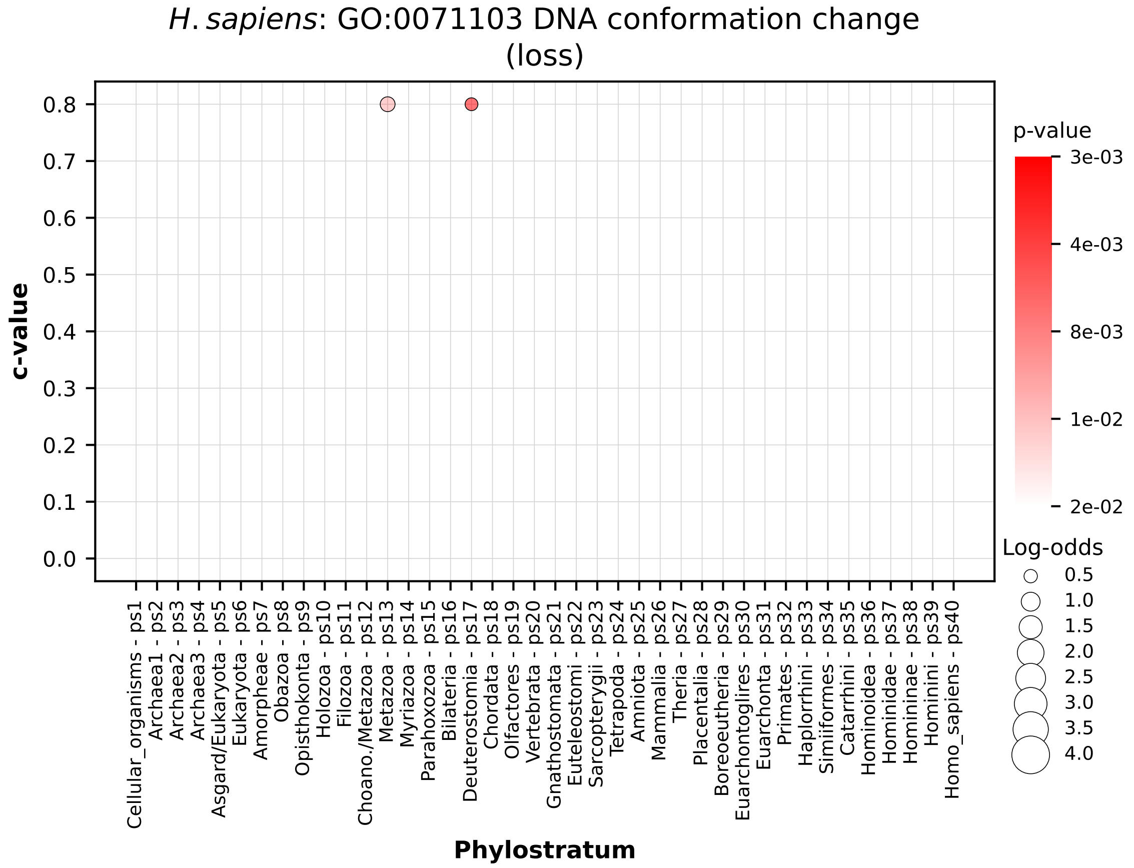

H. sapiens: GO:0071103 DNA conformation change
(loss)
c-value
0.8
0.7
0.6
0.5
0.4
0.3
0.2
0.1
0.0
p-value
3e-03
4e-03
8e-03
1e-02
2e-02
Log-odds
0.5
1.0
1.5
2.0
2.5
3.0
3.5
4.0
Cellular_organisms - ps1
Archaea1 - ps2
Archaea2 - ps3
Archaea3 - ps4
Asgard/Eukaryota - ps5
Eukaryota - ps6
Amorpheae - ps7
Obazoa - ps8
Opisthokonta - ps9
Holozoa - ps10
Filozoa - ps11
Choano./Metazoa - ps12
Metazoa - ps13
Myriazoa - ps14
Parahoxozoa - ps15
Bilateria - ps16
Deuterostomia - ps17
Chordata - ps18
Olfactores - ps19
Vertebrata - ps20
Gnathostomata - ps21
Euteleostomi - ps22
Sarcopterygii - ps23
Tetrapoda - ps24
Amniota - ps25
Mammalia - ps26
Theria - ps27
Placentalia - ps28
Boreoeutheria - ps29
Euarchontoglires - ps30
Euarchonta - ps31
Primates - ps32
Haplorrhini - ps33
Simiiformes - ps34
Catarrhini - ps35
Hominoidea - ps36
Hominidae - ps37
Homininae - ps38
Hominini - ps39
Homo_sapiens - ps40
Phylostratum

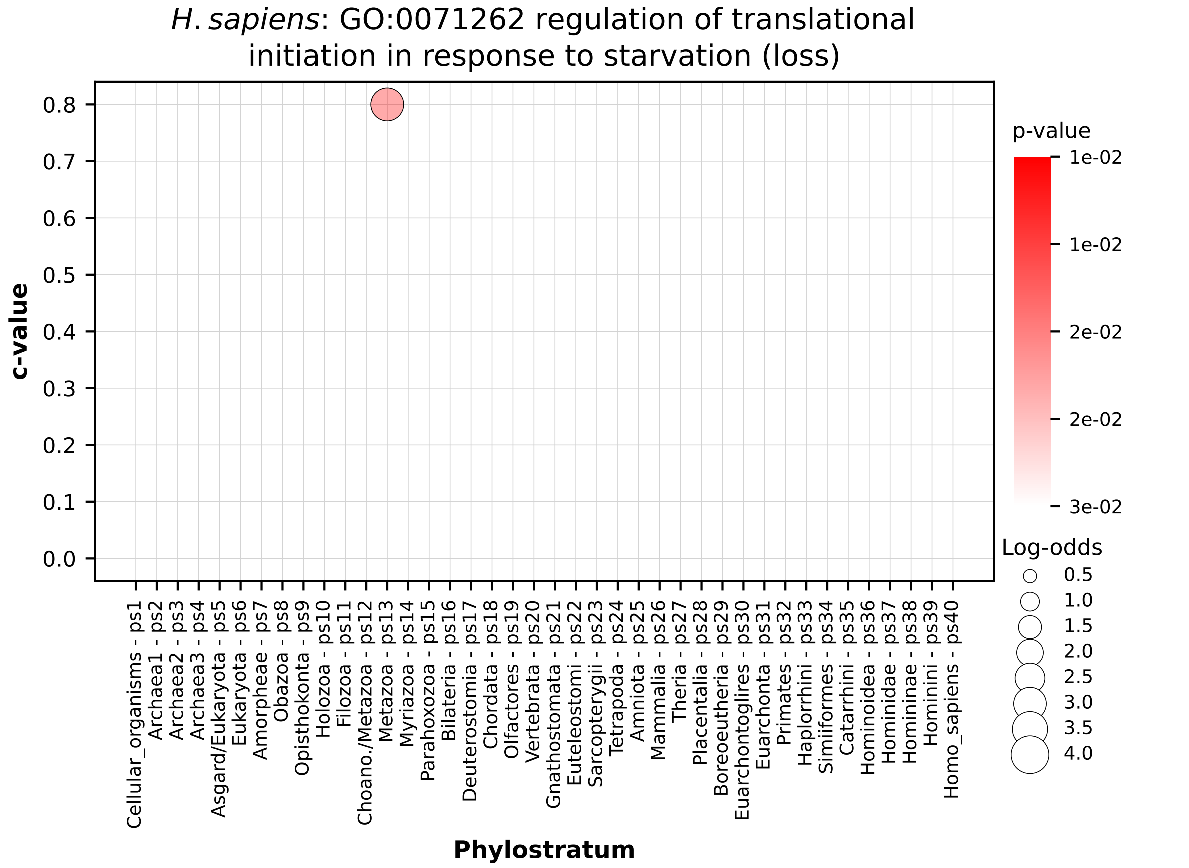

H. sapiens: GO:0071262 regulation of translational initiation in response to starvation (loss)
c-value
0.0
0.1
0.2
0.3
0.4
0.5
0.6
0.7
0.8
Phylostratum
Cellular_organisms - ps1
Archaea1 - ps2
Archaea2 - ps3
Archaea3 - ps4
Asgard/Eukaryota - ps5
Eukaryota - ps6
Amorpheae - ps7
Obazoa - ps8
Opisthokonta - ps9
Holozoa - ps10
Filozoa - ps11
Choano./Metazoa - ps12
Metazoa - ps13
Myriazoa - ps14
Parahoxozoa - ps15
Bilateria - ps16
Deuterostomia - ps17
Chordata - ps18
Olfactores - ps19
Vertebrata - ps20
Gnathostomata - ps21
Euteleostomi - ps22
Sarcopterygii - ps23
Tetrapoda - ps24
Amniota - ps25
Mammalia - ps26
Theria - ps27
Placentalia - ps28
Boreoeutheria - ps29
Euarchontoglires - ps30
Euarchonta - ps31
Primates - ps32
Haplorrhini - ps33
Simiiformes - ps34
Catarrhini - ps35
Hominoidea - ps36
Hominidae - ps37
Homininae - ps38
Hominini - ps39
Homo_sapiens - ps40
p-value
1e-02
1e-02
2e-02
2e-02
3e-02
Log-odds
0.5
1.0
1.5
2.0
2.5
3.0
3.5
4.0

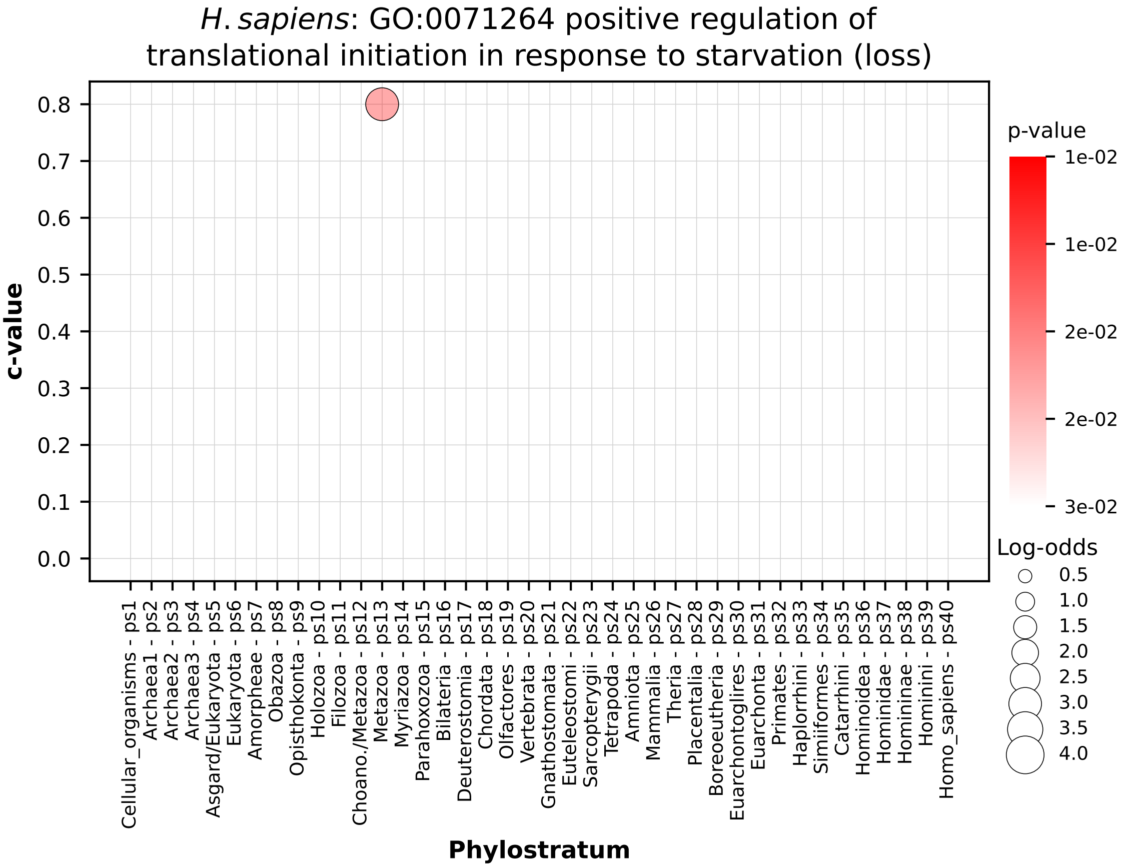

H. sapiens: GO:0071264 positive regulation of translational initiation in response to starvation (loss)
c-value
0.8
0.7
0.6
0.5
0.4
0.3
0.2
0.1
0.0
p-value
1e-02
1e-02
2e-02
2e-02
3e-02
Log-odds
0.5
1.0
1.5
2.0
2.5
3.0
3.5
4.0
Cellular_organisms - ps1
Archaea1 - ps2
Archaea2 - ps3
Archaea3 - ps4
Asgard/Eukaryota - ps5
Eukaryota - ps6
Amorpheae - ps7
Obazoa - ps8
Opisthokonta - ps9
Holozoa - ps10
Filozoa - ps11
Choano./Metazoa - ps12
Metazoa - ps13
Myriazoa - ps14
Parahoxozoa - ps15
Bilateria - ps16
Deuterostomia - ps17
Chordata - ps18
Olfactores - ps19
Vertebrata - ps20
Gnathostomata - ps21
Euteleostomi - ps22
Sarcopterygii - ps23
Tetrapoda - ps24
Amniota - ps25
Mammalia - ps26
Theria - ps27
Placentalia - ps28
Boreoeutheria - ps29
Euarchontoglires - ps30
Euarchonta - ps31
Primates - ps32
Haplorrhini - ps33
Simiiformes - ps34
Catarrhini - ps35
Hominoidea - ps36
Hominidae - ps37
Homininae - ps38
Hominini - ps39
Homo_sapiens - ps40
Phylostratum

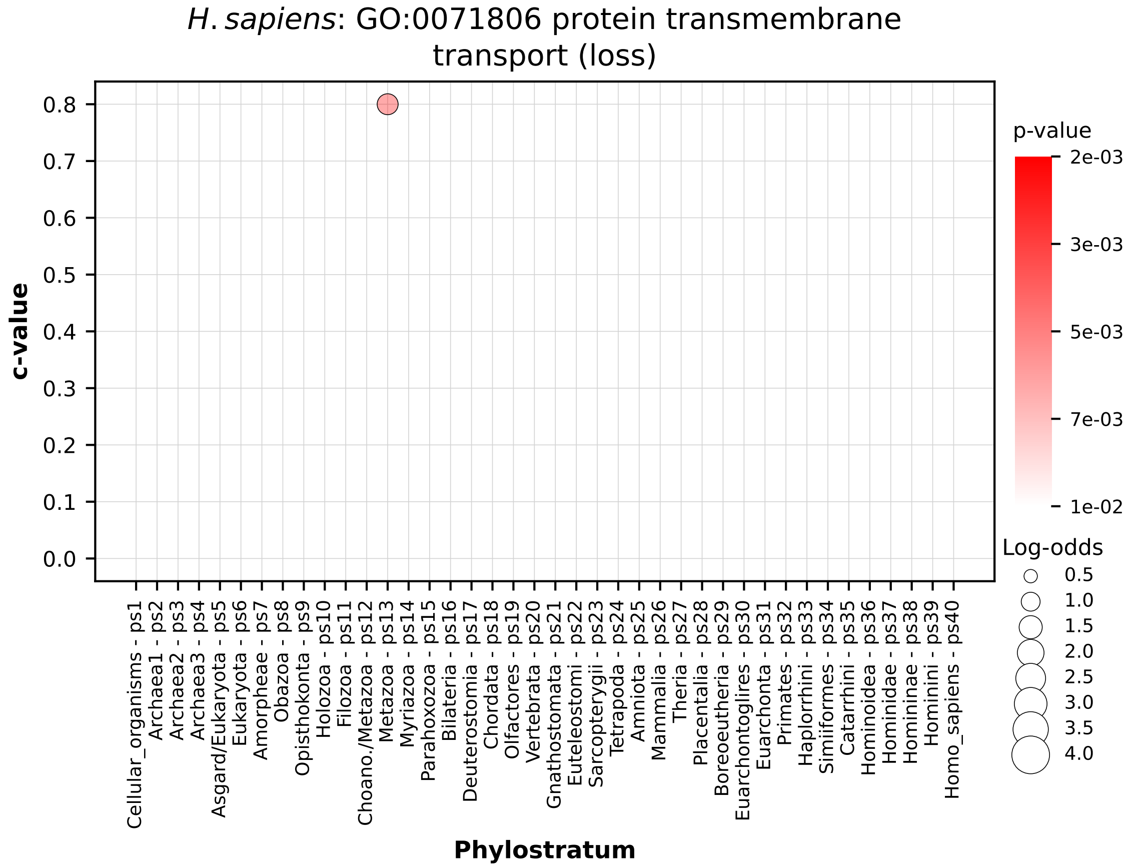

H. sapiens: GO:0071806 protein transmembrane transport (loss)
c-value
0.8
0.7
0.6
0.5
0.4
0.3
0.2
0.1
0.0
p-value
2e-03
3e-03
5e-03
7e-03
1e-02
Log-odds
0.5
1.0
1.5
2.0
2.5
3.0
3.5
4.0
Cellular_organisms - ps1
Archaea1 - ps2
Archaea2 - ps3
Archaea3 - ps4
Asgard/Eukaryota - ps5
Eukaryota - ps6
Amorpheae - ps7
Obazoa - ps8
Opisthokonta - ps9
Holozoa - ps10
Filozoa - ps11
Choano./Metazoa - ps12
Metazoa - ps13
Myriazoa - ps14
Parahoxozoa - ps15
Bilateria - ps16
Deuterostomia - ps17
Chordata - ps18
Olfactores - ps19
Vertebrata - ps20
Gnathostomata - ps21
Euteleostomi - ps22
Sarcopterygii - ps23
Tetrapoda - ps24
Amniota - ps25
Mammalia - ps26
Theria - ps27
Placentalia - ps28
Boreoeutheria - ps29
Euarchontoglires - ps30
Euarchonta - ps31
Primates - ps32
Haplorrhini - ps33
Simiiformes - ps34
Catarrhini - ps35
Hominoidea - ps36
Hominidae - ps37
Homininae - ps38
Hominini - ps39
Homo_sapiens - ps40
Phylostratum

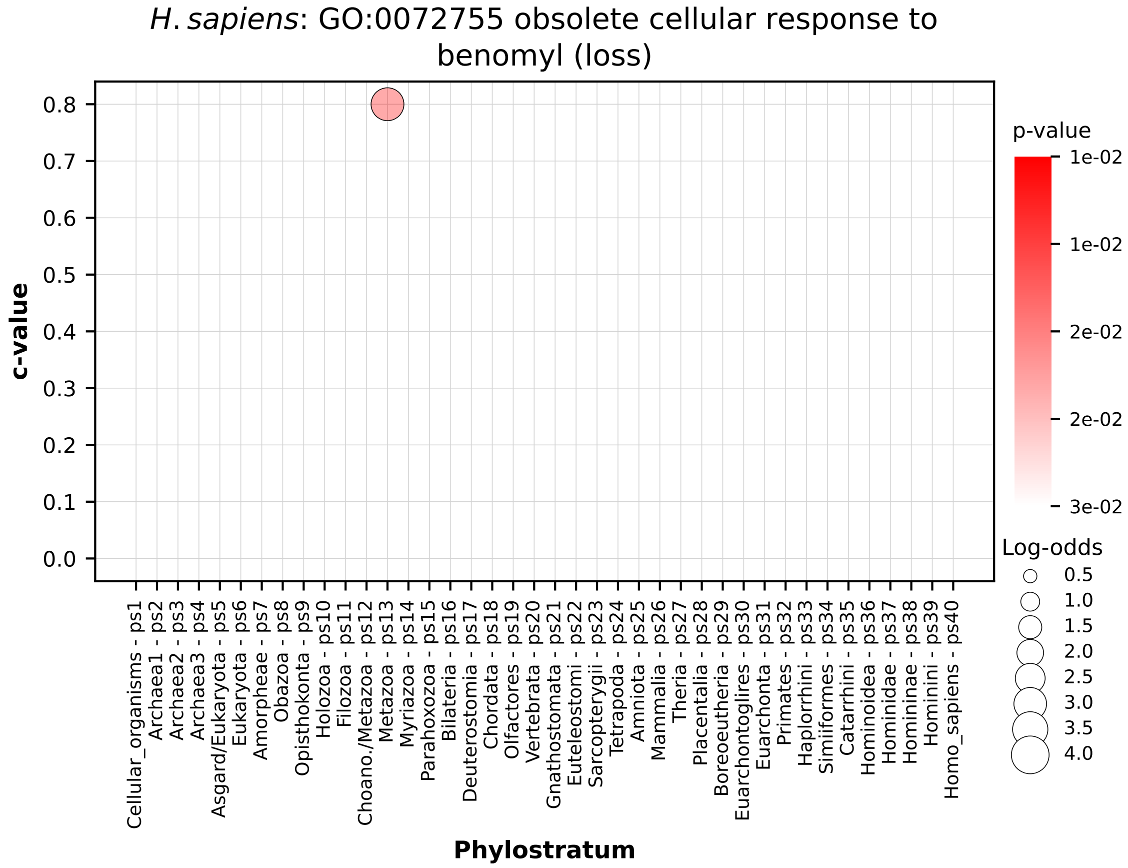

H. sapiens: GO:0072755 obsolete cellular response to benomyl (loss)
c-value
0.0
0.1
0.2
0.3
0.4
0.5
0.6
0.7
0.8
Phylostratum
Cellular_organisms - ps1
Archaea1 - ps2
Archaea2 - ps3
Archaea3 - ps4
Asgard/Eukaryota - ps5
Eukaryota - ps6
Amorpheae - ps7
Obazoa - ps8
Opisthokonta - ps9
Holozoa - ps10
Filozoa - ps11
Choano./Metazoa - ps12
Metazoa - ps13
Myriazoa - ps14
Parahoxozoa - ps15
Bilateria - ps16
Deuterostomia - ps17
Chordata - ps18
Olfactores - ps19
Vertebrata - ps20
Gnathostomata - ps21
Euteleostomi - ps22
Sarcopterygii - ps23
Tetrapoda - ps24
Amniota - ps25
Mammalia - ps26
Theria - ps27
Placentalia - ps28
Boreoeutheria - ps29
Euarchontoglires - ps30
Euarchonta - ps31
Primates - ps32
Haplorrhini - ps33
Simiiformes - ps34
Catarrhini - ps35
Hominoidea - ps36
Hominidae - ps37
Homininae - ps38
Hominini - ps39
Homo_sapiens - ps40
p-value
1e-02
1e-02
2e-02
2e-02
3e-02
Log-odds
0.5
1.0
1.5
2.0
2.5
3.0
3.5
4.0

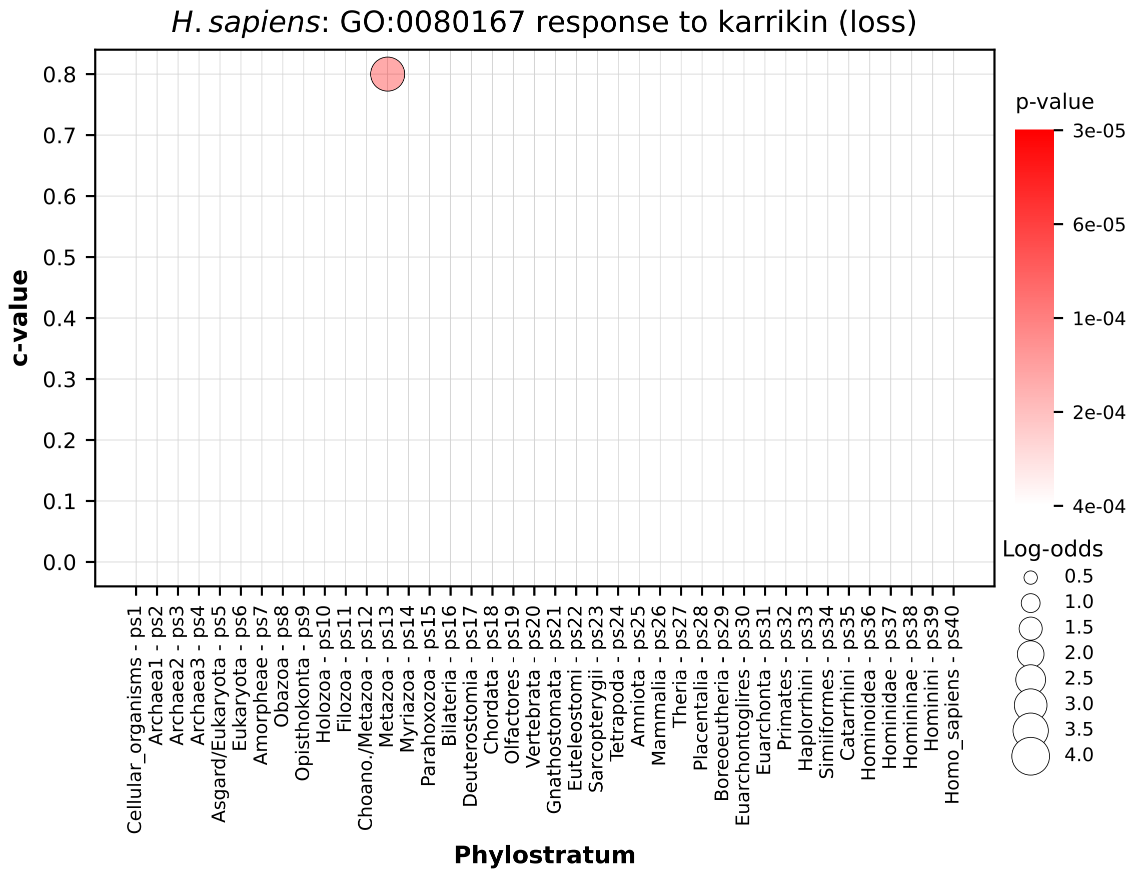

H. sapiens: GO:0080167 response to karrikin (loss)
c-value
0.8
0.7
0.6
0.5
0.4
0.3
0.2
0.1
0.0
p-value
3e-05
6e-05
1e-04
2e-04
4e-04
Log-odds
0.5
1.0
1.5
2.0
2.5
3.0
3.5
4.0
Cellular_organisms - ps1
Archaea1 - ps2
Archaea2 - ps3
Archaea3 - ps4
Asgard/Eukaryota - ps5
Eukaryota - ps6
Amorpheae - ps7
Obazoa - ps8
Opisthokonta - ps9
Holozoa - ps10
Filozoa - ps11
Choano./Metazoa - ps12
Metazoa - ps13
Myriazoa - ps14
Parahoxozoa - ps15
Bilateria - ps16
Deuterostomia - ps17
Chordata - ps18
Olfactores - ps19
Vertebrata - ps20
Gnathostomata - ps21
Euteleostomi - ps22
Sarcopterygii - ps23
Tetrapoda - ps24
Amniota - ps25
Mammalia - ps26
Theria - ps27
Placentalia - ps28
Boreoeutheria - ps29
Euarchontoglires - ps30
Euarchonta - ps31
Primates - ps32
Haplorrhini - ps33
Simiiformes - ps34
Catarrhini - ps35
Hominoidea - ps36
Hominidae - ps37
Homininae - ps38
Hominini - ps39
Homo_sapiens - ps40
Phylostratum

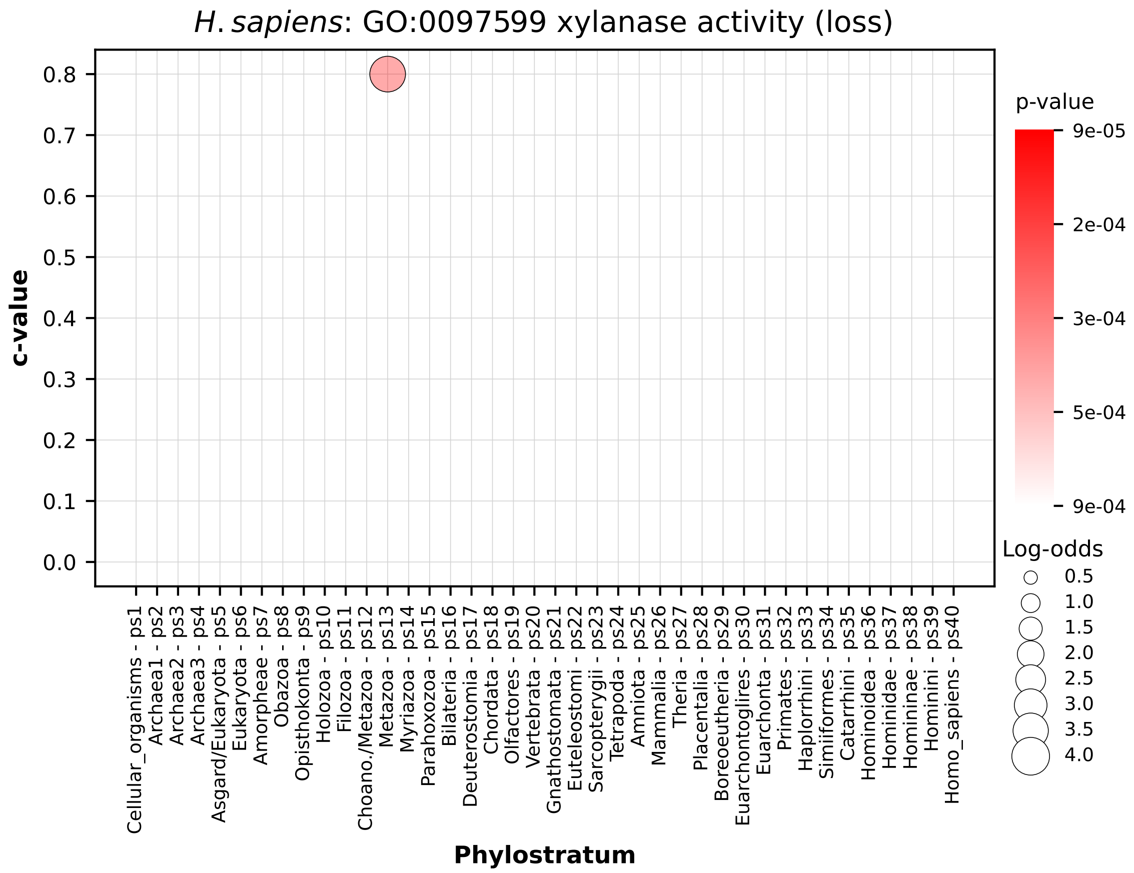

H. sapiens: GO:0097599 xylanase activity (loss)
c-value
0.0
0.1
0.2
0.3
0.4
0.5
0.6
0.7
0.8
Phylostratum
Cellular_organisms - ps1
Archaea1 - ps2
Archaea2 - ps3
Archaea3 - ps4
Asgard/Eukaryota - ps5
Eukaryota - ps6
Amorpheae - ps7
Obazoa - ps8
Opisthokonta - ps9
Holozoa - ps10
Filozoa - ps11
Choano./Metazoa - ps12
Metazoa - ps13
Myriazoa - ps14
Parahoxozoa - ps15
Bilateria - ps16
Deuterostomia - ps17
Chordata - ps18
Olfactores - ps19
Vertebrata - ps20
Gnathostomata - ps21
Euteleostomi - ps22
Sarcopterygii - ps23
Tetrapoda - ps24
Amniota - ps25
Mammalia - ps26
Theria - ps27
Placentalia - ps28
Boreoeutheria - ps29
Euarchontoglires - ps30
Euarchonta - ps31
Primates - ps32
Haplorrhini - ps33
Simiiformes - ps34
Catarrhini - ps35
Hominoidea - ps36
Hominidae - ps37
Homininae - ps38
Hominini - ps39
Homo_sapiens - ps40
p-value
9e-05
2e-04
3e-04
5e-04
9e-04
Log-odds
0.5
1.0
1.5
2.0
2.5
3.0
3.5
4.0

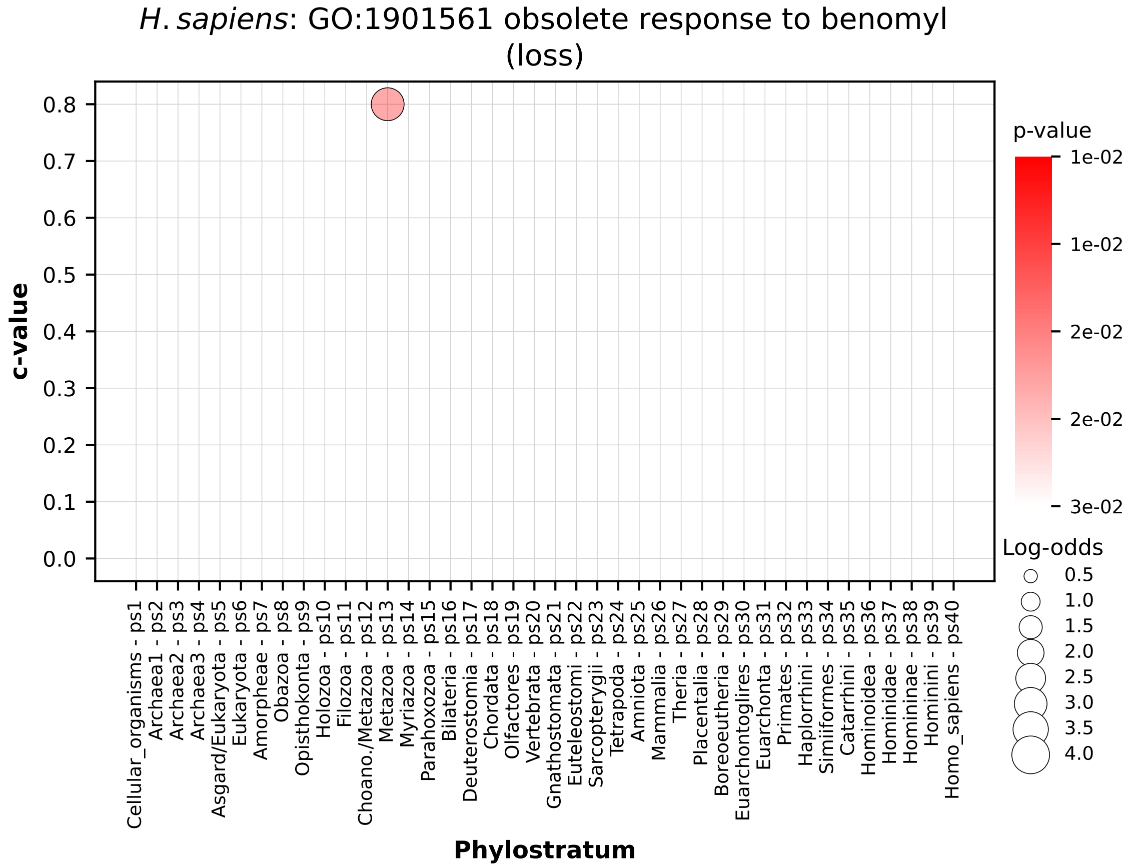

H. sapiens: GO:1901561 obsolete response to benomyl (loss)
c-value
0.8
0.7
0.6
0.5
0.4
0.3
0.2
0.1
0.0
p-value
1e-02
1e-02
2e-02
2e-02
3e-02
Log-odds
0.5
1.0
1.5
2.0
2.5
3.0
3.5
4.0
Cellular_organisms - ps1
Archaea1 - ps2
Archaea2 - ps3
Archaea3 - ps4
Asgard/Eukaryota - ps5
Eukaryota - ps6
Amorpheae - ps7
Obazoa - ps8
Opisthokonta - ps9
Holozoa - ps10
Filozoa - ps11
Choano./Metazoa - ps12
Metazoa - ps13
Myriazoa - ps14
Parahoxozoa - ps15
Bilateria - ps16
Deuterostomia - ps17
Chordata - ps18
Olfactores - ps19
Vertebrata - ps20
Gnathostomata - ps21
Euteleostomi - ps22
Sarcopterygii - ps23
Tetrapoda - ps24
Amniota - ps25
Mammalia - ps26
Theria - ps27
Placentalia - ps28
Boreoeutheria - ps29
Euarchontoglires - ps30
Euarchonta - ps31
Primates - ps32
Haplorrhini - ps33
Simiiformes - ps34
Catarrhini - ps35
Hominoidea - ps36
Hominidae - ps37
Homininae - ps38
Hominini - ps39
Homo_sapiens - ps40
Phylostratum

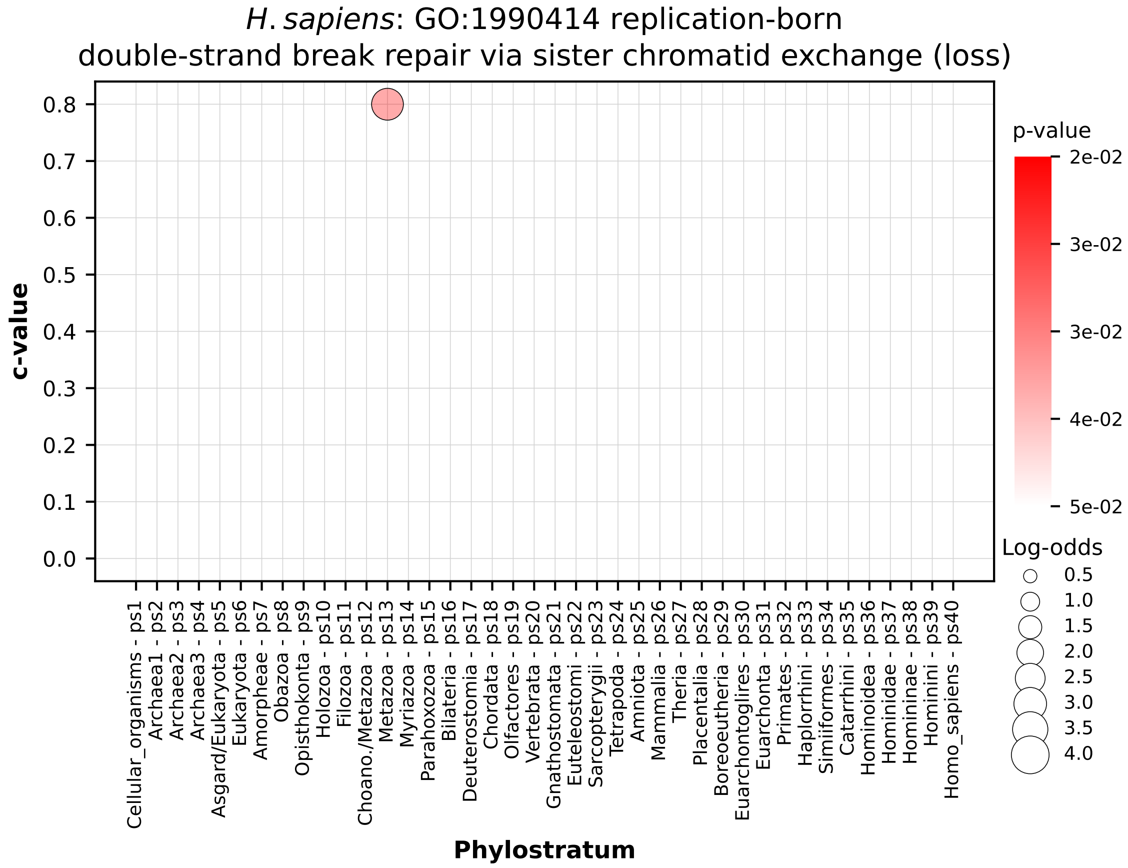
H. sapiens: GO:1990414 replication-born
double-strand break repair via sister chromatid exchange (loss)
c-value
0.0
0.1
0.2
0.3
0.4
0.5
0.6
0.7
0.8
Phylostratum
Cellular_organisms - ps1
Archaea1 - ps2
Archaea2 - ps3
Archaea3 - ps4
Asgard/Eukaryota - ps5
Eukaryota - ps6
Amorpheae - ps7
Obazoa - ps8
Opisthokonta - ps9
Holozoa - ps10
Filozoa - ps11
Choano./Metazoa - ps12
Metazoa - ps13
Myriazoa - ps14
Parahoxozoa - ps15
Bilateria - ps16
Deuterostomia - ps17
Chordata - ps18
Olfactores - ps19
Vertebrata - ps20
Gnathostomata - ps21
Euteleostomi - ps22
Sarcopterygii - ps23
Tetrapoda - ps24
Amniota - ps25
Mammalia - ps26
Theria - ps27
Placentalia - ps28
Boreoeutheria - ps29
Euarchontoglires - ps30
Euarchonta - ps31
Primates - ps32
Haplorrhini - ps33
Simiiformes - ps34
Catarrhini - ps35
Hominoidea - ps36
Hominidae - ps37
Homininae - ps38
Hominini - ps39
Homo_sapiens - ps40
p-value
2e-02
3e-02
3e-02
4e-02
5e-02
Log-odds
0.5
1.0
1.5
2.0
2.5
3.0
3.5
4.0

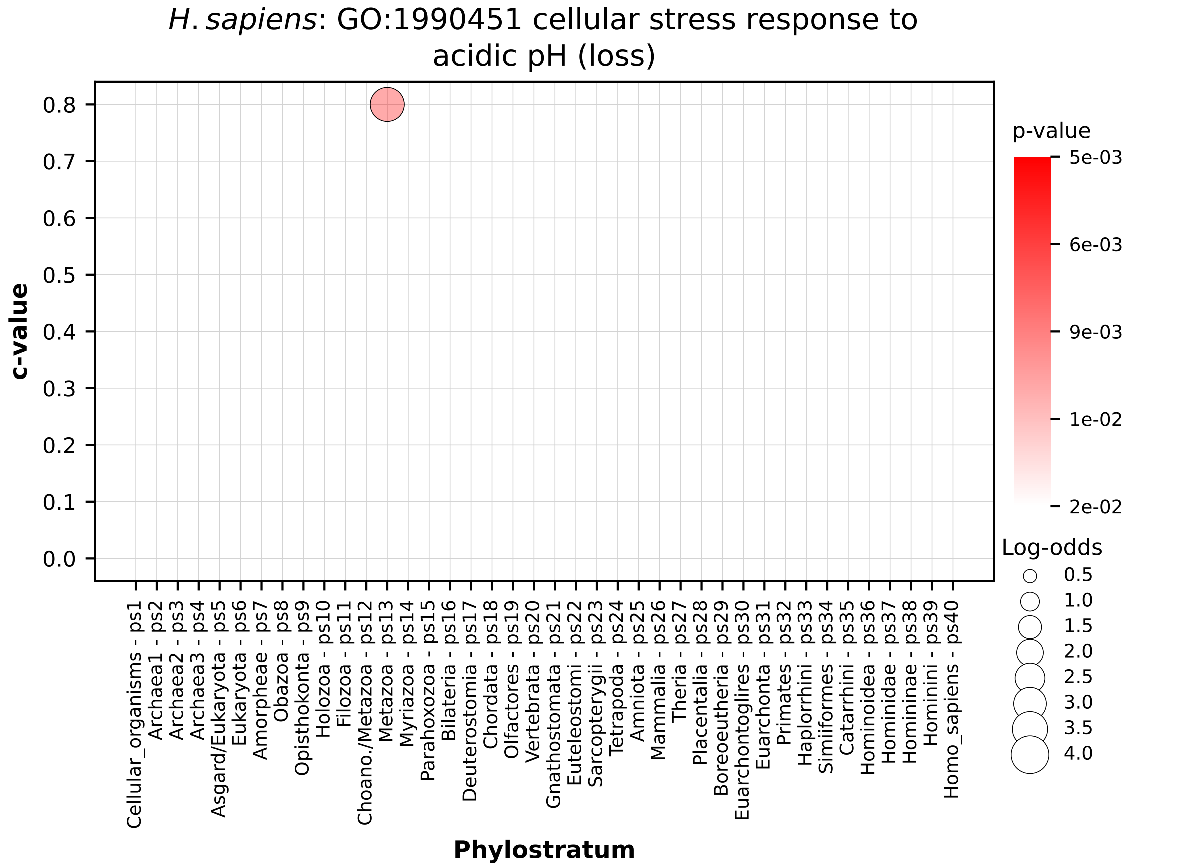

H. sapiens: GO:1990451 cellular stress response to acidic pH (loss)
c-value
0.8
0.7
0.6
0.5
0.4
0.3
0.2
0.1
0.0
p-value
5e-03
6e-03
9e-03
1e-02
2e-02
Log-odds
0.5
1.0
1.5
2.0
2.5
3.0
3.5
4.0
Cellular_organisms - ps1
Archaea1 - ps2
Archaea2 - ps3
Archaea3 - ps4
Asgard/Eukaryota - ps5
Eukaryota - ps6
Amorpheae - ps7
Obazoa - ps8
Opisthokonta - ps9
Holozoa - ps10
Filozoa - ps11
Choano./Metazoa - ps12
Metazoa - ps13
Myriazoa - ps14
Parahoxozoa - ps15
Bilateria - ps16
Deuterostomia - ps17
Chordata - ps18
Olfactores - ps19
Vertebrata - ps20
Gnathostomata - ps21
Euteleostomi - ps22
Sarcopterygii - ps23
Tetrapoda - ps24
Amniota - ps25
Mammalia - ps26
Theria - ps27
Placentalia - ps28
Boreoeutheria - ps29
Euarchontoglires - ps30
Euarchonta - ps31
Primates - ps32
Haplorrhini - ps33
Simiiformes - ps34
Catarrhini - ps35
Hominoidea - ps36
Hominidae - ps37
Homininae - ps38
Hominini - ps39
Homo_sapiens - ps40
Phylostratum

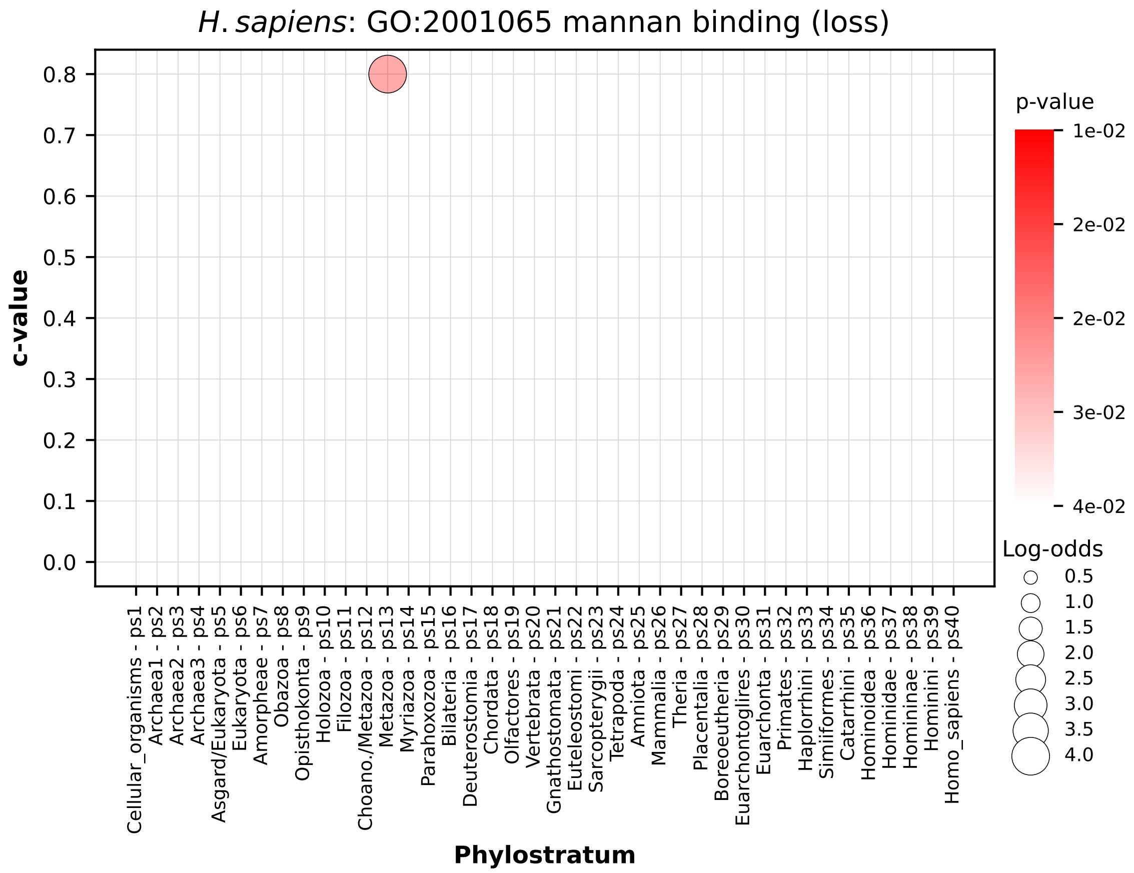

H. sapiens: GO:2001065 mannan binding (loss)
c-value
0.0
0.1
0.2
0.3
0.4
0.5
0.6
0.7
0.8
p-value
1e-02
2e-02
2e-02
3e-02
4e-02
Log-odds
0.5
1.0
1.5
2.0
2.5
3.0
3.5
4.0
Cellular_organisms - ps1
Archaea1 - ps2
Archaea2 - ps3
Archaea3 - ps4
Asgard/Eukaryota - ps5
Eukaryota - ps6
Amorpheae - ps7
Obazoa - ps8
Opisthokonta - ps9
Holozoa - ps10
Filozoa - ps11
Choano./Metazoa - ps12
Metazoa - ps13
Myriazoa - ps14
Parahoxozoa - ps15
Bilateria - ps16
Deuterostomia - ps17
Chordata - ps18
Olfactores - ps19
Vertebrata - ps20
Gnathostomata - ps21
Euteleostomi - ps22
Sarcopterygii - ps23
Tetrapoda - ps24
Amniota - ps25
Mammalia - ps26
Theria - ps27
Placentalia - ps28
Boreoeutheria - ps29
Euarchontoglires - ps30
Euarchonta - ps31
Primates - ps32
Haplorrhini - ps33
Simiiformes - ps34
Catarrhini - ps35
Hominoidea - ps36
Hominidae - ps37
Homininae - ps38
Hominini - ps39
Homo_sapiens - ps40
Phylostratum

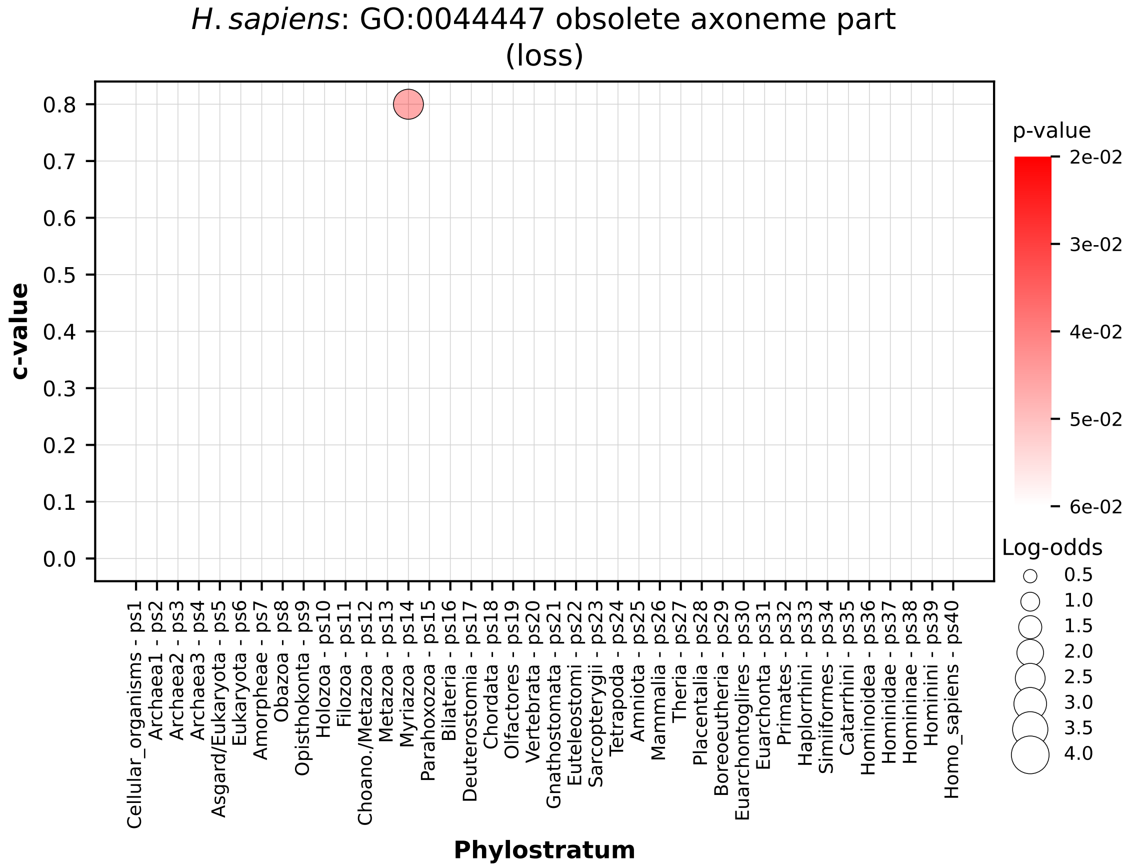

H. sapiens: GO:0044447 obsolete axoneme part
(loss)
c-value
0.0
0.1
0.2
0.3
0.4
0.5
0.6
0.7
0.8
p-value
2e-02
3e-02
4e-02
5e-02
6e-02
Log-odds
0.5
1.0
1.5
2.0
2.5
3.0
3.5
4.0
Cellular_organisms - ps1
Archaea1 - ps2
Archaea2 - ps3
Archaea3 - ps4
Asgard/Eukaryota - ps5
Eukaryota - ps6
Amorpheae - ps7
Obazoa - ps8
Opisthokonta - ps9
Holozoa - ps10
Filozoa - ps11
Choano./Metazoa - ps12
Metazoa - ps13
Myriazoa - ps14
Parahoxozoa - ps15
Bilateria - ps16
Deuterostomia - ps17
Chordata - ps18
Olfactores - ps19
Vertebrata - ps20
Gnathostomata - ps21
Euteleostomi - ps22
Sarcopterygii - ps23
Tetrapoda - ps24
Amniota - ps25
Mammalia - ps26
Theria - ps27
Placentalia - ps28
Boreoeutheria - ps29
Euarchontoglires - ps30
Euarchonta - ps31
Primates - ps32
Haplorrhini - ps33
Simiiformes - ps34
Catarrhini - ps35
Hominoidea - ps36
Hominidae - ps37
Homininae - ps38
Hominini - ps39
Homo_sapiens - ps40
Phylostratum

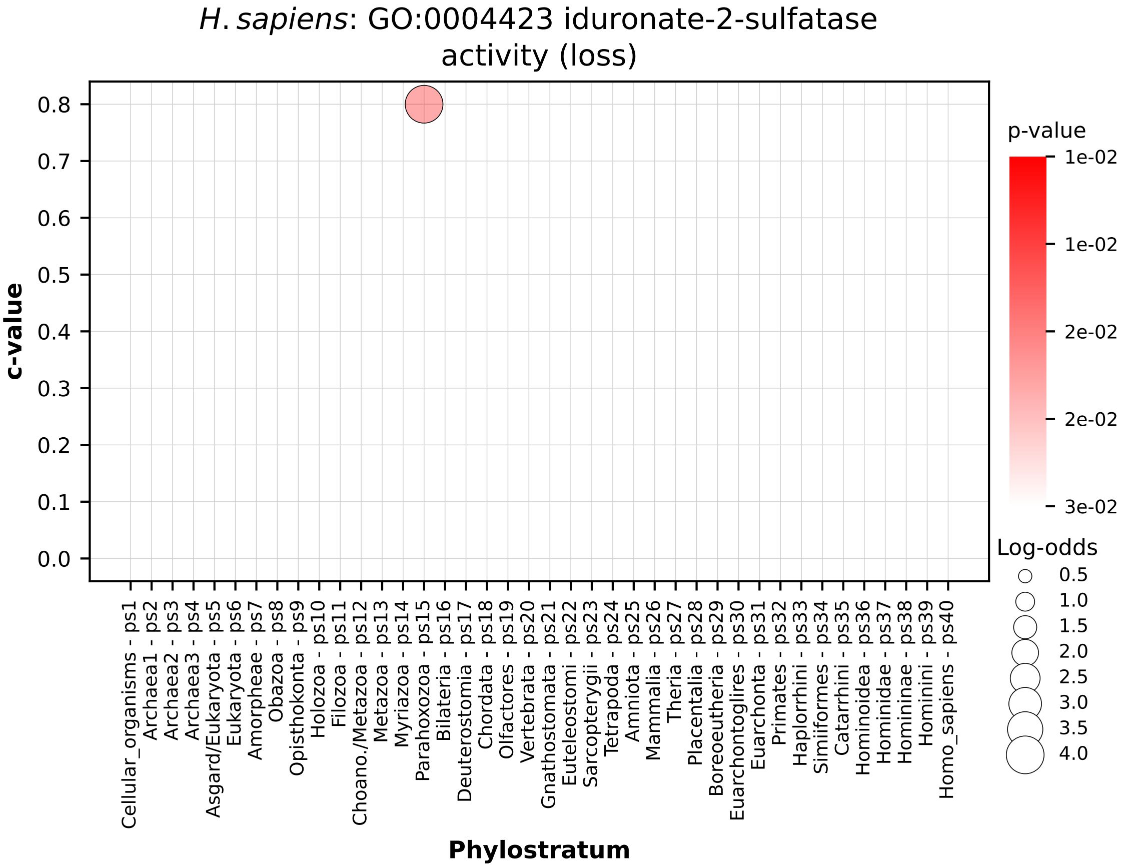

H. sapiens: GO:0004423 iduronate-2-sulfatase activity (loss)
c-value
0.8
0.7
0.6
0.5
0.4
0.3
0.2
0.1
0.0
p-value
1e-02
1e-02
2e-02
2e-02
3e-02
Log-odds
0.5
1.0
1.5
2.0
2.5
3.0
3.5
4.0
Cellular_organisms - ps1
Archaea1 - ps2
Archaea2 - ps3
Archaea3 - ps4
Asgard/Eukaryota - ps5
Eukaryota - ps6
Amorpheae - ps7
Obazoa - ps8
Opisthokonta - ps9
Holozoa - ps10
Filozoa - ps11
Choano./Metazoa - ps12
Metazoa - ps13
Myriazoa - ps14
Parahoxozoa - ps15
Bilateria - ps16
Deuterostomia - ps17
Chordata - ps18
Olfactores - ps19
Vertebrata - ps20
Gnathostomata - ps21
Euteleostomi - ps22
Sarcopterygii - ps23
Tetrapoda - ps24
Amniota - ps25
Mammalia - ps26
Theria - ps27
Placentalia - ps28
Boreoeutheria - ps29
Euarchontoglires - ps30
Euarchonta - ps31
Primates - ps32
Haplorrhini - ps33
Simiiformes - ps34
Catarrhini - ps35
Hominoidea - ps36
Hominidae - ps37
Homininae - ps38
Hominini - ps39
Homo_sapiens - ps40
Phylostratum

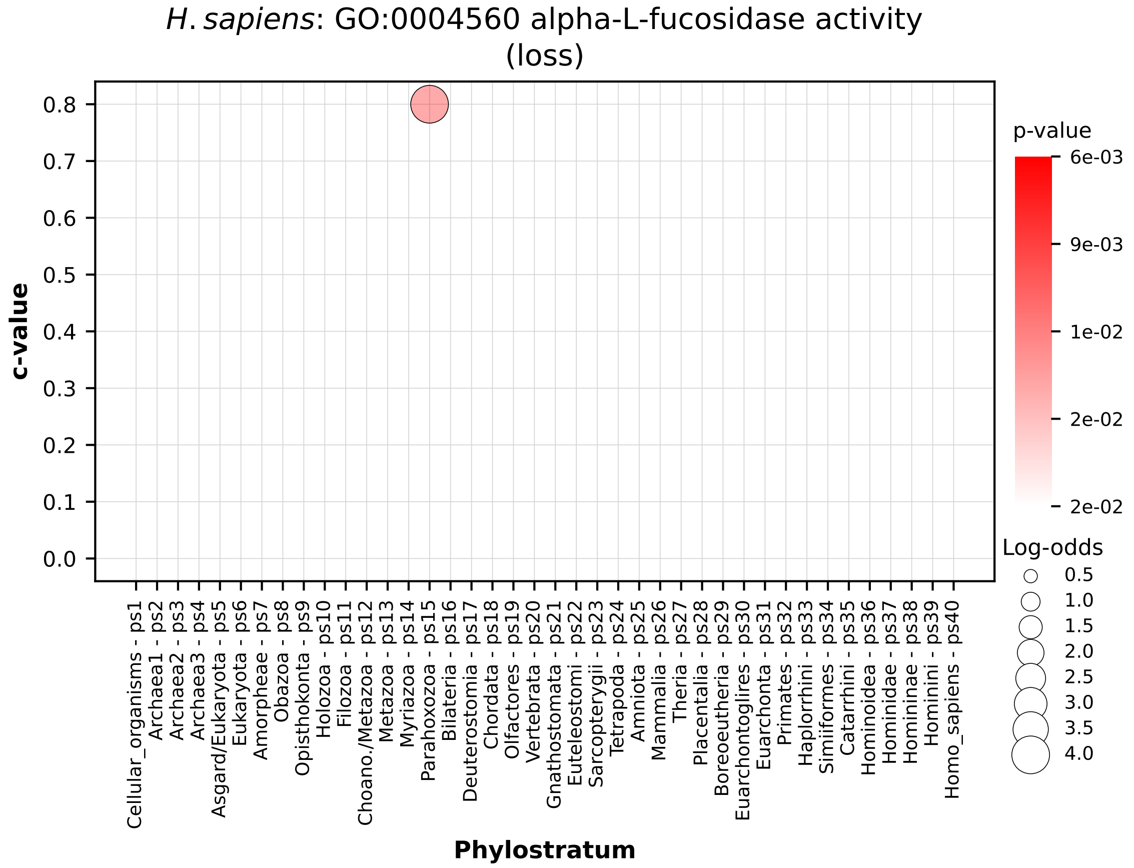
H. sapiens: GO:0004560 alpha-L-fucosidase activity
(loss)
c-value
0.0
0.1
0.2
0.3
0.4
0.5
0.6
0.7
0.8
p-value
6e-03
9e-03
1e-02
2e-02
2e-02
Log-odds
0.5
1.0
1.5
2.0
2.5
3.0
3.5
4.0
Cellular_organisms - ps1
Archaea1 - ps2
Archaea2 - ps3
Archaea3 - ps4
Asgard/Eukaryota - ps5
Eukaryota - ps6
Amorpheae - ps7
Obazoa - ps8
Opisthokonta - ps9
Holozoa - ps10
Filozoa - ps11
Choano./Metazoa - ps12
Metazoa - ps13
Myriazoa - ps14
Parahoxozoa - ps15
Bilateria - ps16
Deuterostomia - ps17
Chordata - ps18
Olfactores - ps19
Vertebrata - ps20
Gnathostomata - ps21
Euteleostomi - ps22
Sarcopterygii - ps23
Tetrapoda - ps24
Amniota - ps25
Mammalia - ps26
Theria - ps27
Placentalia - ps28
Boreoeutheria - ps29
Euarchontoglires - ps30
Euarchonta - ps31
Primates - ps32
Haplorrhini - ps33
Simiiformes - ps34
Catarrhini - ps35
Hominoidea - ps36
Hominidae - ps37
Homininae - ps38
Hominini - ps39
Homo_sapiens - ps40
Phylostratum

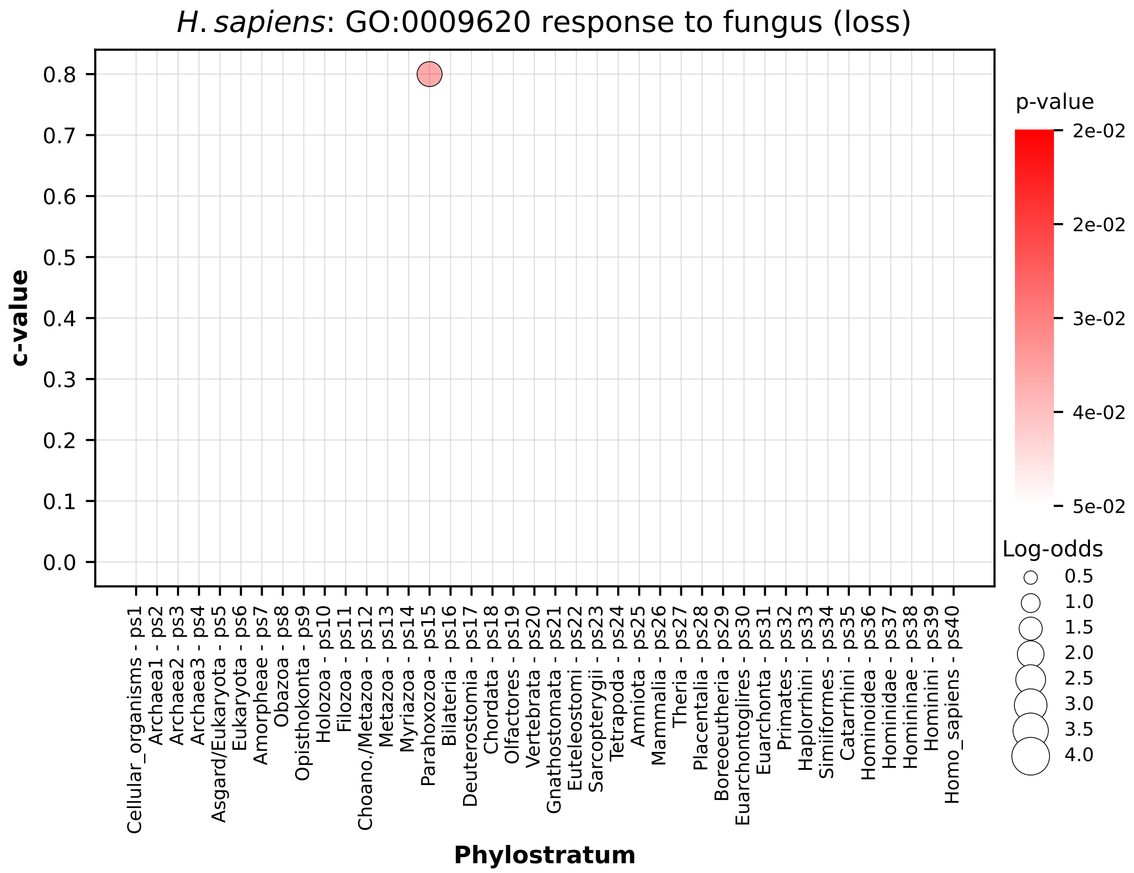

H. sapiens: GO:0009620 response to fungus (loss)
c-value
0.0
0.1
0.2
0.3
0.4
0.5
0.6
0.7
0.8
p-value
2e-02
2e-02
3e-02
4e-02
5e-02
Log-odds
0.5
1.0
1.5
2.0
2.5
3.0
3.5
4.0
Cellular_organisms - ps1
Archaea1 - ps2
Archaea2 - ps3
Archaea3 - ps4
Asgard/Eukaryota - ps5
Eukaryota - ps6
Amorpheae - ps7
Obazoa - ps8
Opisthokonta - ps9
Holozoa - ps10
Filozoa - ps11
Choano./Metazoa - ps12
Metazoa - ps13
Myriazoa - ps14
Parahoxozoa - ps15
Bilateria - ps16
Deuterostomia - ps17
Chordata - ps18
Olfactores - ps19
Vertebrata - ps20
Gnathostomata - ps21
Euteleostomi - ps22
Sarcopterygii - ps23
Tetrapoda - ps24
Amniota - ps25
Mammalia - ps26
Theria - ps27
Placentalia - ps28
Boreoeutheria - ps29
Euarchontoglires - ps30
Euarchonta - ps31
Primates - ps32
Haplorrhini - ps33
Simiiformes - ps34
Catarrhini - ps35
Hominoidea - ps36
Hominidae - ps37
Homininae - ps38
Hominini - ps39
Homo_sapiens - ps40
Phylostratum

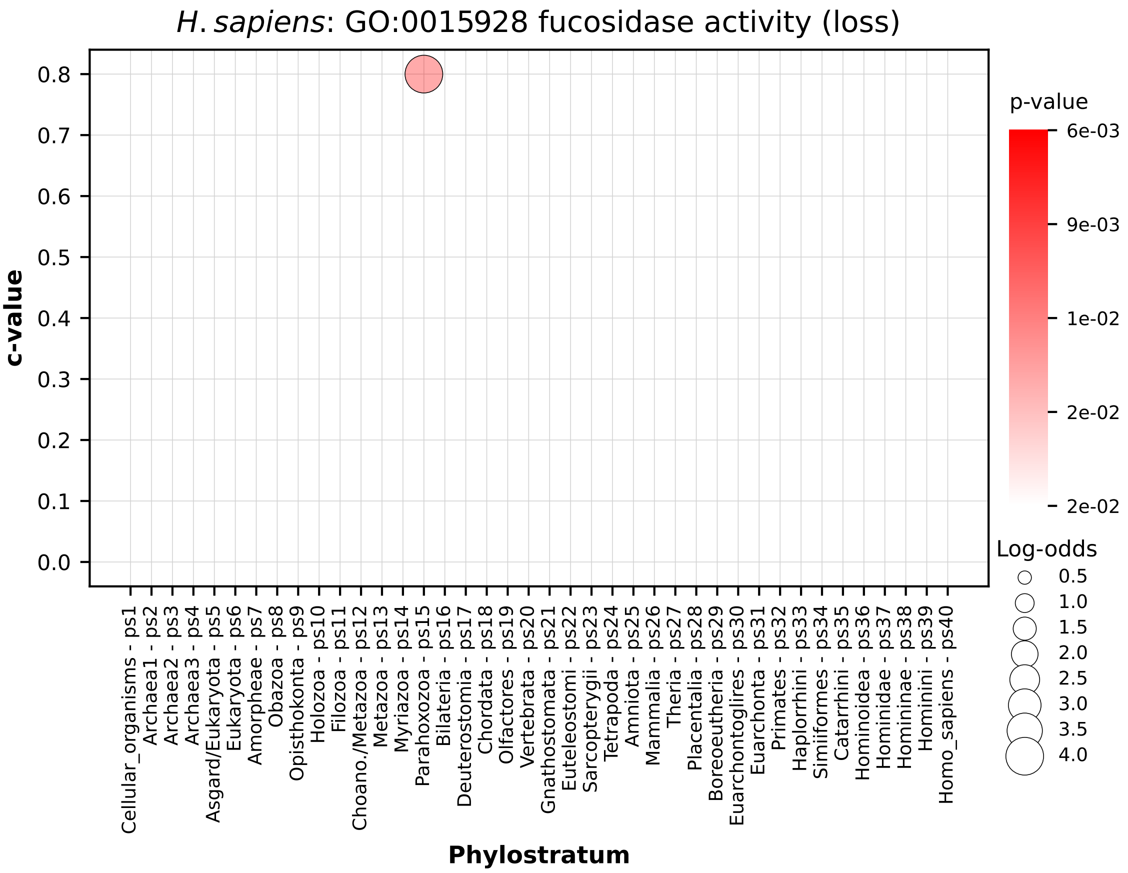

H. sapiens: GO:0015928 fucosidase activity (loss)
c-value
0.0
0.1
0.2
0.3
0.4
0.5
0.6
0.7
0.8
Phylostratum
Cellular_organisms - ps1
Archaea1 - ps2
Archaea2 - ps3
Archaea3 - ps4
Asgard/Eukaryota - ps5
Eukaryota - ps6
Amorpheae - ps7
Obazoa - ps8
Opisthokonta - ps9
Holozoa - ps10
Filozoa - ps11
Choano./Metazoa - ps12
Metazoa - ps13
Myriazoa - ps14
Parahoxozoa - ps15
Bilateria - ps16
Deuterostomia - ps17
Chordata - ps18
Olfactores - ps19
Vertebrata - ps20
Gnathostomata - ps21
Euteleostomi - ps22
Sarcopterygii - ps23
Tetrapoda - ps24
Amniota - ps25
Mammalia - ps26
Theria - ps27
Placentalia - ps28
Boreoeutheria - ps29
Euarchontoglires - ps30
Euarchonta - ps31
Primates - ps32
Haplorrhini - ps33
Simiiformes - ps34
Catarrhini - ps35
Hominoidea - ps36
Hominidae - ps37
Homininae - ps38
Hominini - ps39
Homo_sapiens - ps40
p-value
6e-03
9e-03
1e-02
2e-02
2e-02
Log-odds
0.5
1.0
1.5
2.0
2.5
3.0
3.5
4.0

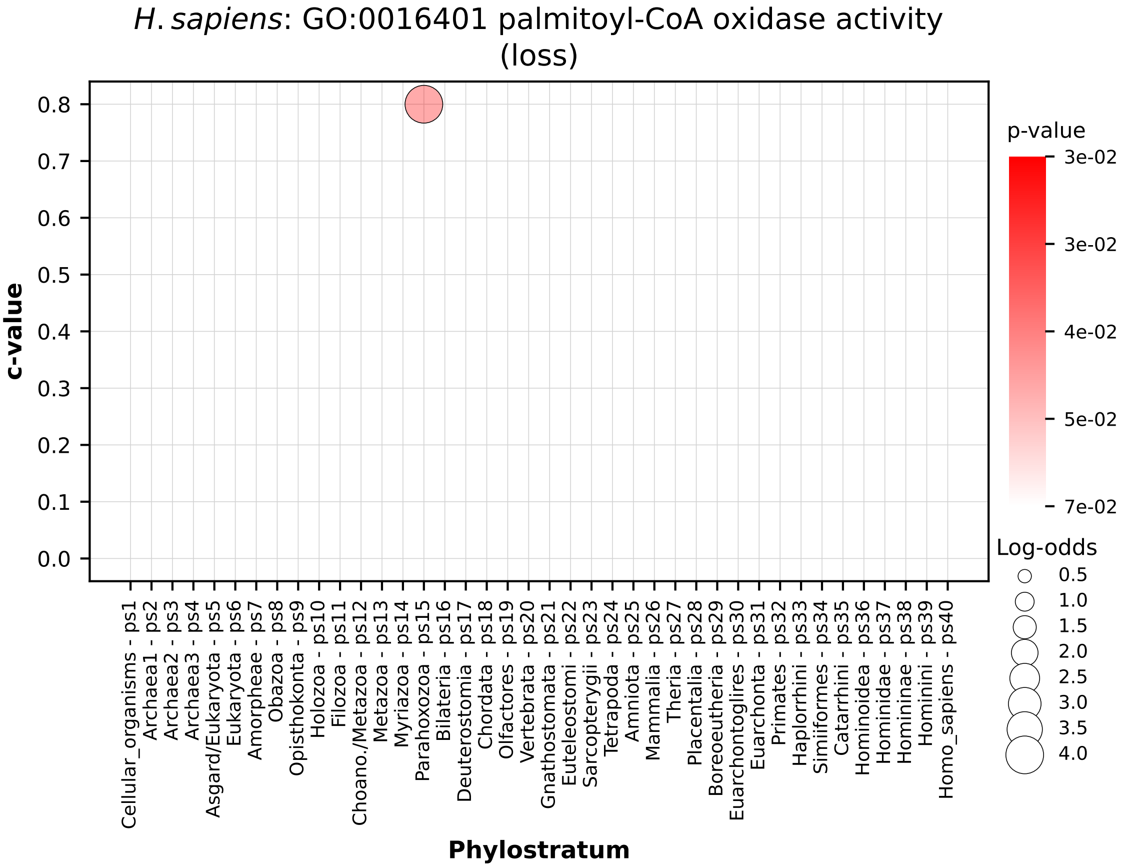
H. sapiens: GO:0016401 palmitoyl-CoA oxidase activity (loss)
c-value
0.8
0.7
0.6
0.5
0.4
0.3
0.2
0.1
0.0
Phylostratum
Cellular_organisms - ps1
Archaea1 - ps2
Archaea2 - ps3
Archaea3 - ps4
Asgard/Eukaryota - ps5
Eukaryota - ps6
Amorpheae - ps7
Obazoa - ps8
Opisthokonta - ps9
Holozoa - ps10
Filozoa - ps11
Choano./Metazoa - ps12
Metazoa - ps13
Myriazoa - ps14
Parahoxozoa - ps15
Bilateria - ps16
Deuterostomia - ps17
Chordata - ps18
Olfactores - ps19
Vertebrata - ps20
Gnathostomata - ps21
Euteleostomi - ps22
Sarcopterygii - ps23
Tetrapoda - ps24
Amniota - ps25
Mammalia - ps26
Theria - ps27
Placentalia - ps28
Boreoeutheria - ps29
Euarchontoglires - ps30
Euarchonta - ps31
Primates - ps32
Haplorrhini - ps33
Simiiformes - ps34
Catarrhini - ps35
Hominoidea - ps36
Hominidae - ps37
Homininae - ps38
Hominini - ps39
Homo_sapiens - ps40
p-value
3e-02
3e-02
4e-02
5e-02
7e-02
Log-odds
0.5
1.0
1.5
2.0
2.5
3.0
3.5
4.0

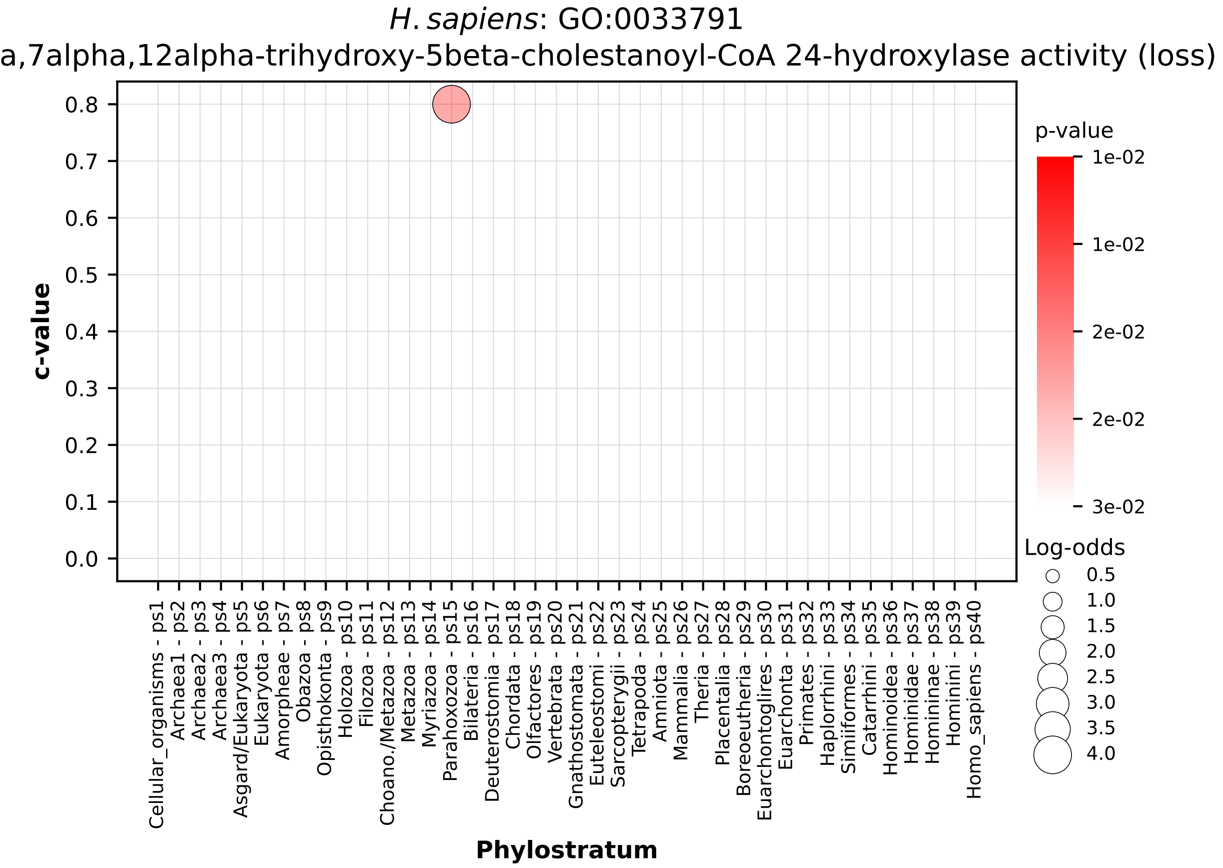

H. sapiens: GO:0033791
a,7alpha,12alpha-trihydroxy-5beta-cholestanoyl-CoA 24-hydroxylase activity (loss)
c-value
0.8
0.7
0.6
0.5
0.4
0.3
0.2
0.1
0.0
p-value
1e-02
1e-02
2e-02
2e-02
3e-02
Log-odds
0.5
1.0
1.5
2.0
2.5
3.0
3.5
4.0
Cellular_organisms - ps1
Archaea1 - ps2
Archaea2 - ps3
Archaea3 - ps4
Asgard/Eukaryota - ps5
Eukaryota - ps6
Amorpheae - ps7
Obazoa - ps8
Opisthokonta - ps9
Holozoa - ps10
Filozoa - ps11
Choano./Metazoa - ps12
Metazoa - ps13
Myriazoa - ps14
Parahoxozoa - ps15
Bilateria - ps16
Deuterostomia - ps17
Chordata - ps18
Olfactores - ps19
Vertebrata - ps20
Gnathostomata - ps21
Euteleostomi - ps22
Sarcopterygii - ps23
Tetrapoda - ps24
Amniota - ps25
Mammalia - ps26
Theria - ps27
Placentalia - ps28
Boreoeutheria - ps29
Euarchontoglires - ps30
Euarchonta - ps31
Primates - ps32
Haplorrhini - ps33
Simiiformes - ps34
Catarrhini - ps35
Hominoidea - ps36
Hominidae - ps37
Homininae - ps38
Hominini - ps39
Homo_sapiens - ps40
Phylostratum

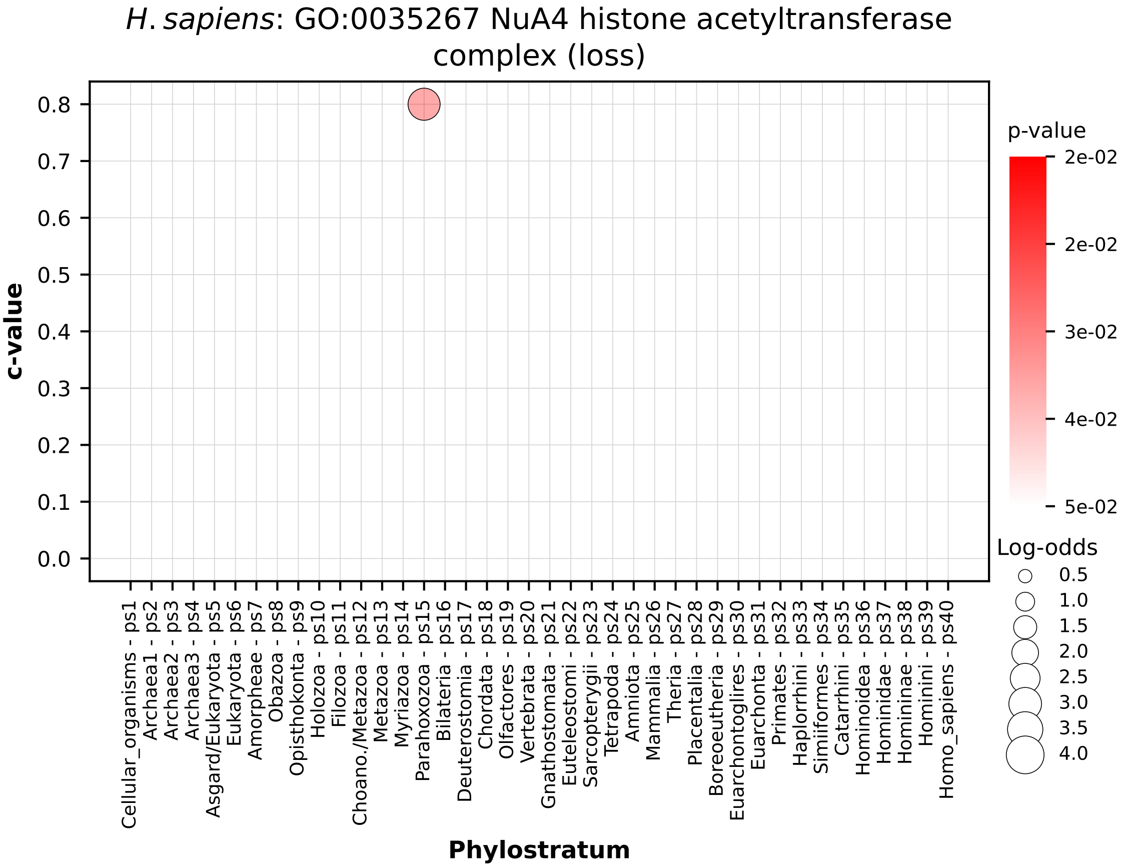

H. sapiens: GO:0035267 NuA4 histone acetyltransferase complex (loss)
c-value
0.8
0.7
0.6
0.5
0.4
0.3
0.2
0.1
0.0
p-value
2e-02
2e-02
3e-02
4e-02
5e-02
Log-odds
0.5
1.0
1.5
2.0
2.5
3.0
3.5
4.0
Cellular_organisms - ps1
Archaea1 - ps2
Archaea2 - ps3
Archaea3 - ps4
Asgard/Eukaryota - ps5
Eukaryota - ps6
Amorpheae - ps7
Obazoa - ps8
Opisthokonta - ps9
Holozoa - ps10
Filozoa - ps11
Choano./Metazoa - ps12
Metazoa - ps13
Myriazoa - ps14
Parahoxozoa - ps15
Bilateria - ps16
Deuterostomia - ps17
Chordata - ps18
Olfactores - ps19
Vertebrata - ps20
Gnathostomata - ps21
Euteleostomi - ps22
Sarcopterygii - ps23
Tetrapoda - ps24
Amniota - ps25
Mammalia - ps26
Theria - ps27
Placentalia - ps28
Boreoeutheria - ps29
Euarchontoglires - ps30
Euarchonta - ps31
Primates - ps32
Haplorrhini - ps33
Simiiformes - ps34
Catarrhini - ps35
Hominoidea - ps36
Hominidae - ps37
Homininae - ps38
Hominini - ps39
Homo_sapiens - ps40
Phylostratum

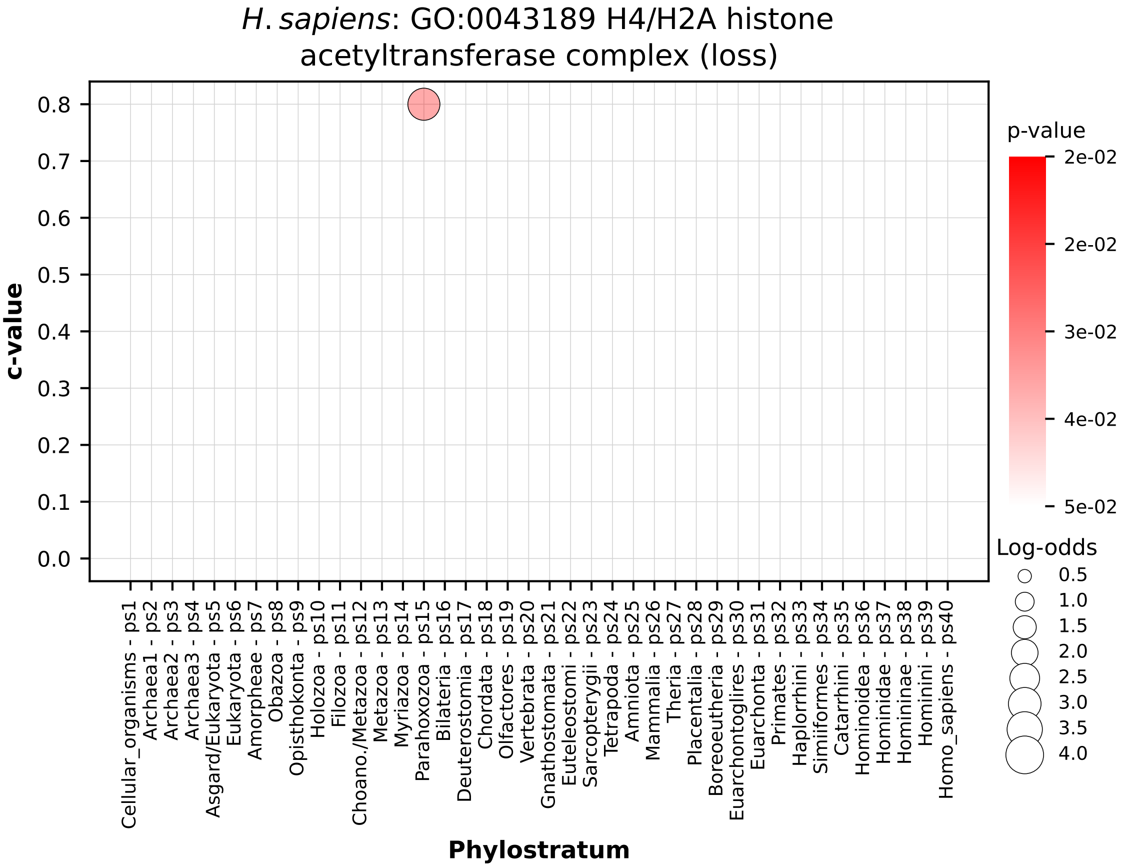

H. sapiens: GO:0043189 H4/H2A histone acetyltransferase complex (loss)
c-value
0.8
0.7
0.6
0.5
0.4
0.3
0.2
0.1
0.0
Phylostratum
Cellular_organisms - ps1
Archaea1 - ps2
Archaea2 - ps3
Archaea3 - ps4
Asgard/Eukaryota - ps5
Eukaryota - ps6
Amorpheae - ps7
Obazoa - ps8
Opisthokonta - ps9
Holozoa - ps10
Filozoa - ps11
Choano./Metazoa - ps12
Metazoa - ps13
Myriazoa - ps14
Parahoxozoa - ps15
Bilateria - ps16
Deuterostomia - ps17
Chordata - ps18
Olfactores - ps19
Vertebrata - ps20
Gnathostomata - ps21
Euteleostomi - ps22
Sarcopterygii - ps23
Tetrapoda - ps24
Amniota - ps25
Mammalia - ps26
Theria - ps27
Placentalia - ps28
Boreoeutheria - ps29
Euarchontoglires - ps30
Euarchonta - ps31
Primates - ps32
Haplorrhini - ps33
Simiiformes - ps34
Catarrhini - ps35
Hominoidea - ps36
Hominidae - ps37
Homininae - ps38
Hominini - ps39
Homo_sapiens - ps40
p-value
2e-02
2e-02
3e-02
4e-02
5e-02
Log-odds
0.5
1.0
1.5
2.0
2.5
3.0
3.5
4.0

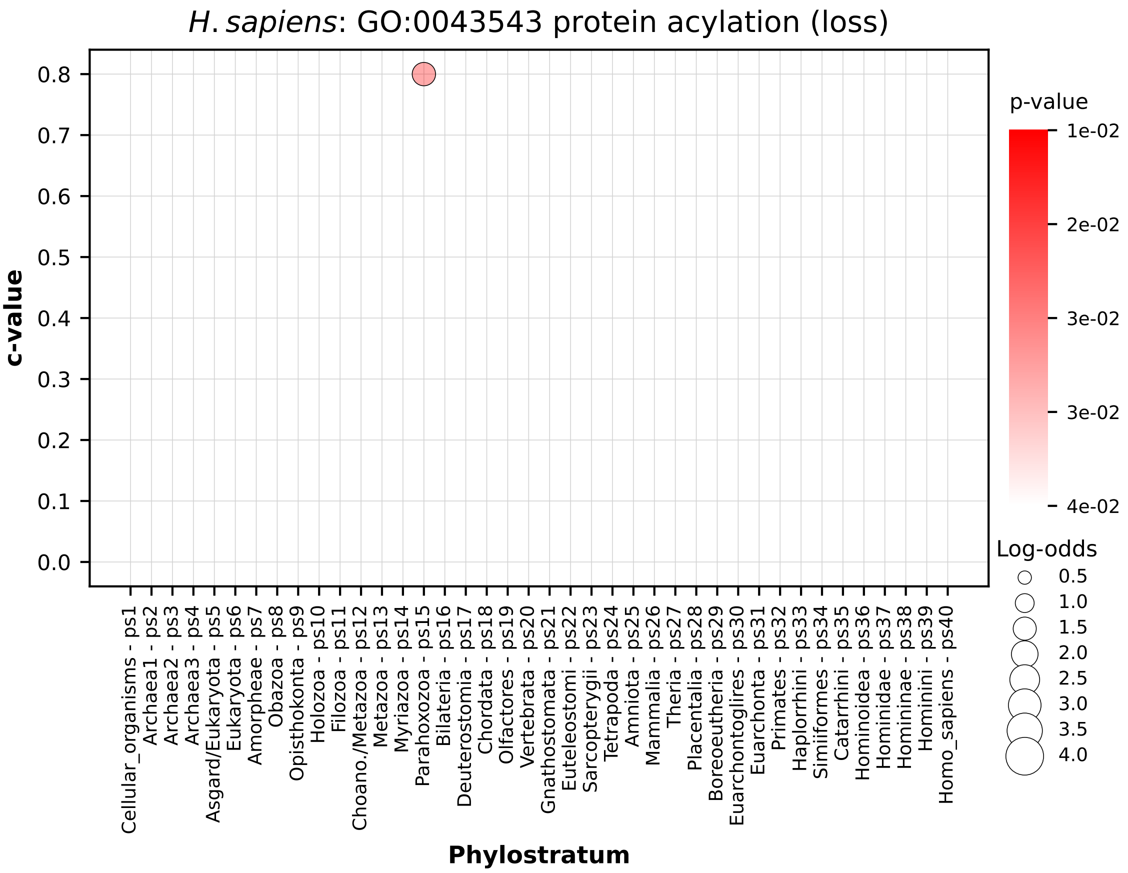

H. sapiens: GO:0043543 protein acylation (loss)
c-value
0.0
0.1
0.2
0.3
0.4
0.5
0.6
0.7
0.8
Phylostratum
Cellular_organisms - ps1
Archaea1 - ps2
Archaea2 - ps3
Archaea3 - ps4
Asgard/Eukaryota - ps5
Eukaryota - ps6
Amorpheae - ps7
Obazoa - ps8
Opisthokonta - ps9
Holozoa - ps10
Filozoa - ps11
Choano./Metazoa - ps12
Metazoa - ps13
Myriazoa - ps14
Parahoxozoa - ps15
Bilateria - ps16
Deuterostomia - ps17
Chordata - ps18
Olfactores - ps19
Vertebrata - ps20
Gnathostomata - ps21
Euteleostomi - ps22
Sarcopterygii - ps23
Tetrapoda - ps24
Amniota - ps25
Mammalia - ps26
Theria - ps27
Placentalia - ps28
Boreoeutheria - ps29
Euarchontoglires - ps30
Euarchonta - ps31
Primates - ps32
Haplorrhini - ps33
Simiiformes - ps34
Catarrhini - ps35
Hominoidea - ps36
Hominidae - ps37
Homininae - ps38
Hominini - ps39
Homo_sapiens - ps40
p-value
1e-02
2e-02
3e-02
3e-02
4e-02
Log-odds
0.5
1.0
1.5
2.0
2.5
3.0
3.5
4.0

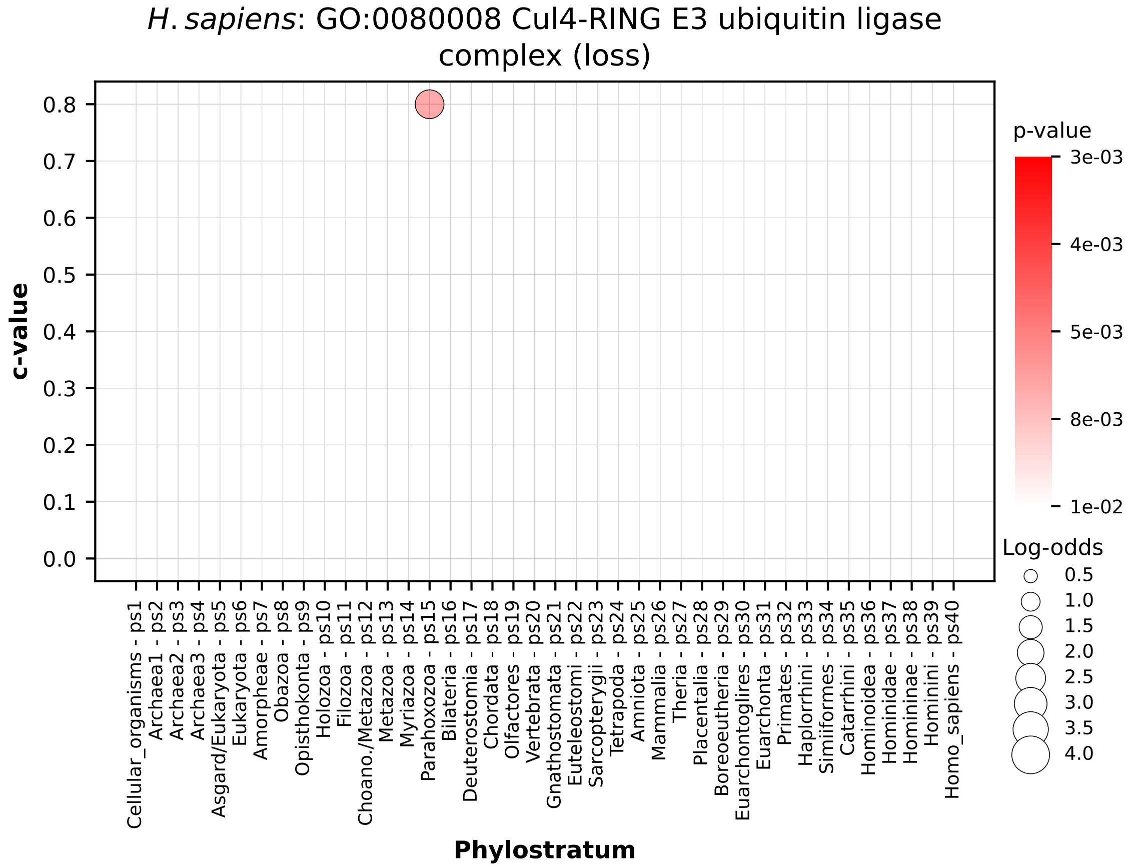

H. sapiens: GO:0080008 Cul4-RING E3 ubiquitin ligase complex (loss)
c-value
0.0
0.1
0.2
0.3
0.4
0.5
0.6
0.7
0.8
Phylostratum
Cellular_organisms - ps1
Archaea1 - ps2
Archaea2 - ps3
Archaea3 - ps4
Asgard/Eukaryota - ps5
Eukaryota - ps6
Amorpheae - ps7
Obazoa - ps8
Opisthokonta - ps9
Holozoa - ps10
Filozoa - ps11
Choano./Metazoa - ps12
Metazoa - ps13
Myriazoa - ps14
Parahoxozoa - ps15
Bilateria - ps16
Deuterostomia - ps17
Chordata - ps18
Olfactores - ps19
Vertebrata - ps20
Gnathostomata - ps21
Euteleostomi - ps22
Sarcopterygii - ps23
Tetrapoda - ps24
Amniota - ps25
Mammalia - ps26
Theria - ps27
Placentalia - ps28
Boreoeutheria - ps29
Euarchontoglires - ps30
Euarchonta - ps31
Primates - ps32
Haplorrhini - ps33
Simiiformes - ps34
Catarrhini - ps35
Hominoidea - ps36
Hominidae - ps37
Homininae - ps38
Hominini - ps39
Homo_sapiens - ps40
p-value
3e-03
4e-03
5e-03
8e-03
1e-02
Log-odds
0.5
1.0
1.5
2.0
2.5
3.0
3.5
4.0

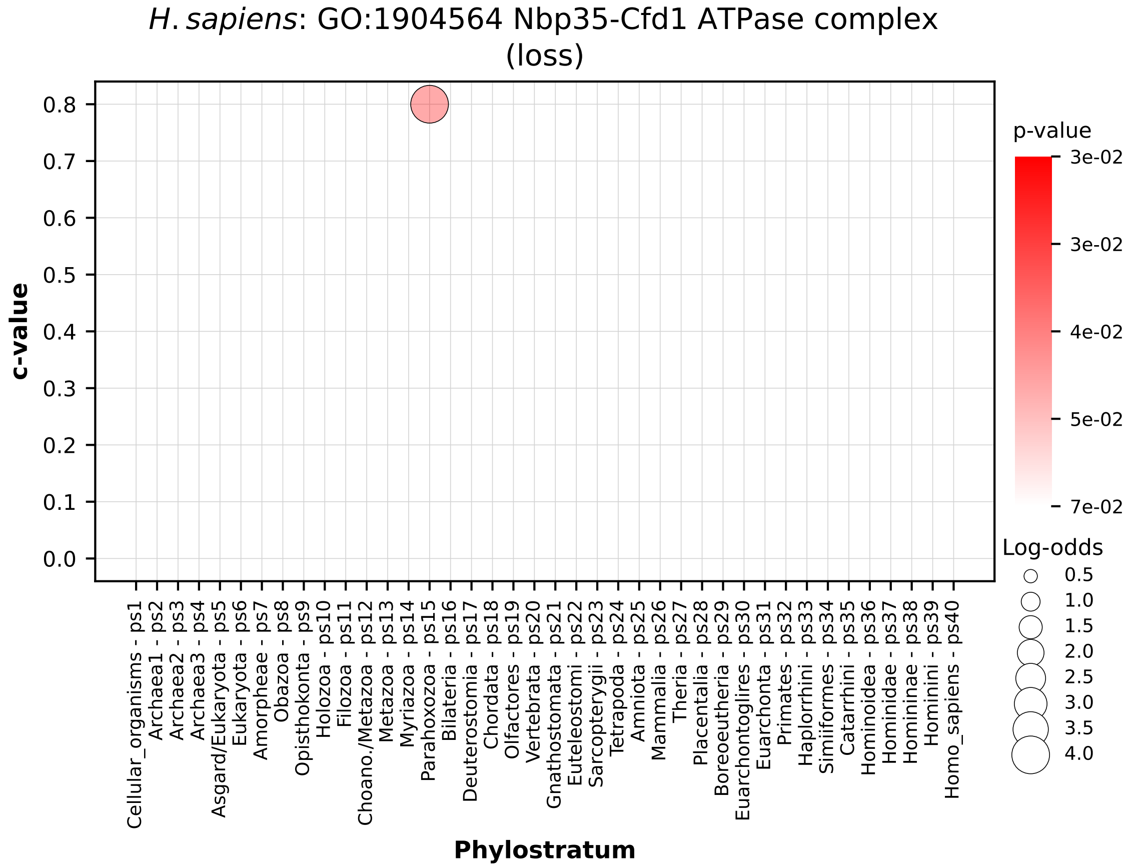

H. sapiens: GO:1904564 Nbp35-Cfd1 ATPase complex
(loss)
c-value
0.0
0.1
0.2
0.3
0.4
0.5
0.6
0.7
0.8
p-value
3e-02
3e-02
4e-02
5e-02
7e-02
Log-odds
0.5
1.0
1.5
2.0
2.5
3.0
3.5
4.0
Cellular_organisms - ps1
Archaea1 - ps2
Archaea2 - ps3
Archaea3 - ps4
Asgard/Eukaryota - ps5
Eukaryota - ps6
Amorpheae - ps7
Obazoa - ps8
Opisthokonta - ps9
Holozoa - ps10
Filozoa - ps11
Choano./Metazoa - ps12
Metazoa - ps13
Myriazoa - ps14
Parahoxozoa - ps15
Bilateria - ps16
Deuterostomia - ps17
Chordata - ps18
Olfactores - ps19
Vertebrata - ps20
Gnathostomata - ps21
Euteleostomi - ps22
Sarcopterygii - ps23
Tetrapoda - ps24
Amniota - ps25
Mammalia - ps26
Theria - ps27
Placentalia - ps28
Boreoeutheria - ps29
Euarchontoglires - ps30
Euarchonta - ps31
Primates - ps32
Haplorrhini - ps33
Simiiformes - ps34
Catarrhini - ps35
Hominoidea - ps36
Hominidae - ps37
Homininae - ps38
Hominini - ps39
Homo_sapiens - ps40
Phylostratum

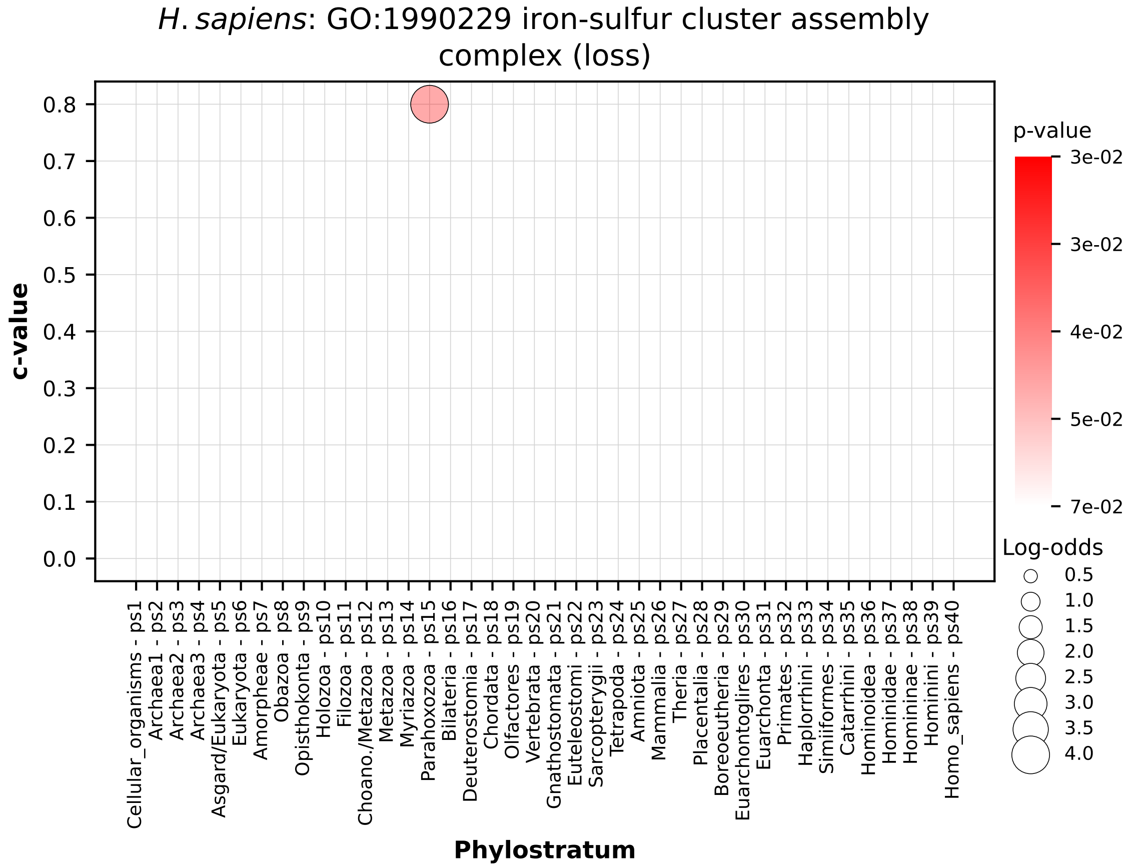
H. sapiens: GO:1990229 iron-sulfur cluster assembly complex (loss)
c-value
0.0
0.1
0.2
0.3
0.4
0.5
0.6
0.7
0.8
Phylostratum
Cellular_organisms - ps1
Archaea1 - ps2
Archaea2 - ps3
Archaea3 - ps4
Asgard/Eukaryota - ps5
Eukaryota - ps6
Amorpheae - ps7
Obazoa - ps8
Opisthokonta - ps9
Holozoa - ps10
Filozoa - ps11
Choano./Metazoa - ps12
Metazoa - ps13
Myriazoa - ps14
Parahoxozoa - ps15
Bilateria - ps16
Deuterostomia - ps17
Chordata - ps18
Olfactores - ps19
Vertebrata - ps20
Gnathostomata - ps21
Euteleostomi - ps22
Sarcopterygii - ps23
Tetrapoda - ps24
Amniota - ps25
Mammalia - ps26
Theria - ps27
Placentalia - ps28
Boreoeutheria - ps29
Euarchontoglires - ps30
Euarchonta - ps31
Primates - ps32
Haplorrhini - ps33
Simiiformes - ps34
Catarrhini - ps35
Hominoidea - ps36
Hominidae - ps37
Homininae - ps38
Hominini - ps39
Homo_sapiens - ps40
p-value
3e-02
3e-02
4e-02
5e-02
7e-02
Log-odds
0.5
1.0
1.5
2.0
2.5
3.0
3.5
4.0

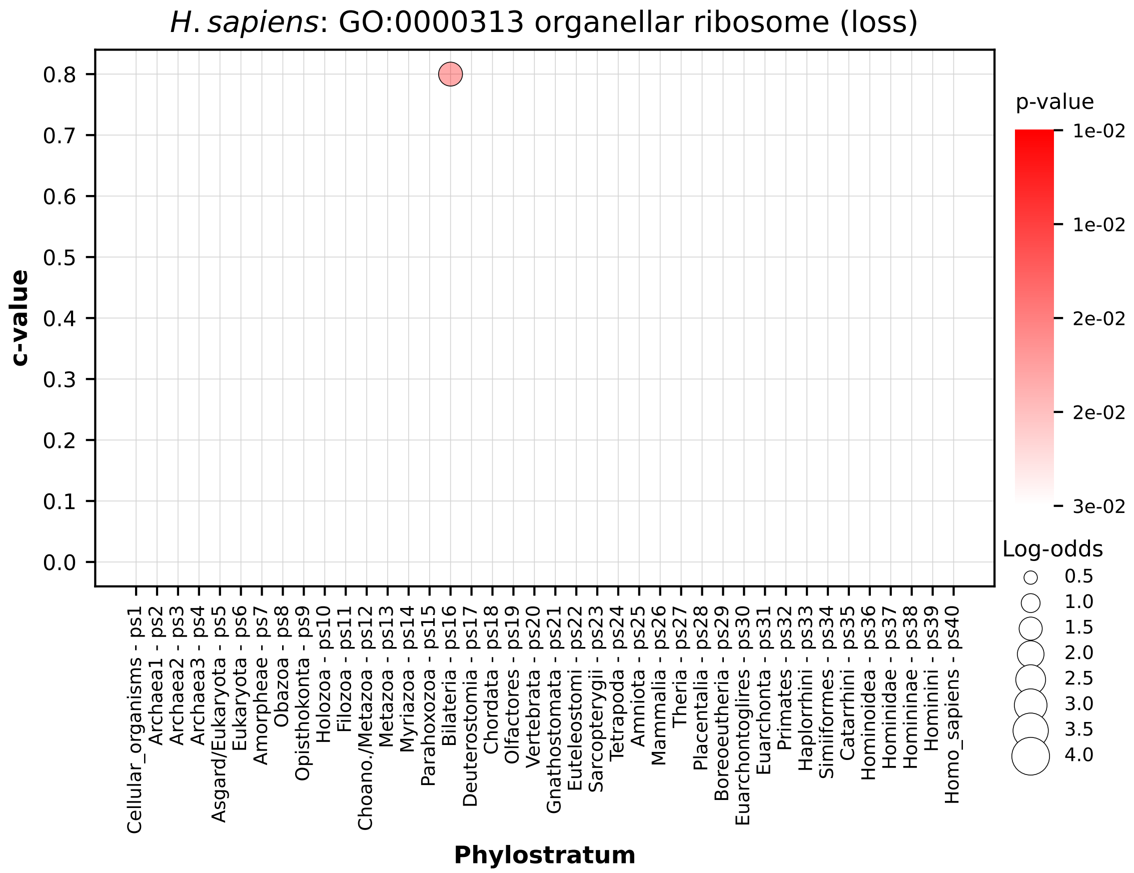

H. sapiens: GO:0000313 organellar ribosome (loss)
c-value
0.8
0.7
0.6
0.5
0.4
0.3
0.2
0.1
0.0
Phylostratum
Cellular_organisms - ps1
Archaea1 - ps2
Archaea2 - ps3
Archaea3 - ps4
Asgard/Eukaryota - ps5
Eukaryota - ps6
Amorpheae - ps7
Obazoa - ps8
Opisthokonta - ps9
Holozoa - ps10
Filozoa - ps11
Choano./Metazoa - ps12
Metazoa - ps13
Myriazoa - ps14
Parahoxozoa - ps15
Bilateria - ps16
Deuterostomia - ps17
Chordata - ps18
Olfactores - ps19
Vertebrata - ps20
Gnathostomata - ps21
Euteleostomi - ps22
Sarcopterygii - ps23
Tetrapoda - ps24
Amniota - ps25
Mammalia - ps26
Theria - ps27
Placentalia - ps28
Boreoeutheria - ps29
Euarchontoglires - ps30
Euarchonta - ps31
Primates - ps32
Haplorrhini - ps33
Simiiformes - ps34
Catarrhini - ps35
Hominoidea - ps36
Hominidae - ps37
Homininae - ps38
Hominini - ps39
Homo_sapiens - ps40
p-value
1e-02
1e-02
2e-02
2e-02
3e-02
Log-odds
0.5
1.0
1.5
2.0
2.5
3.0
3.5
4.0

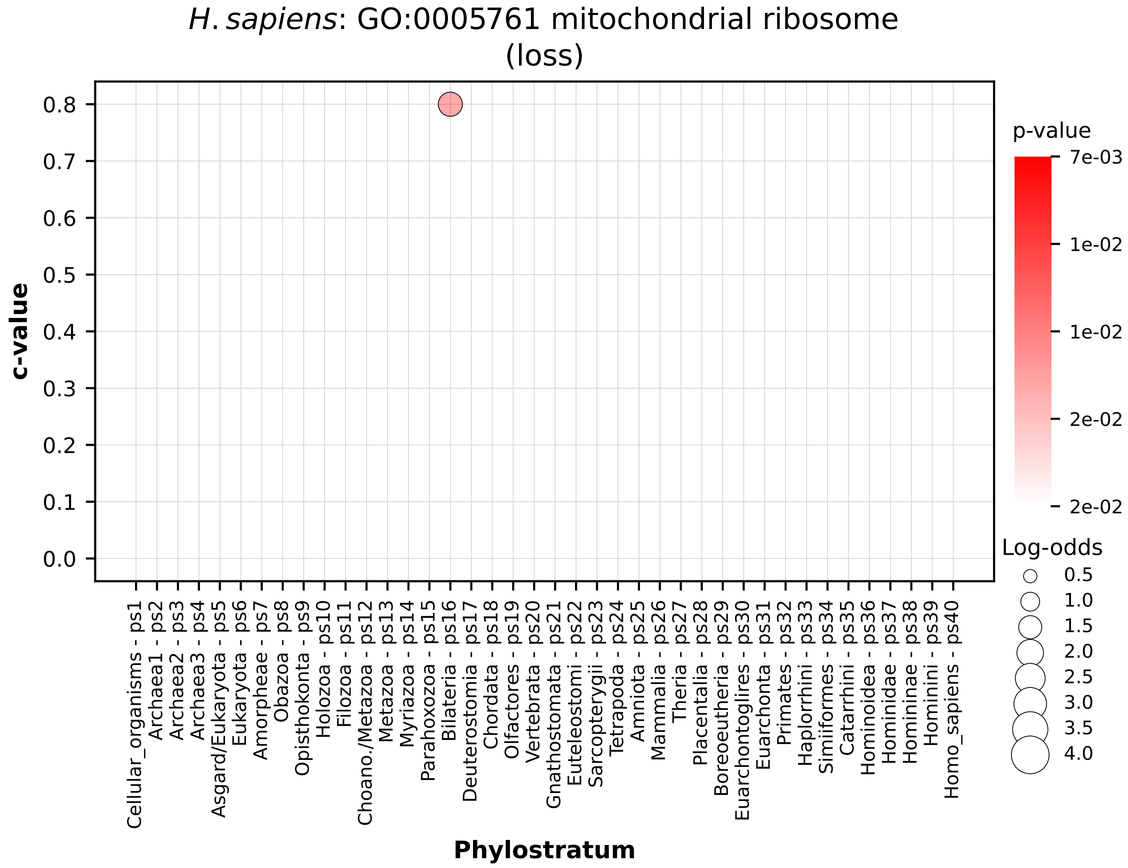

H. sapiens: GO:0005761 mitochondrial ribosome
(loss)
c-value
0.8
0.7
0.6
0.5
0.4
0.3
0.2
0.1
0.0
p-value
7e-03
1e-02
1e-02
2e-02
2e-02
Log-odds
0.5
1.0
1.5
2.0
2.5
3.0
3.5
4.0
Cellular_organisms - ps1
Archaea1 - ps2
Archaea2 - ps3
Archaea3 - ps4
Asgard/Eukaryota - ps5
Eukaryota - ps6
Amorpheae - ps7
Obazoa - ps8
Opisthokonta - ps9
Holozoa - ps10
Filozoa - ps11
Choano./Metazoa - ps12
Metazoa - ps13
Myriazoa - ps14
Parahoxozoa - ps15
Bilateria - ps16
Deuterostomia - ps17
Chordata - ps18
Olfactores - ps19
Vertebrata - ps20
Gnathostomata - ps21
Euteleostomi - ps22
Sarcopterygii - ps23
Tetrapoda - ps24
Amniota - ps25
Mammalia - ps26
Theria - ps27
Placentalia - ps28
Boreoeutheria - ps29
Euarchontoglires - ps30
Euarchonta - ps31
Primates - ps32
Haplorrhini - ps33
Simiiformes - ps34
Catarrhini - ps35
Hominoidea - ps36
Hominidae - ps37
Homininae - ps38
Hominini - ps39
Homo_sapiens - ps40
Phylostratum

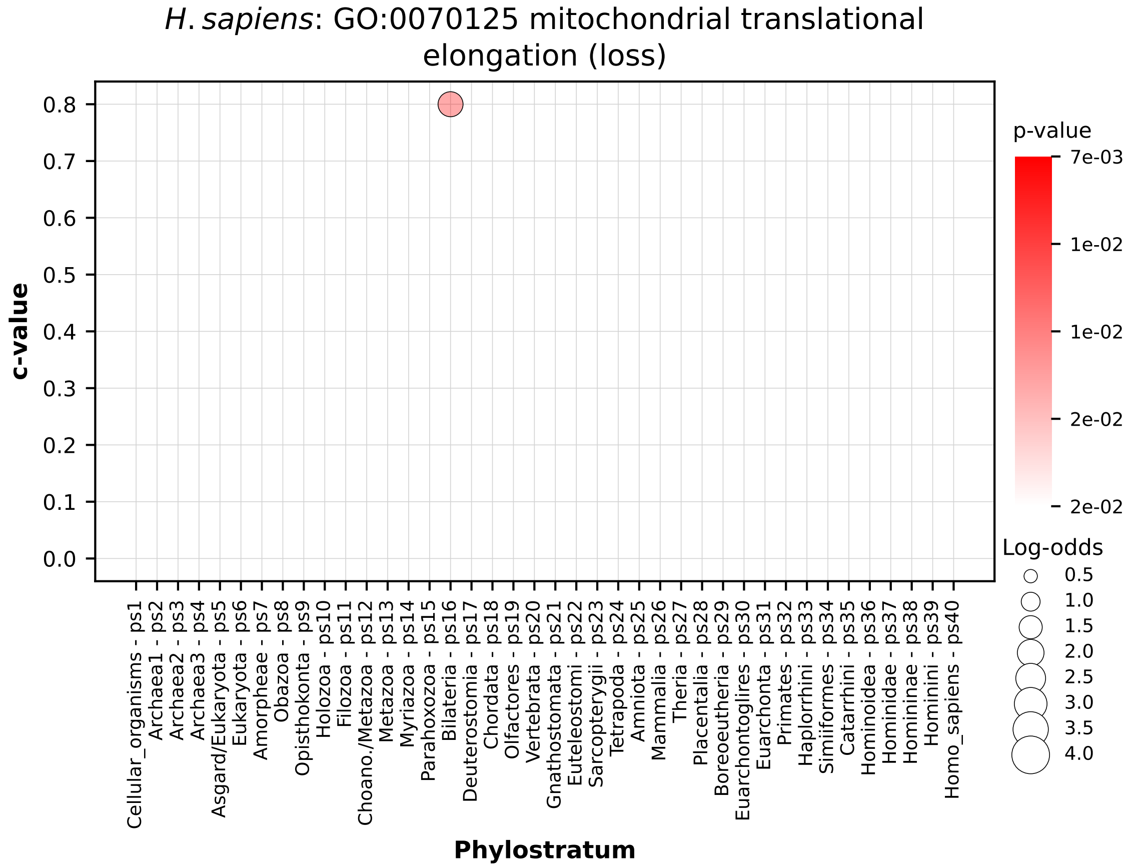

H. sapiens: GO:0070125 mitochondrial translational elongation (loss)
c-value
0.8
0.7
0.6
0.5
0.4
0.3
0.2
0.1
0.0
p-value
7e-03
1e-02
1e-02
2e-02
2e-02
Log-odds
0.5
1.0
1.5
2.0
2.5
3.0
3.5
4.0
Cellular_organisms - ps1
Archaea1 - ps2
Archaea2 - ps3
Archaea3 - ps4
Asgard/Eukaryota - ps5
Eukaryota - ps6
Amorpheae - ps7
Obazoa - ps8
Opisthokonta - ps9
Holozoa - ps10
Filozoa - ps11
Choano./Metazoa - ps12
Metazoa - ps13
Myriazoa - ps14
Parahoxozoa - ps15
Bilateria - ps16
Deuterostomia - ps17
Chordata - ps18
Olfactores - ps19
Vertebrata - ps20
Gnathostomata - ps21
Euteleostomi - ps22
Sarcopterygii - ps23
Tetrapoda - ps24
Amniota - ps25
Mammalia - ps26
Theria - ps27
Placentalia - ps28
Boreoeutheria - ps29
Euarchontoglires - ps30
Euarchonta - ps31
Primates - ps32
Haplorrhini - ps33
Simiiformes - ps34
Catarrhini - ps35
Hominoidea - ps36
Hominidae - ps37
Homininae - ps38
Hominini - ps39
Homo_sapiens - ps40
Phylostratum

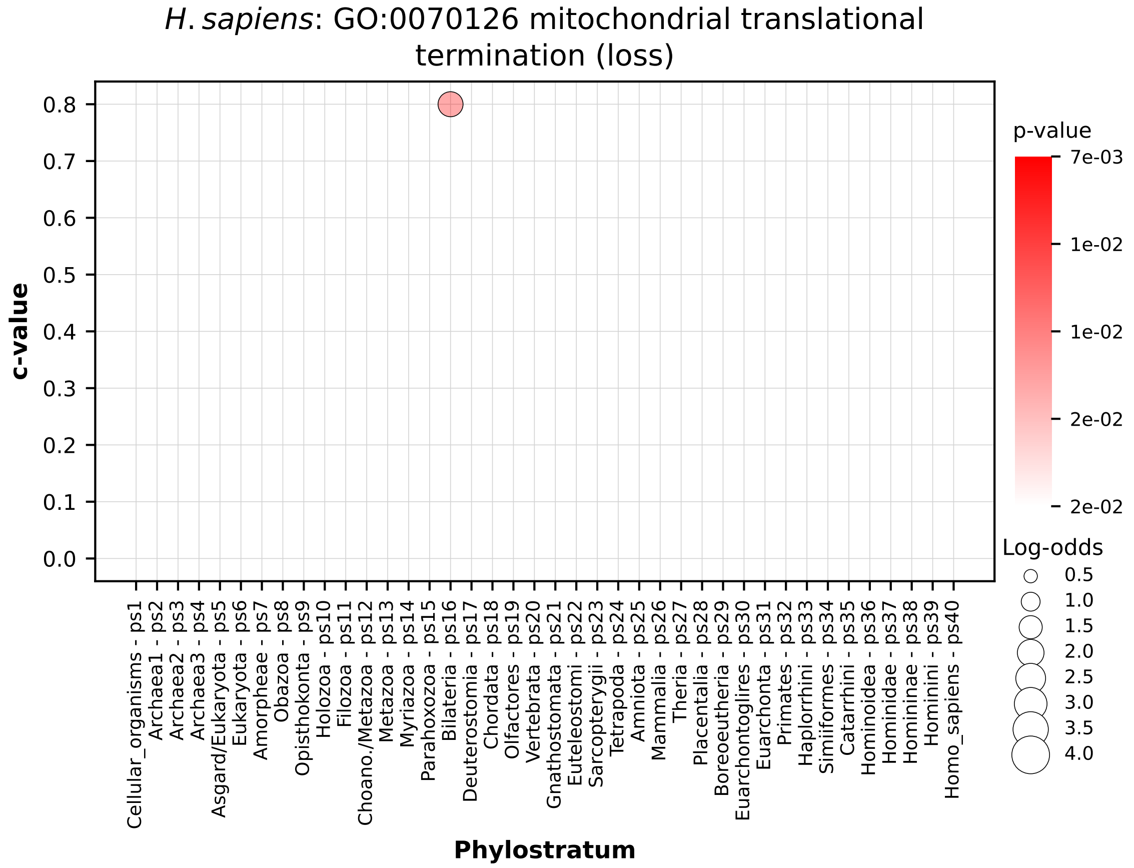

H. sapiens: GO:0070126 mitochondrial translational termination (loss)
c-value
0.8
0.7
0.6
0.5
0.4
0.3
0.2
0.1
0.0
p-value
7e-03
1e-02
1e-02
2e-02
2e-02
Log-odds
0.5
1.0
1.5
2.0
2.5
3.0
3.5
4.0
Cellular_organisms - ps1
Archaea1 - ps2
Archaea2 - ps3
Archaea3 - ps4
Asgard/Eukaryota - ps5
Eukaryota - ps6
Amorpheae - ps7
Obazoa - ps8
Opisthokonta - ps9
Holozoa - ps10
Filozoa - ps11
Choano./Metazoa - ps12
Metazoa - ps13
Myriazoa - ps14
Parahoxozoa - ps15
Bilateria - ps16
Deuterostomia - ps17
Chordata - ps18
Olfactores - ps19
Vertebrata - ps20
Gnathostomata - ps21
Euteleostomi - ps22
Sarcopterygii - ps23
Tetrapoda - ps24
Amniota - ps25
Mammalia - ps26
Theria - ps27
Placentalia - ps28
Boreoeutheria - ps29
Euarchontoglires - ps30
Euarchonta - ps31
Primates - ps32
Haplorrhini - ps33
Simiiformes - ps34
Catarrhini - ps35
Hominoidea - ps36
Hominidae - ps37
Homininae - ps38
Hominini - ps39
Homo_sapiens - ps40
Phylostratum

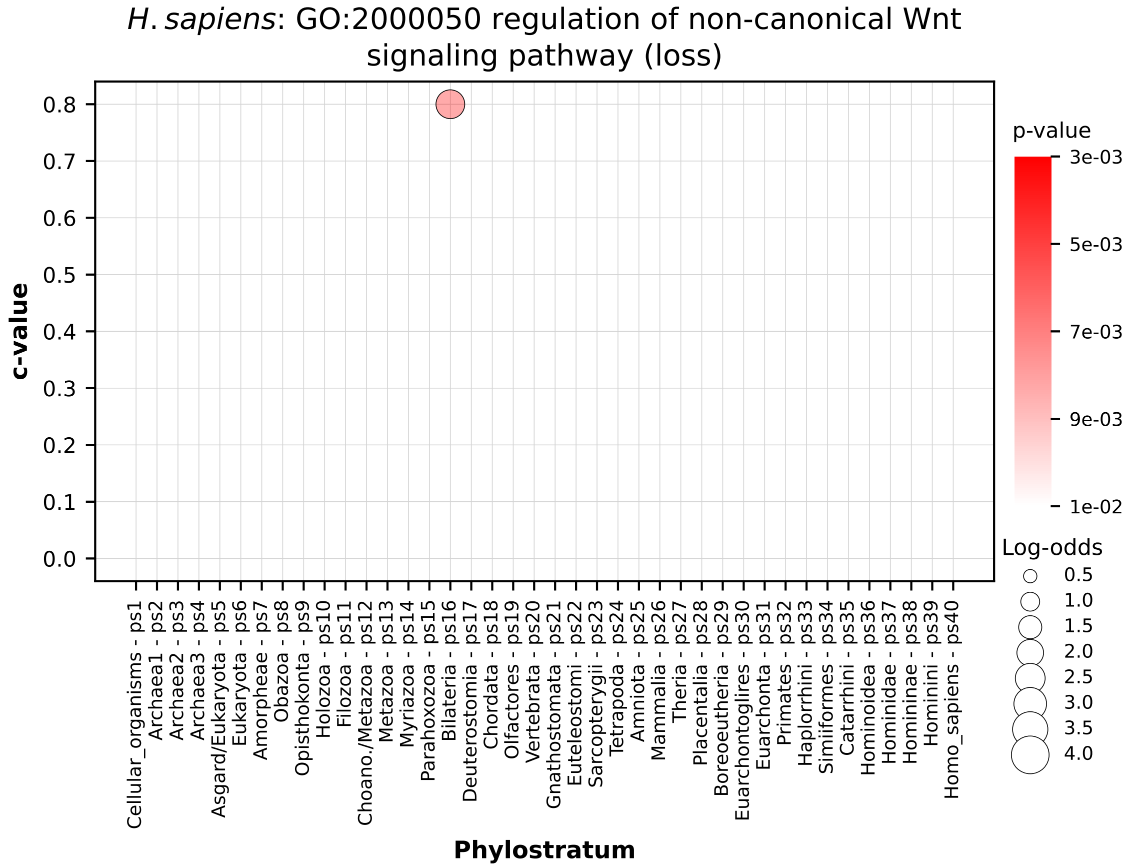
H. sapiens: GO:2000050 regulation of non-canonical Wnt signaling pathway (loss)
c-value
0.0
0.1
0.2
0.3
0.4
0.5
0.6
0.7
0.8
p-value
3e-03
5e-03
7e-03
9e-03
1e-02
Log-odds
0.5
1.0
1.5
2.0
2.5
3.0
3.5
4.0
Cellular_organisms - ps1
Archaea1 - ps2
Archaea2 - ps3
Archaea3 - ps4
Asgard/Eukaryota - ps5
Eukaryota - ps6
Amorpheae - ps7
Obazoa - ps8
Opisthokonta - ps9
Holozoa - ps10
Filozoa - ps11
Choano./Metazoa - ps12
Metazoa - ps13
Myriazoa - ps14
Parahoxozoa - ps15
Bilateria - ps16
Deuterostomia - ps17
Chordata - ps18
Olfactores - ps19
Vertebrata - ps20
Gnathostomata - ps21
Euteleostomi - ps22
Sarcopterygii - ps23
Tetrapoda - ps24
Amniota - ps25
Mammalia - ps26
Theria - ps27
Placentalia - ps28
Boreoeutheria - ps29
Euarchontoglires - ps30
Euarchonta - ps31
Primates - ps32
[truncated: 392,436 more chars]
